# Genome-wide identification, characterization and gene expression of BES1 transcription factor family in grapevine (*Vitis vinifera* L.)

**DOI:** 10.1038/s41598-022-24407-y

**Published:** 2023-01-05

**Authors:** Jiajia Li, Lei Wang, Feng Leng, Chao Ma, Caixi Zhang, Shiping Wang

**Affiliations:** 1 Department of Plant Science, School of Agriculture and Biology, Shanghai Jiao Tong University, Shanghai, China; 2 College of Horticulture and Plant Protection, Yangzhou University, Yangzhou, China

**Keywords:** Plant molecular biology, Plant physiology

## Abstract

BES1, as the most important transcription factor responsible for brassinolide (BR) signaling, has been confirmed to play a significant role in regulating plant growth and the improvement of stress resistance. The transcriptional regulatory mechanism of BES1 has been well elucidated in several plants, such as *Arabidopsis thaliana* (*A. thaliana*), *Triticum aestivum* L. (*T. aestivum*), and *Oryza sativa* L. (*O. sativa*). Nevertheless, the genome-wide analysis of the BES1 family in *Vitis vinifera* L. (*V. vinifera*). has not been comprehensively carried out. Thus, we have conducted a detailed analysis and identification of the BES1 transcription factors family in *V. vinifera*; a total of eight VvBES1 genes was predicted, and the phylogenetic relationships, gene structures, and *Cis*-acting element in their promoters were also analyzed. BES1 genes have been divided into three groups (I, II and III) based on phylogenetic relationship analysis, and most of VvBES1 genes were in group III. Also, we found that VvBES1 genes was located at seven of the total nineteen chromosomes, whereas VvBES1-2 (Vitvi04g01234) and VvBES1-5 (Vitvi18g00924) had a collinearity relationship, and their three copies are well preserved. In addition, the intron–exon model of VvBES1 genes were mostly conserved, and there existed several *Cis*-acting elements related to stress resistance responsive and phytohormones responsive in BES1s genes promoter. Moreover, the BES1 expressions were different in different *V. vinifera* organs, and BES1 expressions were different in different *V. vinifera* varieties under saline-alkali stress and heat stress, the expression of VvBES1 also changed with the prolongation of saline-alkali stress treatment time. The above findings could not only lay a primary foundation for the further validation of VvBES1 function, but could also provide a reference for molecular breeding in *V. vinifera*.

## Introduction

Brassinosteroids (BRs), as a new type of plant endogenous hormones, could not only significantly increase plant nutrient growth and effectively increase chlorophyll content, but could also improve plant stress resistance, including salinity, heat damage, cold damage and water stress^1^. Furthermore, it has been confirmed that BRs play the important roles in regulating fruit ripening and leaf senescence^2^. Therefore, the molecular mechanism and signal transduction of BR anabolism have been systematically studied in *A. thaliana*^3^. It has been reported that the biosynthesis of BR could be divided into the early C6 pathway and the late C6 pathway^4^, and in cattail plants, the main forms of BRs are typhasterol and teasterone, while the main forms of BRs in gramineous *O. sativa* are brassinolide, castasterone and homodolichosterone^5^. BR need to be degraded after performing their functions as plant hormones, and their metabolic pathways can be categorized into hydroxylation, glycosylation and acylation^6^. The signaling transduction of BRs is a complex and delicate process; recent studies have found that their signaling is jointly transmitted by receptor BRI1 and co-receptor BAK1^7^. A variety of BRI1 mutants has been confirmed to show delayed aging and decreased fertility together with BR deletion bodies^8^. Furtherly, BR biosynthesis, metabolism and signal transduction depended on the activity of a series of enzymes, the joint regulation of these enzyme played role in plant growth and development^9^.

BES1 and BZR1 are two key transcription factors that were initially identified as specifically regulating BRs to mediate gene expression^10^. The two genes belong to a family of six genes, including VvBES1, VvBZR1, VvBEH1, VvBEH2, VvBEH3 and VvBEH4. It has been reported that BES1/BZR1 mediate BR signal transduction, and BES1/BZR1 could regulate plant growth and development by regulating the expression of downstream target genes in BR biosynthesis^11^. BES1 has been confirmed to have a significant impact on BR signal transduction, plays an important role in both abscisic acid (ABA) and strigolactone (SL) signaling^12^, and it could interact with REPRESSOR OF GA1-3 (RGA), a member of the DELLA transcriptional regulatory family, by regulating the biosynthesis of gibberellin 3 (GA_3_), and co-controlling cell elongation and plant growth^13^. Moreover, the interaction of BES1 and BZR1 co-regulated plant physiological metabolism through different signaling pathways, thus regulated plant growth development, stress response formation, and promote the stable transmission of BR signal downstream through the dual regulation of phosphorylation, etc^14^. In recent years, BES1 interacts with BIM1 has been widely reported, which recognized as a basic helix-loop-helix protein, will bind to E box (CANNTG) sequences present in many promoters (BR-induced)^15^. And UV-B activated the expression of UVR8 and inhibits the DNA binding activity of BES1/BIM1, thus affecting the occurrence of photomorphogenesis in *A. thaliana*^16^*.* Further investigation illustrated that, BES1 and BZR1 mutant, showed BR-activated phenotypes (enhanced hypocotyl elongation). In contrast, BES1 and BIM mutant, indicated hypocotyl shortened and dwarf phenotypes^17^. It has been reported that there is an interaction between BES1 and other transcription factors, such as the PIF4, WRKY and MYB gene families^18^. For instance, the interaction between PIF4 and BES1 could control cell elongation and plant growth^19^, WRKY46, WRKY54, and WRKY70 interacts with BES1 co-regulate plant stress resistance formation^20^, and the interaction between MYB and BES1 regulates the accumulation of anthocyanins and the improvement of fruit quality^21^.

*Vitis vinifera* L., as a widely planted economic crop around the world, is recognized by consumers because of its sweet, sour taste, as well as its high nutritional value. In recent years, the genome-wide identification of BES1 has been well studied in *A. thaliana* and *T. aestivum*^22,23^, and some genome-wide identifications have also been carried out in horticultural crops including *Cucumis sativus* L. (*C*. *sativus*), *Solanum lycopersicum* L. (*S. lycopersicum*), *Solanum tuberosum* L. (*S*. *tuberosum*), *Citrus reticulata* Blanco. (*C. reticulata*) and *Musa nana* Lour. (*M. nana*)^24–29^. However, the genome-wide identification and characterization, and phylogenetic relationship of the BES1 family has not been systematically studied in *V. vinifera*. There was one triplication event in *V. vinifera* ancestors. Theoretically, there should be a lot of duplicate genes with three copies in *V. vinifera*. However, during the evolution, some copies were lost while some were still retained^30^. It is also of great significance to explore the gain and loss of copies in VvBES1 genes. Thus, in this study, the functions of BES1 of *V. vinifera* were investigated, the phylogenetic relationship of BES1 gene family members were systematically analyze, the structure and promoter elements of BES1 genes were explored. Meanwhile, the interspecific collinearity analysis among *Amborella trichopoda* (*A. trichopoda*), *V. vinifera* and *A. thaliana*, the intraspecific collinearity analysis of VvBES1s of *V. vinifera*, and the search of copies of VvBES1 genes were furtherly conducted. To further explore the differential expression of VvBES1 genes in *V. vinifera* organs as well as their abilities to cope with various stresses, the expression pattern of the VvBES1 genes in different organs, various abiotic and biological stresses of *V. vinifera* were quantified. In all, the above findings could lay a primary foundation for understanding the potential regulatory mechanisms of BES1 in *V. vinifera*, and provide a reference for future *V. vinifera* breeding.

## Materials and methods

### Plant material, growth conditions, and stress treatments

“Muscat Hamburg” (“Muscat of Alexandria” × “Trollinger”) was collected from a greenhouse in the Department of Plant Science, School of Agriculture and Biology, Shanghai Jiao Tong University (121.38° E, 31.11° W). The seedlings of *V. vinifera* were 2-year-self-rooted, came from our laboratory, and were cultivated in a 30 × 36 × 15 cm flowerpot with substrate (Nutrient soil: vermiculite: perlite = 4:3:3), regularly irrigated with Hoagland’s nutrient solution. “A17” (“Milk” × “Royal Autumn”), “Benifuji” (“Golden Muscat” × “Igawa 200”), “Shine Muscat” (“No. 21, Anyunjin” × “White South”) and “Ruby Seedless” (“Emperor” × “Pirovan 075”) were collected from greenhouse in Zhoushan Academy of Agriculture and Forestry Sciences Ma 'ao base (121°30′ E, 29°32′ W). *V. vinifera* for both control groups and treatment groups were used shelter cultivation, supplied the same water and fertilizer condition, and planted in a 50 cm deep and 100 cm wide substrate (nutrient soil: vermiculite: perlite = 4:3:3).

Firstly, at Shanghai Jiao Tong University, the roots, stems, leaves, inflorescences and berries of six “Muscat Hamburg” under conventional cultivation were collected for analysis. At 20 days after anthesis (DAA 20), six “Muscat Hamburg” were subjected to heat stress treatment (40 °C, 8 h) and another six “Muscat Hamburg” continued using normal cultivation. At the stages of green berry, verasion and maturity in both the treatment group and control group, thirty berries were collected randomly from the upper, middle and lower parts of the fruit cluster. At the same time, six “Muscat Hamburg” were subjected to saline-alkali stress treatment, and a 0.5% NaCl and 1% NaHCO_3_ mixed solution (1 L) was applied to the *V. vinifera* every three days; we then chose another six *V. vinifera*, and used normal rain shelter cultivation for the control group. Thirty leaves were collected from each plant every five days from the beginning of treatment for further analysis.

Secondly, at Zhejiang Zhoushan Academy of Agriculture and Forestry Sciences Ma 'ao base (121°30′ E, 29°32′ W), at 20 days after anthesis (DAA 20), eighteen *V. vinifera* of each variety (“A17”, “Benifuji”, “Shine Muscat” and “Ruby Seedless”) were selected; nine were used for saline-alkali treatment and nine were used for control. In saline-alkali treatment group, NaCl and NaHCO_3_ (80 mmol/l) mixed solution (pH  8.5) was used to irrigate the vines every three days. In the control group, Hoagland’s nutrient solution was used to irrigate the vines every 3 days. At maturity stage, 30 berries were collected from the upper, middle and lower parts of the fruit cluster from each treatment group, and immediately mailed back to Shanghai Jiao Tong University for further study.

### Identification of the VvBES1 gene family in *V. vinifera*

The BES1 gene and amino acid sequences of the *V. vinifera*, *A. thaliana*, *A. trichopoda*, *Actinidia chinensis* Planch. (*A. chinensis*), and *Prunus persica* L. (*P. persica*) were obtained from Ensembl Plants (https://plants.ensembl.org/index.html). The BES1 gene and amino acid sequences of the *Fragaria* × *ananassa* Duch. (*F. ananassa*), *Malus pumila* Mill. (*M. pumila*), and *Prunus* spp. (*Prunus*) were obtained from NCBI (https://www.ncbi.nlm.nih.gov/). The BES1 gene and amino acid sequences of *Citrullus lanatus* (Thunb.) Matsum. (C. *lanatus*). was obtained from Watermelon (97103) v2 Genome (http://cucurbitgenomics.org/organism/21). The information of *Cis*-acting elements in BES1 genes promoter were found in Plantcare (http://bioinformatics.psb.ugent.be/webtools/plantcare/html/) and PlantTFDB (http://planttfdb.gao-lab.org/tf.php?sp=Ppe&did=Prupe.I004500.1.p).

### Phylogenetic relationship analysis, sequence alignment and gene structure analysis of BES1 gene family

A total of 61 BES1 protein sequences were identified from nine plants, including *V. vinifera*, *A. thaliana*, *A. trichopoda*, *A. chinensis*, *F. ananassa*, C. *lanatus*, *M. pumila*, *P. persica*, and *Prunus*. The multiple alignment of 61 amino acid sequences of BES1 was performed using ClustalW in the Software MEGA 5.0 (software, USA)^31^; a phylogenetic tree was built according to the maximum likelihood method, and bootstrap (1000 repeats) values were used to assess support for a particular grouping pattern. Meanwhile, based on the obtained BES1 protein sequences of *A. thaliana* and *V. vinifera*, MEME (https://meme-suite.org/meme/tools/meme) was used to analyze their motifs^32^. The ORF length of each VvBES1 gene was searched by ORF Finder (http://www.bioinformatics.org/sms2/orf_find.html). Protparam (https://web.expasy.org/protparam/) was applied to analyze the physicochemical properties of VvBES1 protein sequences, including theoretical molecular weight (MW), isoelectric point (PI) and total hydrophilic mean (GRAVY). GSDS 2.0 (https://mybiosoftware.com/gsds-2-0-gene-structure-display-server.html) was used to analyze the nuclear cytoplasmic localization of eight VvBES1 proteins^33^. To further investigate the tertiary structure of protein family, Swiss-MODEL (https://swissmodel.expasy.org/) was used to model the tertiary structure of VvBES1 genes^34^.

### Interspecific and intraspecific synteny analysis of BES1 genes

The interspecific synteny analysis of BES1 genes among *V. vinifera*, *A. thaliana*, and *A. trichopoda*, as well as the intraspecific synteny analysis of VvBES1 genes were all conducted by MCScanx (https://github.com/wyp1125/MCScanx)^35,36^. Firstly, the genome information and corresponding annotation sequences of these three species were all downloaded from EnsemblPlants (http://plants.ensembl.org/index.html). According to their genome sequences and annotation files, their cds sequences were extracted and then translated into amino acid sequences. Blastp (https://blast.ncbi.nlm.nih.gov/Blast.cgi) was used for the protein comparison, Text Merge for the MCScanx template was used to finish the chromosome colinear analysis. Finally, TBtools (https://github.com/CJ-Chen/TBtools-Manual) was used to integrate the results of collinearity relationship, as well as to draw the collinearity plot. All output data are shown in the Supplementary materials.

### The whole-genome doubling (WGD) analysis of VvBES1 gene family

WGD analysis was also carried out using Blastp (https://blast.ncbi.nlm.nih.gov/Blast.cgi)^37^ and MCScanx (https://github.com/wyp1125/MCScanx)^36^. First, the genomic protein sequences of *V. vinifera* were downloaded from EnsemblPlants (http://plants.ensembl.org/index.html), whole genome alignment was performed using Blastp (https://blast.ncbi.nlm.nih.gov/Blast.cgi). Collinearity analysis was performed combined with gene alignment information and the location of the gene on chromosome. Finally, known genes were searched for in the resulting collinearity list. All output data are shown in the Supplementary materials.

### RNA extraction and qRT-PCR

The RNA prep Pure Plant Plus Kit (TaKaRa, Dalian, China) was used to extract the RNA from berry, root, leaf, stem and inflorescence from different *V. vinifera* varieties under different treatments. A BIO-RADXR gel imaging analysis system (Bio-Rad, CA, USA) was established to analyze the purity and integrality of RNA. According to the manufacturer’s instructions, we used the PrimeScript™ RT reagent kit (TaKaRa, Dalian, China) with 1 μg of total RNA to perform qRT-PCR analysis. The qPCR system employed the following program: 95 °C for 20 s, then followed by 39 cycles of 95 °C for 15 s, finally, 55 °C for 15 s and 60 °C for 15 s. The 2^−ΔΔCt^ assay was used to calculate the final relative expressions of each gene. The primers for qPCR analysis are all listed in Table S1.

### Data analysis and figure drawing

In the matter of data analysis, SPSS 16.0 (IBM, Armonk, NY, USA) was used for analyzing the difference significance of data, which was expressed in the form of mean + standard error (SE). Independent sample T-test, and univariate variance homogeneity of ANOVA were applied to analyze the difference significance among treatments (P < 0.05). GraphPad Prism 9.0 (GraphPad Software Inc., San Diego, CA, USA) was applied for histogram and heat map rendering.

## Results

### Identification and phylogenetic analysis of BES1 gene family in *V. vinifera*

The sequence information of eight genes of VvBES1 was comprehensively identified in this study. The annotation IDs of BES1 genes in *V. vinifera* were Vitvi10g00636 (VvBES1-1), Vitvi04g01234 (VvBES1-2), Vitvi19g00061 (VvBES1-3), Vitvi10g01901 (VvBES1-4), Vitvi18g00924 (VvBES1-5), Vitvi02g01232_t001 (VvBES1-6), Vitvi15g01128_t001 (VvBES1-7) and Vitvi08g00772_t001 (VvBES1-8). As Table 1 indicated, VvBES1s were located on chromosomes 10, 4, 19, 18, 2, 15 and 8. To further understand the characteristics of VvBES1, we analyzed their protein physicochemical parameters. The ORF lengths of the eight VvBES1 genes varied little, from 414 to 2100. Moreover, the length of the protein they coded varied little too, from 137 to 1154; the molecular weight also ranged from 15.13 to 128.30 KDA, and the isoelectric point (pI) from 5.91 to 10.44; the GRAVY range of VvBES1 was from -1.055 to -0.523. Further explore the subcellular localization of VvBES1s, we found except VvBES1-6 and VvBES1-7, which were located in the cytoplasm, almost all of the rest of the VvBES1s were located in the nucleus.

**Table 1:** The characteristic of BZR1/BES1 genes from grape.

Genes	Locus name	Chr	Subcellular localization	ORF length	Protein physicochemical characteristics			
					Length	MW (KDA)	pI	GRAVY
VvBES1-1	Vitvi10g00636	10	Nuclear	1026	341	36.30	8.53	−0.611
VvBES1-2	Vitvi04g01234	4	Cytoplasmic/nuclear	951	316	33.93	9.04	−0.631
VvBES1-3	Vitvi19g00061	19	Cytoplasmic/nuclear	849	326	34.88	8.39	−0.548
VvBES1-4	Vitvi10g01901	10	Cytoplasmic/nuclear	648	215	22.41	9.44	−0.523
VvBES1-5	Vitvi18g00924	18	Cytoplasmic/nuclear	924	307	33.65	9.13	−0.669
VvBES1-6	Vitvi02g01232_t001	2	Cytoplasmic	2013	141	15.13	10.44	−1.055
VvBES1-7	Vitvi15g01128_t001	15	Cytoplasmic	2100	1154	128.30	5.91	−0.574
VvBES1-8	Vitvi08g00772_t001	8	Nucleus	414	137	15.54	9.15	−0.992

**Figure 1:**
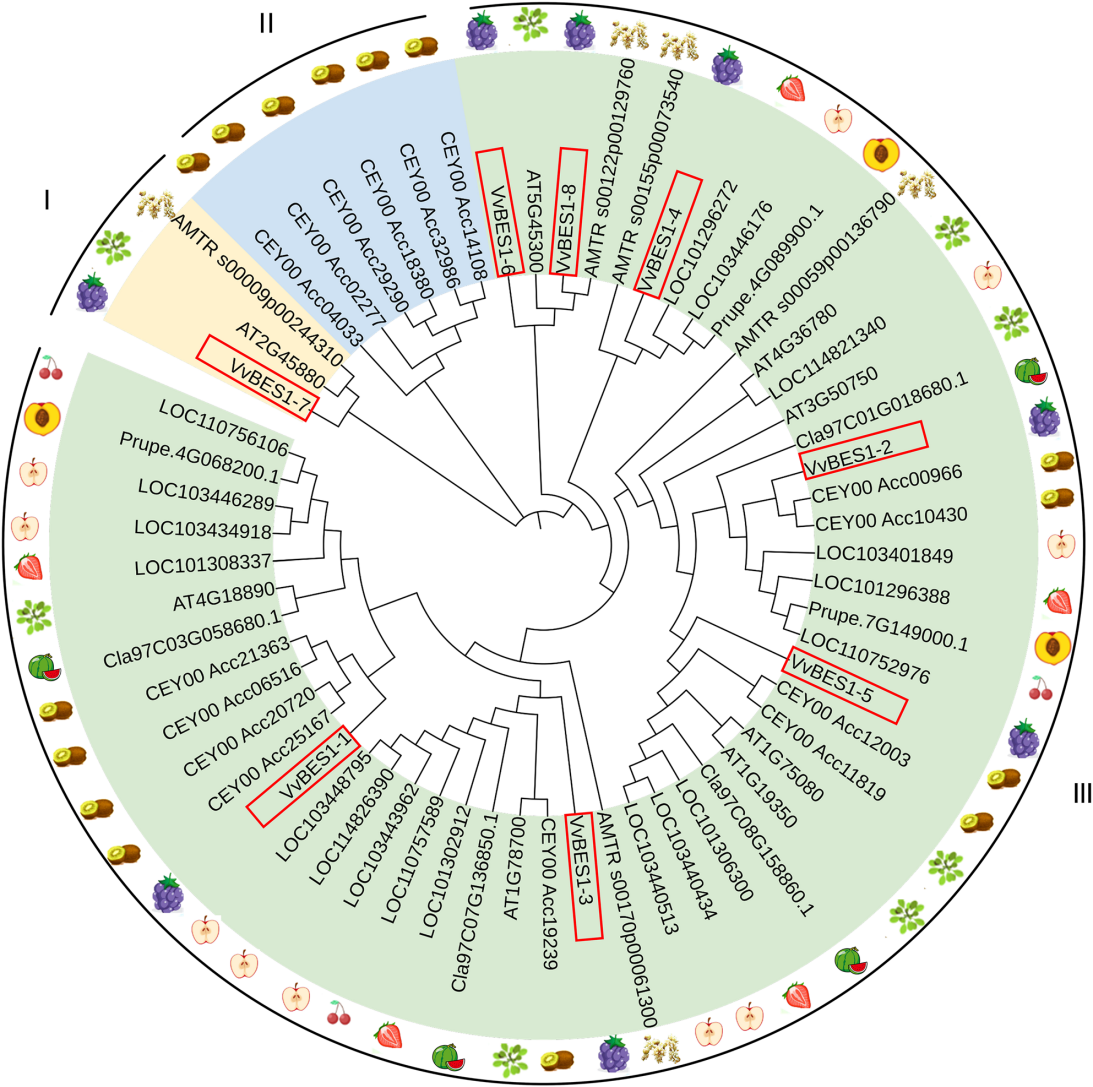
Phylogenetic analysis of BES1 gene family; gene information is from *V. vinifera*, *A. thaliana*, *A. trichopoda*, *A. chinensis*, *F. ananassa*, C. *lanatus*, *M. pumila*, *P. persica*, and *Prunus*. The neighbor-joining tree was generated from eight BES1 genes from *V. vinifera*, eight BES1 genes from *A. thaliana*, five BES1 genes from *A. trichopoda*, fifteen BES1 genes from *A. chinensis*, ten BES1 genes from *M. pumila*, three BES1 genes from *P. persica*, and three BES1 genes from *Prunus*, five BES1 genes from *F. ananassa*, four BES1 genes from C. *lanatus*.

To better study the functional analysis of the VvBES1 gene family, as shown in Table 2, we constructed a phylogenetic evolutionary tree using 61 BES1 genes from *V. vinifera*, *A. thaliana*, *A. trichopoda*, and six other horticultural crops (*A. chinensis*, *F. ananassa*, C. *lanatus*, *M. pumila*, *P. persica*, and *Prunus*). Based on the results obtained, we divided the BES1 proteins into three groups (I, II, III). Among them, the best results were obtained in Fig. 1, showing that there was one VvBES1 gene (VvBES1-7) in Group I, no VvBES1 gene in Group II, and seven genes (VvBES1-1, VvBES1-2, VvBES1-3, VvBES1-4, VvBES1-5, VvBES1-6, VvBES1-8) in group III. We also found that BES1 genes of various species were scattered in different groups, and there was no specific rule: some were related to *A. thaliana* (VvBES1-6, VvBES1-7 and VvBES1-8), VvBES1-4 was related to *F. ananassa*, VvBES1-1, VvBES1-2, VvBES1-3 and VvBES1-5 were related to *A. chinensis*.

**Table 2:** Identified members of the BES1 protein family in diverse plants.

Species	Gene family (number)
*Arabidopsis thaliana*	ATBES1 (8)
*Amborella trichopoda*	AMTRBES1 (5)
*Vitis vinifera* L	VvBES1 (8)
*Prunus* spp.	FUNBES1 (3)
*Citrullus lanatus* (Thunb.) Matsum	ClaBES1 (4)
*Actinidia chinensis* Planch	CEYBES1 (15)
*Fragaria* × *ananassa* Duch	FvBES1 (5)
*Prunus persica* L	PrupeBES1 (3)
*Malus pumila* Mill	MalusBES1 (10)

A total of nine species were discussed in this study, including *V. vinifera*, *A. thaliana*, *A. trichopoda*, *A. chinensis*, *F. ananassa*, C. *lanatus*, *M. pumila*, *P. persica*, and *Prunus.*

### Analysis of gene structure and conserved motifs in *V. vinifera*

**Figure 2:**
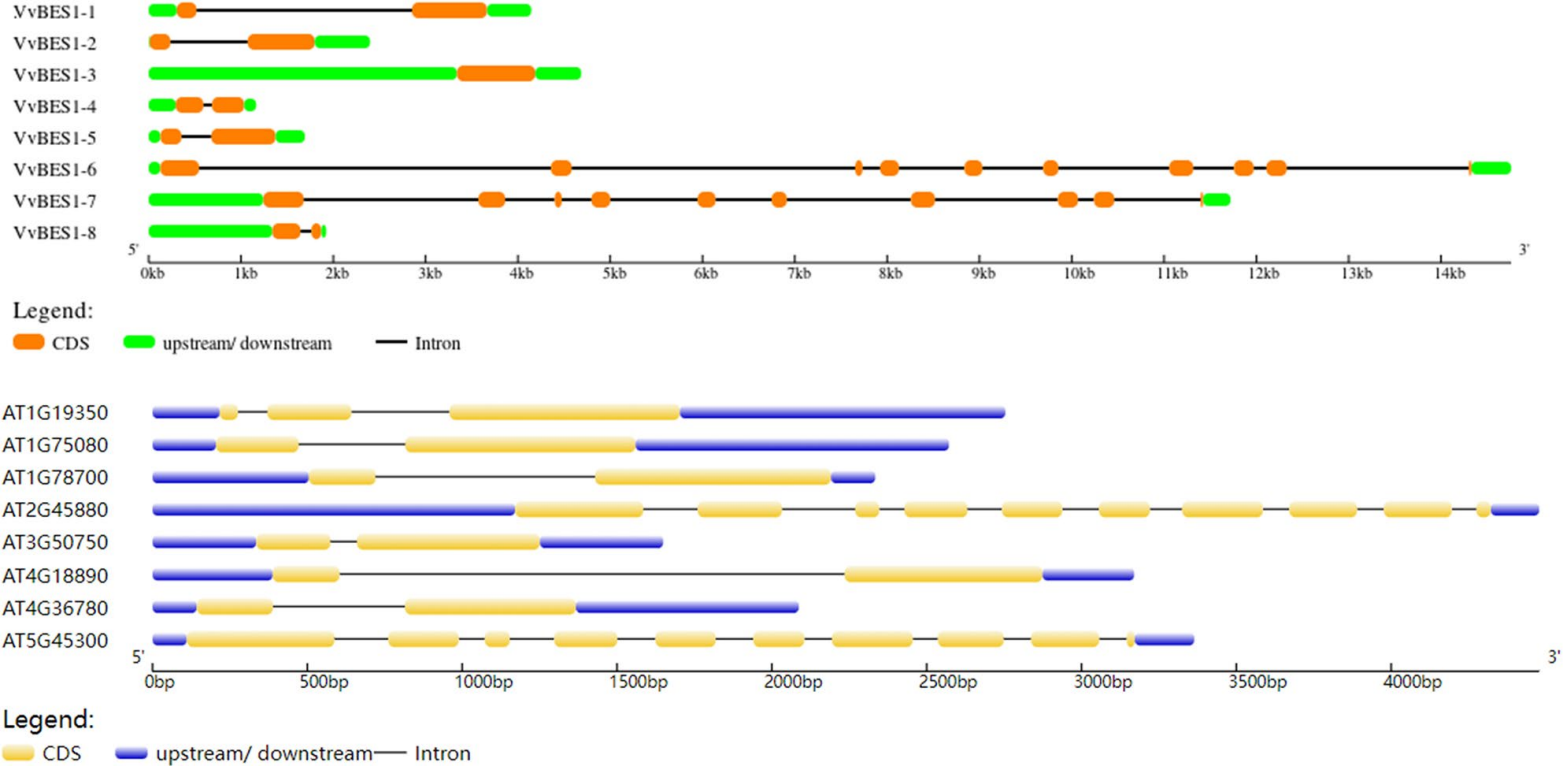
Intron and exon structures of BES1 in *V. vinifera* and *A. thaliana*. The yellow/orange boxes indicate exons and the black lines indicate introns, while blue/green boxes indicate untranslated regions in the upstream and downstream regions. The figure legends are shown at the bottom of each graphic.

Since the distribution of exons and introns is also an aspect of the information of gene, we also analyzed the structural characteristics of eight VvBES1 genes, including the numbers and lengths of their exons and introns (Fig. 2). The results show that every VvBES1s had at least two exons, except for VvBES1-3. Moreover, we found that VvBES1-6 and VvBES1-7 had more introns than the other four VvBES1s genes. The evolution of VvBES1 was accompanied by post-transcriptional gene regulation and modification, resulting in the variation of its gene structures, which also indirectly indicates that BES1 structural differences would lead to the diversification of gene functions. Compared with VvBES1 genes, we found that all ATBES1 genes were conserved, with the lengths ranging from 1600 to 45,000 KB, and AT2G45880 contained more introns, while AT3G50750 contained fewer. Different gene structures would inevitably lead to differences in gene functions.

**Figure 3:**
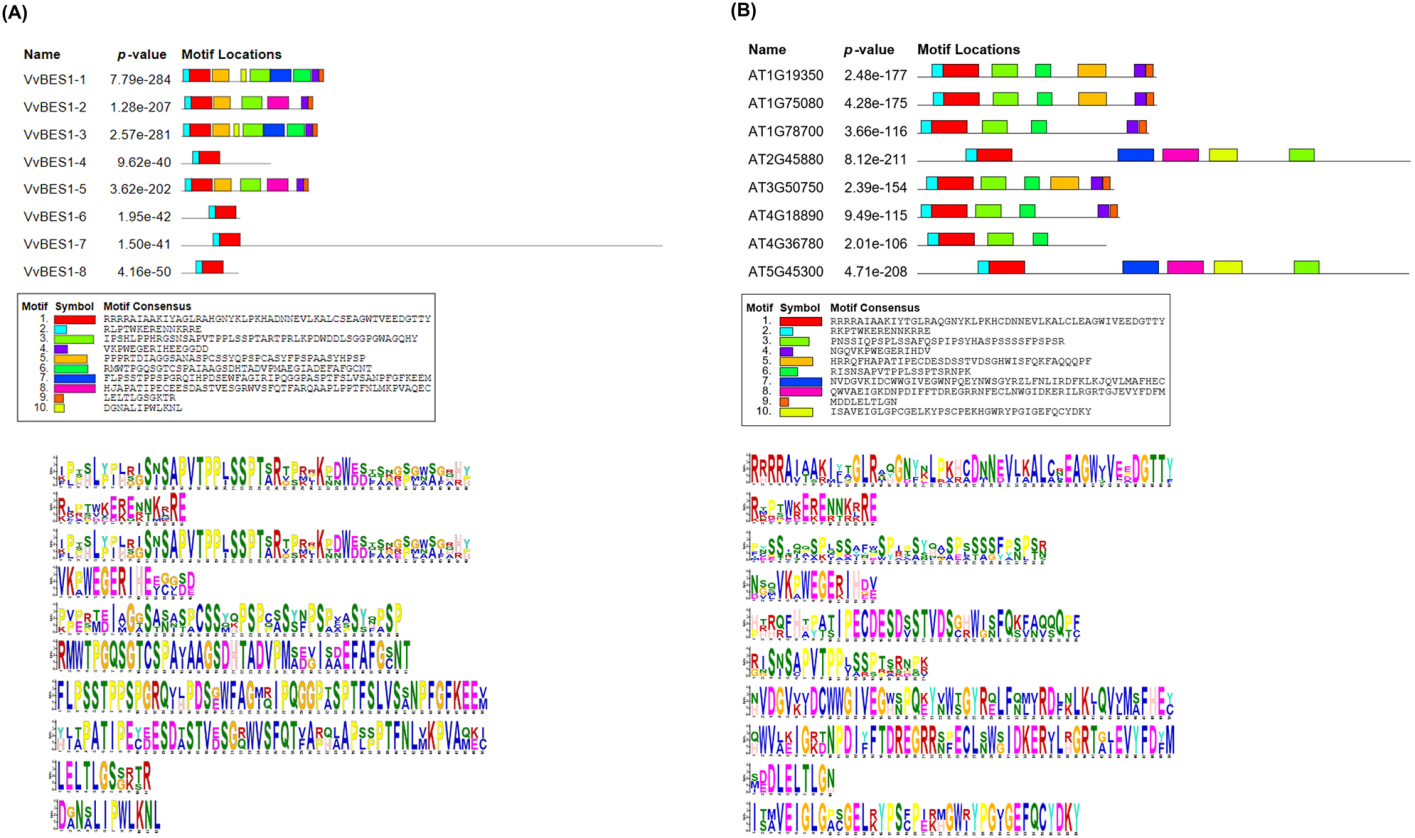
Identification of conserved motifs of BES1 gene family in *V. vinifera* and *A. thaliana*. (**A**) Analysis of conserved motif of BES1 amino acids in *V. vinifera*. (**B**) Analysis of conserved motif of BES1 amino acid in *A. thaliana*. Different boxes describe different conserved motifs. The same conserved motifs are indicated by same color. The representative motifs of VvBES1 and ATBES1 are displayed below the motif graph in the form of SeqLogo.

In order to better understand the diversity and similarities of VvBES1 proteins, as Fig. 3 has shown, there were ten kinds of motifs found in both *V. vinifera* and *A. thaliana*. Specifically, there were night kinds of motifs in VvBES1-1, seven kinds of motifs in VvBES1-2, nine kinds of motifs in VvBES1-3, two kinds of motifs in VvBES1-4, seven kinds of motifs in VvBES1-5, two kinds of motifs in VvBES1-6, two kinds of motifs in VvBES1-7, and two kinds of motifs in VvBES1-8 (Fig. 3A). Concerned with motifs in *A. thaliana*, there were seven kinds of motifs in AT1G19350, seven kinds of motifs in AT1G75080, six kinds of motifs in AT1G78700, six kinds of motifs in AT2G45880, seven kinds of motifs in AT3G50750, six kinds of motifs in AT4G18890, four kinds of motifs in AT4G36780, and six kinds of motifs in AT5G45300 (Fig. 3B).

### *Cis*-acting element analysis of VvBES1 promoters

**Figure 4:**
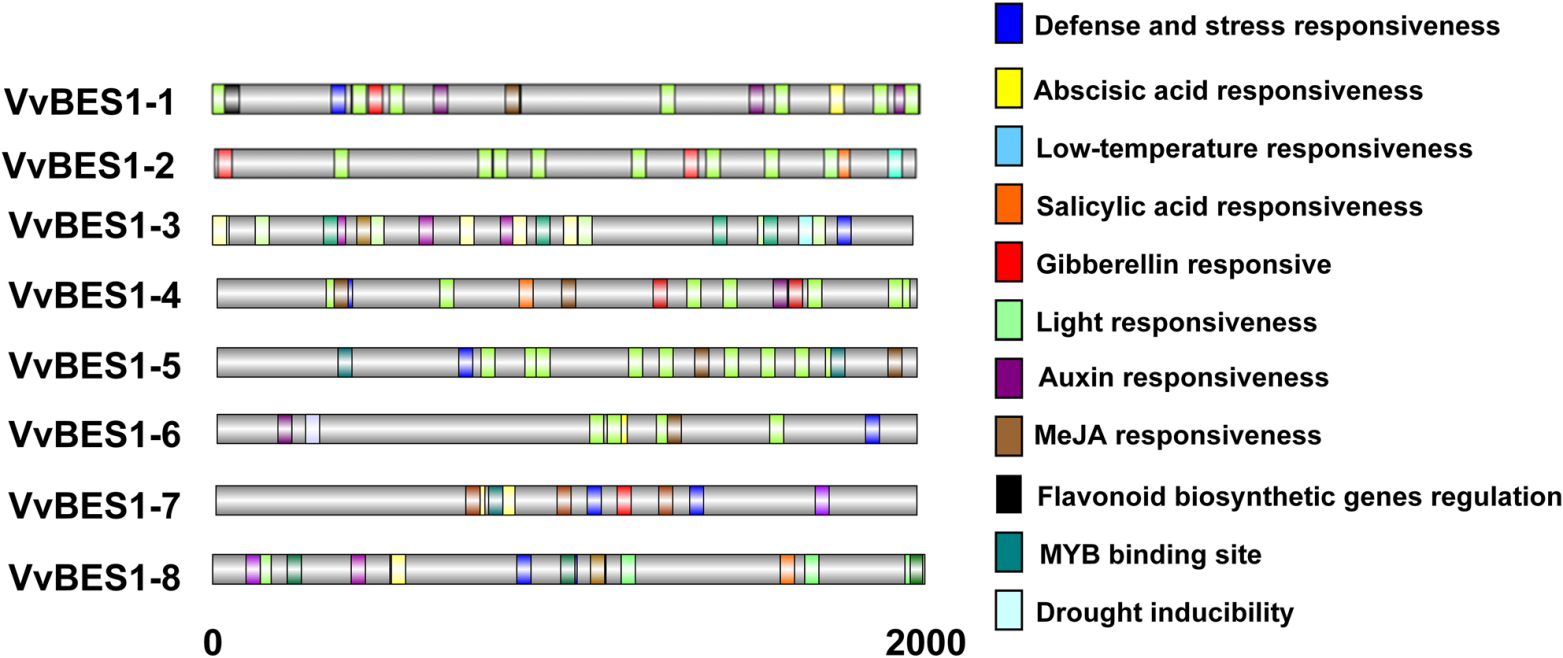
Analysis of *Cis*-acting elements associated with plant hormones responses and stress responses in the promoter region of VvBES1 genes. *Cis*-acting elements associated with plant hormones responsiveness and stress responsiveness are all shown by boxes with different color.

In order to better understand the possible regulatory mechanism of the VvBES1 gene in *V. vinifera* growth and development, we studied the *Cis*-acting elements in the promoter of VvBES1 genes. As shown in Fig. 4, the *Cis*-acting elements, could be divided into stress-response element, light response element, phytohormones elements, and so on. To be specific, there were seven kinds of elements in VvBES1-1 (four for phytohormones, one for light response, one for stress response, and one for flavonoid biosynthesis), four kinds of elements in VvBES1-2 (two for phytohormones, one for light response and one for stress response), seven kinds of elements in VvBES1-3 (three for phytohormones, one for light response, two for stress response and one for MYB binding site), six kinds of elements in VvBES1-4 (four for phytohormones, one for light response and one for stress response), four kinds of elements in VvBES1-5 (one for phytohormones, one for light response, one for stress response and one for MYB binding), and six kinds of elements in VvBES1-6 (three for phytohormones, one for light responsiveness, and two for stress response), six kinds of elements in VvBES1-7 (four for phytohormones, and one for stress response, and one for MYB binding), and eight kinds of elements in VvBES1-8 (four for phytohormones, and one for stress response, one for light response, one for MYB binding, and one for flavonoid biosynthesis). Since the numbers of *Cis*-acting elements of VvBES1s that respond to different stresses and phytohormones were different, it could be inferred that, in VvBES1-5 and VvBES1-6, there were several elements related to stress response, while in VvBES1-1, VvBES1-2, VvBES1-3, VvBES1-4, VvBES1-7 and VvBES1-8, there were more elements related to phytohormone responsiveness. The above findings indicated that different BES1 genes in *V. vinifera* have different biological functions, which reflects the different numbers of *Cis*-acting elements with different biological functions on their promoters.

### Structure prediction of VvBES1 proteins

The structure of a protein determines its biological function. Further analysis of the tertiary structure of the protein indicated that VvBES1 was further coiled and folded on the basis of the secondary structure, forming a tertiary structure maintained by the secondary bonds, on which there were many hydrophobic bonds, salt bonds and hydrogen bonds (Fig. S1). Moreover, the protein structure of VvBES1-7 was different from those of the other seven BES1 genes, which confirms that it might have different biological functions from other BES1s genes.

### Synteny analysis of BES1 gene family

**Figure 5:**
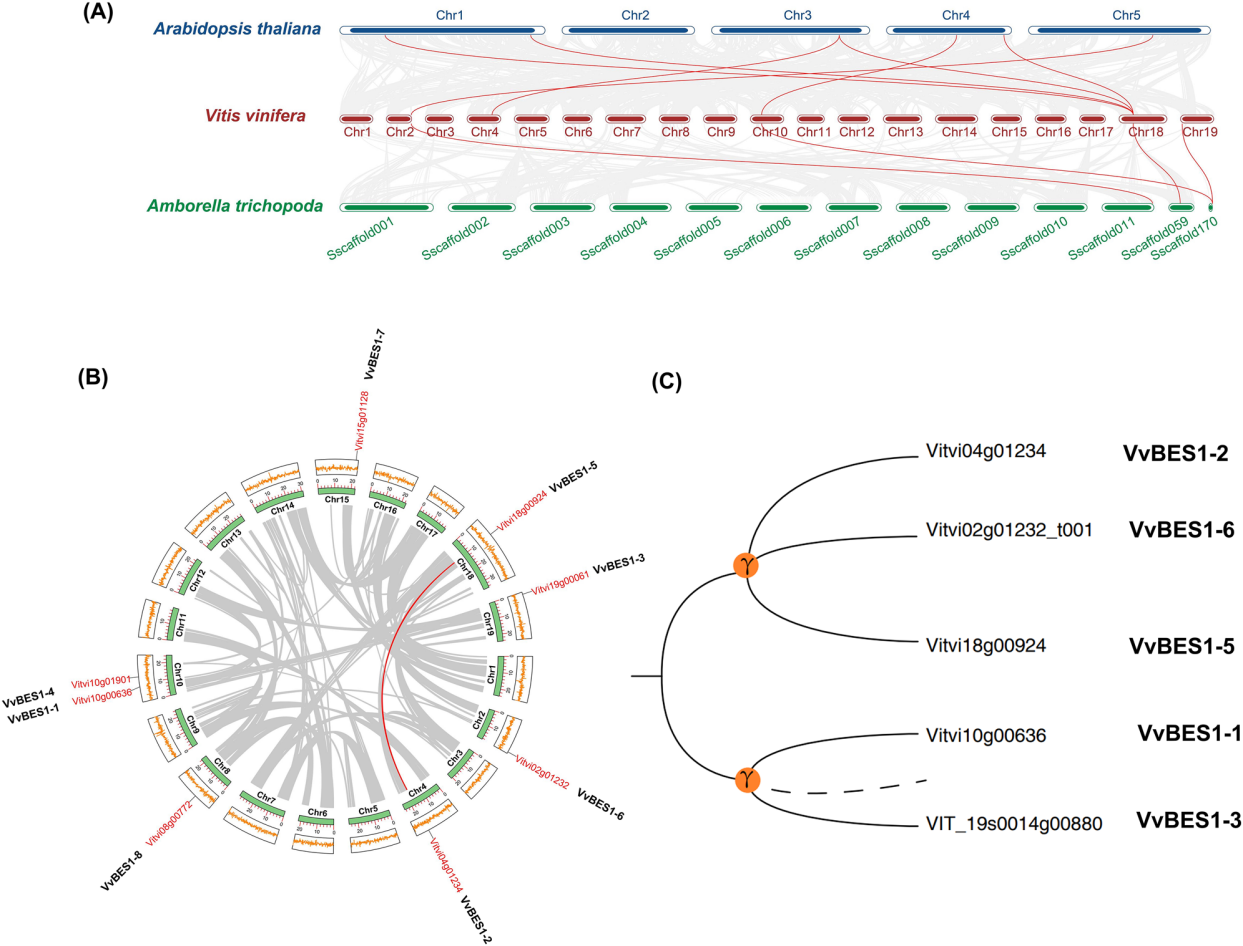
Collinearity analysis of BES1 genes and evolutional analysis of VvBES1 gene family. (**A**) Interspecific syntenic relationships of BES1 gene family among *A. thaliana*, *A. trichopoda* and *V. vinifera* genomes, the red line represents BES1 gene pairs with collinear relationship between *V. vinifera* vs. *A. trichopoda as well as V. vinifera* vs. *A. thaliana*. The serial number is expressed on each chromosome. (**B**) Intraspecies syntenic relationships of VvBES1 gene family in *V. vinifera* genome; the gray line represents the gene pairs with collinearity in the whole *V. vinifera* gene family, and the red line represents the gene pairs with collinearity in the VvBES1 family. Chromosome length (Mb) is indicated by the scale bar on each chromosome, and the yellow boxes upon the chromosomes indicated corresponding GC content of each chromosome. (**C**) Retention and loss of copies in VvBES1 gene family after species evolution. The solid line represents the BES1 gene with three copies, and the dotted line represents the BES1 gene with one copy missing.

In order to further understand the evolution of BES1 gene family, the interspecific and intraspecific collinearity analysis of this gene family were also carried out in this study. For the interspecific analysis of BES1, we selected *A. thaliana* (model plants), *A. trichopoda* (the earliest known angiosperms that evolved separately from other angiosperms) and *V. vinifera* as research object, to test the consistency of their gene sequence. Figure 5A indicates that a total of four pairs of BES1 genes had collinear relationships in *V. vinifera* and *A. trichopoda* (VvBES1-6 and AMTR_s00011p00265640; VvBES1-5 and AMTR_s00059p00136790; VvBES1-1 and AMTR_s00170p00061300; VvBES1-3 and AMTR_s00170p00061300). And seven pairs of BES1 genes had collinear relationships in *V. vinifera* and *A. thaliana* (VvBES1-1 and AT4G18890; VvBES1-5 and AT1G19350; VvBES1-5 and AT1G75080; VvBES1-5 and AT3G50750; VvBES1-6 and AT5G45300; VvBES1-2 and AT3G50750; VvBES1-5 and AT4G36780). Furthermore, for the interspecific analysis of BES1 in *V. vinifera*, as shown in Fig. 5B, we found there were many colinear gene pairs in the whole *V. vinifera* genome, but we only found that VvBES1 genes, located on chromosome 18 (VvBES1-5) had a collinearity relationship with the genes located on chromosome 4 (VvBES1-2).

Finally, we searched known genes in the obtained synteny list for WGD analysis in Vitis Intraspecific. The results demonstrated that in the process of *V. vinifera* evolution, the genes with three copies of VvBES1-2, VvBES1-5, and VvBES1-6 were completely retained, while genes with only two copies were retained in VvBES1-1 and VvBES1-3, while there was no evolutionary relationship in VvBES1-4, VvBES1-7 and VvBES1-8 (Fig. 5C).

### Expression patterns of VvBES1 in diverse organs of *V. vinifera*

**Figure 6:**
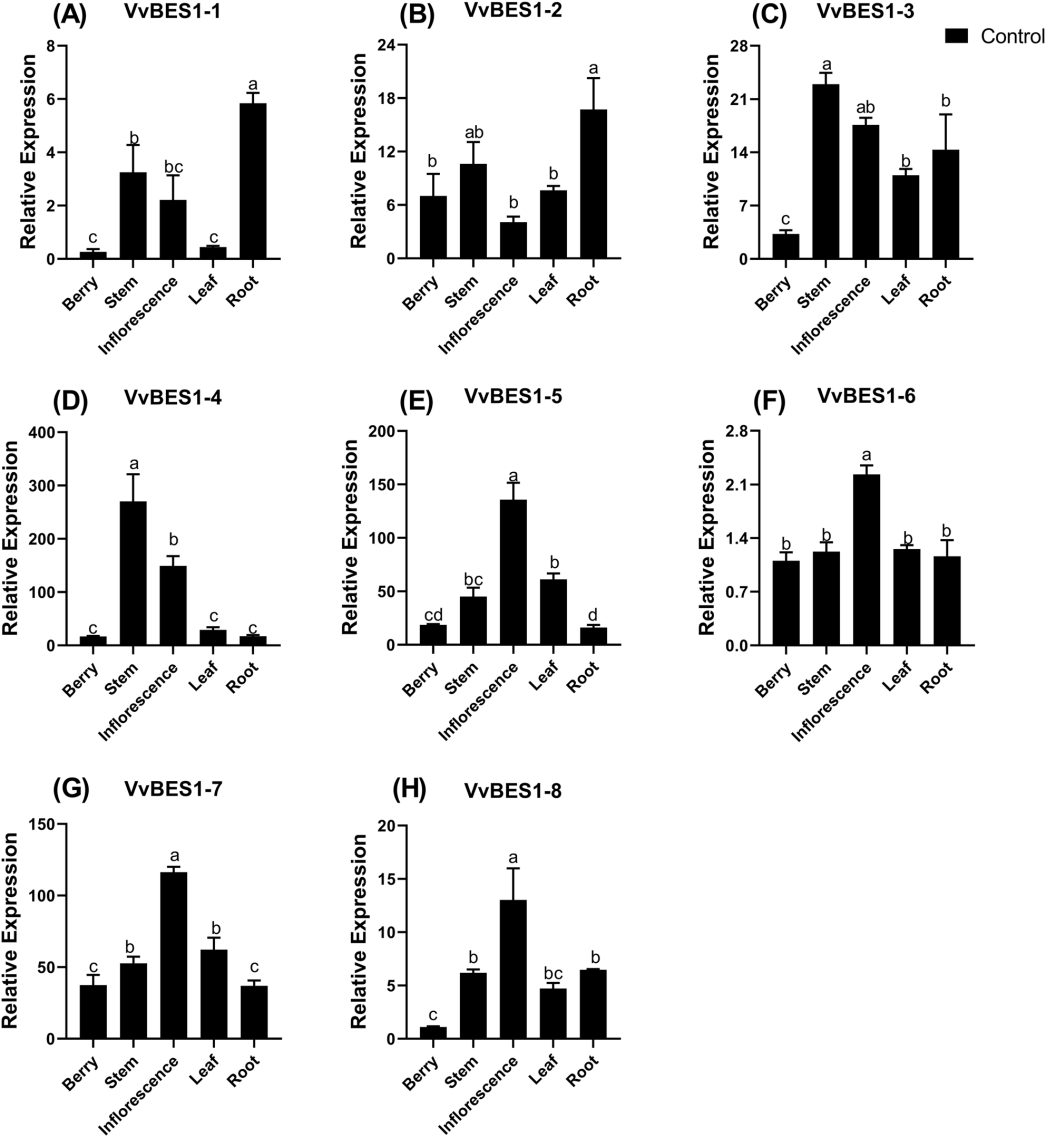
The expression profiles of VvBES1 genes in berry, root, leaf, stem and inflorescence. (**A**) Gene expression quantification of VvBES1-1. (**B**) Gene expression quantification of VvBES1-2. (**C**) Gene expression quantification of VvBES1-3. (**D**) Gene expression quantification of VvBES1-4. (**E**) Gene expression quantification of VvBES1-5. (**F**) Gene expression quantification of VvBES1-6. (**G**) Gene expression quantification of VvBES1-7. (**H**) Gene expression quantification of VvBES1-8. The error bars represent ± SE (n = 3), and the relative expression changes are relative to the control group (VvBES1-1 of berry), which are represented as log_2_ fold change. The one-way Duncan’s new multiple-range test was implemented to assess the significance of differences among treatments (p < 0.05).

In order to explore the possible biological functions of VvBES1, the expression levels of eight VvBES1s in *V. vinifera* roots, stems, leaves, inflorescences and berries were determined by qRT-PCR. As Fig. 6 has shown, VvBES1-1 and VvBES1-2 were expressed at high levels in both the stem and the root (Fig. 6A,B), and the expression of VvBES1-3 was higher in the stem, inflorescence, leaf and root (Fig. 6C), while the expression of VvBES1-4 was higher in both stem and inflorescence (Fig. 6D). Moreover, VvBES1-5, VvBES1-6, VvBES1-7 and VvBES1-8 were expressed at high levels in the inflorescence (Fig. 6E–H). It is worth noting that the expression levels of all the VvBES1 genes in *V. vinifera* berries were very low, while they were generally high in leaves and inflorescences.

### Expression patterns of VvBES1 under heat stress

**Figure 7:**
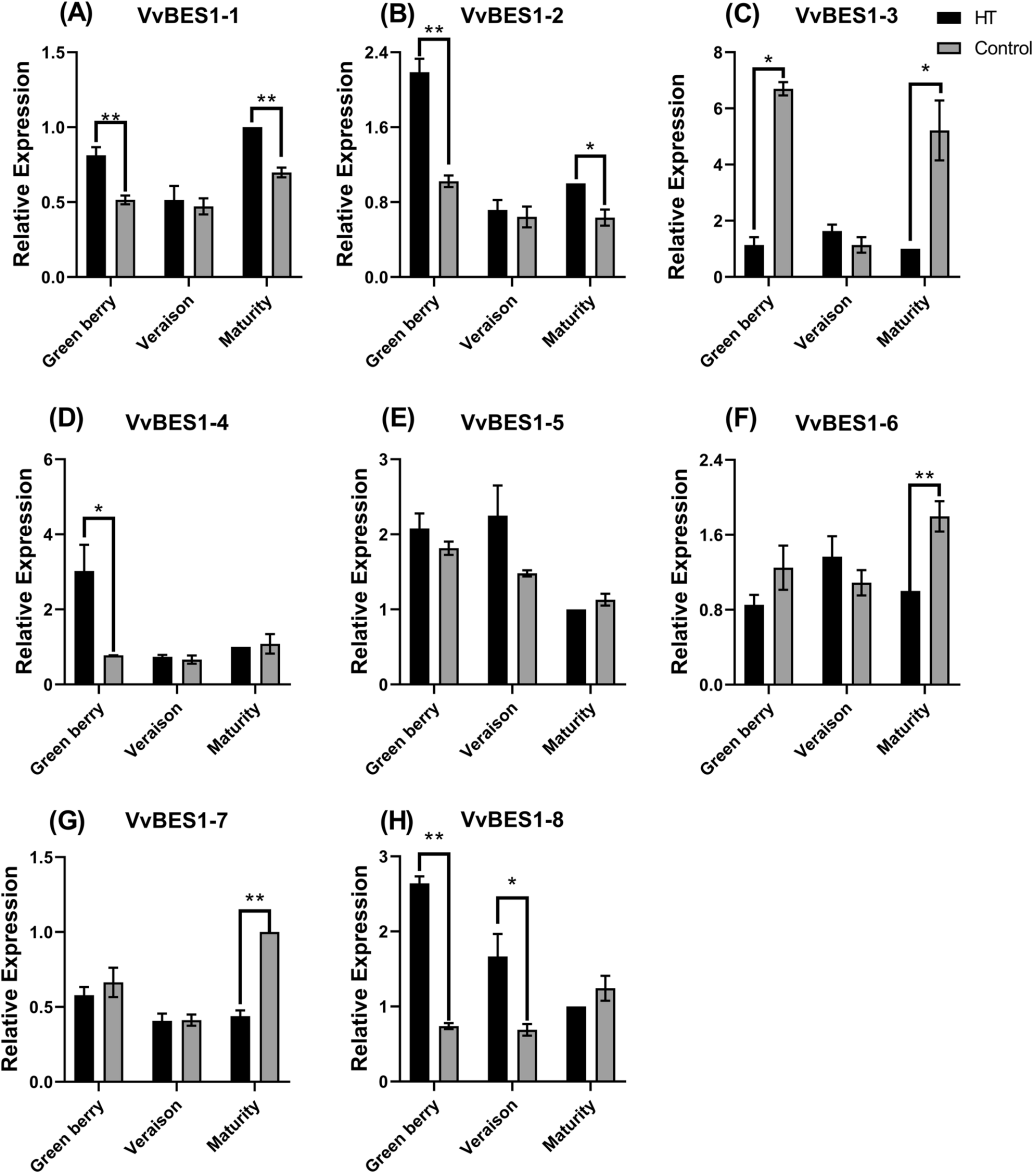
The expression profiles of VvBES1 genes in *V. vinifera* berry in response to heat stress at different developmental stages (green berry stage, veraison stage, maturity stage). (**A**) VvBES1-1 expression level. (**B**) VvBES1-2 expression level. (**C**) VvBES1-3 expression level. (**D**) VvBES1-4 expression level. (**E**) VvBES1-5 expression level. (**F**) VvBES1-6 expression level. (**G**) VvBES1-7 expression level. (**H**) VvBES1-8 expression level. The error bars represent ± SE (n = 3), and the relative expression changes are relative to the control group (maturity stage), which are represented as log_2_ fold change. An independent sample T-test was implemented to assess the significance of differences between treatments (p < 0.05).

Combined with previous results, we found that there were many *Cis*-acting elements related to stress response in VvBES1’s promoter. To further verify the ability of VvBES1 in respond to different abiotic stresses, the expression levels of VvBES1 in berries under heat stress were investigated. The results shown in the Fig. 7 indicate that the expressions of VvBES1-1, VvBES1-2, VvBES1-3 and VvBES1-4 were lower and then higher. At the green berry stage, heat stress treatment increased VvBES1-1, VvBES1-2, and VvBES1-4 expressions (Fig. 7A,B,D), while the expression level of VvBES1-3 was up-regulated in the control group (Fig. 7C). The expression levels of VvBES1-5 and VvBES1-8 in the green berry and in the veraison stages, heat stress treatment increased their expressions (Fig. 7E,H). Interestingly, the variation tendency of VvBES1-6 expression increased and then decreased in the heat stress treatment group, decreased and then increased in the control group, heat stress up-regulated expression of VvBES1-6 at the veraison stage (Fig. 7F). Moreover, the expression of VvBES1-7 decreased then increased, while in the control group, and the expression of VvBES1-7 was significantly up-regulated at the maturity stage (Fig. 7G).

### Expression patterns of VvBES1 under saline-alkali stress

**Figure 8:**
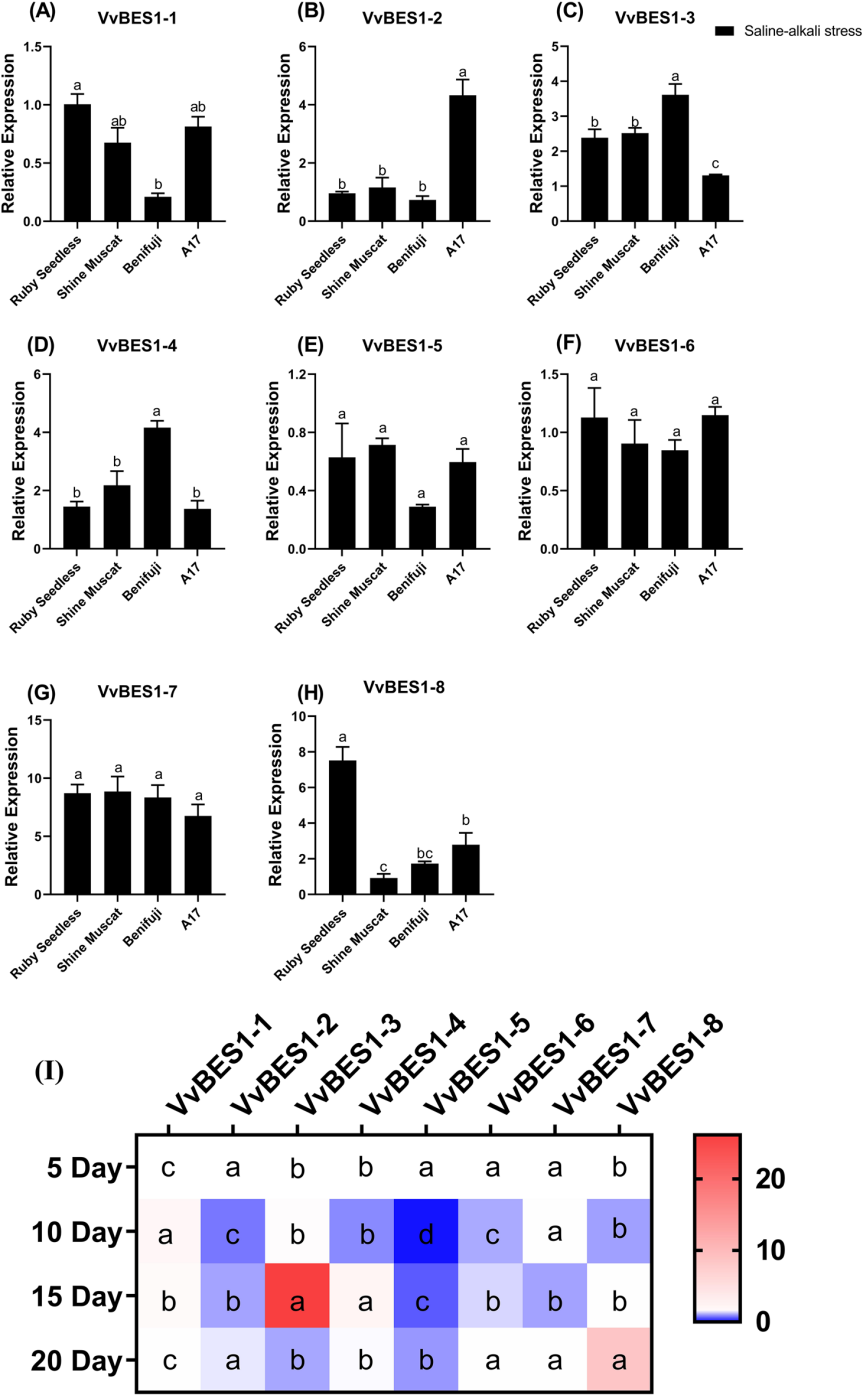
(**A–H**) The expression profiles of the VvBES1 gene in different *V. vinifera* varieties in response to saline–alkali stress; the error bars represent ± SE (n = 3), and the relative expression changes are relative to the control group (VvBES1-1 of Ruby seedless), which are represented as log_2_ fold change. The one-way Duncan’s new multiple-range test was implemented to assess the significance of differences among treatments (p < 0.05), asterisk indicates differences between data, while double asterisk indicates significant differences between data. (**I**) The expression profiles of VvBES1 gene under saline–alkali stress in “Muscat Hamburger” *V. vinifera* leaves; the error bars represent ± SE (n = 3), and the relative expression changes are relative to the VvBES1-1 at 5 days after treatment, which are represented as log_2_ fold change. The one-way Duncan’s new multiple-range test was implemented to assess the significance of differences among treatments (p < 0.05).

The expression patterns of VvBES1 under saline-alkali stress in different *V. vinifera* varieties were also investigated in this study. As the results shown in the Fig. 8, by detecting the expression levels of VvBES1 in ripening berries in different *V. vinifera* varieties under saline-alkali stress, we found that all BES1 genes were expressed at a high level in “A17” while expressed a low level in “Benifuji”, except for VvBES1-3, VvBES1-4 and VvBES1-7 (Fig. 8A–H).

After that, we also investigated the expression levels of the BES1 genes in “Muscat Hamburger” *V. vinifera* leaves under saline-alkali stress, and the results indicated that the variation trends of VVBES1-1, VVBES1-3 and VVBES1-4 were consistent, from 5 to 20 days, rising first and then falling. The trends of the other five genes (VVBES1-2, VVBES1-5, VVBES1-6, VVBES1-7, VVBES1-8) were relatively consistent from 5 to 20 days, falling first and then rising. In addition, VvBES1-1, VvBES1-3, VvBES1-4 expressed at a high level at 15 days after treatment, while VvBES1-2, VvBES1-5, VvBES1-6, VvBES1-7 and VvBES1-8 expressed at a low level 10–15 days after treatment, and stayed at a relatively higher level at 20 days after treatment (Fig. 8I).

## Discussion

The BES1 gene family has previously been reported in a variety of horticulture plants, including *M. pumila*, *A. chinensis*, *C. sativus*, *S. lycopersicum*, *S*. *tuberosum*, *C. reticulata* and *M. nana*^24–29,38,39^. Before now, the transcriptional regulation mechanism of the BES1 transcription factor had been effectively revealed in *A. thaliana*, *O. sativa* and *T. aestivum*^22,25^. However, the research on BES1 genes in *V. vinifera* only offers in simple identification, and is not comprehensive. Thus, this study carried out BES1 genome-wide identification, conserved motifs and *Cis*-acting elements search, interspecific collinearity analysis of BES1 gene family among *V. vinifera*, *A. thaliana*, *A. trichopoda*, and intraspecific collinearity analysis of VvBES1. Moreover, the phylogenetic analysis of the BES1 genes among *V. vinifera*, *A. thaliana*, *A. trichopoda* as well as six other horticultural crops was constructed, in the hope of further studying the evolutionary relationship of BES1 gene family. Through comparing BES1 protein sequences in *V. vinifera*, C. *lanatus*, *F. ananassa*, *A. chinensis*, *Prunus*, *M. pumila*, *A. thaliana* and *A. trichopoda*, the evolutionary tree shows that the BES1 gene family could be divided into three groups in each, and most of the VvBES1 genes were in group III. Previous studies have reported that a change of in gene family size might be beneficial, harmful or neutral, but changes in gene family number are also one of the most important reasons for species specificity^40^. Our results further support the conclusion, determined that the genetic relationship of BES1 gene in different species was different, and the BES1 genes in *V. vinifera* were more closely related to *Prunus*, *A. chinensis* and *A. thaliana*. This had important implications for future studies of their functions and mechanisms of transcriptional regulation. Further studying protein tertiary structure and subcellular localization, we found there were a lot of α-helices and random coils in VvBES1 protein, whereas they essentially located in cytoplasmic and nuclei. Therefore, we could make a reasoned that VvBES1 would fulfil a role in their biological functions in the cytoplasm and nucleus, and different function was decided by different protein structure.

The *Cis*-acting elements included promoters, enhancers, regulatory sequences and inducible elements, which could affect gene expression and regulate gene expression, ultimately leading to differences in plant growth and development^41^. In this study, several kinds of *Cis*-acting elements related to stress and phytohormones were found located in the VvBES1s promoters. Nevertheless, the numbers of these elements varied in different VvBES1 proteins, ultimately resulting in the different biological functions of VvBES1 in relation to regulating the growth and development of *V. vinifera*. As regards GA responsive, it was previously reported that GA signal transduction interacted with the BR signal, while the DELLA protein could reduce AtBZR1 abundance and transcription level via participation in GA signal transduction^42^. The phylogenetic analysis also showed that AT5G45300 and VvBES1-6 were all in Group III, and AT2G45880 and VvBES1-7 were all in Group I, which indicates that VvBES1-6 and VvBES1-7 might be closer to the BES1 transcription factors in *A. thaliana*, and might have similar biological functions, which provides relevant reference for the further study that the functions of these two transcription factors. Furthermore, auxin response factor (ARF) was the key transcription factor regulating the expressions of several auxin response genes^43^. It has been confirmed that a variety of ARF interact with BZR1 to regulate a series of activities, such as the growth and development of plants through signal cross talk with BR^44^. Our results showed that there existed IAA responsive elements in VvBES1-1, VvBES1-3, VvBES1-4, VvBES1-6, VvBES1-7 and VvBES1-8, which further corroborated that BR signal might have crosstalk with IAA signal in *V. vinifera*. In regard with stress resistance elements in VvBES1, we found many low-temperature responsive elements in VvBES1-2 and VvBES1-3, and many stress-related responsive elements in VvBES1-1, VvBES1-4, VvBES1-5, VvBES1-6, VvBES1-7 and VvBES1-8. In addition, VvBES1-2, which possessed more low-temperature responsive elements were in the same group as AT3G50750, AT1G19350, AT4G18890, AT1G75080, AT5G45300, AT4G36780, and AT1G78700, further confirmed the previous finding demonstrated BES1 is closely related to the formation of frost resistance in *A. thaliana*^45^. VvBES1-7, which owned more stress-related responsive elements were in the same group as AT2G45880, which was also consistent with a previous study’s finding that ATBES1 was closely associated with the improvement of stress resistance^10^. The diagram of the tertiary structure pattern of the protein also showed much random crimping in protein structure, which was beneficial to the formation of their biological functions. Collinearity studies the arrangement of homologous genes or sequences. It is more common to evaluate the assembly effect of genomes and the retention and loss of homologous genes, through homology comparisons, to study the evolutionary relationships of diverse materials. Through further colinear analyses and evolutionary relationship exploration of BES1 gene family, we found four pairs of BES1 genes had collinear relationships in *V. vinifera* and *A. trichopoda*, and there existed seven pairs of collinear BES1 genes in *V. vinifera* and *A. thaliana.* Also, in *V. vinifera* genome, many colinear gene pairs were found, but only two VvBES1 genes were highly collinear, and located on different chromosomes (4, 18, respectively). Moreover, we found copies of only three VvBES1 genes (VvBES1-2, VvBES1-5, and VvBES1-6) have been well preserved, two (VvBES1-1and VvBES1-3) have lost one copy, while three (VvBES1-4, VvBES1-7 and VvBES1-8) have no evolutionary relationship. Therefore, we have inferred that gene pairs with collinearity might have the analogous biological functions, while genes without collinearity could regulate the production of different traits and physiological activities in plants due to their different biological functions. Since there is a collinear relationship between VvBES1-2 and VvBES1-5, and their copies have been completely preserved, it is meaningful to extensively study their biological functions in the future.

BR, a plant hormone that was found in recent decades, and has been called the sixth hormone after IAA, cytokinin, GA, ABA and ethylene^46^. BR is tightly involved in various stress responses and the increase in stress resistance^47^. Therefore, the BR signal’s regulation of the key transcription factor BES1 has been demonstrated to be involved in plant stress response, such as water stress, heat damage, cold damage, etc^23,48,49^. To further study the role of the VvBES1 gene in plant responses to stress, we have also carried out several quantitative experiments to characterize the expression profiles of the VvBES1 gene in different *V. vinifera* organs, and the their capacity in response to heat stress and saline-alkali stress. In all, the expressions of different kinds of VvBES1 proteins are all higher in stems, roots and inflorescence, and VvBES1s could respond differently to heat stress and saline-alkali stress. Specifically, VvBES1-1 and VvBES1-2 genes showed relatively consistent change trends, whereas heat stress significantly up-regulated their expressions. Under the heat stress, the increases in the expression level of VvBES1s mainly occurred in the veraison stage and the green berry stage, indirectly proving that VvBES1 is an important transcription factor in response to heat stress at early fruit ripening stage. In terms of saline-alkali stress, we found “A17” was more drought-tolerant than other varieties, and with the extension of treatment time, the VvBES1-1, VvBES1-4 and VvBES1-8 genes showed a gradual upward trend. The above findings illustrated that all VvBES1 proteins might participate in responses to stress, though the expression levels of BES1 were also different in diverse *V. vinifera* varieties and different organs, the up-regulation of most of them could positively regulate the improvement of *V. vinifera* stress resistance. This assertion is verified by studies using *A. thaliana* and *O. sativa* as material, confirming that BES1 played an important role in regulating the development of stress resistance and phytohormones-related activities^50^. In addition, the interaction between BES1 and several transcription factors are also been widely reported. At present, it is known that BES1/BZR1 family transcription factors can affect plant growth and development through a brassinosteroid-dependent pathway and brassinosteroid-independent pathway^51^. Relevant studies have shown that CRY1 not only interacted with BIM1 physically, but also interacted with BES1 in a blue light dependent manner, as which the three together regulated the elongation of *A. thaliana* hypocotyls^50^. It is a pity that our study did not continue to explore whether VvBES1 gene interacts with VvBZR1 or VvBIM1 in regulating the improvement of *V. vinifera* stress resistance or the occurrence of phytohormones responses. However, our experimental results still systematically summarized the basic structure and possible biological functions of VvBES1. As well, we have attempted to clarify the evolutional and collinearity relationships of the VvBES1 gene family, thus laid a theoretical foundation for the development of subsequent molecular experiments.

## Conclusion

In this study, we not only analyzed the basic structures of VvBES1 genes, but also studied their phylogenetic relationships, identified the motifs they embodied, and analyzed their chromosomal synteny and evolutionary relationship among Interspecific and intraspecific. Finally, we obtained the expression profiles of VvBES1 in different *V. vinifera* organs and diverse varieties, and the ability of VvBES1 to cope with heat stress and saline-alkali stress was also assessed. In summary, different VvBES1 genes had different biological functions due to their different structures; during the continuous evolution of *V. vinifera*, copies of some VvBES1 gene were well retained, while some losses occurred in other VvBES1 gene; More BES1 collinear gene pairs were discovered in the *V. vinifera* vs. *A. thaliana* than in the *V. vinifera* vs. *A. trichopoda*. In addition, VvBES1 played significant role in the improvement of stress resistance, and their expressions in *V. vinifera* were not only tissue-specific, but also slightly differed under heat stress and saline-alkali stress. This study provides a reference for further studies on the function and transcriptional regulation mechanism of VvBES1, clarifies the degree of VvBES1s gene respond to stress, so as to speculate the specific role of VvBES1 in plant growth, development and stress resistance formation.

## Supplementary Information

Supplementary Figure S1.

Supplementary Legends.

Supplementary Information.

Supplementary Table S1.

## Data Availability

All data showed in this study are included in the article.

## References

[1] Ahammed GJ, Li X, Liu A, Chen S (2020). Brassinosteroids in plant tolerance to abiotic stress. J. Plant Growth Regul..

[2] Baghel M, Nagaraja A, Srivastav M, Meena NK, Senthil Kumar M, Kumar A, Sharma RR (2019). Pleiotropic influences of brassinosteroids on fruit crops: A review. Plant Growth Regul..

[3] Wu C, Li F, Xu H, Zeng W, Yu R, Wu X, Li J (2019). The potential role of brassinosteroids (BRs) in alleviating antimony (Sb) stress in *Arabidopsis thaliana*. Plant Physiol. Biochem..

[4] Hurski AL, Ermolovich YV, Zhabinskii VN, Khripach VA (2015). The development and use of a general route to brassinolide, its biosynthetic precursors, metabolites and analogues. Org. Biomol. Chem..

[5] Yokota, T., & Mori, K. Molecular structure and biological activity of brassinolide and related brassinosteroids. in *Molecular Structure and Biological Activity of Steroids*. 317–340. (CRC Press, 2018).

[6] Vriet C, Russinova E, Reuzeau C (2013). From squalene to brassinolide: The steroid metabolic and signaling pathways across the plant kingdom. Mol. Plant.

[7] Sun Y, Han Z, Tang J, Hu Z, Chai C, Zhou B, Chai J (2013). Structure reveals that BAK1 as a co-receptor recognizes the BRI1-bound brassinolide. Cell Res..

[8] Bücherl CA, van Esse GW, Kruis A, Luchtenberg J, Westphal AH, Aker J, de Vries SC (2013). Visualization of BRI1 and BAK1 (SERK3) membrane receptor heterooligomers during brassinosteroid signaling. Plant Physiol..

[9] Vardhini BV, Anjum NA (2015). Brassinosteroids make plant life easier under abiotic stresses mainly by modulating major components of antioxidant defense system. Front. Environ. Sci..

[10] Setsungnern A, Muñoz P, Pérez-Llorca M, Müller M, Thiravetyan P, Munné-Bosch S (2020). A defect in BRI1-EMS-SUPPRESSOR 1 (bes1)-mediated brassinosteroid signaling increases photoinhibition and photo-oxidative stress during heat stress in Arabidopsis. Plant Sci..

[11] Li L, Yu X, Thompson A, Guo M, Yoshida S, Asami T, Yin Y (2009). Arabidopsis MYB30 is a direct target of BES1 and cooperates with BES1 to regulate brassinosteroid-induced gene expression. Plant J..

[12] Bulgakov VP, Avramenko TV (2020). Linking brassinosteroid and ABA signaling in the context of stress acclimation. Int. J. Mol. Sci..

[13] Stewart Lilley JL, Gan Y, Graham IA, Nemhauser JL (2013). The effects of DELLA s on growth change with developmental stage and brassinosteroid levels. Plant J..

[14] Li QF, Lu J, Yu JW, Zhang CQ, He JX, Liu QQ (2018). The brassinosteroid-regulated transcription factors BZR1/BES1 function as a coordinator in multisignal-regulated plant growth. Biochim. Biophys. Acta BBA-Gene Regul. Mech..

[15] Yin Y, Vafeados D, Tao Y, Yoshida S, Asami T, Chory J (2005). A new class of transcription factors mediates brassinosteroid-regulated gene expression in Arabidopsis. Cell.

[16] Liang T, Mei S, Shi C, Yang Y, Peng Y, Ma L, Liu H (2018). UVR8 interacts with BES1 and BIM1 to regulate transcription and photomorphogenesis in Arabidopsis. Dev. Cell.

[17] Yin Y, Wang ZY, Mora-Garcia S, Li J, Yoshida S, Asami T, Chory J (2002). BES1 accumulates in the nucleus in response to brassinosteroids to regulate gene expression and promote stem elongation. Cell.

[18] Chen J, Yin Y (2017). WRKY transcription factors are involved in brassinosteroid signaling and mediate the crosstalk between plant growth and drought tolerance. Plant Signal. Behav..

[19] Bernardo-García S, de Lucas M, Martínez C, Espinosa-Ruiz A, Daviere JM, Prat S (2014). BR-dependent phosphorylation modulates PIF4 transcriptional activity and shapes diurnal hypocotyl growth. Genes Dev..

[20] Chen J, Nolan TM, Ye H, Zhang M, Tong H, Xin P, Yin Y (2017). Arabidopsis WRKY46, WRKY54, and WRKY70 transcription factors are involved in brassinosteroid-regulated plant growth and drought responses. Plant Cell.

[21] Nemie-Feyissa D, Olafsdottir SM, Heidari B, Lillo C (2014). Nitrogen depletion and small R3-MYB transcription factors affecting anthocyanin accumulation in Arabidopsis leaves. Phytochemistry.

[22] Yu X, Li L, Zola J, Aluru M, Ye H, Foudree A, Yin Y (2011). A brassinosteroid transcriptional network revealed by genome-wide identification of BESI target genes in *Arabidopsis thaliana*. Plant J..

[23] Liu X, Zhao C, Gao Y, Xu Y, Wang S, Li C, Guan Q (2021). A multifaceted module of BRI1 ETHYLMETHANE SULFONATE SUPRESSOR1 (BES1)-MYB88 in growth and stress tolerance of apple. Plant Physiol..

[24] Ma S, Ji T, Liang M, Li S, Tian Y, Gao L (2020). Genome-wide identification, structural, and gene expression analysis of BRI1-EMS-suppressor 1 transcription factor family in *Cucumis sativus*. Front. Genet..

[25] Liu D, Cui Y, Zhao Z, Li S, Liang D, Wang C, Liu Z (2021). Genome-wide identification and characterization of the BES/BZR gene family in wheat and foxtail millet. BMC Genomics.

[26] Su D, Xiang W, Wen L, Lu W, Shi Y, Liu Y, Li Z (2021). Genome-wide identification, characterization and expression analysis of BES1 gene family in tomato. BMC Plant Biol..

[27] Zhu W, Jiao D, Zhang J, Xue C, Chen M, Yang Q (2020). Genome-wide identification and analysis of BES1/BZR1 transcription factor family in potato (*Solanum tuberosum* L.). Plant Growth Regul..

[28] Izadi F, Zarrini HN, Jelodar NB (2016). In silico analysis of BES1 transcription factors in Citrus senescence. Res. j. life sci. bioinform. pharm. chem. sci..

[29] Guo YF, Shan W, Liang SM, Wu CJ, Wei W, Chen JY, Kuang JF (2019). MaBZR1/2 act as transcriptional repressors of ethylene biosynthetic genes in banana fruit. Physiol. Plant.

[30] Velasco R, Zharkikh A, Troggio M, Cartwright DA, Cestaro A, Pruss D, Viola R (2007). A high quality draft consensus sequence of the genome of a heterozygous *V. vinifera* variety. PLoS ONE.

[31] Hall BG (2013). Building phylogenetic trees from molecular data with MEGA. Mol. Biol. Evol..

[32] Bailey TL, Boden M, Buske FA, Frith M, Grant CE, Clementi L, Noble WS (2009). MEME SUITE: Tools for motif discovery and searching. Nucleic Acids Res..

[33] Guo AY, Zhu QH, Chen X, Luo JC (2007). GSDS: A gene structure display server. Yi Chuan Hereditas..

[34] Guex N, Peitsch MC (1997). SWISS-MODEL and the Swiss-Pdb Viewer: An environment for comparative protein modeling. Electrophoresis.

[35] Krzywinski M, Schein J, Birol I, Connors J, Gascoyne R, Horsman D, Marra MA (2009). Circos: An information aesthetic for comparative genomics. Genome Res..

[36] Wang Y, Tang H, DeBarry JD, Tan X, Li J, Wang X, Paterson AH (2012). MCScanX: A toolkit for detection and evolutionary analysis of gene synteny and collinearity. Nucleic Acids Res..

[37] Camacho C, Coulouris G, Avagyan V, Ma N, Papadopoulos J, Bealer K, Madden TL (2009). BLAST+: Architecture and applications. BMC Bioinform..

[38] Cao X, Khaliq A, Lu S, Xie M, Ma Z, Mao J, Chen B (2020). Genome-wide identification and characterization of the BES1 gene family in apple (*Malus domestica*). Plant Biol..

[39] Tang P, Zhang Q, Yao X (2017). Comparative transcript profiling explores differentially expressed genes associated with sexual phenotype in kiwifruit. PLoS ONE.

[40] Demuth JP, Hahn MW (2009). The life and death of gene families. BioEssays.

[41] Zhao Q, Luo Z, Chen J, Jia H, Ai P, Chen A, Xu G (2021). Characterization of two cis-acting elements, P1BS and W-box, in the regulation of OsPT6 responsive to phosphors deficiency. Plant Growth Regul..

[42] Liu Z, Qanmber G, Lu L, Qin W, Liu J, Li J, Yang Z (2018). Genome-wide analysis of BES1 genes in Gossypium revealed their evolutionary conserved roles in brassinosteroid signaling. Sci. China Life Sci..

[43] Powers SK, Holehouse AS, Korasick DA, Schreiber KH, Clark NM, Jing H, Strader LC (2019). Nucleo-cytoplasmic partitioning of ARF proteins controls auxin responses in *Arabidopsis thaliana*. Mol. Cell..

[44] Xue S, Zou J, Liu Y, Wang M, Zhang C, Le J (2019). Involvement of BIG5 and BIG3 in BRI1 trafficking reveals diverse functions of BIG-subfamily ARF-GEFs in plant growth and gravitropism. Int. J. Mol. Sci..

[45] Sadura I, Janeczko A (2018). Physiological and molecular mechanisms of brassinosteroid-induced tolerance to high and low temperature in plants. Biol. Plant..

[46] Planas-Riverola A, Gupta A, Betegón-Putze I, Bosch N, Ibañes M, Caño-Delgado AI (2019). Brassinosteroid signaling in plant development and adaptation to stress. Development.

[47] Tian B, Liu J (2020). Resveratrol: A review of plant sources, synthesis, stability, modification and food application. J. Sci. Food Agric..

[48] Hayes S (2019). BRacing for water stress: Brassinosteroid signaling promotes drought survival in wheat. Plant Physiol..

[49] Higgins, D. G., Thompson, J. D., & Gibson, T. J. Using CLUSTAL for multiple sequence alignments. in *Methods in Enzymology*. Vol. 266. 383–402. (Academic Press, 1996). 10.1016/s0076-6879(96)66024-8 8743695

[50] Wang W, Lu X, Li L, Lian H, Mao Z, Xu P, Yang HQ (2018). Photoexcited CRYPTOCHROME1 interacts with dephosphorylated BES1 to regulate brassinosteroid signaling and photomorphogenesis in Arabidopsis. Plant Cell.

[51] Shi H, Li X, Lv M, Li J (2022). BES1/BZR1 family transcription factors regulate plant development via brassinosteroid-dependent and independent pathways. Int. J. Mol. Sci..

