# Supplementary figures and images for: Genome-wide identification, characterization and gene expression of BES1 transcription factor family in grapevine (Vitis vinifera L.)
Source: Sci Rep. 2023 Jan 5;13:240. doi: 10.1038/s41598-022-24407-y (PMC9816167; doi:10.1038/s41598-022-24407-y)

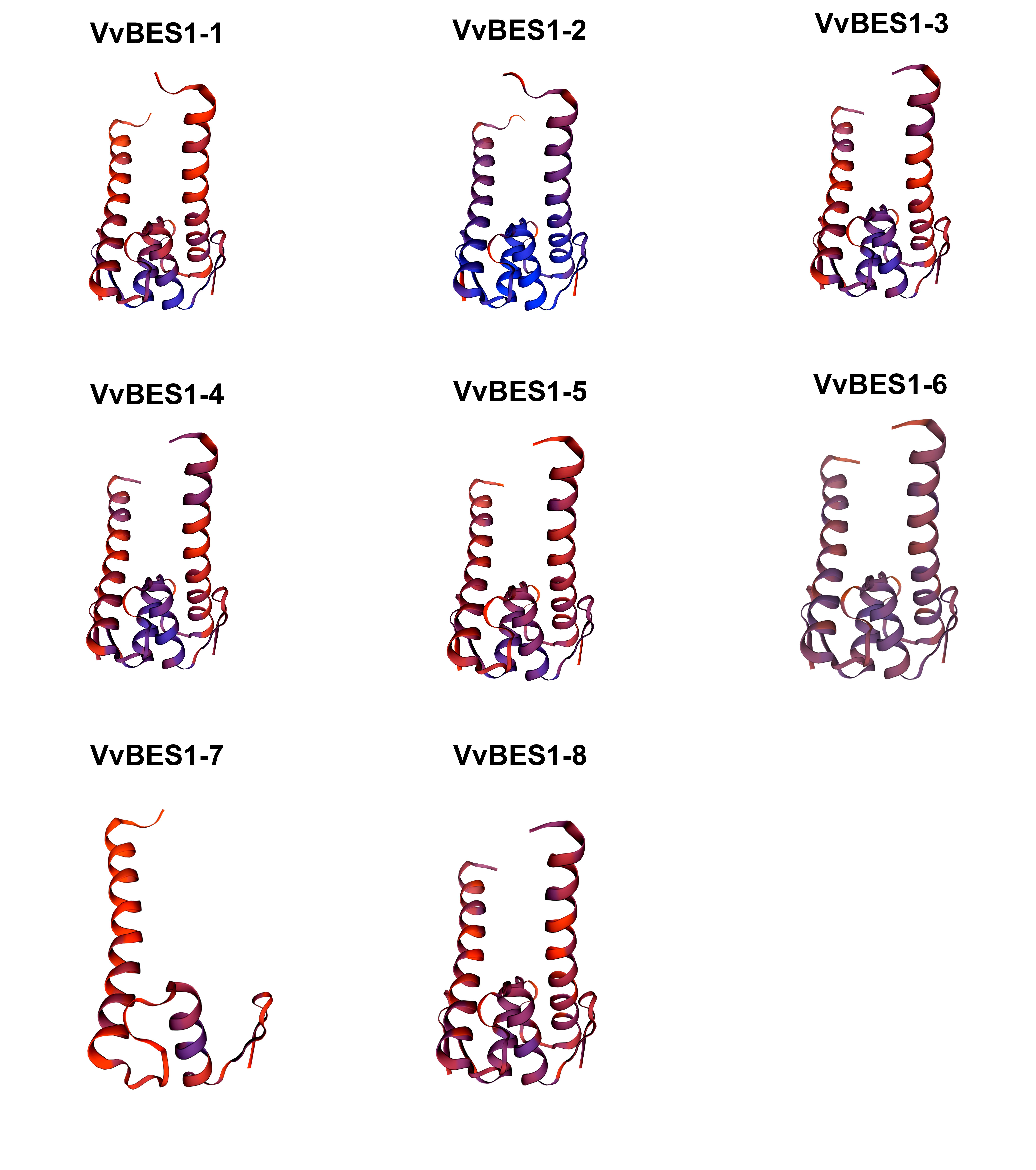

Supplement: Supplementary file 1 — Supplementary Figure S1. [file 41598_2022_24407_MOESM1_ESM.jpg]

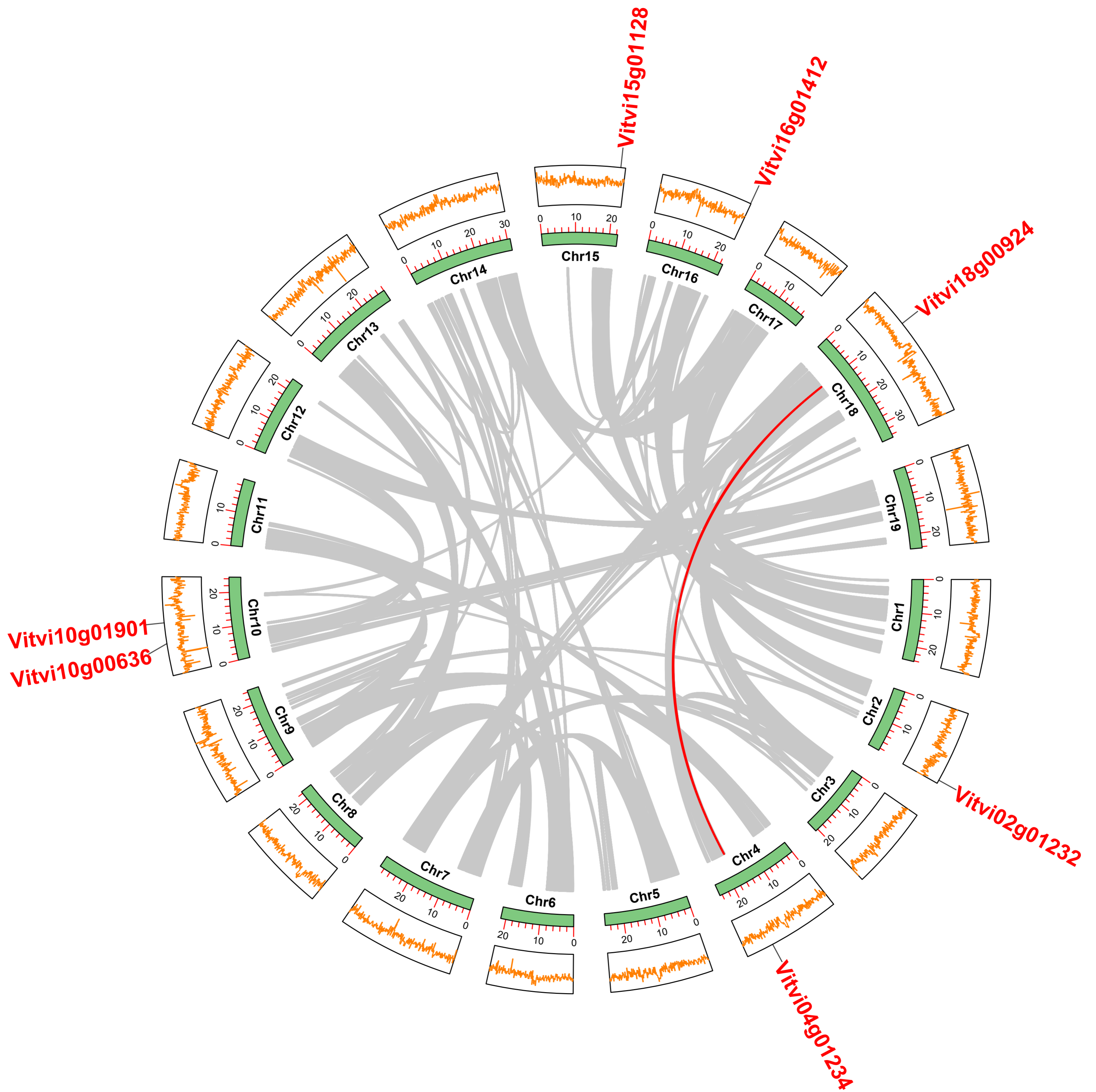

Supplement: Supplementary file 3 — Supplementary Information. [file 41598_2022_24407_MOESM3_ESM.zip › Chromosome collinearity analysis/Circos.pdf]

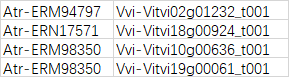

Supplement: Supplementary file 3 — Supplementary Information. [file 41598_2022_24407_MOESM3_ESM.zip › 4E9B@6_JXVMO_L@$B`]`B4U.png]

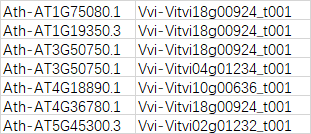

Supplement: Supplementary file 3 — Supplementary Information. [file 41598_2022_24407_MOESM3_ESM.zip › 8WWR[GTU`ZSHV})MUGBLU$2.png]

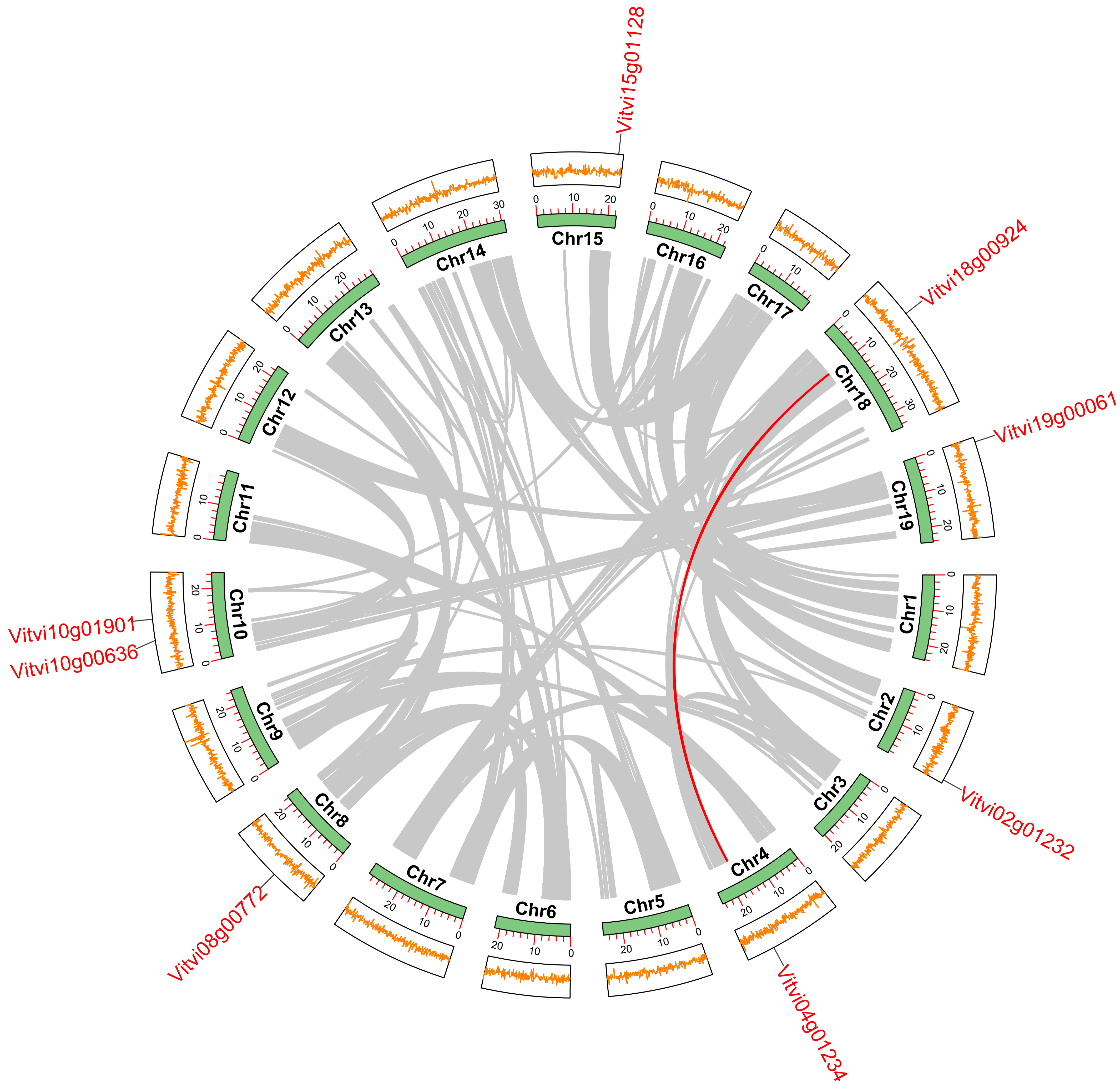

Supplement: Supplementary file 3 — Supplementary Information. [file 41598_2022_24407_MOESM3_ESM.zip › CIrcos.pdf]
