# Supplementary material for: Genome-wide identification, characterization and gene expression of BES1 transcription factor family in grapevine (Vitis vinifera L.)
Source: Sci Rep. 2023 Jan 5;13:240. doi: 10.1038/s41598-022-24407-y (PMC9816167; doi:10.1038/s41598-022-24407-y)
Supplement: Supplementary file 2 — Supplementary Legends. [file 41598_2022_24407_MOESM2_ESM.docx]

**Supplementary legend**

**Figure S1.** Tertiary structure of VvBES1 proteins. The total structures of eight VvBES1 proteins are displayed.

**Table S1.** The primers for qRT-PCR quantification; the sequence information of the genes was all acquired from EnsemblPlants (http://plants.ensembl.org/info/about/index.html).
