# Supplementary material for: Genome-wide identification, characterization and gene expression of BES1 transcription factor family in grapevine (Vitis vinifera L.)
Source: Sci Rep. 2023 Jan 5;13:240. doi: 10.1038/s41598-022-24407-y (PMC9816167; doi:10.1038/s41598-022-24407-y)
Supplement: Supplementary file 3 — Supplementary Information. [file 41598_2022_24407_MOESM3_ESM.zip › Vvi_Ath/Vitis_vinifera.PN40024.v4.dna_sm.toplevel.fa.vs.Arabidopsis_thaliana.TAIR10.dna_sm.toplevel.fa.html/Ath-1.html]

|  |  |  |  |  |  |  |  |  |  |  |  |  |  |  |  |  |  |
| --- | --- | --- | --- | --- | --- | --- | --- | --- | --- | --- | --- | --- | --- | --- | --- | --- | --- |
| Duplication depth | Reference chromosome | Collinear blocks | | | | | | | | | | | | | | | |
| 0 | Ath-AT1G01010.1 |  |  |  |  |  |  |  |  |
| 1 | Ath-AT1G01020.1 |  | Vvi-Vitvi15g01547\_t001 |  |  |  |  |  |  |  |
| 2 | Ath-AT1G01030.1 |  | Vvi-Vitvi15g00863\_t001 |  | Vvi-Vitvi02g00275\_t001 |  |  |  |  |  |  |
| 2 | Ath-AT1G01040.2 |  | Vvi-Vitvi15g00864\_t001 |  | | | |  |  |  |  |  |  |
| 2 | Ath-AT1G01050.1 |  | Vvi-Vitvi15g00867\_t001 |  | Vvi-Vitvi02g00272\_t001 |  |  |  |  |  |  |
| 2 | Ath-AT1G01060.3 |  | Vvi-Vitvi15g00870\_t001.1.6037826c |  | | | |  |  |  |  |  |  |
| 2 | Ath-AT1G01070.1 |  | | | |  | | | |  |  |  |  |  |  |
| 2 | Ath-AT1G01080.2 |  | Vvi-Vitvi15g00881\_t002 |  | | | |  |  |  |  |  |  |
| 2 | Ath-AT1G01090.1 |  | Vvi-Vitvi15g00883\_t001 |  | Vvi-Vitvi02g00252\_t001 |  |  |  |  |  |  |
| 2 | Ath-AT1G01100.2 |  | Vvi-Vitvi15g04470\_t001 |  | Vvi-Vitvi02g00249\_t001 |  |  |  |  |  |  |
| 2 | Ath-AT1G01110.2 |  | Vvi-Vitvi15g00893\_t001 |  | Vvi-Vitvi02g00240\_t001 |  |  |  |  |  |  |
| 2 | Ath-AT1G01120.1 |  | Vvi-Vitvi15g00896\_t001 |  | | | |  |  |  |  |  |  |
| 2 | Ath-AT1G01130.1 |  | | | |  | | | |  |  |  |  |  |  |
| 2 | Ath-AT1G01140.3 |  | Vvi-Vitvi15g00900\_t001 |  | | | |  |  |  |  |  |  |
| 2 | Ath-AT1G01150.1 |  | | | |  | | | |  |  |  |  |  |  |
| 2 | Ath-AT1G01160.2 |  | Vvi-Vitvi15g00903\_t001 |  | | | |  |  |  |  |  |  |
| 2 | Ath-AT1G01170.2 |  | | | |  | | | |  |  |  |  |  |  |
| 2 | Ath-AT1G01180.1 |  | Vvi-Vitvi15g01555\_t001 |  | | | |  |  |  |  |  |  |
| 2 | Ath-AT1G01190.2 |  | Vvi-Vitvi15g00915\_t001 |  | Vvi-Vitvi02g00226\_t001 |  |  |  |  |  |  |
| 1 | Ath-AT1G01200.1 |  | Vvi-Vitvi15g00921\_t001 |  |  |  |  |  |  |  |
| 1 | Ath-AT1G01210.2 |  | Vvi-Vitvi15g00930\_t002 |  |  |  |  |  |  |  |
| 1 | Ath-AT1G01220.1 |  | Vvi-Vitvi15g00932\_t001 |  |  |  |  |  |  |  |
| 1 | Ath-AT1G01225.1 |  | Vvi-Vitvi15g00933\_t001 |  |  |  |  |  |  |  |
| 1 | Ath-AT1G01230.1 |  | Vvi-Vitvi15g00935\_t001 |  |  |  |  |  |  |  |
| 1 | Ath-AT1G01240.4 |  | Vvi-Vitvi15g00945\_t008 |  |  |  |  |  |  |  |
| 1 | Ath-AT1G01250.1 |  | Vvi-Vitvi15g00947\_t001 |  |  |  |  |  |  |  |
| 1 | Ath-AT1G01260.3 |  | Vvi-Vitvi15g00948\_t001 |  |  |  |  |  |  |  |
| 1 | Ath-AT1G01280.1 |  | Vvi-Vitvi15g00949\_t001 |  |  |  |  |  |  |  |
| 1 | Ath-AT1G01290.1 |  | Vvi-Vitvi15g00951\_t001 |  |  |  |  |  |  |  |
| 1 | Ath-AT1G01300.1 |  | Vvi-Vitvi15g00952\_t001 |  |  |  |  |  |  |  |
| 0 | Ath-AT1G01305.1 |  |  |  |  |  |  |  |  |
| 0 | Ath-AT1G01310.1 |  |  |  |  |  |  |  |  |
| 0 | Ath-AT1G01320.1 |  |  |  |  |  |  |  |  |
| 0 | Ath-AT1G01335.1 |  |  |  |  |  |  |  |  |
| 1 | Ath-AT1G01340.2 |  | Vvi-Vitvi15g01617\_t001 |  |  |  |  |  |  |  |
| 1 | Ath-AT1G01350.1 |  | Vvi-Vitvi15g00994\_t001 |  |  |  |  |  |  |  |
| 1 | Ath-AT1G01355.1 |  | | | |  |  |  |  |  |  |  |
| 1 | Ath-AT1G01360.1 |  | Vvi-Vitvi15g00997\_t001 |  |  |  |  |  |  |  |
| 1 | Ath-AT1G01370.1 |  | Vvi-Vitvi15g01621\_t001 |  |  |  |  |  |  |  |
| 1 | Ath-AT1G01380.1 |  | | | |  |  |  |  |  |  |  |
| 1 | Ath-AT1G01390.2 |  | Vvi-Vitvi15g01623\_t001 |  |  |  |  |  |  |  |
| 1 | Ath-AT1G01400.1 |  | | | |  |  |  |  |  |  |  |
| 1 | Ath-AT1G01410.1 |  | | | |  |  |  |  |  |  |  |
| 1 | Ath-AT1G01420.2 |  | | | |  |  |  |  |  |  |  |
| 1 | Ath-AT1G01430.1 |  | Vvi-Vitvi15g01011\_t001 |  |  |  |  |  |  |  |
| 1 | Ath-AT1G01440.1 |  | Vvi-Vitvi15g01013\_t001 |  |  |  |  |  |  |  |
| 1 | Ath-AT1G01450.1 |  | Vvi-Vitvi15g01628\_t001 |  |  |  |  |  |  |  |
| 1 | Ath-AT1G01453.2 |  | Vvi-Vitvi15g01024\_t001 |  |  |  |  |  |  |  |
| 0 | Ath-AT1G01460.1 |  |  |  |  |  |  |  |  |
| 1 | Ath-AT1G01470.1 |  | Vvi-Vitvi15g01646\_t001 |  |  |  |  |  |  |  |
| 1 | Ath-AT1G01480.1 |  | Vvi-Vitvi15g01093\_t001 |  |  |  |  |  |  |  |
| 1 | Ath-AT1G01490.4 |  | Vvi-Vitvi15g01094\_t001 |  |  |  |  |  |  |  |
| 1 | Ath-AT1G01500.1 |  | Vvi-Vitvi15g01095\_t001 |  |  |  |  |  |  |  |
| 1 | Ath-AT1G01510.1 |  | Vvi-Vitvi15g01096\_t001 |  |  |  |  |  |  |  |
| 1 | Ath-AT1G01520.3 |  | Vvi-Vitvi15g01097\_t001 |  |  |  |  |  |  |  |
| 1 | Ath-AT1G01530.1 |  | | | |  |  |  |  |  |  |  |
| 1 | Ath-AT1G01540.2 |  | | | |  |  |  |  |  |  |  |
| 1 | Ath-AT1G01550.1 |  | | | |  |  |  |  |  |  |  |
| 1 | Ath-AT1G01560.2 |  | | | |  |  |  |  |  |  |  |
| 1 | Ath-AT1G01570.2 |  | | | |  |  |  |  |  |  |  |
| 1 | Ath-AT1G01580.2 |  | | | |  |  |  |  |  |  |  |
| 1 | Ath-AT1G01590.1 |  | | | |  |  |  |  |  |  |  |
| 1 | Ath-AT1G01600.1 |  | Vvi-Vitvi15g01106\_t001 |  |  |  |  |  |  |  |
| 1 | Ath-AT1G01610.1 |  | Vvi-Vitvi15g01108\_t001 |  |  |  |  |  |  |  |
| 1 | Ath-AT1G01620.1 |  | Vvi-Vitvi15g01110\_t002 |  |  |  |  |  |  |  |
| 1 | Ath-AT1G01630.1 |  | | | |  |  |  |  |  |  |  |
| 1 | Ath-AT1G01640.1 |  | | | |  |  |  |  |  |  |  |
| 1 | Ath-AT1G01650.1 |  | Vvi-Vitvi15g01114\_t001 |  |  |  |  |  |  |  |
| 1 | Ath-AT1G01660.2 |  | | | |  |  |  |  |  |  |  |
| 1 | Ath-AT1G01670.1 |  | | | |  |  |  |  |  |  |  |
| 1 | Ath-AT1G01680.1 |  | | | |  |  |  |  |  |  |  |
| 1 | Ath-AT1G01690.1 |  | Vvi-Vitvi15g01116\_t001 |  |  |  |  |  |  |  |
| 1 | Ath-AT1G01695.1 |  | Vvi-Vitvi15g01117\_t002 |  |  |  |  |  |  |  |
| 1 | Ath-AT1G01700.4 |  | Vvi-Vitvi15g01121\_t001 |  |  |  |  |  |  |  |
| 1 | Ath-AT1G01710.1 |  | Vvi-Vitvi15g01135\_t001 |  |  |  |  |  |  |  |
| 1 | Ath-AT1G01715.1 |  | Vvi-Vitvi15g04625\_t001 |  |  |  |  |  |  |  |
| 1 | Ath-AT1G01720.1 |  | | | |  |  |  |  |  |  |  |
| 1 | Ath-AT1G01725.1 |  | Vvi-Vitvi15g01672\_t001 |  |  |  |  |  |  |  |
| 1 | Ath-AT1G01730.1 |  | Vvi-Vitvi15g01143\_t001 |  |  |  |  |  |  |  |
| 1 | Ath-AT1G01740.1 |  | | | |  |  |  |  |  |  |  |
| 1 | Ath-AT1G01750.2 |  | Vvi-Vitvi15g01148\_t001 |  |  |  |  |  |  |  |
| 1 | Ath-AT1G01760.3 |  | Vvi-Vitvi15g01159\_t001 |  |  |  |  |  |  |  |
| 1 | Ath-AT1G01770.1 |  | Vvi-Vitvi15g01170\_t001 |  |  |  |  |  |  |  |
| 1 | Ath-AT1G01780.1 |  | Vvi-Vitvi15g01171\_t001 |  |  |  |  |  |  |  |
| 1 | Ath-AT1G01790.1 |  | Vvi-Vitvi15g01172\_t002 |  |  |  |  |  |  |  |
| 1 | Ath-AT1G01800.1 |  | Vvi-Vitvi15g01179\_t001 |  |  |  |  |  |  |  |
| 0 | Ath-AT1G01810.1 |  |  |  |  |  |  |  |  |
| 0 | Ath-AT1G01820.1 |  |  |  |  |  |  |  |  |
| 0 | Ath-AT1G01830.1 |  |  |  |  |  |  |  |  |
| 0 | Ath-AT1G01840.1 |  |  |  |  |  |  |  |  |
| 0 | Ath-AT1G01860.4 |  |  |  |  |  |  |  |  |
| 0 | Ath-AT1G01880.3 |  |  |  |  |  |  |  |  |
| 0 | Ath-AT1G01900.1 |  |  |  |  |  |  |  |  |
| 0 | Ath-AT1G01910.4 |  |  |  |  |  |  |  |  |
| 0 | Ath-AT1G01920.1 |  |  |  |  |  |  |  |  |
| 0 | Ath-AT1G01930.1 |  |  |  |  |  |  |  |  |
| 0 | Ath-AT1G01940.1 |  |  |  |  |  |  |  |  |
| 0 | Ath-AT1G01950.3 |  |  |  |  |  |  |  |  |
| 0 | Ath-AT1G01960.1 |  |  |  |  |  |  |  |  |
| 0 | Ath-AT1G01970.1 |  |  |  |  |  |  |  |  |
| 0 | Ath-AT1G01980.1 |  |  |  |  |  |  |  |  |
| 0 | Ath-AT1G01990.1 |  |  |  |  |  |  |  |  |
| 0 | Ath-AT1G02000.1 |  |  |  |  |  |  |  |  |
| 0 | Ath-AT1G02010.4 |  |  |  |  |  |  |  |  |
| 0 | Ath-AT1G02020.1 |  |  |  |  |  |  |  |  |
| 0 | Ath-AT1G02030.1 |  |  |  |  |  |  |  |  |
| 0 | Ath-AT1G02040.1 |  |  |  |  |  |  |  |  |
| 0 | Ath-AT1G02050.1 |  |  |  |  |  |  |  |  |
| 0 | Ath-AT1G02060.1 |  |  |  |  |  |  |  |  |
| 0 | Ath-AT1G02065.1 |  |  |  |  |  |  |  |  |
| 0 | Ath-AT1G02067.1 |  |  |  |  |  |  |  |  |
| 0 | Ath-AT1G02070.1 |  |  |  |  |  |  |  |  |
| 0 | Ath-AT1G02080.1 |  |  |  |  |  |  |  |  |
| 1 | Ath-AT1G02090.1 |  | Vvi-Vitvi15g00565\_t001 |  |  |  |  |  |  |  |
| 1 | Ath-AT1G02100.1 |  | Vvi-Vitvi15g00564\_t001 |  |  |  |  |  |  |  |
| 1 | Ath-AT1G02110.1 |  | Vvi-Vitvi15g00563\_t001 |  |  |  |  |  |  |  |
| 1 | Ath-AT1G02120.1 |  | Vvi-Vitvi15g04322\_t001 |  |  |  |  |  |  |  |
| 1 | Ath-AT1G02130.1 |  | Vvi-Vitvi15g00548\_t001 |  |  |  |  |  |  |  |
| 1 | Ath-AT1G02140.1 |  | | | |  |  |  |  |  |  |  |
| 1 | Ath-AT1G02145.3 |  | Vvi-Vitvi15g00540\_t001 |  |  |  |  |  |  |  |
| 1 | Ath-AT1G02150.1 |  | Vvi-Vitvi15g00538\_t001 |  |  |  |  |  |  |  |
| 1 | Ath-AT1G02160.2 |  | Vvi-Vitvi15g01420\_t002 |  |  |  |  |  |  |  |
| 1 | Ath-AT1G02170.1 |  | Vvi-Vitvi15g00514\_t001 |  |  |  |  |  |  |  |
| 1 | Ath-AT1G02180.1 |  | Vvi-Vitvi15g00500\_t001 |  |  |  |  |  |  |  |
| 0 | Ath-AT1G02190.1 |  |  |  |  |  |  |  |  |
| 0 | Ath-AT1G02205.3 |  |  |  |  |  |  |  |  |
| 0 | Ath-AT1G02210.1 |  |  |  |  |  |  |  |  |
| 0 | Ath-AT1G02220.1 |  |  |  |  |  |  |  |  |
| 0 | Ath-AT1G02230.1 |  |  |  |  |  |  |  |  |
| 0 | Ath-AT1G02250.1 |  |  |  |  |  |  |  |  |
| 0 | Ath-AT1G02260.1 |  |  |  |  |  |  |  |  |
| 0 | Ath-AT1G02270.1 |  |  |  |  |  |  |  |  |
| 0 | Ath-AT1G02280.1 |  |  |  |  |  |  |  |  |
| 0 | Ath-AT1G02290.1 |  |  |  |  |  |  |  |  |
| 0 | Ath-AT1G02300.1 |  |  |  |  |  |  |  |  |
| 0 | Ath-AT1G02305.1 |  |  |  |  |  |  |  |  |
| 0 | Ath-AT1G02310.2 |  |  |  |  |  |  |  |  |
| 0 | Ath-AT1G02320.1 |  |  |  |  |  |  |  |  |
| 0 | Ath-AT1G02330.1 |  |  |  |  |  |  |  |  |
| 0 | Ath-AT1G02335.1 |  |  |  |  |  |  |  |  |
| 0 | Ath-AT1G02340.1 |  |  |  |  |  |  |  |  |
| 0 | Ath-AT1G02350.1 |  |  |  |  |  |  |  |  |
| 0 | Ath-AT1G02360.3 |  |  |  |  |  |  |  |  |
| 2 | Ath-AT1G02370.1 |  | Vvi-Vitvi07g00427\_t001 |  | Vvi-Vitvi07g00427\_t001 |  |  |  |  |  |  |
| 2 | Ath-AT1G02380.1 |  | Vvi-Vitvi07g00439\_t001 |  | | | |  |  |  |  |  |  |
| 2 | Ath-AT1G02390.1 |  | Vvi-Vitvi07g00441\_t001 |  | | | |  |  |  |  |  |  |
| 2 | Ath-AT1G02400.1 |  | Vvi-Vitvi07g00453\_t001 |  | | | |  |  |  |  |  |  |
| 2 | Ath-AT1G02405.1 |  | | | |  | | | |  |  |  |  |  |  |
| 2 | Ath-AT1G02410.1 |  | | | |  | | | |  |  |  |  |  |  |
| 2 | Ath-AT1G02420.1 |  | Vvi-Vitvi07g00463\_t001 |  | | | |  |  |  |  |  |  |
| 2 | Ath-AT1G02430.1 |  | | | |  | | | |  |  |  |  |  |  |
| 2 | Ath-AT1G02440.1 |  | | | |  | | | |  |  |  |  |  |  |
| 2 | Ath-AT1G02450.1 |  | | | |  | | | |  |  |  |  |  |  |
| 2 | Ath-AT1G02460.1 |  | Vvi-Vitvi07g00471\_t001 |  | | | |  |  |  |  |  |  |
| 2 | Ath-AT1G02470.1 |  | Vvi-Vitvi07g00475\_t001 |  | | | |  |  |  |  |  |  |
| 2 | Ath-AT1G02475.1 |  | | | |  | | | |  |  |  |  |  |  |
| 2 | Ath-AT1G02490.1 |  | | | |  | | | |  |  |  |  |  |  |
| 2 | Ath-AT1G02500.1 |  | Vvi-Vitvi07g02247\_t001 |  | | | |  |  |  |  |  |  |
| 1 | Ath-AT1G02510.1 |  |  |  | | | |  |  |  |  |  |  |
| 1 | Ath-AT1G02520.1 |  |  |  | | | |  |  |  |  |  |  |
| 1 | Ath-AT1G02530.1 |  |  |  | | | |  |  |  |  |  |  |
| 1 | Ath-AT1G02540.1 |  |  |  | | | |  |  |  |  |  |  |
| 1 | Ath-AT1G02550.1 |  |  |  | Vvi-Vitvi07g02228\_t001 |  |  |  |  |  |  |
| 1 | Ath-AT1G02560.1 |  |  |  | Vvi-Vitvi07g00422\_t001 |  |  |  |  |  |  |
| 1 | Ath-AT1G02570.1 |  |  |  | Vvi-Vitvi07g00420\_t001 |  |  |  |  |  |  |
| 1 | Ath-AT1G02575.1 |  |  |  | | | |  |  |  |  |  |  |
| 1 | Ath-AT1G02580.1 |  |  |  | Vvi-Vitvi07g00417\_t001 |  |  |  |  |  |  |
| 1 | Ath-AT1G02590.1 |  |  |  | | | |  |  |  |  |  |  |
| 1 | Ath-AT1G02610.1 |  |  |  | Vvi-Vitvi07g00405\_t001 |  |  |  |  |  |  |
| 1 | Ath-AT1G02620.1 |  |  |  | Vvi-Vitvi07g00402\_t001 |  |  |  |  |  |  |
| 1 | Ath-AT1G02630.1 |  |  |  | | | |  |  |  |  |  |  |
| 1 | Ath-AT1G02640.1 |  |  |  | Vvi-Vitvi07g00401\_t001 |  |  |  |  |  |  |
| 1 | Ath-AT1G02650.1 |  |  |  | Vvi-Vitvi07g00400\_t001 |  |  |  |  |  |  |
| 1 | Ath-AT1G02660.1 |  |  |  | Vvi-Vitvi07g00395\_t001 |  |  |  |  |  |  |
| 1 | Ath-AT1G02670.3 |  |  |  | | | |  |  |  |  |  |  |
| 1 | Ath-AT1G02680.1 |  |  |  | Vvi-Vitvi07g02218\_t003 |  |  |  |  |  |  |
| 1 | Ath-AT1G02690.2 |  |  |  | Vvi-Vitvi07g00382\_t001 |  |  |  |  |  |  |
| 1 | Ath-AT1G02700.1 |  |  |  | Vvi-Vitvi07g00380\_t001 |  |  |  |  |  |  |
| 1 | Ath-AT1G02705.1 |  |  |  | | | |  |  |  |  |  |  |
| 1 | Ath-AT1G02710.1 |  |  |  | | | |  |  |  |  |  |  |
| 1 | Ath-AT1G02720.1 |  |  |  | Vvi-Vitvi07g00378\_t001 |  |  |  |  |  |  |
| 1 | Ath-AT1G02730.1 |  |  |  | Vvi-Vitvi07g00376\_t001 |  |  |  |  |  |  |
| 1 | Ath-AT1G02740.1 |  |  |  | | | |  |  |  |  |  |  |
| 1 | Ath-AT1G02750.1 |  |  |  | | | |  |  |  |  |  |  |
| 1 | Ath-AT1G02770.1 |  |  |  | | | |  |  |  |  |  |  |
| 1 | Ath-AT1G02780.1 |  |  |  | Vvi-Vitvi07g02210\_t001 |  |  |  |  |  |  |
| 1 | Ath-AT1G02790.1 |  |  |  | Vvi-Vitvi07g00354\_t001 |  |  |  |  |  |  |
| 1 | Ath-AT1G02800.1 |  |  |  | Vvi-Vitvi07g00352\_t001 |  |  |  |  |  |  |
| 1 | Ath-AT1G02810.1 |  |  |  | Vvi-Vitvi07g00350\_t001 |  |  |  |  |  |  |
| 1 | Ath-AT1G02813.1 |  |  |  | Vvi-Vitvi07g00342\_t001 |  |  |  |  |  |  |
| 1 | Ath-AT1G02816.1 |  |  |  | | | |  |  |  |  |  |  |
| 1 | Ath-AT1G02820.1 |  |  |  | Vvi-Vitvi07g00341\_t001 |  |  |  |  |  |  |
| 1 | Ath-AT1G02830.1 |  |  |  | Vvi-Vitvi07g00330\_t001 |  |  |  |  |  |  |
| 1 | Ath-AT1G02840.1 |  |  |  | | | |  |  |  |  |  |  |
| 1 | Ath-AT1G02850.4 |  |  |  | Vvi-Vitvi07g02198\_t001 |  |  |  |  |  |  |
| 1 | Ath-AT1G02860.1 |  |  |  | Vvi-Vitvi07g00313\_t001 |  |  |  |  |  |  |
| 1 | Ath-AT1G02870.1 |  |  |  | Vvi-Vitvi07g00312\_t001 |  |  |  |  |  |  |
| 1 | Ath-AT1G02880.6 |  |  |  | Vvi-Vitvi07g00306\_t002 |  |  |  |  |  |  |
| 1 | Ath-AT1G02890.1 |  |  |  | Vvi-Vitvi07g00303\_t001 |  |  |  |  |  |  |
| 1 | Ath-AT1G02900.1 |  |  |  | Vvi-Vitvi07g00295\_t001 |  |  |  |  |  |  |
| 1 | Ath-AT1G02910.1 |  |  |  | Vvi-Vitvi07g00294\_t001 |  |  |  |  |  |  |
| 1 | Ath-AT1G02920.1 |  |  |  | | | |  |  |  |  |  |  |
| 1 | Ath-AT1G02930.1 |  |  |  | | | |  |  |  |  |  |  |
| 1 | Ath-AT1G02940.2 |  |  |  | | | |  |  |  |  |  |  |
| 1 | Ath-AT1G02950.5 |  |  |  | Vvi-Vitvi07g00284\_t001 |  |  |  |  |  |  |
| 1 | Ath-AT1G02960.2 |  |  |  | Vvi-Vitvi07g00277\_t001 |  |  |  |  |  |  |
| 1 | Ath-AT1G02965.1 |  |  |  | | | |  |  |  |  |  |  |
| 1 | Ath-AT1G02970.1 |  |  |  | Vvi-Vitvi07g04071\_t001 |  |  |  |  |  |  |
| 1 | Ath-AT1G02980.1 |  |  |  | | | |  |  |  |  |  |  |
| 1 | Ath-AT1G02990.4 |  |  |  | | | |  |  |  |  |  |  |
| 1 | Ath-AT1G03000.1 |  |  |  | Vvi-Vitvi07g00270\_t001 |  |  |  |  |  |  |
| 1 | Ath-AT1G03010.1 |  |  |  | Vvi-Vitvi07g00260\_t001 |  |  |  |  |  |  |
| 1 | Ath-AT1G03020.1 |  |  |  | Vvi-Vitvi07g00259\_t001 |  |  |  |  |  |  |
| 1 | Ath-AT1G03030.1 |  |  |  | | | |  |  |  |  |  |  |
| 1 | Ath-AT1G03040.3 |  |  |  | | | |  |  |  |  |  |  |
| 1 | Ath-AT1G03050.1 |  |  |  | | | |  |  |  |  |  |  |
| 1 | Ath-AT1G03055.1 |  |  |  | | | |  |  |  |  |  |  |
| 1 | Ath-AT1G03060.1 |  |  |  | | | |  |  |  |  |  |  |
| 2 | Ath-AT1G03070.1 |  | Vvi-Vitvi07g00087\_t001 |  | | | |  |  |  |  |  |  |
| 2 | Ath-AT1G03080.2 |  | Vvi-Vitvi07g00094\_t001 |  | Vvi-Vitvi07g00243\_t001 |  |  |  |  |  |  |
| 1 | Ath-AT1G03090.2 |  | Vvi-Vitvi07g00099\_t001 |  |  |  |  |  |  |  |
| 1 | Ath-AT1G03100.1 |  | Vvi-Vitvi07g00106\_t001 |  |  |  |  |  |  |  |
| 1 | Ath-AT1G03103.1 |  | Vvi-Vitvi07g02131\_t001 |  |  |  |  |  |  |  |
| 1 | Ath-AT1G03106.1 |  | | | |  |  |  |  |  |  |  |
| 1 | Ath-AT1G03110.1 |  | Vvi-Vitvi07g00113\_t001 |  |  |  |  |  |  |  |
| 1 | Ath-AT1G03120.1 |  | Vvi-Vitvi07g00117\_t001 |  |  |  |  |  |  |  |
| 1 | Ath-AT1G03130.1 |  | Vvi-Vitvi07g00125\_t001 |  |  |  |  |  |  |  |
| 1 | Ath-AT1G03140.1 |  | Vvi-Vitvi07g02135\_t001 |  |  |  |  |  |  |  |
| 1 | Ath-AT1G03150.1 |  | Vvi-Vitvi07g00126\_t001 |  |  |  |  |  |  |  |
| 1 | Ath-AT1G03160.1 |  | | | |  |  |  |  |  |  |  |
| 1 | Ath-AT1G03165.1 |  | | | |  |  |  |  |  |  |  |
| 2 | Ath-AT1G03170.1 |  | | | |  | Vvi-Vitvi05g00448\_t001 |  |  |  |  |  |  |
| 2 | Ath-AT1G03180.1 |  | Vvi-Vitvi07g00136\_t001 |  | | | |  |  |  |  |  |  |
| 2 | Ath-AT1G03190.1 |  | Vvi-Vitvi07g00137\_t001 |  | | | |  |  |  |  |  |  |
| 2 | Ath-AT1G03200.1 |  | | | |  | | | |  |  |  |  |  |  |
| 2 | Ath-AT1G03210.2 |  | | | |  | | | |  |  |  |  |  |  |
| 2 | Ath-AT1G03220.1 |  | Vvi-Vitvi07g00146\_t001 |  | Vvi-Vitvi05g00430\_t001 |  |  |  |  |  |  |
| 2 | Ath-AT1G03230.1 |  | | | |  | | | |  |  |  |  |  |  |
| 2 | Ath-AT1G03240.1 |  | | | |  | | | |  |  |  |  |  |  |
| 2 | Ath-AT1G03250.2 |  | Vvi-Vitvi07g00150\_t002 |  | | | |  |  |  |  |  |  |
| 2 | Ath-AT1G03260.1 |  | Vvi-Vitvi07g00152\_t001 |  | Vvi-Vitvi05g01875\_t001 |  |  |  |  |  |  |
| 2 | Ath-AT1G03270.1 |  | Vvi-Vitvi07g00158\_t001 |  | Vvi-Vitvi05g00420\_t001 |  |  |  |  |  |  |
| 2 | Ath-AT1G03280.1 |  | | | |  | | | |  |  |  |  |  |  |
| 2 | Ath-AT1G03290.2 |  | Vvi-Vitvi07g00159\_t005 |  | | | |  |  |  |  |  |  |
| 2 | Ath-AT1G03300.1 |  | | | |  | | | |  |  |  |  |  |  |
| 2 | Ath-AT1G03310.1 |  | Vvi-Vitvi07g00167\_t001 |  | | | |  |  |  |  |  |  |
| 2 | Ath-AT1G03320.1 |  | | | |  | | | |  |  |  |  |  |  |
| 2 | Ath-AT1G03325.1 |  | | | |  | | | |  |  |  |  |  |  |
| 2 | Ath-AT1G03330.1 |  | Vvi-Vitvi07g04046\_t001 |  | | | |  |  |  |  |  |  |
| 2 | Ath-AT1G03340.1 |  | Vvi-Vitvi07g00185\_t001 |  | | | |  |  |  |  |  |  |
| 2 | Ath-AT1G03350.1 |  | Vvi-Vitvi07g00191\_t001 |  | Vvi-Vitvi05g00398\_t001 |  |  |  |  |  |  |
| 2 | Ath-AT1G03360.1 |  | Vvi-Vitvi07g00195\_t001 |  | | | |  |  |  |  |  |  |
| 2 | Ath-AT1G03365.1 |  | | | |  | Vvi-Vitvi05g00388\_t002 |  |  |  |  |  |  |
| 2 | Ath-AT1G03370.1 |  | | | |  | | | |  |  |  |  |  |  |
| 2 | Ath-AT1G03380.1 |  | Vvi-Vitvi07g00210\_t001 |  | | | |  |  |  |  |  |  |
| 2 | Ath-AT1G03390.1 |  | | | |  | | | |  |  |  |  |  |  |
| 2 | Ath-AT1G03395.1 |  | | | |  | | | |  |  |  |  |  |  |
| 2 | Ath-AT1G03400.2 |  | | | |  | | | |  |  |  |  |  |  |
| 2 | Ath-AT1G03410.1 |  | | | |  | | | |  |  |  |  |  |  |
| 2 | Ath-AT1G03430.1 |  | Vvi-Vitvi07g00211\_t001 |  | Vvi-Vitvi05g00382\_t001 |  |  |  |  |  |  |
| 1 | Ath-AT1G03440.1 |  | Vvi-Vitvi07g00213\_t001 |  |  |  |  |  |  |  |
| 1 | Ath-AT1G03445.1 |  | Vvi-Vitvi07g00223\_t001 |  |  |  |  |  |  |  |
| 1 | Ath-AT1G03457.2 |  | Vvi-Vitvi07g00232\_t001 |  |  |  |  |  |  |  |
| 1 | Ath-AT1G03470.2 |  | Vvi-Vitvi07g00243\_t001 |  |  |  |  |  |  |  |
| 0 | Ath-AT1G03475.1 |  |  |  |  |  |  |  |  |
| 0 | Ath-AT1G03490.1 |  |  |  |  |  |  |  |  |
| 0 | Ath-AT1G03495.1 |  |  |  |  |  |  |  |  |
| 0 | Ath-AT1G03510.1 |  |  |  |  |  |  |  |  |
| 1 | Ath-AT1G03520.1 |  | Vvi-Vitvi12g00491\_t001 |  |  |  |  |  |  |  |
| 1 | Ath-AT1G03530.1 |  | Vvi-Vitvi12g00489\_t001 |  |  |  |  |  |  |  |
| 1 | Ath-AT1G03540.1 |  | Vvi-Vitvi12g00486\_t001 |  |  |  |  |  |  |  |
| 1 | Ath-AT1G03550.1 |  | Vvi-Vitvi12g00484\_t001 |  |  |  |  |  |  |  |
| 1 | Ath-AT1G03560.1 |  | Vvi-Vitvi12g00479\_t001 |  |  |  |  |  |  |  |
| 1 | Ath-AT1G03590.1 |  | Vvi-Vitvi12g00477\_t003 |  |  |  |  |  |  |  |
| 1 | Ath-AT1G03600.1 |  | Vvi-Vitvi12g00475\_t001 |  |  |  |  |  |  |  |
| 1 | Ath-AT1G03610.1 |  | Vvi-Vitvi12g00474\_t001 |  |  |  |  |  |  |  |
| 1 | Ath-AT1G03620.12 |  | Vvi-Vitvi12g00473\_t001 |  |  |  |  |  |  |  |
| 0 | Ath-AT1G03630.1 |  |  |  |  |  |  |  |  |
| 0 | Ath-AT1G03650.1 |  |  |  |  |  |  |  |  |
| 0 | Ath-AT1G03660.1 |  |  |  |  |  |  |  |  |
| 1 | Ath-AT1G03670.1 |  | Vvi-Vitvi12g00326\_t001 |  |  |  |  |  |  |  |
| 1 | Ath-AT1G03680.1 |  | Vvi-Vitvi12g00319\_t001 |  |  |  |  |  |  |  |
| 1 | Ath-AT1G03687.1 |  | Vvi-Vitvi12g00317\_t001 |  |  |  |  |  |  |  |
| 1 | Ath-AT1G03700.1 |  | Vvi-Vitvi12g00312\_t001 |  |  |  |  |  |  |  |
| 1 | Ath-AT1G03710.1 |  | | | |  |  |  |  |  |  |  |
| 1 | Ath-AT1G03720.1 |  | | | |  |  |  |  |  |  |  |
| 1 | Ath-AT1G03730.1 |  | Vvi-Vitvi12g02308\_t001 |  |  |  |  |  |  |  |
| 1 | Ath-AT1G03740.1 |  | Vvi-Vitvi12g00301\_t001 |  |  |  |  |  |  |  |
| 1 | Ath-AT1G03750.1 |  | Vvi-Vitvi12g00289\_t001 |  |  |  |  |  |  |  |
| 1 | Ath-AT1G03760.1 |  | Vvi-Vitvi12g00288\_t001 |  |  |  |  |  |  |  |
| 1 | Ath-AT1G03770.2 |  | Vvi-Vitvi12g00279\_t001 |  |  |  |  |  |  |  |
| 1 | Ath-AT1G03780.3 |  | Vvi-Vitvi12g00278\_t001 |  |  |  |  |  |  |  |
| 1 | Ath-AT1G03790.1 |  | Vvi-Vitvi12g00276\_t001 |  |  |  |  |  |  |  |
| 1 | Ath-AT1G03800.1 |  | Vvi-Vitvi12g00274\_t001 |  |  |  |  |  |  |  |
| 1 | Ath-AT1G03810.1 |  | Vvi-Vitvi12g00268\_t001 |  |  |  |  |  |  |  |
| 1 | Ath-AT1G03820.1 |  | | | |  |  |  |  |  |  |  |
| 1 | Ath-AT1G03830.2 |  | | | |  |  |  |  |  |  |  |
| 1 | Ath-AT1G03840.1 |  | Vvi-Vitvi12g00252\_t001 |  |  |  |  |  |  |  |
| 1 | Ath-AT1G03850.1 |  | | | |  |  |  |  |  |  |  |
| 1 | Ath-AT1G03860.1 |  | | | |  |  |  |  |  |  |  |
| 1 | Ath-AT1G03870.1 |  | Vvi-Vitvi12g00248\_t001 |  |  |  |  |  |  |  |
| 1 | Ath-AT1G03880.1 |  | Vvi-Vitvi07g00605\_t001 |  |  |  |  |  |  |  |
| 1 | Ath-AT1G03890.1 |  | | | |  |  |  |  |  |  |  |
| 1 | Ath-AT1G03900.1 |  | Vvi-Vitvi07g00623\_t001 |  |  |  |  |  |  |  |
| 1 | Ath-AT1G03905.1 |  | Vvi-Vitvi07g00625\_t001 |  |  |  |  |  |  |  |
| 1 | Ath-AT1G03910.2 |  | | | |  |  |  |  |  |  |  |
| 1 | Ath-AT1G03920.3 |  | Vvi-Vitvi07g00626\_t001 |  |  |  |  |  |  |  |
| 1 | Ath-AT1G03930.1 |  | Vvi-Vitvi07g00628\_t001 |  |  |  |  |  |  |  |
| 1 | Ath-AT1G03940.1 |  | | | |  |  |  |  |  |  |  |
| 1 | Ath-AT1G03950.1 |  | Vvi-Vitvi07g00629\_t001 |  |  |  |  |  |  |  |
| 1 | Ath-AT1G03960.1 |  | Vvi-Vitvi07g00630\_t002 |  |  |  |  |  |  |  |
| 1 | Ath-AT1G03970.1 |  | | | |  |  |  |  |  |  |  |
| 1 | Ath-AT1G03980.2 |  | Vvi-Vitvi07g00635\_t001 |  |  |  |  |  |  |  |
| 1 | Ath-AT1G03982.1 |  | | | |  |  |  |  |  |  |  |
| 1 | Ath-AT1G03990.1 |  | Vvi-Vitvi07g00636\_t001 |  |  |  |  |  |  |  |
| 1 | Ath-AT1G04000.1 |  | Vvi-Vitvi07g00639\_t001 |  |  |  |  |  |  |  |
| 1 | Ath-AT1G04010.1 |  | Vvi-Vitvi07g00649\_t001 |  |  |  |  |  |  |  |
| 1 | Ath-AT1G04020.1 |  | Vvi-Vitvi07g00653\_t001 |  |  |  |  |  |  |  |
| 1 | Ath-AT1G04030.1 |  | Vvi-Vitvi07g00659\_t001 |  |  |  |  |  |  |  |
| 1 | Ath-AT1G04040.1 |  | Vvi-Vitvi07g00667\_t001 |  |  |  |  |  |  |  |
| 1 | Ath-AT1G04050.1 |  | Vvi-Vitvi07g00675\_t001 |  |  |  |  |  |  |  |
| 1 | Ath-AT1G04070.1 |  | Vvi-Vitvi07g00677\_t001 |  |  |  |  |  |  |  |
| 1 | Ath-AT1G04080.3 |  | Vvi-Vitvi07g00682\_t001 |  |  |  |  |  |  |  |
| 1 | Ath-AT1G04090.1 |  | Vvi-Vitvi07g00685\_t001 |  |  |  |  |  |  |  |
| 1 | Ath-AT1G04100.1 |  | Vvi-Vitvi07g00687\_t001 |  |  |  |  |  |  |  |
| 1 | Ath-AT1G04105.1 |  | | | |  |  |  |  |  |  |  |
| 1 | Ath-AT1G04110.1 |  | Vvi-Vitvi07g02300\_t001 |  |  |  |  |  |  |  |
| 1 | Ath-AT1G04120.1 |  | Vvi-Vitvi07g02301\_t001 |  |  |  |  |  |  |  |
| 1 | Ath-AT1G04130.1 |  | Vvi-Vitvi07g00707\_t001 |  |  |  |  |  |  |  |
| 1 | Ath-AT1G04140.2 |  | Vvi-Vitvi07g00710\_t001 |  |  |  |  |  |  |  |
| 1 | Ath-AT1G04150.1 |  | Vvi-Vitvi07g00722\_t001 |  |  |  |  |  |  |  |
| 1 | Ath-AT1G04160.1 |  | Vvi-Vitvi07g00724\_t001 |  |  |  |  |  |  |  |
| 1 | Ath-AT1G04170.1 |  | | | |  |  |  |  |  |  |  |
| 1 | Ath-AT1G04180.1 |  | Vvi-Vitvi07g00726\_t001 |  |  |  |  |  |  |  |
| 0 | Ath-AT1G04190.1 |  |  |  |  |  |  |  |  |
| 0 | Ath-AT1G04200.1 |  |  |  |  |  |  |  |  |
| 0 | Ath-AT1G04210.1 |  |  |  |  |  |  |  |  |
| 1 | Ath-AT1G04220.1 |  | Vvi-Vitvi05g00616\_t001 |  |  |  |  |  |  |  |
| 1 | Ath-AT1G04230.1 |  | Vvi-Vitvi05g00626\_t001 |  |  |  |  |  |  |  |
| 1 | Ath-AT1G04240.2 |  | | | |  |  |  |  |  |  |  |
| 1 | Ath-AT1G04250.1 |  | Vvi-Vitvi05g00630\_t001 |  |  |  |  |  |  |  |
| 1 | Ath-AT1G04260.1 |  | Vvi-Vitvi05g01906\_t001 |  |  |  |  |  |  |  |
| 1 | Ath-AT1G04270.1 |  | Vvi-Vitvi05g00641\_t001 |  |  |  |  |  |  |  |
| 1 | Ath-AT1G04280.1 |  | Vvi-Vitvi05g00648\_t001 |  |  |  |  |  |  |  |
| 1 | Ath-AT1G04290.1 |  | | | |  |  |  |  |  |  |  |
| 1 | Ath-AT1G04300.3 |  | Vvi-Vitvi05g00666\_t001 |  |  |  |  |  |  |  |
| 1 | Ath-AT1G04310.2 |  | Vvi-Vitvi05g00684\_t001 |  |  |  |  |  |  |  |
| 1 | Ath-AT1G04330.1 |  | Vvi-Vitvi05g01918\_t001 |  |  |  |  |  |  |  |
| 1 | Ath-AT1G04340.1 |  | Vvi-Vitvi05g00692\_t001 |  |  |  |  |  |  |  |
| 1 | Ath-AT1G04350.1 |  | Vvi-Vitvi05g00707\_t001 |  |  |  |  |  |  |  |
| 1 | Ath-AT1G04360.1 |  | Vvi-Vitvi05g00713\_t001 |  |  |  |  |  |  |  |
| 1 | Ath-AT1G04370.1 |  | Vvi-Vitvi05g01722\_t001 |  |  |  |  |  |  |  |
| 0 | Ath-AT1G04380.1 |  |  |  |  |  |  |  |  |
| 0 | Ath-AT1G04390.1 |  |  |  |  |  |  |  |  |
| 1 | Ath-AT1G04400.1 |  | Vvi-Vitvi05g00737\_t002 |  |  |  |  |  |  |  |
| 1 | Ath-AT1G04410.1 |  | | | |  |  |  |  |  |  |  |
| 1 | Ath-AT1G04420.1 |  | Vvi-Vitvi05g00752\_t002 |  |  |  |  |  |  |  |
| 1 | Ath-AT1G04430.1 |  | Vvi-Vitvi05g00759\_t001 |  |  |  |  |  |  |  |
| 1 | Ath-AT1G04440.1 |  | Vvi-Vitvi05g00767\_t001 |  |  |  |  |  |  |  |
| 1 | Ath-AT1G04445.2 |  | | | |  |  |  |  |  |  |  |
| 1 | Ath-AT1G04450.1 |  | Vvi-Vitvi05g01965\_t001 |  |  |  |  |  |  |  |
| 1 | Ath-AT1G04455.1 |  | | | |  |  |  |  |  |  |  |
| 1 | Ath-AT1G04470.1 |  | Vvi-Vitvi05g00803\_t001 |  |  |  |  |  |  |  |
| 1 | Ath-AT1G04480.1 |  | Vvi-Vitvi05g04210\_t001 |  |  |  |  |  |  |  |
| 1 | Ath-AT1G04490.8 |  | Vvi-Vitvi05g00822\_t001 |  |  |  |  |  |  |  |
| 1 | Ath-AT1G04500.1 |  | Vvi-Vitvi05g00823\_t001 |  |  |  |  |  |  |  |
| 1 | Ath-AT1G04510.1 |  | Vvi-Vitvi05g00825\_t001 |  |  |  |  |  |  |  |
| 1 | Ath-AT1G04520.1 |  | Vvi-Vitvi05g00828\_t001 |  |  |  |  |  |  |  |
| 1 | Ath-AT1G04530.1 |  | Vvi-Vitvi05g00836\_t002 |  |  |  |  |  |  |  |
| 1 | Ath-AT1G04540.1 |  | Vvi-Vitvi05g00837\_t001 |  |  |  |  |  |  |  |
| 1 | Ath-AT1G04550.2 |  | Vvi-Vitvi05g00838\_t001 |  |  |  |  |  |  |  |
| 1 | Ath-AT1G04555.1 |  | Vvi-Vitvi05g04220\_t001 |  |  |  |  |  |  |  |
| 1 | Ath-AT1G04560.1 |  | Vvi-Vitvi05g00857\_t001 |  |  |  |  |  |  |  |
| 1 | Ath-AT1G04570.1 |  | Vvi-Vitvi05g04223\_t001 |  |  |  |  |  |  |  |
| 1 | Ath-AT1G04580.1 |  | | | |  |  |  |  |  |  |  |
| 1 | Ath-AT1G04590.2 |  | | | |  |  |  |  |  |  |  |
| 1 | Ath-AT1G04600.1 |  | | | |  |  |  |  |  |  |  |
| 1 | Ath-AT1G04610.1 |  | Vvi-Vitvi05g01998\_t001 |  |  |  |  |  |  |  |
| 1 | Ath-AT1G04620.1 |  | Vvi-Vitvi05g00891\_t001 |  |  |  |  |  |  |  |
| 0 | Ath-AT1G04625.1 |  |  |  |  |  |  |  |  |
| 0 | Ath-AT1G04630.1 |  |  |  |  |  |  |  |  |
| 0 | Ath-AT1G04635.1 |  |  |  |  |  |  |  |  |
| 0 | Ath-AT1G04640.1 |  |  |  |  |  |  |  |  |
| 1 | Ath-AT1G04645.1 |  | Vvi-Vitvi05g00943\_t001 |  |  |  |  |  |  |  |
| 1 | Ath-AT1G04650.1 |  | Vvi-Vitvi05g00944\_t001 |  |  |  |  |  |  |  |
| 1 | Ath-AT1G04660.1 |  | | | |  |  |  |  |  |  |  |
| 1 | Ath-AT1G04670.1 |  | | | |  |  |  |  |  |  |  |
| 1 | Ath-AT1G04680.1 |  | Vvi-Vitvi05g00953\_t001 |  |  |  |  |  |  |  |
| 1 | Ath-AT1G04690.1 |  | Vvi-Vitvi05g00960\_t001 |  |  |  |  |  |  |  |
| 1 | Ath-AT1G04700.1 |  | Vvi-Vitvi05g00961\_t001 |  |  |  |  |  |  |  |
| 1 | Ath-AT1G04710.1 |  | Vvi-Vitvi05g00966\_t001 |  |  |  |  |  |  |  |
| 1 | Ath-AT1G04730.1 |  | | | |  |  |  |  |  |  |  |
| 1 | Ath-AT1G04750.1 |  | | | |  |  |  |  |  |  |  |
| 1 | Ath-AT1G04760.1 |  | | | |  |  |  |  |  |  |  |
| 1 | Ath-AT1G04770.1 |  | | | |  |  |  |  |  |  |  |
| 1 | Ath-AT1G04778.1 |  | | | |  |  |  |  |  |  |  |
| 1 | Ath-AT1G04780.1 |  | | | |  |  |  |  |  |  |  |
| 1 | Ath-AT1G04790.1 |  | Vvi-Vitvi05g02021\_t002 |  |  |  |  |  |  |  |
| 1 | Ath-AT1G04800.1 |  | | | |  |  |  |  |  |  |  |
| 1 | Ath-AT1G04810.1 |  | | | |  |  |  |  |  |  |  |
| 1 | Ath-AT1G04820.1 |  | Vvi-Vitvi05g00973\_t001 |  |  |  |  |  |  |  |
| 0 | Ath-AT1G04830.2 |  |  |  |  |  |  |  |  |
| 0 | Ath-AT1G04840.1 |  |  |  |  |  |  |  |  |
| 0 | Ath-AT1G04850.1 |  |  |  |  |  |  |  |  |
| 0 | Ath-AT1G04860.1 |  |  |  |  |  |  |  |  |
| 0 | Ath-AT1G04870.2 |  |  |  |  |  |  |  |  |
| 0 | Ath-AT1G04880.1 |  |  |  |  |  |  |  |  |
| 0 | Ath-AT1G04890.1 |  |  |  |  |  |  |  |  |
| 0 | Ath-AT1G04895.1 |  |  |  |  |  |  |  |  |
| 0 | Ath-AT1G04900.1 |  |  |  |  |  |  |  |  |
| 0 | Ath-AT1G04910.1 |  |  |  |  |  |  |  |  |
| 0 | Ath-AT1G04920.1 |  |  |  |  |  |  |  |  |
| 0 | Ath-AT1G04930.2 |  |  |  |  |  |  |  |  |
| 0 | Ath-AT1G04945.3 |  |  |  |  |  |  |  |  |
| 0 | Ath-AT1G04940.1 |  |  |  |  |  |  |  |  |
| 0 | Ath-AT1G04950.1 |  |  |  |  |  |  |  |  |
| 1 | Ath-AT1G04960.2 |  | Vvi-Vitvi12g00467\_t001 |  |  |  |  |  |  |  |
| 1 | Ath-AT1G04970.1 |  | Vvi-Vitvi12g00466\_t001 |  |  |  |  |  |  |  |
| 1 | Ath-AT1G04980.1 |  | Vvi-Vitvi12g00453\_t001 |  |  |  |  |  |  |  |
| 1 | Ath-AT1G04985.1 |  | Vvi-Vitvi12g02381\_t001 |  |  |  |  |  |  |  |
| 1 | Ath-AT1G04990.2 |  | Vvi-Vitvi12g00449\_t001 |  |  |  |  |  |  |  |
| 1 | Ath-AT1G05000.2 |  | Vvi-Vitvi12g00446\_t001 |  |  |  |  |  |  |  |
| 1 | Ath-AT1G05005.1 |  | | | |  |  |  |  |  |  |  |
| 1 | Ath-AT1G05010.1 |  | Vvi-Vitvi12g00445\_t001 |  |  |  |  |  |  |  |
| 1 | Ath-AT1G05020.1 |  | Vvi-Vitvi12g00441\_t001 |  |  |  |  |  |  |  |
| 1 | Ath-AT1G05030.1 |  | Vvi-Vitvi12g00431\_t001 |  |  |  |  |  |  |  |
| 1 | Ath-AT1G05035.1 |  | | | |  |  |  |  |  |  |  |
| 1 | Ath-AT1G05040.1 |  | | | |  |  |  |  |  |  |  |
| 1 | Ath-AT1G05055.1 |  | | | |  |  |  |  |  |  |  |
| 1 | Ath-AT1G05060.1 |  | Vvi-Vitvi12g00419\_t001 |  |  |  |  |  |  |  |
| 1 | Ath-AT1G05065.1 |  | | | |  |  |  |  |  |  |  |
| 1 | Ath-AT1G05070.1 |  | Vvi-Vitvi12g00404\_t001 |  |  |  |  |  |  |  |
| 1 | Ath-AT1G05080.1 |  | | | |  |  |  |  |  |  |  |
| 1 | Ath-AT1G05085.1 |  | | | |  |  |  |  |  |  |  |
| 1 | Ath-AT1G05087.1 |  | Vvi-Vitvi12g00403\_t001 |  |  |  |  |  |  |  |
| 1 | Ath-AT1G05090.1 |  | | | |  |  |  |  |  |  |  |
| 1 | Ath-AT1G05100.1 |  | Vvi-Vitvi12g00387\_t001 |  |  |  |  |  |  |  |
| 1 | Ath-AT1G05120.2 |  | Vvi-Vitvi12g00384\_t001 |  |  |  |  |  |  |  |
| 1 | Ath-AT1G05136.1 |  | | | |  |  |  |  |  |  |  |
| 1 | Ath-AT1G05140.1 |  | Vvi-Vitvi12g00377\_t001 |  |  |  |  |  |  |  |
| 1 | Ath-AT1G05150.1 |  | | | |  |  |  |  |  |  |  |
| 1 | Ath-AT1G05160.2 |  | | | |  |  |  |  |  |  |  |
| 1 | Ath-AT1G05170.2 |  | Vvi-Vitvi12g00369\_t001 |  |  |  |  |  |  |  |
| 1 | Ath-AT1G05180.1 |  | Vvi-Vitvi12g00365\_t002 |  |  |  |  |  |  |  |
| 1 | Ath-AT1G05190.1 |  | Vvi-Vitvi12g00515\_t001 |  |  |  |  |  |  |  |
| 1 | Ath-AT1G05200.2 |  | Vvi-Vitvi12g00516\_t001 |  |  |  |  |  |  |  |
| 1 | Ath-AT1G05205.1 |  | | | |  |  |  |  |  |  |  |
| 1 | Ath-AT1G05210.1 |  | Vvi-Vitvi12g00520\_t001 |  |  |  |  |  |  |  |
| 1 | Ath-AT1G05220.1 |  | | | |  |  |  |  |  |  |  |
| 1 | Ath-AT1G05230.1 |  | Vvi-Vitvi12g00522\_t001 |  |  |  |  |  |  |  |
| 1 | Ath-AT1G05240.1 |  | Vvi-Vitvi12g00532\_t001 |  |  |  |  |  |  |  |
| 1 | Ath-AT1G05250.1 |  | | | |  |  |  |  |  |  |  |
| 1 | Ath-AT1G05260.1 |  | | | |  |  |  |  |  |  |  |
| 1 | Ath-AT1G05270.1 |  | Vvi-Vitvi12g00534\_t002 |  |  |  |  |  |  |  |
| 1 | Ath-AT1G05280.1 |  | Vvi-Vitvi12g00544\_t001 |  |  |  |  |  |  |  |
| 0 | Ath-AT1G05290.1 |  |  |  |  |  |  |  |  |
| 0 | Ath-AT1G05291.1 |  |  |  |  |  |  |  |  |
| 0 | Ath-AT1G05300.1 |  |  |  |  |  |  |  |  |
| 0 | Ath-AT1G05310.1 |  |  |  |  |  |  |  |  |
| 0 | Ath-AT1G05320.1 |  |  |  |  |  |  |  |  |
| 0 | Ath-AT1G05330.1 |  |  |  |  |  |  |  |  |
| 0 | Ath-AT1G05340.1 |  |  |  |  |  |  |  |  |
| 0 | Ath-AT1G05350.1 |  |  |  |  |  |  |  |  |
| 1 | Ath-AT1G05360.1 |  | Vvi-Vitvi05g00470\_t001 |  |  |  |  |  |  |  |
| 1 | Ath-AT1G05370.1 |  | Vvi-Vitvi05g00487\_t001 |  |  |  |  |  |  |  |
| 1 | Ath-AT1G05380.2 |  | Vvi-Vitvi05g00496\_t001 |  |  |  |  |  |  |  |
| 1 | Ath-AT1G05385.1 |  | Vvi-Vitvi05g00498\_t001 |  |  |  |  |  |  |  |
| 1 | Ath-AT1G05400.1 |  | | | |  |  |  |  |  |  |  |
| 1 | Ath-AT1G05410.1 |  | | | |  |  |  |  |  |  |  |
| 1 | Ath-AT1G05420.2 |  | Vvi-Vitvi05g00509\_t001 |  |  |  |  |  |  |  |
| 1 | Ath-AT1G05430.1 |  | Vvi-Vitvi05g01891\_t001 |  |  |  |  |  |  |  |
| 1 | Ath-AT1G05440.1 |  | Vvi-Vitvi05g00519\_t001 |  |  |  |  |  |  |  |
| 1 | Ath-AT1G05450.2 |  | Vvi-Vitvi05g00529\_t001 |  |  |  |  |  |  |  |
| 1 | Ath-AT1G05460.1 |  | Vvi-Vitvi05g00530\_t001 |  |  |  |  |  |  |  |
| 1 | Ath-AT1G05470.1 |  | Vvi-Vitvi05g00533\_t001 |  |  |  |  |  |  |  |
| 0 | Ath-AT1G05490.1 |  |  |  |  |  |  |  |  |
| 0 | Ath-AT1G05500.1 |  |  |  |  |  |  |  |  |
| 0 | Ath-AT1G05510.1 |  |  |  |  |  |  |  |  |
| 0 | Ath-AT1G05520.1 |  |  |  |  |  |  |  |  |
| 0 | Ath-AT1G05530.1 |  |  |  |  |  |  |  |  |
| 0 | Ath-AT1G05540.1 |  |  |  |  |  |  |  |  |
| 0 | Ath-AT1G05550.3 |  |  |  |  |  |  |  |  |
| 0 | Ath-AT1G05560.2 |  |  |  |  |  |  |  |  |
| 0 | Ath-AT1G05570.3 |  |  |  |  |  |  |  |  |
| 0 | Ath-AT1G05575.1 |  |  |  |  |  |  |  |  |
| 0 | Ath-AT1G05577.1 |  |  |  |  |  |  |  |  |
| 0 | Ath-AT1G05580.1 |  |  |  |  |  |  |  |  |
| 1 | Ath-AT1G05590.1 |  | Vvi-Vitvi07g04480\_t001 |  |  |  |  |  |  |  |
| 1 | Ath-AT1G05600.1 |  | | | |  |  |  |  |  |  |  |
| 1 | Ath-AT1G05610.2 |  | | | |  |  |  |  |  |  |  |
| 1 | Ath-AT1G05615.1 |  | | | |  |  |  |  |  |  |  |
| 1 | Ath-AT1G05620.1 |  | | | |  |  |  |  |  |  |  |
| 1 | Ath-AT1G05630.1 |  | Vvi-Vitvi07g01242\_t001 |  |  |  |  |  |  |  |
| 1 | Ath-AT1G05640.1 |  | | | |  |  |  |  |  |  |  |
| 1 | Ath-AT1G05650.1 |  | Vvi-Vitvi07g02498\_t001 |  |  |  |  |  |  |  |
| 1 | Ath-AT1G05660.1 |  | | | |  |  |  |  |  |  |  |
| 1 | Ath-AT1G05670.1 |  | Vvi-Vitvi07g01251\_t001 |  |  |  |  |  |  |  |
| 1 | Ath-AT1G05675.1 |  | | | |  |  |  |  |  |  |  |
| 1 | Ath-AT1G05680.1 |  | | | |  |  |  |  |  |  |  |
| 1 | Ath-AT1G05690.1 |  | | | |  |  |  |  |  |  |  |
| 1 | Ath-AT1G05700.2 |  | | | |  |  |  |  |  |  |  |
| 1 | Ath-AT1G05710.2 |  | | | |  |  |  |  |  |  |  |
| 1 | Ath-AT1G05720.1 |  | | | |  |  |  |  |  |  |  |
| 1 | Ath-AT1G05730.1 |  | | | |  |  |  |  |  |  |  |
| 1 | Ath-AT1G05740.1 |  | | | |  |  |  |  |  |  |  |
| 1 | Ath-AT1G05750.2 |  | | | |  |  |  |  |  |  |  |
| 1 | Ath-AT1G05760.2 |  | | | |  |  |  |  |  |  |  |
| 1 | Ath-AT1G05770.1 |  | | | |  |  |  |  |  |  |  |
| 1 | Ath-AT1G05780.1 |  | | | |  |  |  |  |  |  |  |
| 1 | Ath-AT1G05783.1 |  | | | |  |  |  |  |  |  |  |
| 1 | Ath-AT1G05785.6 |  | | | |  |  |  |  |  |  |  |
| 1 | Ath-AT1G05790.3 |  | | | |  |  |  |  |  |  |  |
| 1 | Ath-AT1G05800.1 |  | | | |  |  |  |  |  |  |  |
| 1 | Ath-AT1G05805.1 |  | | | |  |  |  |  |  |  |  |
| 1 | Ath-AT1G05810.2 |  | | | |  |  |  |  |  |  |  |
| 1 | Ath-AT1G05820.1 |  | | | |  |  |  |  |  |  |  |
| 1 | Ath-AT1G05830.3 |  | | | |  |  |  |  |  |  |  |
| 1 | Ath-AT1G05835.1 |  | | | |  |  |  |  |  |  |  |
| 1 | Ath-AT1G05840.1 |  | | | |  |  |  |  |  |  |  |
| 1 | Ath-AT1G05850.2 |  | | | |  |  |  |  |  |  |  |
| 2 | Ath-AT1G05860.2 |  | Vvi-Vitvi07g02500\_t002 |  | Vvi-Vitvi05g01465\_t001 |  |  |  |  |  |  |
| 2 | Ath-AT1G05870.6 |  | | | |  | Vvi-Vitvi05g01470\_t001 |  |  |  |  |  |  |
| 2 | Ath-AT1G05880.2 |  | | | |  | | | |  |  |  |  |  |  |
| 2 | Ath-AT1G05890.2 |  | Vvi-Vitvi07g01261\_t001 |  | Vvi-Vitvi05g01475\_t001 |  |  |  |  |  |  |
| 1 | Ath-AT1G05894.1 |  |  |  | Vvi-Vitvi05g01479\_t001 |  |  |  |  |  |  |
| 1 | Ath-AT1G05900.2 |  |  |  | Vvi-Vitvi05g01484\_t001 |  |  |  |  |  |  |
| 1 | Ath-AT1G05910.1 |  |  |  | Vvi-Vitvi05g01486\_t001 |  |  |  |  |  |  |
| 1 | Ath-AT1G05920.1 |  |  |  | | | |  |  |  |  |  |  |
| 1 | Ath-AT1G05930.1 |  |  |  | | | |  |  |  |  |  |  |
| 1 | Ath-AT1G05940.1 |  |  |  | | | |  |  |  |  |  |  |
| 1 | Ath-AT1G05950.1 |  |  |  | | | |  |  |  |  |  |  |
| 1 | Ath-AT1G05960.2 |  |  |  | Vvi-Vitvi05g01488\_t001 |  |  |  |  |  |  |
| 1 | Ath-AT1G05970.2 |  |  |  | | | |  |  |  |  |  |  |
| 1 | Ath-AT1G05990.1 |  |  |  | Vvi-Vitvi05g01500\_t001 |  |  |  |  |  |  |
| 0 | Ath-AT1G06000.1 |  |  |  |  |  |  |  |  |
| 0 | Ath-AT1G06010.1 |  |  |  |  |  |  |  |  |
| 0 | Ath-AT1G06020.1 |  |  |  |  |  |  |  |  |
| 0 | Ath-AT1G06030.1 |  |  |  |  |  |  |  |  |
| 0 | Ath-AT1G06040.1 |  |  |  |  |  |  |  |  |
| 0 | Ath-AT1G06045.1 |  |  |  |  |  |  |  |  |
| 0 | Ath-AT1G06050.1 |  |  |  |  |  |  |  |  |
| 0 | Ath-AT1G06060.1 |  |  |  |  |  |  |  |  |
| 0 | Ath-AT1G06070.1 |  |  |  |  |  |  |  |  |
| 0 | Ath-AT1G06080.1 |  |  |  |  |  |  |  |  |
| 0 | Ath-AT1G06090.1 |  |  |  |  |  |  |  |  |
| 0 | Ath-AT1G06100.1 |  |  |  |  |  |  |  |  |
| 0 | Ath-AT1G06110.1 |  |  |  |  |  |  |  |  |
| 0 | Ath-AT1G06120.1 |  |  |  |  |  |  |  |  |
| 0 | Ath-AT1G06130.1 |  |  |  |  |  |  |  |  |
| 0 | Ath-AT1G06135.1 |  |  |  |  |  |  |  |  |
| 0 | Ath-AT1G06137.1 |  |  |  |  |  |  |  |  |
| 0 | Ath-AT1G06140.1 |  |  |  |  |  |  |  |  |
| 0 | Ath-AT1G06143.1 |  |  |  |  |  |  |  |  |
| 0 | Ath-AT1G06150.2 |  |  |  |  |  |  |  |  |
| 0 | Ath-AT1G06148.2 |  |  |  |  |  |  |  |  |
| 0 | Ath-AT1G06160.1 |  |  |  |  |  |  |  |  |
| 0 | Ath-AT1G06170.2 |  |  |  |  |  |  |  |  |
| 0 | Ath-AT1G06180.1 |  |  |  |  |  |  |  |  |
| 0 | Ath-AT1G06190.5 |  |  |  |  |  |  |  |  |
| 0 | Ath-AT1G06200.2 |  |  |  |  |  |  |  |  |
| 0 | Ath-AT1G06210.1 |  |  |  |  |  |  |  |  |
| 0 | Ath-AT1G06220.2 |  |  |  |  |  |  |  |  |
| 0 | Ath-AT1G06225.1 |  |  |  |  |  |  |  |  |
| 0 | Ath-AT1G06230.4 |  |  |  |  |  |  |  |  |
| 0 | Ath-AT1G06240.1 |  |  |  |  |  |  |  |  |
| 0 | Ath-AT1G06250.1 |  |  |  |  |  |  |  |  |
| 0 | Ath-AT1G06260.1 |  |  |  |  |  |  |  |  |
| 0 | Ath-AT1G06270.1 |  |  |  |  |  |  |  |  |
| 0 | Ath-AT1G06280.1 |  |  |  |  |  |  |  |  |
| 0 | Ath-AT1G06290.1 |  |  |  |  |  |  |  |  |
| 0 | Ath-AT1G06310.1 |  |  |  |  |  |  |  |  |
| 0 | Ath-AT1G06320.1 |  |  |  |  |  |  |  |  |
| 0 | Ath-AT1G06330.1 |  |  |  |  |  |  |  |  |
| 0 | Ath-AT1G06340.1 |  |  |  |  |  |  |  |  |
| 0 | Ath-AT1G06350.1 |  |  |  |  |  |  |  |  |
| 0 | Ath-AT1G06360.2 |  |  |  |  |  |  |  |  |
| 1 | Ath-AT1G06380.1 |  | Vvi-Vitvi12g00029\_t001 |  |  |  |  |  |  |  |
| 1 | Ath-AT1G06390.1 |  | | | |  |  |  |  |  |  |  |
| 1 | Ath-AT1G06400.1 |  | | | |  |  |  |  |  |  |  |
| 1 | Ath-AT1G06410.1 |  | | | |  |  |  |  |  |  |  |
| 1 | Ath-AT1G06420.1 |  | | | |  |  |  |  |  |  |  |
| 1 | Ath-AT1G06430.2 |  | | | |  |  |  |  |  |  |  |
| 1 | Ath-AT1G06440.1 |  | | | |  |  |  |  |  |  |  |
| 1 | Ath-AT1G06450.1 |  | | | |  |  |  |  |  |  |  |
| 1 | Ath-AT1G06460.1 |  | | | |  |  |  |  |  |  |  |
| 1 | Ath-AT1G06470.2 |  | | | |  |  |  |  |  |  |  |
| 1 | Ath-AT1G06475.1 |  | | | |  |  |  |  |  |  |  |
| 1 | Ath-AT1G06490.1 |  | | | |  |  |  |  |  |  |  |
| 1 | Ath-AT1G06500.4 |  | | | |  |  |  |  |  |  |  |
| 1 | Ath-AT1G06515.2 |  | | | |  |  |  |  |  |  |  |
| 1 | Ath-AT1G06510.1 |  | Vvi-Vitvi12g00045\_t001 |  |  |  |  |  |  |  |
| 1 | Ath-AT1G06520.1 |  | | | |  |  |  |  |  |  |  |
| 1 | Ath-AT1G06530.1 |  | | | |  |  |  |  |  |  |  |
| 1 | Ath-AT1G06540.1 |  | | | |  |  |  |  |  |  |  |
| 1 | Ath-AT1G06550.1 |  | Vvi-Vitvi12g00065\_t001 |  |  |  |  |  |  |  |
| 1 | Ath-AT1G06560.1 |  | Vvi-Vitvi12g00068\_t001 |  |  |  |  |  |  |  |
| 1 | Ath-AT1G06570.1 |  | Vvi-Vitvi12g00071\_t001 |  |  |  |  |  |  |  |
| 1 | Ath-AT1G06580.1 |  | | | |  |  |  |  |  |  |  |
| 1 | Ath-AT1G06590.1 |  | Vvi-Vitvi12g00073\_t001 |  |  |  |  |  |  |  |
| 1 | Ath-AT1G06620.1 |  | | | |  |  |  |  |  |  |  |
| 1 | Ath-AT1G06630.1 |  | | | |  |  |  |  |  |  |  |
| 1 | Ath-AT1G06640.1 |  | | | |  |  |  |  |  |  |  |
| 1 | Ath-AT1G06645.1 |  | | | |  |  |  |  |  |  |  |
| 1 | Ath-AT1G06650.2 |  | | | |  |  |  |  |  |  |  |
| 1 | Ath-AT1G06660.1 |  | Vvi-Vitvi12g00082\_t001 |  |  |  |  |  |  |  |
| 1 | Ath-AT1G06670.1 |  | Vvi-Vitvi12g00088\_t001 |  |  |  |  |  |  |  |
| 1 | Ath-AT1G06680.1 |  | Vvi-Vitvi12g00092\_t001 |  |  |  |  |  |  |  |
| 1 | Ath-AT1G06690.1 |  | Vvi-Vitvi12g00095\_t001 |  |  |  |  |  |  |  |
| 0 | Ath-AT1G06700.2 |  |  |  |  |  |  |  |  |
| 0 | Ath-AT1G06710.6 |  |  |  |  |  |  |  |  |
| 0 | Ath-AT1G06720.2 |  |  |  |  |  |  |  |  |
| 1 | Ath-AT1G06730.1 |  | Vvi-Vitvi07g00016\_t001 |  |  |  |  |  |  |  |
| 1 | Ath-AT1G06740.1 |  | Vvi-Vitvi07g00017\_t001 |  |  |  |  |  |  |  |
| 1 | Ath-AT1G06750.2 |  | Vvi-Vitvi07g00021\_t001 |  |  |  |  |  |  |  |
| 1 | Ath-AT1G06760.1 |  | | | |  |  |  |  |  |  |  |
| 1 | Ath-AT1G06770.1 |  | Vvi-Vitvi07g00031\_t001 |  |  |  |  |  |  |  |
| 1 | Ath-AT1G06780.2 |  | Vvi-Vitvi07g00034\_t001 |  |  |  |  |  |  |  |
| 1 | Ath-AT1G06790.1 |  | Vvi-Vitvi07g04010\_t001 |  |  |  |  |  |  |  |
| 1 | Ath-AT1G06800.1 |  | Vvi-Vitvi07g00039\_t001 |  |  |  |  |  |  |  |
| 0 | Ath-AT1G06810.1 |  |  |  |  |  |  |  |  |
| 0 | Ath-AT1G06820.1 |  |  |  |  |  |  |  |  |
| 0 | Ath-AT1G06830.1 |  |  |  |  |  |  |  |  |
| 1 | Ath-AT1G06840.1 |  | Vvi-Vitvi06g00819\_t002 |  |  |  |  |  |  |  |
| 1 | Ath-AT1G06850.1 |  | Vvi-Vitvi06g00800\_t001 |  |  |  |  |  |  |  |
| 1 | Ath-AT1G06870.1 |  | | | |  |  |  |  |  |  |  |
| 1 | Ath-AT1G06890.1 |  | | | |  |  |  |  |  |  |  |
| 1 | Ath-AT1G06900.1 |  | Vvi-Vitvi06g00791\_t001 |  |  |  |  |  |  |  |
| 1 | Ath-AT1G06910.1 |  | | | |  |  |  |  |  |  |  |
| 1 | Ath-AT1G06920.1 |  | Vvi-Vitvi06g00783\_t001 |  |  |  |  |  |  |  |
| 1 | Ath-AT1G06923.1 |  | Vvi-Vitvi06g00782\_t001 |  |  |  |  |  |  |  |
| 1 | Ath-AT1G06925.1 |  | | | |  |  |  |  |  |  |  |
| 1 | Ath-AT1G06930.1 |  | | | |  |  |  |  |  |  |  |
| 1 | Ath-AT1G06950.1 |  | Vvi-Vitvi06g00767\_t001 |  |  |  |  |  |  |  |
| 1 | Ath-AT1G06960.1 |  | Vvi-Vitvi06g00743\_t001 |  |  |  |  |  |  |  |
| 1 | Ath-AT1G06970.1 |  | Vvi-Vitvi06g00737\_t001 |  |  |  |  |  |  |  |
| 1 | Ath-AT1G06980.1 |  | Vvi-Vitvi06g00736\_t001 |  |  |  |  |  |  |  |
| 1 | Ath-AT1G06990.1 |  | | | |  |  |  |  |  |  |  |
| 1 | Ath-AT1G07000.1 |  | Vvi-Vitvi06g00733\_t001 |  |  |  |  |  |  |  |
| 1 | Ath-AT1G07010.3 |  | Vvi-Vitvi06g00725\_t001 |  |  |  |  |  |  |  |
| 1 | Ath-AT1G07020.1 |  | Vvi-Vitvi06g00722\_t001 |  |  |  |  |  |  |  |
| 1 | Ath-AT1G07025.1 |  | Vvi-Vitvi06g00720\_t001 |  |  |  |  |  |  |  |
| 1 | Ath-AT1G07030.1 |  | | | |  |  |  |  |  |  |  |
| 1 | Ath-AT1G07040.1 |  | | | |  |  |  |  |  |  |  |
| 1 | Ath-AT1G07050.2 |  | Vvi-Vitvi06g00707\_t001 |  |  |  |  |  |  |  |
| 1 | Ath-AT1G07060.2 |  | Vvi-Vitvi06g00705\_t001 |  |  |  |  |  |  |  |
| 1 | Ath-AT1G07070.1 |  | | | |  |  |  |  |  |  |  |
| 1 | Ath-AT1G07080.1 |  | Vvi-Vitvi06g01759\_t001 |  |  |  |  |  |  |  |
| 1 | Ath-AT1G07090.1 |  | Vvi-Vitvi06g00695\_t001 |  |  |  |  |  |  |  |
| 1 | Ath-AT1G07110.1 |  | Vvi-Vitvi06g00676\_t001 |  |  |  |  |  |  |  |
| 1 | Ath-AT1G07120.1 |  | Vvi-Vitvi06g00674\_t001 |  |  |  |  |  |  |  |
| 1 | Ath-AT1G07130.1 |  | | | |  |  |  |  |  |  |  |
| 1 | Ath-AT1G07135.1 |  | | | |  |  |  |  |  |  |  |
| 1 | Ath-AT1G07140.1 |  | Vvi-Vitvi06g00671\_t001 |  |  |  |  |  |  |  |
| 1 | Ath-AT1G07150.1 |  | Vvi-Vitvi06g00668\_t001 |  |  |  |  |  |  |  |
| 1 | Ath-AT1G07160.1 |  | Vvi-Vitvi06g00667\_t001 |  |  |  |  |  |  |  |
| 1 | Ath-AT1G07170.1 |  | Vvi-Vitvi06g00658\_t001 |  |  |  |  |  |  |  |
| 1 | Ath-AT1G07175.1 |  | | | |  |  |  |  |  |  |  |
| 1 | Ath-AT1G07180.1 |  | Vvi-Vitvi06g01751\_t001 |  |  |  |  |  |  |  |
| 1 | Ath-AT1G07190.1 |  | | | |  |  |  |  |  |  |  |
| 1 | Ath-AT1G07200.2 |  | Vvi-Vitvi06g00652\_t001 |  |  |  |  |  |  |  |
| 1 | Ath-AT1G07210.1 |  | Vvi-Vitvi06g00651\_t002 |  |  |  |  |  |  |  |
| 1 | Ath-AT1G07220.1 |  | Vvi-Vitvi06g00645\_t001 |  |  |  |  |  |  |  |
| 1 | Ath-AT1G07230.1 |  | Vvi-Vitvi06g00632\_t001 |  |  |  |  |  |  |  |
| 1 | Ath-AT1G07240.1 |  | Vvi-Vitvi06g00621\_t001 |  |  |  |  |  |  |  |
| 1 | Ath-AT1G07250.1 |  | | | |  |  |  |  |  |  |  |
| 1 | Ath-AT1G07260.1 |  | | | |  |  |  |  |  |  |  |
| 1 | Ath-AT1G07270.1 |  | Vvi-Vitvi06g00614\_t001 |  |  |  |  |  |  |  |
| 1 | Ath-AT1G07280.1 |  | Vvi-Vitvi06g00613\_t001 |  |  |  |  |  |  |  |
| 1 | Ath-AT1G07290.1 |  | Vvi-Vitvi06g00610\_t001 |  |  |  |  |  |  |  |
| 1 | Ath-AT1G07300.3 |  | | | |  |  |  |  |  |  |  |
| 1 | Ath-AT1G07310.1 |  | Vvi-Vitvi06g00599\_t001 |  |  |  |  |  |  |  |
| 1 | Ath-AT1G07320.1 |  | Vvi-Vitvi06g00598\_t001 |  |  |  |  |  |  |  |
| 1 | Ath-AT1G07330.2 |  | Vvi-Vitvi06g00594\_t001 |  |  |  |  |  |  |  |
| 1 | Ath-AT1G07340.1 |  | | | |  |  |  |  |  |  |  |
| 1 | Ath-AT1G07350.1 |  | Vvi-Vitvi06g04198\_t001 |  |  |  |  |  |  |  |
| 1 | Ath-AT1G07360.1 |  | Vvi-Vitvi06g04197\_t001 |  |  |  |  |  |  |  |
| 1 | Ath-AT1G07370.1 |  | Vvi-Vitvi06g00576\_t001 |  |  |  |  |  |  |  |
| 1 | Ath-AT1G07380.2 |  | Vvi-Vitvi06g00573\_t001 |  |  |  |  |  |  |  |
| 1 | Ath-AT1G07390.3 |  | | | |  |  |  |  |  |  |  |
| 1 | Ath-AT1G07400.1 |  | Vvi-Vitvi06g00561\_t001 |  |  |  |  |  |  |  |
| 1 | Ath-AT1G07410.1 |  | Vvi-Vitvi06g00542\_t001 |  |  |  |  |  |  |  |
| 1 | Ath-AT1G07420.1 |  | Vvi-Vitvi06g00534\_t001 |  |  |  |  |  |  |  |
| 1 | Ath-AT1G07430.1 |  | Vvi-Vitvi06g00533\_t001 |  |  |  |  |  |  |  |
| 1 | Ath-AT1G07440.1 |  | Vvi-Vitvi06g04174\_t001 |  |  |  |  |  |  |  |
| 1 | Ath-AT1G07450.1 |  | Vvi-Vitvi06g00522\_t001 |  |  |  |  |  |  |  |
| 1 | Ath-AT1G07460.2 |  | | | |  |  |  |  |  |  |  |
| 1 | Ath-AT1G07470.2 |  | Vvi-Vitvi06g00515\_t001 |  |  |  |  |  |  |  |
| 1 | Ath-AT1G07473.1 |  | | | |  |  |  |  |  |  |  |
| 1 | Ath-AT1G07476.1 |  | | | |  |  |  |  |  |  |  |
| 1 | Ath-AT1G07480.3 |  | | | |  |  |  |  |  |  |  |
| 1 | Ath-AT1G07485.1 |  | | | |  |  |  |  |  |  |  |
| 1 | Ath-AT1G07490.1 |  | | | |  |  |  |  |  |  |  |
| 2 | Ath-AT1G07510.1 |  | Vvi-Vitvi06g00496\_t001 |  | Vvi-Vitvi08g01218\_t001 |  |  |  |  |  |  |
| 2 | Ath-AT1G07500.1 |  | | | |  | | | |  |  |  |  |  |  |
| 2 | Ath-AT1G07520.3 |  | Vvi-Vitvi06g01569\_t001 |  | | | |  |  |  |  |  |  |
| 2 | Ath-AT1G07530.1 |  | | | |  | Vvi-Vitvi08g01214\_t001 |  |  |  |  |  |  |
| 2 | Ath-AT1G07540.1 |  | Vvi-Vitvi06g00488\_t001 |  | Vvi-Vitvi08g01213\_t002 |  |  |  |  |  |  |
| 2 | Ath-AT1G07550.1 |  | | | |  | | | |  |  |  |  |  |  |
| 2 | Ath-AT1G07560.1 |  | | | |  | | | |  |  |  |  |  |  |
| 2 | Ath-AT1G07570.3 |  | Vvi-Vitvi06g00473\_t001 |  | Vvi-Vitvi08g01204\_t004 |  |  |  |  |  |  |
| 2 | Ath-AT1G07590.1 |  | Vvi-Vitvi06g00464\_t001 |  | | | |  |  |  |  |  |  |
| 2 | Ath-AT1G07600.1 |  | | | |  | | | |  |  |  |  |  |  |
| 2 | Ath-AT1G07610.1 |  | | | |  | | | |  |  |  |  |  |  |
| 2 | Ath-AT1G07615.1 |  | Vvi-Vitvi06g00462\_t001 |  | | | |  |  |  |  |  |  |
| 2 | Ath-AT1G07620.2 |  | Vvi-Vitvi06g00460\_t001 |  | Vvi-Vitvi08g01195\_t001 |  |  |  |  |  |  |
| 2 | Ath-AT1G07630.1 |  | Vvi-Vitvi06g00456\_t001 |  | Vvi-Vitvi08g01194\_t001 |  |  |  |  |  |  |
| 2 | Ath-AT1G07640.3 |  | Vvi-Vitvi06g00449\_t001 |  | Vvi-Vitvi08g01186\_t001 |  |  |  |  |  |  |
| 2 | Ath-AT1G07650.2 |  | | | |  | | | |  |  |  |  |  |  |
| 2 | Ath-AT1G07645.1 |  | Vvi-Vitvi06g00438\_t001 |  | | | |  |  |  |  |  |  |
| 2 | Ath-AT1G07660.1 |  | | | |  | | | |  |  |  |  |  |  |
| 3 | Ath-AT1G07670.1 |  | | | |  | | | |  | Vvi-Vitvi06g00386\_t002 |  |  |  |  |  |
| 3 | Ath-AT1G07680.1 |  | | | |  | | | |  | | | |  |  |  |  |  |
| 3 | Ath-AT1G07690.2 |  | | | |  | | | |  | | | |  |  |  |  |  |
| 3 | Ath-AT1G07700.3 |  | | | |  | | | |  | | | |  |  |  |  |  |
| 3 | Ath-AT1G07705.2 |  | | | |  | | | |  | Vvi-Vitvi06g00389\_t001 |  |  |  |  |  |
| 3 | Ath-AT1G07710.1 |  | | | |  | | | |  | Vvi-Vitvi06g00393\_t001 |  |  |  |  |  |
| 3 | Ath-AT1G07720.2 |  | | | |  | | | |  | Vvi-Vitvi06g00396\_t001 |  |  |  |  |  |
| 3 | Ath-AT1G07725.1 |  | | | |  | | | |  | | | |  |  |  |  |  |
| 4 | Ath-AT1G07740.1 |  | | | |  | | | |  | | | |  | Vvi-Vitvi06g00402\_t001 |  |  |  |  |
| 4 | Ath-AT1G07730.2 |  | | | |  | | | |  | Vvi-Vitvi06g00400\_t001 |  | | | |  |  |  |  |
| 4 | Ath-AT1G07745.1 |  | | | |  | | | |  | Vvi-Vitvi06g00403\_t001 |  | | | |  |  |  |  |
| 4 | Ath-AT1G07747.1 |  | | | |  | | | |  | | | |  | | | |  |  |  |  |
| 4 | Ath-AT1G07750.1 |  | | | |  | | | |  | Vvi-Vitvi06g00406\_t001 |  | | | |  |  |  |  |
| 4 | Ath-AT1G07770.1 |  | | | |  | | | |  | | | |  | | | |  |  |  |  |
| 4 | Ath-AT1G07780.5 |  | | | |  | | | |  | | | |  | | | |  |  |  |  |
| 4 | Ath-AT1G07790.1 |  | Vvi-Vitvi06g00426\_t001 |  | | | |  | Vvi-Vitvi06g00422\_t001 |  | | | |  |  |  |  |
| 2 | Ath-AT1G07795.1 |  |  |  | Vvi-Vitvi08g02184\_t001 |  |  |  | | | |  |  |  |  |
| 2 | Ath-AT1G07810.1 |  |  |  | | | |  |  |  | Vvi-Vitvi06g00386\_t002 |  |  |  |  |
| 2 | Ath-AT1G07820.1 |  |  |  | | | |  |  |  | | | |  |  |  |  |
| 2 | Ath-AT1G07830.1 |  |  |  | | | |  |  |  | | | |  |  |  |  |
| 2 | Ath-AT1G07840.1 |  |  |  | | | |  |  |  | | | |  |  |  |  |
| 2 | Ath-AT1G07850.1 |  |  |  | | | |  |  |  | Vvi-Vitvi06g00371\_t001 |  |  |  |  |
| 2 | Ath-AT1G07870.2 |  |  |  | | | |  |  |  | Vvi-Vitvi06g00369\_t001 |  |  |  |  |
| 2 | Ath-AT1G07860.1 |  |  |  | | | |  |  |  | | | |  |  |  |  |
| 2 | Ath-AT1G07880.2 |  |  |  | | | |  |  |  | Vvi-Vitvi06g00365\_t001 |  |  |  |  |
| 2 | Ath-AT1G07885.2 |  |  |  | | | |  |  |  | | | |  |  |  |  |
| 2 | Ath-AT1G07890.1 |  |  |  | Vvi-Vitvi08g01143\_t002 |  |  |  | Vvi-Vitvi06g00358\_t003 |  |  |  |  |
| 1 | Ath-AT1G07900.1 |  |  |  |  |  |  |  | Vvi-Vitvi06g00336\_t001 |  |  |  |  |
| 1 | Ath-AT1G07902.1 |  |  |  |  |  |  |  | | | |  |  |  |  |
| 1 | Ath-AT1G07901.1 |  |  |  |  |  |  |  | | | |  |  |  |  |
| 1 | Ath-AT1G07910.2 |  |  |  |  |  |  |  | Vvi-Vitvi06g00330\_t001 |  |  |  |  |
| 1 | Ath-AT1G07920.1 |  |  |  |  |  |  |  | | | |  |  |  |  |
| 1 | Ath-AT1G07930.1 |  |  |  |  |  |  |  | | | |  |  |  |  |
| 1 | Ath-AT1G07940.3 |  |  |  |  |  |  |  | | | |  |  |  |  |
| 1 | Ath-AT1G07950.2 |  |  |  |  |  |  |  | | | |  |  |  |  |
| 1 | Ath-AT1G07960.3 |  |  |  |  |  |  |  | Vvi-Vitvi06g00322\_t001 |  |  |  |  |
| 1 | Ath-AT1G07970.1 |  |  |  |  |  |  |  | Vvi-Vitvi06g00313\_t001 |  |  |  |  |
| 1 | Ath-AT1G07980.1 |  |  |  |  |  |  |  | Vvi-Vitvi06g01659\_t001 |  |  |  |  |
| 0 | Ath-AT1G07985.1 |  |  |  |  |  |  |  |  |
| 1 | Ath-AT1G07990.1 |  | Vvi-Vitvi06g00274\_t004 |  |  |  |  |  |  |  |
| 1 | Ath-AT1G08000.2 |  | Vvi-Vitvi06g04095\_t001 |  |  |  |  |  |  |  |
| 1 | Ath-AT1G08005.1 |  | | | |  |  |  |  |  |  |  |
| 1 | Ath-AT1G08010.1 |  | | | |  |  |  |  |  |  |  |
| 1 | Ath-AT1G08030.1 |  | | | |  |  |  |  |  |  |  |
| 1 | Ath-AT1G08035.1 |  | | | |  |  |  |  |  |  |  |
| 1 | Ath-AT1G08040.1 |  | Vvi-Vitvi06g00267\_t001 |  |  |  |  |  |  |  |
| 1 | Ath-AT1G08050.1 |  | Vvi-Vitvi06g00258\_t001 |  |  |  |  |  |  |  |
| 1 | Ath-AT1G08060.3 |  | Vvi-Vitvi06g04090\_t001 |  |  |  |  |  |  |  |
| 1 | Ath-AT1G08065.1 |  | Vvi-Vitvi06g00250\_t001 |  |  |  |  |  |  |  |
| 0 | Ath-AT1G08070.1 |  |  |  |  |  |  |  |  |
| 0 | Ath-AT1G08080.1 |  |  |  |  |  |  |  |  |
| 1 | Ath-AT1G08090.1 |  | Vvi-Vitvi06g01299\_t001 |  |  |  |  |  |  |  |
| 1 | Ath-AT1G08100.1 |  | | | |  |  |  |  |  |  |  |
| 1 | Ath-AT1G08110.4 |  | Vvi-Vitvi06g01320\_t002 |  |  |  |  |  |  |  |
| 1 | Ath-AT1G08120.1 |  | | | |  |  |  |  |  |  |  |
| 1 | Ath-AT1G08125.2 |  | Vvi-Vitvi06g01324\_t002 |  |  |  |  |  |  |  |
| 1 | Ath-AT1G08130.1 |  | Vvi-Vitvi06g01341\_t001 |  |  |  |  |  |  |  |
| 1 | Ath-AT1G08135.1 |  | | | |  |  |  |  |  |  |  |
| 1 | Ath-AT1G08140.1 |  | | | |  |  |  |  |  |  |  |
| 1 | Ath-AT1G08150.1 |  | | | |  |  |  |  |  |  |  |
| 1 | Ath-AT1G08160.1 |  | Vvi-Vitvi06g01357\_t001 |  |  |  |  |  |  |  |
| 1 | Ath-AT1G08165.1 |  | | | |  |  |  |  |  |  |  |
| 1 | Ath-AT1G08170.1 |  | Vvi-Vitvi06g01360\_t001 |  |  |  |  |  |  |  |
| 1 | Ath-AT1G08180.1 |  | | | |  |  |  |  |  |  |  |
| 1 | Ath-AT1G08190.1 |  | Vvi-Vitvi06g01364\_t001 |  |  |  |  |  |  |  |
| 2 | Ath-AT1G08200.1 |  | | | |  | Vvi-Vitvi06g01468\_t001 |  |  |  |  |  |  |
| 2 | Ath-AT1G08210.3 |  | | | |  | | | |  |  |  |  |  |  |
| 2 | Ath-AT1G08220.1 |  | Vvi-Vitvi06g01376\_t001 |  | | | |  |  |  |  |  |  |
| 2 | Ath-AT1G08230.2 |  | Vvi-Vitvi06g01396\_t001 |  | | | |  |  |  |  |  |  |
| 2 | Ath-AT1G08250.1 |  | Vvi-Vitvi06g01946\_t001 |  | | | |  |  |  |  |  |  |
| 1 | Ath-AT1G08260.1 |  |  |  | | | |  |  |  |  |  |  |
| 1 | Ath-AT1G08270.2 |  |  |  | | | |  |  |  |  |  |  |
| 1 | Ath-AT1G08280.1 |  |  |  | Vvi-Vitvi06g01469\_t001 |  |  |  |  |  |  |
| 1 | Ath-AT1G08290.1 |  |  |  | Vvi-Vitvi06g01471\_t001 |  |  |  |  |  |  |
| 1 | Ath-AT1G08300.1 |  |  |  | | | |  |  |  |  |  |  |
| 1 | Ath-AT1G08310.2 |  |  |  | Vvi-Vitvi06g01477\_t001 |  |  |  |  |  |  |
| 1 | Ath-AT1G08315.1 |  |  |  | | | |  |  |  |  |  |  |
| 1 | Ath-AT1G08320.4 |  |  |  | Vvi-Vitvi06g01480\_t001 |  |  |  |  |  |  |
| 1 | Ath-AT1G08340.1 |  |  |  | | | |  |  |  |  |  |  |
| 1 | Ath-AT1G08350.2 |  |  |  | Vvi-Vitvi06g01485\_t001 |  |  |  |  |  |  |
| 1 | Ath-AT1G08360.1 |  |  |  | Vvi-Vitvi06g01486\_t002 |  |  |  |  |  |  |
| 0 | Ath-AT1G08370.1 |  |  |  |  |  |  |  |  |
| 0 | Ath-AT1G08380.1 |  |  |  |  |  |  |  |  |
| 0 | Ath-AT1G08390.1 |  |  |  |  |  |  |  |  |
| 0 | Ath-AT1G08400.1 |  |  |  |  |  |  |  |  |
| 0 | Ath-AT1G08410.1 |  |  |  |  |  |  |  |  |
| 0 | Ath-AT1G08420.1 |  |  |  |  |  |  |  |  |
| 0 | Ath-AT1G08430.1 |  |  |  |  |  |  |  |  |
| 0 | Ath-AT1G08440.1 |  |  |  |  |  |  |  |  |
| 0 | Ath-AT1G08450.1 |  |  |  |  |  |  |  |  |
| 0 | Ath-AT1G08460.1 |  |  |  |  |  |  |  |  |
| 0 | Ath-AT1G08465.1 |  |  |  |  |  |  |  |  |
| 0 | Ath-AT1G08470.1 |  |  |  |  |  |  |  |  |
| 0 | Ath-AT1G08480.1 |  |  |  |  |  |  |  |  |
| 0 | Ath-AT1G08490.1 |  |  |  |  |  |  |  |  |
| 0 | Ath-AT1G08500.1 |  |  |  |  |  |  |  |  |
| 0 | Ath-AT1G08510.1 |  |  |  |  |  |  |  |  |
| 0 | Ath-AT1G08520.1 |  |  |  |  |  |  |  |  |
| 0 | Ath-AT1G08530.1 |  |  |  |  |  |  |  |  |
| 0 | Ath-AT1G08540.1 |  |  |  |  |  |  |  |  |
| 0 | Ath-AT1G08550.3 |  |  |  |  |  |  |  |  |
| 0 | Ath-AT1G08560.1 |  |  |  |  |  |  |  |  |
| 0 | Ath-AT1G08570.1 |  |  |  |  |  |  |  |  |
| 0 | Ath-AT1G08580.1 |  |  |  |  |  |  |  |  |
| 0 | Ath-AT1G08590.1 |  |  |  |  |  |  |  |  |
| 0 | Ath-AT1G08600.3 |  |  |  |  |  |  |  |  |
| 0 | Ath-AT1G08610.1 |  |  |  |  |  |  |  |  |
| 0 | Ath-AT1G08620.3 |  |  |  |  |  |  |  |  |
| 0 | Ath-AT1G08630.4 |  |  |  |  |  |  |  |  |
| 0 | Ath-AT1G08640.1 |  |  |  |  |  |  |  |  |
| 0 | Ath-AT1G08645.1 |  |  |  |  |  |  |  |  |
| 0 | Ath-AT1G08650.1 |  |  |  |  |  |  |  |  |
| 0 | Ath-AT1G08660.1 |  |  |  |  |  |  |  |  |
| 0 | Ath-AT1G08670.1 |  |  |  |  |  |  |  |  |
| 0 | Ath-AT1G08680.4 |  |  |  |  |  |  |  |  |
| 0 | Ath-AT1G08695.1 |  |  |  |  |  |  |  |  |
| 0 | Ath-AT1G08700.1 |  |  |  |  |  |  |  |  |
| 0 | Ath-AT1G08710.2 |  |  |  |  |  |  |  |  |
| 0 | Ath-AT1G08720.1 |  |  |  |  |  |  |  |  |
| 0 | Ath-AT1G08730.1 |  |  |  |  |  |  |  |  |
| 0 | Ath-AT1G08750.1 |  |  |  |  |  |  |  |  |
| 0 | Ath-AT1G08760.1 |  |  |  |  |  |  |  |  |
| 0 | Ath-AT1G08770.1 |  |  |  |  |  |  |  |  |
| 0 | Ath-AT1G08780.1 |  |  |  |  |  |  |  |  |
| 0 | Ath-AT1G08790.1 |  |  |  |  |  |  |  |  |
| 0 | Ath-AT1G08800.1 |  |  |  |  |  |  |  |  |
| 0 | Ath-AT1G08810.1 |  |  |  |  |  |  |  |  |
| 1 | Ath-AT1G08820.2 |  | Vvi-Vitvi14g00347\_t001 |  |  |  |  |  |  |  |
| 1 | Ath-AT1G08830.1 |  | | | |  |  |  |  |  |  |  |
| 1 | Ath-AT1G08840.3 |  | | | |  |  |  |  |  |  |  |
| 1 | Ath-AT1G08845.2 |  | Vvi-Vitvi14g00336\_t002 |  |  |  |  |  |  |  |
| 1 | Ath-AT1G08860.1 |  | Vvi-Vitvi14g00327\_t001 |  |  |  |  |  |  |  |
| 1 | Ath-AT1G08880.1 |  | Vvi-Vitvi14g00323\_t001 |  |  |  |  |  |  |  |
| 1 | Ath-AT1G08890.1 |  | | | |  |  |  |  |  |  |  |
| 1 | Ath-AT1G08900.2 |  | Vvi-Vitvi14g00312\_t001 |  |  |  |  |  |  |  |
| 1 | Ath-AT1G08910.1 |  | | | |  |  |  |  |  |  |  |
| 1 | Ath-AT1G08920.2 |  | | | |  |  |  |  |  |  |  |
| 1 | Ath-AT1G08930.1 |  | Vvi-Vitvi14g02582\_t001 |  |  |  |  |  |  |  |
| 1 | Ath-AT1G08940.1 |  | Vvi-Vitvi14g00300\_t001 |  |  |  |  |  |  |  |
| 1 | Ath-AT1G08960.1 |  | Vvi-Vitvi14g00265\_t001 |  |  |  |  |  |  |  |
| 1 | Ath-AT1G08970.2 |  | Vvi-Vitvi14g00264\_t001 |  |  |  |  |  |  |  |
| 1 | Ath-AT1G08980.1 |  | Vvi-Vitvi14g00259\_t001 |  |  |  |  |  |  |  |
| 1 | Ath-AT1G08985.1 |  | | | |  |  |  |  |  |  |  |
| 1 | Ath-AT1G08990.1 |  | Vvi-Vitvi14g00249\_t001 |  |  |  |  |  |  |  |
| 1 | Ath-AT1G09000.1 |  | Vvi-Vitvi14g00248\_t001 |  |  |  |  |  |  |  |
| 1 | Ath-AT1G09010.1 |  | Vvi-Vitvi14g00239\_t001 |  |  |  |  |  |  |  |
| 1 | Ath-AT1G09020.1 |  | Vvi-Vitvi14g00229\_t001 |  |  |  |  |  |  |  |
| 1 | Ath-AT1G09030.1 |  | Vvi-Vitvi14g00217\_t001 |  |  |  |  |  |  |  |
| 1 | Ath-AT1G09040.1 |  | Vvi-Vitvi14g00215\_t002 |  |  |  |  |  |  |  |
| 0 | Ath-AT1G09050.1 |  |  |  |  |  |  |  |  |
| 1 | Ath-AT1G09060.3 |  | Vvi-Vitvi14g00196\_t001 |  |  |  |  |  |  |  |
| 1 | Ath-AT1G09070.1 |  | Vvi-Vitvi14g00193\_t001 |  |  |  |  |  |  |  |
| 1 | Ath-AT1G09080.1 |  | Vvi-Vitvi14g00185\_t001 |  |  |  |  |  |  |  |
| 1 | Ath-AT1G09090.2 |  | Vvi-Vitvi14g00183\_t001 |  |  |  |  |  |  |  |
| 1 | Ath-AT1G09100.1 |  | Vvi-Vitvi14g00178\_t001 |  |  |  |  |  |  |  |
| 1 | Ath-AT1G09130.3 |  | Vvi-Vitvi14g02506\_t002 |  |  |  |  |  |  |  |
| 1 | Ath-AT1G09140.1 |  | | | |  |  |  |  |  |  |  |
| 1 | Ath-AT1G09150.1 |  | | | |  |  |  |  |  |  |  |
| 1 | Ath-AT1G09155.1 |  | Vvi-Vitvi14g00157\_t001 |  |  |  |  |  |  |  |
| 0 | Ath-AT1G09157.1 |  |  |  |  |  |  |  |  |
| 1 | Ath-AT1G09160.1 |  | Vvi-Vitvi14g00128\_t003 |  |  |  |  |  |  |  |
| 1 | Ath-AT1G09170.5 |  | Vvi-Vitvi14g00127\_t001 |  |  |  |  |  |  |  |
| 1 | Ath-AT1G09176.1 |  | | | |  |  |  |  |  |  |  |
| 1 | Ath-AT1G09180.1 |  | | | |  |  |  |  |  |  |  |
| 1 | Ath-AT1G09190.1 |  | | | |  |  |  |  |  |  |  |
| 1 | Ath-AT1G09195.11 |  | Vvi-Vitvi14g00117\_t001 |  |  |  |  |  |  |  |
| 1 | Ath-AT1G09200.1 |  | | | |  |  |  |  |  |  |  |
| 1 | Ath-AT1G09210.1 |  | Vvi-Vitvi14g00102\_t001 |  |  |  |  |  |  |  |
| 1 | Ath-AT1G09220.1 |  | | | |  |  |  |  |  |  |  |
| 1 | Ath-AT1G09230.1 |  | | | |  |  |  |  |  |  |  |
| 1 | Ath-AT1G09240.1 |  | Vvi-Vitvi14g02474\_t001 |  |  |  |  |  |  |  |
| 1 | Ath-AT1G09245.1 |  | | | |  |  |  |  |  |  |  |
| 1 | Ath-AT1G09250.1 |  | Vvi-Vitvi14g04018\_t001 |  |  |  |  |  |  |  |
| 1 | Ath-AT1G09260.1 |  | | | |  |  |  |  |  |  |  |
| 1 | Ath-AT1G09270.1 |  | Vvi-Vitvi14g00075\_t001 |  |  |  |  |  |  |  |
| 1 | Ath-AT1G09280.1 |  | Vvi-Vitvi14g00074\_t001 |  |  |  |  |  |  |  |
| 1 | Ath-AT1G09290.1 |  | | | |  |  |  |  |  |  |  |
| 1 | Ath-AT1G09300.1 |  | Vvi-Vitvi14g00073\_t001 |  |  |  |  |  |  |  |
| 1 | Ath-AT1G09310.1 |  | Vvi-Vitvi14g02463\_t001 |  |  |  |  |  |  |  |
| 1 | Ath-AT1G09320.1 |  | Vvi-Vitvi14g00072\_t001 |  |  |  |  |  |  |  |
| 1 | Ath-AT1G09330.1 |  | Vvi-Vitvi14g00067\_t001 |  |  |  |  |  |  |  |
| 1 | Ath-AT1G09340.2 |  | Vvi-Vitvi14g00066\_t001 |  |  |  |  |  |  |  |
| 1 | Ath-AT1G09350.1 |  | Vvi-Vitvi14g02457\_t001 |  |  |  |  |  |  |  |
| 1 | Ath-AT1G09360.1 |  | | | |  |  |  |  |  |  |  |
| 1 | Ath-AT1G09370.1 |  | Vvi-Vitvi14g02455\_t001 |  |  |  |  |  |  |  |
| 1 | Ath-AT1G09380.1 |  | Vvi-Vitvi14g04009\_t001 |  |  |  |  |  |  |  |
| 1 | Ath-AT1G09390.1 |  | Vvi-Vitvi14g00059\_t001 |  |  |  |  |  |  |  |
| 1 | Ath-AT1G09400.1 |  | | | |  |  |  |  |  |  |  |
| 1 | Ath-AT1G09410.1 |  | Vvi-Vitvi14g00058\_t001 |  |  |  |  |  |  |  |
| 1 | Ath-AT1G09415.1 |  | | | |  |  |  |  |  |  |  |
| 1 | Ath-AT1G09420.2 |  | Vvi-Vitvi14g00057\_t002 |  |  |  |  |  |  |  |
| 1 | Ath-AT1G09430.1 |  | Vvi-Vitvi14g00047\_t002 |  |  |  |  |  |  |  |
| 1 | Ath-AT1G09440.1 |  | Vvi-Vitvi14g00042\_t001 |  |  |  |  |  |  |  |
| 1 | Ath-AT1G09450.1 |  | Vvi-Vitvi14g00041\_t001 |  |  |  |  |  |  |  |
| 1 | Ath-AT1G09460.1 |  | Vvi-Vitvi14g00040\_t001 |  |  |  |  |  |  |  |
| 1 | Ath-AT1G09470.1 |  | Vvi-Vitvi14g00039\_t001 |  |  |  |  |  |  |  |
| 1 | Ath-AT1G09480.1 |  | | | |  |  |  |  |  |  |  |
| 1 | Ath-AT1G09483.1 |  | | | |  |  |  |  |  |  |  |
| 1 | Ath-AT1G09490.1 |  | | | |  |  |  |  |  |  |  |
| 1 | Ath-AT1G09500.1 |  | | | |  |  |  |  |  |  |  |
| 1 | Ath-AT1G09510.1 |  | | | |  |  |  |  |  |  |  |
| 1 | Ath-AT1G09520.1 |  | Vvi-Vitvi14g00034\_t001 |  |  |  |  |  |  |  |
| 0 | Ath-AT1G09530.1 |  |  |  |  |  |  |  |  |
| 0 | Ath-AT1G09540.1 |  |  |  |  |  |  |  |  |
| 0 | Ath-AT1G09550.1 |  |  |  |  |  |  |  |  |
| 0 | Ath-AT1G09560.1 |  |  |  |  |  |  |  |  |
| 0 | Ath-AT1G09570.3 |  |  |  |  |  |  |  |  |
| 0 | Ath-AT1G09575.1 |  |  |  |  |  |  |  |  |
| 0 | Ath-AT1G09580.1 |  |  |  |  |  |  |  |  |
| 0 | Ath-AT1G09590.1 |  |  |  |  |  |  |  |  |
| 1 | Ath-AT1G09600.1 |  | Vvi-Vitvi12g02060\_t001 |  |  |  |  |  |  |  |
| 1 | Ath-AT1G09610.1 |  | Vvi-Vitvi12g02059\_t001 |  |  |  |  |  |  |  |
| 1 | Ath-AT1G09620.1 |  | Vvi-Vitvi12g02058\_t001 |  |  |  |  |  |  |  |
| 1 | Ath-AT1G09625.1 |  | | | |  |  |  |  |  |  |  |
| 1 | Ath-AT1G09630.1 |  | Vvi-Vitvi12g02056\_t001 |  |  |  |  |  |  |  |
| 1 | Ath-AT1G09640.1 |  | Vvi-Vitvi12g02055\_t003 |  |  |  |  |  |  |  |
| 1 | Ath-AT1G09645.1 |  | Vvi-Vitvi12g02723\_t002 |  |  |  |  |  |  |  |
| 1 | Ath-AT1G09650.1 |  | | | |  |  |  |  |  |  |  |
| 1 | Ath-AT1G09660.1 |  | Vvi-Vitvi12g02050\_t001 |  |  |  |  |  |  |  |
| 1 | Ath-AT1G09665.1 |  | | | |  |  |  |  |  |  |  |
| 1 | Ath-AT1G09680.1 |  | | | |  |  |  |  |  |  |  |
| 1 | Ath-AT1G09690.1 |  | | | |  |  |  |  |  |  |  |
| 2 | Ath-AT1G09700.1 |  | | | |  | Vvi-Vitvi18g00531\_t001 |  |  |  |  |  |  |
| 2 | Ath-AT1G09710.2 |  | | | |  | | | |  |  |  |  |  |  |
| 2 | Ath-AT1G09720.1 |  | | | |  | | | |  |  |  |  |  |  |
| 2 | Ath-AT1G09730.1 |  | | | |  | | | |  |  |  |  |  |  |
| 2 | Ath-AT1G09740.1 |  | | | |  | | | |  |  |  |  |  |  |
| 2 | Ath-AT1G09750.1 |  | | | |  | | | |  |  |  |  |  |  |
| 2 | Ath-AT1G09760.1 |  | | | |  | | | |  |  |  |  |  |  |
| 2 | Ath-AT1G09770.1 |  | | | |  | | | |  |  |  |  |  |  |
| 2 | Ath-AT1G09780.1 |  | | | |  | | | |  |  |  |  |  |  |
| 3 | Ath-AT1G09790.1 |  | | | |  | | | |  | Vvi-Vitvi12g01997\_t001 |  |  |  |  |  |
| 3 | Ath-AT1G09794.1 |  | | | |  | | | |  | Vvi-Vitvi12g02009\_t001 |  |  |  |  |  |
| 3 | Ath-AT1G09795.1 |  | | | |  | | | |  | Vvi-Vitvi12g02013\_t001 |  |  |  |  |  |
| 3 | Ath-AT1G09800.1 |  | | | |  | | | |  | Vvi-Vitvi12g02033\_t001 |  |  |  |  |  |
| 3 | Ath-AT1G09810.1 |  | Vvi-Vitvi12g02034\_t001 |  | | | |  | Vvi-Vitvi12g02034\_t001 |  |  |  |  |  |
| 2 | Ath-AT1G09812.1 |  |  |  | | | |  | Vvi-Vitvi12g02041\_t001 |  |  |  |  |  |
| 2 | Ath-AT1G09815.1 |  |  |  | | | |  | | | |  |  |  |  |  |
| 2 | Ath-AT1G09820.1 |  |  |  | | | |  | | | |  |  |  |  |  |
| 2 | Ath-AT1G09830.1 |  |  |  | | | |  | | | |  |  |  |  |  |
| 2 | Ath-AT1G09840.3 |  |  |  | | | |  | Vvi-Vitvi12g02045\_t003 |  |  |  |  |  |
| 1 | Ath-AT1G09850.1 |  |  |  | | | |  |  |  |  |  |  |
| 1 | Ath-AT1G09860.1 |  |  |  | | | |  |  |  |  |  |  |
| 1 | Ath-AT1G09870.1 |  |  |  | | | |  |  |  |  |  |  |
| 1 | Ath-AT1G09880.3 |  |  |  | Vvi-Vitvi18g00549\_t001 |  |  |  |  |  |  |
| 1 | Ath-AT1G09890.3 |  |  |  | | | |  |  |  |  |  |  |
| 1 | Ath-AT1G09900.1 |  |  |  | Vvi-Vitvi18g00556\_t001 |  |  |  |  |  |  |
| 1 | Ath-AT1G09910.1 |  |  |  | | | |  |  |  |  |  |  |
| 1 | Ath-AT1G09920.1 |  |  |  | | | |  |  |  |  |  |  |
| 1 | Ath-AT1G09930.1 |  |  |  | Vvi-Vitvi18g00557\_t001 |  |  |  |  |  |  |
| 1 | Ath-AT1G09932.1 |  |  |  | Vvi-Vitvi18g00566\_t001 |  |  |  |  |  |  |
| 1 | Ath-AT1G09935.1 |  |  |  | | | |  |  |  |  |  |  |
| 1 | Ath-AT1G09940.1 |  |  |  | Vvi-Vitvi18g00573\_t001 |  |  |  |  |  |  |
| 1 | Ath-AT1G09950.1 |  |  |  | | | |  |  |  |  |  |  |
| 1 | Ath-AT1G09960.1 |  |  |  | Vvi-Vitvi18g04132\_t001 |  |  |  |  |  |  |
| 1 | Ath-AT1G09970.2 |  |  |  | Vvi-Vitvi18g00587\_t001 |  |  |  |  |  |  |
| 1 | Ath-AT1G09980.3 |  |  |  | | | |  |  |  |  |  |  |
| 1 | Ath-AT1G09995.3 |  |  |  | | | |  |  |  |  |  |  |
| 1 | Ath-AT1G10000.1 |  |  |  | | | |  |  |  |  |  |  |
| 1 | Ath-AT1G10010.2 |  |  |  | Vvi-Vitvi18g00593\_t001 |  |  |  |  |  |  |
| 1 | Ath-AT1G10020.1 |  |  |  | Vvi-Vitvi18g00598\_t001 |  |  |  |  |  |  |
| 1 | Ath-AT1G10030.1 |  |  |  | Vvi-Vitvi18g00599\_t001 |  |  |  |  |  |  |
| 1 | Ath-AT1G10040.1 |  |  |  | Vvi-Vitvi18g00600\_t001 |  |  |  |  |  |  |
| 1 | Ath-AT1G10050.3 |  |  |  | Vvi-Vitvi18g00601\_t001 |  |  |  |  |  |  |
| 1 | Ath-AT1G10060.5 |  |  |  | Vvi-Vitvi18g02625\_t001 |  |  |  |  |  |  |
| 1 | Ath-AT1G10070.2 |  |  |  | | | |  |  |  |  |  |  |
| 1 | Ath-AT1G10090.1 |  |  |  | Vvi-Vitvi18g00606\_t001 |  |  |  |  |  |  |
| 1 | Ath-AT1G10095.1 |  |  |  | | | |  |  |  |  |  |  |
| 1 | Ath-AT1G10100.1 |  |  |  | | | |  |  |  |  |  |  |
| 1 | Ath-AT1G10110.1 |  |  |  | | | |  |  |  |  |  |  |
| 1 | Ath-AT1G10120.1 |  |  |  | Vvi-Vitvi18g00617\_t001 |  |  |  |  |  |  |
| 1 | Ath-AT1G10130.1 |  |  |  | | | |  |  |  |  |  |  |
| 1 | Ath-AT1G10140.1 |  |  |  | Vvi-Vitvi18g02636\_t001 |  |  |  |  |  |  |
| 1 | Ath-AT1G10150.1 |  |  |  | | | |  |  |  |  |  |  |
| 1 | Ath-AT1G10155.1 |  |  |  | | | |  |  |  |  |  |  |
| 1 | Ath-AT1G10170.4 |  |  |  | | | |  |  |  |  |  |  |
| 1 | Ath-AT1G10180.1 |  |  |  | | | |  |  |  |  |  |  |
| 1 | Ath-AT1G10190.1 |  |  |  | | | |  |  |  |  |  |  |
| 1 | Ath-AT1G10200.1 |  |  |  | Vvi-Vitvi18g00654\_t001 |  |  |  |  |  |  |
| 1 | Ath-AT1G10210.1 |  |  |  | | | |  |  |  |  |  |  |
| 1 | Ath-AT1G10220.2 |  |  |  | Vvi-Vitvi18g00656\_t001 |  |  |  |  |  |  |
| 0 | Ath-AT1G10225.1 |  |  |  |  |  |  |  |  |
| 0 | Ath-AT1G10230.1 |  |  |  |  |  |  |  |  |
| 0 | Ath-AT1G10240.1 |  |  |  |  |  |  |  |  |
| 0 | Ath-AT1G10250.1 |  |  |  |  |  |  |  |  |
| 0 | Ath-AT1G10270.1 |  |  |  |  |  |  |  |  |
| 0 | Ath-AT1G10280.1 |  |  |  |  |  |  |  |  |
| 0 | Ath-AT1G10290.1 |  |  |  |  |  |  |  |  |
| 0 | Ath-AT1G10300.1 |  |  |  |  |  |  |  |  |
| 0 | Ath-AT1G10310.1 |  |  |  |  |  |  |  |  |
| 0 | Ath-AT1G10320.1 |  |  |  |  |  |  |  |  |
| 0 | Ath-AT1G10330.1 |  |  |  |  |  |  |  |  |
| 0 | Ath-AT1G10340.1 |  |  |  |  |  |  |  |  |
| 1 | Ath-AT1G10350.1 |  | Vvi-Vitvi01g00906\_t001 |  |  |  |  |  |  |  |
| 1 | Ath-AT1G10360.1 |  | Vvi-Vitvi01g00888\_t001 |  |  |  |  |  |  |  |
| 1 | Ath-AT1G10370.1 |  | | | |  |  |  |  |  |  |  |
| 1 | Ath-AT1G10380.1 |  | Vvi-Vitvi01g00883\_t001 |  |  |  |  |  |  |  |
| 1 | Ath-AT1G10385.1 |  | Vvi-Vitvi01g00881\_t001 |  |  |  |  |  |  |  |
| 1 | Ath-AT1G10390.2 |  | | | |  |  |  |  |  |  |  |
| 1 | Ath-AT1G10395.1 |  | | | |  |  |  |  |  |  |  |
| 1 | Ath-AT1G10400.1 |  | | | |  |  |  |  |  |  |  |
| 1 | Ath-AT1G10410.1 |  | Vvi-Vitvi01g00878\_t001 |  |  |  |  |  |  |  |
| 1 | Ath-AT1G10417.4 |  | Vvi-Vitvi01g00877\_t001 |  |  |  |  |  |  |  |
| 1 | Ath-AT1G10430.1 |  | Vvi-Vitvi01g00870\_t001 |  |  |  |  |  |  |  |
| 1 | Ath-AT1G10450.3 |  | | | |  |  |  |  |  |  |  |
| 1 | Ath-AT1G10455.1 |  | | | |  |  |  |  |  |  |  |
| 1 | Ath-AT1G10460.1 |  | Vvi-Vitvi01g02097\_t001 |  |  |  |  |  |  |  |
| 1 | Ath-AT1G10470.1 |  | Vvi-Vitvi01g00857\_t001 |  |  |  |  |  |  |  |
| 1 | Ath-AT1G10480.1 |  | Vvi-Vitvi01g00845\_t001 |  |  |  |  |  |  |  |
| 1 | Ath-AT1G10490.1 |  | | | |  |  |  |  |  |  |  |
| 1 | Ath-AT1G10500.1 |  | | | |  |  |  |  |  |  |  |
| 1 | Ath-AT1G10510.1 |  | | | |  |  |  |  |  |  |  |
| 1 | Ath-AT1G10520.1 |  | | | |  |  |  |  |  |  |  |
| 1 | Ath-AT1G10522.1 |  | | | |  |  |  |  |  |  |  |
| 1 | Ath-AT1G10530.1 |  | | | |  |  |  |  |  |  |  |
| 1 | Ath-AT1G10540.1 |  | Vvi-Vitvi01g00833\_t001 |  |  |  |  |  |  |  |
| 1 | Ath-AT1G10550.1 |  | Vvi-Vitvi01g00784\_t001 |  |  |  |  |  |  |  |
| 1 | Ath-AT1G10560.1 |  | Vvi-Vitvi01g00782\_t001 |  |  |  |  |  |  |  |
| 1 | Ath-AT1G10570.1 |  | Vvi-Vitvi01g00776\_t001 |  |  |  |  |  |  |  |
| 1 | Ath-AT1G10580.1 |  | Vvi-Vitvi01g00772\_t001 |  |  |  |  |  |  |  |
| 1 | Ath-AT1G10585.1 |  | Vvi-Vitvi01g00752\_t001 |  |  |  |  |  |  |  |
| 1 | Ath-AT1G10586.1 |  | | | |  |  |  |  |  |  |  |
| 1 | Ath-AT1G10588.1 |  | | | |  |  |  |  |  |  |  |
| 1 | Ath-AT1G10590.3 |  | Vvi-Vitvi01g00744\_t002 |  |  |  |  |  |  |  |
| 1 | Ath-AT1G10600.4 |  | Vvi-Vitvi01g00733\_t002 |  |  |  |  |  |  |  |
| 1 | Ath-AT1G10610.1 |  | | | |  |  |  |  |  |  |  |
| 1 | Ath-AT1G10620.1 |  | Vvi-Vitvi01g04183\_t001 |  |  |  |  |  |  |  |
| 1 | Ath-AT1G10630.1 |  | Vvi-Vitvi01g04177\_t002 |  |  |  |  |  |  |  |
| 1 | Ath-AT1G10640.1 |  | Vvi-Vitvi01g00710\_t001 |  |  |  |  |  |  |  |
| 1 | Ath-AT1G10650.1 |  | Vvi-Vitvi01g00705\_t001 |  |  |  |  |  |  |  |
| 1 | Ath-AT1G10657.1 |  | Vvi-Vitvi01g02051\_t001 |  |  |  |  |  |  |  |
| 1 | Ath-AT1G10660.1 |  | Vvi-Vitvi01g00701\_t001 |  |  |  |  |  |  |  |
| 1 | Ath-AT1G10670.3 |  | Vvi-Vitvi01g00698\_t001 |  |  |  |  |  |  |  |
| 0 | Ath-AT1G10680.1 |  |  |  |  |  |  |  |  |
| 1 | Ath-AT1G10690.1 |  | Vvi-Vitvi01g02039\_t001 |  |  |  |  |  |  |  |
| 1 | Ath-AT1G10700.1 |  | Vvi-Vitvi01g00663\_t001 |  |  |  |  |  |  |  |
| 1 | Ath-AT1G10710.1 |  | Vvi-Vitvi01g00665\_t001 |  |  |  |  |  |  |  |
| 1 | Ath-AT1G10715.1 |  | | | |  |  |  |  |  |  |  |
| 1 | Ath-AT1G10717.1 |  | | | |  |  |  |  |  |  |  |
| 1 | Ath-AT1G10720.1 |  | | | |  |  |  |  |  |  |  |
| 1 | Ath-AT1G10730.1 |  | Vvi-Vitvi01g00669\_t001 |  |  |  |  |  |  |  |
| 1 | Ath-AT1G10740.4 |  | Vvi-Vitvi01g00674\_t001 |  |  |  |  |  |  |  |
| 1 | Ath-AT1G10745.1 |  | | | |  |  |  |  |  |  |  |
| 1 | Ath-AT1G10747.1 |  | | | |  |  |  |  |  |  |  |
| 1 | Ath-AT1G10750.1 |  | Vvi-Vitvi01g00676\_t001 |  |  |  |  |  |  |  |
| 1 | Ath-AT1G10760.1 |  | Vvi-Vitvi01g00681\_t001 |  |  |  |  |  |  |  |
| 1 | Ath-AT1G10770.1 |  | Vvi-Vitvi01g02044\_t001 |  |  |  |  |  |  |  |
| 0 | Ath-AT1G10780.2 |  |  |  |  |  |  |  |  |
| 0 | Ath-AT1G10790.1 |  |  |  |  |  |  |  |  |
| 0 | Ath-AT1G10800.2 |  |  |  |  |  |  |  |  |
| 0 | Ath-AT1G10810.1 |  |  |  |  |  |  |  |  |
| 0 | Ath-AT1G10820.2 |  |  |  |  |  |  |  |  |
| 0 | Ath-AT1G10830.1 |  |  |  |  |  |  |  |  |
| 0 | Ath-AT1G10840.1 |  |  |  |  |  |  |  |  |
| 0 | Ath-AT1G10850.1 |  |  |  |  |  |  |  |  |
| 0 | Ath-AT1G10865.1 |  |  |  |  |  |  |  |  |
| 0 | Ath-AT1G10870.2 |  |  |  |  |  |  |  |  |
| 0 | Ath-AT1G10875.1 |  |  |  |  |  |  |  |  |
| 0 | Ath-AT1G10880.1 |  |  |  |  |  |  |  |  |
| 0 | Ath-AT1G10890.1 |  |  |  |  |  |  |  |  |
| 0 | Ath-AT1G10900.3 |  |  |  |  |  |  |  |  |
| 0 | Ath-AT1G10910.1 |  |  |  |  |  |  |  |  |
| 0 | Ath-AT1G10920.4 |  |  |  |  |  |  |  |  |
| 0 | Ath-AT1G10930.1 |  |  |  |  |  |  |  |  |
| 0 | Ath-AT1G10940.2 |  |  |  |  |  |  |  |  |
| 0 | Ath-AT1G10950.1 |  |  |  |  |  |  |  |  |
| 0 | Ath-AT1G10960.1 |  |  |  |  |  |  |  |  |
| 0 | Ath-AT1G10970.2 |  |  |  |  |  |  |  |  |
| 0 | Ath-AT1G10980.1 |  |  |  |  |  |  |  |  |
| 0 | Ath-AT1G10990.2 |  |  |  |  |  |  |  |  |
| 0 | Ath-AT1G11000.1 |  |  |  |  |  |  |  |  |
| 0 | Ath-AT1G11020.1 |  |  |  |  |  |  |  |  |
| 0 | Ath-AT1G11040.1 |  |  |  |  |  |  |  |  |
| 0 | Ath-AT1G11050.1 |  |  |  |  |  |  |  |  |
| 1 | Ath-AT1G11060.1 |  | Vvi-Vitvi10g02345\_t001 |  |  |  |  |  |  |  |
| 1 | Ath-AT1G11070.4 |  | | | |  |  |  |  |  |  |  |
| 1 | Ath-AT1G11080.2 |  | Vvi-Vitvi10g00427\_t001 |  |  |  |  |  |  |  |
| 1 | Ath-AT1G11090.1 |  | Vvi-Vitvi10g00426\_t001 |  |  |  |  |  |  |  |
| 1 | Ath-AT1G11100.4 |  | Vvi-Vitvi10g00425\_t001 |  |  |  |  |  |  |  |
| 1 | Ath-AT1G11110.1 |  | Vvi-Vitvi10g04277\_t001 |  |  |  |  |  |  |  |
| 1 | Ath-AT1G11112.1 |  | | | |  |  |  |  |  |  |  |
| 1 | Ath-AT1G11120.1 |  | Vvi-Vitvi10g00422\_t001 |  |  |  |  |  |  |  |
| 1 | Ath-AT1G11125.1 |  | Vvi-Vitvi10g01760\_t001 |  |  |  |  |  |  |  |
| 1 | Ath-AT1G11130.1 |  | Vvi-Vitvi10g00417\_t002 |  |  |  |  |  |  |  |
| 1 | Ath-AT1G11145.1 |  | | | |  |  |  |  |  |  |  |
| 1 | Ath-AT1G11160.1 |  | Vvi-Vitvi10g04273\_t002 |  |  |  |  |  |  |  |
| 1 | Ath-AT1G11170.1 |  | Vvi-Vitvi10g04271\_t001 |  |  |  |  |  |  |  |
| 1 | Ath-AT1G11180.2 |  | Vvi-Vitvi10g00397\_t001 |  |  |  |  |  |  |  |
| 1 | Ath-AT1G11190.1 |  | Vvi-Vitvi10g00401\_t001 |  |  |  |  |  |  |  |
| 1 | Ath-AT1G11200.1 |  | Vvi-Vitvi10g00394\_t001 |  |  |  |  |  |  |  |
| 1 | Ath-AT1G11210.1 |  | Vvi-Vitvi10g00390\_t001 |  |  |  |  |  |  |  |
| 1 | Ath-AT1G11220.2 |  | | | |  |  |  |  |  |  |  |
| 1 | Ath-AT1G11230.2 |  | | | |  |  |  |  |  |  |  |
| 1 | Ath-AT1G11240.1 |  | | | |  |  |  |  |  |  |  |
| 1 | Ath-AT1G11250.1 |  | Vvi-Vitvi10g00385\_t001 |  |  |  |  |  |  |  |
| 1 | Ath-AT1G11260.1 |  | Vvi-Vitvi10g00358\_t001 |  |  |  |  |  |  |  |
| 1 | Ath-AT1G11270.2 |  | | | |  |  |  |  |  |  |  |
| 1 | Ath-AT1G11280.1 |  | | | |  |  |  |  |  |  |  |
| 1 | Ath-AT1G11290.1 |  | | | |  |  |  |  |  |  |  |
| 1 | Ath-AT1G11300.1 |  | | | |  |  |  |  |  |  |  |
| 1 | Ath-AT1G11303.1 |  | | | |  |  |  |  |  |  |  |
| 1 | Ath-AT1G11310.1 |  | | | |  |  |  |  |  |  |  |
| 1 | Ath-AT1G11320.1 |  | | | |  |  |  |  |  |  |  |
| 2 | Ath-AT1G11330.2 |  | | | |  | Vvi-Vitvi10g04158\_t001 |  |  |  |  |  |  |
| 2 | Ath-AT1G11340.1 |  | | | |  | Vvi-Vitvi10g04161\_t001 |  |  |  |  |  |  |
| 2 | Ath-AT1G11350.1 |  | | | |  | | | |  |  |  |  |  |  |
| 2 | Ath-AT1G11360.4 |  | | | |  | | | |  |  |  |  |  |  |
| 2 | Ath-AT1G11362.1 |  | | | |  | | | |  |  |  |  |  |  |
| 2 | Ath-AT1G11370.1 |  | | | |  | | | |  |  |  |  |  |  |
| 2 | Ath-AT1G11380.1 |  | Vvi-Vitvi10g04243\_t001 |  | | | |  |  |  |  |  |  |
| 2 | Ath-AT1G11390.1 |  | | | |  | | | |  |  |  |  |  |  |
| 2 | Ath-AT1G11400.2 |  | | | |  | | | |  |  |  |  |  |  |
| 2 | Ath-AT1G11410.4 |  | Vvi-Vitvi10g04227\_t001 |  | Vvi-Vitvi10g02151\_t001 |  |  |  |  |  |  |
| 2 | Ath-AT1G11420.1 |  | | | |  | | | |  |  |  |  |  |  |
| 2 | Ath-AT1G11430.1 |  | | | |  | | | |  |  |  |  |  |  |
| 2 | Ath-AT1G11440.1 |  | Vvi-Vitvi10g04225\_t001 |  | | | |  |  |  |  |  |  |
| 1 | Ath-AT1G11450.3 |  |  |  | | | |  |  |  |  |  |  |
| 1 | Ath-AT1G11460.1 |  |  |  | | | |  |  |  |  |  |  |
| 1 | Ath-AT1G11470.1 |  |  |  | | | |  |  |  |  |  |  |
| 1 | Ath-AT1G11475.1 |  |  |  | | | |  |  |  |  |  |  |
| 1 | Ath-AT1G11480.1 |  |  |  | Vvi-Vitvi10g02178\_t001 |  |  |  |  |  |  |
| 1 | Ath-AT1G11490.2 |  |  |  | Vvi-Vitvi10g00290\_t001 |  |  |  |  |  |  |
| 1 | Ath-AT1G11500.3 |  |  |  | Vvi-Vitvi10g02202\_t001 |  |  |  |  |  |  |
| 1 | Ath-AT1G11510.1 |  |  |  | Vvi-Vitvi10g02200\_t001 |  |  |  |  |  |  |
| 1 | Ath-AT1G11520.1 |  |  |  | | | |  |  |  |  |  |  |
| 1 | Ath-AT1G11530.1 |  |  |  | Vvi-Vitvi10g02216\_t001 |  |  |  |  |  |  |
| 1 | Ath-AT1G11540.2 |  |  |  | Vvi-Vitvi10g02219\_t001 |  |  |  |  |  |  |
| 0 | Ath-AT1G11545.1 |  |  |  |  |  |  |  |  |
| 0 | Ath-AT1G11560.1 |  |  |  |  |  |  |  |  |
| 0 | Ath-AT1G11570.3 |  |  |  |  |  |  |  |  |
| 0 | Ath-AT1G11572.1 |  |  |  |  |  |  |  |  |
| 0 | Ath-AT1G11580.2 |  |  |  |  |  |  |  |  |
| 0 | Ath-AT1G11590.1 |  |  |  |  |  |  |  |  |
| 0 | Ath-AT1G11591.1 |  |  |  |  |  |  |  |  |
| 0 | Ath-AT1G11593.1 |  |  |  |  |  |  |  |  |
| 0 | Ath-AT1G11600.1 |  |  |  |  |  |  |  |  |
| 0 | Ath-AT1G11608.1 |  |  |  |  |  |  |  |  |
| 0 | Ath-AT1G11610.2 |  |  |  |  |  |  |  |  |
| 0 | Ath-AT1G11620.1 |  |  |  |  |  |  |  |  |
| 0 | Ath-AT1G11630.1 |  |  |  |  |  |  |  |  |
| 1 | Ath-AT1G11650.2 |  | Vvi-Vitvi19g00486\_t001 |  |  |  |  |  |  |  |
| 2 | Ath-AT1G11655.1 |  | | | |  | Vvi-Vitvi10g00110\_t001 |  |  |  |  |  |  |
| 2 | Ath-AT1G11660.1 |  | Vvi-Vitvi19g00490\_t001 |  | | | |  |  |  |  |  |  |
| 2 | Ath-AT1G11670.1 |  | | | |  | Vvi-Vitvi10g00107\_t001 |  |  |  |  |  |  |
| 2 | Ath-AT1G11680.1 |  | | | |  | | | |  |  |  |  |  |  |
| 2 | Ath-AT1G11684.1 |  | | | |  | | | |  |  |  |  |  |  |
| 2 | Ath-AT1G11690.1 |  | Vvi-Vitvi19g00492\_t001 |  | Vvi-Vitvi10g00105\_t001 |  |  |  |  |  |  |
| 2 | Ath-AT1G11700.1 |  | | | |  | Vvi-Vitvi10g01642\_t001 |  |  |  |  |  |  |
| 2 | Ath-AT1G11710.1 |  | | | |  | | | |  |  |  |  |  |  |
| 2 | Ath-AT1G11720.2 |  | | | |  | Vvi-Vitvi10g00094\_t001 |  |  |  |  |  |  |
| 2 | Ath-AT1G11730.1 |  | | | |  | Vvi-Vitvi10g00092\_t001 |  |  |  |  |  |  |
| 2 | Ath-AT1G11740.1 |  | | | |  | Vvi-Vitvi10g00090\_t001 |  |  |  |  |  |  |
| 2 | Ath-AT1G11750.2 |  | | | |  | | | |  |  |  |  |  |  |
| 2 | Ath-AT1G11755.1 |  | | | |  | | | |  |  |  |  |  |  |
| 2 | Ath-AT1G11760.1 |  | | | |  | | | |  |  |  |  |  |  |
| 2 | Ath-AT1G11765.1 |  | | | |  | | | |  |  |  |  |  |  |
| 2 | Ath-AT1G11770.1 |  | | | |  | | | |  |  |  |  |  |  |
| 2 | Ath-AT1G11780.1 |  | | | |  | | | |  |  |  |  |  |  |
| 2 | Ath-AT1G11785.1 |  | | | |  | | | |  |  |  |  |  |  |
| 2 | Ath-AT1G11790.1 |  | | | |  | Vvi-Vitvi10g00088\_t001 |  |  |  |  |  |  |
| 2 | Ath-AT1G11800.1 |  | | | |  | | | |  |  |  |  |  |  |
| 2 | Ath-AT1G11810.1 |  | | | |  | | | |  |  |  |  |  |  |
| 2 | Ath-AT1G11820.2 |  | | | |  | Vvi-Vitvi10g00085\_t001 |  |  |  |  |  |  |
| 2 | Ath-AT1G11840.6 |  | Vvi-Vitvi19g02023\_t001 |  | | | |  |  |  |  |  |  |
| 2 | Ath-AT1G11850.2 |  | | | |  | | | |  |  |  |  |  |  |
| 2 | Ath-AT1G11860.3 |  | | | |  | Vvi-Vitvi10g00078\_t001.1.6037826a |  |  |  |  |  |  |
| 2 | Ath-AT1G11870.2 |  | | | |  | | | |  |  |  |  |  |  |
| 2 | Ath-AT1G11880.1 |  | | | |  | | | |  |  |  |  |  |  |
| 2 | Ath-AT1G11890.1 |  | | | |  | | | |  |  |  |  |  |  |
| 2 | Ath-AT1G11900.1 |  | | | |  | | | |  |  |  |  |  |  |
| 2 | Ath-AT1G11905.1 |  | | | |  | Vvi-Vitvi10g04011\_t001 |  |  |  |  |  |  |
| 2 | Ath-AT1G11910.2 |  | Vvi-Vitvi19g00529\_t001 |  | Vvi-Vitvi10g00064\_t001 |  |  |  |  |  |  |
| 2 | Ath-AT1G11915.1 |  | | | |  | Vvi-Vitvi10g00062\_t001 |  |  |  |  |  |  |
| 2 | Ath-AT1G11920.1 |  | | | |  | Vvi-Vitvi10g00061\_t001 |  |  |  |  |  |  |
| 2 | Ath-AT1G11925.1 |  | Vvi-Vitvi19g00534\_t001 |  | Vvi-Vitvi10g01615\_t001 |  |  |  |  |  |  |
| 2 | Ath-AT1G11930.1 |  | Vvi-Vitvi19g00537\_t002 |  | Vvi-Vitvi10g00057\_t001 |  |  |  |  |  |  |
| 1 | Ath-AT1G11940.1 |  |  |  | Vvi-Vitvi10g00055\_t001 |  |  |  |  |  |  |
| 1 | Ath-AT1G11950.1 |  |  |  | Vvi-Vitvi10g00053\_t001 |  |  |  |  |  |  |
| 1 | Ath-AT1G11960.1 |  |  |  | Vvi-Vitvi10g00052\_t004 |  |  |  |  |  |  |
| 0 | Ath-AT1G11970.1 |  |  |  |  |  |  |  |  |
| 0 | Ath-AT1G11980.1 |  |  |  |  |  |  |  |  |
| 1 | Ath-AT1G11990.2 |  | Vvi-Vitvi10g00120\_t001 |  |  |  |  |  |  |  |
| 1 | Ath-AT1G12000.1 |  | | | |  |  |  |  |  |  |  |
| 1 | Ath-AT1G12010.1 |  | Vvi-Vitvi10g04049\_t001 |  |  |  |  |  |  |  |
| 1 | Ath-AT1G12020.1 |  | Vvi-Vitvi10g00159\_t001 |  |  |  |  |  |  |  |
| 1 | Ath-AT1G12030.1 |  | Vvi-Vitvi10g04074\_t001 |  |  |  |  |  |  |  |
| 1 | Ath-AT1G12040.1 |  | | | |  |  |  |  |  |  |  |
| 1 | Ath-AT1G12050.1 |  | Vvi-Vitvi10g04077\_t001 |  |  |  |  |  |  |  |
| 1 | Ath-AT1G12060.1 |  | Vvi-Vitvi10g00185\_t001 |  |  |  |  |  |  |  |
| 1 | Ath-AT1G12064.1 |  | Vvi-Vitvi10g00187\_t001 |  |  |  |  |  |  |  |
| 1 | Ath-AT1G12070.1 |  | Vvi-Vitvi10g00194\_t001 |  |  |  |  |  |  |  |
| 0 | Ath-AT1G12080.2 |  |  |  |  |  |  |  |  |
| 0 | Ath-AT1G12090.1 |  |  |  |  |  |  |  |  |
| 1 | Ath-AT1G12100.1 |  | Vvi-Vitvi02g01438\_t001 |  |  |  |  |  |  |  |
| 1 | Ath-AT1G12110.1 |  | Vvi-Vitvi02g00529\_t001 |  |  |  |  |  |  |  |
| 1 | Ath-AT1G12120.1 |  | Vvi-Vitvi02g01682\_t002 |  |  |  |  |  |  |  |
| 1 | Ath-AT1G12130.1 |  | | | |  |  |  |  |  |  |  |
| 1 | Ath-AT1G12140.1 |  | Vvi-Vitvi02g01685\_t001 |  |  |  |  |  |  |  |
| 1 | Ath-AT1G12150.2 |  | | | |  |  |  |  |  |  |  |
| 1 | Ath-AT1G12160.1 |  | | | |  |  |  |  |  |  |  |
| 1 | Ath-AT1G12170.1 |  | | | |  |  |  |  |  |  |  |
| 1 | Ath-AT1G12180.1 |  | | | |  |  |  |  |  |  |  |
| 1 | Ath-AT1G12190.1 |  | | | |  |  |  |  |  |  |  |
| 1 | Ath-AT1G12200.1 |  | Vvi-Vitvi02g04150\_t001 |  |  |  |  |  |  |  |
| 1 | Ath-AT1G12211.1 |  | | | |  |  |  |  |  |  |  |
| 1 | Ath-AT1G12210.1 |  | | | |  |  |  |  |  |  |  |
| 1 | Ath-AT1G12220.2 |  | | | |  |  |  |  |  |  |  |
| 1 | Ath-AT1G12230.2 |  | Vvi-Vitvi02g04145\_t001 |  |  |  |  |  |  |  |
| 1 | Ath-AT1G12240.1 |  | Vvi-Vitvi02g00512\_t001 |  |  |  |  |  |  |  |
| 1 | Ath-AT1G12244.1 |  | | | |  |  |  |  |  |  |  |
| 1 | Ath-AT1G12250.1 |  | Vvi-Vitvi02g00509\_t004 |  |  |  |  |  |  |  |
| 1 | Ath-AT1G12260.1 |  | Vvi-Vitvi02g00508\_t001 |  |  |  |  |  |  |  |
| 1 | Ath-AT1G12270.1 |  | Vvi-Vitvi02g01852\_t001 |  |  |  |  |  |  |  |
| 0 | Ath-AT1G12280.1 |  |  |  |  |  |  |  |  |
| 0 | Ath-AT1G12290.1 |  |  |  |  |  |  |  |  |
| 0 | Ath-AT1G12300.1 |  |  |  |  |  |  |  |  |
| 1 | Ath-AT1G12320.1 |  | Vvi-Vitvi02g00481\_t001 |  |  |  |  |  |  |  |
| 1 | Ath-AT1G12310.1 |  | | | |  |  |  |  |  |  |  |
| 1 | Ath-AT1G12330.1 |  | Vvi-Vitvi02g00477\_t001 |  |  |  |  |  |  |  |
| 1 | Ath-AT1G12340.1 |  | | | |  |  |  |  |  |  |  |
| 1 | Ath-AT1G12350.1 |  | | | |  |  |  |  |  |  |  |
| 1 | Ath-AT1G12360.1 |  | | | |  |  |  |  |  |  |  |
| 1 | Ath-AT1G12370.2 |  | Vvi-Vitvi02g00475\_t001 |  |  |  |  |  |  |  |
| 1 | Ath-AT1G12380.1 |  | | | |  |  |  |  |  |  |  |
| 1 | Ath-AT1G12390.1 |  | | | |  |  |  |  |  |  |  |
| 1 | Ath-AT1G12400.3 |  | Vvi-Vitvi02g00470\_t001 |  |  |  |  |  |  |  |
| 1 | Ath-AT1G12410.1 |  | Vvi-Vitvi02g00469\_t001 |  |  |  |  |  |  |  |
| 1 | Ath-AT1G12411.1 |  | | | |  |  |  |  |  |  |  |
| 1 | Ath-AT1G12420.1 |  | Vvi-Vitvi02g00467\_t001 |  |  |  |  |  |  |  |
| 1 | Ath-AT1G12430.2 |  | Vvi-Vitvi02g00462\_t001 |  |  |  |  |  |  |  |
| 1 | Ath-AT1G12440.2 |  | Vvi-Vitvi02g00461\_t002 |  |  |  |  |  |  |  |
| 1 | Ath-AT1G12450.1 |  | Vvi-Vitvi02g04119\_t001 |  |  |  |  |  |  |  |
| 1 | Ath-AT1G12460.1 |  | Vvi-Vitvi02g04118\_t001 |  |  |  |  |  |  |  |
| 1 | Ath-AT1G12470.1 |  | | | |  |  |  |  |  |  |  |
| 1 | Ath-AT1G12480.1 |  | Vvi-Vitvi02g00451\_t001 |  |  |  |  |  |  |  |
| 1 | Ath-AT1G12490.1 |  | | | |  |  |  |  |  |  |  |
| 1 | Ath-AT1G12500.1 |  | Vvi-Vitvi02g00450\_t001 |  |  |  |  |  |  |  |
| 1 | Ath-AT1G12520.1 |  | Vvi-Vitvi02g00444\_t001 |  |  |  |  |  |  |  |
| 1 | Ath-AT1G12530.1 |  | Vvi-Vitvi02g00441\_t002 |  |  |  |  |  |  |  |
| 1 | Ath-AT1G12540.1 |  | Vvi-Vitvi02g00439\_t001 |  |  |  |  |  |  |  |
| 1 | Ath-AT1G12550.1 |  | Vvi-Vitvi02g00436\_t001 |  |  |  |  |  |  |  |
| 1 | Ath-AT1G12560.1 |  | Vvi-Vitvi02g00433\_t001 |  |  |  |  |  |  |  |
| 2 | Ath-AT1G12570.1 |  | Vvi-Vitvi02g00416\_t001 |  | Vvi-Vitvi16g01854\_t001 |  |  |  |  |  |  |
| 2 | Ath-AT1G12580.1 |  | Vvi-Vitvi02g00413\_t002 |  | Vvi-Vitvi16g00935\_t001 |  |  |  |  |  |  |
| 2 | Ath-AT1G12600.1 |  | Vvi-Vitvi02g00410\_t001 |  | | | |  |  |  |  |  |  |
| 2 | Ath-AT1G12610.1 |  | Vvi-Vitvi02g00407\_t001 |  | | | |  |  |  |  |  |  |
| 2 | Ath-AT1G12615.1 |  | | | |  | | | |  |  |  |  |  |  |
| 2 | Ath-AT1G12620.1 |  | | | |  | | | |  |  |  |  |  |  |
| 2 | Ath-AT1G12630.1 |  | Vvi-Vitvi02g04095\_t001 |  | Vvi-Vitvi16g00942\_t001 |  |  |  |  |  |  |
| 2 | Ath-AT1G12640.1 |  | Vvi-Vitvi02g00402\_t001 |  | | | |  |  |  |  |  |  |
| 2 | Ath-AT1G12650.2 |  | Vvi-Vitvi02g04094\_t001 |  | | | |  |  |  |  |  |  |
| 2 | Ath-AT1G12660.1 |  | | | |  | | | |  |  |  |  |  |  |
| 2 | Ath-AT1G12663.1 |  | | | |  | | | |  |  |  |  |  |  |
| 2 | Ath-AT1G12665.1 |  | | | |  | | | |  |  |  |  |  |  |
| 2 | Ath-AT1G12672.2 |  | | | |  | | | |  |  |  |  |  |  |
| 2 | Ath-AT1G12680.1 |  | | | |  | | | |  |  |  |  |  |  |
| 2 | Ath-AT1G12700.2 |  | | | |  | | | |  |  |  |  |  |  |
| 2 | Ath-AT1G12710.2 |  | Vvi-Vitvi02g00398\_t001 |  | Vvi-Vitvi16g00957\_t001 |  |  |  |  |  |  |
| 3 | Ath-AT1G12730.1 |  | | | |  | | | |  | Vvi-Vitvi02g00375\_t001 |  |  |  |  |  |
| 3 | Ath-AT1G12740.2 |  | | | |  | Vvi-Vitvi16g00966\_t001 |  | Vvi-Vitvi02g00377\_t001 |  |  |  |  |  |
| 3 | Ath-AT1G12750.1 |  | | | |  | Vvi-Vitvi16g00972\_t001 |  | Vvi-Vitvi02g00382\_t001 |  |  |  |  |  |
| 2 | Ath-AT1G12760.1 |  | | | |  |  |  | Vvi-Vitvi02g00384\_t001 |  |  |  |  |  |
| 2 | Ath-AT1G12770.1 |  | | | |  |  |  | Vvi-Vitvi02g00389\_t001 |  |  |  |  |  |
| 2 | Ath-AT1G12775.1 |  | | | |  |  |  | | | |  |  |  |  |  |
| 2 | Ath-AT1G12780.1 |  | Vvi-Vitvi02g00390\_t001 |  |  |  | Vvi-Vitvi02g00390\_t001 |  |  |  |  |  |
| 1 | Ath-AT1G12790.1 |  | | | |  |  |  |  |  |  |  |
| 1 | Ath-AT1G12800.1 |  | Vvi-Vitvi02g00367\_t001 |  |  |  |  |  |  |  |
| 0 | Ath-AT1G12805.1 |  |  |  |  |  |  |  |  |
| 0 | Ath-AT1G12810.2 |  |  |  |  |  |  |  |  |
| 0 | Ath-AT1G12820.1 |  |  |  |  |  |  |  |  |
| 0 | Ath-AT1G12840.1 |  |  |  |  |  |  |  |  |
| 0 | Ath-AT1G12830.1 |  |  |  |  |  |  |  |  |
| 0 | Ath-AT1G12845.1 |  |  |  |  |  |  |  |  |
| 0 | Ath-AT1G12850.1 |  |  |  |  |  |  |  |  |
| 0 | Ath-AT1G12855.1 |  |  |  |  |  |  |  |  |
| 0 | Ath-AT1G12860.1 |  |  |  |  |  |  |  |  |
| 0 | Ath-AT1G12870.1 |  |  |  |  |  |  |  |  |
| 0 | Ath-AT1G12880.1 |  |  |  |  |  |  |  |  |
| 0 | Ath-AT1G12890.1 |  |  |  |  |  |  |  |  |
| 0 | Ath-AT1G12900.5 |  |  |  |  |  |  |  |  |
| 0 | Ath-AT1G12910.1 |  |  |  |  |  |  |  |  |
| 0 | Ath-AT1G12920.1 |  |  |  |  |  |  |  |  |
| 0 | Ath-AT1G12930.1 |  |  |  |  |  |  |  |  |
| 0 | Ath-AT1G12940.1 |  |  |  |  |  |  |  |  |
| 1 | Ath-AT1G12950.1 |  | Vvi-Vitvi01g00653\_t001 |  |  |  |  |  |  |  |
| 1 | Ath-AT1G12960.1 |  | Vvi-Vitvi01g04157\_t001 |  |  |  |  |  |  |  |
| 1 | Ath-AT1G12970.1 |  | | | |  |  |  |  |  |  |  |
| 1 | Ath-AT1G12980.1 |  | Vvi-Vitvi01g00645\_t001 |  |  |  |  |  |  |  |
| 1 | Ath-AT1G12990.1 |  | Vvi-Vitvi01g00642\_t001 |  |  |  |  |  |  |  |
| 1 | Ath-AT1G13000.1 |  | Vvi-Vitvi01g00641\_t001 |  |  |  |  |  |  |  |
| 1 | Ath-AT1G13020.1 |  | Vvi-Vitvi01g00633\_t001 |  |  |  |  |  |  |  |
| 1 | Ath-AT1G13030.1 |  | Vvi-Vitvi01g00627\_t001 |  |  |  |  |  |  |  |
| 1 | Ath-AT1G13040.1 |  | Vvi-Vitvi01g00625\_t001 |  |  |  |  |  |  |  |
| 1 | Ath-AT1G13050.1 |  | Vvi-Vitvi01g04149\_t001 |  |  |  |  |  |  |  |
| 1 | Ath-AT1G13060.2 |  | | | |  |  |  |  |  |  |  |
| 1 | Ath-AT1G13080.1 |  | | | |  |  |  |  |  |  |  |
| 1 | Ath-AT1G13090.2 |  | | | |  |  |  |  |  |  |  |
| 1 | Ath-AT1G13100.1 |  | | | |  |  |  |  |  |  |  |
| 1 | Ath-AT1G13110.1 |  | | | |  |  |  |  |  |  |  |
| 1 | Ath-AT1G13120.1 |  | Vvi-Vitvi01g00613\_t001 |  |  |  |  |  |  |  |
| 1 | Ath-AT1G13130.1 |  | Vvi-Vitvi01g02024\_t001 |  |  |  |  |  |  |  |
| 1 | Ath-AT1G13140.1 |  | Vvi-Vitvi01g00612\_t001 |  |  |  |  |  |  |  |
| 1 | Ath-AT1G13143.1 |  | | | |  |  |  |  |  |  |  |
| 1 | Ath-AT1G13150.1 |  | | | |  |  |  |  |  |  |  |
| 1 | Ath-AT1G13160.1 |  | Vvi-Vitvi01g00599\_t001 |  |  |  |  |  |  |  |
| 1 | Ath-AT1G13170.2 |  | Vvi-Vitvi01g00598\_t001 |  |  |  |  |  |  |  |
| 1 | Ath-AT1G13180.1 |  | Vvi-Vitvi01g00596\_t001 |  |  |  |  |  |  |  |
| 1 | Ath-AT1G13190.1 |  | Vvi-Vitvi01g00595\_t001 |  |  |  |  |  |  |  |
| 0 | Ath-AT1G13195.1 |  |  |  |  |  |  |  |  |
| 0 | Ath-AT1G13200.1 |  |  |  |  |  |  |  |  |
| 0 | Ath-AT1G13210.1 |  |  |  |  |  |  |  |  |
| 0 | Ath-AT1G13220.3 |  |  |  |  |  |  |  |  |
| 1 | Ath-AT1G13230.1 |  | Vvi-Vitvi01g00218\_t001 |  |  |  |  |  |  |  |
| 1 | Ath-AT1G13245.1 |  | | | |  |  |  |  |  |  |  |
| 1 | Ath-AT1G13250.1 |  | Vvi-Vitvi01g00241\_t001 |  |  |  |  |  |  |  |
| 1 | Ath-AT1G13260.1 |  | Vvi-Vitvi01g00244\_t001 |  |  |  |  |  |  |  |
| 1 | Ath-AT1G13270.1 |  | Vvi-Vitvi01g00245\_t001 |  |  |  |  |  |  |  |
| 1 | Ath-AT1G13280.1 |  | Vvi-Vitvi01g00246\_t001 |  |  |  |  |  |  |  |
| 1 | Ath-AT1G13290.1 |  | Vvi-Vitvi01g00247\_t001 |  |  |  |  |  |  |  |
| 1 | Ath-AT1G13300.1 |  | Vvi-Vitvi01g00249\_t001 |  |  |  |  |  |  |  |
| 1 | Ath-AT1G13310.1 |  | Vvi-Vitvi01g00251\_t001 |  |  |  |  |  |  |  |
| 1 | Ath-AT1G13320.1 |  | Vvi-Vitvi01g00265\_t002 |  |  |  |  |  |  |  |
| 1 | Ath-AT1G13330.1 |  | Vvi-Vitvi01g00270\_t001 |  |  |  |  |  |  |  |
| 1 | Ath-AT1G13340.1 |  | Vvi-Vitvi01g00271\_t001 |  |  |  |  |  |  |  |
| 1 | Ath-AT1G13350.3 |  | Vvi-Vitvi01g00272\_t001 |  |  |  |  |  |  |  |
| 1 | Ath-AT1G13360.1 |  | Vvi-Vitvi01g00279\_t001 |  |  |  |  |  |  |  |
| 1 | Ath-AT1G13370.1 |  | | | |  |  |  |  |  |  |  |
| 1 | Ath-AT1G13380.1 |  | Vvi-Vitvi01g00292\_t001 |  |  |  |  |  |  |  |
| 1 | Ath-AT1G13390.2 |  | Vvi-Vitvi01g01938\_t001 |  |  |  |  |  |  |  |
| 1 | Ath-AT1G13400.1 |  | Vvi-Vitvi01g01939\_t001 |  |  |  |  |  |  |  |
| 1 | Ath-AT1G13410.1 |  | Vvi-Vitvi01g00296\_t001 |  |  |  |  |  |  |  |
| 0 | Ath-AT1G13420.1 |  |  |  |  |  |  |  |  |
| 0 | Ath-AT1G13430.1 |  |  |  |  |  |  |  |  |
| 0 | Ath-AT1G13440.1 |  |  |  |  |  |  |  |  |
| 0 | Ath-AT1G13450.1 |  |  |  |  |  |  |  |  |
| 0 | Ath-AT1G13460.1 |  |  |  |  |  |  |  |  |
| 0 | Ath-AT1G13470.1 |  |  |  |  |  |  |  |  |
| 0 | Ath-AT1G13480.1 |  |  |  |  |  |  |  |  |
| 0 | Ath-AT1G13485.1 |  |  |  |  |  |  |  |  |
| 0 | Ath-AT1G13490.2 |  |  |  |  |  |  |  |  |
| 0 | Ath-AT1G13500.1 |  |  |  |  |  |  |  |  |
| 0 | Ath-AT1G13510.1 |  |  |  |  |  |  |  |  |
| 0 | Ath-AT1G13520.1 |  |  |  |  |  |  |  |  |
| 0 | Ath-AT1G13530.1 |  |  |  |  |  |  |  |  |
| 0 | Ath-AT1G13540.1 |  |  |  |  |  |  |  |  |
| 0 | Ath-AT1G13550.1 |  |  |  |  |  |  |  |  |
| 0 | Ath-AT1G13560.1 |  |  |  |  |  |  |  |  |
| 0 | Ath-AT1G13570.1 |  |  |  |  |  |  |  |  |
| 0 | Ath-AT1G13580.3 |  |  |  |  |  |  |  |  |
| 0 | Ath-AT1G13590.1 |  |  |  |  |  |  |  |  |
| 0 | Ath-AT1G13600.1 |  |  |  |  |  |  |  |  |
| 0 | Ath-AT1G13605.1 |  |  |  |  |  |  |  |  |
| 0 | Ath-AT1G13607.1 |  |  |  |  |  |  |  |  |
| 0 | Ath-AT1G13608.1 |  |  |  |  |  |  |  |  |
| 0 | Ath-AT1G13609.1 |  |  |  |  |  |  |  |  |
| 0 | Ath-AT1G13610.1 |  |  |  |  |  |  |  |  |
| 0 | Ath-AT1G13620.1 |  |  |  |  |  |  |  |  |
| 0 | Ath-AT1G13630.4 |  |  |  |  |  |  |  |  |
| 0 | Ath-AT1G13635.2 |  |  |  |  |  |  |  |  |
| 0 | Ath-AT1G13640.1 |  |  |  |  |  |  |  |  |
| 0 | Ath-AT1G13650.2 |  |  |  |  |  |  |  |  |
| 0 | Ath-AT1G13670.1 |  |  |  |  |  |  |  |  |
| 0 | Ath-AT1G13680.1 |  |  |  |  |  |  |  |  |
| 0 | Ath-AT1G13690.1 |  |  |  |  |  |  |  |  |
| 1 | Ath-AT1G13700.1 |  | Vvi-Vitvi01g00026\_t002 |  |  |  |  |  |  |  |
| 1 | Ath-AT1G13710.1 |  | Vvi-Vitvi01g00031\_t001 |  |  |  |  |  |  |  |
| 1 | Ath-AT1G13730.1 |  | Vvi-Vitvi01g00036\_t002 |  |  |  |  |  |  |  |
| 1 | Ath-AT1G13740.1 |  | Vvi-Vitvi01g00037\_t001 |  |  |  |  |  |  |  |
| 1 | Ath-AT1G13750.1 |  | Vvi-Vitvi01g00042\_t001 |  |  |  |  |  |  |  |
| 1 | Ath-AT1G13755.1 |  | | | |  |  |  |  |  |  |  |
| 1 | Ath-AT1G13760.1 |  | | | |  |  |  |  |  |  |  |
| 1 | Ath-AT1G13770.1 |  | Vvi-Vitvi01g00045\_t001 |  |  |  |  |  |  |  |
| 1 | Ath-AT1G13780.1 |  | | | |  |  |  |  |  |  |  |
| 1 | Ath-AT1G13790.1 |  | | | |  |  |  |  |  |  |  |
| 1 | Ath-AT1G13800.1 |  | | | |  |  |  |  |  |  |  |
| 1 | Ath-AT1G13805.1 |  | | | |  |  |  |  |  |  |  |
| 1 | Ath-AT1G13810.1 |  | | | |  |  |  |  |  |  |  |
| 1 | Ath-AT1G13820.1 |  | Vvi-Vitvi01g00050\_t001 |  |  |  |  |  |  |  |
| 1 | Ath-AT1G13825.1 |  | | | |  |  |  |  |  |  |  |
| 1 | Ath-AT1G13830.2 |  | | | |  |  |  |  |  |  |  |
| 1 | Ath-AT1G13860.5 |  | | | |  |  |  |  |  |  |  |
| 1 | Ath-AT1G13870.1 |  | | | |  |  |  |  |  |  |  |
| 2 | Ath-AT1G13880.2 |  | | | |  | Vvi-Vitvi14g02034\_t001 |  |  |  |  |  |  |
| 2 | Ath-AT1G13890.1 |  | | | |  | | | |  |  |  |  |  |  |
| 2 | Ath-AT1G13900.1 |  | | | |  | | | |  |  |  |  |  |  |
| 2 | Ath-AT1G13910.1 |  | Vvi-Vitvi01g00056\_t001 |  | | | |  |  |  |  |  |  |
| 2 | Ath-AT1G13920.4 |  | Vvi-Vitvi01g01845\_t001 |  | | | |  |  |  |  |  |  |
| 2 | Ath-AT1G13930.1 |  | Vvi-Vitvi01g01846\_t001 |  | | | |  |  |  |  |  |  |
| 2 | Ath-AT1G13940.1 |  | Vvi-Vitvi01g00080\_t001 |  | Vvi-Vitvi14g02014\_t001 |  |  |  |  |  |  |
| 1 | Ath-AT1G13950.1 |  |  |  | | | |  |  |  |  |  |  |
| 1 | Ath-AT1G13960.1 |  |  |  | Vvi-Vitvi14g02007\_t001 |  |  |  |  |  |  |
| 1 | Ath-AT1G13970.1 |  |  |  | Vvi-Vitvi14g02005\_t001 |  |  |  |  |  |  |
| 1 | Ath-AT1G13980.2 |  |  |  | | | |  |  |  |  |  |  |
| 1 | Ath-AT1G13990.3 |  |  |  | | | |  |  |  |  |  |  |
| 2 | Ath-AT1G14000.1 |  | Vvi-Vitvi01g01055\_t001 |  | | | |  |  |  |  |  |  |
| 2 | Ath-AT1G14010.1 |  | Vvi-Vitvi01g01056\_t001 |  | Vvi-Vitvi14g03093\_t001 |  |  |  |  |  |  |
| 2 | Ath-AT1G14020.1 |  | Vvi-Vitvi01g01057\_t001 |  | | | |  |  |  |  |  |  |
| 2 | Ath-AT1G14030.1 |  | Vvi-Vitvi01g01058\_t001 |  | | | |  |  |  |  |  |  |
| 2 | Ath-AT1G14040.1 |  | Vvi-Vitvi01g01077\_t001 |  | Vvi-Vitvi14g01992\_t001 |  |  |  |  |  |  |
| 2 | Ath-AT1G14048.1 |  | | | |  | | | |  |  |  |  |  |  |
| 2 | Ath-AT1G14060.1 |  | | | |  | | | |  |  |  |  |  |  |
| 2 | Ath-AT1G14080.1 |  | Vvi-Vitvi01g01079\_t001 |  | | | |  |  |  |  |  |  |
| 2 | Ath-AT1G14100.1 |  | | | |  | | | |  |  |  |  |  |  |
| 2 | Ath-AT1G14110.1 |  | | | |  | | | |  |  |  |  |  |  |
| 2 | Ath-AT1G14120.1 |  | | | |  | | | |  |  |  |  |  |  |
| 2 | Ath-AT1G14130.1 |  | | | |  | | | |  |  |  |  |  |  |
| 2 | Ath-AT1G14140.1 |  | Vvi-Vitvi01g01049\_t001 |  | | | |  |  |  |  |  |  |
| 2 | Ath-AT1G14150.1 |  | Vvi-Vitvi01g01035\_t001 |  | | | |  |  |  |  |  |  |
| 2 | Ath-AT1G14160.1 |  | | | |  | Vvi-Vitvi14g03091\_t001 |  |  |  |  |  |  |
| 2 | Ath-AT1G14170.3 |  | Vvi-Vitvi01g01034\_t001 |  | Vvi-Vitvi14g01981\_t002 |  |  |  |  |  |  |
| 1 | Ath-AT1G14180.1 |  | | | |  |  |  |  |  |  |  |
| 1 | Ath-AT1G14185.1 |  | | | |  |  |  |  |  |  |  |
| 1 | Ath-AT1G14190.2 |  | | | |  |  |  |  |  |  |  |
| 1 | Ath-AT1G14200.1 |  | Vvi-Vitvi01g01023\_t001 |  |  |  |  |  |  |  |
| 1 | Ath-AT1G14205.1 |  | | | |  |  |  |  |  |  |  |
| 1 | Ath-AT1G14210.1 |  | Vvi-Vitvi01g01021\_t001 |  |  |  |  |  |  |  |
| 1 | Ath-AT1G14220.1 |  | | | |  |  |  |  |  |  |  |
| 1 | Ath-AT1G14230.1 |  | Vvi-Vitvi01g01018\_t001 |  |  |  |  |  |  |  |
| 1 | Ath-AT1G14240.2 |  | | | |  |  |  |  |  |  |  |
| 1 | Ath-AT1G14250.1 |  | | | |  |  |  |  |  |  |  |
| 2 | Ath-AT1G14260.2 |  | Vvi-Vitvi01g01015\_t001 |  | Vvi-Vitvi17g00603\_t001 |  |  |  |  |  |  |
| 2 | Ath-AT1G14270.1 |  | Vvi-Vitvi01g00997\_t001 |  | | | |  |  |  |  |  |  |
| 2 | Ath-AT1G14280.1 |  | Vvi-Vitvi01g00994\_t001 |  | Vvi-Vitvi17g00583\_t001 |  |  |  |  |  |  |
| 2 | Ath-AT1G14290.1 |  | Vvi-Vitvi01g00993\_t001 |  | | | |  |  |  |  |  |  |
| 2 | Ath-AT1G14300.2 |  | Vvi-Vitvi01g00991\_t001 |  | | | |  |  |  |  |  |  |
| 2 | Ath-AT1G14310.1 |  | Vvi-Vitvi01g02129\_t001 |  | | | |  |  |  |  |  |  |
| 2 | Ath-AT1G14315.2 |  | | | |  | | | |  |  |  |  |  |  |
| 2 | Ath-AT1G14320.1 |  | | | |  | | | |  |  |  |  |  |  |
| 2 | Ath-AT1G14330.1 |  | Vvi-Vitvi01g00970\_t001 |  | Vvi-Vitvi17g00570\_t001 |  |  |  |  |  |  |
| 2 | Ath-AT1G14340.1 |  | Vvi-Vitvi01g00960\_t003 |  | | | |  |  |  |  |  |  |
| 2 | Ath-AT1G14345.1 |  | Vvi-Vitvi01g00957\_t001 |  | | | |  |  |  |  |  |  |
| 2 | Ath-AT1G14350.2 |  | Vvi-Vitvi01g00956\_t001 |  | | | |  |  |  |  |  |  |
| 2 | Ath-AT1G14360.1 |  | Vvi-Vitvi01g00955\_t002 |  | | | |  |  |  |  |  |  |
| 2 | Ath-AT1G14370.1 |  | Vvi-Vitvi01g00953\_t001 |  | Vvi-Vitvi17g00568\_t001 |  |  |  |  |  |  |
| 2 | Ath-AT1G14380.3 |  | Vvi-Vitvi01g00945\_t001 |  | Vvi-Vitvi17g00560\_t001 |  |  |  |  |  |  |
| 2 | Ath-AT1G14390.1 |  | Vvi-Vitvi01g00944\_t001 |  | | | |  |  |  |  |  |  |
| 2 | Ath-AT1G14400.1 |  | Vvi-Vitvi01g00943\_t002 |  | Vvi-Vitvi17g04153\_t001 |  |  |  |  |  |  |
| 2 | Ath-AT1G14410.1 |  | Vvi-Vitvi01g00938\_t001 |  | | | |  |  |  |  |  |  |
| 2 | Ath-AT1G14420.1 |  | Vvi-Vitvi01g02109\_t001 |  | Vvi-Vitvi17g00550\_t001 |  |  |  |  |  |  |
| 1 | Ath-AT1G14430.1 |  | Vvi-Vitvi01g00922\_t001 |  |  |  |  |  |  |  |
| 0 | Ath-AT1G14440.1 |  |  |  |  |  |  |  |  |
| 0 | Ath-AT1G14450.2 |  |  |  |  |  |  |  |  |
| 0 | Ath-AT1G14453.1 |  |  |  |  |  |  |  |  |
| 0 | Ath-AT1G14455.1 |  |  |  |  |  |  |  |  |
| 0 | Ath-AT1G14460.1 |  |  |  |  |  |  |  |  |
| 0 | Ath-AT1G14470.1 |  |  |  |  |  |  |  |  |
| 0 | Ath-AT1G14480.1 |  |  |  |  |  |  |  |  |
| 0 | Ath-AT1G14490.2 |  |  |  |  |  |  |  |  |
| 0 | Ath-AT1G14500.1 |  |  |  |  |  |  |  |  |
| 0 | Ath-AT1G14510.1 |  |  |  |  |  |  |  |  |
| 0 | Ath-AT1G14520.3 |  |  |  |  |  |  |  |  |
| 0 | Ath-AT1G14530.2 |  |  |  |  |  |  |  |  |
| 0 | Ath-AT1G14540.1 |  |  |  |  |  |  |  |  |
| 0 | Ath-AT1G14550.1 |  |  |  |  |  |  |  |  |
| 0 | Ath-AT1G14560.1 |  |  |  |  |  |  |  |  |
| 0 | Ath-AT1G14570.1 |  |  |  |  |  |  |  |  |
| 0 | Ath-AT1G14580.3 |  |  |  |  |  |  |  |  |
| 0 | Ath-AT1G14590.1 |  |  |  |  |  |  |  |  |
| 1 | Ath-AT1G14600.1 |  | Vvi-Vitvi01g00578\_t001 |  |  |  |  |  |  |  |
| 1 | Ath-AT1G14610.1 |  | | | |  |  |  |  |  |  |  |
| 1 | Ath-AT1G14620.1 |  | | | |  |  |  |  |  |  |  |
| 1 | Ath-AT1G14630.1 |  | Vvi-Vitvi01g00567\_t001 |  |  |  |  |  |  |  |
| 1 | Ath-AT1G14640.1 |  | | | |  |  |  |  |  |  |  |
| 1 | Ath-AT1G14642.1 |  | | | |  |  |  |  |  |  |  |
| 1 | Ath-AT1G14650.1 |  | | | |  |  |  |  |  |  |  |
| 1 | Ath-AT1G14660.1 |  | Vvi-Vitvi01g00562\_t001 |  |  |  |  |  |  |  |
| 1 | Ath-AT1G14670.1 |  | Vvi-Vitvi01g00561\_t001 |  |  |  |  |  |  |  |
| 1 | Ath-AT1G14680.1 |  | | | |  |  |  |  |  |  |  |
| 1 | Ath-AT1G14685.1 |  | Vvi-Vitvi01g00547\_t001 |  |  |  |  |  |  |  |
| 1 | Ath-AT1G14686.1 |  | Vvi-Vitvi01g00546\_t001 |  |  |  |  |  |  |  |
| 1 | Ath-AT1G14687.1 |  | Vvi-Vitvi01g00545\_t001 |  |  |  |  |  |  |  |
| 1 | Ath-AT1G14688.1 |  | | | |  |  |  |  |  |  |  |
| 1 | Ath-AT1G14690.2 |  | Vvi-Vitvi01g00543\_t001 |  |  |  |  |  |  |  |
| 1 | Ath-AT1G14700.1 |  | Vvi-Vitvi01g00539\_t001 |  |  |  |  |  |  |  |
| 1 | Ath-AT1G14710.1 |  | Vvi-Vitvi01g00537\_t002 |  |  |  |  |  |  |  |
| 1 | Ath-AT1G14720.1 |  | Vvi-Vitvi01g00533\_t001 |  |  |  |  |  |  |  |
| 1 | Ath-AT1G14730.1 |  | Vvi-Vitvi01g02004\_t001 |  |  |  |  |  |  |  |
| 1 | Ath-AT1G14740.1 |  | Vvi-Vitvi01g00530\_t001 |  |  |  |  |  |  |  |
| 1 | Ath-AT1G14750.3 |  | Vvi-Vitvi01g00517\_t001 |  |  |  |  |  |  |  |
| 1 | Ath-AT1G14755.1 |  | | | |  |  |  |  |  |  |  |
| 1 | Ath-AT1G14760.1 |  | Vvi-Vitvi01g00510\_t001 |  |  |  |  |  |  |  |
| 1 | Ath-AT1G14770.1 |  | | | |  |  |  |  |  |  |  |
| 1 | Ath-AT1G14780.1 |  | Vvi-Vitvi01g00508\_t001 |  |  |  |  |  |  |  |
| 1 | Ath-AT1G14790.1 |  | Vvi-Vitvi01g00503\_t002 |  |  |  |  |  |  |  |
| 1 | Ath-AT1G14800.1 |  | | | |  |  |  |  |  |  |  |
| 1 | Ath-AT1G14810.1 |  | Vvi-Vitvi01g00502\_t001 |  |  |  |  |  |  |  |
| 1 | Ath-AT1G14820.3 |  | Vvi-Vitvi01g00501\_t001 |  |  |  |  |  |  |  |
| 1 | Ath-AT1G14830.1 |  | Vvi-Vitvi01g00498\_t001 |  |  |  |  |  |  |  |
| 1 | Ath-AT1G14840.1 |  | Vvi-Vitvi01g00497\_t001 |  |  |  |  |  |  |  |
| 1 | Ath-AT1G14850.1 |  | Vvi-Vitvi01g00488\_t001 |  |  |  |  |  |  |  |
| 1 | Ath-AT1G14860.1 |  | Vvi-Vitvi01g00483\_t001 |  |  |  |  |  |  |  |
| 1 | Ath-AT1G14870.1 |  | Vvi-Vitvi01g01987\_t001 |  |  |  |  |  |  |  |
| 1 | Ath-AT1G14880.1 |  | | | |  |  |  |  |  |  |  |
| 1 | Ath-AT1G14890.1 |  | Vvi-Vitvi01g00457\_t001 |  |  |  |  |  |  |  |
| 1 | Ath-AT1G14900.1 |  | Vvi-Vitvi01g00454\_t001 |  |  |  |  |  |  |  |
| 1 | Ath-AT1G14910.1 |  | Vvi-Vitvi01g00451\_t002 |  |  |  |  |  |  |  |
| 1 | Ath-AT1G14920.1 |  | Vvi-Vitvi01g00446\_t001 |  |  |  |  |  |  |  |
| 1 | Ath-AT1G14930.1 |  | Vvi-Vitvi01g04114\_t001 |  |  |  |  |  |  |  |
| 1 | Ath-AT1G14940.1 |  | | | |  |  |  |  |  |  |  |
| 1 | Ath-AT1G14950.1 |  | | | |  |  |  |  |  |  |  |
| 1 | Ath-AT1G14960.1 |  | | | |  |  |  |  |  |  |  |
| 1 | Ath-AT1G14970.1 |  | Vvi-Vitvi01g00425\_t001 |  |  |  |  |  |  |  |
| 1 | Ath-AT1G14980.1 |  | Vvi-Vitvi01g00424\_t001 |  |  |  |  |  |  |  |
| 1 | Ath-AT1G14990.2 |  | | | |  |  |  |  |  |  |  |
| 1 | Ath-AT1G15000.1 |  | Vvi-Vitvi01g00415\_t001 |  |  |  |  |  |  |  |
| 0 | Ath-AT1G15010.1 |  |  |  |  |  |  |  |  |
| 0 | Ath-AT1G15015.1 |  |  |  |  |  |  |  |  |
| 0 | Ath-AT1G15020.2 |  |  |  |  |  |  |  |  |
| 1 | Ath-AT1G15030.1 |  | Vvi-Vitvi01g00328\_t001 |  |  |  |  |  |  |  |
| 1 | Ath-AT1G15040.1 |  | Vvi-Vitvi01g00329\_t001 |  |  |  |  |  |  |  |
| 1 | Ath-AT1G15050.1 |  | Vvi-Vitvi01g00336\_t001 |  |  |  |  |  |  |  |
| 1 | Ath-AT1G15060.1 |  | Vvi-Vitvi01g00338\_t001 |  |  |  |  |  |  |  |
| 1 | Ath-AT1G15080.1 |  | Vvi-Vitvi01g01956\_t001 |  |  |  |  |  |  |  |
| 1 | Ath-AT1G15085.1 |  | | | |  |  |  |  |  |  |  |
| 1 | Ath-AT1G15100.1 |  | Vvi-Vitvi01g00354\_t001 |  |  |  |  |  |  |  |
| 1 | Ath-AT1G15110.2 |  | Vvi-Vitvi01g00362\_t001.1.60378269 |  |  |  |  |  |  |  |
| 1 | Ath-AT1G15120.2 |  | | | |  |  |  |  |  |  |  |
| 1 | Ath-AT1G15125.1 |  | | | |  |  |  |  |  |  |  |
| 1 | Ath-AT1G15130.1 |  | | | |  |  |  |  |  |  |  |
| 1 | Ath-AT1G15140.1 |  | | | |  |  |  |  |  |  |  |
| 1 | Ath-AT1G15150.1 |  | Vvi-Vitvi01g00370\_t001 |  |  |  |  |  |  |  |
| 0 | Ath-AT1G15160.1 |  |  |  |  |  |  |  |  |
| 0 | Ath-AT1G15165.1 |  |  |  |  |  |  |  |  |
| 0 | Ath-AT1G15170.1 |  |  |  |  |  |  |  |  |
| 0 | Ath-AT1G15180.1 |  |  |  |  |  |  |  |  |
| 0 | Ath-AT1G15190.1 |  |  |  |  |  |  |  |  |
| 0 | Ath-AT1G15200.3 |  |  |  |  |  |  |  |  |
| 0 | Ath-AT1G15210.1 |  |  |  |  |  |  |  |  |
| 0 | Ath-AT1G15215.4 |  |  |  |  |  |  |  |  |
| 1 | Ath-AT1G15220.2 |  | Vvi-Vitvi09g00345\_t001 |  |  |  |  |  |  |  |
| 1 | Ath-AT1G15230.1 |  | Vvi-Vitvi09g01598\_t001 |  |  |  |  |  |  |  |
| 1 | Ath-AT1G15240.2 |  | Vvi-Vitvi09g00369\_t001 |  |  |  |  |  |  |  |
| 1 | Ath-AT1G15250.2 |  | Vvi-Vitvi09g00370\_t001 |  |  |  |  |  |  |  |
| 1 | Ath-AT1G15260.1 |  | Vvi-Vitvi09g00372\_t001 |  |  |  |  |  |  |  |
| 1 | Ath-AT1G15270.1 |  | Vvi-Vitvi09g01606\_t001 |  |  |  |  |  |  |  |
| 0 | Ath-AT1G15280.2 |  |  |  |  |  |  |  |  |
| 1 | Ath-AT1G15290.1 |  | Vvi-Vitvi09g00659\_t001 |  |  |  |  |  |  |  |
| 1 | Ath-AT1G15310.1 |  | Vvi-Vitvi09g00643\_t001.1.6037826f |  |  |  |  |  |  |  |
| 1 | Ath-AT1G15320.1 |  | Vvi-Vitvi09g00627\_t001 |  |  |  |  |  |  |  |
| 1 | Ath-AT1G15330.1 |  | Vvi-Vitvi09g00619\_t001 |  |  |  |  |  |  |  |
| 1 | Ath-AT1G15340.1 |  | Vvi-Vitvi09g00606\_t001 |  |  |  |  |  |  |  |
| 1 | Ath-AT1G15350.2 |  | Vvi-Vitvi09g04185\_t001 |  |  |  |  |  |  |  |
| 1 | Ath-AT1G15360.2 |  | Vvi-Vitvi09g00593\_t001 |  |  |  |  |  |  |  |
| 1 | Ath-AT1G15370.1 |  | Vvi-Vitvi09g00592\_t001 |  |  |  |  |  |  |  |
| 2 | Ath-AT1G15380.1 |  | Vvi-Vitvi11g00443\_t001 |  | Vvi-Vitvi09g00559\_t001 |  |  |  |  |  |  |
| 2 | Ath-AT1G15385.1 |  | | | |  | | | |  |  |  |  |  |  |
| 2 | Ath-AT1G15390.1 |  | | | |  | Vvi-Vitvi09g00557\_t001 |  |  |  |  |  |  |
| 2 | Ath-AT1G15400.3 |  | Vvi-Vitvi11g01429\_t001 |  | Vvi-Vitvi09g01654\_t001 |  |  |  |  |  |  |
| 2 | Ath-AT1G15410.2 |  | Vvi-Vitvi11g00438\_t001 |  | Vvi-Vitvi09g00539\_t001 |  |  |  |  |  |  |
| 2 | Ath-AT1G15415.1 |  | | | |  | Vvi-Vitvi09g00525\_t001 |  |  |  |  |  |  |
| 2 | Ath-AT1G15420.1 |  | | | |  | Vvi-Vitvi09g00523\_t001 |  |  |  |  |  |  |
| 2 | Ath-AT1G15430.1 |  | | | |  | | | |  |  |  |  |  |  |
| 2 | Ath-AT1G15440.1 |  | | | |  | Vvi-Vitvi09g00520\_t001 |  |  |  |  |  |  |
| 2 | Ath-AT1G15460.1 |  | Vvi-Vitvi11g00419\_t001 |  | Vvi-Vitvi09g00500\_t001 |  |  |  |  |  |  |
| 2 | Ath-AT1G15470.1 |  | Vvi-Vitvi11g00416\_t001 |  | Vvi-Vitvi09g00496\_t001 |  |  |  |  |  |  |
| 2 | Ath-AT1G15480.1 |  | | | |  | | | |  |  |  |  |  |  |
| 2 | Ath-AT1G15490.1 |  | | | |  | Vvi-Vitvi09g00492\_t001 |  |  |  |  |  |  |
| 2 | Ath-AT1G15500.1 |  | | | |  | Vvi-Vitvi09g00489\_t001 |  |  |  |  |  |  |
| 2 | Ath-AT1G15510.1 |  | | | |  | Vvi-Vitvi09g00487\_t001 |  |  |  |  |  |  |
| 2 | Ath-AT1G15520.1 |  | Vvi-Vitvi11g00398\_t001 |  | Vvi-Vitvi09g04146\_t001 |  |  |  |  |  |  |
| 1 | Ath-AT1G15530.1 |  |  |  | | | |  |  |  |  |  |  |
| 1 | Ath-AT1G15540.3 |  |  |  | Vvi-Vitvi09g00451\_t001 |  |  |  |  |  |  |
| 1 | Ath-AT1G15550.1 |  |  |  | Vvi-Vitvi09g00448\_t001 |  |  |  |  |  |  |
| 1 | Ath-AT1G15570.1 |  |  |  | Vvi-Vitvi09g00443\_t001 |  |  |  |  |  |  |
| 1 | Ath-AT1G15580.1 |  |  |  | Vvi-Vitvi09g00436\_t001 |  |  |  |  |  |  |
| 1 | Ath-AT1G15590.2 |  |  |  | | | |  |  |  |  |  |  |
| 1 | Ath-AT1G15600.1 |  |  |  | | | |  |  |  |  |  |  |
| 1 | Ath-AT1G15610.1 |  |  |  | | | |  |  |  |  |  |  |
| 1 | Ath-AT1G15620.1 |  |  |  | | | |  |  |  |  |  |  |
| 1 | Ath-AT1G15625.1 |  |  |  | | | |  |  |  |  |  |  |
| 1 | Ath-AT1G15630.1 |  |  |  | | | |  |  |  |  |  |  |
| 1 | Ath-AT1G15640.1 |  |  |  | | | |  |  |  |  |  |  |
| 1 | Ath-AT1G15660.1 |  |  |  | Vvi-Vitvi09g00428\_t001 |  |  |  |  |  |  |
| 1 | Ath-AT1G15670.1 |  |  |  | Vvi-Vitvi09g00427\_t001 |  |  |  |  |  |  |
| 0 | Ath-AT1G15680.1 |  |  |  |  |  |  |  |  |
| 1 | Ath-AT1G15690.1 |  | Vvi-Vitvi09g00693\_t001 |  |  |  |  |  |  |  |
| 1 | Ath-AT1G15700.1 |  | | | |  |  |  |  |  |  |  |
| 1 | Ath-AT1G15710.1 |  | Vvi-Vitvi09g00694\_t001 |  |  |  |  |  |  |  |
| 1 | Ath-AT1G15720.1 |  | | | |  |  |  |  |  |  |  |
| 1 | Ath-AT1G15730.1 |  | Vvi-Vitvi09g00731\_t001 |  |  |  |  |  |  |  |
| 1 | Ath-AT1G15740.1 |  | Vvi-Vitvi09g00734\_t001 |  |  |  |  |  |  |  |
| 2 | Ath-AT1G15750.3 |  | Vvi-Vitvi09g00753\_t002 |  | Vvi-Vitvi04g00567\_t001 |  |  |  |  |  |  |
| 2 | Ath-AT1G15757.1 |  | | | |  | | | |  |  |  |  |  |  |
| 2 | Ath-AT1G15760.1 |  | Vvi-Vitvi09g00759\_t001 |  | | | |  |  |  |  |  |  |
| 1 | Ath-AT1G15770.1 |  |  |  | | | |  |  |  |  |  |  |
| 1 | Ath-AT1G15772.2 |  |  |  | | | |  |  |  |  |  |  |
| 1 | Ath-AT1G15780.2 |  |  |  | | | |  |  |  |  |  |  |
| 1 | Ath-AT1G15790.3 |  |  |  | | | |  |  |  |  |  |  |
| 1 | Ath-AT1G15800.1 |  |  |  | Vvi-Vitvi04g00540\_t001 |  |  |  |  |  |  |
| 1 | Ath-AT1G15810.1 |  |  |  | | | |  |  |  |  |  |  |
| 1 | Ath-AT1G15820.1 |  |  |  | | | |  |  |  |  |  |  |
| 1 | Ath-AT1G15825.1 |  |  |  | | | |  |  |  |  |  |  |
| 1 | Ath-AT1G15830.1 |  |  |  | | | |  |  |  |  |  |  |
| 1 | Ath-AT1G15840.1 |  |  |  | | | |  |  |  |  |  |  |
| 1 | Ath-AT1G15850.1 |  |  |  | | | |  |  |  |  |  |  |
| 1 | Ath-AT1G15860.3 |  |  |  | | | |  |  |  |  |  |  |
| 1 | Ath-AT1G15870.1 |  |  |  | Vvi-Vitvi04g00528\_t002 |  |  |  |  |  |  |
| 1 | Ath-AT1G15880.1 |  |  |  | Vvi-Vitvi04g00526\_t001 |  |  |  |  |  |  |
| 1 | Ath-AT1G15885.1 |  |  |  | | | |  |  |  |  |  |  |
| 1 | Ath-AT1G15890.1 |  |  |  | | | |  |  |  |  |  |  |
| 1 | Ath-AT1G15900.1 |  |  |  | | | |  |  |  |  |  |  |
| 1 | Ath-AT1G15910.1 |  |  |  | | | |  |  |  |  |  |  |
| 1 | Ath-AT1G15920.2 |  |  |  | Vvi-Vitvi04g00520\_t001 |  |  |  |  |  |  |
| 1 | Ath-AT1G15930.2 |  |  |  | | | |  |  |  |  |  |  |
| 1 | Ath-AT1G15940.1 |  |  |  | Vvi-Vitvi04g00514\_t001 |  |  |  |  |  |  |
| 0 | Ath-AT1G15950.1 |  |  |  |  |  |  |  |  |
| 0 | Ath-AT1G15960.1 |  |  |  |  |  |  |  |  |
| 0 | Ath-AT1G15970.1 |  |  |  |  |  |  |  |  |
| 0 | Ath-AT1G15980.1 |  |  |  |  |  |  |  |  |
| 0 | Ath-AT1G15990.1 |  |  |  |  |  |  |  |  |
| 0 | Ath-AT1G16000.1 |  |  |  |  |  |  |  |  |
| 0 | Ath-AT1G16010.2 |  |  |  |  |  |  |  |  |
| 0 | Ath-AT1G16020.1 |  |  |  |  |  |  |  |  |
| 0 | Ath-AT1G16022.1 |  |  |  |  |  |  |  |  |
| 0 | Ath-AT1G16025.1 |  |  |  |  |  |  |  |  |
| 0 | Ath-AT1G16030.1 |  |  |  |  |  |  |  |  |
| 1 | Ath-AT1G16040.1 |  | Vvi-Vitvi09g01278\_t001 |  |  |  |  |  |  |  |
| 1 | Ath-AT1G16060.1 |  | Vvi-Vitvi09g01269\_t001 |  |  |  |  |  |  |  |
| 1 | Ath-AT1G16070.1 |  | Vvi-Vitvi09g01266\_t001 |  |  |  |  |  |  |  |
| 1 | Ath-AT1G16080.1 |  | Vvi-Vitvi09g01263\_t001 |  |  |  |  |  |  |  |
| 1 | Ath-AT1G16090.1 |  | | | |  |  |  |  |  |  |  |
| 1 | Ath-AT1G16110.1 |  | | | |  |  |  |  |  |  |  |
| 1 | Ath-AT1G16120.1 |  | | | |  |  |  |  |  |  |  |
| 1 | Ath-AT1G16130.1 |  | | | |  |  |  |  |  |  |  |
| 1 | Ath-AT1G16150.1 |  | | | |  |  |  |  |  |  |  |
| 1 | Ath-AT1G16160.1 |  | | | |  |  |  |  |  |  |  |
| 1 | Ath-AT1G16170.1 |  | | | |  |  |  |  |  |  |  |
| 1 | Ath-AT1G16180.1 |  | Vvi-Vitvi09g01251\_t001 |  |  |  |  |  |  |  |
| 1 | Ath-AT1G16190.1 |  | Vvi-Vitvi09g01250\_t001 |  |  |  |  |  |  |  |
| 0 | Ath-AT1G16210.1 |  |  |  |  |  |  |  |  |
| 0 | Ath-AT1G16220.1 |  |  |  |  |  |  |  |  |
| 0 | Ath-AT1G16225.2 |  |  |  |  |  |  |  |  |
| 0 | Ath-AT1G16230.1 |  |  |  |  |  |  |  |  |
| 0 | Ath-AT1G16240.1 |  |  |  |  |  |  |  |  |
| 0 | Ath-AT1G16250.1 |  |  |  |  |  |  |  |  |
| 0 | Ath-AT1G16260.1 |  |  |  |  |  |  |  |  |
| 2 | Ath-AT1G16270.2 |  | Vvi-Vitvi19g01707\_t001 |  | Vvi-Vitvi19g01707\_t001 |  |  |  |  |  |  |
| 2 | Ath-AT1G16280.1 |  | Vvi-Vitvi19g01704\_t001 |  | | | |  |  |  |  |  |  |
| 2 | Ath-AT1G16290.2 |  | | | |  | | | |  |  |  |  |  |  |
| 2 | Ath-AT1G16300.1 |  | | | |  | Vvi-Vitvi19g01710\_t001 |  |  |  |  |  |  |
| 2 | Ath-AT1G16310.1 |  | | | |  | Vvi-Vitvi19g01715\_t001 |  |  |  |  |  |  |
| 2 | Ath-AT1G16320.1 |  | | | |  | Vvi-Vitvi19g01718\_t001 |  |  |  |  |  |  |
| 2 | Ath-AT1G16330.1 |  | | | |  | Vvi-Vitvi19g01719\_t001 |  |  |  |  |  |  |
| 2 | Ath-AT1G16340.4 |  | | | |  | Vvi-Vitvi19g01724\_t001 |  |  |  |  |  |  |
| 2 | Ath-AT1G16350.1 |  | | | |  | Vvi-Vitvi19g01733\_t001 |  |  |  |  |  |  |
| 2 | Ath-AT1G16360.2 |  | | | |  | Vvi-Vitvi19g01738\_t002 |  |  |  |  |  |  |
| 2 | Ath-AT1G16370.1 |  | | | |  | Vvi-Vitvi19g01746\_t001 |  |  |  |  |  |  |
| 2 | Ath-AT1G16380.1 |  | | | |  | | | |  |  |  |  |  |  |
| 2 | Ath-AT1G16390.1 |  | | | |  | | | |  |  |  |  |  |  |
| 2 | Ath-AT1G16400.1 |  | | | |  | | | |  |  |  |  |  |  |
| 2 | Ath-AT1G16410.1 |  | | | |  | | | |  |  |  |  |  |  |
| 2 | Ath-AT1G16420.1 |  | | | |  | Vvi-Vitvi19g02355\_t001 |  |  |  |  |  |  |
| 2 | Ath-AT1G16430.1 |  | | | |  | Vvi-Vitvi19g01768\_t001 |  |  |  |  |  |  |
| 2 | Ath-AT1G16440.1 |  | | | |  | Vvi-Vitvi19g01770\_t001 |  |  |  |  |  |  |
| 1 | Ath-AT1G16445.1 |  | Vvi-Vitvi19g01686\_t001 |  |  |  |  |  |  |  |
| 1 | Ath-AT1G16460.2 |  | Vvi-Vitvi19g01681\_t001 |  |  |  |  |  |  |  |
| 1 | Ath-AT1G16470.1 |  | | | |  |  |  |  |  |  |  |
| 1 | Ath-AT1G16480.1 |  | Vvi-Vitvi19g01679\_t001 |  |  |  |  |  |  |  |
| 1 | Ath-AT1G16490.1 |  | Vvi-Vitvi19g01669\_t001 |  |  |  |  |  |  |  |
| 1 | Ath-AT1G16500.1 |  | Vvi-Vitvi19g01667\_t001 |  |  |  |  |  |  |  |
| 1 | Ath-AT1G16510.1 |  | Vvi-Vitvi19g01665\_t001 |  |  |  |  |  |  |  |
| 0 | Ath-AT1G16515.2 |  |  |  |  |  |  |  |  |
| 0 | Ath-AT1G16520.1 |  |  |  |  |  |  |  |  |
| 0 | Ath-AT1G16530.1 |  |  |  |  |  |  |  |  |
| 0 | Ath-AT1G16540.3 |  |  |  |  |  |  |  |  |
| 0 | Ath-AT1G16560.2 |  |  |  |  |  |  |  |  |
| 0 | Ath-AT1G16570.2 |  |  |  |  |  |  |  |  |
| 0 | Ath-AT1G16590.1 |  |  |  |  |  |  |  |  |
| 0 | Ath-AT1G16610.3 |  |  |  |  |  |  |  |  |
| 0 | Ath-AT1G16630.1 |  |  |  |  |  |  |  |  |
| 0 | Ath-AT1G16640.1 |  |  |  |  |  |  |  |  |
| 0 | Ath-AT1G16650.3 |  |  |  |  |  |  |  |  |
| 1 | Ath-AT1G16670.1 |  | Vvi-Vitvi19g00127\_t001 |  |  |  |  |  |  |  |
| 1 | Ath-AT1G16680.1 |  | | | |  |  |  |  |  |  |  |
| 1 | Ath-AT1G16690.1 |  | | | |  |  |  |  |  |  |  |
| 1 | Ath-AT1G16700.1 |  | | | |  |  |  |  |  |  |  |
| 1 | Ath-AT1G16705.2 |  | | | |  |  |  |  |  |  |  |
| 1 | Ath-AT1G16710.1 |  | | | |  |  |  |  |  |  |  |
| 1 | Ath-AT1G16720.2 |  | | | |  |  |  |  |  |  |  |
| 1 | Ath-AT1G16730.1 |  | | | |  |  |  |  |  |  |  |
| 1 | Ath-AT1G16740.1 |  | | | |  |  |  |  |  |  |  |
| 1 | Ath-AT1G16750.1 |  | | | |  |  |  |  |  |  |  |
| 1 | Ath-AT1G16760.2 |  | | | |  |  |  |  |  |  |  |
| 1 | Ath-AT1G16770.1 |  | | | |  |  |  |  |  |  |  |
| 2 | Ath-AT1G16780.1 |  | | | |  | Vvi-Vitvi09g01381\_t001 |  |  |  |  |  |  |
| 2 | Ath-AT1G16790.1 |  | | | |  | Vvi-Vitvi09g01382\_t001 |  |  |  |  |  |  |
| 2 | Ath-AT1G16800.2 |  | | | |  | Vvi-Vitvi09g01386\_t001 |  |  |  |  |  |  |
| 2 | Ath-AT1G16810.3 |  | | | |  | | | |  |  |  |  |  |  |
| 2 | Ath-AT1G16820.1 |  | | | |  | | | |  |  |  |  |  |  |
| 2 | Ath-AT1G16825.1 |  | | | |  | Vvi-Vitvi09g01985\_t001 |  |  |  |  |  |  |
| 2 | Ath-AT1G16830.1 |  | | | |  | Vvi-Vitvi09g01402\_t001 |  |  |  |  |  |  |
| 2 | Ath-AT1G16840.5 |  | | | |  | Vvi-Vitvi09g01405\_t001 |  |  |  |  |  |  |
| 1 | Ath-AT1G16850.1 |  | | | |  |  |  |  |  |  |  |
| 1 | Ath-AT1G16860.1 |  | | | |  |  |  |  |  |  |  |
| 1 | Ath-AT1G16870.1 |  | | | |  |  |  |  |  |  |  |
| 1 | Ath-AT1G16880.1 |  | | | |  |  |  |  |  |  |  |
| 1 | Ath-AT1G16890.3 |  | Vvi-Vitvi19g00121\_t003 |  |  |  |  |  |  |  |
| 1 | Ath-AT1G16900.1 |  | Vvi-Vitvi19g00114\_t001 |  |  |  |  |  |  |  |
| 1 | Ath-AT1G16905.1 |  | | | |  |  |  |  |  |  |  |
| 1 | Ath-AT1G16910.1 |  | Vvi-Vitvi19g00108\_t001 |  |  |  |  |  |  |  |
| 1 | Ath-AT1G16916.2 |  | Vvi-Vitvi19g00096\_t001 |  |  |  |  |  |  |  |
| 1 | Ath-AT1G16920.1 |  | Vvi-Vitvi19g00091\_t001 |  |  |  |  |  |  |  |
| 1 | Ath-AT1G16930.3 |  | | | |  |  |  |  |  |  |  |
| 1 | Ath-AT1G16940.1 |  | | | |  |  |  |  |  |  |  |
| 1 | Ath-AT1G16945.1 |  | | | |  |  |  |  |  |  |  |
| 1 | Ath-AT1G16950.1 |  | Vvi-Vitvi19g04043\_t001 |  |  |  |  |  |  |  |
| 1 | Ath-AT1G16960.1 |  | Vvi-Vitvi19g01829\_t001 |  |  |  |  |  |  |  |
| 0 | Ath-AT1G16970.1 |  |  |  |  |  |  |  |  |
| 0 | Ath-AT1G16980.1 |  |  |  |  |  |  |  |  |
| 0 | Ath-AT1G17010.1 |  |  |  |  |  |  |  |  |
| 0 | Ath-AT1G17020.1 |  |  |  |  |  |  |  |  |
| 0 | Ath-AT1G17030.1 |  |  |  |  |  |  |  |  |
| 0 | Ath-AT1G17040.2 |  |  |  |  |  |  |  |  |
| 0 | Ath-AT1G17050.1 |  |  |  |  |  |  |  |  |
| 0 | Ath-AT1G17060.1 |  |  |  |  |  |  |  |  |
| 0 | Ath-AT1G17065.1 |  |  |  |  |  |  |  |  |
| 0 | Ath-AT1G17070.1 |  |  |  |  |  |  |  |  |
| 0 | Ath-AT1G17080.1 |  |  |  |  |  |  |  |  |
| 0 | Ath-AT1G17090.2 |  |  |  |  |  |  |  |  |
| 0 | Ath-AT1G17100.1 |  |  |  |  |  |  |  |  |
| 0 | Ath-AT1G17110.2 |  |  |  |  |  |  |  |  |
| 0 | Ath-AT1G17120.1 |  |  |  |  |  |  |  |  |
| 0 | Ath-AT1G17130.2 |  |  |  |  |  |  |  |  |
| 0 | Ath-AT1G17140.1 |  |  |  |  |  |  |  |  |
| 0 | Ath-AT1G17145.1 |  |  |  |  |  |  |  |  |
| 0 | Ath-AT1G17147.1 |  |  |  |  |  |  |  |  |
| 0 | Ath-AT1G17150.1 |  |  |  |  |  |  |  |  |
| 0 | Ath-AT1G17160.1 |  |  |  |  |  |  |  |  |
| 0 | Ath-AT1G17170.1 |  |  |  |  |  |  |  |  |
| 0 | Ath-AT1G17180.1 |  |  |  |  |  |  |  |  |
| 0 | Ath-AT1G17190.1 |  |  |  |  |  |  |  |  |
| 0 | Ath-AT1G17200.1 |  |  |  |  |  |  |  |  |
| 0 | Ath-AT1G17210.1 |  |  |  |  |  |  |  |  |
| 0 | Ath-AT1G17220.1 |  |  |  |  |  |  |  |  |
| 0 | Ath-AT1G17230.2 |  |  |  |  |  |  |  |  |
| 0 | Ath-AT1G17235.1 |  |  |  |  |  |  |  |  |
| 1 | Ath-AT1G17240.2 |  | Vvi-Vitvi09g00003\_t001 |  |  |  |  |  |  |  |
| 1 | Ath-AT1G17250.1 |  | | | |  |  |  |  |  |  |  |
| 1 | Ath-AT1G17260.1 |  | Vvi-Vitvi09g00006\_t001 |  |  |  |  |  |  |  |
| 1 | Ath-AT1G17270.2 |  | Vvi-Vitvi09g00010\_t001 |  |  |  |  |  |  |  |
| 1 | Ath-AT1G17280.4 |  | Vvi-Vitvi09g00014\_t002 |  |  |  |  |  |  |  |
| 1 | Ath-AT1G17285.2 |  | | | |  |  |  |  |  |  |  |
| 1 | Ath-AT1G17290.1 |  | Vvi-Vitvi09g00019\_t001 |  |  |  |  |  |  |  |
| 1 | Ath-AT1G17300.2 |  | | | |  |  |  |  |  |  |  |
| 1 | Ath-AT1G17310.2 |  | | | |  |  |  |  |  |  |  |
| 1 | Ath-AT1G17330.1 |  | | | |  |  |  |  |  |  |  |
| 1 | Ath-AT1G17340.1 |  | Vvi-Vitvi09g00042\_t001 |  |  |  |  |  |  |  |
| 1 | Ath-AT1G17345.1 |  | Vvi-Vitvi09g00046\_t001 |  |  |  |  |  |  |  |
| 1 | Ath-AT1G17350.2 |  | Vvi-Vitvi09g00047\_t002 |  |  |  |  |  |  |  |
| 1 | Ath-AT1G17360.1 |  | Vvi-Vitvi09g00052\_t001 |  |  |  |  |  |  |  |
| 1 | Ath-AT1G17370.1 |  | Vvi-Vitvi09g00063\_t001 |  |  |  |  |  |  |  |
| 1 | Ath-AT1G17380.1 |  | Vvi-Vitvi09g00064\_t001 |  |  |  |  |  |  |  |
| 1 | Ath-AT1G17400.1 |  | Vvi-Vitvi09g01501\_t001 |  |  |  |  |  |  |  |
| 1 | Ath-AT1G17410.1 |  | Vvi-Vitvi09g00070\_t001 |  |  |  |  |  |  |  |
| 1 | Ath-AT1G17420.1 |  | Vvi-Vitvi09g00085\_t002 |  |  |  |  |  |  |  |
| 1 | Ath-AT1G17430.1 |  | Vvi-Vitvi09g00095\_t001 |  |  |  |  |  |  |  |
| 1 | Ath-AT1G17440.1 |  | Vvi-Vitvi09g00115\_t001 |  |  |  |  |  |  |  |
| 1 | Ath-AT1G17450.2 |  | Vvi-Vitvi09g00116\_t001 |  |  |  |  |  |  |  |
| 1 | Ath-AT1G17455.1 |  | Vvi-Vitvi09g00117\_t001 |  |  |  |  |  |  |  |
| 1 | Ath-AT1G17460.2 |  | Vvi-Vitvi09g00122\_t001 |  |  |  |  |  |  |  |
| 1 | Ath-AT1G17470.1 |  | Vvi-Vitvi09g00123\_t001 |  |  |  |  |  |  |  |
| 1 | Ath-AT1G17480.1 |  | Vvi-Vitvi09g00143\_t001 |  |  |  |  |  |  |  |
| 1 | Ath-AT1G17490.1 |  | Vvi-Vitvi09g04034\_t001 |  |  |  |  |  |  |  |
| 1 | Ath-AT1G17500.1 |  | Vvi-Vitvi09g00147\_t001 |  |  |  |  |  |  |  |
| 1 | Ath-AT1G17510.1 |  | | | |  |  |  |  |  |  |  |
| 1 | Ath-AT1G17520.2 |  | | | |  |  |  |  |  |  |  |
| 1 | Ath-AT1G17530.1 |  | Vvi-Vitvi09g00152\_t001 |  |  |  |  |  |  |  |
| 1 | Ath-AT1G17540.1 |  | Vvi-Vitvi09g00155\_t001 |  |  |  |  |  |  |  |
| 1 | Ath-AT1G17545.1 |  | | | |  |  |  |  |  |  |  |
| 1 | Ath-AT1G17550.1 |  | Vvi-Vitvi09g00156\_t002 |  |  |  |  |  |  |  |
| 1 | Ath-AT1G17560.1 |  | | | |  |  |  |  |  |  |  |
| 1 | Ath-AT1G17580.1 |  | Vvi-Vitvi09g00162\_t001 |  |  |  |  |  |  |  |
| 1 | Ath-AT1G17590.1 |  | | | |  |  |  |  |  |  |  |
| 1 | Ath-AT1G17600.1 |  | | | |  |  |  |  |  |  |  |
| 1 | Ath-AT1G17610.1 |  | | | |  |  |  |  |  |  |  |
| 1 | Ath-AT1G17615.1 |  | | | |  |  |  |  |  |  |  |
| 2 | Ath-AT1G17620.1 |  | | | |  | Vvi-Vitvi09g00185\_t001 |  |  |  |  |  |  |
| 2 | Ath-AT1G17630.1 |  | | | |  | Vvi-Vitvi09g00187\_t001 |  |  |  |  |  |  |
| 2 | Ath-AT1G17640.1 |  | | | |  | | | |  |  |  |  |  |  |
| 2 | Ath-AT1G17650.1 |  | | | |  | Vvi-Vitvi09g00195\_t001 |  |  |  |  |  |  |
| 2 | Ath-AT1G17665.1 |  | Vvi-Vitvi09g00178\_t001 |  | | | |  |  |  |  |  |  |
| 1 | Ath-AT1G17680.2 |  |  |  | | | |  |  |  |  |  |  |
| 1 | Ath-AT1G17690.1 |  |  |  | | | |  |  |  |  |  |  |
| 1 | Ath-AT1G17700.1 |  |  |  | Vvi-Vitvi09g00201\_t001 |  |  |  |  |  |  |
| 1 | Ath-AT1G17710.1 |  |  |  | Vvi-Vitvi09g01542\_t002 |  |  |  |  |  |  |
| 1 | Ath-AT1G17720.1 |  |  |  | Vvi-Vitvi09g00208\_t001 |  |  |  |  |  |  |
| 1 | Ath-AT1G17730.1 |  |  |  | Vvi-Vitvi09g00218\_t001 |  |  |  |  |  |  |
| 0 | Ath-AT1G17744.1 |  |  |  |  |  |  |  |  |
| 0 | Ath-AT1G17745.2 |  |  |  |  |  |  |  |  |
| 0 | Ath-AT1G17750.1 |  |  |  |  |  |  |  |  |
| 0 | Ath-AT1G17760.1 |  |  |  |  |  |  |  |  |
| 0 | Ath-AT1G17770.1 |  |  |  |  |  |  |  |  |
| 0 | Ath-AT1G17780.2 |  |  |  |  |  |  |  |  |
| 0 | Ath-AT1G17790.1 |  |  |  |  |  |  |  |  |
| 0 | Ath-AT1G17800.1 |  |  |  |  |  |  |  |  |
| 0 | Ath-AT1G17810.1 |  |  |  |  |  |  |  |  |
| 0 | Ath-AT1G17820.1 |  |  |  |  |  |  |  |  |
| 0 | Ath-AT1G17830.1 |  |  |  |  |  |  |  |  |
| 0 | Ath-AT1G17840.1 |  |  |  |  |  |  |  |  |
| 0 | Ath-AT1G17850.2 |  |  |  |  |  |  |  |  |
| 0 | Ath-AT1G17860.1 |  |  |  |  |  |  |  |  |
| 0 | Ath-AT1G17870.1 |  |  |  |  |  |  |  |  |
| 0 | Ath-AT1G17880.1 |  |  |  |  |  |  |  |  |
| 0 | Ath-AT1G17890.1 |  |  |  |  |  |  |  |  |
| 0 | Ath-AT1G17910.1 |  |  |  |  |  |  |  |  |
| 0 | Ath-AT1G17920.1 |  |  |  |  |  |  |  |  |
| 0 | Ath-AT1G17930.1 |  |  |  |  |  |  |  |  |
| 0 | Ath-AT1G17940.3 |  |  |  |  |  |  |  |  |
| 0 | Ath-AT1G17950.1 |  |  |  |  |  |  |  |  |
| 0 | Ath-AT1G17960.1 |  |  |  |  |  |  |  |  |
| 1 | Ath-AT1G17970.1 |  | Vvi-Vitvi17g00277\_t001 |  |  |  |  |  |  |  |
| 1 | Ath-AT1G17980.1 |  | Vvi-Vitvi17g00270\_t001 |  |  |  |  |  |  |  |
| 1 | Ath-AT1G17990.1 |  | | | |  |  |  |  |  |  |  |
| 1 | Ath-AT1G18000.1 |  | | | |  |  |  |  |  |  |  |
| 1 | Ath-AT1G18010.1 |  | | | |  |  |  |  |  |  |  |
| 1 | Ath-AT1G18020.1 |  | | | |  |  |  |  |  |  |  |
| 1 | Ath-AT1G18030.1 |  | Vvi-Vitvi17g00268\_t001 |  |  |  |  |  |  |  |
| 1 | Ath-AT1G18040.1 |  | Vvi-Vitvi17g00255\_t001 |  |  |  |  |  |  |  |
| 1 | Ath-AT1G18050.1 |  | | | |  |  |  |  |  |  |  |
| 1 | Ath-AT1G18060.1 |  | Vvi-Vitvi17g00254\_t001 |  |  |  |  |  |  |  |
| 1 | Ath-AT1G18070.3 |  | Vvi-Vitvi17g00250\_t001 |  |  |  |  |  |  |  |
| 1 | Ath-AT1G18080.1 |  | Vvi-Vitvi17g00239\_t001 |  |  |  |  |  |  |  |
| 1 | Ath-AT1G18090.1 |  | Vvi-Vitvi17g00235\_t001 |  |  |  |  |  |  |  |
| 1 | Ath-AT1G18100.1 |  | Vvi-Vitvi17g00229\_t001 |  |  |  |  |  |  |  |
| 1 | Ath-AT1G18130.1 |  | | | |  |  |  |  |  |  |  |
| 1 | Ath-AT1G18140.1 |  | Vvi-Vitvi17g00227\_t001 |  |  |  |  |  |  |  |
| 1 | Ath-AT1G18150.3 |  | Vvi-Vitvi17g00225\_t001 |  |  |  |  |  |  |  |
| 1 | Ath-AT1G18160.1 |  | Vvi-Vitvi17g00222\_t001 |  |  |  |  |  |  |  |
| 1 | Ath-AT1G18170.1 |  | Vvi-Vitvi17g01370\_t001 |  |  |  |  |  |  |  |
| 1 | Ath-AT1G18180.1 |  | Vvi-Vitvi17g04052\_t001 |  |  |  |  |  |  |  |
| 1 | Ath-AT1G18190.1 |  | Vvi-Vitvi17g00220\_t001 |  |  |  |  |  |  |  |
| 1 | Ath-AT1G18193.1 |  | | | |  |  |  |  |  |  |  |
| 1 | Ath-AT1G18197.2 |  | | | |  |  |  |  |  |  |  |
| 1 | Ath-AT1G18200.1 |  | Vvi-Vitvi17g00219\_t001 |  |  |  |  |  |  |  |
| 1 | Ath-AT1G18210.2 |  | Vvi-Vitvi17g00218\_t001 |  |  |  |  |  |  |  |
| 1 | Ath-AT1G18220.1 |  | | | |  |  |  |  |  |  |  |
| 1 | Ath-AT1G18250.2 |  | Vvi-Vitvi17g00217\_t001 |  |  |  |  |  |  |  |
| 0 | Ath-AT1G18260.1 |  |  |  |  |  |  |  |  |
| 0 | Ath-AT1G18265.1 |  |  |  |  |  |  |  |  |
| 0 | Ath-AT1G18270.3 |  |  |  |  |  |  |  |  |
| 0 | Ath-AT1G18280.1 |  |  |  |  |  |  |  |  |
| 0 | Ath-AT1G18290.1 |  |  |  |  |  |  |  |  |
| 0 | Ath-AT1G18300.1 |  |  |  |  |  |  |  |  |
| 0 | Ath-AT1G18310.1 |  |  |  |  |  |  |  |  |
| 0 | Ath-AT1G18330.2 |  |  |  |  |  |  |  |  |
| 0 | Ath-AT1G18335.2 |  |  |  |  |  |  |  |  |
| 0 | Ath-AT1G18340.1 |  |  |  |  |  |  |  |  |
| 0 | Ath-AT1G18350.1 |  |  |  |  |  |  |  |  |
| 0 | Ath-AT1G18360.1 |  |  |  |  |  |  |  |  |
| 0 | Ath-AT1G18370.1 |  |  |  |  |  |  |  |  |
| 0 | Ath-AT1G18390.2 |  |  |  |  |  |  |  |  |
| 1 | Ath-AT1G18400.1 |  | Vvi-Vitvi17g00311\_t001 |  |  |  |  |  |  |  |
| 1 | Ath-AT1G18410.4 |  | Vvi-Vitvi17g00321\_t001 |  |  |  |  |  |  |  |
| 1 | Ath-AT1G18420.1 |  | Vvi-Vitvi17g00333\_t001 |  |  |  |  |  |  |  |
| 1 | Ath-AT1G18440.1 |  | Vvi-Vitvi17g00349\_t001 |  |  |  |  |  |  |  |
| 1 | Ath-AT1G18450.1 |  | Vvi-Vitvi17g00351\_t002 |  |  |  |  |  |  |  |
| 1 | Ath-AT1G18460.1 |  | Vvi-Vitvi17g00359\_t001 |  |  |  |  |  |  |  |
| 1 | Ath-AT1G18470.1 |  | Vvi-Vitvi17g00372\_t001 |  |  |  |  |  |  |  |
| 1 | Ath-AT1G18480.1 |  | | | |  |  |  |  |  |  |  |
| 1 | Ath-AT1G18485.1 |  | | | |  |  |  |  |  |  |  |
| 1 | Ath-AT1G18486.1 |  | | | |  |  |  |  |  |  |  |
| 1 | Ath-AT1G18490.1 |  | | | |  |  |  |  |  |  |  |
| 1 | Ath-AT1G18500.1 |  | | | |  |  |  |  |  |  |  |
| 1 | Ath-AT1G18510.1 |  | | | |  |  |  |  |  |  |  |
| 1 | Ath-AT1G18520.1 |  | | | |  |  |  |  |  |  |  |
| 1 | Ath-AT1G18530.1 |  | | | |  |  |  |  |  |  |  |
| 1 | Ath-AT1G18540.1 |  | | | |  |  |  |  |  |  |  |
| 1 | Ath-AT1G18550.1 |  | | | |  |  |  |  |  |  |  |
| 1 | Ath-AT1G18560.1 |  | Vvi-Vitvi17g00382\_t003 |  |  |  |  |  |  |  |
| 0 | Ath-AT1G18570.1 |  |  |  |  |  |  |  |  |
| 0 | Ath-AT1G18580.1 |  |  |  |  |  |  |  |  |
| 0 | Ath-AT1G18590.1 |  |  |  |  |  |  |  |  |
| 0 | Ath-AT1G18600.1 |  |  |  |  |  |  |  |  |
| 0 | Ath-AT1G18610.2 |  |  |  |  |  |  |  |  |
| 0 | Ath-AT1G18620.2 |  |  |  |  |  |  |  |  |
| 0 | Ath-AT1G18630.1 |  |  |  |  |  |  |  |  |
| 0 | Ath-AT1G18640.2 |  |  |  |  |  |  |  |  |
| 0 | Ath-AT1G18650.1 |  |  |  |  |  |  |  |  |
| 1 | Ath-AT1G18660.4 |  | Vvi-Vitvi17g00643\_t001 |  |  |  |  |  |  |  |
| 1 | Ath-AT1G18670.3 |  | | | |  |  |  |  |  |  |  |
| 1 | Ath-AT1G18680.1 |  | | | |  |  |  |  |  |  |  |
| 1 | Ath-AT1G18690.2 |  | Vvi-Vitvi17g00642\_t001 |  |  |  |  |  |  |  |
| 1 | Ath-AT1G18700.5 |  | Vvi-Vitvi17g00624\_t001 |  |  |  |  |  |  |  |
| 1 | Ath-AT1G18710.1 |  | | | |  |  |  |  |  |  |  |
| 1 | Ath-AT1G18720.2 |  | Vvi-Vitvi17g00621\_t001 |  |  |  |  |  |  |  |
| 1 | Ath-AT1G18730.1 |  | Vvi-Vitvi17g04171\_t001 |  |  |  |  |  |  |  |
| 1 | Ath-AT1G18740.1 |  | | | |  |  |  |  |  |  |  |
| 1 | Ath-AT1G18750.3 |  | Vvi-Vitvi17g00614\_t001 |  |  |  |  |  |  |  |
| 1 | Ath-AT1G18760.1 |  | | | |  |  |  |  |  |  |  |
| 1 | Ath-AT1G18770.1 |  | | | |  |  |  |  |  |  |  |
| 1 | Ath-AT1G18773.4 |  | | | |  |  |  |  |  |  |  |
| 1 | Ath-AT1G18780.1 |  | | | |  |  |  |  |  |  |  |
| 1 | Ath-AT1G18790.1 |  | Vvi-Vitvi17g00605\_t001 |  |  |  |  |  |  |  |
| 1 | Ath-AT1G18800.1 |  | | | |  |  |  |  |  |  |  |
| 1 | Ath-AT1G18810.1 |  | | | |  |  |  |  |  |  |  |
| 1 | Ath-AT1G18830.1 |  | | | |  |  |  |  |  |  |  |
| 1 | Ath-AT1G18835.1 |  | Vvi-Vitvi17g00600\_t001 |  |  |  |  |  |  |  |
| 0 | Ath-AT1G18840.3 |  |  |  |  |  |  |  |  |
| 0 | Ath-AT1G18850.1 |  |  |  |  |  |  |  |  |
| 0 | Ath-AT1G18860.1 |  |  |  |  |  |  |  |  |
| 0 | Ath-AT1G18870.1 |  |  |  |  |  |  |  |  |
| 0 | Ath-AT1G18871.1 |  |  |  |  |  |  |  |  |
| 1 | Ath-AT1G18880.1 |  | Vvi-Vitvi17g00528\_t001 |  |  |  |  |  |  |  |
| 1 | Ath-AT1G18890.1 |  | Vvi-Vitvi17g00523\_t001 |  |  |  |  |  |  |  |
| 1 | Ath-AT1G18900.3 |  | Vvi-Vitvi17g00522\_t001 |  |  |  |  |  |  |  |
| 1 | Ath-AT1G18910.1 |  | Vvi-Vitvi17g00519\_t001 |  |  |  |  |  |  |  |
| 1 | Ath-AT1G18940.1 |  | Vvi-Vitvi17g00516\_t001 |  |  |  |  |  |  |  |
| 1 | Ath-AT1G18950.2 |  | Vvi-Vitvi17g00512\_t001 |  |  |  |  |  |  |  |
| 1 | Ath-AT1G18960.1 |  | | | |  |  |  |  |  |  |  |
| 1 | Ath-AT1G18970.1 |  | Vvi-Vitvi17g01451\_t001 |  |  |  |  |  |  |  |
| 1 | Ath-AT1G18975.1 |  | | | |  |  |  |  |  |  |  |
| 1 | Ath-AT1G18980.1 |  | | | |  |  |  |  |  |  |  |
| 1 | Ath-AT1G18990.1 |  | Vvi-Vitvi17g00504\_t001 |  |  |  |  |  |  |  |
| 0 | Ath-AT1G19000.2 |  |  |  |  |  |  |  |  |
| 0 | Ath-AT1G19010.1 |  |  |  |  |  |  |  |  |
| 0 | Ath-AT1G19020.1 |  |  |  |  |  |  |  |  |
| 0 | Ath-AT1G19025.1 |  |  |  |  |  |  |  |  |
| 0 | Ath-AT1G19040.1 |  |  |  |  |  |  |  |  |
| 0 | Ath-AT1G19050.1 |  |  |  |  |  |  |  |  |
| 0 | Ath-AT1G19060.1 |  |  |  |  |  |  |  |  |
| 0 | Ath-AT1G19080.2 |  |  |  |  |  |  |  |  |
| 0 | Ath-AT1G19070.1 |  |  |  |  |  |  |  |  |
| 0 | Ath-AT1G19086.1 |  |  |  |  |  |  |  |  |
| 0 | Ath-AT1G19100.1 |  |  |  |  |  |  |  |  |
| 1 | Ath-AT1G19110.1 |  | Vvi-Vitvi11g00061\_t001 |  |  |  |  |  |  |  |
| 1 | Ath-AT1G19115.4 |  | Vvi-Vitvi11g01327\_t001 |  |  |  |  |  |  |  |
| 1 | Ath-AT1G19120.1 |  | Vvi-Vitvi11g00058\_t001 |  |  |  |  |  |  |  |
| 1 | Ath-AT1G19130.1 |  | Vvi-Vitvi11g00057\_t001 |  |  |  |  |  |  |  |
| 1 | Ath-AT1G19140.2 |  | Vvi-Vitvi11g00055\_t001 |  |  |  |  |  |  |  |
| 1 | Ath-AT1G19150.1 |  | Vvi-Vitvi11g00052\_t001 |  |  |  |  |  |  |  |
| 1 | Ath-AT1G19160.1 |  | | | |  |  |  |  |  |  |  |
| 1 | Ath-AT1G19170.1 |  | Vvi-Vitvi11g00051\_t003 |  |  |  |  |  |  |  |
| 1 | Ath-AT1G19180.1 |  | Vvi-Vitvi11g00050\_t001 |  |  |  |  |  |  |  |
| 1 | Ath-AT1G19190.1 |  | | | |  |  |  |  |  |  |  |
| 1 | Ath-AT1G19200.2 |  | Vvi-Vitvi11g00048\_t001 |  |  |  |  |  |  |  |
| 1 | Ath-AT1G19210.1 |  | Vvi-Vitvi11g00045\_t001 |  |  |  |  |  |  |  |
| 1 | Ath-AT1G19220.1 |  | Vvi-Vitvi11g00043\_t001 |  |  |  |  |  |  |  |
| 1 | Ath-AT1G19230.2 |  | | | |  |  |  |  |  |  |  |
| 1 | Ath-AT1G19240.1 |  | | | |  |  |  |  |  |  |  |
| 1 | Ath-AT1G19250.1 |  | Vvi-Vitvi11g00039\_t001 |  |  |  |  |  |  |  |
| 0 | Ath-AT1G19260.1 |  |  |  |  |  |  |  |  |
| 1 | Ath-AT1G19270.1 |  | Vvi-Vitvi18g00903\_t001 |  |  |  |  |  |  |  |
| 1 | Ath-AT1G19290.1 |  | Vvi-Vitvi18g00908\_t001 |  |  |  |  |  |  |  |
| 1 | Ath-AT1G19300.1 |  | Vvi-Vitvi18g00909\_t001 |  |  |  |  |  |  |  |
| 1 | Ath-AT1G19310.1 |  | Vvi-Vitvi18g00912\_t001 |  |  |  |  |  |  |  |
| 1 | Ath-AT1G19320.1 |  | Vvi-Vitvi18g00917\_t001 |  |  |  |  |  |  |  |
| 1 | Ath-AT1G19330.3 |  | Vvi-Vitvi18g00919\_t001 |  |  |  |  |  |  |  |
| 1 | Ath-AT1G19340.1 |  | Vvi-Vitvi18g00920\_t001 |  |  |  |  |  |  |  |
| 1 | Ath-AT1G19350.3 |  | Vvi-Vitvi18g00924\_t001 |  |  |  |  |  |  |  |
| 1 | Ath-AT1G19360.2 |  | Vvi-Vitvi18g00931\_t001 |  |  |  |  |  |  |  |
| 1 | Ath-AT1G19370.1 |  | Vvi-Vitvi18g00941\_t001 |  |  |  |  |  |  |  |
| 1 | Ath-AT1G19380.1 |  | Vvi-Vitvi18g00948\_t001 |  |  |  |  |  |  |  |
| 1 | Ath-AT1G19390.1 |  | | | |  |  |  |  |  |  |  |
| 1 | Ath-AT1G19394.1 |  | | | |  |  |  |  |  |  |  |
| 1 | Ath-AT1G19396.2 |  | | | |  |  |  |  |  |  |  |
| 1 | Ath-AT1G19397.1 |  | | | |  |  |  |  |  |  |  |
| 2 | Ath-AT1G19400.2 |  | Vvi-Vitvi18g00958\_t001 |  | Vvi-Vitvi03g00334\_t001 |  |  |  |  |  |  |
| 2 | Ath-AT1G19410.1 |  | | | |  | | | |  |  |  |  |  |  |
| 2 | Ath-AT1G19430.1 |  | Vvi-Vitvi18g00960\_t001 |  | | | |  |  |  |  |  |  |
| 2 | Ath-AT1G19440.1 |  | Vvi-Vitvi18g00969\_t001 |  | Vvi-Vitvi03g00322\_t001 |  |  |  |  |  |  |
| 2 | Ath-AT1G19450.1 |  | Vvi-Vitvi18g00970\_t002 |  | | | |  |  |  |  |  |  |
| 2 | Ath-AT1G19460.1 |  | | | |  | | | |  |  |  |  |  |  |
| 2 | Ath-AT1G19470.1 |  | | | |  | | | |  |  |  |  |  |  |
| 2 | Ath-AT1G19480.1 |  | Vvi-Vitvi18g00971\_t001 |  | Vvi-Vitvi03g00319\_t001 |  |  |  |  |  |  |
| 2 | Ath-AT1G19485.2 |  | | | |  | | | |  |  |  |  |  |  |
| 2 | Ath-AT1G19490.1 |  | | | |  | | | |  |  |  |  |  |  |
| 2 | Ath-AT1G19500.1 |  | | | |  | | | |  |  |  |  |  |  |
| 2 | Ath-AT1G19510.1 |  | Vvi-Vitvi18g00973\_t001 |  | Vvi-Vitvi03g01495\_t001 |  |  |  |  |  |  |
| 2 | Ath-AT1G19520.1 |  | Vvi-Vitvi18g00975\_t001 |  | | | |  |  |  |  |  |  |
| 2 | Ath-AT1G19530.1 |  | Vvi-Vitvi18g02754\_t002 |  | | | |  |  |  |  |  |  |
| 2 | Ath-AT1G19540.1 |  | Vvi-Vitvi18g00979\_t001 |  | Vvi-Vitvi03g01484\_t001 |  |  |  |  |  |  |
| 2 | Ath-AT1G19550.1 |  | | | |  | | | |  |  |  |  |  |  |
| 2 | Ath-AT1G19565.1 |  | | | |  | | | |  |  |  |  |  |  |
| 2 | Ath-AT1G19570.1 |  | | | |  | | | |  |  |  |  |  |  |
| 2 | Ath-AT1G19580.1 |  | Vvi-Vitvi18g00981\_t001 |  | | | |  |  |  |  |  |  |
| 2 | Ath-AT1G19600.1 |  | Vvi-Vitvi18g02757\_t001 |  | | | |  |  |  |  |  |  |
| 2 | Ath-AT1G19610.1 |  | Vvi-Vitvi18g02760\_t001 |  | | | |  |  |  |  |  |  |
| 2 | Ath-AT1G19630.1 |  | Vvi-Vitvi18g00993\_t001 |  | | | |  |  |  |  |  |  |
| 2 | Ath-AT1G19620.1 |  | | | |  | | | |  |  |  |  |  |  |
| 2 | Ath-AT1G19640.1 |  | Vvi-Vitvi18g02761\_t002 |  | Vvi-Vitvi03g00299\_t001 |  |  |  |  |  |  |
| 2 | Ath-AT1G19650.1 |  | Vvi-Vitvi18g00997\_t001 |  | Vvi-Vitvi03g00294\_t001 |  |  |  |  |  |  |
| 2 | Ath-AT1G19660.1 |  | Vvi-Vitvi18g00998\_t001 |  | | | |  |  |  |  |  |  |
| 2 | Ath-AT1G19670.1 |  | | | |  | | | |  |  |  |  |  |  |
| 2 | Ath-AT1G19680.1 |  | Vvi-Vitvi18g02767\_t001 |  | Vvi-Vitvi03g00288\_t001 |  |  |  |  |  |  |
| 1 | Ath-AT1G19690.1 |  | Vvi-Vitvi18g01008\_t001 |  |  |  |  |  |  |  |
| 1 | Ath-AT1G19700.3 |  | | | |  |  |  |  |  |  |  |
| 1 | Ath-AT1G19710.1 |  | Vvi-Vitvi18g01013\_t002 |  |  |  |  |  |  |  |
| 1 | Ath-AT1G19715.3 |  | Vvi-Vitvi18g02773\_t001 |  |  |  |  |  |  |  |
| 1 | Ath-AT1G19720.1 |  | Vvi-Vitvi18g01020\_t001 |  |  |  |  |  |  |  |
| 1 | Ath-AT1G19730.1 |  | Vvi-Vitvi18g02775\_t001 |  |  |  |  |  |  |  |
| 1 | Ath-AT1G19740.1 |  | Vvi-Vitvi18g01023\_t001 |  |  |  |  |  |  |  |
| 1 | Ath-AT1G19750.1 |  | | | |  |  |  |  |  |  |  |
| 1 | Ath-AT1G19770.1 |  | | | |  |  |  |  |  |  |  |
| 1 | Ath-AT1G19780.1 |  | | | |  |  |  |  |  |  |  |
| 1 | Ath-AT1G19790.1 |  | Vvi-Vitvi18g01041\_t001 |  |  |  |  |  |  |  |
| 1 | Ath-AT1G19800.1 |  | | | |  |  |  |  |  |  |  |
| 1 | Ath-AT1G19830.1 |  | | | |  |  |  |  |  |  |  |
| 2 | Ath-AT1G19835.1 |  | | | |  | Vvi-Vitvi18g01092\_t001.2.6037826c |  |  |  |  |  |  |
| 3 | Ath-AT1G19840.1 |  | | | |  | | | |  | Vvi-Vitvi18g01091\_t001 |  |  |  |  |  |
| 3 | Ath-AT1G19850.1 |  | | | |  | Vvi-Vitvi18g01086\_t001 |  | | | |  |  |  |  |  |
| 3 | Ath-AT1G19860.2 |  | | | |  | Vvi-Vitvi18g01081\_t001 |  | | | |  |  |  |  |  |
| 3 | Ath-AT1G19870.1 |  | | | |  | Vvi-Vitvi18g01080\_t001 |  | | | |  |  |  |  |  |
| 3 | Ath-AT1G19880.1 |  | | | |  | Vvi-Vitvi18g01069\_t001 |  | | | |  |  |  |  |  |
| 3 | Ath-AT1G19890.1 |  | | | |  | | | |  | | | |  |  |  |  |  |
| 3 | Ath-AT1G19900.1 |  | | | |  | Vvi-Vitvi18g01063\_t001 |  | | | |  |  |  |  |  |
| 2 | Ath-AT1G19910.1 |  | Vvi-Vitvi18g01058\_t001 |  |  |  | | | |  |  |  |  |  |
| 1 | Ath-AT1G19920.1 |  |  |  |  |  | Vvi-Vitvi18g01095\_t001 |  |  |  |  |  |
| 1 | Ath-AT1G19930.1 |  |  |  |  |  | | | |  |  |  |  |  |
| 1 | Ath-AT1G19940.1 |  |  |  |  |  | Vvi-Vitvi18g01098\_t001 |  |  |  |  |  |
| 1 | Ath-AT1G19950.1 |  |  |  |  |  | Vvi-Vitvi18g01105\_t002 |  |  |  |  |  |
| 1 | Ath-AT1G19960.1 |  |  |  |  |  | | | |  |  |  |  |  |
| 1 | Ath-AT1G19970.1 |  |  |  |  |  | Vvi-Vitvi18g01116\_t001 |  |  |  |  |  |
| 1 | Ath-AT1G19980.1 |  |  |  |  |  | Vvi-Vitvi18g04267\_t001 |  |  |  |  |  |
| 1 | Ath-AT1G19990.1 |  |  |  |  |  | Vvi-Vitvi18g02799\_t001 |  |  |  |  |  |
| 1 | Ath-AT1G20000.1 |  |  |  |  |  | | | |  |  |  |  |  |
| 1 | Ath-AT1G20010.1 |  |  |  |  |  | Vvi-Vitvi18g01120\_t001 |  |  |  |  |  |
| 1 | Ath-AT1G20020.1 |  |  |  |  |  | Vvi-Vitvi18g01130\_t001 |  |  |  |  |  |
| 2 | Ath-AT1G20030.2 |  | Vvi-Vitvi03g00123\_t001 |  |  |  | Vvi-Vitvi18g01131\_t001 |  |  |  |  |  |
| 2 | Ath-AT1G20050.1 |  | | | |  |  |  | | | |  |  |  |  |  |
| 2 | Ath-AT1G20060.1 |  | | | |  |  |  | Vvi-Vitvi18g01142\_t001 |  |  |  |  |  |
| 2 | Ath-AT1G20065.2 |  | | | |  |  |  | Vvi-Vitvi18g02813\_t001 |  |  |  |  |  |
| 2 | Ath-AT1G20070.1 |  | | | |  |  |  | | | |  |  |  |  |  |
| 2 | Ath-AT1G20080.2 |  | Vvi-Vitvi03g00138\_t001 |  |  |  | | | |  |  |  |  |  |
| 2 | Ath-AT1G20090.1 |  | Vvi-Vitvi03g00143\_t001 |  |  |  | | | |  |  |  |  |  |
| 2 | Ath-AT1G20100.1 |  | | | |  |  |  | | | |  |  |  |  |  |
| 2 | Ath-AT1G20110.1 |  | | | |  |  |  | Vvi-Vitvi18g01157\_t001 |  |  |  |  |  |
| 2 | Ath-AT1G20120.1 |  | | | |  |  |  | | | |  |  |  |  |  |
| 2 | Ath-AT1G20130.1 |  | | | |  |  |  | | | |  |  |  |  |  |
| 2 | Ath-AT1G20132.1 |  | | | |  |  |  | | | |  |  |  |  |  |
| 2 | Ath-AT1G20135.1 |  | | | |  |  |  | | | |  |  |  |  |  |
| 2 | Ath-AT1G20140.1 |  | Vvi-Vitvi03g00152\_t001 |  |  |  | Vvi-Vitvi18g01161\_t001 |  |  |  |  |  |
| 2 | Ath-AT1G20150.1 |  | | | |  |  |  | Vvi-Vitvi18g01162\_t001 |  |  |  |  |  |
| 2 | Ath-AT1G20160.1 |  | | | |  |  |  | | | |  |  |  |  |  |
| 2 | Ath-AT1G20180.1 |  | | | |  |  |  | Vvi-Vitvi18g01168\_t001 |  |  |  |  |  |
| 2 | Ath-AT1G20190.1 |  | | | |  | Vvi-Vitvi18g00189\_t001 |  |  |  |  |  |  |
| 2 | Ath-AT1G20200.1 |  | Vvi-Vitvi03g00161\_t001 |  | Vvi-Vitvi18g00188\_t002 |  |  |  |  |  |  |
| 2 | Ath-AT1G20220.1 |  | | | |  | Vvi-Vitvi18g00184\_t001 |  |  |  |  |  |  |
| 2 | Ath-AT1G20225.1 |  | | | |  | Vvi-Vitvi18g00183\_t001 |  |  |  |  |  |  |
| 2 | Ath-AT1G20230.1 |  | | | |  | Vvi-Vitvi18g00182\_t001 |  |  |  |  |  |  |
| 2 | Ath-AT1G20240.1 |  | | | |  | | | |  |  |  |  |  |  |
| 2 | Ath-AT1G20260.1 |  | Vvi-Vitvi03g00173\_t001 |  | Vvi-Vitvi18g00176\_t001 |  |  |  |  |  |  |
| 2 | Ath-AT1G20270.1 |  | | | |  | Vvi-Vitvi18g00172\_t001.3.6037826c |  |  |  |  |  |  |
| 2 | Ath-AT1G20280.1 |  | | | |  | | | |  |  |  |  |  |  |
| 2 | Ath-AT1G20290.1 |  | | | |  | | | |  |  |  |  |  |  |
| 2 | Ath-AT1G20300.1 |  | | | |  | Vvi-Vitvi18g00169\_t001 |  |  |  |  |  |  |
| 2 | Ath-AT1G20310.1 |  | | | |  | Vvi-Vitvi18g00164\_t001 |  |  |  |  |  |  |
| 2 | Ath-AT1G20320.1 |  | | | |  | | | |  |  |  |  |  |  |
| 2 | Ath-AT1G20330.1 |  | Vvi-Vitvi03g00185\_t001 |  | Vvi-Vitvi18g00163\_t001 |  |  |  |  |  |  |
| 1 | Ath-AT1G20340.1 |  |  |  | Vvi-Vitvi18g00158\_t001 |  |  |  |  |  |  |
| 1 | Ath-AT1G20350.1 |  |  |  | | | |  |  |  |  |  |  |
| 1 | Ath-AT1G20360.1 |  |  |  | | | |  |  |  |  |  |  |
| 1 | Ath-AT1G20370.1 |  |  |  | Vvi-Vitvi18g00146\_t001 |  |  |  |  |  |  |
| 1 | Ath-AT1G20380.1 |  |  |  | Vvi-Vitvi18g00142\_t001 |  |  |  |  |  |  |
| 1 | Ath-AT1G20400.1 |  |  |  | | | |  |  |  |  |  |  |
| 1 | Ath-AT1G20405.1 |  |  |  | | | |  |  |  |  |  |  |
| 1 | Ath-AT1G20410.1 |  |  |  | Vvi-Vitvi18g00140\_t001 |  |  |  |  |  |  |
| 1 | Ath-AT1G20430.1 |  |  |  | Vvi-Vitvi18g02500\_t001 |  |  |  |  |  |  |
| 2 | Ath-AT1G20440.1 |  | Vvi-Vitvi03g01463\_t001 |  | Vvi-Vitvi18g00130\_t001 |  |  |  |  |  |  |
| 2 | Ath-AT1G20450.1 |  | | | |  | | | |  |  |  |  |  |  |
| 2 | Ath-AT1G20460.1 |  | | | |  | Vvi-Vitvi18g02499\_t001 |  |  |  |  |  |  |
| 2 | Ath-AT1G20470.1 |  | | | |  | | | |  |  |  |  |  |  |
| 2 | Ath-AT1G20480.1 |  | | | |  | Vvi-Vitvi18g00124\_t003 |  |  |  |  |  |  |
| 2 | Ath-AT1G20490.1 |  | | | |  | | | |  |  |  |  |  |  |
| 2 | Ath-AT1G20500.1 |  | | | |  | | | |  |  |  |  |  |  |
| 2 | Ath-AT1G20510.1 |  | | | |  | | | |  |  |  |  |  |  |
| 2 | Ath-AT1G20520.1 |  | | | |  | Vvi-Vitvi18g00122\_t001 |  |  |  |  |  |  |
| 2 | Ath-AT1G20530.1 |  | | | |  | Vvi-Vitvi18g00119\_t001 |  |  |  |  |  |  |
| 2 | Ath-AT1G20540.1 |  | | | |  | Vvi-Vitvi18g00116\_t002 |  |  |  |  |  |  |
| 2 | Ath-AT1G20550.1 |  | | | |  | Vvi-Vitvi18g00114\_t001 |  |  |  |  |  |  |
| 2 | Ath-AT1G20560.1 |  | | | |  | | | |  |  |  |  |  |  |
| 2 | Ath-AT1G20570.3 |  | | | |  | | | |  |  |  |  |  |  |
| 2 | Ath-AT1G20575.1 |  | | | |  | Vvi-Vitvi18g00110\_t001 |  |  |  |  |  |  |
| 2 | Ath-AT1G20580.1 |  | Vvi-Vitvi03g00226\_t001 |  | Vvi-Vitvi18g00098\_t001 |  |  |  |  |  |  |
| 2 | Ath-AT1G20590.1 |  | | | |  | | | |  |  |  |  |  |  |
| 2 | Ath-AT1G20600.1 |  | | | |  | | | |  |  |  |  |  |  |
| 2 | Ath-AT1G20610.1 |  | | | |  | | | |  |  |  |  |  |  |
| 2 | Ath-AT1G20620.6 |  | | | |  | Vvi-Vitvi18g00095\_t001 |  |  |  |  |  |  |
| 2 | Ath-AT1G20630.1 |  | | | |  | | | |  |  |  |  |  |  |
| 2 | Ath-AT1G20640.3 |  | Vvi-Vitvi03g00231\_t001 |  | Vvi-Vitvi18g02488\_t001 |  |  |  |  |  |  |
| 2 | Ath-AT1G20650.1 |  | Vvi-Vitvi03g00239\_t001 |  | Vvi-Vitvi18g00090\_t001 |  |  |  |  |  |  |
| 2 | Ath-AT1G20670.1 |  | Vvi-Vitvi03g00240\_t001 |  | Vvi-Vitvi18g00088\_t001 |  |  |  |  |  |  |
| 2 | Ath-AT1G20680.1 |  | | | |  | Vvi-Vitvi18g00087\_t001 |  |  |  |  |  |  |
| 2 | Ath-AT1G20683.1 |  | | | |  | | | |  |  |  |  |  |  |
| 2 | Ath-AT1G20687.1 |  | | | |  | | | |  |  |  |  |  |  |
| 2 | Ath-AT1G20690.1 |  | | | |  | | | |  |  |  |  |  |  |
| 2 | Ath-AT1G20693.1 |  | | | |  | Vvi-Vitvi18g02486\_t001 |  |  |  |  |  |  |
| 2 | Ath-AT1G20696.2 |  | | | |  | | | |  |  |  |  |  |  |
| 2 | Ath-AT1G20700.1 |  | | | |  | Vvi-Vitvi18g00084\_t001 |  |  |  |  |  |  |
| 2 | Ath-AT1G20710.1 |  | | | |  | | | |  |  |  |  |  |  |
| 2 | Ath-AT1G20720.2 |  | | | |  | Vvi-Vitvi18g00082\_t001 |  |  |  |  |  |  |
| 2 | Ath-AT1G20730.1 |  | Vvi-Vitvi03g00241\_t001 |  | | | |  |  |  |  |  |  |
| 2 | Ath-AT1G20735.1 |  | | | |  | | | |  |  |  |  |  |  |
| 2 | Ath-AT1G20740.1 |  | | | |  | | | |  |  |  |  |  |  |
| 2 | Ath-AT1G20750.1 |  | | | |  | | | |  |  |  |  |  |  |
| 2 | Ath-AT1G20760.1 |  | Vvi-Vitvi03g00243\_t001 |  | Vvi-Vitvi18g00079\_t001 |  |  |  |  |  |  |
| 2 | Ath-AT1G20770.1 |  | | | |  | Vvi-Vitvi18g00078\_t001 |  |  |  |  |  |  |
| 2 | Ath-AT1G20780.1 |  | | | |  | Vvi-Vitvi18g00076\_t001 |  |  |  |  |  |  |
| 2 | Ath-AT1G20790.1 |  | | | |  | | | |  |  |  |  |  |  |
| 2 | Ath-AT1G20795.1 |  | | | |  | | | |  |  |  |  |  |  |
| 2 | Ath-AT1G20800.1 |  | | | |  | | | |  |  |  |  |  |  |
| 2 | Ath-AT1G20803.1 |  | | | |  | | | |  |  |  |  |  |  |
| 2 | Ath-AT1G20810.1 |  | | | |  | Vvi-Vitvi18g02483\_t001 |  |  |  |  |  |  |
| 2 | Ath-AT1G20816.1 |  | | | |  | Vvi-Vitvi18g00059\_t001 |  |  |  |  |  |  |
| 2 | Ath-AT1G20823.1 |  | Vvi-Vitvi03g01473\_t001 |  | Vvi-Vitvi18g00058\_t001 |  |  |  |  |  |  |
| 2 | Ath-AT1G20830.1 |  | | | |  | Vvi-Vitvi18g00057\_t001 |  |  |  |  |  |  |
| 2 | Ath-AT1G20840.1 |  | Vvi-Vitvi03g00247\_t001 |  | Vvi-Vitvi18g00056\_t002 |  |  |  |  |  |  |
| 2 | Ath-AT1G20850.1 |  | Vvi-Vitvi03g00253\_t001 |  | Vvi-Vitvi18g00051\_t001 |  |  |  |  |  |  |
| 2 | Ath-AT1G20860.2 |  | | | |  | Vvi-Vitvi18g00050\_t001 |  |  |  |  |  |  |
| 2 | Ath-AT1G20870.1 |  | | | |  | Vvi-Vitvi18g00049\_t001 |  |  |  |  |  |  |
| 2 | Ath-AT1G20875.1 |  | | | |  | | | |  |  |  |  |  |  |
| 2 | Ath-AT1G20880.1 |  | | | |  | Vvi-Vitvi18g00044\_t001 |  |  |  |  |  |  |
| 2 | Ath-AT1G20890.1 |  | | | |  | | | |  |  |  |  |  |  |
| 2 | Ath-AT1G20900.1 |  | Vvi-Vitvi03g00263\_t001 |  | Vvi-Vitvi18g00042\_t001 |  |  |  |  |  |  |
| 2 | Ath-AT1G20910.1 |  | | | |  | Vvi-Vitvi18g00041\_t001 |  |  |  |  |  |  |
| 2 | Ath-AT1G20920.1 |  | Vvi-Vitvi03g00265\_t001 |  | | | |  |  |  |  |  |  |
| 2 | Ath-AT1G20923.1 |  | | | |  | | | |  |  |  |  |  |  |
| 2 | Ath-AT1G20925.1 |  | | | |  | | | |  |  |  |  |  |  |
| 2 | Ath-AT1G20930.1 |  | | | |  | Vvi-Vitvi18g00040\_t001 |  |  |  |  |  |  |
| 2 | Ath-AT1G20940.1 |  | | | |  | | | |  |  |  |  |  |  |
| 2 | Ath-AT1G20950.1 |  | | | |  | Vvi-Vitvi18g00037\_t001 |  |  |  |  |  |  |
| 2 | Ath-AT1G20960.1 |  | | | |  | | | |  |  |  |  |  |  |
| 2 | Ath-AT1G20970.1 |  | | | |  | Vvi-Vitvi18g00030\_t001 |  |  |  |  |  |  |
| 2 | Ath-AT1G20980.1 |  | | | |  | Vvi-Vitvi18g00027\_t001 |  |  |  |  |  |  |
| 2 | Ath-AT1G20990.1 |  | Vvi-Vitvi03g00267\_t001 |  | Vvi-Vitvi18g00023\_t001 |  |  |  |  |  |  |
| 2 | Ath-AT1G21000.1 |  | | | |  | Vvi-Vitvi18g02472\_t001 |  |  |  |  |  |  |
| 2 | Ath-AT1G21010.1 |  | | | |  | Vvi-Vitvi18g00020\_t001 |  |  |  |  |  |  |
| 2 | Ath-AT1G21050.2 |  | | | |  | Vvi-Vitvi18g02471\_t001 |  |  |  |  |  |  |
| 2 | Ath-AT1G21060.1 |  | | | |  | Vvi-Vitvi18g00018\_t001 |  |  |  |  |  |  |
| 2 | Ath-AT1G21065.1 |  | | | |  | Vvi-Vitvi18g00011\_t001 |  |  |  |  |  |  |
| 2 | Ath-AT1G21070.1 |  | | | |  | Vvi-Vitvi18g00004\_t001 |  |  |  |  |  |  |
| 2 | Ath-AT1G21080.3 |  | Vvi-Vitvi03g00289\_t001 |  | Vvi-Vitvi18g00002\_t003 |  |  |  |  |  |  |
| 0 | Ath-AT1G21090.1 |  |  |  |  |  |  |  |  |
| 0 | Ath-AT1G21100.1 |  |  |  |  |  |  |  |  |
| 0 | Ath-AT1G21110.1 |  |  |  |  |  |  |  |  |
| 0 | Ath-AT1G21120.2 |  |  |  |  |  |  |  |  |
| 0 | Ath-AT1G21130.1 |  |  |  |  |  |  |  |  |
| 0 | Ath-AT1G21140.1 |  |  |  |  |  |  |  |  |
| 0 | Ath-AT1G21150.2 |  |  |  |  |  |  |  |  |
| 0 | Ath-AT1G21160.1 |  |  |  |  |  |  |  |  |
| 0 | Ath-AT1G21170.2 |  |  |  |  |  |  |  |  |
| 0 | Ath-AT1G21190.1 |  |  |  |  |  |  |  |  |
| 0 | Ath-AT1G21200.2 |  |  |  |  |  |  |  |  |
| 1 | Ath-AT1G21210.1 |  | Vvi-Vitvi18g02512\_t001 |  |  |  |  |  |  |  |
| 1 | Ath-AT1G21230.1 |  | | | |  |  |  |  |  |  |  |
| 1 | Ath-AT1G21240.1 |  | | | |  |  |  |  |  |  |  |
| 1 | Ath-AT1G21245.1 |  | | | |  |  |  |  |  |  |  |
| 1 | Ath-AT1G21250.1 |  | Vvi-Vitvi18g02513\_t001 |  |  |  |  |  |  |  |
| 1 | Ath-AT1G21270.1 |  | | | |  |  |  |  |  |  |  |
| 1 | Ath-AT1G21280.1 |  | | | |  |  |  |  |  |  |  |
| 1 | Ath-AT1G21310.1 |  | | | |  |  |  |  |  |  |  |
| 1 | Ath-AT1G21313.1 |  | | | |  |  |  |  |  |  |  |
| 1 | Ath-AT1G21320.1 |  | | | |  |  |  |  |  |  |  |
| 1 | Ath-AT1G21323.1 |  | | | |  |  |  |  |  |  |  |
| 1 | Ath-AT1G21326.1 |  | | | |  |  |  |  |  |  |  |
| 1 | Ath-AT1G21340.1 |  | | | |  |  |  |  |  |  |  |
| 1 | Ath-AT1G21350.5 |  | | | |  |  |  |  |  |  |  |
| 1 | Ath-AT1G21360.1 |  | | | |  |  |  |  |  |  |  |
| 1 | Ath-AT1G21370.2 |  | | | |  |  |  |  |  |  |  |
| 1 | Ath-AT1G21380.1 |  | | | |  |  |  |  |  |  |  |
| 2 | Ath-AT1G21390.1 |  | | | |  | Vvi-Vitvi18g01171\_t001 |  |  |  |  |  |  |
| 2 | Ath-AT1G21395.1 |  | | | |  | | | |  |  |  |  |  |  |
| 2 | Ath-AT1G21400.5 |  | | | |  | Vvi-Vitvi18g01174\_t001 |  |  |  |  |  |  |
| 2 | Ath-AT1G21410.1 |  | | | |  | Vvi-Vitvi18g01180\_t001 |  |  |  |  |  |  |
| 2 | Ath-AT1G21430.1 |  | | | |  | Vvi-Vitvi18g01200\_t001 |  |  |  |  |  |  |
| 2 | Ath-AT1G21440.1 |  | | | |  | | | |  |  |  |  |  |  |
| 2 | Ath-AT1G21450.1 |  | | | |  | Vvi-Vitvi18g01210\_t001 |  |  |  |  |  |  |
| 2 | Ath-AT1G21460.1 |  | | | |  | Vvi-Vitvi18g01215\_t001 |  |  |  |  |  |  |
| 2 | Ath-AT1G21470.2 |  | | | |  | | | |  |  |  |  |  |  |
| 2 | Ath-AT1G21480.1 |  | | | |  | Vvi-Vitvi18g01219\_t001 |  |  |  |  |  |  |
| 1 | Ath-AT1G21475.1 |  | | | |  |  |  |  |  |  |  |
| 1 | Ath-AT1G21500.1 |  | | | |  |  |  |  |  |  |  |
| 1 | Ath-AT1G21510.1 |  | Vvi-Vitvi18g02517\_t001 |  |  |  |  |  |  |  |
| 1 | Ath-AT1G21520.1 |  | | | |  |  |  |  |  |  |  |
| 1 | Ath-AT1G21528.1 |  | | | |  |  |  |  |  |  |  |
| 1 | Ath-AT1G21530.2 |  | | | |  |  |  |  |  |  |  |
| 1 | Ath-AT1G21540.1 |  | | | |  |  |  |  |  |  |  |
| 1 | Ath-AT1G21550.1 |  | Vvi-Vitvi18g00214\_t001 |  |  |  |  |  |  |  |
| 1 | Ath-AT1G21560.2 |  | Vvi-Vitvi18g02525\_t001 |  |  |  |  |  |  |  |
| 1 | Ath-AT1G21580.1 |  | Vvi-Vitvi18g00217\_t003 |  |  |  |  |  |  |  |
| 1 | Ath-AT1G21590.1 |  | Vvi-Vitvi18g04039\_t001 |  |  |  |  |  |  |  |
| 1 | Ath-AT1G21600.2 |  | Vvi-Vitvi18g00222\_t001 |  |  |  |  |  |  |  |
| 1 | Ath-AT1G21610.3 |  | Vvi-Vitvi18g00223\_t004 |  |  |  |  |  |  |  |
| 1 | Ath-AT1G21620.1 |  | | | |  |  |  |  |  |  |  |
| 1 | Ath-AT1G21630.2 |  | | | |  |  |  |  |  |  |  |
| 1 | Ath-AT1G21640.2 |  | Vvi-Vitvi18g00226\_t001 |  |  |  |  |  |  |  |
| 1 | Ath-AT1G21650.3 |  | Vvi-Vitvi18g00230\_t001.1.6037826c |  |  |  |  |  |  |  |
| 1 | Ath-AT1G21651.1 |  | Vvi-Vitvi18g00231\_t001 |  |  |  |  |  |  |  |
| 1 | Ath-AT1G21660.1 |  | Vvi-Vitvi18g00234\_t001 |  |  |  |  |  |  |  |
| 1 | Ath-AT1G21670.1 |  | Vvi-Vitvi18g00238\_t001 |  |  |  |  |  |  |  |
| 1 | Ath-AT1G21680.1 |  | | | |  |  |  |  |  |  |  |
| 1 | Ath-AT1G21690.3 |  | Vvi-Vitvi18g00240\_t001 |  |  |  |  |  |  |  |
| 1 | Ath-AT1G21695.1 |  | | | |  |  |  |  |  |  |  |
| 1 | Ath-AT1G21700.1 |  | Vvi-Vitvi18g00243\_t001 |  |  |  |  |  |  |  |
| 1 | Ath-AT1G21710.1 |  | Vvi-Vitvi18g00244\_t001 |  |  |  |  |  |  |  |
| 1 | Ath-AT1G21720.1 |  | Vvi-Vitvi18g00247\_t001 |  |  |  |  |  |  |  |
| 1 | Ath-AT1G21722.1 |  | Vvi-Vitvi18g00248\_t001 |  |  |  |  |  |  |  |
| 1 | Ath-AT1G21730.1 |  | Vvi-Vitvi18g00253\_t001 |  |  |  |  |  |  |  |
| 1 | Ath-AT1G21740.1 |  | Vvi-Vitvi18g00257\_t001 |  |  |  |  |  |  |  |
| 1 | Ath-AT1G21738.1 |  | | | |  |  |  |  |  |  |  |
| 1 | Ath-AT1G21750.1 |  | Vvi-Vitvi18g00258\_t001 |  |  |  |  |  |  |  |
| 1 | Ath-AT1G21760.2 |  | Vvi-Vitvi18g00264\_t001 |  |  |  |  |  |  |  |
| 1 | Ath-AT1G21770.1 |  | Vvi-Vitvi18g00265\_t001 |  |  |  |  |  |  |  |
| 1 | Ath-AT1G21780.2 |  | Vvi-Vitvi18g00267\_t002 |  |  |  |  |  |  |  |
| 1 | Ath-AT1G21790.1 |  | Vvi-Vitvi18g00270\_t001 |  |  |  |  |  |  |  |
| 1 | Ath-AT1G21810.1 |  | Vvi-Vitvi18g00271\_t001 |  |  |  |  |  |  |  |
| 1 | Ath-AT1G21830.1 |  | Vvi-Vitvi18g00274\_t001 |  |  |  |  |  |  |  |
| 1 | Ath-AT1G21835.1 |  | | | |  |  |  |  |  |  |  |
| 1 | Ath-AT1G21840.1 |  | | | |  |  |  |  |  |  |  |
| 1 | Ath-AT1G21850.1 |  | | | |  |  |  |  |  |  |  |
| 1 | Ath-AT1G21860.1 |  | | | |  |  |  |  |  |  |  |
| 1 | Ath-AT1G21864.1 |  | | | |  |  |  |  |  |  |  |
| 1 | Ath-AT1G21866.1 |  | | | |  |  |  |  |  |  |  |
| 1 | Ath-AT1G21870.1 |  | Vvi-Vitvi18g00280\_t001 |  |  |  |  |  |  |  |
| 1 | Ath-AT1G21880.2 |  | Vvi-Vitvi18g00283\_t001 |  |  |  |  |  |  |  |
| 1 | Ath-AT1G21890.1 |  | Vvi-Vitvi18g00289\_t001 |  |  |  |  |  |  |  |
| 1 | Ath-AT1G21900.1 |  | Vvi-Vitvi18g04043\_t001 |  |  |  |  |  |  |  |
| 1 | Ath-AT1G21910.1 |  | Vvi-Vitvi18g00295\_t001 |  |  |  |  |  |  |  |
| 1 | Ath-AT1G21920.1 |  | Vvi-Vitvi18g00298\_t001 |  |  |  |  |  |  |  |
| 0 | Ath-AT1G21925.1 |  |  |  |  |  |  |  |  |
| 0 | Ath-AT1G21928.1 |  |  |  |  |  |  |  |  |
| 0 | Ath-AT1G21930.1 |  |  |  |  |  |  |  |  |
| 0 | Ath-AT1G21940.1 |  |  |  |  |  |  |  |  |
| 0 | Ath-AT1G21950.1 |  |  |  |  |  |  |  |  |
| 0 | Ath-AT1G21960.1 |  |  |  |  |  |  |  |  |
| 0 | Ath-AT1G21970.1 |  |  |  |  |  |  |  |  |
| 1 | Ath-AT1G21980.1 |  | Vvi-Vitvi18g00322\_t001 |  |  |  |  |  |  |  |
| 1 | Ath-AT1G21990.1 |  | | | |  |  |  |  |  |  |  |
| 1 | Ath-AT1G22000.1 |  | | | |  |  |  |  |  |  |  |
| 1 | Ath-AT1G22010.1 |  | | | |  |  |  |  |  |  |  |
| 1 | Ath-AT1G22015.1 |  | Vvi-Vitvi18g00335\_t001 |  |  |  |  |  |  |  |
| 1 | Ath-AT1G22020.2 |  | Vvi-Vitvi18g00341\_t001 |  |  |  |  |  |  |  |
| 1 | Ath-AT1G22030.1 |  | Vvi-Vitvi18g00342\_t001 |  |  |  |  |  |  |  |
| 1 | Ath-AT1G22040.1 |  | | | |  |  |  |  |  |  |  |
| 1 | Ath-AT1G22050.1 |  | | | |  |  |  |  |  |  |  |
| 1 | Ath-AT1G22060.1 |  | | | |  |  |  |  |  |  |  |
| 1 | Ath-AT1G22065.1 |  | | | |  |  |  |  |  |  |  |
| 1 | Ath-AT1G22070.1 |  | Vvi-Vitvi18g00346\_t001 |  |  |  |  |  |  |  |
| 1 | Ath-AT1G22080.1 |  | | | |  |  |  |  |  |  |  |
| 1 | Ath-AT1G22090.1 |  | | | |  |  |  |  |  |  |  |
| 1 | Ath-AT1G22100.1 |  | | | |  |  |  |  |  |  |  |
| 1 | Ath-AT1G22110.3 |  | Vvi-Vitvi18g02557\_t001 |  |  |  |  |  |  |  |
| 1 | Ath-AT1G22120.1 |  | | | |  |  |  |  |  |  |  |
| 1 | Ath-AT1G22130.1 |  | Vvi-Vitvi18g00361\_t001 |  |  |  |  |  |  |  |
| 1 | Ath-AT1G22140.3 |  | Vvi-Vitvi18g02565\_t002 |  |  |  |  |  |  |  |
| 1 | Ath-AT1G22150.1 |  | Vvi-Vitvi18g00363\_t001 |  |  |  |  |  |  |  |
| 1 | Ath-AT1G22160.1 |  | Vvi-Vitvi18g02569\_t001 |  |  |  |  |  |  |  |
| 1 | Ath-AT1G22170.1 |  | Vvi-Vitvi18g02571\_t001 |  |  |  |  |  |  |  |
| 1 | Ath-AT1G22180.2 |  | Vvi-Vitvi18g00372\_t001 |  |  |  |  |  |  |  |
| 1 | Ath-AT1G22190.1 |  | Vvi-Vitvi18g00381\_t001 |  |  |  |  |  |  |  |
| 1 | Ath-AT1G22200.1 |  | Vvi-Vitvi18g00382\_t001 |  |  |  |  |  |  |  |
| 1 | Ath-AT1G22210.1 |  | | | |  |  |  |  |  |  |  |
| 1 | Ath-AT1G22220.1 |  | Vvi-Vitvi18g00386\_t001 |  |  |  |  |  |  |  |
| 1 | Ath-AT1G22230.1 |  | Vvi-Vitvi18g00388\_t001 |  |  |  |  |  |  |  |
| 1 | Ath-AT1G22240.1 |  | Vvi-Vitvi18g02573\_t001 |  |  |  |  |  |  |  |
| 1 | Ath-AT1G22250.1 |  | Vvi-Vitvi18g00398\_t001 |  |  |  |  |  |  |  |
| 1 | Ath-AT1G22260.1 |  | Vvi-Vitvi18g00401\_t001 |  |  |  |  |  |  |  |
| 1 | Ath-AT1G22270.1 |  | | | |  |  |  |  |  |  |  |
| 1 | Ath-AT1G22275.1 |  | | | |  |  |  |  |  |  |  |
| 1 | Ath-AT1G22280.3 |  | Vvi-Vitvi18g00408\_t001 |  |  |  |  |  |  |  |
| 1 | Ath-AT1G22290.2 |  | | | |  |  |  |  |  |  |  |
| 1 | Ath-AT1G22300.1 |  | Vvi-Vitvi18g00409\_t001 |  |  |  |  |  |  |  |
| 1 | Ath-AT1G22310.2 |  | Vvi-Vitvi18g00411\_t001 |  |  |  |  |  |  |  |
| 1 | Ath-AT1G22330.1 |  | Vvi-Vitvi18g00416\_t001 |  |  |  |  |  |  |  |
| 1 | Ath-AT1G22340.1 |  | Vvi-Vitvi18g04082\_t001 |  |  |  |  |  |  |  |
| 1 | Ath-AT1G22360.1 |  | | | |  |  |  |  |  |  |  |
| 1 | Ath-AT1G22370.2 |  | Vvi-Vitvi18g00420\_t001 |  |  |  |  |  |  |  |
| 1 | Ath-AT1G22380.1 |  | | | |  |  |  |  |  |  |  |
| 1 | Ath-AT1G22400.1 |  | | | |  |  |  |  |  |  |  |
| 1 | Ath-AT1G22410.1 |  | Vvi-Vitvi18g00436\_t001 |  |  |  |  |  |  |  |
| 1 | Ath-AT1G22420.1 |  | | | |  |  |  |  |  |  |  |
| 1 | Ath-AT1G22430.1 |  | Vvi-Vitvi18g00444\_t001 |  |  |  |  |  |  |  |
| 1 | Ath-AT1G22440.1 |  | | | |  |  |  |  |  |  |  |
| 1 | Ath-AT1G22450.1 |  | | | |  |  |  |  |  |  |  |
| 1 | Ath-AT1G22460.1 |  | Vvi-Vitvi18g00446\_t001 |  |  |  |  |  |  |  |
| 1 | Ath-AT1G22470.1 |  | | | |  |  |  |  |  |  |  |
| 1 | Ath-AT1G22480.1 |  | Vvi-Vitvi18g04095\_t001 |  |  |  |  |  |  |  |
| 1 | Ath-AT1G22490.2 |  | Vvi-Vitvi18g00463\_t001 |  |  |  |  |  |  |  |
| 1 | Ath-AT1G22500.1 |  | Vvi-Vitvi18g00464\_t001 |  |  |  |  |  |  |  |
| 0 | Ath-AT1G22510.2 |  |  |  |  |  |  |  |  |
| 0 | Ath-AT1G22520.2 |  |  |  |  |  |  |  |  |
| 0 | Ath-AT1G22530.1 |  |  |  |  |  |  |  |  |
| 0 | Ath-AT1G22540.1 |  |  |  |  |  |  |  |  |
| 0 | Ath-AT1G22550.1 |  |  |  |  |  |  |  |  |
| 0 | Ath-AT1G22570.1 |  |  |  |  |  |  |  |  |
| 0 | Ath-AT1G22590.2 |  |  |  |  |  |  |  |  |
| 0 | Ath-AT1G22600.1 |  |  |  |  |  |  |  |  |
| 0 | Ath-AT1G22620.1 |  |  |  |  |  |  |  |  |
| 0 | Ath-AT1G22610.1 |  |  |  |  |  |  |  |  |
| 0 | Ath-AT1G22630.1 |  |  |  |  |  |  |  |  |
| 0 | Ath-AT1G22640.1 |  |  |  |  |  |  |  |  |
| 0 | Ath-AT1G22650.1 |  |  |  |  |  |  |  |  |
| 0 | Ath-AT1G22660.4 |  |  |  |  |  |  |  |  |
| 0 | Ath-AT1G22670.1 |  |  |  |  |  |  |  |  |
| 0 | Ath-AT1G22680.1 |  |  |  |  |  |  |  |  |
| 0 | Ath-AT1G22690.1 |  |  |  |  |  |  |  |  |
| 0 | Ath-AT1G22700.1 |  |  |  |  |  |  |  |  |
| 0 | Ath-AT1G22710.1 |  |  |  |  |  |  |  |  |
| 0 | Ath-AT1G22720.1 |  |  |  |  |  |  |  |  |
| 0 | Ath-AT1G22730.1 |  |  |  |  |  |  |  |  |
| 0 | Ath-AT1G22740.1 |  |  |  |  |  |  |  |  |
| 0 | Ath-AT1G22750.4 |  |  |  |  |  |  |  |  |
| 0 | Ath-AT1G22760.1 |  |  |  |  |  |  |  |  |
| 0 | Ath-AT1G22770.1 |  |  |  |  |  |  |  |  |
| 0 | Ath-AT1G22780.1 |  |  |  |  |  |  |  |  |
| 0 | Ath-AT1G22790.2 |  |  |  |  |  |  |  |  |
| 0 | Ath-AT1G22800.1 |  |  |  |  |  |  |  |  |
| 0 | Ath-AT1G22810.1 |  |  |  |  |  |  |  |  |
| 0 | Ath-AT1G22830.1 |  |  |  |  |  |  |  |  |
| 0 | Ath-AT1G22840.1 |  |  |  |  |  |  |  |  |
| 0 | Ath-AT1G22850.1 |  |  |  |  |  |  |  |  |
| 0 | Ath-AT1G22860.1 |  |  |  |  |  |  |  |  |
| 0 | Ath-AT1G22870.1 |  |  |  |  |  |  |  |  |
| 0 | Ath-AT1G22880.1 |  |  |  |  |  |  |  |  |
| 0 | Ath-AT1G22882.1 |  |  |  |  |  |  |  |  |
| 0 | Ath-AT1G22885.2 |  |  |  |  |  |  |  |  |
| 0 | Ath-AT1G22890.1 |  |  |  |  |  |  |  |  |
| 0 | Ath-AT1G22900.1 |  |  |  |  |  |  |  |  |
| 0 | Ath-AT1G22910.3 |  |  |  |  |  |  |  |  |
| 0 | Ath-AT1G22920.1 |  |  |  |  |  |  |  |  |
| 0 | Ath-AT1G22930.1 |  |  |  |  |  |  |  |  |
| 0 | Ath-AT1G22940.1 |  |  |  |  |  |  |  |  |
| 0 | Ath-AT1G22950.1 |  |  |  |  |  |  |  |  |
| 0 | Ath-AT1G22960.2 |  |  |  |  |  |  |  |  |
| 0 | Ath-AT1G22970.1 |  |  |  |  |  |  |  |  |
| 0 | Ath-AT1G22980.2 |  |  |  |  |  |  |  |  |
| 0 | Ath-AT1G22985.1 |  |  |  |  |  |  |  |  |
| 1 | Ath-AT1G22990.1 |  | Vvi-Vitvi01g00393\_t001 |  |  |  |  |  |  |  |
| 1 | Ath-AT1G23000.2 |  | Vvi-Vitvi01g00394\_t001 |  |  |  |  |  |  |  |
| 1 | Ath-AT1G23010.1 |  | Vvi-Vitvi01g00397\_t001 |  |  |  |  |  |  |  |
| 1 | Ath-AT1G23020.2 |  | | | |  |  |  |  |  |  |  |
| 1 | Ath-AT1G23030.1 |  | Vvi-Vitvi01g00402\_t001 |  |  |  |  |  |  |  |
| 1 | Ath-AT1G23037.1 |  | | | |  |  |  |  |  |  |  |
| 1 | Ath-AT1G23040.1 |  | Vvi-Vitvi01g01971\_t001 |  |  |  |  |  |  |  |
| 1 | Ath-AT1G23050.1 |  | | | |  |  |  |  |  |  |  |
| 1 | Ath-AT1G23060.1 |  | Vvi-Vitvi01g00409\_t001 |  |  |  |  |  |  |  |
| 1 | Ath-AT1G23070.1 |  | | | |  |  |  |  |  |  |  |
| 1 | Ath-AT1G23080.1 |  | Vvi-Vitvi01g00411\_t001 |  |  |  |  |  |  |  |
| 1 | Ath-AT1G23090.1 |  | Vvi-Vitvi01g00423\_t001 |  |  |  |  |  |  |  |
| 1 | Ath-AT1G23100.1 |  | Vvi-Vitvi01g00424\_t001 |  |  |  |  |  |  |  |
| 1 | Ath-AT1G23110.1 |  | Vvi-Vitvi01g00428\_t001 |  |  |  |  |  |  |  |
| 1 | Ath-AT1G23120.2 |  | Vvi-Vitvi01g00436\_t001 |  |  |  |  |  |  |  |
| 1 | Ath-AT1G23130.1 |  | | | |  |  |  |  |  |  |  |
| 1 | Ath-AT1G23140.1 |  | | | |  |  |  |  |  |  |  |
| 1 | Ath-AT1G23145.1 |  | | | |  |  |  |  |  |  |  |
| 1 | Ath-AT1G23147.1 |  | | | |  |  |  |  |  |  |  |
| 1 | Ath-AT1G23150.1 |  | Vvi-Vitvi01g00445\_t001 |  |  |  |  |  |  |  |
| 1 | Ath-AT1G23160.1 |  | | | |  |  |  |  |  |  |  |
| 1 | Ath-AT1G23170.2 |  | Vvi-Vitvi01g00448\_t001 |  |  |  |  |  |  |  |
| 1 | Ath-AT1G23180.1 |  | Vvi-Vitvi01g00450\_t001 |  |  |  |  |  |  |  |
| 1 | Ath-AT1G23190.1 |  | Vvi-Vitvi01g00455\_t002 |  |  |  |  |  |  |  |
| 1 | Ath-AT1G23200.1 |  | Vvi-Vitvi01g00456\_t001 |  |  |  |  |  |  |  |
| 1 | Ath-AT1G23201.2 |  | | | |  |  |  |  |  |  |  |
| 1 | Ath-AT1G23205.1 |  | Vvi-Vitvi01g00457\_t001 |  |  |  |  |  |  |  |
| 1 | Ath-AT1G23210.1 |  | | | |  |  |  |  |  |  |  |
| 1 | Ath-AT1G23220.1 |  | Vvi-Vitvi01g00466\_t001 |  |  |  |  |  |  |  |
| 1 | Ath-AT1G23230.1 |  | Vvi-Vitvi01g04118\_t002 |  |  |  |  |  |  |  |
| 1 | Ath-AT1G23240.4 |  | Vvi-Vitvi01g00478\_t001 |  |  |  |  |  |  |  |
| 1 | Ath-AT1G23250.1 |  | | | |  |  |  |  |  |  |  |
| 1 | Ath-AT1G23260.1 |  | Vvi-Vitvi01g00479\_t002 |  |  |  |  |  |  |  |
| 0 | Ath-AT1G23270.1 |  |  |  |  |  |  |  |  |
| 0 | Ath-AT1G23280.1 |  |  |  |  |  |  |  |  |
| 1 | Ath-AT1G23290.1 |  | Vvi-Vitvi01g04157\_t001 |  |  |  |  |  |  |  |
| 1 | Ath-AT1G23300.1 |  | Vvi-Vitvi01g00653\_t001 |  |  |  |  |  |  |  |
| 1 | Ath-AT1G23310.1 |  | Vvi-Vitvi01g00662\_t004 |  |  |  |  |  |  |  |
| 1 | Ath-AT1G23320.1 |  | Vvi-Vitvi01g00672\_t001 |  |  |  |  |  |  |  |
| 1 | Ath-AT1G23330.1 |  | Vvi-Vitvi01g00674\_t001 |  |  |  |  |  |  |  |
| 1 | Ath-AT1G23340.2 |  | Vvi-Vitvi01g00676\_t001 |  |  |  |  |  |  |  |
| 1 | Ath-AT1G23350.1 |  | Vvi-Vitvi01g02044\_t001 |  |  |  |  |  |  |  |
| 1 | Ath-AT1G23360.1 |  | Vvi-Vitvi01g00688\_t001 |  |  |  |  |  |  |  |
| 1 | Ath-AT1G23380.2 |  | Vvi-Vitvi01g00694\_t001 |  |  |  |  |  |  |  |
| 1 | Ath-AT1G23390.1 |  | Vvi-Vitvi01g00697\_t001 |  |  |  |  |  |  |  |
| 1 | Ath-AT1G23400.1 |  | Vvi-Vitvi01g02047\_t001 |  |  |  |  |  |  |  |
| 1 | Ath-AT1G23410.1 |  | | | |  |  |  |  |  |  |  |
| 1 | Ath-AT1G23420.2 |  | Vvi-Vitvi01g00703\_t001 |  |  |  |  |  |  |  |
| 1 | Ath-AT1G23440.1 |  | Vvi-Vitvi01g00706\_t001 |  |  |  |  |  |  |  |
| 1 | Ath-AT1G23450.1 |  | Vvi-Vitvi01g04174\_t001 |  |  |  |  |  |  |  |
| 1 | Ath-AT1G23460.1 |  | Vvi-Vitvi01g04175\_t001 |  |  |  |  |  |  |  |
| 1 | Ath-AT1G23465.1 |  | | | |  |  |  |  |  |  |  |
| 1 | Ath-AT1G23480.1 |  | | | |  |  |  |  |  |  |  |
| 2 | Ath-AT1G23490.1 |  | Vvi-Vitvi01g04177\_t002 |  | Vvi-Vitvi14g01748\_t001 |  |  |  |  |  |  |
| 2 | Ath-AT1G23500.1 |  | | | |  | | | |  |  |  |  |  |  |
| 2 | Ath-AT1G23510.1 |  | | | |  | | | |  |  |  |  |  |  |
| 2 | Ath-AT1G23520.1 |  | Vvi-Vitvi01g00717\_t001 |  | | | |  |  |  |  |  |  |
| 2 | Ath-AT1G23530.1 |  | Vvi-Vitvi01g02062\_t001 |  | | | |  |  |  |  |  |  |
| 2 | Ath-AT1G23540.1 |  | Vvi-Vitvi01g04183\_t001 |  | | | |  |  |  |  |  |  |
| 2 | Ath-AT1G23550.1 |  | Vvi-Vitvi01g00729\_t001 |  | | | |  |  |  |  |  |  |
| 2 | Ath-AT1G23560.1 |  | | | |  | | | |  |  |  |  |  |  |
| 2 | Ath-AT1G23570.2 |  | | | |  | | | |  |  |  |  |  |  |
| 2 | Ath-AT1G23580.1 |  | | | |  | | | |  |  |  |  |  |  |
| 2 | Ath-AT1G23590.1 |  | | | |  | | | |  |  |  |  |  |  |
| 2 | Ath-AT1G23600.1 |  | | | |  | | | |  |  |  |  |  |  |
| 2 | Ath-AT1G23610.1 |  | | | |  | | | |  |  |  |  |  |  |
| 2 | Ath-AT1G23640.1 |  | | | |  | | | |  |  |  |  |  |  |
| 2 | Ath-AT1G23645.1 |  | | | |  | | | |  |  |  |  |  |  |
| 2 | Ath-AT1G23650.1 |  | | | |  | | | |  |  |  |  |  |  |
| 2 | Ath-AT1G23660.1 |  | | | |  | | | |  |  |  |  |  |  |
| 2 | Ath-AT1G23670.2 |  | | | |  | | | |  |  |  |  |  |  |
| 2 | Ath-AT1G23680.1 |  | | | |  | | | |  |  |  |  |  |  |
| 2 | Ath-AT1G23690.1 |  | | | |  | | | |  |  |  |  |  |  |
| 2 | Ath-AT1G23700.3 |  | | | |  | | | |  |  |  |  |  |  |
| 3 | Ath-AT1G23710.1 |  | Vvi-Vitvi01g00732\_t001 |  | Vvi-Vitvi14g01761\_t001 |  | Vvi-Vitvi17g00815\_t001 |  |  |  |  |  |
| 3 | Ath-AT1G23720.2 |  | | | |  | | | |  | | | |  |  |  |  |  |
| 3 | Ath-AT1G23730.2 |  | Vvi-Vitvi01g00735\_t001 |  | Vvi-Vitvi14g01763\_t001 |  | | | |  |  |  |  |  |
| 3 | Ath-AT1G23740.1 |  | Vvi-Vitvi01g00738\_t001 |  | | | |  | | | |  |  |  |  |  |
| 3 | Ath-AT1G23750.1 |  | Vvi-Vitvi01g00744\_t002 |  | Vvi-Vitvi14g01775\_t001 |  | | | |  |  |  |  |  |
| 3 | Ath-AT1G23760.1 |  | Vvi-Vitvi01g00745\_t001 |  | | | |  | Vvi-Vitvi17g00796\_t001 |  |  |  |  |  |
| 3 | Ath-AT1G23770.1 |  | | | |  | | | |  | | | |  |  |  |  |  |
| 3 | Ath-AT1G23780.1 |  | | | |  | | | |  | | | |  |  |  |  |  |
| 3 | Ath-AT1G23790.1 |  | Vvi-Vitvi01g00770\_t001 |  | | | |  | | | |  |  |  |  |  |
| 3 | Ath-AT1G23800.1 |  | Vvi-Vitvi01g00785\_t001 |  | Vvi-Vitvi14g01796\_t001 |  | Vvi-Vitvi17g00786\_t001 |  |  |  |  |  |
| 3 | Ath-AT1G23810.1 |  | | | |  | | | |  | | | |  |  |  |  |  |
| 3 | Ath-AT1G23820.1 |  | Vvi-Vitvi01g00789\_t001 |  | | | |  | Vvi-Vitvi17g00780\_t001 |  |  |  |  |  |
| 3 | Ath-AT1G23830.1 |  | Vvi-Vitvi01g04200\_t001 |  | Vvi-Vitvi14g04635\_t001 |  | | | |  |  |  |  |  |
| 3 | Ath-AT1G23840.1 |  | | | |  | | | |  | | | |  |  |  |  |  |
| 3 | Ath-AT1G23850.1 |  | | | |  | | | |  | | | |  |  |  |  |  |
| 3 | Ath-AT1G23860.1 |  | Vvi-Vitvi01g02080\_t001 |  | | | |  | | | |  |  |  |  |  |
| 3 | Ath-AT1G23870.1 |  | Vvi-Vitvi01g00793\_t001 |  | | | |  | Vvi-Vitvi17g00778\_t001 |  |  |  |  |  |
| 3 | Ath-AT1G23880.1 |  | | | |  | Vvi-Vitvi14g01799\_t001 |  | | | |  |  |  |  |  |
| 3 | Ath-AT1G23890.2 |  | | | |  | | | |  | | | |  |  |  |  |  |
| 3 | Ath-AT1G23900.2 |  | | | |  | | | |  | | | |  |  |  |  |  |
| 3 | Ath-AT1G23910.1 |  | | | |  | | | |  | | | |  |  |  |  |  |
| 3 | Ath-AT1G23915.1 |  | | | |  | | | |  | | | |  |  |  |  |  |
| 3 | Ath-AT1G23935.1 |  | | | |  | | | |  | | | |  |  |  |  |  |
| 3 | Ath-AT1G23950.2 |  | | | |  | | | |  | | | |  |  |  |  |  |
| 3 | Ath-AT1G23960.1 |  | | | |  | | | |  | | | |  |  |  |  |  |
| 3 | Ath-AT1G23965.1 |  | Vvi-Vitvi01g04204\_t001 |  | | | |  | | | |  |  |  |  |  |
| 3 | Ath-AT1G23970.2 |  | | | |  | | | |  | | | |  |  |  |  |  |
| 3 | Ath-AT1G23980.1 |  | Vvi-Vitvi01g04205\_t001 |  | Vvi-Vitvi14g01801\_t001 |  | Vvi-Vitvi17g00775\_t001 |  |  |  |  |  |
| 3 | Ath-AT1G23985.1 |  | | | |  | | | |  | | | |  |  |  |  |  |
| 3 | Ath-AT1G23995.1 |  | | | |  | | | |  | Vvi-Vitvi17g04225\_t001 |  |  |  |  |  |
| 3 | Ath-AT1G24000.1 |  | | | |  | | | |  | | | |  |  |  |  |  |
| 3 | Ath-AT1G24010.1 |  | | | |  | | | |  | | | |  |  |  |  |  |
| 3 | Ath-AT1G24020.1 |  | Vvi-Vitvi01g00816\_t001 |  | | | |  | | | |  |  |  |  |  |
| 3 | Ath-AT1G24030.1 |  | Vvi-Vitvi01g00817\_t001 |  | | | |  | | | |  |  |  |  |  |
| 3 | Ath-AT1G24040.1 |  | Vvi-Vitvi01g00818\_t001 |  | | | |  | | | |  |  |  |  |  |
| 3 | Ath-AT1G24050.1 |  | | | |  | | | |  | | | |  |  |  |  |  |
| 3 | Ath-AT1G24060.1 |  | | | |  | | | |  | | | |  |  |  |  |  |
| 3 | Ath-AT1G24062.1 |  | | | |  | | | |  | | | |  |  |  |  |  |
| 3 | Ath-AT1G24070.1 |  | | | |  | | | |  | | | |  |  |  |  |  |
| 3 | Ath-AT1G24090.1 |  | | | |  | Vvi-Vitvi14g01818\_t002 |  | | | |  |  |  |  |  |
| 3 | Ath-AT1G24095.1 |  | Vvi-Vitvi01g00826\_t001 |  | | | |  | | | |  |  |  |  |  |
| 3 | Ath-AT1G24100.1 |  | | | |  | | | |  | Vvi-Vitvi17g00753\_t001 |  |  |  |  |  |
| 3 | Ath-AT1G24110.1 |  | Vvi-Vitvi01g00844\_t001 |  | Vvi-Vitvi14g01821\_t001 |  | Vvi-Vitvi17g00747\_t001 |  |  |  |  |  |
| 3 | Ath-AT1G24120.1 |  | Vvi-Vitvi01g02093\_t001 |  | | | |  | | | |  |  |  |  |  |
| 3 | Ath-AT1G24130.1 |  | Vvi-Vitvi01g00849\_t001 |  | | | |  | Vvi-Vitvi17g01528\_t001 |  |  |  |  |  |
| 3 | Ath-AT1G24140.1 |  | Vvi-Vitvi01g00850\_t001 |  | Vvi-Vitvi14g03035\_t001 |  | | | |  |  |  |  |  |
| 2 | Ath-AT1G24145.1 |  | | | |  |  |  | | | |  |  |  |  |  |
| 2 | Ath-AT1G24147.1 |  | | | |  |  |  | | | |  |  |  |  |  |
| 2 | Ath-AT1G24148.1 |  | | | |  |  |  | | | |  |  |  |  |  |
| 2 | Ath-AT1G24150.2 |  | | | |  |  |  | | | |  |  |  |  |  |
| 2 | Ath-AT1G24159.1 |  | | | |  |  |  | | | |  |  |  |  |  |
| 2 | Ath-AT1G24160.1 |  | Vvi-Vitvi01g00859\_t001 |  |  |  | | | |  |  |  |  |  |
| 2 | Ath-AT1G24170.1 |  | Vvi-Vitvi01g00860\_t001 |  |  |  | | | |  |  |  |  |  |
| 2 | Ath-AT1G24180.1 |  | Vvi-Vitvi01g00861\_t001 |  |  |  | | | |  |  |  |  |  |
| 2 | Ath-AT1G24190.3 |  | Vvi-Vitvi01g02098\_t001 |  |  |  | Vvi-Vitvi17g01522\_t001 |  |  |  |  |  |
| 0 | Ath-AT1G24200.1 |  |  |  |  |  |  |  |  |
| 0 | Ath-AT1G24210.1 |  |  |  |  |  |  |  |  |
| 0 | Ath-AT1G24220.2 |  |  |  |  |  |  |  |  |
| 0 | Ath-AT1G24230.1 |  |  |  |  |  |  |  |  |
| 0 | Ath-AT1G24240.1 |  |  |  |  |  |  |  |  |
| 0 | Ath-AT1G24250.1 |  |  |  |  |  |  |  |  |
| 0 | Ath-AT1G24256.1 |  |  |  |  |  |  |  |  |
| 0 | Ath-AT1G24260.2 |  |  |  |  |  |  |  |  |
| 0 | Ath-AT1G24265.3 |  |  |  |  |  |  |  |  |
| 0 | Ath-AT1G24267.2 |  |  |  |  |  |  |  |  |
| 0 | Ath-AT1G24270.1 |  |  |  |  |  |  |  |  |
| 0 | Ath-AT1G24280.1 |  |  |  |  |  |  |  |  |
| 0 | Ath-AT1G24290.1 |  |  |  |  |  |  |  |  |
| 0 | Ath-AT1G24300.1 |  |  |  |  |  |  |  |  |
| 0 | Ath-AT1G24310.1 |  |  |  |  |  |  |  |  |
| 0 | Ath-AT1G24320.1 |  |  |  |  |  |  |  |  |
| 0 | Ath-AT1G24330.2 |  |  |  |  |  |  |  |  |
| 0 | Ath-AT1G24340.1 |  |  |  |  |  |  |  |  |
| 0 | Ath-AT1G24350.3 |  |  |  |  |  |  |  |  |
| 0 | Ath-AT1G24360.1 |  |  |  |  |  |  |  |  |
| 0 | Ath-AT1G24380.1 |  |  |  |  |  |  |  |  |
| 0 | Ath-AT1G24388.1 |  |  |  |  |  |  |  |  |
| 0 | Ath-AT1G24390.1 |  |  |  |  |  |  |  |  |
| 0 | Ath-AT1G24400.1 |  |  |  |  |  |  |  |  |
| 0 | Ath-AT1G24405.1 |  |  |  |  |  |  |  |  |
| 0 | Ath-AT1G24420.1 |  |  |  |  |  |  |  |  |
| 0 | Ath-AT1G24430.1 |  |  |  |  |  |  |  |  |
| 0 | Ath-AT1G24440.1 |  |  |  |  |  |  |  |  |
| 0 | Ath-AT1G24450.1 |  |  |  |  |  |  |  |  |
| 0 | Ath-AT1G24460.1 |  |  |  |  |  |  |  |  |
| 1 | Ath-AT1G24470.1 |  | Vvi-Vitvi01g00580\_t001 |  |  |  |  |  |  |  |
| 1 | Ath-AT1G24475.1 |  | | | |  |  |  |  |  |  |  |
| 1 | Ath-AT1G24480.1 |  | Vvi-Vitvi01g00588\_t001 |  |  |  |  |  |  |  |
| 1 | Ath-AT1G24485.3 |  | | | |  |  |  |  |  |  |  |
| 1 | Ath-AT1G24490.1 |  | Vvi-Vitvi01g00590\_t001 |  |  |  |  |  |  |  |
| 1 | Ath-AT1G24510.1 |  | Vvi-Vitvi01g00594\_t001 |  |  |  |  |  |  |  |
| 1 | Ath-AT1G24520.1 |  | | | |  |  |  |  |  |  |  |
| 1 | Ath-AT1G24530.1 |  | Vvi-Vitvi01g00608\_t001 |  |  |  |  |  |  |  |
| 1 | Ath-AT1G24540.1 |  | Vvi-Vitvi01g00612\_t001 |  |  |  |  |  |  |  |
| 1 | Ath-AT1G24545.1 |  | | | |  |  |  |  |  |  |  |
| 1 | Ath-AT1G24560.1 |  | Vvi-Vitvi01g00616\_t001 |  |  |  |  |  |  |  |
| 0 | Ath-AT1G24570.2 |  |  |  |  |  |  |  |  |
| 0 | Ath-AT1G24580.1 |  |  |  |  |  |  |  |  |
| 0 | Ath-AT1G24575.1 |  |  |  |  |  |  |  |  |
| 0 | Ath-AT1G24577.1 |  |  |  |  |  |  |  |  |
| 0 | Ath-AT1G24590.1 |  |  |  |  |  |  |  |  |
| 0 | Ath-AT1G24600.1 |  |  |  |  |  |  |  |  |
| 0 | Ath-AT1G24610.1 |  |  |  |  |  |  |  |  |
| 0 | Ath-AT1G24620.1 |  |  |  |  |  |  |  |  |
| 0 | Ath-AT1G24625.1 |  |  |  |  |  |  |  |  |
| 0 | Ath-AT1G24650.1 |  |  |  |  |  |  |  |  |
| 0 | Ath-AT1G24660.1 |  |  |  |  |  |  |  |  |
| 0 | Ath-AT1G24706.2 |  |  |  |  |  |  |  |  |
| 0 | Ath-AT1G24735.2 |  |  |  |  |  |  |  |  |
| 0 | Ath-AT1G24764.3 |  |  |  |  |  |  |  |  |
| 0 | Ath-AT1G24793.1 |  |  |  |  |  |  |  |  |
| 0 | Ath-AT1G24800.1 |  |  |  |  |  |  |  |  |
| 0 | Ath-AT1G24807.1 |  |  |  |  |  |  |  |  |
| 0 | Ath-AT1G24822.1 |  |  |  |  |  |  |  |  |
| 0 | Ath-AT1G24880.2 |  |  |  |  |  |  |  |  |
| 0 | Ath-AT1G24851.1 |  |  |  |  |  |  |  |  |
| 0 | Ath-AT1G24881.1 |  |  |  |  |  |  |  |  |
| 0 | Ath-AT1G24909.1 |  |  |  |  |  |  |  |  |
| 0 | Ath-AT1G24996.1 |  |  |  |  |  |  |  |  |
| 0 | Ath-AT1G25025.1 |  |  |  |  |  |  |  |  |
| 0 | Ath-AT1G25054.1 |  |  |  |  |  |  |  |  |
| 0 | Ath-AT1G25055.1 |  |  |  |  |  |  |  |  |
| 0 | Ath-AT1G25083.1 |  |  |  |  |  |  |  |  |
| 0 | Ath-AT1G25097.1 |  |  |  |  |  |  |  |  |
| 0 | Ath-AT1G25112.1 |  |  |  |  |  |  |  |  |
| 0 | Ath-AT1G25141.1 |  |  |  |  |  |  |  |  |
| 0 | Ath-AT1G25145.1 |  |  |  |  |  |  |  |  |
| 0 | Ath-AT1G25150.2 |  |  |  |  |  |  |  |  |
| 0 | Ath-AT1G25155.1 |  |  |  |  |  |  |  |  |
| 0 | Ath-AT1G25180.1 |  |  |  |  |  |  |  |  |
| 0 | Ath-AT1G25210.2 |  |  |  |  |  |  |  |  |
| 0 | Ath-AT1G25211.1 |  |  |  |  |  |  |  |  |
| 0 | Ath-AT1G25220.2 |  |  |  |  |  |  |  |  |
| 0 | Ath-AT1G25230.3 |  |  |  |  |  |  |  |  |
| 0 | Ath-AT1G25240.2 |  |  |  |  |  |  |  |  |
| 0 | Ath-AT1G25250.3 |  |  |  |  |  |  |  |  |
| 0 | Ath-AT1G25260.1 |  |  |  |  |  |  |  |  |
| 0 | Ath-AT1G25270.1 |  |  |  |  |  |  |  |  |
| 0 | Ath-AT1G25275.3 |  |  |  |  |  |  |  |  |
| 0 | Ath-AT1G25277.1 |  |  |  |  |  |  |  |  |
| 0 | Ath-AT1G25280.1 |  |  |  |  |  |  |  |  |
| 0 | Ath-AT1G25290.1 |  |  |  |  |  |  |  |  |
| 0 | Ath-AT1G25300.1 |  |  |  |  |  |  |  |  |
| 0 | Ath-AT1G25310.1 |  |  |  |  |  |  |  |  |
| 0 | Ath-AT1G25320.1 |  |  |  |  |  |  |  |  |
| 2 | Ath-AT1G25330.1 |  | Vvi-Vitvi17g00311\_t001 |  | Vvi-Vitvi01g01946\_t001 |  |  |  |  |  |  |
| 2 | Ath-AT1G25340.3 |  | | | |  | Vvi-Vitvi01g00302\_t001 |  |  |  |  |  |  |
| 2 | Ath-AT1G25350.2 |  | | | |  | Vvi-Vitvi01g00303\_t001 |  |  |  |  |  |  |
| 2 | Ath-AT1G25360.1 |  | | | |  | | | |  |  |  |  |  |  |
| 2 | Ath-AT1G25370.1 |  | | | |  | Vvi-Vitvi01g00306\_t002 |  |  |  |  |  |  |
| 2 | Ath-AT1G25375.1 |  | | | |  | Vvi-Vitvi01g00314\_t002 |  |  |  |  |  |  |
| 2 | Ath-AT1G25380.1 |  | | | |  | Vvi-Vitvi01g00326\_t001 |  |  |  |  |  |  |
| 2 | Ath-AT1G25390.2 |  | | | |  | Vvi-Vitvi01g04083\_t002 |  |  |  |  |  |  |
| 2 | Ath-AT1G25400.1 |  | | | |  | Vvi-Vitvi01g00299\_t001 |  |  |  |  |  |  |
| 2 | Ath-AT1G25410.1 |  | | | |  | Vvi-Vitvi01g00298\_t001 |  |  |  |  |  |  |
| 2 | Ath-AT1G25420.1 |  | | | |  | Vvi-Vitvi01g00294\_t001 |  |  |  |  |  |  |
| 2 | Ath-AT1G25422.1 |  | | | |  | Vvi-Vitvi01g01937\_t001 |  |  |  |  |  |  |
| 2 | Ath-AT1G25425.1 |  | | | |  | Vvi-Vitvi01g01936\_t001 |  |  |  |  |  |  |
| 2 | Ath-AT1G25440.1 |  | Vvi-Vitvi17g00328\_t001 |  | Vvi-Vitvi01g00288\_t001 |  |  |  |  |  |  |
| 2 | Ath-AT1G25450.1 |  | | | |  | Vvi-Vitvi01g00284\_t001 |  |  |  |  |  |  |
| 2 | Ath-AT1G25460.1 |  | | | |  | Vvi-Vitvi01g00282\_t001 |  |  |  |  |  |  |
| 2 | Ath-AT1G25470.2 |  | | | |  | Vvi-Vitvi01g01827\_t001 |  |  |  |  |  |  |
| 2 | Ath-AT1G25480.1 |  | Vvi-Vitvi17g00333\_t001 |  | Vvi-Vitvi01g00266\_t001 |  |  |  |  |  |  |
| 2 | Ath-AT1G25490.1 |  | Vvi-Vitvi17g00335\_t001 |  | Vvi-Vitvi01g00265\_t002 |  |  |  |  |  |  |
| 2 | Ath-AT1G25500.2 |  | | | |  | Vvi-Vitvi01g00259\_t001 |  |  |  |  |  |  |
| 2 | Ath-AT1G25510.1 |  | Vvi-Vitvi17g00350\_t001 |  | Vvi-Vitvi01g00258\_t001 |  |  |  |  |  |  |
| 2 | Ath-AT1G25520.1 |  | | | |  | Vvi-Vitvi01g00255\_t002 |  |  |  |  |  |  |
| 2 | Ath-AT1G25530.1 |  | | | |  | Vvi-Vitvi01g00253\_t001 |  |  |  |  |  |  |
| 2 | Ath-AT1G25540.1 |  | Vvi-Vitvi17g00360\_t001 |  | Vvi-Vitvi01g00252\_t001 |  |  |  |  |  |  |
| 1 | Ath-AT1G25550.1 |  |  |  | Vvi-Vitvi01g00249\_t001 |  |  |  |  |  |  |
| 1 | Ath-AT1G25560.1 |  |  |  | Vvi-Vitvi01g00244\_t001 |  |  |  |  |  |  |
| 1 | Ath-AT1G25570.1 |  |  |  | Vvi-Vitvi01g00243\_t001 |  |  |  |  |  |  |
| 1 | Ath-AT1G25580.1 |  |  |  | Vvi-Vitvi01g00237\_t003 |  |  |  |  |  |  |
| 1 | Ath-AT1G25682.1 |  |  |  | Vvi-Vitvi01g00227\_t001 |  |  |  |  |  |  |
| 1 | Ath-AT1G25988.1 |  |  |  | | | |  |  |  |  |  |  |
| 1 | Ath-AT1G26090.1 |  |  |  | Vvi-Vitvi01g00225\_t001 |  |  |  |  |  |  |
| 1 | Ath-AT1G26100.1 |  |  |  | Vvi-Vitvi01g00222\_t001 |  |  |  |  |  |  |
| 1 | Ath-AT1G26110.1 |  |  |  | Vvi-Vitvi01g00220\_t002 |  |  |  |  |  |  |
| 1 | Ath-AT1G26120.1 |  | Vvi-Vitvi01g00204\_t001 |  |  |  |  |  |  |  |
| 1 | Ath-AT1G26130.3 |  | Vvi-Vitvi01g00199\_t001 |  |  |  |  |  |  |  |
| 1 | Ath-AT1G26140.1 |  | Vvi-Vitvi01g00197\_t001 |  |  |  |  |  |  |  |
| 1 | Ath-AT1G26150.1 |  | Vvi-Vitvi01g00196\_t001 |  |  |  |  |  |  |  |
| 1 | Ath-AT1G26160.1 |  | Vvi-Vitvi01g01903\_t001 |  |  |  |  |  |  |  |
| 1 | Ath-AT1G26170.1 |  | Vvi-Vitvi01g00190\_t001 |  |  |  |  |  |  |  |
| 1 | Ath-AT1G26180.1 |  | Vvi-Vitvi01g00186\_t001 |  |  |  |  |  |  |  |
| 0 | Ath-AT1G26190.1 |  |  |  |  |  |  |  |  |
| 0 | Ath-AT1G26200.3 |  |  |  |  |  |  |  |  |
| 0 | Ath-AT1G26210.1 |  |  |  |  |  |  |  |  |
| 0 | Ath-AT1G26220.1 |  |  |  |  |  |  |  |  |
| 0 | Ath-AT1G26230.1 |  |  |  |  |  |  |  |  |
| 0 | Ath-AT1G26225.1 |  |  |  |  |  |  |  |  |
| 0 | Ath-AT1G26240.1 |  |  |  |  |  |  |  |  |
| 0 | Ath-AT1G26250.1 |  |  |  |  |  |  |  |  |
| 0 | Ath-AT1G26255.3 |  |  |  |  |  |  |  |  |
| 0 | Ath-AT1G26260.1 |  |  |  |  |  |  |  |  |
| 0 | Ath-AT1G26270.1 |  |  |  |  |  |  |  |  |
| 0 | Ath-AT1G26300.1 |  |  |  |  |  |  |  |  |
| 0 | Ath-AT1G26290.1 |  |  |  |  |  |  |  |  |
| 1 | Ath-AT1G26310.1 |  | Vvi-Vitvi01g00008\_t001 |  |  |  |  |  |  |  |
| 1 | Ath-AT1G26320.1 |  | | | |  |  |  |  |  |  |  |
| 1 | Ath-AT1G26330.2 |  | Vvi-Vitvi01g00014\_t001 |  |  |  |  |  |  |  |
| 1 | Ath-AT1G26340.1 |  | Vvi-Vitvi01g00023\_t001 |  |  |  |  |  |  |  |
| 1 | Ath-AT1G26350.1 |  | | | |  |  |  |  |  |  |  |
| 1 | Ath-AT1G26355.1 |  | Vvi-Vitvi01g00033\_t002 |  |  |  |  |  |  |  |
| 1 | Ath-AT1G26360.1 |  | Vvi-Vitvi01g00034\_t001 |  |  |  |  |  |  |  |
| 1 | Ath-AT1G26370.1 |  | | | |  |  |  |  |  |  |  |
| 1 | Ath-AT1G26380.1 |  | | | |  |  |  |  |  |  |  |
| 1 | Ath-AT1G26390.1 |  | | | |  |  |  |  |  |  |  |
| 1 | Ath-AT1G26400.1 |  | | | |  |  |  |  |  |  |  |
| 1 | Ath-AT1G26410.1 |  | | | |  |  |  |  |  |  |  |
| 1 | Ath-AT1G26420.1 |  | | | |  |  |  |  |  |  |  |
| 1 | Ath-AT1G26440.3 |  | Vvi-Vitvi01g00048\_t001 |  |  |  |  |  |  |  |
| 1 | Ath-AT1G26450.1 |  | | | |  |  |  |  |  |  |  |
| 1 | Ath-AT1G26460.1 |  | Vvi-Vitvi01g01841\_t001.1.60378269 |  |  |  |  |  |  |  |
| 1 | Ath-AT1G26470.1 |  | Vvi-Vitvi01g00054\_t002 |  |  |  |  |  |  |  |
| 1 | Ath-AT1G26480.1 |  | Vvi-Vitvi01g00055\_t001 |  |  |  |  |  |  |  |
| 1 | Ath-AT1G26500.1 |  | | | |  |  |  |  |  |  |  |
| 1 | Ath-AT1G26510.1 |  | | | |  |  |  |  |  |  |  |
| 1 | Ath-AT1G26515.1 |  | | | |  |  |  |  |  |  |  |
| 1 | Ath-AT1G26520.1 |  | | | |  |  |  |  |  |  |  |
| 1 | Ath-AT1G26530.1 |  | | | |  |  |  |  |  |  |  |
| 1 | Ath-AT1G26540.1 |  | | | |  |  |  |  |  |  |  |
| 1 | Ath-AT1G26550.1 |  | | | |  |  |  |  |  |  |  |
| 1 | Ath-AT1G26560.1 |  | | | |  |  |  |  |  |  |  |
| 3 | Ath-AT1G26570.1 |  | Vvi-Vitvi01g00061\_t001 |  | Vvi-Vitvi17g00682\_t002 |  | Vvi-Vitvi14g03099\_t002 |  |  |  |  |  |
| 3 | Ath-AT1G26580.1 |  | Vvi-Vitvi01g00062\_t001 |  | | | |  | Vvi-Vitvi14g02034\_t001 |  |  |  |  |  |
| 3 | Ath-AT1G26590.2 |  | Vvi-Vitvi01g00063\_t001 |  | | | |  | Vvi-Vitvi14g02033\_t001 |  |  |  |  |  |
| 3 | Ath-AT1G26600.2 |  | | | |  | | | |  | | | |  |  |  |  |  |
| 3 | Ath-AT1G26610.1 |  | | | |  | Vvi-Vitvi17g00670\_t001 |  | | | |  |  |  |  |  |
| 3 | Ath-AT1G26620.1 |  | Vvi-Vitvi01g00080\_t001 |  | Vvi-Vitvi17g00660\_t001 |  | Vvi-Vitvi14g02014\_t001 |  |  |  |  |  |
| 2 | Ath-AT1G26630.1 |  |  |  | Vvi-Vitvi17g00650\_t001 |  | | | |  |  |  |  |  |
| 2 | Ath-AT1G26640.1 |  |  |  | | | |  | | | |  |  |  |  |  |
| 2 | Ath-AT1G26650.1 |  |  |  | Vvi-Vitvi17g00645\_t001 |  | | | |  |  |  |  |  |
| 2 | Ath-AT1G26660.2 |  |  |  | | | |  | | | |  |  |  |  |  |
| 2 | Ath-AT1G26665.1 |  |  |  | | | |  | | | |  |  |  |  |  |
| 2 | Ath-AT1G26670.1 |  |  |  | Vvi-Vitvi17g00641\_t001 |  | Vvi-Vitvi14g01999\_t001 |  |  |  |  |  |
| 2 | Ath-AT1G26680.4 |  |  |  | | | |  | | | |  |  |  |  |  |
| 2 | Ath-AT1G26690.1 |  |  |  | | | |  | Vvi-Vitvi14g03093\_t001 |  |  |  |  |  |
| 2 | Ath-AT1G26700.1 |  |  |  | | | |  | | | |  |  |  |  |  |
| 2 | Ath-AT1G26710.1 |  |  |  | | | |  | | | |  |  |  |  |  |
| 2 | Ath-AT1G26720.1 |  |  |  | | | |  | | | |  |  |  |  |  |
| 2 | Ath-AT1G26730.1 |  |  |  | | | |  | | | |  |  |  |  |  |
| 2 | Ath-AT1G26740.1 |  |  |  | | | |  | Vvi-Vitvi14g01989\_t001 |  |  |  |  |  |
| 2 | Ath-AT1G26750.1 |  |  |  | | | |  | | | |  |  |  |  |  |
| 2 | Ath-AT1G26760.1 |  |  |  | | | |  | | | |  |  |  |  |  |
| 2 | Ath-AT1G26761.1 |  |  |  | | | |  | | | |  |  |  |  |  |
| 2 | Ath-AT1G26762.1 |  |  |  | | | |  | | | |  |  |  |  |  |
| 3 | Ath-AT1G26770.2 |  | Vvi-Vitvi01g01030\_t001 |  | Vvi-Vitvi17g00616\_t001 |  | Vvi-Vitvi14g01977\_t001 |  |  |  |  |  |
| 3 | Ath-AT1G26773.1 |  | | | |  | | | |  | | | |  |  |  |  |  |
| 3 | Ath-AT1G26780.2 |  | Vvi-Vitvi01g01028\_t001 |  | | | |  | Vvi-Vitvi14g01976\_t001 |  |  |  |  |  |
| 3 | Ath-AT1G26790.1 |  | Vvi-Vitvi01g01026\_t001 |  | Vvi-Vitvi17g00611\_t001 |  | Vvi-Vitvi14g01973\_t001 |  |  |  |  |  |
| 2 | Ath-AT1G26795.1 |  | | | |  |  |  | | | |  |  |  |  |  |
| 2 | Ath-AT1G26796.1 |  | | | |  |  |  | | | |  |  |  |  |  |
| 2 | Ath-AT1G26797.1 |  | | | |  |  |  | | | |  |  |  |  |  |
| 2 | Ath-AT1G26798.1 |  | | | |  |  |  | | | |  |  |  |  |  |
| 2 | Ath-AT1G26799.1 |  | | | |  |  |  | | | |  |  |  |  |  |
| 3 | Ath-AT1G26800.1 |  | Vvi-Vitvi01g01023\_t001 |  | Vvi-Vitvi01g04225\_t001 |  | Vvi-Vitvi14g01971\_t001 |  |  |  |  |  |
| 3 | Ath-AT1G26810.2 |  | Vvi-Vitvi01g01022\_t001 |  | | | |  | Vvi-Vitvi14g01965\_t001 |  |  |  |  |  |
| 3 | Ath-AT1G26815.1 |  | | | |  | | | |  | | | |  |  |  |  |  |
| 3 | Ath-AT1G26820.1 |  | Vvi-Vitvi01g01021\_t001 |  | | | |  | | | |  |  |  |  |  |
| 4 | Ath-AT1G26830.1 |  | | | |  | | | |  | | | |  | Vvi-Vitvi01g00985\_t001 |  |  |  |  |
| 4 | Ath-AT1G26840.1 |  | | | |  | | | |  | | | |  | Vvi-Vitvi01g00992\_t001 |  |  |  |  |
| 4 | Ath-AT1G26850.1 |  | | | |  | | | |  | | | |  | Vvi-Vitvi01g00995\_t001 |  |  |  |  |
| 4 | Ath-AT1G26870.1 |  | | | |  | | | |  | Vvi-Vitvi14g01963\_t001 |  | Vvi-Vitvi01g00999\_t001 |  |  |  |  |
| 4 | Ath-AT1G26880.1 |  | Vvi-Vitvi01g04246\_t001 |  | | | |  | Vvi-Vitvi14g04683\_t001 |  | Vvi-Vitvi01g04246\_t001 |  |  |  |  |
| 3 | Ath-AT1G26890.1 |  | | | |  | | | |  |  |  | | | |  |  |  |  |
| 3 | Ath-AT1G26900.1 |  | Vvi-Vitvi01g00975\_t001 |  | | | |  |  |  | | | |  |  |  |  |
| 3 | Ath-AT1G26910.1 |  | | | |  | | | |  |  |  | | | |  |  |  |  |
| 3 | Ath-AT1G26920.1 |  | Vvi-Vitvi01g02127\_t001 |  | | | |  |  |  | | | |  |  |  |  |
| 3 | Ath-AT1G26921.1 |  | | | |  | | | |  |  |  | | | |  |  |  |  |
| 3 | Ath-AT1G26930.1 |  | Vvi-Vitvi01g00970\_t001 |  | | | |  |  |  | | | |  |  |  |  |
| 3 | Ath-AT1G26940.1 |  | Vvi-Vitvi01g00968\_t002 |  | | | |  |  |  | | | |  |  |  |  |
| 3 | Ath-AT1G26945.1 |  | Vvi-Vitvi01g00964\_t001 |  | | | |  |  |  | | | |  |  |  |  |
| 3 | Ath-AT1G26960.1 |  | Vvi-Vitvi01g00958\_t002 |  | | | |  |  |  | | | |  |  |  |  |
| 3 | Ath-AT1G26970.1 |  | Vvi-Vitvi01g00954\_t001 |  | | | |  |  |  | | | |  |  |  |  |
| 2 | Ath-AT1G26976.1 |  |  |  | | | |  |  |  | | | |  |  |  |  |
| 2 | Ath-AT1G27000.1 |  |  |  | Vvi-Vitvi01g00935\_t001 |  |  |  | | | |  |  |  |  |
| 2 | Ath-AT1G27008.1 |  |  |  | | | |  |  |  | | | |  |  |  |  |
| 2 | Ath-AT1G27020.1 |  |  |  | Vvi-Vitvi01g00928\_t001 |  |  |  | | | |  |  |  |  |
| 2 | Ath-AT1G27030.1 |  |  |  | | | |  |  |  | | | |  |  |  |  |
| 2 | Ath-AT1G27040.1 |  |  |  | Vvi-Vitvi01g00921\_t001 |  |  |  | | | |  |  |  |  |
| 2 | Ath-AT1G27045.4 |  |  |  | | | |  |  |  | | | |  |  |  |  |
| 2 | Ath-AT1G27050.1 |  |  |  | Vvi-Vitvi01g00916\_t001 |  |  |  | | | |  |  |  |  |
| 2 | Ath-AT1G27060.1 |  |  |  | Vvi-Vitvi01g00915\_t001 |  |  |  | | | |  |  |  |  |
| 2 | Ath-AT1G27070.1 |  |  |  | Vvi-Vitvi01g00914\_t001 |  |  |  | | | |  |  |  |  |
| 2 | Ath-AT1G27080.1 |  |  |  | Vvi-Vitvi01g00911\_t001 |  |  |  | | | |  |  |  |  |
| 2 | Ath-AT1G27090.1 |  |  |  | Vvi-Vitvi01g00908\_t001 |  |  |  | | | |  |  |  |  |
| 2 | Ath-AT1G27100.1 |  |  |  | Vvi-Vitvi01g00902\_t004 |  |  |  | | | |  |  |  |  |
| 2 | Ath-AT1G27110.1 |  |  |  | Vvi-Vitvi01g00898\_t001 |  |  |  | | | |  |  |  |  |
| 2 | Ath-AT1G27120.1 |  |  |  | | | |  |  |  | Vvi-Vitvi01g01022\_t001 |  |  |  |  |
| 1 | Ath-AT1G27130.1 |  |  |  | Vvi-Vitvi01g00892\_t001 |  |  |  |  |  |  |
| 1 | Ath-AT1G27135.1 |  |  |  | | | |  |  |  |  |  |  |
| 1 | Ath-AT1G27140.1 |  |  |  | | | |  |  |  |  |  |  |
| 1 | Ath-AT1G27150.1 |  |  |  | | | |  |  |  |  |  |  |
| 1 | Ath-AT1G27160.1 |  |  |  | | | |  |  |  |  |  |  |
| 1 | Ath-AT1G27170.2 |  |  |  | Vvi-Vitvi01g00873\_t001 |  |  |  |  |  |  |
| 1 | Ath-AT1G27180.1 |  |  |  | | | |  |  |  |  |  |  |
| 1 | Ath-AT1G27190.1 |  |  |  | Vvi-Vitvi01g00868\_t001 |  |  |  |  |  |  |
| 1 | Ath-AT1G27200.1 |  |  |  | Vvi-Vitvi01g02099\_t001 |  |  |  |  |  |  |
| 1 | Ath-AT1G27210.1 |  |  |  | Vvi-Vitvi01g00864\_t001 |  |  |  |  |  |  |
| 0 | Ath-AT1G27220.1 |  |  |  |  |  |  |  |  |
| 0 | Ath-AT1G27240.1 |  |  |  |  |  |  |  |  |
| 0 | Ath-AT1G27250.1 |  |  |  |  |  |  |  |  |
| 0 | Ath-AT1G27260.1 |  |  |  |  |  |  |  |  |
| 0 | Ath-AT1G27270.1 |  |  |  |  |  |  |  |  |
| 0 | Ath-AT1G27280.1 |  |  |  |  |  |  |  |  |
| 1 | Ath-AT1G27290.1 |  | Vvi-Vitvi01g04446\_t003 |  |  |  |  |  |  |  |
| 1 | Ath-AT1G27300.1 |  | Vvi-Vitvi01g02272\_t001 |  |  |  |  |  |  |  |
| 1 | Ath-AT1G27310.1 |  | Vvi-Vitvi01g04443\_t002 |  |  |  |  |  |  |  |
| 1 | Ath-AT1G27320.1 |  | Vvi-Vitvi01g01666\_t001 |  |  |  |  |  |  |  |
| 1 | Ath-AT1G27330.1 |  | Vvi-Vitvi01g04442\_t001 |  |  |  |  |  |  |  |
| 1 | Ath-AT1G27340.1 |  | Vvi-Vitvi01g01662\_t001 |  |  |  |  |  |  |  |
| 1 | Ath-AT1G27350.1 |  | | | |  |  |  |  |  |  |  |
| 1 | Ath-AT1G27360.2 |  | Vvi-Vitvi01g01660\_t001 |  |  |  |  |  |  |  |
| 1 | Ath-AT1G27370.5 |  | | | |  |  |  |  |  |  |  |
| 1 | Ath-AT1G27380.4 |  | | | |  |  |  |  |  |  |  |
| 1 | Ath-AT1G27385.4 |  | Vvi-Vitvi01g01653\_t002 |  |  |  |  |  |  |  |
| 0 | Ath-AT1G27390.1 |  |  |  |  |  |  |  |  |
| 0 | Ath-AT1G27400.1 |  |  |  |  |  |  |  |  |
| 0 | Ath-AT1G27410.1 |  |  |  |  |  |  |  |  |
| 0 | Ath-AT1G27420.1 |  |  |  |  |  |  |  |  |
| 0 | Ath-AT1G27430.1 |  |  |  |  |  |  |  |  |
| 0 | Ath-AT1G27435.1 |  |  |  |  |  |  |  |  |
| 0 | Ath-AT1G27440.1 |  |  |  |  |  |  |  |  |
| 0 | Ath-AT1G27450.3 |  |  |  |  |  |  |  |  |
| 0 | Ath-AT1G27460.1 |  |  |  |  |  |  |  |  |
| 0 | Ath-AT1G27461.1 |  |  |  |  |  |  |  |  |
| 0 | Ath-AT1G27470.1 |  |  |  |  |  |  |  |  |
| 0 | Ath-AT1G27480.1 |  |  |  |  |  |  |  |  |
| 0 | Ath-AT1G27490.1 |  |  |  |  |  |  |  |  |
| 0 | Ath-AT1G27500.2 |  |  |  |  |  |  |  |  |
| 0 | Ath-AT1G27510.1 |  |  |  |  |  |  |  |  |
| 0 | Ath-AT1G27520.1 |  |  |  |  |  |  |  |  |
| 0 | Ath-AT1G27530.1 |  |  |  |  |  |  |  |  |
| 0 | Ath-AT1G27535.1 |  |  |  |  |  |  |  |  |
| 0 | Ath-AT1G27540.1 |  |  |  |  |  |  |  |  |
| 0 | Ath-AT1G27550.1 |  |  |  |  |  |  |  |  |
| 0 | Ath-AT1G27555.1 |  |  |  |  |  |  |  |  |
| 0 | Ath-AT1G27565.1 |  |  |  |  |  |  |  |  |
| 0 | Ath-AT1G27570.1 |  |  |  |  |  |  |  |  |
| 0 | Ath-AT1G27580.1 |  |  |  |  |  |  |  |  |
| 0 | Ath-AT1G27595.1 |  |  |  |  |  |  |  |  |
| 0 | Ath-AT1G27600.2 |  |  |  |  |  |  |  |  |
| 0 | Ath-AT1G27610.2 |  |  |  |  |  |  |  |  |
| 0 | Ath-AT1G27620.1 |  |  |  |  |  |  |  |  |
| 0 | Ath-AT1G27630.1 |  |  |  |  |  |  |  |  |
| 0 | Ath-AT1G27640.1 |  |  |  |  |  |  |  |  |
| 0 | Ath-AT1G27650.1 |  |  |  |  |  |  |  |  |
| 0 | Ath-AT1G27660.1 |  |  |  |  |  |  |  |  |
| 0 | Ath-AT1G27670.1 |  |  |  |  |  |  |  |  |
| 0 | Ath-AT1G27680.1 |  |  |  |  |  |  |  |  |
| 1 | Ath-AT1G27690.1 |  | Vvi-Vitvi18g00690\_t001 |  |  |  |  |  |  |  |
| 1 | Ath-AT1G27695.1 |  | | | |  |  |  |  |  |  |  |
| 1 | Ath-AT1G27700.1 |  | Vvi-Vitvi18g00685\_t001 |  |  |  |  |  |  |  |
| 1 | Ath-AT1G27710.1 |  | | | |  |  |  |  |  |  |  |
| 1 | Ath-AT1G27720.1 |  | Vvi-Vitvi18g00680\_t001 |  |  |  |  |  |  |  |
| 1 | Ath-AT1G27730.1 |  | Vvi-Vitvi18g00675\_t001 |  |  |  |  |  |  |  |
| 1 | Ath-AT1G27740.1 |  | Vvi-Vitvi18g00673\_t001 |  |  |  |  |  |  |  |
| 1 | Ath-AT1G27750.1 |  | Vvi-Vitvi18g00672\_t001 |  |  |  |  |  |  |  |
| 1 | Ath-AT1G27752.1 |  | Vvi-Vitvi18g00671\_t001 |  |  |  |  |  |  |  |
| 1 | Ath-AT1G27760.3 |  | Vvi-Vitvi18g00670\_t001 |  |  |  |  |  |  |  |
| 1 | Ath-AT1G27770.1 |  | Vvi-Vitvi18g00668\_t003 |  |  |  |  |  |  |  |
| 1 | Ath-AT1G27820.1 |  | | | |  |  |  |  |  |  |  |
| 1 | Ath-AT1G27840.3 |  | Vvi-Vitvi18g00660\_t001 |  |  |  |  |  |  |  |
| 0 | Ath-AT1G27850.1 |  |  |  |  |  |  |  |  |
| 0 | Ath-AT1G27860.1 |  |  |  |  |  |  |  |  |
| 0 | Ath-AT1G27870.1 |  |  |  |  |  |  |  |  |
| 0 | Ath-AT1G27880.1 |  |  |  |  |  |  |  |  |
| 0 | Ath-AT1G27890.1 |  |  |  |  |  |  |  |  |
| 0 | Ath-AT1G27900.1 |  |  |  |  |  |  |  |  |
| 0 | Ath-AT1G27910.1 |  |  |  |  |  |  |  |  |
| 1 | Ath-AT1G27920.1 |  | Vvi-Vitvi01g01641\_t001 |  |  |  |  |  |  |  |
| 1 | Ath-AT1G27930.1 |  | Vvi-Vitvi01g01655\_t001 |  |  |  |  |  |  |  |
| 1 | Ath-AT1G27940.1 |  | | | |  |  |  |  |  |  |  |
| 1 | Ath-AT1G27950.1 |  | Vvi-Vitvi01g01657\_t001 |  |  |  |  |  |  |  |
| 1 | Ath-AT1G27960.1 |  | Vvi-Vitvi01g01658\_t001 |  |  |  |  |  |  |  |
| 1 | Ath-AT1G27970.2 |  | Vvi-Vitvi01g04443\_t002 |  |  |  |  |  |  |  |
| 1 | Ath-AT1G27980.1 |  | Vvi-Vitvi01g01682\_t001 |  |  |  |  |  |  |  |
| 1 | Ath-AT1G27990.1 |  | Vvi-Vitvi01g01687\_t001 |  |  |  |  |  |  |  |
| 0 | Ath-AT1G28000.1 |  |  |  |  |  |  |  |  |
| 0 | Ath-AT1G28005.1 |  |  |  |  |  |  |  |  |
| 0 | Ath-AT1G28010.1 |  |  |  |  |  |  |  |  |
| 1 | Ath-AT1G28020.1 |  | Vvi-Vitvi10g04309\_t001 |  |  |  |  |  |  |  |
| 1 | Ath-AT1G28030.1 |  | | | |  |  |  |  |  |  |  |
| 1 | Ath-AT1G28040.1 |  | | | |  |  |  |  |  |  |  |
| 2 | Ath-AT1G28050.1 |  | | | |  | Vvi-Vitvi01g01729\_t001 |  |  |  |  |  |  |
| 2 | Ath-AT1G28060.1 |  | | | |  | | | |  |  |  |  |  |  |
| 2 | Ath-AT1G28070.1 |  | | | |  | Vvi-Vitvi01g01733\_t001 |  |  |  |  |  |  |
| 2 | Ath-AT1G28080.2 |  | | | |  | Vvi-Vitvi01g02295\_t001 |  |  |  |  |  |  |
| 2 | Ath-AT1G28090.1 |  | | | |  | Vvi-Vitvi01g01747\_t001 |  |  |  |  |  |  |
| 2 | Ath-AT1G28100.4 |  | | | |  | | | |  |  |  |  |  |  |
| 2 | Ath-AT1G28110.2 |  | | | |  | Vvi-Vitvi01g01753\_t001 |  |  |  |  |  |  |
| 2 | Ath-AT1G28120.1 |  | | | |  | Vvi-Vitvi01g01769\_t001 |  |  |  |  |  |  |
| 2 | Ath-AT1G28130.1 |  | | | |  | Vvi-Vitvi01g01791\_t001 |  |  |  |  |  |  |
| 2 | Ath-AT1G28135.1 |  | | | |  | | | |  |  |  |  |  |  |
| 2 | Ath-AT1G28140.1 |  | | | |  | | | |  |  |  |  |  |  |
| 2 | Ath-AT1G28150.1 |  | | | |  | | | |  |  |  |  |  |  |
| 2 | Ath-AT1G28160.1 |  | | | |  | | | |  |  |  |  |  |  |
| 2 | Ath-AT1G28170.1 |  | | | |  | | | |  |  |  |  |  |  |
| 2 | Ath-AT1G28180.1 |  | | | |  | | | |  |  |  |  |  |  |
| 2 | Ath-AT1G28190.1 |  | | | |  | Vvi-Vitvi01g01801\_t001 |  |  |  |  |  |  |
| 1 | Ath-AT1G28200.2 |  | | | |  |  |  |  |  |  |  |
| 1 | Ath-AT1G28210.2 |  | | | |  |  |  |  |  |  |  |
| 1 | Ath-AT1G28220.1 |  | | | |  |  |  |  |  |  |  |
| 1 | Ath-AT1G28230.1 |  | | | |  |  |  |  |  |  |  |
| 1 | Ath-AT1G28240.1 |  | Vvi-Vitvi10g00455\_t001 |  |  |  |  |  |  |  |
| 1 | Ath-AT1G28250.1 |  | | | |  |  |  |  |  |  |  |
| 1 | Ath-AT1G28260.2 |  | | | |  |  |  |  |  |  |  |
| 1 | Ath-AT1G28265.1 |  | | | |  |  |  |  |  |  |  |
| 1 | Ath-AT1G28270.1 |  | | | |  |  |  |  |  |  |  |
| 1 | Ath-AT1G28280.1 |  | | | |  |  |  |  |  |  |  |
| 1 | Ath-AT1G28290.1 |  | Vvi-Vitvi10g02256\_t001 |  |  |  |  |  |  |  |
| 1 | Ath-AT1G28300.1 |  | | | |  |  |  |  |  |  |  |
| 1 | Ath-AT1G28305.1 |  | | | |  |  |  |  |  |  |  |
| 1 | Ath-AT1G28306.1 |  | | | |  |  |  |  |  |  |  |
| 1 | Ath-AT1G28310.2 |  | Vvi-Vitvi10g00480\_t001 |  |  |  |  |  |  |  |
| 1 | Ath-AT1G28320.1 |  | Vvi-Vitvi10g00483\_t001 |  |  |  |  |  |  |  |
| 2 | Ath-AT1G28327.1 |  | Vvi-Vitvi10g00484\_t001 |  | Vvi-Vitvi12g00279\_t001 |  |  |  |  |  |  |
| 2 | Ath-AT1G28330.3 |  | Vvi-Vitvi10g00485\_t003 |  | | | |  |  |  |  |  |  |
| 2 | Ath-AT1G28335.1 |  | | | |  | | | |  |  |  |  |  |  |
| 2 | Ath-AT1G28340.1 |  | Vvi-Vitvi10g00487\_t001 |  | | | |  |  |  |  |  |  |
| 2 | Ath-AT1G28350.1 |  | | | |  | | | |  |  |  |  |  |  |
| 2 | Ath-AT1G28360.1 |  | Vvi-Vitvi10g00488\_t001 |  | Vvi-Vitvi12g00274\_t001 |  |  |  |  |  |  |
| 2 | Ath-AT1G28370.2 |  | | | |  | | | |  |  |  |  |  |  |
| 2 | Ath-AT1G28375.1 |  | | | |  | | | |  |  |  |  |  |  |
| 2 | Ath-AT1G28380.1 |  | Vvi-Vitvi10g00489\_t001 |  | | | |  |  |  |  |  |  |
| 2 | Ath-AT1G28390.2 |  | | | |  | | | |  |  |  |  |  |  |
| 2 | Ath-AT1G28395.1 |  | Vvi-Vitvi10g01769\_t004 |  | Vvi-Vitvi12g00267\_t004 |  |  |  |  |  |  |
| 2 | Ath-AT1G28400.1 |  | Vvi-Vitvi10g01771\_t001 |  | | | |  |  |  |  |  |  |
| 2 | Ath-AT1G28410.2 |  | Vvi-Vitvi10g01772\_t001 |  | | | |  |  |  |  |  |  |
| 2 | Ath-AT1G28420.1 |  | Vvi-Vitvi10g00497\_t001 |  | Vvi-Vitvi12g00258\_t003 |  |  |  |  |  |  |
| 2 | Ath-AT1G28430.1 |  | | | |  | | | |  |  |  |  |  |  |
| 2 | Ath-AT1G28440.1 |  | Vvi-Vitvi10g00503\_t001 |  | Vvi-Vitvi12g00256\_t001 |  |  |  |  |  |  |
| 2 | Ath-AT1G28450.1 |  | | | |  | | | |  |  |  |  |  |  |
| 2 | Ath-AT1G28460.1 |  | | | |  | | | |  |  |  |  |  |  |
| 2 | Ath-AT1G28470.1 |  | Vvi-Vitvi10g00505\_t001 |  | Vvi-Vitvi12g00255\_t001 |  |  |  |  |  |  |
| 2 | Ath-AT1G28480.1 |  | Vvi-Vitvi10g00508\_t001 |  | Vvi-Vitvi12g00251\_t001 |  |  |  |  |  |  |
| 2 | Ath-AT1G28490.1 |  | | | |  | | | |  |  |  |  |  |  |
| 2 | Ath-AT1G28500.1 |  | | | |  | | | |  |  |  |  |  |  |
| 2 | Ath-AT1G28510.1 |  | Vvi-Vitvi10g00514\_t001 |  | Vvi-Vitvi12g00236\_t001 |  |  |  |  |  |  |
| 2 | Ath-AT1G28520.1 |  | Vvi-Vitvi10g00515\_t001 |  | Vvi-Vitvi12g00235\_t001 |  |  |  |  |  |  |
| 2 | Ath-AT1G28530.1 |  | Vvi-Vitvi10g00517\_t002 |  | | | |  |  |  |  |  |  |
| 2 | Ath-AT1G28540.1 |  | Vvi-Vitvi10g01780\_t001 |  | | | |  |  |  |  |  |  |
| 2 | Ath-AT1G28550.1 |  | Vvi-Vitvi10g01781\_t001 |  | Vvi-Vitvi12g00233\_t001 |  |  |  |  |  |  |
| 2 | Ath-AT1G28560.3 |  | | | |  | | | |  |  |  |  |  |  |
| 3 | Ath-AT1G28570.1 |  | | | |  | | | |  | Vvi-Vitvi10g00555\_t001 |  |  |  |  |  |
| 3 | Ath-AT1G28580.1 |  | | | |  | | | |  | | | |  |  |  |  |  |
| 3 | Ath-AT1G28590.1 |  | | | |  | | | |  | | | |  |  |  |  |  |
| 3 | Ath-AT1G28600.1 |  | | | |  | | | |  | | | |  |  |  |  |  |
| 3 | Ath-AT1G28610.2 |  | | | |  | | | |  | | | |  |  |  |  |  |
| 3 | Ath-AT1G28630.4 |  | | | |  | | | |  | | | |  |  |  |  |  |
| 3 | Ath-AT1G28640.1 |  | | | |  | | | |  | Vvi-Vitvi10g00557\_t001 |  |  |  |  |  |
| 3 | Ath-AT1G28650.1 |  | | | |  | | | |  | | | |  |  |  |  |  |
| 3 | Ath-AT1G28660.1 |  | | | |  | | | |  | | | |  |  |  |  |  |
| 3 | Ath-AT1G28670.1 |  | | | |  | | | |  | | | |  |  |  |  |  |
| 3 | Ath-AT1G28680.1 |  | | | |  | | | |  | | | |  |  |  |  |  |
| 3 | Ath-AT1G28690.1 |  | | | |  | | | |  | | | |  |  |  |  |  |
| 3 | Ath-AT1G28695.1 |  | | | |  | | | |  | | | |  |  |  |  |  |
| 3 | Ath-AT1G28700.1 |  | | | |  | | | |  | | | |  |  |  |  |  |
| 3 | Ath-AT1G28710.3 |  | | | |  | | | |  | | | |  |  |  |  |  |
| 3 | Ath-AT1G28815.1 |  | | | |  | | | |  | | | |  |  |  |  |  |
| 3 | Ath-AT1G28760.1 |  | | | |  | | | |  | | | |  |  |  |  |  |
| 3 | Ath-AT1G28765.2 |  | | | |  | | | |  | | | |  |  |  |  |  |
| 3 | Ath-AT1G28960.5 |  | | | |  | | | |  | | | |  |  |  |  |  |
| 3 | Ath-AT1G29000.1 |  | | | |  | | | |  | | | |  |  |  |  |  |
| 3 | Ath-AT1G29005.1 |  | | | |  | | | |  | | | |  |  |  |  |  |
| 3 | Ath-AT1G29010.1 |  | | | |  | | | |  | | | |  |  |  |  |  |
| 3 | Ath-AT1G29020.2 |  | Vvi-Vitvi10g00537\_t001 |  | Vvi-Vitvi12g04057\_t001 |  | | | |  |  |  |  |  |
| 2 | Ath-AT1G29025.1 |  |  |  | | | |  | | | |  |  |  |  |  |
| 2 | Ath-AT1G29030.1 |  |  |  | | | |  | | | |  |  |  |  |  |
| 2 | Ath-AT1G29040.1 |  |  |  | | | |  | Vvi-Vitvi10g00563\_t001 |  |  |  |  |  |
| 2 | Ath-AT1G29041.1 |  |  |  | | | |  | | | |  |  |  |  |  |
| 2 | Ath-AT1G29050.1 |  |  |  | Vvi-Vitvi12g00209\_t001 |  | Vvi-Vitvi10g00564\_t001 |  |  |  |  |  |
| 1 | Ath-AT1G29060.1 |  |  |  |  |  | | | |  |  |  |  |  |
| 1 | Ath-AT1G29070.1 |  |  |  |  |  | Vvi-Vitvi10g00565\_t001 |  |  |  |  |  |
| 1 | Ath-AT1G29080.1 |  |  |  |  |  | | | |  |  |  |  |  |
| 1 | Ath-AT1G29090.1 |  |  |  |  |  | | | |  |  |  |  |  |
| 1 | Ath-AT1G29100.2 |  |  |  |  |  | Vvi-Vitvi10g00576\_t001 |  |  |  |  |  |
| 1 | Ath-AT1G29110.1 |  |  |  |  |  | | | |  |  |  |  |  |
| 1 | Ath-AT1G29120.5 |  |  |  |  |  | Vvi-Vitvi10g00577\_t001 |  |  |  |  |  |
| 1 | Ath-AT1G29140.1 |  |  |  |  |  | Vvi-Vitvi10g00580\_t001 |  |  |  |  |  |
| 1 | Ath-AT1G29150.1 |  |  |  |  |  | | | |  |  |  |  |  |
| 1 | Ath-AT1G29160.1 |  |  |  |  |  | Vvi-Vitvi10g00581\_t001 |  |  |  |  |  |
| 1 | Ath-AT1G29170.1 |  |  |  |  |  | Vvi-Vitvi10g00582\_t001 |  |  |  |  |  |
| 1 | Ath-AT1G29179.1 |  |  |  |  |  | | | |  |  |  |  |  |
| 1 | Ath-AT1G29180.1 |  |  |  |  |  | | | |  |  |  |  |  |
| 1 | Ath-AT1G29195.1 |  |  |  |  |  | Vvi-Vitvi10g00585\_t001 |  |  |  |  |  |
| 1 | Ath-AT1G29200.2 |  |  |  |  |  | | | |  |  |  |  |  |
| 1 | Ath-AT1G29220.2 |  |  |  |  |  | | | |  |  |  |  |  |
| 1 | Ath-AT1G29230.1 |  |  |  |  |  | Vvi-Vitvi10g00600\_t001 |  |  |  |  |  |
| 1 | Ath-AT1G29240.1 |  |  |  |  |  | | | |  |  |  |  |  |
| 1 | Ath-AT1G29250.1 |  |  |  |  |  | | | |  |  |  |  |  |
| 1 | Ath-AT1G29260.1 |  |  |  |  |  | | | |  |  |  |  |  |
| 1 | Ath-AT1G29270.1 |  |  |  |  |  | Vvi-Vitvi10g00617\_t001 |  |  |  |  |  |
| 1 | Ath-AT1G29280.1 |  |  |  |  |  | Vvi-Vitvi10g00618\_t001 |  |  |  |  |  |
| 1 | Ath-AT1G29290.1 |  |  |  |  |  | Vvi-Vitvi10g01798\_t001 |  |  |  |  |  |
| 1 | Ath-AT1G29300.1 |  |  |  |  |  | Vvi-Vitvi10g00626\_t001 |  |  |  |  |  |
| 1 | Ath-AT1G29310.1 |  |  |  |  |  | | | |  |  |  |  |  |
| 1 | Ath-AT1G29320.2 |  |  |  |  |  | | | |  |  |  |  |  |
| 1 | Ath-AT1G29330.1 |  |  |  |  |  | Vvi-Vitvi10g00627\_t001 |  |  |  |  |  |
| 1 | Ath-AT1G29340.1 |  |  |  |  |  | Vvi-Vitvi10g00630\_t001 |  |  |  |  |  |
| 0 | Ath-AT1G29350.1 |  |  |  |  |  |  |  |  |
| 0 | Ath-AT1G29355.1 |  |  |  |  |  |  |  |  |
| 0 | Ath-AT1G29370.1 |  |  |  |  |  |  |  |  |
| 0 | Ath-AT1G29380.1 |  |  |  |  |  |  |  |  |
| 0 | Ath-AT1G29390.1 |  |  |  |  |  |  |  |  |
| 0 | Ath-AT1G29395.1 |  |  |  |  |  |  |  |  |
| 0 | Ath-AT1G29400.2 |  |  |  |  |  |  |  |  |
| 0 | Ath-AT1G29410.2 |  |  |  |  |  |  |  |  |
| 0 | Ath-AT1G29418.1 |  |  |  |  |  |  |  |  |
| 0 | Ath-AT1G29420.2 |  |  |  |  |  |  |  |  |
| 0 | Ath-AT1G29430.1 |  |  |  |  |  |  |  |  |
| 0 | Ath-AT1G29435.1 |  |  |  |  |  |  |  |  |
| 0 | Ath-AT1G29440.1 |  |  |  |  |  |  |  |  |
| 0 | Ath-AT1G29450.1 |  |  |  |  |  |  |  |  |
| 0 | Ath-AT1G29460.2 |  |  |  |  |  |  |  |  |
| 0 | Ath-AT1G29465.1 |  |  |  |  |  |  |  |  |
| 0 | Ath-AT1G29470.1 |  |  |  |  |  |  |  |  |
| 0 | Ath-AT1G29480.1 |  |  |  |  |  |  |  |  |
| 0 | Ath-AT1G29490.1 |  |  |  |  |  |  |  |  |
| 0 | Ath-AT1G29500.1 |  |  |  |  |  |  |  |  |
| 0 | Ath-AT1G29510.1 |  |  |  |  |  |  |  |  |
| 1 | Ath-AT1G29520.1 |  | Vvi-Vitvi10g01835\_t001 |  |  |  |  |  |  |  |
| 1 | Ath-AT1G29530.1 |  | | | |  |  |  |  |  |  |  |
| 1 | Ath-AT1G29535.1 |  | | | |  |  |  |  |  |  |  |
| 1 | Ath-AT1G29540.1 |  | | | |  |  |  |  |  |  |  |
| 2 | Ath-AT1G29550.1 |  | | | |  | Vvi-Vitvi10g00684\_t001 |  |  |  |  |  |  |
| 2 | Ath-AT1G29560.1 |  | | | |  | | | |  |  |  |  |  |  |
| 2 | Ath-AT1G29570.1 |  | | | |  | | | |  |  |  |  |  |  |
| 2 | Ath-AT1G29580.1 |  | | | |  | | | |  |  |  |  |  |  |
| 2 | Ath-AT1G29590.1 |  | | | |  | | | |  |  |  |  |  |  |
| 2 | Ath-AT1G29600.2 |  | | | |  | | | |  |  |  |  |  |  |
| 2 | Ath-AT1G29620.1 |  | | | |  | | | |  |  |  |  |  |  |
| 2 | Ath-AT1G29630.2 |  | | | |  | Vvi-Vitvi10g00678\_t001 |  |  |  |  |  |  |
| 2 | Ath-AT1G29640.1 |  | | | |  | Vvi-Vitvi10g01813\_t001 |  |  |  |  |  |  |
| 2 | Ath-AT1G29660.1 |  | | | |  | Vvi-Vitvi10g00665\_t001 |  |  |  |  |  |  |
| 2 | Ath-AT1G29670.2 |  | | | |  | | | |  |  |  |  |  |  |
| 2 | Ath-AT1G29680.1 |  | | | |  | Vvi-Vitvi10g00660\_t001 |  |  |  |  |  |  |
| 2 | Ath-AT1G29690.1 |  | | | |  | Vvi-Vitvi10g00657\_t001 |  |  |  |  |  |  |
| 2 | Ath-AT1G29700.1 |  | | | |  | | | |  |  |  |  |  |  |
| 2 | Ath-AT1G29710.1 |  | | | |  | | | |  |  |  |  |  |  |
| 2 | Ath-AT1G29720.1 |  | | | |  | Vvi-Vitvi10g04384\_t001 |  |  |  |  |  |  |
| 2 | Ath-AT1G29730.2 |  | | | |  | | | |  |  |  |  |  |  |
| 2 | Ath-AT1G29740.1 |  | | | |  | | | |  |  |  |  |  |  |
| 2 | Ath-AT1G29750.2 |  | | | |  | | | |  |  |  |  |  |  |
| 2 | Ath-AT1G29760.1 |  | | | |  | Vvi-Vitvi10g00640\_t001 |  |  |  |  |  |  |
| 2 | Ath-AT1G29770.1 |  | Vvi-Vitvi10g00716\_t001 |  | Vvi-Vitvi10g00638\_t001 |  |  |  |  |  |  |
| 2 | Ath-AT1G29775.1 |  | | | |  | | | |  |  |  |  |  |  |
| 2 | Ath-AT1G29780.1 |  | | | |  | | | |  |  |  |  |  |  |
| 2 | Ath-AT1G29790.1 |  | | | |  | | | |  |  |  |  |  |  |
| 2 | Ath-AT1G29800.1 |  | | | |  | Vvi-Vitvi10g00637\_t002 |  |  |  |  |  |  |
| 1 | Ath-AT1G29810.1 |  | | | |  |  |  |  |  |  |  |
| 1 | Ath-AT1G29820.1 |  | Vvi-Vitvi10g00723\_t004 |  |  |  |  |  |  |  |
| 1 | Ath-AT1G29830.3 |  | | | |  |  |  |  |  |  |  |
| 1 | Ath-AT1G29840.1 |  | | | |  |  |  |  |  |  |  |
| 1 | Ath-AT1G29850.3 |  | Vvi-Vitvi10g00728\_t001 |  |  |  |  |  |  |  |
| 1 | Ath-AT1G29860.1 |  | Vvi-Vitvi10g00732\_t001 |  |  |  |  |  |  |  |
| 1 | Ath-AT1G29870.1 |  | | | |  |  |  |  |  |  |  |
| 1 | Ath-AT1G29880.1 |  | | | |  |  |  |  |  |  |  |
| 1 | Ath-AT1G29890.2 |  | Vvi-Vitvi10g00733\_t001 |  |  |  |  |  |  |  |
| 1 | Ath-AT1G29900.1 |  | | | |  |  |  |  |  |  |  |
| 1 | Ath-AT1G29910.1 |  | Vvi-Vitvi10g01839\_t001 |  |  |  |  |  |  |  |
| 1 | Ath-AT1G29920.1 |  | | | |  |  |  |  |  |  |  |
| 1 | Ath-AT1G29930.1 |  | | | |  |  |  |  |  |  |  |
| 1 | Ath-AT1G29940.1 |  | | | |  |  |  |  |  |  |  |
| 1 | Ath-AT1G29950.1 |  | Vvi-Vitvi10g00744\_t001 |  |  |  |  |  |  |  |
| 1 | Ath-AT1G29960.1 |  | Vvi-Vitvi10g00749\_t001 |  |  |  |  |  |  |  |
| 1 | Ath-AT1G29962.1 |  | | | |  |  |  |  |  |  |  |
| 1 | Ath-AT1G29965.1 |  | Vvi-Vitvi10g00761\_t003 |  |  |  |  |  |  |  |
| 1 | Ath-AT1G29970.2 |  | | | |  |  |  |  |  |  |  |
| 1 | Ath-AT1G29980.1 |  | Vvi-Vitvi10g00774\_t001 |  |  |  |  |  |  |  |
| 1 | Ath-AT1G29990.1 |  | Vvi-Vitvi10g00775\_t001 |  |  |  |  |  |  |  |
| 1 | Ath-AT1G30000.2 |  | Vvi-Vitvi10g00778\_t001 |  |  |  |  |  |  |  |
| 1 | Ath-AT1G30010.1 |  | Vvi-Vitvi10g00780\_t001 |  |  |  |  |  |  |  |
| 1 | Ath-AT1G30020.1 |  | Vvi-Vitvi10g00781\_t001 |  |  |  |  |  |  |  |
| 1 | Ath-AT1G30040.1 |  | Vvi-Vitvi10g00796\_t001 |  |  |  |  |  |  |  |
| 1 | Ath-AT1G30050.1 |  | | | |  |  |  |  |  |  |  |
| 1 | Ath-AT1G30060.1 |  | | | |  |  |  |  |  |  |  |
| 1 | Ath-AT1G30070.2 |  | Vvi-Vitvi10g00798\_t001 |  |  |  |  |  |  |  |
| 1 | Ath-AT1G30080.1 |  | Vvi-Vitvi10g00811\_t001 |  |  |  |  |  |  |  |
| 1 | Ath-AT1G30090.1 |  | Vvi-Vitvi10g04439\_t001 |  |  |  |  |  |  |  |
| 1 | Ath-AT1G30100.1 |  | Vvi-Vitvi10g00821\_t001 |  |  |  |  |  |  |  |
| 1 | Ath-AT1G30110.2 |  | | | |  |  |  |  |  |  |  |
| 1 | Ath-AT1G30120.1 |  | Vvi-Vitvi10g04440\_t001 |  |  |  |  |  |  |  |
| 1 | Ath-AT1G30130.1 |  | Vvi-Vitvi10g00825\_t002 |  |  |  |  |  |  |  |
| 1 | Ath-AT1G30135.1 |  | Vvi-Vitvi10g00826\_t001 |  |  |  |  |  |  |  |
| 1 | Ath-AT1G30140.1 |  | | | |  |  |  |  |  |  |  |
| 1 | Ath-AT1G30160.2 |  | | | |  |  |  |  |  |  |  |
| 1 | Ath-AT1G30170.1 |  | | | |  |  |  |  |  |  |  |
| 1 | Ath-AT1G30190.1 |  | | | |  |  |  |  |  |  |  |
| 1 | Ath-AT1G30200.1 |  | Vvi-Vitvi10g00837\_t001 |  |  |  |  |  |  |  |
| 1 | Ath-AT1G30210.1 |  | Vvi-Vitvi10g00838\_t001 |  |  |  |  |  |  |  |
| 1 | Ath-AT1G30220.1 |  | Vvi-Vitvi10g00839\_t001 |  |  |  |  |  |  |  |
| 1 | Ath-AT1G30230.2 |  | | | |  |  |  |  |  |  |  |
| 1 | Ath-AT1G30240.2 |  | | | |  |  |  |  |  |  |  |
| 1 | Ath-AT1G30250.1 |  | | | |  |  |  |  |  |  |  |
| 1 | Ath-AT1G30260.1 |  | Vvi-Vitvi10g04446\_t001 |  |  |  |  |  |  |  |
| 1 | Ath-AT1G30270.1 |  | Vvi-Vitvi10g01887\_t001 |  |  |  |  |  |  |  |
| 1 | Ath-AT1G30280.1 |  | Vvi-Vitvi10g01888\_t001 |  |  |  |  |  |  |  |
| 1 | Ath-AT1G30290.2 |  | Vvi-Vitvi10g00850\_t001 |  |  |  |  |  |  |  |
| 1 | Ath-AT1G30300.2 |  | Vvi-Vitvi10g00851\_t002 |  |  |  |  |  |  |  |
| 1 | Ath-AT1G30320.1 |  | Vvi-Vitvi10g00853\_t001 |  |  |  |  |  |  |  |
| 1 | Ath-AT1G30330.2 |  | Vvi-Vitvi10g00854\_t001 |  |  |  |  |  |  |  |
| 1 | Ath-AT1G30350.1 |  | | | |  |  |  |  |  |  |  |
| 1 | Ath-AT1G30360.1 |  | Vvi-Vitvi10g00862\_t001 |  |  |  |  |  |  |  |
| 1 | Ath-AT1G30370.1 |  | Vvi-Vitvi10g04449\_t001 |  |  |  |  |  |  |  |
| 1 | Ath-AT1G30380.1 |  | Vvi-Vitvi10g00882\_t001 |  |  |  |  |  |  |  |
| 1 | Ath-AT1G30400.1 |  | Vvi-Vitvi10g00886\_t001 |  |  |  |  |  |  |  |
| 1 | Ath-AT1G30410.1 |  | | | |  |  |  |  |  |  |  |
| 1 | Ath-AT1G30420.1 |  | | | |  |  |  |  |  |  |  |
| 1 | Ath-AT1G30440.1 |  | Vvi-Vitvi10g00892\_t001 |  |  |  |  |  |  |  |
| 1 | Ath-AT1G30450.3 |  | Vvi-Vitvi10g00899\_t001 |  |  |  |  |  |  |  |
| 1 | Ath-AT1G30455.1 |  | | | |  |  |  |  |  |  |  |
| 1 | Ath-AT1G30460.1 |  | Vvi-Vitvi10g00907\_t001 |  |  |  |  |  |  |  |
| 1 | Ath-AT1G30470.1 |  | Vvi-Vitvi10g00910\_t001 |  |  |  |  |  |  |  |
| 1 | Ath-AT1G30473.1 |  | | | |  |  |  |  |  |  |  |
| 1 | Ath-AT1G30475.1 |  | | | |  |  |  |  |  |  |  |
| 1 | Ath-AT1G30480.1 |  | Vvi-Vitvi10g00915\_t001 |  |  |  |  |  |  |  |
| 1 | Ath-AT1G30490.1 |  | | | |  |  |  |  |  |  |  |
| 1 | Ath-AT1G30500.2 |  | | | |  |  |  |  |  |  |  |
| 1 | Ath-AT1G30510.2 |  | Vvi-Vitvi10g00934\_t001 |  |  |  |  |  |  |  |
| 1 | Ath-AT1G30515.1 |  | Vvi-Vitvi10g01912\_t001 |  |  |  |  |  |  |  |
| 1 | Ath-AT1G30520.1 |  | Vvi-Vitvi10g00940\_t001 |  |  |  |  |  |  |  |
| 1 | Ath-AT1G30530.1 |  | | | |  |  |  |  |  |  |  |
| 1 | Ath-AT1G30540.1 |  | Vvi-Vitvi10g00944\_t001 |  |  |  |  |  |  |  |
| 1 | Ath-AT1G30545.2 |  | | | |  |  |  |  |  |  |  |
| 1 | Ath-AT1G30550.2 |  | | | |  |  |  |  |  |  |  |
| 1 | Ath-AT1G30560.1 |  | | | |  |  |  |  |  |  |  |
| 1 | Ath-AT1G30570.1 |  | Vvi-Vitvi10g00946\_t001 |  |  |  |  |  |  |  |
| 1 | Ath-AT1G30580.1 |  | Vvi-Vitvi10g00948\_t001 |  |  |  |  |  |  |  |
| 0 | Ath-AT1G30590.2 |  |  |  |  |  |  |  |  |
| 0 | Ath-AT1G30600.1 |  |  |  |  |  |  |  |  |
| 1 | Ath-AT1G30610.1 |  | Vvi-Vitvi10g01068\_t001 |  |  |  |  |  |  |  |
| 1 | Ath-AT1G30620.1 |  | Vvi-Vitvi10g01066\_t001 |  |  |  |  |  |  |  |
| 1 | Ath-AT1G30630.1 |  | Vvi-Vitvi10g01065\_t001 |  |  |  |  |  |  |  |
| 1 | Ath-AT1G30640.1 |  | | | |  |  |  |  |  |  |  |
| 1 | Ath-AT1G30650.1 |  | | | |  |  |  |  |  |  |  |
| 1 | Ath-AT1G30660.1 |  | Vvi-Vitvi10g01935\_t001 |  |  |  |  |  |  |  |
| 1 | Ath-AT1G30670.1 |  | | | |  |  |  |  |  |  |  |
| 1 | Ath-AT1G30680.1 |  | | | |  |  |  |  |  |  |  |
| 1 | Ath-AT1G30690.2 |  | Vvi-Vitvi10g01035\_t001 |  |  |  |  |  |  |  |
| 1 | Ath-AT1G30700.1 |  | Vvi-Vitvi10g01029\_t001 |  |  |  |  |  |  |  |
| 1 | Ath-AT1G30710.1 |  | Vvi-Vitvi10g04509\_t001 |  |  |  |  |  |  |  |
| 1 | Ath-AT1G30720.1 |  | | | |  |  |  |  |  |  |  |
| 1 | Ath-AT1G30730.1 |  | | | |  |  |  |  |  |  |  |
| 1 | Ath-AT1G30740.2 |  | | | |  |  |  |  |  |  |  |
| 1 | Ath-AT1G30750.1 |  | | | |  |  |  |  |  |  |  |
| 1 | Ath-AT1G30755.1 |  | | | |  |  |  |  |  |  |  |
| 1 | Ath-AT1G30757.1 |  | | | |  |  |  |  |  |  |  |
| 1 | Ath-AT1G30760.2 |  | Vvi-Vitvi10g01017\_t001 |  |  |  |  |  |  |  |
| 0 | Ath-AT1G30780.1 |  |  |  |  |  |  |  |  |
| 0 | Ath-AT1G30790.1 |  |  |  |  |  |  |  |  |
| 0 | Ath-AT1G30795.1 |  |  |  |  |  |  |  |  |
| 0 | Ath-AT1G30810.1 |  |  |  |  |  |  |  |  |
| 0 | Ath-AT1G30800.1 |  |  |  |  |  |  |  |  |
| 0 | Ath-AT1G30814.3 |  |  |  |  |  |  |  |  |
| 0 | Ath-AT1G30820.1 |  |  |  |  |  |  |  |  |
| 0 | Ath-AT1G30825.1 |  |  |  |  |  |  |  |  |
| 0 | Ath-AT1G30840.1 |  |  |  |  |  |  |  |  |
| 0 | Ath-AT1G30845.1 |  |  |  |  |  |  |  |  |
| 0 | Ath-AT1G30850.1 |  |  |  |  |  |  |  |  |
| 0 | Ath-AT1G30860.1 |  |  |  |  |  |  |  |  |
| 0 | Ath-AT1G30870.1 |  |  |  |  |  |  |  |  |
| 0 | Ath-AT1G30880.1 |  |  |  |  |  |  |  |  |
| 0 | Ath-AT1G30890.1 |  |  |  |  |  |  |  |  |
| 1 | Ath-AT1G30900.1 |  | Vvi-Vitvi01g01855\_t001 |  |  |  |  |  |  |  |
| 1 | Ath-AT1G30910.1 |  | Vvi-Vitvi01g00087\_t002 |  |  |  |  |  |  |  |
| 1 | Ath-AT1G30920.1 |  | | | |  |  |  |  |  |  |  |
| 1 | Ath-AT1G30925.1 |  | | | |  |  |  |  |  |  |  |
| 1 | Ath-AT1G30930.1 |  | | | |  |  |  |  |  |  |  |
| 1 | Ath-AT1G30950.1 |  | Vvi-Vitvi01g00096\_t001 |  |  |  |  |  |  |  |
| 1 | Ath-AT1G30960.1 |  | Vvi-Vitvi01g00097\_t001 |  |  |  |  |  |  |  |
| 1 | Ath-AT1G30970.3 |  | Vvi-Vitvi01g00098\_t002 |  |  |  |  |  |  |  |
| 1 | Ath-AT1G30972.1 |  | | | |  |  |  |  |  |  |  |
| 1 | Ath-AT1G30974.1 |  | | | |  |  |  |  |  |  |  |
| 1 | Ath-AT1G30990.1 |  | | | |  |  |  |  |  |  |  |
| 1 | Ath-AT1G31000.1 |  | | | |  |  |  |  |  |  |  |
| 1 | Ath-AT1G31010.1 |  | | | |  |  |  |  |  |  |  |
| 1 | Ath-AT1G31020.1 |  | Vvi-Vitvi01g01866\_t001 |  |  |  |  |  |  |  |
| 1 | Ath-AT1G31040.1 |  | Vvi-Vitvi01g00111\_t001 |  |  |  |  |  |  |  |
| 1 | Ath-AT1G31050.6 |  | | | |  |  |  |  |  |  |  |
| 1 | Ath-AT1G31070.2 |  | Vvi-Vitvi01g00112\_t001 |  |  |  |  |  |  |  |
| 1 | Ath-AT1G31080.1 |  | | | |  |  |  |  |  |  |  |
| 1 | Ath-AT1G31090.1 |  | | | |  |  |  |  |  |  |  |
| 1 | Ath-AT1G31095.1 |  | | | |  |  |  |  |  |  |  |
| 1 | Ath-AT1G31120.1 |  | Vvi-Vitvi01g00121\_t001 |  |  |  |  |  |  |  |
| 1 | Ath-AT1G31130.1 |  | Vvi-Vitvi01g00124\_t001 |  |  |  |  |  |  |  |
| 1 | Ath-AT1G31140.2 |  | | | |  |  |  |  |  |  |  |
| 1 | Ath-AT1G31150.1 |  | | | |  |  |  |  |  |  |  |
| 1 | Ath-AT1G31160.1 |  | Vvi-Vitvi01g00132\_t001 |  |  |  |  |  |  |  |
| 1 | Ath-AT1G31163.1 |  | | | |  |  |  |  |  |  |  |
| 1 | Ath-AT1G31170.4 |  | Vvi-Vitvi01g00133\_t001 |  |  |  |  |  |  |  |
| 1 | Ath-AT1G31175.2 |  | | | |  |  |  |  |  |  |  |
| 1 | Ath-AT1G31180.2 |  | | | |  |  |  |  |  |  |  |
| 1 | Ath-AT1G31190.1 |  | | | |  |  |  |  |  |  |  |
| 1 | Ath-AT1G31200.1 |  | | | |  |  |  |  |  |  |  |
| 1 | Ath-AT1G31220.1 |  | | | |  |  |  |  |  |  |  |
| 1 | Ath-AT1G31230.1 |  | | | |  |  |  |  |  |  |  |
| 1 | Ath-AT1G31240.1 |  | Vvi-Vitvi01g01875\_t001 |  |  |  |  |  |  |  |
| 0 | Ath-AT1G31243.1 |  |  |  |  |  |  |  |  |
| 0 | Ath-AT1G31250.1 |  |  |  |  |  |  |  |  |
| 0 | Ath-AT1G31255.1 |  |  |  |  |  |  |  |  |
| 0 | Ath-AT1G31260.1 |  |  |  |  |  |  |  |  |
| 0 | Ath-AT1G31270.1 |  |  |  |  |  |  |  |  |
| 0 | Ath-AT1G31280.1 |  |  |  |  |  |  |  |  |
| 0 | Ath-AT1G31290.1 |  |  |  |  |  |  |  |  |
| 0 | Ath-AT1G31300.2 |  |  |  |  |  |  |  |  |
| 0 | Ath-AT1G31310.1 |  |  |  |  |  |  |  |  |
| 0 | Ath-AT1G31320.1 |  |  |  |  |  |  |  |  |
| 0 | Ath-AT1G31330.1 |  |  |  |  |  |  |  |  |
| 0 | Ath-AT1G31335.1 |  |  |  |  |  |  |  |  |
| 0 | Ath-AT1G31340.1 |  |  |  |  |  |  |  |  |
| 0 | Ath-AT1G31350.2 |  |  |  |  |  |  |  |  |
| 1 | Ath-AT1G31360.3 |  | Vvi-Vitvi02g01191\_t001 |  |  |  |  |  |  |  |
| 1 | Ath-AT1G31370.1 |  | | | |  |  |  |  |  |  |  |
| 1 | Ath-AT1G31380.1 |  | | | |  |  |  |  |  |  |  |
| 1 | Ath-AT1G31390.1 |  | | | |  |  |  |  |  |  |  |
| 1 | Ath-AT1G31400.1 |  | | | |  |  |  |  |  |  |  |
| 1 | Ath-AT1G31410.1 |  | | | |  |  |  |  |  |  |  |
| 1 | Ath-AT1G31420.1 |  | Vvi-Vitvi02g01204\_t001 |  |  |  |  |  |  |  |
| 1 | Ath-AT1G31430.1 |  | | | |  |  |  |  |  |  |  |
| 1 | Ath-AT1G31440.1 |  | Vvi-Vitvi02g04382\_t001 |  |  |  |  |  |  |  |
| 1 | Ath-AT1G31450.1 |  | Vvi-Vitvi02g01207\_t001 |  |  |  |  |  |  |  |
| 1 | Ath-AT1G31460.1 |  | Vvi-Vitvi02g01210\_t001 |  |  |  |  |  |  |  |
| 1 | Ath-AT1G31470.1 |  | Vvi-Vitvi02g01224\_t001 |  |  |  |  |  |  |  |
| 1 | Ath-AT1G31480.1 |  | | | |  |  |  |  |  |  |  |
| 1 | Ath-AT1G31490.1 |  | Vvi-Vitvi02g01227\_t001 |  |  |  |  |  |  |  |
| 1 | Ath-AT1G31500.7 |  | Vvi-Vitvi02g01228\_t001 |  |  |  |  |  |  |  |
| 1 | Ath-AT1G31510.1 |  | | | |  |  |  |  |  |  |  |
| 1 | Ath-AT1G31520.1 |  | | | |  |  |  |  |  |  |  |
| 1 | Ath-AT1G31530.1 |  | | | |  |  |  |  |  |  |  |
| 1 | Ath-AT1G31540.2 |  | | | |  |  |  |  |  |  |  |
| 1 | Ath-AT1G31550.2 |  | | | |  |  |  |  |  |  |  |
| 1 | Ath-AT1G31555.1 |  | | | |  |  |  |  |  |  |  |
| 1 | Ath-AT1G31580.1 |  | | | |  |  |  |  |  |  |  |
| 1 | Ath-AT1G31600.3 |  | Vvi-Vitvi02g01235\_t001 |  |  |  |  |  |  |  |
| 1 | Ath-AT1G31620.1 |  | | | |  |  |  |  |  |  |  |
| 1 | Ath-AT1G31630.1 |  | | | |  |  |  |  |  |  |  |
| 1 | Ath-AT1G31640.1 |  | | | |  |  |  |  |  |  |  |
| 1 | Ath-AT1G31650.1 |  | Vvi-Vitvi02g01236\_t005 |  |  |  |  |  |  |  |
| 0 | Ath-AT1G31660.1 |  |  |  |  |  |  |  |  |
| 0 | Ath-AT1G31670.1 |  |  |  |  |  |  |  |  |
| 0 | Ath-AT1G31690.1 |  |  |  |  |  |  |  |  |
| 0 | Ath-AT1G31710.1 |  |  |  |  |  |  |  |  |
| 0 | Ath-AT1G31720.1 |  |  |  |  |  |  |  |  |
| 0 | Ath-AT1G31730.1 |  |  |  |  |  |  |  |  |
| 0 | Ath-AT1G31740.1 |  |  |  |  |  |  |  |  |
| 0 | Ath-AT1G31750.1 |  |  |  |  |  |  |  |  |
| 0 | Ath-AT1G31760.1 |  |  |  |  |  |  |  |  |
| 0 | Ath-AT1G31770.1 |  |  |  |  |  |  |  |  |
| 0 | Ath-AT1G31772.2 |  |  |  |  |  |  |  |  |
| 0 | Ath-AT1G31780.1 |  |  |  |  |  |  |  |  |
| 0 | Ath-AT1G31790.1 |  |  |  |  |  |  |  |  |
| 0 | Ath-AT1G31800.1 |  |  |  |  |  |  |  |  |
| 0 | Ath-AT1G31810.1 |  |  |  |  |  |  |  |  |
| 0 | Ath-AT1G31812.1 |  |  |  |  |  |  |  |  |
| 0 | Ath-AT1G31814.1 |  |  |  |  |  |  |  |  |
| 0 | Ath-AT1G31817.1 |  |  |  |  |  |  |  |  |
| 0 | Ath-AT1G31820.1 |  |  |  |  |  |  |  |  |
| 0 | Ath-AT1G31830.1 |  |  |  |  |  |  |  |  |
| 0 | Ath-AT1G31835.2 |  |  |  |  |  |  |  |  |
| 0 | Ath-AT1G31840.1 |  |  |  |  |  |  |  |  |
| 0 | Ath-AT1G31850.1 |  |  |  |  |  |  |  |  |
| 0 | Ath-AT1G31860.1 |  |  |  |  |  |  |  |  |
| 0 | Ath-AT1G31870.2 |  |  |  |  |  |  |  |  |
| 0 | Ath-AT1G31880.2 |  |  |  |  |  |  |  |  |
| 0 | Ath-AT1G31885.1 |  |  |  |  |  |  |  |  |
| 0 | Ath-AT1G31910.1 |  |  |  |  |  |  |  |  |
| 0 | Ath-AT1G31920.1 |  |  |  |  |  |  |  |  |
| 0 | Ath-AT1G31930.2 |  |  |  |  |  |  |  |  |
| 0 | Ath-AT1G31940.1 |  |  |  |  |  |  |  |  |
| 0 | Ath-AT1G31950.3 |  |  |  |  |  |  |  |  |
| 0 | Ath-AT1G31960.1 |  |  |  |  |  |  |  |  |
| 0 | Ath-AT1G31970.1 |  |  |  |  |  |  |  |  |
| 0 | Ath-AT1G31990.1 |  |  |  |  |  |  |  |  |
| 0 | Ath-AT1G32000.1 |  |  |  |  |  |  |  |  |
| 0 | Ath-AT1G32010.1 |  |  |  |  |  |  |  |  |
| 0 | Ath-AT1G32020.1 |  |  |  |  |  |  |  |  |
| 0 | Ath-AT1G32030.1 |  |  |  |  |  |  |  |  |
| 0 | Ath-AT1G32050.1 |  |  |  |  |  |  |  |  |
| 0 | Ath-AT1G32060.1 |  |  |  |  |  |  |  |  |
| 0 | Ath-AT1G32070.2 |  |  |  |  |  |  |  |  |
| 0 | Ath-AT1G32080.1 |  |  |  |  |  |  |  |  |
| 0 | Ath-AT1G32090.1 |  |  |  |  |  |  |  |  |
| 0 | Ath-AT1G32100.1 |  |  |  |  |  |  |  |  |
| 0 | Ath-AT1G32120.1 |  |  |  |  |  |  |  |  |
| 0 | Ath-AT1G32127.1 |  |  |  |  |  |  |  |  |
| 0 | Ath-AT1G32130.1 |  |  |  |  |  |  |  |  |
| 0 | Ath-AT1G32140.1 |  |  |  |  |  |  |  |  |
| 1 | Ath-AT1G32150.1 |  | Vvi-Vitvi02g00796\_t003 |  |  |  |  |  |  |  |
| 1 | Ath-AT1G32160.1 |  | Vvi-Vitvi02g00795\_t001 |  |  |  |  |  |  |  |
| 1 | Ath-AT1G32170.1 |  | Vvi-Vitvi02g00792\_t001 |  |  |  |  |  |  |  |
| 1 | Ath-AT1G32180.1 |  | | | |  |  |  |  |  |  |  |
| 1 | Ath-AT1G32190.1 |  | Vvi-Vitvi02g00787\_t001 |  |  |  |  |  |  |  |
| 1 | Ath-AT1G32200.2 |  | Vvi-Vitvi02g00785\_t001 |  |  |  |  |  |  |  |
| 1 | Ath-AT1G32210.1 |  | Vvi-Vitvi02g00780\_t001 |  |  |  |  |  |  |  |
| 1 | Ath-AT1G32220.1 |  | | | |  |  |  |  |  |  |  |
| 1 | Ath-AT1G32225.1 |  | | | |  |  |  |  |  |  |  |
| 1 | Ath-AT1G32230.1 |  | | | |  |  |  |  |  |  |  |
| 1 | Ath-AT1G32240.1 |  | Vvi-Vitvi02g00757\_t002 |  |  |  |  |  |  |  |
| 1 | Ath-AT1G32250.1 |  | | | |  |  |  |  |  |  |  |
| 1 | Ath-AT1G32260.1 |  | | | |  |  |  |  |  |  |  |
| 1 | Ath-AT1G32270.1 |  | Vvi-Vitvi02g00747\_t001 |  |  |  |  |  |  |  |
| 1 | Ath-AT1G32280.1 |  | | | |  |  |  |  |  |  |  |
| 1 | Ath-AT1G32300.1 |  | | | |  |  |  |  |  |  |  |
| 1 | Ath-AT1G32310.1 |  | | | |  |  |  |  |  |  |  |
| 1 | Ath-AT1G32320.1 |  | | | |  |  |  |  |  |  |  |
| 1 | Ath-AT1G32330.1 |  | Vvi-Vitvi02g00739\_t001 |  |  |  |  |  |  |  |
| 1 | Ath-AT1G32337.1 |  | | | |  |  |  |  |  |  |  |
| 1 | Ath-AT1G32340.1 |  | Vvi-Vitvi02g00732\_t001 |  |  |  |  |  |  |  |
| 1 | Ath-AT1G32350.1 |  | | | |  |  |  |  |  |  |  |
| 1 | Ath-AT1G32360.1 |  | Vvi-Vitvi02g00715\_t001 |  |  |  |  |  |  |  |
| 0 | Ath-AT1G32361.1 |  |  |  |  |  |  |  |  |
| 0 | Ath-AT1G32370.2 |  |  |  |  |  |  |  |  |
| 0 | Ath-AT1G32375.1 |  |  |  |  |  |  |  |  |
| 0 | Ath-AT1G32380.1 |  |  |  |  |  |  |  |  |
| 0 | Ath-AT1G32400.1 |  |  |  |  |  |  |  |  |
| 0 | Ath-AT1G32410.3 |  |  |  |  |  |  |  |  |
| 0 | Ath-AT1G32415.1 |  |  |  |  |  |  |  |  |
| 0 | Ath-AT1G32420.1 |  |  |  |  |  |  |  |  |
| 0 | Ath-AT1G32430.1 |  |  |  |  |  |  |  |  |
| 1 | Ath-AT1G32440.1 |  | Vvi-Vitvi02g00684\_t001 |  |  |  |  |  |  |  |
| 1 | Ath-AT1G32450.1 |  | Vvi-Vitvi02g00683\_t001 |  |  |  |  |  |  |  |
| 1 | Ath-AT1G32460.1 |  | | | |  |  |  |  |  |  |  |
| 1 | Ath-AT1G32470.1 |  | Vvi-Vitvi02g00680\_t001 |  |  |  |  |  |  |  |
| 1 | Ath-AT1G32480.1 |  | | | |  |  |  |  |  |  |  |
| 1 | Ath-AT1G32490.1 |  | | | |  |  |  |  |  |  |  |
| 1 | Ath-AT1G32500.1 |  | Vvi-Vitvi02g00674\_t001 |  |  |  |  |  |  |  |
| 1 | Ath-AT1G32510.1 |  | Vvi-Vitvi02g00673\_t001 |  |  |  |  |  |  |  |
| 1 | Ath-AT1G32520.1 |  | Vvi-Vitvi02g00672\_t001 |  |  |  |  |  |  |  |
| 1 | Ath-AT1G32530.1 |  | Vvi-Vitvi02g00670\_t001 |  |  |  |  |  |  |  |
| 1 | Ath-AT1G32540.1 |  | Vvi-Vitvi02g00666\_t003 |  |  |  |  |  |  |  |
| 1 | Ath-AT1G32550.2 |  | Vvi-Vitvi02g00665\_t001 |  |  |  |  |  |  |  |
| 1 | Ath-AT1G32560.1 |  | Vvi-Vitvi02g01498\_t001 |  |  |  |  |  |  |  |
| 0 | Ath-AT1G32570.2 |  |  |  |  |  |  |  |  |
| 0 | Ath-AT1G32575.1 |  |  |  |  |  |  |  |  |
| 0 | Ath-AT1G32580.1 |  |  |  |  |  |  |  |  |
| 0 | Ath-AT1G32583.1 |  |  |  |  |  |  |  |  |
| 0 | Ath-AT1G32585.1 |  |  |  |  |  |  |  |  |
| 0 | Ath-AT1G32600.1 |  |  |  |  |  |  |  |  |
| 0 | Ath-AT1G32610.1 |  |  |  |  |  |  |  |  |
| 0 | Ath-AT1G32630.1 |  |  |  |  |  |  |  |  |
| 1 | Ath-AT1G32640.1 |  | Vvi-Vitvi02g00231\_t001 |  |  |  |  |  |  |  |
| 1 | Ath-AT1G32650.1 |  | | | |  |  |  |  |  |  |  |
| 1 | Ath-AT1G32660.1 |  | | | |  |  |  |  |  |  |  |
| 1 | Ath-AT1G32690.1 |  | | | |  |  |  |  |  |  |  |
| 1 | Ath-AT1G32700.1 |  | | | |  |  |  |  |  |  |  |
| 1 | Ath-AT1G32710.1 |  | | | |  |  |  |  |  |  |  |
| 1 | Ath-AT1G32720.1 |  | | | |  |  |  |  |  |  |  |
| 1 | Ath-AT1G32730.1 |  | | | |  |  |  |  |  |  |  |
| 1 | Ath-AT1G32740.1 |  | | | |  |  |  |  |  |  |  |
| 1 | Ath-AT1G32750.1 |  | | | |  |  |  |  |  |  |  |
| 1 | Ath-AT1G32760.1 |  | Vvi-Vitvi02g00241\_t001 |  |  |  |  |  |  |  |
| 1 | Ath-AT1G32763.1 |  | | | |  |  |  |  |  |  |  |
| 1 | Ath-AT1G32770.1 |  | Vvi-Vitvi02g00242\_t001 |  |  |  |  |  |  |  |
| 1 | Ath-AT1G32780.1 |  | Vvi-Vitvi02g00244\_t001 |  |  |  |  |  |  |  |
| 2 | Ath-AT1G32790.2 |  | Vvi-Vitvi02g00245\_t001 |  | Vvi-Vitvi16g01350\_t001 |  |  |  |  |  |  |
| 2 | Ath-AT1G32810.2 |  | Vvi-Vitvi02g00246\_t001 |  | | | |  |  |  |  |  |  |
| 2 | Ath-AT1G32850.1 |  | Vvi-Vitvi02g00247\_t001 |  | | | |  |  |  |  |  |  |
| 2 | Ath-AT1G32860.1 |  | Vvi-Vitvi02g00248\_t001 |  | | | |  |  |  |  |  |  |
| 2 | Ath-AT1G32870.3 |  | | | |  | | | |  |  |  |  |  |  |
| 2 | Ath-AT1G32880.1 |  | | | |  | | | |  |  |  |  |  |  |
| 2 | Ath-AT1G32900.1 |  | Vvi-Vitvi02g00250\_t001 |  | | | |  |  |  |  |  |  |
| 2 | Ath-AT1G32910.1 |  | | | |  | | | |  |  |  |  |  |  |
| 2 | Ath-AT1G32920.1 |  | Vvi-Vitvi02g04056\_t001 |  | | | |  |  |  |  |  |  |
| 2 | Ath-AT1G32928.1 |  | | | |  | | | |  |  |  |  |  |  |
| 2 | Ath-AT1G32930.1 |  | Vvi-Vitvi02g00256\_t001 |  | | | |  |  |  |  |  |  |
| 2 | Ath-AT1G32940.1 |  | Vvi-Vitvi02g00258\_t001 |  | Vvi-Vitvi16g01344\_t002 |  |  |  |  |  |  |
| 2 | Ath-AT1G32950.1 |  | | | |  | | | |  |  |  |  |  |  |
| 2 | Ath-AT1G32960.1 |  | | | |  | | | |  |  |  |  |  |  |
| 2 | Ath-AT1G32970.1 |  | | | |  | | | |  |  |  |  |  |  |
| 2 | Ath-AT1G32975.1 |  | | | |  | | | |  |  |  |  |  |  |
| 2 | Ath-AT1G32980.1 |  | | | |  | | | |  |  |  |  |  |  |
| 2 | Ath-AT1G32990.1 |  | Vvi-Vitvi02g00261\_t001 |  | | | |  |  |  |  |  |  |
| 2 | Ath-AT1G33010.1 |  | | | |  | | | |  |  |  |  |  |  |
| 2 | Ath-AT1G33020.1 |  | | | |  | | | |  |  |  |  |  |  |
| 2 | Ath-AT1G33030.1 |  | Vvi-Vitvi02g00263\_t001 |  | Vvi-Vitvi16g01334\_t001 |  |  |  |  |  |  |
| 2 | Ath-AT1G33040.1 |  | | | |  | Vvi-Vitvi16g01333\_t001 |  |  |  |  |  |  |
| 2 | Ath-AT1G33050.1 |  | | | |  | | | |  |  |  |  |  |  |
| 2 | Ath-AT1G33055.1 |  | | | |  | | | |  |  |  |  |  |  |
| 2 | Ath-AT1G33060.2 |  | Vvi-Vitvi02g00277\_t001 |  | Vvi-Vitvi16g01327\_t002 |  |  |  |  |  |  |
| 2 | Ath-AT1G33070.1 |  | | | |  | | | |  |  |  |  |  |  |
| 2 | Ath-AT1G33080.1 |  | | | |  | | | |  |  |  |  |  |  |
| 2 | Ath-AT1G33090.1 |  | | | |  | | | |  |  |  |  |  |  |
| 2 | Ath-AT1G33100.1 |  | | | |  | | | |  |  |  |  |  |  |
| 2 | Ath-AT1G33102.1 |  | | | |  | | | |  |  |  |  |  |  |
| 2 | Ath-AT1G33110.1 |  | | | |  | | | |  |  |  |  |  |  |
| 2 | Ath-AT1G33120.1 |  | | | |  | Vvi-Vitvi16g01324\_t001 |  |  |  |  |  |  |
| 1 | Ath-AT1G33140.1 |  | | | |  |  |  |  |  |  |  |
| 1 | Ath-AT1G33170.1 |  | Vvi-Vitvi02g00289\_t001 |  |  |  |  |  |  |  |
| 1 | Ath-AT1G33220.1 |  | | | |  |  |  |  |  |  |  |
| 1 | Ath-AT1G33230.2 |  | Vvi-Vitvi02g00290\_t001 |  |  |  |  |  |  |  |
| 1 | Ath-AT1G33240.1 |  | Vvi-Vitvi02g00291\_t001 |  |  |  |  |  |  |  |
| 1 | Ath-AT1G33250.1 |  | Vvi-Vitvi02g00293\_t001 |  |  |  |  |  |  |  |
| 1 | Ath-AT1G33260.1 |  | Vvi-Vitvi02g04067\_t001 |  |  |  |  |  |  |  |
| 1 | Ath-AT1G33265.1 |  | Vvi-Vitvi02g04068\_t001 |  |  |  |  |  |  |  |
| 0 | Ath-AT1G33270.1 |  |  |  |  |  |  |  |  |
| 0 | Ath-AT1G33280.1 |  |  |  |  |  |  |  |  |
| 0 | Ath-AT1G33290.1 |  |  |  |  |  |  |  |  |
| 0 | Ath-AT1G33320.1 |  |  |  |  |  |  |  |  |
| 1 | Ath-AT1G33330.1 |  | Vvi-Vitvi18g02429\_t002 |  |  |  |  |  |  |  |
| 1 | Ath-AT1G33340.1 |  | Vvi-Vitvi18g02428\_t001 |  |  |  |  |  |  |  |
| 1 | Ath-AT1G33350.1 |  | Vvi-Vitvi18g02420\_t001 |  |  |  |  |  |  |  |
| 1 | Ath-AT1G33355.1 |  | | | |  |  |  |  |  |  |  |
| 1 | Ath-AT1G33360.1 |  | Vvi-Vitvi18g02418\_t001 |  |  |  |  |  |  |  |
| 1 | Ath-AT1G33390.2 |  | | | |  |  |  |  |  |  |  |
| 1 | Ath-AT1G33400.2 |  | | | |  |  |  |  |  |  |  |
| 1 | Ath-AT1G33410.2 |  | | | |  |  |  |  |  |  |  |
| 1 | Ath-AT1G33420.1 |  | Vvi-Vitvi18g02400\_t001 |  |  |  |  |  |  |  |
| 1 | Ath-AT1G33430.2 |  | Vvi-Vitvi18g02399\_t001 |  |  |  |  |  |  |  |
| 1 | Ath-AT1G33440.1 |  | Vvi-Vitvi18g02396\_t001 |  |  |  |  |  |  |  |
| 1 | Ath-AT1G33470.1 |  | Vvi-Vitvi18g02391\_t001 |  |  |  |  |  |  |  |
| 1 | Ath-AT1G33475.1 |  | Vvi-Vitvi18g03258\_t002 |  |  |  |  |  |  |  |
| 1 | Ath-AT1G33480.1 |  | Vvi-Vitvi18g03257\_t001 |  |  |  |  |  |  |  |
| 1 | Ath-AT1G33490.1 |  | Vvi-Vitvi18g02380\_t001 |  |  |  |  |  |  |  |
| 0 | Ath-AT1G33500.1 |  |  |  |  |  |  |  |  |
| 0 | Ath-AT1G33520.1 |  |  |  |  |  |  |  |  |
| 0 | Ath-AT1G33530.1 |  |  |  |  |  |  |  |  |
| 0 | Ath-AT1G33540.1 |  |  |  |  |  |  |  |  |
| 0 | Ath-AT1G33560.1 |  |  |  |  |  |  |  |  |
| 0 | Ath-AT1G33590.2 |  |  |  |  |  |  |  |  |
| 0 | Ath-AT1G33600.1 |  |  |  |  |  |  |  |  |
| 0 | Ath-AT1G33607.1 |  |  |  |  |  |  |  |  |
| 0 | Ath-AT1G33610.1 |  |  |  |  |  |  |  |  |
| 0 | Ath-AT1G33612.1 |  |  |  |  |  |  |  |  |
| 0 | Ath-AT1G33640.1 |  |  |  |  |  |  |  |  |
| 0 | Ath-AT1G33670.1 |  |  |  |  |  |  |  |  |
| 0 | Ath-AT1G33680.1 |  |  |  |  |  |  |  |  |
| 1 | Ath-AT1G33700.1 |  | Vvi-Vitvi12g02063\_t001 |  |  |  |  |  |  |  |
| 1 | Ath-AT1G33710.1 |  | | | |  |  |  |  |  |  |  |
| 1 | Ath-AT1G33720.1 |  | | | |  |  |  |  |  |  |  |
| 1 | Ath-AT1G33730.1 |  | | | |  |  |  |  |  |  |  |
| 1 | Ath-AT1G33750.1 |  | | | |  |  |  |  |  |  |  |
| 1 | Ath-AT1G33760.1 |  | | | |  |  |  |  |  |  |  |
| 1 | Ath-AT1G33770.1 |  | | | |  |  |  |  |  |  |  |
| 1 | Ath-AT1G33780.1 |  | | | |  |  |  |  |  |  |  |
| 1 | Ath-AT1G33790.2 |  | | | |  |  |  |  |  |  |  |
| 1 | Ath-AT1G33800.1 |  | Vvi-Vitvi12g02059\_t001 |  |  |  |  |  |  |  |
| 1 | Ath-AT1G33810.1 |  | | | |  |  |  |  |  |  |  |
| 1 | Ath-AT1G33811.1 |  | | | |  |  |  |  |  |  |  |
| 1 | Ath-AT1G33820.1 |  | | | |  |  |  |  |  |  |  |
| 1 | Ath-AT1G33830.1 |  | | | |  |  |  |  |  |  |  |
| 1 | Ath-AT1G33840.1 |  | | | |  |  |  |  |  |  |  |
| 1 | Ath-AT1G33850.1 |  | | | |  |  |  |  |  |  |  |
| 1 | Ath-AT1G33860.2 |  | | | |  |  |  |  |  |  |  |
| 1 | Ath-AT1G33870.1 |  | | | |  |  |  |  |  |  |  |
| 1 | Ath-AT1G33880.2 |  | | | |  |  |  |  |  |  |  |
| 1 | Ath-AT1G33890.1 |  | | | |  |  |  |  |  |  |  |
| 1 | Ath-AT1G33900.1 |  | | | |  |  |  |  |  |  |  |
| 1 | Ath-AT1G33910.1 |  | | | |  |  |  |  |  |  |  |
| 1 | Ath-AT1G33920.1 |  | | | |  |  |  |  |  |  |  |
| 1 | Ath-AT1G33930.1 |  | | | |  |  |  |  |  |  |  |
| 1 | Ath-AT1G33940.1 |  | | | |  |  |  |  |  |  |  |
| 1 | Ath-AT1G33945.1 |  | | | |  |  |  |  |  |  |  |
| 1 | Ath-AT1G33950.1 |  | | | |  |  |  |  |  |  |  |
| 1 | Ath-AT1G33960.2 |  | | | |  |  |  |  |  |  |  |
| 1 | Ath-AT1G33970.1 |  | | | |  |  |  |  |  |  |  |
| 1 | Ath-AT1G33980.2 |  | Vvi-Vitvi12g02047\_t003 |  |  |  |  |  |  |  |
| 1 | Ath-AT1G33990.1 |  | | | |  |  |  |  |  |  |  |
| 2 | Ath-AT1G34000.1 |  | | | |  | Vvi-Vitvi18g02961\_t001 |  |  |  |  |  |  |
| 2 | Ath-AT1G34010.2 |  | | | |  | Vvi-Vitvi18g01604\_t001 |  |  |  |  |  |  |
| 2 | Ath-AT1G34015.1 |  | | | |  | | | |  |  |  |  |  |  |
| 2 | Ath-AT1G34020.2 |  | | | |  | Vvi-Vitvi18g01602\_t001 |  |  |  |  |  |  |
| 2 | Ath-AT1G34030.1 |  | | | |  | Vvi-Vitvi18g04412\_t001 |  |  |  |  |  |  |
| 2 | Ath-AT1G34040.1 |  | | | |  | | | |  |  |  |  |  |  |
| 2 | Ath-AT1G34042.1 |  | | | |  | | | |  |  |  |  |  |  |
| 2 | Ath-AT1G34046.1 |  | | | |  | | | |  |  |  |  |  |  |
| 2 | Ath-AT1G34047.2 |  | | | |  | | | |  |  |  |  |  |  |
| 2 | Ath-AT1G34049.1 |  | | | |  | | | |  |  |  |  |  |  |
| 2 | Ath-AT1G34050.1 |  | | | |  | | | |  |  |  |  |  |  |
| 2 | Ath-AT1G34060.1 |  | | | |  | Vvi-Vitvi18g01570\_t001 |  |  |  |  |  |  |
| 2 | Ath-AT1G34065.1 |  | | | |  | Vvi-Vitvi18g01571\_t001 |  |  |  |  |  |  |
| 2 | Ath-AT1G34070.1 |  | Vvi-Vitvi12g02717\_t001 |  | | | |  |  |  |  |  |  |
| 2 | Ath-AT1G34095.1 |  | | | |  | | | |  |  |  |  |  |  |
| 2 | Ath-AT1G34110.1 |  | | | |  | Vvi-Vitvi18g01545\_t001 |  |  |  |  |  |  |
| 2 | Ath-AT1G34120.2 |  | | | |  | | | |  |  |  |  |  |  |
| 2 | Ath-AT1G34130.1 |  | | | |  | | | |  |  |  |  |  |  |
| 2 | Ath-AT1G34140.1 |  | | | |  | | | |  |  |  |  |  |  |
| 2 | Ath-AT1G34150.1 |  | | | |  | Vvi-Vitvi18g02917\_t001 |  |  |  |  |  |  |
| 2 | Ath-AT1G34160.1 |  | | | |  | Vvi-Vitvi18g01524\_t001 |  |  |  |  |  |  |
| 2 | Ath-AT1G34170.3 |  | | | |  | | | |  |  |  |  |  |  |
| 2 | Ath-AT1G34180.2 |  | | | |  | Vvi-Vitvi18g01520\_t001 |  |  |  |  |  |  |
| 2 | Ath-AT1G34190.1 |  | | | |  | | | |  |  |  |  |  |  |
| 2 | Ath-AT1G34200.1 |  | | | |  | Vvi-Vitvi18g01501\_t001 |  |  |  |  |  |  |
| 1 | Ath-AT1G34210.1 |  | | | |  |  |  |  |  |  |  |
| 1 | Ath-AT1G34220.1 |  | | | |  |  |  |  |  |  |  |
| 1 | Ath-AT1G34245.1 |  | | | |  |  |  |  |  |  |  |
| 1 | Ath-AT1G34260.2 |  | | | |  |  |  |  |  |  |  |
| 1 | Ath-AT1G34270.1 |  | | | |  |  |  |  |  |  |  |
| 1 | Ath-AT1G34290.1 |  | | | |  |  |  |  |  |  |  |
| 1 | Ath-AT1G34300.1 |  | | | |  |  |  |  |  |  |  |
| 1 | Ath-AT1G34310.1 |  | | | |  |  |  |  |  |  |  |
| 1 | Ath-AT1G34315.1 |  | | | |  |  |  |  |  |  |  |
| 1 | Ath-AT1G34317.1 |  | | | |  |  |  |  |  |  |  |
| 1 | Ath-AT1G34320.1 |  | | | |  |  |  |  |  |  |  |
| 1 | Ath-AT1G34340.1 |  | Vvi-Vitvi12g02716\_t001 |  |  |  |  |  |  |  |
| 1 | Ath-AT1G34350.2 |  | Vvi-Vitvi12g02018\_t001 |  |  |  |  |  |  |  |
| 2 | Ath-AT1G34355.1 |  | | | |  | Vvi-Vitvi18g01661\_t001 |  |  |  |  |  |  |
| 2 | Ath-AT1G34360.1 |  | | | |  | | | |  |  |  |  |  |  |
| 2 | Ath-AT1G34370.1 |  | | | |  | Vvi-Vitvi18g01662\_t003 |  |  |  |  |  |  |
| 2 | Ath-AT1G34380.2 |  | | | |  | Vvi-Vitvi18g01667\_t002 |  |  |  |  |  |  |
| 2 | Ath-AT1G34390.1 |  | | | |  | | | |  |  |  |  |  |  |
| 2 | Ath-AT1G34400.1 |  | | | |  | | | |  |  |  |  |  |  |
| 2 | Ath-AT1G34403.1 |  | | | |  | | | |  |  |  |  |  |  |
| 2 | Ath-AT1G34410.1 |  | | | |  | | | |  |  |  |  |  |  |
| 2 | Ath-AT1G34420.1 |  | | | |  | Vvi-Vitvi18g01675\_t001 |  |  |  |  |  |  |
| 2 | Ath-AT1G34430.1 |  | | | |  | Vvi-Vitvi18g01677\_t001 |  |  |  |  |  |  |
| 2 | Ath-AT1G34440.1 |  | | | |  | | | |  |  |  |  |  |  |
| 2 | Ath-AT1G34460.5 |  | | | |  | | | |  |  |  |  |  |  |
| 2 | Ath-AT1G34470.1 |  | Vvi-Vitvi12g02010\_t001 |  | | | |  |  |  |  |  |  |
| 1 | Ath-AT1G34480.1 |  |  |  | | | |  |  |  |  |  |  |
| 1 | Ath-AT1G34490.1 |  |  |  | | | |  |  |  |  |  |  |
| 1 | Ath-AT1G34500.1 |  |  |  | | | |  |  |  |  |  |  |
| 1 | Ath-AT1G34510.1 |  |  |  | | | |  |  |  |  |  |  |
| 1 | Ath-AT1G34520.1 |  |  |  | | | |  |  |  |  |  |  |
| 1 | Ath-AT1G34540.1 |  |  |  | | | |  |  |  |  |  |  |
| 1 | Ath-AT1G34550.1 |  |  |  | Vvi-Vitvi18g01679\_t001 |  |  |  |  |  |  |
| 0 | Ath-AT1G34560.1 |  |  |  |  |  |  |  |  |
| 0 | Ath-AT1G34570.1 |  |  |  |  |  |  |  |  |
| 0 | Ath-AT1G34575.1 |  |  |  |  |  |  |  |  |
| 1 | Ath-AT1G34580.1 |  | Vvi-Vitvi18g00397\_t001 |  |  |  |  |  |  |  |
| 1 | Ath-AT1G34630.1 |  | Vvi-Vitvi18g00403\_t001 |  |  |  |  |  |  |  |
| 1 | Ath-AT1G34640.1 |  | Vvi-Vitvi18g00405\_t001 |  |  |  |  |  |  |  |
| 1 | Ath-AT1G34650.1 |  | | | |  |  |  |  |  |  |  |
| 1 | Ath-AT1G34670.1 |  | Vvi-Vitvi18g00406\_t001 |  |  |  |  |  |  |  |
| 1 | Ath-AT1G34750.3 |  | Vvi-Vitvi18g00408\_t001 |  |  |  |  |  |  |  |
| 1 | Ath-AT1G34760.1 |  | Vvi-Vitvi18g00409\_t001 |  |  |  |  |  |  |  |
| 1 | Ath-AT1G34770.4 |  | | | |  |  |  |  |  |  |  |
| 1 | Ath-AT1G34780.1 |  | Vvi-Vitvi18g00410\_t001 |  |  |  |  |  |  |  |
| 1 | Ath-AT1G34790.1 |  | Vvi-Vitvi18g00417\_t001 |  |  |  |  |  |  |  |
| 1 | Ath-AT1G34792.1 |  | | | |  |  |  |  |  |  |  |
| 1 | Ath-AT1G34795.1 |  | | | |  |  |  |  |  |  |  |
| 1 | Ath-AT1G34800.1 |  | | | |  |  |  |  |  |  |  |
| 1 | Ath-AT1G34805.1 |  | | | |  |  |  |  |  |  |  |
| 1 | Ath-AT1G34807.1 |  | | | |  |  |  |  |  |  |  |
| 1 | Ath-AT1G34810.1 |  | | | |  |  |  |  |  |  |  |
| 1 | Ath-AT1G34812.1 |  | | | |  |  |  |  |  |  |  |
| 1 | Ath-AT1G34815.1 |  | | | |  |  |  |  |  |  |  |
| 1 | Ath-AT1G34817.1 |  | | | |  |  |  |  |  |  |  |
| 1 | Ath-AT1G34820.1 |  | | | |  |  |  |  |  |  |  |
| 1 | Ath-AT1G34822.1 |  | | | |  |  |  |  |  |  |  |
| 1 | Ath-AT1G34825.1 |  | | | |  |  |  |  |  |  |  |
| 1 | Ath-AT1G34827.1 |  | | | |  |  |  |  |  |  |  |
| 1 | Ath-AT1G34830.1 |  | | | |  |  |  |  |  |  |  |
| 1 | Ath-AT1G34840.1 |  | | | |  |  |  |  |  |  |  |
| 1 | Ath-AT1G34850.1 |  | | | |  |  |  |  |  |  |  |
| 1 | Ath-AT1G34855.1 |  | | | |  |  |  |  |  |  |  |
| 1 | Ath-AT1G34860.1 |  | | | |  |  |  |  |  |  |  |
| 1 | Ath-AT1G34910.1 |  | | | |  |  |  |  |  |  |  |
| 1 | Ath-AT1G34930.1 |  | | | |  |  |  |  |  |  |  |
| 1 | Ath-AT1G35030.1 |  | | | |  |  |  |  |  |  |  |
| 1 | Ath-AT1G35035.1 |  | | | |  |  |  |  |  |  |  |
| 1 | Ath-AT1G35040.1 |  | | | |  |  |  |  |  |  |  |
| 1 | Ath-AT1G35140.1 |  | Vvi-Vitvi18g00431\_t001 |  |  |  |  |  |  |  |
| 1 | Ath-AT1G35150.1 |  | | | |  |  |  |  |  |  |  |
| 1 | Ath-AT1G35160.2 |  | Vvi-Vitvi18g00442\_t001 |  |  |  |  |  |  |  |
| 1 | Ath-AT1G35170.1 |  | | | |  |  |  |  |  |  |  |
| 1 | Ath-AT1G35180.1 |  | | | |  |  |  |  |  |  |  |
| 1 | Ath-AT1G35181.1 |  | | | |  |  |  |  |  |  |  |
| 1 | Ath-AT1G35183.2 |  | | | |  |  |  |  |  |  |  |
| 1 | Ath-AT1G35190.1 |  | | | |  |  |  |  |  |  |  |
| 1 | Ath-AT1G35210.1 |  | Vvi-Vitvi18g02585\_t001 |  |  |  |  |  |  |  |
| 1 | Ath-AT1G35215.1 |  | | | |  |  |  |  |  |  |  |
| 1 | Ath-AT1G35220.2 |  | Vvi-Vitvi18g00452\_t001 |  |  |  |  |  |  |  |
| 1 | Ath-AT1G35230.1 |  | | | |  |  |  |  |  |  |  |
| 1 | Ath-AT1G35240.1 |  | | | |  |  |  |  |  |  |  |
| 1 | Ath-AT1G35242.1 |  | | | |  |  |  |  |  |  |  |
| 1 | Ath-AT1G35250.1 |  | | | |  |  |  |  |  |  |  |
| 1 | Ath-AT1G35255.1 |  | | | |  |  |  |  |  |  |  |
| 1 | Ath-AT1G35260.1 |  | | | |  |  |  |  |  |  |  |
| 1 | Ath-AT1G35290.1 |  | | | |  |  |  |  |  |  |  |
| 1 | Ath-AT1G35310.1 |  | | | |  |  |  |  |  |  |  |
| 1 | Ath-AT1G35320.1 |  | | | |  |  |  |  |  |  |  |
| 1 | Ath-AT1G35330.1 |  | Vvi-Vitvi18g00464\_t001 |  |  |  |  |  |  |  |
| 0 | Ath-AT1G35340.1 |  |  |  |  |  |  |  |  |
| 0 | Ath-AT1G35350.1 |  |  |  |  |  |  |  |  |
| 0 | Ath-AT1G35353.1 |  |  |  |  |  |  |  |  |
| 0 | Ath-AT1G35365.1 |  |  |  |  |  |  |  |  |
| 0 | Ath-AT1G35375.1 |  |  |  |  |  |  |  |  |
| 0 | Ath-AT1G35400.1 |  |  |  |  |  |  |  |  |
| 0 | Ath-AT1G35410.1 |  |  |  |  |  |  |  |  |
| 0 | Ath-AT1G35420.2 |  |  |  |  |  |  |  |  |
| 0 | Ath-AT1G35430.1 |  |  |  |  |  |  |  |  |
| 0 | Ath-AT1G35435.1 |  |  |  |  |  |  |  |  |
| 0 | Ath-AT1G35440.1 |  |  |  |  |  |  |  |  |
| 0 | Ath-AT1G35460.1 |  |  |  |  |  |  |  |  |
| 0 | Ath-AT1G35467.1 |  |  |  |  |  |  |  |  |
| 0 | Ath-AT1G35470.2 |  |  |  |  |  |  |  |  |
| 0 | Ath-AT1G35490.1 |  |  |  |  |  |  |  |  |
| 0 | Ath-AT1G35500.1 |  |  |  |  |  |  |  |  |
| 0 | Ath-AT1G35510.1 |  |  |  |  |  |  |  |  |
| 0 | Ath-AT1G35515.1 |  |  |  |  |  |  |  |  |
| 0 | Ath-AT1G35516.2 |  |  |  |  |  |  |  |  |
| 0 | Ath-AT1G35520.1 |  |  |  |  |  |  |  |  |
| 0 | Ath-AT1G35530.2 |  |  |  |  |  |  |  |  |
| 0 | Ath-AT1G35537.1 |  |  |  |  |  |  |  |  |
| 0 | Ath-AT1G35540.1 |  |  |  |  |  |  |  |  |
| 0 | Ath-AT1G35550.1 |  |  |  |  |  |  |  |  |
| 0 | Ath-AT1G35560.1 |  |  |  |  |  |  |  |  |
| 0 | Ath-AT1G35580.2 |  |  |  |  |  |  |  |  |
| 0 | Ath-AT1G35610.1 |  |  |  |  |  |  |  |  |
| 0 | Ath-AT1G35614.1 |  |  |  |  |  |  |  |  |
| 0 | Ath-AT1G35617.1 |  |  |  |  |  |  |  |  |
| 0 | Ath-AT1G35620.1 |  |  |  |  |  |  |  |  |
| 0 | Ath-AT1G35625.1 |  |  |  |  |  |  |  |  |
| 0 | Ath-AT1G35630.1 |  |  |  |  |  |  |  |  |
| 0 | Ath-AT1G35660.1 |  |  |  |  |  |  |  |  |
| 0 | Ath-AT1G35670.1 |  |  |  |  |  |  |  |  |
| 0 | Ath-AT1G35680.1 |  |  |  |  |  |  |  |  |
| 0 | Ath-AT1G35710.1 |  |  |  |  |  |  |  |  |
| 1 | Ath-AT1G35720.1 |  | Vvi-Vitvi18g00395\_t001 |  |  |  |  |  |  |  |
| 1 | Ath-AT1G35730.2 |  | Vvi-Vitvi18g02573\_t001 |  |  |  |  |  |  |  |
| 1 | Ath-AT1G35750.1 |  | | | |  |  |  |  |  |  |  |
| 1 | Ath-AT1G35780.1 |  | Vvi-Vitvi18g00394\_t001 |  |  |  |  |  |  |  |
| 1 | Ath-AT1G35820.1 |  | | | |  |  |  |  |  |  |  |
| 1 | Ath-AT1G35830.1 |  | Vvi-Vitvi18g00391\_t001 |  |  |  |  |  |  |  |
| 1 | Ath-AT1G35850.1 |  | | | |  |  |  |  |  |  |  |
| 1 | Ath-AT1G35860.1 |  | | | |  |  |  |  |  |  |  |
| 1 | Ath-AT1G35880.1 |  | | | |  |  |  |  |  |  |  |
| 1 | Ath-AT1G35890.1 |  | | | |  |  |  |  |  |  |  |
| 1 | Ath-AT1G35895.1 |  | | | |  |  |  |  |  |  |  |
| 1 | Ath-AT1G35910.1 |  | Vvi-Vitvi18g00384\_t001 |  |  |  |  |  |  |  |
| 1 | Ath-AT1G36000.1 |  | | | |  |  |  |  |  |  |  |
| 1 | Ath-AT1G36005.1 |  | | | |  |  |  |  |  |  |  |
| 1 | Ath-AT1G36020.1 |  | | | |  |  |  |  |  |  |  |
| 1 | Ath-AT1G36030.1 |  | | | |  |  |  |  |  |  |  |
| 1 | Ath-AT1G36050.1 |  | Vvi-Vitvi18g00382\_t001 |  |  |  |  |  |  |  |
| 1 | Ath-AT1G36060.1 |  | | | |  |  |  |  |  |  |  |
| 1 | Ath-AT1G36070.1 |  | Vvi-Vitvi18g00378\_t001 |  |  |  |  |  |  |  |
| 1 | Ath-AT1G36078.1 |  | | | |  |  |  |  |  |  |  |
| 1 | Ath-AT1G36085.1 |  | | | |  |  |  |  |  |  |  |
| 1 | Ath-AT1G36095.1 |  | | | |  |  |  |  |  |  |  |
| 1 | Ath-AT1G36100.1 |  | | | |  |  |  |  |  |  |  |
| 1 | Ath-AT1G36150.1 |  | | | |  |  |  |  |  |  |  |
| 1 | Ath-AT1G36160.2 |  | Vvi-Vitvi18g00368\_t003 |  |  |  |  |  |  |  |
| 1 | Ath-AT1G36180.1 |  | | | |  |  |  |  |  |  |  |
| 1 | Ath-AT1G36230.1 |  | | | |  |  |  |  |  |  |  |
| 1 | Ath-AT1G36240.1 |  | Vvi-Vitvi18g00358\_t002 |  |  |  |  |  |  |  |
| 1 | Ath-AT1G36272.1 |  | | | |  |  |  |  |  |  |  |
| 1 | Ath-AT1G36280.1 |  | | | |  |  |  |  |  |  |  |
| 1 | Ath-AT1G36310.1 |  | Vvi-Vitvi18g00352\_t002 |  |  |  |  |  |  |  |
| 1 | Ath-AT1G36320.1 |  | Vvi-Vitvi18g00349\_t001 |  |  |  |  |  |  |  |
| 1 | Ath-AT1G36325.1 |  | | | |  |  |  |  |  |  |  |
| 1 | Ath-AT1G36340.2 |  | | | |  |  |  |  |  |  |  |
| 1 | Ath-AT1G36370.1 |  | Vvi-Vitvi18g00341\_t001 |  |  |  |  |  |  |  |
| 1 | Ath-AT1G36380.1 |  | Vvi-Vitvi18g04060\_t001 |  |  |  |  |  |  |  |
| 1 | Ath-AT1G36390.2 |  | Vvi-Vitvi18g00338\_t001 |  |  |  |  |  |  |  |
| 1 | Ath-AT1G36510.1 |  | | | |  |  |  |  |  |  |  |
| 1 | Ath-AT1G36580.1 |  | | | |  |  |  |  |  |  |  |
| 1 | Ath-AT1G36622.1 |  | | | |  |  |  |  |  |  |  |
| 1 | Ath-AT1G36623.1 |  | | | |  |  |  |  |  |  |  |
| 1 | Ath-AT1G36627.1 |  | | | |  |  |  |  |  |  |  |
| 1 | Ath-AT1G36640.1 |  | | | |  |  |  |  |  |  |  |
| 1 | Ath-AT1G36675.1 |  | | | |  |  |  |  |  |  |  |
| 1 | Ath-AT1G36730.1 |  | Vvi-Vitvi18g00336\_t001 |  |  |  |  |  |  |  |
| 1 | Ath-AT1G36745.1 |  | | | |  |  |  |  |  |  |  |
| 1 | Ath-AT1G36756.1 |  | | | |  |  |  |  |  |  |  |
| 1 | Ath-AT1G36920.1 |  | | | |  |  |  |  |  |  |  |
| 1 | Ath-AT1G36922.2 |  | | | |  |  |  |  |  |  |  |
| 1 | Ath-AT1G36925.1 |  | | | |  |  |  |  |  |  |  |
| 1 | Ath-AT1G36940.2 |  | | | |  |  |  |  |  |  |  |
| 1 | Ath-AT1G36942.1 |  | | | |  |  |  |  |  |  |  |
| 1 | Ath-AT1G36950.1 |  | | | |  |  |  |  |  |  |  |
| 1 | Ath-AT1G36960.1 |  | | | |  |  |  |  |  |  |  |
| 1 | Ath-AT1G36970.1 |  | | | |  |  |  |  |  |  |  |
| 1 | Ath-AT1G36980.1 |  | Vvi-Vitvi18g02550\_t001 |  |  |  |  |  |  |  |
| 1 | Ath-AT1G36990.1 |  | Vvi-Vitvi18g00330\_t001 |  |  |  |  |  |  |  |
| 1 | Ath-AT1G37000.1 |  | | | |  |  |  |  |  |  |  |
| 1 | Ath-AT1G37010.1 |  | | | |  |  |  |  |  |  |  |
| 1 | Ath-AT1G37020.1 |  | | | |  |  |  |  |  |  |  |
| 1 | Ath-AT1G37113.1 |  | | | |  |  |  |  |  |  |  |
| 1 | Ath-AT1G37130.1 |  | Vvi-Vitvi18g00326\_t001 |  |  |  |  |  |  |  |
| 1 | Ath-AT1G37140.1 |  | Vvi-Vitvi18g02546\_t001 |  |  |  |  |  |  |  |
| 1 | Ath-AT1G37150.9 |  | Vvi-Vitvi18g00320\_t001 |  |  |  |  |  |  |  |
| 1 | Ath-AT1G38065.1 |  | Vvi-Vitvi18g00313\_t002 |  |  |  |  |  |  |  |
| 0 | Ath-AT1G38131.1 |  |  |  |  |  |  |  |  |
| 0 | Ath-AT1G38790.1 |  |  |  |  |  |  |  |  |
| 0 | Ath-AT1G39060.1 |  |  |  |  |  |  |  |  |
| 0 | Ath-AT1G39070.1 |  |  |  |  |  |  |  |  |
| 0 | Ath-AT1G40083.1 |  |  |  |  |  |  |  |  |
| 0 | Ath-AT1G40086.1 |  |  |  |  |  |  |  |  |
| 0 | Ath-AT1G40087.1 |  |  |  |  |  |  |  |  |
| 0 | Ath-AT1G40090.1 |  |  |  |  |  |  |  |  |
| 0 | Ath-AT1G40104.1 |  |  |  |  |  |  |  |  |
| 0 | Ath-AT1G40129.1 |  |  |  |  |  |  |  |  |
| 0 | Ath-AT1G40390.1 |  |  |  |  |  |  |  |  |
| 0 | Ath-AT1G41820.1 |  |  |  |  |  |  |  |  |
| 1 | Ath-AT1G41830.1 |  | Vvi-Vitvi18g00137\_t001 |  |  |  |  |  |  |  |
| 1 | Ath-AT1G41880.2 |  | | | |  |  |  |  |  |  |  |
| 1 | Ath-AT1G41920.1 |  | | | |  |  |  |  |  |  |  |
| 1 | Ath-AT1G42080.1 |  | | | |  |  |  |  |  |  |  |
| 1 | Ath-AT1G42190.1 |  | | | |  |  |  |  |  |  |  |
| 1 | Ath-AT1G42430.1 |  | Vvi-Vitvi18g00115\_t001 |  |  |  |  |  |  |  |
| 1 | Ath-AT1G42440.1 |  | Vvi-Vitvi18g00099\_t001 |  |  |  |  |  |  |  |
| 1 | Ath-AT1G42470.1 |  | Vvi-Vitvi18g00093\_t001 |  |  |  |  |  |  |  |
| 1 | Ath-AT1G42480.1 |  | Vvi-Vitvi18g00073\_t001 |  |  |  |  |  |  |  |
| 1 | Ath-AT1G42525.1 |  | | | |  |  |  |  |  |  |  |
| 1 | Ath-AT1G42540.2 |  | Vvi-Vitvi18g00071\_t001 |  |  |  |  |  |  |  |
| 1 | Ath-AT1G42550.1 |  | | | |  |  |  |  |  |  |  |
| 1 | Ath-AT1G42560.1 |  | | | |  |  |  |  |  |  |  |
| 1 | Ath-AT1G42615.1 |  | | | |  |  |  |  |  |  |  |
| 1 | Ath-AT1G42680.1 |  | | | |  |  |  |  |  |  |  |
| 1 | Ath-AT1G42700.1 |  | | | |  |  |  |  |  |  |  |
| 1 | Ath-AT1G42710.1 |  | | | |  |  |  |  |  |  |  |
| 1 | Ath-AT1G42960.1 |  | Vvi-Vitvi18g00067\_t001 |  |  |  |  |  |  |  |
| 0 | Ath-AT1G42970.1 |  |  |  |  |  |  |  |  |
| 0 | Ath-AT1G42980.1 |  |  |  |  |  |  |  |  |
| 0 | Ath-AT1G42990.1 |  |  |  |  |  |  |  |  |
| 0 | Ath-AT1G43000.2 |  |  |  |  |  |  |  |  |
| 0 | Ath-AT1G43005.1 |  |  |  |  |  |  |  |  |
| 0 | Ath-AT1G43010.1 |  |  |  |  |  |  |  |  |
| 0 | Ath-AT1G43020.1 |  |  |  |  |  |  |  |  |
| 0 | Ath-AT1G43040.2 |  |  |  |  |  |  |  |  |
| 0 | Ath-AT1G43080.1 |  |  |  |  |  |  |  |  |
| 0 | Ath-AT1G43090.1 |  |  |  |  |  |  |  |  |
| 0 | Ath-AT1G43100.1 |  |  |  |  |  |  |  |  |
| 0 | Ath-AT1G43130.1 |  |  |  |  |  |  |  |  |
| 0 | Ath-AT1G43140.1 |  |  |  |  |  |  |  |  |
| 0 | Ath-AT1G43145.1 |  |  |  |  |  |  |  |  |
| 1 | Ath-AT1G43160.1 |  | Vvi-Vitvi18g01617\_t001 |  |  |  |  |  |  |  |
| 1 | Ath-AT1G43170.1 |  | | | |  |  |  |  |  |  |  |
| 1 | Ath-AT1G43171.1 |  | | | |  |  |  |  |  |  |  |
| 1 | Ath-AT1G43190.1 |  | Vvi-Vitvi18g01621\_t001 |  |  |  |  |  |  |  |
| 1 | Ath-AT1G43245.2 |  | Vvi-Vitvi18g01629\_t001 |  |  |  |  |  |  |  |
| 1 | Ath-AT1G43260.1 |  | | | |  |  |  |  |  |  |  |
| 1 | Ath-AT1G43310.1 |  | | | |  |  |  |  |  |  |  |
| 1 | Ath-AT1G43320.1 |  | | | |  |  |  |  |  |  |  |
| 1 | Ath-AT1G43330.1 |  | | | |  |  |  |  |  |  |  |
| 1 | Ath-AT1G43415.1 |  | | | |  |  |  |  |  |  |  |
| 1 | Ath-AT1G43560.1 |  | Vvi-Vitvi18g02974\_t001 |  |  |  |  |  |  |  |
| 1 | Ath-AT1G43580.1 |  | Vvi-Vitvi18g02975\_t001 |  |  |  |  |  |  |  |
| 1 | Ath-AT1G43600.1 |  | | | |  |  |  |  |  |  |  |
| 1 | Ath-AT1G43605.2 |  | | | |  |  |  |  |  |  |  |
| 1 | Ath-AT1G43610.1 |  | | | |  |  |  |  |  |  |  |
| 1 | Ath-AT1G43620.4 |  | Vvi-Vitvi18g01632\_t003 |  |  |  |  |  |  |  |
| 1 | Ath-AT1G43630.1 |  | Vvi-Vitvi18g01639\_t001 |  |  |  |  |  |  |  |
| 1 | Ath-AT1G43640.1 |  | Vvi-Vitvi18g01647\_t004 |  |  |  |  |  |  |  |
| 1 | Ath-AT1G43650.1 |  | Vvi-Vitvi18g01648\_t001 |  |  |  |  |  |  |  |
| 1 | Ath-AT1G43665.1 |  | | | |  |  |  |  |  |  |  |
| 1 | Ath-AT1G43666.1 |  | | | |  |  |  |  |  |  |  |
| 1 | Ath-AT1G43667.1 |  | | | |  |  |  |  |  |  |  |
| 1 | Ath-AT1G43670.1 |  | Vvi-Vitvi18g01657\_t001 |  |  |  |  |  |  |  |
| 1 | Ath-AT1G43680.1 |  | | | |  |  |  |  |  |  |  |
| 1 | Ath-AT1G43690.1 |  | Vvi-Vitvi18g01659\_t001 |  |  |  |  |  |  |  |
| 0 | Ath-AT1G43700.1 |  |  |  |  |  |  |  |  |
| 0 | Ath-AT1G43710.1 |  |  |  |  |  |  |  |  |
| 0 | Ath-AT1G43720.1 |  |  |  |  |  |  |  |  |
| 0 | Ath-AT1G43722.1 |  |  |  |  |  |  |  |  |
| 0 | Ath-AT1G43730.1 |  |  |  |  |  |  |  |  |
| 0 | Ath-AT1G43760.1 |  |  |  |  |  |  |  |  |
| 0 | Ath-AT1G43770.2 |  |  |  |  |  |  |  |  |
| 1 | Ath-AT1G43780.1 |  | Vvi-Vitvi18g01238\_t001 |  |  |  |  |  |  |  |
| 1 | Ath-AT1G43790.1 |  | Vvi-Vitvi18g02833\_t001 |  |  |  |  |  |  |  |
| 1 | Ath-AT1G43800.1 |  | Vvi-Vitvi18g01231\_t001 |  |  |  |  |  |  |  |
| 1 | Ath-AT1G43810.1 |  | | | |  |  |  |  |  |  |  |
| 1 | Ath-AT1G43815.2 |  | | | |  |  |  |  |  |  |  |
| 1 | Ath-AT1G43825.1 |  | | | |  |  |  |  |  |  |  |
| 1 | Ath-AT1G43850.1 |  | Vvi-Vitvi18g01214\_t001 |  |  |  |  |  |  |  |
| 1 | Ath-AT1G43860.1 |  | Vvi-Vitvi18g01212\_t001 |  |  |  |  |  |  |  |
| 1 | Ath-AT1G43890.1 |  | Vvi-Vitvi18g01187\_t001 |  |  |  |  |  |  |  |
| 1 | Ath-AT1G43900.1 |  | Vvi-Vitvi18g01183\_t002 |  |  |  |  |  |  |  |
| 1 | Ath-AT1G43910.1 |  | | | |  |  |  |  |  |  |  |
| 1 | Ath-AT1G43950.1 |  | | | |  |  |  |  |  |  |  |
| 1 | Ath-AT1G43970.1 |  | | | |  |  |  |  |  |  |  |
| 1 | Ath-AT1G43980.1 |  | Vvi-Vitvi18g01173\_t001 |  |  |  |  |  |  |  |
| 0 | Ath-AT1G44000.1 |  |  |  |  |  |  |  |  |
| 0 | Ath-AT1G44010.1 |  |  |  |  |  |  |  |  |
| 0 | Ath-AT1G44020.1 |  |  |  |  |  |  |  |  |
| 0 | Ath-AT1G44030.2 |  |  |  |  |  |  |  |  |
| 0 | Ath-AT1G44050.1 |  |  |  |  |  |  |  |  |
| 0 | Ath-AT1G44080.1 |  |  |  |  |  |  |  |  |
| 0 | Ath-AT1G44085.1 |  |  |  |  |  |  |  |  |
| 0 | Ath-AT1G44090.1 |  |  |  |  |  |  |  |  |
| 1 | Ath-AT1G44100.1 |  | Vvi-Vitvi18g00237\_t001 |  |  |  |  |  |  |  |
| 1 | Ath-AT1G44110.1 |  | Vvi-Vitvi18g00239\_t001 |  |  |  |  |  |  |  |
| 1 | Ath-AT1G44120.2 |  | Vvi-Vitvi18g00251\_t001 |  |  |  |  |  |  |  |
| 1 | Ath-AT1G44130.1 |  | Vvi-Vitvi18g00254\_t001 |  |  |  |  |  |  |  |
| 1 | Ath-AT1G44160.1 |  | | | |  |  |  |  |  |  |  |
| 1 | Ath-AT1G44170.2 |  | Vvi-Vitvi18g00255\_t001 |  |  |  |  |  |  |  |
| 1 | Ath-AT1G44180.1 |  | | | |  |  |  |  |  |  |  |
| 1 | Ath-AT1G44191.1 |  | | | |  |  |  |  |  |  |  |
| 1 | Ath-AT1G44224.1 |  | | | |  |  |  |  |  |  |  |
| 1 | Ath-AT1G44318.1 |  | Vvi-Vitvi18g00261\_t001 |  |  |  |  |  |  |  |
| 1 | Ath-AT1G44350.1 |  | Vvi-Vitvi18g00262\_t001 |  |  |  |  |  |  |  |
| 1 | Ath-AT1G44414.1 |  | Vvi-Vitvi18g00268\_t001 |  |  |  |  |  |  |  |
| 1 | Ath-AT1G44446.1 |  | Vvi-Vitvi18g00269\_t001 |  |  |  |  |  |  |  |
| 1 | Ath-AT1G44478.1 |  | | | |  |  |  |  |  |  |  |
| 1 | Ath-AT1G44542.1 |  | | | |  |  |  |  |  |  |  |
| 1 | Ath-AT1G44575.1 |  | Vvi-Vitvi18g00272\_t001 |  |  |  |  |  |  |  |
| 1 | Ath-AT1G44608.1 |  | Vvi-Vitvi18g00274\_t001 |  |  |  |  |  |  |  |
| 1 | Ath-AT1G44740.1 |  | | | |  |  |  |  |  |  |  |
| 1 | Ath-AT1G44750.1 |  | | | |  |  |  |  |  |  |  |
| 1 | Ath-AT1G44760.2 |  | Vvi-Vitvi18g00281\_t001 |  |  |  |  |  |  |  |
| 1 | Ath-AT1G44770.1 |  | Vvi-Vitvi18g00286\_t001 |  |  |  |  |  |  |  |
| 1 | Ath-AT1G44780.1 |  | Vvi-Vitvi18g00287\_t001 |  |  |  |  |  |  |  |
| 1 | Ath-AT1G44790.1 |  | Vvi-Vitvi18g00288\_t002 |  |  |  |  |  |  |  |
| 1 | Ath-AT1G44800.1 |  | Vvi-Vitvi18g00289\_t001 |  |  |  |  |  |  |  |
| 1 | Ath-AT1G44810.1 |  | | | |  |  |  |  |  |  |  |
| 1 | Ath-AT1G44820.1 |  | Vvi-Vitvi18g00294\_t001 |  |  |  |  |  |  |  |
| 1 | Ath-AT1G44830.1 |  | Vvi-Vitvi18g00295\_t001 |  |  |  |  |  |  |  |
| 1 | Ath-AT1G44835.2 |  | Vvi-Vitvi18g00296\_t001 |  |  |  |  |  |  |  |
| 0 | Ath-AT1G44890.1 |  |  |  |  |  |  |  |  |
| 0 | Ath-AT1G44895.1 |  |  |  |  |  |  |  |  |
| 0 | Ath-AT1G44900.1 |  |  |  |  |  |  |  |  |
| 0 | Ath-AT1G44910.1 |  |  |  |  |  |  |  |  |
| 0 | Ath-AT1G44920.1 |  |  |  |  |  |  |  |  |
| 0 | Ath-AT1G44940.2 |  |  |  |  |  |  |  |  |
| 0 | Ath-AT1G44941.1 |  |  |  |  |  |  |  |  |
| 0 | Ath-AT1G44960.1 |  |  |  |  |  |  |  |  |
| 0 | Ath-AT1G44970.1 |  |  |  |  |  |  |  |  |
| 0 | Ath-AT1G44980.1 |  |  |  |  |  |  |  |  |
| 0 | Ath-AT1G44990.1 |  |  |  |  |  |  |  |  |
| 0 | Ath-AT1G45000.1 |  |  |  |  |  |  |  |  |
| 0 | Ath-AT1G45010.1 |  |  |  |  |  |  |  |  |
| 0 | Ath-AT1G45015.1 |  |  |  |  |  |  |  |  |
| 0 | Ath-AT1G45050.1 |  |  |  |  |  |  |  |  |
| 0 | Ath-AT1G45063.1 |  |  |  |  |  |  |  |  |
| 0 | Ath-AT1G45100.1 |  |  |  |  |  |  |  |  |
| 0 | Ath-AT1G45110.1 |  |  |  |  |  |  |  |  |
| 0 | Ath-AT1G45130.1 |  |  |  |  |  |  |  |  |
| 0 | Ath-AT1G45145.1 |  |  |  |  |  |  |  |  |
| 0 | Ath-AT1G45150.1 |  |  |  |  |  |  |  |  |
| 0 | Ath-AT1G45160.2 |  |  |  |  |  |  |  |  |
| 0 | Ath-AT1G45163.1 |  |  |  |  |  |  |  |  |
| 0 | Ath-AT1G45165.1 |  |  |  |  |  |  |  |  |
| 0 | Ath-AT1G45170.1 |  |  |  |  |  |  |  |  |
| 1 | Ath-AT1G45180.1 |  | Vvi-Vitvi18g00762\_t001 |  |  |  |  |  |  |  |
| 1 | Ath-AT1G45190.1 |  | | | |  |  |  |  |  |  |  |
| 1 | Ath-AT1G45191.1 |  | | | |  |  |  |  |  |  |  |
| 1 | Ath-AT1G45201.3 |  | Vvi-Vitvi18g00780\_t001 |  |  |  |  |  |  |  |
| 1 | Ath-AT1G45207.2 |  | Vvi-Vitvi18g00781\_t002 |  |  |  |  |  |  |  |
| 1 | Ath-AT1G45215.1 |  | | | |  |  |  |  |  |  |  |
| 1 | Ath-AT1G45221.1 |  | | | |  |  |  |  |  |  |  |
| 1 | Ath-AT1G45223.1 |  | | | |  |  |  |  |  |  |  |
| 1 | Ath-AT1G45243.1 |  | | | |  |  |  |  |  |  |  |
| 1 | Ath-AT1G45231.2 |  | Vvi-Vitvi18g00782\_t001 |  |  |  |  |  |  |  |
| 1 | Ath-AT1G45233.2 |  | Vvi-Vitvi18g00783\_t001 |  |  |  |  |  |  |  |
| 1 | Ath-AT1G45229.2 |  | | | |  |  |  |  |  |  |  |
| 1 | Ath-AT1G45248.5 |  | | | |  |  |  |  |  |  |  |
| 1 | Ath-AT1G45249.6 |  | Vvi-Vitvi18g00784\_t001 |  |  |  |  |  |  |  |
| 1 | Ath-AT1G45230.1 |  | Vvi-Vitvi18g00785\_t001 |  |  |  |  |  |  |  |
| 1 | Ath-AT1G45332.1 |  | | | |  |  |  |  |  |  |  |
| 1 | Ath-AT1G45403.1 |  | | | |  |  |  |  |  |  |  |
| 1 | Ath-AT1G45474.2 |  | Vvi-Vitvi18g02680\_t001 |  |  |  |  |  |  |  |
| 1 | Ath-AT1G45545.2 |  | Vvi-Vitvi18g00797\_t001 |  |  |  |  |  |  |  |
| 0 | Ath-AT1G45616.1 |  |  |  |  |  |  |  |  |
| 0 | Ath-AT1G45688.1 |  |  |  |  |  |  |  |  |
| 0 | Ath-AT1G45976.1 |  |  |  |  |  |  |  |  |
| 0 | Ath-AT1G46264.1 |  |  |  |  |  |  |  |  |
| 0 | Ath-AT1G46336.1 |  |  |  |  |  |  |  |  |
| 0 | Ath-AT1G46408.1 |  |  |  |  |  |  |  |  |
| 0 | Ath-AT1G46480.1 |  |  |  |  |  |  |  |  |
| 0 | Ath-AT1G46696.1 |  |  |  |  |  |  |  |  |
| 0 | Ath-AT1G46768.2 |  |  |  |  |  |  |  |  |
| 0 | Ath-AT1G46840.1 |  |  |  |  |  |  |  |  |
| 0 | Ath-AT1G46912.1 |  |  |  |  |  |  |  |  |
| 0 | Ath-AT1G46984.1 |  |  |  |  |  |  |  |  |
| 0 | Ath-AT1G47056.1 |  |  |  |  |  |  |  |  |
| 0 | Ath-AT1G47128.1 |  |  |  |  |  |  |  |  |
| 0 | Ath-AT1G47130.1 |  |  |  |  |  |  |  |  |
| 0 | Ath-AT1G47200.1 |  |  |  |  |  |  |  |  |
| 0 | Ath-AT1G47210.2 |  |  |  |  |  |  |  |  |
| 0 | Ath-AT1G47220.1 |  |  |  |  |  |  |  |  |
| 0 | Ath-AT1G47230.2 |  |  |  |  |  |  |  |  |
| 0 | Ath-AT1G47240.1 |  |  |  |  |  |  |  |  |
| 0 | Ath-AT1G47250.1 |  |  |  |  |  |  |  |  |
| 0 | Ath-AT1G47260.1 |  |  |  |  |  |  |  |  |
| 0 | Ath-AT1G47265.1 |  |  |  |  |  |  |  |  |
| 0 | Ath-AT1G47270.1 |  |  |  |  |  |  |  |  |
| 0 | Ath-AT1G47271.1 |  |  |  |  |  |  |  |  |
| 0 | Ath-AT1G47278.2 |  |  |  |  |  |  |  |  |
| 0 | Ath-AT1G47280.1 |  |  |  |  |  |  |  |  |
| 0 | Ath-AT1G47290.2 |  |  |  |  |  |  |  |  |
| 0 | Ath-AT1G47300.1 |  |  |  |  |  |  |  |  |
| 0 | Ath-AT1G47310.1 |  |  |  |  |  |  |  |  |
| 0 | Ath-AT1G47317.1 |  |  |  |  |  |  |  |  |
| 0 | Ath-AT1G47330.1 |  |  |  |  |  |  |  |  |
| 0 | Ath-AT1G47340.1 |  |  |  |  |  |  |  |  |
| 0 | Ath-AT1G47350.1 |  |  |  |  |  |  |  |  |
| 0 | Ath-AT1G47370.1 |  |  |  |  |  |  |  |  |
| 0 | Ath-AT1G47380.1 |  |  |  |  |  |  |  |  |
| 0 | Ath-AT1G47389.1 |  |  |  |  |  |  |  |  |
| 0 | Ath-AT1G47390.1 |  |  |  |  |  |  |  |  |
| 0 | Ath-AT1G47395.1 |  |  |  |  |  |  |  |  |
| 0 | Ath-AT1G47400.1 |  |  |  |  |  |  |  |  |
| 0 | Ath-AT1G47410.2 |  |  |  |  |  |  |  |  |
| 0 | Ath-AT1G47420.1 |  |  |  |  |  |  |  |  |
| 0 | Ath-AT1G47450.1 |  |  |  |  |  |  |  |  |
| 0 | Ath-AT1G47470.1 |  |  |  |  |  |  |  |  |
| 0 | Ath-AT1G47480.1 |  |  |  |  |  |  |  |  |
| 0 | Ath-AT1G47485.1 |  |  |  |  |  |  |  |  |
| 0 | Ath-AT1G47490.1 |  |  |  |  |  |  |  |  |
| 0 | Ath-AT1G47495.1 |  |  |  |  |  |  |  |  |
| 0 | Ath-AT1G47497.1 |  |  |  |  |  |  |  |  |
| 0 | Ath-AT1G47500.1 |  |  |  |  |  |  |  |  |
| 0 | Ath-AT1G47510.1 |  |  |  |  |  |  |  |  |
| 0 | Ath-AT1G47530.1 |  |  |  |  |  |  |  |  |
| 0 | Ath-AT1G47540.2 |  |  |  |  |  |  |  |  |
| 0 | Ath-AT1G47550.2 |  |  |  |  |  |  |  |  |
| 0 | Ath-AT1G47560.1 |  |  |  |  |  |  |  |  |
| 0 | Ath-AT1G47570.1 |  |  |  |  |  |  |  |  |
| 0 | Ath-AT1G47578.1 |  |  |  |  |  |  |  |  |
| 0 | Ath-AT1G47580.1 |  |  |  |  |  |  |  |  |
| 0 | Ath-AT1G47600.1 |  |  |  |  |  |  |  |  |
| 0 | Ath-AT1G47603.1 |  |  |  |  |  |  |  |  |
| 0 | Ath-AT1G47610.1 |  |  |  |  |  |  |  |  |
| 0 | Ath-AT1G47620.1 |  |  |  |  |  |  |  |  |
| 0 | Ath-AT1G47640.1 |  |  |  |  |  |  |  |  |
| 0 | Ath-AT1G47655.1 |  |  |  |  |  |  |  |  |
| 0 | Ath-AT1G47657.1 |  |  |  |  |  |  |  |  |
| 0 | Ath-AT1G47660.1 |  |  |  |  |  |  |  |  |
| 0 | Ath-AT1G47670.1 |  |  |  |  |  |  |  |  |
| 0 | Ath-AT1G47680.1 |  |  |  |  |  |  |  |  |
| 0 | Ath-AT1G47690.1 |  |  |  |  |  |  |  |  |
| 0 | Ath-AT1G47695.1 |  |  |  |  |  |  |  |  |
| 0 | Ath-AT1G47700.1 |  |  |  |  |  |  |  |  |
| 0 | Ath-AT1G47710.2 |  |  |  |  |  |  |  |  |
| 0 | Ath-AT1G47720.1 |  |  |  |  |  |  |  |  |
| 0 | Ath-AT1G47730.1 |  |  |  |  |  |  |  |  |
| 0 | Ath-AT1G47740.1 |  |  |  |  |  |  |  |  |
| 0 | Ath-AT1G47750.1 |  |  |  |  |  |  |  |  |
| 0 | Ath-AT1G47760.1 |  |  |  |  |  |  |  |  |
| 0 | Ath-AT1G47765.1 |  |  |  |  |  |  |  |  |
| 0 | Ath-AT1G47770.1 |  |  |  |  |  |  |  |  |
| 0 | Ath-AT1G47780.1 |  |  |  |  |  |  |  |  |
| 0 | Ath-AT1G47786.1 |  |  |  |  |  |  |  |  |
| 0 | Ath-AT1G47790.1 |  |  |  |  |  |  |  |  |
| 0 | Ath-AT1G47800.1 |  |  |  |  |  |  |  |  |
| 0 | Ath-AT1G47810.1 |  |  |  |  |  |  |  |  |
| 0 | Ath-AT1G47813.1 |  |  |  |  |  |  |  |  |
| 0 | Ath-AT1G47820.2 |  |  |  |  |  |  |  |  |
| 0 | Ath-AT1G47830.1 |  |  |  |  |  |  |  |  |
| 0 | Ath-AT1G47840.1 |  |  |  |  |  |  |  |  |
| 0 | Ath-AT1G47845.1 |  |  |  |  |  |  |  |  |
| 0 | Ath-AT1G47870.1 |  |  |  |  |  |  |  |  |
| 0 | Ath-AT1G47885.1 |  |  |  |  |  |  |  |  |
| 0 | Ath-AT1G47890.1 |  |  |  |  |  |  |  |  |
| 0 | Ath-AT1G47900.3 |  |  |  |  |  |  |  |  |
| 0 | Ath-AT1G47915.1 |  |  |  |  |  |  |  |  |
| 0 | Ath-AT1G47920.1 |  |  |  |  |  |  |  |  |
| 0 | Ath-AT1G47940.1 |  |  |  |  |  |  |  |  |
| 1 | Ath-AT1G47960.1 |  | Vvi-Vitvi05g01786\_t001 |  |  |  |  |  |  |  |
| 1 | Ath-AT1G47970.1 |  | Vvi-Vitvi05g01790\_t003 |  |  |  |  |  |  |  |
| 1 | Ath-AT1G47980.1 |  | Vvi-Vitvi05g04028\_t001 |  |  |  |  |  |  |  |
| 1 | Ath-AT1G47990.1 |  | Vvi-Vitvi05g00163\_t001 |  |  |  |  |  |  |  |
| 1 | Ath-AT1G48000.1 |  | Vvi-Vitvi05g00166\_t001 |  |  |  |  |  |  |  |
| 1 | Ath-AT1G48010.1 |  | | | |  |  |  |  |  |  |  |
| 1 | Ath-AT1G48020.1 |  | | | |  |  |  |  |  |  |  |
| 1 | Ath-AT1G48030.1 |  | | | |  |  |  |  |  |  |  |
| 1 | Ath-AT1G48040.1 |  | | | |  |  |  |  |  |  |  |
| 1 | Ath-AT1G48050.1 |  | | | |  |  |  |  |  |  |  |
| 1 | Ath-AT1G48060.1 |  | | | |  |  |  |  |  |  |  |
| 1 | Ath-AT1G48070.1 |  | | | |  |  |  |  |  |  |  |
| 1 | Ath-AT1G48090.2 |  | | | |  |  |  |  |  |  |  |
| 1 | Ath-AT1G48095.1 |  | | | |  |  |  |  |  |  |  |
| 1 | Ath-AT1G48100.1 |  | | | |  |  |  |  |  |  |  |
| 1 | Ath-AT1G48110.2 |  | | | |  |  |  |  |  |  |  |
| 1 | Ath-AT1G48120.1 |  | | | |  |  |  |  |  |  |  |
| 2 | Ath-AT1G48130.1 |  | Vvi-Vitvi05g00181\_t001 |  | Vvi-Vitvi05g00235\_t001 |  |  |  |  |  |  |
| 2 | Ath-AT1G48140.1 |  | | | |  | | | |  |  |  |  |  |  |
| 2 | Ath-AT1G48145.1 |  | | | |  | | | |  |  |  |  |  |  |
| 2 | Ath-AT1G48150.1 |  | | | |  | | | |  |  |  |  |  |  |
| 2 | Ath-AT1G48160.1 |  | Vvi-Vitvi05g00231\_t001 |  | | | |  |  |  |  |  |  |
| 2 | Ath-AT1G48170.1 |  | Vvi-Vitvi05g00186\_t002 |  | | | |  |  |  |  |  |  |
| 1 | Ath-AT1G48175.1 |  |  |  | | | |  |  |  |  |  |  |
| 1 | Ath-AT1G48180.2 |  |  |  | | | |  |  |  |  |  |  |
| 1 | Ath-AT1G48190.1 |  |  |  | | | |  |  |  |  |  |  |
| 1 | Ath-AT1G48195.1 |  |  |  | | | |  |  |  |  |  |  |
| 1 | Ath-AT1G48200.1 |  |  |  | | | |  |  |  |  |  |  |
| 1 | Ath-AT1G48210.1 |  |  |  | Vvi-Vitvi05g00246\_t003 |  |  |  |  |  |  |
| 1 | Ath-AT1G48220.1 |  |  |  | | | |  |  |  |  |  |  |
| 1 | Ath-AT1G48230.1 |  |  |  | Vvi-Vitvi05g00248\_t001 |  |  |  |  |  |  |
| 1 | Ath-AT1G48240.1 |  |  |  | Vvi-Vitvi05g00249\_t001 |  |  |  |  |  |  |
| 1 | Ath-AT1G48260.1 |  |  |  | Vvi-Vitvi05g00256\_t001 |  |  |  |  |  |  |
| 1 | Ath-AT1G48270.2 |  |  |  | | | |  |  |  |  |  |  |
| 1 | Ath-AT1G48280.1 |  |  |  | Vvi-Vitvi05g00264\_t001 |  |  |  |  |  |  |
| 1 | Ath-AT1G48285.1 |  |  |  | | | |  |  |  |  |  |  |
| 1 | Ath-AT1G48300.1 |  |  |  | Vvi-Vitvi05g01835\_t001 |  |  |  |  |  |  |
| 1 | Ath-AT1G48310.2 |  |  |  | Vvi-Vitvi05g00265\_t001 |  |  |  |  |  |  |
| 1 | Ath-AT1G48320.1 |  |  |  | Vvi-Vitvi05g00267\_t001 |  |  |  |  |  |  |
| 1 | Ath-AT1G48325.1 |  |  |  | | | |  |  |  |  |  |  |
| 1 | Ath-AT1G48330.1 |  |  |  | Vvi-Vitvi05g01843\_t001 |  |  |  |  |  |  |
| 1 | Ath-AT1G48350.1 |  |  |  | Vvi-Vitvi05g00279\_t001 |  |  |  |  |  |  |
| 0 | Ath-AT1G48355.1 |  |  |  |  |  |  |  |  |
| 0 | Ath-AT1G48360.2 |  |  |  |  |  |  |  |  |
| 0 | Ath-AT1G48370.1 |  |  |  |  |  |  |  |  |
| 0 | Ath-AT1G48380.2 |  |  |  |  |  |  |  |  |
| 0 | Ath-AT1G48390.1 |  |  |  |  |  |  |  |  |
| 0 | Ath-AT1G48400.1 |  |  |  |  |  |  |  |  |
| 0 | Ath-AT1G48405.1 |  |  |  |  |  |  |  |  |
| 0 | Ath-AT1G48410.3 |  |  |  |  |  |  |  |  |
| 0 | Ath-AT1G48420.1 |  |  |  |  |  |  |  |  |
| 0 | Ath-AT1G48430.1 |  |  |  |  |  |  |  |  |
| 1 | Ath-AT1G48440.1 |  | Vvi-Vitvi17g00170\_t001 |  |  |  |  |  |  |  |
| 1 | Ath-AT1G48450.1 |  | | | |  |  |  |  |  |  |  |
| 1 | Ath-AT1G48460.1 |  | | | |  |  |  |  |  |  |  |
| 1 | Ath-AT1G48470.1 |  | | | |  |  |  |  |  |  |  |
| 1 | Ath-AT1G48480.1 |  | | | |  |  |  |  |  |  |  |
| 1 | Ath-AT1G48490.4 |  | | | |  |  |  |  |  |  |  |
| 1 | Ath-AT1G48500.1 |  | | | |  |  |  |  |  |  |  |
| 1 | Ath-AT1G48510.3 |  | | | |  |  |  |  |  |  |  |
| 1 | Ath-AT1G48520.1 |  | | | |  |  |  |  |  |  |  |
| 1 | Ath-AT1G48530.1 |  | | | |  |  |  |  |  |  |  |
| 1 | Ath-AT1G48540.1 |  | Vvi-Vitvi17g00183\_t001 |  |  |  |  |  |  |  |
| 1 | Ath-AT1G48550.1 |  | Vvi-Vitvi17g00194\_t001 |  |  |  |  |  |  |  |
| 1 | Ath-AT1G48560.2 |  | | | |  |  |  |  |  |  |  |
| 1 | Ath-AT1G48570.1 |  | Vvi-Vitvi17g00198\_t001 |  |  |  |  |  |  |  |
| 1 | Ath-AT1G48580.2 |  | | | |  |  |  |  |  |  |  |
| 1 | Ath-AT1G48590.2 |  | | | |  |  |  |  |  |  |  |
| 1 | Ath-AT1G48600.2 |  | Vvi-Vitvi17g00213\_t001 |  |  |  |  |  |  |  |
| 1 | Ath-AT1G48605.1 |  | | | |  |  |  |  |  |  |  |
| 1 | Ath-AT1G48610.1 |  | | | |  |  |  |  |  |  |  |
| 1 | Ath-AT1G48620.1 |  | Vvi-Vitvi17g00223\_t001 |  |  |  |  |  |  |  |
| 1 | Ath-AT1G48630.1 |  | Vvi-Vitvi17g00239\_t001 |  |  |  |  |  |  |  |
| 1 | Ath-AT1G48635.2 |  | Vvi-Vitvi17g00246\_t001 |  |  |  |  |  |  |  |
| 1 | Ath-AT1G48640.1 |  | | | |  |  |  |  |  |  |  |
| 1 | Ath-AT1G48650.2 |  | Vvi-Vitvi17g00262\_t001 |  |  |  |  |  |  |  |
| 1 | Ath-AT1G48660.2 |  | | | |  |  |  |  |  |  |  |
| 1 | Ath-AT1G48670.1 |  | | | |  |  |  |  |  |  |  |
| 1 | Ath-AT1G48690.1 |  | | | |  |  |  |  |  |  |  |
| 1 | Ath-AT1G48700.4 |  | Vvi-Vitvi17g04069\_t001 |  |  |  |  |  |  |  |
| 1 | Ath-AT1G48720.1 |  | | | |  |  |  |  |  |  |  |
| 1 | Ath-AT1G48725.1 |  | | | |  |  |  |  |  |  |  |
| 1 | Ath-AT1G48730.1 |  | | | |  |  |  |  |  |  |  |
| 1 | Ath-AT1G48740.2 |  | | | |  |  |  |  |  |  |  |
| 1 | Ath-AT1G48745.1 |  | | | |  |  |  |  |  |  |  |
| 1 | Ath-AT1G48750.1 |  | Vvi-Vitvi17g00283\_t001 |  |  |  |  |  |  |  |
| 1 | Ath-AT1G48760.2 |  | Vvi-Vitvi17g00298\_t001 |  |  |  |  |  |  |  |
| 1 | Ath-AT1G48770.2 |  | Vvi-Vitvi17g04082\_t001 |  |  |  |  |  |  |  |
| 1 | Ath-AT1G48780.1 |  | Vvi-Vitvi17g00300\_t001 |  |  |  |  |  |  |  |
| 1 | Ath-AT1G48790.1 |  | Vvi-Vitvi17g00301\_t001 |  |  |  |  |  |  |  |
| 1 | Ath-AT1G48800.1 |  | | | |  |  |  |  |  |  |  |
| 1 | Ath-AT1G48820.1 |  | | | |  |  |  |  |  |  |  |
| 1 | Ath-AT1G48830.1 |  | Vvi-Vitvi17g00305\_t001 |  |  |  |  |  |  |  |
| 1 | Ath-AT1G48840.1 |  | Vvi-Vitvi17g00312\_t002 |  |  |  |  |  |  |  |
| 0 | Ath-AT1G48850.1 |  |  |  |  |  |  |  |  |
| 0 | Ath-AT1G48860.1 |  |  |  |  |  |  |  |  |
| 0 | Ath-AT1G48870.1 |  |  |  |  |  |  |  |  |
| 0 | Ath-AT1G48880.1 |  |  |  |  |  |  |  |  |
| 0 | Ath-AT1G48900.1 |  |  |  |  |  |  |  |  |
| 0 | Ath-AT1G48910.1 |  |  |  |  |  |  |  |  |
| 0 | Ath-AT1G48912.1 |  |  |  |  |  |  |  |  |
| 0 | Ath-AT1G48920.1 |  |  |  |  |  |  |  |  |
| 0 | Ath-AT1G48930.1 |  |  |  |  |  |  |  |  |
| 1 | Ath-AT1G48940.1 |  | Vvi-Vitvi17g00386\_t001 |  |  |  |  |  |  |  |
| 1 | Ath-AT1G48950.1 |  | Vvi-Vitvi17g00385\_t001 |  |  |  |  |  |  |  |
| 1 | Ath-AT1G48953.1 |  | | | |  |  |  |  |  |  |  |
| 1 | Ath-AT1G48960.1 |  | Vvi-Vitvi17g00383\_t001 |  |  |  |  |  |  |  |
| 1 | Ath-AT1G48970.1 |  | Vvi-Vitvi17g00381\_t002 |  |  |  |  |  |  |  |
| 1 | Ath-AT1G48980.1 |  | | | |  |  |  |  |  |  |  |
| 1 | Ath-AT1G48990.1 |  | Vvi-Vitvi17g00379\_t001 |  |  |  |  |  |  |  |
| 1 | Ath-AT1G49000.1 |  | Vvi-Vitvi17g01420\_t001 |  |  |  |  |  |  |  |
| 1 | Ath-AT1G49005.1 |  | | | |  |  |  |  |  |  |  |
| 1 | Ath-AT1G49010.1 |  | Vvi-Vitvi17g00366\_t001 |  |  |  |  |  |  |  |
| 1 | Ath-AT1G49015.1 |  | | | |  |  |  |  |  |  |  |
| 1 | Ath-AT1G49030.1 |  | Vvi-Vitvi17g00347\_t001 |  |  |  |  |  |  |  |
| 1 | Ath-AT1G49032.1 |  | | | |  |  |  |  |  |  |  |
| 1 | Ath-AT1G49040.1 |  | Vvi-Vitvi17g00344\_t001 |  |  |  |  |  |  |  |
| 1 | Ath-AT1G49050.1 |  | Vvi-Vitvi17g00336\_t001 |  |  |  |  |  |  |  |
| 1 | Ath-AT1G49100.1 |  | | | |  |  |  |  |  |  |  |
| 1 | Ath-AT1G49110.1 |  | | | |  |  |  |  |  |  |  |
| 1 | Ath-AT1G49120.1 |  | | | |  |  |  |  |  |  |  |
| 1 | Ath-AT1G49130.1 |  | Vvi-Vitvi17g00328\_t001 |  |  |  |  |  |  |  |
| 1 | Ath-AT1G49140.1 |  | Vvi-Vitvi17g00327\_t001 |  |  |  |  |  |  |  |
| 0 | Ath-AT1G49150.1 |  |  |  |  |  |  |  |  |
| 0 | Ath-AT1G49160.5 |  |  |  |  |  |  |  |  |
| 0 | Ath-AT1G49170.1 |  |  |  |  |  |  |  |  |
| 0 | Ath-AT1G49180.1 |  |  |  |  |  |  |  |  |
| 0 | Ath-AT1G49190.2 |  |  |  |  |  |  |  |  |
| 0 | Ath-AT1G49200.1 |  |  |  |  |  |  |  |  |
| 0 | Ath-AT1G49205.1 |  |  |  |  |  |  |  |  |
| 0 | Ath-AT1G49210.1 |  |  |  |  |  |  |  |  |
| 0 | Ath-AT1G49220.1 |  |  |  |  |  |  |  |  |
| 0 | Ath-AT1G49225.1 |  |  |  |  |  |  |  |  |
| 0 | Ath-AT1G49230.1 |  |  |  |  |  |  |  |  |
| 0 | Ath-AT1G49240.1 |  |  |  |  |  |  |  |  |
| 0 | Ath-AT1G49245.1 |  |  |  |  |  |  |  |  |
| 0 | Ath-AT1G49250.1 |  |  |  |  |  |  |  |  |
| 0 | Ath-AT1G49260.1 |  |  |  |  |  |  |  |  |
| 1 | Ath-AT1G49270.1 |  | Vvi-Vitvi03g00345\_t001 |  |  |  |  |  |  |  |
| 1 | Ath-AT1G49290.1 |  | | | |  |  |  |  |  |  |  |
| 1 | Ath-AT1G49300.1 |  | Vvi-Vitvi03g00348\_t001 |  |  |  |  |  |  |  |
| 1 | Ath-AT1G49310.1 |  | | | |  |  |  |  |  |  |  |
| 1 | Ath-AT1G49320.1 |  | Vvi-Vitvi03g00352\_t003 |  |  |  |  |  |  |  |
| 1 | Ath-AT1G49330.1 |  | Vvi-Vitvi03g01507\_t001 |  |  |  |  |  |  |  |
| 1 | Ath-AT1G49340.1 |  | Vvi-Vitvi03g00358\_t001 |  |  |  |  |  |  |  |
| 1 | Ath-AT1G49350.1 |  | | | |  |  |  |  |  |  |  |
| 1 | Ath-AT1G49360.1 |  | | | |  |  |  |  |  |  |  |
| 1 | Ath-AT1G49380.1 |  | Vvi-Vitvi03g00373\_t002 |  |  |  |  |  |  |  |
| 1 | Ath-AT1G49390.1 |  | | | |  |  |  |  |  |  |  |
| 1 | Ath-AT1G49400.1 |  | | | |  |  |  |  |  |  |  |
| 1 | Ath-AT1G49405.1 |  | Vvi-Vitvi03g00384\_t001 |  |  |  |  |  |  |  |
| 1 | Ath-AT1G49410.1 |  | Vvi-Vitvi03g00385\_t001 |  |  |  |  |  |  |  |
| 1 | Ath-AT1G49420.1 |  | | | |  |  |  |  |  |  |  |
| 1 | Ath-AT1G49430.1 |  | Vvi-Vitvi03g00388\_t001 |  |  |  |  |  |  |  |
| 1 | Ath-AT1G49435.1 |  | | | |  |  |  |  |  |  |  |
| 1 | Ath-AT1G49450.1 |  | Vvi-Vitvi03g00397\_t001 |  |  |  |  |  |  |  |
| 1 | Ath-AT1G49470.1 |  | Vvi-Vitvi03g00405\_t001 |  |  |  |  |  |  |  |
| 1 | Ath-AT1G49475.1 |  | Vvi-Vitvi03g00416\_t001 |  |  |  |  |  |  |  |
| 1 | Ath-AT1G49480.2 |  | | | |  |  |  |  |  |  |  |
| 1 | Ath-AT1G49490.2 |  | Vvi-Vitvi03g00427\_t001 |  |  |  |  |  |  |  |
| 1 | Ath-AT1G49500.1 |  | | | |  |  |  |  |  |  |  |
| 1 | Ath-AT1G49510.1 |  | Vvi-Vitvi03g00449\_t003 |  |  |  |  |  |  |  |
| 1 | Ath-AT1G49520.1 |  | Vvi-Vitvi03g04170\_t001 |  |  |  |  |  |  |  |
| 1 | Ath-AT1G49530.1 |  | | | |  |  |  |  |  |  |  |
| 1 | Ath-AT1G49540.2 |  | | | |  |  |  |  |  |  |  |
| 1 | Ath-AT1G49560.1 |  | Vvi-Vitvi03g00454\_t002 |  |  |  |  |  |  |  |
| 1 | Ath-AT1G49570.1 |  | Vvi-Vitvi03g00456\_t001 |  |  |  |  |  |  |  |
| 1 | Ath-AT1G49580.1 |  | Vvi-Vitvi03g00464\_t001 |  |  |  |  |  |  |  |
| 1 | Ath-AT1G49590.1 |  | Vvi-Vitvi03g00472\_t002 |  |  |  |  |  |  |  |
| 1 | Ath-AT1G49600.2 |  | Vvi-Vitvi03g00476\_t002 |  |  |  |  |  |  |  |
| 1 | Ath-AT1G49610.1 |  | | | |  |  |  |  |  |  |  |
| 1 | Ath-AT1G49620.3 |  | | | |  |  |  |  |  |  |  |
| 1 | Ath-AT1G49630.2 |  | Vvi-Vitvi03g00485\_t001 |  |  |  |  |  |  |  |
| 1 | Ath-AT1G49640.1 |  | | | |  |  |  |  |  |  |  |
| 1 | Ath-AT1G49650.1 |  | | | |  |  |  |  |  |  |  |
| 1 | Ath-AT1G49660.1 |  | | | |  |  |  |  |  |  |  |
| 1 | Ath-AT1G49670.2 |  | Vvi-Vitvi03g00491\_t001 |  |  |  |  |  |  |  |
| 1 | Ath-AT1G49680.1 |  | | | |  |  |  |  |  |  |  |
| 1 | Ath-AT1G49700.1 |  | | | |  |  |  |  |  |  |  |
| 1 | Ath-AT1G49710.1 |  | Vvi-Vitvi03g00510\_t001 |  |  |  |  |  |  |  |
| 1 | Ath-AT1G49715.1 |  | | | |  |  |  |  |  |  |  |
| 1 | Ath-AT1G49720.2 |  | Vvi-Vitvi03g01574\_t003 |  |  |  |  |  |  |  |
| 1 | Ath-AT1G49730.1 |  | Vvi-Vitvi03g00516\_t002 |  |  |  |  |  |  |  |
| 1 | Ath-AT1G49740.1 |  | Vvi-Vitvi03g00517\_t001 |  |  |  |  |  |  |  |
| 1 | Ath-AT1G49750.1 |  | | | |  |  |  |  |  |  |  |
| 1 | Ath-AT1G49760.2 |  | Vvi-Vitvi03g00531\_t001 |  |  |  |  |  |  |  |
| 1 | Ath-AT1G49770.1 |  | | | |  |  |  |  |  |  |  |
| 1 | Ath-AT1G49780.1 |  | | | |  |  |  |  |  |  |  |
| 1 | Ath-AT1G49790.2 |  | | | |  |  |  |  |  |  |  |
| 1 | Ath-AT1G49800.1 |  | | | |  |  |  |  |  |  |  |
| 1 | Ath-AT1G49810.1 |  | Vvi-Vitvi03g00550\_t001 |  |  |  |  |  |  |  |
| 1 | Ath-AT1G49820.1 |  | Vvi-Vitvi03g00573\_t001 |  |  |  |  |  |  |  |
| 1 | Ath-AT1G49830.1 |  | Vvi-Vitvi03g04201\_t001 |  |  |  |  |  |  |  |
| 1 | Ath-AT1G49840.1 |  | Vvi-Vitvi03g00597\_t001 |  |  |  |  |  |  |  |
| 1 | Ath-AT1G49850.1 |  | Vvi-Vitvi03g00606\_t001 |  |  |  |  |  |  |  |
| 1 | Ath-AT1G49860.1 |  | | | |  |  |  |  |  |  |  |
| 1 | Ath-AT1G49870.1 |  | Vvi-Vitvi03g00613\_t001 |  |  |  |  |  |  |  |
| 1 | Ath-AT1G49880.1 |  | Vvi-Vitvi03g00621\_t001 |  |  |  |  |  |  |  |
| 1 | Ath-AT1G49890.1 |  | Vvi-Vitvi03g00626\_t001 |  |  |  |  |  |  |  |
| 1 | Ath-AT1G49900.1 |  | | | |  |  |  |  |  |  |  |
| 1 | Ath-AT1G49910.1 |  | Vvi-Vitvi03g00633\_t001 |  |  |  |  |  |  |  |
| 1 | Ath-AT1G49920.1 |  | | | |  |  |  |  |  |  |  |
| 1 | Ath-AT1G49930.1 |  | | | |  |  |  |  |  |  |  |
| 1 | Ath-AT1G49940.1 |  | | | |  |  |  |  |  |  |  |
| 1 | Ath-AT1G49950.1 |  | Vvi-Vitvi03g00668\_t001 |  |  |  |  |  |  |  |
| 1 | Ath-AT1G49960.1 |  | Vvi-Vitvi03g00673\_t001 |  |  |  |  |  |  |  |
| 1 | Ath-AT1G49970.1 |  | | | |  |  |  |  |  |  |  |
| 1 | Ath-AT1G49975.1 |  | Vvi-Vitvi03g01612\_t001 |  |  |  |  |  |  |  |
| 0 | Ath-AT1G49980.1 |  |  |  |  |  |  |  |  |
| 0 | Ath-AT1G49990.1 |  |  |  |  |  |  |  |  |
| 1 | Ath-AT1G50000.1 |  | Vvi-Vitvi03g00710\_t001 |  |  |  |  |  |  |  |
| 1 | Ath-AT1G50010.1 |  | Vvi-Vitvi03g00721\_t001 |  |  |  |  |  |  |  |
| 1 | Ath-AT1G50020.1 |  | Vvi-Vitvi03g01639\_t001 |  |  |  |  |  |  |  |
| 1 | Ath-AT1G50030.1 |  | Vvi-Vitvi03g00724\_t001 |  |  |  |  |  |  |  |
| 1 | Ath-AT1G50040.1 |  | Vvi-Vitvi03g00735\_t001 |  |  |  |  |  |  |  |
| 1 | Ath-AT1G50050.1 |  | | | |  |  |  |  |  |  |  |
| 1 | Ath-AT1G50060.1 |  | Vvi-Vitvi03g00740\_t001 |  |  |  |  |  |  |  |
| 0 | Ath-AT1G50080.1 |  |  |  |  |  |  |  |  |
| 0 | Ath-AT1G50090.1 |  |  |  |  |  |  |  |  |
| 0 | Ath-AT1G50110.1 |  |  |  |  |  |  |  |  |
| 0 | Ath-AT1G50120.2 |  |  |  |  |  |  |  |  |
| 0 | Ath-AT1G50140.1 |  |  |  |  |  |  |  |  |
| 0 | Ath-AT1G50160.1 |  |  |  |  |  |  |  |  |
| 0 | Ath-AT1G50170.1 |  |  |  |  |  |  |  |  |
| 0 | Ath-AT1G50180.1 |  |  |  |  |  |  |  |  |
| 0 | Ath-AT1G50190.1 |  |  |  |  |  |  |  |  |
| 0 | Ath-AT1G50200.2 |  |  |  |  |  |  |  |  |
| 0 | Ath-AT1G50220.1 |  |  |  |  |  |  |  |  |
| 0 | Ath-AT1G50240.2 |  |  |  |  |  |  |  |  |
| 0 | Ath-AT1G50250.1 |  |  |  |  |  |  |  |  |
| 0 | Ath-AT1G50260.1 |  |  |  |  |  |  |  |  |
| 0 | Ath-AT1G50270.1 |  |  |  |  |  |  |  |  |
| 0 | Ath-AT1G50280.1 |  |  |  |  |  |  |  |  |
| 0 | Ath-AT1G50290.1 |  |  |  |  |  |  |  |  |
| 0 | Ath-AT1G50300.1 |  |  |  |  |  |  |  |  |
| 0 | Ath-AT1G50310.1 |  |  |  |  |  |  |  |  |
| 0 | Ath-AT1G50320.1 |  |  |  |  |  |  |  |  |
| 0 | Ath-AT1G50325.1 |  |  |  |  |  |  |  |  |
| 0 | Ath-AT1G50340.1 |  |  |  |  |  |  |  |  |
| 0 | Ath-AT1G50350.1 |  |  |  |  |  |  |  |  |
| 0 | Ath-AT1G50360.1 |  |  |  |  |  |  |  |  |
| 0 | Ath-AT1G50370.1 |  |  |  |  |  |  |  |  |
| 0 | Ath-AT1G50380.2 |  |  |  |  |  |  |  |  |
| 0 | Ath-AT1G50390.1 |  |  |  |  |  |  |  |  |
| 0 | Ath-AT1G50400.1 |  |  |  |  |  |  |  |  |
| 0 | Ath-AT1G50410.2 |  |  |  |  |  |  |  |  |
| 0 | Ath-AT1G50420.1 |  |  |  |  |  |  |  |  |
| 0 | Ath-AT1G50430.1 |  |  |  |  |  |  |  |  |
| 0 | Ath-AT1G50440.1 |  |  |  |  |  |  |  |  |
| 0 | Ath-AT1G50450.1 |  |  |  |  |  |  |  |  |
| 0 | Ath-AT1G50460.1 |  |  |  |  |  |  |  |  |
| 0 | Ath-AT1G50470.1 |  |  |  |  |  |  |  |  |
| 0 | Ath-AT1G50480.1 |  |  |  |  |  |  |  |  |
| 0 | Ath-AT1G50490.1 |  |  |  |  |  |  |  |  |
| 0 | Ath-AT1G50500.2 |  |  |  |  |  |  |  |  |
| 0 | Ath-AT1G50510.1 |  |  |  |  |  |  |  |  |
| 0 | Ath-AT1G50520.1 |  |  |  |  |  |  |  |  |
| 0 | Ath-AT1G50530.1 |  |  |  |  |  |  |  |  |
| 0 | Ath-AT1G50560.1 |  |  |  |  |  |  |  |  |
| 0 | Ath-AT1G50570.1 |  |  |  |  |  |  |  |  |
| 0 | Ath-AT1G50575.1 |  |  |  |  |  |  |  |  |
| 0 | Ath-AT1G50580.1 |  |  |  |  |  |  |  |  |
| 0 | Ath-AT1G50590.1 |  |  |  |  |  |  |  |  |
| 0 | Ath-AT1G50600.2 |  |  |  |  |  |  |  |  |
| 0 | Ath-AT1G50610.1 |  |  |  |  |  |  |  |  |
| 1 | Ath-AT1G50620.1 |  | Vvi-Vitvi19g00575\_t001 |  |  |  |  |  |  |  |
| 1 | Ath-AT1G50630.1 |  | Vvi-Vitvi19g00559\_t001 |  |  |  |  |  |  |  |
| 1 | Ath-AT1G50640.1 |  | Vvi-Vitvi19g00558\_t001 |  |  |  |  |  |  |  |
| 1 | Ath-AT1G50650.1 |  | Vvi-Vitvi19g00550\_t001 |  |  |  |  |  |  |  |
| 2 | Ath-AT1G50660.1 |  | | | |  | Vvi-Vitvi19g00492\_t001 |  |  |  |  |  |  |
| 2 | Ath-AT1G50670.1 |  | | | |  | | | |  |  |  |  |  |  |
| 2 | Ath-AT1G50680.1 |  | | | |  | | | |  |  |  |  |  |  |
| 2 | Ath-AT1G50690.2 |  | | | |  | | | |  |  |  |  |  |  |
| 2 | Ath-AT1G50700.2 |  | | | |  | | | |  |  |  |  |  |  |
| 2 | Ath-AT1G50710.1 |  | | | |  | | | |  |  |  |  |  |  |
| 2 | Ath-AT1G50720.1 |  | Vvi-Vitvi19g00534\_t001 |  | | | |  |  |  |  |  |  |
| 2 | Ath-AT1G50730.1 |  | Vvi-Vitvi19g00533\_t001 |  | | | |  |  |  |  |  |  |
| 1 | Ath-AT1G50732.1 |  |  |  | | | |  |  |  |  |  |  |
| 1 | Ath-AT1G50740.1 |  |  |  | Vvi-Vitvi19g00480\_t001 |  |  |  |  |  |  |
| 1 | Ath-AT1G50745.1 |  |  |  | | | |  |  |  |  |  |  |
| 1 | Ath-AT1G50750.1 |  |  |  | | | |  |  |  |  |  |  |
| 1 | Ath-AT1G50760.1 |  |  |  | | | |  |  |  |  |  |  |
| 1 | Ath-AT1G50770.1 |  |  |  | | | |  |  |  |  |  |  |
| 1 | Ath-AT1G50780.1 |  |  |  | | | |  |  |  |  |  |  |
| 1 | Ath-AT1G50790.1 |  |  |  | Vvi-Vitvi19g00471\_t001 |  |  |  |  |  |  |
| 1 | Ath-AT1G50820.2 |  |  |  | | | |  |  |  |  |  |  |
| 1 | Ath-AT1G50830.1 |  |  |  | | | |  |  |  |  |  |  |
| 1 | Ath-AT1G50840.2 |  |  |  | | | |  |  |  |  |  |  |
| 1 | Ath-AT1G50870.1 |  |  |  | | | |  |  |  |  |  |  |
| 1 | Ath-AT1G50880.1 |  |  |  | | | |  |  |  |  |  |  |
| 1 | Ath-AT1G50890.1 |  |  |  | Vvi-Vitvi19g00469\_t001 |  |  |  |  |  |  |
| 1 | Ath-AT1G50900.1 |  |  |  | Vvi-Vitvi19g02003\_t001 |  |  |  |  |  |  |
| 1 | Ath-AT1G50910.1 |  |  |  | | | |  |  |  |  |  |  |
| 1 | Ath-AT1G50920.1 |  |  |  | | | |  |  |  |  |  |  |
| 1 | Ath-AT1G50930.1 |  |  |  | | | |  |  |  |  |  |  |
| 1 | Ath-AT1G50940.1 |  |  |  | Vvi-Vitvi19g00447\_t001 |  |  |  |  |  |  |
| 1 | Ath-AT1G50950.2 |  |  |  | Vvi-Vitvi19g00446\_t001 |  |  |  |  |  |  |
| 1 | Ath-AT1G50960.1 |  |  |  | Vvi-Vitvi19g00431\_t001 |  |  |  |  |  |  |
| 1 | Ath-AT1G50970.1 |  |  |  | | | |  |  |  |  |  |  |
| 1 | Ath-AT1G50980.1 |  |  |  | | | |  |  |  |  |  |  |
| 1 | Ath-AT1G50990.1 |  |  |  | | | |  |  |  |  |  |  |
| 1 | Ath-AT1G51000.1 |  |  |  | | | |  |  |  |  |  |  |
| 1 | Ath-AT1G51010.1 |  |  |  | | | |  |  |  |  |  |  |
| 1 | Ath-AT1G51020.1 |  |  |  | | | |  |  |  |  |  |  |
| 1 | Ath-AT1G51030.1 |  |  |  | | | |  |  |  |  |  |  |
| 1 | Ath-AT1G51035.1 |  |  |  | | | |  |  |  |  |  |  |
| 1 | Ath-AT1G51040.1 |  |  |  | | | |  |  |  |  |  |  |
| 1 | Ath-AT1G51055.1 |  |  |  | | | |  |  |  |  |  |  |
| 1 | Ath-AT1G51060.1 |  |  |  | | | |  |  |  |  |  |  |
| 1 | Ath-AT1G51070.2 |  |  |  | | | |  |  |  |  |  |  |
| 1 | Ath-AT1G51080.1 |  |  |  | | | |  |  |  |  |  |  |
| 1 | Ath-AT1G51090.1 |  |  |  | | | |  |  |  |  |  |  |
| 1 | Ath-AT1G51100.1 |  |  |  | Vvi-Vitvi19g01986\_t001 |  |  |  |  |  |  |
| 0 | Ath-AT1G51110.1 |  |  |  |  |  |  |  |  |
| 0 | Ath-AT1G51120.1 |  |  |  |  |  |  |  |  |
| 0 | Ath-AT1G51130.1 |  |  |  |  |  |  |  |  |
| 2 | Ath-AT1G51140.1 |  | Vvi-Vitvi06g00181\_t001 |  | Vvi-Vitvi08g01852\_t001 |  |  |  |  |  |  |
| 2 | Ath-AT1G51150.1 |  | | | |  | | | |  |  |  |  |  |  |
| 2 | Ath-AT1G51160.2 |  | | | |  | | | |  |  |  |  |  |  |
| 2 | Ath-AT1G51170.1 |  | Vvi-Vitvi06g00185\_t001 |  | | | |  |  |  |  |  |  |
| 2 | Ath-AT1G51172.1 |  | | | |  | | | |  |  |  |  |  |  |
| 2 | Ath-AT1G51190.1 |  | Vvi-Vitvi06g00187\_t001 |  | Vvi-Vitvi08g01861\_t001 |  |  |  |  |  |  |
| 2 | Ath-AT1G51200.1 |  | Vvi-Vitvi06g00189\_t003 |  | Vvi-Vitvi08g01864\_t001 |  |  |  |  |  |  |
| 2 | Ath-AT1G51210.1 |  | | | |  | | | |  |  |  |  |  |  |
| 2 | Ath-AT1G51220.1 |  | Vvi-Vitvi06g00195\_t001 |  | Vvi-Vitvi08g01869\_t001 |  |  |  |  |  |  |
| 2 | Ath-AT1G51230.1 |  | | | |  | | | |  |  |  |  |  |  |
| 2 | Ath-AT1G51240.1 |  | | | |  | | | |  |  |  |  |  |  |
| 2 | Ath-AT1G51250.1 |  | | | |  | | | |  |  |  |  |  |  |
| 2 | Ath-AT1G51260.1 |  | | | |  | Vvi-Vitvi08g01872\_t001 |  |  |  |  |  |  |
| 2 | Ath-AT1G51270.3 |  | | | |  | | | |  |  |  |  |  |  |
| 2 | Ath-AT1G51290.1 |  | | | |  | | | |  |  |  |  |  |  |
| 2 | Ath-AT1G51300.2 |  | | | |  | | | |  |  |  |  |  |  |
| 2 | Ath-AT1G51310.1 |  | Vvi-Vitvi06g01631\_t001 |  | | | |  |  |  |  |  |  |
| 2 | Ath-AT1G51320.1 |  | | | |  | | | |  |  |  |  |  |  |
| 2 | Ath-AT1G51330.1 |  | | | |  | | | |  |  |  |  |  |  |
| 2 | Ath-AT1G51340.2 |  | Vvi-Vitvi06g00216\_t001 |  | Vvi-Vitvi08g01879\_t001 |  |  |  |  |  |  |
| 2 | Ath-AT1G51350.1 |  | Vvi-Vitvi06g01633\_t001 |  | | | |  |  |  |  |  |  |
| 2 | Ath-AT1G51355.1 |  | Vvi-Vitvi06g01635\_t001 |  | Vvi-Vitvi08g02393\_t001 |  |  |  |  |  |  |
| 1 | Ath-AT1G51360.1 |  | | | |  |  |  |  |  |  |  |
| 1 | Ath-AT1G51370.1 |  | | | |  |  |  |  |  |  |  |
| 1 | Ath-AT1G51380.1 |  | | | |  |  |  |  |  |  |  |
| 1 | Ath-AT1G51390.1 |  | | | |  |  |  |  |  |  |  |
| 1 | Ath-AT1G51400.1 |  | Vvi-Vitvi06g01637\_t001 |  |  |  |  |  |  |  |
| 1 | Ath-AT1G51402.1 |  | | | |  |  |  |  |  |  |  |
| 1 | Ath-AT1G51405.1 |  | | | |  |  |  |  |  |  |  |
| 1 | Ath-AT1G51410.1 |  | Vvi-Vitvi06g00233\_t001 |  |  |  |  |  |  |  |
| 1 | Ath-AT1G51420.4 |  | | | |  |  |  |  |  |  |  |
| 1 | Ath-AT1G51430.1 |  | | | |  |  |  |  |  |  |  |
| 1 | Ath-AT1G51440.1 |  | Vvi-Vitvi06g00234\_t001 |  |  |  |  |  |  |  |
| 1 | Ath-AT1G51450.1 |  | Vvi-Vitvi06g00237\_t001 |  |  |  |  |  |  |  |
| 1 | Ath-AT1G51460.1 |  | | | |  |  |  |  |  |  |  |
| 1 | Ath-AT1G51470.1 |  | | | |  |  |  |  |  |  |  |
| 1 | Ath-AT1G51480.1 |  | | | |  |  |  |  |  |  |  |
| 1 | Ath-AT1G51490.1 |  | | | |  |  |  |  |  |  |  |
| 1 | Ath-AT1G51500.1 |  | Vvi-Vitvi06g00242\_t001 |  |  |  |  |  |  |  |
| 0 | Ath-AT1G51510.1 |  |  |  |  |  |  |  |  |
| 0 | Ath-AT1G51520.2 |  |  |  |  |  |  |  |  |
| 0 | Ath-AT1G51530.2 |  |  |  |  |  |  |  |  |
| 0 | Ath-AT1G51538.1 |  |  |  |  |  |  |  |  |
| 1 | Ath-AT1G51540.1 |  | Vvi-Vitvi09g01374\_t001 |  |  |  |  |  |  |  |
| 1 | Ath-AT1G51550.1 |  | Vvi-Vitvi09g01373\_t001 |  |  |  |  |  |  |  |
| 1 | Ath-AT1G51560.1 |  | Vvi-Vitvi09g01372\_t002 |  |  |  |  |  |  |  |
| 1 | Ath-AT1G51570.1 |  | | | |  |  |  |  |  |  |  |
| 1 | Ath-AT1G51580.1 |  | Vvi-Vitvi09g01354\_t001 |  |  |  |  |  |  |  |
| 1 | Ath-AT1G51590.1 |  | Vvi-Vitvi09g04562\_t001 |  |  |  |  |  |  |  |
| 1 | Ath-AT1G51600.1 |  | | | |  |  |  |  |  |  |  |
| 1 | Ath-AT1G51610.1 |  | Vvi-Vitvi09g04560\_t001 |  |  |  |  |  |  |  |
| 0 | Ath-AT1G51620.2 |  |  |  |  |  |  |  |  |
| 0 | Ath-AT1G51630.1 |  |  |  |  |  |  |  |  |
| 0 | Ath-AT1G51640.1 |  |  |  |  |  |  |  |  |
| 0 | Ath-AT1G51650.1 |  |  |  |  |  |  |  |  |
| 0 | Ath-AT1G51660.1 |  |  |  |  |  |  |  |  |
| 0 | Ath-AT1G51670.1 |  |  |  |  |  |  |  |  |
| 0 | Ath-AT1G51680.1 |  |  |  |  |  |  |  |  |
| 1 | Ath-AT1G51690.5 |  | Vvi-Vitvi09g00208\_t001 |  |  |  |  |  |  |  |
| 1 | Ath-AT1G51700.1 |  | Vvi-Vitvi09g00210\_t001 |  |  |  |  |  |  |  |
| 1 | Ath-AT1G51710.1 |  | Vvi-Vitvi09g00211\_t001 |  |  |  |  |  |  |  |
| 1 | Ath-AT1G51720.1 |  | Vvi-Vitvi09g00214\_t003 |  |  |  |  |  |  |  |
| 1 | Ath-AT1G51730.1 |  | Vvi-Vitvi09g00215\_t002 |  |  |  |  |  |  |  |
| 1 | Ath-AT1G51740.1 |  | | | |  |  |  |  |  |  |  |
| 1 | Ath-AT1G51745.1 |  | Vvi-Vitvi09g00216\_t001 |  |  |  |  |  |  |  |
| 1 | Ath-AT1G51760.1 |  | Vvi-Vitvi09g00220\_t001 |  |  |  |  |  |  |  |
| 1 | Ath-AT1G51770.1 |  | Vvi-Vitvi09g00223\_t001 |  |  |  |  |  |  |  |
| 1 | Ath-AT1G51780.1 |  | | | |  |  |  |  |  |  |  |
| 1 | Ath-AT1G51790.2 |  | | | |  |  |  |  |  |  |  |
| 1 | Ath-AT1G51800.1 |  | Vvi-Vitvi09g00228\_t001 |  |  |  |  |  |  |  |
| 1 | Ath-AT1G51805.1 |  | Vvi-Vitvi09g00230\_t001 |  |  |  |  |  |  |  |
| 1 | Ath-AT1G51810.1 |  | | | |  |  |  |  |  |  |  |
| 1 | Ath-AT1G51820.1 |  | Vvi-Vitvi09g04065\_t001 |  |  |  |  |  |  |  |
| 1 | Ath-AT1G51823.1 |  | | | |  |  |  |  |  |  |  |
| 1 | Ath-AT1G51830.2 |  | | | |  |  |  |  |  |  |  |
| 1 | Ath-AT1G51840.3 |  | | | |  |  |  |  |  |  |  |
| 1 | Ath-AT1G51850.2 |  | | | |  |  |  |  |  |  |  |
| 1 | Ath-AT1G51860.1 |  | Vvi-Vitvi09g00255\_t001 |  |  |  |  |  |  |  |
| 1 | Ath-AT1G51870.2 |  | | | |  |  |  |  |  |  |  |
| 1 | Ath-AT1G51880.3 |  | | | |  |  |  |  |  |  |  |
| 1 | Ath-AT1G51890.2 |  | | | |  |  |  |  |  |  |  |
| 1 | Ath-AT1G51900.1 |  | | | |  |  |  |  |  |  |  |
| 1 | Ath-AT1G51910.1 |  | | | |  |  |  |  |  |  |  |
| 1 | Ath-AT1G51913.1 |  | | | |  |  |  |  |  |  |  |
| 1 | Ath-AT1G51915.1 |  | | | |  |  |  |  |  |  |  |
| 1 | Ath-AT1G51920.1 |  | | | |  |  |  |  |  |  |  |
| 1 | Ath-AT1G51930.1 |  | | | |  |  |  |  |  |  |  |
| 1 | Ath-AT1G51940.1 |  | | | |  |  |  |  |  |  |  |
| 1 | Ath-AT1G51950.1 |  | | | |  |  |  |  |  |  |  |
| 1 | Ath-AT1G51960.1 |  | | | |  |  |  |  |  |  |  |
| 1 | Ath-AT1G51965.1 |  | | | |  |  |  |  |  |  |  |
| 1 | Ath-AT1G51970.1 |  | | | |  |  |  |  |  |  |  |
| 1 | Ath-AT1G51980.1 |  | Vvi-Vitvi09g00271\_t001 |  |  |  |  |  |  |  |
| 0 | Ath-AT1G51990.2 |  |  |  |  |  |  |  |  |
| 0 | Ath-AT1G52000.1 |  |  |  |  |  |  |  |  |
| 0 | Ath-AT1G52030.1 |  |  |  |  |  |  |  |  |
| 0 | Ath-AT1G52040.1 |  |  |  |  |  |  |  |  |
| 0 | Ath-AT1G52050.1 |  |  |  |  |  |  |  |  |
| 0 | Ath-AT1G52060.1 |  |  |  |  |  |  |  |  |
| 0 | Ath-AT1G52070.1 |  |  |  |  |  |  |  |  |
| 0 | Ath-AT1G52080.1 |  |  |  |  |  |  |  |  |
| 0 | Ath-AT1G52100.1 |  |  |  |  |  |  |  |  |
| 0 | Ath-AT1G52110.1 |  |  |  |  |  |  |  |  |
| 0 | Ath-AT1G52120.2 |  |  |  |  |  |  |  |  |
| 0 | Ath-AT1G52130.1 |  |  |  |  |  |  |  |  |
| 0 | Ath-AT1G52140.1 |  |  |  |  |  |  |  |  |
| 0 | Ath-AT1G52150.2 |  |  |  |  |  |  |  |  |
| 1 | Ath-AT1G52155.1 |  | Vvi-Vitvi09g00324\_t001 |  |  |  |  |  |  |  |
| 1 | Ath-AT1G52160.1 |  | Vvi-Vitvi09g00327\_t001 |  |  |  |  |  |  |  |
| 1 | Ath-AT1G52180.1 |  | | | |  |  |  |  |  |  |  |
| 2 | Ath-AT1G52190.1 |  | Vvi-Vitvi09g00340\_t001 |  | Vvi-Vitvi11g00306\_t001 |  |  |  |  |  |  |
| 2 | Ath-AT1G52191.1 |  | Vvi-Vitvi09g01597\_t001 |  | | | |  |  |  |  |  |  |
| 2 | Ath-AT1G52200.1 |  | Vvi-Vitvi09g00343\_t001 |  | | | |  |  |  |  |  |  |
| 3 | Ath-AT1G52220.1 |  | | | |  | | | |  | Vvi-Vitvi09g01601\_t001 |  |  |  |  |  |
| 3 | Ath-AT1G52230.1 |  | | | |  | | | |  | Vvi-Vitvi09g00361\_t001 |  |  |  |  |  |
| 3 | Ath-AT1G52240.1 |  | | | |  | Vvi-Vitvi11g00319\_t001 |  | Vvi-Vitvi09g00359\_t001 |  |  |  |  |  |
| 3 | Ath-AT1G52245.1 |  | | | |  | Vvi-Vitvi11g00322\_t001 |  | Vvi-Vitvi09g00355\_t001 |  |  |  |  |  |
| 3 | Ath-AT1G52260.1 |  | Vvi-Vitvi09g00351\_t001 |  | | | |  | | | |  |  |  |  |  |
| 3 | Ath-AT1G52270.1 |  | | | |  | | | |  | | | |  |  |  |  |  |
| 3 | Ath-AT1G52280.1 |  | | | |  | | | |  | Vvi-Vitvi09g00348\_t001 |  |  |  |  |  |
| 3 | Ath-AT1G52290.1 |  | | | |  | Vvi-Vitvi11g00332\_t001 |  | Vvi-Vitvi09g00347\_t001 |  |  |  |  |  |
| 2 | Ath-AT1G52300.1 |  | Vvi-Vitvi09g00370\_t001 |  | | | |  |  |  |  |  |  |
| 2 | Ath-AT1G52310.1 |  | | | |  | | | |  |  |  |  |  |  |
| 2 | Ath-AT1G52315.1 |  | Vvi-Vitvi09g00385\_t001 |  | Vvi-Vitvi11g00346\_t001 |  |  |  |  |  |  |
| 2 | Ath-AT1G52320.5 |  | Vvi-Vitvi09g00386\_t001 |  | | | |  |  |  |  |  |  |
| 2 | Ath-AT1G52325.1 |  | | | |  | | | |  |  |  |  |  |  |
| 2 | Ath-AT1G52330.2 |  | Vvi-Vitvi09g00388\_t001 |  | Vvi-Vitvi11g00348\_t001 |  |  |  |  |  |  |
| 2 | Ath-AT1G52340.1 |  | | | |  | | | |  |  |  |  |  |  |
| 2 | Ath-AT1G52342.1 |  | | | |  | | | |  |  |  |  |  |  |
| 2 | Ath-AT1G52343.1 |  | Vvi-Vitvi09g00389\_t001 |  | | | |  |  |  |  |  |  |
| 2 | Ath-AT1G52360.2 |  | Vvi-Vitvi09g00400\_t001 |  | Vvi-Vitvi11g00359\_t001 |  |  |  |  |  |  |
| 1 | Ath-AT1G52370.1 |  | | | |  |  |  |  |  |  |  |
| 1 | Ath-AT1G52380.2 |  | Vvi-Vitvi09g00402\_t001 |  |  |  |  |  |  |  |
| 1 | Ath-AT1G52390.1 |  | | | |  |  |  |  |  |  |  |
| 1 | Ath-AT1G52400.1 |  | | | |  |  |  |  |  |  |  |
| 1 | Ath-AT1G52410.2 |  | | | |  |  |  |  |  |  |  |
| 1 | Ath-AT1G52415.1 |  | | | |  |  |  |  |  |  |  |
| 1 | Ath-AT1G52420.1 |  | Vvi-Vitvi09g00406\_t001 |  |  |  |  |  |  |  |
| 1 | Ath-AT1G52430.1 |  | | | |  |  |  |  |  |  |  |
| 1 | Ath-AT1G52440.1 |  | | | |  |  |  |  |  |  |  |
| 1 | Ath-AT1G52450.1 |  | | | |  |  |  |  |  |  |  |
| 1 | Ath-AT1G52460.1 |  | | | |  |  |  |  |  |  |  |
| 1 | Ath-AT1G52470.1 |  | | | |  |  |  |  |  |  |  |
| 1 | Ath-AT1G52490.1 |  | | | |  |  |  |  |  |  |  |
| 1 | Ath-AT1G52495.1 |  | | | |  |  |  |  |  |  |  |
| 1 | Ath-AT1G52500.2 |  | | | |  |  |  |  |  |  |  |
| 1 | Ath-AT1G52510.1 |  | | | |  |  |  |  |  |  |  |
| 1 | Ath-AT1G52520.1 |  | | | |  |  |  |  |  |  |  |
| 1 | Ath-AT1G52530.2 |  | | | |  |  |  |  |  |  |  |
| 1 | Ath-AT1G52540.1 |  | | | |  |  |  |  |  |  |  |
| 1 | Ath-AT1G52550.1 |  | | | |  |  |  |  |  |  |  |
| 2 | Ath-AT1G52560.1 |  | | | |  | Vvi-Vitvi09g00599\_t001 |  |  |  |  |  |  |
| 2 | Ath-AT1G52565.1 |  | | | |  | Vvi-Vitvi09g04184\_t001 |  |  |  |  |  |  |
| 2 | Ath-AT1G52570.1 |  | | | |  | Vvi-Vitvi09g00595\_t001 |  |  |  |  |  |  |
| 2 | Ath-AT1G52580.1 |  | | | |  | Vvi-Vitvi09g00578\_t001 |  |  |  |  |  |  |
| 2 | Ath-AT1G52590.1 |  | | | |  | Vvi-Vitvi09g00577\_t001 |  |  |  |  |  |  |
| 2 | Ath-AT1G52600.1 |  | | | |  | Vvi-Vitvi09g00576\_t001 |  |  |  |  |  |  |
| 2 | Ath-AT1G52603.1 |  | | | |  | | | |  |  |  |  |  |  |
| 2 | Ath-AT1G52618.1 |  | | | |  | | | |  |  |  |  |  |  |
| 2 | Ath-AT1G52620.1 |  | | | |  | Vvi-Vitvi09g00574\_t001 |  |  |  |  |  |  |
| 2 | Ath-AT1G52630.1 |  | | | |  | Vvi-Vitvi09g00570\_t001 |  |  |  |  |  |  |
| 2 | Ath-AT1G52640.1 |  | | | |  | Vvi-Vitvi09g00568\_t001 |  |  |  |  |  |  |
| 1 | Ath-AT1G52650.1 |  | | | |  |  |  |  |  |  |  |
| 1 | Ath-AT1G52660.1 |  | Vvi-Vitvi09g04130\_t001 |  |  |  |  |  |  |  |
| 2 | Ath-AT1G52670.1 |  | | | |  | Vvi-Vitvi09g00529\_t001 |  |  |  |  |  |  |
| 2 | Ath-AT1G52680.1 |  | | | |  | Vvi-Vitvi09g00525\_t001 |  |  |  |  |  |  |
| 2 | Ath-AT1G52690.2 |  | | | |  | Vvi-Vitvi09g00524\_t001 |  |  |  |  |  |  |
| 2 | Ath-AT1G52695.1 |  | | | |  | Vvi-Vitvi09g00518\_t001 |  |  |  |  |  |  |
| 2 | Ath-AT1G52700.2 |  | | | |  | | | |  |  |  |  |  |  |
| 2 | Ath-AT1G52710.1 |  | | | |  | Vvi-Vitvi09g01645\_t001 |  |  |  |  |  |  |
| 2 | Ath-AT1G52720.1 |  | | | |  | Vvi-Vitvi09g01641\_t001 |  |  |  |  |  |  |
| 2 | Ath-AT1G52730.2 |  | | | |  | Vvi-Vitvi09g00496\_t001 |  |  |  |  |  |  |
| 2 | Ath-AT1G52740.1 |  | | | |  | Vvi-Vitvi09g04160\_t001 |  |  |  |  |  |  |
| 2 | Ath-AT1G52750.1 |  | | | |  | Vvi-Vitvi09g00492\_t001 |  |  |  |  |  |  |
| 2 | Ath-AT1G52760.1 |  | | | |  | Vvi-Vitvi09g00491\_t001 |  |  |  |  |  |  |
| 2 | Ath-AT1G52770.1 |  | | | |  | Vvi-Vitvi09g00488\_t001 |  |  |  |  |  |  |
| 2 | Ath-AT1G52780.1 |  | | | |  | Vvi-Vitvi09g00482\_t001 |  |  |  |  |  |  |
| 2 | Ath-AT1G52790.1 |  | | | |  | Vvi-Vitvi09g00449\_t001 |  |  |  |  |  |  |
| 1 | Ath-AT1G52800.1 |  | | | |  |  |  |  |  |  |  |
| 1 | Ath-AT1G52810.1 |  | | | |  |  |  |  |  |  |  |
| 1 | Ath-AT1G52820.1 |  | | | |  |  |  |  |  |  |  |
| 1 | Ath-AT1G52825.1 |  | | | |  |  |  |  |  |  |  |
| 1 | Ath-AT1G52827.1 |  | | | |  |  |  |  |  |  |  |
| 1 | Ath-AT1G52830.1 |  | Vvi-Vitvi09g00436\_t001 |  |  |  |  |  |  |  |
| 0 | Ath-AT1G52857.1 |  |  |  |  |  |  |  |  |
| 0 | Ath-AT1G52855.1 |  |  |  |  |  |  |  |  |
| 1 | Ath-AT1G52870.2 |  | Vvi-Vitvi19g00272\_t001 |  |  |  |  |  |  |  |
| 1 | Ath-AT1G52880.1 |  | Vvi-Vitvi19g00271\_t001 |  |  |  |  |  |  |  |
| 1 | Ath-AT1G52890.1 |  | Vvi-Vitvi19g00270\_t001 |  |  |  |  |  |  |  |
| 1 | Ath-AT1G52900.1 |  | Vvi-Vitvi19g00263\_t001 |  |  |  |  |  |  |  |
| 1 | Ath-AT1G52905.1 |  | | | |  |  |  |  |  |  |  |
| 1 | Ath-AT1G52910.1 |  | Vvi-Vitvi19g00261\_t003 |  |  |  |  |  |  |  |
| 1 | Ath-AT1G52920.1 |  | Vvi-Vitvi19g00256\_t001 |  |  |  |  |  |  |  |
| 1 | Ath-AT1G52930.1 |  | | | |  |  |  |  |  |  |  |
| 1 | Ath-AT1G52940.1 |  | | | |  |  |  |  |  |  |  |
| 1 | Ath-AT1G52950.1 |  | | | |  |  |  |  |  |  |  |
| 1 | Ath-AT1G52970.1 |  | | | |  |  |  |  |  |  |  |
| 1 | Ath-AT1G52980.1 |  | Vvi-Vitvi19g00245\_t001 |  |  |  |  |  |  |  |
| 1 | Ath-AT1G52990.1 |  | | | |  |  |  |  |  |  |  |
| 1 | Ath-AT1G53000.2 |  | Vvi-Vitvi19g00234\_t001 |  |  |  |  |  |  |  |
| 1 | Ath-AT1G53010.1 |  | | | |  |  |  |  |  |  |  |
| 1 | Ath-AT1G53023.1 |  | Vvi-Vitvi19g00228\_t001 |  |  |  |  |  |  |  |
| 1 | Ath-AT1G53025.2 |  | | | |  |  |  |  |  |  |  |
| 1 | Ath-AT1G53030.1 |  | Vvi-Vitvi19g04087\_t001 |  |  |  |  |  |  |  |
| 1 | Ath-AT1G53035.2 |  | | | |  |  |  |  |  |  |  |
| 1 | Ath-AT1G53040.1 |  | Vvi-Vitvi19g00225\_t001 |  |  |  |  |  |  |  |
| 1 | Ath-AT1G53050.1 |  | Vvi-Vitvi19g00221\_t001 |  |  |  |  |  |  |  |
| 1 | Ath-AT1G53060.1 |  | | | |  |  |  |  |  |  |  |
| 1 | Ath-AT1G53070.1 |  | | | |  |  |  |  |  |  |  |
| 1 | Ath-AT1G53080.1 |  | | | |  |  |  |  |  |  |  |
| 1 | Ath-AT1G53090.3 |  | Vvi-Vitvi19g00220\_t001 |  |  |  |  |  |  |  |
| 1 | Ath-AT1G53100.1 |  | Vvi-Vitvi19g01869\_t001.1.6037826c |  |  |  |  |  |  |  |
| 1 | Ath-AT1G53110.1 |  | Vvi-Vitvi19g00219\_t001 |  |  |  |  |  |  |  |
| 1 | Ath-AT1G53120.1 |  | Vvi-Vitvi19g00217\_t001 |  |  |  |  |  |  |  |
| 1 | Ath-AT1G53130.1 |  | | | |  |  |  |  |  |  |  |
| 1 | Ath-AT1G53140.1 |  | Vvi-Vitvi19g00210\_t001 |  |  |  |  |  |  |  |
| 1 | Ath-AT1G53160.1 |  | Vvi-Vitvi19g00200\_t001 |  |  |  |  |  |  |  |
| 1 | Ath-AT1G53163.1 |  | Vvi-Vitvi19g01859\_t001 |  |  |  |  |  |  |  |
| 1 | Ath-AT1G53165.3 |  | Vvi-Vitvi19g00197\_t001 |  |  |  |  |  |  |  |
| 0 | Ath-AT1G53170.1 |  |  |  |  |  |  |  |  |
| 0 | Ath-AT1G53180.2 |  |  |  |  |  |  |  |  |
| 1 | Ath-AT1G53190.1 |  | Vvi-Vitvi19g00155\_t003 |  |  |  |  |  |  |  |
| 1 | Ath-AT1G53200.1 |  | Vvi-Vitvi19g00151\_t003 |  |  |  |  |  |  |  |
| 1 | Ath-AT1G53210.1 |  | Vvi-Vitvi19g00143\_t001 |  |  |  |  |  |  |  |
| 1 | Ath-AT1G53230.1 |  | Vvi-Vitvi19g00140\_t001 |  |  |  |  |  |  |  |
| 1 | Ath-AT1G53240.1 |  | Vvi-Vitvi19g00138\_t001 |  |  |  |  |  |  |  |
| 1 | Ath-AT1G53250.1 |  | Vvi-Vitvi19g00130\_t001 |  |  |  |  |  |  |  |
| 1 | Ath-AT1G53260.1 |  | | | |  |  |  |  |  |  |  |
| 1 | Ath-AT1G53265.1 |  | | | |  |  |  |  |  |  |  |
| 1 | Ath-AT1G53270.1 |  | Vvi-Vitvi19g00128\_t001 |  |  |  |  |  |  |  |
| 1 | Ath-AT1G53280.1 |  | Vvi-Vitvi19g00125\_t001 |  |  |  |  |  |  |  |
| 1 | Ath-AT1G53282.1 |  | | | |  |  |  |  |  |  |  |
| 1 | Ath-AT1G53285.1 |  | | | |  |  |  |  |  |  |  |
| 1 | Ath-AT1G53290.1 |  | Vvi-Vitvi19g00123\_t001 |  |  |  |  |  |  |  |
| 1 | Ath-AT1G53300.1 |  | Vvi-Vitvi19g00122\_t001 |  |  |  |  |  |  |  |
| 1 | Ath-AT1G53310.2 |  | Vvi-Vitvi19g00112\_t001 |  |  |  |  |  |  |  |
| 1 | Ath-AT1G53320.1 |  | Vvi-Vitvi19g00099\_t001 |  |  |  |  |  |  |  |
| 1 | Ath-AT1G53325.1 |  | | | |  |  |  |  |  |  |  |
| 1 | Ath-AT1G53330.1 |  | Vvi-Vitvi19g00089\_t001 |  |  |  |  |  |  |  |
| 1 | Ath-AT1G53340.2 |  | | | |  |  |  |  |  |  |  |
| 1 | Ath-AT1G53345.1 |  | | | |  |  |  |  |  |  |  |
| 1 | Ath-AT1G53350.1 |  | | | |  |  |  |  |  |  |  |
| 1 | Ath-AT1G53360.2 |  | | | |  |  |  |  |  |  |  |
| 1 | Ath-AT1G53366.1 |  | | | |  |  |  |  |  |  |  |
| 1 | Ath-AT1G53370.2 |  | | | |  |  |  |  |  |  |  |
| 1 | Ath-AT1G53380.1 |  | Vvi-Vitvi19g01830\_t002 |  |  |  |  |  |  |  |
| 1 | Ath-AT1G53390.1 |  | | | |  |  |  |  |  |  |  |
| 1 | Ath-AT1G53400.1 |  | Vvi-Vitvi19g01829\_t001 |  |  |  |  |  |  |  |
| 1 | Ath-AT1G53420.1 |  | | | |  |  |  |  |  |  |  |
| 1 | Ath-AT1G53430.1 |  | | | |  |  |  |  |  |  |  |
| 1 | Ath-AT1G53440.1 |  | | | |  |  |  |  |  |  |  |
| 1 | Ath-AT1G53450.2 |  | | | |  |  |  |  |  |  |  |
| 1 | Ath-AT1G53460.1 |  | Vvi-Vitvi19g00071\_t001 |  |  |  |  |  |  |  |
| 0 | Ath-AT1G53470.1 |  |  |  |  |  |  |  |  |
| 0 | Ath-AT1G53480.1 |  |  |  |  |  |  |  |  |
| 0 | Ath-AT1G53490.1 |  |  |  |  |  |  |  |  |
| 0 | Ath-AT1G53500.1 |  |  |  |  |  |  |  |  |
| 0 | Ath-AT1G53510.1 |  |  |  |  |  |  |  |  |
| 0 | Ath-AT1G53520.1 |  |  |  |  |  |  |  |  |
| 0 | Ath-AT1G53530.1 |  |  |  |  |  |  |  |  |
| 0 | Ath-AT1G53540.1 |  |  |  |  |  |  |  |  |
| 0 | Ath-AT1G53541.1 |  |  |  |  |  |  |  |  |
| 0 | Ath-AT1G53542.1 |  |  |  |  |  |  |  |  |
| 0 | Ath-AT1G53550.1 |  |  |  |  |  |  |  |  |
| 0 | Ath-AT1G53560.1 |  |  |  |  |  |  |  |  |
| 0 | Ath-AT1G53570.1 |  |  |  |  |  |  |  |  |
| 0 | Ath-AT1G53580.1 |  |  |  |  |  |  |  |  |
| 0 | Ath-AT1G53590.1 |  |  |  |  |  |  |  |  |
| 0 | Ath-AT1G53600.1 |  |  |  |  |  |  |  |  |
| 0 | Ath-AT1G53610.1 |  |  |  |  |  |  |  |  |
| 0 | Ath-AT1G53620.1 |  |  |  |  |  |  |  |  |
| 0 | Ath-AT1G53625.1 |  |  |  |  |  |  |  |  |
| 0 | Ath-AT1G53633.1 |  |  |  |  |  |  |  |  |
| 0 | Ath-AT1G53635.1 |  |  |  |  |  |  |  |  |
| 0 | Ath-AT1G53640.1 |  |  |  |  |  |  |  |  |
| 0 | Ath-AT1G53645.1 |  |  |  |  |  |  |  |  |
| 0 | Ath-AT1G53650.1 |  |  |  |  |  |  |  |  |
| 0 | Ath-AT1G53655.1 |  |  |  |  |  |  |  |  |
| 0 | Ath-AT1G53660.3 |  |  |  |  |  |  |  |  |
| 0 | Ath-AT1G53670.1 |  |  |  |  |  |  |  |  |
| 0 | Ath-AT1G53680.1 |  |  |  |  |  |  |  |  |
| 0 | Ath-AT1G53690.2 |  |  |  |  |  |  |  |  |
| 0 | Ath-AT1G53700.1 |  |  |  |  |  |  |  |  |
| 0 | Ath-AT1G53705.2 |  |  |  |  |  |  |  |  |
| 0 | Ath-AT1G53708.1 |  |  |  |  |  |  |  |  |
| 0 | Ath-AT1G53710.1 |  |  |  |  |  |  |  |  |
| 0 | Ath-AT1G53720.1 |  |  |  |  |  |  |  |  |
| 1 | Ath-AT1G53730.2 |  | Vvi-Vitvi09g00008\_t001 |  |  |  |  |  |  |  |
| 1 | Ath-AT1G53750.1 |  | Vvi-Vitvi09g00009\_t001 |  |  |  |  |  |  |  |
| 1 | Ath-AT1G53760.1 |  | Vvi-Vitvi09g01491\_t001 |  |  |  |  |  |  |  |
| 1 | Ath-AT1G53770.2 |  | Vvi-Vitvi09g00010\_t001 |  |  |  |  |  |  |  |
| 1 | Ath-AT1G53780.2 |  | | | |  |  |  |  |  |  |  |
| 1 | Ath-AT1G53790.3 |  | | | |  |  |  |  |  |  |  |
| 1 | Ath-AT1G53800.3 |  | Vvi-Vitvi09g00011\_t001 |  |  |  |  |  |  |  |
| 1 | Ath-AT1G53815.1 |  | | | |  |  |  |  |  |  |  |
| 1 | Ath-AT1G53820.1 |  | Vvi-Vitvi09g00012\_t001 |  |  |  |  |  |  |  |
| 1 | Ath-AT1G53830.1 |  | Vvi-Vitvi09g00017\_t001 |  |  |  |  |  |  |  |
| 1 | Ath-AT1G53840.1 |  | Vvi-Vitvi09g00018\_t001 |  |  |  |  |  |  |  |
| 1 | Ath-AT1G53850.2 |  | Vvi-Vitvi09g00020\_t001 |  |  |  |  |  |  |  |
| 1 | Ath-AT1G53860.1 |  | Vvi-Vitvi09g00021\_t001 |  |  |  |  |  |  |  |
| 1 | Ath-AT1G53870.1 |  | Vvi-Vitvi09g00026\_t001 |  |  |  |  |  |  |  |
| 1 | Ath-AT1G53875.1 |  | | | |  |  |  |  |  |  |  |
| 1 | Ath-AT1G53880.1 |  | Vvi-Vitvi09g00027\_t001 |  |  |  |  |  |  |  |
| 1 | Ath-AT1G53885.1 |  | Vvi-Vitvi09g01493\_t001 |  |  |  |  |  |  |  |
| 1 | Ath-AT1G53887.1 |  | | | |  |  |  |  |  |  |  |
| 1 | Ath-AT1G53890.3 |  | | | |  |  |  |  |  |  |  |
| 1 | Ath-AT1G53900.2 |  | | | |  |  |  |  |  |  |  |
| 1 | Ath-AT1G53903.1 |  | | | |  |  |  |  |  |  |  |
| 1 | Ath-AT1G53910.1 |  | Vvi-Vitvi09g00031\_t002 |  |  |  |  |  |  |  |
| 1 | Ath-AT1G53920.1 |  | Vvi-Vitvi09g00033\_t001 |  |  |  |  |  |  |  |
| 1 | Ath-AT1G53930.1 |  | | | |  |  |  |  |  |  |  |
| 1 | Ath-AT1G53935.1 |  | | | |  |  |  |  |  |  |  |
| 1 | Ath-AT1G53940.2 |  | | | |  |  |  |  |  |  |  |
| 1 | Ath-AT1G53945.1 |  | | | |  |  |  |  |  |  |  |
| 1 | Ath-AT1G53950.1 |  | | | |  |  |  |  |  |  |  |
| 1 | Ath-AT1G53970.1 |  | | | |  |  |  |  |  |  |  |
| 1 | Ath-AT1G53980.1 |  | | | |  |  |  |  |  |  |  |
| 1 | Ath-AT1G53990.1 |  | | | |  |  |  |  |  |  |  |
| 1 | Ath-AT1G54000.1 |  | | | |  |  |  |  |  |  |  |
| 1 | Ath-AT1G54010.1 |  | | | |  |  |  |  |  |  |  |
| 1 | Ath-AT1G54020.2 |  | | | |  |  |  |  |  |  |  |
| 1 | Ath-AT1G54030.1 |  | | | |  |  |  |  |  |  |  |
| 1 | Ath-AT1G54040.2 |  | | | |  |  |  |  |  |  |  |
| 1 | Ath-AT1G54050.1 |  | Vvi-Vitvi09g00045\_t001 |  |  |  |  |  |  |  |
| 1 | Ath-AT1G54060.1 |  | | | |  |  |  |  |  |  |  |
| 1 | Ath-AT1G54070.1 |  | Vvi-Vitvi09g00050\_t003 |  |  |  |  |  |  |  |
| 1 | Ath-AT1G54080.2 |  | Vvi-Vitvi09g00063\_t001 |  |  |  |  |  |  |  |
| 1 | Ath-AT1G54090.1 |  | Vvi-Vitvi09g00067\_t001 |  |  |  |  |  |  |  |
| 1 | Ath-AT1G54095.1 |  | Vvi-Vitvi09g00077\_t001 |  |  |  |  |  |  |  |
| 1 | Ath-AT1G54100.2 |  | Vvi-Vitvi09g00078\_t001 |  |  |  |  |  |  |  |
| 1 | Ath-AT1G54110.2 |  | | | |  |  |  |  |  |  |  |
| 1 | Ath-AT1G54115.1 |  | Vvi-Vitvi09g00079\_t001 |  |  |  |  |  |  |  |
| 1 | Ath-AT1G54120.1 |  | Vvi-Vitvi09g00083\_t001 |  |  |  |  |  |  |  |
| 1 | Ath-AT1G54130.1 |  | Vvi-Vitvi09g00093\_t001 |  |  |  |  |  |  |  |
| 1 | Ath-AT1G54140.1 |  | Vvi-Vitvi09g00094\_t001 |  |  |  |  |  |  |  |
| 1 | Ath-AT1G54150.1 |  | Vvi-Vitvi09g00096\_t001 |  |  |  |  |  |  |  |
| 1 | Ath-AT1G54160.1 |  | | | |  |  |  |  |  |  |  |
| 1 | Ath-AT1G54170.1 |  | | | |  |  |  |  |  |  |  |
| 1 | Ath-AT1G54180.1 |  | | | |  |  |  |  |  |  |  |
| 1 | Ath-AT1G54200.1 |  | | | |  |  |  |  |  |  |  |
| 1 | Ath-AT1G54210.1 |  | Vvi-Vitvi09g00111\_t001 |  |  |  |  |  |  |  |
| 0 | Ath-AT1G54215.1 |  |  |  |  |  |  |  |  |
| 0 | Ath-AT1G54217.8 |  |  |  |  |  |  |  |  |
| 0 | Ath-AT1G54220.1 |  |  |  |  |  |  |  |  |
| 0 | Ath-AT1G54230.1 |  |  |  |  |  |  |  |  |
| 0 | Ath-AT1G54240.1 |  |  |  |  |  |  |  |  |
| 0 | Ath-AT1G54250.1 |  |  |  |  |  |  |  |  |
| 0 | Ath-AT1G54260.1 |  |  |  |  |  |  |  |  |
| 0 | Ath-AT1G54270.1 |  |  |  |  |  |  |  |  |
| 0 | Ath-AT1G54280.1 |  |  |  |  |  |  |  |  |
| 0 | Ath-AT1G54290.1 |  |  |  |  |  |  |  |  |
| 0 | Ath-AT1G54300.1 |  |  |  |  |  |  |  |  |
| 0 | Ath-AT1G54310.2 |  |  |  |  |  |  |  |  |
| 0 | Ath-AT1G54320.1 |  |  |  |  |  |  |  |  |
| 0 | Ath-AT1G54330.1 |  |  |  |  |  |  |  |  |
| 0 | Ath-AT1G54340.1 |  |  |  |  |  |  |  |  |
| 0 | Ath-AT1G54350.1 |  |  |  |  |  |  |  |  |
| 0 | Ath-AT1G54360.6 |  |  |  |  |  |  |  |  |
| 0 | Ath-AT1G54370.1 |  |  |  |  |  |  |  |  |
| 0 | Ath-AT1G54380.1 |  |  |  |  |  |  |  |  |
| 0 | Ath-AT1G54385.1 |  |  |  |  |  |  |  |  |
| 0 | Ath-AT1G54390.2 |  |  |  |  |  |  |  |  |
| 0 | Ath-AT1G54400.1 |  |  |  |  |  |  |  |  |
| 0 | Ath-AT1G54410.1 |  |  |  |  |  |  |  |  |
| 0 | Ath-AT1G54420.1 |  |  |  |  |  |  |  |  |
| 0 | Ath-AT1G54440.2 |  |  |  |  |  |  |  |  |
| 0 | Ath-AT1G54445.1 |  |  |  |  |  |  |  |  |
| 0 | Ath-AT1G54450.1 |  |  |  |  |  |  |  |  |
| 0 | Ath-AT1G54460.1 |  |  |  |  |  |  |  |  |
| 0 | Ath-AT1G54470.1 |  |  |  |  |  |  |  |  |
| 0 | Ath-AT1G54475.1 |  |  |  |  |  |  |  |  |
| 0 | Ath-AT1G54490.1 |  |  |  |  |  |  |  |  |
| 0 | Ath-AT1G54500.1 |  |  |  |  |  |  |  |  |
| 0 | Ath-AT1G54510.4 |  |  |  |  |  |  |  |  |
| 0 | Ath-AT1G54520.1 |  |  |  |  |  |  |  |  |
| 0 | Ath-AT1G54530.1 |  |  |  |  |  |  |  |  |
| 0 | Ath-AT1G54540.1 |  |  |  |  |  |  |  |  |
| 0 | Ath-AT1G54550.1 |  |  |  |  |  |  |  |  |
| 0 | Ath-AT1G54560.1 |  |  |  |  |  |  |  |  |
| 0 | Ath-AT1G54570.1 |  |  |  |  |  |  |  |  |
| 0 | Ath-AT1G54575.2 |  |  |  |  |  |  |  |  |
| 0 | Ath-AT1G54580.1 |  |  |  |  |  |  |  |  |
| 0 | Ath-AT1G54590.1 |  |  |  |  |  |  |  |  |
| 0 | Ath-AT1G54610.1 |  |  |  |  |  |  |  |  |
| 0 | Ath-AT1G54620.1 |  |  |  |  |  |  |  |  |
| 0 | Ath-AT1G54630.1 |  |  |  |  |  |  |  |  |
| 0 | Ath-AT1G54640.1 |  |  |  |  |  |  |  |  |
| 0 | Ath-AT1G54650.3 |  |  |  |  |  |  |  |  |
| 0 | Ath-AT1G54680.4 |  |  |  |  |  |  |  |  |
| 0 | Ath-AT1G54690.1 |  |  |  |  |  |  |  |  |
| 0 | Ath-AT1G54700.1 |  |  |  |  |  |  |  |  |
| 0 | Ath-AT1G54710.1 |  |  |  |  |  |  |  |  |
| 0 | Ath-AT1G54720.1 |  |  |  |  |  |  |  |  |
| 0 | Ath-AT1G54730.2 |  |  |  |  |  |  |  |  |
| 0 | Ath-AT1G54740.1 |  |  |  |  |  |  |  |  |
| 0 | Ath-AT1G54760.1 |  |  |  |  |  |  |  |  |
| 0 | Ath-AT1G54770.1 |  |  |  |  |  |  |  |  |
| 0 | Ath-AT1G54773.1 |  |  |  |  |  |  |  |  |
| 0 | Ath-AT1G54775.1 |  |  |  |  |  |  |  |  |
| 1 | Ath-AT1G54780.1 |  | Vvi-Vitvi14g00284\_t001 |  |  |  |  |  |  |  |
| 1 | Ath-AT1G54790.2 |  | Vvi-Vitvi14g00282\_t001 |  |  |  |  |  |  |  |
| 1 | Ath-AT1G54820.1 |  | Vvi-Vitvi14g00273\_t001 |  |  |  |  |  |  |  |
| 1 | Ath-AT1G54830.1 |  | Vvi-Vitvi14g00264\_t001 |  |  |  |  |  |  |  |
| 1 | Ath-AT1G54840.1 |  | Vvi-Vitvi14g00262\_t001 |  |  |  |  |  |  |  |
| 1 | Ath-AT1G54850.1 |  | | | |  |  |  |  |  |  |  |
| 1 | Ath-AT1G54860.1 |  | | | |  |  |  |  |  |  |  |
| 1 | Ath-AT1G54870.2 |  | Vvi-Vitvi14g00257\_t001 |  |  |  |  |  |  |  |
| 1 | Ath-AT1G54890.1 |  | | | |  |  |  |  |  |  |  |
| 1 | Ath-AT1G54920.3 |  | Vvi-Vitvi14g00256\_t001 |  |  |  |  |  |  |  |
| 1 | Ath-AT1G54930.2 |  | | | |  |  |  |  |  |  |  |
| 1 | Ath-AT1G54940.1 |  | Vvi-Vitvi14g00249\_t001 |  |  |  |  |  |  |  |
| 1 | Ath-AT1G54950.1 |  | | | |  |  |  |  |  |  |  |
| 1 | Ath-AT1G54953.1 |  | | | |  |  |  |  |  |  |  |
| 1 | Ath-AT1G54957.1 |  | | | |  |  |  |  |  |  |  |
| 1 | Ath-AT1G54960.1 |  | Vvi-Vitvi14g00248\_t001 |  |  |  |  |  |  |  |
| 1 | Ath-AT1G54970.1 |  | | | |  |  |  |  |  |  |  |
| 1 | Ath-AT1G54980.1 |  | | | |  |  |  |  |  |  |  |
| 1 | Ath-AT1G54990.1 |  | Vvi-Vitvi14g00236\_t001 |  |  |  |  |  |  |  |
| 1 | Ath-AT1G55000.1 |  | Vvi-Vitvi14g00235\_t001 |  |  |  |  |  |  |  |
| 1 | Ath-AT1G55010.1 |  | | | |  |  |  |  |  |  |  |
| 1 | Ath-AT1G55020.1 |  | Vvi-Vitvi14g00234\_t004 |  |  |  |  |  |  |  |
| 1 | Ath-AT1G55030.2 |  | | | |  |  |  |  |  |  |  |
| 1 | Ath-AT1G55040.1 |  | Vvi-Vitvi14g00231\_t001 |  |  |  |  |  |  |  |
| 1 | Ath-AT1G55050.1 |  | Vvi-Vitvi14g00215\_t002 |  |  |  |  |  |  |  |
| 0 | Ath-AT1G55060.1 |  |  |  |  |  |  |  |  |
| 0 | Ath-AT1G55070.1 |  |  |  |  |  |  |  |  |
| 2 | Ath-AT1G55080.1 |  | Vvi-Vitvi09g01540\_t001 |  | Vvi-Vitvi09g01540\_t001 |  |  |  |  |  |  |
| 2 | Ath-AT1G55090.1 |  | | | |  | | | |  |  |  |  |  |  |
| 2 | Ath-AT1G55110.2 |  | Vvi-Vitvi09g00191\_t001 |  | | | |  |  |  |  |  |  |
| 2 | Ath-AT1G55120.1 |  | Vvi-Vitvi09g00193\_t001 |  | | | |  |  |  |  |  |  |
| 2 | Ath-AT1G55130.1 |  | | | |  | Vvi-Vitvi09g00179\_t001 |  |  |  |  |  |  |
| 2 | Ath-AT1G55140.1 |  | | | |  | Vvi-Vitvi09g00176\_t001 |  |  |  |  |  |  |
| 2 | Ath-AT1G55150.2 |  | | | |  | Vvi-Vitvi09g00175\_t001 |  |  |  |  |  |  |
| 2 | Ath-AT1G55152.1 |  | | | |  | | | |  |  |  |  |  |  |
| 2 | Ath-AT1G55160.3 |  | | | |  | Vvi-Vitvi09g00173\_t001 |  |  |  |  |  |  |
| 2 | Ath-AT1G55170.1 |  | | | |  | Vvi-Vitvi09g00171\_t001 |  |  |  |  |  |  |
| 1 | Ath-AT1G55175.1 |  | | | |  |  |  |  |  |  |  |
| 1 | Ath-AT1G55180.1 |  | Vvi-Vitvi09g00200\_t001 |  |  |  |  |  |  |  |
| 1 | Ath-AT1G55190.1 |  | Vvi-Vitvi09g00201\_t001 |  |  |  |  |  |  |  |
| 1 | Ath-AT1G55200.1 |  | Vvi-Vitvi09g00203\_t001 |  |  |  |  |  |  |  |
| 0 | Ath-AT1G55205.2 |  |  |  |  |  |  |  |  |
| 0 | Ath-AT1G55207.1 |  |  |  |  |  |  |  |  |
| 1 | Ath-AT1G55210.1 |  | Vvi-Vitvi06g04029\_t001 |  |  |  |  |  |  |  |
| 1 | Ath-AT1G55220.1 |  | | | |  |  |  |  |  |  |  |
| 1 | Ath-AT1G55230.1 |  | Vvi-Vitvi06g00098\_t001 |  |  |  |  |  |  |  |
| 1 | Ath-AT1G55240.1 |  | | | |  |  |  |  |  |  |  |
| 1 | Ath-AT1G55250.1 |  | Vvi-Vitvi06g00097\_t001 |  |  |  |  |  |  |  |
| 1 | Ath-AT1G55260.1 |  | Vvi-Vitvi06g00089\_t001 |  |  |  |  |  |  |  |
| 1 | Ath-AT1G55265.1 |  | Vvi-Vitvi06g04028\_t001 |  |  |  |  |  |  |  |
| 1 | Ath-AT1G55270.1 |  | Vvi-Vitvi06g00087\_t001 |  |  |  |  |  |  |  |
| 1 | Ath-AT1G55280.2 |  | Vvi-Vitvi06g00083\_t001 |  |  |  |  |  |  |  |
| 1 | Ath-AT1G55290.1 |  | Vvi-Vitvi06g00076\_t001 |  |  |  |  |  |  |  |
| 1 | Ath-AT1G55300.2 |  | | | |  |  |  |  |  |  |  |
| 1 | Ath-AT1G55310.3 |  | | | |  |  |  |  |  |  |  |
| 1 | Ath-AT1G55320.1 |  | Vvi-Vitvi06g01588\_t001 |  |  |  |  |  |  |  |
| 1 | Ath-AT1G55325.2 |  | Vvi-Vitvi06g00048\_t001 |  |  |  |  |  |  |  |
| 1 | Ath-AT1G55330.1 |  | | | |  |  |  |  |  |  |  |
| 1 | Ath-AT1G55335.1 |  | | | |  |  |  |  |  |  |  |
| 1 | Ath-AT1G55340.1 |  | Vvi-Vitvi06g00046\_t002 |  |  |  |  |  |  |  |
| 1 | Ath-AT1G55350.5 |  | Vvi-Vitvi06g00045\_t001 |  |  |  |  |  |  |  |
| 1 | Ath-AT1G55360.1 |  | Vvi-Vitvi06g00044\_t001 |  |  |  |  |  |  |  |
| 1 | Ath-AT1G55365.1 |  | Vvi-Vitvi06g01582\_t001 |  |  |  |  |  |  |  |
| 1 | Ath-AT1G55370.2 |  | | | |  |  |  |  |  |  |  |
| 1 | Ath-AT1G55380.1 |  | | | |  |  |  |  |  |  |  |
| 1 | Ath-AT1G55390.1 |  | | | |  |  |  |  |  |  |  |
| 1 | Ath-AT1G55420.1 |  | | | |  |  |  |  |  |  |  |
| 1 | Ath-AT1G55430.1 |  | | | |  |  |  |  |  |  |  |
| 1 | Ath-AT1G55440.1 |  | | | |  |  |  |  |  |  |  |
| 1 | Ath-AT1G55450.2 |  | | | |  |  |  |  |  |  |  |
| 1 | Ath-AT1G55460.1 |  | | | |  |  |  |  |  |  |  |
| 1 | Ath-AT1G55475.1 |  | Vvi-Vitvi06g01580\_t001 |  |  |  |  |  |  |  |
| 1 | Ath-AT1G55480.1 |  | Vvi-Vitvi06g04007\_t001 |  |  |  |  |  |  |  |
| 1 | Ath-AT1G55490.1 |  | Vvi-Vitvi06g00032\_t001 |  |  |  |  |  |  |  |
| 1 | Ath-AT1G55500.5 |  | Vvi-Vitvi06g00029\_t001 |  |  |  |  |  |  |  |
| 1 | Ath-AT1G55510.1 |  | Vvi-Vitvi06g00028\_t001 |  |  |  |  |  |  |  |
| 1 | Ath-AT1G55520.1 |  | Vvi-Vitvi06g01573\_t001 |  |  |  |  |  |  |  |
| 1 | Ath-AT1G55530.2 |  | Vvi-Vitvi06g00020\_t001 |  |  |  |  |  |  |  |
| 1 | Ath-AT1G55535.1 |  | Vvi-Vitvi06g00019\_t001 |  |  |  |  |  |  |  |
| 1 | Ath-AT1G55540.2 |  | Vvi-Vitvi06g00017\_t001 |  |  |  |  |  |  |  |
| 1 | Ath-AT1G55546.1 |  | Vvi-Vitvi06g00013\_t001 |  |  |  |  |  |  |  |
| 1 | Ath-AT1G55550.1 |  | Vvi-Vitvi06g00012\_t001 |  |  |  |  |  |  |  |
| 1 | Ath-AT1G55560.1 |  | Vvi-Vitvi06g04002\_t001 |  |  |  |  |  |  |  |
| 0 | Ath-AT1G55570.1 |  |  |  |  |  |  |  |  |
| 1 | Ath-AT1G55580.1 |  | Vvi-Vitvi19g00932\_t001 |  |  |  |  |  |  |  |
| 1 | Ath-AT1G55590.1 |  | Vvi-Vitvi19g00930\_t001 |  |  |  |  |  |  |  |
| 1 | Ath-AT1G55600.1 |  | | | |  |  |  |  |  |  |  |
| 1 | Ath-AT1G55610.2 |  | Vvi-Vitvi19g00928\_t001 |  |  |  |  |  |  |  |
| 1 | Ath-AT1G55620.2 |  | Vvi-Vitvi19g00922\_t002 |  |  |  |  |  |  |  |
| 1 | Ath-AT1G55630.1 |  | | | |  |  |  |  |  |  |  |
| 1 | Ath-AT1G55640.1 |  | Vvi-Vitvi19g00916\_t001 |  |  |  |  |  |  |  |
| 1 | Ath-AT1G55650.1 |  | Vvi-Vitvi19g00911\_t001 |  |  |  |  |  |  |  |
| 1 | Ath-AT1G55660.1 |  | | | |  |  |  |  |  |  |  |
| 1 | Ath-AT1G55670.1 |  | Vvi-Vitvi19g00909\_t001 |  |  |  |  |  |  |  |
| 1 | Ath-AT1G55673.1 |  | | | |  |  |  |  |  |  |  |
| 1 | Ath-AT1G55675.1 |  | | | |  |  |  |  |  |  |  |
| 1 | Ath-AT1G55680.1 |  | Vvi-Vitvi19g00908\_t001 |  |  |  |  |  |  |  |
| 3 | Ath-AT1G55690.4 |  | | | |  | Vvi-Vitvi19g00740\_t001 |  | Vvi-Vitvi19g00740\_t001 |  |  |  |  |  |
| 3 | Ath-AT1G55700.1 |  | | | |  | | | |  | | | |  |  |  |  |  |
| 3 | Ath-AT1G55710.1 |  | | | |  | | | |  | | | |  |  |  |  |  |
| 3 | Ath-AT1G55720.1 |  | | | |  | | | |  | Vvi-Vitvi19g00762\_t002 |  |  |  |  |  |
| 3 | Ath-AT1G55730.2 |  | | | |  | | | |  | | | |  |  |  |  |  |
| 3 | Ath-AT1G55740.1 |  | | | |  | | | |  | Vvi-Vitvi19g00768\_t001 |  |  |  |  |  |
| 3 | Ath-AT1G55750.1 |  | | | |  | | | |  | Vvi-Vitvi19g00778\_t001 |  |  |  |  |  |
| 3 | Ath-AT1G55755.1 |  | | | |  | | | |  | | | |  |  |  |  |  |
| 3 | Ath-AT1G55760.1 |  | | | |  | | | |  | Vvi-Vitvi19g00782\_t001 |  |  |  |  |  |
| 3 | Ath-AT1G55770.1 |  | | | |  | | | |  | | | |  |  |  |  |  |
| 3 | Ath-AT1G55775.1 |  | | | |  | | | |  | Vvi-Vitvi19g04298\_t001 |  |  |  |  |  |
| 3 | Ath-AT1G55790.1 |  | | | |  | | | |  | Vvi-Vitvi19g02101\_t001 |  |  |  |  |  |
| 3 | Ath-AT1G55800.1 |  | | | |  | | | |  | | | |  |  |  |  |  |
| 3 | Ath-AT1G55803.1 |  | | | |  | | | |  | | | |  |  |  |  |  |
| 3 | Ath-AT1G55805.1 |  | Vvi-Vitvi19g00900\_t001 |  | | | |  | Vvi-Vitvi19g00900\_t001 |  |  |  |  |  |
| 2 | Ath-AT1G55810.4 |  |  |  | | | |  | Vvi-Vitvi19g00905\_t001 |  |  |  |  |  |
| 1 | Ath-AT1G55820.1 |  |  |  | | | |  |  |  |  |  |  |
| 1 | Ath-AT1G55830.2 |  |  |  | Vvi-Vitvi19g00725\_t001 |  |  |  |  |  |  |
| 1 | Ath-AT1G55840.1 |  |  |  | Vvi-Vitvi19g00706\_t001 |  |  |  |  |  |  |
| 1 | Ath-AT1G55850.1 |  |  |  | Vvi-Vitvi19g00697\_t001 |  |  |  |  |  |  |
| 1 | Ath-AT1G55860.1 |  |  |  | Vvi-Vitvi19g00694\_t002 |  |  |  |  |  |  |
| 1 | Ath-AT1G55865.1 |  |  |  | Vvi-Vitvi19g02068\_t001 |  |  |  |  |  |  |
| 1 | Ath-AT1G55870.1 |  |  |  | Vvi-Vitvi19g00692\_t001 |  |  |  |  |  |  |
| 1 | Ath-AT1G55880.1 |  | Vvi-Vitvi19g00666\_t001 |  |  |  |  |  |  |  |
| 1 | Ath-AT1G55890.1 |  | | | |  |  |  |  |  |  |  |
| 1 | Ath-AT1G55900.1 |  | Vvi-Vitvi19g00660\_t001 |  |  |  |  |  |  |  |
| 1 | Ath-AT1G55910.1 |  | Vvi-Vitvi19g02052\_t001 |  |  |  |  |  |  |  |
| 1 | Ath-AT1G55915.2 |  | Vvi-Vitvi19g00658\_t001 |  |  |  |  |  |  |  |
| 1 | Ath-AT1G55917.1 |  | | | |  |  |  |  |  |  |  |
| 1 | Ath-AT1G55920.1 |  | Vvi-Vitvi19g00654\_t001 |  |  |  |  |  |  |  |
| 1 | Ath-AT1G55928.1 |  | | | |  |  |  |  |  |  |  |
| 1 | Ath-AT1G55930.1 |  | Vvi-Vitvi19g00627\_t001 |  |  |  |  |  |  |  |
| 0 | Ath-AT1G55940.2 |  |  |  |  |  |  |  |  |
| 0 | Ath-AT1G55945.1 |  |  |  |  |  |  |  |  |
| 0 | Ath-AT1G55947.1 |  |  |  |  |  |  |  |  |
| 0 | Ath-AT1G55950.1 |  |  |  |  |  |  |  |  |
| 0 | Ath-AT1G55960.1 |  |  |  |  |  |  |  |  |
| 0 | Ath-AT1G55970.1 |  |  |  |  |  |  |  |  |
| 0 | Ath-AT1G55980.1 |  |  |  |  |  |  |  |  |
| 0 | Ath-AT1G55990.1 |  |  |  |  |  |  |  |  |
| 0 | Ath-AT1G56000.1 |  |  |  |  |  |  |  |  |
| 0 | Ath-AT1G56010.2 |  |  |  |  |  |  |  |  |
| 0 | Ath-AT1G56020.1 |  |  |  |  |  |  |  |  |
| 0 | Ath-AT1G56030.1 |  |  |  |  |  |  |  |  |
| 0 | Ath-AT1G56040.1 |  |  |  |  |  |  |  |  |
| 0 | Ath-AT1G56045.1 |  |  |  |  |  |  |  |  |
| 0 | Ath-AT1G56050.1 |  |  |  |  |  |  |  |  |
| 0 | Ath-AT1G56060.2 |  |  |  |  |  |  |  |  |
| 0 | Ath-AT1G56070.1 |  |  |  |  |  |  |  |  |
| 0 | Ath-AT1G56080.1 |  |  |  |  |  |  |  |  |
| 0 | Ath-AT1G56085.1 |  |  |  |  |  |  |  |  |
| 0 | Ath-AT1G56090.2 |  |  |  |  |  |  |  |  |
| 0 | Ath-AT1G56100.1 |  |  |  |  |  |  |  |  |
| 0 | Ath-AT1G56105.1 |  |  |  |  |  |  |  |  |
| 0 | Ath-AT1G56110.1 |  |  |  |  |  |  |  |  |
| 0 | Ath-AT1G56120.1 |  |  |  |  |  |  |  |  |
| 0 | Ath-AT1G56130.2 |  |  |  |  |  |  |  |  |
| 0 | Ath-AT1G56140.1 |  |  |  |  |  |  |  |  |
| 0 | Ath-AT1G56145.2 |  |  |  |  |  |  |  |  |
| 0 | Ath-AT1G56150.1 |  |  |  |  |  |  |  |  |
| 0 | Ath-AT1G56160.1 |  |  |  |  |  |  |  |  |
| 0 | Ath-AT1G56170.1 |  |  |  |  |  |  |  |  |
| 0 | Ath-AT1G56180.1 |  |  |  |  |  |  |  |  |
| 0 | Ath-AT1G56190.1 |  |  |  |  |  |  |  |  |
| 0 | Ath-AT1G56200.1 |  |  |  |  |  |  |  |  |
| 0 | Ath-AT1G56210.1 |  |  |  |  |  |  |  |  |
| 1 | Ath-AT1G56220.3 |  | Vvi-Vitvi14g00142\_t002 |  |  |  |  |  |  |  |
| 1 | Ath-AT1G56225.1 |  | | | |  |  |  |  |  |  |  |
| 1 | Ath-AT1G56230.1 |  | | | |  |  |  |  |  |  |  |
| 1 | Ath-AT1G56233.1 |  | | | |  |  |  |  |  |  |  |
| 1 | Ath-AT1G56240.1 |  | | | |  |  |  |  |  |  |  |
| 1 | Ath-AT1G56250.1 |  | | | |  |  |  |  |  |  |  |
| 1 | Ath-AT1G56260.1 |  | | | |  |  |  |  |  |  |  |
| 1 | Ath-AT1G56270.1 |  | | | |  |  |  |  |  |  |  |
| 1 | Ath-AT1G56280.2 |  | Vvi-Vitvi14g00126\_t001 |  |  |  |  |  |  |  |
| 1 | Ath-AT1G56290.1 |  | | | |  |  |  |  |  |  |  |
| 1 | Ath-AT1G56300.1 |  | Vvi-Vitvi14g00119\_t001 |  |  |  |  |  |  |  |
| 1 | Ath-AT1G56310.1 |  | Vvi-Vitvi14g00116\_t001 |  |  |  |  |  |  |  |
| 1 | Ath-AT1G56320.1 |  | Vvi-Vitvi14g02477\_t001 |  |  |  |  |  |  |  |
| 1 | Ath-AT1G56330.1 |  | Vvi-Vitvi14g04028\_t002 |  |  |  |  |  |  |  |
| 1 | Ath-AT1G56340.1 |  | Vvi-Vitvi14g00102\_t001 |  |  |  |  |  |  |  |
| 1 | Ath-AT1G56345.1 |  | Vvi-Vitvi14g00100\_t001 |  |  |  |  |  |  |  |
| 1 | Ath-AT1G56350.1 |  | Vvi-Vitvi14g00099\_t001 |  |  |  |  |  |  |  |
| 1 | Ath-AT1G56360.1 |  | | | |  |  |  |  |  |  |  |
| 1 | Ath-AT1G56380.2 |  | | | |  |  |  |  |  |  |  |
| 1 | Ath-AT1G56385.1 |  | | | |  |  |  |  |  |  |  |
| 1 | Ath-AT1G56400.1 |  | | | |  |  |  |  |  |  |  |
| 1 | Ath-AT1G56410.1 |  | | | |  |  |  |  |  |  |  |
| 1 | Ath-AT1G56415.1 |  | | | |  |  |  |  |  |  |  |
| 1 | Ath-AT1G56418.2 |  | | | |  |  |  |  |  |  |  |
| 1 | Ath-AT1G56420.1 |  | Vvi-Vitvi14g00098\_t001 |  |  |  |  |  |  |  |
| 1 | Ath-AT1G56423.1 |  | Vvi-Vitvi14g02475\_t001 |  |  |  |  |  |  |  |
| 1 | Ath-AT1G56430.1 |  | Vvi-Vitvi14g02474\_t001 |  |  |  |  |  |  |  |
| 1 | Ath-AT1G56440.1 |  | Vvi-Vitvi14g00091\_t001 |  |  |  |  |  |  |  |
| 1 | Ath-AT1G56450.1 |  | | | |  |  |  |  |  |  |  |
| 1 | Ath-AT1G56460.3 |  | Vvi-Vitvi14g00090\_t001 |  |  |  |  |  |  |  |
| 1 | Ath-AT1G56500.1 |  | Vvi-Vitvi14g04019\_t001 |  |  |  |  |  |  |  |
| 1 | Ath-AT1G56510.1 |  | | | |  |  |  |  |  |  |  |
| 1 | Ath-AT1G56520.2 |  | | | |  |  |  |  |  |  |  |
| 1 | Ath-AT1G56530.1 |  | | | |  |  |  |  |  |  |  |
| 1 | Ath-AT1G56540.1 |  | | | |  |  |  |  |  |  |  |
| 1 | Ath-AT1G56550.1 |  | | | |  |  |  |  |  |  |  |
| 1 | Ath-AT1G56553.1 |  | | | |  |  |  |  |  |  |  |
| 1 | Ath-AT1G56555.1 |  | | | |  |  |  |  |  |  |  |
| 1 | Ath-AT1G56560.1 |  | | | |  |  |  |  |  |  |  |
| 1 | Ath-AT1G56570.1 |  | | | |  |  |  |  |  |  |  |
| 1 | Ath-AT1G56580.1 |  | Vvi-Vitvi14g02463\_t001 |  |  |  |  |  |  |  |
| 1 | Ath-AT1G56590.1 |  | Vvi-Vitvi14g00068\_t001 |  |  |  |  |  |  |  |
| 1 | Ath-AT1G56600.1 |  | Vvi-Vitvi14g02457\_t001 |  |  |  |  |  |  |  |
| 1 | Ath-AT1G56610.1 |  | | | |  |  |  |  |  |  |  |
| 1 | Ath-AT1G56620.1 |  | | | |  |  |  |  |  |  |  |
| 1 | Ath-AT1G56630.1 |  | | | |  |  |  |  |  |  |  |
| 1 | Ath-AT1G56650.1 |  | | | |  |  |  |  |  |  |  |
| 1 | Ath-AT1G56660.1 |  | | | |  |  |  |  |  |  |  |
| 1 | Ath-AT1G56670.1 |  | Vvi-Vitvi14g00059\_t001 |  |  |  |  |  |  |  |
| 1 | Ath-AT1G56680.2 |  | | | |  |  |  |  |  |  |  |
| 1 | Ath-AT1G56690.1 |  | Vvi-Vitvi14g00058\_t001 |  |  |  |  |  |  |  |
| 1 | Ath-AT1G56700.1 |  | Vvi-Vitvi14g00056\_t001 |  |  |  |  |  |  |  |
| 1 | Ath-AT1G56710.1 |  | Vvi-Vitvi14g00055\_t001 |  |  |  |  |  |  |  |
| 1 | Ath-AT1G56720.3 |  | Vvi-Vitvi14g00042\_t001 |  |  |  |  |  |  |  |
| 1 | Ath-AT1G57540.3 |  | Vvi-Vitvi14g00030\_t002 |  |  |  |  |  |  |  |
| 0 | Ath-AT1G57550.1 |  |  |  |  |  |  |  |  |
| 0 | Ath-AT1G57560.1 |  |  |  |  |  |  |  |  |
| 0 | Ath-AT1G57565.1 |  |  |  |  |  |  |  |  |
| 0 | Ath-AT1G57570.1 |  |  |  |  |  |  |  |  |
| 0 | Ath-AT1G57580.1 |  |  |  |  |  |  |  |  |
| 0 | Ath-AT1G57590.1 |  |  |  |  |  |  |  |  |
| 0 | Ath-AT1G57600.1 |  |  |  |  |  |  |  |  |
| 0 | Ath-AT1G57610.1 |  |  |  |  |  |  |  |  |
| 0 | Ath-AT1G57613.1 |  |  |  |  |  |  |  |  |
| 0 | Ath-AT1G57620.1 |  |  |  |  |  |  |  |  |
| 0 | Ath-AT1G57630.1 |  |  |  |  |  |  |  |  |
| 0 | Ath-AT1G57650.1 |  |  |  |  |  |  |  |  |
| 0 | Ath-AT1G57660.1 |  |  |  |  |  |  |  |  |
| 0 | Ath-AT1G57670.1 |  |  |  |  |  |  |  |  |
| 1 | Ath-AT1G57680.1 |  | Vvi-Vitvi12g02061\_t001 |  |  |  |  |  |  |  |
| 1 | Ath-AT1G57690.1 |  | | | |  |  |  |  |  |  |  |
| 1 | Ath-AT1G57700.1 |  | Vvi-Vitvi12g02060\_t001 |  |  |  |  |  |  |  |
| 1 | Ath-AT1G57720.1 |  | Vvi-Vitvi12g02055\_t003 |  |  |  |  |  |  |  |
| 1 | Ath-AT1G57730.1 |  | | | |  |  |  |  |  |  |  |
| 1 | Ath-AT1G57750.1 |  | | | |  |  |  |  |  |  |  |
| 1 | Ath-AT1G57760.1 |  | | | |  |  |  |  |  |  |  |
| 1 | Ath-AT1G57765.2 |  | Vvi-Vitvi12g02723\_t002 |  |  |  |  |  |  |  |
| 1 | Ath-AT1G57770.1 |  | Vvi-Vitvi12g02046\_t001 |  |  |  |  |  |  |  |
| 1 | Ath-AT1G57775.1 |  | | | |  |  |  |  |  |  |  |
| 1 | Ath-AT1G57777.1 |  | | | |  |  |  |  |  |  |  |
| 1 | Ath-AT1G57780.1 |  | | | |  |  |  |  |  |  |  |
| 1 | Ath-AT1G57790.1 |  | | | |  |  |  |  |  |  |  |
| 1 | Ath-AT1G57800.2 |  | | | |  |  |  |  |  |  |  |
| 1 | Ath-AT1G57820.1 |  | | | |  |  |  |  |  |  |  |
| 1 | Ath-AT1G57830.1 |  | | | |  |  |  |  |  |  |  |
| 1 | Ath-AT1G57850.1 |  | | | |  |  |  |  |  |  |  |
| 1 | Ath-AT1G57860.1 |  | | | |  |  |  |  |  |  |  |
| 1 | Ath-AT1G57870.3 |  | Vvi-Vitvi12g02045\_t003 |  |  |  |  |  |  |  |
| 1 | Ath-AT1G57906.1 |  | | | |  |  |  |  |  |  |  |
| 1 | Ath-AT1G57943.1 |  | | | |  |  |  |  |  |  |  |
| 1 | Ath-AT1G57980.1 |  | | | |  |  |  |  |  |  |  |
| 1 | Ath-AT1G57990.1 |  | | | |  |  |  |  |  |  |  |
| 1 | Ath-AT1G58007.2 |  | Vvi-Vitvi12g02041\_t001 |  |  |  |  |  |  |  |
| 1 | Ath-AT1G58025.2 |  | Vvi-Vitvi12g04585\_t001 |  |  |  |  |  |  |  |
| 1 | Ath-AT1G58030.1 |  | | | |  |  |  |  |  |  |  |
| 1 | Ath-AT1G58037.1 |  | | | |  |  |  |  |  |  |  |
| 1 | Ath-AT1G58050.1 |  | Vvi-Vitvi12g02017\_t001 |  |  |  |  |  |  |  |
| 1 | Ath-AT1G58055.1 |  | | | |  |  |  |  |  |  |  |
| 1 | Ath-AT1G58060.1 |  | | | |  |  |  |  |  |  |  |
| 1 | Ath-AT1G58070.1 |  | Vvi-Vitvi12g02016\_t001 |  |  |  |  |  |  |  |
| 1 | Ath-AT1G58080.1 |  | Vvi-Vitvi12g02013\_t001 |  |  |  |  |  |  |  |
| 1 | Ath-AT1G58090.1 |  | | | |  |  |  |  |  |  |  |
| 1 | Ath-AT1G58100.1 |  | Vvi-Vitvi12g02006\_t001 |  |  |  |  |  |  |  |
| 1 | Ath-AT1G58110.2 |  | Vvi-Vitvi12g01996\_t002 |  |  |  |  |  |  |  |
| 1 | Ath-AT1G58120.1 |  | Vvi-Vitvi18g00509\_t001 |  |  |  |  |  |  |  |
| 1 | Ath-AT1G58150.1 |  | | | |  |  |  |  |  |  |  |
| 1 | Ath-AT1G58160.1 |  | | | |  |  |  |  |  |  |  |
| 1 | Ath-AT1G58170.1 |  | | | |  |  |  |  |  |  |  |
| 1 | Ath-AT1G58180.2 |  | Vvi-Vitvi18g00512\_t001 |  |  |  |  |  |  |  |
| 1 | Ath-AT1G58190.2 |  | | | |  |  |  |  |  |  |  |
| 1 | Ath-AT1G58200.2 |  | Vvi-Vitvi18g02610\_t001.1.6037826d |  |  |  |  |  |  |  |
| 1 | Ath-AT1G58210.1 |  | | | |  |  |  |  |  |  |  |
| 1 | Ath-AT1G58215.1 |  | Vvi-Vitvi18g00525\_t001 |  |  |  |  |  |  |  |
| 1 | Ath-AT1G58220.1 |  | Vvi-Vitvi18g02612\_t001 |  |  |  |  |  |  |  |
| 1 | Ath-AT1G58223.1 |  | | | |  |  |  |  |  |  |  |
| 1 | Ath-AT1G58225.1 |  | | | |  |  |  |  |  |  |  |
| 1 | Ath-AT1G58230.2 |  | Vvi-Vitvi18g00535\_t001 |  |  |  |  |  |  |  |
| 1 | Ath-AT1G58235.1 |  | | | |  |  |  |  |  |  |  |
| 1 | Ath-AT1G58242.1 |  | | | |  |  |  |  |  |  |  |
| 1 | Ath-AT1G58245.1 |  | | | |  |  |  |  |  |  |  |
| 1 | Ath-AT1G58248.1 |  | | | |  |  |  |  |  |  |  |
| 1 | Ath-AT1G58250.2 |  | Vvi-Vitvi18g00539\_t001 |  |  |  |  |  |  |  |
| 0 | Ath-AT1G58260.1 |  |  |  |  |  |  |  |  |
| 0 | Ath-AT1G58265.1 |  |  |  |  |  |  |  |  |
| 0 | Ath-AT1G58270.1 |  |  |  |  |  |  |  |  |
| 1 | Ath-AT1G58280.2 |  | Vvi-Vitvi18g00566\_t001 |  |  |  |  |  |  |  |
| 1 | Ath-AT1G58290.1 |  | Vvi-Vitvi18g00573\_t001 |  |  |  |  |  |  |  |
| 1 | Ath-AT1G58300.1 |  | | | |  |  |  |  |  |  |  |
| 1 | Ath-AT1G58310.1 |  | | | |  |  |  |  |  |  |  |
| 1 | Ath-AT1G58320.1 |  | | | |  |  |  |  |  |  |  |
| 1 | Ath-AT1G58330.1 |  | | | |  |  |  |  |  |  |  |
| 1 | Ath-AT1G58340.1 |  | Vvi-Vitvi18g00583\_t001 |  |  |  |  |  |  |  |
| 1 | Ath-AT1G58350.1 |  | | | |  |  |  |  |  |  |  |
| 1 | Ath-AT1G58360.1 |  | Vvi-Vitvi18g00593\_t001 |  |  |  |  |  |  |  |
| 1 | Ath-AT1G58370.2 |  | Vvi-Vitvi18g00601\_t001 |  |  |  |  |  |  |  |
| 1 | Ath-AT1G58380.1 |  | | | |  |  |  |  |  |  |  |
| 1 | Ath-AT1G58390.1 |  | | | |  |  |  |  |  |  |  |
| 1 | Ath-AT1G58400.2 |  | | | |  |  |  |  |  |  |  |
| 1 | Ath-AT1G58410.4 |  | | | |  |  |  |  |  |  |  |
| 1 | Ath-AT1G58420.1 |  | | | |  |  |  |  |  |  |  |
| 1 | Ath-AT1G58430.1 |  | | | |  |  |  |  |  |  |  |
| 1 | Ath-AT1G58440.1 |  | Vvi-Vitvi18g00612\_t001 |  |  |  |  |  |  |  |
| 1 | Ath-AT1G58450.1 |  | | | |  |  |  |  |  |  |  |
| 1 | Ath-AT1G58460.2 |  | | | |  |  |  |  |  |  |  |
| 1 | Ath-AT1G58470.1 |  | Vvi-Vitvi18g00622\_t001 |  |  |  |  |  |  |  |
| 1 | Ath-AT1G58525.1 |  | | | |  |  |  |  |  |  |  |
| 1 | Ath-AT1G58520.2 |  | | | |  |  |  |  |  |  |  |
| 1 | Ath-AT1G58602.2 |  | | | |  |  |  |  |  |  |  |
| 1 | Ath-AT1G58643.1 |  | | | |  |  |  |  |  |  |  |
| 1 | Ath-AT1G58684.1 |  | | | |  |  |  |  |  |  |  |
| 1 | Ath-AT1G58725.1 |  | | | |  |  |  |  |  |  |  |
| 1 | Ath-AT1G58766.1 |  | | | |  |  |  |  |  |  |  |
| 1 | Ath-AT1G58807.1 |  | | | |  |  |  |  |  |  |  |
| 1 | Ath-AT1G58848.2 |  | | | |  |  |  |  |  |  |  |
| 1 | Ath-AT1G58936.1 |  | | | |  |  |  |  |  |  |  |
| 1 | Ath-AT1G58983.1 |  | | | |  |  |  |  |  |  |  |
| 1 | Ath-AT1G59030.1 |  | | | |  |  |  |  |  |  |  |
| 1 | Ath-AT1G59077.1 |  | | | |  |  |  |  |  |  |  |
| 1 | Ath-AT1G59124.2 |  | | | |  |  |  |  |  |  |  |
| 1 | Ath-AT1G59171.1 |  | | | |  |  |  |  |  |  |  |
| 1 | Ath-AT1G59218.1 |  | | | |  |  |  |  |  |  |  |
| 1 | Ath-AT1G59312.1 |  | | | |  |  |  |  |  |  |  |
| 1 | Ath-AT1G59359.1 |  | | | |  |  |  |  |  |  |  |
| 1 | Ath-AT1G59406.1 |  | | | |  |  |  |  |  |  |  |
| 1 | Ath-AT1G59453.1 |  | | | |  |  |  |  |  |  |  |
| 1 | Ath-AT1G59460.1 |  | | | |  |  |  |  |  |  |  |
| 1 | Ath-AT1G59470.1 |  | | | |  |  |  |  |  |  |  |
| 1 | Ath-AT1G59500.1 |  | | | |  |  |  |  |  |  |  |
| 1 | Ath-AT1G59510.1 |  | Vvi-Vitvi18g00636\_t001 |  |  |  |  |  |  |  |
| 0 | Ath-AT1G59520.4 |  |  |  |  |  |  |  |  |
| 0 | Ath-AT1G59530.1 |  |  |  |  |  |  |  |  |
| 0 | Ath-AT1G59540.1 |  |  |  |  |  |  |  |  |
| 0 | Ath-AT1G59550.1 |  |  |  |  |  |  |  |  |
| 0 | Ath-AT1G59560.1 |  |  |  |  |  |  |  |  |
| 0 | Ath-AT1G59580.1 |  |  |  |  |  |  |  |  |
| 0 | Ath-AT1G59590.1 |  |  |  |  |  |  |  |  |
| 0 | Ath-AT1G59600.1 |  |  |  |  |  |  |  |  |
| 0 | Ath-AT1G59610.1 |  |  |  |  |  |  |  |  |
| 0 | Ath-AT1G59620.2 |  |  |  |  |  |  |  |  |
| 0 | Ath-AT1G59630.1 |  |  |  |  |  |  |  |  |
| 1 | Ath-AT1G59640.2 |  | Vvi-Vitvi01g00876\_t001 |  |  |  |  |  |  |  |
| 1 | Ath-AT1G59650.1 |  | Vvi-Vitvi01g00878\_t001 |  |  |  |  |  |  |  |
| 1 | Ath-AT1G59660.1 |  | | | |  |  |  |  |  |  |  |
| 1 | Ath-AT1G59670.1 |  | Vvi-Vitvi01g00888\_t001 |  |  |  |  |  |  |  |
| 1 | Ath-AT1G59675.1 |  | | | |  |  |  |  |  |  |  |
| 1 | Ath-AT1G59680.2 |  | | | |  |  |  |  |  |  |  |
| 1 | Ath-AT1G59690.1 |  | | | |  |  |  |  |  |  |  |
| 1 | Ath-AT1G59700.1 |  | | | |  |  |  |  |  |  |  |
| 1 | Ath-AT1G59710.2 |  | Vvi-Vitvi01g00902\_t004 |  |  |  |  |  |  |  |
| 1 | Ath-AT1G59720.1 |  | Vvi-Vitvi01g00903\_t001 |  |  |  |  |  |  |  |
| 1 | Ath-AT1G59722.1 |  | | | |  |  |  |  |  |  |  |
| 1 | Ath-AT1G59723.1 |  | | | |  |  |  |  |  |  |  |
| 1 | Ath-AT1G59724.1 |  | | | |  |  |  |  |  |  |  |
| 1 | Ath-AT1G59725.1 |  | Vvi-Vitvi01g00906\_t001 |  |  |  |  |  |  |  |
| 1 | Ath-AT1G59730.1 |  | Vvi-Vitvi01g00907\_t001 |  |  |  |  |  |  |  |
| 1 | Ath-AT1G59740.1 |  | Vvi-Vitvi01g00921\_t001 |  |  |  |  |  |  |  |
| 0 | Ath-AT1G59750.1 |  |  |  |  |  |  |  |  |
| 0 | Ath-AT1G59760.1 |  |  |  |  |  |  |  |  |
| 0 | Ath-AT1G59780.1 |  |  |  |  |  |  |  |  |
| 0 | Ath-AT1G59790.1 |  |  |  |  |  |  |  |  |
| 0 | Ath-AT1G59800.1 |  |  |  |  |  |  |  |  |
| 0 | Ath-AT1G59810.1 |  |  |  |  |  |  |  |  |
| 0 | Ath-AT1G59820.1 |  |  |  |  |  |  |  |  |
| 1 | Ath-AT1G59830.1 |  | Vvi-Vitvi01g00870\_t001 |  |  |  |  |  |  |  |
| 1 | Ath-AT1G59833.1 |  | | | |  |  |  |  |  |  |  |
| 1 | Ath-AT1G59835.1 |  | | | |  |  |  |  |  |  |  |
| 1 | Ath-AT1G59840.1 |  | Vvi-Vitvi01g00865\_t001 |  |  |  |  |  |  |  |
| 1 | Ath-AT1G59850.1 |  | Vvi-Vitvi01g00864\_t001 |  |  |  |  |  |  |  |
| 1 | Ath-AT1G59860.1 |  | | | |  |  |  |  |  |  |  |
| 1 | Ath-AT1G59865.1 |  | | | |  |  |  |  |  |  |  |
| 1 | Ath-AT1G59870.1 |  | | | |  |  |  |  |  |  |  |
| 1 | Ath-AT1G59885.1 |  | | | |  |  |  |  |  |  |  |
| 1 | Ath-AT1G59890.2 |  | Vvi-Vitvi01g02098\_t001 |  |  |  |  |  |  |  |
| 1 | Ath-AT1G59900.1 |  | Vvi-Vitvi01g00861\_t001 |  |  |  |  |  |  |  |
| 1 | Ath-AT1G59910.1 |  | Vvi-Vitvi01g00858\_t001 |  |  |  |  |  |  |  |
| 1 | Ath-AT1G59920.1 |  | | | |  |  |  |  |  |  |  |
| 1 | Ath-AT1G59930.1 |  | | | |  |  |  |  |  |  |  |
| 1 | Ath-AT1G59940.2 |  | Vvi-Vitvi01g00857\_t001 |  |  |  |  |  |  |  |
| 1 | Ath-AT1G59950.1 |  | | | |  |  |  |  |  |  |  |
| 1 | Ath-AT1G59960.1 |  | | | |  |  |  |  |  |  |  |
| 1 | Ath-AT1G59970.1 |  | Vvi-Vitvi01g00850\_t001 |  |  |  |  |  |  |  |
| 1 | Ath-AT1G59980.1 |  | Vvi-Vitvi01g02093\_t001 |  |  |  |  |  |  |  |
| 1 | Ath-AT1G59990.1 |  | | | |  |  |  |  |  |  |  |
| 1 | Ath-AT1G60000.1 |  | | | |  |  |  |  |  |  |  |
| 2 | Ath-AT1G60010.1 |  | | | |  | Vvi-Vitvi17g00758\_t001 |  |  |  |  |  |  |
| 2 | Ath-AT1G60030.1 |  | Vvi-Vitvi01g00833\_t001 |  | Vvi-Vitvi17g00759\_t001 |  |  |  |  |  |  |
| 2 | Ath-AT1G60040.1 |  | | | |  | | | |  |  |  |  |  |  |
| 2 | Ath-AT1G60050.1 |  | Vvi-Vitvi01g00815\_t001 |  | | | |  |  |  |  |  |  |
| 2 | Ath-AT1G60060.1 |  | Vvi-Vitvi01g00801\_t001 |  | Vvi-Vitvi17g00770\_t001 |  |  |  |  |  |  |
| 2 | Ath-AT1G60070.2 |  | Vvi-Vitvi01g00797\_t001 |  | | | |  |  |  |  |  |  |
| 2 | Ath-AT1G60080.1 |  | | | |  | | | |  |  |  |  |  |  |
| 2 | Ath-AT1G60090.1 |  | | | |  | | | |  |  |  |  |  |  |
| 2 | Ath-AT1G60095.1 |  | | | |  | | | |  |  |  |  |  |  |
| 2 | Ath-AT1G60110.2 |  | | | |  | | | |  |  |  |  |  |  |
| 2 | Ath-AT1G60130.1 |  | | | |  | | | |  |  |  |  |  |  |
| 2 | Ath-AT1G60140.6 |  | Vvi-Vitvi01g00793\_t001 |  | Vvi-Vitvi17g00778\_t001 |  |  |  |  |  |  |
| 2 | Ath-AT1G60160.1 |  | Vvi-Vitvi01g00791\_t001 |  | | | |  |  |  |  |  |  |
| 2 | Ath-AT1G60170.1 |  | Vvi-Vitvi01g00788\_t001 |  | | | |  |  |  |  |  |  |
| 2 | Ath-AT1G60190.1 |  | Vvi-Vitvi01g00782\_t001 |  | Vvi-Vitvi17g00787\_t001 |  |  |  |  |  |  |
| 2 | Ath-AT1G60200.1 |  | Vvi-Vitvi01g00777\_t005 |  | | | |  |  |  |  |  |  |
| 2 | Ath-AT1G60220.1 |  | Vvi-Vitvi01g00776\_t001 |  | | | |  |  |  |  |  |  |
| 2 | Ath-AT1G60230.1 |  | Vvi-Vitvi01g00773\_t001 |  | | | |  |  |  |  |  |  |
| 2 | Ath-AT1G60240.1 |  | | | |  | | | |  |  |  |  |  |  |
| 2 | Ath-AT1G60250.1 |  | | | |  | | | |  |  |  |  |  |  |
| 2 | Ath-AT1G60270.1 |  | | | |  | | | |  |  |  |  |  |  |
| 2 | Ath-AT1G60280.1 |  | | | |  | | | |  |  |  |  |  |  |
| 2 | Ath-AT1G60300.1 |  | | | |  | | | |  |  |  |  |  |  |
| 2 | Ath-AT1G60320.1 |  | | | |  | | | |  |  |  |  |  |  |
| 2 | Ath-AT1G60340.1 |  | | | |  | | | |  |  |  |  |  |  |
| 2 | Ath-AT1G60350.1 |  | | | |  | | | |  |  |  |  |  |  |
| 2 | Ath-AT1G60360.1 |  | | | |  | | | |  |  |  |  |  |  |
| 2 | Ath-AT1G60370.1 |  | | | |  | | | |  |  |  |  |  |  |
| 2 | Ath-AT1G60380.1 |  | | | |  | | | |  |  |  |  |  |  |
| 2 | Ath-AT1G60390.1 |  | Vvi-Vitvi01g00745\_t001 |  | Vvi-Vitvi17g00796\_t001 |  |  |  |  |  |  |
| 0 | Ath-AT1G60400.1 |  |  |  |  |  |  |  |  |
| 0 | Ath-AT1G60410.1 |  |  |  |  |  |  |  |  |
| 1 | Ath-AT1G60420.1 |  | Vvi-Vitvi01g00719\_t001 |  |  |  |  |  |  |  |
| 1 | Ath-AT1G60430.2 |  | | | |  |  |  |  |  |  |  |
| 1 | Ath-AT1G60440.1 |  | Vvi-Vitvi01g00716\_t001 |  |  |  |  |  |  |  |
| 1 | Ath-AT1G60450.1 |  | Vvi-Vitvi01g00714\_t001 |  |  |  |  |  |  |  |
| 1 | Ath-AT1G60460.1 |  | | | |  |  |  |  |  |  |  |
| 1 | Ath-AT1G60470.1 |  | | | |  |  |  |  |  |  |  |
| 1 | Ath-AT1G60490.1 |  | Vvi-Vitvi01g00712\_t001 |  |  |  |  |  |  |  |
| 1 | Ath-AT1G60500.1 |  | | | |  |  |  |  |  |  |  |
| 1 | Ath-AT1G60530.1 |  | | | |  |  |  |  |  |  |  |
| 1 | Ath-AT1G60550.1 |  | Vvi-Vitvi01g00711\_t001 |  |  |  |  |  |  |  |
| 1 | Ath-AT1G60560.1 |  | | | |  |  |  |  |  |  |  |
| 1 | Ath-AT1G60570.1 |  | | | |  |  |  |  |  |  |  |
| 1 | Ath-AT1G60590.1 |  | Vvi-Vitvi01g00710\_t001 |  |  |  |  |  |  |  |
| 1 | Ath-AT1G60600.2 |  | | | |  |  |  |  |  |  |  |
| 1 | Ath-AT1G60610.1 |  | Vvi-Vitvi01g00705\_t001 |  |  |  |  |  |  |  |
| 1 | Ath-AT1G60620.1 |  | | | |  |  |  |  |  |  |  |
| 1 | Ath-AT1G60625.1 |  | | | |  |  |  |  |  |  |  |
| 1 | Ath-AT1G60630.1 |  | | | |  |  |  |  |  |  |  |
| 1 | Ath-AT1G60640.2 |  | | | |  |  |  |  |  |  |  |
| 1 | Ath-AT1G60650.1 |  | | | |  |  |  |  |  |  |  |
| 1 | Ath-AT1G60660.1 |  | | | |  |  |  |  |  |  |  |
| 1 | Ath-AT1G60670.2 |  | | | |  |  |  |  |  |  |  |
| 1 | Ath-AT1G60680.1 |  | | | |  |  |  |  |  |  |  |
| 1 | Ath-AT1G60690.1 |  | | | |  |  |  |  |  |  |  |
| 1 | Ath-AT1G60700.1 |  | | | |  |  |  |  |  |  |  |
| 1 | Ath-AT1G60710.1 |  | | | |  |  |  |  |  |  |  |
| 1 | Ath-AT1G60720.1 |  | | | |  |  |  |  |  |  |  |
| 1 | Ath-AT1G60730.3 |  | | | |  |  |  |  |  |  |  |
| 1 | Ath-AT1G60740.1 |  | | | |  |  |  |  |  |  |  |
| 1 | Ath-AT1G60750.1 |  | | | |  |  |  |  |  |  |  |
| 1 | Ath-AT1G60760.1 |  | | | |  |  |  |  |  |  |  |
| 2 | Ath-AT1G60770.1 |  | | | |  | Vvi-Vitvi10g00470\_t001 |  |  |  |  |  |  |
| 2 | Ath-AT1G60780.1 |  | | | |  | | | |  |  |  |  |  |  |
| 2 | Ath-AT1G60783.1 |  | | | |  | | | |  |  |  |  |  |  |
| 2 | Ath-AT1G60787.1 |  | | | |  | | | |  |  |  |  |  |  |
| 2 | Ath-AT1G60790.1 |  | Vvi-Vitvi01g00695\_t001 |  | | | |  |  |  |  |  |  |
| 1 | Ath-AT1G60800.1 |  |  |  | | | |  |  |  |  |  |  |
| 1 | Ath-AT1G60810.2 |  |  |  | | | |  |  |  |  |  |  |
| 1 | Ath-AT1G60815.1 |  |  |  | | | |  |  |  |  |  |  |
| 1 | Ath-AT1G60830.2 |  |  |  | | | |  |  |  |  |  |  |
| 1 | Ath-AT1G60850.2 |  |  |  | | | |  |  |  |  |  |  |
| 1 | Ath-AT1G60860.1 |  |  |  | | | |  |  |  |  |  |  |
| 1 | Ath-AT1G60870.1 |  |  |  | | | |  |  |  |  |  |  |
| 1 | Ath-AT1G60880.1 |  |  |  | | | |  |  |  |  |  |  |
| 1 | Ath-AT1G60890.2 |  |  |  | | | |  |  |  |  |  |  |
| 1 | Ath-AT1G60900.1 |  |  |  | Vvi-Vitvi10g04301\_t001 |  |  |  |  |  |  |
| 1 | Ath-AT1G60913.1 |  |  |  | | | |  |  |  |  |  |  |
| 1 | Ath-AT1G60920.1 |  |  |  | | | |  |  |  |  |  |  |
| 1 | Ath-AT1G60940.2 |  |  |  | | | |  |  |  |  |  |  |
| 1 | Ath-AT1G60950.1 |  |  |  | | | |  |  |  |  |  |  |
| 1 | Ath-AT1G60960.1 |  |  |  | | | |  |  |  |  |  |  |
| 1 | Ath-AT1G60970.1 |  |  |  | | | |  |  |  |  |  |  |
| 1 | Ath-AT1G60980.1 |  |  |  | | | |  |  |  |  |  |  |
| 1 | Ath-AT1G60983.1 |  |  |  | | | |  |  |  |  |  |  |
| 1 | Ath-AT1G60985.1 |  |  |  | | | |  |  |  |  |  |  |
| 1 | Ath-AT1G60986.1 |  |  |  | | | |  |  |  |  |  |  |
| 1 | Ath-AT1G60987.1 |  |  |  | | | |  |  |  |  |  |  |
| 1 | Ath-AT1G60990.2 |  |  |  | | | |  |  |  |  |  |  |
| 1 | Ath-AT1G60989.1 |  |  |  | | | |  |  |  |  |  |  |
| 1 | Ath-AT1G60995.1 |  |  |  | | | |  |  |  |  |  |  |
| 1 | Ath-AT1G61000.1 |  |  |  | | | |  |  |  |  |  |  |
| 1 | Ath-AT1G61010.2 |  |  |  | | | |  |  |  |  |  |  |
| 1 | Ath-AT1G61030.1 |  |  |  | | | |  |  |  |  |  |  |
| 1 | Ath-AT1G61040.1 |  |  |  | | | |  |  |  |  |  |  |
| 1 | Ath-AT1G61050.2 |  |  |  | Vvi-Vitvi10g02340\_t001 |  |  |  |  |  |  |
| 1 | Ath-AT1G61060.1 |  |  |  | | | |  |  |  |  |  |  |
| 1 | Ath-AT1G61065.1 |  |  |  | Vvi-Vitvi10g01762\_t001 |  |  |  |  |  |  |
| 1 | Ath-AT1G61070.1 |  |  |  | | | |  |  |  |  |  |  |
| 1 | Ath-AT1G61080.5 |  |  |  | Vvi-Vitvi10g02333\_t001 |  |  |  |  |  |  |
| 1 | Ath-AT1G61090.1 |  |  |  | | | |  |  |  |  |  |  |
| 1 | Ath-AT1G61093.1 |  |  |  | | | |  |  |  |  |  |  |
| 1 | Ath-AT1G61095.1 |  |  |  | | | |  |  |  |  |  |  |
| 1 | Ath-AT1G61097.1 |  |  |  | | | |  |  |  |  |  |  |
| 1 | Ath-AT1G61100.1 |  |  |  | | | |  |  |  |  |  |  |
| 1 | Ath-AT1G61105.1 |  |  |  | | | |  |  |  |  |  |  |
| 1 | Ath-AT1G61110.1 |  |  |  | Vvi-Vitvi10g00437\_t001 |  |  |  |  |  |  |
| 1 | Ath-AT1G61120.1 |  |  |  | Vvi-Vitvi10g01600\_t001 |  |  |  |  |  |  |
| 1 | Ath-AT1G61130.1 |  |  |  | Vvi-Vitvi10g00427\_t001 |  |  |  |  |  |  |
| 1 | Ath-AT1G61140.1 |  |  |  | Vvi-Vitvi10g00425\_t001 |  |  |  |  |  |  |
| 1 | Ath-AT1G61150.1 |  |  |  | Vvi-Vitvi10g04277\_t001 |  |  |  |  |  |  |
| 1 | Ath-AT1G61160.1 |  |  |  | | | |  |  |  |  |  |  |
| 1 | Ath-AT1G61165.1 |  |  |  | | | |  |  |  |  |  |  |
| 1 | Ath-AT1G61170.1 |  |  |  | | | |  |  |  |  |  |  |
| 1 | Ath-AT1G61180.2 |  |  |  | | | |  |  |  |  |  |  |
| 1 | Ath-AT1G61190.1 |  |  |  | | | |  |  |  |  |  |  |
| 1 | Ath-AT1G61200.1 |  |  |  | | | |  |  |  |  |  |  |
| 1 | Ath-AT1G61210.1 |  |  |  | Vvi-Vitvi10g04273\_t002 |  |  |  |  |  |  |
| 1 | Ath-AT1G61215.1 |  |  |  | Vvi-Vitvi10g04272\_t001 |  |  |  |  |  |  |
| 1 | Ath-AT1G61230.1 |  |  |  | | | |  |  |  |  |  |  |
| 1 | Ath-AT1G61240.4 |  |  |  | Vvi-Vitvi10g04271\_t001 |  |  |  |  |  |  |
| 1 | Ath-AT1G61250.1 |  |  |  | Vvi-Vitvi10g00397\_t001 |  |  |  |  |  |  |
| 1 | Ath-AT1G61255.1 |  |  |  | Vvi-Vitvi10g01753\_t001 |  |  |  |  |  |  |
| 1 | Ath-AT1G61260.1 |  |  |  | Vvi-Vitvi10g00390\_t001 |  |  |  |  |  |  |
| 1 | Ath-AT1G61270.1 |  |  |  | | | |  |  |  |  |  |  |
| 1 | Ath-AT1G61280.2 |  |  |  | | | |  |  |  |  |  |  |
| 1 | Ath-AT1G61290.1 |  |  |  | Vvi-Vitvi10g00385\_t001 |  |  |  |  |  |  |
| 1 | Ath-AT1G61300.1 |  |  |  | | | |  |  |  |  |  |  |
| 1 | Ath-AT1G61310.1 |  |  |  | | | |  |  |  |  |  |  |
| 1 | Ath-AT1G61320.1 |  |  |  | Vvi-Vitvi10g00364\_t001 |  |  |  |  |  |  |
| 1 | Ath-AT1G61330.1 |  |  |  | | | |  |  |  |  |  |  |
| 1 | Ath-AT1G61340.1 |  |  |  | Vvi-Vitvi10g01748\_t001 |  |  |  |  |  |  |
| 1 | Ath-AT1G61350.1 |  |  |  | Vvi-Vitvi10g00359\_t001 |  |  |  |  |  |  |
| 0 | Ath-AT1G61360.1 |  |  |  |  |  |  |  |  |
| 1 | Ath-AT1G61370.1 |  | Vvi-Vitvi10g04229\_t001 |  |  |  |  |  |  |  |
| 1 | Ath-AT1G61380.1 |  | | | |  |  |  |  |  |  |  |
| 1 | Ath-AT1G61390.1 |  | | | |  |  |  |  |  |  |  |
| 1 | Ath-AT1G61400.2 |  | | | |  |  |  |  |  |  |  |
| 1 | Ath-AT1G61410.1 |  | | | |  |  |  |  |  |  |  |
| 1 | Ath-AT1G61415.1 |  | | | |  |  |  |  |  |  |  |
| 1 | Ath-AT1G61420.4 |  | | | |  |  |  |  |  |  |  |
| 1 | Ath-AT1G61430.1 |  | | | |  |  |  |  |  |  |  |
| 1 | Ath-AT1G61440.2 |  | | | |  |  |  |  |  |  |  |
| 1 | Ath-AT1G61450.1 |  | | | |  |  |  |  |  |  |  |
| 1 | Ath-AT1G61460.1 |  | | | |  |  |  |  |  |  |  |
| 1 | Ath-AT1G61470.1 |  | | | |  |  |  |  |  |  |  |
| 1 | Ath-AT1G61475.1 |  | | | |  |  |  |  |  |  |  |
| 1 | Ath-AT1G61480.1 |  | Vvi-Vitvi10g04230\_t001 |  |  |  |  |  |  |  |
| 1 | Ath-AT1G61490.1 |  | | | |  |  |  |  |  |  |  |
| 1 | Ath-AT1G61500.3 |  | Vvi-Vitvi10g04231\_t001 |  |  |  |  |  |  |  |
| 1 | Ath-AT1G61520.3 |  | | | |  |  |  |  |  |  |  |
| 1 | Ath-AT1G61540.1 |  | | | |  |  |  |  |  |  |  |
| 1 | Ath-AT1G61550.1 |  | | | |  |  |  |  |  |  |  |
| 1 | Ath-AT1G61560.1 |  | | | |  |  |  |  |  |  |  |
| 1 | Ath-AT1G61562.1 |  | | | |  |  |  |  |  |  |  |
| 1 | Ath-AT1G61563.1 |  | | | |  |  |  |  |  |  |  |
| 1 | Ath-AT1G61565.1 |  | | | |  |  |  |  |  |  |  |
| 1 | Ath-AT1G61566.1 |  | | | |  |  |  |  |  |  |  |
| 1 | Ath-AT1G61570.1 |  | | | |  |  |  |  |  |  |  |
| 1 | Ath-AT1G61575.1 |  | | | |  |  |  |  |  |  |  |
| 1 | Ath-AT1G61580.1 |  | | | |  |  |  |  |  |  |  |
| 1 | Ath-AT1G61590.1 |  | | | |  |  |  |  |  |  |  |
| 1 | Ath-AT1G61600.1 |  | | | |  |  |  |  |  |  |  |
| 2 | Ath-AT1G61610.1 |  | Vvi-Vitvi10g04237\_t001 |  | Vvi-Vitvi10g00008\_t001 |  |  |  |  |  |  |
| 2 | Ath-AT1G61620.1 |  | Vvi-Vitvi10g00350\_t001 |  | | | |  |  |  |  |  |  |
| 2 | Ath-AT1G61630.1 |  | Vvi-Vitvi10g00342\_t001 |  | | | |  |  |  |  |  |  |
| 2 | Ath-AT1G61640.1 |  | Vvi-Vitvi10g00336\_t001.1.6037826a |  | | | |  |  |  |  |  |  |
| 1 | Ath-AT1G61660.1 |  |  |  | | | |  |  |  |  |  |  |
| 1 | Ath-AT1G61667.2 |  |  |  | | | |  |  |  |  |  |  |
| 1 | Ath-AT1G61670.1 |  |  |  | | | |  |  |  |  |  |  |
| 1 | Ath-AT1G61680.1 |  |  |  | | | |  |  |  |  |  |  |
| 1 | Ath-AT1G61688.1 |  |  |  | | | |  |  |  |  |  |  |
| 1 | Ath-AT1G61690.1 |  |  |  | | | |  |  |  |  |  |  |
| 1 | Ath-AT1G61700.1 |  |  |  | | | |  |  |  |  |  |  |
| 1 | Ath-AT1G61710.1 |  |  |  | | | |  |  |  |  |  |  |
| 1 | Ath-AT1G61720.1 |  |  |  | | | |  |  |  |  |  |  |
| 1 | Ath-AT1G61730.1 |  |  |  | | | |  |  |  |  |  |  |
| 1 | Ath-AT1G61740.1 |  |  |  | | | |  |  |  |  |  |  |
| 1 | Ath-AT1G61750.1 |  |  |  | | | |  |  |  |  |  |  |
| 1 | Ath-AT1G61760.1 |  |  |  | Vvi-Vitvi10g04005\_t001 |  |  |  |  |  |  |
| 1 | Ath-AT1G61770.1 |  |  |  | Vvi-Vitvi10g00022\_t001 |  |  |  |  |  |  |
| 1 | Ath-AT1G61780.1 |  |  |  | Vvi-Vitvi10g00025\_t001 |  |  |  |  |  |  |
| 1 | Ath-AT1G61790.1 |  |  |  | Vvi-Vitvi10g00029\_t001 |  |  |  |  |  |  |
| 1 | Ath-AT1G61795.1 |  |  |  | | | |  |  |  |  |  |  |
| 1 | Ath-AT1G61800.2 |  |  |  | Vvi-Vitvi10g00037\_t001 |  |  |  |  |  |  |
| 1 | Ath-AT1G61810.3 |  |  |  | Vvi-Vitvi10g00039\_t001 |  |  |  |  |  |  |
| 1 | Ath-AT1G61820.1 |  |  |  | | | |  |  |  |  |  |  |
| 1 | Ath-AT1G61840.1 |  |  |  | | | |  |  |  |  |  |  |
| 1 | Ath-AT1G61850.2 |  |  |  | Vvi-Vitvi10g00045\_t001 |  |  |  |  |  |  |
| 1 | Ath-AT1G61860.1 |  |  |  | Vvi-Vitvi10g00046\_t001 |  |  |  |  |  |  |
| 0 | Ath-AT1G61870.1 |  |  |  |  |  |  |  |  |
| 1 | Ath-AT1G61890.1 |  | Vvi-Vitvi10g00107\_t001 |  |  |  |  |  |  |  |
| 1 | Ath-AT1G61900.1 |  | Vvi-Vitvi10g00106\_t001 |  |  |  |  |  |  |  |
| 1 | Ath-AT1G61920.1 |  | | | |  |  |  |  |  |  |  |
| 1 | Ath-AT1G61930.1 |  | Vvi-Vitvi10g01642\_t001 |  |  |  |  |  |  |  |
| 1 | Ath-AT1G61940.2 |  | | | |  |  |  |  |  |  |  |
| 1 | Ath-AT1G61950.1 |  | Vvi-Vitvi10g00102\_t001 |  |  |  |  |  |  |  |
| 1 | Ath-AT1G61960.1 |  | | | |  |  |  |  |  |  |  |
| 1 | Ath-AT1G61970.1 |  | | | |  |  |  |  |  |  |  |
| 1 | Ath-AT1G61980.1 |  | | | |  |  |  |  |  |  |  |
| 1 | Ath-AT1G61990.1 |  | | | |  |  |  |  |  |  |  |
| 1 | Ath-AT1G62000.1 |  | | | |  |  |  |  |  |  |  |
| 1 | Ath-AT1G62010.1 |  | | | |  |  |  |  |  |  |  |
| 1 | Ath-AT1G62020.1 |  | | | |  |  |  |  |  |  |  |
| 1 | Ath-AT1G62030.1 |  | | | |  |  |  |  |  |  |  |
| 1 | Ath-AT1G62040.1 |  | Vvi-Vitvi10g04024\_t001 |  |  |  |  |  |  |  |
| 1 | Ath-AT1G62045.1 |  | | | |  |  |  |  |  |  |  |
| 1 | Ath-AT1G62050.1 |  | Vvi-Vitvi10g00090\_t001 |  |  |  |  |  |  |  |
| 1 | Ath-AT1G62060.1 |  | | | |  |  |  |  |  |  |  |
| 1 | Ath-AT1G62070.1 |  | | | |  |  |  |  |  |  |  |
| 1 | Ath-AT1G62080.1 |  | | | |  |  |  |  |  |  |  |
| 1 | Ath-AT1G62085.1 |  | | | |  |  |  |  |  |  |  |
| 1 | Ath-AT1G62110.1 |  | | | |  |  |  |  |  |  |  |
| 1 | Ath-AT1G62120.1 |  | | | |  |  |  |  |  |  |  |
| 1 | Ath-AT1G62130.1 |  | | | |  |  |  |  |  |  |  |
| 1 | Ath-AT1G62150.1 |  | | | |  |  |  |  |  |  |  |
| 1 | Ath-AT1G62160.1 |  | | | |  |  |  |  |  |  |  |
| 1 | Ath-AT1G62170.2 |  | | | |  |  |  |  |  |  |  |
| 1 | Ath-AT1G62180.1 |  | Vvi-Vitvi10g00086\_t001 |  |  |  |  |  |  |  |
| 1 | Ath-AT1G62190.1 |  | | | |  |  |  |  |  |  |  |
| 1 | Ath-AT1G62200.3 |  | | | |  |  |  |  |  |  |  |
| 1 | Ath-AT1G62210.1 |  | | | |  |  |  |  |  |  |  |
| 1 | Ath-AT1G62220.1 |  | | | |  |  |  |  |  |  |  |
| 1 | Ath-AT1G62225.1 |  | | | |  |  |  |  |  |  |  |
| 1 | Ath-AT1G62250.1 |  | Vvi-Vitvi10g00079\_t001 |  |  |  |  |  |  |  |
| 1 | Ath-AT1G62240.1 |  | | | |  |  |  |  |  |  |  |
| 1 | Ath-AT1G62260.1 |  | | | |  |  |  |  |  |  |  |
| 1 | Ath-AT1G62262.1 |  | Vvi-Vitvi10g00065\_t001 |  |  |  |  |  |  |  |
| 1 | Ath-AT1G62270.1 |  | | | |  |  |  |  |  |  |  |
| 1 | Ath-AT1G62280.1 |  | | | |  |  |  |  |  |  |  |
| 1 | Ath-AT1G62290.2 |  | Vvi-Vitvi10g00064\_t001 |  |  |  |  |  |  |  |
| 1 | Ath-AT1G62300.1 |  | Vvi-Vitvi10g00063\_t001 |  |  |  |  |  |  |  |
| 1 | Ath-AT1G62305.1 |  | Vvi-Vitvi10g00055\_t001 |  |  |  |  |  |  |  |
| 1 | Ath-AT1G62310.1 |  | Vvi-Vitvi10g00053\_t001 |  |  |  |  |  |  |  |
| 1 | Ath-AT1G62320.3 |  | Vvi-Vitvi10g00052\_t004 |  |  |  |  |  |  |  |
| 0 | Ath-AT1G62330.1 |  |  |  |  |  |  |  |  |
| 0 | Ath-AT1G62333.1 |  |  |  |  |  |  |  |  |
| 0 | Ath-AT1G62340.1 |  |  |  |  |  |  |  |  |
| 0 | Ath-AT1G62350.2 |  |  |  |  |  |  |  |  |
| 0 | Ath-AT1G62360.1 |  |  |  |  |  |  |  |  |
| 0 | Ath-AT1G62370.1 |  |  |  |  |  |  |  |  |
| 0 | Ath-AT1G62380.1 |  |  |  |  |  |  |  |  |
| 0 | Ath-AT1G62390.1 |  |  |  |  |  |  |  |  |
| 0 | Ath-AT1G62400.1 |  |  |  |  |  |  |  |  |
| 0 | Ath-AT1G62410.1 |  |  |  |  |  |  |  |  |
| 0 | Ath-AT1G62421.1 |  |  |  |  |  |  |  |  |
| 0 | Ath-AT1G62422.1 |  |  |  |  |  |  |  |  |
| 0 | Ath-AT1G62420.1 |  |  |  |  |  |  |  |  |
| 0 | Ath-AT1G62430.1 |  |  |  |  |  |  |  |  |
| 0 | Ath-AT1G62440.1 |  |  |  |  |  |  |  |  |
| 0 | Ath-AT1G62450.1 |  |  |  |  |  |  |  |  |
| 0 | Ath-AT1G62480.1 |  |  |  |  |  |  |  |  |
| 0 | Ath-AT1G62490.1 |  |  |  |  |  |  |  |  |
| 1 | Ath-AT1G62500.1 |  | Vvi-Vitvi02g00531\_t001 |  |  |  |  |  |  |  |
| 1 | Ath-AT1G62510.1 |  | Vvi-Vitvi02g01439\_t001 |  |  |  |  |  |  |  |
| 1 | Ath-AT1G62515.1 |  | | | |  |  |  |  |  |  |  |
| 1 | Ath-AT1G62520.1 |  | Vvi-Vitvi02g00526\_t001 |  |  |  |  |  |  |  |
| 1 | Ath-AT1G62530.2 |  | Vvi-Vitvi02g01682\_t002 |  |  |  |  |  |  |  |
| 1 | Ath-AT1G62540.2 |  | | | |  |  |  |  |  |  |  |
| 1 | Ath-AT1G62560.1 |  | | | |  |  |  |  |  |  |  |
| 1 | Ath-AT1G62570.1 |  | | | |  |  |  |  |  |  |  |
| 1 | Ath-AT1G62580.1 |  | | | |  |  |  |  |  |  |  |
| 1 | Ath-AT1G62590.1 |  | | | |  |  |  |  |  |  |  |
| 1 | Ath-AT1G62600.1 |  | Vvi-Vitvi02g01685\_t001 |  |  |  |  |  |  |  |
| 1 | Ath-AT1G62610.4 |  | | | |  |  |  |  |  |  |  |
| 1 | Ath-AT1G62620.1 |  | Vvi-Vitvi02g04150\_t001 |  |  |  |  |  |  |  |
| 1 | Ath-AT1G62630.1 |  | | | |  |  |  |  |  |  |  |
| 1 | Ath-AT1G62640.2 |  | Vvi-Vitvi02g01695\_t001 |  |  |  |  |  |  |  |
| 1 | Ath-AT1G62660.1 |  | Vvi-Vitvi02g00512\_t001 |  |  |  |  |  |  |  |
| 1 | Ath-AT1G62670.1 |  | | | |  |  |  |  |  |  |  |
| 1 | Ath-AT1G62680.1 |  | | | |  |  |  |  |  |  |  |
| 1 | Ath-AT1G62690.1 |  | | | |  |  |  |  |  |  |  |
| 1 | Ath-AT1G62700.1 |  | Vvi-Vitvi02g00508\_t001 |  |  |  |  |  |  |  |
| 1 | Ath-AT1G62710.1 |  | Vvi-Vitvi02g00505\_t001 |  |  |  |  |  |  |  |
| 1 | Ath-AT1G62720.1 |  | | | |  |  |  |  |  |  |  |
| 1 | Ath-AT1G62730.1 |  | | | |  |  |  |  |  |  |  |
| 1 | Ath-AT1G62740.1 |  | Vvi-Vitvi02g01852\_t001 |  |  |  |  |  |  |  |
| 1 | Ath-AT1G62750.1 |  | Vvi-Vitvi02g00502\_t001 |  |  |  |  |  |  |  |
| 1 | Ath-AT1G62760.1 |  | Vvi-Vitvi02g00500\_t001 |  |  |  |  |  |  |  |
| 1 | Ath-AT1G62770.1 |  | Vvi-Vitvi02g00499\_t001 |  |  |  |  |  |  |  |
| 1 | Ath-AT1G62780.1 |  | Vvi-Vitvi02g00498\_t001 |  |  |  |  |  |  |  |
| 1 | Ath-AT1G62790.1 |  | Vvi-Vitvi02g04142\_t001 |  |  |  |  |  |  |  |
| 1 | Ath-AT1G62800.2 |  | | | |  |  |  |  |  |  |  |
| 1 | Ath-AT1G62810.1 |  | Vvi-Vitvi02g04136\_t001 |  |  |  |  |  |  |  |
| 1 | Ath-AT1G62820.1 |  | Vvi-Vitvi02g00485\_t001 |  |  |  |  |  |  |  |
| 1 | Ath-AT1G62830.1 |  | | | |  |  |  |  |  |  |  |
| 1 | Ath-AT1G62840.1 |  | Vvi-Vitvi02g00481\_t001 |  |  |  |  |  |  |  |
| 1 | Ath-AT1G62850.2 |  | Vvi-Vitvi02g00480\_t001 |  |  |  |  |  |  |  |
| 1 | Ath-AT1G62870.1 |  | Vvi-Vitvi02g00476\_t002 |  |  |  |  |  |  |  |
| 1 | Ath-AT1G62880.1 |  | Vvi-Vitvi02g00471\_t001 |  |  |  |  |  |  |  |
| 1 | Ath-AT1G62886.1 |  | Vvi-Vitvi02g00470\_t001 |  |  |  |  |  |  |  |
| 1 | Ath-AT1G62895.1 |  | | | |  |  |  |  |  |  |  |
| 1 | Ath-AT1G62900.1 |  | | | |  |  |  |  |  |  |  |
| 1 | Ath-AT1G62910.1 |  | | | |  |  |  |  |  |  |  |
| 1 | Ath-AT1G62914.1 |  | | | |  |  |  |  |  |  |  |
| 1 | Ath-AT1G62915.1 |  | | | |  |  |  |  |  |  |  |
| 1 | Ath-AT1G62920.1 |  | | | |  |  |  |  |  |  |  |
| 1 | Ath-AT1G62930.1 |  | | | |  |  |  |  |  |  |  |
| 1 | Ath-AT1G62935.1 |  | | | |  |  |  |  |  |  |  |
| 1 | Ath-AT1G62940.1 |  | | | |  |  |  |  |  |  |  |
| 1 | Ath-AT1G62950.1 |  | Vvi-Vitvi02g04118\_t001 |  |  |  |  |  |  |  |
| 1 | Ath-AT1G62960.1 |  | Vvi-Vitvi02g04117\_t001 |  |  |  |  |  |  |  |
| 0 | Ath-AT1G62970.1 |  |  |  |  |  |  |  |  |
| 1 | Ath-AT1G62975.1 |  | Vvi-Vitvi02g00439\_t001 |  |  |  |  |  |  |  |
| 1 | Ath-AT1G62978.1 |  | | | |  |  |  |  |  |  |  |
| 1 | Ath-AT1G62980.1 |  | Vvi-Vitvi02g00433\_t001 |  |  |  |  |  |  |  |
| 1 | Ath-AT1G62981.1 |  | Vvi-Vitvi02g00432\_t001 |  |  |  |  |  |  |  |
| 1 | Ath-AT1G62990.1 |  | Vvi-Vitvi02g00425\_t001 |  |  |  |  |  |  |  |
| 1 | Ath-AT1G63000.1 |  | Vvi-Vitvi02g00423\_t001 |  |  |  |  |  |  |  |
| 1 | Ath-AT1G63010.5 |  | Vvi-Vitvi02g00415\_t001 |  |  |  |  |  |  |  |
| 1 | Ath-AT1G63020.2 |  | Vvi-Vitvi02g00414\_t001 |  |  |  |  |  |  |  |
| 1 | Ath-AT1G63030.2 |  | Vvi-Vitvi02g00407\_t001 |  |  |  |  |  |  |  |
| 1 | Ath-AT1G63050.1 |  | Vvi-Vitvi02g00402\_t001 |  |  |  |  |  |  |  |
| 1 | Ath-AT1G63055.1 |  | | | |  |  |  |  |  |  |  |
| 1 | Ath-AT1G63057.2 |  | | | |  |  |  |  |  |  |  |
| 1 | Ath-AT1G63060.1 |  | | | |  |  |  |  |  |  |  |
| 1 | Ath-AT1G63070.1 |  | | | |  |  |  |  |  |  |  |
| 1 | Ath-AT1G63080.1 |  | | | |  |  |  |  |  |  |  |
| 1 | Ath-AT1G63090.1 |  | Vvi-Vitvi02g00398\_t001 |  |  |  |  |  |  |  |
| 2 | Ath-AT1G63100.2 |  | | | |  | Vvi-Vitvi02g00370\_t001 |  |  |  |  |  |  |
| 2 | Ath-AT1G63105.1 |  | | | |  | | | |  |  |  |  |  |  |
| 2 | Ath-AT1G63110.1 |  | | | |  | Vvi-Vitvi02g00375\_t001 |  |  |  |  |  |  |
| 2 | Ath-AT1G63120.1 |  | | | |  | Vvi-Vitvi02g00382\_t001 |  |  |  |  |  |  |
| 2 | Ath-AT1G63130.2 |  | | | |  | | | |  |  |  |  |  |  |
| 2 | Ath-AT1G63140.4 |  | | | |  | | | |  |  |  |  |  |  |
| 2 | Ath-AT1G63150.1 |  | | | |  | | | |  |  |  |  |  |  |
| 2 | Ath-AT1G63160.1 |  | | | |  | Vvi-Vitvi02g00383\_t001 |  |  |  |  |  |  |
| 2 | Ath-AT1G63170.1 |  | Vvi-Vitvi02g00384\_t001 |  | Vvi-Vitvi02g00384\_t001 |  |  |  |  |  |  |
| 2 | Ath-AT1G63180.1 |  | | | |  | Vvi-Vitvi02g00390\_t001 |  |  |  |  |  |  |
| 2 | Ath-AT1G63190.1 |  | | | |  | | | |  |  |  |  |  |  |
| 2 | Ath-AT1G63200.1 |  | | | |  | | | |  |  |  |  |  |  |
| 2 | Ath-AT1G63205.1 |  | | | |  | | | |  |  |  |  |  |  |
| 2 | Ath-AT1G63206.2 |  | | | |  | | | |  |  |  |  |  |  |
| 2 | Ath-AT1G63210.1 |  | | | |  | | | |  |  |  |  |  |  |
| 2 | Ath-AT1G63220.1 |  | | | |  | Vvi-Vitvi02g00394\_t001 |  |  |  |  |  |  |
| 1 | Ath-AT1G63230.1 |  | | | |  |  |  |  |  |  |  |
| 1 | Ath-AT1G63240.1 |  | | | |  |  |  |  |  |  |  |
| 1 | Ath-AT1G63245.1 |  | | | |  |  |  |  |  |  |  |
| 1 | Ath-AT1G63250.1 |  | | | |  |  |  |  |  |  |  |
| 1 | Ath-AT1G63260.1 |  | Vvi-Vitvi02g00366\_t001 |  |  |  |  |  |  |  |
| 1 | Ath-AT1G63270.1 |  | | | |  |  |  |  |  |  |  |
| 1 | Ath-AT1G63280.1 |  | | | |  |  |  |  |  |  |  |
| 1 | Ath-AT1G63290.1 |  | | | |  |  |  |  |  |  |  |
| 1 | Ath-AT1G63295.2 |  | Vvi-Vitvi02g00348\_t001 |  |  |  |  |  |  |  |
| 1 | Ath-AT1G63300.1 |  | Vvi-Vitvi02g00346\_t001 |  |  |  |  |  |  |  |
| 1 | Ath-AT1G63310.1 |  | | | |  |  |  |  |  |  |  |
| 1 | Ath-AT1G63320.1 |  | | | |  |  |  |  |  |  |  |
| 1 | Ath-AT1G63330.1 |  | | | |  |  |  |  |  |  |  |
| 1 | Ath-AT1G63340.1 |  | | | |  |  |  |  |  |  |  |
| 1 | Ath-AT1G63350.2 |  | | | |  |  |  |  |  |  |  |
| 1 | Ath-AT1G63360.1 |  | | | |  |  |  |  |  |  |  |
| 1 | Ath-AT1G63370.1 |  | | | |  |  |  |  |  |  |  |
| 1 | Ath-AT1G63380.2 |  | | | |  |  |  |  |  |  |  |
| 1 | Ath-AT1G63390.1 |  | | | |  |  |  |  |  |  |  |
| 1 | Ath-AT1G63400.1 |  | | | |  |  |  |  |  |  |  |
| 1 | Ath-AT1G63410.2 |  | Vvi-Vitvi02g00344\_t001 |  |  |  |  |  |  |  |
| 1 | Ath-AT1G63420.1 |  | Vvi-Vitvi02g01384\_t001 |  |  |  |  |  |  |  |
| 1 | Ath-AT1G63430.2 |  | Vvi-Vitvi02g00342\_t001 |  |  |  |  |  |  |  |
| 1 | Ath-AT1G63440.1 |  | Vvi-Vitvi02g00337\_t001 |  |  |  |  |  |  |  |
| 1 | Ath-AT1G63450.1 |  | Vvi-Vitvi02g00334\_t001 |  |  |  |  |  |  |  |
| 1 | Ath-AT1G63460.1 |  | Vvi-Vitvi02g00332\_t001 |  |  |  |  |  |  |  |
| 1 | Ath-AT1G63470.1 |  | Vvi-Vitvi02g00331\_t001 |  |  |  |  |  |  |  |
| 1 | Ath-AT1G63480.1 |  | | | |  |  |  |  |  |  |  |
| 1 | Ath-AT1G63490.4 |  | Vvi-Vitvi02g00329\_t001 |  |  |  |  |  |  |  |
| 1 | Ath-AT1G63500.1 |  | Vvi-Vitvi02g00328\_t003 |  |  |  |  |  |  |  |
| 1 | Ath-AT1G63520.1 |  | Vvi-Vitvi02g04072\_t001 |  |  |  |  |  |  |  |
| 1 | Ath-AT1G63522.1 |  | | | |  |  |  |  |  |  |  |
| 1 | Ath-AT1G63530.4 |  | | | |  |  |  |  |  |  |  |
| 1 | Ath-AT1G63535.1 |  | | | |  |  |  |  |  |  |  |
| 1 | Ath-AT1G63540.1 |  | | | |  |  |  |  |  |  |  |
| 1 | Ath-AT1G63550.1 |  | | | |  |  |  |  |  |  |  |
| 1 | Ath-AT1G63570.2 |  | | | |  |  |  |  |  |  |  |
| 1 | Ath-AT1G63580.1 |  | | | |  |  |  |  |  |  |  |
| 1 | Ath-AT1G63590.1 |  | | | |  |  |  |  |  |  |  |
| 1 | Ath-AT1G63600.1 |  | | | |  |  |  |  |  |  |  |
| 1 | Ath-AT1G63610.1 |  | | | |  |  |  |  |  |  |  |
| 1 | Ath-AT1G63615.1 |  | | | |  |  |  |  |  |  |  |
| 1 | Ath-AT1G63630.1 |  | | | |  |  |  |  |  |  |  |
| 1 | Ath-AT1G63640.3 |  | Vvi-Vitvi02g00319\_t001 |  |  |  |  |  |  |  |
| 2 | Ath-AT1G63650.3 |  | Vvi-Vitvi02g00317\_t001 |  | Vvi-Vitvi15g01124\_t001 |  |  |  |  |  |  |
| 2 | Ath-AT1G63660.1 |  | Vvi-Vitvi02g00315\_t001 |  | | | |  |  |  |  |  |  |
| 2 | Ath-AT1G63670.3 |  | | | |  | Vvi-Vitvi15g01117\_t002 |  |  |  |  |  |  |
| 2 | Ath-AT1G63680.2 |  | Vvi-Vitvi02g00313\_t001 |  | | | |  |  |  |  |  |  |
| 2 | Ath-AT1G63690.1 |  | Vvi-Vitvi02g00311\_t001 |  | Vvi-Vitvi15g01114\_t001 |  |  |  |  |  |  |
| 2 | Ath-AT1G63700.1 |  | Vvi-Vitvi02g00308\_t001 |  | | | |  |  |  |  |  |  |
| 2 | Ath-AT1G63710.1 |  | Vvi-Vitvi02g00303\_t001 |  | Vvi-Vitvi15g01106\_t001 |  |  |  |  |  |  |
| 2 | Ath-AT1G63720.1 |  | Vvi-Vitvi02g00297\_t001 |  | | | |  |  |  |  |  |  |
| 2 | Ath-AT1G63730.1 |  | | | |  | | | |  |  |  |  |  |  |
| 2 | Ath-AT1G63740.2 |  | | | |  | | | |  |  |  |  |  |  |
| 2 | Ath-AT1G63750.3 |  | | | |  | | | |  |  |  |  |  |  |
| 2 | Ath-AT1G63770.3 |  | Vvi-Vitvi02g00296\_t001 |  | | | |  |  |  |  |  |  |
| 1 | Ath-AT1G63780.1 |  |  |  | | | |  |  |  |  |  |  |
| 2 | Ath-AT1G63800.1 |  | Vvi-Vitvi02g04001\_t001 |  | Vvi-Vitvi15g01099\_t003 |  |  |  |  |  |  |
| 2 | Ath-AT1G63810.1 |  | | | |  | | | |  |  |  |  |  |  |
| 2 | Ath-AT1G63820.1 |  | Vvi-Vitvi02g00011\_t001 |  | | | |  |  |  |  |  |  |
| 2 | Ath-AT1G63830.3 |  | Vvi-Vitvi02g04003\_t002 |  | | | |  |  |  |  |  |  |
| 2 | Ath-AT1G63840.1 |  | Vvi-Vitvi02g00013\_t001 |  | | | |  |  |  |  |  |  |
| 2 | Ath-AT1G63850.1 |  | Vvi-Vitvi02g00014\_t001 |  | | | |  |  |  |  |  |  |
| 2 | Ath-AT1G63855.4 |  | | | |  | | | |  |  |  |  |  |  |
| 2 | Ath-AT1G63860.1 |  | | | |  | | | |  |  |  |  |  |  |
| 2 | Ath-AT1G63870.1 |  | | | |  | | | |  |  |  |  |  |  |
| 2 | Ath-AT1G63880.1 |  | | | |  | | | |  |  |  |  |  |  |
| 2 | Ath-AT1G63900.1 |  | | | |  | | | |  |  |  |  |  |  |
| 2 | Ath-AT1G63910.1 |  | Vvi-Vitvi02g00028\_t001 |  | | | |  |  |  |  |  |  |
| 2 | Ath-AT1G63930.1 |  | Vvi-Vitvi02g00029\_t001 |  | | | |  |  |  |  |  |  |
| 2 | Ath-AT1G63940.2 |  | Vvi-Vitvi02g00030\_t001 |  | | | |  |  |  |  |  |  |
| 2 | Ath-AT1G63950.2 |  | Vvi-Vitvi02g00031\_t001 |  | Vvi-Vitvi15g01094\_t001 |  |  |  |  |  |  |
| 2 | Ath-AT1G63960.2 |  | | | |  | | | |  |  |  |  |  |  |
| 2 | Ath-AT1G63970.1 |  | Vvi-Vitvi02g00034\_t001 |  | | | |  |  |  |  |  |  |
| 2 | Ath-AT1G63980.1 |  | Vvi-Vitvi02g00037\_t002 |  | | | |  |  |  |  |  |  |
| 2 | Ath-AT1G63990.1 |  | Vvi-Vitvi02g00038\_t001 |  | | | |  |  |  |  |  |  |
| 2 | Ath-AT1G64000.1 |  | | | |  | Vvi-Vitvi15g01087\_t001 |  |  |  |  |  |  |
| 2 | Ath-AT1G64010.2 |  | | | |  | | | |  |  |  |  |  |  |
| 2 | Ath-AT1G64020.1 |  | | | |  | | | |  |  |  |  |  |  |
| 2 | Ath-AT1G64030.1 |  | | | |  | | | |  |  |  |  |  |  |
| 2 | Ath-AT1G64040.1 |  | Vvi-Vitvi02g00044\_t001 |  | | | |  |  |  |  |  |  |
| 2 | Ath-AT1G64050.1 |  | Vvi-Vitvi02g00045\_t001 |  | | | |  |  |  |  |  |  |
| 2 | Ath-AT1G64060.1 |  | Vvi-Vitvi02g00048\_t001 |  | | | |  |  |  |  |  |  |
| 2 | Ath-AT1G64065.1 |  | Vvi-Vitvi02g01317\_t001 |  | Vvi-Vitvi15g01083\_t001 |  |  |  |  |  |  |
| 1 | Ath-AT1G64070.1 |  | | | |  |  |  |  |  |  |  |
| 1 | Ath-AT1G64080.1 |  | Vvi-Vitvi02g00051\_t001 |  |  |  |  |  |  |  |
| 1 | Ath-AT1G64090.2 |  | Vvi-Vitvi02g00059\_t001 |  |  |  |  |  |  |  |
| 1 | Ath-AT1G64100.2 |  | | | |  |  |  |  |  |  |  |
| 1 | Ath-AT1G64105.1 |  | | | |  |  |  |  |  |  |  |
| 1 | Ath-AT1G64107.1 |  | | | |  |  |  |  |  |  |  |
| 1 | Ath-AT1G64110.2 |  | Vvi-Vitvi02g00060\_t001 |  |  |  |  |  |  |  |
| 1 | Ath-AT1G64130.1 |  | | | |  |  |  |  |  |  |  |
| 1 | Ath-AT1G64140.1 |  | Vvi-Vitvi02g00067\_t001 |  |  |  |  |  |  |  |
| 1 | Ath-AT1G64150.1 |  | Vvi-Vitvi02g00068\_t001 |  |  |  |  |  |  |  |
| 1 | Ath-AT1G64160.1 |  | Vvi-Vitvi02g00069\_t001 |  |  |  |  |  |  |  |
| 1 | Ath-AT1G64170.1 |  | Vvi-Vitvi02g00071\_t001 |  |  |  |  |  |  |  |
| 1 | Ath-AT1G64180.1 |  | Vvi-Vitvi02g01329\_t001 |  |  |  |  |  |  |  |
| 1 | Ath-AT1G64185.1 |  | Vvi-Vitvi02g00076\_t001 |  |  |  |  |  |  |  |
| 1 | Ath-AT1G64190.1 |  | Vvi-Vitvi02g00080\_t001 |  |  |  |  |  |  |  |
| 1 | Ath-AT1G64195.1 |  | | | |  |  |  |  |  |  |  |
| 1 | Ath-AT1G64200.2 |  | Vvi-Vitvi02g01333\_t001 |  |  |  |  |  |  |  |
| 1 | Ath-AT1G64210.1 |  | Vvi-Vitvi02g00091\_t003 |  |  |  |  |  |  |  |
| 1 | Ath-AT1G64220.1 |  | | | |  |  |  |  |  |  |  |
| 1 | Ath-AT1G64230.5 |  | | | |  |  |  |  |  |  |  |
| 1 | Ath-AT1G64235.1 |  | | | |  |  |  |  |  |  |  |
| 1 | Ath-AT1G64253.1 |  | | | |  |  |  |  |  |  |  |
| 1 | Ath-AT1G64255.1 |  | | | |  |  |  |  |  |  |  |
| 1 | Ath-AT1G64260.1 |  | | | |  |  |  |  |  |  |  |
| 1 | Ath-AT1G64280.1 |  | | | |  |  |  |  |  |  |  |
| 1 | Ath-AT1G64290.1 |  | | | |  |  |  |  |  |  |  |
| 1 | Ath-AT1G64295.1 |  | | | |  |  |  |  |  |  |  |
| 1 | Ath-AT1G64300.2 |  | Vvi-Vitvi02g00097\_t001 |  |  |  |  |  |  |  |
| 1 | Ath-AT1G64310.1 |  | | | |  |  |  |  |  |  |  |
| 1 | Ath-AT1G64320.1 |  | Vvi-Vitvi02g04015\_t001 |  |  |  |  |  |  |  |
| 1 | Ath-AT1G64330.1 |  | | | |  |  |  |  |  |  |  |
| 1 | Ath-AT1G64340.1 |  | Vvi-Vitvi02g01337\_t001 |  |  |  |  |  |  |  |
| 1 | Ath-AT1G64350.1 |  | Vvi-Vitvi02g00111\_t001 |  |  |  |  |  |  |  |
| 1 | Ath-AT1G64355.1 |  | Vvi-Vitvi02g00113\_t001 |  |  |  |  |  |  |  |
| 1 | Ath-AT1G64360.1 |  | | | |  |  |  |  |  |  |  |
| 1 | Ath-AT1G64370.1 |  | | | |  |  |  |  |  |  |  |
| 1 | Ath-AT1G64380.1 |  | Vvi-Vitvi02g00121\_t001 |  |  |  |  |  |  |  |
| 1 | Ath-AT1G64385.1 |  | Vvi-Vitvi02g00123\_t001 |  |  |  |  |  |  |  |
| 1 | Ath-AT1G64390.1 |  | Vvi-Vitvi02g00125\_t001 |  |  |  |  |  |  |  |
| 1 | Ath-AT1G64400.1 |  | Vvi-Vitvi02g00128\_t001 |  |  |  |  |  |  |  |
| 1 | Ath-AT1G64405.1 |  | Vvi-Vitvi02g01339\_t001 |  |  |  |  |  |  |  |
| 1 | Ath-AT1G64430.1 |  | Vvi-Vitvi02g00139\_t001 |  |  |  |  |  |  |  |
| 1 | Ath-AT1G64440.1 |  | Vvi-Vitvi02g00143\_t001 |  |  |  |  |  |  |  |
| 1 | Ath-AT1G64450.1 |  | Vvi-Vitvi02g00147\_t001 |  |  |  |  |  |  |  |
| 1 | Ath-AT1G64455.1 |  | | | |  |  |  |  |  |  |  |
| 1 | Ath-AT1G64480.1 |  | Vvi-Vitvi02g00152\_t001 |  |  |  |  |  |  |  |
| 1 | Ath-AT1G64490.1 |  | | | |  |  |  |  |  |  |  |
| 1 | Ath-AT1G64500.1 |  | Vvi-Vitvi02g00160\_t001 |  |  |  |  |  |  |  |
| 1 | Ath-AT1G64510.2 |  | Vvi-Vitvi02g00175\_t001 |  |  |  |  |  |  |  |
| 1 | Ath-AT1G64520.1 |  | Vvi-Vitvi02g00176\_t001 |  |  |  |  |  |  |  |
| 1 | Ath-AT1G64530.1 |  | Vvi-Vitvi02g00179\_t001 |  |  |  |  |  |  |  |
| 1 | Ath-AT1G64540.1 |  | | | |  |  |  |  |  |  |  |
| 1 | Ath-AT1G64550.1 |  | Vvi-Vitvi02g00189\_t001 |  |  |  |  |  |  |  |
| 1 | Ath-AT1G64561.1 |  | | | |  |  |  |  |  |  |  |
| 1 | Ath-AT1G64570.1 |  | | | |  |  |  |  |  |  |  |
| 1 | Ath-AT1G64580.1 |  | | | |  |  |  |  |  |  |  |
| 1 | Ath-AT1G64583.1 |  | | | |  |  |  |  |  |  |  |
| 1 | Ath-AT1G64584.1 |  | | | |  |  |  |  |  |  |  |
| 1 | Ath-AT1G64585.1 |  | | | |  |  |  |  |  |  |  |
| 1 | Ath-AT1G64590.1 |  | Vvi-Vitvi02g00192\_t001 |  |  |  |  |  |  |  |
| 1 | Ath-AT1G64600.1 |  | Vvi-Vitvi02g00194\_t001 |  |  |  |  |  |  |  |
| 1 | Ath-AT1G64610.2 |  | Vvi-Vitvi02g00198\_t001 |  |  |  |  |  |  |  |
| 1 | Ath-AT1G64620.1 |  | Vvi-Vitvi02g00199\_t001 |  |  |  |  |  |  |  |
| 1 | Ath-AT1G64625.1 |  | Vvi-Vitvi02g00202\_t001 |  |  |  |  |  |  |  |
| 1 | Ath-AT1G64630.1 |  | Vvi-Vitvi02g00207\_t001 |  |  |  |  |  |  |  |
| 1 | Ath-AT1G64633.1 |  | | | |  |  |  |  |  |  |  |
| 1 | Ath-AT1G64640.1 |  | Vvi-Vitvi02g00211\_t001 |  |  |  |  |  |  |  |
| 1 | Ath-AT1G64650.1 |  | Vvi-Vitvi02g00212\_t001 |  |  |  |  |  |  |  |
| 1 | Ath-AT1G64660.1 |  | Vvi-Vitvi02g00225\_t001 |  |  |  |  |  |  |  |
| 1 | Ath-AT1G64670.2 |  | Vvi-Vitvi02g00232\_t001 |  |  |  |  |  |  |  |
| 1 | Ath-AT1G64680.1 |  | Vvi-Vitvi02g00235\_t001 |  |  |  |  |  |  |  |
| 1 | Ath-AT1G64690.3 |  | Vvi-Vitvi02g00238\_t001 |  |  |  |  |  |  |  |
| 1 | Ath-AT1G64700.1 |  | Vvi-Vitvi02g00243\_t001 |  |  |  |  |  |  |  |
| 2 | Ath-AT1G64710.1 |  | Vvi-Vitvi02g00244\_t001 |  | Vvi-Vitvi16g00018\_t001 |  |  |  |  |  |  |
| 2 | Ath-AT1G64720.1 |  | Vvi-Vitvi02g00255\_t001 |  | | | |  |  |  |  |  |  |
| 1 | Ath-AT1G64740.1 |  |  |  | | | |  |  |  |  |  |  |
| 1 | Ath-AT1G64750.3 |  |  |  | | | |  |  |  |  |  |  |
| 1 | Ath-AT1G64760.1 |  |  |  | | | |  |  |  |  |  |  |
| 1 | Ath-AT1G64770.3 |  |  |  | | | |  |  |  |  |  |  |
| 1 | Ath-AT1G64780.1 |  |  |  | | | |  |  |  |  |  |  |
| 1 | Ath-AT1G64790.2 |  |  |  | | | |  |  |  |  |  |  |
| 1 | Ath-AT1G64795.1 |  |  |  | | | |  |  |  |  |  |  |
| 1 | Ath-AT1G64800.1 |  |  |  | | | |  |  |  |  |  |  |
| 1 | Ath-AT1G64810.2 |  |  |  | | | |  |  |  |  |  |  |
| 1 | Ath-AT1G64820.1 |  |  |  | | | |  |  |  |  |  |  |
| 1 | Ath-AT1G64830.1 |  |  |  | | | |  |  |  |  |  |  |
| 1 | Ath-AT1G64840.1 |  |  |  | | | |  |  |  |  |  |  |
| 1 | Ath-AT1G64850.1 |  |  |  | | | |  |  |  |  |  |  |
| 1 | Ath-AT1G64860.1 |  |  |  | Vvi-Vitvi16g00031\_t002 |  |  |  |  |  |  |
| 1 | Ath-AT1G64870.1 |  |  |  | | | |  |  |  |  |  |  |
| 1 | Ath-AT1G64880.1 |  |  |  | | | |  |  |  |  |  |  |
| 1 | Ath-AT1G64890.1 |  |  |  | Vvi-Vitvi16g00034\_t001 |  |  |  |  |  |  |
| 1 | Ath-AT1G64900.1 |  |  |  | Vvi-Vitvi16g01500\_t001 |  |  |  |  |  |  |
| 1 | Ath-AT1G64910.1 |  |  |  | | | |  |  |  |  |  |  |
| 1 | Ath-AT1G64920.1 |  |  |  | | | |  |  |  |  |  |  |
| 1 | Ath-AT1G64930.1 |  |  |  | | | |  |  |  |  |  |  |
| 1 | Ath-AT1G64940.1 |  |  |  | | | |  |  |  |  |  |  |
| 1 | Ath-AT1G64950.1 |  |  |  | | | |  |  |  |  |  |  |
| 1 | Ath-AT1G64960.2 |  |  |  | | | |  |  |  |  |  |  |
| 1 | Ath-AT1G64970.1 |  |  |  | Vvi-Vitvi16g01509\_t001 |  |  |  |  |  |  |
| 1 | Ath-AT1G64980.2 |  |  |  | Vvi-Vitvi16g00071\_t004 |  |  |  |  |  |  |
| 1 | Ath-AT1G64990.1 |  |  |  | Vvi-Vitvi16g01510\_t001 |  |  |  |  |  |  |
| 1 | Ath-AT1G65000.1 |  |  |  | | | |  |  |  |  |  |  |
| 1 | Ath-AT1G65010.1 |  |  |  | Vvi-Vitvi16g00093\_t003 |  |  |  |  |  |  |
| 1 | Ath-AT1G65020.1 |  |  |  | Vvi-Vitvi16g00094\_t001 |  |  |  |  |  |  |
| 0 | Ath-AT1G65030.1 |  |  |  |  |  |  |  |  |
| 0 | Ath-AT1G65032.1 |  |  |  |  |  |  |  |  |
| 0 | Ath-AT1G65040.2 |  |  |  |  |  |  |  |  |
| 0 | Ath-AT1G65050.1 |  |  |  |  |  |  |  |  |
| 0 | Ath-AT1G65060.1 |  |  |  |  |  |  |  |  |
| 0 | Ath-AT1G65070.2 |  |  |  |  |  |  |  |  |
| 0 | Ath-AT1G65080.1 |  |  |  |  |  |  |  |  |
| 0 | Ath-AT1G65090.2 |  |  |  |  |  |  |  |  |
| 0 | Ath-AT1G65110.2 |  |  |  |  |  |  |  |  |
| 0 | Ath-AT1G65113.1 |  |  |  |  |  |  |  |  |
| 0 | Ath-AT1G65120.2 |  |  |  |  |  |  |  |  |
| 0 | Ath-AT1G65130.1 |  |  |  |  |  |  |  |  |
| 0 | Ath-AT1G65140.1 |  |  |  |  |  |  |  |  |
| 0 | Ath-AT1G65150.1 |  |  |  |  |  |  |  |  |
| 0 | Ath-AT1G65160.1 |  |  |  |  |  |  |  |  |
| 0 | Ath-AT1G65165.1 |  |  |  |  |  |  |  |  |
| 0 | Ath-AT1G65170.1 |  |  |  |  |  |  |  |  |
| 0 | Ath-AT1G65180.1 |  |  |  |  |  |  |  |  |
| 0 | Ath-AT1G65190.1 |  |  |  |  |  |  |  |  |
| 0 | Ath-AT1G65200.1 |  |  |  |  |  |  |  |  |
| 0 | Ath-AT1G65210.1 |  |  |  |  |  |  |  |  |
| 1 | Ath-AT1G65220.1 |  | Vvi-Vitvi04g01182\_t001 |  |  |  |  |  |  |  |
| 1 | Ath-AT1G65230.1 |  | Vvi-Vitvi04g01189\_t002 |  |  |  |  |  |  |  |
| 1 | Ath-AT1G65240.1 |  | Vvi-Vitvi04g01190\_t001 |  |  |  |  |  |  |  |
| 1 | Ath-AT1G65250.1 |  | | | |  |  |  |  |  |  |  |
| 1 | Ath-AT1G65260.1 |  | Vvi-Vitvi04g01195\_t001 |  |  |  |  |  |  |  |
| 1 | Ath-AT1G65270.2 |  | Vvi-Vitvi04g02083\_t001 |  |  |  |  |  |  |  |
| 1 | Ath-AT1G65280.1 |  | | | |  |  |  |  |  |  |  |
| 1 | Ath-AT1G65290.1 |  | Vvi-Vitvi04g01199\_t001 |  |  |  |  |  |  |  |
| 0 | Ath-AT1G65295.1 |  |  |  |  |  |  |  |  |
| 0 | Ath-AT1G65300.1 |  |  |  |  |  |  |  |  |
| 0 | Ath-AT1G65310.2 |  |  |  |  |  |  |  |  |
| 0 | Ath-AT1G65320.1 |  |  |  |  |  |  |  |  |
| 0 | Ath-AT1G65330.1 |  |  |  |  |  |  |  |  |
| 0 | Ath-AT1G65340.1 |  |  |  |  |  |  |  |  |
| 0 | Ath-AT1G65342.1 |  |  |  |  |  |  |  |  |
| 0 | Ath-AT1G65350.1 |  |  |  |  |  |  |  |  |
| 0 | Ath-AT1G65346.1 |  |  |  |  |  |  |  |  |
| 0 | Ath-AT1G65349.1 |  |  |  |  |  |  |  |  |
| 0 | Ath-AT1G65352.1 |  |  |  |  |  |  |  |  |
| 0 | Ath-AT1G65360.1 |  |  |  |  |  |  |  |  |
| 0 | Ath-AT1G65370.1 |  |  |  |  |  |  |  |  |
| 0 | Ath-AT1G65380.1 |  |  |  |  |  |  |  |  |
| 0 | Ath-AT1G65390.1 |  |  |  |  |  |  |  |  |
| 0 | Ath-AT1G65410.1 |  |  |  |  |  |  |  |  |
| 1 | Ath-AT1G65420.1 |  | Vvi-Vitvi07g04490\_t001 |  |  |  |  |  |  |  |
| 1 | Ath-AT1G65425.1 |  | | | |  |  |  |  |  |  |  |
| 1 | Ath-AT1G65430.1 |  | Vvi-Vitvi07g01261\_t001 |  |  |  |  |  |  |  |
| 1 | Ath-AT1G65440.1 |  | | | |  |  |  |  |  |  |  |
| 1 | Ath-AT1G65450.1 |  | Vvi-Vitvi07g01255\_t001 |  |  |  |  |  |  |  |
| 1 | Ath-AT1G65470.1 |  | | | |  |  |  |  |  |  |  |
| 1 | Ath-AT1G65480.2 |  | Vvi-Vitvi07g04487\_t001 |  |  |  |  |  |  |  |
| 1 | Ath-AT1G65481.1 |  | | | |  |  |  |  |  |  |  |
| 1 | Ath-AT1G65483.1 |  | | | |  |  |  |  |  |  |  |
| 1 | Ath-AT1G65484.1 |  | | | |  |  |  |  |  |  |  |
| 1 | Ath-AT1G65486.3 |  | | | |  |  |  |  |  |  |  |
| 1 | Ath-AT1G65490.2 |  | | | |  |  |  |  |  |  |  |
| 1 | Ath-AT1G65500.1 |  | | | |  |  |  |  |  |  |  |
| 1 | Ath-AT1G65510.2 |  | | | |  |  |  |  |  |  |  |
| 1 | Ath-AT1G65520.1 |  | | | |  |  |  |  |  |  |  |
| 1 | Ath-AT1G65540.2 |  | Vvi-Vitvi07g04485\_t002 |  |  |  |  |  |  |  |
| 1 | Ath-AT1G65541.1 |  | | | |  |  |  |  |  |  |  |
| 1 | Ath-AT1G65550.1 |  | | | |  |  |  |  |  |  |  |
| 1 | Ath-AT1G65560.1 |  | Vvi-Vitvi07g01244\_t001 |  |  |  |  |  |  |  |
| 1 | Ath-AT1G65570.1 |  | Vvi-Vitvi07g02498\_t001 |  |  |  |  |  |  |  |
| 1 | Ath-AT1G65580.1 |  | Vvi-Vitvi07g01242\_t001 |  |  |  |  |  |  |  |
| 1 | Ath-AT1G65590.1 |  | Vvi-Vitvi07g04480\_t001 |  |  |  |  |  |  |  |
| 1 | Ath-AT1G65610.1 |  | Vvi-Vitvi07g02990\_t001 |  |  |  |  |  |  |  |
| 1 | Ath-AT1G65620.2 |  | | | |  |  |  |  |  |  |  |
| 1 | Ath-AT1G65630.1 |  | Vvi-Vitvi07g01237\_t001 |  |  |  |  |  |  |  |
| 0 | Ath-AT1G65640.2 |  |  |  |  |  |  |  |  |
| 0 | Ath-AT1G65650.1 |  |  |  |  |  |  |  |  |
| 0 | Ath-AT1G65660.1 |  |  |  |  |  |  |  |  |
| 0 | Ath-AT1G65670.1 |  |  |  |  |  |  |  |  |
| 0 | Ath-AT1G65680.1 |  |  |  |  |  |  |  |  |
| 0 | Ath-AT1G65681.1 |  |  |  |  |  |  |  |  |
| 0 | Ath-AT1G65690.1 |  |  |  |  |  |  |  |  |
| 0 | Ath-AT1G65700.3 |  |  |  |  |  |  |  |  |
| 0 | Ath-AT1G65710.1 |  |  |  |  |  |  |  |  |
| 0 | Ath-AT1G65720.1 |  |  |  |  |  |  |  |  |
| 0 | Ath-AT1G65730.1 |  |  |  |  |  |  |  |  |
| 0 | Ath-AT1G65735.1 |  |  |  |  |  |  |  |  |
| 0 | Ath-AT1G65740.1 |  |  |  |  |  |  |  |  |
| 0 | Ath-AT1G65760.1 |  |  |  |  |  |  |  |  |
| 0 | Ath-AT1G65770.1 |  |  |  |  |  |  |  |  |
| 0 | Ath-AT1G65780.1 |  |  |  |  |  |  |  |  |
| 0 | Ath-AT1G65790.1 |  |  |  |  |  |  |  |  |
| 0 | Ath-AT1G65800.1 |  |  |  |  |  |  |  |  |
| 0 | Ath-AT1G65810.1 |  |  |  |  |  |  |  |  |
| 0 | Ath-AT1G65820.3 |  |  |  |  |  |  |  |  |
| 0 | Ath-AT1G65840.1 |  |  |  |  |  |  |  |  |
| 0 | Ath-AT1G65845.1 |  |  |  |  |  |  |  |  |
| 0 | Ath-AT1G65850.2 |  |  |  |  |  |  |  |  |
| 0 | Ath-AT1G65860.1 |  |  |  |  |  |  |  |  |
| 0 | Ath-AT1G65870.1 |  |  |  |  |  |  |  |  |
| 0 | Ath-AT1G65880.1 |  |  |  |  |  |  |  |  |
| 0 | Ath-AT1G65890.1 |  |  |  |  |  |  |  |  |
| 1 | Ath-AT1G65900.1 |  | Vvi-Vitvi04g00836\_t001 |  |  |  |  |  |  |  |
| 1 | Ath-AT1G65910.1 |  | Vvi-Vitvi04g00837\_t001 |  |  |  |  |  |  |  |
| 1 | Ath-AT1G65920.1 |  | Vvi-Vitvi04g00838\_t001 |  |  |  |  |  |  |  |
| 1 | Ath-AT1G65930.1 |  | Vvi-Vitvi04g00860\_t001 |  |  |  |  |  |  |  |
| 1 | Ath-AT1G65950.1 |  | Vvi-Vitvi04g00861\_t001 |  |  |  |  |  |  |  |
| 1 | Ath-AT1G65960.2 |  | Vvi-Vitvi04g00870\_t001 |  |  |  |  |  |  |  |
| 1 | Ath-AT1G65970.1 |  | | | |  |  |  |  |  |  |  |
| 1 | Ath-AT1G65980.1 |  | | | |  |  |  |  |  |  |  |
| 1 | Ath-AT1G65985.2 |  | | | |  |  |  |  |  |  |  |
| 1 | Ath-AT1G65990.1 |  | | | |  |  |  |  |  |  |  |
| 1 | Ath-AT1G66000.1 |  | | | |  |  |  |  |  |  |  |
| 1 | Ath-AT1G66020.1 |  | | | |  |  |  |  |  |  |  |
| 1 | Ath-AT1G66030.1 |  | | | |  |  |  |  |  |  |  |
| 1 | Ath-AT1G66040.1 |  | | | |  |  |  |  |  |  |  |
| 1 | Ath-AT1G66045.1 |  | | | |  |  |  |  |  |  |  |
| 1 | Ath-AT1G66050.1 |  | | | |  |  |  |  |  |  |  |
| 1 | Ath-AT1G66060.1 |  | | | |  |  |  |  |  |  |  |
| 1 | Ath-AT1G66070.2 |  | | | |  |  |  |  |  |  |  |
| 1 | Ath-AT1G66080.1 |  | | | |  |  |  |  |  |  |  |
| 1 | Ath-AT1G66090.1 |  | | | |  |  |  |  |  |  |  |
| 1 | Ath-AT1G66100.1 |  | | | |  |  |  |  |  |  |  |
| 1 | Ath-AT1G66110.1 |  | | | |  |  |  |  |  |  |  |
| 1 | Ath-AT1G66120.1 |  | Vvi-Vitvi04g00890\_t001 |  |  |  |  |  |  |  |
| 0 | Ath-AT1G66130.1 |  |  |  |  |  |  |  |  |
| 0 | Ath-AT1G66140.1 |  |  |  |  |  |  |  |  |
| 0 | Ath-AT1G66145.1 |  |  |  |  |  |  |  |  |
| 0 | Ath-AT1G66150.1 |  |  |  |  |  |  |  |  |
| 0 | Ath-AT1G66160.1 |  |  |  |  |  |  |  |  |
| 1 | Ath-AT1G66170.1 |  | Vvi-Vitvi14g00762\_t001 |  |  |  |  |  |  |  |
| 1 | Ath-AT1G66180.1 |  | Vvi-Vitvi14g00772\_t001 |  |  |  |  |  |  |  |
| 1 | Ath-AT1G66190.1 |  | | | |  |  |  |  |  |  |  |
| 1 | Ath-AT1G66200.3 |  | Vvi-Vitvi14g00791\_t001 |  |  |  |  |  |  |  |
| 1 | Ath-AT1G66210.1 |  | | | |  |  |  |  |  |  |  |
| 1 | Ath-AT1G66220.1 |  | | | |  |  |  |  |  |  |  |
| 1 | Ath-AT1G66230.1 |  | Vvi-Vitvi14g02430\_t001 |  |  |  |  |  |  |  |
| 1 | Ath-AT1G66235.1 |  | | | |  |  |  |  |  |  |  |
| 1 | Ath-AT1G66240.1 |  | | | |  |  |  |  |  |  |  |
| 1 | Ath-AT1G66245.1 |  | | | |  |  |  |  |  |  |  |
| 1 | Ath-AT1G66250.1 |  | Vvi-Vitvi14g00821\_t001 |  |  |  |  |  |  |  |
| 2 | Ath-AT1G66260.1 |  | | | |  | Vvi-Vitvi14g00893\_t001 |  |  |  |  |  |  |
| 2 | Ath-AT1G66270.1 |  | | | |  | | | |  |  |  |  |  |  |
| 2 | Ath-AT1G66280.1 |  | | | |  | | | |  |  |  |  |  |  |
| 2 | Ath-AT1G66290.1 |  | | | |  | | | |  |  |  |  |  |  |
| 2 | Ath-AT1G66300.1 |  | | | |  | | | |  |  |  |  |  |  |
| 2 | Ath-AT1G66310.1 |  | | | |  | | | |  |  |  |  |  |  |
| 2 | Ath-AT1G66320.1 |  | | | |  | | | |  |  |  |  |  |  |
| 2 | Ath-AT1G66330.1 |  | | | |  | | | |  |  |  |  |  |  |
| 2 | Ath-AT1G66340.1 |  | | | |  | | | |  |  |  |  |  |  |
| 2 | Ath-AT1G66345.1 |  | | | |  | | | |  |  |  |  |  |  |
| 2 | Ath-AT1G66350.1 |  | Vvi-Vitvi14g00841\_t001 |  | | | |  |  |  |  |  |  |
| 1 | Ath-AT1G66360.1 |  |  |  | Vvi-Vitvi14g00920\_t001 |  |  |  |  |  |  |
| 1 | Ath-AT1G66370.1 |  |  |  | Vvi-Vitvi14g00940\_t001 |  |  |  |  |  |  |
| 1 | Ath-AT1G66380.1 |  |  |  | | | |  |  |  |  |  |  |
| 1 | Ath-AT1G66390.1 |  |  |  | | | |  |  |  |  |  |  |
| 1 | Ath-AT1G66400.1 |  |  |  | Vvi-Vitvi14g00949\_t001 |  |  |  |  |  |  |
| 1 | Ath-AT1G66410.2 |  |  |  | | | |  |  |  |  |  |  |
| 1 | Ath-AT1G66420.1 |  |  |  | | | |  |  |  |  |  |  |
| 1 | Ath-AT1G66430.1 |  |  |  | Vvi-Vitvi14g00950\_t001 |  |  |  |  |  |  |
| 1 | Ath-AT1G66440.1 |  |  |  | | | |  |  |  |  |  |  |
| 1 | Ath-AT1G66450.2 |  |  |  | | | |  |  |  |  |  |  |
| 1 | Ath-AT1G66465.1 |  |  |  | | | |  |  |  |  |  |  |
| 1 | Ath-AT1G66460.3 |  |  |  | Vvi-Vitvi14g00952\_t001 |  |  |  |  |  |  |
| 1 | Ath-AT1G66470.1 |  |  |  | Vvi-Vitvi14g00958\_t001 |  |  |  |  |  |  |
| 1 | Ath-AT1G66480.1 |  |  |  | Vvi-Vitvi14g00973\_t001 |  |  |  |  |  |  |
| 1 | Ath-AT1G66490.1 |  |  |  | | | |  |  |  |  |  |  |
| 1 | Ath-AT1G66500.1 |  |  |  | | | |  |  |  |  |  |  |
| 1 | Ath-AT1G66510.4 |  |  |  | Vvi-Vitvi14g00992\_t001 |  |  |  |  |  |  |
| 1 | Ath-AT1G66520.1 |  |  |  | Vvi-Vitvi14g00999\_t001 |  |  |  |  |  |  |
| 1 | Ath-AT1G66530.1 |  |  |  | | | |  |  |  |  |  |  |
| 1 | Ath-AT1G66540.1 |  |  |  | | | |  |  |  |  |  |  |
| 1 | Ath-AT1G66550.1 |  |  |  | | | |  |  |  |  |  |  |
| 1 | Ath-AT1G66560.1 |  |  |  | | | |  |  |  |  |  |  |
| 1 | Ath-AT1G66570.1 |  |  |  | | | |  |  |  |  |  |  |
| 1 | Ath-AT1G66580.1 |  |  |  | | | |  |  |  |  |  |  |
| 1 | Ath-AT1G66590.2 |  |  |  | | | |  |  |  |  |  |  |
| 1 | Ath-AT1G66600.1 |  |  |  | | | |  |  |  |  |  |  |
| 1 | Ath-AT1G66610.1 |  |  |  | | | |  |  |  |  |  |  |
| 1 | Ath-AT1G66620.1 |  |  |  | Vvi-Vitvi14g01003\_t002 |  |  |  |  |  |  |
| 1 | Ath-AT1G66630.1 |  |  |  | | | |  |  |  |  |  |  |
| 1 | Ath-AT1G66640.1 |  |  |  | | | |  |  |  |  |  |  |
| 1 | Ath-AT1G66650.1 |  |  |  | | | |  |  |  |  |  |  |
| 1 | Ath-AT1G66660.2 |  |  |  | | | |  |  |  |  |  |  |
| 1 | Ath-AT1G66670.1 |  |  |  | Vvi-Vitvi14g01006\_t001 |  |  |  |  |  |  |
| 1 | Ath-AT1G66680.1 |  |  |  | Vvi-Vitvi14g01035\_t001 |  |  |  |  |  |  |
| 1 | Ath-AT1G66690.1 |  |  |  | | | |  |  |  |  |  |  |
| 1 | Ath-AT1G66700.1 |  |  |  | | | |  |  |  |  |  |  |
| 1 | Ath-AT1G66720.1 |  |  |  | | | |  |  |  |  |  |  |
| 1 | Ath-AT1G66730.1 |  |  |  | Vvi-Vitvi14g01055\_t001 |  |  |  |  |  |  |
| 1 | Ath-AT1G66740.1 |  |  |  | Vvi-Vitvi14g01057\_t001 |  |  |  |  |  |  |
| 1 | Ath-AT1G66750.1 |  |  |  | Vvi-Vitvi14g01066\_t001 |  |  |  |  |  |  |
| 1 | Ath-AT1G66760.2 |  |  |  | | | |  |  |  |  |  |  |
| 1 | Ath-AT1G66770.1 |  |  |  | | | |  |  |  |  |  |  |
| 1 | Ath-AT1G66780.1 |  |  |  | | | |  |  |  |  |  |  |
| 1 | Ath-AT1G66800.1 |  |  |  | | | |  |  |  |  |  |  |
| 2 | Ath-AT1G66810.3 |  | Vvi-Vitvi01g00347\_t001 |  | Vvi-Vitvi14g01114\_t001 |  |  |  |  |  |  |
| 2 | Ath-AT1G66820.1 |  | | | |  | | | |  |  |  |  |  |  |
| 2 | Ath-AT1G66830.1 |  | Vvi-Vitvi01g00335\_t001 |  | | | |  |  |  |  |  |  |
| 2 | Ath-AT1G66840.1 |  | | | |  | Vvi-Vitvi14g01124\_t001 |  |  |  |  |  |  |
| 2 | Ath-AT1G66850.1 |  | | | |  | Vvi-Vitvi14g01128\_t001 |  |  |  |  |  |  |
| 2 | Ath-AT1G66852.1 |  | | | |  | | | |  |  |  |  |  |  |
| 2 | Ath-AT1G66855.1 |  | | | |  | | | |  |  |  |  |  |  |
| 2 | Ath-AT1G66860.1 |  | Vvi-Vitvi01g00329\_t001 |  | Vvi-Vitvi14g04419\_t001 |  |  |  |  |  |  |
| 2 | Ath-AT1G66870.2 |  | | | |  | | | |  |  |  |  |  |  |
| 3 | Ath-AT1G66880.1 |  | Vvi-Vitvi01g04083\_t002 |  | | | |  | Vvi-Vitvi17g01392\_t001 |  |  |  |  |  |
| 3 | Ath-AT1G66890.1 |  | Vvi-Vitvi01g01949\_t001 |  | Vvi-Vitvi14g01139\_t001 |  | | | |  |  |  |  |  |
| 3 | Ath-AT1G66900.1 |  | | | |  | Vvi-Vitvi14g01144\_t001 |  | | | |  |  |  |  |  |
| 3 | Ath-AT1G66910.1 |  | | | |  | Vvi-Vitvi14g04420\_t001 |  | Vvi-Vitvi17g01394\_t001 |  |  |  |  |  |
| 3 | Ath-AT1G66920.2 |  | | | |  | | | |  | | | |  |  |  |  |  |
| 3 | Ath-AT1G66930.2 |  | | | |  | | | |  | | | |  |  |  |  |  |
| 3 | Ath-AT1G66940.4 |  | | | |  | | | |  | | | |  |  |  |  |  |
| 3 | Ath-AT1G66950.1 |  | | | |  | | | |  | | | |  |  |  |  |  |
| 3 | Ath-AT1G66960.2 |  | | | |  | | | |  | | | |  |  |  |  |  |
| 3 | Ath-AT1G66970.2 |  | | | |  | | | |  | | | |  |  |  |  |  |
| 3 | Ath-AT1G66980.2 |  | | | |  | | | |  | | | |  |  |  |  |  |
| 3 | Ath-AT1G67000.1 |  | | | |  | Vvi-Vitvi14g04425\_t001 |  | | | |  |  |  |  |  |
| 3 | Ath-AT1G67020.1 |  | | | |  | | | |  | | | |  |  |  |  |  |
| 3 | Ath-AT1G67025.1 |  | | | |  | | | |  | | | |  |  |  |  |  |
| 3 | Ath-AT1G67030.1 |  | Vvi-Vitvi01g00309\_t001 |  | Vvi-Vitvi14g02872\_t001 |  | | | |  |  |  |  |  |
| 3 | Ath-AT1G67035.2 |  | | | |  | Vvi-Vitvi14g01161\_t001 |  | | | |  |  |  |  |  |
| 3 | Ath-AT1G67040.2 |  | | | |  | Vvi-Vitvi14g01162\_t001 |  | | | |  |  |  |  |  |
| 3 | Ath-AT1G67050.1 |  | Vvi-Vitvi01g00305\_t001 |  | Vvi-Vitvi14g01164\_t001 |  | Vvi-Vitvi17g00300\_t001 |  |  |  |  |  |
| 3 | Ath-AT1G67060.1 |  | | | |  | Vvi-Vitvi14g01176\_t001 |  | Vvi-Vitvi17g00304\_t001 |  |  |  |  |  |
| 3 | Ath-AT1G67070.1 |  | Vvi-Vitvi01g04074\_t001 |  | Vvi-Vitvi14g01178\_t001 |  | | | |  |  |  |  |  |
| 3 | Ath-AT1G67080.1 |  | | | |  | Vvi-Vitvi14g01185\_t001 |  | | | |  |  |  |  |  |
| 3 | Ath-AT1G67090.1 |  | | | |  | | | |  | Vvi-Vitvi17g00320\_t001 |  |  |  |  |  |
| 3 | Ath-AT1G67100.1 |  | Vvi-Vitvi01g00290\_t001 |  | Vvi-Vitvi14g01193\_t001 |  | Vvi-Vitvi17g00325\_t001 |  |  |  |  |  |
| 2 | Ath-AT1G67110.1 |  | | | |  | Vvi-Vitvi14g01195\_t001 |  |  |  |  |  |  |
| 2 | Ath-AT1G67120.2 |  | | | |  | | | |  |  |  |  |  |  |
| 2 | Ath-AT1G67130.1 |  | | | |  | | | |  |  |  |  |  |  |
| 2 | Ath-AT1G67140.3 |  | | | |  | Vvi-Vitvi14g01221\_t001 |  |  |  |  |  |  |
| 2 | Ath-AT1G67148.1 |  | | | |  | | | |  |  |  |  |  |  |
| 2 | Ath-AT1G67150.2 |  | | | |  | | | |  |  |  |  |  |  |
| 2 | Ath-AT1G67160.1 |  | | | |  | | | |  |  |  |  |  |  |
| 2 | Ath-AT1G67170.1 |  | Vvi-Vitvi01g00287\_t001 |  | | | |  |  |  |  |  |  |
| 1 | Ath-AT1G67180.2 |  |  |  | | | |  |  |  |  |  |  |
| 1 | Ath-AT1G67190.2 |  |  |  | | | |  |  |  |  |  |  |
| 1 | Ath-AT1G67210.1 |  |  |  | | | |  |  |  |  |  |  |
| 1 | Ath-AT1G67220.1 |  |  |  | Vvi-Vitvi14g01230\_t001 |  |  |  |  |  |  |
| 1 | Ath-AT1G67230.1 |  |  |  | Vvi-Vitvi14g01232\_t001 |  |  |  |  |  |  |
| 1 | Ath-AT1G67235.1 |  |  |  | | | |  |  |  |  |  |  |
| 1 | Ath-AT1G67250.1 |  |  |  | | | |  |  |  |  |  |  |
| 1 | Ath-AT1G67260.1 |  |  |  | Vvi-Vitvi14g01253\_t001 |  |  |  |  |  |  |
| 1 | Ath-AT1G67265.1 |  |  |  | | | |  |  |  |  |  |  |
| 1 | Ath-AT1G67270.1 |  |  |  | Vvi-Vitvi14g01258\_t001 |  |  |  |  |  |  |
| 0 | Ath-AT1G67280.1 |  |  |  |  |  |  |  |  |
| 0 | Ath-AT1G67290.1 |  |  |  |  |  |  |  |  |
| 0 | Ath-AT1G67300.2 |  |  |  |  |  |  |  |  |
| 1 | Ath-AT1G67310.1 |  | Vvi-Vitvi01g01671\_t001 |  |  |  |  |  |  |  |
| 1 | Ath-AT1G67320.3 |  | | | |  |  |  |  |  |  |  |
| 1 | Ath-AT1G67325.2 |  | Vvi-Vitvi01g01659\_t002 |  |  |  |  |  |  |  |
| 1 | Ath-AT1G67330.1 |  | Vvi-Vitvi01g01655\_t001 |  |  |  |  |  |  |  |
| 1 | Ath-AT1G67340.1 |  | Vvi-Vitvi01g01650\_t001 |  |  |  |  |  |  |  |
| 1 | Ath-AT1G67350.1 |  | Vvi-Vitvi01g02269\_t001 |  |  |  |  |  |  |  |
| 1 | Ath-AT1G67360.2 |  | Vvi-Vitvi01g02268\_t001 |  |  |  |  |  |  |  |
| 1 | Ath-AT1G67370.1 |  | Vvi-Vitvi01g01646\_t001 |  |  |  |  |  |  |  |
| 1 | Ath-AT1G67390.1 |  | | | |  |  |  |  |  |  |  |
| 1 | Ath-AT1G67400.1 |  | Vvi-Vitvi01g01640\_t001 |  |  |  |  |  |  |  |
| 1 | Ath-AT1G67410.1 |  | Vvi-Vitvi01g04432\_t001 |  |  |  |  |  |  |  |
| 1 | Ath-AT1G67420.4 |  | Vvi-Vitvi01g01629\_t001 |  |  |  |  |  |  |  |
| 1 | Ath-AT1G67430.1 |  | Vvi-Vitvi01g02258\_t004 |  |  |  |  |  |  |  |
| 1 | Ath-AT1G67440.2 |  | Vvi-Vitvi01g01624\_t001 |  |  |  |  |  |  |  |
| 1 | Ath-AT1G67450.1 |  | | | |  |  |  |  |  |  |  |
| 1 | Ath-AT1G67455.1 |  | | | |  |  |  |  |  |  |  |
| 1 | Ath-AT1G67460.1 |  | | | |  |  |  |  |  |  |  |
| 1 | Ath-AT1G67470.1 |  | | | |  |  |  |  |  |  |  |
| 1 | Ath-AT1G67480.2 |  | Vvi-Vitvi01g01621\_t001 |  |  |  |  |  |  |  |
| 1 | Ath-AT1G67490.1 |  | Vvi-Vitvi01g01618\_t001 |  |  |  |  |  |  |  |
| 0 | Ath-AT1G67500.2 |  |  |  |  |  |  |  |  |
| 1 | Ath-AT1G67510.1 |  | Vvi-Vitvi01g01578\_t001 |  |  |  |  |  |  |  |
| 1 | Ath-AT1G67520.6 |  | | | |  |  |  |  |  |  |  |
| 1 | Ath-AT1G67530.1 |  | Vvi-Vitvi01g01576\_t001 |  |  |  |  |  |  |  |
| 1 | Ath-AT1G67540.2 |  | Vvi-Vitvi01g01569\_t001 |  |  |  |  |  |  |  |
| 1 | Ath-AT1G67550.1 |  | Vvi-Vitvi01g01563\_t001 |  |  |  |  |  |  |  |
| 1 | Ath-AT1G67560.1 |  | Vvi-Vitvi01g01562\_t001 |  |  |  |  |  |  |  |
| 2 | Ath-AT1G67570.1 |  | | | |  | Vvi-Vitvi01g01543\_t001 |  |  |  |  |  |  |
| 2 | Ath-AT1G67580.1 |  | | | |  | Vvi-Vitvi01g01546\_t001 |  |  |  |  |  |  |
| 2 | Ath-AT1G67590.1 |  | | | |  | Vvi-Vitvi01g01547\_t001 |  |  |  |  |  |  |
| 2 | Ath-AT1G67600.1 |  | | | |  | Vvi-Vitvi01g01549\_t001 |  |  |  |  |  |  |
| 2 | Ath-AT1G67620.1 |  | | | |  | Vvi-Vitvi01g01554\_t001 |  |  |  |  |  |  |
| 2 | Ath-AT1G67623.1 |  | | | |  | Vvi-Vitvi01g04388\_t001 |  |  |  |  |  |  |
| 2 | Ath-AT1G67630.1 |  | Vvi-Vitvi01g01559\_t001 |  | | | |  |  |  |  |  |  |
| 2 | Ath-AT1G67635.1 |  | | | |  | | | |  |  |  |  |  |  |
| 2 | Ath-AT1G67640.1 |  | Vvi-Vitvi01g01541\_t001 |  | Vvi-Vitvi01g01555\_t001 |  |  |  |  |  |  |
| 1 | Ath-AT1G67650.1 |  | | | |  |  |  |  |  |  |  |
| 1 | Ath-AT1G67660.1 |  | Vvi-Vitvi01g01532\_t002 |  |  |  |  |  |  |  |
| 1 | Ath-AT1G67670.1 |  | Vvi-Vitvi01g04382\_t001 |  |  |  |  |  |  |  |
| 1 | Ath-AT1G67680.1 |  | | | |  |  |  |  |  |  |  |
| 1 | Ath-AT1G67690.1 |  | Vvi-Vitvi01g01523\_t001 |  |  |  |  |  |  |  |
| 1 | Ath-AT1G67700.2 |  | Vvi-Vitvi01g01510\_t001 |  |  |  |  |  |  |  |
| 1 | Ath-AT1G67710.1 |  | Vvi-Vitvi01g01506\_t001 |  |  |  |  |  |  |  |
| 1 | Ath-AT1G67720.1 |  | Vvi-Vitvi01g01504\_t001 |  |  |  |  |  |  |  |
| 1 | Ath-AT1G67730.1 |  | Vvi-Vitvi01g00580\_t001 |  |  |  |  |  |  |  |
| 1 | Ath-AT1G67740.1 |  | Vvi-Vitvi01g00589\_t001 |  |  |  |  |  |  |  |
| 2 | Ath-AT1G67750.1 |  | Vvi-Vitvi01g00593\_t001 |  | Vvi-Vitvi14g01635\_t001 |  |  |  |  |  |  |
| 2 | Ath-AT1G67760.1 |  | | | |  | | | |  |  |  |  |  |  |
| 2 | Ath-AT1G67770.1 |  | Vvi-Vitvi01g00602\_t001 |  | Vvi-Vitvi14g01637\_t001 |  |  |  |  |  |  |
| 2 | Ath-AT1G67775.1 |  | | | |  | | | |  |  |  |  |  |  |
| 2 | Ath-AT1G67780.2 |  | | | |  | | | |  |  |  |  |  |  |
| 2 | Ath-AT1G67785.1 |  | Vvi-Vitvi01g00604\_t001 |  | | | |  |  |  |  |  |  |
| 2 | Ath-AT1G67790.1 |  | Vvi-Vitvi01g00609\_t001 |  | Vvi-Vitvi14g01641\_t001 |  |  |  |  |  |  |
| 2 | Ath-AT1G67800.2 |  | Vvi-Vitvi01g00614\_t001 |  | | | |  |  |  |  |  |  |
| 2 | Ath-AT1G67810.1 |  | Vvi-Vitvi01g00632\_t001 |  | | | |  |  |  |  |  |  |
| 2 | Ath-AT1G67820.1 |  | | | |  | | | |  |  |  |  |  |  |
| 2 | Ath-AT1G67830.1 |  | Vvi-Vitvi01g00634\_t001 |  | Vvi-Vitvi14g04610\_t001 |  |  |  |  |  |  |
| 2 | Ath-AT1G67840.1 |  | Vvi-Vitvi01g00640\_t001 |  | | | |  |  |  |  |  |  |
| 2 | Ath-AT1G67850.1 |  | Vvi-Vitvi01g00641\_t001 |  | Vvi-Vitvi14g01672\_t001 |  |  |  |  |  |  |
| 2 | Ath-AT1G67855.1 |  | | | |  | | | |  |  |  |  |  |  |
| 2 | Ath-AT1G67856.1 |  | Vvi-Vitvi01g02030\_t001 |  | | | |  |  |  |  |  |  |
| 2 | Ath-AT1G67860.1 |  | | | |  | | | |  |  |  |  |  |  |
| 2 | Ath-AT1G67865.1 |  | | | |  | | | |  |  |  |  |  |  |
| 2 | Ath-AT1G67870.1 |  | | | |  | | | |  |  |  |  |  |  |
| 2 | Ath-AT1G67880.1 |  | Vvi-Vitvi01g00642\_t001 |  | Vvi-Vitvi14g01676\_t002 |  |  |  |  |  |  |
| 1 | Ath-AT1G67890.1 |  | | | |  |  |  |  |  |  |  |
| 1 | Ath-AT1G67900.2 |  | Vvi-Vitvi01g00644\_t001 |  |  |  |  |  |  |  |
| 1 | Ath-AT1G67910.2 |  | Vvi-Vitvi01g04155\_t001 |  |  |  |  |  |  |  |
| 0 | Ath-AT1G67920.1 |  |  |  |  |  |  |  |  |
| 0 | Ath-AT1G67930.1 |  |  |  |  |  |  |  |  |
| 0 | Ath-AT1G67940.1 |  |  |  |  |  |  |  |  |
| 1 | Ath-AT1G67950.2 |  | Vvi-Vitvi01g00518\_t001 |  |  |  |  |  |  |  |
| 1 | Ath-AT1G67960.1 |  | Vvi-Vitvi01g00520\_t001 |  |  |  |  |  |  |  |
| 2 | Ath-AT1G67970.1 |  | | | |  | Vvi-Vitvi01g00511\_t001 |  |  |  |  |  |  |
| 2 | Ath-AT1G67980.1 |  | | | |  | | | |  |  |  |  |  |  |
| 2 | Ath-AT1G67990.1 |  | | | |  | | | |  |  |  |  |  |  |
| 2 | Ath-AT1G68000.1 |  | | | |  | | | |  |  |  |  |  |  |
| 2 | Ath-AT1G68010.2 |  | | | |  | | | |  |  |  |  |  |  |
| 2 | Ath-AT1G68020.2 |  | | | |  | Vvi-Vitvi01g00509\_t001 |  |  |  |  |  |  |
| 2 | Ath-AT1G68030.1 |  | | | |  | | | |  |  |  |  |  |  |
| 2 | Ath-AT1G68040.1 |  | | | |  | Vvi-Vitvi01g01997\_t001 |  |  |  |  |  |  |
| 2 | Ath-AT1G68050.1 |  | | | |  | Vvi-Vitvi01g00499\_t001 |  |  |  |  |  |  |
| 2 | Ath-AT1G68060.1 |  | | | |  | Vvi-Vitvi01g00497\_t001 |  |  |  |  |  |  |
| 2 | Ath-AT1G68070.1 |  | | | |  | Vvi-Vitvi01g00492\_t001 |  |  |  |  |  |  |
| 1 | Ath-AT1G68080.1 |  | Vvi-Vitvi01g00538\_t001 |  |  |  |  |  |  |  |
| 1 | Ath-AT1G68090.1 |  | Vvi-Vitvi01g00540\_t001 |  |  |  |  |  |  |  |
| 1 | Ath-AT1G68100.1 |  | Vvi-Vitvi01g00544\_t001 |  |  |  |  |  |  |  |
| 1 | Ath-AT1G68110.1 |  | Vvi-Vitvi01g00546\_t001 |  |  |  |  |  |  |  |
| 1 | Ath-AT1G68120.1 |  | Vvi-Vitvi01g00547\_t001 |  |  |  |  |  |  |  |
| 1 | Ath-AT1G68130.1 |  | Vvi-Vitvi01g00552\_t001 |  |  |  |  |  |  |  |
| 0 | Ath-AT1G68140.1 |  |  |  |  |  |  |  |  |
| 0 | Ath-AT1G68150.1 |  |  |  |  |  |  |  |  |
| 0 | Ath-AT1G68160.1 |  |  |  |  |  |  |  |  |
| 0 | Ath-AT1G68170.2 |  |  |  |  |  |  |  |  |
| 0 | Ath-AT1G68180.1 |  |  |  |  |  |  |  |  |
| 0 | Ath-AT1G68185.2 |  |  |  |  |  |  |  |  |
| 0 | Ath-AT1G68190.2 |  |  |  |  |  |  |  |  |
| 0 | Ath-AT1G68200.1 |  |  |  |  |  |  |  |  |
| 0 | Ath-AT1G68210.1 |  |  |  |  |  |  |  |  |
| 0 | Ath-AT1G68220.1 |  |  |  |  |  |  |  |  |
| 0 | Ath-AT1G68230.2 |  |  |  |  |  |  |  |  |
| 0 | Ath-AT1G68238.1 |  |  |  |  |  |  |  |  |
| 0 | Ath-AT1G68240.1 |  |  |  |  |  |  |  |  |
| 0 | Ath-AT1G68250.1 |  |  |  |  |  |  |  |  |
| 1 | Ath-AT1G68260.1 |  | Vvi-Vitvi01g04072\_t001 |  |  |  |  |  |  |  |
| 1 | Ath-AT1G68270.1 |  | | | |  |  |  |  |  |  |  |
| 1 | Ath-AT1G68280.1 |  | | | |  |  |  |  |  |  |  |
| 2 | Ath-AT1G68290.1 |  | | | |  | Vvi-Vitvi01g00300\_t001 |  |  |  |  |  |  |
| 2 | Ath-AT1G68300.1 |  | | | |  | Vvi-Vitvi01g01942\_t001 |  |  |  |  |  |  |
| 2 | Ath-AT1G68310.1 |  | | | |  | | | |  |  |  |  |  |  |
| 3 | Ath-AT1G68320.1 |  | | | |  | Vvi-Vitvi01g00302\_t001 |  | Vvi-Vitvi17g00309\_t001 |  |  |  |  |  |
| 3 | Ath-AT1G68325.1 |  | | | |  | Vvi-Vitvi01g00303\_t001 |  | | | |  |  |  |  |  |
| 4 | Ath-AT1G68330.1 |  | | | |  | Vvi-Vitvi01g00305\_t001 |  | | | |  | Vvi-Vitvi14g01164\_t001 |  |  |  |  |
| 4 | Ath-AT1G68340.1 |  | | | |  | Vvi-Vitvi01g00306\_t002 |  | | | |  | | | |  |  |  |  |
| 4 | Ath-AT1G68350.1 |  | | | |  | Vvi-Vitvi01g04076\_t001 |  | | | |  | | | |  |  |  |  |
| 4 | Ath-AT1G68360.1 |  | | | |  | Vvi-Vitvi01g00309\_t001 |  | | | |  | | | |  |  |  |  |
| 4 | Ath-AT1G68370.1 |  | | | |  | Vvi-Vitvi01g00310\_t001 |  | | | |  | | | |  |  |  |  |
| 4 | Ath-AT1G68380.1 |  | | | |  | Vvi-Vitvi01g00317\_t001 |  | | | |  | | | |  |  |  |  |
| 4 | Ath-AT1G68390.1 |  | | | |  | | | |  | | | |  | | | |  |  |  |  |
| 4 | Ath-AT1G68400.1 |  | | | |  | Vvi-Vitvi01g00318\_t001 |  | | | |  | | | |  |  |  |  |
| 4 | Ath-AT1G68410.1 |  | | | |  | Vvi-Vitvi01g00325\_t001 |  | | | |  | | | |  |  |  |  |
| 4 | Ath-AT1G68420.1 |  | | | |  | | | |  | | | |  | | | |  |  |  |  |
| 4 | Ath-AT1G68430.1 |  | | | |  | Vvi-Vitvi01g01949\_t001 |  | | | |  | | | |  |  |  |  |
| 3 | Ath-AT1G68440.1 |  | Vvi-Vitvi01g00299\_t001 |  |  |  | | | |  | | | |  |  |  |  |
| 3 | Ath-AT1G68450.1 |  | Vvi-Vitvi01g01941\_t001 |  |  |  | Vvi-Vitvi17g01402\_t001 |  | | | |  |  |  |  |
| 3 | Ath-AT1G68460.1 |  | Vvi-Vitvi01g00298\_t001 |  |  |  | | | |  | | | |  |  |  |  |
| 3 | Ath-AT1G68470.1 |  | | | |  |  |  | | | |  | | | |  |  |  |  |
| 3 | Ath-AT1G68480.1 |  | Vvi-Vitvi01g01939\_t001 |  |  |  | | | |  | | | |  |  |  |  |
| 3 | Ath-AT1G68490.1 |  | Vvi-Vitvi01g01938\_t001 |  |  |  | Vvi-Vitvi17g04085\_t001 |  | Vvi-Vitvi14g01188\_t001 |  |  |  |  |
| 3 | Ath-AT1G68500.1 |  | Vvi-Vitvi01g01937\_t001 |  |  |  | | | |  | | | |  |  |  |  |
| 3 | Ath-AT1G68510.1 |  | Vvi-Vitvi01g00290\_t001 |  |  |  | Vvi-Vitvi17g00325\_t001 |  | Vvi-Vitvi14g01193\_t001 |  |  |  |  |
| 3 | Ath-AT1G68520.1 |  | Vvi-Vitvi01g00288\_t001 |  |  |  | Vvi-Vitvi17g00328\_t001 |  | | | |  |  |  |  |
| 3 | Ath-AT1G68526.1 |  | | | |  |  |  | | | |  | | | |  |  |  |  |
| 3 | Ath-AT1G68530.1 |  | Vvi-Vitvi01g00284\_t001 |  |  |  | | | |  | Vvi-Vitvi14g01198\_t001 |  |  |  |  |
| 3 | Ath-AT1G68540.1 |  | Vvi-Vitvi01g00282\_t001 |  |  |  | | | |  | | | |  |  |  |  |
| 3 | Ath-AT1G68550.2 |  | Vvi-Vitvi01g01827\_t001 |  |  |  | Vvi-Vitvi17g00329\_t001 |  | | | |  |  |  |  |
| 3 | Ath-AT1G68560.1 |  | Vvi-Vitvi01g00280\_t001 |  |  |  | | | |  | | | |  |  |  |  |
| 3 | Ath-AT1G68570.1 |  | Vvi-Vitvi01g00277\_t001 |  |  |  | | | |  | | | |  |  |  |  |
| 3 | Ath-AT1G68580.2 |  | Vvi-Vitvi01g00273\_t001 |  |  |  | | | |  | | | |  |  |  |  |
| 3 | Ath-AT1G68585.1 |  | Vvi-Vitvi01g01932\_t001 |  |  |  | Vvi-Vitvi17g01411\_t001 |  | | | |  |  |  |  |
| 3 | Ath-AT1G68590.1 |  | Vvi-Vitvi01g00268\_t001 |  |  |  | | | |  | Vvi-Vitvi14g01212\_t001 |  |  |  |  |
| 3 | Ath-AT1G68600.1 |  | Vvi-Vitvi01g00266\_t001 |  |  |  | Vvi-Vitvi17g00333\_t001 |  | | | |  |  |  |  |
| 3 | Ath-AT1G68610.1 |  | | | |  |  |  | | | |  | | | |  |  |  |  |
| 3 | Ath-AT1G68620.1 |  | Vvi-Vitvi01g00264\_t001 |  |  |  | | | |  | Vvi-Vitvi14g01222\_t001 |  |  |  |  |
| 3 | Ath-AT1G68630.1 |  | Vvi-Vitvi01g00261\_t001 |  |  |  | | | |  | | | |  |  |  |  |
| 3 | Ath-AT1G68640.1 |  | Vvi-Vitvi01g00260\_t001 |  |  |  | | | |  | | | |  |  |  |  |
| 3 | Ath-AT1G68650.1 |  | Vvi-Vitvi01g00255\_t002 |  |  |  | | | |  | | | |  |  |  |  |
| 3 | Ath-AT1G68660.1 |  | Vvi-Vitvi01g00254\_t001 |  |  |  | Vvi-Vitvi17g00355\_t001 |  | | | |  |  |  |  |
| 3 | Ath-AT1G68670.1 |  | Vvi-Vitvi01g00249\_t001 |  |  |  | | | |  | | | |  |  |  |  |
| 2 | Ath-AT1G68680.1 |  |  |  |  |  | | | |  | | | |  |  |  |  |
| 2 | Ath-AT1G68690.2 |  |  |  |  |  | | | |  | | | |  |  |  |  |
| 3 | Ath-AT1G68700.1 |  | Vvi-Vitvi01g00197\_t001 |  |  |  | | | |  | | | |  |  |  |  |
| 3 | Ath-AT1G68710.3 |  | Vvi-Vitvi01g00199\_t001 |  |  |  | | | |  | | | |  |  |  |  |
| 3 | Ath-AT1G68720.1 |  | Vvi-Vitvi01g00201\_t001 |  |  |  | | | |  | | | |  |  |  |  |
| 3 | Ath-AT1G68723.1 |  | | | |  |  |  | | | |  | | | |  |  |  |  |
| 3 | Ath-AT1G68725.1 |  | | | |  |  |  | | | |  | | | |  |  |  |  |
| 3 | Ath-AT1G68730.1 |  | Vvi-Vitvi01g00202\_t001 |  |  |  | | | |  | | | |  |  |  |  |
| 3 | Ath-AT1G68740.1 |  | Vvi-Vitvi01g00203\_t001 |  |  |  | | | |  | | | |  |  |  |  |
| 3 | Ath-AT1G68750.1 |  | Vvi-Vitvi01g00214\_t001 |  |  |  | | | |  | | | |  |  |  |  |
| 3 | Ath-AT1G68760.1 |  | Vvi-Vitvi01g04058\_t001 |  |  |  | | | |  | | | |  |  |  |  |
| 3 | Ath-AT1G68765.1 |  | | | |  |  |  | | | |  | | | |  |  |  |  |
| 3 | Ath-AT1G68780.1 |  | Vvi-Vitvi01g00218\_t001 |  |  |  | | | |  | | | |  |  |  |  |
| 3 | Ath-AT1G68790.1 |  | Vvi-Vitvi01g00219\_t001 |  |  |  | | | |  | Vvi-Vitvi14g01232\_t001 |  |  |  |  |
| 3 | Ath-AT1G68795.1 |  | Vvi-Vitvi01g01926\_t001 |  |  |  | | | |  | | | |  |  |  |  |
| 3 | Ath-AT1G68800.2 |  | Vvi-Vitvi01g00228\_t001 |  |  |  | Vvi-Vitvi17g00374\_t001 |  | | | |  |  |  |  |
| 2 | Ath-AT1G68810.1 |  | Vvi-Vitvi01g00232\_t001 |  |  |  |  |  | | | |  |  |  |  |
| 2 | Ath-AT1G68820.4 |  | Vvi-Vitvi01g00233\_t001 |  |  |  |  |  | | | |  |  |  |  |
| 2 | Ath-AT1G68825.1 |  | | | |  |  |  |  |  | | | |  |  |  |  |
| 2 | Ath-AT1G68830.1 |  | Vvi-Vitvi01g00238\_t001 |  |  |  |  |  | | | |  |  |  |  |
| 2 | Ath-AT1G68840.1 |  | Vvi-Vitvi01g00244\_t001 |  |  |  |  |  | Vvi-Vitvi14g01248\_t001 |  |  |  |  |
| 0 | Ath-AT1G68845.1 |  |  |  |  |  |  |  |  |
| 1 | Ath-AT1G68850.1 |  | Vvi-Vitvi01g01359\_t001 |  |  |  |  |  |  |  |
| 1 | Ath-AT1G68862.2 |  | | | |  |  |  |  |  |  |  |
| 1 | Ath-AT1G68870.1 |  | | | |  |  |  |  |  |  |  |
| 1 | Ath-AT1G68875.1 |  | | | |  |  |  |  |  |  |  |
| 1 | Ath-AT1G68877.1 |  | | | |  |  |  |  |  |  |  |
| 1 | Ath-AT1G68880.1 |  | | | |  |  |  |  |  |  |  |
| 1 | Ath-AT1G68890.1 |  | Vvi-Vitvi01g01331\_t001 |  |  |  |  |  |  |  |
| 1 | Ath-AT1G68905.1 |  | | | |  |  |  |  |  |  |  |
| 1 | Ath-AT1G68907.1 |  | | | |  |  |  |  |  |  |  |
| 1 | Ath-AT1G68910.1 |  | Vvi-Vitvi01g01317\_t001 |  |  |  |  |  |  |  |
| 1 | Ath-AT1G68920.4 |  | Vvi-Vitvi01g01316\_t002 |  |  |  |  |  |  |  |
| 1 | Ath-AT1G68930.1 |  | Vvi-Vitvi01g01315\_t001 |  |  |  |  |  |  |  |
| 1 | Ath-AT1G68940.2 |  | Vvi-Vitvi01g01313\_t001 |  |  |  |  |  |  |  |
| 1 | Ath-AT1G68935.1 |  | | | |  |  |  |  |  |  |  |
| 1 | Ath-AT1G68937.1 |  | | | |  |  |  |  |  |  |  |
| 1 | Ath-AT1G68945.1 |  | | | |  |  |  |  |  |  |  |
| 1 | Ath-AT1G68960.1 |  | | | |  |  |  |  |  |  |  |
| 1 | Ath-AT1G68980.1 |  | | | |  |  |  |  |  |  |  |
| 1 | Ath-AT1G68990.2 |  | Vvi-Vitvi01g01312\_t001 |  |  |  |  |  |  |  |
| 1 | Ath-AT1G69010.1 |  | | | |  |  |  |  |  |  |  |
| 1 | Ath-AT1G69020.2 |  | Vvi-Vitvi01g01289\_t001 |  |  |  |  |  |  |  |
| 0 | Ath-AT1G69030.1 |  |  |  |  |  |  |  |  |
| 1 | Ath-AT1G69040.2 |  | Vvi-Vitvi01g00002\_t001 |  |  |  |  |  |  |  |
| 1 | Ath-AT1G69050.1 |  | | | |  |  |  |  |  |  |  |
| 1 | Ath-AT1G69060.1 |  | Vvi-Vitvi01g00005\_t001 |  |  |  |  |  |  |  |
| 1 | Ath-AT1G69070.2 |  | Vvi-Vitvi01g00006\_t001 |  |  |  |  |  |  |  |
| 1 | Ath-AT1G69080.1 |  | Vvi-Vitvi01g01835\_t001 |  |  |  |  |  |  |  |
| 1 | Ath-AT1G69085.1 |  | Vvi-Vitvi01g01836\_t001 |  |  |  |  |  |  |  |
| 1 | Ath-AT1G69090.1 |  | | | |  |  |  |  |  |  |  |
| 1 | Ath-AT1G69100.2 |  | | | |  |  |  |  |  |  |  |
| 1 | Ath-AT1G69120.1 |  | Vvi-Vitvi01g00008\_t001 |  |  |  |  |  |  |  |
| 1 | Ath-AT1G69150.1 |  | | | |  |  |  |  |  |  |  |
| 1 | Ath-AT1G69160.1 |  | Vvi-Vitvi01g00012\_t001 |  |  |  |  |  |  |  |
| 1 | Ath-AT1G69170.1 |  | Vvi-Vitvi01g01837\_t003 |  |  |  |  |  |  |  |
| 1 | Ath-AT1G69180.1 |  | Vvi-Vitvi01g00013\_t001 |  |  |  |  |  |  |  |
| 1 | Ath-AT1G69190.1 |  | Vvi-Vitvi01g00024\_t001 |  |  |  |  |  |  |  |
| 1 | Ath-AT1G69200.1 |  | Vvi-Vitvi01g00025\_t001 |  |  |  |  |  |  |  |
| 1 | Ath-AT1G69210.1 |  | Vvi-Vitvi01g00028\_t001 |  |  |  |  |  |  |  |
| 1 | Ath-AT1G69220.1 |  | Vvi-Vitvi01g00029\_t002 |  |  |  |  |  |  |  |
| 1 | Ath-AT1G69230.1 |  | Vvi-Vitvi01g00033\_t002 |  |  |  |  |  |  |  |
| 1 | Ath-AT1G69240.1 |  | Vvi-Vitvi01g00034\_t001 |  |  |  |  |  |  |  |
| 1 | Ath-AT1G69250.1 |  | | | |  |  |  |  |  |  |  |
| 1 | Ath-AT1G69260.1 |  | Vvi-Vitvi01g00037\_t001 |  |  |  |  |  |  |  |
| 1 | Ath-AT1G69270.1 |  | Vvi-Vitvi01g00038\_t001 |  |  |  |  |  |  |  |
| 1 | Ath-AT1G69280.1 |  | | | |  |  |  |  |  |  |  |
| 1 | Ath-AT1G69290.1 |  | Vvi-Vitvi01g00043\_t001 |  |  |  |  |  |  |  |
| 1 | Ath-AT1G69295.1 |  | | | |  |  |  |  |  |  |  |
| 1 | Ath-AT1G69310.1 |  | Vvi-Vitvi01g01844\_t002 |  |  |  |  |  |  |  |
| 1 | Ath-AT1G69320.1 |  | | | |  |  |  |  |  |  |  |
| 1 | Ath-AT1G69325.1 |  | Vvi-Vitvi01g01845\_t001 |  |  |  |  |  |  |  |
| 2 | Ath-AT1G69330.1 |  | | | |  | Vvi-Vitvi14g02020\_t001 |  |  |  |  |  |  |
| 2 | Ath-AT1G69340.1 |  | Vvi-Vitvi01g00076\_t002 |  | | | |  |  |  |  |  |  |
| 2 | Ath-AT1G69350.1 |  | Vvi-Vitvi01g00078\_t001 |  | | | |  |  |  |  |  |  |
| 2 | Ath-AT1G69360.1 |  | Vvi-Vitvi01g00080\_t001 |  | Vvi-Vitvi14g02014\_t001 |  |  |  |  |  |  |
| 1 | Ath-AT1G69370.1 |  |  |  | Vvi-Vitvi14g02011\_t001 |  |  |  |  |  |  |
| 1 | Ath-AT1G69380.1 |  |  |  | | | |  |  |  |  |  |  |
| 1 | Ath-AT1G69390.1 |  |  |  | Vvi-Vitvi14g02009\_t001 |  |  |  |  |  |  |
| 1 | Ath-AT1G69400.1 |  |  |  | | | |  |  |  |  |  |  |
| 1 | Ath-AT1G69410.1 |  |  |  | | | |  |  |  |  |  |  |
| 1 | Ath-AT1G69420.2 |  |  |  | | | |  |  |  |  |  |  |
| 1 | Ath-AT1G69430.1 |  |  |  | | | |  |  |  |  |  |  |
| 1 | Ath-AT1G69440.1 |  |  |  | | | |  |  |  |  |  |  |
| 1 | Ath-AT1G69450.3 |  |  |  | Vvi-Vitvi14g02000\_t001 |  |  |  |  |  |  |
| 1 | Ath-AT1G69460.1 |  |  |  | Vvi-Vitvi14g03093\_t001 |  |  |  |  |  |  |
| 1 | Ath-AT1G69470.1 |  |  |  | | | |  |  |  |  |  |  |
| 1 | Ath-AT1G69480.2 |  |  |  | Vvi-Vitvi14g01992\_t001 |  |  |  |  |  |  |
| 1 | Ath-AT1G69485.1 |  |  |  | Vvi-Vitvi14g01989\_t001 |  |  |  |  |  |  |
| 2 | Ath-AT1G69490.1 |  | Vvi-Vitvi01g01038\_t001 |  | | | |  |  |  |  |  |  |
| 2 | Ath-AT1G69500.1 |  | Vvi-Vitvi01g01037\_t001 |  | | | |  |  |  |  |  |  |
| 2 | Ath-AT1G69510.1 |  | Vvi-Vitvi01g04251\_t001 |  | | | |  |  |  |  |  |  |
| 2 | Ath-AT1G69520.2 |  | Vvi-Vitvi01g01033\_t001 |  | | | |  |  |  |  |  |  |
| 2 | Ath-AT1G69523.1 |  | | | |  | | | |  |  |  |  |  |  |
| 2 | Ath-AT1G69526.2 |  | | | |  | | | |  |  |  |  |  |  |
| 2 | Ath-AT1G69530.3 |  | Vvi-Vitvi01g01030\_t001 |  | Vvi-Vitvi14g01977\_t001 |  |  |  |  |  |  |
| 2 | Ath-AT1G69540.2 |  | | | |  | | | |  |  |  |  |  |  |
| 2 | Ath-AT1G69550.1 |  | | | |  | | | |  |  |  |  |  |  |
| 2 | Ath-AT1G69560.2 |  | Vvi-Vitvi01g01028\_t001 |  | Vvi-Vitvi14g01976\_t001 |  |  |  |  |  |  |
| 2 | Ath-AT1G69570.1 |  | Vvi-Vitvi01g01026\_t001 |  | Vvi-Vitvi14g01973\_t001 |  |  |  |  |  |  |
| 2 | Ath-AT1G69580.2 |  | Vvi-Vitvi01g01024\_t001 |  | | | |  |  |  |  |  |  |
| 2 | Ath-AT1G69588.1 |  | | | |  | | | |  |  |  |  |  |  |
| 2 | Ath-AT1G69600.1 |  | Vvi-Vitvi01g01013\_t001 |  | Vvi-Vitvi14g01955\_t001 |  |  |  |  |  |  |
| 2 | Ath-AT1G69610.2 |  | Vvi-Vitvi01g01009\_t001 |  | | | |  |  |  |  |  |  |
| 2 | Ath-AT1G69620.1 |  | Vvi-Vitvi01g04246\_t001 |  | | | |  |  |  |  |  |  |
| 2 | Ath-AT1G69630.1 |  | | | |  | | | |  |  |  |  |  |  |
| 2 | Ath-AT1G69640.1 |  | Vvi-Vitvi01g00993\_t001 |  | | | |  |  |  |  |  |  |
| 2 | Ath-AT1G69650.1 |  | | | |  | | | |  |  |  |  |  |  |
| 2 | Ath-AT1G69660.1 |  | | | |  | | | |  |  |  |  |  |  |
| 2 | Ath-AT1G69670.1 |  | Vvi-Vitvi01g00985\_t001 |  | Vvi-Vitvi14g01934\_t001 |  |  |  |  |  |  |
| 2 | Ath-AT1G69680.1 |  | Vvi-Vitvi01g04240\_t001 |  | | | |  |  |  |  |  |  |
| 3 | Ath-AT1G69690.1 |  | Vvi-Vitvi01g00980\_t001 |  | | | |  | Vvi-Vitvi17g00581\_t001 |  |  |  |  |  |
| 3 | Ath-AT1G69700.1 |  | Vvi-Vitvi01g00979\_t001 |  | | | |  | Vvi-Vitvi17g00577\_t001 |  |  |  |  |  |
| 3 | Ath-AT1G69710.1 |  | Vvi-Vitvi01g00977\_t001 |  | | | |  | Vvi-Vitvi17g04156\_t001 |  |  |  |  |  |
| 3 | Ath-AT1G69720.1 |  | | | |  | | | |  | | | |  |  |  |  |  |
| 3 | Ath-AT1G69730.1 |  | | | |  | | | |  | | | |  |  |  |  |  |
| 3 | Ath-AT1G69740.1 |  | | | |  | | | |  | | | |  |  |  |  |  |
| 3 | Ath-AT1G69750.3 |  | | | |  | | | |  | | | |  |  |  |  |  |
| 3 | Ath-AT1G69760.1 |  | Vvi-Vitvi01g02127\_t001 |  | | | |  | | | |  |  |  |  |  |
| 3 | Ath-AT1G69770.1 |  | | | |  | | | |  | | | |  |  |  |  |  |
| 3 | Ath-AT1G69780.1 |  | Vvi-Vitvi01g00958\_t002 |  | Vvi-Vitvi14g01922\_t001 |  | | | |  |  |  |  |  |
| 2 | Ath-AT1G69790.1 |  | Vvi-Vitvi01g00954\_t001 |  |  |  | | | |  |  |  |  |  |
| 2 | Ath-AT1G69800.2 |  | Vvi-Vitvi01g00941\_t001 |  |  |  | | | |  |  |  |  |  |
| 2 | Ath-AT1G69810.1 |  | Vvi-Vitvi01g00940\_t001 |  |  |  | Vvi-Vitvi17g00556\_t001 |  |  |  |  |  |
| 2 | Ath-AT1G69818.1 |  | | | |  |  |  | | | |  |  |  |  |  |
| 2 | Ath-AT1G69820.1 |  | | | |  |  |  | | | |  |  |  |  |  |
| 2 | Ath-AT1G69825.1 |  | | | |  |  |  | | | |  |  |  |  |  |
| 2 | Ath-AT1G69828.1 |  | | | |  |  |  | | | |  |  |  |  |  |
| 2 | Ath-AT1G69830.1 |  | Vvi-Vitvi01g00932\_t001 |  |  |  | | | |  |  |  |  |  |
| 2 | Ath-AT1G69840.1 |  | Vvi-Vitvi01g00929\_t001 |  |  |  | Vvi-Vitvi17g00546\_t002 |  |  |  |  |  |
| 2 | Ath-AT1G69850.1 |  | Vvi-Vitvi01g00921\_t001 |  |  |  | Vvi-Vitvi17g00541\_t001 |  |  |  |  |  |
| 2 | Ath-AT1G69860.1 |  | Vvi-Vitvi01g00911\_t001 |  |  |  | Vvi-Vitvi17g04145\_t001 |  |  |  |  |  |
| 2 | Ath-AT1G69870.1 |  | | | |  |  |  | Vvi-Vitvi17g00528\_t001 |  |  |  |  |  |
| 2 | Ath-AT1G69880.1 |  | | | |  |  |  | | | |  |  |  |  |  |
| 2 | Ath-AT1G69890.1 |  | Vvi-Vitvi01g00902\_t004 |  |  |  | | | |  |  |  |  |  |
| 2 | Ath-AT1G69900.1 |  | | | |  |  |  | | | |  |  |  |  |  |
| 2 | Ath-AT1G69910.1 |  | Vvi-Vitvi01g00900\_t001 |  |  |  | | | |  |  |  |  |  |
| 2 | Ath-AT1G69920.1 |  | | | |  |  |  | | | |  |  |  |  |  |
| 2 | Ath-AT1G69930.1 |  | Vvi-Vitvi01g00888\_t001 |  |  |  | | | |  |  |  |  |  |
| 2 | Ath-AT1G69935.1 |  | Vvi-Vitvi01g02101\_t001 |  |  |  | | | |  |  |  |  |  |
| 2 | Ath-AT1G69940.1 |  | | | |  |  |  | | | |  |  |  |  |  |
| 2 | Ath-AT1G69960.1 |  | Vvi-Vitvi01g00870\_t001 |  |  |  | Vvi-Vitvi17g00505\_t001 |  |  |  |  |  |
| 1 | Ath-AT1G69970.1 |  | | | |  |  |  |  |  |  |  |
| 1 | Ath-AT1G69980.1 |  | Vvi-Vitvi01g00869\_t001 |  |  |  |  |  |  |  |
| 1 | Ath-AT1G69990.1 |  | Vvi-Vitvi01g00868\_t001 |  |  |  |  |  |  |  |
| 2 | Ath-AT1G70000.1 |  | Vvi-Vitvi01g00867\_t001 |  | Vvi-Vitvi17g00726\_t001 |  |  |  |  |  |  |
| 2 | Ath-AT1G70020.1 |  | | | |  | | | |  |  |  |  |  |  |
| 2 | Ath-AT1G70030.1 |  | | | |  | Vvi-Vitvi17g01522\_t001 |  |  |  |  |  |  |
| 2 | Ath-AT1G70040.1 |  | | | |  | | | |  |  |  |  |  |  |
| 2 | Ath-AT1G70060.1 |  | Vvi-Vitvi01g02098\_t001 |  | | | |  |  |  |  |  |  |
| 2 | Ath-AT1G70070.1 |  | Vvi-Vitvi01g00862\_t001 |  | | | |  |  |  |  |  |  |
| 2 | Ath-AT1G70080.1 |  | | | |  | | | |  |  |  |  |  |  |
| 2 | Ath-AT1G70090.1 |  | Vvi-Vitvi01g00860\_t001 |  | | | |  |  |  |  |  |  |
| 2 | Ath-AT1G70100.3 |  | Vvi-Vitvi01g00859\_t001 |  | | | |  |  |  |  |  |  |
| 2 | Ath-AT1G70110.1 |  | | | |  | | | |  |  |  |  |  |  |
| 2 | Ath-AT1G70120.1 |  | | | |  | | | |  |  |  |  |  |  |
| 2 | Ath-AT1G70130.1 |  | | | |  | | | |  |  |  |  |  |  |
| 2 | Ath-AT1G70140.1 |  | Vvi-Vitvi01g00858\_t001 |  | Vvi-Vitvi17g00731\_t001 |  |  |  |  |  |  |
| 2 | Ath-AT1G70150.1 |  | Vvi-Vitvi01g00853\_t001 |  | | | |  |  |  |  |  |  |
| 2 | Ath-AT1G70160.1 |  | Vvi-Vitvi01g00852\_t001 |  | | | |  |  |  |  |  |  |
| 2 | Ath-AT1G70170.1 |  | Vvi-Vitvi01g00850\_t001 |  | | | |  |  |  |  |  |  |
| 2 | Ath-AT1G70175.1 |  | | | |  | | | |  |  |  |  |  |  |
| 2 | Ath-AT1G70180.2 |  | Vvi-Vitvi01g00846\_t002 |  | Vvi-Vitvi17g00742\_t001 |  |  |  |  |  |  |
| 2 | Ath-AT1G70190.1 |  | | | |  | | | |  |  |  |  |  |  |
| 2 | Ath-AT1G70200.1 |  | | | |  | | | |  |  |  |  |  |  |
| 2 | Ath-AT1G70210.1 |  | | | |  | | | |  |  |  |  |  |  |
| 2 | Ath-AT1G70209.1 |  | | | |  | | | |  |  |  |  |  |  |
| 2 | Ath-AT1G70220.1 |  | Vvi-Vitvi01g02091\_t001 |  | | | |  |  |  |  |  |  |
| 2 | Ath-AT1G70230.1 |  | | | |  | | | |  |  |  |  |  |  |
| 2 | Ath-AT1G70250.1 |  | | | |  | | | |  |  |  |  |  |  |
| 2 | Ath-AT1G70260.1 |  | Vvi-Vitvi01g00815\_t001 |  | Vvi-Vitvi17g00765\_t001 |  |  |  |  |  |  |
| 2 | Ath-AT1G70270.2 |  | | | |  | | | |  |  |  |  |  |  |
| 2 | Ath-AT1G70280.2 |  | Vvi-Vitvi01g00798\_t001 |  | | | |  |  |  |  |  |  |
| 2 | Ath-AT1G70290.1 |  | Vvi-Vitvi01g00793\_t001 |  | Vvi-Vitvi17g00778\_t001 |  |  |  |  |  |  |
| 2 | Ath-AT1G70300.1 |  | Vvi-Vitvi01g00792\_t002 |  | | | |  |  |  |  |  |  |
| 2 | Ath-AT1G70310.1 |  | Vvi-Vitvi01g00789\_t001 |  | Vvi-Vitvi17g00780\_t001 |  |  |  |  |  |  |
| 2 | Ath-AT1G70320.1 |  | | | |  | | | |  |  |  |  |  |  |
| 2 | Ath-AT1G70330.1 |  | Vvi-Vitvi01g00780\_t001 |  | | | |  |  |  |  |  |  |
| 2 | Ath-AT1G70335.1 |  | | | |  | | | |  |  |  |  |  |  |
| 2 | Ath-AT1G70340.1 |  | Vvi-Vitvi01g00770\_t001 |  | | | |  |  |  |  |  |  |
| 2 | Ath-AT1G70350.1 |  | | | |  | | | |  |  |  |  |  |  |
| 2 | Ath-AT1G70360.1 |  | | | |  | | | |  |  |  |  |  |  |
| 2 | Ath-AT1G70370.2 |  | Vvi-Vitvi01g00745\_t001 |  | Vvi-Vitvi17g00796\_t001 |  |  |  |  |  |  |
| 2 | Ath-AT1G70380.1 |  | | | |  | | | |  |  |  |  |  |  |
| 2 | Ath-AT1G70390.1 |  | | | |  | | | |  |  |  |  |  |  |
| 2 | Ath-AT1G70400.3 |  | | | |  | | | |  |  |  |  |  |  |
| 3 | Ath-AT1G70410.2 |  | Vvi-Vitvi01g00735\_t001 |  | | | |  | Vvi-Vitvi14g01763\_t001 |  |  |  |  |  |
| 3 | Ath-AT1G70420.1 |  | Vvi-Vitvi01g00732\_t001 |  | | | |  | Vvi-Vitvi14g01761\_t001 |  |  |  |  |  |
| 3 | Ath-AT1G70430.3 |  | Vvi-Vitvi01g00730\_t001 |  | | | |  | Vvi-Vitvi14g01758\_t003 |  |  |  |  |  |
| 3 | Ath-AT1G70440.1 |  | Vvi-Vitvi01g00729\_t001 |  | Vvi-Vitvi17g00814\_t001 |  | | | |  |  |  |  |  |
| 3 | Ath-AT1G70450.1 |  | Vvi-Vitvi01g04183\_t001 |  | | | |  | | | |  |  |  |  |  |
| 3 | Ath-AT1G70460.1 |  | | | |  | | | |  | | | |  |  |  |  |  |
| 3 | Ath-AT1G70470.1 |  | | | |  | | | |  | | | |  |  |  |  |  |
| 3 | Ath-AT1G70475.1 |  | | | |  | | | |  | | | |  |  |  |  |  |
| 3 | Ath-AT1G70480.2 |  | Vvi-Vitvi01g00717\_t001 |  | | | |  | | | |  |  |  |  |  |
| 3 | Ath-AT1G70490.1 |  | Vvi-Vitvi01g04177\_t002 |  | | | |  | Vvi-Vitvi14g01748\_t001 |  |  |  |  |  |
| 3 | Ath-AT1G70500.1 |  | Vvi-Vitvi01g04175\_t001 |  | | | |  | | | |  |  |  |  |  |
| 3 | Ath-AT1G70505.1 |  | Vvi-Vitvi01g00701\_t001 |  | Vvi-Vitvi17g00837\_t003 |  | Vvi-Vitvi14g01736\_t001 |  |  |  |  |  |
| 3 | Ath-AT1G70510.2 |  | Vvi-Vitvi01g00694\_t001 |  | Vvi-Vitvi17g00850\_t001 |  | | | |  |  |  |  |  |
| 3 | Ath-AT1G70520.1 |  | Vvi-Vitvi01g00685\_t001 |  | Vvi-Vitvi17g00863\_t001 |  | Vvi-Vitvi14g01727\_t001 |  |  |  |  |  |
| 2 | Ath-AT1G70530.1 |  | | | |  | | | |  |  |  |  |  |  |
| 2 | Ath-AT1G70540.1 |  | Vvi-Vitvi01g02044\_t001 |  | | | |  |  |  |  |  |  |
| 2 | Ath-AT1G70550.1 |  | Vvi-Vitvi01g00676\_t001 |  | Vvi-Vitvi17g00874\_t001 |  |  |  |  |  |  |
| 2 | Ath-AT1G70560.1 |  | Vvi-Vitvi01g00672\_t001 |  | Vvi-Vitvi17g00889\_t001 |  |  |  |  |  |  |
| 2 | Ath-AT1G70570.2 |  | Vvi-Vitvi01g00664\_t001 |  | | | |  |  |  |  |  |  |
| 2 | Ath-AT1G70580.2 |  | Vvi-Vitvi01g00662\_t004 |  | | | |  |  |  |  |  |  |
| 2 | Ath-AT1G70590.1 |  | Vvi-Vitvi01g00660\_t001 |  | | | |  |  |  |  |  |  |
| 2 | Ath-AT1G70600.1 |  | Vvi-Vitvi01g04157\_t001 |  | Vvi-Vitvi17g00899\_t001 |  |  |  |  |  |  |
| 2 | Ath-AT1G70610.1 |  | Vvi-Vitvi01g00650\_t001 |  | | | |  |  |  |  |  |  |
| 2 | Ath-AT1G70620.3 |  | Vvi-Vitvi01g00649\_t001 |  | | | |  |  |  |  |  |  |
| 2 | Ath-AT1G70630.3 |  | Vvi-Vitvi01g00648\_t001 |  | | | |  |  |  |  |  |  |
| 2 | Ath-AT1G70640.1 |  | Vvi-Vitvi01g00646\_t002 |  | Vvi-Vitvi17g04255\_t001 |  |  |  |  |  |  |
| 1 | Ath-AT1G70650.2 |  | Vvi-Vitvi01g00479\_t002 |  |  |  |  |  |  |  |
| 1 | Ath-AT1G70660.1 |  | | | |  |  |  |  |  |  |  |
| 1 | Ath-AT1G70670.1 |  | Vvi-Vitvi01g00478\_t001 |  |  |  |  |  |  |  |
| 1 | Ath-AT1G70680.1 |  | | | |  |  |  |  |  |  |  |
| 1 | Ath-AT1G70690.1 |  | Vvi-Vitvi01g00477\_t001 |  |  |  |  |  |  |  |
| 1 | Ath-AT1G70700.3 |  | Vvi-Vitvi01g00473\_t001 |  |  |  |  |  |  |  |
| 1 | Ath-AT1G70710.1 |  | | | |  |  |  |  |  |  |  |
| 1 | Ath-AT1G70720.2 |  | Vvi-Vitvi01g00457\_t001 |  |  |  |  |  |  |  |
| 1 | Ath-AT1G70730.3 |  | Vvi-Vitvi01g00455\_t002 |  |  |  |  |  |  |  |
| 1 | Ath-AT1G70740.1 |  | Vvi-Vitvi01g00453\_t001 |  |  |  |  |  |  |  |
| 1 | Ath-AT1G70750.1 |  | Vvi-Vitvi01g00452\_t001 |  |  |  |  |  |  |  |
| 1 | Ath-AT1G70760.1 |  | Vvi-Vitvi01g00449\_t001 |  |  |  |  |  |  |  |
| 1 | Ath-AT1G70770.2 |  | Vvi-Vitvi01g00448\_t001 |  |  |  |  |  |  |  |
| 1 | Ath-AT1G70780.1 |  | Vvi-Vitvi01g00445\_t001 |  |  |  |  |  |  |  |
| 1 | Ath-AT1G70790.2 |  | | | |  |  |  |  |  |  |  |
| 1 | Ath-AT1G70800.1 |  | | | |  |  |  |  |  |  |  |
| 1 | Ath-AT1G70810.1 |  | Vvi-Vitvi01g00444\_t001 |  |  |  |  |  |  |  |
| 1 | Ath-AT1G70820.1 |  | Vvi-Vitvi01g00442\_t001 |  |  |  |  |  |  |  |
| 1 | Ath-AT1G70830.1 |  | | | |  |  |  |  |  |  |  |
| 1 | Ath-AT1G70840.1 |  | | | |  |  |  |  |  |  |  |
| 1 | Ath-AT1G70850.3 |  | | | |  |  |  |  |  |  |  |
| 1 | Ath-AT1G70860.2 |  | | | |  |  |  |  |  |  |  |
| 1 | Ath-AT1G70870.1 |  | | | |  |  |  |  |  |  |  |
| 1 | Ath-AT1G70880.1 |  | Vvi-Vitvi01g00436\_t001 |  |  |  |  |  |  |  |
| 1 | Ath-AT1G70890.1 |  | | | |  |  |  |  |  |  |  |
| 1 | Ath-AT1G70895.2 |  | | | |  |  |  |  |  |  |  |
| 1 | Ath-AT1G70900.1 |  | Vvi-Vitvi01g00428\_t001 |  |  |  |  |  |  |  |
| 1 | Ath-AT1G70910.1 |  | | | |  |  |  |  |  |  |  |
| 1 | Ath-AT1G70920.1 |  | Vvi-Vitvi01g00412\_t001 |  |  |  |  |  |  |  |
| 1 | Ath-AT1G70940.1 |  | Vvi-Vitvi01g00411\_t001 |  |  |  |  |  |  |  |
| 1 | Ath-AT1G70944.1 |  | | | |  |  |  |  |  |  |  |
| 1 | Ath-AT1G70949.1 |  | | | |  |  |  |  |  |  |  |
| 1 | Ath-AT1G70950.1 |  | Vvi-Vitvi01g00409\_t001 |  |  |  |  |  |  |  |
| 1 | Ath-AT1G70960.1 |  | | | |  |  |  |  |  |  |  |
| 1 | Ath-AT1G70970.1 |  | | | |  |  |  |  |  |  |  |
| 1 | Ath-AT1G70980.1 |  | | | |  |  |  |  |  |  |  |
| 1 | Ath-AT1G70985.1 |  | | | |  |  |  |  |  |  |  |
| 1 | Ath-AT1G70990.1 |  | | | |  |  |  |  |  |  |  |
| 1 | Ath-AT1G71000.1 |  | Vvi-Vitvi01g00408\_t001 |  |  |  |  |  |  |  |
| 1 | Ath-AT1G71010.1 |  | Vvi-Vitvi01g00404\_t001 |  |  |  |  |  |  |  |
| 1 | Ath-AT1G71015.2 |  | Vvi-Vitvi01g00403\_t001 |  |  |  |  |  |  |  |
| 1 | Ath-AT1G71020.1 |  | Vvi-Vitvi01g00402\_t001 |  |  |  |  |  |  |  |
| 1 | Ath-AT1G71030.1 |  | | | |  |  |  |  |  |  |  |
| 1 | Ath-AT1G71040.1 |  | Vvi-Vitvi01g00397\_t001 |  |  |  |  |  |  |  |
| 1 | Ath-AT1G71050.1 |  | Vvi-Vitvi01g00393\_t001 |  |  |  |  |  |  |  |
| 1 | Ath-AT1G71060.1 |  | Vvi-Vitvi01g00392\_t001 |  |  |  |  |  |  |  |
| 1 | Ath-AT1G71070.1 |  | Vvi-Vitvi01g00391\_t001 |  |  |  |  |  |  |  |
| 1 | Ath-AT1G71080.1 |  | | | |  |  |  |  |  |  |  |
| 1 | Ath-AT1G71090.1 |  | Vvi-Vitvi01g00386\_t001 |  |  |  |  |  |  |  |
| 1 | Ath-AT1G71100.1 |  | Vvi-Vitvi01g00385\_t001 |  |  |  |  |  |  |  |
| 1 | Ath-AT1G71110.1 |  | Vvi-Vitvi01g00384\_t001 |  |  |  |  |  |  |  |
| 1 | Ath-AT1G71120.1 |  | Vvi-Vitvi01g00378\_t001 |  |  |  |  |  |  |  |
| 1 | Ath-AT1G71130.1 |  | | | |  |  |  |  |  |  |  |
| 1 | Ath-AT1G71140.1 |  | Vvi-Vitvi01g00370\_t001 |  |  |  |  |  |  |  |
| 1 | Ath-AT1G71150.1 |  | Vvi-Vitvi01g00369\_t001 |  |  |  |  |  |  |  |
| 0 | Ath-AT1G71160.1 |  |  |  |  |  |  |  |  |
| 0 | Ath-AT1G71170.1 |  |  |  |  |  |  |  |  |
| 0 | Ath-AT1G71180.1 |  |  |  |  |  |  |  |  |
| 0 | Ath-AT1G71190.1 |  |  |  |  |  |  |  |  |
| 0 | Ath-AT1G71200.4 |  |  |  |  |  |  |  |  |
| 0 | Ath-AT1G71210.1 |  |  |  |  |  |  |  |  |
| 0 | Ath-AT1G71220.3 |  |  |  |  |  |  |  |  |
| 0 | Ath-AT1G71230.1 |  |  |  |  |  |  |  |  |
| 0 | Ath-AT1G71235.1 |  |  |  |  |  |  |  |  |
| 0 | Ath-AT1G71240.1 |  |  |  |  |  |  |  |  |
| 0 | Ath-AT1G71250.1 |  |  |  |  |  |  |  |  |
| 0 | Ath-AT1G71260.1 |  |  |  |  |  |  |  |  |
| 0 | Ath-AT1G71270.1 |  |  |  |  |  |  |  |  |
| 0 | Ath-AT1G71280.1 |  |  |  |  |  |  |  |  |
| 0 | Ath-AT1G71290.1 |  |  |  |  |  |  |  |  |
| 0 | Ath-AT1G71300.1 |  |  |  |  |  |  |  |  |
| 0 | Ath-AT1G71310.1 |  |  |  |  |  |  |  |  |
| 0 | Ath-AT1G71320.1 |  |  |  |  |  |  |  |  |
| 0 | Ath-AT1G71330.1 |  |  |  |  |  |  |  |  |
| 0 | Ath-AT1G71340.1 |  |  |  |  |  |  |  |  |
| 0 | Ath-AT1G71350.1 |  |  |  |  |  |  |  |  |
| 0 | Ath-AT1G71360.2 |  |  |  |  |  |  |  |  |
| 1 | Ath-AT1G71370.1 |  | Vvi-Vitvi18g04675\_t001 |  |  |  |  |  |  |  |
| 1 | Ath-AT1G71380.1 |  | | | |  |  |  |  |  |  |  |
| 1 | Ath-AT1G71390.2 |  | | | |  |  |  |  |  |  |  |
| 1 | Ath-AT1G71400.1 |  | | | |  |  |  |  |  |  |  |
| 1 | Ath-AT1G71410.1 |  | | | |  |  |  |  |  |  |  |
| 1 | Ath-AT1G71420.1 |  | | | |  |  |  |  |  |  |  |
| 1 | Ath-AT1G71430.1 |  | | | |  |  |  |  |  |  |  |
| 1 | Ath-AT1G71440.1 |  | Vvi-Vitvi18g02249\_t001 |  |  |  |  |  |  |  |
| 1 | Ath-AT1G71450.1 |  | Vvi-Vitvi18g02240\_t001 |  |  |  |  |  |  |  |
| 1 | Ath-AT1G71460.1 |  | Vvi-Vitvi18g02238\_t001 |  |  |  |  |  |  |  |
| 1 | Ath-AT1G71470.1 |  | | | |  |  |  |  |  |  |  |
| 1 | Ath-AT1G71480.1 |  | Vvi-Vitvi18g02237\_t001 |  |  |  |  |  |  |  |
| 1 | Ath-AT1G71490.1 |  | Vvi-Vitvi18g02236\_t001 |  |  |  |  |  |  |  |
| 0 | Ath-AT1G71500.1 |  |  |  |  |  |  |  |  |
| 0 | Ath-AT1G71520.1 |  |  |  |  |  |  |  |  |
| 1 | Ath-AT1G71530.1 |  | Vvi-Vitvi18g01607\_t001 |  |  |  |  |  |  |  |
| 1 | Ath-AT1G71680.1 |  | | | |  |  |  |  |  |  |  |
| 1 | Ath-AT1G71690.1 |  | | | |  |  |  |  |  |  |  |
| 1 | Ath-AT1G71691.2 |  | | | |  |  |  |  |  |  |  |
| 1 | Ath-AT1G71692.1 |  | | | |  |  |  |  |  |  |  |
| 1 | Ath-AT1G71695.1 |  | | | |  |  |  |  |  |  |  |
| 1 | Ath-AT1G71696.2 |  | Vvi-Vitvi18g01557\_t001 |  |  |  |  |  |  |  |
| 1 | Ath-AT1G71697.1 |  | Vvi-Vitvi18g01572\_t001.1.6037826d |  |  |  |  |  |  |  |
| 1 | Ath-AT1G71710.1 |  | Vvi-Vitvi18g01574\_t005 |  |  |  |  |  |  |  |
| 1 | Ath-AT1G71720.1 |  | Vvi-Vitvi18g01546\_t001 |  |  |  |  |  |  |  |
| 1 | Ath-AT1G71730.1 |  | Vvi-Vitvi18g01544\_t001 |  |  |  |  |  |  |  |
| 1 | Ath-AT1G71740.1 |  | Vvi-Vitvi18g02931\_t001 |  |  |  |  |  |  |  |
| 1 | Ath-AT1G71750.2 |  | Vvi-Vitvi18g01542\_t001 |  |  |  |  |  |  |  |
| 1 | Ath-AT1G71760.2 |  | Vvi-Vitvi18g01541\_t001 |  |  |  |  |  |  |  |
| 1 | Ath-AT1G71770.2 |  | Vvi-Vitvi18g04407\_t001 |  |  |  |  |  |  |  |
| 1 | Ath-AT1G71780.1 |  | Vvi-Vitvi18g02918\_t001 |  |  |  |  |  |  |  |
| 1 | Ath-AT1G71790.1 |  | Vvi-Vitvi18g01527\_t001 |  |  |  |  |  |  |  |
| 1 | Ath-AT1G71800.1 |  | Vvi-Vitvi18g01518\_t001 |  |  |  |  |  |  |  |
| 1 | Ath-AT1G71810.1 |  | Vvi-Vitvi18g01512\_t001 |  |  |  |  |  |  |  |
| 0 | Ath-AT1G71820.2 |  |  |  |  |  |  |  |  |
| 0 | Ath-AT1G71830.1 |  |  |  |  |  |  |  |  |
| 0 | Ath-AT1G71840.1 |  |  |  |  |  |  |  |  |
| 0 | Ath-AT1G71850.1 |  |  |  |  |  |  |  |  |
| 0 | Ath-AT1G71860.1 |  |  |  |  |  |  |  |  |
| 0 | Ath-AT1G71865.1 |  |  |  |  |  |  |  |  |
| 0 | Ath-AT1G71866.1 |  |  |  |  |  |  |  |  |
| 0 | Ath-AT1G71870.1 |  |  |  |  |  |  |  |  |
| 0 | Ath-AT1G71880.1 |  |  |  |  |  |  |  |  |
| 0 | Ath-AT1G71890.1 |  |  |  |  |  |  |  |  |
| 0 | Ath-AT1G71900.2 |  |  |  |  |  |  |  |  |
| 0 | Ath-AT1G71910.2 |  |  |  |  |  |  |  |  |
| 0 | Ath-AT1G71920.2 |  |  |  |  |  |  |  |  |
| 0 | Ath-AT1G71930.2 |  |  |  |  |  |  |  |  |
| 0 | Ath-AT1G71940.2 |  |  |  |  |  |  |  |  |
| 0 | Ath-AT1G71950.1 |  |  |  |  |  |  |  |  |
| 0 | Ath-AT1G71960.1 |  |  |  |  |  |  |  |  |
| 0 | Ath-AT1G71970.1 |  |  |  |  |  |  |  |  |
| 0 | Ath-AT1G71980.1 |  |  |  |  |  |  |  |  |
| 0 | Ath-AT1G71990.1 |  |  |  |  |  |  |  |  |
| 0 | Ath-AT1G72000.1 |  |  |  |  |  |  |  |  |
| 0 | Ath-AT1G72010.1 |  |  |  |  |  |  |  |  |
| 0 | Ath-AT1G72020.1 |  |  |  |  |  |  |  |  |
| 0 | Ath-AT1G72030.1 |  |  |  |  |  |  |  |  |
| 0 | Ath-AT1G72040.1 |  |  |  |  |  |  |  |  |
| 0 | Ath-AT1G72050.3 |  |  |  |  |  |  |  |  |
| 0 | Ath-AT1G72060.1 |  |  |  |  |  |  |  |  |
| 0 | Ath-AT1G72070.1 |  |  |  |  |  |  |  |  |
| 0 | Ath-AT1G72080.1 |  |  |  |  |  |  |  |  |
| 0 | Ath-AT1G72090.1 |  |  |  |  |  |  |  |  |
| 0 | Ath-AT1G72100.1 |  |  |  |  |  |  |  |  |
| 0 | Ath-AT1G72110.1 |  |  |  |  |  |  |  |  |
| 0 | Ath-AT1G72120.1 |  |  |  |  |  |  |  |  |
| 0 | Ath-AT1G72125.1 |  |  |  |  |  |  |  |  |
| 0 | Ath-AT1G72130.1 |  |  |  |  |  |  |  |  |
| 0 | Ath-AT1G72140.1 |  |  |  |  |  |  |  |  |
| 0 | Ath-AT1G72141.1 |  |  |  |  |  |  |  |  |
| 0 | Ath-AT1G72150.1 |  |  |  |  |  |  |  |  |
| 0 | Ath-AT1G72160.1 |  |  |  |  |  |  |  |  |
| 0 | Ath-AT1G72170.1 |  |  |  |  |  |  |  |  |
| 0 | Ath-AT1G72175.1 |  |  |  |  |  |  |  |  |
| 0 | Ath-AT1G72180.1 |  |  |  |  |  |  |  |  |
| 0 | Ath-AT1G72190.1 |  |  |  |  |  |  |  |  |
| 0 | Ath-AT1G72200.1 |  |  |  |  |  |  |  |  |
| 0 | Ath-AT1G72210.1 |  |  |  |  |  |  |  |  |
| 0 | Ath-AT1G72220.1 |  |  |  |  |  |  |  |  |
| 0 | Ath-AT1G72230.1 |  |  |  |  |  |  |  |  |
| 0 | Ath-AT1G72240.1 |  |  |  |  |  |  |  |  |
| 0 | Ath-AT1G72250.3 |  |  |  |  |  |  |  |  |
| 0 | Ath-AT1G72260.1 |  |  |  |  |  |  |  |  |
| 0 | Ath-AT1G72270.1 |  |  |  |  |  |  |  |  |
| 0 | Ath-AT1G72275.1 |  |  |  |  |  |  |  |  |
| 0 | Ath-AT1G72280.1 |  |  |  |  |  |  |  |  |
| 0 | Ath-AT1G72290.1 |  |  |  |  |  |  |  |  |
| 1 | Ath-AT1G72300.1 |  | Vvi-Vitvi09g00003\_t001 |  |  |  |  |  |  |  |
| 1 | Ath-AT1G72310.1 |  | Vvi-Vitvi09g00012\_t001 |  |  |  |  |  |  |  |
| 1 | Ath-AT1G72320.4 |  | Vvi-Vitvi09g00015\_t001 |  |  |  |  |  |  |  |
| 1 | Ath-AT1G72330.3 |  | Vvi-Vitvi09g00019\_t001 |  |  |  |  |  |  |  |
| 1 | Ath-AT1G72340.2 |  | Vvi-Vitvi09g00027\_t001 |  |  |  |  |  |  |  |
| 1 | Ath-AT1G72350.1 |  | | | |  |  |  |  |  |  |  |
| 1 | Ath-AT1G72360.2 |  | Vvi-Vitvi09g00031\_t002 |  |  |  |  |  |  |  |
| 1 | Ath-AT1G72370.1 |  | Vvi-Vitvi09g00043\_t001 |  |  |  |  |  |  |  |
| 1 | Ath-AT1G72390.1 |  | | | |  |  |  |  |  |  |  |
| 1 | Ath-AT1G72410.1 |  | | | |  |  |  |  |  |  |  |
| 1 | Ath-AT1G72416.3 |  | | | |  |  |  |  |  |  |  |
| 1 | Ath-AT1G72420.2 |  | Vvi-Vitvi09g00047\_t002 |  |  |  |  |  |  |  |
| 2 | Ath-AT1G72430.1 |  | | | |  | Vvi-Vitvi11g00033\_t001 |  |  |  |  |  |  |
| 2 | Ath-AT1G72440.1 |  | | | |  | | | |  |  |  |  |  |  |
| 2 | Ath-AT1G72450.1 |  | Vvi-Vitvi09g00064\_t001 |  | Vvi-Vitvi11g00050\_t001 |  |  |  |  |  |  |
| 2 | Ath-AT1G72460.1 |  | Vvi-Vitvi09g00065\_t001 |  | Vvi-Vitvi11g00053\_t001 |  |  |  |  |  |  |
| 2 | Ath-AT1G72470.1 |  | Vvi-Vitvi09g00067\_t001 |  | | | |  |  |  |  |  |  |
| 2 | Ath-AT1G72480.1 |  | Vvi-Vitvi09g00068\_t002 |  | | | |  |  |  |  |  |  |
| 2 | Ath-AT1G72490.3 |  | Vvi-Vitvi09g01501\_t001 |  | Vvi-Vitvi11g01327\_t001 |  |  |  |  |  |  |
| 2 | Ath-AT1G72500.1 |  | Vvi-Vitvi09g00071\_t001 |  | Vvi-Vitvi11g00061\_t001 |  |  |  |  |  |  |
| 2 | Ath-AT1G72510.2 |  | Vvi-Vitvi09g00077\_t001 |  | Vvi-Vitvi11g01331\_t001 |  |  |  |  |  |  |
| 2 | Ath-AT1G72520.1 |  | Vvi-Vitvi09g00085\_t002 |  | | | |  |  |  |  |  |  |
| 2 | Ath-AT1G72530.2 |  | Vvi-Vitvi09g00087\_t001 |  | | | |  |  |  |  |  |  |
| 2 | Ath-AT1G72540.1 |  | Vvi-Vitvi09g00088\_t001 |  | Vvi-Vitvi11g01336\_t001 |  |  |  |  |  |  |
| 2 | Ath-AT1G72545.1 |  | | | |  | | | |  |  |  |  |  |  |
| 2 | Ath-AT1G72550.1 |  | | | |  | | | |  |  |  |  |  |  |
| 2 | Ath-AT1G72560.1 |  | Vvi-Vitvi09g00089\_t002 |  | | | |  |  |  |  |  |  |
| 2 | Ath-AT1G72570.1 |  | | | |  | | | |  |  |  |  |  |  |
| 2 | Ath-AT1G72580.1 |  | | | |  | | | |  |  |  |  |  |  |
| 2 | Ath-AT1G72590.1 |  | | | |  | | | |  |  |  |  |  |  |
| 2 | Ath-AT1G72600.2 |  | | | |  | | | |  |  |  |  |  |  |
| 2 | Ath-AT1G72610.1 |  | Vvi-Vitvi09g01512\_t001 |  | Vvi-Vitvi11g00089\_t001 |  |  |  |  |  |  |
| 2 | Ath-AT1G72620.1 |  | | | |  | | | |  |  |  |  |  |  |
| 2 | Ath-AT1G72630.1 |  | Vvi-Vitvi09g00117\_t001 |  | Vvi-Vitvi11g00102\_t001 |  |  |  |  |  |  |
| 2 | Ath-AT1G72640.1 |  | Vvi-Vitvi09g00118\_t001 |  | | | |  |  |  |  |  |  |
| 2 | Ath-AT1G72645.1 |  | | | |  | | | |  |  |  |  |  |  |
| 2 | Ath-AT1G72650.2 |  | Vvi-Vitvi09g00122\_t001 |  | | | |  |  |  |  |  |  |
| 2 | Ath-AT1G72660.1 |  | Vvi-Vitvi09g00123\_t001 |  | | | |  |  |  |  |  |  |
| 2 | Ath-AT1G72670.1 |  | Vvi-Vitvi09g00143\_t001 |  | | | |  |  |  |  |  |  |
| 2 | Ath-AT1G72680.1 |  | | | |  | | | |  |  |  |  |  |  |
| 3 | Ath-AT1G72690.1 |  | Vvi-Vitvi09g04034\_t001 |  | | | |  | Vvi-Vitvi11g00157\_t002 |  |  |  |  |  |
| 3 | Ath-AT1G72700.1 |  | Vvi-Vitvi09g00147\_t001 |  | | | |  | | | |  |  |  |  |  |
| 3 | Ath-AT1G72710.1 |  | Vvi-Vitvi09g00148\_t001 |  | | | |  | Vvi-Vitvi11g00155\_t001 |  |  |  |  |  |
| 3 | Ath-AT1G72720.1 |  | Vvi-Vitvi09g01521\_t001 |  | | | |  | | | |  |  |  |  |  |
| 3 | Ath-AT1G72730.1 |  | Vvi-Vitvi09g00149\_t001 |  | | | |  | Vvi-Vitvi11g00152\_t001 |  |  |  |  |  |
| 3 | Ath-AT1G72740.1 |  | Vvi-Vitvi09g04036\_t001 |  | | | |  | Vvi-Vitvi11g00149\_t001 |  |  |  |  |  |
| 3 | Ath-AT1G72750.1 |  | Vvi-Vitvi09g00152\_t001 |  | | | |  | | | |  |  |  |  |  |
| 3 | Ath-AT1G72755.1 |  | | | |  | | | |  | | | |  |  |  |  |  |
| 3 | Ath-AT1G72760.2 |  | | | |  | | | |  | | | |  |  |  |  |  |
| 3 | Ath-AT1G72770.1 |  | Vvi-Vitvi09g00156\_t002 |  | | | |  | Vvi-Vitvi11g00137\_t002 |  |  |  |  |  |
[truncated: 57,577 more chars]
