# Supplementary material for: Genome-wide identification, characterization and gene expression of BES1 transcription factor family in grapevine (Vitis vinifera L.)
Source: Sci Rep. 2023 Jan 5;13:240. doi: 10.1038/s41598-022-24407-y (PMC9816167; doi:10.1038/s41598-022-24407-y)
Supplement: Supplementary file 3 — Supplementary Information. [file 41598_2022_24407_MOESM3_ESM.zip › Vvi_Ath/Vitis_vinifera.PN40024.v4.dna_sm.toplevel.fa.vs.Arabidopsis_thaliana.TAIR10.dna_sm.toplevel.fa.html/Ath-2.html]

|  |  |  |  |  |  |  |  |  |  |  |  |  |  |  |  |  |  |
| --- | --- | --- | --- | --- | --- | --- | --- | --- | --- | --- | --- | --- | --- | --- | --- | --- | --- |
| Duplication depth | Reference chromosome | Collinear blocks | | | | | | | | | | | | | | | |
| 0 | Ath-AT2G01008.1 |  |  |  |  |  |  |  |  |
| 0 | Ath-AT2G01021.1 |  |  |  |  |  |  |  |  |
| 0 | Ath-AT2G01023.1 |  |  |  |  |  |  |  |  |
| 0 | Ath-AT2G01035.1 |  |  |  |  |  |  |  |  |
| 0 | Ath-AT2G01045.1 |  |  |  |  |  |  |  |  |
| 0 | Ath-AT2G01050.1 |  |  |  |  |  |  |  |  |
| 0 | Ath-AT2G01060.1 |  |  |  |  |  |  |  |  |
| 0 | Ath-AT2G01070.1 |  |  |  |  |  |  |  |  |
| 0 | Ath-AT2G01080.1 |  |  |  |  |  |  |  |  |
| 0 | Ath-AT2G01090.1 |  |  |  |  |  |  |  |  |
| 1 | Ath-AT2G01100.2 |  | Vvi-Vitvi01g00375\_t001 |  |  |  |  |  |  |  |
| 2 | Ath-AT2G01110.1 |  | | | |  | Vvi-Vitvi01g00368\_t001 |  |  |  |  |  |  |
| 2 | Ath-AT2G01120.2 |  | | | |  | Vvi-Vitvi01g00366\_t001 |  |  |  |  |  |  |
| 2 | Ath-AT2G01130.1 |  | | | |  | Vvi-Vitvi01g04098\_t001 |  |  |  |  |  |  |
| 2 | Ath-AT2G01140.1 |  | | | |  | Vvi-Vitvi01g00360\_t001 |  |  |  |  |  |  |
| 2 | Ath-AT2G01150.2 |  | | | |  | Vvi-Vitvi01g00354\_t001 |  |  |  |  |  |  |
| 2 | Ath-AT2G01170.1 |  | | | |  | Vvi-Vitvi01g00343\_t001 |  |  |  |  |  |  |
| 2 | Ath-AT2G01175.1 |  | | | |  | | | |  |  |  |  |  |  |
| 2 | Ath-AT2G01180.3 |  | | | |  | Vvi-Vitvi01g01956\_t001 |  |  |  |  |  |  |
| 2 | Ath-AT2G01190.1 |  | | | |  | Vvi-Vitvi01g01955\_t001 |  |  |  |  |  |  |
| 2 | Ath-AT2G01200.1 |  | | | |  | Vvi-Vitvi01g00336\_t001 |  |  |  |  |  |  |
| 2 | Ath-AT2G01210.1 |  | | | |  | Vvi-Vitvi01g00335\_t001 |  |  |  |  |  |  |
| 2 | Ath-AT2G01220.2 |  | | | |  | Vvi-Vitvi01g00333\_t001 |  |  |  |  |  |  |
| 2 | Ath-AT2G01240.1 |  | | | |  | | | |  |  |  |  |  |  |
| 2 | Ath-AT2G01250.1 |  | | | |  | | | |  |  |  |  |  |  |
| 2 | Ath-AT2G01260.1 |  | | | |  | Vvi-Vitvi01g00328\_t001 |  |  |  |  |  |  |
| 1 | Ath-AT2G01270.1 |  | Vvi-Vitvi01g00382\_t001 |  |  |  |  |  |  |  |
| 1 | Ath-AT2G01275.5 |  | | | |  |  |  |  |  |  |  |
| 1 | Ath-AT2G01280.1 |  | | | |  |  |  |  |  |  |  |
| 1 | Ath-AT2G01290.1 |  | Vvi-Vitvi01g00385\_t001 |  |  |  |  |  |  |  |
| 1 | Ath-AT2G01300.1 |  | Vvi-Vitvi01g00388\_t001 |  |  |  |  |  |  |  |
| 1 | Ath-AT2G01310.1 |  | | | |  |  |  |  |  |  |  |
| 1 | Ath-AT2G01320.3 |  | Vvi-Vitvi01g00390\_t001 |  |  |  |  |  |  |  |
| 1 | Ath-AT2G01330.1 |  | | | |  |  |  |  |  |  |  |
| 1 | Ath-AT2G01340.1 |  | Vvi-Vitvi01g00403\_t001 |  |  |  |  |  |  |  |
| 1 | Ath-AT2G01350.1 |  | Vvi-Vitvi01g00405\_t006 |  |  |  |  |  |  |  |
| 1 | Ath-AT2G01360.1 |  | | | |  |  |  |  |  |  |  |
| 1 | Ath-AT2G01370.1 |  | | | |  |  |  |  |  |  |  |
| 1 | Ath-AT2G01379.1 |  | Vvi-Vitvi01g04108\_t001 |  |  |  |  |  |  |  |
| 1 | Ath-AT2G01390.1 |  | | | |  |  |  |  |  |  |  |
| 1 | Ath-AT2G01400.1 |  | | | |  |  |  |  |  |  |  |
| 1 | Ath-AT2G01410.1 |  | Vvi-Vitvi01g00407\_t001 |  |  |  |  |  |  |  |
| 1 | Ath-AT2G01420.2 |  | Vvi-Vitvi01g00411\_t001 |  |  |  |  |  |  |  |
| 1 | Ath-AT2G01430.1 |  | Vvi-Vitvi01g00412\_t001 |  |  |  |  |  |  |  |
| 1 | Ath-AT2G01440.1 |  | Vvi-Vitvi01g00413\_t001 |  |  |  |  |  |  |  |
| 1 | Ath-AT2G01450.1 |  | Vvi-Vitvi01g00417\_t001 |  |  |  |  |  |  |  |
| 1 | Ath-AT2G01460.1 |  | Vvi-Vitvi01g00421\_t001 |  |  |  |  |  |  |  |
| 1 | Ath-AT2G01470.1 |  | Vvi-Vitvi01g00422\_t001 |  |  |  |  |  |  |  |
| 1 | Ath-AT2G01480.1 |  | Vvi-Vitvi01g00425\_t001 |  |  |  |  |  |  |  |
| 1 | Ath-AT2G01490.1 |  | Vvi-Vitvi01g00426\_t001 |  |  |  |  |  |  |  |
| 1 | Ath-AT2G01500.1 |  | Vvi-Vitvi01g00427\_t001 |  |  |  |  |  |  |  |
| 1 | Ath-AT2G01505.1 |  | | | |  |  |  |  |  |  |  |
| 1 | Ath-AT2G01510.1 |  | Vvi-Vitvi01g00430\_t001 |  |  |  |  |  |  |  |
| 1 | Ath-AT2G01520.1 |  | | | |  |  |  |  |  |  |  |
| 1 | Ath-AT2G01530.1 |  | | | |  |  |  |  |  |  |  |
| 1 | Ath-AT2G01540.1 |  | Vvi-Vitvi01g00444\_t001 |  |  |  |  |  |  |  |
| 1 | Ath-AT2G01554.1 |  | | | |  |  |  |  |  |  |  |
| 1 | Ath-AT2G01560.1 |  | | | |  |  |  |  |  |  |  |
| 1 | Ath-AT2G01570.1 |  | Vvi-Vitvi01g00446\_t001 |  |  |  |  |  |  |  |
| 1 | Ath-AT2G01580.1 |  | | | |  |  |  |  |  |  |  |
| 1 | Ath-AT2G01590.1 |  | Vvi-Vitvi01g01984\_t001 |  |  |  |  |  |  |  |
| 1 | Ath-AT2G01600.1 |  | Vvi-Vitvi01g00451\_t002 |  |  |  |  |  |  |  |
| 1 | Ath-AT2G01610.1 |  | Vvi-Vitvi01g00457\_t001 |  |  |  |  |  |  |  |
| 1 | Ath-AT2G01620.1 |  | Vvi-Vitvi01g00467\_t001 |  |  |  |  |  |  |  |
| 1 | Ath-AT2G01630.1 |  | Vvi-Vitvi01g00468\_t001 |  |  |  |  |  |  |  |
| 1 | Ath-AT2G01640.1 |  | | | |  |  |  |  |  |  |  |
| 1 | Ath-AT2G01650.1 |  | Vvi-Vitvi01g00474\_t001 |  |  |  |  |  |  |  |
| 1 | Ath-AT2G01660.1 |  | Vvi-Vitvi01g00477\_t001 |  |  |  |  |  |  |  |
| 1 | Ath-AT2G01667.1 |  | | | |  |  |  |  |  |  |  |
| 1 | Ath-AT2G01670.1 |  | Vvi-Vitvi01g00483\_t001 |  |  |  |  |  |  |  |
| 1 | Ath-AT2G01680.1 |  | Vvi-Vitvi01g00486\_t001 |  |  |  |  |  |  |  |
| 1 | Ath-AT2G01690.2 |  | Vvi-Vitvi01g00487\_t001 |  |  |  |  |  |  |  |
| 1 | Ath-AT2G01710.2 |  | | | |  |  |  |  |  |  |  |
| 1 | Ath-AT2G01720.1 |  | | | |  |  |  |  |  |  |  |
| 1 | Ath-AT2G01730.1 |  | | | |  |  |  |  |  |  |  |
| 1 | Ath-AT2G01735.1 |  | Vvi-Vitvi01g00492\_t001 |  |  |  |  |  |  |  |
| 1 | Ath-AT2G01740.1 |  | | | |  |  |  |  |  |  |  |
| 1 | Ath-AT2G01750.2 |  | Vvi-Vitvi01g00497\_t001 |  |  |  |  |  |  |  |
| 1 | Ath-AT2G01755.2 |  | Vvi-Vitvi01g00500\_t001 |  |  |  |  |  |  |  |
| 1 | Ath-AT2G01760.2 |  | Vvi-Vitvi01g01995\_t001 |  |  |  |  |  |  |  |
| 1 | Ath-AT2G01770.1 |  | Vvi-Vitvi01g00512\_t001 |  |  |  |  |  |  |  |
| 1 | Ath-AT2G01780.1 |  | | | |  |  |  |  |  |  |  |
| 1 | Ath-AT2G01790.1 |  | | | |  |  |  |  |  |  |  |
| 1 | Ath-AT2G01800.1 |  | | | |  |  |  |  |  |  |  |
| 1 | Ath-AT2G01810.1 |  | | | |  |  |  |  |  |  |  |
| 1 | Ath-AT2G01818.1 |  | Vvi-Vitvi01g00519\_t001 |  |  |  |  |  |  |  |
| 1 | Ath-AT2G01820.1 |  | Vvi-Vitvi01g00526\_t001 |  |  |  |  |  |  |  |
| 1 | Ath-AT2G01830.2 |  | Vvi-Vitvi01g00528\_t002 |  |  |  |  |  |  |  |
| 1 | Ath-AT2G01850.1 |  | Vvi-Vitvi01g00533\_t001 |  |  |  |  |  |  |  |
| 1 | Ath-AT2G01860.1 |  | | | |  |  |  |  |  |  |  |
| 1 | Ath-AT2G01870.1 |  | | | |  |  |  |  |  |  |  |
| 1 | Ath-AT2G01880.1 |  | Vvi-Vitvi01g00539\_t001 |  |  |  |  |  |  |  |
| 1 | Ath-AT2G01890.1 |  | | | |  |  |  |  |  |  |  |
| 1 | Ath-AT2G01900.3 |  | Vvi-Vitvi01g00541\_t001 |  |  |  |  |  |  |  |
| 1 | Ath-AT2G01905.1 |  | Vvi-Vitvi01g00542\_t001 |  |  |  |  |  |  |  |
| 1 | Ath-AT2G01910.1 |  | Vvi-Vitvi01g00543\_t001 |  |  |  |  |  |  |  |
| 1 | Ath-AT2G01913.1 |  | | | |  |  |  |  |  |  |  |
| 1 | Ath-AT2G01918.1 |  | | | |  |  |  |  |  |  |  |
| 1 | Ath-AT2G01920.1 |  | Vvi-Vitvi01g00546\_t001 |  |  |  |  |  |  |  |
| 1 | Ath-AT2G01930.1 |  | Vvi-Vitvi01g00547\_t001 |  |  |  |  |  |  |  |
| 1 | Ath-AT2G01940.3 |  | Vvi-Vitvi01g00552\_t001 |  |  |  |  |  |  |  |
| 1 | Ath-AT2G01950.1 |  | Vvi-Vitvi01g00553\_t001 |  |  |  |  |  |  |  |
| 1 | Ath-AT2G01960.1 |  | | | |  |  |  |  |  |  |  |
| 1 | Ath-AT2G01970.1 |  | Vvi-Vitvi01g00561\_t001 |  |  |  |  |  |  |  |
| 1 | Ath-AT2G01980.1 |  | Vvi-Vitvi01g00562\_t001 |  |  |  |  |  |  |  |
| 1 | Ath-AT2G01990.2 |  | Vvi-Vitvi01g00567\_t001 |  |  |  |  |  |  |  |
| 1 | Ath-AT2G02000.1 |  | Vvi-Vitvi01g00568\_t001 |  |  |  |  |  |  |  |
| 1 | Ath-AT2G02010.2 |  | | | |  |  |  |  |  |  |  |
| 1 | Ath-AT2G02020.1 |  | Vvi-Vitvi01g00571\_t001 |  |  |  |  |  |  |  |
| 1 | Ath-AT2G02023.1 |  | | | |  |  |  |  |  |  |  |
| 1 | Ath-AT2G02026.1 |  | | | |  |  |  |  |  |  |  |
| 1 | Ath-AT2G02030.1 |  | | | |  |  |  |  |  |  |  |
| 1 | Ath-AT2G02040.1 |  | | | |  |  |  |  |  |  |  |
| 1 | Ath-AT2G02050.1 |  | Vvi-Vitvi01g04133\_t001 |  |  |  |  |  |  |  |
| 1 | Ath-AT2G02060.2 |  | Vvi-Vitvi01g00578\_t001 |  |  |  |  |  |  |  |
| 1 | Ath-AT2G02061.1 |  | Vvi-Vitvi01g01498\_t001 |  |  |  |  |  |  |  |
| 1 | Ath-AT2G02070.1 |  | Vvi-Vitvi01g01492\_t001 |  |  |  |  |  |  |  |
| 1 | Ath-AT2G02080.1 |  | | | |  |  |  |  |  |  |  |
| 1 | Ath-AT2G02090.1 |  | Vvi-Vitvi01g01488\_t001 |  |  |  |  |  |  |  |
| 1 | Ath-AT2G02100.1 |  | Vvi-Vitvi01g01476\_t001 |  |  |  |  |  |  |  |
| 1 | Ath-AT2G02103.1 |  | | | |  |  |  |  |  |  |  |
| 1 | Ath-AT2G02120.1 |  | | | |  |  |  |  |  |  |  |
| 1 | Ath-AT2G02130.1 |  | | | |  |  |  |  |  |  |  |
| 1 | Ath-AT2G02140.1 |  | | | |  |  |  |  |  |  |  |
| 1 | Ath-AT2G02147.1 |  | | | |  |  |  |  |  |  |  |
| 1 | Ath-AT2G02148.3 |  | Vvi-Vitvi01g01456\_t001 |  |  |  |  |  |  |  |
| 1 | Ath-AT2G02150.1 |  | Vvi-Vitvi01g01449\_t001 |  |  |  |  |  |  |  |
| 1 | Ath-AT2G02160.1 |  | Vvi-Vitvi01g01447\_t001 |  |  |  |  |  |  |  |
| 1 | Ath-AT2G02170.2 |  | Vvi-Vitvi01g01446\_t001 |  |  |  |  |  |  |  |
| 1 | Ath-AT2G02180.1 |  | Vvi-Vitvi01g01443\_t001 |  |  |  |  |  |  |  |
| 1 | Ath-AT2G02220.1 |  | Vvi-Vitvi01g01439\_t001 |  |  |  |  |  |  |  |
| 0 | Ath-AT2G02230.1 |  |  |  |  |  |  |  |  |
| 0 | Ath-AT2G02240.1 |  |  |  |  |  |  |  |  |
| 0 | Ath-AT2G02250.1 |  |  |  |  |  |  |  |  |
| 0 | Ath-AT2G02280.1 |  |  |  |  |  |  |  |  |
| 0 | Ath-AT2G02290.1 |  |  |  |  |  |  |  |  |
| 0 | Ath-AT2G02300.1 |  |  |  |  |  |  |  |  |
| 0 | Ath-AT2G02310.1 |  |  |  |  |  |  |  |  |
| 0 | Ath-AT2G02320.1 |  |  |  |  |  |  |  |  |
| 0 | Ath-AT2G02340.1 |  |  |  |  |  |  |  |  |
| 0 | Ath-AT2G02350.2 |  |  |  |  |  |  |  |  |
| 0 | Ath-AT2G02360.1 |  |  |  |  |  |  |  |  |
| 1 | Ath-AT2G02370.1 |  | Vvi-Vitvi12g04645\_t003 |  |  |  |  |  |  |  |
| 1 | Ath-AT2G02380.1 |  | Vvi-Vitvi12g02758\_t001 |  |  |  |  |  |  |  |
| 1 | Ath-AT2G02390.3 |  | | | |  |  |  |  |  |  |  |
| 1 | Ath-AT2G02400.1 |  | Vvi-Vitvi12g02158\_t001 |  |  |  |  |  |  |  |
| 1 | Ath-AT2G02410.6 |  | Vvi-Vitvi12g02152\_t001 |  |  |  |  |  |  |  |
| 1 | Ath-AT2G02440.1 |  | | | |  |  |  |  |  |  |  |
| 1 | Ath-AT2G02450.2 |  | Vvi-Vitvi12g02150\_t001 |  |  |  |  |  |  |  |
| 1 | Ath-AT2G02455.1 |  | | | |  |  |  |  |  |  |  |
| 1 | Ath-AT2G02470.1 |  | Vvi-Vitvi12g02144\_t001 |  |  |  |  |  |  |  |
| 1 | Ath-AT2G02480.1 |  | Vvi-Vitvi12g02142\_t001 |  |  |  |  |  |  |  |
| 1 | Ath-AT2G02490.1 |  | | | |  |  |  |  |  |  |  |
| 1 | Ath-AT2G02493.1 |  | | | |  |  |  |  |  |  |  |
| 1 | Ath-AT2G02497.1 |  | | | |  |  |  |  |  |  |  |
| 1 | Ath-AT2G02498.1 |  | | | |  |  |  |  |  |  |  |
| 1 | Ath-AT2G02500.1 |  | Vvi-Vitvi12g02141\_t001 |  |  |  |  |  |  |  |
| 1 | Ath-AT2G02510.1 |  | Vvi-Vitvi12g02140\_t001 |  |  |  |  |  |  |  |
| 1 | Ath-AT2G02515.1 |  | | | |  |  |  |  |  |  |  |
| 1 | Ath-AT2G02520.1 |  | | | |  |  |  |  |  |  |  |
| 1 | Ath-AT2G02525.1 |  | | | |  |  |  |  |  |  |  |
| 1 | Ath-AT2G02540.1 |  | Vvi-Vitvi12g02135\_t001 |  |  |  |  |  |  |  |
| 1 | Ath-AT2G02550.2 |  | | | |  |  |  |  |  |  |  |
| 1 | Ath-AT2G02560.1 |  | Vvi-Vitvi12g02128\_t001 |  |  |  |  |  |  |  |
| 1 | Ath-AT2G02570.2 |  | Vvi-Vitvi12g02126\_t001 |  |  |  |  |  |  |  |
| 0 | Ath-AT2G02580.1 |  |  |  |  |  |  |  |  |
| 1 | Ath-AT2G02590.1 |  | Vvi-Vitvi01g00917\_t001 |  |  |  |  |  |  |  |
| 1 | Ath-AT2G02610.1 |  | | | |  |  |  |  |  |  |  |
| 1 | Ath-AT2G02620.1 |  | | | |  |  |  |  |  |  |  |
| 1 | Ath-AT2G02630.1 |  | | | |  |  |  |  |  |  |  |
| 1 | Ath-AT2G02635.1 |  | | | |  |  |  |  |  |  |  |
| 1 | Ath-AT2G02640.1 |  | | | |  |  |  |  |  |  |  |
| 1 | Ath-AT2G02650.1 |  | | | |  |  |  |  |  |  |  |
| 1 | Ath-AT2G02660.1 |  | | | |  |  |  |  |  |  |  |
| 1 | Ath-AT2G02680.1 |  | | | |  |  |  |  |  |  |  |
| 1 | Ath-AT2G02690.1 |  | | | |  |  |  |  |  |  |  |
| 1 | Ath-AT2G02695.1 |  | | | |  |  |  |  |  |  |  |
| 1 | Ath-AT2G02700.1 |  | | | |  |  |  |  |  |  |  |
| 1 | Ath-AT2G02710.1 |  | Vvi-Vitvi01g00924\_t005 |  |  |  |  |  |  |  |
| 2 | Ath-AT2G02720.1 |  | Vvi-Vitvi01g02109\_t001 |  | Vvi-Vitvi17g00550\_t001 |  |  |  |  |  |  |
| 2 | Ath-AT2G02730.2 |  | Vvi-Vitvi01g00935\_t001 |  | | | |  |  |  |  |  |  |
| 2 | Ath-AT2G02740.1 |  | Vvi-Vitvi01g00938\_t001 |  | | | |  |  |  |  |  |  |
| 2 | Ath-AT2G02750.1 |  | Vvi-Vitvi01g00942\_t001 |  | | | |  |  |  |  |  |  |
| 2 | Ath-AT2G02760.2 |  | Vvi-Vitvi01g00943\_t002 |  | Vvi-Vitvi17g04153\_t001 |  |  |  |  |  |  |
| 2 | Ath-AT2G02765.1 |  | | | |  | | | |  |  |  |  |  |  |
| 2 | Ath-AT2G02770.1 |  | | | |  | | | |  |  |  |  |  |  |
| 2 | Ath-AT2G02780.1 |  | Vvi-Vitvi01g00944\_t001 |  | | | |  |  |  |  |  |  |
| 2 | Ath-AT2G02790.1 |  | Vvi-Vitvi01g00945\_t001 |  | Vvi-Vitvi17g00560\_t001 |  |  |  |  |  |  |
| 2 | Ath-AT2G02800.2 |  | Vvi-Vitvi01g00953\_t001 |  | Vvi-Vitvi17g00568\_t001 |  |  |  |  |  |  |
| 2 | Ath-AT2G02795.1 |  | | | |  | | | |  |  |  |  |  |  |
| 2 | Ath-AT2G02810.1 |  | Vvi-Vitvi01g00955\_t002 |  | | | |  |  |  |  |  |  |
| 2 | Ath-AT2G02820.2 |  | Vvi-Vitvi01g00956\_t001 |  | | | |  |  |  |  |  |  |
| 2 | Ath-AT2G02835.1 |  | | | |  | | | |  |  |  |  |  |  |
| 2 | Ath-AT2G02840.1 |  | | | |  | | | |  |  |  |  |  |  |
| 2 | Ath-AT2G02850.1 |  | | | |  | | | |  |  |  |  |  |  |
| 2 | Ath-AT2G02860.1 |  | Vvi-Vitvi01g00959\_t001 |  | | | |  |  |  |  |  |  |
| 2 | Ath-AT2G02870.3 |  | Vvi-Vitvi01g00970\_t001 |  | Vvi-Vitvi17g00570\_t001 |  |  |  |  |  |  |
| 2 | Ath-AT2G02880.1 |  | | | |  | | | |  |  |  |  |  |  |
| 2 | Ath-AT2G02890.1 |  | | | |  | | | |  |  |  |  |  |  |
| 2 | Ath-AT2G02910.1 |  | Vvi-Vitvi01g00988\_t001 |  | | | |  |  |  |  |  |  |
| 2 | Ath-AT2G02930.1 |  | | | |  | | | |  |  |  |  |  |  |
| 2 | Ath-AT2G02950.1 |  | Vvi-Vitvi01g00994\_t001 |  | Vvi-Vitvi17g00583\_t001 |  |  |  |  |  |  |
| 2 | Ath-AT2G02955.1 |  | Vvi-Vitvi01g01010\_t001 |  | | | |  |  |  |  |  |  |
| 2 | Ath-AT2G02960.5 |  | Vvi-Vitvi01g01015\_t001 |  | Vvi-Vitvi17g00603\_t001 |  |  |  |  |  |  |
| 2 | Ath-AT2G02970.1 |  | Vvi-Vitvi01g01018\_t001 |  | | | |  |  |  |  |  |  |
| 2 | Ath-AT2G02980.1 |  | Vvi-Vitvi01g01020\_t001 |  | | | |  |  |  |  |  |  |
| 2 | Ath-AT2G02990.1 |  | Vvi-Vitvi01g01021\_t001 |  | | | |  |  |  |  |  |  |
| 2 | Ath-AT2G03000.1 |  | | | |  | | | |  |  |  |  |  |  |
| 2 | Ath-AT2G03010.1 |  | | | |  | | | |  |  |  |  |  |  |
| 2 | Ath-AT2G03020.1 |  | | | |  | | | |  |  |  |  |  |  |
| 2 | Ath-AT2G03030.1 |  | | | |  | | | |  |  |  |  |  |  |
| 2 | Ath-AT2G03040.1 |  | | | |  | | | |  |  |  |  |  |  |
| 2 | Ath-AT2G03050.2 |  | | | |  | | | |  |  |  |  |  |  |
| 2 | Ath-AT2G03060.2 |  | | | |  | Vvi-Vitvi17g00614\_t001 |  |  |  |  |  |  |
| 2 | Ath-AT2G03070.1 |  | | | |  | | | |  |  |  |  |  |  |
| 2 | Ath-AT2G03090.1 |  | Vvi-Vitvi01g01030\_t001 |  | Vvi-Vitvi17g00616\_t001 |  |  |  |  |  |  |
| 2 | Ath-AT2G03110.2 |  | | | |  | | | |  |  |  |  |  |  |
| 2 | Ath-AT2G03120.1 |  | | | |  | | | |  |  |  |  |  |  |
| 2 | Ath-AT2G03130.1 |  | | | |  | | | |  |  |  |  |  |  |
| 2 | Ath-AT2G03140.12 |  | | | |  | | | |  |  |  |  |  |  |
| 2 | Ath-AT2G03150.1 |  | Vvi-Vitvi01g01040\_t001 |  | | | |  |  |  |  |  |  |
| 2 | Ath-AT2G03160.1 |  | | | |  | | | |  |  |  |  |  |  |
| 2 | Ath-AT2G03170.1 |  | | | |  | | | |  |  |  |  |  |  |
| 2 | Ath-AT2G03180.1 |  | | | |  | | | |  |  |  |  |  |  |
| 2 | Ath-AT2G03190.1 |  | | | |  | | | |  |  |  |  |  |  |
| 2 | Ath-AT2G03200.1 |  | | | |  | | | |  |  |  |  |  |  |
| 2 | Ath-AT2G03210.2 |  | Vvi-Vitvi01g01079\_t001 |  | Vvi-Vitvi17g00627\_t001 |  |  |  |  |  |  |
| 2 | Ath-AT2G03220.1 |  | | | |  | | | |  |  |  |  |  |  |
| 2 | Ath-AT2G03240.1 |  | Vvi-Vitvi01g01077\_t001 |  | | | |  |  |  |  |  |  |
| 2 | Ath-AT2G03230.1 |  | | | |  | | | |  |  |  |  |  |  |
| 2 | Ath-AT2G03250.1 |  | | | |  | | | |  |  |  |  |  |  |
| 2 | Ath-AT2G03260.1 |  | | | |  | | | |  |  |  |  |  |  |
| 2 | Ath-AT2G03270.1 |  | | | |  | | | |  |  |  |  |  |  |
| 2 | Ath-AT2G03280.2 |  | Vvi-Vitvi01g01057\_t001 |  | | | |  |  |  |  |  |  |
| 2 | Ath-AT2G03290.1 |  | Vvi-Vitvi01g01056\_t001 |  | | | |  |  |  |  |  |  |
| 1 | Ath-AT2G03300.1 |  |  |  | | | |  |  |  |  |  |  |
| 1 | Ath-AT2G03310.1 |  |  |  | | | |  |  |  |  |  |  |
| 1 | Ath-AT2G03320.1 |  |  |  | | | |  |  |  |  |  |  |
| 1 | Ath-AT2G03330.1 |  |  |  | | | |  |  |  |  |  |  |
| 1 | Ath-AT2G03340.1 |  |  |  | | | |  |  |  |  |  |  |
| 1 | Ath-AT2G03350.1 |  |  |  | Vvi-Vitvi17g00651\_t001 |  |  |  |  |  |  |
| 1 | Ath-AT2G03360.2 |  |  |  | | | |  |  |  |  |  |  |
| 1 | Ath-AT2G03370.2 |  |  |  | | | |  |  |  |  |  |  |
| 2 | Ath-AT2G03380.1 |  | Vvi-Vitvi01g00079\_t001 |  | | | |  |  |  |  |  |  |
| 2 | Ath-AT2G03390.4 |  | Vvi-Vitvi01g00077\_t001 |  | | | |  |  |  |  |  |  |
| 2 | Ath-AT2G03420.1 |  | Vvi-Vitvi01g00075\_t001 |  | | | |  |  |  |  |  |  |
| 2 | Ath-AT2G03410.1 |  | | | |  | | | |  |  |  |  |  |  |
| 2 | Ath-AT2G03430.1 |  | Vvi-Vitvi01g00074\_t001 |  | | | |  |  |  |  |  |  |
| 2 | Ath-AT2G03440.1 |  | Vvi-Vitvi01g01846\_t001 |  | | | |  |  |  |  |  |  |
| 2 | Ath-AT2G03450.1 |  | | | |  | | | |  |  |  |  |  |  |
| 2 | Ath-AT2G03460.1 |  | | | |  | | | |  |  |  |  |  |  |
| 2 | Ath-AT2G03470.1 |  | | | |  | Vvi-Vitvi17g00669\_t001 |  |  |  |  |  |  |
| 1 | Ath-AT2G03480.1 |  | Vvi-Vitvi01g00070\_t001 |  |  |  |  |  |  |  |
| 1 | Ath-AT2G03500.1 |  | | | |  |  |  |  |  |  |  |
| 1 | Ath-AT2G03505.1 |  | Vvi-Vitvi01g00051\_t001 |  |  |  |  |  |  |  |
| 1 | Ath-AT2G03510.1 |  | Vvi-Vitvi01g00049\_t001 |  |  |  |  |  |  |  |
| 1 | Ath-AT2G03520.1 |  | Vvi-Vitvi01g00048\_t001 |  |  |  |  |  |  |  |
| 1 | Ath-AT2G03530.4 |  | | | |  |  |  |  |  |  |  |
| 1 | Ath-AT2G03550.1 |  | | | |  |  |  |  |  |  |  |
| 1 | Ath-AT2G03560.1 |  | | | |  |  |  |  |  |  |  |
| 1 | Ath-AT2G03565.1 |  | | | |  |  |  |  |  |  |  |
| 1 | Ath-AT2G03567.1 |  | | | |  |  |  |  |  |  |  |
| 1 | Ath-AT2G03570.1 |  | | | |  |  |  |  |  |  |  |
| 1 | Ath-AT2G03580.1 |  | | | |  |  |  |  |  |  |  |
| 1 | Ath-AT2G03590.1 |  | | | |  |  |  |  |  |  |  |
| 1 | Ath-AT2G03600.4 |  | | | |  |  |  |  |  |  |  |
| 1 | Ath-AT2G03610.1 |  | | | |  |  |  |  |  |  |  |
| 1 | Ath-AT2G03620.1 |  | Vvi-Vitvi01g00039\_t004 |  |  |  |  |  |  |  |
| 1 | Ath-AT2G03630.1 |  | Vvi-Vitvi01g01840\_t001 |  |  |  |  |  |  |  |
| 2 | Ath-AT2G03640.4 |  | Vvi-Vitvi01g00036\_t002 |  | Vvi-Vitvi17g00496\_t002 |  |  |  |  |  |  |
| 2 | Ath-AT2G03667.1 |  | Vvi-Vitvi01g00035\_t001 |  | | | |  |  |  |  |  |  |
| 2 | Ath-AT2G03670.1 |  | | | |  | | | |  |  |  |  |  |  |
| 2 | Ath-AT2G03680.1 |  | Vvi-Vitvi01g00033\_t002 |  | Vvi-Vitvi17g00489\_t001 |  |  |  |  |  |  |
| 2 | Ath-AT2G03690.1 |  | | | |  | | | |  |  |  |  |  |  |
| 2 | Ath-AT2G03710.1 |  | Vvi-Vitvi01g00011\_t001 |  | Vvi-Vitvi17g00471\_t001 |  |  |  |  |  |  |
| 2 | Ath-AT2G03720.2 |  | Vvi-Vitvi01g01835\_t001 |  | Vvi-Vitvi17g00469\_t001 |  |  |  |  |  |  |
| 2 | Ath-AT2G03730.1 |  | Vvi-Vitvi01g00002\_t001 |  | | | |  |  |  |  |  |  |
| 1 | Ath-AT2G03740.1 |  |  |  | | | |  |  |  |  |  |  |
| 1 | Ath-AT2G03750.1 |  |  |  | | | |  |  |  |  |  |  |
| 1 | Ath-AT2G03760.1 |  |  |  | | | |  |  |  |  |  |  |
| 1 | Ath-AT2G03770.1 |  |  |  | Vvi-Vitvi17g00454\_t001 |  |  |  |  |  |  |
| 1 | Ath-AT2G03780.1 |  |  |  | | | |  |  |  |  |  |  |
| 1 | Ath-AT2G03800.2 |  |  |  | Vvi-Vitvi17g00451\_t001 |  |  |  |  |  |  |
| 1 | Ath-AT2G03810.1 |  |  |  | Vvi-Vitvi17g01434\_t001 |  |  |  |  |  |  |
| 1 | Ath-AT2G03820.1 |  |  |  | | | |  |  |  |  |  |  |
| 1 | Ath-AT2G03821.1 |  |  |  | | | |  |  |  |  |  |  |
| 1 | Ath-AT2G03823.1 |  |  |  | | | |  |  |  |  |  |  |
| 1 | Ath-AT2G03822.1 |  |  |  | | | |  |  |  |  |  |  |
| 1 | Ath-AT2G03830.1 |  |  |  | | | |  |  |  |  |  |  |
| 1 | Ath-AT2G03840.1 |  |  |  | | | |  |  |  |  |  |  |
| 1 | Ath-AT2G03850.1 |  |  |  | | | |  |  |  |  |  |  |
| 1 | Ath-AT2G03870.2 |  |  |  | | | |  |  |  |  |  |  |
| 1 | Ath-AT2G03880.1 |  |  |  | | | |  |  |  |  |  |  |
| 1 | Ath-AT2G03890.1 |  |  |  | Vvi-Vitvi17g00441\_t001 |  |  |  |  |  |  |
| 1 | Ath-AT2G03913.1 |  |  |  | | | |  |  |  |  |  |  |
| 1 | Ath-AT2G03930.2 |  |  |  | | | |  |  |  |  |  |  |
| 1 | Ath-AT2G03932.1 |  |  |  | | | |  |  |  |  |  |  |
| 1 | Ath-AT2G03931.1 |  |  |  | | | |  |  |  |  |  |  |
| 1 | Ath-AT2G03933.1 |  |  |  | | | |  |  |  |  |  |  |
| 1 | Ath-AT2G03936.1 |  |  |  | | | |  |  |  |  |  |  |
| 1 | Ath-AT2G03937.2 |  |  |  | | | |  |  |  |  |  |  |
| 1 | Ath-AT2G03955.1 |  |  |  | | | |  |  |  |  |  |  |
| 1 | Ath-AT2G03965.1 |  |  |  | | | |  |  |  |  |  |  |
| 1 | Ath-AT2G03980.2 |  |  |  | | | |  |  |  |  |  |  |
| 1 | Ath-AT2G04020.2 |  |  |  | | | |  |  |  |  |  |  |
| 1 | Ath-AT2G04025.1 |  |  |  | | | |  |  |  |  |  |  |
| 1 | Ath-AT2G04030.1 |  |  |  | | | |  |  |  |  |  |  |
| 1 | Ath-AT2G04032.1 |  |  |  | | | |  |  |  |  |  |  |
| 1 | Ath-AT2G04034.1 |  |  |  | | | |  |  |  |  |  |  |
| 1 | Ath-AT2G04031.1 |  |  |  | | | |  |  |  |  |  |  |
| 1 | Ath-AT2G04037.1 |  |  |  | | | |  |  |  |  |  |  |
| 1 | Ath-AT2G04041.1 |  |  |  | | | |  |  |  |  |  |  |
| 1 | Ath-AT2G04045.1 |  |  |  | | | |  |  |  |  |  |  |
| 1 | Ath-AT2G04046.1 |  |  |  | | | |  |  |  |  |  |  |
| 1 | Ath-AT2G04038.1 |  |  |  | Vvi-Vitvi17g00434\_t001 |  |  |  |  |  |  |
| 0 | Ath-AT2G04137.1 |  |  |  |  |  |  |  |  |
| 0 | Ath-AT2G04039.3 |  |  |  |  |  |  |  |  |
| 0 | Ath-AT2G04040.1 |  |  |  |  |  |  |  |  |
| 0 | Ath-AT2G04050.1 |  |  |  |  |  |  |  |  |
| 0 | Ath-AT2G04060.1 |  |  |  |  |  |  |  |  |
| 0 | Ath-AT2G04062.1 |  |  |  |  |  |  |  |  |
| 0 | Ath-AT2G04063.1 |  |  |  |  |  |  |  |  |
| 0 | Ath-AT2G04066.1 |  |  |  |  |  |  |  |  |
| 0 | Ath-AT2G04070.1 |  |  |  |  |  |  |  |  |
| 0 | Ath-AT2G04080.1 |  |  |  |  |  |  |  |  |
| 0 | Ath-AT2G04090.1 |  |  |  |  |  |  |  |  |
| 0 | Ath-AT2G04100.1 |  |  |  |  |  |  |  |  |
| 0 | Ath-AT2G04115.1 |  |  |  |  |  |  |  |  |
| 1 | Ath-AT2G04160.1 |  | Vvi-Vitvi12g00766\_t001 |  |  |  |  |  |  |  |
| 1 | Ath-AT2G04170.1 |  | | | |  |  |  |  |  |  |  |
| 1 | Ath-AT2G04190.1 |  | | | |  |  |  |  |  |  |  |
| 1 | Ath-AT2G04220.1 |  | Vvi-Vitvi12g00762\_t001 |  |  |  |  |  |  |  |
| 1 | Ath-AT2G04230.1 |  | | | |  |  |  |  |  |  |  |
| 1 | Ath-AT2G04235.1 |  | Vvi-Vitvi12g00759\_t001 |  |  |  |  |  |  |  |
| 1 | Ath-AT2G04240.1 |  | Vvi-Vitvi12g00755\_t001 |  |  |  |  |  |  |  |
| 1 | Ath-AT2G04270.5 |  | Vvi-Vitvi12g00754\_t001 |  |  |  |  |  |  |  |
| 1 | Ath-AT2G04280.1 |  | Vvi-Vitvi12g00753\_t001 |  |  |  |  |  |  |  |
| 1 | Ath-AT2G04300.2 |  | | | |  |  |  |  |  |  |  |
| 1 | Ath-AT2G04305.1 |  | | | |  |  |  |  |  |  |  |
| 1 | Ath-AT2G04340.1 |  | | | |  |  |  |  |  |  |  |
| 1 | Ath-AT2G04350.1 |  | Vvi-Vitvi12g02505\_t001 |  |  |  |  |  |  |  |
| 1 | Ath-AT2G04360.2 |  | Vvi-Vitvi12g00747\_t001 |  |  |  |  |  |  |  |
| 1 | Ath-AT2G04378.2 |  | | | |  |  |  |  |  |  |  |
| 1 | Ath-AT2G04380.1 |  | | | |  |  |  |  |  |  |  |
| 1 | Ath-AT2G04390.1 |  | | | |  |  |  |  |  |  |  |
| 1 | Ath-AT2G04395.1 |  | | | |  |  |  |  |  |  |  |
| 1 | Ath-AT2G04400.1 |  | Vvi-Vitvi12g00745\_t001 |  |  |  |  |  |  |  |
| 1 | Ath-AT2G04410.1 |  | Vvi-Vitvi12g04252\_t001 |  |  |  |  |  |  |  |
| 1 | Ath-AT2G04420.1 |  | | | |  |  |  |  |  |  |  |
| 1 | Ath-AT2G04425.1 |  | | | |  |  |  |  |  |  |  |
| 1 | Ath-AT2G04430.1 |  | Vvi-Vitvi12g04246\_t001 |  |  |  |  |  |  |  |
| 1 | Ath-AT2G04440.1 |  | | | |  |  |  |  |  |  |  |
| 1 | Ath-AT2G04450.1 |  | | | |  |  |  |  |  |  |  |
| 1 | Ath-AT2G04480.1 |  | Vvi-Vitvi12g02494\_t001 |  |  |  |  |  |  |  |
| 0 | Ath-AT2G04495.1 |  |  |  |  |  |  |  |  |
| 0 | Ath-AT2G04500.1 |  |  |  |  |  |  |  |  |
| 0 | Ath-AT2G04515.1 |  |  |  |  |  |  |  |  |
| 0 | Ath-AT2G04520.1 |  |  |  |  |  |  |  |  |
| 0 | Ath-AT2G04530.1 |  |  |  |  |  |  |  |  |
| 0 | Ath-AT2G04540.1 |  |  |  |  |  |  |  |  |
| 0 | Ath-AT2G04550.1 |  |  |  |  |  |  |  |  |
| 0 | Ath-AT2G04560.1 |  |  |  |  |  |  |  |  |
| 0 | Ath-AT2G04570.1 |  |  |  |  |  |  |  |  |
| 0 | Ath-AT2G04620.1 |  |  |  |  |  |  |  |  |
| 0 | Ath-AT2G04621.1 |  |  |  |  |  |  |  |  |
| 0 | Ath-AT2G04622.1 |  |  |  |  |  |  |  |  |
| 0 | Ath-AT2G04630.1 |  |  |  |  |  |  |  |  |
| 0 | Ath-AT2G04650.1 |  |  |  |  |  |  |  |  |
| 0 | Ath-AT2G04660.1 |  |  |  |  |  |  |  |  |
| 0 | Ath-AT2G04675.1 |  |  |  |  |  |  |  |  |
| 0 | Ath-AT2G04680.1 |  |  |  |  |  |  |  |  |
| 0 | Ath-AT2G04690.5 |  |  |  |  |  |  |  |  |
| 0 | Ath-AT2G04700.3 |  |  |  |  |  |  |  |  |
| 0 | Ath-AT2G04740.1 |  |  |  |  |  |  |  |  |
| 0 | Ath-AT2G04750.1 |  |  |  |  |  |  |  |  |
| 0 | Ath-AT2G04780.1 |  |  |  |  |  |  |  |  |
| 0 | Ath-AT2G04790.10 |  |  |  |  |  |  |  |  |
| 0 | Ath-AT2G04795.1 |  |  |  |  |  |  |  |  |
| 0 | Ath-AT2G04800.1 |  |  |  |  |  |  |  |  |
| 0 | Ath-AT2G04810.1 |  |  |  |  |  |  |  |  |
| 0 | Ath-AT2G04830.1 |  |  |  |  |  |  |  |  |
| 0 | Ath-AT2G04840.1 |  |  |  |  |  |  |  |  |
| 1 | Ath-AT2G04842.1 |  | Vvi-Vitvi12g00647\_t001 |  |  |  |  |  |  |  |
| 1 | Ath-AT2G04845.1 |  | Vvi-Vitvi12g00651\_t001 |  |  |  |  |  |  |  |
| 1 | Ath-AT2G04850.1 |  | Vvi-Vitvi12g00654\_t001 |  |  |  |  |  |  |  |
| 1 | Ath-AT2G04860.1 |  | | | |  |  |  |  |  |  |  |
| 1 | Ath-AT2G04865.2 |  | | | |  |  |  |  |  |  |  |
| 1 | Ath-AT2G04870.1 |  | | | |  |  |  |  |  |  |  |
| 1 | Ath-AT2G04880.1 |  | Vvi-Vitvi12g00664\_t003 |  |  |  |  |  |  |  |
| 1 | Ath-AT2G04890.1 |  | Vvi-Vitvi12g00665\_t002 |  |  |  |  |  |  |  |
| 1 | Ath-AT2G04900.1 |  | | | |  |  |  |  |  |  |  |
| 1 | Ath-AT2G04910.1 |  | | | |  |  |  |  |  |  |  |
| 1 | Ath-AT2G04920.1 |  | | | |  |  |  |  |  |  |  |
| 1 | Ath-AT2G04925.1 |  | | | |  |  |  |  |  |  |  |
| 1 | Ath-AT2G04930.1 |  | | | |  |  |  |  |  |  |  |
| 1 | Ath-AT2G04940.1 |  | | | |  |  |  |  |  |  |  |
| 1 | Ath-AT2G05050.1 |  | | | |  |  |  |  |  |  |  |
| 1 | Ath-AT2G05060.1 |  | | | |  |  |  |  |  |  |  |
| 1 | Ath-AT2G05070.1 |  | | | |  |  |  |  |  |  |  |
| 1 | Ath-AT2G05100.2 |  | | | |  |  |  |  |  |  |  |
| 1 | Ath-AT2G05105.1 |  | | | |  |  |  |  |  |  |  |
| 1 | Ath-AT2G05117.1 |  | | | |  |  |  |  |  |  |  |
| 1 | Ath-AT2G05120.2 |  | | | |  |  |  |  |  |  |  |
| 1 | Ath-AT2G05140.1 |  | | | |  |  |  |  |  |  |  |
| 1 | Ath-AT2G05160.1 |  | Vvi-Vitvi12g00667\_t001 |  |  |  |  |  |  |  |
| 1 | Ath-AT2G05170.1 |  | Vvi-Vitvi12g04221\_t001 |  |  |  |  |  |  |  |
| 1 | Ath-AT2G05180.1 |  | | | |  |  |  |  |  |  |  |
| 1 | Ath-AT2G05185.1 |  | | | |  |  |  |  |  |  |  |
| 1 | Ath-AT2G05210.2 |  | | | |  |  |  |  |  |  |  |
| 1 | Ath-AT2G05220.1 |  | | | |  |  |  |  |  |  |  |
| 1 | Ath-AT2G05230.1 |  | Vvi-Vitvi12g00687\_t001 |  |  |  |  |  |  |  |
| 0 | Ath-AT2G05250.1 |  |  |  |  |  |  |  |  |
| 0 | Ath-AT2G05260.1 |  |  |  |  |  |  |  |  |
| 0 | Ath-AT2G05270.1 |  |  |  |  |  |  |  |  |
| 0 | Ath-AT2G05294.1 |  |  |  |  |  |  |  |  |
| 0 | Ath-AT2G05310.1 |  |  |  |  |  |  |  |  |
| 0 | Ath-AT2G05320.1 |  |  |  |  |  |  |  |  |
| 0 | Ath-AT2G05330.1 |  |  |  |  |  |  |  |  |
| 0 | Ath-AT2G05335.1 |  |  |  |  |  |  |  |  |
| 0 | Ath-AT2G05350.1 |  |  |  |  |  |  |  |  |
| 0 | Ath-AT2G05360.1 |  |  |  |  |  |  |  |  |
| 0 | Ath-AT2G05370.1 |  |  |  |  |  |  |  |  |
| 0 | Ath-AT2G05380.3 |  |  |  |  |  |  |  |  |
| 0 | Ath-AT2G05400.3 |  |  |  |  |  |  |  |  |
| 0 | Ath-AT2G05410.2 |  |  |  |  |  |  |  |  |
| 0 | Ath-AT2G05420.1 |  |  |  |  |  |  |  |  |
| 0 | Ath-AT2G05430.1 |  |  |  |  |  |  |  |  |
| 0 | Ath-AT2G05440.2 |  |  |  |  |  |  |  |  |
| 0 | Ath-AT2G05510.1 |  |  |  |  |  |  |  |  |
| 0 | Ath-AT2G05520.1 |  |  |  |  |  |  |  |  |
| 0 | Ath-AT2G05530.1 |  |  |  |  |  |  |  |  |
| 0 | Ath-AT2G05540.1 |  |  |  |  |  |  |  |  |
| 0 | Ath-AT2G05580.1 |  |  |  |  |  |  |  |  |
| 0 | Ath-AT2G05590.2 |  |  |  |  |  |  |  |  |
| 0 | Ath-AT2G05600.1 |  |  |  |  |  |  |  |  |
| 0 | Ath-AT2G05620.1 |  |  |  |  |  |  |  |  |
| 0 | Ath-AT2G05630.2 |  |  |  |  |  |  |  |  |
| 0 | Ath-AT2G05632.1 |  |  |  |  |  |  |  |  |
| 0 | Ath-AT2G05635.2 |  |  |  |  |  |  |  |  |
| 0 | Ath-AT2G05642.1 |  |  |  |  |  |  |  |  |
| 0 | Ath-AT2G05645.1 |  |  |  |  |  |  |  |  |
| 0 | Ath-AT2G05655.2 |  |  |  |  |  |  |  |  |
| 0 | Ath-AT2G05710.1 |  |  |  |  |  |  |  |  |
| 0 | Ath-AT2G05720.1 |  |  |  |  |  |  |  |  |
| 0 | Ath-AT2G05752.1 |  |  |  |  |  |  |  |  |
| 0 | Ath-AT2G05755.1 |  |  |  |  |  |  |  |  |
| 0 | Ath-AT2G05753.1 |  |  |  |  |  |  |  |  |
| 0 | Ath-AT2G05760.1 |  |  |  |  |  |  |  |  |
| 0 | Ath-AT2G05786.1 |  |  |  |  |  |  |  |  |
| 0 | Ath-AT2G05790.1 |  |  |  |  |  |  |  |  |
| 0 | Ath-AT2G05810.1 |  |  |  |  |  |  |  |  |
| 1 | Ath-AT2G05830.1 |  | Vvi-Vitvi11g00063\_t002 |  |  |  |  |  |  |  |
| 1 | Ath-AT2G05840.1 |  | | | |  |  |  |  |  |  |  |
| 1 | Ath-AT2G05850.1 |  | | | |  |  |  |  |  |  |  |
| 1 | Ath-AT2G05900.1 |  | | | |  |  |  |  |  |  |  |
| 1 | Ath-AT2G05910.1 |  | Vvi-Vitvi11g00069\_t001 |  |  |  |  |  |  |  |
| 1 | Ath-AT2G05915.1 |  | | | |  |  |  |  |  |  |  |
| 1 | Ath-AT2G05920.1 |  | Vvi-Vitvi11g00071\_t001 |  |  |  |  |  |  |  |
| 1 | Ath-AT2G05940.1 |  | Vvi-Vitvi11g01336\_t001 |  |  |  |  |  |  |  |
| 1 | Ath-AT2G05970.1 |  | | | |  |  |  |  |  |  |  |
| 1 | Ath-AT2G05990.1 |  | | | |  |  |  |  |  |  |  |
| 1 | Ath-AT2G06000.2 |  | Vvi-Vitvi11g00077\_t001 |  |  |  |  |  |  |  |
| 1 | Ath-AT2G06005.1 |  | Vvi-Vitvi11g00078\_t001 |  |  |  |  |  |  |  |
| 1 | Ath-AT2G06010.1 |  | Vvi-Vitvi11g04017\_t001 |  |  |  |  |  |  |  |
| 1 | Ath-AT2G06020.1 |  | | | |  |  |  |  |  |  |  |
| 1 | Ath-AT2G06025.1 |  | Vvi-Vitvi11g00083\_t001 |  |  |  |  |  |  |  |
| 1 | Ath-AT2G06040.1 |  | Vvi-Vitvi11g00085\_t001 |  |  |  |  |  |  |  |
| 1 | Ath-AT2G06050.1 |  | Vvi-Vitvi11g00090\_t001 |  |  |  |  |  |  |  |
| 1 | Ath-AT2G06090.1 |  | | | |  |  |  |  |  |  |  |
| 1 | Ath-AT2G06095.1 |  | | | |  |  |  |  |  |  |  |
| 1 | Ath-AT2G06105.1 |  | | | |  |  |  |  |  |  |  |
| 1 | Ath-AT2G06166.1 |  | | | |  |  |  |  |  |  |  |
| 1 | Ath-AT2G06200.2 |  | Vvi-Vitvi11g00092\_t001 |  |  |  |  |  |  |  |
| 1 | Ath-AT2G06255.1 |  | Vvi-Vitvi11g00102\_t001 |  |  |  |  |  |  |  |
| 1 | Ath-AT2G06265.1 |  | | | |  |  |  |  |  |  |  |
| 1 | Ath-AT2G06420.1 |  | | | |  |  |  |  |  |  |  |
| 1 | Ath-AT2G06425.1 |  | | | |  |  |  |  |  |  |  |
| 1 | Ath-AT2G06500.1 |  | | | |  |  |  |  |  |  |  |
| 1 | Ath-AT2G06510.1 |  | Vvi-Vitvi11g00104\_t001 |  |  |  |  |  |  |  |
| 1 | Ath-AT2G06520.1 |  | Vvi-Vitvi11g01345\_t001 |  |  |  |  |  |  |  |
| 1 | Ath-AT2G06530.1 |  | Vvi-Vitvi11g00105\_t001 |  |  |  |  |  |  |  |
| 0 | Ath-AT2G06541.1 |  |  |  |  |  |  |  |  |
| 0 | Ath-AT2G06555.1 |  |  |  |  |  |  |  |  |
| 0 | Ath-AT2G06565.1 |  |  |  |  |  |  |  |  |
| 0 | Ath-AT2G06570.1 |  |  |  |  |  |  |  |  |
| 0 | Ath-AT2G06645.1 |  |  |  |  |  |  |  |  |
| 0 | Ath-AT2G06667.1 |  |  |  |  |  |  |  |  |
| 0 | Ath-AT2G06675.1 |  |  |  |  |  |  |  |  |
| 0 | Ath-AT2G06845.1 |  |  |  |  |  |  |  |  |
| 0 | Ath-AT2G06850.1 |  |  |  |  |  |  |  |  |
| 0 | Ath-AT2G06904.1 |  |  |  |  |  |  |  |  |
| 0 | Ath-AT2G06906.1 |  |  |  |  |  |  |  |  |
| 0 | Ath-AT2G06908.1 |  |  |  |  |  |  |  |  |
| 0 | Ath-AT2G06925.1 |  |  |  |  |  |  |  |  |
| 0 | Ath-AT2G06960.1 |  |  |  |  |  |  |  |  |
| 0 | Ath-AT2G06983.1 |  |  |  |  |  |  |  |  |
| 0 | Ath-AT2G06990.1 |  |  |  |  |  |  |  |  |
| 0 | Ath-AT2G07000.1 |  |  |  |  |  |  |  |  |
| 0 | Ath-AT2G07020.1 |  |  |  |  |  |  |  |  |
| 0 | Ath-AT2G07040.1 |  |  |  |  |  |  |  |  |
| 0 | Ath-AT2G07050.1 |  |  |  |  |  |  |  |  |
| 0 | Ath-AT2G07110.1 |  |  |  |  |  |  |  |  |
| 0 | Ath-AT2G07120.1 |  |  |  |  |  |  |  |  |
| 0 | Ath-AT2G07140.1 |  |  |  |  |  |  |  |  |
| 0 | Ath-AT2G07170.1 |  |  |  |  |  |  |  |  |
| 0 | Ath-AT2G07180.1 |  |  |  |  |  |  |  |  |
| 0 | Ath-AT2G07190.1 |  |  |  |  |  |  |  |  |
| 0 | Ath-AT2G07200.1 |  |  |  |  |  |  |  |  |
| 0 | Ath-AT2G07215.1 |  |  |  |  |  |  |  |  |
| 0 | Ath-AT2G07240.1 |  |  |  |  |  |  |  |  |
| 0 | Ath-AT2G07280.1 |  |  |  |  |  |  |  |  |
| 0 | Ath-AT2G07290.1 |  |  |  |  |  |  |  |  |
| 0 | Ath-AT2G07310.1 |  |  |  |  |  |  |  |  |
| 0 | Ath-AT2G07340.1 |  |  |  |  |  |  |  |  |
| 0 | Ath-AT2G07360.2 |  |  |  |  |  |  |  |  |
| 0 | Ath-AT2G07440.1 |  |  |  |  |  |  |  |  |
| 0 | Ath-AT2G07505.1 |  |  |  |  |  |  |  |  |
| 0 | Ath-AT2G07560.1 |  |  |  |  |  |  |  |  |
| 0 | Ath-AT2G07565.1 |  |  |  |  |  |  |  |  |
| 0 | Ath-AT2G07640.1 |  |  |  |  |  |  |  |  |
| 0 | Ath-AT2G07771.2 |  |  |  |  |  |  |  |  |
| 0 | Ath-AT2G07773.1 |  |  |  |  |  |  |  |  |
| 0 | Ath-AT2G07655.1 |  |  |  |  |  |  |  |  |
| 0 | Ath-AT2G07776.2 |  |  |  |  |  |  |  |  |
| 0 | Ath-AT2G07749.1 |  |  |  |  |  |  |  |  |
| 0 | Ath-AT2G07777.1 |  |  |  |  |  |  |  |  |
| 0 | Ath-AT2G07671.1 |  |  |  |  |  |  |  |  |
| 0 | Ath-AT2G07779.1 |  |  |  |  |  |  |  |  |
| 0 | Ath-AT2G07672.1 |  |  |  |  |  |  |  |  |
| 0 | Ath-AT2G07613.1 |  |  |  |  |  |  |  |  |
| 0 | Ath-AT2G07684.1 |  |  |  |  |  |  |  |  |
| 0 | Ath-AT2G07617.1 |  |  |  |  |  |  |  |  |
| 0 | Ath-AT2G07621.1 |  |  |  |  |  |  |  |  |
| 0 | Ath-AT2G07673.1 |  |  |  |  |  |  |  |  |
| 0 | Ath-AT2G07674.2 |  |  |  |  |  |  |  |  |
| 0 | Ath-AT2G07751.1 |  |  |  |  |  |  |  |  |
| 0 | Ath-AT2G07675.1 |  |  |  |  |  |  |  |  |
| 0 | Ath-AT2G07676.1 |  |  |  |  |  |  |  |  |
| 0 | Ath-AT2G07768.1 |  |  |  |  |  |  |  |  |
| 0 | Ath-AT2G07623.1 |  |  |  |  |  |  |  |  |
| 0 | Ath-AT2G07678.1 |  |  |  |  |  |  |  |  |
| 0 | Ath-AT2G07669.1 |  |  |  |  |  |  |  |  |
| 0 | Ath-AT2G07625.1 |  |  |  |  |  |  |  |  |
| 0 | Ath-AT2G07681.1 |  |  |  |  |  |  |  |  |
| 0 | Ath-AT2G07626.1 |  |  |  |  |  |  |  |  |
| 0 | Ath-AT2G07627.1 |  |  |  |  |  |  |  |  |
| 0 | Ath-AT2G07772.1 |  |  |  |  |  |  |  |  |
| 0 | Ath-AT2G07628.1 |  |  |  |  |  |  |  |  |
| 0 | Ath-AT2G07774.1 |  |  |  |  |  |  |  |  |
| 0 | Ath-AT2G07687.1 |  |  |  |  |  |  |  |  |
| 0 | Ath-AT2G07629.1 |  |  |  |  |  |  |  |  |
| 0 | Ath-AT2G07631.1 |  |  |  |  |  |  |  |  |
| 0 | Ath-AT2G07632.1 |  |  |  |  |  |  |  |  |
| 0 | Ath-AT2G07633.1 |  |  |  |  |  |  |  |  |
| 0 | Ath-AT2G07689.1 |  |  |  |  |  |  |  |  |
| 0 | Ath-AT2G07634.1 |  |  |  |  |  |  |  |  |
| 0 | Ath-AT2G07691.1 |  |  |  |  |  |  |  |  |
| 0 | Ath-AT2G07636.1 |  |  |  |  |  |  |  |  |
| 0 | Ath-AT2G07692.1 |  |  |  |  |  |  |  |  |
| 0 | Ath-AT2G07637.1 |  |  |  |  |  |  |  |  |
| 0 | Ath-AT2G07695.1 |  |  |  |  |  |  |  |  |
| 0 | Ath-AT2G07785.1 |  |  |  |  |  |  |  |  |
| 0 | Ath-AT2G07599.2 |  |  |  |  |  |  |  |  |
| 0 | Ath-AT2G07696.1 |  |  |  |  |  |  |  |  |
| 0 | Ath-AT2G07798.1 |  |  |  |  |  |  |  |  |
| 0 | Ath-AT2G07698.1 |  |  |  |  |  |  |  |  |
| 0 | Ath-AT2G07667.1 |  |  |  |  |  |  |  |  |
| 0 | Ath-AT2G07701.1 |  |  |  |  |  |  |  |  |
| 0 | Ath-AT2G07702.1 |  |  |  |  |  |  |  |  |
| 0 | Ath-AT2G07638.1 |  |  |  |  |  |  |  |  |
| 0 | Ath-AT2G07705.1 |  |  |  |  |  |  |  |  |
| 0 | Ath-AT2G07706.1 |  |  |  |  |  |  |  |  |
| 0 | Ath-AT2G07707.1 |  |  |  |  |  |  |  |  |
| 0 | Ath-AT2G07708.1 |  |  |  |  |  |  |  |  |
| 0 | Ath-AT2G07641.1 |  |  |  |  |  |  |  |  |
| 0 | Ath-AT2G07642.1 |  |  |  |  |  |  |  |  |
| 0 | Ath-AT2G07643.1 |  |  |  |  |  |  |  |  |
| 0 | Ath-AT2G07644.1 |  |  |  |  |  |  |  |  |
| 0 | Ath-AT2G07713.1 |  |  |  |  |  |  |  |  |
| 0 | Ath-AT2G07714.1 |  |  |  |  |  |  |  |  |
| 0 | Ath-AT2G07646.1 |  |  |  |  |  |  |  |  |
| 0 | Ath-AT2G07715.1 |  |  |  |  |  |  |  |  |
| 0 | Ath-AT2G07718.1 |  |  |  |  |  |  |  |  |
| 0 | Ath-AT2G07648.1 |  |  |  |  |  |  |  |  |
| 0 | Ath-AT2G07719.1 |  |  |  |  |  |  |  |  |
| 0 | Ath-AT2G07721.1 |  |  |  |  |  |  |  |  |
| 0 | Ath-AT2G07722.1 |  |  |  |  |  |  |  |  |
| 0 | Ath-AT2G07652.1 |  |  |  |  |  |  |  |  |
| 0 | Ath-AT2G07815.1 |  |  |  |  |  |  |  |  |
| 0 | Ath-AT2G07724.1 |  |  |  |  |  |  |  |  |
| 0 | Ath-AT2G07725.1 |  |  |  |  |  |  |  |  |
| 0 | Ath-AT2G07727.1 |  |  |  |  |  |  |  |  |
| 0 | Ath-AT2G07728.1 |  |  |  |  |  |  |  |  |
| 0 | Ath-AT2G07654.1 |  |  |  |  |  |  |  |  |
| 0 | Ath-AT2G07820.1 |  |  |  |  |  |  |  |  |
| 0 | Ath-AT2G07656.1 |  |  |  |  |  |  |  |  |
| 0 | Ath-AT2G07732.1 |  |  |  |  |  |  |  |  |
| 0 | Ath-AT2G07734.1 |  |  |  |  |  |  |  |  |
| 0 | Ath-AT2G07825.1 |  |  |  |  |  |  |  |  |
| 0 | Ath-AT2G07827.2 |  |  |  |  |  |  |  |  |
| 0 | Ath-AT2G07830.1 |  |  |  |  |  |  |  |  |
| 0 | Ath-AT2G07787.1 |  |  |  |  |  |  |  |  |
| 0 | Ath-AT2G07658.1 |  |  |  |  |  |  |  |  |
| 0 | Ath-AT2G07659.1 |  |  |  |  |  |  |  |  |
| 0 | Ath-AT2G07775.1 |  |  |  |  |  |  |  |  |
| 0 | Ath-AT2G07661.1 |  |  |  |  |  |  |  |  |
| 0 | Ath-AT2G07806.2 |  |  |  |  |  |  |  |  |
| 0 | Ath-AT2G07738.1 |  |  |  |  |  |  |  |  |
| 0 | Ath-AT2G07795.1 |  |  |  |  |  |  |  |  |
| 0 | Ath-AT2G07739.1 |  |  |  |  |  |  |  |  |
| 0 | Ath-AT2G07662.1 |  |  |  |  |  |  |  |  |
| 0 | Ath-AT2G07665.1 |  |  |  |  |  |  |  |  |
| 0 | Ath-AT2G07835.1 |  |  |  |  |  |  |  |  |
| 0 | Ath-AT2G07741.1 |  |  |  |  |  |  |  |  |
| 0 | Ath-AT2G07680.3 |  |  |  |  |  |  |  |  |
| 0 | Ath-AT2G07690.1 |  |  |  |  |  |  |  |  |
| 0 | Ath-AT2G07750.1 |  |  |  |  |  |  |  |  |
| 0 | Ath-AT2G07760.1 |  |  |  |  |  |  |  |  |
| 0 | Ath-AT2G07800.1 |  |  |  |  |  |  |  |  |
| 0 | Ath-AT2G07810.1 |  |  |  |  |  |  |  |  |
| 0 | Ath-AT2G07981.1 |  |  |  |  |  |  |  |  |
| 0 | Ath-AT2G08986.1 |  |  |  |  |  |  |  |  |
| 0 | Ath-AT2G09388.1 |  |  |  |  |  |  |  |  |
| 0 | Ath-AT2G09838.1 |  |  |  |  |  |  |  |  |
| 0 | Ath-AT2G09840.1 |  |  |  |  |  |  |  |  |
| 0 | Ath-AT2G09970.1 |  |  |  |  |  |  |  |  |
| 0 | Ath-AT2G09990.1 |  |  |  |  |  |  |  |  |
| 0 | Ath-AT2G10020.1 |  |  |  |  |  |  |  |  |
| 0 | Ath-AT2G10025.1 |  |  |  |  |  |  |  |  |
| 0 | Ath-AT2G10260.1 |  |  |  |  |  |  |  |  |
| 0 | Ath-AT2G10440.1 |  |  |  |  |  |  |  |  |
| 0 | Ath-AT2G10450.1 |  |  |  |  |  |  |  |  |
| 0 | Ath-AT2G10455.1 |  |  |  |  |  |  |  |  |
| 0 | Ath-AT2G10535.1 |  |  |  |  |  |  |  |  |
| 0 | Ath-AT2G10545.1 |  |  |  |  |  |  |  |  |
| 0 | Ath-AT2G10550.1 |  |  |  |  |  |  |  |  |
| 0 | Ath-AT2G10553.1 |  |  |  |  |  |  |  |  |
| 0 | Ath-AT2G10556.1 |  |  |  |  |  |  |  |  |
| 0 | Ath-AT2G10557.1 |  |  |  |  |  |  |  |  |
| 0 | Ath-AT2G10560.1 |  |  |  |  |  |  |  |  |
| 0 | Ath-AT2G10602.1 |  |  |  |  |  |  |  |  |
| 0 | Ath-AT2G10608.1 |  |  |  |  |  |  |  |  |
| 0 | Ath-AT2G10615.1 |  |  |  |  |  |  |  |  |
| 0 | Ath-AT2G10625.1 |  |  |  |  |  |  |  |  |
| 0 | Ath-AT2G10920.1 |  |  |  |  |  |  |  |  |
| 0 | Ath-AT2G10930.1 |  |  |  |  |  |  |  |  |
| 0 | Ath-AT2G10931.1 |  |  |  |  |  |  |  |  |
| 1 | Ath-AT2G10940.1 |  | Vvi-Vitvi11g00518\_t001 |  |  |  |  |  |  |  |
| 1 | Ath-AT2G10950.1 |  | Vvi-Vitvi11g01454\_t001 |  |  |  |  |  |  |  |
| 1 | Ath-AT2G10955.1 |  | | | |  |  |  |  |  |  |  |
| 1 | Ath-AT2G10965.1 |  | | | |  |  |  |  |  |  |  |
| 1 | Ath-AT2G10970.1 |  | | | |  |  |  |  |  |  |  |
| 1 | Ath-AT2G10975.1 |  | | | |  |  |  |  |  |  |  |
| 1 | Ath-AT2G11000.1 |  | Vvi-Vitvi11g00524\_t001 |  |  |  |  |  |  |  |
| 1 | Ath-AT2G11005.1 |  | | | |  |  |  |  |  |  |  |
| 1 | Ath-AT2G11010.1 |  | | | |  |  |  |  |  |  |  |
| 1 | Ath-AT2G11015.1 |  | | | |  |  |  |  |  |  |  |
| 1 | Ath-AT2G11025.1 |  | | | |  |  |  |  |  |  |  |
| 1 | Ath-AT2G11035.1 |  | | | |  |  |  |  |  |  |  |
| 1 | Ath-AT2G11045.1 |  | | | |  |  |  |  |  |  |  |
| 1 | Ath-AT2G11200.1 |  | | | |  |  |  |  |  |  |  |
| 1 | Ath-AT2G11205.1 |  | | | |  |  |  |  |  |  |  |
| 1 | Ath-AT2G11215.1 |  | | | |  |  |  |  |  |  |  |
| 1 | Ath-AT2G11225.1 |  | | | |  |  |  |  |  |  |  |
| 1 | Ath-AT2G11270.1 |  | | | |  |  |  |  |  |  |  |
| 1 | Ath-AT2G11271.1 |  | | | |  |  |  |  |  |  |  |
| 1 | Ath-AT2G11405.1 |  | | | |  |  |  |  |  |  |  |
| 1 | Ath-AT2G11462.1 |  | | | |  |  |  |  |  |  |  |
| 1 | Ath-AT2G11520.1 |  | Vvi-Vitvi11g00543\_t001 |  |  |  |  |  |  |  |
| 1 | Ath-AT2G11522.1 |  | | | |  |  |  |  |  |  |  |
| 1 | Ath-AT2G11570.1 |  | | | |  |  |  |  |  |  |  |
| 1 | Ath-AT2G11620.1 |  | | | |  |  |  |  |  |  |  |
| 1 | Ath-AT2G11623.1 |  | | | |  |  |  |  |  |  |  |
| 1 | Ath-AT2G11626.1 |  | | | |  |  |  |  |  |  |  |
| 1 | Ath-AT2G11773.1 |  | | | |  |  |  |  |  |  |  |
| 1 | Ath-AT2G11778.1 |  | | | |  |  |  |  |  |  |  |
| 1 | Ath-AT2G11810.1 |  | Vvi-Vitvi11g00555\_t001 |  |  |  |  |  |  |  |
| 1 | Ath-AT2G11815.1 |  | | | |  |  |  |  |  |  |  |
| 1 | Ath-AT2G11851.1 |  | | | |  |  |  |  |  |  |  |
| 1 | Ath-AT2G11890.1 |  | Vvi-Vitvi11g00571\_t002 |  |  |  |  |  |  |  |
| 1 | Ath-AT2G11891.1 |  | | | |  |  |  |  |  |  |  |
| 1 | Ath-AT2G11910.1 |  | | | |  |  |  |  |  |  |  |
| 1 | Ath-AT2G12170.1 |  | | | |  |  |  |  |  |  |  |
| 1 | Ath-AT2G12190.1 |  | | | |  |  |  |  |  |  |  |
| 1 | Ath-AT2G12200.1 |  | | | |  |  |  |  |  |  |  |
| 1 | Ath-AT2G12205.1 |  | | | |  |  |  |  |  |  |  |
| 1 | Ath-AT2G12280.1 |  | | | |  |  |  |  |  |  |  |
| 1 | Ath-AT2G12290.1 |  | | | |  |  |  |  |  |  |  |
| 1 | Ath-AT2G12400.1 |  | | | |  |  |  |  |  |  |  |
| 1 | Ath-AT2G12405.1 |  | | | |  |  |  |  |  |  |  |
| 1 | Ath-AT2G12461.1 |  | | | |  |  |  |  |  |  |  |
| 1 | Ath-AT2G12462.1 |  | Vvi-Vitvi11g00580\_t001 |  |  |  |  |  |  |  |
| 1 | Ath-AT2G12465.1 |  | | | |  |  |  |  |  |  |  |
| 1 | Ath-AT2G12475.1 |  | | | |  |  |  |  |  |  |  |
| 1 | Ath-AT2G12480.3 |  | | | |  |  |  |  |  |  |  |
| 1 | Ath-AT2G12550.1 |  | | | |  |  |  |  |  |  |  |
| 1 | Ath-AT2G12646.1 |  | Vvi-Vitvi11g00585\_t001 |  |  |  |  |  |  |  |
| 1 | Ath-AT2G12875.1 |  | | | |  |  |  |  |  |  |  |
| 1 | Ath-AT2G12880.1 |  | | | |  |  |  |  |  |  |  |
| 1 | Ath-AT2G12900.1 |  | | | |  |  |  |  |  |  |  |
| 1 | Ath-AT2G12905.1 |  | | | |  |  |  |  |  |  |  |
| 1 | Ath-AT2G12935.1 |  | | | |  |  |  |  |  |  |  |
| 1 | Ath-AT2G12940.1 |  | | | |  |  |  |  |  |  |  |
| 1 | Ath-AT2G12945.1 |  | | | |  |  |  |  |  |  |  |
| 1 | Ath-AT2G13100.1 |  | Vvi-Vitvi11g00593\_t001 |  |  |  |  |  |  |  |
| 0 | Ath-AT2G13105.1 |  |  |  |  |  |  |  |  |
| 0 | Ath-AT2G13125.1 |  |  |  |  |  |  |  |  |
| 0 | Ath-AT2G13126.1 |  |  |  |  |  |  |  |  |
| 0 | Ath-AT2G13150.1 |  |  |  |  |  |  |  |  |
| 0 | Ath-AT2G13275.1 |  |  |  |  |  |  |  |  |
| 0 | Ath-AT2G13290.1 |  |  |  |  |  |  |  |  |
| 0 | Ath-AT2G13295.1 |  |  |  |  |  |  |  |  |
| 0 | Ath-AT2G13350.1 |  |  |  |  |  |  |  |  |
| 0 | Ath-AT2G13360.1 |  |  |  |  |  |  |  |  |
| 0 | Ath-AT2G13370.2 |  |  |  |  |  |  |  |  |
| 0 | Ath-AT2G13422.1 |  |  |  |  |  |  |  |  |
| 0 | Ath-AT2G13430.1 |  |  |  |  |  |  |  |  |
| 0 | Ath-AT2G13440.1 |  |  |  |  |  |  |  |  |
| 0 | Ath-AT2G13450.1 |  |  |  |  |  |  |  |  |
| 0 | Ath-AT2G13463.1 |  |  |  |  |  |  |  |  |
| 0 | Ath-AT2G13500.1 |  |  |  |  |  |  |  |  |
| 0 | Ath-AT2G13510.1 |  |  |  |  |  |  |  |  |
| 0 | Ath-AT2G13540.1 |  |  |  |  |  |  |  |  |
| 0 | Ath-AT2G13542.1 |  |  |  |  |  |  |  |  |
| 0 | Ath-AT2G13547.1 |  |  |  |  |  |  |  |  |
| 0 | Ath-AT2G13550.2 |  |  |  |  |  |  |  |  |
| 0 | Ath-AT2G13555.1 |  |  |  |  |  |  |  |  |
| 0 | Ath-AT2G13560.1 |  |  |  |  |  |  |  |  |
| 0 | Ath-AT2G13570.1 |  |  |  |  |  |  |  |  |
| 0 | Ath-AT2G13600.1 |  |  |  |  |  |  |  |  |
| 0 | Ath-AT2G13610.1 |  |  |  |  |  |  |  |  |
| 0 | Ath-AT2G13620.1 |  |  |  |  |  |  |  |  |
| 0 | Ath-AT2G13630.1 |  |  |  |  |  |  |  |  |
| 0 | Ath-AT2G13640.1 |  |  |  |  |  |  |  |  |
| 0 | Ath-AT2G13650.1 |  |  |  |  |  |  |  |  |
| 0 | Ath-AT2G13660.1 |  |  |  |  |  |  |  |  |
| 0 | Ath-AT2G13680.1 |  |  |  |  |  |  |  |  |
| 0 | Ath-AT2G13690.1 |  |  |  |  |  |  |  |  |
| 0 | Ath-AT2G13720.1 |  |  |  |  |  |  |  |  |
| 0 | Ath-AT2G13760.1 |  |  |  |  |  |  |  |  |
| 0 | Ath-AT2G13770.1 |  |  |  |  |  |  |  |  |
| 0 | Ath-AT2G13790.1 |  |  |  |  |  |  |  |  |
| 0 | Ath-AT2G13800.3 |  |  |  |  |  |  |  |  |
| 0 | Ath-AT2G13810.1 |  |  |  |  |  |  |  |  |
| 0 | Ath-AT2G13820.3 |  |  |  |  |  |  |  |  |
| 0 | Ath-AT2G13840.1 |  |  |  |  |  |  |  |  |
| 0 | Ath-AT2G13845.1 |  |  |  |  |  |  |  |  |
| 0 | Ath-AT2G13895.1 |  |  |  |  |  |  |  |  |
| 0 | Ath-AT2G13900.1 |  |  |  |  |  |  |  |  |
| 0 | Ath-AT2G13905.1 |  |  |  |  |  |  |  |  |
| 0 | Ath-AT2G13950.1 |  |  |  |  |  |  |  |  |
| 0 | Ath-AT2G13960.1 |  |  |  |  |  |  |  |  |
| 0 | Ath-AT2G13965.1 |  |  |  |  |  |  |  |  |
| 0 | Ath-AT2G13980.1 |  |  |  |  |  |  |  |  |
| 0 | Ath-AT2G13985.1 |  |  |  |  |  |  |  |  |
| 0 | Ath-AT2G14000.1 |  |  |  |  |  |  |  |  |
| 0 | Ath-AT2G14045.1 |  |  |  |  |  |  |  |  |
| 0 | Ath-AT2G14050.1 |  |  |  |  |  |  |  |  |
| 0 | Ath-AT2G14060.1 |  |  |  |  |  |  |  |  |
| 0 | Ath-AT2G14070.1 |  |  |  |  |  |  |  |  |
| 0 | Ath-AT2G14080.1 |  |  |  |  |  |  |  |  |
| 0 | Ath-AT2G14095.1 |  |  |  |  |  |  |  |  |
| 0 | Ath-AT2G14100.1 |  |  |  |  |  |  |  |  |
| 0 | Ath-AT2G14110.1 |  |  |  |  |  |  |  |  |
| 0 | Ath-AT2G14120.3 |  |  |  |  |  |  |  |  |
| 0 | Ath-AT2G14160.1 |  |  |  |  |  |  |  |  |
| 0 | Ath-AT2G14170.1 |  |  |  |  |  |  |  |  |
| 1 | Ath-AT2G14210.2 |  | Vvi-Vitvi03g00819\_t001 |  |  |  |  |  |  |  |
| 1 | Ath-AT2G14247.1 |  | | | |  |  |  |  |  |  |  |
| 1 | Ath-AT2G14255.1 |  | Vvi-Vitvi03g00793\_t001 |  |  |  |  |  |  |  |
| 1 | Ath-AT2G14260.1 |  | Vvi-Vitvi03g00782\_t001 |  |  |  |  |  |  |  |
| 1 | Ath-AT2G14265.1 |  | | | |  |  |  |  |  |  |  |
| 1 | Ath-AT2G14270.1 |  | | | |  |  |  |  |  |  |  |
| 1 | Ath-AT2G14282.1 |  | | | |  |  |  |  |  |  |  |
| 1 | Ath-AT2G14285.1 |  | | | |  |  |  |  |  |  |  |
| 1 | Ath-AT2G14288.1 |  | | | |  |  |  |  |  |  |  |
| 1 | Ath-AT2G14289.1 |  | | | |  |  |  |  |  |  |  |
| 1 | Ath-AT2G14290.1 |  | | | |  |  |  |  |  |  |  |
| 1 | Ath-AT2G14365.1 |  | | | |  |  |  |  |  |  |  |
| 1 | Ath-AT2G14378.1 |  | | | |  |  |  |  |  |  |  |
| 1 | Ath-AT2G14390.1 |  | | | |  |  |  |  |  |  |  |
| 1 | Ath-AT2G14395.1 |  | | | |  |  |  |  |  |  |  |
| 1 | Ath-AT2G14440.1 |  | | | |  |  |  |  |  |  |  |
| 1 | Ath-AT2G14460.1 |  | | | |  |  |  |  |  |  |  |
| 1 | Ath-AT2G14500.1 |  | | | |  |  |  |  |  |  |  |
| 1 | Ath-AT2G14510.2 |  | | | |  |  |  |  |  |  |  |
| 1 | Ath-AT2G14520.1 |  | Vvi-Vitvi03g00766\_t001 |  |  |  |  |  |  |  |
| 1 | Ath-AT2G14530.1 |  | Vvi-Vitvi03g01659\_t001 |  |  |  |  |  |  |  |
| 1 | Ath-AT2G14540.1 |  | | | |  |  |  |  |  |  |  |
| 1 | Ath-AT2G14560.3 |  | | | |  |  |  |  |  |  |  |
| 1 | Ath-AT2G14580.1 |  | Vvi-Vitvi03g01649\_t001 |  |  |  |  |  |  |  |
| 1 | Ath-AT2G14610.1 |  | | | |  |  |  |  |  |  |  |
| 1 | Ath-AT2G14620.1 |  | Vvi-Vitvi03g00737\_t001 |  |  |  |  |  |  |  |
| 0 | Ath-AT2G14635.1 |  |  |  |  |  |  |  |  |
| 0 | Ath-AT2G14660.1 |  |  |  |  |  |  |  |  |
| 0 | Ath-AT2G14670.1 |  |  |  |  |  |  |  |  |
| 1 | Ath-AT2G14680.2 |  | Vvi-Vitvi03g00667\_t001 |  |  |  |  |  |  |  |
| 1 | Ath-AT2G14690.1 |  | | | |  |  |  |  |  |  |  |
| 1 | Ath-AT2G14692.1 |  | | | |  |  |  |  |  |  |  |
| 1 | Ath-AT2G14695.1 |  | | | |  |  |  |  |  |  |  |
| 1 | Ath-AT2G14700.1 |  | | | |  |  |  |  |  |  |  |
| 1 | Ath-AT2G14710.1 |  | | | |  |  |  |  |  |  |  |
| 1 | Ath-AT2G14720.2 |  | | | |  |  |  |  |  |  |  |
| 1 | Ath-AT2G14740.1 |  | | | |  |  |  |  |  |  |  |
| 1 | Ath-AT2G14750.1 |  | Vvi-Vitvi03g00651\_t001 |  |  |  |  |  |  |  |
| 1 | Ath-AT2G14755.1 |  | | | |  |  |  |  |  |  |  |
| 1 | Ath-AT2G14760.3 |  | Vvi-Vitvi03g00635\_t001 |  |  |  |  |  |  |  |
| 1 | Ath-AT2G14765.1 |  | | | |  |  |  |  |  |  |  |
| 1 | Ath-AT2G14775.1 |  | | | |  |  |  |  |  |  |  |
| 1 | Ath-AT2G14800.1 |  | | | |  |  |  |  |  |  |  |
| 1 | Ath-AT2G14810.1 |  | | | |  |  |  |  |  |  |  |
| 1 | Ath-AT2G14820.1 |  | Vvi-Vitvi03g00629\_t001 |  |  |  |  |  |  |  |
| 1 | Ath-AT2G14825.1 |  | Vvi-Vitvi03g00623\_t001 |  |  |  |  |  |  |  |
| 1 | Ath-AT2G14830.1 |  | | | |  |  |  |  |  |  |  |
| 1 | Ath-AT2G14835.2 |  | | | |  |  |  |  |  |  |  |
| 1 | Ath-AT2G14846.1 |  | | | |  |  |  |  |  |  |  |
| 1 | Ath-AT2G14850.1 |  | Vvi-Vitvi03g00618\_t001 |  |  |  |  |  |  |  |
| 1 | Ath-AT2G14860.1 |  | Vvi-Vitvi03g01605\_t002 |  |  |  |  |  |  |  |
| 1 | Ath-AT2G14870.1 |  | | | |  |  |  |  |  |  |  |
| 1 | Ath-AT2G14880.1 |  | Vvi-Vitvi03g00599\_t001 |  |  |  |  |  |  |  |
| 1 | Ath-AT2G14890.1 |  | | | |  |  |  |  |  |  |  |
| 1 | Ath-AT2G14900.1 |  | Vvi-Vitvi03g00593\_t001 |  |  |  |  |  |  |  |
| 1 | Ath-AT2G14910.1 |  | Vvi-Vitvi03g00589\_t001 |  |  |  |  |  |  |  |
| 1 | Ath-AT2G14920.1 |  | | | |  |  |  |  |  |  |  |
| 1 | Ath-AT2G14935.1 |  | | | |  |  |  |  |  |  |  |
| 1 | Ath-AT2G14945.1 |  | | | |  |  |  |  |  |  |  |
| 1 | Ath-AT2G14960.1 |  | Vvi-Vitvi03g00586\_t001 |  |  |  |  |  |  |  |
| 1 | Ath-AT2G15000.6 |  | Vvi-Vitvi03g01599\_t001 |  |  |  |  |  |  |  |
| 1 | Ath-AT2G15010.1 |  | | | |  |  |  |  |  |  |  |
| 1 | Ath-AT2G15020.1 |  | Vvi-Vitvi03g00580\_t001 |  |  |  |  |  |  |  |
| 1 | Ath-AT2G15025.1 |  | | | |  |  |  |  |  |  |  |
| 1 | Ath-AT2G15029.1 |  | | | |  |  |  |  |  |  |  |
| 1 | Ath-AT2G15042.1 |  | | | |  |  |  |  |  |  |  |
| 1 | Ath-AT2G15050.1 |  | | | |  |  |  |  |  |  |  |
| 1 | Ath-AT2G15080.1 |  | | | |  |  |  |  |  |  |  |
| 1 | Ath-AT2G15090.1 |  | | | |  |  |  |  |  |  |  |
| 1 | Ath-AT2G15110.1 |  | | | |  |  |  |  |  |  |  |
| 1 | Ath-AT2G15130.1 |  | | | |  |  |  |  |  |  |  |
| 1 | Ath-AT2G15170.1 |  | Vvi-Vitvi03g04198\_t001 |  |  |  |  |  |  |  |
| 1 | Ath-AT2G15180.1 |  | | | |  |  |  |  |  |  |  |
| 1 | Ath-AT2G15185.1 |  | | | |  |  |  |  |  |  |  |
| 1 | Ath-AT2G15220.1 |  | | | |  |  |  |  |  |  |  |
| 1 | Ath-AT2G15230.1 |  | Vvi-Vitvi03g00570\_t001 |  |  |  |  |  |  |  |
| 1 | Ath-AT2G15240.1 |  | Vvi-Vitvi03g00560\_t001 |  |  |  |  |  |  |  |
| 1 | Ath-AT2G15260.1 |  | | | |  |  |  |  |  |  |  |
| 1 | Ath-AT2G15270.1 |  | Vvi-Vitvi03g01581\_t001 |  |  |  |  |  |  |  |
| 1 | Ath-AT2G15280.1 |  | Vvi-Vitvi03g01580\_t001 |  |  |  |  |  |  |  |
| 1 | Ath-AT2G15290.1 |  | Vvi-Vitvi03g00556\_t001 |  |  |  |  |  |  |  |
| 1 | Ath-AT2G15300.1 |  | Vvi-Vitvi03g00553\_t001 |  |  |  |  |  |  |  |
| 1 | Ath-AT2G15310.1 |  | | | |  |  |  |  |  |  |  |
| 1 | Ath-AT2G15318.1 |  | | | |  |  |  |  |  |  |  |
| 1 | Ath-AT2G15320.1 |  | Vvi-Vitvi03g00548\_t001 |  |  |  |  |  |  |  |
| 1 | Ath-AT2G15325.1 |  | | | |  |  |  |  |  |  |  |
| 1 | Ath-AT2G15327.1 |  | | | |  |  |  |  |  |  |  |
| 1 | Ath-AT2G15340.1 |  | | | |  |  |  |  |  |  |  |
| 1 | Ath-AT2G15345.1 |  | | | |  |  |  |  |  |  |  |
| 1 | Ath-AT2G15350.1 |  | | | |  |  |  |  |  |  |  |
| 1 | Ath-AT2G15360.1 |  | | | |  |  |  |  |  |  |  |
| 1 | Ath-AT2G15370.1 |  | | | |  |  |  |  |  |  |  |
| 1 | Ath-AT2G15390.2 |  | | | |  |  |  |  |  |  |  |
| 1 | Ath-AT2G15400.1 |  | Vvi-Vitvi03g00543\_t001 |  |  |  |  |  |  |  |
| 1 | Ath-AT2G15420.1 |  | | | |  |  |  |  |  |  |  |
| 1 | Ath-AT2G15430.1 |  | | | |  |  |  |  |  |  |  |
| 1 | Ath-AT2G15440.1 |  | Vvi-Vitvi03g00535\_t001 |  |  |  |  |  |  |  |
| 1 | Ath-AT2G15450.1 |  | | | |  |  |  |  |  |  |  |
| 1 | Ath-AT2G15460.1 |  | | | |  |  |  |  |  |  |  |
| 1 | Ath-AT2G15470.1 |  | | | |  |  |  |  |  |  |  |
| 1 | Ath-AT2G15480.2 |  | Vvi-Vitvi03g00533\_t001 |  |  |  |  |  |  |  |
| 1 | Ath-AT2G15490.1 |  | | | |  |  |  |  |  |  |  |
| 1 | Ath-AT2G15500.1 |  | | | |  |  |  |  |  |  |  |
| 1 | Ath-AT2G15530.4 |  | Vvi-Vitvi03g00521\_t001 |  |  |  |  |  |  |  |
| 1 | Ath-AT2G15535.1 |  | | | |  |  |  |  |  |  |  |
| 1 | Ath-AT2G15560.1 |  | Vvi-Vitvi03g00515\_t003 |  |  |  |  |  |  |  |
| 1 | Ath-AT2G15570.2 |  | Vvi-Vitvi03g00514\_t001 |  |  |  |  |  |  |  |
| 1 | Ath-AT2G15580.1 |  | | | |  |  |  |  |  |  |  |
| 1 | Ath-AT2G15590.2 |  | | | |  |  |  |  |  |  |  |
| 1 | Ath-AT2G15610.1 |  | | | |  |  |  |  |  |  |  |
| 1 | Ath-AT2G15620.1 |  | Vvi-Vitvi03g00509\_t001 |  |  |  |  |  |  |  |
| 1 | Ath-AT2G15630.1 |  | | | |  |  |  |  |  |  |  |
| 1 | Ath-AT2G15640.1 |  | | | |  |  |  |  |  |  |  |
| 1 | Ath-AT2G15660.1 |  | | | |  |  |  |  |  |  |  |
| 1 | Ath-AT2G15670.1 |  | | | |  |  |  |  |  |  |  |
| 1 | Ath-AT2G15680.1 |  | Vvi-Vitvi03g00496\_t001 |  |  |  |  |  |  |  |
| 1 | Ath-AT2G15690.1 |  | Vvi-Vitvi03g00495\_t001 |  |  |  |  |  |  |  |
| 1 | Ath-AT2G15695.1 |  | Vvi-Vitvi03g00486\_t001 |  |  |  |  |  |  |  |
| 1 | Ath-AT2G15710.1 |  | | | |  |  |  |  |  |  |  |
| 1 | Ath-AT2G15730.1 |  | Vvi-Vitvi03g00477\_t001 |  |  |  |  |  |  |  |
| 1 | Ath-AT2G15740.1 |  | | | |  |  |  |  |  |  |  |
| 1 | Ath-AT2G15760.1 |  | Vvi-Vitvi03g00473\_t001 |  |  |  |  |  |  |  |
| 1 | Ath-AT2G15770.1 |  | Vvi-Vitvi03g00459\_t001 |  |  |  |  |  |  |  |
| 1 | Ath-AT2G15780.1 |  | | | |  |  |  |  |  |  |  |
| 1 | Ath-AT2G15790.1 |  | Vvi-Vitvi03g00457\_t001 |  |  |  |  |  |  |  |
| 0 | Ath-AT2G15820.1 |  |  |  |  |  |  |  |  |
| 0 | Ath-AT2G15830.1 |  |  |  |  |  |  |  |  |
| 0 | Ath-AT2G15860.2 |  |  |  |  |  |  |  |  |
| 0 | Ath-AT2G15880.1 |  |  |  |  |  |  |  |  |
| 0 | Ath-AT2G15890.1 |  |  |  |  |  |  |  |  |
| 0 | Ath-AT2G15900.1 |  |  |  |  |  |  |  |  |
| 1 | Ath-AT2G15910.1 |  | Vvi-Vitvi03g00403\_t001 |  |  |  |  |  |  |  |
| 1 | Ath-AT2G15960.1 |  | | | |  |  |  |  |  |  |  |
| 1 | Ath-AT2G15970.1 |  | Vvi-Vitvi03g00389\_t001 |  |  |  |  |  |  |  |
| 1 | Ath-AT2G15980.1 |  | Vvi-Vitvi03g00383\_t001 |  |  |  |  |  |  |  |
| 1 | Ath-AT2G16005.1 |  | | | |  |  |  |  |  |  |  |
| 1 | Ath-AT2G16015.1 |  | | | |  |  |  |  |  |  |  |
| 1 | Ath-AT2G16016.1 |  | | | |  |  |  |  |  |  |  |
| 1 | Ath-AT2G16018.1 |  | | | |  |  |  |  |  |  |  |
| 1 | Ath-AT2G16019.1 |  | | | |  |  |  |  |  |  |  |
| 1 | Ath-AT2G16020.1 |  | | | |  |  |  |  |  |  |  |
| 1 | Ath-AT2G16030.1 |  | | | |  |  |  |  |  |  |  |
| 1 | Ath-AT2G16040.1 |  | | | |  |  |  |  |  |  |  |
| 1 | Ath-AT2G16050.1 |  | Vvi-Vitvi03g00381\_t001 |  |  |  |  |  |  |  |
| 1 | Ath-AT2G16060.1 |  | Vvi-Vitvi03g00379\_t002 |  |  |  |  |  |  |  |
| 1 | Ath-AT2G16070.2 |  | Vvi-Vitvi03g04133\_t001 |  |  |  |  |  |  |  |
| 1 | Ath-AT2G16090.1 |  | Vvi-Vitvi03g00377\_t001 |  |  |  |  |  |  |  |
| 1 | Ath-AT2G16120.1 |  | Vvi-Vitvi03g00359\_t001 |  |  |  |  |  |  |  |
| 1 | Ath-AT2G16130.1 |  | | | |  |  |  |  |  |  |  |
| 1 | Ath-AT2G16190.1 |  | Vvi-Vitvi03g01507\_t001 |  |  |  |  |  |  |  |
| 1 | Ath-AT2G16200.2 |  | Vvi-Vitvi03g00344\_t001 |  |  |  |  |  |  |  |
| 1 | Ath-AT2G16210.1 |  | | | |  |  |  |  |  |  |  |
| 1 | Ath-AT2G16220.1 |  | | | |  |  |  |  |  |  |  |
| 1 | Ath-AT2G16225.1 |  | | | |  |  |  |  |  |  |  |
| 1 | Ath-AT2G16230.1 |  | Vvi-Vitvi03g00340\_t001 |  |  |  |  |  |  |  |
| 1 | Ath-AT2G16250.1 |  | Vvi-Vitvi03g00328\_t002 |  |  |  |  |  |  |  |
| 1 | Ath-AT2G16270.1 |  | | | |  |  |  |  |  |  |  |
| 1 | Ath-AT2G16280.1 |  | Vvi-Vitvi03g00322\_t001 |  |  |  |  |  |  |  |
| 1 | Ath-AT2G16290.1 |  | | | |  |  |  |  |  |  |  |
| 1 | Ath-AT2G16300.1 |  | | | |  |  |  |  |  |  |  |
| 1 | Ath-AT2G16340.1 |  | | | |  |  |  |  |  |  |  |
| 1 | Ath-AT2G16365.1 |  | Vvi-Vitvi03g00306\_t001 |  |  |  |  |  |  |  |
| 1 | Ath-AT2G16360.1 |  | Vvi-Vitvi03g00303\_t001 |  |  |  |  |  |  |  |
| 1 | Ath-AT2G16370.1 |  | Vvi-Vitvi03g04109\_t001 |  |  |  |  |  |  |  |
| 1 | Ath-AT2G16380.1 |  | | | |  |  |  |  |  |  |  |
| 1 | Ath-AT2G16385.1 |  | Vvi-Vitvi03g01482\_t001 |  |  |  |  |  |  |  |
| 2 | Ath-AT2G16390.2 |  | Vvi-Vitvi03g04102\_t001 |  | Vvi-Vitvi03g00003\_t001 |  |  |  |  |  |  |
| 1 | Ath-AT2G16400.1 |  |  |  | Vvi-Vitvi03g00004\_t001 |  |  |  |  |  |  |
| 1 | Ath-AT2G16405.1 |  |  |  | Vvi-Vitvi03g00012\_t001 |  |  |  |  |  |  |
| 1 | Ath-AT2G16430.2 |  |  |  | Vvi-Vitvi03g00015\_t001 |  |  |  |  |  |  |
| 1 | Ath-AT2G16440.1 |  |  |  | Vvi-Vitvi03g00023\_t001 |  |  |  |  |  |  |
| 1 | Ath-AT2G16450.1 |  |  |  | | | |  |  |  |  |  |  |
| 1 | Ath-AT2G16460.1 |  |  |  | Vvi-Vitvi03g01326\_t001 |  |  |  |  |  |  |
| 1 | Ath-AT2G16485.1 |  |  |  | Vvi-Vitvi03g00042\_t001 |  |  |  |  |  |  |
| 1 | Ath-AT2G16490.1 |  |  |  | | | |  |  |  |  |  |  |
| 1 | Ath-AT2G16500.1 |  |  |  | Vvi-Vitvi03g00054\_t001 |  |  |  |  |  |  |
| 1 | Ath-AT2G16505.1 |  |  |  | | | |  |  |  |  |  |  |
| 1 | Ath-AT2G16510.1 |  |  |  | Vvi-Vitvi03g04022\_t001 |  |  |  |  |  |  |
| 1 | Ath-AT2G16520.1 |  |  |  | | | |  |  |  |  |  |  |
| 1 | Ath-AT2G16530.3 |  |  |  | Vvi-Vitvi03g00057\_t001 |  |  |  |  |  |  |
| 1 | Ath-AT2G16535.1 |  |  |  | | | |  |  |  |  |  |  |
| 1 | Ath-AT2G16570.1 |  |  |  | Vvi-Vitvi03g00061\_t001 |  |  |  |  |  |  |
| 1 | Ath-AT2G16580.1 |  |  |  | Vvi-Vitvi03g00064\_t001 |  |  |  |  |  |  |
| 0 | Ath-AT2G16575.1 |  |  |  |  |  |  |  |  |
| 0 | Ath-AT2G16586.1 |  |  |  |  |  |  |  |  |
| 0 | Ath-AT2G16592.1 |  |  |  |  |  |  |  |  |
| 0 | Ath-AT2G16594.1 |  |  |  |  |  |  |  |  |
| 1 | Ath-AT2G16595.1 |  | Vvi-Vitvi03g00100\_t001 |  |  |  |  |  |  |  |
| 1 | Ath-AT2G16600.1 |  | Vvi-Vitvi03g04055\_t001 |  |  |  |  |  |  |  |
| 1 | Ath-AT2G16620.1 |  | | | |  |  |  |  |  |  |  |
| 1 | Ath-AT2G16630.1 |  | Vvi-Vitvi03g01391\_t001 |  |  |  |  |  |  |  |
| 1 | Ath-AT2G16640.1 |  | Vvi-Vitvi03g00114\_t001 |  |  |  |  |  |  |  |
| 1 | Ath-AT2G16650.1 |  | | | |  |  |  |  |  |  |  |
| 1 | Ath-AT2G16660.1 |  | Vvi-Vitvi03g00126\_t001 |  |  |  |  |  |  |  |
| 1 | Ath-AT2G16676.1 |  | | | |  |  |  |  |  |  |  |
| 1 | Ath-AT2G16700.1 |  | Vvi-Vitvi03g00129\_t001 |  |  |  |  |  |  |  |
| 1 | Ath-AT2G16710.3 |  | | | |  |  |  |  |  |  |  |
| 1 | Ath-AT2G16720.1 |  | Vvi-Vitvi03g00136\_t001 |  |  |  |  |  |  |  |
| 1 | Ath-AT2G16730.1 |  | Vvi-Vitvi03g00141\_t001 |  |  |  |  |  |  |  |
| 1 | Ath-AT2G16740.1 |  | | | |  |  |  |  |  |  |  |
| 1 | Ath-AT2G16750.2 |  | Vvi-Vitvi03g00144\_t001 |  |  |  |  |  |  |  |
| 1 | Ath-AT2G16760.1 |  | | | |  |  |  |  |  |  |  |
| 1 | Ath-AT2G16770.1 |  | Vvi-Vitvi03g00146\_t003 |  |  |  |  |  |  |  |
| 1 | Ath-AT2G16780.1 |  | Vvi-Vitvi03g00147\_t001 |  |  |  |  |  |  |  |
| 1 | Ath-AT2G16790.3 |  | | | |  |  |  |  |  |  |  |
| 1 | Ath-AT2G16800.1 |  | Vvi-Vitvi03g00151\_t001 |  |  |  |  |  |  |  |
| 1 | Ath-AT2G16810.1 |  | | | |  |  |  |  |  |  |  |
| 1 | Ath-AT2G16835.1 |  | | | |  |  |  |  |  |  |  |
| 1 | Ath-AT2G16850.1 |  | Vvi-Vitvi03g00155\_t001 |  |  |  |  |  |  |  |
| 1 | Ath-AT2G16860.1 |  | Vvi-Vitvi03g00156\_t001 |  |  |  |  |  |  |  |
| 1 | Ath-AT2G16870.1 |  | | | |  |  |  |  |  |  |  |
| 1 | Ath-AT2G16880.1 |  | | | |  |  |  |  |  |  |  |
| 1 | Ath-AT2G16890.2 |  | | | |  |  |  |  |  |  |  |
| 1 | Ath-AT2G16900.5 |  | | | |  |  |  |  |  |  |  |
| 1 | Ath-AT2G16910.1 |  | Vvi-Vitvi03g00157\_t001 |  |  |  |  |  |  |  |
| 1 | Ath-AT2G16920.1 |  | Vvi-Vitvi03g00159\_t001 |  |  |  |  |  |  |  |
| 1 | Ath-AT2G16930.2 |  | | | |  |  |  |  |  |  |  |
| 1 | Ath-AT2G16940.2 |  | Vvi-Vitvi03g04068\_t001 |  |  |  |  |  |  |  |
| 1 | Ath-AT2G16950.1 |  | Vvi-Vitvi03g00180\_t001 |  |  |  |  |  |  |  |
| 1 | Ath-AT2G16953.1 |  | | | |  |  |  |  |  |  |  |
| 1 | Ath-AT2G16960.1 |  | | | |  |  |  |  |  |  |  |
| 1 | Ath-AT2G16970.2 |  | Vvi-Vitvi03g00186\_t001 |  |  |  |  |  |  |  |
| 1 | Ath-AT2G16980.4 |  | | | |  |  |  |  |  |  |  |
| 1 | Ath-AT2G16990.2 |  | | | |  |  |  |  |  |  |  |
| 1 | Ath-AT2G17000.1 |  | Vvi-Vitvi03g00191\_t001 |  |  |  |  |  |  |  |
| 1 | Ath-AT2G17010.1 |  | | | |  |  |  |  |  |  |  |
| 1 | Ath-AT2G17020.1 |  | Vvi-Vitvi03g01420\_t001 |  |  |  |  |  |  |  |
| 2 | Ath-AT2G17030.1 |  | | | |  | Vvi-Vitvi03g04094\_t001 |  |  |  |  |  |  |
| 2 | Ath-AT2G17033.2 |  | | | |  | | | |  |  |  |  |  |  |
| 2 | Ath-AT2G17036.1 |  | | | |  | | | |  |  |  |  |  |  |
| 2 | Ath-AT2G17040.1 |  | | | |  | | | |  |  |  |  |  |  |
| 2 | Ath-AT2G17043.1 |  | | | |  | | | |  |  |  |  |  |  |
| 2 | Ath-AT2G17050.2 |  | | | |  | | | |  |  |  |  |  |  |
| 2 | Ath-AT2G17055.1 |  | | | |  | | | |  |  |  |  |  |  |
| 2 | Ath-AT2G17060.1 |  | | | |  | | | |  |  |  |  |  |  |
| 2 | Ath-AT2G17070.1 |  | | | |  | | | |  |  |  |  |  |  |
| 2 | Ath-AT2G17080.1 |  | | | |  | | | |  |  |  |  |  |  |
| 2 | Ath-AT2G17090.1 |  | | | |  | | | |  |  |  |  |  |  |
| 2 | Ath-AT2G17110.1 |  | | | |  | | | |  |  |  |  |  |  |
| 2 | Ath-AT2G17120.1 |  | | | |  | | | |  |  |  |  |  |  |
| 2 | Ath-AT2G17130.1 |  | Vvi-Vitvi03g00197\_t001 |  | | | |  |  |  |  |  |  |
| 1 | Ath-AT2G17140.1 |  |  |  | | | |  |  |  |  |  |  |
| 1 | Ath-AT2G17150.1 |  |  |  | Vvi-Vitvi03g00231\_t001 |  |  |  |  |  |  |
| 1 | Ath-AT2G17160.1 |  |  |  | | | |  |  |  |  |  |  |
| 1 | Ath-AT2G17170.1 |  |  |  | | | |  |  |  |  |  |  |
| 1 | Ath-AT2G17180.1 |  |  |  | | | |  |  |  |  |  |  |
| 1 | Ath-AT2G17190.1 |  |  |  | | | |  |  |  |  |  |  |
| 1 | Ath-AT2G17200.1 |  |  |  | | | |  |  |  |  |  |  |
| 1 | Ath-AT2G17210.1 |  |  |  | Vvi-Vitvi03g00234\_t001 |  |  |  |  |  |  |
| 1 | Ath-AT2G17220.1 |  |  |  | Vvi-Vitvi03g00238\_t001 |  |  |  |  |  |  |
| 1 | Ath-AT2G17230.1 |  |  |  | Vvi-Vitvi03g00242\_t001 |  |  |  |  |  |  |
| 1 | Ath-AT2G17240.1 |  |  |  | | | |  |  |  |  |  |  |
| 1 | Ath-AT2G17250.1 |  |  |  | | | |  |  |  |  |  |  |
| 1 | Ath-AT2G17260.1 |  |  |  | Vvi-Vitvi03g00246\_t001 |  |  |  |  |  |  |
| 1 | Ath-AT2G17265.1 |  |  |  | | | |  |  |  |  |  |  |
| 1 | Ath-AT2G17270.1 |  |  |  | Vvi-Vitvi03g00248\_t001 |  |  |  |  |  |  |
| 1 | Ath-AT2G17280.2 |  |  |  | | | |  |  |  |  |  |  |
| 1 | Ath-AT2G17290.2 |  |  |  | Vvi-Vitvi03g00249\_t001 |  |  |  |  |  |  |
| 1 | Ath-AT2G17300.1 |  |  |  | Vvi-Vitvi03g00251\_t001 |  |  |  |  |  |  |
| 1 | Ath-AT2G17305.1 |  |  |  | | | |  |  |  |  |  |  |
| 1 | Ath-AT2G17310.1 |  |  |  | | | |  |  |  |  |  |  |
| 1 | Ath-AT2G17320.1 |  |  |  | Vvi-Vitvi03g00254\_t001 |  |  |  |  |  |  |
| 1 | Ath-AT2G17340.1 |  |  |  | | | |  |  |  |  |  |  |
| 1 | Ath-AT2G17350.1 |  |  |  | | | |  |  |  |  |  |  |
| 1 | Ath-AT2G17360.1 |  |  |  | | | |  |  |  |  |  |  |
| 1 | Ath-AT2G17370.1 |  |  |  | Vvi-Vitvi03g00262\_t001 |  |  |  |  |  |  |
| 1 | Ath-AT2G17380.1 |  |  |  | | | |  |  |  |  |  |  |
| 1 | Ath-AT2G17390.1 |  |  |  | Vvi-Vitvi03g00277\_t003 |  |  |  |  |  |  |
| 0 | Ath-AT2G17410.1 |  |  |  |  |  |  |  |  |
| 0 | Ath-AT2G17420.1 |  |  |  |  |  |  |  |  |
| 0 | Ath-AT2G17430.1 |  |  |  |  |  |  |  |  |
| 1 | Ath-AT2G17440.1 |  | Vvi-Vitvi07g01903\_t001 |  |  |  |  |  |  |  |
| 1 | Ath-AT2G17442.7 |  | | | |  |  |  |  |  |  |  |
| 1 | Ath-AT2G17450.1 |  | Vvi-Vitvi07g02702\_t001 |  |  |  |  |  |  |  |
| 1 | Ath-AT2G17470.2 |  | | | |  |  |  |  |  |  |  |
| 1 | Ath-AT2G17480.1 |  | Vvi-Vitvi07g01896\_t001 |  |  |  |  |  |  |  |
| 1 | Ath-AT2G17500.2 |  | Vvi-Vitvi07g01893\_t001 |  |  |  |  |  |  |  |
| 1 | Ath-AT2G17510.2 |  | Vvi-Vitvi07g01892\_t001 |  |  |  |  |  |  |  |
| 1 | Ath-AT2G17520.1 |  | Vvi-Vitvi07g01891\_t001 |  |  |  |  |  |  |  |
| 1 | Ath-AT2G17525.1 |  | | | |  |  |  |  |  |  |  |
| 1 | Ath-AT2G17530.1 |  | Vvi-Vitvi07g01886\_t002 |  |  |  |  |  |  |  |
| 1 | Ath-AT2G17540.2 |  | Vvi-Vitvi07g01885\_t001 |  |  |  |  |  |  |  |
| 1 | Ath-AT2G17550.1 |  | Vvi-Vitvi04g01577\_t001 |  |  |  |  |  |  |  |
| 1 | Ath-AT2G17560.2 |  | Vvi-Vitvi04g02203\_t004 |  |  |  |  |  |  |  |
| 1 | Ath-AT2G17570.1 |  | Vvi-Vitvi04g04451\_t001 |  |  |  |  |  |  |  |
| 1 | Ath-AT2G17580.1 |  | Vvi-Vitvi04g04444\_t001 |  |  |  |  |  |  |  |
| 1 | Ath-AT2G17590.1 |  | | | |  |  |  |  |  |  |  |
| 1 | Ath-AT2G17600.1 |  | | | |  |  |  |  |  |  |  |
| 1 | Ath-AT2G17620.1 |  | Vvi-Vitvi04g04443\_t001 |  |  |  |  |  |  |  |
| 1 | Ath-AT2G17630.1 |  | Vvi-Vitvi04g04442\_t001 |  |  |  |  |  |  |  |
| 1 | Ath-AT2G17640.1 |  | Vvi-Vitvi04g01558\_t001 |  |  |  |  |  |  |  |
| 1 | Ath-AT2G17650.1 |  | Vvi-Vitvi04g04440\_t001 |  |  |  |  |  |  |  |
| 1 | Ath-AT2G17660.1 |  | | | |  |  |  |  |  |  |  |
| 1 | Ath-AT2G17670.1 |  | Vvi-Vitvi04g04435\_t001 |  |  |  |  |  |  |  |
| 1 | Ath-AT2G17680.1 |  | Vvi-Vitvi04g02299\_t001 |  |  |  |  |  |  |  |
| 1 | Ath-AT2G17690.1 |  | | | |  |  |  |  |  |  |  |
| 1 | Ath-AT2G17695.3 |  | Vvi-Vitvi04g02179\_t001 |  |  |  |  |  |  |  |
| 1 | Ath-AT2G17700.1 |  | Vvi-Vitvi04g04409\_t001 |  |  |  |  |  |  |  |
| 1 | Ath-AT2G17705.1 |  | Vvi-Vitvi04g01534\_t001 |  |  |  |  |  |  |  |
| 1 | Ath-AT2G17710.1 |  | Vvi-Vitvi04g02184\_t001 |  |  |  |  |  |  |  |
| 1 | Ath-AT2G17720.1 |  | Vvi-Vitvi04g02185\_t001 |  |  |  |  |  |  |  |
| 1 | Ath-AT2G17723.1 |  | | | |  |  |  |  |  |  |  |
| 1 | Ath-AT2G17730.2 |  | Vvi-Vitvi04g01536\_t001 |  |  |  |  |  |  |  |
| 1 | Ath-AT2G17740.1 |  | | | |  |  |  |  |  |  |  |
| 1 | Ath-AT2G17750.1 |  | | | |  |  |  |  |  |  |  |
| 1 | Ath-AT2G17760.1 |  | Vvi-Vitvi04g04401\_t001 |  |  |  |  |  |  |  |
| 2 | Ath-AT2G17770.3 |  | Vvi-Vitvi04g01505\_t001 |  | Vvi-Vitvi18g01165\_t001 |  |  |  |  |  |  |
| 2 | Ath-AT2G17780.5 |  | Vvi-Vitvi04g01503\_t001 |  | | | |  |  |  |  |  |  |
| 2 | Ath-AT2G17785.1 |  | | | |  | | | |  |  |  |  |  |  |
| 2 | Ath-AT2G17787.1 |  | | | |  | Vvi-Vitvi18g02814\_t002 |  |  |  |  |  |  |
| 2 | Ath-AT2G17790.1 |  | Vvi-Vitvi04g01499\_t001 |  | Vvi-Vitvi18g01150\_t001 |  |  |  |  |  |  |
| 2 | Ath-AT2G17800.1 |  | Vvi-Vitvi04g01498\_t001 |  | Vvi-Vitvi18g01149\_t001 |  |  |  |  |  |  |
| 2 | Ath-AT2G17820.1 |  | Vvi-Vitvi04g01483\_t001 |  | | | |  |  |  |  |  |  |
| 2 | Ath-AT2G17830.1 |  | | | |  | | | |  |  |  |  |  |  |
| 2 | Ath-AT2G17840.1 |  | Vvi-Vitvi04g01482\_t001 |  | | | |  |  |  |  |  |  |
| 2 | Ath-AT2G17845.1 |  | | | |  | | | |  |  |  |  |  |  |
| 2 | Ath-AT2G17850.3 |  | Vvi-Vitvi04g02158\_t001 |  | | | |  |  |  |  |  |  |
| 2 | Ath-AT2G17860.1 |  | Vvi-Vitvi04g01470\_t001 |  | Vvi-Vitvi18g01131\_t001 |  |  |  |  |  |  |
| 2 | Ath-AT2G17870.1 |  | Vvi-Vitvi04g01469\_t001 |  | | | |  |  |  |  |  |  |
| 2 | Ath-AT2G17880.1 |  | Vvi-Vitvi04g01466\_t001 |  | Vvi-Vitvi18g01129\_t001 |  |  |  |  |  |  |
| 2 | Ath-AT2G17890.1 |  | Vvi-Vitvi04g01462\_t001 |  | | | |  |  |  |  |  |  |
| 2 | Ath-AT2G17900.1 |  | | | |  | | | |  |  |  |  |  |  |
| 2 | Ath-AT2G17905.1 |  | | | |  | | | |  |  |  |  |  |  |
| 2 | Ath-AT2G17920.1 |  | | | |  | | | |  |  |  |  |  |  |
| 2 | Ath-AT2G17930.1 |  | Vvi-Vitvi04g01452\_t001 |  | | | |  |  |  |  |  |  |
| 2 | Ath-AT2G17940.1 |  | Vvi-Vitvi04g02150\_t001 |  | Vvi-Vitvi18g02792\_t001 |  |  |  |  |  |  |
| 1 | Ath-AT2G17950.1 |  | Vvi-Vitvi04g01449\_t001 |  |  |  |  |  |  |  |
| 1 | Ath-AT2G17960.1 |  | | | |  |  |  |  |  |  |  |
| 1 | Ath-AT2G17970.1 |  | Vvi-Vitvi04g01448\_t001 |  |  |  |  |  |  |  |
| 1 | Ath-AT2G17972.1 |  | Vvi-Vitvi04g02148\_t001 |  |  |  |  |  |  |  |
| 1 | Ath-AT2G17975.1 |  | Vvi-Vitvi04g01447\_t001 |  |  |  |  |  |  |  |
| 1 | Ath-AT2G17980.1 |  | | | |  |  |  |  |  |  |  |
| 1 | Ath-AT2G17990.3 |  | Vvi-Vitvi04g01443\_t001 |  |  |  |  |  |  |  |
| 1 | Ath-AT2G18000.2 |  | Vvi-Vitvi04g01442\_t001 |  |  |  |  |  |  |  |
| 1 | Ath-AT2G18010.1 |  | | | |  |  |  |  |  |  |  |
| 1 | Ath-AT2G18020.1 |  | Vvi-Vitvi04g01439\_t001 |  |  |  |  |  |  |  |
| 1 | Ath-AT2G18025.1 |  | | | |  |  |  |  |  |  |  |
| 1 | Ath-AT2G18030.1 |  | Vvi-Vitvi04g01438\_t001 |  |  |  |  |  |  |  |
| 1 | Ath-AT2G18040.1 |  | Vvi-Vitvi04g01437\_t001 |  |  |  |  |  |  |  |
| 1 | Ath-AT2G18050.1 |  | Vvi-Vitvi04g01432\_t001 |  |  |  |  |  |  |  |
| 1 | Ath-AT2G18060.1 |  | Vvi-Vitvi04g01430\_t001 |  |  |  |  |  |  |  |
| 1 | Ath-AT2G18070.1 |  | | | |  |  |  |  |  |  |  |
| 1 | Ath-AT2G18080.1 |  | Vvi-Vitvi04g01422\_t002 |  |  |  |  |  |  |  |
| 1 | Ath-AT2G18090.1 |  | Vvi-Vitvi04g01414\_t002 |  |  |  |  |  |  |  |
| 1 | Ath-AT2G18100.1 |  | Vvi-Vitvi04g01413\_t001 |  |  |  |  |  |  |  |
| 0 | Ath-AT2G18110.1 |  |  |  |  |  |  |  |  |
| 0 | Ath-AT2G18120.1 |  |  |  |  |  |  |  |  |
| 0 | Ath-AT2G18130.1 |  |  |  |  |  |  |  |  |
| 0 | Ath-AT2G18140.1 |  |  |  |  |  |  |  |  |
| 0 | Ath-AT2G18150.1 |  |  |  |  |  |  |  |  |
| 1 | Ath-AT2G18160.1 |  | Vvi-Vitvi03g00292\_t001 |  |  |  |  |  |  |  |
| 1 | Ath-AT2G18170.1 |  | | | |  |  |  |  |  |  |  |
| 1 | Ath-AT2G18180.1 |  | Vvi-Vitvi03g00295\_t001 |  |  |  |  |  |  |  |
| 1 | Ath-AT2G18190.1 |  | | | |  |  |  |  |  |  |  |
| 1 | Ath-AT2G18193.1 |  | Vvi-Vitvi03g00298\_t001 |  |  |  |  |  |  |  |
| 1 | Ath-AT2G18196.1 |  | | | |  |  |  |  |  |  |  |
| 1 | Ath-AT2G18200.1 |  | | | |  |  |  |  |  |  |  |
| 2 | Ath-AT2G18210.1 |  | | | |  | Vvi-Vitvi04g04348\_t001 |  |  |  |  |  |  |
| 2 | Ath-AT2G18220.1 |  | | | |  | | | |  |  |  |  |  |  |
| 2 | Ath-AT2G18230.1 |  | | | |  | Vvi-Vitvi04g01324\_t002 |  |  |  |  |  |  |
| 3 | Ath-AT2G18240.1 |  | Vvi-Vitvi03g00307\_t003 |  | Vvi-Vitvi04g01323\_t002 |  | Vvi-Vitvi18g00980\_t001 |  |  |  |  |  |
| 3 | Ath-AT2G18245.1 |  | | | |  | Vvi-Vitvi04g01321\_t001 |  | | | |  |  |  |  |  |
| 3 | Ath-AT2G18250.1 |  | | | |  | Vvi-Vitvi04g01320\_t003 |  | | | |  |  |  |  |  |
| 3 | Ath-AT2G18260.1 |  | Vvi-Vitvi03g00311\_t001 |  | Vvi-Vitvi04g01319\_t001 |  | | | |  |  |  |  |  |
| 3 | Ath-AT2G18280.2 |  | | | |  | Vvi-Vitvi04g01315\_t001 |  | Vvi-Vitvi18g00976\_t001 |  |  |  |  |  |
| 3 | Ath-AT2G18270.1 |  | | | |  | | | |  | | | |  |  |  |  |  |
| 3 | Ath-AT2G18290.1 |  | | | |  | Vvi-Vitvi04g01313\_t001 |  | | | |  |  |  |  |  |
| 3 | Ath-AT2G18300.3 |  | Vvi-Vitvi03g00315\_t001 |  | Vvi-Vitvi04g01312\_t001 |  | | | |  |  |  |  |  |
| 3 | Ath-AT2G18320.1 |  | | | |  | | | |  | | | |  |  |  |  |  |
| 3 | Ath-AT2G18328.1 |  | Vvi-Vitvi03g01495\_t001 |  | Vvi-Vitvi04g01309\_t001 |  | Vvi-Vitvi18g00973\_t001 |  |  |  |  |  |
| 3 | Ath-AT2G18330.1 |  | | | |  | Vvi-Vitvi04g01307\_t001 |  | | | |  |  |  |  |  |
| 3 | Ath-AT2G18340.1 |  | | | |  | Vvi-Vitvi04g01306\_t001 |  | | | |  |  |  |  |  |
| 3 | Ath-AT2G18350.1 |  | | | |  | Vvi-Vitvi04g01304\_t001 |  | Vvi-Vitvi18g00972\_t001 |  |  |  |  |  |
| 3 | Ath-AT2G18360.1 |  | Vvi-Vitvi03g00321\_t001 |  | Vvi-Vitvi04g01300\_t001 |  | | | |  |  |  |  |  |
| 2 | Ath-AT2G18370.1 |  |  |  | | | |  | | | |  |  |  |  |  |
| 2 | Ath-AT2G18380.1 |  |  |  | Vvi-Vitvi04g01299\_t001 |  | | | |  |  |  |  |  |
| 2 | Ath-AT2G18390.1 |  |  |  | Vvi-Vitvi04g01297\_t002 |  | | | |  |  |  |  |  |
| 2 | Ath-AT2G18400.1 |  |  |  | Vvi-Vitvi04g04344\_t001 |  | | | |  |  |  |  |  |
| 2 | Ath-AT2G18410.1 |  |  |  | | | |  | | | |  |  |  |  |  |
| 2 | Ath-AT2G18420.1 |  |  |  | | | |  | | | |  |  |  |  |  |
| 2 | Ath-AT2G18450.1 |  |  |  | Vvi-Vitvi04g01280\_t001 |  | | | |  |  |  |  |  |
| 2 | Ath-AT2G18460.1 |  |  |  | | | |  | Vvi-Vitvi18g00954\_t001 |  |  |  |  |  |
| 2 | Ath-AT2G18465.1 |  |  |  | | | |  | | | |  |  |  |  |  |
| 2 | Ath-AT2G18470.1 |  |  |  | Vvi-Vitvi04g01269\_t001 |  | | | |  |  |  |  |  |
| 2 | Ath-AT2G18480.1 |  |  |  | Vvi-Vitvi04g01263\_t001 |  | | | |  |  |  |  |  |
| 2 | Ath-AT2G18490.2 |  |  |  | | | |  | | | |  |  |  |  |  |
| 2 | Ath-AT2G18500.1 |  |  |  | Vvi-Vitvi04g01262\_t001 |  | Vvi-Vitvi18g00946\_t001 |  |  |  |  |  |
| 1 | Ath-AT2G18510.1 |  |  |  | Vvi-Vitvi04g01260\_t001 |  |  |  |  |  |  |
| 1 | Ath-AT2G18520.1 |  |  |  | Vvi-Vitvi04g01258\_t001 |  |  |  |  |  |  |
| 1 | Ath-AT2G18530.1 |  |  |  | | | |  |  |  |  |  |  |
| 1 | Ath-AT2G18540.2 |  |  |  | Vvi-Vitvi04g01248\_t001 |  |  |  |  |  |  |
| 1 | Ath-AT2G18550.1 |  |  |  | Vvi-Vitvi04g01244\_t001 |  |  |  |  |  |  |
| 1 | Ath-AT2G18560.1 |  |  |  | | | |  |  |  |  |  |  |
| 1 | Ath-AT2G18570.1 |  |  |  | Vvi-Vitvi04g01237\_t001 |  |  |  |  |  |  |
| 1 | Ath-AT2G18590.1 |  |  |  | Vvi-Vitvi04g01235\_t001 |  |  |  |  |  |  |
| 1 | Ath-AT2G18600.2 |  |  |  | | | |  |  |  |  |  |  |
| 1 | Ath-AT2G18620.1 |  |  |  | Vvi-Vitvi04g01230\_t001 |  |  |  |  |  |  |
| 1 | Ath-AT2G18630.1 |  |  |  | Vvi-Vitvi04g01228\_t001 |  |  |  |  |  |  |
| 0 | Ath-AT2G18640.1 |  |  |  |  |  |  |  |  |
| 0 | Ath-AT2G18650.1 |  |  |  |  |  |  |  |  |
| 0 | Ath-AT2G18660.1 |  |  |  |  |  |  |  |  |
| 0 | Ath-AT2G18670.1 |  |  |  |  |  |  |  |  |
| 0 | Ath-AT2G18680.1 |  |  |  |  |  |  |  |  |
| 0 | Ath-AT2G18685.1 |  |  |  |  |  |  |  |  |
| 0 | Ath-AT2G18690.1 |  |  |  |  |  |  |  |  |
| 0 | Ath-AT2G18700.1 |  |  |  |  |  |  |  |  |
| 0 | Ath-AT2G18710.1 |  |  |  |  |  |  |  |  |
| 0 | Ath-AT2G18720.1 |  |  |  |  |  |  |  |  |
| 0 | Ath-AT2G18721.1 |  |  |  |  |  |  |  |  |
| 0 | Ath-AT2G18730.1 |  |  |  |  |  |  |  |  |
| 0 | Ath-AT2G18740.1 |  |  |  |  |  |  |  |  |
| 0 | Ath-AT2G18750.1 |  |  |  |  |  |  |  |  |
| 0 | Ath-AT2G18760.3 |  |  |  |  |  |  |  |  |
| 0 | Ath-AT2G18770.2 |  |  |  |  |  |  |  |  |
| 0 | Ath-AT2G18780.1 |  |  |  |  |  |  |  |  |
| 0 | Ath-AT2G18790.1 |  |  |  |  |  |  |  |  |
| 0 | Ath-AT2G18800.1 |  |  |  |  |  |  |  |  |
| 0 | Ath-AT2G18810.1 |  |  |  |  |  |  |  |  |
| 0 | Ath-AT2G18830.1 |  |  |  |  |  |  |  |  |
| 1 | Ath-AT2G18840.1 |  | Vvi-Vitvi11g01244\_t001 |  |  |  |  |  |  |  |
| 1 | Ath-AT2G18850.1 |  | | | |  |  |  |  |  |  |  |
| 1 | Ath-AT2G18860.1 |  | Vvi-Vitvi11g04343\_t001 |  |  |  |  |  |  |  |
| 1 | Ath-AT2G18870.1 |  | | | |  |  |  |  |  |  |  |
| 1 | Ath-AT2G18876.1 |  | Vvi-Vitvi11g01238\_t002 |  |  |  |  |  |  |  |
| 1 | Ath-AT2G18880.1 |  | | | |  |  |  |  |  |  |  |
| 1 | Ath-AT2G18890.1 |  | Vvi-Vitvi11g01228\_t001 |  |  |  |  |  |  |  |
| 1 | Ath-AT2G18900.1 |  | | | |  |  |  |  |  |  |  |
| 1 | Ath-AT2G18910.1 |  | | | |  |  |  |  |  |  |  |
| 1 | Ath-AT2G18915.2 |  | Vvi-Vitvi11g01220\_t001 |  |  |  |  |  |  |  |
| 1 | Ath-AT2G18920.1 |  | | | |  |  |  |  |  |  |  |
| 1 | Ath-AT2G18938.1 |  | | | |  |  |  |  |  |  |  |
| 2 | Ath-AT2G18940.1 |  | | | |  | Vvi-Vitvi11g01203\_t001 |  |  |  |  |  |  |
| 2 | Ath-AT2G18950.1 |  | | | |  | Vvi-Vitvi11g01205\_t001 |  |  |  |  |  |  |
| 2 | Ath-AT2G18960.1 |  | | | |  | Vvi-Vitvi11g01208\_t001 |  |  |  |  |  |  |
| 2 | Ath-AT2G18969.1 |  | | | |  | Vvi-Vitvi11g01650\_t002 |  |  |  |  |  |  |
| 2 | Ath-AT2G18970.1 |  | | | |  | | | |  |  |  |  |  |  |
| 2 | Ath-AT2G18980.1 |  | Vvi-Vitvi11g01210\_t001 |  | Vvi-Vitvi11g01210\_t001 |  |  |  |  |  |  |
| 1 | Ath-AT2G18990.1 |  |  |  | | | |  |  |  |  |  |  |
| 1 | Ath-AT2G19000.1 |  |  |  | | | |  |  |  |  |  |  |
| 1 | Ath-AT2G19010.2 |  |  |  | | | |  |  |  |  |  |  |
| 1 | Ath-AT2G19020.1 |  |  |  | | | |  |  |  |  |  |  |
| 1 | Ath-AT2G19030.1 |  |  |  | | | |  |  |  |  |  |  |
| 1 | Ath-AT2G19040.1 |  |  |  | | | |  |  |  |  |  |  |
| 1 | Ath-AT2G19045.1 |  |  |  | | | |  |  |  |  |  |  |
| 1 | Ath-AT2G19050.1 |  |  |  | | | |  |  |  |  |  |  |
| 1 | Ath-AT2G19060.1 |  |  |  | | | |  |  |  |  |  |  |
| 2 | Ath-AT2G19070.1 |  | Vvi-Vitvi11g01099\_t001 |  | | | |  |  |  |  |  |  |
| 2 | Ath-AT2G19080.1 |  | Vvi-Vitvi11g01106\_t001 |  | | | |  |  |  |  |  |  |
| 2 | Ath-AT2G19090.1 |  | Vvi-Vitvi11g01107\_t002 |  | | | |  |  |  |  |  |  |
| 2 | Ath-AT2G19110.3 |  | Vvi-Vitvi11g04307\_t002 |  | | | |  |  |  |  |  |  |
| 2 | Ath-AT2G19120.1 |  | | | |  | | | |  |  |  |  |  |  |
| 2 | Ath-AT2G19130.1 |  | | | |  | Vvi-Vitvi11g04337\_t001 |  |  |  |  |  |  |
| 1 | Ath-AT2G19146.1 |  | | | |  |  |  |  |  |  |  |
| 1 | Ath-AT2G19150.1 |  | | | |  |  |  |  |  |  |  |
| 1 | Ath-AT2G19160.1 |  | Vvi-Vitvi11g01128\_t001 |  |  |  |  |  |  |  |
| 1 | Ath-AT2G19170.1 |  | Vvi-Vitvi11g01134\_t006 |  |  |  |  |  |  |  |
| 1 | Ath-AT2G19180.2 |  | | | |  |  |  |  |  |  |  |
| 1 | Ath-AT2G19190.1 |  | | | |  |  |  |  |  |  |  |
| 1 | Ath-AT2G19210.1 |  | | | |  |  |  |  |  |  |  |
| 1 | Ath-AT2G19220.1 |  | | | |  |  |  |  |  |  |  |
| 1 | Ath-AT2G19230.1 |  | Vvi-Vitvi11g01136\_t001 |  |  |  |  |  |  |  |
| 1 | Ath-AT2G19240.2 |  | | | |  |  |  |  |  |  |  |
| 1 | Ath-AT2G19260.1 |  | Vvi-Vitvi11g01141\_t001 |  |  |  |  |  |  |  |
| 1 | Ath-AT2G19270.1 |  | Vvi-Vitvi11g01150\_t001 |  |  |  |  |  |  |  |
| 0 | Ath-AT2G19280.3 |  |  |  |  |  |  |  |  |
| 0 | Ath-AT2G19290.1 |  |  |  |  |  |  |  |  |
| 0 | Ath-AT2G19300.1 |  |  |  |  |  |  |  |  |
| 0 | Ath-AT2G19310.1 |  |  |  |  |  |  |  |  |
| 0 | Ath-AT2G19320.1 |  |  |  |  |  |  |  |  |
| 0 | Ath-AT2G19330.1 |  |  |  |  |  |  |  |  |
| 0 | Ath-AT2G19340.1 |  |  |  |  |  |  |  |  |
| 0 | Ath-AT2G19350.1 |  |  |  |  |  |  |  |  |
| 0 | Ath-AT2G19360.1 |  |  |  |  |  |  |  |  |
| 0 | Ath-AT2G19365.1 |  |  |  |  |  |  |  |  |
| 1 | Ath-AT2G19380.1 |  | Vvi-Vitvi11g00133\_t001 |  |  |  |  |  |  |  |
| 1 | Ath-AT2G19385.1 |  | | | |  |  |  |  |  |  |  |
| 1 | Ath-AT2G19390.1 |  | Vvi-Vitvi11g00138\_t001 |  |  |  |  |  |  |  |
| 1 | Ath-AT2G19400.1 |  | Vvi-Vitvi11g00139\_t001 |  |  |  |  |  |  |  |
| 1 | Ath-AT2G19410.2 |  | Vvi-Vitvi11g00142\_t001 |  |  |  |  |  |  |  |
| 1 | Ath-AT2G19420.1 |  | | | |  |  |  |  |  |  |  |
| 1 | Ath-AT2G19430.1 |  | Vvi-Vitvi11g00151\_t001 |  |  |  |  |  |  |  |
| 1 | Ath-AT2G19440.1 |  | | | |  |  |  |  |  |  |  |
| 1 | Ath-AT2G19450.1 |  | Vvi-Vitvi11g00153\_t001 |  |  |  |  |  |  |  |
| 1 | Ath-AT2G19460.2 |  | Vvi-Vitvi11g00154\_t001 |  |  |  |  |  |  |  |
| 1 | Ath-AT2G19470.1 |  | Vvi-Vitvi11g00155\_t001 |  |  |  |  |  |  |  |
| 2 | Ath-AT2G19480.1 |  | Vvi-Vitvi11g00163\_t001 |  | Vvi-Vitvi09g00194\_t001 |  |  |  |  |  |  |
| 2 | Ath-AT2G19490.1 |  | Vvi-Vitvi11g00164\_t001 |  | | | |  |  |  |  |  |  |
| 2 | Ath-AT2G19500.1 |  | Vvi-Vitvi11g01371\_t001 |  | | | |  |  |  |  |  |  |
| 2 | Ath-AT2G19510.1 |  | | | |  | | | |  |  |  |  |  |  |
| 2 | Ath-AT2G19520.1 |  | Vvi-Vitvi11g00174\_t001 |  | | | |  |  |  |  |  |  |
| 2 | Ath-AT2G19530.1 |  | Vvi-Vitvi11g04046\_t001 |  | | | |  |  |  |  |  |  |
| 2 | Ath-AT2G19540.1 |  | | | |  | | | |  |  |  |  |  |  |
| 2 | Ath-AT2G19550.1 |  | | | |  | | | |  |  |  |  |  |  |
| 2 | Ath-AT2G19560.1 |  | Vvi-Vitvi11g00187\_t001 |  | | | |  |  |  |  |  |  |
| 2 | Ath-AT2G19570.1 |  | Vvi-Vitvi11g01376\_t001 |  | | | |  |  |  |  |  |  |
| 2 | Ath-AT2G19580.1 |  | Vvi-Vitvi11g00193\_t001 |  | | | |  |  |  |  |  |  |
| 2 | Ath-AT2G19590.1 |  | Vvi-Vitvi11g00195\_t001 |  | | | |  |  |  |  |  |  |
| 2 | Ath-AT2G19600.1 |  | Vvi-Vitvi11g00196\_t001 |  | | | |  |  |  |  |  |  |
| 2 | Ath-AT2G19610.2 |  | | | |  | | | |  |  |  |  |  |  |
| 2 | Ath-AT2G19620.1 |  | Vvi-Vitvi11g00203\_t001 |  | | | |  |  |  |  |  |  |
| 2 | Ath-AT2G19630.1 |  | | | |  | | | |  |  |  |  |  |  |
| 2 | Ath-AT2G19640.2 |  | | | |  | | | |  |  |  |  |  |  |
| 2 | Ath-AT2G19650.1 |  | | | |  | | | |  |  |  |  |  |  |
| 2 | Ath-AT2G19660.2 |  | | | |  | | | |  |  |  |  |  |  |
| 2 | Ath-AT2G19670.1 |  | Vvi-Vitvi11g00208\_t001 |  | | | |  |  |  |  |  |  |
| 2 | Ath-AT2G19680.1 |  | | | |  | Vvi-Vitvi09g00209\_t001 |  |  |  |  |  |  |
| 2 | Ath-AT2G19690.2 |  | Vvi-Vitvi11g01379\_t001 |  | | | |  |  |  |  |  |  |
| 2 | Ath-AT2G19700.1 |  | | | |  | | | |  |  |  |  |  |  |
| 2 | Ath-AT2G19710.1 |  | | | |  | | | |  |  |  |  |  |  |
| 2 | Ath-AT2G19720.1 |  | | | |  | Vvi-Vitvi09g00213\_t002 |  |  |  |  |  |  |
| 2 | Ath-AT2G19730.2 |  | | | |  | Vvi-Vitvi09g01545\_t001 |  |  |  |  |  |  |
| 2 | Ath-AT2G19740.1 |  | Vvi-Vitvi11g00222\_t001 |  | | | |  |  |  |  |  |  |
| 2 | Ath-AT2G19750.1 |  | Vvi-Vitvi11g04055\_t001 |  | Vvi-Vitvi09g04055\_t001 |  |  |  |  |  |  |
| 2 | Ath-AT2G19760.1 |  | | | |  | | | |  |  |  |  |  |  |
| 2 | Ath-AT2G19770.1 |  | | | |  | | | |  |  |  |  |  |  |
| 2 | Ath-AT2G19780.1 |  | | | |  | | | |  |  |  |  |  |  |
| 2 | Ath-AT2G19790.1 |  | Vvi-Vitvi11g00235\_t001 |  | | | |  |  |  |  |  |  |
| 2 | Ath-AT2G19800.1 |  | | | |  | | | |  |  |  |  |  |  |
| 2 | Ath-AT2G19802.1 |  | | | |  | | | |  |  |  |  |  |  |
| 2 | Ath-AT2G19810.1 |  | | | |  | | | |  |  |  |  |  |  |
| 2 | Ath-AT2G19820.1 |  | | | |  | | | |  |  |  |  |  |  |
| 2 | Ath-AT2G19830.2 |  | | | |  | Vvi-Vitvi09g01550\_t006 |  |  |  |  |  |  |
| 1 | Ath-AT2G19850.2 |  | | | |  |  |  |  |  |  |  |
| 1 | Ath-AT2G19860.1 |  | Vvi-Vitvi11g00260\_t001 |  |  |  |  |  |  |  |
| 1 | Ath-AT2G19870.1 |  | Vvi-Vitvi11g00265\_t001 |  |  |  |  |  |  |  |
| 1 | Ath-AT2G19880.2 |  | Vvi-Vitvi11g00266\_t001 |  |  |  |  |  |  |  |
| 1 | Ath-AT2G19890.1 |  | | | |  |  |  |  |  |  |  |
| 1 | Ath-AT2G19893.1 |  | | | |  |  |  |  |  |  |  |
| 1 | Ath-AT2G19900.1 |  | Vvi-Vitvi11g00272\_t001 |  |  |  |  |  |  |  |
| 1 | Ath-AT2G19910.1 |  | Vvi-Vitvi11g00273\_t002 |  |  |  |  |  |  |  |
| 1 | Ath-AT2G19920.2 |  | | | |  |  |  |  |  |  |  |
| 1 | Ath-AT2G19930.1 |  | | | |  |  |  |  |  |  |  |
| 1 | Ath-AT2G19940.1 |  | | | |  |  |  |  |  |  |  |
| 1 | Ath-AT2G19950.2 |  | Vvi-Vitvi11g00274\_t001 |  |  |  |  |  |  |  |
| 1 | Ath-AT2G19960.3 |  | | | |  |  |  |  |  |  |  |
| 1 | Ath-AT2G19970.1 |  | | | |  |  |  |  |  |  |  |
| 1 | Ath-AT2G19980.1 |  | | | |  |  |  |  |  |  |  |
| 1 | Ath-AT2G19990.1 |  | | | |  |  |  |  |  |  |  |
| 1 | Ath-AT2G20000.1 |  | Vvi-Vitvi11g00277\_t001 |  |  |  |  |  |  |  |
| 1 | Ath-AT2G20010.2 |  | Vvi-Vitvi11g00283\_t001 |  |  |  |  |  |  |  |
| 1 | Ath-AT2G20020.1 |  | Vvi-Vitvi11g00288\_t001 |  |  |  |  |  |  |  |
| 1 | Ath-AT2G20030.1 |  | Vvi-Vitvi11g00293\_t001 |  |  |  |  |  |  |  |
| 1 | Ath-AT2G20050.1 |  | Vvi-Vitvi11g00294\_t001 |  |  |  |  |  |  |  |
| 1 | Ath-AT2G20060.1 |  | | | |  |  |  |  |  |  |  |
| 1 | Ath-AT2G20070.1 |  | | | |  |  |  |  |  |  |  |
| 1 | Ath-AT2G20080.1 |  | Vvi-Vitvi11g01399\_t001 |  |  |  |  |  |  |  |
| 1 | Ath-AT2G20100.3 |  | Vvi-Vitvi11g00303\_t001 |  |  |  |  |  |  |  |
| 1 | Ath-AT2G20110.2 |  | Vvi-Vitvi11g00314\_t001 |  |  |  |  |  |  |  |
| 1 | Ath-AT2G20120.1 |  | Vvi-Vitvi11g00323\_t001 |  |  |  |  |  |  |  |
| 0 | Ath-AT2G20130.1 |  |  |  |  |  |  |  |  |
| 1 | Ath-AT2G20140.1 |  | Vvi-Vitvi07g00765\_t001 |  |  |  |  |  |  |  |
| 1 | Ath-AT2G20142.1 |  | | | |  |  |  |  |  |  |  |
| 1 | Ath-AT2G20150.1 |  | | | |  |  |  |  |  |  |  |
| 1 | Ath-AT2G20160.1 |  | | | |  |  |  |  |  |  |  |
| 1 | Ath-AT2G20170.2 |  | | | |  |  |  |  |  |  |  |
| 1 | Ath-AT2G20180.2 |  | Vvi-Vitvi07g00762\_t001 |  |  |  |  |  |  |  |
| 1 | Ath-AT2G20190.1 |  | Vvi-Vitvi07g00744\_t001 |  |  |  |  |  |  |  |
| 1 | Ath-AT2G20208.1 |  | | | |  |  |  |  |  |  |  |
| 1 | Ath-AT2G20210.1 |  | Vvi-Vitvi07g00743\_t001 |  |  |  |  |  |  |  |
| 1 | Ath-AT2G20230.1 |  | Vvi-Vitvi07g00740\_t001 |  |  |  |  |  |  |  |
| 1 | Ath-AT2G20240.1 |  | Vvi-Vitvi07g00739\_t001 |  |  |  |  |  |  |  |
| 1 | Ath-AT2G20250.2 |  | | | |  |  |  |  |  |  |  |
| 1 | Ath-AT2G20260.1 |  | Vvi-Vitvi07g02309\_t001 |  |  |  |  |  |  |  |
| 1 | Ath-AT2G20270.2 |  | Vvi-Vitvi07g00733\_t001 |  |  |  |  |  |  |  |
| 1 | Ath-AT2G20280.1 |  | | | |  |  |  |  |  |  |  |
| 1 | Ath-AT2G20290.1 |  | Vvi-Vitvi07g00724\_t001 |  |  |  |  |  |  |  |
| 1 | Ath-AT2G20300.1 |  | Vvi-Vitvi07g00719\_t001 |  |  |  |  |  |  |  |
| 1 | Ath-AT2G20310.1 |  | Vvi-Vitvi07g00705\_t001 |  |  |  |  |  |  |  |
| 1 | Ath-AT2G20320.1 |  | Vvi-Vitvi07g00698\_t001 |  |  |  |  |  |  |  |
| 1 | Ath-AT2G20330.1 |  | Vvi-Vitvi07g00697\_t001 |  |  |  |  |  |  |  |
| 1 | Ath-AT2G20340.1 |  | Vvi-Vitvi07g00696\_t001 |  |  |  |  |  |  |  |
| 1 | Ath-AT2G20350.1 |  | | | |  |  |  |  |  |  |  |
| 1 | Ath-AT2G20360.1 |  | Vvi-Vitvi07g00693\_t001 |  |  |  |  |  |  |  |
| 1 | Ath-AT2G20362.1 |  | | | |  |  |  |  |  |  |  |
| 1 | Ath-AT2G20370.1 |  | Vvi-Vitvi07g00691\_t001 |  |  |  |  |  |  |  |
| 1 | Ath-AT2G20380.1 |  | | | |  |  |  |  |  |  |  |
| 1 | Ath-AT2G20390.1 |  | Vvi-Vitvi07g00686\_t001 |  |  |  |  |  |  |  |
| 1 | Ath-AT2G20400.2 |  | Vvi-Vitvi07g00666\_t001 |  |  |  |  |  |  |  |
| 0 | Ath-AT2G20410.1 |  |  |  |  |  |  |  |  |
| 1 | Ath-AT2G20420.1 |  | Vvi-Vitvi07g00637\_t001 |  |  |  |  |  |  |  |
| 1 | Ath-AT2G20430.1 |  | Vvi-Vitvi07g02274\_t001 |  |  |  |  |  |  |  |
| 1 | Ath-AT2G20440.2 |  | Vvi-Vitvi07g00633\_t002 |  |  |  |  |  |  |  |
| 1 | Ath-AT2G20450.1 |  | | | |  |  |  |  |  |  |  |
| 1 | Ath-AT2G20463.1 |  | | | |  |  |  |  |  |  |  |
| 1 | Ath-AT2G20465.1 |  | | | |  |  |  |  |  |  |  |
| 1 | Ath-AT2G20470.1 |  | Vvi-Vitvi07g00626\_t001 |  |  |  |  |  |  |  |
| 1 | Ath-AT2G20480.1 |  | | | |  |  |  |  |  |  |  |
| 1 | Ath-AT2G20490.1 |  | | | |  |  |  |  |  |  |  |
| 1 | Ath-AT2G20495.3 |  | Vvi-Vitvi07g00620\_t001 |  |  |  |  |  |  |  |
| 1 | Ath-AT2G20500.1 |  | | | |  |  |  |  |  |  |  |
| 1 | Ath-AT2G20510.2 |  | | | |  |  |  |  |  |  |  |
| 1 | Ath-AT2G20515.1 |  | Vvi-Vitvi07g00604\_t001 |  |  |  |  |  |  |  |
| 1 | Ath-AT2G20520.1 |  | Vvi-Vitvi12g00248\_t001 |  |  |  |  |  |  |  |
| 1 | Ath-AT2G20530.1 |  | | | |  |  |  |  |  |  |  |
| 1 | Ath-AT2G20540.1 |  | | | |  |  |  |  |  |  |  |
| 1 | Ath-AT2G20550.1 |  | | | |  |  |  |  |  |  |  |
| 1 | Ath-AT2G20560.1 |  | | | |  |  |  |  |  |  |  |
| 1 | Ath-AT2G20562.1 |  | | | |  |  |  |  |  |  |  |
| 1 | Ath-AT2G20570.2 |  | Vvi-Vitvi12g00260\_t001 |  |  |  |  |  |  |  |
| 1 | Ath-AT2G20580.1 |  | Vvi-Vitvi12g00263\_t001 |  |  |  |  |  |  |  |
| 1 | Ath-AT2G20585.7 |  | Vvi-Vitvi12g00267\_t004 |  |  |  |  |  |  |  |
| 1 | Ath-AT2G20590.1 |  | Vvi-Vitvi12g00269\_t001 |  |  |  |  |  |  |  |
| 1 | Ath-AT2G20595.1 |  | | | |  |  |  |  |  |  |  |
| 1 | Ath-AT2G20597.1 |  | | | |  |  |  |  |  |  |  |
| 1 | Ath-AT2G20605.1 |  | | | |  |  |  |  |  |  |  |
| 2 | Ath-AT2G20610.1 |  | Vvi-Vitvi12g00270\_t001 |  | Vvi-Vitvi12g00335\_t001 |  |  |  |  |  |  |
| 2 | Ath-AT2G20613.1 |  | | | |  | | | |  |  |  |  |  |  |
| 2 | Ath-AT2G20616.1 |  | | | |  | | | |  |  |  |  |  |  |
| 2 | Ath-AT2G20618.1 |  | | | |  | | | |  |  |  |  |  |  |
| 2 | Ath-AT2G20619.1 |  | | | |  | | | |  |  |  |  |  |  |
| 2 | Ath-AT2G20620.1 |  | | | |  | | | |  |  |  |  |  |  |
| 2 | Ath-AT2G20625.2 |  | | | |  | | | |  |  |  |  |  |  |
| 2 | Ath-AT2G20630.2 |  | Vvi-Vitvi12g00277\_t001 |  | | | |  |  |  |  |  |  |
| 2 | Ath-AT2G20635.1 |  | Vvi-Vitvi12g00281\_t001 |  | | | |  |  |  |  |  |  |
| 2 | Ath-AT2G20650.2 |  | Vvi-Vitvi12g00286\_t001 |  | | | |  |  |  |  |  |  |
| 2 | Ath-AT2G20660.1 |  | | | |  | | | |  |  |  |  |  |  |
| 2 | Ath-AT2G20670.1 |  | Vvi-Vitvi12g00298\_t001 |  | | | |  |  |  |  |  |  |
| 2 | Ath-AT2G20680.1 |  | Vvi-Vitvi12g00303\_t004 |  | | | |  |  |  |  |  |  |
| 2 | Ath-AT2G20690.1 |  | Vvi-Vitvi12g00314\_t001 |  | | | |  |  |  |  |  |  |
| 2 | Ath-AT2G20700.1 |  | Vvi-Vitvi12g00316\_t001 |  | | | |  |  |  |  |  |  |
| 2 | Ath-AT2G20710.1 |  | Vvi-Vitvi12g00322\_t001 |  | | | |  |  |  |  |  |  |
| 2 | Ath-AT2G20720.1 |  | | | |  | | | |  |  |  |  |  |  |
| 2 | Ath-AT2G20725.1 |  | Vvi-Vitvi12g00325\_t001 |  | | | |  |  |  |  |  |  |
| 1 | Ath-AT2G20740.1 |  |  |  | Vvi-Vitvi12g00341\_t001 |  |  |  |  |  |  |
| 1 | Ath-AT2G20750.1 |  |  |  | Vvi-Vitvi12g00342\_t001 |  |  |  |  |  |  |
| 1 | Ath-AT2G20760.1 |  |  |  | | | |  |  |  |  |  |  |
| 1 | Ath-AT2G20770.1 |  |  |  | Vvi-Vitvi12g00343\_t001 |  |  |  |  |  |  |
| 1 | Ath-AT2G20780.1 |  |  |  | Vvi-Vitvi12g00344\_t001 |  |  |  |  |  |  |
| 1 | Ath-AT2G20790.1 |  |  |  | | | |  |  |  |  |  |  |
| 1 | Ath-AT2G20784.1 |  |  |  | | | |  |  |  |  |  |  |
| 1 | Ath-AT2G20800.1 |  |  |  | | | |  |  |  |  |  |  |
| 1 | Ath-AT2G20805.1 |  |  |  | | | |  |  |  |  |  |  |
| 2 | Ath-AT2G20810.1 |  | Vvi-Vitvi12g00502\_t001 |  | | | |  |  |  |  |  |  |
| 2 | Ath-AT2G20815.3 |  | Vvi-Vitvi12g00501\_t001 |  | | | |  |  |  |  |  |  |
| 2 | Ath-AT2G20820.2 |  | Vvi-Vitvi12g02402\_t001 |  | | | |  |  |  |  |  |  |
| 2 | Ath-AT2G20825.1 |  | | | |  | | | |  |  |  |  |  |  |
| 2 | Ath-AT2G20830.2 |  | Vvi-Vitvi12g00493\_t002 |  | | | |  |  |  |  |  |  |
| 2 | Ath-AT2G20835.1 |  | | | |  | | | |  |  |  |  |  |  |
| 2 | Ath-AT2G20840.1 |  | Vvi-Vitvi12g00484\_t001 |  | | | |  |  |  |  |  |  |
| 2 | Ath-AT2G20850.1 |  | Vvi-Vitvi12g00483\_t003 |  | | | |  |  |  |  |  |  |
| 2 | Ath-AT2G20860.2 |  | | | |  | | | |  |  |  |  |  |  |
| 2 | Ath-AT2G20870.1 |  | Vvi-Vitvi12g02394\_t001 |  | | | |  |  |  |  |  |  |
| 2 | Ath-AT2G20873.1 |  | | | |  | | | |  |  |  |  |  |  |
| 2 | Ath-AT2G20875.1 |  | Vvi-Vitvi12g00472\_t001 |  | | | |  |  |  |  |  |  |
| 1 | Ath-AT2G20880.1 |  |  |  | Vvi-Vitvi12g00348\_t001 |  |  |  |  |  |  |
| 0 | Ath-AT2G20890.1 |  |  |  |  |  |  |  |  |
| 0 | Ath-AT2G20900.4 |  |  |  |  |  |  |  |  |
| 1 | Ath-AT2G20920.1 |  | Vvi-Vitvi03g00169\_t001 |  |  |  |  |  |  |  |
| 1 | Ath-AT2G20921.1 |  | | | |  |  |  |  |  |  |  |
| 1 | Ath-AT2G20930.1 |  | Vvi-Vitvi03g00160\_t001 |  |  |  |  |  |  |  |
| 1 | Ath-AT2G20940.1 |  | Vvi-Vitvi03g00158\_t001 |  |  |  |  |  |  |  |
| 1 | Ath-AT2G20950.7 |  | | | |  |  |  |  |  |  |  |
| 1 | Ath-AT2G20960.1 |  | | | |  |  |  |  |  |  |  |
| 1 | Ath-AT2G20970.2 |  | | | |  |  |  |  |  |  |  |
| 1 | Ath-AT2G20980.1 |  | Vvi-Vitvi03g04064\_t001 |  |  |  |  |  |  |  |
| 1 | Ath-AT2G20990.3 |  | Vvi-Vitvi03g00138\_t001 |  |  |  |  |  |  |  |
| 1 | Ath-AT2G21010.1 |  | | | |  |  |  |  |  |  |  |
| 1 | Ath-AT2G21030.1 |  | | | |  |  |  |  |  |  |  |
| 1 | Ath-AT2G21040.1 |  | | | |  |  |  |  |  |  |  |
| 1 | Ath-AT2G21045.1 |  | | | |  |  |  |  |  |  |  |
| 1 | Ath-AT2G21050.1 |  | Vvi-Vitvi03g00122\_t001 |  |  |  |  |  |  |  |
| 1 | Ath-AT2G21060.1 |  | | | |  |  |  |  |  |  |  |
| 1 | Ath-AT2G21070.3 |  | Vvi-Vitvi03g00118\_t001 |  |  |  |  |  |  |  |
| 1 | Ath-AT2G21080.1 |  | | | |  |  |  |  |  |  |  |
| 1 | Ath-AT2G21090.1 |  | Vvi-Vitvi03g00116\_t001 |  |  |  |  |  |  |  |
| 1 | Ath-AT2G21100.1 |  | Vvi-Vitvi03g01395\_t001 |  |  |  |  |  |  |  |
| 1 | Ath-AT2G21105.1 |  | | | |  |  |  |  |  |  |  |
| 1 | Ath-AT2G21110.1 |  | Vvi-Vitvi03g01394\_t001 |  |  |  |  |  |  |  |
| 1 | Ath-AT2G21120.1 |  | Vvi-Vitvi03g00106\_t001 |  |  |  |  |  |  |  |
| 1 | Ath-AT2G21130.1 |  | Vvi-Vitvi03g04055\_t001 |  |  |  |  |  |  |  |
| 1 | Ath-AT2G21140.1 |  | Vvi-Vitvi03g01388\_t001 |  |  |  |  |  |  |  |
| 1 | Ath-AT2G21150.1 |  | | | |  |  |  |  |  |  |  |
| 1 | Ath-AT2G21160.1 |  | Vvi-Vitvi03g00100\_t001 |  |  |  |  |  |  |  |
| 1 | Ath-AT2G21170.1 |  | Vvi-Vitvi03g00097\_t001 |  |  |  |  |  |  |  |
| 1 | Ath-AT2G21180.1 |  | Vvi-Vitvi03g04038\_t001 |  |  |  |  |  |  |  |
| 1 | Ath-AT2G21185.1 |  | | | |  |  |  |  |  |  |  |
| 1 | Ath-AT2G21190.1 |  | Vvi-Vitvi03g00087\_t001 |  |  |  |  |  |  |  |
| 1 | Ath-AT2G21195.4 |  | | | |  |  |  |  |  |  |  |
| 1 | Ath-AT2G21200.1 |  | Vvi-Vitvi03g01369\_t001 |  |  |  |  |  |  |  |
| 1 | Ath-AT2G21210.2 |  | Vvi-Vitvi03g01347\_t001 |  |  |  |  |  |  |  |
| 2 | Ath-AT2G21220.1 |  | Vvi-Vitvi03g00064\_t001 |  | Vvi-Vitvi18g01093\_t001 |  |  |  |  |  |  |
| 2 | Ath-AT2G21230.3 |  | Vvi-Vitvi03g00059\_t001 |  | Vvi-Vitvi18g01068\_t001 |  |  |  |  |  |  |
| 2 | Ath-AT2G21235.1 |  | | | |  | | | |  |  |  |  |  |  |
| 2 | Ath-AT2G21237.1 |  | | | |  | | | |  |  |  |  |  |  |
| 2 | Ath-AT2G21240.1 |  | Vvi-Vitvi03g00058\_t001 |  | Vvi-Vitvi18g04249\_t001 |  |  |  |  |  |  |
| 2 | Ath-AT2G21250.1 |  | Vvi-Vitvi03g00056\_t001 |  | | | |  |  |  |  |  |  |
| 2 | Ath-AT2G21260.1 |  | | | |  | | | |  |  |  |  |  |  |
| 2 | Ath-AT2G21270.3 |  | Vvi-Vitvi03g00053\_t001 |  | | | |  |  |  |  |  |  |
| 2 | Ath-AT2G21280.2 |  | Vvi-Vitvi03g00052\_t001 |  | | | |  |  |  |  |  |  |
| 2 | Ath-AT2G21290.1 |  | Vvi-Vitvi03g01334\_t001 |  | | | |  |  |  |  |  |  |
| 2 | Ath-AT2G21300.3 |  | Vvi-Vitvi03g00050\_t001 |  | Vvi-Vitvi18g01055\_t001 |  |  |  |  |  |  |
| 2 | Ath-AT2G21320.1 |  | Vvi-Vitvi03g00049\_t001 |  | | | |  |  |  |  |  |  |
| 2 | Ath-AT2G21330.1 |  | Vvi-Vitvi03g00048\_t001 |  | | | |  |  |  |  |  |  |
| 2 | Ath-AT2G21340.1 |  | Vvi-Vitvi03g00032\_t001 |  | | | |  |  |  |  |  |  |
| 2 | Ath-AT2G21350.1 |  | Vvi-Vitvi03g00031\_t001 |  | | | |  |  |  |  |  |  |
| 2 | Ath-AT2G21370.1 |  | Vvi-Vitvi03g00029\_t001 |  | | | |  |  |  |  |  |  |
| 2 | Ath-AT2G21380.2 |  | Vvi-Vitvi03g00028\_t001 |  | | | |  |  |  |  |  |  |
| 2 | Ath-AT2G21385.1 |  | Vvi-Vitvi03g00025\_t001 |  | | | |  |  |  |  |  |  |
| 2 | Ath-AT2G21390.1 |  | | | |  | Vvi-Vitvi18g01042\_t001 |  |  |  |  |  |  |
| 2 | Ath-AT2G21400.2 |  | Vvi-Vitvi03g00024\_t001 |  | | | |  |  |  |  |  |  |
| 2 | Ath-AT2G21410.1 |  | Vvi-Vitvi03g00022\_t001 |  | Vvi-Vitvi18g01038\_t001 |  |  |  |  |  |  |
| 2 | Ath-AT2G21420.1 |  | | | |  | | | |  |  |  |  |  |  |
| 2 | Ath-AT2G21430.1 |  | Vvi-Vitvi03g00021\_t001 |  | Vvi-Vitvi18g01036\_t001 |  |  |  |  |  |  |
| 1 | Ath-AT2G21440.1 |  | Vvi-Vitvi03g00007\_t001 |  |  |  |  |  |  |  |
| 2 | Ath-AT2G21450.2 |  | Vvi-Vitvi03g00003\_t001 |  | Vvi-Vitvi03g04102\_t001 |  |  |  |  |  |  |
| 1 | Ath-AT2G21455.1 |  |  |  | | | |  |  |  |  |  |  |
| 1 | Ath-AT2G21465.1 |  |  |  | | | |  |  |  |  |  |  |
| 1 | Ath-AT2G21470.2 |  |  |  | Vvi-Vitvi03g00283\_t001 |  |  |  |  |  |  |
| 1 | Ath-AT2G21480.1 |  |  |  | Vvi-Vitvi03g00284\_t001 |  |  |  |  |  |  |
| 1 | Ath-AT2G21490.1 |  |  |  | Vvi-Vitvi03g01481\_t001 |  |  |  |  |  |  |
| 2 | Ath-AT2G21500.1 |  | Vvi-Vitvi18g02767\_t001 |  | Vvi-Vitvi03g00288\_t001 |  |  |  |  |  |  |
| 2 | Ath-AT2G21510.2 |  | | | |  | Vvi-Vitvi03g00289\_t001 |  |  |  |  |  |  |
| 2 | Ath-AT2G21520.2 |  | Vvi-Vitvi18g00997\_t001 |  | Vvi-Vitvi03g00294\_t001 |  |  |  |  |  |  |
| 2 | Ath-AT2G21530.1 |  | | | |  | | | |  |  |  |  |  |  |
| 2 | Ath-AT2G21540.1 |  | | | |  | | | |  |  |  |  |  |  |
| 2 | Ath-AT2G21550.1 |  | Vvi-Vitvi18g00996\_t001 |  | Vvi-Vitvi03g04109\_t001 |  |  |  |  |  |  |
| 2 | Ath-AT2G21560.1 |  | | | |  | Vvi-Vitvi03g00302\_t001 |  |  |  |  |  |  |
| 2 | Ath-AT2G21580.1 |  | | | |  | Vvi-Vitvi03g00303\_t001 |  |  |  |  |  |  |
| 2 | Ath-AT2G21590.1 |  | Vvi-Vitvi18g02758\_t002 |  | Vvi-Vitvi03g00304\_t001 |  |  |  |  |  |  |
| 2 | Ath-AT2G21595.1 |  | | | |  | | | |  |  |  |  |  |  |
| 2 | Ath-AT2G21600.1 |  | Vvi-Vitvi18g00980\_t001 |  | Vvi-Vitvi03g00307\_t003 |  |  |  |  |  |  |
| 2 | Ath-AT2G21610.2 |  | Vvi-Vitvi18g00977\_t001 |  | Vvi-Vitvi03g00313\_t001 |  |  |  |  |  |  |
| 2 | Ath-AT2G21620.2 |  | | | |  | Vvi-Vitvi03g00314\_t001 |  |  |  |  |  |  |
| 2 | Ath-AT2G21630.1 |  | | | |  | | | |  |  |  |  |  |  |
| 2 | Ath-AT2G21640.1 |  | | | |  | | | |  |  |  |  |  |  |
| 2 | Ath-AT2G21650.1 |  | Vvi-Vitvi18g00973\_t001 |  | Vvi-Vitvi03g01495\_t001 |  |  |  |  |  |  |
| 1 | Ath-AT2G21655.1 |  |  |  | | | |  |  |  |  |  |  |
| 1 | Ath-AT2G21660.1 |  |  |  | Vvi-Vitvi03g00327\_t001 |  |  |  |  |  |  |
| 1 | Ath-AT2G21680.1 |  |  |  | | | |  |  |  |  |  |  |
| 1 | Ath-AT2G21690.1 |  |  |  | | | |  |  |  |  |  |  |
| 1 | Ath-AT2G21710.1 |  |  |  | Vvi-Vitvi03g00329\_t001 |  |  |  |  |  |  |
| 0 | Ath-AT2G21720.2 |  |  |  |  |  |  |  |  |
| 0 | Ath-AT2G21725.1 |  |  |  |  |  |  |  |  |
| 0 | Ath-AT2G21727.1 |  |  |  |  |  |  |  |  |
| 0 | Ath-AT2G21730.1 |  |  |  |  |  |  |  |  |
| 1 | Ath-AT2G21740.1 |  | Vvi-Vitvi07g01882\_t001 |  |  |  |  |  |  |  |
| 1 | Ath-AT2G21750.1 |  | | | |  |  |  |  |  |  |  |
| 1 | Ath-AT2G21770.1 |  | Vvi-Vitvi07g01881\_t001 |  |  |  |  |  |  |  |
| 1 | Ath-AT2G21780.1 |  | | | |  |  |  |  |  |  |  |
| 1 | Ath-AT2G21790.1 |  | Vvi-Vitvi07g01875\_t001 |  |  |  |  |  |  |  |
| 1 | Ath-AT2G21800.2 |  | Vvi-Vitvi07g01867\_t001 |  |  |  |  |  |  |  |
| 1 | Ath-AT2G21810.1 |  | | | |  |  |  |  |  |  |  |
| 1 | Ath-AT2G21820.1 |  | | | |  |  |  |  |  |  |  |
| 1 | Ath-AT2G21830.1 |  | | | |  |  |  |  |  |  |  |
| 1 | Ath-AT2G21840.1 |  | | | |  |  |  |  |  |  |  |
| 1 | Ath-AT2G21850.1 |  | | | |  |  |  |  |  |  |  |
| 1 | Ath-AT2G21860.1 |  | Vvi-Vitvi07g01853\_t001 |  |  |  |  |  |  |  |
| 1 | Ath-AT2G21870.1 |  | Vvi-Vitvi07g01850\_t001 |  |  |  |  |  |  |  |
| 1 | Ath-AT2G21880.1 |  | Vvi-Vitvi07g01848\_t001 |  |  |  |  |  |  |  |
| 1 | Ath-AT2G21890.1 |  | | | |  |  |  |  |  |  |  |
| 1 | Ath-AT2G21900.1 |  | Vvi-Vitvi07g01847\_t001 |  |  |  |  |  |  |  |
| 1 | Ath-AT2G21910.1 |  | Vvi-Vitvi07g02692\_t001 |  |  |  |  |  |  |  |
| 1 | Ath-AT2G21920.1 |  | | | |  |  |  |  |  |  |  |
| 1 | Ath-AT2G21930.2 |  | | | |  |  |  |  |  |  |  |
| 1 | Ath-AT2G21940.4 |  | Vvi-Vitvi07g01835\_t001 |  |  |  |  |  |  |  |
| 1 | Ath-AT2G21950.1 |  | Vvi-Vitvi07g02689\_t001 |  |  |  |  |  |  |  |
| 1 | Ath-AT2G21960.1 |  | Vvi-Vitvi07g01831\_t002 |  |  |  |  |  |  |  |
| 1 | Ath-AT2G21970.1 |  | Vvi-Vitvi07g01829\_t001 |  |  |  |  |  |  |  |
| 1 | Ath-AT2G21980.1 |  | | | |  |  |  |  |  |  |  |
| 1 | Ath-AT2G21990.1 |  | Vvi-Vitvi07g01828\_t001 |  |  |  |  |  |  |  |
| 1 | Ath-AT2G22000.1 |  | | | |  |  |  |  |  |  |  |
| 1 | Ath-AT2G22010.2 |  | Vvi-Vitvi07g01826\_t001 |  |  |  |  |  |  |  |
| 1 | Ath-AT2G22030.1 |  | | | |  |  |  |  |  |  |  |
| 1 | Ath-AT2G22040.1 |  | | | |  |  |  |  |  |  |  |
| 1 | Ath-AT2G22050.1 |  | | | |  |  |  |  |  |  |  |
| 1 | Ath-AT2G22055.1 |  | | | |  |  |  |  |  |  |  |
| 1 | Ath-AT2G22060.1 |  | | | |  |  |  |  |  |  |  |
| 1 | Ath-AT2G22070.1 |  | Vvi-Vitvi07g01809\_t001 |  |  |  |  |  |  |  |
| 1 | Ath-AT2G22080.1 |  | | | |  |  |  |  |  |  |  |
| 1 | Ath-AT2G22088.1 |  | | | |  |  |  |  |  |  |  |
| 1 | Ath-AT2G22090.2 |  | Vvi-Vitvi07g02683\_t001 |  |  |  |  |  |  |  |
| 1 | Ath-AT2G22100.1 |  | | | |  |  |  |  |  |  |  |
| 1 | Ath-AT2G22120.2 |  | Vvi-Vitvi07g01806\_t001 |  |  |  |  |  |  |  |
| 1 | Ath-AT2G22121.1 |  | | | |  |  |  |  |  |  |  |
| 1 | Ath-AT2G22122.1 |  | | | |  |  |  |  |  |  |  |
| 1 | Ath-AT2G22125.1 |  | Vvi-Vitvi07g01796\_t001 |  |  |  |  |  |  |  |
| 1 | Ath-AT2G22140.1 |  | | | |  |  |  |  |  |  |  |
| 1 | Ath-AT2G22145.1 |  | | | |  |  |  |  |  |  |  |
| 1 | Ath-AT2G22155.1 |  | | | |  |  |  |  |  |  |  |
| 1 | Ath-AT2G22160.1 |  | | | |  |  |  |  |  |  |  |
| 2 | Ath-AT2G22170.1 |  | | | |  | Vvi-Vitvi07g01364\_t001 |  |  |  |  |  |  |
| 2 | Ath-AT2G22180.1 |  | | | |  | | | |  |  |  |  |  |  |
| 2 | Ath-AT2G22190.1 |  | | | |  | | | |  |  |  |  |  |  |
| 2 | Ath-AT2G22200.1 |  | | | |  | | | |  |  |  |  |  |  |
| 2 | Ath-AT2G22230.1 |  | | | |  | | | |  |  |  |  |  |  |
| 2 | Ath-AT2G22240.1 |  | | | |  | | | |  |  |  |  |  |  |
| 2 | Ath-AT2G22241.1 |  | | | |  | | | |  |  |  |  |  |  |
| 2 | Ath-AT2G22250.2 |  | Vvi-Vitvi07g01783\_t002 |  | | | |  |  |  |  |  |  |
| 1 | Ath-AT2G22260.6 |  |  |  | | | |  |  |  |  |  |  |
| 1 | Ath-AT2G22270.1 |  |  |  | | | |  |  |  |  |  |  |
| 1 | Ath-AT2G22290.1 |  |  |  | Vvi-Vitvi07g04554\_t001 |  |  |  |  |  |  |
| 1 | Ath-AT2G22300.2 |  |  |  | | | |  |  |  |  |  |  |
| 1 | Ath-AT2G22310.1 |  |  |  | | | |  |  |  |  |  |  |
| 1 | Ath-AT2G22320.1 |  |  |  | | | |  |  |  |  |  |  |
| 1 | Ath-AT2G22330.2 |  |  |  | | | |  |  |  |  |  |  |
| 1 | Ath-AT2G22340.1 |  |  |  | | | |  |  |  |  |  |  |
| 1 | Ath-AT2G22345.1 |  |  |  | | | |  |  |  |  |  |  |
| 2 | Ath-AT2G22360.1 |  | Vvi-Vitvi18g00483\_t001 |  | Vvi-Vitvi07g01393\_t001 |  |  |  |  |  |  |
| 2 | Ath-AT2G22370.1 |  | | | |  | | | |  |  |  |  |  |  |
| 2 | Ath-AT2G22400.1 |  | | | |  | | | |  |  |  |  |  |  |
| 2 | Ath-AT2G22410.1 |  | | | |  | Vvi-Vitvi07g02560\_t001 |  |  |  |  |  |  |
| 2 | Ath-AT2G22420.1 |  | | | |  | Vvi-Vitvi07g01502\_t001 |  |  |  |  |  |  |
| 2 | Ath-AT2G22425.1 |  | | | |  | | | |  |  |  |  |  |  |
| 2 | Ath-AT2G22426.1 |  | | | |  | | | |  |  |  |  |  |  |
| 2 | Ath-AT2G22430.1 |  | | | |  | Vvi-Vitvi07g01488\_t001 |  |  |  |  |  |  |
| 2 | Ath-AT2G22440.1 |  | | | |  | | | |  |  |  |  |  |  |
| 2 | Ath-AT2G22450.1 |  | | | |  | Vvi-Vitvi07g01482\_t001 |  |  |  |  |  |  |
| 2 | Ath-AT2G22460.1 |  | | | |  | Vvi-Vitvi07g01472\_t001 |  |  |  |  |  |  |
| 2 | Ath-AT2G22465.1 |  | | | |  | | | |  |  |  |  |  |  |
| 2 | Ath-AT2G22470.1 |  | | | |  | | | |  |  |  |  |  |  |
| 2 | Ath-AT2G22475.1 |  | Vvi-Vitvi18g00492\_t001 |  | Vvi-Vitvi07g04592\_t001 |  |  |  |  |  |  |
| 2 | Ath-AT2G22480.1 |  | | | |  | Vvi-Vitvi07g01462\_t001 |  |  |  |  |  |  |
| 2 | Ath-AT2G22490.2 |  | Vvi-Vitvi18g00499\_t001 |  | Vvi-Vitvi07g01460\_t001 |  |  |  |  |  |  |
| 2 | Ath-AT2G22500.1 |  | Vvi-Vitvi18g00508\_t001 |  | Vvi-Vitvi07g04600\_t001 |  |  |  |  |  |  |
| 2 | Ath-AT2G22510.1 |  | | | |  | | | |  |  |  |  |  |  |
| 2 | Ath-AT2G22520.1 |  | | | |  | | | |  |  |  |  |  |  |
| 2 | Ath-AT2G22530.1 |  | | | |  | Vvi-Vitvi07g04604\_t001 |  |  |  |  |  |  |
| 2 | Ath-AT2G22540.1 |  | Vvi-Vitvi18g04114\_t001 |  | Vvi-Vitvi07g01441\_t001 |  |  |  |  |  |  |
| 2 | Ath-AT2G22560.1 |  | Vvi-Vitvi18g00525\_t001 |  | Vvi-Vitvi07g04608\_t001 |  |  |  |  |  |  |
| 2 | Ath-AT2G22570.1 |  | | | |  | Vvi-Vitvi07g01428\_t001 |  |  |  |  |  |  |
| 2 | Ath-AT2G22590.1 |  | | | |  | Vvi-Vitvi07g04617\_t001 |  |  |  |  |  |  |
| 2 | Ath-AT2G22600.1 |  | | | |  | | | |  |  |  |  |  |  |
| 2 | Ath-AT2G22610.3 |  | Vvi-Vitvi18g00544\_t001 |  | Vvi-Vitvi07g01402\_t001 |  |  |  |  |  |  |
| 2 | Ath-AT2G22620.1 |  | Vvi-Vitvi18g00549\_t001 |  | Vvi-Vitvi07g01388\_t001 |  |  |  |  |  |  |
| 2 | Ath-AT2G22630.2 |  | Vvi-Vitvi18g00553\_t001 |  | Vvi-Vitvi07g01520\_t001 |  |  |  |  |  |  |
| 2 | Ath-AT2G22640.1 |  | | | |  | Vvi-Vitvi07g04636\_t002 |  |  |  |  |  |  |
| 2 | Ath-AT2G22650.1 |  | | | |  | | | |  |  |  |  |  |  |
| 2 | Ath-AT2G22660.2 |  | Vvi-Vitvi18g00562\_t001 |  | Vvi-Vitvi07g01523\_t001 |  |  |  |  |  |  |
| 2 | Ath-AT2G22670.4 |  | Vvi-Vitvi18g00571\_t003 |  | Vvi-Vitvi07g04642\_t003 |  |  |  |  |  |  |
| 2 | Ath-AT2G22680.1 |  | Vvi-Vitvi18g00572\_t001 |  | Vvi-Vitvi07g04644\_t001 |  |  |  |  |  |  |
| 1 | Ath-AT2G22690.2 |  |  |  | Vvi-Vitvi07g01541\_t001 |  |  |  |  |  |  |
| 1 | Ath-AT2G22720.2 |  |  |  | Vvi-Vitvi07g01538\_t001 |  |  |  |  |  |  |
| 1 | Ath-AT2G22730.2 |  |  |  | Vvi-Vitvi07g01536\_t001 |  |  |  |  |  |  |
| 1 | Ath-AT2G22740.2 |  |  |  | | | |  |  |  |  |  |  |
| 1 | Ath-AT2G22750.3 |  |  |  | Vvi-Vitvi07g01532\_t001 |  |  |  |  |  |  |
| 1 | Ath-AT2G22760.1 |  |  |  | | | |  |  |  |  |  |  |
| 1 | Ath-AT2G22770.1 |  |  |  | | | |  |  |  |  |  |  |
| 1 | Ath-AT2G22780.1 |  |  |  | Vvi-Vitvi07g03070\_t001 |  |  |  |  |  |  |
| 1 | Ath-AT2G22790.1 |  |  |  | Vvi-Vitvi07g04658\_t001 |  |  |  |  |  |  |
| 2 | Ath-AT2G22795.1 |  | Vvi-Vitvi18g02621\_t001 |  | | | |  |  |  |  |  |  |
| 2 | Ath-AT2G22800.1 |  | Vvi-Vitvi18g00603\_t001 |  | Vvi-Vitvi07g03056\_t002 |  |  |  |  |  |  |
| 2 | Ath-AT2G22805.1 |  | | | |  | | | |  |  |  |  |  |  |
| 2 | Ath-AT2G22807.1 |  | | | |  | | | |  |  |  |  |  |  |
| 2 | Ath-AT2G22810.1 |  | Vvi-Vitvi18g00609\_t001 |  | Vvi-Vitvi07g03132\_t001 |  |  |  |  |  |  |
| 2 | Ath-AT2G22820.1 |  | | | |  | | | |  |  |  |  |  |  |
| 3 | Ath-AT2G22830.1 |  | Vvi-Vitvi18g00612\_t001 |  | | | |  | Vvi-Vitvi15g04526\_t001 |  |  |  |  |  |
| 3 | Ath-AT2G22840.1 |  | Vvi-Vitvi18g00623\_t001 |  | | | |  | Vvi-Vitvi15g04517\_t001 |  |  |  |  |  |
| 3 | Ath-AT2G22850.2 |  | Vvi-Vitvi18g00628\_t001 |  | | | |  | Vvi-Vitvi15g04514\_t001 |  |  |  |  |  |
| 3 | Ath-AT2G22860.1 |  | Vvi-Vitvi18g00635\_t001 |  | | | |  | Vvi-Vitvi15g04511\_t001 |  |  |  |  |  |
| 2 | Ath-AT2G22870.1 |  |  |  | | | |  | Vvi-Vitvi15g04505\_t001 |  |  |  |  |  |
| 2 | Ath-AT2G22880.1 |  |  |  | | | |  | Vvi-Vitvi15g04504\_t001 |  |  |  |  |  |
| 2 | Ath-AT2G22890.1 |  |  |  | | | |  | | | |  |  |  |  |  |
| 2 | Ath-AT2G22900.1 |  |  |  | | | |  | Vvi-Vitvi15g04497\_t001 |  |  |  |  |  |
| 1 | Ath-AT2G22905.1 |  |  |  | | | |  |  |  |  |  |  |
| 1 | Ath-AT2G22910.1 |  |  |  | Vvi-Vitvi07g04669\_t001 |  |  |  |  |  |  |
| 1 | Ath-AT2G22920.2 |  |  |  | | | |  |  |  |  |  |  |
| 1 | Ath-AT2G22930.1 |  |  |  | | | |  |  |  |  |  |  |
| 1 | Ath-AT2G22940.1 |  |  |  | | | |  |  |  |  |  |  |
| 1 | Ath-AT2G22941.1 |  |  |  | | | |  |  |  |  |  |  |
| 1 | Ath-AT2G22942.1 |  |  |  | | | |  |  |  |  |  |  |
| 1 | Ath-AT2G22950.1 |  |  |  | Vvi-Vitvi07g01595\_t001 |  |  |  |  |  |  |
| 1 | Ath-AT2G22960.1 |  |  |  | | | |  |  |  |  |  |  |
| 1 | Ath-AT2G22970.3 |  |  |  | | | |  |  |  |  |  |  |
| 1 | Ath-AT2G22980.4 |  |  |  | | | |  |  |  |  |  |  |
| 1 | Ath-AT2G22990.3 |  |  |  | | | |  |  |  |  |  |  |
| 1 | Ath-AT2G23000.2 |  |  |  | | | |  |  |  |  |  |  |
| 1 | Ath-AT2G23010.1 |  |  |  | | | |  |  |  |  |  |  |
| 1 | Ath-AT2G23030.1 |  |  |  | | | |  |  |  |  |  |  |
| 2 | Ath-AT2G23050.1 |  | Vvi-Vitvi03g00629\_t001 |  | Vvi-Vitvi07g01603\_t003 |  |  |  |  |  |  |
| 2 | Ath-AT2G23060.1 |  | | | |  | Vvi-Vitvi07g01604\_t001 |  |  |  |  |  |  |
| 2 | Ath-AT2G23067.1 |  | | | |  | | | |  |  |  |  |  |  |
| 2 | Ath-AT2G23070.1 |  | | | |  | Vvi-Vitvi07g01605\_t001 |  |  |  |  |  |  |
| 2 | Ath-AT2G23080.1 |  | | | |  | | | |  |  |  |  |  |  |
| 2 | Ath-AT2G23090.1 |  | | | |  | Vvi-Vitvi07g01621\_t001 |  |  |  |  |  |  |
| 2 | Ath-AT2G23093.1 |  | | | |  | Vvi-Vitvi07g01623\_t001 |  |  |  |  |  |  |
| 2 | Ath-AT2G23096.1 |  | Vvi-Vitvi03g00609\_t001 |  | Vvi-Vitvi07g01624\_t001 |  |  |  |  |  |  |
| 2 | Ath-AT2G23100.1 |  | | | |  | | | |  |  |  |  |  |  |
| 2 | Ath-AT2G23110.1 |  | | | |  | Vvi-Vitvi07g01630\_t001 |  |  |  |  |  |  |
| 2 | Ath-AT2G23120.1 |  | | | |  | | | |  |  |  |  |  |  |
| 2 | Ath-AT2G23118.1 |  | | | |  | | | |  |  |  |  |  |  |
| 2 | Ath-AT2G23130.1 |  | | | |  | | | |  |  |  |  |  |  |
| 2 | Ath-AT2G23140.1 |  | | | |  | Vvi-Vitvi07g04684\_t001 |  |  |  |  |  |  |
| 2 | Ath-AT2G23142.1 |  | | | |  | | | |  |  |  |  |  |  |
| 2 | Ath-AT2G23148.1 |  | | | |  | | | |  |  |  |  |  |  |
| 2 | Ath-AT2G23150.1 |  | | | |  | Vvi-Vitvi07g01642\_t001 |  |  |  |  |  |  |
| 2 | Ath-AT2G23160.1 |  | | | |  | | | |  |  |  |  |  |  |
| 2 | Ath-AT2G23170.1 |  | Vvi-Vitvi03g00586\_t001 |  | Vvi-Vitvi07g01644\_t001 |  |  |  |  |  |  |
| 2 | Ath-AT2G23180.1 |  | | | |  | | | |  |  |  |  |  |  |
| 2 | Ath-AT2G23190.1 |  | | | |  | Vvi-Vitvi07g04690\_t001 |  |  |  |  |  |  |
| 2 | Ath-AT2G23200.1 |  | | | |  | Vvi-Vitvi07g01663\_t001 |  |  |  |  |  |  |
| 2 | Ath-AT2G23210.1 |  | | | |  | | | |  |  |  |  |  |  |
| 2 | Ath-AT2G23220.1 |  | | | |  | | | |  |  |  |  |  |  |
| 2 | Ath-AT2G23230.2 |  | | | |  | | | |  |  |  |  |  |  |
| 2 | Ath-AT2G23240.1 |  | | | |  | | | |  |  |  |  |  |  |
| 2 | Ath-AT2G23250.1 |  | | | |  | | | |  |  |  |  |  |  |
| 2 | Ath-AT2G23260.1 |  | Vvi-Vitvi03g00569\_t001 |  | | | |  |  |  |  |  |  |
| 2 | Ath-AT2G23270.1 |  | | | |  | Vvi-Vitvi07g02650\_t001 |  |  |  |  |  |  |
| 2 | Ath-AT2G23290.1 |  | Vvi-Vitvi03g00559\_t001 |  | Vvi-Vitvi07g01676\_t001 |  |  |  |  |  |  |
| 2 | Ath-AT2G23300.1 |  | Vvi-Vitvi03g00553\_t001 |  | Vvi-Vitvi07g01678\_t001 |  |  |  |  |  |  |
| 2 | Ath-AT2G23310.1 |  | | | |  | Vvi-Vitvi07g01688\_t001 |  |  |  |  |  |  |
| 2 | Ath-AT2G23320.1 |  | | | |  | Vvi-Vitvi07g01694\_t001 |  |  |  |  |  |  |
| 2 | Ath-AT2G23321.1 |  | | | |  | | | |  |  |  |  |  |  |
| 2 | Ath-AT2G23340.1 |  | | | |  | Vvi-Vitvi07g01702\_t001 |  |  |  |  |  |  |
| 2 | Ath-AT2G23348.2 |  | | | |  | | | |  |  |  |  |  |  |
| 2 | Ath-AT2G23350.1 |  | Vvi-Vitvi03g00531\_t001 |  | Vvi-Vitvi07g01705\_t001 |  |  |  |  |  |  |
| 2 | Ath-AT2G23360.1 |  | Vvi-Vitvi03g00529\_t002 |  | Vvi-Vitvi07g01709\_t001 |  |  |  |  |  |  |
| 2 | Ath-AT2G23370.1 |  | Vvi-Vitvi03g00528\_t001 |  | Vvi-Vitvi07g01710\_t001 |  |  |  |  |  |  |
| 1 | Ath-AT2G23380.1 |  |  |  | Vvi-Vitvi07g01721\_t001 |  |  |  |  |  |  |
| 1 | Ath-AT2G23390.1 |  |  |  | | | |  |  |  |  |  |  |
| 1 | Ath-AT2G23400.1 |  |  |  | | | |  |  |  |  |  |  |
| 1 | Ath-AT2G23410.1 |  |  |  | | | |  |  |  |  |  |  |
| 1 | Ath-AT2G23420.1 |  |  |  | Vvi-Vitvi07g01730\_t002 |  |  |  |  |  |  |
| 0 | Ath-AT2G23430.1 |  |  |  |  |  |  |  |  |
| 0 | Ath-AT2G23440.1 |  |  |  |  |  |  |  |  |
| 0 | Ath-AT2G23445.1 |  |  |  |  |  |  |  |  |
| 2 | Ath-AT2G23450.1 |  | Vvi-Vitvi18g00885\_t001 |  | Vvi-Vitvi07g01280\_t001 |  |  |  |  |  |  |
| 2 | Ath-AT2G23460.1 |  | | | |  | Vvi-Vitvi07g01287\_t001 |  |  |  |  |  |  |
| 2 | Ath-AT2G23470.1 |  | | | |  | | | |  |  |  |  |  |  |
| 2 | Ath-AT2G23510.1 |  | | | |  | | | |  |  |  |  |  |  |
| 2 | Ath-AT2G23520.1 |  | | | |  | Vvi-Vitvi07g01295\_t001 |  |  |  |  |  |  |
| 2 | Ath-AT2G23530.1 |  | | | |  | Vvi-Vitvi07g01296\_t001 |  |  |  |  |  |  |
| 2 | Ath-AT2G23540.1 |  | | | |  | Vvi-Vitvi07g01297\_t001 |  |  |  |  |  |  |
| 2 | Ath-AT2G23550.4 |  | | | |  | | | |  |  |  |  |  |  |
| 2 | Ath-AT2G23560.1 |  | | | |  | Vvi-Vitvi07g02512\_t001 |  |  |  |  |  |  |
| 2 | Ath-AT2G23570.1 |  | | | |  | | | |  |  |  |  |  |  |
| 2 | Ath-AT2G23580.1 |  | | | |  | Vvi-Vitvi07g02513\_t001 |  |  |  |  |  |  |
| 2 | Ath-AT2G23590.1 |  | | | |  | | | |  |  |  |  |  |  |
| 2 | Ath-AT2G23600.2 |  | | | |  | | | |  |  |  |  |  |  |
| 2 | Ath-AT2G23610.1 |  | | | |  | | | |  |  |  |  |  |  |
| 2 | Ath-AT2G23620.1 |  | | | |  | | | |  |  |  |  |  |  |
| 2 | Ath-AT2G23630.2 |  | | | |  | Vvi-Vitvi07g01308\_t002 |  |  |  |  |  |  |
| 2 | Ath-AT2G23640.1 |  | | | |  | Vvi-Vitvi07g01309\_t001 |  |  |  |  |  |  |
| 1 | Ath-AT2G23660.2 |  | | | |  |  |  |  |  |  |  |
| 1 | Ath-AT2G23670.1 |  | | | |  |  |  |  |  |  |  |
| 1 | Ath-AT2G23680.1 |  | | | |  |  |  |  |  |  |  |
| 1 | Ath-AT2G23690.1 |  | | | |  |  |  |  |  |  |  |
| 1 | Ath-AT2G23700.1 |  | | | |  |  |  |  |  |  |  |
| 2 | Ath-AT2G23740.3 |  | | | |  | Vvi-Vitvi04g01208\_t002 |  |  |  |  |  |  |
| 2 | Ath-AT2G23755.1 |  | | | |  | Vvi-Vitvi04g02090\_t001 |  |  |  |  |  |  |
| 2 | Ath-AT2G23760.4 |  | Vvi-Vitvi18g00902\_t001 |  | Vvi-Vitvi04g01210\_t001 |  |  |  |  |  |  |
| 2 | Ath-AT2G23770.1 |  | Vvi-Vitvi18g00904\_t001 |  | Vvi-Vitvi04g01214\_t001 |  |  |  |  |  |  |
| 2 | Ath-AT2G23780.1 |  | Vvi-Vitvi18g00912\_t001 |  | Vvi-Vitvi04g01223\_t001 |  |  |  |  |  |  |
| 2 | Ath-AT2G23790.1 |  | Vvi-Vitvi18g02730\_t001 |  | Vvi-Vitvi04g01225\_t001 |  |  |  |  |  |  |
| 2 | Ath-AT2G23800.1 |  | Vvi-Vitvi18g00922\_t001 |  | Vvi-Vitvi04g01230\_t001 |  |  |  |  |  |  |
| 1 | Ath-AT2G23810.1 |  | Vvi-Vitvi11g00828\_t001 |  |  |  |  |  |  |  |
| 1 | Ath-AT2G23820.2 |  | Vvi-Vitvi11g00823\_t001 |  |  |  |  |  |  |  |
| 1 | Ath-AT2G23830.1 |  | | | |  |  |  |  |  |  |  |
| 1 | Ath-AT2G23834.1 |  | | | |  |  |  |  |  |  |  |
| 1 | Ath-AT2G23840.1 |  | | | |  |  |  |  |  |  |  |
| 1 | Ath-AT2G23890.2 |  | Vvi-Vitvi11g00822\_t001 |  |  |  |  |  |  |  |
| 1 | Ath-AT2G23900.1 |  | | | |  |  |  |  |  |  |  |
| 1 | Ath-AT2G23910.1 |  | Vvi-Vitvi11g00821\_t001 |  |  |  |  |  |  |  |
| 1 | Ath-AT2G23930.1 |  | | | |  |  |  |  |  |  |  |
| 1 | Ath-AT2G23940.1 |  | | | |  |  |  |  |  |  |  |
| 1 | Ath-AT2G23945.1 |  | | | |  |  |  |  |  |  |  |
| 1 | Ath-AT2G23950.1 |  | Vvi-Vitvi11g00812\_t001 |  |  |  |  |  |  |  |
| 1 | Ath-AT2G23960.1 |  | Vvi-Vitvi11g00805\_t001 |  |  |  |  |  |  |  |
| 1 | Ath-AT2G23970.1 |  | | | |  |  |  |  |  |  |  |
| 2 | Ath-AT2G23980.8 |  | | | |  | Vvi-Vitvi11g00701\_t002 |  |  |  |  |  |  |
| 2 | Ath-AT2G23985.13 |  | | | |  | | | |  |  |  |  |  |  |
| 2 | Ath-AT2G23987.1 |  | | | |  | | | |  |  |  |  |  |  |
| 2 | Ath-AT2G23990.2 |  | | | |  | | | |  |  |  |  |  |  |
| 2 | Ath-AT2G24000.1 |  | | | |  | Vvi-Vitvi11g00712\_t001 |  |  |  |  |  |  |
| 2 | Ath-AT2G24010.2 |  | | | |  | | | |  |  |  |  |  |  |
| 2 | Ath-AT2G24020.1 |  | | | |  | Vvi-Vitvi11g00713\_t001 |  |  |  |  |  |  |
| 2 | Ath-AT2G24030.1 |  | | | |  | Vvi-Vitvi11g00715\_t001 |  |  |  |  |  |  |
| 2 | Ath-AT2G24040.1 |  | | | |  | Vvi-Vitvi11g00718\_t001 |  |  |  |  |  |  |
| 2 | Ath-AT2G24050.1 |  | | | |  | Vvi-Vitvi11g00723\_t001 |  |  |  |  |  |  |
| 2 | Ath-AT2G24060.1 |  | | | |  | Vvi-Vitvi11g00725\_t001 |  |  |  |  |  |  |
| 2 | Ath-AT2G24070.2 |  | | | |  | Vvi-Vitvi11g00728\_t002 |  |  |  |  |  |  |
| 2 | Ath-AT2G24080.1 |  | | | |  | | | |  |  |  |  |  |  |
| 2 | Ath-AT2G24090.1 |  | | | |  | | | |  |  |  |  |  |  |
| 2 | Ath-AT2G24100.1 |  | Vvi-Vitvi11g00786\_t001 |  | | | |  |  |  |  |  |  |
| 1 | Ath-AT2G24120.1 |  |  |  | | | |  |  |  |  |  |  |
| 1 | Ath-AT2G24130.2 |  |  |  | | | |  |  |  |  |  |  |
| 1 | Ath-AT2G24140.3 |  |  |  | Vvi-Vitvi11g00745\_t001 |  |  |  |  |  |  |
| 0 | Ath-AT2G24150.1 |  |  |  |  |  |  |  |  |
| 1 | Ath-AT2G24170.1 |  | Vvi-Vitvi11g00700\_t001 |  |  |  |  |  |  |  |
| 1 | Ath-AT2G24180.1 |  | | | |  |  |  |  |  |  |  |
| 1 | Ath-AT2G24190.2 |  | | | |  |  |  |  |  |  |  |
| 1 | Ath-AT2G24195.1 |  | | | |  |  |  |  |  |  |  |
| 1 | Ath-AT2G24200.2 |  | Vvi-Vitvi11g00698\_t001 |  |  |  |  |  |  |  |
| 1 | Ath-AT2G24205.1 |  | | | |  |  |  |  |  |  |  |
| 1 | Ath-AT2G24210.1 |  | | | |  |  |  |  |  |  |  |
| 1 | Ath-AT2G24220.2 |  | | | |  |  |  |  |  |  |  |
| 1 | Ath-AT2G24230.1 |  | | | |  |  |  |  |  |  |  |
| 1 | Ath-AT2G24240.1 |  | | | |  |  |  |  |  |  |  |
| 1 | Ath-AT2G24250.2 |  | | | |  |  |  |  |  |  |  |
| 1 | Ath-AT2G24255.1 |  | | | |  |  |  |  |  |  |  |
| 1 | Ath-AT2G24260.1 |  | Vvi-Vitvi11g00680\_t001 |  |  |  |  |  |  |  |
| 1 | Ath-AT2G24270.2 |  | | | |  |  |  |  |  |  |  |
| 1 | Ath-AT2G24280.1 |  | Vvi-Vitvi11g04164\_t001 |  |  |  |  |  |  |  |
| 1 | Ath-AT2G24285.2 |  | | | |  |  |  |  |  |  |  |
| 1 | Ath-AT2G24290.1 |  | Vvi-Vitvi11g00655\_t002 |  |  |  |  |  |  |  |
| 1 | Ath-AT2G24300.3 |  | Vvi-Vitvi11g00652\_t002 |  |  |  |  |  |  |  |
| 1 | Ath-AT2G24310.1 |  | | | |  |  |  |  |  |  |  |
| 1 | Ath-AT2G24320.2 |  | Vvi-Vitvi11g04161\_t001 |  |  |  |  |  |  |  |
| 0 | Ath-AT2G24330.1 |  |  |  |  |  |  |  |  |
| 0 | Ath-AT2G24340.1 |  |  |  |  |  |  |  |  |
| 1 | Ath-AT2G24350.1 |  | Vvi-Vitvi04g01834\_t001 |  |  |  |  |  |  |  |
| 1 | Ath-AT2G24360.1 |  | Vvi-Vitvi04g00266\_t001 |  |  |  |  |  |  |  |
| 1 | Ath-AT2G24370.1 |  | Vvi-Vitvi04g00257\_t001 |  |  |  |  |  |  |  |
| 1 | Ath-AT2G24390.1 |  | Vvi-Vitvi04g00250\_t001 |  |  |  |  |  |  |  |
| 1 | Ath-AT2G24395.1 |  | Vvi-Vitvi04g00247\_t001 |  |  |  |  |  |  |  |
| 1 | Ath-AT2G24400.1 |  | Vvi-Vitvi04g01831\_t001 |  |  |  |  |  |  |  |
| 1 | Ath-AT2G24410.1 |  | | | |  |  |  |  |  |  |  |
| 1 | Ath-AT2G24420.1 |  | Vvi-Vitvi04g00237\_t001 |  |  |  |  |  |  |  |
| 1 | Ath-AT2G24430.1 |  | Vvi-Vitvi04g00236\_t001 |  |  |  |  |  |  |  |
| 1 | Ath-AT2G24440.1 |  | | | |  |  |  |  |  |  |  |
| 1 | Ath-AT2G24450.1 |  | Vvi-Vitvi04g00225\_t001 |  |  |  |  |  |  |  |
| 1 | Ath-AT2G24460.1 |  | | | |  |  |  |  |  |  |  |
| 1 | Ath-AT2G24470.1 |  | | | |  |  |  |  |  |  |  |
| 1 | Ath-AT2G24490.2 |  | Vvi-Vitvi04g00223\_t001 |  |  |  |  |  |  |  |
| 1 | Ath-AT2G24480.1 |  | | | |  |  |  |  |  |  |  |
| 1 | Ath-AT2G24500.1 |  | Vvi-Vitvi04g00219\_t001 |  |  |  |  |  |  |  |
| 1 | Ath-AT2G24510.1 |  | | | |  |  |  |  |  |  |  |
| 1 | Ath-AT2G24520.2 |  | Vvi-Vitvi04g00211\_t001 |  |  |  |  |  |  |  |
| 1 | Ath-AT2G24530.1 |  | Vvi-Vitvi04g00206\_t001 |  |  |  |  |  |  |  |
| 1 | Ath-AT2G24535.2 |  | | | |  |  |  |  |  |  |  |
| 1 | Ath-AT2G24540.1 |  | Vvi-Vitvi04g00202\_t001 |  |  |  |  |  |  |  |
| 1 | Ath-AT2G24550.1 |  | Vvi-Vitvi04g01819\_t001 |  |  |  |  |  |  |  |
| 0 | Ath-AT2G24560.1 |  |  |  |  |  |  |  |  |
| 1 | Ath-AT2G24570.1 |  | Vvi-Vitvi04g00756\_t001 |  |  |  |  |  |  |  |
| 1 | Ath-AT2G24580.1 |  | Vvi-Vitvi04g00747\_t001 |  |  |  |  |  |  |  |
| 1 | Ath-AT2G24590.1 |  | Vvi-Vitvi04g04188\_t003 |  |  |  |  |  |  |  |
| 1 | Ath-AT2G24600.4 |  | | | |  |  |  |  |  |  |  |
| 1 | Ath-AT2G24610.2 |  | Vvi-Vitvi04g00736\_t001 |  |  |  |  |  |  |  |
| 1 | Ath-AT2G24615.1 |  | | | |  |  |  |  |  |  |  |
| 1 | Ath-AT2G24617.1 |  | | | |  |  |  |  |  |  |  |
| 1 | Ath-AT2G24620.1 |  | | | |  |  |  |  |  |  |  |
| 1 | Ath-AT2G24625.1 |  | | | |  |  |  |  |  |  |  |
| 1 | Ath-AT2G24630.2 |  | Vvi-Vitvi04g00735\_t003 |  |  |  |  |  |  |  |
| 1 | Ath-AT2G24640.1 |  | Vvi-Vitvi04g00732\_t001 |  |  |  |  |  |  |  |
| 1 | Ath-AT2G24645.3 |  | | | |  |  |  |  |  |  |  |
| 1 | Ath-AT2G24650.1 |  | | | |  |  |  |  |  |  |  |
| 1 | Ath-AT2G24670.1 |  | | | |  |  |  |  |  |  |  |
| 1 | Ath-AT2G24680.6 |  | | | |  |  |  |  |  |  |  |
| 1 | Ath-AT2G24681.1 |  | | | |  |  |  |  |  |  |  |
| 1 | Ath-AT2G24683.1 |  | | | |  |  |  |  |  |  |  |
| 1 | Ath-AT2G24690.2 |  | | | |  |  |  |  |  |  |  |
| 1 | Ath-AT2G24692.1 |  | | | |  |  |  |  |  |  |  |
| 1 | Ath-AT2G24693.1 |  | | | |  |  |  |  |  |  |  |
| 1 | Ath-AT2G24696.1 |  | | | |  |  |  |  |  |  |  |
| 1 | Ath-AT2G24700.1 |  | | | |  |  |  |  |  |  |  |
| 1 | Ath-AT2G24710.3 |  | | | |  |  |  |  |  |  |  |
| 1 | Ath-AT2G24720.1 |  | Vvi-Vitvi04g00715\_t001 |  |  |  |  |  |  |  |
| 0 | Ath-AT2G24740.1 |  |  |  |  |  |  |  |  |
| 0 | Ath-AT2G24762.1 |  |  |  |  |  |  |  |  |
| 0 | Ath-AT2G24765.1 |  |  |  |  |  |  |  |  |
| 0 | Ath-AT2G24780.1 |  |  |  |  |  |  |  |  |
| 0 | Ath-AT2G24790.1 |  |  |  |  |  |  |  |  |
| 1 | Ath-AT2G24800.1 |  | Vvi-Vitvi04g00635\_t001 |  |  |  |  |  |  |  |
| 1 | Ath-AT2G24810.1 |  | | | |  |  |  |  |  |  |  |
| 1 | Ath-AT2G24820.1 |  | Vvi-Vitvi04g00633\_t001 |  |  |  |  |  |  |  |
| 1 | Ath-AT2G24830.1 |  | Vvi-Vitvi04g00629\_t001 |  |  |  |  |  |  |  |
| 1 | Ath-AT2G24840.1 |  | | | |  |  |  |  |  |  |  |
| 1 | Ath-AT2G24850.1 |  | | | |  |  |  |  |  |  |  |
| 1 | Ath-AT2G24860.1 |  | Vvi-Vitvi04g00626\_t001 |  |  |  |  |  |  |  |
| 1 | Ath-AT2G24870.1 |  | | | |  |  |  |  |  |  |  |
| 1 | Ath-AT2G24880.1 |  | | | |  |  |  |  |  |  |  |
| 1 | Ath-AT2G24940.1 |  | Vvi-Vitvi04g00618\_t001 |  |  |  |  |  |  |  |
| 1 | Ath-AT2G24945.1 |  | | | |  |  |  |  |  |  |  |
| 1 | Ath-AT2G24950.1 |  | | | |  |  |  |  |  |  |  |
| 1 | Ath-AT2G24960.1 |  | Vvi-Vitvi04g00614\_t001 |  |  |  |  |  |  |  |
| 1 | Ath-AT2G24970.1 |  | | | |  |  |  |  |  |  |  |
| 1 | Ath-AT2G24980.3 |  | | | |  |  |  |  |  |  |  |
| 1 | Ath-AT2G24990.1 |  | | | |  |  |  |  |  |  |  |
| 2 | Ath-AT2G25000.1 |  | | | |  | Vvi-Vitvi04g00510\_t001 |  |  |  |  |  |  |
| 2 | Ath-AT2G25010.1 |  | | | |  | | | |  |  |  |  |  |  |
| 2 | Ath-AT2G25050.1 |  | | | |  | | | |  |  |  |  |  |  |
| 2 | Ath-AT2G25055.1 |  | | | |  | | | |  |  |  |  |  |  |
| 2 | Ath-AT2G25060.1 |  | | | |  | | | |  |  |  |  |  |  |
| 2 | Ath-AT2G25070.1 |  | Vvi-Vitvi04g00606\_t001 |  | | | |  |  |  |  |  |  |
| 1 | Ath-AT2G25080.1 |  |  |  | | | |  |  |  |  |  |  |
| 1 | Ath-AT2G25090.1 |  |  |  | Vvi-Vitvi04g00512\_t001 |  |  |  |  |  |  |
| 1 | Ath-AT2G25100.1 |  |  |  | | | |  |  |  |  |  |  |
| 1 | Ath-AT2G25110.1 |  |  |  | Vvi-Vitvi04g04130\_t001 |  |  |  |  |  |  |
| 1 | Ath-AT2G25120.1 |  |  |  | | | |  |  |  |  |  |  |
| 1 | Ath-AT2G25130.1 |  |  |  | Vvi-Vitvi04g00517\_t001 |  |  |  |  |  |  |
| 1 | Ath-AT2G25140.1 |  |  |  | Vvi-Vitvi04g00522\_t001 |  |  |  |  |  |  |
| 1 | Ath-AT2G25150.1 |  |  |  | | | |  |  |  |  |  |  |
| 1 | Ath-AT2G25160.1 |  |  |  | | | |  |  |  |  |  |  |
| 1 | Ath-AT2G25169.1 |  |  |  | | | |  |  |  |  |  |  |
| 1 | Ath-AT2G25170.3 |  |  |  | Vvi-Vitvi04g00523\_t001 |  |  |  |  |  |  |
| 1 | Ath-AT2G25180.1 |  |  |  | Vvi-Vitvi04g00525\_t001 |  |  |  |  |  |  |
| 1 | Ath-AT2G25185.1 |  |  |  | | | |  |  |  |  |  |  |
| 1 | Ath-AT2G25190.1 |  |  |  | Vvi-Vitvi04g00530\_t002 |  |  |  |  |  |  |
| 1 | Ath-AT2G25200.1 |  |  |  | Vvi-Vitvi04g00531\_t001 |  |  |  |  |  |  |
| 1 | Ath-AT2G25210.1 |  |  |  | Vvi-Vitvi04g00536\_t001 |  |  |  |  |  |  |
| 1 | Ath-AT2G25215.1 |  |  |  | | | |  |  |  |  |  |  |
| 1 | Ath-AT2G25125.1 |  |  |  | | | |  |  |  |  |  |  |
| 1 | Ath-AT2G25220.2 |  |  |  | Vvi-Vitvi04g00538\_t001 |  |  |  |  |  |  |
| 1 | Ath-AT2G25230.1 |  |  |  | | | |  |  |  |  |  |  |
| 1 | Ath-AT2G25240.1 |  |  |  | | | |  |  |  |  |  |  |
| 1 | Ath-AT2G25250.1 |  |  |  | | | |  |  |  |  |  |  |
| 1 | Ath-AT2G25260.1 |  |  |  | Vvi-Vitvi04g00549\_t001 |  |  |  |  |  |  |
| 1 | Ath-AT2G25270.1 |  |  |  | Vvi-Vitvi04g00556\_t001 |  |  |  |  |  |  |
| 1 | Ath-AT2G25280.1 |  |  |  | | | |  |  |  |  |  |  |
| 1 | Ath-AT2G25290.2 |  |  |  | Vvi-Vitvi04g00565\_t001 |  |  |  |  |  |  |
| 1 | Ath-AT2G25295.1 |  |  |  | | | |  |  |  |  |  |  |
| 1 | Ath-AT2G25297.2 |  |  |  | | | |  |  |  |  |  |  |
| 1 | Ath-AT2G25300.1 |  |  |  | Vvi-Vitvi04g00581\_t001 |  |  |  |  |  |  |
| 1 | Ath-AT2G25305.1 |  |  |  | | | |  |  |  |  |  |  |
| 1 | Ath-AT2G25310.1 |  |  |  | Vvi-Vitvi04g00582\_t001 |  |  |  |  |  |  |
| 0 | Ath-AT2G25312.1 |  |  |  |  |  |  |  |  |
| 1 | Ath-AT2G25320.3 |  | Vvi-Vitvi04g00505\_t001 |  |  |  |  |  |  |  |
| 1 | Ath-AT2G25330.1 |  | | | |  |  |  |  |  |  |  |
| 1 | Ath-AT2G25340.1 |  | Vvi-Vitvi04g00503\_t001 |  |  |  |  |  |  |  |
| 1 | Ath-AT2G25344.1 |  | | | |  |  |  |  |  |  |  |
| 1 | Ath-AT2G25350.2 |  | Vvi-Vitvi04g00500\_t001 |  |  |  |  |  |  |  |
| 1 | Ath-AT2G25355.1 |  | | | |  |  |  |  |  |  |  |
| 1 | Ath-AT2G25360.1 |  | | | |  |  |  |  |  |  |  |
| 1 | Ath-AT2G25370.1 |  | | | |  |  |  |  |  |  |  |
| 1 | Ath-AT2G25410.1 |  | | | |  |  |  |  |  |  |  |
| 1 | Ath-AT2G25409.1 |  | | | |  |  |  |  |  |  |  |
| 1 | Ath-AT2G25420.4 |  | | | |  |  |  |  |  |  |  |
| 1 | Ath-AT2G25430.1 |  | Vvi-Vitvi04g00490\_t001 |  |  |  |  |  |  |  |
| 1 | Ath-AT2G25440.1 |  | | | |  |  |  |  |  |  |  |
| 1 | Ath-AT2G25450.1 |  | | | |  |  |  |  |  |  |  |
| 1 | Ath-AT2G25460.2 |  | Vvi-Vitvi04g00486\_t001 |  |  |  |  |  |  |  |
| 1 | Ath-AT2G25470.3 |  | | | |  |  |  |  |  |  |  |
| 1 | Ath-AT2G25480.2 |  | Vvi-Vitvi04g00483\_t002 |  |  |  |  |  |  |  |
| 1 | Ath-AT2G25482.1 |  | | | |  |  |  |  |  |  |  |
| 1 | Ath-AT2G25490.1 |  | Vvi-Vitvi04g00482\_t001 |  |  |  |  |  |  |  |
| 1 | Ath-AT2G25500.1 |  | | | |  |  |  |  |  |  |  |
| 1 | Ath-AT2G25510.1 |  | | | |  |  |  |  |  |  |  |
| 1 | Ath-AT2G25520.1 |  | Vvi-Vitvi04g00471\_t001 |  |  |  |  |  |  |  |
| 1 | Ath-AT2G25530.1 |  | Vvi-Vitvi04g00469\_t002 |  |  |  |  |  |  |  |
| 1 | Ath-AT2G25540.1 |  | Vvi-Vitvi04g00465\_t001 |  |  |  |  |  |  |  |
| 1 | Ath-AT2G25560.1 |  | | | |  |  |  |  |  |  |  |
| 1 | Ath-AT2G25565.1 |  | | | |  |  |  |  |  |  |  |
| 1 | Ath-AT2G25570.3 |  | | | |  |  |  |  |  |  |  |
| 1 | Ath-AT2G25580.1 |  | Vvi-Vitvi04g00447\_t001 |  |  |  |  |  |  |  |
| 1 | Ath-AT2G25590.1 |  | Vvi-Vitvi04g00444\_t001 |  |  |  |  |  |  |  |
| 1 | Ath-AT2G25600.1 |  | Vvi-Vitvi04g00440\_t001 |  |  |  |  |  |  |  |
| 1 | Ath-AT2G25605.1 |  | | | |  |  |  |  |  |  |  |
| 1 | Ath-AT2G25610.2 |  | | | |  |  |  |  |  |  |  |
| 1 | Ath-AT2G25620.2 |  | Vvi-Vitvi04g00419\_t001 |  |  |  |  |  |  |  |
| 1 | Ath-AT2G25625.1 |  | Vvi-Vitvi04g01887\_t001 |  |  |  |  |  |  |  |
| 0 | Ath-AT2G25630.1 |  |  |  |  |  |  |  |  |
| 1 | Ath-AT2G25640.1 |  | Vvi-Vitvi04g00373\_t001 |  |  |  |  |  |  |  |
| 1 | Ath-AT2G25650.1 |  | | | |  |  |  |  |  |  |  |
| 1 | Ath-AT2G25660.4 |  | Vvi-Vitvi04g00377\_t001 |  |  |  |  |  |  |  |
| 1 | Ath-AT2G25670.1 |  | Vvi-Vitvi04g01874\_t001 |  |  |  |  |  |  |  |
| 1 | Ath-AT2G25680.1 |  | Vvi-Vitvi04g00385\_t001 |  |  |  |  |  |  |  |
| 1 | Ath-AT2G25685.1 |  | | | |  |  |  |  |  |  |  |
| 1 | Ath-AT2G25690.1 |  | Vvi-Vitvi04g00390\_t003 |  |  |  |  |  |  |  |
| 1 | Ath-AT2G25700.1 |  | | | |  |  |  |  |  |  |  |
| 1 | Ath-AT2G25710.1 |  | | | |  |  |  |  |  |  |  |
| 1 | Ath-AT2G25720.1 |  | | | |  |  |  |  |  |  |  |
| 1 | Ath-AT2G25730.3 |  | Vvi-Vitvi04g00392\_t001 |  |  |  |  |  |  |  |
| 0 | Ath-AT2G25735.1 |  |  |  |  |  |  |  |  |
| 0 | Ath-AT2G25737.1 |  |  |  |  |  |  |  |  |
| 1 | Ath-AT2G25740.1 |  | Vvi-Vitvi04g00010\_t001 |  |  |  |  |  |  |  |
| 1 | Ath-AT2G25760.2 |  | | | |  |  |  |  |  |  |  |
| 1 | Ath-AT2G25770.1 |  | | | |  |  |  |  |  |  |  |
| 1 | Ath-AT2G25780.1 |  | | | |  |  |  |  |  |  |  |
| 1 | Ath-AT2G25790.1 |  | Vvi-Vitvi04g04016\_t001 |  |  |  |  |  |  |  |
| 1 | Ath-AT2G25800.1 |  | | | |  |  |  |  |  |  |  |
| 1 | Ath-AT2G25810.1 |  | | | |  |  |  |  |  |  |  |
| 1 | Ath-AT2G25820.1 |  | | | |  |  |  |  |  |  |  |
| 1 | Ath-AT2G25830.1 |  | | | |  |  |  |  |  |  |  |
| 1 | Ath-AT2G25840.2 |  | | | |  |  |  |  |  |  |  |
| 1 | Ath-AT2G25850.2 |  | | | |  |  |  |  |  |  |  |
| 1 | Ath-AT2G25870.1 |  | Vvi-Vitvi04g00035\_t001 |  |  |  |  |  |  |  |
| 1 | Ath-AT2G25880.3 |  | Vvi-Vitvi04g00041\_t001 |  |  |  |  |  |  |  |
| 1 | Ath-AT2G25890.2 |  | Vvi-Vitvi04g00042\_t001 |  |  |  |  |  |  |  |
| 1 | Ath-AT2G25900.2 |  | Vvi-Vitvi04g00047\_t001 |  |  |  |  |  |  |  |
| 1 | Ath-AT2G25905.1 |  | | | |  |  |  |  |  |  |  |
| 1 | Ath-AT2G25910.2 |  | Vvi-Vitvi04g00051\_t001 |  |  |  |  |  |  |  |
| 1 | Ath-AT2G25920.1 |  | | | |  |  |  |  |  |  |  |
| 1 | Ath-AT2G25930.1 |  | Vvi-Vitvi04g00052\_t001 |  |  |  |  |  |  |  |
| 1 | Ath-AT2G25940.1 |  | Vvi-Vitvi04g04024\_t001 |  |  |  |  |  |  |  |
| 1 | Ath-AT2G25950.1 |  | Vvi-Vitvi04g00062\_t001 |  |  |  |  |  |  |  |
| 1 | Ath-AT2G25964.1 |  | | | |  |  |  |  |  |  |  |
| 1 | Ath-AT2G25970.1 |  | Vvi-Vitvi04g00066\_t001 |  |  |  |  |  |  |  |
| 1 | Ath-AT2G25980.1 |  | | | |  |  |  |  |  |  |  |
| 1 | Ath-AT2G25990.1 |  | | | |  |  |  |  |  |  |  |
| 1 | Ath-AT2G26000.2 |  | | | |  |  |  |  |  |  |  |
| 1 | Ath-AT2G26010.1 |  | | | |  |  |  |  |  |  |  |
| 1 | Ath-AT2G26020.1 |  | | | |  |  |  |  |  |  |  |
| 1 | Ath-AT2G26030.4 |  | | | |  |  |  |  |  |  |  |
| 1 | Ath-AT2G26040.1 |  | Vvi-Vitvi04g00075\_t001 |  |  |  |  |  |  |  |
| 1 | Ath-AT2G26050.1 |  | | | |  |  |  |  |  |  |  |
| 1 | Ath-AT2G26060.1 |  | | | |  |  |  |  |  |  |  |
| 2 | Ath-AT2G26070.2 |  | | | |  | Vvi-Vitvi04g00115\_t002 |  |  |  |  |  |  |
| 2 | Ath-AT2G26080.1 |  | | | |  | Vvi-Vitvi04g00112\_t001 |  |  |  |  |  |  |
| 2 | Ath-AT2G26100.1 |  | | | |  | Vvi-Vitvi04g00110\_t001 |  |  |  |  |  |  |
| 2 | Ath-AT2G26110.1 |  | | | |  | Vvi-Vitvi04g00107\_t001 |  |  |  |  |  |  |
| 2 | Ath-AT2G26120.1 |  | | | |  | | | |  |  |  |  |  |  |
| 2 | Ath-AT2G26130.1 |  | | | |  | | | |  |  |  |  |  |  |
| 2 | Ath-AT2G26135.1 |  | | | |  | | | |  |  |  |  |  |  |
| 2 | Ath-AT2G26140.1 |  | | | |  | Vvi-Vitvi04g00098\_t001 |  |  |  |  |  |  |
| 2 | Ath-AT2G26150.1 |  | | | |  | Vvi-Vitvi04g00092\_t001 |  |  |  |  |  |  |
| 2 | Ath-AT2G26160.1 |  | | | |  | | | |  |  |  |  |  |  |
| 2 | Ath-AT2G26170.1 |  | | | |  | Vvi-Vitvi04g00090\_t001 |  |  |  |  |  |  |
| 2 | Ath-AT2G26180.1 |  | | | |  | Vvi-Vitvi04g00089\_t001 |  |  |  |  |  |  |
| 1 | Ath-AT2G26190.1 |  | Vvi-Vitvi04g00088\_t001 |  |  |  |  |  |  |  |
| 0 | Ath-AT2G26200.1 |  |  |  |  |  |  |  |  |
| 0 | Ath-AT2G26210.6 |  |  |  |  |  |  |  |  |
| 0 | Ath-AT2G26230.1 |  |  |  |  |  |  |  |  |
| 0 | Ath-AT2G26240.1 |  |  |  |  |  |  |  |  |
| 1 | Ath-AT2G26250.1 |  | Vvi-Vitvi04g00192\_t001 |  |  |  |  |  |  |  |
| 1 | Ath-AT2G26260.1 |  | Vvi-Vitvi04g04048\_t001 |  |  |  |  |  |  |  |
| 1 | Ath-AT2G26270.1 |  | Vvi-Vitvi04g00187\_t001 |  |  |  |  |  |  |  |
| 1 | Ath-AT2G26280.1 |  | Vvi-Vitvi04g00183\_t001 |  |  |  |  |  |  |  |
| 1 | Ath-AT2G26290.1 |  | Vvi-Vitvi04g00179\_t001 |  |  |  |  |  |  |  |
| 1 | Ath-AT2G26300.1 |  | Vvi-Vitvi04g00176\_t002 |  |  |  |  |  |  |  |
| 1 | Ath-AT2G26310.1 |  | Vvi-Vitvi04g00175\_t002 |  |  |  |  |  |  |  |
| 1 | Ath-AT2G26320.1 |  | Vvi-Vitvi04g00171\_t001 |  |  |  |  |  |  |  |
| 1 | Ath-AT2G26330.1 |  | Vvi-Vitvi04g00168\_t001 |  |  |  |  |  |  |  |
| 1 | Ath-AT2G26340.2 |  | Vvi-Vitvi04g00163\_t001 |  |  |  |  |  |  |  |
| 1 | Ath-AT2G26350.1 |  | Vvi-Vitvi04g00150\_t001 |  |  |  |  |  |  |  |
| 0 | Ath-AT2G26360.1 |  |  |  |  |  |  |  |  |
| 0 | Ath-AT2G26370.1 |  |  |  |  |  |  |  |  |
| 0 | Ath-AT2G26380.1 |  |  |  |  |  |  |  |  |
| 0 | Ath-AT2G26390.1 |  |  |  |  |  |  |  |  |
| 0 | Ath-AT2G26400.2 |  |  |  |  |  |  |  |  |
| 0 | Ath-AT2G26410.1 |  |  |  |  |  |  |  |  |
| 0 | Ath-AT2G26420.2 |  |  |  |  |  |  |  |  |
| 0 | Ath-AT2G26430.1 |  |  |  |  |  |  |  |  |
| 0 | Ath-AT2G26440.1 |  |  |  |  |  |  |  |  |
| 0 | Ath-AT2G26450.1 |  |  |  |  |  |  |  |  |
| 0 | Ath-AT2G26460.1 |  |  |  |  |  |  |  |  |
| 0 | Ath-AT2G26470.1 |  |  |  |  |  |  |  |  |
| 0 | Ath-AT2G26480.1 |  |  |  |  |  |  |  |  |
| 1 | Ath-AT2G26490.1 |  | Vvi-Vitvi18g00882\_t001 |  |  |  |  |  |  |  |
| 1 | Ath-AT2G26500.1 |  | Vvi-Vitvi18g00867\_t002 |  |  |  |  |  |  |  |
| 1 | Ath-AT2G26510.1 |  | Vvi-Vitvi18g00851\_t001 |  |  |  |  |  |  |  |
| 1 | Ath-AT2G26515.1 |  | | | |  |  |  |  |  |  |  |
| 1 | Ath-AT2G26520.1 |  | Vvi-Vitvi18g00845\_t001 |  |  |  |  |  |  |  |
| 1 | Ath-AT2G26530.1 |  | Vvi-Vitvi18g00839\_t001 |  |  |  |  |  |  |  |
| 1 | Ath-AT2G26540.1 |  | Vvi-Vitvi18g00835\_t002 |  |  |  |  |  |  |  |
| 1 | Ath-AT2G26550.1 |  | Vvi-Vitvi18g00830\_t001 |  |  |  |  |  |  |  |
| 1 | Ath-AT2G26560.1 |  | Vvi-Vitvi18g02692\_t001 |  |  |  |  |  |  |  |
| 0 | Ath-AT2G26570.1 |  |  |  |  |  |  |  |  |
| 1 | Ath-AT2G26580.1 |  | Vvi-Vitvi11g00492\_t001 |  |  |  |  |  |  |  |
| 1 | Ath-AT2G26590.2 |  | | | |  |  |  |  |  |  |  |
| 1 | Ath-AT2G26600.1 |  | Vvi-Vitvi11g00487\_t001 |  |  |  |  |  |  |  |
| 1 | Ath-AT2G26610.1 |  | Vvi-Vitvi11g00483\_t002 |  |  |  |  |  |  |  |
| 1 | Ath-AT2G26620.1 |  | | | |  |  |  |  |  |  |  |
| 1 | Ath-AT2G26640.1 |  | | | |  |  |  |  |  |  |  |
| 1 | Ath-AT2G26650.1 |  | | | |  |  |  |  |  |  |  |
| 1 | Ath-AT2G26660.1 |  | Vvi-Vitvi11g00466\_t001 |  |  |  |  |  |  |  |
| 1 | Ath-AT2G26670.1 |  | Vvi-Vitvi11g04106\_t001 |  |  |  |  |  |  |  |
| 1 | Ath-AT2G26680.1 |  | Vvi-Vitvi11g00463\_t001 |  |  |  |  |  |  |  |
| 1 | Ath-AT2G26690.1 |  | Vvi-Vitvi11g00454\_t002 |  |  |  |  |  |  |  |
| 1 | Ath-AT2G26695.2 |  | Vvi-Vitvi11g04099\_t001 |  |  |  |  |  |  |  |
| 1 | Ath-AT2G26700.1 |  | Vvi-Vitvi11g00435\_t001 |  |  |  |  |  |  |  |
| 1 | Ath-AT2G26710.1 |  | Vvi-Vitvi11g00424\_t001 |  |  |  |  |  |  |  |
| 0 | Ath-AT2G26720.1 |  |  |  |  |  |  |  |  |
| 0 | Ath-AT2G26730.1 |  |  |  |  |  |  |  |  |
| 0 | Ath-AT2G26740.1 |  |  |  |  |  |  |  |  |
| 0 | Ath-AT2G26750.1 |  |  |  |  |  |  |  |  |
| 1 | Ath-AT2G26760.1 |  | Vvi-Vitvi06g01106\_t001 |  |  |  |  |  |  |  |
| 1 | Ath-AT2G26770.1 |  | Vvi-Vitvi06g01104\_t001 |  |  |  |  |  |  |  |
| 1 | Ath-AT2G26780.1 |  | | | |  |  |  |  |  |  |  |
| 1 | Ath-AT2G26790.1 |  | | | |  |  |  |  |  |  |  |
| 1 | Ath-AT2G26800.2 |  | | | |  |  |  |  |  |  |  |
| 1 | Ath-AT2G26810.2 |  | | | |  |  |  |  |  |  |  |
| 1 | Ath-AT2G26820.3 |  | | | |  |  |  |  |  |  |  |
| 1 | Ath-AT2G26830.1 |  | | | |  |  |  |  |  |  |  |
| 1 | Ath-AT2G26840.2 |  | | | |  |  |  |  |  |  |  |
| 1 | Ath-AT2G26850.1 |  | | | |  |  |  |  |  |  |  |
| 1 | Ath-AT2G26860.1 |  | | | |  |  |  |  |  |  |  |
| 1 | Ath-AT2G26865.1 |  | | | |  |  |  |  |  |  |  |
| 1 | Ath-AT2G26870.1 |  | | | |  |  |  |  |  |  |  |
| 1 | Ath-AT2G26880.1 |  | | | |  |  |  |  |  |  |  |
| 1 | Ath-AT2G26890.1 |  | | | |  |  |  |  |  |  |  |
| 1 | Ath-AT2G26900.1 |  | | | |  |  |  |  |  |  |  |
| 1 | Ath-AT2G26910.1 |  | | | |  |  |  |  |  |  |  |
| 1 | Ath-AT2G26920.1 |  | | | |  |  |  |  |  |  |  |
| 1 | Ath-AT2G26930.1 |  | | | |  |  |  |  |  |  |  |
| 1 | Ath-AT2G26940.1 |  | | | |  |  |  |  |  |  |  |
| 1 | Ath-AT2G26950.1 |  | | | |  |  |  |  |  |  |  |
| 1 | Ath-AT2G26960.1 |  | | | |  |  |  |  |  |  |  |
| 1 | Ath-AT2G26970.1 |  | Vvi-Vitvi06g01087\_t001 |  |  |  |  |  |  |  |
| 1 | Ath-AT2G26975.1 |  | | | |  |  |  |  |  |  |  |
| 1 | Ath-AT2G26980.4 |  | Vvi-Vitvi06g01084\_t003 |  |  |  |  |  |  |  |
| 1 | Ath-AT2G26990.1 |  | | | |  |  |  |  |  |  |  |
| 1 | Ath-AT2G27000.1 |  | | | |  |  |  |  |  |  |  |
| 1 | Ath-AT2G27010.1 |  | | | |  |  |  |  |  |  |  |
| 1 | Ath-AT2G27020.2 |  | | | |  |  |  |  |  |  |  |
| 1 | Ath-AT2G27030.3 |  | | | |  |  |  |  |  |  |  |
| 1 | Ath-AT2G27035.1 |  | | | |  |  |  |  |  |  |  |
| 1 | Ath-AT2G27040.1 |  | | | |  |  |  |  |  |  |  |
| 1 | Ath-AT2G27050.1 |  | | | |  |  |  |  |  |  |  |
| 1 | Ath-AT2G27060.1 |  | Vvi-Vitvi06g01057\_t001 |  |  |  |  |  |  |  |
| 1 | Ath-AT2G27070.1 |  | | | |  |  |  |  |  |  |  |
| 1 | Ath-AT2G27080.2 |  | Vvi-Vitvi06g01055\_t001 |  |  |  |  |  |  |  |
| 1 | Ath-AT2G27090.2 |  | Vvi-Vitvi06g01053\_t001 |  |  |  |  |  |  |  |
| 1 | Ath-AT2G27100.1 |  | Vvi-Vitvi06g01050\_t001 |  |  |  |  |  |  |  |
| 1 | Ath-AT2G27110.1 |  | Vvi-Vitvi06g01048\_t001 |  |  |  |  |  |  |  |
| 1 | Ath-AT2G27120.2 |  | | | |  |  |  |  |  |  |  |
| 1 | Ath-AT2G27130.1 |  | Vvi-Vitvi06g01830\_t002 |  |  |  |  |  |  |  |
| 1 | Ath-AT2G27140.1 |  | Vvi-Vitvi06g01821\_t001 |  |  |  |  |  |  |  |
| 1 | Ath-AT2G27145.1 |  | | | |  |  |  |  |  |  |  |
| 1 | Ath-AT2G27150.1 |  | | | |  |  |  |  |  |  |  |
| 1 | Ath-AT2G27160.2 |  | | | |  |  |  |  |  |  |  |
| 1 | Ath-AT2G27170.1 |  | | | |  |  |  |  |  |  |  |
| 1 | Ath-AT2G27180.1 |  | Vvi-Vitvi06g01819\_t001 |  |  |  |  |  |  |  |
| 0 | Ath-AT2G27190.1 |  |  |  |  |  |  |  |  |
| 0 | Ath-AT2G27200.1 |  |  |  |  |  |  |  |  |
| 0 | Ath-AT2G27210.1 |  |  |  |  |  |  |  |  |
| 0 | Ath-AT2G27220.2 |  |  |  |  |  |  |  |  |
| 0 | Ath-AT2G27229.1 |  |  |  |  |  |  |  |  |
| 0 | Ath-AT2G27230.1 |  |  |  |  |  |  |  |  |
| 0 | Ath-AT2G27240.1 |  |  |  |  |  |  |  |  |
| 0 | Ath-AT2G27250.3 |  |  |  |  |  |  |  |  |
| 1 | Ath-AT2G27260.1 |  | Vvi-Vitvi06g01560\_t001 |  |  |  |  |  |  |  |
| 1 | Ath-AT2G27270.1 |  | | | |  |  |  |  |  |  |  |
| 1 | Ath-AT2G27280.1 |  | Vvi-Vitvi06g01555\_t001 |  |  |  |  |  |  |  |
| 1 | Ath-AT2G27285.1 |  | | | |  |  |  |  |  |  |  |
| 1 | Ath-AT2G27290.1 |  | Vvi-Vitvi06g01551\_t001 |  |  |  |  |  |  |  |
| 1 | Ath-AT2G27300.1 |  | Vvi-Vitvi06g01536\_t001 |  |  |  |  |  |  |  |
| 1 | Ath-AT2G27310.1 |  | Vvi-Vitvi06g01530\_t001 |  |  |  |  |  |  |  |
| 1 | Ath-AT2G27313.1 |  | | | |  |  |  |  |  |  |  |
| 1 | Ath-AT2G27315.1 |  | | | |  |  |  |  |  |  |  |
| 1 | Ath-AT2G27320.1 |  | | | |  |  |  |  |  |  |  |
| 1 | Ath-AT2G27330.1 |  | Vvi-Vitvi06g01974\_t001 |  |  |  |  |  |  |  |
| 1 | Ath-AT2G27340.4 |  | | | |  |  |  |  |  |  |  |
| 1 | Ath-AT2G27350.3 |  | Vvi-Vitvi06g01523\_t001 |  |  |  |  |  |  |  |
| 1 | Ath-AT2G27360.1 |  | | | |  |  |  |  |  |  |  |
| 1 | Ath-AT2G27370.1 |  | Vvi-Vitvi06g01522\_t001 |  |  |  |  |  |  |  |
| 1 | Ath-AT2G27380.1 |  | | | |  |  |  |  |  |  |  |
| 1 | Ath-AT2G27385.3 |  | Vvi-Vitvi06g01513\_t001 |  |  |  |  |  |  |  |
| 1 | Ath-AT2G27389.1 |  | | | |  |  |  |  |  |  |  |
| 1 | Ath-AT2G27390.1 |  | | | |  |  |  |  |  |  |  |
| 1 | Ath-AT2G27402.1 |  | | | |  |  |  |  |  |  |  |
| 1 | Ath-AT2G27410.1 |  | | | |  |  |  |  |  |  |  |
| 1 | Ath-AT2G27420.1 |  | | | |  |  |  |  |  |  |  |
| 1 | Ath-AT2G27430.1 |  | | | |  |  |  |  |  |  |  |
| 1 | Ath-AT2G27450.2 |  | Vvi-Vitvi06g01502\_t001 |  |  |  |  |  |  |  |
| 1 | Ath-AT2G27460.1 |  | Vvi-Vitvi06g01498\_t001 |  |  |  |  |  |  |  |
| 1 | Ath-AT2G27470.1 |  | Vvi-Vitvi06g01492\_t001 |  |  |  |  |  |  |  |
| 1 | Ath-AT2G27480.1 |  | Vvi-Vitvi06g01961\_t001 |  |  |  |  |  |  |  |
| 1 | Ath-AT2G27490.1 |  | | | |  |  |  |  |  |  |  |
| 1 | Ath-AT2G27500.1 |  | Vvi-Vitvi06g01489\_t001 |  |  |  |  |  |  |  |
| 1 | Ath-AT2G27505.1 |  | | | |  |  |  |  |  |  |  |
| 1 | Ath-AT2G27507.1 |  | | | |  |  |  |  |  |  |  |
| 1 | Ath-AT2G27510.1 |  | Vvi-Vitvi06g01487\_t002 |  |  |  |  |  |  |  |
| 1 | Ath-AT2G27520.1 |  | | | |  |  |  |  |  |  |  |
| 2 | Ath-AT2G27530.2 |  | Vvi-Vitvi06g01486\_t002 |  | Vvi-Vitvi08g01462\_t001 |  |  |  |  |  |  |
| 2 | Ath-AT2G27535.1 |  | | | |  | | | |  |  |  |  |  |  |
| 2 | Ath-AT2G27540.1 |  | | | |  | | | |  |  |  |  |  |  |
| 2 | Ath-AT2G27550.1 |  | Vvi-Vitvi06g01473\_t001 |  | Vvi-Vitvi08g01473\_t001 |  |  |  |  |  |  |
| 2 | Ath-AT2G27570.1 |  | | | |  | | | |  |  |  |  |  |  |
| 2 | Ath-AT2G27580.1 |  | | | |  | Vvi-Vitvi08g02270\_t001 |  |  |  |  |  |  |
| 2 | Ath-AT2G27590.1 |  | Vvi-Vitvi06g01467\_t001 |  | | | |  |  |  |  |  |  |
| 2 | Ath-AT2G27600.1 |  | Vvi-Vitvi06g01460\_t001 |  | | | |  |  |  |  |  |  |
| 2 | Ath-AT2G27610.1 |  | Vvi-Vitvi06g01458\_t001 |  | | | |  |  |  |  |  |  |
| 2 | Ath-AT2G27630.1 |  | | | |  | | | |  |  |  |  |  |  |
| 2 | Ath-AT2G27650.1 |  | | | |  | | | |  |  |  |  |  |  |
| 2 | Ath-AT2G27660.1 |  | | | |  | | | |  |  |  |  |  |  |
| 2 | Ath-AT2G27670.1 |  | | | |  | | | |  |  |  |  |  |  |
| 2 | Ath-AT2G27680.1 |  | Vvi-Vitvi06g01444\_t001 |  | | | |  |  |  |  |  |  |
| 2 | Ath-AT2G27690.1 |  | | | |  | Vvi-Vitvi08g01487\_t001 |  |  |  |  |  |  |
| 2 | Ath-AT2G27700.2 |  | | | |  | | | |  |  |  |  |  |  |
| 2 | Ath-AT2G27710.2 |  | Vvi-Vitvi06g01433\_t001 |  | | | |  |  |  |  |  |  |
| 2 | Ath-AT2G27720.2 |  | | | |  | | | |  |  |  |  |  |  |
| 2 | Ath-AT2G27730.1 |  | | | |  | | | |  |  |  |  |  |  |
| 2 | Ath-AT2G27740.1 |  | Vvi-Vitvi06g01418\_t001 |  | Vvi-Vitvi08g01499\_t001 |  |  |  |  |  |  |
| 2 | Ath-AT2G27750.1 |  | | | |  | | | |  |  |  |  |  |  |
| 2 | Ath-AT2G27760.1 |  | Vvi-Vitvi06g01415\_t001 |  | | | |  |  |  |  |  |  |
| 2 | Ath-AT2G27770.1 |  | Vvi-Vitvi06g01408\_t001 |  | Vvi-Vitvi08g01507\_t001 |  |  |  |  |  |  |
| 2 | Ath-AT2G27775.2 |  | | | |  | | | |  |  |  |  |  |  |
| 2 | Ath-AT2G27780.1 |  | | | |  | | | |  |  |  |  |  |  |
| 2 | Ath-AT2G27790.2 |  | | | |  | | | |  |  |  |  |  |  |
| 2 | Ath-AT2G27800.1 |  | | | |  | | | |  |  |  |  |  |  |
| 2 | Ath-AT2G27810.4 |  | Vvi-Vitvi06g01404\_t001 |  | | | |  |  |  |  |  |  |
| 2 | Ath-AT2G27820.1 |  | Vvi-Vitvi06g01946\_t001 |  | | | |  |  |  |  |  |  |
| 2 | Ath-AT2G27830.1 |  | Vvi-Vitvi06g01400\_t001 |  | | | |  |  |  |  |  |  |
| 2 | Ath-AT2G27840.3 |  | Vvi-Vitvi06g01399\_t001 |  | Vvi-Vitvi08g01518\_t002 |  |  |  |  |  |  |
| 1 | Ath-AT2G27860.1 |  | Vvi-Vitvi06g01389\_t001 |  |  |  |  |  |  |  |
| 1 | Ath-AT2G27880.1 |  | Vvi-Vitvi06g01378\_t001 |  |  |  |  |  |  |  |
| 1 | Ath-AT2G27900.1 |  | Vvi-Vitvi06g04416\_t001 |  |  |  |  |  |  |  |
| 1 | Ath-AT2G27920.1 |  | Vvi-Vitvi06g01362\_t001 |  |  |  |  |  |  |  |
| 1 | Ath-AT2G27930.6 |  | | | |  |  |  |  |  |  |  |
| 1 | Ath-AT2G27940.1 |  | Vvi-Vitvi06g01359\_t001 |  |  |  |  |  |  |  |
| 1 | Ath-AT2G27950.1 |  | Vvi-Vitvi06g01358\_t001 |  |  |  |  |  |  |  |
| 1 | Ath-AT2G27960.1 |  | | | |  |  |  |  |  |  |  |
| 1 | Ath-AT2G27970.1 |  | | | |  |  |  |  |  |  |  |
| 1 | Ath-AT2G27980.1 |  | Vvi-Vitvi06g01933\_t001 |  |  |  |  |  |  |  |
| 2 | Ath-AT2G27990.1 |  | | | |  | Vvi-Vitvi06g00248\_t001 |  |  |  |  |  |  |
| 3 | Ath-AT2G28000.1 |  | | | |  | | | |  | Vvi-Vitvi06g01295\_t001 |  |  |  |  |  |
| 3 | Ath-AT2G28010.1 |  | | | |  | | | |  | | | |  |  |  |  |  |
| 3 | Ath-AT2G28020.1 |  | | | |  | | | |  | | | |  |  |  |  |  |
| 3 | Ath-AT2G28030.1 |  | | | |  | | | |  | | | |  |  |  |  |  |
| 3 | Ath-AT2G28040.1 |  | | | |  | | | |  | | | |  |  |  |  |  |
| 3 | Ath-AT2G28050.1 |  | | | |  | | | |  | | | |  |  |  |  |  |
| 3 | Ath-AT2G28060.1 |  | | | |  | | | |  | Vvi-Vitvi06g01298\_t001 |  |  |  |  |  |
| 3 | Ath-AT2G28070.1 |  | | | |  | | | |  | Vvi-Vitvi06g01306\_t001 |  |  |  |  |  |
| 3 | Ath-AT2G28080.1 |  | | | |  | | | |  | Vvi-Vitvi06g01307\_t001 |  |  |  |  |  |
| 3 | Ath-AT2G28085.1 |  | | | |  | | | |  | Vvi-Vitvi06g01310\_t001 |  |  |  |  |  |
| 3 | Ath-AT2G28090.1 |  | | | |  | | | |  | Vvi-Vitvi06g01315\_t001 |  |  |  |  |  |
| 3 | Ath-AT2G28100.1 |  | | | |  | | | |  | Vvi-Vitvi06g01323\_t001 |  |  |  |  |  |
| 3 | Ath-AT2G28105.1 |  | | | |  | | | |  | Vvi-Vitvi06g01327\_t001 |  |  |  |  |  |
| 3 | Ath-AT2G28110.1 |  | | | |  | | | |  | Vvi-Vitvi06g01330\_t001 |  |  |  |  |  |
| 3 | Ath-AT2G28120.1 |  | | | |  | | | |  | Vvi-Vitvi06g01334\_t001 |  |  |  |  |  |
| 3 | Ath-AT2G28130.1 |  | | | |  | | | |  | Vvi-Vitvi06g01335\_t001 |  |  |  |  |  |
| 3 | Ath-AT2G28140.1 |  | Vvi-Vitvi06g01340\_t001 |  | | | |  | Vvi-Vitvi06g01340\_t001 |  |  |  |  |  |
| 2 | Ath-AT2G28150.1 |  |  |  | | | |  | Vvi-Vitvi06g01343\_t001 |  |  |  |  |  |
| 2 | Ath-AT2G28160.1 |  |  |  | | | |  | Vvi-Vitvi06g01345\_t001 |  |  |  |  |  |
| 2 | Ath-AT2G28170.1 |  |  |  | | | |  | | | |  |  |  |  |  |
| 2 | Ath-AT2G28180.1 |  |  |  | | | |  | | | |  |  |  |  |  |
| 2 | Ath-AT2G28190.1 |  |  |  | | | |  | Vvi-Vitvi06g01349\_t001 |  |  |  |  |  |
| 2 | Ath-AT2G28200.1 |  |  |  | | | |  | Vvi-Vitvi06g01350\_t001 |  |  |  |  |  |
| 1 | Ath-AT2G28210.1 |  |  |  | Vvi-Vitvi06g00250\_t001 |  |  |  |  |  |  |
| 1 | Ath-AT2G28220.1 |  |  |  | | | |  |  |  |  |  |  |
| 1 | Ath-AT2G28225.1 |  |  |  | | | |  |  |  |  |  |  |
| 1 | Ath-AT2G28230.1 |  |  |  | | | |  |  |  |  |  |  |
| 1 | Ath-AT2G28240.1 |  |  |  | | | |  |  |  |  |  |  |
| 1 | Ath-AT2G28250.3 |  |  |  | Vvi-Vitvi06g00259\_t001 |  |  |  |  |  |  |
| 1 | Ath-AT2G28260.1 |  |  |  | Vvi-Vitvi06g00262\_t001 |  |  |  |  |  |  |
| 1 | Ath-AT2G28270.1 |  |  |  | | | |  |  |  |  |  |  |
| 1 | Ath-AT2G28290.5 |  |  |  | | | |  |  |  |  |  |  |
| 1 | Ath-AT2G28305.1 |  |  |  | Vvi-Vitvi06g00263\_t001 |  |  |  |  |  |  |
| 1 | Ath-AT2G28310.1 |  |  |  | Vvi-Vitvi06g00267\_t001 |  |  |  |  |  |  |
| 1 | Ath-AT2G28315.1 |  |  |  | Vvi-Vitvi06g00268\_t001 |  |  |  |  |  |  |
| 1 | Ath-AT2G28320.1 |  |  |  | Vvi-Vitvi06g00269\_t001 |  |  |  |  |  |  |
| 1 | Ath-AT2G28330.1 |  |  |  | | | |  |  |  |  |  |  |
| 1 | Ath-AT2G28340.1 |  |  |  | | | |  |  |  |  |  |  |
| 1 | Ath-AT2G28350.1 |  |  |  | Vvi-Vitvi06g00272\_t002 |  |  |  |  |  |  |
| 1 | Ath-AT2G28355.1 |  |  |  | | | |  |  |  |  |  |  |
| 1 | Ath-AT2G28360.1 |  |  |  | Vvi-Vitvi06g00274\_t004 |  |  |  |  |  |  |
| 1 | Ath-AT2G28370.1 |  |  |  | Vvi-Vitvi06g00275\_t001 |  |  |  |  |  |  |
| 1 | Ath-AT2G28380.1 |  |  |  | Vvi-Vitvi06g00277\_t001 |  |  |  |  |  |  |
| 1 | Ath-AT2G28390.1 |  |  |  | Vvi-Vitvi06g00278\_t001 |  |  |  |  |  |  |
| 1 | Ath-AT2G28400.1 |  |  |  | Vvi-Vitvi06g00279\_t001 |  |  |  |  |  |  |
| 1 | Ath-AT2G28405.1 |  |  |  | | | |  |  |  |  |  |  |
| 1 | Ath-AT2G28410.1 |  |  |  | Vvi-Vitvi06g01658\_t001 |  |  |  |  |  |  |
| 1 | Ath-AT2G28420.1 |  |  |  | | | |  |  |  |  |  |  |
| 1 | Ath-AT2G28426.1 |  |  |  | | | |  |  |  |  |  |  |
| 1 | Ath-AT2G28430.1 |  |  |  | Vvi-Vitvi06g00282\_t001 |  |  |  |  |  |  |
| 1 | Ath-AT2G28440.1 |  |  |  | Vvi-Vitvi06g00285\_t001 |  |  |  |  |  |  |
| 1 | Ath-AT2G28450.1 |  |  |  | Vvi-Vitvi06g00290\_t001 |  |  |  |  |  |  |
| 1 | Ath-AT2G28460.1 |  |  |  | | | |  |  |  |  |  |  |
| 1 | Ath-AT2G28470.1 |  |  |  | Vvi-Vitvi06g00300\_t001 |  |  |  |  |  |  |
| 0 | Ath-AT2G28480.1 |  |  |  |  |  |  |  |  |
| 1 | Ath-AT2G28490.1 |  | Vvi-Vitvi06g00323\_t001 |  |  |  |  |  |  |  |
| 1 | Ath-AT2G28500.1 |  | Vvi-Vitvi06g00336\_t001 |  |  |  |  |  |  |  |
| 1 | Ath-AT2G28510.1 |  | Vvi-Vitvi06g00345\_t001 |  |  |  |  |  |  |  |
| 1 | Ath-AT2G28520.1 |  | Vvi-Vitvi06g00348\_t001 |  |  |  |  |  |  |  |
| 2 | Ath-AT2G28540.2 |  | Vvi-Vitvi06g00359\_t002 |  | Vvi-Vitvi08g02180\_t001 |  |  |  |  |  |  |
| 2 | Ath-AT2G28550.3 |  | Vvi-Vitvi06g00360\_t001 |  | Vvi-Vitvi08g01146\_t001 |  |  |  |  |  |  |
| 2 | Ath-AT2G28560.1 |  | Vvi-Vitvi06g00362\_t001 |  | | | |  |  |  |  |  |  |
| 2 | Ath-AT2G28570.2 |  | | | |  | | | |  |  |  |  |  |  |
| 2 | Ath-AT2G28580.2 |  | | | |  | | | |  |  |  |  |  |  |
| 2 | Ath-AT2G28590.1 |  | Vvi-Vitvi06g00369\_t001 |  | | | |  |  |  |  |  |  |
| 2 | Ath-AT2G28600.1 |  | | | |  | | | |  |  |  |  |  |  |
| 2 | Ath-AT2G28605.1 |  | | | |  | | | |  |  |  |  |  |  |
| 2 | Ath-AT2G28610.1 |  | Vvi-Vitvi06g00380\_t001 |  | | | |  |  |  |  |  |  |
| 2 | Ath-AT2G28620.2 |  | Vvi-Vitvi06g00383\_t001 |  | | | |  |  |  |  |  |  |
| 1 | Ath-AT2G28625.1 |  |  |  | | | |  |  |  |  |  |  |
| 2 | Ath-AT2G28630.1 |  | Vvi-Vitvi06g00396\_t001 |  | | | |  |  |  |  |  |  |
| 2 | Ath-AT2G28640.1 |  | | | |  | | | |  |  |  |  |  |  |
| 2 | Ath-AT2G28650.1 |  | | | |  | | | |  |  |  |  |  |  |
| 2 | Ath-AT2G28660.1 |  | | | |  | | | |  |  |  |  |  |  |
| 2 | Ath-AT2G28671.1 |  | | | |  | | | |  |  |  |  |  |  |
| 2 | Ath-AT2G28670.1 |  | Vvi-Vitvi06g00400\_t001 |  | | | |  |  |  |  |  |  |
| 2 | Ath-AT2G28680.1 |  | Vvi-Vitvi06g00406\_t001 |  | | | |  |  |  |  |  |  |
| 2 | Ath-AT2G28690.1 |  | Vvi-Vitvi06g00411\_t001 |  | | | |  |  |  |  |  |  |
| 2 | Ath-AT2G28700.1 |  | | | |  | | | |  |  |  |  |  |  |
| 2 | Ath-AT2G28710.1 |  | Vvi-Vitvi06g01682\_t001 |  | | | |  |  |  |  |  |  |
| 2 | Ath-AT2G28720.1 |  | Vvi-Vitvi06g00426\_t001 |  | | | |  |  |  |  |  |  |
| 2 | Ath-AT2G28725.1 |  | | | |  | Vvi-Vitvi08g02184\_t001 |  |  |  |  |  |  |
| 2 | Ath-AT2G28740.1 |  | | | |  | | | |  |  |  |  |  |  |
| 2 | Ath-AT2G28755.1 |  | | | |  | | | |  |  |  |  |  |  |
| 2 | Ath-AT2G28760.4 |  | Vvi-Vitvi06g00435\_t001 |  | | | |  |  |  |  |  |  |
| 2 | Ath-AT2G28780.1 |  | Vvi-Vitvi06g00436\_t001 |  | Vvi-Vitvi08g01179\_t001 |  |  |  |  |  |  |
| 2 | Ath-AT2G28790.2 |  | Vvi-Vitvi06g00440\_t001 |  | | | |  |  |  |  |  |  |
| 2 | Ath-AT2G28800.1 |  | Vvi-Vitvi06g00442\_t001 |  | | | |  |  |  |  |  |  |
| 3 | Ath-AT2G28810.1 |  | Vvi-Vitvi06g00449\_t001 |  | Vvi-Vitvi08g01186\_t001 |  | Vvi-Vitvi13g00298\_t001 |  |  |  |  |  |
| 3 | Ath-AT2G28815.1 |  | | | |  | | | |  | | | |  |  |  |  |  |
| 3 | Ath-AT2G28830.1 |  | Vvi-Vitvi06g00450\_t001 |  | | | |  | | | |  |  |  |  |  |
| 3 | Ath-AT2G28840.1 |  | Vvi-Vitvi06g00451\_t001 |  | Vvi-Vitvi08g01188\_t001 |  | | | |  |  |  |  |  |
| 3 | Ath-AT2G28850.1 |  | | | |  | | | |  | | | |  |  |  |  |  |
| 3 | Ath-AT2G28860.1 |  | | | |  | | | |  | | | |  |  |  |  |  |
| 3 | Ath-AT2G28870.1 |  | | | |  | | | |  | | | |  |  |  |  |  |
| 3 | Ath-AT2G28880.1 |  | | | |  | | | |  | | | |  |  |  |  |  |
| 3 | Ath-AT2G28890.1 |  | Vvi-Vitvi06g00456\_t001 |  | Vvi-Vitvi08g01194\_t001 |  | | | |  |  |  |  |  |
| 3 | Ath-AT2G28900.1 |  | Vvi-Vitvi06g00465\_t001 |  | | | |  | | | |  |  |  |  |  |
| 3 | Ath-AT2G28910.3 |  | Vvi-Vitvi06g01700\_t001 |  | | | |  | | | |  |  |  |  |  |
| 3 | Ath-AT2G28920.1 |  | | | |  | | | |  | | | |  |  |  |  |  |
| 3 | Ath-AT2G28930.1 |  | Vvi-Vitvi06g00473\_t001 |  | Vvi-Vitvi08g01204\_t004 |  | | | |  |  |  |  |  |
| 3 | Ath-AT2G28940.2 |  | Vvi-Vitvi06g00474\_t001 |  | | | |  | | | |  |  |  |  |  |
| 3 | Ath-AT2G28950.1 |  | Vvi-Vitvi06g00481\_t001 |  | Vvi-Vitvi08g01206\_t001 |  | Vvi-Vitvi13g00309\_t001 |  |  |  |  |  |
| 3 | Ath-AT2G28960.2 |  | | | |  | | | |  | | | |  |  |  |  |  |
| 3 | Ath-AT2G28970.1 |  | | | |  | | | |  | | | |  |  |  |  |  |
| 3 | Ath-AT2G28990.2 |  | | | |  | | | |  | | | |  |  |  |  |  |
| 3 | Ath-AT2G29000.1 |  | | | |  | | | |  | | | |  |  |  |  |  |
| 3 | Ath-AT2G29020.1 |  | Vvi-Vitvi06g00486\_t001 |  | | | |  | | | |  |  |  |  |  |
| 3 | Ath-AT2G29040.1 |  | | | |  | | | |  | | | |  |  |  |  |  |
| 3 | Ath-AT2G29045.1 |  | | | |  | | | |  | | | |  |  |  |  |  |
| 3 | Ath-AT2G29050.1 |  | | | |  | Vvi-Vitvi08g01209\_t001 |  | | | |  |  |  |  |  |
| 3 | Ath-AT2G29060.1 |  | Vvi-Vitvi06g01569\_t001 |  | Vvi-Vitvi08g01214\_t001 |  | Vvi-Vitvi13g00311\_t001 |  |  |  |  |  |
| 3 | Ath-AT2G29065.1 |  | | | |  | | | |  | | | |  |  |  |  |  |
| 3 | Ath-AT2G29070.2 |  | Vvi-Vitvi06g00494\_t001 |  | | | |  | | | |  |  |  |  |  |
| 3 | Ath-AT2G29080.1 |  | Vvi-Vitvi06g00496\_t001 |  | Vvi-Vitvi08g01218\_t001 |  | | | |  |  |  |  |  |
| 3 | Ath-AT2G29090.1 |  | Vvi-Vitvi06g00498\_t001 |  | | | |  | | | |  |  |  |  |  |
| 3 | Ath-AT2G29100.2 |  | | | |  | | | |  | | | |  |  |  |  |  |
| 3 | Ath-AT2G29110.2 |  | | | |  | | | |  | | | |  |  |  |  |  |
| 3 | Ath-AT2G29120.1 |  | | | |  | | | |  | | | |  |  |  |  |  |
| 3 | Ath-AT2G29125.1 |  | Vvi-Vitvi06g04166\_t001 |  | | | |  | Vvi-Vitvi13g01992\_t001 |  |  |  |  |  |
| 3 | Ath-AT2G29130.1 |  | | | |  | Vvi-Vitvi08g01223\_t001 |  | Vvi-Vitvi13g00321\_t001 |  |  |  |  |  |
| 2 | Ath-AT2G29140.2 |  | Vvi-Vitvi06g00503\_t001 |  |  |  | Vvi-Vitvi13g00328\_t001 |  |  |  |  |  |
| 2 | Ath-AT2G29150.1 |  | | | |  |  |  | | | |  |  |  |  |  |
| 2 | Ath-AT2G29170.1 |  | | | |  |  |  | | | |  |  |  |  |  |
| 2 | Ath-AT2G29180.1 |  | Vvi-Vitvi06g01708\_t001 |  |  |  | | | |  |  |  |  |  |
| 2 | Ath-AT2G29190.1 |  | | | |  |  |  | | | |  |  |  |  |  |
| 2 | Ath-AT2G29200.1 |  | | | |  |  |  | | | |  |  |  |  |  |
| 2 | Ath-AT2G29210.1 |  | Vvi-Vitvi06g00516\_t001 |  |  |  | | | |  |  |  |  |  |
| 2 | Ath-AT2G29220.1 |  | | | |  |  |  | | | |  |  |  |  |  |
| 2 | Ath-AT2G29250.1 |  | | | |  |  |  | | | |  |  |  |  |  |
| 2 | Ath-AT2G29260.1 |  | Vvi-Vitvi06g00522\_t001 |  |  |  | Vvi-Vitvi13g01997\_t001 |  |  |  |  |  |
| 2 | Ath-AT2G29263.1 |  | | | |  |  |  | | | |  |  |  |  |  |
| 2 | Ath-AT2G29290.2 |  | | | |  |  |  | | | |  |  |  |  |  |
| 2 | Ath-AT2G29300.2 |  | | | |  |  |  | | | |  |  |  |  |  |
| 2 | Ath-AT2G29310.1 |  | | | |  |  |  | | | |  |  |  |  |  |
| 2 | Ath-AT2G29320.1 |  | | | |  |  |  | | | |  |  |  |  |  |
| 2 | Ath-AT2G29330.1 |  | | | |  |  |  | | | |  |  |  |  |  |
| 2 | Ath-AT2G29340.1 |  | | | |  |  |  | | | |  |  |  |  |  |
| 2 | Ath-AT2G29350.1 |  | Vvi-Vitvi06g04170\_t001 |  |  |  | Vvi-Vitvi13g01999\_t001 |  |  |  |  |  |
| 2 | Ath-AT2G29360.1 |  | | | |  |  |  | Vvi-Vitvi13g00343\_t001 |  |  |  |  |  |
| 2 | Ath-AT2G29370.1 |  | Vvi-Vitvi06g01718\_t001 |  |  |  | | | |  |  |  |  |  |
| 2 | Ath-AT2G29380.1 |  | Vvi-Vitvi06g00533\_t001 |  |  |  | Vvi-Vitvi13g00344\_t001 |  |  |  |  |  |
| 2 | Ath-AT2G29390.1 |  | Vvi-Vitvi06g00534\_t001 |  |  |  | Vvi-Vitvi13g00345\_t001 |  |  |  |  |  |
| 2 | Ath-AT2G29400.1 |  | Vvi-Vitvi06g00540\_t003 |  |  |  | Vvi-Vitvi13g00362\_t003 |  |  |  |  |  |
| 1 | Ath-AT2G29410.1 |  | Vvi-Vitvi06g00541\_t001 |  |  |  |  |  |  |  |
| 1 | Ath-AT2G29420.1 |  | Vvi-Vitvi06g01724\_t001 |  |  |  |  |  |  |  |
| 1 | Ath-AT2G29430.1 |  | | | |  |  |  |  |  |  |  |
| 1 | Ath-AT2G29440.1 |  | | | |  |  |  |  |  |  |  |
| 1 | Ath-AT2G29450.1 |  | | | |  |  |  |  |  |  |  |
| 1 | Ath-AT2G29452.1 |  | | | |  |  |  |  |  |  |  |
| 1 | Ath-AT2G29460.1 |  | Vvi-Vitvi06g04184\_t001 |  |  |  |  |  |  |  |
| 1 | Ath-AT2G29470.1 |  | Vvi-Vitvi06g04186\_t001 |  |  |  |  |  |  |  |
| 1 | Ath-AT2G29480.1 |  | | | |  |  |  |  |  |  |  |
| 1 | Ath-AT2G29485.1 |  | | | |  |  |  |  |  |  |  |
| 1 | Ath-AT2G29490.1 |  | | | |  |  |  |  |  |  |  |
| 1 | Ath-AT2G29500.1 |  | Vvi-Vitvi06g00561\_t001 |  |  |  |  |  |  |  |
| 1 | Ath-AT2G29510.1 |  | Vvi-Vitvi06g00563\_t001 |  |  |  |  |  |  |  |
| 1 | Ath-AT2G29525.1 |  | Vvi-Vitvi06g00564\_t001 |  |  |  |  |  |  |  |
| 1 | Ath-AT2G29530.3 |  | Vvi-Vitvi06g04192\_t001 |  |  |  |  |  |  |  |
| 1 | Ath-AT2G29540.3 |  | Vvi-Vitvi06g01732\_t001 |  |  |  |  |  |  |  |
| 1 | Ath-AT2G29550.1 |  | Vvi-Vitvi06g00569\_t001 |  |  |  |  |  |  |  |
| 1 | Ath-AT2G29560.1 |  | Vvi-Vitvi06g00574\_t001 |  |  |  |  |  |  |  |
| 1 | Ath-AT2G29570.1 |  | Vvi-Vitvi06g00576\_t001 |  |  |  |  |  |  |  |
| 1 | Ath-AT2G29580.1 |  | Vvi-Vitvi06g04197\_t001 |  |  |  |  |  |  |  |
| 1 | Ath-AT2G29590.1 |  | Vvi-Vitvi06g00586\_t001 |  |  |  |  |  |  |  |
| 1 | Ath-AT2G29600.1 |  | | | |  |  |  |  |  |  |  |
| 1 | Ath-AT2G29605.1 |  | | | |  |  |  |  |  |  |  |
| 1 | Ath-AT2G29620.2 |  | Vvi-Vitvi06g00594\_t001 |  |  |  |  |  |  |  |
| 1 | Ath-AT2G29628.1 |  | | | |  |  |  |  |  |  |  |
| 1 | Ath-AT2G29630.3 |  | Vvi-Vitvi06g01739\_t001 |  |  |  |  |  |  |  |
| 1 | Ath-AT2G29640.1 |  | Vvi-Vitvi06g00600\_t001 |  |  |  |  |  |  |  |
| 1 | Ath-AT2G29650.1 |  | Vvi-Vitvi06g00606\_t001 |  |  |  |  |  |  |  |
| 1 | Ath-AT2G29654.1 |  | | | |  |  |  |  |  |  |  |
| 1 | Ath-AT2G29660.1 |  | Vvi-Vitvi06g00607\_t001 |  |  |  |  |  |  |  |
| 1 | Ath-AT2G29670.1 |  | Vvi-Vitvi06g00613\_t001 |  |  |  |  |  |  |  |
| 1 | Ath-AT2G29679.1 |  | | | |  |  |  |  |  |  |  |
| 1 | Ath-AT2G29680.1 |  | Vvi-Vitvi06g00614\_t001 |  |  |  |  |  |  |  |
| 1 | Ath-AT2G29690.1 |  | Vvi-Vitvi06g00617\_t001 |  |  |  |  |  |  |  |
| 1 | Ath-AT2G29700.1 |  | Vvi-Vitvi06g00619\_t001 |  |  |  |  |  |  |  |
| 1 | Ath-AT2G29710.1 |  | Vvi-Vitvi06g00621\_t001 |  |  |  |  |  |  |  |
| 1 | Ath-AT2G29720.1 |  | | | |  |  |  |  |  |  |  |
| 1 | Ath-AT2G29730.1 |  | | | |  |  |  |  |  |  |  |
| 1 | Ath-AT2G29735.1 |  | | | |  |  |  |  |  |  |  |
| 1 | Ath-AT2G29740.1 |  | Vvi-Vitvi06g04208\_t001 |  |  |  |  |  |  |  |
| 1 | Ath-AT2G29750.1 |  | | | |  |  |  |  |  |  |  |
| 1 | Ath-AT2G29760.1 |  | Vvi-Vitvi06g04209\_t001 |  |  |  |  |  |  |  |
| 1 | Ath-AT2G29770.1 |  | | | |  |  |  |  |  |  |  |
| 1 | Ath-AT2G29780.1 |  | | | |  |  |  |  |  |  |  |
| 1 | Ath-AT2G29790.1 |  | | | |  |  |  |  |  |  |  |
| 1 | Ath-AT2G29800.1 |  | | | |  |  |  |  |  |  |  |
| 1 | Ath-AT2G29810.1 |  | | | |  |  |  |  |  |  |  |
| 1 | Ath-AT2G29820.1 |  | | | |  |  |  |  |  |  |  |
| 1 | Ath-AT2G29830.1 |  | | | |  |  |  |  |  |  |  |
| 1 | Ath-AT2G29840.1 |  | | | |  |  |  |  |  |  |  |
| 1 | Ath-AT2G29850.1 |  | | | |  |  |  |  |  |  |  |
| 1 | Ath-AT2G29860.1 |  | | | |  |  |  |  |  |  |  |
| 1 | Ath-AT2G29870.1 |  | | | |  |  |  |  |  |  |  |
| 1 | Ath-AT2G29880.1 |  | | | |  |  |  |  |  |  |  |
| 1 | Ath-AT2G29890.3 |  | Vvi-Vitvi06g00631\_t001 |  |  |  |  |  |  |  |
| 1 | Ath-AT2G29900.1 |  | | | |  |  |  |  |  |  |  |
| 1 | Ath-AT2G29910.6 |  | | | |  |  |  |  |  |  |  |
| 1 | Ath-AT2G29920.1 |  | | | |  |  |  |  |  |  |  |
| 1 | Ath-AT2G29925.1 |  | | | |  |  |  |  |  |  |  |
| 1 | Ath-AT2G29930.1 |  | | | |  |  |  |  |  |  |  |
| 1 | Ath-AT2G29940.1 |  | Vvi-Vitvi06g04214\_t001 |  |  |  |  |  |  |  |
| 2 | Ath-AT2G29950.1 |  | Vvi-Vitvi06g00642\_t001 |  | Vvi-Vitvi13g01892\_t001 |  |  |  |  |  |  |
| 2 | Ath-AT2G29960.1 |  | Vvi-Vitvi06g00643\_t001 |  | Vvi-Vitvi13g00046\_t001 |  |  |  |  |  |  |
| 2 | Ath-AT2G29970.1 |  | Vvi-Vitvi06g00652\_t001 |  | Vvi-Vitvi13g00053\_t001 |  |  |  |  |  |  |
| 2 | Ath-AT2G29980.1 |  | Vvi-Vitvi06g00654\_t001 |  | Vvi-Vitvi13g00060\_t001 |  |  |  |  |  |  |
| 2 | Ath-AT2G29990.1 |  | Vvi-Vitvi06g01751\_t001 |  | | | |  |  |  |  |  |  |
| 2 | Ath-AT2G29995.1 |  | | | |  | | | |  |  |  |  |  |  |
| 2 | Ath-AT2G30000.1 |  | Vvi-Vitvi06g00658\_t001 |  | | | |  |  |  |  |  |  |
| 2 | Ath-AT2G30010.2 |  | Vvi-Vitvi06g00665\_t001 |  | | | |  |  |  |  |  |  |
| 2 | Ath-AT2G30020.1 |  | Vvi-Vitvi06g00667\_t001 |  | Vvi-Vitvi13g00071\_t001 |  |  |  |  |  |  |
| 2 | Ath-AT2G30040.1 |  | Vvi-Vitvi06g00668\_t001 |  | | | |  |  |  |  |  |  |
| 2 | Ath-AT2G30050.1 |  | | | |  | | | |  |  |  |  |  |  |
| 2 | Ath-AT2G30060.1 |  | Vvi-Vitvi06g00671\_t001 |  | | | |  |  |  |  |  |  |
| 2 | Ath-AT2G30070.1 |  | Vvi-Vitvi06g00672\_t001 |  | | | |  |  |  |  |  |  |
| 2 | Ath-AT2G30080.1 |  | Vvi-Vitvi06g00678\_t001 |  | Vvi-Vitvi13g01860\_t001 |  |  |  |  |  |  |
| 2 | Ath-AT2G30090.1 |  | Vvi-Vitvi06g00679\_t001 |  | | | |  |  |  |  |  |  |
| 2 | Ath-AT2G30100.1 |  | Vvi-Vitvi06g00687\_t001 |  | | | |  |  |  |  |  |  |
| 2 | Ath-AT2G30105.1 |  | Vvi-Vitvi06g00691\_t001 |  | | | |  |  |  |  |  |  |
| 2 | Ath-AT2G30110.1 |  | Vvi-Vitvi06g00692\_t003 |  | | | |  |  |  |  |  |  |
| 2 | Ath-AT2G30120.5 |  | Vvi-Vitvi06g00702\_t001 |  | | | |  |  |  |  |  |  |
| 2 | Ath-AT2G30130.1 |  | Vvi-Vitvi06g00706\_t001 |  | Vvi-Vitvi13g00085\_t001 |  |  |  |  |  |  |
| 1 | Ath-AT2G30140.1 |  | Vvi-Vitvi06g00708\_t001 |  |  |  |  |  |  |  |
| 1 | Ath-AT2G30150.2 |  | | | |  |  |  |  |  |  |  |
| 1 | Ath-AT2G30160.1 |  | Vvi-Vitvi06g00720\_t001 |  |  |  |  |  |  |  |
| 1 | Ath-AT2G30170.1 |  | Vvi-Vitvi06g00721\_t001 |  |  |  |  |  |  |  |
| 1 | Ath-AT2G30200.1 |  | Vvi-Vitvi06g00723\_t001 |  |  |  |  |  |  |  |
| 1 | Ath-AT2G30210.1 |  | Vvi-Vitvi06g00728\_t001 |  |  |  |  |  |  |  |
| 1 | Ath-AT2G30220.1 |  | Vvi-Vitvi06g00734\_t001 |  |  |  |  |  |  |  |
| 1 | Ath-AT2G30230.1 |  | Vvi-Vitvi06g00736\_t001 |  |  |  |  |  |  |  |
| 1 | Ath-AT2G30240.1 |  | Vvi-Vitvi06g00737\_t001 |  |  |  |  |  |  |  |
| 1 | Ath-AT2G30250.1 |  | Vvi-Vitvi06g00741\_t001 |  |  |  |  |  |  |  |
| 1 | Ath-AT2G30260.1 |  | Vvi-Vitvi06g00743\_t001 |  |  |  |  |  |  |  |
| 1 | Ath-AT2G30270.1 |  | Vvi-Vitvi06g01772\_t001 |  |  |  |  |  |  |  |
| 1 | Ath-AT2G30280.1 |  | Vvi-Vitvi06g01773\_t002 |  |  |  |  |  |  |  |
| 1 | Ath-AT2G30290.2 |  | | | |  |  |  |  |  |  |  |
| 1 | Ath-AT2G30300.1 |  | Vvi-Vitvi06g00761\_t001 |  |  |  |  |  |  |  |
| 1 | Ath-AT2G30310.1 |  | | | |  |  |  |  |  |  |  |
| 1 | Ath-AT2G30320.1 |  | Vvi-Vitvi06g00764\_t001 |  |  |  |  |  |  |  |
| 1 | Ath-AT2G30330.1 |  | Vvi-Vitvi06g00770\_t001 |  |  |  |  |  |  |  |
| 1 | Ath-AT2G30340.2 |  | Vvi-Vitvi06g00772\_t001 |  |  |  |  |  |  |  |
| 1 | Ath-AT2G30350.2 |  | Vvi-Vitvi06g00776\_t001 |  |  |  |  |  |  |  |
| 1 | Ath-AT2G30360.1 |  | Vvi-Vitvi06g00779\_t001 |  |  |  |  |  |  |  |
| 1 | Ath-AT2G30370.1 |  | Vvi-Vitvi06g00780\_t001 |  |  |  |  |  |  |  |
| 1 | Ath-AT2G30380.1 |  | Vvi-Vitvi06g00781\_t001 |  |  |  |  |  |  |  |
| 1 | Ath-AT2G30385.1 |  | | | |  |  |  |  |  |  |  |
| 1 | Ath-AT2G30390.2 |  | | | |  |  |  |  |  |  |  |
| 1 | Ath-AT2G30395.1 |  | Vvi-Vitvi06g00782\_t001 |  |  |  |  |  |  |  |
| 1 | Ath-AT2G30400.1 |  | Vvi-Vitvi06g00783\_t001 |  |  |  |  |  |  |  |
| 1 | Ath-AT2G30410.1 |  | | | |  |  |  |  |  |  |  |
| 1 | Ath-AT2G30420.1 |  | | | |  |  |  |  |  |  |  |
| 1 | Ath-AT2G30424.1 |  | | | |  |  |  |  |  |  |  |
| 1 | Ath-AT2G30430.1 |  | | | |  |  |  |  |  |  |  |
| 1 | Ath-AT2G30432.1 |  | | | |  |  |  |  |  |  |  |
| 1 | Ath-AT2G30440.1 |  | | | |  |  |  |  |  |  |  |
| 1 | Ath-AT2G30460.2 |  | Vvi-Vitvi06g00789\_t002 |  |  |  |  |  |  |  |
| 1 | Ath-AT2G30470.1 |  | Vvi-Vitvi06g04253\_t001 |  |  |  |  |  |  |  |
| 1 | Ath-AT2G30480.5 |  | Vvi-Vitvi06g01781\_t001 |  |  |  |  |  |  |  |
| 1 | Ath-AT2G30490.1 |  | Vvi-Vitvi06g00803\_t001 |  |  |  |  |  |  |  |
| 1 | Ath-AT2G30500.1 |  | Vvi-Vitvi06g00805\_t001 |  |  |  |  |  |  |  |
| 1 | Ath-AT2G30505.1 |  | | | |  |  |  |  |  |  |  |
| 1 | Ath-AT2G30520.1 |  | Vvi-Vitvi06g00814\_t001 |  |  |  |  |  |  |  |
| 1 | Ath-AT2G30530.1 |  | Vvi-Vitvi06g00827\_t001 |  |  |  |  |  |  |  |
| 0 | Ath-AT2G30540.1 |  |  |  |  |  |  |  |  |
| 1 | Ath-AT2G30550.2 |  | Vvi-Vitvi07g00039\_t001 |  |  |  |  |  |  |  |
| 1 | Ath-AT2G30560.1 |  | | | |  |  |  |  |  |  |  |
| 1 | Ath-AT2G30570.1 |  | Vvi-Vitvi07g00035\_t001 |  |  |  |  |  |  |  |
| 1 | Ath-AT2G30575.1 |  | Vvi-Vitvi07g00034\_t001 |  |  |  |  |  |  |  |
| 1 | Ath-AT2G30580.2 |  | Vvi-Vitvi07g00031\_t001 |  |  |  |  |  |  |  |
| 1 | Ath-AT2G30590.1 |  | Vvi-Vitvi07g00026\_t001 |  |  |  |  |  |  |  |
| 1 | Ath-AT2G30600.5 |  | | | |  |  |  |  |  |  |  |
| 1 | Ath-AT2G30615.1 |  | | | |  |  |  |  |  |  |  |
| 1 | Ath-AT2G30620.1 |  | Vvi-Vitvi07g00024\_t001 |  |  |  |  |  |  |  |
| 1 | Ath-AT2G30630.2 |  | Vvi-Vitvi07g00021\_t001 |  |  |  |  |  |  |  |
| 1 | Ath-AT2G30650.1 |  | | | |  |  |  |  |  |  |  |
| 1 | Ath-AT2G30660.1 |  | | | |  |  |  |  |  |  |  |
| 1 | Ath-AT2G30670.1 |  | | | |  |  |  |  |  |  |  |
| 1 | Ath-AT2G30680.1 |  | | | |  |  |  |  |  |  |  |
| 1 | Ath-AT2G30690.1 |  | Vvi-Vitvi07g00013\_t001 |  |  |  |  |  |  |  |
| 0 | Ath-AT2G30695.3 |  |  |  |  |  |  |  |  |
| 0 | Ath-AT2G30700.1 |  |  |  |  |  |  |  |  |
| 0 | Ath-AT2G30710.1 |  |  |  |  |  |  |  |  |
| 0 | Ath-AT2G30720.1 |  |  |  |  |  |  |  |  |
| 0 | Ath-AT2G30730.2 |  |  |  |  |  |  |  |  |
| 0 | Ath-AT2G30740.3 |  |  |  |  |  |  |  |  |
| 0 | Ath-AT2G30750.1 |  |  |  |  |  |  |  |  |
| 0 | Ath-AT2G30760.1 |  |  |  |  |  |  |  |  |
| 0 | Ath-AT2G30766.1 |  |  |  |  |  |  |  |  |
| 0 | Ath-AT2G30770.1 |  |  |  |  |  |  |  |  |
| 0 | Ath-AT2G30780.1 |  |  |  |  |  |  |  |  |
| 1 | Ath-AT2G30790.1 |  | Vvi-Vitvi12g00092\_t001 |  |  |  |  |  |  |  |
| 1 | Ath-AT2G30800.1 |  | Vvi-Vitvi12g00088\_t001 |  |  |  |  |  |  |  |
| 1 | Ath-AT2G30810.1 |  | | | |  |  |  |  |  |  |  |
| 1 | Ath-AT2G30820.1 |  | Vvi-Vitvi12g00082\_t001 |  |  |  |  |  |  |  |
| 1 | Ath-AT2G30830.1 |  | | | |  |  |  |  |  |  |  |
| 1 | Ath-AT2G30840.1 |  | | | |  |  |  |  |  |  |  |
| 1 | Ath-AT2G30860.1 |  | Vvi-Vitvi12g00080\_t001 |  |  |  |  |  |  |  |
| 1 | Ath-AT2G30870.1 |  | | | |  |  |  |  |  |  |  |
| 1 | Ath-AT2G30880.1 |  | Vvi-Vitvi12g00075\_t001 |  |  |  |  |  |  |  |
| 1 | Ath-AT2G30890.1 |  | Vvi-Vitvi12g00062\_t001 |  |  |  |  |  |  |  |
| 1 | Ath-AT2G30900.1 |  | | | |  |  |  |  |  |  |  |
| 1 | Ath-AT2G30910.3 |  | | | |  |  |  |  |  |  |  |
| 1 | Ath-AT2G30920.1 |  | | | |  |  |  |  |  |  |  |
| 1 | Ath-AT2G30925.1 |  | | | |  |  |  |  |  |  |  |
| 1 | Ath-AT2G30930.1 |  | | | |  |  |  |  |  |  |  |
| 1 | Ath-AT2G30933.1 |  | Vvi-Vitvi12g00038\_t001 |  |  |  |  |  |  |  |
| 0 | Ath-AT2G30940.2 |  |  |  |  |  |  |  |  |
| 0 | Ath-AT2G30942.1 |  |  |  |  |  |  |  |  |
| 0 | Ath-AT2G30950.4 |  |  |  |  |  |  |  |  |
| 0 | Ath-AT2G30960.1 |  |  |  |  |  |  |  |  |
| 0 | Ath-AT2G30970.1 |  |  |  |  |  |  |  |  |
| 0 | Ath-AT2G30980.1 |  |  |  |  |  |  |  |  |
| 0 | Ath-AT2G30985.1 |  |  |  |  |  |  |  |  |
| 0 | Ath-AT2G30990.2 |  |  |  |  |  |  |  |  |
| 0 | Ath-AT2G31005.1 |  |  |  |  |  |  |  |  |
| 0 | Ath-AT2G31010.2 |  |  |  |  |  |  |  |  |
| 0 | Ath-AT2G31020.1 |  |  |  |  |  |  |  |  |
| 0 | Ath-AT2G31018.1 |  |  |  |  |  |  |  |  |
| 0 | Ath-AT2G31030.1 |  |  |  |  |  |  |  |  |
| 0 | Ath-AT2G31040.1 |  |  |  |  |  |  |  |  |
| 0 | Ath-AT2G31035.1 |  |  |  |  |  |  |  |  |
| 0 | Ath-AT2G31050.1 |  |  |  |  |  |  |  |  |
| 0 | Ath-AT2G31060.3 |  |  |  |  |  |  |  |  |
| 0 | Ath-AT2G31070.1 |  |  |  |  |  |  |  |  |
| 0 | Ath-AT2G31081.1 |  |  |  |  |  |  |  |  |
| 0 | Ath-AT2G31082.1 |  |  |  |  |  |  |  |  |
| 0 | Ath-AT2G31083.2 |  |  |  |  |  |  |  |  |
| 0 | Ath-AT2G31085.1 |  |  |  |  |  |  |  |  |
| 0 | Ath-AT2G31090.1 |  |  |  |  |  |  |  |  |
| 0 | Ath-AT2G31110.2 |  |  |  |  |  |  |  |  |
| 0 | Ath-AT2G31130.2 |  |  |  |  |  |  |  |  |
| 1 | Ath-AT2G31140.1 |  | Vvi-Vitvi07g00612\_t001 |  |  |  |  |  |  |  |
| 1 | Ath-AT2G31141.1 |  | | | |  |  |  |  |  |  |  |
| 1 | Ath-AT2G31150.1 |  | | | |  |  |  |  |  |  |  |
| 1 | Ath-AT2G31160.1 |  | | | |  |  |  |  |  |  |  |
| 1 | Ath-AT2G31170.1 |  | | | |  |  |  |  |  |  |  |
| 1 | Ath-AT2G31180.1 |  | Vvi-Vitvi07g00598\_t001 |  |  |  |  |  |  |  |
| 1 | Ath-AT2G31190.1 |  | Vvi-Vitvi07g00597\_t001 |  |  |  |  |  |  |  |
| 1 | Ath-AT2G31200.1 |  | Vvi-Vitvi07g00595\_t002 |  |  |  |  |  |  |  |
| 1 | Ath-AT2G31210.1 |  | | | |  |  |  |  |  |  |  |
| 1 | Ath-AT2G31215.1 |  | | | |  |  |  |  |  |  |  |
| 1 | Ath-AT2G31220.2 |  | | | |  |  |  |  |  |  |  |
| 1 | Ath-AT2G31230.1 |  | Vvi-Vitvi07g02063\_t001 |  |  |  |  |  |  |  |
| 1 | Ath-AT2G31240.1 |  | Vvi-Vitvi07g00581\_t001 |  |  |  |  |  |  |  |
| 1 | Ath-AT2G31250.1 |  | | | |  |  |  |  |  |  |  |
| 1 | Ath-AT2G31260.1 |  | Vvi-Vitvi07g00580\_t002 |  |  |  |  |  |  |  |
| 1 | Ath-AT2G31265.1 |  | | | |  |  |  |  |  |  |  |
| 1 | Ath-AT2G31270.1 |  | Vvi-Vitvi07g00579\_t001 |  |  |  |  |  |  |  |
| 1 | Ath-AT2G31280.3 |  | Vvi-Vitvi07g00577\_t001 |  |  |  |  |  |  |  |
| 1 | Ath-AT2G31290.2 |  | | | |  |  |  |  |  |  |  |
| 1 | Ath-AT2G31300.2 |  | | | |  |  |  |  |  |  |  |
| 1 | Ath-AT2G31305.1 |  | | | |  |  |  |  |  |  |  |
| 1 | Ath-AT2G31310.1 |  | Vvi-Vitvi07g00572\_t001 |  |  |  |  |  |  |  |
| 0 | Ath-AT2G31320.1 |  |  |  |  |  |  |  |  |
| 0 | Ath-AT2G31335.1 |  |  |  |  |  |  |  |  |
| 0 | Ath-AT2G31340.1 |  |  |  |  |  |  |  |  |
| 0 | Ath-AT2G31345.1 |  |  |  |  |  |  |  |  |
| 0 | Ath-AT2G31350.1 |  |  |  |  |  |  |  |  |
| 0 | Ath-AT2G31360.1 |  |  |  |  |  |  |  |  |
| 0 | Ath-AT2G31370.6 |  |  |  |  |  |  |  |  |
| 0 | Ath-AT2G31380.1 |  |  |  |  |  |  |  |  |
| 0 | Ath-AT2G31390.1 |  |  |  |  |  |  |  |  |
| 0 | Ath-AT2G31400.1 |  |  |  |  |  |  |  |  |
| 0 | Ath-AT2G31410.1 |  |  |  |  |  |  |  |  |
| 0 | Ath-AT2G31420.1 |  |  |  |  |  |  |  |  |
| 0 | Ath-AT2G31425.1 |  |  |  |  |  |  |  |  |
| 0 | Ath-AT2G31430.1 |  |  |  |  |  |  |  |  |
| 0 | Ath-AT2G31432.2 |  |  |  |  |  |  |  |  |
| 0 | Ath-AT2G31440.1 |  |  |  |  |  |  |  |  |
| 1 | Ath-AT2G31450.1 |  | Vvi-Vitvi05g01484\_t001 |  |  |  |  |  |  |  |
| 1 | Ath-AT2G31460.1 |  | | | |  |  |  |  |  |  |  |
| 1 | Ath-AT2G31470.1 |  | | | |  |  |  |  |  |  |  |
| 1 | Ath-AT2G31480.1 |  | Vvi-Vitvi05g01479\_t001 |  |  |  |  |  |  |  |
| 1 | Ath-AT2G31490.1 |  | | | |  |  |  |  |  |  |  |
| 1 | Ath-AT2G31500.1 |  | Vvi-Vitvi05g01477\_t001 |  |  |  |  |  |  |  |
| 1 | Ath-AT2G31510.1 |  | Vvi-Vitvi05g01475\_t001 |  |  |  |  |  |  |  |
| 1 | Ath-AT2G31530.1 |  | | | |  |  |  |  |  |  |  |
| 1 | Ath-AT2G31540.1 |  | | | |  |  |  |  |  |  |  |
| 1 | Ath-AT2G31550.1 |  | | | |  |  |  |  |  |  |  |
| 1 | Ath-AT2G31560.1 |  | Vvi-Vitvi05g01470\_t001 |  |  |  |  |  |  |  |
| 1 | Ath-AT2G31570.1 |  | Vvi-Vitvi05g01469\_t001 |  |  |  |  |  |  |  |
| 1 | Ath-AT2G31580.1 |  | | | |  |  |  |  |  |  |  |
| 1 | Ath-AT2G31590.1 |  | | | |  |  |  |  |  |  |  |
| 1 | Ath-AT2G31600.1 |  | Vvi-Vitvi05g01465\_t001 |  |  |  |  |  |  |  |
| 0 | Ath-AT2G31610.1 |  |  |  |  |  |  |  |  |
| 0 | Ath-AT2G31620.1 |  |  |  |  |  |  |  |  |
| 0 | Ath-AT2G31650.1 |  |  |  |  |  |  |  |  |
| 0 | Ath-AT2G31660.1 |  |  |  |  |  |  |  |  |
| 0 | Ath-AT2G31670.1 |  |  |  |  |  |  |  |  |
| 0 | Ath-AT2G31680.1 |  |  |  |  |  |  |  |  |
| 0 | Ath-AT2G31690.1 |  |  |  |  |  |  |  |  |
| 0 | Ath-AT2G31700.1 |  |  |  |  |  |  |  |  |
| 0 | Ath-AT2G31710.1 |  |  |  |  |  |  |  |  |
| 0 | Ath-AT2G31720.1 |  |  |  |  |  |  |  |  |
| 0 | Ath-AT2G31725.1 |  |  |  |  |  |  |  |  |
| 0 | Ath-AT2G31730.1 |  |  |  |  |  |  |  |  |
| 0 | Ath-AT2G31740.1 |  |  |  |  |  |  |  |  |
| 0 | Ath-AT2G31750.2 |  |  |  |  |  |  |  |  |
| 0 | Ath-AT2G31760.1 |  |  |  |  |  |  |  |  |
| 0 | Ath-AT2G31770.1 |  |  |  |  |  |  |  |  |
| 0 | Ath-AT2G31780.1 |  |  |  |  |  |  |  |  |
| 0 | Ath-AT2G31790.1 |  |  |  |  |  |  |  |  |
| 0 | Ath-AT2G31800.1 |  |  |  |  |  |  |  |  |
| 0 | Ath-AT2G31810.1 |  |  |  |  |  |  |  |  |
| 0 | Ath-AT2G31820.1 |  |  |  |  |  |  |  |  |
| 0 | Ath-AT2G31830.2 |  |  |  |  |  |  |  |  |
| 0 | Ath-AT2G31840.2 |  |  |  |  |  |  |  |  |
| 0 | Ath-AT2G31850.1 |  |  |  |  |  |  |  |  |
| 0 | Ath-AT2G31862.1 |  |  |  |  |  |  |  |  |
| 0 | Ath-AT2G31865.2 |  |  |  |  |  |  |  |  |
| 0 | Ath-AT2G31870.1 |  |  |  |  |  |  |  |  |
| 0 | Ath-AT2G31880.1 |  |  |  |  |  |  |  |  |
| 0 | Ath-AT2G31890.1 |  |  |  |  |  |  |  |  |
| 0 | Ath-AT2G31900.2 |  |  |  |  |  |  |  |  |
| 0 | Ath-AT2G31910.1 |  |  |  |  |  |  |  |  |
| 0 | Ath-AT2G31920.1 |  |  |  |  |  |  |  |  |
| 0 | Ath-AT2G31930.1 |  |  |  |  |  |  |  |  |
| 0 | Ath-AT2G31940.1 |  |  |  |  |  |  |  |  |
| 0 | Ath-AT2G31945.1 |  |  |  |  |  |  |  |  |
| 0 | Ath-AT2G31953.1 |  |  |  |  |  |  |  |  |
| 0 | Ath-AT2G31955.1 |  |  |  |  |  |  |  |  |
| 0 | Ath-AT2G31957.1 |  |  |  |  |  |  |  |  |
| 0 | Ath-AT2G31960.2 |  |  |  |  |  |  |  |  |
| 0 | Ath-AT2G31970.1 |  |  |  |  |  |  |  |  |
| 0 | Ath-AT2G31980.1 |  |  |  |  |  |  |  |  |
| 0 | Ath-AT2G31981.1 |  |  |  |  |  |  |  |  |
| 0 | Ath-AT2G31990.2 |  |  |  |  |  |  |  |  |
| 0 | Ath-AT2G31985.1 |  |  |  |  |  |  |  |  |
| 0 | Ath-AT2G32000.1 |  |  |  |  |  |  |  |  |
| 1 | Ath-AT2G32010.1 |  | Vvi-Vitvi05g00533\_t001 |  |  |  |  |  |  |  |
| 1 | Ath-AT2G32020.1 |  | Vvi-Vitvi05g00523\_t001 |  |  |  |  |  |  |  |
| 1 | Ath-AT2G32030.1 |  | | | |  |  |  |  |  |  |  |
| 1 | Ath-AT2G32040.1 |  | Vvi-Vitvi05g00521\_t001 |  |  |  |  |  |  |  |
| 1 | Ath-AT2G32050.1 |  | | | |  |  |  |  |  |  |  |
| 1 | Ath-AT2G32060.2 |  | | | |  |  |  |  |  |  |  |
| 1 | Ath-AT2G32070.1 |  | | | |  |  |  |  |  |  |  |
| 1 | Ath-AT2G32080.1 |  | | | |  |  |  |  |  |  |  |
| 1 | Ath-AT2G32090.1 |  | Vvi-Vitvi05g00512\_t001 |  |  |  |  |  |  |  |
| 1 | Ath-AT2G32100.1 |  | Vvi-Vitvi05g00509\_t001 |  |  |  |  |  |  |  |
| 1 | Ath-AT2G32120.2 |  | Vvi-Vitvi05g00486\_t001 |  |  |  |  |  |  |  |
| 1 | Ath-AT2G32130.1 |  | | | |  |  |  |  |  |  |  |
| 1 | Ath-AT2G32140.1 |  | | | |  |  |  |  |  |  |  |
| 1 | Ath-AT2G32150.1 |  | Vvi-Vitvi05g00475\_t001 |  |  |  |  |  |  |  |
| 1 | Ath-AT2G32160.3 |  | | | |  |  |  |  |  |  |  |
| 1 | Ath-AT2G32170.2 |  | | | |  |  |  |  |  |  |  |
| 1 | Ath-AT2G32180.1 |  | | | |  |  |  |  |  |  |  |
| 1 | Ath-AT2G32190.1 |  | | | |  |  |  |  |  |  |  |
| 1 | Ath-AT2G32200.2 |  | | | |  |  |  |  |  |  |  |
| 1 | Ath-AT2G32210.1 |  | | | |  |  |  |  |  |  |  |
| 1 | Ath-AT2G32220.1 |  | | | |  |  |  |  |  |  |  |
| 1 | Ath-AT2G32230.1 |  | | | |  |  |  |  |  |  |  |
| 1 | Ath-AT2G32235.1 |  | | | |  |  |  |  |  |  |  |
| 1 | Ath-AT2G32240.1 |  | | | |  |  |  |  |  |  |  |
| 1 | Ath-AT2G32250.3 |  | | | |  |  |  |  |  |  |  |
| 1 | Ath-AT2G32260.1 |  | | | |  |  |  |  |  |  |  |
| 1 | Ath-AT2G32270.1 |  | | | |  |  |  |  |  |  |  |
| 1 | Ath-AT2G32275.1 |  | | | |  |  |  |  |  |  |  |
| 2 | Ath-AT2G32280.1 |  | | | |  | Vvi-Vitvi12g00566\_t001 |  |  |  |  |  |  |
| 2 | Ath-AT2G32290.1 |  | | | |  | Vvi-Vitvi12g00558\_t001 |  |  |  |  |  |  |
| 2 | Ath-AT2G32295.1 |  | | | |  | Vvi-Vitvi12g00541\_t001 |  |  |  |  |  |  |
| 2 | Ath-AT2G32300.2 |  | Vvi-Vitvi05g00467\_t001 |  | | | |  |  |  |  |  |  |
| 1 | Ath-AT2G32310.1 |  |  |  | | | |  |  |  |  |  |  |
| 1 | Ath-AT2G32320.2 |  |  |  | | | |  |  |  |  |  |  |
| 1 | Ath-AT2G32340.1 |  |  |  | Vvi-Vitvi12g00534\_t002 |  |  |  |  |  |  |
| 1 | Ath-AT2G32350.1 |  |  |  | Vvi-Vitvi12g00531\_t001 |  |  |  |  |  |  |
| 1 | Ath-AT2G32360.1 |  |  |  | Vvi-Vitvi12g04160\_t001 |  |  |  |  |  |  |
| 1 | Ath-AT2G32370.1 |  |  |  | Vvi-Vitvi12g00522\_t001 |  |  |  |  |  |  |
| 1 | Ath-AT2G32380.1 |  |  |  | Vvi-Vitvi12g00520\_t001 |  |  |  |  |  |  |
| 1 | Ath-AT2G32390.5 |  |  |  | Vvi-Vitvi12g00517\_t001 |  |  |  |  |  |  |
| 1 | Ath-AT2G32400.1 |  |  |  | Vvi-Vitvi12g00516\_t001 |  |  |  |  |  |  |
| 1 | Ath-AT2G32410.1 |  | Vvi-Vitvi12g00365\_t002 |  |  |  |  |  |  |  |
| 1 | Ath-AT2G32415.3 |  | Vvi-Vitvi12g00367\_t001 |  |  |  |  |  |  |  |
| 1 | Ath-AT2G32430.1 |  | Vvi-Vitvi12g00369\_t001 |  |  |  |  |  |  |  |
| 1 | Ath-AT2G32440.1 |  | | | |  |  |  |  |  |  |  |
| 1 | Ath-AT2G32450.1 |  | | | |  |  |  |  |  |  |  |
| 1 | Ath-AT2G32460.1 |  | Vvi-Vitvi12g00376\_t001 |  |  |  |  |  |  |  |
| 1 | Ath-AT2G32470.1 |  | | | |  |  |  |  |  |  |  |
| 1 | Ath-AT2G32480.1 |  | Vvi-Vitvi12g00377\_t001 |  |  |  |  |  |  |  |
| 1 | Ath-AT2G32487.2 |  | | | |  |  |  |  |  |  |  |
| 1 | Ath-AT2G32500.2 |  | | | |  |  |  |  |  |  |  |
| 1 | Ath-AT2G32510.1 |  | Vvi-Vitvi12g00387\_t001 |  |  |  |  |  |  |  |
| 1 | Ath-AT2G32520.2 |  | Vvi-Vitvi12g02368\_t001 |  |  |  |  |  |  |  |
| 1 | Ath-AT2G32530.1 |  | Vvi-Vitvi12g02369\_t001 |  |  |  |  |  |  |  |
| 1 | Ath-AT2G32540.1 |  | | | |  |  |  |  |  |  |  |
| 1 | Ath-AT2G32550.2 |  | Vvi-Vitvi12g00402\_t001 |  |  |  |  |  |  |  |
| 1 | Ath-AT2G32560.1 |  | | | |  |  |  |  |  |  |  |
| 1 | Ath-AT2G32580.1 |  | Vvi-Vitvi12g00404\_t001 |  |  |  |  |  |  |  |
| 1 | Ath-AT2G32590.1 |  | Vvi-Vitvi12g00406\_t001 |  |  |  |  |  |  |  |
| 1 | Ath-AT2G32600.1 |  | | | |  |  |  |  |  |  |  |
| 1 | Ath-AT2G32610.1 |  | | | |  |  |  |  |  |  |  |
| 1 | Ath-AT2G32620.1 |  | | | |  |  |  |  |  |  |  |
| 1 | Ath-AT2G32630.1 |  | | | |  |  |  |  |  |  |  |
| 1 | Ath-AT2G32640.1 |  | Vvi-Vitvi12g00420\_t001 |  |  |  |  |  |  |  |
| 1 | Ath-AT2G32650.1 |  | Vvi-Vitvi12g00421\_t001 |  |  |  |  |  |  |  |
| 0 | Ath-AT2G32645.1 |  |  |  |  |  |  |  |  |
| 0 | Ath-AT2G32660.2 |  |  |  |  |  |  |  |  |
| 0 | Ath-AT2G32670.1 |  |  |  |  |  |  |  |  |
| 0 | Ath-AT2G32680.1 |  |  |  |  |  |  |  |  |
| 0 | Ath-AT2G32690.1 |  |  |  |  |  |  |  |  |
| 0 | Ath-AT2G32700.7 |  |  |  |  |  |  |  |  |
| 0 | Ath-AT2G32710.1 |  |  |  |  |  |  |  |  |
| 0 | Ath-AT2G32720.1 |  |  |  |  |  |  |  |  |
| 0 | Ath-AT2G32730.1 |  |  |  |  |  |  |  |  |
| 0 | Ath-AT2G32740.1 |  |  |  |  |  |  |  |  |
| 0 | Ath-AT2G32750.2 |  |  |  |  |  |  |  |  |
| 0 | Ath-AT2G32760.1 |  |  |  |  |  |  |  |  |
| 0 | Ath-AT2G32765.1 |  |  |  |  |  |  |  |  |
| 0 | Ath-AT2G32770.3 |  |  |  |  |  |  |  |  |
| 0 | Ath-AT2G32780.1 |  |  |  |  |  |  |  |  |
| 0 | Ath-AT2G32785.1 |  |  |  |  |  |  |  |  |
| 0 | Ath-AT2G32788.1 |  |  |  |  |  |  |  |  |
| 0 | Ath-AT2G32790.1 |  |  |  |  |  |  |  |  |
| 0 | Ath-AT2G32800.1 |  |  |  |  |  |  |  |  |
| 0 | Ath-AT2G32810.1 |  |  |  |  |  |  |  |  |
| 0 | Ath-AT2G32820.1 |  |  |  |  |  |  |  |  |
| 0 | Ath-AT2G32830.1 |  |  |  |  |  |  |  |  |
| 0 | Ath-AT2G32835.1 |  |  |  |  |  |  |  |  |
| 0 | Ath-AT2G32840.1 |  |  |  |  |  |  |  |  |
| 0 | Ath-AT2G32850.2 |  |  |  |  |  |  |  |  |
| 0 | Ath-AT2G32860.2 |  |  |  |  |  |  |  |  |
| 0 | Ath-AT2G32870.1 |  |  |  |  |  |  |  |  |
| 0 | Ath-AT2G32880.1 |  |  |  |  |  |  |  |  |
| 0 | Ath-AT2G32885.1 |  |  |  |  |  |  |  |  |
| 0 | Ath-AT2G32890.1 |  |  |  |  |  |  |  |  |
| 1 | Ath-AT2G32900.1 |  | Vvi-Vitvi12g00454\_t001 |  |  |  |  |  |  |  |
| 1 | Ath-AT2G32905.1 |  | | | |  |  |  |  |  |  |  |
| 1 | Ath-AT2G32910.1 |  | | | |  |  |  |  |  |  |  |
| 1 | Ath-AT2G32920.1 |  | Vvi-Vitvi12g00453\_t001 |  |  |  |  |  |  |  |
| 1 | Ath-AT2G32930.2 |  | Vvi-Vitvi12g00449\_t001 |  |  |  |  |  |  |  |
| 1 | Ath-AT2G32940.1 |  | Vvi-Vitvi12g00448\_t001 |  |  |  |  |  |  |  |
| 1 | Ath-AT2G32950.1 |  | Vvi-Vitvi12g00447\_t001 |  |  |  |  |  |  |  |
| 1 | Ath-AT2G32960.1 |  | Vvi-Vitvi12g00446\_t001 |  |  |  |  |  |  |  |
| 1 | Ath-AT2G32970.2 |  | Vvi-Vitvi12g00438\_t001 |  |  |  |  |  |  |  |
| 1 | Ath-AT2G32980.1 |  | Vvi-Vitvi12g00437\_t001 |  |  |  |  |  |  |  |
| 1 | Ath-AT2G32990.1 |  | Vvi-Vitvi12g00430\_t001 |  |  |  |  |  |  |  |
| 0 | Ath-AT2G33000.1 |  |  |  |  |  |  |  |  |
| 0 | Ath-AT2G33010.1 |  |  |  |  |  |  |  |  |
| 0 | Ath-AT2G33020.1 |  |  |  |  |  |  |  |  |
| 0 | Ath-AT2G33030.1 |  |  |  |  |  |  |  |  |
| 0 | Ath-AT2G33040.1 |  |  |  |  |  |  |  |  |
| 0 | Ath-AT2G33050.1 |  |  |  |  |  |  |  |  |
| 0 | Ath-AT2G33060.1 |  |  |  |  |  |  |  |  |
| 0 | Ath-AT2G33070.3 |  |  |  |  |  |  |  |  |
| 0 | Ath-AT2G33080.1 |  |  |  |  |  |  |  |  |
| 0 | Ath-AT2G33090.1 |  |  |  |  |  |  |  |  |
| 0 | Ath-AT2G33100.1 |  |  |  |  |  |  |  |  |
| 0 | Ath-AT2G33110.1 |  |  |  |  |  |  |  |  |
| 0 | Ath-AT2G33120.2 |  |  |  |  |  |  |  |  |
| 0 | Ath-AT2G33130.1 |  |  |  |  |  |  |  |  |
| 1 | Ath-AT2G33150.1 |  | Vvi-Vitvi05g00966\_t001 |  |  |  |  |  |  |  |
| 1 | Ath-AT2G33160.1 |  | | | |  |  |  |  |  |  |  |
| 1 | Ath-AT2G33170.1 |  | Vvi-Vitvi05g00939\_t001 |  |  |  |  |  |  |  |
| 1 | Ath-AT2G33175.1 |  | | | |  |  |  |  |  |  |  |
| 1 | Ath-AT2G33180.1 |  | | | |  |  |  |  |  |  |  |
| 1 | Ath-AT2G33190.1 |  | | | |  |  |  |  |  |  |  |
| 1 | Ath-AT2G33200.1 |  | | | |  |  |  |  |  |  |  |
| 1 | Ath-AT2G33205.5 |  | Vvi-Vitvi05g00928\_t001 |  |  |  |  |  |  |  |
| 1 | Ath-AT2G33210.1 |  | Vvi-Vitvi05g00924\_t001 |  |  |  |  |  |  |  |
| 1 | Ath-AT2G33220.2 |  | | | |  |  |  |  |  |  |  |
| 1 | Ath-AT2G33230.1 |  | Vvi-Vitvi05g01998\_t001 |  |  |  |  |  |  |  |
| 1 | Ath-AT2G33233.1 |  | | | |  |  |  |  |  |  |  |
| 1 | Ath-AT2G33240.1 |  | | | |  |  |  |  |  |  |  |
| 1 | Ath-AT2G33250.1 |  | Vvi-Vitvi05g00875\_t001 |  |  |  |  |  |  |  |
| 1 | Ath-AT2G33255.1 |  | Vvi-Vitvi05g00867\_t001 |  |  |  |  |  |  |  |
| 1 | Ath-AT2G33260.2 |  | | | |  |  |  |  |  |  |  |
| 1 | Ath-AT2G33270.1 |  | | | |  |  |  |  |  |  |  |
| 1 | Ath-AT2G33280.1 |  | Vvi-Vitvi05g04223\_t001 |  |  |  |  |  |  |  |
| 1 | Ath-AT2G33290.2 |  | Vvi-Vitvi05g00855\_t001 |  |  |  |  |  |  |  |
| 1 | Ath-AT2G33300.1 |  | | | |  |  |  |  |  |  |  |
| 1 | Ath-AT2G33310.2 |  | Vvi-Vitvi05g00838\_t001 |  |  |  |  |  |  |  |
| 1 | Ath-AT2G33320.1 |  | Vvi-Vitvi05g00837\_t001 |  |  |  |  |  |  |  |
| 1 | Ath-AT2G33330.1 |  | Vvi-Vitvi05g00828\_t001 |  |  |  |  |  |  |  |
| 1 | Ath-AT2G33340.1 |  | Vvi-Vitvi05g00825\_t001 |  |  |  |  |  |  |  |
| 1 | Ath-AT2G33350.5 |  | Vvi-Vitvi05g00823\_t001 |  |  |  |  |  |  |  |
| 1 | Ath-AT2G33360.1 |  | Vvi-Vitvi05g00822\_t001 |  |  |  |  |  |  |  |
| 1 | Ath-AT2G33370.1 |  | Vvi-Vitvi05g04210\_t001 |  |  |  |  |  |  |  |
| 1 | Ath-AT2G33380.1 |  | Vvi-Vitvi05g00819\_t001 |  |  |  |  |  |  |  |
| 1 | Ath-AT2G33385.2 |  | Vvi-Vitvi05g01981\_t001 |  |  |  |  |  |  |  |
| 1 | Ath-AT2G33390.1 |  | Vvi-Vitvi05g04209\_t001 |  |  |  |  |  |  |  |
| 1 | Ath-AT2G33400.1 |  | Vvi-Vitvi05g00815\_t001 |  |  |  |  |  |  |  |
| 1 | Ath-AT2G33410.1 |  | Vvi-Vitvi05g00812\_t002 |  |  |  |  |  |  |  |
| 1 | Ath-AT2G33420.1 |  | Vvi-Vitvi05g00803\_t001 |  |  |  |  |  |  |  |
| 1 | Ath-AT2G33435.1 |  | Vvi-Vitvi05g00796\_t001 |  |  |  |  |  |  |  |
| 1 | Ath-AT2G33450.1 |  | Vvi-Vitvi05g00795\_t001 |  |  |  |  |  |  |  |
| 1 | Ath-AT2G33460.1 |  | Vvi-Vitvi05g01965\_t001 |  |  |  |  |  |  |  |
| 1 | Ath-AT2G33470.1 |  | Vvi-Vitvi05g00779\_t001 |  |  |  |  |  |  |  |
| 1 | Ath-AT2G33480.1 |  | Vvi-Vitvi01g01722\_t001 |  |  |  |  |  |  |  |
| 1 | Ath-AT2G33490.1 |  | Vvi-Vitvi01g01726\_t002 |  |  |  |  |  |  |  |
| 1 | Ath-AT2G33500.1 |  | Vvi-Vitvi01g01729\_t001 |  |  |  |  |  |  |  |
| 1 | Ath-AT2G33510.2 |  | Vvi-Vitvi01g01733\_t001 |  |  |  |  |  |  |  |
| 1 | Ath-AT2G33520.1 |  | | | |  |  |  |  |  |  |  |
| 1 | Ath-AT2G33530.1 |  | | | |  |  |  |  |  |  |  |
| 1 | Ath-AT2G33540.1 |  | Vvi-Vitvi01g01749\_t001 |  |  |  |  |  |  |  |
| 1 | Ath-AT2G33550.1 |  | Vvi-Vitvi01g02298\_t001 |  |  |  |  |  |  |  |
| 2 | Ath-AT2G33560.2 |  | | | |  | Vvi-Vitvi18g00034\_t001 |  |  |  |  |  |  |
| 2 | Ath-AT2G33570.1 |  | | | |  | | | |  |  |  |  |  |  |
| 2 | Ath-AT2G33580.1 |  | | | |  | | | |  |  |  |  |  |  |
| 2 | Ath-AT2G33585.1 |  | | | |  | Vvi-Vitvi18g04010\_t001 |  |  |  |  |  |  |
| 2 | Ath-AT2G33590.1 |  | | | |  | Vvi-Vitvi18g02476\_t001 |  |  |  |  |  |  |
| 2 | Ath-AT2G33600.1 |  | | | |  | | | |  |  |  |  |  |  |
| 2 | Ath-AT2G33610.1 |  | | | |  | Vvi-Vitvi18g00045\_t001 |  |  |  |  |  |  |
| 2 | Ath-AT2G33620.4 |  | | | |  | Vvi-Vitvi18g00047\_t004 |  |  |  |  |  |  |
| 2 | Ath-AT2G33630.1 |  | | | |  | Vvi-Vitvi18g00048\_t001 |  |  |  |  |  |  |
| 2 | Ath-AT2G33640.1 |  | | | |  | | | |  |  |  |  |  |  |
| 2 | Ath-AT2G33655.1 |  | | | |  | | | |  |  |  |  |  |  |
| 2 | Ath-AT2G33670.1 |  | | | |  | | | |  |  |  |  |  |  |
| 2 | Ath-AT2G33680.5 |  | | | |  | | | |  |  |  |  |  |  |
| 2 | Ath-AT2G33685.1 |  | | | |  | | | |  |  |  |  |  |  |
| 2 | Ath-AT2G33690.1 |  | | | |  | Vvi-Vitvi18g00053\_t001 |  |  |  |  |  |  |
| 2 | Ath-AT2G33700.1 |  | | | |  | Vvi-Vitvi18g00072\_t005 |  |  |  |  |  |  |
| 1 | Ath-AT2G33705.1 |  | | | |  |  |  |  |  |  |  |
| 1 | Ath-AT2G33710.2 |  | Vvi-Vitvi01g01826\_t001 |  |  |  |  |  |  |  |
| 0 | Ath-AT2G33720.1 |  |  |  |  |  |  |  |  |
| 0 | Ath-AT2G33730.1 |  |  |  |  |  |  |  |  |
| 0 | Ath-AT2G33735.1 |  |  |  |  |  |  |  |  |
| 0 | Ath-AT2G33740.2 |  |  |  |  |  |  |  |  |
| 0 | Ath-AT2G33750.1 |  |  |  |  |  |  |  |  |
| 1 | Ath-AT2G33760.1 |  | Vvi-Vitvi10g00465\_t001 |  |  |  |  |  |  |  |
| 1 | Ath-AT2G33770.1 |  | Vvi-Vitvi10g01764\_t001 |  |  |  |  |  |  |  |
| 1 | Ath-AT2G33775.1 |  | | | |  |  |  |  |  |  |  |
| 1 | Ath-AT2G33780.1 |  | | | |  |  |  |  |  |  |  |
| 1 | Ath-AT2G33790.1 |  | Vvi-Vitvi10g02256\_t001 |  |  |  |  |  |  |  |
| 1 | Ath-AT2G33793.1 |  | | | |  |  |  |  |  |  |  |
| 1 | Ath-AT2G33796.1 |  | | | |  |  |  |  |  |  |  |
| 1 | Ath-AT2G33800.1 |  | Vvi-Vitvi10g01767\_t001 |  |  |  |  |  |  |  |
| 1 | Ath-AT2G33810.1 |  | Vvi-Vitvi10g04328\_t002 |  |  |  |  |  |  |  |
| 1 | Ath-AT2G33820.3 |  | Vvi-Vitvi10g00482\_t001 |  |  |  |  |  |  |  |
| 1 | Ath-AT2G33830.2 |  | Vvi-Vitvi10g00485\_t003 |  |  |  |  |  |  |  |
| 1 | Ath-AT2G33835.1 |  | Vvi-Vitvi10g00486\_t001 |  |  |  |  |  |  |  |
| 1 | Ath-AT2G33840.1 |  | | | |  |  |  |  |  |  |  |
| 1 | Ath-AT2G33845.1 |  | Vvi-Vitvi10g00492\_t001 |  |  |  |  |  |  |  |
| 1 | Ath-AT2G33847.1 |  | Vvi-Vitvi10g01769\_t004 |  |  |  |  |  |  |  |
| 1 | Ath-AT2G33850.1 |  | | | |  |  |  |  |  |  |  |
| 1 | Ath-AT2G33855.1 |  | | | |  |  |  |  |  |  |  |
| 1 | Ath-AT2G33860.1 |  | Vvi-Vitvi10g00510\_t002 |  |  |  |  |  |  |  |
| 1 | Ath-AT2G33870.1 |  | Vvi-Vitvi10g01781\_t001 |  |  |  |  |  |  |  |
| 1 | Ath-AT2G33880.2 |  | Vvi-Vitvi10g00519\_t001 |  |  |  |  |  |  |  |
| 1 | Ath-AT2G33980.3 |  | | | |  |  |  |  |  |  |  |
| 1 | Ath-AT2G33990.1 |  | | | |  |  |  |  |  |  |  |
| 1 | Ath-AT2G34000.1 |  | | | |  |  |  |  |  |  |  |
| 1 | Ath-AT2G34010.1 |  | | | |  |  |  |  |  |  |  |
| 1 | Ath-AT2G34020.2 |  | | | |  |  |  |  |  |  |  |
| 1 | Ath-AT2G34030.1 |  | | | |  |  |  |  |  |  |  |
| 1 | Ath-AT2G34040.1 |  | | | |  |  |  |  |  |  |  |
| 1 | Ath-AT2G34050.1 |  | | | |  |  |  |  |  |  |  |
| 1 | Ath-AT2G34060.1 |  | Vvi-Vitvi10g00527\_t001 |  |  |  |  |  |  |  |
| 2 | Ath-AT2G34070.1 |  | | | |  | Vvi-Vitvi10g00564\_t001 |  |  |  |  |  |  |
| 2 | Ath-AT2G34110.1 |  | | | |  | | | |  |  |  |  |  |  |
| 2 | Ath-AT2G34080.1 |  | | | |  | | | |  |  |  |  |  |  |
| 2 | Ath-AT2G34090.4 |  | | | |  | Vvi-Vitvi10g00568\_t001 |  |  |  |  |  |  |
| 2 | Ath-AT2G34100.1 |  | | | |  | | | |  |  |  |  |  |  |
| 2 | Ath-AT2G34120.1 |  | | | |  | | | |  |  |  |  |  |  |
| 2 | Ath-AT2G34123.1 |  | | | |  | | | |  |  |  |  |  |  |
| 2 | Ath-AT2G34140.1 |  | | | |  | Vvi-Vitvi10g00581\_t001 |  |  |  |  |  |  |
| 2 | Ath-AT2G34150.2 |  | | | |  | Vvi-Vitvi10g00582\_t001 |  |  |  |  |  |  |
| 2 | Ath-AT2G34160.1 |  | | | |  | | | |  |  |  |  |  |  |
| 2 | Ath-AT2G34170.3 |  | | | |  | Vvi-Vitvi10g00592\_t001 |  |  |  |  |  |  |
| 2 | Ath-AT2G34180.1 |  | | | |  | Vvi-Vitvi10g00600\_t001 |  |  |  |  |  |  |
| 2 | Ath-AT2G34185.2 |  | | | |  | | | |  |  |  |  |  |  |
| 2 | Ath-AT2G34186.1 |  | | | |  | | | |  |  |  |  |  |  |
| 2 | Ath-AT2G34190.1 |  | | | |  | Vvi-Vitvi10g00602\_t001 |  |  |  |  |  |  |
| 2 | Ath-AT2G34200.1 |  | | | |  | Vvi-Vitvi10g00607\_t001 |  |  |  |  |  |  |
| 1 | Ath-AT2G34210.1 |  | | | |  |  |  |  |  |  |  |
| 1 | Ath-AT2G34220.2 |  | | | |  |  |  |  |  |  |  |
| 1 | Ath-AT2G34224.1 |  | | | |  |  |  |  |  |  |  |
| 1 | Ath-AT2G34230.1 |  | | | |  |  |  |  |  |  |  |
| 1 | Ath-AT2G34238.1 |  | | | |  |  |  |  |  |  |  |
| 1 | Ath-AT2G34240.1 |  | | | |  |  |  |  |  |  |  |
| 1 | Ath-AT2G34250.1 |  | Vvi-Vitvi10g00531\_t001 |  |  |  |  |  |  |  |
| 0 | Ath-AT2G34260.1 |  |  |  |  |  |  |  |  |
| 0 | Ath-AT2G34270.1 |  |  |  |  |  |  |  |  |
| 0 | Ath-AT2G34280.1 |  |  |  |  |  |  |  |  |
| 0 | Ath-AT2G34290.1 |  |  |  |  |  |  |  |  |
| 0 | Ath-AT2G34300.1 |  |  |  |  |  |  |  |  |
| 0 | Ath-AT2G34310.3 |  |  |  |  |  |  |  |  |
| 0 | Ath-AT2G34315.1 |  |  |  |  |  |  |  |  |
| 0 | Ath-AT2G34317.1 |  |  |  |  |  |  |  |  |
| 0 | Ath-AT2G34320.1 |  |  |  |  |  |  |  |  |
| 0 | Ath-AT2G34325.1 |  |  |  |  |  |  |  |  |
| 0 | Ath-AT2G34330.1 |  |  |  |  |  |  |  |  |
| 0 | Ath-AT2G34340.1 |  |  |  |  |  |  |  |  |
| 0 | Ath-AT2G34350.1 |  |  |  |  |  |  |  |  |
| 0 | Ath-AT2G34355.1 |  |  |  |  |  |  |  |  |
| 0 | Ath-AT2G34357.1 |  |  |  |  |  |  |  |  |
| 0 | Ath-AT2G34360.1 |  |  |  |  |  |  |  |  |
| 0 | Ath-AT2G34370.1 |  |  |  |  |  |  |  |  |
| 0 | Ath-AT2G34380.1 |  |  |  |  |  |  |  |  |
| 0 | Ath-AT2G34390.1 |  |  |  |  |  |  |  |  |
| 0 | Ath-AT2G34400.1 |  |  |  |  |  |  |  |  |
| 1 | Ath-AT2G34410.2 |  | Vvi-Vitvi10g00733\_t001 |  |  |  |  |  |  |  |
| 1 | Ath-AT2G34420.1 |  | Vvi-Vitvi10g01839\_t001 |  |  |  |  |  |  |  |
| 1 | Ath-AT2G34430.1 |  | | | |  |  |  |  |  |  |  |
| 1 | Ath-AT2G34440.1 |  | Vvi-Vitvi10g01588\_t001 |  |  |  |  |  |  |  |
| 1 | Ath-AT2G34450.2 |  | Vvi-Vitvi10g01844\_t001 |  |  |  |  |  |  |  |
| 1 | Ath-AT2G34460.1 |  | Vvi-Vitvi10g00755\_t001 |  |  |  |  |  |  |  |
| 1 | Ath-AT2G34470.2 |  | Vvi-Vitvi10g00757\_t001 |  |  |  |  |  |  |  |
| 1 | Ath-AT2G34480.2 |  | Vvi-Vitvi10g00761\_t003 |  |  |  |  |  |  |  |
| 1 | Ath-AT2G34490.1 |  | Vvi-Vitvi10g00764\_t001 |  |  |  |  |  |  |  |
| 1 | Ath-AT2G34500.1 |  | | | |  |  |  |  |  |  |  |
| 1 | Ath-AT2G34510.1 |  | Vvi-Vitvi10g00774\_t001 |  |  |  |  |  |  |  |
| 1 | Ath-AT2G34520.1 |  | | | |  |  |  |  |  |  |  |
| 1 | Ath-AT2G34530.3 |  | Vvi-Vitvi10g01857\_t001 |  |  |  |  |  |  |  |
| 1 | Ath-AT2G34540.2 |  | | | |  |  |  |  |  |  |  |
| 1 | Ath-AT2G34555.1 |  | Vvi-Vitvi10g00796\_t001 |  |  |  |  |  |  |  |
| 1 | Ath-AT2G34560.2 |  | Vvi-Vitvi10g00797\_t002 |  |  |  |  |  |  |  |
| 1 | Ath-AT2G34570.1 |  | | | |  |  |  |  |  |  |  |
| 1 | Ath-AT2G34580.1 |  | Vvi-Vitvi10g00809\_t001 |  |  |  |  |  |  |  |
| 1 | Ath-AT2G34585.1 |  | | | |  |  |  |  |  |  |  |
| 1 | Ath-AT2G34590.1 |  | Vvi-Vitvi10g04440\_t001 |  |  |  |  |  |  |  |
| 1 | Ath-AT2G34600.1 |  | Vvi-Vitvi10g00826\_t001 |  |  |  |  |  |  |  |
| 0 | Ath-AT2G34610.1 |  |  |  |  |  |  |  |  |
| 0 | Ath-AT2G34620.1 |  |  |  |  |  |  |  |  |
| 0 | Ath-AT2G34630.2 |  |  |  |  |  |  |  |  |
| 0 | Ath-AT2G34640.1 |  |  |  |  |  |  |  |  |
| 1 | Ath-AT2G34650.1 |  | Vvi-Vitvi10g00879\_t001 |  |  |  |  |  |  |  |
| 1 | Ath-AT2G34655.1 |  | | | |  |  |  |  |  |  |  |
| 1 | Ath-AT2G34660.2 |  | Vvi-Vitvi10g00886\_t001 |  |  |  |  |  |  |  |
| 1 | Ath-AT2G34670.2 |  | Vvi-Vitvi10g00889\_t001 |  |  |  |  |  |  |  |
| 1 | Ath-AT2G34680.2 |  | Vvi-Vitvi10g00905\_t001 |  |  |  |  |  |  |  |
| 1 | Ath-AT2G34690.1 |  | Vvi-Vitvi10g00906\_t001 |  |  |  |  |  |  |  |
| 1 | Ath-AT2G34700.1 |  | | | |  |  |  |  |  |  |  |
| 1 | Ath-AT2G34710.1 |  | | | |  |  |  |  |  |  |  |
| 1 | Ath-AT2G34720.1 |  | Vvi-Vitvi10g00911\_t001 |  |  |  |  |  |  |  |
| 0 | Ath-AT2G34730.1 |  |  |  |  |  |  |  |  |
| 0 | Ath-AT2G34740.1 |  |  |  |  |  |  |  |  |
| 0 | Ath-AT2G34750.1 |  |  |  |  |  |  |  |  |
| 0 | Ath-AT2G34770.1 |  |  |  |  |  |  |  |  |
| 0 | Ath-AT2G34780.1 |  |  |  |  |  |  |  |  |
| 0 | Ath-AT2G34790.1 |  |  |  |  |  |  |  |  |
| 0 | Ath-AT2G34810.1 |  |  |  |  |  |  |  |  |
| 0 | Ath-AT2G34820.1 |  |  |  |  |  |  |  |  |
| 0 | Ath-AT2G34825.1 |  |  |  |  |  |  |  |  |
| 0 | Ath-AT2G34830.2 |  |  |  |  |  |  |  |  |
| 0 | Ath-AT2G34840.1 |  |  |  |  |  |  |  |  |
| 0 | Ath-AT2G34850.1 |  |  |  |  |  |  |  |  |
| 0 | Ath-AT2G34860.1 |  |  |  |  |  |  |  |  |
| 0 | Ath-AT2G34870.1 |  |  |  |  |  |  |  |  |
| 0 | Ath-AT2G34880.1 |  |  |  |  |  |  |  |  |
| 0 | Ath-AT2G34890.1 |  |  |  |  |  |  |  |  |
| 0 | Ath-AT2G34900.1 |  |  |  |  |  |  |  |  |
| 0 | Ath-AT2G34910.1 |  |  |  |  |  |  |  |  |
| 0 | Ath-AT2G34920.2 |  |  |  |  |  |  |  |  |
| 0 | Ath-AT2G34925.1 |  |  |  |  |  |  |  |  |
| 0 | Ath-AT2G34930.1 |  |  |  |  |  |  |  |  |
| 1 | Ath-AT2G34940.1 |  | Vvi-Vitvi01g01855\_t001 |  |  |  |  |  |  |  |
| 1 | Ath-AT2G34960.1 |  | Vvi-Vitvi01g01859\_t001 |  |  |  |  |  |  |  |
| 1 | Ath-AT2G34970.1 |  | | | |  |  |  |  |  |  |  |
| 1 | Ath-AT2G34980.1 |  | Vvi-Vitvi01g00099\_t001 |  |  |  |  |  |  |  |
| 1 | Ath-AT2G34990.2 |  | | | |  |  |  |  |  |  |  |
| 2 | Ath-AT2G35000.1 |  | | | |  | Vvi-Vitvi02g00714\_t001 |  |  |  |  |  |  |
| 2 | Ath-AT2G35010.1 |  | Vvi-Vitvi01g01866\_t001 |  | | | |  |  |  |  |  |  |
| 2 | Ath-AT2G35020.1 |  | Vvi-Vitvi01g00112\_t001 |  | | | |  |  |  |  |  |  |
| 2 | Ath-AT2G35030.1 |  | | | |  | | | |  |  |  |  |  |  |
| 2 | Ath-AT2G35035.1 |  | Vvi-Vitvi01g00113\_t001 |  | | | |  |  |  |  |  |  |
| 2 | Ath-AT2G35040.1 |  | Vvi-Vitvi01g00115\_t001 |  | | | |  |  |  |  |  |  |
| 2 | Ath-AT2G35050.1 |  | Vvi-Vitvi01g00120\_t001 |  | | | |  |  |  |  |  |  |
| 2 | Ath-AT2G35060.2 |  | Vvi-Vitvi01g00121\_t001 |  | | | |  |  |  |  |  |  |
| 2 | Ath-AT2G35070.1 |  | | | |  | | | |  |  |  |  |  |  |
| 2 | Ath-AT2G35075.1 |  | | | |  | | | |  |  |  |  |  |  |
| 2 | Ath-AT2G35080.2 |  | | | |  | | | |  |  |  |  |  |  |
| 2 | Ath-AT2G35090.1 |  | | | |  | | | |  |  |  |  |  |  |
| 2 | Ath-AT2G35100.1 |  | Vvi-Vitvi01g00131\_t001 |  | | | |  |  |  |  |  |  |
| 2 | Ath-AT2G35110.1 |  | Vvi-Vitvi01g00134\_t001 |  | | | |  |  |  |  |  |  |
| 2 | Ath-AT2G35120.1 |  | | | |  | | | |  |  |  |  |  |  |
| 2 | Ath-AT2G35130.2 |  | | | |  | | | |  |  |  |  |  |  |
| 2 | Ath-AT2G35140.6 |  | | | |  | | | |  |  |  |  |  |  |
| 2 | Ath-AT2G35150.1 |  | | | |  | | | |  |  |  |  |  |  |
| 2 | Ath-AT2G35155.1 |  | Vvi-Vitvi01g00146\_t001 |  | | | |  |  |  |  |  |  |
| 2 | Ath-AT2G35160.1 |  | | | |  | | | |  |  |  |  |  |  |
| 2 | Ath-AT2G35170.1 |  | | | |  | | | |  |  |  |  |  |  |
| 2 | Ath-AT2G35190.1 |  | | | |  | | | |  |  |  |  |  |  |
| 2 | Ath-AT2G35200.1 |  | | | |  | Vvi-Vitvi02g00703\_t001 |  |  |  |  |  |  |
| 2 | Ath-AT2G35210.1 |  | | | |  | Vvi-Vitvi02g00699\_t001 |  |  |  |  |  |  |
| 2 | Ath-AT2G35215.1 |  | | | |  | Vvi-Vitvi02g01505\_t001 |  |  |  |  |  |  |
| 2 | Ath-AT2G35230.3 |  | | | |  | Vvi-Vitvi02g00696\_t001 |  |  |  |  |  |  |
| 2 | Ath-AT2G35240.1 |  | | | |  | | | |  |  |  |  |  |  |
| 2 | Ath-AT2G35250.1 |  | | | |  | | | |  |  |  |  |  |  |
| 2 | Ath-AT2G35260.1 |  | | | |  | Vvi-Vitvi02g00689\_t001 |  |  |  |  |  |  |
| 3 | Ath-AT2G35270.1 |  | | | |  | | | |  | Vvi-Vitvi02g00658\_t001 |  |  |  |  |  |
| 3 | Ath-AT2G35280.1 |  | | | |  | | | |  | | | |  |  |  |  |  |
| 3 | Ath-AT2G35290.1 |  | | | |  | | | |  | Vvi-Vitvi02g00659\_t001 |  |  |  |  |  |
| 3 | Ath-AT2G35300.1 |  | | | |  | | | |  | Vvi-Vitvi02g01498\_t001 |  |  |  |  |  |
| 3 | Ath-AT2G35310.1 |  | | | |  | | | |  | | | |  |  |  |  |  |
| 3 | Ath-AT2G35320.1 |  | | | |  | | | |  | Vvi-Vitvi02g00667\_t001 |  |  |  |  |  |
| 3 | Ath-AT2G35330.1 |  | | | |  | | | |  | Vvi-Vitvi02g00670\_t001 |  |  |  |  |  |
| 3 | Ath-AT2G35340.1 |  | | | |  | | | |  | | | |  |  |  |  |  |
| 3 | Ath-AT2G35343.1 |  | | | |  | | | |  | | | |  |  |  |  |  |
| 3 | Ath-AT2G35345.1 |  | | | |  | | | |  | | | |  |  |  |  |  |
| 3 | Ath-AT2G35350.1 |  | | | |  | Vvi-Vitvi02g00678\_t001 |  | Vvi-Vitvi02g00678\_t001 |  |  |  |  |  |
| 2 | Ath-AT2G35360.1 |  | | | |  |  |  | Vvi-Vitvi02g00679\_t002 |  |  |  |  |  |
| 2 | Ath-AT2G35370.1 |  | Vvi-Vitvi01g00157\_t001 |  |  |  | Vvi-Vitvi02g00680\_t001 |  |  |  |  |  |
| 1 | Ath-AT2G35380.1 |  |  |  |  |  | | | |  |  |  |  |  |
| 1 | Ath-AT2G35390.2 |  |  |  |  |  | | | |  |  |  |  |  |
| 1 | Ath-AT2G35410.1 |  |  |  |  |  | Vvi-Vitvi02g00706\_t001 |  |  |  |  |  |
| 1 | Ath-AT2G35420.1 |  |  |  |  |  | Vvi-Vitvi02g00714\_t001 |  |  |  |  |  |
| 1 | Ath-AT2G35430.1 |  |  |  |  |  | Vvi-Vitvi02g00715\_t001 |  |  |  |  |  |
| 1 | Ath-AT2G35450.1 |  |  |  |  |  | | | |  |  |  |  |  |
| 1 | Ath-AT2G35460.1 |  |  |  |  |  | | | |  |  |  |  |  |
| 1 | Ath-AT2G35470.1 |  |  |  |  |  | | | |  |  |  |  |  |
| 1 | Ath-AT2G35480.1 |  |  |  |  |  | | | |  |  |  |  |  |
| 1 | Ath-AT2G35490.1 |  |  |  |  |  | | | |  |  |  |  |  |
| 1 | Ath-AT2G35500.1 |  |  |  |  |  | Vvi-Vitvi02g00738\_t001 |  |  |  |  |  |
| 1 | Ath-AT2G35510.3 |  |  |  |  |  | Vvi-Vitvi02g00751\_t001 |  |  |  |  |  |
| 1 | Ath-AT2G35520.2 |  |  |  |  |  | Vvi-Vitvi02g00780\_t001 |  |  |  |  |  |
| 1 | Ath-AT2G35530.1 |  |  |  |  |  | Vvi-Vitvi02g00796\_t003 |  |  |  |  |  |
| 1 | Ath-AT2G35540.1 |  |  |  |  |  | Vvi-Vitvi02g00802\_t001 |  |  |  |  |  |
| 0 | Ath-AT2G35550.1 |  |  |  |  |  |  |  |  |
| 0 | Ath-AT2G35555.1 |  |  |  |  |  |  |  |  |
| 0 | Ath-AT2G35580.1 |  |  |  |  |  |  |  |  |
| 0 | Ath-AT2G35585.1 |  |  |  |  |  |  |  |  |
| 0 | Ath-AT2G35600.1 |  |  |  |  |  |  |  |  |
| 0 | Ath-AT2G35605.1 |  |  |  |  |  |  |  |  |
| 0 | Ath-AT2G35610.1 |  |  |  |  |  |  |  |  |
| 0 | Ath-AT2G35612.1 |  |  |  |  |  |  |  |  |
| 0 | Ath-AT2G35615.1 |  |  |  |  |  |  |  |  |
| 0 | Ath-AT2G35620.1 |  |  |  |  |  |  |  |  |
| 0 | Ath-AT2G35630.1 |  |  |  |  |  |  |  |  |
| 0 | Ath-AT2G35635.1 |  |  |  |  |  |  |  |  |
| 0 | Ath-AT2G35640.1 |  |  |  |  |  |  |  |  |
| 0 | Ath-AT2G35650.1 |  |  |  |  |  |  |  |  |
| 0 | Ath-AT2G35660.1 |  |  |  |  |  |  |  |  |
| 0 | Ath-AT2G35658.2 |  |  |  |  |  |  |  |  |
| 0 | Ath-AT2G35670.1 |  |  |  |  |  |  |  |  |
| 0 | Ath-AT2G35680.1 |  |  |  |  |  |  |  |  |
| 0 | Ath-AT2G35690.1 |  |  |  |  |  |  |  |  |
| 0 | Ath-AT2G35700.1 |  |  |  |  |  |  |  |  |
| 0 | Ath-AT2G35710.1 |  |  |  |  |  |  |  |  |
| 0 | Ath-AT2G35720.1 |  |  |  |  |  |  |  |  |
| 0 | Ath-AT2G35730.1 |  |  |  |  |  |  |  |  |
| 0 | Ath-AT2G35736.2 |  |  |  |  |  |  |  |  |
| 0 | Ath-AT2G35733.1 |  |  |  |  |  |  |  |  |
| 0 | Ath-AT2G35740.1 |  |  |  |  |  |  |  |  |
| 0 | Ath-AT2G35750.1 |  |  |  |  |  |  |  |  |
| 0 | Ath-AT2G35760.1 |  |  |  |  |  |  |  |  |
| 0 | Ath-AT2G35765.1 |  |  |  |  |  |  |  |  |
| 1 | Ath-AT2G35770.1 |  | Vvi-Vitvi13g00576\_t001 |  |  |  |  |  |  |  |
| 1 | Ath-AT2G35780.1 |  | Vvi-Vitvi13g00575\_t001 |  |  |  |  |  |  |  |
| 1 | Ath-AT2G35790.1 |  | Vvi-Vitvi13g00565\_t001 |  |  |  |  |  |  |  |
| 1 | Ath-AT2G35795.1 |  | Vvi-Vitvi13g02052\_t001 |  |  |  |  |  |  |  |
| 1 | Ath-AT2G35800.1 |  | Vvi-Vitvi13g00562\_t001 |  |  |  |  |  |  |  |
| 1 | Ath-AT2G35810.1 |  | Vvi-Vitvi13g00541\_t001 |  |  |  |  |  |  |  |
| 0 | Ath-AT2G35820.2 |  |  |  |  |  |  |  |  |
| 0 | Ath-AT2G35830.2 |  |  |  |  |  |  |  |  |
| 0 | Ath-AT2G35840.4 |  |  |  |  |  |  |  |  |
| 0 | Ath-AT2G35850.1 |  |  |  |  |  |  |  |  |
| 0 | Ath-AT2G35860.1 |  |  |  |  |  |  |  |  |
| 0 | Ath-AT2G35880.2 |  |  |  |  |  |  |  |  |
| 0 | Ath-AT2G35890.1 |  |  |  |  |  |  |  |  |
| 0 | Ath-AT2G35900.1 |  |  |  |  |  |  |  |  |
| 0 | Ath-AT2G35910.1 |  |  |  |  |  |  |  |  |
| 0 | Ath-AT2G35920.3 |  |  |  |  |  |  |  |  |
| 0 | Ath-AT2G35930.1 |  |  |  |  |  |  |  |  |
| 0 | Ath-AT2G35935.1 |  |  |  |  |  |  |  |  |
| 0 | Ath-AT2G35940.2 |  |  |  |  |  |  |  |  |
| 0 | Ath-AT2G35950.2 |  |  |  |  |  |  |  |  |
| 2 | Ath-AT2G35960.1 |  | Vvi-Vitvi08g01376\_t001 |  | Vvi-Vitvi06g01559\_t001 |  |  |  |  |  |  |
| 2 | Ath-AT2G35965.1 |  | | | |  | | | |  |  |  |  |  |  |
| 2 | Ath-AT2G35970.1 |  | | | |  | | | |  |  |  |  |  |  |
| 2 | Ath-AT2G35980.1 |  | Vvi-Vitvi08g01377\_t001 |  | | | |  |  |  |  |  |  |
| 2 | Ath-AT2G35990.1 |  | Vvi-Vitvi08g01385\_t001 |  | | | |  |  |  |  |  |  |
| 2 | Ath-AT2G36000.1 |  | Vvi-Vitvi08g01387\_t001 |  | | | |  |  |  |  |  |  |
| 2 | Ath-AT2G36010.2 |  | Vvi-Vitvi08g01388\_t001 |  | Vvi-Vitvi06g01552\_t001 |  |  |  |  |  |  |
| 2 | Ath-AT2G36020.1 |  | Vvi-Vitvi08g02251\_t001.1.6037826f |  | | | |  |  |  |  |  |  |
| 2 | Ath-AT2G36026.1 |  | Vvi-Vitvi08g01391\_t001 |  | Vvi-Vitvi06g01549\_t001 |  |  |  |  |  |  |
| 2 | Ath-AT2G36030.1 |  | | | |  | | | |  |  |  |  |  |  |
| 2 | Ath-AT2G36050.1 |  | Vvi-Vitvi08g01392\_t001 |  | Vvi-Vitvi06g01548\_t001 |  |  |  |  |  |  |
| 2 | Ath-AT2G36060.2 |  | Vvi-Vitvi08g01394\_t001 |  | Vvi-Vitvi06g01545\_t001 |  |  |  |  |  |  |
| 2 | Ath-AT2G36070.1 |  | | | |  | | | |  |  |  |  |  |  |
| 2 | Ath-AT2G36080.1 |  | Vvi-Vitvi08g01412\_t001 |  | | | |  |  |  |  |  |  |
| 2 | Ath-AT2G36090.1 |  | Vvi-Vitvi08g01416\_t001 |  | Vvi-Vitvi06g01530\_t001 |  |  |  |  |  |  |
| 2 | Ath-AT2G36100.1 |  | Vvi-Vitvi08g01418\_t001 |  | Vvi-Vitvi06g01522\_t001 |  |  |  |  |  |  |
| 2 | Ath-AT2G36110.1 |  | | | |  | | | |  |  |  |  |  |  |
| 2 | Ath-AT2G36120.1 |  | | | |  | | | |  |  |  |  |  |  |
| 3 | Ath-AT2G36130.1 |  | Vvi-Vitvi08g01421\_t001 |  | | | |  | Vvi-Vitvi06g04434\_t001 |  |  |  |  |  |
| 3 | Ath-AT2G36145.1 |  | Vvi-Vitvi08g02258\_t001 |  | | | |  | | | |  |  |  |  |  |
| 3 | Ath-AT2G36160.1 |  | Vvi-Vitvi08g04305\_t001 |  | Vvi-Vitvi06g04445\_t001 |  | | | |  |  |  |  |  |
| 2 | Ath-AT2G36170.1 |  | Vvi-Vitvi08g01432\_t001 |  |  |  | | | |  |  |  |  |  |
| 2 | Ath-AT2G36180.1 |  | | | |  |  |  | | | |  |  |  |  |  |
| 2 | Ath-AT2G36190.1 |  | Vvi-Vitvi08g01434\_t001 |  |  |  | | | |  |  |  |  |  |
| 2 | Ath-AT2G36200.2 |  | Vvi-Vitvi08g01436\_t001 |  |  |  | | | |  |  |  |  |  |
| 2 | Ath-AT2G36210.1 |  | Vvi-Vitvi08g01439\_t001 |  |  |  | | | |  |  |  |  |  |
| 2 | Ath-AT2G36220.1 |  | Vvi-Vitvi08g01452\_t001 |  |  |  | | | |  |  |  |  |  |
| 2 | Ath-AT2G36230.1 |  | Vvi-Vitvi08g01457\_t001 |  |  |  | | | |  |  |  |  |  |
| 2 | Ath-AT2G36240.1 |  | Vvi-Vitvi08g01458\_t001 |  |  |  | | | |  |  |  |  |  |
| 2 | Ath-AT2G36250.2 |  | Vvi-Vitvi08g01467\_t001 |  |  |  | | | |  |  |  |  |  |
| 2 | Ath-AT2G36255.1 |  | | | |  |  |  | | | |  |  |  |  |  |
| 2 | Ath-AT2G36260.1 |  | Vvi-Vitvi08g01468\_t001 |  |  |  | | | |  |  |  |  |  |
| 2 | Ath-AT2G36270.3 |  | Vvi-Vitvi08g01470\_t001 |  |  |  | | | |  |  |  |  |  |
| 2 | Ath-AT2G36290.1 |  | Vvi-Vitvi08g01472\_t001 |  |  |  | | | |  |  |  |  |  |
| 2 | Ath-AT2G36295.1 |  | Vvi-Vitvi08g04311\_t001 |  |  |  | | | |  |  |  |  |  |
| 2 | Ath-AT2G36300.1 |  | Vvi-Vitvi08g01474\_t001 |  |  |  | | | |  |  |  |  |  |
| 2 | Ath-AT2G36305.1 |  | Vvi-Vitvi08g01475\_t001 |  |  |  | | | |  |  |  |  |  |
| 2 | Ath-AT2G36310.1 |  | | | |  |  |  | | | |  |  |  |  |  |
| 2 | Ath-AT2G36320.1 |  | Vvi-Vitvi08g02270\_t001 |  |  |  | | | |  |  |  |  |  |
| 2 | Ath-AT2G36325.1 |  | | | |  |  |  | | | |  |  |  |  |  |
| 2 | Ath-AT2G36330.1 |  | | | |  |  |  | | | |  |  |  |  |  |
| 2 | Ath-AT2G36340.1 |  | | | |  |  |  | | | |  |  |  |  |  |
| 2 | Ath-AT2G36350.1 |  | Vvi-Vitvi08g01482\_t001 |  |  |  | Vvi-Vitvi06g01429\_t001 |  |  |  |  |  |
| 2 | Ath-AT2G36355.1 |  | | | |  |  |  | | | |  |  |  |  |  |
| 2 | Ath-AT2G36360.4 |  | | | |  |  |  | | | |  |  |  |  |  |
| 2 | Ath-AT2G36370.1 |  | | | |  |  |  | | | |  |  |  |  |  |
| 2 | Ath-AT2G36380.1 |  | Vvi-Vitvi08g01493\_t001 |  |  |  | Vvi-Vitvi06g01420\_t001 |  |  |  |  |  |
| 2 | Ath-AT2G36390.1 |  | Vvi-Vitvi08g01497\_t001 |  |  |  | | | |  |  |  |  |  |
| 2 | Ath-AT2G36400.1 |  | Vvi-Vitvi08g01498\_t001 |  |  |  | | | |  |  |  |  |  |
| 2 | Ath-AT2G36410.1 |  | Vvi-Vitvi08g01499\_t001 |  |  |  | Vvi-Vitvi06g01418\_t001 |  |  |  |  |  |
| 2 | Ath-AT2G36420.1 |  | Vvi-Vitvi08g01500\_t001 |  |  |  | | | |  |  |  |  |  |
| 2 | Ath-AT2G36430.1 |  | Vvi-Vitvi08g01502\_t001 |  |  |  | | | |  |  |  |  |  |
| 2 | Ath-AT2G36440.1 |  | | | |  |  |  | | | |  |  |  |  |  |
| 2 | Ath-AT2G36450.1 |  | Vvi-Vitvi08g04320\_t001 |  |  |  | | | |  |  |  |  |  |
| 2 | Ath-AT2G36460.1 |  | Vvi-Vitvi08g01506\_t001 |  |  |  | | | |  |  |  |  |  |
| 2 | Ath-AT2G36470.1 |  | Vvi-Vitvi08g01507\_t001 |  |  |  | Vvi-Vitvi06g01408\_t001 |  |  |  |  |  |
| 2 | Ath-AT2G36480.1 |  | Vvi-Vitvi08g01511\_t001 |  |  |  | | | |  |  |  |  |  |
| 2 | Ath-AT2G36490.1 |  | Vvi-Vitvi08g01515\_t001 |  |  |  | Vvi-Vitvi06g01402\_t001 |  |  |  |  |  |
| 1 | Ath-AT2G36500.1 |  | | | |  |  |  |  |  |  |  |
| 1 | Ath-AT2G36530.1 |  | Vvi-Vitvi08g01520\_t002 |  |  |  |  |  |  |  |
| 1 | Ath-AT2G36540.1 |  | Vvi-Vitvi08g01524\_t001 |  |  |  |  |  |  |  |
| 1 | Ath-AT2G36550.1 |  | | | |  |  |  |  |  |  |  |
| 1 | Ath-AT2G36560.1 |  | | | |  |  |  |  |  |  |  |
| 1 | Ath-AT2G36570.1 |  | Vvi-Vitvi08g01537\_t001 |  |  |  |  |  |  |  |
| 1 | Ath-AT2G36580.1 |  | Vvi-Vitvi08g01539\_t001 |  |  |  |  |  |  |  |
| 1 | Ath-AT2G36590.1 |  | | | |  |  |  |  |  |  |  |
| 1 | Ath-AT2G36610.1 |  | Vvi-Vitvi08g01543\_t001 |  |  |  |  |  |  |  |
| 1 | Ath-AT2G36620.1 |  | Vvi-Vitvi08g01544\_t001 |  |  |  |  |  |  |  |
| 1 | Ath-AT2G36630.1 |  | Vvi-Vitvi08g01546\_t001 |  |  |  |  |  |  |  |
| 1 | Ath-AT2G36640.1 |  | Vvi-Vitvi08g01547\_t001 |  |  |  |  |  |  |  |
| 1 | Ath-AT2G36650.1 |  | Vvi-Vitvi08g01555\_t001 |  |  |  |  |  |  |  |
| 1 | Ath-AT2G36660.1 |  | Vvi-Vitvi08g01561\_t001 |  |  |  |  |  |  |  |
| 1 | Ath-AT2G36670.1 |  | Vvi-Vitvi08g01562\_t001 |  |  |  |  |  |  |  |
| 1 | Ath-AT2G36680.1 |  | Vvi-Vitvi08g01568\_t001 |  |  |  |  |  |  |  |
| 1 | Ath-AT2G36690.1 |  | | | |  |  |  |  |  |  |  |
| 1 | Ath-AT2G36695.1 |  | | | |  |  |  |  |  |  |  |
| 1 | Ath-AT2G36700.1 |  | Vvi-Vitvi08g01570\_t001 |  |  |  |  |  |  |  |
| 1 | Ath-AT2G36710.1 |  | | | |  |  |  |  |  |  |  |
| 2 | Ath-AT2G36720.2 |  | Vvi-Vitvi08g01571\_t001 |  | Vvi-Vitvi06g01933\_t001 |  |  |  |  |  |  |
| 2 | Ath-AT2G36724.1 |  | | | |  | | | |  |  |  |  |  |  |
| 2 | Ath-AT2G36730.1 |  | | | |  | | | |  |  |  |  |  |  |
| 2 | Ath-AT2G36740.1 |  | | | |  | | | |  |  |  |  |  |  |
| 2 | Ath-AT2G36750.1 |  | Vvi-Vitvi08g01580\_t001 |  | | | |  |  |  |  |  |  |
| 2 | Ath-AT2G36760.1 |  | Vvi-Vitvi08g02288\_t001 |  | | | |  |  |  |  |  |  |
| 2 | Ath-AT2G36770.1 |  | | | |  | | | |  |  |  |  |  |  |
| 2 | Ath-AT2G36780.1 |  | | | |  | | | |  |  |  |  |  |  |
| 2 | Ath-AT2G36790.1 |  | | | |  | | | |  |  |  |  |  |  |
| 2 | Ath-AT2G36800.1 |  | | | |  | | | |  |  |  |  |  |  |
| 2 | Ath-AT2G36810.1 |  | Vvi-Vitvi08g01583\_t001 |  | | | |  |  |  |  |  |  |
| 2 | Ath-AT2G36815.1 |  | | | |  | | | |  |  |  |  |  |  |
| 2 | Ath-AT2G36830.1 |  | Vvi-Vitvi08g01602\_t001 |  | Vvi-Vitvi06g01346\_t001 |  |  |  |  |  |  |
| 2 | Ath-AT2G36835.1 |  | Vvi-Vitvi08g01608\_t001 |  | | | |  |  |  |  |  |  |
| 2 | Ath-AT2G36840.1 |  | Vvi-Vitvi08g01614\_t001 |  | | | |  |  |  |  |  |  |
| 2 | Ath-AT2G36850.1 |  | | | |  | | | |  |  |  |  |  |  |
| 2 | Ath-AT2G36854.1 |  | | | |  | | | |  |  |  |  |  |  |
| 2 | Ath-AT2G36870.1 |  | Vvi-Vitvi08g01617\_t001 |  | Vvi-Vitvi06g01329\_t001 |  |  |  |  |  |  |
| 2 | Ath-AT2G36880.1 |  | Vvi-Vitvi08g04349\_t001 |  | Vvi-Vitvi06g01325\_t001 |  |  |  |  |  |  |
| 2 | Ath-AT2G36885.1 |  | Vvi-Vitvi08g01621\_t001 |  | | | |  |  |  |  |  |  |
| 2 | Ath-AT2G36890.2 |  | Vvi-Vitvi08g01623\_t001 |  | Vvi-Vitvi06g01321\_t001 |  |  |  |  |  |  |
| 2 | Ath-AT2G36895.3 |  | Vvi-Vitvi08g01624\_t001 |  | | | |  |  |  |  |  |  |
| 2 | Ath-AT2G36900.1 |  | | | |  | | | |  |  |  |  |  |  |
| 2 | Ath-AT2G36910.1 |  | Vvi-Vitvi08g01627\_t001 |  | | | |  |  |  |  |  |  |
| 2 | Ath-AT2G36920.1 |  | | | |  | | | |  |  |  |  |  |  |
| 2 | Ath-AT2G36930.1 |  | Vvi-Vitvi08g04352\_t001 |  | | | |  |  |  |  |  |  |
| 2 | Ath-AT2G36950.1 |  | Vvi-Vitvi08g00992\_t001 |  | Vvi-Vitvi06g01315\_t001 |  |  |  |  |  |  |
| 2 | Ath-AT2G36960.3 |  | Vvi-Vitvi08g02136\_t001 |  | | | |  |  |  |  |  |  |
| 2 | Ath-AT2G36970.1 |  | Vvi-Vitvi08g01001\_t001 |  | Vvi-Vitvi06g01307\_t001 |  |  |  |  |  |  |
| 1 | Ath-AT2G36980.1 |  | | | |  |  |  |  |  |  |  |
| 1 | Ath-AT2G36985.1 |  | Vvi-Vitvi08g02137\_t001 |  |  |  |  |  |  |  |
| 1 | Ath-AT2G36990.1 |  | Vvi-Vitvi08g01008\_t001 |  |  |  |  |  |  |  |
| 1 | Ath-AT2G37000.1 |  | Vvi-Vitvi08g01011\_t001 |  |  |  |  |  |  |  |
| 1 | Ath-AT2G37010.1 |  | Vvi-Vitvi08g01013\_t001 |  |  |  |  |  |  |  |
| 1 | Ath-AT2G37020.3 |  | Vvi-Vitvi08g01014\_t001 |  |  |  |  |  |  |  |
| 1 | Ath-AT2G37025.1 |  | Vvi-Vitvi08g02140\_t003 |  |  |  |  |  |  |  |
| 1 | Ath-AT2G37030.1 |  | Vvi-Vitvi08g01016\_t001 |  |  |  |  |  |  |  |
| 1 | Ath-AT2G37035.1 |  | Vvi-Vitvi08g01018\_t001 |  |  |  |  |  |  |  |
| 1 | Ath-AT2G37040.1 |  | | | |  |  |  |  |  |  |  |
| 2 | Ath-AT2G37050.3 |  | | | |  | Vvi-Vitvi08g01142\_t003 |  |  |  |  |  |  |
| 2 | Ath-AT2G37060.1 |  | | | |  | | | |  |  |  |  |  |  |
| 3 | Ath-AT2G37070.1 |  | | | |  | | | |  | Vvi-Vitvi08g01077\_t001 |  |  |  |  |  |
| 3 | Ath-AT2G37080.2 |  | | | |  | | | |  | Vvi-Vitvi08g01075\_t003 |  |  |  |  |  |
| 3 | Ath-AT2G37090.1 |  | | | |  | | | |  | Vvi-Vitvi08g01070\_t001 |  |  |  |  |  |
| 3 | Ath-AT2G37100.1 |  | | | |  | | | |  | Vvi-Vitvi08g01061\_t001 |  |  |  |  |  |
| 3 | Ath-AT2G37110.1 |  | | | |  | | | |  | Vvi-Vitvi08g01060\_t002 |  |  |  |  |  |
| 3 | Ath-AT2G37120.1 |  | | | |  | | | |  | Vvi-Vitvi08g02161\_t003 |  |  |  |  |  |
| 3 | Ath-AT2G37125.1 |  | | | |  | | | |  | | | |  |  |  |  |  |
| 3 | Ath-AT2G37130.1 |  | | | |  | | | |  | Vvi-Vitvi08g01058\_t001 |  |  |  |  |  |
| 3 | Ath-AT2G37140.1 |  | | | |  | | | |  | | | |  |  |  |  |  |
| 3 | Ath-AT2G37150.3 |  | | | |  | | | |  | Vvi-Vitvi08g01054\_t001 |  |  |  |  |  |
| 3 | Ath-AT2G37160.2 |  | | | |  | | | |  | Vvi-Vitvi08g01044\_t001 |  |  |  |  |  |
| 3 | Ath-AT2G37170.2 |  | | | |  | | | |  | Vvi-Vitvi08g01038\_t001 |  |  |  |  |  |
| 3 | Ath-AT2G37180.1 |  | | | |  | | | |  | | | |  |  |  |  |  |
| 3 | Ath-AT2G37190.1 |  | | | |  | | | |  | Vvi-Vitvi08g01037\_t001 |  |  |  |  |  |
| 3 | Ath-AT2G37195.2 |  | Vvi-Vitvi08g02145\_t001 |  | | | |  | | | |  |  |  |  |  |
| 2 | Ath-AT2G37200.2 |  |  |  | | | |  | Vvi-Vitvi08g01035\_t001 |  |  |  |  |  |
| 2 | Ath-AT2G37210.2 |  |  |  | | | |  | Vvi-Vitvi08g01030\_t001 |  |  |  |  |  |
| 2 | Ath-AT2G37220.1 |  |  |  | | | |  | Vvi-Vitvi08g01027\_t001 |  |  |  |  |  |
| 2 | Ath-AT2G37230.1 |  |  |  | | | |  | Vvi-Vitvi08g01026\_t001 |  |  |  |  |  |
| 2 | Ath-AT2G37240.1 |  | Vvi-Vitvi08g01140\_t002 |  | | | |  |  |  |  |  |  |
| 3 | Ath-AT2G37250.1 |  | | | |  | | | |  | Vvi-Vitvi13g00202\_t001 |  |  |  |  |  |
| 3 | Ath-AT2G37260.1 |  | | | |  | Vvi-Vitvi08g01134\_t001 |  | | | |  |  |  |  |  |
| 3 | Ath-AT2G37270.2 |  | | | |  | | | |  | | | |  |  |  |  |  |
| 3 | Ath-AT2G37280.3 |  | | | |  | | | |  | | | |  |  |  |  |  |
| 3 | Ath-AT2G37290.2 |  | | | |  | Vvi-Vitvi08g01130\_t001 |  | Vvi-Vitvi13g00206\_t001 |  |  |  |  |  |
| 3 | Ath-AT2G37300.2 |  | | | |  | Vvi-Vitvi08g02176\_t001 |  | | | |  |  |  |  |  |
| 3 | Ath-AT2G37310.1 |  | | | |  | Vvi-Vitvi08g01122\_t001 |  | | | |  |  |  |  |  |
| 3 | Ath-AT2G37320.1 |  | | | |  | Vvi-Vitvi08g01119\_t001 |  | | | |  |  |  |  |  |
| 3 | Ath-AT2G37330.1 |  | | | |  | Vvi-Vitvi08g01117\_t001 |  | | | |  |  |  |  |  |
| 3 | Ath-AT2G37340.1 |  | | | |  | Vvi-Vitvi08g01113\_t001 |  | Vvi-Vitvi13g00214\_t001 |  |  |  |  |  |
| 3 | Ath-AT2G37360.1 |  | | | |  | Vvi-Vitvi08g01112\_t001 |  | Vvi-Vitvi13g00221\_t001 |  |  |  |  |  |
| 3 | Ath-AT2G37370.2 |  | | | |  | Vvi-Vitvi08g01107\_t001 |  | Vvi-Vitvi13g00227\_t002 |  |  |  |  |  |
| 3 | Ath-AT2G37380.1 |  | | | |  | Vvi-Vitvi08g01105\_t001 |  | Vvi-Vitvi13g01929\_t001 |  |  |  |  |  |
| 2 | Ath-AT2G37390.1 |  | | | |  | Vvi-Vitvi08g01097\_t001 |  |  |  |  |  |  |
| 2 | Ath-AT2G37400.1 |  | | | |  | Vvi-Vitvi08g01095\_t001 |  |  |  |  |  |  |
| 2 | Ath-AT2G37410.1 |  | | | |  | | | |  |  |  |  |  |  |
| 2 | Ath-AT2G37420.1 |  | | | |  | Vvi-Vitvi08g01087\_t001 |  |  |  |  |  |  |
| 2 | Ath-AT2G37430.1 |  | | | |  | | | |  |  |  |  |  |  |
| 2 | Ath-AT2G37435.1 |  | | | |  | | | |  |  |  |  |  |  |
| 2 | Ath-AT2G37440.1 |  | | | |  | Vvi-Vitvi08g04188\_t001 |  |  |  |  |  |  |
| 2 | Ath-AT2G37450.2 |  | | | |  | Vvi-Vitvi08g01083\_t001 |  |  |  |  |  |  |
| 1 | Ath-AT2G37460.1 |  | | | |  |  |  |  |  |  |  |
| 1 | Ath-AT2G37470.1 |  | | | |  |  |  |  |  |  |  |
| 1 | Ath-AT2G37480.1 |  | Vvi-Vitvi08g01161\_t003 |  |  |  |  |  |  |  |
| 1 | Ath-AT2G37500.1 |  | Vvi-Vitvi08g01162\_t001 |  |  |  |  |  |  |  |
| 1 | Ath-AT2G37510.2 |  | Vvi-Vitvi08g01163\_t002 |  |  |  |  |  |  |  |
| 1 | Ath-AT2G37520.1 |  | Vvi-Vitvi08g01164\_t001 |  |  |  |  |  |  |  |
| 1 | Ath-AT2G37530.1 |  | Vvi-Vitvi08g02184\_t001 |  |  |  |  |  |  |  |
| 1 | Ath-AT2G37540.1 |  | Vvi-Vitvi08g01168\_t001 |  |  |  |  |  |  |  |
| 1 | Ath-AT2G37550.1 |  | Vvi-Vitvi08g01171\_t001 |  |  |  |  |  |  |  |
| 1 | Ath-AT2G37560.1 |  | | | |  |  |  |  |  |  |  |
| 1 | Ath-AT2G37570.1 |  | Vvi-Vitvi08g01182\_t001 |  |  |  |  |  |  |  |
| 1 | Ath-AT2G37580.1 |  | Vvi-Vitvi08g04216\_t001 |  |  |  |  |  |  |  |
| 1 | Ath-AT2G37585.1 |  | Vvi-Vitvi08g01184\_t001 |  |  |  |  |  |  |  |
| 2 | Ath-AT2G37590.1 |  | Vvi-Vitvi08g01186\_t001 |  | Vvi-Vitvi13g00298\_t001 |  |  |  |  |  |  |
| 2 | Ath-AT2G37600.1 |  | | | |  | | | |  |  |  |  |  |  |
| 2 | Ath-AT2G37610.1 |  | | | |  | | | |  |  |  |  |  |  |
| 2 | Ath-AT2G37620.1 |  | | | |  | | | |  |  |  |  |  |  |
| 2 | Ath-AT2G37630.1 |  | Vvi-Vitvi08g04220\_t001 |  | | | |  |  |  |  |  |  |
| 2 | Ath-AT2G37640.1 |  | Vvi-Vitvi08g01206\_t001 |  | Vvi-Vitvi13g00309\_t001 |  |  |  |  |  |  |
| 2 | Ath-AT2G37650.1 |  | Vvi-Vitvi08g01214\_t001 |  | Vvi-Vitvi13g00311\_t001 |  |  |  |  |  |  |
| 2 | Ath-AT2G37660.1 |  | Vvi-Vitvi08g01216\_t001 |  | | | |  |  |  |  |  |  |
| 2 | Ath-AT2G37670.2 |  | | | |  | | | |  |  |  |  |  |  |
| 2 | Ath-AT2G37678.1 |  | Vvi-Vitvi08g02198\_t001 |  | | | |  |  |  |  |  |  |
| 2 | Ath-AT2G37680.2 |  | Vvi-Vitvi08g01240\_t001 |  | | | |  |  |  |  |  |  |
| 2 | Ath-AT2G37690.1 |  | | | |  | | | |  |  |  |  |  |  |
| 2 | Ath-AT2G37700.3 |  | | | |  | | | |  |  |  |  |  |  |
| 2 | Ath-AT2G37710.1 |  | Vvi-Vitvi08g01241\_t001 |  | Vvi-Vitvi13g00332\_t001 |  |  |  |  |  |  |
| 2 | Ath-AT2G37720.1 |  | | | |  | | | |  |  |  |  |  |  |
| 2 | Ath-AT2G37730.1 |  | Vvi-Vitvi08g01248\_t001 |  | Vvi-Vitvi13g01996\_t001 |  |  |  |  |  |  |
| 2 | Ath-AT2G37740.1 |  | Vvi-Vitvi08g02203\_t001 |  | Vvi-Vitvi13g00340\_t001 |  |  |  |  |  |  |
| 1 | Ath-AT2G37750.1 |  | | | |  |  |  |  |  |  |  |
| 1 | Ath-AT2G37760.1 |  | Vvi-Vitvi08g04241\_t001 |  |  |  |  |  |  |  |
| 1 | Ath-AT2G37770.2 |  | | | |  |  |  |  |  |  |  |
| 1 | Ath-AT2G37780.1 |  | Vvi-Vitvi08g01262\_t001 |  |  |  |  |  |  |  |
| 1 | Ath-AT2G37790.1 |  | | | |  |  |  |  |  |  |  |
| 1 | Ath-AT2G37800.1 |  | | | |  |  |  |  |  |  |  |
| 1 | Ath-AT2G37805.1 |  | | | |  |  |  |  |  |  |  |
| 1 | Ath-AT2G37810.1 |  | | | |  |  |  |  |  |  |  |
| 1 | Ath-AT2G37820.1 |  | | | |  |  |  |  |  |  |  |
| 1 | Ath-AT2G37840.1 |  | Vvi-Vitvi08g01267\_t001 |  |  |  |  |  |  |  |
| 1 | Ath-AT2G37860.3 |  | Vvi-Vitvi08g01270\_t001 |  |  |  |  |  |  |  |
| 1 | Ath-AT2G37870.1 |  | | | |  |  |  |  |  |  |  |
| 1 | Ath-AT2G37880.1 |  | Vvi-Vitvi08g01272\_t001 |  |  |  |  |  |  |  |
| 1 | Ath-AT2G37890.1 |  | Vvi-Vitvi08g01273\_t001 |  |  |  |  |  |  |  |
| 1 | Ath-AT2G37900.1 |  | Vvi-Vitvi08g04251\_t001 |  |  |  |  |  |  |  |
| 1 | Ath-AT2G37920.1 |  | Vvi-Vitvi08g01278\_t001 |  |  |  |  |  |  |  |
| 1 | Ath-AT2G37910.1 |  | | | |  |  |  |  |  |  |  |
| 2 | Ath-AT2G37925.1 |  | Vvi-Vitvi08g02230\_t001 |  | Vvi-Vitvi06g01729\_t001 |  |  |  |  |  |  |
| 2 | Ath-AT2G37930.3 |  | Vvi-Vitvi08g01293\_t001 |  | Vvi-Vitvi06g00563\_t001 |  |  |  |  |  |  |
| 2 | Ath-AT2G37940.2 |  | Vvi-Vitvi08g01295\_t001 |  | Vvi-Vitvi06g00564\_t001 |  |  |  |  |  |  |
| 2 | Ath-AT2G37950.1 |  | Vvi-Vitvi08g01297\_t001 |  | Vvi-Vitvi06g00567\_t001 |  |  |  |  |  |  |
| 2 | Ath-AT2G37960.1 |  | Vvi-Vitvi08g01303\_t001 |  | | | |  |  |  |  |  |  |
| 2 | Ath-AT2G37970.1 |  | Vvi-Vitvi08g01307\_t001 |  | | | |  |  |  |  |  |  |
| 2 | Ath-AT2G37975.1 |  | | | |  | | | |  |  |  |  |  |  |
| 2 | Ath-AT2G37980.1 |  | Vvi-Vitvi08g01308\_t001 |  | | | |  |  |  |  |  |  |
| 2 | Ath-AT2G37990.1 |  | | | |  | | | |  |  |  |  |  |  |
| 2 | Ath-AT2G38000.1 |  | Vvi-Vitvi08g01309\_t002 |  | | | |  |  |  |  |  |  |
| 2 | Ath-AT2G38010.2 |  | Vvi-Vitvi08g01311\_t001 |  | Vvi-Vitvi06g00573\_t001 |  |  |  |  |  |  |
| 2 | Ath-AT2G38020.1 |  | | | |  | | | |  |  |  |  |  |  |
| 2 | Ath-AT2G38025.1 |  | Vvi-Vitvi08g01318\_t001 |  | | | |  |  |  |  |  |  |
| 2 | Ath-AT2G38040.1 |  | Vvi-Vitvi08g01319\_t001 |  | | | |  |  |  |  |  |  |
| 2 | Ath-AT2G38050.1 |  | Vvi-Vitvi08g01320\_t001 |  | | | |  |  |  |  |  |  |
| 2 | Ath-AT2G38060.2 |  | Vvi-Vitvi08g01325\_t001 |  | Vvi-Vitvi06g00585\_t001 |  |  |  |  |  |  |
| 2 | Ath-AT2G38070.1 |  | Vvi-Vitvi08g01327\_t001 |  | Vvi-Vitvi06g00587\_t001 |  |  |  |  |  |  |
| 2 | Ath-AT2G38080.1 |  | Vvi-Vitvi08g01335\_t001 |  | Vvi-Vitvi06g00591\_t001 |  |  |  |  |  |  |
| 2 | Ath-AT2G38090.1 |  | Vvi-Vitvi08g01336\_t001 |  | Vvi-Vitvi06g00592\_t001 |  |  |  |  |  |  |
| 1 | Ath-AT2G38100.3 |  | Vvi-Vitvi08g04275\_t001 |  |  |  |  |  |  |  |
| 1 | Ath-AT2G38110.1 |  | Vvi-Vitvi08g01342\_t001 |  |  |  |  |  |  |  |
| 1 | Ath-AT2G38120.1 |  | Vvi-Vitvi08g01346\_t001 |  |  |  |  |  |  |  |
| 1 | Ath-AT2G38130.1 |  | | | |  |  |  |  |  |  |  |
| 1 | Ath-AT2G38140.1 |  | Vvi-Vitvi08g02243\_t002 |  |  |  |  |  |  |  |
| 1 | Ath-AT2G38150.1 |  | Vvi-Vitvi08g01349\_t001 |  |  |  |  |  |  |  |
| 1 | Ath-AT2G38152.1 |  | | | |  |  |  |  |  |  |  |
| 1 | Ath-AT2G38160.3 |  | Vvi-Vitvi08g01357\_t001 |  |  |  |  |  |  |  |
| 1 | Ath-AT2G38170.3 |  | Vvi-Vitvi08g02245\_t002 |  |  |  |  |  |  |  |
| 1 | Ath-AT2G38180.1 |  | Vvi-Vitvi08g01366\_t001 |  |  |  |  |  |  |  |
| 0 | Ath-AT2G38185.4 |  |  |  |  |  |  |  |  |
| 0 | Ath-AT2G38195.1 |  |  |  |  |  |  |  |  |
| 0 | Ath-AT2G38210.1 |  |  |  |  |  |  |  |  |
| 0 | Ath-AT2G38220.1 |  |  |  |  |  |  |  |  |
| 0 | Ath-AT2G38230.1 |  |  |  |  |  |  |  |  |
| 0 | Ath-AT2G38240.1 |  |  |  |  |  |  |  |  |
| 0 | Ath-AT2G38250.1 |  |  |  |  |  |  |  |  |
| 0 | Ath-AT2G38255.1 |  |  |  |  |  |  |  |  |
| 0 | Ath-AT2G38270.1 |  |  |  |  |  |  |  |  |
| 0 | Ath-AT2G38280.2 |  |  |  |  |  |  |  |  |
| 0 | Ath-AT2G38290.1 |  |  |  |  |  |  |  |  |
| 0 | Ath-AT2G38300.1 |  |  |  |  |  |  |  |  |
| 0 | Ath-AT2G38310.1 |  |  |  |  |  |  |  |  |
| 0 | Ath-AT2G38320.1 |  |  |  |  |  |  |  |  |
| 0 | Ath-AT2G38330.1 |  |  |  |  |  |  |  |  |
| 0 | Ath-AT2G38340.1 |  |  |  |  |  |  |  |  |
| 0 | Ath-AT2G38350.1 |  |  |  |  |  |  |  |  |
| 0 | Ath-AT2G38360.1 |  |  |  |  |  |  |  |  |
| 0 | Ath-AT2G38365.1 |  |  |  |  |  |  |  |  |
| 1 | Ath-AT2G38370.2 |  | Vvi-Vitvi08g00825\_t002 |  |  |  |  |  |  |  |
| 1 | Ath-AT2G38380.1 |  | | | |  |  |  |  |  |  |  |
| 1 | Ath-AT2G38390.1 |  | | | |  |  |  |  |  |  |  |
| 2 | Ath-AT2G38400.2 |  | | | |  | Vvi-Vitvi08g00816\_t001 |  |  |  |  |  |  |
| 2 | Ath-AT2G38410.1 |  | | | |  | Vvi-Vitvi08g00812\_t001 |  |  |  |  |  |  |
| 2 | Ath-AT2G38420.1 |  | | | |  | Vvi-Vitvi08g00808\_t001 |  |  |  |  |  |  |
| 2 | Ath-AT2G38430.1 |  | | | |  | | | |  |  |  |  |  |  |
| 2 | Ath-AT2G38440.1 |  | | | |  | Vvi-Vitvi08g00799\_t001 |  |  |  |  |  |  |
| 2 | Ath-AT2G38450.1 |  | | | |  | Vvi-Vitvi08g00797\_t002 |  |  |  |  |  |  |
| 2 | Ath-AT2G38460.1 |  | | | |  | Vvi-Vitvi08g00795\_t002 |  |  |  |  |  |  |
| 2 | Ath-AT2G38465.1 |  | | | |  | | | |  |  |  |  |  |  |
| 2 | Ath-AT2G38470.1 |  | | | |  | Vvi-Vitvi08g00793\_t001 |  |  |  |  |  |  |
| 2 | Ath-AT2G38480.1 |  | | | |  | Vvi-Vitvi08g00792\_t001 |  |  |  |  |  |  |
| 1 | Ath-AT2G38490.1 |  | Vvi-Vitvi08g00835\_t001 |  |  |  |  |  |  |  |
| 1 | Ath-AT2G38500.1 |  | Vvi-Vitvi08g00841\_t001 |  |  |  |  |  |  |  |
| 1 | Ath-AT2G38510.1 |  | Vvi-Vitvi08g00847\_t001 |  |  |  |  |  |  |  |
| 1 | Ath-AT2G38530.1 |  | Vvi-Vitvi08g00853\_t001 |  |  |  |  |  |  |  |
| 1 | Ath-AT2G38540.1 |  | | | |  |  |  |  |  |  |  |
| 1 | Ath-AT2G38550.1 |  | Vvi-Vitvi08g00864\_t001 |  |  |  |  |  |  |  |
| 1 | Ath-AT2G38560.1 |  | Vvi-Vitvi08g00870\_t001 |  |  |  |  |  |  |  |
| 1 | Ath-AT2G38570.1 |  | Vvi-Vitvi08g00872\_t001 |  |  |  |  |  |  |  |
| 1 | Ath-AT2G38580.1 |  | Vvi-Vitvi08g00881\_t001 |  |  |  |  |  |  |  |
| 0 | Ath-AT2G38590.1 |  |  |  |  |  |  |  |  |
| 1 | Ath-AT2G38600.1 |  | Vvi-Vitvi08g00054\_t001 |  |  |  |  |  |  |  |
| 1 | Ath-AT2G38610.2 |  | Vvi-Vitvi08g00042\_t001 |  |  |  |  |  |  |  |
| 1 | Ath-AT2G38620.2 |  | | | |  |  |  |  |  |  |  |
| 1 | Ath-AT2G38630.1 |  | Vvi-Vitvi08g00021\_t001 |  |  |  |  |  |  |  |
| 1 | Ath-AT2G38640.1 |  | Vvi-Vitvi08g00014\_t001 |  |  |  |  |  |  |  |
| 1 | Ath-AT2G38646.1 |  | | | |  |  |  |  |  |  |  |
| 1 | Ath-AT2G38650.1 |  | Vvi-Vitvi08g00009\_t001 |  |  |  |  |  |  |  |
| 1 | Ath-AT2G38660.3 |  | Vvi-Vitvi08g00008\_t001 |  |  |  |  |  |  |  |
| 0 | Ath-AT2G38670.1 |  |  |  |  |  |  |  |  |
| 0 | Ath-AT2G38680.1 |  |  |  |  |  |  |  |  |
| 0 | Ath-AT2G38690.1 |  |  |  |  |  |  |  |  |
| 0 | Ath-AT2G38695.3 |  |  |  |  |  |  |  |  |
| 0 | Ath-AT2G38700.1 |  |  |  |  |  |  |  |  |
| 0 | Ath-AT2G38710.1 |  |  |  |  |  |  |  |  |
| 0 | Ath-AT2G38720.1 |  |  |  |  |  |  |  |  |
| 0 | Ath-AT2G38730.1 |  |  |  |  |  |  |  |  |
| 0 | Ath-AT2G38740.1 |  |  |  |  |  |  |  |  |
| 0 | Ath-AT2G38750.1 |  |  |  |  |  |  |  |  |
| 0 | Ath-AT2G38760.1 |  |  |  |  |  |  |  |  |
| 0 | Ath-AT2G38770.1 |  |  |  |  |  |  |  |  |
| 0 | Ath-AT2G38780.6 |  |  |  |  |  |  |  |  |
| 0 | Ath-AT2G38790.1 |  |  |  |  |  |  |  |  |
| 0 | Ath-AT2G38800.1 |  |  |  |  |  |  |  |  |
| 0 | Ath-AT2G38810.3 |  |  |  |  |  |  |  |  |
| 0 | Ath-AT2G38820.2 |  |  |  |  |  |  |  |  |
| 0 | Ath-AT2G38823.1 |  |  |  |  |  |  |  |  |
| 0 | Ath-AT2G38830.1 |  |  |  |  |  |  |  |  |
| 0 | Ath-AT2G38840.1 |  |  |  |  |  |  |  |  |
| 0 | Ath-AT2G38860.2 |  |  |  |  |  |  |  |  |
| 0 | Ath-AT2G38870.1 |  |  |  |  |  |  |  |  |
| 0 | Ath-AT2G38880.8 |  |  |  |  |  |  |  |  |
| 0 | Ath-AT2G38890.3 |  |  |  |  |  |  |  |  |
| 0 | Ath-AT2G38900.2 |  |  |  |  |  |  |  |  |
| 0 | Ath-AT2G38905.1 |  |  |  |  |  |  |  |  |
| 0 | Ath-AT2G38910.1 |  |  |  |  |  |  |  |  |
| 0 | Ath-AT2G38920.6 |  |  |  |  |  |  |  |  |
| 0 | Ath-AT2G38940.1 |  |  |  |  |  |  |  |  |
| 0 | Ath-AT2G38950.1 |  |  |  |  |  |  |  |  |
| 0 | Ath-AT2G38960.3 |  |  |  |  |  |  |  |  |
| 1 | Ath-AT2G38970.1 |  | Vvi-Vitvi13g00620\_t001 |  |  |  |  |  |  |  |
| 1 | Ath-AT2G38995.2 |  | | | |  |  |  |  |  |  |  |
| 1 | Ath-AT2G39000.1 |  | | | |  |  |  |  |  |  |  |
| 1 | Ath-AT2G39010.1 |  | | | |  |  |  |  |  |  |  |
| 1 | Ath-AT2G39020.1 |  | Vvi-Vitvi13g00631\_t001 |  |  |  |  |  |  |  |
| 1 | Ath-AT2G39030.1 |  | | | |  |  |  |  |  |  |  |
| 1 | Ath-AT2G39040.1 |  | | | |  |  |  |  |  |  |  |
| 1 | Ath-AT2G39050.1 |  | | | |  |  |  |  |  |  |  |
| 1 | Ath-AT2G39060.1 |  | | | |  |  |  |  |  |  |  |
| 1 | Ath-AT2G39080.1 |  | | | |  |  |  |  |  |  |  |
| 1 | Ath-AT2G39090.1 |  | | | |  |  |  |  |  |  |  |
| 1 | Ath-AT2G39100.1 |  | | | |  |  |  |  |  |  |  |
| 1 | Ath-AT2G39110.1 |  | Vvi-Vitvi13g00639\_t001 |  |  |  |  |  |  |  |
| 1 | Ath-AT2G39120.1 |  | Vvi-Vitvi13g00643\_t001 |  |  |  |  |  |  |  |
| 1 | Ath-AT2G39130.1 |  | Vvi-Vitvi13g00646\_t001 |  |  |  |  |  |  |  |
| 1 | Ath-AT2G39140.1 |  | Vvi-Vitvi13g00656\_t001 |  |  |  |  |  |  |  |
| 1 | Ath-AT2G39160.1 |  | | | |  |  |  |  |  |  |  |
| 1 | Ath-AT2G39170.1 |  | Vvi-Vitvi13g00662\_t003 |  |  |  |  |  |  |  |
| 1 | Ath-AT2G39180.1 |  | | | |  |  |  |  |  |  |  |
| 1 | Ath-AT2G39190.2 |  | | | |  |  |  |  |  |  |  |
| 1 | Ath-AT2G39200.1 |  | | | |  |  |  |  |  |  |  |
| 1 | Ath-AT2G39210.1 |  | Vvi-Vitvi13g00675\_t001 |  |  |  |  |  |  |  |
| 0 | Ath-AT2G39220.1 |  |  |  |  |  |  |  |  |
| 0 | Ath-AT2G39230.1 |  |  |  |  |  |  |  |  |
| 0 | Ath-AT2G39240.1 |  |  |  |  |  |  |  |  |
| 0 | Ath-AT2G39250.1 |  |  |  |  |  |  |  |  |
| 0 | Ath-AT2G39260.1 |  |  |  |  |  |  |  |  |
| 1 | Ath-AT2G39270.1 |  | Vvi-Vitvi13g00202\_t001 |  |  |  |  |  |  |  |
| 2 | Ath-AT2G39280.2 |  | Vvi-Vitvi13g00206\_t001 |  | Vvi-Vitvi08g01130\_t001 |  |  |  |  |  |  |
| 2 | Ath-AT2G39290.1 |  | Vvi-Vitvi13g00207\_t001 |  | | | |  |  |  |  |  |  |
| 2 | Ath-AT2G39300.4 |  | Vvi-Vitvi13g00212\_t001 |  | | | |  |  |  |  |  |  |
| 2 | Ath-AT2G39310.4 |  | | | |  | | | |  |  |  |  |  |  |
| 2 | Ath-AT2G39320.1 |  | | | |  | | | |  |  |  |  |  |  |
| 2 | Ath-AT2G39330.2 |  | | | |  | | | |  |  |  |  |  |  |
| 2 | Ath-AT2G39340.1 |  | Vvi-Vitvi13g00219\_t001 |  | | | |  |  |  |  |  |  |
| 2 | Ath-AT2G39350.1 |  | Vvi-Vitvi13g00221\_t001 |  | Vvi-Vitvi08g01112\_t001 |  |  |  |  |  |  |
| 2 | Ath-AT2G39355.1 |  | | | |  | | | |  |  |  |  |  |  |
| 3 | Ath-AT2G39360.1 |  | Vvi-Vitvi13g00223\_t001 |  | | | |  | Vvi-Vitvi06g00388\_t001 |  |  |  |  |  |
| 3 | Ath-AT2G39370.1 |  | Vvi-Vitvi13g01929\_t001 |  | Vvi-Vitvi08g01105\_t001 |  | | | |  |  |  |  |  |
| 3 | Ath-AT2G39375.1 |  | | | |  | | | |  | | | |  |  |  |  |  |
| 3 | Ath-AT2G39380.1 |  | Vvi-Vitvi13g00233\_t001 |  | Vvi-Vitvi08g01101\_t001 |  | Vvi-Vitvi06g00397\_t001 |  |  |  |  |  |
| 3 | Ath-AT2G39390.1 |  | Vvi-Vitvi13g00234\_t001 |  | | | |  | Vvi-Vitvi06g00398\_t001 |  |  |  |  |  |
| 3 | Ath-AT2G39400.1 |  | Vvi-Vitvi13g00235\_t001 |  | | | |  | | | |  |  |  |  |  |
| 3 | Ath-AT2G39410.2 |  | | | |  | | | |  | | | |  |  |  |  |  |
| 3 | Ath-AT2G39415.1 |  | | | |  | | | |  | | | |  |  |  |  |  |
| 3 | Ath-AT2G39420.1 |  | | | |  | | | |  | | | |  |  |  |  |  |
| 3 | Ath-AT2G39430.1 |  | Vvi-Vitvi13g00238\_t001 |  | | | |  | Vvi-Vitvi06g00400\_t001 |  |  |  |  |  |
| 3 | Ath-AT2G39435.2 |  | Vvi-Vitvi13g00240\_t001 |  | Vvi-Vitvi08g01096\_t001 |  | | | |  |  |  |  |  |
| 3 | Ath-AT2G39440.1 |  | | | |  | | | |  | | | |  |  |  |  |  |
| 3 | Ath-AT2G39445.1 |  | | | |  | | | |  | | | |  |  |  |  |  |
| 3 | Ath-AT2G39450.1 |  | Vvi-Vitvi13g00244\_t001 |  | | | |  | | | |  |  |  |  |  |
| 3 | Ath-AT2G39460.2 |  | Vvi-Vitvi13g00249\_t001 |  | Vvi-Vitvi08g01090\_t001 |  | Vvi-Vitvi06g00408\_t001 |  |  |  |  |  |
| 3 | Ath-AT2G39470.1 |  | Vvi-Vitvi13g00250\_t001 |  | | | |  | | | |  |  |  |  |  |
| 3 | Ath-AT2G39480.1 |  | Vvi-Vitvi13g00252\_t001 |  | | | |  | Vvi-Vitvi06g00409\_t001 |  |  |  |  |  |
| 2 | Ath-AT2G39490.1 |  | Vvi-Vitvi13g01950\_t001 |  | Vvi-Vitvi08g02166\_t001 |  |  |  |  |  |  |
| 2 | Ath-AT2G39500.1 |  | | | |  | | | |  |  |  |  |  |  |
| 2 | Ath-AT2G39510.1 |  | Vvi-Vitvi13g00264\_t001 |  | Vvi-Vitvi08g01083\_t001 |  |  |  |  |  |  |
| 3 | Ath-AT2G39518.1 |  | | | |  | | | |  | Vvi-Vitvi13g01982\_t001 |  |  |  |  |  |
| 3 | Ath-AT2G39520.1 |  | | | |  | | | |  | | | |  |  |  |  |  |
| 3 | Ath-AT2G39530.1 |  | | | |  | | | |  | | | |  |  |  |  |  |
| 3 | Ath-AT2G39540.1 |  | | | |  | | | |  | | | |  |  |  |  |  |
| 3 | Ath-AT2G39550.1 |  | | | |  | | | |  | Vvi-Vitvi13g00302\_t001 |  |  |  |  |  |
| 3 | Ath-AT2G39560.1 |  | | | |  | | | |  | | | |  |  |  |  |  |
| 3 | Ath-AT2G39570.1 |  | | | |  | | | |  | | | |  |  |  |  |  |
| 3 | Ath-AT2G39580.2 |  | | | |  | | | |  | | | |  |  |  |  |  |
| 3 | Ath-AT2G39590.1 |  | Vvi-Vitvi13g04080\_t001 |  | Vvi-Vitvi08g04187\_t001 |  | | | |  |  |  |  |  |
| 2 | Ath-AT2G39620.1 |  | Vvi-Vitvi13g00270\_t001 |  |  |  | | | |  |  |  |  |  |
| 1 | Ath-AT2G39630.1 |  |  |  |  |  | | | |  |  |  |  |  |
| 2 | Ath-AT2G39640.1 |  | Vvi-Vitvi06g00468\_t001 |  |  |  | | | |  |  |  |  |  |
| 2 | Ath-AT2G39650.1 |  | | | |  |  |  | | | |  |  |  |  |  |
| 2 | Ath-AT2G39660.1 |  | Vvi-Vitvi06g00473\_t001 |  |  |  | | | |  |  |  |  |  |
| 2 | Ath-AT2G39670.2 |  | | | |  |  |  | | | |  |  |  |  |  |
| 2 | Ath-AT2G39690.5 |  | | | |  |  |  | Vvi-Vitvi13g00306\_t001 |  |  |  |  |  |
| 2 | Ath-AT2G39700.1 |  | Vvi-Vitvi06g00481\_t001 |  |  |  | Vvi-Vitvi13g00309\_t001 |  |  |  |  |  |
| 2 | Ath-AT2G39705.1 |  | Vvi-Vitvi06g04166\_t001 |  |  |  | Vvi-Vitvi13g01992\_t001 |  |  |  |  |  |
| 2 | Ath-AT2G39710.1 |  | | | |  |  |  | Vvi-Vitvi13g00325\_t001 |  |  |  |  |  |
| 2 | Ath-AT2G39720.1 |  | Vvi-Vitvi06g00502\_t001 |  |  |  | Vvi-Vitvi13g00326\_t001 |  |  |  |  |  |
| 2 | Ath-AT2G39725.2 |  | | | |  |  |  | | | |  |  |  |  |  |
| 2 | Ath-AT2G39730.1 |  | Vvi-Vitvi06g00513\_t001 |  |  |  | Vvi-Vitvi13g01994\_t001 |  |  |  |  |  |
| 1 | Ath-AT2G39740.2 |  |  |  |  |  | Vvi-Vitvi13g00346\_t001 |  |  |  |  |  |
| 1 | Ath-AT2G39750.1 |  |  |  |  |  | Vvi-Vitvi13g00348\_t002 |  |  |  |  |  |
| 1 | Ath-AT2G39760.1 |  |  |  |  |  | Vvi-Vitvi13g00349\_t001 |  |  |  |  |  |
| 1 | Ath-AT2G39770.1 |  |  |  |  |  | Vvi-Vitvi13g00352\_t001 |  |  |  |  |  |
| 1 | Ath-AT2G39780.1 |  |  |  |  |  | | | |  |  |  |  |  |
| 1 | Ath-AT2G39782.1 |  |  |  |  |  | | | |  |  |  |  |  |
| 1 | Ath-AT2G39790.1 |  |  |  |  |  | Vvi-Vitvi13g00354\_t001 |  |  |  |  |  |
| 1 | Ath-AT2G39795.1 |  |  |  |  |  | | | |  |  |  |  |  |
| 1 | Ath-AT2G39800.4 |  |  |  |  |  | Vvi-Vitvi13g00355\_t001 |  |  |  |  |  |
| 1 | Ath-AT2G39805.2 |  |  |  |  |  | Vvi-Vitvi13g00356\_t001 |  |  |  |  |  |
| 1 | Ath-AT2G39810.1 |  |  |  |  |  | | | |  |  |  |  |  |
| 1 | Ath-AT2G39820.1 |  |  |  |  |  | | | |  |  |  |  |  |
| 1 | Ath-AT2G39830.2 |  |  |  |  |  | | | |  |  |  |  |  |
| 1 | Ath-AT2G39840.1 |  |  |  |  |  | Vvi-Vitvi13g00362\_t003 |  |  |  |  |  |
| 0 | Ath-AT2G39850.1 |  |  |  |  |  |  |  |  |
| 0 | Ath-AT2G39851.1 |  |  |  |  |  |  |  |  |
| 0 | Ath-AT2G39855.1 |  |  |  |  |  |  |  |  |
| 0 | Ath-AT2G39865.1 |  |  |  |  |  |  |  |  |
| 0 | Ath-AT2G39870.1 |  |  |  |  |  |  |  |  |
| 1 | Ath-AT2G39880.1 |  | Vvi-Vitvi13g00494\_t001 |  |  |  |  |  |  |  |
| 1 | Ath-AT2G39890.1 |  | Vvi-Vitvi13g00496\_t001 |  |  |  |  |  |  |  |
| 1 | Ath-AT2G39900.1 |  | Vvi-Vitvi13g02031\_t001 |  |  |  |  |  |  |  |
| 1 | Ath-AT2G39910.1 |  | Vvi-Vitvi13g00503\_t001 |  |  |  |  |  |  |  |
| 1 | Ath-AT2G39920.1 |  | Vvi-Vitvi13g02033\_t005 |  |  |  |  |  |  |  |
| 1 | Ath-AT2G39930.1 |  | | | |  |  |  |  |  |  |  |
| 1 | Ath-AT2G39940.1 |  | Vvi-Vitvi13g00512\_t001 |  |  |  |  |  |  |  |
| 1 | Ath-AT2G39950.1 |  | Vvi-Vitvi13g00513\_t001 |  |  |  |  |  |  |  |
| 1 | Ath-AT2G39960.1 |  | Vvi-Vitvi13g02037\_t001 |  |  |  |  |  |  |  |
| 1 | Ath-AT2G39970.1 |  | Vvi-Vitvi13g00515\_t001 |  |  |  |  |  |  |  |
| 2 | Ath-AT2G39980.1 |  | Vvi-Vitvi13g00517\_t001 |  | Vvi-Vitvi08g01337\_t001 |  |  |  |  |  |  |
| 2 | Ath-AT2G39975.1 |  | | | |  | | | |  |  |  |  |  |  |
| 2 | Ath-AT2G39990.1 |  | Vvi-Vitvi13g00522\_t001 |  | Vvi-Vitvi08g01340\_t001 |  |  |  |  |  |  |
| 2 | Ath-AT2G40000.1 |  | Vvi-Vitvi13g00015\_t001 |  | | | |  |  |  |  |  |  |
| 2 | Ath-AT2G40004.1 |  | | | |  | | | |  |  |  |  |  |  |
| 2 | Ath-AT2G40010.1 |  | Vvi-Vitvi13g00017\_t001 |  | | | |  |  |  |  |  |  |
| 2 | Ath-AT2G40020.4 |  | | | |  | | | |  |  |  |  |  |  |
| 2 | Ath-AT2G40030.1 |  | | | |  | | | |  |  |  |  |  |  |
| 2 | Ath-AT2G40050.1 |  | | | |  | | | |  |  |  |  |  |  |
| 2 | Ath-AT2G40060.1 |  | Vvi-Vitvi13g00027\_t001 |  | Vvi-Vitvi08g01355\_t001 |  |  |  |  |  |  |
| 2 | Ath-AT2G40070.1 |  | Vvi-Vitvi13g00028\_t001 |  | Vvi-Vitvi08g01357\_t001 |  |  |  |  |  |  |
| 3 | Ath-AT2G40080.1 |  | | | |  | | | |  | Vvi-Vitvi06g00642\_t001 |  |  |  |  |  |
| 3 | Ath-AT2G40085.1 |  | | | |  | | | |  | | | |  |  |  |  |  |
| 3 | Ath-AT2G40090.1 |  | | | |  | | | |  | | | |  |  |  |  |  |
| 3 | Ath-AT2G40095.2 |  | Vvi-Vitvi13g00035\_t001 |  | | | |  | | | |  |  |  |  |  |
| 3 | Ath-AT2G40100.1 |  | | | |  | Vvi-Vitvi08g01360\_t001 |  | | | |  |  |  |  |  |
| 3 | Ath-AT2G40110.1 |  | | | |  | | | |  | | | |  |  |  |  |  |
| 3 | Ath-AT2G40113.1 |  | | | |  | | | |  | | | |  |  |  |  |  |
| 3 | Ath-AT2G40116.1 |  | Vvi-Vitvi13g00047\_t001 |  | Vvi-Vitvi08g01369\_t001 |  | Vvi-Vitvi06g00647\_t001 |  |  |  |  |  |
| 2 | Ath-AT2G40120.1 |  | Vvi-Vitvi13g00052\_t001 |  |  |  | | | |  |  |  |  |  |
| 2 | Ath-AT2G40130.2 |  | Vvi-Vitvi13g00053\_t001 |  |  |  | Vvi-Vitvi06g00652\_t001 |  |  |  |  |  |
| 2 | Ath-AT2G40140.1 |  | Vvi-Vitvi13g00058\_t001 |  |  |  | Vvi-Vitvi06g00653\_t002 |  |  |  |  |  |
| 2 | Ath-AT2G40150.1 |  | Vvi-Vitvi13g00066\_t001 |  |  |  | | | |  |  |  |  |  |
| 2 | Ath-AT2G40160.2 |  | | | |  |  |  | | | |  |  |  |  |  |
| 2 | Ath-AT2G40165.1 |  | | | |  |  |  | | | |  |  |  |  |  |
| 2 | Ath-AT2G40170.1 |  | Vvi-Vitvi13g00070\_t001 |  |  |  | | | |  |  |  |  |  |
| 2 | Ath-AT2G40180.1 |  | Vvi-Vitvi13g00071\_t001 |  |  |  | Vvi-Vitvi06g00667\_t001 |  |  |  |  |  |
| 2 | Ath-AT2G40190.1 |  | Vvi-Vitvi13g00073\_t001 |  |  |  | | | |  |  |  |  |  |
| 2 | Ath-AT2G40200.1 |  | Vvi-Vitvi13g00075\_t001 |  |  |  | | | |  |  |  |  |  |
| 2 | Ath-AT2G40205.1 |  | | | |  |  |  | | | |  |  |  |  |  |
| 2 | Ath-AT2G40210.1 |  | | | |  |  |  | | | |  |  |  |  |  |
| 2 | Ath-AT2G40220.1 |  | Vvi-Vitvi13g00080\_t001 |  |  |  | | | |  |  |  |  |  |
| 2 | Ath-AT2G40230.1 |  | Vvi-Vitvi13g00081\_t001 |  |  |  | | | |  |  |  |  |  |
| 2 | Ath-AT2G40240.1 |  | Vvi-Vitvi13g00083\_t001 |  |  |  | | | |  |  |  |  |  |
| 2 | Ath-AT2G40250.1 |  | | | |  |  |  | | | |  |  |  |  |  |
| 2 | Ath-AT2G40260.1 |  | Vvi-Vitvi13g00088\_t001 |  |  |  | Vvi-Vitvi06g00686\_t001 |  |  |  |  |  |
| 2 | Ath-AT2G40270.1 |  | Vvi-Vitvi13g00092\_t001 |  |  |  | Vvi-Vitvi06g00689\_t001 |  |  |  |  |  |
| 2 | Ath-AT2G40280.1 |  | Vvi-Vitvi13g00100\_t001 |  |  |  | | | |  |  |  |  |  |
| 2 | Ath-AT2G40290.1 |  | Vvi-Vitvi13g00101\_t001 |  |  |  | Vvi-Vitvi06g00696\_t001 |  |  |  |  |  |
| 2 | Ath-AT2G40300.1 |  | Vvi-Vitvi13g00107\_t001 |  |  |  | Vvi-Vitvi06g01761\_t001 |  |  |  |  |  |
| 2 | Ath-AT2G40310.1 |  | | | |  |  |  | | | |  |  |  |  |  |
| 2 | Ath-AT2G40316.1 |  | Vvi-Vitvi13g00112\_t001 |  |  |  | | | |  |  |  |  |  |
| 2 | Ath-AT2G40320.1 |  | Vvi-Vitvi13g00113\_t001 |  |  |  | | | |  |  |  |  |  |
| 2 | Ath-AT2G40330.1 |  | Vvi-Vitvi13g00114\_t001 |  |  |  | Vvi-Vitvi06g00724\_t001 |  |  |  |  |  |
| 2 | Ath-AT2G40340.6 |  | Vvi-Vitvi13g00116\_t001 |  |  |  | | | |  |  |  |  |  |
| 2 | Ath-AT2G40350.2 |  | | | |  |  |  | | | |  |  |  |  |  |
| 2 | Ath-AT2G40360.1 |  | | | |  |  |  | | | |  |  |  |  |  |
| 2 | Ath-AT2G40370.1 |  | Vvi-Vitvi13g00117\_t001 |  |  |  | Vvi-Vitvi06g00728\_t001 |  |  |  |  |  |
| 1 | Ath-AT2G40380.1 |  | Vvi-Vitvi13g00121\_t001 |  |  |  |  |  |  |  |
| 1 | Ath-AT2G40390.1 |  | Vvi-Vitvi13g00122\_t001 |  |  |  |  |  |  |  |
| 1 | Ath-AT2G40400.1 |  | Vvi-Vitvi13g00126\_t001 |  |  |  |  |  |  |  |
| 1 | Ath-AT2G40410.2 |  | Vvi-Vitvi13g00128\_t001 |  |  |  |  |  |  |  |
| 1 | Ath-AT2G40420.1 |  | Vvi-Vitvi13g00134\_t001 |  |  |  |  |  |  |  |
| 1 | Ath-AT2G40430.2 |  | Vvi-Vitvi13g00136\_t001 |  |  |  |  |  |  |  |
| 3 | Ath-AT2G40435.1 |  | Vvi-Vitvi13g00139\_t002 |  | Vvi-Vitvi08g00820\_t001 |  | Vvi-Vitvi06g00763\_t001 |  |  |  |  |  |
| 3 | Ath-AT2G40440.1 |  | | | |  | | | |  | | | |  |  |  |  |  |
| 3 | Ath-AT2G40450.1 |  | Vvi-Vitvi13g00140\_t001 |  | | | |  | | | |  |  |  |  |  |
| 3 | Ath-AT2G40460.1 |  | Vvi-Vitvi13g00142\_t001 |  | | | |  | | | |  |  |  |  |  |
| 3 | Ath-AT2G40470.1 |  | Vvi-Vitvi13g00144\_t001 |  | | | |  | Vvi-Vitvi06g00772\_t001 |  |  |  |  |  |
| 3 | Ath-AT2G40475.1 |  | Vvi-Vitvi13g01903\_t001 |  | | | |  | | | |  |  |  |  |  |
| 3 | Ath-AT2G40480.1 |  | Vvi-Vitvi13g00145\_t001 |  | Vvi-Vitvi08g00825\_t002 |  | | | |  |  |  |  |  |
| 3 | Ath-AT2G40490.1 |  | Vvi-Vitvi13g00147\_t001 |  | | | |  | | | |  |  |  |  |  |
| 3 | Ath-AT2G40500.1 |  | | | |  | | | |  | | | |  |  |  |  |  |
| 3 | Ath-AT2G40510.1 |  | | | |  | | | |  | | | |  |  |  |  |  |
| 3 | Ath-AT2G40520.2 |  | | | |  | Vvi-Vitvi08g00844\_t001 |  | Vvi-Vitvi06g00785\_t001 |  |  |  |  |  |
| 3 | Ath-AT2G40530.1 |  | | | |  | | | |  | | | |  |  |  |  |  |
| 3 | Ath-AT2G40540.3 |  | | | |  | | | |  | | | |  |  |  |  |  |
| 3 | Ath-AT2G40550.1 |  | Vvi-Vitvi13g00153\_t001 |  | | | |  | | | |  |  |  |  |  |
| 3 | Ath-AT2G40560.1 |  | | | |  | | | |  | | | |  |  |  |  |  |
| 3 | Ath-AT2G40570.1 |  | | | |  | | | |  | | | |  |  |  |  |  |
| 3 | Ath-AT2G40580.1 |  | | | |  | | | |  | | | |  |  |  |  |  |
| 3 | Ath-AT2G40590.1 |  | Vvi-Vitvi13g00160\_t001 |  | | | |  | Vvi-Vitvi06g00786\_t001 |  |  |  |  |  |
| 3 | Ath-AT2G40600.1 |  | | | |  | | | |  | | | |  |  |  |  |  |
| 3 | Ath-AT2G40610.1 |  | | | |  | Vvi-Vitvi08g00852\_t001 |  | Vvi-Vitvi06g00790\_t001 |  |  |  |  |  |
| 3 | Ath-AT2G40620.1 |  | Vvi-Vitvi13g00171\_t001 |  | | | |  | Vvi-Vitvi06g00800\_t001 |  |  |  |  |  |
| 2 | Ath-AT2G40630.1 |  | Vvi-Vitvi13g00179\_t001 |  | | | |  |  |  |  |  |  |
| 2 | Ath-AT2G40640.1 |  | Vvi-Vitvi13g00180\_t001 |  | | | |  |  |  |  |  |  |
| 2 | Ath-AT2G40650.1 |  | | | |  | | | |  |  |  |  |  |  |
| 2 | Ath-AT2G40660.1 |  | Vvi-Vitvi13g01915\_t001 |  | | | |  |  |  |  |  |  |
| 2 | Ath-AT2G40670.2 |  | Vvi-Vitvi13g00183\_t001 |  | | | |  |  |  |  |  |  |
| 2 | Ath-AT2G40690.1 |  | Vvi-Vitvi13g00185\_t001 |  | | | |  |  |  |  |  |  |
| 2 | Ath-AT2G40700.1 |  | | | |  | | | |  |  |  |  |  |  |
| 2 | Ath-AT2G40710.1 |  | | | |  | | | |  |  |  |  |  |  |
| 2 | Ath-AT2G40711.1 |  | | | |  | | | |  |  |  |  |  |  |
| 2 | Ath-AT2G40715.1 |  | | | |  | | | |  |  |  |  |  |  |
| 2 | Ath-AT2G40720.1 |  | Vvi-Vitvi13g00186\_t001 |  | | | |  |  |  |  |  |  |
| 2 | Ath-AT2G40730.1 |  | Vvi-Vitvi13g00188\_t001 |  | | | |  |  |  |  |  |  |
| 2 | Ath-AT2G40740.3 |  | Vvi-Vitvi13g00189\_t001 |  | | | |  |  |  |  |  |  |
| 2 | Ath-AT2G40750.1 |  | Vvi-Vitvi13g01916\_t001 |  | Vvi-Vitvi08g00868\_t001 |  |  |  |  |  |  |
| 2 | Ath-AT2G40745.1 |  | | | |  | | | |  |  |  |  |  |  |
| 2 | Ath-AT2G40760.1 |  | Vvi-Vitvi13g00190\_t001 |  | | | |  |  |  |  |  |  |
| 2 | Ath-AT2G40765.1 |  | | | |  | | | |  |  |  |  |  |  |
| 2 | Ath-AT2G40770.1 |  | Vvi-Vitvi13g00192\_t001 |  | | | |  |  |  |  |  |  |
| 1 | Ath-AT2G40780.1 |  |  |  | | | |  |  |  |  |  |  |
| 1 | Ath-AT2G40790.1 |  |  |  | Vvi-Vitvi08g04141\_t001 |  |  |  |  |  |  |
| 1 | Ath-AT2G40800.1 |  |  |  | | | |  |  |  |  |  |  |
| 1 | Ath-AT2G40810.1 |  |  |  | | | |  |  |  |  |  |  |
| 1 | Ath-AT2G40815.1 |  |  |  | | | |  |  |  |  |  |  |
| 1 | Ath-AT2G40820.4 |  |  |  | | | |  |  |  |  |  |  |
| 2 | Ath-AT2G40830.2 |  | Vvi-Vitvi08g00923\_t001 |  | | | |  |  |  |  |  |  |
| 2 | Ath-AT2G40840.1 |  | Vvi-Vitvi08g00930\_t003 |  | | | |  |  |  |  |  |  |
| 2 | Ath-AT2G40850.1 |  | Vvi-Vitvi08g00931\_t001 |  | | | |  |  |  |  |  |  |
| 2 | Ath-AT2G40860.1 |  | Vvi-Vitvi08g00937\_t001 |  | | | |  |  |  |  |  |  |
| 2 | Ath-AT2G40880.1 |  | Vvi-Vitvi08g00939\_t001 |  | | | |  |  |  |  |  |  |
| 2 | Ath-AT2G40890.1 |  | Vvi-Vitvi08g00940\_t001 |  | | | |  |  |  |  |  |  |
| 2 | Ath-AT2G40900.1 |  | Vvi-Vitvi08g00942\_t001 |  | | | |  |  |  |  |  |  |
| 2 | Ath-AT2G40910.1 |  | | | |  | | | |  |  |  |  |  |  |
| 2 | Ath-AT2G40920.1 |  | | | |  | | | |  |  |  |  |  |  |
| 2 | Ath-AT2G40925.1 |  | | | |  | | | |  |  |  |  |  |  |
| 2 | Ath-AT2G40930.2 |  | | | |  | | | |  |  |  |  |  |  |
| 2 | Ath-AT2G40935.5 |  | | | |  | Vvi-Vitvi08g00887\_t001 |  |  |  |  |  |  |
| 2 | Ath-AT2G40940.1 |  | | | |  | | | |  |  |  |  |  |  |
| 2 | Ath-AT2G40950.1 |  | Vvi-Vitvi08g00950\_t001 |  | | | |  |  |  |  |  |  |
| 2 | Ath-AT2G40955.1 |  | | | |  | | | |  |  |  |  |  |  |
| 2 | Ath-AT2G40960.1 |  | Vvi-Vitvi08g00954\_t003 |  | | | |  |  |  |  |  |  |
| 2 | Ath-AT2G40970.1 |  | Vvi-Vitvi08g00955\_t001 |  | | | |  |  |  |  |  |  |
| 1 | Ath-AT2G40980.1 |  |  |  | | | |  |  |  |  |  |  |
| 1 | Ath-AT2G40990.1 |  |  |  | Vvi-Vitvi08g00901\_t001 |  |  |  |  |  |  |
| 1 | Ath-AT2G40995.2 |  |  |  | | | |  |  |  |  |  |  |
| 1 | Ath-AT2G41997.1 |  |  |  | | | |  |  |  |  |  |  |
| 1 | Ath-AT2G41000.2 |  |  |  | | | |  |  |  |  |  |  |
| 1 | Ath-AT2G41010.1 |  |  |  | Vvi-Vitvi08g00916\_t001 |  |  |  |  |  |  |
| 1 | Ath-AT2G41020.1 |  |  |  | Vvi-Vitvi08g00922\_t001 |  |  |  |  |  |  |
| 0 | Ath-AT2G41040.1 |  |  |  |  |  |  |  |  |
| 0 | Ath-AT2G41050.3 |  |  |  |  |  |  |  |  |
| 0 | Ath-AT2G41060.1 |  |  |  |  |  |  |  |  |
| 0 | Ath-AT2G41070.4 |  |  |  |  |  |  |  |  |
| 0 | Ath-AT2G41080.1 |  |  |  |  |  |  |  |  |
| 0 | Ath-AT2G41082.1 |  |  |  |  |  |  |  |  |
| 0 | Ath-AT2G41090.1 |  |  |  |  |  |  |  |  |
| 0 | Ath-AT2G41100.4 |  |  |  |  |  |  |  |  |
| 0 | Ath-AT2G41105.1 |  |  |  |  |  |  |  |  |
| 0 | Ath-AT2G41110.2 |  |  |  |  |  |  |  |  |
| 0 | Ath-AT2G41120.1 |  |  |  |  |  |  |  |  |
| 0 | Ath-AT2G41130.1 |  |  |  |  |  |  |  |  |
| 0 | Ath-AT2G41140.1 |  |  |  |  |  |  |  |  |
| 0 | Ath-AT2G41150.2 |  |  |  |  |  |  |  |  |
| 0 | Ath-AT2G41160.1 |  |  |  |  |  |  |  |  |
| 0 | Ath-AT2G41170.2 |  |  |  |  |  |  |  |  |
| 1 | Ath-AT2G41180.1 |  | Vvi-Vitvi08g01639\_t001 |  |  |  |  |  |  |  |
| 1 | Ath-AT2G41190.1 |  | Vvi-Vitvi08g01641\_t001 |  |  |  |  |  |  |  |
| 1 | Ath-AT2G41200.1 |  | Vvi-Vitvi08g02304\_t001 |  |  |  |  |  |  |  |
| 1 | Ath-AT2G41210.1 |  | Vvi-Vitvi08g01644\_t001 |  |  |  |  |  |  |  |
| 1 | Ath-AT2G41220.1 |  | Vvi-Vitvi08g01646\_t001 |  |  |  |  |  |  |  |
| 1 | Ath-AT2G41225.1 |  | | | |  |  |  |  |  |  |  |
| 1 | Ath-AT2G41230.1 |  | | | |  |  |  |  |  |  |  |
| 1 | Ath-AT2G41231.1 |  | | | |  |  |  |  |  |  |  |
| 1 | Ath-AT2G41240.1 |  | Vvi-Vitvi08g01649\_t001 |  |  |  |  |  |  |  |
| 1 | Ath-AT2G41250.1 |  | Vvi-Vitvi08g01655\_t002 |  |  |  |  |  |  |  |
| 1 | Ath-AT2G41260.2 |  | | | |  |  |  |  |  |  |  |
| 1 | Ath-AT2G41280.1 |  | | | |  |  |  |  |  |  |  |
| 1 | Ath-AT2G41290.1 |  | Vvi-Vitvi08g01656\_t001 |  |  |  |  |  |  |  |
| 1 | Ath-AT2G41300.1 |  | Vvi-Vitvi08g01657\_t001 |  |  |  |  |  |  |  |
| 1 | Ath-AT2G41310.1 |  | Vvi-Vitvi08g02307\_t001 |  |  |  |  |  |  |  |
| 1 | Ath-AT2G41330.1 |  | Vvi-Vitvi08g01668\_t001 |  |  |  |  |  |  |  |
| 1 | Ath-AT2G41340.1 |  | Vvi-Vitvi08g02313\_t001 |  |  |  |  |  |  |  |
| 1 | Ath-AT2G41342.1 |  | | | |  |  |  |  |  |  |  |
| 1 | Ath-AT2G41350.2 |  | | | |  |  |  |  |  |  |  |
| 1 | Ath-AT2G41355.1 |  | | | |  |  |  |  |  |  |  |
| 1 | Ath-AT2G41360.1 |  | | | |  |  |  |  |  |  |  |
| 2 | Ath-AT2G41370.1 |  | | | |  | Vvi-Vitvi08g01678\_t001 |  |  |  |  |  |  |
| 2 | Ath-AT2G41375.1 |  | | | |  | | | |  |  |  |  |  |  |
| 2 | Ath-AT2G41380.1 |  | Vvi-Vitvi08g01669\_t001 |  | | | |  |  |  |  |  |  |
| 1 | Ath-AT2G41390.1 |  |  |  | | | |  |  |  |  |  |  |
| 1 | Ath-AT2G41400.1 |  |  |  | | | |  |  |  |  |  |  |
| 1 | Ath-AT2G41410.1 |  |  |  | Vvi-Vitvi08g01683\_t001 |  |  |  |  |  |  |
| 1 | Ath-AT2G41415.1 |  |  |  | | | |  |  |  |  |  |  |
| 1 | Ath-AT2G41417.1 |  |  |  | | | |  |  |  |  |  |  |
| 1 | Ath-AT2G41420.1 |  |  |  | | | |  |  |  |  |  |  |
| 1 | Ath-AT2G41430.2 |  |  |  | | | |  |  |  |  |  |  |
| 1 | Ath-AT2G41440.1 |  |  |  | | | |  |  |  |  |  |  |
| 1 | Ath-AT2G41445.2 |  |  |  | | | |  |  |  |  |  |  |
| 1 | Ath-AT2G41450.1 |  |  |  | Vvi-Vitvi08g01691\_t001 |  |  |  |  |  |  |
| 1 | Ath-AT2G41451.1 |  |  |  | | | |  |  |  |  |  |  |
| 1 | Ath-AT2G41460.1 |  |  |  | Vvi-Vitvi08g01694\_t001 |  |  |  |  |  |  |
| 1 | Ath-AT2G41470.1 |  |  |  | | | |  |  |  |  |  |  |
| 1 | Ath-AT2G41473.1 |  |  |  | | | |  |  |  |  |  |  |
| 1 | Ath-AT2G41475.1 |  |  |  | | | |  |  |  |  |  |  |
| 1 | Ath-AT2G41480.1 |  |  |  | | | |  |  |  |  |  |  |
| 1 | Ath-AT2G41490.1 |  |  |  | Vvi-Vitvi08g01695\_t001 |  |  |  |  |  |  |
| 1 | Ath-AT2G41500.1 |  |  |  | Vvi-Vitvi08g01696\_t001 |  |  |  |  |  |  |
| 0 | Ath-AT2G41505.1 |  |  |  |  |  |  |  |  |
| 1 | Ath-AT2G41510.2 |  | Vvi-Vitvi08g01962\_t001 |  |  |  |  |  |  |  |
| 1 | Ath-AT2G41515.1 |  | | | |  |  |  |  |  |  |  |
| 1 | Ath-AT2G41520.1 |  | | | |  |  |  |  |  |  |  |
| 1 | Ath-AT2G41530.1 |  | Vvi-Vitvi08g01951\_t002 |  |  |  |  |  |  |  |
| 1 | Ath-AT2G41540.4 |  | Vvi-Vitvi08g01950\_t001 |  |  |  |  |  |  |  |
| 1 | Ath-AT2G41550.1 |  | | | |  |  |  |  |  |  |  |
| 1 | Ath-AT2G41560.1 |  | Vvi-Vitvi08g02413\_t001 |  |  |  |  |  |  |  |
| 1 | Ath-AT2G41590.1 |  | | | |  |  |  |  |  |  |  |
| 1 | Ath-AT2G41600.7 |  | Vvi-Vitvi08g01947\_t001 |  |  |  |  |  |  |  |
| 1 | Ath-AT2G41610.1 |  | | | |  |  |  |  |  |  |  |
| 1 | Ath-AT2G41620.1 |  | Vvi-Vitvi08g01946\_t001 |  |  |  |  |  |  |  |
| 1 | Ath-AT2G41630.1 |  | Vvi-Vitvi08g01943\_t001 |  |  |  |  |  |  |  |
| 1 | Ath-AT2G41640.1 |  | Vvi-Vitvi08g01940\_t001 |  |  |  |  |  |  |  |
| 1 | Ath-AT2G41650.1 |  | | | |  |  |  |  |  |  |  |
| 1 | Ath-AT2G41660.1 |  | Vvi-Vitvi08g01934\_t001 |  |  |  |  |  |  |  |
| 1 | Ath-AT2G41670.1 |  | Vvi-Vitvi08g01933\_t001 |  |  |  |  |  |  |  |
| 2 | Ath-AT2G41680.1 |  | Vvi-Vitvi08g01932\_t001 |  | Vvi-Vitvi08g04425\_t001 |  |  |  |  |  |  |
| 2 | Ath-AT2G41690.1 |  | Vvi-Vitvi08g01931\_t001 |  | | | |  |  |  |  |  |  |
| 2 | Ath-AT2G41700.1 |  | Vvi-Vitvi08g01929\_t001 |  | | | |  |  |  |  |  |  |
| 2 | Ath-AT2G41705.1 |  | | | |  | | | |  |  |  |  |  |  |
| 2 | Ath-AT2G41710.4 |  | | | |  | Vvi-Vitvi08g01919\_t001 |  |  |  |  |  |  |
| 2 | Ath-AT2G41720.1 |  | Vvi-Vitvi08g01920\_t001 |  | Vvi-Vitvi08g01920\_t001 |  |  |  |  |  |  |
| 2 | Ath-AT2G41730.1 |  | | | |  | | | |  |  |  |  |  |  |
| 2 | Ath-AT2G41740.1 |  | | | |  | Vvi-Vitvi08g01924\_t001 |  |  |  |  |  |  |
| 2 | Ath-AT2G41750.1 |  | | | |  | Vvi-Vitvi08g01925\_t001 |  |  |  |  |  |  |
| 2 | Ath-AT2G41760.1 |  | | | |  | Vvi-Vitvi08g01926\_t001 |  |  |  |  |  |  |
| 1 | Ath-AT2G41770.1 |  | Vvi-Vitvi08g01914\_t001 |  |  |  |  |  |  |  |
| 1 | Ath-AT2G41780.1 |  | | | |  |  |  |  |  |  |  |
| 1 | Ath-AT2G41790.1 |  | Vvi-Vitvi08g01906\_t001 |  |  |  |  |  |  |  |
| 1 | Ath-AT2G41800.1 |  | Vvi-Vitvi08g01904\_t001 |  |  |  |  |  |  |  |
| 1 | Ath-AT2G41810.1 |  | | | |  |  |  |  |  |  |  |
| 1 | Ath-AT2G41820.1 |  | Vvi-Vitvi08g01901\_t001 |  |  |  |  |  |  |  |
| 1 | Ath-AT2G41830.1 |  | | | |  |  |  |  |  |  |  |
| 1 | Ath-AT2G41835.1 |  | | | |  |  |  |  |  |  |  |
| 1 | Ath-AT2G41840.1 |  | Vvi-Vitvi08g01896\_t001 |  |  |  |  |  |  |  |
| 1 | Ath-AT2G41850.1 |  | Vvi-Vitvi08g02394\_t001 |  |  |  |  |  |  |  |
| 1 | Ath-AT2G41860.1 |  | Vvi-Vitvi08g01889\_t002 |  |  |  |  |  |  |  |
| 1 | Ath-AT2G41870.1 |  | Vvi-Vitvi08g01887\_t001 |  |  |  |  |  |  |  |
| 1 | Ath-AT2G41880.5 |  | Vvi-Vitvi08g01884\_t001 |  |  |  |  |  |  |  |
| 1 | Ath-AT2G41890.1 |  | Vvi-Vitvi08g01881\_t001 |  |  |  |  |  |  |  |
| 1 | Ath-AT2G41900.1 |  | Vvi-Vitvi08g01880\_t002 |  |  |  |  |  |  |  |
| 0 | Ath-AT2G41905.1 |  |  |  |  |  |  |  |  |
| 0 | Ath-AT2G41910.1 |  |  |  |  |  |  |  |  |
| 0 | Ath-AT2G41920.1 |  |  |  |  |  |  |  |  |
| 0 | Ath-AT2G41930.1 |  |  |  |  |  |  |  |  |
| 1 | Ath-AT2G41940.1 |  | Vvi-Vitvi08g01771\_t001 |  |  |  |  |  |  |  |
| 1 | Ath-AT2G41945.3 |  | | | |  |  |  |  |  |  |  |
| 1 | Ath-AT2G41950.1 |  | Vvi-Vitvi08g01765\_t001 |  |  |  |  |  |  |  |
| 1 | Ath-AT2G41960.1 |  | Vvi-Vitvi08g01764\_t001 |  |  |  |  |  |  |  |
| 1 | Ath-AT2G41970.1 |  | Vvi-Vitvi08g01761\_t001 |  |  |  |  |  |  |  |
| 1 | Ath-AT2G41980.1 |  | Vvi-Vitvi08g01760\_t001 |  |  |  |  |  |  |  |
| 1 | Ath-AT2G41990.1 |  | | | |  |  |  |  |  |  |  |
| 1 | Ath-AT2G42000.2 |  | | | |  |  |  |  |  |  |  |
| 1 | Ath-AT2G42005.1 |  | | | |  |  |  |  |  |  |  |
| 1 | Ath-AT2G42010.2 |  | | | |  |  |  |  |  |  |  |
| 1 | Ath-AT2G42030.1 |  | Vvi-Vitvi08g01751\_t001 |  |  |  |  |  |  |  |
| 1 | Ath-AT2G42040.1 |  | Vvi-Vitvi08g01749\_t001 |  |  |  |  |  |  |  |
| 1 | Ath-AT2G42060.1 |  | Vvi-Vitvi08g02349\_t001 |  |  |  |  |  |  |  |
| 1 | Ath-AT2G42070.1 |  | Vvi-Vitvi08g01742\_t001 |  |  |  |  |  |  |  |
| 1 | Ath-AT2G42065.1 |  | Vvi-Vitvi08g01741\_t001 |  |  |  |  |  |  |  |
| 1 | Ath-AT2G42080.1 |  | | | |  |  |  |  |  |  |  |
| 1 | Ath-AT2G42090.1 |  | | | |  |  |  |  |  |  |  |
| 1 | Ath-AT2G42100.1 |  | | | |  |  |  |  |  |  |  |
| 1 | Ath-AT2G42110.1 |  | Vvi-Vitvi08g01738\_t001 |  |  |  |  |  |  |  |
| 1 | Ath-AT2G42120.1 |  | Vvi-Vitvi08g01737\_t002 |  |  |  |  |  |  |  |
| 1 | Ath-AT2G42130.4 |  | Vvi-Vitvi08g02346\_t001 |  |  |  |  |  |  |  |
| 1 | Ath-AT2G42140.1 |  | Vvi-Vitvi08g01736\_t001 |  |  |  |  |  |  |  |
| 1 | Ath-AT2G42150.1 |  | | | |  |  |  |  |  |  |  |
| 1 | Ath-AT2G42160.1 |  | | | |  |  |  |  |  |  |  |
| 1 | Ath-AT2G42170.3 |  | | | |  |  |  |  |  |  |  |
| 1 | Ath-AT2G42180.1 |  | Vvi-Vitvi08g01724\_t001 |  |  |  |  |  |  |  |
| 1 | Ath-AT2G42190.2 |  | Vvi-Vitvi08g02342\_t001 |  |  |  |  |  |  |  |
| 1 | Ath-AT2G42200.1 |  | Vvi-Vitvi08g01720\_t001 |  |  |  |  |  |  |  |
| 1 | Ath-AT2G42210.2 |  | Vvi-Vitvi08g01716\_t002 |  |  |  |  |  |  |  |
| 1 | Ath-AT2G42220.1 |  | Vvi-Vitvi08g01714\_t001 |  |  |  |  |  |  |  |
| 1 | Ath-AT2G42230.2 |  | Vvi-Vitvi08g01713\_t001 |  |  |  |  |  |  |  |
| 0 | Ath-AT2G42240.4 |  |  |  |  |  |  |  |  |
| 1 | Ath-AT2G42250.1 |  | Vvi-Vitvi08g02372\_t001 |  |  |  |  |  |  |  |
| 1 | Ath-AT2G42260.1 |  | Vvi-Vitvi08g01851\_t001 |  |  |  |  |  |  |  |
| 1 | Ath-AT2G42270.1 |  | | | |  |  |  |  |  |  |  |
| 1 | Ath-AT2G42280.3 |  | Vvi-Vitvi08g01852\_t001 |  |  |  |  |  |  |  |
| 1 | Ath-AT2G42290.1 |  | Vvi-Vitvi08g01853\_t001 |  |  |  |  |  |  |  |
| 1 | Ath-AT2G42300.1 |  | Vvi-Vitvi08g01856\_t001 |  |  |  |  |  |  |  |
| 1 | Ath-AT2G42310.1 |  | Vvi-Vitvi08g01859\_t001 |  |  |  |  |  |  |  |
| 1 | Ath-AT2G42320.2 |  | Vvi-Vitvi08g01860\_t003 |  |  |  |  |  |  |  |
| 0 | Ath-AT2G42330.1 |  |  |  |  |  |  |  |  |
| 0 | Ath-AT2G42340.1 |  |  |  |  |  |  |  |  |
| 0 | Ath-AT2G42350.1 |  |  |  |  |  |  |  |  |
| 0 | Ath-AT2G42360.1 |  |  |  |  |  |  |  |  |
| 1 | Ath-AT2G42370.1 |  | Vvi-Vitvi12g00225\_t002 |  |  |  |  |  |  |  |
| 1 | Ath-AT2G42380.2 |  | | | |  |  |  |  |  |  |  |
| 1 | Ath-AT2G42390.1 |  | | | |  |  |  |  |  |  |  |
| 1 | Ath-AT2G42395.1 |  | | | |  |  |  |  |  |  |  |
| 1 | Ath-AT2G42400.1 |  | | | |  |  |  |  |  |  |  |
| 1 | Ath-AT2G42410.3 |  | | | |  |  |  |  |  |  |  |
| 1 | Ath-AT2G42430.1 |  | | | |  |  |  |  |  |  |  |
| 1 | Ath-AT2G42440.1 |  | | | |  |  |  |  |  |  |  |
| 1 | Ath-AT2G42450.1 |  | | | |  |  |  |  |  |  |  |
| 1 | Ath-AT2G42455.1 |  | | | |  |  |  |  |  |  |  |
| 1 | Ath-AT2G42460.1 |  | | | |  |  |  |  |  |  |  |
| 1 | Ath-AT2G42465.1 |  | | | |  |  |  |  |  |  |  |
| 1 | Ath-AT2G42470.1 |  | | | |  |  |  |  |  |  |  |
| 1 | Ath-AT2G42475.1 |  | | | |  |  |  |  |  |  |  |
| 1 | Ath-AT2G42480.1 |  | | | |  |  |  |  |  |  |  |
| 1 | Ath-AT2G42490.1 |  | | | |  |  |  |  |  |  |  |
| 1 | Ath-AT2G42500.1 |  | | | |  |  |  |  |  |  |  |
| 1 | Ath-AT2G42510.2 |  | | | |  |  |  |  |  |  |  |
| 1 | Ath-AT2G42520.1 |  | | | |  |  |  |  |  |  |  |
| 1 | Ath-AT2G42530.1 |  | | | |  |  |  |  |  |  |  |
| 1 | Ath-AT2G42540.2 |  | | | |  |  |  |  |  |  |  |
| 1 | Ath-AT2G42550.1 |  | | | |  |  |  |  |  |  |  |
| 1 | Ath-AT2G42560.1 |  | Vvi-Vitvi12g00210\_t001 |  |  |  |  |  |  |  |
| 2 | Ath-AT2G42570.1 |  | Vvi-Vitvi12g00209\_t001 |  | Vvi-Vitvi10g00564\_t001 |  |  |  |  |  |  |
| 2 | Ath-AT2G42580.1 |  | Vvi-Vitvi12g00193\_t001 |  | | | |  |  |  |  |  |  |
| 2 | Ath-AT2G42590.3 |  | Vvi-Vitvi12g00187\_t001 |  | Vvi-Vitvi10g00579\_t003 |  |  |  |  |  |  |
| 2 | Ath-AT2G42600.1 |  | Vvi-Vitvi12g00185\_t001 |  | | | |  |  |  |  |  |  |
| 2 | Ath-AT2G42610.1 |  | Vvi-Vitvi12g00183\_t001 |  | Vvi-Vitvi10g00583\_t001 |  |  |  |  |  |  |
| 2 | Ath-AT2G42620.1 |  | Vvi-Vitvi12g02250\_t001 |  | Vvi-Vitvi10g04364\_t001 |  |  |  |  |  |  |
| 2 | Ath-AT2G42640.1 |  | Vvi-Vitvi12g00181\_t002 |  | | | |  |  |  |  |  |  |
| 2 | Ath-AT2G42650.1 |  | | | |  | | | |  |  |  |  |  |  |
| 2 | Ath-AT2G42660.1 |  | Vvi-Vitvi12g00177\_t001 |  | Vvi-Vitvi10g01793\_t001 |  |  |  |  |  |  |
| 2 | Ath-AT2G42670.2 |  | Vvi-Vitvi12g00175\_t001 |  | Vvi-Vitvi10g00593\_t001 |  |  |  |  |  |  |
| 2 | Ath-AT2G42680.1 |  | Vvi-Vitvi12g00170\_t001 |  | | | |  |  |  |  |  |  |
| 2 | Ath-AT2G42690.1 |  | Vvi-Vitvi12g00167\_t001 |  | | | |  |  |  |  |  |  |
| 2 | Ath-AT2G42700.2 |  | Vvi-Vitvi12g00165\_t002 |  | | | |  |  |  |  |  |  |
| 2 | Ath-AT2G42710.1 |  | Vvi-Vitvi12g00164\_t001 |  | | | |  |  |  |  |  |  |
| 2 | Ath-AT2G42720.1 |  | | | |  | | | |  |  |  |  |  |  |
| 2 | Ath-AT2G42725.1 |  | | | |  | | | |  |  |  |  |  |  |
| 2 | Ath-AT2G42730.1 |  | | | |  | | | |  |  |  |  |  |  |
| 2 | Ath-AT2G42740.1 |  | Vvi-Vitvi12g00155\_t001 |  | Vvi-Vitvi10g00609\_t001 |  |  |  |  |  |  |
| 2 | Ath-AT2G42750.1 |  | Vvi-Vitvi12g00151\_t001 |  | | | |  |  |  |  |  |  |
| 2 | Ath-AT2G42760.1 |  | Vvi-Vitvi12g02233\_t001 |  | Vvi-Vitvi10g00615\_t001 |  |  |  |  |  |  |
| 2 | Ath-AT2G42770.1 |  | Vvi-Vitvi12g00137\_t001 |  | | | |  |  |  |  |  |  |
| 1 | Ath-AT2G42780.1 |  |  |  | | | |  |  |  |  |  |  |
| 2 | Ath-AT2G42790.1 |  | Vvi-Vitvi12g00008\_t001 |  | | | |  |  |  |  |  |  |
| 2 | Ath-AT2G42800.1 |  | | | |  | | | |  |  |  |  |  |  |
| 2 | Ath-AT2G42810.2 |  | | | |  | | | |  |  |  |  |  |  |
| 2 | Ath-AT2G42820.1 |  | Vvi-Vitvi12g00015\_t001 |  | | | |  |  |  |  |  |  |
| 2 | Ath-AT2G42830.2 |  | Vvi-Vitvi12g00019\_t002 |  | | | |  |  |  |  |  |  |
| 2 | Ath-AT2G42840.1 |  | Vvi-Vitvi12g00025\_t001 |  | | | |  |  |  |  |  |  |
| 2 | Ath-AT2G42850.1 |  | Vvi-Vitvi12g04008\_t001 |  | | | |  |  |  |  |  |  |
| 2 | Ath-AT2G42860.1 |  | | | |  | | | |  |  |  |  |  |  |
| 2 | Ath-AT2G42865.1 |  | | | |  | | | |  |  |  |  |  |  |
| 2 | Ath-AT2G42870.1 |  | Vvi-Vitvi12g02185\_t001 |  | | | |  |  |  |  |  |  |
| 2 | Ath-AT2G42880.1 |  | Vvi-Vitvi12g00032\_t001 |  | | | |  |  |  |  |  |  |
| 2 | Ath-AT2G42885.1 |  | | | |  | | | |  |  |  |  |  |  |
| 2 | Ath-AT2G42890.1 |  | Vvi-Vitvi12g00033\_t001 |  | | | |  |  |  |  |  |  |
| 2 | Ath-AT2G42900.1 |  | Vvi-Vitvi12g00037\_t001 |  | | | |  |  |  |  |  |  |
| 2 | Ath-AT2G42910.1 |  | | | |  | | | |  |  |  |  |  |  |
| 2 | Ath-AT2G42920.2 |  | Vvi-Vitvi12g00039\_t001 |  | Vvi-Vitvi10g00634\_t001 |  |  |  |  |  |  |
| 1 | Ath-AT2G42930.1 |  | | | |  |  |  |  |  |  |  |
| 1 | Ath-AT2G42940.1 |  | Vvi-Vitvi12g00040\_t001 |  |  |  |  |  |  |  |
| 1 | Ath-AT2G42950.2 |  | | | |  |  |  |  |  |  |  |
| 1 | Ath-AT2G42955.1 |  | | | |  |  |  |  |  |  |  |
| 1 | Ath-AT2G42960.4 |  | Vvi-Vitvi12g00049\_t001 |  |  |  |  |  |  |  |
| 1 | Ath-AT2G42975.1 |  | Vvi-Vitvi12g00061\_t001 |  |  |  |  |  |  |  |
| 1 | Ath-AT2G42980.1 |  | Vvi-Vitvi12g00066\_t001 |  |  |  |  |  |  |  |
| 1 | Ath-AT2G42990.1 |  | | | |  |  |  |  |  |  |  |
| 1 | Ath-AT2G43000.1 |  | Vvi-Vitvi12g00076\_t001 |  |  |  |  |  |  |  |
| 1 | Ath-AT2G43010.5 |  | Vvi-Vitvi12g00096\_t001 |  |  |  |  |  |  |  |
| 1 | Ath-AT2G43020.1 |  | Vvi-Vitvi12g00097\_t001 |  |  |  |  |  |  |  |
| 0 | Ath-AT2G43030.1 |  |  |  |  |  |  |  |  |
| 0 | Ath-AT2G43040.2 |  |  |  |  |  |  |  |  |
| 0 | Ath-AT2G43050.1 |  |  |  |  |  |  |  |  |
| 0 | Ath-AT2G43060.1 |  |  |  |  |  |  |  |  |
| 0 | Ath-AT2G43070.4 |  |  |  |  |  |  |  |  |
| 0 | Ath-AT2G43080.1 |  |  |  |  |  |  |  |  |
| 0 | Ath-AT2G43090.1 |  |  |  |  |  |  |  |  |
| 0 | Ath-AT2G43100.1 |  |  |  |  |  |  |  |  |
| 0 | Ath-AT2G43110.1 |  |  |  |  |  |  |  |  |
| 0 | Ath-AT2G43120.2 |  |  |  |  |  |  |  |  |
| 0 | Ath-AT2G43130.1 |  |  |  |  |  |  |  |  |
| 0 | Ath-AT2G43140.2 |  |  |  |  |  |  |  |  |
| 0 | Ath-AT2G43150.1 |  |  |  |  |  |  |  |  |
| 0 | Ath-AT2G43160.1 |  |  |  |  |  |  |  |  |
| 0 | Ath-AT2G43180.1 |  |  |  |  |  |  |  |  |
| 0 | Ath-AT2G43190.1 |  |  |  |  |  |  |  |  |
| 0 | Ath-AT2G43200.1 |  |  |  |  |  |  |  |  |
| 0 | Ath-AT2G43210.2 |  |  |  |  |  |  |  |  |
| 0 | Ath-AT2G43220.1 |  |  |  |  |  |  |  |  |
| 0 | Ath-AT2G43230.2 |  |  |  |  |  |  |  |  |
| 0 | Ath-AT2G43235.1 |  |  |  |  |  |  |  |  |
| 0 | Ath-AT2G43240.1 |  |  |  |  |  |  |  |  |
| 0 | Ath-AT2G43250.1 |  |  |  |  |  |  |  |  |
| 0 | Ath-AT2G43255.2 |  |  |  |  |  |  |  |  |
| 0 | Ath-AT2G43260.2 |  |  |  |  |  |  |  |  |
| 0 | Ath-AT2G43261.1 |  |  |  |  |  |  |  |  |
| 0 | Ath-AT2G43270.1 |  |  |  |  |  |  |  |  |
| 1 | Ath-AT2G43280.1 |  | Vvi-Vitvi05g04445\_t001 |  |  |  |  |  |  |  |
| 1 | Ath-AT2G43290.1 |  | Vvi-Vitvi05g01500\_t001 |  |  |  |  |  |  |  |
| 1 | Ath-AT2G43310.1 |  | Vvi-Vitvi05g01489\_t001 |  |  |  |  |  |  |  |
| 1 | Ath-AT2G43320.1 |  | Vvi-Vitvi05g01480\_t001 |  |  |  |  |  |  |  |
| 1 | Ath-AT2G43330.1 |  | Vvi-Vitvi05g01471\_t001 |  |  |  |  |  |  |  |
| 1 | Ath-AT2G43340.1 |  | Vvi-Vitvi05g01470\_t001 |  |  |  |  |  |  |  |
| 1 | Ath-AT2G43350.2 |  | Vvi-Vitvi05g01469\_t001 |  |  |  |  |  |  |  |
| 1 | Ath-AT2G43360.1 |  | Vvi-Vitvi05g01467\_t001 |  |  |  |  |  |  |  |
| 1 | Ath-AT2G43370.1 |  | Vvi-Vitvi05g01466\_t001 |  |  |  |  |  |  |  |
| 0 | Ath-AT2G43386.1 |  |  |  |  |  |  |  |  |
| 0 | Ath-AT2G43390.1 |  |  |  |  |  |  |  |  |
| 1 | Ath-AT2G43400.1 |  | Vvi-Vitvi05g01536\_t001 |  |  |  |  |  |  |  |
| 1 | Ath-AT2G43410.2 |  | Vvi-Vitvi05g01542\_t001 |  |  |  |  |  |  |  |
| 1 | Ath-AT2G43420.1 |  | Vvi-Vitvi05g01543\_t001 |  |  |  |  |  |  |  |
| 1 | Ath-AT2G43430.1 |  | Vvi-Vitvi05g01544\_t001 |  |  |  |  |  |  |  |
| 1 | Ath-AT2G43440.1 |  | | | |  |  |  |  |  |  |  |
| 1 | Ath-AT2G43445.1 |  | | | |  |  |  |  |  |  |  |
| 1 | Ath-AT2G43450.1 |  | | | |  |  |  |  |  |  |  |
| 1 | Ath-AT2G43460.1 |  | Vvi-Vitvi05g01548\_t001 |  |  |  |  |  |  |  |
| 1 | Ath-AT2G43465.1 |  | Vvi-Vitvi05g01553\_t001 |  |  |  |  |  |  |  |
| 1 | Ath-AT2G43470.1 |  | Vvi-Vitvi05g04512\_t001 |  |  |  |  |  |  |  |
| 1 | Ath-AT2G43480.1 |  | | | |  |  |  |  |  |  |  |
| 1 | Ath-AT2G43490.7 |  | Vvi-Vitvi05g01558\_t001 |  |  |  |  |  |  |  |
| 1 | Ath-AT2G43500.4 |  | Vvi-Vitvi05g01560\_t001 |  |  |  |  |  |  |  |
| 1 | Ath-AT2G43510.1 |  | | | |  |  |  |  |  |  |  |
| 1 | Ath-AT2G43520.1 |  | | | |  |  |  |  |  |  |  |
| 1 | Ath-AT2G43530.1 |  | | | |  |  |  |  |  |  |  |
| 1 | Ath-AT2G43535.1 |  | | | |  |  |  |  |  |  |  |
| 1 | Ath-AT2G43540.1 |  | Vvi-Vitvi05g02239\_t001 |  |  |  |  |  |  |  |
| 1 | Ath-AT2G43550.1 |  | | | |  |  |  |  |  |  |  |
| 1 | Ath-AT2G43560.1 |  | Vvi-Vitvi05g01566\_t001 |  |  |  |  |  |  |  |
| 1 | Ath-AT2G43570.1 |  | Vvi-Vitvi05g01573\_t001 |  |  |  |  |  |  |  |
| 1 | Ath-AT2G43580.1 |  | | | |  |  |  |  |  |  |  |
| 1 | Ath-AT2G43590.1 |  | | | |  |  |  |  |  |  |  |
| 1 | Ath-AT2G43600.2 |  | | | |  |  |  |  |  |  |  |
| 1 | Ath-AT2G43610.1 |  | | | |  |  |  |  |  |  |  |
| 1 | Ath-AT2G43620.1 |  | Vvi-Vitvi05g02251\_t001 |  |  |  |  |  |  |  |
| 1 | Ath-AT2G43630.1 |  | Vvi-Vitvi05g01585\_t001 |  |  |  |  |  |  |  |
| 1 | Ath-AT2G43640.2 |  | Vvi-Vitvi05g01587\_t001 |  |  |  |  |  |  |  |
| 1 | Ath-AT2G43650.1 |  | Vvi-Vitvi05g01593\_t002 |  |  |  |  |  |  |  |
| 1 | Ath-AT2G43660.2 |  | | | |  |  |  |  |  |  |  |
| 1 | Ath-AT2G43670.1 |  | | | |  |  |  |  |  |  |  |
| 1 | Ath-AT2G43680.1 |  | Vvi-Vitvi05g01603\_t001 |  |  |  |  |  |  |  |
| 1 | Ath-AT2G43690.1 |  | | | |  |  |  |  |  |  |  |
| 1 | Ath-AT2G43700.1 |  | | | |  |  |  |  |  |  |  |
| 1 | Ath-AT2G43710.1 |  | Vvi-Vitvi05g01619\_t001 |  |  |  |  |  |  |  |
| 1 | Ath-AT2G43720.1 |  | | | |  |  |  |  |  |  |  |
| 1 | Ath-AT2G43730.1 |  | | | |  |  |  |  |  |  |  |
| 1 | Ath-AT2G43740.1 |  | | | |  |  |  |  |  |  |  |
| 1 | Ath-AT2G43745.1 |  | | | |  |  |  |  |  |  |  |
| 1 | Ath-AT2G43750.2 |  | Vvi-Vitvi05g01624\_t001 |  |  |  |  |  |  |  |
| 1 | Ath-AT2G43760.3 |  | | | |  |  |  |  |  |  |  |
| 1 | Ath-AT2G43770.1 |  | Vvi-Vitvi05g01626\_t001 |  |  |  |  |  |  |  |
| 1 | Ath-AT2G43780.4 |  | | | |  |  |  |  |  |  |  |
| 1 | Ath-AT2G43790.1 |  | Vvi-Vitvi05g01634\_t001 |  |  |  |  |  |  |  |
| 1 | Ath-AT2G43795.2 |  | Vvi-Vitvi05g01636\_t001 |  |  |  |  |  |  |  |
| 1 | Ath-AT2G43800.1 |  | Vvi-Vitvi05g01638\_t001 |  |  |  |  |  |  |  |
| 1 | Ath-AT2G43810.2 |  | Vvi-Vitvi05g01640\_t001 |  |  |  |  |  |  |  |
| 1 | Ath-AT2G43820.1 |  | Vvi-Vitvi05g01642\_t001 |  |  |  |  |  |  |  |
| 1 | Ath-AT2G43840.2 |  | | | |  |  |  |  |  |  |  |
| 1 | Ath-AT2G43850.2 |  | Vvi-Vitvi05g01656\_t001 |  |  |  |  |  |  |  |
| 2 | Ath-AT2G43860.1 |  | | | |  | Vvi-Vitvi05g01679\_t001 |  |  |  |  |  |  |
| 2 | Ath-AT2G43865.1 |  | | | |  | | | |  |  |  |  |  |  |
| 2 | Ath-AT2G43870.1 |  | | | |  | | | |  |  |  |  |  |  |
| 2 | Ath-AT2G43880.1 |  | | | |  | Vvi-Vitvi05g01681\_t001 |  |  |  |  |  |  |
| 2 | Ath-AT2G43890.1 |  | | | |  | | | |  |  |  |  |  |  |
| 2 | Ath-AT2G43900.2 |  | | | |  | Vvi-Vitvi05g01683\_t001 |  |  |  |  |  |  |
| 2 | Ath-AT2G43910.1 |  | | | |  | Vvi-Vitvi05g02272\_t001 |  |  |  |  |  |  |
| 2 | Ath-AT2G43920.1 |  | | | |  | | | |  |  |  |  |  |  |
| 2 | Ath-AT2G43930.1 |  | | | |  | | | |  |  |  |  |  |  |
| 2 | Ath-AT2G43940.1 |  | | | |  | | | |  |  |  |  |  |  |
| 2 | Ath-AT2G43945.1 |  | | | |  | Vvi-Vitvi05g02275\_t002 |  |  |  |  |  |  |
| 2 | Ath-AT2G43950.1 |  | | | |  | Vvi-Vitvi05g01685\_t001 |  |  |  |  |  |  |
| 2 | Ath-AT2G43960.1 |  | | | |  | | | |  |  |  |  |  |  |
| 2 | Ath-AT2G43970.1 |  | | | |  | Vvi-Vitvi05g02276\_t001 |  |  |  |  |  |  |
| 2 | Ath-AT2G43980.1 |  | | | |  | Vvi-Vitvi05g01686\_t001 |  |  |  |  |  |  |
| 2 | Ath-AT2G43990.1 |  | | | |  | Vvi-Vitvi05g01688\_t001 |  |  |  |  |  |  |
| 2 | Ath-AT2G44000.1 |  | | | |  | | | |  |  |  |  |  |  |
| 2 | Ath-AT2G44010.1 |  | | | |  | Vvi-Vitvi05g04575\_t001 |  |  |  |  |  |  |
| 2 | Ath-AT2G44020.1 |  | | | |  | | | |  |  |  |  |  |  |
| 2 | Ath-AT2G44030.1 |  | | | |  | | | |  |  |  |  |  |  |
| 2 | Ath-AT2G44040.1 |  | | | |  | Vvi-Vitvi05g01692\_t001 |  |  |  |  |  |  |
| 2 | Ath-AT2G44050.1 |  | | | |  | Vvi-Vitvi05g01693\_t001 |  |  |  |  |  |  |
| 2 | Ath-AT2G44060.1 |  | | | |  | Vvi-Vitvi05g01697\_t001 |  |  |  |  |  |  |
| 2 | Ath-AT2G44065.2 |  | Vvi-Vitvi05g01659\_t001 |  | | | |  |  |  |  |  |  |
| 1 | Ath-AT2G44070.1 |  |  |  | | | |  |  |  |  |  |  |
| 1 | Ath-AT2G44080.1 |  |  |  | Vvi-Vitvi05g02279\_t001 |  |  |  |  |  |  |
| 1 | Ath-AT2G44090.1 |  |  |  | Vvi-Vitvi05g04576\_t001 |  |  |  |  |  |  |
| 1 | Ath-AT2G44100.1 |  |  |  | Vvi-Vitvi05g01700\_t001 |  |  |  |  |  |  |
| 1 | Ath-AT2G44110.2 |  |  |  | Vvi-Vitvi05g01702\_t001 |  |  |  |  |  |  |
| 1 | Ath-AT2G44120.2 |  |  |  | | | |  |  |  |  |  |  |
| 1 | Ath-AT2G44140.1 |  |  |  | | | |  |  |  |  |  |  |
| 1 | Ath-AT2G44130.1 |  |  |  | Vvi-Vitvi05g01703\_t001 |  |  |  |  |  |  |
| 1 | Ath-AT2G44150.1 |  |  |  | | | |  |  |  |  |  |  |
| 1 | Ath-AT2G44160.1 |  |  |  | Vvi-Vitvi05g01704\_t001 |  |  |  |  |  |  |
| 0 | Ath-AT2G44175.1 |  |  |  |  |  |  |  |  |
| 0 | Ath-AT2G44180.1 |  |  |  |  |  |  |  |  |
| 0 | Ath-AT2G44190.1 |  |  |  |  |  |  |  |  |
| 0 | Ath-AT2G44195.1 |  |  |  |  |  |  |  |  |
| 0 | Ath-AT2G44198.1 |  |  |  |  |  |  |  |  |
| 0 | Ath-AT2G44200.1 |  |  |  |  |  |  |  |  |
| 0 | Ath-AT2G44210.2 |  |  |  |  |  |  |  |  |
| 0 | Ath-AT2G44220.1 |  |  |  |  |  |  |  |  |
| 0 | Ath-AT2G44230.1 |  |  |  |  |  |  |  |  |
| 0 | Ath-AT2G44240.1 |  |  |  |  |  |  |  |  |
| 0 | Ath-AT2G44250.1 |  |  |  |  |  |  |  |  |
| 0 | Ath-AT2G44260.2 |  |  |  |  |  |  |  |  |
| 0 | Ath-AT2G44270.1 |  |  |  |  |  |  |  |  |
| 1 | Ath-AT2G44280.2 |  | Vvi-Vitvi13g01840\_t001 |  |  |  |  |  |  |  |
| 1 | Ath-AT2G44290.1 |  | Vvi-Vitvi13g01839\_t001 |  |  |  |  |  |  |  |
| 1 | Ath-AT2G44300.1 |  | | | |  |  |  |  |  |  |  |
| 1 | Ath-AT2G44310.1 |  | Vvi-Vitvi13g01836\_t001 |  |  |  |  |  |  |  |
| 1 | Ath-AT2G44330.1 |  | | | |  |  |  |  |  |  |  |
| 1 | Ath-AT2G44340.1 |  | Vvi-Vitvi13g02571\_t001 |  |  |  |  |  |  |  |
| 1 | Ath-AT2G44350.2 |  | Vvi-Vitvi13g01832\_t001 |  |  |  |  |  |  |  |
| 1 | Ath-AT2G44360.1 |  | Vvi-Vitvi13g01831\_t001 |  |  |  |  |  |  |  |
| 1 | Ath-AT2G44370.1 |  | Vvi-Vitvi13g04765\_t001 |  |  |  |  |  |  |  |
| 1 | Ath-AT2G44380.1 |  | | | |  |  |  |  |  |  |  |
| 1 | Ath-AT2G44390.1 |  | | | |  |  |  |  |  |  |  |
| 1 | Ath-AT2G44400.1 |  | Vvi-Vitvi13g01825\_t001 |  |  |  |  |  |  |  |
| 1 | Ath-AT2G44410.1 |  | Vvi-Vitvi13g01824\_t001 |  |  |  |  |  |  |  |
| 1 | Ath-AT2G44420.2 |  | Vvi-Vitvi13g01820\_t002 |  |  |  |  |  |  |  |
| 1 | Ath-AT2G44430.1 |  | Vvi-Vitvi13g02569\_t001 |  |  |  |  |  |  |  |
| 1 | Ath-AT2G44440.1 |  | Vvi-Vitvi13g01816\_t001 |  |  |  |  |  |  |  |
| 0 | Ath-AT2G44450.1 |  |  |  |  |  |  |  |  |
| 0 | Ath-AT2G44460.1 |  |  |  |  |  |  |  |  |
| 0 | Ath-AT2G44470.3 |  |  |  |  |  |  |  |  |
| 0 | Ath-AT2G44480.3 |  |  |  |  |  |  |  |  |
| 0 | Ath-AT2G44490.1 |  |  |  |  |  |  |  |  |
| 0 | Ath-AT2G44500.1 |  |  |  |  |  |  |  |  |
| 0 | Ath-AT2G44510.1 |  |  |  |  |  |  |  |  |
| 1 | Ath-AT2G44520.1 |  | Vvi-Vitvi15g00440\_t001 |  |  |  |  |  |  |  |
| 1 | Ath-AT2G44525.1 |  | Vvi-Vitvi15g00443\_t001 |  |  |  |  |  |  |  |
| 2 | Ath-AT2G44530.1 |  | Vvi-Vitvi15g00445\_t001 |  | Vvi-Vitvi02g00633\_t001 |  |  |  |  |  |  |
| 2 | Ath-AT2G44540.1 |  | | | |  | | | |  |  |  |  |  |  |
| 2 | Ath-AT2G44550.1 |  | | | |  | | | |  |  |  |  |  |  |
| 2 | Ath-AT2G44560.1 |  | | | |  | | | |  |  |  |  |  |  |
| 2 | Ath-AT2G44570.1 |  | | | |  | | | |  |  |  |  |  |  |
| 2 | Ath-AT2G44581.1 |  | | | |  | | | |  |  |  |  |  |  |
| 2 | Ath-AT2G44578.1 |  | | | |  | | | |  |  |  |  |  |  |
| 2 | Ath-AT2G44580.1 |  | | | |  | | | |  |  |  |  |  |  |
| 2 | Ath-AT2G44590.3 |  | Vvi-Vitvi15g00448\_t001 |  | | | |  |  |  |  |  |  |
| 2 | Ath-AT2G44600.1 |  | Vvi-Vitvi15g00457\_t001 |  | | | |  |  |  |  |  |  |
| 2 | Ath-AT2G44610.1 |  | | | |  | | | |  |  |  |  |  |  |
| 2 | Ath-AT2G44620.1 |  | Vvi-Vitvi15g00463\_t001 |  | | | |  |  |  |  |  |  |
| 2 | Ath-AT2G44630.1 |  | | | |  | | | |  |  |  |  |  |  |
| 2 | Ath-AT2G44640.1 |  | | | |  | | | |  |  |  |  |  |  |
| 2 | Ath-AT2G44650.1 |  | Vvi-Vitvi15g00492\_t001 |  | | | |  |  |  |  |  |  |
| 2 | Ath-AT2G44660.1 |  | Vvi-Vitvi15g00502\_t001 |  | | | |  |  |  |  |  |  |
| 2 | Ath-AT2G44670.1 |  | Vvi-Vitvi15g04309\_t001 |  | Vvi-Vitvi02g00614\_t001 |  |  |  |  |  |  |
| 2 | Ath-AT2G44680.1 |  | Vvi-Vitvi15g00518\_t001 |  | Vvi-Vitvi02g00597\_t001 |  |  |  |  |  |  |
| 2 | Ath-AT2G44690.1 |  | Vvi-Vitvi15g00526\_t001 |  | | | |  |  |  |  |  |  |
| 2 | Ath-AT2G44700.1 |  | | | |  | | | |  |  |  |  |  |  |
| 2 | Ath-AT2G44710.1 |  | Vvi-Vitvi15g01421\_t001 |  | | | |  |  |  |  |  |  |
| 2 | Ath-AT2G44730.1 |  | Vvi-Vitvi15g00532\_t001 |  | | | |  |  |  |  |  |  |
| 2 | Ath-AT2G44735.1 |  | | | |  | | | |  |  |  |  |  |  |
| 2 | Ath-AT2G44740.1 |  | Vvi-Vitvi15g00537\_t001 |  | | | |  |  |  |  |  |  |
| 2 | Ath-AT2G44745.1 |  | Vvi-Vitvi15g00539\_t001 |  | | | |  |  |  |  |  |  |
| 2 | Ath-AT2G44750.2 |  | | | |  | | | |  |  |  |  |  |  |
| 2 | Ath-AT2G44760.1 |  | | | |  | | | |  |  |  |  |  |  |
| 2 | Ath-AT2G44770.1 |  | Vvi-Vitvi15g00542\_t001 |  | | | |  |  |  |  |  |  |
| 2 | Ath-AT2G44790.1 |  | Vvi-Vitvi15g00547\_t001 |  | | | |  |  |  |  |  |  |
| 2 | Ath-AT2G44800.1 |  | | | |  | | | |  |  |  |  |  |  |
| 2 | Ath-AT2G44810.2 |  | Vvi-Vitvi15g00556\_t001 |  | | | |  |  |  |  |  |  |
| 2 | Ath-AT2G44820.1 |  | | | |  | | | |  |  |  |  |  |  |
| 2 | Ath-AT2G44830.2 |  | Vvi-Vitvi15g00562\_t001 |  | | | |  |  |  |  |  |  |
| 2 | Ath-AT2G44840.1 |  | Vvi-Vitvi15g01202\_t001 |  | | | |  |  |  |  |  |  |
| 2 | Ath-AT2G44850.1 |  | Vvi-Vitvi15g01426\_t001 |  | | | |  |  |  |  |  |  |
| 2 | Ath-AT2G44860.2 |  | | | |  | | | |  |  |  |  |  |  |
| 2 | Ath-AT2G44870.1 |  | | | |  | | | |  |  |  |  |  |  |
| 2 | Ath-AT2G44880.2 |  | Vvi-Vitvi15g00568\_t001 |  | | | |  |  |  |  |  |  |
| 2 | Ath-AT2G44890.1 |  | | | |  | | | |  |  |  |  |  |  |
| 2 | Ath-AT2G44900.2 |  | Vvi-Vitvi15g00572\_t001 |  | | | |  |  |  |  |  |  |
| 2 | Ath-AT2G44910.1 |  | Vvi-Vitvi15g00579\_t001 |  | Vvi-Vitvi02g01717\_t001 |  |  |  |  |  |  |
| 2 | Ath-AT2G44920.2 |  | | | |  | | | |  |  |  |  |  |  |
| 2 | Ath-AT2G44925.1 |  | Vvi-Vitvi15g01454\_t001 |  | | | |  |  |  |  |  |  |
| 2 | Ath-AT2G44930.1 |  | | | |  | | | |  |  |  |  |  |  |
| 2 | Ath-AT2G44940.1 |  | Vvi-Vitvi15g00601\_t001 |  | | | |  |  |  |  |  |  |
| 2 | Ath-AT2G44950.1 |  | Vvi-Vitvi15g00606\_t001 |  | | | |  |  |  |  |  |  |
| 2 | Ath-AT2G44970.1 |  | Vvi-Vitvi15g00607\_t001 |  | | | |  |  |  |  |  |  |
| 2 | Ath-AT2G44980.2 |  | Vvi-Vitvi15g00611\_t001 |  | | | |  |  |  |  |  |  |
| 2 | Ath-AT2G44990.2 |  | Vvi-Vitvi15g00612\_t001 |  | | | |  |  |  |  |  |  |
| 2 | Ath-AT2G44993.1 |  | | | |  | | | |  |  |  |  |  |  |
| 2 | Ath-AT2G45000.1 |  | | | |  | | | |  |  |  |  |  |  |
| 2 | Ath-AT2G45010.1 |  | | | |  | | | |  |  |  |  |  |  |
| 2 | Ath-AT2G45030.1 |  | | | |  | | | |  |  |  |  |  |  |
| 2 | Ath-AT2G45040.1 |  | Vvi-Vitvi15g00618\_t001 |  | Vvi-Vitvi02g00564\_t001 |  |  |  |  |  |  |
| 2 | Ath-AT2G45050.1 |  | Vvi-Vitvi15g00636\_t001 |  | | | |  |  |  |  |  |  |
| 2 | Ath-AT2G45060.1 |  | Vvi-Vitvi15g01469\_t001 |  | | | |  |  |  |  |  |  |
| 2 | Ath-AT2G45070.3 |  | | | |  | | | |  |  |  |  |  |  |
| 2 | Ath-AT2G45080.1 |  | | | |  | | | |  |  |  |  |  |  |
| 2 | Ath-AT2G45100.1 |  | | | |  | | | |  |  |  |  |  |  |
| 2 | Ath-AT2G45110.1 |  | Vvi-Vitvi15g00640\_t001 |  | | | |  |  |  |  |  |  |
| 2 | Ath-AT2G45120.1 |  | Vvi-Vitvi15g01472\_t001 |  | Vvi-Vitvi02g00560\_t001 |  |  |  |  |  |  |
| 2 | Ath-AT2G45130.1 |  | Vvi-Vitvi15g00674\_t001 |  | | | |  |  |  |  |  |  |
| 2 | Ath-AT2G45135.2 |  | | | |  | | | |  |  |  |  |  |  |
| 2 | Ath-AT2G45140.1 |  | Vvi-Vitvi15g00677\_t001 |  | Vvi-Vitvi02g00545\_t001 |  |  |  |  |  |  |
| 2 | Ath-AT2G45150.1 |  | Vvi-Vitvi15g00679\_t001 |  | | | |  |  |  |  |  |  |
| 2 | Ath-AT2G45160.1 |  | Vvi-Vitvi15g00680\_t001 |  | Vvi-Vitvi02g00536\_t002 |  |  |  |  |  |  |
| 3 | Ath-AT2G45170.1 |  | | | |  | Vvi-Vitvi02g00535\_t001 |  | Vvi-Vitvi15g00718\_t002 |  |  |  |  |  |
| 3 | Ath-AT2G45180.1 |  | | | |  | Vvi-Vitvi02g01440\_t001 |  | Vvi-Vitvi15g00714\_t001 |  |  |  |  |  |
| 3 | Ath-AT2G45190.1 |  | | | |  | Vvi-Vitvi02g00510\_t001 |  | Vvi-Vitvi15g00708\_t002 |  |  |  |  |  |
| 3 | Ath-AT2G45200.2 |  | | | |  | | | |  | Vvi-Vitvi15g04418\_t001 |  |  |  |  |  |
| 3 | Ath-AT2G45210.1 |  | | | |  | Vvi-Vitvi02g00507\_t001 |  | Vvi-Vitvi15g00706\_t001 |  |  |  |  |  |
| 3 | Ath-AT2G45220.1 |  | Vvi-Vitvi15g00701\_t001 |  | | | |  | | | |  |  |  |  |  |
| 3 | Ath-AT2G45240.1 |  | Vvi-Vitvi15g00702\_t001 |  | | | |  | | | |  |  |  |  |  |
| 3 | Ath-AT2G45243.1 |  | | | |  | | | |  | | | |  |  |  |  |  |
| 3 | Ath-AT2G45250.2 |  | | | |  | | | |  | | | |  |  |  |  |  |
| 3 | Ath-AT2G45260.1 |  | Vvi-Vitvi15g00709\_t001 |  | | | |  | Vvi-Vitvi15g00697\_t001 |  |  |  |  |  |
| 3 | Ath-AT2G45270.1 |  | | | |  | | | |  | Vvi-Vitvi15g00692\_t001 |  |  |  |  |  |
| 3 | Ath-AT2G45280.2 |  | | | |  | | | |  | Vvi-Vitvi15g00691\_t001 |  |  |  |  |  |
| 3 | Ath-AT2G45290.1 |  | | | |  | | | |  | Vvi-Vitvi15g00690\_t001 |  |  |  |  |  |
| 3 | Ath-AT2G45300.4 |  | | | |  | | | |  | Vvi-Vitvi15g01490\_t001 |  |  |  |  |  |
| 3 | Ath-AT2G45310.1 |  | | | |  | Vvi-Vitvi02g04132\_t001 |  | Vvi-Vitvi15g00684\_t001 |  |  |  |  |  |
| 3 | Ath-AT2G45320.1 |  | | | |  | | | |  | Vvi-Vitvi15g00682\_t001 |  |  |  |  |  |
| 3 | Ath-AT2G45330.1 |  | | | |  | | | |  | Vvi-Vitvi15g01488\_t002 |  |  |  |  |  |
| 2 | Ath-AT2G45340.1 |  | Vvi-Vitvi15g00721\_t001 |  | Vvi-Vitvi02g00483\_t001 |  |  |  |  |  |  |
| 2 | Ath-AT2G45350.1 |  | Vvi-Vitvi15g00723\_t001 |  | | | |  |  |  |  |  |  |
| 2 | Ath-AT2G45360.1 |  | Vvi-Vitvi15g00724\_t001 |  | Vvi-Vitvi02g00481\_t001 |  |  |  |  |  |  |
| 2 | Ath-AT2G45380.1 |  | | | |  | Vvi-Vitvi02g00479\_t001 |  |  |  |  |  |  |
| 2 | Ath-AT2G45400.1 |  | | | |  | | | |  |  |  |  |  |  |
| 2 | Ath-AT2G45403.1 |  | | | |  | | | |  |  |  |  |  |  |
| 2 | Ath-AT2G45405.1 |  | | | |  | | | |  |  |  |  |  |  |
| 2 | Ath-AT2G45406.1 |  | | | |  | | | |  |  |  |  |  |  |
| 2 | Ath-AT2G45410.1 |  | | | |  | | | |  |  |  |  |  |  |
| 2 | Ath-AT2G45420.1 |  | | | |  | | | |  |  |  |  |  |  |
| 2 | Ath-AT2G45430.1 |  | Vvi-Vitvi15g00732\_t001 |  | Vvi-Vitvi02g00465\_t001 |  |  |  |  |  |  |
| 0 | Ath-AT2G45440.1 |  |  |  |  |  |  |  |  |
| 0 | Ath-AT2G45450.1 |  |  |  |  |  |  |  |  |
| 0 | Ath-AT2G45460.3 |  |  |  |  |  |  |  |  |
| 1 | Ath-AT2G45470.1 |  | Vvi-Vitvi15g00816\_t001 |  |  |  |  |  |  |  |
| 1 | Ath-AT2G45480.3 |  | Vvi-Vitvi15g00815\_t001 |  |  |  |  |  |  |  |
| 1 | Ath-AT2G45490.1 |  | Vvi-Vitvi15g00814\_t001 |  |  |  |  |  |  |  |
| 1 | Ath-AT2G45500.5 |  | Vvi-Vitvi15g00813\_t003 |  |  |  |  |  |  |  |
| 3 | Ath-AT2G45510.1 |  | Vvi-Vitvi15g01524\_t001 |  | Vvi-Vitvi15g01524\_t001 |  | Vvi-Vitvi16g00872\_t001 |  |  |  |  |  |
| 3 | Ath-AT2G45520.1 |  | Vvi-Vitvi15g04449\_t001 |  | | | |  | | | |  |  |  |  |  |
| 3 | Ath-AT2G45530.1 |  | | | |  | | | |  | | | |  |  |  |  |  |
| 3 | Ath-AT2G45540.2 |  | Vvi-Vitvi15g00810\_t001 |  | | | |  | | | |  |  |  |  |  |
| 4 | Ath-AT2G45550.1 |  | | | |  | | | |  | | | |  | Vvi-Vitvi02g00395\_t001 |  |  |  |  |
| 4 | Ath-AT2G45560.1 |  | | | |  | | | |  | | | |  | | | |  |  |  |  |
| 4 | Ath-AT2G45570.1 |  | | | |  | | | |  | | | |  | | | |  |  |  |  |
| 4 | Ath-AT2G45580.1 |  | Vvi-Vitvi15g00796\_t001 |  | | | |  | | | |  | | | |  |  |  |  |
| 4 | Ath-AT2G45590.1 |  | Vvi-Vitvi15g00790\_t001 |  | | | |  | Vvi-Vitvi16g00880\_t001 |  | | | |  |  |  |  |
| 4 | Ath-AT2G45600.1 |  | Vvi-Vitvi15g00786\_t001 |  | | | |  | Vvi-Vitvi16g00889\_t001 |  | | | |  |  |  |  |
| 4 | Ath-AT2G45610.1 |  | | | |  | | | |  | | | |  | | | |  |  |  |  |
| 4 | Ath-AT2G45620.1 |  | Vvi-Vitvi15g00785\_t001 |  | | | |  | | | |  | | | |  |  |  |  |
| 4 | Ath-AT2G45630.2 |  | Vvi-Vitvi15g00783\_t002 |  | | | |  | | | |  | | | |  |  |  |  |
| 4 | Ath-AT2G45640.1 |  | | | |  | | | |  | | | |  | | | |  |  |  |  |
| 4 | Ath-AT2G45650.1 |  | Vvi-Vitvi15g00776\_t001 |  | | | |  | Vvi-Vitvi16g00894\_t001 |  | | | |  |  |  |  |
| 4 | Ath-AT2G45660.1 |  | Vvi-Vitvi15g00774\_t002 |  | | | |  | Vvi-Vitvi16g00898\_t001 |  | | | |  |  |  |  |
| 4 | Ath-AT2G45670.1 |  | Vvi-Vitvi15g00773\_t002 |  | | | |  | | | |  | | | |  |  |  |  |
| 4 | Ath-AT2G45680.1 |  | Vvi-Vitvi15g00765\_t001 |  | | | |  | Vvi-Vitvi16g01846\_t001 |  | | | |  |  |  |  |
| 3 | Ath-AT2G45690.1 |  | Vvi-Vitvi15g00764\_t001 |  | | | |  |  |  | | | |  |  |  |  |
| 3 | Ath-AT2G45695.1 |  | Vvi-Vitvi15g00761\_t001 |  | | | |  |  |  | | | |  |  |  |  |
| 2 | Ath-AT2G45700.1 |  |  |  | | | |  |  |  | | | |  |  |  |  |
| 2 | Ath-AT2G45710.1 |  |  |  | | | |  |  |  | | | |  |  |  |  |
| 2 | Ath-AT2G45720.1 |  |  |  | Vvi-Vitvi15g00828\_t008 |  |  |  | | | |  |  |  |  |
| 2 | Ath-AT2G45730.1 |  |  |  | | | |  |  |  | | | |  |  |  |  |
| 2 | Ath-AT2G45740.2 |  |  |  | Vvi-Vitvi15g00829\_t003 |  |  |  | | | |  |  |  |  |
| 2 | Ath-AT2G45750.1 |  |  |  | Vvi-Vitvi15g00833\_t001 |  |  |  | | | |  |  |  |  |
| 2 | Ath-AT2G45760.1 |  |  |  | Vvi-Vitvi15g00844\_t001 |  |  |  | Vvi-Vitvi02g00386\_t001 |  |  |  |  |
| 2 | Ath-AT2G45770.1 |  |  |  | Vvi-Vitvi15g00845\_t001.1.6037826b |  |  |  | | | |  |  |  |  |
| 2 | Ath-AT2G45790.1 |  | Vvi-Vitvi15g01178\_t002 |  |  |  |  |  | | | |  |  |  |  |
| 2 | Ath-AT2G45800.1 |  | Vvi-Vitvi15g01171\_t001 |  |  |  |  |  | Vvi-Vitvi02g00368\_t001 |  |  |  |  |
| 2 | Ath-AT2G45810.1 |  | Vvi-Vitvi15g01162\_t002 |  |  |  |  |  | Vvi-Vitvi02g00349\_t001 |  |  |  |  |
| 2 | Ath-AT2G45820.1 |  | Vvi-Vitvi15g01160\_t001 |  |  |  |  |  | Vvi-Vitvi02g00348\_t001 |  |  |  |  |
| 2 | Ath-AT2G45830.1 |  | | | |  |  |  |  |  | Vvi-Vitvi02g01384\_t001 |  |  |  |  |
| 2 | Ath-AT2G45840.1 |  | Vvi-Vitvi15g01682\_t001 |  |  |  |  |  | | | |  |  |  |  |
| 2 | Ath-AT2G45850.2 |  | Vvi-Vitvi15g01145\_t001 |  |  |  |  |  | Vvi-Vitvi02g00331\_t001 |  |  |  |  |
| 2 | Ath-AT2G45860.1 |  | Vvi-Vitvi15g01674\_t001 |  |  |  |  |  | | | |  |  |  |  |
| 2 | Ath-AT2G45870.1 |  | Vvi-Vitvi15g01142\_t001 |  |  |  |  |  | | | |  |  |  |  |
| 2 | Ath-AT2G45880.1 |  | Vvi-Vitvi15g01127\_t001 |  |  |  |  |  | | | |  |  |  |  |
| 2 | Ath-AT2G45890.1 |  | Vvi-Vitvi15g01121\_t001 |  |  |  |  |  | | | |  |  |  |  |
| 2 | Ath-AT2G45900.1 |  | Vvi-Vitvi15g01117\_t002 |  |  |  |  |  | Vvi-Vitvi02g00314\_t002 |  |  |  |  |
| 2 | Ath-AT2G45910.2 |  | Vvi-Vitvi15g01115\_t002 |  |  |  |  |  | | | |  |  |  |  |
| 2 | Ath-AT2G45920.1 |  | | | |  |  |  |  |  | | | |  |  |  |  |
| 2 | Ath-AT2G45930.1 |  | | | |  |  |  |  |  | | | |  |  |  |  |
| 2 | Ath-AT2G45940.2 |  | | | |  |  |  |  |  | | | |  |  |  |  |
| 2 | Ath-AT2G45950.2 |  | Vvi-Vitvi15g01113\_t001 |  |  |  |  |  | | | |  |  |  |  |
| 2 | Ath-AT2G45960.3 |  | Vvi-Vitvi15g01110\_t002 |  |  |  |  |  | Vvi-Vitvi02g00310\_t001 |  |  |  |  |
| 2 | Ath-AT2G45970.1 |  | Vvi-Vitvi15g01106\_t001 |  |  |  |  |  | Vvi-Vitvi02g00303\_t001 |  |  |  |  |
| 1 | Ath-AT2G45980.1 |  | Vvi-Vitvi15g04613\_t001 |  |  |  |  |  |  |  |
| 1 | Ath-AT2G45990.1 |  | Vvi-Vitvi15g01104\_t001 |  |  |  |  |  |  |  |
| 1 | Ath-AT2G46000.1 |  | Vvi-Vitvi15g01103\_t001 |  |  |  |  |  |  |  |
| 1 | Ath-AT2G46020.3 |  | Vvi-Vitvi15g01100\_t001 |  |  |  |  |  |  |  |
| 2 | Ath-AT2G46030.3 |  | Vvi-Vitvi15g01099\_t003 |  | Vvi-Vitvi02g04001\_t001 |  |  |  |  |  |  |
| 3 | Ath-AT2G46040.2 |  | | | |  | | | |  | Vvi-Vitvi15g01056\_t001 |  |  |  |  |  |
| 3 | Ath-AT2G46050.1 |  | | | |  | | | |  | Vvi-Vitvi15g01061\_t001 |  |  |  |  |  |
| 3 | Ath-AT2G46060.1 |  | | | |  | | | |  | Vvi-Vitvi15g01062\_t001 |  |  |  |  |  |
| 3 | Ath-AT2G46070.3 |  | | | |  | Vvi-Vitvi02g00023\_t001 |  | Vvi-Vitvi15g01077\_t001 |  |  |  |  |  |
| 3 | Ath-AT2G46080.1 |  | | | |  | | | |  | Vvi-Vitvi15g01644\_t003 |  |  |  |  |  |
| 4 | Ath-AT2G46090.1 |  | | | |  | | | |  | | | |  | Vvi-Vitvi15g01054\_t002 |  |  |  |  |
| 4 | Ath-AT2G46100.1 |  | | | |  | | | |  | Vvi-Vitvi15g01080\_t001 |  | | | |  |  |  |  |
| 4 | Ath-AT2G46110.1 |  | Vvi-Vitvi15g01089\_t001 |  | | | |  | | | |  | | | |  |  |  |  |
| 4 | Ath-AT2G46130.1 |  | Vvi-Vitvi15g01087\_t001 |  | | | |  | | | |  | | | |  |  |  |  |
| 4 | Ath-AT2G46140.1 |  | Vvi-Vitvi15g01646\_t001 |  | | | |  | | | |  | | | |  |  |  |  |
| 4 | Ath-AT2G46150.1 |  | | | |  | Vvi-Vitvi02g01317\_t001 |  | Vvi-Vitvi15g01083\_t001 |  | | | |  |  |  |  |
| 3 | Ath-AT2G46160.1 |  | Vvi-Vitvi15g01082\_t001 |  | | | |  |  |  | | | |  |  |  |  |
| 2 | Ath-AT2G46170.1 |  |  |  | Vvi-Vitvi02g00059\_t001 |  |  |  | Vvi-Vitvi15g01640\_t001.1.6037826c |  |  |  |  |
| 2 | Ath-AT2G46180.1 |  |  |  | | | |  |  |  | Vvi-Vitvi15g01053\_t001 |  |  |  |  |
| 2 | Ath-AT2G46190.1 |  |  |  | | | |  |  |  | | | |  |  |  |  |
| 2 | Ath-AT2G46200.1 |  |  |  | | | |  |  |  | Vvi-Vitvi15g01052\_t001 |  |  |  |  |
| 2 | Ath-AT2G46210.1 |  |  |  | | | |  |  |  | Vvi-Vitvi15g01049\_t001 |  |  |  |  |
| 2 | Ath-AT2G46220.1 |  |  |  | | | |  |  |  | Vvi-Vitvi15g01048\_t001 |  |  |  |  |
| 2 | Ath-AT2G46225.2 |  |  |  | | | |  |  |  | Vvi-Vitvi15g01047\_t001 |  |  |  |  |
| 2 | Ath-AT2G46230.1 |  |  |  | | | |  |  |  | Vvi-Vitvi15g04598\_t001 |  |  |  |  |
| 2 | Ath-AT2G46240.1 |  |  |  | | | |  |  |  | Vvi-Vitvi15g01034\_t002 |  |  |  |  |
| 2 | Ath-AT2G46250.1 |  |  |  | Vvi-Vitvi02g01329\_t001 |  |  |  | Vvi-Vitvi15g01032\_t001 |  |  |  |  |
| 2 | Ath-AT2G46260.1 |  |  |  | Vvi-Vitvi02g00078\_t001 |  |  |  | Vvi-Vitvi15g01031\_t001 |  |  |  |  |
| 2 | Ath-AT2G46270.1 |  |  |  | Vvi-Vitvi02g00089\_t001 |  |  |  | Vvi-Vitvi15g01027\_t001 |  |  |  |  |
| 2 | Ath-AT2G46280.1 |  |  |  | | | |  |  |  | | | |  |  |  |  |
| 2 | Ath-AT2G46290.1 |  |  |  | | | |  |  |  | | | |  |  |  |  |
| 2 | Ath-AT2G46300.1 |  |  |  | | | |  |  |  | Vvi-Vitvi15g01024\_t001 |  |  |  |  |
| 2 | Ath-AT2G46308.1 |  |  |  | | | |  |  |  | | | |  |  |  |  |
| 2 | Ath-AT2G46310.1 |  |  |  | | | |  |  |  | Vvi-Vitvi15g01021\_t001 |  |  |  |  |
| 2 | Ath-AT2G46320.1 |  |  |  | | | |  |  |  | Vvi-Vitvi15g01020\_t001 |  |  |  |  |
| 2 | Ath-AT2G46330.1 |  |  |  | Vvi-Vitvi02g01335\_t001 |  |  |  | Vvi-Vitvi15g01019\_t001 |  |  |  |  |
| 2 | Ath-AT2G46340.1 |  |  |  | Vvi-Vitvi02g00098\_t001 |  |  |  | Vvi-Vitvi15g01016\_t002 |  |  |  |  |
| 2 | Ath-AT2G46360.1 |  |  |  | | | |  |  |  | | | |  |  |  |  |
| 2 | Ath-AT2G46370.4 |  |  |  | | | |  |  |  | Vvi-Vitvi15g01014\_t003 |  |  |  |  |
| 2 | Ath-AT2G46375.1 |  |  |  | | | |  |  |  | | | |  |  |  |  |
| 2 | Ath-AT2G46380.1 |  |  |  | Vvi-Vitvi02g00105\_t001 |  |  |  | Vvi-Vitvi15g01013\_t001 |  |  |  |  |
| 2 | Ath-AT2G46390.1 |  |  |  | | | |  |  |  | | | |  |  |  |  |
| 2 | Ath-AT2G46400.1 |  |  |  | Vvi-Vitvi02g00114\_t001 |  |  |  | Vvi-Vitvi15g01003\_t001 |  |  |  |  |
| 2 | Ath-AT2G46410.1 |  |  |  | | | |  |  |  | Vvi-Vitvi15g01002\_t001 |  |  |  |  |
| 2 | Ath-AT2G46420.1 |  |  |  | | | |  |  |  | Vvi-Vitvi15g00991\_t001 |  |  |  |  |
| 2 | Ath-AT2G46430.2 |  |  |  | | | |  |  |  | Vvi-Vitvi15g01617\_t001 |  |  |  |  |
| 2 | Ath-AT2G46440.1 |  |  |  | | | |  |  |  | | | |  |  |  |  |
| 2 | Ath-AT2G46450.1 |  |  |  | | | |  |  |  | | | |  |  |  |  |
| 2 | Ath-AT2G46455.2 |  |  |  | | | |  |  |  | | | |  |  |  |  |
| 2 | Ath-AT2G46460.1 |  |  |  | | | |  |  |  | | | |  |  |  |  |
| 2 | Ath-AT2G46470.1 |  |  |  | Vvi-Vitvi02g00129\_t001 |  |  |  | | | |  |  |  |  |
| 2 | Ath-AT2G46480.4 |  |  |  | | | |  |  |  | | | |  |  |  |  |
| 2 | Ath-AT2G46490.1 |  |  |  | | | |  |  |  | | | |  |  |  |  |
| 2 | Ath-AT2G46493.1 |  |  |  | | | |  |  |  | | | |  |  |  |  |
| 2 | Ath-AT2G46494.1 |  |  |  | Vvi-Vitvi02g00130\_t001 |  |  |  | Vvi-Vitvi15g01611\_t001 |  |  |  |  |
| 1 | Ath-AT2G46495.1 |  |  |  | | | |  |  |  |  |  |  |
| 3 | Ath-AT2G46500.2 |  | Vvi-Vitvi16g01417\_t003 |  | Vvi-Vitvi02g00149\_t001 |  | Vvi-Vitvi15g01576\_t001 |  |  |  |  |  |
| 3 | Ath-AT2G46505.1 |  | | | |  | | | |  | | | |  |  |  |  |  |
| 3 | Ath-AT2G46510.1 |  | | | |  | | | |  | Vvi-Vitvi15g00948\_t001 |  |  |  |  |  |
| 3 | Ath-AT2G46520.1 |  | | | |  | | | |  | Vvi-Vitvi15g01571\_t002 |  |  |  |  |  |
| 3 | Ath-AT2G46530.3 |  | | | |  | Vvi-Vitvi02g00163\_t001 |  | Vvi-Vitvi15g00946\_t001 |  |  |  |  |  |
| 2 | Ath-AT2G46535.1 |  | | | |  |  |  | | | |  |  |  |  |  |
| 2 | Ath-AT2G46540.1 |  | | | |  |  |  | Vvi-Vitvi15g01569\_t001 |  |  |  |  |  |
| 2 | Ath-AT2G46550.1 |  | Vvi-Vitvi16g02085\_t001 |  |  |  | Vvi-Vitvi15g00945\_t008 |  |  |  |  |  |
| 2 | Ath-AT2G46560.1 |  | | | |  |  |  | Vvi-Vitvi15g00944\_t001 |  |  |  |  |  |
| 2 | Ath-AT2G46570.1 |  | | | |  |  |  | Vvi-Vitvi15g00941\_t001 |  |  |  |  |  |
| 2 | Ath-AT2G46567.1 |  | | | |  |  |  | | | |  |  |  |  |  |
| 2 | Ath-AT2G46580.1 |  | | | |  |  |  | Vvi-Vitvi15g00940\_t001 |  |  |  |  |  |
| 2 | Ath-AT2G46590.2 |  | Vvi-Vitvi16g01384\_t001 |  |  |  | Vvi-Vitvi15g00936\_t001 |  |  |  |  |  |
| 2 | Ath-AT2G46600.1 |  | | | |  |  |  | Vvi-Vitvi15g00928\_t001 |  |  |  |  |  |
| 2 | Ath-AT2G46610.1 |  | | | |  |  |  | Vvi-Vitvi15g00926\_t003 |  |  |  |  |  |
| 2 | Ath-AT2G46620.1 |  | Vvi-Vitvi16g01374\_t001 |  | Vvi-Vitvi15g00920\_t001 |  |  |  |  |  |  |
| 2 | Ath-AT2G46630.1 |  | | | |  | Vvi-Vitvi15g01559\_t001 |  |  |  |  |  |  |
| 2 | Ath-AT2G46640.4 |  | | | |  | Vvi-Vitvi15g00916\_t001 |  |  |  |  |  |  |
| 2 | Ath-AT2G46650.1 |  | Vvi-Vitvi16g01367\_t001 |  | | | |  |  |  |  |  |  |
| 3 | Ath-AT2G46660.1 |  | | | |  | Vvi-Vitvi15g00915\_t001 |  | Vvi-Vitvi02g00226\_t001 |  |  |  |  |  |
| 3 | Ath-AT2G46670.1 |  | | | |  | | | |  | | | |  |  |  |  |  |
| 3 | Ath-AT2G46680.1 |  | Vvi-Vitvi16g01362\_t001 |  | Vvi-Vitvi15g00912\_t001 |  | Vvi-Vitvi02g00228\_t001 |  |  |  |  |  |
| 3 | Ath-AT2G46690.1 |  | Vvi-Vitvi16g01359\_t001 |  | Vvi-Vitvi15g00910\_t001 |  | | | |  |  |  |  |  |
| 3 | Ath-AT2G46700.1 |  | Vvi-Vitvi16g01358\_t001 |  | Vvi-Vitvi15g00908\_t001 |  | | | |  |  |  |  |  |
| 3 | Ath-AT2G46710.1 |  | | | |  | Vvi-Vitvi15g00907\_t001 |  | | | |  |  |  |  |  |
| 3 | Ath-AT2G46720.1 |  | | | |  | | | |  | | | |  |  |  |  |  |
| 3 | Ath-AT2G46735.1 |  | | | |  | Vvi-Vitvi15g01553\_t001 |  | | | |  |  |  |  |  |
| 3 | Ath-AT2G46740.1 |  | | | |  | Vvi-Vitvi15g00894\_t001 |  | | | |  |  |  |  |  |
| 3 | Ath-AT2G46750.1 |  | | | |  | | | |  | | | |  |  |  |  |  |
| 3 | Ath-AT2G46760.1 |  | | | |  | | | |  | | | |  |  |  |  |  |
| 3 | Ath-AT2G46765.1 |  | | | |  | | | |  | | | |  |  |  |  |  |
| 3 | Ath-AT2G46770.1 |  | | | |  | Vvi-Vitvi15g00889\_t001 |  | Vvi-Vitvi02g00242\_t001 |  |  |  |  |  |
| 3 | Ath-AT2G46780.1 |  | Vvi-Vitvi16g02069\_t001 |  | Vvi-Vitvi15g00887\_t001 |  | | | |  |  |  |  |  |
| 3 | Ath-AT2G46790.1 |  | Vvi-Vitvi16g01340\_t001 |  | Vvi-Vitvi15g00879\_t001 |  | | | |  |  |  |  |  |
| 2 | Ath-AT2G46800.2 |  |  |  | Vvi-Vitvi15g00877\_t001 |  | Vvi-Vitvi02g01369\_t001 |  |  |  |  |  |
| 2 | Ath-AT2G46810.1 |  |  |  | Vvi-Vitvi15g00876\_t001 |  | | | |  |  |  |  |  |
| 2 | Ath-AT2G46820.1 |  |  |  | Vvi-Vitvi15g01549\_t001 |  | | | |  |  |  |  |  |
| 2 | Ath-AT2G46830.1 |  |  |  | Vvi-Vitvi15g00870\_t001.1.6037826c |  | | | |  |  |  |  |  |
| 2 | Ath-AT2G46840.1 |  |  |  | | | |  | | | |  |  |  |  |  |
| 2 | Ath-AT2G46850.1 |  |  |  | Vvi-Vitvi15g00868\_t001 |  | | | |  |  |  |  |  |
| 2 | Ath-AT2G46860.1 |  |  |  | Vvi-Vitvi15g00867\_t001 |  | Vvi-Vitvi02g00272\_t001 |  |  |  |  |  |
| 2 | Ath-AT2G46870.1 |  |  |  | Vvi-Vitvi15g00863\_t001 |  | Vvi-Vitvi02g00275\_t001 |  |  |  |  |  |
| 1 | Ath-AT2G46880.1 |  |  |  | Vvi-Vitvi15g01538\_t004 |  |  |  |  |  |  |
| 1 | Ath-AT2G46890.1 |  |  |  | Vvi-Vitvi15g00857\_t001 |  |  |  |  |  |  |
| 1 | Ath-AT2G46900.1 |  |  |  | Vvi-Vitvi15g00856\_t001 |  |  |  |  |  |  |
| 1 | Ath-AT2G46910.1 |  |  |  | Vvi-Vitvi15g00854\_t002 |  |  |  |  |  |  |
| 1 | Ath-AT2G46915.1 |  |  |  | Vvi-Vitvi15g00853\_t001 |  |  |  |  |  |  |
| 1 | Ath-AT2G46920.2 |  | Vvi-Vitvi07g00506\_t002 |  |  |  |  |  |  |  |
| 1 | Ath-AT2G46930.1 |  | Vvi-Vitvi07g02249\_t003 |  |  |  |  |  |  |  |
| 1 | Ath-AT2G46940.1 |  | Vvi-Vitvi07g02074\_t001 |  |  |  |  |  |  |  |
| 1 | Ath-AT2G46950.1 |  | Vvi-Vitvi07g00516\_t001 |  |  |  |  |  |  |  |
| 1 | Ath-AT2G46960.2 |  | | | |  |  |  |  |  |  |  |
| 1 | Ath-AT2G46970.1 |  | Vvi-Vitvi07g02251\_t002 |  |  |  |  |  |  |  |
| 1 | Ath-AT2G46980.2 |  | Vvi-Vitvi07g00518\_t001 |  |  |  |  |  |  |  |
| 1 | Ath-AT2G46990.1 |  | Vvi-Vitvi07g00521\_t001 |  |  |  |  |  |  |  |
| 1 | Ath-AT2G46995.1 |  | | | |  |  |  |  |  |  |  |
| 1 | Ath-AT2G47000.5 |  | Vvi-Vitvi07g00534\_t001 |  |  |  |  |  |  |  |
| 1 | Ath-AT2G47010.1 |  | Vvi-Vitvi07g00538\_t001 |  |  |  |  |  |  |  |
| 1 | Ath-AT2G47020.3 |  | Vvi-Vitvi07g00539\_t001 |  |  |  |  |  |  |  |
| 0 | Ath-AT2G47030.1 |  |  |  |  |  |  |  |  |
| 0 | Ath-AT2G47040.1 |  |  |  |  |  |  |  |  |
| 0 | Ath-AT2G47050.1 |  |  |  |  |  |  |  |  |
| 1 | Ath-AT2G47060.4 |  | Vvi-Vitvi07g00492\_t001 |  |  |  |  |  |  |  |
| 1 | Ath-AT2G47070.1 |  | Vvi-Vitvi07g00491\_t001 |  |  |  |  |  |  |  |
| 1 | Ath-AT2G47090.1 |  | Vvi-Vitvi07g00484\_t002 |  |  |  |  |  |  |  |
| 1 | Ath-AT2G47110.1 |  | | | |  |  |  |  |  |  |  |
| 1 | Ath-AT2G47115.1 |  | | | |  |  |  |  |  |  |  |
| 1 | Ath-AT2G47120.2 |  | Vvi-Vitvi07g02245\_t001 |  |  |  |  |  |  |  |
| 1 | Ath-AT2G47130.1 |  | | | |  |  |  |  |  |  |  |
| 1 | Ath-AT2G47140.1 |  | | | |  |  |  |  |  |  |  |
| 1 | Ath-AT2G47150.1 |  | | | |  |  |  |  |  |  |  |
| 1 | Ath-AT2G47160.2 |  | Vvi-Vitvi07g00470\_t001 |  |  |  |  |  |  |  |
| 1 | Ath-AT2G47170.1 |  | Vvi-Vitvi07g00464\_t001 |  |  |  |  |  |  |  |
| 2 | Ath-AT2G47180.1 |  | Vvi-Vitvi07g00457\_t001 |  | Vvi-Vitvi14g02457\_t001 |  |  |  |  |  |  |
| 2 | Ath-AT2G47190.1 |  | Vvi-Vitvi07g00455\_t001 |  | | | |  |  |  |  |  |  |
| 2 | Ath-AT2G47200.1 |  | | | |  | | | |  |  |  |  |  |  |
| 2 | Ath-AT2G47210.2 |  | Vvi-Vitvi07g00452\_t003 |  | | | |  |  |  |  |  |  |
| 2 | Ath-AT2G47220.1 |  | | | |  | | | |  |  |  |  |  |  |
| 2 | Ath-AT2G47230.2 |  | Vvi-Vitvi07g00446\_t001 |  | Vvi-Vitvi14g00072\_t001 |  |  |  |  |  |  |
| 2 | Ath-AT2G47240.1 |  | Vvi-Vitvi07g00445\_t001 |  | | | |  |  |  |  |  |  |
| 2 | Ath-AT2G47245.1 |  | | | |  | | | |  |  |  |  |  |  |
| 2 | Ath-AT2G47250.1 |  | Vvi-Vitvi07g00442\_t001 |  | | | |  |  |  |  |  |  |
| 2 | Ath-AT2G47260.1 |  | Vvi-Vitvi07g00434\_t001 |  | | | |  |  |  |  |  |  |
| 2 | Ath-AT2G47270.1 |  | Vvi-Vitvi07g02230\_t001 |  | | | |  |  |  |  |  |  |
| 2 | Ath-AT2G47280.1 |  | | | |  | Vvi-Vitvi14g04020\_t001 |  |  |  |  |  |  |
| 2 | Ath-AT2G47300.2 |  | | | |  | | | |  |  |  |  |  |  |
| 2 | Ath-AT2G47310.1 |  | | | |  | | | |  |  |  |  |  |  |
| 3 | Ath-AT2G47320.1 |  | | | |  | | | |  | Vvi-Vitvi05g00120\_t001 |  |  |  |  |  |
| 3 | Ath-AT2G47330.1 |  | Vvi-Vitvi07g00425\_t001 |  | | | |  | | | |  |  |  |  |  |
| 3 | Ath-AT2G47340.1 |  | Vvi-Vitvi07g02228\_t001 |  | | | |  | Vvi-Vitvi05g01772\_t001 |  |  |  |  |  |
| 3 | Ath-AT2G47350.1 |  | Vvi-Vitvi07g00423\_t001.3.6037826e |  | Vvi-Vitvi14g00090\_t001 |  | Vvi-Vitvi05g00114\_t001 |  |  |  |  |  |
| 3 | Ath-AT2G47360.1 |  | Vvi-Vitvi07g00420\_t001 |  | | | |  | | | |  |  |  |  |  |
| 3 | Ath-AT2G47370.1 |  | Vvi-Vitvi07g00415\_t001 |  | | | |  | | | |  |  |  |  |  |
| 3 | Ath-AT2G47380.1 |  | Vvi-Vitvi07g02224\_t001 |  | Vvi-Vitvi14g02473\_t001 |  | | | |  |  |  |  |  |
| 3 | Ath-AT2G47390.1 |  | Vvi-Vitvi07g00414\_t003 |  | | | |  | | | |  |  |  |  |  |
| 3 | Ath-AT2G47400.1 |  | | | |  | | | |  | | | |  |  |  |  |  |
| 3 | Ath-AT2G47410.5 |  | Vvi-Vitvi07g00410\_t002 |  | | | |  | Vvi-Vitvi05g00106\_t001 |  |  |  |  |  |
| 3 | Ath-AT2G47420.1 |  | Vvi-Vitvi07g00409\_t001 |  | | | |  | | | |  |  |  |  |  |
| 3 | Ath-AT2G47430.1 |  | Vvi-Vitvi07g00407\_t001 |  | | | |  | | | |  |  |  |  |  |
| 3 | Ath-AT2G47440.1 |  | Vvi-Vitvi07g00400\_t001 |  | | | |  | | | |  |  |  |  |  |
| 3 | Ath-AT2G47450.1 |  | | | |  | | | |  | | | |  |  |  |  |  |
| 3 | Ath-AT2G47460.1 |  | Vvi-Vitvi07g00393\_t001 |  | | | |  | Vvi-Vitvi05g00084\_t001 |  |  |  |  |  |
| 3 | Ath-AT2G47470.1 |  | | | |  | Vvi-Vitvi14g00111\_t001 |  | | | |  |  |  |  |  |
| 3 | Ath-AT2G47480.1 |  | Vvi-Vitvi07g02216\_t001 |  | Vvi-Vitvi14g02479\_t001 |  | Vvi-Vitvi05g01766\_t001 |  |  |  |  |  |
| 2 | Ath-AT2G47485.1 |  | Vvi-Vitvi07g02211\_t001 |  | | | |  |  |  |  |  |  |
| 2 | Ath-AT2G47490.1 |  | Vvi-Vitvi07g00369\_t001 |  | | | |  |  |  |  |  |  |
| 2 | Ath-AT2G47500.1 |  | Vvi-Vitvi07g00368\_t001 |  | Vvi-Vitvi14g00127\_t001 |  |  |  |  |  |  |
| 2 | Ath-AT2G47510.2 |  | Vvi-Vitvi07g00362\_t001 |  | Vvi-Vitvi14g00138\_t001 |  |  |  |  |  |  |
| 2 | Ath-AT2G47520.1 |  | Vvi-Vitvi07g00357\_t001 |  | | | |  |  |  |  |  |  |
| 2 | Ath-AT2G47530.1 |  | Vvi-Vitvi07g02208\_t001 |  | | | |  |  |  |  |  |  |
| 2 | Ath-AT2G47540.1 |  | | | |  | | | |  |  |  |  |  |  |
| 2 | Ath-AT2G47550.1 |  | Vvi-Vitvi07g00350\_t001 |  | | | |  |  |  |  |  |  |
| 2 | Ath-AT2G47560.1 |  | Vvi-Vitvi07g00348\_t001 |  | | | |  |  |  |  |  |  |
| 2 | Ath-AT2G47570.1 |  | Vvi-Vitvi07g00346\_t001 |  | Vvi-Vitvi14g00151\_t001 |  |  |  |  |  |  |
| 2 | Ath-AT2G47580.1 |  | Vvi-Vitvi07g00340\_t001 |  | | | |  |  |  |  |  |  |
| 2 | Ath-AT2G47590.1 |  | Vvi-Vitvi07g00333\_t001 |  | | | |  |  |  |  |  |  |
| 2 | Ath-AT2G47600.1 |  | Vvi-Vitvi07g00332\_t001 |  | | | |  |  |  |  |  |  |
| 2 | Ath-AT2G47610.1 |  | | | |  | | | |  |  |  |  |  |  |
| 2 | Ath-AT2G47620.1 |  | | | |  | | | |  |  |  |  |  |  |
| 2 | Ath-AT2G47630.1 |  | Vvi-Vitvi07g00327\_t001 |  | | | |  |  |  |  |  |  |
| 2 | Ath-AT2G47640.1 |  | Vvi-Vitvi07g04082\_t001 |  | | | |  |  |  |  |  |  |
| 2 | Ath-AT2G47650.2 |  | Vvi-Vitvi07g00325\_t001 |  | | | |  |  |  |  |  |  |
| 2 | Ath-AT2G47670.1 |  | Vvi-Vitvi07g00324\_t001 |  | | | |  |  |  |  |  |  |
| 2 | Ath-AT2G47680.1 |  | | | |  | | | |  |  |  |  |  |  |
| 2 | Ath-AT2G47690.1 |  | | | |  | | | |  |  |  |  |  |  |
| 2 | Ath-AT2G47700.1 |  | Vvi-Vitvi07g02202\_t001 |  | | | |  |  |  |  |  |  |
| 2 | Ath-AT2G47710.1 |  | Vvi-Vitvi07g00314\_t001 |  | | | |  |  |  |  |  |  |
| 2 | Ath-AT2G47730.1 |  | Vvi-Vitvi07g02188\_t003 |  | | | |  |  |  |  |  |  |
| 2 | Ath-AT2G47720.1 |  | | | |  | | | |  |  |  |  |  |  |
| 2 | Ath-AT2G47750.1 |  | Vvi-Vitvi07g00293\_t001 |  | | | |  |  |  |  |  |  |
| 2 | Ath-AT2G47760.5 |  | Vvi-Vitvi07g00296\_t001 |  | | | |  |  |  |  |  |  |
| 2 | Ath-AT2G47770.1 |  | Vvi-Vitvi07g00298\_t001 |  | | | |  |  |  |  |  |  |
| 2 | Ath-AT2G47780.1 |  | Vvi-Vitvi07g00301\_t001 |  | Vvi-Vitvi14g00167\_t001 |  |  |  |  |  |  |
| 1 | Ath-AT2G47790.1 |  | Vvi-Vitvi07g00309\_t001 |  |  |  |  |  |  |  |
| 1 | Ath-AT2G47800.1 |  | Vvi-Vitvi07g00311\_t001 |  |  |  |  |  |  |  |
| 1 | Ath-AT2G47810.1 |  | Vvi-Vitvi07g00273\_t001 |  |  |  |  |  |  |  |
| 1 | Ath-AT2G47820.2 |  | Vvi-Vitvi07g00272\_t001 |  |  |  |  |  |  |  |
| 1 | Ath-AT2G47830.1 |  | Vvi-Vitvi07g00266\_t001 |  |  |  |  |  |  |  |
| 1 | Ath-AT2G47840.1 |  | | | |  |  |  |  |  |  |  |
| 1 | Ath-AT2G47844.1 |  | | | |  |  |  |  |  |  |  |
| 1 | Ath-AT2G47850.1 |  | Vvi-Vitvi07g00262\_t002 |  |  |  |  |  |  |  |
| 1 | Ath-AT2G47860.3 |  | Vvi-Vitvi07g00260\_t001 |  |  |  |  |  |  |  |
| 1 | Ath-AT2G47870.1 |  | Vvi-Vitvi07g00256\_t001 |  |  |  |  |  |  |  |
| 1 | Ath-AT2G47880.1 |  | Vvi-Vitvi07g00255\_t001 |  |  |  |  |  |  |  |
| 1 | Ath-AT2G47885.1 |  | | | |  |  |  |  |  |  |  |
| 1 | Ath-AT2G47890.1 |  | Vvi-Vitvi07g00252\_t001 |  |  |  |  |  |  |  |
| 1 | Ath-AT2G47900.3 |  | Vvi-Vitvi07g02159\_t002 |  |  |  |  |  |  |  |
| 1 | Ath-AT2G47910.1 |  | | | |  |  |  |  |  |  |  |
| 1 | Ath-AT2G47920.1 |  | Vvi-Vitvi07g00243\_t001 |  |  |  |  |  |  |  |
| 1 | Ath-AT2G47930.1 |  | | | |  |  |  |  |  |  |  |
| 1 | Ath-AT2G47935.1 |  | | | |  |  |  |  |  |  |  |
| 1 | Ath-AT2G47940.1 |  | Vvi-Vitvi07g00240\_t001 |  |  |  |  |  |  |  |
| 1 | Ath-AT2G47950.2 |  | | | |  |  |  |  |  |  |  |
| 1 | Ath-AT2G47960.1 |  | | | |  |  |  |  |  |  |  |
| 1 | Ath-AT2G47970.1 |  | Vvi-Vitvi07g00228\_t001 |  |  |  |  |  |  |  |
| 1 | Ath-AT2G47980.1 |  | Vvi-Vitvi07g00227\_t001 |  |  |  |  |  |  |  |
| 1 | Ath-AT2G47990.1 |  | Vvi-Vitvi07g00226\_t001 |  |  |  |  |  |  |  |
| 1 | Ath-AT2G48000.1 |  | Vvi-Vitvi07g00216\_t001 |  |  |  |  |  |  |  |
| 1 | Ath-AT2G48010.1 |  | Vvi-Vitvi07g00208\_t001 |  |  |  |  |  |  |  |
| 1 | Ath-AT2G48020.1 |  | Vvi-Vitvi07g00207\_t001 |  |  |  |  |  |  |  |
| 1 | Ath-AT2G48030.1 |  | Vvi-Vitvi07g00202\_t001 |  |  |  |  |  |  |  |
| 1 | Ath-AT2G48060.2 |  | Vvi-Vitvi07g00199\_t001 |  |  |  |  |  |  |  |
| 1 | Ath-AT2G48070.3 |  | Vvi-Vitvi07g00198\_t001 |  |  |  |  |  |  |  |
| 1 | Ath-AT2G48075.1 |  | | | |  |  |  |  |  |  |  |
| 1 | Ath-AT2G48080.1 |  | Vvi-Vitvi07g00190\_t001 |  |  |  |  |  |  |  |
| 1 | Ath-AT2G48090.2 |  | | | |  |  |  |  |  |  |  |
| 1 | Ath-AT2G48100.1 |  | Vvi-Vitvi07g00171\_t003 |  |  |  |  |  |  |  |
| 1 | Ath-AT2G48110.1 |  | Vvi-Vitvi07g00169\_t001 |  |  |  |  |  |  |  |
| 1 | Ath-AT2G48120.1 |  | Vvi-Vitvi07g00168\_t001 |  |  |  |  |  |  |  |
| 1 | Ath-AT2G48121.2 |  | | | |  |  |  |  |  |  |  |
| 1 | Ath-AT2G48130.3 |  | | | |  |  |  |  |  |  |  |
| 1 | Ath-AT2G48140.1 |  | | | |  |  |  |  |  |  |  |
| 1 | Ath-AT2G48150.1 |  | | | |  |  |  |  |  |  |  |
| 1 | Ath-AT2G48160.2 |  | Vvi-Vitvi07g00162\_t001 |  |  |  |  |  |  |  |
