# Supplementary material for: Genome-wide identification, characterization and gene expression of BES1 transcription factor family in grapevine (Vitis vinifera L.)
Source: Sci Rep. 2023 Jan 5;13:240. doi: 10.1038/s41598-022-24407-y (PMC9816167; doi:10.1038/s41598-022-24407-y)
Supplement: Supplementary file 3 — Supplementary Information. [file 41598_2022_24407_MOESM3_ESM.zip › Vvi_Ath/Vitis_vinifera.PN40024.v4.dna_sm.toplevel.fa.vs.Arabidopsis_thaliana.TAIR10.dna_sm.toplevel.fa.html/Ath-3.html]

|  |  |  |  |  |  |  |  |  |  |  |  |  |  |  |  |  |  |
| --- | --- | --- | --- | --- | --- | --- | --- | --- | --- | --- | --- | --- | --- | --- | --- | --- | --- |
| Duplication depth | Reference chromosome | Collinear blocks | | | | | | | | | | | | | | | |
| 1 | Ath-AT3G01015.1 |  | Vvi-Vitvi14g02039\_t001 |  |  |  |  |  |  |  |
| 1 | Ath-AT3G01010.1 |  | Vvi-Vitvi14g03099\_t002 |  |  |  |  |  |  |  |
| 1 | Ath-AT3G01020.1 |  | | | |  |  |  |  |  |  |  |
| 1 | Ath-AT3G01030.1 |  | | | |  |  |  |  |  |  |  |
| 1 | Ath-AT3G01040.2 |  | Vvi-Vitvi14g02028\_t001 |  |  |  |  |  |  |  |
| 1 | Ath-AT3G01050.2 |  | Vvi-Vitvi14g02027\_t001 |  |  |  |  |  |  |  |
| 1 | Ath-AT3G01060.1 |  | Vvi-Vitvi14g02021\_t001 |  |  |  |  |  |  |  |
| 1 | Ath-AT3G01070.1 |  | Vvi-Vitvi14g03096\_t001 |  |  |  |  |  |  |  |
| 1 | Ath-AT3G01080.2 |  | Vvi-Vitvi14g02007\_t001 |  |  |  |  |  |  |  |
| 1 | Ath-AT3G01085.3 |  | Vvi-Vitvi14g02006\_t001 |  |  |  |  |  |  |  |
| 1 | Ath-AT3G01090.2 |  | Vvi-Vitvi14g02002\_t003 |  |  |  |  |  |  |  |
| 1 | Ath-AT3G01100.4 |  | Vvi-Vitvi14g02000\_t001 |  |  |  |  |  |  |  |
| 1 | Ath-AT3G01120.1 |  | Vvi-Vitvi14g01990\_t001 |  |  |  |  |  |  |  |
| 1 | Ath-AT3G01130.8 |  | | | |  |  |  |  |  |  |  |
| 1 | Ath-AT3G01140.1 |  | Vvi-Vitvi14g01987\_t001 |  |  |  |  |  |  |  |
| 1 | Ath-AT3G01150.1 |  | Vvi-Vitvi14g01978\_t001 |  |  |  |  |  |  |  |
| 1 | Ath-AT3G01160.1 |  | | | |  |  |  |  |  |  |  |
| 1 | Ath-AT3G01170.1 |  | Vvi-Vitvi14g01972\_t001 |  |  |  |  |  |  |  |
| 1 | Ath-AT3G01175.1 |  | | | |  |  |  |  |  |  |  |
| 1 | Ath-AT3G01180.1 |  | Vvi-Vitvi14g01968\_t001 |  |  |  |  |  |  |  |
| 0 | Ath-AT3G01185.1 |  |  |  |  |  |  |  |  |
| 0 | Ath-AT3G01190.1 |  |  |  |  |  |  |  |  |
| 0 | Ath-AT3G01200.1 |  |  |  |  |  |  |  |  |
| 1 | Ath-AT3G01210.1 |  | Vvi-Vitvi14g03076\_t001 |  |  |  |  |  |  |  |
| 1 | Ath-AT3G01220.1 |  | Vvi-Vitvi14g01922\_t001 |  |  |  |  |  |  |  |
| 1 | Ath-AT3G01230.1 |  | | | |  |  |  |  |  |  |  |
| 1 | Ath-AT3G01240.1 |  | | | |  |  |  |  |  |  |  |
| 1 | Ath-AT3G01250.1 |  | | | |  |  |  |  |  |  |  |
| 1 | Ath-AT3G01260.1 |  | Vvi-Vitvi14g01911\_t001 |  |  |  |  |  |  |  |
| 1 | Ath-AT3G01270.1 |  | Vvi-Vitvi14g01900\_t001 |  |  |  |  |  |  |  |
| 1 | Ath-AT3G01280.1 |  | Vvi-Vitvi14g01898\_t001 |  |  |  |  |  |  |  |
| 1 | Ath-AT3G01290.1 |  | | | |  |  |  |  |  |  |  |
| 1 | Ath-AT3G01300.1 |  | | | |  |  |  |  |  |  |  |
| 1 | Ath-AT3G01310.1 |  | Vvi-Vitvi14g01887\_t001 |  |  |  |  |  |  |  |
| 1 | Ath-AT3G01311.1 |  | Vvi-Vitvi14g01879\_t001 |  |  |  |  |  |  |  |
| 1 | Ath-AT3G01320.1 |  | | | |  |  |  |  |  |  |  |
| 1 | Ath-AT3G01319.1 |  | | | |  |  |  |  |  |  |  |
| 1 | Ath-AT3G01322.1 |  | | | |  |  |  |  |  |  |  |
| 1 | Ath-AT3G01326.1 |  | | | |  |  |  |  |  |  |  |
| 1 | Ath-AT3G01327.1 |  | | | |  |  |  |  |  |  |  |
| 1 | Ath-AT3G01328.1 |  | | | |  |  |  |  |  |  |  |
| 1 | Ath-AT3G01329.1 |  | | | |  |  |  |  |  |  |  |
| 1 | Ath-AT3G01331.1 |  | | | |  |  |  |  |  |  |  |
| 1 | Ath-AT3G01323.1 |  | | | |  |  |  |  |  |  |  |
| 1 | Ath-AT3G01324.1 |  | | | |  |  |  |  |  |  |  |
| 1 | Ath-AT3G01325.1 |  | | | |  |  |  |  |  |  |  |
| 1 | Ath-AT3G01330.1 |  | Vvi-Vitvi14g01870\_t001 |  |  |  |  |  |  |  |
| 0 | Ath-AT3G01340.1 |  |  |  |  |  |  |  |  |
| 0 | Ath-AT3G01345.1 |  |  |  |  |  |  |  |  |
| 1 | Ath-AT3G01350.1 |  | Vvi-Vitvi14g01832\_t001 |  |  |  |  |  |  |  |
| 1 | Ath-AT3G01360.1 |  | Vvi-Vitvi14g01829\_t001 |  |  |  |  |  |  |  |
| 1 | Ath-AT3G01370.1 |  | Vvi-Vitvi14g01827\_t002 |  |  |  |  |  |  |  |
| 1 | Ath-AT3G01380.2 |  | Vvi-Vitvi14g01820\_t001 |  |  |  |  |  |  |  |
| 1 | Ath-AT3G01390.3 |  | | | |  |  |  |  |  |  |  |
| 1 | Ath-AT3G01400.1 |  | | | |  |  |  |  |  |  |  |
| 1 | Ath-AT3G01410.1 |  | Vvi-Vitvi14g01818\_t002 |  |  |  |  |  |  |  |
| 1 | Ath-AT3G01415.1 |  | | | |  |  |  |  |  |  |  |
| 1 | Ath-AT3G01420.1 |  | Vvi-Vitvi14g01808\_t001 |  |  |  |  |  |  |  |
| 1 | Ath-AT3G01430.1 |  | Vvi-Vitvi14g01800\_t001 |  |  |  |  |  |  |  |
| 1 | Ath-AT3G01435.2 |  | | | |  |  |  |  |  |  |  |
| 1 | Ath-AT3G01440.1 |  | | | |  |  |  |  |  |  |  |
| 1 | Ath-AT3G01450.2 |  | Vvi-Vitvi14g04630\_t001 |  |  |  |  |  |  |  |
| 1 | Ath-AT3G01460.1 |  | Vvi-Vitvi14g01787\_t001 |  |  |  |  |  |  |  |
| 1 | Ath-AT3G01470.1 |  | Vvi-Vitvi14g01786\_t001 |  |  |  |  |  |  |  |
| 1 | Ath-AT3G01475.1 |  | | | |  |  |  |  |  |  |  |
| 1 | Ath-AT3G01480.1 |  | Vvi-Vitvi14g01782\_t001 |  |  |  |  |  |  |  |
| 1 | Ath-AT3G01490.1 |  | Vvi-Vitvi14g01781\_t001 |  |  |  |  |  |  |  |
| 1 | Ath-AT3G01500.2 |  | Vvi-Vitvi14g01763\_t001 |  |  |  |  |  |  |  |
| 1 | Ath-AT3G01510.1 |  | Vvi-Vitvi14g01762\_t002 |  |  |  |  |  |  |  |
| 1 | Ath-AT3G01513.1 |  | | | |  |  |  |  |  |  |  |
| 1 | Ath-AT3G01516.1 |  | Vvi-Vitvi14g03018\_t001 |  |  |  |  |  |  |  |
| 1 | Ath-AT3G01520.1 |  | Vvi-Vitvi14g03017\_t003 |  |  |  |  |  |  |  |
| 1 | Ath-AT3G01530.1 |  | Vvi-Vitvi14g01750\_t001 |  |  |  |  |  |  |  |
| 1 | Ath-AT3G01540.4 |  | Vvi-Vitvi14g01741\_t001 |  |  |  |  |  |  |  |
| 1 | Ath-AT3G01550.1 |  | Vvi-Vitvi14g01739\_t001 |  |  |  |  |  |  |  |
| 1 | Ath-AT3G01560.1 |  | Vvi-Vitvi14g01715\_t001 |  |  |  |  |  |  |  |
| 1 | Ath-AT3G01570.1 |  | Vvi-Vitvi14g03008\_t001 |  |  |  |  |  |  |  |
| 1 | Ath-AT3G01572.1 |  | | | |  |  |  |  |  |  |  |
| 1 | Ath-AT3G01580.1 |  | Vvi-Vitvi14g01685\_t001 |  |  |  |  |  |  |  |
| 1 | Ath-AT3G01590.1 |  | Vvi-Vitvi14g01684\_t001.2.6037826b |  |  |  |  |  |  |  |
| 1 | Ath-AT3G01600.1 |  | Vvi-Vitvi14g01678\_t001 |  |  |  |  |  |  |  |
| 1 | Ath-AT3G01610.2 |  | | | |  |  |  |  |  |  |  |
| 1 | Ath-AT3G01620.1 |  | Vvi-Vitvi14g01676\_t002 |  |  |  |  |  |  |  |
| 1 | Ath-AT3G01630.2 |  | | | |  |  |  |  |  |  |  |
| 1 | Ath-AT3G01640.2 |  | Vvi-Vitvi14g01675\_t001 |  |  |  |  |  |  |  |
| 1 | Ath-AT3G01650.1 |  | Vvi-Vitvi14g01671\_t001 |  |  |  |  |  |  |  |
| 1 | Ath-AT3G01660.1 |  | Vvi-Vitvi14g01656\_t001 |  |  |  |  |  |  |  |
| 1 | Ath-AT3G01670.2 |  | Vvi-Vitvi14g01641\_t001 |  |  |  |  |  |  |  |
| 1 | Ath-AT3G01680.1 |  | | | |  |  |  |  |  |  |  |
| 1 | Ath-AT3G01690.1 |  | Vvi-Vitvi14g01640\_t001 |  |  |  |  |  |  |  |
| 1 | Ath-AT3G01700.1 |  | | | |  |  |  |  |  |  |  |
| 1 | Ath-AT3G01710.3 |  | Vvi-Vitvi14g01621\_t001 |  |  |  |  |  |  |  |
| 1 | Ath-AT3G01720.1 |  | Vvi-Vitvi14g01620\_t001 |  |  |  |  |  |  |  |
| 1 | Ath-AT3G01730.1 |  | | | |  |  |  |  |  |  |  |
| 1 | Ath-AT3G01740.1 |  | Vvi-Vitvi14g01598\_t002 |  |  |  |  |  |  |  |
| 1 | Ath-AT3G01750.1 |  | Vvi-Vitvi14g01597\_t001 |  |  |  |  |  |  |  |
| 1 | Ath-AT3G01760.1 |  | | | |  |  |  |  |  |  |  |
| 2 | Ath-AT3G01770.1 |  | Vvi-Vitvi14g01594\_t002 |  | Vvi-Vitvi17g00153\_t002 |  |  |  |  |  |  |
| 2 | Ath-AT3G01780.1 |  | Vvi-Vitvi14g04595\_t001 |  | | | |  |  |  |  |  |  |
| 2 | Ath-AT3G01790.2 |  | Vvi-Vitvi14g01584\_t001 |  | | | |  |  |  |  |  |  |
| 2 | Ath-AT3G01800.1 |  | Vvi-Vitvi14g01582\_t001 |  | | | |  |  |  |  |  |  |
| 2 | Ath-AT3G01810.1 |  | Vvi-Vitvi14g01575\_t001 |  | | | |  |  |  |  |  |  |
| 2 | Ath-AT3G01820.1 |  | Vvi-Vitvi14g01574\_t001 |  | Vvi-Vitvi17g00137\_t001 |  |  |  |  |  |  |
| 2 | Ath-AT3G01830.1 |  | Vvi-Vitvi14g01573\_t001 |  | Vvi-Vitvi17g00131\_t001 |  |  |  |  |  |  |
| 2 | Ath-AT3G01840.1 |  | Vvi-Vitvi14g02422\_t001 |  | | | |  |  |  |  |  |  |
| 2 | Ath-AT3G01850.2 |  | Vvi-Vitvi14g01571\_t001 |  | | | |  |  |  |  |  |  |
| 2 | Ath-AT3G01860.1 |  | Vvi-Vitvi14g02985\_t001 |  | | | |  |  |  |  |  |  |
| 1 | Ath-AT3G01870.1 |  |  |  | | | |  |  |  |  |  |  |
| 1 | Ath-AT3G01880.1 |  |  |  | | | |  |  |  |  |  |  |
| 2 | Ath-AT3G01890.1 |  | Vvi-Vitvi14g01554\_t001 |  | Vvi-Vitvi17g00120\_t001 |  |  |  |  |  |  |
| 2 | Ath-AT3G01900.1 |  | Vvi-Vitvi14g01552\_t001 |  | Vvi-Vitvi17g00119\_t001 |  |  |  |  |  |  |
| 2 | Ath-AT3G01910.1 |  | Vvi-Vitvi14g01551\_t001 |  | | | |  |  |  |  |  |  |
| 2 | Ath-AT3G01920.2 |  | Vvi-Vitvi14g01544\_t001 |  | | | |  |  |  |  |  |  |
| 2 | Ath-AT3G01930.2 |  | Vvi-Vitvi14g01530\_t004 |  | | | |  |  |  |  |  |  |
| 2 | Ath-AT3G01940.1 |  | | | |  | | | |  |  |  |  |  |  |
| 2 | Ath-AT3G01960.1 |  | | | |  | | | |  |  |  |  |  |  |
| 2 | Ath-AT3G01950.1 |  | Vvi-Vitvi14g01527\_t001 |  | | | |  |  |  |  |  |  |
| 2 | Ath-AT3G01961.1 |  | | | |  | | | |  |  |  |  |  |  |
| 2 | Ath-AT3G01970.1 |  | Vvi-Vitvi14g01523\_t001 |  | | | |  |  |  |  |  |  |
| 2 | Ath-AT3G01980.3 |  | Vvi-Vitvi14g01522\_t001 |  | | | |  |  |  |  |  |  |
| 2 | Ath-AT3G01990.5 |  | Vvi-Vitvi14g01517\_t001 |  | | | |  |  |  |  |  |  |
| 2 | Ath-AT3G02000.1 |  | Vvi-Vitvi14g01507\_t001 |  | | | |  |  |  |  |  |  |
| 2 | Ath-AT3G02020.1 |  | | | |  | | | |  |  |  |  |  |  |
| 2 | Ath-AT3G02010.1 |  | | | |  | Vvi-Vitvi17g00114\_t001 |  |  |  |  |  |  |
| 2 | Ath-AT3G02030.2 |  | | | |  | | | |  |  |  |  |  |  |
| 2 | Ath-AT3G02040.1 |  | Vvi-Vitvi14g01500\_t001 |  | | | |  |  |  |  |  |  |
| 2 | Ath-AT3G02050.1 |  | Vvi-Vitvi14g01440\_t001 |  | | | |  |  |  |  |  |  |
| 2 | Ath-AT3G02060.1 |  | Vvi-Vitvi14g01436\_t001 |  | | | |  |  |  |  |  |  |
| 2 | Ath-AT3G02065.2 |  | Vvi-Vitvi14g01427\_t002 |  | | | |  |  |  |  |  |  |
| 2 | Ath-AT3G02070.2 |  | Vvi-Vitvi14g01425\_t001 |  | | | |  |  |  |  |  |  |
| 3 | Ath-AT3G02080.1 |  | | | |  | | | |  | Vvi-Vitvi01g00060\_t001 |  |  |  |  |  |
| 3 | Ath-AT3G02090.2 |  | | | |  | | | |  | | | |  |  |  |  |  |
| 3 | Ath-AT3G02100.1 |  | Vvi-Vitvi14g01409\_t001 |  | | | |  | | | |  |  |  |  |  |
| 4 | Ath-AT3G02110.1 |  | | | |  | | | |  | | | |  | Vvi-Vitvi14g01390\_t001 |  |  |  |  |
| 4 | Ath-AT3G02120.1 |  | | | |  | | | |  | | | |  | Vvi-Vitvi14g04527\_t001 |  |  |  |  |
| 4 | Ath-AT3G02125.1 |  | | | |  | | | |  | | | |  | Vvi-Vitvi14g02938\_t001 |  |  |  |  |
| 4 | Ath-AT3G02130.1 |  | | | |  | | | |  | Vvi-Vitvi01g00038\_t001 |  | Vvi-Vitvi14g01392\_t001 |  |  |  |  |
| 5 | Ath-AT3G02140.1 |  | | | |  | | | |  | Vvi-Vitvi01g00037\_t001 |  | Vvi-Vitvi14g01394\_t001 |  | Vvi-Vitvi17g01448\_t001 |  |  |  |
| 5 | Ath-AT3G02150.2 |  | | | |  | | | |  | | | |  | Vvi-Vitvi14g01398\_t001 |  | Vvi-Vitvi17g00495\_t001 |  |  |  |
| 5 | Ath-AT3G02160.1 |  | | | |  | | | |  | | | |  | Vvi-Vitvi14g01400\_t001 |  | | | |  |  |  |
| 5 | Ath-AT3G02170.1 |  | | | |  | | | |  | | | |  | Vvi-Vitvi14g01401\_t001 |  | Vvi-Vitvi17g00494\_t001 |  |  |  |
| 4 | Ath-AT3G02180.1 |  | Vvi-Vitvi14g04526\_t001 |  | | | |  | Vvi-Vitvi01g00033\_t002 |  |  |  | Vvi-Vitvi17g00489\_t001 |  |  |  |
| 4 | Ath-AT3G02190.1 |  | | | |  | | | |  | | | |  |  |  | | | |  |  |  |
| 4 | Ath-AT3G02200.2 |  | Vvi-Vitvi14g01385\_t001 |  | | | |  | | | |  |  |  | | | |  |  |  |
| 4 | Ath-AT3G02210.1 |  | Vvi-Vitvi14g01357\_t001 |  | | | |  | | | |  |  |  | Vvi-Vitvi17g04133\_t001 |  |  |  |
| 4 | Ath-AT3G02220.1 |  | | | |  | | | |  | | | |  |  |  | | | |  |  |  |
| 4 | Ath-AT3G02230.1 |  | Vvi-Vitvi14g01350\_t001 |  | Vvi-Vitvi17g00103\_t002 |  | Vvi-Vitvi01g01838\_t001 |  |  |  | Vvi-Vitvi17g01440\_t001 |  |  |  |
| 3 | Ath-AT3G02240.1 |  | | | |  |  |  | | | |  |  |  | | | |  |  |  |
| 3 | Ath-AT3G02242.1 |  | | | |  |  |  | | | |  |  |  | | | |  |  |  |
| 3 | Ath-AT3G02245.1 |  | | | |  |  |  | | | |  |  |  | | | |  |  |  |
| 4 | Ath-AT3G02250.1 |  | | | |  | Vvi-Vitvi14g01327\_t001 |  | | | |  |  |  | | | |  |  |  |
| 4 | Ath-AT3G02255.1 |  | | | |  | | | |  | | | |  |  |  | | | |  |  |  |
| 4 | Ath-AT3G02260.1 |  | Vvi-Vitvi14g01330\_t001 |  | Vvi-Vitvi14g01330\_t001 |  | | | |  |  |  | | | |  |  |  |
| 4 | Ath-AT3G02270.1 |  | | | |  | | | |  | | | |  |  |  | | | |  |  |  |
| 4 | Ath-AT3G02280.3 |  | | | |  | | | |  | | | |  |  |  | | | |  |  |  |
| 4 | Ath-AT3G02290.5 |  | | | |  | Vvi-Vitvi14g01338\_t001 |  | Vvi-Vitvi01g01836\_t001 |  |  |  | | | |  |  |  |
| 3 | Ath-AT3G02300.2 |  | | | |  | Vvi-Vitvi14g01340\_t002 |  |  |  |  |  | | | |  |  |  |
| 3 | Ath-AT3G02310.1 |  | | | |  | Vvi-Vitvi14g01344\_t001 |  |  |  |  |  | Vvi-Vitvi17g00471\_t001 |  |  |  |
| 2 | Ath-AT3G02320.1 |  | | | |  | Vvi-Vitvi14g01349\_t001 |  |  |  |  |  |  |
| 1 | Ath-AT3G02330.1 |  | Vvi-Vitvi14g01305\_t001 |  |  |  |  |  |  |  |
| 1 | Ath-AT3G02340.1 |  | Vvi-Vitvi14g01303\_t001 |  |  |  |  |  |  |  |
| 1 | Ath-AT3G02350.1 |  | Vvi-Vitvi14g01301\_t002 |  |  |  |  |  |  |  |
| 1 | Ath-AT3G02360.1 |  | | | |  |  |  |  |  |  |  |
| 1 | Ath-AT3G02370.4 |  | | | |  |  |  |  |  |  |  |
| 1 | Ath-AT3G02380.1 |  | Vvi-Vitvi14g01296\_t001 |  |  |  |  |  |  |  |
| 1 | Ath-AT3G02390.2 |  | | | |  |  |  |  |  |  |  |
| 1 | Ath-AT3G02410.1 |  | | | |  |  |  |  |  |  |  |
| 1 | Ath-AT3G02400.1 |  | | | |  |  |  |  |  |  |  |
| 3 | Ath-AT3G02420.1 |  | | | |  | Vvi-Vitvi14g01263\_t001 |  | Vvi-Vitvi14g01263\_t001 |  |  |  |  |  |
| 3 | Ath-AT3G02430.1 |  | | | |  | | | |  | | | |  |  |  |  |  |
| 3 | Ath-AT3G02440.2 |  | | | |  | | | |  | Vvi-Vitvi14g01268\_t001 |  |  |  |  |  |
| 3 | Ath-AT3G02450.1 |  | | | |  | | | |  | Vvi-Vitvi14g01286\_t001 |  |  |  |  |  |
| 3 | Ath-AT3G02460.1 |  | | | |  | | | |  | Vvi-Vitvi14g01287\_t002 |  |  |  |  |  |
| 3 | Ath-AT3G02470.4 |  | Vvi-Vitvi14g01289\_t001 |  | | | |  | Vvi-Vitvi14g01289\_t001 |  |  |  |  |  |
| 2 | Ath-AT3G02480.1 |  |  |  | | | |  | Vvi-Vitvi14g01291\_t001 |  |  |  |  |  |
| 2 | Ath-AT3G02490.1 |  |  |  | | | |  | Vvi-Vitvi14g01292\_t001 |  |  |  |  |  |
| 1 | Ath-AT3G02493.1 |  |  |  | | | |  |  |  |  |  |  |
| 1 | Ath-AT3G02500.3 |  |  |  | Vvi-Vitvi14g01240\_t001 |  |  |  |  |  |  |
| 1 | Ath-AT3G02510.1 |  |  |  | Vvi-Vitvi14g01227\_t002 |  |  |  |  |  |  |
| 1 | Ath-AT3G02520.2 |  |  |  | Vvi-Vitvi14g01226\_t001 |  |  |  |  |  |  |
| 1 | Ath-AT3G02530.1 |  |  |  | Vvi-Vitvi14g01224\_t001 |  |  |  |  |  |  |
| 1 | Ath-AT3G02540.1 |  |  |  | Vvi-Vitvi14g01208\_t001 |  |  |  |  |  |  |
| 1 | Ath-AT3G02550.1 |  |  |  | Vvi-Vitvi14g01193\_t001 |  |  |  |  |  |  |
| 1 | Ath-AT3G02555.1 |  |  |  | Vvi-Vitvi14g01188\_t001 |  |  |  |  |  |  |
| 1 | Ath-AT3G02560.1 |  |  |  | Vvi-Vitvi14g01179\_t001 |  |  |  |  |  |  |
| 1 | Ath-AT3G02570.1 |  |  |  | Vvi-Vitvi14g01178\_t001 |  |  |  |  |  |  |
| 1 | Ath-AT3G02580.1 |  |  |  | Vvi-Vitvi14g01152\_t001 |  |  |  |  |  |  |
| 0 | Ath-AT3G02590.1 |  |  |  |  |  |  |  |  |
| 0 | Ath-AT3G02600.1 |  |  |  |  |  |  |  |  |
| 0 | Ath-AT3G02610.2 |  |  |  |  |  |  |  |  |
| 0 | Ath-AT3G02620.2 |  |  |  |  |  |  |  |  |
| 0 | Ath-AT3G02630.1 |  |  |  |  |  |  |  |  |
| 0 | Ath-AT3G02640.1 |  |  |  |  |  |  |  |  |
| 0 | Ath-AT3G02645.1 |  |  |  |  |  |  |  |  |
| 0 | Ath-AT3G02650.1 |  |  |  |  |  |  |  |  |
| 0 | Ath-AT3G02660.2 |  |  |  |  |  |  |  |  |
| 0 | Ath-AT3G02670.1 |  |  |  |  |  |  |  |  |
| 0 | Ath-AT3G02673.1 |  |  |  |  |  |  |  |  |
| 0 | Ath-AT3G02677.1 |  |  |  |  |  |  |  |  |
| 0 | Ath-AT3G02680.1 |  |  |  |  |  |  |  |  |
| 0 | Ath-AT3G02690.1 |  |  |  |  |  |  |  |  |
| 0 | Ath-AT3G02700.1 |  |  |  |  |  |  |  |  |
| 0 | Ath-AT3G02710.1 |  |  |  |  |  |  |  |  |
| 0 | Ath-AT3G02720.1 |  |  |  |  |  |  |  |  |
| 1 | Ath-AT3G02730.1 |  | Vvi-Vitvi04g04319\_t001 |  |  |  |  |  |  |  |
| 1 | Ath-AT3G02740.1 |  | Vvi-Vitvi04g01190\_t001 |  |  |  |  |  |  |  |
| 1 | Ath-AT3G02750.3 |  | Vvi-Vitvi04g01184\_t002 |  |  |  |  |  |  |  |
| 1 | Ath-AT3G02760.1 |  | | | |  |  |  |  |  |  |  |
| 1 | Ath-AT3G02770.1 |  | Vvi-Vitvi04g01174\_t001 |  |  |  |  |  |  |  |
| 1 | Ath-AT3G02780.1 |  | | | |  |  |  |  |  |  |  |
| 1 | Ath-AT3G02790.1 |  | Vvi-Vitvi04g01161\_t001 |  |  |  |  |  |  |  |
| 1 | Ath-AT3G02800.1 |  | Vvi-Vitvi04g01160\_t001 |  |  |  |  |  |  |  |
| 1 | Ath-AT3G02810.1 |  | | | |  |  |  |  |  |  |  |
| 1 | Ath-AT3G02820.2 |  | | | |  |  |  |  |  |  |  |
| 1 | Ath-AT3G02830.1 |  | Vvi-Vitvi04g01157\_t002 |  |  |  |  |  |  |  |
| 0 | Ath-AT3G02840.1 |  |  |  |  |  |  |  |  |
| 0 | Ath-AT3G02850.1 |  |  |  |  |  |  |  |  |
| 0 | Ath-AT3G02860.2 |  |  |  |  |  |  |  |  |
| 0 | Ath-AT3G02870.1 |  |  |  |  |  |  |  |  |
| 0 | Ath-AT3G02875.1 |  |  |  |  |  |  |  |  |
| 0 | Ath-AT3G02880.1 |  |  |  |  |  |  |  |  |
| 0 | Ath-AT3G02885.1 |  |  |  |  |  |  |  |  |
| 1 | Ath-AT3G02890.1 |  | Vvi-Vitvi16g00076\_t001 |  |  |  |  |  |  |  |
| 1 | Ath-AT3G02900.2 |  | Vvi-Vitvi16g00077\_t001 |  |  |  |  |  |  |  |
| 1 | Ath-AT3G02910.1 |  | Vvi-Vitvi16g00082\_t001 |  |  |  |  |  |  |  |
| 1 | Ath-AT3G02920.1 |  | | | |  |  |  |  |  |  |  |
| 1 | Ath-AT3G02930.1 |  | Vvi-Vitvi16g00093\_t003 |  |  |  |  |  |  |  |
| 1 | Ath-AT3G02940.1 |  | Vvi-Vitvi16g00106\_t001 |  |  |  |  |  |  |  |
| 1 | Ath-AT3G02950.1 |  | | | |  |  |  |  |  |  |  |
| 1 | Ath-AT3G02960.1 |  | | | |  |  |  |  |  |  |  |
| 1 | Ath-AT3G02970.1 |  | Vvi-Vitvi16g00110\_t001 |  |  |  |  |  |  |  |
| 1 | Ath-AT3G02975.1 |  | | | |  |  |  |  |  |  |  |
| 1 | Ath-AT3G02980.1 |  | Vvi-Vitvi16g00111\_t001 |  |  |  |  |  |  |  |
| 1 | Ath-AT3G02990.1 |  | Vvi-Vitvi16g00114\_t001 |  |  |  |  |  |  |  |
| 1 | Ath-AT3G03000.1 |  | Vvi-Vitvi16g00119\_t001 |  |  |  |  |  |  |  |
| 1 | Ath-AT3G03010.3 |  | Vvi-Vitvi16g00135\_t002 |  |  |  |  |  |  |  |
| 1 | Ath-AT3G03020.2 |  | | | |  |  |  |  |  |  |  |
| 1 | Ath-AT3G03030.1 |  | | | |  |  |  |  |  |  |  |
| 1 | Ath-AT3G03040.1 |  | | | |  |  |  |  |  |  |  |
| 1 | Ath-AT3G03050.1 |  | Vvi-Vitvi16g00137\_t001 |  |  |  |  |  |  |  |
| 1 | Ath-AT3G03060.1 |  | Vvi-Vitvi16g00146\_t001 |  |  |  |  |  |  |  |
| 1 | Ath-AT3G03070.1 |  | Vvi-Vitvi16g00147\_t001 |  |  |  |  |  |  |  |
| 1 | Ath-AT3G03080.1 |  | | | |  |  |  |  |  |  |  |
| 1 | Ath-AT3G03090.1 |  | Vvi-Vitvi16g01538\_t001 |  |  |  |  |  |  |  |
| 1 | Ath-AT3G03100.1 |  | Vvi-Vitvi16g00151\_t001 |  |  |  |  |  |  |  |
| 1 | Ath-AT3G03110.1 |  | Vvi-Vitvi16g00155\_t001 |  |  |  |  |  |  |  |
| 1 | Ath-AT3G03120.1 |  | Vvi-Vitvi16g01539\_t001 |  |  |  |  |  |  |  |
| 1 | Ath-AT3G03130.1 |  | Vvi-Vitvi16g00170\_t001 |  |  |  |  |  |  |  |
| 1 | Ath-AT3G03140.1 |  | Vvi-Vitvi16g00173\_t001 |  |  |  |  |  |  |  |
| 1 | Ath-AT3G03150.1 |  | Vvi-Vitvi16g00182\_t001 |  |  |  |  |  |  |  |
| 0 | Ath-AT3G03160.1 |  |  |  |  |  |  |  |  |
| 0 | Ath-AT3G03170.1 |  |  |  |  |  |  |  |  |
| 0 | Ath-AT3G03180.3 |  |  |  |  |  |  |  |  |
| 0 | Ath-AT3G03190.1 |  |  |  |  |  |  |  |  |
| 0 | Ath-AT3G03200.1 |  |  |  |  |  |  |  |  |
| 0 | Ath-AT3G03210.1 |  |  |  |  |  |  |  |  |
| 0 | Ath-AT3G03220.1 |  |  |  |  |  |  |  |  |
| 0 | Ath-AT3G03230.1 |  |  |  |  |  |  |  |  |
| 0 | Ath-AT3G03240.1 |  |  |  |  |  |  |  |  |
| 0 | Ath-AT3G03250.2 |  |  |  |  |  |  |  |  |
| 0 | Ath-AT3G03260.1 |  |  |  |  |  |  |  |  |
| 0 | Ath-AT3G03270.1 |  |  |  |  |  |  |  |  |
| 0 | Ath-AT3G03272.1 |  |  |  |  |  |  |  |  |
| 0 | Ath-AT3G03280.1 |  |  |  |  |  |  |  |  |
| 0 | Ath-AT3G03290.1 |  |  |  |  |  |  |  |  |
| 0 | Ath-AT3G03300.3 |  |  |  |  |  |  |  |  |
| 0 | Ath-AT3G03305.1 |  |  |  |  |  |  |  |  |
| 0 | Ath-AT3G03310.1 |  |  |  |  |  |  |  |  |
| 0 | Ath-AT3G03320.1 |  |  |  |  |  |  |  |  |
| 0 | Ath-AT3G03330.1 |  |  |  |  |  |  |  |  |
| 0 | Ath-AT3G03340.2 |  |  |  |  |  |  |  |  |
| 0 | Ath-AT3G03341.1 |  |  |  |  |  |  |  |  |
| 0 | Ath-AT3G03350.2 |  |  |  |  |  |  |  |  |
| 0 | Ath-AT3G03360.1 |  |  |  |  |  |  |  |  |
| 0 | Ath-AT3G03370.1 |  |  |  |  |  |  |  |  |
| 0 | Ath-AT3G03380.1 |  |  |  |  |  |  |  |  |
| 0 | Ath-AT3G03400.1 |  |  |  |  |  |  |  |  |
| 0 | Ath-AT3G03405.1 |  |  |  |  |  |  |  |  |
| 0 | Ath-AT3G03410.1 |  |  |  |  |  |  |  |  |
| 0 | Ath-AT3G03420.1 |  |  |  |  |  |  |  |  |
| 0 | Ath-AT3G03430.1 |  |  |  |  |  |  |  |  |
| 0 | Ath-AT3G03440.1 |  |  |  |  |  |  |  |  |
| 0 | Ath-AT3G03450.1 |  |  |  |  |  |  |  |  |
| 0 | Ath-AT3G03460.1 |  |  |  |  |  |  |  |  |
| 0 | Ath-AT3G03470.1 |  |  |  |  |  |  |  |  |
| 0 | Ath-AT3G03480.1 |  |  |  |  |  |  |  |  |
| 0 | Ath-AT3G03490.1 |  |  |  |  |  |  |  |  |
| 0 | Ath-AT3G03500.1 |  |  |  |  |  |  |  |  |
| 0 | Ath-AT3G03510.1 |  |  |  |  |  |  |  |  |
| 0 | Ath-AT3G03520.1 |  |  |  |  |  |  |  |  |
| 0 | Ath-AT3G03530.1 |  |  |  |  |  |  |  |  |
| 0 | Ath-AT3G03540.1 |  |  |  |  |  |  |  |  |
| 0 | Ath-AT3G03550.1 |  |  |  |  |  |  |  |  |
| 0 | Ath-AT3G03560.1 |  |  |  |  |  |  |  |  |
| 0 | Ath-AT3G03570.1 |  |  |  |  |  |  |  |  |
| 0 | Ath-AT3G03580.1 |  |  |  |  |  |  |  |  |
| 0 | Ath-AT3G03590.1 |  |  |  |  |  |  |  |  |
| 0 | Ath-AT3G03600.1 |  |  |  |  |  |  |  |  |
| 1 | Ath-AT3G03610.4 |  | Vvi-Vitvi08g00068\_t002 |  |  |  |  |  |  |  |
| 1 | Ath-AT3G03620.2 |  | Vvi-Vitvi08g00085\_t002 |  |  |  |  |  |  |  |
| 1 | Ath-AT3G03630.1 |  | | | |  |  |  |  |  |  |  |
| 1 | Ath-AT3G03640.1 |  | | | |  |  |  |  |  |  |  |
| 1 | Ath-AT3G03650.1 |  | Vvi-Vitvi08g00101\_t001 |  |  |  |  |  |  |  |
| 1 | Ath-AT3G03660.3 |  | Vvi-Vitvi08g00108\_t001 |  |  |  |  |  |  |  |
| 1 | Ath-AT3G03670.1 |  | | | |  |  |  |  |  |  |  |
| 1 | Ath-AT3G03680.1 |  | | | |  |  |  |  |  |  |  |
| 1 | Ath-AT3G03690.1 |  | | | |  |  |  |  |  |  |  |
| 1 | Ath-AT3G03700.1 |  | | | |  |  |  |  |  |  |  |
| 1 | Ath-AT3G03710.1 |  | Vvi-Vitvi08g00126\_t001 |  |  |  |  |  |  |  |
| 1 | Ath-AT3G03720.1 |  | | | |  |  |  |  |  |  |  |
| 1 | Ath-AT3G03726.1 |  | | | |  |  |  |  |  |  |  |
| 1 | Ath-AT3G03730.1 |  | | | |  |  |  |  |  |  |  |
| 1 | Ath-AT3G03740.1 |  | | | |  |  |  |  |  |  |  |
| 1 | Ath-AT3G03750.2 |  | | | |  |  |  |  |  |  |  |
| 1 | Ath-AT3G03760.1 |  | Vvi-Vitvi08g00144\_t001 |  |  |  |  |  |  |  |
| 1 | Ath-AT3G03770.1 |  | | | |  |  |  |  |  |  |  |
| 1 | Ath-AT3G03773.2 |  | | | |  |  |  |  |  |  |  |
| 1 | Ath-AT3G03776.2 |  | | | |  |  |  |  |  |  |  |
| 1 | Ath-AT3G03780.2 |  | | | |  |  |  |  |  |  |  |
| 1 | Ath-AT3G03790.3 |  | Vvi-Vitvi08g00173\_t001 |  |  |  |  |  |  |  |
| 1 | Ath-AT3G03800.1 |  | Vvi-Vitvi08g00187\_t001 |  |  |  |  |  |  |  |
| 0 | Ath-AT3G03810.1 |  |  |  |  |  |  |  |  |
| 0 | Ath-AT3G03820.1 |  |  |  |  |  |  |  |  |
| 0 | Ath-AT3G03826.1 |  |  |  |  |  |  |  |  |
| 0 | Ath-AT3G03828.1 |  |  |  |  |  |  |  |  |
| 0 | Ath-AT3G03830.1 |  |  |  |  |  |  |  |  |
| 0 | Ath-AT3G03840.1 |  |  |  |  |  |  |  |  |
| 0 | Ath-AT3G03847.1 |  |  |  |  |  |  |  |  |
| 0 | Ath-AT3G03850.1 |  |  |  |  |  |  |  |  |
| 1 | Ath-AT3G03860.1 |  | Vvi-Vitvi08g01804\_t001 |  |  |  |  |  |  |  |
| 1 | Ath-AT3G03870.2 |  | Vvi-Vitvi08g01805\_t001 |  |  |  |  |  |  |  |
| 1 | Ath-AT3G03880.1 |  | Vvi-Vitvi08g01807\_t001 |  |  |  |  |  |  |  |
| 1 | Ath-AT3G03890.1 |  | Vvi-Vitvi08g01808\_t002 |  |  |  |  |  |  |  |
| 1 | Ath-AT3G03900.1 |  | Vvi-Vitvi08g01809\_t001 |  |  |  |  |  |  |  |
| 1 | Ath-AT3G03910.1 |  | Vvi-Vitvi08g01812\_t002 |  |  |  |  |  |  |  |
| 1 | Ath-AT3G03920.1 |  | Vvi-Vitvi08g01813\_t001 |  |  |  |  |  |  |  |
| 1 | Ath-AT3G03930.1 |  | Vvi-Vitvi08g01814\_t001 |  |  |  |  |  |  |  |
| 1 | Ath-AT3G03940.1 |  | | | |  |  |  |  |  |  |  |
| 1 | Ath-AT3G03950.3 |  | Vvi-Vitvi08g01817\_t003 |  |  |  |  |  |  |  |
| 1 | Ath-AT3G03960.1 |  | Vvi-Vitvi08g01818\_t001 |  |  |  |  |  |  |  |
| 1 | Ath-AT3G03970.2 |  | Vvi-Vitvi08g01822\_t001 |  |  |  |  |  |  |  |
| 1 | Ath-AT3G03980.1 |  | Vvi-Vitvi08g01826\_t001 |  |  |  |  |  |  |  |
| 1 | Ath-AT3G03990.1 |  | | | |  |  |  |  |  |  |  |
| 1 | Ath-AT3G04000.1 |  | | | |  |  |  |  |  |  |  |
| 1 | Ath-AT3G04010.1 |  | Vvi-Vitvi08g01832\_t001 |  |  |  |  |  |  |  |
| 1 | Ath-AT3G04020.1 |  | | | |  |  |  |  |  |  |  |
| 1 | Ath-AT3G04030.3 |  | Vvi-Vitvi08g01834\_t001 |  |  |  |  |  |  |  |
| 1 | Ath-AT3G04040.1 |  | Vvi-Vitvi08g01835\_t001 |  |  |  |  |  |  |  |
| 1 | Ath-AT3G04050.1 |  | Vvi-Vitvi08g01836\_t001 |  |  |  |  |  |  |  |
| 1 | Ath-AT3G04060.1 |  | Vvi-Vitvi08g01841\_t001 |  |  |  |  |  |  |  |
| 1 | Ath-AT3G04070.1 |  | Vvi-Vitvi08g01843\_t001 |  |  |  |  |  |  |  |
| 1 | Ath-AT3G04080.1 |  | | | |  |  |  |  |  |  |  |
| 1 | Ath-AT3G04090.1 |  | Vvi-Vitvi08g02371\_t001 |  |  |  |  |  |  |  |
| 0 | Ath-AT3G04100.1 |  |  |  |  |  |  |  |  |
| 0 | Ath-AT3G04110.1 |  |  |  |  |  |  |  |  |
| 0 | Ath-AT3G04120.1 |  |  |  |  |  |  |  |  |
| 0 | Ath-AT3G04130.2 |  |  |  |  |  |  |  |  |
| 0 | Ath-AT3G04140.1 |  |  |  |  |  |  |  |  |
| 0 | Ath-AT3G04150.2 |  |  |  |  |  |  |  |  |
| 1 | Ath-AT3G04160.2 |  | Vvi-Vitvi04g01041\_t001 |  |  |  |  |  |  |  |
| 1 | Ath-AT3G04170.1 |  | | | |  |  |  |  |  |  |  |
| 1 | Ath-AT3G04180.1 |  | | | |  |  |  |  |  |  |  |
| 1 | Ath-AT3G04181.1 |  | | | |  |  |  |  |  |  |  |
| 1 | Ath-AT3G04184.1 |  | | | |  |  |  |  |  |  |  |
| 1 | Ath-AT3G04190.1 |  | | | |  |  |  |  |  |  |  |
| 1 | Ath-AT3G04200.1 |  | | | |  |  |  |  |  |  |  |
| 1 | Ath-AT3G04210.1 |  | | | |  |  |  |  |  |  |  |
| 1 | Ath-AT3G04220.3 |  | | | |  |  |  |  |  |  |  |
| 1 | Ath-AT3G04230.1 |  | | | |  |  |  |  |  |  |  |
| 1 | Ath-AT3G04240.1 |  | Vvi-Vitvi04g01024\_t001 |  |  |  |  |  |  |  |
| 1 | Ath-AT3G04250.1 |  | | | |  |  |  |  |  |  |  |
| 1 | Ath-AT3G04260.1 |  | Vvi-Vitvi04g01013\_t001 |  |  |  |  |  |  |  |
| 1 | Ath-AT3G04270.1 |  | | | |  |  |  |  |  |  |  |
| 1 | Ath-AT3G04280.2 |  | Vvi-Vitvi04g01011\_t001 |  |  |  |  |  |  |  |
| 1 | Ath-AT3G04290.1 |  | Vvi-Vitvi04g00997\_t001 |  |  |  |  |  |  |  |
| 2 | Ath-AT3G04300.1 |  | | | |  | Vvi-Vitvi07g00709\_t001 |  |  |  |  |  |  |
| 2 | Ath-AT3G04310.2 |  | Vvi-Vitvi04g00972\_t001 |  | | | |  |  |  |  |  |  |
| 2 | Ath-AT3G04320.2 |  | | | |  | | | |  |  |  |  |  |  |
| 2 | Ath-AT3G04330.1 |  | | | |  | | | |  |  |  |  |  |  |
| 2 | Ath-AT3G04340.1 |  | | | |  | | | |  |  |  |  |  |  |
| 2 | Ath-AT3G04350.1 |  | | | |  | Vvi-Vitvi07g00685\_t001 |  |  |  |  |  |  |
| 2 | Ath-AT3G04360.1 |  | | | |  | | | |  |  |  |  |  |  |
| 2 | Ath-AT3G04370.1 |  | Vvi-Vitvi04g02024\_t001 |  | Vvi-Vitvi07g00676\_t001 |  |  |  |  |  |  |
| 1 | Ath-AT3G04380.1 |  |  |  | Vvi-Vitvi07g00675\_t001 |  |  |  |  |  |  |
| 1 | Ath-AT3G04390.1 |  |  |  | | | |  |  |  |  |  |  |
| 1 | Ath-AT3G04400.1 |  |  |  | Vvi-Vitvi07g04166\_t001 |  |  |  |  |  |  |
| 1 | Ath-AT3G04410.1 |  |  |  | | | |  |  |  |  |  |  |
| 1 | Ath-AT3G04420.1 |  |  |  | | | |  |  |  |  |  |  |
| 1 | Ath-AT3G04430.1 |  |  |  | | | |  |  |  |  |  |  |
| 1 | Ath-AT3G04443.1 |  |  |  | | | |  |  |  |  |  |  |
| 1 | Ath-AT3G04440.1 |  |  |  | | | |  |  |  |  |  |  |
| 1 | Ath-AT3G04450.1 |  |  |  | Vvi-Vitvi07g00666\_t001 |  |  |  |  |  |  |
| 1 | Ath-AT3G04460.2 |  | Vvi-Vitvi14g00696\_t001 |  |  |  |  |  |  |  |
| 1 | Ath-AT3G04470.1 |  | Vvi-Vitvi14g00674\_t001 |  |  |  |  |  |  |  |
| 1 | Ath-AT3G04480.1 |  | Vvi-Vitvi14g00648\_t001 |  |  |  |  |  |  |  |
| 1 | Ath-AT3G04490.2 |  | Vvi-Vitvi14g00632\_t001 |  |  |  |  |  |  |  |
| 1 | Ath-AT3G04500.1 |  | Vvi-Vitvi14g04267\_t001 |  |  |  |  |  |  |  |
| 1 | Ath-AT3G04510.1 |  | Vvi-Vitvi14g00623\_t001 |  |  |  |  |  |  |  |
| 1 | Ath-AT3G04520.1 |  | Vvi-Vitvi14g00619\_t003 |  |  |  |  |  |  |  |
| 1 | Ath-AT3G04530.1 |  | Vvi-Vitvi14g00605\_t001 |  |  |  |  |  |  |  |
| 0 | Ath-AT3G04540.1 |  |  |  |  |  |  |  |  |
| 0 | Ath-AT3G04545.1 |  |  |  |  |  |  |  |  |
| 1 | Ath-AT3G04550.1 |  | Vvi-Vitvi14g00561\_t001 |  |  |  |  |  |  |  |
| 1 | Ath-AT3G04560.1 |  | Vvi-Vitvi14g00557\_t001 |  |  |  |  |  |  |  |
| 1 | Ath-AT3G04570.1 |  | Vvi-Vitvi14g00555\_t001 |  |  |  |  |  |  |  |
| 1 | Ath-AT3G04580.1 |  | Vvi-Vitvi14g00547\_t001 |  |  |  |  |  |  |  |
| 1 | Ath-AT3G04590.2 |  | Vvi-Vitvi14g00546\_t001 |  |  |  |  |  |  |  |
| 1 | Ath-AT3G04600.1 |  | | | |  |  |  |  |  |  |  |
| 1 | Ath-AT3G04610.1 |  | | | |  |  |  |  |  |  |  |
| 1 | Ath-AT3G04620.1 |  | | | |  |  |  |  |  |  |  |
| 1 | Ath-AT3G04630.3 |  | | | |  |  |  |  |  |  |  |
| 1 | Ath-AT3G04640.1 |  | | | |  |  |  |  |  |  |  |
| 1 | Ath-AT3G04650.1 |  | | | |  |  |  |  |  |  |  |
| 1 | Ath-AT3G04660.1 |  | | | |  |  |  |  |  |  |  |
| 1 | Ath-AT3G04670.1 |  | Vvi-Vitvi14g00540\_t001 |  |  |  |  |  |  |  |
| 0 | Ath-AT3G04680.3 |  |  |  |  |  |  |  |  |
| 0 | Ath-AT3G04690.1 |  |  |  |  |  |  |  |  |
| 0 | Ath-AT3G04700.1 |  |  |  |  |  |  |  |  |
| 0 | Ath-AT3G04710.3 |  |  |  |  |  |  |  |  |
| 0 | Ath-AT3G04720.1 |  |  |  |  |  |  |  |  |
| 0 | Ath-AT3G04730.1 |  |  |  |  |  |  |  |  |
| 0 | Ath-AT3G04735.1 |  |  |  |  |  |  |  |  |
| 0 | Ath-AT3G04740.1 |  |  |  |  |  |  |  |  |
| 0 | Ath-AT3G04750.1 |  |  |  |  |  |  |  |  |
| 0 | Ath-AT3G04760.1 |  |  |  |  |  |  |  |  |
| 0 | Ath-AT3G04770.1 |  |  |  |  |  |  |  |  |
| 1 | Ath-AT3G04780.1 |  | Vvi-Vitvi14g00440\_t002 |  |  |  |  |  |  |  |
| 1 | Ath-AT3G04790.1 |  | Vvi-Vitvi14g00439\_t001 |  |  |  |  |  |  |  |
| 1 | Ath-AT3G04800.1 |  | | | |  |  |  |  |  |  |  |
| 1 | Ath-AT3G04810.2 |  | Vvi-Vitvi14g00434\_t001 |  |  |  |  |  |  |  |
| 1 | Ath-AT3G04820.1 |  | Vvi-Vitvi14g00432\_t002 |  |  |  |  |  |  |  |
| 1 | Ath-AT3G04830.1 |  | Vvi-Vitvi14g00430\_t001 |  |  |  |  |  |  |  |
| 1 | Ath-AT3G04840.1 |  | Vvi-Vitvi14g00429\_t001 |  |  |  |  |  |  |  |
| 1 | Ath-AT3G04850.1 |  | Vvi-Vitvi14g00428\_t001 |  |  |  |  |  |  |  |
| 1 | Ath-AT3G04854.1 |  | | | |  |  |  |  |  |  |  |
| 1 | Ath-AT3G04855.1 |  | | | |  |  |  |  |  |  |  |
| 1 | Ath-AT3G04860.1 |  | Vvi-Vitvi14g00425\_t001 |  |  |  |  |  |  |  |
| 1 | Ath-AT3G04870.1 |  | Vvi-Vitvi14g00424\_t001 |  |  |  |  |  |  |  |
| 1 | Ath-AT3G04880.1 |  | Vvi-Vitvi14g02653\_t003 |  |  |  |  |  |  |  |
| 1 | Ath-AT3G04890.4 |  | Vvi-Vitvi14g00422\_t001 |  |  |  |  |  |  |  |
| 1 | Ath-AT3G04900.1 |  | | | |  |  |  |  |  |  |  |
| 1 | Ath-AT3G04903.1 |  | | | |  |  |  |  |  |  |  |
| 1 | Ath-AT3G04910.1 |  | Vvi-Vitvi14g00417\_t001 |  |  |  |  |  |  |  |
| 1 | Ath-AT3G04920.1 |  | Vvi-Vitvi14g00415\_t001 |  |  |  |  |  |  |  |
| 1 | Ath-AT3G04930.1 |  | Vvi-Vitvi14g04171\_t001 |  |  |  |  |  |  |  |
| 1 | Ath-AT3G04940.1 |  | Vvi-Vitvi14g04165\_t001 |  |  |  |  |  |  |  |
| 1 | Ath-AT3G04943.1 |  | | | |  |  |  |  |  |  |  |
| 1 | Ath-AT3G04945.1 |  | | | |  |  |  |  |  |  |  |
| 1 | Ath-AT3G04950.1 |  | Vvi-Vitvi14g00396\_t001 |  |  |  |  |  |  |  |
| 1 | Ath-AT3G04960.3 |  | | | |  |  |  |  |  |  |  |
| 2 | Ath-AT3G04970.1 |  | | | |  | Vvi-Vitvi14g00326\_t001 |  |  |  |  |  |  |
| 2 | Ath-AT3G04980.2 |  | | | |  | | | |  |  |  |  |  |  |
| 2 | Ath-AT3G04990.1 |  | | | |  | | | |  |  |  |  |  |  |
| 2 | Ath-AT3G05000.1 |  | | | |  | | | |  |  |  |  |  |  |
| 2 | Ath-AT3G05010.1 |  | | | |  | | | |  |  |  |  |  |  |
| 2 | Ath-AT3G05020.1 |  | | | |  | | | |  |  |  |  |  |  |
| 3 | Ath-AT3G05030.1 |  | | | |  | | | |  | Vvi-Vitvi14g00288\_t001 |  |  |  |  |  |
| 3 | Ath-AT3G05035.2 |  | | | |  | | | |  | | | |  |  |  |  |  |
| 3 | Ath-AT3G05040.2 |  | | | |  | | | |  | | | |  |  |  |  |  |
| 3 | Ath-AT3G05050.2 |  | | | |  | | | |  | | | |  |  |  |  |  |
| 3 | Ath-AT3G05060.1 |  | | | |  | | | |  | | | |  |  |  |  |  |
| 3 | Ath-AT3G05070.1 |  | | | |  | | | |  | | | |  |  |  |  |  |
| 3 | Ath-AT3G05090.2 |  | Vvi-Vitvi14g00389\_t002 |  | | | |  | | | |  |  |  |  |  |
| 2 | Ath-AT3G05080.1 |  |  |  | | | |  | | | |  |  |  |  |  |
| 2 | Ath-AT3G05100.1 |  |  |  | | | |  | | | |  |  |  |  |  |
| 2 | Ath-AT3G05110.1 |  |  |  | | | |  | | | |  |  |  |  |  |
| 2 | Ath-AT3G05120.1 |  |  |  | Vvi-Vitvi14g00322\_t001 |  | | | |  |  |  |  |  |
| 2 | Ath-AT3G05130.1 |  |  |  | Vvi-Vitvi14g00321\_t001 |  | | | |  |  |  |  |  |
| 2 | Ath-AT3G05140.2 |  |  |  | Vvi-Vitvi14g00316\_t001 |  | | | |  |  |  |  |  |
| 2 | Ath-AT3G05150.2 |  |  |  | Vvi-Vitvi14g04108\_t001 |  | | | |  |  |  |  |  |
| 2 | Ath-AT3G05155.1 |  |  |  | | | |  | | | |  |  |  |  |  |
| 2 | Ath-AT3G05160.1 |  |  |  | Vvi-Vitvi14g00304\_t001 |  | | | |  |  |  |  |  |
| 2 | Ath-AT3G05165.6 |  |  |  | | | |  | | | |  |  |  |  |  |
| 2 | Ath-AT3G05170.1 |  |  |  | Vvi-Vitvi14g00300\_t001 |  | | | |  |  |  |  |  |
| 1 | Ath-AT3G05180.1 |  |  |  |  |  | Vvi-Vitvi14g00282\_t001 |  |  |  |  |  |
| 1 | Ath-AT3G05190.1 |  |  |  |  |  | Vvi-Vitvi14g00281\_t001 |  |  |  |  |  |
| 1 | Ath-AT3G05200.1 |  |  |  |  |  | Vvi-Vitvi14g00275\_t001 |  |  |  |  |  |
| 1 | Ath-AT3G05210.1 |  |  |  |  |  | Vvi-Vitvi14g00274\_t001 |  |  |  |  |  |
| 1 | Ath-AT3G05220.1 |  |  |  |  |  | Vvi-Vitvi14g00270\_t001 |  |  |  |  |  |
| 1 | Ath-AT3G05230.1 |  |  |  |  |  | Vvi-Vitvi14g00267\_t001 |  |  |  |  |  |
| 1 | Ath-AT3G05240.1 |  |  |  |  |  | Vvi-Vitvi14g00261\_t001 |  |  |  |  |  |
| 1 | Ath-AT3G05250.1 |  |  |  |  |  | Vvi-Vitvi14g00260\_t001 |  |  |  |  |  |
| 1 | Ath-AT3G05260.1 |  |  |  |  |  | Vvi-Vitvi14g00257\_t001 |  |  |  |  |  |
| 1 | Ath-AT3G05270.3 |  |  |  |  |  | | | |  |  |  |  |  |
| 1 | Ath-AT3G05280.1 |  |  |  |  |  | Vvi-Vitvi14g00254\_t001 |  |  |  |  |  |
| 1 | Ath-AT3G05290.1 |  |  |  |  |  | Vvi-Vitvi14g00253\_t001 |  |  |  |  |  |
| 1 | Ath-AT3G05300.1 |  |  |  |  |  | | | |  |  |  |  |  |
| 1 | Ath-AT3G05310.1 |  |  |  |  |  | Vvi-Vitvi14g00251\_t001 |  |  |  |  |  |
| 1 | Ath-AT3G05320.2 |  |  |  |  |  | | | |  |  |  |  |  |
| 1 | Ath-AT3G05327.1 |  |  |  |  |  | Vvi-Vitvi14g00250\_t001 |  |  |  |  |  |
| 1 | Ath-AT3G05330.1 |  |  |  |  |  | Vvi-Vitvi14g00247\_t001 |  |  |  |  |  |
| 1 | Ath-AT3G05345.1 |  |  |  |  |  | Vvi-Vitvi14g00244\_t001 |  |  |  |  |  |
| 1 | Ath-AT3G05340.1 |  |  |  |  |  | Vvi-Vitvi14g00240\_t001 |  |  |  |  |  |
| 1 | Ath-AT3G05350.1 |  |  |  |  |  | Vvi-Vitvi14g00237\_t001 |  |  |  |  |  |
| 1 | Ath-AT3G05360.1 |  |  |  |  |  | | | |  |  |  |  |  |
| 1 | Ath-AT3G05365.3 |  |  |  |  |  | | | |  |  |  |  |  |
| 1 | Ath-AT3G05370.1 |  |  |  |  |  | | | |  |  |  |  |  |
| 1 | Ath-AT3G05380.4 |  |  |  |  |  | Vvi-Vitvi14g00230\_t001 |  |  |  |  |  |
| 1 | Ath-AT3G05390.1 |  |  |  |  |  | Vvi-Vitvi14g00216\_t001 |  |  |  |  |  |
| 0 | Ath-AT3G05400.1 |  |  |  |  |  |  |  |  |
| 1 | Ath-AT3G05410.2 |  | Vvi-Vitvi14g02511\_t001 |  |  |  |  |  |  |  |
| 1 | Ath-AT3G05420.2 |  | Vvi-Vitvi14g00195\_t001 |  |  |  |  |  |  |  |
| 1 | Ath-AT3G05425.1 |  | | | |  |  |  |  |  |  |  |
| 1 | Ath-AT3G05430.1 |  | Vvi-Vitvi14g00194\_t001 |  |  |  |  |  |  |  |
| 1 | Ath-AT3G05440.1 |  | | | |  |  |  |  |  |  |  |
| 1 | Ath-AT3G05450.1 |  | | | |  |  |  |  |  |  |  |
| 1 | Ath-AT3G05460.1 |  | | | |  |  |  |  |  |  |  |
| 1 | Ath-AT3G05470.1 |  | Vvi-Vitvi14g00184\_t001 |  |  |  |  |  |  |  |
| 2 | Ath-AT3G05480.3 |  | | | |  | Vvi-Vitvi14g00166\_t001 |  |  |  |  |  |  |
| 2 | Ath-AT3G05490.1 |  | | | |  | Vvi-Vitvi14g00168\_t001 |  |  |  |  |  |  |
| 2 | Ath-AT3G05500.1 |  | Vvi-Vitvi14g00167\_t001 |  | | | |  |  |  |  |  |  |
| 2 | Ath-AT3G05510.1 |  | | | |  | Vvi-Vitvi14g00172\_t001 |  |  |  |  |  |  |
| 2 | Ath-AT3G05520.2 |  | | | |  | Vvi-Vitvi14g00174\_t001 |  |  |  |  |  |  |
| 2 | Ath-AT3G05530.1 |  | | | |  | Vvi-Vitvi14g00178\_t001 |  |  |  |  |  |  |
| 2 | Ath-AT3G05540.1 |  | | | |  | Vvi-Vitvi14g00179\_t001 |  |  |  |  |  |  |
| 2 | Ath-AT3G05545.1 |  | | | |  | Vvi-Vitvi14g00180\_t003 |  |  |  |  |  |  |
| 3 | Ath-AT3G05550.1 |  | | | |  | Vvi-Vitvi14g02510\_t001 |  | Vvi-Vitvi05g00007\_t001 |  |  |  |  |  |
| 3 | Ath-AT3G05560.1 |  | Vvi-Vitvi14g00159\_t001 |  | Vvi-Vitvi14g00182\_t001 |  | Vvi-Vitvi05g00010\_t001 |  |  |  |  |  |
| 3 | Ath-AT3G05570.1 |  | | | |  | | | |  | | | |  |  |  |  |  |
| 5 | Ath-AT3G05580.1 |  | | | |  | | | |  | | | |  | Vvi-Vitvi14g00144\_t001 |  | Vvi-Vitvi14g00144\_t001 |  |  |  |
| 5 | Ath-AT3G05590.1 |  | | | |  | | | |  | Vvi-Vitvi05g00033\_t001 |  | | | |  | Vvi-Vitvi14g00151\_t001 |  |  |  |
| 5 | Ath-AT3G05600.1 |  | | | |  | | | |  | Vvi-Vitvi05g01742\_t002 |  | | | |  | Vvi-Vitvi14g00152\_t001 |  |  |  |
| 5 | Ath-AT3G05610.1 |  | | | |  | | | |  | Vvi-Vitvi05g00043\_t001 |  | | | |  | Vvi-Vitvi14g02501\_t001 |  |  |  |
| 5 | Ath-AT3G05620.1 |  | Vvi-Vitvi14g04048\_t001 |  | | | |  | | | |  | | | |  | Vvi-Vitvi14g04048\_t001 |  |  |  |
| 4 | Ath-AT3G05625.1 |  |  |  | | | |  | | | |  | | | |  | Vvi-Vitvi14g00160\_t001 |  |  |  |
| 4 | Ath-AT3G05630.1 |  |  |  | | | |  | Vvi-Vitvi05g00054\_t001 |  | | | |  | | | |  |  |  |
| 4 | Ath-AT3G05640.1 |  |  |  | | | |  | Vvi-Vitvi05g00055\_t001 |  | | | |  | Vvi-Vitvi14g00161\_t003 |  |  |  |
| 2 | Ath-AT3G05650.1 |  |  |  | | | |  |  |  | | | |  |  |  |  |
| 2 | Ath-AT3G05660.1 |  |  |  | | | |  |  |  | | | |  |  |  |  |
| 2 | Ath-AT3G05670.1 |  |  |  | | | |  |  |  | Vvi-Vitvi14g02481\_t001 |  |  |  |  |
| 2 | Ath-AT3G05675.1 |  |  |  | | | |  |  |  | | | |  |  |  |  |
| 2 | Ath-AT3G05680.2 |  |  |  | | | |  |  |  | | | |  |  |  |  |
| 2 | Ath-AT3G05685.1 |  |  |  | | | |  |  |  | | | |  |  |  |  |
| 2 | Ath-AT3G05690.2 |  |  |  | | | |  |  |  | | | |  |  |  |  |
| 2 | Ath-AT3G05700.1 |  |  |  | | | |  |  |  | Vvi-Vitvi14g00126\_t001 |  |  |  |  |
| 2 | Ath-AT3G05710.2 |  |  |  | | | |  |  |  | Vvi-Vitvi14g00125\_t001 |  |  |  |  |
| 2 | Ath-AT3G05720.1 |  |  |  | | | |  |  |  | | | |  |  |  |  |
| 2 | Ath-AT3G05725.1 |  |  |  | | | |  |  |  | Vvi-Vitvi14g02479\_t001 |  |  |  |  |
| 2 | Ath-AT3G05727.1 |  |  |  | | | |  |  |  | | | |  |  |  |  |
| 2 | Ath-AT3G05730.1 |  |  |  | | | |  |  |  | | | |  |  |  |  |
| 2 | Ath-AT3G05740.1 |  |  |  | Vvi-Vitvi14g00186\_t001 |  |  |  | Vvi-Vitvi14g00115\_t001 |  |  |  |  |
| 1 | Ath-AT3G05741.1 |  |  |  |  |  |  |  | | | |  |  |  |  |
| 1 | Ath-AT3G05746.1 |  |  |  |  |  |  |  | | | |  |  |  |  |
| 1 | Ath-AT3G05750.1 |  |  |  |  |  |  |  | Vvi-Vitvi14g00114\_t001 |  |  |  |  |
| 1 | Ath-AT3G05760.1 |  |  |  |  |  |  |  | Vvi-Vitvi14g00110\_t001 |  |  |  |  |
| 1 | Ath-AT3G05770.1 |  |  |  |  |  |  |  | Vvi-Vitvi14g00097\_t001 |  |  |  |  |
| 1 | Ath-AT3G05775.1 |  |  |  |  |  |  |  | Vvi-Vitvi14g00096\_t001 |  |  |  |  |
| 1 | Ath-AT3G05780.1 |  |  |  |  |  |  |  | | | |  |  |  |  |
| 1 | Ath-AT3G05790.1 |  |  |  |  |  |  |  | | | |  |  |  |  |
| 1 | Ath-AT3G05800.1 |  |  |  |  |  |  |  | Vvi-Vitvi14g04018\_t001 |  |  |  |  |
| 1 | Ath-AT3G05810.1 |  |  |  |  |  |  |  | Vvi-Vitvi14g00079\_t001 |  |  |  |  |
| 1 | Ath-AT3G05820.2 |  |  |  |  |  |  |  | Vvi-Vitvi14g00070\_t001 |  |  |  |  |
| 0 | Ath-AT3G05830.2 |  |  |  |  |  |  |  |  |
| 0 | Ath-AT3G05840.1 |  |  |  |  |  |  |  |  |
| 0 | Ath-AT3G05850.1 |  |  |  |  |  |  |  |  |
| 0 | Ath-AT3G05858.1 |  |  |  |  |  |  |  |  |
| 0 | Ath-AT3G05860.1 |  |  |  |  |  |  |  |  |
| 0 | Ath-AT3G05870.3 |  |  |  |  |  |  |  |  |
| 0 | Ath-AT3G05880.1 |  |  |  |  |  |  |  |  |
| 0 | Ath-AT3G05890.1 |  |  |  |  |  |  |  |  |
| 0 | Ath-AT3G05900.1 |  |  |  |  |  |  |  |  |
| 0 | Ath-AT3G05910.1 |  |  |  |  |  |  |  |  |
| 0 | Ath-AT3G05920.1 |  |  |  |  |  |  |  |  |
| 0 | Ath-AT3G05930.1 |  |  |  |  |  |  |  |  |
| 0 | Ath-AT3G05935.2 |  |  |  |  |  |  |  |  |
| 0 | Ath-AT3G05936.1 |  |  |  |  |  |  |  |  |
| 0 | Ath-AT3G05937.1 |  |  |  |  |  |  |  |  |
| 0 | Ath-AT3G05940.1 |  |  |  |  |  |  |  |  |
| 0 | Ath-AT3G05950.1 |  |  |  |  |  |  |  |  |
| 1 | Ath-AT3G05960.1 |  | Vvi-Vitvi05g00465\_t001 |  |  |  |  |  |  |  |
| 1 | Ath-AT3G05970.1 |  | Vvi-Vitvi05g00463\_t001 |  |  |  |  |  |  |  |
| 1 | Ath-AT3G05975.1 |  | | | |  |  |  |  |  |  |  |
| 1 | Ath-AT3G05980.1 |  | Vvi-Vitvi05g00460\_t001 |  |  |  |  |  |  |  |
| 1 | Ath-AT3G05990.1 |  | Vvi-Vitvi05g00459\_t001 |  |  |  |  |  |  |  |
| 1 | Ath-AT3G06000.1 |  | Vvi-Vitvi05g00454\_t001 |  |  |  |  |  |  |  |
| 1 | Ath-AT3G06010.1 |  | Vvi-Vitvi05g00453\_t001 |  |  |  |  |  |  |  |
| 1 | Ath-AT3G06019.1 |  | | | |  |  |  |  |  |  |  |
| 1 | Ath-AT3G06020.1 |  | Vvi-Vitvi05g00448\_t001 |  |  |  |  |  |  |  |
| 1 | Ath-AT3G06030.1 |  | Vvi-Vitvi05g00444\_t001 |  |  |  |  |  |  |  |
| 1 | Ath-AT3G06035.1 |  | Vvi-Vitvi05g01879\_t001 |  |  |  |  |  |  |  |
| 1 | Ath-AT3G06040.1 |  | | | |  |  |  |  |  |  |  |
| 1 | Ath-AT3G06050.1 |  | Vvi-Vitvi05g00439\_t001 |  |  |  |  |  |  |  |
| 1 | Ath-AT3G06060.2 |  | Vvi-Vitvi05g01878\_t002 |  |  |  |  |  |  |  |
| 1 | Ath-AT3G06070.1 |  | Vvi-Vitvi05g01877\_t001 |  |  |  |  |  |  |  |
| 1 | Ath-AT3G06080.2 |  | Vvi-Vitvi05g00437\_t001 |  |  |  |  |  |  |  |
| 1 | Ath-AT3G06085.1 |  | | | |  |  |  |  |  |  |  |
| 1 | Ath-AT3G06090.1 |  | | | |  |  |  |  |  |  |  |
| 1 | Ath-AT3G06100.1 |  | Vvi-Vitvi05g00432\_t001 |  |  |  |  |  |  |  |
| 1 | Ath-AT3G06110.3 |  | Vvi-Vitvi05g00429\_t001 |  |  |  |  |  |  |  |
| 1 | Ath-AT3G06120.1 |  | Vvi-Vitvi05g00427\_t001 |  |  |  |  |  |  |  |
| 1 | Ath-AT3G06130.1 |  | Vvi-Vitvi05g00426\_t001 |  |  |  |  |  |  |  |
| 1 | Ath-AT3G06140.1 |  | Vvi-Vitvi05g00425\_t002 |  |  |  |  |  |  |  |
| 1 | Ath-AT3G06145.1 |  | Vvi-Vitvi05g00424\_t001 |  |  |  |  |  |  |  |
| 1 | Ath-AT3G06150.1 |  | Vvi-Vitvi05g00423\_t001 |  |  |  |  |  |  |  |
| 1 | Ath-AT3G06160.2 |  | | | |  |  |  |  |  |  |  |
| 1 | Ath-AT3G06170.1 |  | Vvi-Vitvi05g00419\_t001 |  |  |  |  |  |  |  |
| 1 | Ath-AT3G06180.1 |  | Vvi-Vitvi05g00418\_t001 |  |  |  |  |  |  |  |
| 1 | Ath-AT3G06190.1 |  | Vvi-Vitvi05g00408\_t001 |  |  |  |  |  |  |  |
| 1 | Ath-AT3G06200.1 |  | Vvi-Vitvi05g00407\_t001 |  |  |  |  |  |  |  |
| 1 | Ath-AT3G06210.1 |  | Vvi-Vitvi05g00406\_t001 |  |  |  |  |  |  |  |
| 1 | Ath-AT3G06220.1 |  | | | |  |  |  |  |  |  |  |
| 1 | Ath-AT3G06230.1 |  | | | |  |  |  |  |  |  |  |
| 1 | Ath-AT3G06240.1 |  | | | |  |  |  |  |  |  |  |
| 1 | Ath-AT3G06250.2 |  | Vvi-Vitvi05g01873\_t001 |  |  |  |  |  |  |  |
| 1 | Ath-AT3G06260.1 |  | Vvi-Vitvi05g00394\_t001 |  |  |  |  |  |  |  |
| 1 | Ath-AT3G06270.1 |  | Vvi-Vitvi05g00392\_t001 |  |  |  |  |  |  |  |
| 1 | Ath-AT3G06280.1 |  | | | |  |  |  |  |  |  |  |
| 1 | Ath-AT3G06290.1 |  | Vvi-Vitvi05g00391\_t001 |  |  |  |  |  |  |  |
| 1 | Ath-AT3G06300.1 |  | Vvi-Vitvi05g00383\_t001 |  |  |  |  |  |  |  |
| 1 | Ath-AT3G06310.3 |  | Vvi-Vitvi05g00374\_t002 |  |  |  |  |  |  |  |
| 1 | Ath-AT3G06320.1 |  | Vvi-Vitvi05g04095\_t001 |  |  |  |  |  |  |  |
| 1 | Ath-AT3G06330.1 |  | Vvi-Vitvi05g00368\_t002 |  |  |  |  |  |  |  |
| 1 | Ath-AT3G06340.3 |  | Vvi-Vitvi05g00366\_t001 |  |  |  |  |  |  |  |
| 1 | Ath-AT3G06350.1 |  | Vvi-Vitvi05g00364\_t001 |  |  |  |  |  |  |  |
| 1 | Ath-AT3G06360.1 |  | | | |  |  |  |  |  |  |  |
| 1 | Ath-AT3G06370.3 |  | Vvi-Vitvi05g00359\_t001 |  |  |  |  |  |  |  |
| 1 | Ath-AT3G06380.1 |  | Vvi-Vitvi05g01862\_t002 |  |  |  |  |  |  |  |
| 1 | Ath-AT3G06390.1 |  | Vvi-Vitvi05g00349\_t001 |  |  |  |  |  |  |  |
| 1 | Ath-AT3G06400.3 |  | Vvi-Vitvi05g00345\_t001 |  |  |  |  |  |  |  |
| 1 | Ath-AT3G06410.1 |  | Vvi-Vitvi05g00333\_t001 |  |  |  |  |  |  |  |
| 0 | Ath-AT3G06420.1 |  |  |  |  |  |  |  |  |
| 0 | Ath-AT3G06430.1 |  |  |  |  |  |  |  |  |
| 0 | Ath-AT3G06435.1 |  |  |  |  |  |  |  |  |
| 0 | Ath-AT3G06436.1 |  |  |  |  |  |  |  |  |
| 0 | Ath-AT3G06440.1 |  |  |  |  |  |  |  |  |
| 0 | Ath-AT3G06450.2 |  |  |  |  |  |  |  |  |
| 0 | Ath-AT3G06455.1 |  |  |  |  |  |  |  |  |
| 0 | Ath-AT3G06460.1 |  |  |  |  |  |  |  |  |
| 0 | Ath-AT3G06470.1 |  |  |  |  |  |  |  |  |
| 0 | Ath-AT3G06480.1 |  |  |  |  |  |  |  |  |
| 0 | Ath-AT3G06483.1 |  |  |  |  |  |  |  |  |
| 1 | Ath-AT3G06490.1 |  | Vvi-Vitvi05g00166\_t001 |  |  |  |  |  |  |  |
| 1 | Ath-AT3G06500.1 |  | Vvi-Vitvi05g00164\_t001 |  |  |  |  |  |  |  |
| 1 | Ath-AT3G06510.2 |  | Vvi-Vitvi05g00161\_t001 |  |  |  |  |  |  |  |
| 1 | Ath-AT3G06520.1 |  | | | |  |  |  |  |  |  |  |
| 1 | Ath-AT3G06530.4 |  | | | |  |  |  |  |  |  |  |
| 1 | Ath-AT3G06540.1 |  | | | |  |  |  |  |  |  |  |
| 1 | Ath-AT3G06545.1 |  | | | |  |  |  |  |  |  |  |
| 1 | Ath-AT3G06550.2 |  | Vvi-Vitvi05g00153\_t001 |  |  |  |  |  |  |  |
| 1 | Ath-AT3G06560.1 |  | Vvi-Vitvi05g00151\_t001 |  |  |  |  |  |  |  |
| 1 | Ath-AT3G06570.1 |  | | | |  |  |  |  |  |  |  |
| 1 | Ath-AT3G06580.1 |  | Vvi-Vitvi05g00149\_t001 |  |  |  |  |  |  |  |
| 1 | Ath-AT3G06590.2 |  | Vvi-Vitvi05g00143\_t001 |  |  |  |  |  |  |  |
| 1 | Ath-AT3G06600.3 |  | | | |  |  |  |  |  |  |  |
| 1 | Ath-AT3G06610.1 |  | Vvi-Vitvi05g01774\_t001 |  |  |  |  |  |  |  |
| 1 | Ath-AT3G06620.1 |  | Vvi-Vitvi05g00132\_t001 |  |  |  |  |  |  |  |
| 1 | Ath-AT3G06630.2 |  | | | |  |  |  |  |  |  |  |
| 1 | Ath-AT3G06640.1 |  | | | |  |  |  |  |  |  |  |
| 1 | Ath-AT3G06650.2 |  | Vvi-Vitvi05g00129\_t002 |  |  |  |  |  |  |  |
| 1 | Ath-AT3G66652.2 |  | Vvi-Vitvi05g00125\_t001 |  |  |  |  |  |  |  |
| 1 | Ath-AT3G66654.3 |  | Vvi-Vitvi05g00120\_t001 |  |  |  |  |  |  |  |
| 1 | Ath-AT3G66656.1 |  | | | |  |  |  |  |  |  |  |
| 1 | Ath-AT3G66658.2 |  | Vvi-Vitvi05g00117\_t001 |  |  |  |  |  |  |  |
| 1 | Ath-AT3G36659.1 |  | Vvi-Vitvi05g01772\_t001 |  |  |  |  |  |  |  |
| 1 | Ath-AT3G06660.1 |  | Vvi-Vitvi05g00114\_t001 |  |  |  |  |  |  |  |
| 1 | Ath-AT3G06670.2 |  | Vvi-Vitvi05g00101\_t002 |  |  |  |  |  |  |  |
| 1 | Ath-AT3G06680.1 |  | Vvi-Vitvi05g00098\_t001 |  |  |  |  |  |  |  |
| 1 | Ath-AT3G06690.1 |  | | | |  |  |  |  |  |  |  |
| 1 | Ath-AT3G06700.1 |  | | | |  |  |  |  |  |  |  |
| 1 | Ath-AT3G06710.1 |  | | | |  |  |  |  |  |  |  |
| 1 | Ath-AT3G06720.1 |  | Vvi-Vitvi05g01765\_t001 |  |  |  |  |  |  |  |
| 1 | Ath-AT3G06730.1 |  | | | |  |  |  |  |  |  |  |
| 1 | Ath-AT3G06740.1 |  | Vvi-Vitvi05g00077\_t001 |  |  |  |  |  |  |  |
| 1 | Ath-AT3G06750.1 |  | | | |  |  |  |  |  |  |  |
| 1 | Ath-AT3G06760.2 |  | Vvi-Vitvi05g01762\_t001 |  |  |  |  |  |  |  |
| 1 | Ath-AT3G06770.5 |  | Vvi-Vitvi05g00061\_t001 |  |  |  |  |  |  |  |
| 1 | Ath-AT3G06778.1 |  | Vvi-Vitvi05g01747\_t001 |  |  |  |  |  |  |  |
| 1 | Ath-AT3G06780.1 |  | Vvi-Vitvi05g01745\_t001 |  |  |  |  |  |  |  |
| 1 | Ath-AT3G06790.1 |  | Vvi-Vitvi05g00049\_t001 |  |  |  |  |  |  |  |
| 1 | Ath-AT3G06810.1 |  | Vvi-Vitvi05g00044\_t001 |  |  |  |  |  |  |  |
| 1 | Ath-AT3G06820.3 |  | | | |  |  |  |  |  |  |  |
| 1 | Ath-AT3G06830.1 |  | Vvi-Vitvi05g00043\_t001 |  |  |  |  |  |  |  |
| 1 | Ath-AT3G06840.1 |  | Vvi-Vitvi05g01743\_t001 |  |  |  |  |  |  |  |
| 1 | Ath-AT3G06850.1 |  | Vvi-Vitvi05g00032\_t001 |  |  |  |  |  |  |  |
| 1 | Ath-AT3G06860.1 |  | Vvi-Vitvi05g00020\_t002 |  |  |  |  |  |  |  |
| 1 | Ath-AT3G06868.1 |  | Vvi-Vitvi05g00012\_t001 |  |  |  |  |  |  |  |
| 1 | Ath-AT3G06870.1 |  | | | |  |  |  |  |  |  |  |
| 1 | Ath-AT3G06880.4 |  | Vvi-Vitvi05g00006\_t002 |  |  |  |  |  |  |  |
| 1 | Ath-AT3G06890.1 |  | Vvi-Vitvi05g01734\_t001 |  |  |  |  |  |  |  |
| 0 | Ath-AT3G06895.1 |  |  |  |  |  |  |  |  |
| 0 | Ath-AT3G06910.1 |  |  |  |  |  |  |  |  |
| 0 | Ath-AT3G06920.3 |  |  |  |  |  |  |  |  |
| 0 | Ath-AT3G06930.2 |  |  |  |  |  |  |  |  |
| 0 | Ath-AT3G06950.1 |  |  |  |  |  |  |  |  |
| 0 | Ath-AT3G06960.1 |  |  |  |  |  |  |  |  |
| 0 | Ath-AT3G06970.1 |  |  |  |  |  |  |  |  |
| 0 | Ath-AT3G06980.1 |  |  |  |  |  |  |  |  |
| 0 | Ath-AT3G06985.1 |  |  |  |  |  |  |  |  |
| 0 | Ath-AT3G06990.1 |  |  |  |  |  |  |  |  |
| 0 | Ath-AT3G07000.1 |  |  |  |  |  |  |  |  |
| 0 | Ath-AT3G07005.1 |  |  |  |  |  |  |  |  |
| 0 | Ath-AT3G07010.1 |  |  |  |  |  |  |  |  |
| 0 | Ath-AT3G07020.1 |  |  |  |  |  |  |  |  |
| 0 | Ath-AT3G07030.5 |  |  |  |  |  |  |  |  |
| 0 | Ath-AT3G07040.1 |  |  |  |  |  |  |  |  |
| 0 | Ath-AT3G07050.1 |  |  |  |  |  |  |  |  |
| 0 | Ath-AT3G07060.1 |  |  |  |  |  |  |  |  |
| 0 | Ath-AT3G07070.1 |  |  |  |  |  |  |  |  |
| 0 | Ath-AT3G07080.1 |  |  |  |  |  |  |  |  |
| 0 | Ath-AT3G07090.1 |  |  |  |  |  |  |  |  |
| 0 | Ath-AT3G07100.1 |  |  |  |  |  |  |  |  |
| 0 | Ath-AT3G07110.2 |  |  |  |  |  |  |  |  |
| 0 | Ath-AT3G07120.1 |  |  |  |  |  |  |  |  |
| 0 | Ath-AT3G07130.2 |  |  |  |  |  |  |  |  |
| 0 | Ath-AT3G07140.1 |  |  |  |  |  |  |  |  |
| 0 | Ath-AT3G07150.1 |  |  |  |  |  |  |  |  |
| 0 | Ath-AT3G07160.2 |  |  |  |  |  |  |  |  |
| 0 | Ath-AT3G07170.1 |  |  |  |  |  |  |  |  |
| 0 | Ath-AT3G07180.1 |  |  |  |  |  |  |  |  |
| 0 | Ath-AT3G07190.1 |  |  |  |  |  |  |  |  |
| 0 | Ath-AT3G07195.2 |  |  |  |  |  |  |  |  |
| 0 | Ath-AT3G07200.1 |  |  |  |  |  |  |  |  |
| 0 | Ath-AT3G07210.1 |  |  |  |  |  |  |  |  |
| 0 | Ath-AT3G07220.1 |  |  |  |  |  |  |  |  |
| 0 | Ath-AT3G07230.1 |  |  |  |  |  |  |  |  |
| 0 | Ath-AT3G07250.1 |  |  |  |  |  |  |  |  |
| 0 | Ath-AT3G07255.1 |  |  |  |  |  |  |  |  |
| 0 | Ath-AT3G07260.1 |  |  |  |  |  |  |  |  |
| 0 | Ath-AT3G07270.2 |  |  |  |  |  |  |  |  |
| 0 | Ath-AT3G07273.1 |  |  |  |  |  |  |  |  |
| 0 | Ath-AT3G07290.1 |  |  |  |  |  |  |  |  |
| 0 | Ath-AT3G07300.3 |  |  |  |  |  |  |  |  |
| 0 | Ath-AT3G07310.1 |  |  |  |  |  |  |  |  |
| 0 | Ath-AT3G07320.1 |  |  |  |  |  |  |  |  |
| 0 | Ath-AT3G07330.2 |  |  |  |  |  |  |  |  |
| 0 | Ath-AT3G07340.1 |  |  |  |  |  |  |  |  |
| 0 | Ath-AT3G07350.1 |  |  |  |  |  |  |  |  |
| 0 | Ath-AT3G07360.1 |  |  |  |  |  |  |  |  |
| 0 | Ath-AT3G07370.1 |  |  |  |  |  |  |  |  |
| 0 | Ath-AT3G07380.1 |  |  |  |  |  |  |  |  |
| 0 | Ath-AT3G07390.1 |  |  |  |  |  |  |  |  |
| 0 | Ath-AT3G07400.1 |  |  |  |  |  |  |  |  |
| 0 | Ath-AT3G07410.1 |  |  |  |  |  |  |  |  |
| 0 | Ath-AT3G07420.1 |  |  |  |  |  |  |  |  |
| 0 | Ath-AT3G07425.1 |  |  |  |  |  |  |  |  |
| 0 | Ath-AT3G07430.1 |  |  |  |  |  |  |  |  |
| 0 | Ath-AT3G07440.1 |  |  |  |  |  |  |  |  |
| 0 | Ath-AT3G07450.1 |  |  |  |  |  |  |  |  |
| 0 | Ath-AT3G07460.2 |  |  |  |  |  |  |  |  |
| 0 | Ath-AT3G07470.1 |  |  |  |  |  |  |  |  |
| 0 | Ath-AT3G07480.1 |  |  |  |  |  |  |  |  |
| 0 | Ath-AT3G07490.1 |  |  |  |  |  |  |  |  |
| 0 | Ath-AT3G07500.1 |  |  |  |  |  |  |  |  |
| 0 | Ath-AT3G07510.3 |  |  |  |  |  |  |  |  |
| 0 | Ath-AT3G07520.1 |  |  |  |  |  |  |  |  |
| 0 | Ath-AT3G07522.1 |  |  |  |  |  |  |  |  |
| 0 | Ath-AT3G07525.2 |  |  |  |  |  |  |  |  |
| 0 | Ath-AT3G07530.4 |  |  |  |  |  |  |  |  |
| 0 | Ath-AT3G07540.1 |  |  |  |  |  |  |  |  |
| 0 | Ath-AT3G07550.1 |  |  |  |  |  |  |  |  |
| 0 | Ath-AT3G07560.1 |  |  |  |  |  |  |  |  |
| 0 | Ath-AT3G07565.4 |  |  |  |  |  |  |  |  |
| 0 | Ath-AT3G07568.1 |  |  |  |  |  |  |  |  |
| 0 | Ath-AT3G07570.1 |  |  |  |  |  |  |  |  |
| 0 | Ath-AT3G07580.1 |  |  |  |  |  |  |  |  |
| 0 | Ath-AT3G07590.2 |  |  |  |  |  |  |  |  |
| 1 | Ath-AT3G07600.1 |  | Vvi-Vitvi12g02508\_t001 |  |  |  |  |  |  |  |
| 1 | Ath-AT3G07610.3 |  | | | |  |  |  |  |  |  |  |
| 1 | Ath-AT3G07620.1 |  | Vvi-Vitvi12g00764\_t001 |  |  |  |  |  |  |  |
| 1 | Ath-AT3G07630.1 |  | | | |  |  |  |  |  |  |  |
| 1 | Ath-AT3G07640.1 |  | Vvi-Vitvi12g00760\_t001 |  |  |  |  |  |  |  |
| 1 | Ath-AT3G07650.4 |  | Vvi-Vitvi12g00757\_t001 |  |  |  |  |  |  |  |
| 1 | Ath-AT3G07660.1 |  | Vvi-Vitvi12g00751\_t001 |  |  |  |  |  |  |  |
| 1 | Ath-AT3G07670.1 |  | Vvi-Vitvi12g00746\_t001 |  |  |  |  |  |  |  |
| 1 | Ath-AT3G07680.1 |  | Vvi-Vitvi12g00741\_t001 |  |  |  |  |  |  |  |
| 1 | Ath-AT3G07690.1 |  | | | |  |  |  |  |  |  |  |
| 1 | Ath-AT3G07700.3 |  | Vvi-Vitvi12g02500\_t001 |  |  |  |  |  |  |  |
| 1 | Ath-AT3G07710.1 |  | | | |  |  |  |  |  |  |  |
| 1 | Ath-AT3G07720.1 |  | Vvi-Vitvi12g00727\_t001 |  |  |  |  |  |  |  |
| 1 | Ath-AT3G07730.1 |  | | | |  |  |  |  |  |  |  |
| 1 | Ath-AT3G07740.4 |  | Vvi-Vitvi12g00709\_t001 |  |  |  |  |  |  |  |
| 0 | Ath-AT3G07750.2 |  |  |  |  |  |  |  |  |
| 0 | Ath-AT3G07760.3 |  |  |  |  |  |  |  |  |
| 0 | Ath-AT3G07770.1 |  |  |  |  |  |  |  |  |
| 0 | Ath-AT3G07780.2 |  |  |  |  |  |  |  |  |
| 0 | Ath-AT3G07790.1 |  |  |  |  |  |  |  |  |
| 0 | Ath-AT3G07800.1 |  |  |  |  |  |  |  |  |
| 0 | Ath-AT3G07810.2 |  |  |  |  |  |  |  |  |
| 0 | Ath-AT3G07820.1 |  |  |  |  |  |  |  |  |
| 0 | Ath-AT3G07830.1 |  |  |  |  |  |  |  |  |
| 0 | Ath-AT3G07840.1 |  |  |  |  |  |  |  |  |
| 0 | Ath-AT3G07850.1 |  |  |  |  |  |  |  |  |
| 0 | Ath-AT3G07860.3 |  |  |  |  |  |  |  |  |
| 0 | Ath-AT3G07870.1 |  |  |  |  |  |  |  |  |
| 0 | Ath-AT3G07880.1 |  |  |  |  |  |  |  |  |
| 0 | Ath-AT3G07890.3 |  |  |  |  |  |  |  |  |
| 0 | Ath-AT3G07900.1 |  |  |  |  |  |  |  |  |
| 0 | Ath-AT3G07910.1 |  |  |  |  |  |  |  |  |
| 0 | Ath-AT3G07920.1 |  |  |  |  |  |  |  |  |
| 0 | Ath-AT3G07930.3 |  |  |  |  |  |  |  |  |
| 0 | Ath-AT3G07940.1 |  |  |  |  |  |  |  |  |
| 0 | Ath-AT3G07950.1 |  |  |  |  |  |  |  |  |
| 0 | Ath-AT3G07960.1 |  |  |  |  |  |  |  |  |
| 0 | Ath-AT3G07970.1 |  |  |  |  |  |  |  |  |
| 0 | Ath-AT3G07980.1 |  |  |  |  |  |  |  |  |
| 0 | Ath-AT3G07990.1 |  |  |  |  |  |  |  |  |
| 0 | Ath-AT3G08000.1 |  |  |  |  |  |  |  |  |
| 0 | Ath-AT3G08010.1 |  |  |  |  |  |  |  |  |
| 0 | Ath-AT3G08020.1 |  |  |  |  |  |  |  |  |
| 0 | Ath-AT3G08030.1 |  |  |  |  |  |  |  |  |
| 0 | Ath-AT3G08040.1 |  |  |  |  |  |  |  |  |
| 0 | Ath-AT3G08490.1 |  |  |  |  |  |  |  |  |
| 0 | Ath-AT3G08500.1 |  |  |  |  |  |  |  |  |
| 0 | Ath-AT3G08505.1 |  |  |  |  |  |  |  |  |
| 0 | Ath-AT3G08510.1 |  |  |  |  |  |  |  |  |
| 0 | Ath-AT3G08520.1 |  |  |  |  |  |  |  |  |
| 0 | Ath-AT3G08530.1 |  |  |  |  |  |  |  |  |
| 0 | Ath-AT3G08550.1 |  |  |  |  |  |  |  |  |
| 0 | Ath-AT3G08560.1 |  |  |  |  |  |  |  |  |
| 0 | Ath-AT3G08570.1 |  |  |  |  |  |  |  |  |
| 0 | Ath-AT3G08580.2 |  |  |  |  |  |  |  |  |
| 1 | Ath-AT3G08590.1 |  | Vvi-Vitvi08g00018\_t001 |  |  |  |  |  |  |  |
| 1 | Ath-AT3G08600.1 |  | Vvi-Vitvi08g00041\_t001 |  |  |  |  |  |  |  |
| 1 | Ath-AT3G08610.1 |  | | | |  |  |  |  |  |  |  |
| 1 | Ath-AT3G08620.1 |  | Vvi-Vitvi08g00042\_t001 |  |  |  |  |  |  |  |
| 1 | Ath-AT3G08630.1 |  | Vvi-Vitvi08g01983\_t001 |  |  |  |  |  |  |  |
| 1 | Ath-AT3G08636.1 |  | | | |  |  |  |  |  |  |  |
| 1 | Ath-AT3G08640.1 |  | | | |  |  |  |  |  |  |  |
| 1 | Ath-AT3G08650.2 |  | | | |  |  |  |  |  |  |  |
| 1 | Ath-AT3G08660.1 |  | Vvi-Vitvi08g00052\_t001 |  |  |  |  |  |  |  |
| 1 | Ath-AT3G08670.1 |  | Vvi-Vitvi08g00055\_t001 |  |  |  |  |  |  |  |
| 1 | Ath-AT3G08680.2 |  | Vvi-Vitvi08g00883\_t004 |  |  |  |  |  |  |  |
| 1 | Ath-AT3G08690.1 |  | Vvi-Vitvi08g04142\_t002 |  |  |  |  |  |  |  |
| 1 | Ath-AT3G08700.1 |  | | | |  |  |  |  |  |  |  |
| 1 | Ath-AT3G08710.1 |  | Vvi-Vitvi08g04141\_t001 |  |  |  |  |  |  |  |
| 1 | Ath-AT3G08720.2 |  | Vvi-Vitvi08g00874\_t001 |  |  |  |  |  |  |  |
| 1 | Ath-AT3G08730.1 |  | | | |  |  |  |  |  |  |  |
| 1 | Ath-AT3G08740.1 |  | Vvi-Vitvi08g00860\_t001 |  |  |  |  |  |  |  |
| 1 | Ath-AT3G08750.1 |  | | | |  |  |  |  |  |  |  |
| 1 | Ath-AT3G08760.2 |  | Vvi-Vitvi08g00858\_t001 |  |  |  |  |  |  |  |
| 0 | Ath-AT3G08770.2 |  |  |  |  |  |  |  |  |
| 1 | Ath-AT3G08780.1 |  | Vvi-Vitvi08g00780\_t001 |  |  |  |  |  |  |  |
| 1 | Ath-AT3G08790.1 |  | | | |  |  |  |  |  |  |  |
| 1 | Ath-AT3G08800.1 |  | Vvi-Vitvi08g00785\_t001 |  |  |  |  |  |  |  |
| 1 | Ath-AT3G08810.1 |  | | | |  |  |  |  |  |  |  |
| 1 | Ath-AT3G08820.1 |  | Vvi-Vitvi08g00798\_t001 |  |  |  |  |  |  |  |
| 1 | Ath-AT3G08840.7 |  | Vvi-Vitvi08g00805\_t001 |  |  |  |  |  |  |  |
| 1 | Ath-AT3G08850.1 |  | Vvi-Vitvi08g02087\_t001 |  |  |  |  |  |  |  |
| 1 | Ath-AT3G08860.1 |  | Vvi-Vitvi08g00816\_t001 |  |  |  |  |  |  |  |
| 0 | Ath-AT3G08870.2 |  |  |  |  |  |  |  |  |
| 0 | Ath-AT3G08880.1 |  |  |  |  |  |  |  |  |
| 0 | Ath-AT3G08890.1 |  |  |  |  |  |  |  |  |
| 0 | Ath-AT3G08900.1 |  |  |  |  |  |  |  |  |
| 0 | Ath-AT3G08910.1 |  |  |  |  |  |  |  |  |
| 0 | Ath-AT3G08920.1 |  |  |  |  |  |  |  |  |
| 1 | Ath-AT3G08930.1 |  | Vvi-Vitvi08g01367\_t001 |  |  |  |  |  |  |  |
| 1 | Ath-AT3G08940.2 |  | Vvi-Vitvi08g01360\_t001 |  |  |  |  |  |  |  |
| 1 | Ath-AT3G08947.1 |  | | | |  |  |  |  |  |  |  |
| 1 | Ath-AT3G08950.1 |  | | | |  |  |  |  |  |  |  |
| 1 | Ath-AT3G08960.1 |  | | | |  |  |  |  |  |  |  |
| 1 | Ath-AT3G08970.1 |  | | | |  |  |  |  |  |  |  |
| 1 | Ath-AT3G08980.2 |  | Vvi-Vitvi08g01359\_t003 |  |  |  |  |  |  |  |
| 1 | Ath-AT3G08990.2 |  | Vvi-Vitvi08g01358\_t001 |  |  |  |  |  |  |  |
| 1 | Ath-AT3G09000.1 |  | Vvi-Vitvi08g01357\_t001 |  |  |  |  |  |  |  |
| 1 | Ath-AT3G09010.1 |  | Vvi-Vitvi08g01356\_t001 |  |  |  |  |  |  |  |
| 1 | Ath-AT3G09020.1 |  | Vvi-Vitvi08g01349\_t001 |  |  |  |  |  |  |  |
| 1 | Ath-AT3G09030.1 |  | Vvi-Vitvi08g01341\_t001 |  |  |  |  |  |  |  |
| 1 | Ath-AT3G09032.1 |  | Vvi-Vitvi08g02235\_t001 |  |  |  |  |  |  |  |
| 1 | Ath-AT3G09035.1 |  | | | |  |  |  |  |  |  |  |
| 1 | Ath-AT3G09040.2 |  | | | |  |  |  |  |  |  |  |
| 1 | Ath-AT3G09050.1 |  | Vvi-Vitvi08g01332\_t001 |  |  |  |  |  |  |  |
| 1 | Ath-AT3G09055.1 |  | | | |  |  |  |  |  |  |  |
| 1 | Ath-AT3G09060.1 |  | Vvi-Vitvi08g04270\_t001 |  |  |  |  |  |  |  |
| 1 | Ath-AT3G09070.1 |  | Vvi-Vitvi08g01327\_t001 |  |  |  |  |  |  |  |
| 1 | Ath-AT3G09080.3 |  | Vvi-Vitvi08g01326\_t001 |  |  |  |  |  |  |  |
| 1 | Ath-AT3G09085.1 |  | Vvi-Vitvi08g04267\_t001 |  |  |  |  |  |  |  |
| 1 | Ath-AT3G09090.1 |  | Vvi-Vitvi08g01323\_t001 |  |  |  |  |  |  |  |
| 1 | Ath-AT3G09100.2 |  | Vvi-Vitvi08g01322\_t001 |  |  |  |  |  |  |  |
| 1 | Ath-AT3G09110.1 |  | | | |  |  |  |  |  |  |  |
| 1 | Ath-AT3G09120.1 |  | | | |  |  |  |  |  |  |  |
| 1 | Ath-AT3G09130.1 |  | | | |  |  |  |  |  |  |  |
| 1 | Ath-AT3G09140.1 |  | | | |  |  |  |  |  |  |  |
| 1 | Ath-AT3G09150.2 |  | | | |  |  |  |  |  |  |  |
| 1 | Ath-AT3G09160.1 |  | | | |  |  |  |  |  |  |  |
| 1 | Ath-AT3G09162.1 |  | | | |  |  |  |  |  |  |  |
| 1 | Ath-AT3G09180.1 |  | | | |  |  |  |  |  |  |  |
| 1 | Ath-AT3G09190.1 |  | Vvi-Vitvi08g01305\_t001 |  |  |  |  |  |  |  |
| 1 | Ath-AT3G09200.1 |  | | | |  |  |  |  |  |  |  |
| 1 | Ath-AT3G09210.1 |  | Vvi-Vitvi08g01304\_t001 |  |  |  |  |  |  |  |
| 1 | Ath-AT3G09220.1 |  | Vvi-Vitvi08g01299\_t001 |  |  |  |  |  |  |  |
| 1 | Ath-AT3G09230.1 |  | Vvi-Vitvi08g01298\_t001 |  |  |  |  |  |  |  |
| 1 | Ath-AT3G09240.3 |  | | | |  |  |  |  |  |  |  |
| 1 | Ath-AT3G09250.2 |  | Vvi-Vitvi08g01294\_t001 |  |  |  |  |  |  |  |
| 1 | Ath-AT3G09260.1 |  | | | |  |  |  |  |  |  |  |
| 1 | Ath-AT3G09270.1 |  | Vvi-Vitvi08g02226\_t002 |  |  |  |  |  |  |  |
| 1 | Ath-AT3G09280.1 |  | Vvi-Vitvi08g02223\_t001 |  |  |  |  |  |  |  |
| 0 | Ath-AT3G09290.1 |  |  |  |  |  |  |  |  |
| 1 | Ath-AT3G09300.1 |  | Vvi-Vitvi08g01211\_t001 |  |  |  |  |  |  |  |
| 1 | Ath-AT3G09310.1 |  | | | |  |  |  |  |  |  |  |
| 1 | Ath-AT3G09320.1 |  | | | |  |  |  |  |  |  |  |
| 1 | Ath-AT3G09330.1 |  | | | |  |  |  |  |  |  |  |
| 1 | Ath-AT3G09340.1 |  | | | |  |  |  |  |  |  |  |
| 1 | Ath-AT3G09350.1 |  | | | |  |  |  |  |  |  |  |
| 1 | Ath-AT3G09360.1 |  | | | |  |  |  |  |  |  |  |
| 1 | Ath-AT3G09370.3 |  | Vvi-Vitvi08g02196\_t001 |  |  |  |  |  |  |  |
| 1 | Ath-AT3G09380.1 |  | | | |  |  |  |  |  |  |  |
| 1 | Ath-AT3G09390.1 |  | Vvi-Vitvi08g01196\_t001 |  |  |  |  |  |  |  |
| 1 | Ath-AT3G09400.1 |  | Vvi-Vitvi08g01194\_t001 |  |  |  |  |  |  |  |
| 1 | Ath-AT3G09405.1 |  | Vvi-Vitvi08g01193\_t001 |  |  |  |  |  |  |  |
| 1 | Ath-AT3G09410.1 |  | | | |  |  |  |  |  |  |  |
| 1 | Ath-AT3G09430.1 |  | Vvi-Vitvi08g01189\_t001 |  |  |  |  |  |  |  |
| 1 | Ath-AT3G09440.2 |  | Vvi-Vitvi08g02189\_t001 |  |  |  |  |  |  |  |
| 1 | Ath-AT3G09450.1 |  | Vvi-Vitvi08g01179\_t001 |  |  |  |  |  |  |  |
| 1 | Ath-AT3G09455.2 |  | Vvi-Vitvi08g04214\_t001 |  |  |  |  |  |  |  |
| 1 | Ath-AT3G09470.2 |  | Vvi-Vitvi08g01167\_t001 |  |  |  |  |  |  |  |
| 1 | Ath-AT3G09480.1 |  | | | |  |  |  |  |  |  |  |
| 3 | Ath-AT3G09490.1 |  | | | |  | Vvi-Vitvi08g01095\_t001 |  | Vvi-Vitvi08g01095\_t001 |  |  |  |  |  |
| 3 | Ath-AT3G09500.1 |  | | | |  | | | |  | | | |  |  |  |  |  |
| 3 | Ath-AT3G09510.1 |  | | | |  | | | |  | | | |  |  |  |  |  |
| 3 | Ath-AT3G09520.1 |  | | | |  | Vvi-Vitvi08g01101\_t001 |  | | | |  |  |  |  |  |
| 3 | Ath-AT3G09530.1 |  | | | |  | | | |  | | | |  |  |  |  |  |
| 3 | Ath-AT3G09540.1 |  | | | |  | Vvi-Vitvi08g01102\_t001 |  | | | |  |  |  |  |  |
| 3 | Ath-AT3G09550.2 |  | | | |  | Vvi-Vitvi08g01103\_t001 |  | | | |  |  |  |  |  |
| 3 | Ath-AT3G09560.2 |  | | | |  | Vvi-Vitvi08g01108\_t001 |  | | | |  |  |  |  |  |
| 3 | Ath-AT3G09570.1 |  | | | |  | Vvi-Vitvi08g01109\_t001 |  | | | |  |  |  |  |  |
| 2 | Ath-AT3G09580.1 |  | | | |  |  |  | | | |  |  |  |  |  |
| 2 | Ath-AT3G09590.1 |  | | | |  |  |  | | | |  |  |  |  |  |
| 2 | Ath-AT3G09600.1 |  | Vvi-Vitvi08g01149\_t002 |  |  |  | | | |  |  |  |  |  |
| 2 | Ath-AT3G09620.2 |  | | | |  |  |  | | | |  |  |  |  |  |
| 2 | Ath-AT3G09630.1 |  | Vvi-Vitvi08g01147\_t001 |  |  |  | | | |  |  |  |  |  |
| 2 | Ath-AT3G09640.1 |  | Vvi-Vitvi08g01143\_t002 |  |  |  | | | |  |  |  |  |  |
| 1 | Ath-AT3G09650.1 |  |  |  |  |  | | | |  |  |  |  |  |
| 1 | Ath-AT3G09660.1 |  |  |  |  |  | | | |  |  |  |  |  |
| 1 | Ath-AT3G09670.1 |  |  |  |  |  | Vvi-Vitvi08g01078\_t001 |  |  |  |  |  |
| 1 | Ath-AT3G09680.1 |  |  |  |  |  | Vvi-Vitvi08g01076\_t001 |  |  |  |  |  |
| 1 | Ath-AT3G09690.1 |  |  |  |  |  | Vvi-Vitvi08g01073\_t001 |  |  |  |  |  |
| 1 | Ath-AT3G09700.1 |  |  |  |  |  | Vvi-Vitvi08g01072\_t001 |  |  |  |  |  |
| 1 | Ath-AT3G09710.2 |  |  |  |  |  | Vvi-Vitvi08g01071\_t001 |  |  |  |  |  |
| 1 | Ath-AT3G09720.1 |  |  |  |  |  | Vvi-Vitvi08g01067\_t001 |  |  |  |  |  |
| 1 | Ath-AT3G09730.3 |  |  |  |  |  | Vvi-Vitvi08g01062\_t001 |  |  |  |  |  |
| 1 | Ath-AT3G09735.1 |  |  |  |  |  | | | |  |  |  |  |  |
| 1 | Ath-AT3G09740.1 |  |  |  |  |  | Vvi-Vitvi08g01053\_t001 |  |  |  |  |  |
| 1 | Ath-AT3G09750.1 |  |  |  |  |  | | | |  |  |  |  |  |
| 1 | Ath-AT3G09760.1 |  |  |  |  |  | Vvi-Vitvi08g01043\_t001 |  |  |  |  |  |
| 1 | Ath-AT3G09770.1 |  |  |  |  |  | Vvi-Vitvi08g01042\_t001 |  |  |  |  |  |
| 1 | Ath-AT3G09780.1 |  |  |  |  |  | Vvi-Vitvi08g01040\_t001 |  |  |  |  |  |
| 1 | Ath-AT3G09790.1 |  |  |  |  |  | | | |  |  |  |  |  |
| 1 | Ath-AT3G09800.6 |  |  |  |  |  | | | |  |  |  |  |  |
| 1 | Ath-AT3G09810.1 |  |  |  |  |  | Vvi-Vitvi08g01021\_t001 |  |  |  |  |  |
| 1 | Ath-AT3G09820.1 |  |  |  |  |  | | | |  |  |  |  |  |
| 1 | Ath-AT3G09830.1 |  |  |  |  |  | Vvi-Vitvi08g01009\_t001 |  |  |  |  |  |
| 1 | Ath-AT3G09840.1 |  |  |  |  |  | Vvi-Vitvi08g01002\_t001 |  |  |  |  |  |
| 1 | Ath-AT3G09850.1 |  |  |  |  |  | | | |  |  |  |  |  |
| 1 | Ath-AT3G09860.1 |  |  |  |  |  | Vvi-Vitvi08g00996\_t001 |  |  |  |  |  |
| 1 | Ath-AT3G09863.1 |  |  |  |  |  | | | |  |  |  |  |  |
| 1 | Ath-AT3G09870.1 |  |  |  |  |  | Vvi-Vitvi08g02133\_t001 |  |  |  |  |  |
| 0 | Ath-AT3G09880.1 |  |  |  |  |  |  |  |  |
| 0 | Ath-AT3G09890.1 |  |  |  |  |  |  |  |  |
| 0 | Ath-AT3G09900.1 |  |  |  |  |  |  |  |  |
| 0 | Ath-AT3G09910.1 |  |  |  |  |  |  |  |  |
| 0 | Ath-AT3G09920.3 |  |  |  |  |  |  |  |  |
| 0 | Ath-AT3G09922.1 |  |  |  |  |  |  |  |  |
| 0 | Ath-AT3G09925.1 |  |  |  |  |  |  |  |  |
| 1 | Ath-AT3G09930.1 |  | Vvi-Vitvi08g01488\_t001 |  |  |  |  |  |  |  |
| 1 | Ath-AT3G09940.1 |  | | | |  |  |  |  |  |  |  |
| 1 | Ath-AT3G09950.1 |  | | | |  |  |  |  |  |  |  |
| 1 | Ath-AT3G09960.1 |  | Vvi-Vitvi08g01494\_t001 |  |  |  |  |  |  |  |
| 1 | Ath-AT3G09970.1 |  | | | |  |  |  |  |  |  |  |
| 1 | Ath-AT3G09980.1 |  | Vvi-Vitvi08g01499\_t001 |  |  |  |  |  |  |  |
| 1 | Ath-AT3G09990.1 |  | | | |  |  |  |  |  |  |  |
| 1 | Ath-AT3G10000.1 |  | Vvi-Vitvi08g01505\_t001 |  |  |  |  |  |  |  |
| 1 | Ath-AT3G10010.2 |  | Vvi-Vitvi08g01515\_t001 |  |  |  |  |  |  |  |
| 1 | Ath-AT3G10020.1 |  | Vvi-Vitvi08g02274\_t001 |  |  |  |  |  |  |  |
| 1 | Ath-AT3G10030.1 |  | Vvi-Vitvi08g01538\_t002 |  |  |  |  |  |  |  |
| 1 | Ath-AT3G10040.1 |  | Vvi-Vitvi08g01540\_t001 |  |  |  |  |  |  |  |
| 1 | Ath-AT3G10050.1 |  | Vvi-Vitvi08g01554\_t001 |  |  |  |  |  |  |  |
| 1 | Ath-AT3G10060.1 |  | Vvi-Vitvi08g01556\_t001 |  |  |  |  |  |  |  |
| 0 | Ath-AT3G10070.1 |  |  |  |  |  |  |  |  |
| 0 | Ath-AT3G10080.2 |  |  |  |  |  |  |  |  |
| 0 | Ath-AT3G10090.1 |  |  |  |  |  |  |  |  |
| 0 | Ath-AT3G10110.1 |  |  |  |  |  |  |  |  |
| 0 | Ath-AT3G10113.1 |  |  |  |  |  |  |  |  |
| 0 | Ath-AT3G10116.1 |  |  |  |  |  |  |  |  |
| 0 | Ath-AT3G10130.1 |  |  |  |  |  |  |  |  |
| 0 | Ath-AT3G10120.1 |  |  |  |  |  |  |  |  |
| 0 | Ath-AT3G10140.1 |  |  |  |  |  |  |  |  |
| 1 | Ath-AT3G10150.2 |  | Vvi-Vitvi08g01698\_t001 |  |  |  |  |  |  |  |
| 1 | Ath-AT3G10160.1 |  | Vvi-Vitvi08g01693\_t001 |  |  |  |  |  |  |  |
| 1 | Ath-AT3G10185.1 |  | Vvi-Vitvi08g01686\_t001 |  |  |  |  |  |  |  |
| 1 | Ath-AT3G10180.1 |  | | | |  |  |  |  |  |  |  |
| 1 | Ath-AT3G10190.1 |  | Vvi-Vitvi08g01683\_t001 |  |  |  |  |  |  |  |
| 1 | Ath-AT3G10195.1 |  | | | |  |  |  |  |  |  |  |
| 1 | Ath-AT3G10200.2 |  | Vvi-Vitvi08g01679\_t001 |  |  |  |  |  |  |  |
| 1 | Ath-AT3G10210.1 |  | Vvi-Vitvi08g01675\_t002 |  |  |  |  |  |  |  |
| 1 | Ath-AT3G10220.1 |  | | | |  |  |  |  |  |  |  |
| 1 | Ath-AT3G10230.1 |  | Vvi-Vitvi08g01674\_t001 |  |  |  |  |  |  |  |
| 0 | Ath-AT3G10240.1 |  |  |  |  |  |  |  |  |
| 0 | Ath-AT3G10250.1 |  |  |  |  |  |  |  |  |
| 0 | Ath-AT3G10260.3 |  |  |  |  |  |  |  |  |
| 0 | Ath-AT3G10270.2 |  |  |  |  |  |  |  |  |
| 0 | Ath-AT3G10280.1 |  |  |  |  |  |  |  |  |
| 0 | Ath-AT3G10290.1 |  |  |  |  |  |  |  |  |
| 0 | Ath-AT3G10300.6 |  |  |  |  |  |  |  |  |
| 0 | Ath-AT3G10310.2 |  |  |  |  |  |  |  |  |
| 0 | Ath-AT3G10320.1 |  |  |  |  |  |  |  |  |
| 0 | Ath-AT3G10330.1 |  |  |  |  |  |  |  |  |
| 1 | Ath-AT3G10340.1 |  | Vvi-Vitvi13g00622\_t001 |  |  |  |  |  |  |  |
| 1 | Ath-AT3G10350.2 |  | Vvi-Vitvi13g00637\_t001.1.6037826b |  |  |  |  |  |  |  |
| 1 | Ath-AT3G10360.1 |  | Vvi-Vitvi13g00644\_t002 |  |  |  |  |  |  |  |
| 1 | Ath-AT3G10370.1 |  | Vvi-Vitvi13g00654\_t001 |  |  |  |  |  |  |  |
| 1 | Ath-AT3G10380.1 |  | Vvi-Vitvi13g00659\_t001 |  |  |  |  |  |  |  |
| 1 | Ath-AT3G10390.3 |  | Vvi-Vitvi13g00661\_t001 |  |  |  |  |  |  |  |
| 1 | Ath-AT3G10405.1 |  | Vvi-Vitvi13g00663\_t001 |  |  |  |  |  |  |  |
| 1 | Ath-AT3G10400.1 |  | | | |  |  |  |  |  |  |  |
| 1 | Ath-AT3G10410.1 |  | | | |  |  |  |  |  |  |  |
| 1 | Ath-AT3G10420.2 |  | Vvi-Vitvi13g00685\_t001 |  |  |  |  |  |  |  |
| 1 | Ath-AT3G10430.1 |  | | | |  |  |  |  |  |  |  |
| 1 | Ath-AT3G10439.1 |  | | | |  |  |  |  |  |  |  |
| 1 | Ath-AT3G10440.1 |  | | | |  |  |  |  |  |  |  |
| 1 | Ath-AT3G10450.1 |  | | | |  |  |  |  |  |  |  |
| 1 | Ath-AT3G10455.1 |  | | | |  |  |  |  |  |  |  |
| 1 | Ath-AT3G10460.1 |  | | | |  |  |  |  |  |  |  |
| 1 | Ath-AT3G10470.1 |  | Vvi-Vitvi13g00694\_t001 |  |  |  |  |  |  |  |
| 1 | Ath-AT3G10480.3 |  | Vvi-Vitvi13g00698\_t001 |  |  |  |  |  |  |  |
| 1 | Ath-AT3G10490.2 |  | Vvi-Vitvi13g00699\_t001 |  |  |  |  |  |  |  |
| 0 | Ath-AT3G10500.1 |  |  |  |  |  |  |  |  |
| 0 | Ath-AT3G10510.1 |  |  |  |  |  |  |  |  |
| 0 | Ath-AT3G10520.1 |  |  |  |  |  |  |  |  |
| 0 | Ath-AT3G10525.1 |  |  |  |  |  |  |  |  |
| 0 | Ath-AT3G10527.1 |  |  |  |  |  |  |  |  |
| 0 | Ath-AT3G10530.1 |  |  |  |  |  |  |  |  |
| 0 | Ath-AT3G10540.1 |  |  |  |  |  |  |  |  |
| 0 | Ath-AT3G10550.1 |  |  |  |  |  |  |  |  |
| 0 | Ath-AT3G10560.1 |  |  |  |  |  |  |  |  |
| 0 | Ath-AT3G10570.1 |  |  |  |  |  |  |  |  |
| 0 | Ath-AT3G10572.1 |  |  |  |  |  |  |  |  |
| 0 | Ath-AT3G10580.1 |  |  |  |  |  |  |  |  |
| 0 | Ath-AT3G10585.1 |  |  |  |  |  |  |  |  |
| 0 | Ath-AT3G10590.1 |  |  |  |  |  |  |  |  |
| 0 | Ath-AT3G10595.1 |  |  |  |  |  |  |  |  |
| 1 | Ath-AT3G10600.1 |  | Vvi-Vitvi13g00991\_t001 |  |  |  |  |  |  |  |
| 1 | Ath-AT3G10605.1 |  | | | |  |  |  |  |  |  |  |
| 1 | Ath-AT3G10610.1 |  | Vvi-Vitvi13g00996\_t001 |  |  |  |  |  |  |  |
| 1 | Ath-AT3G10620.1 |  | | | |  |  |  |  |  |  |  |
| 1 | Ath-AT3G10630.1 |  | Vvi-Vitvi13g01021\_t001 |  |  |  |  |  |  |  |
| 1 | Ath-AT3G10640.1 |  | Vvi-Vitvi13g01031\_t001 |  |  |  |  |  |  |  |
| 1 | Ath-AT3G10650.1 |  | Vvi-Vitvi13g01060\_t001 |  |  |  |  |  |  |  |
| 1 | Ath-AT3G10660.2 |  | Vvi-Vitvi13g04335\_t001 |  |  |  |  |  |  |  |
| 1 | Ath-AT3G10670.1 |  | | | |  |  |  |  |  |  |  |
| 1 | Ath-AT3G10680.1 |  | Vvi-Vitvi13g01074\_t001 |  |  |  |  |  |  |  |
| 0 | Ath-AT3G10690.1 |  |  |  |  |  |  |  |  |
| 0 | Ath-AT3G10700.1 |  |  |  |  |  |  |  |  |
| 0 | Ath-AT3G10710.1 |  |  |  |  |  |  |  |  |
| 0 | Ath-AT3G10720.2 |  |  |  |  |  |  |  |  |
| 0 | Ath-AT3G10730.1 |  |  |  |  |  |  |  |  |
| 0 | Ath-AT3G10740.2 |  |  |  |  |  |  |  |  |
| 0 | Ath-AT3G10750.1 |  |  |  |  |  |  |  |  |
| 0 | Ath-AT3G10760.1 |  |  |  |  |  |  |  |  |
| 0 | Ath-AT3G10770.1 |  |  |  |  |  |  |  |  |
| 0 | Ath-AT3G10780.1 |  |  |  |  |  |  |  |  |
| 0 | Ath-AT3G10790.1 |  |  |  |  |  |  |  |  |
| 0 | Ath-AT3G10800.1 |  |  |  |  |  |  |  |  |
| 0 | Ath-AT3G10810.1 |  |  |  |  |  |  |  |  |
| 0 | Ath-AT3G10815.1 |  |  |  |  |  |  |  |  |
| 0 | Ath-AT3G10820.2 |  |  |  |  |  |  |  |  |
| 0 | Ath-AT3G10840.1 |  |  |  |  |  |  |  |  |
| 0 | Ath-AT3G10845.1 |  |  |  |  |  |  |  |  |
| 0 | Ath-AT3G10850.1 |  |  |  |  |  |  |  |  |
| 0 | Ath-AT3G10860.1 |  |  |  |  |  |  |  |  |
| 0 | Ath-AT3G10870.1 |  |  |  |  |  |  |  |  |
| 0 | Ath-AT3G10880.3 |  |  |  |  |  |  |  |  |
| 0 | Ath-AT3G10890.1 |  |  |  |  |  |  |  |  |
| 0 | Ath-AT3G10900.1 |  |  |  |  |  |  |  |  |
| 0 | Ath-AT3G10910.1 |  |  |  |  |  |  |  |  |
| 0 | Ath-AT3G10915.7 |  |  |  |  |  |  |  |  |
| 0 | Ath-AT3G10920.1 |  |  |  |  |  |  |  |  |
| 0 | Ath-AT3G10930.1 |  |  |  |  |  |  |  |  |
| 1 | Ath-AT3G10940.1 |  | Vvi-Vitvi13g01905\_t001 |  |  |  |  |  |  |  |
| 1 | Ath-AT3G10950.1 |  | | | |  |  |  |  |  |  |  |
| 1 | Ath-AT3G10960.1 |  | Vvi-Vitvi13g00150\_t001 |  |  |  |  |  |  |  |
| 1 | Ath-AT3G10970.1 |  | Vvi-Vitvi13g00146\_t001 |  |  |  |  |  |  |  |
| 1 | Ath-AT3G10974.1 |  | | | |  |  |  |  |  |  |  |
| 2 | Ath-AT3G10980.1 |  | Vvi-Vitvi13g00132\_t001 |  | Vvi-Vitvi08g00803\_t001 |  |  |  |  |  |  |
| 2 | Ath-AT3G10985.1 |  | Vvi-Vitvi13g00131\_t001 |  | Vvi-Vitvi08g00802\_t001 |  |  |  |  |  |  |
| 2 | Ath-AT3G10986.1 |  | | | |  | | | |  |  |  |  |  |  |
| 2 | Ath-AT3G10990.1 |  | | | |  | | | |  |  |  |  |  |  |
| 2 | Ath-AT3G11000.2 |  | Vvi-Vitvi13g00124\_t001 |  | Vvi-Vitvi08g00786\_t002 |  |  |  |  |  |  |
| 2 | Ath-AT3G11010.2 |  | | | |  | | | |  |  |  |  |  |  |
| 2 | Ath-AT3G11020.1 |  | Vvi-Vitvi13g00116\_t001 |  | | | |  |  |  |  |  |  |
| 2 | Ath-AT3G11030.1 |  | Vvi-Vitvi13g00113\_t001 |  | Vvi-Vitvi08g00769\_t001 |  |  |  |  |  |  |
| 2 | Ath-AT3G11040.3 |  | Vvi-Vitvi13g00110\_t001 |  | | | |  |  |  |  |  |  |
| 2 | Ath-AT3G11050.1 |  | Vvi-Vitvi13g00107\_t001 |  | Vvi-Vitvi08g02076\_t001 |  |  |  |  |  |  |
| 1 | Ath-AT3G11060.1 |  |  |  | | | |  |  |  |  |  |  |
| 2 | Ath-AT3G11070.1 |  | Vvi-Vitvi13g00089\_t001 |  | | | |  |  |  |  |  |  |
| 2 | Ath-AT3G11080.1 |  | | | |  | | | |  |  |  |  |  |  |
| 2 | Ath-AT3G11090.1 |  | Vvi-Vitvi13g00085\_t001 |  | | | |  |  |  |  |  |  |
| 2 | Ath-AT3G11100.1 |  | Vvi-Vitvi13g00084\_t001 |  | Vvi-Vitvi08g00730\_t001 |  |  |  |  |  |  |
| 2 | Ath-AT3G11110.1 |  | Vvi-Vitvi13g00079\_t001 |  | Vvi-Vitvi08g00718\_t001 |  |  |  |  |  |  |
| 1 | Ath-AT3G11120.1 |  | | | |  |  |  |  |  |  |  |
| 1 | Ath-AT3G11130.1 |  | Vvi-Vitvi13g00072\_t001 |  |  |  |  |  |  |  |
| 1 | Ath-AT3G11150.1 |  | Vvi-Vitvi13g00062\_t001 |  |  |  |  |  |  |  |
| 1 | Ath-AT3G11160.1 |  | | | |  |  |  |  |  |  |  |
| 1 | Ath-AT3G11165.1 |  | | | |  |  |  |  |  |  |  |
| 1 | Ath-AT3G11170.1 |  | Vvi-Vitvi13g00060\_t001 |  |  |  |  |  |  |  |
| 1 | Ath-AT3G11180.2 |  | Vvi-Vitvi13g00055\_t001 |  |  |  |  |  |  |  |
| 1 | Ath-AT3G11200.1 |  | Vvi-Vitvi13g00054\_t001 |  |  |  |  |  |  |  |
| 1 | Ath-AT3G11210.1 |  | Vvi-Vitvi13g00037\_t001 |  |  |  |  |  |  |  |
| 1 | Ath-AT3G11220.1 |  | | | |  |  |  |  |  |  |  |
| 1 | Ath-AT3G11230.2 |  | Vvi-Vitvi13g01886\_t002 |  |  |  |  |  |  |  |
| 1 | Ath-AT3G11240.1 |  | Vvi-Vitvi13g00029\_t002 |  |  |  |  |  |  |  |
| 1 | Ath-AT3G11250.1 |  | Vvi-Vitvi13g00017\_t001 |  |  |  |  |  |  |  |
| 0 | Ath-AT3G11260.1 |  |  |  |  |  |  |  |  |
| 0 | Ath-AT3G11270.1 |  |  |  |  |  |  |  |  |
| 0 | Ath-AT3G11280.2 |  |  |  |  |  |  |  |  |
| 0 | Ath-AT3G11290.1 |  |  |  |  |  |  |  |  |
| 0 | Ath-AT3G11300.1 |  |  |  |  |  |  |  |  |
| 0 | Ath-AT3G11310.1 |  |  |  |  |  |  |  |  |
| 0 | Ath-AT3G11320.1 |  |  |  |  |  |  |  |  |
| 0 | Ath-AT3G11325.2 |  |  |  |  |  |  |  |  |
| 0 | Ath-AT3G11330.1 |  |  |  |  |  |  |  |  |
| 0 | Ath-AT3G11340.1 |  |  |  |  |  |  |  |  |
| 0 | Ath-AT3G11350.1 |  |  |  |  |  |  |  |  |
| 0 | Ath-AT3G11370.1 |  |  |  |  |  |  |  |  |
| 0 | Ath-AT3G11380.1 |  |  |  |  |  |  |  |  |
| 0 | Ath-AT3G11385.1 |  |  |  |  |  |  |  |  |
| 0 | Ath-AT3G11390.1 |  |  |  |  |  |  |  |  |
| 0 | Ath-AT3G11402.1 |  |  |  |  |  |  |  |  |
| 0 | Ath-AT3G11397.1 |  |  |  |  |  |  |  |  |
| 0 | Ath-AT3G11400.2 |  |  |  |  |  |  |  |  |
| 0 | Ath-AT3G11405.1 |  |  |  |  |  |  |  |  |
| 0 | Ath-AT3G11410.1 |  |  |  |  |  |  |  |  |
| 0 | Ath-AT3G11420.1 |  |  |  |  |  |  |  |  |
| 0 | Ath-AT3G11430.1 |  |  |  |  |  |  |  |  |
| 0 | Ath-AT3G11440.1 |  |  |  |  |  |  |  |  |
| 0 | Ath-AT3G11450.1 |  |  |  |  |  |  |  |  |
| 1 | Ath-AT3G11460.1 |  | Vvi-Vitvi08g01443\_t001 |  |  |  |  |  |  |  |
| 1 | Ath-AT3G11470.1 |  | | | |  |  |  |  |  |  |  |
| 1 | Ath-AT3G11480.1 |  | | | |  |  |  |  |  |  |  |
| 2 | Ath-AT3G11490.1 |  | Vvi-Vitvi08g01442\_t001 |  | Vvi-Vitvi06g01504\_t001 |  |  |  |  |  |  |
| 2 | Ath-AT3G11500.1 |  | Vvi-Vitvi08g01441\_t001 |  | | | |  |  |  |  |  |  |
| 2 | Ath-AT3G11510.1 |  | Vvi-Vitvi08g04305\_t001 |  | Vvi-Vitvi06g04445\_t001 |  |  |  |  |  |  |
| 2 | Ath-AT3G11520.1 |  | | | |  | | | |  |  |  |  |  |  |
| 2 | Ath-AT3G11530.2 |  | Vvi-Vitvi08g01425\_t001 |  | | | |  |  |  |  |  |  |
| 2 | Ath-AT3G11540.1 |  | Vvi-Vitvi08g01422\_t001 |  | | | |  |  |  |  |  |  |
| 2 | Ath-AT3G11550.1 |  | Vvi-Vitvi08g01418\_t001 |  | Vvi-Vitvi06g01522\_t001 |  |  |  |  |  |  |
| 2 | Ath-AT3G11560.2 |  | Vvi-Vitvi08g01415\_t001 |  | | | |  |  |  |  |  |  |
| 2 | Ath-AT3G11570.1 |  | Vvi-Vitvi08g01414\_t001 |  | | | |  |  |  |  |  |  |
| 2 | Ath-AT3G11580.3 |  | Vvi-Vitvi08g01412\_t001 |  | | | |  |  |  |  |  |  |
| 2 | Ath-AT3G11590.1 |  | Vvi-Vitvi08g01410\_t002 |  | Vvi-Vitvi06g01532\_t001 |  |  |  |  |  |  |
| 2 | Ath-AT3G11591.1 |  | | | |  | | | |  |  |  |  |  |  |
| 2 | Ath-AT3G11600.1 |  | Vvi-Vitvi08g01395\_t001 |  | Vvi-Vitvi06g01543\_t001 |  |  |  |  |  |  |
| 2 | Ath-AT3G11620.1 |  | Vvi-Vitvi08g01390\_t001 |  | | | |  |  |  |  |  |  |
| 2 | Ath-AT3G11630.1 |  | Vvi-Vitvi08g01386\_t001 |  | | | |  |  |  |  |  |  |
| 2 | Ath-AT3G11640.1 |  | | | |  | | | |  |  |  |  |  |  |
| 2 | Ath-AT3G11650.1 |  | Vvi-Vitvi08g01377\_t001 |  | | | |  |  |  |  |  |  |
| 2 | Ath-AT3G11660.1 |  | Vvi-Vitvi08g01376\_t001 |  | Vvi-Vitvi06g01559\_t001 |  |  |  |  |  |  |
| 1 | Ath-AT3G11670.1 |  |  |  | | | |  |  |  |  |  |  |
| 1 | Ath-AT3G11680.2 |  |  |  | | | |  |  |  |  |  |  |
| 2 | Ath-AT3G11690.1 |  | Vvi-Vitvi08g02035\_t001 |  | | | |  |  |  |  |  |  |
| 2 | Ath-AT3G11700.1 |  | Vvi-Vitvi08g00235\_t003 |  | | | |  |  |  |  |  |  |
| 2 | Ath-AT3G11710.1 |  | | | |  | | | |  |  |  |  |  |  |
| 2 | Ath-AT3G11720.3 |  | Vvi-Vitvi08g00227\_t001 |  | | | |  |  |  |  |  |  |
| 2 | Ath-AT3G11730.1 |  | | | |  | | | |  |  |  |  |  |  |
| 2 | Ath-AT3G11740.1 |  | | | |  | | | |  |  |  |  |  |  |
| 2 | Ath-AT3G11745.1 |  | | | |  | | | |  |  |  |  |  |  |
| 2 | Ath-AT3G11750.1 |  | | | |  | | | |  |  |  |  |  |  |
| 2 | Ath-AT3G11760.1 |  | | | |  | | | |  |  |  |  |  |  |
| 2 | Ath-AT3G11770.1 |  | | | |  | | | |  |  |  |  |  |  |
| 2 | Ath-AT3G11773.2 |  | | | |  | | | |  |  |  |  |  |  |
| 2 | Ath-AT3G11780.2 |  | | | |  | Vvi-Vitvi06g01563\_t001 |  |  |  |  |  |  |
| 1 | Ath-AT3G11800.1 |  | | | |  |  |  |  |  |  |  |
| 1 | Ath-AT3G11810.1 |  | | | |  |  |  |  |  |  |  |
| 1 | Ath-AT3G11820.1 |  | | | |  |  |  |  |  |  |  |
| 1 | Ath-AT3G11825.1 |  | | | |  |  |  |  |  |  |  |
| 2 | Ath-AT3G11830.1 |  | | | |  | Vvi-Vitvi08g01818\_t001 |  |  |  |  |  |  |
| 2 | Ath-AT3G11840.1 |  | | | |  | | | |  |  |  |  |  |  |
| 2 | Ath-AT3G11850.2 |  | Vvi-Vitvi08g00215\_t001 |  | | | |  |  |  |  |  |  |
| 2 | Ath-AT3G11860.1 |  | | | |  | | | |  |  |  |  |  |  |
| 2 | Ath-AT3G11870.1 |  | | | |  | | | |  |  |  |  |  |  |
| 2 | Ath-AT3G11880.2 |  | | | |  | | | |  |  |  |  |  |  |
| 2 | Ath-AT3G11890.2 |  | Vvi-Vitvi08g00203\_t002 |  | | | |  |  |  |  |  |  |
| 2 | Ath-AT3G11900.1 |  | | | |  | | | |  |  |  |  |  |  |
| 2 | Ath-AT3G11910.3 |  | Vvi-Vitvi08g00200\_t001 |  | | | |  |  |  |  |  |  |
| 2 | Ath-AT3G11920.1 |  | | | |  | | | |  |  |  |  |  |  |
| 2 | Ath-AT3G11930.3 |  | Vvi-Vitvi08g04047\_t001 |  | | | |  |  |  |  |  |  |
| 1 | Ath-AT3G11940.2 |  |  |  | Vvi-Vitvi08g01796\_t002 |  |  |  |  |  |  |
| 1 | Ath-AT3G11945.2 |  |  |  | Vvi-Vitvi08g01795\_t001 |  |  |  |  |  |  |
| 1 | Ath-AT3G11950.4 |  |  |  | Vvi-Vitvi08g01792\_t002 |  |  |  |  |  |  |
| 1 | Ath-AT3G11960.3 |  |  |  | Vvi-Vitvi08g01790\_t001 |  |  |  |  |  |  |
| 1 | Ath-AT3G11964.2 |  |  |  | Vvi-Vitvi08g01788\_t001 |  |  |  |  |  |  |
| 1 | Ath-AT3G11980.1 |  |  |  | Vvi-Vitvi08g01784\_t001 |  |  |  |  |  |  |
| 1 | Ath-AT3G11990.1 |  |  |  | | | |  |  |  |  |  |  |
| 1 | Ath-AT3G12000.1 |  |  |  | | | |  |  |  |  |  |  |
| 1 | Ath-AT3G12010.1 |  |  |  | Vvi-Vitvi08g01769\_t001 |  |  |  |  |  |  |
| 1 | Ath-AT3G12020.3 |  |  |  | Vvi-Vitvi08g01768\_t001 |  |  |  |  |  |  |
| 1 | Ath-AT3G12030.1 |  |  |  | Vvi-Vitvi08g01766\_t001 |  |  |  |  |  |  |
| 1 | Ath-AT3G12040.1 |  |  |  | Vvi-Vitvi08g01758\_t001 |  |  |  |  |  |  |
| 1 | Ath-AT3G12050.1 |  |  |  | Vvi-Vitvi08g01757\_t001 |  |  |  |  |  |  |
| 1 | Ath-AT3G12060.1 |  |  |  | Vvi-Vitvi08g01753\_t001 |  |  |  |  |  |  |
| 1 | Ath-AT3G12070.1 |  |  |  | | | |  |  |  |  |  |  |
| 1 | Ath-AT3G12080.1 |  |  |  | | | |  |  |  |  |  |  |
| 1 | Ath-AT3G12090.1 |  |  |  | | | |  |  |  |  |  |  |
| 1 | Ath-AT3G12100.1 |  |  |  | Vvi-Vitvi08g01743\_t001 |  |  |  |  |  |  |
| 1 | Ath-AT3G12110.1 |  |  |  | Vvi-Vitvi08g01740\_t001 |  |  |  |  |  |  |
| 1 | Ath-AT3G12120.1 |  |  |  | Vvi-Vitvi08g02345\_t001 |  |  |  |  |  |  |
| 1 | Ath-AT3G12130.1 |  |  |  | Vvi-Vitvi08g01732\_t001 |  |  |  |  |  |  |
| 1 | Ath-AT3G12140.3 |  |  |  | Vvi-Vitvi08g01725\_t001 |  |  |  |  |  |  |
| 1 | Ath-AT3G12145.1 |  |  |  | | | |  |  |  |  |  |  |
| 1 | Ath-AT3G12150.1 |  |  |  | | | |  |  |  |  |  |  |
| 1 | Ath-AT3G12160.1 |  |  |  | | | |  |  |  |  |  |  |
| 1 | Ath-AT3G12170.2 |  |  |  | | | |  |  |  |  |  |  |
| 1 | Ath-AT3G12180.1 |  |  |  | | | |  |  |  |  |  |  |
| 1 | Ath-AT3G12190.1 |  |  |  | | | |  |  |  |  |  |  |
| 1 | Ath-AT3G12200.2 |  |  |  | | | |  |  |  |  |  |  |
| 1 | Ath-AT3G12203.1 |  |  |  | | | |  |  |  |  |  |  |
| 1 | Ath-AT3G12210.2 |  |  |  | | | |  |  |  |  |  |  |
| 1 | Ath-AT3G12220.1 |  |  |  | | | |  |  |  |  |  |  |
| 1 | Ath-AT3G12230.1 |  |  |  | | | |  |  |  |  |  |  |
| 1 | Ath-AT3G12240.1 |  |  |  | | | |  |  |  |  |  |  |
| 1 | Ath-AT3G12250.4 |  |  |  | Vvi-Vitvi08g01710\_t001 |  |  |  |  |  |  |
| 0 | Ath-AT3G12260.1 |  |  |  |  |  |  |  |  |
| 0 | Ath-AT3G12270.1 |  |  |  |  |  |  |  |  |
| 0 | Ath-AT3G12280.1 |  |  |  |  |  |  |  |  |
| 0 | Ath-AT3G12290.1 |  |  |  |  |  |  |  |  |
| 0 | Ath-AT3G12300.1 |  |  |  |  |  |  |  |  |
| 0 | Ath-AT3G12320.1 |  |  |  |  |  |  |  |  |
| 0 | Ath-AT3G12340.1 |  |  |  |  |  |  |  |  |
| 0 | Ath-AT3G12345.1 |  |  |  |  |  |  |  |  |
| 0 | Ath-AT3G12350.1 |  |  |  |  |  |  |  |  |
| 0 | Ath-AT3G12360.1 |  |  |  |  |  |  |  |  |
| 0 | Ath-AT3G12370.1 |  |  |  |  |  |  |  |  |
| 0 | Ath-AT3G12380.2 |  |  |  |  |  |  |  |  |
| 0 | Ath-AT3G12390.1 |  |  |  |  |  |  |  |  |
| 0 | Ath-AT3G12400.2 |  |  |  |  |  |  |  |  |
| 0 | Ath-AT3G12410.1 |  |  |  |  |  |  |  |  |
| 0 | Ath-AT3G12420.1 |  |  |  |  |  |  |  |  |
| 0 | Ath-AT3G12430.1 |  |  |  |  |  |  |  |  |
| 0 | Ath-AT3G12440.1 |  |  |  |  |  |  |  |  |
| 0 | Ath-AT3G12460.1 |  |  |  |  |  |  |  |  |
| 0 | Ath-AT3G12470.1 |  |  |  |  |  |  |  |  |
| 0 | Ath-AT3G12480.1 |  |  |  |  |  |  |  |  |
| 0 | Ath-AT3G12490.2 |  |  |  |  |  |  |  |  |
| 0 | Ath-AT3G12500.1 |  |  |  |  |  |  |  |  |
| 0 | Ath-AT3G12510.1 |  |  |  |  |  |  |  |  |
| 0 | Ath-AT3G12520.1 |  |  |  |  |  |  |  |  |
| 0 | Ath-AT3G12530.3 |  |  |  |  |  |  |  |  |
| 0 | Ath-AT3G12540.1 |  |  |  |  |  |  |  |  |
| 0 | Ath-AT3G12545.1 |  |  |  |  |  |  |  |  |
| 0 | Ath-AT3G12550.4 |  |  |  |  |  |  |  |  |
| 0 | Ath-AT3G12560.2 |  |  |  |  |  |  |  |  |
| 0 | Ath-AT3G12570.2 |  |  |  |  |  |  |  |  |
| 0 | Ath-AT3G12580.1 |  |  |  |  |  |  |  |  |
| 0 | Ath-AT3G12587.1 |  |  |  |  |  |  |  |  |
| 0 | Ath-AT3G12590.1 |  |  |  |  |  |  |  |  |
| 0 | Ath-AT3G12600.1 |  |  |  |  |  |  |  |  |
| 0 | Ath-AT3G12610.1 |  |  |  |  |  |  |  |  |
| 0 | Ath-AT3G12620.1 |  |  |  |  |  |  |  |  |
| 0 | Ath-AT3G12630.1 |  |  |  |  |  |  |  |  |
| 0 | Ath-AT3G12640.2 |  |  |  |  |  |  |  |  |
| 0 | Ath-AT3G12650.1 |  |  |  |  |  |  |  |  |
| 1 | Ath-AT3G12670.1 |  | Vvi-Vitvi19g01776\_t001 |  |  |  |  |  |  |  |
| 1 | Ath-AT3G12660.1 |  | | | |  |  |  |  |  |  |  |
| 1 | Ath-AT3G12680.1 |  | Vvi-Vitvi19g01773\_t001 |  |  |  |  |  |  |  |
| 1 | Ath-AT3G12685.1 |  | Vvi-Vitvi19g01772\_t001 |  |  |  |  |  |  |  |
| 1 | Ath-AT3G12690.3 |  | Vvi-Vitvi19g01770\_t001 |  |  |  |  |  |  |  |
| 1 | Ath-AT3G12700.1 |  | Vvi-Vitvi19g01767\_t001 |  |  |  |  |  |  |  |
| 1 | Ath-AT3G12710.1 |  | | | |  |  |  |  |  |  |  |
| 1 | Ath-AT3G12720.1 |  | Vvi-Vitvi19g01749\_t001 |  |  |  |  |  |  |  |
| 1 | Ath-AT3G12730.1 |  | Vvi-Vitvi19g01742\_t002 |  |  |  |  |  |  |  |
| 1 | Ath-AT3G12740.1 |  | Vvi-Vitvi19g01738\_t002 |  |  |  |  |  |  |  |
| 1 | Ath-AT3G12750.1 |  | Vvi-Vitvi19g01726\_t001 |  |  |  |  |  |  |  |
| 1 | Ath-AT3G12760.1 |  | Vvi-Vitvi19g01712\_t001 |  |  |  |  |  |  |  |
| 1 | Ath-AT3G12770.1 |  | Vvi-Vitvi19g01708\_t001 |  |  |  |  |  |  |  |
| 1 | Ath-AT3G12775.1 |  | | | |  |  |  |  |  |  |  |
| 1 | Ath-AT3G12780.1 |  | | | |  |  |  |  |  |  |  |
| 1 | Ath-AT3G12800.1 |  | Vvi-Vitvi19g01703\_t001 |  |  |  |  |  |  |  |
| 0 | Ath-AT3G12810.1 |  |  |  |  |  |  |  |  |
| 0 | Ath-AT3G12820.1 |  |  |  |  |  |  |  |  |
| 0 | Ath-AT3G12830.1 |  |  |  |  |  |  |  |  |
| 0 | Ath-AT3G12833.1 |  |  |  |  |  |  |  |  |
| 0 | Ath-AT3G12835.1 |  |  |  |  |  |  |  |  |
| 0 | Ath-AT3G12840.1 |  |  |  |  |  |  |  |  |
| 0 | Ath-AT3G12850.1 |  |  |  |  |  |  |  |  |
| 0 | Ath-AT3G12860.1 |  |  |  |  |  |  |  |  |
| 0 | Ath-AT3G12870.1 |  |  |  |  |  |  |  |  |
| 0 | Ath-AT3G12880.1 |  |  |  |  |  |  |  |  |
| 0 | Ath-AT3G12890.1 |  |  |  |  |  |  |  |  |
| 0 | Ath-AT3G12900.1 |  |  |  |  |  |  |  |  |
| 1 | Ath-AT3G12910.1 |  | Vvi-Vitvi19g01561\_t001 |  |  |  |  |  |  |  |
| 1 | Ath-AT3G12915.2 |  | Vvi-Vitvi19g01552\_t001 |  |  |  |  |  |  |  |
| 1 | Ath-AT3G12920.1 |  | Vvi-Vitvi19g01550\_t001 |  |  |  |  |  |  |  |
| 1 | Ath-AT3G12930.1 |  | Vvi-Vitvi19g01548\_t001 |  |  |  |  |  |  |  |
| 1 | Ath-AT3G12940.2 |  | Vvi-Vitvi19g01533\_t007 |  |  |  |  |  |  |  |
| 1 | Ath-AT3G12950.1 |  | Vvi-Vitvi19g01524\_t001 |  |  |  |  |  |  |  |
| 1 | Ath-AT3G12955.1 |  | Vvi-Vitvi19g02261\_t001 |  |  |  |  |  |  |  |
| 1 | Ath-AT3G12960.1 |  | | | |  |  |  |  |  |  |  |
| 1 | Ath-AT3G12970.1 |  | Vvi-Vitvi19g01500\_t001 |  |  |  |  |  |  |  |
| 1 | Ath-AT3G12977.1 |  | Vvi-Vitvi19g01484\_t001 |  |  |  |  |  |  |  |
| 2 | Ath-AT3G12980.1 |  | | | |  | Vvi-Vitvi09g01474\_t001 |  |  |  |  |  |  |
| 2 | Ath-AT3G12990.3 |  | | | |  | | | |  |  |  |  |  |  |
| 2 | Ath-AT3G13000.2 |  | | | |  | Vvi-Vitvi09g01464\_t002 |  |  |  |  |  |  |
| 2 | Ath-AT3G13010.1 |  | | | |  | Vvi-Vitvi09g04604\_t001 |  |  |  |  |  |  |
| 2 | Ath-AT3G13020.1 |  | | | |  | | | |  |  |  |  |  |  |
| 2 | Ath-AT3G13030.4 |  | | | |  | | | |  |  |  |  |  |  |
| 2 | Ath-AT3G13040.1 |  | | | |  | Vvi-Vitvi09g01459\_t001 |  |  |  |  |  |  |
| 2 | Ath-AT3G13050.1 |  | | | |  | Vvi-Vitvi09g01458\_t001 |  |  |  |  |  |  |
| 2 | Ath-AT3G13060.2 |  | | | |  | Vvi-Vitvi09g01457\_t001 |  |  |  |  |  |  |
| 2 | Ath-AT3G13062.2 |  | | | |  | Vvi-Vitvi09g01456\_t001 |  |  |  |  |  |  |
| 2 | Ath-AT3G13065.1 |  | | | |  | Vvi-Vitvi09g01455\_t001 |  |  |  |  |  |  |
| 2 | Ath-AT3G13070.1 |  | | | |  | Vvi-Vitvi19g00627\_t001 |  |  |  |  |  |  |
| 2 | Ath-AT3G13075.1 |  | | | |  | | | |  |  |  |  |  |  |
| 2 | Ath-AT3G13080.1 |  | Vvi-Vitvi19g04473\_t001 |  | Vvi-Vitvi19g00631\_t001 |  |  |  |  |  |  |
| 1 | Ath-AT3G13090.1 |  |  |  | | | |  |  |  |  |  |  |
| 1 | Ath-AT3G13100.1 |  |  |  | | | |  |  |  |  |  |  |
| 1 | Ath-AT3G13110.1 |  |  |  | Vvi-Vitvi19g00654\_t001 |  |  |  |  |  |  |
| 1 | Ath-AT3G13120.1 |  |  |  | Vvi-Vitvi19g00655\_t001 |  |  |  |  |  |  |
| 1 | Ath-AT3G13130.1 |  |  |  | Vvi-Vitvi19g02058\_t001 |  |  |  |  |  |  |
| 1 | Ath-AT3G13140.1 |  |  |  | | | |  |  |  |  |  |  |
| 1 | Ath-AT3G13150.1 |  |  |  | | | |  |  |  |  |  |  |
| 1 | Ath-AT3G13160.1 |  |  |  | | | |  |  |  |  |  |  |
| 1 | Ath-AT3G13170.1 |  |  |  | Vvi-Vitvi19g00662\_t001 |  |  |  |  |  |  |
| 1 | Ath-AT3G13175.1 |  |  |  | Vvi-Vitvi19g00665\_t001 |  |  |  |  |  |  |
| 1 | Ath-AT3G13180.1 |  | Vvi-Vitvi19g04281\_t003 |  |  |  |  |  |  |  |
| 1 | Ath-AT3G13190.3 |  | | | |  |  |  |  |  |  |  |
| 1 | Ath-AT3G13200.1 |  | | | |  |  |  |  |  |  |  |
| 1 | Ath-AT3G13210.1 |  | | | |  |  |  |  |  |  |  |
| 1 | Ath-AT3G13220.1 |  | | | |  |  |  |  |  |  |  |
| 1 | Ath-AT3G13222.1 |  | | | |  |  |  |  |  |  |  |
| 1 | Ath-AT3G13224.2 |  | Vvi-Vitvi19g00722\_t001 |  |  |  |  |  |  |  |
| 1 | Ath-AT3G13227.1 |  | | | |  |  |  |  |  |  |  |
| 1 | Ath-AT3G13225.2 |  | Vvi-Vitvi19g00730\_t001 |  |  |  |  |  |  |  |
| 1 | Ath-AT3G13226.1 |  | Vvi-Vitvi19g00736\_t001 |  |  |  |  |  |  |  |
| 1 | Ath-AT3G13228.1 |  | | | |  |  |  |  |  |  |  |
| 1 | Ath-AT3G13229.1 |  | Vvi-Vitvi19g00738\_t001 |  |  |  |  |  |  |  |
| 1 | Ath-AT3G13230.1 |  | | | |  |  |  |  |  |  |  |
| 1 | Ath-AT3G13235.2 |  | | | |  |  |  |  |  |  |  |
| 1 | Ath-AT3G13240.2 |  | | | |  |  |  |  |  |  |  |
| 1 | Ath-AT3G13275.1 |  | | | |  |  |  |  |  |  |  |
| 1 | Ath-AT3G13276.1 |  | | | |  |  |  |  |  |  |  |
| 1 | Ath-AT3G13280.1 |  | | | |  |  |  |  |  |  |  |
| 1 | Ath-AT3G13290.1 |  | | | |  |  |  |  |  |  |  |
| 1 | Ath-AT3G13300.1 |  | | | |  |  |  |  |  |  |  |
| 1 | Ath-AT3G13310.1 |  | | | |  |  |  |  |  |  |  |
| 1 | Ath-AT3G13320.1 |  | | | |  |  |  |  |  |  |  |
| 1 | Ath-AT3G13330.1 |  | Vvi-Vitvi19g00757\_t001 |  |  |  |  |  |  |  |
| 0 | Ath-AT3G13340.3 |  |  |  |  |  |  |  |  |
| 0 | Ath-AT3G13350.1 |  |  |  |  |  |  |  |  |
| 0 | Ath-AT3G13360.2 |  |  |  |  |  |  |  |  |
| 0 | Ath-AT3G13370.1 |  |  |  |  |  |  |  |  |
| 0 | Ath-AT3G13380.1 |  |  |  |  |  |  |  |  |
| 1 | Ath-AT3G13390.1 |  | Vvi-Vitvi06g04002\_t001 |  |  |  |  |  |  |  |
| 1 | Ath-AT3G13400.1 |  | | | |  |  |  |  |  |  |  |
| 1 | Ath-AT3G13404.1 |  | | | |  |  |  |  |  |  |  |
| 1 | Ath-AT3G13403.1 |  | | | |  |  |  |  |  |  |  |
| 1 | Ath-AT3G13410.1 |  | Vvi-Vitvi06g00013\_t001 |  |  |  |  |  |  |  |
| 1 | Ath-AT3G13420.3 |  | Vvi-Vitvi06g00019\_t001 |  |  |  |  |  |  |  |
| 1 | Ath-AT3G13430.3 |  | Vvi-Vitvi06g00020\_t001 |  |  |  |  |  |  |  |
| 1 | Ath-AT3G13432.1 |  | | | |  |  |  |  |  |  |  |
| 1 | Ath-AT3G13433.1 |  | | | |  |  |  |  |  |  |  |
| 1 | Ath-AT3G13435.1 |  | | | |  |  |  |  |  |  |  |
| 1 | Ath-AT3G13437.2 |  | | | |  |  |  |  |  |  |  |
| 1 | Ath-AT3G13440.1 |  | | | |  |  |  |  |  |  |  |
| 1 | Ath-AT3G13445.1 |  | Vvi-Vitvi06g01573\_t001 |  |  |  |  |  |  |  |
| 1 | Ath-AT3G13450.1 |  | Vvi-Vitvi06g00028\_t001 |  |  |  |  |  |  |  |
| 1 | Ath-AT3G13460.1 |  | Vvi-Vitvi06g00029\_t001 |  |  |  |  |  |  |  |
| 1 | Ath-AT3G13470.1 |  | Vvi-Vitvi06g00032\_t001 |  |  |  |  |  |  |  |
| 1 | Ath-AT3G13480.2 |  | Vvi-Vitvi06g01580\_t001 |  |  |  |  |  |  |  |
| 1 | Ath-AT3G13490.1 |  | Vvi-Vitvi06g00043\_t001 |  |  |  |  |  |  |  |
| 1 | Ath-AT3G13500.1 |  | | | |  |  |  |  |  |  |  |
| 1 | Ath-AT3G13510.1 |  | Vvi-Vitvi06g00044\_t001 |  |  |  |  |  |  |  |
| 1 | Ath-AT3G13520.1 |  | Vvi-Vitvi06g04015\_t001 |  |  |  |  |  |  |  |
| 1 | Ath-AT3G13530.1 |  | | | |  |  |  |  |  |  |  |
| 1 | Ath-AT3G13540.1 |  | Vvi-Vitvi06g00059\_t001 |  |  |  |  |  |  |  |
| 1 | Ath-AT3G13550.1 |  | | | |  |  |  |  |  |  |  |
| 1 | Ath-AT3G13560.3 |  | | | |  |  |  |  |  |  |  |
| 1 | Ath-AT3G13570.1 |  | | | |  |  |  |  |  |  |  |
| 1 | Ath-AT3G13580.1 |  | | | |  |  |  |  |  |  |  |
| 1 | Ath-AT3G13590.1 |  | | | |  |  |  |  |  |  |  |
| 1 | Ath-AT3G13600.1 |  | Vvi-Vitvi06g00071\_t001 |  |  |  |  |  |  |  |
| 1 | Ath-AT3G13610.1 |  | Vvi-Vitvi06g00076\_t001 |  |  |  |  |  |  |  |
| 1 | Ath-AT3G13620.1 |  | Vvi-Vitvi06g00079\_t001 |  |  |  |  |  |  |  |
| 1 | Ath-AT3G13630.1 |  | | | |  |  |  |  |  |  |  |
| 1 | Ath-AT3G13640.1 |  | | | |  |  |  |  |  |  |  |
| 1 | Ath-AT3G13650.1 |  | Vvi-Vitvi06g04029\_t001 |  |  |  |  |  |  |  |
| 1 | Ath-AT3G13660.1 |  | | | |  |  |  |  |  |  |  |
| 1 | Ath-AT3G13662.1 |  | Vvi-Vitvi06g01596\_t001 |  |  |  |  |  |  |  |
| 1 | Ath-AT3G13670.1 |  | Vvi-Vitvi06g00104\_t001 |  |  |  |  |  |  |  |
| 0 | Ath-AT3G13672.2 |  |  |  |  |  |  |  |  |
| 0 | Ath-AT3G13674.1 |  |  |  |  |  |  |  |  |
| 0 | Ath-AT3G13677.1 |  |  |  |  |  |  |  |  |
| 0 | Ath-AT3G13680.1 |  |  |  |  |  |  |  |  |
| 0 | Ath-AT3G13682.1 |  |  |  |  |  |  |  |  |
| 2 | Ath-AT3G13690.1 |  | Vvi-Vitvi09g00203\_t001 |  | Vvi-Vitvi11g00199\_t002 |  |  |  |  |  |  |
| 2 | Ath-AT3G13710.1 |  | | | |  | Vvi-Vitvi11g00191\_t001 |  |  |  |  |  |  |
| 2 | Ath-AT3G13700.1 |  | Vvi-Vitvi09g00202\_t001 |  | | | |  |  |  |  |  |  |
| 2 | Ath-AT3G13720.1 |  | | | |  | | | |  |  |  |  |  |  |
| 2 | Ath-AT3G13730.1 |  | | | |  | | | |  |  |  |  |  |  |
| 2 | Ath-AT3G13740.3 |  | | | |  | | | |  |  |  |  |  |  |
| 2 | Ath-AT3G13750.1 |  | | | |  | Vvi-Vitvi11g00178\_t001 |  |  |  |  |  |  |
| 2 | Ath-AT3G13760.1 |  | | | |  | | | |  |  |  |  |  |  |
| 2 | Ath-AT3G13770.1 |  | | | |  | | | |  |  |  |  |  |  |
| 2 | Ath-AT3G13772.1 |  | | | |  | | | |  |  |  |  |  |  |
| 2 | Ath-AT3G13780.1 |  | Vvi-Vitvi09g00197\_t001 |  | | | |  |  |  |  |  |  |
| 2 | Ath-AT3G13782.1 |  | Vvi-Vitvi09g00194\_t001 |  | | | |  |  |  |  |  |  |
| 2 | Ath-AT3G13784.2 |  | Vvi-Vitvi09g00193\_t001 |  | | | |  |  |  |  |  |  |
| 2 | Ath-AT3G13790.1 |  | | | |  | | | |  |  |  |  |  |  |
| 2 | Ath-AT3G13800.1 |  | Vvi-Vitvi09g00192\_t001 |  | | | |  |  |  |  |  |  |
| 2 | Ath-AT3G13810.2 |  | Vvi-Vitvi09g00191\_t001 |  | | | |  |  |  |  |  |  |
| 2 | Ath-AT3G13820.1 |  | | | |  | | | |  |  |  |  |  |  |
| 2 | Ath-AT3G13830.1 |  | | | |  | | | |  |  |  |  |  |  |
| 3 | Ath-AT3G13840.1 |  | | | |  | | | |  | Vvi-Vitvi09g01487\_t001 |  |  |  |  |  |
| 3 | Ath-AT3G13845.1 |  | | | |  | | | |  | | | |  |  |  |  |  |
| 3 | Ath-AT3G13850.1 |  | Vvi-Vitvi09g00188\_t001 |  | Vvi-Vitvi11g00169\_t001 |  | | | |  |  |  |  |  |
| 3 | Ath-AT3G13857.1 |  | | | |  | | | |  | | | |  |  |  |  |  |
| 3 | Ath-AT3G13860.1 |  | Vvi-Vitvi09g00186\_t001 |  | | | |  | | | |  |  |  |  |  |
| 3 | Ath-AT3G13870.1 |  | Vvi-Vitvi09g00182\_t001 |  | | | |  | | | |  |  |  |  |  |
| 2 | Ath-AT3G13880.1 |  |  |  | | | |  | | | |  |  |  |  |  |
| 3 | Ath-AT3G13882.2 |  | Vvi-Vitvi09g00141\_t001 |  | | | |  | | | |  |  |  |  |  |
| 3 | Ath-AT3G13890.1 |  | Vvi-Vitvi09g00142\_t001 |  | | | |  | | | |  |  |  |  |  |
| 3 | Ath-AT3G13898.1 |  | Vvi-Vitvi09g04034\_t001 |  | Vvi-Vitvi11g01366\_t001 |  | | | |  |  |  |  |  |
| 3 | Ath-AT3G13900.1 |  | Vvi-Vitvi09g00147\_t001 |  | | | |  | | | |  |  |  |  |  |
| 3 | Ath-AT3G13910.2 |  | Vvi-Vitvi09g01521\_t001 |  | Vvi-Vitvi11g00154\_t001 |  | | | |  |  |  |  |  |
| 3 | Ath-AT3G13920.5 |  | Vvi-Vitvi09g00149\_t001 |  | Vvi-Vitvi11g00152\_t001 |  | | | |  |  |  |  |  |
| 3 | Ath-AT3G13930.1 |  | Vvi-Vitvi09g00151\_t002 |  | Vvi-Vitvi11g00147\_t001 |  | | | |  |  |  |  |  |
| 3 | Ath-AT3G13940.1 |  | Vvi-Vitvi09g00157\_t001 |  | | | |  | | | |  |  |  |  |  |
| 2 | Ath-AT3G13950.1 |  |  |  | Vvi-Vitvi11g01362\_t001 |  | | | |  |  |  |  |  |
| 2 | Ath-AT3G13960.1 |  | Vvi-Vitvi09g00107\_t001 |  |  |  | Vvi-Vitvi09g00107\_t001 |  |  |  |  |  |
| 2 | Ath-AT3G13970.1 |  | | | |  |  |  | Vvi-Vitvi09g00111\_t001 |  |  |  |  |  |
| 2 | Ath-AT3G13980.1 |  | | | |  |  |  | Vvi-Vitvi09g00126\_t001 |  |  |  |  |  |
| 2 | Ath-AT3G13990.1 |  | | | |  |  |  | Vvi-Vitvi09g00127\_t001 |  |  |  |  |  |
| 2 | Ath-AT3G14000.1 |  | | | |  |  |  | Vvi-Vitvi09g00128\_t001 |  |  |  |  |  |
| 2 | Ath-AT3G14010.5 |  | | | |  |  |  | Vvi-Vitvi09g00130\_t004 |  |  |  |  |  |
| 2 | Ath-AT3G14020.1 |  | | | |  |  |  | Vvi-Vitvi09g00133\_t001 |  |  |  |  |  |
| 1 | Ath-AT3G14030.1 |  | | | |  |  |  |  |  |  |  |
| 1 | Ath-AT3G14040.1 |  | | | |  |  |  |  |  |  |  |
| 1 | Ath-AT3G14050.1 |  | Vvi-Vitvi09g00093\_t001 |  |  |  |  |  |  |  |
| 1 | Ath-AT3G14060.1 |  | Vvi-Vitvi09g00083\_t001 |  |  |  |  |  |  |  |
| 1 | Ath-AT3G14067.1 |  | Vvi-Vitvi09g00081\_t001 |  |  |  |  |  |  |  |
| 1 | Ath-AT3G14070.1 |  | Vvi-Vitvi09g00079\_t001 |  |  |  |  |  |  |  |
| 1 | Ath-AT3G14075.2 |  | Vvi-Vitvi09g00076\_t001 |  |  |  |  |  |  |  |
| 1 | Ath-AT3G14080.2 |  | Vvi-Vitvi09g00069\_t001 |  |  |  |  |  |  |  |
| 1 | Ath-AT3G14090.1 |  | Vvi-Vitvi09g00067\_t001 |  |  |  |  |  |  |  |
| 1 | Ath-AT3G14100.1 |  | Vvi-Vitvi09g00063\_t001 |  |  |  |  |  |  |  |
| 1 | Ath-AT3G14110.3 |  | Vvi-Vitvi09g00062\_t001 |  |  |  |  |  |  |  |
| 1 | Ath-AT3G14120.1 |  | Vvi-Vitvi09g00061\_t001 |  |  |  |  |  |  |  |
| 1 | Ath-AT3G14130.1 |  | Vvi-Vitvi09g01499\_t001 |  |  |  |  |  |  |  |
| 1 | Ath-AT3G14140.1 |  | Vvi-Vitvi09g00056\_t001 |  |  |  |  |  |  |  |
| 1 | Ath-AT3G14150.1 |  | | | |  |  |  |  |  |  |  |
| 1 | Ath-AT3G14160.1 |  | | | |  |  |  |  |  |  |  |
| 1 | Ath-AT3G14170.2 |  | Vvi-Vitvi09g00055\_t001 |  |  |  |  |  |  |  |
| 1 | Ath-AT3G14172.1 |  | Vvi-Vitvi09g00052\_t001 |  |  |  |  |  |  |  |
| 1 | Ath-AT3G14180.1 |  | Vvi-Vitvi09g01496\_t001 |  |  |  |  |  |  |  |
| 1 | Ath-AT3G14190.2 |  | | | |  |  |  |  |  |  |  |
| 1 | Ath-AT3G14200.1 |  | Vvi-Vitvi09g00049\_t001 |  |  |  |  |  |  |  |
| 1 | Ath-AT3G14205.1 |  | Vvi-Vitvi09g00042\_t001 |  |  |  |  |  |  |  |
| 1 | Ath-AT3G14210.1 |  | | | |  |  |  |  |  |  |  |
| 1 | Ath-AT3G14220.1 |  | | | |  |  |  |  |  |  |  |
| 1 | Ath-AT3G14225.1 |  | Vvi-Vitvi09g00033\_t001 |  |  |  |  |  |  |  |
| 1 | Ath-AT3G14230.1 |  | Vvi-Vitvi09g00031\_t002 |  |  |  |  |  |  |  |
| 1 | Ath-AT3G14240.1 |  | Vvi-Vitvi09g00030\_t001 |  |  |  |  |  |  |  |
| 1 | Ath-AT3G14250.1 |  | | | |  |  |  |  |  |  |  |
| 1 | Ath-AT3G14260.1 |  | Vvi-Vitvi09g00026\_t001 |  |  |  |  |  |  |  |
| 1 | Ath-AT3G14270.2 |  | Vvi-Vitvi09g00023\_t001 |  |  |  |  |  |  |  |
| 1 | Ath-AT3G14280.1 |  | Vvi-Vitvi09g00022\_t001 |  |  |  |  |  |  |  |
| 1 | Ath-AT3G14290.1 |  | Vvi-Vitvi09g00020\_t001 |  |  |  |  |  |  |  |
| 1 | Ath-AT3G14300.1 |  | Vvi-Vitvi09g00018\_t001 |  |  |  |  |  |  |  |
| 1 | Ath-AT3G14310.1 |  | Vvi-Vitvi09g00017\_t001 |  |  |  |  |  |  |  |
| 1 | Ath-AT3G14320.1 |  | | | |  |  |  |  |  |  |  |
| 1 | Ath-AT3G14330.1 |  | | | |  |  |  |  |  |  |  |
| 1 | Ath-AT3G14340.1 |  | | | |  |  |  |  |  |  |  |
| 1 | Ath-AT3G14350.1 |  | Vvi-Vitvi09g00008\_t001 |  |  |  |  |  |  |  |
| 1 | Ath-AT3G14360.1 |  | Vvi-Vitvi09g00007\_t001 |  |  |  |  |  |  |  |
| 0 | Ath-AT3G14362.1 |  |  |  |  |  |  |  |  |
| 0 | Ath-AT3G14370.1 |  |  |  |  |  |  |  |  |
| 0 | Ath-AT3G14380.1 |  |  |  |  |  |  |  |  |
| 0 | Ath-AT3G14390.1 |  |  |  |  |  |  |  |  |
| 0 | Ath-AT3G14395.1 |  |  |  |  |  |  |  |  |
| 0 | Ath-AT3G14400.1 |  |  |  |  |  |  |  |  |
| 0 | Ath-AT3G14410.1 |  |  |  |  |  |  |  |  |
| 0 | Ath-AT3G14415.2 |  |  |  |  |  |  |  |  |
| 0 | Ath-AT3G14420.1 |  |  |  |  |  |  |  |  |
| 0 | Ath-AT3G14430.1 |  |  |  |  |  |  |  |  |
| 0 | Ath-AT3G14440.1 |  |  |  |  |  |  |  |  |
| 0 | Ath-AT3G14450.1 |  |  |  |  |  |  |  |  |
| 0 | Ath-AT3G14452.1 |  |  |  |  |  |  |  |  |
| 0 | Ath-AT3G14460.1 |  |  |  |  |  |  |  |  |
| 0 | Ath-AT3G14463.1 |  |  |  |  |  |  |  |  |
| 0 | Ath-AT3G14467.1 |  |  |  |  |  |  |  |  |
| 0 | Ath-AT3G14470.1 |  |  |  |  |  |  |  |  |
| 0 | Ath-AT3G14480.1 |  |  |  |  |  |  |  |  |
| 0 | Ath-AT3G14490.1 |  |  |  |  |  |  |  |  |
| 0 | Ath-AT3G14510.1 |  |  |  |  |  |  |  |  |
| 0 | Ath-AT3G14520.1 |  |  |  |  |  |  |  |  |
| 0 | Ath-AT3G14530.1 |  |  |  |  |  |  |  |  |
| 0 | Ath-AT3G14540.1 |  |  |  |  |  |  |  |  |
| 0 | Ath-AT3G14550.2 |  |  |  |  |  |  |  |  |
| 0 | Ath-AT3G14560.1 |  |  |  |  |  |  |  |  |
| 0 | Ath-AT3G14570.1 |  |  |  |  |  |  |  |  |
| 0 | Ath-AT3G14580.1 |  |  |  |  |  |  |  |  |
| 0 | Ath-AT3G14590.2 |  |  |  |  |  |  |  |  |
| 1 | Ath-AT3G14595.1 |  | Vvi-Vitvi19g01391\_t001 |  |  |  |  |  |  |  |
| 1 | Ath-AT3G14600.1 |  | Vvi-Vitvi19g01389\_t001 |  |  |  |  |  |  |  |
| 1 | Ath-AT3G14610.1 |  | | | |  |  |  |  |  |  |  |
| 1 | Ath-AT3G14620.1 |  | Vvi-Vitvi19g04398\_t001 |  |  |  |  |  |  |  |
| 1 | Ath-AT3G14630.2 |  | Vvi-Vitvi19g04395\_t001 |  |  |  |  |  |  |  |
| 1 | Ath-AT3G14640.2 |  | Vvi-Vitvi19g01371\_t001 |  |  |  |  |  |  |  |
| 1 | Ath-AT3G14650.1 |  | Vvi-Vitvi19g02164\_t001 |  |  |  |  |  |  |  |
| 0 | Ath-AT3G14660.1 |  |  |  |  |  |  |  |  |
| 0 | Ath-AT3G14670.3 |  |  |  |  |  |  |  |  |
| 0 | Ath-AT3G14680.1 |  |  |  |  |  |  |  |  |
| 0 | Ath-AT3G14690.2 |  |  |  |  |  |  |  |  |
| 0 | Ath-AT3G14700.2 |  |  |  |  |  |  |  |  |
| 0 | Ath-AT3G14710.1 |  |  |  |  |  |  |  |  |
| 1 | Ath-AT3G14720.1 |  | Vvi-Vitvi19g00020\_t001 |  |  |  |  |  |  |  |
| 1 | Ath-AT3G14730.1 |  | | | |  |  |  |  |  |  |  |
| 1 | Ath-AT3G14740.2 |  | Vvi-Vitvi19g00021\_t001 |  |  |  |  |  |  |  |
| 1 | Ath-AT3G14750.1 |  | Vvi-Vitvi19g00023\_t001 |  |  |  |  |  |  |  |
| 1 | Ath-AT3G14760.1 |  | Vvi-Vitvi19g01798\_t001 |  |  |  |  |  |  |  |
| 1 | Ath-AT3G14770.1 |  | Vvi-Vitvi19g00024\_t001 |  |  |  |  |  |  |  |
| 1 | Ath-AT3G14780.1 |  | | | |  |  |  |  |  |  |  |
| 1 | Ath-AT3G14790.2 |  | Vvi-Vitvi19g00025\_t002 |  |  |  |  |  |  |  |
| 1 | Ath-AT3G14810.1 |  | | | |  |  |  |  |  |  |  |
| 1 | Ath-AT3G14820.1 |  | | | |  |  |  |  |  |  |  |
| 2 | Ath-AT3G14830.1 |  | | | |  | Vvi-Vitvi19g00059\_t001 |  |  |  |  |  |  |
| 2 | Ath-AT3G14840.2 |  | | | |  | | | |  |  |  |  |  |  |
| 2 | Ath-AT3G14850.2 |  | Vvi-Vitvi19g00041\_t001 |  | | | |  |  |  |  |  |  |
| 1 | Ath-AT3G14860.2 |  |  |  | Vvi-Vitvi19g00075\_t002 |  |  |  |  |  |  |
| 1 | Ath-AT3G14870.1 |  |  |  | Vvi-Vitvi19g01830\_t002 |  |  |  |  |  |  |
| 1 | Ath-AT3G14880.1 |  |  |  | Vvi-Vitvi19g00097\_t001 |  |  |  |  |  |  |
| 1 | Ath-AT3G14890.1 |  |  |  | Vvi-Vitvi19g00103\_t001 |  |  |  |  |  |  |
| 1 | Ath-AT3G14900.1 |  |  |  | Vvi-Vitvi19g00104\_t001 |  |  |  |  |  |  |
| 1 | Ath-AT3G14910.1 |  |  |  | Vvi-Vitvi19g00105\_t001 |  |  |  |  |  |  |
| 1 | Ath-AT3G14920.1 |  |  |  | Vvi-Vitvi19g00106\_t001 |  |  |  |  |  |  |
| 1 | Ath-AT3G14930.1 |  |  |  | Vvi-Vitvi19g00107\_t002 |  |  |  |  |  |  |
| 1 | Ath-AT3G14940.1 |  |  |  | Vvi-Vitvi19g00112\_t001 |  |  |  |  |  |  |
| 1 | Ath-AT3G14950.1 |  |  |  | Vvi-Vitvi19g00122\_t001 |  |  |  |  |  |  |
| 1 | Ath-AT3G14960.1 |  |  |  | Vvi-Vitvi19g00123\_t001 |  |  |  |  |  |  |
| 1 | Ath-AT3G14970.1 |  |  |  | | | |  |  |  |  |  |  |
| 1 | Ath-AT3G14980.2 |  |  |  | Vvi-Vitvi19g00124\_t001 |  |  |  |  |  |  |
| 1 | Ath-AT3G14981.1 |  |  |  | | | |  |  |  |  |  |  |
| 1 | Ath-AT3G14990.1 |  |  |  | Vvi-Vitvi19g00125\_t001 |  |  |  |  |  |  |
| 1 | Ath-AT3G15000.1 |  |  |  | Vvi-Vitvi19g00129\_t001 |  |  |  |  |  |  |
| 1 | Ath-AT3G15010.2 |  |  |  | Vvi-Vitvi19g00131\_t001 |  |  |  |  |  |  |
| 1 | Ath-AT3G15020.1 |  |  |  | Vvi-Vitvi19g00138\_t001 |  |  |  |  |  |  |
| 1 | Ath-AT3G15030.2 |  |  |  | Vvi-Vitvi19g00140\_t001 |  |  |  |  |  |  |
| 1 | Ath-AT3G15040.1 |  |  |  | | | |  |  |  |  |  |  |
| 1 | Ath-AT3G15050.1 |  |  |  | Vvi-Vitvi19g00141\_t001 |  |  |  |  |  |  |
| 1 | Ath-AT3G15060.1 |  |  |  | Vvi-Vitvi19g00153\_t001 |  |  |  |  |  |  |
| 1 | Ath-AT3G15070.1 |  |  |  | Vvi-Vitvi19g00155\_t003 |  |  |  |  |  |  |
| 1 | Ath-AT3G15080.1 |  |  |  | Vvi-Vitvi19g00156\_t001 |  |  |  |  |  |  |
| 1 | Ath-AT3G15090.1 |  |  |  | Vvi-Vitvi19g00158\_t001 |  |  |  |  |  |  |
| 1 | Ath-AT3G15095.1 |  |  |  | Vvi-Vitvi19g00159\_t001 |  |  |  |  |  |  |
| 1 | Ath-AT3G15110.1 |  |  |  | Vvi-Vitvi19g00162\_t001 |  |  |  |  |  |  |
| 1 | Ath-AT3G15111.1 |  |  |  | | | |  |  |  |  |  |  |
| 1 | Ath-AT3G15115.1 |  |  |  | Vvi-Vitvi19g00164\_t001 |  |  |  |  |  |  |
| 1 | Ath-AT3G15120.2 |  |  |  | Vvi-Vitvi19g00178\_t001 |  |  |  |  |  |  |
| 1 | Ath-AT3G15130.1 |  |  |  | | | |  |  |  |  |  |  |
| 1 | Ath-AT3G15140.1 |  |  |  | Vvi-Vitvi19g00179\_t001 |  |  |  |  |  |  |
| 1 | Ath-AT3G15150.1 |  |  |  | Vvi-Vitvi19g00180\_t001 |  |  |  |  |  |  |
| 1 | Ath-AT3G15160.1 |  |  |  | | | |  |  |  |  |  |  |
| 1 | Ath-AT3G15170.1 |  |  |  | Vvi-Vitvi19g00188\_t001 |  |  |  |  |  |  |
| 1 | Ath-AT3G15180.2 |  |  |  | Vvi-Vitvi19g00189\_t001 |  |  |  |  |  |  |
| 1 | Ath-AT3G15190.1 |  |  |  | | | |  |  |  |  |  |  |
| 1 | Ath-AT3G15200.1 |  |  |  | Vvi-Vitvi19g00190\_t001 |  |  |  |  |  |  |
| 3 | Ath-AT3G15210.1 |  | Vvi-Vitvi10g00488\_t001 |  | Vvi-Vitvi19g01784\_t001 |  | Vvi-Vitvi12g00274\_t001 |  |  |  |  |  |
| 3 | Ath-AT3G15220.1 |  | | | |  | Vvi-Vitvi19g00197\_t001 |  | | | |  |  |  |  |  |
| 3 | Ath-AT3G15240.2 |  | | | |  | Vvi-Vitvi19g00198\_t001 |  | | | |  |  |  |  |  |
| 3 | Ath-AT3G15250.1 |  | | | |  | Vvi-Vitvi19g01859\_t001 |  | | | |  |  |  |  |  |
| 3 | Ath-AT3G15251.1 |  | | | |  | | | |  | | | |  |  |  |  |  |
| 3 | Ath-AT3G15260.1 |  | | | |  | Vvi-Vitvi19g00199\_t001 |  | Vvi-Vitvi12g00277\_t001 |  |  |  |  |  |
| 3 | Ath-AT3G15270.1 |  | Vvi-Vitvi10g04328\_t002 |  | Vvi-Vitvi19g00200\_t001 |  | Vvi-Vitvi12g00280\_t001 |  |  |  |  |  |
| 3 | Ath-AT3G15280.1 |  | | | |  | | | |  | | | |  |  |  |  |  |
| 3 | Ath-AT3G15290.1 |  | | | |  | Vvi-Vitvi19g00202\_t001 |  | | | |  |  |  |  |  |
| 3 | Ath-AT3G15300.1 |  | Vvi-Vitvi10g02257\_t001 |  | Vvi-Vitvi19g00209\_t001 |  | | | |  |  |  |  |  |
| 3 | Ath-AT3G15340.3 |  | | | |  | Vvi-Vitvi19g00219\_t001 |  | | | |  |  |  |  |  |
| 3 | Ath-AT3G15350.2 |  | | | |  | Vvi-Vitvi19g01869\_t001.1.6037826c |  | | | |  |  |  |  |  |
| 3 | Ath-AT3G15354.3 |  | | | |  | Vvi-Vitvi19g00220\_t001 |  | Vvi-Vitvi12g00299\_t002 |  |  |  |  |  |
| 3 | Ath-AT3G15359.1 |  | | | |  | | | |  | | | |  |  |  |  |  |
| 3 | Ath-AT3G15356.1 |  | | | |  | | | |  | | | |  |  |  |  |  |
| 3 | Ath-AT3G15351.1 |  | | | |  | | | |  | | | |  |  |  |  |  |
| 3 | Ath-AT3G15358.1 |  | Vvi-Vitvi10g00454\_t001 |  | Vvi-Vitvi19g04087\_t001 |  | Vvi-Vitvi12g00302\_t001 |  |  |  |  |  |
| 3 | Ath-AT3G15352.4 |  | | | |  | | | |  | | | |  |  |  |  |  |
| 3 | Ath-AT3G15353.1 |  | | | |  | Vvi-Vitvi19g01871\_t001 |  | | | |  |  |  |  |  |
| 3 | Ath-AT3G15355.1 |  | Vvi-Vitvi10g01764\_t001 |  | Vvi-Vitvi19g00228\_t001 |  | Vvi-Vitvi12g00304\_t001 |  |  |  |  |  |
| 3 | Ath-AT3G15357.1 |  | | | |  | | | |  | | | |  |  |  |  |  |
| 3 | Ath-AT3G15360.1 |  | | | |  | Vvi-Vitvi19g01880\_t001 |  | Vvi-Vitvi12g00319\_t001 |  |  |  |  |  |
| 2 | Ath-AT3G15370.2 |  | | | |  | Vvi-Vitvi19g00240\_t001 |  |  |  |  |  |  |
| 2 | Ath-AT3G15380.1 |  | | | |  | Vvi-Vitvi19g00242\_t001 |  |  |  |  |  |  |
| 2 | Ath-AT3G15390.1 |  | Vvi-Vitvi10g00473\_t003 |  | | | |  |  |  |  |  |  |
| 2 | Ath-AT3G15395.4 |  | | | |  | Vvi-Vitvi19g04091\_t001 |  |  |  |  |  |  |
| 2 | Ath-AT3G15400.1 |  | | | |  | | | |  |  |  |  |  |  |
| 2 | Ath-AT3G15410.2 |  | | | |  | Vvi-Vitvi19g00246\_t001 |  |  |  |  |  |  |
| 2 | Ath-AT3G15420.1 |  | | | |  | Vvi-Vitvi19g04103\_t001 |  |  |  |  |  |  |
| 2 | Ath-AT3G15430.2 |  | | | |  | Vvi-Vitvi19g00251\_t001 |  |  |  |  |  |  |
| 2 | Ath-AT3G15440.1 |  | | | |  | | | |  |  |  |  |  |  |
| 2 | Ath-AT3G15450.1 |  | Vvi-Vitvi10g00451\_t001 |  | Vvi-Vitvi19g00255\_t001 |  |  |  |  |  |  |
| 2 | Ath-AT3G15460.1 |  | | | |  | | | |  |  |  |  |  |  |
| 2 | Ath-AT3G15470.1 |  | | | |  | Vvi-Vitvi19g00258\_t001 |  |  |  |  |  |  |
| 2 | Ath-AT3G15480.1 |  | Vvi-Vitvi10g01762\_t001 |  | Vvi-Vitvi19g00261\_t003 |  |  |  |  |  |  |
| 2 | Ath-AT3G15490.1 |  | | | |  | | | |  |  |  |  |  |  |
| 2 | Ath-AT3G15500.1 |  | | | |  | Vvi-Vitvi19g00270\_t001 |  |  |  |  |  |  |
| 2 | Ath-AT3G15510.1 |  | Vvi-Vitvi10g00437\_t001 |  | Vvi-Vitvi19g00271\_t001 |  |  |  |  |  |  |
| 1 | Ath-AT3G15518.1 |  |  |  | Vvi-Vitvi19g01897\_t001 |  |  |  |  |  |  |
| 1 | Ath-AT3G15520.1 |  |  |  | Vvi-Vitvi19g04107\_t001 |  |  |  |  |  |  |
| 1 | Ath-AT3G15530.1 |  |  |  | Vvi-Vitvi19g00275\_t001 |  |  |  |  |  |  |
| 0 | Ath-AT3G15534.1 |  |  |  |  |  |  |  |  |
| 1 | Ath-AT3G15540.1 |  | Vvi-Vitvi09g00436\_t001 |  |  |  |  |  |  |  |
| 1 | Ath-AT3G15548.1 |  | | | |  |  |  |  |  |  |  |
| 2 | Ath-AT3G15550.1 |  | | | |  | Vvi-Vitvi09g00486\_t001 |  |  |  |  |  |  |
| 2 | Ath-AT3G15570.1 |  | | | |  | Vvi-Vitvi09g00488\_t001 |  |  |  |  |  |  |
| 2 | Ath-AT3G15580.1 |  | | | |  | | | |  |  |  |  |  |  |
| 2 | Ath-AT3G15578.1 |  | | | |  | | | |  |  |  |  |  |  |
| 2 | Ath-AT3G15590.1 |  | | | |  | | | |  |  |  |  |  |  |
| 2 | Ath-AT3G15604.1 |  | | | |  | | | |  |  |  |  |  |  |
| 2 | Ath-AT3G15605.4 |  | | | |  | Vvi-Vitvi09g00493\_t001 |  |  |  |  |  |  |
| 2 | Ath-AT3G15610.1 |  | | | |  | Vvi-Vitvi09g00496\_t001 |  |  |  |  |  |  |
| 2 | Ath-AT3G15620.1 |  | | | |  | | | |  |  |  |  |  |  |
| 2 | Ath-AT3G15630.1 |  | | | |  | Vvi-Vitvi09g01641\_t001 |  |  |  |  |  |  |
| 2 | Ath-AT3G15635.1 |  | | | |  | | | |  |  |  |  |  |  |
| 2 | Ath-AT3G15640.1 |  | | | |  | Vvi-Vitvi09g01645\_t001 |  |  |  |  |  |  |
| 2 | Ath-AT3G15650.2 |  | | | |  | Vvi-Vitvi09g00518\_t001 |  |  |  |  |  |  |
| 2 | Ath-AT3G15660.1 |  | | | |  | Vvi-Vitvi09g00521\_t002 |  |  |  |  |  |  |
| 2 | Ath-AT3G15670.1 |  | | | |  | Vvi-Vitvi09g00524\_t001 |  |  |  |  |  |  |
| 2 | Ath-AT3G15680.1 |  | | | |  | Vvi-Vitvi09g04170\_t001 |  |  |  |  |  |  |
| 2 | Ath-AT3G15690.2 |  | | | |  | Vvi-Vitvi09g00529\_t001 |  |  |  |  |  |  |
| 1 | Ath-AT3G15700.1 |  | Vvi-Vitvi09g04127\_t001 |  |  |  |  |  |  |  |
| 2 | Ath-AT3G15710.1 |  | | | |  | Vvi-Vitvi09g00576\_t001 |  |  |  |  |  |  |
| 2 | Ath-AT3G15720.1 |  | | | |  | Vvi-Vitvi09g00579\_t001 |  |  |  |  |  |  |
| 2 | Ath-AT3G15730.1 |  | | | |  | Vvi-Vitvi09g00595\_t001 |  |  |  |  |  |  |
| 2 | Ath-AT3G15740.1 |  | | | |  | | | |  |  |  |  |  |  |
| 2 | Ath-AT3G15750.1 |  | | | |  | | | |  |  |  |  |  |  |
| 2 | Ath-AT3G15760.1 |  | | | |  | Vvi-Vitvi09g04184\_t001 |  |  |  |  |  |  |
| 2 | Ath-AT3G15770.1 |  | | | |  | Vvi-Vitvi09g04185\_t001 |  |  |  |  |  |  |
| 2 | Ath-AT3G15780.1 |  | | | |  | | | |  |  |  |  |  |  |
| 2 | Ath-AT3G15790.1 |  | | | |  | Vvi-Vitvi09g00606\_t001 |  |  |  |  |  |  |
| 2 | Ath-AT3G15800.1 |  | | | |  | Vvi-Vitvi09g00607\_t002 |  |  |  |  |  |  |
| 2 | Ath-AT3G15810.1 |  | | | |  | Vvi-Vitvi09g00610\_t001 |  |  |  |  |  |  |
| 2 | Ath-AT3G15820.1 |  | | | |  | | | |  |  |  |  |  |  |
| 2 | Ath-AT3G15830.1 |  | | | |  | | | |  |  |  |  |  |  |
| 2 | Ath-AT3G15840.1 |  | | | |  | | | |  |  |  |  |  |  |
| 2 | Ath-AT3G15850.1 |  | | | |  | Vvi-Vitvi09g00616\_t001 |  |  |  |  |  |  |
| 2 | Ath-AT3G15860.1 |  | | | |  | | | |  |  |  |  |  |  |
| 2 | Ath-AT3G15870.1 |  | | | |  | | | |  |  |  |  |  |  |
| 2 | Ath-AT3G15880.2 |  | | | |  | Vvi-Vitvi09g00620\_t001 |  |  |  |  |  |  |
| 2 | Ath-AT3G15890.1 |  | | | |  | Vvi-Vitvi09g00622\_t001 |  |  |  |  |  |  |
| 2 | Ath-AT3G15900.1 |  | | | |  | Vvi-Vitvi09g01670\_t001 |  |  |  |  |  |  |
| 2 | Ath-AT3G15909.1 |  | | | |  | | | |  |  |  |  |  |  |
| 2 | Ath-AT3G15910.1 |  | | | |  | | | |  |  |  |  |  |  |
| 2 | Ath-AT3G15920.1 |  | | | |  | Vvi-Vitvi09g00644\_t001 |  |  |  |  |  |  |
| 2 | Ath-AT3G15930.1 |  | | | |  | Vvi-Vitvi09g00647\_t001 |  |  |  |  |  |  |
| 1 | Ath-AT3G15940.2 |  | Vvi-Vitvi09g00406\_t001 |  |  |  |  |  |  |  |
| 1 | Ath-AT3G15950.1 |  | | | |  |  |  |  |  |  |  |
| 1 | Ath-AT3G15960.1 |  | | | |  |  |  |  |  |  |  |
| 1 | Ath-AT3G15970.1 |  | Vvi-Vitvi09g00402\_t001 |  |  |  |  |  |  |  |
| 2 | Ath-AT3G15980.5 |  | Vvi-Vitvi09g00400\_t001 |  | Vvi-Vitvi11g00359\_t001 |  |  |  |  |  |  |
| 2 | Ath-AT3G15990.1 |  | Vvi-Vitvi09g00399\_t001 |  | Vvi-Vitvi11g00355\_t001 |  |  |  |  |  |  |
| 2 | Ath-AT3G16000.1 |  | Vvi-Vitvi09g00395\_t003 |  | | | |  |  |  |  |  |  |
| 2 | Ath-AT3G16010.1 |  | Vvi-Vitvi09g01610\_t001 |  | | | |  |  |  |  |  |  |
| 2 | Ath-AT3G16020.1 |  | | | |  | | | |  |  |  |  |  |  |
| 2 | Ath-AT3G16030.6 |  | Vvi-Vitvi09g04110\_t001 |  | | | |  |  |  |  |  |  |
| 2 | Ath-AT3G16040.1 |  | Vvi-Vitvi09g01606\_t001 |  | | | |  |  |  |  |  |  |
| 2 | Ath-AT3G16050.1 |  | Vvi-Vitvi09g00375\_t001 |  | | | |  |  |  |  |  |  |
| 2 | Ath-AT3G16060.1 |  | Vvi-Vitvi09g00373\_t001 |  | Vvi-Vitvi11g00341\_t001 |  |  |  |  |  |  |
| 2 | Ath-AT3G16070.1 |  | Vvi-Vitvi09g00372\_t001 |  | Vvi-Vitvi11g01403\_t001 |  |  |  |  |  |  |
| 2 | Ath-AT3G16080.1 |  | Vvi-Vitvi09g00370\_t001 |  | | | |  |  |  |  |  |  |
| 2 | Ath-AT3G16090.1 |  | | | |  | | | |  |  |  |  |  |  |
| 2 | Ath-AT3G16100.1 |  | | | |  | | | |  |  |  |  |  |  |
| 2 | Ath-AT3G16110.1 |  | | | |  | | | |  |  |  |  |  |  |
| 2 | Ath-AT3G16117.1 |  | | | |  | | | |  |  |  |  |  |  |
| 2 | Ath-AT3G16120.1 |  | | | |  | Vvi-Vitvi11g00322\_t001 |  |  |  |  |  |  |
| 2 | Ath-AT3G16130.1 |  | | | |  | | | |  |  |  |  |  |  |
| 2 | Ath-AT3G16140.1 |  | Vvi-Vitvi09g00361\_t001 |  | | | |  |  |  |  |  |  |
| 2 | Ath-AT3G16150.1 |  | | | |  | | | |  |  |  |  |  |  |
| 2 | Ath-AT3G16160.1 |  | | | |  | Vvi-Vitvi11g00314\_t001 |  |  |  |  |  |  |
| 2 | Ath-AT3G16170.1 |  | Vvi-Vitvi09g00342\_t001 |  | | | |  |  |  |  |  |  |
| 2 | Ath-AT3G16175.1 |  | Vvi-Vitvi09g01597\_t001 |  | | | |  |  |  |  |  |  |
| 2 | Ath-AT3G16180.1 |  | | | |  | Vvi-Vitvi11g00306\_t001 |  |  |  |  |  |  |
| 2 | Ath-AT3G16190.1 |  | Vvi-Vitvi09g00341\_t001 |  | | | |  |  |  |  |  |  |
| 2 | Ath-AT3G16200.1 |  | Vvi-Vitvi09g00335\_t001 |  | | | |  |  |  |  |  |  |
| 2 | Ath-AT3G16210.1 |  | | | |  | | | |  |  |  |  |  |  |
| 2 | Ath-AT3G16220.1 |  | Vvi-Vitvi09g00333\_t001 |  | | | |  |  |  |  |  |  |
| 2 | Ath-AT3G16230.1 |  | | | |  | | | |  |  |  |  |  |  |
| 2 | Ath-AT3G16240.1 |  | Vvi-Vitvi09g00329\_t001 |  | | | |  |  |  |  |  |  |
| 2 | Ath-AT3G16250.1 |  | Vvi-Vitvi09g00328\_t001 |  | | | |  |  |  |  |  |  |
| 2 | Ath-AT3G16260.1 |  | Vvi-Vitvi09g00327\_t001 |  | | | |  |  |  |  |  |  |
| 2 | Ath-AT3G16270.1 |  | Vvi-Vitvi09g00326\_t001 |  | | | |  |  |  |  |  |  |
| 2 | Ath-AT3G16280.2 |  | Vvi-Vitvi09g00323\_t001 |  | Vvi-Vitvi11g00285\_t001 |  |  |  |  |  |  |
| 2 | Ath-AT3G16290.1 |  | Vvi-Vitvi09g00319\_t002 |  | | | |  |  |  |  |  |  |
| 2 | Ath-AT3G16300.1 |  | Vvi-Vitvi09g00314\_t001 |  | | | |  |  |  |  |  |  |
| 2 | Ath-AT3G16310.1 |  | Vvi-Vitvi09g00312\_t001 |  | | | |  |  |  |  |  |  |
| 2 | Ath-AT3G16320.1 |  | | | |  | Vvi-Vitvi11g00277\_t001 |  |  |  |  |  |  |
| 2 | Ath-AT3G16330.1 |  | Vvi-Vitvi09g00307\_t001 |  | Vvi-Vitvi11g00275\_t001 |  |  |  |  |  |  |
| 2 | Ath-AT3G16340.1 |  | Vvi-Vitvi09g04084\_t001 |  | | | |  |  |  |  |  |  |
| 2 | Ath-AT3G16350.1 |  | Vvi-Vitvi09g00293\_t001 |  | | | |  |  |  |  |  |  |
| 2 | Ath-AT3G16360.2 |  | Vvi-Vitvi09g00290\_t001 |  | Vvi-Vitvi11g01392\_t001 |  |  |  |  |  |  |
| 2 | Ath-AT3G16370.1 |  | | | |  | | | |  |  |  |  |  |  |
| 2 | Ath-AT3G16380.1 |  | | | |  | | | |  |  |  |  |  |  |
| 2 | Ath-AT3G16390.2 |  | | | |  | | | |  |  |  |  |  |  |
| 2 | Ath-AT3G16400.1 |  | | | |  | | | |  |  |  |  |  |  |
| 2 | Ath-AT3G16410.1 |  | | | |  | | | |  |  |  |  |  |  |
| 2 | Ath-AT3G16420.1 |  | | | |  | | | |  |  |  |  |  |  |
| 2 | Ath-AT3G16430.1 |  | | | |  | | | |  |  |  |  |  |  |
| 2 | Ath-AT3G16432.1 |  | | | |  | | | |  |  |  |  |  |  |
| 2 | Ath-AT3G16440.2 |  | | | |  | | | |  |  |  |  |  |  |
| 2 | Ath-AT3G16450.2 |  | | | |  | | | |  |  |  |  |  |  |
| 2 | Ath-AT3G16460.1 |  | | | |  | | | |  |  |  |  |  |  |
| 2 | Ath-AT3G16470.1 |  | | | |  | | | |  |  |  |  |  |  |
| 2 | Ath-AT3G16480.1 |  | | | |  | | | |  |  |  |  |  |  |
| 2 | Ath-AT3G16490.1 |  | Vvi-Vitvi09g00276\_t002 |  | Vvi-Vitvi11g00257\_t001 |  |  |  |  |  |  |
| 0 | Ath-AT3G16500.1 |  |  |  |  |  |  |  |  |
| 0 | Ath-AT3G16510.1 |  |  |  |  |  |  |  |  |
| 0 | Ath-AT3G16520.3 |  |  |  |  |  |  |  |  |
| 0 | Ath-AT3G16525.1 |  |  |  |  |  |  |  |  |
| 0 | Ath-AT3G16530.1 |  |  |  |  |  |  |  |  |
| 0 | Ath-AT3G16540.1 |  |  |  |  |  |  |  |  |
| 0 | Ath-AT3G16550.1 |  |  |  |  |  |  |  |  |
| 0 | Ath-AT3G16555.1 |  |  |  |  |  |  |  |  |
| 0 | Ath-AT3G16560.2 |  |  |  |  |  |  |  |  |
| 0 | Ath-AT3G16565.3 |  |  |  |  |  |  |  |  |
| 0 | Ath-AT3G16570.1 |  |  |  |  |  |  |  |  |
| 0 | Ath-AT3G16580.1 |  |  |  |  |  |  |  |  |
| 0 | Ath-AT3G16590.1 |  |  |  |  |  |  |  |  |
| 0 | Ath-AT3G16600.1 |  |  |  |  |  |  |  |  |
| 0 | Ath-AT3G16610.1 |  |  |  |  |  |  |  |  |
| 0 | Ath-AT3G16620.1 |  |  |  |  |  |  |  |  |
| 0 | Ath-AT3G16630.1 |  |  |  |  |  |  |  |  |
| 0 | Ath-AT3G16640.1 |  |  |  |  |  |  |  |  |
| 1 | Ath-AT3G16650.1 |  | Vvi-Vitvi05g00008\_t001 |  |  |  |  |  |  |  |
| 1 | Ath-AT3G16660.2 |  | Vvi-Vitvi05g00011\_t001 |  |  |  |  |  |  |  |
| 1 | Ath-AT3G16670.1 |  | | | |  |  |  |  |  |  |  |
| 1 | Ath-AT3G16680.1 |  | | | |  |  |  |  |  |  |  |
| 1 | Ath-AT3G16690.3 |  | Vvi-Vitvi05g04001\_t001 |  |  |  |  |  |  |  |
| 1 | Ath-AT3G16700.1 |  | Vvi-Vitvi05g00035\_t001 |  |  |  |  |  |  |  |
| 1 | Ath-AT3G16710.1 |  | | | |  |  |  |  |  |  |  |
| 1 | Ath-AT3G16712.1 |  | | | |  |  |  |  |  |  |  |
| 2 | Ath-AT3G16720.1 |  | Vvi-Vitvi05g00041\_t001 |  | Vvi-Vitvi07g00348\_t001 |  |  |  |  |  |  |
| 2 | Ath-AT3G16730.1 |  | Vvi-Vitvi05g00042\_t001 |  | | | |  |  |  |  |  |  |
| 2 | Ath-AT3G16740.1 |  | | | |  | | | |  |  |  |  |  |  |
| 2 | Ath-AT3G16750.2 |  | | | |  | | | |  |  |  |  |  |  |
| 2 | Ath-AT3G16760.1 |  | Vvi-Vitvi05g00050\_t001 |  | | | |  |  |  |  |  |  |
| 2 | Ath-AT3G16770.1 |  | Vvi-Vitvi05g01724\_t001 |  | Vvi-Vitvi07g00357\_t001 |  |  |  |  |  |  |
| 2 | Ath-AT3G16780.1 |  | Vvi-Vitvi05g00053\_t001 |  | Vvi-Vitvi07g02210\_t001 |  |  |  |  |  |  |
| 2 | Ath-AT3G16785.4 |  | Vvi-Vitvi05g00054\_t001 |  | | | |  |  |  |  |  |  |
| 2 | Ath-AT3G16800.2 |  | Vvi-Vitvi05g00055\_t001 |  | | | |  |  |  |  |  |  |
| 2 | Ath-AT3G16810.1 |  | Vvi-Vitvi05g00060\_t001 |  | | | |  |  |  |  |  |  |
| 2 | Ath-AT3G16820.1 |  | | | |  | | | |  |  |  |  |  |  |
| 2 | Ath-AT3G16830.1 |  | | | |  | | | |  |  |  |  |  |  |
| 2 | Ath-AT3G16840.2 |  | | | |  | | | |  |  |  |  |  |  |
| 2 | Ath-AT3G16850.1 |  | Vvi-Vitvi05g00061\_t001 |  | Vvi-Vitvi07g00363\_t001 |  |  |  |  |  |  |
| 2 | Ath-AT3G16857.2 |  | Vvi-Vitvi05g00075\_t001 |  | Vvi-Vitvi07g00374\_t001 |  |  |  |  |  |  |
| 2 | Ath-AT3G16860.1 |  | Vvi-Vitvi05g00076\_t001 |  | Vvi-Vitvi07g00375\_t001 |  |  |  |  |  |  |
| 2 | Ath-AT3G16870.1 |  | Vvi-Vitvi05g00077\_t001 |  | Vvi-Vitvi07g02214\_t001 |  |  |  |  |  |  |
| 1 | Ath-AT3G16880.1 |  | | | |  |  |  |  |  |  |  |
| 1 | Ath-AT3G16890.1 |  | Vvi-Vitvi05g00087\_t001 |  |  |  |  |  |  |  |
| 1 | Ath-AT3G16895.1 |  | | | |  |  |  |  |  |  |  |
| 1 | Ath-AT3G16900.1 |  | | | |  |  |  |  |  |  |  |
| 1 | Ath-AT3G16910.1 |  | Vvi-Vitvi05g00092\_t001 |  |  |  |  |  |  |  |
| 1 | Ath-AT3G16920.2 |  | Vvi-Vitvi05g00094\_t001 |  |  |  |  |  |  |  |
| 1 | Ath-AT3G16930.1 |  | | | |  |  |  |  |  |  |  |
| 1 | Ath-AT3G16940.1 |  | Vvi-Vitvi05g00096\_t001 |  |  |  |  |  |  |  |
| 1 | Ath-AT3G16950.2 |  | Vvi-Vitvi05g00100\_t001 |  |  |  |  |  |  |  |
| 1 | Ath-AT3G16960.1 |  | | | |  |  |  |  |  |  |  |
| 1 | Ath-AT3G16970.1 |  | | | |  |  |  |  |  |  |  |
| 1 | Ath-AT3G16980.1 |  | Vvi-Vitvi05g00105\_t001 |  |  |  |  |  |  |  |
| 1 | Ath-AT3G16990.1 |  | Vvi-Vitvi05g00109\_t001 |  |  |  |  |  |  |  |
| 1 | Ath-AT3G17000.1 |  | Vvi-Vitvi05g00112\_t001 |  |  |  |  |  |  |  |
| 1 | Ath-AT3G17010.1 |  | | | |  |  |  |  |  |  |  |
| 1 | Ath-AT3G17020.1 |  | Vvi-Vitvi05g00115\_t001 |  |  |  |  |  |  |  |
| 1 | Ath-AT3G17030.1 |  | Vvi-Vitvi05g00118\_t001 |  |  |  |  |  |  |  |
| 1 | Ath-AT3G17040.1 |  | Vvi-Vitvi05g00124\_t001 |  |  |  |  |  |  |  |
| 1 | Ath-AT3G17060.1 |  | Vvi-Vitvi05g00134\_t001 |  |  |  |  |  |  |  |
| 1 | Ath-AT3G17070.1 |  | Vvi-Vitvi05g00135\_t001 |  |  |  |  |  |  |  |
| 1 | Ath-AT3G17080.1 |  | | | |  |  |  |  |  |  |  |
| 1 | Ath-AT3G17090.1 |  | Vvi-Vitvi05g00141\_t001 |  |  |  |  |  |  |  |
| 1 | Ath-AT3G17100.2 |  | Vvi-Vitvi05g00143\_t001 |  |  |  |  |  |  |  |
| 1 | Ath-AT3G17120.3 |  | Vvi-Vitvi05g00150\_t001 |  |  |  |  |  |  |  |
| 1 | Ath-AT3G17130.1 |  | Vvi-Vitvi05g01786\_t001 |  |  |  |  |  |  |  |
| 1 | Ath-AT3G17140.1 |  | | | |  |  |  |  |  |  |  |
| 1 | Ath-AT3G17150.1 |  | | | |  |  |  |  |  |  |  |
| 1 | Ath-AT3G17152.1 |  | | | |  |  |  |  |  |  |  |
| 1 | Ath-AT3G17155.1 |  | | | |  |  |  |  |  |  |  |
| 1 | Ath-AT3G17160.1 |  | Vvi-Vitvi05g01790\_t003 |  |  |  |  |  |  |  |
| 1 | Ath-AT3G17170.1 |  | Vvi-Vitvi05g00157\_t001 |  |  |  |  |  |  |  |
| 1 | Ath-AT3G17180.1 |  | Vvi-Vitvi05g00160\_t001 |  |  |  |  |  |  |  |
| 1 | Ath-AT3G17190.1 |  | | | |  |  |  |  |  |  |  |
| 1 | Ath-AT3G17205.1 |  | Vvi-Vitvi05g00167\_t001 |  |  |  |  |  |  |  |
| 2 | Ath-AT3G17210.1 |  | Vvi-Vitvi05g04031\_t001 |  | Vvi-Vitvi05g01828\_t001 |  |  |  |  |  |  |
| 2 | Ath-AT3G17220.1 |  | | | |  | Vvi-Vitvi05g00205\_t001 |  |  |  |  |  |  |
| 2 | Ath-AT3G17225.1 |  | | | |  | | | |  |  |  |  |  |  |
| 2 | Ath-AT3G17227.1 |  | | | |  | | | |  |  |  |  |  |  |
| 2 | Ath-AT3G17230.1 |  | | | |  | | | |  |  |  |  |  |  |
| 2 | Ath-AT3G17240.1 |  | | | |  | Vvi-Vitvi05g00207\_t001 |  |  |  |  |  |  |
| 2 | Ath-AT3G17250.1 |  | | | |  | Vvi-Vitvi05g00208\_t001 |  |  |  |  |  |  |
| 2 | Ath-AT3G17261.1 |  | | | |  | | | |  |  |  |  |  |  |
| 2 | Ath-AT3G17265.1 |  | | | |  | | | |  |  |  |  |  |  |
| 2 | Ath-AT3G17270.1 |  | | | |  | | | |  |  |  |  |  |  |
| 2 | Ath-AT3G17280.1 |  | | | |  | | | |  |  |  |  |  |  |
| 2 | Ath-AT3G17300.1 |  | | | |  | Vvi-Vitvi05g00213\_t001 |  |  |  |  |  |  |
| 2 | Ath-AT3G17310.2 |  | | | |  | Vvi-Vitvi05g00215\_t001 |  |  |  |  |  |  |
| 2 | Ath-AT3G17320.1 |  | | | |  | | | |  |  |  |  |  |  |
| 2 | Ath-AT3G17330.3 |  | | | |  | Vvi-Vitvi05g01830\_t001 |  |  |  |  |  |  |
| 2 | Ath-AT3G17340.2 |  | Vvi-Vitvi05g04044\_t001 |  | Vvi-Vitvi05g04072\_t001 |  |  |  |  |  |  |
| 1 | Ath-AT3G17350.1 |  |  |  | Vvi-Vitvi05g00236\_t001 |  |  |  |  |  |  |
| 1 | Ath-AT3G17360.3 |  |  |  | Vvi-Vitvi05g00237\_t001 |  |  |  |  |  |  |
| 1 | Ath-AT3G17365.2 |  |  |  | Vvi-Vitvi05g00241\_t001 |  |  |  |  |  |  |
| 1 | Ath-AT3G17370.1 |  |  |  | | | |  |  |  |  |  |  |
| 1 | Ath-AT3G17380.1 |  |  |  | | | |  |  |  |  |  |  |
| 1 | Ath-AT3G17390.1 |  |  |  | Vvi-Vitvi05g00242\_t001 |  |  |  |  |  |  |
| 1 | Ath-AT3G17400.1 |  |  |  | | | |  |  |  |  |  |  |
| 1 | Ath-AT3G17410.2 |  |  |  | Vvi-Vitvi05g00246\_t003 |  |  |  |  |  |  |
| 1 | Ath-AT3G17420.1 |  |  |  | | | |  |  |  |  |  |  |
| 1 | Ath-AT3G17430.1 |  |  |  | Vvi-Vitvi05g00248\_t001 |  |  |  |  |  |  |
| 1 | Ath-AT3G17440.1 |  |  |  | Vvi-Vitvi05g00249\_t001 |  |  |  |  |  |  |
| 1 | Ath-AT3G17450.1 |  |  |  | Vvi-Vitvi05g00252\_t001 |  |  |  |  |  |  |
| 1 | Ath-AT3G17460.1 |  |  |  | Vvi-Vitvi05g01832\_t001 |  |  |  |  |  |  |
| 1 | Ath-AT3G17465.1 |  |  |  | Vvi-Vitvi05g00254\_t001 |  |  |  |  |  |  |
| 1 | Ath-AT3G17470.2 |  |  |  | Vvi-Vitvi05g00255\_t001 |  |  |  |  |  |  |
| 1 | Ath-AT3G17480.1 |  |  |  | | | |  |  |  |  |  |  |
| 1 | Ath-AT3G17490.1 |  |  |  | | | |  |  |  |  |  |  |
| 1 | Ath-AT3G17500.1 |  |  |  | | | |  |  |  |  |  |  |
| 1 | Ath-AT3G17510.1 |  |  |  | Vvi-Vitvi05g00256\_t001 |  |  |  |  |  |  |
| 1 | Ath-AT3G17520.1 |  |  |  | | | |  |  |  |  |  |  |
| 1 | Ath-AT3G17530.1 |  |  |  | | | |  |  |  |  |  |  |
| 1 | Ath-AT3G17540.1 |  |  |  | | | |  |  |  |  |  |  |
| 1 | Ath-AT3G17550.1 |  |  |  | | | |  |  |  |  |  |  |
| 1 | Ath-AT3G17560.1 |  |  |  | | | |  |  |  |  |  |  |
| 1 | Ath-AT3G17570.1 |  |  |  | | | |  |  |  |  |  |  |
| 1 | Ath-AT3G17580.1 |  |  |  | Vvi-Vitvi05g01843\_t001 |  |  |  |  |  |  |
| 1 | Ath-AT3G17590.2 |  |  |  | Vvi-Vitvi05g00270\_t001 |  |  |  |  |  |  |
| 1 | Ath-AT3G17600.1 |  |  |  | Vvi-Vitvi05g00271\_t001 |  |  |  |  |  |  |
| 1 | Ath-AT3G17609.2 |  |  |  | Vvi-Vitvi05g00274\_t001 |  |  |  |  |  |  |
| 1 | Ath-AT3G17611.1 |  |  |  | Vvi-Vitvi05g00277\_t001 |  |  |  |  |  |  |
| 1 | Ath-AT3G17620.1 |  |  |  | | | |  |  |  |  |  |  |
| 1 | Ath-AT3G17626.1 |  |  |  | Vvi-Vitvi05g00279\_t001 |  |  |  |  |  |  |
| 1 | Ath-AT3G17630.1 |  |  |  | Vvi-Vitvi05g00280\_t001 |  |  |  |  |  |  |
| 0 | Ath-AT3G17640.1 |  |  |  |  |  |  |  |  |
| 0 | Ath-AT3G17650.1 |  |  |  |  |  |  |  |  |
| 0 | Ath-AT3G17655.1 |  |  |  |  |  |  |  |  |
| 0 | Ath-AT3G17660.2 |  |  |  |  |  |  |  |  |
| 0 | Ath-AT3G17668.1 |  |  |  |  |  |  |  |  |
| 0 | Ath-AT3G17670.2 |  |  |  |  |  |  |  |  |
| 0 | Ath-AT3G17675.1 |  |  |  |  |  |  |  |  |
| 0 | Ath-AT3G17680.2 |  |  |  |  |  |  |  |  |
| 0 | Ath-AT3G17690.1 |  |  |  |  |  |  |  |  |
| 0 | Ath-AT3G17700.1 |  |  |  |  |  |  |  |  |
| 0 | Ath-AT3G17710.1 |  |  |  |  |  |  |  |  |
| 0 | Ath-AT3G17712.2 |  |  |  |  |  |  |  |  |
| 0 | Ath-AT3G17717.1 |  |  |  |  |  |  |  |  |
| 0 | Ath-AT3G17720.1 |  |  |  |  |  |  |  |  |
| 0 | Ath-AT3G17730.1 |  |  |  |  |  |  |  |  |
| 0 | Ath-AT3G17740.1 |  |  |  |  |  |  |  |  |
| 0 | Ath-AT3G17750.1 |  |  |  |  |  |  |  |  |
| 0 | Ath-AT3G17760.1 |  |  |  |  |  |  |  |  |
| 0 | Ath-AT3G17765.1 |  |  |  |  |  |  |  |  |
| 0 | Ath-AT3G17770.1 |  |  |  |  |  |  |  |  |
| 1 | Ath-AT3G17780.1 |  | Vvi-Vitvi17g00170\_t001 |  |  |  |  |  |  |  |
| 1 | Ath-AT3G17790.1 |  | | | |  |  |  |  |  |  |  |
| 1 | Ath-AT3G17800.2 |  | | | |  |  |  |  |  |  |  |
| 1 | Ath-AT3G17810.1 |  | | | |  |  |  |  |  |  |  |
| 1 | Ath-AT3G17820.1 |  | | | |  |  |  |  |  |  |  |
| 1 | Ath-AT3G17830.1 |  | Vvi-Vitvi17g00171\_t001 |  |  |  |  |  |  |  |
| 1 | Ath-AT3G17840.1 |  | | | |  |  |  |  |  |  |  |
| 1 | Ath-AT3G17850.1 |  | | | |  |  |  |  |  |  |  |
| 1 | Ath-AT3G17860.1 |  | | | |  |  |  |  |  |  |  |
| 1 | Ath-AT3G17880.1 |  | | | |  |  |  |  |  |  |  |
| 1 | Ath-AT3G17890.1 |  | | | |  |  |  |  |  |  |  |
| 1 | Ath-AT3G17900.1 |  | | | |  |  |  |  |  |  |  |
| 1 | Ath-AT3G17910.1 |  | | | |  |  |  |  |  |  |  |
| 1 | Ath-AT3G17920.1 |  | | | |  |  |  |  |  |  |  |
| 1 | Ath-AT3G17930.1 |  | Vvi-Vitvi17g00181\_t001 |  |  |  |  |  |  |  |
| 1 | Ath-AT3G17940.1 |  | Vvi-Vitvi17g00192\_t001 |  |  |  |  |  |  |  |
| 1 | Ath-AT3G17950.1 |  | | | |  |  |  |  |  |  |  |
| 1 | Ath-AT3G17970.1 |  | | | |  |  |  |  |  |  |  |
| 1 | Ath-AT3G17980.1 |  | Vvi-Vitvi17g00196\_t002 |  |  |  |  |  |  |  |
| 1 | Ath-AT3G18000.1 |  | Vvi-Vitvi17g00213\_t001 |  |  |  |  |  |  |  |
| 1 | Ath-AT3G18010.1 |  | Vvi-Vitvi17g00216\_t001 |  |  |  |  |  |  |  |
| 1 | Ath-AT3G18020.1 |  | | | |  |  |  |  |  |  |  |
| 1 | Ath-AT3G18030.1 |  | | | |  |  |  |  |  |  |  |
| 1 | Ath-AT3G18035.1 |  | Vvi-Vitvi17g00223\_t001 |  |  |  |  |  |  |  |
| 1 | Ath-AT3G18040.3 |  | Vvi-Vitvi17g00225\_t001 |  |  |  |  |  |  |  |
| 1 | Ath-AT3G18050.1 |  | Vvi-Vitvi17g00228\_t001 |  |  |  |  |  |  |  |
| 1 | Ath-AT3G18060.1 |  | Vvi-Vitvi17g00233\_t001 |  |  |  |  |  |  |  |
| 1 | Ath-AT3G18070.1 |  | Vvi-Vitvi17g00234\_t001 |  |  |  |  |  |  |  |
| 1 | Ath-AT3G18080.1 |  | | | |  |  |  |  |  |  |  |
| 1 | Ath-AT3G18090.1 |  | | | |  |  |  |  |  |  |  |
| 1 | Ath-AT3G18100.1 |  | Vvi-Vitvi17g00238\_t001 |  |  |  |  |  |  |  |
| 1 | Ath-AT3G18110.1 |  | | | |  |  |  |  |  |  |  |
| 1 | Ath-AT3G18120.1 |  | | | |  |  |  |  |  |  |  |
| 1 | Ath-AT3G18130.1 |  | Vvi-Vitvi17g00239\_t001 |  |  |  |  |  |  |  |
| 1 | Ath-AT3G18140.1 |  | | | |  |  |  |  |  |  |  |
| 1 | Ath-AT3G18160.3 |  | Vvi-Vitvi17g00246\_t001 |  |  |  |  |  |  |  |
| 1 | Ath-AT3G18165.1 |  | Vvi-Vitvi17g00247\_t001 |  |  |  |  |  |  |  |
| 1 | Ath-AT3G18170.2 |  | Vvi-Vitvi17g00260\_t001 |  |  |  |  |  |  |  |
| 1 | Ath-AT3G18180.1 |  | | | |  |  |  |  |  |  |  |
| 1 | Ath-AT3G18190.1 |  | Vvi-Vitvi17g00263\_t001 |  |  |  |  |  |  |  |
| 1 | Ath-AT3G18200.2 |  | Vvi-Vitvi17g00264\_t001 |  |  |  |  |  |  |  |
| 2 | Ath-AT3G18210.1 |  | Vvi-Vitvi17g04069\_t001 |  | Vvi-Vitvi01g00357\_t001 |  |  |  |  |  |  |
| 2 | Ath-AT3G18215.1 |  | Vvi-Vitvi17g00271\_t001 |  | | | |  |  |  |  |  |  |
| 2 | Ath-AT3G18220.1 |  | Vvi-Vitvi17g00273\_t001 |  | Vvi-Vitvi01g01956\_t001 |  |  |  |  |  |  |
| 2 | Ath-AT3G18230.1 |  | Vvi-Vitvi17g00274\_t001 |  | Vvi-Vitvi01g01955\_t001 |  |  |  |  |  |  |
| 2 | Ath-AT3G18235.1 |  | | | |  | | | |  |  |  |  |  |  |
| 2 | Ath-AT3G18240.2 |  | Vvi-Vitvi17g00280\_t001 |  | | | |  |  |  |  |  |  |
| 2 | Ath-AT3G18250.1 |  | | | |  | | | |  |  |  |  |  |  |
| 2 | Ath-AT3G18260.1 |  | Vvi-Vitvi17g00282\_t001 |  | Vvi-Vitvi01g00332\_t001 |  |  |  |  |  |  |
| 2 | Ath-AT3G18270.1 |  | Vvi-Vitvi17g01391\_t001 |  | | | |  |  |  |  |  |  |
| 2 | Ath-AT3G18280.2 |  | Vvi-Vitvi17g00283\_t001 |  | Vvi-Vitvi01g00331\_t001 |  |  |  |  |  |  |
| 2 | Ath-AT3G18282.1 |  | | | |  | | | |  |  |  |  |  |  |
| 2 | Ath-AT3G18290.1 |  | Vvi-Vitvi17g00299\_t001 |  | Vvi-Vitvi01g00313\_t001 |  |  |  |  |  |  |
| 2 | Ath-AT3G18291.1 |  | | | |  | | | |  |  |  |  |  |  |
| 2 | Ath-AT3G18295.1 |  | Vvi-Vitvi17g04082\_t001 |  | Vvi-Vitvi01g00306\_t002 |  |  |  |  |  |  |
| 2 | Ath-AT3G18300.1 |  | Vvi-Vitvi17g00300\_t001 |  | Vvi-Vitvi01g00305\_t001 |  |  |  |  |  |  |
| 2 | Ath-AT3G18310.1 |  | | | |  | | | |  |  |  |  |  |  |
| 2 | Ath-AT3G18320.1 |  | | | |  | | | |  |  |  |  |  |  |
| 2 | Ath-AT3G18330.1 |  | | | |  | | | |  |  |  |  |  |  |
| 2 | Ath-AT3G18340.1 |  | | | |  | | | |  |  |  |  |  |  |
| 2 | Ath-AT3G18350.2 |  | Vvi-Vitvi17g00312\_t002 |  | | | |  |  |  |  |  |  |
| 2 | Ath-AT3G18360.1 |  | Vvi-Vitvi17g01402\_t001 |  | Vvi-Vitvi01g01941\_t001 |  |  |  |  |  |  |
| 1 | Ath-AT3G18370.1 |  | Vvi-Vitvi17g00313\_t001 |  |  |  |  |  |  |  |
| 1 | Ath-AT3G18380.2 |  | | | |  |  |  |  |  |  |  |
| 1 | Ath-AT3G18390.1 |  | Vvi-Vitvi17g00314\_t001 |  |  |  |  |  |  |  |
| 1 | Ath-AT3G18400.1 |  | Vvi-Vitvi17g00316\_t001 |  |  |  |  |  |  |  |
| 1 | Ath-AT3G18410.1 |  | Vvi-Vitvi17g00327\_t001 |  |  |  |  |  |  |  |
| 1 | Ath-AT3G18420.1 |  | | | |  |  |  |  |  |  |  |
| 1 | Ath-AT3G18430.1 |  | | | |  |  |  |  |  |  |  |
| 1 | Ath-AT3G18440.1 |  | Vvi-Vitvi17g00333\_t001 |  |  |  |  |  |  |  |
| 1 | Ath-AT3G18450.1 |  | Vvi-Vitvi17g00347\_t001 |  |  |  |  |  |  |  |
| 1 | Ath-AT3G18460.1 |  | | | |  |  |  |  |  |  |  |
| 1 | Ath-AT3G18470.1 |  | | | |  |  |  |  |  |  |  |
| 1 | Ath-AT3G18480.1 |  | Vvi-Vitvi17g00348\_t001 |  |  |  |  |  |  |  |
| 1 | Ath-AT3G18485.1 |  | | | |  |  |  |  |  |  |  |
| 1 | Ath-AT3G18490.1 |  | Vvi-Vitvi17g00350\_t001 |  |  |  |  |  |  |  |
| 1 | Ath-AT3G18500.3 |  | Vvi-Vitvi17g00356\_t001 |  |  |  |  |  |  |  |
| 1 | Ath-AT3G18510.1 |  | Vvi-Vitvi17g04092\_t001 |  |  |  |  |  |  |  |
| 1 | Ath-AT3G18518.1 |  | | | |  |  |  |  |  |  |  |
| 1 | Ath-AT3G18520.2 |  | Vvi-Vitvi17g00365\_t002 |  |  |  |  |  |  |  |
| 1 | Ath-AT3G18524.1 |  | Vvi-Vitvi17g00373\_t001 |  |  |  |  |  |  |  |
| 1 | Ath-AT3G18530.1 |  | | | |  |  |  |  |  |  |  |
| 1 | Ath-AT3G18535.3 |  | | | |  |  |  |  |  |  |  |
| 1 | Ath-AT3G18550.3 |  | Vvi-Vitvi17g00374\_t001 |  |  |  |  |  |  |  |
| 1 | Ath-AT3G18560.1 |  | Vvi-Vitvi17g01420\_t001 |  |  |  |  |  |  |  |
| 1 | Ath-AT3G18570.1 |  | Vvi-Vitvi17g00379\_t001 |  |  |  |  |  |  |  |
| 1 | Ath-AT3G18580.1 |  | | | |  |  |  |  |  |  |  |
| 1 | Ath-AT3G18590.1 |  | Vvi-Vitvi17g00386\_t001 |  |  |  |  |  |  |  |
| 1 | Ath-AT3G18600.1 |  | Vvi-Vitvi17g00388\_t001 |  |  |  |  |  |  |  |
| 0 | Ath-AT3G18610.1 |  |  |  |  |  |  |  |  |
| 0 | Ath-AT3G18620.1 |  |  |  |  |  |  |  |  |
| 0 | Ath-AT3G18630.1 |  |  |  |  |  |  |  |  |
| 1 | Ath-AT3G18640.1 |  | Vvi-Vitvi04g01661\_t001 |  |  |  |  |  |  |  |
| 1 | Ath-AT3G18650.1 |  | | | |  |  |  |  |  |  |  |
| 1 | Ath-AT3G18660.2 |  | Vvi-Vitvi04g01657\_t001 |  |  |  |  |  |  |  |
| 1 | Ath-AT3G18670.2 |  | Vvi-Vitvi04g01648\_t001 |  |  |  |  |  |  |  |
| 1 | Ath-AT3G18680.2 |  | Vvi-Vitvi04g01645\_t001 |  |  |  |  |  |  |  |
| 1 | Ath-AT3G18690.1 |  | | | |  |  |  |  |  |  |  |
| 1 | Ath-AT3G18700.1 |  | | | |  |  |  |  |  |  |  |
| 1 | Ath-AT3G18710.1 |  | Vvi-Vitvi04g02243\_t001 |  |  |  |  |  |  |  |
| 1 | Ath-AT3G18715.1 |  | Vvi-Vitvi04g02242\_t001 |  |  |  |  |  |  |  |
| 1 | Ath-AT3G18720.1 |  | | | |  |  |  |  |  |  |  |
| 1 | Ath-AT3G18730.1 |  | Vvi-Vitvi04g01641\_t001 |  |  |  |  |  |  |  |
| 1 | Ath-AT3G18740.1 |  | | | |  |  |  |  |  |  |  |
| 1 | Ath-AT3G18750.3 |  | Vvi-Vitvi04g01640\_t001 |  |  |  |  |  |  |  |
| 0 | Ath-AT3G18760.1 |  |  |  |  |  |  |  |  |
| 0 | Ath-AT3G18770.1 |  |  |  |  |  |  |  |  |
| 0 | Ath-AT3G18773.1 |  |  |  |  |  |  |  |  |
| 0 | Ath-AT3G18775.1 |  |  |  |  |  |  |  |  |
| 0 | Ath-AT3G18780.2 |  |  |  |  |  |  |  |  |
| 0 | Ath-AT3G18790.1 |  |  |  |  |  |  |  |  |
| 0 | Ath-AT3G18800.1 |  |  |  |  |  |  |  |  |
| 1 | Ath-AT3G18810.1 |  | Vvi-Vitvi03g00345\_t001 |  |  |  |  |  |  |  |
| 1 | Ath-AT3G18820.1 |  | Vvi-Vitvi03g00348\_t001 |  |  |  |  |  |  |  |
| 1 | Ath-AT3G18830.1 |  | Vvi-Vitvi03g00359\_t001 |  |  |  |  |  |  |  |
| 1 | Ath-AT3G18840.2 |  | Vvi-Vitvi03g00361\_t001 |  |  |  |  |  |  |  |
| 1 | Ath-AT3G18850.3 |  | Vvi-Vitvi03g00370\_t003 |  |  |  |  |  |  |  |
| 1 | Ath-AT3G18860.1 |  | Vvi-Vitvi03g00371\_t003 |  |  |  |  |  |  |  |
| 1 | Ath-AT3G18870.1 |  | | | |  |  |  |  |  |  |  |
| 1 | Ath-AT3G18880.1 |  | | | |  |  |  |  |  |  |  |
| 1 | Ath-AT3G18890.1 |  | Vvi-Vitvi03g00375\_t002 |  |  |  |  |  |  |  |
| 1 | Ath-AT3G18900.4 |  | Vvi-Vitvi03g00378\_t001 |  |  |  |  |  |  |  |
| 1 | Ath-AT3G18905.1 |  | | | |  |  |  |  |  |  |  |
| 1 | Ath-AT3G18910.1 |  | | | |  |  |  |  |  |  |  |
| 1 | Ath-AT3G18930.1 |  | Vvi-Vitvi03g00386\_t001 |  |  |  |  |  |  |  |
| 1 | Ath-AT3G18915.1 |  | | | |  |  |  |  |  |  |  |
| 1 | Ath-AT3G18940.1 |  | Vvi-Vitvi03g00396\_t001 |  |  |  |  |  |  |  |
| 1 | Ath-AT3G18950.1 |  | Vvi-Vitvi03g00397\_t001 |  |  |  |  |  |  |  |
| 0 | Ath-AT3G18957.1 |  |  |  |  |  |  |  |  |
| 1 | Ath-AT3G18960.1 |  | Vvi-Vitvi03g00416\_t001 |  |  |  |  |  |  |  |
| 1 | Ath-AT3G18970.1 |  | | | |  |  |  |  |  |  |  |
| 1 | Ath-AT3G18980.1 |  | | | |  |  |  |  |  |  |  |
| 1 | Ath-AT3G18990.2 |  | | | |  |  |  |  |  |  |  |
| 1 | Ath-AT3G19000.1 |  | | | |  |  |  |  |  |  |  |
| 1 | Ath-AT3G19010.1 |  | | | |  |  |  |  |  |  |  |
| 1 | Ath-AT3G19020.1 |  | Vvi-Vitvi03g00427\_t001 |  |  |  |  |  |  |  |
| 1 | Ath-AT3G19030.1 |  | | | |  |  |  |  |  |  |  |
| 1 | Ath-AT3G19035.1 |  | | | |  |  |  |  |  |  |  |
| 1 | Ath-AT3G19040.2 |  | | | |  |  |  |  |  |  |  |
| 1 | Ath-AT3G19050.1 |  | Vvi-Vitvi03g00447\_t001 |  |  |  |  |  |  |  |
| 1 | Ath-AT3G19055.1 |  | | | |  |  |  |  |  |  |  |
| 1 | Ath-AT3G19070.1 |  | | | |  |  |  |  |  |  |  |
| 1 | Ath-AT3G19080.3 |  | Vvi-Vitvi03g04170\_t001 |  |  |  |  |  |  |  |
| 1 | Ath-AT3G19085.2 |  | | | |  |  |  |  |  |  |  |
| 1 | Ath-AT3G19090.2 |  | Vvi-Vitvi03g00455\_t001 |  |  |  |  |  |  |  |
| 1 | Ath-AT3G19100.1 |  | Vvi-Vitvi03g00464\_t001 |  |  |  |  |  |  |  |
| 1 | Ath-AT3G19120.1 |  | Vvi-Vitvi03g00469\_t001 |  |  |  |  |  |  |  |
| 1 | Ath-AT3G19130.1 |  | Vvi-Vitvi03g00476\_t002 |  |  |  |  |  |  |  |
| 1 | Ath-AT3G19140.1 |  | | | |  |  |  |  |  |  |  |
| 1 | Ath-AT3G19150.1 |  | | | |  |  |  |  |  |  |  |
| 1 | Ath-AT3G19160.1 |  | | | |  |  |  |  |  |  |  |
| 1 | Ath-AT3G19170.1 |  | Vvi-Vitvi03g00485\_t001 |  |  |  |  |  |  |  |
| 1 | Ath-AT3G19180.1 |  | | | |  |  |  |  |  |  |  |
| 1 | Ath-AT3G19184.2 |  | Vvi-Vitvi03g04176\_t001 |  |  |  |  |  |  |  |
| 1 | Ath-AT3G19190.2 |  | Vvi-Vitvi03g00492\_t001 |  |  |  |  |  |  |  |
| 1 | Ath-AT3G19200.1 |  | | | |  |  |  |  |  |  |  |
| 1 | Ath-AT3G19210.1 |  | Vvi-Vitvi03g00493\_t001 |  |  |  |  |  |  |  |
| 1 | Ath-AT3G19220.1 |  | Vvi-Vitvi03g01568\_t001 |  |  |  |  |  |  |  |
| 1 | Ath-AT3G19230.1 |  | Vvi-Vitvi03g00497\_t001 |  |  |  |  |  |  |  |
| 1 | Ath-AT3G19240.1 |  | Vvi-Vitvi03g00499\_t001 |  |  |  |  |  |  |  |
| 1 | Ath-AT3G19250.1 |  | | | |  |  |  |  |  |  |  |
| 1 | Ath-AT3G19260.1 |  | Vvi-Vitvi03g00505\_t001 |  |  |  |  |  |  |  |
| 1 | Ath-AT3G19270.2 |  | Vvi-Vitvi03g00508\_t001 |  |  |  |  |  |  |  |
| 1 | Ath-AT3G19274.1 |  | | | |  |  |  |  |  |  |  |
| 1 | Ath-AT3G19280.1 |  | Vvi-Vitvi03g00510\_t001 |  |  |  |  |  |  |  |
| 1 | Ath-AT3G19290.3 |  | Vvi-Vitvi03g01574\_t003 |  |  |  |  |  |  |  |
| 1 | Ath-AT3G19300.1 |  | Vvi-Vitvi03g00516\_t002 |  |  |  |  |  |  |  |
| 1 | Ath-AT3G19310.1 |  | Vvi-Vitvi03g00517\_t001 |  |  |  |  |  |  |  |
| 1 | Ath-AT3G19320.1 |  | | | |  |  |  |  |  |  |  |
| 1 | Ath-AT3G19330.1 |  | | | |  |  |  |  |  |  |  |
| 1 | Ath-AT3G19340.1 |  | | | |  |  |  |  |  |  |  |
| 1 | Ath-AT3G19350.1 |  | | | |  |  |  |  |  |  |  |
| 1 | Ath-AT3G19360.1 |  | | | |  |  |  |  |  |  |  |
| 1 | Ath-AT3G19370.1 |  | Vvi-Vitvi03g00529\_t002 |  |  |  |  |  |  |  |
| 1 | Ath-AT3G19380.1 |  | | | |  |  |  |  |  |  |  |
| 1 | Ath-AT3G19390.1 |  | Vvi-Vitvi03g00539\_t001 |  |  |  |  |  |  |  |
| 1 | Ath-AT3G19400.1 |  | | | |  |  |  |  |  |  |  |
| 1 | Ath-AT3G19410.1 |  | | | |  |  |  |  |  |  |  |
| 1 | Ath-AT3G19420.1 |  | Vvi-Vitvi03g00540\_t001 |  |  |  |  |  |  |  |
| 1 | Ath-AT3G19430.2 |  | Vvi-Vitvi03g04187\_t001 |  |  |  |  |  |  |  |
| 1 | Ath-AT3G19440.1 |  | | | |  |  |  |  |  |  |  |
| 1 | Ath-AT3G19450.1 |  | Vvi-Vitvi03g00561\_t001 |  |  |  |  |  |  |  |
| 1 | Ath-AT3G19460.3 |  | | | |  |  |  |  |  |  |  |
| 1 | Ath-AT3G19470.2 |  | | | |  |  |  |  |  |  |  |
| 1 | Ath-AT3G19480.1 |  | | | |  |  |  |  |  |  |  |
| 1 | Ath-AT3G19490.1 |  | | | |  |  |  |  |  |  |  |
| 1 | Ath-AT3G19500.1 |  | Vvi-Vitvi03g04201\_t001 |  |  |  |  |  |  |  |
| 1 | Ath-AT3G19508.1 |  | Vvi-Vitvi03g00579\_t001 |  |  |  |  |  |  |  |
| 1 | Ath-AT3G19510.1 |  | Vvi-Vitvi03g01600\_t001 |  |  |  |  |  |  |  |
| 1 | Ath-AT3G19515.2 |  | | | |  |  |  |  |  |  |  |
| 1 | Ath-AT3G19520.3 |  | | | |  |  |  |  |  |  |  |
| 1 | Ath-AT3G19530.1 |  | | | |  |  |  |  |  |  |  |
| 1 | Ath-AT3G19540.1 |  | Vvi-Vitvi03g00597\_t001 |  |  |  |  |  |  |  |
| 1 | Ath-AT3G19550.1 |  | Vvi-Vitvi03g04204\_t001 |  |  |  |  |  |  |  |
| 1 | Ath-AT3G19552.1 |  | | | |  |  |  |  |  |  |  |
| 1 | Ath-AT3G19553.1 |  | Vvi-Vitvi03g00619\_t001 |  |  |  |  |  |  |  |
| 1 | Ath-AT3G19560.1 |  | | | |  |  |  |  |  |  |  |
| 1 | Ath-AT3G19565.1 |  | | | |  |  |  |  |  |  |  |
| 1 | Ath-AT3G19570.2 |  | Vvi-Vitvi03g00626\_t001 |  |  |  |  |  |  |  |
| 1 | Ath-AT3G19580.1 |  | Vvi-Vitvi03g00630\_t001 |  |  |  |  |  |  |  |
| 1 | Ath-AT3G19590.1 |  | Vvi-Vitvi03g00633\_t001 |  |  |  |  |  |  |  |
| 1 | Ath-AT3G19595.1 |  | | | |  |  |  |  |  |  |  |
| 1 | Ath-AT3G19600.1 |  | | | |  |  |  |  |  |  |  |
| 1 | Ath-AT3G19610.1 |  | | | |  |  |  |  |  |  |  |
| 1 | Ath-AT3G19613.1 |  | | | |  |  |  |  |  |  |  |
| 1 | Ath-AT3G19615.1 |  | | | |  |  |  |  |  |  |  |
| 1 | Ath-AT3G19620.1 |  | Vvi-Vitvi03g00638\_t001 |  |  |  |  |  |  |  |
| 1 | Ath-AT3G19630.1 |  | Vvi-Vitvi03g00646\_t001 |  |  |  |  |  |  |  |
| 1 | Ath-AT3G19640.1 |  | | | |  |  |  |  |  |  |  |
| 1 | Ath-AT3G19650.1 |  | Vvi-Vitvi03g00647\_t001 |  |  |  |  |  |  |  |
| 0 | Ath-AT3G19660.1 |  |  |  |  |  |  |  |  |
| 0 | Ath-AT3G19663.1 |  |  |  |  |  |  |  |  |
| 0 | Ath-AT3G19670.2 |  |  |  |  |  |  |  |  |
| 0 | Ath-AT3G19680.1 |  |  |  |  |  |  |  |  |
| 0 | Ath-AT3G19690.1 |  |  |  |  |  |  |  |  |
| 0 | Ath-AT3G19700.1 |  |  |  |  |  |  |  |  |
| 0 | Ath-AT3G19710.1 |  |  |  |  |  |  |  |  |
| 0 | Ath-AT3G19720.1 |  |  |  |  |  |  |  |  |
| 0 | Ath-AT3G19740.1 |  |  |  |  |  |  |  |  |
| 0 | Ath-AT3G19760.1 |  |  |  |  |  |  |  |  |
| 0 | Ath-AT3G19770.1 |  |  |  |  |  |  |  |  |
| 0 | Ath-AT3G19780.1 |  |  |  |  |  |  |  |  |
| 0 | Ath-AT3G19790.2 |  |  |  |  |  |  |  |  |
| 0 | Ath-AT3G19800.1 |  |  |  |  |  |  |  |  |
| 0 | Ath-AT3G19810.1 |  |  |  |  |  |  |  |  |
| 0 | Ath-AT3G19820.2 |  |  |  |  |  |  |  |  |
| 0 | Ath-AT3G19830.3 |  |  |  |  |  |  |  |  |
| 0 | Ath-AT3G19840.1 |  |  |  |  |  |  |  |  |
| 0 | Ath-AT3G19850.1 |  |  |  |  |  |  |  |  |
| 0 | Ath-AT3G19860.2 |  |  |  |  |  |  |  |  |
| 0 | Ath-AT3G19870.2 |  |  |  |  |  |  |  |  |
| 0 | Ath-AT3G19880.1 |  |  |  |  |  |  |  |  |
| 0 | Ath-AT3G19890.1 |  |  |  |  |  |  |  |  |
| 0 | Ath-AT3G19895.1 |  |  |  |  |  |  |  |  |
| 0 | Ath-AT3G19900.1 |  |  |  |  |  |  |  |  |
| 0 | Ath-AT3G19910.1 |  |  |  |  |  |  |  |  |
| 0 | Ath-AT3G19920.2 |  |  |  |  |  |  |  |  |
| 0 | Ath-AT3G19930.1 |  |  |  |  |  |  |  |  |
| 0 | Ath-AT3G19940.1 |  |  |  |  |  |  |  |  |
| 0 | Ath-AT3G19950.3 |  |  |  |  |  |  |  |  |
| 0 | Ath-AT3G19960.2 |  |  |  |  |  |  |  |  |
| 0 | Ath-AT3G19970.1 |  |  |  |  |  |  |  |  |
| 0 | Ath-AT3G19980.1 |  |  |  |  |  |  |  |  |
| 0 | Ath-AT3G19990.2 |  |  |  |  |  |  |  |  |
| 0 | Ath-AT3G20000.1 |  |  |  |  |  |  |  |  |
| 0 | Ath-AT3G20010.1 |  |  |  |  |  |  |  |  |
| 0 | Ath-AT3G20015.1 |  |  |  |  |  |  |  |  |
| 0 | Ath-AT3G20020.3 |  |  |  |  |  |  |  |  |
| 0 | Ath-AT3G20030.1 |  |  |  |  |  |  |  |  |
| 0 | Ath-AT3G20040.1 |  |  |  |  |  |  |  |  |
| 0 | Ath-AT3G20050.1 |  |  |  |  |  |  |  |  |
| 0 | Ath-AT3G20060.1 |  |  |  |  |  |  |  |  |
| 0 | Ath-AT3G20070.1 |  |  |  |  |  |  |  |  |
| 0 | Ath-AT3G20080.1 |  |  |  |  |  |  |  |  |
| 0 | Ath-AT3G20090.2 |  |  |  |  |  |  |  |  |
| 0 | Ath-AT3G20100.1 |  |  |  |  |  |  |  |  |
| 0 | Ath-AT3G20110.1 |  |  |  |  |  |  |  |  |
| 0 | Ath-AT3G20120.4 |  |  |  |  |  |  |  |  |
| 0 | Ath-AT3G20130.1 |  |  |  |  |  |  |  |  |
| 0 | Ath-AT3G20140.1 |  |  |  |  |  |  |  |  |
| 0 | Ath-AT3G20150.1 |  |  |  |  |  |  |  |  |
| 0 | Ath-AT3G20155.1 |  |  |  |  |  |  |  |  |
| 0 | Ath-AT3G20160.1 |  |  |  |  |  |  |  |  |
| 0 | Ath-AT3G20170.1 |  |  |  |  |  |  |  |  |
| 0 | Ath-AT3G20180.2 |  |  |  |  |  |  |  |  |
| 0 | Ath-AT3G20190.1 |  |  |  |  |  |  |  |  |
| 0 | Ath-AT3G20200.1 |  |  |  |  |  |  |  |  |
| 1 | Ath-AT3G20210.1 |  | Vvi-Vitvi09g01380\_t001 |  |  |  |  |  |  |  |
| 1 | Ath-AT3G20220.1 |  | Vvi-Vitvi09g01385\_t001 |  |  |  |  |  |  |  |
| 1 | Ath-AT3G20230.1 |  | | | |  |  |  |  |  |  |  |
| 1 | Ath-AT3G20240.1 |  | Vvi-Vitvi09g01388\_t002 |  |  |  |  |  |  |  |
| 1 | Ath-AT3G20250.2 |  | Vvi-Vitvi09g01389\_t001 |  |  |  |  |  |  |  |
| 1 | Ath-AT3G20260.1 |  | Vvi-Vitvi09g01390\_t001 |  |  |  |  |  |  |  |
| 2 | Ath-AT3G20270.3 |  | Vvi-Vitvi09g04580\_t001 |  | Vvi-Vitvi19g00576\_t001 |  |  |  |  |  |  |
| 1 | Ath-AT3G20280.1 |  |  |  | Vvi-Vitvi19g00575\_t001 |  |  |  |  |  |  |
| 1 | Ath-AT3G20290.2 |  |  |  | Vvi-Vitvi19g00561\_t001 |  |  |  |  |  |  |
| 1 | Ath-AT3G20300.1 |  |  |  | Vvi-Vitvi19g00559\_t001 |  |  |  |  |  |  |
| 1 | Ath-AT3G20310.1 |  |  |  | Vvi-Vitvi19g00558\_t001 |  |  |  |  |  |  |
| 1 | Ath-AT3G20320.1 |  |  |  | | | |  |  |  |  |  |  |
| 1 | Ath-AT3G20330.1 |  |  |  | Vvi-Vitvi19g00552\_t001 |  |  |  |  |  |  |
| 2 | Ath-AT3G20340.1 |  | Vvi-Vitvi19g02012\_t001 |  | | | |  |  |  |  |  |  |
| 2 | Ath-AT3G20350.1 |  | Vvi-Vitvi19g00492\_t001 |  | | | |  |  |  |  |  |  |
| 2 | Ath-AT3G20360.1 |  | | | |  | | | |  |  |  |  |  |  |
| 2 | Ath-AT3G20362.1 |  | | | |  | | | |  |  |  |  |  |  |
| 2 | Ath-AT3G20370.1 |  | | | |  | | | |  |  |  |  |  |  |
| 2 | Ath-AT3G20380.1 |  | | | |  | | | |  |  |  |  |  |  |
| 2 | Ath-AT3G20390.2 |  | Vvi-Vitvi19g02013\_t001 |  | | | |  |  |  |  |  |  |
| 2 | Ath-AT3G20395.1 |  | Vvi-Vitvi19g00494\_t001 |  | | | |  |  |  |  |  |  |
| 2 | Ath-AT3G20400.1 |  | | | |  | | | |  |  |  |  |  |  |
| 2 | Ath-AT3G20410.1 |  | Vvi-Vitvi19g00497\_t001 |  | | | |  |  |  |  |  |  |
| 2 | Ath-AT3G20420.1 |  | | | |  | Vvi-Vitvi19g00541\_t001 |  |  |  |  |  |  |
| 2 | Ath-AT3G20430.1 |  | | | |  | | | |  |  |  |  |  |  |
| 2 | Ath-AT3G20440.2 |  | | | |  | Vvi-Vitvi19g00538\_t001 |  |  |  |  |  |  |
| 2 | Ath-AT3G20450.1 |  | | | |  | | | |  |  |  |  |  |  |
| 2 | Ath-AT3G20460.1 |  | | | |  | | | |  |  |  |  |  |  |
| 2 | Ath-AT3G20470.1 |  | | | |  | | | |  |  |  |  |  |  |
| 2 | Ath-AT3G20475.1 |  | | | |  | Vvi-Vitvi19g00515\_t001 |  |  |  |  |  |  |
| 1 | Ath-AT3G20480.1 |  | Vvi-Vitvi19g00512\_t001 |  |  |  |  |  |  |  |
| 1 | Ath-AT3G20490.1 |  | Vvi-Vitvi19g00491\_t002 |  |  |  |  |  |  |  |
| 1 | Ath-AT3G20500.1 |  | Vvi-Vitvi19g00483\_t001 |  |  |  |  |  |  |  |
| 1 | Ath-AT3G20510.1 |  | Vvi-Vitvi19g00480\_t001 |  |  |  |  |  |  |  |
| 1 | Ath-AT3G20520.1 |  | | | |  |  |  |  |  |  |  |
| 1 | Ath-AT3G20530.1 |  | Vvi-Vitvi19g00470\_t001 |  |  |  |  |  |  |  |
| 1 | Ath-AT3G20540.2 |  | | | |  |  |  |  |  |  |  |
| 1 | Ath-AT3G20550.1 |  | | | |  |  |  |  |  |  |  |
| 1 | Ath-AT3G20555.1 |  | | | |  |  |  |  |  |  |  |
| 1 | Ath-AT3G20557.1 |  | | | |  |  |  |  |  |  |  |
| 1 | Ath-AT3G20560.1 |  | Vvi-Vitvi19g00446\_t001 |  |  |  |  |  |  |  |
| 1 | Ath-AT3G20570.1 |  | Vvi-Vitvi19g00445\_t001 |  |  |  |  |  |  |  |
| 1 | Ath-AT3G20580.1 |  | Vvi-Vitvi19g00442\_t001 |  |  |  |  |  |  |  |
| 1 | Ath-AT3G20590.1 |  | | | |  |  |  |  |  |  |  |
| 1 | Ath-AT3G20600.1 |  | | | |  |  |  |  |  |  |  |
| 1 | Ath-AT3G20610.1 |  | | | |  |  |  |  |  |  |  |
| 1 | Ath-AT3G20620.1 |  | Vvi-Vitvi19g04215\_t001 |  |  |  |  |  |  |  |
| 0 | Ath-AT3G20630.1 |  |  |  |  |  |  |  |  |
| 1 | Ath-AT3G20640.1 |  | Vvi-Vitvi19g00367\_t001 |  |  |  |  |  |  |  |
| 1 | Ath-AT3G20650.1 |  | Vvi-Vitvi19g00372\_t001 |  |  |  |  |  |  |  |
| 1 | Ath-AT3G20660.1 |  | Vvi-Vitvi19g00378\_t002 |  |  |  |  |  |  |  |
| 1 | Ath-AT3G20670.1 |  | Vvi-Vitvi19g00401\_t001 |  |  |  |  |  |  |  |
| 1 | Ath-AT3G20680.1 |  | Vvi-Vitvi19g00407\_t001 |  |  |  |  |  |  |  |
| 1 | Ath-AT3G20690.1 |  | | | |  |  |  |  |  |  |  |
| 1 | Ath-AT3G20700.1 |  | | | |  |  |  |  |  |  |  |
| 1 | Ath-AT3G20710.1 |  | | | |  |  |  |  |  |  |  |
| 1 | Ath-AT3G20720.2 |  | Vvi-Vitvi19g00416\_t001 |  |  |  |  |  |  |  |
| 1 | Ath-AT3G20730.1 |  | | | |  |  |  |  |  |  |  |
| 1 | Ath-AT3G20740.1 |  | Vvi-Vitvi19g00417\_t001 |  |  |  |  |  |  |  |
| 0 | Ath-AT3G20750.1 |  |  |  |  |  |  |  |  |
| 0 | Ath-AT3G20760.1 |  |  |  |  |  |  |  |  |
| 0 | Ath-AT3G20770.1 |  |  |  |  |  |  |  |  |
| 1 | Ath-AT3G20780.1 |  | Vvi-Vitvi06g00160\_t001 |  |  |  |  |  |  |  |
| 1 | Ath-AT3G20790.1 |  | Vvi-Vitvi06g00161\_t001 |  |  |  |  |  |  |  |
| 1 | Ath-AT3G20800.1 |  | | | |  |  |  |  |  |  |  |
| 1 | Ath-AT3G20810.2 |  | Vvi-Vitvi06g00167\_t001 |  |  |  |  |  |  |  |
| 1 | Ath-AT3G20820.1 |  | Vvi-Vitvi06g00182\_t001 |  |  |  |  |  |  |  |
| 1 | Ath-AT3G20830.1 |  | Vvi-Vitvi06g00185\_t001 |  |  |  |  |  |  |  |
| 1 | Ath-AT3G20840.1 |  | Vvi-Vitvi06g00187\_t001 |  |  |  |  |  |  |  |
| 1 | Ath-AT3G20850.1 |  | | | |  |  |  |  |  |  |  |
| 1 | Ath-AT3G20860.1 |  | Vvi-Vitvi06g00191\_t002 |  |  |  |  |  |  |  |
| 1 | Ath-AT3G20865.1 |  | | | |  |  |  |  |  |  |  |
| 1 | Ath-AT3G20870.1 |  | Vvi-Vitvi06g00193\_t001 |  |  |  |  |  |  |  |
| 1 | Ath-AT3G20880.1 |  | Vvi-Vitvi06g00195\_t001 |  |  |  |  |  |  |  |
| 1 | Ath-AT3G20890.1 |  | Vvi-Vitvi06g00223\_t001 |  |  |  |  |  |  |  |
| 1 | Ath-AT3G20898.1 |  | Vvi-Vitvi06g01635\_t001 |  |  |  |  |  |  |  |
| 1 | Ath-AT3G20900.1 |  | | | |  |  |  |  |  |  |  |
| 1 | Ath-AT3G20910.1 |  | Vvi-Vitvi06g00224\_t001 |  |  |  |  |  |  |  |
| 1 | Ath-AT3G20920.1 |  | Vvi-Vitvi06g00225\_t001 |  |  |  |  |  |  |  |
| 1 | Ath-AT3G20930.2 |  | | | |  |  |  |  |  |  |  |
| 1 | Ath-AT3G20935.1 |  | | | |  |  |  |  |  |  |  |
| 1 | Ath-AT3G20940.1 |  | | | |  |  |  |  |  |  |  |
| 1 | Ath-AT3G20950.1 |  | | | |  |  |  |  |  |  |  |
| 1 | Ath-AT3G20960.2 |  | | | |  |  |  |  |  |  |  |
| 1 | Ath-AT3G20970.1 |  | | | |  |  |  |  |  |  |  |
| 1 | Ath-AT3G20980.1 |  | | | |  |  |  |  |  |  |  |
| 1 | Ath-AT3G20993.1 |  | | | |  |  |  |  |  |  |  |
| 1 | Ath-AT3G20997.1 |  | | | |  |  |  |  |  |  |  |
| 1 | Ath-AT3G21000.1 |  | | | |  |  |  |  |  |  |  |
| 1 | Ath-AT3G21055.2 |  | Vvi-Vitvi06g01637\_t001 |  |  |  |  |  |  |  |
| 1 | Ath-AT3G21060.1 |  | | | |  |  |  |  |  |  |  |
| 1 | Ath-AT3G21070.3 |  | Vvi-Vitvi06g00236\_t001 |  |  |  |  |  |  |  |
| 1 | Ath-AT3G21080.1 |  | | | |  |  |  |  |  |  |  |
| 1 | Ath-AT3G21090.1 |  | Vvi-Vitvi06g00242\_t001 |  |  |  |  |  |  |  |
| 0 | Ath-AT3G21100.3 |  |  |  |  |  |  |  |  |
| 0 | Ath-AT3G21110.1 |  |  |  |  |  |  |  |  |
| 0 | Ath-AT3G21120.1 |  |  |  |  |  |  |  |  |
| 0 | Ath-AT3G21130.1 |  |  |  |  |  |  |  |  |
| 0 | Ath-AT3G21140.1 |  |  |  |  |  |  |  |  |
| 0 | Ath-AT3G21150.1 |  |  |  |  |  |  |  |  |
| 0 | Ath-AT3G21160.1 |  |  |  |  |  |  |  |  |
| 0 | Ath-AT3G21165.1 |  |  |  |  |  |  |  |  |
| 0 | Ath-AT3G21170.1 |  |  |  |  |  |  |  |  |
| 0 | Ath-AT3G21175.1 |  |  |  |  |  |  |  |  |
| 0 | Ath-AT3G21180.1 |  |  |  |  |  |  |  |  |
| 0 | Ath-AT3G21190.1 |  |  |  |  |  |  |  |  |
| 0 | Ath-AT3G21200.1 |  |  |  |  |  |  |  |  |
| 0 | Ath-AT3G21210.1 |  |  |  |  |  |  |  |  |
| 1 | Ath-AT3G21215.1 |  | Vvi-Vitvi09g00202\_t001 |  |  |  |  |  |  |  |
| 1 | Ath-AT3G21220.1 |  | | | |  |  |  |  |  |  |  |
| 1 | Ath-AT3G21230.1 |  | | | |  |  |  |  |  |  |  |
| 1 | Ath-AT3G21240.1 |  | | | |  |  |  |  |  |  |  |
| 1 | Ath-AT3G21250.4 |  | Vvi-Vitvi09g04050\_t001 |  |  |  |  |  |  |  |
| 1 | Ath-AT3G21260.3 |  | | | |  |  |  |  |  |  |  |
| 1 | Ath-AT3G21270.1 |  | Vvi-Vitvi09g00210\_t001 |  |  |  |  |  |  |  |
| 1 | Ath-AT3G21280.1 |  | Vvi-Vitvi09g00211\_t001 |  |  |  |  |  |  |  |
| 1 | Ath-AT3G21290.1 |  | Vvi-Vitvi09g00212\_t001 |  |  |  |  |  |  |  |
| 1 | Ath-AT3G21295.1 |  | Vvi-Vitvi09g00216\_t001 |  |  |  |  |  |  |  |
| 1 | Ath-AT3G21300.1 |  | | | |  |  |  |  |  |  |  |
| 1 | Ath-AT3G21305.1 |  | | | |  |  |  |  |  |  |  |
| 1 | Ath-AT3G21310.1 |  | Vvi-Vitvi09g00223\_t001 |  |  |  |  |  |  |  |
| 1 | Ath-AT3G21320.1 |  | Vvi-Vitvi09g00225\_t001 |  |  |  |  |  |  |  |
| 1 | Ath-AT3G21330.1 |  | Vvi-Vitvi09g00227\_t001 |  |  |  |  |  |  |  |
| 0 | Ath-AT3G21340.1 |  |  |  |  |  |  |  |  |
| 0 | Ath-AT3G21350.2 |  |  |  |  |  |  |  |  |
| 0 | Ath-AT3G21351.1 |  |  |  |  |  |  |  |  |
| 0 | Ath-AT3G21352.1 |  |  |  |  |  |  |  |  |
| 0 | Ath-AT3G21360.1 |  |  |  |  |  |  |  |  |
| 0 | Ath-AT3G21370.1 |  |  |  |  |  |  |  |  |
| 0 | Ath-AT3G21371.1 |  |  |  |  |  |  |  |  |
| 0 | Ath-AT3G21380.1 |  |  |  |  |  |  |  |  |
| 0 | Ath-AT3G21390.2 |  |  |  |  |  |  |  |  |
| 0 | Ath-AT3G21400.1 |  |  |  |  |  |  |  |  |
| 0 | Ath-AT3G21405.1 |  |  |  |  |  |  |  |  |
| 0 | Ath-AT3G21410.1 |  |  |  |  |  |  |  |  |
| 0 | Ath-AT3G21420.1 |  |  |  |  |  |  |  |  |
| 0 | Ath-AT3G21430.2 |  |  |  |  |  |  |  |  |
| 0 | Ath-AT3G21450.1 |  |  |  |  |  |  |  |  |
| 0 | Ath-AT3G21460.1 |  |  |  |  |  |  |  |  |
| 0 | Ath-AT3G21465.2 |  |  |  |  |  |  |  |  |
| 0 | Ath-AT3G21470.1 |  |  |  |  |  |  |  |  |
| 0 | Ath-AT3G21473.1 |  |  |  |  |  |  |  |  |
| 1 | Ath-AT3G21480.1 |  | Vvi-Vitvi05g00367\_t001 |  |  |  |  |  |  |  |
| 1 | Ath-AT3G21490.1 |  | Vvi-Vitvi05g01867\_t001 |  |  |  |  |  |  |  |
| 1 | Ath-AT3G21500.3 |  | Vvi-Vitvi05g00372\_t001 |  |  |  |  |  |  |  |
| 1 | Ath-AT3G21510.1 |  | Vvi-Vitvi05g00382\_t001 |  |  |  |  |  |  |  |
| 1 | Ath-AT3G21520.1 |  | Vvi-Vitvi05g00385\_t001 |  |  |  |  |  |  |  |
| 1 | Ath-AT3G21530.1 |  | Vvi-Vitvi05g00386\_t001 |  |  |  |  |  |  |  |
| 1 | Ath-AT3G21540.1 |  | | | |  |  |  |  |  |  |  |
| 1 | Ath-AT3G21550.1 |  | | | |  |  |  |  |  |  |  |
| 1 | Ath-AT3G21560.1 |  | | | |  |  |  |  |  |  |  |
| 1 | Ath-AT3G21570.1 |  | | | |  |  |  |  |  |  |  |
| 1 | Ath-AT3G21580.1 |  | Vvi-Vitvi05g00401\_t001 |  |  |  |  |  |  |  |
| 0 | Ath-AT3G21590.3 |  |  |  |  |  |  |  |  |
| 0 | Ath-AT3G21600.1 |  |  |  |  |  |  |  |  |
| 1 | Ath-AT3G21610.1 |  | Vvi-Vitvi12g00409\_t001 |  |  |  |  |  |  |  |
| 1 | Ath-AT3G21620.1 |  | Vvi-Vitvi12g00410\_t001 |  |  |  |  |  |  |  |
| 1 | Ath-AT3G21630.1 |  | Vvi-Vitvi12g00415\_t001 |  |  |  |  |  |  |  |
| 1 | Ath-AT3G21640.1 |  | | | |  |  |  |  |  |  |  |
| 1 | Ath-AT3G21650.2 |  | Vvi-Vitvi12g00422\_t001 |  |  |  |  |  |  |  |
| 1 | Ath-AT3G21660.2 |  | Vvi-Vitvi12g00425\_t001 |  |  |  |  |  |  |  |
| 1 | Ath-AT3G21670.1 |  | Vvi-Vitvi12g00429\_t001 |  |  |  |  |  |  |  |
| 0 | Ath-AT3G21680.1 |  |  |  |  |  |  |  |  |
| 1 | Ath-AT3G21690.1 |  | Vvi-Vitvi12g00506\_t001 |  |  |  |  |  |  |  |
| 1 | Ath-AT3G21700.3 |  | Vvi-Vitvi12g00519\_t002 |  |  |  |  |  |  |  |
| 1 | Ath-AT3G21710.2 |  | Vvi-Vitvi12g02414\_t001 |  |  |  |  |  |  |  |
| 1 | Ath-AT3G21720.1 |  | Vvi-Vitvi12g00526\_t001 |  |  |  |  |  |  |  |
| 1 | Ath-AT3G21730.1 |  | | | |  |  |  |  |  |  |  |
| 1 | Ath-AT3G21740.1 |  | Vvi-Vitvi12g00529\_t001 |  |  |  |  |  |  |  |
| 1 | Ath-AT3G21750.1 |  | | | |  |  |  |  |  |  |  |
| 1 | Ath-AT3G21760.1 |  | | | |  |  |  |  |  |  |  |
| 1 | Ath-AT3G21770.1 |  | Vvi-Vitvi12g00533\_t001 |  |  |  |  |  |  |  |
| 1 | Ath-AT3G21780.1 |  | | | |  |  |  |  |  |  |  |
| 1 | Ath-AT3G21790.1 |  | | | |  |  |  |  |  |  |  |
| 1 | Ath-AT3G21800.1 |  | | | |  |  |  |  |  |  |  |
| 1 | Ath-AT3G21810.1 |  | Vvi-Vitvi12g00535\_t001 |  |  |  |  |  |  |  |
| 1 | Ath-AT3G21820.1 |  | Vvi-Vitvi12g00537\_t001 |  |  |  |  |  |  |  |
| 1 | Ath-AT3G21825.1 |  | | | |  |  |  |  |  |  |  |
| 1 | Ath-AT3G21830.1 |  | | | |  |  |  |  |  |  |  |
| 1 | Ath-AT3G21840.1 |  | | | |  |  |  |  |  |  |  |
| 1 | Ath-AT3G21850.1 |  | | | |  |  |  |  |  |  |  |
| 1 | Ath-AT3G21860.1 |  | | | |  |  |  |  |  |  |  |
| 1 | Ath-AT3G21865.1 |  | Vvi-Vitvi12g00538\_t001 |  |  |  |  |  |  |  |
| 1 | Ath-AT3G21870.1 |  | Vvi-Vitvi12g00540\_t001 |  |  |  |  |  |  |  |
| 1 | Ath-AT3G21880.2 |  | Vvi-Vitvi12g00542\_t001 |  |  |  |  |  |  |  |
| 1 | Ath-AT3G21890.1 |  | Vvi-Vitvi12g00543\_t001 |  |  |  |  |  |  |  |
| 1 | Ath-AT3G21900.2 |  | | | |  |  |  |  |  |  |  |
| 1 | Ath-AT3G21910.1 |  | | | |  |  |  |  |  |  |  |
| 1 | Ath-AT3G21920.2 |  | | | |  |  |  |  |  |  |  |
| 1 | Ath-AT3G21930.1 |  | | | |  |  |  |  |  |  |  |
| 1 | Ath-AT3G21940.1 |  | Vvi-Vitvi12g02417\_t001 |  |  |  |  |  |  |  |
| 1 | Ath-AT3G21945.1 |  | | | |  |  |  |  |  |  |  |
| 1 | Ath-AT3G21950.2 |  | | | |  |  |  |  |  |  |  |
| 1 | Ath-AT3G21960.1 |  | | | |  |  |  |  |  |  |  |
| 1 | Ath-AT3G21970.1 |  | | | |  |  |  |  |  |  |  |
| 1 | Ath-AT3G21980.1 |  | | | |  |  |  |  |  |  |  |
| 1 | Ath-AT3G21990.1 |  | | | |  |  |  |  |  |  |  |
| 1 | Ath-AT3G22000.1 |  | | | |  |  |  |  |  |  |  |
| 1 | Ath-AT3G22010.1 |  | | | |  |  |  |  |  |  |  |
| 1 | Ath-AT3G22020.1 |  | | | |  |  |  |  |  |  |  |
| 1 | Ath-AT3G22022.1 |  | | | |  |  |  |  |  |  |  |
| 1 | Ath-AT3G22030.1 |  | | | |  |  |  |  |  |  |  |
| 1 | Ath-AT3G22040.1 |  | | | |  |  |  |  |  |  |  |
| 1 | Ath-AT3G22050.1 |  | | | |  |  |  |  |  |  |  |
| 1 | Ath-AT3G22053.1 |  | | | |  |  |  |  |  |  |  |
| 1 | Ath-AT3G22057.1 |  | | | |  |  |  |  |  |  |  |
| 1 | Ath-AT3G22060.1 |  | | | |  |  |  |  |  |  |  |
| 1 | Ath-AT3G22070.1 |  | Vvi-Vitvi12g02420\_t001 |  |  |  |  |  |  |  |
| 1 | Ath-AT3G22080.1 |  | | | |  |  |  |  |  |  |  |
| 1 | Ath-AT3G22085.1 |  | | | |  |  |  |  |  |  |  |
| 1 | Ath-AT3G22090.1 |  | | | |  |  |  |  |  |  |  |
| 1 | Ath-AT3G22100.1 |  | Vvi-Vitvi12g00567\_t001 |  |  |  |  |  |  |  |
| 1 | Ath-AT3G22104.1 |  | Vvi-Vitvi12g00572\_t001 |  |  |  |  |  |  |  |
| 0 | Ath-AT3G22110.1 |  |  |  |  |  |  |  |  |
| 0 | Ath-AT3G22120.2 |  |  |  |  |  |  |  |  |
| 0 | Ath-AT3G22142.1 |  |  |  |  |  |  |  |  |
| 0 | Ath-AT3G22125.3 |  |  |  |  |  |  |  |  |
| 0 | Ath-AT3G22150.2 |  |  |  |  |  |  |  |  |
| 0 | Ath-AT3G22160.1 |  |  |  |  |  |  |  |  |
| 0 | Ath-AT3G22170.2 |  |  |  |  |  |  |  |  |
| 0 | Ath-AT3G22180.1 |  |  |  |  |  |  |  |  |
| 0 | Ath-AT3G22190.2 |  |  |  |  |  |  |  |  |
| 0 | Ath-AT3G22183.1 |  |  |  |  |  |  |  |  |
| 0 | Ath-AT3G22200.2 |  |  |  |  |  |  |  |  |
| 0 | Ath-AT3G22210.1 |  |  |  |  |  |  |  |  |
| 0 | Ath-AT3G22220.1 |  |  |  |  |  |  |  |  |
| 0 | Ath-AT3G22230.1 |  |  |  |  |  |  |  |  |
| 0 | Ath-AT3G22231.1 |  |  |  |  |  |  |  |  |
| 0 | Ath-AT3G22235.1 |  |  |  |  |  |  |  |  |
| 0 | Ath-AT3G22240.1 |  |  |  |  |  |  |  |  |
| 0 | Ath-AT3G22250.1 |  |  |  |  |  |  |  |  |
| 0 | Ath-AT3G22260.4 |  |  |  |  |  |  |  |  |
| 0 | Ath-AT3G22270.1 |  |  |  |  |  |  |  |  |
| 0 | Ath-AT3G22275.1 |  |  |  |  |  |  |  |  |
| 0 | Ath-AT3G22290.1 |  |  |  |  |  |  |  |  |
| 0 | Ath-AT3G22300.1 |  |  |  |  |  |  |  |  |
| 0 | Ath-AT3G22310.1 |  |  |  |  |  |  |  |  |
| 0 | Ath-AT3G22320.1 |  |  |  |  |  |  |  |  |
| 0 | Ath-AT3G22330.1 |  |  |  |  |  |  |  |  |
| 0 | Ath-AT3G22333.1 |  |  |  |  |  |  |  |  |
| 0 | Ath-AT3G22337.1 |  |  |  |  |  |  |  |  |
| 0 | Ath-AT3G22345.1 |  |  |  |  |  |  |  |  |
| 0 | Ath-AT3G22350.1 |  |  |  |  |  |  |  |  |
| 0 | Ath-AT3G22360.1 |  |  |  |  |  |  |  |  |
| 0 | Ath-AT3G22370.1 |  |  |  |  |  |  |  |  |
| 2 | Ath-AT3G22380.2 |  | Vvi-Vitvi07g00131\_t001 |  | Vvi-Vitvi05g00469\_t001 |  |  |  |  |  |  |
| 2 | Ath-AT3G22400.1 |  | | | |  | Vvi-Vitvi05g00472\_t001 |  |  |  |  |  |  |
| 2 | Ath-AT3G22410.1 |  | | | |  | Vvi-Vitvi05g00487\_t001 |  |  |  |  |  |  |
| 2 | Ath-AT3G22415.1 |  | | | |  | | | |  |  |  |  |  |  |
| 2 | Ath-AT3G22420.3 |  | | | |  | Vvi-Vitvi05g00492\_t001 |  |  |  |  |  |  |
| 2 | Ath-AT3G22421.1 |  | | | |  | | | |  |  |  |  |  |  |
| 2 | Ath-AT3G22425.2 |  | | | |  | Vvi-Vitvi05g00497\_t001 |  |  |  |  |  |  |
| 2 | Ath-AT3G22430.1 |  | | | |  | Vvi-Vitvi05g00500\_t001 |  |  |  |  |  |  |
| 2 | Ath-AT3G22436.1 |  | | | |  | | | |  |  |  |  |  |  |
| 2 | Ath-AT3G22440.1 |  | | | |  | Vvi-Vitvi05g00503\_t001 |  |  |  |  |  |  |
| 2 | Ath-AT3G22450.1 |  | | | |  | Vvi-Vitvi05g00504\_t001 |  |  |  |  |  |  |
| 2 | Ath-AT3G22460.1 |  | | | |  | | | |  |  |  |  |  |  |
| 2 | Ath-AT3G22470.1 |  | | | |  | | | |  |  |  |  |  |  |
| 2 | Ath-AT3G22480.1 |  | | | |  | Vvi-Vitvi05g00506\_t002 |  |  |  |  |  |  |
| 2 | Ath-AT3G22490.1 |  | Vvi-Vitvi07g00117\_t001 |  | Vvi-Vitvi05g00510\_t001 |  |  |  |  |  |  |
| 2 | Ath-AT3G22500.1 |  | | | |  | | | |  |  |  |  |  |  |
| 2 | Ath-AT3G22510.1 |  | | | |  | Vvi-Vitvi05g01890\_t001 |  |  |  |  |  |  |
| 2 | Ath-AT3G22520.1 |  | | | |  | Vvi-Vitvi05g04120\_t001 |  |  |  |  |  |  |
| 2 | Ath-AT3G22530.1 |  | Vvi-Vitvi07g00116\_t001 |  | Vvi-Vitvi05g00517\_t001 |  |  |  |  |  |  |
| 2 | Ath-AT3G22540.1 |  | | | |  | Vvi-Vitvi05g00520\_t001 |  |  |  |  |  |  |
| 2 | Ath-AT3G22550.1 |  | Vvi-Vitvi07g00114\_t001 |  | Vvi-Vitvi05g04122\_t001 |  |  |  |  |  |  |
| 2 | Ath-AT3G22560.1 |  | | | |  | Vvi-Vitvi05g00523\_t001 |  |  |  |  |  |  |
| 2 | Ath-AT3G22565.2 |  | | | |  | | | |  |  |  |  |  |  |
| 2 | Ath-AT3G22570.1 |  | | | |  | | | |  |  |  |  |  |  |
| 2 | Ath-AT3G22580.1 |  | | | |  | | | |  |  |  |  |  |  |
| 2 | Ath-AT3G22590.1 |  | | | |  | Vvi-Vitvi05g00526\_t001 |  |  |  |  |  |  |
| 2 | Ath-AT3G22600.1 |  | Vvi-Vitvi07g00110\_t001 |  | Vvi-Vitvi05g00527\_t002 |  |  |  |  |  |  |
| 2 | Ath-AT3G22620.1 |  | | | |  | Vvi-Vitvi05g00529\_t001 |  |  |  |  |  |  |
| 2 | Ath-AT3G22630.1 |  | Vvi-Vitvi07g00109\_t001 |  | | | |  |  |  |  |  |  |
| 2 | Ath-AT3G22640.1 |  | Vvi-Vitvi07g00107\_t001 |  | | | |  |  |  |  |  |  |
| 2 | Ath-AT3G22650.2 |  | | | |  | | | |  |  |  |  |  |  |
| 2 | Ath-AT3G22660.1 |  | | | |  | | | |  |  |  |  |  |  |
| 2 | Ath-AT3G22680.1 |  | | | |  | | | |  |  |  |  |  |  |
| 2 | Ath-AT3G22670.1 |  | | | |  | Vvi-Vitvi05g00535\_t001 |  |  |  |  |  |  |
| 2 | Ath-AT3G22690.1 |  | | | |  | Vvi-Vitvi05g00538\_t001 |  |  |  |  |  |  |
| 2 | Ath-AT3G22700.1 |  | | | |  | | | |  |  |  |  |  |  |
| 2 | Ath-AT3G22710.1 |  | | | |  | | | |  |  |  |  |  |  |
| 2 | Ath-AT3G22720.1 |  | | | |  | | | |  |  |  |  |  |  |
| 2 | Ath-AT3G22723.1 |  | | | |  | | | |  |  |  |  |  |  |
| 2 | Ath-AT3G22730.1 |  | | | |  | | | |  |  |  |  |  |  |
| 2 | Ath-AT3G22740.1 |  | | | |  | Vvi-Vitvi05g00539\_t003 |  |  |  |  |  |  |
| 2 | Ath-AT3G22750.1 |  | Vvi-Vitvi07g04032\_t001 |  | Vvi-Vitvi05g00540\_t001 |  |  |  |  |  |  |
| 2 | Ath-AT3G22760.1 |  | | | |  | Vvi-Vitvi05g00541\_t001 |  |  |  |  |  |  |
| 2 | Ath-AT3G22770.1 |  | | | |  | | | |  |  |  |  |  |  |
| 2 | Ath-AT3G22780.1 |  | | | |  | | | |  |  |  |  |  |  |
| 2 | Ath-AT3G22790.1 |  | Vvi-Vitvi07g00097\_t002 |  | Vvi-Vitvi05g00542\_t001 |  |  |  |  |  |  |
| 2 | Ath-AT3G22800.1 |  | | | |  | Vvi-Vitvi05g00544\_t001 |  |  |  |  |  |  |
| 2 | Ath-AT3G22810.1 |  | Vvi-Vitvi07g00088\_t001 |  | Vvi-Vitvi05g00554\_t001 |  |  |  |  |  |  |
| 1 | Ath-AT3G22820.1 |  |  |  | Vvi-Vitvi05g00557\_t001 |  |  |  |  |  |  |
| 2 | Ath-AT3G22830.1 |  | Vvi-Vitvi07g00078\_t001 |  | Vvi-Vitvi05g00561\_t001 |  |  |  |  |  |  |
| 2 | Ath-AT3G22840.1 |  | | | |  | Vvi-Vitvi05g00563\_t001 |  |  |  |  |  |  |
| 2 | Ath-AT3G22845.1 |  | Vvi-Vitvi07g00074\_t001 |  | Vvi-Vitvi05g00568\_t001 |  |  |  |  |  |  |
| 2 | Ath-AT3G22850.1 |  | | | |  | Vvi-Vitvi05g00569\_t001 |  |  |  |  |  |  |
| 2 | Ath-AT3G22860.1 |  | | | |  | | | |  |  |  |  |  |  |
| 2 | Ath-AT3G22870.1 |  | | | |  | | | |  |  |  |  |  |  |
| 2 | Ath-AT3G22880.1 |  | | | |  | Vvi-Vitvi05g00570\_t001 |  |  |  |  |  |  |
| 2 | Ath-AT3G22890.1 |  | | | |  | Vvi-Vitvi05g00576\_t001 |  |  |  |  |  |  |
| 2 | Ath-AT3G22900.1 |  | | | |  | | | |  |  |  |  |  |  |
| 2 | Ath-AT3G22910.1 |  | Vvi-Vitvi07g00071\_t001 |  | Vvi-Vitvi05g00577\_t001 |  |  |  |  |  |  |
| 1 | Ath-AT3G22920.1 |  | | | |  |  |  |  |  |  |  |
| 2 | Ath-AT3G22930.1 |  | Vvi-Vitvi07g00068\_t001 |  | Vvi-Vitvi05g00605\_t001 |  |  |  |  |  |  |
| 2 | Ath-AT3G22935.1 |  | | | |  | | | |  |  |  |  |  |  |
| 2 | Ath-AT3G22940.1 |  | | | |  | | | |  |  |  |  |  |  |
| 2 | Ath-AT3G22942.1 |  | | | |  | Vvi-Vitvi05g00609\_t001 |  |  |  |  |  |  |
| 2 | Ath-AT3G22950.2 |  | | | |  | Vvi-Vitvi05g00611\_t001 |  |  |  |  |  |  |
| 2 | Ath-AT3G22960.1 |  | Vvi-Vitvi07g00062\_t001 |  | Vvi-Vitvi05g00612\_t001 |  |  |  |  |  |  |
| 2 | Ath-AT3G22961.1 |  | | | |  | | | |  |  |  |  |  |  |
| 2 | Ath-AT3G22970.1 |  | Vvi-Vitvi07g00061\_t002 |  | Vvi-Vitvi05g04150\_t001 |  |  |  |  |  |  |
| 2 | Ath-AT3G22980.2 |  | | | |  | | | |  |  |  |  |  |  |
| 2 | Ath-AT3G22990.1 |  | | | |  | Vvi-Vitvi05g00615\_t002 |  |  |  |  |  |  |
| 2 | Ath-AT3G23000.1 |  | | | |  | Vvi-Vitvi05g00618\_t001 |  |  |  |  |  |  |
| 2 | Ath-AT3G23010.1 |  | | | |  | | | |  |  |  |  |  |  |
| 2 | Ath-AT3G23020.1 |  | | | |  | Vvi-Vitvi05g00619\_t001 |  |  |  |  |  |  |
| 2 | Ath-AT3G23030.2 |  | | | |  | | | |  |  |  |  |  |  |
| 2 | Ath-AT3G23050.1 |  | Vvi-Vitvi07g00042\_t001 |  | Vvi-Vitvi05g00630\_t001 |  |  |  |  |  |  |
| 2 | Ath-AT3G23060.1 |  | Vvi-Vitvi07g00031\_t001 |  | Vvi-Vitvi05g00634\_t001 |  |  |  |  |  |  |
| 1 | Ath-AT3G23070.1 |  |  |  | Vvi-Vitvi05g00639\_t001 |  |  |  |  |  |  |
| 1 | Ath-AT3G23080.1 |  |  |  | Vvi-Vitvi05g00651\_t001 |  |  |  |  |  |  |
| 1 | Ath-AT3G23090.3 |  |  |  | Vvi-Vitvi05g00652\_t001 |  |  |  |  |  |  |
| 1 | Ath-AT3G23100.1 |  |  |  | Vvi-Vitvi05g00655\_t001 |  |  |  |  |  |  |
| 1 | Ath-AT3G23110.1 |  |  |  | | | |  |  |  |  |  |  |
| 1 | Ath-AT3G23120.1 |  |  |  | | | |  |  |  |  |  |  |
| 1 | Ath-AT3G23122.1 |  |  |  | | | |  |  |  |  |  |  |
| 1 | Ath-AT3G23123.1 |  |  |  | Vvi-Vitvi05g04160\_t001 |  |  |  |  |  |  |
| 2 | Ath-AT3G23130.1 |  | Vvi-Vitvi07g02261\_t001 |  | Vvi-Vitvi05g01913\_t001 |  |  |  |  |  |  |
| 2 | Ath-AT3G23140.1 |  | | | |  | | | |  |  |  |  |  |  |
| 2 | Ath-AT3G23145.1 |  | | | |  | | | |  |  |  |  |  |  |
| 2 | Ath-AT3G23150.1 |  | | | |  | Vvi-Vitvi05g00684\_t001 |  |  |  |  |  |  |
| 2 | Ath-AT3G23160.1 |  | | | |  | Vvi-Vitvi05g00686\_t001 |  |  |  |  |  |  |
| 2 | Ath-AT3G23165.1 |  | | | |  | | | |  |  |  |  |  |  |
| 2 | Ath-AT3G23167.1 |  | | | |  | | | |  |  |  |  |  |  |
| 2 | Ath-AT3G23170.1 |  | | | |  | Vvi-Vitvi05g01918\_t001 |  |  |  |  |  |  |
| 2 | Ath-AT3G23172.1 |  | | | |  | | | |  |  |  |  |  |  |
| 2 | Ath-AT3G23175.1 |  | | | |  | | | |  |  |  |  |  |  |
| 2 | Ath-AT3G23180.1 |  | | | |  | Vvi-Vitvi05g00692\_t001 |  |  |  |  |  |  |
| 2 | Ath-AT3G23190.1 |  | | | |  | | | |  |  |  |  |  |  |
| 2 | Ath-AT3G23200.1 |  | | | |  | Vvi-Vitvi05g00709\_t001 |  |  |  |  |  |  |
| 2 | Ath-AT3G23210.1 |  | | | |  | Vvi-Vitvi05g00711\_t001 |  |  |  |  |  |  |
| 2 | Ath-AT3G23220.1 |  | | | |  | | | |  |  |  |  |  |  |
| 2 | Ath-AT3G23230.1 |  | Vvi-Vitvi07g02062\_t001 |  | Vvi-Vitvi05g01722\_t001 |  |  |  |  |  |  |
| 1 | Ath-AT3G23240.1 |  | | | |  |  |  |  |  |  |  |
| 1 | Ath-AT3G23245.1 |  | | | |  |  |  |  |  |  |  |
| 2 | Ath-AT3G23250.1 |  | Vvi-Vitvi07g00598\_t001 |  | Vvi-Vitvi05g01732\_t001 |  |  |  |  |  |  |
| 2 | Ath-AT3G23255.2 |  | | | |  | | | |  |  |  |  |  |  |
| 2 | Ath-AT3G23260.1 |  | | | |  | | | |  |  |  |  |  |  |
| 2 | Ath-AT3G23270.2 |  | | | |  | Vvi-Vitvi05g00753\_t001 |  |  |  |  |  |  |
| 2 | Ath-AT3G23280.1 |  | | | |  | Vvi-Vitvi05g00755\_t002 |  |  |  |  |  |  |
| 2 | Ath-AT3G23290.2 |  | Vvi-Vitvi07g00615\_t001 |  | Vvi-Vitvi05g00757\_t001 |  |  |  |  |  |  |
| 2 | Ath-AT3G23300.1 |  | Vvi-Vitvi07g00621\_t002 |  | Vvi-Vitvi05g00759\_t001 |  |  |  |  |  |  |
| 2 | Ath-AT3G23310.1 |  | Vvi-Vitvi07g00626\_t001 |  | Vvi-Vitvi05g00764\_t001 |  |  |  |  |  |  |
| 2 | Ath-AT3G23320.1 |  | | | |  | | | |  |  |  |  |  |  |
| 2 | Ath-AT3G23325.1 |  | | | |  | Vvi-Vitvi05g00765\_t001 |  |  |  |  |  |  |
| 2 | Ath-AT3G23330.1 |  | | | |  | | | |  |  |  |  |  |  |
| 2 | Ath-AT3G23340.1 |  | Vvi-Vitvi07g00628\_t001 |  | Vvi-Vitvi05g00767\_t001 |  |  |  |  |  |  |
| 2 | Ath-AT3G23350.2 |  | | | |  | Vvi-Vitvi05g00769\_t001 |  |  |  |  |  |  |
| 2 | Ath-AT3G23360.1 |  | | | |  | Vvi-Vitvi05g00772\_t001 |  |  |  |  |  |  |
| 2 | Ath-AT3G23370.1 |  | | | |  | Vvi-Vitvi05g00774\_t001 |  |  |  |  |  |  |
| 2 | Ath-AT3G23380.1 |  | Vvi-Vitvi07g02274\_t001 |  | Vvi-Vitvi05g01965\_t001 |  |  |  |  |  |  |
| 2 | Ath-AT3G23390.1 |  | | | |  | | | |  |  |  |  |  |  |
| 2 | Ath-AT3G23400.1 |  | | | |  | Vvi-Vitvi05g00781\_t001 |  |  |  |  |  |  |
| 2 | Ath-AT3G23410.1 |  | Vvi-Vitvi07g00636\_t001 |  | Vvi-Vitvi05g00784\_t001 |  |  |  |  |  |  |
| 2 | Ath-AT3G23420.1 |  | | | |  | | | |  |  |  |  |  |  |
| 2 | Ath-AT3G23430.1 |  | | | |  | Vvi-Vitvi05g00785\_t001 |  |  |  |  |  |  |
| 2 | Ath-AT3G23440.1 |  | Vvi-Vitvi07g00639\_t001 |  | Vvi-Vitvi05g00788\_t001 |  |  |  |  |  |  |
| 1 | Ath-AT3G23450.1 |  |  |  | | | |  |  |  |  |  |  |
| 1 | Ath-AT3G23460.1 |  |  |  | | | |  |  |  |  |  |  |
| 1 | Ath-AT3G23470.2 |  |  |  | | | |  |  |  |  |  |  |
| 1 | Ath-AT3G23480.2 |  |  |  | | | |  |  |  |  |  |  |
| 1 | Ath-AT3G23490.1 |  |  |  | Vvi-Vitvi05g00800\_t001 |  |  |  |  |  |  |
| 1 | Ath-AT3G23510.1 |  |  |  | Vvi-Vitvi05g04205\_t001 |  |  |  |  |  |  |
| 0 | Ath-AT3G23530.1 |  |  |  |  |  |  |  |  |
| 0 | Ath-AT3G23540.1 |  |  |  |  |  |  |  |  |
| 0 | Ath-AT3G23550.1 |  |  |  |  |  |  |  |  |
| 0 | Ath-AT3G23560.1 |  |  |  |  |  |  |  |  |
| 0 | Ath-AT3G23570.1 |  |  |  |  |  |  |  |  |
| 0 | Ath-AT3G23580.1 |  |  |  |  |  |  |  |  |
| 0 | Ath-AT3G23590.1 |  |  |  |  |  |  |  |  |
| 0 | Ath-AT3G23600.1 |  |  |  |  |  |  |  |  |
| 0 | Ath-AT3G23605.1 |  |  |  |  |  |  |  |  |
| 0 | Ath-AT3G23610.3 |  |  |  |  |  |  |  |  |
| 0 | Ath-AT3G23620.1 |  |  |  |  |  |  |  |  |
| 0 | Ath-AT3G23630.1 |  |  |  |  |  |  |  |  |
| 0 | Ath-AT3G23633.1 |  |  |  |  |  |  |  |  |
| 0 | Ath-AT3G23635.1 |  |  |  |  |  |  |  |  |
| 0 | Ath-AT3G23637.1 |  |  |  |  |  |  |  |  |
| 0 | Ath-AT3G23640.3 |  |  |  |  |  |  |  |  |
| 0 | Ath-AT3G23650.1 |  |  |  |  |  |  |  |  |
| 0 | Ath-AT3G23660.2 |  |  |  |  |  |  |  |  |
| 0 | Ath-AT3G23670.1 |  |  |  |  |  |  |  |  |
| 0 | Ath-AT3G23680.1 |  |  |  |  |  |  |  |  |
| 0 | Ath-AT3G23685.1 |  |  |  |  |  |  |  |  |
| 0 | Ath-AT3G23690.1 |  |  |  |  |  |  |  |  |
| 0 | Ath-AT3G23700.1 |  |  |  |  |  |  |  |  |
| 0 | Ath-AT3G23710.1 |  |  |  |  |  |  |  |  |
| 0 | Ath-AT3G23715.1 |  |  |  |  |  |  |  |  |
| 0 | Ath-AT3G23727.1 |  |  |  |  |  |  |  |  |
| 0 | Ath-AT3G23730.1 |  |  |  |  |  |  |  |  |
| 0 | Ath-AT3G23740.1 |  |  |  |  |  |  |  |  |
| 0 | Ath-AT3G23750.1 |  |  |  |  |  |  |  |  |
| 0 | Ath-AT3G23760.1 |  |  |  |  |  |  |  |  |
| 0 | Ath-AT3G23770.1 |  |  |  |  |  |  |  |  |
| 0 | Ath-AT3G23780.1 |  |  |  |  |  |  |  |  |
| 0 | Ath-AT3G23790.1 |  |  |  |  |  |  |  |  |
| 1 | Ath-AT3G23800.1 |  | Vvi-Vitvi05g01088\_t001 |  |  |  |  |  |  |  |
| 1 | Ath-AT3G23805.1 |  | | | |  |  |  |  |  |  |  |
| 1 | Ath-AT3G23810.1 |  | Vvi-Vitvi05g01094\_t001 |  |  |  |  |  |  |  |
| 1 | Ath-AT3G23820.1 |  | Vvi-Vitvi05g01108\_t001 |  |  |  |  |  |  |  |
| 1 | Ath-AT3G23830.1 |  | Vvi-Vitvi05g01112\_t001 |  |  |  |  |  |  |  |
| 1 | Ath-AT3G23840.1 |  | Vvi-Vitvi05g01116\_t001 |  |  |  |  |  |  |  |
| 1 | Ath-AT3G23860.2 |  | | | |  |  |  |  |  |  |  |
| 1 | Ath-AT3G23870.1 |  | Vvi-Vitvi05g01128\_t001 |  |  |  |  |  |  |  |
| 1 | Ath-AT3G23880.1 |  | | | |  |  |  |  |  |  |  |
| 1 | Ath-AT3G23890.1 |  | | | |  |  |  |  |  |  |  |
| 1 | Ath-AT3G23900.1 |  | | | |  |  |  |  |  |  |  |
| 1 | Ath-AT3G23910.1 |  | Vvi-Vitvi05g02056\_t001 |  |  |  |  |  |  |  |
| 1 | Ath-AT3G23920.1 |  | Vvi-Vitvi05g00883\_t001 |  |  |  |  |  |  |  |
| 1 | Ath-AT3G23930.1 |  | Vvi-Vitvi05g00885\_t001 |  |  |  |  |  |  |  |
| 1 | Ath-AT3G23940.1 |  | | | |  |  |  |  |  |  |  |
| 1 | Ath-AT3G23950.1 |  | | | |  |  |  |  |  |  |  |
| 1 | Ath-AT3G23955.2 |  | | | |  |  |  |  |  |  |  |
| 1 | Ath-AT3G23960.1 |  | | | |  |  |  |  |  |  |  |
| 1 | Ath-AT3G23970.1 |  | | | |  |  |  |  |  |  |  |
| 1 | Ath-AT3G23980.1 |  | Vvi-Vitvi05g00903\_t001 |  |  |  |  |  |  |  |
| 1 | Ath-AT3G23990.1 |  | Vvi-Vitvi05g00924\_t001 |  |  |  |  |  |  |  |
| 1 | Ath-AT3G24000.1 |  | | | |  |  |  |  |  |  |  |
| 1 | Ath-AT3G24010.1 |  | Vvi-Vitvi05g00925\_t001 |  |  |  |  |  |  |  |
| 1 | Ath-AT3G24020.1 |  | Vvi-Vitvi05g00929\_t001 |  |  |  |  |  |  |  |
| 1 | Ath-AT3G24030.1 |  | Vvi-Vitvi05g00930\_t001 |  |  |  |  |  |  |  |
| 1 | Ath-AT3G24040.1 |  | Vvi-Vitvi05g00932\_t001 |  |  |  |  |  |  |  |
| 1 | Ath-AT3G24050.1 |  | Vvi-Vitvi05g00938\_t002 |  |  |  |  |  |  |  |
| 1 | Ath-AT3G24060.1 |  | Vvi-Vitvi05g00941\_t001 |  |  |  |  |  |  |  |
| 1 | Ath-AT3G24065.1 |  | | | |  |  |  |  |  |  |  |
| 1 | Ath-AT3G24068.1 |  | | | |  |  |  |  |  |  |  |
| 1 | Ath-AT3G24070.1 |  | | | |  |  |  |  |  |  |  |
| 1 | Ath-AT3G24080.1 |  | | | |  |  |  |  |  |  |  |
| 1 | Ath-AT3G24090.1 |  | | | |  |  |  |  |  |  |  |
| 1 | Ath-AT3G24093.1 |  | | | |  |  |  |  |  |  |  |
| 1 | Ath-AT3G24100.1 |  | | | |  |  |  |  |  |  |  |
| 2 | Ath-AT3G24110.1 |  | | | |  | Vvi-Vitvi05g01068\_t001 |  |  |  |  |  |  |
| 2 | Ath-AT3G24120.2 |  | | | |  | Vvi-Vitvi05g01066\_t002 |  |  |  |  |  |  |
| 2 | Ath-AT3G24130.1 |  | | | |  | | | |  |  |  |  |  |  |
| 2 | Ath-AT3G24140.1 |  | | | |  | Vvi-Vitvi05g01063\_t001 |  |  |  |  |  |  |
| 2 | Ath-AT3G24150.2 |  | | | |  | | | |  |  |  |  |  |  |
| 2 | Ath-AT3G24160.1 |  | | | |  | Vvi-Vitvi05g01054\_t002 |  |  |  |  |  |  |
| 2 | Ath-AT3G24170.1 |  | | | |  | | | |  |  |  |  |  |  |
| 2 | Ath-AT3G24180.1 |  | | | |  | | | |  |  |  |  |  |  |
| 2 | Ath-AT3G24190.1 |  | | | |  | Vvi-Vitvi05g01038\_t001 |  |  |  |  |  |  |
| 2 | Ath-AT3G24200.2 |  | | | |  | Vvi-Vitvi05g01036\_t001 |  |  |  |  |  |  |
| 1 | Ath-AT3G24210.1 |  | | | |  |  |  |  |  |  |  |
| 1 | Ath-AT3G24220.1 |  | Vvi-Vitvi05g00963\_t001 |  |  |  |  |  |  |  |
| 0 | Ath-AT3G24225.1 |  |  |  |  |  |  |  |  |
| 0 | Ath-AT3G24230.1 |  |  |  |  |  |  |  |  |
| 0 | Ath-AT3G24240.1 |  |  |  |  |  |  |  |  |
| 0 | Ath-AT3G24250.1 |  |  |  |  |  |  |  |  |
| 0 | Ath-AT3G24255.7 |  |  |  |  |  |  |  |  |
| 0 | Ath-AT3G24260.1 |  |  |  |  |  |  |  |  |
| 0 | Ath-AT3G24270.1 |  |  |  |  |  |  |  |  |
| 0 | Ath-AT3G24280.1 |  |  |  |  |  |  |  |  |
| 0 | Ath-AT3G24290.1 |  |  |  |  |  |  |  |  |
| 0 | Ath-AT3G24300.1 |  |  |  |  |  |  |  |  |
| 0 | Ath-AT3G24310.1 |  |  |  |  |  |  |  |  |
| 0 | Ath-AT3G24315.1 |  |  |  |  |  |  |  |  |
| 0 | Ath-AT3G24320.1 |  |  |  |  |  |  |  |  |
| 1 | Ath-AT3G24330.1 |  | Vvi-Vitvi11g00380\_t001 |  |  |  |  |  |  |  |
| 1 | Ath-AT3G24340.1 |  | | | |  |  |  |  |  |  |  |
| 1 | Ath-AT3G24350.2 |  | | | |  |  |  |  |  |  |  |
| 1 | Ath-AT3G24360.1 |  | Vvi-Vitvi11g00373\_t001 |  |  |  |  |  |  |  |
| 1 | Ath-AT3G24420.1 |  | Vvi-Vitvi11g00370\_t001 |  |  |  |  |  |  |  |
| 1 | Ath-AT3G24430.1 |  | Vvi-Vitvi11g00368\_t001 |  |  |  |  |  |  |  |
| 1 | Ath-AT3G24440.1 |  | Vvi-Vitvi11g00367\_t001 |  |  |  |  |  |  |  |
| 1 | Ath-AT3G24450.1 |  | Vvi-Vitvi11g00365\_t001 |  |  |  |  |  |  |  |
| 1 | Ath-AT3G24460.1 |  | Vvi-Vitvi11g00362\_t001 |  |  |  |  |  |  |  |
| 1 | Ath-AT3G24463.1 |  | | | |  |  |  |  |  |  |  |
| 1 | Ath-AT3G24465.1 |  | | | |  |  |  |  |  |  |  |
| 1 | Ath-AT3G24480.1 |  | Vvi-Vitvi11g00360\_t001 |  |  |  |  |  |  |  |
| 1 | Ath-AT3G24490.1 |  | Vvi-Vitvi11g00354\_t002 |  |  |  |  |  |  |  |
| 1 | Ath-AT3G24495.1 |  | Vvi-Vitvi11g00352\_t001 |  |  |  |  |  |  |  |
| 1 | Ath-AT3G24500.1 |  | Vvi-Vitvi11g00351\_t001 |  |  |  |  |  |  |  |
| 1 | Ath-AT3G24503.1 |  | | | |  |  |  |  |  |  |  |
| 1 | Ath-AT3G24506.1 |  | Vvi-Vitvi11g00343\_t001 |  |  |  |  |  |  |  |
| 1 | Ath-AT3G24508.1 |  | | | |  |  |  |  |  |  |  |
| 1 | Ath-AT3G24510.1 |  | | | |  |  |  |  |  |  |  |
| 1 | Ath-AT3G24513.1 |  | | | |  |  |  |  |  |  |  |
| 1 | Ath-AT3G24515.3 |  | Vvi-Vitvi11g00342\_t001 |  |  |  |  |  |  |  |
| 1 | Ath-AT3G24514.1 |  | | | |  |  |  |  |  |  |  |
| 1 | Ath-AT3G24516.1 |  | | | |  |  |  |  |  |  |  |
| 1 | Ath-AT3G24517.1 |  | | | |  |  |  |  |  |  |  |
| 1 | Ath-AT3G24520.1 |  | Vvi-Vitvi11g00339\_t001 |  |  |  |  |  |  |  |
| 1 | Ath-AT3G24530.1 |  | Vvi-Vitvi11g00334\_t001 |  |  |  |  |  |  |  |
| 1 | Ath-AT3G24535.1 |  | | | |  |  |  |  |  |  |  |
| 1 | Ath-AT3G24540.1 |  | Vvi-Vitvi11g00332\_t001 |  |  |  |  |  |  |  |
| 1 | Ath-AT3G24542.1 |  | | | |  |  |  |  |  |  |  |
| 1 | Ath-AT3G24550.1 |  | | | |  |  |  |  |  |  |  |
| 1 | Ath-AT3G24560.2 |  | Vvi-Vitvi11g00330\_t001 |  |  |  |  |  |  |  |
| 1 | Ath-AT3G24570.1 |  | Vvi-Vitvi11g00329\_t001 |  |  |  |  |  |  |  |
| 1 | Ath-AT3G24575.1 |  | | | |  |  |  |  |  |  |  |
| 1 | Ath-AT3G24580.1 |  | | | |  |  |  |  |  |  |  |
| 1 | Ath-AT3G24590.2 |  | Vvi-Vitvi11g00327\_t001 |  |  |  |  |  |  |  |
| 1 | Ath-AT3G24600.1 |  | Vvi-Vitvi11g00326\_t001 |  |  |  |  |  |  |  |
| 1 | Ath-AT3G24605.1 |  | | | |  |  |  |  |  |  |  |
| 1 | Ath-AT3G24610.1 |  | | | |  |  |  |  |  |  |  |
| 1 | Ath-AT3G24620.1 |  | | | |  |  |  |  |  |  |  |
| 1 | Ath-AT3G24630.2 |  | Vvi-Vitvi11g00321\_t001 |  |  |  |  |  |  |  |
| 0 | Ath-AT3G24640.1 |  |  |  |  |  |  |  |  |
| 1 | Ath-AT3G24650.1 |  | Vvi-Vitvi07g00781\_t001 |  |  |  |  |  |  |  |
| 1 | Ath-AT3G24660.1 |  | Vvi-Vitvi07g00786\_t001 |  |  |  |  |  |  |  |
| 1 | Ath-AT3G24670.1 |  | Vvi-Vitvi07g00787\_t001 |  |  |  |  |  |  |  |
| 1 | Ath-AT3G24690.1 |  | | | |  |  |  |  |  |  |  |
| 1 | Ath-AT3G24700.2 |  | | | |  |  |  |  |  |  |  |
| 1 | Ath-AT3G24710.1 |  | | | |  |  |  |  |  |  |  |
| 1 | Ath-AT3G24715.1 |  | | | |  |  |  |  |  |  |  |
| 1 | Ath-AT3G24730.1 |  | Vvi-Vitvi07g00796\_t001 |  |  |  |  |  |  |  |
| 1 | Ath-AT3G24740.4 |  | Vvi-Vitvi07g00801\_t003 |  |  |  |  |  |  |  |
| 1 | Ath-AT3G24750.2 |  | Vvi-Vitvi07g02345\_t001 |  |  |  |  |  |  |  |
| 1 | Ath-AT3G24760.1 |  | Vvi-Vitvi07g00803\_t001 |  |  |  |  |  |  |  |
| 0 | Ath-AT3G24770.1 |  |  |  |  |  |  |  |  |
| 0 | Ath-AT3G24780.1 |  |  |  |  |  |  |  |  |
| 0 | Ath-AT3G24790.2 |  |  |  |  |  |  |  |  |
| 0 | Ath-AT3G24800.1 |  |  |  |  |  |  |  |  |
| 0 | Ath-AT3G24810.1 |  |  |  |  |  |  |  |  |
| 0 | Ath-AT3G24820.1 |  |  |  |  |  |  |  |  |
| 0 | Ath-AT3G24830.1 |  |  |  |  |  |  |  |  |
| 0 | Ath-AT3G24840.3 |  |  |  |  |  |  |  |  |
| 0 | Ath-AT3G24850.1 |  |  |  |  |  |  |  |  |
| 0 | Ath-AT3G24860.1 |  |  |  |  |  |  |  |  |
| 0 | Ath-AT3G24870.1 |  |  |  |  |  |  |  |  |
| 0 | Ath-AT3G24880.1 |  |  |  |  |  |  |  |  |
| 0 | Ath-AT3G24890.2 |  |  |  |  |  |  |  |  |
| 0 | Ath-AT3G24900.2 |  |  |  |  |  |  |  |  |
| 0 | Ath-AT3G24929.1 |  |  |  |  |  |  |  |  |
| 0 | Ath-AT3G24982.1 |  |  |  |  |  |  |  |  |
| 0 | Ath-AT3G25010.2 |  |  |  |  |  |  |  |  |
| 0 | Ath-AT3G25012.1 |  |  |  |  |  |  |  |  |
| 0 | Ath-AT3G25011.1 |  |  |  |  |  |  |  |  |
| 0 | Ath-AT3G25013.1 |  |  |  |  |  |  |  |  |
| 0 | Ath-AT3G25014.1 |  |  |  |  |  |  |  |  |
| 0 | Ath-AT3G25020.1 |  |  |  |  |  |  |  |  |
| 1 | Ath-AT3G25030.4 |  | Vvi-Vitvi07g02789\_t001 |  |  |  |  |  |  |  |
| 1 | Ath-AT3G25040.1 |  | | | |  |  |  |  |  |  |  |
| 1 | Ath-AT3G25050.1 |  | | | |  |  |  |  |  |  |  |
| 1 | Ath-AT3G25060.1 |  | | | |  |  |  |  |  |  |  |
| 1 | Ath-AT3G25070.2 |  | | | |  |  |  |  |  |  |  |
| 1 | Ath-AT3G25080.1 |  | | | |  |  |  |  |  |  |  |
| 1 | Ath-AT3G25090.1 |  | | | |  |  |  |  |  |  |  |
| 1 | Ath-AT3G25100.1 |  | | | |  |  |  |  |  |  |  |
| 1 | Ath-AT3G25110.1 |  | | | |  |  |  |  |  |  |  |
| 1 | Ath-AT3G25120.1 |  | | | |  |  |  |  |  |  |  |
| 1 | Ath-AT3G25130.1 |  | Vvi-Vitvi07g01078\_t001 |  |  |  |  |  |  |  |
| 1 | Ath-AT3G25140.1 |  | Vvi-Vitvi07g01113\_t001 |  |  |  |  |  |  |  |
| 1 | Ath-AT3G25150.2 |  | Vvi-Vitvi07g01114\_t001 |  |  |  |  |  |  |  |
| 1 | Ath-AT3G25160.1 |  | Vvi-Vitvi07g01119\_t001 |  |  |  |  |  |  |  |
| 1 | Ath-AT3G25165.1 |  | | | |  |  |  |  |  |  |  |
| 1 | Ath-AT3G25170.1 |  | | | |  |  |  |  |  |  |  |
| 1 | Ath-AT3G25180.1 |  | | | |  |  |  |  |  |  |  |
| 1 | Ath-AT3G25190.1 |  | | | |  |  |  |  |  |  |  |
| 1 | Ath-AT3G25200.1 |  | | | |  |  |  |  |  |  |  |
| 1 | Ath-AT3G25210.1 |  | | | |  |  |  |  |  |  |  |
| 1 | Ath-AT3G25220.1 |  | | | |  |  |  |  |  |  |  |
| 1 | Ath-AT3G25221.1 |  | | | |  |  |  |  |  |  |  |
| 1 | Ath-AT3G25230.2 |  | | | |  |  |  |  |  |  |  |
| 1 | Ath-AT3G25240.1 |  | Vvi-Vitvi07g02951\_t001 |  |  |  |  |  |  |  |
| 0 | Ath-AT3G25250.1 |  |  |  |  |  |  |  |  |
| 0 | Ath-AT3G25260.1 |  |  |  |  |  |  |  |  |
| 0 | Ath-AT3G25265.1 |  |  |  |  |  |  |  |  |
| 0 | Ath-AT3G25270.1 |  |  |  |  |  |  |  |  |
| 0 | Ath-AT3G25280.1 |  |  |  |  |  |  |  |  |
| 0 | Ath-AT3G25290.1 |  |  |  |  |  |  |  |  |
| 0 | Ath-AT3G25400.1 |  |  |  |  |  |  |  |  |
| 0 | Ath-AT3G25410.1 |  |  |  |  |  |  |  |  |
| 0 | Ath-AT3G25420.1 |  |  |  |  |  |  |  |  |
| 0 | Ath-AT3G25430.1 |  |  |  |  |  |  |  |  |
| 0 | Ath-AT3G25440.1 |  |  |  |  |  |  |  |  |
| 0 | Ath-AT3G25460.1 |  |  |  |  |  |  |  |  |
| 0 | Ath-AT3G25470.1 |  |  |  |  |  |  |  |  |
| 0 | Ath-AT3G25480.1 |  |  |  |  |  |  |  |  |
| 0 | Ath-AT3G25490.1 |  |  |  |  |  |  |  |  |
| 0 | Ath-AT3G25493.1 |  |  |  |  |  |  |  |  |
| 0 | Ath-AT3G25500.1 |  |  |  |  |  |  |  |  |
| 0 | Ath-AT3G25505.1 |  |  |  |  |  |  |  |  |
| 0 | Ath-AT3G25510.2 |  |  |  |  |  |  |  |  |
| 0 | Ath-AT3G25520.1 |  |  |  |  |  |  |  |  |
| 0 | Ath-AT3G25530.1 |  |  |  |  |  |  |  |  |
| 0 | Ath-AT3G25540.3 |  |  |  |  |  |  |  |  |
| 0 | Ath-AT3G25545.1 |  |  |  |  |  |  |  |  |
| 0 | Ath-AT3G25550.1 |  |  |  |  |  |  |  |  |
| 0 | Ath-AT3G25560.3 |  |  |  |  |  |  |  |  |
| 0 | Ath-AT3G25570.2 |  |  |  |  |  |  |  |  |
| 0 | Ath-AT3G25573.1 |  |  |  |  |  |  |  |  |
| 0 | Ath-AT3G25577.1 |  |  |  |  |  |  |  |  |
| 0 | Ath-AT3G25580.1 |  |  |  |  |  |  |  |  |
| 0 | Ath-AT3G25585.1 |  |  |  |  |  |  |  |  |
| 1 | Ath-AT3G25590.1 |  | Vvi-Vitvi01g00187\_t001 |  |  |  |  |  |  |  |
| 1 | Ath-AT3G25597.1 |  | Vvi-Vitvi01g00197\_t001 |  |  |  |  |  |  |  |
| 1 | Ath-AT3G25600.1 |  | Vvi-Vitvi01g00198\_t001 |  |  |  |  |  |  |  |
| 1 | Ath-AT3G25610.1 |  | Vvi-Vitvi01g00199\_t001 |  |  |  |  |  |  |  |
| 1 | Ath-AT3G25620.2 |  | Vvi-Vitvi01g00211\_t001 |  |  |  |  |  |  |  |
| 1 | Ath-AT3G25640.2 |  | Vvi-Vitvi01g00217\_t001 |  |  |  |  |  |  |  |
| 1 | Ath-AT3G25650.1 |  | | | |  |  |  |  |  |  |  |
| 1 | Ath-AT3G25655.1 |  | | | |  |  |  |  |  |  |  |
| 1 | Ath-AT3G25660.1 |  | | | |  |  |  |  |  |  |  |
| 1 | Ath-AT3G25670.1 |  | Vvi-Vitvi01g00218\_t001 |  |  |  |  |  |  |  |
| 1 | Ath-AT3G25680.1 |  | Vvi-Vitvi01g00223\_t001 |  |  |  |  |  |  |  |
| 1 | Ath-AT3G25690.5 |  | Vvi-Vitvi01g00224\_t001 |  |  |  |  |  |  |  |
| 1 | Ath-AT3G25700.1 |  | Vvi-Vitvi01g00230\_t001 |  |  |  |  |  |  |  |
| 1 | Ath-AT3G25710.1 |  | Vvi-Vitvi01g00232\_t001 |  |  |  |  |  |  |  |
| 1 | Ath-AT3G25716.1 |  | | | |  |  |  |  |  |  |  |
| 1 | Ath-AT3G25717.1 |  | | | |  |  |  |  |  |  |  |
| 1 | Ath-AT3G25719.1 |  | | | |  |  |  |  |  |  |  |
| 1 | Ath-AT3G25720.1 |  | | | |  |  |  |  |  |  |  |
| 1 | Ath-AT3G25727.1 |  | | | |  |  |  |  |  |  |  |
| 1 | Ath-AT3G25730.1 |  | Vvi-Vitvi01g00244\_t001 |  |  |  |  |  |  |  |
| 1 | Ath-AT3G25740.1 |  | Vvi-Vitvi01g00245\_t001 |  |  |  |  |  |  |  |
| 1 | Ath-AT3G25750.2 |  | | | |  |  |  |  |  |  |  |
| 1 | Ath-AT3G25760.1 |  | Vvi-Vitvi01g00246\_t001 |  |  |  |  |  |  |  |
| 1 | Ath-AT3G25770.1 |  | | | |  |  |  |  |  |  |  |
| 1 | Ath-AT3G25780.1 |  | | | |  |  |  |  |  |  |  |
| 1 | Ath-AT3G25790.1 |  | Vvi-Vitvi01g00249\_t001 |  |  |  |  |  |  |  |
| 1 | Ath-AT3G25800.1 |  | Vvi-Vitvi01g00265\_t002 |  |  |  |  |  |  |  |
| 1 | Ath-AT3G25805.1 |  | Vvi-Vitvi01g00269\_t001 |  |  |  |  |  |  |  |
| 1 | Ath-AT3G25810.1 |  | | | |  |  |  |  |  |  |  |
| 1 | Ath-AT3G25820.1 |  | | | |  |  |  |  |  |  |  |
| 1 | Ath-AT3G25830.1 |  | | | |  |  |  |  |  |  |  |
| 1 | Ath-AT3G25840.1 |  | Vvi-Vitvi01g00272\_t001 |  |  |  |  |  |  |  |
| 1 | Ath-AT3G25850.1 |  | | | |  |  |  |  |  |  |  |
| 1 | Ath-AT3G25855.1 |  | | | |  |  |  |  |  |  |  |
| 1 | Ath-AT3G25860.1 |  | Vvi-Vitvi01g00274\_t001 |  |  |  |  |  |  |  |
| 1 | Ath-AT3G25870.1 |  | Vvi-Vitvi01g00279\_t001 |  |  |  |  |  |  |  |
| 1 | Ath-AT3G25880.2 |  | | | |  |  |  |  |  |  |  |
| 1 | Ath-AT3G25882.1 |  | | | |  |  |  |  |  |  |  |
| 1 | Ath-AT3G25890.2 |  | Vvi-Vitvi01g01827\_t001 |  |  |  |  |  |  |  |
| 1 | Ath-AT3G25900.1 |  | Vvi-Vitvi01g00286\_t001 |  |  |  |  |  |  |  |
| 1 | Ath-AT3G25905.1 |  | Vvi-Vitvi01g01936\_t001 |  |  |  |  |  |  |  |
| 1 | Ath-AT3G25910.1 |  | | | |  |  |  |  |  |  |  |
| 1 | Ath-AT3G25920.1 |  | Vvi-Vitvi01g00293\_t001 |  |  |  |  |  |  |  |
| 1 | Ath-AT3G25930.1 |  | Vvi-Vitvi01g01942\_t001 |  |  |  |  |  |  |  |
| 0 | Ath-AT3G25940.1 |  |  |  |  |  |  |  |  |
| 1 | Ath-AT3G25950.1 |  | Vvi-Vitvi14g01595\_t001 |  |  |  |  |  |  |  |
| 1 | Ath-AT3G25960.1 |  | | | |  |  |  |  |  |  |  |
| 1 | Ath-AT3G25970.1 |  | | | |  |  |  |  |  |  |  |
| 2 | Ath-AT3G25980.1 |  | | | |  | Vvi-Vitvi01g01534\_t001 |  |  |  |  |  |  |
| 2 | Ath-AT3G25990.1 |  | Vvi-Vitvi14g01602\_t001 |  | Vvi-Vitvi01g01533\_t002 |  |  |  |  |  |  |
| 2 | Ath-AT3G26000.1 |  | Vvi-Vitvi14g01604\_t001 |  | Vvi-Vitvi01g01529\_t001 |  |  |  |  |  |  |
| 2 | Ath-AT3G26010.1 |  | | | |  | | | |  |  |  |  |  |  |
| 2 | Ath-AT3G26020.4 |  | Vvi-Vitvi14g04601\_t001 |  | Vvi-Vitvi01g01521\_t001 |  |  |  |  |  |  |
| 2 | Ath-AT3G26030.1 |  | | | |  | | | |  |  |  |  |  |  |
| 2 | Ath-AT3G26040.1 |  | | | |  | Vvi-Vitvi01g02213\_t001 |  |  |  |  |  |  |
| 2 | Ath-AT3G26050.1 |  | Vvi-Vitvi14g01621\_t001 |  | Vvi-Vitvi01g01507\_t001 |  |  |  |  |  |  |
| 2 | Ath-AT3G26060.2 |  | | | |  | | | |  |  |  |  |  |  |
| 2 | Ath-AT3G26070.1 |  | | | |  | | | |  |  |  |  |  |  |
| 2 | Ath-AT3G26080.2 |  | | | |  | | | |  |  |  |  |  |  |
| 2 | Ath-AT3G26085.2 |  | | | |  | Vvi-Vitvi01g01500\_t001 |  |  |  |  |  |  |
| 2 | Ath-AT3G26090.1 |  | | | |  | Vvi-Vitvi01g01499\_t001 |  |  |  |  |  |  |
| 2 | Ath-AT3G26100.2 |  | | | |  | Vvi-Vitvi01g00591\_t007 |  |  |  |  |  |  |
| 2 | Ath-AT3G26110.1 |  | | | |  | | | |  |  |  |  |  |  |
| 2 | Ath-AT3G26115.1 |  | | | |  | Vvi-Vitvi01g00600\_t001 |  |  |  |  |  |  |
| 2 | Ath-AT3G26120.1 |  | Vvi-Vitvi14g01637\_t001 |  | Vvi-Vitvi01g00602\_t001 |  |  |  |  |  |  |
| 1 | Ath-AT3G26130.2 |  |  |  | | | |  |  |  |  |  |  |
| 1 | Ath-AT3G26125.1 |  |  |  | Vvi-Vitvi01g00612\_t001 |  |  |  |  |  |  |
| 1 | Ath-AT3G26135.1 |  |  |  | | | |  |  |  |  |  |  |
| 1 | Ath-AT3G26140.3 |  |  |  | | | |  |  |  |  |  |  |
| 1 | Ath-AT3G26147.1 |  |  |  | | | |  |  |  |  |  |  |
| 1 | Ath-AT3G26150.1 |  |  |  | | | |  |  |  |  |  |  |
| 1 | Ath-AT3G26160.1 |  |  |  | | | |  |  |  |  |  |  |
| 1 | Ath-AT3G26170.1 |  |  |  | | | |  |  |  |  |  |  |
| 1 | Ath-AT3G26180.1 |  |  |  | | | |  |  |  |  |  |  |
| 1 | Ath-AT3G26190.1 |  |  |  | | | |  |  |  |  |  |  |
| 1 | Ath-AT3G26200.1 |  |  |  | | | |  |  |  |  |  |  |
| 1 | Ath-AT3G26210.1 |  |  |  | | | |  |  |  |  |  |  |
| 1 | Ath-AT3G26220.1 |  |  |  | | | |  |  |  |  |  |  |
| 1 | Ath-AT3G26230.1 |  |  |  | | | |  |  |  |  |  |  |
| 1 | Ath-AT3G26235.1 |  |  |  | | | |  |  |  |  |  |  |
| 1 | Ath-AT3G26237.1 |  |  |  | | | |  |  |  |  |  |  |
| 1 | Ath-AT3G26240.1 |  |  |  | | | |  |  |  |  |  |  |
| 1 | Ath-AT3G26250.1 |  |  |  | | | |  |  |  |  |  |  |
| 1 | Ath-AT3G26270.1 |  |  |  | | | |  |  |  |  |  |  |
| 1 | Ath-AT3G26280.2 |  |  |  | | | |  |  |  |  |  |  |
| 1 | Ath-AT3G26290.2 |  |  |  | | | |  |  |  |  |  |  |
| 1 | Ath-AT3G26300.1 |  |  |  | Vvi-Vitvi01g00617\_t001 |  |  |  |  |  |  |
| 1 | Ath-AT3G26310.1 |  |  |  | | | |  |  |  |  |  |  |
| 1 | Ath-AT3G26320.1 |  |  |  | | | |  |  |  |  |  |  |
| 1 | Ath-AT3G26330.1 |  |  |  | | | |  |  |  |  |  |  |
| 1 | Ath-AT3G26340.1 |  |  |  | | | |  |  |  |  |  |  |
| 1 | Ath-AT3G26350.1 |  |  |  | Vvi-Vitvi01g04149\_t001 |  |  |  |  |  |  |
| 1 | Ath-AT3G26355.1 |  |  |  | | | |  |  |  |  |  |  |
| 1 | Ath-AT3G26360.1 |  |  |  | | | |  |  |  |  |  |  |
| 1 | Ath-AT3G26370.1 |  |  |  | Vvi-Vitvi01g00628\_t001 |  |  |  |  |  |  |
| 1 | Ath-AT3G26380.1 |  |  |  | Vvi-Vitvi01g00629\_t001 |  |  |  |  |  |  |
| 1 | Ath-AT3G26390.1 |  |  |  | | | |  |  |  |  |  |  |
| 1 | Ath-AT3G26395.1 |  |  |  | | | |  |  |  |  |  |  |
| 1 | Ath-AT3G26400.1 |  |  |  | Vvi-Vitvi01g00633\_t001 |  |  |  |  |  |  |
| 1 | Ath-AT3G26410.1 |  |  |  | | | |  |  |  |  |  |  |
| 1 | Ath-AT3G26420.1 |  |  |  | | | |  |  |  |  |  |  |
| 1 | Ath-AT3G26430.1 |  |  |  | Vvi-Vitvi01g00634\_t001 |  |  |  |  |  |  |
| 1 | Ath-AT3G26440.5 |  |  |  | Vvi-Vitvi01g00641\_t001 |  |  |  |  |  |  |
| 1 | Ath-AT3G26445.1 |  |  |  | | | |  |  |  |  |  |  |
| 1 | Ath-AT3G26450.1 |  |  |  | | | |  |  |  |  |  |  |
| 1 | Ath-AT3G26460.1 |  |  |  | | | |  |  |  |  |  |  |
| 1 | Ath-AT3G26470.1 |  |  |  | | | |  |  |  |  |  |  |
| 1 | Ath-AT3G26480.1 |  |  |  | | | |  |  |  |  |  |  |
| 1 | Ath-AT3G26490.1 |  |  |  | Vvi-Vitvi01g00644\_t001 |  |  |  |  |  |  |
| 1 | Ath-AT3G26500.1 |  |  |  | | | |  |  |  |  |  |  |
| 1 | Ath-AT3G26510.7 |  |  |  | Vvi-Vitvi01g00646\_t002 |  |  |  |  |  |  |
| 1 | Ath-AT3G26512.1 |  |  |  | | | |  |  |  |  |  |  |
| 1 | Ath-AT3G26520.1 |  |  |  | | | |  |  |  |  |  |  |
| 1 | Ath-AT3G26539.1 |  |  |  | | | |  |  |  |  |  |  |
| 1 | Ath-AT3G26540.1 |  |  |  | | | |  |  |  |  |  |  |
| 1 | Ath-AT3G26550.1 |  |  |  | | | |  |  |  |  |  |  |
| 1 | Ath-AT3G26560.1 |  |  |  | | | |  |  |  |  |  |  |
| 1 | Ath-AT3G26570.1 |  |  |  | Vvi-Vitvi01g00647\_t001 |  |  |  |  |  |  |
| 1 | Ath-AT3G26580.1 |  |  |  | Vvi-Vitvi01g00652\_t001 |  |  |  |  |  |  |
| 1 | Ath-AT3G26590.1 |  |  |  | Vvi-Vitvi01g00653\_t001 |  |  |  |  |  |  |
| 1 | Ath-AT3G26600.1 |  |  |  | Vvi-Vitvi01g00659\_t001 |  |  |  |  |  |  |
| 1 | Ath-AT3G26610.1 |  |  |  | Vvi-Vitvi01g00684\_t001 |  |  |  |  |  |  |
| 0 | Ath-AT3G26616.1 |  |  |  |  |  |  |  |  |
| 0 | Ath-AT3G26618.1 |  |  |  |  |  |  |  |  |
| 0 | Ath-AT3G26620.1 |  |  |  |  |  |  |  |  |
| 0 | Ath-AT3G26630.1 |  |  |  |  |  |  |  |  |
| 1 | Ath-AT3G26640.1 |  | Vvi-Vitvi14g01422\_t001 |  |  |  |  |  |  |  |
| 1 | Ath-AT3G26650.1 |  | Vvi-Vitvi14g01424\_t001 |  |  |  |  |  |  |  |
| 1 | Ath-AT3G26660.1 |  | | | |  |  |  |  |  |  |  |
| 1 | Ath-AT3G26670.3 |  | Vvi-Vitvi14g01433\_t001 |  |  |  |  |  |  |  |
| 1 | Ath-AT3G26680.2 |  | | | |  |  |  |  |  |  |  |
| 1 | Ath-AT3G26690.2 |  | Vvi-Vitvi14g01442\_t001 |  |  |  |  |  |  |  |
| 1 | Ath-AT3G26700.1 |  | Vvi-Vitvi14g01450\_t001 |  |  |  |  |  |  |  |
| 1 | Ath-AT3G26710.1 |  | Vvi-Vitvi14g01456\_t001 |  |  |  |  |  |  |  |
| 2 | Ath-AT3G26720.1 |  | Vvi-Vitvi14g01465\_t002 |  | Vvi-Vitvi17g00031\_t001 |  |  |  |  |  |  |
| 2 | Ath-AT3G26730.1 |  | Vvi-Vitvi14g01466\_t001 |  | | | |  |  |  |  |  |  |
| 3 | Ath-AT3G26740.1 |  | Vvi-Vitvi14g01469\_t001 |  | | | |  | Vvi-Vitvi01g02296\_t001 |  |  |  |  |  |
| 3 | Ath-AT3G26742.1 |  | | | |  | | | |  | | | |  |  |  |  |  |
| 3 | Ath-AT3G26744.2 |  | Vvi-Vitvi14g01473\_t001 |  | Vvi-Vitvi17g00037\_t001 |  | Vvi-Vitvi01g01757\_t001 |  |  |  |  |  |
| 3 | Ath-AT3G26750.1 |  | | | |  | | | |  | | | |  |  |  |  |  |
| 3 | Ath-AT3G26760.1 |  | Vvi-Vitvi14g01474\_t001 |  | | | |  | | | |  |  |  |  |  |
| 3 | Ath-AT3G26770.1 |  | | | |  | | | |  | | | |  |  |  |  |  |
| 3 | Ath-AT3G26780.1 |  | Vvi-Vitvi14g01476\_t001 |  | | | |  | | | |  |  |  |  |  |
| 3 | Ath-AT3G26782.1 |  | Vvi-Vitvi14g01477\_t001 |  | | | |  | | | |  |  |  |  |  |
| 3 | Ath-AT3G26790.1 |  | Vvi-Vitvi14g01480\_t001 |  | | | |  | Vvi-Vitvi01g01743\_t001 |  |  |  |  |  |
| 3 | Ath-AT3G26800.1 |  | | | |  | | | |  | | | |  |  |  |  |  |
| 3 | Ath-AT3G26810.1 |  | Vvi-Vitvi14g01482\_t001 |  | | | |  | Vvi-Vitvi01g01738\_t001 |  |  |  |  |  |
| 3 | Ath-AT3G26820.1 |  | Vvi-Vitvi14g01491\_t001 |  | | | |  | | | |  |  |  |  |  |
| 3 | Ath-AT3G26830.1 |  | | | |  | | | |  | | | |  |  |  |  |  |
| 3 | Ath-AT3G26840.1 |  | | | |  | | | |  | | | |  |  |  |  |  |
| 3 | Ath-AT3G26850.2 |  | | | |  | | | |  | | | |  |  |  |  |  |
| 3 | Ath-AT3G26855.1 |  | | | |  | | | |  | | | |  |  |  |  |  |
| 3 | Ath-AT3G26860.2 |  | | | |  | | | |  | | | |  |  |  |  |  |
| 3 | Ath-AT3G26870.1 |  | | | |  | | | |  | | | |  |  |  |  |  |
| 3 | Ath-AT3G26880.1 |  | | | |  | | | |  | | | |  |  |  |  |  |
| 3 | Ath-AT3G26890.2 |  | Vvi-Vitvi14g01496\_t003 |  | Vvi-Vitvi17g00054\_t001 |  | | | |  |  |  |  |  |
| 3 | Ath-AT3G26900.2 |  | Vvi-Vitvi14g01497\_t001 |  | | | |  | | | |  |  |  |  |  |
| 3 | Ath-AT3G26910.3 |  | Vvi-Vitvi14g01498\_t002 |  | | | |  | Vvi-Vitvi01g01726\_t002 |  |  |  |  |  |
| 3 | Ath-AT3G26920.1 |  | | | |  | | | |  | | | |  |  |  |  |  |
| 3 | Ath-AT3G26922.2 |  | | | |  | | | |  | | | |  |  |  |  |  |
| 3 | Ath-AT3G26930.2 |  | | | |  | | | |  | | | |  |  |  |  |  |
| 3 | Ath-AT3G26934.1 |  | | | |  | | | |  | | | |  |  |  |  |  |
| 3 | Ath-AT3G26932.2 |  | Vvi-Vitvi14g01502\_t001 |  | | | |  | | | |  |  |  |  |  |
| 3 | Ath-AT3G26935.1 |  | Vvi-Vitvi14g01503\_t001 |  | Vvi-Vitvi17g00068\_t001 |  | | | |  |  |  |  |  |
| 3 | Ath-AT3G26940.1 |  | | | |  | | | |  | | | |  |  |  |  |  |
| 3 | Ath-AT3G26950.1 |  | Vvi-Vitvi14g01505\_t001 |  | Vvi-Vitvi17g00071\_t001 |  | | | |  |  |  |  |  |
| 3 | Ath-AT3G26960.1 |  | | | |  | | | |  | Vvi-Vitvi01g01714\_t001 |  |  |  |  |  |
| 2 | Ath-AT3G26980.1 |  | Vvi-Vitvi14g01509\_t001 |  | | | |  |  |  |  |  |  |
| 2 | Ath-AT3G26990.1 |  | | | |  | Vvi-Vitvi17g00072\_t001 |  |  |  |  |  |  |
| 1 | Ath-AT3G27000.1 |  | Vvi-Vitvi14g01513\_t001 |  |  |  |  |  |  |  |
| 1 | Ath-AT3G27010.2 |  | Vvi-Vitvi14g01519\_t001 |  |  |  |  |  |  |  |
| 1 | Ath-AT3G27020.1 |  | Vvi-Vitvi14g01520\_t001 |  |  |  |  |  |  |  |
| 1 | Ath-AT3G27025.1 |  | | | |  |  |  |  |  |  |  |
| 1 | Ath-AT3G27027.1 |  | Vvi-Vitvi14g02964\_t001 |  |  |  |  |  |  |  |
| 1 | Ath-AT3G27030.1 |  | | | |  |  |  |  |  |  |  |
| 1 | Ath-AT3G27040.1 |  | | | |  |  |  |  |  |  |  |
| 1 | Ath-AT3G27050.1 |  | Vvi-Vitvi14g02971\_t001 |  |  |  |  |  |  |  |
| 1 | Ath-AT3G27060.1 |  | Vvi-Vitvi14g01542\_t001 |  |  |  |  |  |  |  |
| 1 | Ath-AT3G27070.2 |  | Vvi-Vitvi14g01545\_t001 |  |  |  |  |  |  |  |
| 1 | Ath-AT3G27080.1 |  | | | |  |  |  |  |  |  |  |
| 1 | Ath-AT3G27090.1 |  | Vvi-Vitvi14g01546\_t001 |  |  |  |  |  |  |  |
| 1 | Ath-AT3G27100.1 |  | Vvi-Vitvi14g04569\_t001 |  |  |  |  |  |  |  |
| 1 | Ath-AT3G27110.2 |  | Vvi-Vitvi14g01548\_t001 |  |  |  |  |  |  |  |
| 1 | Ath-AT3G27120.1 |  | Vvi-Vitvi14g01553\_t001 |  |  |  |  |  |  |  |
| 1 | Ath-AT3G27140.1 |  | | | |  |  |  |  |  |  |  |
| 1 | Ath-AT3G27150.2 |  | Vvi-Vitvi14g01555\_t002 |  |  |  |  |  |  |  |
| 1 | Ath-AT3G27160.2 |  | Vvi-Vitvi14g04570\_t001 |  |  |  |  |  |  |  |
| 1 | Ath-AT3G27170.1 |  | Vvi-Vitvi14g01560\_t001 |  |  |  |  |  |  |  |
| 1 | Ath-AT3G27180.1 |  | Vvi-Vitvi14g04581\_t001 |  |  |  |  |  |  |  |
| 1 | Ath-AT3G27185.1 |  | | | |  |  |  |  |  |  |  |
| 1 | Ath-AT3G27190.1 |  | Vvi-Vitvi14g02984\_t001 |  |  |  |  |  |  |  |
| 1 | Ath-AT3G27200.1 |  | Vvi-Vitvi14g01568\_t001 |  |  |  |  |  |  |  |
| 1 | Ath-AT3G27210.1 |  | Vvi-Vitvi14g02985\_t001 |  |  |  |  |  |  |  |
| 1 | Ath-AT3G27220.1 |  | Vvi-Vitvi14g01591\_t001 |  |  |  |  |  |  |  |
| 1 | Ath-AT3G27230.1 |  | | | |  |  |  |  |  |  |  |
| 1 | Ath-AT3G27240.1 |  | Vvi-Vitvi14g04596\_t001 |  |  |  |  |  |  |  |
| 1 | Ath-AT3G27250.1 |  | Vvi-Vitvi14g02990\_t001 |  |  |  |  |  |  |  |
| 1 | Ath-AT3G27260.3 |  | Vvi-Vitvi14g01594\_t002 |  |  |  |  |  |  |  |
| 1 | Ath-AT3G27270.2 |  | Vvi-Vitvi14g01595\_t001 |  |  |  |  |  |  |  |
| 1 | Ath-AT3G27280.1 |  | Vvi-Vitvi14g02991\_t001 |  |  |  |  |  |  |  |
| 1 | Ath-AT3G27290.1 |  | Vvi-Vitvi14g01604\_t001 |  |  |  |  |  |  |  |
| 1 | Ath-AT3G27300.4 |  | Vvi-Vitvi14g01605\_t005 |  |  |  |  |  |  |  |
| 1 | Ath-AT3G27310.1 |  | Vvi-Vitvi14g01606\_t002 |  |  |  |  |  |  |  |
| 1 | Ath-AT3G27320.1 |  | Vvi-Vitvi14g01610\_t001 |  |  |  |  |  |  |  |
| 1 | Ath-AT3G27325.2 |  | | | |  |  |  |  |  |  |  |
| 1 | Ath-AT3G27329.1 |  | | | |  |  |  |  |  |  |  |
| 1 | Ath-AT3G27331.1 |  | | | |  |  |  |  |  |  |  |
| 1 | Ath-AT3G27330.1 |  | Vvi-Vitvi14g01611\_t001 |  |  |  |  |  |  |  |
| 1 | Ath-AT3G27340.1 |  | Vvi-Vitvi14g01616\_t001 |  |  |  |  |  |  |  |
| 1 | Ath-AT3G27350.2 |  | Vvi-Vitvi14g01621\_t001 |  |  |  |  |  |  |  |
| 1 | Ath-AT3G27355.1 |  | | | |  |  |  |  |  |  |  |
| 1 | Ath-AT3G27360.1 |  | | | |  |  |  |  |  |  |  |
| 1 | Ath-AT3G27380.2 |  | Vvi-Vitvi14g01627\_t001 |  |  |  |  |  |  |  |
| 1 | Ath-AT3G27390.1 |  | Vvi-Vitvi14g01631\_t001 |  |  |  |  |  |  |  |
| 1 | Ath-AT3G27400.2 |  | Vvi-Vitvi14g01635\_t001 |  |  |  |  |  |  |  |
| 1 | Ath-AT3G27410.1 |  | | | |  |  |  |  |  |  |  |
| 1 | Ath-AT3G27415.1 |  | | | |  |  |  |  |  |  |  |
| 1 | Ath-AT3G27416.1 |  | | | |  |  |  |  |  |  |  |
| 1 | Ath-AT3G27420.1 |  | Vvi-Vitvi14g02998\_t001 |  |  |  |  |  |  |  |
| 1 | Ath-AT3G27425.1 |  | | | |  |  |  |  |  |  |  |
| 1 | Ath-AT3G27430.2 |  | | | |  |  |  |  |  |  |  |
| 1 | Ath-AT3G27440.1 |  | | | |  |  |  |  |  |  |  |
| 1 | Ath-AT3G27460.4 |  | Vvi-Vitvi14g01661\_t001 |  |  |  |  |  |  |  |
| 1 | Ath-AT3G27470.5 |  | Vvi-Vitvi14g01672\_t001 |  |  |  |  |  |  |  |
| 1 | Ath-AT3G27473.1 |  | | | |  |  |  |  |  |  |  |
| 1 | Ath-AT3G27475.2 |  | | | |  |  |  |  |  |  |  |
| 1 | Ath-AT3G27480.1 |  | | | |  |  |  |  |  |  |  |
| 1 | Ath-AT3G27490.1 |  | | | |  |  |  |  |  |  |  |
| 1 | Ath-AT3G27495.1 |  | | | |  |  |  |  |  |  |  |
| 1 | Ath-AT3G27500.1 |  | | | |  |  |  |  |  |  |  |
| 1 | Ath-AT3G27503.1 |  | | | |  |  |  |  |  |  |  |
| 1 | Ath-AT3G27510.1 |  | | | |  |  |  |  |  |  |  |
| 1 | Ath-AT3G27520.1 |  | Vvi-Vitvi14g01674\_t001 |  |  |  |  |  |  |  |
| 1 | Ath-AT3G27530.1 |  | | | |  |  |  |  |  |  |  |
| 1 | Ath-AT3G27540.1 |  | Vvi-Vitvi14g01676\_t002 |  |  |  |  |  |  |  |
| 1 | Ath-AT3G27550.2 |  | Vvi-Vitvi14g01677\_t001 |  |  |  |  |  |  |  |
| 1 | Ath-AT3G27560.1 |  | Vvi-Vitvi14g01679\_t001 |  |  |  |  |  |  |  |
| 1 | Ath-AT3G27570.1 |  | Vvi-Vitvi14g01692\_t001 |  |  |  |  |  |  |  |
| 1 | Ath-AT3G27580.1 |  | Vvi-Vitvi14g01693\_t002 |  |  |  |  |  |  |  |
| 1 | Ath-AT3G27590.1 |  | | | |  |  |  |  |  |  |  |
| 1 | Ath-AT3G27600.1 |  | | | |  |  |  |  |  |  |  |
| 1 | Ath-AT3G27610.1 |  | | | |  |  |  |  |  |  |  |
| 1 | Ath-AT3G27620.1 |  | | | |  |  |  |  |  |  |  |
| 1 | Ath-AT3G27630.1 |  | Vvi-Vitvi14g03003\_t001 |  |  |  |  |  |  |  |
| 1 | Ath-AT3G27640.1 |  | Vvi-Vitvi14g01704\_t001 |  |  |  |  |  |  |  |
| 1 | Ath-AT3G27650.1 |  | Vvi-Vitvi14g01707\_t001 |  |  |  |  |  |  |  |
| 1 | Ath-AT3G27660.1 |  | Vvi-Vitvi14g03008\_t001 |  |  |  |  |  |  |  |
| 1 | Ath-AT3G27670.1 |  | Vvi-Vitvi14g04618\_t001 |  |  |  |  |  |  |  |
| 1 | Ath-AT3G27680.1 |  | | | |  |  |  |  |  |  |  |
| 1 | Ath-AT3G27690.2 |  | | | |  |  |  |  |  |  |  |
| 1 | Ath-AT3G27700.1 |  | Vvi-Vitvi14g01714\_t001 |  |  |  |  |  |  |  |
| 1 | Ath-AT3G27710.1 |  | | | |  |  |  |  |  |  |  |
| 1 | Ath-AT3G27720.1 |  | | | |  |  |  |  |  |  |  |
| 1 | Ath-AT3G27730.2 |  | Vvi-Vitvi14g01723\_t001 |  |  |  |  |  |  |  |
| 1 | Ath-AT3G27740.1 |  | | | |  |  |  |  |  |  |  |
| 1 | Ath-AT3G27750.1 |  | | | |  |  |  |  |  |  |  |
| 2 | Ath-AT3G27770.1 |  | Vvi-Vitvi14g01736\_t001 |  | Vvi-Vitvi17g00837\_t003 |  |  |  |  |  |  |
| 2 | Ath-AT3G27785.1 |  | Vvi-Vitvi14g01740\_t001 |  | Vvi-Vitvi17g00832\_t001 |  |  |  |  |  |  |
| 2 | Ath-AT3G27810.1 |  | Vvi-Vitvi14g01750\_t001 |  | | | |  |  |  |  |  |  |
| 2 | Ath-AT3G27809.1 |  | | | |  | | | |  |  |  |  |  |  |
| 2 | Ath-AT3G27820.1 |  | Vvi-Vitvi14g01751\_t001 |  | | | |  |  |  |  |  |  |
| 2 | Ath-AT3G27830.1 |  | Vvi-Vitvi14g01754\_t001 |  | | | |  |  |  |  |  |  |
| 2 | Ath-AT3G27831.1 |  | | | |  | | | |  |  |  |  |  |  |
| 2 | Ath-AT3G27835.1 |  | | | |  | | | |  |  |  |  |  |  |
| 2 | Ath-AT3G27840.1 |  | | | |  | | | |  |  |  |  |  |  |
| 2 | Ath-AT3G27850.1 |  | | | |  | | | |  |  |  |  |  |  |
| 2 | Ath-AT3G27860.1 |  | Vvi-Vitvi14g01756\_t001 |  | Vvi-Vitvi17g00825\_t001 |  |  |  |  |  |  |
| 2 | Ath-AT3G27870.1 |  | Vvi-Vitvi14g01760\_t001 |  | | | |  |  |  |  |  |  |
| 2 | Ath-AT3G27880.1 |  | Vvi-Vitvi14g01761\_t001 |  | Vvi-Vitvi17g00815\_t001 |  |  |  |  |  |  |
| 2 | Ath-AT3G27890.1 |  | | | |  | | | |  |  |  |  |  |  |
| 2 | Ath-AT3G27900.2 |  | | | |  | | | |  |  |  |  |  |  |
| 2 | Ath-AT3G27906.1 |  | | | |  | | | |  |  |  |  |  |  |
| 2 | Ath-AT3G27910.1 |  | | | |  | | | |  |  |  |  |  |  |
| 2 | Ath-AT3G27920.1 |  | Vvi-Vitvi14g03020\_t001 |  | | | |  |  |  |  |  |  |
| 2 | Ath-AT3G27925.2 |  | | | |  | | | |  |  |  |  |  |  |
| 2 | Ath-AT3G27930.1 |  | Vvi-Vitvi14g01764\_t001 |  | | | |  |  |  |  |  |  |
| 2 | Ath-AT3G27940.1 |  | | | |  | | | |  |  |  |  |  |  |
| 2 | Ath-AT3G27950.1 |  | Vvi-Vitvi14g01766\_t001 |  | | | |  |  |  |  |  |  |
| 2 | Ath-AT3G27960.1 |  | Vvi-Vitvi14g01769\_t001.1.6037826b |  | Vvi-Vitvi17g00807\_t002 |  |  |  |  |  |  |
| 2 | Ath-AT3G27970.1 |  | Vvi-Vitvi14g01770\_t001 |  | | | |  |  |  |  |  |  |
| 2 | Ath-AT3G27980.1 |  | | | |  | | | |  |  |  |  |  |  |
| 2 | Ath-AT3G27999.1 |  | | | |  | | | |  |  |  |  |  |  |
| 2 | Ath-AT3G28007.1 |  | Vvi-Vitvi14g01783\_t001 |  | Vvi-Vitvi17g00791\_t001 |  |  |  |  |  |  |
| 2 | Ath-AT3G28020.1 |  | | | |  | | | |  |  |  |  |  |  |
| 2 | Ath-AT3G28030.3 |  | Vvi-Vitvi14g01804\_t001 |  | | | |  |  |  |  |  |  |
| 2 | Ath-AT3G28040.1 |  | | | |  | | | |  |  |  |  |  |  |
| 3 | Ath-AT3G28050.1 |  | Vvi-Vitvi14g01805\_t001 |  | Vvi-Vitvi17g00765\_t001 |  | Vvi-Vitvi01g00810\_t001 |  |  |  |  |  |
| 3 | Ath-AT3G28060.1 |  | | | |  | | | |  | | | |  |  |  |  |  |
| 3 | Ath-AT3G28070.1 |  | | | |  | | | |  | | | |  |  |  |  |  |
| 3 | Ath-AT3G28080.1 |  | | | |  | | | |  | | | |  |  |  |  |  |
| 3 | Ath-AT3G28100.1 |  | | | |  | | | |  | Vvi-Vitvi01g00812\_t001 |  |  |  |  |  |
| 3 | Ath-AT3G28120.1 |  | | | |  | | | |  | | | |  |  |  |  |  |
| 3 | Ath-AT3G28130.2 |  | | | |  | | | |  | Vvi-Vitvi01g00814\_t002 |  |  |  |  |  |
| 3 | Ath-AT3G28140.1 |  | Vvi-Vitvi14g04639\_t001 |  | | | |  | | | |  |  |  |  |  |
| 3 | Ath-AT3G28150.1 |  | Vvi-Vitvi14g01811\_t001 |  | | | |  | | | |  |  |  |  |  |
| 3 | Ath-AT3G28155.1 |  | | | |  | | | |  | | | |  |  |  |  |  |
| 3 | Ath-AT3G28170.1 |  | | | |  | | | |  | | | |  |  |  |  |  |
| 3 | Ath-AT3G28180.1 |  | Vvi-Vitvi14g01815\_t001 |  | | | |  | | | |  |  |  |  |  |
| 3 | Ath-AT3G28190.1 |  | | | |  | | | |  | | | |  |  |  |  |  |
| 3 | Ath-AT3G28193.1 |  | | | |  | | | |  | | | |  |  |  |  |  |
| 3 | Ath-AT3G28200.1 |  | Vvi-Vitvi14g01821\_t001 |  | Vvi-Vitvi17g00747\_t001 |  | Vvi-Vitvi01g00844\_t001 |  |  |  |  |  |
| 2 | Ath-AT3G28210.1 |  | Vvi-Vitvi14g01824\_t001 |  |  |  | | | |  |  |  |  |  |
| 1 | Ath-AT3G28216.1 |  |  |  |  |  | | | |  |  |  |  |  |
| 1 | Ath-AT3G28220.1 |  |  |  |  |  | | | |  |  |  |  |  |
| 1 | Ath-AT3G28223.1 |  |  |  |  |  | | | |  |  |  |  |  |
| 1 | Ath-AT3G28230.2 |  |  |  |  |  | | | |  |  |  |  |  |
| 1 | Ath-AT3G28243.1 |  |  |  |  |  | | | |  |  |  |  |  |
| 1 | Ath-AT3G28250.1 |  |  |  |  |  | | | |  |  |  |  |  |
| 1 | Ath-AT3G28270.2 |  |  |  |  |  | | | |  |  |  |  |  |
| 1 | Ath-AT3G28280.1 |  |  |  |  |  | | | |  |  |  |  |  |
| 1 | Ath-AT3G28290.1 |  |  |  |  |  | | | |  |  |  |  |  |
| 1 | Ath-AT3G28291.1 |  |  |  |  |  | | | |  |  |  |  |  |
| 1 | Ath-AT3G28300.1 |  |  |  |  |  | | | |  |  |  |  |  |
| 1 | Ath-AT3G28310.1 |  |  |  |  |  | | | |  |  |  |  |  |
| 1 | Ath-AT3G28320.1 |  |  |  |  |  | | | |  |  |  |  |  |
| 1 | Ath-AT3G28330.1 |  |  |  |  |  | | | |  |  |  |  |  |
| 1 | Ath-AT3G28335.1 |  |  |  |  |  | | | |  |  |  |  |  |
| 2 | Ath-AT3G28340.1 |  | Vvi-Vitvi14g01868\_t001 |  |  |  | Vvi-Vitvi01g00860\_t001 |  |  |  |  |  |
| 3 | Ath-AT3G28345.1 |  | | | |  | Vvi-Vitvi14g01858\_t001 |  | | | |  |  |  |  |  |
| 3 | Ath-AT3G28360.1 |  | | | |  | | | |  | | | |  |  |  |  |  |
| 3 | Ath-AT3G28370.5 |  | Vvi-Vitvi14g01857\_t001 |  | Vvi-Vitvi14g04658\_t001 |  | | | |  |  |  |  |  |
| 3 | Ath-AT3G28380.1 |  | | | |  | | | |  | | | |  |  |  |  |  |
| 3 | Ath-AT3G28390.1 |  | | | |  | | | |  | | | |  |  |  |  |  |
| 3 | Ath-AT3G28410.1 |  | | | |  | | | |  | | | |  |  |  |  |  |
| 3 | Ath-AT3G28415.2 |  | | | |  | | | |  | | | |  |  |  |  |  |
| 3 | Ath-AT3G28420.1 |  | | | |  | | | |  | | | |  |  |  |  |  |
| 3 | Ath-AT3G28430.1 |  | | | |  | | | |  | | | |  |  |  |  |  |
| 3 | Ath-AT3G28450.1 |  | Vvi-Vitvi14g01853\_t001 |  | | | |  | Vvi-Vitvi01g00868\_t001 |  |  |  |  |  |
| 3 | Ath-AT3G28455.1 |  | Vvi-Vitvi14g04650\_t001 |  | | | |  | Vvi-Vitvi01g02100\_t001 |  |  |  |  |  |
| 3 | Ath-AT3G28460.1 |  | Vvi-Vitvi14g01847\_t001 |  | | | |  | | | |  |  |  |  |  |
| 3 | Ath-AT3G28470.1 |  | Vvi-Vitvi14g01845\_t001 |  | | | |  | | | |  |  |  |  |  |
| 3 | Ath-AT3G28480.2 |  | Vvi-Vitvi14g03047\_t001 |  | | | |  | | | |  |  |  |  |  |
| 3 | Ath-AT3G28490.1 |  | | | |  | | | |  | | | |  |  |  |  |  |
| 3 | Ath-AT3G28500.1 |  | | | |  | | | |  | | | |  |  |  |  |  |
| 3 | Ath-AT3G28510.1 |  | | | |  | Vvi-Vitvi14g03049\_t001 |  | | | |  |  |  |  |  |
| 3 | Ath-AT3G28520.1 |  | | | |  | | | |  | | | |  |  |  |  |  |
| 3 | Ath-AT3G28530.1 |  | | | |  | | | |  | | | |  |  |  |  |  |
| 3 | Ath-AT3G28540.1 |  | | | |  | Vvi-Vitvi14g01875\_t001 |  | | | |  |  |  |  |  |
| 3 | Ath-AT3G28550.1 |  | | | |  | | | |  | | | |  |  |  |  |  |
| 3 | Ath-AT3G28560.1 |  | | | |  | | | |  | | | |  |  |  |  |  |
| 3 | Ath-AT3G28570.2 |  | Vvi-Vitvi14g01840\_t001 |  | | | |  | | | |  |  |  |  |  |
| 3 | Ath-AT3G28580.1 |  | Vvi-Vitvi14g01835\_t001 |  | | | |  | | | |  |  |  |  |  |
| 2 | Ath-AT3G28590.1 |  |  |  | | | |  | | | |  |  |  |  |  |
| 2 | Ath-AT3G28600.1 |  |  |  | | | |  | | | |  |  |  |  |  |
| 2 | Ath-AT3G28610.1 |  |  |  | | | |  | | | |  |  |  |  |  |
| 2 | Ath-AT3G28620.1 |  |  |  | | | |  | | | |  |  |  |  |  |
| 2 | Ath-AT3G28630.1 |  |  |  | Vvi-Vitvi14g01879\_t001 |  | Vvi-Vitvi01g00902\_t004 |  |  |  |  |  |
| 2 | Ath-AT3G28635.1 |  |  |  | | | |  | | | |  |  |  |  |  |
| 2 | Ath-AT3G28640.1 |  |  |  | Vvi-Vitvi14g03051\_t001 |  | | | |  |  |  |  |  |
| 2 | Ath-AT3G28650.1 |  |  |  | | | |  | | | |  |  |  |  |  |
| 2 | Ath-AT3G28660.1 |  |  |  | | | |  | | | |  |  |  |  |  |
| 2 | Ath-AT3G28670.2 |  |  |  | | | |  | | | |  |  |  |  |  |
| 2 | Ath-AT3G28674.1 |  |  |  | | | |  | | | |  |  |  |  |  |
| 2 | Ath-AT3G28680.1 |  |  |  | | | |  | | | |  |  |  |  |  |
| 2 | Ath-AT3G28690.2 |  |  |  | | | |  | Vvi-Vitvi01g00927\_t001 |  |  |  |  |  |
| 1 | Ath-AT3G28700.1 |  |  |  | Vvi-Vitvi14g01894\_t001 |  |  |  |  |  |  |
| 1 | Ath-AT3G28710.1 |  |  |  | | | |  |  |  |  |  |  |
| 1 | Ath-AT3G28715.1 |  |  |  | | | |  |  |  |  |  |  |
| 1 | Ath-AT3G28720.2 |  |  |  | | | |  |  |  |  |  |  |
| 1 | Ath-AT3G28730.1 |  |  |  | Vvi-Vitvi14g01901\_t001 |  |  |  |  |  |  |
| 0 | Ath-AT3G28740.1 |  |  |  |  |  |  |  |  |
| 0 | Ath-AT3G28750.1 |  |  |  |  |  |  |  |  |
| 1 | Ath-AT3G28760.2 |  | Vvi-Vitvi14g01919\_t001 |  |  |  |  |  |  |  |
| 1 | Ath-AT3G28770.1 |  | | | |  |  |  |  |  |  |  |
| 1 | Ath-AT3G28780.1 |  | | | |  |  |  |  |  |  |  |
| 1 | Ath-AT3G28785.1 |  | | | |  |  |  |  |  |  |  |
| 1 | Ath-AT3G28790.1 |  | | | |  |  |  |  |  |  |  |
| 1 | Ath-AT3G28810.1 |  | | | |  |  |  |  |  |  |  |
| 1 | Ath-AT3G28820.1 |  | | | |  |  |  |  |  |  |  |
| 1 | Ath-AT3G28830.1 |  | | | |  |  |  |  |  |  |  |
| 1 | Ath-AT3G28840.1 |  | | | |  |  |  |  |  |  |  |
| 1 | Ath-AT3G28850.1 |  | Vvi-Vitvi14g01923\_t001 |  |  |  |  |  |  |  |
| 1 | Ath-AT3G28853.1 |  | | | |  |  |  |  |  |  |  |
| 1 | Ath-AT3G28857.1 |  | Vvi-Vitvi14g01926\_t001 |  |  |  |  |  |  |  |
| 1 | Ath-AT3G28860.1 |  | Vvi-Vitvi14g01928\_t001 |  |  |  |  |  |  |  |
| 1 | Ath-AT3G28870.1 |  | | | |  |  |  |  |  |  |  |
| 1 | Ath-AT3G28875.1 |  | Vvi-Vitvi14g01939\_t001 |  |  |  |  |  |  |  |
| 1 | Ath-AT3G28880.1 |  | | | |  |  |  |  |  |  |  |
| 1 | Ath-AT3G28890.1 |  | | | |  |  |  |  |  |  |  |
| 2 | Ath-AT3G28900.1 |  | | | |  | Vvi-Vitvi01g04246\_t001 |  |  |  |  |  |  |
| 2 | Ath-AT3G28899.1 |  | | | |  | | | |  |  |  |  |  |  |
| 3 | Ath-AT3G28910.1 |  | | | |  | | | |  | Vvi-Vitvi17g00598\_t001 |  |  |  |  |  |
| 3 | Ath-AT3G28917.1 |  | | | |  | Vvi-Vitvi01g01012\_t001 |  | Vvi-Vitvi17g00600\_t001 |  |  |  |  |  |
| 3 | Ath-AT3G28918.1 |  | | | |  | | | |  | | | |  |  |  |  |  |
| 3 | Ath-AT3G28920.1 |  | Vvi-Vitvi14g01955\_t001 |  | Vvi-Vitvi01g01013\_t001 |  | | | |  |  |  |  |  |
| 3 | Ath-AT3G28922.1 |  | | | |  | | | |  | | | |  |  |  |  |  |
| 3 | Ath-AT3G28925.1 |  | | | |  | | | |  | | | |  |  |  |  |  |
| 3 | Ath-AT3G28930.1 |  | | | |  | | | |  | | | |  |  |  |  |  |
| 3 | Ath-AT3G28940.1 |  | | | |  | | | |  | | | |  |  |  |  |  |
| 3 | Ath-AT3G28950.1 |  | | | |  | | | |  | | | |  |  |  |  |  |
| 3 | Ath-AT3G28956.3 |  | | | |  | | | |  | | | |  |  |  |  |  |
| 3 | Ath-AT3G28958.1 |  | | | |  | | | |  | | | |  |  |  |  |  |
| 3 | Ath-AT3G28960.3 |  | | | |  | Vvi-Vitvi01g01019\_t001 |  | | | |  |  |  |  |  |
| 3 | Ath-AT3G28970.1 |  | Vvi-Vitvi14g01970\_t001 |  | | | |  | | | |  |  |  |  |  |
| 3 | Ath-AT3G28980.1 |  | | | |  | | | |  | | | |  |  |  |  |  |
| 3 | Ath-AT3G28990.1 |  | | | |  | | | |  | | | |  |  |  |  |  |
| 3 | Ath-AT3G29000.1 |  | Vvi-Vitvi14g01975\_t001 |  | Vvi-Vitvi01g01027\_t001 |  | Vvi-Vitvi17g01471\_t001 |  |  |  |  |  |
| 3 | Ath-AT3G29010.1 |  | | | |  | | | |  | Vvi-Vitvi17g00613\_t001 |  |  |  |  |  |
| 3 | Ath-AT3G29020.2 |  | Vvi-Vitvi14g01976\_t001 |  | Vvi-Vitvi01g01028\_t001 |  | | | |  |  |  |  |  |
| 2 | Ath-AT3G29030.1 |  | Vvi-Vitvi14g01977\_t001 |  |  |  | | | |  |  |  |  |  |
| 2 | Ath-AT3G29033.1 |  | | | |  |  |  | | | |  |  |  |  |  |
| 2 | Ath-AT3G29034.1 |  | | | |  |  |  | | | |  |  |  |  |  |
| 2 | Ath-AT3G29035.1 |  | Vvi-Vitvi14g01985\_t001 |  |  |  | Vvi-Vitvi17g00622\_t001 |  |  |  |  |  |
| 2 | Ath-AT3G29040.1 |  | | | |  |  |  | | | |  |  |  |  |  |
| 2 | Ath-AT3G29050.2 |  | | | |  |  |  | | | |  |  |  |  |  |
| 2 | Ath-AT3G29060.1 |  | Vvi-Vitvi14g01992\_t001 |  |  |  | | | |  |  |  |  |  |
| 2 | Ath-AT3G29070.1 |  | Vvi-Vitvi14g03093\_t001 |  |  |  | | | |  |  |  |  |  |
| 2 | Ath-AT3G29075.1 |  | Vvi-Vitvi14g01994\_t001 |  |  |  | | | |  |  |  |  |  |
| 2 | Ath-AT3G29080.1 |  | | | |  |  |  | | | |  |  |  |  |  |
| 2 | Ath-AT3G29090.1 |  | Vvi-Vitvi14g01995\_t001 |  |  |  | | | |  |  |  |  |  |
| 2 | Ath-AT3G29100.3 |  | Vvi-Vitvi14g01999\_t001 |  |  |  | Vvi-Vitvi17g00641\_t001 |  |  |  |  |  |
| 1 | Ath-AT3G29110.1 |  | | | |  |  |  |  |  |  |  |
| 1 | Ath-AT3G29130.2 |  | | | |  |  |  |  |  |  |  |
| 1 | Ath-AT3G29140.1 |  | | | |  |  |  |  |  |  |  |
| 1 | Ath-AT3G29152.1 |  | | | |  |  |  |  |  |  |  |
| 1 | Ath-AT3G29160.2 |  | Vvi-Vitvi14g02002\_t003 |  |  |  |  |  |  |  |
| 1 | Ath-AT3G29170.1 |  | Vvi-Vitvi14g04692\_t001 |  |  |  |  |  |  |  |
| 1 | Ath-AT3G29173.1 |  | | | |  |  |  |  |  |  |  |
| 1 | Ath-AT3G29180.1 |  | Vvi-Vitvi14g02005\_t001 |  |  |  |  |  |  |  |
| 1 | Ath-AT3G29185.1 |  | Vvi-Vitvi14g02008\_t001 |  |  |  |  |  |  |  |
| 1 | Ath-AT3G29190.1 |  | | | |  |  |  |  |  |  |  |
| 1 | Ath-AT3G29200.1 |  | Vvi-Vitvi14g02011\_t001 |  |  |  |  |  |  |  |
| 1 | Ath-AT3G29230.1 |  | Vvi-Vitvi14g02012\_t001 |  |  |  |  |  |  |  |
| 1 | Ath-AT3G29240.2 |  | Vvi-Vitvi14g02013\_t001 |  |  |  |  |  |  |  |
| 1 | Ath-AT3G29250.1 |  | | | |  |  |  |  |  |  |  |
| 1 | Ath-AT3G29255.1 |  | | | |  |  |  |  |  |  |  |
| 1 | Ath-AT3G29260.1 |  | | | |  |  |  |  |  |  |  |
| 1 | Ath-AT3G29270.1 |  | Vvi-Vitvi14g02020\_t001 |  |  |  |  |  |  |  |
| 1 | Ath-AT3G29280.2 |  | Vvi-Vitvi14g02024\_t002 |  |  |  |  |  |  |  |
| 1 | Ath-AT3G29290.1 |  | Vvi-Vitvi14g02025\_t001.1.6037826b |  |  |  |  |  |  |  |
| 1 | Ath-AT3G29300.2 |  | | | |  |  |  |  |  |  |  |
| 1 | Ath-AT3G29305.1 |  | | | |  |  |  |  |  |  |  |
| 1 | Ath-AT3G29310.1 |  | Vvi-Vitvi14g02031\_t001 |  |  |  |  |  |  |  |
| 1 | Ath-AT3G29320.1 |  | Vvi-Vitvi14g02032\_t001 |  |  |  |  |  |  |  |
| 1 | Ath-AT3G29330.2 |  | | | |  |  |  |  |  |  |  |
| 1 | Ath-AT3G29340.1 |  | | | |  |  |  |  |  |  |  |
| 1 | Ath-AT3G29350.1 |  | | | |  |  |  |  |  |  |  |
| 1 | Ath-AT3G29360.1 |  | Vvi-Vitvi14g03099\_t002 |  |  |  |  |  |  |  |
| 0 | Ath-AT3G29370.1 |  |  |  |  |  |  |  |  |
| 0 | Ath-AT3G29375.1 |  |  |  |  |  |  |  |  |
| 0 | Ath-AT3G29380.1 |  |  |  |  |  |  |  |  |
| 0 | Ath-AT3G29385.1 |  |  |  |  |  |  |  |  |
| 0 | Ath-AT3G29390.1 |  |  |  |  |  |  |  |  |
| 0 | Ath-AT3G29400.1 |  |  |  |  |  |  |  |  |
| 0 | Ath-AT3G29410.1 |  |  |  |  |  |  |  |  |
| 0 | Ath-AT3G29430.1 |  |  |  |  |  |  |  |  |
| 0 | Ath-AT3G29450.1 |  |  |  |  |  |  |  |  |
| 0 | Ath-AT3G29455.1 |  |  |  |  |  |  |  |  |
| 0 | Ath-AT3G29560.1 |  |  |  |  |  |  |  |  |
| 0 | Ath-AT3G29570.1 |  |  |  |  |  |  |  |  |
| 0 | Ath-AT3G29575.1 |  |  |  |  |  |  |  |  |
| 0 | Ath-AT3G29580.1 |  |  |  |  |  |  |  |  |
| 0 | Ath-AT3G29590.1 |  |  |  |  |  |  |  |  |
| 0 | Ath-AT3G29630.1 |  |  |  |  |  |  |  |  |
| 0 | Ath-AT3G29633.1 |  |  |  |  |  |  |  |  |
| 0 | Ath-AT3G29635.1 |  |  |  |  |  |  |  |  |
| 0 | Ath-AT3G29636.1 |  |  |  |  |  |  |  |  |
| 0 | Ath-AT3G29631.1 |  |  |  |  |  |  |  |  |
| 0 | Ath-AT3G29637.2 |  |  |  |  |  |  |  |  |
| 0 | Ath-AT3G29638.1 |  |  |  |  |  |  |  |  |
| 0 | Ath-AT3G29639.1 |  |  |  |  |  |  |  |  |
| 0 | Ath-AT3G29645.1 |  |  |  |  |  |  |  |  |
| 1 | Ath-AT3G29670.1 |  | Vvi-Vitvi13g02515\_t001 |  |  |  |  |  |  |  |
| 1 | Ath-AT3G29680.1 |  | | | |  |  |  |  |  |  |  |
| 1 | Ath-AT3G29690.1 |  | | | |  |  |  |  |  |  |  |
| 1 | Ath-AT3G29720.1 |  | | | |  |  |  |  |  |  |  |
| 1 | Ath-AT3G29740.1 |  | | | |  |  |  |  |  |  |  |
| 1 | Ath-AT3G29750.1 |  | | | |  |  |  |  |  |  |  |
| 1 | Ath-AT3G29760.2 |  | | | |  |  |  |  |  |  |  |
| 1 | Ath-AT3G29763.1 |  | | | |  |  |  |  |  |  |  |
| 1 | Ath-AT3G29765.1 |  | | | |  |  |  |  |  |  |  |
| 1 | Ath-AT3G29785.1 |  | | | |  |  |  |  |  |  |  |
| 1 | Ath-AT3G29770.1 |  | | | |  |  |  |  |  |  |  |
| 1 | Ath-AT3G29780.1 |  | | | |  |  |  |  |  |  |  |
| 1 | Ath-AT3G29790.1 |  | | | |  |  |  |  |  |  |  |
| 1 | Ath-AT3G29791.1 |  | | | |  |  |  |  |  |  |  |
| 1 | Ath-AT3G29796.1 |  | | | |  |  |  |  |  |  |  |
| 1 | Ath-AT3G29797.1 |  | | | |  |  |  |  |  |  |  |
| 1 | Ath-AT3G29800.1 |  | | | |  |  |  |  |  |  |  |
| 1 | Ath-AT3G29810.1 |  | Vvi-Vitvi14g01357\_t001 |  |  |  |  |  |  |  |
| 1 | Ath-AT3G29830.1 |  | | | |  |  |  |  |  |  |  |
| 1 | Ath-AT3G29970.1 |  | Vvi-Vitvi14g01354\_t001 |  |  |  |  |  |  |  |
| 1 | Ath-AT3G30160.1 |  | | | |  |  |  |  |  |  |  |
| 1 | Ath-AT3G30165.1 |  | | | |  |  |  |  |  |  |  |
| 1 | Ath-AT3G30180.1 |  | Vvi-Vitvi14g01351\_t001 |  |  |  |  |  |  |  |
| 1 | Ath-AT3G30200.1 |  | | | |  |  |  |  |  |  |  |
| 1 | Ath-AT3G30210.1 |  | Vvi-Vitvi14g01346\_t001 |  |  |  |  |  |  |  |
| 1 | Ath-AT3G30220.1 |  | | | |  |  |  |  |  |  |  |
| 1 | Ath-AT3G30222.1 |  | | | |  |  |  |  |  |  |  |
| 1 | Ath-AT3G30230.1 |  | | | |  |  |  |  |  |  |  |
| 1 | Ath-AT3G30235.1 |  | | | |  |  |  |  |  |  |  |
| 1 | Ath-AT3G30247.1 |  | | | |  |  |  |  |  |  |  |
| 1 | Ath-AT3G30260.1 |  | Vvi-Vitvi14g01341\_t001 |  |  |  |  |  |  |  |
| 1 | Ath-AT3G30280.1 |  | | | |  |  |  |  |  |  |  |
| 1 | Ath-AT3G30290.1 |  | | | |  |  |  |  |  |  |  |
| 1 | Ath-AT3G30300.1 |  | Vvi-Vitvi14g01325\_t001 |  |  |  |  |  |  |  |
| 1 | Ath-AT3G30305.1 |  | | | |  |  |  |  |  |  |  |
| 1 | Ath-AT3G30320.1 |  | | | |  |  |  |  |  |  |  |
| 1 | Ath-AT3G30340.1 |  | | | |  |  |  |  |  |  |  |
| 1 | Ath-AT3G30350.2 |  | | | |  |  |  |  |  |  |  |
| 1 | Ath-AT3G30370.1 |  | | | |  |  |  |  |  |  |  |
| 1 | Ath-AT3G30380.1 |  | Vvi-Vitvi14g01312\_t001 |  |  |  |  |  |  |  |
| 1 | Ath-AT3G30390.3 |  | Vvi-Vitvi14g01307\_t001 |  |  |  |  |  |  |  |
| 1 | Ath-AT3G30383.1 |  | | | |  |  |  |  |  |  |  |
| 1 | Ath-AT3G30385.1 |  | | | |  |  |  |  |  |  |  |
| 1 | Ath-AT3G30387.1 |  | | | |  |  |  |  |  |  |  |
| 1 | Ath-AT3G30430.1 |  | | | |  |  |  |  |  |  |  |
| 1 | Ath-AT3G30460.2 |  | | | |  |  |  |  |  |  |  |
| 1 | Ath-AT3G30520.1 |  | | | |  |  |  |  |  |  |  |
| 1 | Ath-AT3G30525.1 |  | | | |  |  |  |  |  |  |  |
| 1 | Ath-AT3G30530.1 |  | Vvi-Vitvi14g01302\_t001 |  |  |  |  |  |  |  |
| 1 | Ath-AT3G30540.1 |  | | | |  |  |  |  |  |  |  |
| 1 | Ath-AT3G30580.1 |  | | | |  |  |  |  |  |  |  |
| 1 | Ath-AT3G30705.1 |  | | | |  |  |  |  |  |  |  |
| 1 | Ath-AT3G30720.1 |  | | | |  |  |  |  |  |  |  |
| 1 | Ath-AT3G30725.1 |  | | | |  |  |  |  |  |  |  |
| 1 | Ath-AT3G30730.2 |  | | | |  |  |  |  |  |  |  |
| 1 | Ath-AT3G30739.1 |  | | | |  |  |  |  |  |  |  |
| 1 | Ath-AT3G30770.1 |  | | | |  |  |  |  |  |  |  |
| 1 | Ath-AT3G30775.2 |  | Vvi-Vitvi14g01283\_t001 |  |  |  |  |  |  |  |
| 1 | Ath-AT3G30805.1 |  | | | |  |  |  |  |  |  |  |
| 1 | Ath-AT3G30820.1 |  | | | |  |  |  |  |  |  |  |
| 1 | Ath-AT3G30823.1 |  | | | |  |  |  |  |  |  |  |
| 1 | Ath-AT3G30840.1 |  | | | |  |  |  |  |  |  |  |
| 1 | Ath-AT3G30841.1 |  | Vvi-Vitvi14g04457\_t001 |  |  |  |  |  |  |  |
| 0 | Ath-AT3G30842.1 |  |  |  |  |  |  |  |  |
| 0 | Ath-AT3G30845.1 |  |  |  |  |  |  |  |  |
| 0 | Ath-AT3G31350.1 |  |  |  |  |  |  |  |  |
| 0 | Ath-AT3G31402.1 |  |  |  |  |  |  |  |  |
| 0 | Ath-AT3G31430.1 |  |  |  |  |  |  |  |  |
| 0 | Ath-AT3G31550.1 |  |  |  |  |  |  |  |  |
| 0 | Ath-AT3G31560.1 |  |  |  |  |  |  |  |  |
| 0 | Ath-AT3G31900.1 |  |  |  |  |  |  |  |  |
| 0 | Ath-AT3G31910.1 |  |  |  |  |  |  |  |  |
| 0 | Ath-AT3G31925.1 |  |  |  |  |  |  |  |  |
| 0 | Ath-AT3G31950.1 |  |  |  |  |  |  |  |  |
| 0 | Ath-AT3G32030.1 |  |  |  |  |  |  |  |  |
| 0 | Ath-AT3G32040.1 |  |  |  |  |  |  |  |  |
| 0 | Ath-AT3G32047.1 |  |  |  |  |  |  |  |  |
| 0 | Ath-AT3G32050.1 |  |  |  |  |  |  |  |  |
| 0 | Ath-AT3G32090.1 |  |  |  |  |  |  |  |  |
| 0 | Ath-AT3G32130.1 |  |  |  |  |  |  |  |  |
| 0 | Ath-AT3G32150.1 |  |  |  |  |  |  |  |  |
| 0 | Ath-AT3G32160.1 |  |  |  |  |  |  |  |  |
| 0 | Ath-AT3G32180.1 |  |  |  |  |  |  |  |  |
| 0 | Ath-AT3G32190.1 |  |  |  |  |  |  |  |  |
| 0 | Ath-AT3G32200.1 |  |  |  |  |  |  |  |  |
| 0 | Ath-AT3G32260.1 |  |  |  |  |  |  |  |  |
| 0 | Ath-AT3G32280.1 |  |  |  |  |  |  |  |  |
| 0 | Ath-AT3G32330.1 |  |  |  |  |  |  |  |  |
| 0 | Ath-AT3G32380.1 |  |  |  |  |  |  |  |  |
| 0 | Ath-AT3G32400.2 |  |  |  |  |  |  |  |  |
| 0 | Ath-AT3G32410.1 |  |  |  |  |  |  |  |  |
| 0 | Ath-AT3G32420.1 |  |  |  |  |  |  |  |  |
| 0 | Ath-AT3G32896.1 |  |  |  |  |  |  |  |  |
| 0 | Ath-AT3G32904.1 |  |  |  |  |  |  |  |  |
| 0 | Ath-AT3G32920.1 |  |  |  |  |  |  |  |  |
| 0 | Ath-AT3G32930.1 |  |  |  |  |  |  |  |  |
| 0 | Ath-AT3G32940.1 |  |  |  |  |  |  |  |  |
| 0 | Ath-AT3G32960.1 |  |  |  |  |  |  |  |  |
| 0 | Ath-AT3G32980.1 |  |  |  |  |  |  |  |  |
| 0 | Ath-AT3G33187.1 |  |  |  |  |  |  |  |  |
| 0 | Ath-AT3G33293.1 |  |  |  |  |  |  |  |  |
| 0 | Ath-AT3G33393.1 |  |  |  |  |  |  |  |  |
| 0 | Ath-AT3G33494.1 |  |  |  |  |  |  |  |  |
| 0 | Ath-AT3G33528.1 |  |  |  |  |  |  |  |  |
| 0 | Ath-AT3G33530.3 |  |  |  |  |  |  |  |  |
| 0 | Ath-AT3G33520.1 |  |  |  |  |  |  |  |  |
| 0 | Ath-AT3G41762.1 |  |  |  |  |  |  |  |  |
| 0 | Ath-AT3G42050.1 |  |  |  |  |  |  |  |  |
| 0 | Ath-AT3G42060.1 |  |  |  |  |  |  |  |  |
| 0 | Ath-AT3G42075.1 |  |  |  |  |  |  |  |  |
| 0 | Ath-AT3G42130.1 |  |  |  |  |  |  |  |  |
| 0 | Ath-AT3G42140.1 |  |  |  |  |  |  |  |  |
| 0 | Ath-AT3G42148.1 |  |  |  |  |  |  |  |  |
| 0 | Ath-AT3G42150.1 |  |  |  |  |  |  |  |  |
| 0 | Ath-AT3G42153.1 |  |  |  |  |  |  |  |  |
| 0 | Ath-AT3G42155.1 |  |  |  |  |  |  |  |  |
| 0 | Ath-AT3G42160.1 |  |  |  |  |  |  |  |  |
| 0 | Ath-AT3G42170.1 |  |  |  |  |  |  |  |  |
| 0 | Ath-AT3G42180.1 |  |  |  |  |  |  |  |  |
| 0 | Ath-AT3G42310.1 |  |  |  |  |  |  |  |  |
| 0 | Ath-AT3G42390.1 |  |  |  |  |  |  |  |  |
| 0 | Ath-AT3G42473.1 |  |  |  |  |  |  |  |  |
| 0 | Ath-AT3G42550.1 |  |  |  |  |  |  |  |  |
| 0 | Ath-AT3G42560.1 |  |  |  |  |  |  |  |  |
| 0 | Ath-AT3G42565.1 |  |  |  |  |  |  |  |  |
| 0 | Ath-AT3G42570.1 |  |  |  |  |  |  |  |  |
| 0 | Ath-AT3G42628.1 |  |  |  |  |  |  |  |  |
| 0 | Ath-AT3G42630.1 |  |  |  |  |  |  |  |  |
| 0 | Ath-AT3G42640.1 |  |  |  |  |  |  |  |  |
| 0 | Ath-AT3G42660.1 |  |  |  |  |  |  |  |  |
| 0 | Ath-AT3G42670.4 |  |  |  |  |  |  |  |  |
| 0 | Ath-AT3G42723.1 |  |  |  |  |  |  |  |  |
| 1 | Ath-AT3G42725.1 |  | Vvi-Vitvi11g01341\_t001 |  |  |  |  |  |  |  |
| 1 | Ath-AT3G42770.1 |  | | | |  |  |  |  |  |  |  |
| 1 | Ath-AT3G42780.1 |  | | | |  |  |  |  |  |  |  |
| 1 | Ath-AT3G42786.1 |  | | | |  |  |  |  |  |  |  |
| 1 | Ath-AT3G42790.1 |  | | | |  |  |  |  |  |  |  |
| 1 | Ath-AT3G29794.1 |  | | | |  |  |  |  |  |  |  |
| 1 | Ath-AT3G42800.1 |  | | | |  |  |  |  |  |  |  |
| 1 | Ath-AT3G42830.1 |  | Vvi-Vitvi11g00072\_t001 |  |  |  |  |  |  |  |
| 1 | Ath-AT3G42850.2 |  | | | |  |  |  |  |  |  |  |
| 1 | Ath-AT3G42860.1 |  | | | |  |  |  |  |  |  |  |
| 1 | Ath-AT3G42870.1 |  | | | |  |  |  |  |  |  |  |
| 1 | Ath-AT3G42880.1 |  | Vvi-Vitvi11g00053\_t001 |  |  |  |  |  |  |  |
| 1 | Ath-AT3G42940.1 |  | | | |  |  |  |  |  |  |  |
| 1 | Ath-AT3G42950.1 |  | Vvi-Vitvi11g00051\_t003 |  |  |  |  |  |  |  |
| 1 | Ath-AT3G42960.1 |  | | | |  |  |  |  |  |  |  |
| 1 | Ath-AT3G42990.1 |  | | | |  |  |  |  |  |  |  |
| 1 | Ath-AT3G42995.1 |  | | | |  |  |  |  |  |  |  |
| 1 | Ath-AT3G43083.1 |  | | | |  |  |  |  |  |  |  |
| 1 | Ath-AT3G43110.1 |  | Vvi-Vitvi11g01325\_t001 |  |  |  |  |  |  |  |
| 1 | Ath-AT3G43120.1 |  | Vvi-Vitvi11g00035\_t001 |  |  |  |  |  |  |  |
| 1 | Ath-AT3G43148.1 |  | | | |  |  |  |  |  |  |  |
| 1 | Ath-AT3G43150.1 |  | | | |  |  |  |  |  |  |  |
| 1 | Ath-AT3G43153.1 |  | | | |  |  |  |  |  |  |  |
| 1 | Ath-AT3G43160.1 |  | | | |  |  |  |  |  |  |  |
| 1 | Ath-AT3G43170.1 |  | | | |  |  |  |  |  |  |  |
| 1 | Ath-AT3G43180.1 |  | | | |  |  |  |  |  |  |  |
| 1 | Ath-AT3G43190.1 |  | Vvi-Vitvi11g00030\_t001 |  |  |  |  |  |  |  |
| 1 | Ath-AT3G43210.1 |  | Vvi-Vitvi11g00028\_t001 |  |  |  |  |  |  |  |
| 1 | Ath-AT3G43220.1 |  | Vvi-Vitvi11g00026\_t001 |  |  |  |  |  |  |  |
| 1 | Ath-AT3G43230.1 |  | Vvi-Vitvi11g00023\_t001 |  |  |  |  |  |  |  |
| 1 | Ath-AT3G43240.1 |  | Vvi-Vitvi11g00020\_t001 |  |  |  |  |  |  |  |
| 1 | Ath-AT3G43250.1 |  | | | |  |  |  |  |  |  |  |
| 1 | Ath-AT3G43260.1 |  | | | |  |  |  |  |  |  |  |
| 1 | Ath-AT3G43270.1 |  | | | |  |  |  |  |  |  |  |
| 1 | Ath-AT3G43280.1 |  | | | |  |  |  |  |  |  |  |
| 1 | Ath-AT3G43290.1 |  | | | |  |  |  |  |  |  |  |
| 1 | Ath-AT3G43291.1 |  | | | |  |  |  |  |  |  |  |
| 1 | Ath-AT3G43300.1 |  | Vvi-Vitvi11g00007\_t001 |  |  |  |  |  |  |  |
| 1 | Ath-AT3G43340.1 |  | | | |  |  |  |  |  |  |  |
| 1 | Ath-AT3G43400.1 |  | | | |  |  |  |  |  |  |  |
| 1 | Ath-AT3G43410.1 |  | | | |  |  |  |  |  |  |  |
| 1 | Ath-AT3G43415.1 |  | | | |  |  |  |  |  |  |  |
| 1 | Ath-AT3G43420.1 |  | | | |  |  |  |  |  |  |  |
| 1 | Ath-AT3G43430.1 |  | Vvi-Vitvi11g00004\_t001 |  |  |  |  |  |  |  |
| 0 | Ath-AT3G43432.1 |  |  |  |  |  |  |  |  |
| 0 | Ath-AT3G43440.1 |  |  |  |  |  |  |  |  |
| 0 | Ath-AT3G43470.1 |  |  |  |  |  |  |  |  |
| 0 | Ath-AT3G43480.1 |  |  |  |  |  |  |  |  |
| 0 | Ath-AT3G43490.1 |  |  |  |  |  |  |  |  |
| 0 | Ath-AT3G43500.1 |  |  |  |  |  |  |  |  |
| 0 | Ath-AT3G43503.1 |  |  |  |  |  |  |  |  |
| 0 | Ath-AT3G43505.1 |  |  |  |  |  |  |  |  |
| 0 | Ath-AT3G43520.1 |  |  |  |  |  |  |  |  |
| 0 | Ath-AT3G43540.1 |  |  |  |  |  |  |  |  |
| 0 | Ath-AT3G43550.1 |  |  |  |  |  |  |  |  |
| 0 | Ath-AT3G43570.1 |  |  |  |  |  |  |  |  |
| 0 | Ath-AT3G43572.1 |  |  |  |  |  |  |  |  |
| 0 | Ath-AT3G43574.1 |  |  |  |  |  |  |  |  |
| 0 | Ath-AT3G43580.1 |  |  |  |  |  |  |  |  |
| 0 | Ath-AT3G43583.1 |  |  |  |  |  |  |  |  |
| 0 | Ath-AT3G43590.1 |  |  |  |  |  |  |  |  |
| 0 | Ath-AT3G43600.1 |  |  |  |  |  |  |  |  |
| 0 | Ath-AT3G43610.2 |  |  |  |  |  |  |  |  |
| 0 | Ath-AT3G43630.1 |  |  |  |  |  |  |  |  |
| 0 | Ath-AT3G43645.1 |  |  |  |  |  |  |  |  |
| 0 | Ath-AT3G43660.1 |  |  |  |  |  |  |  |  |
| 0 | Ath-AT3G43670.1 |  |  |  |  |  |  |  |  |
| 0 | Ath-AT3G43682.1 |  |  |  |  |  |  |  |  |
| 0 | Ath-AT3G43700.1 |  |  |  |  |  |  |  |  |
| 0 | Ath-AT3G43710.1 |  |  |  |  |  |  |  |  |
| 0 | Ath-AT3G43720.1 |  |  |  |  |  |  |  |  |
| 0 | Ath-AT3G43740.2 |  |  |  |  |  |  |  |  |
| 0 | Ath-AT3G43750.1 |  |  |  |  |  |  |  |  |
| 0 | Ath-AT3G43790.2 |  |  |  |  |  |  |  |  |
| 0 | Ath-AT3G43800.1 |  |  |  |  |  |  |  |  |
| 0 | Ath-AT3G43810.2 |  |  |  |  |  |  |  |  |
| 0 | Ath-AT3G43833.1 |  |  |  |  |  |  |  |  |
| 0 | Ath-AT3G43837.1 |  |  |  |  |  |  |  |  |
| 0 | Ath-AT3G43840.2 |  |  |  |  |  |  |  |  |
| 0 | Ath-AT3G43850.1 |  |  |  |  |  |  |  |  |
| 0 | Ath-AT3G43860.1 |  |  |  |  |  |  |  |  |
| 0 | Ath-AT3G43870.1 |  |  |  |  |  |  |  |  |
| 0 | Ath-AT3G43880.1 |  |  |  |  |  |  |  |  |
| 0 | Ath-AT3G43890.1 |  |  |  |  |  |  |  |  |
| 0 | Ath-AT3G43910.1 |  |  |  |  |  |  |  |  |
| 0 | Ath-AT3G43920.2 |  |  |  |  |  |  |  |  |
| 0 | Ath-AT3G43930.1 |  |  |  |  |  |  |  |  |
| 0 | Ath-AT3G43940.1 |  |  |  |  |  |  |  |  |
| 0 | Ath-AT3G43950.1 |  |  |  |  |  |  |  |  |
| 0 | Ath-AT3G43960.1 |  |  |  |  |  |  |  |  |
| 0 | Ath-AT3G43970.1 |  |  |  |  |  |  |  |  |
| 0 | Ath-AT3G43980.1 |  |  |  |  |  |  |  |  |
| 0 | Ath-AT3G43990.1 |  |  |  |  |  |  |  |  |
| 0 | Ath-AT3G44006.1 |  |  |  |  |  |  |  |  |
| 0 | Ath-AT3G44010.1 |  |  |  |  |  |  |  |  |
| 0 | Ath-AT3G44020.1 |  |  |  |  |  |  |  |  |
| 0 | Ath-AT3G44050.1 |  |  |  |  |  |  |  |  |
| 0 | Ath-AT3G44060.1 |  |  |  |  |  |  |  |  |
| 0 | Ath-AT3G44070.1 |  |  |  |  |  |  |  |  |
| 0 | Ath-AT3G44080.1 |  |  |  |  |  |  |  |  |
| 0 | Ath-AT3G44090.1 |  |  |  |  |  |  |  |  |
| 1 | Ath-AT3G44100.1 |  | Vvi-Vitvi06g01563\_t001 |  |  |  |  |  |  |  |
| 1 | Ath-AT3G44110.1 |  | Vvi-Vitvi06g01562\_t001 |  |  |  |  |  |  |  |
| 1 | Ath-AT3G44115.1 |  | | | |  |  |  |  |  |  |  |
| 1 | Ath-AT3G44120.1 |  | | | |  |  |  |  |  |  |  |
| 1 | Ath-AT3G44130.1 |  | | | |  |  |  |  |  |  |  |
| 1 | Ath-AT3G44140.1 |  | | | |  |  |  |  |  |  |  |
| 1 | Ath-AT3G44150.1 |  | | | |  |  |  |  |  |  |  |
| 1 | Ath-AT3G44160.1 |  | | | |  |  |  |  |  |  |  |
| 1 | Ath-AT3G44170.1 |  | | | |  |  |  |  |  |  |  |
| 1 | Ath-AT3G44180.1 |  | | | |  |  |  |  |  |  |  |
| 1 | Ath-AT3G44190.1 |  | | | |  |  |  |  |  |  |  |
| 1 | Ath-AT3G44200.1 |  | | | |  |  |  |  |  |  |  |
| 1 | Ath-AT3G44210.1 |  | | | |  |  |  |  |  |  |  |
| 1 | Ath-AT3G44220.1 |  | Vvi-Vitvi06g01559\_t001 |  |  |  |  |  |  |  |
| 1 | Ath-AT3G44230.1 |  | | | |  |  |  |  |  |  |  |
| 1 | Ath-AT3G44235.1 |  | | | |  |  |  |  |  |  |  |
| 1 | Ath-AT3G44240.1 |  | | | |  |  |  |  |  |  |  |
| 1 | Ath-AT3G44250.1 |  | | | |  |  |  |  |  |  |  |
| 1 | Ath-AT3G44260.1 |  | Vvi-Vitvi06g01546\_t001 |  |  |  |  |  |  |  |
| 1 | Ath-AT3G44261.1 |  | | | |  |  |  |  |  |  |  |
| 1 | Ath-AT3G44265.1 |  | | | |  |  |  |  |  |  |  |
| 1 | Ath-AT3G44280.1 |  | Vvi-Vitvi06g04446\_t001 |  |  |  |  |  |  |  |
| 1 | Ath-AT3G44290.1 |  | Vvi-Vitvi06g01536\_t001 |  |  |  |  |  |  |  |
| 1 | Ath-AT3G44300.1 |  | Vvi-Vitvi06g01533\_t001 |  |  |  |  |  |  |  |
| 1 | Ath-AT3G44310.1 |  | | | |  |  |  |  |  |  |  |
| 1 | Ath-AT3G44320.1 |  | | | |  |  |  |  |  |  |  |
| 1 | Ath-AT3G44326.1 |  | Vvi-Vitvi06g01530\_t001 |  |  |  |  |  |  |  |
| 1 | Ath-AT3G44330.1 |  | Vvi-Vitvi06g01526\_t001 |  |  |  |  |  |  |  |
| 1 | Ath-AT3G44340.3 |  | | | |  |  |  |  |  |  |  |
| 1 | Ath-AT3G44350.2 |  | Vvi-Vitvi06g01515\_t001 |  |  |  |  |  |  |  |
| 1 | Ath-AT3G44370.1 |  | | | |  |  |  |  |  |  |  |
| 1 | Ath-AT3G44380.1 |  | Vvi-Vitvi06g01505\_t001 |  |  |  |  |  |  |  |
| 1 | Ath-AT3G44400.3 |  | | | |  |  |  |  |  |  |  |
| 1 | Ath-AT3G44405.4 |  | | | |  |  |  |  |  |  |  |
| 1 | Ath-AT3G44428.1 |  | | | |  |  |  |  |  |  |  |
| 1 | Ath-AT3G44430.1 |  | | | |  |  |  |  |  |  |  |
| 1 | Ath-AT3G44435.1 |  | | | |  |  |  |  |  |  |  |
| 1 | Ath-AT3G44440.1 |  | | | |  |  |  |  |  |  |  |
| 2 | Ath-AT3G44450.1 |  | Vvi-Vitvi06g01959\_t001 |  | Vvi-Vitvi08g01465\_t001 |  |  |  |  |  |  |
| 2 | Ath-AT3G44460.1 |  | Vvi-Vitvi06g01478\_t001 |  | Vvi-Vitvi08g01470\_t001 |  |  |  |  |  |  |
| 2 | Ath-AT3G44480.3 |  | | | |  | | | |  |  |  |  |  |  |
| 2 | Ath-AT3G44485.1 |  | | | |  | | | |  |  |  |  |  |  |
| 2 | Ath-AT3G44490.1 |  | | | |  | | | |  |  |  |  |  |  |
| 2 | Ath-AT3G44510.2 |  | Vvi-Vitvi06g01476\_t001 |  | | | |  |  |  |  |  |  |
| 2 | Ath-AT3G44530.1 |  | Vvi-Vitvi06g01457\_t002 |  | | | |  |  |  |  |  |  |
| 2 | Ath-AT3G44540.1 |  | Vvi-Vitvi06g01455\_t001 |  | | | |  |  |  |  |  |  |
| 2 | Ath-AT3G44550.1 |  | | | |  | | | |  |  |  |  |  |  |
| 2 | Ath-AT3G44555.1 |  | | | |  | | | |  |  |  |  |  |  |
| 2 | Ath-AT3G44560.1 |  | | | |  | | | |  |  |  |  |  |  |
| 2 | Ath-AT3G44570.1 |  | | | |  | | | |  |  |  |  |  |  |
| 2 | Ath-AT3G44580.1 |  | | | |  | | | |  |  |  |  |  |  |
| 2 | Ath-AT3G44590.1 |  | Vvi-Vitvi06g01433\_t001 |  | Vvi-Vitvi08g02271\_t001 |  |  |  |  |  |  |
| 2 | Ath-AT3G44600.1 |  | Vvi-Vitvi06g04434\_t001 |  | | | |  |  |  |  |  |  |
| 2 | Ath-AT3G44610.1 |  | Vvi-Vitvi06g01430\_t001 |  | | | |  |  |  |  |  |  |
| 2 | Ath-AT3G44620.2 |  | Vvi-Vitvi06g01428\_t001 |  | | | |  |  |  |  |  |  |
| 2 | Ath-AT3G44630.3 |  | | | |  | | | |  |  |  |  |  |  |
| 2 | Ath-AT3G44635.1 |  | | | |  | | | |  |  |  |  |  |  |
| 2 | Ath-AT3G44660.1 |  | | | |  | | | |  |  |  |  |  |  |
| 2 | Ath-AT3G44665.1 |  | | | |  | | | |  |  |  |  |  |  |
| 2 | Ath-AT3G44670.2 |  | | | |  | | | |  |  |  |  |  |  |
| 2 | Ath-AT3G44680.1 |  | Vvi-Vitvi06g04432\_t001 |  | | | |  |  |  |  |  |  |
| 2 | Ath-AT3G44690.1 |  | | | |  | | | |  |  |  |  |  |  |
| 2 | Ath-AT3G44695.1 |  | | | |  | | | |  |  |  |  |  |  |
| 2 | Ath-AT3G44700.1 |  | | | |  | | | |  |  |  |  |  |  |
| 2 | Ath-AT3G44703.3 |  | | | |  | | | |  |  |  |  |  |  |
| 2 | Ath-AT3G44704.1 |  | | | |  | | | |  |  |  |  |  |  |
| 2 | Ath-AT3G44710.1 |  | | | |  | Vvi-Vitvi08g01502\_t001 |  |  |  |  |  |  |
| 2 | Ath-AT3G44713.1 |  | | | |  | | | |  |  |  |  |  |  |
| 2 | Ath-AT3G44716.1 |  | Vvi-Vitvi06g01409\_t001 |  | | | |  |  |  |  |  |  |
| 2 | Ath-AT3G44718.1 |  | | | |  | | | |  |  |  |  |  |  |
| 2 | Ath-AT3G44720.1 |  | Vvi-Vitvi06g01946\_t001 |  | | | |  |  |  |  |  |  |
| 2 | Ath-AT3G44730.2 |  | | | |  | | | |  |  |  |  |  |  |
| 2 | Ath-AT3G44735.1 |  | Vvi-Vitvi06g01945\_t001 |  | Vvi-Vitvi08g01510\_t001 |  |  |  |  |  |  |
| 2 | Ath-AT3G44740.1 |  | | | |  | | | |  |  |  |  |  |  |
| 2 | Ath-AT3G44750.1 |  | Vvi-Vitvi06g01399\_t001 |  | Vvi-Vitvi08g01518\_t002 |  |  |  |  |  |  |
| 2 | Ath-AT3G44753.1 |  | | | |  | | | |  |  |  |  |  |  |
| 2 | Ath-AT3G44755.1 |  | | | |  | | | |  |  |  |  |  |  |
| 2 | Ath-AT3G44760.1 |  | | | |  | | | |  |  |  |  |  |  |
| 2 | Ath-AT3G44763.1 |  | | | |  | | | |  |  |  |  |  |  |
| 2 | Ath-AT3G44766.1 |  | | | |  | | | |  |  |  |  |  |  |
| 2 | Ath-AT3G44769.1 |  | | | |  | | | |  |  |  |  |  |  |
| 2 | Ath-AT3G44770.1 |  | | | |  | | | |  |  |  |  |  |  |
| 2 | Ath-AT3G44780.1 |  | | | |  | | | |  |  |  |  |  |  |
| 2 | Ath-AT3G44785.1 |  | | | |  | Vvi-Vitvi08g01519\_t003 |  |  |  |  |  |  |
| 1 | Ath-AT3G44790.1 |  | | | |  |  |  |  |  |  |  |
| 1 | Ath-AT3G44800.1 |  | | | |  |  |  |  |  |  |  |
| 1 | Ath-AT3G44805.1 |  | | | |  |  |  |  |  |  |  |
| 1 | Ath-AT3G44810.1 |  | | | |  |  |  |  |  |  |  |
| 1 | Ath-AT3G44820.1 |  | Vvi-Vitvi06g01942\_t001 |  |  |  |  |  |  |  |
| 1 | Ath-AT3G44830.1 |  | Vvi-Vitvi06g01388\_t001 |  |  |  |  |  |  |  |
| 1 | Ath-AT3G44840.1 |  | | | |  |  |  |  |  |  |  |
| 1 | Ath-AT3G44850.1 |  | Vvi-Vitvi06g01380\_t001 |  |  |  |  |  |  |  |
| 1 | Ath-AT3G44860.1 |  | | | |  |  |  |  |  |  |  |
| 1 | Ath-AT3G44870.1 |  | | | |  |  |  |  |  |  |  |
| 2 | Ath-AT3G44880.1 |  | | | |  | Vvi-Vitvi06g01354\_t001 |  |  |  |  |  |  |
| 2 | Ath-AT3G44890.1 |  | | | |  | Vvi-Vitvi06g01351\_t001 |  |  |  |  |  |  |
| 2 | Ath-AT3G44900.1 |  | | | |  | | | |  |  |  |  |  |  |
| 2 | Ath-AT3G44910.1 |  | | | |  | | | |  |  |  |  |  |  |
| 2 | Ath-AT3G44920.1 |  | | | |  | | | |  |  |  |  |  |  |
| 2 | Ath-AT3G44930.1 |  | | | |  | | | |  |  |  |  |  |  |
| 2 | Ath-AT3G44935.1 |  | | | |  | | | |  |  |  |  |  |  |
| 2 | Ath-AT3G44940.1 |  | | | |  | Vvi-Vitvi06g01340\_t001 |  |  |  |  |  |  |
| 2 | Ath-AT3G44950.1 |  | | | |  | | | |  |  |  |  |  |  |
| 2 | Ath-AT3G44960.2 |  | | | |  | Vvi-Vitvi06g01928\_t001 |  |  |  |  |  |  |
| 2 | Ath-AT3G44970.1 |  | | | |  | | | |  |  |  |  |  |  |
| 2 | Ath-AT3G44980.1 |  | | | |  | | | |  |  |  |  |  |  |
| 2 | Ath-AT3G44990.1 |  | | | |  | Vvi-Vitvi06g01329\_t001 |  |  |  |  |  |  |
| 2 | Ath-AT3G45000.1 |  | | | |  | Vvi-Vitvi06g01328\_t002 |  |  |  |  |  |  |
| 2 | Ath-AT3G45010.1 |  | Vvi-Vitvi06g01362\_t001 |  | Vvi-Vitvi06g01924\_t001 |  |  |  |  |  |  |
| 1 | Ath-AT3G45020.1 |  |  |  | Vvi-Vitvi06g01314\_t001 |  |  |  |  |  |  |
| 1 | Ath-AT3G45030.1 |  |  |  | | | |  |  |  |  |  |  |
| 1 | Ath-AT3G45040.1 |  |  |  | Vvi-Vitvi06g01309\_t001 |  |  |  |  |  |  |
| 1 | Ath-AT3G45050.4 |  |  |  | Vvi-Vitvi06g01302\_t001 |  |  |  |  |  |  |
| 1 | Ath-AT3G45060.1 |  |  |  | Vvi-Vitvi06g01299\_t001 |  |  |  |  |  |  |
| 1 | Ath-AT3G45070.2 |  |  |  | | | |  |  |  |  |  |  |
| 1 | Ath-AT3G45080.1 |  |  |  | | | |  |  |  |  |  |  |
| 1 | Ath-AT3G45090.1 |  |  |  | Vvi-Vitvi06g01297\_t001 |  |  |  |  |  |  |
| 0 | Ath-AT3G45093.1 |  |  |  |  |  |  |  |  |
| 0 | Ath-AT3G45100.3 |  |  |  |  |  |  |  |  |
| 0 | Ath-AT3G45110.1 |  |  |  |  |  |  |  |  |
| 0 | Ath-AT3G45130.3 |  |  |  |  |  |  |  |  |
| 0 | Ath-AT3G45140.1 |  |  |  |  |  |  |  |  |
| 0 | Ath-AT3G45150.1 |  |  |  |  |  |  |  |  |
| 0 | Ath-AT3G45160.1 |  |  |  |  |  |  |  |  |
| 0 | Ath-AT3G45170.1 |  |  |  |  |  |  |  |  |
| 0 | Ath-AT3G45180.1 |  |  |  |  |  |  |  |  |
| 1 | Ath-AT3G45190.1 |  | Vvi-Vitvi06g00274\_t004 |  |  |  |  |  |  |  |
| 1 | Ath-AT3G45200.1 |  | | | |  |  |  |  |  |  |  |
| 1 | Ath-AT3G45210.1 |  | Vvi-Vitvi06g00279\_t001 |  |  |  |  |  |  |  |
| 1 | Ath-AT3G45220.1 |  | | | |  |  |  |  |  |  |  |
| 1 | Ath-AT3G45230.1 |  | Vvi-Vitvi06g00285\_t001 |  |  |  |  |  |  |  |
| 1 | Ath-AT3G45240.1 |  | Vvi-Vitvi06g00296\_t002 |  |  |  |  |  |  |  |
| 1 | Ath-AT3G45243.1 |  | | | |  |  |  |  |  |  |  |
| 1 | Ath-AT3G45245.1 |  | | | |  |  |  |  |  |  |  |
| 1 | Ath-AT3G45248.1 |  | | | |  |  |  |  |  |  |  |
| 1 | Ath-AT3G45252.1 |  | | | |  |  |  |  |  |  |  |
| 1 | Ath-AT3G45260.1 |  | Vvi-Vitvi06g00304\_t001 |  |  |  |  |  |  |  |
| 1 | Ath-AT3G45275.1 |  | | | |  |  |  |  |  |  |  |
| 1 | Ath-AT3G45280.1 |  | Vvi-Vitvi06g00306\_t001 |  |  |  |  |  |  |  |
| 1 | Ath-AT3G45285.1 |  | | | |  |  |  |  |  |  |  |
| 1 | Ath-AT3G45290.1 |  | Vvi-Vitvi06g00310\_t001 |  |  |  |  |  |  |  |
| 1 | Ath-AT3G45300.1 |  | Vvi-Vitvi06g00312\_t001 |  |  |  |  |  |  |  |
| 1 | Ath-AT3G45310.1 |  | | | |  |  |  |  |  |  |  |
| 1 | Ath-AT3G45320.1 |  | | | |  |  |  |  |  |  |  |
| 1 | Ath-AT3G45330.1 |  | | | |  |  |  |  |  |  |  |
| 1 | Ath-AT3G45390.1 |  | | | |  |  |  |  |  |  |  |
| 1 | Ath-AT3G45400.1 |  | | | |  |  |  |  |  |  |  |
| 1 | Ath-AT3G45410.1 |  | | | |  |  |  |  |  |  |  |
| 1 | Ath-AT3G45420.1 |  | | | |  |  |  |  |  |  |  |
| 1 | Ath-AT3G45430.1 |  | | | |  |  |  |  |  |  |  |
| 1 | Ath-AT3G45440.2 |  | | | |  |  |  |  |  |  |  |
| 1 | Ath-AT3G45443.1 |  | | | |  |  |  |  |  |  |  |
| 1 | Ath-AT3G45450.1 |  | | | |  |  |  |  |  |  |  |
| 1 | Ath-AT3G45460.1 |  | | | |  |  |  |  |  |  |  |
| 1 | Ath-AT3G45470.1 |  | | | |  |  |  |  |  |  |  |
| 1 | Ath-AT3G45480.1 |  | | | |  |  |  |  |  |  |  |
| 1 | Ath-AT3G45490.1 |  | | | |  |  |  |  |  |  |  |
| 1 | Ath-AT3G45500.1 |  | | | |  |  |  |  |  |  |  |
| 1 | Ath-AT3G45510.1 |  | | | |  |  |  |  |  |  |  |
| 1 | Ath-AT3G45525.1 |  | | | |  |  |  |  |  |  |  |
| 1 | Ath-AT3G45530.1 |  | | | |  |  |  |  |  |  |  |
| 1 | Ath-AT3G45540.1 |  | | | |  |  |  |  |  |  |  |
| 1 | Ath-AT3G45555.1 |  | | | |  |  |  |  |  |  |  |
| 1 | Ath-AT3G45560.1 |  | | | |  |  |  |  |  |  |  |
| 1 | Ath-AT3G45570.1 |  | | | |  |  |  |  |  |  |  |
| 1 | Ath-AT3G45577.1 |  | | | |  |  |  |  |  |  |  |
| 1 | Ath-AT3G45580.1 |  | Vvi-Vitvi06g00335\_t001 |  |  |  |  |  |  |  |
| 1 | Ath-AT3G45590.1 |  | | | |  |  |  |  |  |  |  |
| 1 | Ath-AT3G45600.1 |  | Vvi-Vitvi06g00342\_t001 |  |  |  |  |  |  |  |
| 1 | Ath-AT3G45610.1 |  | Vvi-Vitvi06g00345\_t001 |  |  |  |  |  |  |  |
| 1 | Ath-AT3G45620.2 |  | Vvi-Vitvi06g00349\_t001 |  |  |  |  |  |  |  |
| 1 | Ath-AT3G45630.1 |  | | | |  |  |  |  |  |  |  |
| 1 | Ath-AT3G45640.1 |  | Vvi-Vitvi06g00356\_t001 |  |  |  |  |  |  |  |
| 1 | Ath-AT3G45645.1 |  | | | |  |  |  |  |  |  |  |
| 1 | Ath-AT3G45650.2 |  | | | |  |  |  |  |  |  |  |
| 1 | Ath-AT3G45660.2 |  | | | |  |  |  |  |  |  |  |
| 1 | Ath-AT3G45670.1 |  | | | |  |  |  |  |  |  |  |
| 1 | Ath-AT3G45673.1 |  | | | |  |  |  |  |  |  |  |
| 1 | Ath-AT3G45680.1 |  | | | |  |  |  |  |  |  |  |
| 1 | Ath-AT3G45690.1 |  | | | |  |  |  |  |  |  |  |
| 1 | Ath-AT3G45700.1 |  | | | |  |  |  |  |  |  |  |
| 1 | Ath-AT3G45710.1 |  | | | |  |  |  |  |  |  |  |
| 1 | Ath-AT3G45720.1 |  | | | |  |  |  |  |  |  |  |
| 1 | Ath-AT3G45730.1 |  | | | |  |  |  |  |  |  |  |
| 1 | Ath-AT3G45740.1 |  | Vvi-Vitvi06g00366\_t001 |  |  |  |  |  |  |  |
| 1 | Ath-AT3G45750.1 |  | | | |  |  |  |  |  |  |  |
| 1 | Ath-AT3G45760.1 |  | | | |  |  |  |  |  |  |  |
| 1 | Ath-AT3G45770.1 |  | Vvi-Vitvi06g00367\_t001.1.6037826e |  |  |  |  |  |  |  |
| 1 | Ath-AT3G45780.1 |  | Vvi-Vitvi06g00374\_t001 |  |  |  |  |  |  |  |
| 1 | Ath-AT3G45790.1 |  | | | |  |  |  |  |  |  |  |
| 1 | Ath-AT3G45800.1 |  | | | |  |  |  |  |  |  |  |
| 1 | Ath-AT3G45810.1 |  | Vvi-Vitvi06g00381\_t001 |  |  |  |  |  |  |  |
| 1 | Ath-AT3G45820.1 |  | | | |  |  |  |  |  |  |  |
| 1 | Ath-AT3G45830.1 |  | Vvi-Vitvi06g00382\_t001 |  |  |  |  |  |  |  |
| 1 | Ath-AT3G45840.1 |  | | | |  |  |  |  |  |  |  |
| 1 | Ath-AT3G45850.2 |  | Vvi-Vitvi06g00383\_t001 |  |  |  |  |  |  |  |
| 1 | Ath-AT3G45851.1 |  | | | |  |  |  |  |  |  |  |
| 1 | Ath-AT3G45860.1 |  | | | |  |  |  |  |  |  |  |
| 1 | Ath-AT3G45870.1 |  | | | |  |  |  |  |  |  |  |
| 1 | Ath-AT3G45880.1 |  | | | |  |  |  |  |  |  |  |
| 1 | Ath-AT3G45890.1 |  | | | |  |  |  |  |  |  |  |
| 1 | Ath-AT3G45900.1 |  | | | |  |  |  |  |  |  |  |
| 1 | Ath-AT3G45910.1 |  | | | |  |  |  |  |  |  |  |
| 1 | Ath-AT3G45920.1 |  | | | |  |  |  |  |  |  |  |
| 3 | Ath-AT3G45930.1 |  | Vvi-Vitvi06g04126\_t001 |  | Vvi-Vitvi06g04151\_t001 |  | Vvi-Vitvi13g04084\_t001 |  |  |  |  |  |
| 2 | Ath-AT3G45940.1 |  |  |  | | | |  | | | |  |  |  |  |  |
| 2 | Ath-AT3G45950.1 |  |  |  | | | |  | | | |  |  |  |  |  |
| 2 | Ath-AT3G45960.2 |  |  |  | | | |  | | | |  |  |  |  |  |
| 2 | Ath-AT3G45970.1 |  |  |  | | | |  | | | |  |  |  |  |  |
| 2 | Ath-AT3G45980.1 |  |  |  | Vvi-Vitvi06g00426\_t001 |  | | | |  |  |  |  |  |
| 2 | Ath-AT3G45990.1 |  |  |  | | | |  | | | |  |  |  |  |  |
| 2 | Ath-AT3G46000.2 |  |  |  | Vvi-Vitvi06g00424\_t001 |  | Vvi-Vitvi13g00269\_t003 |  |  |  |  |  |
| 2 | Ath-AT3G46010.2 |  |  |  | | | |  | | | |  |  |  |  |  |
| 2 | Ath-AT3G46020.1 |  |  |  | Vvi-Vitvi06g01688\_t001 |  | | | |  |  |  |  |  |
| 2 | Ath-AT3G46030.1 |  |  |  | | | |  | | | |  |  |  |  |  |
| 2 | Ath-AT3G46040.1 |  |  |  | | | |  | Vvi-Vitvi13g04080\_t001 |  |  |  |  |  |
| 2 | Ath-AT3G46050.1 |  |  |  | | | |  | | | |  |  |  |  |  |
| 3 | Ath-AT3G46060.1 |  | Vvi-Vitvi06g00420\_t001 |  | | | |  | Vvi-Vitvi13g00266\_t001 |  |  |  |  |  |
| 3 | Ath-AT3G46070.1 |  | | | |  | | | |  | Vvi-Vitvi13g00262\_t001 |  |  |  |  |  |
| 3 | Ath-AT3G46080.1 |  | | | |  | Vvi-Vitvi06g01682\_t001 |  | | | |  |  |  |  |  |
| 3 | Ath-AT3G46086.1 |  | | | |  | | | |  | | | |  |  |  |  |  |
| 3 | Ath-AT3G46090.1 |  | | | |  | | | |  | | | |  |  |  |  |  |
| 3 | Ath-AT3G46100.1 |  | | | |  | | | |  | | | |  |  |  |  |  |
| 3 | Ath-AT3G46110.1 |  | | | |  | Vvi-Vitvi06g00415\_t001 |  | Vvi-Vitvi13g00260\_t002 |  |  |  |  |  |
| 2 | Ath-AT3G46120.1 |  | | | |  | | | |  |  |  |  |  |  |
| 2 | Ath-AT3G46130.1 |  | | | |  | Vvi-Vitvi06g00414\_t002 |  |  |  |  |  |  |
| 2 | Ath-AT3G46140.1 |  | | | |  | | | |  |  |  |  |  |  |
| 2 | Ath-AT3G46150.1 |  | | | |  | | | |  |  |  |  |  |  |
| 2 | Ath-AT3G46160.1 |  | | | |  | | | |  |  |  |  |  |  |
| 2 | Ath-AT3G46170.1 |  | | | |  | | | |  |  |  |  |  |  |
| 2 | Ath-AT3G46180.1 |  | | | |  | Vvi-Vitvi06g01681\_t001 |  |  |  |  |  |  |
| 2 | Ath-AT3G46190.2 |  | | | |  | | | |  |  |  |  |  |  |
| 2 | Ath-AT3G46200.1 |  | | | |  | Vvi-Vitvi06g00394\_t001 |  |  |  |  |  |  |
| 2 | Ath-AT3G46210.5 |  | | | |  | | | |  |  |  |  |  |  |
| 2 | Ath-AT3G46220.2 |  | | | |  | Vvi-Vitvi06g00392\_t001 |  |  |  |  |  |  |
| 2 | Ath-AT3G46230.1 |  | | | |  | | | |  |  |  |  |  |  |
| 2 | Ath-AT3G46240.1 |  | | | |  | | | |  |  |  |  |  |  |
| 2 | Ath-AT3G46260.1 |  | | | |  | Vvi-Vitvi06g04129\_t001 |  |  |  |  |  |  |
| 2 | Ath-AT3G46270.2 |  | | | |  | | | |  |  |  |  |  |  |
| 2 | Ath-AT3G46280.1 |  | | | |  | | | |  |  |  |  |  |  |
| 2 | Ath-AT3G46290.1 |  | | | |  | Vvi-Vitvi06g00388\_t001 |  |  |  |  |  |  |
| 2 | Ath-AT3G46300.1 |  | | | |  | Vvi-Vitvi06g00387\_t001 |  |  |  |  |  |  |
| 2 | Ath-AT3G46310.1 |  | | | |  | | | |  |  |  |  |  |  |
| 2 | Ath-AT3G46320.1 |  | Vvi-Vitvi06g04151\_t001 |  | Vvi-Vitvi06g04126\_t001 |  |  |  |  |  |  |
| 1 | Ath-AT3G46330.1 |  | | | |  |  |  |  |  |  |  |
| 1 | Ath-AT3G46340.1 |  | | | |  |  |  |  |  |  |  |
| 1 | Ath-AT3G46350.1 |  | | | |  |  |  |  |  |  |  |
| 1 | Ath-AT3G46355.1 |  | | | |  |  |  |  |  |  |  |
| 1 | Ath-AT3G46360.1 |  | | | |  |  |  |  |  |  |  |
| 1 | Ath-AT3G46370.1 |  | | | |  |  |  |  |  |  |  |
| 1 | Ath-AT3G46380.1 |  | | | |  |  |  |  |  |  |  |
| 1 | Ath-AT3G46390.1 |  | | | |  |  |  |  |  |  |  |
| 1 | Ath-AT3G46400.1 |  | | | |  |  |  |  |  |  |  |
| 1 | Ath-AT3G46410.1 |  | | | |  |  |  |  |  |  |  |
| 1 | Ath-AT3G46420.1 |  | | | |  |  |  |  |  |  |  |
| 1 | Ath-AT3G46430.1 |  | Vvi-Vitvi06g00434\_t001 |  |  |  |  |  |  |  |
| 1 | Ath-AT3G46440.1 |  | Vvi-Vitvi06g00435\_t001 |  |  |  |  |  |  |  |
| 1 | Ath-AT3G46450.2 |  | Vvi-Vitvi06g00437\_t001 |  |  |  |  |  |  |  |
| 1 | Ath-AT3G46460.1 |  | Vvi-Vitvi06g00439\_t003 |  |  |  |  |  |  |  |
| 1 | Ath-AT3G46470.1 |  | | | |  |  |  |  |  |  |  |
| 1 | Ath-AT3G46480.1 |  | | | |  |  |  |  |  |  |  |
| 1 | Ath-AT3G46490.1 |  | | | |  |  |  |  |  |  |  |
| 1 | Ath-AT3G46500.5 |  | | | |  |  |  |  |  |  |  |
| 1 | Ath-AT3G46510.1 |  | Vvi-Vitvi06g00450\_t001 |  |  |  |  |  |  |  |
| 1 | Ath-AT3G46520.1 |  | Vvi-Vitvi06g00453\_t003 |  |  |  |  |  |  |  |
| 1 | Ath-AT3G46530.1 |  | | | |  |  |  |  |  |  |  |
| 1 | Ath-AT3G46540.1 |  | Vvi-Vitvi06g00455\_t001.1.6037826f |  |  |  |  |  |  |  |
| 1 | Ath-AT3G46550.1 |  | Vvi-Vitvi06g00459\_t001 |  |  |  |  |  |  |  |
| 1 | Ath-AT3G46560.1 |  | Vvi-Vitvi06g00466\_t001 |  |  |  |  |  |  |  |
| 1 | Ath-AT3G46565.1 |  | | | |  |  |  |  |  |  |  |
| 1 | Ath-AT3G46570.1 |  | | | |  |  |  |  |  |  |  |
| 1 | Ath-AT3G46580.1 |  | Vvi-Vitvi06g01703\_t001 |  |  |  |  |  |  |  |
| 1 | Ath-AT3G46590.2 |  | Vvi-Vitvi06g00488\_t001 |  |  |  |  |  |  |  |
| 1 | Ath-AT3G46600.1 |  | | | |  |  |  |  |  |  |  |
| 1 | Ath-AT3G46610.1 |  | | | |  |  |  |  |  |  |  |
| 1 | Ath-AT3G46613.1 |  | | | |  |  |  |  |  |  |  |
| 1 | Ath-AT3G46616.2 |  | | | |  |  |  |  |  |  |  |
| 1 | Ath-AT3G46617.1 |  | | | |  |  |  |  |  |  |  |
| 1 | Ath-AT3G46620.1 |  | Vvi-Vitvi06g00502\_t001 |  |  |  |  |  |  |  |
| 1 | Ath-AT3G46630.1 |  | | | |  |  |  |  |  |  |  |
| 1 | Ath-AT3G46640.3 |  | Vvi-Vitvi06g00505\_t001 |  |  |  |  |  |  |  |
| 1 | Ath-AT3G46650.1 |  | | | |  |  |  |  |  |  |  |
| 1 | Ath-AT3G46660.1 |  | | | |  |  |  |  |  |  |  |
| 1 | Ath-AT3G46666.1 |  | | | |  |  |  |  |  |  |  |
| 1 | Ath-AT3G46670.1 |  | | | |  |  |  |  |  |  |  |
| 1 | Ath-AT3G46680.1 |  | | | |  |  |  |  |  |  |  |
| 1 | Ath-AT3G46690.1 |  | | | |  |  |  |  |  |  |  |
| 1 | Ath-AT3G46700.1 |  | | | |  |  |  |  |  |  |  |
| 1 | Ath-AT3G46710.1 |  | | | |  |  |  |  |  |  |  |
| 1 | Ath-AT3G46720.1 |  | | | |  |  |  |  |  |  |  |
| 1 | Ath-AT3G46730.1 |  | | | |  |  |  |  |  |  |  |
| 1 | Ath-AT3G46735.1 |  | | | |  |  |  |  |  |  |  |
| 1 | Ath-AT3G46740.1 |  | Vvi-Vitvi06g00510\_t001 |  |  |  |  |  |  |  |
| 1 | Ath-AT3G46750.1 |  | | | |  |  |  |  |  |  |  |
| 1 | Ath-AT3G46760.1 |  | | | |  |  |  |  |  |  |  |
| 1 | Ath-AT3G46770.1 |  | | | |  |  |  |  |  |  |  |
| 1 | Ath-AT3G46780.1 |  | Vvi-Vitvi06g00517\_t001 |  |  |  |  |  |  |  |
| 1 | Ath-AT3G46790.1 |  | Vvi-Vitvi06g00538\_t001 |  |  |  |  |  |  |  |
| 1 | Ath-AT3G46800.1 |  | | | |  |  |  |  |  |  |  |
| 1 | Ath-AT3G46810.1 |  | | | |  |  |  |  |  |  |  |
| 1 | Ath-AT3G46820.1 |  | Vvi-Vitvi06g00540\_t003 |  |  |  |  |  |  |  |
| 1 | Ath-AT3G46830.1 |  | Vvi-Vitvi06g00542\_t001 |  |  |  |  |  |  |  |
| 1 | Ath-AT3G46840.1 |  | Vvi-Vitvi06g04180\_t001 |  |  |  |  |  |  |  |
| 1 | Ath-AT3G46845.1 |  | | | |  |  |  |  |  |  |  |
| 1 | Ath-AT3G46850.1 |  | Vvi-Vitvi06g00547\_t001 |  |  |  |  |  |  |  |
| 1 | Ath-AT3G46860.1 |  | | | |  |  |  |  |  |  |  |
| 1 | Ath-AT3G46870.2 |  | Vvi-Vitvi06g01722\_t003 |  |  |  |  |  |  |  |
| 1 | Ath-AT3G46880.1 |  | | | |  |  |  |  |  |  |  |
| 1 | Ath-AT3G46890.1 |  | Vvi-Vitvi06g00555\_t001 |  |  |  |  |  |  |  |
| 1 | Ath-AT3G46900.1 |  | Vvi-Vitvi06g01729\_t001 |  |  |  |  |  |  |  |
| 0 | Ath-AT3G46910.1 |  |  |  |  |  |  |  |  |
| 0 | Ath-AT3G46920.2 |  |  |  |  |  |  |  |  |
| 0 | Ath-AT3G46930.2 |  |  |  |  |  |  |  |  |
| 0 | Ath-AT3G46940.2 |  |  |  |  |  |  |  |  |
| 0 | Ath-AT3G46950.1 |  |  |  |  |  |  |  |  |
| 1 | Ath-AT3G46960.1 |  | Vvi-Vitvi06g00580\_t001 |  |  |  |  |  |  |  |
| 1 | Ath-AT3G46970.1 |  | Vvi-Vitvi06g00583\_t001 |  |  |  |  |  |  |  |
| 1 | Ath-AT3G46980.4 |  | Vvi-Vitvi06g00585\_t001 |  |  |  |  |  |  |  |
| 1 | Ath-AT3G46990.1 |  | Vvi-Vitvi06g00587\_t001 |  |  |  |  |  |  |  |
| 1 | Ath-AT3G47000.1 |  | Vvi-Vitvi06g00593\_t001 |  |  |  |  |  |  |  |
| 1 | Ath-AT3G47010.1 |  | | | |  |  |  |  |  |  |  |
| 1 | Ath-AT3G47020.1 |  | | | |  |  |  |  |  |  |  |
| 1 | Ath-AT3G47030.1 |  | | | |  |  |  |  |  |  |  |
| 1 | Ath-AT3G47040.2 |  | | | |  |  |  |  |  |  |  |
| 1 | Ath-AT3G47050.1 |  | | | |  |  |  |  |  |  |  |
| 1 | Ath-AT3G47060.1 |  | Vvi-Vitvi06g00595\_t001 |  |  |  |  |  |  |  |
| 1 | Ath-AT3G47070.1 |  | Vvi-Vitvi06g01740\_t001 |  |  |  |  |  |  |  |
| 1 | Ath-AT3G47080.1 |  | Vvi-Vitvi06g00613\_t001 |  |  |  |  |  |  |  |
| 1 | Ath-AT3G47090.1 |  | | | |  |  |  |  |  |  |  |
| 1 | Ath-AT3G47100.1 |  | | | |  |  |  |  |  |  |  |
| 1 | Ath-AT3G47110.1 |  | | | |  |  |  |  |  |  |  |
| 1 | Ath-AT3G47120.1 |  | Vvi-Vitvi06g00627\_t001 |  |  |  |  |  |  |  |
| 1 | Ath-AT3G47130.1 |  | | | |  |  |  |  |  |  |  |
| 1 | Ath-AT3G47140.1 |  | | | |  |  |  |  |  |  |  |
| 1 | Ath-AT3G47150.1 |  | | | |  |  |  |  |  |  |  |
| 1 | Ath-AT3G47160.2 |  | Vvi-Vitvi06g01748\_t001 |  |  |  |  |  |  |  |
| 0 | Ath-AT3G47170.1 |  |  |  |  |  |  |  |  |
| 0 | Ath-AT3G47180.1 |  |  |  |  |  |  |  |  |
| 0 | Ath-AT3G47190.1 |  |  |  |  |  |  |  |  |
| 0 | Ath-AT3G47200.2 |  |  |  |  |  |  |  |  |
| 0 | Ath-AT3G47210.1 |  |  |  |  |  |  |  |  |
| 0 | Ath-AT3G47220.1 |  |  |  |  |  |  |  |  |
| 0 | Ath-AT3G47250.2 |  |  |  |  |  |  |  |  |
| 0 | Ath-AT3G47290.2 |  |  |  |  |  |  |  |  |
| 0 | Ath-AT3G47295.1 |  |  |  |  |  |  |  |  |
| 0 | Ath-AT3G47300.1 |  |  |  |  |  |  |  |  |
| 0 | Ath-AT3G47340.1 |  |  |  |  |  |  |  |  |
| 0 | Ath-AT3G47341.1 |  |  |  |  |  |  |  |  |
| 0 | Ath-AT3G47350.2 |  |  |  |  |  |  |  |  |
| 0 | Ath-AT3G47360.1 |  |  |  |  |  |  |  |  |
| 0 | Ath-AT3G47370.1 |  |  |  |  |  |  |  |  |
| 1 | Ath-AT3G47380.1 |  | Vvi-Vitvi16g04295\_t001 |  |  |  |  |  |  |  |
| 1 | Ath-AT3G47390.1 |  | | | |  |  |  |  |  |  |  |
| 1 | Ath-AT3G47400.1 |  | Vvi-Vitvi16g00717\_t001 |  |  |  |  |  |  |  |
| 1 | Ath-AT3G47410.1 |  | | | |  |  |  |  |  |  |  |
| 1 | Ath-AT3G47420.3 |  | Vvi-Vitvi16g00692\_t002 |  |  |  |  |  |  |  |
| 1 | Ath-AT3G47430.1 |  | Vvi-Vitvi16g00673\_t001 |  |  |  |  |  |  |  |
| 1 | Ath-AT3G47440.1 |  | Vvi-Vitvi16g00655\_t001 |  |  |  |  |  |  |  |
| 1 | Ath-AT3G47450.1 |  | Vvi-Vitvi16g00648\_t001 |  |  |  |  |  |  |  |
| 1 | Ath-AT3G47460.2 |  | Vvi-Vitvi16g04271\_t001 |  |  |  |  |  |  |  |
| 1 | Ath-AT3G47470.1 |  | Vvi-Vitvi17g00615\_t001 |  |  |  |  |  |  |  |
| 1 | Ath-AT3G47480.1 |  | Vvi-Vitvi17g01471\_t001 |  |  |  |  |  |  |  |
| 1 | Ath-AT3G47490.4 |  | Vvi-Vitvi17g00612\_t001 |  |  |  |  |  |  |  |
| 1 | Ath-AT3G47500.1 |  | Vvi-Vitvi17g00611\_t001 |  |  |  |  |  |  |  |
| 1 | Ath-AT3G47510.1 |  | | | |  |  |  |  |  |  |  |
| 1 | Ath-AT3G47520.1 |  | Vvi-Vitvi17g00607\_t001 |  |  |  |  |  |  |  |
| 1 | Ath-AT3G47530.1 |  | Vvi-Vitvi17g04165\_t001 |  |  |  |  |  |  |  |
| 1 | Ath-AT3G47540.2 |  | | | |  |  |  |  |  |  |  |
| 1 | Ath-AT3G47550.3 |  | Vvi-Vitvi17g00603\_t001 |  |  |  |  |  |  |  |
| 1 | Ath-AT3G47560.2 |  | | | |  |  |  |  |  |  |  |
| 1 | Ath-AT3G47570.1 |  | | | |  |  |  |  |  |  |  |
| 1 | Ath-AT3G47580.1 |  | | | |  |  |  |  |  |  |  |
| 1 | Ath-AT3G47590.1 |  | | | |  |  |  |  |  |  |  |
| 1 | Ath-AT3G47600.1 |  | Vvi-Vitvi17g00598\_t001 |  |  |  |  |  |  |  |
| 1 | Ath-AT3G47610.1 |  | Vvi-Vitvi17g00587\_t001 |  |  |  |  |  |  |  |
| 1 | Ath-AT3G47620.1 |  | Vvi-Vitvi17g00581\_t001 |  |  |  |  |  |  |  |
| 1 | Ath-AT3G47630.3 |  | | | |  |  |  |  |  |  |  |
| 1 | Ath-AT3G47640.2 |  | Vvi-Vitvi17g00578\_t001 |  |  |  |  |  |  |  |
| 1 | Ath-AT3G47650.1 |  | | | |  |  |  |  |  |  |  |
| 1 | Ath-AT3G47660.4 |  | | | |  |  |  |  |  |  |  |
| 1 | Ath-AT3G47670.1 |  | Vvi-Vitvi17g00574\_t001 |  |  |  |  |  |  |  |
| 1 | Ath-AT3G47675.1 |  | | | |  |  |  |  |  |  |  |
| 1 | Ath-AT3G47680.1 |  | | | |  |  |  |  |  |  |  |
| 1 | Ath-AT3G47690.1 |  | | | |  |  |  |  |  |  |  |
| 1 | Ath-AT3G47700.1 |  | Vvi-Vitvi17g00571\_t001 |  |  |  |  |  |  |  |
| 1 | Ath-AT3G47710.1 |  | Vvi-Vitvi17g00569\_t001 |  |  |  |  |  |  |  |
| 1 | Ath-AT3G47720.1 |  | | | |  |  |  |  |  |  |  |
| 1 | Ath-AT3G47730.1 |  | | | |  |  |  |  |  |  |  |
| 1 | Ath-AT3G47740.1 |  | Vvi-Vitvi17g00563\_t001 |  |  |  |  |  |  |  |
| 0 | Ath-AT3G47750.1 |  |  |  |  |  |  |  |  |
| 0 | Ath-AT3G47760.3 |  |  |  |  |  |  |  |  |
| 0 | Ath-AT3G47770.2 |  |  |  |  |  |  |  |  |
| 0 | Ath-AT3G47780.1 |  |  |  |  |  |  |  |  |
| 0 | Ath-AT3G47790.1 |  |  |  |  |  |  |  |  |
| 0 | Ath-AT3G47800.1 |  |  |  |  |  |  |  |  |
| 0 | Ath-AT3G47810.1 |  |  |  |  |  |  |  |  |
| 0 | Ath-AT3G47820.1 |  |  |  |  |  |  |  |  |
| 0 | Ath-AT3G47830.1 |  |  |  |  |  |  |  |  |
| 0 | Ath-AT3G47833.1 |  |  |  |  |  |  |  |  |
| 0 | Ath-AT3G47836.1 |  |  |  |  |  |  |  |  |
| 0 | Ath-AT3G47840.1 |  |  |  |  |  |  |  |  |
| 1 | Ath-AT3G47850.1 |  | Vvi-Vitvi17g00509\_t001 |  |  |  |  |  |  |  |
| 1 | Ath-AT3G47860.1 |  | | | |  |  |  |  |  |  |  |
| 1 | Ath-AT3G47870.1 |  | Vvi-Vitvi17g00520\_t001 |  |  |  |  |  |  |  |
| 1 | Ath-AT3G47890.1 |  | Vvi-Vitvi17g00521\_t001 |  |  |  |  |  |  |  |
| 1 | Ath-AT3G47910.2 |  | | | |  |  |  |  |  |  |  |
| 1 | Ath-AT3G47920.1 |  | | | |  |  |  |  |  |  |  |
| 1 | Ath-AT3G47930.1 |  | | | |  |  |  |  |  |  |  |
| 1 | Ath-AT3G47940.1 |  | Vvi-Vitvi17g00524\_t002 |  |  |  |  |  |  |  |
| 1 | Ath-AT3G47950.1 |  | Vvi-Vitvi17g00527\_t001 |  |  |  |  |  |  |  |
| 1 | Ath-AT3G47960.1 |  | Vvi-Vitvi17g00528\_t001 |  |  |  |  |  |  |  |
| 1 | Ath-AT3G47965.1 |  | | | |  |  |  |  |  |  |  |
| 1 | Ath-AT3G47980.1 |  | Vvi-Vitvi17g00539\_t001 |  |  |  |  |  |  |  |
| 2 | Ath-AT3G47990.1 |  | | | |  | Vvi-Vitvi17g00801\_t001 |  |  |  |  |  |  |
| 3 | Ath-AT3G48000.1 |  | Vvi-Vitvi17g00555\_t001 |  | Vvi-Vitvi17g00786\_t001 |  | Vvi-Vitvi14g01796\_t001 |  |  |  |  |  |
| 2 | Ath-AT3G48010.1 |  |  |  | Vvi-Vitvi17g00781\_t001 |  | Vvi-Vitvi14g01797\_t001 |  |  |  |  |  |
| 2 | Ath-AT3G48020.1 |  |  |  | Vvi-Vitvi17g01535\_t001 |  | Vvi-Vitvi14g01800\_t001 |  |  |  |  |  |
| 2 | Ath-AT3G48030.1 |  |  |  | Vvi-Vitvi17g00767\_t001 |  | Vvi-Vitvi14g01801\_t001 |  |  |  |  |  |
| 2 | Ath-AT3G48040.1 |  |  |  | Vvi-Vitvi17g00762\_t001 |  | | | |  |  |  |  |  |
| 2 | Ath-AT3G48050.2 |  |  |  | Vvi-Vitvi17g00761\_t001 |  | Vvi-Vitvi14g01812\_t002 |  |  |  |  |  |
| 1 | Ath-AT3G48060.2 |  |  |  |  |  | | | |  |  |  |  |  |
| 2 | Ath-AT3G48070.2 |  | Vvi-Vitvi17g00730\_t002 |  |  |  | | | |  |  |  |  |  |
| 2 | Ath-AT3G48080.1 |  | Vvi-Vitvi17g01523\_t001 |  |  |  | Vvi-Vitvi14g03030\_t001 |  |  |  |  |  |
| 1 | Ath-AT3G48090.1 |  | | | |  |  |  |  |  |  |  |
| 1 | Ath-AT3G48100.1 |  | Vvi-Vitvi17g00732\_t001 |  |  |  |  |  |  |  |
| 1 | Ath-AT3G48110.1 |  | | | |  |  |  |  |  |  |  |
| 1 | Ath-AT3G48120.1 |  | Vvi-Vitvi17g00733\_t002 |  |  |  |  |  |  |  |
| 1 | Ath-AT3G48131.1 |  | | | |  |  |  |  |  |  |  |
| 1 | Ath-AT3G48140.1 |  | Vvi-Vitvi17g00734\_t001 |  |  |  |  |  |  |  |
| 1 | Ath-AT3G48150.1 |  | | | |  |  |  |  |  |  |  |
| 1 | Ath-AT3G48160.2 |  | Vvi-Vitvi17g00736\_t002 |  |  |  |  |  |  |  |
| 1 | Ath-AT3G48170.1 |  | Vvi-Vitvi17g01525\_t001 |  |  |  |  |  |  |  |
| 1 | Ath-AT3G48180.1 |  | Vvi-Vitvi17g01527\_t001 |  |  |  |  |  |  |  |
| 0 | Ath-AT3G48185.1 |  |  |  |  |  |  |  |  |
| 0 | Ath-AT3G48187.1 |  |  |  |  |  |  |  |  |
| 0 | Ath-AT3G48190.2 |  |  |  |  |  |  |  |  |
| 0 | Ath-AT3G48195.1 |  |  |  |  |  |  |  |  |
| 0 | Ath-AT3G48200.1 |  |  |  |  |  |  |  |  |
| 0 | Ath-AT3G48205.2 |  |  |  |  |  |  |  |  |
| 0 | Ath-AT3G48208.1 |  |  |  |  |  |  |  |  |
| 0 | Ath-AT3G48209.1 |  |  |  |  |  |  |  |  |
| 0 | Ath-AT3G48210.1 |  |  |  |  |  |  |  |  |
| 0 | Ath-AT3G48220.1 |  |  |  |  |  |  |  |  |
| 0 | Ath-AT3G48230.1 |  |  |  |  |  |  |  |  |
| 0 | Ath-AT3G48231.1 |  |  |  |  |  |  |  |  |
| 1 | Ath-AT3G48240.1 |  | Vvi-Vitvi17g04255\_t001 |  |  |  |  |  |  |  |
| 1 | Ath-AT3G48250.1 |  | Vvi-Vitvi17g00921\_t001 |  |  |  |  |  |  |  |
| 1 | Ath-AT3G48260.1 |  | Vvi-Vitvi17g00929\_t002 |  |  |  |  |  |  |  |
| 1 | Ath-AT3G48270.1 |  | Vvi-Vitvi17g00944\_t001 |  |  |  |  |  |  |  |
| 1 | Ath-AT3G48280.1 |  | | | |  |  |  |  |  |  |  |
| 1 | Ath-AT3G48290.2 |  | | | |  |  |  |  |  |  |  |
| 1 | Ath-AT3G48300.2 |  | | | |  |  |  |  |  |  |  |
| 1 | Ath-AT3G48298.1 |  | | | |  |  |  |  |  |  |  |
| 1 | Ath-AT3G48310.1 |  | | | |  |  |  |  |  |  |  |
| 1 | Ath-AT3G48320.1 |  | Vvi-Vitvi17g04269\_t001 |  |  |  |  |  |  |  |
| 1 | Ath-AT3G48330.4 |  | Vvi-Vitvi17g00959\_t001 |  |  |  |  |  |  |  |
| 1 | Ath-AT3G48340.1 |  | Vvi-Vitvi17g00973\_t001 |  |  |  |  |  |  |  |
| 1 | Ath-AT3G48343.1 |  | | | |  |  |  |  |  |  |  |
| 1 | Ath-AT3G48344.1 |  | | | |  |  |  |  |  |  |  |
| 1 | Ath-AT3G48346.1 |  | | | |  |  |  |  |  |  |  |
| 1 | Ath-AT3G48350.1 |  | | | |  |  |  |  |  |  |  |
| 1 | Ath-AT3G48360.1 |  | Vvi-Vitvi17g00975\_t001 |  |  |  |  |  |  |  |
| 1 | Ath-AT3G48380.3 |  | Vvi-Vitvi17g00985\_t001 |  |  |  |  |  |  |  |
| 1 | Ath-AT3G48390.1 |  | Vvi-Vitvi17g00987\_t001 |  |  |  |  |  |  |  |
| 1 | Ath-AT3G48410.2 |  | | | |  |  |  |  |  |  |  |
| 1 | Ath-AT3G48400.1 |  | | | |  |  |  |  |  |  |  |
| 1 | Ath-AT3G48420.1 |  | Vvi-Vitvi17g01585\_t001 |  |  |  |  |  |  |  |
| 1 | Ath-AT3G48425.1 |  | Vvi-Vitvi17g01000\_t001 |  |  |  |  |  |  |  |
| 1 | Ath-AT3G48430.1 |  | Vvi-Vitvi17g01001\_t001 |  |  |  |  |  |  |  |
| 1 | Ath-AT3G48440.1 |  | Vvi-Vitvi17g01002\_t001 |  |  |  |  |  |  |  |
| 1 | Ath-AT3G48450.1 |  | | | |  |  |  |  |  |  |  |
| 1 | Ath-AT3G48460.1 |  | Vvi-Vitvi17g01012\_t001 |  |  |  |  |  |  |  |
| 1 | Ath-AT3G48470.1 |  | Vvi-Vitvi17g01034\_t001 |  |  |  |  |  |  |  |
| 1 | Ath-AT3G48475.1 |  | | | |  |  |  |  |  |  |  |
| 1 | Ath-AT3G48480.1 |  | Vvi-Vitvi17g01036\_t001 |  |  |  |  |  |  |  |
| 0 | Ath-AT3G48490.1 |  |  |  |  |  |  |  |  |
| 0 | Ath-AT3G48500.2 |  |  |  |  |  |  |  |  |
| 0 | Ath-AT3G48510.1 |  |  |  |  |  |  |  |  |
| 1 | Ath-AT3G48520.1 |  | Vvi-Vitvi17g00119\_t001 |  |  |  |  |  |  |  |
| 1 | Ath-AT3G48530.1 |  | Vvi-Vitvi17g00116\_t001 |  |  |  |  |  |  |  |
| 1 | Ath-AT3G48540.1 |  | Vvi-Vitvi17g00109\_t001 |  |  |  |  |  |  |  |
| 1 | Ath-AT3G48550.1 |  | Vvi-Vitvi17g01347\_t001 |  |  |  |  |  |  |  |
| 1 | Ath-AT3G48560.1 |  | Vvi-Vitvi17g00104\_t001 |  |  |  |  |  |  |  |
| 1 | Ath-AT3G48570.1 |  | Vvi-Vitvi17g01346\_t001 |  |  |  |  |  |  |  |
| 1 | Ath-AT3G48580.1 |  | | | |  |  |  |  |  |  |  |
| 1 | Ath-AT3G48590.1 |  | | | |  |  |  |  |  |  |  |
| 1 | Ath-AT3G48600.2 |  | | | |  |  |  |  |  |  |  |
| 1 | Ath-AT3G48610.1 |  | Vvi-Vitvi17g00095\_t001 |  |  |  |  |  |  |  |
| 1 | Ath-AT3G48620.1 |  | | | |  |  |  |  |  |  |  |
| 1 | Ath-AT3G48630.1 |  | | | |  |  |  |  |  |  |  |
| 1 | Ath-AT3G48640.2 |  | | | |  |  |  |  |  |  |  |
| 1 | Ath-AT3G48660.1 |  | | | |  |  |  |  |  |  |  |
| 1 | Ath-AT3G48670.3 |  | | | |  |  |  |  |  |  |  |
| 1 | Ath-AT3G48675.1 |  | | | |  |  |  |  |  |  |  |
| 1 | Ath-AT3G48680.1 |  | | | |  |  |  |  |  |  |  |
| 1 | Ath-AT3G48690.1 |  | | | |  |  |  |  |  |  |  |
| 1 | Ath-AT3G48700.1 |  | | | |  |  |  |  |  |  |  |
| 1 | Ath-AT3G48710.1 |  | Vvi-Vitvi17g00079\_t001 |  |  |  |  |  |  |  |
| 1 | Ath-AT3G48720.1 |  | Vvi-Vitvi17g00078\_t001 |  |  |  |  |  |  |  |
| 1 | Ath-AT3G48730.1 |  | Vvi-Vitvi17g00074\_t001 |  |  |  |  |  |  |  |
| 1 | Ath-AT3G48740.1 |  | Vvi-Vitvi17g00069\_t001 |  |  |  |  |  |  |  |
| 1 | Ath-AT3G48750.1 |  | | | |  |  |  |  |  |  |  |
| 1 | Ath-AT3G48760.1 |  | Vvi-Vitvi17g00068\_t001 |  |  |  |  |  |  |  |
| 1 | Ath-AT3G48770.1 |  | Vvi-Vitvi17g00061\_t001 |  |  |  |  |  |  |  |
| 1 | Ath-AT3G48780.1 |  | Vvi-Vitvi17g00051\_t001 |  |  |  |  |  |  |  |
| 1 | Ath-AT3G48790.1 |  | | | |  |  |  |  |  |  |  |
| 1 | Ath-AT3G48800.1 |  | Vvi-Vitvi17g00050\_t001 |  |  |  |  |  |  |  |
| 1 | Ath-AT3G48810.1 |  | | | |  |  |  |  |  |  |  |
| 1 | Ath-AT3G48820.1 |  | Vvi-Vitvi17g00048\_t001 |  |  |  |  |  |  |  |
| 1 | Ath-AT3G48830.1 |  | | | |  |  |  |  |  |  |  |
| 1 | Ath-AT3G48835.1 |  | | | |  |  |  |  |  |  |  |
| 1 | Ath-AT3G48840.1 |  | | | |  |  |  |  |  |  |  |
| 1 | Ath-AT3G48850.1 |  | Vvi-Vitvi17g00044\_t001 |  |  |  |  |  |  |  |
| 1 | Ath-AT3G48860.2 |  | Vvi-Vitvi17g00041\_t001 |  |  |  |  |  |  |  |
| 1 | Ath-AT3G48870.3 |  | Vvi-Vitvi17g00038\_t001 |  |  |  |  |  |  |  |
| 1 | Ath-AT3G48880.2 |  | Vvi-Vitvi16g01003\_t001 |  |  |  |  |  |  |  |
| 1 | Ath-AT3G48890.1 |  | Vvi-Vitvi16g01004\_t001 |  |  |  |  |  |  |  |
| 1 | Ath-AT3G48900.2 |  | Vvi-Vitvi16g01007\_t001 |  |  |  |  |  |  |  |
| 1 | Ath-AT3G48920.1 |  | Vvi-Vitvi16g01015\_t001 |  |  |  |  |  |  |  |
| 1 | Ath-AT3G48930.1 |  | | | |  |  |  |  |  |  |  |
| 1 | Ath-AT3G48940.1 |  | Vvi-Vitvi16g01018\_t001 |  |  |  |  |  |  |  |
| 1 | Ath-AT3G48950.1 |  | | | |  |  |  |  |  |  |  |
| 1 | Ath-AT3G48960.1 |  | | | |  |  |  |  |  |  |  |
| 1 | Ath-AT3G48970.1 |  | Vvi-Vitvi16g01023\_t001 |  |  |  |  |  |  |  |
| 1 | Ath-AT3G48980.2 |  | Vvi-Vitvi16g01024\_t001 |  |  |  |  |  |  |  |
| 1 | Ath-AT3G48990.1 |  | Vvi-Vitvi16g01036\_t001 |  |  |  |  |  |  |  |
| 1 | Ath-AT3G49000.1 |  | Vvi-Vitvi16g01041\_t002 |  |  |  |  |  |  |  |
| 1 | Ath-AT3G49010.2 |  | | | |  |  |  |  |  |  |  |
| 1 | Ath-AT3G49020.1 |  | | | |  |  |  |  |  |  |  |
| 1 | Ath-AT3G49030.1 |  | | | |  |  |  |  |  |  |  |
| 1 | Ath-AT3G49040.1 |  | | | |  |  |  |  |  |  |  |
| 1 | Ath-AT3G49045.1 |  | | | |  |  |  |  |  |  |  |
| 1 | Ath-AT3G49050.1 |  | Vvi-Vitvi16g01055\_t002 |  |  |  |  |  |  |  |
| 1 | Ath-AT3G49055.1 |  | Vvi-Vitvi16g01065\_t001 |  |  |  |  |  |  |  |
| 1 | Ath-AT3G49060.1 |  | Vvi-Vitvi16g01066\_t001 |  |  |  |  |  |  |  |
| 1 | Ath-AT3G49070.1 |  | Vvi-Vitvi16g01079\_t001 |  |  |  |  |  |  |  |
| 0 | Ath-AT3G49080.1 |  |  |  |  |  |  |  |  |
| 0 | Ath-AT3G49100.2 |  |  |  |  |  |  |  |  |
| 0 | Ath-AT3G49110.1 |  |  |  |  |  |  |  |  |
| 0 | Ath-AT3G49115.1 |  |  |  |  |  |  |  |  |
| 0 | Ath-AT3G49120.1 |  |  |  |  |  |  |  |  |
| 0 | Ath-AT3G49130.1 |  |  |  |  |  |  |  |  |
| 0 | Ath-AT3G49140.1 |  |  |  |  |  |  |  |  |
| 0 | Ath-AT3G49142.1 |  |  |  |  |  |  |  |  |
| 0 | Ath-AT3G49150.1 |  |  |  |  |  |  |  |  |
| 0 | Ath-AT3G49155.1 |  |  |  |  |  |  |  |  |
| 0 | Ath-AT3G49160.1 |  |  |  |  |  |  |  |  |
| 0 | Ath-AT3G49170.1 |  |  |  |  |  |  |  |  |
| 0 | Ath-AT3G49180.1 |  |  |  |  |  |  |  |  |
| 0 | Ath-AT3G49190.1 |  |  |  |  |  |  |  |  |
| 0 | Ath-AT3G49200.1 |  |  |  |  |  |  |  |  |
| 0 | Ath-AT3G49210.1 |  |  |  |  |  |  |  |  |
| 0 | Ath-AT3G49220.3 |  |  |  |  |  |  |  |  |
| 0 | Ath-AT3G49230.1 |  |  |  |  |  |  |  |  |
| 0 | Ath-AT3G49250.1 |  |  |  |  |  |  |  |  |
| 0 | Ath-AT3G49240.1 |  |  |  |  |  |  |  |  |
| 0 | Ath-AT3G49260.3 |  |  |  |  |  |  |  |  |
| 0 | Ath-AT3G49270.3 |  |  |  |  |  |  |  |  |
| 0 | Ath-AT3G49290.1 |  |  |  |  |  |  |  |  |
| 0 | Ath-AT3G49300.1 |  |  |  |  |  |  |  |  |
| 0 | Ath-AT3G49305.1 |  |  |  |  |  |  |  |  |
| 0 | Ath-AT3G49307.1 |  |  |  |  |  |  |  |  |
| 1 | Ath-AT3G49310.1 |  | Vvi-Vitvi16g02074\_t001 |  |  |  |  |  |  |  |
| 1 | Ath-AT3G49320.1 |  | | | |  |  |  |  |  |  |  |
| 1 | Ath-AT3G49330.2 |  | | | |  |  |  |  |  |  |  |
| 1 | Ath-AT3G49340.1 |  | | | |  |  |  |  |  |  |  |
| 1 | Ath-AT3G49350.1 |  | Vvi-Vitvi16g01366\_t002 |  |  |  |  |  |  |  |
| 1 | Ath-AT3G49360.1 |  | Vvi-Vitvi16g01365\_t001 |  |  |  |  |  |  |  |
| 1 | Ath-AT3G49370.1 |  | Vvi-Vitvi16g01358\_t001 |  |  |  |  |  |  |  |
| 1 | Ath-AT3G49380.1 |  | Vvi-Vitvi16g01353\_t001 |  |  |  |  |  |  |  |
| 1 | Ath-AT3G49390.1 |  | Vvi-Vitvi16g01350\_t001 |  |  |  |  |  |  |  |
| 1 | Ath-AT3G49400.1 |  | Vvi-Vitvi16g01349\_t001 |  |  |  |  |  |  |  |
| 1 | Ath-AT3G49410.1 |  | | | |  |  |  |  |  |  |  |
| 1 | Ath-AT3G49420.1 |  | | | |  |  |  |  |  |  |  |
| 1 | Ath-AT3G49430.6 |  | Vvi-Vitvi16g01348\_t005 |  |  |  |  |  |  |  |
| 1 | Ath-AT3G49440.1 |  | | | |  |  |  |  |  |  |  |
| 1 | Ath-AT3G49450.1 |  | | | |  |  |  |  |  |  |  |
| 1 | Ath-AT3G49460.1 |  | | | |  |  |  |  |  |  |  |
| 1 | Ath-AT3G49470.2 |  | Vvi-Vitvi16g01333\_t001 |  |  |  |  |  |  |  |
| 1 | Ath-AT3G49480.2 |  | | | |  |  |  |  |  |  |  |
| 1 | Ath-AT3G49490.1 |  | Vvi-Vitvi16g01330\_t002 |  |  |  |  |  |  |  |
| 1 | Ath-AT3G49500.2 |  | | | |  |  |  |  |  |  |  |
| 1 | Ath-AT3G49510.1 |  | | | |  |  |  |  |  |  |  |
| 1 | Ath-AT3G49520.1 |  | | | |  |  |  |  |  |  |  |
| 1 | Ath-AT3G49530.1 |  | Vvi-Vitvi16g01327\_t002 |  |  |  |  |  |  |  |
| 1 | Ath-AT3G49540.1 |  | | | |  |  |  |  |  |  |  |
| 1 | Ath-AT3G49550.1 |  | | | |  |  |  |  |  |  |  |
| 1 | Ath-AT3G49551.1 |  | | | |  |  |  |  |  |  |  |
| 1 | Ath-AT3G49560.1 |  | Vvi-Vitvi16g01311\_t001 |  |  |  |  |  |  |  |
| 1 | Ath-AT3G49570.1 |  | Vvi-Vitvi16g01310\_t001 |  |  |  |  |  |  |  |
| 1 | Ath-AT3G49580.1 |  | | | |  |  |  |  |  |  |  |
| 1 | Ath-AT3G49590.3 |  | Vvi-Vitvi16g01309\_t001 |  |  |  |  |  |  |  |
| 0 | Ath-AT3G49600.1 |  |  |  |  |  |  |  |  |
| 0 | Ath-AT3G49601.1 |  |  |  |  |  |  |  |  |
| 0 | Ath-AT3G49610.1 |  |  |  |  |  |  |  |  |
| 0 | Ath-AT3G49620.1 |  |  |  |  |  |  |  |  |
| 0 | Ath-AT3G49630.1 |  |  |  |  |  |  |  |  |
| 1 | Ath-AT3G49640.1 |  | Vvi-Vitvi07g04645\_t001 |  |  |  |  |  |  |  |
| 1 | Ath-AT3G49645.2 |  | | | |  |  |  |  |  |  |  |
| 1 | Ath-AT3G49650.1 |  | Vvi-Vitvi07g01535\_t001 |  |  |  |  |  |  |  |
| 1 | Ath-AT3G49660.1 |  | Vvi-Vitvi07g01534\_t001 |  |  |  |  |  |  |  |
| 1 | Ath-AT3G49670.1 |  | Vvi-Vitvi07g04652\_t001 |  |  |  |  |  |  |  |
| 2 | Ath-AT3G49680.1 |  | | | |  | Vvi-Vitvi18g02625\_t001 |  |  |  |  |  |  |
| 2 | Ath-AT3G49690.1 |  | | | |  | Vvi-Vitvi18g00605\_t001 |  |  |  |  |  |  |
| 2 | Ath-AT3G49700.1 |  | | | |  | Vvi-Vitvi18g00609\_t001 |  |  |  |  |  |  |
| 2 | Ath-AT3G49710.1 |  | | | |  | | | |  |  |  |  |  |  |
| 3 | Ath-AT3G49720.2 |  | Vvi-Vitvi07g03099\_t001 |  | Vvi-Vitvi18g00610\_t001 |  | Vvi-Vitvi15g04535\_t001 |  |  |  |  |  |
| 3 | Ath-AT3G49725.1 |  | | | |  | | | |  | Vvi-Vitvi15g04533\_t001 |  |  |  |  |  |
| 3 | Ath-AT3G49730.1 |  | | | |  | | | |  | Vvi-Vitvi15g04529\_t001 |  |  |  |  |  |
| 3 | Ath-AT3G49740.1 |  | | | |  | | | |  | Vvi-Vitvi15g04528\_t001 |  |  |  |  |  |
| 3 | Ath-AT3G49744.1 |  | | | |  | | | |  | | | |  |  |  |  |  |
| 3 | Ath-AT3G49750.1 |  | | | |  | | | |  | Vvi-Vitvi15g04523\_t001 |  |  |  |  |  |
| 3 | Ath-AT3G49760.1 |  | | | |  | Vvi-Vitvi18g00628\_t001 |  | Vvi-Vitvi15g04514\_t001 |  |  |  |  |  |
| 3 | Ath-AT3G49770.1 |  | | | |  | | | |  | | | |  |  |  |  |  |
| 3 | Ath-AT3G49780.1 |  | | | |  | Vvi-Vitvi18g00635\_t001 |  | Vvi-Vitvi15g04511\_t001 |  |  |  |  |  |
| 3 | Ath-AT3G49790.1 |  | | | |  | Vvi-Vitvi18g00636\_t001 |  | Vvi-Vitvi15g04508\_t001 |  |  |  |  |  |
| 3 | Ath-AT3G49796.1 |  | | | |  | | | |  | | | |  |  |  |  |  |
| 3 | Ath-AT3G49800.1 |  | | | |  | Vvi-Vitvi18g00637\_t001 |  | Vvi-Vitvi15g04506\_t001 |  |  |  |  |  |
| 3 | Ath-AT3G49810.1 |  | | | |  | Vvi-Vitvi18g00638\_t001 |  | Vvi-Vitvi15g04503\_t001 |  |  |  |  |  |
| 2 | Ath-AT3G49820.1 |  | | | |  |  |  | | | |  |  |  |  |  |
| 2 | Ath-AT3G49830.1 |  | | | |  |  |  | Vvi-Vitvi15g04498\_t001 |  |  |  |  |  |
| 2 | Ath-AT3G49840.2 |  | | | |  |  |  | Vvi-Vitvi15g04490\_t001 |  |  |  |  |  |
| 1 | Ath-AT3G49845.1 |  | | | |  |  |  |  |  |  |  |
| 1 | Ath-AT3G49850.1 |  | Vvi-Vitvi07g04670\_t004 |  |  |  |  |  |  |  |
| 1 | Ath-AT3G49860.1 |  | Vvi-Vitvi07g01577\_t001 |  |  |  |  |  |  |  |
| 1 | Ath-AT3G49870.1 |  | | | |  |  |  |  |  |  |  |
| 1 | Ath-AT3G49880.1 |  | Vvi-Vitvi07g01580\_t002 |  |  |  |  |  |  |  |
| 1 | Ath-AT3G49890.2 |  | Vvi-Vitvi07g02633\_t001 |  |  |  |  |  |  |  |
| 1 | Ath-AT3G49900.2 |  | Vvi-Vitvi07g01591\_t001 |  |  |  |  |  |  |  |
| 1 | Ath-AT3G49910.1 |  | Vvi-Vitvi07g04673\_t001 |  |  |  |  |  |  |  |
| 1 | Ath-AT3G49920.1 |  | Vvi-Vitvi07g01593\_t001 |  |  |  |  |  |  |  |
| 1 | Ath-AT3G49930.1 |  | Vvi-Vitvi07g01602\_t001 |  |  |  |  |  |  |  |
| 1 | Ath-AT3G49940.1 |  | Vvi-Vitvi07g01610\_t001 |  |  |  |  |  |  |  |
| 1 | Ath-AT3G49950.1 |  | Vvi-Vitvi07g01612\_t001 |  |  |  |  |  |  |  |
| 1 | Ath-AT3G49960.1 |  | Vvi-Vitvi07g01614\_t001 |  |  |  |  |  |  |  |
| 1 | Ath-AT3G49970.2 |  | Vvi-Vitvi07g01617\_t001 |  |  |  |  |  |  |  |
| 1 | Ath-AT3G49980.2 |  | | | |  |  |  |  |  |  |  |
| 1 | Ath-AT3G49990.1 |  | | | |  |  |  |  |  |  |  |
| 1 | Ath-AT3G50000.1 |  | Vvi-Vitvi07g01620\_t002 |  |  |  |  |  |  |  |
| 1 | Ath-AT3G50010.1 |  | | | |  |  |  |  |  |  |  |
| 1 | Ath-AT3G50020.1 |  | | | |  |  |  |  |  |  |  |
| 1 | Ath-AT3G50030.1 |  | Vvi-Vitvi07g01635\_t001 |  |  |  |  |  |  |  |
| 1 | Ath-AT3G50040.2 |  | | | |  |  |  |  |  |  |  |
| 1 | Ath-AT3G50050.1 |  | Vvi-Vitvi07g04684\_t001 |  |  |  |  |  |  |  |
| 2 | Ath-AT3G50060.1 |  | Vvi-Vitvi07g01676\_t001 |  | Vvi-Vitvi18g00725\_t001 |  |  |  |  |  |  |
| 2 | Ath-AT3G50070.1 |  | Vvi-Vitvi07g01683\_t001 |  | Vvi-Vitvi18g00731\_t001 |  |  |  |  |  |  |
| 2 | Ath-AT3G50080.1 |  | Vvi-Vitvi07g01684\_t001 |  | Vvi-Vitvi18g00734\_t001 |  |  |  |  |  |  |
| 2 | Ath-AT3G50090.1 |  | Vvi-Vitvi07g01685\_t001 |  | | | |  |  |  |  |  |  |
| 2 | Ath-AT3G50100.1 |  | | | |  | | | |  |  |  |  |  |  |
| 2 | Ath-AT3G50110.1 |  | Vvi-Vitvi07g01686\_t001 |  | | | |  |  |  |  |  |  |
| 2 | Ath-AT3G50120.1 |  | Vvi-Vitvi07g01689\_t001 |  | | | |  |  |  |  |  |  |
| 2 | Ath-AT3G50123.1 |  | | | |  | | | |  |  |  |  |  |  |
| 2 | Ath-AT3G50130.1 |  | | | |  | | | |  |  |  |  |  |  |
| 2 | Ath-AT3G50140.1 |  | | | |  | | | |  |  |  |  |  |  |
| 2 | Ath-AT3G50150.1 |  | | | |  | | | |  |  |  |  |  |  |
| 2 | Ath-AT3G50160.1 |  | | | |  | | | |  |  |  |  |  |  |
| 2 | Ath-AT3G50170.1 |  | | | |  | | | |  |  |  |  |  |  |
| 2 | Ath-AT3G50180.1 |  | | | |  | | | |  |  |  |  |  |  |
| 2 | Ath-AT3G50190.1 |  | | | |  | | | |  |  |  |  |  |  |
| 2 | Ath-AT3G50200.1 |  | | | |  | | | |  |  |  |  |  |  |
| 2 | Ath-AT3G50210.3 |  | Vvi-Vitvi07g01696\_t002 |  | | | |  |  |  |  |  |  |
| 2 | Ath-AT3G50220.1 |  | Vvi-Vitvi07g01697\_t001 |  | | | |  |  |  |  |  |  |
| 2 | Ath-AT3G50230.2 |  | Vvi-Vitvi07g01698\_t001 |  | Vvi-Vitvi18g00747\_t001 |  |  |  |  |  |  |
| 2 | Ath-AT3G50240.1 |  | Vvi-Vitvi07g01699\_t002 |  | Vvi-Vitvi18g00749\_t002 |  |  |  |  |  |  |
| 2 | Ath-AT3G50250.1 |  | | | |  | | | |  |  |  |  |  |  |
| 2 | Ath-AT3G50260.1 |  | Vvi-Vitvi07g01702\_t001 |  | Vvi-Vitvi18g04174\_t001 |  |  |  |  |  |  |
| 1 | Ath-AT3G50270.1 |  | Vvi-Vitvi07g04707\_t001 |  |  |  |  |  |  |  |
| 1 | Ath-AT3G50280.1 |  | | | |  |  |  |  |  |  |  |
| 1 | Ath-AT3G50290.1 |  | Vvi-Vitvi07g01713\_t001 |  |  |  |  |  |  |  |
| 0 | Ath-AT3G50300.1 |  |  |  |  |  |  |  |  |
| 1 | Ath-AT3G50310.1 |  | Vvi-Vitvi07g01737\_t001 |  |  |  |  |  |  |  |
| 1 | Ath-AT3G50320.1 |  | | | |  |  |  |  |  |  |  |
| 1 | Ath-AT3G50330.1 |  | Vvi-Vitvi07g01740\_t001 |  |  |  |  |  |  |  |
| 1 | Ath-AT3G50340.1 |  | Vvi-Vitvi07g01746\_t001 |  |  |  |  |  |  |  |
| 1 | Ath-AT3G50350.1 |  | Vvi-Vitvi07g01748\_t001 |  |  |  |  |  |  |  |
| 1 | Ath-AT3G50360.1 |  | Vvi-Vitvi07g01752\_t001 |  |  |  |  |  |  |  |
| 1 | Ath-AT3G50370.2 |  | Vvi-Vitvi07g01756\_t001 |  |  |  |  |  |  |  |
| 1 | Ath-AT3G50373.1 |  | | | |  |  |  |  |  |  |  |
| 2 | Ath-AT3G50380.2 |  | | | |  | Vvi-Vitvi07g04496\_t001 |  |  |  |  |  |  |
| 2 | Ath-AT3G50390.1 |  | | | |  | Vvi-Vitvi07g01286\_t001 |  |  |  |  |  |  |
| 2 | Ath-AT3G50400.1 |  | | | |  | Vvi-Vitvi07g01297\_t001 |  |  |  |  |  |  |
| 2 | Ath-AT3G50410.1 |  | | | |  | Vvi-Vitvi07g01298\_t001 |  |  |  |  |  |  |
| 2 | Ath-AT3G50420.1 |  | | | |  | Vvi-Vitvi07g04506\_t001 |  |  |  |  |  |  |
| 2 | Ath-AT3G50430.1 |  | | | |  | Vvi-Vitvi07g04507\_t001 |  |  |  |  |  |  |
| 2 | Ath-AT3G50440.1 |  | | | |  | Vvi-Vitvi07g02512\_t001 |  |  |  |  |  |  |
| 1 | Ath-AT3G50450.1 |  | | | |  |  |  |  |  |  |  |
| 1 | Ath-AT3G50460.1 |  | | | |  |  |  |  |  |  |  |
| 1 | Ath-AT3G50470.1 |  | | | |  |  |  |  |  |  |  |
| 1 | Ath-AT3G50480.1 |  | | | |  |  |  |  |  |  |  |
| 1 | Ath-AT3G50500.2 |  | | | |  |  |  |  |  |  |  |
| 1 | Ath-AT3G50510.2 |  | | | |  |  |  |  |  |  |  |
| 1 | Ath-AT3G50520.1 |  | | | |  |  |  |  |  |  |  |
| 1 | Ath-AT3G50530.2 |  | | | |  |  |  |  |  |  |  |
| 1 | Ath-AT3G50540.1 |  | | | |  |  |  |  |  |  |  |
| 1 | Ath-AT3G50550.1 |  | | | |  |  |  |  |  |  |  |
| 1 | Ath-AT3G50560.1 |  | | | |  |  |  |  |  |  |  |
| 1 | Ath-AT3G50570.2 |  | | | |  |  |  |  |  |  |  |
| 1 | Ath-AT3G50580.1 |  | | | |  |  |  |  |  |  |  |
| 1 | Ath-AT3G50590.1 |  | | | |  |  |  |  |  |  |  |
| 1 | Ath-AT3G50610.2 |  | Vvi-Vitvi07g02668\_t001 |  |  |  |  |  |  |  |
| 0 | Ath-AT3G50620.1 |  |  |  |  |  |  |  |  |
| 0 | Ath-AT3G50630.1 |  |  |  |  |  |  |  |  |
| 0 | Ath-AT3G50640.1 |  |  |  |  |  |  |  |  |
| 1 | Ath-AT3G50650.1 |  | Vvi-Vitvi04g01281\_t001 |  |  |  |  |  |  |  |
| 1 | Ath-AT3G50660.1 |  | Vvi-Vitvi04g01278\_t001 |  |  |  |  |  |  |  |
| 1 | Ath-AT3G50670.1 |  | Vvi-Vitvi04g01271\_t001 |  |  |  |  |  |  |  |
| 1 | Ath-AT3G50685.1 |  | Vvi-Vitvi04g01267\_t001 |  |  |  |  |  |  |  |
| 2 | Ath-AT3G50690.1 |  | Vvi-Vitvi04g02101\_t001 |  | Vvi-Vitvi04g02101\_t001 |  |  |  |  |  |  |
| 3 | Ath-AT3G50700.1 |  | Vvi-Vitvi04g01252\_t001 |  | | | |  | Vvi-Vitvi18g00935\_t001 |  |  |  |  |  |
| 3 | Ath-AT3G50710.1 |  | | | |  | | | |  | | | |  |  |  |  |  |
| 3 | Ath-AT3G50720.1 |  | Vvi-Vitvi04g01246\_t001 |  | | | |  | | | |  |  |  |  |  |
| 3 | Ath-AT3G50730.1 |  | | | |  | | | |  | | | |  |  |  |  |  |
| 3 | Ath-AT3G50740.1 |  | Vvi-Vitvi04g01237\_t001 |  | | | |  | Vvi-Vitvi18g04218\_t001 |  |  |  |  |  |
| 3 | Ath-AT3G50750.1 |  | Vvi-Vitvi04g01234\_t001 |  | | | |  | Vvi-Vitvi18g00924\_t001 |  |  |  |  |  |
| 3 | Ath-AT3G50760.1 |  | Vvi-Vitvi04g01222\_t001 |  | | | |  | Vvi-Vitvi18g00909\_t001 |  |  |  |  |  |
| 3 | Ath-AT3G50770.1 |  | Vvi-Vitvi04g01220\_t001 |  | | | |  | Vvi-Vitvi18g00905\_t001 |  |  |  |  |  |
| 3 | Ath-AT3G50780.2 |  | | | |  | | | |  | | | |  |  |  |  |  |
| 3 | Ath-AT3G50790.1 |  | | | |  | | | |  | | | |  |  |  |  |  |
| 3 | Ath-AT3G50800.1 |  | | | |  | | | |  | | | |  |  |  |  |  |
| 3 | Ath-AT3G50808.1 |  | | | |  | | | |  | | | |  |  |  |  |  |
| 3 | Ath-AT3G50810.2 |  | | | |  | | | |  | | | |  |  |  |  |  |
| 3 | Ath-AT3G50820.1 |  | | | |  | | | |  | Vvi-Vitvi18g00894\_t001 |  |  |  |  |  |
| 2 | Ath-AT3G50830.1 |  | Vvi-Vitvi04g02094\_t001 |  | | | |  |  |  |  |  |  |
| 1 | Ath-AT3G50840.1 |  |  |  | | | |  |  |  |  |  |  |
| 1 | Ath-AT3G50845.1 |  |  |  | Vvi-Vitvi04g04343\_t001 |  |  |  |  |  |  |
| 1 | Ath-AT3G50850.1 |  |  |  | Vvi-Vitvi04g01290\_t001 |  |  |  |  |  |  |
| 1 | Ath-AT3G50860.1 |  |  |  | Vvi-Vitvi04g01291\_t001 |  |  |  |  |  |  |
| 1 | Ath-AT3G50870.1 |  |  |  | Vvi-Vitvi04g01299\_t001 |  |  |  |  |  |  |
| 1 | Ath-AT3G50880.1 |  |  |  | Vvi-Vitvi04g01303\_t001 |  |  |  |  |  |  |
| 1 | Ath-AT3G50890.1 |  |  |  | Vvi-Vitvi04g01304\_t001 |  |  |  |  |  |  |
| 1 | Ath-AT3G50900.1 |  |  |  | Vvi-Vitvi04g04348\_t001 |  |  |  |  |  |  |
| 1 | Ath-AT3G50910.1 |  |  |  | Vvi-Vitvi04g01330\_t001 |  |  |  |  |  |  |
| 1 | Ath-AT3G50920.1 |  |  |  | Vvi-Vitvi04g01336\_t001 |  |  |  |  |  |  |
| 0 | Ath-AT3G50925.1 |  |  |  |  |  |  |  |  |
| 1 | Ath-AT3G50930.1 |  | Vvi-Vitvi04g01353\_t001 |  |  |  |  |  |  |  |
| 1 | Ath-AT3G50940.1 |  | | | |  |  |  |  |  |  |  |
| 1 | Ath-AT3G50950.1 |  | Vvi-Vitvi04g01358\_t001 |  |  |  |  |  |  |  |
| 1 | Ath-AT3G50960.1 |  | Vvi-Vitvi04g01359\_t001 |  |  |  |  |  |  |  |
| 1 | Ath-AT3G50970.1 |  | | | |  |  |  |  |  |  |  |
| 1 | Ath-AT3G50980.1 |  | Vvi-Vitvi04g01368\_t001 |  |  |  |  |  |  |  |
| 1 | Ath-AT3G50990.1 |  | Vvi-Vitvi04g01378\_t001 |  |  |  |  |  |  |  |
| 2 | Ath-AT3G51000.1 |  | Vvi-Vitvi04g01382\_t001 |  | Vvi-Vitvi03g04008\_t001 |  |  |  |  |  |  |
| 2 | Ath-AT3G51010.1 |  | Vvi-Vitvi04g01386\_t001 |  | | | |  |  |  |  |  |  |
| 2 | Ath-AT3G51020.1 |  | | | |  | | | |  |  |  |  |  |  |
| 2 | Ath-AT3G51030.1 |  | Vvi-Vitvi04g01390\_t001 |  | | | |  |  |  |  |  |  |
| 2 | Ath-AT3G51040.1 |  | Vvi-Vitvi04g01391\_t003 |  | | | |  |  |  |  |  |  |
| 2 | Ath-AT3G51050.1 |  | Vvi-Vitvi04g01396\_t001 |  | | | |  |  |  |  |  |  |
| 2 | Ath-AT3G51060.1 |  | Vvi-Vitvi04g01399\_t001 |  | Vvi-Vitvi03g00024\_t001 |  |  |  |  |  |  |
| 2 | Ath-AT3G51070.1 |  | Vvi-Vitvi04g01400\_t001 |  | | | |  |  |  |  |  |  |
| 2 | Ath-AT3G51080.1 |  | Vvi-Vitvi04g01410\_t001 |  | Vvi-Vitvi03g00037\_t001 |  |  |  |  |  |  |
| 2 | Ath-AT3G51090.1 |  | Vvi-Vitvi04g02143\_t002 |  | Vvi-Vitvi03g01326\_t001 |  |  |  |  |  |  |
| 2 | Ath-AT3G51100.1 |  | | | |  | Vvi-Vitvi03g00041\_t001 |  |  |  |  |  |  |
| 2 | Ath-AT3G51110.1 |  | | | |  | | | |  |  |  |  |  |  |
| 2 | Ath-AT3G51120.1 |  | Vvi-Vitvi04g01414\_t002 |  | Vvi-Vitvi03g00042\_t001 |  |  |  |  |  |  |
| 2 | Ath-AT3G51130.1 |  | Vvi-Vitvi04g01418\_t001 |  | | | |  |  |  |  |  |  |
| 2 | Ath-AT3G51140.1 |  | Vvi-Vitvi04g01420\_t001 |  | | | |  |  |  |  |  |  |
| 2 | Ath-AT3G51150.2 |  | Vvi-Vitvi04g01425\_t003 |  | Vvi-Vitvi03g00050\_t001 |  |  |  |  |  |  |
| 1 | Ath-AT3G51160.1 |  | Vvi-Vitvi04g02147\_t001 |  |  |  |  |  |  |  |
| 1 | Ath-AT3G51171.1 |  | | | |  |  |  |  |  |  |  |
| 1 | Ath-AT3G51180.1 |  | Vvi-Vitvi04g01436\_t001 |  |  |  |  |  |  |  |
| 1 | Ath-AT3G51190.1 |  | Vvi-Vitvi04g01439\_t001 |  |  |  |  |  |  |  |
| 1 | Ath-AT3G51200.1 |  | | | |  |  |  |  |  |  |  |
| 1 | Ath-AT3G51210.1 |  | | | |  |  |  |  |  |  |  |
| 1 | Ath-AT3G51220.1 |  | Vvi-Vitvi04g02150\_t001 |  |  |  |  |  |  |  |
| 1 | Ath-AT3G51230.1 |  | Vvi-Vitvi04g01453\_t001 |  |  |  |  |  |  |  |
| 1 | Ath-AT3G51240.1 |  | Vvi-Vitvi04g01454\_t001 |  |  |  |  |  |  |  |
| 1 | Ath-AT3G51250.1 |  | Vvi-Vitvi04g01482\_t001 |  |  |  |  |  |  |  |
| 1 | Ath-AT3G51260.1 |  | Vvi-Vitvi04g01484\_t001 |  |  |  |  |  |  |  |
| 1 | Ath-AT3G51270.1 |  | | | |  |  |  |  |  |  |  |
| 1 | Ath-AT3G51280.1 |  | Vvi-Vitvi04g02163\_t001 |  |  |  |  |  |  |  |
| 1 | Ath-AT3G51290.2 |  | Vvi-Vitvi04g04392\_t001 |  |  |  |  |  |  |  |
| 1 | Ath-AT3G51300.1 |  | Vvi-Vitvi04g01498\_t001 |  |  |  |  |  |  |  |
| 1 | Ath-AT3G51310.1 |  | Vvi-Vitvi04g01499\_t001 |  |  |  |  |  |  |  |
| 1 | Ath-AT3G51320.1 |  | Vvi-Vitvi04g04400\_t001 |  |  |  |  |  |  |  |
| 1 | Ath-AT3G51325.1 |  | Vvi-Vitvi04g02174\_t001 |  |  |  |  |  |  |  |
| 1 | Ath-AT3G51330.1 |  | Vvi-Vitvi04g04401\_t001 |  |  |  |  |  |  |  |
| 1 | Ath-AT3G51340.1 |  | | | |  |  |  |  |  |  |  |
| 1 | Ath-AT3G51350.1 |  | | | |  |  |  |  |  |  |  |
| 1 | Ath-AT3G51360.9 |  | | | |  |  |  |  |  |  |  |
| 1 | Ath-AT3G51370.1 |  | Vvi-Vitvi04g02280\_t001 |  |  |  |  |  |  |  |
| 1 | Ath-AT3G51380.1 |  | | | |  |  |  |  |  |  |  |
| 1 | Ath-AT3G51390.1 |  | Vvi-Vitvi04g04411\_t001 |  |  |  |  |  |  |  |
| 1 | Ath-AT3G51400.1 |  | Vvi-Vitvi04g04419\_t001 |  |  |  |  |  |  |  |
| 1 | Ath-AT3G51410.1 |  | | | |  |  |  |  |  |  |  |
| 1 | Ath-AT3G51420.1 |  | Vvi-Vitvi04g02190\_t001 |  |  |  |  |  |  |  |
| 1 | Ath-AT3G51430.1 |  | | | |  |  |  |  |  |  |  |
| 1 | Ath-AT3G51440.1 |  | | | |  |  |  |  |  |  |  |
| 1 | Ath-AT3G51450.1 |  | | | |  |  |  |  |  |  |  |
| 1 | Ath-AT3G51460.1 |  | Vvi-Vitvi04g01565\_t001 |  |  |  |  |  |  |  |
| 0 | Ath-AT3G51470.1 |  |  |  |  |  |  |  |  |
| 0 | Ath-AT3G51480.1 |  |  |  |  |  |  |  |  |
| 0 | Ath-AT3G51490.2 |  |  |  |  |  |  |  |  |
| 0 | Ath-AT3G51500.1 |  |  |  |  |  |  |  |  |
| 0 | Ath-AT3G51510.1 |  |  |  |  |  |  |  |  |
| 0 | Ath-AT3G51520.1 |  |  |  |  |  |  |  |  |
| 0 | Ath-AT3G51530.1 |  |  |  |  |  |  |  |  |
| 0 | Ath-AT3G51540.1 |  |  |  |  |  |  |  |  |
| 1 | Ath-AT3G51550.1 |  | Vvi-Vitvi08g04140\_t001 |  |  |  |  |  |  |  |
| 1 | Ath-AT3G51560.1 |  | | | |  |  |  |  |  |  |  |
| 1 | Ath-AT3G51570.1 |  | | | |  |  |  |  |  |  |  |
| 1 | Ath-AT3G51580.2 |  | Vvi-Vitvi08g00862\_t001 |  |  |  |  |  |  |  |
| 1 | Ath-AT3G51590.1 |  | | | |  |  |  |  |  |  |  |
| 1 | Ath-AT3G51600.1 |  | Vvi-Vitvi08g00854\_t001 |  |  |  |  |  |  |  |
| 1 | Ath-AT3G51610.1 |  | Vvi-Vitvi08g00845\_t001 |  |  |  |  |  |  |  |
| 1 | Ath-AT3G51620.2 |  | Vvi-Vitvi08g00844\_t001 |  |  |  |  |  |  |  |
| 1 | Ath-AT3G51630.2 |  | Vvi-Vitvi08g00843\_t001 |  |  |  |  |  |  |  |
| 1 | Ath-AT3G51640.2 |  | Vvi-Vitvi08g00829\_t001 |  |  |  |  |  |  |  |
| 1 | Ath-AT3G51642.1 |  | | | |  |  |  |  |  |  |  |
| 1 | Ath-AT3G51644.1 |  | | | |  |  |  |  |  |  |  |
| 1 | Ath-AT3G51650.2 |  | | | |  |  |  |  |  |  |  |
| 2 | Ath-AT3G51660.1 |  | | | |  | Vvi-Vitvi08g00783\_t003 |  |  |  |  |  |  |
| 2 | Ath-AT3G51670.1 |  | | | |  | Vvi-Vitvi08g00788\_t002 |  |  |  |  |  |  |
| 2 | Ath-AT3G51680.1 |  | | | |  | Vvi-Vitvi08g00801\_t001 |  |  |  |  |  |  |
| 2 | Ath-AT3G51690.1 |  | | | |  | | | |  |  |  |  |  |  |
| 2 | Ath-AT3G51700.1 |  | | | |  | | | |  |  |  |  |  |  |
| 2 | Ath-AT3G51710.1 |  | | | |  | Vvi-Vitvi08g00819\_t001 |  |  |  |  |  |  |
| 2 | Ath-AT3G51720.1 |  | Vvi-Vitvi08g00825\_t002 |  | Vvi-Vitvi08g00825\_t002 |  |  |  |  |  |  |
| 1 | Ath-AT3G51730.1 |  |  |  | Vvi-Vitvi08g00826\_t002 |  |  |  |  |  |  |
| 0 | Ath-AT3G51740.1 |  |  |  |  |  |  |  |  |
| 0 | Ath-AT3G51750.3 |  |  |  |  |  |  |  |  |
| 0 | Ath-AT3G51760.2 |  |  |  |  |  |  |  |  |
| 0 | Ath-AT3G51770.2 |  |  |  |  |  |  |  |  |
| 0 | Ath-AT3G51780.1 |  |  |  |  |  |  |  |  |
| 0 | Ath-AT3G51790.1 |  |  |  |  |  |  |  |  |
| 1 | Ath-AT3G51800.2 |  | Vvi-Vitvi08g00685\_t001 |  |  |  |  |  |  |  |
| 1 | Ath-AT3G51810.1 |  | Vvi-Vitvi08g00679\_t001 |  |  |  |  |  |  |  |
| 1 | Ath-AT3G51820.1 |  | Vvi-Vitvi08g00678\_t001 |  |  |  |  |  |  |  |
| 1 | Ath-AT3G51830.1 |  | Vvi-Vitvi08g00671\_t001 |  |  |  |  |  |  |  |
| 1 | Ath-AT3G51840.1 |  | Vvi-Vitvi08g00667\_t001 |  |  |  |  |  |  |  |
| 1 | Ath-AT3G51850.1 |  | Vvi-Vitvi08g02055\_t001 |  |  |  |  |  |  |  |
| 0 | Ath-AT3G51860.2 |  |  |  |  |  |  |  |  |
| 0 | Ath-AT3G51870.1 |  |  |  |  |  |  |  |  |
| 0 | Ath-AT3G51880.2 |  |  |  |  |  |  |  |  |
| 0 | Ath-AT3G51890.1 |  |  |  |  |  |  |  |  |
| 0 | Ath-AT3G51895.2 |  |  |  |  |  |  |  |  |
| 0 | Ath-AT3G51910.1 |  |  |  |  |  |  |  |  |
| 0 | Ath-AT3G51920.1 |  |  |  |  |  |  |  |  |
| 1 | Ath-AT3G51930.1 |  | Vvi-Vitvi13g01570\_t001 |  |  |  |  |  |  |  |
| 1 | Ath-AT3G51940.2 |  | Vvi-Vitvi13g01575\_t002 |  |  |  |  |  |  |  |
| 1 | Ath-AT3G51950.3 |  | | | |  |  |  |  |  |  |  |
| 1 | Ath-AT3G51960.2 |  | Vvi-Vitvi13g01609\_t001 |  |  |  |  |  |  |  |
| 1 | Ath-AT3G51970.1 |  | | | |  |  |  |  |  |  |  |
| 1 | Ath-AT3G51980.1 |  | | | |  |  |  |  |  |  |  |
| 1 | Ath-AT3G51990.1 |  | Vvi-Vitvi13g01626\_t001 |  |  |  |  |  |  |  |
| 1 | Ath-AT3G52000.1 |  | | | |  |  |  |  |  |  |  |
| 1 | Ath-AT3G52010.1 |  | | | |  |  |  |  |  |  |  |
| 1 | Ath-AT3G52020.1 |  | Vvi-Vitvi13g01633\_t001 |  |  |  |  |  |  |  |
| 1 | Ath-AT3G52030.1 |  | Vvi-Vitvi13g01640\_t001 |  |  |  |  |  |  |  |
| 1 | Ath-AT3G52040.1 |  | Vvi-Vitvi13g01643\_t001 |  |  |  |  |  |  |  |
| 0 | Ath-AT3G52050.3 |  |  |  |  |  |  |  |  |
| 0 | Ath-AT3G52060.1 |  |  |  |  |  |  |  |  |
| 0 | Ath-AT3G52070.1 |  |  |  |  |  |  |  |  |
| 0 | Ath-AT3G52075.1 |  |  |  |  |  |  |  |  |
| 0 | Ath-AT3G52080.1 |  |  |  |  |  |  |  |  |
| 0 | Ath-AT3G52090.2 |  |  |  |  |  |  |  |  |
| 0 | Ath-AT3G52100.1 |  |  |  |  |  |  |  |  |
| 0 | Ath-AT3G52105.2 |  |  |  |  |  |  |  |  |
| 0 | Ath-AT3G52110.1 |  |  |  |  |  |  |  |  |
| 0 | Ath-AT3G52115.1 |  |  |  |  |  |  |  |  |
| 0 | Ath-AT3G52120.3 |  |  |  |  |  |  |  |  |
| 0 | Ath-AT3G52130.1 |  |  |  |  |  |  |  |  |
| 0 | Ath-AT3G52140.4 |  |  |  |  |  |  |  |  |
| 0 | Ath-AT3G52150.1 |  |  |  |  |  |  |  |  |
| 0 | Ath-AT3G52155.1 |  |  |  |  |  |  |  |  |
| 0 | Ath-AT3G52160.1 |  |  |  |  |  |  |  |  |
| 1 | Ath-AT3G52170.2 |  | Vvi-Vitvi13g01244\_t001 |  |  |  |  |  |  |  |
| 1 | Ath-AT3G52180.1 |  | Vvi-Vitvi13g01242\_t001 |  |  |  |  |  |  |  |
| 1 | Ath-AT3G52190.1 |  | Vvi-Vitvi13g00710\_t001 |  |  |  |  |  |  |  |
| 1 | Ath-AT3G52200.2 |  | Vvi-Vitvi13g00719\_t001 |  |  |  |  |  |  |  |
| 1 | Ath-AT3G52210.3 |  | Vvi-Vitvi13g00722\_t001 |  |  |  |  |  |  |  |
| 1 | Ath-AT3G52220.1 |  | | | |  |  |  |  |  |  |  |
| 1 | Ath-AT3G52230.1 |  | Vvi-Vitvi13g00725\_t001 |  |  |  |  |  |  |  |
| 0 | Ath-AT3G52240.1 |  |  |  |  |  |  |  |  |
| 1 | Ath-AT3G52250.1 |  | Vvi-Vitvi13g00573\_t001 |  |  |  |  |  |  |  |
| 1 | Ath-AT3G52260.3 |  | Vvi-Vitvi13g00571\_t001 |  |  |  |  |  |  |  |
| 1 | Ath-AT3G52270.1 |  | Vvi-Vitvi13g00569\_t001 |  |  |  |  |  |  |  |
| 1 | Ath-AT3G52280.2 |  | Vvi-Vitvi13g00566\_t001 |  |  |  |  |  |  |  |
| 1 | Ath-AT3G52290.1 |  | Vvi-Vitvi13g00563\_t002 |  |  |  |  |  |  |  |
| 1 | Ath-AT3G52300.1 |  | Vvi-Vitvi13g00561\_t001 |  |  |  |  |  |  |  |
| 0 | Ath-AT3G52320.1 |  |  |  |  |  |  |  |  |
| 0 | Ath-AT3G52330.1 |  |  |  |  |  |  |  |  |
| 0 | Ath-AT3G52340.6 |  |  |  |  |  |  |  |  |
| 0 | Ath-AT3G52350.1 |  |  |  |  |  |  |  |  |
| 0 | Ath-AT3G52360.1 |  |  |  |  |  |  |  |  |
| 0 | Ath-AT3G52370.2 |  |  |  |  |  |  |  |  |
| 0 | Ath-AT3G52380.1 |  |  |  |  |  |  |  |  |
| 0 | Ath-AT3G52390.2 |  |  |  |  |  |  |  |  |
| 0 | Ath-AT3G52400.1 |  |  |  |  |  |  |  |  |
| 0 | Ath-AT3G52420.1 |  |  |  |  |  |  |  |  |
| 0 | Ath-AT3G52430.1 |  |  |  |  |  |  |  |  |
| 0 | Ath-AT3G52440.2 |  |  |  |  |  |  |  |  |
| 0 | Ath-AT3G52450.1 |  |  |  |  |  |  |  |  |
| 1 | Ath-AT3G52460.1 |  | Vvi-Vitvi06g01560\_t001 |  |  |  |  |  |  |  |
| 2 | Ath-AT3G52470.1 |  | Vvi-Vitvi06g01559\_t001 |  | Vvi-Vitvi08g01376\_t001 |  |  |  |  |  |  |
| 2 | Ath-AT3G52480.1 |  | | | |  | Vvi-Vitvi08g02246\_t001 |  |  |  |  |  |  |
| 2 | Ath-AT3G52490.1 |  | | | |  | Vvi-Vitvi08g01378\_t001 |  |  |  |  |  |  |
| 2 | Ath-AT3G52500.1 |  | Vvi-Vitvi06g01556\_t001 |  | Vvi-Vitvi08g04284\_t001 |  |  |  |  |  |  |
| 2 | Ath-AT3G52510.1 |  | | | |  | | | |  |  |  |  |  |  |
| 2 | Ath-AT3G52520.1 |  | | | |  | | | |  |  |  |  |  |  |
| 2 | Ath-AT3G52525.1 |  | Vvi-Vitvi06g01549\_t001 |  | Vvi-Vitvi08g01391\_t001 |  |  |  |  |  |  |
| 2 | Ath-AT3G52526.2 |  | | | |  | | | |  |  |  |  |  |  |
| 2 | Ath-AT3G52530.1 |  | | | |  | | | |  |  |  |  |  |  |
| 2 | Ath-AT3G52540.1 |  | Vvi-Vitvi06g01548\_t001 |  | Vvi-Vitvi08g01392\_t001 |  |  |  |  |  |  |
| 2 | Ath-AT3G52550.1 |  | | | |  | | | |  |  |  |  |  |  |
| 2 | Ath-AT3G52560.4 |  | Vvi-Vitvi06g01545\_t001 |  | Vvi-Vitvi08g01394\_t001 |  |  |  |  |  |  |
| 0 | Ath-AT3G52561.1 |  |  |  |  |  |  |  |  |
| 1 | Ath-AT3G52570.1 |  | Vvi-Vitvi08g01430\_t001 |  |  |  |  |  |  |  |
| 1 | Ath-AT3G52580.1 |  | Vvi-Vitvi08g04305\_t001 |  |  |  |  |  |  |  |
| 1 | Ath-AT3G52590.1 |  | Vvi-Vitvi08g01432\_t001 |  |  |  |  |  |  |  |
| 1 | Ath-AT3G52600.1 |  | Vvi-Vitvi08g01434\_t001 |  |  |  |  |  |  |  |
| 1 | Ath-AT3G52610.1 |  | Vvi-Vitvi08g01437\_t001 |  |  |  |  |  |  |  |
| 1 | Ath-AT3G52620.1 |  | Vvi-Vitvi08g01445\_t001 |  |  |  |  |  |  |  |
| 1 | Ath-AT3G52630.2 |  | Vvi-Vitvi08g04306\_t001 |  |  |  |  |  |  |  |
| 1 | Ath-AT3G52640.2 |  | | | |  |  |  |  |  |  |  |
| 1 | Ath-AT3G52660.2 |  | Vvi-Vitvi08g01450\_t002 |  |  |  |  |  |  |  |
| 1 | Ath-AT3G52680.1 |  | | | |  |  |  |  |  |  |  |
| 1 | Ath-AT3G52690.1 |  | | | |  |  |  |  |  |  |  |
| 1 | Ath-AT3G52700.1 |  | | | |  |  |  |  |  |  |  |
| 1 | Ath-AT3G52710.1 |  | Vvi-Vitvi08g01452\_t001 |  |  |  |  |  |  |  |
| 1 | Ath-AT3G52720.1 |  | Vvi-Vitvi08g01454\_t001 |  |  |  |  |  |  |  |
| 1 | Ath-AT3G52730.2 |  | Vvi-Vitvi08g01463\_t001 |  |  |  |  |  |  |  |
| 1 | Ath-AT3G52740.1 |  | Vvi-Vitvi08g01465\_t001 |  |  |  |  |  |  |  |
| 1 | Ath-AT3G52750.2 |  | Vvi-Vitvi08g01467\_t001 |  |  |  |  |  |  |  |
| 1 | Ath-AT3G52760.1 |  | Vvi-Vitvi08g01474\_t001 |  |  |  |  |  |  |  |
| 1 | Ath-AT3G52770.1 |  | Vvi-Vitvi08g02266\_t001 |  |  |  |  |  |  |  |
| 1 | Ath-AT3G52780.1 |  | Vvi-Vitvi08g02268\_t001 |  |  |  |  |  |  |  |
| 1 | Ath-AT3G52790.1 |  | | | |  |  |  |  |  |  |  |
| 1 | Ath-AT3G52800.1 |  | Vvi-Vitvi08g02270\_t001 |  |  |  |  |  |  |  |
| 1 | Ath-AT3G52810.1 |  | | | |  |  |  |  |  |  |  |
| 1 | Ath-AT3G52820.1 |  | | | |  |  |  |  |  |  |  |
| 1 | Ath-AT3G52830.1 |  | | | |  |  |  |  |  |  |  |
| 1 | Ath-AT3G52840.2 |  | | | |  |  |  |  |  |  |  |
| 1 | Ath-AT3G52850.1 |  | | | |  |  |  |  |  |  |  |
| 1 | Ath-AT3G52860.1 |  | | | |  |  |  |  |  |  |  |
| 1 | Ath-AT3G52870.1 |  | Vvi-Vitvi08g01484\_t001 |  |  |  |  |  |  |  |
| 1 | Ath-AT3G52880.2 |  | | | |  |  |  |  |  |  |  |
| 1 | Ath-AT3G52890.2 |  | | | |  |  |  |  |  |  |  |
| 1 | Ath-AT3G52900.1 |  | | | |  |  |  |  |  |  |  |
| 1 | Ath-AT3G52905.1 |  | | | |  |  |  |  |  |  |  |
| 1 | Ath-AT3G52910.1 |  | Vvi-Vitvi08g01498\_t001 |  |  |  |  |  |  |  |
| 1 | Ath-AT3G52920.1 |  | Vvi-Vitvi08g01499\_t001 |  |  |  |  |  |  |  |
| 1 | Ath-AT3G52930.1 |  | Vvi-Vitvi08g01506\_t001 |  |  |  |  |  |  |  |
| 1 | Ath-AT3G52940.1 |  | Vvi-Vitvi08g01517\_t001 |  |  |  |  |  |  |  |
| 1 | Ath-AT3G52941.1 |  | | | |  |  |  |  |  |  |  |
| 1 | Ath-AT3G52950.1 |  | | | |  |  |  |  |  |  |  |
| 1 | Ath-AT3G52960.1 |  | Vvi-Vitvi08g01521\_t001 |  |  |  |  |  |  |  |
| 1 | Ath-AT3G52970.2 |  | Vvi-Vitvi08g01528\_t001 |  |  |  |  |  |  |  |
| 1 | Ath-AT3G52980.1 |  | | | |  |  |  |  |  |  |  |
| 1 | Ath-AT3G52990.1 |  | Vvi-Vitvi08g01539\_t001 |  |  |  |  |  |  |  |
| 1 | Ath-AT3G53000.1 |  | Vvi-Vitvi08g01542\_t001 |  |  |  |  |  |  |  |
| 1 | Ath-AT3G53010.1 |  | | | |  |  |  |  |  |  |  |
| 1 | Ath-AT3G53020.1 |  | Vvi-Vitvi08g01544\_t001 |  |  |  |  |  |  |  |
| 1 | Ath-AT3G53030.1 |  | Vvi-Vitvi08g01545\_t001 |  |  |  |  |  |  |  |
| 1 | Ath-AT3G53040.1 |  | Vvi-Vitvi08g01547\_t001 |  |  |  |  |  |  |  |
| 1 | Ath-AT3G53050.1 |  | | | |  |  |  |  |  |  |  |
| 1 | Ath-AT3G53060.1 |  | | | |  |  |  |  |  |  |  |
| 1 | Ath-AT3G53065.1 |  | | | |  |  |  |  |  |  |  |
| 1 | Ath-AT3G53070.1 |  | | | |  |  |  |  |  |  |  |
| 1 | Ath-AT3G53075.2 |  | | | |  |  |  |  |  |  |  |
| 1 | Ath-AT3G53080.1 |  | | | |  |  |  |  |  |  |  |
| 1 | Ath-AT3G53090.1 |  | Vvi-Vitvi08g01551\_t001.1.6037826f |  |  |  |  |  |  |  |
| 1 | Ath-AT3G53100.1 |  | Vvi-Vitvi08g01553\_t001 |  |  |  |  |  |  |  |
| 1 | Ath-AT3G53110.1 |  | Vvi-Vitvi08g02283\_t002 |  |  |  |  |  |  |  |
| 1 | Ath-AT3G53120.1 |  | Vvi-Vitvi08g01568\_t001 |  |  |  |  |  |  |  |
| 1 | Ath-AT3G53130.1 |  | | | |  |  |  |  |  |  |  |
| 1 | Ath-AT3G53140.1 |  | Vvi-Vitvi08g01573\_t001 |  |  |  |  |  |  |  |
| 1 | Ath-AT3G53150.1 |  | Vvi-Vitvi08g01580\_t001 |  |  |  |  |  |  |  |
| 1 | Ath-AT3G53160.1 |  | | | |  |  |  |  |  |  |  |
| 1 | Ath-AT3G53170.2 |  | | | |  |  |  |  |  |  |  |
| 1 | Ath-AT3G53180.1 |  | Vvi-Vitvi08g01586\_t001 |  |  |  |  |  |  |  |
| 1 | Ath-AT3G53190.1 |  | Vvi-Vitvi08g01606\_t001 |  |  |  |  |  |  |  |
| 1 | Ath-AT3G53200.1 |  | Vvi-Vitvi08g01607\_t001 |  |  |  |  |  |  |  |
| 1 | Ath-AT3G53210.1 |  | | | |  |  |  |  |  |  |  |
| 1 | Ath-AT3G53220.1 |  | Vvi-Vitvi08g01619\_t001 |  |  |  |  |  |  |  |
| 0 | Ath-AT3G53230.1 |  |  |  |  |  |  |  |  |
| 0 | Ath-AT3G53232.1 |  |  |  |  |  |  |  |  |
| 0 | Ath-AT3G53235.1 |  |  |  |  |  |  |  |  |
| 0 | Ath-AT3G53240.2 |  |  |  |  |  |  |  |  |
| 0 | Ath-AT3G53250.2 |  |  |  |  |  |  |  |  |
| 0 | Ath-AT3G53260.1 |  |  |  |  |  |  |  |  |
| 0 | Ath-AT3G53270.1 |  |  |  |  |  |  |  |  |
| 0 | Ath-AT3G53280.1 |  |  |  |  |  |  |  |  |
| 0 | Ath-AT3G53290.1 |  |  |  |  |  |  |  |  |
| 0 | Ath-AT3G53294.1 |  |  |  |  |  |  |  |  |
| 0 | Ath-AT3G53300.1 |  |  |  |  |  |  |  |  |
| 0 | Ath-AT3G53305.1 |  |  |  |  |  |  |  |  |
| 0 | Ath-AT3G53310.1 |  |  |  |  |  |  |  |  |
| 0 | Ath-AT3G53320.1 |  |  |  |  |  |  |  |  |
| 0 | Ath-AT3G53330.1 |  |  |  |  |  |  |  |  |
| 0 | Ath-AT3G53340.5 |  |  |  |  |  |  |  |  |
| 0 | Ath-AT3G53342.1 |  |  |  |  |  |  |  |  |
| 0 | Ath-AT3G53350.9 |  |  |  |  |  |  |  |  |
| 0 | Ath-AT3G53360.1 |  |  |  |  |  |  |  |  |
| 0 | Ath-AT3G53370.1 |  |  |  |  |  |  |  |  |
| 0 | Ath-AT3G53380.1 |  |  |  |  |  |  |  |  |
| 1 | Ath-AT3G53390.1 |  | Vvi-Vitvi08g01044\_t001 |  |  |  |  |  |  |  |
| 1 | Ath-AT3G53400.1 |  | | | |  |  |  |  |  |  |  |
| 1 | Ath-AT3G53410.1 |  | Vvi-Vitvi08g01042\_t001 |  |  |  |  |  |  |  |
| 1 | Ath-AT3G53420.1 |  | Vvi-Vitvi08g01038\_t001 |  |  |  |  |  |  |  |
| 1 | Ath-AT3G53430.1 |  | Vvi-Vitvi08g01037\_t001 |  |  |  |  |  |  |  |
| 1 | Ath-AT3G53440.2 |  | | | |  |  |  |  |  |  |  |
| 1 | Ath-AT3G53450.1 |  | Vvi-Vitvi08g01030\_t001 |  |  |  |  |  |  |  |
| 1 | Ath-AT3G53460.4 |  | Vvi-Vitvi08g01027\_t001 |  |  |  |  |  |  |  |
| 1 | Ath-AT3G53470.2 |  | Vvi-Vitvi08g01150\_t001 |  |  |  |  |  |  |  |
| 1 | Ath-AT3G53480.1 |  | | | |  |  |  |  |  |  |  |
| 2 | Ath-AT3G53490.1 |  | | | |  | Vvi-Vitvi08g04200\_t001 |  |  |  |  |  |  |
| 2 | Ath-AT3G53500.2 |  | | | |  | Vvi-Vitvi08g01113\_t001 |  |  |  |  |  |  |
| 2 | Ath-AT3G53510.1 |  | | | |  | Vvi-Vitvi08g01112\_t001 |  |  |  |  |  |  |
| 2 | Ath-AT3G53520.4 |  | | | |  | Vvi-Vitvi08g01098\_t001 |  |  |  |  |  |  |
| 2 | Ath-AT3G53530.2 |  | | | |  | Vvi-Vitvi08g01097\_t001 |  |  |  |  |  |  |
| 2 | Ath-AT3G53540.2 |  | | | |  | Vvi-Vitvi08g01096\_t001 |  |  |  |  |  |  |
| 2 | Ath-AT3G53550.1 |  | | | |  | | | |  |  |  |  |  |  |
| 2 | Ath-AT3G53560.2 |  | | | |  | Vvi-Vitvi08g01095\_t001 |  |  |  |  |  |  |
| 2 | Ath-AT3G53570.2 |  | | | |  | Vvi-Vitvi08g01091\_t002 |  |  |  |  |  |  |
| 2 | Ath-AT3G53580.1 |  | | | |  | Vvi-Vitvi08g01085\_t001 |  |  |  |  |  |  |
| 2 | Ath-AT3G53590.1 |  | | | |  | | | |  |  |  |  |  |  |
| 2 | Ath-AT3G53600.1 |  | | | |  | | | |  |  |  |  |  |  |
| 2 | Ath-AT3G53610.2 |  | | | |  | Vvi-Vitvi08g01082\_t004 |  |  |  |  |  |  |
| 2 | Ath-AT3G53611.1 |  | | | |  | | | |  |  |  |  |  |  |
| 2 | Ath-AT3G53620.1 |  | | | |  | Vvi-Vitvi08g01081\_t001 |  |  |  |  |  |  |
| 2 | Ath-AT3G53630.2 |  | | | |  | Vvi-Vitvi08g01079\_t001 |  |  |  |  |  |  |
| 1 | Ath-AT3G53640.1 |  | | | |  |  |  |  |  |  |  |
| 1 | Ath-AT3G53650.1 |  | | | |  |  |  |  |  |  |  |
| 1 | Ath-AT3G53670.2 |  | Vvi-Vitvi08g01161\_t003 |  |  |  |  |  |  |  |
| 1 | Ath-AT3G53680.2 |  | Vvi-Vitvi08g01164\_t001 |  |  |  |  |  |  |  |
| 1 | Ath-AT3G53690.1 |  | | | |  |  |  |  |  |  |  |
| 1 | Ath-AT3G53700.1 |  | Vvi-Vitvi08g01166\_t001 |  |  |  |  |  |  |  |
| 1 | Ath-AT3G53710.2 |  | Vvi-Vitvi08g01171\_t001 |  |  |  |  |  |  |  |
| 1 | Ath-AT3G53720.1 |  | Vvi-Vitvi08g01172\_t001 |  |  |  |  |  |  |  |
| 1 | Ath-AT3G53730.1 |  | Vvi-Vitvi08g04213\_t001 |  |  |  |  |  |  |  |
| 0 | Ath-AT3G53740.2 |  |  |  |  |  |  |  |  |
| 1 | Ath-AT3G53750.2 |  | Vvi-Vitvi13g00317\_t001 |  |  |  |  |  |  |  |
| 1 | Ath-AT3G53760.1 |  | | | |  |  |  |  |  |  |  |
| 1 | Ath-AT3G53770.1 |  | | | |  |  |  |  |  |  |  |
| 1 | Ath-AT3G53780.2 |  | | | |  |  |  |  |  |  |  |
| 1 | Ath-AT3G53790.1 |  | | | |  |  |  |  |  |  |  |
| 2 | Ath-AT3G53800.1 |  | | | |  | Vvi-Vitvi08g01234\_t001 |  |  |  |  |  |  |
| 2 | Ath-AT3G53810.1 |  | Vvi-Vitvi13g00332\_t001 |  | Vvi-Vitvi08g01241\_t001 |  |  |  |  |  |  |
| 2 | Ath-AT3G53820.1 |  | | | |  | Vvi-Vitvi08g02203\_t001 |  |  |  |  |  |  |
| 2 | Ath-AT3G53830.4 |  | Vvi-Vitvi13g00350\_t002 |  | Vvi-Vitvi08g02205\_t001 |  |  |  |  |  |  |
| 2 | Ath-AT3G53840.1 |  | Vvi-Vitvi13g02001\_t001 |  | Vvi-Vitvi08g01255\_t001 |  |  |  |  |  |  |
| 2 | Ath-AT3G53850.1 |  | Vvi-Vitvi13g00353\_t001 |  | Vvi-Vitvi08g01257\_t001 |  |  |  |  |  |  |
| 2 | Ath-AT3G53860.1 |  | | | |  | | | |  |  |  |  |  |  |
| 2 | Ath-AT3G53870.1 |  | | | |  | | | |  |  |  |  |  |  |
| 2 | Ath-AT3G53880.1 |  | | | |  | Vvi-Vitvi08g04241\_t001 |  |  |  |  |  |  |
| 2 | Ath-AT3G53890.2 |  | | | |  | | | |  |  |  |  |  |  |
| 2 | Ath-AT3G53900.2 |  | | | |  | Vvi-Vitvi08g01265\_t001 |  |  |  |  |  |  |
| 2 | Ath-AT3G53910.1 |  | | | |  | | | |  |  |  |  |  |  |
| 2 | Ath-AT3G53920.1 |  | | | |  | | | |  |  |  |  |  |  |
| 2 | Ath-AT3G53930.2 |  | | | |  | Vvi-Vitvi08g01267\_t001 |  |  |  |  |  |  |
| 2 | Ath-AT3G53940.1 |  | Vvi-Vitvi13g00365\_t001 |  | Vvi-Vitvi08g01273\_t001 |  |  |  |  |  |  |
| 2 | Ath-AT3G53950.1 |  | | | |  | Vvi-Vitvi08g01276\_t001 |  |  |  |  |  |  |
| 2 | Ath-AT3G53960.1 |  | Vvi-Vitvi13g00367\_t001 |  | Vvi-Vitvi08g04251\_t001 |  |  |  |  |  |  |
| 1 | Ath-AT3G53970.1 |  |  |  | Vvi-Vitvi08g01280\_t001 |  |  |  |  |  |  |
| 1 | Ath-AT3G53980.1 |  |  |  | Vvi-Vitvi08g01281\_t001 |  |  |  |  |  |  |
| 2 | Ath-AT3G53990.1 |  | Vvi-Vitvi06g00556\_t001 |  | Vvi-Vitvi08g01285\_t001 |  |  |  |  |  |  |
| 2 | Ath-AT3G54000.1 |  | Vvi-Vitvi06g00558\_t001 |  | Vvi-Vitvi08g01287\_t001 |  |  |  |  |  |  |
| 2 | Ath-AT3G54010.1 |  | | | |  | Vvi-Vitvi08g01288\_t002 |  |  |  |  |  |  |
| 2 | Ath-AT3G54020.1 |  | Vvi-Vitvi06g00564\_t001 |  | Vvi-Vitvi08g01295\_t001 |  |  |  |  |  |  |
| 2 | Ath-AT3G54030.1 |  | Vvi-Vitvi06g00565\_t001 |  | Vvi-Vitvi08g01296\_t001 |  |  |  |  |  |  |
| 2 | Ath-AT3G54040.2 |  | Vvi-Vitvi06g01734\_t001 |  | Vvi-Vitvi08g01300\_t001 |  |  |  |  |  |  |
| 2 | Ath-AT3G54050.1 |  | | | |  | Vvi-Vitvi08g01301\_t001 |  |  |  |  |  |  |
| 2 | Ath-AT3G54060.1 |  | | | |  | Vvi-Vitvi08g01303\_t001 |  |  |  |  |  |  |
| 2 | Ath-AT3G54065.1 |  | | | |  | | | |  |  |  |  |  |  |
| 2 | Ath-AT3G54070.1 |  | | | |  | | | |  |  |  |  |  |  |
| 2 | Ath-AT3G54080.1 |  | | | |  | Vvi-Vitvi08g01305\_t001 |  |  |  |  |  |  |
| 2 | Ath-AT3G54085.2 |  | | | |  | | | |  |  |  |  |  |  |
| 2 | Ath-AT3G54090.1 |  | | | |  | Vvi-Vitvi08g02233\_t001 |  |  |  |  |  |  |
| 2 | Ath-AT3G54100.1 |  | | | |  | Vvi-Vitvi08g01308\_t001 |  |  |  |  |  |  |
| 2 | Ath-AT3G54110.1 |  | Vvi-Vitvi06g00577\_t001.1.6037826e |  | Vvi-Vitvi08g01313\_t001 |  |  |  |  |  |  |
| 2 | Ath-AT3G54120.1 |  | | | |  | Vvi-Vitvi08g01315\_t001 |  |  |  |  |  |  |
| 2 | Ath-AT3G54130.1 |  | | | |  | Vvi-Vitvi08g01316\_t001 |  |  |  |  |  |  |
| 2 | Ath-AT3G54140.1 |  | Vvi-Vitvi06g00590\_t001 |  | Vvi-Vitvi08g01333\_t001 |  |  |  |  |  |  |
| 0 | Ath-AT3G54150.1 |  |  |  |  |  |  |  |  |
| 0 | Ath-AT3G54160.1 |  |  |  |  |  |  |  |  |
| 0 | Ath-AT3G54170.1 |  |  |  |  |  |  |  |  |
| 0 | Ath-AT3G54180.1 |  |  |  |  |  |  |  |  |
| 0 | Ath-AT3G54190.1 |  |  |  |  |  |  |  |  |
| 0 | Ath-AT3G54200.1 |  |  |  |  |  |  |  |  |
| 0 | Ath-AT3G54210.1 |  |  |  |  |  |  |  |  |
| 0 | Ath-AT3G54220.1 |  |  |  |  |  |  |  |  |
| 0 | Ath-AT3G54230.2 |  |  |  |  |  |  |  |  |
| 0 | Ath-AT3G54240.1 |  |  |  |  |  |  |  |  |
| 0 | Ath-AT3G54250.1 |  |  |  |  |  |  |  |  |
| 0 | Ath-AT3G54260.1 |  |  |  |  |  |  |  |  |
| 0 | Ath-AT3G54270.1 |  |  |  |  |  |  |  |  |
| 0 | Ath-AT3G54280.2 |  |  |  |  |  |  |  |  |
| 0 | Ath-AT3G54290.1 |  |  |  |  |  |  |  |  |
| 0 | Ath-AT3G54300.1 |  |  |  |  |  |  |  |  |
| 0 | Ath-AT3G54310.1 |  |  |  |  |  |  |  |  |
| 0 | Ath-AT3G54320.1 |  |  |  |  |  |  |  |  |
| 0 | Ath-AT3G54340.1 |  |  |  |  |  |  |  |  |
| 0 | Ath-AT3G54350.1 |  |  |  |  |  |  |  |  |
| 0 | Ath-AT3G54360.2 |  |  |  |  |  |  |  |  |
| 0 | Ath-AT3G54363.1 |  |  |  |  |  |  |  |  |
| 0 | Ath-AT3G54380.1 |  |  |  |  |  |  |  |  |
| 0 | Ath-AT3G54390.2 |  |  |  |  |  |  |  |  |
| 0 | Ath-AT3G54400.1 |  |  |  |  |  |  |  |  |
| 0 | Ath-AT3G54410.1 |  |  |  |  |  |  |  |  |
| 0 | Ath-AT3G54420.1 |  |  |  |  |  |  |  |  |
| 0 | Ath-AT3G54430.1 |  |  |  |  |  |  |  |  |
| 0 | Ath-AT3G54440.3 |  |  |  |  |  |  |  |  |
| 0 | Ath-AT3G54450.1 |  |  |  |  |  |  |  |  |
| 0 | Ath-AT3G54460.1 |  |  |  |  |  |  |  |  |
| 0 | Ath-AT3G54470.1 |  |  |  |  |  |  |  |  |
| 0 | Ath-AT3G54480.1 |  |  |  |  |  |  |  |  |
| 0 | Ath-AT3G54490.1 |  |  |  |  |  |  |  |  |
| 0 | Ath-AT3G54500.3 |  |  |  |  |  |  |  |  |
| 0 | Ath-AT3G54510.2 |  |  |  |  |  |  |  |  |
| 0 | Ath-AT3G54520.1 |  |  |  |  |  |  |  |  |
| 0 | Ath-AT3G54530.1 |  |  |  |  |  |  |  |  |
| 0 | Ath-AT3G54540.1 |  |  |  |  |  |  |  |  |
| 1 | Ath-AT3G54550.1 |  | Vvi-Vitvi07g00061\_t002 |  |  |  |  |  |  |  |
| 1 | Ath-AT3G54560.1 |  | | | |  |  |  |  |  |  |  |
| 1 | Ath-AT3G54570.1 |  | | | |  |  |  |  |  |  |  |
| 1 | Ath-AT3G54580.1 |  | | | |  |  |  |  |  |  |  |
| 1 | Ath-AT3G54590.3 |  | | | |  |  |  |  |  |  |  |
| 1 | Ath-AT3G54600.1 |  | | | |  |  |  |  |  |  |  |
| 1 | Ath-AT3G54610.1 |  | Vvi-Vitvi07g00050\_t001 |  |  |  |  |  |  |  |
| 1 | Ath-AT3G54620.1 |  | Vvi-Vitvi07g00049\_t001 |  |  |  |  |  |  |  |
| 1 | Ath-AT3G54630.1 |  | | | |  |  |  |  |  |  |  |
| 1 | Ath-AT3G54640.1 |  | Vvi-Vitvi07g00047\_t001 |  |  |  |  |  |  |  |
| 1 | Ath-AT3G54650.1 |  | Vvi-Vitvi07g00040\_t001 |  |  |  |  |  |  |  |
| 1 | Ath-AT3G54660.1 |  | Vvi-Vitvi07g00037\_t002 |  |  |  |  |  |  |  |
| 0 | Ath-AT3G54670.1 |  |  |  |  |  |  |  |  |
| 0 | Ath-AT3G54680.1 |  |  |  |  |  |  |  |  |
| 0 | Ath-AT3G54690.1 |  |  |  |  |  |  |  |  |
| 0 | Ath-AT3G54700.1 |  |  |  |  |  |  |  |  |
| 0 | Ath-AT3G54710.1 |  |  |  |  |  |  |  |  |
| 0 | Ath-AT3G54720.1 |  |  |  |  |  |  |  |  |
| 0 | Ath-AT3G54730.1 |  |  |  |  |  |  |  |  |
| 0 | Ath-AT3G54740.2 |  |  |  |  |  |  |  |  |
| 0 | Ath-AT3G54750.3 |  |  |  |  |  |  |  |  |
| 0 | Ath-AT3G54760.1 |  |  |  |  |  |  |  |  |
| 0 | Ath-AT3G54770.1 |  |  |  |  |  |  |  |  |
| 1 | Ath-AT3G54780.4 |  | Vvi-Vitvi13g00620\_t001 |  |  |  |  |  |  |  |
| 1 | Ath-AT3G54790.1 |  | | | |  |  |  |  |  |  |  |
| 1 | Ath-AT3G54800.1 |  | | | |  |  |  |  |  |  |  |
| 1 | Ath-AT3G54810.2 |  | | | |  |  |  |  |  |  |  |
| 1 | Ath-AT3G54820.1 |  | | | |  |  |  |  |  |  |  |
| 1 | Ath-AT3G54826.1 |  | Vvi-Vitvi13g00636\_t001 |  |  |  |  |  |  |  |
| 1 | Ath-AT3G54830.1 |  | Vvi-Vitvi13g00646\_t001 |  |  |  |  |  |  |  |
| 1 | Ath-AT3G54840.1 |  | Vvi-Vitvi13g00650\_t001 |  |  |  |  |  |  |  |
| 1 | Ath-AT3G54850.1 |  | Vvi-Vitvi13g00651\_t001 |  |  |  |  |  |  |  |
| 1 | Ath-AT3G54860.2 |  | Vvi-Vitvi13g00652\_t001 |  |  |  |  |  |  |  |
| 1 | Ath-AT3G54870.2 |  | Vvi-Vitvi13g00657\_t001 |  |  |  |  |  |  |  |
| 1 | Ath-AT3G54880.1 |  | | | |  |  |  |  |  |  |  |
| 1 | Ath-AT3G54890.1 |  | | | |  |  |  |  |  |  |  |
| 1 | Ath-AT3G54900.1 |  | | | |  |  |  |  |  |  |  |
| 1 | Ath-AT3G54910.1 |  | | | |  |  |  |  |  |  |  |
| 1 | Ath-AT3G54920.1 |  | Vvi-Vitvi13g00667\_t001 |  |  |  |  |  |  |  |
| 1 | Ath-AT3G54925.1 |  | | | |  |  |  |  |  |  |  |
| 1 | Ath-AT3G54930.2 |  | Vvi-Vitvi13g00670\_t001 |  |  |  |  |  |  |  |
| 1 | Ath-AT3G54940.2 |  | Vvi-Vitvi13g00688\_t001 |  |  |  |  |  |  |  |
| 0 | Ath-AT3G54950.1 |  |  |  |  |  |  |  |  |
| 0 | Ath-AT3G54960.1 |  |  |  |  |  |  |  |  |
| 0 | Ath-AT3G54970.1 |  |  |  |  |  |  |  |  |
| 0 | Ath-AT3G54980.1 |  |  |  |  |  |  |  |  |
| 0 | Ath-AT3G54990.1 |  |  |  |  |  |  |  |  |
| 1 | Ath-AT3G55000.1 |  | Vvi-Vitvi13g00200\_t001 |  |  |  |  |  |  |  |
| 1 | Ath-AT3G55005.1 |  | | | |  |  |  |  |  |  |  |
| 1 | Ath-AT3G55010.1 |  | | | |  |  |  |  |  |  |  |
| 2 | Ath-AT3G55020.1 |  | Vvi-Vitvi13g00206\_t001 |  | Vvi-Vitvi08g01130\_t001 |  |  |  |  |  |  |
| 2 | Ath-AT3G55030.1 |  | Vvi-Vitvi13g00207\_t001 |  | | | |  |  |  |  |  |  |
| 2 | Ath-AT3G55040.1 |  | Vvi-Vitvi13g00208\_t001 |  | Vvi-Vitvi08g01129\_t001 |  |  |  |  |  |  |
| 2 | Ath-AT3G55050.2 |  | Vvi-Vitvi13g00210\_t002 |  | Vvi-Vitvi08g01124\_t001 |  |  |  |  |  |  |
| 2 | Ath-AT3G55060.2 |  | Vvi-Vitvi13g00212\_t001 |  | | | |  |  |  |  |  |  |
| 2 | Ath-AT3G55070.1 |  | Vvi-Vitvi13g00213\_t001 |  | | | |  |  |  |  |  |  |
| 2 | Ath-AT3G55080.1 |  | Vvi-Vitvi13g00215\_t001 |  | | | |  |  |  |  |  |  |
| 2 | Ath-AT3G55090.1 |  | Vvi-Vitvi13g00221\_t001 |  | Vvi-Vitvi08g01112\_t001 |  |  |  |  |  |  |
| 2 | Ath-AT3G55100.1 |  | | | |  | | | |  |  |  |  |  |  |
| 2 | Ath-AT3G55110.1 |  | | | |  | | | |  |  |  |  |  |  |
| 2 | Ath-AT3G55120.1 |  | Vvi-Vitvi13g00225\_t001 |  | | | |  |  |  |  |  |  |
| 2 | Ath-AT3G55130.1 |  | | | |  | | | |  |  |  |  |  |  |
| 2 | Ath-AT3G55140.1 |  | Vvi-Vitvi13g00232\_t001 |  | Vvi-Vitvi08g01102\_t001 |  |  |  |  |  |  |
| 2 | Ath-AT3G55150.1 |  | Vvi-Vitvi13g00233\_t001 |  | Vvi-Vitvi08g01101\_t001 |  |  |  |  |  |  |
| 2 | Ath-AT3G55160.3 |  | | | |  | | | |  |  |  |  |  |  |
| 2 | Ath-AT3G55170.5 |  | Vvi-Vitvi13g00234\_t001 |  | | | |  |  |  |  |  |  |
| 2 | Ath-AT3G55180.1 |  | Vvi-Vitvi13g00235\_t001 |  | | | |  |  |  |  |  |  |
| 2 | Ath-AT3G55190.1 |  | | | |  | | | |  |  |  |  |  |  |
| 2 | Ath-AT3G55200.2 |  | Vvi-Vitvi13g00236\_t001 |  | | | |  |  |  |  |  |  |
| 2 | Ath-AT3G55210.1 |  | | | |  | | | |  |  |  |  |  |  |
| 2 | Ath-AT3G55220.1 |  | | | |  | | | |  |  |  |  |  |  |
| 2 | Ath-AT3G55230.1 |  | Vvi-Vitvi13g00237\_t001 |  | | | |  |  |  |  |  |  |
| 2 | Ath-AT3G55240.1 |  | Vvi-Vitvi13g01932\_t001 |  | Vvi-Vitvi08g02169\_t001 |  |  |  |  |  |  |
| 2 | Ath-AT3G55250.1 |  | | | |  | | | |  |  |  |  |  |  |
| 2 | Ath-AT3G55252.1 |  | | | |  | | | |  |  |  |  |  |  |
| 2 | Ath-AT3G55254.1 |  | | | |  | | | |  |  |  |  |  |  |
| 2 | Ath-AT3G55260.1 |  | Vvi-Vitvi13g00245\_t001 |  | | | |  |  |  |  |  |  |
| 2 | Ath-AT3G55280.1 |  | Vvi-Vitvi13g00249\_t001 |  | Vvi-Vitvi08g01090\_t001 |  |  |  |  |  |  |
| 1 | Ath-AT3G55290.1 |  | | | |  |  |  |  |  |  |  |
| 1 | Ath-AT3G55310.1 |  | | | |  |  |  |  |  |  |  |
| 1 | Ath-AT3G55320.1 |  | Vvi-Vitvi13g00252\_t001 |  |  |  |  |  |  |  |
| 1 | Ath-AT3G55330.1 |  | | | |  |  |  |  |  |  |  |
| 1 | Ath-AT3G55340.1 |  | Vvi-Vitvi13g00259\_t001 |  |  |  |  |  |  |  |
| 1 | Ath-AT3G55350.1 |  | | | |  |  |  |  |  |  |  |
| 2 | Ath-AT3G55360.1 |  | | | |  | Vvi-Vitvi13g00295\_t001 |  |  |  |  |  |  |
| 2 | Ath-AT3G55370.3 |  | | | |  | Vvi-Vitvi13g00298\_t001 |  |  |  |  |  |  |
| 2 | Ath-AT3G55380.2 |  | | | |  | | | |  |  |  |  |  |  |
| 2 | Ath-AT3G55390.1 |  | | | |  | | | |  |  |  |  |  |  |
| 2 | Ath-AT3G55400.1 |  | | | |  | | | |  |  |  |  |  |  |
| 2 | Ath-AT3G55410.1 |  | Vvi-Vitvi13g04079\_t001 |  | | | |  |  |  |  |  |  |
| 1 | Ath-AT3G55420.1 |  |  |  | | | |  |  |  |  |  |  |
| 1 | Ath-AT3G55430.1 |  |  |  | | | |  |  |  |  |  |  |
| 1 | Ath-AT3G55440.1 |  |  |  | | | |  |  |  |  |  |  |
| 1 | Ath-AT3G55450.2 |  |  |  | | | |  |  |  |  |  |  |
| 1 | Ath-AT3G55460.1 |  |  |  | | | |  |  |  |  |  |  |
| 1 | Ath-AT3G55470.1 |  |  |  | Vvi-Vitvi13g00305\_t001 |  |  |  |  |  |  |
| 1 | Ath-AT3G55480.2 |  |  |  | Vvi-Vitvi13g00307\_t001 |  |  |  |  |  |  |
| 1 | Ath-AT3G55490.1 |  |  |  | | | |  |  |  |  |  |  |
| 1 | Ath-AT3G55500.1 |  |  |  | Vvi-Vitvi13g00309\_t001 |  |  |  |  |  |  |
| 1 | Ath-AT3G55510.1 |  |  |  | | | |  |  |  |  |  |  |
| 1 | Ath-AT3G55513.1 |  |  |  | | | |  |  |  |  |  |  |
| 1 | Ath-AT3G55515.1 |  |  |  | Vvi-Vitvi13g01992\_t001 |  |  |  |  |  |  |
| 1 | Ath-AT3G55520.3 |  |  |  | Vvi-Vitvi13g00320\_t003 |  |  |  |  |  |  |
| 1 | Ath-AT3G55530.1 |  |  |  | Vvi-Vitvi13g00324\_t001 |  |  |  |  |  |  |
| 1 | Ath-AT3G55540.1 |  |  |  | | | |  |  |  |  |  |  |
| 2 | Ath-AT3G55550.1 |  | Vvi-Vitvi08g01243\_t001 |  | Vvi-Vitvi13g00332\_t001 |  |  |  |  |  |  |
| 2 | Ath-AT3G55560.1 |  | | | |  | Vvi-Vitvi13g00338\_t001 |  |  |  |  |  |  |
| 2 | Ath-AT3G55566.1 |  | | | |  | | | |  |  |  |  |  |  |
| 2 | Ath-AT3G55570.1 |  | | | |  | Vvi-Vitvi13g02000\_t001 |  |  |  |  |  |  |
| 2 | Ath-AT3G55580.1 |  | Vvi-Vitvi08g02205\_t001 |  | Vvi-Vitvi13g00350\_t002 |  |  |  |  |  |  |
| 2 | Ath-AT3G55590.1 |  | | | |  | Vvi-Vitvi13g00352\_t001 |  |  |  |  |  |  |
| 2 | Ath-AT3G55600.1 |  | | | |  | | | |  |  |  |  |  |  |
| 2 | Ath-AT3G55605.1 |  | | | |  | Vvi-Vitvi13g00354\_t001 |  |  |  |  |  |  |
| 2 | Ath-AT3G55610.1 |  | Vvi-Vitvi08g01261\_t001 |  | Vvi-Vitvi13g00355\_t001 |  |  |  |  |  |  |
| 2 | Ath-AT3G55620.1 |  | | | |  | | | |  |  |  |  |  |  |
| 2 | Ath-AT3G55630.3 |  | | | |  | Vvi-Vitvi13g00359\_t001 |  |  |  |  |  |  |
| 2 | Ath-AT3G55640.1 |  | Vvi-Vitvi08g01273\_t001 |  | Vvi-Vitvi13g00365\_t001 |  |  |  |  |  |  |
| 2 | Ath-AT3G55646.1 |  | Vvi-Vitvi08g04253\_t006 |  | Vvi-Vitvi13g00387\_t001 |  |  |  |  |  |  |
| 2 | Ath-AT3G55650.1 |  | | | |  | | | |  |  |  |  |  |  |
| 2 | Ath-AT3G55660.1 |  | Vvi-Vitvi08g01282\_t001 |  | Vvi-Vitvi13g00390\_t001 |  |  |  |  |  |  |
| 2 | Ath-AT3G55665.1 |  | | | |  | | | |  |  |  |  |  |  |
| 2 | Ath-AT3G55670.1 |  | | | |  | | | |  |  |  |  |  |  |
| 2 | Ath-AT3G55672.1 |  | | | |  | | | |  |  |  |  |  |  |
| 2 | Ath-AT3G55677.1 |  | | | |  | | | |  |  |  |  |  |  |
| 2 | Ath-AT3G55680.1 |  | | | |  | | | |  |  |  |  |  |  |
| 2 | Ath-AT3G55690.1 |  | | | |  | | | |  |  |  |  |  |  |
| 2 | Ath-AT3G55700.1 |  | | | |  | Vvi-Vitvi13g04127\_t001 |  |  |  |  |  |  |
| 2 | Ath-AT3G55710.1 |  | | | |  | | | |  |  |  |  |  |  |
| 2 | Ath-AT3G55720.1 |  | | | |  | Vvi-Vitvi13g00492\_t001 |  |  |  |  |  |  |
| 2 | Ath-AT3G55730.1 |  | Vvi-Vitvi08g01298\_t001 |  | Vvi-Vitvi13g00494\_t001 |  |  |  |  |  |  |
| 2 | Ath-AT3G55740.1 |  | | | |  | Vvi-Vitvi13g00496\_t001 |  |  |  |  |  |  |
| 2 | Ath-AT3G55750.1 |  | | | |  | Vvi-Vitvi13g04142\_t001 |  |  |  |  |  |  |
| 2 | Ath-AT3G55760.1 |  | | | |  | | | |  |  |  |  |  |  |
| 2 | Ath-AT3G55770.7 |  | | | |  | Vvi-Vitvi13g02031\_t001 |  |  |  |  |  |  |
| 2 | Ath-AT3G55780.1 |  | | | |  | Vvi-Vitvi13g00504\_t001 |  |  |  |  |  |  |
| 2 | Ath-AT3G55790.1 |  | | | |  | | | |  |  |  |  |  |  |
| 2 | Ath-AT3G55800.1 |  | Vvi-Vitvi08g01301\_t001 |  | Vvi-Vitvi13g00507\_t001 |  |  |  |  |  |  |
| 1 | Ath-AT3G55810.1 |  |  |  | | | |  |  |  |  |  |  |
| 1 | Ath-AT3G55820.1 |  |  |  | Vvi-Vitvi13g00521\_t001 |  |  |  |  |  |  |
| 1 | Ath-AT3G55830.1 |  | Vvi-Vitvi13g00013\_t001 |  |  |  |  |  |  |  |
| 1 | Ath-AT3G55840.1 |  | Vvi-Vitvi13g00015\_t001 |  |  |  |  |  |  |  |
| 1 | Ath-AT3G55850.5 |  | | | |  |  |  |  |  |  |  |
| 1 | Ath-AT3G55860.1 |  | | | |  |  |  |  |  |  |  |
| 1 | Ath-AT3G55870.3 |  | Vvi-Vitvi13g00022\_t001 |  |  |  |  |  |  |  |
| 1 | Ath-AT3G55880.3 |  | | | |  |  |  |  |  |  |  |
| 1 | Ath-AT3G55890.1 |  | Vvi-Vitvi13g01886\_t002 |  |  |  |  |  |  |  |
| 1 | Ath-AT3G55900.1 |  | | | |  |  |  |  |  |  |  |
| 1 | Ath-AT3G55910.1 |  | | | |  |  |  |  |  |  |  |
| 1 | Ath-AT3G55920.1 |  | Vvi-Vitvi13g00046\_t001 |  |  |  |  |  |  |  |
| 1 | Ath-AT3G55930.1 |  | | | |  |  |  |  |  |  |  |
| 1 | Ath-AT3G55935.1 |  | | | |  |  |  |  |  |  |  |
| 1 | Ath-AT3G55940.1 |  | Vvi-Vitvi13g00047\_t001 |  |  |  |  |  |  |  |
| 1 | Ath-AT3G55950.1 |  | Vvi-Vitvi13g00050\_t001 |  |  |  |  |  |  |  |
| 1 | Ath-AT3G55960.1 |  | Vvi-Vitvi13g00051\_t001 |  |  |  |  |  |  |  |
| 1 | Ath-AT3G55970.1 |  | Vvi-Vitvi13g00055\_t001 |  |  |  |  |  |  |  |
| 1 | Ath-AT3G55980.2 |  | Vvi-Vitvi13g00058\_t001 |  |  |  |  |  |  |  |
| 1 | Ath-AT3G55990.1 |  | Vvi-Vitvi13g00066\_t001 |  |  |  |  |  |  |  |
| 1 | Ath-AT3G56000.1 |  | | | |  |  |  |  |  |  |  |
| 1 | Ath-AT3G56010.1 |  | Vvi-Vitvi13g00074\_t001 |  |  |  |  |  |  |  |
| 1 | Ath-AT3G56020.1 |  | | | |  |  |  |  |  |  |  |
| 1 | Ath-AT3G56030.1 |  | Vvi-Vitvi13g00083\_t001 |  |  |  |  |  |  |  |
| 1 | Ath-AT3G56040.1 |  | Vvi-Vitvi13g00087\_t001 |  |  |  |  |  |  |  |
| 1 | Ath-AT3G56050.1 |  | Vvi-Vitvi13g00092\_t001 |  |  |  |  |  |  |  |
| 1 | Ath-AT3G56060.1 |  | | | |  |  |  |  |  |  |  |
| 1 | Ath-AT3G56070.1 |  | | | |  |  |  |  |  |  |  |
| 1 | Ath-AT3G56080.1 |  | Vvi-Vitvi13g00100\_t001 |  |  |  |  |  |  |  |
| 1 | Ath-AT3G56090.1 |  | Vvi-Vitvi13g00107\_t001 |  |  |  |  |  |  |  |
| 1 | Ath-AT3G56100.2 |  | Vvi-Vitvi13g00120\_t001 |  |  |  |  |  |  |  |
| 1 | Ath-AT3G56110.2 |  | Vvi-Vitvi13g00121\_t001 |  |  |  |  |  |  |  |
| 1 | Ath-AT3G56120.1 |  | | | |  |  |  |  |  |  |  |
| 1 | Ath-AT3G56130.1 |  | Vvi-Vitvi13g00123\_t001 |  |  |  |  |  |  |  |
| 1 | Ath-AT3G56140.1 |  | Vvi-Vitvi13g00126\_t001 |  |  |  |  |  |  |  |
| 1 | Ath-AT3G56150.1 |  | | | |  |  |  |  |  |  |  |
| 1 | Ath-AT3G56160.1 |  | | | |  |  |  |  |  |  |  |
| 1 | Ath-AT3G56170.1 |  | Vvi-Vitvi13g00128\_t001 |  |  |  |  |  |  |  |
| 1 | Ath-AT3G56180.2 |  | | | |  |  |  |  |  |  |  |
| 2 | Ath-AT3G56190.1 |  | Vvi-Vitvi13g00133\_t001 |  | Vvi-Vitvi08g00806\_t001 |  |  |  |  |  |  |
| 2 | Ath-AT3G56200.1 |  | Vvi-Vitvi13g00134\_t001 |  | | | |  |  |  |  |  |  |
| 2 | Ath-AT3G56210.5 |  | Vvi-Vitvi13g00135\_t001 |  | | | |  |  |  |  |  |  |
| 2 | Ath-AT3G56220.1 |  | Vvi-Vitvi13g00139\_t002 |  | Vvi-Vitvi08g00820\_t001 |  |  |  |  |  |  |
| 2 | Ath-AT3G56230.1 |  | Vvi-Vitvi13g00140\_t001 |  | | | |  |  |  |  |  |  |
| 2 | Ath-AT3G56240.1 |  | | | |  | | | |  |  |  |  |  |  |
| 2 | Ath-AT3G56250.3 |  | Vvi-Vitvi13g00141\_t001 |  | | | |  |  |  |  |  |  |
| 2 | Ath-AT3G56260.2 |  | | | |  | | | |  |  |  |  |  |  |
| 2 | Ath-AT3G56270.1 |  | Vvi-Vitvi13g00145\_t001 |  | Vvi-Vitvi08g00825\_t002 |  |  |  |  |  |  |
| 2 | Ath-AT3G56290.1 |  | Vvi-Vitvi13g00155\_t001 |  | | | |  |  |  |  |  |  |
| 2 | Ath-AT3G56300.1 |  | | | |  | | | |  |  |  |  |  |  |
| 2 | Ath-AT3G56310.1 |  | Vvi-Vitvi13g00157\_t001 |  | | | |  |  |  |  |  |  |
| 2 | Ath-AT3G56320.1 |  | Vvi-Vitvi13g00158\_t001 |  | Vvi-Vitvi08g00844\_t001 |  |  |  |  |  |  |
| 2 | Ath-AT3G56330.1 |  | Vvi-Vitvi13g00159\_t001 |  | | | |  |  |  |  |  |  |
| 2 | Ath-AT3G56340.1 |  | Vvi-Vitvi13g00160\_t001 |  | | | |  |  |  |  |  |  |
| 2 | Ath-AT3G56350.1 |  | Vvi-Vitvi13g00177\_t001 |  | | | |  |  |  |  |  |  |
| 2 | Ath-AT3G56360.1 |  | Vvi-Vitvi13g00178\_t001 |  | | | |  |  |  |  |  |  |
| 2 | Ath-AT3G56370.1 |  | Vvi-Vitvi13g00182\_t001 |  | Vvi-Vitvi08g00863\_t001 |  |  |  |  |  |  |
| 2 | Ath-AT3G56380.2 |  | Vvi-Vitvi13g00183\_t001 |  | | | |  |  |  |  |  |  |
| 2 | Ath-AT3G56390.1 |  | | | |  | | | |  |  |  |  |  |  |
| 2 | Ath-AT3G56400.1 |  | Vvi-Vitvi13g00189\_t001 |  | Vvi-Vitvi08g00868\_t001 |  |  |  |  |  |  |
| 2 | Ath-AT3G56410.2 |  | Vvi-Vitvi13g00194\_t001 |  | | | |  |  |  |  |  |  |
| 1 | Ath-AT3G56420.3 |  |  |  | Vvi-Vitvi08g04141\_t001 |  |  |  |  |  |  |
| 0 | Ath-AT3G56430.1 |  |  |  |  |  |  |  |  |
| 0 | Ath-AT3G56440.1 |  |  |  |  |  |  |  |  |
| 0 | Ath-AT3G56450.1 |  |  |  |  |  |  |  |  |
| 0 | Ath-AT3G56460.1 |  |  |  |  |  |  |  |  |
| 0 | Ath-AT3G56470.1 |  |  |  |  |  |  |  |  |
| 0 | Ath-AT3G56480.1 |  |  |  |  |  |  |  |  |
| 0 | Ath-AT3G56490.1 |  |  |  |  |  |  |  |  |
| 0 | Ath-AT3G56500.1 |  |  |  |  |  |  |  |  |
| 0 | Ath-AT3G56510.2 |  |  |  |  |  |  |  |  |
| 0 | Ath-AT3G56520.1 |  |  |  |  |  |  |  |  |
| 0 | Ath-AT3G56530.1 |  |  |  |  |  |  |  |  |
| 0 | Ath-AT3G56540.1 |  |  |  |  |  |  |  |  |
| 0 | Ath-AT3G56550.1 |  |  |  |  |  |  |  |  |
| 0 | Ath-AT3G56560.1 |  |  |  |  |  |  |  |  |
| 0 | Ath-AT3G56570.2 |  |  |  |  |  |  |  |  |
| 1 | Ath-AT3G56580.1 |  | Vvi-Vitvi08g00923\_t001 |  |  |  |  |  |  |  |
| 1 | Ath-AT3G56590.2 |  | | | |  |  |  |  |  |  |  |
| 1 | Ath-AT3G56600.1 |  | Vvi-Vitvi08g00931\_t001 |  |  |  |  |  |  |  |
| 1 | Ath-AT3G56610.1 |  | | | |  |  |  |  |  |  |  |
| 1 | Ath-AT3G56620.1 |  | Vvi-Vitvi08g00942\_t001 |  |  |  |  |  |  |  |
| 1 | Ath-AT3G56630.1 |  | Vvi-Vitvi08g00944\_t001 |  |  |  |  |  |  |  |
| 1 | Ath-AT3G56640.1 |  | Vvi-Vitvi08g00946\_t001 |  |  |  |  |  |  |  |
| 1 | Ath-AT3G56650.1 |  | Vvi-Vitvi08g00947\_t001 |  |  |  |  |  |  |  |
| 1 | Ath-AT3G56660.1 |  | Vvi-Vitvi08g00950\_t001 |  |  |  |  |  |  |  |
| 1 | Ath-AT3G56670.1 |  | | | |  |  |  |  |  |  |  |
| 1 | Ath-AT3G56680.1 |  | Vvi-Vitvi08g00954\_t003 |  |  |  |  |  |  |  |
| 1 | Ath-AT3G56690.1 |  | Vvi-Vitvi08g00956\_t001 |  |  |  |  |  |  |  |
| 0 | Ath-AT3G56700.2 |  |  |  |  |  |  |  |  |
| 0 | Ath-AT3G56710.1 |  |  |  |  |  |  |  |  |
| 1 | Ath-AT3G56720.3 |  | Vvi-Vitvi08g01629\_t002 |  |  |  |  |  |  |  |
| 1 | Ath-AT3G56730.3 |  | | | |  |  |  |  |  |  |  |
| 1 | Ath-AT3G56740.1 |  | | | |  |  |  |  |  |  |  |
| 1 | Ath-AT3G56750.1 |  | | | |  |  |  |  |  |  |  |
| 1 | Ath-AT3G56760.1 |  | | | |  |  |  |  |  |  |  |
| 1 | Ath-AT3G56770.1 |  | | | |  |  |  |  |  |  |  |
| 1 | Ath-AT3G56780.2 |  | | | |  |  |  |  |  |  |  |
| 1 | Ath-AT3G56790.1 |  | | | |  |  |  |  |  |  |  |
| 1 | Ath-AT3G56800.1 |  | | | |  |  |  |  |  |  |  |
| 1 | Ath-AT3G56810.1 |  | | | |  |  |  |  |  |  |  |
| 1 | Ath-AT3G56820.1 |  | | | |  |  |  |  |  |  |  |
| 1 | Ath-AT3G56830.3 |  | | | |  |  |  |  |  |  |  |
| 1 | Ath-AT3G56840.1 |  | | | |  |  |  |  |  |  |  |
| 1 | Ath-AT3G56850.1 |  | | | |  |  |  |  |  |  |  |
| 1 | Ath-AT3G56860.4 |  | | | |  |  |  |  |  |  |  |
| 1 | Ath-AT3G56870.2 |  | | | |  |  |  |  |  |  |  |
| 1 | Ath-AT3G56880.1 |  | | | |  |  |  |  |  |  |  |
| 1 | Ath-AT3G56890.1 |  | | | |  |  |  |  |  |  |  |
| 1 | Ath-AT3G56891.1 |  | | | |  |  |  |  |  |  |  |
| 1 | Ath-AT3G56900.1 |  | | | |  |  |  |  |  |  |  |
| 1 | Ath-AT3G56910.1 |  | | | |  |  |  |  |  |  |  |
| 1 | Ath-AT3G56920.1 |  | | | |  |  |  |  |  |  |  |
| 1 | Ath-AT3G56930.1 |  | | | |  |  |  |  |  |  |  |
| 1 | Ath-AT3G56940.1 |  | | | |  |  |  |  |  |  |  |
| 1 | Ath-AT3G56950.2 |  | | | |  |  |  |  |  |  |  |
| 1 | Ath-AT3G56960.1 |  | Vvi-Vitvi08g01644\_t001 |  |  |  |  |  |  |  |
| 1 | Ath-AT3G56970.1 |  | Vvi-Vitvi08g01649\_t001 |  |  |  |  |  |  |  |
| 1 | Ath-AT3G56980.1 |  | | | |  |  |  |  |  |  |  |
| 1 | Ath-AT3G56990.1 |  | Vvi-Vitvi08g01652\_t001 |  |  |  |  |  |  |  |
| 1 | Ath-AT3G57000.1 |  | | | |  |  |  |  |  |  |  |
| 1 | Ath-AT3G57010.1 |  | Vvi-Vitvi08g01656\_t001 |  |  |  |  |  |  |  |
| 1 | Ath-AT3G57020.1 |  | Vvi-Vitvi08g01657\_t001 |  |  |  |  |  |  |  |
| 1 | Ath-AT3G57030.1 |  | | | |  |  |  |  |  |  |  |
| 1 | Ath-AT3G57040.2 |  | Vvi-Vitvi08g02307\_t001 |  |  |  |  |  |  |  |
| 1 | Ath-AT3G57050.1 |  | Vvi-Vitvi08g01660\_t001 |  |  |  |  |  |  |  |
| 1 | Ath-AT3G57060.2 |  | Vvi-Vitvi08g01666\_t001 |  |  |  |  |  |  |  |
| 1 | Ath-AT3G57062.1 |  | | | |  |  |  |  |  |  |  |
| 1 | Ath-AT3G57070.1 |  | Vvi-Vitvi08g01668\_t001 |  |  |  |  |  |  |  |
| 1 | Ath-AT3G57072.1 |  | | | |  |  |  |  |  |  |  |
| 1 | Ath-AT3G57080.1 |  | Vvi-Vitvi08g02313\_t001 |  |  |  |  |  |  |  |
| 1 | Ath-AT3G57090.1 |  | Vvi-Vitvi08g02314\_t001 |  |  |  |  |  |  |  |
| 0 | Ath-AT3G57100.1 |  |  |  |  |  |  |  |  |
| 0 | Ath-AT3G57110.1 |  |  |  |  |  |  |  |  |
| 0 | Ath-AT3G57120.1 |  |  |  |  |  |  |  |  |
| 1 | Ath-AT3G57130.1 |  | Vvi-Vitvi08g01678\_t001 |  |  |  |  |  |  |  |
| 1 | Ath-AT3G57140.1 |  | Vvi-Vitvi08g01684\_t001 |  |  |  |  |  |  |  |
| 1 | Ath-AT3G57150.1 |  | | | |  |  |  |  |  |  |  |
| 1 | Ath-AT3G57160.2 |  | | | |  |  |  |  |  |  |  |
| 1 | Ath-AT3G57170.1 |  | Vvi-Vitvi08g01687\_t001 |  |  |  |  |  |  |  |
| 1 | Ath-AT3G57190.1 |  | Vvi-Vitvi08g01692\_t001 |  |  |  |  |  |  |  |
| 1 | Ath-AT3G57200.1 |  | | | |  |  |  |  |  |  |  |
| 1 | Ath-AT3G57210.1 |  | | | |  |  |  |  |  |  |  |
| 1 | Ath-AT3G57220.1 |  | Vvi-Vitvi08g01695\_t001 |  |  |  |  |  |  |  |
| 1 | Ath-AT3G57230.1 |  | | | |  |  |  |  |  |  |  |
| 1 | Ath-AT3G57240.1 |  | Vvi-Vitvi08g01699\_t001 |  |  |  |  |  |  |  |
| 0 | Ath-AT3G57250.1 |  |  |  |  |  |  |  |  |
| 0 | Ath-AT3G57260.2 |  |  |  |  |  |  |  |  |
| 0 | Ath-AT3G57270.1 |  |  |  |  |  |  |  |  |
| 1 | Ath-AT3G57280.1 |  | Vvi-Vitvi08g02419\_t001 |  |  |  |  |  |  |  |
| 1 | Ath-AT3G57290.1 |  | | | |  |  |  |  |  |  |  |
| 1 | Ath-AT3G57300.2 |  | Vvi-Vitvi08g01952\_t001 |  |  |  |  |  |  |  |
| 1 | Ath-AT3G57310.1 |  | | | |  |  |  |  |  |  |  |
| 1 | Ath-AT3G57320.2 |  | | | |  |  |  |  |  |  |  |
| 1 | Ath-AT3G57330.1 |  | Vvi-Vitvi08g02413\_t001 |  |  |  |  |  |  |  |
| 1 | Ath-AT3G57340.1 |  | | | |  |  |  |  |  |  |  |
| 1 | Ath-AT3G57350.1 |  | Vvi-Vitvi08g01946\_t001 |  |  |  |  |  |  |  |
| 1 | Ath-AT3G57360.1 |  | Vvi-Vitvi08g02408\_t001 |  |  |  |  |  |  |  |
| 1 | Ath-AT3G57370.1 |  | Vvi-Vitvi08g01943\_t001 |  |  |  |  |  |  |  |
| 1 | Ath-AT3G57380.1 |  | Vvi-Vitvi08g01940\_t001 |  |  |  |  |  |  |  |
| 1 | Ath-AT3G57390.1 |  | Vvi-Vitvi08g01935\_t001 |  |  |  |  |  |  |  |
| 1 | Ath-AT3G57400.1 |  | | | |  |  |  |  |  |  |  |
| 1 | Ath-AT3G57410.2 |  | Vvi-Vitvi08g01924\_t001 |  |  |  |  |  |  |  |
| 1 | Ath-AT3G57420.1 |  | Vvi-Vitvi08g01914\_t001 |  |  |  |  |  |  |  |
| 1 | Ath-AT3G57430.1 |  | | | |  |  |  |  |  |  |  |
| 1 | Ath-AT3G57440.1 |  | | | |  |  |  |  |  |  |  |
| 1 | Ath-AT3G57450.1 |  | Vvi-Vitvi08g02401\_t001 |  |  |  |  |  |  |  |
| 1 | Ath-AT3G57460.1 |  | Vvi-Vitvi08g01906\_t001 |  |  |  |  |  |  |  |
| 1 | Ath-AT3G57465.1 |  | | | |  |  |  |  |  |  |  |
| 1 | Ath-AT3G57470.2 |  | | | |  |  |  |  |  |  |  |
| 1 | Ath-AT3G57480.1 |  | | | |  |  |  |  |  |  |  |
| 1 | Ath-AT3G57490.1 |  | | | |  |  |  |  |  |  |  |
| 1 | Ath-AT3G57500.1 |  | Vvi-Vitvi08g02395\_t001 |  |  |  |  |  |  |  |
| 1 | Ath-AT3G57510.1 |  | Vvi-Vitvi08g02394\_t001 |  |  |  |  |  |  |  |
| 1 | Ath-AT3G57520.1 |  | Vvi-Vitvi08g01890\_t003 |  |  |  |  |  |  |  |
| 1 | Ath-AT3G57530.1 |  | Vvi-Vitvi08g01889\_t002 |  |  |  |  |  |  |  |
| 1 | Ath-AT3G57540.1 |  | Vvi-Vitvi08g01887\_t001 |  |  |  |  |  |  |  |
| 1 | Ath-AT3G57550.2 |  | Vvi-Vitvi08g01884\_t001 |  |  |  |  |  |  |  |
| 1 | Ath-AT3G57560.1 |  | | | |  |  |  |  |  |  |  |
| 1 | Ath-AT3G57570.1 |  | Vvi-Vitvi08g01876\_t002 |  |  |  |  |  |  |  |
| 1 | Ath-AT3G57580.1 |  | | | |  |  |  |  |  |  |  |
| 1 | Ath-AT3G57587.1 |  | | | |  |  |  |  |  |  |  |
| 1 | Ath-AT3G57590.1 |  | | | |  |  |  |  |  |  |  |
| 1 | Ath-AT3G57600.1 |  | Vvi-Vitvi08g04423\_t001 |  |  |  |  |  |  |  |
| 1 | Ath-AT3G57610.1 |  | Vvi-Vitvi08g01874\_t001 |  |  |  |  |  |  |  |
| 1 | Ath-AT3G57620.1 |  | | | |  |  |  |  |  |  |  |
| 1 | Ath-AT3G57630.1 |  | Vvi-Vitvi08g01873\_t001 |  |  |  |  |  |  |  |
| 1 | Ath-AT3G57640.1 |  | | | |  |  |  |  |  |  |  |
| 1 | Ath-AT3G57650.1 |  | Vvi-Vitvi08g01872\_t001 |  |  |  |  |  |  |  |
| 1 | Ath-AT3G57660.1 |  | Vvi-Vitvi08g01871\_t001 |  |  |  |  |  |  |  |
| 1 | Ath-AT3G57670.1 |  | Vvi-Vitvi08g01869\_t001 |  |  |  |  |  |  |  |
| 1 | Ath-AT3G57680.1 |  | Vvi-Vitvi08g01867\_t001 |  |  |  |  |  |  |  |
| 1 | Ath-AT3G57690.1 |  | Vvi-Vitvi08g02381\_t001 |  |  |  |  |  |  |  |
| 1 | Ath-AT3G57710.1 |  | | | |  |  |  |  |  |  |  |
| 1 | Ath-AT3G57700.1 |  | | | |  |  |  |  |  |  |  |
| 1 | Ath-AT3G57720.1 |  | | | |  |  |  |  |  |  |  |
| 1 | Ath-AT3G57730.1 |  | | | |  |  |  |  |  |  |  |
| 1 | Ath-AT3G57740.2 |  | | | |  |  |  |  |  |  |  |
| 1 | Ath-AT3G57750.1 |  | | | |  |  |  |  |  |  |  |
| 1 | Ath-AT3G57760.1 |  | | | |  |  |  |  |  |  |  |
| 1 | Ath-AT3G57770.1 |  | | | |  |  |  |  |  |  |  |
| 1 | Ath-AT3G57780.1 |  | Vvi-Vitvi08g01860\_t003 |  |  |  |  |  |  |  |
| 1 | Ath-AT3G57785.1 |  | Vvi-Vitvi08g01859\_t001 |  |  |  |  |  |  |  |
| 1 | Ath-AT3G57787.1 |  | | | |  |  |  |  |  |  |  |
| 1 | Ath-AT3G57790.1 |  | Vvi-Vitvi08g01857\_t001 |  |  |  |  |  |  |  |
| 1 | Ath-AT3G57800.1 |  | Vvi-Vitvi08g01856\_t001 |  |  |  |  |  |  |  |
| 1 | Ath-AT3G57810.3 |  | Vvi-Vitvi08g01854\_t001 |  |  |  |  |  |  |  |
| 1 | Ath-AT3G57830.1 |  | Vvi-Vitvi08g01853\_t001 |  |  |  |  |  |  |  |
| 1 | Ath-AT3G57840.1 |  | | | |  |  |  |  |  |  |  |
| 1 | Ath-AT3G57850.1 |  | | | |  |  |  |  |  |  |  |
| 1 | Ath-AT3G57860.1 |  | Vvi-Vitvi08g01851\_t001 |  |  |  |  |  |  |  |
| 0 | Ath-AT3G57870.1 |  |  |  |  |  |  |  |  |
| 1 | Ath-AT3G57880.2 |  | Vvi-Vitvi08g01708\_t001 |  |  |  |  |  |  |  |
| 1 | Ath-AT3G57890.2 |  | Vvi-Vitvi08g01713\_t001 |  |  |  |  |  |  |  |
| 1 | Ath-AT3G57900.1 |  | | | |  |  |  |  |  |  |  |
| 1 | Ath-AT3G57910.1 |  | Vvi-Vitvi08g01719\_t001 |  |  |  |  |  |  |  |
| 1 | Ath-AT3G57920.1 |  | Vvi-Vitvi08g01720\_t001 |  |  |  |  |  |  |  |
| 1 | Ath-AT3G57930.2 |  | Vvi-Vitvi08g02342\_t001 |  |  |  |  |  |  |  |
| 1 | Ath-AT3G57940.1 |  | | | |  |  |  |  |  |  |  |
| 1 | Ath-AT3G57950.1 |  | Vvi-Vitvi08g01724\_t001 |  |  |  |  |  |  |  |
| 1 | Ath-AT3G57960.1 |  | | | |  |  |  |  |  |  |  |
| 1 | Ath-AT3G57970.1 |  | | | |  |  |  |  |  |  |  |
| 1 | Ath-AT3G57980.2 |  | | | |  |  |  |  |  |  |  |
| 1 | Ath-AT3G57990.1 |  | Vvi-Vitvi08g01728\_t001 |  |  |  |  |  |  |  |
| 1 | Ath-AT3G58000.1 |  | Vvi-Vitvi08g01736\_t001 |  |  |  |  |  |  |  |
| 1 | Ath-AT3G58010.1 |  | Vvi-Vitvi08g02346\_t001 |  |  |  |  |  |  |  |
| 1 | Ath-AT3G58020.1 |  | Vvi-Vitvi08g01741\_t001 |  |  |  |  |  |  |  |
| 1 | Ath-AT3G58030.4 |  | Vvi-Vitvi08g01751\_t001 |  |  |  |  |  |  |  |
| 1 | Ath-AT3G58040.3 |  | Vvi-Vitvi08g01760\_t001 |  |  |  |  |  |  |  |
| 1 | Ath-AT3G58050.2 |  | | | |  |  |  |  |  |  |  |
| 1 | Ath-AT3G58060.1 |  | Vvi-Vitvi08g01762\_t001 |  |  |  |  |  |  |  |
| 1 | Ath-AT3G58070.1 |  | Vvi-Vitvi08g01771\_t001 |  |  |  |  |  |  |  |
| 0 | Ath-AT3G58090.1 |  |  |  |  |  |  |  |  |
| 0 | Ath-AT3G58100.2 |  |  |  |  |  |  |  |  |
| 0 | Ath-AT3G58110.1 |  |  |  |  |  |  |  |  |
| 0 | Ath-AT3G58120.1 |  |  |  |  |  |  |  |  |
| 0 | Ath-AT3G58130.2 |  |  |  |  |  |  |  |  |
| 0 | Ath-AT3G58140.1 |  |  |  |  |  |  |  |  |
| 0 | Ath-AT3G58150.1 |  |  |  |  |  |  |  |  |
| 0 | Ath-AT3G58160.1 |  |  |  |  |  |  |  |  |
| 0 | Ath-AT3G58170.1 |  |  |  |  |  |  |  |  |
| 0 | Ath-AT3G58180.1 |  |  |  |  |  |  |  |  |
| 0 | Ath-AT3G58190.1 |  |  |  |  |  |  |  |  |
| 0 | Ath-AT3G58200.1 |  |  |  |  |  |  |  |  |
| 0 | Ath-AT3G58210.1 |  |  |  |  |  |  |  |  |
| 0 | Ath-AT3G58220.2 |  |  |  |  |  |  |  |  |
| 0 | Ath-AT3G58230.1 |  |  |  |  |  |  |  |  |
| 0 | Ath-AT3G58240.1 |  |  |  |  |  |  |  |  |
| 0 | Ath-AT3G58250.1 |  |  |  |  |  |  |  |  |
| 0 | Ath-AT3G58260.1 |  |  |  |  |  |  |  |  |
| 0 | Ath-AT3G58270.2 |  |  |  |  |  |  |  |  |
| 0 | Ath-AT3G58280.1 |  |  |  |  |  |  |  |  |
| 0 | Ath-AT3G58290.1 |  |  |  |  |  |  |  |  |
| 0 | Ath-AT3G58300.1 |  |  |  |  |  |  |  |  |
| 0 | Ath-AT3G58310.1 |  |  |  |  |  |  |  |  |
| 0 | Ath-AT3G58320.1 |  |  |  |  |  |  |  |  |
| 0 | Ath-AT3G58330.1 |  |  |  |  |  |  |  |  |
| 0 | Ath-AT3G58340.1 |  |  |  |  |  |  |  |  |
| 0 | Ath-AT3G58350.1 |  |  |  |  |  |  |  |  |
| 0 | Ath-AT3G58360.1 |  |  |  |  |  |  |  |  |
| 0 | Ath-AT3G58370.1 |  |  |  |  |  |  |  |  |
| 0 | Ath-AT3G58380.1 |  |  |  |  |  |  |  |  |
| 0 | Ath-AT3G58390.1 |  |  |  |  |  |  |  |  |
| 0 | Ath-AT3G58400.1 |  |  |  |  |  |  |  |  |
| 0 | Ath-AT3G58410.1 |  |  |  |  |  |  |  |  |
| 0 | Ath-AT3G58415.2 |  |  |  |  |  |  |  |  |
| 0 | Ath-AT3G58420.1 |  |  |  |  |  |  |  |  |
| 0 | Ath-AT3G58430.1 |  |  |  |  |  |  |  |  |
| 0 | Ath-AT3G58440.1 |  |  |  |  |  |  |  |  |
| 0 | Ath-AT3G58450.1 |  |  |  |  |  |  |  |  |
| 0 | Ath-AT3G58460.2 |  |  |  |  |  |  |  |  |
| 0 | Ath-AT3G58470.1 |  |  |  |  |  |  |  |  |
| 1 | Ath-AT3G58480.1 |  | Vvi-Vitvi08g01794\_t001 |  |  |  |  |  |  |  |
| 1 | Ath-AT3G58490.1 |  | Vvi-Vitvi08g01783\_t001 |  |  |  |  |  |  |  |
| 1 | Ath-AT3G58500.1 |  | | | |  |  |  |  |  |  |  |
| 1 | Ath-AT3G58510.1 |  | Vvi-Vitvi08g01782\_t001 |  |  |  |  |  |  |  |
| 1 | Ath-AT3G58520.1 |  | Vvi-Vitvi08g01781\_t001 |  |  |  |  |  |  |  |
| 1 | Ath-AT3G58530.1 |  | Vvi-Vitvi08g01779\_t002 |  |  |  |  |  |  |  |
| 1 | Ath-AT3G58540.1 |  | | | |  |  |  |  |  |  |  |
| 1 | Ath-AT3G58550.1 |  | | | |  |  |  |  |  |  |  |
| 1 | Ath-AT3G58560.1 |  | Vvi-Vitvi08g02363\_t004 |  |  |  |  |  |  |  |
| 0 | Ath-AT3G58570.1 |  |  |  |  |  |  |  |  |
| 0 | Ath-AT3G58580.1 |  |  |  |  |  |  |  |  |
| 0 | Ath-AT3G58585.1 |  |  |  |  |  |  |  |  |
| 1 | Ath-AT3G58590.1 |  | Vvi-Vitvi12g00217\_t001 |  |  |  |  |  |  |  |
| 1 | Ath-AT3G58600.1 |  | Vvi-Vitvi12g00204\_t001 |  |  |  |  |  |  |  |
| 1 | Ath-AT3G58610.1 |  | Vvi-Vitvi12g00198\_t001 |  |  |  |  |  |  |  |
| 1 | Ath-AT3G58620.1 |  | Vvi-Vitvi12g00193\_t001 |  |  |  |  |  |  |  |
| 1 | Ath-AT3G58630.1 |  | Vvi-Vitvi12g00182\_t001 |  |  |  |  |  |  |  |
| 1 | Ath-AT3G58640.2 |  | Vvi-Vitvi12g00181\_t002 |  |  |  |  |  |  |  |
| 1 | Ath-AT3G58650.1 |  | | | |  |  |  |  |  |  |  |
| 1 | Ath-AT3G58660.1 |  | | | |  |  |  |  |  |  |  |
| 1 | Ath-AT3G58670.1 |  | Vvi-Vitvi12g00175\_t001 |  |  |  |  |  |  |  |
| 1 | Ath-AT3G58680.1 |  | Vvi-Vitvi12g00170\_t001 |  |  |  |  |  |  |  |
| 1 | Ath-AT3G58690.1 |  | Vvi-Vitvi12g00160\_t001 |  |  |  |  |  |  |  |
| 1 | Ath-AT3G58700.1 |  | Vvi-Vitvi12g00155\_t001 |  |  |  |  |  |  |  |
| 1 | Ath-AT3G58710.1 |  | Vvi-Vitvi12g00148\_t001 |  |  |  |  |  |  |  |
| 1 | Ath-AT3G58720.3 |  | Vvi-Vitvi12g02228\_t001 |  |  |  |  |  |  |  |
| 0 | Ath-AT3G58730.1 |  |  |  |  |  |  |  |  |
| 1 | Ath-AT3G58740.1 |  | Vvi-Vitvi12g00008\_t001 |  |  |  |  |  |  |  |
| 1 | Ath-AT3G58750.1 |  | | | |  |  |  |  |  |  |  |
| 1 | Ath-AT3G58760.3 |  | | | |  |  |  |  |  |  |  |
| 1 | Ath-AT3G58770.1 |  | Vvi-Vitvi12g00014\_t001 |  |  |  |  |  |  |  |
| 1 | Ath-AT3G58780.4 |  | Vvi-Vitvi12g00019\_t002 |  |  |  |  |  |  |  |
| 1 | Ath-AT3G58790.1 |  | Vvi-Vitvi12g00020\_t002 |  |  |  |  |  |  |  |
| 1 | Ath-AT3G58800.1 |  | Vvi-Vitvi12g00024\_t001 |  |  |  |  |  |  |  |
| 1 | Ath-AT3G58810.1 |  | | | |  |  |  |  |  |  |  |
| 1 | Ath-AT3G58820.1 |  | | | |  |  |  |  |  |  |  |
| 1 | Ath-AT3G58830.2 |  | Vvi-Vitvi12g00027\_t001 |  |  |  |  |  |  |  |
| 1 | Ath-AT3G58840.2 |  | Vvi-Vitvi12g02184\_t001 |  |  |  |  |  |  |  |
| 1 | Ath-AT3G58850.1 |  | Vvi-Vitvi12g02185\_t001 |  |  |  |  |  |  |  |
| 1 | Ath-AT3G58860.1 |  | | | |  |  |  |  |  |  |  |
| 1 | Ath-AT3G58875.1 |  | | | |  |  |  |  |  |  |  |
| 1 | Ath-AT3G58877.1 |  | | | |  |  |  |  |  |  |  |
| 1 | Ath-AT3G58880.1 |  | | | |  |  |  |  |  |  |  |
| 1 | Ath-AT3G58890.1 |  | | | |  |  |  |  |  |  |  |
| 1 | Ath-AT3G58900.4 |  | | | |  |  |  |  |  |  |  |
| 1 | Ath-AT3G58910.1 |  | | | |  |  |  |  |  |  |  |
| 1 | Ath-AT3G58920.1 |  | | | |  |  |  |  |  |  |  |
| 1 | Ath-AT3G58930.5 |  | | | |  |  |  |  |  |  |  |
| 1 | Ath-AT3G58940.2 |  | | | |  |  |  |  |  |  |  |
| 1 | Ath-AT3G58950.1 |  | | | |  |  |  |  |  |  |  |
| 1 | Ath-AT3G58960.1 |  | | | |  |  |  |  |  |  |  |
| 1 | Ath-AT3G58970.1 |  | | | |  |  |  |  |  |  |  |
| 1 | Ath-AT3G58980.1 |  | | | |  |  |  |  |  |  |  |
| 1 | Ath-AT3G58990.1 |  | | | |  |  |  |  |  |  |  |
| 1 | Ath-AT3G59000.1 |  | | | |  |  |  |  |  |  |  |
| 1 | Ath-AT3G59010.1 |  | | | |  |  |  |  |  |  |  |
| 1 | Ath-AT3G59020.2 |  | | | |  |  |  |  |  |  |  |
| 1 | Ath-AT3G59030.1 |  | | | |  |  |  |  |  |  |  |
| 1 | Ath-AT3G59040.2 |  | | | |  |  |  |  |  |  |  |
| 1 | Ath-AT3G59050.1 |  | | | |  |  |  |  |  |  |  |
| 1 | Ath-AT3G59060.2 |  | | | |  |  |  |  |  |  |  |
| 1 | Ath-AT3G59070.1 |  | | | |  |  |  |  |  |  |  |
| 1 | Ath-AT3G59080.1 |  | | | |  |  |  |  |  |  |  |
| 1 | Ath-AT3G59090.2 |  | | | |  |  |  |  |  |  |  |
| 1 | Ath-AT3G59100.1 |  | Vvi-Vitvi12g00056\_t001 |  |  |  |  |  |  |  |
| 0 | Ath-AT3G59110.1 |  |  |  |  |  |  |  |  |
| 0 | Ath-AT3G59120.1 |  |  |  |  |  |  |  |  |
| 0 | Ath-AT3G59130.1 |  |  |  |  |  |  |  |  |
| 0 | Ath-AT3G59140.1 |  |  |  |  |  |  |  |  |
| 0 | Ath-AT3G59150.1 |  |  |  |  |  |  |  |  |
| 0 | Ath-AT3G59160.1 |  |  |  |  |  |  |  |  |
| 0 | Ath-AT3G59170.1 |  |  |  |  |  |  |  |  |
| 0 | Ath-AT3G59180.1 |  |  |  |  |  |  |  |  |
| 0 | Ath-AT3G59190.2 |  |  |  |  |  |  |  |  |
| 0 | Ath-AT3G59200.1 |  |  |  |  |  |  |  |  |
| 0 | Ath-AT3G59210.3 |  |  |  |  |  |  |  |  |
| 0 | Ath-AT3G59220.1 |  |  |  |  |  |  |  |  |
| 0 | Ath-AT3G59230.1 |  |  |  |  |  |  |  |  |
| 0 | Ath-AT3G59240.1 |  |  |  |  |  |  |  |  |
| 0 | Ath-AT3G59245.1 |  |  |  |  |  |  |  |  |
| 0 | Ath-AT3G59250.1 |  |  |  |  |  |  |  |  |
| 0 | Ath-AT3G59260.1 |  |  |  |  |  |  |  |  |
| 0 | Ath-AT3G59270.1 |  |  |  |  |  |  |  |  |
| 0 | Ath-AT3G59280.1 |  |  |  |  |  |  |  |  |
| 0 | Ath-AT3G59290.1 |  |  |  |  |  |  |  |  |
| 0 | Ath-AT3G59295.1 |  |  |  |  |  |  |  |  |
| 0 | Ath-AT3G59300.3 |  |  |  |  |  |  |  |  |
| 0 | Ath-AT3G59310.2 |  |  |  |  |  |  |  |  |
| 0 | Ath-AT3G59320.1 |  |  |  |  |  |  |  |  |
| 0 | Ath-AT3G59330.3 |  |  |  |  |  |  |  |  |
| 0 | Ath-AT3G59340.2 |  |  |  |  |  |  |  |  |
| 0 | Ath-AT3G59350.6 |  |  |  |  |  |  |  |  |
| 0 | Ath-AT3G59360.2 |  |  |  |  |  |  |  |  |
| 0 | Ath-AT3G59370.1 |  |  |  |  |  |  |  |  |
| 1 | Ath-AT3G59380.1 |  | Vvi-Vitvi05g01482\_t001 |  |  |  |  |  |  |  |
| 1 | Ath-AT3G59390.2 |  | Vvi-Vitvi05g01490\_t001 |  |  |  |  |  |  |  |
| 1 | Ath-AT3G59400.1 |  | Vvi-Vitvi05g01492\_t001 |  |  |  |  |  |  |  |
| 1 | Ath-AT3G59410.2 |  | Vvi-Vitvi05g01493\_t001 |  |  |  |  |  |  |  |
| 1 | Ath-AT3G59420.1 |  | Vvi-Vitvi05g01494\_t001 |  |  |  |  |  |  |  |
| 1 | Ath-AT3G59430.2 |  | Vvi-Vitvi05g01495\_t001 |  |  |  |  |  |  |  |
| 1 | Ath-AT3G59435.1 |  | | | |  |  |  |  |  |  |  |
| 1 | Ath-AT3G59440.1 |  | Vvi-Vitvi05g01500\_t001 |  |  |  |  |  |  |  |
| 1 | Ath-AT3G59450.1 |  | | | |  |  |  |  |  |  |  |
| 1 | Ath-AT3G59455.1 |  | | | |  |  |  |  |  |  |  |
| 1 | Ath-AT3G59460.1 |  | | | |  |  |  |  |  |  |  |
| 1 | Ath-AT3G59470.2 |  | Vvi-Vitvi05g01510\_t001 |  |  |  |  |  |  |  |
| 1 | Ath-AT3G59480.1 |  | Vvi-Vitvi05g01516\_t001 |  |  |  |  |  |  |  |
| 1 | Ath-AT3G59490.2 |  | Vvi-Vitvi05g01517\_t001 |  |  |  |  |  |  |  |
| 1 | Ath-AT3G59500.1 |  | Vvi-Vitvi05g01518\_t001 |  |  |  |  |  |  |  |
| 1 | Ath-AT3G59510.1 |  | Vvi-Vitvi05g04459\_t001 |  |  |  |  |  |  |  |
| 1 | Ath-AT3G59520.1 |  | Vvi-Vitvi05g01520\_t001 |  |  |  |  |  |  |  |
| 0 | Ath-AT3G59530.2 |  |  |  |  |  |  |  |  |
| 0 | Ath-AT3G59540.1 |  |  |  |  |  |  |  |  |
| 0 | Ath-AT3G59550.1 |  |  |  |  |  |  |  |  |
| 0 | Ath-AT3G59570.3 |  |  |  |  |  |  |  |  |
| 0 | Ath-AT3G59580.2 |  |  |  |  |  |  |  |  |
| 0 | Ath-AT3G59590.1 |  |  |  |  |  |  |  |  |
| 0 | Ath-AT3G59600.1 |  |  |  |  |  |  |  |  |
| 0 | Ath-AT3G59610.1 |  |  |  |  |  |  |  |  |
| 0 | Ath-AT3G59620.1 |  |  |  |  |  |  |  |  |
| 1 | Ath-AT3G59630.2 |  | Vvi-Vitvi05g01584\_t001 |  |  |  |  |  |  |  |
| 1 | Ath-AT3G59640.1 |  | Vvi-Vitvi05g01585\_t001 |  |  |  |  |  |  |  |
| 1 | Ath-AT3G59650.2 |  | Vvi-Vitvi05g01594\_t002 |  |  |  |  |  |  |  |
| 1 | Ath-AT3G59660.1 |  | Vvi-Vitvi05g01600\_t001 |  |  |  |  |  |  |  |
| 1 | Ath-AT3G59670.1 |  | Vvi-Vitvi05g01601\_t001 |  |  |  |  |  |  |  |
| 1 | Ath-AT3G59680.2 |  | Vvi-Vitvi05g01602\_t001 |  |  |  |  |  |  |  |
| 1 | Ath-AT3G59690.2 |  | Vvi-Vitvi05g01603\_t001 |  |  |  |  |  |  |  |
| 0 | Ath-AT3G59700.1 |  |  |  |  |  |  |  |  |
| 1 | Ath-AT3G59710.2 |  | Vvi-Vitvi05g01623\_t001 |  |  |  |  |  |  |  |
| 1 | Ath-AT3G59730.1 |  | | | |  |  |  |  |  |  |  |
| 1 | Ath-AT3G59740.1 |  | | | |  |  |  |  |  |  |  |
| 1 | Ath-AT3G59750.1 |  | | | |  |  |  |  |  |  |  |
| 1 | Ath-AT3G59760.1 |  | Vvi-Vitvi05g01624\_t001 |  |  |  |  |  |  |  |
| 1 | Ath-AT3G59770.3 |  | | | |  |  |  |  |  |  |  |
| 1 | Ath-AT3G59780.1 |  | Vvi-Vitvi05g01629\_t001 |  |  |  |  |  |  |  |
| 1 | Ath-AT3G59790.1 |  | Vvi-Vitvi05g01634\_t001 |  |  |  |  |  |  |  |
| 1 | Ath-AT3G59800.1 |  | Vvi-Vitvi05g01636\_t001 |  |  |  |  |  |  |  |
| 1 | Ath-AT3G59810.1 |  | Vvi-Vitvi05g01640\_t001 |  |  |  |  |  |  |  |
| 1 | Ath-AT3G59820.2 |  | Vvi-Vitvi05g01655\_t001 |  |  |  |  |  |  |  |
| 1 | Ath-AT3G59830.1 |  | Vvi-Vitvi05g01656\_t001 |  |  |  |  |  |  |  |
| 0 | Ath-AT3G59840.1 |  |  |  |  |  |  |  |  |
| 0 | Ath-AT3G59845.1 |  |  |  |  |  |  |  |  |
| 1 | Ath-AT3G59850.2 |  | Vvi-Vitvi05g01679\_t001 |  |  |  |  |  |  |  |
| 1 | Ath-AT3G59870.1 |  | Vvi-Vitvi05g02275\_t002 |  |  |  |  |  |  |  |
| 1 | Ath-AT3G59880.1 |  | Vvi-Vitvi05g04575\_t001 |  |  |  |  |  |  |  |
| 1 | Ath-AT3G59890.1 |  | Vvi-Vitvi05g01692\_t001 |  |  |  |  |  |  |  |
| 1 | Ath-AT3G59900.1 |  | Vvi-Vitvi05g02279\_t001 |  |  |  |  |  |  |  |
| 1 | Ath-AT3G59910.2 |  | Vvi-Vitvi05g04576\_t001 |  |  |  |  |  |  |  |
| 1 | Ath-AT3G59920.1 |  | Vvi-Vitvi05g01700\_t001 |  |  |  |  |  |  |  |
| 1 | Ath-AT3G59930.1 |  | | | |  |  |  |  |  |  |  |
| 1 | Ath-AT3G59940.1 |  | Vvi-Vitvi05g01703\_t001 |  |  |  |  |  |  |  |
| 1 | Ath-AT3G59950.1 |  | | | |  |  |  |  |  |  |  |
| 1 | Ath-AT3G59960.1 |  | | | |  |  |  |  |  |  |  |
| 1 | Ath-AT3G59970.3 |  | Vvi-Vitvi05g01704\_t001 |  |  |  |  |  |  |  |
| 1 | Ath-AT3G59980.1 |  | Vvi-Vitvi05g04578\_t001 |  |  |  |  |  |  |  |
| 0 | Ath-AT3G59990.4 |  |  |  |  |  |  |  |  |
| 0 | Ath-AT3G60000.2 |  |  |  |  |  |  |  |  |
| 0 | Ath-AT3G60010.1 |  |  |  |  |  |  |  |  |
| 0 | Ath-AT3G60020.1 |  |  |  |  |  |  |  |  |
| 0 | Ath-AT3G60030.1 |  |  |  |  |  |  |  |  |
| 0 | Ath-AT3G60040.3 |  |  |  |  |  |  |  |  |
| 0 | Ath-AT3G60050.1 |  |  |  |  |  |  |  |  |
| 0 | Ath-AT3G60060.1 |  |  |  |  |  |  |  |  |
| 0 | Ath-AT3G60070.4 |  |  |  |  |  |  |  |  |
| 0 | Ath-AT3G60080.1 |  |  |  |  |  |  |  |  |
| 0 | Ath-AT3G60090.1 |  |  |  |  |  |  |  |  |
| 0 | Ath-AT3G60100.2 |  |  |  |  |  |  |  |  |
| 0 | Ath-AT3G60110.1 |  |  |  |  |  |  |  |  |
| 0 | Ath-AT3G60120.1 |  |  |  |  |  |  |  |  |
| 0 | Ath-AT3G60130.1 |  |  |  |  |  |  |  |  |
| 0 | Ath-AT3G60140.1 |  |  |  |  |  |  |  |  |
| 0 | Ath-AT3G60150.1 |  |  |  |  |  |  |  |  |
| 0 | Ath-AT3G60160.1 |  |  |  |  |  |  |  |  |
| 0 | Ath-AT3G60180.1 |  |  |  |  |  |  |  |  |
| 0 | Ath-AT3G60190.1 |  |  |  |  |  |  |  |  |
| 0 | Ath-AT3G60200.1 |  |  |  |  |  |  |  |  |
| 0 | Ath-AT3G60210.1 |  |  |  |  |  |  |  |  |
| 0 | Ath-AT3G60220.1 |  |  |  |  |  |  |  |  |
| 0 | Ath-AT3G60240.4 |  |  |  |  |  |  |  |  |
| 1 | Ath-AT3G60245.1 |  | Vvi-Vitvi02g00599\_t001 |  |  |  |  |  |  |  |
| 1 | Ath-AT3G60250.1 |  | Vvi-Vitvi02g00597\_t001 |  |  |  |  |  |  |  |
| 2 | Ath-AT3G60260.1 |  | | | |  | Vvi-Vitvi15g00542\_t001 |  |  |  |  |  |  |
| 2 | Ath-AT3G60270.1 |  | | | |  | Vvi-Vitvi15g00547\_t001 |  |  |  |  |  |  |
| 2 | Ath-AT3G60280.1 |  | | | |  | | | |  |  |  |  |  |  |
| 2 | Ath-AT3G60286.2 |  | | | |  | | | |  |  |  |  |  |  |
| 2 | Ath-AT3G60290.1 |  | | | |  | | | |  |  |  |  |  |  |
| 2 | Ath-AT3G60300.2 |  | | | |  | Vvi-Vitvi15g00555\_t001 |  |  |  |  |  |  |
| 2 | Ath-AT3G60310.1 |  | | | |  | | | |  |  |  |  |  |  |
| 2 | Ath-AT3G60320.1 |  | | | |  | Vvi-Vitvi15g00563\_t001 |  |  |  |  |  |  |
| 2 | Ath-AT3G60328.1 |  | | | |  | | | |  |  |  |  |  |  |
| 2 | Ath-AT3G60330.2 |  | | | |  | Vvi-Vitvi15g00567\_t001 |  |  |  |  |  |  |
| 2 | Ath-AT3G60340.2 |  | | | |  | Vvi-Vitvi15g00571\_t001 |  |  |  |  |  |  |
| 2 | Ath-AT3G60350.1 |  | | | |  | Vvi-Vitvi15g00572\_t001 |  |  |  |  |  |  |
| 2 | Ath-AT3G60360.1 |  | | | |  | | | |  |  |  |  |  |  |
| 2 | Ath-AT3G60370.2 |  | | | |  | Vvi-Vitvi15g00574\_t001 |  |  |  |  |  |  |
| 2 | Ath-AT3G60380.1 |  | | | |  | Vvi-Vitvi15g00577\_t001 |  |  |  |  |  |  |
| 2 | Ath-AT3G60390.1 |  | | | |  | Vvi-Vitvi15g00579\_t001 |  |  |  |  |  |  |
| 2 | Ath-AT3G60400.1 |  | | | |  | Vvi-Vitvi15g00582\_t001 |  |  |  |  |  |  |
| 2 | Ath-AT3G60410.2 |  | Vvi-Vitvi02g01718\_t001 |  | Vvi-Vitvi15g01445\_t001 |  |  |  |  |  |  |
| 2 | Ath-AT3G60415.1 |  | | | |  | | | |  |  |  |  |  |  |
| 2 | Ath-AT3G60420.1 |  | | | |  | | | |  |  |  |  |  |  |
| 2 | Ath-AT3G60440.1 |  | | | |  | | | |  |  |  |  |  |  |
| 2 | Ath-AT3G60450.2 |  | | | |  | | | |  |  |  |  |  |  |
| 2 | Ath-AT3G60460.1 |  | | | |  | | | |  |  |  |  |  |  |
| 2 | Ath-AT3G60470.1 |  | | | |  | | | |  |  |  |  |  |  |
| 2 | Ath-AT3G60480.3 |  | | | |  | Vvi-Vitvi15g00593\_t001 |  |  |  |  |  |  |
| 2 | Ath-AT3G60490.1 |  | | | |  | Vvi-Vitvi15g00601\_t001 |  |  |  |  |  |  |
| 2 | Ath-AT3G60500.2 |  | | | |  | | | |  |  |  |  |  |  |
| 2 | Ath-AT3G60510.3 |  | Vvi-Vitvi02g00569\_t001 |  | Vvi-Vitvi15g00615\_t001 |  |  |  |  |  |  |
| 2 | Ath-AT3G60520.1 |  | | | |  | Vvi-Vitvi15g00617\_t001 |  |  |  |  |  |  |
| 2 | Ath-AT3G60530.1 |  | | | |  | | | |  |  |  |  |  |  |
| 2 | Ath-AT3G60540.1 |  | | | |  | Vvi-Vitvi15g01468\_t001 |  |  |  |  |  |  |
| 2 | Ath-AT3G60550.1 |  | | | |  | | | |  |  |  |  |  |  |
| 2 | Ath-AT3G60560.1 |  | | | |  | | | |  |  |  |  |  |  |
| 2 | Ath-AT3G60570.1 |  | | | |  | Vvi-Vitvi15g00640\_t001 |  |  |  |  |  |  |
| 2 | Ath-AT3G60580.1 |  | Vvi-Vitvi02g00560\_t001 |  | Vvi-Vitvi15g01472\_t001 |  |  |  |  |  |  |
| 2 | Ath-AT3G60590.3 |  | | | |  | Vvi-Vitvi15g00676\_t001 |  |  |  |  |  |  |
| 2 | Ath-AT3G60600.1 |  | Vvi-Vitvi02g00545\_t001 |  | Vvi-Vitvi15g00677\_t001 |  |  |  |  |  |  |
| 2 | Ath-AT3G60620.1 |  | | | |  | Vvi-Vitvi15g00679\_t001 |  |  |  |  |  |  |
| 2 | Ath-AT3G60630.1 |  | Vvi-Vitvi02g00536\_t002 |  | Vvi-Vitvi15g00680\_t001 |  |  |  |  |  |  |
| 3 | Ath-AT3G60640.1 |  | Vvi-Vitvi02g00535\_t001 |  | | | |  | Vvi-Vitvi15g00718\_t002 |  |  |  |  |  |
| 2 | Ath-AT3G60650.1 |  |  |  | | | |  | | | |  |  |  |  |  |
| 2 | Ath-AT3G60660.1 |  |  |  | | | |  | Vvi-Vitvi15g00716\_t001 |  |  |  |  |  |
| 2 | Ath-AT3G60670.1 |  |  |  | | | |  | Vvi-Vitvi15g00710\_t001 |  |  |  |  |  |
| 2 | Ath-AT3G60680.1 |  |  |  | | | |  | Vvi-Vitvi15g00709\_t001 |  |  |  |  |  |
| 2 | Ath-AT3G60690.1 |  |  |  | | | |  | Vvi-Vitvi15g00706\_t001 |  |  |  |  |  |
| 2 | Ath-AT3G60700.1 |  |  |  | | | |  | | | |  |  |  |  |  |
| 2 | Ath-AT3G60710.1 |  |  |  | | | |  | | | |  |  |  |  |  |
| 2 | Ath-AT3G60720.3 |  |  |  | | | |  | Vvi-Vitvi15g00705\_t001 |  |  |  |  |  |
| 2 | Ath-AT3G60730.1 |  |  |  | | | |  | Vvi-Vitvi15g00701\_t001 |  |  |  |  |  |
| 2 | Ath-AT3G60740.1 |  |  |  | Vvi-Vitvi15g00699\_t001 |  | | | |  |  |  |  |  |
| 2 | Ath-AT3G60750.1 |  |  |  | | | |  | Vvi-Vitvi15g00690\_t001 |  |  |  |  |  |
| 1 | Ath-AT3G60760.1 |  |  |  | | | |  |  |  |  |  |  |
| 1 | Ath-AT3G60770.1 |  |  |  | Vvi-Vitvi15g00719\_t001 |  |  |  |  |  |  |
| 1 | Ath-AT3G60780.1 |  |  |  | Vvi-Vitvi15g00724\_t001 |  |  |  |  |  |  |
| 1 | Ath-AT3G60790.1 |  |  |  | | | |  |  |  |  |  |  |
| 1 | Ath-AT3G60800.1 |  |  |  | Vvi-Vitvi15g00725\_t001 |  |  |  |  |  |  |
| 2 | Ath-AT3G60810.1 |  | Vvi-Vitvi15g00743\_t001 |  | | | |  |  |  |  |  |  |
| 2 | Ath-AT3G60820.1 |  | Vvi-Vitvi15g00742\_t001 |  | | | |  |  |  |  |  |  |
| 2 | Ath-AT3G60830.1 |  | | | |  | | | |  |  |  |  |  |  |
| 2 | Ath-AT3G60840.1 |  | Vvi-Vitvi15g00739\_t001 |  | | | |  |  |  |  |  |  |
| 2 | Ath-AT3G60850.1 |  | Vvi-Vitvi15g00737\_t001 |  | | | |  |  |  |  |  |  |
| 2 | Ath-AT3G60860.1 |  | | | |  | Vvi-Vitvi15g00733\_t001 |  |  |  |  |  |  |
| 1 | Ath-AT3G60870.1 |  | Vvi-Vitvi15g00732\_t001 |  |  |  |  |  |  |  |
| 1 | Ath-AT3G60880.2 |  | Vvi-Vitvi15g00728\_t002 |  |  |  |  |  |  |  |
| 1 | Ath-AT3G60890.2 |  | Vvi-Vitvi15g00727\_t001 |  |  |  |  |  |  |  |
| 1 | Ath-AT3G60900.1 |  | Vvi-Vitvi15g00816\_t001 |  |  |  |  |  |  |  |
| 1 | Ath-AT3G60910.1 |  | | | |  |  |  |  |  |  |  |
| 1 | Ath-AT3G60920.2 |  | | | |  |  |  |  |  |  |  |
| 1 | Ath-AT3G60940.2 |  | | | |  |  |  |  |  |  |  |
| 1 | Ath-AT3G60950.1 |  | | | |  |  |  |  |  |  |  |
| 1 | Ath-AT3G60960.1 |  | | | |  |  |  |  |  |  |  |
| 1 | Ath-AT3G60961.1 |  | | | |  |  |  |  |  |  |  |
| 1 | Ath-AT3G60966.1 |  | | | |  |  |  |  |  |  |  |
| 1 | Ath-AT3G60970.1 |  | | | |  |  |  |  |  |  |  |
| 1 | Ath-AT3G60975.1 |  | | | |  |  |  |  |  |  |  |
| 1 | Ath-AT3G60980.1 |  | | | |  |  |  |  |  |  |  |
| 1 | Ath-AT3G60990.1 |  | | | |  |  |  |  |  |  |  |
| 1 | Ath-AT3G61010.3 |  | | | |  |  |  |  |  |  |  |
| 1 | Ath-AT3G61028.2 |  | | | |  |  |  |  |  |  |  |
| 1 | Ath-AT3G61030.1 |  | | | |  |  |  |  |  |  |  |
| 1 | Ath-AT3G61035.1 |  | | | |  |  |  |  |  |  |  |
| 2 | Ath-AT3G61040.1 |  | | | |  | Vvi-Vitvi02g00395\_t001 |  |  |  |  |  |  |
| 2 | Ath-AT3G61050.2 |  | | | |  | | | |  |  |  |  |  |  |
| 2 | Ath-AT3G61060.2 |  | | | |  | | | |  |  |  |  |  |  |
| 2 | Ath-AT3G61070.3 |  | Vvi-Vitvi15g00829\_t003 |  | | | |  |  |  |  |  |  |
| 2 | Ath-AT3G61080.2 |  | | | |  | | | |  |  |  |  |  |  |
| 2 | Ath-AT3G61090.1 |  | | | |  | | | |  |  |  |  |  |  |
| 2 | Ath-AT3G61100.1 |  | | | |  | | | |  |  |  |  |  |  |
| 2 | Ath-AT3G61110.1 |  | | | |  | | | |  |  |  |  |  |  |
| 2 | Ath-AT3G61111.1 |  | | | |  | | | |  |  |  |  |  |  |
| 2 | Ath-AT3G61113.1 |  | | | |  | | | |  |  |  |  |  |  |
| 2 | Ath-AT3G61117.1 |  | | | |  | | | |  |  |  |  |  |  |
| 2 | Ath-AT3G61120.1 |  | | | |  | | | |  |  |  |  |  |  |
| 2 | Ath-AT3G61130.1 |  | | | |  | | | |  |  |  |  |  |  |
| 2 | Ath-AT3G61140.1 |  | Vvi-Vitvi15g00837\_t001 |  | | | |  |  |  |  |  |  |
| 2 | Ath-AT3G61150.1 |  | Vvi-Vitvi15g00839\_t001 |  | | | |  |  |  |  |  |  |
| 2 | Ath-AT3G61160.2 |  | Vvi-Vitvi15g00840\_t001 |  | | | |  |  |  |  |  |  |
| 2 | Ath-AT3G61170.1 |  | Vvi-Vitvi15g00841\_t001 |  | | | |  |  |  |  |  |  |
| 2 | Ath-AT3G61172.1 |  | | | |  | | | |  |  |  |  |  |  |
| 2 | Ath-AT3G61175.1 |  | | | |  | | | |  |  |  |  |  |  |
| 2 | Ath-AT3G61177.1 |  | | | |  | | | |  |  |  |  |  |  |
| 2 | Ath-AT3G61180.1 |  | Vvi-Vitvi15g00842\_t001 |  | Vvi-Vitvi02g00384\_t001 |  |  |  |  |  |  |
| 2 | Ath-AT3G61182.1 |  | | | |  | | | |  |  |  |  |  |  |
| 2 | Ath-AT3G61190.2 |  | Vvi-Vitvi15g00844\_t001 |  | | | |  |  |  |  |  |  |
| 2 | Ath-AT3G61200.1 |  | Vvi-Vitvi15g00846\_t001 |  | | | |  |  |  |  |  |  |
| 1 | Ath-AT3G61210.1 |  |  |  | | | |  |  |  |  |  |  |
| 2 | Ath-AT3G61220.3 |  | Vvi-Vitvi15g01179\_t001 |  | | | |  |  |  |  |  |  |
| 2 | Ath-AT3G61230.1 |  | Vvi-Vitvi15g01171\_t001 |  | Vvi-Vitvi02g00368\_t001 |  |  |  |  |  |  |
| 2 | Ath-AT3G61240.1 |  | Vvi-Vitvi15g01162\_t002 |  | Vvi-Vitvi02g00349\_t001 |  |  |  |  |  |  |
| 2 | Ath-AT3G61250.1 |  | Vvi-Vitvi15g01161\_t001 |  | | | |  |  |  |  |  |  |
| 2 | Ath-AT3G61260.1 |  | Vvi-Vitvi15g01160\_t001 |  | Vvi-Vitvi02g00348\_t001 |  |  |  |  |  |  |
| 2 | Ath-AT3G61270.1 |  | | | |  | Vvi-Vitvi02g01384\_t001 |  |  |  |  |  |  |
| 2 | Ath-AT3G61280.1 |  | Vvi-Vitvi15g01682\_t001 |  | | | |  |  |  |  |  |  |
| 2 | Ath-AT3G61290.1 |  | | | |  | | | |  |  |  |  |  |  |
| 2 | Ath-AT3G61300.1 |  | Vvi-Vitvi15g01147\_t001 |  | | | |  |  |  |  |  |  |
| 2 | Ath-AT3G61310.1 |  | Vvi-Vitvi15g01145\_t001 |  | Vvi-Vitvi02g00331\_t001 |  |  |  |  |  |  |
| 2 | Ath-AT3G61320.3 |  | Vvi-Vitvi15g01142\_t001 |  | | | |  |  |  |  |  |  |
| 2 | Ath-AT3G61340.2 |  | | | |  | | | |  |  |  |  |  |  |
| 2 | Ath-AT3G61350.1 |  | Vvi-Vitvi15g01659\_t001 |  | | | |  |  |  |  |  |  |
| 2 | Ath-AT3G61360.1 |  | | | |  | | | |  |  |  |  |  |  |
| 2 | Ath-AT3G61370.1 |  | | | |  | | | |  |  |  |  |  |  |
| 2 | Ath-AT3G61380.1 |  | Vvi-Vitvi15g01117\_t002 |  | | | |  |  |  |  |  |  |
| 2 | Ath-AT3G61390.2 |  | | | |  | | | |  |  |  |  |  |  |
| 2 | Ath-AT3G61400.1 |  | | | |  | | | |  |  |  |  |  |  |
| 2 | Ath-AT3G61410.3 |  | | | |  | | | |  |  |  |  |  |  |
| 2 | Ath-AT3G61415.1 |  | Vvi-Vitvi15g01113\_t001 |  | | | |  |  |  |  |  |  |
| 2 | Ath-AT3G61420.1 |  | | | |  | | | |  |  |  |  |  |  |
| 2 | Ath-AT3G61430.1 |  | Vvi-Vitvi15g01110\_t002 |  | Vvi-Vitvi02g00310\_t001 |  |  |  |  |  |  |
| 1 | Ath-AT3G61440.1 |  | Vvi-Vitvi15g01101\_t002 |  |  |  |  |  |  |  |
| 2 | Ath-AT3G61450.2 |  | | | |  | Vvi-Vitvi15g01063\_t001 |  |  |  |  |  |  |
| 2 | Ath-AT3G61460.1 |  | | | |  | Vvi-Vitvi15g01067\_t001 |  |  |  |  |  |  |
| 2 | Ath-AT3G61470.1 |  | | | |  | | | |  |  |  |  |  |  |
| 2 | Ath-AT3G61480.2 |  | | | |  | Vvi-Vitvi15g01068\_t001 |  |  |  |  |  |  |
| 2 | Ath-AT3G61490.4 |  | | | |  | Vvi-Vitvi15g04607\_t001 |  |  |  |  |  |  |
| 2 | Ath-AT3G61500.2 |  | | | |  | Vvi-Vitvi15g01644\_t003 |  |  |  |  |  |  |
| 2 | Ath-AT3G61510.1 |  | Vvi-Vitvi15g01093\_t001 |  | | | |  |  |  |  |  |  |
| 2 | Ath-AT3G61520.1 |  | Vvi-Vitvi15g01649\_t001 |  | | | |  |  |  |  |  |  |
| 2 | Ath-AT3G61530.2 |  | Vvi-Vitvi15g01089\_t001 |  | | | |  |  |  |  |  |  |
| 2 | Ath-AT3G61540.1 |  | Vvi-Vitvi15g01088\_t001 |  | | | |  |  |  |  |  |  |
| 1 | Ath-AT3G61550.1 |  |  |  | Vvi-Vitvi15g01082\_t001 |  |  |  |  |  |  |
| 1 | Ath-AT3G61560.1 |  | Vvi-Vitvi15g01640\_t001.1.6037826c |  |  |  |  |  |  |  |
| 1 | Ath-AT3G61570.1 |  | Vvi-Vitvi15g01053\_t001 |  |  |  |  |  |  |  |
| 1 | Ath-AT3G61571.1 |  | | | |  |  |  |  |  |  |  |
| 1 | Ath-AT3G61580.1 |  | Vvi-Vitvi15g01049\_t001 |  |  |  |  |  |  |  |
| 2 | Ath-AT3G61590.4 |  | Vvi-Vitvi15g01033\_t003 |  | Vvi-Vitvi02g00075\_t001 |  |  |  |  |  |  |
| 2 | Ath-AT3G61600.2 |  | Vvi-Vitvi15g01031\_t001 |  | Vvi-Vitvi02g00078\_t001 |  |  |  |  |  |  |
| 2 | Ath-AT3G61610.1 |  | Vvi-Vitvi15g01026\_t001 |  | Vvi-Vitvi02g00090\_t001 |  |  |  |  |  |  |
| 2 | Ath-AT3G61620.1 |  | Vvi-Vitvi15g01025\_t001 |  | | | |  |  |  |  |  |  |
| 2 | Ath-AT3G61630.1 |  | Vvi-Vitvi15g01021\_t001 |  | Vvi-Vitvi02g00093\_t001 |  |  |  |  |  |  |
| 2 | Ath-AT3G61640.1 |  | Vvi-Vitvi15g01019\_t001 |  | Vvi-Vitvi02g01335\_t001 |  |  |  |  |  |  |
| 2 | Ath-AT3G61650.1 |  | Vvi-Vitvi15g01018\_t001 |  | | | |  |  |  |  |  |  |
| 2 | Ath-AT3G61660.2 |  | Vvi-Vitvi15g01626\_t001 |  | | | |  |  |  |  |  |  |
| 2 | Ath-AT3G61670.1 |  | Vvi-Vitvi15g01013\_t001 |  | Vvi-Vitvi02g00105\_t001 |  |  |  |  |  |  |
| 1 | Ath-AT3G61680.1 |  | Vvi-Vitvi15g00995\_t001 |  |  |  |  |  |  |  |
| 1 | Ath-AT3G61690.3 |  | Vvi-Vitvi15g01618\_t001 |  |  |  |  |  |  |  |
| 1 | Ath-AT3G61700.2 |  | Vvi-Vitvi15g00991\_t001 |  |  |  |  |  |  |  |
| 1 | Ath-AT3G61710.1 |  | Vvi-Vitvi15g00986\_t001 |  |  |  |  |  |  |  |
| 1 | Ath-AT3G61720.1 |  | | | |  |  |  |  |  |  |  |
| 1 | Ath-AT3G61723.1 |  | | | |  |  |  |  |  |  |  |
| 1 | Ath-AT3G61730.1 |  | Vvi-Vitvi15g04578\_t001 |  |  |  |  |  |  |  |
| 1 | Ath-AT3G61740.1 |  | Vvi-Vitvi15g00976\_t001 |  |  |  |  |  |  |  |
| 1 | Ath-AT3G61750.1 |  | Vvi-Vitvi15g00973\_t001 |  |  |  |  |  |  |  |
| 1 | Ath-AT3G61760.1 |  | Vvi-Vitvi15g00958\_t001 |  |  |  |  |  |  |  |
| 1 | Ath-AT3G61770.1 |  | Vvi-Vitvi15g00956\_t001 |  |  |  |  |  |  |  |
| 1 | Ath-AT3G61780.1 |  | Vvi-Vitvi15g00954\_t002 |  |  |  |  |  |  |  |
| 1 | Ath-AT3G61790.1 |  | Vvi-Vitvi15g04556\_t001 |  |  |  |  |  |  |  |
| 1 | Ath-AT3G61800.1 |  | Vvi-Vitvi15g00953\_t001 |  |  |  |  |  |  |  |
| 1 | Ath-AT3G61810.1 |  | | | |  |  |  |  |  |  |  |
| 1 | Ath-AT3G61820.1 |  | Vvi-Vitvi15g00952\_t001 |  |  |  |  |  |  |  |
| 1 | Ath-AT3G61826.1 |  | | | |  |  |  |  |  |  |  |
| 1 | Ath-AT3G61829.1 |  | | | |  |  |  |  |  |  |  |
| 1 | Ath-AT3G61830.1 |  | Vvi-Vitvi15g00946\_t001 |  |  |  |  |  |  |  |
| 1 | Ath-AT3G61840.1 |  | | | |  |  |  |  |  |  |  |
| 1 | Ath-AT3G61850.4 |  | Vvi-Vitvi15g00936\_t001 |  |  |  |  |  |  |  |
| 1 | Ath-AT3G61860.1 |  | Vvi-Vitvi15g00926\_t003 |  |  |  |  |  |  |  |
| 1 | Ath-AT3G61870.1 |  | Vvi-Vitvi15g00919\_t001 |  |  |  |  |  |  |  |
| 2 | Ath-AT3G61880.2 |  | Vvi-Vitvi15g00915\_t001 |  | Vvi-Vitvi02g00226\_t001 |  |  |  |  |  |  |
| 2 | Ath-AT3G61890.1 |  | Vvi-Vitvi15g00912\_t001 |  | Vvi-Vitvi02g00228\_t001 |  |  |  |  |  |  |
| 2 | Ath-AT3G61898.1 |  | | | |  | | | |  |  |  |  |  |  |
| 2 | Ath-AT3G61900.1 |  | Vvi-Vitvi15g00910\_t001 |  | | | |  |  |  |  |  |  |
| 2 | Ath-AT3G61910.1 |  | Vvi-Vitvi15g00889\_t001 |  | Vvi-Vitvi02g00242\_t001 |  |  |  |  |  |  |
| 2 | Ath-AT3G61920.1 |  | Vvi-Vitvi15g00888\_t001 |  | Vvi-Vitvi02g00243\_t001 |  |  |  |  |  |  |
| 2 | Ath-AT3G61930.1 |  | | | |  | | | |  |  |  |  |  |  |
| 2 | Ath-AT3G61940.1 |  | Vvi-Vitvi15g00877\_t001 |  | Vvi-Vitvi02g01369\_t001 |  |  |  |  |  |  |
| 2 | Ath-AT3G61950.1 |  | Vvi-Vitvi15g00876\_t001 |  | | | |  |  |  |  |  |  |
| 2 | Ath-AT3G61960.1 |  | Vvi-Vitvi15g00869\_t001 |  | | | |  |  |  |  |  |  |
| 2 | Ath-AT3G61962.1 |  | | | |  | | | |  |  |  |  |  |  |
| 2 | Ath-AT3G61970.1 |  | Vvi-Vitvi15g00863\_t001 |  | Vvi-Vitvi02g00275\_t001 |  |  |  |  |  |  |
| 1 | Ath-AT3G61980.1 |  | | | |  |  |  |  |  |  |  |
| 1 | Ath-AT3G61990.1 |  | Vvi-Vitvi15g00855\_t001 |  |  |  |  |  |  |  |
| 0 | Ath-AT3G62000.2 |  |  |  |  |  |  |  |  |
| 0 | Ath-AT3G62010.1 |  |  |  |  |  |  |  |  |
| 1 | Ath-AT3G62020.1 |  | Vvi-Vitvi07g00502\_t001 |  |  |  |  |  |  |  |
| 1 | Ath-AT3G62030.2 |  | Vvi-Vitvi07g00508\_t001 |  |  |  |  |  |  |  |
| 1 | Ath-AT3G62040.1 |  | Vvi-Vitvi07g00509\_t001 |  |  |  |  |  |  |  |
| 1 | Ath-AT3G62050.1 |  | | | |  |  |  |  |  |  |  |
| 1 | Ath-AT3G62060.2 |  | Vvi-Vitvi07g02249\_t003 |  |  |  |  |  |  |  |
| 1 | Ath-AT3G62070.1 |  | Vvi-Vitvi07g02074\_t001 |  |  |  |  |  |  |  |
| 1 | Ath-AT3G62080.2 |  | Vvi-Vitvi07g00517\_t001 |  |  |  |  |  |  |  |
| 1 | Ath-AT3G62090.2 |  | Vvi-Vitvi07g02251\_t002 |  |  |  |  |  |  |  |
| 1 | Ath-AT3G62100.1 |  | Vvi-Vitvi07g00521\_t001 |  |  |  |  |  |  |  |
| 1 | Ath-AT3G62110.1 |  | Vvi-Vitvi07g00525\_t001 |  |  |  |  |  |  |  |
| 1 | Ath-AT3G62120.2 |  | Vvi-Vitvi07g00529\_t002 |  |  |  |  |  |  |  |
| 1 | Ath-AT3G62130.1 |  | Vvi-Vitvi07g00531\_t001 |  |  |  |  |  |  |  |
| 1 | Ath-AT3G62140.1 |  | Vvi-Vitvi07g00533\_t001 |  |  |  |  |  |  |  |
| 1 | Ath-AT3G62150.2 |  | Vvi-Vitvi07g00534\_t001 |  |  |  |  |  |  |  |
| 1 | Ath-AT3G62160.1 |  | Vvi-Vitvi07g00535\_t001 |  |  |  |  |  |  |  |
| 1 | Ath-AT3G62170.1 |  | | | |  |  |  |  |  |  |  |
| 1 | Ath-AT3G62180.2 |  | | | |  |  |  |  |  |  |  |
| 1 | Ath-AT3G62190.1 |  | Vvi-Vitvi07g00540\_t001 |  |  |  |  |  |  |  |
| 1 | Ath-AT3G62200.1 |  | Vvi-Vitvi07g00495\_t001 |  |  |  |  |  |  |  |
| 1 | Ath-AT3G62210.1 |  | | | |  |  |  |  |  |  |  |
| 1 | Ath-AT3G62220.1 |  | Vvi-Vitvi07g00492\_t001 |  |  |  |  |  |  |  |
| 1 | Ath-AT3G62230.1 |  | | | |  |  |  |  |  |  |  |
| 1 | Ath-AT3G62240.1 |  | Vvi-Vitvi07g00484\_t002 |  |  |  |  |  |  |  |
| 1 | Ath-AT3G62250.1 |  | Vvi-Vitvi07g00479\_t001 |  |  |  |  |  |  |  |
| 1 | Ath-AT3G62260.2 |  | Vvi-Vitvi07g00474\_t001 |  |  |  |  |  |  |  |
| 1 | Ath-AT3G62270.1 |  | Vvi-Vitvi07g00470\_t001 |  |  |  |  |  |  |  |
| 1 | Ath-AT3G62280.1 |  | Vvi-Vitvi07g04113\_t001 |  |  |  |  |  |  |  |
| 1 | Ath-AT3G62290.2 |  | Vvi-Vitvi07g00464\_t001 |  |  |  |  |  |  |  |
| 1 | Ath-AT3G62300.2 |  | Vvi-Vitvi07g00448\_t001 |  |  |  |  |  |  |  |
| 1 | Ath-AT3G62310.1 |  | Vvi-Vitvi07g00442\_t001 |  |  |  |  |  |  |  |
| 1 | Ath-AT3G62320.1 |  | | | |  |  |  |  |  |  |  |
| 1 | Ath-AT3G62330.1 |  | Vvi-Vitvi07g00436\_t001 |  |  |  |  |  |  |  |
| 1 | Ath-AT3G62340.1 |  | Vvi-Vitvi07g00434\_t001 |  |  |  |  |  |  |  |
| 1 | Ath-AT3G62350.1 |  | | | |  |  |  |  |  |  |  |
| 1 | Ath-AT3G62360.1 |  | Vvi-Vitvi07g00429\_t001 |  |  |  |  |  |  |  |
| 2 | Ath-AT3G62370.1 |  | | | |  | Vvi-Vitvi14g00088\_t001 |  |  |  |  |  |  |
| 2 | Ath-AT3G62380.1 |  | | | |  | | | |  |  |  |  |  |  |
| 2 | Ath-AT3G62390.1 |  | Vvi-Vitvi07g00416\_t001 |  | | | |  |  |  |  |  |  |
| 2 | Ath-AT3G62400.2 |  | | | |  | | | |  |  |  |  |  |  |
| 2 | Ath-AT3G62410.1 |  | | | |  | | | |  |  |  |  |  |  |
| 2 | Ath-AT3G62420.1 |  | Vvi-Vitvi07g00413\_t001 |  | Vvi-Vitvi14g00094\_t001 |  |  |  |  |  |  |
| 2 | Ath-AT3G62430.1 |  | | | |  | | | |  |  |  |  |  |  |
| 2 | Ath-AT3G62440.1 |  | | | |  | | | |  |  |  |  |  |  |
| 2 | Ath-AT3G62450.1 |  | | | |  | | | |  |  |  |  |  |  |
| 2 | Ath-AT3G62460.1 |  | | | |  | | | |  |  |  |  |  |  |
| 2 | Ath-AT3G62470.1 |  | | | |  | | | |  |  |  |  |  |  |
| 2 | Ath-AT3G62499.1 |  | | | |  | | | |  |  |  |  |  |  |
| 2 | Ath-AT3G62500.2 |  | | | |  | | | |  |  |  |  |  |  |
| 2 | Ath-AT3G62510.2 |  | | | |  | | | |  |  |  |  |  |  |
| 2 | Ath-AT3G62528.1 |  | | | |  | | | |  |  |  |  |  |  |
| 2 | Ath-AT3G62530.1 |  | | | |  | | | |  |  |  |  |  |  |
| 2 | Ath-AT3G62540.1 |  | | | |  | | | |  |  |  |  |  |  |
| 2 | Ath-AT3G62550.1 |  | Vvi-Vitvi07g00404\_t001 |  | Vvi-Vitvi14g02476\_t001 |  |  |  |  |  |  |
| 2 | Ath-AT3G62560.1 |  | Vvi-Vitvi07g00402\_t001 |  | Vvi-Vitvi14g04028\_t002 |  |  |  |  |  |  |
| 2 | Ath-AT3G62570.1 |  | Vvi-Vitvi07g00400\_t001 |  | | | |  |  |  |  |  |  |
| 2 | Ath-AT3G62580.1 |  | Vvi-Vitvi07g00396\_t001 |  | | | |  |  |  |  |  |  |
| 2 | Ath-AT3G62590.1 |  | Vvi-Vitvi07g00395\_t001 |  | | | |  |  |  |  |  |  |
| 2 | Ath-AT3G62600.1 |  | Vvi-Vitvi07g00394\_t001 |  | | | |  |  |  |  |  |  |
| 2 | Ath-AT3G62610.1 |  | Vvi-Vitvi07g00393\_t001 |  | | | |  |  |  |  |  |  |
| 2 | Ath-AT3G62615.1 |  | | | |  | | | |  |  |  |  |  |  |
| 2 | Ath-AT3G62620.1 |  | Vvi-Vitvi07g00392\_t001 |  | | | |  |  |  |  |  |  |
| 2 | Ath-AT3G62630.1 |  | Vvi-Vitvi07g00390\_t001 |  | Vvi-Vitvi14g00113\_t001 |  |  |  |  |  |  |
| 2 | Ath-AT3G62640.1 |  | Vvi-Vitvi07g02216\_t001 |  | Vvi-Vitvi14g02479\_t001 |  |  |  |  |  |  |
| 1 | Ath-AT3G62650.2 |  | Vvi-Vitvi07g02211\_t001 |  |  |  |  |  |  |  |
| 1 | Ath-AT3G62660.1 |  | Vvi-Vitvi07g00378\_t001 |  |  |  |  |  |  |  |
| 0 | Ath-AT3G62670.1 |  |  |  |  |  |  |  |  |
| 0 | Ath-AT3G62680.1 |  |  |  |  |  |  |  |  |
| 0 | Ath-AT3G62690.1 |  |  |  |  |  |  |  |  |
| 0 | Ath-AT3G62695.1 |  |  |  |  |  |  |  |  |
| 1 | Ath-AT3G62700.1 |  | Vvi-Vitvi07g00311\_t001 |  |  |  |  |  |  |  |
| 1 | Ath-AT3G62710.1 |  | | | |  |  |  |  |  |  |  |
| 2 | Ath-AT3G62720.2 |  | | | |  | Vvi-Vitvi07g00299\_t001 |  |  |  |  |  |  |
| 2 | Ath-AT3G62730.1 |  | | | |  | Vvi-Vitvi07g00292\_t001 |  |  |  |  |  |  |
| 2 | Ath-AT3G62740.1 |  | | | |  | | | |  |  |  |  |  |  |
| 2 | Ath-AT3G62750.2 |  | Vvi-Vitvi07g04076\_t001 |  | | | |  |  |  |  |  |  |
| 2 | Ath-AT3G62760.1 |  | | | |  | Vvi-Vitvi07g02188\_t003 |  |  |  |  |  |  |
| 2 | Ath-AT3G62770.1 |  | | | |  | Vvi-Vitvi07g00280\_t001 |  |  |  |  |  |  |
| 2 | Ath-AT3G62780.1 |  | | | |  | Vvi-Vitvi07g00278\_t001 |  |  |  |  |  |  |
| 2 | Ath-AT3G62790.1 |  | | | |  | | | |  |  |  |  |  |  |
| 2 | Ath-AT3G62800.1 |  | | | |  | | | |  |  |  |  |  |  |
| 2 | Ath-AT3G62810.1 |  | Vvi-Vitvi07g00322\_t001 |  | | | |  |  |  |  |  |  |
| 2 | Ath-AT3G62820.1 |  | Vvi-Vitvi07g00324\_t001 |  | | | |  |  |  |  |  |  |
| 2 | Ath-AT3G62830.1 |  | Vvi-Vitvi07g00325\_t001 |  | | | |  |  |  |  |  |  |
| 2 | Ath-AT3G62840.1 |  | Vvi-Vitvi07g04082\_t001 |  | | | |  |  |  |  |  |  |
| 2 | Ath-AT3G62850.1 |  | | | |  | | | |  |  |  |  |  |  |
| 2 | Ath-AT3G62860.1 |  | Vvi-Vitvi07g00327\_t001 |  | | | |  |  |  |  |  |  |
| 2 | Ath-AT3G62870.1 |  | | | |  | | | |  |  |  |  |  |  |
| 2 | Ath-AT3G62880.1 |  | Vvi-Vitvi07g00328\_t001 |  | | | |  |  |  |  |  |  |
| 2 | Ath-AT3G62890.1 |  | Vvi-Vitvi07g00331\_t001 |  | | | |  |  |  |  |  |  |
| 1 | Ath-AT3G62900.2 |  |  |  | Vvi-Vitvi07g00269\_t001 |  |  |  |  |  |  |
| 1 | Ath-AT3G62910.1 |  |  |  | Vvi-Vitvi07g00264\_t001 |  |  |  |  |  |  |
| 1 | Ath-AT3G62920.1 |  |  |  | Vvi-Vitvi07g02164\_t001 |  |  |  |  |  |  |
| 1 | Ath-AT3G62930.1 |  |  |  | Vvi-Vitvi07g00259\_t001 |  |  |  |  |  |  |
| 1 | Ath-AT3G62940.2 |  |  |  | Vvi-Vitvi07g00257\_t002 |  |  |  |  |  |  |
| 1 | Ath-AT3G62950.1 |  |  |  | Vvi-Vitvi07g00256\_t001 |  |  |  |  |  |  |
| 1 | Ath-AT3G62960.1 |  |  |  | Vvi-Vitvi07g00255\_t001 |  |  |  |  |  |  |
| 1 | Ath-AT3G62970.1 |  |  |  | Vvi-Vitvi07g00253\_t001 |  |  |  |  |  |  |
| 1 | Ath-AT3G62980.1 |  |  |  | Vvi-Vitvi07g00248\_t002 |  |  |  |  |  |  |
| 1 | Ath-AT3G62990.1 |  |  |  | | | |  |  |  |  |  |  |
| 1 | Ath-AT3G63000.1 |  |  |  | Vvi-Vitvi07g00228\_t001 |  |  |  |  |  |  |
| 1 | Ath-AT3G63010.1 |  |  |  | Vvi-Vitvi07g00217\_t001 |  |  |  |  |  |  |
| 1 | Ath-AT3G63020.1 |  |  |  | | | |  |  |  |  |  |  |
| 1 | Ath-AT3G63030.1 |  |  |  | Vvi-Vitvi07g00200\_t001 |  |  |  |  |  |  |
| 1 | Ath-AT3G63040.1 |  |  |  | | | |  |  |  |  |  |  |
| 1 | Ath-AT3G63050.1 |  |  |  | | | |  |  |  |  |  |  |
| 1 | Ath-AT3G63052.1 |  |  |  | | | |  |  |  |  |  |  |
| 2 | Ath-AT3G63060.1 |  | Vvi-Vitvi07g00164\_t001 |  | | | |  |  |  |  |  |  |
| 2 | Ath-AT3G63070.1 |  | Vvi-Vitvi07g00162\_t001 |  | | | |  |  |  |  |  |  |
| 2 | Ath-AT3G63080.1 |  | Vvi-Vitvi07g00160\_t001 |  | | | |  |  |  |  |  |  |
| 2 | Ath-AT3G63088.1 |  | | | |  | | | |  |  |  |  |  |  |
| 2 | Ath-AT3G63090.1 |  | Vvi-Vitvi07g00157\_t001 |  | | | |  |  |  |  |  |  |
| 2 | Ath-AT3G63093.1 |  | | | |  | | | |  |  |  |  |  |  |
| 2 | Ath-AT3G63095.1 |  | Vvi-Vitvi07g00155\_t001 |  | | | |  |  |  |  |  |  |
| 2 | Ath-AT3G63110.1 |  | Vvi-Vitvi07g00154\_t001 |  | | | |  |  |  |  |  |  |
| 2 | Ath-AT3G63120.2 |  | | | |  | Vvi-Vitvi07g00181\_t001 |  |  |  |  |  |  |
| 2 | Ath-AT3G63130.1 |  | | | |  | Vvi-Vitvi07g00179\_t001.1.6037826e |  |  |  |  |  |  |
| 2 | Ath-AT3G63140.1 |  | | | |  | Vvi-Vitvi07g00178\_t001 |  |  |  |  |  |  |
| 2 | Ath-AT3G63150.1 |  | | | |  | Vvi-Vitvi07g00173\_t003 |  |  |  |  |  |  |
| 1 | Ath-AT3G63160.1 |  | | | |  |  |  |  |  |  |  |
| 1 | Ath-AT3G63170.1 |  | | | |  |  |  |  |  |  |  |
| 1 | Ath-AT3G63180.1 |  | | | |  |  |  |  |  |  |  |
| 1 | Ath-AT3G63190.1 |  | | | |  |  |  |  |  |  |  |
| 1 | Ath-AT3G63200.1 |  | Vvi-Vitvi07g02137\_t001 |  |  |  |  |  |  |  |
| 2 | Ath-AT3G63210.1 |  | Vvi-Vitvi07g00114\_t001 |  | Vvi-Vitvi05g04122\_t001 |  |  |  |  |  |  |
| 2 | Ath-AT3G63215.1 |  | | | |  | | | |  |  |  |  |  |  |
| 2 | Ath-AT3G63220.2 |  | Vvi-Vitvi07g00112\_t001 |  | | | |  |  |  |  |  |  |
| 2 | Ath-AT3G63230.2 |  | | | |  | | | |  |  |  |  |  |  |
| 2 | Ath-AT3G63240.1 |  | Vvi-Vitvi07g00105\_t001 |  | Vvi-Vitvi05g00533\_t001 |  |  |  |  |  |  |
| 2 | Ath-AT3G63250.1 |  | | | |  | Vvi-Vitvi05g00539\_t003 |  |  |  |  |  |  |
| 2 | Ath-AT3G63255.1 |  | | | |  | | | |  |  |  |  |  |  |
| 2 | Ath-AT3G63260.1 |  | Vvi-Vitvi07g04032\_t001 |  | Vvi-Vitvi05g00540\_t001 |  |  |  |  |  |  |
| 2 | Ath-AT3G63270.1 |  | | | |  | | | |  |  |  |  |  |  |
| 2 | Ath-AT3G63280.4 |  | Vvi-Vitvi07g00090\_t001 |  | | | |  |  |  |  |  |  |
| 2 | Ath-AT3G63290.1 |  | Vvi-Vitvi07g04029\_t001 |  | | | |  |  |  |  |  |  |
| 2 | Ath-AT3G63300.1 |  | Vvi-Vitvi07g00088\_t001 |  | Vvi-Vitvi05g00554\_t001 |  |  |  |  |  |  |
| 2 | Ath-AT3G63310.1 |  | Vvi-Vitvi07g00087\_t001 |  | Vvi-Vitvi05g00555\_t001 |  |  |  |  |  |  |
| 2 | Ath-AT3G63320.1 |  | Vvi-Vitvi07g00079\_t001 |  | | | |  |  |  |  |  |  |
| 2 | Ath-AT3G63340.2 |  | | | |  | | | |  |  |  |  |  |  |
| 2 | Ath-AT3G63350.1 |  | Vvi-Vitvi07g00078\_t001 |  | | | |  |  |  |  |  |  |
| 2 | Ath-AT3G63360.1 |  | | | |  | | | |  |  |  |  |  |  |
| 2 | Ath-AT3G63370.1 |  | Vvi-Vitvi07g00072\_t001 |  | | | |  |  |  |  |  |  |
| 2 | Ath-AT3G63380.1 |  | Vvi-Vitvi07g00071\_t001 |  | Vvi-Vitvi05g00577\_t001 |  |  |  |  |  |  |
| 1 | Ath-AT3G63390.1 |  | Vvi-Vitvi07g00069\_t001 |  |  |  |  |  |  |  |
| 1 | Ath-AT3G63400.4 |  | Vvi-Vitvi07g00067\_t001 |  |  |  |  |  |  |  |
| 1 | Ath-AT3G63410.1 |  | Vvi-Vitvi07g00066\_t001 |  |  |  |  |  |  |  |
| 1 | Ath-AT3G63420.3 |  | | | |  |  |  |  |  |  |  |
| 1 | Ath-AT3G63430.1 |  | Vvi-Vitvi07g00064\_t001 |  |  |  |  |  |  |  |
| 0 | Ath-AT3G63440.1 |  |  |  |  |  |  |  |  |
| 0 | Ath-AT3G63450.4 |  |  |  |  |  |  |  |  |
| 0 | Ath-AT3G63460.1 |  |  |  |  |  |  |  |  |
| 0 | Ath-AT3G63470.1 |  |  |  |  |  |  |  |  |
| 0 | Ath-AT3G63480.1 |  |  |  |  |  |  |  |  |
| 0 | Ath-AT3G63490.1 |  |  |  |  |  |  |  |  |
| 0 | Ath-AT3G63500.2 |  |  |  |  |  |  |  |  |
| 0 | Ath-AT3G63510.1 |  |  |  |  |  |  |  |  |
| 0 | Ath-AT3G63520.1 |  |  |  |  |  |  |  |  |
| 0 | Ath-AT3G63530.2 |  |  |  |  |  |  |  |  |
| 0 | Ath-AT3G63540.1 |  |  |  |  |  |  |  |  |
