# Supplementary material for: Genome-wide identification, characterization and gene expression of BES1 transcription factor family in grapevine (Vitis vinifera L.)
Source: Sci Rep. 2023 Jan 5;13:240. doi: 10.1038/s41598-022-24407-y (PMC9816167; doi:10.1038/s41598-022-24407-y)
Supplement: Supplementary file 3 — Supplementary Information. [file 41598_2022_24407_MOESM3_ESM.zip › Vvi_Ath/Vitis_vinifera.PN40024.v4.dna_sm.toplevel.fa.vs.Arabidopsis_thaliana.TAIR10.dna_sm.toplevel.fa.html/Ath-4.html]

|  |  |  |  |  |  |  |  |  |  |  |  |  |  |  |  |  |  |
| --- | --- | --- | --- | --- | --- | --- | --- | --- | --- | --- | --- | --- | --- | --- | --- | --- | --- |
| Duplication depth | Reference chromosome | Collinear blocks | | | | | | | | | | | | | | | |
| 0 | Ath-AT4G00005.1 |  |  |  |  |  |  |  |  |
| 0 | Ath-AT4G00020.2 |  |  |  |  |  |  |  |  |
| 0 | Ath-AT4G00026.1 |  |  |  |  |  |  |  |  |
| 0 | Ath-AT4G00030.1 |  |  |  |  |  |  |  |  |
| 0 | Ath-AT4G00040.1 |  |  |  |  |  |  |  |  |
| 0 | Ath-AT4G00050.1 |  |  |  |  |  |  |  |  |
| 0 | Ath-AT4G00060.1 |  |  |  |  |  |  |  |  |
| 0 | Ath-AT4G00070.1 |  |  |  |  |  |  |  |  |
| 0 | Ath-AT4G00080.1 |  |  |  |  |  |  |  |  |
| 0 | Ath-AT4G00090.1 |  |  |  |  |  |  |  |  |
| 0 | Ath-AT4G00100.1 |  |  |  |  |  |  |  |  |
| 0 | Ath-AT4G00110.1 |  |  |  |  |  |  |  |  |
| 0 | Ath-AT4G00120.1 |  |  |  |  |  |  |  |  |
| 0 | Ath-AT4G00130.1 |  |  |  |  |  |  |  |  |
| 0 | Ath-AT4G00140.1 |  |  |  |  |  |  |  |  |
| 0 | Ath-AT4G00150.1 |  |  |  |  |  |  |  |  |
| 0 | Ath-AT4G00155.1 |  |  |  |  |  |  |  |  |
| 0 | Ath-AT4G00160.1 |  |  |  |  |  |  |  |  |
| 0 | Ath-AT4G00163.1 |  |  |  |  |  |  |  |  |
| 0 | Ath-AT4G00165.1 |  |  |  |  |  |  |  |  |
| 0 | Ath-AT4G00170.1 |  |  |  |  |  |  |  |  |
| 0 | Ath-AT4G00180.1 |  |  |  |  |  |  |  |  |
| 0 | Ath-AT4G00190.1 |  |  |  |  |  |  |  |  |
| 0 | Ath-AT4G00200.1 |  |  |  |  |  |  |  |  |
| 0 | Ath-AT4G00210.1 |  |  |  |  |  |  |  |  |
| 0 | Ath-AT4G00220.1 |  |  |  |  |  |  |  |  |
| 0 | Ath-AT4G00230.1 |  |  |  |  |  |  |  |  |
| 0 | Ath-AT4G00231.1 |  |  |  |  |  |  |  |  |
| 0 | Ath-AT4G00232.1 |  |  |  |  |  |  |  |  |
| 0 | Ath-AT4G00234.1 |  |  |  |  |  |  |  |  |
| 0 | Ath-AT4G00238.1 |  |  |  |  |  |  |  |  |
| 0 | Ath-AT4G00240.1 |  |  |  |  |  |  |  |  |
| 0 | Ath-AT4G00250.1 |  |  |  |  |  |  |  |  |
| 0 | Ath-AT4G00260.1 |  |  |  |  |  |  |  |  |
| 0 | Ath-AT4G00270.1 |  |  |  |  |  |  |  |  |
| 0 | Ath-AT4G00280.1 |  |  |  |  |  |  |  |  |
| 1 | Ath-AT4G00290.1 |  | Vvi-Vitvi15g01072\_t001 |  |  |  |  |  |  |  |
| 1 | Ath-AT4G00295.1 |  | | | |  |  |  |  |  |  |  |
| 1 | Ath-AT4G00300.1 |  | Vvi-Vitvi15g01071\_t001 |  |  |  |  |  |  |  |
| 1 | Ath-AT4G00305.1 |  | | | |  |  |  |  |  |  |  |
| 1 | Ath-AT4G00310.1 |  | Vvi-Vitvi15g01065\_t001 |  |  |  |  |  |  |  |
| 1 | Ath-AT4G00315.1 |  | | | |  |  |  |  |  |  |  |
| 1 | Ath-AT4G00320.1 |  | | | |  |  |  |  |  |  |  |
| 1 | Ath-AT4G00330.1 |  | Vvi-Vitvi15g01060\_t001 |  |  |  |  |  |  |  |
| 1 | Ath-AT4G00335.3 |  | Vvi-Vitvi15g01059\_t001 |  |  |  |  |  |  |  |
| 1 | Ath-AT4G00340.1 |  | Vvi-Vitvi15g01058\_t001 |  |  |  |  |  |  |  |
| 1 | Ath-AT4G00342.1 |  | | | |  |  |  |  |  |  |  |
| 1 | Ath-AT4G00350.1 |  | Vvi-Vitvi15g01057\_t001 |  |  |  |  |  |  |  |
| 1 | Ath-AT4G00355.1 |  | Vvi-Vitvi15g04613\_t001 |  |  |  |  |  |  |  |
| 1 | Ath-AT4G00360.1 |  | Vvi-Vitvi15g01106\_t001 |  |  |  |  |  |  |  |
| 1 | Ath-AT4G00370.1 |  | Vvi-Vitvi15g01107\_t001 |  |  |  |  |  |  |  |
| 1 | Ath-AT4G00380.1 |  | | | |  |  |  |  |  |  |  |
| 1 | Ath-AT4G00390.1 |  | | | |  |  |  |  |  |  |  |
| 1 | Ath-AT4G00400.1 |  | Vvi-Vitvi15g01108\_t001 |  |  |  |  |  |  |  |
| 1 | Ath-AT4G00416.1 |  | | | |  |  |  |  |  |  |  |
| 1 | Ath-AT4G00420.4 |  | | | |  |  |  |  |  |  |  |
| 1 | Ath-AT4G00430.1 |  | Vvi-Vitvi15g01110\_t002 |  |  |  |  |  |  |  |
| 1 | Ath-AT4G00440.2 |  | Vvi-Vitvi15g01117\_t002 |  |  |  |  |  |  |  |
| 1 | Ath-AT4G00450.3 |  | Vvi-Vitvi15g01118\_t005 |  |  |  |  |  |  |  |
| 1 | Ath-AT4G00460.2 |  | Vvi-Vitvi15g01121\_t001 |  |  |  |  |  |  |  |
| 1 | Ath-AT4G00467.1 |  | Vvi-Vitvi15g04616\_t001 |  |  |  |  |  |  |  |
| 1 | Ath-AT4G00480.2 |  | Vvi-Vitvi15g01124\_t001 |  |  |  |  |  |  |  |
| 1 | Ath-AT4G00490.1 |  | Vvi-Vitvi15g01127\_t001 |  |  |  |  |  |  |  |
| 1 | Ath-AT4G00500.2 |  | Vvi-Vitvi15g01129\_t002 |  |  |  |  |  |  |  |
| 1 | Ath-AT4G00520.2 |  | Vvi-Vitvi15g01135\_t001 |  |  |  |  |  |  |  |
| 1 | Ath-AT4G00525.1 |  | Vvi-Vitvi15g04625\_t001 |  |  |  |  |  |  |  |
| 1 | Ath-AT4G00530.1 |  | Vvi-Vitvi15g01672\_t001 |  |  |  |  |  |  |  |
| 2 | Ath-AT4G00540.1 |  | | | |  | Vvi-Vitvi15g00847\_t001 |  |  |  |  |  |  |
| 3 | Ath-AT4G00550.2 |  | | | |  | | | |  | Vvi-Vitvi15g01200\_t001 |  |  |  |  |  |
| 3 | Ath-AT4G00560.4 |  | | | |  | | | |  | Vvi-Vitvi15g01191\_t002 |  |  |  |  |  |
| 3 | Ath-AT4G00570.1 |  | | | |  | | | |  | Vvi-Vitvi15g01188\_t001 |  |  |  |  |  |
| 3 | Ath-AT4G00580.1 |  | | | |  | | | |  | | | |  |  |  |  |  |
| 3 | Ath-AT4G00585.1 |  | | | |  | | | |  | | | |  |  |  |  |  |
| 3 | Ath-AT4G00590.1 |  | | | |  | | | |  | Vvi-Vitvi15g01174\_t001 |  |  |  |  |  |
| 3 | Ath-AT4G00600.1 |  | | | |  | | | |  | Vvi-Vitvi15g01173\_t001 |  |  |  |  |  |
| 3 | Ath-AT4G00610.1 |  | | | |  | | | |  | | | |  |  |  |  |  |
| 3 | Ath-AT4G00620.1 |  | | | |  | | | |  | | | |  |  |  |  |  |
| 3 | Ath-AT4G00630.2 |  | | | |  | | | |  | Vvi-Vitvi15g01172\_t002 |  |  |  |  |  |
| 3 | Ath-AT4G00650.1 |  | | | |  | | | |  | | | |  |  |  |  |  |
| 3 | Ath-AT4G00651.1 |  | | | |  | | | |  | | | |  |  |  |  |  |
| 3 | Ath-AT4G00660.2 |  | | | |  | | | |  | Vvi-Vitvi15g01162\_t002 |  |  |  |  |  |
| 3 | Ath-AT4G00670.1 |  | | | |  | | | |  | Vvi-Vitvi15g01160\_t001 |  |  |  |  |  |
| 3 | Ath-AT4G00680.2 |  | Vvi-Vitvi15g01148\_t001 |  | | | |  | | | |  |  |  |  |  |
| 2 | Ath-AT4G00690.1 |  |  |  | | | |  | | | |  |  |  |  |  |
| 2 | Ath-AT4G00695.3 |  |  |  | | | |  | | | |  |  |  |  |  |
| 2 | Ath-AT4G00700.2 |  |  |  | | | |  | Vvi-Vitvi15g01147\_t001 |  |  |  |  |  |
| 2 | Ath-AT4G00710.1 |  |  |  | | | |  | Vvi-Vitvi15g01144\_t001 |  |  |  |  |  |
| 1 | Ath-AT4G00720.1 |  |  |  | Vvi-Vitvi15g00840\_t001 |  |  |  |  |  |  |
| 1 | Ath-AT4G00730.1 |  |  |  | Vvi-Vitvi15g00839\_t001 |  |  |  |  |  |  |
| 1 | Ath-AT4G00740.1 |  |  |  | Vvi-Vitvi15g00838\_t003 |  |  |  |  |  |  |
| 1 | Ath-AT4G00750.1 |  |  |  | Vvi-Vitvi15g00833\_t001 |  |  |  |  |  |  |
| 1 | Ath-AT4G00752.1 |  |  |  | | | |  |  |  |  |  |  |
| 1 | Ath-AT4G00755.2 |  |  |  | Vvi-Vitvi15g00831\_t002 |  |  |  |  |  |  |
| 0 | Ath-AT4G00760.1 |  |  |  |  |  |  |  |  |
| 0 | Ath-AT4G00770.1 |  |  |  |  |  |  |  |  |
| 0 | Ath-AT4G00780.1 |  |  |  |  |  |  |  |  |
| 1 | Ath-AT4G00800.1 |  | Vvi-Vitvi15g00884\_t001 |  |  |  |  |  |  |  |
| 1 | Ath-AT4G00810.1 |  | Vvi-Vitvi15g04470\_t001 |  |  |  |  |  |  |  |
| 1 | Ath-AT4G00820.1 |  | Vvi-Vitvi15g00893\_t001 |  |  |  |  |  |  |  |
| 1 | Ath-AT4G00830.1 |  | Vvi-Vitvi15g00895\_t001 |  |  |  |  |  |  |  |
| 1 | Ath-AT4G00840.1 |  | Vvi-Vitvi15g00902\_t001 |  |  |  |  |  |  |  |
| 1 | Ath-AT4G00850.1 |  | Vvi-Vitvi15g00903\_t001 |  |  |  |  |  |  |  |
| 1 | Ath-AT4G00860.1 |  | | | |  |  |  |  |  |  |  |
| 1 | Ath-AT4G00870.1 |  | Vvi-Vitvi15g00906\_t001 |  |  |  |  |  |  |  |
| 1 | Ath-AT4G00872.1 |  | | | |  |  |  |  |  |  |  |
| 1 | Ath-AT4G00880.1 |  | Vvi-Vitvi15g00910\_t001 |  |  |  |  |  |  |  |
| 0 | Ath-AT4G00883.1 |  |  |  |  |  |  |  |  |
| 0 | Ath-AT4G00890.1 |  |  |  |  |  |  |  |  |
| 0 | Ath-AT4G00889.1 |  |  |  |  |  |  |  |  |
| 0 | Ath-AT4G00893.1 |  |  |  |  |  |  |  |  |
| 0 | Ath-AT4G00895.1 |  |  |  |  |  |  |  |  |
| 0 | Ath-AT4G00900.2 |  |  |  |  |  |  |  |  |
| 0 | Ath-AT4G00910.1 |  |  |  |  |  |  |  |  |
| 0 | Ath-AT4G00905.1 |  |  |  |  |  |  |  |  |
| 0 | Ath-AT4G00920.1 |  |  |  |  |  |  |  |  |
| 0 | Ath-AT4G00925.1 |  |  |  |  |  |  |  |  |
| 0 | Ath-AT4G00930.1 |  |  |  |  |  |  |  |  |
| 0 | Ath-AT4G00940.2 |  |  |  |  |  |  |  |  |
| 0 | Ath-AT4G00950.1 |  |  |  |  |  |  |  |  |
| 0 | Ath-AT4G00953.1 |  |  |  |  |  |  |  |  |
| 0 | Ath-AT4G00955.2 |  |  |  |  |  |  |  |  |
| 0 | Ath-AT4G00960.1 |  |  |  |  |  |  |  |  |
| 0 | Ath-AT4G00970.1 |  |  |  |  |  |  |  |  |
| 0 | Ath-AT4G00980.1 |  |  |  |  |  |  |  |  |
| 0 | Ath-AT4G00990.1 |  |  |  |  |  |  |  |  |
| 1 | Ath-AT4G01000.1 |  | Vvi-Vitvi15g00985\_t001 |  |  |  |  |  |  |  |
| 1 | Ath-AT4G01010.1 |  | Vvi-Vitvi15g01617\_t001 |  |  |  |  |  |  |  |
| 1 | Ath-AT4G01020.1 |  | | | |  |  |  |  |  |  |  |
| 1 | Ath-AT4G01023.2 |  | Vvi-Vitvi15g00994\_t001 |  |  |  |  |  |  |  |
| 2 | Ath-AT4G01026.1 |  | Vvi-Vitvi15g00997\_t001 |  | Vvi-Vitvi02g00119\_t001 |  |  |  |  |  |  |
| 2 | Ath-AT4G01030.1 |  | Vvi-Vitvi15g00998\_t001 |  | | | |  |  |  |  |  |  |
| 2 | Ath-AT4G01037.1 |  | Vvi-Vitvi15g00999\_t001 |  | | | |  |  |  |  |  |  |
| 2 | Ath-AT4G01040.1 |  | Vvi-Vitvi15g01000\_t002 |  | | | |  |  |  |  |  |  |
| 2 | Ath-AT4G01050.2 |  | Vvi-Vitvi15g01619\_t001 |  | | | |  |  |  |  |  |  |
| 2 | Ath-AT4G01060.1 |  | Vvi-Vitvi15g01002\_t001 |  | | | |  |  |  |  |  |  |
| 2 | Ath-AT4G01070.1 |  | Vvi-Vitvi15g01623\_t001 |  | Vvi-Vitvi02g00110\_t001 |  |  |  |  |  |  |
| 2 | Ath-AT4G01080.1 |  | Vvi-Vitvi15g01011\_t001 |  | Vvi-Vitvi02g00106\_t001 |  |  |  |  |  |  |
| 2 | Ath-AT4G01090.1 |  | Vvi-Vitvi15g01013\_t001 |  | | | |  |  |  |  |  |  |
| 2 | Ath-AT4G01100.2 |  | Vvi-Vitvi15g01015\_t001 |  | Vvi-Vitvi02g00103\_t001 |  |  |  |  |  |  |
| 2 | Ath-AT4G01110.1 |  | Vvi-Vitvi15g01024\_t001 |  | | | |  |  |  |  |  |  |
| 2 | Ath-AT4G01120.1 |  | Vvi-Vitvi15g01027\_t001 |  | Vvi-Vitvi02g00089\_t001 |  |  |  |  |  |  |
| 2 | Ath-AT4G01130.1 |  | Vvi-Vitvi15g01028\_t001 |  | | | |  |  |  |  |  |  |
| 2 | Ath-AT4G01140.1 |  | Vvi-Vitvi15g01029\_t001 |  | Vvi-Vitvi02g00079\_t001 |  |  |  |  |  |  |
| 2 | Ath-AT4G01150.1 |  | Vvi-Vitvi15g01030\_t001 |  | | | |  |  |  |  |  |  |
| 2 | Ath-AT4G01160.2 |  | Vvi-Vitvi15g01031\_t001 |  | Vvi-Vitvi02g00078\_t001 |  |  |  |  |  |  |
| 1 | Ath-AT4G01170.1 |  | | | |  |  |  |  |  |  |  |
| 1 | Ath-AT4G01180.1 |  | | | |  |  |  |  |  |  |  |
| 1 | Ath-AT4G01190.2 |  | | | |  |  |  |  |  |  |  |
| 1 | Ath-AT4G01200.1 |  | Vvi-Vitvi15g01634\_t001 |  |  |  |  |  |  |  |
| 1 | Ath-AT4G01210.1 |  | Vvi-Vitvi15g01045\_t001 |  |  |  |  |  |  |  |
| 1 | Ath-AT4G01220.1 |  | Vvi-Vitvi15g01046\_t001 |  |  |  |  |  |  |  |
| 1 | Ath-AT4G01230.2 |  | | | |  |  |  |  |  |  |  |
| 1 | Ath-AT4G01240.1 |  | | | |  |  |  |  |  |  |  |
| 1 | Ath-AT4G01250.1 |  | | | |  |  |  |  |  |  |  |
| 1 | Ath-AT4G01245.1 |  | | | |  |  |  |  |  |  |  |
| 1 | Ath-AT4G01260.1 |  | | | |  |  |  |  |  |  |  |
| 1 | Ath-AT4G01270.1 |  | | | |  |  |  |  |  |  |  |
| 1 | Ath-AT4G01280.2 |  | | | |  |  |  |  |  |  |  |
| 1 | Ath-AT4G01290.1 |  | | | |  |  |  |  |  |  |  |
| 1 | Ath-AT4G01310.1 |  | Vvi-Vitvi15g01055\_t001 |  |  |  |  |  |  |  |
| 0 | Ath-AT4G01320.1 |  |  |  |  |  |  |  |  |
| 0 | Ath-AT4G01330.2 |  |  |  |  |  |  |  |  |
| 0 | Ath-AT4G01335.1 |  |  |  |  |  |  |  |  |
| 0 | Ath-AT4G01340.1 |  |  |  |  |  |  |  |  |
| 0 | Ath-AT4G01350.1 |  |  |  |  |  |  |  |  |
| 0 | Ath-AT4G01360.1 |  |  |  |  |  |  |  |  |
| 0 | Ath-AT4G01370.1 |  |  |  |  |  |  |  |  |
| 0 | Ath-AT4G01380.1 |  |  |  |  |  |  |  |  |
| 0 | Ath-AT4G01390.1 |  |  |  |  |  |  |  |  |
| 0 | Ath-AT4G01395.1 |  |  |  |  |  |  |  |  |
| 0 | Ath-AT4G01400.1 |  |  |  |  |  |  |  |  |
| 0 | Ath-AT4G01410.1 |  |  |  |  |  |  |  |  |
| 0 | Ath-AT4G01420.1 |  |  |  |  |  |  |  |  |
| 0 | Ath-AT4G01430.1 |  |  |  |  |  |  |  |  |
| 0 | Ath-AT4G01435.1 |  |  |  |  |  |  |  |  |
| 0 | Ath-AT4G01440.1 |  |  |  |  |  |  |  |  |
| 0 | Ath-AT4G01450.2 |  |  |  |  |  |  |  |  |
| 0 | Ath-AT4G01460.1 |  |  |  |  |  |  |  |  |
| 0 | Ath-AT4G01470.1 |  |  |  |  |  |  |  |  |
| 0 | Ath-AT4G01480.1 |  |  |  |  |  |  |  |  |
| 0 | Ath-AT4G01500.1 |  |  |  |  |  |  |  |  |
| 0 | Ath-AT4G01510.5 |  |  |  |  |  |  |  |  |
| 0 | Ath-AT4G01516.1 |  |  |  |  |  |  |  |  |
| 0 | Ath-AT4G01520.1 |  |  |  |  |  |  |  |  |
| 0 | Ath-AT4G01535.1 |  |  |  |  |  |  |  |  |
| 0 | Ath-AT4G01540.1 |  |  |  |  |  |  |  |  |
| 0 | Ath-AT4G01550.1 |  |  |  |  |  |  |  |  |
| 0 | Ath-AT4G01560.1 |  |  |  |  |  |  |  |  |
| 0 | Ath-AT4G01570.1 |  |  |  |  |  |  |  |  |
| 0 | Ath-AT4G01575.1 |  |  |  |  |  |  |  |  |
| 0 | Ath-AT4G01580.1 |  |  |  |  |  |  |  |  |
| 0 | Ath-AT4G01590.3 |  |  |  |  |  |  |  |  |
| 0 | Ath-AT4G01595.1 |  |  |  |  |  |  |  |  |
| 0 | Ath-AT4G01600.1 |  |  |  |  |  |  |  |  |
| 0 | Ath-AT4G01610.1 |  |  |  |  |  |  |  |  |
| 1 | Ath-AT4G01630.1 |  | Vvi-Vitvi07g00496\_t001 |  |  |  |  |  |  |  |
| 1 | Ath-AT4G01640.1 |  | | | |  |  |  |  |  |  |  |
| 2 | Ath-AT4G01650.1 |  | Vvi-Vitvi07g00507\_t001 |  | Vvi-Vitvi07g00507\_t001 |  |  |  |  |  |  |
| 2 | Ath-AT4G01660.1 |  | Vvi-Vitvi07g00510\_t001 |  | | | |  |  |  |  |  |  |
| 2 | Ath-AT4G01670.1 |  | Vvi-Vitvi07g02074\_t001 |  | | | |  |  |  |  |  |  |
| 2 | Ath-AT4G01671.1 |  | | | |  | | | |  |  |  |  |  |  |
| 2 | Ath-AT4G01680.2 |  | Vvi-Vitvi07g00515\_t001 |  | | | |  |  |  |  |  |  |
| 2 | Ath-AT4G01690.1 |  | Vvi-Vitvi07g00520\_t001 |  | | | |  |  |  |  |  |  |
| 2 | Ath-AT4G01700.1 |  | Vvi-Vitvi07g00522\_t001 |  | | | |  |  |  |  |  |  |
| 2 | Ath-AT4G01703.1 |  | | | |  | | | |  |  |  |  |  |  |
| 2 | Ath-AT4G01710.1 |  | | | |  | | | |  |  |  |  |  |  |
| 2 | Ath-AT4G01720.1 |  | Vvi-Vitvi07g00523\_t001 |  | | | |  |  |  |  |  |  |
| 2 | Ath-AT4G01730.1 |  | Vvi-Vitvi07g00524\_t001 |  | | | |  |  |  |  |  |  |
| 2 | Ath-AT4G01735.1 |  | | | |  | | | |  |  |  |  |  |  |
| 2 | Ath-AT4G01740.1 |  | | | |  | | | |  |  |  |  |  |  |
| 2 | Ath-AT4G01750.1 |  | | | |  | | | |  |  |  |  |  |  |
| 2 | Ath-AT4G01760.1 |  | | | |  | | | |  |  |  |  |  |  |
| 2 | Ath-AT4G01770.2 |  | | | |  | | | |  |  |  |  |  |  |
| 2 | Ath-AT4G01780.1 |  | | | |  | | | |  |  |  |  |  |  |
| 2 | Ath-AT4G01790.1 |  | Vvi-Vitvi07g00526\_t001 |  | | | |  |  |  |  |  |  |
| 2 | Ath-AT4G01800.2 |  | Vvi-Vitvi07g00527\_t001 |  | | | |  |  |  |  |  |  |
| 2 | Ath-AT4G01810.1 |  | Vvi-Vitvi07g00530\_t001 |  | | | |  |  |  |  |  |  |
| 2 | Ath-AT4G01820.1 |  | | | |  | | | |  |  |  |  |  |  |
| 2 | Ath-AT4G01830.1 |  | Vvi-Vitvi07g00534\_t001 |  | | | |  |  |  |  |  |  |
| 2 | Ath-AT4G01840.1 |  | Vvi-Vitvi07g00537\_t001 |  | | | |  |  |  |  |  |  |
| 1 | Ath-AT4G01850.1 |  |  |  | Vvi-Vitvi07g02247\_t001 |  |  |  |  |  |  |
| 1 | Ath-AT4G01860.2 |  |  |  | Vvi-Vitvi07g00486\_t001 |  |  |  |  |  |  |
| 1 | Ath-AT4G01870.2 |  |  |  | | | |  |  |  |  |  |  |
| 1 | Ath-AT4G01880.1 |  |  |  | Vvi-Vitvi07g00477\_t001 |  |  |  |  |  |  |
| 1 | Ath-AT4G01883.1 |  |  |  | Vvi-Vitvi07g00475\_t001 |  |  |  |  |  |  |
| 1 | Ath-AT4G01890.1 |  |  |  | Vvi-Vitvi07g00471\_t001 |  |  |  |  |  |  |
| 1 | Ath-AT4G01895.1 |  |  |  | | | |  |  |  |  |  |  |
| 1 | Ath-AT4G01897.1 |  |  |  | Vvi-Vitvi07g04112\_t001 |  |  |  |  |  |  |
| 1 | Ath-AT4G01900.1 |  |  |  | Vvi-Vitvi07g00465\_t001 |  |  |  |  |  |  |
| 1 | Ath-AT4G01910.1 |  |  |  | | | |  |  |  |  |  |  |
| 1 | Ath-AT4G01915.3 |  |  |  | | | |  |  |  |  |  |  |
| 1 | Ath-AT4G01920.1 |  |  |  | | | |  |  |  |  |  |  |
| 1 | Ath-AT4G01925.1 |  |  |  | | | |  |  |  |  |  |  |
| 1 | Ath-AT4G01930.1 |  |  |  | | | |  |  |  |  |  |  |
| 1 | Ath-AT4G01935.1 |  |  |  | Vvi-Vitvi07g00450\_t001 |  |  |  |  |  |  |
| 1 | Ath-AT4G01940.1 |  |  |  | Vvi-Vitvi07g00449\_t001 |  |  |  |  |  |  |
| 1 | Ath-AT4G01950.2 |  |  |  | Vvi-Vitvi07g00441\_t001 |  |  |  |  |  |  |
| 1 | Ath-AT4G01960.2 |  |  |  | Vvi-Vitvi07g00439\_t001 |  |  |  |  |  |  |
| 1 | Ath-AT4G01970.2 |  |  |  | Vvi-Vitvi07g00431\_t001 |  |  |  |  |  |  |
| 1 | Ath-AT4G01985.1 |  |  |  | | | |  |  |  |  |  |  |
| 1 | Ath-AT4G01990.1 |  |  |  | Vvi-Vitvi07g00427\_t001 |  |  |  |  |  |  |
| 1 | Ath-AT4G01995.1 |  |  |  | Vvi-Vitvi07g00426\_t001 |  |  |  |  |  |  |
| 1 | Ath-AT4G02000.1 |  |  |  | | | |  |  |  |  |  |  |
| 1 | Ath-AT4G02010.1 |  |  |  | Vvi-Vitvi07g00424\_t001 |  |  |  |  |  |  |
| 1 | Ath-AT4G02020.1 |  |  |  | Vvi-Vitvi07g00417\_t001 |  |  |  |  |  |  |
| 1 | Ath-AT4G02030.2 |  |  |  | Vvi-Vitvi07g00412\_t001 |  |  |  |  |  |  |
| 1 | Ath-AT4G02040.1 |  |  |  | | | |  |  |  |  |  |  |
| 1 | Ath-AT4G02050.1 |  |  |  | | | |  |  |  |  |  |  |
| 1 | Ath-AT4G02060.2 |  |  |  | Vvi-Vitvi07g00411\_t001 |  |  |  |  |  |  |
| 1 | Ath-AT4G02070.1 |  |  |  | Vvi-Vitvi07g00406\_t001 |  |  |  |  |  |  |
| 1 | Ath-AT4G02075.1 |  |  |  | Vvi-Vitvi07g00405\_t001 |  |  |  |  |  |  |
| 1 | Ath-AT4G02080.1 |  |  |  | Vvi-Vitvi07g00402\_t001 |  |  |  |  |  |  |
| 1 | Ath-AT4G02090.1 |  |  |  | Vvi-Vitvi07g02220\_t001 |  |  |  |  |  |  |
| 1 | Ath-AT4G02100.1 |  |  |  | Vvi-Vitvi07g00400\_t001 |  |  |  |  |  |  |
| 1 | Ath-AT4G02110.1 |  |  |  | Vvi-Vitvi07g00399\_t001 |  |  |  |  |  |  |
| 1 | Ath-AT4G02120.2 |  |  |  | Vvi-Vitvi07g00387\_t001 |  |  |  |  |  |  |
| 1 | Ath-AT4G02130.1 |  |  |  | | | |  |  |  |  |  |  |
| 1 | Ath-AT4G02140.1 |  |  |  | | | |  |  |  |  |  |  |
| 1 | Ath-AT4G02150.1 |  |  |  | Vvi-Vitvi07g00382\_t001 |  |  |  |  |  |  |
| 1 | Ath-AT4G02160.1 |  |  |  | | | |  |  |  |  |  |  |
| 1 | Ath-AT4G02170.1 |  |  |  | | | |  |  |  |  |  |  |
| 1 | Ath-AT4G02180.1 |  |  |  | | | |  |  |  |  |  |  |
| 1 | Ath-AT4G02190.1 |  |  |  | | | |  |  |  |  |  |  |
| 1 | Ath-AT4G02195.1 |  |  |  | Vvi-Vitvi07g00373\_t001 |  |  |  |  |  |  |
| 1 | Ath-AT4G02200.3 |  |  |  | | | |  |  |  |  |  |  |
| 1 | Ath-AT4G02210.2 |  |  |  | Vvi-Vitvi07g00371\_t001 |  |  |  |  |  |  |
| 1 | Ath-AT4G02220.1 |  |  |  | Vvi-Vitvi07g00364\_t001 |  |  |  |  |  |  |
| 1 | Ath-AT4G02230.1 |  |  |  | Vvi-Vitvi07g02210\_t001 |  |  |  |  |  |  |
| 1 | Ath-AT4G02235.1 |  |  |  | | | |  |  |  |  |  |  |
| 1 | Ath-AT4G02250.1 |  |  |  | | | |  |  |  |  |  |  |
| 1 | Ath-AT4G02260.1 |  |  |  | | | |  |  |  |  |  |  |
| 1 | Ath-AT4G02270.1 |  |  |  | Vvi-Vitvi07g02208\_t001 |  |  |  |  |  |  |
| 1 | Ath-AT4G02280.1 |  |  |  | Vvi-Vitvi07g00353\_t001 |  |  |  |  |  |  |
| 1 | Ath-AT4G02290.1 |  |  |  | Vvi-Vitvi07g00352\_t001 |  |  |  |  |  |  |
| 1 | Ath-AT4G02300.1 |  |  |  | Vvi-Vitvi07g00351\_t001 |  |  |  |  |  |  |
| 1 | Ath-AT4G02310.1 |  |  |  | | | |  |  |  |  |  |  |
| 1 | Ath-AT4G02320.1 |  |  |  | | | |  |  |  |  |  |  |
| 1 | Ath-AT4G02330.1 |  |  |  | Vvi-Vitvi07g00350\_t001 |  |  |  |  |  |  |
| 1 | Ath-AT4G02340.1 |  |  |  | Vvi-Vitvi07g00347\_t001 |  |  |  |  |  |  |
| 1 | Ath-AT4G02350.2 |  |  |  | Vvi-Vitvi07g00343\_t001 |  |  |  |  |  |  |
| 1 | Ath-AT4G02360.1 |  |  |  | Vvi-Vitvi07g00342\_t001 |  |  |  |  |  |  |
| 1 | Ath-AT4G02370.1 |  |  |  | | | |  |  |  |  |  |  |
| 1 | Ath-AT4G02380.3 |  |  |  | Vvi-Vitvi07g00341\_t001 |  |  |  |  |  |  |
| 1 | Ath-AT4G02390.1 |  |  |  | Vvi-Vitvi07g00337\_t001 |  |  |  |  |  |  |
| 1 | Ath-AT4G02400.1 |  |  |  | Vvi-Vitvi07g00336\_t001 |  |  |  |  |  |  |
| 1 | Ath-AT4G02405.1 |  |  |  | Vvi-Vitvi07g00334\_t001 |  |  |  |  |  |  |
| 1 | Ath-AT4G02410.1 |  |  |  | | | |  |  |  |  |  |  |
| 1 | Ath-AT4G02420.1 |  |  |  | | | |  |  |  |  |  |  |
| 1 | Ath-AT4G02425.1 |  |  |  | Vvi-Vitvi07g02203\_t001 |  |  |  |  |  |  |
| 1 | Ath-AT4G02430.4 |  |  |  | | | |  |  |  |  |  |  |
| 1 | Ath-AT4G02440.1 |  |  |  | Vvi-Vitvi07g00317\_t001 |  |  |  |  |  |  |
| 1 | Ath-AT4G02450.1 |  |  |  | Vvi-Vitvi07g00310\_t003 |  |  |  |  |  |  |
| 1 | Ath-AT4G02460.1 |  |  |  | Vvi-Vitvi07g00307\_t001 |  |  |  |  |  |  |
| 1 | Ath-AT4G02465.1 |  |  |  | | | |  |  |  |  |  |  |
| 1 | Ath-AT4G02480.1 |  |  |  | Vvi-Vitvi07g00303\_t001 |  |  |  |  |  |  |
| 1 | Ath-AT4G02482.1 |  |  |  | | | |  |  |  |  |  |  |
| 1 | Ath-AT4G02485.1 |  |  |  | Vvi-Vitvi07g00300\_t001 |  |  |  |  |  |  |
| 1 | Ath-AT4G02489.1 |  |  |  | | | |  |  |  |  |  |  |
| 1 | Ath-AT4G02500.1 |  |  |  | Vvi-Vitvi07g00299\_t001 |  |  |  |  |  |  |
| 1 | Ath-AT4G02510.1 |  |  |  | | | |  |  |  |  |  |  |
| 1 | Ath-AT4G02520.1 |  |  |  | Vvi-Vitvi07g02188\_t003 |  |  |  |  |  |  |
| 0 | Ath-AT4G02530.2 |  |  |  |  |  |  |  |  |
| 0 | Ath-AT4G02540.1 |  |  |  |  |  |  |  |  |
| 0 | Ath-AT4G02541.1 |  |  |  |  |  |  |  |  |
| 0 | Ath-AT4G02550.3 |  |  |  |  |  |  |  |  |
| 0 | Ath-AT4G02560.1 |  |  |  |  |  |  |  |  |
| 0 | Ath-AT4G02570.4 |  |  |  |  |  |  |  |  |
| 1 | Ath-AT4G02580.1 |  | Vvi-Vitvi07g00045\_t001 |  |  |  |  |  |  |  |
| 1 | Ath-AT4G02590.1 |  | Vvi-Vitvi07g00046\_t001 |  |  |  |  |  |  |  |
| 1 | Ath-AT4G02600.1 |  | | | |  |  |  |  |  |  |  |
| 1 | Ath-AT4G02610.1 |  | Vvi-Vitvi07g00047\_t001 |  |  |  |  |  |  |  |
| 1 | Ath-AT4G02620.1 |  | | | |  |  |  |  |  |  |  |
| 1 | Ath-AT4G02630.1 |  | | | |  |  |  |  |  |  |  |
| 1 | Ath-AT4G02640.2 |  | Vvi-Vitvi07g00049\_t001 |  |  |  |  |  |  |  |
| 1 | Ath-AT4G02650.1 |  | | | |  |  |  |  |  |  |  |
| 1 | Ath-AT4G02655.2 |  | | | |  |  |  |  |  |  |  |
| 1 | Ath-AT4G02660.2 |  | Vvi-Vitvi07g00073\_t002 |  |  |  |  |  |  |  |
| 1 | Ath-AT4G02670.2 |  | Vvi-Vitvi07g00077\_t001 |  |  |  |  |  |  |  |
| 1 | Ath-AT4G02680.1 |  | Vvi-Vitvi07g00081\_t001 |  |  |  |  |  |  |  |
| 1 | Ath-AT4G02690.1 |  | Vvi-Vitvi07g00087\_t001 |  |  |  |  |  |  |  |
| 1 | Ath-AT4G02700.1 |  | Vvi-Vitvi07g00091\_t001 |  |  |  |  |  |  |  |
| 1 | Ath-AT4G02710.1 |  | Vvi-Vitvi07g00097\_t002 |  |  |  |  |  |  |  |
| 1 | Ath-AT4G02715.1 |  | Vvi-Vitvi07g00115\_t001 |  |  |  |  |  |  |  |
| 1 | Ath-AT4G02725.1 |  | | | |  |  |  |  |  |  |  |
| 1 | Ath-AT4G02720.1 |  | Vvi-Vitvi07g00122\_t001 |  |  |  |  |  |  |  |
| 1 | Ath-AT4G02730.1 |  | Vvi-Vitvi07g02133\_t001 |  |  |  |  |  |  |  |
| 1 | Ath-AT4G02733.1 |  | Vvi-Vitvi07g02134\_t001 |  |  |  |  |  |  |  |
| 1 | Ath-AT4G02735.1 |  | | | |  |  |  |  |  |  |  |
| 1 | Ath-AT4G02740.2 |  | | | |  |  |  |  |  |  |  |
| 1 | Ath-AT4G02750.1 |  | | | |  |  |  |  |  |  |  |
| 1 | Ath-AT4G02760.7 |  | | | |  |  |  |  |  |  |  |
| 1 | Ath-AT4G02770.1 |  | Vvi-Vitvi07g00125\_t001 |  |  |  |  |  |  |  |
| 1 | Ath-AT4G02780.1 |  | Vvi-Vitvi07g00127\_t001 |  |  |  |  |  |  |  |
| 1 | Ath-AT4G02790.1 |  | | | |  |  |  |  |  |  |  |
| 1 | Ath-AT4G02800.1 |  | | | |  |  |  |  |  |  |  |
| 1 | Ath-AT4G02810.1 |  | | | |  |  |  |  |  |  |  |
| 1 | Ath-AT4G02820.1 |  | | | |  |  |  |  |  |  |  |
| 1 | Ath-AT4G02830.1 |  | | | |  |  |  |  |  |  |  |
| 1 | Ath-AT4G02840.2 |  | Vvi-Vitvi07g00145\_t002 |  |  |  |  |  |  |  |
| 1 | Ath-AT4G02850.1 |  | Vvi-Vitvi07g02140\_t002 |  |  |  |  |  |  |  |
| 1 | Ath-AT4G02860.1 |  | | | |  |  |  |  |  |  |  |
| 1 | Ath-AT4G02870.1 |  | | | |  |  |  |  |  |  |  |
| 1 | Ath-AT4G02880.2 |  | Vvi-Vitvi07g00159\_t005 |  |  |  |  |  |  |  |
| 1 | Ath-AT4G02890.3 |  | | | |  |  |  |  |  |  |  |
| 1 | Ath-AT4G02900.1 |  | Vvi-Vitvi07g00166\_t001 |  |  |  |  |  |  |  |
| 1 | Ath-AT4G02910.1 |  | | | |  |  |  |  |  |  |  |
| 1 | Ath-AT4G02920.2 |  | Vvi-Vitvi07g00185\_t001 |  |  |  |  |  |  |  |
| 1 | Ath-AT4G02930.1 |  | Vvi-Vitvi07g00186\_t001 |  |  |  |  |  |  |  |
| 1 | Ath-AT4G02940.1 |  | Vvi-Vitvi07g00190\_t001 |  |  |  |  |  |  |  |
| 1 | Ath-AT4G02950.1 |  | | | |  |  |  |  |  |  |  |
| 1 | Ath-AT4G02970.1 |  | | | |  |  |  |  |  |  |  |
| 1 | Ath-AT4G02980.1 |  | Vvi-Vitvi07g00196\_t001 |  |  |  |  |  |  |  |
| 1 | Ath-AT4G02990.1 |  | Vvi-Vitvi07g00197\_t001 |  |  |  |  |  |  |  |
| 1 | Ath-AT4G03000.1 |  | | | |  |  |  |  |  |  |  |
| 1 | Ath-AT4G03010.1 |  | Vvi-Vitvi07g00213\_t001 |  |  |  |  |  |  |  |
| 1 | Ath-AT4G03020.3 |  | Vvi-Vitvi07g00214\_t002 |  |  |  |  |  |  |  |
| 1 | Ath-AT4G03030.1 |  | | | |  |  |  |  |  |  |  |
| 1 | Ath-AT4G03040.1 |  | | | |  |  |  |  |  |  |  |
| 1 | Ath-AT4G03050.1 |  | | | |  |  |  |  |  |  |  |
| 1 | Ath-AT4G03070.1 |  | | | |  |  |  |  |  |  |  |
| 1 | Ath-AT4G03080.1 |  | Vvi-Vitvi07g00223\_t001 |  |  |  |  |  |  |  |
| 1 | Ath-AT4G03090.1 |  | Vvi-Vitvi07g00225\_t001 |  |  |  |  |  |  |  |
| 1 | Ath-AT4G03100.1 |  | Vvi-Vitvi07g00231\_t001 |  |  |  |  |  |  |  |
| 1 | Ath-AT4G03110.1 |  | Vvi-Vitvi07g00232\_t001 |  |  |  |  |  |  |  |
| 1 | Ath-AT4G03115.3 |  | Vvi-Vitvi07g00233\_t001 |  |  |  |  |  |  |  |
| 1 | Ath-AT4G03113.1 |  | | | |  |  |  |  |  |  |  |
| 1 | Ath-AT4G03120.1 |  | Vvi-Vitvi07g00234\_t001 |  |  |  |  |  |  |  |
| 1 | Ath-AT4G03130.2 |  | | | |  |  |  |  |  |  |  |
| 1 | Ath-AT4G03140.1 |  | | | |  |  |  |  |  |  |  |
| 1 | Ath-AT4G03150.1 |  | Vvi-Vitvi07g00237\_t001 |  |  |  |  |  |  |  |
| 1 | Ath-AT4G03153.1 |  | | | |  |  |  |  |  |  |  |
| 1 | Ath-AT4G03156.1 |  | | | |  |  |  |  |  |  |  |
| 1 | Ath-AT4G03157.1 |  | | | |  |  |  |  |  |  |  |
| 1 | Ath-AT4G03160.1 |  | | | |  |  |  |  |  |  |  |
| 1 | Ath-AT4G03165.1 |  | | | |  |  |  |  |  |  |  |
| 1 | Ath-AT4G03170.1 |  | | | |  |  |  |  |  |  |  |
| 1 | Ath-AT4G03175.1 |  | | | |  |  |  |  |  |  |  |
| 1 | Ath-AT4G03180.1 |  | | | |  |  |  |  |  |  |  |
| 1 | Ath-AT4G03190.1 |  | Vvi-Vitvi07g00248\_t002 |  |  |  |  |  |  |  |
| 1 | Ath-AT4G03200.1 |  | Vvi-Vitvi07g00265\_t001 |  |  |  |  |  |  |  |
| 0 | Ath-AT4G03205.2 |  |  |  |  |  |  |  |  |
| 1 | Ath-AT4G03210.1 |  | Vvi-Vitvi12g00586\_t001 |  |  |  |  |  |  |  |
| 1 | Ath-AT4G03220.1 |  | Vvi-Vitvi12g00592\_t001 |  |  |  |  |  |  |  |
| 1 | Ath-AT4G03230.4 |  | Vvi-Vitvi12g00595\_t001 |  |  |  |  |  |  |  |
| 1 | Ath-AT4G03240.1 |  | Vvi-Vitvi12g02442\_t001 |  |  |  |  |  |  |  |
| 1 | Ath-AT4G03250.1 |  | | | |  |  |  |  |  |  |  |
| 1 | Ath-AT4G03260.1 |  | Vvi-Vitvi12g00619\_t001 |  |  |  |  |  |  |  |
| 1 | Ath-AT4G03270.1 |  | Vvi-Vitvi12g00627\_t002 |  |  |  |  |  |  |  |
| 1 | Ath-AT4G03280.1 |  | Vvi-Vitvi12g00628\_t002 |  |  |  |  |  |  |  |
| 0 | Ath-AT4G03290.1 |  |  |  |  |  |  |  |  |
| 0 | Ath-AT4G03292.1 |  |  |  |  |  |  |  |  |
| 0 | Ath-AT4G03298.1 |  |  |  |  |  |  |  |  |
| 0 | Ath-AT4G03320.1 |  |  |  |  |  |  |  |  |
| 1 | Ath-AT4G03330.1 |  | Vvi-Vitvi12g00498\_t001 |  |  |  |  |  |  |  |
| 1 | Ath-AT4G03340.1 |  | Vvi-Vitvi12g00491\_t001 |  |  |  |  |  |  |  |
| 1 | Ath-AT4G03350.1 |  | | | |  |  |  |  |  |  |  |
| 1 | Ath-AT4G03360.1 |  | | | |  |  |  |  |  |  |  |
| 1 | Ath-AT4G03370.1 |  | | | |  |  |  |  |  |  |  |
| 1 | Ath-AT4G03380.1 |  | | | |  |  |  |  |  |  |  |
| 1 | Ath-AT4G03390.1 |  | Vvi-Vitvi12g00483\_t003 |  |  |  |  |  |  |  |
| 1 | Ath-AT4G03400.2 |  | Vvi-Vitvi12g00480\_t001 |  |  |  |  |  |  |  |
| 1 | Ath-AT4G03410.2 |  | Vvi-Vitvi12g00478\_t001 |  |  |  |  |  |  |  |
| 1 | Ath-AT4G03415.3 |  | Vvi-Vitvi12g00477\_t003 |  |  |  |  |  |  |  |
| 1 | Ath-AT4G03420.1 |  | Vvi-Vitvi12g00474\_t001 |  |  |  |  |  |  |  |
| 1 | Ath-AT4G03430.2 |  | Vvi-Vitvi12g00345\_t001 |  |  |  |  |  |  |  |
| 1 | Ath-AT4G03440.1 |  | Vvi-Vitvi12g02324\_t001 |  |  |  |  |  |  |  |
| 1 | Ath-AT4G03443.1 |  | | | |  |  |  |  |  |  |  |
| 1 | Ath-AT4G03450.1 |  | | | |  |  |  |  |  |  |  |
| 1 | Ath-AT4G03460.1 |  | | | |  |  |  |  |  |  |  |
| 1 | Ath-AT4G03470.1 |  | Vvi-Vitvi12g00334\_t001 |  |  |  |  |  |  |  |
| 1 | Ath-AT4G03480.1 |  | Vvi-Vitvi12g04082\_t001 |  |  |  |  |  |  |  |
| 1 | Ath-AT4G03490.3 |  | | | |  |  |  |  |  |  |  |
| 1 | Ath-AT4G03495.1 |  | | | |  |  |  |  |  |  |  |
| 1 | Ath-AT4G03500.1 |  | | | |  |  |  |  |  |  |  |
| 1 | Ath-AT4G03505.1 |  | | | |  |  |  |  |  |  |  |
| 1 | Ath-AT4G03510.1 |  | Vvi-Vitvi12g00320\_t001 |  |  |  |  |  |  |  |
| 1 | Ath-AT4G03520.1 |  | Vvi-Vitvi12g00319\_t001 |  |  |  |  |  |  |  |
| 1 | Ath-AT4G03540.1 |  | Vvi-Vitvi12g00312\_t001 |  |  |  |  |  |  |  |
| 1 | Ath-AT4G03550.1 |  | Vvi-Vitvi12g04069\_t001 |  |  |  |  |  |  |  |
| 1 | Ath-AT4G03560.1 |  | Vvi-Vitvi12g00306\_t001 |  |  |  |  |  |  |  |
| 1 | Ath-AT4G03565.1 |  | | | |  |  |  |  |  |  |  |
| 1 | Ath-AT4G03566.1 |  | | | |  |  |  |  |  |  |  |
| 1 | Ath-AT4G03570.1 |  | | | |  |  |  |  |  |  |  |
| 1 | Ath-AT4G03580.1 |  | | | |  |  |  |  |  |  |  |
| 1 | Ath-AT4G03590.1 |  | | | |  |  |  |  |  |  |  |
| 1 | Ath-AT4G03600.1 |  | Vvi-Vitvi12g02308\_t001 |  |  |  |  |  |  |  |
| 0 | Ath-AT4G03610.4 |  |  |  |  |  |  |  |  |
| 0 | Ath-AT4G03620.1 |  |  |  |  |  |  |  |  |
| 0 | Ath-AT4G03625.1 |  |  |  |  |  |  |  |  |
| 0 | Ath-AT4G03630.1 |  |  |  |  |  |  |  |  |
| 0 | Ath-AT4G03635.1 |  |  |  |  |  |  |  |  |
| 0 | Ath-AT4G03645.1 |  |  |  |  |  |  |  |  |
| 0 | Ath-AT4G03728.1 |  |  |  |  |  |  |  |  |
| 0 | Ath-AT4G03740.1 |  |  |  |  |  |  |  |  |
| 0 | Ath-AT4G03820.4 |  |  |  |  |  |  |  |  |
| 0 | Ath-AT4G03830.1 |  |  |  |  |  |  |  |  |
| 0 | Ath-AT4G03930.1 |  |  |  |  |  |  |  |  |
| 0 | Ath-AT4G03940.1 |  |  |  |  |  |  |  |  |
| 0 | Ath-AT4G03945.1 |  |  |  |  |  |  |  |  |
| 1 | Ath-AT4G03950.1 |  | Vvi-Vitvi10g00037\_t001 |  |  |  |  |  |  |  |
| 2 | Ath-AT4G03960.1 |  | | | |  | Vvi-Vitvi12g00446\_t001 |  |  |  |  |  |  |
| 2 | Ath-AT4G03965.1 |  | | | |  | Vvi-Vitvi12g00444\_t001 |  |  |  |  |  |  |
| 2 | Ath-AT4G04020.1 |  | | | |  | | | |  |  |  |  |  |  |
| 2 | Ath-AT4G04025.1 |  | | | |  | | | |  |  |  |  |  |  |
| 2 | Ath-AT4G04030.2 |  | | | |  | | | |  |  |  |  |  |  |
| 2 | Ath-AT4G04040.1 |  | | | |  | Vvi-Vitvi12g00427\_t001 |  |  |  |  |  |  |
| 2 | Ath-AT4G04078.1 |  | | | |  | | | |  |  |  |  |  |  |
| 2 | Ath-AT4G04080.1 |  | | | |  | | | |  |  |  |  |  |  |
| 2 | Ath-AT4G04090.1 |  | | | |  | | | |  |  |  |  |  |  |
| 2 | Ath-AT4G04110.1 |  | | | |  | | | |  |  |  |  |  |  |
| 2 | Ath-AT4G04180.1 |  | | | |  | | | |  |  |  |  |  |  |
| 2 | Ath-AT4G04190.1 |  | | | |  | | | |  |  |  |  |  |  |
| 2 | Ath-AT4G04200.1 |  | | | |  | | | |  |  |  |  |  |  |
| 2 | Ath-AT4G04210.1 |  | | | |  | Vvi-Vitvi12g00425\_t001 |  |  |  |  |  |  |
| 2 | Ath-AT4G04220.1 |  | | | |  | | | |  |  |  |  |  |  |
| 2 | Ath-AT4G04260.2 |  | | | |  | Vvi-Vitvi12g00423\_t001 |  |  |  |  |  |  |
| 2 | Ath-AT4G04265.1 |  | | | |  | | | |  |  |  |  |  |  |
| 2 | Ath-AT4G04320.1 |  | Vvi-Vitvi10g00048\_t002 |  | | | |  |  |  |  |  |  |
| 2 | Ath-AT4G04330.1 |  | Vvi-Vitvi10g00049\_t001 |  | | | |  |  |  |  |  |  |
| 2 | Ath-AT4G04340.1 |  | Vvi-Vitvi10g00052\_t004 |  | Vvi-Vitvi12g00410\_t001 |  |  |  |  |  |  |
| 2 | Ath-AT4G04350.1 |  | Vvi-Vitvi10g00054\_t001 |  | | | |  |  |  |  |  |  |
| 2 | Ath-AT4G04360.1 |  | Vvi-Vitvi10g04010\_t001 |  | Vvi-Vitvi12g00404\_t001 |  |  |  |  |  |  |
| 2 | Ath-AT4G04370.1 |  | Vvi-Vitvi10g00056\_t001 |  | | | |  |  |  |  |  |  |
| 2 | Ath-AT4G04402.1 |  | | | |  | | | |  |  |  |  |  |  |
| 2 | Ath-AT4G04404.1 |  | | | |  | | | |  |  |  |  |  |  |
| 2 | Ath-AT4G04415.1 |  | | | |  | | | |  |  |  |  |  |  |
| 2 | Ath-AT4G04423.1 |  | | | |  | | | |  |  |  |  |  |  |
| 3 | Ath-AT4G04450.1 |  | Vvi-Vitvi10g00063\_t001 |  | Vvi-Vitvi12g00388\_t001 |  | Vvi-Vitvi19g00530\_t001 |  |  |  |  |  |
| 3 | Ath-AT4G04460.1 |  | Vvi-Vitvi10g00064\_t001 |  | | | |  | Vvi-Vitvi19g00529\_t001 |  |  |  |  |  |
| 3 | Ath-AT4G04470.1 |  | Vvi-Vitvi10g00070\_t002 |  | | | |  | | | |  |  |  |  |  |
| 3 | Ath-AT4G04480.1 |  | Vvi-Vitvi10g04017\_t001 |  | | | |  | | | |  |  |  |  |  |
| 3 | Ath-AT4G04490.1 |  | | | |  | | | |  | | | |  |  |  |  |  |
| 3 | Ath-AT4G04500.1 |  | | | |  | | | |  | | | |  |  |  |  |  |
| 3 | Ath-AT4G04510.2 |  | | | |  | | | |  | | | |  |  |  |  |  |
| 3 | Ath-AT4G04540.1 |  | | | |  | | | |  | | | |  |  |  |  |  |
| 3 | Ath-AT4G04555.1 |  | | | |  | | | |  | | | |  |  |  |  |  |
| 3 | Ath-AT4G04570.1 |  | | | |  | | | |  | | | |  |  |  |  |  |
| 3 | Ath-AT4G04580.1 |  | | | |  | | | |  | | | |  |  |  |  |  |
| 3 | Ath-AT4G04601.1 |  | | | |  | | | |  | | | |  |  |  |  |  |
| 3 | Ath-AT4G04605.1 |  | Vvi-Vitvi10g04022\_t001 |  | | | |  | | | |  |  |  |  |  |
| 3 | Ath-AT4G04610.1 |  | Vvi-Vitvi10g00086\_t001 |  | Vvi-Vitvi12g00381\_t001 |  | | | |  |  |  |  |  |
| 2 | Ath-AT4G04614.2 |  | | | |  |  |  | | | |  |  |  |  |  |
| 2 | Ath-AT4G04620.2 |  | Vvi-Vitvi10g04024\_t001 |  |  |  | Vvi-Vitvi19g02021\_t001 |  |  |  |  |  |
| 2 | Ath-AT4G04630.1 |  | Vvi-Vitvi10g00093\_t001 |  |  |  | Vvi-Vitvi19g04237\_t001 |  |  |  |  |  |
| 2 | Ath-AT4G04632.1 |  | | | |  |  |  | | | |  |  |  |  |  |
| 2 | Ath-AT4G04640.1 |  | Vvi-Vitvi10g00095\_t001 |  |  |  | Vvi-Vitvi19g00504\_t001 |  |  |  |  |  |
| 2 | Ath-AT4G04650.1 |  | | | |  |  |  | | | |  |  |  |  |  |
| 2 | Ath-AT4G04670.1 |  | Vvi-Vitvi10g00098\_t001 |  |  |  | | | |  |  |  |  |  |
| 2 | Ath-AT4G04680.1 |  | | | |  |  |  | | | |  |  |  |  |  |
| 2 | Ath-AT4G04690.1 |  | | | |  |  |  | | | |  |  |  |  |  |
| 2 | Ath-AT4G04695.1 |  | | | |  |  |  | | | |  |  |  |  |  |
| 2 | Ath-AT4G04700.1 |  | | | |  |  |  | | | |  |  |  |  |  |
| 2 | Ath-AT4G04710.4 |  | | | |  |  |  | | | |  |  |  |  |  |
| 2 | Ath-AT4G04720.1 |  | Vvi-Vitvi10g00102\_t001 |  |  |  | Vvi-Vitvi19g00497\_t001 |  |  |  |  |  |
| 1 | Ath-AT4G04730.1 |  | | | |  |  |  |  |  |  |  |
| 1 | Ath-AT4G04740.2 |  | | | |  |  |  |  |  |  |  |
| 1 | Ath-AT4G04745.1 |  | Vvi-Vitvi10g00110\_t001 |  |  |  |  |  |  |  |
| 1 | Ath-AT4G04750.3 |  | | | |  |  |  |  |  |  |  |
| 1 | Ath-AT4G04760.1 |  | | | |  |  |  |  |  |  |  |
| 1 | Ath-AT4G04770.1 |  | Vvi-Vitvi10g00111\_t001 |  |  |  |  |  |  |  |
| 1 | Ath-AT4G04775.1 |  | | | |  |  |  |  |  |  |  |
| 1 | Ath-AT4G04780.1 |  | | | |  |  |  |  |  |  |  |
| 1 | Ath-AT4G04790.1 |  | | | |  |  |  |  |  |  |  |
| 1 | Ath-AT4G04800.1 |  | | | |  |  |  |  |  |  |  |
| 1 | Ath-AT4G04810.1 |  | | | |  |  |  |  |  |  |  |
| 1 | Ath-AT4G04830.1 |  | | | |  |  |  |  |  |  |  |
| 1 | Ath-AT4G04840.1 |  | | | |  |  |  |  |  |  |  |
| 1 | Ath-AT4G04850.2 |  | | | |  |  |  |  |  |  |  |
| 1 | Ath-AT4G04860.1 |  | Vvi-Vitvi10g04026\_t001 |  |  |  |  |  |  |  |
| 0 | Ath-AT4G04870.1 |  |  |  |  |  |  |  |  |
| 0 | Ath-AT4G04880.2 |  |  |  |  |  |  |  |  |
| 0 | Ath-AT4G04885.1 |  |  |  |  |  |  |  |  |
| 0 | Ath-AT4G04890.2 |  |  |  |  |  |  |  |  |
| 0 | Ath-AT4G04900.1 |  |  |  |  |  |  |  |  |
| 0 | Ath-AT4G04910.1 |  |  |  |  |  |  |  |  |
| 0 | Ath-AT4G04920.1 |  |  |  |  |  |  |  |  |
| 0 | Ath-AT4G04925.1 |  |  |  |  |  |  |  |  |
| 0 | Ath-AT4G04930.1 |  |  |  |  |  |  |  |  |
| 0 | Ath-AT4G04940.1 |  |  |  |  |  |  |  |  |
| 0 | Ath-AT4G04950.1 |  |  |  |  |  |  |  |  |
| 0 | Ath-AT4G04955.1 |  |  |  |  |  |  |  |  |
| 0 | Ath-AT4G04957.1 |  |  |  |  |  |  |  |  |
| 0 | Ath-AT4G04960.1 |  |  |  |  |  |  |  |  |
| 0 | Ath-AT4G04970.1 |  |  |  |  |  |  |  |  |
| 0 | Ath-AT4G04972.1 |  |  |  |  |  |  |  |  |
| 0 | Ath-AT4G04980.1 |  |  |  |  |  |  |  |  |
| 0 | Ath-AT4G04985.1 |  |  |  |  |  |  |  |  |
| 0 | Ath-AT4G04990.1 |  |  |  |  |  |  |  |  |
| 1 | Ath-AT4G05000.2 |  | Vvi-Vitvi10g00386\_t002 |  |  |  |  |  |  |  |
| 1 | Ath-AT4G05010.1 |  | Vvi-Vitvi10g01748\_t001 |  |  |  |  |  |  |  |
| 1 | Ath-AT4G05018.1 |  | Vvi-Vitvi10g01746\_t001 |  |  |  |  |  |  |  |
| 1 | Ath-AT4G05020.2 |  | Vvi-Vitvi10g04257\_t001 |  |  |  |  |  |  |  |
| 1 | Ath-AT4G05030.1 |  | | | |  |  |  |  |  |  |  |
| 1 | Ath-AT4G05040.6 |  | | | |  |  |  |  |  |  |  |
| 1 | Ath-AT4G05050.3 |  | | | |  |  |  |  |  |  |  |
| 1 | Ath-AT4G05060.1 |  | Vvi-Vitvi10g04245\_t001 |  |  |  |  |  |  |  |
| 1 | Ath-AT4G05070.1 |  | | | |  |  |  |  |  |  |  |
| 1 | Ath-AT4G05071.1 |  | | | |  |  |  |  |  |  |  |
| 1 | Ath-AT4G05080.1 |  | | | |  |  |  |  |  |  |  |
| 1 | Ath-AT4G05090.1 |  | | | |  |  |  |  |  |  |  |
| 1 | Ath-AT4G05091.1 |  | | | |  |  |  |  |  |  |  |
| 1 | Ath-AT4G05095.1 |  | | | |  |  |  |  |  |  |  |
| 1 | Ath-AT4G05097.1 |  | | | |  |  |  |  |  |  |  |
| 1 | Ath-AT4G05100.1 |  | | | |  |  |  |  |  |  |  |
| 1 | Ath-AT4G05110.2 |  | Vvi-Vitvi10g00342\_t001 |  |  |  |  |  |  |  |
| 1 | Ath-AT4G05120.1 |  | | | |  |  |  |  |  |  |  |
| 1 | Ath-AT4G05130.1 |  | | | |  |  |  |  |  |  |  |
| 1 | Ath-AT4G05140.1 |  | | | |  |  |  |  |  |  |  |
| 1 | Ath-AT4G05150.1 |  | Vvi-Vitvi10g00343\_t001 |  |  |  |  |  |  |  |
| 0 | Ath-AT4G05160.1 |  |  |  |  |  |  |  |  |
| 0 | Ath-AT4G05170.1 |  |  |  |  |  |  |  |  |
| 0 | Ath-AT4G05180.1 |  |  |  |  |  |  |  |  |
| 0 | Ath-AT4G05190.1 |  |  |  |  |  |  |  |  |
| 0 | Ath-AT4G05200.2 |  |  |  |  |  |  |  |  |
| 0 | Ath-AT4G05210.1 |  |  |  |  |  |  |  |  |
| 0 | Ath-AT4G05220.1 |  |  |  |  |  |  |  |  |
| 0 | Ath-AT4G05230.1 |  |  |  |  |  |  |  |  |
| 0 | Ath-AT4G05240.1 |  |  |  |  |  |  |  |  |
| 0 | Ath-AT4G05250.1 |  |  |  |  |  |  |  |  |
| 0 | Ath-AT4G05260.1 |  |  |  |  |  |  |  |  |
| 0 | Ath-AT4G05270.1 |  |  |  |  |  |  |  |  |
| 0 | Ath-AT4G05310.1 |  |  |  |  |  |  |  |  |
| 0 | Ath-AT4G05320.2 |  |  |  |  |  |  |  |  |
| 0 | Ath-AT4G05330.1 |  |  |  |  |  |  |  |  |
| 0 | Ath-AT4G05340.1 |  |  |  |  |  |  |  |  |
| 0 | Ath-AT4G05350.1 |  |  |  |  |  |  |  |  |
| 0 | Ath-AT4G05360.1 |  |  |  |  |  |  |  |  |
| 0 | Ath-AT4G05380.1 |  |  |  |  |  |  |  |  |
| 0 | Ath-AT4G05390.1 |  |  |  |  |  |  |  |  |
| 0 | Ath-AT4G05400.2 |  |  |  |  |  |  |  |  |
| 0 | Ath-AT4G05410.1 |  |  |  |  |  |  |  |  |
| 0 | Ath-AT4G05420.1 |  |  |  |  |  |  |  |  |
| 0 | Ath-AT4G05430.3 |  |  |  |  |  |  |  |  |
| 0 | Ath-AT4G05440.1 |  |  |  |  |  |  |  |  |
| 0 | Ath-AT4G05450.1 |  |  |  |  |  |  |  |  |
| 0 | Ath-AT4G05460.1 |  |  |  |  |  |  |  |  |
| 0 | Ath-AT4G05470.1 |  |  |  |  |  |  |  |  |
| 0 | Ath-AT4G05475.1 |  |  |  |  |  |  |  |  |
| 0 | Ath-AT4G05490.1 |  |  |  |  |  |  |  |  |
| 0 | Ath-AT4G05497.1 |  |  |  |  |  |  |  |  |
| 0 | Ath-AT4G05520.1 |  |  |  |  |  |  |  |  |
| 0 | Ath-AT4G05523.1 |  |  |  |  |  |  |  |  |
| 0 | Ath-AT4G05530.1 |  |  |  |  |  |  |  |  |
| 0 | Ath-AT4G05540.1 |  |  |  |  |  |  |  |  |
| 0 | Ath-AT4G05553.1 |  |  |  |  |  |  |  |  |
| 0 | Ath-AT4G05555.1 |  |  |  |  |  |  |  |  |
| 0 | Ath-AT4G05590.2 |  |  |  |  |  |  |  |  |
| 0 | Ath-AT4G05612.1 |  |  |  |  |  |  |  |  |
| 0 | Ath-AT4G05620.1 |  |  |  |  |  |  |  |  |
| 0 | Ath-AT4G05630.1 |  |  |  |  |  |  |  |  |
| 0 | Ath-AT4G05631.1 |  |  |  |  |  |  |  |  |
| 0 | Ath-AT4G05632.1 |  |  |  |  |  |  |  |  |
| 0 | Ath-AT4G06479.1 |  |  |  |  |  |  |  |  |
| 0 | Ath-AT4G06490.1 |  |  |  |  |  |  |  |  |
| 0 | Ath-AT4G06526.1 |  |  |  |  |  |  |  |  |
| 0 | Ath-AT4G06534.1 |  |  |  |  |  |  |  |  |
| 0 | Ath-AT4G06536.1 |  |  |  |  |  |  |  |  |
| 0 | Ath-AT4G06639.1 |  |  |  |  |  |  |  |  |
| 0 | Ath-AT4G06583.2 |  |  |  |  |  |  |  |  |
| 0 | Ath-AT4G06598.1 |  |  |  |  |  |  |  |  |
| 0 | Ath-AT4G06599.1 |  |  |  |  |  |  |  |  |
| 0 | Ath-AT4G06643.1 |  |  |  |  |  |  |  |  |
| 0 | Ath-AT4G06634.1 |  |  |  |  |  |  |  |  |
| 0 | Ath-AT4G06655.1 |  |  |  |  |  |  |  |  |
| 0 | Ath-AT4G06676.1 |  |  |  |  |  |  |  |  |
| 0 | Ath-AT4G06688.1 |  |  |  |  |  |  |  |  |
| 0 | Ath-AT4G06744.1 |  |  |  |  |  |  |  |  |
| 0 | Ath-AT4G06746.1 |  |  |  |  |  |  |  |  |
| 0 | Ath-AT4G07325.1 |  |  |  |  |  |  |  |  |
| 0 | Ath-AT4G07350.1 |  |  |  |  |  |  |  |  |
| 0 | Ath-AT4G07380.1 |  |  |  |  |  |  |  |  |
| 0 | Ath-AT4G07390.1 |  |  |  |  |  |  |  |  |
| 0 | Ath-AT4G07400.1 |  |  |  |  |  |  |  |  |
| 0 | Ath-AT4G07410.1 |  |  |  |  |  |  |  |  |
| 0 | Ath-AT4G07408.1 |  |  |  |  |  |  |  |  |
| 0 | Ath-AT4G07445.1 |  |  |  |  |  |  |  |  |
| 0 | Ath-AT4G07455.1 |  |  |  |  |  |  |  |  |
| 0 | Ath-AT4G07515.1 |  |  |  |  |  |  |  |  |
| 0 | Ath-AT4G07524.1 |  |  |  |  |  |  |  |  |
| 0 | Ath-AT4G07526.1 |  |  |  |  |  |  |  |  |
| 0 | Ath-AT4G07666.1 |  |  |  |  |  |  |  |  |
| 0 | Ath-AT4G07670.1 |  |  |  |  |  |  |  |  |
| 0 | Ath-AT4G07675.1 |  |  |  |  |  |  |  |  |
| 0 | Ath-AT4G07740.1 |  |  |  |  |  |  |  |  |
| 0 | Ath-AT4G07820.1 |  |  |  |  |  |  |  |  |
| 0 | Ath-AT4G07825.1 |  |  |  |  |  |  |  |  |
| 0 | Ath-AT4G07835.1 |  |  |  |  |  |  |  |  |
| 0 | Ath-AT4G07868.1 |  |  |  |  |  |  |  |  |
| 0 | Ath-AT4G07932.1 |  |  |  |  |  |  |  |  |
| 0 | Ath-AT4G07940.1 |  |  |  |  |  |  |  |  |
| 0 | Ath-AT4G07950.1 |  |  |  |  |  |  |  |  |
| 1 | Ath-AT4G07960.1 |  | Vvi-Vitvi18g00616\_t001 |  |  |  |  |  |  |  |
| 1 | Ath-AT4G07965.1 |  | | | |  |  |  |  |  |  |  |
| 1 | Ath-AT4G07990.1 |  | Vvi-Vitvi18g00611\_t002 |  |  |  |  |  |  |  |
| 1 | Ath-AT4G07995.1 |  | | | |  |  |  |  |  |  |  |
| 1 | Ath-AT4G08025.1 |  | | | |  |  |  |  |  |  |  |
| 1 | Ath-AT4G08028.1 |  | | | |  |  |  |  |  |  |  |
| 1 | Ath-AT4G08039.1 |  | | | |  |  |  |  |  |  |  |
| 1 | Ath-AT4G08040.1 |  | Vvi-Vitvi18g00609\_t001 |  |  |  |  |  |  |  |
| 1 | Ath-AT4G08097.1 |  | | | |  |  |  |  |  |  |  |
| 1 | Ath-AT4G08140.1 |  | | | |  |  |  |  |  |  |  |
| 1 | Ath-AT4G08150.1 |  | Vvi-Vitvi18g00602\_t001 |  |  |  |  |  |  |  |
| 1 | Ath-AT4G08160.1 |  | Vvi-Vitvi18g00601\_t001 |  |  |  |  |  |  |  |
| 1 | Ath-AT4G08170.2 |  | Vvi-Vitvi18g00597\_t002 |  |  |  |  |  |  |  |
| 1 | Ath-AT4G08180.1 |  | Vvi-Vitvi18g00321\_t001 |  |  |  |  |  |  |  |
| 1 | Ath-AT4G08190.1 |  | | | |  |  |  |  |  |  |  |
| 2 | Ath-AT4G08210.1 |  | | | |  | Vvi-Vitvi18g00315\_t001 |  |  |  |  |  |  |
| 2 | Ath-AT4G08230.1 |  | Vvi-Vitvi18g02540\_t002 |  | | | |  |  |  |  |  |  |
| 2 | Ath-AT4G08240.2 |  | Vvi-Vitvi18g00301\_t001 |  | | | |  |  |  |  |  |  |
| 2 | Ath-AT4G08250.1 |  | Vvi-Vitvi18g00300\_t001 |  | | | |  |  |  |  |  |  |
| 2 | Ath-AT4G08260.1 |  | | | |  | | | |  |  |  |  |  |  |
| 2 | Ath-AT4G08263.1 |  | | | |  | | | |  |  |  |  |  |  |
| 2 | Ath-AT4G08267.1 |  | | | |  | | | |  |  |  |  |  |  |
| 2 | Ath-AT4G08270.1 |  | | | |  | | | |  |  |  |  |  |  |
| 2 | Ath-AT4G08280.2 |  | Vvi-Vitvi18g00293\_t001 |  | | | |  |  |  |  |  |  |
| 2 | Ath-AT4G08290.1 |  | Vvi-Vitvi18g00289\_t001 |  | | | |  |  |  |  |  |  |
| 2 | Ath-AT4G08300.1 |  | | | |  | | | |  |  |  |  |  |  |
| 2 | Ath-AT4G08310.1 |  | Vvi-Vitvi18g00287\_t001 |  | | | |  |  |  |  |  |  |
| 2 | Ath-AT4G08320.2 |  | | | |  | | | |  |  |  |  |  |  |
| 2 | Ath-AT4G08330.1 |  | Vvi-Vitvi18g02532\_t001 |  | | | |  |  |  |  |  |  |
| 2 | Ath-AT4G08350.1 |  | | | |  | | | |  |  |  |  |  |  |
| 2 | Ath-AT4G08360.1 |  | | | |  | | | |  |  |  |  |  |  |
| 2 | Ath-AT4G08370.1 |  | | | |  | | | |  |  |  |  |  |  |
| 2 | Ath-AT4G08380.1 |  | | | |  | | | |  |  |  |  |  |  |
| 2 | Ath-AT4G08390.1 |  | Vvi-Vitvi18g00256\_t001 |  | | | |  |  |  |  |  |  |
| 1 | Ath-AT4G08395.1 |  |  |  | | | |  |  |  |  |  |  |
| 1 | Ath-AT4G08400.1 |  |  |  | | | |  |  |  |  |  |  |
| 1 | Ath-AT4G08406.1 |  |  |  | | | |  |  |  |  |  |  |
| 1 | Ath-AT4G08410.1 |  |  |  | | | |  |  |  |  |  |  |
| 1 | Ath-AT4G08430.1 |  |  |  | | | |  |  |  |  |  |  |
| 1 | Ath-AT4G08450.1 |  |  |  | | | |  |  |  |  |  |  |
| 1 | Ath-AT4G08455.1 |  |  |  | | | |  |  |  |  |  |  |
| 1 | Ath-AT4G08460.4 |  |  |  | Vvi-Vitvi18g00328\_t001 |  |  |  |  |  |  |
| 1 | Ath-AT4G08470.1 |  |  |  | Vvi-Vitvi18g00329\_t001 |  |  |  |  |  |  |
| 1 | Ath-AT4G08480.1 |  |  |  | | | |  |  |  |  |  |  |
| 1 | Ath-AT4G08485.1 |  |  |  | | | |  |  |  |  |  |  |
| 1 | Ath-AT4G08500.1 |  |  |  | | | |  |  |  |  |  |  |
| 1 | Ath-AT4G08510.1 |  |  |  | Vvi-Vitvi18g00330\_t001 |  |  |  |  |  |  |
| 1 | Ath-AT4G08520.1 |  |  |  | | | |  |  |  |  |  |  |
| 1 | Ath-AT4G08530.1 |  |  |  | | | |  |  |  |  |  |  |
| 1 | Ath-AT4G08535.1 |  |  |  | | | |  |  |  |  |  |  |
| 1 | Ath-AT4G08540.1 |  |  |  | Vvi-Vitvi18g00348\_t001 |  |  |  |  |  |  |
| 1 | Ath-AT4G08545.1 |  |  |  | | | |  |  |  |  |  |  |
| 1 | Ath-AT4G08550.2 |  |  |  | Vvi-Vitvi18g00351\_t001 |  |  |  |  |  |  |
| 1 | Ath-AT4G08555.1 |  |  |  | | | |  |  |  |  |  |  |
| 1 | Ath-AT4G08560.1 |  |  |  | | | |  |  |  |  |  |  |
| 1 | Ath-AT4G08570.1 |  |  |  | Vvi-Vitvi18g02559\_t001 |  |  |  |  |  |  |
| 1 | Ath-AT4G08580.1 |  |  |  | | | |  |  |  |  |  |  |
| 1 | Ath-AT4G08590.1 |  |  |  | | | |  |  |  |  |  |  |
| 1 | Ath-AT4G08593.1 |  |  |  | | | |  |  |  |  |  |  |
| 1 | Ath-AT4G08620.1 |  |  |  | Vvi-Vitvi18g00363\_t001 |  |  |  |  |  |  |
| 1 | Ath-AT4G08630.1 |  |  |  | | | |  |  |  |  |  |  |
| 1 | Ath-AT4G08640.1 |  |  |  | | | |  |  |  |  |  |  |
| 1 | Ath-AT4G08670.1 |  |  |  | | | |  |  |  |  |  |  |
| 1 | Ath-AT4G08685.1 |  |  |  | Vvi-Vitvi18g00371\_t001 |  |  |  |  |  |  |
| 1 | Ath-AT4G08690.2 |  |  |  | Vvi-Vitvi18g00372\_t001 |  |  |  |  |  |  |
| 1 | Ath-AT4G08691.1 |  |  |  | | | |  |  |  |  |  |  |
| 1 | Ath-AT4G08700.1 |  |  |  | | | |  |  |  |  |  |  |
| 1 | Ath-AT4G08730.1 |  |  |  | | | |  |  |  |  |  |  |
| 1 | Ath-AT4G08740.1 |  |  |  | | | |  |  |  |  |  |  |
| 1 | Ath-AT4G08760.1 |  |  |  | | | |  |  |  |  |  |  |
| 1 | Ath-AT4G08770.1 |  |  |  | | | |  |  |  |  |  |  |
| 1 | Ath-AT4G08780.1 |  |  |  | | | |  |  |  |  |  |  |
| 1 | Ath-AT4G08790.1 |  |  |  | | | |  |  |  |  |  |  |
| 1 | Ath-AT4G08800.1 |  |  |  | | | |  |  |  |  |  |  |
| 1 | Ath-AT4G08810.1 |  |  |  | | | |  |  |  |  |  |  |
| 1 | Ath-AT4G08840.1 |  |  |  | Vvi-Vitvi18g02573\_t001 |  |  |  |  |  |  |
| 1 | Ath-AT4G08850.1 |  |  |  | | | |  |  |  |  |  |  |
| 1 | Ath-AT4G08870.1 |  |  |  | | | |  |  |  |  |  |  |
| 1 | Ath-AT4G08869.1 |  |  |  | | | |  |  |  |  |  |  |
| 1 | Ath-AT4G08867.1 |  |  |  | | | |  |  |  |  |  |  |
| 1 | Ath-AT4G08868.1 |  |  |  | | | |  |  |  |  |  |  |
| 1 | Ath-AT4G08874.2 |  |  |  | | | |  |  |  |  |  |  |
| 1 | Ath-AT4G08875.1 |  |  |  | | | |  |  |  |  |  |  |
| 1 | Ath-AT4G08876.1 |  |  |  | | | |  |  |  |  |  |  |
| 1 | Ath-AT4G08878.1 |  |  |  | | | |  |  |  |  |  |  |
| 1 | Ath-AT4G08895.1 |  |  |  | | | |  |  |  |  |  |  |
| 1 | Ath-AT4G08900.1 |  |  |  | | | |  |  |  |  |  |  |
| 1 | Ath-AT4G08910.1 |  |  |  | Vvi-Vitvi18g00398\_t001 |  |  |  |  |  |  |
| 1 | Ath-AT4G08920.1 |  |  |  | Vvi-Vitvi18g00407\_t001 |  |  |  |  |  |  |
| 1 | Ath-AT4G08930.1 |  |  |  | Vvi-Vitvi18g00410\_t001 |  |  |  |  |  |  |
| 1 | Ath-AT4G08940.1 |  |  |  | Vvi-Vitvi18g00413\_t001 |  |  |  |  |  |  |
| 1 | Ath-AT4G08950.1 |  |  |  | Vvi-Vitvi18g00431\_t001 |  |  |  |  |  |  |
| 1 | Ath-AT4G08960.1 |  |  |  | Vvi-Vitvi18g00434\_t001 |  |  |  |  |  |  |
| 1 | Ath-AT4G08980.4 |  |  |  | Vvi-Vitvi18g00435\_t002 |  |  |  |  |  |  |
| 1 | Ath-AT4G08990.1 |  |  |  | | | |  |  |  |  |  |  |
| 1 | Ath-AT4G09000.2 |  |  |  | Vvi-Vitvi18g00442\_t001 |  |  |  |  |  |  |
| 1 | Ath-AT4G09010.3 |  |  |  | Vvi-Vitvi18g00445\_t001 |  |  |  |  |  |  |
| 1 | Ath-AT4G09012.1 |  |  |  | Vvi-Vitvi18g00447\_t001 |  |  |  |  |  |  |
| 1 | Ath-AT4G09020.1 |  |  |  | Vvi-Vitvi18g00454\_t001 |  |  |  |  |  |  |
| 1 | Ath-AT4G09030.1 |  |  |  | | | |  |  |  |  |  |  |
| 1 | Ath-AT4G09035.1 |  |  |  | | | |  |  |  |  |  |  |
| 1 | Ath-AT4G09040.1 |  |  |  | Vvi-Vitvi18g00456\_t001 |  |  |  |  |  |  |
| 1 | Ath-AT4G09060.2 |  |  |  | Vvi-Vitvi18g00457\_t001 |  |  |  |  |  |  |
| 0 | Ath-AT4G09070.1 |  |  |  |  |  |  |  |  |
| 0 | Ath-AT4G09080.2 |  |  |  |  |  |  |  |  |
| 0 | Ath-AT4G09090.1 |  |  |  |  |  |  |  |  |
| 0 | Ath-AT4G09100.1 |  |  |  |  |  |  |  |  |
| 0 | Ath-AT4G09110.1 |  |  |  |  |  |  |  |  |
| 0 | Ath-AT4G09120.1 |  |  |  |  |  |  |  |  |
| 0 | Ath-AT4G09130.1 |  |  |  |  |  |  |  |  |
| 0 | Ath-AT4G09140.1 |  |  |  |  |  |  |  |  |
| 0 | Ath-AT4G09150.2 |  |  |  |  |  |  |  |  |
| 0 | Ath-AT4G09153.1 |  |  |  |  |  |  |  |  |
| 0 | Ath-AT4G09160.1 |  |  |  |  |  |  |  |  |
| 0 | Ath-AT4G09170.1 |  |  |  |  |  |  |  |  |
| 0 | Ath-AT4G09180.1 |  |  |  |  |  |  |  |  |
| 0 | Ath-AT4G09190.1 |  |  |  |  |  |  |  |  |
| 0 | Ath-AT4G09200.1 |  |  |  |  |  |  |  |  |
| 0 | Ath-AT4G09210.1 |  |  |  |  |  |  |  |  |
| 0 | Ath-AT4G09250.1 |  |  |  |  |  |  |  |  |
| 0 | Ath-AT4G09260.1 |  |  |  |  |  |  |  |  |
| 0 | Ath-AT4G09300.4 |  |  |  |  |  |  |  |  |
| 0 | Ath-AT4G09310.1 |  |  |  |  |  |  |  |  |
| 0 | Ath-AT4G09320.1 |  |  |  |  |  |  |  |  |
| 0 | Ath-AT4G09340.1 |  |  |  |  |  |  |  |  |
| 0 | Ath-AT4G09350.1 |  |  |  |  |  |  |  |  |
| 0 | Ath-AT4G09355.1 |  |  |  |  |  |  |  |  |
| 0 | Ath-AT4G09360.1 |  |  |  |  |  |  |  |  |
| 0 | Ath-AT4G09420.1 |  |  |  |  |  |  |  |  |
| 0 | Ath-AT4G09430.1 |  |  |  |  |  |  |  |  |
| 0 | Ath-AT4G09435.1 |  |  |  |  |  |  |  |  |
| 0 | Ath-AT4G09440.1 |  |  |  |  |  |  |  |  |
| 0 | Ath-AT4G09450.1 |  |  |  |  |  |  |  |  |
| 0 | Ath-AT4G09460.1 |  |  |  |  |  |  |  |  |
| 0 | Ath-AT4G09462.1 |  |  |  |  |  |  |  |  |
| 0 | Ath-AT4G09464.1 |  |  |  |  |  |  |  |  |
| 0 | Ath-AT4G09465.1 |  |  |  |  |  |  |  |  |
| 0 | Ath-AT4G09466.1 |  |  |  |  |  |  |  |  |
| 0 | Ath-AT4G09467.1 |  |  |  |  |  |  |  |  |
| 0 | Ath-AT4G09490.1 |  |  |  |  |  |  |  |  |
| 0 | Ath-AT4G09500.2 |  |  |  |  |  |  |  |  |
| 0 | Ath-AT4G09510.1 |  |  |  |  |  |  |  |  |
| 0 | Ath-AT4G09520.1 |  |  |  |  |  |  |  |  |
| 0 | Ath-AT4G09530.1 |  |  |  |  |  |  |  |  |
| 0 | Ath-AT4G09545.1 |  |  |  |  |  |  |  |  |
| 0 | Ath-AT4G09550.1 |  |  |  |  |  |  |  |  |
| 0 | Ath-AT4G09560.1 |  |  |  |  |  |  |  |  |
| 0 | Ath-AT4G09570.1 |  |  |  |  |  |  |  |  |
| 0 | Ath-AT4G09580.1 |  |  |  |  |  |  |  |  |
| 0 | Ath-AT4G09585.1 |  |  |  |  |  |  |  |  |
| 0 | Ath-AT4G09589.1 |  |  |  |  |  |  |  |  |
| 0 | Ath-AT4G09590.1 |  |  |  |  |  |  |  |  |
| 0 | Ath-AT4G09600.1 |  |  |  |  |  |  |  |  |
| 0 | Ath-AT4G09610.1 |  |  |  |  |  |  |  |  |
| 0 | Ath-AT4G09620.1 |  |  |  |  |  |  |  |  |
| 0 | Ath-AT4G09630.1 |  |  |  |  |  |  |  |  |
| 0 | Ath-AT4G09640.1 |  |  |  |  |  |  |  |  |
| 0 | Ath-AT4G09647.1 |  |  |  |  |  |  |  |  |
| 0 | Ath-AT4G09649.1 |  |  |  |  |  |  |  |  |
| 0 | Ath-AT4G09650.1 |  |  |  |  |  |  |  |  |
| 0 | Ath-AT4G09660.1 |  |  |  |  |  |  |  |  |
| 1 | Ath-AT4G09670.1 |  | Vvi-Vitvi18g01501\_t001 |  |  |  |  |  |  |  |
| 1 | Ath-AT4G09680.1 |  | | | |  |  |  |  |  |  |  |
| 1 | Ath-AT4G09690.1 |  | | | |  |  |  |  |  |  |  |
| 1 | Ath-AT4G09720.3 |  | Vvi-Vitvi18g01535\_t001 |  |  |  |  |  |  |  |
| 1 | Ath-AT4G09730.1 |  | Vvi-Vitvi18g01543\_t001 |  |  |  |  |  |  |  |
| 1 | Ath-AT4G09731.1 |  | | | |  |  |  |  |  |  |  |
| 1 | Ath-AT4G09740.1 |  | | | |  |  |  |  |  |  |  |
| 1 | Ath-AT4G09750.1 |  | Vvi-Vitvi18g01573\_t001 |  |  |  |  |  |  |  |
| 1 | Ath-AT4G09760.2 |  | Vvi-Vitvi18g01572\_t001.1.6037826d |  |  |  |  |  |  |  |
| 1 | Ath-AT4G09770.4 |  | | | |  |  |  |  |  |  |  |
| 1 | Ath-AT4G09775.1 |  | | | |  |  |  |  |  |  |  |
| 1 | Ath-AT4G09780.1 |  | | | |  |  |  |  |  |  |  |
| 1 | Ath-AT4G09784.1 |  | | | |  |  |  |  |  |  |  |
| 1 | Ath-AT4G09795.1 |  | | | |  |  |  |  |  |  |  |
| 1 | Ath-AT4G09800.1 |  | Vvi-Vitvi18g04412\_t001 |  |  |  |  |  |  |  |
| 1 | Ath-AT4G09810.1 |  | Vvi-Vitvi18g01602\_t001 |  |  |  |  |  |  |  |
| 1 | Ath-AT4G09820.1 |  | | | |  |  |  |  |  |  |  |
| 1 | Ath-AT4G09830.1 |  | Vvi-Vitvi18g01603\_t001 |  |  |  |  |  |  |  |
| 1 | Ath-AT4G09840.1 |  | | | |  |  |  |  |  |  |  |
| 1 | Ath-AT4G09850.1 |  | | | |  |  |  |  |  |  |  |
| 1 | Ath-AT4G09860.1 |  | | | |  |  |  |  |  |  |  |
| 1 | Ath-AT4G09870.1 |  | | | |  |  |  |  |  |  |  |
| 1 | Ath-AT4G09880.1 |  | | | |  |  |  |  |  |  |  |
| 1 | Ath-AT4G09890.1 |  | Vvi-Vitvi18g04416\_t001 |  |  |  |  |  |  |  |
| 0 | Ath-AT4G09900.1 |  |  |  |  |  |  |  |  |
| 0 | Ath-AT4G09920.1 |  |  |  |  |  |  |  |  |
| 0 | Ath-AT4G09930.1 |  |  |  |  |  |  |  |  |
| 0 | Ath-AT4G09940.2 |  |  |  |  |  |  |  |  |
| 0 | Ath-AT4G09950.1 |  |  |  |  |  |  |  |  |
| 0 | Ath-AT4G09960.4 |  |  |  |  |  |  |  |  |
| 0 | Ath-AT4G09965.1 |  |  |  |  |  |  |  |  |
| 0 | Ath-AT4G09970.1 |  |  |  |  |  |  |  |  |
| 0 | Ath-AT4G09980.1 |  |  |  |  |  |  |  |  |
| 0 | Ath-AT4G09984.1 |  |  |  |  |  |  |  |  |
| 0 | Ath-AT4G09990.1 |  |  |  |  |  |  |  |  |
| 0 | Ath-AT4G10000.1 |  |  |  |  |  |  |  |  |
| 0 | Ath-AT4G10010.2 |  |  |  |  |  |  |  |  |
| 0 | Ath-AT4G10020.1 |  |  |  |  |  |  |  |  |
| 0 | Ath-AT4G10030.1 |  |  |  |  |  |  |  |  |
| 0 | Ath-AT4G10040.1 |  |  |  |  |  |  |  |  |
| 0 | Ath-AT4G10050.1 |  |  |  |  |  |  |  |  |
| 0 | Ath-AT4G10060.1 |  |  |  |  |  |  |  |  |
| 0 | Ath-AT4G10070.1 |  |  |  |  |  |  |  |  |
| 1 | Ath-AT4G10080.1 |  | Vvi-Vitvi18g02324\_t001 |  |  |  |  |  |  |  |
| 1 | Ath-AT4G10090.1 |  | | | |  |  |  |  |  |  |  |
| 1 | Ath-AT4G10100.3 |  | Vvi-Vitvi18g04729\_t001 |  |  |  |  |  |  |  |
| 1 | Ath-AT4G10110.1 |  | Vvi-Vitvi18g02346\_t001 |  |  |  |  |  |  |  |
| 1 | Ath-AT4G10115.1 |  | | | |  |  |  |  |  |  |  |
| 1 | Ath-AT4G10120.1 |  | Vvi-Vitvi18g02365\_t001 |  |  |  |  |  |  |  |
| 1 | Ath-AT4G10130.2 |  | | | |  |  |  |  |  |  |  |
| 1 | Ath-AT4G10140.1 |  | Vvi-Vitvi18g02380\_t001 |  |  |  |  |  |  |  |
| 1 | Ath-AT4G10150.1 |  | Vvi-Vitvi18g03257\_t001 |  |  |  |  |  |  |  |
| 1 | Ath-AT4G10160.1 |  | | | |  |  |  |  |  |  |  |
| 1 | Ath-AT4G10170.2 |  | Vvi-Vitvi18g03258\_t002 |  |  |  |  |  |  |  |
| 0 | Ath-AT4G10180.1 |  |  |  |  |  |  |  |  |
| 0 | Ath-AT4G10190.1 |  |  |  |  |  |  |  |  |
| 0 | Ath-AT4G10200.1 |  |  |  |  |  |  |  |  |
| 0 | Ath-AT4G10210.1 |  |  |  |  |  |  |  |  |
| 0 | Ath-AT4G10220.1 |  |  |  |  |  |  |  |  |
| 0 | Ath-AT4G10230.1 |  |  |  |  |  |  |  |  |
| 1 | Ath-AT4G10240.1 |  | Vvi-Vitvi18g02424\_t001 |  |  |  |  |  |  |  |
| 1 | Ath-AT4G10250.1 |  | Vvi-Vitvi18g02423\_t001 |  |  |  |  |  |  |  |
| 1 | Ath-AT4G10260.1 |  | Vvi-Vitvi18g02417\_t001 |  |  |  |  |  |  |  |
| 1 | Ath-AT4G10265.1 |  | | | |  |  |  |  |  |  |  |
| 1 | Ath-AT4G10270.1 |  | | | |  |  |  |  |  |  |  |
| 1 | Ath-AT4G10280.1 |  | Vvi-Vitvi18g02415\_t001 |  |  |  |  |  |  |  |
| 1 | Ath-AT4G10290.1 |  | | | |  |  |  |  |  |  |  |
| 1 | Ath-AT4G10300.1 |  | | | |  |  |  |  |  |  |  |
| 1 | Ath-AT4G10305.1 |  | | | |  |  |  |  |  |  |  |
| 1 | Ath-AT4G10310.1 |  | | | |  |  |  |  |  |  |  |
| 1 | Ath-AT4G10320.1 |  | | | |  |  |  |  |  |  |  |
| 1 | Ath-AT4G10330.1 |  | Vvi-Vitvi18g02409\_t001 |  |  |  |  |  |  |  |
| 1 | Ath-AT4G10340.1 |  | Vvi-Vitvi18g02408\_t001 |  |  |  |  |  |  |  |
| 1 | Ath-AT4G10350.1 |  | Vvi-Vitvi18g02404\_t001 |  |  |  |  |  |  |  |
| 0 | Ath-AT4G10360.2 |  |  |  |  |  |  |  |  |
| 0 | Ath-AT4G10370.1 |  |  |  |  |  |  |  |  |
| 1 | Ath-AT4G10380.1 |  | Vvi-Vitvi02g00295\_t001 |  |  |  |  |  |  |  |
| 1 | Ath-AT4G10390.1 |  | Vvi-Vitvi02g04067\_t001 |  |  |  |  |  |  |  |
| 1 | Ath-AT4G10400.1 |  | | | |  |  |  |  |  |  |  |
| 1 | Ath-AT4G10410.1 |  | | | |  |  |  |  |  |  |  |
| 1 | Ath-AT4G10420.1 |  | | | |  |  |  |  |  |  |  |
| 1 | Ath-AT4G10430.3 |  | Vvi-Vitvi02g00290\_t001 |  |  |  |  |  |  |  |
| 1 | Ath-AT4G10440.1 |  | Vvi-Vitvi02g00289\_t001 |  |  |  |  |  |  |  |
| 2 | Ath-AT4G10450.1 |  | | | |  | Vvi-Vitvi16g01324\_t001 |  |  |  |  |  |  |
| 2 | Ath-AT4G10457.1 |  | | | |  | | | |  |  |  |  |  |  |
| 2 | Ath-AT4G10465.1 |  | Vvi-Vitvi02g00276\_t001 |  | | | |  |  |  |  |  |  |
| 2 | Ath-AT4G10470.2 |  | | | |  | | | |  |  |  |  |  |  |
| 2 | Ath-AT4G10480.1 |  | | | |  | Vvi-Vitvi16g01333\_t001 |  |  |  |  |  |  |
| 2 | Ath-AT4G10490.1 |  | Vvi-Vitvi02g04059\_t001 |  | Vvi-Vitvi16g01336\_t001 |  |  |  |  |  |  |
| 2 | Ath-AT4G10500.1 |  | | | |  | | | |  |  |  |  |  |  |
| 2 | Ath-AT4G10510.3 |  | | | |  | Vvi-Vitvi16g01344\_t002 |  |  |  |  |  |  |
| 2 | Ath-AT4G10520.1 |  | Vvi-Vitvi02g00260\_t001 |  | | | |  |  |  |  |  |  |
| 2 | Ath-AT4G10530.1 |  | | | |  | | | |  |  |  |  |  |  |
| 2 | Ath-AT4G10540.1 |  | | | |  | | | |  |  |  |  |  |  |
| 2 | Ath-AT4G10550.3 |  | | | |  | | | |  |  |  |  |  |  |
| 2 | Ath-AT4G10560.1 |  | | | |  | | | |  |  |  |  |  |  |
| 2 | Ath-AT4G10570.1 |  | Vvi-Vitvi02g00247\_t001 |  | | | |  |  |  |  |  |  |
| 2 | Ath-AT4G10590.2 |  | | | |  | | | |  |  |  |  |  |  |
| 2 | Ath-AT4G10595.1 |  | | | |  | | | |  |  |  |  |  |  |
| 2 | Ath-AT4G10600.1 |  | Vvi-Vitvi02g00246\_t001 |  | | | |  |  |  |  |  |  |
| 2 | Ath-AT4G10603.1 |  | | | |  | | | |  |  |  |  |  |  |
| 2 | Ath-AT4G10610.1 |  | Vvi-Vitvi02g00245\_t001 |  | Vvi-Vitvi16g01350\_t001 |  |  |  |  |  |  |
| 2 | Ath-AT4G10613.1 |  | | | |  | | | |  |  |  |  |  |  |
| 2 | Ath-AT4G10620.1 |  | | | |  | | | |  |  |  |  |  |  |
| 2 | Ath-AT4G10630.1 |  | Vvi-Vitvi02g00241\_t001 |  | | | |  |  |  |  |  |  |
| 2 | Ath-AT4G10640.1 |  | Vvi-Vitvi02g00240\_t001 |  | Vvi-Vitvi16g01353\_t001 |  |  |  |  |  |  |
| 1 | Ath-AT4G10650.1 |  | | | |  |  |  |  |  |  |  |
| 1 | Ath-AT4G10660.1 |  | | | |  |  |  |  |  |  |  |
| 1 | Ath-AT4G10670.1 |  | | | |  |  |  |  |  |  |  |
| 1 | Ath-AT4G10680.1 |  | | | |  |  |  |  |  |  |  |
| 1 | Ath-AT4G10695.2 |  | | | |  |  |  |  |  |  |  |
| 1 | Ath-AT4G10700.1 |  | | | |  |  |  |  |  |  |  |
| 1 | Ath-AT4G10710.1 |  | | | |  |  |  |  |  |  |  |
| 1 | Ath-AT4G10720.1 |  | | | |  |  |  |  |  |  |  |
| 1 | Ath-AT4G10730.1 |  | Vvi-Vitvi02g00218\_t003 |  |  |  |  |  |  |  |
| 1 | Ath-AT4G10740.1 |  | | | |  |  |  |  |  |  |  |
| 1 | Ath-AT4G10750.1 |  | Vvi-Vitvi02g00213\_t001 |  |  |  |  |  |  |  |
| 1 | Ath-AT4G10760.1 |  | Vvi-Vitvi02g00210\_t001 |  |  |  |  |  |  |  |
| 1 | Ath-AT4G10767.1 |  | | | |  |  |  |  |  |  |  |
| 1 | Ath-AT4G10770.1 |  | Vvi-Vitvi02g00209\_t001 |  |  |  |  |  |  |  |
| 1 | Ath-AT4G10780.1 |  | | | |  |  |  |  |  |  |  |
| 1 | Ath-AT4G10790.1 |  | Vvi-Vitvi02g00205\_t001 |  |  |  |  |  |  |  |
| 1 | Ath-AT4G10800.1 |  | Vvi-Vitvi02g00201\_t001 |  |  |  |  |  |  |  |
| 1 | Ath-AT4G10810.1 |  | | | |  |  |  |  |  |  |  |
| 1 | Ath-AT4G10820.1 |  | | | |  |  |  |  |  |  |  |
| 1 | Ath-AT4G10840.1 |  | Vvi-Vitvi02g00182\_t001 |  |  |  |  |  |  |  |
| 1 | Ath-AT4G10843.3 |  | | | |  |  |  |  |  |  |  |
| 1 | Ath-AT4G10850.1 |  | Vvi-Vitvi02g00181\_t001 |  |  |  |  |  |  |  |
| 1 | Ath-AT4G10860.1 |  | | | |  |  |  |  |  |  |  |
| 1 | Ath-AT4G10870.1 |  | | | |  |  |  |  |  |  |  |
| 1 | Ath-AT4G10880.1 |  | | | |  |  |  |  |  |  |  |
| 1 | Ath-AT4G10890.1 |  | Vvi-Vitvi02g00178\_t001 |  |  |  |  |  |  |  |
| 0 | Ath-AT4G10895.1 |  |  |  |  |  |  |  |  |
| 0 | Ath-AT4G10910.1 |  |  |  |  |  |  |  |  |
| 1 | Ath-AT4G10920.2 |  | Vvi-Vitvi02g00159\_t001 |  |  |  |  |  |  |  |
| 1 | Ath-AT4G10925.1 |  | Vvi-Vitvi02g00157\_t001 |  |  |  |  |  |  |  |
| 1 | Ath-AT4G10930.1 |  | Vvi-Vitvi02g00153\_t001 |  |  |  |  |  |  |  |
| 1 | Ath-AT4G10950.2 |  | Vvi-Vitvi02g00151\_t001 |  |  |  |  |  |  |  |
| 1 | Ath-AT4G10955.1 |  | Vvi-Vitvi02g00146\_t003 |  |  |  |  |  |  |  |
| 1 | Ath-AT4G10960.1 |  | Vvi-Vitvi02g00143\_t001 |  |  |  |  |  |  |  |
| 1 | Ath-AT4G10970.6 |  | Vvi-Vitvi02g00138\_t001 |  |  |  |  |  |  |  |
| 1 | Ath-AT4G11000.1 |  | | | |  |  |  |  |  |  |  |
| 1 | Ath-AT4G11010.1 |  | Vvi-Vitvi02g00137\_t001 |  |  |  |  |  |  |  |
| 1 | Ath-AT4G11020.1 |  | | | |  |  |  |  |  |  |  |
| 1 | Ath-AT4G11030.1 |  | Vvi-Vitvi02g00128\_t001 |  |  |  |  |  |  |  |
| 1 | Ath-AT4G11040.1 |  | | | |  |  |  |  |  |  |  |
| 1 | Ath-AT4G11050.1 |  | Vvi-Vitvi02g00125\_t001 |  |  |  |  |  |  |  |
| 1 | Ath-AT4G11060.1 |  | | | |  |  |  |  |  |  |  |
| 1 | Ath-AT4G11070.1 |  | Vvi-Vitvi02g00114\_t001 |  |  |  |  |  |  |  |
| 1 | Ath-AT4G11080.1 |  | Vvi-Vitvi02g00108\_t001 |  |  |  |  |  |  |  |
| 1 | Ath-AT4G11090.1 |  | Vvi-Vitvi02g00106\_t001 |  |  |  |  |  |  |  |
| 1 | Ath-AT4G11100.1 |  | | | |  |  |  |  |  |  |  |
| 1 | Ath-AT4G11110.1 |  | Vvi-Vitvi02g00098\_t001 |  |  |  |  |  |  |  |
| 1 | Ath-AT4G11120.1 |  | | | |  |  |  |  |  |  |  |
| 1 | Ath-AT4G11130.1 |  | | | |  |  |  |  |  |  |  |
| 1 | Ath-AT4G11140.1 |  | Vvi-Vitvi02g00093\_t001 |  |  |  |  |  |  |  |
| 1 | Ath-AT4G11150.1 |  | Vvi-Vitvi02g01333\_t001 |  |  |  |  |  |  |  |
| 1 | Ath-AT4G11160.2 |  | | | |  |  |  |  |  |  |  |
| 1 | Ath-AT4G11170.1 |  | | | |  |  |  |  |  |  |  |
| 1 | Ath-AT4G11175.1 |  | | | |  |  |  |  |  |  |  |
| 1 | Ath-AT4G11180.1 |  | Vvi-Vitvi02g00069\_t001 |  |  |  |  |  |  |  |
| 1 | Ath-AT4G11190.1 |  | | | |  |  |  |  |  |  |  |
| 1 | Ath-AT4G11210.1 |  | | | |  |  |  |  |  |  |  |
| 1 | Ath-AT4G11211.1 |  | | | |  |  |  |  |  |  |  |
| 1 | Ath-AT4G11220.1 |  | Vvi-Vitvi02g00059\_t001 |  |  |  |  |  |  |  |
| 1 | Ath-AT4G11230.1 |  | Vvi-Vitvi02g00048\_t001 |  |  |  |  |  |  |  |
| 1 | Ath-AT4G11240.1 |  | Vvi-Vitvi02g00044\_t001 |  |  |  |  |  |  |  |
| 1 | Ath-AT4G11250.1 |  | | | |  |  |  |  |  |  |  |
| 1 | Ath-AT4G11260.1 |  | Vvi-Vitvi02g00042\_t001 |  |  |  |  |  |  |  |
| 1 | Ath-AT4G11270.1 |  | Vvi-Vitvi02g00041\_t001 |  |  |  |  |  |  |  |
| 1 | Ath-AT4G11280.1 |  | Vvi-Vitvi02g00032\_t001 |  |  |  |  |  |  |  |
| 1 | Ath-AT4G11290.1 |  | | | |  |  |  |  |  |  |  |
| 1 | Ath-AT4G11300.1 |  | Vvi-Vitvi02g00029\_t001 |  |  |  |  |  |  |  |
| 1 | Ath-AT4G11310.1 |  | | | |  |  |  |  |  |  |  |
| 1 | Ath-AT4G11320.1 |  | | | |  |  |  |  |  |  |  |
| 1 | Ath-AT4G11330.1 |  | Vvi-Vitvi02g00023\_t001 |  |  |  |  |  |  |  |
| 1 | Ath-AT4G11340.1 |  | | | |  |  |  |  |  |  |  |
| 2 | Ath-AT4G11350.1 |  | Vvi-Vitvi02g00017\_t001 |  | Vvi-Vitvi02g00293\_t001 |  |  |  |  |  |  |
| 2 | Ath-AT4G11360.1 |  | Vvi-Vitvi02g00013\_t001 |  | | | |  |  |  |  |  |  |
| 2 | Ath-AT4G11370.1 |  | | | |  | | | |  |  |  |  |  |  |
| 2 | Ath-AT4G11373.1 |  | | | |  | | | |  |  |  |  |  |  |
| 2 | Ath-AT4G11380.2 |  | Vvi-Vitvi02g00008\_t001 |  | | | |  |  |  |  |  |  |
| 2 | Ath-AT4G11385.1 |  | | | |  | | | |  |  |  |  |  |  |
| 2 | Ath-AT4G11390.2 |  | | | |  | | | |  |  |  |  |  |  |
| 2 | Ath-AT4G11393.1 |  | | | |  | | | |  |  |  |  |  |  |
| 2 | Ath-AT4G11400.1 |  | Vvi-Vitvi02g00002\_t001 |  | | | |  |  |  |  |  |  |
| 1 | Ath-AT4G11402.1 |  |  |  | | | |  |  |  |  |  |  |
| 1 | Ath-AT4G11410.1 |  |  |  | Vvi-Vitvi02g00309\_t001 |  |  |  |  |  |  |
| 1 | Ath-AT4G11420.1 |  |  |  | | | |  |  |  |  |  |  |
| 1 | Ath-AT4G11430.1 |  |  |  | | | |  |  |  |  |  |  |
| 1 | Ath-AT4G11440.1 |  |  |  | Vvi-Vitvi02g00320\_t001 |  |  |  |  |  |  |
| 1 | Ath-AT4G11450.1 |  |  |  | Vvi-Vitvi02g04072\_t001 |  |  |  |  |  |  |
| 1 | Ath-AT4G11460.1 |  |  |  | | | |  |  |  |  |  |  |
| 1 | Ath-AT4G11470.2 |  |  |  | | | |  |  |  |  |  |  |
| 1 | Ath-AT4G11480.1 |  |  |  | | | |  |  |  |  |  |  |
| 1 | Ath-AT4G11485.1 |  |  |  | | | |  |  |  |  |  |  |
| 1 | Ath-AT4G11490.1 |  |  |  | | | |  |  |  |  |  |  |
| 1 | Ath-AT4G11510.1 |  |  |  | | | |  |  |  |  |  |  |
| 1 | Ath-AT4G11521.1 |  |  |  | | | |  |  |  |  |  |  |
| 1 | Ath-AT4G11530.1 |  |  |  | | | |  |  |  |  |  |  |
| 1 | Ath-AT4G11540.1 |  |  |  | | | |  |  |  |  |  |  |
| 1 | Ath-AT4G11543.1 |  |  |  | | | |  |  |  |  |  |  |
| 1 | Ath-AT4G11547.1 |  |  |  | | | |  |  |  |  |  |  |
| 1 | Ath-AT4G11550.1 |  |  |  | | | |  |  |  |  |  |  |
| 1 | Ath-AT4G11560.1 |  |  |  | Vvi-Vitvi02g04073\_t001 |  |  |  |  |  |  |
| 1 | Ath-AT4G11570.2 |  |  |  | Vvi-Vitvi02g00324\_t001 |  |  |  |  |  |  |
| 1 | Ath-AT4G11580.1 |  |  |  | | | |  |  |  |  |  |  |
| 1 | Ath-AT4G11590.1 |  |  |  | | | |  |  |  |  |  |  |
| 1 | Ath-AT4G11600.1 |  |  |  | Vvi-Vitvi02g00332\_t001 |  |  |  |  |  |  |
| 1 | Ath-AT4G11610.1 |  |  |  | Vvi-Vitvi02g01381\_t001 |  |  |  |  |  |  |
| 1 | Ath-AT4G11630.1 |  |  |  | | | |  |  |  |  |  |  |
| 1 | Ath-AT4G11640.1 |  |  |  | Vvi-Vitvi02g00339\_t001 |  |  |  |  |  |  |
| 1 | Ath-AT4G11650.1 |  | Vvi-Vitvi02g00391\_t001 |  |  |  |  |  |  |  |
| 1 | Ath-AT4G11653.1 |  | | | |  |  |  |  |  |  |  |
| 2 | Ath-AT4G11655.1 |  | | | |  | Vvi-Vitvi02g01399\_t001 |  |  |  |  |  |  |
| 2 | Ath-AT4G11660.1 |  | | | |  | Vvi-Vitvi02g00387\_t001 |  |  |  |  |  |  |
| 2 | Ath-AT4G11670.2 |  | | | |  | Vvi-Vitvi02g00385\_t001 |  |  |  |  |  |  |
| 2 | Ath-AT4G11680.2 |  | | | |  | Vvi-Vitvi02g00384\_t001 |  |  |  |  |  |  |
| 2 | Ath-AT4G11690.1 |  | | | |  | | | |  |  |  |  |  |  |
| 2 | Ath-AT4G11700.1 |  | | | |  | | | |  |  |  |  |  |  |
| 2 | Ath-AT4G11720.1 |  | | | |  | Vvi-Vitvi02g00378\_t001 |  |  |  |  |  |  |
| 2 | Ath-AT4G11730.1 |  | | | |  | | | |  |  |  |  |  |  |
| 2 | Ath-AT4G11740.1 |  | | | |  | Vvi-Vitvi02g00373\_t001 |  |  |  |  |  |  |
| 1 | Ath-AT4G11745.1 |  | | | |  |  |  |  |  |  |  |
| 1 | Ath-AT4G11750.1 |  | | | |  |  |  |  |  |  |  |
| 1 | Ath-AT4G11760.1 |  | | | |  |  |  |  |  |  |  |
| 1 | Ath-AT4G11770.1 |  | | | |  |  |  |  |  |  |  |
| 1 | Ath-AT4G11780.1 |  | Vvi-Vitvi02g00409\_t001 |  |  |  |  |  |  |  |
| 1 | Ath-AT4G11790.1 |  | Vvi-Vitvi02g00411\_t001 |  |  |  |  |  |  |  |
| 1 | Ath-AT4G11800.1 |  | | | |  |  |  |  |  |  |  |
| 1 | Ath-AT4G11810.1 |  | Vvi-Vitvi02g00415\_t001 |  |  |  |  |  |  |  |
| 1 | Ath-AT4G11820.2 |  | Vvi-Vitvi02g00420\_t002 |  |  |  |  |  |  |  |
| 1 | Ath-AT4G11830.2 |  | Vvi-Vitvi02g00424\_t001 |  |  |  |  |  |  |  |
| 1 | Ath-AT4G11840.1 |  | | | |  |  |  |  |  |  |  |
| 1 | Ath-AT4G11845.1 |  | | | |  |  |  |  |  |  |  |
| 1 | Ath-AT4G11850.1 |  | | | |  |  |  |  |  |  |  |
| 1 | Ath-AT4G11860.1 |  | Vvi-Vitvi02g00426\_t001 |  |  |  |  |  |  |  |
| 1 | Ath-AT4G11870.1 |  | | | |  |  |  |  |  |  |  |
| 1 | Ath-AT4G11880.1 |  | Vvi-Vitvi02g00427\_t001 |  |  |  |  |  |  |  |
| 1 | Ath-AT4G11890.3 |  | | | |  |  |  |  |  |  |  |
| 1 | Ath-AT4G11900.1 |  | | | |  |  |  |  |  |  |  |
| 1 | Ath-AT4G11910.1 |  | Vvi-Vitvi02g00429\_t002 |  |  |  |  |  |  |  |
| 1 | Ath-AT4G11911.1 |  | | | |  |  |  |  |  |  |  |
| 1 | Ath-AT4G11920.1 |  | Vvi-Vitvi02g00431\_t001 |  |  |  |  |  |  |  |
| 1 | Ath-AT4G11930.1 |  | | | |  |  |  |  |  |  |  |
| 1 | Ath-AT4G11940.1 |  | | | |  |  |  |  |  |  |  |
| 1 | Ath-AT4G11950.1 |  | Vvi-Vitvi02g00432\_t001 |  |  |  |  |  |  |  |
| 1 | Ath-AT4G11960.1 |  | Vvi-Vitvi02g00434\_t001 |  |  |  |  |  |  |  |
| 1 | Ath-AT4G11970.4 |  | Vvi-Vitvi02g00440\_t002 |  |  |  |  |  |  |  |
| 1 | Ath-AT4G11980.1 |  | Vvi-Vitvi02g00445\_t001 |  |  |  |  |  |  |  |
| 1 | Ath-AT4G11990.1 |  | Vvi-Vitvi02g00454\_t001 |  |  |  |  |  |  |  |
| 1 | Ath-AT4G12000.2 |  | Vvi-Vitvi02g04119\_t001 |  |  |  |  |  |  |  |
| 1 | Ath-AT4G12005.1 |  | | | |  |  |  |  |  |  |  |
| 1 | Ath-AT4G12010.1 |  | | | |  |  |  |  |  |  |  |
| 1 | Ath-AT4G12020.2 |  | | | |  |  |  |  |  |  |  |
| 1 | Ath-AT4G12030.2 |  | Vvi-Vitvi02g04120\_t001 |  |  |  |  |  |  |  |
| 2 | Ath-AT4G12040.1 |  | Vvi-Vitvi02g00461\_t002 |  | Vvi-Vitvi16g00816\_t001 |  |  |  |  |  |  |
| 2 | Ath-AT4G12050.1 |  | Vvi-Vitvi02g00465\_t001 |  | | | |  |  |  |  |  |  |
| 2 | Ath-AT4G12060.1 |  | | | |  | | | |  |  |  |  |  |  |
| 2 | Ath-AT4G12070.1 |  | | | |  | | | |  |  |  |  |  |  |
| 2 | Ath-AT4G12080.1 |  | Vvi-Vitvi02g00468\_t001 |  | | | |  |  |  |  |  |  |
| 2 | Ath-AT4G12090.1 |  | Vvi-Vitvi02g00471\_t001 |  | | | |  |  |  |  |  |  |
| 2 | Ath-AT4G12100.1 |  | | | |  | | | |  |  |  |  |  |  |
| 2 | Ath-AT4G12110.1 |  | Vvi-Vitvi02g04126\_t001 |  | | | |  |  |  |  |  |  |
| 2 | Ath-AT4G12120.1 |  | | | |  | | | |  |  |  |  |  |  |
| 2 | Ath-AT4G12130.1 |  | Vvi-Vitvi02g00482\_t001 |  | | | |  |  |  |  |  |  |
| 2 | Ath-AT4G12140.1 |  | | | |  | | | |  |  |  |  |  |  |
| 2 | Ath-AT4G12150.1 |  | | | |  | | | |  |  |  |  |  |  |
| 2 | Ath-AT4G12170.1 |  | | | |  | | | |  |  |  |  |  |  |
| 2 | Ath-AT4G12190.1 |  | | | |  | | | |  |  |  |  |  |  |
| 2 | Ath-AT4G12210.1 |  | | | |  | | | |  |  |  |  |  |  |
| 2 | Ath-AT4G12220.1 |  | | | |  | | | |  |  |  |  |  |  |
| 2 | Ath-AT4G12230.1 |  | Vvi-Vitvi02g00486\_t001 |  | | | |  |  |  |  |  |  |
| 2 | Ath-AT4G12240.1 |  | Vvi-Vitvi02g04130\_t001 |  | | | |  |  |  |  |  |  |
| 2 | Ath-AT4G12250.1 |  | Vvi-Vitvi02g04132\_t001 |  | Vvi-Vitvi16g00797\_t001 |  |  |  |  |  |  |
| 2 | Ath-AT4G12270.1 |  | | | |  | | | |  |  |  |  |  |  |
| 2 | Ath-AT4G12280.1 |  | | | |  | | | |  |  |  |  |  |  |
| 2 | Ath-AT4G12290.1 |  | Vvi-Vitvi02g04136\_t001 |  | | | |  |  |  |  |  |  |
| 2 | Ath-AT4G12300.1 |  | | | |  | Vvi-Vitvi16g00789\_t001 |  |  |  |  |  |  |
| 2 | Ath-AT4G12310.1 |  | | | |  | | | |  |  |  |  |  |  |
| 2 | Ath-AT4G12320.1 |  | | | |  | | | |  |  |  |  |  |  |
| 2 | Ath-AT4G12330.1 |  | | | |  | | | |  |  |  |  |  |  |
| 2 | Ath-AT4G12334.1 |  | | | |  | | | |  |  |  |  |  |  |
| 2 | Ath-AT4G12340.1 |  | Vvi-Vitvi02g00496\_t001 |  | | | |  |  |  |  |  |  |
| 2 | Ath-AT4G12350.1 |  | | | |  | Vvi-Vitvi16g00775\_t001 |  |  |  |  |  |  |
| 2 | Ath-AT4G12360.1 |  | | | |  | | | |  |  |  |  |  |  |
| 2 | Ath-AT4G12370.1 |  | | | |  | | | |  |  |  |  |  |  |
| 2 | Ath-AT4G12380.1 |  | | | |  | | | |  |  |  |  |  |  |
| 2 | Ath-AT4G12382.1 |  | | | |  | | | |  |  |  |  |  |  |
| 2 | Ath-AT4G12390.1 |  | Vvi-Vitvi02g00499\_t001 |  | Vvi-Vitvi16g01800\_t001 |  |  |  |  |  |  |
| 2 | Ath-AT4G12400.2 |  | Vvi-Vitvi02g01852\_t001 |  | | | |  |  |  |  |  |  |
| 2 | Ath-AT4G12410.1 |  | Vvi-Vitvi02g00507\_t001 |  | | | |  |  |  |  |  |  |
| 2 | Ath-AT4G12420.2 |  | Vvi-Vitvi02g00511\_t001 |  | Vvi-Vitvi16g00715\_t001 |  |  |  |  |  |  |
| 2 | Ath-AT4G12430.1 |  | Vvi-Vitvi02g04146\_t001 |  | Vvi-Vitvi16g00712\_t001 |  |  |  |  |  |  |
| 1 | Ath-AT4G12440.2 |  | Vvi-Vitvi02g04155\_t001 |  |  |  |  |  |  |  |
| 1 | Ath-AT4G12450.1 |  | Vvi-Vitvi02g00526\_t001 |  |  |  |  |  |  |  |
| 1 | Ath-AT4G12460.7 |  | Vvi-Vitvi02g04156\_t002 |  |  |  |  |  |  |  |
| 1 | Ath-AT4G12470.1 |  | | | |  |  |  |  |  |  |  |
| 1 | Ath-AT4G12480.1 |  | | | |  |  |  |  |  |  |  |
| 1 | Ath-AT4G12485.1 |  | | | |  |  |  |  |  |  |  |
| 1 | Ath-AT4G12490.1 |  | Vvi-Vitvi02g01438\_t001 |  |  |  |  |  |  |  |
| 0 | Ath-AT4G12495.1 |  |  |  |  |  |  |  |  |
| 0 | Ath-AT4G12500.1 |  |  |  |  |  |  |  |  |
| 0 | Ath-AT4G12510.1 |  |  |  |  |  |  |  |  |
| 0 | Ath-AT4G12520.1 |  |  |  |  |  |  |  |  |
| 0 | Ath-AT4G12530.1 |  |  |  |  |  |  |  |  |
| 0 | Ath-AT4G12540.1 |  |  |  |  |  |  |  |  |
| 0 | Ath-AT4G12543.1 |  |  |  |  |  |  |  |  |
| 0 | Ath-AT4G12545.1 |  |  |  |  |  |  |  |  |
| 0 | Ath-AT4G12550.1 |  |  |  |  |  |  |  |  |
| 0 | Ath-AT4G12555.1 |  |  |  |  |  |  |  |  |
| 0 | Ath-AT4G12560.3 |  |  |  |  |  |  |  |  |
| 0 | Ath-AT4G12570.1 |  |  |  |  |  |  |  |  |
| 0 | Ath-AT4G12580.1 |  |  |  |  |  |  |  |  |
| 0 | Ath-AT4G12590.1 |  |  |  |  |  |  |  |  |
| 0 | Ath-AT4G12600.2 |  |  |  |  |  |  |  |  |
| 0 | Ath-AT4G12610.2 |  |  |  |  |  |  |  |  |
| 0 | Ath-AT4G12617.1 |  |  |  |  |  |  |  |  |
| 0 | Ath-AT4G12620.1 |  |  |  |  |  |  |  |  |
| 1 | Ath-AT4G12640.1 |  | Vvi-Vitvi12g00773\_t001 |  |  |  |  |  |  |  |
| 1 | Ath-AT4G12650.1 |  | | | |  |  |  |  |  |  |  |
| 1 | Ath-AT4G12670.2 |  | Vvi-Vitvi12g00771\_t001 |  |  |  |  |  |  |  |
| 1 | Ath-AT4G12680.1 |  | Vvi-Vitvi12g00763\_t001 |  |  |  |  |  |  |  |
| 1 | Ath-AT4G12690.2 |  | Vvi-Vitvi12g00762\_t001 |  |  |  |  |  |  |  |
| 1 | Ath-AT4G12700.1 |  | Vvi-Vitvi12g00753\_t001 |  |  |  |  |  |  |  |
| 1 | Ath-AT4G12710.1 |  | Vvi-Vitvi12g00752\_t001 |  |  |  |  |  |  |  |
| 1 | Ath-AT4G12720.4 |  | Vvi-Vitvi12g04246\_t001 |  |  |  |  |  |  |  |
| 1 | Ath-AT4G12730.1 |  | Vvi-Vitvi12g00721\_t001 |  |  |  |  |  |  |  |
| 0 | Ath-AT4G12731.1 |  |  |  |  |  |  |  |  |
| 0 | Ath-AT4G12735.1 |  |  |  |  |  |  |  |  |
| 0 | Ath-AT4G12740.1 |  |  |  |  |  |  |  |  |
| 0 | Ath-AT4G12750.1 |  |  |  |  |  |  |  |  |
| 0 | Ath-AT4G12760.1 |  |  |  |  |  |  |  |  |
| 0 | Ath-AT4G12770.1 |  |  |  |  |  |  |  |  |
| 0 | Ath-AT4G12780.1 |  |  |  |  |  |  |  |  |
| 0 | Ath-AT4G12790.6 |  |  |  |  |  |  |  |  |
| 0 | Ath-AT4G12800.2 |  |  |  |  |  |  |  |  |
| 0 | Ath-AT4G12810.1 |  |  |  |  |  |  |  |  |
| 0 | Ath-AT4G12820.1 |  |  |  |  |  |  |  |  |
| 0 | Ath-AT4G12825.1 |  |  |  |  |  |  |  |  |
| 0 | Ath-AT4G12830.1 |  |  |  |  |  |  |  |  |
| 0 | Ath-AT4G12840.1 |  |  |  |  |  |  |  |  |
| 0 | Ath-AT4G12850.1 |  |  |  |  |  |  |  |  |
| 0 | Ath-AT4G12860.1 |  |  |  |  |  |  |  |  |
| 0 | Ath-AT4G12870.1 |  |  |  |  |  |  |  |  |
| 0 | Ath-AT4G12880.1 |  |  |  |  |  |  |  |  |
| 0 | Ath-AT4G12890.1 |  |  |  |  |  |  |  |  |
| 0 | Ath-AT4G12900.1 |  |  |  |  |  |  |  |  |
| 0 | Ath-AT4G12910.1 |  |  |  |  |  |  |  |  |
| 0 | Ath-AT4G12920.1 |  |  |  |  |  |  |  |  |
| 0 | Ath-AT4G12930.1 |  |  |  |  |  |  |  |  |
| 0 | Ath-AT4G12940.1 |  |  |  |  |  |  |  |  |
| 0 | Ath-AT4G12950.1 |  |  |  |  |  |  |  |  |
| 0 | Ath-AT4G12960.2 |  |  |  |  |  |  |  |  |
| 0 | Ath-AT4G12970.1 |  |  |  |  |  |  |  |  |
| 0 | Ath-AT4G12980.1 |  |  |  |  |  |  |  |  |
| 0 | Ath-AT4G12990.2 |  |  |  |  |  |  |  |  |
| 0 | Ath-AT4G13000.1 |  |  |  |  |  |  |  |  |
| 0 | Ath-AT4G13010.1 |  |  |  |  |  |  |  |  |
| 0 | Ath-AT4G13020.5 |  |  |  |  |  |  |  |  |
| 0 | Ath-AT4G13030.3 |  |  |  |  |  |  |  |  |
| 0 | Ath-AT4G13040.3 |  |  |  |  |  |  |  |  |
| 0 | Ath-AT4G13050.1 |  |  |  |  |  |  |  |  |
| 0 | Ath-AT4G13060.1 |  |  |  |  |  |  |  |  |
| 0 | Ath-AT4G13070.1 |  |  |  |  |  |  |  |  |
| 0 | Ath-AT4G13075.1 |  |  |  |  |  |  |  |  |
| 0 | Ath-AT4G13080.1 |  |  |  |  |  |  |  |  |
| 0 | Ath-AT4G13090.1 |  |  |  |  |  |  |  |  |
| 0 | Ath-AT4G13095.1 |  |  |  |  |  |  |  |  |
| 0 | Ath-AT4G13100.2 |  |  |  |  |  |  |  |  |
| 0 | Ath-AT4G13110.1 |  |  |  |  |  |  |  |  |
| 0 | Ath-AT4G13130.1 |  |  |  |  |  |  |  |  |
| 0 | Ath-AT4G13150.1 |  |  |  |  |  |  |  |  |
| 0 | Ath-AT4G13160.1 |  |  |  |  |  |  |  |  |
| 0 | Ath-AT4G13170.1 |  |  |  |  |  |  |  |  |
| 0 | Ath-AT4G13180.1 |  |  |  |  |  |  |  |  |
| 0 | Ath-AT4G13190.1 |  |  |  |  |  |  |  |  |
| 0 | Ath-AT4G13195.1 |  |  |  |  |  |  |  |  |
| 0 | Ath-AT4G13200.1 |  |  |  |  |  |  |  |  |
| 0 | Ath-AT4G13210.2 |  |  |  |  |  |  |  |  |
| 0 | Ath-AT4G13220.1 |  |  |  |  |  |  |  |  |
| 0 | Ath-AT4G13230.1 |  |  |  |  |  |  |  |  |
| 0 | Ath-AT4G13235.1 |  |  |  |  |  |  |  |  |
| 1 | Ath-AT4G13240.1 |  | Vvi-Vitvi11g00319\_t001 |  |  |  |  |  |  |  |
| 1 | Ath-AT4G13250.1 |  | Vvi-Vitvi11g00333\_t001 |  |  |  |  |  |  |  |
| 1 | Ath-AT4G13266.1 |  | | | |  |  |  |  |  |  |  |
| 1 | Ath-AT4G13261.1 |  | | | |  |  |  |  |  |  |  |
| 1 | Ath-AT4G13263.1 |  | | | |  |  |  |  |  |  |  |
| 1 | Ath-AT4G13260.1 |  | Vvi-Vitvi11g00338\_t001 |  |  |  |  |  |  |  |
| 1 | Ath-AT4G13270.1 |  | Vvi-Vitvi11g00348\_t001 |  |  |  |  |  |  |  |
| 1 | Ath-AT4G13280.2 |  | | | |  |  |  |  |  |  |  |
| 1 | Ath-AT4G13290.1 |  | | | |  |  |  |  |  |  |  |
| 1 | Ath-AT4G13300.1 |  | | | |  |  |  |  |  |  |  |
| 1 | Ath-AT4G13310.1 |  | | | |  |  |  |  |  |  |  |
| 2 | Ath-AT4G13330.2 |  | | | |  | Vvi-Vitvi05g00830\_t001 |  |  |  |  |  |  |
| 2 | Ath-AT4G13320.1 |  | | | |  | | | |  |  |  |  |  |  |
| 2 | Ath-AT4G13340.1 |  | Vvi-Vitvi11g00360\_t001 |  | | | |  |  |  |  |  |  |
| 2 | Ath-AT4G13345.4 |  | Vvi-Vitvi11g00362\_t001 |  | | | |  |  |  |  |  |  |
| 2 | Ath-AT4G13350.2 |  | Vvi-Vitvi11g00363\_t001 |  | | | |  |  |  |  |  |  |
| 2 | Ath-AT4G13360.1 |  | Vvi-Vitvi11g00373\_t001 |  | | | |  |  |  |  |  |  |
| 2 | Ath-AT4G13370.1 |  | | | |  | | | |  |  |  |  |  |  |
| 2 | Ath-AT4G13380.1 |  | | | |  | | | |  |  |  |  |  |  |
| 2 | Ath-AT4G13390.1 |  | | | |  | | | |  |  |  |  |  |  |
| 2 | Ath-AT4G13395.1 |  | | | |  | | | |  |  |  |  |  |  |
| 2 | Ath-AT4G13400.1 |  | Vvi-Vitvi11g01414\_t001 |  | | | |  |  |  |  |  |  |
| 1 | Ath-AT4G13410.1 |  |  |  | | | |  |  |  |  |  |  |
| 1 | Ath-AT4G13420.1 |  |  |  | | | |  |  |  |  |  |  |
| 1 | Ath-AT4G13430.1 |  |  |  | Vvi-Vitvi05g00839\_t001 |  |  |  |  |  |  |
| 1 | Ath-AT4G13440.2 |  |  |  | Vvi-Vitvi05g00845\_t001 |  |  |  |  |  |  |
| 1 | Ath-AT4G13450.1 |  |  |  | Vvi-Vitvi05g00852\_t001 |  |  |  |  |  |  |
| 1 | Ath-AT4G13460.1 |  |  |  | Vvi-Vitvi05g00855\_t001 |  |  |  |  |  |  |
| 1 | Ath-AT4G13480.1 |  |  |  | Vvi-Vitvi05g00861\_t001 |  |  |  |  |  |  |
| 1 | Ath-AT4G13490.1 |  |  |  | | | |  |  |  |  |  |  |
| 1 | Ath-AT4G13500.1 |  |  |  | | | |  |  |  |  |  |  |
| 1 | Ath-AT4G13510.1 |  |  |  | Vvi-Vitvi05g00873\_t001 |  |  |  |  |  |  |
| 1 | Ath-AT4G13520.1 |  |  |  | | | |  |  |  |  |  |  |
| 1 | Ath-AT4G13530.1 |  |  |  | | | |  |  |  |  |  |  |
| 1 | Ath-AT4G13540.1 |  |  |  | | | |  |  |  |  |  |  |
| 1 | Ath-AT4G13550.2 |  |  |  | Vvi-Vitvi05g00884\_t001 |  |  |  |  |  |  |
| 0 | Ath-AT4G13560.2 |  |  |  |  |  |  |  |  |
| 0 | Ath-AT4G13570.2 |  |  |  |  |  |  |  |  |
| 0 | Ath-AT4G13572.1 |  |  |  |  |  |  |  |  |
| 0 | Ath-AT4G13575.2 |  |  |  |  |  |  |  |  |
| 0 | Ath-AT4G13577.1 |  |  |  |  |  |  |  |  |
| 0 | Ath-AT4G13580.1 |  |  |  |  |  |  |  |  |
| 0 | Ath-AT4G13590.1 |  |  |  |  |  |  |  |  |
| 0 | Ath-AT4G13600.1 |  |  |  |  |  |  |  |  |
| 0 | Ath-AT4G13610.1 |  |  |  |  |  |  |  |  |
| 0 | Ath-AT4G13615.1 |  |  |  |  |  |  |  |  |
| 0 | Ath-AT4G13620.1 |  |  |  |  |  |  |  |  |
| 0 | Ath-AT4G13630.1 |  |  |  |  |  |  |  |  |
| 0 | Ath-AT4G13640.2 |  |  |  |  |  |  |  |  |
| 0 | Ath-AT4G13650.1 |  |  |  |  |  |  |  |  |
| 0 | Ath-AT4G13660.1 |  |  |  |  |  |  |  |  |
| 0 | Ath-AT4G13670.1 |  |  |  |  |  |  |  |  |
| 0 | Ath-AT4G13680.1 |  |  |  |  |  |  |  |  |
| 0 | Ath-AT4G13690.1 |  |  |  |  |  |  |  |  |
| 0 | Ath-AT4G13700.1 |  |  |  |  |  |  |  |  |
| 0 | Ath-AT4G13710.1 |  |  |  |  |  |  |  |  |
| 0 | Ath-AT4G13720.2 |  |  |  |  |  |  |  |  |
| 0 | Ath-AT4G13730.1 |  |  |  |  |  |  |  |  |
| 0 | Ath-AT4G13750.1 |  |  |  |  |  |  |  |  |
| 0 | Ath-AT4G13760.1 |  |  |  |  |  |  |  |  |
| 0 | Ath-AT4G13770.1 |  |  |  |  |  |  |  |  |
| 0 | Ath-AT4G13780.1 |  |  |  |  |  |  |  |  |
| 0 | Ath-AT4G13790.1 |  |  |  |  |  |  |  |  |
| 1 | Ath-AT4G13800.1 |  | Vvi-Vitvi05g01128\_t001 |  |  |  |  |  |  |  |
| 1 | Ath-AT4G13810.2 |  | | | |  |  |  |  |  |  |  |
| 1 | Ath-AT4G13820.1 |  | | | |  |  |  |  |  |  |  |
| 1 | Ath-AT4G13830.2 |  | Vvi-Vitvi05g01118\_t001 |  |  |  |  |  |  |  |
| 1 | Ath-AT4G13840.1 |  | Vvi-Vitvi05g01116\_t001 |  |  |  |  |  |  |  |
| 1 | Ath-AT4G13850.1 |  | Vvi-Vitvi05g01112\_t001 |  |  |  |  |  |  |  |
| 1 | Ath-AT4G13860.1 |  | | | |  |  |  |  |  |  |  |
| 1 | Ath-AT4G13870.2 |  | Vvi-Vitvi05g01106\_t001 |  |  |  |  |  |  |  |
| 1 | Ath-AT4G13880.1 |  | | | |  |  |  |  |  |  |  |
| 1 | Ath-AT4G13885.1 |  | | | |  |  |  |  |  |  |  |
| 1 | Ath-AT4G13890.1 |  | | | |  |  |  |  |  |  |  |
| 1 | Ath-AT4G13920.1 |  | | | |  |  |  |  |  |  |  |
| 1 | Ath-AT4G13930.1 |  | | | |  |  |  |  |  |  |  |
| 1 | Ath-AT4G13940.1 |  | | | |  |  |  |  |  |  |  |
| 1 | Ath-AT4G13950.1 |  | Vvi-Vitvi05g02048\_t001 |  |  |  |  |  |  |  |
| 1 | Ath-AT4G13955.1 |  | | | |  |  |  |  |  |  |  |
| 1 | Ath-AT4G13960.1 |  | | | |  |  |  |  |  |  |  |
| 1 | Ath-AT4G13965.1 |  | | | |  |  |  |  |  |  |  |
| 1 | Ath-AT4G13968.1 |  | | | |  |  |  |  |  |  |  |
| 1 | Ath-AT4G13970.1 |  | | | |  |  |  |  |  |  |  |
| 1 | Ath-AT4G13980.1 |  | Vvi-Vitvi05g01097\_t001 |  |  |  |  |  |  |  |
| 1 | Ath-AT4G13985.1 |  | | | |  |  |  |  |  |  |  |
| 1 | Ath-AT4G13990.1 |  | | | |  |  |  |  |  |  |  |
| 1 | Ath-AT4G13992.1 |  | | | |  |  |  |  |  |  |  |
| 1 | Ath-AT4G14000.1 |  | | | |  |  |  |  |  |  |  |
| 1 | Ath-AT4G14010.1 |  | | | |  |  |  |  |  |  |  |
| 1 | Ath-AT4G14020.1 |  | | | |  |  |  |  |  |  |  |
| 1 | Ath-AT4G14030.2 |  | Vvi-Vitvi05g01088\_t001 |  |  |  |  |  |  |  |
| 1 | Ath-AT4G14040.1 |  | | | |  |  |  |  |  |  |  |
| 1 | Ath-AT4G14050.1 |  | Vvi-Vitvi05g01055\_t001 |  |  |  |  |  |  |  |
| 0 | Ath-AT4G14060.1 |  |  |  |  |  |  |  |  |
| 0 | Ath-AT4G14070.1 |  |  |  |  |  |  |  |  |
| 0 | Ath-AT4G14080.1 |  |  |  |  |  |  |  |  |
| 0 | Ath-AT4G14100.1 |  |  |  |  |  |  |  |  |
| 0 | Ath-AT4G14090.1 |  |  |  |  |  |  |  |  |
| 0 | Ath-AT4G14096.1 |  |  |  |  |  |  |  |  |
| 0 | Ath-AT4G14103.2 |  |  |  |  |  |  |  |  |
| 0 | Ath-AT4G14104.1 |  |  |  |  |  |  |  |  |
| 0 | Ath-AT4G14105.1 |  |  |  |  |  |  |  |  |
| 0 | Ath-AT4G14110.1 |  |  |  |  |  |  |  |  |
| 0 | Ath-AT4G14120.1 |  |  |  |  |  |  |  |  |
| 0 | Ath-AT4G14130.1 |  |  |  |  |  |  |  |  |
| 0 | Ath-AT4G14140.2 |  |  |  |  |  |  |  |  |
| 0 | Ath-AT4G14145.1 |  |  |  |  |  |  |  |  |
| 0 | Ath-AT4G14147.2 |  |  |  |  |  |  |  |  |
| 0 | Ath-AT4G14150.1 |  |  |  |  |  |  |  |  |
| 0 | Ath-AT4G14160.1 |  |  |  |  |  |  |  |  |
| 0 | Ath-AT4G14165.1 |  |  |  |  |  |  |  |  |
| 0 | Ath-AT4G14170.1 |  |  |  |  |  |  |  |  |
| 0 | Ath-AT4G14180.1 |  |  |  |  |  |  |  |  |
| 0 | Ath-AT4G14190.1 |  |  |  |  |  |  |  |  |
| 0 | Ath-AT4G14200.1 |  |  |  |  |  |  |  |  |
| 0 | Ath-AT4G14210.1 |  |  |  |  |  |  |  |  |
| 0 | Ath-AT4G14220.1 |  |  |  |  |  |  |  |  |
| 0 | Ath-AT4G14225.1 |  |  |  |  |  |  |  |  |
| 0 | Ath-AT4G14226.1 |  |  |  |  |  |  |  |  |
| 0 | Ath-AT4G14230.1 |  |  |  |  |  |  |  |  |
| 0 | Ath-AT4G14240.1 |  |  |  |  |  |  |  |  |
| 0 | Ath-AT4G14245.1 |  |  |  |  |  |  |  |  |
| 0 | Ath-AT4G14250.1 |  |  |  |  |  |  |  |  |
| 0 | Ath-AT4G14260.1 |  |  |  |  |  |  |  |  |
| 0 | Ath-AT4G14270.1 |  |  |  |  |  |  |  |  |
| 0 | Ath-AT4G14272.1 |  |  |  |  |  |  |  |  |
| 0 | Ath-AT4G14276.1 |  |  |  |  |  |  |  |  |
| 0 | Ath-AT4G14280.1 |  |  |  |  |  |  |  |  |
| 0 | Ath-AT4G14290.1 |  |  |  |  |  |  |  |  |
| 1 | Ath-AT4G14300.2 |  | Vvi-Vitvi05g00812\_t002 |  |  |  |  |  |  |  |
| 1 | Ath-AT4G14301.1 |  | | | |  |  |  |  |  |  |  |
| 1 | Ath-AT4G14305.1 |  | Vvi-Vitvi05g00787\_t001 |  |  |  |  |  |  |  |
| 1 | Ath-AT4G14310.2 |  | Vvi-Vitvi05g00783\_t001 |  |  |  |  |  |  |  |
| 1 | Ath-AT4G14315.1 |  | | | |  |  |  |  |  |  |  |
| 1 | Ath-AT4G14320.2 |  | | | |  |  |  |  |  |  |  |
| 1 | Ath-AT4G14330.1 |  | Vvi-Vitvi05g00780\_t001 |  |  |  |  |  |  |  |
| 1 | Ath-AT4G14340.1 |  | Vvi-Vitvi05g00767\_t001 |  |  |  |  |  |  |  |
| 1 | Ath-AT4G14342.2 |  | Vvi-Vitvi05g00765\_t001 |  |  |  |  |  |  |  |
| 1 | Ath-AT4G14350.1 |  | Vvi-Vitvi05g00764\_t001 |  |  |  |  |  |  |  |
| 1 | Ath-AT4G14360.2 |  | Vvi-Vitvi05g00759\_t001 |  |  |  |  |  |  |  |
| 1 | Ath-AT4G14358.1 |  | | | |  |  |  |  |  |  |  |
| 1 | Ath-AT4G14365.1 |  | Vvi-Vitvi05g00755\_t002 |  |  |  |  |  |  |  |
| 1 | Ath-AT4G14368.1 |  | Vvi-Vitvi05g00753\_t001 |  |  |  |  |  |  |  |
| 1 | Ath-AT4G14370.2 |  | | | |  |  |  |  |  |  |  |
| 1 | Ath-AT4G14380.1 |  | Vvi-Vitvi05g01958\_t001 |  |  |  |  |  |  |  |
| 1 | Ath-AT4G14385.1 |  | Vvi-Vitvi05g00742\_t001 |  |  |  |  |  |  |  |
| 0 | Ath-AT4G14390.1 |  |  |  |  |  |  |  |  |
| 0 | Ath-AT4G14400.1 |  |  |  |  |  |  |  |  |
| 1 | Ath-AT4G14410.1 |  | Vvi-Vitvi05g00711\_t001 |  |  |  |  |  |  |  |
| 1 | Ath-AT4G14420.1 |  | Vvi-Vitvi05g00692\_t001 |  |  |  |  |  |  |  |
| 1 | Ath-AT4G14430.1 |  | Vvi-Vitvi05g00690\_t001 |  |  |  |  |  |  |  |
| 1 | Ath-AT4G14440.1 |  | | | |  |  |  |  |  |  |  |
| 1 | Ath-AT4G14450.1 |  | Vvi-Vitvi05g01918\_t001 |  |  |  |  |  |  |  |
| 1 | Ath-AT4G14455.1 |  | | | |  |  |  |  |  |  |  |
| 1 | Ath-AT4G14465.1 |  | Vvi-Vitvi05g01916\_t001 |  |  |  |  |  |  |  |
| 1 | Ath-AT4G14480.1 |  | Vvi-Vitvi05g01912\_t001 |  |  |  |  |  |  |  |
| 1 | Ath-AT4G14490.1 |  | Vvi-Vitvi05g00663\_t001 |  |  |  |  |  |  |  |
| 1 | Ath-AT4G14500.1 |  | Vvi-Vitvi05g00651\_t001 |  |  |  |  |  |  |  |
| 1 | Ath-AT4G14510.1 |  | Vvi-Vitvi05g00639\_t001 |  |  |  |  |  |  |  |
| 1 | Ath-AT4G14520.2 |  | Vvi-Vitvi05g00638\_t001.1.6037826e |  |  |  |  |  |  |  |
| 1 | Ath-AT4G14530.1 |  | | | |  |  |  |  |  |  |  |
| 1 | Ath-AT4G14540.1 |  | | | |  |  |  |  |  |  |  |
| 2 | Ath-AT4G14550.2 |  | Vvi-Vitvi05g00630\_t001 |  | Vvi-Vitvi07g00042\_t001 |  |  |  |  |  |  |
| 2 | Ath-AT4G14560.1 |  | | | |  | | | |  |  |  |  |  |  |
| 2 | Ath-AT4G14570.1 |  | Vvi-Vitvi05g00620\_t001 |  | | | |  |  |  |  |  |  |
| 2 | Ath-AT4G14580.1 |  | Vvi-Vitvi05g00618\_t001 |  | | | |  |  |  |  |  |  |
| 2 | Ath-AT4G14590.1 |  | | | |  | | | |  |  |  |  |  |  |
| 2 | Ath-AT4G14600.1 |  | | | |  | | | |  |  |  |  |  |  |
| 2 | Ath-AT4G14605.1 |  | | | |  | | | |  |  |  |  |  |  |
| 2 | Ath-AT4G14615.1 |  | Vvi-Vitvi05g01904\_t001 |  | | | |  |  |  |  |  |  |
| 2 | Ath-AT4G14620.1 |  | Vvi-Vitvi05g04150\_t001 |  | Vvi-Vitvi07g00061\_t002 |  |  |  |  |  |  |
| 2 | Ath-AT4G14630.1 |  | | | |  | | | |  |  |  |  |  |  |
| 2 | Ath-AT4G14640.1 |  | Vvi-Vitvi05g00605\_t001 |  | Vvi-Vitvi07g00068\_t001 |  |  |  |  |  |  |
| 1 | Ath-AT4G14650.1 |  |  |  | | | |  |  |  |  |  |  |
| 1 | Ath-AT4G14660.1 |  |  |  | | | |  |  |  |  |  |  |
| 1 | Ath-AT4G14670.1 |  |  |  | | | |  |  |  |  |  |  |
| 1 | Ath-AT4G14675.1 |  |  |  | | | |  |  |  |  |  |  |
| 2 | Ath-AT4G14680.2 |  | Vvi-Vitvi05g00576\_t001 |  | | | |  |  |  |  |  |  |
| 2 | Ath-AT4G14690.1 |  | Vvi-Vitvi05g00563\_t001 |  | | | |  |  |  |  |  |  |
| 2 | Ath-AT4G14695.1 |  | | | |  | | | |  |  |  |  |  |  |
| 2 | Ath-AT4G14700.1 |  | | | |  | | | |  |  |  |  |  |  |
| 2 | Ath-AT4G14710.5 |  | Vvi-Vitvi05g00559\_t002 |  | | | |  |  |  |  |  |  |
| 2 | Ath-AT4G14713.1 |  | Vvi-Vitvi05g00558\_t002 |  | Vvi-Vitvi07g00082\_t001 |  |  |  |  |  |  |
| 2 | Ath-AT4G14716.1 |  | | | |  | | | |  |  |  |  |  |  |
| 2 | Ath-AT4G14720.2 |  | | | |  | | | |  |  |  |  |  |  |
| 2 | Ath-AT4G14723.1 |  | Vvi-Vitvi05g00557\_t001 |  | | | |  |  |  |  |  |  |
| 2 | Ath-AT4G14730.1 |  | Vvi-Vitvi05g00555\_t001 |  | Vvi-Vitvi07g00087\_t001 |  |  |  |  |  |  |
| 2 | Ath-AT4G14740.2 |  | Vvi-Vitvi05g00554\_t001 |  | Vvi-Vitvi07g00088\_t001 |  |  |  |  |  |  |
| 2 | Ath-AT4G14746.1 |  | Vvi-Vitvi05g00553\_t001 |  | | | |  |  |  |  |  |  |
| 2 | Ath-AT4G14750.3 |  | Vvi-Vitvi05g00549\_t001 |  | | | |  |  |  |  |  |  |
| 2 | Ath-AT4G14760.2 |  | Vvi-Vitvi05g00542\_t001 |  | Vvi-Vitvi07g00097\_t002 |  |  |  |  |  |  |
| 2 | Ath-AT4G14770.1 |  | Vvi-Vitvi05g00541\_t001 |  | | | |  |  |  |  |  |  |
| 2 | Ath-AT4G14780.1 |  | Vvi-Vitvi05g00540\_t001 |  | Vvi-Vitvi07g04032\_t001 |  |  |  |  |  |  |
| 2 | Ath-AT4G14785.1 |  | | | |  | | | |  |  |  |  |  |  |
| 2 | Ath-AT4G14790.2 |  | Vvi-Vitvi05g00534\_t001 |  | | | |  |  |  |  |  |  |
| 2 | Ath-AT4G14800.2 |  | | | |  | Vvi-Vitvi07g00109\_t001 |  |  |  |  |  |  |
| 2 | Ath-AT4G14805.1 |  | Vvi-Vitvi05g01894\_t001 |  | | | |  |  |  |  |  |  |
| 2 | Ath-AT4G14810.1 |  | | | |  | | | |  |  |  |  |  |  |
| 2 | Ath-AT4G14815.1 |  | Vvi-Vitvi05g00527\_t002 |  | Vvi-Vitvi07g02131\_t001 |  |  |  |  |  |  |
| 2 | Ath-AT4G14819.1 |  | Vvi-Vitvi05g00520\_t001 |  | | | |  |  |  |  |  |  |
| 2 | Ath-AT4G14820.1 |  | Vvi-Vitvi05g00518\_t001 |  | | | |  |  |  |  |  |  |
| 2 | Ath-AT4G14830.1 |  | Vvi-Vitvi05g00517\_t001 |  | Vvi-Vitvi07g00116\_t001 |  |  |  |  |  |  |
| 2 | Ath-AT4G14840.1 |  | Vvi-Vitvi05g04120\_t001 |  | | | |  |  |  |  |  |  |
| 2 | Ath-AT4G14850.1 |  | Vvi-Vitvi05g00513\_t001 |  | | | |  |  |  |  |  |  |
| 2 | Ath-AT4G14860.1 |  | Vvi-Vitvi05g00509\_t001 |  | Vvi-Vitvi07g04040\_t001 |  |  |  |  |  |  |
| 2 | Ath-AT4G14870.1 |  | Vvi-Vitvi05g00508\_t001 |  | | | |  |  |  |  |  |  |
| 2 | Ath-AT4G14880.2 |  | | | |  | | | |  |  |  |  |  |  |
| 2 | Ath-AT4G14900.1 |  | Vvi-Vitvi05g00503\_t001 |  | | | |  |  |  |  |  |  |
| 2 | Ath-AT4G14890.1 |  | | | |  | | | |  |  |  |  |  |  |
| 2 | Ath-AT4G14905.1 |  | | | |  | | | |  |  |  |  |  |  |
| 2 | Ath-AT4G14910.2 |  | Vvi-Vitvi05g00497\_t001 |  | | | |  |  |  |  |  |  |
| 2 | Ath-AT4G14920.3 |  | Vvi-Vitvi05g00496\_t001 |  | Vvi-Vitvi07g00120\_t001 |  |  |  |  |  |  |
| 1 | Ath-AT4G14930.1 |  | Vvi-Vitvi05g00490\_t001 |  |  |  |  |  |  |  |
| 1 | Ath-AT4G14940.1 |  | Vvi-Vitvi05g00481\_t001 |  |  |  |  |  |  |  |
| 1 | Ath-AT4G14950.1 |  | Vvi-Vitvi05g00470\_t001 |  |  |  |  |  |  |  |
| 0 | Ath-AT4G14960.2 |  |  |  |  |  |  |  |  |
| 0 | Ath-AT4G14965.1 |  |  |  |  |  |  |  |  |
| 0 | Ath-AT4G14970.1 |  |  |  |  |  |  |  |  |
| 0 | Ath-AT4G14980.1 |  |  |  |  |  |  |  |  |
| 0 | Ath-AT4G14990.1 |  |  |  |  |  |  |  |  |
| 0 | Ath-AT4G15000.1 |  |  |  |  |  |  |  |  |
| 0 | Ath-AT4G15010.3 |  |  |  |  |  |  |  |  |
| 0 | Ath-AT4G15020.2 |  |  |  |  |  |  |  |  |
| 0 | Ath-AT4G15025.1 |  |  |  |  |  |  |  |  |
| 0 | Ath-AT4G15030.3 |  |  |  |  |  |  |  |  |
| 0 | Ath-AT4G15040.2 |  |  |  |  |  |  |  |  |
| 0 | Ath-AT4G15050.1 |  |  |  |  |  |  |  |  |
| 0 | Ath-AT4G15053.1 |  |  |  |  |  |  |  |  |
| 0 | Ath-AT4G15056.1 |  |  |  |  |  |  |  |  |
| 0 | Ath-AT4G15060.1 |  |  |  |  |  |  |  |  |
| 0 | Ath-AT4G15070.1 |  |  |  |  |  |  |  |  |
| 0 | Ath-AT4G15075.1 |  |  |  |  |  |  |  |  |
| 0 | Ath-AT4G15080.1 |  |  |  |  |  |  |  |  |
| 0 | Ath-AT4G15090.2 |  |  |  |  |  |  |  |  |
| 0 | Ath-AT4G15093.1 |  |  |  |  |  |  |  |  |
| 0 | Ath-AT4G15096.1 |  |  |  |  |  |  |  |  |
| 0 | Ath-AT4G15100.1 |  |  |  |  |  |  |  |  |
| 0 | Ath-AT4G15110.1 |  |  |  |  |  |  |  |  |
| 0 | Ath-AT4G15120.1 |  |  |  |  |  |  |  |  |
| 0 | Ath-AT4G15140.1 |  |  |  |  |  |  |  |  |
| 0 | Ath-AT4G15150.1 |  |  |  |  |  |  |  |  |
| 0 | Ath-AT4G15160.1 |  |  |  |  |  |  |  |  |
| 0 | Ath-AT4G15165.1 |  |  |  |  |  |  |  |  |
| 0 | Ath-AT4G15180.1 |  |  |  |  |  |  |  |  |
| 0 | Ath-AT4G15200.2 |  |  |  |  |  |  |  |  |
| 0 | Ath-AT4G15210.1 |  |  |  |  |  |  |  |  |
| 0 | Ath-AT4G15215.11 |  |  |  |  |  |  |  |  |
| 0 | Ath-AT4G15230.3 |  |  |  |  |  |  |  |  |
| 0 | Ath-AT4G15233.1 |  |  |  |  |  |  |  |  |
| 0 | Ath-AT4G15236.1 |  |  |  |  |  |  |  |  |
| 0 | Ath-AT4G15240.1 |  |  |  |  |  |  |  |  |
| 0 | Ath-AT4G15245.1 |  |  |  |  |  |  |  |  |
| 0 | Ath-AT4G15248.1 |  |  |  |  |  |  |  |  |
| 0 | Ath-AT4G15250.1 |  |  |  |  |  |  |  |  |
| 0 | Ath-AT4G15260.1 |  |  |  |  |  |  |  |  |
| 0 | Ath-AT4G15270.3 |  |  |  |  |  |  |  |  |
| 0 | Ath-AT4G15280.2 |  |  |  |  |  |  |  |  |
| 0 | Ath-AT4G15290.1 |  |  |  |  |  |  |  |  |
| 0 | Ath-AT4G15300.1 |  |  |  |  |  |  |  |  |
| 0 | Ath-AT4G15310.1 |  |  |  |  |  |  |  |  |
| 0 | Ath-AT4G15320.1 |  |  |  |  |  |  |  |  |
| 0 | Ath-AT4G15330.1 |  |  |  |  |  |  |  |  |
| 0 | Ath-AT4G15340.1 |  |  |  |  |  |  |  |  |
| 0 | Ath-AT4G15350.1 |  |  |  |  |  |  |  |  |
| 0 | Ath-AT4G15360.1 |  |  |  |  |  |  |  |  |
| 0 | Ath-AT4G15370.1 |  |  |  |  |  |  |  |  |
| 0 | Ath-AT4G15380.1 |  |  |  |  |  |  |  |  |
| 0 | Ath-AT4G15390.1 |  |  |  |  |  |  |  |  |
| 0 | Ath-AT4G15393.2 |  |  |  |  |  |  |  |  |
| 0 | Ath-AT4G15396.1 |  |  |  |  |  |  |  |  |
| 0 | Ath-AT4G15400.1 |  |  |  |  |  |  |  |  |
| 1 | Ath-AT4G15410.1 |  | Vvi-Vitvi12g00425\_t001 |  |  |  |  |  |  |  |
| 1 | Ath-AT4G15415.2 |  | Vvi-Vitvi12g00422\_t001 |  |  |  |  |  |  |  |
| 1 | Ath-AT4G15417.2 |  | Vvi-Vitvi12g00414\_t001 |  |  |  |  |  |  |  |
| 1 | Ath-AT4G15420.2 |  | Vvi-Vitvi12g00411\_t001 |  |  |  |  |  |  |  |
| 1 | Ath-AT4G15430.1 |  | Vvi-Vitvi12g00410\_t001 |  |  |  |  |  |  |  |
| 1 | Ath-AT4G15440.1 |  | Vvi-Vitvi12g00405\_t001 |  |  |  |  |  |  |  |
| 0 | Ath-AT4G15450.1 |  |  |  |  |  |  |  |  |
| 0 | Ath-AT4G15460.1 |  |  |  |  |  |  |  |  |
| 0 | Ath-AT4G15470.1 |  |  |  |  |  |  |  |  |
| 0 | Ath-AT4G15475.1 |  |  |  |  |  |  |  |  |
| 0 | Ath-AT4G15480.1 |  |  |  |  |  |  |  |  |
| 0 | Ath-AT4G15490.1 |  |  |  |  |  |  |  |  |
| 0 | Ath-AT4G15500.1 |  |  |  |  |  |  |  |  |
| 0 | Ath-AT4G15510.1 |  |  |  |  |  |  |  |  |
| 0 | Ath-AT4G15520.1 |  |  |  |  |  |  |  |  |
| 0 | Ath-AT4G15530.6 |  |  |  |  |  |  |  |  |
| 0 | Ath-AT4G15540.1 |  |  |  |  |  |  |  |  |
| 0 | Ath-AT4G15545.1 |  |  |  |  |  |  |  |  |
| 0 | Ath-AT4G15550.1 |  |  |  |  |  |  |  |  |
| 0 | Ath-AT4G15560.1 |  |  |  |  |  |  |  |  |
| 0 | Ath-AT4G15562.1 |  |  |  |  |  |  |  |  |
| 1 | Ath-AT4G15563.7 |  | Vvi-Vitvi05g00352\_t001 |  |  |  |  |  |  |  |
| 1 | Ath-AT4G15570.1 |  | | | |  |  |  |  |  |  |  |
| 1 | Ath-AT4G15610.1 |  | Vvi-Vitvi05g00349\_t001 |  |  |  |  |  |  |  |
| 1 | Ath-AT4G15620.1 |  | | | |  |  |  |  |  |  |  |
| 1 | Ath-AT4G15630.1 |  | | | |  |  |  |  |  |  |  |
| 1 | Ath-AT4G15640.1 |  | Vvi-Vitvi05g00348\_t001 |  |  |  |  |  |  |  |
| 1 | Ath-AT4G15650.1 |  | | | |  |  |  |  |  |  |  |
| 1 | Ath-AT4G15660.1 |  | | | |  |  |  |  |  |  |  |
| 1 | Ath-AT4G15670.1 |  | Vvi-Vitvi05g00342\_t001 |  |  |  |  |  |  |  |
| 1 | Ath-AT4G15680.1 |  | | | |  |  |  |  |  |  |  |
| 1 | Ath-AT4G15690.1 |  | | | |  |  |  |  |  |  |  |
| 1 | Ath-AT4G15700.1 |  | | | |  |  |  |  |  |  |  |
| 1 | Ath-AT4G15710.1 |  | | | |  |  |  |  |  |  |  |
| 1 | Ath-AT4G15715.1 |  | | | |  |  |  |  |  |  |  |
| 1 | Ath-AT4G15720.1 |  | | | |  |  |  |  |  |  |  |
| 1 | Ath-AT4G15730.1 |  | Vvi-Vitvi05g00326\_t001 |  |  |  |  |  |  |  |
| 1 | Ath-AT4G15733.1 |  | | | |  |  |  |  |  |  |  |
| 1 | Ath-AT4G15735.1 |  | | | |  |  |  |  |  |  |  |
| 1 | Ath-AT4G15740.1 |  | | | |  |  |  |  |  |  |  |
| 1 | Ath-AT4G15750.1 |  | | | |  |  |  |  |  |  |  |
| 1 | Ath-AT4G15755.1 |  | | | |  |  |  |  |  |  |  |
| 1 | Ath-AT4G15760.2 |  | | | |  |  |  |  |  |  |  |
| 1 | Ath-AT4G15765.1 |  | | | |  |  |  |  |  |  |  |
| 1 | Ath-AT4G15770.1 |  | | | |  |  |  |  |  |  |  |
| 1 | Ath-AT4G15780.1 |  | | | |  |  |  |  |  |  |  |
| 1 | Ath-AT4G15790.2 |  | Vvi-Vitvi05g00312\_t001 |  |  |  |  |  |  |  |
| 1 | Ath-AT4G15800.1 |  | | | |  |  |  |  |  |  |  |
| 1 | Ath-AT4G15802.1 |  | | | |  |  |  |  |  |  |  |
| 1 | Ath-AT4G15810.1 |  | Vvi-Vitvi05g00294\_t001 |  |  |  |  |  |  |  |
| 1 | Ath-AT4G15820.1 |  | Vvi-Vitvi05g00293\_t001 |  |  |  |  |  |  |  |
| 1 | Ath-AT4G15830.1 |  | Vvi-Vitvi05g00292\_t001 |  |  |  |  |  |  |  |
| 1 | Ath-AT4G15840.1 |  | Vvi-Vitvi05g00290\_t001 |  |  |  |  |  |  |  |
| 1 | Ath-AT4G15850.1 |  | | | |  |  |  |  |  |  |  |
| 1 | Ath-AT4G15870.1 |  | | | |  |  |  |  |  |  |  |
| 1 | Ath-AT4G15880.1 |  | Vvi-Vitvi05g00288\_t001 |  |  |  |  |  |  |  |
| 1 | Ath-AT4G15885.1 |  | Vvi-Vitvi05g00287\_t001.1.6037826e |  |  |  |  |  |  |  |
| 1 | Ath-AT4G15890.1 |  | Vvi-Vitvi05g00281\_t001 |  |  |  |  |  |  |  |
| 1 | Ath-AT4G15900.1 |  | Vvi-Vitvi05g00008\_t001 |  |  |  |  |  |  |  |
| 2 | Ath-AT4G15910.1 |  | | | |  | Vvi-Vitvi07g00341\_t001 |  |  |  |  |  |  |
| 2 | Ath-AT4G15920.1 |  | Vvi-Vitvi05g04001\_t001 |  | | | |  |  |  |  |  |  |
| 2 | Ath-AT4G15930.1 |  | Vvi-Vitvi05g00027\_t001 |  | | | |  |  |  |  |  |  |
| 2 | Ath-AT4G15940.1 |  | Vvi-Vitvi05g00035\_t001 |  | | | |  |  |  |  |  |  |
| 2 | Ath-AT4G15953.1 |  | | | |  | | | |  |  |  |  |  |  |
| 2 | Ath-AT4G15955.3 |  | Vvi-Vitvi05g00039\_t001 |  | | | |  |  |  |  |  |  |
| 2 | Ath-AT4G15960.1 |  | | | |  | Vvi-Vitvi07g00347\_t001 |  |  |  |  |  |  |
| 2 | Ath-AT4G15970.1 |  | | | |  | | | |  |  |  |  |  |  |
| 2 | Ath-AT4G15975.1 |  | Vvi-Vitvi05g00041\_t001 |  | Vvi-Vitvi07g00348\_t001 |  |  |  |  |  |  |
| 2 | Ath-AT4G15980.2 |  | | | |  | | | |  |  |  |  |  |  |
| 2 | Ath-AT4G15990.1 |  | | | |  | | | |  |  |  |  |  |  |
| 2 | Ath-AT4G16000.1 |  | | | |  | | | |  |  |  |  |  |  |
| 2 | Ath-AT4G16008.1 |  | | | |  | | | |  |  |  |  |  |  |
| 2 | Ath-AT4G16015.1 |  | | | |  | | | |  |  |  |  |  |  |
| 2 | Ath-AT4G16024.1 |  | | | |  | | | |  |  |  |  |  |  |
| 2 | Ath-AT4G16030.1 |  | Vvi-Vitvi05g00053\_t001 |  | Vvi-Vitvi07g02210\_t001 |  |  |  |  |  |  |
| 2 | Ath-AT4G16040.2 |  | | | |  | | | |  |  |  |  |  |  |
| 2 | Ath-AT4G16045.1 |  | | | |  | | | |  |  |  |  |  |  |
| 2 | Ath-AT4G16050.1 |  | | | |  | | | |  |  |  |  |  |  |
| 2 | Ath-AT4G16060.1 |  | Vvi-Vitvi05g00056\_t001 |  | | | |  |  |  |  |  |  |
| 2 | Ath-AT4G16070.3 |  | Vvi-Vitvi05g01746\_t001 |  | | | |  |  |  |  |  |  |
| 2 | Ath-AT4G16080.1 |  | | | |  | | | |  |  |  |  |  |  |
| 2 | Ath-AT4G16090.1 |  | | | |  | | | |  |  |  |  |  |  |
| 2 | Ath-AT4G16095.1 |  | | | |  | | | |  |  |  |  |  |  |
| 2 | Ath-AT4G16100.1 |  | Vvi-Vitvi05g00073\_t002 |  | Vvi-Vitvi07g00370\_t001 |  |  |  |  |  |  |
| 2 | Ath-AT4G16120.1 |  | Vvi-Vitvi05g00076\_t001 |  | Vvi-Vitvi07g00375\_t001 |  |  |  |  |  |  |
| 2 | Ath-AT4G16130.1 |  | | | |  | | | |  |  |  |  |  |  |
| 2 | Ath-AT4G16140.2 |  | | | |  | | | |  |  |  |  |  |  |
| 2 | Ath-AT4G16141.1 |  | Vvi-Vitvi05g00077\_t001 |  | Vvi-Vitvi07g02214\_t001 |  |  |  |  |  |  |
| 2 | Ath-AT4G16143.1 |  | Vvi-Vitvi05g01765\_t001 |  | Vvi-Vitvi07g00382\_t001 |  |  |  |  |  |  |
| 1 | Ath-AT4G16144.1 |  | Vvi-Vitvi05g00085\_t001 |  |  |  |  |  |  |  |
| 1 | Ath-AT4G16146.1 |  | Vvi-Vitvi05g04019\_t001 |  |  |  |  |  |  |  |
| 1 | Ath-AT4G16155.1 |  | Vvi-Vitvi05g00100\_t001 |  |  |  |  |  |  |  |
| 1 | Ath-AT4G16160.2 |  | Vvi-Vitvi05g00102\_t001 |  |  |  |  |  |  |  |
| 1 | Ath-AT4G16162.2 |  | | | |  |  |  |  |  |  |  |
| 1 | Ath-AT4G16165.1 |  | | | |  |  |  |  |  |  |  |
| 1 | Ath-AT4G16180.2 |  | | | |  |  |  |  |  |  |  |
| 1 | Ath-AT4G16190.1 |  | | | |  |  |  |  |  |  |  |
| 1 | Ath-AT4G16195.1 |  | | | |  |  |  |  |  |  |  |
| 1 | Ath-AT4G16200.1 |  | | | |  |  |  |  |  |  |  |
| 1 | Ath-AT4G16210.1 |  | | | |  |  |  |  |  |  |  |
| 1 | Ath-AT4G16215.1 |  | | | |  |  |  |  |  |  |  |
| 1 | Ath-AT4G16220.1 |  | | | |  |  |  |  |  |  |  |
| 1 | Ath-AT4G16230.1 |  | | | |  |  |  |  |  |  |  |
| 1 | Ath-AT4G16233.1 |  | | | |  |  |  |  |  |  |  |
| 1 | Ath-AT4G16240.1 |  | | | |  |  |  |  |  |  |  |
| 1 | Ath-AT4G16250.1 |  | | | |  |  |  |  |  |  |  |
| 1 | Ath-AT4G16260.1 |  | Vvi-Vitvi05g00107\_t001 |  |  |  |  |  |  |  |
| 0 | Ath-AT4G16265.2 |  |  |  |  |  |  |  |  |
| 0 | Ath-AT4G16270.1 |  |  |  |  |  |  |  |  |
| 0 | Ath-AT4G16280.2 |  |  |  |  |  |  |  |  |
| 0 | Ath-AT4G16295.1 |  |  |  |  |  |  |  |  |
| 0 | Ath-AT4G16310.3 |  |  |  |  |  |  |  |  |
| 0 | Ath-AT4G16330.2 |  |  |  |  |  |  |  |  |
| 0 | Ath-AT4G16340.2 |  |  |  |  |  |  |  |  |
| 0 | Ath-AT4G16350.2 |  |  |  |  |  |  |  |  |
| 1 | Ath-AT4G16370.1 |  | Vvi-Vitvi10g04119\_t001 |  |  |  |  |  |  |  |
| 1 | Ath-AT4G16380.3 |  | Vvi-Vitvi10g00233\_t001 |  |  |  |  |  |  |  |
| 1 | Ath-AT4G16390.1 |  | Vvi-Vitvi10g00232\_t001 |  |  |  |  |  |  |  |
| 1 | Ath-AT4G16400.1 |  | Vvi-Vitvi10g04101\_t001 |  |  |  |  |  |  |  |
| 1 | Ath-AT4G16410.1 |  | Vvi-Vitvi10g00227\_t001 |  |  |  |  |  |  |  |
| 1 | Ath-AT4G16420.1 |  | Vvi-Vitvi10g00225\_t003 |  |  |  |  |  |  |  |
| 1 | Ath-AT4G16430.1 |  | | | |  |  |  |  |  |  |  |
| 1 | Ath-AT4G16440.1 |  | | | |  |  |  |  |  |  |  |
| 1 | Ath-AT4G16442.1 |  | | | |  |  |  |  |  |  |  |
| 1 | Ath-AT4G16444.1 |  | | | |  |  |  |  |  |  |  |
| 1 | Ath-AT4G16447.1 |  | | | |  |  |  |  |  |  |  |
| 1 | Ath-AT4G16450.1 |  | | | |  |  |  |  |  |  |  |
| 1 | Ath-AT4G16451.1 |  | | | |  |  |  |  |  |  |  |
| 1 | Ath-AT4G16460.1 |  | | | |  |  |  |  |  |  |  |
| 1 | Ath-AT4G16470.1 |  | | | |  |  |  |  |  |  |  |
| 1 | Ath-AT4G16480.2 |  | | | |  |  |  |  |  |  |  |
| 1 | Ath-AT4G16490.1 |  | | | |  |  |  |  |  |  |  |
| 1 | Ath-AT4G16500.1 |  | Vvi-Vitvi10g00209\_t001 |  |  |  |  |  |  |  |
| 1 | Ath-AT4G16510.1 |  | Vvi-Vitvi02g00534\_t001 |  |  |  |  |  |  |  |
| 1 | Ath-AT4G16515.2 |  | | | |  |  |  |  |  |  |  |
| 1 | Ath-AT4G16520.1 |  | Vvi-Vitvi02g00535\_t001 |  |  |  |  |  |  |  |
| 1 | Ath-AT4G16530.1 |  | | | |  |  |  |  |  |  |  |
| 1 | Ath-AT4G16535.1 |  | Vvi-Vitvi02g00542\_t001 |  |  |  |  |  |  |  |
| 1 | Ath-AT4G16540.1 |  | | | |  |  |  |  |  |  |  |
| 1 | Ath-AT4G16545.1 |  | | | |  |  |  |  |  |  |  |
| 1 | Ath-AT4G16550.1 |  | | | |  |  |  |  |  |  |  |
| 1 | Ath-AT4G16555.1 |  | | | |  |  |  |  |  |  |  |
| 1 | Ath-AT4G16560.1 |  | | | |  |  |  |  |  |  |  |
| 1 | Ath-AT4G16563.1 |  | Vvi-Vitvi02g00547\_t001 |  |  |  |  |  |  |  |
| 1 | Ath-AT4G16566.1 |  | Vvi-Vitvi02g00548\_t001 |  |  |  |  |  |  |  |
| 1 | Ath-AT4G16570.1 |  | Vvi-Vitvi02g00549\_t001 |  |  |  |  |  |  |  |
| 1 | Ath-AT4G16580.1 |  | | | |  |  |  |  |  |  |  |
| 1 | Ath-AT4G16590.1 |  | | | |  |  |  |  |  |  |  |
| 1 | Ath-AT4G16600.1 |  | Vvi-Vitvi02g04164\_t001 |  |  |  |  |  |  |  |
| 1 | Ath-AT4G16610.1 |  | Vvi-Vitvi02g00560\_t001 |  |  |  |  |  |  |  |
| 1 | Ath-AT4G16620.1 |  | Vvi-Vitvi02g04169\_t001 |  |  |  |  |  |  |  |
| 1 | Ath-AT4G16630.1 |  | | | |  |  |  |  |  |  |  |
| 1 | Ath-AT4G16640.1 |  | Vvi-Vitvi02g00564\_t001 |  |  |  |  |  |  |  |
| 1 | Ath-AT4G16650.1 |  | Vvi-Vitvi02g00565\_t001 |  |  |  |  |  |  |  |
| 1 | Ath-AT4G16660.2 |  | | | |  |  |  |  |  |  |  |
| 1 | Ath-AT4G16670.1 |  | Vvi-Vitvi02g04171\_t001 |  |  |  |  |  |  |  |
| 1 | Ath-AT4G16680.2 |  | | | |  |  |  |  |  |  |  |
| 1 | Ath-AT4G16690.1 |  | | | |  |  |  |  |  |  |  |
| 1 | Ath-AT4G16695.5 |  | Vvi-Vitvi02g04172\_t001 |  |  |  |  |  |  |  |
| 1 | Ath-AT4G16700.1 |  | Vvi-Vitvi02g00573\_t001 |  |  |  |  |  |  |  |
| 1 | Ath-AT4G16710.3 |  | | | |  |  |  |  |  |  |  |
| 1 | Ath-AT4G16720.1 |  | Vvi-Vitvi02g01712\_t001 |  |  |  |  |  |  |  |
| 1 | Ath-AT4G16730.1 |  | | | |  |  |  |  |  |  |  |
| 1 | Ath-AT4G16740.1 |  | | | |  |  |  |  |  |  |  |
| 1 | Ath-AT4G16745.2 |  | | | |  |  |  |  |  |  |  |
| 1 | Ath-AT4G16750.1 |  | | | |  |  |  |  |  |  |  |
| 1 | Ath-AT4G16760.1 |  | | | |  |  |  |  |  |  |  |
| 1 | Ath-AT4G16765.1 |  | | | |  |  |  |  |  |  |  |
| 1 | Ath-AT4G16770.1 |  | | | |  |  |  |  |  |  |  |
| 1 | Ath-AT4G16780.1 |  | Vvi-Vitvi02g01717\_t001 |  |  |  |  |  |  |  |
| 0 | Ath-AT4G16790.1 |  |  |  |  |  |  |  |  |
| 0 | Ath-AT4G16800.1 |  |  |  |  |  |  |  |  |
| 0 | Ath-AT4G16807.1 |  |  |  |  |  |  |  |  |
| 0 | Ath-AT4G16810.1 |  |  |  |  |  |  |  |  |
| 1 | Ath-AT4G16820.1 |  | Vvi-Vitvi15g00556\_t001 |  |  |  |  |  |  |  |
| 1 | Ath-AT4G16830.1 |  | Vvi-Vitvi15g00549\_t001 |  |  |  |  |  |  |  |
| 1 | Ath-AT4G16835.1 |  | | | |  |  |  |  |  |  |  |
| 1 | Ath-AT4G16840.1 |  | | | |  |  |  |  |  |  |  |
| 1 | Ath-AT4G16845.1 |  | | | |  |  |  |  |  |  |  |
| 1 | Ath-AT4G16850.1 |  | | | |  |  |  |  |  |  |  |
| 1 | Ath-AT4G16855.1 |  | | | |  |  |  |  |  |  |  |
| 1 | Ath-AT4G16857.2 |  | | | |  |  |  |  |  |  |  |
| 1 | Ath-AT4G16860.1 |  | | | |  |  |  |  |  |  |  |
| 1 | Ath-AT4G16880.1 |  | | | |  |  |  |  |  |  |  |
| 1 | Ath-AT4G16890.1 |  | | | |  |  |  |  |  |  |  |
| 1 | Ath-AT4G16900.1 |  | | | |  |  |  |  |  |  |  |
| 1 | Ath-AT4G16915.1 |  | | | |  |  |  |  |  |  |  |
| 1 | Ath-AT4G16920.2 |  | | | |  |  |  |  |  |  |  |
| 1 | Ath-AT4G16930.1 |  | | | |  |  |  |  |  |  |  |
| 1 | Ath-AT4G16940.2 |  | | | |  |  |  |  |  |  |  |
| 1 | Ath-AT4G16950.1 |  | | | |  |  |  |  |  |  |  |
| 1 | Ath-AT4G16955.1 |  | | | |  |  |  |  |  |  |  |
| 1 | Ath-AT4G16957.1 |  | | | |  |  |  |  |  |  |  |
| 1 | Ath-AT4G16960.1 |  | | | |  |  |  |  |  |  |  |
| 1 | Ath-AT4G16970.1 |  | Vvi-Vitvi15g00545\_t001 |  |  |  |  |  |  |  |
| 1 | Ath-AT4G16980.1 |  | | | |  |  |  |  |  |  |  |
| 1 | Ath-AT4G16983.1 |  | | | |  |  |  |  |  |  |  |
| 1 | Ath-AT4G16990.7 |  | | | |  |  |  |  |  |  |  |
| 1 | Ath-AT4G17000.1 |  | | | |  |  |  |  |  |  |  |
| 1 | Ath-AT4G17010.1 |  | | | |  |  |  |  |  |  |  |
| 1 | Ath-AT4G17020.3 |  | | | |  |  |  |  |  |  |  |
| 1 | Ath-AT4G17030.1 |  | | | |  |  |  |  |  |  |  |
| 1 | Ath-AT4G17040.1 |  | | | |  |  |  |  |  |  |  |
| 1 | Ath-AT4G17050.1 |  | | | |  |  |  |  |  |  |  |
| 1 | Ath-AT4G17060.1 |  | Vvi-Vitvi15g00532\_t001 |  |  |  |  |  |  |  |
| 1 | Ath-AT4G17070.1 |  | Vvi-Vitvi15g00531\_t001 |  |  |  |  |  |  |  |
| 1 | Ath-AT4G17080.1 |  | Vvi-Vitvi15g00517\_t001 |  |  |  |  |  |  |  |
| 0 | Ath-AT4G17085.1 |  |  |  |  |  |  |  |  |
| 0 | Ath-AT4G17090.1 |  |  |  |  |  |  |  |  |
| 0 | Ath-AT4G17100.2 |  |  |  |  |  |  |  |  |
| 0 | Ath-AT4G17140.3 |  |  |  |  |  |  |  |  |
| 0 | Ath-AT4G17150.1 |  |  |  |  |  |  |  |  |
| 0 | Ath-AT4G17160.1 |  |  |  |  |  |  |  |  |
| 0 | Ath-AT4G17170.1 |  |  |  |  |  |  |  |  |
| 0 | Ath-AT4G17180.1 |  |  |  |  |  |  |  |  |
| 0 | Ath-AT4G17190.1 |  |  |  |  |  |  |  |  |
| 0 | Ath-AT4G17200.1 |  |  |  |  |  |  |  |  |
| 0 | Ath-AT4G17210.1 |  |  |  |  |  |  |  |  |
| 0 | Ath-AT4G17215.1 |  |  |  |  |  |  |  |  |
| 0 | Ath-AT4G17220.1 |  |  |  |  |  |  |  |  |
| 0 | Ath-AT4G17230.1 |  |  |  |  |  |  |  |  |
| 0 | Ath-AT4G17240.1 |  |  |  |  |  |  |  |  |
| 0 | Ath-AT4G17243.1 |  |  |  |  |  |  |  |  |
| 0 | Ath-AT4G17245.1 |  |  |  |  |  |  |  |  |
| 1 | Ath-AT4G17250.1 |  | Vvi-Vitvi02g00538\_t001 |  |  |  |  |  |  |  |
| 1 | Ath-AT4G17260.1 |  | | | |  |  |  |  |  |  |  |
| 1 | Ath-AT4G17270.1 |  | | | |  |  |  |  |  |  |  |
| 1 | Ath-AT4G17280.1 |  | | | |  |  |  |  |  |  |  |
| 1 | Ath-AT4G17300.2 |  | Vvi-Vitvi02g00558\_t001 |  |  |  |  |  |  |  |
| 1 | Ath-AT4G17310.3 |  | Vvi-Vitvi02g01706\_t006 |  |  |  |  |  |  |  |
| 1 | Ath-AT4G17330.1 |  | Vvi-Vitvi02g00566\_t001 |  |  |  |  |  |  |  |
| 1 | Ath-AT4G17340.1 |  | Vvi-Vitvi02g00568\_t001 |  |  |  |  |  |  |  |
| 1 | Ath-AT4G17350.1 |  | Vvi-Vitvi02g04171\_t001 |  |  |  |  |  |  |  |
| 1 | Ath-AT4G17360.1 |  | Vvi-Vitvi02g00572\_t001 |  |  |  |  |  |  |  |
| 1 | Ath-AT4G17370.1 |  | Vvi-Vitvi02g00575\_t001 |  |  |  |  |  |  |  |
| 1 | Ath-AT4G17380.1 |  | Vvi-Vitvi02g04173\_t001 |  |  |  |  |  |  |  |
| 1 | Ath-AT4G17390.1 |  | Vvi-Vitvi02g01712\_t001 |  |  |  |  |  |  |  |
| 1 | Ath-AT4G17410.2 |  | Vvi-Vitvi02g01714\_t001 |  |  |  |  |  |  |  |
| 1 | Ath-AT4G17420.1 |  | | | |  |  |  |  |  |  |  |
| 1 | Ath-AT4G17430.1 |  | | | |  |  |  |  |  |  |  |
| 1 | Ath-AT4G17440.3 |  | | | |  |  |  |  |  |  |  |
| 1 | Ath-AT4G17460.1 |  | Vvi-Vitvi02g01717\_t001 |  |  |  |  |  |  |  |
| 1 | Ath-AT4G17470.1 |  | | | |  |  |  |  |  |  |  |
| 1 | Ath-AT4G17480.1 |  | | | |  |  |  |  |  |  |  |
| 1 | Ath-AT4G17483.1 |  | | | |  |  |  |  |  |  |  |
| 1 | Ath-AT4G17486.1 |  | | | |  |  |  |  |  |  |  |
| 1 | Ath-AT4G17490.1 |  | | | |  |  |  |  |  |  |  |
| 1 | Ath-AT4G17500.1 |  | | | |  |  |  |  |  |  |  |
| 1 | Ath-AT4G17505.1 |  | | | |  |  |  |  |  |  |  |
| 1 | Ath-AT4G17510.1 |  | | | |  |  |  |  |  |  |  |
| 1 | Ath-AT4G17520.1 |  | | | |  |  |  |  |  |  |  |
| 1 | Ath-AT4G17530.1 |  | | | |  |  |  |  |  |  |  |
| 1 | Ath-AT4G17540.1 |  | | | |  |  |  |  |  |  |  |
| 1 | Ath-AT4G17550.1 |  | | | |  |  |  |  |  |  |  |
| 1 | Ath-AT4G17560.1 |  | | | |  |  |  |  |  |  |  |
| 1 | Ath-AT4G17565.1 |  | | | |  |  |  |  |  |  |  |
| 1 | Ath-AT4G17570.2 |  | | | |  |  |  |  |  |  |  |
| 1 | Ath-AT4G17580.1 |  | | | |  |  |  |  |  |  |  |
| 1 | Ath-AT4G17585.1 |  | | | |  |  |  |  |  |  |  |
| 1 | Ath-AT4G17590.1 |  | | | |  |  |  |  |  |  |  |
| 1 | Ath-AT4G17600.1 |  | | | |  |  |  |  |  |  |  |
| 1 | Ath-AT4G17610.1 |  | | | |  |  |  |  |  |  |  |
| 1 | Ath-AT4G17615.3 |  | | | |  |  |  |  |  |  |  |
| 1 | Ath-AT4G17616.1 |  | | | |  |  |  |  |  |  |  |
| 1 | Ath-AT4G17620.1 |  | Vvi-Vitvi02g00590\_t001 |  |  |  |  |  |  |  |
| 1 | Ath-AT4G17640.1 |  | Vvi-Vitvi02g00597\_t001 |  |  |  |  |  |  |  |
| 1 | Ath-AT4G17650.1 |  | | | |  |  |  |  |  |  |  |
| 1 | Ath-AT4G17660.1 |  | Vvi-Vitvi02g00604\_t001 |  |  |  |  |  |  |  |
| 1 | Ath-AT4G17670.1 |  | Vvi-Vitvi02g00614\_t001 |  |  |  |  |  |  |  |
| 1 | Ath-AT4G17680.1 |  | Vvi-Vitvi02g01471\_t001 |  |  |  |  |  |  |  |
| 0 | Ath-AT4G17690.1 |  |  |  |  |  |  |  |  |
| 1 | Ath-AT4G17695.1 |  | Vvi-Vitvi02g00757\_t002 |  |  |  |  |  |  |  |
| 1 | Ath-AT4G17700.1 |  | | | |  |  |  |  |  |  |  |
| 1 | Ath-AT4G17710.1 |  | | | |  |  |  |  |  |  |  |
| 1 | Ath-AT4G17713.1 |  | | | |  |  |  |  |  |  |  |
| 1 | Ath-AT4G17718.1 |  | | | |  |  |  |  |  |  |  |
| 1 | Ath-AT4G17720.1 |  | Vvi-Vitvi02g00749\_t001 |  |  |  |  |  |  |  |
| 1 | Ath-AT4G17730.2 |  | Vvi-Vitvi02g00747\_t001 |  |  |  |  |  |  |  |
| 1 | Ath-AT4G17740.1 |  | | | |  |  |  |  |  |  |  |
| 1 | Ath-AT4G17750.1 |  | Vvi-Vitvi02g00739\_t001 |  |  |  |  |  |  |  |
| 1 | Ath-AT4G17760.1 |  | Vvi-Vitvi02g00734\_t001 |  |  |  |  |  |  |  |
| 1 | Ath-AT4G17770.2 |  | Vvi-Vitvi02g00729\_t003 |  |  |  |  |  |  |  |
| 1 | Ath-AT4G17780.1 |  | | | |  |  |  |  |  |  |  |
| 1 | Ath-AT4G17785.1 |  | Vvi-Vitvi02g00725\_t001 |  |  |  |  |  |  |  |
| 1 | Ath-AT4G17790.1 |  | Vvi-Vitvi02g00723\_t001 |  |  |  |  |  |  |  |
| 1 | Ath-AT4G17800.1 |  | | | |  |  |  |  |  |  |  |
| 2 | Ath-AT4G17810.1 |  | | | |  | Vvi-Vitvi02g00686\_t001 |  |  |  |  |  |  |
| 2 | Ath-AT4G17830.2 |  | | | |  | Vvi-Vitvi02g00688\_t001 |  |  |  |  |  |  |
| 2 | Ath-AT4G17840.1 |  | | | |  | Vvi-Vitvi02g00689\_t001 |  |  |  |  |  |  |
| 2 | Ath-AT4G17850.1 |  | | | |  | Vvi-Vitvi02g00691\_t003 |  |  |  |  |  |  |
| 2 | Ath-AT4G17860.1 |  | | | |  | | | |  |  |  |  |  |  |
| 2 | Ath-AT4G17870.1 |  | | | |  | Vvi-Vitvi02g00695\_t001 |  |  |  |  |  |  |
| 2 | Ath-AT4G17880.1 |  | | | |  | Vvi-Vitvi02g00698\_t001 |  |  |  |  |  |  |
| 2 | Ath-AT4G17890.1 |  | | | |  | Vvi-Vitvi02g00699\_t001 |  |  |  |  |  |  |
| 2 | Ath-AT4G17895.1 |  | | | |  | Vvi-Vitvi02g00700\_t001 |  |  |  |  |  |  |
| 2 | Ath-AT4G17900.1 |  | | | |  | Vvi-Vitvi02g00704\_t001 |  |  |  |  |  |  |
| 2 | Ath-AT4G17905.2 |  | | | |  | Vvi-Vitvi02g00708\_t001 |  |  |  |  |  |  |
| 2 | Ath-AT4G17910.4 |  | | | |  | Vvi-Vitvi02g00710\_t001 |  |  |  |  |  |  |
| 2 | Ath-AT4G17920.1 |  | Vvi-Vitvi02g00714\_t001 |  | Vvi-Vitvi02g00714\_t001 |  |  |  |  |  |  |
| 1 | Ath-AT4G17940.1 |  |  |  | Vvi-Vitvi02g00719\_t001 |  |  |  |  |  |  |
| 0 | Ath-AT4G17950.1 |  |  |  |  |  |  |  |  |
| 0 | Ath-AT4G17960.1 |  |  |  |  |  |  |  |  |
| 0 | Ath-AT4G17970.1 |  |  |  |  |  |  |  |  |
| 0 | Ath-AT4G17980.2 |  |  |  |  |  |  |  |  |
| 0 | Ath-AT4G17990.2 |  |  |  |  |  |  |  |  |
| 0 | Ath-AT4G18010.1 |  |  |  |  |  |  |  |  |
| 0 | Ath-AT4G18020.1 |  |  |  |  |  |  |  |  |
| 0 | Ath-AT4G18030.1 |  |  |  |  |  |  |  |  |
| 0 | Ath-AT4G18040.1 |  |  |  |  |  |  |  |  |
| 1 | Ath-AT4G18050.2 |  | Vvi-Vitvi10g00702\_t001 |  |  |  |  |  |  |  |
| 1 | Ath-AT4G18060.1 |  | Vvi-Vitvi10g00707\_t001 |  |  |  |  |  |  |  |
| 1 | Ath-AT4G18070.1 |  | | | |  |  |  |  |  |  |  |
| 1 | Ath-AT4G18080.1 |  | | | |  |  |  |  |  |  |  |
| 1 | Ath-AT4G18090.1 |  | | | |  |  |  |  |  |  |  |
| 1 | Ath-AT4G18100.1 |  | Vvi-Vitvi10g00712\_t001 |  |  |  |  |  |  |  |
| 1 | Ath-AT4G18110.1 |  | | | |  |  |  |  |  |  |  |
| 1 | Ath-AT4G18130.1 |  | Vvi-Vitvi10g00715\_t001 |  |  |  |  |  |  |  |
| 1 | Ath-AT4G18140.2 |  | Vvi-Vitvi10g00716\_t001 |  |  |  |  |  |  |  |
| 1 | Ath-AT4G18150.1 |  | Vvi-Vitvi10g00722\_t001 |  |  |  |  |  |  |  |
| 1 | Ath-AT4G18160.1 |  | Vvi-Vitvi10g04409\_t001 |  |  |  |  |  |  |  |
| 1 | Ath-AT4G18170.1 |  | Vvi-Vitvi10g00732\_t001 |  |  |  |  |  |  |  |
| 1 | Ath-AT4G18180.1 |  | | | |  |  |  |  |  |  |  |
| 1 | Ath-AT4G18190.1 |  | Vvi-Vitvi10g00734\_t001 |  |  |  |  |  |  |  |
| 1 | Ath-AT4G18195.1 |  | | | |  |  |  |  |  |  |  |
| 1 | Ath-AT4G18197.1 |  | Vvi-Vitvi10g00736\_t001 |  |  |  |  |  |  |  |
| 1 | Ath-AT4G18203.1 |  | | | |  |  |  |  |  |  |  |
| 1 | Ath-AT4G18205.1 |  | | | |  |  |  |  |  |  |  |
| 1 | Ath-AT4G18210.1 |  | | | |  |  |  |  |  |  |  |
| 1 | Ath-AT4G18215.1 |  | | | |  |  |  |  |  |  |  |
| 1 | Ath-AT4G18220.1 |  | | | |  |  |  |  |  |  |  |
| 1 | Ath-AT4G18230.3 |  | Vvi-Vitvi10g00738\_t001 |  |  |  |  |  |  |  |
| 1 | Ath-AT4G18240.1 |  | Vvi-Vitvi10g00739\_t001 |  |  |  |  |  |  |  |
| 0 | Ath-AT4G18250.1 |  |  |  |  |  |  |  |  |
| 0 | Ath-AT4G18253.1 |  |  |  |  |  |  |  |  |
| 0 | Ath-AT4G18260.1 |  |  |  |  |  |  |  |  |
| 0 | Ath-AT4G18270.1 |  |  |  |  |  |  |  |  |
| 0 | Ath-AT4G18280.1 |  |  |  |  |  |  |  |  |
| 0 | Ath-AT4G18290.2 |  |  |  |  |  |  |  |  |
| 0 | Ath-AT4G18300.1 |  |  |  |  |  |  |  |  |
| 0 | Ath-AT4G18310.1 |  |  |  |  |  |  |  |  |
| 0 | Ath-AT4G18320.2 |  |  |  |  |  |  |  |  |
| 0 | Ath-AT4G18330.2 |  |  |  |  |  |  |  |  |
| 0 | Ath-AT4G18335.1 |  |  |  |  |  |  |  |  |
| 1 | Ath-AT4G18340.1 |  | Vvi-Vitvi10g00811\_t001 |  |  |  |  |  |  |  |
| 1 | Ath-AT4G18350.1 |  | Vvi-Vitvi10g00821\_t001 |  |  |  |  |  |  |  |
| 1 | Ath-AT4G18360.1 |  | Vvi-Vitvi10g00829\_t001 |  |  |  |  |  |  |  |
| 1 | Ath-AT4G18370.1 |  | Vvi-Vitvi10g00832\_t001 |  |  |  |  |  |  |  |
| 1 | Ath-AT4G18372.1 |  | | | |  |  |  |  |  |  |  |
| 1 | Ath-AT4G18375.2 |  | Vvi-Vitvi10g00834\_t001 |  |  |  |  |  |  |  |
| 1 | Ath-AT4G18380.1 |  | Vvi-Vitvi10g00837\_t001 |  |  |  |  |  |  |  |
| 1 | Ath-AT4G18390.1 |  | Vvi-Vitvi10g00838\_t001 |  |  |  |  |  |  |  |
| 1 | Ath-AT4G18395.1 |  | | | |  |  |  |  |  |  |  |
| 1 | Ath-AT4G18400.1 |  | Vvi-Vitvi10g01885\_t001 |  |  |  |  |  |  |  |
| 0 | Ath-AT4G18422.1 |  |  |  |  |  |  |  |  |
| 1 | Ath-AT4G18425.1 |  | Vvi-Vitvi10g00501\_t002 |  |  |  |  |  |  |  |
| 1 | Ath-AT4G18430.1 |  | Vvi-Vitvi10g01781\_t001 |  |  |  |  |  |  |  |
| 1 | Ath-AT4G18440.1 |  | | | |  |  |  |  |  |  |  |
| 1 | Ath-AT4G18450.1 |  | Vvi-Vitvi10g00522\_t001 |  |  |  |  |  |  |  |
| 1 | Ath-AT4G18470.1 |  | Vvi-Vitvi10g00525\_t001 |  |  |  |  |  |  |  |
| 1 | Ath-AT4G18460.1 |  | Vvi-Vitvi10g00526\_t001 |  |  |  |  |  |  |  |
| 1 | Ath-AT4G18465.1 |  | Vvi-Vitvi10g00536\_t001 |  |  |  |  |  |  |  |
| 1 | Ath-AT4G18480.1 |  | Vvi-Vitvi10g00543\_t001 |  |  |  |  |  |  |  |
| 1 | Ath-AT4G18490.3 |  | Vvi-Vitvi10g01789\_t001 |  |  |  |  |  |  |  |
| 1 | Ath-AT4G18500.1 |  | | | |  |  |  |  |  |  |  |
| 1 | Ath-AT4G18501.1 |  | | | |  |  |  |  |  |  |  |
| 1 | Ath-AT4G18510.1 |  | | | |  |  |  |  |  |  |  |
| 1 | Ath-AT4G18520.1 |  | | | |  |  |  |  |  |  |  |
| 1 | Ath-AT4G18530.1 |  | | | |  |  |  |  |  |  |  |
| 1 | Ath-AT4G18540.1 |  | Vvi-Vitvi10g04358\_t001 |  |  |  |  |  |  |  |
| 1 | Ath-AT4G18550.2 |  | Vvi-Vitvi10g00561\_t001 |  |  |  |  |  |  |  |
| 1 | Ath-AT4G18570.1 |  | Vvi-Vitvi10g00562\_t001 |  |  |  |  |  |  |  |
| 1 | Ath-AT4G18580.2 |  | | | |  |  |  |  |  |  |  |
| 1 | Ath-AT4G18590.1 |  | | | |  |  |  |  |  |  |  |
| 1 | Ath-AT4G18593.1 |  | | | |  |  |  |  |  |  |  |
| 1 | Ath-AT4G18596.2 |  | Vvi-Vitvi10g00580\_t001 |  |  |  |  |  |  |  |
| 1 | Ath-AT4G18600.1 |  | Vvi-Vitvi10g00582\_t001 |  |  |  |  |  |  |  |
| 1 | Ath-AT4G18610.1 |  | Vvi-Vitvi10g00583\_t001 |  |  |  |  |  |  |  |
| 1 | Ath-AT4G18620.1 |  | | | |  |  |  |  |  |  |  |
| 1 | Ath-AT4G18630.1 |  | Vvi-Vitvi10g00592\_t001 |  |  |  |  |  |  |  |
| 1 | Ath-AT4G18640.1 |  | Vvi-Vitvi10g00597\_t001 |  |  |  |  |  |  |  |
| 1 | Ath-AT4G18650.1 |  | Vvi-Vitvi10g00598\_t001 |  |  |  |  |  |  |  |
| 1 | Ath-AT4G18660.1 |  | | | |  |  |  |  |  |  |  |
| 1 | Ath-AT4G18670.1 |  | | | |  |  |  |  |  |  |  |
| 1 | Ath-AT4G18680.1 |  | | | |  |  |  |  |  |  |  |
| 1 | Ath-AT4G18690.1 |  | | | |  |  |  |  |  |  |  |
| 1 | Ath-AT4G18692.1 |  | | | |  |  |  |  |  |  |  |
| 1 | Ath-AT4G18700.1 |  | Vvi-Vitvi10g00599\_t001 |  |  |  |  |  |  |  |
| 1 | Ath-AT4G18710.1 |  | Vvi-Vitvi10g00604\_t001 |  |  |  |  |  |  |  |
| 1 | Ath-AT4G18720.1 |  | | | |  |  |  |  |  |  |  |
| 1 | Ath-AT4G18730.1 |  | Vvi-Vitvi10g00609\_t001 |  |  |  |  |  |  |  |
| 1 | Ath-AT4G18740.1 |  | Vvi-Vitvi10g01796\_t001 |  |  |  |  |  |  |  |
| 1 | Ath-AT4G18750.1 |  | Vvi-Vitvi10g00611\_t001 |  |  |  |  |  |  |  |
| 1 | Ath-AT4G18760.1 |  | Vvi-Vitvi10g00612\_t001 |  |  |  |  |  |  |  |
| 1 | Ath-AT4G18770.1 |  | | | |  |  |  |  |  |  |  |
| 1 | Ath-AT4G18780.1 |  | Vvi-Vitvi10g00613\_t001 |  |  |  |  |  |  |  |
| 1 | Ath-AT4G18790.1 |  | | | |  |  |  |  |  |  |  |
| 1 | Ath-AT4G18800.1 |  | Vvi-Vitvi10g00619\_t001 |  |  |  |  |  |  |  |
| 1 | Ath-AT4G18810.2 |  | Vvi-Vitvi10g00622\_t001 |  |  |  |  |  |  |  |
| 1 | Ath-AT4G18820.1 |  | Vvi-Vitvi10g00629\_t001 |  |  |  |  |  |  |  |
| 1 | Ath-AT4G18823.1 |  | | | |  |  |  |  |  |  |  |
| 1 | Ath-AT4G18830.1 |  | Vvi-Vitvi10g00632\_t001 |  |  |  |  |  |  |  |
| 1 | Ath-AT4G18840.2 |  | Vvi-Vitvi10g00634\_t001 |  |  |  |  |  |  |  |
| 1 | Ath-AT4G18860.1 |  | | | |  |  |  |  |  |  |  |
| 1 | Ath-AT4G18870.1 |  | | | |  |  |  |  |  |  |  |
| 1 | Ath-AT4G18880.1 |  | Vvi-Vitvi10g00635\_t002 |  |  |  |  |  |  |  |
| 1 | Ath-AT4G18890.1 |  | Vvi-Vitvi10g00636\_t001 |  |  |  |  |  |  |  |
| 1 | Ath-AT4G18900.1 |  | | | |  |  |  |  |  |  |  |
| 1 | Ath-AT4G18905.2 |  | | | |  |  |  |  |  |  |  |
| 1 | Ath-AT4G18910.1 |  | Vvi-Vitvi10g00639\_t001 |  |  |  |  |  |  |  |
| 1 | Ath-AT4G18920.1 |  | Vvi-Vitvi10g00660\_t001 |  |  |  |  |  |  |  |
| 1 | Ath-AT4G18930.1 |  | Vvi-Vitvi10g00661\_t002 |  |  |  |  |  |  |  |
| 1 | Ath-AT4G18940.1 |  | | | |  |  |  |  |  |  |  |
| 1 | Ath-AT4G18950.1 |  | Vvi-Vitvi10g00662\_t001 |  |  |  |  |  |  |  |
| 1 | Ath-AT4G18960.1 |  | Vvi-Vitvi10g00663\_t001 |  |  |  |  |  |  |  |
| 1 | Ath-AT4G18970.2 |  | Vvi-Vitvi10g00665\_t001 |  |  |  |  |  |  |  |
| 1 | Ath-AT4G18975.1 |  | | | |  |  |  |  |  |  |  |
| 1 | Ath-AT4G18980.1 |  | Vvi-Vitvi10g01813\_t001 |  |  |  |  |  |  |  |
| 0 | Ath-AT4G18990.1 |  |  |  |  |  |  |  |  |
| 0 | Ath-AT4G19000.1 |  |  |  |  |  |  |  |  |
| 0 | Ath-AT4G19003.1 |  |  |  |  |  |  |  |  |
| 0 | Ath-AT4G19006.2 |  |  |  |  |  |  |  |  |
| 0 | Ath-AT4G19010.1 |  |  |  |  |  |  |  |  |
| 0 | Ath-AT4G19020.1 |  |  |  |  |  |  |  |  |
| 0 | Ath-AT4G19030.1 |  |  |  |  |  |  |  |  |
| 0 | Ath-AT4G19035.1 |  |  |  |  |  |  |  |  |
| 0 | Ath-AT4G19038.1 |  |  |  |  |  |  |  |  |
| 0 | Ath-AT4G19040.2 |  |  |  |  |  |  |  |  |
| 0 | Ath-AT4G19045.1 |  |  |  |  |  |  |  |  |
| 0 | Ath-AT4G19050.1 |  |  |  |  |  |  |  |  |
| 0 | Ath-AT4G19060.1 |  |  |  |  |  |  |  |  |
| 0 | Ath-AT4G19070.1 |  |  |  |  |  |  |  |  |
| 0 | Ath-AT4G19080.1 |  |  |  |  |  |  |  |  |
| 0 | Ath-AT4G19090.1 |  |  |  |  |  |  |  |  |
| 0 | Ath-AT4G19095.1 |  |  |  |  |  |  |  |  |
| 0 | Ath-AT4G19100.1 |  |  |  |  |  |  |  |  |
| 0 | Ath-AT4G19110.2 |  |  |  |  |  |  |  |  |
| 0 | Ath-AT4G19120.1 |  |  |  |  |  |  |  |  |
| 0 | Ath-AT4G19130.2 |  |  |  |  |  |  |  |  |
| 0 | Ath-AT4G19140.1 |  |  |  |  |  |  |  |  |
| 0 | Ath-AT4G19150.1 |  |  |  |  |  |  |  |  |
| 0 | Ath-AT4G19160.2 |  |  |  |  |  |  |  |  |
| 1 | Ath-AT4G19170.1 |  | Vvi-Vitvi02g01286\_t001 |  |  |  |  |  |  |  |
| 1 | Ath-AT4G19180.1 |  | | | |  |  |  |  |  |  |  |
| 1 | Ath-AT4G19185.1 |  | Vvi-Vitvi02g01284\_t001 |  |  |  |  |  |  |  |
| 1 | Ath-AT4G19190.1 |  | Vvi-Vitvi02g01278\_t001 |  |  |  |  |  |  |  |
| 1 | Ath-AT4G19191.1 |  | | | |  |  |  |  |  |  |  |
| 1 | Ath-AT4G19200.1 |  | | | |  |  |  |  |  |  |  |
| 1 | Ath-AT4G19210.1 |  | Vvi-Vitvi02g01669\_t001 |  |  |  |  |  |  |  |
| 1 | Ath-AT4G19220.1 |  | Vvi-Vitvi02g01274\_t001 |  |  |  |  |  |  |  |
| 1 | Ath-AT4G19230.2 |  | Vvi-Vitvi02g01269\_t001 |  |  |  |  |  |  |  |
| 1 | Ath-AT4G19240.1 |  | | | |  |  |  |  |  |  |  |
| 1 | Ath-AT4G19250.1 |  | | | |  |  |  |  |  |  |  |
| 1 | Ath-AT4G19260.1 |  | | | |  |  |  |  |  |  |  |
| 1 | Ath-AT4G19270.1 |  | | | |  |  |  |  |  |  |  |
| 1 | Ath-AT4G19275.1 |  | Vvi-Vitvi02g01267\_t001 |  |  |  |  |  |  |  |
| 1 | Ath-AT4G19330.1 |  | | | |  |  |  |  |  |  |  |
| 1 | Ath-AT4G19340.1 |  | | | |  |  |  |  |  |  |  |
| 1 | Ath-AT4G19350.1 |  | | | |  |  |  |  |  |  |  |
| 1 | Ath-AT4G19360.1 |  | | | |  |  |  |  |  |  |  |
| 1 | Ath-AT4G19370.1 |  | Vvi-Vitvi02g01266\_t001 |  |  |  |  |  |  |  |
| 1 | Ath-AT4G19380.2 |  | Vvi-Vitvi02g01264\_t001 |  |  |  |  |  |  |  |
| 1 | Ath-AT4G19390.1 |  | Vvi-Vitvi02g01261\_t001 |  |  |  |  |  |  |  |
| 0 | Ath-AT4G19400.1 |  |  |  |  |  |  |  |  |
| 0 | Ath-AT4G19410.2 |  |  |  |  |  |  |  |  |
| 0 | Ath-AT4G19420.4 |  |  |  |  |  |  |  |  |
| 0 | Ath-AT4G19430.1 |  |  |  |  |  |  |  |  |
| 0 | Ath-AT4G19440.1 |  |  |  |  |  |  |  |  |
| 0 | Ath-AT4G19450.1 |  |  |  |  |  |  |  |  |
| 0 | Ath-AT4G19460.1 |  |  |  |  |  |  |  |  |
| 0 | Ath-AT4G19470.1 |  |  |  |  |  |  |  |  |
| 0 | Ath-AT4G19480.1 |  |  |  |  |  |  |  |  |
| 0 | Ath-AT4G19485.1 |  |  |  |  |  |  |  |  |
| 0 | Ath-AT4G19490.2 |  |  |  |  |  |  |  |  |
| 0 | Ath-AT4G19500.1 |  |  |  |  |  |  |  |  |
| 0 | Ath-AT4G19510.4 |  |  |  |  |  |  |  |  |
| 0 | Ath-AT4G19520.1 |  |  |  |  |  |  |  |  |
| 0 | Ath-AT4G19530.2 |  |  |  |  |  |  |  |  |
| 0 | Ath-AT4G19540.1 |  |  |  |  |  |  |  |  |
| 0 | Ath-AT4G19550.2 |  |  |  |  |  |  |  |  |
| 0 | Ath-AT4G19560.1 |  |  |  |  |  |  |  |  |
| 0 | Ath-AT4G19570.1 |  |  |  |  |  |  |  |  |
| 0 | Ath-AT4G19580.1 |  |  |  |  |  |  |  |  |
| 0 | Ath-AT4G19590.2 |  |  |  |  |  |  |  |  |
| 0 | Ath-AT4G19600.1 |  |  |  |  |  |  |  |  |
| 0 | Ath-AT4G19610.1 |  |  |  |  |  |  |  |  |
| 0 | Ath-AT4G19620.1 |  |  |  |  |  |  |  |  |
| 0 | Ath-AT4G19630.1 |  |  |  |  |  |  |  |  |
| 0 | Ath-AT4G19640.1 |  |  |  |  |  |  |  |  |
| 1 | Ath-AT4G19645.1 |  | Vvi-Vitvi10g01325\_t001 |  |  |  |  |  |  |  |
| 1 | Ath-AT4G19650.2 |  | Vvi-Vitvi10g01334\_t001 |  |  |  |  |  |  |  |
| 1 | Ath-AT4G19660.2 |  | Vvi-Vitvi10g01335\_t001 |  |  |  |  |  |  |  |
| 1 | Ath-AT4G19670.3 |  | Vvi-Vitvi10g01351\_t001 |  |  |  |  |  |  |  |
| 1 | Ath-AT4G19680.2 |  | Vvi-Vitvi10g04616\_t001 |  |  |  |  |  |  |  |
| 1 | Ath-AT4G19690.2 |  | | | |  |  |  |  |  |  |  |
| 1 | Ath-AT4G19700.1 |  | Vvi-Vitvi10g01359\_t001 |  |  |  |  |  |  |  |
| 0 | Ath-AT4G19710.2 |  |  |  |  |  |  |  |  |
| 0 | Ath-AT4G19720.1 |  |  |  |  |  |  |  |  |
| 0 | Ath-AT4G19730.1 |  |  |  |  |  |  |  |  |
| 0 | Ath-AT4G19740.1 |  |  |  |  |  |  |  |  |
| 0 | Ath-AT4G19750.1 |  |  |  |  |  |  |  |  |
| 0 | Ath-AT4G19760.1 |  |  |  |  |  |  |  |  |
| 0 | Ath-AT4G19770.1 |  |  |  |  |  |  |  |  |
| 0 | Ath-AT4G19800.1 |  |  |  |  |  |  |  |  |
| 0 | Ath-AT4G19810.1 |  |  |  |  |  |  |  |  |
| 0 | Ath-AT4G19820.1 |  |  |  |  |  |  |  |  |
| 2 | Ath-AT4G19830.1 |  | Vvi-Vitvi01g01874\_t001 |  | Vvi-Vitvi01g01874\_t001 |  |  |  |  |  |  |
| 2 | Ath-AT4G19840.1 |  | | | |  | Vvi-Vitvi01g01884\_t001 |  |  |  |  |  |  |
| 2 | Ath-AT4G19850.2 |  | | | |  | | | |  |  |  |  |  |  |
| 2 | Ath-AT4G19860.1 |  | | | |  | Vvi-Vitvi01g00143\_t001 |  |  |  |  |  |  |
| 2 | Ath-AT4G19865.1 |  | | | |  | | | |  |  |  |  |  |  |
| 2 | Ath-AT4G19870.1 |  | | | |  | | | |  |  |  |  |  |  |
| 2 | Ath-AT4G19880.2 |  | | | |  | Vvi-Vitvi01g00148\_t001 |  |  |  |  |  |  |
| 2 | Ath-AT4G19890.1 |  | | | |  | Vvi-Vitvi01g00150\_t001 |  |  |  |  |  |  |
| 2 | Ath-AT4G19900.1 |  | | | |  | Vvi-Vitvi01g00151\_t001 |  |  |  |  |  |  |
| 1 | Ath-AT4G19905.1 |  | | | |  |  |  |  |  |  |  |
| 1 | Ath-AT4G19910.1 |  | | | |  |  |  |  |  |  |  |
| 1 | Ath-AT4G19920.1 |  | | | |  |  |  |  |  |  |  |
| 1 | Ath-AT4G19925.1 |  | | | |  |  |  |  |  |  |  |
| 1 | Ath-AT4G19930.1 |  | | | |  |  |  |  |  |  |  |
| 1 | Ath-AT4G19940.1 |  | | | |  |  |  |  |  |  |  |
| 1 | Ath-AT4G19950.1 |  | Vvi-Vitvi01g00124\_t001 |  |  |  |  |  |  |  |
| 1 | Ath-AT4G19960.3 |  | Vvi-Vitvi01g00121\_t001 |  |  |  |  |  |  |  |
| 1 | Ath-AT4G19970.1 |  | | | |  |  |  |  |  |  |  |
| 1 | Ath-AT4G19975.1 |  | | | |  |  |  |  |  |  |  |
| 1 | Ath-AT4G19980.1 |  | Vvi-Vitvi01g01870\_t001 |  |  |  |  |  |  |  |
| 1 | Ath-AT4G19985.4 |  | | | |  |  |  |  |  |  |  |
| 1 | Ath-AT4G19990.2 |  | Vvi-Vitvi01g00116\_t001 |  |  |  |  |  |  |  |
| 1 | Ath-AT4G20000.1 |  | | | |  |  |  |  |  |  |  |
| 1 | Ath-AT4G20010.1 |  | | | |  |  |  |  |  |  |  |
| 1 | Ath-AT4G20020.1 |  | | | |  |  |  |  |  |  |  |
| 1 | Ath-AT4G20030.1 |  | Vvi-Vitvi01g04027\_t001 |  |  |  |  |  |  |  |
| 1 | Ath-AT4G20040.1 |  | Vvi-Vitvi01g00104\_t001 |  |  |  |  |  |  |  |
| 1 | Ath-AT4G20050.5 |  | | | |  |  |  |  |  |  |  |
| 1 | Ath-AT4G20060.1 |  | | | |  |  |  |  |  |  |  |
| 1 | Ath-AT4G20070.1 |  | Vvi-Vitvi01g00103\_t001 |  |  |  |  |  |  |  |
| 1 | Ath-AT4G20080.1 |  | | | |  |  |  |  |  |  |  |
| 1 | Ath-AT4G20090.1 |  | Vvi-Vitvi01g00089\_t001 |  |  |  |  |  |  |  |
| 1 | Ath-AT4G20095.3 |  | | | |  |  |  |  |  |  |  |
| 1 | Ath-AT4G20100.1 |  | | | |  |  |  |  |  |  |  |
| 1 | Ath-AT4G20110.2 |  | | | |  |  |  |  |  |  |  |
| 1 | Ath-AT4G20130.1 |  | Vvi-Vitvi01g00086\_t001 |  |  |  |  |  |  |  |
| 0 | Ath-AT4G20140.1 |  |  |  |  |  |  |  |  |
| 0 | Ath-AT4G20150.1 |  |  |  |  |  |  |  |  |
| 0 | Ath-AT4G20160.2 |  |  |  |  |  |  |  |  |
| 0 | Ath-AT4G20170.1 |  |  |  |  |  |  |  |  |
| 0 | Ath-AT4G20190.1 |  |  |  |  |  |  |  |  |
| 0 | Ath-AT4G20200.1 |  |  |  |  |  |  |  |  |
| 0 | Ath-AT4G20210.1 |  |  |  |  |  |  |  |  |
| 0 | Ath-AT4G20220.1 |  |  |  |  |  |  |  |  |
| 0 | Ath-AT4G20230.2 |  |  |  |  |  |  |  |  |
| 0 | Ath-AT4G20235.1 |  |  |  |  |  |  |  |  |
| 0 | Ath-AT4G20240.1 |  |  |  |  |  |  |  |  |
| 0 | Ath-AT4G20250.1 |  |  |  |  |  |  |  |  |
| 0 | Ath-AT4G20260.4 |  |  |  |  |  |  |  |  |
| 0 | Ath-AT4G20270.1 |  |  |  |  |  |  |  |  |
| 0 | Ath-AT4G20280.1 |  |  |  |  |  |  |  |  |
| 0 | Ath-AT4G20290.1 |  |  |  |  |  |  |  |  |
| 0 | Ath-AT4G20300.2 |  |  |  |  |  |  |  |  |
| 0 | Ath-AT4G20310.4 |  |  |  |  |  |  |  |  |
| 0 | Ath-AT4G20320.2 |  |  |  |  |  |  |  |  |
| 0 | Ath-AT4G20325.1 |  |  |  |  |  |  |  |  |
| 0 | Ath-AT4G20330.1 |  |  |  |  |  |  |  |  |
| 0 | Ath-AT4G20340.1 |  |  |  |  |  |  |  |  |
| 0 | Ath-AT4G20350.2 |  |  |  |  |  |  |  |  |
| 0 | Ath-AT4G20360.1 |  |  |  |  |  |  |  |  |
| 0 | Ath-AT4G20370.1 |  |  |  |  |  |  |  |  |
| 0 | Ath-AT4G20380.8 |  |  |  |  |  |  |  |  |
| 0 | Ath-AT4G20390.1 |  |  |  |  |  |  |  |  |
| 0 | Ath-AT4G20400.1 |  |  |  |  |  |  |  |  |
| 0 | Ath-AT4G20410.2 |  |  |  |  |  |  |  |  |
| 0 | Ath-AT4G20430.1 |  |  |  |  |  |  |  |  |
| 0 | Ath-AT4G20420.1 |  |  |  |  |  |  |  |  |
| 0 | Ath-AT4G20440.1 |  |  |  |  |  |  |  |  |
| 0 | Ath-AT4G20450.1 |  |  |  |  |  |  |  |  |
| 0 | Ath-AT4G20460.1 |  |  |  |  |  |  |  |  |
| 0 | Ath-AT4G20470.1 |  |  |  |  |  |  |  |  |
| 0 | Ath-AT4G20480.1 |  |  |  |  |  |  |  |  |
| 0 | Ath-AT4G20520.1 |  |  |  |  |  |  |  |  |
| 0 | Ath-AT4G20530.1 |  |  |  |  |  |  |  |  |
| 0 | Ath-AT4G20535.1 |  |  |  |  |  |  |  |  |
| 0 | Ath-AT4G20540.1 |  |  |  |  |  |  |  |  |
| 0 | Ath-AT4G20545.1 |  |  |  |  |  |  |  |  |
| 0 | Ath-AT4G20550.1 |  |  |  |  |  |  |  |  |
| 0 | Ath-AT4G20555.1 |  |  |  |  |  |  |  |  |
| 0 | Ath-AT4G20560.1 |  |  |  |  |  |  |  |  |
| 0 | Ath-AT4G20565.1 |  |  |  |  |  |  |  |  |
| 0 | Ath-AT4G20570.1 |  |  |  |  |  |  |  |  |
| 0 | Ath-AT4G20575.1 |  |  |  |  |  |  |  |  |
| 0 | Ath-AT4G20580.1 |  |  |  |  |  |  |  |  |
| 0 | Ath-AT4G20590.1 |  |  |  |  |  |  |  |  |
| 0 | Ath-AT4G20600.1 |  |  |  |  |  |  |  |  |
| 0 | Ath-AT4G20610.1 |  |  |  |  |  |  |  |  |
| 0 | Ath-AT4G20620.1 |  |  |  |  |  |  |  |  |
| 0 | Ath-AT4G20630.1 |  |  |  |  |  |  |  |  |
| 0 | Ath-AT4G20640.1 |  |  |  |  |  |  |  |  |
| 0 | Ath-AT4G20645.1 |  |  |  |  |  |  |  |  |
| 0 | Ath-AT4G20650.1 |  |  |  |  |  |  |  |  |
| 0 | Ath-AT4G20670.1 |  |  |  |  |  |  |  |  |
| 0 | Ath-AT4G20680.1 |  |  |  |  |  |  |  |  |
| 0 | Ath-AT4G20690.1 |  |  |  |  |  |  |  |  |
| 0 | Ath-AT4G20700.1 |  |  |  |  |  |  |  |  |
| 0 | Ath-AT4G20703.1 |  |  |  |  |  |  |  |  |
| 0 | Ath-AT4G20707.1 |  |  |  |  |  |  |  |  |
| 0 | Ath-AT4G20720.1 |  |  |  |  |  |  |  |  |
| 0 | Ath-AT4G20735.1 |  |  |  |  |  |  |  |  |
| 0 | Ath-AT4G20740.1 |  |  |  |  |  |  |  |  |
| 1 | Ath-AT4G20760.2 |  | Vvi-Vitvi10g01084\_t001 |  |  |  |  |  |  |  |
| 1 | Ath-AT4G20770.1 |  | | | |  |  |  |  |  |  |  |
| 1 | Ath-AT4G20780.1 |  | | | |  |  |  |  |  |  |  |
| 1 | Ath-AT4G20790.1 |  | | | |  |  |  |  |  |  |  |
| 1 | Ath-AT4G20800.1 |  | | | |  |  |  |  |  |  |  |
| 1 | Ath-AT4G20810.1 |  | | | |  |  |  |  |  |  |  |
| 1 | Ath-AT4G20820.1 |  | Vvi-Vitvi10g01045\_t001 |  |  |  |  |  |  |  |
| 1 | Ath-AT4G20830.1 |  | Vvi-Vitvi10g04513\_t001 |  |  |  |  |  |  |  |
| 1 | Ath-AT4G20835.1 |  | | | |  |  |  |  |  |  |  |
| 1 | Ath-AT4G20840.1 |  | | | |  |  |  |  |  |  |  |
| 1 | Ath-AT4G20850.1 |  | | | |  |  |  |  |  |  |  |
| 1 | Ath-AT4G20860.1 |  | Vvi-Vitvi10g01017\_t001 |  |  |  |  |  |  |  |
| 1 | Ath-AT4G20870.1 |  | Vvi-Vitvi10g01015\_t001 |  |  |  |  |  |  |  |
| 1 | Ath-AT4G20880.1 |  | Vvi-Vitvi10g01931\_t001 |  |  |  |  |  |  |  |
| 1 | Ath-AT4G20890.1 |  | | | |  |  |  |  |  |  |  |
| 1 | Ath-AT4G20900.1 |  | Vvi-Vitvi10g00996\_t001 |  |  |  |  |  |  |  |
| 0 | Ath-AT4G20910.2 |  |  |  |  |  |  |  |  |
| 0 | Ath-AT4G20920.2 |  |  |  |  |  |  |  |  |
| 0 | Ath-AT4G20930.1 |  |  |  |  |  |  |  |  |
| 0 | Ath-AT4G20935.1 |  |  |  |  |  |  |  |  |
| 0 | Ath-AT4G20940.1 |  |  |  |  |  |  |  |  |
| 0 | Ath-AT4G20947.1 |  |  |  |  |  |  |  |  |
| 0 | Ath-AT4G20953.1 |  |  |  |  |  |  |  |  |
| 0 | Ath-AT4G20960.1 |  |  |  |  |  |  |  |  |
| 0 | Ath-AT4G20970.1 |  |  |  |  |  |  |  |  |
| 0 | Ath-AT4G20980.4 |  |  |  |  |  |  |  |  |
| 0 | Ath-AT4G20990.1 |  |  |  |  |  |  |  |  |
| 0 | Ath-AT4G21000.1 |  |  |  |  |  |  |  |  |
| 0 | Ath-AT4G21010.1 |  |  |  |  |  |  |  |  |
| 0 | Ath-AT4G21020.1 |  |  |  |  |  |  |  |  |
| 0 | Ath-AT4G21030.1 |  |  |  |  |  |  |  |  |
| 0 | Ath-AT4G21040.1 |  |  |  |  |  |  |  |  |
| 0 | Ath-AT4G21050.1 |  |  |  |  |  |  |  |  |
| 0 | Ath-AT4G21060.1 |  |  |  |  |  |  |  |  |
| 0 | Ath-AT4G21063.1 |  |  |  |  |  |  |  |  |
| 0 | Ath-AT4G21065.1 |  |  |  |  |  |  |  |  |
| 0 | Ath-AT4G21070.1 |  |  |  |  |  |  |  |  |
| 0 | Ath-AT4G21080.1 |  |  |  |  |  |  |  |  |
| 0 | Ath-AT4G21090.2 |  |  |  |  |  |  |  |  |
| 0 | Ath-AT4G21100.1 |  |  |  |  |  |  |  |  |
| 0 | Ath-AT4G21105.3 |  |  |  |  |  |  |  |  |
| 0 | Ath-AT4G21110.1 |  |  |  |  |  |  |  |  |
| 0 | Ath-AT4G21120.1 |  |  |  |  |  |  |  |  |
| 0 | Ath-AT4G21130.1 |  |  |  |  |  |  |  |  |
| 0 | Ath-AT4G21140.1 |  |  |  |  |  |  |  |  |
| 0 | Ath-AT4G21150.3 |  |  |  |  |  |  |  |  |
| 0 | Ath-AT4G21160.1 |  |  |  |  |  |  |  |  |
| 1 | Ath-AT4G21170.1 |  | Vvi-Vitvi10g00028\_t001 |  |  |  |  |  |  |  |
| 1 | Ath-AT4G21180.1 |  | Vvi-Vitvi10g00022\_t001 |  |  |  |  |  |  |  |
| 1 | Ath-AT4G21190.1 |  | | | |  |  |  |  |  |  |  |
| 1 | Ath-AT4G21192.2 |  | | | |  |  |  |  |  |  |  |
| 1 | Ath-AT4G21200.1 |  | Vvi-Vitvi10g00020\_t001 |  |  |  |  |  |  |  |
| 1 | Ath-AT4G21210.1 |  | Vvi-Vitvi10g00019\_t001 |  |  |  |  |  |  |  |
| 1 | Ath-AT4G21213.1 |  | | | |  |  |  |  |  |  |  |
| 1 | Ath-AT4G21215.2 |  | Vvi-Vitvi10g01606\_t001 |  |  |  |  |  |  |  |
| 1 | Ath-AT4G21220.1 |  | Vvi-Vitvi10g00012\_t001 |  |  |  |  |  |  |  |
| 2 | Ath-AT4G21230.1 |  | | | |  | Vvi-Vitvi10g04213\_t001 |  |  |  |  |  |  |
| 2 | Ath-AT4G21240.1 |  | | | |  | | | |  |  |  |  |  |  |
| 2 | Ath-AT4G21250.1 |  | | | |  | | | |  |  |  |  |  |  |
| 2 | Ath-AT4G21260.1 |  | | | |  | | | |  |  |  |  |  |  |
| 2 | Ath-AT4G21270.1 |  | | | |  | | | |  |  |  |  |  |  |
| 2 | Ath-AT4G21280.2 |  | | | |  | | | |  |  |  |  |  |  |
| 2 | Ath-AT4G21300.1 |  | | | |  | | | |  |  |  |  |  |  |
| 2 | Ath-AT4G21310.1 |  | | | |  | | | |  |  |  |  |  |  |
| 2 | Ath-AT4G21320.1 |  | | | |  | | | |  |  |  |  |  |  |
| 2 | Ath-AT4G21323.1 |  | | | |  | | | |  |  |  |  |  |  |
| 2 | Ath-AT4G21326.1 |  | | | |  | | | |  |  |  |  |  |  |
| 2 | Ath-AT4G21330.1 |  | | | |  | Vvi-Vitvi10g02164\_t001 |  |  |  |  |  |  |
| 2 | Ath-AT4G21340.1 |  | | | |  | Vvi-Vitvi10g00323\_t001 |  |  |  |  |  |  |
| 2 | Ath-AT4G21350.1 |  | | | |  | Vvi-Vitvi10g04226\_t001 |  |  |  |  |  |  |
| 2 | Ath-AT4G21366.1 |  | | | |  | | | |  |  |  |  |  |  |
| 2 | Ath-AT4G21380.1 |  | | | |  | Vvi-Vitvi10g04227\_t001 |  |  |  |  |  |  |
| 2 | Ath-AT4G21390.1 |  | Vvi-Vitvi10g00008\_t001 |  | Vvi-Vitvi10g04232\_t001 |  |  |  |  |  |  |
| 1 | Ath-AT4G21400.1 |  |  |  | | | |  |  |  |  |  |  |
| 1 | Ath-AT4G21410.3 |  |  |  | | | |  |  |  |  |  |  |
| 1 | Ath-AT4G21430.1 |  |  |  | | | |  |  |  |  |  |  |
| 1 | Ath-AT4G21440.1 |  |  |  | Vvi-Vitvi10g00345\_t001 |  |  |  |  |  |  |
| 1 | Ath-AT4G21445.1 |  |  |  | | | |  |  |  |  |  |  |
| 1 | Ath-AT4G21450.3 |  |  |  | Vvi-Vitvi10g04245\_t001 |  |  |  |  |  |  |
| 1 | Ath-AT4G21460.2 |  |  |  | | | |  |  |  |  |  |  |
| 1 | Ath-AT4G21470.1 |  |  |  | | | |  |  |  |  |  |  |
| 1 | Ath-AT4G21480.1 |  |  |  | Vvi-Vitvi10g00358\_t001 |  |  |  |  |  |  |
| 1 | Ath-AT4G21490.1 |  |  |  | Vvi-Vitvi10g04257\_t001 |  |  |  |  |  |  |
| 1 | Ath-AT4G21500.1 |  |  |  | Vvi-Vitvi10g01746\_t001 |  |  |  |  |  |  |
| 1 | Ath-AT4G21510.1 |  |  |  | Vvi-Vitvi10g01748\_t001 |  |  |  |  |  |  |
| 1 | Ath-AT4G21515.1 |  |  |  | | | |  |  |  |  |  |  |
| 1 | Ath-AT4G21520.1 |  |  |  | Vvi-Vitvi10g00365\_t001 |  |  |  |  |  |  |
| 1 | Ath-AT4G21530.1 |  |  |  | Vvi-Vitvi10g00379\_t001 |  |  |  |  |  |  |
| 1 | Ath-AT4G21534.1 |  |  |  | Vvi-Vitvi10g00383\_t001 |  |  |  |  |  |  |
| 1 | Ath-AT4G21540.1 |  |  |  | | | |  |  |  |  |  |  |
| 1 | Ath-AT4G21550.1 |  |  |  | Vvi-Vitvi10g04259\_t002 |  |  |  |  |  |  |
| 1 | Ath-AT4G21560.1 |  |  |  | Vvi-Vitvi10g00386\_t002 |  |  |  |  |  |  |
| 1 | Ath-AT4G21570.2 |  |  |  | Vvi-Vitvi10g00394\_t001 |  |  |  |  |  |  |
| 1 | Ath-AT4G21580.1 |  |  |  | Vvi-Vitvi10g01757\_t001 |  |  |  |  |  |  |
| 1 | Ath-AT4G21585.6 |  |  |  | Vvi-Vitvi10g00401\_t001 |  |  |  |  |  |  |
| 1 | Ath-AT4G21590.1 |  |  |  | | | |  |  |  |  |  |  |
| 1 | Ath-AT4G21600.1 |  |  |  | | | |  |  |  |  |  |  |
| 1 | Ath-AT4G21610.1 |  |  |  | | | |  |  |  |  |  |  |
| 1 | Ath-AT4G21620.1 |  |  |  | Vvi-Vitvi10g01753\_t001 |  |  |  |  |  |  |
| 0 | Ath-AT4G21630.1 |  |  |  |  |  |  |  |  |
| 0 | Ath-AT4G21640.1 |  |  |  |  |  |  |  |  |
| 0 | Ath-AT4G21650.1 |  |  |  |  |  |  |  |  |
| 0 | Ath-AT4G21660.3 |  |  |  |  |  |  |  |  |
| 0 | Ath-AT4G21670.1 |  |  |  |  |  |  |  |  |
| 0 | Ath-AT4G21680.1 |  |  |  |  |  |  |  |  |
| 0 | Ath-AT4G21690.1 |  |  |  |  |  |  |  |  |
| 0 | Ath-AT4G21700.1 |  |  |  |  |  |  |  |  |
| 0 | Ath-AT4G21705.1 |  |  |  |  |  |  |  |  |
| 0 | Ath-AT4G21710.1 |  |  |  |  |  |  |  |  |
| 0 | Ath-AT4G21720.1 |  |  |  |  |  |  |  |  |
| 0 | Ath-AT4G21740.1 |  |  |  |  |  |  |  |  |
| 0 | Ath-AT4G21745.1 |  |  |  |  |  |  |  |  |
| 0 | Ath-AT4G21750.2 |  |  |  |  |  |  |  |  |
| 0 | Ath-AT4G21760.2 |  |  |  |  |  |  |  |  |
| 0 | Ath-AT4G21770.1 |  |  |  |  |  |  |  |  |
| 0 | Ath-AT4G21780.1 |  |  |  |  |  |  |  |  |
| 1 | Ath-AT4G21790.1 |  | Vvi-Vitvi10g00116\_t001 |  |  |  |  |  |  |  |
| 1 | Ath-AT4G21800.2 |  | | | |  |  |  |  |  |  |  |
| 2 | Ath-AT4G21810.1 |  | Vvi-Vitvi10g04039\_t001 |  | Vvi-Vitvi10g04026\_t001 |  |  |  |  |  |  |
| 2 | Ath-AT4G21820.3 |  | Vvi-Vitvi10g00131\_t001 |  | | | |  |  |  |  |  |  |
| 2 | Ath-AT4G21830.1 |  | | | |  | | | |  |  |  |  |  |  |
| 2 | Ath-AT4G21840.1 |  | | | |  | | | |  |  |  |  |  |  |
| 2 | Ath-AT4G21850.1 |  | | | |  | | | |  |  |  |  |  |  |
| 2 | Ath-AT4G21860.3 |  | | | |  | | | |  |  |  |  |  |  |
| 2 | Ath-AT4G21865.1 |  | | | |  | | | |  |  |  |  |  |  |
| 2 | Ath-AT4G21870.1 |  | Vvi-Vitvi10g00134\_t001 |  | | | |  |  |  |  |  |  |
| 2 | Ath-AT4G21880.1 |  | | | |  | | | |  |  |  |  |  |  |
| 2 | Ath-AT4G21890.1 |  | Vvi-Vitvi10g04040\_t001 |  | | | |  |  |  |  |  |  |
| 3 | Ath-AT4G21895.1 |  | Vvi-Vitvi10g04043\_t001 |  | | | |  | Vvi-Vitvi19g00489\_t002 |  |  |  |  |  |
| 2 | Ath-AT4G21900.1 |  |  |  | | | |  | | | |  |  |  |  |  |
| 2 | Ath-AT4G21902.1 |  |  |  | Vvi-Vitvi10g00110\_t001 |  | | | |  |  |  |  |  |
| 2 | Ath-AT4G21903.2 |  |  |  | Vvi-Vitvi10g00107\_t001 |  | | | |  |  |  |  |  |
| 2 | Ath-AT4G21910.4 |  |  |  | | | |  | | | |  |  |  |  |  |
| 2 | Ath-AT4G21920.1 |  |  |  | Vvi-Vitvi10g01643\_t001 |  | | | |  |  |  |  |  |
| 2 | Ath-AT4G21926.1 |  |  |  | | | |  | | | |  |  |  |  |  |
| 2 | Ath-AT4G21930.1 |  |  |  | Vvi-Vitvi10g01642\_t001 |  | | | |  |  |  |  |  |
| 2 | Ath-AT4G21940.2 |  |  |  | Vvi-Vitvi10g00102\_t001 |  | Vvi-Vitvi19g00497\_t001 |  |  |  |  |  |
| 2 | Ath-AT4G21950.1 |  |  |  | | | |  | | | |  |  |  |  |  |
| 2 | Ath-AT4G21960.1 |  |  |  | Vvi-Vitvi10g00100\_t001 |  | | | |  |  |  |  |  |
| 2 | Ath-AT4G21970.1 |  |  |  | Vvi-Vitvi10g00093\_t001 |  | Vvi-Vitvi19g04237\_t001 |  |  |  |  |  |
| 2 | Ath-AT4G21980.2 |  |  |  | Vvi-Vitvi10g04024\_t001 |  | Vvi-Vitvi19g02021\_t001 |  |  |  |  |  |
| 2 | Ath-AT4G21990.1 |  |  |  | Vvi-Vitvi10g00086\_t001 |  | | | |  |  |  |  |  |
| 2 | Ath-AT4G22000.1 |  |  |  | | | |  | | | |  |  |  |  |  |
| 2 | Ath-AT4G22010.1 |  |  |  | Vvi-Vitvi10g00082\_t001 |  | | | |  |  |  |  |  |
| 2 | Ath-AT4G22030.1 |  |  |  | Vvi-Vitvi10g04012\_t001 |  | | | |  |  |  |  |  |
| 2 | Ath-AT4G22035.1 |  |  |  | | | |  | | | |  |  |  |  |  |
| 2 | Ath-AT4G22050.1 |  |  |  | Vvi-Vitvi10g00064\_t001 |  | Vvi-Vitvi19g00529\_t001 |  |  |  |  |  |
| 2 | Ath-AT4G22060.1 |  |  |  | | | |  | | | |  |  |  |  |  |
| 2 | Ath-AT4G22070.1 |  |  |  | Vvi-Vitvi10g00063\_t001 |  | Vvi-Vitvi19g00530\_t001 |  |  |  |  |  |
| 1 | Ath-AT4G22080.1 |  |  |  | Vvi-Vitvi10g00061\_t001 |  |  |  |  |  |  |
| 1 | Ath-AT4G22090.1 |  |  |  | | | |  |  |  |  |  |  |
| 1 | Ath-AT4G22100.1 |  |  |  | | | |  |  |  |  |  |  |
| 1 | Ath-AT4G22105.1 |  |  |  | | | |  |  |  |  |  |  |
| 1 | Ath-AT4G22110.2 |  |  |  | | | |  |  |  |  |  |  |
| 1 | Ath-AT4G22115.1 |  |  |  | | | |  |  |  |  |  |  |
| 1 | Ath-AT4G22120.6 |  |  |  | Vvi-Vitvi10g00052\_t004 |  |  |  |  |  |  |
| 1 | Ath-AT4G22130.1 |  | Vvi-Vitvi10g00119\_t002 |  |  |  |  |  |  |  |
| 1 | Ath-AT4G22140.3 |  | | | |  |  |  |  |  |  |  |
| 1 | Ath-AT4G22150.1 |  | | | |  |  |  |  |  |  |  |
| 1 | Ath-AT4G22160.2 |  | | | |  |  |  |  |  |  |  |
| 1 | Ath-AT4G22165.1 |  | | | |  |  |  |  |  |  |  |
| 1 | Ath-AT4G22170.1 |  | | | |  |  |  |  |  |  |  |
| 1 | Ath-AT4G22180.1 |  | | | |  |  |  |  |  |  |  |
| 1 | Ath-AT4G22190.1 |  | | | |  |  |  |  |  |  |  |
| 1 | Ath-AT4G22200.1 |  | | | |  |  |  |  |  |  |  |
| 1 | Ath-AT4G22210.2 |  | | | |  |  |  |  |  |  |  |
| 1 | Ath-AT4G22212.1 |  | | | |  |  |  |  |  |  |  |
| 1 | Ath-AT4G22214.1 |  | | | |  |  |  |  |  |  |  |
| 1 | Ath-AT4G22217.1 |  | | | |  |  |  |  |  |  |  |
| 1 | Ath-AT4G22220.1 |  | | | |  |  |  |  |  |  |  |
| 1 | Ath-AT4G22230.1 |  | | | |  |  |  |  |  |  |  |
| 1 | Ath-AT4G22235.1 |  | | | |  |  |  |  |  |  |  |
| 1 | Ath-AT4G22240.1 |  | | | |  |  |  |  |  |  |  |
| 1 | Ath-AT4G22250.1 |  | Vvi-Vitvi10g04050\_t001 |  |  |  |  |  |  |  |
| 1 | Ath-AT4G22260.1 |  | | | |  |  |  |  |  |  |  |
| 1 | Ath-AT4G22270.2 |  | | | |  |  |  |  |  |  |  |
| 1 | Ath-AT4G22280.1 |  | | | |  |  |  |  |  |  |  |
| 1 | Ath-AT4G22285.1 |  | | | |  |  |  |  |  |  |  |
| 1 | Ath-AT4G22290.1 |  | | | |  |  |  |  |  |  |  |
| 1 | Ath-AT4G22300.1 |  | Vvi-Vitvi10g04063\_t001 |  |  |  |  |  |  |  |
| 1 | Ath-AT4G22305.1 |  | | | |  |  |  |  |  |  |  |
| 1 | Ath-AT4G22310.1 |  | | | |  |  |  |  |  |  |  |
| 1 | Ath-AT4G22320.1 |  | Vvi-Vitvi10g04073\_t001 |  |  |  |  |  |  |  |
| 1 | Ath-AT4G22330.1 |  | Vvi-Vitvi10g04075\_t001 |  |  |  |  |  |  |  |
| 1 | Ath-AT4G22340.3 |  | Vvi-Vitvi10g00174\_t002 |  |  |  |  |  |  |  |
| 1 | Ath-AT4G22350.2 |  | | | |  |  |  |  |  |  |  |
| 1 | Ath-AT4G22360.1 |  | Vvi-Vitvi10g00191\_t001 |  |  |  |  |  |  |  |
| 1 | Ath-AT4G22370.2 |  | Vvi-Vitvi10g04081\_t001 |  |  |  |  |  |  |  |
| 0 | Ath-AT4G22380.1 |  |  |  |  |  |  |  |  |
| 0 | Ath-AT4G22390.1 |  |  |  |  |  |  |  |  |
| 0 | Ath-AT4G22400.1 |  |  |  |  |  |  |  |  |
| 0 | Ath-AT4G22410.1 |  |  |  |  |  |  |  |  |
| 0 | Ath-AT4G22430.1 |  |  |  |  |  |  |  |  |
| 0 | Ath-AT4G22440.1 |  |  |  |  |  |  |  |  |
| 0 | Ath-AT4G22460.1 |  |  |  |  |  |  |  |  |
| 0 | Ath-AT4G22463.1 |  |  |  |  |  |  |  |  |
| 0 | Ath-AT4G22467.1 |  |  |  |  |  |  |  |  |
| 0 | Ath-AT4G22470.1 |  |  |  |  |  |  |  |  |
| 0 | Ath-AT4G22475.2 |  |  |  |  |  |  |  |  |
| 0 | Ath-AT4G22485.1 |  |  |  |  |  |  |  |  |
| 0 | Ath-AT4G22490.1 |  |  |  |  |  |  |  |  |
| 0 | Ath-AT4G22495.1 |  |  |  |  |  |  |  |  |
| 0 | Ath-AT4G22505.1 |  |  |  |  |  |  |  |  |
| 0 | Ath-AT4G22510.2 |  |  |  |  |  |  |  |  |
| 0 | Ath-AT4G22513.1 |  |  |  |  |  |  |  |  |
| 0 | Ath-AT4G22517.1 |  |  |  |  |  |  |  |  |
| 0 | Ath-AT4G22530.2 |  |  |  |  |  |  |  |  |
| 0 | Ath-AT4G22520.1 |  |  |  |  |  |  |  |  |
| 1 | Ath-AT4G22540.1 |  | Vvi-Vitvi02g04156\_t002 |  |  |  |  |  |  |  |
| 1 | Ath-AT4G22550.1 |  | Vvi-Vitvi02g00527\_t001 |  |  |  |  |  |  |  |
| 1 | Ath-AT4G22560.1 |  | Vvi-Vitvi02g00526\_t001 |  |  |  |  |  |  |  |
| 1 | Ath-AT4G22570.1 |  | Vvi-Vitvi02g04155\_t001 |  |  |  |  |  |  |  |
| 1 | Ath-AT4G22580.1 |  | Vvi-Vitvi02g01696\_t001 |  |  |  |  |  |  |  |
| 1 | Ath-AT4G22590.1 |  | Vvi-Vitvi02g04146\_t001 |  |  |  |  |  |  |  |
| 1 | Ath-AT4G22600.1 |  | | | |  |  |  |  |  |  |  |
| 1 | Ath-AT4G22610.1 |  | | | |  |  |  |  |  |  |  |
| 1 | Ath-AT4G22620.1 |  | Vvi-Vitvi02g00507\_t001 |  |  |  |  |  |  |  |
| 1 | Ath-AT4G22630.1 |  | | | |  |  |  |  |  |  |  |
| 1 | Ath-AT4G22640.2 |  | | | |  |  |  |  |  |  |  |
| 1 | Ath-AT4G22650.1 |  | | | |  |  |  |  |  |  |  |
| 1 | Ath-AT4G22660.1 |  | | | |  |  |  |  |  |  |  |
| 1 | Ath-AT4G22666.1 |  | | | |  |  |  |  |  |  |  |
| 1 | Ath-AT4G22670.1 |  | Vvi-Vitvi02g01429\_t003 |  |  |  |  |  |  |  |
| 1 | Ath-AT4G22680.1 |  | Vvi-Vitvi02g01823\_t001 |  |  |  |  |  |  |  |
| 1 | Ath-AT4G22690.1 |  | | | |  |  |  |  |  |  |  |
| 1 | Ath-AT4G22700.1 |  | | | |  |  |  |  |  |  |  |
| 1 | Ath-AT4G22710.1 |  | | | |  |  |  |  |  |  |  |
| 1 | Ath-AT4G22720.1 |  | | | |  |  |  |  |  |  |  |
| 1 | Ath-AT4G22730.1 |  | Vvi-Vitvi02g00483\_t001 |  |  |  |  |  |  |  |
| 1 | Ath-AT4G22740.1 |  | Vvi-Vitvi02g00479\_t001 |  |  |  |  |  |  |  |
| 1 | Ath-AT4G22745.1 |  | | | |  |  |  |  |  |  |  |
| 1 | Ath-AT4G22750.1 |  | Vvi-Vitvi02g04129\_t001 |  |  |  |  |  |  |  |
| 1 | Ath-AT4G22755.1 |  | Vvi-Vitvi02g04126\_t001 |  |  |  |  |  |  |  |
| 1 | Ath-AT4G22756.1 |  | | | |  |  |  |  |  |  |  |
| 1 | Ath-AT4G22758.1 |  | Vvi-Vitvi02g01423\_t001 |  |  |  |  |  |  |  |
| 1 | Ath-AT4G22760.1 |  | Vvi-Vitvi02g00474\_t001 |  |  |  |  |  |  |  |
| 1 | Ath-AT4G22770.1 |  | Vvi-Vitvi02g00468\_t001 |  |  |  |  |  |  |  |
| 1 | Ath-AT4G22780.1 |  | Vvi-Vitvi02g00467\_t001 |  |  |  |  |  |  |  |
| 1 | Ath-AT4G22790.1 |  | Vvi-Vitvi02g00466\_t001 |  |  |  |  |  |  |  |
| 1 | Ath-AT4G22810.1 |  | Vvi-Vitvi02g00465\_t001 |  |  |  |  |  |  |  |
| 1 | Ath-AT4G22820.1 |  | Vvi-Vitvi02g00461\_t002 |  |  |  |  |  |  |  |
| 1 | Ath-AT4G22830.2 |  | Vvi-Vitvi02g00459\_t001 |  |  |  |  |  |  |  |
| 1 | Ath-AT4G22840.1 |  | Vvi-Vitvi02g04120\_t001 |  |  |  |  |  |  |  |
| 1 | Ath-AT4G22850.1 |  | Vvi-Vitvi02g04119\_t001 |  |  |  |  |  |  |  |
| 1 | Ath-AT4G22860.1 |  | Vvi-Vitvi02g00454\_t001 |  |  |  |  |  |  |  |
| 0 | Ath-AT4G22870.2 |  |  |  |  |  |  |  |  |
| 1 | Ath-AT4G22880.2 |  | Vvi-Vitvi02g00435\_t001 |  |  |  |  |  |  |  |
| 1 | Ath-AT4G22890.3 |  | Vvi-Vitvi02g00434\_t001 |  |  |  |  |  |  |  |
| 1 | Ath-AT4G22900.1 |  | Vvi-Vitvi02g00432\_t001 |  |  |  |  |  |  |  |
| 1 | Ath-AT4G22910.1 |  | Vvi-Vitvi02g00431\_t001 |  |  |  |  |  |  |  |
| 1 | Ath-AT4G22920.1 |  | Vvi-Vitvi02g00429\_t002 |  |  |  |  |  |  |  |
| 1 | Ath-AT4G22930.2 |  | | | |  |  |  |  |  |  |  |
| 1 | Ath-AT4G22940.2 |  | | | |  |  |  |  |  |  |  |
| 1 | Ath-AT4G22950.1 |  | Vvi-Vitvi02g00427\_t001 |  |  |  |  |  |  |  |
| 1 | Ath-AT4G22960.1 |  | Vvi-Vitvi02g00426\_t001 |  |  |  |  |  |  |  |
| 1 | Ath-AT4G22970.3 |  | | | |  |  |  |  |  |  |  |
| 1 | Ath-AT4G22980.1 |  | Vvi-Vitvi02g00419\_t001 |  |  |  |  |  |  |  |
| 1 | Ath-AT4G22990.2 |  | Vvi-Vitvi02g00415\_t001 |  |  |  |  |  |  |  |
| 1 | Ath-AT4G23000.2 |  | | | |  |  |  |  |  |  |  |
| 1 | Ath-AT4G23010.3 |  | Vvi-Vitvi02g00410\_t001 |  |  |  |  |  |  |  |
| 1 | Ath-AT4G23015.1 |  | | | |  |  |  |  |  |  |  |
| 1 | Ath-AT4G23020.2 |  | Vvi-Vitvi02g00409\_t001 |  |  |  |  |  |  |  |
| 1 | Ath-AT4G23030.1 |  | Vvi-Vitvi02g00403\_t001 |  |  |  |  |  |  |  |
| 0 | Ath-AT4G23040.1 |  |  |  |  |  |  |  |  |
| 0 | Ath-AT4G23050.2 |  |  |  |  |  |  |  |  |
| 0 | Ath-AT4G23060.1 |  |  |  |  |  |  |  |  |
| 0 | Ath-AT4G23070.1 |  |  |  |  |  |  |  |  |
| 0 | Ath-AT4G23080.1 |  |  |  |  |  |  |  |  |
| 0 | Ath-AT4G23090.2 |  |  |  |  |  |  |  |  |
| 1 | Ath-AT4G23100.3 |  | Vvi-Vitvi02g00326\_t002 |  |  |  |  |  |  |  |
| 1 | Ath-AT4G23110.1 |  | | | |  |  |  |  |  |  |  |
| 1 | Ath-AT4G23120.1 |  | Vvi-Vitvi02g04073\_t001 |  |  |  |  |  |  |  |
| 1 | Ath-AT4G23130.2 |  | | | |  |  |  |  |  |  |  |
| 1 | Ath-AT4G23140.2 |  | | | |  |  |  |  |  |  |  |
| 1 | Ath-AT4G23150.1 |  | | | |  |  |  |  |  |  |  |
| 1 | Ath-AT4G23160.3 |  | | | |  |  |  |  |  |  |  |
| 1 | Ath-AT4G23170.1 |  | | | |  |  |  |  |  |  |  |
| 1 | Ath-AT4G23180.1 |  | | | |  |  |  |  |  |  |  |
| 1 | Ath-AT4G23190.1 |  | | | |  |  |  |  |  |  |  |
| 1 | Ath-AT4G23200.1 |  | | | |  |  |  |  |  |  |  |
| 1 | Ath-AT4G23210.3 |  | | | |  |  |  |  |  |  |  |
| 1 | Ath-AT4G23220.1 |  | | | |  |  |  |  |  |  |  |
| 1 | Ath-AT4G23230.1 |  | | | |  |  |  |  |  |  |  |
| 1 | Ath-AT4G23240.1 |  | | | |  |  |  |  |  |  |  |
| 1 | Ath-AT4G23250.2 |  | | | |  |  |  |  |  |  |  |
| 1 | Ath-AT4G23260.1 |  | | | |  |  |  |  |  |  |  |
| 1 | Ath-AT4G23270.3 |  | | | |  |  |  |  |  |  |  |
| 1 | Ath-AT4G23271.1 |  | | | |  |  |  |  |  |  |  |
| 1 | Ath-AT4G23280.1 |  | | | |  |  |  |  |  |  |  |
| 1 | Ath-AT4G23290.2 |  | | | |  |  |  |  |  |  |  |
| 1 | Ath-AT4G23300.1 |  | | | |  |  |  |  |  |  |  |
| 1 | Ath-AT4G23310.1 |  | | | |  |  |  |  |  |  |  |
| 1 | Ath-AT4G23320.3 |  | | | |  |  |  |  |  |  |  |
| 1 | Ath-AT4G23330.1 |  | Vvi-Vitvi02g01379\_t001 |  |  |  |  |  |  |  |
| 1 | Ath-AT4G23340.1 |  | Vvi-Vitvi02g00316\_t001 |  |  |  |  |  |  |  |
| 1 | Ath-AT4G23350.1 |  | | | |  |  |  |  |  |  |  |
| 1 | Ath-AT4G23355.1 |  | | | |  |  |  |  |  |  |  |
| 1 | Ath-AT4G23360.1 |  | | | |  |  |  |  |  |  |  |
| 1 | Ath-AT4G23365.1 |  | | | |  |  |  |  |  |  |  |
| 1 | Ath-AT4G23370.1 |  | | | |  |  |  |  |  |  |  |
| 1 | Ath-AT4G23373.1 |  | | | |  |  |  |  |  |  |  |
| 1 | Ath-AT4G23380.1 |  | | | |  |  |  |  |  |  |  |
| 1 | Ath-AT4G23390.1 |  | | | |  |  |  |  |  |  |  |
| 1 | Ath-AT4G23400.1 |  | Vvi-Vitvi02g00310\_t001 |  |  |  |  |  |  |  |
| 1 | Ath-AT4G23410.1 |  | | | |  |  |  |  |  |  |  |
| 1 | Ath-AT4G23420.3 |  | Vvi-Vitvi02g00309\_t001 |  |  |  |  |  |  |  |
| 1 | Ath-AT4G23430.2 |  | | | |  |  |  |  |  |  |  |
| 1 | Ath-AT4G23440.1 |  | | | |  |  |  |  |  |  |  |
| 1 | Ath-AT4G23450.2 |  | | | |  |  |  |  |  |  |  |
| 2 | Ath-AT4G23460.1 |  | | | |  | Vvi-Vitvi02g00008\_t001 |  |  |  |  |  |  |
| 3 | Ath-AT4G23470.1 |  | | | |  | Vvi-Vitvi02g04003\_t002 |  | Vvi-Vitvi15g01066\_t001 |  |  |  |  |  |
| 3 | Ath-AT4G23490.1 |  | Vvi-Vitvi02g00293\_t001 |  | Vvi-Vitvi02g00017\_t001 |  | | | |  |  |  |  |  |
| 2 | Ath-AT4G23493.1 |  |  |  | | | |  | | | |  |  |  |  |  |
| 2 | Ath-AT4G23496.1 |  |  |  | Vvi-Vitvi02g00021\_t001 |  | | | |  |  |  |  |  |
| 2 | Ath-AT4G23500.1 |  |  |  | Vvi-Vitvi02g00022\_t001 |  | | | |  |  |  |  |  |
| 2 | Ath-AT4G23510.1 |  |  |  | | | |  | | | |  |  |  |  |  |
| 2 | Ath-AT4G23515.1 |  |  |  | | | |  | | | |  |  |  |  |  |
| 2 | Ath-AT4G23520.1 |  |  |  | | | |  | | | |  |  |  |  |  |
| 2 | Ath-AT4G23530.1 |  |  |  | Vvi-Vitvi02g00029\_t001 |  | | | |  |  |  |  |  |
| 2 | Ath-AT4G23540.1 |  |  |  | | | |  | | | |  |  |  |  |  |
| 2 | Ath-AT4G23550.1 |  |  |  | Vvi-Vitvi02g00039\_t001 |  | | | |  |  |  |  |  |
| 2 | Ath-AT4G23560.1 |  |  |  | Vvi-Vitvi02g00040\_t001 |  | | | |  |  |  |  |  |
| 2 | Ath-AT4G23570.3 |  |  |  | Vvi-Vitvi02g00042\_t001 |  | | | |  |  |  |  |  |
| 2 | Ath-AT4G23580.1 |  |  |  | | | |  | | | |  |  |  |  |  |
| 2 | Ath-AT4G23590.1 |  |  |  | | | |  | | | |  |  |  |  |  |
| 2 | Ath-AT4G23600.1 |  |  |  | | | |  | | | |  |  |  |  |  |
| 2 | Ath-AT4G23610.1 |  |  |  | | | |  | | | |  |  |  |  |  |
| 2 | Ath-AT4G23620.2 |  |  |  | Vvi-Vitvi02g01321\_t001 |  | | | |  |  |  |  |  |
| 2 | Ath-AT4G23630.1 |  |  |  | Vvi-Vitvi02g00059\_t001 |  | Vvi-Vitvi15g01640\_t001.1.6037826c |  |  |  |  |  |
| 2 | Ath-AT4G23640.1 |  |  |  | Vvi-Vitvi02g00063\_t001 |  | | | |  |  |  |  |  |
| 2 | Ath-AT4G23650.1 |  |  |  | Vvi-Vitvi02g00065\_t002 |  | | | |  |  |  |  |  |
| 2 | Ath-AT4G23660.3 |  |  |  | | | |  | | | |  |  |  |  |  |
| 2 | Ath-AT4G23670.1 |  |  |  | | | |  | | | |  |  |  |  |  |
| 2 | Ath-AT4G23680.1 |  |  |  | | | |  | | | |  |  |  |  |  |
| 2 | Ath-AT4G23690.1 |  |  |  | Vvi-Vitvi02g00069\_t001 |  | | | |  |  |  |  |  |
| 2 | Ath-AT4G23700.2 |  |  |  | Vvi-Vitvi02g00071\_t001 |  | | | |  |  |  |  |  |
| 2 | Ath-AT4G23710.1 |  |  |  | | | |  | | | |  |  |  |  |  |
| 2 | Ath-AT4G23720.1 |  |  |  | Vvi-Vitvi02g00079\_t001 |  | Vvi-Vitvi15g01029\_t001 |  |  |  |  |  |
| 2 | Ath-AT4G23730.3 |  |  |  | Vvi-Vitvi02g00090\_t001 |  | Vvi-Vitvi15g01026\_t001 |  |  |  |  |  |
| 2 | Ath-AT4G23740.1 |  |  |  | Vvi-Vitvi02g00091\_t003 |  | | | |  |  |  |  |  |
| 2 | Ath-AT4G23750.1 |  |  |  | Vvi-Vitvi02g00093\_t001 |  | Vvi-Vitvi15g01021\_t001 |  |  |  |  |  |
| 2 | Ath-AT4G23760.1 |  |  |  | Vvi-Vitvi02g00095\_t001 |  | | | |  |  |  |  |  |
| 2 | Ath-AT4G23770.1 |  |  |  | | | |  | | | |  |  |  |  |  |
| 2 | Ath-AT4G23780.1 |  |  |  | | | |  | | | |  |  |  |  |  |
| 2 | Ath-AT4G23790.1 |  |  |  | Vvi-Vitvi02g00106\_t001 |  | Vvi-Vitvi15g01011\_t001 |  |  |  |  |  |
| 2 | Ath-AT4G23800.1 |  |  |  | Vvi-Vitvi02g00108\_t001 |  | | | |  |  |  |  |  |
| 2 | Ath-AT4G23810.1 |  |  |  | Vvi-Vitvi02g00114\_t001 |  | Vvi-Vitvi15g01003\_t001 |  |  |  |  |  |
| 1 | Ath-AT4G23820.1 |  |  |  | Vvi-Vitvi02g00118\_t003 |  |  |  |  |  |  |
| 1 | Ath-AT4G23840.1 |  |  |  | Vvi-Vitvi02g00127\_t001 |  |  |  |  |  |  |
| 1 | Ath-AT4G23850.1 |  |  |  | Vvi-Vitvi02g00128\_t001 |  |  |  |  |  |  |
| 1 | Ath-AT4G23860.3 |  |  |  | Vvi-Vitvi02g00131\_t001 |  |  |  |  |  |  |
| 1 | Ath-AT4G23870.1 |  |  |  | | | |  |  |  |  |  |  |
| 1 | Ath-AT4G23880.1 |  |  |  | | | |  |  |  |  |  |  |
| 1 | Ath-AT4G23882.1 |  |  |  | | | |  |  |  |  |  |  |
| 1 | Ath-AT4G23885.1 |  |  |  | Vvi-Vitvi02g00132\_t001 |  |  |  |  |  |  |
| 1 | Ath-AT4G23890.1 |  |  |  | Vvi-Vitvi02g00133\_t001 |  |  |  |  |  |  |
| 1 | Ath-AT4G23895.3 |  |  |  | Vvi-Vitvi02g04022\_t001 |  |  |  |  |  |  |
| 1 | Ath-AT4G23900.1 |  |  |  | Vvi-Vitvi02g00137\_t001 |  |  |  |  |  |  |
| 1 | Ath-AT4G23910.1 |  |  |  | Vvi-Vitvi02g00138\_t001 |  |  |  |  |  |  |
| 1 | Ath-AT4G23920.1 |  |  |  | Vvi-Vitvi02g00143\_t001 |  |  |  |  |  |  |
| 1 | Ath-AT4G23930.1 |  |  |  | Vvi-Vitvi02g00147\_t001 |  |  |  |  |  |  |
| 1 | Ath-AT4G23940.1 |  |  |  | | | |  |  |  |  |  |  |
| 1 | Ath-AT4G23950.2 |  |  |  | Vvi-Vitvi02g00156\_t001 |  |  |  |  |  |  |
| 1 | Ath-AT4G23960.2 |  |  |  | Vvi-Vitvi02g00157\_t001 |  |  |  |  |  |  |
| 1 | Ath-AT4G23970.1 |  |  |  | | | |  |  |  |  |  |  |
| 1 | Ath-AT4G23980.1 |  |  |  | Vvi-Vitvi02g00163\_t001 |  |  |  |  |  |  |
| 1 | Ath-AT4G23990.1 |  |  |  | Vvi-Vitvi02g01341\_t001 |  |  |  |  |  |  |
| 1 | Ath-AT4G24000.1 |  |  |  | | | |  |  |  |  |  |  |
| 1 | Ath-AT4G24010.1 |  |  |  | | | |  |  |  |  |  |  |
| 1 | Ath-AT4G24015.1 |  |  |  | Vvi-Vitvi02g04038\_t001 |  |  |  |  |  |  |
| 1 | Ath-AT4G24020.1 |  |  |  | Vvi-Vitvi02g00179\_t001 |  |  |  |  |  |  |
| 1 | Ath-AT4G24030.1 |  |  |  | | | |  |  |  |  |  |  |
| 1 | Ath-AT4G24026.1 |  |  |  | | | |  |  |  |  |  |  |
| 1 | Ath-AT4G24040.1 |  |  |  | Vvi-Vitvi02g00191\_t001 |  |  |  |  |  |  |
| 1 | Ath-AT4G24050.1 |  |  |  | Vvi-Vitvi02g00192\_t001 |  |  |  |  |  |  |
| 1 | Ath-AT4G24060.1 |  |  |  | Vvi-Vitvi02g00199\_t001 |  |  |  |  |  |  |
| 1 | Ath-AT4G24070.1 |  |  |  | Vvi-Vitvi02g00213\_t001 |  |  |  |  |  |  |
| 1 | Ath-AT4G24080.1 |  |  |  | | | |  |  |  |  |  |  |
| 1 | Ath-AT4G24090.1 |  |  |  | Vvi-Vitvi02g04049\_t001 |  |  |  |  |  |  |
| 1 | Ath-AT4G24100.4 |  |  |  | Vvi-Vitvi02g00218\_t003 |  |  |  |  |  |  |
| 1 | Ath-AT4G24110.1 |  |  |  | Vvi-Vitvi02g00219\_t001 |  |  |  |  |  |  |
| 1 | Ath-AT4G24120.1 |  |  |  | Vvi-Vitvi02g01365\_t002 |  |  |  |  |  |  |
| 1 | Ath-AT4G24130.1 |  |  |  | Vvi-Vitvi02g00223\_t001 |  |  |  |  |  |  |
| 1 | Ath-AT4G24140.1 |  |  |  | Vvi-Vitvi02g00232\_t001 |  |  |  |  |  |  |
| 1 | Ath-AT4G24150.1 |  |  |  | Vvi-Vitvi02g00239\_t001 |  |  |  |  |  |  |
| 0 | Ath-AT4G24160.1 |  |  |  |  |  |  |  |  |
| 0 | Ath-AT4G24170.1 |  |  |  |  |  |  |  |  |
| 0 | Ath-AT4G24175.1 |  |  |  |  |  |  |  |  |
| 0 | Ath-AT4G24180.3 |  |  |  |  |  |  |  |  |
| 0 | Ath-AT4G24190.1 |  |  |  |  |  |  |  |  |
| 0 | Ath-AT4G24200.1 |  |  |  |  |  |  |  |  |
| 0 | Ath-AT4G24204.3 |  |  |  |  |  |  |  |  |
| 0 | Ath-AT4G24210.1 |  |  |  |  |  |  |  |  |
| 0 | Ath-AT4G24220.1 |  |  |  |  |  |  |  |  |
| 0 | Ath-AT4G24230.6 |  |  |  |  |  |  |  |  |
| 0 | Ath-AT4G24231.1 |  |  |  |  |  |  |  |  |
| 0 | Ath-AT4G24240.1 |  |  |  |  |  |  |  |  |
| 0 | Ath-AT4G24250.1 |  |  |  |  |  |  |  |  |
| 0 | Ath-AT4G24260.1 |  |  |  |  |  |  |  |  |
| 0 | Ath-AT4G24265.2 |  |  |  |  |  |  |  |  |
| 0 | Ath-AT4G24270.2 |  |  |  |  |  |  |  |  |
| 0 | Ath-AT4G24275.1 |  |  |  |  |  |  |  |  |
| 0 | Ath-AT4G24280.1 |  |  |  |  |  |  |  |  |
| 0 | Ath-AT4G24290.2 |  |  |  |  |  |  |  |  |
| 0 | Ath-AT4G24300.1 |  |  |  |  |  |  |  |  |
| 0 | Ath-AT4G24310.1 |  |  |  |  |  |  |  |  |
| 0 | Ath-AT4G24320.1 |  |  |  |  |  |  |  |  |
| 0 | Ath-AT4G24330.1 |  |  |  |  |  |  |  |  |
| 0 | Ath-AT4G24340.1 |  |  |  |  |  |  |  |  |
| 0 | Ath-AT4G24350.1 |  |  |  |  |  |  |  |  |
| 0 | Ath-AT4G24370.1 |  |  |  |  |  |  |  |  |
| 0 | Ath-AT4G24380.3 |  |  |  |  |  |  |  |  |
| 0 | Ath-AT4G24390.1 |  |  |  |  |  |  |  |  |
| 1 | Ath-AT4G24400.1 |  | Vvi-Vitvi18g00561\_t001 |  |  |  |  |  |  |  |
| 1 | Ath-AT4G24410.1 |  | | | |  |  |  |  |  |  |  |
| 1 | Ath-AT4G24413.1 |  | | | |  |  |  |  |  |  |  |
| 1 | Ath-AT4G24420.2 |  | | | |  |  |  |  |  |  |  |
| 1 | Ath-AT4G24430.1 |  | Vvi-Vitvi18g00549\_t001 |  |  |  |  |  |  |  |
| 1 | Ath-AT4G24440.1 |  | Vvi-Vitvi18g00547\_t001 |  |  |  |  |  |  |  |
| 1 | Ath-AT4G24450.2 |  | Vvi-Vitvi18g02616\_t001 |  |  |  |  |  |  |  |
| 1 | Ath-AT4G24460.1 |  | Vvi-Vitvi18g00541\_t001 |  |  |  |  |  |  |  |
| 1 | Ath-AT4G24470.3 |  | Vvi-Vitvi18g00537\_t001 |  |  |  |  |  |  |  |
| 1 | Ath-AT4G24480.1 |  | Vvi-Vitvi18g00534\_t001 |  |  |  |  |  |  |  |
| 1 | Ath-AT4G24490.1 |  | Vvi-Vitvi18g00532\_t001 |  |  |  |  |  |  |  |
| 1 | Ath-AT4G24500.1 |  | Vvi-Vitvi18g02613\_t003 |  |  |  |  |  |  |  |
| 1 | Ath-AT4G24510.1 |  | Vvi-Vitvi18g00529\_t001 |  |  |  |  |  |  |  |
| 1 | Ath-AT4G24520.1 |  | Vvi-Vitvi18g00528\_t001 |  |  |  |  |  |  |  |
| 2 | Ath-AT4G24530.1 |  | Vvi-Vitvi18g00518\_t002 |  | Vvi-Vitvi07g01437\_t001 |  |  |  |  |  |  |
| 2 | Ath-AT4G24540.1 |  | Vvi-Vitvi18g04114\_t001 |  | Vvi-Vitvi07g01441\_t001 |  |  |  |  |  |  |
| 2 | Ath-AT4G24550.2 |  | | | |  | | | |  |  |  |  |  |  |
| 2 | Ath-AT4G24560.1 |  | Vvi-Vitvi18g00511\_t001 |  | Vvi-Vitvi07g01449\_t001 |  |  |  |  |  |  |
| 2 | Ath-AT4G24570.1 |  | Vvi-Vitvi18g00508\_t001 |  | Vvi-Vitvi07g04600\_t001 |  |  |  |  |  |  |
| 2 | Ath-AT4G24580.1 |  | Vvi-Vitvi18g00507\_t001 |  | Vvi-Vitvi07g04599\_t001 |  |  |  |  |  |  |
| 2 | Ath-AT4G24590.4 |  | Vvi-Vitvi18g00506\_t001 |  | | | |  |  |  |  |  |  |
| 2 | Ath-AT4G24600.1 |  | | | |  | | | |  |  |  |  |  |  |
| 2 | Ath-AT4G24615.1 |  | | | |  | | | |  |  |  |  |  |  |
| 2 | Ath-AT4G24610.2 |  | Vvi-Vitvi18g00505\_t002 |  | Vvi-Vitvi07g01451\_t001 |  |  |  |  |  |  |
| 2 | Ath-AT4G24620.1 |  | Vvi-Vitvi18g00504\_t001 |  | | | |  |  |  |  |  |  |
| 2 | Ath-AT4G24630.1 |  | | | |  | | | |  |  |  |  |  |  |
| 2 | Ath-AT4G24640.1 |  | | | |  | | | |  |  |  |  |  |  |
| 2 | Ath-AT4G24644.1 |  | | | |  | | | |  |  |  |  |  |  |
| 2 | Ath-AT4G24650.1 |  | | | |  | | | |  |  |  |  |  |  |
| 2 | Ath-AT4G24660.2 |  | Vvi-Vitvi18g00493\_t001 |  | Vvi-Vitvi07g01465\_t001 |  |  |  |  |  |  |
| 0 | Ath-AT4G24670.1 |  |  |  |  |  |  |  |  |
| 0 | Ath-AT4G24680.1 |  |  |  |  |  |  |  |  |
| 1 | Ath-AT4G24690.1 |  | Vvi-Vitvi17g00916\_t003 |  |  |  |  |  |  |  |
| 1 | Ath-AT4G24700.1 |  | Vvi-Vitvi17g04259\_t001 |  |  |  |  |  |  |  |
| 1 | Ath-AT4G24710.3 |  | Vvi-Vitvi17g00920\_t001 |  |  |  |  |  |  |  |
| 1 | Ath-AT4G24715.1 |  | Vvi-Vitvi17g00936\_t001 |  |  |  |  |  |  |  |
| 1 | Ath-AT4G24730.1 |  | Vvi-Vitvi17g00937\_t001 |  |  |  |  |  |  |  |
| 1 | Ath-AT4G24740.1 |  | Vvi-Vitvi17g00942\_t001 |  |  |  |  |  |  |  |
| 1 | Ath-AT4G24750.1 |  | Vvi-Vitvi17g00953\_t001 |  |  |  |  |  |  |  |
| 1 | Ath-AT4G24760.1 |  | | | |  |  |  |  |  |  |  |
| 1 | Ath-AT4G24770.1 |  | Vvi-Vitvi17g00958\_t001 |  |  |  |  |  |  |  |
| 1 | Ath-AT4G24780.1 |  | Vvi-Vitvi17g00977\_t001 |  |  |  |  |  |  |  |
| 1 | Ath-AT4G24790.2 |  | Vvi-Vitvi17g00979\_t001 |  |  |  |  |  |  |  |
| 1 | Ath-AT4G24800.2 |  | Vvi-Vitvi17g00987\_t001 |  |  |  |  |  |  |  |
| 1 | Ath-AT4G24805.1 |  | Vvi-Vitvi17g00990\_t001 |  |  |  |  |  |  |  |
| 0 | Ath-AT4G24810.2 |  |  |  |  |  |  |  |  |
| 0 | Ath-AT4G24820.2 |  |  |  |  |  |  |  |  |
| 1 | Ath-AT4G24830.1 |  | Vvi-Vitvi17g00138\_t001 |  |  |  |  |  |  |  |
| 1 | Ath-AT4G24840.1 |  | Vvi-Vitvi17g00125\_t001 |  |  |  |  |  |  |  |
| 1 | Ath-AT4G24860.1 |  | Vvi-Vitvi17g00124\_t002 |  |  |  |  |  |  |  |
| 1 | Ath-AT4G24880.1 |  | Vvi-Vitvi17g00113\_t001 |  |  |  |  |  |  |  |
| 1 | Ath-AT4G24890.1 |  | Vvi-Vitvi17g01350\_t001 |  |  |  |  |  |  |  |
| 1 | Ath-AT4G24900.1 |  | Vvi-Vitvi17g00108\_t001 |  |  |  |  |  |  |  |
| 1 | Ath-AT4G24910.1 |  | Vvi-Vitvi17g00106\_t001 |  |  |  |  |  |  |  |
| 1 | Ath-AT4G24920.2 |  | | | |  |  |  |  |  |  |  |
| 1 | Ath-AT4G24930.1 |  | | | |  |  |  |  |  |  |  |
| 1 | Ath-AT4G24940.1 |  | Vvi-Vitvi17g00101\_t001 |  |  |  |  |  |  |  |
| 1 | Ath-AT4G24950.1 |  | | | |  |  |  |  |  |  |  |
| 1 | Ath-AT4G24960.1 |  | Vvi-Vitvi17g00085\_t001 |  |  |  |  |  |  |  |
| 1 | Ath-AT4G24970.1 |  | Vvi-Vitvi17g00076\_t001 |  |  |  |  |  |  |  |
| 1 | Ath-AT4G24972.1 |  | Vvi-Vitvi17g00075\_t001 |  |  |  |  |  |  |  |
| 1 | Ath-AT4G24973.1 |  | | | |  |  |  |  |  |  |  |
| 1 | Ath-AT4G24974.1 |  | | | |  |  |  |  |  |  |  |
| 1 | Ath-AT4G24975.1 |  | | | |  |  |  |  |  |  |  |
| 1 | Ath-AT4G24980.1 |  | | | |  |  |  |  |  |  |  |
| 1 | Ath-AT4G24990.2 |  | Vvi-Vitvi17g01331\_t001 |  |  |  |  |  |  |  |
| 1 | Ath-AT4G25000.1 |  | | | |  |  |  |  |  |  |  |
| 1 | Ath-AT4G25010.1 |  | Vvi-Vitvi17g00070\_t001 |  |  |  |  |  |  |  |
| 1 | Ath-AT4G25020.1 |  | | | |  |  |  |  |  |  |  |
| 1 | Ath-AT4G25030.1 |  | | | |  |  |  |  |  |  |  |
| 1 | Ath-AT4G25040.1 |  | Vvi-Vitvi17g00053\_t001 |  |  |  |  |  |  |  |
| 1 | Ath-AT4G25050.2 |  | | | |  |  |  |  |  |  |  |
| 1 | Ath-AT4G25070.2 |  | Vvi-Vitvi17g00041\_t001 |  |  |  |  |  |  |  |
| 1 | Ath-AT4G25080.6 |  | Vvi-Vitvi17g00030\_t001 |  |  |  |  |  |  |  |
| 0 | Ath-AT4G25090.1 |  |  |  |  |  |  |  |  |
| 0 | Ath-AT4G25100.1 |  |  |  |  |  |  |  |  |
| 0 | Ath-AT4G25110.1 |  |  |  |  |  |  |  |  |
| 0 | Ath-AT4G25120.1 |  |  |  |  |  |  |  |  |
| 0 | Ath-AT4G25130.1 |  |  |  |  |  |  |  |  |
| 0 | Ath-AT4G25140.1 |  |  |  |  |  |  |  |  |
| 0 | Ath-AT4G25150.1 |  |  |  |  |  |  |  |  |
| 0 | Ath-AT4G25160.1 |  |  |  |  |  |  |  |  |
| 0 | Ath-AT4G25170.2 |  |  |  |  |  |  |  |  |
| 0 | Ath-AT4G25180.1 |  |  |  |  |  |  |  |  |
| 0 | Ath-AT4G25190.2 |  |  |  |  |  |  |  |  |
| 1 | Ath-AT4G25200.1 |  | Vvi-Vitvi16g00681\_t001 |  |  |  |  |  |  |  |
| 1 | Ath-AT4G25210.1 |  | | | |  |  |  |  |  |  |  |
| 1 | Ath-AT4G25220.1 |  | Vvi-Vitvi16g00692\_t002 |  |  |  |  |  |  |  |
| 1 | Ath-AT4G25225.2 |  | | | |  |  |  |  |  |  |  |
| 1 | Ath-AT4G25230.2 |  | Vvi-Vitvi16g00703\_t001 |  |  |  |  |  |  |  |
| 1 | Ath-AT4G25240.1 |  | Vvi-Vitvi16g00715\_t001 |  |  |  |  |  |  |  |
| 1 | Ath-AT4G25250.1 |  | | | |  |  |  |  |  |  |  |
| 1 | Ath-AT4G25260.1 |  | Vvi-Vitvi16g04295\_t001 |  |  |  |  |  |  |  |
| 1 | Ath-AT4G25270.1 |  | Vvi-Vitvi16g00745\_t001 |  |  |  |  |  |  |  |
| 1 | Ath-AT4G25280.2 |  | Vvi-Vitvi16g00746\_t003 |  |  |  |  |  |  |  |
| 0 | Ath-AT4G25290.1 |  |  |  |  |  |  |  |  |
| 0 | Ath-AT4G25300.1 |  |  |  |  |  |  |  |  |
| 0 | Ath-AT4G25310.1 |  |  |  |  |  |  |  |  |
| 0 | Ath-AT4G25315.1 |  |  |  |  |  |  |  |  |
| 0 | Ath-AT4G25320.1 |  |  |  |  |  |  |  |  |
| 0 | Ath-AT4G25330.1 |  |  |  |  |  |  |  |  |
| 0 | Ath-AT4G25340.1 |  |  |  |  |  |  |  |  |
| 0 | Ath-AT4G25350.1 |  |  |  |  |  |  |  |  |
| 0 | Ath-AT4G25360.1 |  |  |  |  |  |  |  |  |
| 0 | Ath-AT4G25370.1 |  |  |  |  |  |  |  |  |
| 0 | Ath-AT4G25380.1 |  |  |  |  |  |  |  |  |
| 1 | Ath-AT4G25390.1 |  | Vvi-Vitvi16g00880\_t001 |  |  |  |  |  |  |  |
| 1 | Ath-AT4G25400.2 |  | Vvi-Vitvi16g00882\_t001 |  |  |  |  |  |  |  |
| 1 | Ath-AT4G25410.1 |  | | | |  |  |  |  |  |  |  |
| 1 | Ath-AT4G25420.1 |  | Vvi-Vitvi16g00890\_t001 |  |  |  |  |  |  |  |
| 1 | Ath-AT4G25430.1 |  | Vvi-Vitvi16g00897\_t001 |  |  |  |  |  |  |  |
| 1 | Ath-AT4G25433.1 |  | | | |  |  |  |  |  |  |  |
| 1 | Ath-AT4G25434.8 |  | | | |  |  |  |  |  |  |  |
| 1 | Ath-AT4G25440.1 |  | Vvi-Vitvi16g04337\_t001 |  |  |  |  |  |  |  |
| 1 | Ath-AT4G25450.1 |  | Vvi-Vitvi16g00936\_t003 |  |  |  |  |  |  |  |
| 1 | Ath-AT4G25470.1 |  | Vvi-Vitvi16g04340\_t001 |  |  |  |  |  |  |  |
| 1 | Ath-AT4G25480.1 |  | | | |  |  |  |  |  |  |  |
| 1 | Ath-AT4G25490.1 |  | | | |  |  |  |  |  |  |  |
| 1 | Ath-AT4G25500.1 |  | Vvi-Vitvi16g00949\_t004 |  |  |  |  |  |  |  |
| 1 | Ath-AT4G25510.1 |  | | | |  |  |  |  |  |  |  |
| 1 | Ath-AT4G25515.1 |  | Vvi-Vitvi16g00956\_t001 |  |  |  |  |  |  |  |
| 0 | Ath-AT4G25520.2 |  |  |  |  |  |  |  |  |
| 0 | Ath-AT4G25530.1 |  |  |  |  |  |  |  |  |
| 1 | Ath-AT4G25540.1 |  | Vvi-Vitvi16g01009\_t001 |  |  |  |  |  |  |  |
| 1 | Ath-AT4G25550.1 |  | Vvi-Vitvi16g01012\_t001 |  |  |  |  |  |  |  |
| 1 | Ath-AT4G25560.1 |  | Vvi-Vitvi16g01015\_t001 |  |  |  |  |  |  |  |
| 1 | Ath-AT4G25570.2 |  | Vvi-Vitvi16g01019\_t001 |  |  |  |  |  |  |  |
| 1 | Ath-AT4G25580.1 |  | Vvi-Vitvi16g01022\_t001 |  |  |  |  |  |  |  |
| 1 | Ath-AT4G25590.1 |  | Vvi-Vitvi16g01026\_t001 |  |  |  |  |  |  |  |
| 1 | Ath-AT4G25600.1 |  | Vvi-Vitvi16g01056\_t001 |  |  |  |  |  |  |  |
| 1 | Ath-AT4G25610.1 |  | Vvi-Vitvi16g01067\_t001 |  |  |  |  |  |  |  |
| 1 | Ath-AT4G25620.1 |  | Vvi-Vitvi16g01074\_t001 |  |  |  |  |  |  |  |
| 1 | Ath-AT4G25630.1 |  | Vvi-Vitvi16g01083\_t001 |  |  |  |  |  |  |  |
| 1 | Ath-AT4G25640.2 |  | Vvi-Vitvi16g01911\_t001 |  |  |  |  |  |  |  |
| 1 | Ath-AT4G25650.2 |  | | | |  |  |  |  |  |  |  |
| 1 | Ath-AT4G25660.1 |  | Vvi-Vitvi16g01095\_t001 |  |  |  |  |  |  |  |
| 1 | Ath-AT4G25670.2 |  | Vvi-Vitvi16g01096\_t001 |  |  |  |  |  |  |  |
| 1 | Ath-AT4G25680.1 |  | | | |  |  |  |  |  |  |  |
| 1 | Ath-AT4G25690.2 |  | | | |  |  |  |  |  |  |  |
| 1 | Ath-AT4G25700.1 |  | Vvi-Vitvi16g01099\_t001 |  |  |  |  |  |  |  |
| 1 | Ath-AT4G25707.1 |  | | | |  |  |  |  |  |  |  |
| 1 | Ath-AT4G25710.1 |  | | | |  |  |  |  |  |  |  |
| 1 | Ath-AT4G25720.1 |  | Vvi-Vitvi16g01102\_t001 |  |  |  |  |  |  |  |
| 1 | Ath-AT4G25730.1 |  | | | |  |  |  |  |  |  |  |
| 1 | Ath-AT4G25740.1 |  | Vvi-Vitvi16g01104\_t001 |  |  |  |  |  |  |  |
| 0 | Ath-AT4G25750.1 |  |  |  |  |  |  |  |  |
| 1 | Ath-AT4G25760.1 |  | Vvi-Vitvi11g01702\_t001 |  |  |  |  |  |  |  |
| 1 | Ath-AT4G25770.2 |  | Vvi-Vitvi11g01297\_t001.1.6037826a |  |  |  |  |  |  |  |
| 1 | Ath-AT4G25780.1 |  | Vvi-Vitvi11g01293\_t001 |  |  |  |  |  |  |  |
| 1 | Ath-AT4G25790.1 |  | | | |  |  |  |  |  |  |  |
| 1 | Ath-AT4G25800.1 |  | Vvi-Vitvi11g01272\_t001 |  |  |  |  |  |  |  |
| 1 | Ath-AT4G25810.1 |  | Vvi-Vitvi11g04355\_t001 |  |  |  |  |  |  |  |
| 1 | Ath-AT4G25820.1 |  | | | |  |  |  |  |  |  |  |
| 1 | Ath-AT4G25830.1 |  | Vvi-Vitvi11g01671\_t001 |  |  |  |  |  |  |  |
| 1 | Ath-AT4G25835.1 |  | Vvi-Vitvi11g01243\_t001 |  |  |  |  |  |  |  |
| 1 | Ath-AT4G25840.1 |  | Vvi-Vitvi11g01233\_t001 |  |  |  |  |  |  |  |
| 1 | Ath-AT4G25845.1 |  | | | |  |  |  |  |  |  |  |
| 2 | Ath-AT4G25850.2 |  | | | |  | Vvi-Vitvi11g01132\_t001 |  |  |  |  |  |  |
| 2 | Ath-AT4G25860.1 |  | | | |  | | | |  |  |  |  |  |  |
| 2 | Ath-AT4G25870.1 |  | | | |  | | | |  |  |  |  |  |  |
| 2 | Ath-AT4G25880.4 |  | | | |  | | | |  |  |  |  |  |  |
| 2 | Ath-AT4G25890.1 |  | | | |  | | | |  |  |  |  |  |  |
| 2 | Ath-AT4G25900.1 |  | | | |  | | | |  |  |  |  |  |  |
| 2 | Ath-AT4G25910.1 |  | Vvi-Vitvi11g01209\_t001 |  | | | |  |  |  |  |  |  |
| 1 | Ath-AT4G25920.1 |  |  |  | | | |  |  |  |  |  |  |
| 1 | Ath-AT4G25930.1 |  |  |  | | | |  |  |  |  |  |  |
| 1 | Ath-AT4G25940.1 |  |  |  | Vvi-Vitvi11g01139\_t001 |  |  |  |  |  |  |
| 1 | Ath-AT4G25950.1 |  |  |  | | | |  |  |  |  |  |  |
| 1 | Ath-AT4G25960.1 |  |  |  | | | |  |  |  |  |  |  |
| 1 | Ath-AT4G25970.1 |  |  |  | | | |  |  |  |  |  |  |
| 1 | Ath-AT4G25980.1 |  |  |  | | | |  |  |  |  |  |  |
| 1 | Ath-AT4G25990.2 |  |  |  | Vvi-Vitvi11g01143\_t001 |  |  |  |  |  |  |
| 1 | Ath-AT4G26000.1 |  |  |  | Vvi-Vitvi11g01145\_t001 |  |  |  |  |  |  |
| 1 | Ath-AT4G26010.2 |  |  |  | | | |  |  |  |  |  |  |
| 1 | Ath-AT4G26020.2 |  |  |  | Vvi-Vitvi11g01155\_t001 |  |  |  |  |  |  |
| 1 | Ath-AT4G26030.1 |  |  |  | | | |  |  |  |  |  |  |
| 1 | Ath-AT4G26040.1 |  |  |  | | | |  |  |  |  |  |  |
| 1 | Ath-AT4G26050.1 |  |  |  | | | |  |  |  |  |  |  |
| 1 | Ath-AT4G26055.1 |  |  |  | | | |  |  |  |  |  |  |
| 2 | Ath-AT4G26060.1 |  | Vvi-Vitvi11g00134\_t002 |  | | | |  |  |  |  |  |  |
| 2 | Ath-AT4G26070.3 |  | Vvi-Vitvi11g00136\_t002 |  | | | |  |  |  |  |  |  |
| 2 | Ath-AT4G26080.1 |  | Vvi-Vitvi11g00137\_t002 |  | | | |  |  |  |  |  |  |
| 2 | Ath-AT4G26090.1 |  | Vvi-Vitvi11g00146\_t001 |  | Vvi-Vitvi11g01630\_t003 |  |  |  |  |  |  |
| 1 | Ath-AT4G26100.1 |  | Vvi-Vitvi11g00155\_t001 |  |  |  |  |  |  |  |
| 1 | Ath-AT4G26110.1 |  | | | |  |  |  |  |  |  |  |
| 1 | Ath-AT4G26120.2 |  | Vvi-Vitvi11g00158\_t001 |  |  |  |  |  |  |  |
| 1 | Ath-AT4G26130.1 |  | Vvi-Vitvi11g01372\_t001 |  |  |  |  |  |  |  |
| 1 | Ath-AT4G26140.7 |  | Vvi-Vitvi11g00178\_t001 |  |  |  |  |  |  |  |
| 1 | Ath-AT4G26145.1 |  | | | |  |  |  |  |  |  |  |
| 1 | Ath-AT4G26150.1 |  | Vvi-Vitvi11g00180\_t001 |  |  |  |  |  |  |  |
| 1 | Ath-AT4G26160.1 |  | Vvi-Vitvi11g00189\_t001 |  |  |  |  |  |  |  |
| 1 | Ath-AT4G26170.1 |  | Vvi-Vitvi11g00200\_t001 |  |  |  |  |  |  |  |
| 1 | Ath-AT4G26180.1 |  | Vvi-Vitvi11g00201\_t001 |  |  |  |  |  |  |  |
| 1 | Ath-AT4G26190.1 |  | | | |  |  |  |  |  |  |  |
| 1 | Ath-AT4G26200.1 |  | Vvi-Vitvi11g00212\_t001 |  |  |  |  |  |  |  |
| 1 | Ath-AT4G26210.1 |  | | | |  |  |  |  |  |  |  |
| 1 | Ath-AT4G26220.1 |  | Vvi-Vitvi11g04050\_t001 |  |  |  |  |  |  |  |
| 1 | Ath-AT4G26230.1 |  | Vvi-Vitvi11g00222\_t001 |  |  |  |  |  |  |  |
| 1 | Ath-AT4G26240.1 |  | Vvi-Vitvi11g00223\_t001 |  |  |  |  |  |  |  |
| 1 | Ath-AT4G26250.1 |  | | | |  |  |  |  |  |  |  |
| 1 | Ath-AT4G26260.2 |  | Vvi-Vitvi11g00231\_t001 |  |  |  |  |  |  |  |
| 1 | Ath-AT4G26270.1 |  | Vvi-Vitvi11g00237\_t001 |  |  |  |  |  |  |  |
| 1 | Ath-AT4G26280.1 |  | | | |  |  |  |  |  |  |  |
| 1 | Ath-AT4G26288.1 |  | Vvi-Vitvi11g00251\_t001 |  |  |  |  |  |  |  |
| 1 | Ath-AT4G26290.1 |  | | | |  |  |  |  |  |  |  |
| 1 | Ath-AT4G26300.5 |  | Vvi-Vitvi11g00252\_t001 |  |  |  |  |  |  |  |
| 0 | Ath-AT4G26310.2 |  |  |  |  |  |  |  |  |
| 0 | Ath-AT4G26320.1 |  |  |  |  |  |  |  |  |
| 1 | Ath-AT4G26330.1 |  | Vvi-Vitvi06g00030\_t001 |  |  |  |  |  |  |  |
| 1 | Ath-AT4G26340.1 |  | | | |  |  |  |  |  |  |  |
| 1 | Ath-AT4G26350.1 |  | | | |  |  |  |  |  |  |  |
| 1 | Ath-AT4G26370.1 |  | Vvi-Vitvi06g00027\_t001 |  |  |  |  |  |  |  |
| 1 | Ath-AT4G26380.1 |  | | | |  |  |  |  |  |  |  |
| 1 | Ath-AT4G26390.1 |  | Vvi-Vitvi06g00021\_t001 |  |  |  |  |  |  |  |
| 1 | Ath-AT4G26400.1 |  | Vvi-Vitvi06g00020\_t001 |  |  |  |  |  |  |  |
| 1 | Ath-AT4G26410.1 |  | Vvi-Vitvi06g00018\_t002 |  |  |  |  |  |  |  |
| 1 | Ath-AT4G26415.1 |  | Vvi-Vitvi06g00015\_t001 |  |  |  |  |  |  |  |
| 1 | Ath-AT4G26420.1 |  | Vvi-Vitvi06g00008\_t001 |  |  |  |  |  |  |  |
| 1 | Ath-AT4G26430.1 |  | Vvi-Vitvi19g00935\_t001 |  |  |  |  |  |  |  |
| 1 | Ath-AT4G26440.1 |  | Vvi-Vitvi19g00927\_t001 |  |  |  |  |  |  |  |
| 1 | Ath-AT4G26450.2 |  | Vvi-Vitvi19g02112\_t003 |  |  |  |  |  |  |  |
| 1 | Ath-AT4G26455.1 |  | Vvi-Vitvi19g00913\_t001 |  |  |  |  |  |  |  |
| 1 | Ath-AT4G26466.1 |  | | | |  |  |  |  |  |  |  |
| 1 | Ath-AT4G26460.2 |  | | | |  |  |  |  |  |  |  |
| 2 | Ath-AT4G26470.3 |  | | | |  | Vvi-Vitvi19g00745\_t001 |  |  |  |  |  |  |
| 2 | Ath-AT4G26480.1 |  | | | |  | | | |  |  |  |  |  |  |
| 2 | Ath-AT4G26483.1 |  | | | |  | | | |  |  |  |  |  |  |
| 2 | Ath-AT4G26485.1 |  | | | |  | | | |  |  |  |  |  |  |
| 2 | Ath-AT4G26490.1 |  | | | |  | | | |  |  |  |  |  |  |
| 2 | Ath-AT4G26510.2 |  | Vvi-Vitvi19g00905\_t001 |  | | | |  |  |  |  |  |  |
| 2 | Ath-AT4G26500.1 |  | | | |  | | | |  |  |  |  |  |  |
| 2 | Ath-AT4G26520.1 |  | Vvi-Vitvi19g00904\_t001 |  | | | |  |  |  |  |  |  |
| 1 | Ath-AT4G26530.3 |  |  |  | | | |  |  |  |  |  |  |
| 1 | Ath-AT4G26540.1 |  |  |  | Vvi-Vitvi19g00735\_t001 |  |  |  |  |  |  |
| 1 | Ath-AT4G26550.1 |  |  |  | Vvi-Vitvi19g00734\_t001 |  |  |  |  |  |  |
| 1 | Ath-AT4G26555.1 |  |  |  | Vvi-Vitvi19g00732\_t001 |  |  |  |  |  |  |
| 1 | Ath-AT4G26560.1 |  |  |  | Vvi-Vitvi19g00731\_t001 |  |  |  |  |  |  |
| 1 | Ath-AT4G26570.2 |  |  |  | | | |  |  |  |  |  |  |
| 1 | Ath-AT4G26580.1 |  |  |  | Vvi-Vitvi19g00728\_t001 |  |  |  |  |  |  |
| 1 | Ath-AT4G26590.1 |  |  |  | Vvi-Vitvi19g00712\_t001 |  |  |  |  |  |  |
| 1 | Ath-AT4G26600.5 |  |  |  | Vvi-Vitvi19g04281\_t003 |  |  |  |  |  |  |
| 1 | Ath-AT4G26610.1 |  |  |  | Vvi-Vitvi19g00707\_t001 |  |  |  |  |  |  |
| 1 | Ath-AT4G26620.1 |  |  |  | Vvi-Vitvi19g00704\_t001 |  |  |  |  |  |  |
| 0 | Ath-AT4G26630.1 |  |  |  |  |  |  |  |  |
| 1 | Ath-AT4G26640.2 |  | Vvi-Vitvi19g00617\_t001 |  |  |  |  |  |  |  |
| 1 | Ath-AT4G26650.1 |  | Vvi-Vitvi19g00612\_t001 |  |  |  |  |  |  |  |
| 1 | Ath-AT4G26660.1 |  | Vvi-Vitvi19g00609\_t001 |  |  |  |  |  |  |  |
| 1 | Ath-AT4G26670.1 |  | Vvi-Vitvi19g00608\_t001 |  |  |  |  |  |  |  |
| 1 | Ath-AT4G26680.2 |  | | | |  |  |  |  |  |  |  |
| 1 | Ath-AT4G26690.1 |  | Vvi-Vitvi19g00596\_t001 |  |  |  |  |  |  |  |
| 1 | Ath-AT4G26700.4 |  | Vvi-Vitvi19g00589\_t001 |  |  |  |  |  |  |  |
| 1 | Ath-AT4G26701.1 |  | | | |  |  |  |  |  |  |  |
| 1 | Ath-AT4G26710.1 |  | Vvi-Vitvi19g00580\_t001 |  |  |  |  |  |  |  |
| 1 | Ath-AT4G26720.1 |  | Vvi-Vitvi19g00577\_t003 |  |  |  |  |  |  |  |
| 1 | Ath-AT4G26730.1 |  | | | |  |  |  |  |  |  |  |
| 1 | Ath-AT4G26740.1 |  | | | |  |  |  |  |  |  |  |
| 1 | Ath-AT4G26750.1 |  | Vvi-Vitvi19g00573\_t001 |  |  |  |  |  |  |  |
| 1 | Ath-AT4G26760.1 |  | Vvi-Vitvi19g00572\_t001 |  |  |  |  |  |  |  |
| 1 | Ath-AT4G26770.1 |  | Vvi-Vitvi19g00570\_t001 |  |  |  |  |  |  |  |
| 1 | Ath-AT4G26780.1 |  | Vvi-Vitvi19g00567\_t001 |  |  |  |  |  |  |  |
| 1 | Ath-AT4G26790.1 |  | Vvi-Vitvi19g00564\_t001 |  |  |  |  |  |  |  |
| 1 | Ath-AT4G26800.2 |  | | | |  |  |  |  |  |  |  |
| 1 | Ath-AT4G26810.1 |  | Vvi-Vitvi19g00563\_t001 |  |  |  |  |  |  |  |
| 1 | Ath-AT4G26820.1 |  | | | |  |  |  |  |  |  |  |
| 1 | Ath-AT4G26830.2 |  | Vvi-Vitvi19g00557\_t001 |  |  |  |  |  |  |  |
| 1 | Ath-AT4G26840.1 |  | | | |  |  |  |  |  |  |  |
| 1 | Ath-AT4G26850.1 |  | Vvi-Vitvi19g00549\_t001 |  |  |  |  |  |  |  |
| 2 | Ath-AT4G26860.2 |  | Vvi-Vitvi19g00537\_t002 |  | Vvi-Vitvi10g00057\_t001 |  |  |  |  |  |  |
| 2 | Ath-AT4G26870.1 |  | | | |  | | | |  |  |  |  |  |  |
| 2 | Ath-AT4G26880.1 |  | Vvi-Vitvi19g00534\_t001 |  | Vvi-Vitvi10g01615\_t001 |  |  |  |  |  |  |
| 2 | Ath-AT4G26890.1 |  | Vvi-Vitvi19g00528\_t001 |  | Vvi-Vitvi10g01621\_t001 |  |  |  |  |  |  |
| 2 | Ath-AT4G26900.1 |  | Vvi-Vitvi19g00527\_t001 |  | | | |  |  |  |  |  |  |
| 2 | Ath-AT4G26910.1 |  | Vvi-Vitvi19g00521\_t001 |  | Vvi-Vitvi10g00073\_t002 |  |  |  |  |  |  |
| 2 | Ath-AT4G26920.1 |  | | | |  | | | |  |  |  |  |  |  |
| 2 | Ath-AT4G26930.1 |  | Vvi-Vitvi19g00508\_t001 |  | | | |  |  |  |  |  |  |
| 2 | Ath-AT4G26940.1 |  | Vvi-Vitvi19g02020\_t001 |  | Vvi-Vitvi10g00092\_t001 |  |  |  |  |  |  |
| 2 | Ath-AT4G26950.2 |  | | | |  | Vvi-Vitvi10g00093\_t001 |  |  |  |  |  |  |
| 1 | Ath-AT4G26960.1 |  | Vvi-Vitvi19g04236\_t001 |  |  |  |  |  |  |  |
| 1 | Ath-AT4G26965.1 |  | Vvi-Vitvi19g00503\_t001 |  |  |  |  |  |  |  |
| 1 | Ath-AT4G26970.1 |  | Vvi-Vitvi19g00502\_t001 |  |  |  |  |  |  |  |
| 1 | Ath-AT4G26980.1 |  | Vvi-Vitvi19g00501\_t001 |  |  |  |  |  |  |  |
| 1 | Ath-AT4G26990.1 |  | Vvi-Vitvi19g00488\_t001 |  |  |  |  |  |  |  |
| 1 | Ath-AT4G27000.1 |  | Vvi-Vitvi19g00486\_t001 |  |  |  |  |  |  |  |
| 1 | Ath-AT4G27010.2 |  | | | |  |  |  |  |  |  |  |
| 1 | Ath-AT4G27020.1 |  | Vvi-Vitvi19g00476\_t001 |  |  |  |  |  |  |  |
| 1 | Ath-AT4G27030.1 |  | Vvi-Vitvi19g04230\_t001 |  |  |  |  |  |  |  |
| 1 | Ath-AT4G27040.3 |  | Vvi-Vitvi19g00474\_t001 |  |  |  |  |  |  |  |
| 1 | Ath-AT4G27050.1 |  | | | |  |  |  |  |  |  |  |
| 1 | Ath-AT4G27060.1 |  | Vvi-Vitvi19g00469\_t001 |  |  |  |  |  |  |  |
| 1 | Ath-AT4G27070.1 |  | Vvi-Vitvi19g00458\_t001 |  |  |  |  |  |  |  |
| 1 | Ath-AT4G27080.2 |  | Vvi-Vitvi19g00446\_t001 |  |  |  |  |  |  |  |
| 1 | Ath-AT4G27090.1 |  | | | |  |  |  |  |  |  |  |
| 1 | Ath-AT4G27100.1 |  | Vvi-Vitvi19g00443\_t001 |  |  |  |  |  |  |  |
| 1 | Ath-AT4G27110.1 |  | Vvi-Vitvi19g00442\_t001 |  |  |  |  |  |  |  |
| 1 | Ath-AT4G27120.1 |  | Vvi-Vitvi19g01998\_t002 |  |  |  |  |  |  |  |
| 1 | Ath-AT4G27130.1 |  | Vvi-Vitvi19g00436\_t001 |  |  |  |  |  |  |  |
| 0 | Ath-AT4G27140.1 |  |  |  |  |  |  |  |  |
| 0 | Ath-AT4G27150.1 |  |  |  |  |  |  |  |  |
| 0 | Ath-AT4G27160.1 |  |  |  |  |  |  |  |  |
| 0 | Ath-AT4G27170.1 |  |  |  |  |  |  |  |  |
| 0 | Ath-AT4G27180.1 |  |  |  |  |  |  |  |  |
| 0 | Ath-AT4G27190.1 |  |  |  |  |  |  |  |  |
| 0 | Ath-AT4G27220.2 |  |  |  |  |  |  |  |  |
| 0 | Ath-AT4G27230.1 |  |  |  |  |  |  |  |  |
| 0 | Ath-AT4G27240.1 |  |  |  |  |  |  |  |  |
| 0 | Ath-AT4G27250.1 |  |  |  |  |  |  |  |  |
| 0 | Ath-AT4G27260.1 |  |  |  |  |  |  |  |  |
| 0 | Ath-AT4G27270.1 |  |  |  |  |  |  |  |  |
| 0 | Ath-AT4G27280.1 |  |  |  |  |  |  |  |  |
| 0 | Ath-AT4G27290.2 |  |  |  |  |  |  |  |  |
| 0 | Ath-AT4G27300.1 |  |  |  |  |  |  |  |  |
| 0 | Ath-AT4G27310.1 |  |  |  |  |  |  |  |  |
| 0 | Ath-AT4G27320.1 |  |  |  |  |  |  |  |  |
| 0 | Ath-AT4G27330.1 |  |  |  |  |  |  |  |  |
| 0 | Ath-AT4G27340.1 |  |  |  |  |  |  |  |  |
| 1 | Ath-AT4G27350.1 |  | Vvi-Vitvi19g00303\_t001 |  |  |  |  |  |  |  |
| 1 | Ath-AT4G27360.1 |  | Vvi-Vitvi19g00302\_t001 |  |  |  |  |  |  |  |
| 1 | Ath-AT4G27370.2 |  | Vvi-Vitvi19g00297\_t001 |  |  |  |  |  |  |  |
| 1 | Ath-AT4G27380.1 |  | | | |  |  |  |  |  |  |  |
| 1 | Ath-AT4G27390.1 |  | Vvi-Vitvi19g01901\_t001.2.6037826c |  |  |  |  |  |  |  |
| 1 | Ath-AT4G27400.1 |  | Vvi-Vitvi19g00282\_t001 |  |  |  |  |  |  |  |
| 1 | Ath-AT4G27410.3 |  | Vvi-Vitvi19g00270\_t001 |  |  |  |  |  |  |  |
| 1 | Ath-AT4G27415.2 |  | | | |  |  |  |  |  |  |  |
| 1 | Ath-AT4G27420.1 |  | Vvi-Vitvi19g04106\_t001 |  |  |  |  |  |  |  |
| 1 | Ath-AT4G27430.1 |  | Vvi-Vitvi19g00262\_t001 |  |  |  |  |  |  |  |
| 1 | Ath-AT4G27435.1 |  | Vvi-Vitvi19g00261\_t003 |  |  |  |  |  |  |  |
| 1 | Ath-AT4G27440.2 |  | Vvi-Vitvi19g00257\_t001 |  |  |  |  |  |  |  |
| 1 | Ath-AT4G27450.1 |  | Vvi-Vitvi19g00255\_t001 |  |  |  |  |  |  |  |
| 1 | Ath-AT4G27460.1 |  | Vvi-Vitvi19g00252\_t001 |  |  |  |  |  |  |  |
| 1 | Ath-AT4G27470.1 |  | Vvi-Vitvi19g00239\_t001 |  |  |  |  |  |  |  |
| 1 | Ath-AT4G27480.1 |  | Vvi-Vitvi19g01869\_t001.1.6037826c |  |  |  |  |  |  |  |
| 1 | Ath-AT4G27490.1 |  | | | |  |  |  |  |  |  |  |
| 1 | Ath-AT4G27500.1 |  | Vvi-Vitvi19g04086\_t001 |  |  |  |  |  |  |  |
| 1 | Ath-AT4G27510.1 |  | | | |  |  |  |  |  |  |  |
| 1 | Ath-AT4G27520.1 |  | Vvi-Vitvi19g00206\_t001 |  |  |  |  |  |  |  |
| 1 | Ath-AT4G27530.1 |  | | | |  |  |  |  |  |  |  |
| 1 | Ath-AT4G27540.1 |  | Vvi-Vitvi19g00196\_t001 |  |  |  |  |  |  |  |
| 0 | Ath-AT4G27550.1 |  |  |  |  |  |  |  |  |
| 0 | Ath-AT4G27560.1 |  |  |  |  |  |  |  |  |
| 0 | Ath-AT4G27565.1 |  |  |  |  |  |  |  |  |
| 0 | Ath-AT4G27570.1 |  |  |  |  |  |  |  |  |
| 0 | Ath-AT4G27580.1 |  |  |  |  |  |  |  |  |
| 0 | Ath-AT4G27585.1 |  |  |  |  |  |  |  |  |
| 0 | Ath-AT4G27590.2 |  |  |  |  |  |  |  |  |
| 0 | Ath-AT4G27595.1 |  |  |  |  |  |  |  |  |
| 0 | Ath-AT4G27600.1 |  |  |  |  |  |  |  |  |
| 0 | Ath-AT4G27610.4 |  |  |  |  |  |  |  |  |
| 0 | Ath-AT4G27620.1 |  |  |  |  |  |  |  |  |
| 0 | Ath-AT4G27630.3 |  |  |  |  |  |  |  |  |
| 0 | Ath-AT4G27640.1 |  |  |  |  |  |  |  |  |
| 0 | Ath-AT4G27650.2 |  |  |  |  |  |  |  |  |
| 0 | Ath-AT4G27652.1 |  |  |  |  |  |  |  |  |
| 0 | Ath-AT4G27654.1 |  |  |  |  |  |  |  |  |
| 0 | Ath-AT4G27657.1 |  |  |  |  |  |  |  |  |
| 0 | Ath-AT4G27660.1 |  |  |  |  |  |  |  |  |
| 1 | Ath-AT4G27670.1 |  | Vvi-Vitvi16g01352\_t001 |  |  |  |  |  |  |  |
| 1 | Ath-AT4G27680.1 |  | Vvi-Vitvi16g01373\_t001 |  |  |  |  |  |  |  |
| 1 | Ath-AT4G27690.1 |  | Vvi-Vitvi16g01375\_t001 |  |  |  |  |  |  |  |
| 1 | Ath-AT4G27700.1 |  | Vvi-Vitvi16g02073\_t001 |  |  |  |  |  |  |  |
| 1 | Ath-AT4G27710.1 |  | | | |  |  |  |  |  |  |  |
| 1 | Ath-AT4G27720.1 |  | Vvi-Vitvi16g02074\_t001 |  |  |  |  |  |  |  |
| 1 | Ath-AT4G27730.1 |  | Vvi-Vitvi16g04514\_t001 |  |  |  |  |  |  |  |
| 1 | Ath-AT4G27740.1 |  | Vvi-Vitvi16g04515\_t001 |  |  |  |  |  |  |  |
| 1 | Ath-AT4G27745.1 |  | | | |  |  |  |  |  |  |  |
| 1 | Ath-AT4G27750.1 |  | Vvi-Vitvi16g01383\_t001 |  |  |  |  |  |  |  |
| 1 | Ath-AT4G27760.1 |  | | | |  |  |  |  |  |  |  |
| 1 | Ath-AT4G27780.1 |  | Vvi-Vitvi16g01390\_t001 |  |  |  |  |  |  |  |
| 1 | Ath-AT4G27790.1 |  | Vvi-Vitvi16g02081\_t001 |  |  |  |  |  |  |  |
| 1 | Ath-AT4G27800.1 |  | Vvi-Vitvi16g01395\_t001 |  |  |  |  |  |  |  |
| 1 | Ath-AT4G27810.1 |  | Vvi-Vitvi16g02087\_t001 |  |  |  |  |  |  |  |
| 1 | Ath-AT4G27820.1 |  | | | |  |  |  |  |  |  |  |
| 1 | Ath-AT4G27830.1 |  | | | |  |  |  |  |  |  |  |
| 1 | Ath-AT4G27840.1 |  | Vvi-Vitvi16g02088\_t001 |  |  |  |  |  |  |  |
| 1 | Ath-AT4G27845.1 |  | | | |  |  |  |  |  |  |  |
| 1 | Ath-AT4G27850.1 |  | | | |  |  |  |  |  |  |  |
| 1 | Ath-AT4G27860.3 |  | | | |  |  |  |  |  |  |  |
| 1 | Ath-AT4G27870.2 |  | | | |  |  |  |  |  |  |  |
| 1 | Ath-AT4G27880.1 |  | Vvi-Vitvi16g01412\_t001 |  |  |  |  |  |  |  |
| 0 | Ath-AT4G27890.1 |  |  |  |  |  |  |  |  |
| 0 | Ath-AT4G27900.1 |  |  |  |  |  |  |  |  |
| 0 | Ath-AT4G27910.1 |  |  |  |  |  |  |  |  |
| 0 | Ath-AT4G27920.1 |  |  |  |  |  |  |  |  |
| 0 | Ath-AT4G27940.1 |  |  |  |  |  |  |  |  |
| 0 | Ath-AT4G27950.1 |  |  |  |  |  |  |  |  |
| 0 | Ath-AT4G27960.2 |  |  |  |  |  |  |  |  |
| 0 | Ath-AT4G27970.1 |  |  |  |  |  |  |  |  |
| 0 | Ath-AT4G27980.2 |  |  |  |  |  |  |  |  |
| 0 | Ath-AT4G27990.1 |  |  |  |  |  |  |  |  |
| 0 | Ath-AT4G28000.2 |  |  |  |  |  |  |  |  |
| 0 | Ath-AT4G28005.1 |  |  |  |  |  |  |  |  |
| 0 | Ath-AT4G28010.1 |  |  |  |  |  |  |  |  |
| 0 | Ath-AT4G28020.1 |  |  |  |  |  |  |  |  |
| 0 | Ath-AT4G28025.1 |  |  |  |  |  |  |  |  |
| 1 | Ath-AT4G28030.1 |  | Vvi-Vitvi11g00847\_t001 |  |  |  |  |  |  |  |
| 1 | Ath-AT4G28040.1 |  | Vvi-Vitvi11g04210\_t001 |  |  |  |  |  |  |  |
| 1 | Ath-AT4G28050.1 |  | Vvi-Vitvi11g00828\_t001 |  |  |  |  |  |  |  |
| 1 | Ath-AT4G28060.1 |  | Vvi-Vitvi11g00826\_t001 |  |  |  |  |  |  |  |
| 1 | Ath-AT4G28068.1 |  | | | |  |  |  |  |  |  |  |
| 1 | Ath-AT4G28070.2 |  | Vvi-Vitvi11g00814\_t001 |  |  |  |  |  |  |  |
| 1 | Ath-AT4G28080.1 |  | Vvi-Vitvi11g00795\_t001 |  |  |  |  |  |  |  |
| 0 | Ath-AT4G28085.1 |  |  |  |  |  |  |  |  |
| 0 | Ath-AT4G28088.1 |  |  |  |  |  |  |  |  |
| 0 | Ath-AT4G28090.1 |  |  |  |  |  |  |  |  |
| 0 | Ath-AT4G28100.1 |  |  |  |  |  |  |  |  |
| 0 | Ath-AT4G28110.1 |  |  |  |  |  |  |  |  |
| 0 | Ath-AT4G28130.1 |  |  |  |  |  |  |  |  |
| 0 | Ath-AT4G28140.1 |  |  |  |  |  |  |  |  |
| 0 | Ath-AT4G28150.1 |  |  |  |  |  |  |  |  |
| 0 | Ath-AT4G28160.1 |  |  |  |  |  |  |  |  |
| 0 | Ath-AT4G28170.1 |  |  |  |  |  |  |  |  |
| 0 | Ath-AT4G28180.1 |  |  |  |  |  |  |  |  |
| 0 | Ath-AT4G28190.2 |  |  |  |  |  |  |  |  |
| 0 | Ath-AT4G28200.1 |  |  |  |  |  |  |  |  |
| 0 | Ath-AT4G28210.1 |  |  |  |  |  |  |  |  |
| 0 | Ath-AT4G28220.1 |  |  |  |  |  |  |  |  |
| 0 | Ath-AT4G28230.1 |  |  |  |  |  |  |  |  |
| 0 | Ath-AT4G28240.1 |  |  |  |  |  |  |  |  |
| 0 | Ath-AT4G28250.1 |  |  |  |  |  |  |  |  |
| 1 | Ath-AT4G28260.1 |  | Vvi-Vitvi12g00324\_t001 |  |  |  |  |  |  |  |
| 1 | Ath-AT4G28270.1 |  | Vvi-Vitvi12g00320\_t001 |  |  |  |  |  |  |  |
| 1 | Ath-AT4G28280.2 |  | Vvi-Vitvi12g00316\_t001 |  |  |  |  |  |  |  |
| 1 | Ath-AT4G28290.2 |  | | | |  |  |  |  |  |  |  |
| 1 | Ath-AT4G28300.1 |  | Vvi-Vitvi12g00309\_t001 |  |  |  |  |  |  |  |
| 1 | Ath-AT4G28310.1 |  | Vvi-Vitvi12g02309\_t001 |  |  |  |  |  |  |  |
| 1 | Ath-AT4G28320.1 |  | Vvi-Vitvi12g00303\_t004 |  |  |  |  |  |  |  |
| 1 | Ath-AT4G28330.1 |  | Vvi-Vitvi12g02308\_t001 |  |  |  |  |  |  |  |
| 1 | Ath-AT4G28340.1 |  | | | |  |  |  |  |  |  |  |
| 1 | Ath-AT4G28350.1 |  | Vvi-Vitvi12g00300\_t001 |  |  |  |  |  |  |  |
| 1 | Ath-AT4G28360.1 |  | | | |  |  |  |  |  |  |  |
| 1 | Ath-AT4G28365.1 |  | Vvi-Vitvi12g00290\_t001 |  |  |  |  |  |  |  |
| 1 | Ath-AT4G28370.3 |  | Vvi-Vitvi12g00286\_t001 |  |  |  |  |  |  |  |
| 1 | Ath-AT4G28380.1 |  | Vvi-Vitvi12g00285\_t001 |  |  |  |  |  |  |  |
| 1 | Ath-AT4G28390.1 |  | Vvi-Vitvi12g00284\_t001 |  |  |  |  |  |  |  |
| 1 | Ath-AT4G28395.1 |  | | | |  |  |  |  |  |  |  |
| 1 | Ath-AT4G28397.1 |  | | | |  |  |  |  |  |  |  |
| 1 | Ath-AT4G28400.1 |  | Vvi-Vitvi12g00277\_t001 |  |  |  |  |  |  |  |
| 1 | Ath-AT4G28405.1 |  | | | |  |  |  |  |  |  |  |
| 1 | Ath-AT4G28410.1 |  | Vvi-Vitvi12g00270\_t001 |  |  |  |  |  |  |  |
| 1 | Ath-AT4G28420.2 |  | | | |  |  |  |  |  |  |  |
| 1 | Ath-AT4G28430.1 |  | Vvi-Vitvi12g00269\_t001 |  |  |  |  |  |  |  |
| 1 | Ath-AT4G28440.1 |  | Vvi-Vitvi12g00268\_t001 |  |  |  |  |  |  |  |
| 1 | Ath-AT4G28450.1 |  | Vvi-Vitvi12g00264\_t001 |  |  |  |  |  |  |  |
| 1 | Ath-AT4G28460.1 |  | | | |  |  |  |  |  |  |  |
| 1 | Ath-AT4G28470.1 |  | Vvi-Vitvi12g00263\_t001 |  |  |  |  |  |  |  |
| 1 | Ath-AT4G28480.1 |  | | | |  |  |  |  |  |  |  |
| 1 | Ath-AT4G28485.2 |  | Vvi-Vitvi12g00257\_t001 |  |  |  |  |  |  |  |
| 1 | Ath-AT4G28490.1 |  | Vvi-Vitvi12g00256\_t001 |  |  |  |  |  |  |  |
| 1 | Ath-AT4G28500.1 |  | Vvi-Vitvi12g00255\_t001 |  |  |  |  |  |  |  |
| 0 | Ath-AT4G28510.1 |  |  |  |  |  |  |  |  |
| 1 | Ath-AT4G28520.1 |  | Vvi-Vitvi07g00605\_t001 |  |  |  |  |  |  |  |
| 1 | Ath-AT4G28530.1 |  | Vvi-Vitvi07g00619\_t001 |  |  |  |  |  |  |  |
| 1 | Ath-AT4G28540.1 |  | Vvi-Vitvi07g00628\_t001 |  |  |  |  |  |  |  |
| 1 | Ath-AT4G28550.1 |  | Vvi-Vitvi07g00633\_t002 |  |  |  |  |  |  |  |
| 1 | Ath-AT4G28556.1 |  | Vvi-Vitvi07g02274\_t001 |  |  |  |  |  |  |  |
| 1 | Ath-AT4G28560.1 |  | Vvi-Vitvi07g00634\_t001 |  |  |  |  |  |  |  |
| 1 | Ath-AT4G28570.1 |  | Vvi-Vitvi07g00636\_t001 |  |  |  |  |  |  |  |
| 1 | Ath-AT4G28580.1 |  | | | |  |  |  |  |  |  |  |
| 1 | Ath-AT4G28590.1 |  | Vvi-Vitvi07g02276\_t001 |  |  |  |  |  |  |  |
| 1 | Ath-AT4G28600.3 |  | Vvi-Vitvi07g00643\_t001 |  |  |  |  |  |  |  |
| 1 | Ath-AT4G28610.1 |  | Vvi-Vitvi07g00666\_t001 |  |  |  |  |  |  |  |
| 1 | Ath-AT4G28620.1 |  | | | |  |  |  |  |  |  |  |
| 1 | Ath-AT4G28630.1 |  | | | |  |  |  |  |  |  |  |
| 1 | Ath-AT4G28640.2 |  | Vvi-Vitvi07g00687\_t001 |  |  |  |  |  |  |  |
| 1 | Ath-AT4G28650.1 |  | Vvi-Vitvi07g00688\_t001 |  |  |  |  |  |  |  |
| 1 | Ath-AT4G28660.2 |  | Vvi-Vitvi07g00690\_t001 |  |  |  |  |  |  |  |
| 1 | Ath-AT4G28670.1 |  | | | |  |  |  |  |  |  |  |
| 1 | Ath-AT4G28680.5 |  | Vvi-Vitvi07g00696\_t001 |  |  |  |  |  |  |  |
| 1 | Ath-AT4G28690.1 |  | Vvi-Vitvi07g00705\_t001 |  |  |  |  |  |  |  |
| 1 | Ath-AT4G28700.1 |  | | | |  |  |  |  |  |  |  |
| 1 | Ath-AT4G28703.1 |  | Vvi-Vitvi07g00709\_t001 |  |  |  |  |  |  |  |
| 1 | Ath-AT4G28706.4 |  | Vvi-Vitvi07g00721\_t001 |  |  |  |  |  |  |  |
| 1 | Ath-AT4G28710.1 |  | Vvi-Vitvi07g00724\_t001 |  |  |  |  |  |  |  |
| 1 | Ath-AT4G28720.1 |  | Vvi-Vitvi07g00726\_t001 |  |  |  |  |  |  |  |
| 1 | Ath-AT4G28730.1 |  | Vvi-Vitvi07g00733\_t001 |  |  |  |  |  |  |  |
| 1 | Ath-AT4G28740.1 |  | Vvi-Vitvi07g00737\_t001 |  |  |  |  |  |  |  |
| 1 | Ath-AT4G28750.1 |  | Vvi-Vitvi07g02309\_t001 |  |  |  |  |  |  |  |
| 1 | Ath-AT4G28755.1 |  | | | |  |  |  |  |  |  |  |
| 1 | Ath-AT4G28760.2 |  | Vvi-Vitvi07g00739\_t001 |  |  |  |  |  |  |  |
| 1 | Ath-AT4G28770.2 |  | Vvi-Vitvi07g00740\_t001 |  |  |  |  |  |  |  |
| 1 | Ath-AT4G28775.1 |  | | | |  |  |  |  |  |  |  |
| 1 | Ath-AT4G28780.1 |  | Vvi-Vitvi07g00741\_t001 |  |  |  |  |  |  |  |
| 1 | Ath-AT4G28790.1 |  | Vvi-Vitvi07g00762\_t001 |  |  |  |  |  |  |  |
| 1 | Ath-AT4G28800.2 |  | | | |  |  |  |  |  |  |  |
| 1 | Ath-AT4G28811.1 |  | | | |  |  |  |  |  |  |  |
| 1 | Ath-AT4G28815.1 |  | | | |  |  |  |  |  |  |  |
| 1 | Ath-AT4G28820.3 |  | Vvi-Vitvi07g02313\_t001 |  |  |  |  |  |  |  |
| 2 | Ath-AT4G28830.3 |  | | | |  | Vvi-Vitvi11g00299\_t001 |  |  |  |  |  |  |
| 2 | Ath-AT4G28840.1 |  | | | |  | Vvi-Vitvi11g01399\_t001 |  |  |  |  |  |  |
| 3 | Ath-AT4G28850.1 |  | | | |  | | | |  | Vvi-Vitvi11g01398\_t001 |  |  |  |  |  |
| 3 | Ath-AT4G28860.1 |  | | | |  | Vvi-Vitvi11g01396\_t001 |  | | | |  |  |  |  |  |
| 3 | Ath-AT4G28870.1 |  | | | |  | | | |  | | | |  |  |  |  |  |
| 3 | Ath-AT4G28880.1 |  | | | |  | | | |  | | | |  |  |  |  |  |
| 3 | Ath-AT4G28890.1 |  | | | |  | Vvi-Vitvi11g00293\_t001 |  | | | |  |  |  |  |  |
| 3 | Ath-AT4G28910.2 |  | | | |  | Vvi-Vitvi11g00292\_t001 |  | | | |  |  |  |  |  |
| 3 | Ath-AT4G28920.1 |  | | | |  | | | |  | | | |  |  |  |  |  |
| 3 | Ath-AT4G28930.1 |  | | | |  | | | |  | | | |  |  |  |  |  |
| 3 | Ath-AT4G28940.1 |  | | | |  | | | |  | Vvi-Vitvi11g00304\_t001 |  |  |  |  |  |
| 3 | Ath-AT4G28950.1 |  | | | |  | | | |  | Vvi-Vitvi11g00309\_t001 |  |  |  |  |  |
| 3 | Ath-AT4G28980.2 |  | | | |  | | | |  | Vvi-Vitvi11g00310\_t001 |  |  |  |  |  |
| 3 | Ath-AT4G28990.2 |  | | | |  | | | |  | Vvi-Vitvi11g00312\_t001 |  |  |  |  |  |
| 3 | Ath-AT4G29000.1 |  | | | |  | | | |  | Vvi-Vitvi11g00314\_t001 |  |  |  |  |  |
| 3 | Ath-AT4G29010.1 |  | | | |  | | | |  | Vvi-Vitvi11g00315\_t001 |  |  |  |  |  |
| 2 | Ath-AT4G29020.1 |  | | | |  | | | |  |  |  |  |  |  |
| 2 | Ath-AT4G29030.1 |  | | | |  | | | |  |  |  |  |  |  |
| 2 | Ath-AT4G29033.1 |  | | | |  | | | |  |  |  |  |  |  |
| 2 | Ath-AT4G29035.1 |  | | | |  | | | |  |  |  |  |  |  |
| 2 | Ath-AT4G29037.1 |  | | | |  | | | |  |  |  |  |  |  |
| 2 | Ath-AT4G29040.1 |  | Vvi-Vitvi07g00765\_t001 |  | | | |  |  |  |  |  |  |
| 1 | Ath-AT4G29050.2 |  |  |  | | | |  |  |  |  |  |  |
| 1 | Ath-AT4G29060.1 |  |  |  | | | |  |  |  |  |  |  |
| 1 | Ath-AT4G29070.1 |  |  |  | | | |  |  |  |  |  |  |
| 1 | Ath-AT4G29080.1 |  |  |  | | | |  |  |  |  |  |  |
| 1 | Ath-AT4G29090.1 |  |  |  | | | |  |  |  |  |  |  |
| 1 | Ath-AT4G29100.1 |  |  |  | | | |  |  |  |  |  |  |
| 1 | Ath-AT4G29103.1 |  |  |  | | | |  |  |  |  |  |  |
| 1 | Ath-AT4G29110.1 |  |  |  | Vvi-Vitvi11g00275\_t001 |  |  |  |  |  |  |
| 1 | Ath-AT4G29120.1 |  |  |  | Vvi-Vitvi11g00262\_t001 |  |  |  |  |  |  |
| 1 | Ath-AT4G29130.1 |  |  |  | Vvi-Vitvi11g00260\_t001 |  |  |  |  |  |  |
| 1 | Ath-AT4G29140.1 |  |  |  | Vvi-Vitvi11g00259\_t001 |  |  |  |  |  |  |
| 1 | Ath-AT4G29150.1 |  |  |  | Vvi-Vitvi11g00257\_t001 |  |  |  |  |  |  |
| 2 | Ath-AT4G29160.1 |  | Vvi-Vitvi11g01381\_t001 |  | Vvi-Vitvi09g01550\_t006 |  |  |  |  |  |  |
| 2 | Ath-AT4G29170.1 |  | Vvi-Vitvi11g00227\_t001 |  | | | |  |  |  |  |  |  |
| 2 | Ath-AT4G29180.2 |  | | | |  | | | |  |  |  |  |  |  |
| 2 | Ath-AT4G29190.1 |  | Vvi-Vitvi11g00229\_t001 |  | | | |  |  |  |  |  |  |
| 2 | Ath-AT4G29200.1 |  | | | |  | | | |  |  |  |  |  |  |
| 2 | Ath-AT4G29210.1 |  | Vvi-Vitvi11g00234\_t001 |  | | | |  |  |  |  |  |  |
| 2 | Ath-AT4G29220.1 |  | Vvi-Vitvi11g00237\_t001 |  | | | |  |  |  |  |  |  |
| 2 | Ath-AT4G29230.1 |  | Vvi-Vitvi11g00241\_t001 |  | | | |  |  |  |  |  |  |
| 2 | Ath-AT4G29240.1 |  | Vvi-Vitvi11g00243\_t001 |  | | | |  |  |  |  |  |  |
| 2 | Ath-AT4G29250.1 |  | | | |  | | | |  |  |  |  |  |  |
| 2 | Ath-AT4G29260.1 |  | Vvi-Vitvi11g00244\_t001 |  | | | |  |  |  |  |  |  |
| 2 | Ath-AT4G29270.1 |  | | | |  | | | |  |  |  |  |  |  |
| 2 | Ath-AT4G29273.1 |  | | | |  | | | |  |  |  |  |  |  |
| 2 | Ath-AT4G29280.1 |  | | | |  | | | |  |  |  |  |  |  |
| 2 | Ath-AT4G29283.1 |  | | | |  | | | |  |  |  |  |  |  |
| 2 | Ath-AT4G29285.1 |  | | | |  | | | |  |  |  |  |  |  |
| 2 | Ath-AT4G29290.1 |  | | | |  | | | |  |  |  |  |  |  |
| 2 | Ath-AT4G29300.1 |  | | | |  | | | |  |  |  |  |  |  |
| 2 | Ath-AT4G29305.1 |  | | | |  | | | |  |  |  |  |  |  |
| 2 | Ath-AT4G29310.1 |  | Vvi-Vitvi11g00245\_t001 |  | | | |  |  |  |  |  |  |
| 2 | Ath-AT4G29330.1 |  | | | |  | | | |  |  |  |  |  |  |
| 2 | Ath-AT4G29340.1 |  | Vvi-Vitvi11g00247\_t001 |  | | | |  |  |  |  |  |  |
| 2 | Ath-AT4G29350.1 |  | | | |  | | | |  |  |  |  |  |  |
| 2 | Ath-AT4G29360.2 |  | Vvi-Vitvi11g00249\_t001 |  | | | |  |  |  |  |  |  |
| 2 | Ath-AT4G29370.1 |  | | | |  | | | |  |  |  |  |  |  |
| 2 | Ath-AT4G29380.1 |  | Vvi-Vitvi11g00254\_t001 |  | | | |  |  |  |  |  |  |
| 2 | Ath-AT4G29390.1 |  | Vvi-Vitvi11g04055\_t001 |  | Vvi-Vitvi09g04055\_t001 |  |  |  |  |  |  |
| 2 | Ath-AT4G29400.1 |  | Vvi-Vitvi11g00220\_t001 |  | | | |  |  |  |  |  |  |
| 2 | Ath-AT4G29410.1 |  | | | |  | Vvi-Vitvi09g01545\_t001 |  |  |  |  |  |  |
| 2 | Ath-AT4G29420.1 |  | | | |  | | | |  |  |  |  |  |  |
| 2 | Ath-AT4G29430.1 |  | | | |  | Vvi-Vitvi09g00213\_t002 |  |  |  |  |  |  |
| 2 | Ath-AT4G29440.1 |  | Vvi-Vitvi11g00213\_t001 |  | | | |  |  |  |  |  |  |
| 2 | Ath-AT4G29450.1 |  | | | |  | | | |  |  |  |  |  |  |
| 2 | Ath-AT4G29460.1 |  | Vvi-Vitvi11g01379\_t001 |  | | | |  |  |  |  |  |  |
| 2 | Ath-AT4G29470.2 |  | | | |  | | | |  |  |  |  |  |  |
| 2 | Ath-AT4G29480.1 |  | | | |  | Vvi-Vitvi09g00209\_t001 |  |  |  |  |  |  |
| 2 | Ath-AT4G29490.1 |  | Vvi-Vitvi11g00209\_t001 |  | | | |  |  |  |  |  |  |
| 2 | Ath-AT4G29510.1 |  | Vvi-Vitvi11g00208\_t001 |  | | | |  |  |  |  |  |  |
| 2 | Ath-AT4G29520.1 |  | Vvi-Vitvi11g00207\_t001 |  | | | |  |  |  |  |  |  |
| 2 | Ath-AT4G29530.1 |  | Vvi-Vitvi11g00202\_t001 |  | Vvi-Vitvi09g01542\_t002 |  |  |  |  |  |  |
| 2 | Ath-AT4G29540.2 |  | Vvi-Vitvi11g00198\_t001 |  | | | |  |  |  |  |  |  |
| 2 | Ath-AT4G29548.1 |  | | | |  | | | |  |  |  |  |  |  |
| 2 | Ath-AT4G29550.1 |  | | | |  | | | |  |  |  |  |  |  |
| 2 | Ath-AT4G29560.1 |  | Vvi-Vitvi11g00194\_t001 |  | | | |  |  |  |  |  |  |
| 2 | Ath-AT4G29570.1 |  | | | |  | | | |  |  |  |  |  |  |
| 2 | Ath-AT4G29580.2 |  | | | |  | | | |  |  |  |  |  |  |
| 2 | Ath-AT4G29590.1 |  | Vvi-Vitvi11g00192\_t001 |  | | | |  |  |  |  |  |  |
| 2 | Ath-AT4G29600.1 |  | Vvi-Vitvi11g01376\_t001 |  | | | |  |  |  |  |  |  |
| 2 | Ath-AT4G29610.1 |  | | | |  | | | |  |  |  |  |  |  |
| 2 | Ath-AT4G29620.1 |  | | | |  | | | |  |  |  |  |  |  |
| 2 | Ath-AT4G29630.1 |  | | | |  | | | |  |  |  |  |  |  |
| 2 | Ath-AT4G29640.1 |  | | | |  | | | |  |  |  |  |  |  |
| 2 | Ath-AT4G29650.1 |  | | | |  | | | |  |  |  |  |  |  |
| 2 | Ath-AT4G29654.1 |  | | | |  | | | |  |  |  |  |  |  |
| 2 | Ath-AT4G29658.2 |  | | | |  | | | |  |  |  |  |  |  |
| 2 | Ath-AT4G29660.1 |  | Vvi-Vitvi11g00190\_t001 |  | | | |  |  |  |  |  |  |
| 2 | Ath-AT4G29670.2 |  | Vvi-Vitvi11g00189\_t001 |  | Vvi-Vitvi09g00199\_t002 |  |  |  |  |  |  |
| 1 | Ath-AT4G29680.1 |  | Vvi-Vitvi11g00188\_t001 |  |  |  |  |  |  |  |
| 1 | Ath-AT4G29690.1 |  | | | |  |  |  |  |  |  |  |
| 1 | Ath-AT4G29700.1 |  | | | |  |  |  |  |  |  |  |
| 1 | Ath-AT4G29710.1 |  | | | |  |  |  |  |  |  |  |
| 1 | Ath-AT4G29720.1 |  | Vvi-Vitvi11g00175\_t001 |  |  |  |  |  |  |  |
| 1 | Ath-AT4G29730.1 |  | Vvi-Vitvi11g00174\_t001 |  |  |  |  |  |  |  |
| 1 | Ath-AT4G29735.2 |  | Vvi-Vitvi11g01373\_t001 |  |  |  |  |  |  |  |
| 1 | Ath-AT4G29740.2 |  | Vvi-Vitvi11g01371\_t001 |  |  |  |  |  |  |  |
| 1 | Ath-AT4G29750.1 |  | Vvi-Vitvi11g00167\_t001 |  |  |  |  |  |  |  |
| 0 | Ath-AT4G29760.1 |  |  |  |  |  |  |  |  |
| 0 | Ath-AT4G29770.2 |  |  |  |  |  |  |  |  |
| 1 | Ath-AT4G29780.1 |  | Vvi-Vitvi11g00140\_t001 |  |  |  |  |  |  |  |
| 1 | Ath-AT4G29790.1 |  | Vvi-Vitvi11g00138\_t001 |  |  |  |  |  |  |  |
| 1 | Ath-AT4G29800.2 |  | | | |  |  |  |  |  |  |  |
| 1 | Ath-AT4G29810.2 |  | Vvi-Vitvi11g00136\_t002 |  |  |  |  |  |  |  |
| 1 | Ath-AT4G29820.1 |  | Vvi-Vitvi11g00135\_t001 |  |  |  |  |  |  |  |
| 1 | Ath-AT4G29830.1 |  | Vvi-Vitvi11g00131\_t001 |  |  |  |  |  |  |  |
| 1 | Ath-AT4G29840.1 |  | Vvi-Vitvi11g00130\_t001 |  |  |  |  |  |  |  |
| 1 | Ath-AT4G29850.1 |  | Vvi-Vitvi11g01202\_t001 |  |  |  |  |  |  |  |
| 1 | Ath-AT4G29860.1 |  | | | |  |  |  |  |  |  |  |
| 1 | Ath-AT4G29870.1 |  | Vvi-Vitvi11g01183\_t001 |  |  |  |  |  |  |  |
| 1 | Ath-AT4G29880.1 |  | Vvi-Vitvi11g01182\_t001 |  |  |  |  |  |  |  |
| 1 | Ath-AT4G29890.1 |  | Vvi-Vitvi11g01177\_t001 |  |  |  |  |  |  |  |
| 1 | Ath-AT4G29900.1 |  | Vvi-Vitvi11g01176\_t001 |  |  |  |  |  |  |  |
| 1 | Ath-AT4G29905.1 |  | Vvi-Vitvi11g01629\_t001 |  |  |  |  |  |  |  |
| 1 | Ath-AT4G29910.2 |  | | | |  |  |  |  |  |  |  |
| 1 | Ath-AT4G29920.2 |  | Vvi-Vitvi11g01162\_t001 |  |  |  |  |  |  |  |
| 1 | Ath-AT4G29930.3 |  | Vvi-Vitvi11g01153\_t001 |  |  |  |  |  |  |  |
| 1 | Ath-AT4G29940.1 |  | Vvi-Vitvi11g01140\_t001 |  |  |  |  |  |  |  |
| 1 | Ath-AT4G29950.1 |  | | | |  |  |  |  |  |  |  |
| 1 | Ath-AT4G29960.1 |  | Vvi-Vitvi11g01137\_t001 |  |  |  |  |  |  |  |
| 1 | Ath-AT4G29970.1 |  | | | |  |  |  |  |  |  |  |
| 1 | Ath-AT4G29980.1 |  | | | |  |  |  |  |  |  |  |
| 1 | Ath-AT4G29990.1 |  | | | |  |  |  |  |  |  |  |
| 1 | Ath-AT4G30000.2 |  | | | |  |  |  |  |  |  |  |
| 1 | Ath-AT4G30010.1 |  | | | |  |  |  |  |  |  |  |
| 1 | Ath-AT4G30020.1 |  | Vvi-Vitvi11g01134\_t006 |  |  |  |  |  |  |  |
| 1 | Ath-AT4G30030.1 |  | Vvi-Vitvi11g01130\_t001 |  |  |  |  |  |  |  |
| 1 | Ath-AT4G30040.1 |  | | | |  |  |  |  |  |  |  |
| 1 | Ath-AT4G30050.1 |  | | | |  |  |  |  |  |  |  |
| 1 | Ath-AT4G30060.1 |  | Vvi-Vitvi11g01128\_t001 |  |  |  |  |  |  |  |
| 1 | Ath-AT4G30064.1 |  | | | |  |  |  |  |  |  |  |
| 1 | Ath-AT4G30067.1 |  | | | |  |  |  |  |  |  |  |
| 1 | Ath-AT4G30070.1 |  | | | |  |  |  |  |  |  |  |
| 1 | Ath-AT4G30074.1 |  | | | |  |  |  |  |  |  |  |
| 1 | Ath-AT4G30080.1 |  | | | |  |  |  |  |  |  |  |
| 1 | Ath-AT4G30090.1 |  | | | |  |  |  |  |  |  |  |
| 1 | Ath-AT4G30100.1 |  | | | |  |  |  |  |  |  |  |
| 1 | Ath-AT4G30097.1 |  | | | |  |  |  |  |  |  |  |
| 1 | Ath-AT4G30110.1 |  | Vvi-Vitvi11g04307\_t002 |  |  |  |  |  |  |  |
| 1 | Ath-AT4G30120.1 |  | | | |  |  |  |  |  |  |  |
| 1 | Ath-AT4G30130.1 |  | Vvi-Vitvi11g01107\_t002 |  |  |  |  |  |  |  |
| 0 | Ath-AT4G30140.1 |  |  |  |  |  |  |  |  |
| 0 | Ath-AT4G30150.1 |  |  |  |  |  |  |  |  |
| 1 | Ath-AT4G30160.2 |  | Vvi-Vitvi11g01212\_t001 |  |  |  |  |  |  |  |
| 1 | Ath-AT4G30170.1 |  | | | |  |  |  |  |  |  |  |
| 1 | Ath-AT4G30180.1 |  | | | |  |  |  |  |  |  |  |
| 1 | Ath-AT4G30190.2 |  | | | |  |  |  |  |  |  |  |
| 1 | Ath-AT4G30200.2 |  | Vvi-Vitvi11g01230\_t001 |  |  |  |  |  |  |  |
| 1 | Ath-AT4G30210.3 |  | Vvi-Vitvi11g01236\_t001 |  |  |  |  |  |  |  |
| 1 | Ath-AT4G30220.2 |  | | | |  |  |  |  |  |  |  |
| 1 | Ath-AT4G30230.1 |  | Vvi-Vitvi11g01658\_t001 |  |  |  |  |  |  |  |
| 1 | Ath-AT4G30240.1 |  | Vvi-Vitvi11g04344\_t001 |  |  |  |  |  |  |  |
| 1 | Ath-AT4G30250.2 |  | Vvi-Vitvi11g01243\_t001 |  |  |  |  |  |  |  |
| 1 | Ath-AT4G30260.2 |  | Vvi-Vitvi11g01244\_t001 |  |  |  |  |  |  |  |
| 0 | Ath-AT4G30270.1 |  |  |  |  |  |  |  |  |
| 0 | Ath-AT4G30280.1 |  |  |  |  |  |  |  |  |
| 0 | Ath-AT4G30290.1 |  |  |  |  |  |  |  |  |
| 0 | Ath-AT4G30300.1 |  |  |  |  |  |  |  |  |
| 0 | Ath-AT4G30310.2 |  |  |  |  |  |  |  |  |
| 0 | Ath-AT4G30320.1 |  |  |  |  |  |  |  |  |
| 0 | Ath-AT4G30330.1 |  |  |  |  |  |  |  |  |
| 0 | Ath-AT4G30340.1 |  |  |  |  |  |  |  |  |
| 0 | Ath-AT4G30350.1 |  |  |  |  |  |  |  |  |
| 0 | Ath-AT4G30360.1 |  |  |  |  |  |  |  |  |
| 0 | Ath-AT4G30370.1 |  |  |  |  |  |  |  |  |
| 0 | Ath-AT4G30380.2 |  |  |  |  |  |  |  |  |
| 0 | Ath-AT4G30390.1 |  |  |  |  |  |  |  |  |
| 1 | Ath-AT4G30400.1 |  | Vvi-Vitvi11g00869\_t001 |  |  |  |  |  |  |  |
| 1 | Ath-AT4G30410.1 |  | Vvi-Vitvi11g00838\_t001 |  |  |  |  |  |  |  |
| 1 | Ath-AT4G30420.2 |  | Vvi-Vitvi11g04210\_t001 |  |  |  |  |  |  |  |
| 1 | Ath-AT4G30430.1 |  | Vvi-Vitvi11g00828\_t001 |  |  |  |  |  |  |  |
| 1 | Ath-AT4G30440.1 |  | | | |  |  |  |  |  |  |  |
| 1 | Ath-AT4G30450.1 |  | | | |  |  |  |  |  |  |  |
| 1 | Ath-AT4G30460.1 |  | | | |  |  |  |  |  |  |  |
| 1 | Ath-AT4G30470.1 |  | Vvi-Vitvi11g00821\_t001 |  |  |  |  |  |  |  |
| 1 | Ath-AT4G30480.2 |  | Vvi-Vitvi11g00818\_t001 |  |  |  |  |  |  |  |
| 1 | Ath-AT4G30490.1 |  | Vvi-Vitvi11g00814\_t001 |  |  |  |  |  |  |  |
| 1 | Ath-AT4G30500.1 |  | | | |  |  |  |  |  |  |  |
| 1 | Ath-AT4G30510.1 |  | Vvi-Vitvi11g00813\_t001 |  |  |  |  |  |  |  |
| 1 | Ath-AT4G30520.1 |  | Vvi-Vitvi11g00812\_t001 |  |  |  |  |  |  |  |
| 1 | Ath-AT4G30530.1 |  | Vvi-Vitvi11g00805\_t001 |  |  |  |  |  |  |  |
| 1 | Ath-AT4G30540.1 |  | | | |  |  |  |  |  |  |  |
| 1 | Ath-AT4G30550.1 |  | | | |  |  |  |  |  |  |  |
| 2 | Ath-AT4G30560.1 |  | | | |  | Vvi-Vitvi11g00701\_t002 |  |  |  |  |  |  |
| 2 | Ath-AT4G30570.1 |  | | | |  | | | |  |  |  |  |  |  |
| 2 | Ath-AT4G30580.1 |  | | | |  | Vvi-Vitvi11g00704\_t001 |  |  |  |  |  |  |
| 2 | Ath-AT4G30590.1 |  | | | |  | Vvi-Vitvi11g00708\_t001 |  |  |  |  |  |  |
| 2 | Ath-AT4G30600.2 |  | | | |  | Vvi-Vitvi11g00710\_t001 |  |  |  |  |  |  |
| 2 | Ath-AT4G30610.1 |  | Vvi-Vitvi11g00778\_t001 |  | Vvi-Vitvi11g00712\_t001 |  |  |  |  |  |  |
| 1 | Ath-AT4G30620.1 |  |  |  | Vvi-Vitvi11g00713\_t001 |  |  |  |  |  |  |
| 1 | Ath-AT4G30630.1 |  |  |  | Vvi-Vitvi11g01505\_t001 |  |  |  |  |  |  |
| 1 | Ath-AT4G30640.1 |  |  |  | | | |  |  |  |  |  |  |
| 1 | Ath-AT4G30650.1 |  |  |  | Vvi-Vitvi11g00718\_t001 |  |  |  |  |  |  |
| 1 | Ath-AT4G30660.1 |  |  |  | | | |  |  |  |  |  |  |
| 1 | Ath-AT4G30662.1 |  |  |  | | | |  |  |  |  |  |  |
| 1 | Ath-AT4G30670.1 |  |  |  | | | |  |  |  |  |  |  |
| 1 | Ath-AT4G30680.1 |  |  |  | Vvi-Vitvi11g00723\_t001 |  |  |  |  |  |  |
| 1 | Ath-AT4G30690.1 |  |  |  | Vvi-Vitvi11g00725\_t001 |  |  |  |  |  |  |
| 1 | Ath-AT4G30700.1 |  |  |  | Vvi-Vitvi11g00727\_t001 |  |  |  |  |  |  |
| 1 | Ath-AT4G30710.1 |  |  |  | Vvi-Vitvi11g00728\_t002 |  |  |  |  |  |  |
| 1 | Ath-AT4G30720.1 |  |  |  | | | |  |  |  |  |  |  |
| 1 | Ath-AT4G30730.2 |  |  |  | | | |  |  |  |  |  |  |
| 1 | Ath-AT4G30740.1 |  |  |  | | | |  |  |  |  |  |  |
| 1 | Ath-AT4G30750.1 |  |  |  | | | |  |  |  |  |  |  |
| 1 | Ath-AT4G30760.1 |  |  |  | | | |  |  |  |  |  |  |
| 1 | Ath-AT4G30770.1 |  |  |  | | | |  |  |  |  |  |  |
| 1 | Ath-AT4G30780.1 |  |  |  | | | |  |  |  |  |  |  |
| 2 | Ath-AT4G30790.1 |  | Vvi-Vitvi11g00767\_t001 |  | | | |  |  |  |  |  |  |
| 2 | Ath-AT4G30800.1 |  | | | |  | | | |  |  |  |  |  |  |
| 2 | Ath-AT4G30810.1 |  | Vvi-Vitvi11g00778\_t001 |  | | | |  |  |  |  |  |  |
| 2 | Ath-AT4G30820.16 |  | Vvi-Vitvi11g01514\_t001 |  | | | |  |  |  |  |  |  |
| 2 | Ath-AT4G30825.1 |  | | | |  | | | |  |  |  |  |  |  |
| 2 | Ath-AT4G30830.1 |  | | | |  | Vvi-Vitvi11g00745\_t001 |  |  |  |  |  |  |
| 1 | Ath-AT4G30840.1 |  | Vvi-Vitvi11g00787\_t001 |  |  |  |  |  |  |  |
| 1 | Ath-AT4G30845.1 |  | Vvi-Vitvi11g00788\_t001 |  |  |  |  |  |  |  |
| 1 | Ath-AT4G30850.2 |  | Vvi-Vitvi11g00796\_t001 |  |  |  |  |  |  |  |
| 1 | Ath-AT4G30860.1 |  | Vvi-Vitvi11g00800\_t001 |  |  |  |  |  |  |  |
| 0 | Ath-AT4G30870.1 |  |  |  |  |  |  |  |  |
| 0 | Ath-AT4G30880.1 |  |  |  |  |  |  |  |  |
| 0 | Ath-AT4G30890.2 |  |  |  |  |  |  |  |  |
| 1 | Ath-AT4G30900.2 |  | Vvi-Vitvi11g00699\_t001 |  |  |  |  |  |  |  |
| 1 | Ath-AT4G30910.2 |  | Vvi-Vitvi11g00698\_t001 |  |  |  |  |  |  |  |
| 1 | Ath-AT4G30920.1 |  | | | |  |  |  |  |  |  |  |
| 1 | Ath-AT4G30930.1 |  | | | |  |  |  |  |  |  |  |
| 1 | Ath-AT4G30935.1 |  | Vvi-Vitvi11g00694\_t002 |  |  |  |  |  |  |  |
| 1 | Ath-AT4G30940.1 |  | | | |  |  |  |  |  |  |  |
| 1 | Ath-AT4G30950.1 |  | | | |  |  |  |  |  |  |  |
| 1 | Ath-AT4G30960.1 |  | | | |  |  |  |  |  |  |  |
| 1 | Ath-AT4G30970.1 |  | | | |  |  |  |  |  |  |  |
| 1 | Ath-AT4G30980.2 |  | Vvi-Vitvi11g00680\_t001 |  |  |  |  |  |  |  |
| 1 | Ath-AT4G30990.3 |  | | | |  |  |  |  |  |  |  |
| 1 | Ath-AT4G30993.2 |  | Vvi-Vitvi11g00661\_t003 |  |  |  |  |  |  |  |
| 1 | Ath-AT4G30996.1 |  | Vvi-Vitvi11g00655\_t002 |  |  |  |  |  |  |  |
| 1 | Ath-AT4G31000.1 |  | Vvi-Vitvi11g00652\_t002 |  |  |  |  |  |  |  |
| 1 | Ath-AT4G31010.2 |  | | | |  |  |  |  |  |  |  |
| 1 | Ath-AT4G31020.2 |  | Vvi-Vitvi11g04161\_t001 |  |  |  |  |  |  |  |
| 0 | Ath-AT4G31030.1 |  |  |  |  |  |  |  |  |
| 0 | Ath-AT4G31040.1 |  |  |  |  |  |  |  |  |
| 0 | Ath-AT4G31050.1 |  |  |  |  |  |  |  |  |
| 0 | Ath-AT4G31060.1 |  |  |  |  |  |  |  |  |
| 0 | Ath-AT4G31070.2 |  |  |  |  |  |  |  |  |
| 0 | Ath-AT4G31073.1 |  |  |  |  |  |  |  |  |
| 0 | Ath-AT4G31080.2 |  |  |  |  |  |  |  |  |
| 0 | Ath-AT4G31100.1 |  |  |  |  |  |  |  |  |
| 0 | Ath-AT4G31110.1 |  |  |  |  |  |  |  |  |
| 1 | Ath-AT4G31115.2 |  | Vvi-Vitvi04g00277\_t001.1.6037826d |  |  |  |  |  |  |  |
| 1 | Ath-AT4G31120.1 |  | Vvi-Vitvi04g00275\_t001 |  |  |  |  |  |  |  |
| 1 | Ath-AT4G31130.1 |  | Vvi-Vitvi04g00270\_t001 |  |  |  |  |  |  |  |
| 1 | Ath-AT4G31140.1 |  | | | |  |  |  |  |  |  |  |
| 1 | Ath-AT4G31150.4 |  | Vvi-Vitvi04g00269\_t001 |  |  |  |  |  |  |  |
| 1 | Ath-AT4G31160.1 |  | Vvi-Vitvi04g00268\_t001 |  |  |  |  |  |  |  |
| 1 | Ath-AT4G31170.3 |  | Vvi-Vitvi04g00266\_t001 |  |  |  |  |  |  |  |
| 1 | Ath-AT4G31180.1 |  | Vvi-Vitvi04g00265\_t002 |  |  |  |  |  |  |  |
| 1 | Ath-AT4G31196.1 |  | | | |  |  |  |  |  |  |  |
| 1 | Ath-AT4G31200.2 |  | | | |  |  |  |  |  |  |  |
| 1 | Ath-AT4G31210.1 |  | Vvi-Vitvi04g00261\_t001 |  |  |  |  |  |  |  |
| 1 | Ath-AT4G31230.1 |  | Vvi-Vitvi04g00257\_t001 |  |  |  |  |  |  |  |
| 1 | Ath-AT4G31240.2 |  | Vvi-Vitvi04g00256\_t002 |  |  |  |  |  |  |  |
| 1 | Ath-AT4G31250.1 |  | Vvi-Vitvi04g00255\_t001 |  |  |  |  |  |  |  |
| 1 | Ath-AT4G31260.1 |  | | | |  |  |  |  |  |  |  |
| 1 | Ath-AT4G31270.1 |  | Vvi-Vitvi04g00253\_t003 |  |  |  |  |  |  |  |
| 1 | Ath-AT4G31290.1 |  | Vvi-Vitvi04g00252\_t001 |  |  |  |  |  |  |  |
| 1 | Ath-AT4G31280.1 |  | | | |  |  |  |  |  |  |  |
| 1 | Ath-AT4G31300.2 |  | Vvi-Vitvi04g00251\_t001 |  |  |  |  |  |  |  |
| 1 | Ath-AT4G31310.2 |  | Vvi-Vitvi04g00250\_t001 |  |  |  |  |  |  |  |
| 1 | Ath-AT4G31320.1 |  | Vvi-Vitvi04g01831\_t001 |  |  |  |  |  |  |  |
| 1 | Ath-AT4G31330.1 |  | Vvi-Vitvi04g00241\_t001 |  |  |  |  |  |  |  |
| 1 | Ath-AT4G31340.1 |  | Vvi-Vitvi04g00237\_t001 |  |  |  |  |  |  |  |
| 1 | Ath-AT4G31350.2 |  | Vvi-Vitvi04g00228\_t001 |  |  |  |  |  |  |  |
| 1 | Ath-AT4G31351.1 |  | | | |  |  |  |  |  |  |  |
| 1 | Ath-AT4G31354.1 |  | | | |  |  |  |  |  |  |  |
| 1 | Ath-AT4G31355.1 |  | | | |  |  |  |  |  |  |  |
| 1 | Ath-AT4G31360.1 |  | Vvi-Vitvi04g01825\_t001 |  |  |  |  |  |  |  |
| 1 | Ath-AT4G31370.1 |  | | | |  |  |  |  |  |  |  |
| 1 | Ath-AT4G31380.1 |  | | | |  |  |  |  |  |  |  |
| 1 | Ath-AT4G31390.1 |  | Vvi-Vitvi04g00221\_t001 |  |  |  |  |  |  |  |
| 1 | Ath-AT4G31400.1 |  | | | |  |  |  |  |  |  |  |
| 1 | Ath-AT4G31405.1 |  | | | |  |  |  |  |  |  |  |
| 1 | Ath-AT4G31410.1 |  | Vvi-Vitvi04g00220\_t001 |  |  |  |  |  |  |  |
| 1 | Ath-AT4G31420.2 |  | Vvi-Vitvi04g00219\_t001 |  |  |  |  |  |  |  |
| 1 | Ath-AT4G31430.2 |  | Vvi-Vitvi04g00212\_t001 |  |  |  |  |  |  |  |
| 1 | Ath-AT4G31440.1 |  | Vvi-Vitvi04g00206\_t001 |  |  |  |  |  |  |  |
| 1 | Ath-AT4G31441.1 |  | | | |  |  |  |  |  |  |  |
| 1 | Ath-AT4G31450.2 |  | Vvi-Vitvi04g00205\_t002 |  |  |  |  |  |  |  |
| 1 | Ath-AT4G31460.1 |  | | | |  |  |  |  |  |  |  |
| 1 | Ath-AT4G31470.1 |  | | | |  |  |  |  |  |  |  |
| 1 | Ath-AT4G31480.6 |  | | | |  |  |  |  |  |  |  |
| 1 | Ath-AT4G31490.2 |  | | | |  |  |  |  |  |  |  |
| 1 | Ath-AT4G31500.1 |  | | | |  |  |  |  |  |  |  |
| 1 | Ath-AT4G31510.1 |  | Vvi-Vitvi04g01819\_t001 |  |  |  |  |  |  |  |
| 0 | Ath-AT4G31520.1 |  |  |  |  |  |  |  |  |
| 0 | Ath-AT4G31530.2 |  |  |  |  |  |  |  |  |
| 0 | Ath-AT4G31540.1 |  |  |  |  |  |  |  |  |
| 1 | Ath-AT4G31550.1 |  | Vvi-Vitvi04g00756\_t001 |  |  |  |  |  |  |  |
| 1 | Ath-AT4G31560.1 |  | | | |  |  |  |  |  |  |  |
| 1 | Ath-AT4G31570.1 |  | Vvi-Vitvi04g00748\_t002 |  |  |  |  |  |  |  |
| 1 | Ath-AT4G31580.1 |  | Vvi-Vitvi04g04188\_t003 |  |  |  |  |  |  |  |
| 1 | Ath-AT4G31590.1 |  | Vvi-Vitvi04g00735\_t003 |  |  |  |  |  |  |  |
| 2 | Ath-AT4G31600.1 |  | Vvi-Vitvi04g00734\_t001 |  | Vvi-Vitvi04g00494\_t001 |  |  |  |  |  |  |
| 2 | Ath-AT4G31610.2 |  | | | |  | | | |  |  |  |  |  |  |
| 2 | Ath-AT4G31615.1 |  | | | |  | | | |  |  |  |  |  |  |
| 2 | Ath-AT4G31620.1 |  | | | |  | | | |  |  |  |  |  |  |
| 2 | Ath-AT4G31630.1 |  | | | |  | | | |  |  |  |  |  |  |
| 2 | Ath-AT4G31640.1 |  | | | |  | | | |  |  |  |  |  |  |
| 2 | Ath-AT4G31650.2 |  | | | |  | | | |  |  |  |  |  |  |
| 2 | Ath-AT4G31660.1 |  | | | |  | | | |  |  |  |  |  |  |
| 2 | Ath-AT4G31670.1 |  | Vvi-Vitvi04g00732\_t001 |  | | | |  |  |  |  |  |  |
| 1 | Ath-AT4G31680.1 |  |  |  | | | |  |  |  |  |  |  |
| 1 | Ath-AT4G31685.1 |  |  |  | | | |  |  |  |  |  |  |
| 1 | Ath-AT4G31690.1 |  |  |  | | | |  |  |  |  |  |  |
| 1 | Ath-AT4G31700.1 |  |  |  | | | |  |  |  |  |  |  |
| 1 | Ath-AT4G31710.2 |  |  |  | | | |  |  |  |  |  |  |
| 1 | Ath-AT4G31715.1 |  |  |  | | | |  |  |  |  |  |  |
| 1 | Ath-AT4G31720.3 |  |  |  | | | |  |  |  |  |  |  |
| 1 | Ath-AT4G31730.1 |  |  |  | | | |  |  |  |  |  |  |
| 1 | Ath-AT4G31750.1 |  |  |  | | | |  |  |  |  |  |  |
| 1 | Ath-AT4G31740.1 |  |  |  | | | |  |  |  |  |  |  |
| 1 | Ath-AT4G31760.1 |  |  |  | | | |  |  |  |  |  |  |
| 1 | Ath-AT4G31770.1 |  |  |  | | | |  |  |  |  |  |  |
| 1 | Ath-AT4G31780.3 |  |  |  | | | |  |  |  |  |  |  |
| 1 | Ath-AT4G31790.1 |  |  |  | | | |  |  |  |  |  |  |
| 1 | Ath-AT4G31800.1 |  |  |  | Vvi-Vitvi04g00510\_t001 |  |  |  |  |  |  |
| 2 | Ath-AT4G31805.1 |  | Vvi-Vitvi04g00590\_t001 |  | | | |  |  |  |  |  |  |
| 2 | Ath-AT4G31810.1 |  | Vvi-Vitvi04g00597\_t001 |  | | | |  |  |  |  |  |  |
| 2 | Ath-AT4G31820.1 |  | Vvi-Vitvi04g00599\_t001 |  | | | |  |  |  |  |  |  |
| 2 | Ath-AT4G31830.1 |  | Vvi-Vitvi04g00601\_t001 |  | | | |  |  |  |  |  |  |
| 2 | Ath-AT4G31840.1 |  | Vvi-Vitvi04g00602\_t001 |  | | | |  |  |  |  |  |  |
| 2 | Ath-AT4G31850.1 |  | Vvi-Vitvi04g00605\_t001 |  | | | |  |  |  |  |  |  |
| 2 | Ath-AT4G31860.1 |  | Vvi-Vitvi04g00606\_t001 |  | | | |  |  |  |  |  |  |
| 2 | Ath-AT4G31870.1 |  | Vvi-Vitvi04g00610\_t001 |  | | | |  |  |  |  |  |  |
| 1 | Ath-AT4G31875.1 |  |  |  | | | |  |  |  |  |  |  |
| 1 | Ath-AT4G31880.1 |  |  |  | Vvi-Vitvi04g00514\_t001 |  |  |  |  |  |  |
| 1 | Ath-AT4G31890.2 |  |  |  | Vvi-Vitvi04g00517\_t001 |  |  |  |  |  |  |
| 1 | Ath-AT4G31900.1 |  |  |  | Vvi-Vitvi04g00523\_t001 |  |  |  |  |  |  |
| 1 | Ath-AT4G31910.1 |  |  |  | Vvi-Vitvi04g00524\_t001 |  |  |  |  |  |  |
| 1 | Ath-AT4G31920.1 |  |  |  | Vvi-Vitvi04g00525\_t001 |  |  |  |  |  |  |
| 1 | Ath-AT4G31930.1 |  |  |  | Vvi-Vitvi04g00528\_t002 |  |  |  |  |  |  |
| 1 | Ath-AT4G31940.1 |  |  |  | | | |  |  |  |  |  |  |
| 1 | Ath-AT4G31950.1 |  |  |  | | | |  |  |  |  |  |  |
| 1 | Ath-AT4G31960.1 |  |  |  | | | |  |  |  |  |  |  |
| 1 | Ath-AT4G31970.1 |  |  |  | | | |  |  |  |  |  |  |
| 1 | Ath-AT4G31980.1 |  |  |  | Vvi-Vitvi04g00530\_t002 |  |  |  |  |  |  |
| 1 | Ath-AT4G31985.1 |  |  |  | Vvi-Vitvi04g00536\_t001 |  |  |  |  |  |  |
| 1 | Ath-AT4G31990.3 |  |  |  | Vvi-Vitvi04g00537\_t001 |  |  |  |  |  |  |
| 1 | Ath-AT4G32000.3 |  |  |  | Vvi-Vitvi04g00538\_t001 |  |  |  |  |  |  |
| 1 | Ath-AT4G32010.1 |  |  |  | | | |  |  |  |  |  |  |
| 1 | Ath-AT4G32020.1 |  |  |  | | | |  |  |  |  |  |  |
| 1 | Ath-AT4G32030.1 |  |  |  | Vvi-Vitvi04g00540\_t001 |  |  |  |  |  |  |
| 1 | Ath-AT4G32040.1 |  |  |  | Vvi-Vitvi04g00546\_t001 |  |  |  |  |  |  |
| 1 | Ath-AT4G32050.1 |  |  |  | Vvi-Vitvi04g00548\_t002 |  |  |  |  |  |  |
| 1 | Ath-AT4G32060.1 |  |  |  | Vvi-Vitvi04g00559\_t001 |  |  |  |  |  |  |
| 1 | Ath-AT4G32070.1 |  |  |  | Vvi-Vitvi04g00565\_t001 |  |  |  |  |  |  |
| 1 | Ath-AT4G32080.3 |  |  |  | | | |  |  |  |  |  |  |
| 1 | Ath-AT4G32090.1 |  |  |  | Vvi-Vitvi04g04139\_t001 |  |  |  |  |  |  |
| 1 | Ath-AT4G32100.1 |  |  |  | | | |  |  |  |  |  |  |
| 1 | Ath-AT4G32105.1 |  |  |  | | | |  |  |  |  |  |  |
| 1 | Ath-AT4G32110.1 |  |  |  | | | |  |  |  |  |  |  |
| 1 | Ath-AT4G32120.1 |  |  |  | Vvi-Vitvi04g00581\_t001 |  |  |  |  |  |  |
| 1 | Ath-AT4G32130.1 |  |  |  | Vvi-Vitvi04g00582\_t001 |  |  |  |  |  |  |
| 1 | Ath-AT4G32140.1 |  |  |  | Vvi-Vitvi04g00583\_t001 |  |  |  |  |  |  |
| 2 | Ath-AT4G32150.1 |  | Vvi-Vitvi04g00503\_t001 |  | Vvi-Vitvi11g04113\_t001 |  |  |  |  |  |  |
| 3 | Ath-AT4G32160.1 |  | Vvi-Vitvi04g00500\_t001 |  | | | |  | Vvi-Vitvi09g00644\_t001 |  |  |  |  |  |
| 3 | Ath-AT4G32170.1 |  | | | |  | | | |  | | | |  |  |  |  |  |
| 3 | Ath-AT4G32175.1 |  | | | |  | | | |  | | | |  |  |  |  |  |
| 3 | Ath-AT4G32180.1 |  | | | |  | | | |  | | | |  |  |  |  |  |
| 3 | Ath-AT4G32190.1 |  | Vvi-Vitvi04g00498\_t001 |  | | | |  | | | |  |  |  |  |  |
| 3 | Ath-AT4G32200.1 |  | | | |  | | | |  | | | |  |  |  |  |  |
| 3 | Ath-AT4G32210.1 |  | | | |  | | | |  | | | |  |  |  |  |  |
| 3 | Ath-AT4G32208.1 |  | | | |  | | | |  | | | |  |  |  |  |  |
| 3 | Ath-AT4G32230.1 |  | | | |  | | | |  | | | |  |  |  |  |  |
| 3 | Ath-AT4G32240.1 |  | | | |  | | | |  | | | |  |  |  |  |  |
| 3 | Ath-AT4G32250.3 |  | Vvi-Vitvi04g00497\_t001 |  | | | |  | Vvi-Vitvi09g00627\_t001 |  |  |  |  |  |
| 3 | Ath-AT4G32260.1 |  | Vvi-Vitvi04g00496\_t001 |  | | | |  | | | |  |  |  |  |  |
| 3 | Ath-AT4G32270.1 |  | Vvi-Vitvi04g00495\_t001 |  | Vvi-Vitvi11g01447\_t001 |  | Vvi-Vitvi09g00625\_t001 |  |  |  |  |  |
| 3 | Ath-AT4G32272.2 |  | Vvi-Vitvi04g00494\_t001 |  | Vvi-Vitvi11g00505\_t001 |  | | | |  |  |  |  |  |
| 3 | Ath-AT4G32280.1 |  | Vvi-Vitvi04g00493\_t001 |  | Vvi-Vitvi11g00497\_t001 |  | | | |  |  |  |  |  |
| 3 | Ath-AT4G32285.2 |  | Vvi-Vitvi04g00490\_t001 |  | | | |  | | | |  |  |  |  |  |
| 3 | Ath-AT4G32290.1 |  | Vvi-Vitvi04g00489\_t001 |  | | | |  | | | |  |  |  |  |  |
| 3 | Ath-AT4G32295.1 |  | Vvi-Vitvi04g00487\_t001 |  | Vvi-Vitvi11g00484\_t001 |  | | | |  |  |  |  |  |
| 4 | Ath-AT4G32300.1 |  | Vvi-Vitvi04g00485\_t001 |  | | | |  | Vvi-Vitvi09g00604\_t001 |  | Vvi-Vitvi11g00427\_t001 |  |  |  |  |
| 4 | Ath-AT4G32320.1 |  | Vvi-Vitvi04g00484\_t001 |  | | | |  | | | |  | | | |  |  |  |  |
| 4 | Ath-AT4G32330.3 |  | Vvi-Vitvi04g00483\_t002 |  | Vvi-Vitvi11g00480\_t001 |  | | | |  | | | |  |  |  |  |
| 4 | Ath-AT4G32340.1 |  | | | |  | Vvi-Vitvi11g00477\_t001 |  | Vvi-Vitvi09g00601\_t001 |  | | | |  |  |  |  |
| 4 | Ath-AT4G32342.2 |  | Vvi-Vitvi04g00481\_t003 |  | Vvi-Vitvi11g00474\_t001 |  | Vvi-Vitvi09g04185\_t001 |  | | | |  |  |  |  |
| 4 | Ath-AT4G32350.1 |  | | | |  | | | |  | | | |  | | | |  |  |  |  |
| 4 | Ath-AT4G32360.1 |  | | | |  | | | |  | | | |  | | | |  |  |  |  |
| 4 | Ath-AT4G32370.1 |  | | | |  | | | |  | | | |  | | | |  |  |  |  |
| 4 | Ath-AT4G32375.1 |  | | | |  | | | |  | | | |  | | | |  |  |  |  |
| 4 | Ath-AT4G32380.1 |  | | | |  | | | |  | Vvi-Vitvi09g00579\_t001 |  | | | |  |  |  |  |
| 3 | Ath-AT4G32390.1 |  | Vvi-Vitvi04g00471\_t001 |  | Vvi-Vitvi11g01435\_t001 |  |  |  | | | |  |  |  |  |
| 3 | Ath-AT4G32400.1 |  | Vvi-Vitvi04g00466\_t001 |  | | | |  |  |  | | | |  |  |  |  |
| 3 | Ath-AT4G32410.1 |  | Vvi-Vitvi04g00465\_t001 |  | | | |  |  |  | | | |  |  |  |  |
| 3 | Ath-AT4G32420.1 |  | | | |  | | | |  |  |  | | | |  |  |  |  |
| 3 | Ath-AT4G32430.1 |  | | | |  | Vvi-Vitvi11g00453\_t001 |  |  |  | | | |  |  |  |  |
| 2 | Ath-AT4G32440.2 |  | | | |  |  |  |  |  | Vvi-Vitvi11g00425\_t003 |  |  |  |  |
| 2 | Ath-AT4G32450.1 |  | Vvi-Vitvi04g00447\_t001 |  |  |  |  |  | | | |  |  |  |  |
| 2 | Ath-AT4G32460.2 |  | Vvi-Vitvi04g00442\_t001 |  |  |  |  |  | | | |  |  |  |  |
| 2 | Ath-AT4G32470.1 |  | Vvi-Vitvi04g00441\_t001 |  |  |  |  |  | | | |  |  |  |  |
| 2 | Ath-AT4G32480.1 |  | | | |  |  |  |  |  | | | |  |  |  |  |
| 2 | Ath-AT4G32490.1 |  | | | |  |  |  |  |  | | | |  |  |  |  |
| 3 | Ath-AT4G32500.1 |  | Vvi-Vitvi04g00440\_t001 |  | Vvi-Vitvi04g00391\_t001 |  |  |  | Vvi-Vitvi11g00420\_t001 |  |  |  |  |
| 3 | Ath-AT4G32510.2 |  | Vvi-Vitvi04g00439\_t001 |  | | | |  |  |  | Vvi-Vitvi11g00419\_t001 |  |  |  |  |
| 3 | Ath-AT4G32520.1 |  | | | |  | | | |  |  |  | | | |  |  |  |  |
| 3 | Ath-AT4G32530.2 |  | Vvi-Vitvi04g00416\_t001 |  | | | |  |  |  | | | |  |  |  |  |
| 3 | Ath-AT4G32535.1 |  | | | |  | | | |  |  |  | | | |  |  |  |  |
| 3 | Ath-AT4G32540.2 |  | | | |  | | | |  |  |  | | | |  |  |  |  |
| 3 | Ath-AT4G32551.2 |  | | | |  | | | |  |  |  | Vvi-Vitvi11g00399\_t001 |  |  |  |  |
| 3 | Ath-AT4G32560.2 |  | | | |  | | | |  |  |  | | | |  |  |  |  |
| 3 | Ath-AT4G32570.1 |  | | | |  | | | |  |  |  | | | |  |  |  |  |
| 3 | Ath-AT4G32580.1 |  | | | |  | | | |  |  |  | | | |  |  |  |  |
| 4 | Ath-AT4G32590.3 |  | | | |  | | | |  | Vvi-Vitvi04g00366\_t001 |  | | | |  |  |  |  |
| 4 | Ath-AT4G32600.1 |  | | | |  | | | |  | Vvi-Vitvi04g00372\_t001 |  | Vvi-Vitvi11g00390\_t001 |  |  |  |  |
| 4 | Ath-AT4G32605.1 |  | | | |  | | | |  | | | |  | | | |  |  |  |  |
| 4 | Ath-AT4G32610.1 |  | | | |  | Vvi-Vitvi04g01874\_t001 |  | Vvi-Vitvi04g01874\_t001 |  | Vvi-Vitvi11g00385\_t001 |  |  |  |  |
| 4 | Ath-AT4G32620.2 |  | | | |  | | | |  | Vvi-Vitvi04g00380\_t001 |  | | | |  |  |  |  |
| 4 | Ath-AT4G32630.2 |  | | | |  | | | |  | Vvi-Vitvi04g00384\_t001 |  | Vvi-Vitvi11g00363\_t001 |  |  |  |  |
| 3 | Ath-AT4G32640.2 |  | | | |  | | | |  | | | |  |  |  |  |  |
| 3 | Ath-AT4G32650.1 |  | | | |  | | | |  | Vvi-Vitvi04g00391\_t001 |  |  |  |  |  |
| 3 | Ath-AT4G32660.1 |  | | | |  | Vvi-Vitvi04g04090\_t002 |  | | | |  |  |  |  |  |
| 3 | Ath-AT4G32670.1 |  | | | |  | | | |  | | | |  |  |  |  |  |
| 3 | Ath-AT4G32680.1 |  | Vvi-Vitvi04g01880\_t001 |  | | | |  | Vvi-Vitvi04g01880\_t001 |  |  |  |  |  |
| 1 | Ath-AT4G32690.1 |  |  |  | Vvi-Vitvi04g04089\_t001 |  |  |  |  |  |  |
| 1 | Ath-AT4G32700.2 |  |  |  | | | |  |  |  |  |  |  |
| 1 | Ath-AT4G32710.1 |  |  |  | | | |  |  |  |  |  |  |
| 1 | Ath-AT4G32714.1 |  |  |  | | | |  |  |  |  |  |  |
| 1 | Ath-AT4G32717.1 |  |  |  | | | |  |  |  |  |  |  |
| 1 | Ath-AT4G32720.1 |  |  |  | Vvi-Vitvi04g00346\_t001 |  |  |  |  |  |  |
| 1 | Ath-AT4G32730.2 |  |  |  | Vvi-Vitvi04g01855\_t001 |  |  |  |  |  |  |
| 1 | Ath-AT4G32750.1 |  |  |  | Vvi-Vitvi04g00326\_t001 |  |  |  |  |  |  |
| 1 | Ath-AT4G32760.2 |  |  |  | Vvi-Vitvi04g00324\_t001 |  |  |  |  |  |  |
| 1 | Ath-AT4G32770.1 |  |  |  | Vvi-Vitvi04g00313\_t001 |  |  |  |  |  |  |
| 1 | Ath-AT4G32780.2 |  |  |  | Vvi-Vitvi04g00308\_t001 |  |  |  |  |  |  |
| 1 | Ath-AT4G32790.1 |  |  |  | Vvi-Vitvi04g01841\_t001 |  |  |  |  |  |  |
| 1 | Ath-AT4G32800.1 |  |  |  | Vvi-Vitvi04g00300\_t001 |  |  |  |  |  |  |
| 1 | Ath-AT4G32810.2 |  |  |  | Vvi-Vitvi04g00298\_t001 |  |  |  |  |  |  |
| 1 | Ath-AT4G32820.2 |  |  |  | | | |  |  |  |  |  |  |
| 1 | Ath-AT4G32830.1 |  |  |  | | | |  |  |  |  |  |  |
| 2 | Ath-AT4G32840.1 |  | Vvi-Vitvi04g00040\_t003 |  | | | |  |  |  |  |  |  |
| 2 | Ath-AT4G32850.8 |  | | | |  | | | |  |  |  |  |  |  |
| 2 | Ath-AT4G32860.1 |  | | | |  | | | |  |  |  |  |  |  |
| 2 | Ath-AT4G32870.1 |  | | | |  | | | |  |  |  |  |  |  |
| 2 | Ath-AT4G32880.1 |  | | | |  | | | |  |  |  |  |  |  |
| 2 | Ath-AT4G32890.1 |  | | | |  | | | |  |  |  |  |  |  |
| 2 | Ath-AT4G32900.2 |  | | | |  | Vvi-Vitvi04g00290\_t001 |  |  |  |  |  |  |
| 1 | Ath-AT4G32910.1 |  | | | |  |  |  |  |  |  |  |
| 1 | Ath-AT4G32915.1 |  | Vvi-Vitvi04g01777\_t001 |  |  |  |  |  |  |  |
| 1 | Ath-AT4G32920.2 |  | Vvi-Vitvi04g00045\_t001 |  |  |  |  |  |  |  |
| 1 | Ath-AT4G32930.2 |  | Vvi-Vitvi04g00055\_t001 |  |  |  |  |  |  |  |
| 1 | Ath-AT4G32940.1 |  | Vvi-Vitvi04g04024\_t001 |  |  |  |  |  |  |  |
| 1 | Ath-AT4G32950.1 |  | Vvi-Vitvi04g00060\_t001 |  |  |  |  |  |  |  |
| 1 | Ath-AT4G32960.1 |  | Vvi-Vitvi04g04025\_t001 |  |  |  |  |  |  |  |
| 1 | Ath-AT4G32970.1 |  | | | |  |  |  |  |  |  |  |
| 1 | Ath-AT4G32980.1 |  | Vvi-Vitvi04g00072\_t001 |  |  |  |  |  |  |  |
| 1 | Ath-AT4G32990.1 |  | | | |  |  |  |  |  |  |  |
| 1 | Ath-AT4G33000.1 |  | Vvi-Vitvi04g00080\_t001 |  |  |  |  |  |  |  |
| 1 | Ath-AT4G33010.1 |  | | | |  |  |  |  |  |  |  |
| 1 | Ath-AT4G33020.2 |  | | | |  |  |  |  |  |  |  |
| 1 | Ath-AT4G33030.1 |  | Vvi-Vitvi04g04036\_t001 |  |  |  |  |  |  |  |
| 1 | Ath-AT4G33040.1 |  | | | |  |  |  |  |  |  |  |
| 1 | Ath-AT4G33050.5 |  | | | |  |  |  |  |  |  |  |
| 1 | Ath-AT4G33060.1 |  | Vvi-Vitvi04g00119\_t001 |  |  |  |  |  |  |  |
| 1 | Ath-AT4G33070.1 |  | | | |  |  |  |  |  |  |  |
| 1 | Ath-AT4G33080.1 |  | Vvi-Vitvi04g00124\_t001 |  |  |  |  |  |  |  |
| 1 | Ath-AT4G33090.1 |  | Vvi-Vitvi04g00128\_t001 |  |  |  |  |  |  |  |
| 1 | Ath-AT4G33100.1 |  | Vvi-Vitvi04g00129\_t001 |  |  |  |  |  |  |  |
| 1 | Ath-AT4G33110.1 |  | Vvi-Vitvi04g00130\_t001 |  |  |  |  |  |  |  |
| 0 | Ath-AT4G33120.1 |  |  |  |  |  |  |  |  |
| 0 | Ath-AT4G33130.3 |  |  |  |  |  |  |  |  |
| 1 | Ath-AT4G33140.1 |  | Vvi-Vitvi11g00011\_t001 |  |  |  |  |  |  |  |
| 1 | Ath-AT4G33145.1 |  | | | |  |  |  |  |  |  |  |
| 1 | Ath-AT4G33150.2 |  | Vvi-Vitvi11g00012\_t001 |  |  |  |  |  |  |  |
| 1 | Ath-AT4G33160.2 |  | | | |  |  |  |  |  |  |  |
| 1 | Ath-AT4G33170.1 |  | | | |  |  |  |  |  |  |  |
| 1 | Ath-AT4G33180.1 |  | | | |  |  |  |  |  |  |  |
| 1 | Ath-AT4G33200.4 |  | | | |  |  |  |  |  |  |  |
| 1 | Ath-AT4G33210.1 |  | | | |  |  |  |  |  |  |  |
| 1 | Ath-AT4G33220.1 |  | Vvi-Vitvi11g00014\_t001 |  |  |  |  |  |  |  |
| 1 | Ath-AT4G33230.1 |  | Vvi-Vitvi11g00015\_t001 |  |  |  |  |  |  |  |
| 1 | Ath-AT4G33240.1 |  | Vvi-Vitvi11g04003\_t001 |  |  |  |  |  |  |  |
| 1 | Ath-AT4G33250.1 |  | Vvi-Vitvi11g00027\_t001 |  |  |  |  |  |  |  |
| 0 | Ath-AT4G33260.1 |  |  |  |  |  |  |  |  |
| 0 | Ath-AT4G33270.1 |  |  |  |  |  |  |  |  |
| 0 | Ath-AT4G33280.3 |  |  |  |  |  |  |  |  |
| 0 | Ath-AT4G33290.1 |  |  |  |  |  |  |  |  |
| 0 | Ath-AT4G33300.2 |  |  |  |  |  |  |  |  |
| 0 | Ath-AT4G33310.1 |  |  |  |  |  |  |  |  |
| 0 | Ath-AT4G33320.1 |  |  |  |  |  |  |  |  |
| 0 | Ath-AT4G33330.2 |  |  |  |  |  |  |  |  |
| 0 | Ath-AT4G33350.1 |  |  |  |  |  |  |  |  |
| 0 | Ath-AT4G33355.1 |  |  |  |  |  |  |  |  |
| 0 | Ath-AT4G33360.1 |  |  |  |  |  |  |  |  |
| 0 | Ath-AT4G33370.1 |  |  |  |  |  |  |  |  |
| 0 | Ath-AT4G33380.1 |  |  |  |  |  |  |  |  |
| 0 | Ath-AT4G33390.1 |  |  |  |  |  |  |  |  |
| 0 | Ath-AT4G33400.1 |  |  |  |  |  |  |  |  |
| 0 | Ath-AT4G33410.1 |  |  |  |  |  |  |  |  |
| 0 | Ath-AT4G33420.2 |  |  |  |  |  |  |  |  |
| 0 | Ath-AT4G33430.2 |  |  |  |  |  |  |  |  |
| 0 | Ath-AT4G33440.1 |  |  |  |  |  |  |  |  |
| 0 | Ath-AT4G33450.1 |  |  |  |  |  |  |  |  |
| 0 | Ath-AT4G33460.1 |  |  |  |  |  |  |  |  |
| 0 | Ath-AT4G33465.2 |  |  |  |  |  |  |  |  |
| 0 | Ath-AT4G33467.1 |  |  |  |  |  |  |  |  |
| 0 | Ath-AT4G33470.1 |  |  |  |  |  |  |  |  |
| 0 | Ath-AT4G33480.1 |  |  |  |  |  |  |  |  |
| 0 | Ath-AT4G33490.2 |  |  |  |  |  |  |  |  |
| 0 | Ath-AT4G33495.1 |  |  |  |  |  |  |  |  |
| 0 | Ath-AT4G33500.1 |  |  |  |  |  |  |  |  |
| 0 | Ath-AT4G33510.1 |  |  |  |  |  |  |  |  |
| 0 | Ath-AT4G33520.2 |  |  |  |  |  |  |  |  |
| 0 | Ath-AT4G33530.1 |  |  |  |  |  |  |  |  |
| 0 | Ath-AT4G33540.1 |  |  |  |  |  |  |  |  |
| 0 | Ath-AT4G33550.1 |  |  |  |  |  |  |  |  |
| 0 | Ath-AT4G33560.1 |  |  |  |  |  |  |  |  |
| 0 | Ath-AT4G33565.1 |  |  |  |  |  |  |  |  |
| 0 | Ath-AT4G33580.2 |  |  |  |  |  |  |  |  |
| 0 | Ath-AT4G33585.1 |  |  |  |  |  |  |  |  |
| 0 | Ath-AT4G33590.1 |  |  |  |  |  |  |  |  |
| 0 | Ath-AT4G33600.1 |  |  |  |  |  |  |  |  |
| 0 | Ath-AT4G33610.1 |  |  |  |  |  |  |  |  |
| 0 | Ath-AT4G33620.1 |  |  |  |  |  |  |  |  |
| 0 | Ath-AT4G33625.1 |  |  |  |  |  |  |  |  |
| 0 | Ath-AT4G33630.2 |  |  |  |  |  |  |  |  |
| 0 | Ath-AT4G33640.2 |  |  |  |  |  |  |  |  |
| 0 | Ath-AT4G33650.2 |  |  |  |  |  |  |  |  |
| 0 | Ath-AT4G33660.1 |  |  |  |  |  |  |  |  |
| 0 | Ath-AT4G33666.1 |  |  |  |  |  |  |  |  |
| 0 | Ath-AT4G33670.1 |  |  |  |  |  |  |  |  |
| 0 | Ath-AT4G33680.1 |  |  |  |  |  |  |  |  |
| 0 | Ath-AT4G33690.1 |  |  |  |  |  |  |  |  |
| 0 | Ath-AT4G33700.1 |  |  |  |  |  |  |  |  |
| 0 | Ath-AT4G33710.1 |  |  |  |  |  |  |  |  |
| 0 | Ath-AT4G33720.1 |  |  |  |  |  |  |  |  |
| 0 | Ath-AT4G33730.1 |  |  |  |  |  |  |  |  |
| 0 | Ath-AT4G33735.1 |  |  |  |  |  |  |  |  |
| 0 | Ath-AT4G33740.1 |  |  |  |  |  |  |  |  |
| 0 | Ath-AT4G33760.1 |  |  |  |  |  |  |  |  |
| 0 | Ath-AT4G33770.1 |  |  |  |  |  |  |  |  |
| 0 | Ath-AT4G33780.1 |  |  |  |  |  |  |  |  |
| 0 | Ath-AT4G33790.1 |  |  |  |  |  |  |  |  |
| 1 | Ath-AT4G33800.1 |  | Vvi-Vitvi03g00677\_t001 |  |  |  |  |  |  |  |
| 1 | Ath-AT4G33810.2 |  | | | |  |  |  |  |  |  |  |
| 1 | Ath-AT4G33820.1 |  | Vvi-Vitvi03g00654\_t001 |  |  |  |  |  |  |  |
| 1 | Ath-AT4G33830.1 |  | | | |  |  |  |  |  |  |  |
| 1 | Ath-AT4G33840.1 |  | | | |  |  |  |  |  |  |  |
| 1 | Ath-AT4G33860.1 |  | | | |  |  |  |  |  |  |  |
| 1 | Ath-AT4G33865.1 |  | | | |  |  |  |  |  |  |  |
| 1 | Ath-AT4G33870.1 |  | | | |  |  |  |  |  |  |  |
| 1 | Ath-AT4G33880.1 |  | Vvi-Vitvi03g00635\_t001 |  |  |  |  |  |  |  |
| 1 | Ath-AT4G33885.1 |  | | | |  |  |  |  |  |  |  |
| 1 | Ath-AT4G33890.1 |  | Vvi-Vitvi03g00618\_t001 |  |  |  |  |  |  |  |
| 1 | Ath-AT4G33900.1 |  | | | |  |  |  |  |  |  |  |
| 1 | Ath-AT4G33905.1 |  | Vvi-Vitvi03g01605\_t002 |  |  |  |  |  |  |  |
| 1 | Ath-AT4G33910.1 |  | Vvi-Vitvi03g00609\_t001 |  |  |  |  |  |  |  |
| 1 | Ath-AT4G33920.1 |  | Vvi-Vitvi03g00605\_t001 |  |  |  |  |  |  |  |
| 1 | Ath-AT4G33925.1 |  | Vvi-Vitvi03g00602\_t001 |  |  |  |  |  |  |  |
| 0 | Ath-AT4G33930.1 |  |  |  |  |  |  |  |  |
| 0 | Ath-AT4G33940.1 |  |  |  |  |  |  |  |  |
| 0 | Ath-AT4G33945.1 |  |  |  |  |  |  |  |  |
| 0 | Ath-AT4G33950.1 |  |  |  |  |  |  |  |  |
| 0 | Ath-AT4G33960.1 |  |  |  |  |  |  |  |  |
| 0 | Ath-AT4G33970.1 |  |  |  |  |  |  |  |  |
| 1 | Ath-AT4G33980.2 |  | Vvi-Vitvi03g01573\_t001 |  |  |  |  |  |  |  |
| 1 | Ath-AT4G33985.1 |  | | | |  |  |  |  |  |  |  |
| 1 | Ath-AT4G33990.1 |  | Vvi-Vitvi03g00511\_t001 |  |  |  |  |  |  |  |
| 2 | Ath-AT4G34000.2 |  | Vvi-Vitvi03g01574\_t003 |  | Vvi-Vitvi18g00784\_t001 |  |  |  |  |  |  |
| 2 | Ath-AT4G34020.1 |  | Vvi-Vitvi03g00512\_t001 |  | | | |  |  |  |  |  |  |
| 2 | Ath-AT4G34030.1 |  | Vvi-Vitvi03g00513\_t001 |  | | | |  |  |  |  |  |  |
| 3 | Ath-AT4G34040.1 |  | Vvi-Vitvi03g00521\_t001 |  | Vvi-Vitvi18g00762\_t001 |  | Vvi-Vitvi07g02658\_t001 |  |  |  |  |  |
| 3 | Ath-AT4G34050.3 |  | Vvi-Vitvi03g00524\_t001 |  | | | |  | Vvi-Vitvi07g01723\_t001 |  |  |  |  |  |
| 3 | Ath-AT4G34060.3 |  | | | |  | | | |  | | | |  |  |  |  |  |
| 3 | Ath-AT4G34070.1 |  | | | |  | | | |  | | | |  |  |  |  |  |
| 3 | Ath-AT4G34080.1 |  | | | |  | | | |  | | | |  |  |  |  |  |
| 3 | Ath-AT4G34090.3 |  | Vvi-Vitvi03g00528\_t001 |  | | | |  | Vvi-Vitvi07g01710\_t001 |  |  |  |  |  |
| 3 | Ath-AT4G34100.1 |  | Vvi-Vitvi03g00530\_t002 |  | | | |  | Vvi-Vitvi07g01707\_t002 |  |  |  |  |  |
| 3 | Ath-AT4G34110.1 |  | Vvi-Vitvi03g00531\_t001 |  | Vvi-Vitvi18g00758\_t001 |  | Vvi-Vitvi07g01705\_t001 |  |  |  |  |  |
| 3 | Ath-AT4G34120.1 |  | Vvi-Vitvi03g00532\_t001 |  | | | |  | Vvi-Vitvi07g01703\_t001 |  |  |  |  |  |
| 3 | Ath-AT4G34131.1 |  | Vvi-Vitvi03g00533\_t001 |  | Vvi-Vitvi18g00753\_t001 |  | | | |  |  |  |  |  |
| 3 | Ath-AT4G34135.1 |  | | | |  | | | |  | | | |  |  |  |  |  |
| 3 | Ath-AT4G34138.1 |  | | | |  | | | |  | | | |  |  |  |  |  |
| 3 | Ath-AT4G34139.1 |  | | | |  | | | |  | | | |  |  |  |  |  |
| 3 | Ath-AT4G34140.1 |  | | | |  | | | |  | | | |  |  |  |  |  |
| 3 | Ath-AT4G34150.1 |  | Vvi-Vitvi03g00541\_t001 |  | | | |  | | | |  |  |  |  |  |
| 3 | Ath-AT4G34160.1 |  | Vvi-Vitvi03g00542\_t001 |  | Vvi-Vitvi18g00731\_t001 |  | Vvi-Vitvi07g01683\_t001 |  |  |  |  |  |
| 3 | Ath-AT4G34170.1 |  | | | |  | | | |  | | | |  |  |  |  |  |
| 3 | Ath-AT4G34180.1 |  | Vvi-Vitvi03g00545\_t001 |  | | | |  | | | |  |  |  |  |  |
| 3 | Ath-AT4G34190.1 |  | Vvi-Vitvi03g04189\_t001 |  | | | |  | | | |  |  |  |  |  |
| 3 | Ath-AT4G34200.1 |  | Vvi-Vitvi03g00551\_t001 |  | Vvi-Vitvi18g00729\_t001 |  | | | |  |  |  |  |  |
| 3 | Ath-AT4G34210.1 |  | | | |  | | | |  | | | |  |  |  |  |  |
| 3 | Ath-AT4G34215.2 |  | Vvi-Vitvi03g00552\_t001 |  | Vvi-Vitvi18g00727\_t002 |  | | | |  |  |  |  |  |
| 2 | Ath-AT4G34220.1 |  | Vvi-Vitvi03g00553\_t001 |  |  |  | Vvi-Vitvi07g01678\_t001 |  |  |  |  |  |
| 2 | Ath-AT4G34230.1 |  | Vvi-Vitvi03g00561\_t001 |  |  |  | Vvi-Vitvi07g01673\_t001 |  |  |  |  |  |
| 0 | Ath-AT4G34240.1 |  |  |  |  |  |  |  |  |
| 0 | Ath-AT4G34250.1 |  |  |  |  |  |  |  |  |
| 0 | Ath-AT4G34260.1 |  |  |  |  |  |  |  |  |
| 0 | Ath-AT4G34265.3 |  |  |  |  |  |  |  |  |
| 0 | Ath-AT4G34270.1 |  |  |  |  |  |  |  |  |
| 0 | Ath-AT4G34280.1 |  |  |  |  |  |  |  |  |
| 0 | Ath-AT4G34290.1 |  |  |  |  |  |  |  |  |
| 0 | Ath-AT4G34300.1 |  |  |  |  |  |  |  |  |
| 0 | Ath-AT4G34310.1 |  |  |  |  |  |  |  |  |
| 1 | Ath-AT4G34320.2 |  | Vvi-Vitvi03g00369\_t001 |  |  |  |  |  |  |  |
| 1 | Ath-AT4G34330.1 |  | | | |  |  |  |  |  |  |  |
| 1 | Ath-AT4G34340.1 |  | | | |  |  |  |  |  |  |  |
| 1 | Ath-AT4G34345.1 |  | | | |  |  |  |  |  |  |  |
| 1 | Ath-AT4G34350.1 |  | Vvi-Vitvi03g00374\_t001 |  |  |  |  |  |  |  |
| 1 | Ath-AT4G34360.1 |  | Vvi-Vitvi03g00376\_t001 |  |  |  |  |  |  |  |
| 1 | Ath-AT4G34370.1 |  | Vvi-Vitvi03g00377\_t001 |  |  |  |  |  |  |  |
| 1 | Ath-AT4G34380.1 |  | Vvi-Vitvi03g00397\_t001 |  |  |  |  |  |  |  |
| 1 | Ath-AT4G34390.1 |  | Vvi-Vitvi03g00398\_t001 |  |  |  |  |  |  |  |
| 0 | Ath-AT4G34400.1 |  |  |  |  |  |  |  |  |
| 0 | Ath-AT4G34410.1 |  |  |  |  |  |  |  |  |
| 0 | Ath-AT4G34412.1 |  |  |  |  |  |  |  |  |
| 0 | Ath-AT4G34419.1 |  |  |  |  |  |  |  |  |
| 0 | Ath-AT4G34420.1 |  |  |  |  |  |  |  |  |
| 1 | Ath-AT4G34430.4 |  | Vvi-Vitvi03g00349\_t001 |  |  |  |  |  |  |  |
| 1 | Ath-AT4G34440.1 |  | Vvi-Vitvi03g00345\_t001 |  |  |  |  |  |  |  |
| 1 | Ath-AT4G34450.1 |  | Vvi-Vitvi03g00344\_t001 |  |  |  |  |  |  |  |
| 1 | Ath-AT4G34460.1 |  | Vvi-Vitvi03g00343\_t001 |  |  |  |  |  |  |  |
| 1 | Ath-AT4G34470.1 |  | | | |  |  |  |  |  |  |  |
| 1 | Ath-AT4G34480.1 |  | Vvi-Vitvi03g00340\_t001 |  |  |  |  |  |  |  |
| 1 | Ath-AT4G34490.2 |  | Vvi-Vitvi03g00339\_t001 |  |  |  |  |  |  |  |
| 1 | Ath-AT4G34500.1 |  | Vvi-Vitvi03g00338\_t001 |  |  |  |  |  |  |  |
| 1 | Ath-AT4G34510.1 |  | Vvi-Vitvi03g00322\_t001 |  |  |  |  |  |  |  |
| 1 | Ath-AT4G34520.1 |  | | | |  |  |  |  |  |  |  |
| 1 | Ath-AT4G34530.1 |  | Vvi-Vitvi03g00315\_t001 |  |  |  |  |  |  |  |
| 1 | Ath-AT4G34540.1 |  | Vvi-Vitvi03g01492\_t001 |  |  |  |  |  |  |  |
| 1 | Ath-AT4G34550.1 |  | Vvi-Vitvi03g00306\_t001 |  |  |  |  |  |  |  |
| 1 | Ath-AT4G34555.1 |  | Vvi-Vitvi03g00303\_t001 |  |  |  |  |  |  |  |
| 1 | Ath-AT4G34560.1 |  | Vvi-Vitvi03g00302\_t001 |  |  |  |  |  |  |  |
| 1 | Ath-AT4G34570.1 |  | Vvi-Vitvi03g04109\_t001 |  |  |  |  |  |  |  |
| 1 | Ath-AT4G34580.2 |  | Vvi-Vitvi03g00295\_t001 |  |  |  |  |  |  |  |
| 1 | Ath-AT4G34590.1 |  | Vvi-Vitvi03g00292\_t001 |  |  |  |  |  |  |  |
| 1 | Ath-AT4G34600.1 |  | Vvi-Vitvi03g01482\_t001 |  |  |  |  |  |  |  |
| 0 | Ath-AT4G34610.2 |  |  |  |  |  |  |  |  |
| 0 | Ath-AT4G34620.1 |  |  |  |  |  |  |  |  |
| 0 | Ath-AT4G34630.1 |  |  |  |  |  |  |  |  |
| 1 | Ath-AT4G34640.1 |  | Vvi-Vitvi03g00034\_t004 |  |  |  |  |  |  |  |
| 1 | Ath-AT4G34650.1 |  | | | |  |  |  |  |  |  |  |
| 1 | Ath-AT4G34660.1 |  | Vvi-Vitvi03g00035\_t001 |  |  |  |  |  |  |  |
| 1 | Ath-AT4G34670.1 |  | | | |  |  |  |  |  |  |  |
| 1 | Ath-AT4G34680.2 |  | Vvi-Vitvi03g00037\_t001 |  |  |  |  |  |  |  |
| 1 | Ath-AT4G34690.1 |  | | | |  |  |  |  |  |  |  |
| 1 | Ath-AT4G34700.1 |  | Vvi-Vitvi03g00051\_t001 |  |  |  |  |  |  |  |
| 1 | Ath-AT4G34710.1 |  | Vvi-Vitvi03g00054\_t001 |  |  |  |  |  |  |  |
| 1 | Ath-AT4G34720.1 |  | Vvi-Vitvi03g04022\_t001 |  |  |  |  |  |  |  |
| 1 | Ath-AT4G34730.1 |  | | | |  |  |  |  |  |  |  |
| 1 | Ath-AT4G34740.1 |  | Vvi-Vitvi03g00061\_t001 |  |  |  |  |  |  |  |
| 1 | Ath-AT4G34750.2 |  | Vvi-Vitvi03g01336\_t001 |  |  |  |  |  |  |  |
| 2 | Ath-AT4G34760.1 |  | Vvi-Vitvi03g00064\_t001 |  | Vvi-Vitvi04g01441\_t001 |  |  |  |  |  |  |
| 2 | Ath-AT4G34770.1 |  | Vvi-Vitvi03g01347\_t001 |  | | | |  |  |  |  |  |  |
| 2 | Ath-AT4G34780.1 |  | | | |  | | | |  |  |  |  |  |  |
| 2 | Ath-AT4G34790.1 |  | | | |  | | | |  |  |  |  |  |  |
| 2 | Ath-AT4G34800.1 |  | | | |  | | | |  |  |  |  |  |  |
| 2 | Ath-AT4G34810.1 |  | Vvi-Vitvi03g00075\_t001 |  | | | |  |  |  |  |  |  |
| 2 | Ath-AT4G34830.1 |  | Vvi-Vitvi03g00076\_t001 |  | | | |  |  |  |  |  |  |
| 2 | Ath-AT4G34840.1 |  | Vvi-Vitvi03g00085\_t004 |  | | | |  |  |  |  |  |  |
| 2 | Ath-AT4G34850.1 |  | Vvi-Vitvi03g00086\_t001 |  | | | |  |  |  |  |  |  |
| 2 | Ath-AT4G34860.2 |  | Vvi-Vitvi03g00088\_t001 |  | | | |  |  |  |  |  |  |
| 2 | Ath-AT4G34870.1 |  | Vvi-Vitvi03g04055\_t001 |  | | | |  |  |  |  |  |  |
| 2 | Ath-AT4G34880.2 |  | Vvi-Vitvi03g04056\_t001 |  | | | |  |  |  |  |  |  |
| 2 | Ath-AT4G34881.1 |  | | | |  | | | |  |  |  |  |  |  |
| 2 | Ath-AT4G34890.1 |  | | | |  | | | |  |  |  |  |  |  |
| 2 | Ath-AT4G34900.1 |  | | | |  | | | |  |  |  |  |  |  |
| 2 | Ath-AT4G34910.1 |  | | | |  | | | |  |  |  |  |  |  |
| 2 | Ath-AT4G34920.1 |  | Vvi-Vitvi03g01396\_t001 |  | | | |  |  |  |  |  |  |
| 2 | Ath-AT4G34930.1 |  | | | |  | | | |  |  |  |  |  |  |
| 2 | Ath-AT4G34940.1 |  | Vvi-Vitvi03g00120\_t001 |  | Vvi-Vitvi04g01467\_t001 |  |  |  |  |  |  |
| 2 | Ath-AT4G34950.1 |  | Vvi-Vitvi03g00126\_t001 |  | | | |  |  |  |  |  |  |
| 2 | Ath-AT4G34960.1 |  | Vvi-Vitvi03g00127\_t002 |  | | | |  |  |  |  |  |  |
| 2 | Ath-AT4G34970.1 |  | Vvi-Vitvi03g00129\_t001 |  | | | |  |  |  |  |  |  |
| 2 | Ath-AT4G34980.1 |  | Vvi-Vitvi03g00130\_t001 |  | | | |  |  |  |  |  |  |
| 2 | Ath-AT4G34990.1 |  | Vvi-Vitvi03g00136\_t001 |  | Vvi-Vitvi04g01486\_t001 |  |  |  |  |  |  |
| 2 | Ath-AT4G35000.1 |  | Vvi-Vitvi03g00137\_t001 |  | Vvi-Vitvi04g04391\_t001 |  |  |  |  |  |  |
| 2 | Ath-AT4G35010.1 |  | Vvi-Vitvi03g00141\_t001 |  | Vvi-Vitvi04g04394\_t001 |  |  |  |  |  |  |
| 2 | Ath-AT4G35020.2 |  | Vvi-Vitvi03g00143\_t001 |  | Vvi-Vitvi04g01498\_t001 |  |  |  |  |  |  |
| 2 | Ath-AT4G35030.3 |  | Vvi-Vitvi03g00144\_t001 |  | | | |  |  |  |  |  |  |
| 2 | Ath-AT4G35025.1 |  | | | |  | | | |  |  |  |  |  |  |
| 2 | Ath-AT4G35040.2 |  | Vvi-Vitvi03g00146\_t003 |  | | | |  |  |  |  |  |  |
| 2 | Ath-AT4G35050.1 |  | Vvi-Vitvi03g00147\_t001 |  | | | |  |  |  |  |  |  |
| 2 | Ath-AT4G35060.1 |  | Vvi-Vitvi03g00148\_t001 |  | Vvi-Vitvi04g02170\_t001 |  |  |  |  |  |  |
| 1 | Ath-AT4G35070.1 |  | Vvi-Vitvi03g00149\_t001 |  |  |  |  |  |  |  |
| 1 | Ath-AT4G35080.3 |  | Vvi-Vitvi03g00151\_t001 |  |  |  |  |  |  |  |
| 1 | Ath-AT4G35090.1 |  | | | |  |  |  |  |  |  |  |
| 1 | Ath-AT4G35100.1 |  | Vvi-Vitvi03g00155\_t001 |  |  |  |  |  |  |  |
| 0 | Ath-AT4G35110.3 |  |  |  |  |  |  |  |  |
| 0 | Ath-AT4G35120.1 |  |  |  |  |  |  |  |  |
| 0 | Ath-AT4G35130.1 |  |  |  |  |  |  |  |  |
| 0 | Ath-AT4G35140.2 |  |  |  |  |  |  |  |  |
| 0 | Ath-AT4G35150.1 |  |  |  |  |  |  |  |  |
| 0 | Ath-AT4G35160.1 |  |  |  |  |  |  |  |  |
| 0 | Ath-AT4G35165.1 |  |  |  |  |  |  |  |  |
| 1 | Ath-AT4G35170.1 |  | Vvi-Vitvi03g00215\_t001 |  |  |  |  |  |  |  |
| 1 | Ath-AT4G35180.1 |  | | | |  |  |  |  |  |  |  |
| 2 | Ath-AT4G35190.1 |  | | | |  | Vvi-Vitvi03g00208\_t001 |  |  |  |  |  |  |
| 2 | Ath-AT4G35200.1 |  | | | |  | Vvi-Vitvi03g01436\_t001 |  |  |  |  |  |  |
| 2 | Ath-AT4G35210.1 |  | | | |  | | | |  |  |  |  |  |  |
| 2 | Ath-AT4G35220.1 |  | | | |  | | | |  |  |  |  |  |  |
| 2 | Ath-AT4G35230.1 |  | | | |  | Vvi-Vitvi03g00205\_t001 |  |  |  |  |  |  |
| 2 | Ath-AT4G35240.1 |  | | | |  | Vvi-Vitvi03g00204\_t001 |  |  |  |  |  |  |
| 2 | Ath-AT4G35250.1 |  | | | |  | Vvi-Vitvi03g00203\_t001 |  |  |  |  |  |  |
| 2 | Ath-AT4G35260.1 |  | | | |  | Vvi-Vitvi03g00197\_t001 |  |  |  |  |  |  |
| 1 | Ath-AT4G35270.4 |  | | | |  |  |  |  |  |  |  |
| 1 | Ath-AT4G35280.1 |  | Vvi-Vitvi03g00230\_t001 |  |  |  |  |  |  |  |
| 1 | Ath-AT4G35290.2 |  | Vvi-Vitvi03g00246\_t001 |  |  |  |  |  |  |  |
| 1 | Ath-AT4G35295.1 |  | | | |  |  |  |  |  |  |  |
| 1 | Ath-AT4G35300.6 |  | Vvi-Vitvi03g00247\_t001 |  |  |  |  |  |  |  |
| 1 | Ath-AT4G35310.1 |  | Vvi-Vitvi03g00249\_t001 |  |  |  |  |  |  |  |
| 1 | Ath-AT4G35320.1 |  | Vvi-Vitvi03g00251\_t001 |  |  |  |  |  |  |  |
| 2 | Ath-AT4G35350.1 |  | Vvi-Vitvi03g00253\_t001 |  | Vvi-Vitvi18g00051\_t001 |  |  |  |  |  |  |
| 2 | Ath-AT4G35360.1 |  | Vvi-Vitvi03g00254\_t001 |  | | | |  |  |  |  |  |  |
| 2 | Ath-AT4G35370.1 |  | | | |  | | | |  |  |  |  |  |  |
| 2 | Ath-AT4G35375.1 |  | | | |  | | | |  |  |  |  |  |  |
| 2 | Ath-AT4G35380.1 |  | Vvi-Vitvi03g00261\_t003 |  | | | |  |  |  |  |  |  |
| 2 | Ath-AT4G35390.1 |  | Vvi-Vitvi03g00263\_t001 |  | | | |  |  |  |  |  |  |
| 2 | Ath-AT4G35410.2 |  | | | |  | | | |  |  |  |  |  |  |
| 2 | Ath-AT4G35420.1 |  | Vvi-Vitvi03g01477\_t001 |  | | | |  |  |  |  |  |  |
| 2 | Ath-AT4G35440.2 |  | Vvi-Vitvi03g00275\_t001 |  | | | |  |  |  |  |  |  |
| 2 | Ath-AT4G35430.1 |  | | | |  | | | |  |  |  |  |  |  |
| 2 | Ath-AT4G35450.5 |  | Vvi-Vitvi03g00277\_t003 |  | | | |  |  |  |  |  |  |
| 1 | Ath-AT4G35460.1 |  |  |  | | | |  |  |  |  |  |  |
| 1 | Ath-AT4G35470.1 |  |  |  | | | |  |  |  |  |  |  |
| 1 | Ath-AT4G35480.1 |  |  |  | Vvi-Vitvi18g00058\_t001 |  |  |  |  |  |  |
| 1 | Ath-AT4G35485.1 |  |  |  | | | |  |  |  |  |  |  |
| 1 | Ath-AT4G35490.1 |  |  |  | | | |  |  |  |  |  |  |
| 1 | Ath-AT4G35500.2 |  |  |  | | | |  |  |  |  |  |  |
| 1 | Ath-AT4G35510.1 |  |  |  | | | |  |  |  |  |  |  |
| 1 | Ath-AT4G35519.1 |  |  |  | | | |  |  |  |  |  |  |
| 2 | Ath-AT4G35520.2 |  | Vvi-Vitvi04g01580\_t001 |  | | | |  |  |  |  |  |  |
| 2 | Ath-AT4G35530.1 |  | | | |  | Vvi-Vitvi18g00080\_t001 |  |  |  |  |  |  |
| 2 | Ath-AT4G35540.1 |  | | | |  | | | |  |  |  |  |  |  |
| 2 | Ath-AT4G35550.1 |  | Vvi-Vitvi04g01578\_t001 |  | Vvi-Vitvi18g00084\_t001 |  |  |  |  |  |  |
| 2 | Ath-AT4G35560.2 |  | Vvi-Vitvi04g01576\_t001 |  | | | |  |  |  |  |  |  |
| 2 | Ath-AT4G35570.1 |  | Vvi-Vitvi04g02203\_t004 |  | Vvi-Vitvi18g02486\_t001 |  |  |  |  |  |  |
| 2 | Ath-AT4G35580.2 |  | | | |  | | | |  |  |  |  |  |  |
| 2 | Ath-AT4G35590.1 |  | Vvi-Vitvi04g04452\_t001 |  | | | |  |  |  |  |  |  |
| 2 | Ath-AT4G35600.2 |  | Vvi-Vitvi04g01568\_t001 |  | Vvi-Vitvi18g00091\_t002 |  |  |  |  |  |  |
| 1 | Ath-AT4G35610.1 |  | Vvi-Vitvi04g02200\_t001 |  |  |  |  |  |  |  |
| 1 | Ath-AT4G35620.1 |  | Vvi-Vitvi04g04443\_t001 |  |  |  |  |  |  |  |
| 1 | Ath-AT4G35630.1 |  | Vvi-Vitvi04g04442\_t001 |  |  |  |  |  |  |  |
| 1 | Ath-AT4G35640.1 |  | Vvi-Vitvi04g01558\_t001 |  |  |  |  |  |  |  |
| 1 | Ath-AT4G35650.1 |  | Vvi-Vitvi04g01557\_t001 |  |  |  |  |  |  |  |
| 1 | Ath-AT4G35655.1 |  | | | |  |  |  |  |  |  |  |
| 1 | Ath-AT4G35660.1 |  | Vvi-Vitvi04g04428\_t001 |  |  |  |  |  |  |  |
| 1 | Ath-AT4G35670.1 |  | | | |  |  |  |  |  |  |  |
| 1 | Ath-AT4G35680.1 |  | | | |  |  |  |  |  |  |  |
| 1 | Ath-AT4G35685.1 |  | | | |  |  |  |  |  |  |  |
| 1 | Ath-AT4G35690.1 |  | | | |  |  |  |  |  |  |  |
| 1 | Ath-AT4G35700.1 |  | | | |  |  |  |  |  |  |  |
| 1 | Ath-AT4G35710.1 |  | Vvi-Vitvi04g02299\_t001 |  |  |  |  |  |  |  |
| 1 | Ath-AT4G35720.1 |  | | | |  |  |  |  |  |  |  |
| 1 | Ath-AT4G35725.1 |  | | | |  |  |  |  |  |  |  |
| 1 | Ath-AT4G35730.1 |  | Vvi-Vitvi04g02297\_t001 |  |  |  |  |  |  |  |
| 1 | Ath-AT4G35733.1 |  | | | |  |  |  |  |  |  |  |
| 1 | Ath-AT4G35740.1 |  | | | |  |  |  |  |  |  |  |
| 1 | Ath-AT4G35750.1 |  | | | |  |  |  |  |  |  |  |
| 1 | Ath-AT4G35760.2 |  | Vvi-Vitvi04g01541\_t001 |  |  |  |  |  |  |  |
| 1 | Ath-AT4G35770.1 |  | | | |  |  |  |  |  |  |  |
| 1 | Ath-AT4G35780.1 |  | Vvi-Vitvi04g04409\_t001 |  |  |  |  |  |  |  |
| 1 | Ath-AT4G35783.1 |  | | | |  |  |  |  |  |  |  |
| 1 | Ath-AT4G35785.2 |  | Vvi-Vitvi04g01527\_t001 |  |  |  |  |  |  |  |
| 1 | Ath-AT4G35790.1 |  | Vvi-Vitvi04g01530\_t002 |  |  |  |  |  |  |  |
| 1 | Ath-AT4G35800.1 |  | Vvi-Vitvi04g04403\_t001 |  |  |  |  |  |  |  |
| 1 | Ath-AT4G35810.2 |  | Vvi-Vitvi04g02185\_t001 |  |  |  |  |  |  |  |
| 1 | Ath-AT4G35820.1 |  | | | |  |  |  |  |  |  |  |
| 1 | Ath-AT4G35830.1 |  | Vvi-Vitvi04g01535\_t001 |  |  |  |  |  |  |  |
| 1 | Ath-AT4G35840.2 |  | Vvi-Vitvi04g01536\_t001 |  |  |  |  |  |  |  |
| 1 | Ath-AT4G35850.1 |  | | | |  |  |  |  |  |  |  |
| 1 | Ath-AT4G35860.1 |  | | | |  |  |  |  |  |  |  |
| 1 | Ath-AT4G35870.1 |  | Vvi-Vitvi04g02278\_t001 |  |  |  |  |  |  |  |
| 1 | Ath-AT4G35880.1 |  | Vvi-Vitvi04g04401\_t001 |  |  |  |  |  |  |  |
| 1 | Ath-AT4G35890.1 |  | Vvi-Vitvi04g01506\_t001 |  |  |  |  |  |  |  |
| 1 | Ath-AT4G35900.1 |  | Vvi-Vitvi04g01505\_t001 |  |  |  |  |  |  |  |
| 1 | Ath-AT4G35905.1 |  | Vvi-Vitvi04g04399\_t001 |  |  |  |  |  |  |  |
| 1 | Ath-AT4G35910.1 |  | | | |  |  |  |  |  |  |  |
| 1 | Ath-AT4G35920.5 |  | Vvi-Vitvi04g01503\_t001 |  |  |  |  |  |  |  |
| 1 | Ath-AT4G35930.4 |  | Vvi-Vitvi04g04397\_t001 |  |  |  |  |  |  |  |
| 1 | Ath-AT4G35940.1 |  | | | |  |  |  |  |  |  |  |
| 1 | Ath-AT4G35950.1 |  | Vvi-Vitvi04g01498\_t001 |  |  |  |  |  |  |  |
| 2 | Ath-AT4G35970.1 |  | Vvi-Vitvi04g04391\_t001 |  | Vvi-Vitvi03g00137\_t001 |  |  |  |  |  |  |
| 2 | Ath-AT4G35980.1 |  | Vvi-Vitvi04g01485\_t001 |  | | | |  |  |  |  |  |  |
| 2 | Ath-AT4G35985.1 |  | Vvi-Vitvi04g01482\_t001 |  | Vvi-Vitvi03g00133\_t001 |  |  |  |  |  |  |
| 2 | Ath-AT4G35987.1 |  | Vvi-Vitvi04g01472\_t001 |  | | | |  |  |  |  |  |  |
| 2 | Ath-AT4G36000.1 |  | | | |  | | | |  |  |  |  |  |  |
| 2 | Ath-AT4G36010.2 |  | Vvi-Vitvi04g01470\_t001 |  | Vvi-Vitvi03g00123\_t001 |  |  |  |  |  |  |
| 2 | Ath-AT4G36020.1 |  | Vvi-Vitvi04g01469\_t001 |  | Vvi-Vitvi03g00121\_t001 |  |  |  |  |  |  |
| 2 | Ath-AT4G36030.1 |  | Vvi-Vitvi04g01467\_t001 |  | Vvi-Vitvi03g00120\_t001 |  |  |  |  |  |  |
| 2 | Ath-AT4G36040.1 |  | Vvi-Vitvi04g01466\_t001 |  | Vvi-Vitvi03g00119\_t001 |  |  |  |  |  |  |
| 1 | Ath-AT4G36050.2 |  | Vvi-Vitvi04g01464\_t001 |  |  |  |  |  |  |  |
| 1 | Ath-AT4G36060.3 |  | Vvi-Vitvi04g01463\_t001 |  |  |  |  |  |  |  |
| 1 | Ath-AT4G36070.2 |  | Vvi-Vitvi04g01462\_t001 |  |  |  |  |  |  |  |
| 1 | Ath-AT4G36080.1 |  | Vvi-Vitvi04g01452\_t001 |  |  |  |  |  |  |  |
| 1 | Ath-AT4G36090.3 |  | Vvi-Vitvi04g01448\_t001 |  |  |  |  |  |  |  |
| 1 | Ath-AT4G36100.1 |  | | | |  |  |  |  |  |  |  |
| 1 | Ath-AT4G36105.2 |  | Vvi-Vitvi04g01443\_t001 |  |  |  |  |  |  |  |
| 1 | Ath-AT4G36110.1 |  | | | |  |  |  |  |  |  |  |
| 1 | Ath-AT4G36120.1 |  | Vvi-Vitvi04g01440\_t001 |  |  |  |  |  |  |  |
| 1 | Ath-AT4G36130.1 |  | Vvi-Vitvi04g01439\_t001 |  |  |  |  |  |  |  |
| 1 | Ath-AT4G36140.1 |  | | | |  |  |  |  |  |  |  |
| 1 | Ath-AT4G36150.1 |  | | | |  |  |  |  |  |  |  |
| 1 | Ath-AT4G36160.2 |  | Vvi-Vitvi04g01430\_t001 |  |  |  |  |  |  |  |
| 1 | Ath-AT4G36170.1 |  | | | |  |  |  |  |  |  |  |
| 2 | Ath-AT4G36180.1 |  | Vvi-Vitvi04g01426\_t001 |  | Vvi-Vitvi18g01056\_t001 |  |  |  |  |  |  |
| 2 | Ath-AT4G36190.1 |  | Vvi-Vitvi04g01422\_t002 |  | | | |  |  |  |  |  |  |
| 2 | Ath-AT4G36195.3 |  | | | |  | | | |  |  |  |  |  |  |
| 2 | Ath-AT4G36210.3 |  | Vvi-Vitvi04g01413\_t001 |  | | | |  |  |  |  |  |  |
| 2 | Ath-AT4G36220.1 |  | Vvi-Vitvi04g01412\_t001 |  | | | |  |  |  |  |  |  |
| 2 | Ath-AT4G36230.1 |  | | | |  | | | |  |  |  |  |  |  |
| 2 | Ath-AT4G36240.1 |  | Vvi-Vitvi04g01410\_t001 |  | | | |  |  |  |  |  |  |
| 2 | Ath-AT4G36250.1 |  | Vvi-Vitvi04g01402\_t001 |  | | | |  |  |  |  |  |  |
| 2 | Ath-AT4G36260.1 |  | Vvi-Vitvi04g01399\_t001 |  | Vvi-Vitvi18g01041\_t001 |  |  |  |  |  |  |
| 2 | Ath-AT4G36270.1 |  | Vvi-Vitvi04g01393\_t001 |  | | | |  |  |  |  |  |  |
| 2 | Ath-AT4G36280.1 |  | | | |  | | | |  |  |  |  |  |  |
| 2 | Ath-AT4G36290.1 |  | | | |  | | | |  |  |  |  |  |  |
| 2 | Ath-AT4G36350.1 |  | | | |  | Vvi-Vitvi18g02777\_t001 |  |  |  |  |  |  |
| 2 | Ath-AT4G36360.1 |  | Vvi-Vitvi04g01389\_t001 |  | Vvi-Vitvi18g01022\_t002 |  |  |  |  |  |  |
| 2 | Ath-AT4G36370.1 |  | | | |  | | | |  |  |  |  |  |  |
| 2 | Ath-AT4G36380.1 |  | Vvi-Vitvi04g01385\_t001 |  | | | |  |  |  |  |  |  |
| 2 | Ath-AT4G36390.1 |  | Vvi-Vitvi04g01384\_t001 |  | | | |  |  |  |  |  |  |
| 2 | Ath-AT4G36400.2 |  | Vvi-Vitvi04g01383\_t001 |  | | | |  |  |  |  |  |  |
| 2 | Ath-AT4G36410.1 |  | Vvi-Vitvi04g02133\_t001 |  | Vvi-Vitvi18g04240\_t001 |  |  |  |  |  |  |
| 2 | Ath-AT4G36420.1 |  | Vvi-Vitvi04g02132\_t001 |  | | | |  |  |  |  |  |  |
| 2 | Ath-AT4G36430.1 |  | Vvi-Vitvi04g01378\_t001 |  | Vvi-Vitvi18g01014\_t001 |  |  |  |  |  |  |
| 2 | Ath-AT4G36440.1 |  | Vvi-Vitvi04g01377\_t002 |  | | | |  |  |  |  |  |  |
| 2 | Ath-AT4G36450.1 |  | Vvi-Vitvi04g01362\_t001 |  | Vvi-Vitvi18g01004\_t001 |  |  |  |  |  |  |
| 2 | Ath-AT4G36460.1 |  | | | |  | | | |  |  |  |  |  |  |
| 2 | Ath-AT4G36470.1 |  | Vvi-Vitvi04g04356\_t001 |  | Vvi-Vitvi18g02761\_t002 |  |  |  |  |  |  |
| 2 | Ath-AT4G36480.1 |  | | | |  | | | |  |  |  |  |  |  |
| 2 | Ath-AT4G36490.1 |  | | | |  | | | |  |  |  |  |  |  |
| 2 | Ath-AT4G36500.1 |  | Vvi-Vitvi04g04348\_t001 |  | | | |  |  |  |  |  |  |
| 2 | Ath-AT4G36510.1 |  | | | |  | | | |  |  |  |  |  |  |
| 2 | Ath-AT4G36515.1 |  | | | |  | | | |  |  |  |  |  |  |
| 2 | Ath-AT4G36520.1 |  | Vvi-Vitvi04g01327\_t002 |  | Vvi-Vitvi18g00983\_t001 |  |  |  |  |  |  |
| 2 | Ath-AT4G36530.2 |  | Vvi-Vitvi04g01322\_t001 |  | | | |  |  |  |  |  |  |
| 2 | Ath-AT4G36540.1 |  | Vvi-Vitvi04g01312\_t001 |  | | | |  |  |  |  |  |  |
| 2 | Ath-AT4G36550.1 |  | Vvi-Vitvi04g01311\_t001 |  | Vvi-Vitvi18g00974\_t001 |  |  |  |  |  |  |
| 2 | Ath-AT4G36560.1 |  | | | |  | | | |  |  |  |  |  |  |
| 2 | Ath-AT4G36570.1 |  | | | |  | | | |  |  |  |  |  |  |
| 2 | Ath-AT4G36580.1 |  | Vvi-Vitvi04g01307\_t001 |  | | | |  |  |  |  |  |  |
| 2 | Ath-AT4G36590.1 |  | | | |  | | | |  |  |  |  |  |  |
| 2 | Ath-AT4G36600.2 |  | Vvi-Vitvi04g01306\_t001 |  | | | |  |  |  |  |  |  |
| 2 | Ath-AT4G36610.1 |  | Vvi-Vitvi04g01300\_t001 |  | | | |  |  |  |  |  |  |
| 2 | Ath-AT4G36620.1 |  | Vvi-Vitvi04g01299\_t001 |  | | | |  |  |  |  |  |  |
| 2 | Ath-AT4G36630.1 |  | Vvi-Vitvi04g01289\_t001 |  | | | |  |  |  |  |  |  |
| 2 | Ath-AT4G36640.3 |  | Vvi-Vitvi04g01287\_t001 |  | Vvi-Vitvi18g00957\_t003 |  |  |  |  |  |  |
| 2 | Ath-AT4G36650.1 |  | Vvi-Vitvi04g01266\_t001 |  | | | |  |  |  |  |  |  |
| 2 | Ath-AT4G36660.1 |  | Vvi-Vitvi04g01265\_t001 |  | Vvi-Vitvi18g00948\_t001 |  |  |  |  |  |  |
| 2 | Ath-AT4G36670.1 |  | Vvi-Vitvi04g01263\_t001 |  | | | |  |  |  |  |  |  |
| 2 | Ath-AT4G36680.1 |  | Vvi-Vitvi04g01258\_t001 |  | | | |  |  |  |  |  |  |
| 2 | Ath-AT4G36690.1 |  | Vvi-Vitvi04g01257\_t001 |  | | | |  |  |  |  |  |  |
| 2 | Ath-AT4G36700.1 |  | Vvi-Vitvi04g01248\_t001 |  | | | |  |  |  |  |  |  |
| 2 | Ath-AT4G36710.1 |  | Vvi-Vitvi04g01247\_t001 |  | | | |  |  |  |  |  |  |
| 2 | Ath-AT4G36720.1 |  | Vvi-Vitvi04g02099\_t001 |  | | | |  |  |  |  |  |  |
| 2 | Ath-AT4G36730.1 |  | Vvi-Vitvi04g02098\_t001 |  | Vvi-Vitvi18g00930\_t003 |  |  |  |  |  |  |
| 2 | Ath-AT4G36740.2 |  | Vvi-Vitvi04g01244\_t001 |  | | | |  |  |  |  |  |  |
| 2 | Ath-AT4G36750.1 |  | Vvi-Vitvi04g01243\_t001 |  | | | |  |  |  |  |  |  |
| 2 | Ath-AT4G36760.2 |  | Vvi-Vitvi04g01238\_t001 |  | | | |  |  |  |  |  |  |
| 2 | Ath-AT4G36770.1 |  | Vvi-Vitvi04g01237\_t001 |  | Vvi-Vitvi18g04219\_t001 |  |  |  |  |  |  |
| 2 | Ath-AT4G36780.1 |  | | | |  | Vvi-Vitvi18g00924\_t001 |  |  |  |  |  |  |
| 2 | Ath-AT4G36790.1 |  | Vvi-Vitvi04g01235\_t001 |  | | | |  |  |  |  |  |  |
| 2 | Ath-AT4G36791.1 |  | | | |  | | | |  |  |  |  |  |  |
| 2 | Ath-AT4G36795.1 |  | | | |  | | | |  |  |  |  |  |  |
| 2 | Ath-AT4G36800.1 |  | | | |  | Vvi-Vitvi18g00923\_t001 |  |  |  |  |  |  |
| 2 | Ath-AT4G36810.1 |  | Vvi-Vitvi04g01230\_t001 |  | Vvi-Vitvi18g00922\_t001 |  |  |  |  |  |  |
| 2 | Ath-AT4G36820.1 |  | Vvi-Vitvi04g01225\_t001 |  | Vvi-Vitvi18g02730\_t001 |  |  |  |  |  |  |
| 2 | Ath-AT4G36830.2 |  | Vvi-Vitvi04g01224\_t001 |  | Vvi-Vitvi18g00913\_t001 |  |  |  |  |  |  |
| 2 | Ath-AT4G36840.1 |  | | | |  | | | |  |  |  |  |  |  |
| 2 | Ath-AT4G36850.1 |  | Vvi-Vitvi04g01221\_t002 |  | | | |  |  |  |  |  |  |
| 2 | Ath-AT4G36860.3 |  | Vvi-Vitvi04g02091\_t001 |  | Vvi-Vitvi18g00903\_t001 |  |  |  |  |  |  |
| 2 | Ath-AT4G36870.2 |  | Vvi-Vitvi04g01210\_t001 |  | Vvi-Vitvi18g00902\_t001 |  |  |  |  |  |  |
| 1 | Ath-AT4G36880.1 |  | Vvi-Vitvi07g01690\_t001 |  |  |  |  |  |  |  |
| 1 | Ath-AT4G36890.1 |  | Vvi-Vitvi07g01692\_t001 |  |  |  |  |  |  |  |
| 1 | Ath-AT4G36900.1 |  | Vvi-Vitvi07g01702\_t001 |  |  |  |  |  |  |  |
| 1 | Ath-AT4G36910.1 |  | Vvi-Vitvi07g01703\_t001 |  |  |  |  |  |  |  |
| 1 | Ath-AT4G36920.2 |  | Vvi-Vitvi07g01706\_t001 |  |  |  |  |  |  |  |
| 1 | Ath-AT4G36925.2 |  | | | |  |  |  |  |  |  |  |
| 1 | Ath-AT4G36930.1 |  | | | |  |  |  |  |  |  |  |
| 1 | Ath-AT4G36940.1 |  | | | |  |  |  |  |  |  |  |
| 1 | Ath-AT4G36945.1 |  | Vvi-Vitvi07g01722\_t001 |  |  |  |  |  |  |  |
| 1 | Ath-AT4G36950.1 |  | Vvi-Vitvi07g01737\_t001 |  |  |  |  |  |  |  |
| 1 | Ath-AT4G36960.1 |  | Vvi-Vitvi07g01743\_t004 |  |  |  |  |  |  |  |
| 1 | Ath-AT4G36970.1 |  | Vvi-Vitvi07g01744\_t001 |  |  |  |  |  |  |  |
| 1 | Ath-AT4G36980.4 |  | Vvi-Vitvi07g01747\_t001 |  |  |  |  |  |  |  |
| 1 | Ath-AT4G36990.1 |  | Vvi-Vitvi07g01749\_t001 |  |  |  |  |  |  |  |
| 1 | Ath-AT4G37000.1 |  | Vvi-Vitvi07g01750\_t001 |  |  |  |  |  |  |  |
| 1 | Ath-AT4G37010.2 |  | Vvi-Vitvi07g01752\_t001 |  |  |  |  |  |  |  |
| 1 | Ath-AT4G37020.2 |  | Vvi-Vitvi07g01757\_t001 |  |  |  |  |  |  |  |
| 1 | Ath-AT4G37022.1 |  | | | |  |  |  |  |  |  |  |
| 1 | Ath-AT4G37030.1 |  | Vvi-Vitvi07g01763\_t001 |  |  |  |  |  |  |  |
| 1 | Ath-AT4G37040.1 |  | Vvi-Vitvi07g01765\_t001 |  |  |  |  |  |  |  |
| 1 | Ath-AT4G37050.1 |  | Vvi-Vitvi07g01766\_t001 |  |  |  |  |  |  |  |
| 0 | Ath-AT4G37060.2 |  |  |  |  |  |  |  |  |
| 0 | Ath-AT4G37070.2 |  |  |  |  |  |  |  |  |
| 0 | Ath-AT4G37080.3 |  |  |  |  |  |  |  |  |
| 2 | Ath-AT4G37090.1 |  | Vvi-Vitvi07g01281\_t001 |  | Vvi-Vitvi07g01281\_t001 |  |  |  |  |  |  |
| 2 | Ath-AT4G37095.1 |  | | | |  | | | |  |  |  |  |  |  |
| 2 | Ath-AT4G37100.1 |  | | | |  | Vvi-Vitvi07g01295\_t001 |  |  |  |  |  |  |
| 2 | Ath-AT4G37110.1 |  | | | |  | Vvi-Vitvi07g01296\_t001 |  |  |  |  |  |  |
| 2 | Ath-AT4G37120.1 |  | | | |  | | | |  |  |  |  |  |  |
| 2 | Ath-AT4G37130.1 |  | | | |  | Vvi-Vitvi07g04505\_t001 |  |  |  |  |  |  |
| 2 | Ath-AT4G37140.1 |  | | | |  | | | |  |  |  |  |  |  |
| 2 | Ath-AT4G37150.1 |  | | | |  | Vvi-Vitvi07g01300\_t001 |  |  |  |  |  |  |
| 2 | Ath-AT4G37160.1 |  | | | |  | | | |  |  |  |  |  |  |
| 2 | Ath-AT4G37170.1 |  | | | |  | Vvi-Vitvi07g01312\_t001 |  |  |  |  |  |  |
| 2 | Ath-AT4G37180.2 |  | | | |  | Vvi-Vitvi07g01325\_t001 |  |  |  |  |  |  |
| 2 | Ath-AT4G37190.1 |  | | | |  | Vvi-Vitvi07g02529\_t001.1.6037826f |  |  |  |  |  |  |
| 1 | Ath-AT4G37200.1 |  | Vvi-Vitvi07g01279\_t001 |  |  |  |  |  |  |  |
| 1 | Ath-AT4G37210.1 |  | Vvi-Vitvi07g01278\_t002 |  |  |  |  |  |  |  |
| 1 | Ath-AT4G37220.1 |  | Vvi-Vitvi07g01276\_t001 |  |  |  |  |  |  |  |
| 1 | Ath-AT4G37230.1 |  | Vvi-Vitvi07g01275\_t001 |  |  |  |  |  |  |  |
| 1 | Ath-AT4G37235.1 |  | Vvi-Vitvi07g01274\_t001 |  |  |  |  |  |  |  |
| 1 | Ath-AT4G37240.1 |  | Vvi-Vitvi07g01267\_t001 |  |  |  |  |  |  |  |
| 1 | Ath-AT4G37250.1 |  | Vvi-Vitvi07g01678\_t001 |  |  |  |  |  |  |  |
| 1 | Ath-AT4G37260.1 |  | Vvi-Vitvi07g01676\_t001 |  |  |  |  |  |  |  |
| 1 | Ath-AT4G37270.1 |  | Vvi-Vitvi07g01674\_t001 |  |  |  |  |  |  |  |
| 1 | Ath-AT4G37280.1 |  | Vvi-Vitvi07g01672\_t001 |  |  |  |  |  |  |  |
| 1 | Ath-AT4G37290.1 |  | Vvi-Vitvi07g02650\_t001 |  |  |  |  |  |  |  |
| 1 | Ath-AT4G37295.1 |  | | | |  |  |  |  |  |  |  |
| 1 | Ath-AT4G37300.1 |  | Vvi-Vitvi07g02645\_t001 |  |  |  |  |  |  |  |
| 1 | Ath-AT4G37310.1 |  | Vvi-Vitvi07g04690\_t001 |  |  |  |  |  |  |  |
| 1 | Ath-AT4G37320.1 |  | Vvi-Vitvi07g01648\_t001 |  |  |  |  |  |  |  |
| 1 | Ath-AT4G37330.1 |  | | | |  |  |  |  |  |  |  |
| 1 | Ath-AT4G37340.1 |  | | | |  |  |  |  |  |  |  |
| 1 | Ath-AT4G37360.1 |  | | | |  |  |  |  |  |  |  |
| 1 | Ath-AT4G37370.1 |  | | | |  |  |  |  |  |  |  |
| 1 | Ath-AT4G37380.1 |  | Vvi-Vitvi07g01646\_t001 |  |  |  |  |  |  |  |
| 1 | Ath-AT4G37390.1 |  | Vvi-Vitvi07g01644\_t001 |  |  |  |  |  |  |  |
| 1 | Ath-AT4G37400.1 |  | | | |  |  |  |  |  |  |  |
| 1 | Ath-AT4G37409.1 |  | | | |  |  |  |  |  |  |  |
| 1 | Ath-AT4G37410.1 |  | | | |  |  |  |  |  |  |  |
| 1 | Ath-AT4G37420.1 |  | | | |  |  |  |  |  |  |  |
| 1 | Ath-AT4G37430.1 |  | | | |  |  |  |  |  |  |  |
| 1 | Ath-AT4G37435.1 |  | | | |  |  |  |  |  |  |  |
| 1 | Ath-AT4G37440.1 |  | | | |  |  |  |  |  |  |  |
| 1 | Ath-AT4G37445.1 |  | | | |  |  |  |  |  |  |  |
| 1 | Ath-AT4G37450.2 |  | Vvi-Vitvi07g01636\_t001 |  |  |  |  |  |  |  |
| 1 | Ath-AT4G37460.1 |  | Vvi-Vitvi07g01634\_t001 |  |  |  |  |  |  |  |
| 1 | Ath-AT4G37470.1 |  | Vvi-Vitvi07g01633\_t001 |  |  |  |  |  |  |  |
| 1 | Ath-AT4G37480.1 |  | Vvi-Vitvi07g01626\_t001 |  |  |  |  |  |  |  |
| 1 | Ath-AT4G37483.1 |  | | | |  |  |  |  |  |  |  |
| 1 | Ath-AT4G37490.1 |  | | | |  |  |  |  |  |  |  |
| 1 | Ath-AT4G37500.1 |  | | | |  |  |  |  |  |  |  |
| 1 | Ath-AT4G37510.1 |  | Vvi-Vitvi07g01619\_t001 |  |  |  |  |  |  |  |
| 1 | Ath-AT4G37520.1 |  | Vvi-Vitvi07g01614\_t001 |  |  |  |  |  |  |  |
| 1 | Ath-AT4G37530.1 |  | | | |  |  |  |  |  |  |  |
| 1 | Ath-AT4G37540.1 |  | Vvi-Vitvi07g01610\_t001 |  |  |  |  |  |  |  |
| 1 | Ath-AT4G37550.5 |  | Vvi-Vitvi07g01608\_t001 |  |  |  |  |  |  |  |
| 1 | Ath-AT4G37560.1 |  | | | |  |  |  |  |  |  |  |
| 1 | Ath-AT4G37580.1 |  | Vvi-Vitvi07g01604\_t001 |  |  |  |  |  |  |  |
| 1 | Ath-AT4G37590.1 |  | Vvi-Vitvi07g01603\_t003 |  |  |  |  |  |  |  |
| 1 | Ath-AT4G37608.1 |  | | | |  |  |  |  |  |  |  |
| 1 | Ath-AT4G37610.1 |  | Vvi-Vitvi07g01598\_t001 |  |  |  |  |  |  |  |
| 1 | Ath-AT4G37630.1 |  | Vvi-Vitvi07g04675\_t001 |  |  |  |  |  |  |  |
| 1 | Ath-AT4G37640.1 |  | Vvi-Vitvi07g01595\_t001 |  |  |  |  |  |  |  |
| 1 | Ath-AT4G37650.1 |  | Vvi-Vitvi07g02073\_t001 |  |  |  |  |  |  |  |
| 1 | Ath-AT4G37660.1 |  | Vvi-Vitvi07g01572\_t001 |  |  |  |  |  |  |  |
| 1 | Ath-AT4G37670.2 |  | Vvi-Vitvi07g04669\_t001 |  |  |  |  |  |  |  |
| 3 | Ath-AT4G37680.4 |  | | | |  | Vvi-Vitvi18g00645\_t003 |  | Vvi-Vitvi15g04494\_t001 |  |  |  |  |  |
| 3 | Ath-AT4G37682.1 |  | | | |  | | | |  | | | |  |  |  |  |  |
| 3 | Ath-AT4G37685.1 |  | | | |  | | | |  | | | |  |  |  |  |  |
| 3 | Ath-AT4G37690.1 |  | | | |  | | | |  | Vvi-Vitvi15g04497\_t001 |  |  |  |  |  |
| 3 | Ath-AT4G37700.1 |  | | | |  | Vvi-Vitvi18g02636\_t001 |  | Vvi-Vitvi15g04501\_t001 |  |  |  |  |  |
| 3 | Ath-AT4G37710.1 |  | | | |  | | | |  | Vvi-Vitvi15g04504\_t001 |  |  |  |  |  |
| 3 | Ath-AT4G37720.1 |  | | | |  | Vvi-Vitvi18g00635\_t001 |  | Vvi-Vitvi15g04511\_t001 |  |  |  |  |  |
| 3 | Ath-AT4G37730.1 |  | | | |  | Vvi-Vitvi18g00628\_t001 |  | Vvi-Vitvi15g04514\_t001 |  |  |  |  |  |
| 3 | Ath-AT4G37740.1 |  | | | |  | Vvi-Vitvi18g00623\_t001 |  | Vvi-Vitvi15g04517\_t001 |  |  |  |  |  |
| 3 | Ath-AT4G37750.1 |  | | | |  | Vvi-Vitvi18g00618\_t001 |  | Vvi-Vitvi15g04521\_t001 |  |  |  |  |  |
| 3 | Ath-AT4G37760.1 |  | | | |  | Vvi-Vitvi18g00612\_t001 |  | Vvi-Vitvi15g04526\_t001 |  |  |  |  |  |
| 2 | Ath-AT4G37770.1 |  | Vvi-Vitvi07g03132\_t001 |  | Vvi-Vitvi18g00609\_t001 |  |  |  |  |  |  |
| 2 | Ath-AT4G37780.1 |  | | | |  | Vvi-Vitvi18g00605\_t001 |  |  |  |  |  |  |
| 2 | Ath-AT4G37790.1 |  | Vvi-Vitvi07g03056\_t002 |  | Vvi-Vitvi18g00603\_t001 |  |  |  |  |  |  |
| 2 | Ath-AT4G37800.1 |  | Vvi-Vitvi07g04661\_t001 |  | | | |  |  |  |  |  |  |
| 2 | Ath-AT4G37810.1 |  | Vvi-Vitvi07g03061\_t001 |  | Vvi-Vitvi18g02622\_t001 |  |  |  |  |  |  |
| 2 | Ath-AT4G37820.1 |  | Vvi-Vitvi07g03063\_t002 |  | Vvi-Vitvi18g02621\_t001 |  |  |  |  |  |  |
| 1 | Ath-AT4G37830.3 |  | Vvi-Vitvi07g03064\_t001 |  |  |  |  |  |  |  |
| 1 | Ath-AT4G37840.1 |  | Vvi-Vitvi07g04651\_t001 |  |  |  |  |  |  |  |
| 1 | Ath-AT4G37850.2 |  | Vvi-Vitvi07g01532\_t001 |  |  |  |  |  |  |  |
| 1 | Ath-AT4G37860.1 |  | Vvi-Vitvi07g01538\_t001 |  |  |  |  |  |  |  |
| 1 | Ath-AT4G37870.1 |  | Vvi-Vitvi07g04646\_t001 |  |  |  |  |  |  |  |
| 1 | Ath-AT4G37880.1 |  | Vvi-Vitvi07g01541\_t001 |  |  |  |  |  |  |  |
| 1 | Ath-AT4G37890.1 |  | Vvi-Vitvi07g04644\_t001 |  |  |  |  |  |  |  |
| 1 | Ath-AT4G37900.2 |  | Vvi-Vitvi07g01523\_t001 |  |  |  |  |  |  |  |
| 1 | Ath-AT4G37910.2 |  | Vvi-Vitvi07g01524\_t001 |  |  |  |  |  |  |  |
| 1 | Ath-AT4G37920.1 |  | Vvi-Vitvi07g01506\_t001 |  |  |  |  |  |  |  |
| 1 | Ath-AT4G37925.1 |  | | | |  |  |  |  |  |  |  |
| 1 | Ath-AT4G37930.1 |  | Vvi-Vitvi07g02610\_t001 |  |  |  |  |  |  |  |
| 1 | Ath-AT4G37940.1 |  | Vvi-Vitvi07g01520\_t001 |  |  |  |  |  |  |  |
| 1 | Ath-AT4G37950.1 |  | Vvi-Vitvi07g01388\_t001 |  |  |  |  |  |  |  |
| 1 | Ath-AT4G37970.1 |  | Vvi-Vitvi07g04623\_t001 |  |  |  |  |  |  |  |
| 1 | Ath-AT4G37980.1 |  | | | |  |  |  |  |  |  |  |
| 1 | Ath-AT4G37990.1 |  | | | |  |  |  |  |  |  |  |
| 1 | Ath-AT4G38000.1 |  | | | |  |  |  |  |  |  |  |
| 1 | Ath-AT4G38005.1 |  | | | |  |  |  |  |  |  |  |
| 1 | Ath-AT4G38010.1 |  | | | |  |  |  |  |  |  |  |
| 1 | Ath-AT4G38020.1 |  | Vvi-Vitvi07g01405\_t001 |  |  |  |  |  |  |  |
| 1 | Ath-AT4G38030.1 |  | | | |  |  |  |  |  |  |  |
| 1 | Ath-AT4G38040.1 |  | Vvi-Vitvi07g01406\_t001 |  |  |  |  |  |  |  |
| 1 | Ath-AT4G38050.1 |  | Vvi-Vitvi07g04611\_t002 |  |  |  |  |  |  |  |
| 1 | Ath-AT4G38060.3 |  | Vvi-Vitvi07g01431\_t001 |  |  |  |  |  |  |  |
| 1 | Ath-AT4G38062.1 |  | Vvi-Vitvi07g01433\_t001 |  |  |  |  |  |  |  |
| 1 | Ath-AT4G38065.1 |  | | | |  |  |  |  |  |  |  |
| 1 | Ath-AT4G38070.1 |  | Vvi-Vitvi07g04603\_t001 |  |  |  |  |  |  |  |
| 1 | Ath-AT4G38080.1 |  | | | |  |  |  |  |  |  |  |
| 1 | Ath-AT4G38090.1 |  | Vvi-Vitvi07g02582\_t001 |  |  |  |  |  |  |  |
| 1 | Ath-AT4G38092.1 |  | | | |  |  |  |  |  |  |  |
| 1 | Ath-AT4G38100.1 |  | Vvi-Vitvi07g02589\_t001 |  |  |  |  |  |  |  |
| 0 | Ath-AT4G38120.3 |  |  |  |  |  |  |  |  |
| 1 | Ath-AT4G38130.1 |  | Vvi-Vitvi03g00273\_t002 |  |  |  |  |  |  |  |
| 1 | Ath-AT4G38140.1 |  | Vvi-Vitvi03g00271\_t001 |  |  |  |  |  |  |  |
| 1 | Ath-AT4G38150.1 |  | Vvi-Vitvi03g00270\_t001 |  |  |  |  |  |  |  |
| 1 | Ath-AT4G38160.3 |  | Vvi-Vitvi03g00269\_t001 |  |  |  |  |  |  |  |
| 1 | Ath-AT4G38170.1 |  | Vvi-Vitvi03g01475\_t003 |  |  |  |  |  |  |  |
| 1 | Ath-AT4G38180.1 |  | | | |  |  |  |  |  |  |  |
| 1 | Ath-AT4G38190.1 |  | Vvi-Vitvi03g00264\_t001 |  |  |  |  |  |  |  |
| 1 | Ath-AT4G38200.1 |  | Vvi-Vitvi03g00261\_t003 |  |  |  |  |  |  |  |
| 1 | Ath-AT4G38210.1 |  | Vvi-Vitvi03g00258\_t001 |  |  |  |  |  |  |  |
| 1 | Ath-AT4G38213.1 |  | Vvi-Vitvi03g01474\_t001 |  |  |  |  |  |  |  |
| 1 | Ath-AT4G38220.2 |  | Vvi-Vitvi03g00257\_t002 |  |  |  |  |  |  |  |
| 1 | Ath-AT4G38225.3 |  | Vvi-Vitvi03g00250\_t001 |  |  |  |  |  |  |  |
| 1 | Ath-AT4G38230.2 |  | Vvi-Vitvi03g00249\_t001 |  |  |  |  |  |  |  |
| 1 | Ath-AT4G38240.2 |  | | | |  |  |  |  |  |  |  |
| 1 | Ath-AT4G38250.1 |  | Vvi-Vitvi03g00244\_t001 |  |  |  |  |  |  |  |
| 1 | Ath-AT4G38260.1 |  | Vvi-Vitvi03g00241\_t001 |  |  |  |  |  |  |  |
| 1 | Ath-AT4G38270.1 |  | Vvi-Vitvi03g00235\_t001 |  |  |  |  |  |  |  |
| 1 | Ath-AT4G38280.1 |  | | | |  |  |  |  |  |  |  |
| 1 | Ath-AT4G38290.1 |  | | | |  |  |  |  |  |  |  |
| 1 | Ath-AT4G38300.1 |  | | | |  |  |  |  |  |  |  |
| 1 | Ath-AT4G38310.1 |  | | | |  |  |  |  |  |  |  |
| 1 | Ath-AT4G38320.1 |  | | | |  |  |  |  |  |  |  |
| 1 | Ath-AT4G38330.1 |  | | | |  |  |  |  |  |  |  |
| 1 | Ath-AT4G38340.1 |  | | | |  |  |  |  |  |  |  |
| 1 | Ath-AT4G38350.2 |  | Vvi-Vitvi03g00233\_t001 |  |  |  |  |  |  |  |
| 1 | Ath-AT4G38360.2 |  | Vvi-Vitvi03g00228\_t001 |  |  |  |  |  |  |  |
| 1 | Ath-AT4G38370.1 |  | Vvi-Vitvi03g00225\_t001 |  |  |  |  |  |  |  |
| 2 | Ath-AT4G38380.2 |  | | | |  | Vvi-Vitvi03g00199\_t003 |  |  |  |  |  |  |
| 2 | Ath-AT4G38390.1 |  | | | |  | | | |  |  |  |  |  |  |
| 2 | Ath-AT4G38400.1 |  | | | |  | | | |  |  |  |  |  |  |
| 2 | Ath-AT4G38401.1 |  | | | |  | | | |  |  |  |  |  |  |
| 2 | Ath-AT4G38405.1 |  | | | |  | | | |  |  |  |  |  |  |
| 2 | Ath-AT4G38410.1 |  | | | |  | | | |  |  |  |  |  |  |
| 2 | Ath-AT4G38420.1 |  | Vvi-Vitvi03g00217\_t001 |  | | | |  |  |  |  |  |  |
| 1 | Ath-AT4G38430.1 |  |  |  | | | |  |  |  |  |  |  |
| 1 | Ath-AT4G38440.1 |  |  |  | Vvi-Vitvi03g00195\_t001 |  |  |  |  |  |  |
| 1 | Ath-AT4G38460.1 |  |  |  | Vvi-Vitvi03g01421\_t001 |  |  |  |  |  |  |
| 2 | Ath-AT4G38470.1 |  | Vvi-Vitvi04g04409\_t001 |  | Vvi-Vitvi03g01416\_t001 |  |  |  |  |  |  |
| 2 | Ath-AT4G38480.1 |  | | | |  | Vvi-Vitvi03g00187\_t001 |  |  |  |  |  |  |
| 2 | Ath-AT4G38490.1 |  | | | |  | Vvi-Vitvi03g01410\_t001 |  |  |  |  |  |  |
| 2 | Ath-AT4G38495.1 |  | | | |  | | | |  |  |  |  |  |  |
| 2 | Ath-AT4G38500.1 |  | | | |  | Vvi-Vitvi03g00179\_t001 |  |  |  |  |  |  |
| 2 | Ath-AT4G38510.5 |  | | | |  | Vvi-Vitvi03g00173\_t001 |  |  |  |  |  |  |
| 2 | Ath-AT4G38520.1 |  | Vvi-Vitvi04g02280\_t001 |  | Vvi-Vitvi03g00167\_t001 |  |  |  |  |  |  |
| 2 | Ath-AT4G38530.1 |  | | | |  | | | |  |  |  |  |  |  |
| 2 | Ath-AT4G38540.1 |  | | | |  | Vvi-Vitvi03g04066\_t001 |  |  |  |  |  |  |
| 2 | Ath-AT4G38550.2 |  | | | |  | | | |  |  |  |  |  |  |
| 2 | Ath-AT4G38560.2 |  | | | |  | | | |  |  |  |  |  |  |
| 2 | Ath-AT4G38570.1 |  | | | |  | Vvi-Vitvi03g00150\_t001 |  |  |  |  |  |  |
| 2 | Ath-AT4G38580.1 |  | Vvi-Vitvi04g02170\_t001 |  | Vvi-Vitvi03g00148\_t001 |  |  |  |  |  |  |
| 2 | Ath-AT4G38590.2 |  | Vvi-Vitvi04g04394\_t001 |  | Vvi-Vitvi03g00141\_t001 |  |  |  |  |  |  |
| 2 | Ath-AT4G38600.1 |  | Vvi-Vitvi04g01493\_t001 |  | Vvi-Vitvi03g00139\_t002 |  |  |  |  |  |  |
| 2 | Ath-AT4G38620.1 |  | Vvi-Vitvi04g01486\_t001 |  | Vvi-Vitvi03g00136\_t001 |  |  |  |  |  |  |
| 2 | Ath-AT4G38630.1 |  | Vvi-Vitvi04g01476\_t001 |  | | | |  |  |  |  |  |  |
| 2 | Ath-AT4G38640.1 |  | | | |  | Vvi-Vitvi03g00128\_t001 |  |  |  |  |  |  |
| 2 | Ath-AT4G38650.1 |  | | | |  | Vvi-Vitvi03g00125\_t001 |  |  |  |  |  |  |
| 2 | Ath-AT4G38660.1 |  | Vvi-Vitvi04g01471\_t001 |  | Vvi-Vitvi03g00124\_t001 |  |  |  |  |  |  |
| 2 | Ath-AT4G38670.1 |  | Vvi-Vitvi04g01470\_t001 |  | Vvi-Vitvi03g00123\_t001 |  |  |  |  |  |  |
| 1 | Ath-AT4G38680.1 |  |  |  | Vvi-Vitvi03g00121\_t001 |  |  |  |  |  |  |
| 1 | Ath-AT4G38690.1 |  |  |  | Vvi-Vitvi03g01396\_t001 |  |  |  |  |  |  |
| 1 | Ath-AT4G38700.1 |  |  |  | Vvi-Vitvi03g01394\_t001 |  |  |  |  |  |  |
| 1 | Ath-AT4G38710.2 |  |  |  | Vvi-Vitvi03g00107\_t001 |  |  |  |  |  |  |
| 1 | Ath-AT4G38730.1 |  |  |  | Vvi-Vitvi03g00106\_t001 |  |  |  |  |  |  |
| 1 | Ath-AT4G38740.1 |  |  |  | Vvi-Vitvi03g04055\_t001 |  |  |  |  |  |  |
| 1 | Ath-AT4G38760.1 |  |  |  | Vvi-Vitvi03g00103\_t001 |  |  |  |  |  |  |
| 1 | Ath-AT4G38770.1 |  |  |  | Vvi-Vitvi03g01388\_t001 |  |  |  |  |  |  |
| 0 | Ath-AT4G38775.1 |  |  |  |  |  |  |  |  |
| 0 | Ath-AT4G38780.1 |  |  |  |  |  |  |  |  |
| 0 | Ath-AT4G38781.1 |  |  |  |  |  |  |  |  |
| 0 | Ath-AT4G38790.1 |  |  |  |  |  |  |  |  |
| 0 | Ath-AT4G38800.1 |  |  |  |  |  |  |  |  |
| 0 | Ath-AT4G38810.2 |  |  |  |  |  |  |  |  |
| 0 | Ath-AT4G38825.1 |  |  |  |  |  |  |  |  |
| 0 | Ath-AT4G38820.1 |  |  |  |  |  |  |  |  |
| 0 | Ath-AT4G38830.1 |  |  |  |  |  |  |  |  |
| 2 | Ath-AT4G38840.1 |  | Vvi-Vitvi18g01094\_t001 |  | Vvi-Vitvi03g01346\_t001 |  |  |  |  |  |  |
| 2 | Ath-AT4G38850.1 |  | | | |  | | | |  |  |  |  |  |  |
| 2 | Ath-AT4G38860.1 |  | Vvi-Vitvi18g01093\_t001 |  | Vvi-Vitvi03g00064\_t001 |  |  |  |  |  |  |
| 2 | Ath-AT4G38870.1 |  | | | |  | | | |  |  |  |  |  |  |
| 2 | Ath-AT4G38880.1 |  | | | |  | Vvi-Vitvi03g00061\_t001 |  |  |  |  |  |  |
| 2 | Ath-AT4G38890.1 |  | | | |  | Vvi-Vitvi03g00060\_t001 |  |  |  |  |  |  |
| 2 | Ath-AT4G38900.1 |  | Vvi-Vitvi18g01068\_t001 |  | Vvi-Vitvi03g00059\_t001 |  |  |  |  |  |  |
| 2 | Ath-AT4G38905.1 |  | | | |  | | | |  |  |  |  |  |  |
| 2 | Ath-AT4G38910.2 |  | Vvi-Vitvi18g04249\_t001 |  | Vvi-Vitvi03g00058\_t001 |  |  |  |  |  |  |
| 2 | Ath-AT4G38920.1 |  | Vvi-Vitvi18g01058\_t001 |  | Vvi-Vitvi03g04022\_t001 |  |  |  |  |  |  |
| 2 | Ath-AT4G38930.3 |  | | | |  | Vvi-Vitvi03g00053\_t001 |  |  |  |  |  |  |
| 2 | Ath-AT4G38940.1 |  | | | |  | | | |  |  |  |  |  |  |
| 2 | Ath-AT4G38950.4 |  | Vvi-Vitvi18g01055\_t001 |  | Vvi-Vitvi03g00050\_t001 |  |  |  |  |  |  |
| 2 | Ath-AT4G38960.3 |  | | | |  | Vvi-Vitvi03g00049\_t001 |  |  |  |  |  |  |
| 2 | Ath-AT4G38970.1 |  | | | |  | Vvi-Vitvi03g00048\_t001 |  |  |  |  |  |  |
| 2 | Ath-AT4G38980.1 |  | | | |  | | | |  |  |  |  |  |  |
| 2 | Ath-AT4G38990.2 |  | | | |  | | | |  |  |  |  |  |  |
| 2 | Ath-AT4G39000.1 |  | | | |  | Vvi-Vitvi03g00044\_t001 |  |  |  |  |  |  |
| 2 | Ath-AT4G39010.1 |  | | | |  | | | |  |  |  |  |  |  |
| 2 | Ath-AT4G39020.1 |  | Vvi-Vitvi18g01049\_t001 |  | Vvi-Vitvi03g00035\_t001 |  |  |  |  |  |  |
| 2 | Ath-AT4G39030.1 |  | | | |  | Vvi-Vitvi03g00032\_t001 |  |  |  |  |  |  |
| 2 | Ath-AT4G39040.1 |  | | | |  | Vvi-Vitvi03g00031\_t001 |  |  |  |  |  |  |
| 2 | Ath-AT4G39050.1 |  | | | |  | Vvi-Vitvi03g00028\_t001 |  |  |  |  |  |  |
| 2 | Ath-AT4G39060.1 |  | | | |  | | | |  |  |  |  |  |  |
| 2 | Ath-AT4G39070.1 |  | Vvi-Vitvi18g01048\_t001 |  | Vvi-Vitvi03g00026\_t001 |  |  |  |  |  |  |
| 2 | Ath-AT4G39080.1 |  | Vvi-Vitvi18g01038\_t001 |  | Vvi-Vitvi03g00022\_t001 |  |  |  |  |  |  |
| 2 | Ath-AT4G39090.1 |  | Vvi-Vitvi18g01036\_t001 |  | Vvi-Vitvi03g00021\_t001 |  |  |  |  |  |  |
| 1 | Ath-AT4G39100.1 |  |  |  | Vvi-Vitvi03g00011\_t001 |  |  |  |  |  |  |
| 1 | Ath-AT4G39110.1 |  | Vvi-Vitvi03g00284\_t001 |  |  |  |  |  |  |  |
| 1 | Ath-AT4G39120.2 |  | Vvi-Vitvi03g00287\_t001 |  |  |  |  |  |  |  |
| 1 | Ath-AT4G39130.1 |  | Vvi-Vitvi03g01481\_t001 |  |  |  |  |  |  |  |
| 2 | Ath-AT4G39140.4 |  | Vvi-Vitvi03g00288\_t001 |  | Vvi-Vitvi18g02767\_t001 |  |  |  |  |  |  |
| 2 | Ath-AT4G39150.1 |  | Vvi-Vitvi03g00289\_t001 |  | | | |  |  |  |  |  |  |
| 2 | Ath-AT4G39160.2 |  | Vvi-Vitvi03g00293\_t001 |  | | | |  |  |  |  |  |  |
| 2 | Ath-AT4G39170.1 |  | Vvi-Vitvi03g00294\_t001 |  | Vvi-Vitvi18g00997\_t001 |  |  |  |  |  |  |
| 2 | Ath-AT4G39180.1 |  | Vvi-Vitvi03g00295\_t001 |  | | | |  |  |  |  |  |  |
| 2 | Ath-AT4G39190.1 |  | | | |  | | | |  |  |  |  |  |  |
| 2 | Ath-AT4G39200.1 |  | Vvi-Vitvi03g00303\_t001 |  | | | |  |  |  |  |  |  |
| 2 | Ath-AT4G39210.1 |  | Vvi-Vitvi03g00304\_t001 |  | Vvi-Vitvi18g02758\_t002 |  |  |  |  |  |  |
| 2 | Ath-AT4G39220.1 |  | Vvi-Vitvi03g00307\_t003 |  | Vvi-Vitvi18g00980\_t001 |  |  |  |  |  |  |
| 2 | Ath-AT4G39230.1 |  | Vvi-Vitvi03g01484\_t001 |  | Vvi-Vitvi18g00979\_t001 |  |  |  |  |  |  |
| 2 | Ath-AT4G39235.1 |  | | | |  | | | |  |  |  |  |  |  |
| 2 | Ath-AT4G39240.1 |  | | | |  | | | |  |  |  |  |  |  |
| 2 | Ath-AT4G39250.1 |  | Vvi-Vitvi03g01495\_t001 |  | Vvi-Vitvi18g00973\_t001 |  |  |  |  |  |  |
| 2 | Ath-AT4G39260.1 |  | Vvi-Vitvi03g00327\_t001 |  | | | |  |  |  |  |  |  |
| 2 | Ath-AT4G39270.1 |  | Vvi-Vitvi03g00328\_t002 |  | Vvi-Vitvi18g00966\_t001 |  |  |  |  |  |  |
| 1 | Ath-AT4G39280.2 |  | Vvi-Vitvi03g00331\_t001 |  |  |  |  |  |  |  |
| 1 | Ath-AT4G39290.1 |  | | | |  |  |  |  |  |  |  |
| 1 | Ath-AT4G39300.2 |  | Vvi-Vitvi03g00333\_t001 |  |  |  |  |  |  |  |
| 0 | Ath-AT4G39320.1 |  |  |  |  |  |  |  |  |
| 0 | Ath-AT4G39330.1 |  |  |  |  |  |  |  |  |
| 1 | Ath-AT4G39340.1 |  | Vvi-Vitvi07g01882\_t001 |  |  |  |  |  |  |  |
| 1 | Ath-AT4G39350.1 |  | Vvi-Vitvi07g01881\_t001 |  |  |  |  |  |  |  |
| 1 | Ath-AT4G39360.1 |  | | | |  |  |  |  |  |  |  |
| 1 | Ath-AT4G39370.3 |  | | | |  |  |  |  |  |  |  |
| 1 | Ath-AT4G39380.2 |  | Vvi-Vitvi07g01870\_t001 |  |  |  |  |  |  |  |
| 1 | Ath-AT4G39390.2 |  | Vvi-Vitvi07g01862\_t001 |  |  |  |  |  |  |  |
| 1 | Ath-AT4G39400.1 |  | Vvi-Vitvi07g01861\_t001 |  |  |  |  |  |  |  |
| 1 | Ath-AT4G39403.1 |  | | | |  |  |  |  |  |  |  |
| 1 | Ath-AT4G39410.1 |  | Vvi-Vitvi07g01860\_t001 |  |  |  |  |  |  |  |
| 1 | Ath-AT4G39420.2 |  | Vvi-Vitvi07g01858\_t002 |  |  |  |  |  |  |  |
| 1 | Ath-AT4G39460.2 |  | Vvi-Vitvi07g01857\_t001 |  |  |  |  |  |  |  |
| 1 | Ath-AT4G39470.1 |  | Vvi-Vitvi07g01852\_t001 |  |  |  |  |  |  |  |
| 1 | Ath-AT4G39480.1 |  | Vvi-Vitvi07g01842\_t001 |  |  |  |  |  |  |  |
| 1 | Ath-AT4G39490.1 |  | | | |  |  |  |  |  |  |  |
| 1 | Ath-AT4G39500.2 |  | | | |  |  |  |  |  |  |  |
| 1 | Ath-AT4G39510.1 |  | | | |  |  |  |  |  |  |  |
| 1 | Ath-AT4G39520.1 |  | Vvi-Vitvi07g01838\_t001 |  |  |  |  |  |  |  |
| 1 | Ath-AT4G39530.1 |  | Vvi-Vitvi07g01836\_t001 |  |  |  |  |  |  |  |
| 1 | Ath-AT4G39540.3 |  | Vvi-Vitvi07g01835\_t001 |  |  |  |  |  |  |  |
| 1 | Ath-AT4G39550.1 |  | Vvi-Vitvi07g02689\_t001 |  |  |  |  |  |  |  |
| 1 | Ath-AT4G39560.1 |  | | | |  |  |  |  |  |  |  |
| 1 | Ath-AT4G39570.1 |  | | | |  |  |  |  |  |  |  |
| 1 | Ath-AT4G39580.1 |  | | | |  |  |  |  |  |  |  |
| 1 | Ath-AT4G39590.1 |  | | | |  |  |  |  |  |  |  |
| 1 | Ath-AT4G39600.2 |  | | | |  |  |  |  |  |  |  |
| 1 | Ath-AT4G39610.1 |  | Vvi-Vitvi07g01828\_t001 |  |  |  |  |  |  |  |
| 1 | Ath-AT4G39620.1 |  | Vvi-Vitvi07g01811\_t001 |  |  |  |  |  |  |  |
| 1 | Ath-AT4G39630.1 |  | Vvi-Vitvi07g01810\_t001 |  |  |  |  |  |  |  |
| 1 | Ath-AT4G39640.1 |  | | | |  |  |  |  |  |  |  |
| 1 | Ath-AT4G39650.1 |  | | | |  |  |  |  |  |  |  |
| 1 | Ath-AT4G39660.1 |  | Vvi-Vitvi07g01808\_t001 |  |  |  |  |  |  |  |
| 1 | Ath-AT4G39670.1 |  | | | |  |  |  |  |  |  |  |
| 1 | Ath-AT4G39675.1 |  | | | |  |  |  |  |  |  |  |
| 1 | Ath-AT4G39680.2 |  | Vvi-Vitvi07g01800\_t001 |  |  |  |  |  |  |  |
| 1 | Ath-AT4G39690.1 |  | Vvi-Vitvi07g01797\_t001 |  |  |  |  |  |  |  |
| 1 | Ath-AT4G39700.1 |  | Vvi-Vitvi07g01794\_t001 |  |  |  |  |  |  |  |
| 1 | Ath-AT4G39710.3 |  | Vvi-Vitvi07g04732\_t003 |  |  |  |  |  |  |  |
| 2 | Ath-AT4G39720.2 |  | | | |  | Vvi-Vitvi07g01359\_t001 |  |  |  |  |  |  |
| 3 | Ath-AT4G39730.1 |  | | | |  | | | |  | Vvi-Vitvi07g01364\_t001 |  |  |  |  |  |
| 3 | Ath-AT4G39740.1 |  | | | |  | Vvi-Vitvi07g01361\_t001 |  | Vvi-Vitvi07g01361\_t001 |  |  |  |  |  |
| 3 | Ath-AT4G39745.1 |  | | | |  | | | |  | | | |  |  |  |  |  |
| 3 | Ath-AT4G39750.1 |  | | | |  | | | |  | | | |  |  |  |  |  |
| 3 | Ath-AT4G39753.1 |  | | | |  | | | |  | | | |  |  |  |  |  |
| 3 | Ath-AT4G39756.1 |  | | | |  | | | |  | | | |  |  |  |  |  |
| 3 | Ath-AT4G39760.1 |  | | | |  | | | |  | | | |  |  |  |  |  |
| 3 | Ath-AT4G39770.1 |  | | | |  | Vvi-Vitvi07g01365\_t001 |  | | | |  |  |  |  |  |
| 3 | Ath-AT4G39780.1 |  | | | |  | Vvi-Vitvi07g03030\_t001 |  | | | |  |  |  |  |  |
| 3 | Ath-AT4G39790.1 |  | | | |  | Vvi-Vitvi07g03131\_t001 |  | | | |  |  |  |  |  |
| 3 | Ath-AT4G39795.1 |  | | | |  | Vvi-Vitvi07g04543\_t001 |  | | | |  |  |  |  |  |
| 2 | Ath-AT4G39800.1 |  | | | |  |  |  | | | |  |  |  |  |  |
| 2 | Ath-AT4G39810.1 |  | | | |  |  |  | | | |  |  |  |  |  |
| 2 | Ath-AT4G39820.2 |  | Vvi-Vitvi07g02672\_t001 |  |  |  | | | |  |  |  |  |  |
| 1 | Ath-AT4G39830.1 |  |  |  |  |  | | | |  |  |  |  |  |
| 1 | Ath-AT4G39840.1 |  |  |  |  |  | | | |  |  |  |  |  |
| 2 | Ath-AT4G39850.3 |  | Vvi-Vitvi07g04559\_t001 |  |  |  | | | |  |  |  |  |  |
| 2 | Ath-AT4G39860.1 |  | Vvi-Vitvi07g04557\_t001 |  |  |  | Vvi-Vitvi07g04557\_t001 |  |  |  |  |  |
| 2 | Ath-AT4G39870.2 |  | Vvi-Vitvi07g04556\_t002 |  |  |  | | | |  |  |  |  |  |
| 2 | Ath-AT4G39880.1 |  | Vvi-Vitvi07g02548\_t001 |  |  |  | | | |  |  |  |  |  |
| 2 | Ath-AT4G39890.1 |  | Vvi-Vitvi07g04554\_t001 |  |  |  | | | |  |  |  |  |  |
| 2 | Ath-AT4G39900.1 |  | Vvi-Vitvi07g04553\_t001 |  |  |  | | | |  |  |  |  |  |
| 1 | Ath-AT4G39910.1 |  |  |  |  |  | | | |  |  |  |  |  |
| 1 | Ath-AT4G39917.1 |  |  |  |  |  | | | |  |  |  |  |  |
| 1 | Ath-AT4G39920.1 |  |  |  |  |  | | | |  |  |  |  |  |
| 1 | Ath-AT4G39925.1 |  |  |  |  |  | | | |  |  |  |  |  |
| 1 | Ath-AT4G39930.1 |  |  |  |  |  | | | |  |  |  |  |  |
| 1 | Ath-AT4G39940.1 |  |  |  |  |  | | | |  |  |  |  |  |
| 1 | Ath-AT4G39950.2 |  |  |  |  |  | | | |  |  |  |  |  |
| 1 | Ath-AT4G39952.1 |  |  |  |  |  | | | |  |  |  |  |  |
| 1 | Ath-AT4G39955.1 |  |  |  |  |  | Vvi-Vitvi07g04569\_t001 |  |  |  |  |  |
| 1 | Ath-AT4G39960.1 |  |  |  |  |  | Vvi-Vitvi07g01393\_t001 |  |  |  |  |  |
| 1 | Ath-AT4G39970.1 |  |  |  |  |  | Vvi-Vitvi07g03044\_t001 |  |  |  |  |  |
| 1 | Ath-AT4G39980.1 |  |  |  |  |  | Vvi-Vitvi07g03049\_t001 |  |  |  |  |  |
| 1 | Ath-AT4G39990.1 |  |  |  |  |  | Vvi-Vitvi07g01504\_t001 |  |  |  |  |  |
| 1 | Ath-AT4G40000.1 |  |  |  |  |  | Vvi-Vitvi07g01503\_t001 |  |  |  |  |  |
| 1 | Ath-AT4G40010.1 |  |  |  |  |  | Vvi-Vitvi07g01500\_t001 |  |  |  |  |  |
| 1 | Ath-AT4G40011.1 |  |  |  |  |  | | | |  |  |  |  |  |
| 1 | Ath-AT4G40020.1 |  |  |  |  |  | Vvi-Vitvi07g04587\_t001 |  |  |  |  |  |
| 1 | Ath-AT4G40030.2 |  |  |  |  |  | | | |  |  |  |  |  |
| 1 | Ath-AT4G40040.3 |  |  |  |  |  | | | |  |  |  |  |  |
| 1 | Ath-AT4G40042.1 |  |  |  |  |  | | | |  |  |  |  |  |
| 1 | Ath-AT4G40045.1 |  |  |  |  |  | | | |  |  |  |  |  |
| 1 | Ath-AT4G40050.1 |  |  |  |  |  | Vvi-Vitvi07g01492\_t001 |  |  |  |  |  |
| 1 | Ath-AT4G40060.1 |  |  |  |  |  | Vvi-Vitvi07g01488\_t001 |  |  |  |  |  |
| 1 | Ath-AT4G40063.1 |  |  |  |  |  | | | |  |  |  |  |  |
| 1 | Ath-AT4G40070.1 |  |  |  |  |  | | | |  |  |  |  |  |
| 1 | Ath-AT4G40080.1 |  |  |  |  |  | Vvi-Vitvi07g01471\_t001 |  |  |  |  |  |
| 1 | Ath-AT4G40090.1 |  |  |  |  |  | | | |  |  |  |  |  |
| 1 | Ath-AT4G40100.1 |  |  |  |  |  | Vvi-Vitvi07g04592\_t001 |  |  |  |  |  |
