# Supplementary material for: Genome-wide identification, characterization and gene expression of BES1 transcription factor family in grapevine (Vitis vinifera L.)
Source: Sci Rep. 2023 Jan 5;13:240. doi: 10.1038/s41598-022-24407-y (PMC9816167; doi:10.1038/s41598-022-24407-y)
Supplement: Supplementary file 3 — Supplementary Information. [file 41598_2022_24407_MOESM3_ESM.zip › Vvi_Ath/Vitis_vinifera.PN40024.v4.dna_sm.toplevel.fa.vs.Arabidopsis_thaliana.TAIR10.dna_sm.toplevel.fa.html/Ath-5.html]

|  |  |  |  |  |  |  |  |  |  |  |  |  |  |  |  |  |  |
| --- | --- | --- | --- | --- | --- | --- | --- | --- | --- | --- | --- | --- | --- | --- | --- | --- | --- |
| Duplication depth | Reference chromosome | Collinear blocks | | | | | | | | | | | | | | | |
| 0 | Ath-AT5G01010.2 |  |  |  |  |  |  |  |  |
| 0 | Ath-AT5G01015.1 |  |  |  |  |  |  |  |  |
| 0 | Ath-AT5G01017.1 |  |  |  |  |  |  |  |  |
| 0 | Ath-AT5G01020.1 |  |  |  |  |  |  |  |  |
| 2 | Ath-AT5G01030.2 |  | Vvi-Vitvi06g00563\_t001 |  | Vvi-Vitvi08g01293\_t001 |  |  |  |  |  |  |
| 2 | Ath-AT5G01040.1 |  | | | |  | | | |  |  |  |  |  |  |
| 2 | Ath-AT5G01050.1 |  | | | |  | | | |  |  |  |  |  |  |
| 2 | Ath-AT5G01060.1 |  | | | |  | | | |  |  |  |  |  |  |
| 3 | Ath-AT5G01070.1 |  | Vvi-Vitvi06g00567\_t001 |  | Vvi-Vitvi08g01297\_t001 |  | Vvi-Vitvi13g04140\_t003 |  |  |  |  |  |
| 3 | Ath-AT5G01075.1 |  | | | |  | | | |  | | | |  |  |  |  |  |
| 3 | Ath-AT5G01080.1 |  | | | |  | | | |  | | | |  |  |  |  |  |
| 3 | Ath-AT5G01090.1 |  | | | |  | Vvi-Vitvi08g01305\_t001 |  | Vvi-Vitvi13g00495\_t001 |  |  |  |  |  |
| 3 | Ath-AT5G01100.1 |  | | | |  | Vvi-Vitvi08g01308\_t001 |  | Vvi-Vitvi13g00499\_t001 |  |  |  |  |  |
| 3 | Ath-AT5G01110.2 |  | | | |  | | | |  | | | |  |  |  |  |  |
| 3 | Ath-AT5G01120.1 |  | | | |  | | | |  | | | |  |  |  |  |  |
| 3 | Ath-AT5G01130.1 |  | | | |  | | | |  | | | |  |  |  |  |  |
| 3 | Ath-AT5G01140.1 |  | | | |  | | | |  | | | |  |  |  |  |  |
| 3 | Ath-AT5G01150.1 |  | | | |  | | | |  | | | |  |  |  |  |  |
| 3 | Ath-AT5G01160.1 |  | | | |  | Vvi-Vitvi08g01314\_t001 |  | | | |  |  |  |  |  |
| 3 | Ath-AT5G01170.1 |  | Vvi-Vitvi06g00587\_t001 |  | Vvi-Vitvi08g01327\_t001 |  | | | |  |  |  |  |  |
| 3 | Ath-AT5G01180.1 |  | Vvi-Vitvi06g00590\_t001 |  | Vvi-Vitvi08g01333\_t001 |  | | | |  |  |  |  |  |
| 3 | Ath-AT5G01190.1 |  | Vvi-Vitvi06g00591\_t001 |  | Vvi-Vitvi08g01335\_t001 |  | Vvi-Vitvi13g00509\_t001 |  |  |  |  |  |
| 3 | Ath-AT5G01200.1 |  | Vvi-Vitvi06g00592\_t001 |  | Vvi-Vitvi08g01336\_t001 |  | Vvi-Vitvi13g00510\_t001 |  |  |  |  |  |
| 2 | Ath-AT5G01210.1 |  |  |  | Vvi-Vitvi08g01337\_t001 |  | Vvi-Vitvi13g00517\_t001 |  |  |  |  |  |
| 1 | Ath-AT5G01220.1 |  |  |  | Vvi-Vitvi08g01338\_t001 |  |  |  |  |  |  |
| 1 | Ath-AT5G01225.1 |  |  |  | Vvi-Vitvi08g02235\_t001 |  |  |  |  |  |  |
| 1 | Ath-AT5G01230.1 |  |  |  | Vvi-Vitvi08g01339\_t001 |  |  |  |  |  |  |
| 1 | Ath-AT5G01240.1 |  |  |  | Vvi-Vitvi08g01346\_t001 |  |  |  |  |  |  |
| 1 | Ath-AT5G01250.1 |  |  |  | Vvi-Vitvi08g01349\_t001 |  |  |  |  |  |  |
| 1 | Ath-AT5G01260.2 |  |  |  | Vvi-Vitvi08g01353\_t001 |  |  |  |  |  |  |
| 1 | Ath-AT5G01270.2 |  |  |  | Vvi-Vitvi08g01354\_t001 |  |  |  |  |  |  |
| 1 | Ath-AT5G01280.1 |  |  |  | Vvi-Vitvi08g01357\_t001 |  |  |  |  |  |  |
| 1 | Ath-AT5G01290.1 |  |  |  | | | |  |  |  |  |  |  |
| 1 | Ath-AT5G01300.1 |  |  |  | | | |  |  |  |  |  |  |
| 2 | Ath-AT5G01305.1 |  | Vvi-Vitvi08g00719\_t001 |  | | | |  |  |  |  |  |  |
| 3 | Ath-AT5G01310.1 |  | | | |  | | | |  | Vvi-Vitvi08g00691\_t001 |  |  |  |  |  |
| 4 | Ath-AT5G01320.1 |  | | | |  | | | |  | Vvi-Vitvi08g00690\_t001 |  | Vvi-Vitvi06g00673\_t001 |  |  |  |  |
| 4 | Ath-AT5G01330.1 |  | | | |  | | | |  | | | |  | | | |  |  |  |  |
| 4 | Ath-AT5G01340.1 |  | | | |  | | | |  | Vvi-Vitvi08g00684\_t001 |  | | | |  |  |  |  |
| 4 | Ath-AT5G01350.1 |  | | | |  | | | |  | Vvi-Vitvi08g00674\_t002 |  | | | |  |  |  |  |
| 4 | Ath-AT5G01360.1 |  | | | |  | | | |  | Vvi-Vitvi08g00673\_t001 |  | | | |  |  |  |  |
| 4 | Ath-AT5G01370.1 |  | | | |  | | | |  | Vvi-Vitvi08g02056\_t001 |  | Vvi-Vitvi06g01749\_t001 |  |  |  |  |
| 4 | Ath-AT5G01380.1 |  | | | |  | | | |  | Vvi-Vitvi08g00658\_t001 |  | | | |  |  |  |  |
| 4 | Ath-AT5G01390.1 |  | | | |  | | | |  | Vvi-Vitvi08g00650\_t002 |  | | | |  |  |  |  |
| 4 | Ath-AT5G01400.1 |  | | | |  | | | |  | Vvi-Vitvi08g00644\_t001 |  | | | |  |  |  |  |
| 4 | Ath-AT5G01410.1 |  | | | |  | | | |  | | | |  | Vvi-Vitvi06g00646\_t001 |  |  |  |  |
| 4 | Ath-AT5G01420.1 |  | | | |  | | | |  | | | |  | | | |  |  |  |  |
| 4 | Ath-AT5G01430.1 |  | | | |  | | | |  | | | |  | | | |  |  |  |  |
| 4 | Ath-AT5G01440.1 |  | | | |  | | | |  | | | |  | | | |  |  |  |  |
| 4 | Ath-AT5G01445.1 |  | | | |  | | | |  | | | |  | | | |  |  |  |  |
| 4 | Ath-AT5G01450.1 |  | | | |  | | | |  | Vvi-Vitvi08g00641\_t001 |  | Vvi-Vitvi06g00644\_t001 |  |  |  |  |
| 3 | Ath-AT5G01460.1 |  | | | |  | Vvi-Vitvi08g01367\_t001 |  |  |  | | | |  |  |  |  |
| 3 | Ath-AT5G01470.6 |  | | | |  | Vvi-Vitvi08g01365\_t001 |  |  |  | | | |  |  |  |  |
| 3 | Ath-AT5G01480.1 |  | | | |  | | | |  |  |  | | | |  |  |  |  |
| 3 | Ath-AT5G01490.2 |  | | | |  | Vvi-Vitvi08g02245\_t002 |  |  |  | Vvi-Vitvi06g00639\_t001 |  |  |  |  |
| 3 | Ath-AT5G01500.1 |  | | | |  | Vvi-Vitvi08g01364\_t001 |  |  |  | | | |  |  |  |  |
| 3 | Ath-AT5G01510.1 |  | | | |  | Vvi-Vitvi08g01363\_t001 |  |  |  | | | |  |  |  |  |
| 3 | Ath-AT5G01520.1 |  | | | |  | Vvi-Vitvi08g01361\_t006 |  |  |  | Vvi-Vitvi06g01748\_t001 |  |  |  |  |
| 2 | Ath-AT5G01530.1 |  | | | |  | Vvi-Vitvi08g01360\_t001 |  |  |  |  |  |  |
| 1 | Ath-AT5G01540.1 |  | Vvi-Vitvi08g00744\_t001 |  |  |  |  |  |  |  |
| 1 | Ath-AT5G01550.1 |  | | | |  |  |  |  |  |  |  |
| 1 | Ath-AT5G01560.1 |  | | | |  |  |  |  |  |  |  |
| 1 | Ath-AT5G01570.1 |  | Vvi-Vitvi08g02074\_t001 |  |  |  |  |  |  |  |
| 1 | Ath-AT5G01580.1 |  | Vvi-Vitvi08g00754\_t001 |  |  |  |  |  |  |  |
| 1 | Ath-AT5G01590.1 |  | Vvi-Vitvi08g00755\_t002 |  |  |  |  |  |  |  |
| 2 | Ath-AT5G01600.1 |  | Vvi-Vitvi08g02076\_t001 |  | Vvi-Vitvi13g00107\_t001 |  |  |  |  |  |  |
| 2 | Ath-AT5G01610.1 |  | Vvi-Vitvi08g00767\_t001 |  | | | |  |  |  |  |  |  |
| 2 | Ath-AT5G01620.3 |  | Vvi-Vitvi08g00769\_t001 |  | Vvi-Vitvi13g00113\_t001 |  |  |  |  |  |  |
| 2 | Ath-AT5G01630.1 |  | Vvi-Vitvi08g00773\_t001 |  | | | |  |  |  |  |  |  |
| 2 | Ath-AT5G01640.1 |  | Vvi-Vitvi08g00782\_t001 |  | Vvi-Vitvi13g00121\_t001 |  |  |  |  |  |  |
| 2 | Ath-AT5G01650.3 |  | Vvi-Vitvi08g00783\_t003 |  | | | |  |  |  |  |  |  |
| 2 | Ath-AT5G01660.1 |  | Vvi-Vitvi08g00786\_t002 |  | Vvi-Vitvi13g00124\_t001 |  |  |  |  |  |  |
| 2 | Ath-AT5G01670.2 |  | Vvi-Vitvi08g00789\_t001 |  | | | |  |  |  |  |  |  |
| 2 | Ath-AT5G01680.1 |  | | | |  | | | |  |  |  |  |  |  |
| 2 | Ath-AT5G01690.1 |  | | | |  | | | |  |  |  |  |  |  |
| 2 | Ath-AT5G01700.2 |  | Vvi-Vitvi08g02080\_t001 |  | | | |  |  |  |  |  |  |
| 3 | Ath-AT5G01710.1 |  | Vvi-Vitvi08g00791\_t001 |  | | | |  | Vvi-Vitvi06g00740\_t001 |  |  |  |  |  |
| 3 | Ath-AT5G01720.1 |  | Vvi-Vitvi08g00794\_t001 |  | | | |  | Vvi-Vitvi06g00742\_t001 |  |  |  |  |  |
| 3 | Ath-AT5G01730.1 |  | Vvi-Vitvi08g00799\_t001 |  | | | |  | | | |  |  |  |  |  |
| 3 | Ath-AT5G01740.1 |  | Vvi-Vitvi08g00802\_t001 |  | Vvi-Vitvi13g00131\_t001 |  | Vvi-Vitvi06g00745\_t001 |  |  |  |  |  |
| 3 | Ath-AT5G01750.2 |  | Vvi-Vitvi08g02086\_t001 |  | | | |  | Vvi-Vitvi06g01772\_t001 |  |  |  |  |  |
| 3 | Ath-AT5G01760.1 |  | Vvi-Vitvi08g00812\_t001 |  | | | |  | | | |  |  |  |  |  |
| 3 | Ath-AT5G01770.1 |  | Vvi-Vitvi08g02087\_t001 |  | | | |  | | | |  |  |  |  |  |
| 3 | Ath-AT5G01780.2 |  | Vvi-Vitvi08g00817\_t001 |  | | | |  | | | |  |  |  |  |  |
| 3 | Ath-AT5G01790.1 |  | | | |  | | | |  | | | |  |  |  |  |  |
| 3 | Ath-AT5G01800.1 |  | Vvi-Vitvi08g00826\_t002 |  | | | |  | | | |  |  |  |  |  |
| 3 | Ath-AT5G01810.1 |  | Vvi-Vitvi08g00828\_t001 |  | Vvi-Vitvi13g00149\_t001 |  | Vvi-Vitvi06g00775\_t003 |  |  |  |  |  |
| 2 | Ath-AT5G01820.1 |  | Vvi-Vitvi08g00835\_t001 |  |  |  | Vvi-Vitvi06g00779\_t001 |  |  |  |  |  |
| 2 | Ath-AT5G01830.1 |  | Vvi-Vitvi08g00836\_t001 |  |  |  | | | |  |  |  |  |  |
| 2 | Ath-AT5G01840.1 |  | Vvi-Vitvi08g00840\_t001 |  |  |  | Vvi-Vitvi06g00783\_t001 |  |  |  |  |  |
| 2 | Ath-AT5G01850.2 |  | Vvi-Vitvi08g00850\_t001 |  |  |  | | | |  |  |  |  |  |
| 2 | Ath-AT5G01860.1 |  | | | |  |  |  | | | |  |  |  |  |  |
| 2 | Ath-AT5G01870.1 |  | | | |  |  |  | | | |  |  |  |  |  |
| 2 | Ath-AT5G01880.1 |  | Vvi-Vitvi08g00857\_t001 |  |  |  | Vvi-Vitvi06g00801\_t001 |  |  |  |  |  |
| 2 | Ath-AT5G01881.1 |  | | | |  |  |  | | | |  |  |  |  |  |
| 2 | Ath-AT5G01890.1 |  | Vvi-Vitvi08g00863\_t001 |  |  |  | | | |  |  |  |  |  |
| 2 | Ath-AT5G01895.1 |  | | | |  |  |  | | | |  |  |  |  |  |
| 2 | Ath-AT5G01900.1 |  | Vvi-Vitvi08g00868\_t001 |  |  |  | | | |  |  |  |  |  |
| 2 | Ath-AT5G01910.1 |  | Vvi-Vitvi08g00871\_t001 |  |  |  | | | |  |  |  |  |  |
| 2 | Ath-AT5G01920.2 |  | Vvi-Vitvi08g02097\_t001 |  |  |  | | | |  |  |  |  |  |
| 2 | Ath-AT5G01930.1 |  | Vvi-Vitvi08g00877\_t001 |  |  |  | | | |  |  |  |  |  |
| 1 | Ath-AT5G01940.3 |  |  |  |  |  | Vvi-Vitvi06g01782\_t001 |  |  |  |  |  |
| 1 | Ath-AT5G01950.7 |  |  |  |  |  | Vvi-Vitvi06g00819\_t002 |  |  |  |  |  |
| 1 | Ath-AT5G01960.1 |  |  |  |  |  | Vvi-Vitvi06g00826\_t001 |  |  |  |  |  |
| 1 | Ath-AT5G01970.1 |  |  |  |  |  | Vvi-Vitvi06g00827\_t001 |  |  |  |  |  |
| 0 | Ath-AT5G01980.1 |  |  |  |  |  |  |  |  |
| 0 | Ath-AT5G01990.1 |  |  |  |  |  |  |  |  |
| 0 | Ath-AT5G02000.1 |  |  |  |  |  |  |  |  |
| 1 | Ath-AT5G02010.1 |  | Vvi-Vitvi08g01282\_t001 |  |  |  |  |  |  |  |
| 1 | Ath-AT5G02020.1 |  | Vvi-Vitvi08g04253\_t006 |  |  |  |  |  |  |  |
| 1 | Ath-AT5G02030.1 |  | Vvi-Vitvi08g01274\_t001 |  |  |  |  |  |  |  |
| 1 | Ath-AT5G02040.2 |  | Vvi-Vitvi08g01266\_t002 |  |  |  |  |  |  |  |
| 1 | Ath-AT5G02050.1 |  | | | |  |  |  |  |  |  |  |
| 1 | Ath-AT5G02060.1 |  | Vvi-Vitvi08g01257\_t001 |  |  |  |  |  |  |  |
| 1 | Ath-AT5G02065.1 |  | | | |  |  |  |  |  |  |  |
| 1 | Ath-AT5G02070.1 |  | Vvi-Vitvi08g01255\_t001 |  |  |  |  |  |  |  |
| 1 | Ath-AT5G02080.5 |  | | | |  |  |  |  |  |  |  |
| 1 | Ath-AT5G02090.1 |  | | | |  |  |  |  |  |  |  |
| 1 | Ath-AT5G02100.1 |  | | | |  |  |  |  |  |  |  |
| 1 | Ath-AT5G02110.1 |  | Vvi-Vitvi08g02202\_t001 |  |  |  |  |  |  |  |
| 1 | Ath-AT5G02120.1 |  | Vvi-Vitvi08g01239\_t001 |  |  |  |  |  |  |  |
| 1 | Ath-AT5G02130.1 |  | Vvi-Vitvi08g01238\_t001 |  |  |  |  |  |  |  |
| 1 | Ath-AT5G02140.1 |  | Vvi-Vitvi08g01237\_t001 |  |  |  |  |  |  |  |
| 1 | Ath-AT5G02150.1 |  | Vvi-Vitvi08g01234\_t001 |  |  |  |  |  |  |  |
| 1 | Ath-AT5G02160.1 |  | Vvi-Vitvi08g01231\_t001 |  |  |  |  |  |  |  |
| 1 | Ath-AT5G02170.1 |  | | | |  |  |  |  |  |  |  |
| 1 | Ath-AT5G02180.1 |  | | | |  |  |  |  |  |  |  |
| 1 | Ath-AT5G02190.1 |  | Vvi-Vitvi08g01230\_t001 |  |  |  |  |  |  |  |
| 1 | Ath-AT5G02200.2 |  | Vvi-Vitvi08g02198\_t001 |  |  |  |  |  |  |  |
| 1 | Ath-AT5G02210.1 |  | | | |  |  |  |  |  |  |  |
| 2 | Ath-AT5G02220.1 |  | Vvi-Vitvi08g01222\_t001 |  | Vvi-Vitvi06g01706\_t001 |  |  |  |  |  |  |
| 2 | Ath-AT5G02230.3 |  | Vvi-Vitvi08g01217\_t001 |  | Vvi-Vitvi06g00495\_t001 |  |  |  |  |  |  |
| 2 | Ath-AT5G02240.1 |  | Vvi-Vitvi08g01216\_t001 |  | | | |  |  |  |  |  |  |
| 2 | Ath-AT5G02250.1 |  | Vvi-Vitvi08g01208\_t001 |  | | | |  |  |  |  |  |  |
| 2 | Ath-AT5G02260.1 |  | Vvi-Vitvi08g01206\_t001 |  | Vvi-Vitvi06g00481\_t001 |  |  |  |  |  |  |
| 2 | Ath-AT5G02270.2 |  | | | |  | | | |  |  |  |  |  |  |
| 2 | Ath-AT5G02280.1 |  | | | |  | | | |  |  |  |  |  |  |
| 2 | Ath-AT5G02290.1 |  | Vvi-Vitvi08g01204\_t004 |  | Vvi-Vitvi06g00473\_t001 |  |  |  |  |  |  |
| 2 | Ath-AT5G02310.1 |  | Vvi-Vitvi08g01201\_t003 |  | | | |  |  |  |  |  |  |
| 2 | Ath-AT5G02320.2 |  | Vvi-Vitvi08g02196\_t001 |  | | | |  |  |  |  |  |  |
| 2 | Ath-AT5G02330.1 |  | | | |  | | | |  |  |  |  |  |  |
| 2 | Ath-AT5G02340.1 |  | | | |  | | | |  |  |  |  |  |  |
| 2 | Ath-AT5G02350.1 |  | | | |  | | | |  |  |  |  |  |  |
| 2 | Ath-AT5G02360.1 |  | | | |  | | | |  |  |  |  |  |  |
| 2 | Ath-AT5G02370.1 |  | Vvi-Vitvi08g01197\_t001 |  | | | |  |  |  |  |  |  |
| 2 | Ath-AT5G02380.1 |  | | | |  | | | |  |  |  |  |  |  |
| 2 | Ath-AT5G02390.1 |  | Vvi-Vitvi08g01195\_t001 |  | Vvi-Vitvi06g00460\_t001 |  |  |  |  |  |  |
| 2 | Ath-AT5G02400.1 |  | Vvi-Vitvi08g01194\_t001 |  | Vvi-Vitvi06g00456\_t001 |  |  |  |  |  |  |
| 2 | Ath-AT5G02410.1 |  | Vvi-Vitvi08g01192\_t001 |  | | | |  |  |  |  |  |  |
| 2 | Ath-AT5G02420.1 |  | | | |  | | | |  |  |  |  |  |  |
| 2 | Ath-AT5G02430.1 |  | Vvi-Vitvi08g02192\_t001 |  | | | |  |  |  |  |  |  |
| 2 | Ath-AT5G02440.1 |  | Vvi-Vitvi08g02191\_t001 |  | Vvi-Vitvi06g00452\_t001 |  |  |  |  |  |  |
| 2 | Ath-AT5G02450.1 |  | | | |  | | | |  |  |  |  |  |  |
| 2 | Ath-AT5G02460.1 |  | Vvi-Vitvi08g01186\_t001 |  | Vvi-Vitvi06g00449\_t001 |  |  |  |  |  |  |
| 2 | Ath-AT5G02470.3 |  | Vvi-Vitvi08g01185\_t001 |  | | | |  |  |  |  |  |  |
| 2 | Ath-AT5G02480.1 |  | Vvi-Vitvi08g01182\_t001 |  | Vvi-Vitvi06g00444\_t001 |  |  |  |  |  |  |
| 2 | Ath-AT5G02490.1 |  | Vvi-Vitvi08g02189\_t001 |  | Vvi-Vitvi06g00443\_t001 |  |  |  |  |  |  |
| 2 | Ath-AT5G02500.1 |  | | | |  | | | |  |  |  |  |  |  |
| 2 | Ath-AT5G02502.1 |  | Vvi-Vitvi08g04214\_t001 |  | | | |  |  |  |  |  |  |
| 2 | Ath-AT5G02510.2 |  | Vvi-Vitvi08g01178\_t001 |  | | | |  |  |  |  |  |  |
| 2 | Ath-AT5G02520.2 |  | Vvi-Vitvi08g02185\_t001 |  | | | |  |  |  |  |  |  |
| 2 | Ath-AT5G02530.1 |  | | | |  | Vvi-Vitvi06g00429\_t002 |  |  |  |  |  |  |
| 2 | Ath-AT5G02540.1 |  | Vvi-Vitvi08g01168\_t001 |  | | | |  |  |  |  |  |  |
| 2 | Ath-AT5G02550.1 |  | | | |  | | | |  |  |  |  |  |  |
| 2 | Ath-AT5G02560.2 |  | Vvi-Vitvi08g01159\_t001 |  | Vvi-Vitvi06g00423\_t001 |  |  |  |  |  |  |
| 1 | Ath-AT5G02570.1 |  | | | |  |  |  |  |  |  |  |
| 3 | Ath-AT5G02580.1 |  | | | |  | Vvi-Vitvi13g01932\_t001 |  | Vvi-Vitvi08g02169\_t001 |  |  |  |  |  |
| 3 | Ath-AT5G02590.1 |  | | | |  | | | |  | Vvi-Vitvi08g01095\_t001 |  |  |  |  |  |
| 4 | Ath-AT5G02600.2 |  | | | |  | | | |  | Vvi-Vitvi08g01097\_t001 |  | Vvi-Vitvi06g00399\_t001 |  |  |  |  |
| 4 | Ath-AT5G02610.2 |  | | | |  | Vvi-Vitvi13g00234\_t001 |  | | | |  | Vvi-Vitvi06g00398\_t001 |  |  |  |  |
| 4 | Ath-AT5G02620.2 |  | | | |  | | | |  | Vvi-Vitvi08g01104\_t003 |  | Vvi-Vitvi06g00393\_t001 |  |  |  |  |
| 4 | Ath-AT5G02630.1 |  | | | |  | Vvi-Vitvi13g00226\_t001 |  | Vvi-Vitvi08g01109\_t001 |  | | | |  |  |  |  |
| 4 | Ath-AT5G02640.1 |  | | | |  | | | |  | Vvi-Vitvi08g04198\_t001 |  | Vvi-Vitvi06g00387\_t001 |  |  |  |  |
| 4 | Ath-AT5G02650.1 |  | | | |  | | | |  | | | |  | | | |  |  |  |  |
| 4 | Ath-AT5G02660.1 |  | | | |  | | | |  | | | |  | | | |  |  |  |  |
| 4 | Ath-AT5G02670.1 |  | | | |  | | | |  | | | |  | | | |  |  |  |  |
| 4 | Ath-AT5G02680.1 |  | | | |  | | | |  | | | |  | | | |  |  |  |  |
| 4 | Ath-AT5G02690.1 |  | | | |  | | | |  | | | |  | | | |  |  |  |  |
| 4 | Ath-AT5G02700.1 |  | | | |  | | | |  | | | |  | | | |  |  |  |  |
| 4 | Ath-AT5G02710.1 |  | | | |  | | | |  | Vvi-Vitvi08g01114\_t001 |  | | | |  |  |  |  |
| 4 | Ath-AT5G02720.2 |  | | | |  | | | |  | Vvi-Vitvi08g04200\_t001 |  | | | |  |  |  |  |
| 4 | Ath-AT5G02730.1 |  | | | |  | | | |  | | | |  | | | |  |  |  |  |
| 4 | Ath-AT5G02740.1 |  | | | |  | | | |  | Vvi-Vitvi08g01118\_t001 |  | | | |  |  |  |  |
| 4 | Ath-AT5G02750.1 |  | | | |  | | | |  | Vvi-Vitvi08g01123\_t001 |  | | | |  |  |  |  |
| 4 | Ath-AT5G02760.1 |  | | | |  | | | |  | | | |  | | | |  |  |  |  |
| 4 | Ath-AT5G02770.1 |  | | | |  | | | |  | Vvi-Vitvi08g01125\_t001 |  | | | |  |  |  |  |
| 4 | Ath-AT5G02780.1 |  | | | |  | Vvi-Vitvi13g00208\_t001 |  | Vvi-Vitvi08g01129\_t001 |  | Vvi-Vitvi06g00372\_t001 |  |  |  |  |
| 4 | Ath-AT5G02790.1 |  | | | |  | | | |  | | | |  | | | |  |  |  |  |
| 4 | Ath-AT5G02800.1 |  | | | |  | Vvi-Vitvi13g00205\_t001 |  | Vvi-Vitvi08g01133\_t002 |  | Vvi-Vitvi06g00369\_t001 |  |  |  |  |
| 4 | Ath-AT5G02810.1 |  | | | |  | Vvi-Vitvi13g00203\_t004 |  | Vvi-Vitvi08g01139\_t001 |  | Vvi-Vitvi06g00368\_t001 |  |  |  |  |
| 2 | Ath-AT5G02820.1 |  | Vvi-Vitvi08g01153\_t001 |  |  |  | | | |  |  |  |  |  |
| 2 | Ath-AT5G02830.1 |  | Vvi-Vitvi08g01152\_t003 |  |  |  | | | |  |  |  |  |  |
| 2 | Ath-AT5G02840.1 |  | Vvi-Vitvi08g01149\_t002 |  |  |  | | | |  |  |  |  |  |
| 2 | Ath-AT5G02850.1 |  | Vvi-Vitvi08g01148\_t001 |  |  |  | | | |  |  |  |  |  |
| 1 | Ath-AT5G02860.3 |  |  |  |  |  | | | |  |  |  |  |  |
| 1 | Ath-AT5G02870.1 |  |  |  |  |  | Vvi-Vitvi08g01147\_t001 |  |  |  |  |  |
| 1 | Ath-AT5G02880.1 |  |  |  |  |  | Vvi-Vitvi08g01155\_t001 |  |  |  |  |  |
| 0 | Ath-AT5G02890.1 |  |  |  |  |  |  |  |  |
| 0 | Ath-AT5G02900.1 |  |  |  |  |  |  |  |  |
| 0 | Ath-AT5G02910.1 |  |  |  |  |  |  |  |  |
| 0 | Ath-AT5G02920.1 |  |  |  |  |  |  |  |  |
| 0 | Ath-AT5G02930.1 |  |  |  |  |  |  |  |  |
| 0 | Ath-AT5G02940.2 |  |  |  |  |  |  |  |  |
| 1 | Ath-AT5G02950.2 |  | Vvi-Vitvi08g01078\_t001 |  |  |  |  |  |  |  |
| 1 | Ath-AT5G02960.1 |  | Vvi-Vitvi08g01076\_t001 |  |  |  |  |  |  |  |
| 1 | Ath-AT5G02970.1 |  | Vvi-Vitvi08g01073\_t001 |  |  |  |  |  |  |  |
| 1 | Ath-AT5G02980.1 |  | | | |  |  |  |  |  |  |  |
| 1 | Ath-AT5G02990.1 |  | | | |  |  |  |  |  |  |  |
| 1 | Ath-AT5G02995.1 |  | | | |  |  |  |  |  |  |  |
| 1 | Ath-AT5G03000.1 |  | | | |  |  |  |  |  |  |  |
| 1 | Ath-AT5G03010.1 |  | | | |  |  |  |  |  |  |  |
| 1 | Ath-AT5G03020.1 |  | | | |  |  |  |  |  |  |  |
| 1 | Ath-AT5G03030.2 |  | Vvi-Vitvi08g01072\_t001 |  |  |  |  |  |  |  |
| 1 | Ath-AT5G03040.1 |  | Vvi-Vitvi08g01071\_t001 |  |  |  |  |  |  |  |
| 1 | Ath-AT5G03050.1 |  | | | |  |  |  |  |  |  |  |
| 1 | Ath-AT5G03060.1 |  | | | |  |  |  |  |  |  |  |
| 1 | Ath-AT5G03070.1 |  | Vvi-Vitvi08g01065\_t001 |  |  |  |  |  |  |  |
| 1 | Ath-AT5G03080.1 |  | Vvi-Vitvi08g01063\_t001 |  |  |  |  |  |  |  |
| 1 | Ath-AT5G03090.1 |  | | | |  |  |  |  |  |  |  |
| 1 | Ath-AT5G03100.1 |  | | | |  |  |  |  |  |  |  |
| 1 | Ath-AT5G03110.1 |  | Vvi-Vitvi08g01061\_t001 |  |  |  |  |  |  |  |
| 1 | Ath-AT5G03120.2 |  | Vvi-Vitvi08g02162\_t001 |  |  |  |  |  |  |  |
| 1 | Ath-AT5G03130.1 |  | | | |  |  |  |  |  |  |  |
| 1 | Ath-AT5G03140.1 |  | Vvi-Vitvi08g01059\_t001 |  |  |  |  |  |  |  |
| 1 | Ath-AT5G03150.1 |  | Vvi-Vitvi08g01050\_t003 |  |  |  |  |  |  |  |
| 1 | Ath-AT5G03160.1 |  | Vvi-Vitvi08g01049\_t001 |  |  |  |  |  |  |  |
| 1 | Ath-AT5G03170.1 |  | Vvi-Vitvi08g02147\_t001 |  |  |  |  |  |  |  |
| 1 | Ath-AT5G03180.2 |  | Vvi-Vitvi08g01043\_t001 |  |  |  |  |  |  |  |
| 1 | Ath-AT5G03190.2 |  | | | |  |  |  |  |  |  |  |
| 1 | Ath-AT5G03200.1 |  | Vvi-Vitvi08g01042\_t001 |  |  |  |  |  |  |  |
| 1 | Ath-AT5G03204.1 |  | | | |  |  |  |  |  |  |  |
| 1 | Ath-AT5G03210.2 |  | | | |  |  |  |  |  |  |  |
| 1 | Ath-AT5G03220.1 |  | | | |  |  |  |  |  |  |  |
| 1 | Ath-AT5G03230.1 |  | Vvi-Vitvi08g01036\_t001 |  |  |  |  |  |  |  |
| 1 | Ath-AT5G03240.1 |  | | | |  |  |  |  |  |  |  |
| 1 | Ath-AT5G03250.1 |  | Vvi-Vitvi08g01032\_t001 |  |  |  |  |  |  |  |
| 1 | Ath-AT5G03260.1 |  | Vvi-Vitvi08g01031\_t001 |  |  |  |  |  |  |  |
| 1 | Ath-AT5G03270.1 |  | Vvi-Vitvi08g01030\_t001 |  |  |  |  |  |  |  |
| 1 | Ath-AT5G03280.1 |  | Vvi-Vitvi08g01023\_t001 |  |  |  |  |  |  |  |
| 1 | Ath-AT5G03290.1 |  | Vvi-Vitvi08g01021\_t001 |  |  |  |  |  |  |  |
| 1 | Ath-AT5G03300.1 |  | | | |  |  |  |  |  |  |  |
| 1 | Ath-AT5G03310.1 |  | Vvi-Vitvi08g01016\_t001 |  |  |  |  |  |  |  |
| 1 | Ath-AT5G03320.2 |  | | | |  |  |  |  |  |  |  |
| 1 | Ath-AT5G03330.1 |  | Vvi-Vitvi08g01010\_t005 |  |  |  |  |  |  |  |
| 1 | Ath-AT5G03340.1 |  | Vvi-Vitvi08g01002\_t001 |  |  |  |  |  |  |  |
| 1 | Ath-AT5G03345.2 |  | Vvi-Vitvi08g02135\_t001 |  |  |  |  |  |  |  |
| 1 | Ath-AT5G03350.1 |  | | | |  |  |  |  |  |  |  |
| 1 | Ath-AT5G03355.1 |  | | | |  |  |  |  |  |  |  |
| 1 | Ath-AT5G03360.1 |  | | | |  |  |  |  |  |  |  |
| 1 | Ath-AT5G03370.1 |  | Vvi-Vitvi08g00993\_t001 |  |  |  |  |  |  |  |
| 1 | Ath-AT5G03380.1 |  | Vvi-Vitvi08g00992\_t001 |  |  |  |  |  |  |  |
| 1 | Ath-AT5G03390.1 |  | | | |  |  |  |  |  |  |  |
| 1 | Ath-AT5G03400.1 |  | | | |  |  |  |  |  |  |  |
| 1 | Ath-AT5G03406.1 |  | Vvi-Vitvi08g00989\_t001 |  |  |  |  |  |  |  |
| 1 | Ath-AT5G03415.1 |  | Vvi-Vitvi08g00987\_t001 |  |  |  |  |  |  |  |
| 1 | Ath-AT5G03420.1 |  | Vvi-Vitvi08g01622\_t001 |  |  |  |  |  |  |  |
| 1 | Ath-AT5G03430.1 |  | Vvi-Vitvi08g01618\_t001 |  |  |  |  |  |  |  |
| 1 | Ath-AT5G03435.1 |  | | | |  |  |  |  |  |  |  |
| 1 | Ath-AT5G03440.1 |  | Vvi-Vitvi08g04348\_t001 |  |  |  |  |  |  |  |
| 1 | Ath-AT5G03450.1 |  | Vvi-Vitvi08g01616\_t001 |  |  |  |  |  |  |  |
| 1 | Ath-AT5G03455.1 |  | Vvi-Vitvi08g01615\_t001 |  |  |  |  |  |  |  |
| 1 | Ath-AT5G03460.1 |  | Vvi-Vitvi08g02296\_t002 |  |  |  |  |  |  |  |
| 1 | Ath-AT5G03470.1 |  | Vvi-Vitvi08g01609\_t001 |  |  |  |  |  |  |  |
| 1 | Ath-AT5G03480.1 |  | | | |  |  |  |  |  |  |  |
| 1 | Ath-AT5G03490.1 |  | | | |  |  |  |  |  |  |  |
| 1 | Ath-AT5G03495.1 |  | | | |  |  |  |  |  |  |  |
| 1 | Ath-AT5G03500.6 |  | | | |  |  |  |  |  |  |  |
| 1 | Ath-AT5G03510.1 |  | Vvi-Vitvi08g01600\_t001 |  |  |  |  |  |  |  |
| 1 | Ath-AT5G03520.1 |  | Vvi-Vitvi08g01597\_t001 |  |  |  |  |  |  |  |
| 1 | Ath-AT5G03530.1 |  | Vvi-Vitvi08g01596\_t001 |  |  |  |  |  |  |  |
| 1 | Ath-AT5G03540.3 |  | | | |  |  |  |  |  |  |  |
| 1 | Ath-AT5G03545.1 |  | | | |  |  |  |  |  |  |  |
| 1 | Ath-AT5G03550.1 |  | | | |  |  |  |  |  |  |  |
| 1 | Ath-AT5G03553.1 |  | | | |  |  |  |  |  |  |  |
| 1 | Ath-AT5G03555.1 |  | Vvi-Vitvi08g01577\_t001 |  |  |  |  |  |  |  |
| 0 | Ath-AT5G03560.2 |  |  |  |  |  |  |  |  |
| 0 | Ath-AT5G03570.3 |  |  |  |  |  |  |  |  |
| 0 | Ath-AT5G03580.1 |  |  |  |  |  |  |  |  |
| 1 | Ath-AT5G03590.3 |  | Vvi-Vitvi08g01488\_t001 |  |  |  |  |  |  |  |
| 1 | Ath-AT5G03600.2 |  | | | |  |  |  |  |  |  |  |
| 1 | Ath-AT5G03610.1 |  | | | |  |  |  |  |  |  |  |
| 2 | Ath-AT5G03620.1 |  | | | |  | Vvi-Vitvi08g01485\_t001 |  |  |  |  |  |  |
| 2 | Ath-AT5G03630.1 |  | | | |  | Vvi-Vitvi08g01483\_t001 |  |  |  |  |  |  |
| 2 | Ath-AT5G03640.1 |  | | | |  | Vvi-Vitvi08g01482\_t001 |  |  |  |  |  |  |
| 2 | Ath-AT5G03650.1 |  | Vvi-Vitvi08g01497\_t001 |  | | | |  |  |  |  |  |  |
| 2 | Ath-AT5G03660.1 |  | Vvi-Vitvi08g01499\_t001 |  | | | |  |  |  |  |  |  |
| 2 | Ath-AT5G03670.1 |  | Vvi-Vitvi08g01500\_t001 |  | | | |  |  |  |  |  |  |
| 2 | Ath-AT5G03680.1 |  | Vvi-Vitvi08g01505\_t001 |  | | | |  |  |  |  |  |  |
| 2 | Ath-AT5G03690.1 |  | Vvi-Vitvi08g01506\_t001 |  | | | |  |  |  |  |  |  |
| 2 | Ath-AT5G03700.1 |  | Vvi-Vitvi08g01509\_t001 |  | | | |  |  |  |  |  |  |
| 2 | Ath-AT5G03710.1 |  | | | |  | | | |  |  |  |  |  |  |
| 2 | Ath-AT5G03720.1 |  | Vvi-Vitvi08g01513\_t001 |  | | | |  |  |  |  |  |  |
| 2 | Ath-AT5G03730.1 |  | Vvi-Vitvi08g01514\_t001 |  | | | |  |  |  |  |  |  |
| 2 | Ath-AT5G03740.1 |  | Vvi-Vitvi08g01518\_t002 |  | | | |  |  |  |  |  |  |
| 2 | Ath-AT5G03750.1 |  | | | |  | | | |  |  |  |  |  |  |
| 2 | Ath-AT5G03760.1 |  | Vvi-Vitvi08g01523\_t002 |  | | | |  |  |  |  |  |  |
| 2 | Ath-AT5G03770.1 |  | Vvi-Vitvi08g01536\_t001 |  | | | |  |  |  |  |  |  |
| 2 | Ath-AT5G03780.1 |  | | | |  | | | |  |  |  |  |  |  |
| 2 | Ath-AT5G03790.1 |  | Vvi-Vitvi08g01543\_t001 |  | | | |  |  |  |  |  |  |
| 2 | Ath-AT5G03795.1 |  | Vvi-Vitvi08g01548\_t001 |  | | | |  |  |  |  |  |  |
| 2 | Ath-AT5G03800.1 |  | Vvi-Vitvi08g01550\_t001 |  | | | |  |  |  |  |  |  |
| 2 | Ath-AT5G03810.1 |  | Vvi-Vitvi08g01553\_t001 |  | | | |  |  |  |  |  |  |
| 1 | Ath-AT5G03820.2 |  |  |  | | | |  |  |  |  |  |  |
| 1 | Ath-AT5G03830.2 |  |  |  | | | |  |  |  |  |  |  |
| 1 | Ath-AT5G03840.1 |  |  |  | Vvi-Vitvi08g01473\_t001 |  |  |  |  |  |  |
| 1 | Ath-AT5G03850.1 |  |  |  | | | |  |  |  |  |  |  |
| 1 | Ath-AT5G03860.1 |  |  |  | | | |  |  |  |  |  |  |
| 1 | Ath-AT5G03870.1 |  |  |  | | | |  |  |  |  |  |  |
| 1 | Ath-AT5G03880.1 |  |  |  | | | |  |  |  |  |  |  |
| 1 | Ath-AT5G03885.1 |  |  |  | | | |  |  |  |  |  |  |
| 1 | Ath-AT5G03890.1 |  |  |  | | | |  |  |  |  |  |  |
| 1 | Ath-AT5G03900.2 |  |  |  | | | |  |  |  |  |  |  |
| 1 | Ath-AT5G03905.1 |  |  |  | Vvi-Vitvi08g01461\_t001 |  |  |  |  |  |  |
| 1 | Ath-AT5G03910.1 |  |  |  | Vvi-Vitvi08g01448\_t001 |  |  |  |  |  |  |
| 1 | Ath-AT5G03920.1 |  |  |  | | | |  |  |  |  |  |  |
| 1 | Ath-AT5G03930.1 |  |  |  | | | |  |  |  |  |  |  |
| 1 | Ath-AT5G03940.1 |  |  |  | Vvi-Vitvi08g01444\_t001 |  |  |  |  |  |  |
| 0 | Ath-AT5G03944.1 |  |  |  |  |  |  |  |  |
| 1 | Ath-AT5G03960.3 |  | Vvi-Vitvi08g02330\_t001 |  |  |  |  |  |  |  |
| 1 | Ath-AT5G03970.1 |  | | | |  |  |  |  |  |  |  |
| 1 | Ath-AT5G03980.1 |  | | | |  |  |  |  |  |  |  |
| 1 | Ath-AT5G03990.1 |  | Vvi-Vitvi08g01697\_t001 |  |  |  |  |  |  |  |
| 1 | Ath-AT5G03995.1 |  | | | |  |  |  |  |  |  |  |
| 1 | Ath-AT5G04000.2 |  | | | |  |  |  |  |  |  |  |
| 1 | Ath-AT5G04010.1 |  | Vvi-Vitvi08g01690\_t001 |  |  |  |  |  |  |  |
| 1 | Ath-AT5G04020.2 |  | Vvi-Vitvi08g01685\_t001 |  |  |  |  |  |  |  |
| 1 | Ath-AT5G04030.1 |  | | | |  |  |  |  |  |  |  |
| 1 | Ath-AT5G04040.1 |  | Vvi-Vitvi08g01684\_t001 |  |  |  |  |  |  |  |
| 1 | Ath-AT5G04045.1 |  | | | |  |  |  |  |  |  |  |
| 1 | Ath-AT5G04047.1 |  | | | |  |  |  |  |  |  |  |
| 1 | Ath-AT5G04050.2 |  | Vvi-Vitvi08g01682\_t001 |  |  |  |  |  |  |  |
| 1 | Ath-AT5G04060.1 |  | Vvi-Vitvi08g01679\_t001 |  |  |  |  |  |  |  |
| 0 | Ath-AT5G04070.1 |  |  |  |  |  |  |  |  |
| 0 | Ath-AT5G04080.1 |  |  |  |  |  |  |  |  |
| 0 | Ath-AT5G04090.6 |  |  |  |  |  |  |  |  |
| 0 | Ath-AT5G04110.2 |  |  |  |  |  |  |  |  |
| 0 | Ath-AT5G04120.1 |  |  |  |  |  |  |  |  |
| 0 | Ath-AT5G04130.1 |  |  |  |  |  |  |  |  |
| 0 | Ath-AT5G04140.2 |  |  |  |  |  |  |  |  |
| 0 | Ath-AT5G04150.1 |  |  |  |  |  |  |  |  |
| 0 | Ath-AT5G04160.1 |  |  |  |  |  |  |  |  |
| 0 | Ath-AT5G04170.1 |  |  |  |  |  |  |  |  |
| 0 | Ath-AT5G04180.1 |  |  |  |  |  |  |  |  |
| 0 | Ath-AT5G04190.1 |  |  |  |  |  |  |  |  |
| 0 | Ath-AT5G04200.1 |  |  |  |  |  |  |  |  |
| 0 | Ath-AT5G04210.1 |  |  |  |  |  |  |  |  |
| 1 | Ath-AT5G04220.2 |  | Vvi-Vitvi13g00621\_t001 |  |  |  |  |  |  |  |
| 1 | Ath-AT5G04230.2 |  | Vvi-Vitvi13g00622\_t001 |  |  |  |  |  |  |  |
| 1 | Ath-AT5G04240.1 |  | Vvi-Vitvi13g04179\_t002 |  |  |  |  |  |  |  |
| 1 | Ath-AT5G04238.1 |  | | | |  |  |  |  |  |  |  |
| 1 | Ath-AT5G04250.2 |  | Vvi-Vitvi13g00640\_t001 |  |  |  |  |  |  |  |
| 1 | Ath-AT5G04260.1 |  | Vvi-Vitvi13g04181\_t001 |  |  |  |  |  |  |  |
| 1 | Ath-AT5G04267.1 |  | | | |  |  |  |  |  |  |  |
| 1 | Ath-AT5G04270.1 |  | Vvi-Vitvi13g00647\_t001 |  |  |  |  |  |  |  |
| 1 | Ath-AT5G04280.1 |  | | | |  |  |  |  |  |  |  |
| 1 | Ath-AT5G04290.1 |  | Vvi-Vitvi13g00674\_t001 |  |  |  |  |  |  |  |
| 1 | Ath-AT5G04310.1 |  | | | |  |  |  |  |  |  |  |
| 1 | Ath-AT5G04320.5 |  | | | |  |  |  |  |  |  |  |
| 1 | Ath-AT5G04330.1 |  | | | |  |  |  |  |  |  |  |
| 1 | Ath-AT5G04340.1 |  | | | |  |  |  |  |  |  |  |
| 1 | Ath-AT5G04347.2 |  | | | |  |  |  |  |  |  |  |
| 1 | Ath-AT5G04350.1 |  | | | |  |  |  |  |  |  |  |
| 1 | Ath-AT5G04360.1 |  | Vvi-Vitvi13g00692\_t001 |  |  |  |  |  |  |  |
| 1 | Ath-AT5G04370.2 |  | | | |  |  |  |  |  |  |  |
| 1 | Ath-AT5G04380.3 |  | | | |  |  |  |  |  |  |  |
| 1 | Ath-AT5G04390.1 |  | Vvi-Vitvi13g00694\_t001 |  |  |  |  |  |  |  |
| 1 | Ath-AT5G04395.1 |  | | | |  |  |  |  |  |  |  |
| 1 | Ath-AT5G04400.1 |  | | | |  |  |  |  |  |  |  |
| 1 | Ath-AT5G04410.1 |  | Vvi-Vitvi13g00698\_t001 |  |  |  |  |  |  |  |
| 1 | Ath-AT5G04420.2 |  | Vvi-Vitvi13g00703\_t001 |  |  |  |  |  |  |  |
| 1 | Ath-AT5G04430.2 |  | Vvi-Vitvi13g00705\_t002 |  |  |  |  |  |  |  |
| 1 | Ath-AT5G04440.1 |  | Vvi-Vitvi13g00707\_t001 |  |  |  |  |  |  |  |
| 1 | Ath-AT5G04460.1 |  | Vvi-Vitvi13g00708\_t001 |  |  |  |  |  |  |  |
| 1 | Ath-AT5G04470.1 |  | | | |  |  |  |  |  |  |  |
| 1 | Ath-AT5G04475.1 |  | | | |  |  |  |  |  |  |  |
| 1 | Ath-AT5G04480.1 |  | Vvi-Vitvi13g01243\_t001 |  |  |  |  |  |  |  |
| 1 | Ath-AT5G04490.1 |  | Vvi-Vitvi13g00712\_t001 |  |  |  |  |  |  |  |
| 1 | Ath-AT5G04500.1 |  | Vvi-Vitvi13g00714\_t001 |  |  |  |  |  |  |  |
| 1 | Ath-AT5G04510.1 |  | Vvi-Vitvi13g00715\_t001 |  |  |  |  |  |  |  |
| 1 | Ath-AT5G04520.1 |  | Vvi-Vitvi13g00720\_t001 |  |  |  |  |  |  |  |
| 1 | Ath-AT5G04530.1 |  | | | |  |  |  |  |  |  |  |
| 1 | Ath-AT5G04540.1 |  | Vvi-Vitvi13g00731\_t001 |  |  |  |  |  |  |  |
| 1 | Ath-AT5G04550.2 |  | | | |  |  |  |  |  |  |  |
| 1 | Ath-AT5G04560.2 |  | | | |  |  |  |  |  |  |  |
| 1 | Ath-AT5G04590.1 |  | | | |  |  |  |  |  |  |  |
| 1 | Ath-AT5G04600.1 |  | | | |  |  |  |  |  |  |  |
| 1 | Ath-AT5G04610.1 |  | | | |  |  |  |  |  |  |  |
| 1 | Ath-AT5G04620.2 |  | | | |  |  |  |  |  |  |  |
| 1 | Ath-AT5G04630.1 |  | Vvi-Vitvi13g00738\_t001 |  |  |  |  |  |  |  |
| 0 | Ath-AT5G04640.1 |  |  |  |  |  |  |  |  |
| 0 | Ath-AT5G04670.1 |  |  |  |  |  |  |  |  |
| 0 | Ath-AT5G04660.1 |  |  |  |  |  |  |  |  |
| 0 | Ath-AT5G04680.1 |  |  |  |  |  |  |  |  |
| 0 | Ath-AT5G04690.2 |  |  |  |  |  |  |  |  |
| 0 | Ath-AT5G04700.1 |  |  |  |  |  |  |  |  |
| 0 | Ath-AT5G04710.1 |  |  |  |  |  |  |  |  |
| 0 | Ath-AT5G04720.1 |  |  |  |  |  |  |  |  |
| 0 | Ath-AT5G04730.1 |  |  |  |  |  |  |  |  |
| 0 | Ath-AT5G04740.1 |  |  |  |  |  |  |  |  |
| 1 | Ath-AT5G04750.1 |  | Vvi-Vitvi13g04316\_t001 |  |  |  |  |  |  |  |
| 1 | Ath-AT5G04760.1 |  | | | |  |  |  |  |  |  |  |
| 1 | Ath-AT5G04770.1 |  | Vvi-Vitvi13g00991\_t001 |  |  |  |  |  |  |  |
| 1 | Ath-AT5G04780.1 |  | Vvi-Vitvi13g00994\_t001 |  |  |  |  |  |  |  |
| 1 | Ath-AT5G04800.2 |  | Vvi-Vitvi13g00996\_t001 |  |  |  |  |  |  |  |
| 1 | Ath-AT5G04790.1 |  | | | |  |  |  |  |  |  |  |
| 1 | Ath-AT5G04810.1 |  | Vvi-Vitvi13g00998\_t001 |  |  |  |  |  |  |  |
| 1 | Ath-AT5G04820.1 |  | Vvi-Vitvi13g01001\_t001 |  |  |  |  |  |  |  |
| 1 | Ath-AT5G04830.2 |  | Vvi-Vitvi13g01007\_t001 |  |  |  |  |  |  |  |
| 1 | Ath-AT5G04840.1 |  | | | |  |  |  |  |  |  |  |
| 1 | Ath-AT5G04850.2 |  | Vvi-Vitvi13g01031\_t001 |  |  |  |  |  |  |  |
| 1 | Ath-AT5G04853.1 |  | | | |  |  |  |  |  |  |  |
| 1 | Ath-AT5G04860.1 |  | | | |  |  |  |  |  |  |  |
| 1 | Ath-AT5G04870.1 |  | | | |  |  |  |  |  |  |  |
| 1 | Ath-AT5G04885.2 |  | Vvi-Vitvi13g01050\_t001 |  |  |  |  |  |  |  |
| 1 | Ath-AT5G04890.1 |  | Vvi-Vitvi13g01074\_t001 |  |  |  |  |  |  |  |
| 1 | Ath-AT5G04895.1 |  | | | |  |  |  |  |  |  |  |
| 1 | Ath-AT5G04900.1 |  | | | |  |  |  |  |  |  |  |
| 1 | Ath-AT5G04910.1 |  | | | |  |  |  |  |  |  |  |
| 1 | Ath-AT5G04920.1 |  | | | |  |  |  |  |  |  |  |
| 1 | Ath-AT5G04930.1 |  | | | |  |  |  |  |  |  |  |
| 1 | Ath-AT5G04933.1 |  | | | |  |  |  |  |  |  |  |
| 1 | Ath-AT5G04937.1 |  | | | |  |  |  |  |  |  |  |
| 1 | Ath-AT5G04940.2 |  | Vvi-Vitvi13g01110\_t002 |  |  |  |  |  |  |  |
| 1 | Ath-AT5G04950.1 |  | | | |  |  |  |  |  |  |  |
| 1 | Ath-AT5G04960.1 |  | | | |  |  |  |  |  |  |  |
| 1 | Ath-AT5G04970.1 |  | Vvi-Vitvi13g01123\_t001 |  |  |  |  |  |  |  |
| 0 | Ath-AT5G04980.2 |  |  |  |  |  |  |  |  |
| 0 | Ath-AT5G04990.1 |  |  |  |  |  |  |  |  |
| 0 | Ath-AT5G05000.1 |  |  |  |  |  |  |  |  |
| 0 | Ath-AT5G05010.1 |  |  |  |  |  |  |  |  |
| 0 | Ath-AT5G05020.1 |  |  |  |  |  |  |  |  |
| 0 | Ath-AT5G05030.1 |  |  |  |  |  |  |  |  |
| 0 | Ath-AT5G05040.1 |  |  |  |  |  |  |  |  |
| 0 | Ath-AT5G05050.1 |  |  |  |  |  |  |  |  |
| 0 | Ath-AT5G05060.1 |  |  |  |  |  |  |  |  |
| 0 | Ath-AT5G05070.1 |  |  |  |  |  |  |  |  |
| 0 | Ath-AT5G05080.1 |  |  |  |  |  |  |  |  |
| 0 | Ath-AT5G05085.1 |  |  |  |  |  |  |  |  |
| 0 | Ath-AT5G05090.1 |  |  |  |  |  |  |  |  |
| 0 | Ath-AT5G05100.1 |  |  |  |  |  |  |  |  |
| 0 | Ath-AT5G05110.1 |  |  |  |  |  |  |  |  |
| 0 | Ath-AT5G05113.1 |  |  |  |  |  |  |  |  |
| 0 | Ath-AT5G05120.1 |  |  |  |  |  |  |  |  |
| 0 | Ath-AT5G05130.1 |  |  |  |  |  |  |  |  |
| 0 | Ath-AT5G05140.1 |  |  |  |  |  |  |  |  |
| 0 | Ath-AT5G05150.1 |  |  |  |  |  |  |  |  |
| 0 | Ath-AT5G05160.1 |  |  |  |  |  |  |  |  |
| 0 | Ath-AT5G05170.1 |  |  |  |  |  |  |  |  |
| 1 | Ath-AT5G05180.1 |  | Vvi-Vitvi13g00195\_t001 |  |  |  |  |  |  |  |
| 1 | Ath-AT5G05190.1 |  | Vvi-Vitvi13g00194\_t001 |  |  |  |  |  |  |  |
| 1 | Ath-AT5G05200.1 |  | Vvi-Vitvi13g00193\_t001 |  |  |  |  |  |  |  |
| 1 | Ath-AT5G05210.1 |  | | | |  |  |  |  |  |  |  |
| 1 | Ath-AT5G05220.1 |  | Vvi-Vitvi13g01918\_t001 |  |  |  |  |  |  |  |
| 1 | Ath-AT5G05230.1 |  | Vvi-Vitvi13g00180\_t001 |  |  |  |  |  |  |  |
| 1 | Ath-AT5G05240.1 |  | Vvi-Vitvi13g00179\_t001 |  |  |  |  |  |  |  |
| 1 | Ath-AT5G05250.1 |  | Vvi-Vitvi13g00178\_t001 |  |  |  |  |  |  |  |
| 1 | Ath-AT5G05260.2 |  | | | |  |  |  |  |  |  |  |
| 1 | Ath-AT5G05270.1 |  | | | |  |  |  |  |  |  |  |
| 1 | Ath-AT5G05280.1 |  | Vvi-Vitvi13g01912\_t001 |  |  |  |  |  |  |  |
| 1 | Ath-AT5G05285.1 |  | | | |  |  |  |  |  |  |  |
| 1 | Ath-AT5G05290.1 |  | | | |  |  |  |  |  |  |  |
| 1 | Ath-AT5G05300.1 |  | | | |  |  |  |  |  |  |  |
| 1 | Ath-AT5G05310.7 |  | Vvi-Vitvi13g00151\_t001 |  |  |  |  |  |  |  |
| 1 | Ath-AT5G05320.1 |  | | | |  |  |  |  |  |  |  |
| 1 | Ath-AT5G05330.1 |  | | | |  |  |  |  |  |  |  |
| 2 | Ath-AT5G05340.1 |  | Vvi-Vitvi13g00143\_t001 |  | Vvi-Vitvi08g00821\_t001 |  |  |  |  |  |  |
| 2 | Ath-AT5G05350.1 |  | Vvi-Vitvi13g00132\_t001 |  | Vvi-Vitvi08g00803\_t001 |  |  |  |  |  |  |
| 2 | Ath-AT5G05360.1 |  | Vvi-Vitvi13g00129\_t001.1.6037826b |  | Vvi-Vitvi08g00797\_t002 |  |  |  |  |  |  |
| 2 | Ath-AT5G05365.1 |  | Vvi-Vitvi13g01901\_t001 |  | | | |  |  |  |  |  |  |
| 2 | Ath-AT5G05370.1 |  | | | |  | | | |  |  |  |  |  |  |
| 2 | Ath-AT5G05380.2 |  | Vvi-Vitvi13g00121\_t001 |  | Vvi-Vitvi08g00782\_t001 |  |  |  |  |  |  |
| 2 | Ath-AT5G05390.1 |  | Vvi-Vitvi13g00117\_t001 |  | | | |  |  |  |  |  |  |
| 2 | Ath-AT5G05400.1 |  | | | |  | | | |  |  |  |  |  |  |
| 2 | Ath-AT5G05410.1 |  | Vvi-Vitvi13g00116\_t001 |  | Vvi-Vitvi08g00778\_t001 |  |  |  |  |  |  |
| 2 | Ath-AT5G05420.1 |  | | | |  | | | |  |  |  |  |  |  |
| 2 | Ath-AT5G05430.2 |  | | | |  | | | |  |  |  |  |  |  |
| 2 | Ath-AT5G05440.1 |  | Vvi-Vitvi13g00114\_t001 |  | Vvi-Vitvi08g00768\_t001 |  |  |  |  |  |  |
| 1 | Ath-AT5G05450.1 |  | Vvi-Vitvi13g00111\_t001 |  |  |  |  |  |  |  |
| 1 | Ath-AT5G05460.1 |  | Vvi-Vitvi13g00110\_t001 |  |  |  |  |  |  |  |
| 1 | Ath-AT5G05470.1 |  | Vvi-Vitvi13g00101\_t001 |  |  |  |  |  |  |  |
| 1 | Ath-AT5G05480.1 |  | Vvi-Vitvi13g01897\_t001 |  |  |  |  |  |  |  |
| 1 | Ath-AT5G05490.1 |  | Vvi-Vitvi13g00098\_t001 |  |  |  |  |  |  |  |
| 1 | Ath-AT5G05500.1 |  | Vvi-Vitvi13g00091\_t001 |  |  |  |  |  |  |  |
| 1 | Ath-AT5G05510.1 |  | Vvi-Vitvi13g00090\_t001 |  |  |  |  |  |  |  |
| 1 | Ath-AT5G05520.1 |  | Vvi-Vitvi13g00089\_t001 |  |  |  |  |  |  |  |
| 1 | Ath-AT5G05530.1 |  | | | |  |  |  |  |  |  |  |
| 1 | Ath-AT5G05540.1 |  | | | |  |  |  |  |  |  |  |
| 1 | Ath-AT5G05550.2 |  | Vvi-Vitvi13g00084\_t001 |  |  |  |  |  |  |  |
| 1 | Ath-AT5G05560.2 |  | Vvi-Vitvi13g00069\_t001 |  |  |  |  |  |  |  |
| 1 | Ath-AT5G05570.1 |  | Vvi-Vitvi13g00061\_t001 |  |  |  |  |  |  |  |
| 1 | Ath-AT5G05580.1 |  | Vvi-Vitvi13g00060\_t001 |  |  |  |  |  |  |  |
| 1 | Ath-AT5G05590.1 |  | Vvi-Vitvi13g00059\_t001 |  |  |  |  |  |  |  |
| 1 | Ath-AT5G05598.1 |  | | | |  |  |  |  |  |  |  |
| 1 | Ath-AT5G05600.1 |  | Vvi-Vitvi13g00055\_t001 |  |  |  |  |  |  |  |
| 1 | Ath-AT5G05610.1 |  | Vvi-Vitvi13g00054\_t001 |  |  |  |  |  |  |  |
| 1 | Ath-AT5G05620.1 |  | | | |  |  |  |  |  |  |  |
| 1 | Ath-AT5G05630.1 |  | | | |  |  |  |  |  |  |  |
| 1 | Ath-AT5G05635.1 |  | | | |  |  |  |  |  |  |  |
| 1 | Ath-AT5G05640.1 |  | | | |  |  |  |  |  |  |  |
| 1 | Ath-AT5G05650.1 |  | | | |  |  |  |  |  |  |  |
| 1 | Ath-AT5G05653.1 |  | | | |  |  |  |  |  |  |  |
| 1 | Ath-AT5G05657.1 |  | | | |  |  |  |  |  |  |  |
| 1 | Ath-AT5G05660.1 |  | Vvi-Vitvi13g00049\_t001 |  |  |  |  |  |  |  |
| 1 | Ath-AT5G05670.1 |  | | | |  |  |  |  |  |  |  |
| 1 | Ath-AT5G05675.1 |  | | | |  |  |  |  |  |  |  |
| 1 | Ath-AT5G05680.1 |  | Vvi-Vitvi13g00034\_t001 |  |  |  |  |  |  |  |
| 1 | Ath-AT5G05690.1 |  | Vvi-Vitvi13g00033\_t002 |  |  |  |  |  |  |  |
| 1 | Ath-AT5G05700.1 |  | Vvi-Vitvi13g00029\_t002 |  |  |  |  |  |  |  |
| 1 | Ath-AT5G05710.1 |  | Vvi-Vitvi13g00023\_t001 |  |  |  |  |  |  |  |
| 1 | Ath-AT5G05720.1 |  | | | |  |  |  |  |  |  |  |
| 1 | Ath-AT5G05730.2 |  | Vvi-Vitvi13g00022\_t001 |  |  |  |  |  |  |  |
| 1 | Ath-AT5G05735.1 |  | | | |  |  |  |  |  |  |  |
| 1 | Ath-AT5G05740.1 |  | Vvi-Vitvi13g00021\_t001 |  |  |  |  |  |  |  |
| 1 | Ath-AT5G05750.1 |  | | | |  |  |  |  |  |  |  |
| 1 | Ath-AT5G05760.1 |  | Vvi-Vitvi13g00018\_t003 |  |  |  |  |  |  |  |
| 1 | Ath-AT5G05770.1 |  | Vvi-Vitvi13g02039\_t001 |  |  |  |  |  |  |  |
| 1 | Ath-AT5G05780.1 |  | | | |  |  |  |  |  |  |  |
| 2 | Ath-AT5G05790.1 |  | Vvi-Vitvi13g00510\_t001 |  | Vvi-Vitvi06g00592\_t001 |  |  |  |  |  |  |
| 2 | Ath-AT5G05800.2 |  | Vvi-Vitvi13g02032\_t001 |  | | | |  |  |  |  |  |  |
| 2 | Ath-AT5G05810.1 |  | | | |  | | | |  |  |  |  |  |  |
| 3 | Ath-AT5G05820.1 |  | Vvi-Vitvi13g00501\_t001 |  | Vvi-Vitvi06g00575\_t001 |  | Vvi-Vitvi08g01312\_t001 |  |  |  |  |  |
| 3 | Ath-AT5G05830.1 |  | Vvi-Vitvi13g04140\_t003 |  | Vvi-Vitvi06g00567\_t001 |  | Vvi-Vitvi08g01297\_t001 |  |  |  |  |  |
| 3 | Ath-AT5G05840.1 |  | Vvi-Vitvi13g00492\_t001 |  | | | |  | | | |  |  |  |  |  |
| 3 | Ath-AT5G05850.1 |  | | | |  | | | |  | | | |  |  |  |  |  |
| 3 | Ath-AT5G05860.1 |  | Vvi-Vitvi13g04126\_t001 |  | | | |  | | | |  |  |  |  |  |
| 3 | Ath-AT5G05870.1 |  | | | |  | | | |  | | | |  |  |  |  |  |
| 3 | Ath-AT5G05880.1 |  | Vvi-Vitvi13g00397\_t001 |  | | | |  | | | |  |  |  |  |  |
| 3 | Ath-AT5G05890.1 |  | | | |  | | | |  | | | |  |  |  |  |  |
| 3 | Ath-AT5G05900.1 |  | | | |  | | | |  | | | |  |  |  |  |  |
| 3 | Ath-AT5G05910.1 |  | | | |  | | | |  | | | |  |  |  |  |  |
| 3 | Ath-AT5G05920.1 |  | | | |  | | | |  | | | |  |  |  |  |  |
| 3 | Ath-AT5G05930.1 |  | Vvi-Vitvi13g00391\_t001 |  | | | |  | | | |  |  |  |  |  |
| 3 | Ath-AT5G05940.1 |  | Vvi-Vitvi13g00390\_t001 |  | | | |  | Vvi-Vitvi08g01282\_t001 |  |  |  |  |  |
| 3 | Ath-AT5G05950.1 |  | Vvi-Vitvi13g00389\_t001 |  | Vvi-Vitvi06g00555\_t001 |  | | | |  |  |  |  |  |
| 3 | Ath-AT5G05960.1 |  | Vvi-Vitvi13g00388\_t001 |  | | | |  | Vvi-Vitvi08g01281\_t001 |  |  |  |  |  |
| 2 | Ath-AT5G05965.1 |  |  |  | | | |  | | | |  |  |  |  |  |
| 3 | Ath-AT5G05970.2 |  | Vvi-Vitvi13g00361\_t001 |  | | | |  | | | |  |  |  |  |  |
| 3 | Ath-AT5G05980.1 |  | Vvi-Vitvi13g00359\_t001 |  | | | |  | | | |  |  |  |  |  |
| 3 | Ath-AT5G05987.1 |  | | | |  | | | |  | Vvi-Vitvi08g01266\_t002 |  |  |  |  |  |
| 3 | Ath-AT5G05990.1 |  | Vvi-Vitvi13g00354\_t001 |  | | | |  | | | |  |  |  |  |  |
| 3 | Ath-AT5G06000.1 |  | Vvi-Vitvi13g02003\_t001 |  | Vvi-Vitvi06g00535\_t001 |  | Vvi-Vitvi08g01256\_t001 |  |  |  |  |  |
| 3 | Ath-AT5G06010.1 |  | | | |  | | | |  | | | |  |  |  |  |  |
| 3 | Ath-AT5G06020.1 |  | | | |  | | | |  | | | |  |  |  |  |  |
| 3 | Ath-AT5G06030.1 |  | | | |  | | | |  | | | |  |  |  |  |  |
| 3 | Ath-AT5G06040.1 |  | | | |  | | | |  | | | |  |  |  |  |  |
| 3 | Ath-AT5G06043.1 |  | | | |  | | | |  | | | |  |  |  |  |  |
| 3 | Ath-AT5G06050.1 |  | Vvi-Vitvi13g00348\_t002 |  | | | |  | | | |  |  |  |  |  |
| 3 | Ath-AT5G06060.1 |  | Vvi-Vitvi13g01997\_t001 |  | Vvi-Vitvi06g00522\_t001 |  | Vvi-Vitvi08g01250\_t001 |  |  |  |  |  |
| 3 | Ath-AT5G06070.1 |  | Vvi-Vitvi13g00340\_t001 |  | Vvi-Vitvi06g00521\_t001 |  | Vvi-Vitvi08g01249\_t001 |  |  |  |  |  |
| 1 | Ath-AT5G06080.1 |  | Vvi-Vitvi13g00333\_t001 |  |  |  |  |  |  |  |
| 1 | Ath-AT5G06090.1 |  | Vvi-Vitvi13g00327\_t001 |  |  |  |  |  |  |  |
| 0 | Ath-AT5G06100.3 |  |  |  |  |  |  |  |  |
| 0 | Ath-AT5G06110.1 |  |  |  |  |  |  |  |  |
| 0 | Ath-AT5G06120.1 |  |  |  |  |  |  |  |  |
| 1 | Ath-AT5G06130.2 |  | Vvi-Vitvi08g01429\_t001 |  |  |  |  |  |  |  |
| 1 | Ath-AT5G06140.1 |  | Vvi-Vitvi08g01428\_t001 |  |  |  |  |  |  |  |
| 1 | Ath-AT5G06150.1 |  | | | |  |  |  |  |  |  |  |
| 1 | Ath-AT5G06160.1 |  | | | |  |  |  |  |  |  |  |
| 1 | Ath-AT5G06170.1 |  | | | |  |  |  |  |  |  |  |
| 1 | Ath-AT5G06180.1 |  | Vvi-Vitvi08g01419\_t001 |  |  |  |  |  |  |  |
| 1 | Ath-AT5G06190.2 |  | | | |  |  |  |  |  |  |  |
| 1 | Ath-AT5G06200.1 |  | Vvi-Vitvi08g01418\_t001 |  |  |  |  |  |  |  |
| 1 | Ath-AT5G06210.1 |  | Vvi-Vitvi08g02257\_t001 |  |  |  |  |  |  |  |
| 1 | Ath-AT5G06220.2 |  | Vvi-Vitvi08g01415\_t001 |  |  |  |  |  |  |  |
| 1 | Ath-AT5G06230.1 |  | Vvi-Vitvi08g01414\_t001 |  |  |  |  |  |  |  |
| 1 | Ath-AT5G06240.1 |  | Vvi-Vitvi08g01413\_t001 |  |  |  |  |  |  |  |
| 1 | Ath-AT5G06250.4 |  | Vvi-Vitvi08g01412\_t001 |  |  |  |  |  |  |  |
| 1 | Ath-AT5G06260.1 |  | Vvi-Vitvi08g01396\_t001 |  |  |  |  |  |  |  |
| 1 | Ath-AT5G06265.4 |  | | | |  |  |  |  |  |  |  |
| 1 | Ath-AT5G06270.2 |  | Vvi-Vitvi08g01395\_t001 |  |  |  |  |  |  |  |
| 1 | Ath-AT5G06280.1 |  | Vvi-Vitvi08g01389\_t001 |  |  |  |  |  |  |  |
| 1 | Ath-AT5G06290.1 |  | Vvi-Vitvi08g01386\_t001 |  |  |  |  |  |  |  |
| 1 | Ath-AT5G06300.1 |  | Vvi-Vitvi08g01385\_t001 |  |  |  |  |  |  |  |
| 1 | Ath-AT5G06310.1 |  | | | |  |  |  |  |  |  |  |
| 1 | Ath-AT5G06320.1 |  | Vvi-Vitvi08g01377\_t001 |  |  |  |  |  |  |  |
| 1 | Ath-AT5G06330.1 |  | Vvi-Vitvi08g01376\_t001 |  |  |  |  |  |  |  |
| 1 | Ath-AT5G06340.1 |  | | | |  |  |  |  |  |  |  |
| 1 | Ath-AT5G06350.1 |  | Vvi-Vitvi08g01372\_t001 |  |  |  |  |  |  |  |
| 0 | Ath-AT5G06360.1 |  |  |  |  |  |  |  |  |
| 0 | Ath-AT5G06370.1 |  |  |  |  |  |  |  |  |
| 1 | Ath-AT5G06380.1 |  | Vvi-Vitvi08g02035\_t001 |  |  |  |  |  |  |  |
| 1 | Ath-AT5G06390.1 |  | Vvi-Vitvi08g00235\_t003 |  |  |  |  |  |  |  |
| 1 | Ath-AT5G06400.1 |  | | | |  |  |  |  |  |  |  |
| 1 | Ath-AT5G06410.1 |  | | | |  |  |  |  |  |  |  |
| 1 | Ath-AT5G06420.2 |  | | | |  |  |  |  |  |  |  |
| 1 | Ath-AT5G06430.1 |  | | | |  |  |  |  |  |  |  |
| 1 | Ath-AT5G06440.4 |  | Vvi-Vitvi08g00227\_t001 |  |  |  |  |  |  |  |
| 1 | Ath-AT5G06450.1 |  | | | |  |  |  |  |  |  |  |
| 1 | Ath-AT5G06460.1 |  | | | |  |  |  |  |  |  |  |
| 1 | Ath-AT5G06470.1 |  | | | |  |  |  |  |  |  |  |
| 1 | Ath-AT5G06480.1 |  | | | |  |  |  |  |  |  |  |
| 1 | Ath-AT5G06490.1 |  | | | |  |  |  |  |  |  |  |
| 1 | Ath-AT5G06500.1 |  | | | |  |  |  |  |  |  |  |
| 1 | Ath-AT5G06510.6 |  | | | |  |  |  |  |  |  |  |
| 1 | Ath-AT5G06520.1 |  | | | |  |  |  |  |  |  |  |
| 1 | Ath-AT5G06530.1 |  | Vvi-Vitvi08g00222\_t001 |  |  |  |  |  |  |  |
| 1 | Ath-AT5G06540.1 |  | Vvi-Vitvi08g00217\_t001 |  |  |  |  |  |  |  |
| 1 | Ath-AT5G06550.1 |  | Vvi-Vitvi08g00216\_t001 |  |  |  |  |  |  |  |
| 1 | Ath-AT5G06560.1 |  | Vvi-Vitvi08g00215\_t001 |  |  |  |  |  |  |  |
| 1 | Ath-AT5G06570.3 |  | Vvi-Vitvi08g00212\_t001 |  |  |  |  |  |  |  |
| 1 | Ath-AT5G06580.1 |  | Vvi-Vitvi08g00209\_t001 |  |  |  |  |  |  |  |
| 1 | Ath-AT5G06590.1 |  | Vvi-Vitvi08g00202\_t001 |  |  |  |  |  |  |  |
| 1 | Ath-AT5G06600.1 |  | Vvi-Vitvi08g00200\_t001 |  |  |  |  |  |  |  |
| 1 | Ath-AT5G06610.1 |  | Vvi-Vitvi08g02366\_t001 |  |  |  |  |  |  |  |
| 1 | Ath-AT5G06620.1 |  | Vvi-Vitvi08g01786\_t001 |  |  |  |  |  |  |  |
| 1 | Ath-AT5G06630.1 |  | | | |  |  |  |  |  |  |  |
| 1 | Ath-AT5G06640.1 |  | | | |  |  |  |  |  |  |  |
| 1 | Ath-AT5G06645.1 |  | | | |  |  |  |  |  |  |  |
| 1 | Ath-AT5G06650.1 |  | Vvi-Vitvi08g01771\_t001 |  |  |  |  |  |  |  |
| 1 | Ath-AT5G06660.1 |  | | | |  |  |  |  |  |  |  |
| 1 | Ath-AT5G06670.6 |  | Vvi-Vitvi08g01768\_t001 |  |  |  |  |  |  |  |
| 1 | Ath-AT5G06680.1 |  | Vvi-Vitvi08g01759\_t001 |  |  |  |  |  |  |  |
| 1 | Ath-AT5G06690.2 |  | Vvi-Vitvi08g01756\_t001 |  |  |  |  |  |  |  |
| 1 | Ath-AT5G06700.1 |  | Vvi-Vitvi08g01753\_t001 |  |  |  |  |  |  |  |
| 1 | Ath-AT5G06710.1 |  | Vvi-Vitvi08g01752\_t001 |  |  |  |  |  |  |  |
| 1 | Ath-AT5G06720.1 |  | Vvi-Vitvi08g01750\_t001 |  |  |  |  |  |  |  |
| 1 | Ath-AT5G06730.1 |  | | | |  |  |  |  |  |  |  |
| 1 | Ath-AT5G06740.2 |  | Vvi-Vitvi08g01744\_t001 |  |  |  |  |  |  |  |
| 1 | Ath-AT5G06750.1 |  | Vvi-Vitvi08g01739\_t002 |  |  |  |  |  |  |  |
| 1 | Ath-AT5G06755.1 |  | | | |  |  |  |  |  |  |  |
| 1 | Ath-AT5G06760.1 |  | Vvi-Vitvi08g02343\_t001 |  |  |  |  |  |  |  |
| 1 | Ath-AT5G06770.1 |  | Vvi-Vitvi08g01732\_t001 |  |  |  |  |  |  |  |
| 1 | Ath-AT5G06780.1 |  | Vvi-Vitvi08g01725\_t001 |  |  |  |  |  |  |  |
| 1 | Ath-AT5G06790.1 |  | Vvi-Vitvi08g01724\_t001 |  |  |  |  |  |  |  |
| 1 | Ath-AT5G06800.2 |  | Vvi-Vitvi08g01723\_t002 |  |  |  |  |  |  |  |
| 1 | Ath-AT5G06810.1 |  | Vvi-Vitvi08g01721\_t001 |  |  |  |  |  |  |  |
| 1 | Ath-AT5G06811.1 |  | | | |  |  |  |  |  |  |  |
| 1 | Ath-AT5G06820.1 |  | Vvi-Vitvi08g01718\_t001 |  |  |  |  |  |  |  |
| 1 | Ath-AT5G06830.1 |  | Vvi-Vitvi08g01717\_t001 |  |  |  |  |  |  |  |
| 1 | Ath-AT5G06839.3 |  | Vvi-Vitvi08g01710\_t001 |  |  |  |  |  |  |  |
| 1 | Ath-AT5G06850.1 |  | Vvi-Vitvi08g01708\_t001 |  |  |  |  |  |  |  |
| 0 | Ath-AT5G06860.1 |  |  |  |  |  |  |  |  |
| 0 | Ath-AT5G06870.1 |  |  |  |  |  |  |  |  |
| 0 | Ath-AT5G06890.1 |  |  |  |  |  |  |  |  |
| 0 | Ath-AT5G06900.1 |  |  |  |  |  |  |  |  |
| 0 | Ath-AT5G06905.1 |  |  |  |  |  |  |  |  |
| 0 | Ath-AT5G06910.1 |  |  |  |  |  |  |  |  |
| 0 | Ath-AT5G06920.1 |  |  |  |  |  |  |  |  |
| 0 | Ath-AT5G06930.2 |  |  |  |  |  |  |  |  |
| 0 | Ath-AT5G06940.1 |  |  |  |  |  |  |  |  |
| 0 | Ath-AT5G06950.1 |  |  |  |  |  |  |  |  |
| 0 | Ath-AT5G06960.1 |  |  |  |  |  |  |  |  |
| 0 | Ath-AT5G06970.1 |  |  |  |  |  |  |  |  |
| 1 | Ath-AT5G06980.4 |  | Vvi-Vitvi13g02316\_t001 |  |  |  |  |  |  |  |
| 1 | Ath-AT5G06990.1 |  | Vvi-Vitvi13g01376\_t001 |  |  |  |  |  |  |  |
| 1 | Ath-AT5G07000.1 |  | Vvi-Vitvi13g01379\_t001 |  |  |  |  |  |  |  |
| 1 | Ath-AT5G07010.1 |  | | | |  |  |  |  |  |  |  |
| 1 | Ath-AT5G07020.1 |  | Vvi-Vitvi13g01385\_t001 |  |  |  |  |  |  |  |
| 1 | Ath-AT5G07030.1 |  | | | |  |  |  |  |  |  |  |
| 1 | Ath-AT5G07040.1 |  | Vvi-Vitvi13g01387\_t001 |  |  |  |  |  |  |  |
| 1 | Ath-AT5G07050.1 |  | Vvi-Vitvi13g01395\_t001 |  |  |  |  |  |  |  |
| 0 | Ath-AT5G07060.1 |  |  |  |  |  |  |  |  |
| 0 | Ath-AT5G07070.1 |  |  |  |  |  |  |  |  |
| 0 | Ath-AT5G07080.1 |  |  |  |  |  |  |  |  |
| 0 | Ath-AT5G07090.1 |  |  |  |  |  |  |  |  |
| 0 | Ath-AT5G07100.1 |  |  |  |  |  |  |  |  |
| 0 | Ath-AT5G07110.1 |  |  |  |  |  |  |  |  |
| 0 | Ath-AT5G07120.1 |  |  |  |  |  |  |  |  |
| 0 | Ath-AT5G07130.1 |  |  |  |  |  |  |  |  |
| 0 | Ath-AT5G07140.1 |  |  |  |  |  |  |  |  |
| 0 | Ath-AT5G07150.2 |  |  |  |  |  |  |  |  |
| 0 | Ath-AT5G07160.1 |  |  |  |  |  |  |  |  |
| 0 | Ath-AT5G07165.1 |  |  |  |  |  |  |  |  |
| 0 | Ath-AT5G07170.1 |  |  |  |  |  |  |  |  |
| 0 | Ath-AT5G07175.1 |  |  |  |  |  |  |  |  |
| 0 | Ath-AT5G07180.1 |  |  |  |  |  |  |  |  |
| 0 | Ath-AT5G07190.1 |  |  |  |  |  |  |  |  |
| 0 | Ath-AT5G07200.1 |  |  |  |  |  |  |  |  |
| 1 | Ath-AT5G07210.1 |  | Vvi-Vitvi16g00946\_t001 |  |  |  |  |  |  |  |
| 1 | Ath-AT5G07220.1 |  | Vvi-Vitvi16g00952\_t001 |  |  |  |  |  |  |  |
| 1 | Ath-AT5G07225.1 |  | | | |  |  |  |  |  |  |  |
| 1 | Ath-AT5G07230.1 |  | Vvi-Vitvi16g01864\_t001 |  |  |  |  |  |  |  |
| 1 | Ath-AT5G07240.2 |  | Vvi-Vitvi16g00970\_t001 |  |  |  |  |  |  |  |
| 1 | Ath-AT5G07250.1 |  | Vvi-Vitvi16g00972\_t001 |  |  |  |  |  |  |  |
| 1 | Ath-AT5G07260.1 |  | | | |  |  |  |  |  |  |  |
| 1 | Ath-AT5G07270.1 |  | Vvi-Vitvi16g00976\_t001 |  |  |  |  |  |  |  |
| 1 | Ath-AT5G07280.1 |  | Vvi-Vitvi16g00979\_t001 |  |  |  |  |  |  |  |
| 1 | Ath-AT5G07290.1 |  | Vvi-Vitvi17g00028\_t001 |  |  |  |  |  |  |  |
| 1 | Ath-AT5G07300.1 |  | Vvi-Vitvi17g00026\_t001 |  |  |  |  |  |  |  |
| 1 | Ath-AT5G07310.1 |  | Vvi-Vitvi17g00025\_t001 |  |  |  |  |  |  |  |
| 1 | Ath-AT5G07320.1 |  | | | |  |  |  |  |  |  |  |
| 1 | Ath-AT5G07330.1 |  | Vvi-Vitvi17g01318\_t001 |  |  |  |  |  |  |  |
| 1 | Ath-AT5G07340.2 |  | Vvi-Vitvi17g01311\_t001 |  |  |  |  |  |  |  |
| 1 | Ath-AT5G07350.2 |  | Vvi-Vitvi17g00002\_t001 |  |  |  |  |  |  |  |
| 1 | Ath-AT5G07360.1 |  | Vvi-Vitvi17g00001\_t001 |  |  |  |  |  |  |  |
| 0 | Ath-AT5G07370.2 |  |  |  |  |  |  |  |  |
| 0 | Ath-AT5G07380.5 |  |  |  |  |  |  |  |  |
| 0 | Ath-AT5G07390.1 |  |  |  |  |  |  |  |  |
| 0 | Ath-AT5G07400.1 |  |  |  |  |  |  |  |  |
| 0 | Ath-AT5G07410.1 |  |  |  |  |  |  |  |  |
| 0 | Ath-AT5G07420.1 |  |  |  |  |  |  |  |  |
| 0 | Ath-AT5G07430.1 |  |  |  |  |  |  |  |  |
| 0 | Ath-AT5G07440.1 |  |  |  |  |  |  |  |  |
| 0 | Ath-AT5G07450.1 |  |  |  |  |  |  |  |  |
| 0 | Ath-AT5G07460.1 |  |  |  |  |  |  |  |  |
| 0 | Ath-AT5G07470.1 |  |  |  |  |  |  |  |  |
| 0 | Ath-AT5G07475.1 |  |  |  |  |  |  |  |  |
| 0 | Ath-AT5G07480.1 |  |  |  |  |  |  |  |  |
| 0 | Ath-AT5G07490.1 |  |  |  |  |  |  |  |  |
| 0 | Ath-AT5G07500.1 |  |  |  |  |  |  |  |  |
| 0 | Ath-AT5G07510.1 |  |  |  |  |  |  |  |  |
| 0 | Ath-AT5G07520.1 |  |  |  |  |  |  |  |  |
| 0 | Ath-AT5G07530.1 |  |  |  |  |  |  |  |  |
| 0 | Ath-AT5G07540.1 |  |  |  |  |  |  |  |  |
| 0 | Ath-AT5G07545.1 |  |  |  |  |  |  |  |  |
| 0 | Ath-AT5G07550.1 |  |  |  |  |  |  |  |  |
| 0 | Ath-AT5G07560.1 |  |  |  |  |  |  |  |  |
| 0 | Ath-AT5G07570.1 |  |  |  |  |  |  |  |  |
| 0 | Ath-AT5G07571.1 |  |  |  |  |  |  |  |  |
| 0 | Ath-AT5G07572.1 |  |  |  |  |  |  |  |  |
| 0 | Ath-AT5G07580.1 |  |  |  |  |  |  |  |  |
| 0 | Ath-AT5G07590.1 |  |  |  |  |  |  |  |  |
| 0 | Ath-AT5G07600.1 |  |  |  |  |  |  |  |  |
| 0 | Ath-AT5G07610.1 |  |  |  |  |  |  |  |  |
| 0 | Ath-AT5G07620.1 |  |  |  |  |  |  |  |  |
| 0 | Ath-AT5G07630.1 |  |  |  |  |  |  |  |  |
| 0 | Ath-AT5G07640.1 |  |  |  |  |  |  |  |  |
| 0 | Ath-AT5G07650.1 |  |  |  |  |  |  |  |  |
| 0 | Ath-AT5G07660.1 |  |  |  |  |  |  |  |  |
| 0 | Ath-AT5G07670.1 |  |  |  |  |  |  |  |  |
| 1 | Ath-AT5G07680.1 |  | Vvi-Vitvi17g00622\_t001 |  |  |  |  |  |  |  |
| 1 | Ath-AT5G07690.1 |  | | | |  |  |  |  |  |  |  |
| 1 | Ath-AT5G07700.1 |  | | | |  |  |  |  |  |  |  |
| 1 | Ath-AT5G07710.1 |  | Vvi-Vitvi17g00635\_t001 |  |  |  |  |  |  |  |
| 1 | Ath-AT5G07720.1 |  | Vvi-Vitvi17g00642\_t001 |  |  |  |  |  |  |  |
| 1 | Ath-AT5G07730.1 |  | | | |  |  |  |  |  |  |  |
| 1 | Ath-AT5G07740.2 |  | Vvi-Vitvi17g00646\_t001 |  |  |  |  |  |  |  |
| 1 | Ath-AT5G07760.2 |  | | | |  |  |  |  |  |  |  |
| 1 | Ath-AT5G07770.3 |  | | | |  |  |  |  |  |  |  |
| 1 | Ath-AT5G07780.1 |  | | | |  |  |  |  |  |  |  |
| 1 | Ath-AT5G07790.1 |  | | | |  |  |  |  |  |  |  |
| 1 | Ath-AT5G07800.1 |  | Vvi-Vitvi17g04186\_t001 |  |  |  |  |  |  |  |
| 1 | Ath-AT5G07810.1 |  | | | |  |  |  |  |  |  |  |
| 1 | Ath-AT5G07820.1 |  | Vvi-Vitvi17g00666\_t001 |  |  |  |  |  |  |  |
| 1 | Ath-AT5G07830.1 |  | | | |  |  |  |  |  |  |  |
| 1 | Ath-AT5G07840.1 |  | | | |  |  |  |  |  |  |  |
| 1 | Ath-AT5G07850.1 |  | | | |  |  |  |  |  |  |  |
| 1 | Ath-AT5G07860.1 |  | | | |  |  |  |  |  |  |  |
| 1 | Ath-AT5G07870.1 |  | | | |  |  |  |  |  |  |  |
| 1 | Ath-AT5G07880.1 |  | Vvi-Vitvi17g01490\_t001 |  |  |  |  |  |  |  |
| 1 | Ath-AT5G07890.3 |  | | | |  |  |  |  |  |  |  |
| 1 | Ath-AT5G07900.1 |  | | | |  |  |  |  |  |  |  |
| 1 | Ath-AT5G07910.1 |  | Vvi-Vitvi17g00680\_t001 |  |  |  |  |  |  |  |
| 1 | Ath-AT5G07920.1 |  | Vvi-Vitvi17g00683\_t001 |  |  |  |  |  |  |  |
| 1 | Ath-AT5G07930.3 |  | | | |  |  |  |  |  |  |  |
| 1 | Ath-AT5G07940.1 |  | | | |  |  |  |  |  |  |  |
| 1 | Ath-AT5G07950.1 |  | | | |  |  |  |  |  |  |  |
| 1 | Ath-AT5G07960.1 |  | Vvi-Vitvi17g00690\_t001 |  |  |  |  |  |  |  |
| 1 | Ath-AT5G07970.2 |  | | | |  |  |  |  |  |  |  |
| 1 | Ath-AT5G07980.1 |  | | | |  |  |  |  |  |  |  |
| 1 | Ath-AT5G07990.1 |  | Vvi-Vitvi17g00700\_t002 |  |  |  |  |  |  |  |
| 1 | Ath-AT5G08000.2 |  | Vvi-Vitvi17g01511\_t001 |  |  |  |  |  |  |  |
| 1 | Ath-AT5G08005.1 |  | Vvi-Vitvi17g00704\_t002 |  |  |  |  |  |  |  |
| 1 | Ath-AT5G08010.1 |  | Vvi-Vitvi17g00711\_t001 |  |  |  |  |  |  |  |
| 1 | Ath-AT5G08020.1 |  | | | |  |  |  |  |  |  |  |
| 1 | Ath-AT5G08030.2 |  | Vvi-Vitvi17g01521\_t001 |  |  |  |  |  |  |  |
| 0 | Ath-AT5G08040.1 |  |  |  |  |  |  |  |  |
| 0 | Ath-AT5G08050.1 |  |  |  |  |  |  |  |  |
| 0 | Ath-AT5G08055.1 |  |  |  |  |  |  |  |  |
| 0 | Ath-AT5G08060.1 |  |  |  |  |  |  |  |  |
| 0 | Ath-AT5G08070.1 |  |  |  |  |  |  |  |  |
| 0 | Ath-AT5G08080.3 |  |  |  |  |  |  |  |  |
| 0 | Ath-AT5G08090.1 |  |  |  |  |  |  |  |  |
| 1 | Ath-AT5G08100.1 |  | Vvi-Vitvi17g00453\_t001 |  |  |  |  |  |  |  |
| 1 | Ath-AT5G08110.3 |  | Vvi-Vitvi17g00452\_t001 |  |  |  |  |  |  |  |
| 1 | Ath-AT5G08120.1 |  | Vvi-Vitvi17g01435\_t001 |  |  |  |  |  |  |  |
| 1 | Ath-AT5G08130.8 |  | Vvi-Vitvi17g00442\_t001 |  |  |  |  |  |  |  |
| 1 | Ath-AT5G08139.1 |  | Vvi-Vitvi17g00435\_t001 |  |  |  |  |  |  |  |
| 1 | Ath-AT5G08141.1 |  | | | |  |  |  |  |  |  |  |
| 1 | Ath-AT5G08150.1 |  | | | |  |  |  |  |  |  |  |
| 1 | Ath-AT5G08160.1 |  | Vvi-Vitvi17g00428\_t001 |  |  |  |  |  |  |  |
| 1 | Ath-AT5G08170.1 |  | Vvi-Vitvi17g00427\_t001 |  |  |  |  |  |  |  |
| 1 | Ath-AT5G08180.2 |  | Vvi-Vitvi17g00425\_t001 |  |  |  |  |  |  |  |
| 0 | Ath-AT5G08190.1 |  |  |  |  |  |  |  |  |
| 0 | Ath-AT5G08200.1 |  |  |  |  |  |  |  |  |
| 0 | Ath-AT5G08230.1 |  |  |  |  |  |  |  |  |
| 0 | Ath-AT5G08240.1 |  |  |  |  |  |  |  |  |
| 0 | Ath-AT5G08250.2 |  |  |  |  |  |  |  |  |
| 0 | Ath-AT5G08260.1 |  |  |  |  |  |  |  |  |
| 0 | Ath-AT5G08270.1 |  |  |  |  |  |  |  |  |
| 0 | Ath-AT5G08280.1 |  |  |  |  |  |  |  |  |
| 0 | Ath-AT5G08290.1 |  |  |  |  |  |  |  |  |
| 0 | Ath-AT5G08300.1 |  |  |  |  |  |  |  |  |
| 0 | Ath-AT5G08305.1 |  |  |  |  |  |  |  |  |
| 0 | Ath-AT5G08310.1 |  |  |  |  |  |  |  |  |
| 0 | Ath-AT5G08315.1 |  |  |  |  |  |  |  |  |
| 0 | Ath-AT5G08320.1 |  |  |  |  |  |  |  |  |
| 0 | Ath-AT5G08330.1 |  |  |  |  |  |  |  |  |
| 0 | Ath-AT5G08335.1 |  |  |  |  |  |  |  |  |
| 0 | Ath-AT5G08340.1 |  |  |  |  |  |  |  |  |
| 0 | Ath-AT5G08350.1 |  |  |  |  |  |  |  |  |
| 0 | Ath-AT5G08360.1 |  |  |  |  |  |  |  |  |
| 0 | Ath-AT5G08370.1 |  |  |  |  |  |  |  |  |
| 0 | Ath-AT5G08380.1 |  |  |  |  |  |  |  |  |
| 0 | Ath-AT5G08391.1 |  |  |  |  |  |  |  |  |
| 0 | Ath-AT5G08390.1 |  |  |  |  |  |  |  |  |
| 0 | Ath-AT5G08400.1 |  |  |  |  |  |  |  |  |
| 0 | Ath-AT5G08410.2 |  |  |  |  |  |  |  |  |
| 0 | Ath-AT5G08415.1 |  |  |  |  |  |  |  |  |
| 0 | Ath-AT5G08420.1 |  |  |  |  |  |  |  |  |
| 1 | Ath-AT5G08430.1 |  | Vvi-Vitvi07g02055\_t001 |  |  |  |  |  |  |  |
| 1 | Ath-AT5G08440.3 |  | Vvi-Vitvi07g02053\_t001 |  |  |  |  |  |  |  |
| 1 | Ath-AT5G08450.2 |  | Vvi-Vitvi07g02036\_t001 |  |  |  |  |  |  |  |
| 1 | Ath-AT5G08460.1 |  | Vvi-Vitvi07g04772\_t001 |  |  |  |  |  |  |  |
| 1 | Ath-AT5G08470.1 |  | | | |  |  |  |  |  |  |  |
| 1 | Ath-AT5G08480.3 |  | Vvi-Vitvi07g02723\_t001 |  |  |  |  |  |  |  |
| 1 | Ath-AT5G08490.1 |  | | | |  |  |  |  |  |  |  |
| 1 | Ath-AT5G08500.1 |  | | | |  |  |  |  |  |  |  |
| 1 | Ath-AT5G08505.1 |  | | | |  |  |  |  |  |  |  |
| 1 | Ath-AT5G08510.1 |  | | | |  |  |  |  |  |  |  |
| 2 | Ath-AT5G08520.1 |  | | | |  | Vvi-Vitvi10g01533\_t001 |  |  |  |  |  |  |
| 2 | Ath-AT5G08530.1 |  | | | |  | | | |  |  |  |  |  |  |
| 2 | Ath-AT5G08535.1 |  | | | |  | Vvi-Vitvi10g02083\_t001 |  |  |  |  |  |  |
| 2 | Ath-AT5G08540.1 |  | | | |  | Vvi-Vitvi10g01563\_t001 |  |  |  |  |  |  |
| 2 | Ath-AT5G08550.1 |  | | | |  | | | |  |  |  |  |  |  |
| 2 | Ath-AT5G08560.1 |  | | | |  | Vvi-Vitvi10g01565\_t007 |  |  |  |  |  |  |
| 2 | Ath-AT5G08565.2 |  | | | |  | Vvi-Vitvi10g01566\_t001 |  |  |  |  |  |  |
| 2 | Ath-AT5G08570.1 |  | | | |  | Vvi-Vitvi10g01568\_t001 |  |  |  |  |  |  |
| 1 | Ath-AT5G08580.2 |  | | | |  |  |  |  |  |  |  |
| 1 | Ath-AT5G08590.1 |  | Vvi-Vitvi07g02005\_t001 |  |  |  |  |  |  |  |
| 1 | Ath-AT5G08600.2 |  | | | |  |  |  |  |  |  |  |
| 1 | Ath-AT5G08610.1 |  | | | |  |  |  |  |  |  |  |
| 1 | Ath-AT5G08620.1 |  | | | |  |  |  |  |  |  |  |
| 1 | Ath-AT5G08630.2 |  | Vvi-Vitvi07g01996\_t004 |  |  |  |  |  |  |  |
| 1 | Ath-AT5G08640.1 |  | | | |  |  |  |  |  |  |  |
| 1 | Ath-AT5G08650.1 |  | Vvi-Vitvi07g01988\_t001 |  |  |  |  |  |  |  |
| 1 | Ath-AT5G08660.1 |  | Vvi-Vitvi07g01979\_t001 |  |  |  |  |  |  |  |
| 1 | Ath-AT5G08670.1 |  | | | |  |  |  |  |  |  |  |
| 1 | Ath-AT5G08680.1 |  | | | |  |  |  |  |  |  |  |
| 1 | Ath-AT5G08690.1 |  | | | |  |  |  |  |  |  |  |
| 1 | Ath-AT5G08695.1 |  | | | |  |  |  |  |  |  |  |
| 1 | Ath-AT5G08710.1 |  | Vvi-Vitvi07g01964\_t001 |  |  |  |  |  |  |  |
| 1 | Ath-AT5G08720.1 |  | Vvi-Vitvi07g01956\_t001 |  |  |  |  |  |  |  |
| 1 | Ath-AT5G08730.1 |  | | | |  |  |  |  |  |  |  |
| 1 | Ath-AT5G08740.1 |  | Vvi-Vitvi07g01947\_t001 |  |  |  |  |  |  |  |
| 1 | Ath-AT5G08750.7 |  | Vvi-Vitvi07g01937\_t002 |  |  |  |  |  |  |  |
| 1 | Ath-AT5G08760.1 |  | | | |  |  |  |  |  |  |  |
| 1 | Ath-AT5G08770.1 |  | Vvi-Vitvi07g01936\_t001 |  |  |  |  |  |  |  |
| 1 | Ath-AT5G08780.1 |  | | | |  |  |  |  |  |  |  |
| 1 | Ath-AT5G08790.1 |  | Vvi-Vitvi07g01929\_t001 |  |  |  |  |  |  |  |
| 1 | Ath-AT5G09210.1 |  | | | |  |  |  |  |  |  |  |
| 1 | Ath-AT5G09220.1 |  | | | |  |  |  |  |  |  |  |
| 1 | Ath-AT5G09225.1 |  | | | |  |  |  |  |  |  |  |
| 1 | Ath-AT5G09230.7 |  | Vvi-Vitvi07g01918\_t001 |  |  |  |  |  |  |  |
| 1 | Ath-AT5G09240.2 |  | Vvi-Vitvi07g02708\_t001 |  |  |  |  |  |  |  |
| 0 | Ath-AT5G09250.4 |  |  |  |  |  |  |  |  |
| 0 | Ath-AT5G09260.1 |  |  |  |  |  |  |  |  |
| 0 | Ath-AT5G09270.1 |  |  |  |  |  |  |  |  |
| 0 | Ath-AT5G09280.1 |  |  |  |  |  |  |  |  |
| 0 | Ath-AT5G09290.1 |  |  |  |  |  |  |  |  |
| 0 | Ath-AT5G09300.1 |  |  |  |  |  |  |  |  |
| 0 | Ath-AT5G09310.1 |  |  |  |  |  |  |  |  |
| 0 | Ath-AT5G09315.1 |  |  |  |  |  |  |  |  |
| 0 | Ath-AT5G09320.1 |  |  |  |  |  |  |  |  |
| 0 | Ath-AT5G09330.1 |  |  |  |  |  |  |  |  |
| 0 | Ath-AT5G09340.1 |  |  |  |  |  |  |  |  |
| 0 | Ath-AT5G09350.1 |  |  |  |  |  |  |  |  |
| 0 | Ath-AT5G09360.1 |  |  |  |  |  |  |  |  |
| 0 | Ath-AT5G09370.1 |  |  |  |  |  |  |  |  |
| 0 | Ath-AT5G09380.2 |  |  |  |  |  |  |  |  |
| 0 | Ath-AT5G09390.1 |  |  |  |  |  |  |  |  |
| 0 | Ath-AT5G09400.1 |  |  |  |  |  |  |  |  |
| 0 | Ath-AT5G09410.3 |  |  |  |  |  |  |  |  |
| 0 | Ath-AT5G09420.1 |  |  |  |  |  |  |  |  |
| 0 | Ath-AT5G09430.1 |  |  |  |  |  |  |  |  |
| 0 | Ath-AT5G09440.1 |  |  |  |  |  |  |  |  |
| 0 | Ath-AT5G09445.1 |  |  |  |  |  |  |  |  |
| 0 | Ath-AT5G09450.1 |  |  |  |  |  |  |  |  |
| 0 | Ath-AT5G09460.1 |  |  |  |  |  |  |  |  |
| 0 | Ath-AT5G09470.1 |  |  |  |  |  |  |  |  |
| 0 | Ath-AT5G09480.1 |  |  |  |  |  |  |  |  |
| 0 | Ath-AT5G09490.1 |  |  |  |  |  |  |  |  |
| 0 | Ath-AT5G09500.1 |  |  |  |  |  |  |  |  |
| 0 | Ath-AT5G09510.1 |  |  |  |  |  |  |  |  |
| 0 | Ath-AT5G09520.1 |  |  |  |  |  |  |  |  |
| 0 | Ath-AT5G09530.1 |  |  |  |  |  |  |  |  |
| 0 | Ath-AT5G09540.1 |  |  |  |  |  |  |  |  |
| 0 | Ath-AT5G09550.1 |  |  |  |  |  |  |  |  |
| 0 | Ath-AT5G09560.1 |  |  |  |  |  |  |  |  |
| 0 | Ath-AT5G09570.1 |  |  |  |  |  |  |  |  |
| 0 | Ath-AT5G09580.1 |  |  |  |  |  |  |  |  |
| 0 | Ath-AT5G09590.1 |  |  |  |  |  |  |  |  |
| 0 | Ath-AT5G09600.3 |  |  |  |  |  |  |  |  |
| 0 | Ath-AT5G09610.1 |  |  |  |  |  |  |  |  |
| 0 | Ath-AT5G09620.1 |  |  |  |  |  |  |  |  |
| 0 | Ath-AT5G09630.1 |  |  |  |  |  |  |  |  |
| 0 | Ath-AT5G09640.1 |  |  |  |  |  |  |  |  |
| 0 | Ath-AT5G09650.1 |  |  |  |  |  |  |  |  |
| 0 | Ath-AT5G09660.4 |  |  |  |  |  |  |  |  |
| 0 | Ath-AT5G09670.1 |  |  |  |  |  |  |  |  |
| 0 | Ath-AT5G09680.3 |  |  |  |  |  |  |  |  |
| 0 | Ath-AT5G09690.2 |  |  |  |  |  |  |  |  |
| 0 | Ath-AT5G09700.1 |  |  |  |  |  |  |  |  |
| 0 | Ath-AT5G09711.1 |  |  |  |  |  |  |  |  |
| 0 | Ath-AT5G09720.1 |  |  |  |  |  |  |  |  |
| 0 | Ath-AT5G09730.1 |  |  |  |  |  |  |  |  |
| 1 | Ath-AT5G09740.1 |  | Vvi-Vitvi18g00333\_t001 |  |  |  |  |  |  |  |
| 2 | Ath-AT5G09750.1 |  | | | |  | Vvi-Vitvi04g01662\_t001 |  |  |  |  |  |  |
| 2 | Ath-AT5G09760.2 |  | | | |  | Vvi-Vitvi04g02246\_t001 |  |  |  |  |  |  |
| 2 | Ath-AT5G09770.1 |  | | | |  | Vvi-Vitvi04g01655\_t001 |  |  |  |  |  |  |
| 2 | Ath-AT5G09780.1 |  | | | |  | | | |  |  |  |  |  |  |
| 2 | Ath-AT5G09790.2 |  | | | |  | Vvi-Vitvi04g01653\_t001 |  |  |  |  |  |  |
| 2 | Ath-AT5G09800.1 |  | | | |  | Vvi-Vitvi04g02243\_t001 |  |  |  |  |  |  |
| 2 | Ath-AT5G09805.1 |  | | | |  | | | |  |  |  |  |  |  |
| 2 | Ath-AT5G09810.1 |  | | | |  | | | |  |  |  |  |  |  |
| 2 | Ath-AT5G09820.2 |  | | | |  | | | |  |  |  |  |  |  |
| 2 | Ath-AT5G09830.1 |  | | | |  | | | |  |  |  |  |  |  |
| 2 | Ath-AT5G09840.1 |  | | | |  | | | |  |  |  |  |  |  |
| 2 | Ath-AT5G09850.2 |  | | | |  | | | |  |  |  |  |  |  |
| 2 | Ath-AT5G09860.1 |  | | | |  | | | |  |  |  |  |  |  |
| 2 | Ath-AT5G09870.1 |  | | | |  | Vvi-Vitvi04g02233\_t001 |  |  |  |  |  |  |
| 1 | Ath-AT5G09876.1 |  | | | |  |  |  |  |  |  |  |
| 1 | Ath-AT5G09880.1 |  | | | |  |  |  |  |  |  |  |
| 1 | Ath-AT5G09890.2 |  | | | |  |  |  |  |  |  |  |
| 1 | Ath-AT5G09900.3 |  | | | |  |  |  |  |  |  |  |
| 2 | Ath-AT5G09910.1 |  | | | |  | Vvi-Vitvi07g01846\_t001 |  |  |  |  |  |  |
| 2 | Ath-AT5G09920.1 |  | | | |  | | | |  |  |  |  |  |  |
| 2 | Ath-AT5G09930.1 |  | | | |  | Vvi-Vitvi07g01840\_t001 |  |  |  |  |  |  |
| 2 | Ath-AT5G09940.1 |  | | | |  | | | |  |  |  |  |  |  |
| 2 | Ath-AT5G09950.1 |  | Vvi-Vitvi18g00339\_t001 |  | | | |  |  |  |  |  |  |
| 2 | Ath-AT5G09960.1 |  | | | |  | Vvi-Vitvi07g02691\_t001 |  |  |  |  |  |  |
| 2 | Ath-AT5G09970.1 |  | | | |  | Vvi-Vitvi07g01832\_t001 |  |  |  |  |  |  |
| 2 | Ath-AT5G09976.1 |  | | | |  | | | |  |  |  |  |  |  |
| 2 | Ath-AT5G09978.1 |  | | | |  | | | |  |  |  |  |  |  |
| 2 | Ath-AT5G09980.1 |  | | | |  | | | |  |  |  |  |  |  |
| 2 | Ath-AT5G09990.1 |  | | | |  | | | |  |  |  |  |  |  |
| 2 | Ath-AT5G09995.2 |  | | | |  | Vvi-Vitvi07g01827\_t001 |  |  |  |  |  |  |
| 2 | Ath-AT5G10000.1 |  | | | |  | | | |  |  |  |  |  |  |
| 2 | Ath-AT5G10010.1 |  | | | |  | Vvi-Vitvi07g01825\_t001 |  |  |  |  |  |  |
| 2 | Ath-AT5G10020.1 |  | | | |  | Vvi-Vitvi07g01821\_t001 |  |  |  |  |  |  |
| 2 | Ath-AT5G10030.1 |  | Vvi-Vitvi18g00346\_t001 |  | Vvi-Vitvi07g01807\_t001 |  |  |  |  |  |  |
| 2 | Ath-AT5G10040.1 |  | | | |  | | | |  |  |  |  |  |  |
| 2 | Ath-AT5G10050.1 |  | | | |  | Vvi-Vitvi07g01804\_t001 |  |  |  |  |  |  |
| 2 | Ath-AT5G10060.1 |  | | | |  | Vvi-Vitvi07g01803\_t002 |  |  |  |  |  |  |
| 2 | Ath-AT5G10070.2 |  | | | |  | Vvi-Vitvi07g01798\_t001 |  |  |  |  |  |  |
| 2 | Ath-AT5G10080.1 |  | Vvi-Vitvi18g00360\_t001 |  | Vvi-Vitvi07g01795\_t001 |  |  |  |  |  |  |
| 3 | Ath-AT5G10090.1 |  | | | |  | | | |  | Vvi-Vitvi07g01363\_t001 |  |  |  |  |  |
| 4 | Ath-AT5G10100.1 |  | | | |  | | | |  | Vvi-Vitvi07g01365\_t001 |  | Vvi-Vitvi07g01365\_t001 |  |  |  |  |
| 4 | Ath-AT5G10110.1 |  | | | |  | | | |  | Vvi-Vitvi07g03031\_t001 |  | | | |  |  |  |  |
| 4 | Ath-AT5G10120.1 |  | | | |  | | | |  | Vvi-Vitvi07g01353\_t001 |  | | | |  |  |  |  |
| 4 | Ath-AT5G10130.1 |  | Vvi-Vitvi18g00371\_t001 |  | | | |  | Vvi-Vitvi07g01351\_t001 |  | | | |  |  |  |  |
| 4 | Ath-AT5G10140.1 |  | | | |  | | | |  | | | |  | | | |  |  |  |  |
| 4 | Ath-AT5G10150.1 |  | | | |  | | | |  | Vvi-Vitvi07g01350\_t001 |  | | | |  |  |  |  |
| 4 | Ath-AT5G10160.1 |  | | | |  | | | |  | Vvi-Vitvi07g02535\_t001 |  | | | |  |  |  |  |
| 3 | Ath-AT5G10170.1 |  | | | |  | | | |  |  |  | | | |  |  |  |  |
| 3 | Ath-AT5G10180.1 |  | | | |  | Vvi-Vitvi07g01779\_t001 |  |  |  | | | |  |  |  |  |
| 2 | Ath-AT5G10190.1 |  | Vvi-Vitvi18g00392\_t001 |  |  |  |  |  | Vvi-Vitvi07g01358\_t001 |  |  |  |  |
| 2 | Ath-AT5G10200.2 |  | | | |  |  |  |  |  | Vvi-Vitvi07g04551\_t001 |  |  |  |  |
| 2 | Ath-AT5G10210.1 |  | | | |  |  |  |  |  | | | |  |  |  |  |
| 2 | Ath-AT5G10220.1 |  | Vvi-Vitvi18g00395\_t001 |  |  |  |  |  | | | |  |  |  |  |
| 2 | Ath-AT5G10230.1 |  | | | |  |  |  |  |  | | | |  |  |  |  |
| 2 | Ath-AT5G10240.1 |  | | | |  |  |  |  |  | Vvi-Vitvi07g04555\_t001 |  |  |  |  |
| 2 | Ath-AT5G10250.2 |  | | | |  |  |  |  |  | | | |  |  |  |  |
| 2 | Ath-AT5G10260.1 |  | | | |  |  |  |  |  | | | |  |  |  |  |
| 2 | Ath-AT5G10270.1 |  | | | |  |  |  |  |  | | | |  |  |  |  |
| 2 | Ath-AT5G10280.1 |  | Vvi-Vitvi18g00406\_t001 |  |  |  |  |  | | | |  |  |  |  |
| 1 | Ath-AT5G10290.1 |  |  |  |  |  |  |  | Vvi-Vitvi07g04561\_t001 |  |  |  |  |
| 1 | Ath-AT5G10300.1 |  |  |  |  |  |  |  | | | |  |  |  |  |
| 1 | Ath-AT5G10310.2 |  |  |  |  |  |  |  | | | |  |  |  |  |
| 1 | Ath-AT5G10320.1 |  |  |  |  |  |  |  | Vvi-Vitvi07g04577\_t001 |  |  |  |  |
| 1 | Ath-AT5G10330.2 |  |  |  |  |  |  |  | | | |  |  |  |  |
| 1 | Ath-AT5G10336.1 |  |  |  |  |  |  |  | | | |  |  |  |  |
| 1 | Ath-AT5G10340.1 |  |  |  |  |  |  |  | | | |  |  |  |  |
| 1 | Ath-AT5G10350.1 |  |  |  |  |  |  |  | Vvi-Vitvi07g04579\_t001 |  |  |  |  |
| 1 | Ath-AT5G10360.1 |  |  |  |  |  |  |  | Vvi-Vitvi07g01501\_t001 |  |  |  |  |
| 1 | Ath-AT5G10370.1 |  |  |  |  |  |  |  | Vvi-Vitvi07g01480\_t001 |  |  |  |  |
| 1 | Ath-AT5G10380.1 |  |  |  |  |  |  |  | Vvi-Vitvi07g01476\_t001 |  |  |  |  |
| 1 | Ath-AT5G10390.1 |  |  |  |  |  |  |  | | | |  |  |  |  |
| 1 | Ath-AT5G10400.1 |  |  |  |  |  |  |  | | | |  |  |  |  |
| 1 | Ath-AT5G10410.1 |  |  |  |  |  |  |  | Vvi-Vitvi07g01471\_t001 |  |  |  |  |
| 1 | Ath-AT5G10420.1 |  |  |  |  |  |  |  | Vvi-Vitvi07g04591\_t001 |  |  |  |  |
| 1 | Ath-AT5G10430.1 |  |  |  |  |  |  |  | | | |  |  |  |  |
| 1 | Ath-AT5G10435.1 |  |  |  |  |  |  |  | | | |  |  |  |  |
| 1 | Ath-AT5G10440.1 |  |  |  |  |  |  |  | Vvi-Vitvi07g01460\_t001 |  |  |  |  |
| 1 | Ath-AT5G10450.4 |  |  |  |  |  |  |  | Vvi-Vitvi07g01457\_t001 |  |  |  |  |
| 1 | Ath-AT5G10460.1 |  |  |  |  |  |  |  | | | |  |  |  |  |
| 1 | Ath-AT5G10470.2 |  |  |  |  |  |  |  | Vvi-Vitvi07g01442\_t001 |  |  |  |  |
| 1 | Ath-AT5G10480.3 |  |  |  |  |  |  |  | Vvi-Vitvi07g01439\_t001 |  |  |  |  |
| 1 | Ath-AT5G10490.1 |  |  |  |  |  |  |  | Vvi-Vitvi07g04607\_t001 |  |  |  |  |
| 1 | Ath-AT5G10500.1 |  |  |  |  |  |  |  | Vvi-Vitvi07g04608\_t001 |  |  |  |  |
| 1 | Ath-AT5G10510.3 |  |  |  |  |  |  |  | Vvi-Vitvi07g01419\_t001 |  |  |  |  |
| 1 | Ath-AT5G10520.1 |  |  |  |  |  |  |  | Vvi-Vitvi07g04612\_t001 |  |  |  |  |
| 0 | Ath-AT5G10540.1 |  |  |  |  |  |  |  |  |
| 0 | Ath-AT5G10530.1 |  |  |  |  |  |  |  |  |
| 0 | Ath-AT5G10550.1 |  |  |  |  |  |  |  |  |
| 0 | Ath-AT5G10560.1 |  |  |  |  |  |  |  |  |
| 0 | Ath-AT5G10570.1 |  |  |  |  |  |  |  |  |
| 1 | Ath-AT5G10580.3 |  | Vvi-Vitvi04g00241\_t001 |  |  |  |  |  |  |  |
| 1 | Ath-AT5G10590.1 |  | | | |  |  |  |  |  |  |  |
| 1 | Ath-AT5G10600.1 |  | | | |  |  |  |  |  |  |  |
| 1 | Ath-AT5G10605.1 |  | Vvi-Vitvi04g00230\_t001 |  |  |  |  |  |  |  |
| 1 | Ath-AT5G10610.1 |  | | | |  |  |  |  |  |  |  |
| 1 | Ath-AT5G10620.1 |  | | | |  |  |  |  |  |  |  |
| 1 | Ath-AT5G10625.1 |  | Vvi-Vitvi04g00226\_t001 |  |  |  |  |  |  |  |
| 1 | Ath-AT5G10630.4 |  | | | |  |  |  |  |  |  |  |
| 1 | Ath-AT5G10650.1 |  | Vvi-Vitvi04g00205\_t002 |  |  |  |  |  |  |  |
| 1 | Ath-AT5G10660.2 |  | | | |  |  |  |  |  |  |  |
| 1 | Ath-AT5G10680.1 |  | | | |  |  |  |  |  |  |  |
| 1 | Ath-AT5G10690.2 |  | Vvi-Vitvi04g01821\_t001 |  |  |  |  |  |  |  |
| 1 | Ath-AT5G10695.1 |  | | | |  |  |  |  |  |  |  |
| 1 | Ath-AT5G10700.1 |  | Vvi-Vitvi04g00200\_t001 |  |  |  |  |  |  |  |
| 0 | Ath-AT5G10710.1 |  |  |  |  |  |  |  |  |
| 0 | Ath-AT5G10720.1 |  |  |  |  |  |  |  |  |
| 1 | Ath-AT5G10730.1 |  | Vvi-Vitvi04g00673\_t001 |  |  |  |  |  |  |  |
| 1 | Ath-AT5G10740.1 |  | Vvi-Vitvi04g00662\_t001 |  |  |  |  |  |  |  |
| 1 | Ath-AT5G10745.1 |  | | | |  |  |  |  |  |  |  |
| 1 | Ath-AT5G10750.1 |  | Vvi-Vitvi04g00658\_t001 |  |  |  |  |  |  |  |
| 1 | Ath-AT5G10760.1 |  | Vvi-Vitvi04g00641\_t001 |  |  |  |  |  |  |  |
| 1 | Ath-AT5G10770.1 |  | | | |  |  |  |  |  |  |  |
| 1 | Ath-AT5G10780.2 |  | Vvi-Vitvi04g00619\_t001 |  |  |  |  |  |  |  |
| 1 | Ath-AT5G10790.1 |  | Vvi-Vitvi04g00617\_t001 |  |  |  |  |  |  |  |
| 1 | Ath-AT5G10800.1 |  | Vvi-Vitvi04g00613\_t001 |  |  |  |  |  |  |  |
| 1 | Ath-AT5G10810.1 |  | Vvi-Vitvi04g00612\_t001 |  |  |  |  |  |  |  |
| 1 | Ath-AT5G10820.1 |  | Vvi-Vitvi04g00611\_t001 |  |  |  |  |  |  |  |
| 1 | Ath-AT5G10830.1 |  | | | |  |  |  |  |  |  |  |
| 1 | Ath-AT5G10840.1 |  | Vvi-Vitvi04g00600\_t001 |  |  |  |  |  |  |  |
| 1 | Ath-AT5G10860.1 |  | | | |  |  |  |  |  |  |  |
| 1 | Ath-AT5G10870.1 |  | Vvi-Vitvi04g00594\_t001 |  |  |  |  |  |  |  |
| 1 | Ath-AT5G10880.1 |  | | | |  |  |  |  |  |  |  |
| 1 | Ath-AT5G10890.2 |  | Vvi-Vitvi04g00590\_t001 |  |  |  |  |  |  |  |
| 0 | Ath-AT5G10900.2 |  |  |  |  |  |  |  |  |
| 0 | Ath-AT5G10910.1 |  |  |  |  |  |  |  |  |
| 0 | Ath-AT5G10920.1 |  |  |  |  |  |  |  |  |
| 1 | Ath-AT5G10930.1 |  | Vvi-Vitvi04g00512\_t001 |  |  |  |  |  |  |  |
| 1 | Ath-AT5G10940.1 |  | | | |  |  |  |  |  |  |  |
| 1 | Ath-AT5G10946.2 |  | | | |  |  |  |  |  |  |  |
| 1 | Ath-AT5G10950.1 |  | | | |  |  |  |  |  |  |  |
| 1 | Ath-AT5G10960.1 |  | Vvi-Vitvi04g00520\_t001 |  |  |  |  |  |  |  |
| 1 | Ath-AT5G10970.1 |  | Vvi-Vitvi04g00527\_t001 |  |  |  |  |  |  |  |
| 1 | Ath-AT5G10980.1 |  | | | |  |  |  |  |  |  |  |
| 1 | Ath-AT5G10990.1 |  | | | |  |  |  |  |  |  |  |
| 1 | Ath-AT5G11000.1 |  | Vvi-Vitvi04g00531\_t001 |  |  |  |  |  |  |  |
| 1 | Ath-AT5G11010.4 |  | | | |  |  |  |  |  |  |  |
| 1 | Ath-AT5G11020.1 |  | Vvi-Vitvi04g00538\_t001 |  |  |  |  |  |  |  |
| 1 | Ath-AT5G11030.2 |  | Vvi-Vitvi04g00541\_t001 |  |  |  |  |  |  |  |
| 1 | Ath-AT5G11040.1 |  | Vvi-Vitvi04g00543\_t003 |  |  |  |  |  |  |  |
| 1 | Ath-AT5G11050.1 |  | | | |  |  |  |  |  |  |  |
| 1 | Ath-AT5G11060.1 |  | Vvi-Vitvi04g00546\_t001 |  |  |  |  |  |  |  |
| 1 | Ath-AT5G11070.1 |  | | | |  |  |  |  |  |  |  |
| 1 | Ath-AT5G11080.2 |  | Vvi-Vitvi04g00555\_t001 |  |  |  |  |  |  |  |
| 1 | Ath-AT5G11090.1 |  | Vvi-Vitvi04g00557\_t001 |  |  |  |  |  |  |  |
| 1 | Ath-AT5G11110.1 |  | Vvi-Vitvi04g00508\_t001 |  |  |  |  |  |  |  |
| 1 | Ath-AT5G11130.1 |  | Vvi-Vitvi04g00507\_t001 |  |  |  |  |  |  |  |
| 1 | Ath-AT5G11140.1 |  | | | |  |  |  |  |  |  |  |
| 1 | Ath-AT5G11150.2 |  | Vvi-Vitvi04g00503\_t001 |  |  |  |  |  |  |  |
| 1 | Ath-AT5G11160.1 |  | Vvi-Vitvi04g00499\_t001 |  |  |  |  |  |  |  |
| 1 | Ath-AT5G11170.1 |  | | | |  |  |  |  |  |  |  |
| 1 | Ath-AT5G11180.2 |  | | | |  |  |  |  |  |  |  |
| 1 | Ath-AT5G11190.1 |  | Vvi-Vitvi04g00479\_t001 |  |  |  |  |  |  |  |
| 1 | Ath-AT5G11200.2 |  | | | |  |  |  |  |  |  |  |
| 1 | Ath-AT5G11210.5 |  | | | |  |  |  |  |  |  |  |
| 1 | Ath-AT5G11220.1 |  | | | |  |  |  |  |  |  |  |
| 1 | Ath-AT5G11230.1 |  | Vvi-Vitvi04g00471\_t001 |  |  |  |  |  |  |  |
| 1 | Ath-AT5G11240.1 |  | | | |  |  |  |  |  |  |  |
| 1 | Ath-AT5G11250.2 |  | | | |  |  |  |  |  |  |  |
| 1 | Ath-AT5G11260.2 |  | Vvi-Vitvi04g00464\_t001 |  |  |  |  |  |  |  |
| 1 | Ath-AT5G11270.1 |  | Vvi-Vitvi04g00463\_t001 |  |  |  |  |  |  |  |
| 1 | Ath-AT5G11280.1 |  | Vvi-Vitvi04g00462\_t001 |  |  |  |  |  |  |  |
| 1 | Ath-AT5G11290.2 |  | Vvi-Vitvi04g01895\_t001 |  |  |  |  |  |  |  |
| 1 | Ath-AT5G11300.1 |  | | | |  |  |  |  |  |  |  |
| 1 | Ath-AT5G11310.1 |  | Vvi-Vitvi04g00437\_t001 |  |  |  |  |  |  |  |
| 1 | Ath-AT5G11320.1 |  | Vvi-Vitvi04g00430\_t001 |  |  |  |  |  |  |  |
| 1 | Ath-AT5G11330.1 |  | Vvi-Vitvi04g00415\_t001 |  |  |  |  |  |  |  |
| 1 | Ath-AT5G11340.1 |  | Vvi-Vitvi04g00414\_t001 |  |  |  |  |  |  |  |
| 1 | Ath-AT5G11350.4 |  | Vvi-Vitvi04g00411\_t001 |  |  |  |  |  |  |  |
| 1 | Ath-AT5G11360.1 |  | | | |  |  |  |  |  |  |  |
| 1 | Ath-AT5G11370.1 |  | | | |  |  |  |  |  |  |  |
| 1 | Ath-AT5G11380.1 |  | | | |  |  |  |  |  |  |  |
| 1 | Ath-AT5G11390.1 |  | | | |  |  |  |  |  |  |  |
| 1 | Ath-AT5G11400.2 |  | | | |  |  |  |  |  |  |  |
| 1 | Ath-AT5G11410.1 |  | | | |  |  |  |  |  |  |  |
| 1 | Ath-AT5G11412.1 |  | | | |  |  |  |  |  |  |  |
| 1 | Ath-AT5G11416.1 |  | | | |  |  |  |  |  |  |  |
| 1 | Ath-AT5G11420.1 |  | | | |  |  |  |  |  |  |  |
| 1 | Ath-AT5G11430.1 |  | | | |  |  |  |  |  |  |  |
| 1 | Ath-AT5G11425.1 |  | | | |  |  |  |  |  |  |  |
| 1 | Ath-AT5G11440.2 |  | | | |  |  |  |  |  |  |  |
| 1 | Ath-AT5G11450.2 |  | | | |  |  |  |  |  |  |  |
| 1 | Ath-AT5G11460.1 |  | Vvi-Vitvi04g00390\_t003 |  |  |  |  |  |  |  |
| 0 | Ath-AT5G11470.1 |  |  |  |  |  |  |  |  |
| 1 | Ath-AT5G11480.1 |  | Vvi-Vitvi04g00339\_t001 |  |  |  |  |  |  |  |
| 1 | Ath-AT5G11490.2 |  | Vvi-Vitvi04g00338\_t001 |  |  |  |  |  |  |  |
| 1 | Ath-AT5G11500.1 |  | Vvi-Vitvi04g00333\_t001 |  |  |  |  |  |  |  |
| 1 | Ath-AT5G11510.1 |  | Vvi-Vitvi04g01855\_t001 |  |  |  |  |  |  |  |
| 1 | Ath-AT5G11520.1 |  | Vvi-Vitvi04g00328\_t001 |  |  |  |  |  |  |  |
| 1 | Ath-AT5G11530.2 |  | Vvi-Vitvi04g01852\_t001 |  |  |  |  |  |  |  |
| 1 | Ath-AT5G11540.1 |  | Vvi-Vitvi04g00318\_t001 |  |  |  |  |  |  |  |
| 1 | Ath-AT5G11550.1 |  | Vvi-Vitvi04g00317\_t001 |  |  |  |  |  |  |  |
| 1 | Ath-AT5G11560.1 |  | Vvi-Vitvi04g00316\_t001 |  |  |  |  |  |  |  |
| 1 | Ath-AT5G11565.1 |  | | | |  |  |  |  |  |  |  |
| 1 | Ath-AT5G11570.1 |  | | | |  |  |  |  |  |  |  |
| 1 | Ath-AT5G11580.1 |  | | | |  |  |  |  |  |  |  |
| 1 | Ath-AT5G11590.1 |  | | | |  |  |  |  |  |  |  |
| 1 | Ath-AT5G11600.1 |  | | | |  |  |  |  |  |  |  |
| 1 | Ath-AT5G11610.1 |  | Vvi-Vitvi04g00301\_t001 |  |  |  |  |  |  |  |
| 1 | Ath-AT5G11620.1 |  | Vvi-Vitvi04g01838\_t001 |  |  |  |  |  |  |  |
| 1 | Ath-AT5G11630.3 |  | Vvi-Vitvi04g01837\_t001 |  |  |  |  |  |  |  |
| 1 | Ath-AT5G11640.1 |  | Vvi-Vitvi04g00288\_t001 |  |  |  |  |  |  |  |
| 1 | Ath-AT5G11650.1 |  | Vvi-Vitvi04g00283\_t001 |  |  |  |  |  |  |  |
| 0 | Ath-AT5G11660.1 |  |  |  |  |  |  |  |  |
| 0 | Ath-AT5G11670.1 |  |  |  |  |  |  |  |  |
| 0 | Ath-AT5G11680.1 |  |  |  |  |  |  |  |  |
| 0 | Ath-AT5G11690.1 |  |  |  |  |  |  |  |  |
| 1 | Ath-AT5G11700.2 |  | Vvi-Vitvi04g00045\_t001 |  |  |  |  |  |  |  |
| 1 | Ath-AT5G11710.1 |  | Vvi-Vitvi04g00050\_t002 |  |  |  |  |  |  |  |
| 1 | Ath-AT5G11720.1 |  | | | |  |  |  |  |  |  |  |
| 1 | Ath-AT5G11730.1 |  | Vvi-Vitvi04g00056\_t001 |  |  |  |  |  |  |  |
| 1 | Ath-AT5G11740.1 |  | | | |  |  |  |  |  |  |  |
| 1 | Ath-AT5G11750.2 |  | | | |  |  |  |  |  |  |  |
| 1 | Ath-AT5G11760.1 |  | Vvi-Vitvi04g01782\_t001 |  |  |  |  |  |  |  |
| 1 | Ath-AT5G11770.1 |  | Vvi-Vitvi04g00067\_t001 |  |  |  |  |  |  |  |
| 1 | Ath-AT5G11780.1 |  | Vvi-Vitvi04g00069\_t001 |  |  |  |  |  |  |  |
| 1 | Ath-AT5G11790.1 |  | Vvi-Vitvi04g00070\_t001 |  |  |  |  |  |  |  |
| 1 | Ath-AT5G11800.1 |  | | | |  |  |  |  |  |  |  |
| 2 | Ath-AT5G11810.1 |  | | | |  | Vvi-Vitvi04g00116\_t001 |  |  |  |  |  |  |
| 2 | Ath-AT5G11820.1 |  | | | |  | | | |  |  |  |  |  |  |
| 2 | Ath-AT5G11830.1 |  | | | |  | | | |  |  |  |  |  |  |
| 2 | Ath-AT5G11840.1 |  | | | |  | Vvi-Vitvi04g00114\_t001 |  |  |  |  |  |  |
| 2 | Ath-AT5G11850.1 |  | | | |  | Vvi-Vitvi04g00113\_t001 |  |  |  |  |  |  |
| 2 | Ath-AT5G11860.3 |  | | | |  | | | |  |  |  |  |  |  |
| 2 | Ath-AT5G11870.2 |  | | | |  | Vvi-Vitvi04g00106\_t001 |  |  |  |  |  |  |
| 2 | Ath-AT5G11880.1 |  | | | |  | | | |  |  |  |  |  |  |
| 2 | Ath-AT5G11890.1 |  | | | |  | Vvi-Vitvi04g00105\_t001 |  |  |  |  |  |  |
| 2 | Ath-AT5G11900.1 |  | | | |  | Vvi-Vitvi04g04036\_t001 |  |  |  |  |  |  |
| 2 | Ath-AT5G11910.4 |  | | | |  | Vvi-Vitvi04g00099\_t001 |  |  |  |  |  |  |
| 2 | Ath-AT5G11920.1 |  | | | |  | Vvi-Vitvi04g00094\_t001 |  |  |  |  |  |  |
| 2 | Ath-AT5G11930.1 |  | | | |  | Vvi-Vitvi04g01788\_t001 |  |  |  |  |  |  |
| 2 | Ath-AT5G11940.1 |  | | | |  | | | |  |  |  |  |  |  |
| 2 | Ath-AT5G11950.3 |  | | | |  | Vvi-Vitvi04g00085\_t001 |  |  |  |  |  |  |
| 2 | Ath-AT5G11960.1 |  | Vvi-Vitvi04g00084\_t001 |  | | | |  |  |  |  |  |  |
| 1 | Ath-AT5G11970.1 |  |  |  | Vvi-Vitvi04g01786\_t001 |  |  |  |  |  |  |
| 0 | Ath-AT5G11975.1 |  |  |  |  |  |  |  |  |
| 0 | Ath-AT5G11980.1 |  |  |  |  |  |  |  |  |
| 0 | Ath-AT5G11990.1 |  |  |  |  |  |  |  |  |
| 0 | Ath-AT5G12000.1 |  |  |  |  |  |  |  |  |
| 0 | Ath-AT5G12010.1 |  |  |  |  |  |  |  |  |
| 0 | Ath-AT5G12020.1 |  |  |  |  |  |  |  |  |
| 0 | Ath-AT5G12030.1 |  |  |  |  |  |  |  |  |
| 0 | Ath-AT5G12040.1 |  |  |  |  |  |  |  |  |
| 0 | Ath-AT5G12043.1 |  |  |  |  |  |  |  |  |
| 0 | Ath-AT5G12050.1 |  |  |  |  |  |  |  |  |
| 0 | Ath-AT5G12060.1 |  |  |  |  |  |  |  |  |
| 0 | Ath-AT5G12070.1 |  |  |  |  |  |  |  |  |
| 0 | Ath-AT5G12080.1 |  |  |  |  |  |  |  |  |
| 0 | Ath-AT5G12090.1 |  |  |  |  |  |  |  |  |
| 0 | Ath-AT5G12100.1 |  |  |  |  |  |  |  |  |
| 0 | Ath-AT5G12110.1 |  |  |  |  |  |  |  |  |
| 0 | Ath-AT5G12120.1 |  |  |  |  |  |  |  |  |
| 0 | Ath-AT5G12130.1 |  |  |  |  |  |  |  |  |
| 0 | Ath-AT5G12140.1 |  |  |  |  |  |  |  |  |
| 1 | Ath-AT5G12150.1 |  | Vvi-Vitvi06g01217\_t002 |  |  |  |  |  |  |  |
| 1 | Ath-AT5G12170.2 |  | | | |  |  |  |  |  |  |  |
| 1 | Ath-AT5G12180.1 |  | | | |  |  |  |  |  |  |  |
| 1 | Ath-AT5G12190.1 |  | Vvi-Vitvi06g01220\_t001 |  |  |  |  |  |  |  |
| 1 | Ath-AT5G12200.1 |  | | | |  |  |  |  |  |  |  |
| 1 | Ath-AT5G12210.1 |  | | | |  |  |  |  |  |  |  |
| 1 | Ath-AT5G12220.1 |  | | | |  |  |  |  |  |  |  |
| 1 | Ath-AT5G12230.1 |  | | | |  |  |  |  |  |  |  |
| 1 | Ath-AT5G12235.1 |  | | | |  |  |  |  |  |  |  |
| 1 | Ath-AT5G12236.1 |  | | | |  |  |  |  |  |  |  |
| 1 | Ath-AT5G12240.2 |  | | | |  |  |  |  |  |  |  |
| 1 | Ath-AT5G12250.1 |  | | | |  |  |  |  |  |  |  |
| 1 | Ath-AT5G12260.1 |  | | | |  |  |  |  |  |  |  |
| 1 | Ath-AT5G12270.1 |  | | | |  |  |  |  |  |  |  |
| 1 | Ath-AT5G12280.1 |  | | | |  |  |  |  |  |  |  |
| 1 | Ath-AT5G12290.1 |  | | | |  |  |  |  |  |  |  |
| 1 | Ath-AT5G12300.1 |  | Vvi-Vitvi06g01236\_t001 |  |  |  |  |  |  |  |
| 1 | Ath-AT5G12310.1 |  | Vvi-Vitvi06g01237\_t001 |  |  |  |  |  |  |  |
| 1 | Ath-AT5G12320.1 |  | Vvi-Vitvi06g01238\_t001 |  |  |  |  |  |  |  |
| 1 | Ath-AT5G12330.4 |  | Vvi-Vitvi06g01239\_t001 |  |  |  |  |  |  |  |
| 1 | Ath-AT5G12340.2 |  | Vvi-Vitvi06g01240\_t001 |  |  |  |  |  |  |  |
| 1 | Ath-AT5G12350.1 |  | Vvi-Vitvi06g01245\_t001 |  |  |  |  |  |  |  |
| 1 | Ath-AT5G12360.1 |  | Vvi-Vitvi06g01911\_t001 |  |  |  |  |  |  |  |
| 1 | Ath-AT5G12370.2 |  | | | |  |  |  |  |  |  |  |
| 1 | Ath-AT5G12380.1 |  | Vvi-Vitvi06g01252\_t001 |  |  |  |  |  |  |  |
| 1 | Ath-AT5G12390.1 |  | Vvi-Vitvi06g01254\_t001 |  |  |  |  |  |  |  |
| 0 | Ath-AT5G12400.1 |  |  |  |  |  |  |  |  |
| 0 | Ath-AT5G12410.1 |  |  |  |  |  |  |  |  |
| 0 | Ath-AT5G12420.1 |  |  |  |  |  |  |  |  |
| 0 | Ath-AT5G12430.1 |  |  |  |  |  |  |  |  |
| 0 | Ath-AT5G12440.3 |  |  |  |  |  |  |  |  |
| 0 | Ath-AT5G12450.1 |  |  |  |  |  |  |  |  |
| 0 | Ath-AT5G12460.1 |  |  |  |  |  |  |  |  |
| 1 | Ath-AT5G12470.1 |  | Vvi-Vitvi06g00235\_t001 |  |  |  |  |  |  |  |
| 1 | Ath-AT5G12480.1 |  | Vvi-Vitvi06g00227\_t001 |  |  |  |  |  |  |  |
| 1 | Ath-AT5G12840.1 |  | Vvi-Vitvi06g00224\_t001 |  |  |  |  |  |  |  |
| 1 | Ath-AT5G12850.1 |  | Vvi-Vitvi06g00218\_t003 |  |  |  |  |  |  |  |
| 1 | Ath-AT5G12860.1 |  | Vvi-Vitvi06g00215\_t001 |  |  |  |  |  |  |  |
| 1 | Ath-AT5G12870.1 |  | Vvi-Vitvi06g00214\_t001 |  |  |  |  |  |  |  |
| 0 | Ath-AT5G12880.1 |  |  |  |  |  |  |  |  |
| 1 | Ath-AT5G12890.1 |  | Vvi-Vitvi06g00169\_t001 |  |  |  |  |  |  |  |
| 1 | Ath-AT5G12900.1 |  | Vvi-Vitvi06g00173\_t001 |  |  |  |  |  |  |  |
| 1 | Ath-AT5G12910.1 |  | | | |  |  |  |  |  |  |  |
| 1 | Ath-AT5G12920.2 |  | Vvi-Vitvi06g00177\_t001 |  |  |  |  |  |  |  |
| 1 | Ath-AT5G12930.1 |  | Vvi-Vitvi06g00180\_t001 |  |  |  |  |  |  |  |
| 1 | Ath-AT5G12940.1 |  | Vvi-Vitvi06g00182\_t001 |  |  |  |  |  |  |  |
| 1 | Ath-AT5G12950.1 |  | Vvi-Vitvi06g00184\_t001 |  |  |  |  |  |  |  |
| 1 | Ath-AT5G12960.1 |  | | | |  |  |  |  |  |  |  |
| 1 | Ath-AT5G12970.1 |  | | | |  |  |  |  |  |  |  |
| 1 | Ath-AT5G12980.1 |  | | | |  |  |  |  |  |  |  |
| 1 | Ath-AT5G12990.1 |  | | | |  |  |  |  |  |  |  |
| 1 | Ath-AT5G13000.1 |  | | | |  |  |  |  |  |  |  |
| 1 | Ath-AT5G13010.1 |  | | | |  |  |  |  |  |  |  |
| 1 | Ath-AT5G13020.1 |  | | | |  |  |  |  |  |  |  |
| 1 | Ath-AT5G13030.1 |  | | | |  |  |  |  |  |  |  |
| 1 | Ath-AT5G13050.1 |  | | | |  |  |  |  |  |  |  |
| 1 | Ath-AT5G13060.1 |  | | | |  |  |  |  |  |  |  |
| 1 | Ath-AT5G13070.1 |  | Vvi-Vitvi06g00188\_t001 |  |  |  |  |  |  |  |
| 3 | Ath-AT5G13080.1 |  | Vvi-Vitvi01g01680\_t001 |  | Vvi-Vitvi14g01523\_t001 |  | Vvi-Vitvi17g00102\_t001 |  |  |  |  |  |
| 3 | Ath-AT5G13090.1 |  | Vvi-Vitvi01g01686\_t001 |  | | | |  | Vvi-Vitvi17g01341\_t001 |  |  |  |  |  |
| 3 | Ath-AT5G13100.1 |  | Vvi-Vitvi01g04453\_t001 |  | | | |  | | | |  |  |  |  |  |
| 3 | Ath-AT5G13110.1 |  | Vvi-Vitvi01g01700\_t001 |  | | | |  | | | |  |  |  |  |  |
| 3 | Ath-AT5G13120.1 |  | Vvi-Vitvi01g01705\_t001 |  | | | |  | | | |  |  |  |  |  |
| 3 | Ath-AT5G13130.1 |  | | | |  | | | |  | Vvi-Vitvi17g00076\_t001 |  |  |  |  |  |
| 3 | Ath-AT5G13140.1 |  | | | |  | Vvi-Vitvi14g01511\_t001 |  | | | |  |  |  |  |  |
| 3 | Ath-AT5G13150.1 |  | Vvi-Vitvi01g01709\_t001 |  | | | |  | | | |  |  |  |  |  |
| 3 | Ath-AT5G13160.1 |  | Vvi-Vitvi01g01718\_t001 |  | Vvi-Vitvi14g01504\_t001 |  | | | |  |  |  |  |  |
| 3 | Ath-AT5G13170.1 |  | Vvi-Vitvi01g01719\_t001 |  | | | |  | Vvi-Vitvi17g00069\_t001 |  |  |  |  |  |
| 3 | Ath-AT5G13180.1 |  | Vvi-Vitvi01g01722\_t001 |  | Vvi-Vitvi14g01499\_t001 |  | Vvi-Vitvi17g00066\_t001 |  |  |  |  |  |
| 3 | Ath-AT5G13181.1 |  | | | |  | | | |  | | | |  |  |  |  |  |
| 3 | Ath-AT5G13190.2 |  | Vvi-Vitvi01g01731\_t001 |  | | | |  | | | |  |  |  |  |  |
| 3 | Ath-AT5G13200.1 |  | Vvi-Vitvi01g01734\_t001 |  | Vvi-Vitvi14g01485\_t001 |  | | | |  |  |  |  |  |
| 3 | Ath-AT5G13210.1 |  | Vvi-Vitvi01g01737\_t001 |  | | | |  | | | |  |  |  |  |  |
| 3 | Ath-AT5G13220.7 |  | Vvi-Vitvi01g02293\_t001 |  | | | |  | | | |  |  |  |  |  |
| 3 | Ath-AT5G13230.1 |  | Vvi-Vitvi01g01740\_t001 |  | | | |  | | | |  |  |  |  |  |
| 3 | Ath-AT5G13240.3 |  | | | |  | | | |  | | | |  |  |  |  |  |
| 3 | Ath-AT5G13250.1 |  | Vvi-Vitvi01g02295\_t001 |  | | | |  | | | |  |  |  |  |  |
| 3 | Ath-AT5G13260.1 |  | Vvi-Vitvi01g01750\_t001 |  | | | |  | Vvi-Vitvi17g00041\_t001 |  |  |  |  |  |
| 3 | Ath-AT5G13270.1 |  | Vvi-Vitvi01g01756\_t001 |  | | | |  | | | |  |  |  |  |  |
| 3 | Ath-AT5G13280.1 |  | Vvi-Vitvi01g01758\_t001 |  | Vvi-Vitvi14g01472\_t001 |  | | | |  |  |  |  |  |
| 3 | Ath-AT5G13290.2 |  | Vvi-Vitvi01g01761\_t001 |  | | | |  | | | |  |  |  |  |  |
| 3 | Ath-AT5G13295.1 |  | | | |  | | | |  | | | |  |  |  |  |  |
| 3 | Ath-AT5G13300.1 |  | Vvi-Vitvi01g01762\_t002 |  | | | |  | Vvi-Vitvi17g00033\_t001 |  |  |  |  |  |
| 3 | Ath-AT5G13310.1 |  | Vvi-Vitvi01g01763\_t001 |  | Vvi-Vitvi14g01463\_t001 |  | | | |  |  |  |  |  |
| 3 | Ath-AT5G13320.2 |  | | | |  | | | |  | | | |  |  |  |  |  |
| 3 | Ath-AT5G13330.1 |  | Vvi-Vitvi01g01826\_t001 |  | Vvi-Vitvi14g01458\_t001 |  | Vvi-Vitvi17g00025\_t001 |  |  |  |  |  |
| 2 | Ath-AT5G13340.1 |  | | | |  | | | |  |  |  |  |  |  |
| 2 | Ath-AT5G13350.1 |  | | | |  | | | |  |  |  |  |  |  |
| 2 | Ath-AT5G13360.3 |  | | | |  | | | |  |  |  |  |  |  |
| 2 | Ath-AT5G13370.1 |  | | | |  | | | |  |  |  |  |  |  |
| 2 | Ath-AT5G13380.1 |  | | | |  | | | |  |  |  |  |  |  |
| 2 | Ath-AT5G13390.2 |  | | | |  | | | |  |  |  |  |  |  |
| 2 | Ath-AT5G13400.1 |  | | | |  | | | |  |  |  |  |  |  |
| 2 | Ath-AT5G13410.1 |  | Vvi-Vitvi01g01780\_t001 |  | | | |  |  |  |  |  |  |
| 1 | Ath-AT5G13420.1 |  |  |  | | | |  |  |  |  |  |  |
| 1 | Ath-AT5G13430.1 |  |  |  | Vvi-Vitvi14g01457\_t001 |  |  |  |  |  |  |
| 0 | Ath-AT5G13440.1 |  |  |  |  |  |  |  |  |
| 0 | Ath-AT5G13450.1 |  |  |  |  |  |  |  |  |
| 0 | Ath-AT5G13460.1 |  |  |  |  |  |  |  |  |
| 0 | Ath-AT5G13470.1 |  |  |  |  |  |  |  |  |
| 0 | Ath-AT5G13480.2 |  |  |  |  |  |  |  |  |
| 0 | Ath-AT5G13490.1 |  |  |  |  |  |  |  |  |
| 0 | Ath-AT5G13500.1 |  |  |  |  |  |  |  |  |
| 0 | Ath-AT5G13510.1 |  |  |  |  |  |  |  |  |
| 0 | Ath-AT5G13520.1 |  |  |  |  |  |  |  |  |
| 0 | Ath-AT5G13530.1 |  |  |  |  |  |  |  |  |
| 0 | Ath-AT5G13550.1 |  |  |  |  |  |  |  |  |
| 0 | Ath-AT5G13560.1 |  |  |  |  |  |  |  |  |
| 0 | Ath-AT5G13565.1 |  |  |  |  |  |  |  |  |
| 0 | Ath-AT5G13570.2 |  |  |  |  |  |  |  |  |
| 0 | Ath-AT5G13580.1 |  |  |  |  |  |  |  |  |
| 0 | Ath-AT5G13590.5 |  |  |  |  |  |  |  |  |
| 0 | Ath-AT5G13600.1 |  |  |  |  |  |  |  |  |
| 0 | Ath-AT5G13610.1 |  |  |  |  |  |  |  |  |
| 0 | Ath-AT5G13620.1 |  |  |  |  |  |  |  |  |
| 0 | Ath-AT5G13630.1 |  |  |  |  |  |  |  |  |
| 0 | Ath-AT5G13640.1 |  |  |  |  |  |  |  |  |
| 0 | Ath-AT5G13650.2 |  |  |  |  |  |  |  |  |
| 0 | Ath-AT5G13660.2 |  |  |  |  |  |  |  |  |
| 0 | Ath-AT5G13670.1 |  |  |  |  |  |  |  |  |
| 0 | Ath-AT5G13680.1 |  |  |  |  |  |  |  |  |
| 0 | Ath-AT5G13690.1 |  |  |  |  |  |  |  |  |
| 0 | Ath-AT5G13700.1 |  |  |  |  |  |  |  |  |
| 0 | Ath-AT5G13710.1 |  |  |  |  |  |  |  |  |
| 0 | Ath-AT5G13720.1 |  |  |  |  |  |  |  |  |
| 0 | Ath-AT5G13730.1 |  |  |  |  |  |  |  |  |
| 0 | Ath-AT5G13740.1 |  |  |  |  |  |  |  |  |
| 0 | Ath-AT5G13750.1 |  |  |  |  |  |  |  |  |
| 0 | Ath-AT5G13760.1 |  |  |  |  |  |  |  |  |
| 0 | Ath-AT5G13770.1 |  |  |  |  |  |  |  |  |
| 0 | Ath-AT5G13780.1 |  |  |  |  |  |  |  |  |
| 0 | Ath-AT5G13790.2 |  |  |  |  |  |  |  |  |
| 0 | Ath-AT5G13800.3 |  |  |  |  |  |  |  |  |
| 0 | Ath-AT5G13810.1 |  |  |  |  |  |  |  |  |
| 0 | Ath-AT5G13820.1 |  |  |  |  |  |  |  |  |
| 0 | Ath-AT5G13825.1 |  |  |  |  |  |  |  |  |
| 0 | Ath-AT5G13830.1 |  |  |  |  |  |  |  |  |
| 0 | Ath-AT5G13840.1 |  |  |  |  |  |  |  |  |
| 0 | Ath-AT5G13850.1 |  |  |  |  |  |  |  |  |
| 0 | Ath-AT5G13860.1 |  |  |  |  |  |  |  |  |
| 0 | Ath-AT5G13870.3 |  |  |  |  |  |  |  |  |
| 1 | Ath-AT5G13880.2 |  | Vvi-Vitvi14g01429\_t001 |  |  |  |  |  |  |  |
| 1 | Ath-AT5G13890.1 |  | Vvi-Vitvi14g01434\_t001 |  |  |  |  |  |  |  |
| 1 | Ath-AT5G13900.1 |  | Vvi-Vitvi14g01437\_t001 |  |  |  |  |  |  |  |
| 1 | Ath-AT5G13910.1 |  | Vvi-Vitvi14g01441\_t001 |  |  |  |  |  |  |  |
| 1 | Ath-AT5G13920.2 |  | Vvi-Vitvi14g01446\_t001 |  |  |  |  |  |  |  |
| 1 | Ath-AT5G13930.1 |  | Vvi-Vitvi14g01449\_t003 |  |  |  |  |  |  |  |
| 1 | Ath-AT5G13940.1 |  | Vvi-Vitvi14g01452\_t001 |  |  |  |  |  |  |  |
| 1 | Ath-AT5G13950.3 |  | Vvi-Vitvi14g01455\_t003 |  |  |  |  |  |  |  |
| 1 | Ath-AT5G13960.1 |  | Vvi-Vitvi14g01462\_t001 |  |  |  |  |  |  |  |
| 1 | Ath-AT5G13970.1 |  | Vvi-Vitvi14g01463\_t001 |  |  |  |  |  |  |  |
| 1 | Ath-AT5G13980.1 |  | Vvi-Vitvi14g01465\_t002 |  |  |  |  |  |  |  |
| 1 | Ath-AT5G13990.1 |  | | | |  |  |  |  |  |  |  |
| 1 | Ath-AT5G14000.3 |  | | | |  |  |  |  |  |  |  |
| 2 | Ath-AT5G14010.1 |  | | | |  | Vvi-Vitvi14g02957\_t001 |  |  |  |  |  |  |
| 2 | Ath-AT5G14020.1 |  | | | |  | | | |  |  |  |  |  |  |
| 2 | Ath-AT5G14030.1 |  | | | |  | | | |  |  |  |  |  |  |
| 2 | Ath-AT5G14040.1 |  | | | |  | | | |  |  |  |  |  |  |
| 2 | Ath-AT5G14050.1 |  | | | |  | | | |  |  |  |  |  |  |
| 2 | Ath-AT5G14060.1 |  | Vvi-Vitvi14g01472\_t001 |  | | | |  |  |  |  |  |  |
| 1 | Ath-AT5G14070.1 |  |  |  | Vvi-Vitvi14g01507\_t001 |  |  |  |  |  |  |
| 1 | Ath-AT5G14080.1 |  |  |  | Vvi-Vitvi14g01516\_t001 |  |  |  |  |  |  |
| 1 | Ath-AT5G14090.1 |  |  |  | Vvi-Vitvi14g02961\_t001 |  |  |  |  |  |  |
| 1 | Ath-AT5G14100.1 |  |  |  | Vvi-Vitvi14g01521\_t001 |  |  |  |  |  |  |
| 1 | Ath-AT5G14105.1 |  |  |  | | | |  |  |  |  |  |  |
| 1 | Ath-AT5G14110.1 |  |  |  | | | |  |  |  |  |  |  |
| 1 | Ath-AT5G14120.1 |  |  |  | Vvi-Vitvi14g01530\_t004 |  |  |  |  |  |  |
| 1 | Ath-AT5G14130.2 |  |  |  | Vvi-Vitvi14g04568\_t001 |  |  |  |  |  |  |
| 1 | Ath-AT5G14140.1 |  |  |  | Vvi-Vitvi14g01533\_t001 |  |  |  |  |  |  |
| 1 | Ath-AT5G14150.1 |  |  |  | Vvi-Vitvi14g01549\_t001 |  |  |  |  |  |  |
| 1 | Ath-AT5G14160.1 |  |  |  | | | |  |  |  |  |  |  |
| 1 | Ath-AT5G14170.1 |  |  |  | Vvi-Vitvi14g01554\_t001 |  |  |  |  |  |  |
| 1 | Ath-AT5G14180.1 |  | Vvi-Vitvi14g01569\_t001 |  |  |  |  |  |  |  |
| 1 | Ath-AT5G14200.1 |  | | | |  |  |  |  |  |  |  |
| 1 | Ath-AT5G14210.2 |  | Vvi-Vitvi14g01576\_t001 |  |  |  |  |  |  |  |
| 1 | Ath-AT5G14220.4 |  | Vvi-Vitvi14g01579\_t001 |  |  |  |  |  |  |  |
| 1 | Ath-AT5G14230.1 |  | Vvi-Vitvi14g01581\_t001 |  |  |  |  |  |  |  |
| 1 | Ath-AT5G14240.1 |  | Vvi-Vitvi14g01585\_t001 |  |  |  |  |  |  |  |
| 1 | Ath-AT5G14250.1 |  | Vvi-Vitvi14g01586\_t001 |  |  |  |  |  |  |  |
| 1 | Ath-AT5G14260.1 |  | Vvi-Vitvi14g01590\_t001 |  |  |  |  |  |  |  |
| 1 | Ath-AT5G14270.2 |  | Vvi-Vitvi14g01594\_t002 |  |  |  |  |  |  |  |
| 1 | Ath-AT5G14275.1 |  | | | |  |  |  |  |  |  |  |
| 1 | Ath-AT5G14280.1 |  | | | |  |  |  |  |  |  |  |
| 1 | Ath-AT5G14285.1 |  | Vvi-Vitvi14g01595\_t001 |  |  |  |  |  |  |  |
| 1 | Ath-AT5G14290.1 |  | Vvi-Vitvi14g01598\_t002 |  |  |  |  |  |  |  |
| 1 | Ath-AT5G14300.1 |  | Vvi-Vitvi14g02991\_t001 |  |  |  |  |  |  |  |
| 1 | Ath-AT5G14310.1 |  | Vvi-Vitvi14g01610\_t001 |  |  |  |  |  |  |  |
| 1 | Ath-AT5G14320.1 |  | | | |  |  |  |  |  |  |  |
| 1 | Ath-AT5G14330.1 |  | | | |  |  |  |  |  |  |  |
| 1 | Ath-AT5G14340.1 |  | Vvi-Vitvi14g01615\_t001 |  |  |  |  |  |  |  |
| 1 | Ath-AT5G14345.1 |  | Vvi-Vitvi14g01630\_t001 |  |  |  |  |  |  |  |
| 1 | Ath-AT5G14350.1 |  | Vvi-Vitvi14g01632\_t001 |  |  |  |  |  |  |  |
| 1 | Ath-AT5G14360.1 |  | Vvi-Vitvi14g01633\_t001 |  |  |  |  |  |  |  |
| 1 | Ath-AT5G14370.1 |  | Vvi-Vitvi14g01634\_t001 |  |  |  |  |  |  |  |
| 1 | Ath-AT5G14380.1 |  | | | |  |  |  |  |  |  |  |
| 1 | Ath-AT5G14390.1 |  | Vvi-Vitvi14g01640\_t001 |  |  |  |  |  |  |  |
| 1 | Ath-AT5G14400.2 |  | Vvi-Vitvi14g01657\_t001 |  |  |  |  |  |  |  |
| 1 | Ath-AT5G14410.2 |  | | | |  |  |  |  |  |  |  |
| 1 | Ath-AT5G14420.4 |  | | | |  |  |  |  |  |  |  |
| 1 | Ath-AT5G14430.1 |  | | | |  |  |  |  |  |  |  |
| 1 | Ath-AT5G14440.1 |  | | | |  |  |  |  |  |  |  |
| 1 | Ath-AT5G14450.1 |  | Vvi-Vitvi14g04610\_t001 |  |  |  |  |  |  |  |
| 1 | Ath-AT5G14460.1 |  | Vvi-Vitvi14g01673\_t001 |  |  |  |  |  |  |  |
| 1 | Ath-AT5G14470.1 |  | Vvi-Vitvi14g01675\_t001 |  |  |  |  |  |  |  |
| 1 | Ath-AT5G14480.1 |  | Vvi-Vitvi14g01676\_t002 |  |  |  |  |  |  |  |
| 1 | Ath-AT5G14490.3 |  | Vvi-Vitvi14g01678\_t001 |  |  |  |  |  |  |  |
| 1 | Ath-AT5G14500.2 |  | Vvi-Vitvi14g01684\_t001.2.6037826b |  |  |  |  |  |  |  |
| 1 | Ath-AT5G14510.1 |  | Vvi-Vitvi14g01691\_t001 |  |  |  |  |  |  |  |
| 1 | Ath-AT5G14515.1 |  | | | |  |  |  |  |  |  |  |
| 1 | Ath-AT5G14520.1 |  | Vvi-Vitvi14g01703\_t002 |  |  |  |  |  |  |  |
| 1 | Ath-AT5G14530.1 |  | Vvi-Vitvi14g01709\_t001 |  |  |  |  |  |  |  |
| 1 | Ath-AT5G14540.1 |  | Vvi-Vitvi14g01715\_t001 |  |  |  |  |  |  |  |
| 1 | Ath-AT5G14550.1 |  | Vvi-Vitvi14g01718\_t001 |  |  |  |  |  |  |  |
| 1 | Ath-AT5G14560.1 |  | | | |  |  |  |  |  |  |  |
| 1 | Ath-AT5G14570.1 |  | Vvi-Vitvi14g01720\_t001 |  |  |  |  |  |  |  |
| 1 | Ath-AT5G14580.1 |  | Vvi-Vitvi14g01730\_t001 |  |  |  |  |  |  |  |
| 1 | Ath-AT5G14590.1 |  | Vvi-Vitvi14g01732\_t001 |  |  |  |  |  |  |  |
| 1 | Ath-AT5G14600.1 |  | Vvi-Vitvi14g01737\_t001 |  |  |  |  |  |  |  |
| 1 | Ath-AT5G14602.1 |  | | | |  |  |  |  |  |  |  |
| 1 | Ath-AT5G14610.1 |  | Vvi-Vitvi14g01741\_t001 |  |  |  |  |  |  |  |
| 1 | Ath-AT5G14620.1 |  | Vvi-Vitvi14g01743\_t001 |  |  |  |  |  |  |  |
| 1 | Ath-AT5G14640.1 |  | Vvi-Vitvi14g01744\_t001 |  |  |  |  |  |  |  |
| 1 | Ath-AT5G14650.1 |  | Vvi-Vitvi14g01745\_t001 |  |  |  |  |  |  |  |
| 1 | Ath-AT5G14660.1 |  | Vvi-Vitvi14g01746\_t001 |  |  |  |  |  |  |  |
| 1 | Ath-AT5G14670.1 |  | Vvi-Vitvi14g01748\_t001 |  |  |  |  |  |  |  |
| 1 | Ath-AT5G14680.1 |  | Vvi-Vitvi14g03017\_t003 |  |  |  |  |  |  |  |
| 1 | Ath-AT5G14690.1 |  | Vvi-Vitvi14g03018\_t001 |  |  |  |  |  |  |  |
| 1 | Ath-AT5G14700.1 |  | Vvi-Vitvi14g01757\_t001 |  |  |  |  |  |  |  |
| 1 | Ath-AT5G14710.1 |  | Vvi-Vitvi14g03019\_t001 |  |  |  |  |  |  |  |
| 1 | Ath-AT5G14720.1 |  | Vvi-Vitvi14g01758\_t003 |  |  |  |  |  |  |  |
| 2 | Ath-AT5G14730.1 |  | | | |  | Vvi-Vitvi17g00815\_t001 |  |  |  |  |  |  |
| 2 | Ath-AT5G14740.1 |  | Vvi-Vitvi14g01763\_t001 |  | | | |  |  |  |  |  |  |
| 2 | Ath-AT5G14750.1 |  | Vvi-Vitvi14g03020\_t001 |  | | | |  |  |  |  |  |  |
| 2 | Ath-AT5G14760.1 |  | Vvi-Vitvi14g01765\_t001 |  | | | |  |  |  |  |  |  |
| 2 | Ath-AT5G14770.3 |  | | | |  | | | |  |  |  |  |  |  |
| 2 | Ath-AT5G14780.1 |  | Vvi-Vitvi14g01773\_t001 |  | | | |  |  |  |  |  |  |
| 2 | Ath-AT5G14790.1 |  | Vvi-Vitvi14g04630\_t001 |  | | | |  |  |  |  |  |  |
| 2 | Ath-AT5G14800.1 |  | | | |  | | | |  |  |  |  |  |  |
| 2 | Ath-AT5G14820.1 |  | | | |  | | | |  |  |  |  |  |  |
| 2 | Ath-AT5G14850.1 |  | | | |  | | | |  |  |  |  |  |  |
| 2 | Ath-AT5G14860.1 |  | Vvi-Vitvi14g03025\_t001 |  | Vvi-Vitvi17g00788\_t001 |  |  |  |  |  |  |
| 2 | Ath-AT5G14870.1 |  | Vvi-Vitvi14g01797\_t001 |  | Vvi-Vitvi17g00781\_t001 |  |  |  |  |  |  |
| 2 | Ath-AT5G14880.1 |  | Vvi-Vitvi14g01798\_t001 |  | | | |  |  |  |  |  |  |
| 2 | Ath-AT5G14890.1 |  | Vvi-Vitvi14g01799\_t001 |  | | | |  |  |  |  |  |  |
| 2 | Ath-AT5G14895.1 |  | Vvi-Vitvi14g01800\_t001 |  | Vvi-Vitvi17g01535\_t001 |  |  |  |  |  |  |
| 2 | Ath-AT5G14900.1 |  | | | |  | | | |  |  |  |  |  |  |
| 2 | Ath-AT5G14910.1 |  | Vvi-Vitvi14g03029\_t001 |  | | | |  |  |  |  |  |  |
| 2 | Ath-AT5G14920.1 |  | Vvi-Vitvi14g01819\_t001 |  | Vvi-Vitvi17g00750\_t001 |  |  |  |  |  |  |
| 2 | Ath-AT5G14930.2 |  | Vvi-Vitvi14g03030\_t001 |  | | | |  |  |  |  |  |  |
| 2 | Ath-AT5G14940.1 |  | Vvi-Vitvi14g01832\_t001 |  | | | |  |  |  |  |  |  |
| 1 | Ath-AT5G14950.1 |  |  |  | | | |  |  |  |  |  |  |
| 1 | Ath-AT5G14960.1 |  |  |  | Vvi-Vitvi17g00736\_t002 |  |  |  |  |  |  |
| 1 | Ath-AT5G14970.1 |  |  |  | | | |  |  |  |  |  |  |
| 1 | Ath-AT5G14980.1 |  |  |  | | | |  |  |  |  |  |  |
| 1 | Ath-AT5G14990.1 |  |  |  | | | |  |  |  |  |  |  |
| 1 | Ath-AT5G14995.1 |  |  |  | | | |  |  |  |  |  |  |
| 1 | Ath-AT5G15000.1 |  |  |  | | | |  |  |  |  |  |  |
| 1 | Ath-AT5G15008.1 |  |  |  | | | |  |  |  |  |  |  |
| 1 | Ath-AT5G15010.1 |  |  |  | | | |  |  |  |  |  |  |
| 2 | Ath-AT5G15020.1 |  | Vvi-Vitvi14g01866\_t001 |  | Vvi-Vitvi17g01522\_t001 |  |  |  |  |  |  |
| 1 | Ath-AT5G15025.1 |  | | | |  |  |  |  |  |  |  |
| 1 | Ath-AT5G15030.1 |  | | | |  |  |  |  |  |  |  |
| 1 | Ath-AT5G15040.2 |  | | | |  |  |  |  |  |  |  |
| 1 | Ath-AT5G15050.1 |  | Vvi-Vitvi14g03050\_t001 |  |  |  |  |  |  |  |
| 1 | Ath-AT5G15060.1 |  | | | |  |  |  |  |  |  |  |
| 1 | Ath-AT5G15070.2 |  | Vvi-Vitvi14g01887\_t001 |  |  |  |  |  |  |  |
| 1 | Ath-AT5G15080.1 |  | | | |  |  |  |  |  |  |  |
| 2 | Ath-AT5G15090.2 |  | Vvi-Vitvi14g01898\_t001 |  | Vvi-Vitvi17g00547\_t001 |  |  |  |  |  |  |
| 2 | Ath-AT5G15100.1 |  | Vvi-Vitvi14g01899\_t001 |  | | | |  |  |  |  |  |  |
| 2 | Ath-AT5G15110.1 |  | Vvi-Vitvi14g01900\_t001 |  | Vvi-Vitvi17g00550\_t001 |  |  |  |  |  |  |
| 2 | Ath-AT5G15120.1 |  | Vvi-Vitvi14g01906\_t001 |  | Vvi-Vitvi17g00554\_t001 |  |  |  |  |  |  |
| 2 | Ath-AT5G15130.1 |  | Vvi-Vitvi14g01907\_t001 |  | Vvi-Vitvi17g00556\_t001 |  |  |  |  |  |  |
| 2 | Ath-AT5G15140.1 |  | Vvi-Vitvi14g01916\_t001 |  | Vvi-Vitvi17g00565\_t001 |  |  |  |  |  |  |
| 2 | Ath-AT5G15150.1 |  | Vvi-Vitvi14g01922\_t001 |  | | | |  |  |  |  |  |  |
| 2 | Ath-AT5G15160.1 |  | Vvi-Vitvi14g01926\_t001 |  | | | |  |  |  |  |  |  |
| 2 | Ath-AT5G15170.1 |  | | | |  | | | |  |  |  |  |  |  |
| 2 | Ath-AT5G15180.1 |  | | | |  | | | |  |  |  |  |  |  |
| 2 | Ath-AT5G15190.1 |  | Vvi-Vitvi14g03078\_t001 |  | | | |  |  |  |  |  |  |
| 2 | Ath-AT5G15200.1 |  | Vvi-Vitvi14g04680\_t001 |  | Vvi-Vitvi17g04155\_t001 |  |  |  |  |  |  |
| 1 | Ath-AT5G15210.1 |  | | | |  |  |  |  |  |  |  |
| 1 | Ath-AT5G15220.2 |  | | | |  |  |  |  |  |  |  |
| 1 | Ath-AT5G15230.1 |  | Vvi-Vitvi14g03084\_t001 |  |  |  |  |  |  |  |
| 1 | Ath-AT5G15240.1 |  | | | |  |  |  |  |  |  |  |
| 1 | Ath-AT5G15250.1 |  | | | |  |  |  |  |  |  |  |
| 1 | Ath-AT5G15260.2 |  | Vvi-Vitvi14g01972\_t001 |  |  |  |  |  |  |  |
| 1 | Ath-AT5G15265.1 |  | | | |  |  |  |  |  |  |  |
| 1 | Ath-AT5G15270.1 |  | Vvi-Vitvi14g01981\_t002 |  |  |  |  |  |  |  |
| 1 | Ath-AT5G15280.1 |  | Vvi-Vitvi14g01983\_t001 |  |  |  |  |  |  |  |
| 1 | Ath-AT5G15290.1 |  | Vvi-Vitvi14g03091\_t001 |  |  |  |  |  |  |  |
| 1 | Ath-AT5G15300.1 |  | Vvi-Vitvi14g01984\_t001 |  |  |  |  |  |  |  |
| 1 | Ath-AT5G15310.1 |  | Vvi-Vitvi14g01987\_t001 |  |  |  |  |  |  |  |
| 1 | Ath-AT5G15320.1 |  | | | |  |  |  |  |  |  |  |
| 1 | Ath-AT5G15330.2 |  | Vvi-Vitvi14g01991\_t001 |  |  |  |  |  |  |  |
| 1 | Ath-AT5G15340.1 |  | Vvi-Vitvi14g01997\_t001 |  |  |  |  |  |  |  |
| 1 | Ath-AT5G15350.1 |  | Vvi-Vitvi14g03096\_t001 |  |  |  |  |  |  |  |
| 1 | Ath-AT5G15360.1 |  | | | |  |  |  |  |  |  |  |
| 1 | Ath-AT5G15380.1 |  | | | |  |  |  |  |  |  |  |
| 1 | Ath-AT5G15390.1 |  | Vvi-Vitvi14g03097\_t001 |  |  |  |  |  |  |  |
| 1 | Ath-AT5G15400.1 |  | Vvi-Vitvi14g02016\_t001 |  |  |  |  |  |  |  |
| 1 | Ath-AT5G15410.1 |  | Vvi-Vitvi14g02018\_t001 |  |  |  |  |  |  |  |
| 1 | Ath-AT5G15420.1 |  | | | |  |  |  |  |  |  |  |
| 1 | Ath-AT5G15430.1 |  | Vvi-Vitvi14g02022\_t001 |  |  |  |  |  |  |  |
| 1 | Ath-AT5G15440.1 |  | Vvi-Vitvi14g02023\_t003 |  |  |  |  |  |  |  |
| 1 | Ath-AT5G15450.1 |  | Vvi-Vitvi14g02026\_t001 |  |  |  |  |  |  |  |
| 1 | Ath-AT5G15460.1 |  | Vvi-Vitvi14g02027\_t001 |  |  |  |  |  |  |  |
| 1 | Ath-AT5G15470.1 |  | Vvi-Vitvi14g02028\_t001 |  |  |  |  |  |  |  |
| 1 | Ath-AT5G15480.1 |  | | | |  |  |  |  |  |  |  |
| 1 | Ath-AT5G15490.1 |  | Vvi-Vitvi14g03099\_t002 |  |  |  |  |  |  |  |
| 1 | Ath-AT5G15500.2 |  | | | |  |  |  |  |  |  |  |
| 1 | Ath-AT5G15510.2 |  | Vvi-Vitvi14g02039\_t001 |  |  |  |  |  |  |  |
| 1 | Ath-AT5G15520.1 |  | | | |  |  |  |  |  |  |  |
| 1 | Ath-AT5G15530.1 |  | Vvi-Vitvi14g02043\_t001 |  |  |  |  |  |  |  |
| 1 | Ath-AT5G15533.1 |  | | | |  |  |  |  |  |  |  |
| 1 | Ath-AT5G15537.2 |  | | | |  |  |  |  |  |  |  |
| 1 | Ath-AT5G15540.1 |  | Vvi-Vitvi14g02046\_t001 |  |  |  |  |  |  |  |
| 0 | Ath-AT5G15550.3 |  |  |  |  |  |  |  |  |
| 0 | Ath-AT5G15560.1 |  |  |  |  |  |  |  |  |
| 0 | Ath-AT5G15570.1 |  |  |  |  |  |  |  |  |
| 0 | Ath-AT5G15580.1 |  |  |  |  |  |  |  |  |
| 0 | Ath-AT5G15581.1 |  |  |  |  |  |  |  |  |
| 0 | Ath-AT5G15600.1 |  |  |  |  |  |  |  |  |
| 0 | Ath-AT5G15610.1 |  |  |  |  |  |  |  |  |
| 0 | Ath-AT5G15620.1 |  |  |  |  |  |  |  |  |
| 0 | Ath-AT5G15630.1 |  |  |  |  |  |  |  |  |
| 0 | Ath-AT5G15640.1 |  |  |  |  |  |  |  |  |
| 0 | Ath-AT5G15650.1 |  |  |  |  |  |  |  |  |
| 0 | Ath-AT5G15660.1 |  |  |  |  |  |  |  |  |
| 0 | Ath-AT5G15670.1 |  |  |  |  |  |  |  |  |
| 1 | Ath-AT5G15680.1 |  | Vvi-Vitvi14g01308\_t001 |  |  |  |  |  |  |  |
| 1 | Ath-AT5G15685.1 |  | | | |  |  |  |  |  |  |  |
| 1 | Ath-AT5G15690.1 |  | | | |  |  |  |  |  |  |  |
| 1 | Ath-AT5G15700.2 |  | Vvi-Vitvi14g01311\_t001 |  |  |  |  |  |  |  |
| 1 | Ath-AT5G15710.2 |  | Vvi-Vitvi14g01313\_t001 |  |  |  |  |  |  |  |
| 1 | Ath-AT5G15720.1 |  | Vvi-Vitvi14g01314\_t001 |  |  |  |  |  |  |  |
| 1 | Ath-AT5G15725.1 |  | | | |  |  |  |  |  |  |  |
| 1 | Ath-AT5G15730.2 |  | Vvi-Vitvi14g01319\_t001 |  |  |  |  |  |  |  |
| 1 | Ath-AT5G15740.1 |  | Vvi-Vitvi14g01327\_t001 |  |  |  |  |  |  |  |
| 1 | Ath-AT5G15745.1 |  | | | |  |  |  |  |  |  |  |
| 1 | Ath-AT5G15750.1 |  | Vvi-Vitvi14g01329\_t001 |  |  |  |  |  |  |  |
| 1 | Ath-AT5G15760.1 |  | | | |  |  |  |  |  |  |  |
| 1 | Ath-AT5G15770.1 |  | | | |  |  |  |  |  |  |  |
| 1 | Ath-AT5G15780.1 |  | Vvi-Vitvi14g01336\_t001 |  |  |  |  |  |  |  |
| 1 | Ath-AT5G15790.4 |  | Vvi-Vitvi14g01338\_t001 |  |  |  |  |  |  |  |
| 1 | Ath-AT5G15800.2 |  | Vvi-Vitvi14g01344\_t001 |  |  |  |  |  |  |  |
| 1 | Ath-AT5G15802.1 |  | | | |  |  |  |  |  |  |  |
| 1 | Ath-AT5G15810.1 |  | Vvi-Vitvi14g01349\_t001 |  |  |  |  |  |  |  |
| 0 | Ath-AT5G15820.1 |  |  |  |  |  |  |  |  |
| 0 | Ath-AT5G15830.1 |  |  |  |  |  |  |  |  |
| 0 | Ath-AT5G15840.1 |  |  |  |  |  |  |  |  |
| 0 | Ath-AT5G15843.1 |  |  |  |  |  |  |  |  |
| 0 | Ath-AT5G15850.1 |  |  |  |  |  |  |  |  |
| 0 | Ath-AT5G15853.1 |  |  |  |  |  |  |  |  |
| 0 | Ath-AT5G15860.1 |  |  |  |  |  |  |  |  |
| 1 | Ath-AT5G15870.1 |  | Vvi-Vitvi14g02897\_t001 |  |  |  |  |  |  |  |
| 1 | Ath-AT5G15880.1 |  | Vvi-Vitvi14g01267\_t001 |  |  |  |  |  |  |  |
| 2 | Ath-AT5G15890.1 |  | Vvi-Vitvi14g01268\_t001 |  | Vvi-Vitvi14g01268\_t001 |  |  |  |  |  |  |
| 2 | Ath-AT5G15900.2 |  | | | |  | | | |  |  |  |  |  |  |
| 2 | Ath-AT5G15910.1 |  | | | |  | | | |  |  |  |  |  |  |
| 2 | Ath-AT5G15920.1 |  | | | |  | | | |  |  |  |  |  |  |
| 2 | Ath-AT5G15930.1 |  | Vvi-Vitvi14g01287\_t002 |  | | | |  |  |  |  |  |  |
| 2 | Ath-AT5G15940.2 |  | | | |  | | | |  |  |  |  |  |  |
| 2 | Ath-AT5G15950.1 |  | Vvi-Vitvi14g01289\_t001 |  | | | |  |  |  |  |  |  |
| 2 | Ath-AT5G15960.1 |  | | | |  | | | |  |  |  |  |  |  |
| 2 | Ath-AT5G15970.1 |  | | | |  | | | |  |  |  |  |  |  |
| 2 | Ath-AT5G15980.1 |  | Vvi-Vitvi14g01292\_t001 |  | | | |  |  |  |  |  |  |
| 2 | Ath-AT5G16000.1 |  | Vvi-Vitvi14g01294\_t001 |  | | | |  |  |  |  |  |  |
| 1 | Ath-AT5G16010.1 |  |  |  | | | |  |  |  |  |  |  |
| 1 | Ath-AT5G16020.1 |  |  |  | Vvi-Vitvi14g01260\_t001 |  |  |  |  |  |  |
| 1 | Ath-AT5G16023.1 |  |  |  | | | |  |  |  |  |  |  |
| 1 | Ath-AT5G16030.3 |  |  |  | Vvi-Vitvi14g01240\_t001 |  |  |  |  |  |  |
| 1 | Ath-AT5G16040.1 |  |  |  | Vvi-Vitvi14g01227\_t002 |  |  |  |  |  |  |
| 1 | Ath-AT5G16050.1 |  |  |  | Vvi-Vitvi14g01226\_t001 |  |  |  |  |  |  |
| 1 | Ath-AT5G16060.1 |  |  |  | Vvi-Vitvi14g02888\_t001 |  |  |  |  |  |  |
| 1 | Ath-AT5G16070.1 |  |  |  | Vvi-Vitvi14g01224\_t001 |  |  |  |  |  |  |
| 1 | Ath-AT5G16080.1 |  |  |  | Vvi-Vitvi14g01222\_t001 |  |  |  |  |  |  |
| 1 | Ath-AT5G16090.1 |  |  |  | Vvi-Vitvi14g01208\_t001 |  |  |  |  |  |  |
| 1 | Ath-AT5G16100.2 |  |  |  | | | |  |  |  |  |  |  |
| 1 | Ath-AT5G16110.1 |  |  |  | Vvi-Vitvi14g01188\_t001 |  |  |  |  |  |  |
| 1 | Ath-AT5G16120.4 |  |  |  | Vvi-Vitvi14g01187\_t001 |  |  |  |  |  |  |
| 1 | Ath-AT5G16130.1 |  |  |  | Vvi-Vitvi14g01179\_t001 |  |  |  |  |  |  |
| 1 | Ath-AT5G16140.1 |  |  |  | Vvi-Vitvi14g01160\_t001 |  |  |  |  |  |  |
| 1 | Ath-AT5G16150.2 |  |  |  | Vvi-Vitvi14g01157\_t001 |  |  |  |  |  |  |
| 1 | Ath-AT5G16160.1 |  |  |  | Vvi-Vitvi14g02871\_t001 |  |  |  |  |  |  |
| 1 | Ath-AT5G16170.1 |  |  |  | Vvi-Vitvi14g01156\_t001 |  |  |  |  |  |  |
| 1 | Ath-AT5G16180.1 |  |  |  | Vvi-Vitvi14g01151\_t001 |  |  |  |  |  |  |
| 1 | Ath-AT5G16190.4 |  |  |  | | | |  |  |  |  |  |  |
| 1 | Ath-AT5G16200.1 |  |  |  | Vvi-Vitvi14g01139\_t001 |  |  |  |  |  |  |
| 1 | Ath-AT5G16210.1 |  |  |  | Vvi-Vitvi14g01125\_t001 |  |  |  |  |  |  |
| 1 | Ath-AT5G16220.1 |  |  |  | Vvi-Vitvi14g01120\_t002 |  |  |  |  |  |  |
| 0 | Ath-AT5G16230.1 |  |  |  |  |  |  |  |  |
| 1 | Ath-AT5G16240.1 |  | Vvi-Vitvi07g02982\_t001 |  |  |  |  |  |  |  |
| 1 | Ath-AT5G16250.1 |  | Vvi-Vitvi07g02983\_t001 |  |  |  |  |  |  |  |
| 1 | Ath-AT5G16260.1 |  | Vvi-Vitvi07g02984\_t001 |  |  |  |  |  |  |  |
| 1 | Ath-AT5G16270.1 |  | Vvi-Vitvi07g01249\_t001 |  |  |  |  |  |  |  |
| 1 | Ath-AT5G16280.1 |  | | | |  |  |  |  |  |  |  |
| 1 | Ath-AT5G16285.1 |  | | | |  |  |  |  |  |  |  |
| 1 | Ath-AT5G16290.1 |  | Vvi-Vitvi07g01247\_t001 |  |  |  |  |  |  |  |
| 1 | Ath-AT5G16300.1 |  | Vvi-Vitvi07g02992\_t001 |  |  |  |  |  |  |  |
| 0 | Ath-AT5G16310.1 |  |  |  |  |  |  |  |  |
| 0 | Ath-AT5G16320.1 |  |  |  |  |  |  |  |  |
| 0 | Ath-AT5G16330.1 |  |  |  |  |  |  |  |  |
| 0 | Ath-AT5G16340.1 |  |  |  |  |  |  |  |  |
| 0 | Ath-AT5G16350.1 |  |  |  |  |  |  |  |  |
| 0 | Ath-AT5G16360.1 |  |  |  |  |  |  |  |  |
| 0 | Ath-AT5G16370.1 |  |  |  |  |  |  |  |  |
| 1 | Ath-AT5G16380.1 |  | Vvi-Vitvi04g01194\_t001 |  |  |  |  |  |  |  |
| 1 | Ath-AT5G16390.1 |  | Vvi-Vitvi04g01193\_t002 |  |  |  |  |  |  |  |
| 1 | Ath-AT5G16400.1 |  | Vvi-Vitvi04g04319\_t001 |  |  |  |  |  |  |  |
| 1 | Ath-AT5G16410.1 |  | | | |  |  |  |  |  |  |  |
| 1 | Ath-AT5G16420.2 |  | | | |  |  |  |  |  |  |  |
| 1 | Ath-AT5G16430.1 |  | | | |  |  |  |  |  |  |  |
| 1 | Ath-AT5G16440.1 |  | Vvi-Vitvi04g01175\_t001 |  |  |  |  |  |  |  |
| 1 | Ath-AT5G16450.1 |  | Vvi-Vitvi04g01174\_t001 |  |  |  |  |  |  |  |
| 1 | Ath-AT5G16453.1 |  | | | |  |  |  |  |  |  |  |
| 1 | Ath-AT5G16460.1 |  | Vvi-Vitvi04g01164\_t001 |  |  |  |  |  |  |  |
| 1 | Ath-AT5G16470.1 |  | Vvi-Vitvi04g01161\_t001 |  |  |  |  |  |  |  |
| 1 | Ath-AT5G16480.1 |  | Vvi-Vitvi04g01160\_t001 |  |  |  |  |  |  |  |
| 1 | Ath-AT5G16486.1 |  | | | |  |  |  |  |  |  |  |
| 1 | Ath-AT5G16490.1 |  | Vvi-Vitvi04g02062\_t001 |  |  |  |  |  |  |  |
| 1 | Ath-AT5G16500.1 |  | | | |  |  |  |  |  |  |  |
| 1 | Ath-AT5G16510.2 |  | | | |  |  |  |  |  |  |  |
| 1 | Ath-AT5G16520.1 |  | | | |  |  |  |  |  |  |  |
| 1 | Ath-AT5G16530.1 |  | | | |  |  |  |  |  |  |  |
| 1 | Ath-AT5G16540.1 |  | Vvi-Vitvi04g01157\_t002 |  |  |  |  |  |  |  |
| 0 | Ath-AT5G16550.1 |  |  |  |  |  |  |  |  |
| 0 | Ath-AT5G16560.1 |  |  |  |  |  |  |  |  |
| 0 | Ath-AT5G16570.1 |  |  |  |  |  |  |  |  |
| 0 | Ath-AT5G16580.1 |  |  |  |  |  |  |  |  |
| 0 | Ath-AT5G16590.1 |  |  |  |  |  |  |  |  |
| 0 | Ath-AT5G16600.1 |  |  |  |  |  |  |  |  |
| 0 | Ath-AT5G16610.2 |  |  |  |  |  |  |  |  |
| 0 | Ath-AT5G16620.1 |  |  |  |  |  |  |  |  |
| 0 | Ath-AT5G16630.2 |  |  |  |  |  |  |  |  |
| 0 | Ath-AT5G16640.1 |  |  |  |  |  |  |  |  |
| 1 | Ath-AT5G16650.2 |  | Vvi-Vitvi16g00080\_t004 |  |  |  |  |  |  |  |
| 1 | Ath-AT5G16660.1 |  | | | |  |  |  |  |  |  |  |
| 1 | Ath-AT5G16680.1 |  | | | |  |  |  |  |  |  |  |
| 1 | Ath-AT5G16690.1 |  | | | |  |  |  |  |  |  |  |
| 1 | Ath-AT5G16700.1 |  | | | |  |  |  |  |  |  |  |
| 1 | Ath-AT5G16710.1 |  | | | |  |  |  |  |  |  |  |
| 1 | Ath-AT5G16715.1 |  | | | |  |  |  |  |  |  |  |
| 1 | Ath-AT5G16720.1 |  | | | |  |  |  |  |  |  |  |
| 1 | Ath-AT5G16730.1 |  | Vvi-Vitvi16g00093\_t003 |  |  |  |  |  |  |  |
| 1 | Ath-AT5G16740.1 |  | | | |  |  |  |  |  |  |  |
| 1 | Ath-AT5G16750.1 |  | Vvi-Vitvi16g00096\_t001 |  |  |  |  |  |  |  |
| 1 | Ath-AT5G16760.1 |  | Vvi-Vitvi16g00097\_t001 |  |  |  |  |  |  |  |
| 1 | Ath-AT5G16770.2 |  | Vvi-Vitvi16g00106\_t001 |  |  |  |  |  |  |  |
| 1 | Ath-AT5G16780.2 |  | | | |  |  |  |  |  |  |  |
| 1 | Ath-AT5G16790.1 |  | | | |  |  |  |  |  |  |  |
| 1 | Ath-AT5G16800.3 |  | Vvi-Vitvi16g00111\_t001 |  |  |  |  |  |  |  |
| 1 | Ath-AT5G16810.1 |  | Vvi-Vitvi16g00112\_t001 |  |  |  |  |  |  |  |
| 1 | Ath-AT5G16820.2 |  | Vvi-Vitvi16g00114\_t001 |  |  |  |  |  |  |  |
| 1 | Ath-AT5G16830.1 |  | Vvi-Vitvi16g00115\_t001 |  |  |  |  |  |  |  |
| 1 | Ath-AT5G16840.2 |  | Vvi-Vitvi16g00116\_t001 |  |  |  |  |  |  |  |
| 1 | Ath-AT5G16850.1 |  | | | |  |  |  |  |  |  |  |
| 1 | Ath-AT5G16860.1 |  | | | |  |  |  |  |  |  |  |
| 1 | Ath-AT5G16870.1 |  | Vvi-Vitvi16g00135\_t002 |  |  |  |  |  |  |  |
| 1 | Ath-AT5G16880.1 |  | Vvi-Vitvi16g00136\_t001 |  |  |  |  |  |  |  |
| 1 | Ath-AT5G16890.1 |  | | | |  |  |  |  |  |  |  |
| 1 | Ath-AT5G16900.1 |  | | | |  |  |  |  |  |  |  |
| 1 | Ath-AT5G16910.1 |  | Vvi-Vitvi16g00137\_t001 |  |  |  |  |  |  |  |
| 1 | Ath-AT5G16920.1 |  | Vvi-Vitvi16g00138\_t001 |  |  |  |  |  |  |  |
| 1 | Ath-AT5G16930.1 |  | Vvi-Vitvi16g00146\_t001 |  |  |  |  |  |  |  |
| 1 | Ath-AT5G16940.1 |  | | | |  |  |  |  |  |  |  |
| 1 | Ath-AT5G16950.1 |  | | | |  |  |  |  |  |  |  |
| 1 | Ath-AT5G16960.1 |  | | | |  |  |  |  |  |  |  |
| 1 | Ath-AT5G16970.1 |  | | | |  |  |  |  |  |  |  |
| 1 | Ath-AT5G16980.2 |  | | | |  |  |  |  |  |  |  |
| 1 | Ath-AT5G16990.1 |  | | | |  |  |  |  |  |  |  |
| 1 | Ath-AT5G17000.1 |  | | | |  |  |  |  |  |  |  |
| 1 | Ath-AT5G17010.4 |  | Vvi-Vitvi16g01538\_t001 |  |  |  |  |  |  |  |
| 1 | Ath-AT5G17020.1 |  | Vvi-Vitvi16g00155\_t001 |  |  |  |  |  |  |  |
| 1 | Ath-AT5G17030.1 |  | Vvi-Vitvi16g00156\_t001 |  |  |  |  |  |  |  |
| 1 | Ath-AT5G17040.1 |  | | | |  |  |  |  |  |  |  |
| 1 | Ath-AT5G17050.1 |  | | | |  |  |  |  |  |  |  |
| 1 | Ath-AT5G17060.1 |  | Vvi-Vitvi16g01539\_t001 |  |  |  |  |  |  |  |
| 1 | Ath-AT5G17070.1 |  | Vvi-Vitvi16g01540\_t003 |  |  |  |  |  |  |  |
| 1 | Ath-AT5G17080.1 |  | | | |  |  |  |  |  |  |  |
| 1 | Ath-AT5G17090.1 |  | | | |  |  |  |  |  |  |  |
| 1 | Ath-AT5G17100.1 |  | | | |  |  |  |  |  |  |  |
| 1 | Ath-AT5G17110.1 |  | | | |  |  |  |  |  |  |  |
| 1 | Ath-AT5G17120.1 |  | | | |  |  |  |  |  |  |  |
| 1 | Ath-AT5G17130.1 |  | | | |  |  |  |  |  |  |  |
| 1 | Ath-AT5G17140.1 |  | | | |  |  |  |  |  |  |  |
| 1 | Ath-AT5G17150.1 |  | | | |  |  |  |  |  |  |  |
| 1 | Ath-AT5G17160.1 |  | Vvi-Vitvi16g00170\_t001 |  |  |  |  |  |  |  |
| 1 | Ath-AT5G17165.1 |  | | | |  |  |  |  |  |  |  |
| 1 | Ath-AT5G17167.1 |  | | | |  |  |  |  |  |  |  |
| 1 | Ath-AT5G17170.1 |  | | | |  |  |  |  |  |  |  |
| 1 | Ath-AT5G17180.1 |  | | | |  |  |  |  |  |  |  |
| 1 | Ath-AT5G17190.1 |  | | | |  |  |  |  |  |  |  |
| 1 | Ath-AT5G17200.1 |  | | | |  |  |  |  |  |  |  |
| 1 | Ath-AT5G17210.1 |  | Vvi-Vitvi16g00180\_t001 |  |  |  |  |  |  |  |
| 0 | Ath-AT5G17220.1 |  |  |  |  |  |  |  |  |
| 0 | Ath-AT5G17230.3 |  |  |  |  |  |  |  |  |
| 0 | Ath-AT5G17240.1 |  |  |  |  |  |  |  |  |
| 0 | Ath-AT5G17250.1 |  |  |  |  |  |  |  |  |
| 0 | Ath-AT5G17260.1 |  |  |  |  |  |  |  |  |
| 0 | Ath-AT5G17270.1 |  |  |  |  |  |  |  |  |
| 0 | Ath-AT5G17290.1 |  |  |  |  |  |  |  |  |
| 0 | Ath-AT5G17280.1 |  |  |  |  |  |  |  |  |
| 0 | Ath-AT5G17300.1 |  |  |  |  |  |  |  |  |
| 0 | Ath-AT5G17305.1 |  |  |  |  |  |  |  |  |
| 0 | Ath-AT5G17310.2 |  |  |  |  |  |  |  |  |
| 0 | Ath-AT5G17320.1 |  |  |  |  |  |  |  |  |
| 0 | Ath-AT5G17330.1 |  |  |  |  |  |  |  |  |
| 0 | Ath-AT5G17340.1 |  |  |  |  |  |  |  |  |
| 0 | Ath-AT5G17345.1 |  |  |  |  |  |  |  |  |
| 0 | Ath-AT5G17350.1 |  |  |  |  |  |  |  |  |
| 0 | Ath-AT5G17360.1 |  |  |  |  |  |  |  |  |
| 0 | Ath-AT5G17370.2 |  |  |  |  |  |  |  |  |
| 0 | Ath-AT5G17380.1 |  |  |  |  |  |  |  |  |
| 0 | Ath-AT5G17390.1 |  |  |  |  |  |  |  |  |
| 0 | Ath-AT5G17400.1 |  |  |  |  |  |  |  |  |
| 0 | Ath-AT5G17410.2 |  |  |  |  |  |  |  |  |
| 0 | Ath-AT5G17420.1 |  |  |  |  |  |  |  |  |
| 0 | Ath-AT5G17430.1 |  |  |  |  |  |  |  |  |
| 0 | Ath-AT5G17440.1 |  |  |  |  |  |  |  |  |
| 0 | Ath-AT5G17450.1 |  |  |  |  |  |  |  |  |
| 0 | Ath-AT5G17460.2 |  |  |  |  |  |  |  |  |
| 0 | Ath-AT5G17470.1 |  |  |  |  |  |  |  |  |
| 0 | Ath-AT5G17480.1 |  |  |  |  |  |  |  |  |
| 0 | Ath-AT5G17490.1 |  |  |  |  |  |  |  |  |
| 0 | Ath-AT5G17500.1 |  |  |  |  |  |  |  |  |
| 0 | Ath-AT5G17510.1 |  |  |  |  |  |  |  |  |
| 0 | Ath-AT5G17520.1 |  |  |  |  |  |  |  |  |
| 0 | Ath-AT5G17522.1 |  |  |  |  |  |  |  |  |
| 0 | Ath-AT5G17523.1 |  |  |  |  |  |  |  |  |
| 0 | Ath-AT5G17530.4 |  |  |  |  |  |  |  |  |
| 0 | Ath-AT5G17540.1 |  |  |  |  |  |  |  |  |
| 0 | Ath-AT5G17550.1 |  |  |  |  |  |  |  |  |
| 0 | Ath-AT5G17560.1 |  |  |  |  |  |  |  |  |
| 0 | Ath-AT5G17570.1 |  |  |  |  |  |  |  |  |
| 0 | Ath-AT5G17580.2 |  |  |  |  |  |  |  |  |
| 0 | Ath-AT5G17590.1 |  |  |  |  |  |  |  |  |
| 0 | Ath-AT5G17600.1 |  |  |  |  |  |  |  |  |
| 0 | Ath-AT5G17610.1 |  |  |  |  |  |  |  |  |
| 0 | Ath-AT5G17620.1 |  |  |  |  |  |  |  |  |
| 0 | Ath-AT5G17630.1 |  |  |  |  |  |  |  |  |
| 0 | Ath-AT5G17640.1 |  |  |  |  |  |  |  |  |
| 0 | Ath-AT5G17650.1 |  |  |  |  |  |  |  |  |
| 0 | Ath-AT5G17660.1 |  |  |  |  |  |  |  |  |
| 0 | Ath-AT5G17670.1 |  |  |  |  |  |  |  |  |
| 0 | Ath-AT5G17680.2 |  |  |  |  |  |  |  |  |
| 0 | Ath-AT5G17690.3 |  |  |  |  |  |  |  |  |
| 1 | Ath-AT5G17700.1 |  | Vvi-Vitvi08g00085\_t002 |  |  |  |  |  |  |  |
| 1 | Ath-AT5G17710.2 |  | | | |  |  |  |  |  |  |  |
| 1 | Ath-AT5G17720.1 |  | | | |  |  |  |  |  |  |  |
| 1 | Ath-AT5G17730.1 |  | | | |  |  |  |  |  |  |  |
| 1 | Ath-AT5G17740.1 |  | | | |  |  |  |  |  |  |  |
| 1 | Ath-AT5G17750.1 |  | | | |  |  |  |  |  |  |  |
| 1 | Ath-AT5G17760.1 |  | | | |  |  |  |  |  |  |  |
| 1 | Ath-AT5G17770.1 |  | Vvi-Vitvi08g00102\_t001 |  |  |  |  |  |  |  |
| 1 | Ath-AT5G17780.2 |  | Vvi-Vitvi08g00103\_t001 |  |  |  |  |  |  |  |
| 1 | Ath-AT5G17790.1 |  | Vvi-Vitvi08g00104\_t001 |  |  |  |  |  |  |  |
| 1 | Ath-AT5G17800.1 |  | Vvi-Vitvi08g00107\_t001 |  |  |  |  |  |  |  |
| 1 | Ath-AT5G17810.1 |  | Vvi-Vitvi08g00108\_t001 |  |  |  |  |  |  |  |
| 1 | Ath-AT5G17820.2 |  | | | |  |  |  |  |  |  |  |
| 1 | Ath-AT5G17830.1 |  | | | |  |  |  |  |  |  |  |
| 1 | Ath-AT5G17840.1 |  | | | |  |  |  |  |  |  |  |
| 1 | Ath-AT5G17847.1 |  | | | |  |  |  |  |  |  |  |
| 1 | Ath-AT5G17850.1 |  | Vvi-Vitvi08g02000\_t001 |  |  |  |  |  |  |  |
| 1 | Ath-AT5G17860.1 |  | | | |  |  |  |  |  |  |  |
| 1 | Ath-AT5G17870.2 |  | | | |  |  |  |  |  |  |  |
| 1 | Ath-AT5G17880.1 |  | | | |  |  |  |  |  |  |  |
| 1 | Ath-AT5G17890.1 |  | | | |  |  |  |  |  |  |  |
| 1 | Ath-AT5G17900.1 |  | | | |  |  |  |  |  |  |  |
| 1 | Ath-AT5G17910.1 |  | | | |  |  |  |  |  |  |  |
| 1 | Ath-AT5G17920.1 |  | Vvi-Vitvi08g00135\_t003 |  |  |  |  |  |  |  |
| 0 | Ath-AT5G17930.1 |  |  |  |  |  |  |  |  |
| 0 | Ath-AT5G17950.1 |  |  |  |  |  |  |  |  |
| 0 | Ath-AT5G17960.1 |  |  |  |  |  |  |  |  |
| 0 | Ath-AT5G17970.1 |  |  |  |  |  |  |  |  |
| 0 | Ath-AT5G17980.1 |  |  |  |  |  |  |  |  |
| 0 | Ath-AT5G17990.1 |  |  |  |  |  |  |  |  |
| 0 | Ath-AT5G18000.1 |  |  |  |  |  |  |  |  |
| 0 | Ath-AT5G18010.1 |  |  |  |  |  |  |  |  |
| 0 | Ath-AT5G18020.1 |  |  |  |  |  |  |  |  |
| 0 | Ath-AT5G18030.1 |  |  |  |  |  |  |  |  |
| 0 | Ath-AT5G18037.1 |  |  |  |  |  |  |  |  |
| 0 | Ath-AT5G18040.1 |  |  |  |  |  |  |  |  |
| 0 | Ath-AT5G18050.1 |  |  |  |  |  |  |  |  |
| 0 | Ath-AT5G18060.1 |  |  |  |  |  |  |  |  |
| 0 | Ath-AT5G18065.1 |  |  |  |  |  |  |  |  |
| 0 | Ath-AT5G18070.1 |  |  |  |  |  |  |  |  |
| 0 | Ath-AT5G18080.1 |  |  |  |  |  |  |  |  |
| 0 | Ath-AT5G18090.1 |  |  |  |  |  |  |  |  |
| 1 | Ath-AT5G18100.1 |  | Vvi-Vitvi08g01802\_t001 |  |  |  |  |  |  |  |
| 1 | Ath-AT5G18110.1 |  | Vvi-Vitvi08g01803\_t001 |  |  |  |  |  |  |  |
| 1 | Ath-AT5G18120.1 |  | Vvi-Vitvi08g01804\_t001 |  |  |  |  |  |  |  |
| 1 | Ath-AT5G18130.1 |  | Vvi-Vitvi08g01805\_t001 |  |  |  |  |  |  |  |
| 1 | Ath-AT5G18140.1 |  | Vvi-Vitvi08g01810\_t001 |  |  |  |  |  |  |  |
| 1 | Ath-AT5G18150.1 |  | Vvi-Vitvi08g01811\_t001 |  |  |  |  |  |  |  |
| 1 | Ath-AT5G18160.1 |  | | | |  |  |  |  |  |  |  |
| 1 | Ath-AT5G18170.1 |  | Vvi-Vitvi08g01812\_t002 |  |  |  |  |  |  |  |
| 1 | Ath-AT5G18180.1 |  | Vvi-Vitvi08g01813\_t001 |  |  |  |  |  |  |  |
| 1 | Ath-AT5G18190.1 |  | Vvi-Vitvi08g01814\_t001 |  |  |  |  |  |  |  |
| 1 | Ath-AT5G18200.1 |  | Vvi-Vitvi08g01815\_t001 |  |  |  |  |  |  |  |
| 1 | Ath-AT5G18210.2 |  | Vvi-Vitvi08g01826\_t001 |  |  |  |  |  |  |  |
| 1 | Ath-AT5G18220.1 |  | Vvi-Vitvi08g01832\_t001 |  |  |  |  |  |  |  |
| 1 | Ath-AT5G18230.4 |  | Vvi-Vitvi08g01833\_t004 |  |  |  |  |  |  |  |
| 1 | Ath-AT5G18240.4 |  | Vvi-Vitvi08g01834\_t001 |  |  |  |  |  |  |  |
| 1 | Ath-AT5G18250.1 |  | Vvi-Vitvi08g01835\_t001 |  |  |  |  |  |  |  |
| 1 | Ath-AT5G18260.1 |  | Vvi-Vitvi08g01837\_t001 |  |  |  |  |  |  |  |
| 1 | Ath-AT5G18270.2 |  | Vvi-Vitvi08g01841\_t001 |  |  |  |  |  |  |  |
| 1 | Ath-AT5G18280.1 |  | | | |  |  |  |  |  |  |  |
| 1 | Ath-AT5G18290.1 |  | Vvi-Vitvi08g02371\_t001 |  |  |  |  |  |  |  |
| 0 | Ath-AT5G18300.1 |  |  |  |  |  |  |  |  |
| 1 | Ath-AT5G18310.2 |  | Vvi-Vitvi04g01045\_t001 |  |  |  |  |  |  |  |
| 1 | Ath-AT5G18320.1 |  | Vvi-Vitvi04g01044\_t001 |  |  |  |  |  |  |  |
| 1 | Ath-AT5G18330.1 |  | | | |  |  |  |  |  |  |  |
| 1 | Ath-AT5G18340.1 |  | | | |  |  |  |  |  |  |  |
| 1 | Ath-AT5G18350.1 |  | | | |  |  |  |  |  |  |  |
| 1 | Ath-AT5G18360.1 |  | | | |  |  |  |  |  |  |  |
| 1 | Ath-AT5G18370.1 |  | | | |  |  |  |  |  |  |  |
| 1 | Ath-AT5G18380.1 |  | | | |  |  |  |  |  |  |  |
| 1 | Ath-AT5G18390.1 |  | Vvi-Vitvi04g02039\_t001 |  |  |  |  |  |  |  |
| 1 | Ath-AT5G18400.2 |  | | | |  |  |  |  |  |  |  |
| 1 | Ath-AT5G18403.1 |  | | | |  |  |  |  |  |  |  |
| 1 | Ath-AT5G18404.1 |  | | | |  |  |  |  |  |  |  |
| 1 | Ath-AT5G18407.1 |  | | | |  |  |  |  |  |  |  |
| 1 | Ath-AT5G18410.1 |  | Vvi-Vitvi04g02038\_t001 |  |  |  |  |  |  |  |
| 1 | Ath-AT5G18420.2 |  | | | |  |  |  |  |  |  |  |
| 1 | Ath-AT5G18430.1 |  | Vvi-Vitvi04g00997\_t001 |  |  |  |  |  |  |  |
| 1 | Ath-AT5G18440.2 |  | | | |  |  |  |  |  |  |  |
| 1 | Ath-AT5G18450.1 |  | Vvi-Vitvi04g00994\_t001 |  |  |  |  |  |  |  |
| 1 | Ath-AT5G18460.1 |  | Vvi-Vitvi04g00983\_t001 |  |  |  |  |  |  |  |
| 1 | Ath-AT5G18470.1 |  | | | |  |  |  |  |  |  |  |
| 1 | Ath-AT5G18475.1 |  | | | |  |  |  |  |  |  |  |
| 1 | Ath-AT5G18480.1 |  | | | |  |  |  |  |  |  |  |
| 1 | Ath-AT5G18490.1 |  | | | |  |  |  |  |  |  |  |
| 1 | Ath-AT5G18500.1 |  | | | |  |  |  |  |  |  |  |
| 1 | Ath-AT5G18510.1 |  | | | |  |  |  |  |  |  |  |
| 1 | Ath-AT5G18520.1 |  | Vvi-Vitvi04g00970\_t001 |  |  |  |  |  |  |  |
| 0 | Ath-AT5G18525.1 |  |  |  |  |  |  |  |  |
| 1 | Ath-AT5G18540.1 |  | Vvi-Vitvi05g00329\_t001 |  |  |  |  |  |  |  |
| 1 | Ath-AT5G18550.1 |  | Vvi-Vitvi05g00333\_t001 |  |  |  |  |  |  |  |
| 1 | Ath-AT5G18560.1 |  | Vvi-Vitvi05g00334\_t001 |  |  |  |  |  |  |  |
| 1 | Ath-AT5G18570.1 |  | Vvi-Vitvi05g00335\_t001 |  |  |  |  |  |  |  |
| 1 | Ath-AT5G18580.1 |  | Vvi-Vitvi05g00338\_t001 |  |  |  |  |  |  |  |
| 1 | Ath-AT5G18590.2 |  | Vvi-Vitvi05g00341\_t001 |  |  |  |  |  |  |  |
| 1 | Ath-AT5G18600.1 |  | Vvi-Vitvi05g00342\_t001 |  |  |  |  |  |  |  |
| 1 | Ath-AT5G18610.2 |  | Vvi-Vitvi05g00344\_t001 |  |  |  |  |  |  |  |
| 1 | Ath-AT5G18620.2 |  | Vvi-Vitvi05g00345\_t001 |  |  |  |  |  |  |  |
| 1 | Ath-AT5G18630.1 |  | Vvi-Vitvi05g00346\_t001 |  |  |  |  |  |  |  |
| 1 | Ath-AT5G18636.1 |  | | | |  |  |  |  |  |  |  |
| 1 | Ath-AT5G18640.2 |  | | | |  |  |  |  |  |  |  |
| 1 | Ath-AT5G18650.1 |  | Vvi-Vitvi05g00347\_t004 |  |  |  |  |  |  |  |
| 1 | Ath-AT5G18660.1 |  | Vvi-Vitvi05g00350\_t001 |  |  |  |  |  |  |  |
| 1 | Ath-AT5G18661.1 |  | | | |  |  |  |  |  |  |  |
| 1 | Ath-AT5G18670.1 |  | Vvi-Vitvi05g00357\_t001 |  |  |  |  |  |  |  |
| 1 | Ath-AT5G18680.1 |  | Vvi-Vitvi05g01862\_t002 |  |  |  |  |  |  |  |
| 1 | Ath-AT5G18690.1 |  | | | |  |  |  |  |  |  |  |
| 1 | Ath-AT5G18700.1 |  | Vvi-Vitvi05g00363\_t001 |  |  |  |  |  |  |  |
| 1 | Ath-AT5G18705.1 |  | | | |  |  |  |  |  |  |  |
| 1 | Ath-AT5G18720.3 |  | | | |  |  |  |  |  |  |  |
| 1 | Ath-AT5G18730.1 |  | | | |  |  |  |  |  |  |  |
| 1 | Ath-AT5G18735.1 |  | | | |  |  |  |  |  |  |  |
| 1 | Ath-AT5G18740.1 |  | | | |  |  |  |  |  |  |  |
| 1 | Ath-AT5G18748.1 |  | | | |  |  |  |  |  |  |  |
| 1 | Ath-AT5G18750.1 |  | Vvi-Vitvi05g00366\_t001 |  |  |  |  |  |  |  |
| 1 | Ath-AT5G18760.1 |  | Vvi-Vitvi05g00368\_t002 |  |  |  |  |  |  |  |
| 1 | Ath-AT5G18770.1 |  | | | |  |  |  |  |  |  |  |
| 1 | Ath-AT5G18780.1 |  | | | |  |  |  |  |  |  |  |
| 1 | Ath-AT5G18790.1 |  | Vvi-Vitvi05g04095\_t001 |  |  |  |  |  |  |  |
| 1 | Ath-AT5G18800.2 |  | Vvi-Vitvi05g00374\_t002 |  |  |  |  |  |  |  |
| 1 | Ath-AT5G18810.1 |  | | | |  |  |  |  |  |  |  |
| 1 | Ath-AT5G18820.1 |  | | | |  |  |  |  |  |  |  |
| 1 | Ath-AT5G18830.3 |  | Vvi-Vitvi05g00376\_t001 |  |  |  |  |  |  |  |
| 1 | Ath-AT5G18840.1 |  | Vvi-Vitvi05g00377\_t001 |  |  |  |  |  |  |  |
| 1 | Ath-AT5G18850.1 |  | Vvi-Vitvi05g00380\_t001 |  |  |  |  |  |  |  |
| 1 | Ath-AT5G18860.1 |  | Vvi-Vitvi05g00381\_t003 |  |  |  |  |  |  |  |
| 1 | Ath-AT5G18870.1 |  | | | |  |  |  |  |  |  |  |
| 1 | Ath-AT5G18880.1 |  | | | |  |  |  |  |  |  |  |
| 1 | Ath-AT5G18890.1 |  | | | |  |  |  |  |  |  |  |
| 1 | Ath-AT5G18900.1 |  | Vvi-Vitvi05g00383\_t001 |  |  |  |  |  |  |  |
| 1 | Ath-AT5G18910.1 |  | Vvi-Vitvi05g00384\_t001 |  |  |  |  |  |  |  |
| 1 | Ath-AT5G18920.2 |  | Vvi-Vitvi05g00389\_t001 |  |  |  |  |  |  |  |
| 1 | Ath-AT5G18930.1 |  | Vvi-Vitvi05g00393\_t001 |  |  |  |  |  |  |  |
| 1 | Ath-AT5G18933.1 |  | | | |  |  |  |  |  |  |  |
| 1 | Ath-AT5G18937.1 |  | | | |  |  |  |  |  |  |  |
| 1 | Ath-AT5G18940.1 |  | Vvi-Vitvi05g00397\_t001 |  |  |  |  |  |  |  |
| 1 | Ath-AT5G18950.1 |  | Vvi-Vitvi05g00400\_t001 |  |  |  |  |  |  |  |
| 1 | Ath-AT5G18960.2 |  | Vvi-Vitvi05g01873\_t001 |  |  |  |  |  |  |  |
| 1 | Ath-AT5G18970.1 |  | Vvi-Vitvi05g00402\_t001 |  |  |  |  |  |  |  |
| 1 | Ath-AT5G18980.1 |  | Vvi-Vitvi05g00406\_t001 |  |  |  |  |  |  |  |
| 1 | Ath-AT5G18990.1 |  | | | |  |  |  |  |  |  |  |
| 1 | Ath-AT5G19000.2 |  | Vvi-Vitvi05g00408\_t001 |  |  |  |  |  |  |  |
| 1 | Ath-AT5G19010.1 |  | Vvi-Vitvi05g00409\_t001 |  |  |  |  |  |  |  |
| 1 | Ath-AT5G19020.1 |  | Vvi-Vitvi05g00416\_t001 |  |  |  |  |  |  |  |
| 1 | Ath-AT5G19025.2 |  | Vvi-Vitvi05g00418\_t001 |  |  |  |  |  |  |  |
| 1 | Ath-AT5G19030.1 |  | Vvi-Vitvi05g01874\_t001 |  |  |  |  |  |  |  |
| 1 | Ath-AT5G19040.1 |  | Vvi-Vitvi05g00421\_t001 |  |  |  |  |  |  |  |
| 1 | Ath-AT5G19050.1 |  | Vvi-Vitvi05g00422\_t001 |  |  |  |  |  |  |  |
| 1 | Ath-AT5G19060.1 |  | Vvi-Vitvi05g00423\_t001 |  |  |  |  |  |  |  |
| 1 | Ath-AT5G19070.1 |  | Vvi-Vitvi05g01875\_t001 |  |  |  |  |  |  |  |
| 1 | Ath-AT5G19080.1 |  | Vvi-Vitvi05g00425\_t002 |  |  |  |  |  |  |  |
| 1 | Ath-AT5G19090.1 |  | Vvi-Vitvi05g00426\_t001 |  |  |  |  |  |  |  |
| 1 | Ath-AT5G19100.1 |  | Vvi-Vitvi05g00430\_t001 |  |  |  |  |  |  |  |
| 1 | Ath-AT5G19110.2 |  | | | |  |  |  |  |  |  |  |
| 1 | Ath-AT5G19120.1 |  | | | |  |  |  |  |  |  |  |
| 1 | Ath-AT5G19130.1 |  | | | |  |  |  |  |  |  |  |
| 1 | Ath-AT5G19140.1 |  | Vvi-Vitvi05g00434\_t001 |  |  |  |  |  |  |  |
| 1 | Ath-AT5G19150.2 |  | Vvi-Vitvi05g00436\_t001 |  |  |  |  |  |  |  |
| 1 | Ath-AT5G19151.1 |  | | | |  |  |  |  |  |  |  |
| 1 | Ath-AT5G19160.1 |  | Vvi-Vitvi05g00437\_t001 |  |  |  |  |  |  |  |
| 1 | Ath-AT5G19170.1 |  | | | |  |  |  |  |  |  |  |
| 1 | Ath-AT5G19172.1 |  | | | |  |  |  |  |  |  |  |
| 1 | Ath-AT5G19175.1 |  | | | |  |  |  |  |  |  |  |
| 1 | Ath-AT5G19180.1 |  | Vvi-Vitvi05g00438\_t001 |  |  |  |  |  |  |  |
| 1 | Ath-AT5G19190.1 |  | Vvi-Vitvi05g01877\_t001 |  |  |  |  |  |  |  |
| 1 | Ath-AT5G19200.1 |  | Vvi-Vitvi05g01878\_t002 |  |  |  |  |  |  |  |
| 1 | Ath-AT5G19210.2 |  | Vvi-Vitvi05g00441\_t001 |  |  |  |  |  |  |  |
| 1 | Ath-AT5G19220.1 |  | Vvi-Vitvi05g00442\_t001 |  |  |  |  |  |  |  |
| 1 | Ath-AT5G19230.2 |  | Vvi-Vitvi05g01879\_t001 |  |  |  |  |  |  |  |
| 1 | Ath-AT5G19240.1 |  | | | |  |  |  |  |  |  |  |
| 1 | Ath-AT5G19250.1 |  | | | |  |  |  |  |  |  |  |
| 1 | Ath-AT5G19260.1 |  | Vvi-Vitvi05g00448\_t001 |  |  |  |  |  |  |  |
| 1 | Ath-AT5G19270.1 |  | | | |  |  |  |  |  |  |  |
| 1 | Ath-AT5G19280.2 |  | Vvi-Vitvi05g00451\_t001 |  |  |  |  |  |  |  |
| 1 | Ath-AT5G19290.1 |  | | | |  |  |  |  |  |  |  |
| 1 | Ath-AT5G19300.1 |  | Vvi-Vitvi05g00452\_t001 |  |  |  |  |  |  |  |
| 1 | Ath-AT5G19310.1 |  | Vvi-Vitvi05g00453\_t001 |  |  |  |  |  |  |  |
| 1 | Ath-AT5G19315.1 |  | | | |  |  |  |  |  |  |  |
| 1 | Ath-AT5G19320.1 |  | Vvi-Vitvi05g00454\_t001 |  |  |  |  |  |  |  |
| 1 | Ath-AT5G19330.1 |  | Vvi-Vitvi05g00458\_t002 |  |  |  |  |  |  |  |
| 1 | Ath-AT5G19340.1 |  | Vvi-Vitvi05g00460\_t001 |  |  |  |  |  |  |  |
| 1 | Ath-AT5G19350.1 |  | | | |  |  |  |  |  |  |  |
| 2 | Ath-AT5G19360.1 |  | | | |  | Vvi-Vitvi06g01207\_t001 |  |  |  |  |  |  |
| 2 | Ath-AT5G19370.1 |  | | | |  | Vvi-Vitvi06g01211\_t001 |  |  |  |  |  |  |
| 2 | Ath-AT5G19380.2 |  | | | |  | Vvi-Vitvi06g01212\_t001 |  |  |  |  |  |  |
| 2 | Ath-AT5G19390.2 |  | | | |  | Vvi-Vitvi06g01217\_t002 |  |  |  |  |  |  |
| 2 | Ath-AT5G19400.3 |  | | | |  | Vvi-Vitvi06g01225\_t001 |  |  |  |  |  |  |
| 2 | Ath-AT5G19410.2 |  | | | |  | Vvi-Vitvi06g01231\_t001 |  |  |  |  |  |  |
| 2 | Ath-AT5G19420.2 |  | | | |  | | | |  |  |  |  |  |  |
| 2 | Ath-AT5G19430.5 |  | | | |  | Vvi-Vitvi06g01237\_t001 |  |  |  |  |  |  |
| 1 | Ath-AT5G19440.1 |  | | | |  |  |  |  |  |  |  |
| 1 | Ath-AT5G19450.2 |  | | | |  |  |  |  |  |  |  |
| 1 | Ath-AT5G19460.1 |  | | | |  |  |  |  |  |  |  |
| 1 | Ath-AT5G19470.2 |  | | | |  |  |  |  |  |  |  |
| 1 | Ath-AT5G19473.2 |  | | | |  |  |  |  |  |  |  |
| 1 | Ath-AT5G19480.1 |  | | | |  |  |  |  |  |  |  |
| 1 | Ath-AT5G19485.1 |  | | | |  |  |  |  |  |  |  |
| 1 | Ath-AT5G19490.1 |  | | | |  |  |  |  |  |  |  |
| 1 | Ath-AT5G19500.1 |  | | | |  |  |  |  |  |  |  |
| 1 | Ath-AT5G19510.1 |  | | | |  |  |  |  |  |  |  |
| 1 | Ath-AT5G19520.1 |  | | | |  |  |  |  |  |  |  |
| 1 | Ath-AT5G19530.1 |  | Vvi-Vitvi05g00476\_t001 |  |  |  |  |  |  |  |
| 1 | Ath-AT5G19540.1 |  | Vvi-Vitvi11g00335\_t001 |  |  |  |  |  |  |  |
| 1 | Ath-AT5G19550.1 |  | | | |  |  |  |  |  |  |  |
| 1 | Ath-AT5G19560.4 |  | Vvi-Vitvi11g00319\_t001 |  |  |  |  |  |  |  |
| 1 | Ath-AT5G19570.1 |  | | | |  |  |  |  |  |  |  |
| 1 | Ath-AT5G19580.1 |  | Vvi-Vitvi11g00297\_t001 |  |  |  |  |  |  |  |
| 1 | Ath-AT5G19590.2 |  | | | |  |  |  |  |  |  |  |
| 1 | Ath-AT5G19595.1 |  | | | |  |  |  |  |  |  |  |
| 1 | Ath-AT5G19600.1 |  | | | |  |  |  |  |  |  |  |
| 1 | Ath-AT5G19610.1 |  | | | |  |  |  |  |  |  |  |
| 1 | Ath-AT5G19620.1 |  | | | |  |  |  |  |  |  |  |
| 1 | Ath-AT5G19630.1 |  | | | |  |  |  |  |  |  |  |
| 1 | Ath-AT5G19633.1 |  | | | |  |  |  |  |  |  |  |
| 1 | Ath-AT5G19640.1 |  | | | |  |  |  |  |  |  |  |
| 1 | Ath-AT5G19650.1 |  | | | |  |  |  |  |  |  |  |
| 1 | Ath-AT5G19660.1 |  | | | |  |  |  |  |  |  |  |
| 1 | Ath-AT5G19670.1 |  | Vvi-Vitvi11g01394\_t001 |  |  |  |  |  |  |  |
| 1 | Ath-AT5G19675.1 |  | | | |  |  |  |  |  |  |  |
| 1 | Ath-AT5G19680.1 |  | | | |  |  |  |  |  |  |  |
| 1 | Ath-AT5G19690.1 |  | Vvi-Vitvi11g00282\_t001 |  |  |  |  |  |  |  |
| 1 | Ath-AT5G19700.1 |  | Vvi-Vitvi11g00259\_t001 |  |  |  |  |  |  |  |
| 1 | Ath-AT5G19710.1 |  | | | |  |  |  |  |  |  |  |
| 1 | Ath-AT5G19720.1 |  | | | |  |  |  |  |  |  |  |
| 1 | Ath-AT5G19730.1 |  | Vvi-Vitvi11g00256\_t001 |  |  |  |  |  |  |  |
| 1 | Ath-AT5G19740.1 |  | Vvi-Vitvi11g00255\_t001 |  |  |  |  |  |  |  |
| 0 | Ath-AT5G19750.1 |  |  |  |  |  |  |  |  |
| 1 | Ath-AT5G19760.1 |  | Vvi-Vitvi06g00050\_t001 |  |  |  |  |  |  |  |
| 1 | Ath-AT5G19770.1 |  | Vvi-Vitvi06g00051\_t002 |  |  |  |  |  |  |  |
| 1 | Ath-AT5G19780.1 |  | | | |  |  |  |  |  |  |  |
| 1 | Ath-AT5G19790.1 |  | Vvi-Vitvi06g00052\_t001 |  |  |  |  |  |  |  |
| 1 | Ath-AT5G19800.1 |  | | | |  |  |  |  |  |  |  |
| 1 | Ath-AT5G19810.1 |  | | | |  |  |  |  |  |  |  |
| 1 | Ath-AT5G19820.1 |  | Vvi-Vitvi06g00053\_t001 |  |  |  |  |  |  |  |
| 1 | Ath-AT5G19830.1 |  | Vvi-Vitvi06g00058\_t001 |  |  |  |  |  |  |  |
| 1 | Ath-AT5G19840.2 |  | | | |  |  |  |  |  |  |  |
| 1 | Ath-AT5G19850.1 |  | Vvi-Vitvi06g00060\_t001 |  |  |  |  |  |  |  |
| 0 | Ath-AT5G19855.1 |  |  |  |  |  |  |  |  |
| 0 | Ath-AT5G19860.1 |  |  |  |  |  |  |  |  |
| 0 | Ath-AT5G19870.1 |  |  |  |  |  |  |  |  |
| 0 | Ath-AT5G19875.1 |  |  |  |  |  |  |  |  |
| 0 | Ath-AT5G19880.1 |  |  |  |  |  |  |  |  |
| 0 | Ath-AT5G19890.1 |  |  |  |  |  |  |  |  |
| 0 | Ath-AT5G19900.1 |  |  |  |  |  |  |  |  |
| 0 | Ath-AT5G19910.2 |  |  |  |  |  |  |  |  |
| 0 | Ath-AT5G19920.1 |  |  |  |  |  |  |  |  |
| 1 | Ath-AT5G19930.1 |  | Vvi-Vitvi11g00396\_t001 |  |  |  |  |  |  |  |
| 1 | Ath-AT5G19940.1 |  | | | |  |  |  |  |  |  |  |
| 1 | Ath-AT5G19950.1 |  | Vvi-Vitvi11g00397\_t001 |  |  |  |  |  |  |  |
| 1 | Ath-AT5G19960.1 |  | Vvi-Vitvi11g00406\_t001 |  |  |  |  |  |  |  |
| 1 | Ath-AT5G19970.1 |  | Vvi-Vitvi11g00410\_t001 |  |  |  |  |  |  |  |
| 1 | Ath-AT5G19980.1 |  | Vvi-Vitvi11g00412\_t001 |  |  |  |  |  |  |  |
| 1 | Ath-AT5G19990.1 |  | | | |  |  |  |  |  |  |  |
| 1 | Ath-AT5G20000.1 |  | | | |  |  |  |  |  |  |  |
| 1 | Ath-AT5G20010.1 |  | Vvi-Vitvi11g04093\_t001 |  |  |  |  |  |  |  |
| 1 | Ath-AT5G20020.1 |  | | | |  |  |  |  |  |  |  |
| 1 | Ath-AT5G20030.2 |  | Vvi-Vitvi11g00425\_t003 |  |  |  |  |  |  |  |
| 1 | Ath-AT5G20040.3 |  | | | |  |  |  |  |  |  |  |
| 1 | Ath-AT5G20045.1 |  | Vvi-Vitvi11g04095\_t001 |  |  |  |  |  |  |  |
| 1 | Ath-AT5G20050.1 |  | Vvi-Vitvi11g00427\_t001 |  |  |  |  |  |  |  |
| 1 | Ath-AT5G20060.1 |  | Vvi-Vitvi11g00428\_t001 |  |  |  |  |  |  |  |
| 1 | Ath-AT5G20070.1 |  | Vvi-Vitvi11g00437\_t001 |  |  |  |  |  |  |  |
| 1 | Ath-AT5G20080.1 |  | Vvi-Vitvi11g00439\_t001 |  |  |  |  |  |  |  |
| 1 | Ath-AT5G20090.1 |  | | | |  |  |  |  |  |  |  |
| 1 | Ath-AT5G20100.1 |  | Vvi-Vitvi11g01429\_t001 |  |  |  |  |  |  |  |
| 1 | Ath-AT5G20110.1 |  | Vvi-Vitvi11g00442\_t001 |  |  |  |  |  |  |  |
| 1 | Ath-AT5G20120.1 |  | Vvi-Vitvi11g00445\_t001 |  |  |  |  |  |  |  |
| 1 | Ath-AT5G20130.2 |  | | | |  |  |  |  |  |  |  |
| 1 | Ath-AT5G20140.2 |  | | | |  |  |  |  |  |  |  |
| 1 | Ath-AT5G20150.1 |  | | | |  |  |  |  |  |  |  |
| 1 | Ath-AT5G20160.2 |  | Vvi-Vitvi11g00452\_t001 |  |  |  |  |  |  |  |
| 1 | Ath-AT5G20165.3 |  | | | |  |  |  |  |  |  |  |
| 1 | Ath-AT5G20170.1 |  | | | |  |  |  |  |  |  |  |
| 1 | Ath-AT5G20180.3 |  | Vvi-Vitvi11g00473\_t001 |  |  |  |  |  |  |  |
| 1 | Ath-AT5G20190.1 |  | Vvi-Vitvi11g00477\_t001 |  |  |  |  |  |  |  |
| 1 | Ath-AT5G20200.1 |  | Vvi-Vitvi11g00481\_t001 |  |  |  |  |  |  |  |
| 1 | Ath-AT5G20220.4 |  | | | |  |  |  |  |  |  |  |
| 1 | Ath-AT5G20230.1 |  | Vvi-Vitvi11g01445\_t001 |  |  |  |  |  |  |  |
| 1 | Ath-AT5G20240.1 |  | | | |  |  |  |  |  |  |  |
| 1 | Ath-AT5G20250.4 |  | Vvi-Vitvi11g00513\_t001 |  |  |  |  |  |  |  |
| 1 | Ath-AT5G20260.2 |  | Vvi-Vitvi11g00525\_t001 |  |  |  |  |  |  |  |
| 1 | Ath-AT5G20270.1 |  | Vvi-Vitvi11g00539\_t001 |  |  |  |  |  |  |  |
| 1 | Ath-AT5G20280.1 |  | Vvi-Vitvi11g00542\_t001 |  |  |  |  |  |  |  |
| 1 | Ath-AT5G20290.1 |  | | | |  |  |  |  |  |  |  |
| 1 | Ath-AT5G20300.1 |  | Vvi-Vitvi11g00550\_t001 |  |  |  |  |  |  |  |
| 1 | Ath-AT5G20310.1 |  | | | |  |  |  |  |  |  |  |
| 1 | Ath-AT5G20320.1 |  | | | |  |  |  |  |  |  |  |
| 1 | Ath-AT5G20330.1 |  | | | |  |  |  |  |  |  |  |
| 1 | Ath-AT5G20340.1 |  | | | |  |  |  |  |  |  |  |
| 1 | Ath-AT5G20350.1 |  | Vvi-Vitvi11g00573\_t001 |  |  |  |  |  |  |  |
| 1 | Ath-AT5G20360.2 |  | Vvi-Vitvi11g00575\_t001 |  |  |  |  |  |  |  |
| 1 | Ath-AT5G20370.1 |  | Vvi-Vitvi11g00582\_t001 |  |  |  |  |  |  |  |
| 0 | Ath-AT5G20380.2 |  |  |  |  |  |  |  |  |
| 0 | Ath-AT5G20390.1 |  |  |  |  |  |  |  |  |
| 0 | Ath-AT5G20400.1 |  |  |  |  |  |  |  |  |
| 0 | Ath-AT5G20410.1 |  |  |  |  |  |  |  |  |
| 0 | Ath-AT5G20420.1 |  |  |  |  |  |  |  |  |
| 1 | Ath-AT5G20430.1 |  | Vvi-Vitvi11g00087\_t001 |  |  |  |  |  |  |  |
| 1 | Ath-AT5G20440.1 |  | | | |  |  |  |  |  |  |  |
| 1 | Ath-AT5G20447.1 |  | | | |  |  |  |  |  |  |  |
| 1 | Ath-AT5G20450.10 |  | | | |  |  |  |  |  |  |  |
| 1 | Ath-AT5G20460.1 |  | | | |  |  |  |  |  |  |  |
| 1 | Ath-AT5G20470.1 |  | | | |  |  |  |  |  |  |  |
| 1 | Ath-AT5G20480.2 |  | | | |  |  |  |  |  |  |  |
| 1 | Ath-AT5G20490.2 |  | | | |  |  |  |  |  |  |  |
| 1 | Ath-AT5G20500.1 |  | | | |  |  |  |  |  |  |  |
| 1 | Ath-AT5G20510.1 |  | | | |  |  |  |  |  |  |  |
| 1 | Ath-AT5G20520.1 |  | | | |  |  |  |  |  |  |  |
| 1 | Ath-AT5G20540.1 |  | | | |  |  |  |  |  |  |  |
| 1 | Ath-AT5G20550.1 |  | | | |  |  |  |  |  |  |  |
| 1 | Ath-AT5G20560.1 |  | | | |  |  |  |  |  |  |  |
| 1 | Ath-AT5G20570.2 |  | | | |  |  |  |  |  |  |  |
| 1 | Ath-AT5G20580.2 |  | Vvi-Vitvi11g00078\_t001 |  |  |  |  |  |  |  |
| 1 | Ath-AT5G20590.1 |  | | | |  |  |  |  |  |  |  |
| 1 | Ath-AT5G20600.1 |  | | | |  |  |  |  |  |  |  |
| 1 | Ath-AT5G20610.1 |  | | | |  |  |  |  |  |  |  |
| 1 | Ath-AT5G20620.1 |  | | | |  |  |  |  |  |  |  |
| 1 | Ath-AT5G20630.1 |  | | | |  |  |  |  |  |  |  |
| 1 | Ath-AT5G20635.1 |  | Vvi-Vitvi11g04014\_t001 |  |  |  |  |  |  |  |
| 1 | Ath-AT5G20640.1 |  | Vvi-Vitvi11g00069\_t001 |  |  |  |  |  |  |  |
| 1 | Ath-AT5G20650.1 |  | Vvi-Vitvi11g00068\_t001 |  |  |  |  |  |  |  |
| 1 | Ath-AT5G20660.1 |  | | | |  |  |  |  |  |  |  |
| 1 | Ath-AT5G20670.1 |  | Vvi-Vitvi11g01331\_t001 |  |  |  |  |  |  |  |
| 1 | Ath-AT5G20680.3 |  | Vvi-Vitvi11g00062\_t001 |  |  |  |  |  |  |  |
| 1 | Ath-AT5G20690.1 |  | Vvi-Vitvi11g00053\_t001 |  |  |  |  |  |  |  |
| 1 | Ath-AT5G20700.1 |  | Vvi-Vitvi11g00048\_t001 |  |  |  |  |  |  |  |
| 1 | Ath-AT5G20710.1 |  | Vvi-Vitvi11g00047\_t001 |  |  |  |  |  |  |  |
| 1 | Ath-AT5G20720.1 |  | Vvi-Vitvi11g00044\_t002 |  |  |  |  |  |  |  |
| 1 | Ath-AT5G20730.1 |  | Vvi-Vitvi11g00043\_t001 |  |  |  |  |  |  |  |
| 1 | Ath-AT5G20740.2 |  | Vvi-Vitvi11g00040\_t001 |  |  |  |  |  |  |  |
| 1 | Ath-AT5G20790.1 |  | Vvi-Vitvi11g01325\_t001 |  |  |  |  |  |  |  |
| 1 | Ath-AT5G20810.2 |  | Vvi-Vitvi11g00035\_t001 |  |  |  |  |  |  |  |
| 1 | Ath-AT5G20820.1 |  | Vvi-Vitvi11g00033\_t001 |  |  |  |  |  |  |  |
| 1 | Ath-AT5G20830.1 |  | Vvi-Vitvi11g00030\_t001 |  |  |  |  |  |  |  |
| 1 | Ath-AT5G20840.1 |  | Vvi-Vitvi11g00026\_t001 |  |  |  |  |  |  |  |
| 1 | Ath-AT5G20850.1 |  | Vvi-Vitvi11g00017\_t001 |  |  |  |  |  |  |  |
| 1 | Ath-AT5G20860.2 |  | Vvi-Vitvi11g00016\_t001 |  |  |  |  |  |  |  |
| 1 | Ath-AT5G20870.1 |  | Vvi-Vitvi11g00009\_t001 |  |  |  |  |  |  |  |
| 1 | Ath-AT5G20885.1 |  | Vvi-Vitvi11g00004\_t001 |  |  |  |  |  |  |  |
| 0 | Ath-AT5G20890.1 |  |  |  |  |  |  |  |  |
| 0 | Ath-AT5G20900.1 |  |  |  |  |  |  |  |  |
| 0 | Ath-AT5G20910.1 |  |  |  |  |  |  |  |  |
| 0 | Ath-AT5G20920.1 |  |  |  |  |  |  |  |  |
| 1 | Ath-AT5G20930.1 |  | Vvi-Vitvi06g00993\_t001 |  |  |  |  |  |  |  |
| 1 | Ath-AT5G20935.1 |  | Vvi-Vitvi06g00994\_t001 |  |  |  |  |  |  |  |
| 1 | Ath-AT5G20940.1 |  | | | |  |  |  |  |  |  |  |
| 1 | Ath-AT5G20950.3 |  | | | |  |  |  |  |  |  |  |
| 1 | Ath-AT5G20960.1 |  | | | |  |  |  |  |  |  |  |
| 1 | Ath-AT5G20970.1 |  | Vvi-Vitvi06g01821\_t001 |  |  |  |  |  |  |  |
| 1 | Ath-AT5G20980.2 |  | | | |  |  |  |  |  |  |  |
| 1 | Ath-AT5G20990.1 |  | Vvi-Vitvi06g01004\_t002 |  |  |  |  |  |  |  |
| 1 | Ath-AT5G20995.1 |  | | | |  |  |  |  |  |  |  |
| 1 | Ath-AT5G21010.1 |  | | | |  |  |  |  |  |  |  |
| 1 | Ath-AT5G21020.2 |  | | | |  |  |  |  |  |  |  |
| 1 | Ath-AT5G21030.2 |  | Vvi-Vitvi06g01020\_t001 |  |  |  |  |  |  |  |
| 1 | Ath-AT5G21040.1 |  | | | |  |  |  |  |  |  |  |
| 1 | Ath-AT5G21050.1 |  | Vvi-Vitvi06g01829\_t001 |  |  |  |  |  |  |  |
| 1 | Ath-AT5G21060.3 |  | Vvi-Vitvi06g01044\_t001 |  |  |  |  |  |  |  |
| 1 | Ath-AT5G21070.1 |  | Vvi-Vitvi06g01051\_t001 |  |  |  |  |  |  |  |
| 1 | Ath-AT5G21080.1 |  | | | |  |  |  |  |  |  |  |
| 1 | Ath-AT5G21090.1 |  | | | |  |  |  |  |  |  |  |
| 2 | Ath-AT5G21100.1 |  | | | |  | Vvi-Vitvi06g01033\_t001 |  |  |  |  |  |  |
| 2 | Ath-AT5G21105.1 |  | | | |  | | | |  |  |  |  |  |  |
| 2 | Ath-AT5G21120.2 |  | | | |  | | | |  |  |  |  |  |  |
| 2 | Ath-AT5G21125.1 |  | | | |  | | | |  |  |  |  |  |  |
| 2 | Ath-AT5G21130.1 |  | Vvi-Vitvi06g01055\_t001 |  | | | |  |  |  |  |  |  |
| 2 | Ath-AT5G21140.1 |  | | | |  | | | |  |  |  |  |  |  |
| 2 | Ath-AT5G21150.1 |  | | | |  | Vvi-Vitvi06g01020\_t001 |  |  |  |  |  |  |
| 2 | Ath-AT5G21160.3 |  | | | |  | Vvi-Vitvi06g01011\_t001 |  |  |  |  |  |  |
| 2 | Ath-AT5G21170.2 |  | | | |  | | | |  |  |  |  |  |  |
| 2 | Ath-AT5G21222.3 |  | | | |  | | | |  |  |  |  |  |  |
| 2 | Ath-AT5G21274.1 |  | | | |  | | | |  |  |  |  |  |  |
| 2 | Ath-AT5G21326.1 |  | Vvi-Vitvi06g01084\_t003 |  | | | |  |  |  |  |  |  |
| 2 | Ath-AT5G21430.1 |  | Vvi-Vitvi04g00151\_t001 |  | | | |  |  |  |  |  |  |
| 2 | Ath-AT5G21482.1 |  | Vvi-Vitvi04g00161\_t001 |  | | | |  |  |  |  |  |  |
| 2 | Ath-AT5G21900.1 |  | Vvi-Vitvi04g00165\_t001 |  | | | |  |  |  |  |  |  |
| 2 | Ath-AT5G21910.1 |  | Vvi-Vitvi04g01809\_t001 |  | | | |  |  |  |  |  |  |
| 2 | Ath-AT5G21920.1 |  | Vvi-Vitvi04g00166\_t001 |  | | | |  |  |  |  |  |  |
| 2 | Ath-AT5G21930.1 |  | Vvi-Vitvi04g00167\_t001 |  | | | |  |  |  |  |  |  |
| 2 | Ath-AT5G21940.1 |  | Vvi-Vitvi04g00173\_t001 |  | | | |  |  |  |  |  |  |
| 2 | Ath-AT5G21950.6 |  | Vvi-Vitvi04g00178\_t001 |  | | | |  |  |  |  |  |  |
| 2 | Ath-AT5G21960.1 |  | | | |  | | | |  |  |  |  |  |  |
| 2 | Ath-AT5G21970.2 |  | Vvi-Vitvi04g01817\_t002 |  | | | |  |  |  |  |  |  |
| 2 | Ath-AT5G21280.1 |  | Vvi-Vitvi04g00194\_t001 |  | | | |  |  |  |  |  |  |
| 1 | Ath-AT5G21990.1 |  |  |  | Vvi-Vitvi06g00990\_t001 |  |  |  |  |  |  |
| 1 | Ath-AT5G22000.1 |  |  |  | Vvi-Vitvi06g00988\_t001 |  |  |  |  |  |  |
| 1 | Ath-AT5G22010.1 |  |  |  | | | |  |  |  |  |  |  |
| 1 | Ath-AT5G22020.1 |  |  |  | | | |  |  |  |  |  |  |
| 1 | Ath-AT5G22030.2 |  |  |  | | | |  |  |  |  |  |  |
| 1 | Ath-AT5G22040.2 |  |  |  | Vvi-Vitvi06g00975\_t001 |  |  |  |  |  |  |
| 2 | Ath-AT5G22050.2 |  | Vvi-Vitvi06g01564\_t001 |  | | | |  |  |  |  |  |  |
| 2 | Ath-AT5G22060.1 |  | Vvi-Vitvi06g01562\_t001 |  | | | |  |  |  |  |  |  |
| 2 | Ath-AT5G22070.1 |  | | | |  | | | |  |  |  |  |  |  |
| 2 | Ath-AT5G22080.1 |  | | | |  | | | |  |  |  |  |  |  |
| 2 | Ath-AT5G22090.1 |  | | | |  | | | |  |  |  |  |  |  |
| 2 | Ath-AT5G22100.1 |  | | | |  | | | |  |  |  |  |  |  |
| 2 | Ath-AT5G22110.1 |  | | | |  | | | |  |  |  |  |  |  |
| 2 | Ath-AT5G22120.1 |  | | | |  | | | |  |  |  |  |  |  |
| 2 | Ath-AT5G22130.2 |  | | | |  | | | |  |  |  |  |  |  |
| 2 | Ath-AT5G22140.1 |  | | | |  | Vvi-Vitvi06g00946\_t001 |  |  |  |  |  |  |
| 1 | Ath-AT5G22145.1 |  | | | |  |  |  |  |  |  |  |
| 1 | Ath-AT5G22150.1 |  | | | |  |  |  |  |  |  |  |
| 1 | Ath-AT5G22160.1 |  | | | |  |  |  |  |  |  |  |
| 1 | Ath-AT5G22170.1 |  | | | |  |  |  |  |  |  |  |
| 1 | Ath-AT5G22180.1 |  | | | |  |  |  |  |  |  |  |
| 1 | Ath-AT5G22190.1 |  | | | |  |  |  |  |  |  |  |
| 1 | Ath-AT5G22200.1 |  | Vvi-Vitvi06g01559\_t001 |  |  |  |  |  |  |  |
| 1 | Ath-AT5G22210.1 |  | Vvi-Vitvi06g01553\_t001 |  |  |  |  |  |  |  |
| 2 | Ath-AT5G22220.2 |  | Vvi-Vitvi06g01552\_t001 |  | Vvi-Vitvi08g01388\_t001 |  |  |  |  |  |  |
| 2 | Ath-AT5G22240.1 |  | | | |  | | | |  |  |  |  |  |  |
| 2 | Ath-AT5G22250.1 |  | Vvi-Vitvi06g01546\_t001 |  | Vvi-Vitvi08g01393\_t001 |  |  |  |  |  |  |
| 2 | Ath-AT5G22260.1 |  | | | |  | | | |  |  |  |  |  |  |
| 2 | Ath-AT5G22270.1 |  | | | |  | Vvi-Vitvi08g01395\_t001 |  |  |  |  |  |  |
| 2 | Ath-AT5G22280.3 |  | | | |  | | | |  |  |  |  |  |  |
| 2 | Ath-AT5G22290.1 |  | Vvi-Vitvi06g01536\_t001 |  | | | |  |  |  |  |  |  |
| 2 | Ath-AT5G22300.1 |  | Vvi-Vitvi06g01533\_t001 |  | | | |  |  |  |  |  |  |
| 2 | Ath-AT5G22310.1 |  | Vvi-Vitvi06g01532\_t001 |  | Vvi-Vitvi08g01410\_t002 |  |  |  |  |  |  |
| 2 | Ath-AT5G22320.1 |  | Vvi-Vitvi06g01529\_t002 |  | | | |  |  |  |  |  |  |
| 2 | Ath-AT5G22330.1 |  | | | |  | | | |  |  |  |  |  |  |
| 2 | Ath-AT5G22340.2 |  | Vvi-Vitvi06g01527\_t001 |  | | | |  |  |  |  |  |  |
| 2 | Ath-AT5G22350.1 |  | Vvi-Vitvi06g01521\_t001 |  | Vvi-Vitvi08g01419\_t001 |  |  |  |  |  |  |
| 2 | Ath-AT5G22355.1 |  | | | |  | | | |  |  |  |  |  |  |
| 2 | Ath-AT5G22360.1 |  | Vvi-Vitvi06g01517\_t001 |  | | | |  |  |  |  |  |  |
| 2 | Ath-AT5G22370.1 |  | | | |  | | | |  |  |  |  |  |  |
| 2 | Ath-AT5G22380.1 |  | Vvi-Vitvi06g01515\_t001 |  | Vvi-Vitvi08g01426\_t001 |  |  |  |  |  |  |
| 2 | Ath-AT5G22390.1 |  | | | |  | | | |  |  |  |  |  |  |
| 2 | Ath-AT5G22400.1 |  | | | |  | Vvi-Vitvi08g01442\_t001 |  |  |  |  |  |  |
| 2 | Ath-AT5G22410.1 |  | Vvi-Vitvi06g01510\_t001 |  | | | |  |  |  |  |  |  |
| 2 | Ath-AT5G22420.1 |  | | | |  | | | |  |  |  |  |  |  |
| 2 | Ath-AT5G22430.1 |  | | | |  | | | |  |  |  |  |  |  |
| 2 | Ath-AT5G22440.1 |  | Vvi-Vitvi06g01486\_t002 |  | Vvi-Vitvi08g01462\_t001 |  |  |  |  |  |  |
| 1 | Ath-AT5G22450.2 |  | Vvi-Vitvi06g01479\_t001 |  |  |  |  |  |  |  |
| 1 | Ath-AT5G22460.1 |  | Vvi-Vitvi06g04438\_t001 |  |  |  |  |  |  |  |
| 1 | Ath-AT5G22470.1 |  | Vvi-Vitvi06g01470\_t001 |  |  |  |  |  |  |  |
| 1 | Ath-AT5G22480.1 |  | | | |  |  |  |  |  |  |  |
| 1 | Ath-AT5G22490.1 |  | | | |  |  |  |  |  |  |  |
| 1 | Ath-AT5G22500.1 |  | Vvi-Vitvi06g01455\_t001 |  |  |  |  |  |  |  |
| 1 | Ath-AT5G22505.1 |  | | | |  |  |  |  |  |  |  |
| 1 | Ath-AT5G22510.1 |  | Vvi-Vitvi06g01427\_t001 |  |  |  |  |  |  |  |
| 1 | Ath-AT5G22520.1 |  | | | |  |  |  |  |  |  |  |
| 1 | Ath-AT5G22530.1 |  | | | |  |  |  |  |  |  |  |
| 1 | Ath-AT5G22540.1 |  | | | |  |  |  |  |  |  |  |
| 1 | Ath-AT5G22545.1 |  | | | |  |  |  |  |  |  |  |
| 1 | Ath-AT5G22550.2 |  | | | |  |  |  |  |  |  |  |
| 1 | Ath-AT5G22555.1 |  | | | |  |  |  |  |  |  |  |
| 1 | Ath-AT5G22560.1 |  | | | |  |  |  |  |  |  |  |
| 1 | Ath-AT5G22570.1 |  | | | |  |  |  |  |  |  |  |
| 1 | Ath-AT5G22580.1 |  | Vvi-Vitvi06g01410\_t001 |  |  |  |  |  |  |  |
| 1 | Ath-AT5G22590.1 |  | | | |  |  |  |  |  |  |  |
| 1 | Ath-AT5G22600.1 |  | | | |  |  |  |  |  |  |  |
| 1 | Ath-AT5G22608.2 |  | | | |  |  |  |  |  |  |  |
| 1 | Ath-AT5G22610.2 |  | | | |  |  |  |  |  |  |  |
| 1 | Ath-AT5G22620.5 |  | Vvi-Vitvi06g01407\_t001 |  |  |  |  |  |  |  |
| 1 | Ath-AT5G22630.1 |  | Vvi-Vitvi06g01946\_t001 |  |  |  |  |  |  |  |
| 1 | Ath-AT5G22640.1 |  | Vvi-Vitvi06g01401\_t001 |  |  |  |  |  |  |  |
| 1 | Ath-AT5G22650.1 |  | Vvi-Vitvi06g01399\_t001 |  |  |  |  |  |  |  |
| 1 | Ath-AT5G22660.2 |  | | | |  |  |  |  |  |  |  |
| 1 | Ath-AT5G22670.1 |  | | | |  |  |  |  |  |  |  |
| 1 | Ath-AT5G22680.1 |  | | | |  |  |  |  |  |  |  |
| 1 | Ath-AT5G22690.2 |  | | | |  |  |  |  |  |  |  |
| 1 | Ath-AT5G22700.5 |  | | | |  |  |  |  |  |  |  |
| 1 | Ath-AT5G22720.1 |  | | | |  |  |  |  |  |  |  |
| 1 | Ath-AT5G22730.1 |  | | | |  |  |  |  |  |  |  |
| 1 | Ath-AT5G22740.1 |  | Vvi-Vitvi06g01398\_t001 |  |  |  |  |  |  |  |
| 1 | Ath-AT5G22750.1 |  | Vvi-Vitvi06g01395\_t001 |  |  |  |  |  |  |  |
| 1 | Ath-AT5G22760.1 |  | Vvi-Vitvi06g01394\_t003 |  |  |  |  |  |  |  |
| 1 | Ath-AT5G22765.1 |  | | | |  |  |  |  |  |  |  |
| 1 | Ath-AT5G22770.4 |  | Vvi-Vitvi06g01392\_t001 |  |  |  |  |  |  |  |
| 1 | Ath-AT5G22780.1 |  | | | |  |  |  |  |  |  |  |
| 1 | Ath-AT5G22785.1 |  | | | |  |  |  |  |  |  |  |
| 1 | Ath-AT5G22790.1 |  | Vvi-Vitvi06g01391\_t001 |  |  |  |  |  |  |  |
| 1 | Ath-AT5G22791.2 |  | | | |  |  |  |  |  |  |  |
| 1 | Ath-AT5G22794.2 |  | | | |  |  |  |  |  |  |  |
| 1 | Ath-AT5G22796.1 |  | | | |  |  |  |  |  |  |  |
| 1 | Ath-AT5G22799.1 |  | | | |  |  |  |  |  |  |  |
| 1 | Ath-AT5G22800.2 |  | | | |  |  |  |  |  |  |  |
| 1 | Ath-AT5G22810.1 |  | Vvi-Vitvi06g01387\_t001 |  |  |  |  |  |  |  |
| 1 | Ath-AT5G22820.3 |  | Vvi-Vitvi06g01386\_t001 |  |  |  |  |  |  |  |
| 1 | Ath-AT5G22830.2 |  | Vvi-Vitvi06g01383\_t003 |  |  |  |  |  |  |  |
| 1 | Ath-AT5G22840.1 |  | Vvi-Vitvi06g01380\_t001 |  |  |  |  |  |  |  |
| 1 | Ath-AT5G22850.1 |  | Vvi-Vitvi06g01377\_t001 |  |  |  |  |  |  |  |
| 1 | Ath-AT5G22860.1 |  | Vvi-Vitvi06g01365\_t001 |  |  |  |  |  |  |  |
| 1 | Ath-AT5G22870.1 |  | Vvi-Vitvi06g01357\_t001 |  |  |  |  |  |  |  |
| 1 | Ath-AT5G22875.2 |  | Vvi-Vitvi06g04410\_t001 |  |  |  |  |  |  |  |
| 1 | Ath-AT5G22880.1 |  | | | |  |  |  |  |  |  |  |
| 1 | Ath-AT5G22890.1 |  | Vvi-Vitvi06g01353\_t001 |  |  |  |  |  |  |  |
| 1 | Ath-AT5G22900.1 |  | | | |  |  |  |  |  |  |  |
| 1 | Ath-AT5G22910.1 |  | | | |  |  |  |  |  |  |  |
| 1 | Ath-AT5G22920.1 |  | Vvi-Vitvi06g01344\_t001 |  |  |  |  |  |  |  |
| 1 | Ath-AT5G22930.1 |  | Vvi-Vitvi06g01340\_t001 |  |  |  |  |  |  |  |
| 1 | Ath-AT5G22940.2 |  | Vvi-Vitvi06g01330\_t001 |  |  |  |  |  |  |  |
| 1 | Ath-AT5G22950.1 |  | Vvi-Vitvi06g01328\_t002 |  |  |  |  |  |  |  |
| 1 | Ath-AT5G22960.1 |  | Vvi-Vitvi06g01924\_t001 |  |  |  |  |  |  |  |
| 1 | Ath-AT5G22970.3 |  | | | |  |  |  |  |  |  |  |
| 1 | Ath-AT5G22980.1 |  | | | |  |  |  |  |  |  |  |
| 1 | Ath-AT5G22990.1 |  | | | |  |  |  |  |  |  |  |
| 1 | Ath-AT5G23000.1 |  | Vvi-Vitvi06g01321\_t001 |  |  |  |  |  |  |  |
| 0 | Ath-AT5G23010.2 |  |  |  |  |  |  |  |  |
| 0 | Ath-AT5G23020.1 |  |  |  |  |  |  |  |  |
| 0 | Ath-AT5G23030.1 |  |  |  |  |  |  |  |  |
| 0 | Ath-AT5G23035.1 |  |  |  |  |  |  |  |  |
| 1 | Ath-AT5G23040.1 |  | Vvi-Vitvi17g00411\_t001 |  |  |  |  |  |  |  |
| 1 | Ath-AT5G23050.1 |  | Vvi-Vitvi17g00409\_t001 |  |  |  |  |  |  |  |
| 1 | Ath-AT5G23060.1 |  | Vvi-Vitvi17g00407\_t001 |  |  |  |  |  |  |  |
| 1 | Ath-AT5G23070.1 |  | | | |  |  |  |  |  |  |  |
| 1 | Ath-AT5G23080.1 |  | Vvi-Vitvi17g00398\_t001 |  |  |  |  |  |  |  |
| 1 | Ath-AT5G23090.1 |  | Vvi-Vitvi17g01421\_t001 |  |  |  |  |  |  |  |
| 1 | Ath-AT5G23100.1 |  | Vvi-Vitvi17g00391\_t001 |  |  |  |  |  |  |  |
| 1 | Ath-AT5G23110.1 |  | Vvi-Vitvi01g00171\_t001 |  |  |  |  |  |  |  |
| 1 | Ath-AT5G23115.1 |  | | | |  |  |  |  |  |  |  |
| 1 | Ath-AT5G23120.2 |  | Vvi-Vitvi01g00170\_t001 |  |  |  |  |  |  |  |
| 1 | Ath-AT5G23130.1 |  | Vvi-Vitvi01g00169\_t001 |  |  |  |  |  |  |  |
| 1 | Ath-AT5G23140.1 |  | Vvi-Vitvi01g00166\_t002 |  |  |  |  |  |  |  |
| 1 | Ath-AT5G23150.1 |  | | | |  |  |  |  |  |  |  |
| 1 | Ath-AT5G23160.1 |  | Vvi-Vitvi01g00163\_t001 |  |  |  |  |  |  |  |
| 1 | Ath-AT5G23170.1 |  | | | |  |  |  |  |  |  |  |
| 1 | Ath-AT5G23180.1 |  | | | |  |  |  |  |  |  |  |
| 1 | Ath-AT5G23190.1 |  | Vvi-Vitvi01g00162\_t001 |  |  |  |  |  |  |  |
| 2 | Ath-AT5G23200.1 |  | | | |  | Vvi-Vitvi10g01360\_t001 |  |  |  |  |  |  |
| 2 | Ath-AT5G23210.1 |  | Vvi-Vitvi01g00160\_t001 |  | Vvi-Vitvi10g01361\_t001 |  |  |  |  |  |  |
| 2 | Ath-AT5G23212.1 |  | | | |  | | | |  |  |  |  |  |  |
| 2 | Ath-AT5G23220.1 |  | | | |  | Vvi-Vitvi10g01366\_t001 |  |  |  |  |  |  |
| 2 | Ath-AT5G23230.1 |  | | | |  | | | |  |  |  |  |  |  |
| 2 | Ath-AT5G23240.1 |  | | | |  | Vvi-Vitvi10g01384\_t001 |  |  |  |  |  |  |
| 2 | Ath-AT5G23250.1 |  | | | |  | Vvi-Vitvi10g01386\_t001 |  |  |  |  |  |  |
| 2 | Ath-AT5G23260.4 |  | Vvi-Vitvi01g01885\_t001 |  | Vvi-Vitvi10g01395\_t001 |  |  |  |  |  |  |
| 0 | Ath-AT5G23270.1 |  |  |  |  |  |  |  |  |
| 0 | Ath-AT5G23280.1 |  |  |  |  |  |  |  |  |
| 0 | Ath-AT5G23290.1 |  |  |  |  |  |  |  |  |
| 0 | Ath-AT5G23300.1 |  |  |  |  |  |  |  |  |
| 0 | Ath-AT5G23310.1 |  |  |  |  |  |  |  |  |
| 0 | Ath-AT5G23320.2 |  |  |  |  |  |  |  |  |
| 0 | Ath-AT5G23330.1 |  |  |  |  |  |  |  |  |
| 0 | Ath-AT5G23340.1 |  |  |  |  |  |  |  |  |
| 0 | Ath-AT5G23350.1 |  |  |  |  |  |  |  |  |
| 0 | Ath-AT5G23360.1 |  |  |  |  |  |  |  |  |
| 0 | Ath-AT5G23370.1 |  |  |  |  |  |  |  |  |
| 0 | Ath-AT5G23380.1 |  |  |  |  |  |  |  |  |
| 0 | Ath-AT5G23390.1 |  |  |  |  |  |  |  |  |
| 0 | Ath-AT5G23395.1 |  |  |  |  |  |  |  |  |
| 0 | Ath-AT5G23400.1 |  |  |  |  |  |  |  |  |
| 0 | Ath-AT5G23405.1 |  |  |  |  |  |  |  |  |
| 0 | Ath-AT5G23411.1 |  |  |  |  |  |  |  |  |
| 0 | Ath-AT5G23420.1 |  |  |  |  |  |  |  |  |
| 0 | Ath-AT5G23430.1 |  |  |  |  |  |  |  |  |
| 0 | Ath-AT5G23440.1 |  |  |  |  |  |  |  |  |
| 0 | Ath-AT5G23450.3 |  |  |  |  |  |  |  |  |
| 0 | Ath-AT5G23460.1 |  |  |  |  |  |  |  |  |
| 0 | Ath-AT5G23470.1 |  |  |  |  |  |  |  |  |
| 1 | Ath-AT5G23480.4 |  | Vvi-Vitvi07g02055\_t001 |  |  |  |  |  |  |  |
| 1 | Ath-AT5G23490.2 |  | Vvi-Vitvi07g02053\_t001 |  |  |  |  |  |  |  |
| 1 | Ath-AT5G23510.2 |  | | | |  |  |  |  |  |  |  |
| 1 | Ath-AT5G23520.1 |  | Vvi-Vitvi07g02045\_t001 |  |  |  |  |  |  |  |
| 1 | Ath-AT5G23530.1 |  | Vvi-Vitvi07g02041\_t001 |  |  |  |  |  |  |  |
| 1 | Ath-AT5G23535.1 |  | | | |  |  |  |  |  |  |  |
| 1 | Ath-AT5G23540.1 |  | | | |  |  |  |  |  |  |  |
| 1 | Ath-AT5G23550.1 |  | Vvi-Vitvi07g02031\_t001 |  |  |  |  |  |  |  |
| 1 | Ath-AT5G23570.3 |  | Vvi-Vitvi07g04772\_t001 |  |  |  |  |  |  |  |
| 1 | Ath-AT5G23575.1 |  | Vvi-Vitvi07g02023\_t001 |  |  |  |  |  |  |  |
| 1 | Ath-AT5G23580.1 |  | Vvi-Vitvi07g02019\_t001 |  |  |  |  |  |  |  |
| 1 | Ath-AT5G23590.2 |  | | | |  |  |  |  |  |  |  |
| 1 | Ath-AT5G23600.1 |  | | | |  |  |  |  |  |  |  |
| 1 | Ath-AT5G23610.2 |  | Vvi-Vitvi07g02722\_t001 |  |  |  |  |  |  |  |
| 1 | Ath-AT5G23630.1 |  | Vvi-Vitvi07g02013\_t001 |  |  |  |  |  |  |  |
| 0 | Ath-AT5G23650.1 |  |  |  |  |  |  |  |  |
| 0 | Ath-AT5G23660.1 |  |  |  |  |  |  |  |  |
| 0 | Ath-AT5G23670.1 |  |  |  |  |  |  |  |  |
| 0 | Ath-AT5G23680.1 |  |  |  |  |  |  |  |  |
| 0 | Ath-AT5G23690.1 |  |  |  |  |  |  |  |  |
| 0 | Ath-AT5G23700.3 |  |  |  |  |  |  |  |  |
| 0 | Ath-AT5G23710.1 |  |  |  |  |  |  |  |  |
| 0 | Ath-AT5G23720.1 |  |  |  |  |  |  |  |  |
| 1 | Ath-AT5G23730.1 |  | Vvi-Vitvi16g01011\_t001 |  |  |  |  |  |  |  |
| 1 | Ath-AT5G23740.1 |  | | | |  |  |  |  |  |  |  |
| 1 | Ath-AT5G23750.3 |  | Vvi-Vitvi16g01018\_t001 |  |  |  |  |  |  |  |
| 1 | Ath-AT5G23760.1 |  | Vvi-Vitvi16g01876\_t001 |  |  |  |  |  |  |  |
| 1 | Ath-AT5G23770.2 |  | | | |  |  |  |  |  |  |  |
| 1 | Ath-AT5G23780.1 |  | | | |  |  |  |  |  |  |  |
| 1 | Ath-AT5G23790.1 |  | | | |  |  |  |  |  |  |  |
| 1 | Ath-AT5G23800.2 |  | | | |  |  |  |  |  |  |  |
| 1 | Ath-AT5G23810.1 |  | | | |  |  |  |  |  |  |  |
| 1 | Ath-AT5G23820.1 |  | | | |  |  |  |  |  |  |  |
| 1 | Ath-AT5G23830.1 |  | | | |  |  |  |  |  |  |  |
| 1 | Ath-AT5G23840.1 |  | | | |  |  |  |  |  |  |  |
| 1 | Ath-AT5G23850.1 |  | Vvi-Vitvi16g01024\_t001 |  |  |  |  |  |  |  |
| 1 | Ath-AT5G23860.1 |  | Vvi-Vitvi16g01034\_t001 |  |  |  |  |  |  |  |
| 1 | Ath-AT5G23870.3 |  | Vvi-Vitvi16g04372\_t001 |  |  |  |  |  |  |  |
| 1 | Ath-AT5G23880.1 |  | Vvi-Vitvi16g01052\_t001 |  |  |  |  |  |  |  |
| 1 | Ath-AT5G23890.1 |  | Vvi-Vitvi16g01053\_t001 |  |  |  |  |  |  |  |
| 1 | Ath-AT5G23900.1 |  | | | |  |  |  |  |  |  |  |
| 1 | Ath-AT5G23903.1 |  | | | |  |  |  |  |  |  |  |
| 1 | Ath-AT5G23908.1 |  | | | |  |  |  |  |  |  |  |
| 1 | Ath-AT5G23910.2 |  | Vvi-Vitvi16g01063\_t002 |  |  |  |  |  |  |  |
| 1 | Ath-AT5G23920.1 |  | Vvi-Vitvi16g01904\_t001 |  |  |  |  |  |  |  |
| 1 | Ath-AT5G23930.1 |  | | | |  |  |  |  |  |  |  |
| 1 | Ath-AT5G23940.1 |  | Vvi-Vitvi16g01078\_t001 |  |  |  |  |  |  |  |
| 1 | Ath-AT5G23950.2 |  | Vvi-Vitvi16g01080\_t001 |  |  |  |  |  |  |  |
| 1 | Ath-AT5G23960.1 |  | | | |  |  |  |  |  |  |  |
| 1 | Ath-AT5G23970.1 |  | | | |  |  |  |  |  |  |  |
| 1 | Ath-AT5G23980.1 |  | Vvi-Vitvi16g01090\_t001 |  |  |  |  |  |  |  |
| 1 | Ath-AT5G23990.2 |  | | | |  |  |  |  |  |  |  |
| 1 | Ath-AT5G24000.1 |  | Vvi-Vitvi16g01093\_t001 |  |  |  |  |  |  |  |
| 1 | Ath-AT5G24010.1 |  | Vvi-Vitvi16g01094\_t001 |  |  |  |  |  |  |  |
| 1 | Ath-AT5G24020.1 |  | Vvi-Vitvi16g01097\_t001 |  |  |  |  |  |  |  |
| 0 | Ath-AT5G24030.1 |  |  |  |  |  |  |  |  |
| 0 | Ath-AT5G24040.1 |  |  |  |  |  |  |  |  |
| 0 | Ath-AT5G24050.1 |  |  |  |  |  |  |  |  |
| 0 | Ath-AT5G24060.2 |  |  |  |  |  |  |  |  |
| 0 | Ath-AT5G24070.1 |  |  |  |  |  |  |  |  |
| 0 | Ath-AT5G24080.2 |  |  |  |  |  |  |  |  |
| 0 | Ath-AT5G24090.1 |  |  |  |  |  |  |  |  |
| 0 | Ath-AT5G24100.1 |  |  |  |  |  |  |  |  |
| 0 | Ath-AT5G24105.1 |  |  |  |  |  |  |  |  |
| 0 | Ath-AT5G24110.1 |  |  |  |  |  |  |  |  |
| 0 | Ath-AT5G24120.2 |  |  |  |  |  |  |  |  |
| 0 | Ath-AT5G24130.1 |  |  |  |  |  |  |  |  |
| 0 | Ath-AT5G24140.1 |  |  |  |  |  |  |  |  |
| 0 | Ath-AT5G24150.1 |  |  |  |  |  |  |  |  |
| 0 | Ath-AT5G24155.1 |  |  |  |  |  |  |  |  |
| 0 | Ath-AT5G24160.1 |  |  |  |  |  |  |  |  |
| 0 | Ath-AT5G24165.1 |  |  |  |  |  |  |  |  |
| 0 | Ath-AT5G24170.1 |  |  |  |  |  |  |  |  |
| 0 | Ath-AT5G24180.1 |  |  |  |  |  |  |  |  |
| 0 | Ath-AT5G24190.1 |  |  |  |  |  |  |  |  |
| 0 | Ath-AT5G24200.1 |  |  |  |  |  |  |  |  |
| 0 | Ath-AT5G24210.1 |  |  |  |  |  |  |  |  |
| 0 | Ath-AT5G24215.1 |  |  |  |  |  |  |  |  |
| 0 | Ath-AT5G24220.1 |  |  |  |  |  |  |  |  |
| 0 | Ath-AT5G24230.1 |  |  |  |  |  |  |  |  |
| 1 | Ath-AT5G24240.1 |  | Vvi-Vitvi16g01417\_t003 |  |  |  |  |  |  |  |
| 1 | Ath-AT5G24260.2 |  | Vvi-Vitvi16g01416\_t002 |  |  |  |  |  |  |  |
| 1 | Ath-AT5G24270.4 |  | Vvi-Vitvi16g01415\_t001 |  |  |  |  |  |  |  |
| 1 | Ath-AT5G24275.1 |  | Vvi-Vitvi16g01407\_t001 |  |  |  |  |  |  |  |
| 1 | Ath-AT5G24280.1 |  | | | |  |  |  |  |  |  |  |
| 1 | Ath-AT5G24290.1 |  | | | |  |  |  |  |  |  |  |
| 1 | Ath-AT5G24300.1 |  | Vvi-Vitvi16g01405\_t001 |  |  |  |  |  |  |  |
| 1 | Ath-AT5G24310.1 |  | Vvi-Vitvi16g01401\_t001.2.6037826c |  |  |  |  |  |  |  |
| 1 | Ath-AT5G24313.1 |  | | | |  |  |  |  |  |  |  |
| 1 | Ath-AT5G24314.2 |  | | | |  |  |  |  |  |  |  |
| 1 | Ath-AT5G24316.1 |  | | | |  |  |  |  |  |  |  |
| 1 | Ath-AT5G24318.1 |  | Vvi-Vitvi16g01389\_t001 |  |  |  |  |  |  |  |
| 1 | Ath-AT5G24320.2 |  | Vvi-Vitvi16g01387\_t001 |  |  |  |  |  |  |  |
| 1 | Ath-AT5G24330.1 |  | Vvi-Vitvi16g02079\_t001 |  |  |  |  |  |  |  |
| 1 | Ath-AT5G24340.2 |  | Vvi-Vitvi16g01386\_t001 |  |  |  |  |  |  |  |
| 1 | Ath-AT5G24350.2 |  | Vvi-Vitvi16g01377\_t001 |  |  |  |  |  |  |  |
| 1 | Ath-AT5G24352.1 |  | | | |  |  |  |  |  |  |  |
| 1 | Ath-AT5G24355.1 |  | | | |  |  |  |  |  |  |  |
| 1 | Ath-AT5G24360.2 |  | | | |  |  |  |  |  |  |  |
| 1 | Ath-AT5G24370.1 |  | | | |  |  |  |  |  |  |  |
| 1 | Ath-AT5G24380.1 |  | Vvi-Vitvi16g01371\_t001 |  |  |  |  |  |  |  |
| 1 | Ath-AT5G24390.1 |  | Vvi-Vitvi16g01366\_t002 |  |  |  |  |  |  |  |
| 1 | Ath-AT5G24400.1 |  | Vvi-Vitvi16g01365\_t001 |  |  |  |  |  |  |  |
| 1 | Ath-AT5G24410.1 |  | | | |  |  |  |  |  |  |  |
| 1 | Ath-AT5G24420.1 |  | | | |  |  |  |  |  |  |  |
| 1 | Ath-AT5G24430.1 |  | Vvi-Vitvi16g01358\_t001 |  |  |  |  |  |  |  |
| 1 | Ath-AT5G24440.1 |  | | | |  |  |  |  |  |  |  |
| 1 | Ath-AT5G24450.1 |  | | | |  |  |  |  |  |  |  |
| 1 | Ath-AT5G24460.1 |  | | | |  |  |  |  |  |  |  |
| 1 | Ath-AT5G24470.1 |  | | | |  |  |  |  |  |  |  |
| 1 | Ath-AT5G24480.1 |  | | | |  |  |  |  |  |  |  |
| 1 | Ath-AT5G24490.1 |  | | | |  |  |  |  |  |  |  |
| 1 | Ath-AT5G24500.1 |  | | | |  |  |  |  |  |  |  |
| 1 | Ath-AT5G24510.1 |  | Vvi-Vitvi16g02068\_t001 |  |  |  |  |  |  |  |
| 1 | Ath-AT5G24520.1 |  | Vvi-Vitvi16g01337\_t001 |  |  |  |  |  |  |  |
| 1 | Ath-AT5G24530.1 |  | Vvi-Vitvi16g01336\_t001 |  |  |  |  |  |  |  |
| 1 | Ath-AT5G24540.1 |  | | | |  |  |  |  |  |  |  |
| 1 | Ath-AT5G24550.1 |  | | | |  |  |  |  |  |  |  |
| 1 | Ath-AT5G24560.1 |  | | | |  |  |  |  |  |  |  |
| 1 | Ath-AT5G24570.1 |  | | | |  |  |  |  |  |  |  |
| 1 | Ath-AT5G24575.1 |  | | | |  |  |  |  |  |  |  |
| 1 | Ath-AT5G24580.1 |  | Vvi-Vitvi16g02059\_t001 |  |  |  |  |  |  |  |
| 1 | Ath-AT5G24590.2 |  | Vvi-Vitvi16g01327\_t002 |  |  |  |  |  |  |  |
| 1 | Ath-AT5G24600.1 |  | | | |  |  |  |  |  |  |  |
| 1 | Ath-AT5G24610.1 |  | | | |  |  |  |  |  |  |  |
| 1 | Ath-AT5G24620.3 |  | Vvi-Vitvi16g01314\_t001 |  |  |  |  |  |  |  |
| 1 | Ath-AT5G24630.6 |  | Vvi-Vitvi16g01313\_t001 |  |  |  |  |  |  |  |
| 1 | Ath-AT5G24640.1 |  | | | |  |  |  |  |  |  |  |
| 1 | Ath-AT5G24650.1 |  | Vvi-Vitvi16g01311\_t001 |  |  |  |  |  |  |  |
| 1 | Ath-AT5G24655.1 |  | Vvi-Vitvi16g01310\_t001 |  |  |  |  |  |  |  |
| 1 | Ath-AT5G24660.1 |  | | | |  |  |  |  |  |  |  |
| 1 | Ath-AT5G24670.5 |  | | | |  |  |  |  |  |  |  |
| 1 | Ath-AT5G24680.1 |  | Vvi-Vitvi16g01308\_t001 |  |  |  |  |  |  |  |
| 0 | Ath-AT5G24690.1 |  |  |  |  |  |  |  |  |
| 0 | Ath-AT5G24710.1 |  |  |  |  |  |  |  |  |
| 0 | Ath-AT5G24740.3 |  |  |  |  |  |  |  |  |
| 0 | Ath-AT5G24750.1 |  |  |  |  |  |  |  |  |
| 0 | Ath-AT5G24760.1 |  |  |  |  |  |  |  |  |
| 0 | Ath-AT5G24770.1 |  |  |  |  |  |  |  |  |
| 0 | Ath-AT5G24780.1 |  |  |  |  |  |  |  |  |
| 1 | Ath-AT5G24790.1 |  | Vvi-Vitvi04g00241\_t001 |  |  |  |  |  |  |  |
| 1 | Ath-AT5G24800.1 |  | Vvi-Vitvi04g00240\_t001 |  |  |  |  |  |  |  |
| 1 | Ath-AT5G24810.2 |  | Vvi-Vitvi04g00239\_t001 |  |  |  |  |  |  |  |
| 1 | Ath-AT5G24820.1 |  | | | |  |  |  |  |  |  |  |
| 1 | Ath-AT5G24830.1 |  | | | |  |  |  |  |  |  |  |
| 1 | Ath-AT5G24840.1 |  | Vvi-Vitvi04g00235\_t001 |  |  |  |  |  |  |  |
| 1 | Ath-AT5G24850.1 |  | Vvi-Vitvi04g00232\_t001 |  |  |  |  |  |  |  |
| 1 | Ath-AT5G24860.2 |  | Vvi-Vitvi04g00226\_t001 |  |  |  |  |  |  |  |
| 1 | Ath-AT5G24870.3 |  | Vvi-Vitvi04g00205\_t002 |  |  |  |  |  |  |  |
| 1 | Ath-AT5G24879.1 |  | | | |  |  |  |  |  |  |  |
| 1 | Ath-AT5G24880.1 |  | | | |  |  |  |  |  |  |  |
| 1 | Ath-AT5G24890.1 |  | Vvi-Vitvi04g01819\_t001 |  |  |  |  |  |  |  |
| 0 | Ath-AT5G24900.1 |  |  |  |  |  |  |  |  |
| 0 | Ath-AT5G24910.1 |  |  |  |  |  |  |  |  |
| 0 | Ath-AT5G24920.1 |  |  |  |  |  |  |  |  |
| 0 | Ath-AT5G24930.1 |  |  |  |  |  |  |  |  |
| 0 | Ath-AT5G24940.1 |  |  |  |  |  |  |  |  |
| 0 | Ath-AT5G24950.1 |  |  |  |  |  |  |  |  |
| 0 | Ath-AT5G24960.1 |  |  |  |  |  |  |  |  |
| 0 | Ath-AT5G24970.2 |  |  |  |  |  |  |  |  |
| 0 | Ath-AT5G24980.1 |  |  |  |  |  |  |  |  |
| 0 | Ath-AT5G24990.1 |  |  |  |  |  |  |  |  |
| 0 | Ath-AT5G25000.1 |  |  |  |  |  |  |  |  |
| 0 | Ath-AT5G25010.1 |  |  |  |  |  |  |  |  |
| 0 | Ath-AT5G25020.1 |  |  |  |  |  |  |  |  |
| 0 | Ath-AT5G25030.1 |  |  |  |  |  |  |  |  |
| 0 | Ath-AT5G25040.2 |  |  |  |  |  |  |  |  |
| 0 | Ath-AT5G25050.1 |  |  |  |  |  |  |  |  |
| 0 | Ath-AT5G25060.1 |  |  |  |  |  |  |  |  |
| 0 | Ath-AT5G25070.1 |  |  |  |  |  |  |  |  |
| 0 | Ath-AT5G25080.1 |  |  |  |  |  |  |  |  |
| 0 | Ath-AT5G25090.1 |  |  |  |  |  |  |  |  |
| 0 | Ath-AT5G25100.2 |  |  |  |  |  |  |  |  |
| 1 | Ath-AT5G25110.1 |  | Vvi-Vitvi04g00512\_t001 |  |  |  |  |  |  |  |
| 1 | Ath-AT5G25120.1 |  | | | |  |  |  |  |  |  |  |
| 1 | Ath-AT5G25130.1 |  | | | |  |  |  |  |  |  |  |
| 1 | Ath-AT5G25140.1 |  | | | |  |  |  |  |  |  |  |
| 1 | Ath-AT5G25150.1 |  | Vvi-Vitvi04g00513\_t001 |  |  |  |  |  |  |  |
| 1 | Ath-AT5G25160.1 |  | Vvi-Vitvi04g00527\_t001 |  |  |  |  |  |  |  |
| 1 | Ath-AT5G25170.1 |  | Vvi-Vitvi04g00530\_t002 |  |  |  |  |  |  |  |
| 1 | Ath-AT5G25180.1 |  | | | |  |  |  |  |  |  |  |
| 1 | Ath-AT5G25190.1 |  | Vvi-Vitvi04g00533\_t001 |  |  |  |  |  |  |  |
| 1 | Ath-AT5G25195.1 |  | | | |  |  |  |  |  |  |  |
| 1 | Ath-AT5G25200.1 |  | | | |  |  |  |  |  |  |  |
| 1 | Ath-AT5G25210.2 |  | | | |  |  |  |  |  |  |  |
| 1 | Ath-AT5G25220.1 |  | Vvi-Vitvi04g00546\_t001 |  |  |  |  |  |  |  |
| 1 | Ath-AT5G25230.1 |  | | | |  |  |  |  |  |  |  |
| 1 | Ath-AT5G25240.1 |  | | | |  |  |  |  |  |  |  |
| 1 | Ath-AT5G25250.1 |  | | | |  |  |  |  |  |  |  |
| 1 | Ath-AT5G25260.1 |  | | | |  |  |  |  |  |  |  |
| 1 | Ath-AT5G25265.1 |  | Vvi-Vitvi04g00549\_t001 |  |  |  |  |  |  |  |
| 1 | Ath-AT5G25270.1 |  | Vvi-Vitvi04g00555\_t001 |  |  |  |  |  |  |  |
| 1 | Ath-AT5G25280.1 |  | Vvi-Vitvi04g00557\_t001 |  |  |  |  |  |  |  |
| 0 | Ath-AT5G25290.1 |  |  |  |  |  |  |  |  |
| 0 | Ath-AT5G25300.1 |  |  |  |  |  |  |  |  |
| 1 | Ath-AT5G25310.1 |  | Vvi-Vitvi04g00507\_t001 |  |  |  |  |  |  |  |
| 1 | Ath-AT5G25320.1 |  | Vvi-Vitvi04g00506\_t001 |  |  |  |  |  |  |  |
| 1 | Ath-AT5G25330.1 |  | | | |  |  |  |  |  |  |  |
| 1 | Ath-AT5G25340.1 |  | Vvi-Vitvi04g00495\_t001 |  |  |  |  |  |  |  |
| 1 | Ath-AT5G25350.1 |  | Vvi-Vitvi04g00482\_t001 |  |  |  |  |  |  |  |
| 1 | Ath-AT5G25360.3 |  | Vvi-Vitvi04g00481\_t003 |  |  |  |  |  |  |  |
| 1 | Ath-AT5G25370.2 |  | Vvi-Vitvi04g00480\_t001 |  |  |  |  |  |  |  |
| 2 | Ath-AT5G25380.4 |  | | | |  | Vvi-Vitvi04g00369\_t001 |  |  |  |  |  |  |
| 2 | Ath-AT5G25390.2 |  | Vvi-Vitvi04g00479\_t001 |  | | | |  |  |  |  |  |  |
| 2 | Ath-AT5G25400.1 |  | Vvi-Vitvi04g00471\_t001 |  | | | |  |  |  |  |  |  |
| 2 | Ath-AT5G25410.1 |  | | | |  | | | |  |  |  |  |  |  |
| 2 | Ath-AT5G25415.1 |  | | | |  | | | |  |  |  |  |  |  |
| 2 | Ath-AT5G25420.1 |  | | | |  | | | |  |  |  |  |  |  |
| 2 | Ath-AT5G25425.1 |  | | | |  | | | |  |  |  |  |  |  |
| 2 | Ath-AT5G25430.1 |  | | | |  | | | |  |  |  |  |  |  |
| 2 | Ath-AT5G25440.2 |  | | | |  | | | |  |  |  |  |  |  |
| 2 | Ath-AT5G25450.1 |  | | | |  | | | |  |  |  |  |  |  |
| 2 | Ath-AT5G25460.1 |  | | | |  | | | |  |  |  |  |  |  |
| 2 | Ath-AT5G25470.1 |  | | | |  | | | |  |  |  |  |  |  |
| 2 | Ath-AT5G25475.4 |  | | | |  | | | |  |  |  |  |  |  |
| 2 | Ath-AT5G25480.1 |  | | | |  | | | |  |  |  |  |  |  |
| 2 | Ath-AT5G25490.1 |  | Vvi-Vitvi04g01894\_t001 |  | | | |  |  |  |  |  |  |
| 1 | Ath-AT5G25500.1 |  |  |  | | | |  |  |  |  |  |  |
| 1 | Ath-AT5G25510.1 |  |  |  | Vvi-Vitvi04g00371\_t001 |  |  |  |  |  |  |
| 1 | Ath-AT5G25520.6 |  |  |  | Vvi-Vitvi04g00373\_t001 |  |  |  |  |  |  |
| 1 | Ath-AT5G25530.1 |  |  |  | Vvi-Vitvi04g00374\_t001 |  |  |  |  |  |  |
| 1 | Ath-AT5G25540.1 |  |  |  | | | |  |  |  |  |  |  |
| 1 | Ath-AT5G25550.1 |  |  |  | | | |  |  |  |  |  |  |
| 1 | Ath-AT5G25560.3 |  |  |  | Vvi-Vitvi04g00388\_t003 |  |  |  |  |  |  |
| 1 | Ath-AT5G25570.3 |  |  |  | Vvi-Vitvi04g01877\_t001 |  |  |  |  |  |  |
| 1 | Ath-AT5G25580.1 |  |  |  | Vvi-Vitvi04g00394\_t001 |  |  |  |  |  |  |
| 0 | Ath-AT5G25590.1 |  |  |  |  |  |  |  |  |
| 0 | Ath-AT5G25600.1 |  |  |  |  |  |  |  |  |
| 1 | Ath-AT5G25610.1 |  | Vvi-Vitvi04g00341\_t001 |  |  |  |  |  |  |  |
| 1 | Ath-AT5G25620.2 |  | Vvi-Vitvi04g00340\_t001 |  |  |  |  |  |  |  |
| 1 | Ath-AT5G25630.2 |  | Vvi-Vitvi04g00336\_t001 |  |  |  |  |  |  |  |
| 1 | Ath-AT5G25640.1 |  | Vvi-Vitvi04g00335\_t001 |  |  |  |  |  |  |  |
| 1 | Ath-AT5G25750.1 |  | | | |  |  |  |  |  |  |  |
| 1 | Ath-AT5G25754.1 |  | Vvi-Vitvi04g00327\_t001 |  |  |  |  |  |  |  |
| 1 | Ath-AT5G25755.1 |  | | | |  |  |  |  |  |  |  |
| 1 | Ath-AT5G25756.1 |  | | | |  |  |  |  |  |  |  |
| 1 | Ath-AT5G25757.1 |  | | | |  |  |  |  |  |  |  |
| 1 | Ath-AT5G25760.1 |  | Vvi-Vitvi04g00322\_t002 |  |  |  |  |  |  |  |
| 1 | Ath-AT5G25770.3 |  | Vvi-Vitvi04g00321\_t001 |  |  |  |  |  |  |  |
| 1 | Ath-AT5G25780.1 |  | | | |  |  |  |  |  |  |  |
| 1 | Ath-AT5G25790.2 |  | Vvi-Vitvi04g00320\_t001 |  |  |  |  |  |  |  |
| 0 | Ath-AT5G25800.2 |  |  |  |  |  |  |  |  |
| 0 | Ath-AT5G25810.1 |  |  |  |  |  |  |  |  |
| 0 | Ath-AT5G25820.1 |  |  |  |  |  |  |  |  |
| 0 | Ath-AT5G25830.1 |  |  |  |  |  |  |  |  |
| 0 | Ath-AT5G25840.1 |  |  |  |  |  |  |  |  |
| 0 | Ath-AT5G25850.1 |  |  |  |  |  |  |  |  |
| 0 | Ath-AT5G25860.1 |  |  |  |  |  |  |  |  |
| 0 | Ath-AT5G25870.1 |  |  |  |  |  |  |  |  |
| 1 | Ath-AT5G25880.1 |  | Vvi-Vitvi04g00009\_t001 |  |  |  |  |  |  |  |
| 1 | Ath-AT5G25890.1 |  | Vvi-Vitvi04g00013\_t002 |  |  |  |  |  |  |  |
| 1 | Ath-AT5G25900.1 |  | | | |  |  |  |  |  |  |  |
| 1 | Ath-AT5G25910.1 |  | Vvi-Vitvi04g04012\_t001 |  |  |  |  |  |  |  |
| 1 | Ath-AT5G25920.1 |  | | | |  |  |  |  |  |  |  |
| 1 | Ath-AT5G25930.1 |  | | | |  |  |  |  |  |  |  |
| 1 | Ath-AT5G25940.1 |  | Vvi-Vitvi04g01775\_t001 |  |  |  |  |  |  |  |
| 1 | Ath-AT5G25950.1 |  | Vvi-Vitvi04g00053\_t001 |  |  |  |  |  |  |  |
| 1 | Ath-AT5G25960.1 |  | | | |  |  |  |  |  |  |  |
| 1 | Ath-AT5G25970.2 |  | Vvi-Vitvi04g00056\_t001 |  |  |  |  |  |  |  |
| 1 | Ath-AT5G25980.2 |  | | | |  |  |  |  |  |  |  |
| 1 | Ath-AT5G25990.1 |  | | | |  |  |  |  |  |  |  |
| 1 | Ath-AT5G25995.1 |  | | | |  |  |  |  |  |  |  |
| 1 | Ath-AT5G26000.1 |  | | | |  |  |  |  |  |  |  |
| 1 | Ath-AT5G26010.1 |  | Vvi-Vitvi04g00060\_t001 |  |  |  |  |  |  |  |
| 1 | Ath-AT5G26030.1 |  | Vvi-Vitvi04g00065\_t001 |  |  |  |  |  |  |  |
| 1 | Ath-AT5G26040.2 |  | Vvi-Vitvi04g00077\_t001 |  |  |  |  |  |  |  |
| 0 | Ath-AT5G26050.1 |  |  |  |  |  |  |  |  |
| 0 | Ath-AT5G26060.1 |  |  |  |  |  |  |  |  |
| 0 | Ath-AT5G26070.1 |  |  |  |  |  |  |  |  |
| 0 | Ath-AT5G26080.1 |  |  |  |  |  |  |  |  |
| 0 | Ath-AT5G26090.2 |  |  |  |  |  |  |  |  |
| 0 | Ath-AT5G26100.1 |  |  |  |  |  |  |  |  |
| 0 | Ath-AT5G26110.3 |  |  |  |  |  |  |  |  |
| 0 | Ath-AT5G26120.1 |  |  |  |  |  |  |  |  |
| 0 | Ath-AT5G26130.1 |  |  |  |  |  |  |  |  |
| 0 | Ath-AT5G26140.2 |  |  |  |  |  |  |  |  |
| 1 | Ath-AT5G26150.2 |  | Vvi-Vitvi04g00118\_t001 |  |  |  |  |  |  |  |
| 1 | Ath-AT5G26160.2 |  | Vvi-Vitvi04g00132\_t002 |  |  |  |  |  |  |  |
| 1 | Ath-AT5G26170.1 |  | Vvi-Vitvi04g00133\_t001 |  |  |  |  |  |  |  |
| 1 | Ath-AT5G26180.1 |  | Vvi-Vitvi04g00134\_t001 |  |  |  |  |  |  |  |
| 1 | Ath-AT5G26190.1 |  | | | |  |  |  |  |  |  |  |
| 1 | Ath-AT5G26200.1 |  | Vvi-Vitvi04g01805\_t001 |  |  |  |  |  |  |  |
| 1 | Ath-AT5G26210.1 |  | Vvi-Vitvi04g00140\_t001 |  |  |  |  |  |  |  |
| 0 | Ath-AT5G26220.1 |  |  |  |  |  |  |  |  |
| 0 | Ath-AT5G26230.1 |  |  |  |  |  |  |  |  |
| 0 | Ath-AT5G26240.1 |  |  |  |  |  |  |  |  |
| 0 | Ath-AT5G26250.1 |  |  |  |  |  |  |  |  |
| 0 | Ath-AT5G26260.1 |  |  |  |  |  |  |  |  |
| 0 | Ath-AT5G26262.1 |  |  |  |  |  |  |  |  |
| 0 | Ath-AT5G26270.1 |  |  |  |  |  |  |  |  |
| 0 | Ath-AT5G26280.3 |  |  |  |  |  |  |  |  |
| 0 | Ath-AT5G26290.1 |  |  |  |  |  |  |  |  |
| 0 | Ath-AT5G26300.1 |  |  |  |  |  |  |  |  |
| 0 | Ath-AT5G26310.1 |  |  |  |  |  |  |  |  |
| 0 | Ath-AT5G26320.1 |  |  |  |  |  |  |  |  |
| 0 | Ath-AT5G26330.1 |  |  |  |  |  |  |  |  |
| 0 | Ath-AT5G26340.1 |  |  |  |  |  |  |  |  |
| 0 | Ath-AT5G26360.1 |  |  |  |  |  |  |  |  |
| 0 | Ath-AT5G26570.1 |  |  |  |  |  |  |  |  |
| 0 | Ath-AT5G26594.1 |  |  |  |  |  |  |  |  |
| 0 | Ath-AT5G26667.1 |  |  |  |  |  |  |  |  |
| 0 | Ath-AT5G26673.1 |  |  |  |  |  |  |  |  |
| 0 | Ath-AT5G26692.1 |  |  |  |  |  |  |  |  |
| 1 | Ath-AT5G26742.2 |  | Vvi-Vitvi15g04674\_t001 |  |  |  |  |  |  |  |
| 1 | Ath-AT5G26717.1 |  | | | |  |  |  |  |  |  |  |
| 1 | Ath-AT5G26740.1 |  | | | |  |  |  |  |  |  |  |
| 1 | Ath-AT5G26731.1 |  | Vvi-Vitvi15g04671\_t001 |  |  |  |  |  |  |  |
| 1 | Ath-AT5G26730.1 |  | | | |  |  |  |  |  |  |  |
| 1 | Ath-AT5G26720.1 |  | | | |  |  |  |  |  |  |  |
| 1 | Ath-AT5G26710.1 |  | Vvi-Vitvi15g04665\_t001 |  |  |  |  |  |  |  |
| 1 | Ath-AT5G26700.1 |  | Vvi-Vitvi15g04663\_t001 |  |  |  |  |  |  |  |
| 1 | Ath-AT5G26690.1 |  | Vvi-Vitvi15g04662\_t001 |  |  |  |  |  |  |  |
| 1 | Ath-AT5G26680.1 |  | Vvi-Vitvi15g04658\_t001 |  |  |  |  |  |  |  |
| 1 | Ath-AT5G26670.1 |  | Vvi-Vitvi15g04656\_t001 |  |  |  |  |  |  |  |
| 1 | Ath-AT5G26660.1 |  | Vvi-Vitvi15g04655\_t001 |  |  |  |  |  |  |  |
| 1 | Ath-AT5G26650.1 |  | | | |  |  |  |  |  |  |  |
| 1 | Ath-AT5G26640.1 |  | Vvi-Vitvi15g04650\_t001 |  |  |  |  |  |  |  |
| 0 | Ath-AT5G26630.1 |  |  |  |  |  |  |  |  |
| 0 | Ath-AT5G26622.1 |  |  |  |  |  |  |  |  |
| 0 | Ath-AT5G26617.1 |  |  |  |  |  |  |  |  |
| 0 | Ath-AT5G26620.1 |  |  |  |  |  |  |  |  |
| 0 | Ath-AT5G26610.1 |  |  |  |  |  |  |  |  |
| 0 | Ath-AT5G26600.1 |  |  |  |  |  |  |  |  |
| 0 | Ath-AT5G26580.1 |  |  |  |  |  |  |  |  |
| 0 | Ath-AT5G26749.2 |  |  |  |  |  |  |  |  |
| 0 | Ath-AT5G26751.1 |  |  |  |  |  |  |  |  |
| 0 | Ath-AT5G26760.2 |  |  |  |  |  |  |  |  |
| 0 | Ath-AT5G26770.5 |  |  |  |  |  |  |  |  |
| 0 | Ath-AT5G26780.3 |  |  |  |  |  |  |  |  |
| 0 | Ath-AT5G26790.1 |  |  |  |  |  |  |  |  |
| 1 | Ath-AT5G26805.1 |  | Vvi-Vitvi14g02470\_t001 |  |  |  |  |  |  |  |
| 1 | Ath-AT5G26810.1 |  | Vvi-Vitvi14g04020\_t001 |  |  |  |  |  |  |  |
| 1 | Ath-AT5G26820.1 |  | Vvi-Vitvi14g00086\_t001 |  |  |  |  |  |  |  |
| 1 | Ath-AT5G26830.1 |  | | | |  |  |  |  |  |  |  |
| 1 | Ath-AT5G26840.1 |  | | | |  |  |  |  |  |  |  |
| 1 | Ath-AT5G26850.2 |  | Vvi-Vitvi14g00092\_t002 |  |  |  |  |  |  |  |
| 1 | Ath-AT5G26860.1 |  | Vvi-Vitvi14g00096\_t001 |  |  |  |  |  |  |  |
| 1 | Ath-AT5G26865.1 |  | | | |  |  |  |  |  |  |  |
| 1 | Ath-AT5G26880.1 |  | | | |  |  |  |  |  |  |  |
| 1 | Ath-AT5G26890.1 |  | | | |  |  |  |  |  |  |  |
| 1 | Ath-AT5G26900.1 |  | | | |  |  |  |  |  |  |  |
| 1 | Ath-AT5G26910.1 |  | Vvi-Vitvi14g00114\_t001 |  |  |  |  |  |  |  |
| 1 | Ath-AT5G26920.1 |  | | | |  |  |  |  |  |  |  |
| 1 | Ath-AT5G26930.1 |  | Vvi-Vitvi14g00123\_t001 |  |  |  |  |  |  |  |
| 1 | Ath-AT5G26940.4 |  | Vvi-Vitvi14g00124\_t001 |  |  |  |  |  |  |  |
| 1 | Ath-AT5G26950.1 |  | | | |  |  |  |  |  |  |  |
| 1 | Ath-AT5G26955.1 |  | | | |  |  |  |  |  |  |  |
| 1 | Ath-AT5G26960.1 |  | | | |  |  |  |  |  |  |  |
| 1 | Ath-AT5G26970.1 |  | | | |  |  |  |  |  |  |  |
| 1 | Ath-AT5G26980.1 |  | Vvi-Vitvi14g00125\_t001 |  |  |  |  |  |  |  |
| 1 | Ath-AT5G26990.1 |  | Vvi-Vitvi14g00126\_t001 |  |  |  |  |  |  |  |
| 1 | Ath-AT5G27000.1 |  | Vvi-Vitvi14g00127\_t001 |  |  |  |  |  |  |  |
| 1 | Ath-AT5G27010.1 |  | | | |  |  |  |  |  |  |  |
| 1 | Ath-AT5G27020.1 |  | | | |  |  |  |  |  |  |  |
| 1 | Ath-AT5G27030.2 |  | Vvi-Vitvi14g00137\_t001 |  |  |  |  |  |  |  |
| 0 | Ath-AT5G27050.1 |  |  |  |  |  |  |  |  |
| 0 | Ath-AT5G27060.2 |  |  |  |  |  |  |  |  |
| 0 | Ath-AT5G27065.1 |  |  |  |  |  |  |  |  |
| 0 | Ath-AT5G27070.1 |  |  |  |  |  |  |  |  |
| 0 | Ath-AT5G27080.1 |  |  |  |  |  |  |  |  |
| 0 | Ath-AT5G27090.1 |  |  |  |  |  |  |  |  |
| 0 | Ath-AT5G27093.1 |  |  |  |  |  |  |  |  |
| 0 | Ath-AT5G27100.1 |  |  |  |  |  |  |  |  |
| 0 | Ath-AT5G27110.1 |  |  |  |  |  |  |  |  |
| 0 | Ath-AT5G27120.1 |  |  |  |  |  |  |  |  |
| 0 | Ath-AT5G27130.1 |  |  |  |  |  |  |  |  |
| 0 | Ath-AT5G27140.2 |  |  |  |  |  |  |  |  |
| 0 | Ath-AT5G27150.1 |  |  |  |  |  |  |  |  |
| 0 | Ath-AT5G27170.1 |  |  |  |  |  |  |  |  |
| 0 | Ath-AT5G27200.1 |  |  |  |  |  |  |  |  |
| 0 | Ath-AT5G27210.1 |  |  |  |  |  |  |  |  |
| 0 | Ath-AT5G27220.1 |  |  |  |  |  |  |  |  |
| 0 | Ath-AT5G27230.1 |  |  |  |  |  |  |  |  |
| 0 | Ath-AT5G27238.1 |  |  |  |  |  |  |  |  |
| 0 | Ath-AT5G27240.1 |  |  |  |  |  |  |  |  |
| 0 | Ath-AT5G27247.1 |  |  |  |  |  |  |  |  |
| 0 | Ath-AT5G27260.1 |  |  |  |  |  |  |  |  |
| 0 | Ath-AT5G27270.1 |  |  |  |  |  |  |  |  |
| 0 | Ath-AT5G27280.1 |  |  |  |  |  |  |  |  |
| 1 | Ath-AT5G27290.1 |  | Vvi-Vitvi14g04113\_t001 |  |  |  |  |  |  |  |
| 1 | Ath-AT5G27300.1 |  | | | |  |  |  |  |  |  |  |
| 1 | Ath-AT5G27310.1 |  | | | |  |  |  |  |  |  |  |
| 1 | Ath-AT5G27320.1 |  | Vvi-Vitvi14g00322\_t001 |  |  |  |  |  |  |  |
| 1 | Ath-AT5G27330.1 |  | Vvi-Vitvi14g00321\_t001 |  |  |  |  |  |  |  |
| 1 | Ath-AT5G27340.1 |  | | | |  |  |  |  |  |  |  |
| 1 | Ath-AT5G27350.1 |  | Vvi-Vitvi14g04106\_t001 |  |  |  |  |  |  |  |
| 1 | Ath-AT5G27360.3 |  | Vvi-Vitvi14g00304\_t001 |  |  |  |  |  |  |  |
| 1 | Ath-AT5G27370.1 |  | | | |  |  |  |  |  |  |  |
| 1 | Ath-AT5G27380.1 |  | Vvi-Vitvi14g00291\_t001 |  |  |  |  |  |  |  |
| 1 | Ath-AT5G27395.1 |  | Vvi-Vitvi14g00289\_t001 |  |  |  |  |  |  |  |
| 1 | Ath-AT5G27390.2 |  | Vvi-Vitvi14g00286\_t001 |  |  |  |  |  |  |  |
| 1 | Ath-AT5G27400.1 |  | Vvi-Vitvi14g00281\_t001 |  |  |  |  |  |  |  |
| 1 | Ath-AT5G27410.2 |  | | | |  |  |  |  |  |  |  |
| 1 | Ath-AT5G27420.1 |  | Vvi-Vitvi14g00275\_t001 |  |  |  |  |  |  |  |
| 1 | Ath-AT5G27430.1 |  | Vvi-Vitvi14g00267\_t001 |  |  |  |  |  |  |  |
| 1 | Ath-AT5G27440.1 |  | Vvi-Vitvi14g04089\_t001 |  |  |  |  |  |  |  |
| 1 | Ath-AT5G27450.2 |  | Vvi-Vitvi14g00258\_t001 |  |  |  |  |  |  |  |
| 1 | Ath-AT5G27460.1 |  | | | |  |  |  |  |  |  |  |
| 1 | Ath-AT5G27470.1 |  | Vvi-Vitvi14g00255\_t001 |  |  |  |  |  |  |  |
| 1 | Ath-AT5G27490.1 |  | Vvi-Vitvi14g00254\_t001 |  |  |  |  |  |  |  |
| 1 | Ath-AT5G27493.1 |  | | | |  |  |  |  |  |  |  |
| 1 | Ath-AT5G27495.1 |  | | | |  |  |  |  |  |  |  |
| 1 | Ath-AT5G27510.1 |  | | | |  |  |  |  |  |  |  |
| 1 | Ath-AT5G27520.1 |  | Vvi-Vitvi14g00253\_t001 |  |  |  |  |  |  |  |
| 1 | Ath-AT5G27530.1 |  | | | |  |  |  |  |  |  |  |
| 1 | Ath-AT5G27540.2 |  | Vvi-Vitvi14g00251\_t001 |  |  |  |  |  |  |  |
| 1 | Ath-AT5G27550.1 |  | Vvi-Vitvi14g02558\_t001 |  |  |  |  |  |  |  |
| 1 | Ath-AT5G27560.1 |  | Vvi-Vitvi14g00246\_t001 |  |  |  |  |  |  |  |
| 1 | Ath-AT5G27570.1 |  | | | |  |  |  |  |  |  |  |
| 1 | Ath-AT5G27580.1 |  | | | |  |  |  |  |  |  |  |
| 1 | Ath-AT5G27600.1 |  | Vvi-Vitvi14g04084\_t001 |  |  |  |  |  |  |  |
| 1 | Ath-AT5G27606.1 |  | | | |  |  |  |  |  |  |  |
| 1 | Ath-AT5G27607.1 |  | | | |  |  |  |  |  |  |  |
| 1 | Ath-AT5G27610.1 |  | Vvi-Vitvi14g00230\_t001 |  |  |  |  |  |  |  |
| 1 | Ath-AT5G27620.2 |  | Vvi-Vitvi14g02526\_t001 |  |  |  |  |  |  |  |
| 1 | Ath-AT5G27630.3 |  | Vvi-Vitvi14g00195\_t001 |  |  |  |  |  |  |  |
| 1 | Ath-AT5G27640.2 |  | | | |  |  |  |  |  |  |  |
| 1 | Ath-AT5G27650.1 |  | Vvi-Vitvi14g00194\_t001 |  |  |  |  |  |  |  |
| 1 | Ath-AT5G27660.2 |  | Vvi-Vitvi14g00190\_t001 |  |  |  |  |  |  |  |
| 1 | Ath-AT5G27670.1 |  | Vvi-Vitvi14g00189\_t001 |  |  |  |  |  |  |  |
| 1 | Ath-AT5G27680.2 |  | Vvi-Vitvi14g00186\_t001 |  |  |  |  |  |  |  |
| 2 | Ath-AT5G27690.1 |  | | | |  | Vvi-Vitvi14g00163\_t001 |  |  |  |  |  |  |
| 2 | Ath-AT5G27700.1 |  | | | |  | Vvi-Vitvi14g00165\_t001 |  |  |  |  |  |  |
| 2 | Ath-AT5G27710.1 |  | | | |  | Vvi-Vitvi14g00171\_t001 |  |  |  |  |  |  |
| 2 | Ath-AT5G27720.1 |  | | | |  | Vvi-Vitvi14g00175\_t001 |  |  |  |  |  |  |
| 2 | Ath-AT5G27730.1 |  | Vvi-Vitvi14g00176\_t001 |  | Vvi-Vitvi14g00176\_t001 |  |  |  |  |  |  |
| 2 | Ath-AT5G27740.1 |  | | | |  | | | |  |  |  |  |  |  |
| 2 | Ath-AT5G27750.1 |  | | | |  | | | |  |  |  |  |  |  |
| 2 | Ath-AT5G27760.1 |  | | | |  | Vvi-Vitvi14g02510\_t001 |  |  |  |  |  |  |
| 2 | Ath-AT5G27765.1 |  | | | |  | | | |  |  |  |  |  |  |
| 2 | Ath-AT5G27770.1 |  | Vvi-Vitvi14g00159\_t001 |  | Vvi-Vitvi14g00182\_t001 |  |  |  |  |  |  |
| 1 | Ath-AT5G27780.1 |  | | | |  |  |  |  |  |  |  |
| 1 | Ath-AT5G27790.1 |  | | | |  |  |  |  |  |  |  |
| 1 | Ath-AT5G27800.1 |  | | | |  |  |  |  |  |  |  |
| 1 | Ath-AT5G27810.1 |  | | | |  |  |  |  |  |  |  |
| 2 | Ath-AT5G27820.1 |  | | | |  | Vvi-Vitvi14g00141\_t001 |  |  |  |  |  |  |
| 2 | Ath-AT5G27830.4 |  | | | |  | Vvi-Vitvi14g00143\_t001 |  |  |  |  |  |  |
| 2 | Ath-AT5G27840.4 |  | | | |  | Vvi-Vitvi14g00144\_t001 |  |  |  |  |  |  |
| 2 | Ath-AT5G27850.1 |  | | | |  | Vvi-Vitvi14g00151\_t001 |  |  |  |  |  |  |
| 2 | Ath-AT5G27860.1 |  | Vvi-Vitvi14g02500\_t001 |  | Vvi-Vitvi14g02500\_t001 |  |  |  |  |  |  |
| 1 | Ath-AT5G27870.1 |  |  |  | Vvi-Vitvi14g02501\_t001 |  |  |  |  |  |  |
| 1 | Ath-AT5G27880.1 |  |  |  | | | |  |  |  |  |  |  |
| 1 | Ath-AT5G27889.1 |  |  |  | | | |  |  |  |  |  |  |
| 1 | Ath-AT5G27890.1 |  |  |  | | | |  |  |  |  |  |  |
| 1 | Ath-AT5G27893.1 |  |  |  | | | |  |  |  |  |  |  |
| 1 | Ath-AT5G27910.1 |  |  |  | | | |  |  |  |  |  |  |
| 1 | Ath-AT5G27920.1 |  |  |  | Vvi-Vitvi14g00155\_t001 |  |  |  |  |  |  |
| 1 | Ath-AT5G27930.2 |  |  |  | Vvi-Vitvi14g00161\_t003 |  |  |  |  |  |  |
| 0 | Ath-AT5G27940.1 |  |  |  |  |  |  |  |  |
| 0 | Ath-AT5G27944.1 |  |  |  |  |  |  |  |  |
| 0 | Ath-AT5G27945.1 |  |  |  |  |  |  |  |  |
| 1 | Ath-AT5G27950.1 |  | Vvi-Vitvi14g00391\_t001 |  |  |  |  |  |  |  |
| 1 | Ath-AT5G27960.1 |  | | | |  |  |  |  |  |  |  |
| 1 | Ath-AT5G27970.2 |  | | | |  |  |  |  |  |  |  |
| 1 | Ath-AT5G27980.1 |  | | | |  |  |  |  |  |  |  |
| 1 | Ath-AT5G27990.1 |  | Vvi-Vitvi14g00399\_t001 |  |  |  |  |  |  |  |
| 1 | Ath-AT5G28000.1 |  | | | |  |  |  |  |  |  |  |
| 1 | Ath-AT5G28010.1 |  | | | |  |  |  |  |  |  |  |
| 1 | Ath-AT5G28020.1 |  | Vvi-Vitvi14g04165\_t001 |  |  |  |  |  |  |  |
| 1 | Ath-AT5G28030.4 |  | | | |  |  |  |  |  |  |  |
| 1 | Ath-AT5G28040.1 |  | Vvi-Vitvi14g04171\_t001 |  |  |  |  |  |  |  |
| 1 | Ath-AT5G28050.3 |  | Vvi-Vitvi14g00413\_t001 |  |  |  |  |  |  |  |
| 1 | Ath-AT5G28060.1 |  | Vvi-Vitvi14g00415\_t001 |  |  |  |  |  |  |  |
| 1 | Ath-AT5G28080.2 |  | Vvi-Vitvi14g00417\_t001 |  |  |  |  |  |  |  |
| 1 | Ath-AT5G28090.1 |  | | | |  |  |  |  |  |  |  |
| 1 | Ath-AT5G28150.1 |  | Vvi-Vitvi14g00425\_t001 |  |  |  |  |  |  |  |
| 1 | Ath-AT5G28160.1 |  | | | |  |  |  |  |  |  |  |
| 1 | Ath-AT5G28180.1 |  | | | |  |  |  |  |  |  |  |
| 1 | Ath-AT5G28190.1 |  | | | |  |  |  |  |  |  |  |
| 1 | Ath-AT5G28210.1 |  | | | |  |  |  |  |  |  |  |
| 1 | Ath-AT5G28220.1 |  | Vvi-Vitvi14g00430\_t001 |  |  |  |  |  |  |  |
| 1 | Ath-AT5G28235.1 |  | | | |  |  |  |  |  |  |  |
| 1 | Ath-AT5G28237.1 |  | | | |  |  |  |  |  |  |  |
| 1 | Ath-AT5G28288.1 |  | | | |  |  |  |  |  |  |  |
| 1 | Ath-AT5G28290.1 |  | Vvi-Vitvi14g00434\_t001 |  |  |  |  |  |  |  |
| 0 | Ath-AT5G28295.1 |  |  |  |  |  |  |  |  |
| 0 | Ath-AT5G28300.1 |  |  |  |  |  |  |  |  |
| 0 | Ath-AT5G28310.1 |  |  |  |  |  |  |  |  |
| 0 | Ath-AT5G28320.1 |  |  |  |  |  |  |  |  |
| 0 | Ath-AT5G28340.1 |  |  |  |  |  |  |  |  |
| 0 | Ath-AT5G28345.1 |  |  |  |  |  |  |  |  |
| 0 | Ath-AT5G28350.1 |  |  |  |  |  |  |  |  |
| 0 | Ath-AT5G28370.1 |  |  |  |  |  |  |  |  |
| 0 | Ath-AT5G28380.1 |  |  |  |  |  |  |  |  |
| 0 | Ath-AT5G28390.1 |  |  |  |  |  |  |  |  |
| 0 | Ath-AT5G28400.1 |  |  |  |  |  |  |  |  |
| 0 | Ath-AT5G28410.1 |  |  |  |  |  |  |  |  |
| 0 | Ath-AT5G28420.1 |  |  |  |  |  |  |  |  |
| 0 | Ath-AT5G28442.1 |  |  |  |  |  |  |  |  |
| 0 | Ath-AT5G28450.1 |  |  |  |  |  |  |  |  |
| 0 | Ath-AT5G28460.1 |  |  |  |  |  |  |  |  |
| 0 | Ath-AT5G28463.1 |  |  |  |  |  |  |  |  |
| 0 | Ath-AT5G28462.1 |  |  |  |  |  |  |  |  |
| 0 | Ath-AT5G28470.2 |  |  |  |  |  |  |  |  |
| 0 | Ath-AT5G28490.1 |  |  |  |  |  |  |  |  |
| 0 | Ath-AT5G28491.1 |  |  |  |  |  |  |  |  |
| 0 | Ath-AT5G28500.1 |  |  |  |  |  |  |  |  |
| 0 | Ath-AT5G28510.1 |  |  |  |  |  |  |  |  |
| 0 | Ath-AT5G28520.2 |  |  |  |  |  |  |  |  |
| 0 | Ath-AT5G28530.1 |  |  |  |  |  |  |  |  |
| 0 | Ath-AT5G28540.1 |  |  |  |  |  |  |  |  |
| 0 | Ath-AT5G28550.1 |  |  |  |  |  |  |  |  |
| 0 | Ath-AT5G28560.1 |  |  |  |  |  |  |  |  |
| 0 | Ath-AT5G28590.1 |  |  |  |  |  |  |  |  |
| 0 | Ath-AT5G28610.1 |  |  |  |  |  |  |  |  |
| 0 | Ath-AT5G28615.1 |  |  |  |  |  |  |  |  |
| 0 | Ath-AT5G28620.1 |  |  |  |  |  |  |  |  |
| 0 | Ath-AT5G28630.1 |  |  |  |  |  |  |  |  |
| 0 | Ath-AT5G28640.1 |  |  |  |  |  |  |  |  |
| 0 | Ath-AT5G28646.2 |  |  |  |  |  |  |  |  |
| 0 | Ath-AT5G28650.1 |  |  |  |  |  |  |  |  |
| 0 | Ath-AT5G28660.1 |  |  |  |  |  |  |  |  |
| 0 | Ath-AT5G28680.1 |  |  |  |  |  |  |  |  |
| 0 | Ath-AT5G28690.1 |  |  |  |  |  |  |  |  |
| 0 | Ath-AT5G28720.1 |  |  |  |  |  |  |  |  |
| 0 | Ath-AT5G28730.1 |  |  |  |  |  |  |  |  |
| 0 | Ath-AT5G28740.1 |  |  |  |  |  |  |  |  |
| 0 | Ath-AT5G28750.1 |  |  |  |  |  |  |  |  |
| 0 | Ath-AT5G28770.2 |  |  |  |  |  |  |  |  |
| 0 | Ath-AT5G28780.1 |  |  |  |  |  |  |  |  |
| 0 | Ath-AT5G28810.1 |  |  |  |  |  |  |  |  |
| 0 | Ath-AT5G28820.1 |  |  |  |  |  |  |  |  |
| 0 | Ath-AT5G28823.1 |  |  |  |  |  |  |  |  |
| 0 | Ath-AT5G28830.1 |  |  |  |  |  |  |  |  |
| 0 | Ath-AT5G28840.1 |  |  |  |  |  |  |  |  |
| 0 | Ath-AT5G28850.2 |  |  |  |  |  |  |  |  |
| 0 | Ath-AT5G28885.1 |  |  |  |  |  |  |  |  |
| 0 | Ath-AT5G28900.1 |  |  |  |  |  |  |  |  |
| 0 | Ath-AT5G28910.2 |  |  |  |  |  |  |  |  |
| 0 | Ath-AT5G28919.1 |  |  |  |  |  |  |  |  |
| 0 | Ath-AT5G28920.1 |  |  |  |  |  |  |  |  |
| 0 | Ath-AT5G28950.1 |  |  |  |  |  |  |  |  |
| 0 | Ath-AT5G28960.1 |  |  |  |  |  |  |  |  |
| 0 | Ath-AT5G29000.2 |  |  |  |  |  |  |  |  |
| 0 | Ath-AT5G29050.1 |  |  |  |  |  |  |  |  |
| 0 | Ath-AT5G29054.1 |  |  |  |  |  |  |  |  |
| 0 | Ath-AT5G29070.1 |  |  |  |  |  |  |  |  |
| 0 | Ath-AT5G29210.1 |  |  |  |  |  |  |  |  |
| 0 | Ath-AT5G29560.1 |  |  |  |  |  |  |  |  |
| 0 | Ath-AT5G29613.1 |  |  |  |  |  |  |  |  |
| 0 | Ath-AT5G29624.1 |  |  |  |  |  |  |  |  |
| 0 | Ath-AT5G30341.1 |  |  |  |  |  |  |  |  |
| 0 | Ath-AT5G30495.2 |  |  |  |  |  |  |  |  |
| 0 | Ath-AT5G31412.1 |  |  |  |  |  |  |  |  |
| 0 | Ath-AT5G30360.1 |  |  |  |  |  |  |  |  |
| 0 | Ath-AT5G30490.1 |  |  |  |  |  |  |  |  |
| 0 | Ath-AT5G30500.1 |  |  |  |  |  |  |  |  |
| 0 | Ath-AT5G30510.1 |  |  |  |  |  |  |  |  |
| 0 | Ath-AT5G30520.1 |  |  |  |  |  |  |  |  |
| 0 | Ath-AT5G32440.3 |  |  |  |  |  |  |  |  |
| 0 | Ath-AT5G32450.1 |  |  |  |  |  |  |  |  |
| 0 | Ath-AT5G32460.1 |  |  |  |  |  |  |  |  |
| 0 | Ath-AT5G32470.1 |  |  |  |  |  |  |  |  |
| 0 | Ath-AT5G32590.1 |  |  |  |  |  |  |  |  |
| 0 | Ath-AT5G32613.1 |  |  |  |  |  |  |  |  |
| 0 | Ath-AT5G32619.1 |  |  |  |  |  |  |  |  |
| 0 | Ath-AT5G32670.1 |  |  |  |  |  |  |  |  |
| 0 | Ath-AT5G33210.1 |  |  |  |  |  |  |  |  |
| 0 | Ath-AT5G33280.1 |  |  |  |  |  |  |  |  |
| 0 | Ath-AT5G33290.1 |  |  |  |  |  |  |  |  |
| 0 | Ath-AT5G33300.1 |  |  |  |  |  |  |  |  |
| 0 | Ath-AT5G33320.1 |  |  |  |  |  |  |  |  |
| 0 | Ath-AT5G33330.1 |  |  |  |  |  |  |  |  |
| 0 | Ath-AT5G33340.1 |  |  |  |  |  |  |  |  |
| 0 | Ath-AT5G33355.1 |  |  |  |  |  |  |  |  |
| 0 | Ath-AT5G33370.1 |  |  |  |  |  |  |  |  |
| 0 | Ath-AT5G33393.1 |  |  |  |  |  |  |  |  |
| 0 | Ath-AT5G33390.1 |  |  |  |  |  |  |  |  |
| 0 | Ath-AT5G33406.1 |  |  |  |  |  |  |  |  |
| 0 | Ath-AT5G33806.1 |  |  |  |  |  |  |  |  |
| 0 | Ath-AT5G33898.1 |  |  |  |  |  |  |  |  |
| 0 | Ath-AT5G34581.1 |  |  |  |  |  |  |  |  |
| 0 | Ath-AT5G34780.1 |  |  |  |  |  |  |  |  |
| 0 | Ath-AT5G34830.1 |  |  |  |  |  |  |  |  |
| 0 | Ath-AT5G34829.1 |  |  |  |  |  |  |  |  |
| 0 | Ath-AT5G34869.1 |  |  |  |  |  |  |  |  |
| 0 | Ath-AT5G34828.1 |  |  |  |  |  |  |  |  |
| 0 | Ath-AT5G34850.1 |  |  |  |  |  |  |  |  |
| 0 | Ath-AT5G34870.1 |  |  |  |  |  |  |  |  |
| 0 | Ath-AT5G34882.1 |  |  |  |  |  |  |  |  |
| 0 | Ath-AT5G34881.1 |  |  |  |  |  |  |  |  |
| 0 | Ath-AT5G34883.1 |  |  |  |  |  |  |  |  |
| 0 | Ath-AT5G34885.1 |  |  |  |  |  |  |  |  |
| 0 | Ath-AT5G34887.1 |  |  |  |  |  |  |  |  |
| 0 | Ath-AT5G34905.1 |  |  |  |  |  |  |  |  |
| 0 | Ath-AT5G34908.1 |  |  |  |  |  |  |  |  |
| 0 | Ath-AT5G34930.2 |  |  |  |  |  |  |  |  |
| 0 | Ath-AT5G34940.2 |  |  |  |  |  |  |  |  |
| 0 | Ath-AT5G35050.1 |  |  |  |  |  |  |  |  |
| 0 | Ath-AT5G35067.1 |  |  |  |  |  |  |  |  |
| 0 | Ath-AT5G35069.1 |  |  |  |  |  |  |  |  |
| 0 | Ath-AT5G35080.1 |  |  |  |  |  |  |  |  |
| 1 | Ath-AT5G35090.1 |  | Vvi-Vitvi11g01474\_t001 |  |  |  |  |  |  |  |
| 1 | Ath-AT5G35100.1 |  | Vvi-Vitvi11g00603\_t001 |  |  |  |  |  |  |  |
| 1 | Ath-AT5G35110.1 |  | Vvi-Vitvi11g04147\_t001 |  |  |  |  |  |  |  |
| 1 | Ath-AT5G35120.1 |  | | | |  |  |  |  |  |  |  |
| 1 | Ath-AT5G35160.3 |  | Vvi-Vitvi11g00608\_t001 |  |  |  |  |  |  |  |
| 1 | Ath-AT5G35170.1 |  | Vvi-Vitvi11g04151\_t001 |  |  |  |  |  |  |  |
| 1 | Ath-AT5G35180.4 |  | Vvi-Vitvi11g00615\_t001 |  |  |  |  |  |  |  |
| 0 | Ath-AT5G35190.2 |  |  |  |  |  |  |  |  |
| 0 | Ath-AT5G35195.1 |  |  |  |  |  |  |  |  |
| 0 | Ath-AT5G35200.2 |  |  |  |  |  |  |  |  |
| 0 | Ath-AT5G35210.1 |  |  |  |  |  |  |  |  |
| 0 | Ath-AT5G35220.1 |  |  |  |  |  |  |  |  |
| 0 | Ath-AT5G35230.1 |  |  |  |  |  |  |  |  |
| 0 | Ath-AT5G35300.1 |  |  |  |  |  |  |  |  |
| 0 | Ath-AT5G35320.1 |  |  |  |  |  |  |  |  |
| 0 | Ath-AT5G35330.3 |  |  |  |  |  |  |  |  |
| 0 | Ath-AT5G35338.2 |  |  |  |  |  |  |  |  |
| 0 | Ath-AT5G35360.3 |  |  |  |  |  |  |  |  |
| 0 | Ath-AT5G35370.1 |  |  |  |  |  |  |  |  |
| 0 | Ath-AT5G35375.1 |  |  |  |  |  |  |  |  |
| 0 | Ath-AT5G35380.1 |  |  |  |  |  |  |  |  |
| 0 | Ath-AT5G35390.1 |  |  |  |  |  |  |  |  |
| 0 | Ath-AT5G35400.2 |  |  |  |  |  |  |  |  |
| 0 | Ath-AT5G35405.1 |  |  |  |  |  |  |  |  |
| 0 | Ath-AT5G35410.1 |  |  |  |  |  |  |  |  |
| 0 | Ath-AT5G35430.1 |  |  |  |  |  |  |  |  |
| 0 | Ath-AT5G35450.1 |  |  |  |  |  |  |  |  |
| 0 | Ath-AT5G35460.1 |  |  |  |  |  |  |  |  |
| 0 | Ath-AT5G35475.1 |  |  |  |  |  |  |  |  |
| 0 | Ath-AT5G35480.2 |  |  |  |  |  |  |  |  |
| 0 | Ath-AT5G35490.1 |  |  |  |  |  |  |  |  |
| 0 | Ath-AT5G35510.2 |  |  |  |  |  |  |  |  |
| 0 | Ath-AT5G35520.1 |  |  |  |  |  |  |  |  |
| 0 | Ath-AT5G35525.1 |  |  |  |  |  |  |  |  |
| 0 | Ath-AT5G35530.1 |  |  |  |  |  |  |  |  |
| 0 | Ath-AT5G35540.1 |  |  |  |  |  |  |  |  |
| 0 | Ath-AT5G35550.2 |  |  |  |  |  |  |  |  |
| 0 | Ath-AT5G35560.1 |  |  |  |  |  |  |  |  |
| 0 | Ath-AT5G35570.1 |  |  |  |  |  |  |  |  |
| 0 | Ath-AT5G35580.2 |  |  |  |  |  |  |  |  |
| 0 | Ath-AT5G35590.1 |  |  |  |  |  |  |  |  |
| 0 | Ath-AT5G35600.1 |  |  |  |  |  |  |  |  |
| 0 | Ath-AT5G35603.2 |  |  |  |  |  |  |  |  |
| 0 | Ath-AT5G35604.1 |  |  |  |  |  |  |  |  |
| 0 | Ath-AT5G35610.1 |  |  |  |  |  |  |  |  |
| 0 | Ath-AT5G35620.3 |  |  |  |  |  |  |  |  |
| 0 | Ath-AT5G35630.1 |  |  |  |  |  |  |  |  |
| 0 | Ath-AT5G35640.1 |  |  |  |  |  |  |  |  |
| 0 | Ath-AT5G35660.1 |  |  |  |  |  |  |  |  |
| 0 | Ath-AT5G35670.1 |  |  |  |  |  |  |  |  |
| 0 | Ath-AT5G35680.3 |  |  |  |  |  |  |  |  |
| 0 | Ath-AT5G35688.2 |  |  |  |  |  |  |  |  |
| 0 | Ath-AT5G35690.1 |  |  |  |  |  |  |  |  |
| 0 | Ath-AT5G35695.1 |  |  |  |  |  |  |  |  |
| 0 | Ath-AT5G35698.1 |  |  |  |  |  |  |  |  |
| 0 | Ath-AT5G35700.1 |  |  |  |  |  |  |  |  |
| 0 | Ath-AT5G35715.1 |  |  |  |  |  |  |  |  |
| 0 | Ath-AT5G35730.1 |  |  |  |  |  |  |  |  |
| 0 | Ath-AT5G35732.2 |  |  |  |  |  |  |  |  |
| 0 | Ath-AT5G35735.1 |  |  |  |  |  |  |  |  |
| 0 | Ath-AT5G35737.1 |  |  |  |  |  |  |  |  |
| 0 | Ath-AT5G35740.1 |  |  |  |  |  |  |  |  |
| 0 | Ath-AT5G35750.1 |  |  |  |  |  |  |  |  |
| 0 | Ath-AT5G35753.1 |  |  |  |  |  |  |  |  |
| 0 | Ath-AT5G35760.1 |  |  |  |  |  |  |  |  |
| 0 | Ath-AT5G35770.1 |  |  |  |  |  |  |  |  |
| 0 | Ath-AT5G35790.1 |  |  |  |  |  |  |  |  |
| 0 | Ath-AT5G35810.1 |  |  |  |  |  |  |  |  |
| 0 | Ath-AT5G35830.1 |  |  |  |  |  |  |  |  |
| 0 | Ath-AT5G35840.1 |  |  |  |  |  |  |  |  |
| 0 | Ath-AT5G35870.1 |  |  |  |  |  |  |  |  |
| 0 | Ath-AT5G35890.1 |  |  |  |  |  |  |  |  |
| 0 | Ath-AT5G35900.1 |  |  |  |  |  |  |  |  |
| 0 | Ath-AT5G35910.1 |  |  |  |  |  |  |  |  |
| 0 | Ath-AT5G35913.1 |  |  |  |  |  |  |  |  |
| 0 | Ath-AT5G35917.1 |  |  |  |  |  |  |  |  |
| 0 | Ath-AT5G35920.1 |  |  |  |  |  |  |  |  |
| 0 | Ath-AT5G35926.2 |  |  |  |  |  |  |  |  |
| 0 | Ath-AT5G35927.1 |  |  |  |  |  |  |  |  |
| 0 | Ath-AT5G35930.4 |  |  |  |  |  |  |  |  |
| 0 | Ath-AT5G35940.1 |  |  |  |  |  |  |  |  |
| 0 | Ath-AT5G35945.1 |  |  |  |  |  |  |  |  |
| 0 | Ath-AT5G35950.1 |  |  |  |  |  |  |  |  |
| 0 | Ath-AT5G35960.1 |  |  |  |  |  |  |  |  |
| 0 | Ath-AT5G35970.1 |  |  |  |  |  |  |  |  |
| 0 | Ath-AT5G35980.1 |  |  |  |  |  |  |  |  |
| 0 | Ath-AT5G35995.1 |  |  |  |  |  |  |  |  |
| 0 | Ath-AT5G36000.1 |  |  |  |  |  |  |  |  |
| 0 | Ath-AT5G36001.1 |  |  |  |  |  |  |  |  |
| 0 | Ath-AT5G36080.1 |  |  |  |  |  |  |  |  |
| 0 | Ath-AT5G36100.1 |  |  |  |  |  |  |  |  |
| 0 | Ath-AT5G36110.1 |  |  |  |  |  |  |  |  |
| 0 | Ath-AT5G36120.1 |  |  |  |  |  |  |  |  |
| 0 | Ath-AT5G36130.1 |  |  |  |  |  |  |  |  |
| 0 | Ath-AT5G36140.1 |  |  |  |  |  |  |  |  |
| 0 | Ath-AT5G36150.1 |  |  |  |  |  |  |  |  |
| 0 | Ath-AT5G36160.1 |  |  |  |  |  |  |  |  |
| 0 | Ath-AT5G36170.1 |  |  |  |  |  |  |  |  |
| 0 | Ath-AT5G36180.1 |  |  |  |  |  |  |  |  |
| 0 | Ath-AT5G36190.1 |  |  |  |  |  |  |  |  |
| 0 | Ath-AT5G36200.1 |  |  |  |  |  |  |  |  |
| 0 | Ath-AT5G36210.1 |  |  |  |  |  |  |  |  |
| 0 | Ath-AT5G36220.1 |  |  |  |  |  |  |  |  |
| 0 | Ath-AT5G36225.1 |  |  |  |  |  |  |  |  |
| 0 | Ath-AT5G36228.1 |  |  |  |  |  |  |  |  |
| 0 | Ath-AT5G36230.2 |  |  |  |  |  |  |  |  |
| 0 | Ath-AT5G36240.1 |  |  |  |  |  |  |  |  |
| 0 | Ath-AT5G36250.2 |  |  |  |  |  |  |  |  |
| 0 | Ath-AT5G36260.1 |  |  |  |  |  |  |  |  |
| 0 | Ath-AT5G36280.1 |  |  |  |  |  |  |  |  |
| 0 | Ath-AT5G36290.2 |  |  |  |  |  |  |  |  |
| 0 | Ath-AT5G36300.2 |  |  |  |  |  |  |  |  |
| 0 | Ath-AT5G36310.1 |  |  |  |  |  |  |  |  |
| 0 | Ath-AT5G36320.1 |  |  |  |  |  |  |  |  |
| 0 | Ath-AT5G36330.1 |  |  |  |  |  |  |  |  |
| 0 | Ath-AT5G36340.1 |  |  |  |  |  |  |  |  |
| 0 | Ath-AT5G36350.1 |  |  |  |  |  |  |  |  |
| 0 | Ath-AT5G36360.1 |  |  |  |  |  |  |  |  |
| 0 | Ath-AT5G36370.1 |  |  |  |  |  |  |  |  |
| 0 | Ath-AT5G36380.1 |  |  |  |  |  |  |  |  |
| 0 | Ath-AT5G36390.1 |  |  |  |  |  |  |  |  |
| 0 | Ath-AT5G36400.1 |  |  |  |  |  |  |  |  |
| 0 | Ath-AT5G36410.1 |  |  |  |  |  |  |  |  |
| 0 | Ath-AT5G36420.1 |  |  |  |  |  |  |  |  |
| 0 | Ath-AT5G36430.1 |  |  |  |  |  |  |  |  |
| 0 | Ath-AT5G36440.1 |  |  |  |  |  |  |  |  |
| 0 | Ath-AT5G36450.1 |  |  |  |  |  |  |  |  |
| 0 | Ath-AT5G36460.1 |  |  |  |  |  |  |  |  |
| 0 | Ath-AT5G36470.1 |  |  |  |  |  |  |  |  |
| 0 | Ath-AT5G36480.1 |  |  |  |  |  |  |  |  |
| 0 | Ath-AT5G36490.1 |  |  |  |  |  |  |  |  |
| 0 | Ath-AT5G36500.1 |  |  |  |  |  |  |  |  |
| 0 | Ath-AT5G36520.1 |  |  |  |  |  |  |  |  |
| 0 | Ath-AT5G36540.1 |  |  |  |  |  |  |  |  |
| 0 | Ath-AT5G36550.1 |  |  |  |  |  |  |  |  |
| 0 | Ath-AT5G36657.1 |  |  |  |  |  |  |  |  |
| 0 | Ath-AT5G36658.1 |  |  |  |  |  |  |  |  |
| 0 | Ath-AT5G36659.1 |  |  |  |  |  |  |  |  |
| 0 | Ath-AT5G36661.1 |  |  |  |  |  |  |  |  |
| 0 | Ath-AT5G36662.1 |  |  |  |  |  |  |  |  |
| 0 | Ath-AT5G36670.1 |  |  |  |  |  |  |  |  |
| 0 | Ath-AT5G36680.1 |  |  |  |  |  |  |  |  |
| 0 | Ath-AT5G36690.1 |  |  |  |  |  |  |  |  |
| 0 | Ath-AT5G36700.4 |  |  |  |  |  |  |  |  |
| 0 | Ath-AT5G36710.2 |  |  |  |  |  |  |  |  |
| 0 | Ath-AT5G36720.1 |  |  |  |  |  |  |  |  |
| 0 | Ath-AT5G36722.1 |  |  |  |  |  |  |  |  |
| 0 | Ath-AT5G36730.1 |  |  |  |  |  |  |  |  |
| 0 | Ath-AT5G36738.1 |  |  |  |  |  |  |  |  |
| 0 | Ath-AT5G36739.1 |  |  |  |  |  |  |  |  |
| 0 | Ath-AT5G36740.1 |  |  |  |  |  |  |  |  |
| 0 | Ath-AT5G36770.1 |  |  |  |  |  |  |  |  |
| 0 | Ath-AT5G36780.1 |  |  |  |  |  |  |  |  |
| 0 | Ath-AT5G36790.2 |  |  |  |  |  |  |  |  |
| 0 | Ath-AT5G36800.2 |  |  |  |  |  |  |  |  |
| 0 | Ath-AT5G36805.1 |  |  |  |  |  |  |  |  |
| 0 | Ath-AT5G36810.1 |  |  |  |  |  |  |  |  |
| 0 | Ath-AT5G36820.1 |  |  |  |  |  |  |  |  |
| 0 | Ath-AT5G36870.1 |  |  |  |  |  |  |  |  |
| 0 | Ath-AT5G36880.2 |  |  |  |  |  |  |  |  |
| 0 | Ath-AT5G36890.1 |  |  |  |  |  |  |  |  |
| 0 | Ath-AT5G36900.1 |  |  |  |  |  |  |  |  |
| 0 | Ath-AT5G36907.1 |  |  |  |  |  |  |  |  |
| 0 | Ath-AT5G36910.1 |  |  |  |  |  |  |  |  |
| 0 | Ath-AT5G36920.1 |  |  |  |  |  |  |  |  |
| 0 | Ath-AT5G36925.1 |  |  |  |  |  |  |  |  |
| 0 | Ath-AT5G36930.2 |  |  |  |  |  |  |  |  |
| 0 | Ath-AT5G36940.1 |  |  |  |  |  |  |  |  |
| 0 | Ath-AT5G36950.1 |  |  |  |  |  |  |  |  |
| 0 | Ath-AT5G36960.1 |  |  |  |  |  |  |  |  |
| 0 | Ath-AT5G36970.1 |  |  |  |  |  |  |  |  |
| 0 | Ath-AT5G36980.1 |  |  |  |  |  |  |  |  |
| 0 | Ath-AT5G37000.1 |  |  |  |  |  |  |  |  |
| 0 | Ath-AT5G37010.1 |  |  |  |  |  |  |  |  |
| 0 | Ath-AT5G37020.1 |  |  |  |  |  |  |  |  |
| 0 | Ath-AT5G37030.1 |  |  |  |  |  |  |  |  |
| 0 | Ath-AT5G37040.1 |  |  |  |  |  |  |  |  |
| 0 | Ath-AT5G37050.1 |  |  |  |  |  |  |  |  |
| 0 | Ath-AT5G37055.1 |  |  |  |  |  |  |  |  |
| 0 | Ath-AT5G37060.1 |  |  |  |  |  |  |  |  |
| 0 | Ath-AT5G37070.1 |  |  |  |  |  |  |  |  |
| 0 | Ath-AT5G37130.1 |  |  |  |  |  |  |  |  |
| 0 | Ath-AT5G37140.1 |  |  |  |  |  |  |  |  |
| 0 | Ath-AT5G37150.1 |  |  |  |  |  |  |  |  |
| 0 | Ath-AT5G37160.1 |  |  |  |  |  |  |  |  |
| 0 | Ath-AT5G37165.1 |  |  |  |  |  |  |  |  |
| 0 | Ath-AT5G37170.1 |  |  |  |  |  |  |  |  |
| 0 | Ath-AT5G37180.1 |  |  |  |  |  |  |  |  |
| 0 | Ath-AT5G37190.1 |  |  |  |  |  |  |  |  |
| 0 | Ath-AT5G37200.1 |  |  |  |  |  |  |  |  |
| 0 | Ath-AT5G37210.1 |  |  |  |  |  |  |  |  |
| 0 | Ath-AT5G37220.1 |  |  |  |  |  |  |  |  |
| 0 | Ath-AT5G37230.1 |  |  |  |  |  |  |  |  |
| 0 | Ath-AT5G37240.1 |  |  |  |  |  |  |  |  |
| 0 | Ath-AT5G37247.1 |  |  |  |  |  |  |  |  |
| 0 | Ath-AT5G37250.2 |  |  |  |  |  |  |  |  |
| 0 | Ath-AT5G37260.1 |  |  |  |  |  |  |  |  |
| 0 | Ath-AT5G37270.1 |  |  |  |  |  |  |  |  |
| 0 | Ath-AT5G37280.1 |  |  |  |  |  |  |  |  |
| 0 | Ath-AT5G37290.1 |  |  |  |  |  |  |  |  |
| 0 | Ath-AT5G37300.4 |  |  |  |  |  |  |  |  |
| 0 | Ath-AT5G37310.1 |  |  |  |  |  |  |  |  |
| 0 | Ath-AT5G37320.1 |  |  |  |  |  |  |  |  |
| 0 | Ath-AT5G37340.2 |  |  |  |  |  |  |  |  |
| 0 | Ath-AT5G37350.1 |  |  |  |  |  |  |  |  |
| 0 | Ath-AT5G37360.1 |  |  |  |  |  |  |  |  |
| 0 | Ath-AT5G37370.1 |  |  |  |  |  |  |  |  |
| 0 | Ath-AT5G37380.3 |  |  |  |  |  |  |  |  |
| 0 | Ath-AT5G37400.2 |  |  |  |  |  |  |  |  |
| 0 | Ath-AT5G37410.2 |  |  |  |  |  |  |  |  |
| 0 | Ath-AT5G37415.1 |  |  |  |  |  |  |  |  |
| 0 | Ath-AT5G37420.1 |  |  |  |  |  |  |  |  |
| 0 | Ath-AT5G37430.1 |  |  |  |  |  |  |  |  |
| 0 | Ath-AT5G37440.1 |  |  |  |  |  |  |  |  |
| 0 | Ath-AT5G37450.2 |  |  |  |  |  |  |  |  |
| 0 | Ath-AT5G37460.1 |  |  |  |  |  |  |  |  |
| 0 | Ath-AT5G37470.2 |  |  |  |  |  |  |  |  |
| 0 | Ath-AT5G37475.2 |  |  |  |  |  |  |  |  |
| 0 | Ath-AT5G37473.1 |  |  |  |  |  |  |  |  |
| 0 | Ath-AT5G37474.1 |  |  |  |  |  |  |  |  |
| 0 | Ath-AT5G37478.1 |  |  |  |  |  |  |  |  |
| 0 | Ath-AT5G37480.1 |  |  |  |  |  |  |  |  |
| 0 | Ath-AT5G37490.1 |  |  |  |  |  |  |  |  |
| 0 | Ath-AT5G37500.2 |  |  |  |  |  |  |  |  |
| 0 | Ath-AT5G37510.2 |  |  |  |  |  |  |  |  |
| 0 | Ath-AT5G37520.1 |  |  |  |  |  |  |  |  |
| 0 | Ath-AT5G37530.1 |  |  |  |  |  |  |  |  |
| 0 | Ath-AT5G37540.1 |  |  |  |  |  |  |  |  |
| 0 | Ath-AT5G37550.1 |  |  |  |  |  |  |  |  |
| 0 | Ath-AT5G37560.1 |  |  |  |  |  |  |  |  |
| 0 | Ath-AT5G37570.1 |  |  |  |  |  |  |  |  |
| 0 | Ath-AT5G37590.1 |  |  |  |  |  |  |  |  |
| 0 | Ath-AT5G37600.1 |  |  |  |  |  |  |  |  |
| 0 | Ath-AT5G37610.1 |  |  |  |  |  |  |  |  |
| 0 | Ath-AT5G37620.1 |  |  |  |  |  |  |  |  |
| 0 | Ath-AT5G37630.1 |  |  |  |  |  |  |  |  |
| 0 | Ath-AT5G37640.1 |  |  |  |  |  |  |  |  |
| 0 | Ath-AT5G37650.1 |  |  |  |  |  |  |  |  |
| 0 | Ath-AT5G37660.2 |  |  |  |  |  |  |  |  |
| 0 | Ath-AT5G37670.1 |  |  |  |  |  |  |  |  |
| 1 | Ath-AT5G37680.1 |  | Vvi-Vitvi14g00901\_t001 |  |  |  |  |  |  |  |
| 1 | Ath-AT5G37690.1 |  | Vvi-Vitvi14g00902\_t001 |  |  |  |  |  |  |  |
| 1 | Ath-AT5G37710.1 |  | | | |  |  |  |  |  |  |  |
| 1 | Ath-AT5G37715.1 |  | | | |  |  |  |  |  |  |  |
| 1 | Ath-AT5G37720.1 |  | | | |  |  |  |  |  |  |  |
| 1 | Ath-AT5G37730.1 |  | Vvi-Vitvi14g00914\_t001 |  |  |  |  |  |  |  |
| 1 | Ath-AT5G37740.2 |  | Vvi-Vitvi14g00920\_t001 |  |  |  |  |  |  |  |
| 1 | Ath-AT5G37750.1 |  | | | |  |  |  |  |  |  |  |
| 1 | Ath-AT5G37760.1 |  | | | |  |  |  |  |  |  |  |
| 1 | Ath-AT5G37770.1 |  | Vvi-Vitvi14g00949\_t001 |  |  |  |  |  |  |  |
| 1 | Ath-AT5G37780.3 |  | | | |  |  |  |  |  |  |  |
| 1 | Ath-AT5G37790.1 |  | Vvi-Vitvi14g00952\_t001 |  |  |  |  |  |  |  |
| 1 | Ath-AT5G37793.1 |  | | | |  |  |  |  |  |  |  |
| 1 | Ath-AT5G37800.1 |  | Vvi-Vitvi14g00958\_t001 |  |  |  |  |  |  |  |
| 1 | Ath-AT5G37810.1 |  | Vvi-Vitvi14g00966\_t001 |  |  |  |  |  |  |  |
| 1 | Ath-AT5G37820.2 |  | | | |  |  |  |  |  |  |  |
| 1 | Ath-AT5G37830.1 |  | | | |  |  |  |  |  |  |  |
| 1 | Ath-AT5G37840.1 |  | Vvi-Vitvi14g00973\_t001 |  |  |  |  |  |  |  |
| 1 | Ath-AT5G37850.1 |  | Vvi-Vitvi14g00980\_t001 |  |  |  |  |  |  |  |
| 1 | Ath-AT5G37860.1 |  | | | |  |  |  |  |  |  |  |
| 1 | Ath-AT5G37870.1 |  | Vvi-Vitvi14g01003\_t002 |  |  |  |  |  |  |  |
| 1 | Ath-AT5G37890.1 |  | Vvi-Vitvi14g01005\_t001 |  |  |  |  |  |  |  |
| 1 | Ath-AT5G37900.1 |  | | | |  |  |  |  |  |  |  |
| 1 | Ath-AT5G37910.1 |  | | | |  |  |  |  |  |  |  |
| 1 | Ath-AT5G37920.1 |  | | | |  |  |  |  |  |  |  |
| 1 | Ath-AT5G37930.1 |  | | | |  |  |  |  |  |  |  |
| 1 | Ath-AT5G37940.1 |  | | | |  |  |  |  |  |  |  |
| 1 | Ath-AT5G37950.1 |  | | | |  |  |  |  |  |  |  |
| 1 | Ath-AT5G37960.1 |  | | | |  |  |  |  |  |  |  |
| 1 | Ath-AT5G37970.1 |  | | | |  |  |  |  |  |  |  |
| 1 | Ath-AT5G37980.1 |  | | | |  |  |  |  |  |  |  |
| 1 | Ath-AT5G37990.1 |  | | | |  |  |  |  |  |  |  |
| 1 | Ath-AT5G38000.1 |  | | | |  |  |  |  |  |  |  |
| 1 | Ath-AT5G38010.1 |  | | | |  |  |  |  |  |  |  |
| 1 | Ath-AT5G38020.1 |  | | | |  |  |  |  |  |  |  |
| 1 | Ath-AT5G38030.1 |  | | | |  |  |  |  |  |  |  |
| 1 | Ath-AT5G38040.1 |  | | | |  |  |  |  |  |  |  |
| 1 | Ath-AT5G38050.1 |  | Vvi-Vitvi14g01007\_t001 |  |  |  |  |  |  |  |
| 1 | Ath-AT5G38060.1 |  | Vvi-Vitvi14g01011\_t002 |  |  |  |  |  |  |  |
| 1 | Ath-AT5G38070.1 |  | Vvi-Vitvi14g01025\_t001 |  |  |  |  |  |  |  |
| 1 | Ath-AT5G38080.1 |  | | | |  |  |  |  |  |  |  |
| 1 | Ath-AT5G38090.1 |  | | | |  |  |  |  |  |  |  |
| 1 | Ath-AT5G38100.1 |  | Vvi-Vitvi14g01051\_t001 |  |  |  |  |  |  |  |
| 1 | Ath-AT5G38110.1 |  | Vvi-Vitvi14g01057\_t001 |  |  |  |  |  |  |  |
| 1 | Ath-AT5G38120.1 |  | | | |  |  |  |  |  |  |  |
| 1 | Ath-AT5G38130.1 |  | | | |  |  |  |  |  |  |  |
| 1 | Ath-AT5G38140.1 |  | | | |  |  |  |  |  |  |  |
| 1 | Ath-AT5G38150.1 |  | Vvi-Vitvi14g01124\_t001 |  |  |  |  |  |  |  |
| 1 | Ath-AT5G38160.1 |  | | | |  |  |  |  |  |  |  |
| 1 | Ath-AT5G38170.1 |  | Vvi-Vitvi14g02869\_t001 |  |  |  |  |  |  |  |
| 1 | Ath-AT5G38180.1 |  | | | |  |  |  |  |  |  |  |
| 1 | Ath-AT5G38190.1 |  | | | |  |  |  |  |  |  |  |
| 1 | Ath-AT5G38195.1 |  | | | |  |  |  |  |  |  |  |
| 1 | Ath-AT5G38197.1 |  | | | |  |  |  |  |  |  |  |
| 1 | Ath-AT5G38200.1 |  | Vvi-Vitvi14g04419\_t001 |  |  |  |  |  |  |  |
| 1 | Ath-AT5G38210.2 |  | | | |  |  |  |  |  |  |  |
| 1 | Ath-AT5G38220.1 |  | Vvi-Vitvi14g01144\_t001 |  |  |  |  |  |  |  |
| 1 | Ath-AT5G38240.1 |  | | | |  |  |  |  |  |  |  |
| 1 | Ath-AT5G38250.1 |  | | | |  |  |  |  |  |  |  |
| 1 | Ath-AT5G38260.1 |  | | | |  |  |  |  |  |  |  |
| 1 | Ath-AT5G38270.1 |  | | | |  |  |  |  |  |  |  |
| 1 | Ath-AT5G38280.2 |  | | | |  |  |  |  |  |  |  |
| 1 | Ath-AT5G38290.2 |  | Vvi-Vitvi14g01160\_t001 |  |  |  |  |  |  |  |
| 1 | Ath-AT5G38300.1 |  | Vvi-Vitvi14g01161\_t001 |  |  |  |  |  |  |  |
| 1 | Ath-AT5G38310.1 |  | | | |  |  |  |  |  |  |  |
| 1 | Ath-AT5G38317.1 |  | | | |  |  |  |  |  |  |  |
| 1 | Ath-AT5G38320.1 |  | | | |  |  |  |  |  |  |  |
| 1 | Ath-AT5G38330.1 |  | | | |  |  |  |  |  |  |  |
| 1 | Ath-AT5G38340.1 |  | | | |  |  |  |  |  |  |  |
| 1 | Ath-AT5G38344.1 |  | | | |  |  |  |  |  |  |  |
| 1 | Ath-AT5G38350.1 |  | | | |  |  |  |  |  |  |  |
| 1 | Ath-AT5G38360.1 |  | Vvi-Vitvi14g01180\_t001 |  |  |  |  |  |  |  |
| 1 | Ath-AT5G38378.1 |  | | | |  |  |  |  |  |  |  |
| 1 | Ath-AT5G38380.1 |  | Vvi-Vitvi14g02875\_t001 |  |  |  |  |  |  |  |
| 1 | Ath-AT5G38386.2 |  | | | |  |  |  |  |  |  |  |
| 1 | Ath-AT5G38390.1 |  | | | |  |  |  |  |  |  |  |
| 1 | Ath-AT5G38391.1 |  | | | |  |  |  |  |  |  |  |
| 1 | Ath-AT5G38392.1 |  | | | |  |  |  |  |  |  |  |
| 1 | Ath-AT5G38396.1 |  | | | |  |  |  |  |  |  |  |
| 1 | Ath-AT5G38397.1 |  | | | |  |  |  |  |  |  |  |
| 1 | Ath-AT5G38400.1 |  | | | |  |  |  |  |  |  |  |
| 1 | Ath-AT5G38410.3 |  | | | |  |  |  |  |  |  |  |
| 1 | Ath-AT5G38420.1 |  | | | |  |  |  |  |  |  |  |
| 1 | Ath-AT5G38430.1 |  | | | |  |  |  |  |  |  |  |
| 1 | Ath-AT5G38435.1 |  | | | |  |  |  |  |  |  |  |
| 1 | Ath-AT5G38440.1 |  | | | |  |  |  |  |  |  |  |
| 1 | Ath-AT5G38450.1 |  | Vvi-Vitvi14g01195\_t001 |  |  |  |  |  |  |  |
| 1 | Ath-AT5G38460.2 |  | Vvi-Vitvi14g01201\_t001 |  |  |  |  |  |  |  |
| 1 | Ath-AT5G38470.1 |  | Vvi-Vitvi14g01208\_t001 |  |  |  |  |  |  |  |
| 1 | Ath-AT5G38480.1 |  | Vvi-Vitvi14g01226\_t001 |  |  |  |  |  |  |  |
| 1 | Ath-AT5G38490.1 |  | | | |  |  |  |  |  |  |  |
| 1 | Ath-AT5G38500.1 |  | | | |  |  |  |  |  |  |  |
| 1 | Ath-AT5G38510.2 |  | Vvi-Vitvi14g01228\_t001 |  |  |  |  |  |  |  |
| 1 | Ath-AT5G38520.2 |  | | | |  |  |  |  |  |  |  |
| 2 | Ath-AT5G38530.1 |  | | | |  | Vvi-Vitvi14g01281\_t001 |  |  |  |  |  |  |
| 2 | Ath-AT5G38540.1 |  | | | |  | | | |  |  |  |  |  |  |
| 2 | Ath-AT5G38550.1 |  | | | |  | | | |  |  |  |  |  |  |
| 3 | Ath-AT5G38560.1 |  | | | |  | | | |  | Vvi-Vitvi14g01265\_t001 |  |  |  |  |  |
| 3 | Ath-AT5G38565.1 |  | | | |  | | | |  | | | |  |  |  |  |  |
| 3 | Ath-AT5G38570.1 |  | | | |  | | | |  | | | |  |  |  |  |  |
| 3 | Ath-AT5G38580.1 |  | | | |  | | | |  | | | |  |  |  |  |  |
| 3 | Ath-AT5G38590.2 |  | | | |  | | | |  | | | |  |  |  |  |  |
| 3 | Ath-AT5G38600.1 |  | | | |  | | | |  | Vvi-Vitvi14g01259\_t001 |  |  |  |  |  |
| 3 | Ath-AT5G38610.1 |  | | | |  | | | |  | | | |  |  |  |  |  |
| 3 | Ath-AT5G38620.1 |  | | | |  | | | |  | | | |  |  |  |  |  |
| 3 | Ath-AT5G38630.1 |  | | | |  | | | |  | Vvi-Vitvi14g01256\_t001 |  |  |  |  |  |
| 3 | Ath-AT5G38640.1 |  | | | |  | | | |  | Vvi-Vitvi14g01255\_t001 |  |  |  |  |  |
| 3 | Ath-AT5G38660.2 |  | Vvi-Vitvi14g01254\_t001 |  | | | |  | Vvi-Vitvi14g01239\_t002 |  |  |  |  |  |
| 3 | Ath-AT5G38650.1 |  | | | |  | | | |  | | | |  |  |  |  |  |
| 3 | Ath-AT5G38670.1 |  | | | |  | | | |  | | | |  |  |  |  |  |
| 3 | Ath-AT5G38680.1 |  | | | |  | | | |  | | | |  |  |  |  |  |
| 3 | Ath-AT5G38690.1 |  | Vvi-Vitvi14g01258\_t001 |  | | | |  | Vvi-Vitvi14g01238\_t001 |  |  |  |  |  |
| 1 | Ath-AT5G38700.1 |  |  |  | | | |  |  |  |  |  |  |
| 1 | Ath-AT5G38710.1 |  |  |  | Vvi-Vitvi14g01283\_t001 |  |  |  |  |  |  |
| 1 | Ath-AT5G38720.2 |  |  |  | | | |  |  |  |  |  |  |
| 1 | Ath-AT5G38730.1 |  |  |  | Vvi-Vitvi14g01288\_t001 |  |  |  |  |  |  |
| 1 | Ath-AT5G38740.1 |  |  |  | | | |  |  |  |  |  |  |
| 1 | Ath-AT5G38743.1 |  |  |  | | | |  |  |  |  |  |  |
| 1 | Ath-AT5G38747.1 |  |  |  | | | |  |  |  |  |  |  |
| 1 | Ath-AT5G38750.1 |  |  |  | | | |  |  |  |  |  |  |
| 1 | Ath-AT5G38760.1 |  |  |  | Vvi-Vitvi14g01291\_t001 |  |  |  |  |  |  |
| 1 | Ath-AT5G38770.1 |  |  |  | | | |  |  |  |  |  |  |
| 1 | Ath-AT5G38780.1 |  |  |  | | | |  |  |  |  |  |  |
| 1 | Ath-AT5G38790.1 |  |  |  | | | |  |  |  |  |  |  |
| 1 | Ath-AT5G38800.1 |  |  |  | Vvi-Vitvi14g01302\_t001 |  |  |  |  |  |  |
| 1 | Ath-AT5G38810.2 |  |  |  | | | |  |  |  |  |  |  |
| 1 | Ath-AT5G38820.2 |  |  |  | Vvi-Vitvi14g01307\_t001 |  |  |  |  |  |  |
| 1 | Ath-AT5G38830.1 |  |  |  | | | |  |  |  |  |  |  |
| 1 | Ath-AT5G38840.1 |  |  |  | Vvi-Vitvi14g01310\_t001 |  |  |  |  |  |  |
| 1 | Ath-AT5G38850.1 |  |  |  | | | |  |  |  |  |  |  |
| 1 | Ath-AT5G38860.1 |  |  |  | Vvi-Vitvi14g01326\_t001 |  |  |  |  |  |  |
| 1 | Ath-AT5G38865.1 |  |  |  | | | |  |  |  |  |  |  |
| 1 | Ath-AT5G38880.1 |  |  |  | Vvi-Vitvi14g01337\_t001 |  |  |  |  |  |  |
| 1 | Ath-AT5G38890.1 |  |  |  | | | |  |  |  |  |  |  |
| 1 | Ath-AT5G38895.3 |  |  |  | Vvi-Vitvi14g01338\_t001 |  |  |  |  |  |  |
| 1 | Ath-AT5G38900.2 |  |  |  | Vvi-Vitvi14g01339\_t001 |  |  |  |  |  |  |
| 1 | Ath-AT5G38910.1 |  |  |  | | | |  |  |  |  |  |  |
| 1 | Ath-AT5G38920.1 |  |  |  | | | |  |  |  |  |  |  |
| 1 | Ath-AT5G38930.1 |  |  |  | | | |  |  |  |  |  |  |
| 1 | Ath-AT5G38940.2 |  |  |  | | | |  |  |  |  |  |  |
| 1 | Ath-AT5G38950.1 |  |  |  | | | |  |  |  |  |  |  |
| 1 | Ath-AT5G38960.1 |  |  |  | | | |  |  |  |  |  |  |
| 1 | Ath-AT5G38970.1 |  |  |  | Vvi-Vitvi14g01351\_t001 |  |  |  |  |  |  |
| 1 | Ath-AT5G38980.1 |  |  |  | | | |  |  |  |  |  |  |
| 2 | Ath-AT5G38990.1 |  | Vvi-Vitvi14g01368\_t001 |  | | | |  |  |  |  |  |  |
| 2 | Ath-AT5G39000.1 |  | | | |  | | | |  |  |  |  |  |  |
| 2 | Ath-AT5G39010.1 |  | | | |  | | | |  |  |  |  |  |  |
| 2 | Ath-AT5G39020.1 |  | Vvi-Vitvi14g01379\_t001 |  | | | |  |  |  |  |  |  |
| 2 | Ath-AT5G39024.1 |  | | | |  | | | |  |  |  |  |  |  |
| 2 | Ath-AT5G39030.1 |  | | | |  | | | |  |  |  |  |  |  |
| 2 | Ath-AT5G39040.1 |  | Vvi-Vitvi14g02934\_t001 |  | | | |  |  |  |  |  |  |
| 2 | Ath-AT5G39050.1 |  | | | |  | | | |  |  |  |  |  |  |
| 2 | Ath-AT5G39080.1 |  | | | |  | Vvi-Vitvi13g02515\_t001 |  |  |  |  |  |  |
| 1 | Ath-AT5G39090.1 |  | | | |  |  |  |  |  |  |  |
| 1 | Ath-AT5G39110.1 |  | | | |  |  |  |  |  |  |  |
| 1 | Ath-AT5G39120.1 |  | | | |  |  |  |  |  |  |  |
| 1 | Ath-AT5G39130.1 |  | | | |  |  |  |  |  |  |  |
| 1 | Ath-AT5G39140.1 |  | | | |  |  |  |  |  |  |  |
| 1 | Ath-AT5G39150.1 |  | | | |  |  |  |  |  |  |  |
| 1 | Ath-AT5G39160.1 |  | | | |  |  |  |  |  |  |  |
| 1 | Ath-AT5G39170.1 |  | | | |  |  |  |  |  |  |  |
| 1 | Ath-AT5G39180.1 |  | | | |  |  |  |  |  |  |  |
| 1 | Ath-AT5G39190.1 |  | | | |  |  |  |  |  |  |  |
| 1 | Ath-AT5G39200.1 |  | Vvi-Vitvi14g02938\_t001 |  |  |  |  |  |  |  |
| 1 | Ath-AT5G39210.1 |  | | | |  |  |  |  |  |  |  |
| 1 | Ath-AT5G39220.1 |  | Vvi-Vitvi14g01404\_t001 |  |  |  |  |  |  |  |
| 1 | Ath-AT5G39230.1 |  | | | |  |  |  |  |  |  |  |
| 1 | Ath-AT5G39240.1 |  | Vvi-Vitvi14g02946\_t001 |  |  |  |  |  |  |  |
| 1 | Ath-AT5G39250.1 |  | Vvi-Vitvi14g01413\_t001 |  |  |  |  |  |  |  |
| 1 | Ath-AT5G39260.1 |  | | | |  |  |  |  |  |  |  |
| 1 | Ath-AT5G39270.1 |  | Vvi-Vitvi14g01415\_t001 |  |  |  |  |  |  |  |
| 0 | Ath-AT5G39280.1 |  |  |  |  |  |  |  |  |
| 0 | Ath-AT5G39290.1 |  |  |  |  |  |  |  |  |
| 0 | Ath-AT5G39300.1 |  |  |  |  |  |  |  |  |
| 0 | Ath-AT5G39310.1 |  |  |  |  |  |  |  |  |
| 1 | Ath-AT5G39320.1 |  | Vvi-Vitvi14g03099\_t002 |  |  |  |  |  |  |  |
| 1 | Ath-AT5G39330.1 |  | | | |  |  |  |  |  |  |  |
| 1 | Ath-AT5G39340.2 |  | | | |  |  |  |  |  |  |  |
| 1 | Ath-AT5G39350.1 |  | Vvi-Vitvi14g02029\_t001 |  |  |  |  |  |  |  |
| 1 | Ath-AT5G39360.1 |  | Vvi-Vitvi14g02023\_t003 |  |  |  |  |  |  |  |
| 1 | Ath-AT5G39365.1 |  | | | |  |  |  |  |  |  |  |
| 1 | Ath-AT5G39370.1 |  | | | |  |  |  |  |  |  |  |
| 1 | Ath-AT5G39380.5 |  | Vvi-Vitvi14g02022\_t001 |  |  |  |  |  |  |  |
| 1 | Ath-AT5G39390.1 |  | | | |  |  |  |  |  |  |  |
| 1 | Ath-AT5G39400.1 |  | Vvi-Vitvi14g04693\_t001 |  |  |  |  |  |  |  |
| 1 | Ath-AT5G39410.1 |  | Vvi-Vitvi14g02015\_t001 |  |  |  |  |  |  |  |
| 1 | Ath-AT5G39420.1 |  | Vvi-Vitvi14g02006\_t001 |  |  |  |  |  |  |  |
| 1 | Ath-AT5G39430.1 |  | Vvi-Vitvi14g02005\_t001 |  |  |  |  |  |  |  |
| 1 | Ath-AT5G39440.1 |  | Vvi-Vitvi14g02002\_t003 |  |  |  |  |  |  |  |
| 1 | Ath-AT5G39450.1 |  | | | |  |  |  |  |  |  |  |
| 1 | Ath-AT5G39460.1 |  | | | |  |  |  |  |  |  |  |
| 1 | Ath-AT5G39470.1 |  | | | |  |  |  |  |  |  |  |
| 1 | Ath-AT5G39471.1 |  | | | |  |  |  |  |  |  |  |
| 1 | Ath-AT5G39480.1 |  | | | |  |  |  |  |  |  |  |
| 1 | Ath-AT5G39490.1 |  | | | |  |  |  |  |  |  |  |
| 1 | Ath-AT5G39493.1 |  | | | |  |  |  |  |  |  |  |
| 1 | Ath-AT5G39500.1 |  | | | |  |  |  |  |  |  |  |
| 1 | Ath-AT5G39510.1 |  | Vvi-Vitvi14g01999\_t001 |  |  |  |  |  |  |  |
| 1 | Ath-AT5G39520.1 |  | Vvi-Vitvi14g01996\_t001 |  |  |  |  |  |  |  |
| 1 | Ath-AT5G39530.1 |  | | | |  |  |  |  |  |  |  |
| 1 | Ath-AT5G39540.1 |  | | | |  |  |  |  |  |  |  |
| 1 | Ath-AT5G39550.1 |  | | | |  |  |  |  |  |  |  |
| 1 | Ath-AT5G39560.1 |  | | | |  |  |  |  |  |  |  |
| 1 | Ath-AT5G39570.2 |  | | | |  |  |  |  |  |  |  |
| 1 | Ath-AT5G39580.1 |  | | | |  |  |  |  |  |  |  |
| 1 | Ath-AT5G39590.1 |  | | | |  |  |  |  |  |  |  |
| 1 | Ath-AT5G39600.1 |  | | | |  |  |  |  |  |  |  |
| 1 | Ath-AT5G39610.1 |  | Vvi-Vitvi14g01985\_t001 |  |  |  |  |  |  |  |
| 1 | Ath-AT5G39620.1 |  | | | |  |  |  |  |  |  |  |
| 1 | Ath-AT5G39630.1 |  | | | |  |  |  |  |  |  |  |
| 1 | Ath-AT5G39640.1 |  | | | |  |  |  |  |  |  |  |
| 1 | Ath-AT5G39645.1 |  | | | |  |  |  |  |  |  |  |
| 1 | Ath-AT5G39650.1 |  | | | |  |  |  |  |  |  |  |
| 1 | Ath-AT5G39660.1 |  | | | |  |  |  |  |  |  |  |
| 1 | Ath-AT5G39670.1 |  | Vvi-Vitvi14g01975\_t001 |  |  |  |  |  |  |  |
| 1 | Ath-AT5G39680.1 |  | | | |  |  |  |  |  |  |  |
| 1 | Ath-AT5G39690.1 |  | | | |  |  |  |  |  |  |  |
| 1 | Ath-AT5G39700.1 |  | | | |  |  |  |  |  |  |  |
| 1 | Ath-AT5G39710.1 |  | | | |  |  |  |  |  |  |  |
| 1 | Ath-AT5G39715.1 |  | | | |  |  |  |  |  |  |  |
| 1 | Ath-AT5G39720.1 |  | | | |  |  |  |  |  |  |  |
| 1 | Ath-AT5G39730.1 |  | | | |  |  |  |  |  |  |  |
| 1 | Ath-AT5G39740.1 |  | | | |  |  |  |  |  |  |  |
| 1 | Ath-AT5G39750.1 |  | | | |  |  |  |  |  |  |  |
| 1 | Ath-AT5G39760.1 |  | | | |  |  |  |  |  |  |  |
| 1 | Ath-AT5G39770.1 |  | | | |  |  |  |  |  |  |  |
| 1 | Ath-AT5G39775.1 |  | | | |  |  |  |  |  |  |  |
| 1 | Ath-AT5G39785.4 |  | Vvi-Vitvi14g01967\_t001 |  |  |  |  |  |  |  |
| 1 | Ath-AT5G39790.3 |  | | | |  |  |  |  |  |  |  |
| 1 | Ath-AT5G39800.1 |  | | | |  |  |  |  |  |  |  |
| 1 | Ath-AT5G39810.1 |  | | | |  |  |  |  |  |  |  |
| 1 | Ath-AT5G39820.1 |  | Vvi-Vitvi14g01963\_t001 |  |  |  |  |  |  |  |
| 1 | Ath-AT5G39830.1 |  | Vvi-Vitvi14g01941\_t002 |  |  |  |  |  |  |  |
| 1 | Ath-AT5G39840.1 |  | Vvi-Vitvi14g01940\_t001 |  |  |  |  |  |  |  |
| 1 | Ath-AT5G39850.1 |  | Vvi-Vitvi14g04680\_t001 |  |  |  |  |  |  |  |
| 1 | Ath-AT5G39860.1 |  | Vvi-Vitvi14g01926\_t001 |  |  |  |  |  |  |  |
| 1 | Ath-AT5G39865.1 |  | Vvi-Vitvi14g01923\_t001 |  |  |  |  |  |  |  |
| 0 | Ath-AT5G39870.2 |  |  |  |  |  |  |  |  |
| 0 | Ath-AT5G39880.1 |  |  |  |  |  |  |  |  |
| 1 | Ath-AT5G39890.1 |  | Vvi-Vitvi14g01906\_t001 |  |  |  |  |  |  |  |
| 1 | Ath-AT5G39900.1 |  | | | |  |  |  |  |  |  |  |
| 1 | Ath-AT5G39910.1 |  | | | |  |  |  |  |  |  |  |
| 1 | Ath-AT5G39920.1 |  | | | |  |  |  |  |  |  |  |
| 1 | Ath-AT5G39930.1 |  | Vvi-Vitvi14g01896\_t001 |  |  |  |  |  |  |  |
| 1 | Ath-AT5G39940.1 |  | | | |  |  |  |  |  |  |  |
| 1 | Ath-AT5G39950.1 |  | Vvi-Vitvi14g01889\_t001.1.6037826b |  |  |  |  |  |  |  |
| 1 | Ath-AT5G39960.1 |  | | | |  |  |  |  |  |  |  |
| 1 | Ath-AT5G39970.1 |  | | | |  |  |  |  |  |  |  |
| 1 | Ath-AT5G39980.1 |  | Vvi-Vitvi14g01877\_t001 |  |  |  |  |  |  |  |
| 1 | Ath-AT5G39990.1 |  | Vvi-Vitvi14g03050\_t001 |  |  |  |  |  |  |  |
| 1 | Ath-AT5G40000.1 |  | | | |  |  |  |  |  |  |  |
| 2 | Ath-AT5G40010.1 |  | Vvi-Vitvi14g03049\_t001 |  | Vvi-Vitvi14g01835\_t001 |  |  |  |  |  |  |
| 1 | Ath-AT5G40020.1 |  |  |  | | | |  |  |  |  |  |  |
| 1 | Ath-AT5G40030.1 |  |  |  | | | |  |  |  |  |  |  |
| 1 | Ath-AT5G40040.1 |  |  |  | | | |  |  |  |  |  |  |
| 1 | Ath-AT5G40050.1 |  |  |  | | | |  |  |  |  |  |  |
| 1 | Ath-AT5G40060.1 |  |  |  | | | |  |  |  |  |  |  |
| 1 | Ath-AT5G40070.1 |  |  |  | | | |  |  |  |  |  |  |
| 1 | Ath-AT5G40080.1 |  |  |  | | | |  |  |  |  |  |  |
| 1 | Ath-AT5G40090.1 |  |  |  | | | |  |  |  |  |  |  |
| 1 | Ath-AT5G40100.1 |  |  |  | | | |  |  |  |  |  |  |
| 1 | Ath-AT5G40120.1 |  |  |  | | | |  |  |  |  |  |  |
| 2 | Ath-AT5G40140.1 |  | Vvi-Vitvi17g00743\_t001 |  | Vvi-Vitvi14g01823\_t001 |  |  |  |  |  |  |
| 2 | Ath-AT5G40150.1 |  | Vvi-Vitvi17g00747\_t001 |  | Vvi-Vitvi14g01821\_t001 |  |  |  |  |  |  |
| 2 | Ath-AT5G40153.1 |  | | | |  | | | |  |  |  |  |  |  |
| 2 | Ath-AT5G40155.1 |  | | | |  | | | |  |  |  |  |  |  |
| 2 | Ath-AT5G40160.1 |  | | | |  | Vvi-Vitvi14g01817\_t001 |  |  |  |  |  |  |
| 2 | Ath-AT5G40170.1 |  | | | |  | | | |  |  |  |  |  |  |
| 2 | Ath-AT5G40180.1 |  | | | |  | | | |  |  |  |  |  |  |
| 2 | Ath-AT5G40190.1 |  | | | |  | Vvi-Vitvi14g04639\_t001 |  |  |  |  |  |  |
| 2 | Ath-AT5G40200.1 |  | Vvi-Vitvi17g00764\_t001 |  | Vvi-Vitvi14g01807\_t001 |  |  |  |  |  |  |
| 2 | Ath-AT5G40210.1 |  | Vvi-Vitvi17g00765\_t001 |  | Vvi-Vitvi14g01805\_t001 |  |  |  |  |  |  |
| 2 | Ath-AT5G40220.1 |  | | | |  | | | |  |  |  |  |  |  |
| 2 | Ath-AT5G40230.1 |  | | | |  | | | |  |  |  |  |  |  |
| 2 | Ath-AT5G40240.2 |  | | | |  | | | |  |  |  |  |  |  |
| 2 | Ath-AT5G40250.1 |  | Vvi-Vitvi17g00775\_t001 |  | Vvi-Vitvi14g01801\_t001 |  |  |  |  |  |  |
| 2 | Ath-AT5G40260.1 |  | Vvi-Vitvi17g00791\_t001 |  | Vvi-Vitvi14g01783\_t001 |  |  |  |  |  |  |
| 2 | Ath-AT5G40270.1 |  | | | |  | Vvi-Vitvi14g01780\_t001 |  |  |  |  |  |  |
| 2 | Ath-AT5G40280.2 |  | | | |  | Vvi-Vitvi14g01772\_t001 |  |  |  |  |  |  |
| 2 | Ath-AT5G40290.1 |  | | | |  | | | |  |  |  |  |  |  |
| 2 | Ath-AT5G40300.1 |  | Vvi-Vitvi17g00803\_t001 |  | Vvi-Vitvi14g01771\_t001 |  |  |  |  |  |  |
| 2 | Ath-AT5G40310.2 |  | | | |  | Vvi-Vitvi14g01770\_t001 |  |  |  |  |  |  |
| 2 | Ath-AT5G40315.1 |  | | | |  | | | |  |  |  |  |  |  |
| 2 | Ath-AT5G40320.1 |  | | | |  | | | |  |  |  |  |  |  |
| 2 | Ath-AT5G40330.1 |  | Vvi-Vitvi17g00822\_t001 |  | Vvi-Vitvi14g03020\_t001 |  |  |  |  |  |  |
| 2 | Ath-AT5G40340.1 |  | Vvi-Vitvi17g00825\_t001 |  | Vvi-Vitvi14g01756\_t001 |  |  |  |  |  |  |
| 2 | Ath-AT5G40350.1 |  | | | |  | Vvi-Vitvi14g01750\_t001 |  |  |  |  |  |  |
| 2 | Ath-AT5G40360.1 |  | Vvi-Vitvi17g00832\_t001 |  | Vvi-Vitvi14g01740\_t001 |  |  |  |  |  |  |
| 2 | Ath-AT5G40370.2 |  | Vvi-Vitvi17g00844\_t001 |  | Vvi-Vitvi14g01734\_t001 |  |  |  |  |  |  |
| 2 | Ath-AT5G40380.1 |  | Vvi-Vitvi17g00863\_t001 |  | Vvi-Vitvi14g01727\_t001 |  |  |  |  |  |  |
| 2 | Ath-AT5G40382.2 |  | | | |  | Vvi-Vitvi14g01726\_t001 |  |  |  |  |  |  |
| 2 | Ath-AT5G40390.1 |  | Vvi-Vitvi17g00885\_t001 |  | Vvi-Vitvi14g01717\_t001 |  |  |  |  |  |  |
| 2 | Ath-AT5G40400.2 |  | | | |  | | | |  |  |  |  |  |  |
| 2 | Ath-AT5G40405.1 |  | | | |  | | | |  |  |  |  |  |  |
| 2 | Ath-AT5G40410.1 |  | | | |  | | | |  |  |  |  |  |  |
| 2 | Ath-AT5G40420.1 |  | Vvi-Vitvi17g01554\_t001 |  | Vvi-Vitvi14g03008\_t001 |  |  |  |  |  |  |
| 1 | Ath-AT5G40430.1 |  |  |  | | | |  |  |  |  |  |  |
| 1 | Ath-AT5G40440.1 |  |  |  | Vvi-Vitvi14g01706\_t001 |  |  |  |  |  |  |
| 1 | Ath-AT5G40450.2 |  |  |  | Vvi-Vitvi14g01701\_t001 |  |  |  |  |  |  |
| 1 | Ath-AT5G40460.1 |  |  |  | Vvi-Vitvi14g03003\_t001 |  |  |  |  |  |  |
| 1 | Ath-AT5G40470.1 |  |  |  | Vvi-Vitvi14g01700\_t001 |  |  |  |  |  |  |
| 1 | Ath-AT5G40480.1 |  |  |  | Vvi-Vitvi14g01695\_t001 |  |  |  |  |  |  |
| 1 | Ath-AT5G40490.1 |  |  |  | Vvi-Vitvi14g01694\_t001 |  |  |  |  |  |  |
| 1 | Ath-AT5G40500.1 |  |  |  | Vvi-Vitvi14g03001\_t001 |  |  |  |  |  |  |
| 1 | Ath-AT5G40510.2 |  |  |  | Vvi-Vitvi14g01692\_t001 |  |  |  |  |  |  |
| 1 | Ath-AT5G40520.2 |  |  |  | | | |  |  |  |  |  |  |
| 1 | Ath-AT5G40530.3 |  |  |  | Vvi-Vitvi14g01682\_t001 |  |  |  |  |  |  |
| 1 | Ath-AT5G40540.1 |  |  |  | Vvi-Vitvi14g01679\_t001 |  |  |  |  |  |  |
| 1 | Ath-AT5G40550.3 |  |  |  | | | |  |  |  |  |  |  |
| 1 | Ath-AT5G40560.1 |  |  |  | | | |  |  |  |  |  |  |
| 1 | Ath-AT5G40570.2 |  |  |  | Vvi-Vitvi14g01666\_t001 |  |  |  |  |  |  |
| 1 | Ath-AT5G40580.1 |  |  |  | | | |  |  |  |  |  |  |
| 1 | Ath-AT5G40590.1 |  |  |  | | | |  |  |  |  |  |  |
| 1 | Ath-AT5G40600.1 |  |  |  | Vvi-Vitvi14g02998\_t001 |  |  |  |  |  |  |
| 1 | Ath-AT5G40595.1 |  |  |  | | | |  |  |  |  |  |  |
| 1 | Ath-AT5G40610.1 |  |  |  | Vvi-Vitvi14g01639\_t001 |  |  |  |  |  |  |
| 1 | Ath-AT5G40620.1 |  |  |  | | | |  |  |  |  |  |  |
| 1 | Ath-AT5G40630.1 |  |  |  | Vvi-Vitvi14g01633\_t001 |  |  |  |  |  |  |
| 1 | Ath-AT5G40640.1 |  |  |  | Vvi-Vitvi14g01631\_t001 |  |  |  |  |  |  |
| 1 | Ath-AT5G40645.1 |  |  |  | Vvi-Vitvi14g01628\_t001 |  |  |  |  |  |  |
| 1 | Ath-AT5G40650.1 |  |  |  | Vvi-Vitvi14g01627\_t001 |  |  |  |  |  |  |
| 1 | Ath-AT5G40660.1 |  |  |  | Vvi-Vitvi14g01626\_t001 |  |  |  |  |  |  |
| 1 | Ath-AT5G40670.1 |  |  |  | Vvi-Vitvi14g01624\_t003 |  |  |  |  |  |  |
| 1 | Ath-AT5G40680.1 |  |  |  | | | |  |  |  |  |  |  |
| 1 | Ath-AT5G40690.1 |  |  |  | Vvi-Vitvi14g01623\_t001 |  |  |  |  |  |  |
| 1 | Ath-AT5G40700.2 |  |  |  | Vvi-Vitvi14g01621\_t001 |  |  |  |  |  |  |
| 1 | Ath-AT5G40710.1 |  |  |  | Vvi-Vitvi14g01617\_t001 |  |  |  |  |  |  |
| 1 | Ath-AT5G40720.2 |  |  |  | Vvi-Vitvi14g01614\_t001 |  |  |  |  |  |  |
| 1 | Ath-AT5G40730.1 |  |  |  | | | |  |  |  |  |  |  |
| 1 | Ath-AT5G40740.1 |  |  |  | Vvi-Vitvi14g01607\_t002 |  |  |  |  |  |  |
| 1 | Ath-AT5G40750.1 |  |  |  | | | |  |  |  |  |  |  |
| 1 | Ath-AT5G40760.1 |  |  |  | Vvi-Vitvi14g01605\_t005 |  |  |  |  |  |  |
| 1 | Ath-AT5G40770.1 |  |  |  | Vvi-Vitvi14g02991\_t001 |  |  |  |  |  |  |
| 1 | Ath-AT5G40780.1 |  |  |  | Vvi-Vitvi14g01596\_t001 |  |  |  |  |  |  |
| 1 | Ath-AT5G40790.1 |  |  |  | | | |  |  |  |  |  |  |
| 1 | Ath-AT5G40800.1 |  |  |  | Vvi-Vitvi14g02990\_t001 |  |  |  |  |  |  |
| 1 | Ath-AT5G40810.1 |  |  |  | Vvi-Vitvi14g04596\_t001 |  |  |  |  |  |  |
| 1 | Ath-AT5G40820.2 |  |  |  | | | |  |  |  |  |  |  |
| 1 | Ath-AT5G40830.1 |  |  |  | | | |  |  |  |  |  |  |
| 1 | Ath-AT5G40840.2 |  |  |  | Vvi-Vitvi14g02987\_t001 |  |  |  |  |  |  |
| 1 | Ath-AT5G40850.1 |  |  |  | | | |  |  |  |  |  |  |
| 1 | Ath-AT5G40855.1 |  |  |  | | | |  |  |  |  |  |  |
| 1 | Ath-AT5G40860.1 |  |  |  | Vvi-Vitvi14g02985\_t001 |  |  |  |  |  |  |
| 1 | Ath-AT5G40870.1 |  |  |  | Vvi-Vitvi14g02984\_t001 |  |  |  |  |  |  |
| 1 | Ath-AT5G40880.1 |  |  |  | | | |  |  |  |  |  |  |
| 1 | Ath-AT5G40890.1 |  |  |  | Vvi-Vitvi14g01560\_t001 |  |  |  |  |  |  |
| 1 | Ath-AT5G40900.1 |  |  |  | | | |  |  |  |  |  |  |
| 1 | Ath-AT5G40910.1 |  |  |  | | | |  |  |  |  |  |  |
| 1 | Ath-AT5G40930.1 |  |  |  | Vvi-Vitvi14g01545\_t001 |  |  |  |  |  |  |
| 1 | Ath-AT5G40940.1 |  |  |  | Vvi-Vitvi14g02972\_t001 |  |  |  |  |  |  |
| 1 | Ath-AT5G40950.1 |  |  |  | Vvi-Vitvi14g01535\_t001 |  |  |  |  |  |  |
| 1 | Ath-AT5G40960.1 |  |  |  | | | |  |  |  |  |  |  |
| 1 | Ath-AT5G40970.1 |  |  |  | | | |  |  |  |  |  |  |
| 1 | Ath-AT5G40980.1 |  |  |  | Vvi-Vitvi14g02965\_t001 |  |  |  |  |  |  |
| 1 | Ath-AT5G40981.1 |  |  |  | | | |  |  |  |  |  |  |
| 1 | Ath-AT5G40990.1 |  |  |  | | | |  |  |  |  |  |  |
| 1 | Ath-AT5G41000.1 |  |  |  | Vvi-Vitvi14g01520\_t001 |  |  |  |  |  |  |
| 1 | Ath-AT5G41010.1 |  |  |  | | | |  |  |  |  |  |  |
| 1 | Ath-AT5G41020.1 |  |  |  | | | |  |  |  |  |  |  |
| 1 | Ath-AT5G41025.1 |  |  |  | | | |  |  |  |  |  |  |
| 1 | Ath-AT5G41030.1 |  |  |  | Vvi-Vitvi14g01519\_t001 |  |  |  |  |  |  |
| 1 | Ath-AT5G41040.1 |  |  |  | | | |  |  |  |  |  |  |
| 1 | Ath-AT5G41050.1 |  |  |  | Vvi-Vitvi14g01511\_t001 |  |  |  |  |  |  |
| 1 | Ath-AT5G41060.1 |  |  |  | Vvi-Vitvi14g01503\_t001 |  |  |  |  |  |  |
| 1 | Ath-AT5G41070.1 |  |  |  | Vvi-Vitvi14g01502\_t001 |  |  |  |  |  |  |
| 1 | Ath-AT5G41071.1 |  |  |  | | | |  |  |  |  |  |  |
| 1 | Ath-AT5G41080.1 |  |  |  | Vvi-Vitvi14g01500\_t001 |  |  |  |  |  |  |
| 1 | Ath-AT5G41090.1 |  |  |  | | | |  |  |  |  |  |  |
| 1 | Ath-AT5G41100.1 |  |  |  | Vvi-Vitvi14g01498\_t002 |  |  |  |  |  |  |
| 1 | Ath-AT5G41110.1 |  |  |  | Vvi-Vitvi14g01496\_t003 |  |  |  |  |  |  |
| 1 | Ath-AT5G41109.1 |  |  |  | | | |  |  |  |  |  |  |
| 1 | Ath-AT5G41120.3 |  |  |  | Vvi-Vitvi14g01493\_t001 |  |  |  |  |  |  |
| 1 | Ath-AT5G41130.3 |  |  |  | Vvi-Vitvi14g01491\_t001 |  |  |  |  |  |  |
| 1 | Ath-AT5G41140.1 |  | Vvi-Vitvi02g00346\_t001 |  |  |  |  |  |  |  |
| 1 | Ath-AT5G41150.1 |  | | | |  |  |  |  |  |  |  |
| 1 | Ath-AT5G41160.1 |  | | | |  |  |  |  |  |  |  |
| 1 | Ath-AT5G41170.1 |  | | | |  |  |  |  |  |  |  |
| 1 | Ath-AT5G41180.1 |  | Vvi-Vitvi02g00342\_t001 |  |  |  |  |  |  |  |
| 1 | Ath-AT5G41190.1 |  | Vvi-Vitvi02g00341\_t001 |  |  |  |  |  |  |  |
| 1 | Ath-AT5G41200.1 |  | | | |  |  |  |  |  |  |  |
| 1 | Ath-AT5G41210.1 |  | Vvi-Vitvi02g00335\_t001 |  |  |  |  |  |  |  |
| 1 | Ath-AT5G41220.1 |  | | | |  |  |  |  |  |  |  |
| 1 | Ath-AT5G41240.1 |  | | | |  |  |  |  |  |  |  |
| 1 | Ath-AT5G41250.1 |  | Vvi-Vitvi02g00334\_t001 |  |  |  |  |  |  |  |
| 1 | Ath-AT5G41260.1 |  | Vvi-Vitvi02g00328\_t003 |  |  |  |  |  |  |  |
| 1 | Ath-AT5G41270.1 |  | | | |  |  |  |  |  |  |  |
| 1 | Ath-AT5G41280.1 |  | | | |  |  |  |  |  |  |  |
| 1 | Ath-AT5G41290.1 |  | | | |  |  |  |  |  |  |  |
| 1 | Ath-AT5G41300.1 |  | | | |  |  |  |  |  |  |  |
| 1 | Ath-AT5G41310.1 |  | Vvi-Vitvi02g00319\_t001 |  |  |  |  |  |  |  |
| 1 | Ath-AT5G41315.2 |  | Vvi-Vitvi02g00317\_t001 |  |  |  |  |  |  |  |
| 1 | Ath-AT5G41320.1 |  | | | |  |  |  |  |  |  |  |
| 1 | Ath-AT5G41330.1 |  | Vvi-Vitvi02g00298\_t001 |  |  |  |  |  |  |  |
| 2 | Ath-AT5G41340.1 |  | | | |  | Vvi-Vitvi02g04001\_t001 |  |  |  |  |  |  |
| 2 | Ath-AT5G41350.2 |  | | | |  | | | |  |  |  |  |  |  |
| 2 | Ath-AT5G41360.1 |  | | | |  | Vvi-Vitvi02g00009\_t001 |  |  |  |  |  |  |
| 2 | Ath-AT5G41370.1 |  | | | |  | | | |  |  |  |  |  |  |
| 2 | Ath-AT5G41380.1 |  | | | |  | Vvi-Vitvi02g00011\_t001 |  |  |  |  |  |  |
| 2 | Ath-AT5G41390.1 |  | | | |  | Vvi-Vitvi02g04003\_t002 |  |  |  |  |  |  |
| 2 | Ath-AT5G41400.1 |  | | | |  | Vvi-Vitvi02g00013\_t001 |  |  |  |  |  |  |
| 2 | Ath-AT5G41401.1 |  | | | |  | | | |  |  |  |  |  |  |
| 2 | Ath-AT5G41410.1 |  | | | |  | Vvi-Vitvi02g00016\_t001 |  |  |  |  |  |  |
| 2 | Ath-AT5G41420.1 |  | | | |  | | | |  |  |  |  |  |  |
| 2 | Ath-AT5G41430.1 |  | | | |  | | | |  |  |  |  |  |  |
| 2 | Ath-AT5G41440.1 |  | | | |  | | | |  |  |  |  |  |  |
| 2 | Ath-AT5G41450.1 |  | | | |  | | | |  |  |  |  |  |  |
| 2 | Ath-AT5G41460.1 |  | Vvi-Vitvi02g00293\_t001 |  | Vvi-Vitvi02g00017\_t001 |  |  |  |  |  |  |
| 1 | Ath-AT5G41470.1 |  |  |  | | | |  |  |  |  |  |  |
| 1 | Ath-AT5G41480.1 |  |  |  | | | |  |  |  |  |  |  |
| 1 | Ath-AT5G41490.1 |  |  |  | | | |  |  |  |  |  |  |
| 1 | Ath-AT5G41500.1 |  |  |  | | | |  |  |  |  |  |  |
| 1 | Ath-AT5G41510.1 |  |  |  | | | |  |  |  |  |  |  |
| 1 | Ath-AT5G41520.1 |  |  |  | Vvi-Vitvi02g01315\_t001 |  |  |  |  |  |  |
| 1 | Ath-AT5G41530.1 |  |  |  | | | |  |  |  |  |  |  |
| 1 | Ath-AT5G41540.1 |  |  |  | | | |  |  |  |  |  |  |
| 1 | Ath-AT5G41550.1 |  |  |  | | | |  |  |  |  |  |  |
| 1 | Ath-AT5G41560.1 |  |  |  | Vvi-Vitvi02g00035\_t001 |  |  |  |  |  |  |
| 1 | Ath-AT5G41570.1 |  |  |  | | | |  |  |  |  |  |  |
| 1 | Ath-AT5G41580.1 |  |  |  | Vvi-Vitvi02g00043\_t001 |  |  |  |  |  |  |
| 1 | Ath-AT5G41590.1 |  |  |  | Vvi-Vitvi02g00046\_t001 |  |  |  |  |  |  |
| 1 | Ath-AT5G41600.1 |  |  |  | Vvi-Vitvi02g00059\_t001 |  |  |  |  |  |  |
| 1 | Ath-AT5G41610.1 |  |  |  | Vvi-Vitvi02g00071\_t001 |  |  |  |  |  |  |
| 1 | Ath-AT5G41620.1 |  |  |  | Vvi-Vitvi02g01329\_t001 |  |  |  |  |  |  |
| 1 | Ath-AT5G41630.1 |  |  |  | | | |  |  |  |  |  |  |
| 1 | Ath-AT5G41640.1 |  |  |  | | | |  |  |  |  |  |  |
| 1 | Ath-AT5G41650.1 |  |  |  | Vvi-Vitvi02g00076\_t001 |  |  |  |  |  |  |
| 1 | Ath-AT5G41660.1 |  |  |  | | | |  |  |  |  |  |  |
| 1 | Ath-AT5G41670.2 |  |  |  | Vvi-Vitvi02g00080\_t001 |  |  |  |  |  |  |
| 1 | Ath-AT5G41680.1 |  |  |  | | | |  |  |  |  |  |  |
| 1 | Ath-AT5G41685.1 |  |  |  | | | |  |  |  |  |  |  |
| 1 | Ath-AT5G41690.2 |  |  |  | | | |  |  |  |  |  |  |
| 1 | Ath-AT5G41700.4 |  |  |  | Vvi-Vitvi02g04013\_t001 |  |  |  |  |  |  |
| 1 | Ath-AT5G41720.1 |  |  |  | | | |  |  |  |  |  |  |
| 1 | Ath-AT5G41730.1 |  |  |  | | | |  |  |  |  |  |  |
| 1 | Ath-AT5G41740.2 |  |  |  | | | |  |  |  |  |  |  |
| 1 | Ath-AT5G41750.1 |  |  |  | | | |  |  |  |  |  |  |
| 1 | Ath-AT5G41760.1 |  |  |  | | | |  |  |  |  |  |  |
| 1 | Ath-AT5G41761.1 |  |  |  | Vvi-Vitvi02g00096\_t001 |  |  |  |  |  |  |
| 1 | Ath-AT5G41763.1 |  |  |  | | | |  |  |  |  |  |  |
| 1 | Ath-AT5G41765.1 |  |  |  | | | |  |  |  |  |  |  |
| 1 | Ath-AT5G41770.1 |  |  |  | Vvi-Vitvi02g00100\_t001 |  |  |  |  |  |  |
| 1 | Ath-AT5G41780.2 |  |  |  | Vvi-Vitvi02g04015\_t001 |  |  |  |  |  |  |
| 1 | Ath-AT5G41790.2 |  |  |  | | | |  |  |  |  |  |  |
| 1 | Ath-AT5G41800.1 |  |  |  | Vvi-Vitvi02g00109\_t001 |  |  |  |  |  |  |
| 1 | Ath-AT5G41810.1 |  |  |  | Vvi-Vitvi02g01337\_t001 |  |  |  |  |  |  |
| 1 | Ath-AT5G41820.2 |  |  |  | | | |  |  |  |  |  |  |
| 1 | Ath-AT5G41830.2 |  |  |  | | | |  |  |  |  |  |  |
| 1 | Ath-AT5G41840.1 |  |  |  | | | |  |  |  |  |  |  |
| 1 | Ath-AT5G41850.1 |  |  |  | Vvi-Vitvi02g00115\_t001 |  |  |  |  |  |  |
| 1 | Ath-AT5G41860.1 |  |  |  | | | |  |  |  |  |  |  |
| 1 | Ath-AT5G41870.1 |  |  |  | Vvi-Vitvi02g00118\_t003 |  |  |  |  |  |  |
| 1 | Ath-AT5G41880.1 |  |  |  | Vvi-Vitvi02g00120\_t001 |  |  |  |  |  |  |
| 1 | Ath-AT5G41890.1 |  | Vvi-Vitvi02g00234\_t001 |  |  |  |  |  |  |  |
| 1 | Ath-AT5G41900.1 |  | Vvi-Vitvi02g00232\_t001 |  |  |  |  |  |  |  |
| 1 | Ath-AT5G41908.1 |  | | | |  |  |  |  |  |  |  |
| 1 | Ath-AT5G41910.2 |  | Vvi-Vitvi02g00227\_t001 |  |  |  |  |  |  |  |
| 1 | Ath-AT5G41920.1 |  | | | |  |  |  |  |  |  |  |
| 1 | Ath-AT5G41940.1 |  | Vvi-Vitvi02g00222\_t004 |  |  |  |  |  |  |  |
| 1 | Ath-AT5G41950.1 |  | Vvi-Vitvi02g00221\_t001 |  |  |  |  |  |  |  |
| 1 | Ath-AT5G41960.1 |  | Vvi-Vitvi02g01364\_t001 |  |  |  |  |  |  |  |
| 1 | Ath-AT5G41970.1 |  | Vvi-Vitvi02g00214\_t001 |  |  |  |  |  |  |  |
| 1 | Ath-AT5G41980.1 |  | | | |  |  |  |  |  |  |  |
| 1 | Ath-AT5G41990.1 |  | Vvi-Vitvi02g00207\_t001 |  |  |  |  |  |  |  |
| 1 | Ath-AT5G42000.1 |  | Vvi-Vitvi02g00200\_t001 |  |  |  |  |  |  |  |
| 1 | Ath-AT5G42010.1 |  | Vvi-Vitvi02g00198\_t001 |  |  |  |  |  |  |  |
| 1 | Ath-AT5G42020.3 |  | Vvi-Vitvi02g00187\_t001 |  |  |  |  |  |  |  |
| 1 | Ath-AT5G42030.2 |  | Vvi-Vitvi02g00177\_t001 |  |  |  |  |  |  |  |
| 1 | Ath-AT5G42040.1 |  | Vvi-Vitvi02g00176\_t001 |  |  |  |  |  |  |  |
| 0 | Ath-AT5G42050.1 |  |  |  |  |  |  |  |  |
| 0 | Ath-AT5G42053.1 |  |  |  |  |  |  |  |  |
| 0 | Ath-AT5G42060.1 |  |  |  |  |  |  |  |  |
| 0 | Ath-AT5G42070.1 |  |  |  |  |  |  |  |  |
| 0 | Ath-AT5G42080.4 |  |  |  |  |  |  |  |  |
| 0 | Ath-AT5G42090.1 |  |  |  |  |  |  |  |  |
| 0 | Ath-AT5G42100.1 |  |  |  |  |  |  |  |  |
| 0 | Ath-AT5G42110.1 |  |  |  |  |  |  |  |  |
| 0 | Ath-AT5G42120.1 |  |  |  |  |  |  |  |  |
| 0 | Ath-AT5G42130.1 |  |  |  |  |  |  |  |  |
| 0 | Ath-AT5G42140.2 |  |  |  |  |  |  |  |  |
| 0 | Ath-AT5G42146.1 |  |  |  |  |  |  |  |  |
| 0 | Ath-AT5G42150.1 |  |  |  |  |  |  |  |  |
| 0 | Ath-AT5G42170.1 |  |  |  |  |  |  |  |  |
| 0 | Ath-AT5G42180.1 |  |  |  |  |  |  |  |  |
| 0 | Ath-AT5G42190.1 |  |  |  |  |  |  |  |  |
| 0 | Ath-AT5G42200.1 |  |  |  |  |  |  |  |  |
| 0 | Ath-AT5G42210.1 |  |  |  |  |  |  |  |  |
| 0 | Ath-AT5G42220.1 |  |  |  |  |  |  |  |  |
| 0 | Ath-AT5G42223.1 |  |  |  |  |  |  |  |  |
| 0 | Ath-AT5G42230.2 |  |  |  |  |  |  |  |  |
| 0 | Ath-AT5G42232.1 |  |  |  |  |  |  |  |  |
| 0 | Ath-AT5G42235.1 |  |  |  |  |  |  |  |  |
| 0 | Ath-AT5G42240.1 |  |  |  |  |  |  |  |  |
| 0 | Ath-AT5G42242.1 |  |  |  |  |  |  |  |  |
| 0 | Ath-AT5G42250.1 |  |  |  |  |  |  |  |  |
| 0 | Ath-AT5G42260.1 |  |  |  |  |  |  |  |  |
| 0 | Ath-AT5G42270.1 |  |  |  |  |  |  |  |  |
| 0 | Ath-AT5G42280.1 |  |  |  |  |  |  |  |  |
| 0 | Ath-AT5G42300.1 |  |  |  |  |  |  |  |  |
| 0 | Ath-AT5G42290.1 |  |  |  |  |  |  |  |  |
| 0 | Ath-AT5G42310.1 |  |  |  |  |  |  |  |  |
| 0 | Ath-AT5G42320.2 |  |  |  |  |  |  |  |  |
| 0 | Ath-AT5G42325.1 |  |  |  |  |  |  |  |  |
| 0 | Ath-AT5G42330.1 |  |  |  |  |  |  |  |  |
| 0 | Ath-AT5G42340.1 |  |  |  |  |  |  |  |  |
| 0 | Ath-AT5G42350.1 |  |  |  |  |  |  |  |  |
| 0 | Ath-AT5G42360.1 |  |  |  |  |  |  |  |  |
| 0 | Ath-AT5G42370.2 |  |  |  |  |  |  |  |  |
| 0 | Ath-AT5G42380.1 |  |  |  |  |  |  |  |  |
| 0 | Ath-AT5G42390.1 |  |  |  |  |  |  |  |  |
| 0 | Ath-AT5G42400.2 |  |  |  |  |  |  |  |  |
| 0 | Ath-AT5G42410.1 |  |  |  |  |  |  |  |  |
| 0 | Ath-AT5G42420.1 |  |  |  |  |  |  |  |  |
| 0 | Ath-AT5G42430.1 |  |  |  |  |  |  |  |  |
| 0 | Ath-AT5G42440.1 |  |  |  |  |  |  |  |  |
| 0 | Ath-AT5G42450.1 |  |  |  |  |  |  |  |  |
| 0 | Ath-AT5G42460.1 |  |  |  |  |  |  |  |  |
| 0 | Ath-AT5G42470.1 |  |  |  |  |  |  |  |  |
| 0 | Ath-AT5G42480.1 |  |  |  |  |  |  |  |  |
| 0 | Ath-AT5G42490.1 |  |  |  |  |  |  |  |  |
| 0 | Ath-AT5G42500.1 |  |  |  |  |  |  |  |  |
| 0 | Ath-AT5G42504.1 |  |  |  |  |  |  |  |  |
| 0 | Ath-AT5G42510.1 |  |  |  |  |  |  |  |  |
| 0 | Ath-AT5G42520.1 |  |  |  |  |  |  |  |  |
| 0 | Ath-AT5G42530.1 |  |  |  |  |  |  |  |  |
| 0 | Ath-AT5G42540.2 |  |  |  |  |  |  |  |  |
| 0 | Ath-AT5G42560.1 |  |  |  |  |  |  |  |  |
| 0 | Ath-AT5G42567.1 |  |  |  |  |  |  |  |  |
| 0 | Ath-AT5G42570.1 |  |  |  |  |  |  |  |  |
| 0 | Ath-AT5G42580.1 |  |  |  |  |  |  |  |  |
| 0 | Ath-AT5G42590.1 |  |  |  |  |  |  |  |  |
| 0 | Ath-AT5G42600.1 |  |  |  |  |  |  |  |  |
| 0 | Ath-AT5G42610.1 |  |  |  |  |  |  |  |  |
| 0 | Ath-AT5G42620.2 |  |  |  |  |  |  |  |  |
| 0 | Ath-AT5G42630.1 |  |  |  |  |  |  |  |  |
| 0 | Ath-AT5G42635.1 |  |  |  |  |  |  |  |  |
| 0 | Ath-AT5G42640.1 |  |  |  |  |  |  |  |  |
| 0 | Ath-AT5G42650.1 |  |  |  |  |  |  |  |  |
| 0 | Ath-AT5G42655.2 |  |  |  |  |  |  |  |  |
| 0 | Ath-AT5G42660.1 |  |  |  |  |  |  |  |  |
| 0 | Ath-AT5G42670.1 |  |  |  |  |  |  |  |  |
| 0 | Ath-AT5G42680.2 |  |  |  |  |  |  |  |  |
| 0 | Ath-AT5G42690.4 |  |  |  |  |  |  |  |  |
| 1 | Ath-AT5G42700.1 |  | Vvi-Vitvi18g00807\_t001 |  |  |  |  |  |  |  |
| 2 | Ath-AT5G42710.1 |  | | | |  | Vvi-Vitvi18g00956\_t001 |  |  |  |  |  |  |
| 2 | Ath-AT5G42720.1 |  | | | |  | | | |  |  |  |  |  |  |
| 2 | Ath-AT5G42740.2 |  | | | |  | | | |  |  |  |  |  |  |
| 2 | Ath-AT5G42750.1 |  | Vvi-Vitvi18g02690\_t001 |  | | | |  |  |  |  |  |  |
| 2 | Ath-AT5G42760.2 |  | | | |  | Vvi-Vitvi18g00962\_t001 |  |  |  |  |  |  |
| 2 | Ath-AT5G42765.1 |  | | | |  | Vvi-Vitvi18g00963\_t001 |  |  |  |  |  |  |
| 2 | Ath-AT5G42770.2 |  | | | |  | Vvi-Vitvi18g00968\_t002 |  |  |  |  |  |  |
| 2 | Ath-AT5G42780.1 |  | | | |  | | | |  |  |  |  |  |  |
| 2 | Ath-AT5G42785.1 |  | | | |  | | | |  |  |  |  |  |  |
| 2 | Ath-AT5G42790.1 |  | | | |  | Vvi-Vitvi18g00982\_t001 |  |  |  |  |  |  |
| 2 | Ath-AT5G42797.1 |  | | | |  | | | |  |  |  |  |  |  |
| 2 | Ath-AT5G42800.1 |  | | | |  | Vvi-Vitvi18g04232\_t001 |  |  |  |  |  |  |
| 2 | Ath-AT5G42810.1 |  | | | |  | Vvi-Vitvi18g00994\_t001 |  |  |  |  |  |  |
| 2 | Ath-AT5G42820.2 |  | | | |  | | | |  |  |  |  |  |  |
| 2 | Ath-AT5G42825.1 |  | | | |  | | | |  |  |  |  |  |  |
| 2 | Ath-AT5G42830.1 |  | | | |  | | | |  |  |  |  |  |  |
| 2 | Ath-AT5G42840.1 |  | | | |  | | | |  |  |  |  |  |  |
| 2 | Ath-AT5G42850.1 |  | | | |  | | | |  |  |  |  |  |  |
| 2 | Ath-AT5G42860.1 |  | | | |  | | | |  |  |  |  |  |  |
| 2 | Ath-AT5G42870.1 |  | Vvi-Vitvi18g00804\_t002 |  | | | |  |  |  |  |  |  |
| 2 | Ath-AT5G42880.1 |  | Vvi-Vitvi18g00797\_t001 |  | | | |  |  |  |  |  |  |
| 2 | Ath-AT5G42890.1 |  | Vvi-Vitvi18g00794\_t001 |  | | | |  |  |  |  |  |  |
| 2 | Ath-AT5G42895.1 |  | | | |  | | | |  |  |  |  |  |  |
| 2 | Ath-AT5G42900.1 |  | Vvi-Vitvi18g00789\_t001 |  | | | |  |  |  |  |  |  |
| 2 | Ath-AT5G42905.1 |  | | | |  | | | |  |  |  |  |  |  |
| 2 | Ath-AT5G42910.1 |  | Vvi-Vitvi18g00784\_t001 |  | | | |  |  |  |  |  |  |
| 2 | Ath-AT5G42920.2 |  | Vvi-Vitvi18g00783\_t001 |  | | | |  |  |  |  |  |  |
| 2 | Ath-AT5G42930.1 |  | Vvi-Vitvi18g00780\_t001 |  | | | |  |  |  |  |  |  |
| 2 | Ath-AT5G42940.1 |  | Vvi-Vitvi18g00762\_t001 |  | | | |  |  |  |  |  |  |
| 2 | Ath-AT5G42950.1 |  | | | |  | | | |  |  |  |  |  |  |
| 2 | Ath-AT5G42955.1 |  | | | |  | | | |  |  |  |  |  |  |
| 2 | Ath-AT5G42957.1 |  | | | |  | | | |  |  |  |  |  |  |
| 2 | Ath-AT5G42960.1 |  | | | |  | | | |  |  |  |  |  |  |
| 2 | Ath-AT5G42965.1 |  | | | |  | | | |  |  |  |  |  |  |
| 2 | Ath-AT5G42970.1 |  | | | |  | | | |  |  |  |  |  |  |
| 2 | Ath-AT5G42980.1 |  | | | |  | | | |  |  |  |  |  |  |
| 2 | Ath-AT5G42990.1 |  | | | |  | | | |  |  |  |  |  |  |
| 2 | Ath-AT5G43000.1 |  | | | |  | | | |  |  |  |  |  |  |
| 2 | Ath-AT5G43010.1 |  | | | |  | Vvi-Vitvi18g01012\_t001 |  |  |  |  |  |  |
| 2 | Ath-AT5G43020.1 |  | Vvi-Vitvi18g00747\_t001 |  | | | |  |  |  |  |  |  |
| 2 | Ath-AT5G43030.1 |  | | | |  | | | |  |  |  |  |  |  |
| 2 | Ath-AT5G43040.1 |  | | | |  | | | |  |  |  |  |  |  |
| 2 | Ath-AT5G43050.1 |  | | | |  | | | |  |  |  |  |  |  |
| 2 | Ath-AT5G43060.1 |  | Vvi-Vitvi18g00740\_t002 |  | Vvi-Vitvi18g01024\_t001 |  |  |  |  |  |  |
| 0 | Ath-AT5G43064.1 |  |  |  |  |  |  |  |  |
| 0 | Ath-AT5G43066.1 |  |  |  |  |  |  |  |  |
| 0 | Ath-AT5G43068.1 |  |  |  |  |  |  |  |  |
| 1 | Ath-AT5G43070.1 |  | Vvi-Vitvi18g00718\_t001 |  |  |  |  |  |  |  |
| 1 | Ath-AT5G43080.1 |  | Vvi-Vitvi18g00707\_t001 |  |  |  |  |  |  |  |
| 1 | Ath-AT5G43090.1 |  | | | |  |  |  |  |  |  |  |
| 1 | Ath-AT5G43100.1 |  | Vvi-Vitvi18g00693\_t001 |  |  |  |  |  |  |  |
| 1 | Ath-AT5G43110.1 |  | | | |  |  |  |  |  |  |  |
| 1 | Ath-AT5G43120.2 |  | Vvi-Vitvi18g00689\_t001 |  |  |  |  |  |  |  |
| 1 | Ath-AT5G43130.2 |  | Vvi-Vitvi18g00680\_t001 |  |  |  |  |  |  |  |
| 1 | Ath-AT5G43140.1 |  | Vvi-Vitvi18g00679\_t001 |  |  |  |  |  |  |  |
| 1 | Ath-AT5G43150.1 |  | Vvi-Vitvi18g04153\_t002 |  |  |  |  |  |  |  |
| 1 | Ath-AT5G43155.1 |  | | | |  |  |  |  |  |  |  |
| 1 | Ath-AT5G43160.2 |  | Vvi-Vitvi18g00678\_t001 |  |  |  |  |  |  |  |
| 1 | Ath-AT5G43170.1 |  | Vvi-Vitvi18g00675\_t001 |  |  |  |  |  |  |  |
| 1 | Ath-AT5G43175.1 |  | Vvi-Vitvi18g00673\_t001 |  |  |  |  |  |  |  |
| 1 | Ath-AT5G43180.1 |  | | | |  |  |  |  |  |  |  |
| 1 | Ath-AT5G43185.1 |  | | | |  |  |  |  |  |  |  |
| 1 | Ath-AT5G43190.1 |  | Vvi-Vitvi18g00661\_t001 |  |  |  |  |  |  |  |
| 0 | Ath-AT5G43200.1 |  |  |  |  |  |  |  |  |
| 0 | Ath-AT5G43210.1 |  |  |  |  |  |  |  |  |
| 0 | Ath-AT5G43211.1 |  |  |  |  |  |  |  |  |
| 0 | Ath-AT5G43230.1 |  |  |  |  |  |  |  |  |
| 0 | Ath-AT5G43240.1 |  |  |  |  |  |  |  |  |
| 0 | Ath-AT5G43250.1 |  |  |  |  |  |  |  |  |
| 0 | Ath-AT5G43260.1 |  |  |  |  |  |  |  |  |
| 0 | Ath-AT5G43270.1 |  |  |  |  |  |  |  |  |
| 0 | Ath-AT5G43280.1 |  |  |  |  |  |  |  |  |
| 0 | Ath-AT5G43285.1 |  |  |  |  |  |  |  |  |
| 0 | Ath-AT5G43290.1 |  |  |  |  |  |  |  |  |
| 0 | Ath-AT5G43300.1 |  |  |  |  |  |  |  |  |
| 0 | Ath-AT5G43310.1 |  |  |  |  |  |  |  |  |
| 0 | Ath-AT5G43320.1 |  |  |  |  |  |  |  |  |
| 0 | Ath-AT5G43330.1 |  |  |  |  |  |  |  |  |
| 0 | Ath-AT5G43340.1 |  |  |  |  |  |  |  |  |
| 1 | Ath-AT5G43350.1 |  | Vvi-Vitvi05g00733\_t001 |  |  |  |  |  |  |  |
| 1 | Ath-AT5G43360.1 |  | | | |  |  |  |  |  |  |  |
| 1 | Ath-AT5G43370.1 |  | | | |  |  |  |  |  |  |  |
| 1 | Ath-AT5G43380.1 |  | Vvi-Vitvi05g00731\_t002 |  |  |  |  |  |  |  |
| 1 | Ath-AT5G43390.1 |  | Vvi-Vitvi05g00718\_t001 |  |  |  |  |  |  |  |
| 1 | Ath-AT5G43400.1 |  | | | |  |  |  |  |  |  |  |
| 1 | Ath-AT5G43401.1 |  | | | |  |  |  |  |  |  |  |
| 1 | Ath-AT5G43410.1 |  | Vvi-Vitvi05g01722\_t001 |  |  |  |  |  |  |  |
| 1 | Ath-AT5G43420.1 |  | Vvi-Vitvi05g00713\_t001 |  |  |  |  |  |  |  |
| 1 | Ath-AT5G43430.4 |  | Vvi-Vitvi05g00712\_t001 |  |  |  |  |  |  |  |
| 1 | Ath-AT5G43440.1 |  | Vvi-Vitvi05g04172\_t001 |  |  |  |  |  |  |  |
| 1 | Ath-AT5G43450.1 |  | | | |  |  |  |  |  |  |  |
| 1 | Ath-AT5G43460.2 |  | Vvi-Vitvi05g00692\_t001 |  |  |  |  |  |  |  |
| 1 | Ath-AT5G43470.2 |  | | | |  |  |  |  |  |  |  |
| 1 | Ath-AT5G43480.1 |  | | | |  |  |  |  |  |  |  |
| 1 | Ath-AT5G43490.1 |  | | | |  |  |  |  |  |  |  |
| 1 | Ath-AT5G43500.1 |  | Vvi-Vitvi05g00691\_t001 |  |  |  |  |  |  |  |
| 1 | Ath-AT5G43510.2 |  | | | |  |  |  |  |  |  |  |
| 1 | Ath-AT5G43513.1 |  | | | |  |  |  |  |  |  |  |
| 1 | Ath-AT5G43518.1 |  | | | |  |  |  |  |  |  |  |
| 1 | Ath-AT5G43520.1 |  | | | |  |  |  |  |  |  |  |
| 1 | Ath-AT5G43525.1 |  | | | |  |  |  |  |  |  |  |
| 1 | Ath-AT5G43530.1 |  | Vvi-Vitvi05g00688\_t001 |  |  |  |  |  |  |  |
| 1 | Ath-AT5G43540.1 |  | Vvi-Vitvi05g01915\_t001 |  |  |  |  |  |  |  |
| 1 | Ath-AT5G43550.1 |  | | | |  |  |  |  |  |  |  |
| 1 | Ath-AT5G43560.2 |  | | | |  |  |  |  |  |  |  |
| 1 | Ath-AT5G43570.2 |  | Vvi-Vitvi05g00669\_t001 |  |  |  |  |  |  |  |
| 1 | Ath-AT5G43580.1 |  | Vvi-Vitvi05g01911\_t001 |  |  |  |  |  |  |  |
| 1 | Ath-AT5G43590.2 |  | | | |  |  |  |  |  |  |  |
| 1 | Ath-AT5G43600.1 |  | Vvi-Vitvi05g00654\_t001 |  |  |  |  |  |  |  |
| 1 | Ath-AT5G43610.1 |  | | | |  |  |  |  |  |  |  |
| 1 | Ath-AT5G43620.1 |  | | | |  |  |  |  |  |  |  |
| 1 | Ath-AT5G43630.2 |  | Vvi-Vitvi05g00650\_t001 |  |  |  |  |  |  |  |
| 1 | Ath-AT5G43640.1 |  | Vvi-Vitvi05g00641\_t001 |  |  |  |  |  |  |  |
| 1 | Ath-AT5G43650.1 |  | Vvi-Vitvi05g00640\_t001 |  |  |  |  |  |  |  |
| 1 | Ath-AT5G43660.1 |  | | | |  |  |  |  |  |  |  |
| 1 | Ath-AT5G43670.1 |  | Vvi-Vitvi05g00632\_t001 |  |  |  |  |  |  |  |
| 1 | Ath-AT5G43680.1 |  | Vvi-Vitvi05g00631\_t001 |  |  |  |  |  |  |  |
| 1 | Ath-AT5G43690.1 |  | | | |  |  |  |  |  |  |  |
| 1 | Ath-AT5G43695.1 |  | | | |  |  |  |  |  |  |  |
| 1 | Ath-AT5G43700.1 |  | | | |  |  |  |  |  |  |  |
| 1 | Ath-AT5G43710.1 |  | Vvi-Vitvi05g00627\_t001 |  |  |  |  |  |  |  |
| 1 | Ath-AT5G43720.1 |  | Vvi-Vitvi05g00626\_t001 |  |  |  |  |  |  |  |
| 1 | Ath-AT5G43730.1 |  | | | |  |  |  |  |  |  |  |
| 1 | Ath-AT5G43740.2 |  | | | |  |  |  |  |  |  |  |
| 1 | Ath-AT5G43745.1 |  | Vvi-Vitvi05g00625\_t001 |  |  |  |  |  |  |  |
| 1 | Ath-AT5G43750.1 |  | Vvi-Vitvi05g00621\_t001 |  |  |  |  |  |  |  |
| 1 | Ath-AT5G43755.1 |  | | | |  |  |  |  |  |  |  |
| 1 | Ath-AT5G43760.1 |  | Vvi-Vitvi05g00616\_t001 |  |  |  |  |  |  |  |
| 0 | Ath-AT5G43770.1 |  |  |  |  |  |  |  |  |
| 1 | Ath-AT5G43780.1 |  | Vvi-Vitvi05g00576\_t001 |  |  |  |  |  |  |  |
| 1 | Ath-AT5G43790.1 |  | Vvi-Vitvi05g00575\_t001 |  |  |  |  |  |  |  |
| 1 | Ath-AT5G43810.1 |  | Vvi-Vitvi05g00574\_t002 |  |  |  |  |  |  |  |
| 1 | Ath-AT5G43822.1 |  | | | |  |  |  |  |  |  |  |
| 1 | Ath-AT5G43820.1 |  | | | |  |  |  |  |  |  |  |
| 1 | Ath-AT5G43830.1 |  | Vvi-Vitvi05g00569\_t001 |  |  |  |  |  |  |  |
| 1 | Ath-AT5G43840.1 |  | | | |  |  |  |  |  |  |  |
| 1 | Ath-AT5G43850.1 |  | Vvi-Vitvi05g00559\_t002 |  |  |  |  |  |  |  |
| 1 | Ath-AT5G43860.1 |  | Vvi-Vitvi05g00556\_t001 |  |  |  |  |  |  |  |
| 1 | Ath-AT5G43870.1 |  | Vvi-Vitvi05g00554\_t001 |  |  |  |  |  |  |  |
| 1 | Ath-AT5G43880.1 |  | Vvi-Vitvi07g00739\_t001 |  |  |  |  |  |  |  |
| 1 | Ath-AT5G43890.1 |  | Vvi-Vitvi07g00726\_t001 |  |  |  |  |  |  |  |
| 1 | Ath-AT5G43900.3 |  | Vvi-Vitvi07g00724\_t001 |  |  |  |  |  |  |  |
| 1 | Ath-AT5G43910.2 |  | Vvi-Vitvi07g00721\_t001 |  |  |  |  |  |  |  |
| 1 | Ath-AT5G43920.1 |  | Vvi-Vitvi07g00711\_t001 |  |  |  |  |  |  |  |
| 1 | Ath-AT5G43930.4 |  | Vvi-Vitvi07g00710\_t001 |  |  |  |  |  |  |  |
| 1 | Ath-AT5G43935.1 |  | | | |  |  |  |  |  |  |  |
| 1 | Ath-AT5G43940.2 |  | Vvi-Vitvi07g04173\_t001 |  |  |  |  |  |  |  |
| 1 | Ath-AT5G43950.1 |  | Vvi-Vitvi07g00685\_t001 |  |  |  |  |  |  |  |
| 1 | Ath-AT5G43960.1 |  | Vvi-Vitvi07g00683\_t001 |  |  |  |  |  |  |  |
| 1 | Ath-AT5G43970.1 |  | Vvi-Vitvi07g00677\_t001 |  |  |  |  |  |  |  |
| 1 | Ath-AT5G43980.1 |  | Vvi-Vitvi07g00676\_t001 |  |  |  |  |  |  |  |
| 1 | Ath-AT5G43990.9 |  | Vvi-Vitvi07g00675\_t001 |  |  |  |  |  |  |  |
| 1 | Ath-AT5G44000.1 |  | Vvi-Vitvi07g00674\_t001 |  |  |  |  |  |  |  |
| 1 | Ath-AT5G44005.1 |  | | | |  |  |  |  |  |  |  |
| 1 | Ath-AT5G44010.4 |  | Vvi-Vitvi07g00668\_t001 |  |  |  |  |  |  |  |
| 1 | Ath-AT5G44020.1 |  | Vvi-Vitvi07g00667\_t001 |  |  |  |  |  |  |  |
| 1 | Ath-AT5G44030.2 |  | Vvi-Vitvi07g00665\_t001 |  |  |  |  |  |  |  |
| 1 | Ath-AT5G44040.1 |  | Vvi-Vitvi07g00659\_t001 |  |  |  |  |  |  |  |
| 1 | Ath-AT5G44050.1 |  | | | |  |  |  |  |  |  |  |
| 1 | Ath-AT5G44060.1 |  | Vvi-Vitvi07g00639\_t001 |  |  |  |  |  |  |  |
| 1 | Ath-AT5G44063.1 |  | | | |  |  |  |  |  |  |  |
| 1 | Ath-AT5G44065.1 |  | | | |  |  |  |  |  |  |  |
| 1 | Ath-AT5G44070.1 |  | Vvi-Vitvi07g00635\_t001 |  |  |  |  |  |  |  |
| 1 | Ath-AT5G44080.1 |  | | | |  |  |  |  |  |  |  |
| 1 | Ath-AT5G44090.2 |  | Vvi-Vitvi07g00630\_t002 |  |  |  |  |  |  |  |
| 1 | Ath-AT5G44100.1 |  | | | |  |  |  |  |  |  |  |
| 1 | Ath-AT5G44110.1 |  | Vvi-Vitvi07g00625\_t001 |  |  |  |  |  |  |  |
| 1 | Ath-AT5G44120.3 |  | Vvi-Vitvi07g00605\_t001 |  |  |  |  |  |  |  |
| 1 | Ath-AT5G44130.1 |  | Vvi-Vitvi12g00248\_t001 |  |  |  |  |  |  |  |
| 1 | Ath-AT5G44140.1 |  | | | |  |  |  |  |  |  |  |
| 1 | Ath-AT5G44150.1 |  | Vvi-Vitvi12g02295\_t001 |  |  |  |  |  |  |  |
| 1 | Ath-AT5G44160.1 |  | Vvi-Vitvi12g00252\_t001 |  |  |  |  |  |  |  |
| 1 | Ath-AT5G44170.1 |  | Vvi-Vitvi12g02296\_t001 |  |  |  |  |  |  |  |
| 1 | Ath-AT5G44180.1 |  | Vvi-Vitvi12g00258\_t003 |  |  |  |  |  |  |  |
| 1 | Ath-AT5G44190.1 |  | Vvi-Vitvi12g00260\_t001 |  |  |  |  |  |  |  |
| 1 | Ath-AT5G44200.2 |  | Vvi-Vitvi12g02297\_t001 |  |  |  |  |  |  |  |
| 1 | Ath-AT5G44210.1 |  | Vvi-Vitvi12g00274\_t001 |  |  |  |  |  |  |  |
| 1 | Ath-AT5G44220.1 |  | | | |  |  |  |  |  |  |  |
| 1 | Ath-AT5G44230.1 |  | | | |  |  |  |  |  |  |  |
| 1 | Ath-AT5G44240.1 |  | Vvi-Vitvi12g00275\_t002 |  |  |  |  |  |  |  |
| 1 | Ath-AT5G44250.1 |  | | | |  |  |  |  |  |  |  |
| 1 | Ath-AT5G44260.1 |  | Vvi-Vitvi12g00276\_t001 |  |  |  |  |  |  |  |
| 1 | Ath-AT5G44265.1 |  | | | |  |  |  |  |  |  |  |
| 1 | Ath-AT5G44270.1 |  | Vvi-Vitvi12g00278\_t001 |  |  |  |  |  |  |  |
| 1 | Ath-AT5G44280.2 |  | Vvi-Vitvi12g00279\_t001 |  |  |  |  |  |  |  |
| 1 | Ath-AT5G44290.1 |  | Vvi-Vitvi12g00301\_t001 |  |  |  |  |  |  |  |
| 0 | Ath-AT5G44300.1 |  |  |  |  |  |  |  |  |
| 0 | Ath-AT5G44306.1 |  |  |  |  |  |  |  |  |
| 0 | Ath-AT5G44310.2 |  |  |  |  |  |  |  |  |
| 0 | Ath-AT5G44316.1 |  |  |  |  |  |  |  |  |
| 0 | Ath-AT5G44320.1 |  |  |  |  |  |  |  |  |
| 1 | Ath-AT5G44330.1 |  | Vvi-Vitvi10g00996\_t001 |  |  |  |  |  |  |  |
| 1 | Ath-AT5G44340.1 |  | | | |  |  |  |  |  |  |  |
| 1 | Ath-AT5G44345.1 |  | | | |  |  |  |  |  |  |  |
| 1 | Ath-AT5G44350.1 |  | Vvi-Vitvi10g01931\_t001 |  |  |  |  |  |  |  |
| 1 | Ath-AT5G44360.2 |  | Vvi-Vitvi10g01017\_t001 |  |  |  |  |  |  |  |
| 1 | Ath-AT5G44370.1 |  | | | |  |  |  |  |  |  |  |
| 1 | Ath-AT5G44380.2 |  | Vvi-Vitvi10g01020\_t001 |  |  |  |  |  |  |  |
| 1 | Ath-AT5G44390.1 |  | Vvi-Vitvi10g04513\_t001 |  |  |  |  |  |  |  |
| 1 | Ath-AT5G44400.1 |  | | | |  |  |  |  |  |  |  |
| 1 | Ath-AT5G44410.1 |  | Vvi-Vitvi10g01060\_t001 |  |  |  |  |  |  |  |
| 1 | Ath-AT5G44420.1 |  | | | |  |  |  |  |  |  |  |
| 1 | Ath-AT5G44430.1 |  | | | |  |  |  |  |  |  |  |
| 1 | Ath-AT5G44440.2 |  | | | |  |  |  |  |  |  |  |
| 1 | Ath-AT5G44450.2 |  | Vvi-Vitvi10g04519\_t002 |  |  |  |  |  |  |  |
| 1 | Ath-AT5G44460.1 |  | | | |  |  |  |  |  |  |  |
| 1 | Ath-AT5G44470.1 |  | | | |  |  |  |  |  |  |  |
| 1 | Ath-AT5G44480.1 |  | | | |  |  |  |  |  |  |  |
| 1 | Ath-AT5G44490.1 |  | | | |  |  |  |  |  |  |  |
| 1 | Ath-AT5G44495.1 |  | | | |  |  |  |  |  |  |  |
| 1 | Ath-AT5G44500.2 |  | | | |  |  |  |  |  |  |  |
| 1 | Ath-AT5G44510.1 |  | | | |  |  |  |  |  |  |  |
| 1 | Ath-AT5G44520.2 |  | Vvi-Vitvi10g01098\_t001 |  |  |  |  |  |  |  |
| 1 | Ath-AT5G44530.2 |  | Vvi-Vitvi10g01110\_t001 |  |  |  |  |  |  |  |
| 1 | Ath-AT5G44540.1 |  | | | |  |  |  |  |  |  |  |
| 1 | Ath-AT5G44550.1 |  | Vvi-Vitvi10g01121\_t001 |  |  |  |  |  |  |  |
| 1 | Ath-AT5G44560.1 |  | Vvi-Vitvi10g01136\_t001 |  |  |  |  |  |  |  |
| 0 | Ath-AT5G44563.1 |  |  |  |  |  |  |  |  |
| 0 | Ath-AT5G44565.2 |  |  |  |  |  |  |  |  |
| 0 | Ath-AT5G44567.1 |  |  |  |  |  |  |  |  |
| 0 | Ath-AT5G44568.1 |  |  |  |  |  |  |  |  |
| 0 | Ath-AT5G44566.1 |  |  |  |  |  |  |  |  |
| 0 | Ath-AT5G44570.2 |  |  |  |  |  |  |  |  |
| 0 | Ath-AT5G44572.1 |  |  |  |  |  |  |  |  |
| 0 | Ath-AT5G44574.1 |  |  |  |  |  |  |  |  |
| 0 | Ath-AT5G44575.1 |  |  |  |  |  |  |  |  |
| 0 | Ath-AT5G44578.1 |  |  |  |  |  |  |  |  |
| 0 | Ath-AT5G44580.1 |  |  |  |  |  |  |  |  |
| 0 | Ath-AT5G44582.1 |  |  |  |  |  |  |  |  |
| 0 | Ath-AT5G44585.1 |  |  |  |  |  |  |  |  |
| 0 | Ath-AT5G44590.1 |  |  |  |  |  |  |  |  |
| 0 | Ath-AT5G44600.1 |  |  |  |  |  |  |  |  |
| 0 | Ath-AT5G44610.1 |  |  |  |  |  |  |  |  |
| 0 | Ath-AT5G44620.1 |  |  |  |  |  |  |  |  |
| 0 | Ath-AT5G44630.1 |  |  |  |  |  |  |  |  |
| 0 | Ath-AT5G44635.2 |  |  |  |  |  |  |  |  |
| 0 | Ath-AT5G44640.1 |  |  |  |  |  |  |  |  |
| 0 | Ath-AT5G44650.1 |  |  |  |  |  |  |  |  |
| 0 | Ath-AT5G44660.1 |  |  |  |  |  |  |  |  |
| 0 | Ath-AT5G44670.1 |  |  |  |  |  |  |  |  |
| 0 | Ath-AT5G44680.1 |  |  |  |  |  |  |  |  |
| 0 | Ath-AT5G44690.1 |  |  |  |  |  |  |  |  |
| 0 | Ath-AT5G44700.1 |  |  |  |  |  |  |  |  |
| 1 | Ath-AT5G44710.1 |  | Vvi-Vitvi01g01857\_t001 |  |  |  |  |  |  |  |
| 1 | Ath-AT5G44720.1 |  | Vvi-Vitvi01g00087\_t002 |  |  |  |  |  |  |  |
| 1 | Ath-AT5G44730.1 |  | Vvi-Vitvi01g00091\_t001 |  |  |  |  |  |  |  |
| 1 | Ath-AT5G44740.2 |  | Vvi-Vitvi01g00092\_t001 |  |  |  |  |  |  |  |
| 1 | Ath-AT5G44750.2 |  | | | |  |  |  |  |  |  |  |
| 1 | Ath-AT5G44760.1 |  | | | |  |  |  |  |  |  |  |
| 1 | Ath-AT5G44770.1 |  | | | |  |  |  |  |  |  |  |
| 1 | Ath-AT5G44780.1 |  | | | |  |  |  |  |  |  |  |
| 1 | Ath-AT5G44785.2 |  | | | |  |  |  |  |  |  |  |
| 1 | Ath-AT5G44790.1 |  | Vvi-Vitvi01g00108\_t001 |  |  |  |  |  |  |  |
| 1 | Ath-AT5G44800.1 |  | Vvi-Vitvi01g00119\_t001 |  |  |  |  |  |  |  |
| 1 | Ath-AT5G44820.1 |  | | | |  |  |  |  |  |  |  |
| 1 | Ath-AT5G44830.1 |  | | | |  |  |  |  |  |  |  |
| 1 | Ath-AT5G44840.1 |  | | | |  |  |  |  |  |  |  |
| 1 | Ath-AT5G44850.1 |  | | | |  |  |  |  |  |  |  |
| 1 | Ath-AT5G44860.2 |  | Vvi-Vitvi01g00124\_t001 |  |  |  |  |  |  |  |
| 1 | Ath-AT5G44870.1 |  | | | |  |  |  |  |  |  |  |
| 1 | Ath-AT5G44900.1 |  | | | |  |  |  |  |  |  |  |
| 1 | Ath-AT5G44910.1 |  | | | |  |  |  |  |  |  |  |
| 1 | Ath-AT5G44920.1 |  | | | |  |  |  |  |  |  |  |
| 1 | Ath-AT5G44930.1 |  | Vvi-Vitvi01g00131\_t001 |  |  |  |  |  |  |  |
| 1 | Ath-AT5G44940.1 |  | | | |  |  |  |  |  |  |  |
| 1 | Ath-AT5G44950.1 |  | | | |  |  |  |  |  |  |  |
| 1 | Ath-AT5G44960.1 |  | | | |  |  |  |  |  |  |  |
| 1 | Ath-AT5G44970.1 |  | | | |  |  |  |  |  |  |  |
| 1 | Ath-AT5G44973.1 |  | | | |  |  |  |  |  |  |  |
| 1 | Ath-AT5G44980.1 |  | | | |  |  |  |  |  |  |  |
| 1 | Ath-AT5G44990.1 |  | | | |  |  |  |  |  |  |  |
| 1 | Ath-AT5G45000.1 |  | | | |  |  |  |  |  |  |  |
| 1 | Ath-AT5G45010.1 |  | | | |  |  |  |  |  |  |  |
| 1 | Ath-AT5G45020.1 |  | | | |  |  |  |  |  |  |  |
| 1 | Ath-AT5G45030.2 |  | | | |  |  |  |  |  |  |  |
| 1 | Ath-AT5G45040.1 |  | Vvi-Vitvi01g00145\_t001 |  |  |  |  |  |  |  |
| 0 | Ath-AT5G45050.1 |  |  |  |  |  |  |  |  |
| 0 | Ath-AT5G45060.1 |  |  |  |  |  |  |  |  |
| 0 | Ath-AT5G45070.1 |  |  |  |  |  |  |  |  |
| 0 | Ath-AT5G45080.1 |  |  |  |  |  |  |  |  |
| 0 | Ath-AT5G45090.1 |  |  |  |  |  |  |  |  |
| 0 | Ath-AT5G45095.1 |  |  |  |  |  |  |  |  |
| 0 | Ath-AT5G45100.1 |  |  |  |  |  |  |  |  |
| 0 | Ath-AT5G45110.1 |  |  |  |  |  |  |  |  |
| 0 | Ath-AT5G45113.1 |  |  |  |  |  |  |  |  |
| 0 | Ath-AT5G45115.1 |  |  |  |  |  |  |  |  |
| 0 | Ath-AT5G45120.1 |  |  |  |  |  |  |  |  |
| 0 | Ath-AT5G45130.2 |  |  |  |  |  |  |  |  |
| 0 | Ath-AT5G45140.1 |  |  |  |  |  |  |  |  |
| 0 | Ath-AT5G45150.1 |  |  |  |  |  |  |  |  |
| 0 | Ath-AT5G45160.1 |  |  |  |  |  |  |  |  |
| 0 | Ath-AT5G45170.1 |  |  |  |  |  |  |  |  |
| 0 | Ath-AT5G45180.1 |  |  |  |  |  |  |  |  |
| 0 | Ath-AT5G45190.2 |  |  |  |  |  |  |  |  |
| 0 | Ath-AT5G45200.1 |  |  |  |  |  |  |  |  |
| 0 | Ath-AT5G45210.4 |  |  |  |  |  |  |  |  |
| 0 | Ath-AT5G45220.1 |  |  |  |  |  |  |  |  |
| 0 | Ath-AT5G45230.1 |  |  |  |  |  |  |  |  |
| 0 | Ath-AT5G45240.1 |  |  |  |  |  |  |  |  |
| 0 | Ath-AT5G45250.1 |  |  |  |  |  |  |  |  |
| 0 | Ath-AT5G45260.1 |  |  |  |  |  |  |  |  |
| 1 | Ath-AT5G45275.1 |  | Vvi-Vitvi02g01224\_t001 |  |  |  |  |  |  |  |
| 1 | Ath-AT5G45277.1 |  | | | |  |  |  |  |  |  |  |
| 1 | Ath-AT5G45280.2 |  | Vvi-Vitvi02g01230\_t001 |  |  |  |  |  |  |  |
| 1 | Ath-AT5G45290.2 |  | Vvi-Vitvi02g01231\_t002 |  |  |  |  |  |  |  |
| 1 | Ath-AT5G45300.3 |  | Vvi-Vitvi02g01232\_t001 |  |  |  |  |  |  |  |
| 1 | Ath-AT5G45310.2 |  | Vvi-Vitvi02g01248\_t001 |  |  |  |  |  |  |  |
| 1 | Ath-AT5G45320.1 |  | Vvi-Vitvi02g01252\_t001 |  |  |  |  |  |  |  |
| 1 | Ath-AT5G45330.1 |  | Vvi-Vitvi02g01267\_t001 |  |  |  |  |  |  |  |
| 1 | Ath-AT5G45340.1 |  | Vvi-Vitvi02g01269\_t001 |  |  |  |  |  |  |  |
| 1 | Ath-AT5G45350.1 |  | | | |  |  |  |  |  |  |  |
| 1 | Ath-AT5G45360.1 |  | Vvi-Vitvi02g01275\_t001 |  |  |  |  |  |  |  |
| 1 | Ath-AT5G45370.4 |  | Vvi-Vitvi02g01284\_t001 |  |  |  |  |  |  |  |
| 0 | Ath-AT5G45380.1 |  |  |  |  |  |  |  |  |
| 0 | Ath-AT5G45390.1 |  |  |  |  |  |  |  |  |
| 0 | Ath-AT5G45400.1 |  |  |  |  |  |  |  |  |
| 0 | Ath-AT5G45410.3 |  |  |  |  |  |  |  |  |
| 0 | Ath-AT5G45420.1 |  |  |  |  |  |  |  |  |
| 0 | Ath-AT5G45430.1 |  |  |  |  |  |  |  |  |
| 0 | Ath-AT5G45440.1 |  |  |  |  |  |  |  |  |
| 0 | Ath-AT5G45450.1 |  |  |  |  |  |  |  |  |
| 0 | Ath-AT5G45455.1 |  |  |  |  |  |  |  |  |
| 0 | Ath-AT5G45460.1 |  |  |  |  |  |  |  |  |
| 0 | Ath-AT5G45469.1 |  |  |  |  |  |  |  |  |
| 0 | Ath-AT5G45470.1 |  |  |  |  |  |  |  |  |
| 0 | Ath-AT5G45480.1 |  |  |  |  |  |  |  |  |
| 0 | Ath-AT5G45490.2 |  |  |  |  |  |  |  |  |
| 0 | Ath-AT5G45500.6 |  |  |  |  |  |  |  |  |
| 0 | Ath-AT5G45510.1 |  |  |  |  |  |  |  |  |
| 0 | Ath-AT5G45520.1 |  |  |  |  |  |  |  |  |
| 0 | Ath-AT5G45530.1 |  |  |  |  |  |  |  |  |
| 0 | Ath-AT5G45540.1 |  |  |  |  |  |  |  |  |
| 0 | Ath-AT5G45550.1 |  |  |  |  |  |  |  |  |
| 0 | Ath-AT5G45560.1 |  |  |  |  |  |  |  |  |
| 0 | Ath-AT5G45570.2 |  |  |  |  |  |  |  |  |
| 0 | Ath-AT5G45573.1 |  |  |  |  |  |  |  |  |
| 0 | Ath-AT5G45580.2 |  |  |  |  |  |  |  |  |
| 0 | Ath-AT5G45590.1 |  |  |  |  |  |  |  |  |
| 0 | Ath-AT5G45600.1 |  |  |  |  |  |  |  |  |
| 0 | Ath-AT5G45610.1 |  |  |  |  |  |  |  |  |
| 0 | Ath-AT5G45620.1 |  |  |  |  |  |  |  |  |
| 1 | Ath-AT5G45630.1 |  | Vvi-Vitvi10g01813\_t001 |  |  |  |  |  |  |  |
| 1 | Ath-AT5G45640.1 |  | Vvi-Vitvi10g01812\_t001 |  |  |  |  |  |  |  |
| 1 | Ath-AT5G45650.2 |  | | | |  |  |  |  |  |  |  |
| 1 | Ath-AT5G45660.1 |  | Vvi-Vitvi10g04386\_t001 |  |  |  |  |  |  |  |
| 1 | Ath-AT5G45670.1 |  | Vvi-Vitvi10g00665\_t001 |  |  |  |  |  |  |  |
| 1 | Ath-AT5G45680.1 |  | | | |  |  |  |  |  |  |  |
| 1 | Ath-AT5G45690.1 |  | Vvi-Vitvi10g00660\_t001 |  |  |  |  |  |  |  |
| 1 | Ath-AT5G45700.1 |  | Vvi-Vitvi10g00638\_t001 |  |  |  |  |  |  |  |
| 1 | Ath-AT5G45710.1 |  | Vvi-Vitvi10g00635\_t002 |  |  |  |  |  |  |  |
| 1 | Ath-AT5G45720.1 |  | Vvi-Vitvi10g00629\_t001 |  |  |  |  |  |  |  |
| 1 | Ath-AT5G45730.1 |  | | | |  |  |  |  |  |  |  |
| 1 | Ath-AT5G45740.1 |  | Vvi-Vitvi10g00628\_t001 |  |  |  |  |  |  |  |
| 1 | Ath-AT5G45750.1 |  | Vvi-Vitvi10g00619\_t001 |  |  |  |  |  |  |  |
| 1 | Ath-AT5G45760.1 |  | Vvi-Vitvi10g00614\_t001 |  |  |  |  |  |  |  |
| 1 | Ath-AT5G45770.1 |  | Vvi-Vitvi10g00612\_t001 |  |  |  |  |  |  |  |
| 1 | Ath-AT5G45775.2 |  | Vvi-Vitvi10g00609\_t001 |  |  |  |  |  |  |  |
| 1 | Ath-AT5G45780.1 |  | Vvi-Vitvi10g00605\_t001 |  |  |  |  |  |  |  |
| 1 | Ath-AT5G45790.2 |  | | | |  |  |  |  |  |  |  |
| 1 | Ath-AT5G45800.1 |  | Vvi-Vitvi10g00601\_t002 |  |  |  |  |  |  |  |
| 1 | Ath-AT5G45810.1 |  | Vvi-Vitvi10g00599\_t001 |  |  |  |  |  |  |  |
| 1 | Ath-AT5G45820.1 |  | | | |  |  |  |  |  |  |  |
| 1 | Ath-AT5G45830.1 |  | Vvi-Vitvi10g00598\_t001 |  |  |  |  |  |  |  |
| 1 | Ath-AT5G45840.1 |  | Vvi-Vitvi10g00597\_t001 |  |  |  |  |  |  |  |
| 1 | Ath-AT5G45850.1 |  | | | |  |  |  |  |  |  |  |
| 1 | Ath-AT5G45860.1 |  | Vvi-Vitvi10g00587\_t001 |  |  |  |  |  |  |  |
| 1 | Ath-AT5G45870.1 |  | | | |  |  |  |  |  |  |  |
| 1 | Ath-AT5G45875.1 |  | | | |  |  |  |  |  |  |  |
| 1 | Ath-AT5G45880.1 |  | Vvi-Vitvi10g00580\_t001 |  |  |  |  |  |  |  |
| 1 | Ath-AT5G45890.1 |  | | | |  |  |  |  |  |  |  |
| 1 | Ath-AT5G45900.1 |  | Vvi-Vitvi10g00571\_t001 |  |  |  |  |  |  |  |
| 1 | Ath-AT5G45910.1 |  | Vvi-Vitvi10g00557\_t001 |  |  |  |  |  |  |  |
| 1 | Ath-AT5G45920.1 |  | Vvi-Vitvi10g00551\_t002 |  |  |  |  |  |  |  |
| 1 | Ath-AT5G45930.1 |  | Vvi-Vitvi10g00543\_t001 |  |  |  |  |  |  |  |
| 1 | Ath-AT5G45940.1 |  | Vvi-Vitvi10g00541\_t001 |  |  |  |  |  |  |  |
| 1 | Ath-AT5G45950.1 |  | Vvi-Vitvi10g00524\_t001 |  |  |  |  |  |  |  |
| 1 | Ath-AT5G45960.1 |  | Vvi-Vitvi10g00523\_t001 |  |  |  |  |  |  |  |
| 1 | Ath-AT5G45970.1 |  | Vvi-Vitvi10g00520\_t001 |  |  |  |  |  |  |  |
| 1 | Ath-AT5G45980.1 |  | Vvi-Vitvi10g00519\_t001 |  |  |  |  |  |  |  |
| 1 | Ath-AT5G45990.2 |  | | | |  |  |  |  |  |  |  |
| 1 | Ath-AT5G46000.1 |  | | | |  |  |  |  |  |  |  |
| 1 | Ath-AT5G46010.1 |  | | | |  |  |  |  |  |  |  |
| 1 | Ath-AT5G46020.1 |  | Vvi-Vitvi10g00518\_t001 |  |  |  |  |  |  |  |
| 1 | Ath-AT5G46025.1 |  | | | |  |  |  |  |  |  |  |
| 1 | Ath-AT5G46030.1 |  | Vvi-Vitvi10g04335\_t001 |  |  |  |  |  |  |  |
| 1 | Ath-AT5G46040.1 |  | | | |  |  |  |  |  |  |  |
| 1 | Ath-AT5G46050.1 |  | | | |  |  |  |  |  |  |  |
| 1 | Ath-AT5G46060.1 |  | Vvi-Vitvi10g00511\_t001 |  |  |  |  |  |  |  |
| 1 | Ath-AT5G46070.1 |  | Vvi-Vitvi10g00506\_t001 |  |  |  |  |  |  |  |
| 1 | Ath-AT5G46080.1 |  | Vvi-Vitvi10g00504\_t001 |  |  |  |  |  |  |  |
| 1 | Ath-AT5G46085.1 |  | | | |  |  |  |  |  |  |  |
| 1 | Ath-AT5G46090.1 |  | Vvi-Vitvi10g00501\_t002 |  |  |  |  |  |  |  |
| 1 | Ath-AT5G46100.1 |  | | | |  |  |  |  |  |  |  |
| 1 | Ath-AT5G46110.4 |  | Vvi-Vitvi10g00499\_t003 |  |  |  |  |  |  |  |
| 0 | Ath-AT5G46115.1 |  |  |  |  |  |  |  |  |
| 0 | Ath-AT5G46130.1 |  |  |  |  |  |  |  |  |
| 0 | Ath-AT5G46140.2 |  |  |  |  |  |  |  |  |
| 1 | Ath-AT5G46150.2 |  | Vvi-Vitvi10g00844\_t001 |  |  |  |  |  |  |  |
| 1 | Ath-AT5G46160.1 |  | Vvi-Vitvi10g01883\_t001 |  |  |  |  |  |  |  |
| 1 | Ath-AT5G46170.1 |  | Vvi-Vitvi10g00837\_t001 |  |  |  |  |  |  |  |
| 1 | Ath-AT5G46180.1 |  | Vvi-Vitvi10g00835\_t001 |  |  |  |  |  |  |  |
| 1 | Ath-AT5G46190.2 |  | Vvi-Vitvi10g00834\_t001 |  |  |  |  |  |  |  |
| 1 | Ath-AT5G46200.1 |  | | | |  |  |  |  |  |  |  |
| 1 | Ath-AT5G46210.1 |  | Vvi-Vitvi10g00816\_t001 |  |  |  |  |  |  |  |
| 1 | Ath-AT5G46220.1 |  | Vvi-Vitvi10g00782\_t001 |  |  |  |  |  |  |  |
| 1 | Ath-AT5G46230.1 |  | Vvi-Vitvi10g00781\_t001 |  |  |  |  |  |  |  |
| 1 | Ath-AT5G46240.1 |  | Vvi-Vitvi10g00777\_t001 |  |  |  |  |  |  |  |
| 1 | Ath-AT5G46250.1 |  | Vvi-Vitvi10g00767\_t002 |  |  |  |  |  |  |  |
| 1 | Ath-AT5G46260.1 |  | | | |  |  |  |  |  |  |  |
| 1 | Ath-AT5G46270.4 |  | | | |  |  |  |  |  |  |  |
| 1 | Ath-AT5G46280.1 |  | Vvi-Vitvi10g00754\_t001 |  |  |  |  |  |  |  |
| 1 | Ath-AT5G46290.3 |  | Vvi-Vitvi10g00753\_t001 |  |  |  |  |  |  |  |
| 1 | Ath-AT5G46295.1 |  | | | |  |  |  |  |  |  |  |
| 1 | Ath-AT5G46297.1 |  | | | |  |  |  |  |  |  |  |
| 1 | Ath-AT5G46300.1 |  | | | |  |  |  |  |  |  |  |
| 1 | Ath-AT5G46310.1 |  | | | |  |  |  |  |  |  |  |
| 1 | Ath-AT5G46320.1 |  | | | |  |  |  |  |  |  |  |
| 1 | Ath-AT5G46330.1 |  | Vvi-Vitvi10g00742\_t001 |  |  |  |  |  |  |  |
| 1 | Ath-AT5G46340.1 |  | Vvi-Vitvi10g00733\_t001 |  |  |  |  |  |  |  |
| 1 | Ath-AT5G46350.1 |  | Vvi-Vitvi10g00732\_t001 |  |  |  |  |  |  |  |
| 1 | Ath-AT5G46360.2 |  | | | |  |  |  |  |  |  |  |
| 1 | Ath-AT5G46370.1 |  | Vvi-Vitvi10g04409\_t001 |  |  |  |  |  |  |  |
| 1 | Ath-AT5G46380.2 |  | Vvi-Vitvi10g00722\_t001 |  |  |  |  |  |  |  |
| 1 | Ath-AT5G46390.2 |  | Vvi-Vitvi10g01837\_t001 |  |  |  |  |  |  |  |
| 1 | Ath-AT5G46395.1 |  | | | |  |  |  |  |  |  |  |
| 1 | Ath-AT5G46400.1 |  | Vvi-Vitvi10g00717\_t001 |  |  |  |  |  |  |  |
| 1 | Ath-AT5G46410.2 |  | Vvi-Vitvi10g00716\_t001 |  |  |  |  |  |  |  |
| 1 | Ath-AT5G46420.1 |  | Vvi-Vitvi10g00713\_t001 |  |  |  |  |  |  |  |
| 1 | Ath-AT5G46430.1 |  | Vvi-Vitvi10g00712\_t001 |  |  |  |  |  |  |  |
| 1 | Ath-AT5G46440.1 |  | | | |  |  |  |  |  |  |  |
| 1 | Ath-AT5G46450.1 |  | | | |  |  |  |  |  |  |  |
| 1 | Ath-AT5G46460.1 |  | Vvi-Vitvi10g00711\_t001 |  |  |  |  |  |  |  |
| 1 | Ath-AT5G46470.1 |  | | | |  |  |  |  |  |  |  |
| 1 | Ath-AT5G46490.2 |  | | | |  |  |  |  |  |  |  |
| 1 | Ath-AT5G46500.3 |  | | | |  |  |  |  |  |  |  |
| 1 | Ath-AT5G46510.1 |  | | | |  |  |  |  |  |  |  |
| 1 | Ath-AT5G46520.2 |  | | | |  |  |  |  |  |  |  |
| 1 | Ath-AT5G46530.1 |  | Vvi-Vitvi10g01835\_t001 |  |  |  |  |  |  |  |
| 1 | Ath-AT5G46540.1 |  | Vvi-Vitvi10g00702\_t001 |  |  |  |  |  |  |  |
| 0 | Ath-AT5G46550.1 |  |  |  |  |  |  |  |  |
| 0 | Ath-AT5G46560.1 |  |  |  |  |  |  |  |  |
| 1 | Ath-AT5G46570.1 |  | Vvi-Vitvi02g00682\_t001 |  |  |  |  |  |  |  |
| 2 | Ath-AT5G46580.1 |  | | | |  | Vvi-Vitvi02g00677\_t001 |  |  |  |  |  |  |
| 2 | Ath-AT5G46590.1 |  | | | |  | Vvi-Vitvi02g00673\_t001 |  |  |  |  |  |  |
| 2 | Ath-AT5G46600.1 |  | | | |  | Vvi-Vitvi02g00671\_t001 |  |  |  |  |  |  |
| 2 | Ath-AT5G46610.1 |  | | | |  | | | |  |  |  |  |  |  |
| 2 | Ath-AT5G46620.1 |  | | | |  | Vvi-Vitvi02g00663\_t001 |  |  |  |  |  |  |
| 2 | Ath-AT5G46630.2 |  | | | |  | Vvi-Vitvi02g00661\_t001 |  |  |  |  |  |  |
| 2 | Ath-AT5G46640.1 |  | | | |  | Vvi-Vitvi02g00660\_t001 |  |  |  |  |  |  |
| 2 | Ath-AT5G46650.1 |  | | | |  | Vvi-Vitvi02g00714\_t001 |  |  |  |  |  |  |
| 2 | Ath-AT5G46660.1 |  | | | |  | | | |  |  |  |  |  |  |
| 2 | Ath-AT5G46670.1 |  | | | |  | | | |  |  |  |  |  |  |
| 2 | Ath-AT5G46680.1 |  | | | |  | | | |  |  |  |  |  |  |
| 2 | Ath-AT5G46690.1 |  | | | |  | Vvi-Vitvi02g00709\_t001 |  |  |  |  |  |  |
| 2 | Ath-AT5G46700.1 |  | | | |  | Vvi-Vitvi02g00705\_t001 |  |  |  |  |  |  |
| 2 | Ath-AT5G46710.1 |  | Vvi-Vitvi02g00704\_t001 |  | | | |  |  |  |  |  |  |
| 2 | Ath-AT5G46720.1 |  | | | |  | | | |  |  |  |  |  |  |
| 2 | Ath-AT5G46730.1 |  | | | |  | | | |  |  |  |  |  |  |
| 2 | Ath-AT5G46740.1 |  | | | |  | Vvi-Vitvi02g00700\_t001 |  |  |  |  |  |  |
| 2 | Ath-AT5G46750.1 |  | | | |  | Vvi-Vitvi02g00699\_t001 |  |  |  |  |  |  |
| 2 | Ath-AT5G46760.1 |  | | | |  | Vvi-Vitvi02g00698\_t001 |  |  |  |  |  |  |
| 2 | Ath-AT5G46770.1 |  | | | |  | | | |  |  |  |  |  |  |
| 2 | Ath-AT5G46780.1 |  | | | |  | Vvi-Vitvi02g00696\_t001 |  |  |  |  |  |  |
| 2 | Ath-AT5G46790.1 |  | | | |  | Vvi-Vitvi02g00695\_t001 |  |  |  |  |  |  |
| 2 | Ath-AT5G46795.1 |  | | | |  | Vvi-Vitvi02g00694\_t001 |  |  |  |  |  |  |
| 2 | Ath-AT5G46800.1 |  | | | |  | Vvi-Vitvi02g00693\_t001 |  |  |  |  |  |  |
| 1 | Ath-AT5G46810.1 |  | | | |  |  |  |  |  |  |  |
| 1 | Ath-AT5G46820.1 |  | | | |  |  |  |  |  |  |  |
| 1 | Ath-AT5G46825.1 |  | | | |  |  |  |  |  |  |  |
| 1 | Ath-AT5G46830.1 |  | | | |  |  |  |  |  |  |  |
| 1 | Ath-AT5G46840.1 |  | Vvi-Vitvi02g00722\_t001 |  |  |  |  |  |  |  |
| 1 | Ath-AT5G46850.2 |  | Vvi-Vitvi02g00746\_t001 |  |  |  |  |  |  |  |
| 1 | Ath-AT5G46860.1 |  | Vvi-Vitvi02g00747\_t001 |  |  |  |  |  |  |  |
| 1 | Ath-AT5G46870.1 |  | Vvi-Vitvi02g00749\_t001 |  |  |  |  |  |  |  |
| 1 | Ath-AT5G46871.1 |  | | | |  |  |  |  |  |  |  |
| 1 | Ath-AT5G46873.1 |  | | | |  |  |  |  |  |  |  |
| 1 | Ath-AT5G46874.1 |  | | | |  |  |  |  |  |  |  |
| 1 | Ath-AT5G46877.1 |  | | | |  |  |  |  |  |  |  |
| 1 | Ath-AT5G46880.2 |  | | | |  |  |  |  |  |  |  |
| 1 | Ath-AT5G46890.1 |  | | | |  |  |  |  |  |  |  |
| 1 | Ath-AT5G46900.1 |  | | | |  |  |  |  |  |  |  |
| 1 | Ath-AT5G46910.1 |  | Vvi-Vitvi02g00759\_t001 |  |  |  |  |  |  |  |
| 0 | Ath-AT5G46915.1 |  |  |  |  |  |  |  |  |
| 0 | Ath-AT5G46920.1 |  |  |  |  |  |  |  |  |
| 1 | Ath-AT5G46930.1 |  | Vvi-Vitvi02g01481\_t001 |  |  |  |  |  |  |  |
| 1 | Ath-AT5G46940.1 |  | | | |  |  |  |  |  |  |  |
| 1 | Ath-AT5G46950.1 |  | | | |  |  |  |  |  |  |  |
| 1 | Ath-AT5G46960.1 |  | | | |  |  |  |  |  |  |  |
| 1 | Ath-AT5G46970.1 |  | | | |  |  |  |  |  |  |  |
| 1 | Ath-AT5G46980.1 |  | | | |  |  |  |  |  |  |  |
| 1 | Ath-AT5G46990.1 |  | | | |  |  |  |  |  |  |  |
| 1 | Ath-AT5G47000.1 |  | | | |  |  |  |  |  |  |  |
| 1 | Ath-AT5G47010.1 |  | Vvi-Vitvi02g00627\_t001 |  |  |  |  |  |  |  |
| 1 | Ath-AT5G47020.1 |  | Vvi-Vitvi02g00626\_t001 |  |  |  |  |  |  |  |
| 1 | Ath-AT5G47030.1 |  | | | |  |  |  |  |  |  |  |
| 1 | Ath-AT5G47040.1 |  | Vvi-Vitvi02g00617\_t001 |  |  |  |  |  |  |  |
| 1 | Ath-AT5G47050.1 |  | Vvi-Vitvi02g01471\_t001 |  |  |  |  |  |  |  |
| 1 | Ath-AT5G47060.1 |  | Vvi-Vitvi02g00614\_t001 |  |  |  |  |  |  |  |
| 1 | Ath-AT5G47070.1 |  | Vvi-Vitvi02g00604\_t001 |  |  |  |  |  |  |  |
| 1 | Ath-AT5G47075.1 |  | | | |  |  |  |  |  |  |  |
| 1 | Ath-AT5G47077.1 |  | | | |  |  |  |  |  |  |  |
| 1 | Ath-AT5G47080.1 |  | Vvi-Vitvi02g00597\_t001 |  |  |  |  |  |  |  |
| 1 | Ath-AT5G47090.1 |  | Vvi-Vitvi02g00593\_t001 |  |  |  |  |  |  |  |
| 0 | Ath-AT5G47100.1 |  |  |  |  |  |  |  |  |
| 0 | Ath-AT5G47110.1 |  |  |  |  |  |  |  |  |
| 0 | Ath-AT5G47120.1 |  |  |  |  |  |  |  |  |
| 0 | Ath-AT5G47130.2 |  |  |  |  |  |  |  |  |
| 0 | Ath-AT5G47140.1 |  |  |  |  |  |  |  |  |
| 0 | Ath-AT5G47150.1 |  |  |  |  |  |  |  |  |
| 0 | Ath-AT5G47160.1 |  |  |  |  |  |  |  |  |
| 0 | Ath-AT5G47170.1 |  |  |  |  |  |  |  |  |
| 0 | Ath-AT5G47175.1 |  |  |  |  |  |  |  |  |
| 0 | Ath-AT5G47180.1 |  |  |  |  |  |  |  |  |
| 0 | Ath-AT5G47190.1 |  |  |  |  |  |  |  |  |
| 0 | Ath-AT5G47200.1 |  |  |  |  |  |  |  |  |
| 0 | Ath-AT5G47210.1 |  |  |  |  |  |  |  |  |
| 1 | Ath-AT5G47220.1 |  | Vvi-Vitvi02g01780\_t001 |  |  |  |  |  |  |  |
| 1 | Ath-AT5G47229.1 |  | | | |  |  |  |  |  |  |  |
| 1 | Ath-AT5G47230.1 |  | | | |  |  |  |  |  |  |  |
| 1 | Ath-AT5G47240.2 |  | Vvi-Vitvi00g04041\_t002 |  |  |  |  |  |  |  |
| 1 | Ath-AT5G47250.1 |  | | | |  |  |  |  |  |  |  |
| 1 | Ath-AT5G47260.1 |  | | | |  |  |  |  |  |  |  |
| 1 | Ath-AT5G47280.1 |  | | | |  |  |  |  |  |  |  |
| 1 | Ath-AT5G47290.1 |  | | | |  |  |  |  |  |  |  |
| 1 | Ath-AT5G47300.1 |  | | | |  |  |  |  |  |  |  |
| 1 | Ath-AT5G47310.1 |  | Vvi-Vitvi00g04042\_t004 |  |  |  |  |  |  |  |
| 1 | Ath-AT5G47320.1 |  | | | |  |  |  |  |  |  |  |
| 1 | Ath-AT5G47330.1 |  | Vvi-Vitvi02g01739\_t001 |  |  |  |  |  |  |  |
| 1 | Ath-AT5G47340.1 |  | | | |  |  |  |  |  |  |  |
| 1 | Ath-AT5G47350.1 |  | | | |  |  |  |  |  |  |  |
| 1 | Ath-AT5G47360.1 |  | Vvi-Vitvi02g01738\_t001 |  |  |  |  |  |  |  |
| 2 | Ath-AT5G47370.1 |  | | | |  | Vvi-Vitvi02g01717\_t001 |  |  |  |  |  |  |
| 2 | Ath-AT5G47380.1 |  | | | |  | | | |  |  |  |  |  |  |
| 2 | Ath-AT5G47390.1 |  | | | |  | | | |  |  |  |  |  |  |
| 2 | Ath-AT5G47400.1 |  | Vvi-Vitvi00g04048\_t001 |  | | | |  |  |  |  |  |  |
| 2 | Ath-AT5G47420.1 |  | Vvi-Vitvi00g04051\_t001 |  | | | |  |  |  |  |  |  |
| 1 | Ath-AT5G47430.6 |  |  |  | Vvi-Vitvi02g01714\_t001 |  |  |  |  |  |  |
| 1 | Ath-AT5G47435.1 |  |  |  | Vvi-Vitvi02g00572\_t001 |  |  |  |  |  |  |
| 1 | Ath-AT5G47440.1 |  |  |  | Vvi-Vitvi02g04171\_t001 |  |  |  |  |  |  |
| 1 | Ath-AT5G47450.1 |  |  |  | Vvi-Vitvi02g00568\_t001 |  |  |  |  |  |  |
| 1 | Ath-AT5G47455.4 |  |  |  | Vvi-Vitvi02g01706\_t006 |  |  |  |  |  |  |
| 1 | Ath-AT5G47460.1 |  |  |  | Vvi-Vitvi02g00563\_t001 |  |  |  |  |  |  |
| 1 | Ath-AT5G47470.2 |  |  |  | Vvi-Vitvi02g04169\_t001 |  |  |  |  |  |  |
| 1 | Ath-AT5G47480.1 |  |  |  | Vvi-Vitvi02g04167\_t001 |  |  |  |  |  |  |
| 1 | Ath-AT5G47490.2 |  |  |  | | | |  |  |  |  |  |  |
| 1 | Ath-AT5G47500.1 |  |  |  | Vvi-Vitvi02g00553\_t001 |  |  |  |  |  |  |
| 1 | Ath-AT5G47510.4 |  |  |  | Vvi-Vitvi02g00546\_t001 |  |  |  |  |  |  |
| 1 | Ath-AT5G47520.1 |  |  |  | | | |  |  |  |  |  |  |
| 1 | Ath-AT5G47530.1 |  |  |  | | | |  |  |  |  |  |  |
| 1 | Ath-AT5G47540.1 |  |  |  | | | |  |  |  |  |  |  |
| 1 | Ath-AT5G47550.1 |  |  |  | | | |  |  |  |  |  |  |
| 1 | Ath-AT5G47560.1 |  |  |  | | | |  |  |  |  |  |  |
| 1 | Ath-AT5G47570.1 |  |  |  | | | |  |  |  |  |  |  |
| 1 | Ath-AT5G47580.1 |  |  |  | Vvi-Vitvi02g00538\_t001 |  |  |  |  |  |  |
| 0 | Ath-AT5G47590.1 |  |  |  |  |  |  |  |  |
| 0 | Ath-AT5G47600.1 |  |  |  |  |  |  |  |  |
| 0 | Ath-AT5G47610.1 |  |  |  |  |  |  |  |  |
| 0 | Ath-AT5G47620.4 |  |  |  |  |  |  |  |  |
| 1 | Ath-AT5G47630.1 |  | Vvi-Vitvi10g00249\_t001 |  |  |  |  |  |  |  |
| 1 | Ath-AT5G47635.1 |  | Vvi-Vitvi10g01710\_t001 |  |  |  |  |  |  |  |
| 1 | Ath-AT5G47640.1 |  | Vvi-Vitvi10g00251\_t001 |  |  |  |  |  |  |  |
| 1 | Ath-AT5G47650.2 |  | Vvi-Vitvi10g00252\_t001 |  |  |  |  |  |  |  |
| 1 | Ath-AT5G47660.1 |  | Vvi-Vitvi10g02271\_t001 |  |  |  |  |  |  |  |
| 1 | Ath-AT5G47670.3 |  | Vvi-Vitvi10g02273\_t001 |  |  |  |  |  |  |  |
| 1 | Ath-AT5G47680.1 |  | Vvi-Vitvi10g04126\_t001 |  |  |  |  |  |  |  |
| 1 | Ath-AT5G47690.3 |  | Vvi-Vitvi10g04127\_t001 |  |  |  |  |  |  |  |
| 1 | Ath-AT5G47700.1 |  | | | |  |  |  |  |  |  |  |
| 1 | Ath-AT5G47710.1 |  | Vvi-Vitvi10g02267\_t001 |  |  |  |  |  |  |  |
| 1 | Ath-AT5G47720.2 |  | Vvi-Vitvi10g02269\_t001 |  |  |  |  |  |  |  |
| 0 | Ath-AT5G47730.1 |  |  |  |  |  |  |  |  |
| 0 | Ath-AT5G47740.2 |  |  |  |  |  |  |  |  |
| 0 | Ath-AT5G47750.1 |  |  |  |  |  |  |  |  |
| 0 | Ath-AT5G47760.1 |  |  |  |  |  |  |  |  |
| 0 | Ath-AT5G47770.1 |  |  |  |  |  |  |  |  |
| 0 | Ath-AT5G47780.1 |  |  |  |  |  |  |  |  |
| 0 | Ath-AT5G47790.1 |  |  |  |  |  |  |  |  |
| 0 | Ath-AT5G47800.2 |  |  |  |  |  |  |  |  |
| 0 | Ath-AT5G47810.1 |  |  |  |  |  |  |  |  |
| 0 | Ath-AT5G47820.1 |  |  |  |  |  |  |  |  |
| 0 | Ath-AT5G47830.1 |  |  |  |  |  |  |  |  |
| 0 | Ath-AT5G47840.1 |  |  |  |  |  |  |  |  |
| 0 | Ath-AT5G47850.1 |  |  |  |  |  |  |  |  |
| 1 | Ath-AT5G47860.1 |  | Vvi-Vitvi01g01821\_t001 |  |  |  |  |  |  |  |
| 1 | Ath-AT5G47870.1 |  | Vvi-Vitvi01g01820\_t001 |  |  |  |  |  |  |  |
| 1 | Ath-AT5G47880.1 |  | | | |  |  |  |  |  |  |  |
| 1 | Ath-AT5G47890.1 |  | Vvi-Vitvi01g01813\_t003 |  |  |  |  |  |  |  |
| 1 | Ath-AT5G47900.9 |  | Vvi-Vitvi01g01811\_t001 |  |  |  |  |  |  |  |
| 1 | Ath-AT5G47910.1 |  | Vvi-Vitvi01g01803\_t001 |  |  |  |  |  |  |  |
| 1 | Ath-AT5G47920.1 |  | Vvi-Vitvi01g01798\_t001 |  |  |  |  |  |  |  |
| 1 | Ath-AT5G47930.1 |  | Vvi-Vitvi01g01792\_t001 |  |  |  |  |  |  |  |
| 0 | Ath-AT5G47940.1 |  |  |  |  |  |  |  |  |
| 0 | Ath-AT5G47950.1 |  |  |  |  |  |  |  |  |
| 0 | Ath-AT5G47960.1 |  |  |  |  |  |  |  |  |
| 0 | Ath-AT5G47970.1 |  |  |  |  |  |  |  |  |
| 0 | Ath-AT5G47980.1 |  |  |  |  |  |  |  |  |
| 0 | Ath-AT5G47990.1 |  |  |  |  |  |  |  |  |
| 0 | Ath-AT5G48000.1 |  |  |  |  |  |  |  |  |
| 0 | Ath-AT5G48010.2 |  |  |  |  |  |  |  |  |
| 0 | Ath-AT5G48020.1 |  |  |  |  |  |  |  |  |
| 0 | Ath-AT5G48030.1 |  |  |  |  |  |  |  |  |
| 0 | Ath-AT5G48040.1 |  |  |  |  |  |  |  |  |
| 0 | Ath-AT5G48050.1 |  |  |  |  |  |  |  |  |
| 0 | Ath-AT5G48060.1 |  |  |  |  |  |  |  |  |
| 0 | Ath-AT5G48070.1 |  |  |  |  |  |  |  |  |
| 0 | Ath-AT5G48090.1 |  |  |  |  |  |  |  |  |
| 0 | Ath-AT5G48100.1 |  |  |  |  |  |  |  |  |
| 0 | Ath-AT5G48110.1 |  |  |  |  |  |  |  |  |
| 0 | Ath-AT5G48120.1 |  |  |  |  |  |  |  |  |
| 0 | Ath-AT5G48130.1 |  |  |  |  |  |  |  |  |
| 0 | Ath-AT5G48140.1 |  |  |  |  |  |  |  |  |
| 0 | Ath-AT5G48150.3 |  |  |  |  |  |  |  |  |
| 0 | Ath-AT5G48160.1 |  |  |  |  |  |  |  |  |
| 0 | Ath-AT5G48170.1 |  |  |  |  |  |  |  |  |
| 0 | Ath-AT5G48175.1 |  |  |  |  |  |  |  |  |
| 1 | Ath-AT5G48180.1 |  | Vvi-Vitvi12g00727\_t001 |  |  |  |  |  |  |  |
| 1 | Ath-AT5G48190.1 |  | | | |  |  |  |  |  |  |  |
| 1 | Ath-AT5G48200.1 |  | | | |  |  |  |  |  |  |  |
| 1 | Ath-AT5G48205.1 |  | | | |  |  |  |  |  |  |  |
| 1 | Ath-AT5G48210.1 |  | | | |  |  |  |  |  |  |  |
| 1 | Ath-AT5G48220.1 |  | | | |  |  |  |  |  |  |  |
| 1 | Ath-AT5G48230.2 |  | Vvi-Vitvi12g02504\_t002 |  |  |  |  |  |  |  |
| 1 | Ath-AT5G48240.3 |  | Vvi-Vitvi12g00756\_t001 |  |  |  |  |  |  |  |
| 1 | Ath-AT5G48250.1 |  | Vvi-Vitvi12g00757\_t001 |  |  |  |  |  |  |  |
| 1 | Ath-AT5G48270.1 |  | Vvi-Vitvi12g00762\_t001 |  |  |  |  |  |  |  |
| 1 | Ath-AT5G48280.1 |  | | | |  |  |  |  |  |  |  |
| 1 | Ath-AT5G48290.3 |  | Vvi-Vitvi12g02509\_t001 |  |  |  |  |  |  |  |
| 1 | Ath-AT5G48300.1 |  | Vvi-Vitvi12g00770\_t001 |  |  |  |  |  |  |  |
| 1 | Ath-AT5G48310.1 |  | Vvi-Vitvi12g00774\_t001 |  |  |  |  |  |  |  |
| 1 | Ath-AT5G48320.1 |  | | | |  |  |  |  |  |  |  |
| 1 | Ath-AT5G48330.1 |  | Vvi-Vitvi12g00779\_t001 |  |  |  |  |  |  |  |
| 0 | Ath-AT5G48335.1 |  |  |  |  |  |  |  |  |
| 0 | Ath-AT5G48340.1 |  |  |  |  |  |  |  |  |
| 0 | Ath-AT5G48350.1 |  |  |  |  |  |  |  |  |
| 0 | Ath-AT5G48360.1 |  |  |  |  |  |  |  |  |
| 0 | Ath-AT5G48370.1 |  |  |  |  |  |  |  |  |
| 0 | Ath-AT5G48375.1 |  |  |  |  |  |  |  |  |
| 1 | Ath-AT5G48380.1 |  | Vvi-Vitvi07g03153\_t001 |  |  |  |  |  |  |  |
| 1 | Ath-AT5G48385.1 |  | | | |  |  |  |  |  |  |  |
| 1 | Ath-AT5G48390.1 |  | | | |  |  |  |  |  |  |  |
| 1 | Ath-AT5G48400.2 |  | | | |  |  |  |  |  |  |  |
| 1 | Ath-AT5G48410.1 |  | | | |  |  |  |  |  |  |  |
| 1 | Ath-AT5G48420.1 |  | | | |  |  |  |  |  |  |  |
| 1 | Ath-AT5G48430.1 |  | | | |  |  |  |  |  |  |  |
| 1 | Ath-AT5G48440.1 |  | Vvi-Vitvi07g04407\_t001 |  |  |  |  |  |  |  |
| 1 | Ath-AT5G48450.1 |  | Vvi-Vitvi07g01163\_t001 |  |  |  |  |  |  |  |
| 1 | Ath-AT5G48460.1 |  | Vvi-Vitvi07g04400\_t001 |  |  |  |  |  |  |  |
| 1 | Ath-AT5G48470.1 |  | Vvi-Vitvi07g01137\_t002 |  |  |  |  |  |  |  |
| 1 | Ath-AT5G48480.1 |  | Vvi-Vitvi07g01139\_t001 |  |  |  |  |  |  |  |
| 1 | Ath-AT5G48485.1 |  | | | |  |  |  |  |  |  |  |
| 1 | Ath-AT5G48490.1 |  | | | |  |  |  |  |  |  |  |
| 1 | Ath-AT5G48500.1 |  | Vvi-Vitvi07g01140\_t001 |  |  |  |  |  |  |  |
| 1 | Ath-AT5G48510.1 |  | | | |  |  |  |  |  |  |  |
| 1 | Ath-AT5G48515.1 |  | | | |  |  |  |  |  |  |  |
| 1 | Ath-AT5G48520.2 |  | Vvi-Vitvi07g04397\_t002 |  |  |  |  |  |  |  |
| 1 | Ath-AT5G48530.1 |  | | | |  |  |  |  |  |  |  |
| 1 | Ath-AT5G48540.1 |  | Vvi-Vitvi07g01133\_t001 |  |  |  |  |  |  |  |
| 0 | Ath-AT5G48543.1 |  |  |  |  |  |  |  |  |
| 0 | Ath-AT5G48545.1 |  |  |  |  |  |  |  |  |
| 0 | Ath-AT5G48550.1 |  |  |  |  |  |  |  |  |
| 0 | Ath-AT5G48560.1 |  |  |  |  |  |  |  |  |
| 0 | Ath-AT5G48570.1 |  |  |  |  |  |  |  |  |
| 0 | Ath-AT5G48575.1 |  |  |  |  |  |  |  |  |
| 0 | Ath-AT5G48580.1 |  |  |  |  |  |  |  |  |
| 0 | Ath-AT5G48590.1 |  |  |  |  |  |  |  |  |
| 0 | Ath-AT5G48595.1 |  |  |  |  |  |  |  |  |
| 0 | Ath-AT5G48600.2 |  |  |  |  |  |  |  |  |
| 0 | Ath-AT5G48605.1 |  |  |  |  |  |  |  |  |
| 0 | Ath-AT5G48610.3 |  |  |  |  |  |  |  |  |
| 1 | Ath-AT5G48620.2 |  | Vvi-Vitvi07g02386\_t001 |  |  |  |  |  |  |  |
| 1 | Ath-AT5G48630.2 |  | | | |  |  |  |  |  |  |  |
| 1 | Ath-AT5G48640.1 |  | | | |  |  |  |  |  |  |  |
| 1 | Ath-AT5G48650.1 |  | | | |  |  |  |  |  |  |  |
| 1 | Ath-AT5G48655.3 |  | | | |  |  |  |  |  |  |  |
| 1 | Ath-AT5G48657.5 |  | | | |  |  |  |  |  |  |  |
| 1 | Ath-AT5G48660.1 |  | | | |  |  |  |  |  |  |  |
| 1 | Ath-AT5G48670.1 |  | | | |  |  |  |  |  |  |  |
| 1 | Ath-AT5G48680.1 |  | | | |  |  |  |  |  |  |  |
| 1 | Ath-AT5G48690.1 |  | | | |  |  |  |  |  |  |  |
| 1 | Ath-AT5G48700.1 |  | | | |  |  |  |  |  |  |  |
| 1 | Ath-AT5G48710.3 |  | | | |  |  |  |  |  |  |  |
| 1 | Ath-AT5G48720.2 |  | | | |  |  |  |  |  |  |  |
| 1 | Ath-AT5G48730.1 |  | Vvi-Vitvi07g00949\_t001 |  |  |  |  |  |  |  |
| 1 | Ath-AT5G48740.1 |  | Vvi-Vitvi07g00924\_t001 |  |  |  |  |  |  |  |
| 1 | Ath-AT5G48750.1 |  | | | |  |  |  |  |  |  |  |
| 1 | Ath-AT5G48760.1 |  | Vvi-Vitvi07g00923\_t001 |  |  |  |  |  |  |  |
| 1 | Ath-AT5G48770.1 |  | | | |  |  |  |  |  |  |  |
| 1 | Ath-AT5G48780.1 |  | | | |  |  |  |  |  |  |  |
| 1 | Ath-AT5G48790.1 |  | | | |  |  |  |  |  |  |  |
| 1 | Ath-AT5G48800.1 |  | Vvi-Vitvi07g00905\_t001 |  |  |  |  |  |  |  |
| 1 | Ath-AT5G48810.1 |  | Vvi-Vitvi07g00903\_t001 |  |  |  |  |  |  |  |
| 1 | Ath-AT5G48820.2 |  | | | |  |  |  |  |  |  |  |
| 1 | Ath-AT5G48830.2 |  | Vvi-Vitvi07g00884\_t001 |  |  |  |  |  |  |  |
| 1 | Ath-AT5G48840.1 |  | Vvi-Vitvi07g00824\_t001 |  |  |  |  |  |  |  |
| 1 | Ath-AT5G48850.1 |  | Vvi-Vitvi07g00807\_t001 |  |  |  |  |  |  |  |
| 1 | Ath-AT5G48860.1 |  | | | |  |  |  |  |  |  |  |
| 1 | Ath-AT5G48870.1 |  | | | |  |  |  |  |  |  |  |
| 1 | Ath-AT5G48880.2 |  | Vvi-Vitvi07g00795\_t001 |  |  |  |  |  |  |  |
| 1 | Ath-AT5G48890.1 |  | Vvi-Vitvi07g02342\_t001 |  |  |  |  |  |  |  |
| 1 | Ath-AT5G48900.1 |  | Vvi-Vitvi07g00787\_t001 |  |  |  |  |  |  |  |
| 1 | Ath-AT5G48905.1 |  | | | |  |  |  |  |  |  |  |
| 1 | Ath-AT5G48910.1 |  | | | |  |  |  |  |  |  |  |
| 1 | Ath-AT5G48920.1 |  | | | |  |  |  |  |  |  |  |
| 1 | Ath-AT5G48930.1 |  | | | |  |  |  |  |  |  |  |
| 1 | Ath-AT5G48940.1 |  | Vvi-Vitvi07g02330\_t001 |  |  |  |  |  |  |  |
| 0 | Ath-AT5G48945.1 |  |  |  |  |  |  |  |  |
| 0 | Ath-AT5G48950.1 |  |  |  |  |  |  |  |  |
| 0 | Ath-AT5G48953.1 |  |  |  |  |  |  |  |  |
| 0 | Ath-AT5G48960.1 |  |  |  |  |  |  |  |  |
| 0 | Ath-AT5G48970.1 |  |  |  |  |  |  |  |  |
| 0 | Ath-AT5G48980.1 |  |  |  |  |  |  |  |  |
| 0 | Ath-AT5G48990.1 |  |  |  |  |  |  |  |  |
| 0 | Ath-AT5G49000.2 |  |  |  |  |  |  |  |  |
| 0 | Ath-AT5G49010.1 |  |  |  |  |  |  |  |  |
| 0 | Ath-AT5G49015.2 |  |  |  |  |  |  |  |  |
| 0 | Ath-AT5G49020.1 |  |  |  |  |  |  |  |  |
| 0 | Ath-AT5G49030.3 |  |  |  |  |  |  |  |  |
| 0 | Ath-AT5G49040.1 |  |  |  |  |  |  |  |  |
| 0 | Ath-AT5G49050.1 |  |  |  |  |  |  |  |  |
| 1 | Ath-AT5G49060.1 |  | Vvi-Vitvi05g00001\_t002 |  |  |  |  |  |  |  |
| 1 | Ath-AT5G49070.1 |  | | | |  |  |  |  |  |  |  |
| 1 | Ath-AT5G49100.1 |  | Vvi-Vitvi05g00012\_t001 |  |  |  |  |  |  |  |
| 1 | Ath-AT5G49110.2 |  | Vvi-Vitvi05g00026\_t001 |  |  |  |  |  |  |  |
| 1 | Ath-AT5G49120.1 |  | Vvi-Vitvi05g00028\_t001 |  |  |  |  |  |  |  |
| 1 | Ath-AT5G49130.1 |  | Vvi-Vitvi05g00031\_t001 |  |  |  |  |  |  |  |
| 1 | Ath-AT5G49140.1 |  | | | |  |  |  |  |  |  |  |
| 1 | Ath-AT5G49150.2 |  | Vvi-Vitvi05g00034\_t001 |  |  |  |  |  |  |  |
| 1 | Ath-AT5G49160.1 |  | | | |  |  |  |  |  |  |  |
| 1 | Ath-AT5G49170.1 |  | Vvi-Vitvi05g01743\_t001 |  |  |  |  |  |  |  |
| 1 | Ath-AT5G49180.1 |  | Vvi-Vitvi05g00043\_t001 |  |  |  |  |  |  |  |
| 2 | Ath-AT5G49190.1 |  | Vvi-Vitvi05g00045\_t001 |  | Vvi-Vitvi07g00353\_t001 |  |  |  |  |  |  |
| 2 | Ath-AT5G49200.1 |  | | | |  | | | |  |  |  |  |  |  |
| 2 | Ath-AT5G49210.1 |  | | | |  | | | |  |  |  |  |  |  |
| 2 | Ath-AT5G49215.1 |  | Vvi-Vitvi05g00061\_t001 |  | Vvi-Vitvi07g00363\_t001 |  |  |  |  |  |  |
| 2 | Ath-AT5G49220.1 |  | Vvi-Vitvi05g00073\_t002 |  | Vvi-Vitvi07g00370\_t001 |  |  |  |  |  |  |
| 2 | Ath-AT5G49230.1 |  | Vvi-Vitvi05g01762\_t001 |  | Vvi-Vitvi07g04089\_t001 |  |  |  |  |  |  |
| 2 | Ath-AT5G49240.1 |  | | | |  | | | |  |  |  |  |  |  |
| 2 | Ath-AT5G49250.1 |  | | | |  | | | |  |  |  |  |  |  |
| 2 | Ath-AT5G49260.1 |  | | | |  | | | |  |  |  |  |  |  |
| 2 | Ath-AT5G49270.1 |  | Vvi-Vitvi05g00076\_t001 |  | Vvi-Vitvi07g00375\_t001 |  |  |  |  |  |  |
| 2 | Ath-AT5G49280.1 |  | | | |  | | | |  |  |  |  |  |  |
| 2 | Ath-AT5G49290.2 |  | | | |  | | | |  |  |  |  |  |  |
| 2 | Ath-AT5G49300.1 |  | Vvi-Vitvi05g00077\_t001 |  | Vvi-Vitvi07g02214\_t001 |  |  |  |  |  |  |
| 2 | Ath-AT5G49305.1 |  | | | |  | | | |  |  |  |  |  |  |
| 2 | Ath-AT5G49310.1 |  | | | |  | | | |  |  |  |  |  |  |
| 2 | Ath-AT5G49320.1 |  | Vvi-Vitvi05g00079\_t001 |  | | | |  |  |  |  |  |  |
| 2 | Ath-AT5G49330.1 |  | Vvi-Vitvi05g00084\_t001 |  | Vvi-Vitvi07g00393\_t001 |  |  |  |  |  |  |
| 2 | Ath-AT5G49340.1 |  | Vvi-Vitvi05g00086\_t001 |  | | | |  |  |  |  |  |  |
| 2 | Ath-AT5G49350.1 |  | Vvi-Vitvi05g01767\_t001 |  | | | |  |  |  |  |  |  |
| 2 | Ath-AT5G49360.1 |  | Vvi-Vitvi05g00089\_t001 |  | Vvi-Vitvi07g00401\_t001 |  |  |  |  |  |  |
| 2 | Ath-AT5G49390.1 |  | Vvi-Vitvi05g00101\_t002 |  | | | |  |  |  |  |  |  |
| 2 | Ath-AT5G49400.1 |  | Vvi-Vitvi05g00104\_t001 |  | | | |  |  |  |  |  |  |
| 2 | Ath-AT5G49410.1 |  | | | |  | | | |  |  |  |  |  |  |
| 2 | Ath-AT5G49420.1 |  | | | |  | | | |  |  |  |  |  |  |
| 2 | Ath-AT5G49430.1 |  | Vvi-Vitvi05g00106\_t001 |  | Vvi-Vitvi07g00410\_t002 |  |  |  |  |  |  |
| 1 | Ath-AT5G49440.1 |  | | | |  |  |  |  |  |  |  |
| 1 | Ath-AT5G49450.1 |  | | | |  |  |  |  |  |  |  |
| 1 | Ath-AT5G49460.1 |  | Vvi-Vitvi05g00129\_t002 |  |  |  |  |  |  |  |
| 1 | Ath-AT5G49470.2 |  | Vvi-Vitvi05g00132\_t001 |  |  |  |  |  |  |  |
| 1 | Ath-AT5G49480.1 |  | Vvi-Vitvi05g01776\_t001 |  |  |  |  |  |  |  |
| 1 | Ath-AT5G49490.1 |  | | | |  |  |  |  |  |  |  |
| 1 | Ath-AT5G49500.1 |  | | | |  |  |  |  |  |  |  |
| 1 | Ath-AT5G49510.1 |  | Vvi-Vitvi05g00144\_t001 |  |  |  |  |  |  |  |
| 1 | Ath-AT5G49520.1 |  | Vvi-Vitvi05g00145\_t001 |  |  |  |  |  |  |  |
| 1 | Ath-AT5G49525.1 |  | | | |  |  |  |  |  |  |  |
| 1 | Ath-AT5G49530.1 |  | | | |  |  |  |  |  |  |  |
| 1 | Ath-AT5G49540.1 |  | Vvi-Vitvi05g04028\_t001 |  |  |  |  |  |  |  |
| 1 | Ath-AT5G49550.1 |  | | | |  |  |  |  |  |  |  |
| 1 | Ath-AT5G49555.1 |  | | | |  |  |  |  |  |  |  |
| 1 | Ath-AT5G49560.1 |  | | | |  |  |  |  |  |  |  |
| 1 | Ath-AT5G49570.1 |  | | | |  |  |  |  |  |  |  |
| 1 | Ath-AT5G49580.1 |  | Vvi-Vitvi05g00158\_t002 |  |  |  |  |  |  |  |
| 1 | Ath-AT5G49590.1 |  | | | |  |  |  |  |  |  |  |
| 1 | Ath-AT5G49600.1 |  | | | |  |  |  |  |  |  |  |
| 1 | Ath-AT5G49610.1 |  | Vvi-Vitvi05g00159\_t001 |  |  |  |  |  |  |  |
| 1 | Ath-AT5G49620.2 |  | Vvi-Vitvi05g00166\_t001 |  |  |  |  |  |  |  |
| 0 | Ath-AT5G49630.1 |  |  |  |  |  |  |  |  |
| 0 | Ath-AT5G49640.1 |  |  |  |  |  |  |  |  |
| 0 | Ath-AT5G49645.1 |  |  |  |  |  |  |  |  |
| 0 | Ath-AT5G49650.1 |  |  |  |  |  |  |  |  |
| 0 | Ath-AT5G49660.1 |  |  |  |  |  |  |  |  |
| 0 | Ath-AT5G49665.1 |  |  |  |  |  |  |  |  |
| 0 | Ath-AT5G49680.2 |  |  |  |  |  |  |  |  |
| 0 | Ath-AT5G49690.1 |  |  |  |  |  |  |  |  |
| 0 | Ath-AT5G49700.1 |  |  |  |  |  |  |  |  |
| 0 | Ath-AT5G49710.3 |  |  |  |  |  |  |  |  |
| 1 | Ath-AT5G49720.1 |  | Vvi-Vitvi12g02167\_t001 |  |  |  |  |  |  |  |
| 1 | Ath-AT5G49730.1 |  | Vvi-Vitvi12g02162\_t001 |  |  |  |  |  |  |  |
| 1 | Ath-AT5G49740.1 |  | | | |  |  |  |  |  |  |  |
| 1 | Ath-AT5G49750.1 |  | | | |  |  |  |  |  |  |  |
| 1 | Ath-AT5G49760.1 |  | Vvi-Vitvi12g02757\_t001 |  |  |  |  |  |  |  |
| 1 | Ath-AT5G49770.1 |  | | | |  |  |  |  |  |  |  |
| 1 | Ath-AT5G49780.2 |  | | | |  |  |  |  |  |  |  |
| 1 | Ath-AT5G49800.1 |  | Vvi-Vitvi12g02755\_t001 |  |  |  |  |  |  |  |
| 1 | Ath-AT5G49810.1 |  | Vvi-Vitvi12g02139\_t001 |  |  |  |  |  |  |  |
| 1 | Ath-AT5G49820.1 |  | Vvi-Vitvi12g02134\_t002 |  |  |  |  |  |  |  |
| 1 | Ath-AT5G49830.2 |  | | | |  |  |  |  |  |  |  |
| 1 | Ath-AT5G49840.3 |  | Vvi-Vitvi12g02130\_t001 |  |  |  |  |  |  |  |
| 1 | Ath-AT5G49850.1 |  | | | |  |  |  |  |  |  |  |
| 1 | Ath-AT5G49860.1 |  | | | |  |  |  |  |  |  |  |
| 1 | Ath-AT5G49870.1 |  | | | |  |  |  |  |  |  |  |
| 1 | Ath-AT5G49880.1 |  | Vvi-Vitvi12g02124\_t001 |  |  |  |  |  |  |  |
| 1 | Ath-AT5G49890.1 |  | Vvi-Vitvi12g02110\_t001 |  |  |  |  |  |  |  |
| 0 | Ath-AT5G49900.1 |  |  |  |  |  |  |  |  |
| 0 | Ath-AT5G49910.1 |  |  |  |  |  |  |  |  |
| 0 | Ath-AT5G49920.1 |  |  |  |  |  |  |  |  |
| 0 | Ath-AT5G49930.1 |  |  |  |  |  |  |  |  |
| 0 | Ath-AT5G49940.1 |  |  |  |  |  |  |  |  |
| 0 | Ath-AT5G49945.1 |  |  |  |  |  |  |  |  |
| 0 | Ath-AT5G49950.1 |  |  |  |  |  |  |  |  |
| 0 | Ath-AT5G49960.1 |  |  |  |  |  |  |  |  |
| 0 | Ath-AT5G49970.1 |  |  |  |  |  |  |  |  |
| 0 | Ath-AT5G49980.1 |  |  |  |  |  |  |  |  |
| 0 | Ath-AT5G49990.1 |  |  |  |  |  |  |  |  |
| 0 | Ath-AT5G50000.1 |  |  |  |  |  |  |  |  |
| 0 | Ath-AT5G50010.1 |  |  |  |  |  |  |  |  |
| 0 | Ath-AT5G50020.2 |  |  |  |  |  |  |  |  |
| 0 | Ath-AT5G50030.1 |  |  |  |  |  |  |  |  |
| 0 | Ath-AT5G50040.1 |  |  |  |  |  |  |  |  |
| 0 | Ath-AT5G50050.1 |  |  |  |  |  |  |  |  |
| 0 | Ath-AT5G50060.1 |  |  |  |  |  |  |  |  |
| 0 | Ath-AT5G50070.1 |  |  |  |  |  |  |  |  |
| 0 | Ath-AT5G50080.2 |  |  |  |  |  |  |  |  |
| 0 | Ath-AT5G50090.1 |  |  |  |  |  |  |  |  |
| 0 | Ath-AT5G50100.1 |  |  |  |  |  |  |  |  |
| 0 | Ath-AT5G50110.1 |  |  |  |  |  |  |  |  |
| 0 | Ath-AT5G50115.1 |  |  |  |  |  |  |  |  |
| 0 | Ath-AT5G50120.1 |  |  |  |  |  |  |  |  |
| 1 | Ath-AT5G50130.1 |  | Vvi-Vitvi17g00881\_t001 |  |  |  |  |  |  |  |
| 1 | Ath-AT5G50140.1 |  | | | |  |  |  |  |  |  |  |
| 1 | Ath-AT5G50150.1 |  | | | |  |  |  |  |  |  |  |
| 1 | Ath-AT5G50160.1 |  | Vvi-Vitvi17g00904\_t001 |  |  |  |  |  |  |  |
| 1 | Ath-AT5G50170.1 |  | Vvi-Vitvi17g00909\_t001 |  |  |  |  |  |  |  |
| 1 | Ath-AT5G50175.1 |  | | | |  |  |  |  |  |  |  |
| 1 | Ath-AT5G50180.1 |  | Vvi-Vitvi17g00917\_t001 |  |  |  |  |  |  |  |
| 1 | Ath-AT5G50200.3 |  | Vvi-Vitvi17g00936\_t001 |  |  |  |  |  |  |  |
| 1 | Ath-AT5G50210.1 |  | Vvi-Vitvi17g00938\_t001 |  |  |  |  |  |  |  |
| 1 | Ath-AT5G50220.1 |  | | | |  |  |  |  |  |  |  |
| 1 | Ath-AT5G50230.1 |  | | | |  |  |  |  |  |  |  |
| 1 | Ath-AT5G50240.1 |  | | | |  |  |  |  |  |  |  |
| 1 | Ath-AT5G50250.1 |  | Vvi-Vitvi17g00958\_t001 |  |  |  |  |  |  |  |
| 1 | Ath-AT5G50260.1 |  | Vvi-Vitvi17g00973\_t001 |  |  |  |  |  |  |  |
| 1 | Ath-AT5G50270.1 |  | | | |  |  |  |  |  |  |  |
| 1 | Ath-AT5G50280.1 |  | Vvi-Vitvi17g00986\_t001 |  |  |  |  |  |  |  |
| 1 | Ath-AT5G50290.1 |  | | | |  |  |  |  |  |  |  |
| 1 | Ath-AT5G50300.2 |  | Vvi-Vitvi17g01016\_t001 |  |  |  |  |  |  |  |
| 1 | Ath-AT5G50310.1 |  | Vvi-Vitvi17g01021\_t001 |  |  |  |  |  |  |  |
| 1 | Ath-AT5G50320.1 |  | Vvi-Vitvi17g01026\_t001 |  |  |  |  |  |  |  |
| 1 | Ath-AT5G50330.1 |  | Vvi-Vitvi17g01592\_t001 |  |  |  |  |  |  |  |
| 1 | Ath-AT5G50335.1 |  | Vvi-Vitvi17g01596\_t001 |  |  |  |  |  |  |  |
| 1 | Ath-AT5G50340.1 |  | | | |  |  |  |  |  |  |  |
| 1 | Ath-AT5G50345.1 |  | | | |  |  |  |  |  |  |  |
| 1 | Ath-AT5G50350.1 |  | Vvi-Vitvi17g01278\_t001 |  |  |  |  |  |  |  |
| 1 | Ath-AT5G50360.1 |  | Vvi-Vitvi17g00152\_t001 |  |  |  |  |  |  |  |
| 1 | Ath-AT5G50361.1 |  | | | |  |  |  |  |  |  |  |
| 1 | Ath-AT5G50365.1 |  | | | |  |  |  |  |  |  |  |
| 1 | Ath-AT5G50370.1 |  | Vvi-Vitvi17g00137\_t001 |  |  |  |  |  |  |  |
| 1 | Ath-AT5G50375.2 |  | Vvi-Vitvi17g00135\_t001 |  |  |  |  |  |  |  |
| 1 | Ath-AT5G50380.1 |  | Vvi-Vitvi17g00115\_t001 |  |  |  |  |  |  |  |
| 1 | Ath-AT5G50390.1 |  | Vvi-Vitvi17g00114\_t001 |  |  |  |  |  |  |  |
| 1 | Ath-AT5G50400.1 |  | Vvi-Vitvi17g01350\_t001 |  |  |  |  |  |  |  |
| 1 | Ath-AT5G50410.1 |  | Vvi-Vitvi17g00112\_t001 |  |  |  |  |  |  |  |
| 1 | Ath-AT5G50420.1 |  | | | |  |  |  |  |  |  |  |
| 1 | Ath-AT5G50423.1 |  | | | |  |  |  |  |  |  |  |
| 1 | Ath-AT5G50430.1 |  | | | |  |  |  |  |  |  |  |
| 1 | Ath-AT5G50440.1 |  | | | |  |  |  |  |  |  |  |
| 1 | Ath-AT5G50443.1 |  | | | |  |  |  |  |  |  |  |
| 1 | Ath-AT5G50450.1 |  | | | |  |  |  |  |  |  |  |
| 1 | Ath-AT5G50460.1 |  | Vvi-Vitvi17g01346\_t001 |  |  |  |  |  |  |  |
| 1 | Ath-AT5G50470.1 |  | | | |  |  |  |  |  |  |  |
| 1 | Ath-AT5G50480.1 |  | | | |  |  |  |  |  |  |  |
| 1 | Ath-AT5G50490.1 |  | | | |  |  |  |  |  |  |  |
| 1 | Ath-AT5G50500.1 |  | | | |  |  |  |  |  |  |  |
| 1 | Ath-AT5G50510.1 |  | | | |  |  |  |  |  |  |  |
| 1 | Ath-AT5G50520.1 |  | | | |  |  |  |  |  |  |  |
| 1 | Ath-AT5G50530.1 |  | Vvi-Vitvi17g00096\_t002 |  |  |  |  |  |  |  |
| 1 | Ath-AT5G50540.1 |  | | | |  |  |  |  |  |  |  |
| 1 | Ath-AT5G50550.1 |  | | | |  |  |  |  |  |  |  |
| 1 | Ath-AT5G50560.1 |  | | | |  |  |  |  |  |  |  |
| 1 | Ath-AT5G50565.6 |  | | | |  |  |  |  |  |  |  |
| 1 | Ath-AT5G50562.1 |  | | | |  |  |  |  |  |  |  |
| 1 | Ath-AT5G50570.2 |  | | | |  |  |  |  |  |  |  |
| 1 | Ath-AT5G50580.2 |  | | | |  |  |  |  |  |  |  |
| 2 | Ath-AT5G50590.1 |  | | | |  | Vvi-Vitvi17g04015\_t001 |  |  |  |  |  |  |
| 2 | Ath-AT5G50600.1 |  | | | |  | | | |  |  |  |  |  |  |
| 2 | Ath-AT5G50610.1 |  | Vvi-Vitvi17g01338\_t001 |  | Vvi-Vitvi17g01338\_t001 |  |  |  |  |  |  |
| 2 | Ath-AT5G50620.1 |  | | | |  | | | |  |  |  |  |  |  |
| 2 | Ath-AT5G50630.1 |  | | | |  | Vvi-Vitvi17g00091\_t001 |  |  |  |  |  |  |
| 2 | Ath-AT5G50640.1 |  | | | |  | Vvi-Vitvi17g00096\_t002 |  |  |  |  |  |  |
| 2 | Ath-AT5G50650.1 |  | | | |  | | | |  |  |  |  |  |  |
| 2 | Ath-AT5G50660.1 |  | | | |  | | | |  |  |  |  |  |  |
| 2 | Ath-AT5G50665.2 |  | | | |  | | | |  |  |  |  |  |  |
| 3 | Ath-AT5G50670.2 |  | | | |  | Vvi-Vitvi17g00100\_t001 |  | Vvi-Vitvi01g01678\_t001 |  |  |  |  |  |
| 3 | Ath-AT5G50680.1 |  | | | |  | Vvi-Vitvi17g00101\_t001 |  | | | |  |  |  |  |  |
| 3 | Ath-AT5G50690.1 |  | Vvi-Vitvi17g04015\_t001 |  | | | |  | | | |  |  |  |  |  |
| 3 | Ath-AT5G50700.1 |  | | | |  | | | |  | | | |  |  |  |  |  |
| 3 | Ath-AT5G50710.1 |  | | | |  | | | |  | | | |  |  |  |  |  |
| 3 | Ath-AT5G50720.1 |  | | | |  | | | |  | | | |  |  |  |  |  |
| 3 | Ath-AT5G50740.3 |  | | | |  | | | |  | Vvi-Vitvi01g01695\_t001 |  |  |  |  |  |
| 3 | Ath-AT5G50750.1 |  | | | |  | Vvi-Vitvi17g00103\_t002 |  | | | |  |  |  |  |  |
| 2 | Ath-AT5G50760.1 |  | Vvi-Vitvi17g01333\_t001 |  |  |  | | | |  |  |  |  |  |
| 2 | Ath-AT5G50770.1 |  | | | |  |  |  | Vvi-Vitvi01g01701\_t001 |  |  |  |  |  |
| 2 | Ath-AT5G50780.1 |  | Vvi-Vitvi17g00076\_t001 |  |  |  | | | |  |  |  |  |  |
| 2 | Ath-AT5G50790.1 |  | Vvi-Vitvi17g00069\_t001 |  |  |  | Vvi-Vitvi01g01719\_t001 |  |  |  |  |  |
| 2 | Ath-AT5G50800.1 |  | | | |  |  |  | | | |  |  |  |  |  |
| 2 | Ath-AT5G50810.1 |  | Vvi-Vitvi17g00067\_t001 |  |  |  | | | |  |  |  |  |  |
| 2 | Ath-AT5G50820.1 |  | | | |  |  |  | | | |  |  |  |  |  |
| 2 | Ath-AT5G50830.2 |  | | | |  |  |  | | | |  |  |  |  |  |
| 2 | Ath-AT5G50840.2 |  | | | |  |  |  | | | |  |  |  |  |  |
| 2 | Ath-AT5G50850.1 |  | Vvi-Vitvi17g00060\_t001 |  |  |  | Vvi-Vitvi01g01724\_t002 |  |  |  |  |  |
| 2 | Ath-AT5G50860.1 |  | Vvi-Vitvi17g00057\_t001 |  |  |  | | | |  |  |  |  |  |
| 2 | Ath-AT5G50870.2 |  | Vvi-Vitvi17g00056\_t001 |  |  |  | | | |  |  |  |  |  |
| 2 | Ath-AT5G50890.1 |  | | | |  |  |  | | | |  |  |  |  |  |
| 2 | Ath-AT5G50900.1 |  | Vvi-Vitvi17g00052\_t001 |  |  |  | Vvi-Vitvi01g01730\_t001 |  |  |  |  |  |
| 2 | Ath-AT5G50910.1 |  | | | |  |  |  | | | |  |  |  |  |  |
| 2 | Ath-AT5G50915.2 |  | Vvi-Vitvi17g00046\_t001 |  |  |  | Vvi-Vitvi01g01745\_t001 |  |  |  |  |  |
| 1 | Ath-AT5G50920.1 |  | Vvi-Vitvi17g00038\_t001 |  |  |  |  |  |  |  |
| 1 | Ath-AT5G50930.1 |  | Vvi-Vitvi17g01322\_t001 |  |  |  |  |  |  |  |
| 1 | Ath-AT5G50940.1 |  | | | |  |  |  |  |  |  |  |
| 1 | Ath-AT5G50950.1 |  | | | |  |  |  |  |  |  |  |
| 1 | Ath-AT5G50960.1 |  | | | |  |  |  |  |  |  |  |
| 1 | Ath-AT5G50970.1 |  | Vvi-Vitvi17g00027\_t002 |  |  |  |  |  |  |  |
| 1 | Ath-AT5G50990.1 |  | Vvi-Vitvi17g00020\_t001 |  |  |  |  |  |  |  |
| 1 | Ath-AT5G51000.1 |  | | | |  |  |  |  |  |  |  |
| 1 | Ath-AT5G51010.1 |  | Vvi-Vitvi17g00016\_t001 |  |  |  |  |  |  |  |
| 1 | Ath-AT5G51020.1 |  | Vvi-Vitvi17g00015\_t001 |  |  |  |  |  |  |  |
| 1 | Ath-AT5G51030.1 |  | Vvi-Vitvi17g00005\_t001 |  |  |  |  |  |  |  |
| 1 | Ath-AT5G51040.3 |  | Vvi-Vitvi17g00004\_t002 |  |  |  |  |  |  |  |
| 0 | Ath-AT5G51050.1 |  |  |  |  |  |  |  |  |
| 0 | Ath-AT5G51060.1 |  |  |  |  |  |  |  |  |
| 0 | Ath-AT5G51070.1 |  |  |  |  |  |  |  |  |
| 0 | Ath-AT5G51080.2 |  |  |  |  |  |  |  |  |
| 0 | Ath-AT5G51090.1 |  |  |  |  |  |  |  |  |
| 0 | Ath-AT5G51100.1 |  |  |  |  |  |  |  |  |
| 0 | Ath-AT5G51110.1 |  |  |  |  |  |  |  |  |
| 0 | Ath-AT5G51105.1 |  |  |  |  |  |  |  |  |
| 0 | Ath-AT5G51120.2 |  |  |  |  |  |  |  |  |
| 0 | Ath-AT5G51130.1 |  |  |  |  |  |  |  |  |
| 0 | Ath-AT5G51140.2 |  |  |  |  |  |  |  |  |
| 0 | Ath-AT5G51150.1 |  |  |  |  |  |  |  |  |
| 0 | Ath-AT5G51160.1 |  |  |  |  |  |  |  |  |
| 0 | Ath-AT5G51170.1 |  |  |  |  |  |  |  |  |
| 0 | Ath-AT5G51180.1 |  |  |  |  |  |  |  |  |
| 0 | Ath-AT5G51190.1 |  |  |  |  |  |  |  |  |
| 0 | Ath-AT5G51195.1 |  |  |  |  |  |  |  |  |
| 0 | Ath-AT5G51200.2 |  |  |  |  |  |  |  |  |
| 0 | Ath-AT5G51210.1 |  |  |  |  |  |  |  |  |
| 0 | Ath-AT5G51220.1 |  |  |  |  |  |  |  |  |
| 0 | Ath-AT5G51230.1 |  |  |  |  |  |  |  |  |
| 0 | Ath-AT5G51250.1 |  |  |  |  |  |  |  |  |
| 0 | Ath-AT5G51260.1 |  |  |  |  |  |  |  |  |
| 0 | Ath-AT5G51270.1 |  |  |  |  |  |  |  |  |
| 0 | Ath-AT5G51280.1 |  |  |  |  |  |  |  |  |
| 0 | Ath-AT5G51290.1 |  |  |  |  |  |  |  |  |
| 0 | Ath-AT5G51300.2 |  |  |  |  |  |  |  |  |
| 0 | Ath-AT5G51310.1 |  |  |  |  |  |  |  |  |
| 0 | Ath-AT5G51330.1 |  |  |  |  |  |  |  |  |
| 0 | Ath-AT5G51340.1 |  |  |  |  |  |  |  |  |
| 0 | Ath-AT5G51350.1 |  |  |  |  |  |  |  |  |
| 0 | Ath-AT5G51360.1 |  |  |  |  |  |  |  |  |
| 0 | Ath-AT5G51370.2 |  |  |  |  |  |  |  |  |
| 0 | Ath-AT5G51380.1 |  |  |  |  |  |  |  |  |
| 0 | Ath-AT5G51390.1 |  |  |  |  |  |  |  |  |
| 1 | Ath-AT5G51400.1 |  | Vvi-Vitvi16g00672\_t002 |  |  |  |  |  |  |  |
| 1 | Ath-AT5G51410.2 |  | Vvi-Vitvi16g00675\_t001 |  |  |  |  |  |  |  |
| 1 | Ath-AT5G51420.1 |  | | | |  |  |  |  |  |  |  |
| 1 | Ath-AT5G51430.1 |  | Vvi-Vitvi16g00676\_t001 |  |  |  |  |  |  |  |
| 1 | Ath-AT5G51440.1 |  | Vvi-Vitvi16g00681\_t001 |  |  |  |  |  |  |  |
| 1 | Ath-AT5G51450.2 |  | Vvi-Vitvi16g00703\_t001 |  |  |  |  |  |  |  |
| 1 | Ath-AT5G51451.1 |  | | | |  |  |  |  |  |  |  |
| 1 | Ath-AT5G51460.1 |  | Vvi-Vitvi16g00712\_t001 |  |  |  |  |  |  |  |
| 1 | Ath-AT5G51465.1 |  | | | |  |  |  |  |  |  |  |
| 1 | Ath-AT5G51470.1 |  | | | |  |  |  |  |  |  |  |
| 1 | Ath-AT5G51480.1 |  | Vvi-Vitvi16g00715\_t001 |  |  |  |  |  |  |  |
| 1 | Ath-AT5G51490.1 |  | Vvi-Vitvi16g00717\_t001 |  |  |  |  |  |  |  |
| 1 | Ath-AT5G51500.1 |  | | | |  |  |  |  |  |  |  |
| 1 | Ath-AT5G51510.1 |  | Vvi-Vitvi16g01791\_t001 |  |  |  |  |  |  |  |
| 1 | Ath-AT5G51520.1 |  | Vvi-Vitvi16g00744\_t001 |  |  |  |  |  |  |  |
| 1 | Ath-AT5G51530.1 |  | | | |  |  |  |  |  |  |  |
| 1 | Ath-AT5G51540.1 |  | Vvi-Vitvi16g00783\_t001 |  |  |  |  |  |  |  |
| 1 | Ath-AT5G51545.1 |  | Vvi-Vitvi16g00792\_t001 |  |  |  |  |  |  |  |
| 1 | Ath-AT5G51550.1 |  | Vvi-Vitvi16g00793\_t001 |  |  |  |  |  |  |  |
| 1 | Ath-AT5G51560.1 |  | Vvi-Vitvi16g00808\_t001 |  |  |  |  |  |  |  |
| 1 | Ath-AT5G51570.1 |  | Vvi-Vitvi16g00817\_t002 |  |  |  |  |  |  |  |
| 1 | Ath-AT5G51580.1 |  | | | |  |  |  |  |  |  |  |
| 1 | Ath-AT5G51585.1 |  | | | |  |  |  |  |  |  |  |
| 1 | Ath-AT5G51590.1 |  | Vvi-Vitvi16g00835\_t001 |  |  |  |  |  |  |  |
| 1 | Ath-AT5G51600.1 |  | Vvi-Vitvi16g00836\_t001 |  |  |  |  |  |  |  |
| 1 | Ath-AT5G51610.1 |  | | | |  |  |  |  |  |  |  |
| 1 | Ath-AT5G51620.4 |  | | | |  |  |  |  |  |  |  |
| 1 | Ath-AT5G51630.1 |  | | | |  |  |  |  |  |  |  |
| 1 | Ath-AT5G51640.1 |  | Vvi-Vitvi16g00837\_t002 |  |  |  |  |  |  |  |
| 1 | Ath-AT5G51650.1 |  | | | |  |  |  |  |  |  |  |
| 1 | Ath-AT5G51660.1 |  | Vvi-Vitvi16g00841\_t001 |  |  |  |  |  |  |  |
| 1 | Ath-AT5G51670.1 |  | Vvi-Vitvi16g00843\_t001 |  |  |  |  |  |  |  |
| 1 | Ath-AT5G51680.1 |  | Vvi-Vitvi16g00852\_t001 |  |  |  |  |  |  |  |
| 1 | Ath-AT5G51690.1 |  | Vvi-Vitvi16g00853\_t001 |  |  |  |  |  |  |  |
| 1 | Ath-AT5G51700.1 |  | Vvi-Vitvi16g00857\_t001 |  |  |  |  |  |  |  |
| 1 | Ath-AT5G51710.1 |  | Vvi-Vitvi16g00860\_t003 |  |  |  |  |  |  |  |
| 1 | Ath-AT5G51720.1 |  | Vvi-Vitvi16g00863\_t001 |  |  |  |  |  |  |  |
| 1 | Ath-AT5G51730.1 |  | | | |  |  |  |  |  |  |  |
| 1 | Ath-AT5G51740.2 |  | Vvi-Vitvi16g00870\_t001 |  |  |  |  |  |  |  |
| 1 | Ath-AT5G51750.1 |  | Vvi-Vitvi16g00878\_t001 |  |  |  |  |  |  |  |
| 1 | Ath-AT5G51760.1 |  | Vvi-Vitvi16g00879\_t001 |  |  |  |  |  |  |  |
| 1 | Ath-AT5G51770.1 |  | Vvi-Vitvi16g00880\_t001 |  |  |  |  |  |  |  |
| 2 | Ath-AT5G51780.2 |  | Vvi-Vitvi16g00882\_t001 |  | Vvi-Vitvi02g00439\_t001 |  |  |  |  |  |  |
| 2 | Ath-AT5G51790.3 |  | | | |  | | | |  |  |  |  |  |  |
| 2 | Ath-AT5G51795.1 |  | | | |  | | | |  |  |  |  |  |  |
| 2 | Ath-AT5G51800.1 |  | Vvi-Vitvi16g00887\_t001 |  | | | |  |  |  |  |  |  |
| 2 | Ath-AT5G51810.1 |  | Vvi-Vitvi16g00890\_t001 |  | | | |  |  |  |  |  |  |
| 2 | Ath-AT5G51820.1 |  | Vvi-Vitvi16g00891\_t001 |  | | | |  |  |  |  |  |  |
| 2 | Ath-AT5G51830.1 |  | Vvi-Vitvi16g00895\_t001 |  | | | |  |  |  |  |  |  |
| 2 | Ath-AT5G51840.1 |  | Vvi-Vitvi16g00896\_t002 |  | | | |  |  |  |  |  |  |
| 2 | Ath-AT5G51845.1 |  | | | |  | | | |  |  |  |  |  |  |
| 2 | Ath-AT5G51850.1 |  | Vvi-Vitvi16g00897\_t001 |  | | | |  |  |  |  |  |  |
| 2 | Ath-AT5G51860.1 |  | Vvi-Vitvi16g00898\_t001 |  | Vvi-Vitvi02g00427\_t001 |  |  |  |  |  |  |
| 2 | Ath-AT5G51870.3 |  | | | |  | | | |  |  |  |  |  |  |
| 2 | Ath-AT5G51880.1 |  | Vvi-Vitvi16g01827\_t001 |  | | | |  |  |  |  |  |  |
| 2 | Ath-AT5G51890.1 |  | Vvi-Vitvi16g01844\_t001 |  | | | |  |  |  |  |  |  |
| 2 | Ath-AT5G51900.1 |  | | | |  | | | |  |  |  |  |  |  |
| 2 | Ath-AT5G51910.1 |  | Vvi-Vitvi16g01846\_t001 |  | Vvi-Vitvi02g00421\_t001 |  |  |  |  |  |  |
| 2 | Ath-AT5G51915.1 |  | | | |  | | | |  |  |  |  |  |  |
| 2 | Ath-AT5G51920.1 |  | Vvi-Vitvi16g00922\_t001 |  | Vvi-Vitvi02g00419\_t001 |  |  |  |  |  |  |
| 2 | Ath-AT5G51930.1 |  | Vvi-Vitvi16g01854\_t001 |  | Vvi-Vitvi02g00416\_t001 |  |  |  |  |  |  |
| 2 | Ath-AT5G51940.1 |  | | | |  | | | |  |  |  |  |  |  |
| 2 | Ath-AT5G51950.1 |  | | | |  | | | |  |  |  |  |  |  |
| 2 | Ath-AT5G51960.1 |  | | | |  | | | |  |  |  |  |  |  |
| 2 | Ath-AT5G51970.1 |  | Vvi-Vitvi16g01857\_t006 |  | | | |  |  |  |  |  |  |
| 2 | Ath-AT5G51980.1 |  | Vvi-Vitvi16g00933\_t001 |  | | | |  |  |  |  |  |  |
| 2 | Ath-AT5G51990.1 |  | Vvi-Vitvi16g04340\_t001 |  | Vvi-Vitvi02g04095\_t001 |  |  |  |  |  |  |
| 2 | Ath-AT5G52000.1 |  | | | |  | | | |  |  |  |  |  |  |
| 2 | Ath-AT5G52010.1 |  | | | |  | | | |  |  |  |  |  |  |
| 2 | Ath-AT5G52020.1 |  | Vvi-Vitvi16g01860\_t001 |  | | | |  |  |  |  |  |  |
| 2 | Ath-AT5G52030.2 |  | Vvi-Vitvi16g00944\_t001 |  | | | |  |  |  |  |  |  |
| 2 | Ath-AT5G52040.2 |  | Vvi-Vitvi16g00949\_t004 |  | | | |  |  |  |  |  |  |
| 2 | Ath-AT5G52045.1 |  | | | |  | | | |  |  |  |  |  |  |
| 2 | Ath-AT5G52050.1 |  | Vvi-Vitvi16g00951\_t001 |  | Vvi-Vitvi02g00403\_t001 |  |  |  |  |  |  |
| 2 | Ath-AT5G52060.1 |  | Vvi-Vitvi16g00952\_t001 |  | | | |  |  |  |  |  |  |
| 2 | Ath-AT5G52070.1 |  | | | |  | | | |  |  |  |  |  |  |
| 2 | Ath-AT5G52080.1 |  | | | |  | | | |  |  |  |  |  |  |
| 2 | Ath-AT5G52090.1 |  | | | |  | | | |  |  |  |  |  |  |
| 2 | Ath-AT5G52100.1 |  | Vvi-Vitvi16g00953\_t001 |  | | | |  |  |  |  |  |  |
| 2 | Ath-AT5G52110.2 |  | Vvi-Vitvi16g00955\_t001 |  | | | |  |  |  |  |  |  |
| 2 | Ath-AT5G52115.1 |  | | | |  | | | |  |  |  |  |  |  |
| 2 | Ath-AT5G52120.1 |  | Vvi-Vitvi16g00957\_t001 |  | Vvi-Vitvi02g00398\_t001 |  |  |  |  |  |  |
| 2 | Ath-AT5G52130.1 |  | | | |  | | | |  |  |  |  |  |  |
| 2 | Ath-AT5G52140.1 |  | | | |  | | | |  |  |  |  |  |  |
| 2 | Ath-AT5G52150.1 |  | | | |  | | | |  |  |  |  |  |  |
| 2 | Ath-AT5G52155.1 |  | | | |  | | | |  |  |  |  |  |  |
| 2 | Ath-AT5G52160.1 |  | Vvi-Vitvi16g01864\_t001 |  | | | |  |  |  |  |  |  |
| 2 | Ath-AT5G52170.2 |  | Vvi-Vitvi16g00974\_t001 |  | | | |  |  |  |  |  |  |
| 2 | Ath-AT5G52180.1 |  | Vvi-Vitvi16g00984\_t001 |  | | | |  |  |  |  |  |  |
| 2 | Ath-AT5G52190.1 |  | Vvi-Vitvi16g01000\_t001 |  | | | |  |  |  |  |  |  |
| 2 | Ath-AT5G52200.1 |  | Vvi-Vitvi16g01871\_t003 |  | Vvi-Vitvi02g00388\_t001 |  |  |  |  |  |  |
| 2 | Ath-AT5G52210.1 |  | | | |  | | | |  |  |  |  |  |  |
| 2 | Ath-AT5G52220.1 |  | Vvi-Vitvi16g01001\_t001 |  | | | |  |  |  |  |  |  |
| 2 | Ath-AT5G52230.1 |  | Vvi-Vitvi16g01872\_t001 |  | Vvi-Vitvi02g01398\_t001 |  |  |  |  |  |  |
| 1 | Ath-AT5G52240.1 |  | Vvi-Vitvi16g01004\_t001 |  |  |  |  |  |  |  |
| 1 | Ath-AT5G52250.1 |  | Vvi-Vitvi16g01011\_t001 |  |  |  |  |  |  |  |
| 1 | Ath-AT5G52260.1 |  | Vvi-Vitvi16g01015\_t001 |  |  |  |  |  |  |  |
| 1 | Ath-AT5G52270.2 |  | Vvi-Vitvi16g01016\_t001 |  |  |  |  |  |  |  |
| 1 | Ath-AT5G52280.1 |  | Vvi-Vitvi16g01874\_t001 |  |  |  |  |  |  |  |
| 1 | Ath-AT5G52290.1 |  | Vvi-Vitvi16g01021\_t001 |  |  |  |  |  |  |  |
| 1 | Ath-AT5G52300.1 |  | Vvi-Vitvi16g01022\_t001 |  |  |  |  |  |  |  |
| 1 | Ath-AT5G52310.1 |  | | | |  |  |  |  |  |  |  |
| 1 | Ath-AT5G52320.1 |  | | | |  |  |  |  |  |  |  |
| 1 | Ath-AT5G52330.3 |  | | | |  |  |  |  |  |  |  |
| 1 | Ath-AT5G52340.1 |  | | | |  |  |  |  |  |  |  |
| 1 | Ath-AT5G52350.1 |  | | | |  |  |  |  |  |  |  |
| 1 | Ath-AT5G52360.1 |  | Vvi-Vitvi16g01026\_t001 |  |  |  |  |  |  |  |
| 1 | Ath-AT5G52370.1 |  | | | |  |  |  |  |  |  |  |
| 1 | Ath-AT5G52380.1 |  | Vvi-Vitvi16g01027\_t001 |  |  |  |  |  |  |  |
| 1 | Ath-AT5G52390.1 |  | Vvi-Vitvi16g04365\_t001 |  |  |  |  |  |  |  |
| 1 | Ath-AT5G52400.1 |  | Vvi-Vitvi16g01046\_t001 |  |  |  |  |  |  |  |
| 1 | Ath-AT5G52410.2 |  | Vvi-Vitvi16g01053\_t001 |  |  |  |  |  |  |  |
| 1 | Ath-AT5G52420.1 |  | Vvi-Vitvi16g01904\_t001 |  |  |  |  |  |  |  |
| 1 | Ath-AT5G52430.1 |  | Vvi-Vitvi16g01074\_t001 |  |  |  |  |  |  |  |
| 1 | Ath-AT5G52440.1 |  | Vvi-Vitvi16g01909\_t001 |  |  |  |  |  |  |  |
| 1 | Ath-AT5G52450.1 |  | | | |  |  |  |  |  |  |  |
| 1 | Ath-AT5G52460.1 |  | | | |  |  |  |  |  |  |  |
| 1 | Ath-AT5G52470.1 |  | Vvi-Vitvi16g01083\_t001 |  |  |  |  |  |  |  |
| 1 | Ath-AT5G52480.1 |  | | | |  |  |  |  |  |  |  |
| 1 | Ath-AT5G52490.1 |  | | | |  |  |  |  |  |  |  |
| 1 | Ath-AT5G52500.1 |  | | | |  |  |  |  |  |  |  |
| 1 | Ath-AT5G52510.1 |  | Vvi-Vitvi16g01086\_t001 |  |  |  |  |  |  |  |
| 1 | Ath-AT5G52520.1 |  | Vvi-Vitvi16g01087\_t001 |  |  |  |  |  |  |  |
| 2 | Ath-AT5G52530.3 |  | Vvi-Vitvi16g01089\_t001 |  | Vvi-Vitvi02g01312\_t001 |  |  |  |  |  |  |
| 2 | Ath-AT5G52540.1 |  | Vvi-Vitvi16g01093\_t001 |  | | | |  |  |  |  |  |  |
| 2 | Ath-AT5G52545.2 |  | Vvi-Vitvi16g01916\_t001 |  | | | |  |  |  |  |  |  |
| 2 | Ath-AT5G52547.3 |  | | | |  | | | |  |  |  |  |  |  |
| 2 | Ath-AT5G52550.1 |  | Vvi-Vitvi16g01096\_t001 |  | | | |  |  |  |  |  |  |
| 2 | Ath-AT5G52560.1 |  | | | |  | | | |  |  |  |  |  |  |
| 2 | Ath-AT5G52570.1 |  | Vvi-Vitvi16g01099\_t001 |  | Vvi-Vitvi02g00020\_t001 |  |  |  |  |  |  |
| 2 | Ath-AT5G52580.2 |  | | | |  | | | |  |  |  |  |  |  |
| 2 | Ath-AT5G52600.1 |  | | | |  | | | |  |  |  |  |  |  |
| 2 | Ath-AT5G52605.1 |  | | | |  | | | |  |  |  |  |  |  |
| 2 | Ath-AT5G52610.1 |  | | | |  | | | |  |  |  |  |  |  |
| 2 | Ath-AT5G52620.1 |  | | | |  | | | |  |  |  |  |  |  |
| 2 | Ath-AT5G52630.1 |  | | | |  | | | |  |  |  |  |  |  |
| 2 | Ath-AT5G52640.1 |  | Vvi-Vitvi16g01103\_t001 |  | Vvi-Vitvi02g00025\_t001 |  |  |  |  |  |  |
| 2 | Ath-AT5G52650.1 |  | Vvi-Vitvi16g01104\_t001 |  | Vvi-Vitvi02g01315\_t001 |  |  |  |  |  |  |
| 2 | Ath-AT5G52655.1 |  | | | |  | | | |  |  |  |  |  |  |
| 2 | Ath-AT5G52660.2 |  | Vvi-Vitvi16g01106\_t001 |  | | | |  |  |  |  |  |  |
| 2 | Ath-AT5G52670.1 |  | | | |  | | | |  |  |  |  |  |  |
| 2 | Ath-AT5G52680.1 |  | | | |  | | | |  |  |  |  |  |  |
| 2 | Ath-AT5G52690.1 |  | | | |  | | | |  |  |  |  |  |  |
| 2 | Ath-AT5G52700.1 |  | | | |  | | | |  |  |  |  |  |  |
| 2 | Ath-AT5G52710.2 |  | | | |  | | | |  |  |  |  |  |  |
| 2 | Ath-AT5G52720.2 |  | | | |  | | | |  |  |  |  |  |  |
| 2 | Ath-AT5G52730.1 |  | Vvi-Vitvi16g04379\_t001 |  | | | |  |  |  |  |  |  |
| 2 | Ath-AT5G52740.1 |  | | | |  | Vvi-Vitvi02g00031\_t001 |  |  |  |  |  |  |
| 2 | Ath-AT5G52750.1 |  | | | |  | | | |  |  |  |  |  |  |
| 2 | Ath-AT5G52760.1 |  | | | |  | | | |  |  |  |  |  |  |
| 2 | Ath-AT5G52770.1 |  | Vvi-Vitvi16g01926\_t001 |  | | | |  |  |  |  |  |  |
| 2 | Ath-AT5G52780.1 |  | Vvi-Vitvi16g01112\_t001 |  | | | |  |  |  |  |  |  |
| 2 | Ath-AT5G52790.1 |  | Vvi-Vitvi16g01113\_t001 |  | | | |  |  |  |  |  |  |
| 2 | Ath-AT5G52800.3 |  | Vvi-Vitvi16g01126\_t001 |  | | | |  |  |  |  |  |  |
| 2 | Ath-AT5G52810.1 |  | Vvi-Vitvi16g01127\_t001 |  | | | |  |  |  |  |  |  |
| 2 | Ath-AT5G52820.1 |  | | | |  | | | |  |  |  |  |  |  |
| 2 | Ath-AT5G52830.1 |  | Vvi-Vitvi16g01132\_t001 |  | Vvi-Vitvi02g00039\_t001 |  |  |  |  |  |  |
| 2 | Ath-AT5G52840.1 |  | Vvi-Vitvi16g01134\_t001 |  | | | |  |  |  |  |  |  |
| 2 | Ath-AT5G52850.1 |  | Vvi-Vitvi16g01135\_t001 |  | | | |  |  |  |  |  |  |
| 2 | Ath-AT5G52860.1 |  | Vvi-Vitvi16g01144\_t001 |  | | | |  |  |  |  |  |  |
| 2 | Ath-AT5G52870.1 |  | Vvi-Vitvi16g04397\_t001 |  | Vvi-Vitvi02g00051\_t001 |  |  |  |  |  |  |
| 2 | Ath-AT5G52880.1 |  | Vvi-Vitvi16g01162\_t002 |  | | | |  |  |  |  |  |  |
| 2 | Ath-AT5G52882.1 |  | Vvi-Vitvi16g01957\_t001 |  | Vvi-Vitvi02g00060\_t001 |  |  |  |  |  |  |
| 1 | Ath-AT5G52890.2 |  | | | |  |  |  |  |  |  |  |
| 1 | Ath-AT5G52900.1 |  | Vvi-Vitvi16g01959\_t001 |  |  |  |  |  |  |  |
| 1 | Ath-AT5G52910.2 |  | | | |  |  |  |  |  |  |  |
| 1 | Ath-AT5G52920.1 |  | | | |  |  |  |  |  |  |  |
| 1 | Ath-AT5G52930.1 |  | | | |  |  |  |  |  |  |  |
| 1 | Ath-AT5G52940.1 |  | | | |  |  |  |  |  |  |  |
| 1 | Ath-AT5G52950.1 |  | | | |  |  |  |  |  |  |  |
| 1 | Ath-AT5G52960.1 |  | | | |  |  |  |  |  |  |  |
| 1 | Ath-AT5G52965.1 |  | | | |  |  |  |  |  |  |  |
| 1 | Ath-AT5G52970.1 |  | | | |  |  |  |  |  |  |  |
| 1 | Ath-AT5G52975.1 |  | | | |  |  |  |  |  |  |  |
| 1 | Ath-AT5G52980.1 |  | | | |  |  |  |  |  |  |  |
| 2 | Ath-AT5G52990.1 |  | | | |  | Vvi-Vitvi16g02088\_t001 |  |  |  |  |  |  |
| 2 | Ath-AT5G53000.1 |  | | | |  | Vvi-Vitvi16g01404\_t001 |  |  |  |  |  |  |
| 2 | Ath-AT5G53010.1 |  | | | |  | | | |  |  |  |  |  |  |
| 2 | Ath-AT5G53020.7 |  | | | |  | Vvi-Vitvi16g01403\_t002 |  |  |  |  |  |  |
| 2 | Ath-AT5G53030.1 |  | | | |  | Vvi-Vitvi16g02087\_t001 |  |  |  |  |  |  |
| 2 | Ath-AT5G53040.1 |  | | | |  | Vvi-Vitvi16g01402\_t001 |  |  |  |  |  |  |
| 2 | Ath-AT5G53045.1 |  | | | |  | Vvi-Vitvi16g04519\_t002 |  |  |  |  |  |  |
| 2 | Ath-AT5G53050.4 |  | | | |  | Vvi-Vitvi16g01399\_t002 |  |  |  |  |  |  |
| 2 | Ath-AT5G53060.1 |  | | | |  | | | |  |  |  |  |  |  |
| 2 | Ath-AT5G53070.1 |  | | | |  | Vvi-Vitvi16g04518\_t001 |  |  |  |  |  |  |
| 1 | Ath-AT5G53080.1 |  | | | |  |  |  |  |  |  |  |
| 1 | Ath-AT5G53090.1 |  | | | |  |  |  |  |  |  |  |
| 1 | Ath-AT5G53100.1 |  | | | |  |  |  |  |  |  |  |
| 3 | Ath-AT5G53110.1 |  | Vvi-Vitvi16g04412\_t001 |  | Vvi-Vitvi15g01611\_t001 |  | Vvi-Vitvi16g02122\_t001 |  |  |  |  |  |
| 2 | Ath-AT5G53120.6 |  |  |  | | | |  | Vvi-Vitvi16g01233\_t002 |  |  |  |  |  |
| 3 | Ath-AT5G53130.1 |  | Vvi-Vitvi16g01218\_t001 |  | Vvi-Vitvi15g01617\_t001 |  | | | |  |  |  |  |  |
| 3 | Ath-AT5G53140.1 |  | Vvi-Vitvi16g01220\_t001 |  | | | |  | | | |  |  |  |  |  |
| 3 | Ath-AT5G53150.2 |  | Vvi-Vitvi16g01222\_t001 |  | | | |  | | | |  |  |  |  |  |
| 3 | Ath-AT5G53160.2 |  | Vvi-Vitvi16g01226\_t001 |  | Vvi-Vitvi15g00997\_t001 |  | Vvi-Vitvi16g01226\_t001 |  |  |  |  |  |
| 3 | Ath-AT5G53170.1 |  | Vvi-Vitvi16g01229\_t001 |  | | | |  | | | |  |  |  |  |  |
| 3 | Ath-AT5G53180.1 |  | Vvi-Vitvi16g01230\_t001 |  | | | |  | | | |  |  |  |  |  |
| 2 | Ath-AT5G53190.1 |  |  |  | | | |  | Vvi-Vitvi16g01984\_t001 |  |  |  |  |  |
| 2 | Ath-AT5G53200.1 |  |  |  | Vvi-Vitvi15g01002\_t001 |  | Vvi-Vitvi16g01215\_t001 |  |  |  |  |  |
| 2 | Ath-AT5G53210.1 |  |  |  | | | |  | Vvi-Vitvi16g01212\_t001 |  |  |  |  |  |
| 3 | Ath-AT5G53220.2 |  | Vvi-Vitvi16g01190\_t001 |  | | | |  | | | |  |  |  |  |  |
| 3 | Ath-AT5G53230.1 |  | | | |  | | | |  | | | |  |  |  |  |  |
| 3 | Ath-AT5G53240.1 |  | | | |  | | | |  | | | |  |  |  |  |  |
| 3 | Ath-AT5G53250.1 |  | Vvi-Vitvi16g01191\_t001 |  | Vvi-Vitvi15g01019\_t001 |  | | | |  |  |  |  |  |
| 3 | Ath-AT5G53260.1 |  | | | |  | | | |  | | | |  |  |  |  |  |
| 3 | Ath-AT5G53270.1 |  | | | |  | | | |  | | | |  |  |  |  |  |
| 3 | Ath-AT5G53280.1 |  | Vvi-Vitvi16g01983\_t001 |  | Vvi-Vitvi15g01630\_t001 |  | | | |  |  |  |  |  |
| 3 | Ath-AT5G53290.1 |  | Vvi-Vitvi16g01201\_t001 |  | Vvi-Vitvi15g01021\_t001 |  | | | |  |  |  |  |  |
| 3 | Ath-AT5G53300.4 |  | Vvi-Vitvi16g04430\_t001 |  | Vvi-Vitvi15g04595\_t001 |  | | | |  |  |  |  |  |
| 2 | Ath-AT5G53310.1 |  | Vvi-Vitvi16g01203\_t002 |  |  |  | Vvi-Vitvi16g01203\_t002 |  |  |  |  |  |
| 1 | Ath-AT5G53320.1 |  | Vvi-Vitvi16g01204\_t001 |  |  |  |  |  |  |  |
| 1 | Ath-AT5G53330.1 |  | Vvi-Vitvi16g01206\_t001 |  |  |  |  |  |  |  |
| 1 | Ath-AT5G53340.1 |  | Vvi-Vitvi16g01207\_t002 |  |  |  |  |  |  |  |
| 1 | Ath-AT5G53350.1 |  | Vvi-Vitvi16g01413\_t001 |  |  |  |  |  |  |  |
| 1 | Ath-AT5G53360.1 |  | Vvi-Vitvi16g01412\_t001 |  |  |  |  |  |  |  |
| 1 | Ath-AT5G53370.2 |  | | | |  |  |  |  |  |  |  |
| 1 | Ath-AT5G53380.1 |  | | | |  |  |  |  |  |  |  |
| 2 | Ath-AT5G53390.1 |  | | | |  | Vvi-Vitvi16g01291\_t001 |  |  |  |  |  |  |
| 2 | Ath-AT5G53400.1 |  | | | |  | Vvi-Vitvi16g01290\_t002 |  |  |  |  |  |  |
| 2 | Ath-AT5G53410.1 |  | | | |  | | | |  |  |  |  |  |  |
| 2 | Ath-AT5G53420.1 |  | | | |  | Vvi-Vitvi16g01289\_t001 |  |  |  |  |  |  |
| 2 | Ath-AT5G53430.1 |  | | | |  | Vvi-Vitvi16g01288\_t001 |  |  |  |  |  |  |
| 2 | Ath-AT5G53440.1 |  | | | |  | Vvi-Vitvi16g01286\_t001 |  |  |  |  |  |  |
| 2 | Ath-AT5G53450.1 |  | | | |  | Vvi-Vitvi16g01285\_t002 |  |  |  |  |  |  |
| 2 | Ath-AT5G53460.1 |  | | | |  | Vvi-Vitvi16g01282\_t003 |  |  |  |  |  |  |
| 1 | Ath-AT5G53470.1 |  | | | |  |  |  |  |  |  |  |
| 1 | Ath-AT5G53480.1 |  | Vvi-Vitvi16g01394\_t001 |  |  |  |  |  |  |  |
| 1 | Ath-AT5G53486.4 |  | | | |  |  |  |  |  |  |  |
| 1 | Ath-AT5G53490.4 |  | Vvi-Vitvi16g01388\_t001 |  |  |  |  |  |  |  |
| 1 | Ath-AT5G53500.1 |  | Vvi-Vitvi16g01387\_t001 |  |  |  |  |  |  |  |
| 1 | Ath-AT5G53510.1 |  | | | |  |  |  |  |  |  |  |
| 1 | Ath-AT5G53520.1 |  | Vvi-Vitvi16g01380\_t001 |  |  |  |  |  |  |  |
| 1 | Ath-AT5G53530.1 |  | Vvi-Vitvi16g01375\_t001 |  |  |  |  |  |  |  |
| 1 | Ath-AT5G53540.1 |  | Vvi-Vitvi16g01373\_t001 |  |  |  |  |  |  |  |
| 1 | Ath-AT5G53550.2 |  | Vvi-Vitvi16g01371\_t001 |  |  |  |  |  |  |  |
| 1 | Ath-AT5G53560.1 |  | Vvi-Vitvi16g01367\_t001 |  |  |  |  |  |  |  |
| 1 | Ath-AT5G53570.2 |  | Vvi-Vitvi16g01366\_t002 |  |  |  |  |  |  |  |
| 1 | Ath-AT5G53580.1 |  | Vvi-Vitvi16g01363\_t001 |  |  |  |  |  |  |  |
| 1 | Ath-AT5G53590.1 |  | Vvi-Vitvi16g01359\_t001 |  |  |  |  |  |  |  |
| 1 | Ath-AT5G53592.1 |  | | | |  |  |  |  |  |  |  |
| 1 | Ath-AT5G53600.1 |  | | | |  |  |  |  |  |  |  |
| 1 | Ath-AT5G53610.1 |  | | | |  |  |  |  |  |  |  |
| 1 | Ath-AT5G53620.3 |  | Vvi-Vitvi16g02071\_t001 |  |  |  |  |  |  |  |
| 1 | Ath-AT5G53635.1 |  | | | |  |  |  |  |  |  |  |
| 1 | Ath-AT5G53637.1 |  | | | |  |  |  |  |  |  |  |
| 1 | Ath-AT5G53640.1 |  | | | |  |  |  |  |  |  |  |
| 1 | Ath-AT5G53650.1 |  | Vvi-Vitvi16g01356\_t001 |  |  |  |  |  |  |  |
| 1 | Ath-AT5G53660.1 |  | Vvi-Vitvi16g01354\_t001 |  |  |  |  |  |  |  |
| 1 | Ath-AT5G53670.1 |  | | | |  |  |  |  |  |  |  |
| 1 | Ath-AT5G53680.1 |  | | | |  |  |  |  |  |  |  |
| 1 | Ath-AT5G53700.1 |  | | | |  |  |  |  |  |  |  |
| 1 | Ath-AT5G53710.2 |  | | | |  |  |  |  |  |  |  |
| 1 | Ath-AT5G53720.1 |  | | | |  |  |  |  |  |  |  |
| 1 | Ath-AT5G53730.1 |  | Vvi-Vitvi16g01339\_t001 |  |  |  |  |  |  |  |
| 0 | Ath-AT5G53740.1 |  |  |  |  |  |  |  |  |
| 0 | Ath-AT5G53742.1 |  |  |  |  |  |  |  |  |
| 0 | Ath-AT5G53750.1 |  |  |  |  |  |  |  |  |
| 0 | Ath-AT5G53760.1 |  |  |  |  |  |  |  |  |
| 0 | Ath-AT5G53770.1 |  |  |  |  |  |  |  |  |
| 0 | Ath-AT5G53780.1 |  |  |  |  |  |  |  |  |
| 0 | Ath-AT5G53790.1 |  |  |  |  |  |  |  |  |
| 0 | Ath-AT5G53800.1 |  |  |  |  |  |  |  |  |
| 0 | Ath-AT5G53810.1 |  |  |  |  |  |  |  |  |
| 0 | Ath-AT5G53820.1 |  |  |  |  |  |  |  |  |
| 1 | Ath-AT5G53830.1 |  | Vvi-Vitvi19g00209\_t001 |  |  |  |  |  |  |  |
| 1 | Ath-AT5G53840.1 |  | | | |  |  |  |  |  |  |  |
| 1 | Ath-AT5G53850.5 |  | Vvi-Vitvi19g00208\_t001 |  |  |  |  |  |  |  |
| 1 | Ath-AT5G53860.4 |  | Vvi-Vitvi19g00207\_t004 |  |  |  |  |  |  |  |
| 1 | Ath-AT5G53870.1 |  | Vvi-Vitvi19g00206\_t001 |  |  |  |  |  |  |  |
| 1 | Ath-AT5G53880.1 |  | | | |  |  |  |  |  |  |  |
| 1 | Ath-AT5G53890.1 |  | Vvi-Vitvi19g00201\_t001 |  |  |  |  |  |  |  |
| 1 | Ath-AT5G53895.1 |  | | | |  |  |  |  |  |  |  |
| 1 | Ath-AT5G53900.2 |  | Vvi-Vitvi19g00198\_t001 |  |  |  |  |  |  |  |
| 1 | Ath-AT5G53905.1 |  | | | |  |  |  |  |  |  |  |
| 1 | Ath-AT5G53910.1 |  | | | |  |  |  |  |  |  |  |
| 1 | Ath-AT5G53920.1 |  | Vvi-Vitvi19g00195\_t001 |  |  |  |  |  |  |  |
| 1 | Ath-AT5G53930.1 |  | Vvi-Vitvi19g01858\_t003 |  |  |  |  |  |  |  |
| 1 | Ath-AT5G53940.1 |  | Vvi-Vitvi19g00194\_t001 |  |  |  |  |  |  |  |
| 1 | Ath-AT5G53950.1 |  | Vvi-Vitvi19g00188\_t001 |  |  |  |  |  |  |  |
| 1 | Ath-AT5G53960.1 |  | | | |  |  |  |  |  |  |  |
| 1 | Ath-AT5G53970.1 |  | Vvi-Vitvi19g00186\_t001 |  |  |  |  |  |  |  |
| 1 | Ath-AT5G53980.1 |  | Vvi-Vitvi19g01855\_t001 |  |  |  |  |  |  |  |
| 1 | Ath-AT5G53990.1 |  | | | |  |  |  |  |  |  |  |
| 1 | Ath-AT5G54000.2 |  | | | |  |  |  |  |  |  |  |
| 1 | Ath-AT5G54010.1 |  | Vvi-Vitvi19g04072\_t001 |  |  |  |  |  |  |  |
| 1 | Ath-AT5G54020.2 |  | | | |  |  |  |  |  |  |  |
| 1 | Ath-AT5G54030.1 |  | | | |  |  |  |  |  |  |  |
| 1 | Ath-AT5G54035.1 |  | | | |  |  |  |  |  |  |  |
| 1 | Ath-AT5G54040.1 |  | | | |  |  |  |  |  |  |  |
| 1 | Ath-AT5G54043.1 |  | | | |  |  |  |  |  |  |  |
| 1 | Ath-AT5G54050.1 |  | | | |  |  |  |  |  |  |  |
| 1 | Ath-AT5G54060.1 |  | Vvi-Vitvi19g04067\_t001 |  |  |  |  |  |  |  |
| 1 | Ath-AT5G54062.1 |  | | | |  |  |  |  |  |  |  |
| 1 | Ath-AT5G54067.1 |  | | | |  |  |  |  |  |  |  |
| 1 | Ath-AT5G54070.1 |  | | | |  |  |  |  |  |  |  |
| 1 | Ath-AT5G54080.1 |  | Vvi-Vitvi19g00150\_t001 |  |  |  |  |  |  |  |
| 1 | Ath-AT5G54090.1 |  | Vvi-Vitvi16g01316\_t001 |  |  |  |  |  |  |  |
| 1 | Ath-AT5G54095.1 |  | | | |  |  |  |  |  |  |  |
| 1 | Ath-AT5G54100.1 |  | | | |  |  |  |  |  |  |  |
| 1 | Ath-AT5G54110.1 |  | | | |  |  |  |  |  |  |  |
| 1 | Ath-AT5G54130.2 |  | Vvi-Vitvi16g01326\_t001 |  |  |  |  |  |  |  |
| 1 | Ath-AT5G54140.1 |  | Vvi-Vitvi16g02057\_t001 |  |  |  |  |  |  |  |
| 1 | Ath-AT5G54145.1 |  | | | |  |  |  |  |  |  |  |
| 1 | Ath-AT5G54148.1 |  | Vvi-Vitvi16g02060\_t001 |  |  |  |  |  |  |  |
| 1 | Ath-AT5G54150.1 |  | | | |  |  |  |  |  |  |  |
| 1 | Ath-AT5G54160.1 |  | Vvi-Vitvi16g01334\_t001 |  |  |  |  |  |  |  |
| 1 | Ath-AT5G54165.1 |  | | | |  |  |  |  |  |  |  |
| 1 | Ath-AT5G54170.1 |  | | | |  |  |  |  |  |  |  |
| 1 | Ath-AT5G54180.1 |  | Vvi-Vitvi16g01346\_t001 |  |  |  |  |  |  |  |
| 0 | Ath-AT5G54190.1 |  |  |  |  |  |  |  |  |
| 0 | Ath-AT5G54200.1 |  |  |  |  |  |  |  |  |
| 0 | Ath-AT5G54210.1 |  |  |  |  |  |  |  |  |
| 0 | Ath-AT5G54215.1 |  |  |  |  |  |  |  |  |
| 0 | Ath-AT5G54220.1 |  |  |  |  |  |  |  |  |
| 0 | Ath-AT5G54225.1 |  |  |  |  |  |  |  |  |
| 1 | Ath-AT5G54230.1 |  | Vvi-Vitvi19g00306\_t001 |  |  |  |  |  |  |  |
| 1 | Ath-AT5G54240.1 |  | Vvi-Vitvi19g00303\_t001 |  |  |  |  |  |  |  |
| 1 | Ath-AT5G54250.2 |  | Vvi-Vitvi19g00300\_t001 |  |  |  |  |  |  |  |
| 1 | Ath-AT5G54260.2 |  | Vvi-Vitvi19g01910\_t001 |  |  |  |  |  |  |  |
| 1 | Ath-AT5G54270.1 |  | Vvi-Vitvi19g00298\_t001 |  |  |  |  |  |  |  |
| 1 | Ath-AT5G54280.2 |  | Vvi-Vitvi19g00297\_t001 |  |  |  |  |  |  |  |
| 1 | Ath-AT5G54290.2 |  | Vvi-Vitvi19g00292\_t001 |  |  |  |  |  |  |  |
| 1 | Ath-AT5G54300.1 |  | Vvi-Vitvi19g00291\_t001 |  |  |  |  |  |  |  |
| 1 | Ath-AT5G54310.1 |  | Vvi-Vitvi19g00290\_t001 |  |  |  |  |  |  |  |
| 1 | Ath-AT5G54320.1 |  | | | |  |  |  |  |  |  |  |
| 1 | Ath-AT5G54330.1 |  | | | |  |  |  |  |  |  |  |
| 1 | Ath-AT5G54340.1 |  | | | |  |  |  |  |  |  |  |
| 1 | Ath-AT5G54350.1 |  | | | |  |  |  |  |  |  |  |
| 1 | Ath-AT5G54360.1 |  | | | |  |  |  |  |  |  |  |
| 1 | Ath-AT5G54370.1 |  | Vvi-Vitvi19g00282\_t001 |  |  |  |  |  |  |  |
| 1 | Ath-AT5G54380.1 |  | Vvi-Vitvi19g00280\_t001 |  |  |  |  |  |  |  |
| 1 | Ath-AT5G54390.1 |  | Vvi-Vitvi19g00276\_t001 |  |  |  |  |  |  |  |
| 1 | Ath-AT5G54400.1 |  | Vvi-Vitvi19g00275\_t001 |  |  |  |  |  |  |  |
| 1 | Ath-AT5G54410.1 |  | | | |  |  |  |  |  |  |  |
| 1 | Ath-AT5G54420.1 |  | | | |  |  |  |  |  |  |  |
| 1 | Ath-AT5G54430.3 |  | | | |  |  |  |  |  |  |  |
| 1 | Ath-AT5G54440.1 |  | Vvi-Vitvi19g00274\_t001 |  |  |  |  |  |  |  |
| 1 | Ath-AT5G54450.1 |  | | | |  |  |  |  |  |  |  |
| 1 | Ath-AT5G54460.1 |  | | | |  |  |  |  |  |  |  |
| 1 | Ath-AT5G54470.1 |  | | | |  |  |  |  |  |  |  |
| 1 | Ath-AT5G54480.1 |  | | | |  |  |  |  |  |  |  |
| 2 | Ath-AT5G54490.1 |  | | | |  | Vvi-Vitvi19g00364\_t001 |  |  |  |  |  |  |
| 2 | Ath-AT5G54500.2 |  | | | |  | Vvi-Vitvi19g00366\_t001 |  |  |  |  |  |  |
| 2 | Ath-AT5G54510.1 |  | | | |  | | | |  |  |  |  |  |  |
| 2 | Ath-AT5G54520.1 |  | | | |  | Vvi-Vitvi19g00368\_t001 |  |  |  |  |  |  |
| 2 | Ath-AT5G54530.1 |  | | | |  | Vvi-Vitvi19g01972\_t001 |  |  |  |  |  |  |
| 2 | Ath-AT5G54540.1 |  | | | |  | Vvi-Vitvi19g00371\_t001 |  |  |  |  |  |  |
| 2 | Ath-AT5G54550.1 |  | | | |  | | | |  |  |  |  |  |  |
| 2 | Ath-AT5G54560.1 |  | | | |  | | | |  |  |  |  |  |  |
| 2 | Ath-AT5G54570.1 |  | Vvi-Vitvi19g00265\_t001 |  | Vvi-Vitvi19g00373\_t001 |  |  |  |  |  |  |
| 1 | Ath-AT5G54580.1 |  |  |  | Vvi-Vitvi19g00376\_t001 |  |  |  |  |  |  |
| 1 | Ath-AT5G54585.1 |  |  |  | | | |  |  |  |  |  |  |
| 1 | Ath-AT5G54590.2 |  |  |  | Vvi-Vitvi19g00394\_t001 |  |  |  |  |  |  |
| 1 | Ath-AT5G54600.1 |  |  |  | Vvi-Vitvi19g00395\_t001 |  |  |  |  |  |  |
| 1 | Ath-AT5G54610.1 |  |  |  | | | |  |  |  |  |  |  |
| 1 | Ath-AT5G54620.1 |  |  |  | | | |  |  |  |  |  |  |
| 1 | Ath-AT5G54630.1 |  |  |  | Vvi-Vitvi19g00397\_t001 |  |  |  |  |  |  |
| 1 | Ath-AT5G54640.1 |  |  |  | Vvi-Vitvi19g00401\_t001 |  |  |  |  |  |  |
| 1 | Ath-AT5G54650.1 |  |  |  | Vvi-Vitvi19g00402\_t001 |  |  |  |  |  |  |
| 1 | Ath-AT5G54660.1 |  |  |  | Vvi-Vitvi19g00403\_t001 |  |  |  |  |  |  |
| 1 | Ath-AT5G54670.2 |  |  |  | Vvi-Vitvi19g00404\_t001 |  |  |  |  |  |  |
| 1 | Ath-AT5G54680.1 |  |  |  | Vvi-Vitvi19g00406\_t001 |  |  |  |  |  |  |
| 1 | Ath-AT5G54690.1 |  |  |  | Vvi-Vitvi19g00410\_t001 |  |  |  |  |  |  |
| 1 | Ath-AT5G54700.1 |  |  |  | | | |  |  |  |  |  |  |
| 1 | Ath-AT5G54710.2 |  |  |  | | | |  |  |  |  |  |  |
| 1 | Ath-AT5G54720.1 |  |  |  | | | |  |  |  |  |  |  |
| 1 | Ath-AT5G54730.1 |  |  |  | Vvi-Vitvi19g01985\_t003 |  |  |  |  |  |  |
| 1 | Ath-AT5G54740.1 |  | Vvi-Vitvi19g00435\_t001 |  |  |  |  |  |  |  |
| 1 | Ath-AT5G54745.1 |  | | | |  |  |  |  |  |  |  |
| 1 | Ath-AT5G54750.2 |  | | | |  |  |  |  |  |  |  |
| 2 | Ath-AT5G54760.3 |  | Vvi-Vitvi19g00436\_t001 |  | Vvi-Vitvi12g00528\_t002 |  |  |  |  |  |  |
| 2 | Ath-AT5G54770.1 |  | Vvi-Vitvi19g00441\_t001 |  | | | |  |  |  |  |  |  |
| 2 | Ath-AT5G54780.1 |  | Vvi-Vitvi19g00443\_t001 |  | | | |  |  |  |  |  |  |
| 2 | Ath-AT5G54790.1 |  | Vvi-Vitvi19g02000\_t001 |  | Vvi-Vitvi12g02414\_t001 |  |  |  |  |  |  |
| 2 | Ath-AT5G54800.1 |  | Vvi-Vitvi19g00457\_t001 |  | | | |  |  |  |  |  |  |
| 2 | Ath-AT5G54810.1 |  | Vvi-Vitvi19g00458\_t001 |  | | | |  |  |  |  |  |  |
| 2 | Ath-AT5G54820.1 |  | Vvi-Vitvi19g00459\_t001 |  | | | |  |  |  |  |  |  |
| 2 | Ath-AT5G54830.1 |  | | | |  | | | |  |  |  |  |  |  |
| 2 | Ath-AT5G54840.1 |  | | | |  | Vvi-Vitvi12g00519\_t002 |  |  |  |  |  |  |
| 2 | Ath-AT5G54850.1 |  | Vvi-Vitvi19g00463\_t001 |  | Vvi-Vitvi12g02413\_t001 |  |  |  |  |  |  |
| 2 | Ath-AT5G54855.1 |  | Vvi-Vitvi19g00472\_t001 |  | | | |  |  |  |  |  |  |
| 2 | Ath-AT5G54860.1 |  | Vvi-Vitvi19g00473\_t001 |  | | | |  |  |  |  |  |  |
| 2 | Ath-AT5G54870.1 |  | Vvi-Vitvi19g00476\_t001 |  | | | |  |  |  |  |  |  |
| 2 | Ath-AT5G54880.1 |  | Vvi-Vitvi19g00481\_t001 |  | | | |  |  |  |  |  |  |
| 2 | Ath-AT5G54890.1 |  | Vvi-Vitvi19g00482\_t001 |  | | | |  |  |  |  |  |  |
| 2 | Ath-AT5G54900.1 |  | Vvi-Vitvi19g00486\_t001 |  | | | |  |  |  |  |  |  |
| 2 | Ath-AT5G54910.1 |  | Vvi-Vitvi19g00487\_t001 |  | | | |  |  |  |  |  |  |
| 2 | Ath-AT5G54920.2 |  | Vvi-Vitvi19g00488\_t001 |  | | | |  |  |  |  |  |  |
| 2 | Ath-AT5G54930.1 |  | Vvi-Vitvi19g00489\_t002 |  | | | |  |  |  |  |  |  |
| 2 | Ath-AT5G54940.2 |  | Vvi-Vitvi19g00498\_t001 |  | | | |  |  |  |  |  |  |
| 2 | Ath-AT5G54950.1 |  | Vvi-Vitvi19g00502\_t001 |  | Vvi-Vitvi12g00505\_t001 |  |  |  |  |  |  |
| 2 | Ath-AT5G54960.1 |  | | | |  | | | |  |  |  |  |  |  |
| 2 | Ath-AT5G54970.1 |  | Vvi-Vitvi19g04236\_t001 |  | Vvi-Vitvi12g02407\_t001 |  |  |  |  |  |  |
| 1 | Ath-AT5G54980.1 |  | Vvi-Vitvi19g00507\_t001 |  |  |  |  |  |  |  |
| 1 | Ath-AT5G54990.1 |  | | | |  |  |  |  |  |  |  |
| 1 | Ath-AT5G55000.2 |  | | | |  |  |  |  |  |  |  |
| 1 | Ath-AT5G55010.1 |  | | | |  |  |  |  |  |  |  |
| 1 | Ath-AT5G55020.1 |  | Vvi-Vitvi19g00508\_t001 |  |  |  |  |  |  |  |
| 1 | Ath-AT5G55040.1 |  | Vvi-Vitvi19g00509\_t001 |  |  |  |  |  |  |  |
| 1 | Ath-AT5G55050.1 |  | Vvi-Vitvi19g00514\_t001 |  |  |  |  |  |  |  |
| 1 | Ath-AT5G55060.1 |  | Vvi-Vitvi19g00518\_t001 |  |  |  |  |  |  |  |
| 1 | Ath-AT5G55070.1 |  | Vvi-Vitvi19g00521\_t001 |  |  |  |  |  |  |  |
| 1 | Ath-AT5G55080.1 |  | | | |  |  |  |  |  |  |  |
| 1 | Ath-AT5G55090.2 |  | Vvi-Vitvi19g00528\_t001 |  |  |  |  |  |  |  |
| 1 | Ath-AT5G55100.2 |  | Vvi-Vitvi19g04242\_t001 |  |  |  |  |  |  |  |
| 1 | Ath-AT5G55110.1 |  | Vvi-Vitvi19g00534\_t001 |  |  |  |  |  |  |  |
| 1 | Ath-AT5G55120.1 |  | Vvi-Vitvi19g00549\_t001 |  |  |  |  |  |  |  |
| 1 | Ath-AT5G55125.1 |  | | | |  |  |  |  |  |  |  |
| 1 | Ath-AT5G55130.1 |  | Vvi-Vitvi19g00551\_t001 |  |  |  |  |  |  |  |
| 1 | Ath-AT5G55131.1 |  | | | |  |  |  |  |  |  |  |
| 1 | Ath-AT5G55132.1 |  | | | |  |  |  |  |  |  |  |
| 1 | Ath-AT5G55135.1 |  | | | |  |  |  |  |  |  |  |
| 1 | Ath-AT5G55140.1 |  | | | |  |  |  |  |  |  |  |
| 1 | Ath-AT5G55150.1 |  | | | |  |  |  |  |  |  |  |
| 1 | Ath-AT5G55160.2 |  | | | |  |  |  |  |  |  |  |
| 1 | Ath-AT5G55170.1 |  | | | |  |  |  |  |  |  |  |
| 1 | Ath-AT5G55180.2 |  | Vvi-Vitvi19g00557\_t001 |  |  |  |  |  |  |  |
| 1 | Ath-AT5G55190.1 |  | | | |  |  |  |  |  |  |  |
| 1 | Ath-AT5G55200.1 |  | Vvi-Vitvi19g00567\_t001 |  |  |  |  |  |  |  |
| 1 | Ath-AT5G55210.1 |  | Vvi-Vitvi19g00568\_t001 |  |  |  |  |  |  |  |
| 1 | Ath-AT5G55220.1 |  | Vvi-Vitvi19g00569\_t001 |  |  |  |  |  |  |  |
| 1 | Ath-AT5G55230.2 |  | Vvi-Vitvi19g00572\_t001 |  |  |  |  |  |  |  |
| 1 | Ath-AT5G55240.1 |  | | | |  |  |  |  |  |  |  |
| 1 | Ath-AT5G55250.1 |  | Vvi-Vitvi19g00574\_t001 |  |  |  |  |  |  |  |
| 1 | Ath-AT5G55260.2 |  | Vvi-Vitvi19g00577\_t003 |  |  |  |  |  |  |  |
| 1 | Ath-AT5G55270.1 |  | | | |  |  |  |  |  |  |  |
| 1 | Ath-AT5G55280.1 |  | Vvi-Vitvi19g00579\_t001 |  |  |  |  |  |  |  |
| 1 | Ath-AT5G55290.1 |  | Vvi-Vitvi19g00580\_t001 |  |  |  |  |  |  |  |
| 1 | Ath-AT5G55300.2 |  | | | |  |  |  |  |  |  |  |
| 1 | Ath-AT5G55310.1 |  | | | |  |  |  |  |  |  |  |
| 1 | Ath-AT5G55320.1 |  | | | |  |  |  |  |  |  |  |
| 1 | Ath-AT5G55330.1 |  | | | |  |  |  |  |  |  |  |
| 1 | Ath-AT5G55340.1 |  | Vvi-Vitvi19g00585\_t001 |  |  |  |  |  |  |  |
| 1 | Ath-AT5G55350.1 |  | | | |  |  |  |  |  |  |  |
| 1 | Ath-AT5G55360.1 |  | | | |  |  |  |  |  |  |  |
| 1 | Ath-AT5G55370.1 |  | | | |  |  |  |  |  |  |  |
| 1 | Ath-AT5G55380.1 |  | | | |  |  |  |  |  |  |  |
| 1 | Ath-AT5G55390.3 |  | Vvi-Vitvi19g00588\_t001 |  |  |  |  |  |  |  |
| 1 | Ath-AT5G55400.1 |  | Vvi-Vitvi19g00589\_t001 |  |  |  |  |  |  |  |
| 1 | Ath-AT5G55410.2 |  | Vvi-Vitvi19g00591\_t001 |  |  |  |  |  |  |  |
| 1 | Ath-AT5G55430.1 |  | | | |  |  |  |  |  |  |  |
| 1 | Ath-AT5G55440.1 |  | | | |  |  |  |  |  |  |  |
| 1 | Ath-AT5G55450.1 |  | | | |  |  |  |  |  |  |  |
| 1 | Ath-AT5G55460.1 |  | | | |  |  |  |  |  |  |  |
| 1 | Ath-AT5G55470.1 |  | Vvi-Vitvi19g00592\_t001 |  |  |  |  |  |  |  |
| 1 | Ath-AT5G55480.1 |  | Vvi-Vitvi19g00596\_t001 |  |  |  |  |  |  |  |
| 1 | Ath-AT5G55490.9 |  | | | |  |  |  |  |  |  |  |
| 1 | Ath-AT5G55500.1 |  | Vvi-Vitvi19g00602\_t001 |  |  |  |  |  |  |  |
| 1 | Ath-AT5G55507.1 |  | | | |  |  |  |  |  |  |  |
| 1 | Ath-AT5G55508.1 |  | | | |  |  |  |  |  |  |  |
| 1 | Ath-AT5G55510.1 |  | Vvi-Vitvi19g00608\_t001 |  |  |  |  |  |  |  |
| 1 | Ath-AT5G55520.1 |  | Vvi-Vitvi19g00609\_t001 |  |  |  |  |  |  |  |
| 1 | Ath-AT5G55530.1 |  | Vvi-Vitvi19g00610\_t001 |  |  |  |  |  |  |  |
| 1 | Ath-AT5G55540.1 |  | Vvi-Vitvi19g00611\_t001 |  |  |  |  |  |  |  |
| 1 | Ath-AT5G55550.10 |  | Vvi-Vitvi19g00612\_t001 |  |  |  |  |  |  |  |
| 1 | Ath-AT5G55560.1 |  | Vvi-Vitvi19g00614\_t001 |  |  |  |  |  |  |  |
| 1 | Ath-AT5G55565.1 |  | | | |  |  |  |  |  |  |  |
| 1 | Ath-AT5G55570.1 |  | Vvi-Vitvi19g04255\_t001 |  |  |  |  |  |  |  |
| 1 | Ath-AT5G55580.1 |  | Vvi-Vitvi19g00621\_t001 |  |  |  |  |  |  |  |
| 1 | Ath-AT5G55590.1 |  | Vvi-Vitvi19g00656\_t001 |  |  |  |  |  |  |  |
| 1 | Ath-AT5G55600.2 |  | Vvi-Vitvi19g00657\_t002 |  |  |  |  |  |  |  |
| 1 | Ath-AT5G55610.1 |  | Vvi-Vitvi19g00659\_t001 |  |  |  |  |  |  |  |
| 1 | Ath-AT5G55620.1 |  | | | |  |  |  |  |  |  |  |
| 1 | Ath-AT5G55630.2 |  | Vvi-Vitvi19g02059\_t001 |  |  |  |  |  |  |  |
| 1 | Ath-AT5G55640.1 |  | Vvi-Vitvi19g00668\_t001 |  |  |  |  |  |  |  |
| 1 | Ath-AT5G55650.1 |  | | | |  |  |  |  |  |  |  |
| 1 | Ath-AT5G55660.1 |  | Vvi-Vitvi19g00672\_t001 |  |  |  |  |  |  |  |
| 1 | Ath-AT5G55670.1 |  | Vvi-Vitvi19g00674\_t001 |  |  |  |  |  |  |  |
| 1 | Ath-AT5G55680.1 |  | | | |  |  |  |  |  |  |  |
| 1 | Ath-AT5G55690.1 |  | | | |  |  |  |  |  |  |  |
| 1 | Ath-AT5G55700.1 |  | Vvi-Vitvi19g00678\_t001 |  |  |  |  |  |  |  |
| 1 | Ath-AT5G55710.1 |  | | | |  |  |  |  |  |  |  |
| 1 | Ath-AT5G55720.1 |  | Vvi-Vitvi19g00680\_t001 |  |  |  |  |  |  |  |
| 1 | Ath-AT5G55730.1 |  | Vvi-Vitvi19g00682\_t001 |  |  |  |  |  |  |  |
| 1 | Ath-AT5G55740.1 |  | | | |  |  |  |  |  |  |  |
| 1 | Ath-AT5G55750.1 |  | | | |  |  |  |  |  |  |  |
| 1 | Ath-AT5G55760.1 |  | Vvi-Vitvi19g00685\_t001 |  |  |  |  |  |  |  |
| 1 | Ath-AT5G55770.1 |  | | | |  |  |  |  |  |  |  |
| 1 | Ath-AT5G55780.1 |  | | | |  |  |  |  |  |  |  |
| 1 | Ath-AT5G55790.3 |  | | | |  |  |  |  |  |  |  |
| 1 | Ath-AT5G55800.1 |  | | | |  |  |  |  |  |  |  |
| 1 | Ath-AT5G55810.2 |  | Vvi-Vitvi19g00687\_t007 |  |  |  |  |  |  |  |
| 1 | Ath-AT5G55820.1 |  | Vvi-Vitvi19g00688\_t001 |  |  |  |  |  |  |  |
| 1 | Ath-AT5G55830.1 |  | Vvi-Vitvi19g00690\_t001 |  |  |  |  |  |  |  |
| 1 | Ath-AT5G55840.1 |  | | | |  |  |  |  |  |  |  |
| 1 | Ath-AT5G55850.2 |  | Vvi-Vitvi19g00695\_t001 |  |  |  |  |  |  |  |
| 1 | Ath-AT5G55855.2 |  | | | |  |  |  |  |  |  |  |
| 1 | Ath-AT5G55856.1 |  | | | |  |  |  |  |  |  |  |
| 1 | Ath-AT5G55860.1 |  | Vvi-Vitvi19g00703\_t001 |  |  |  |  |  |  |  |
| 1 | Ath-AT5G55870.1 |  | | | |  |  |  |  |  |  |  |
| 1 | Ath-AT5G55880.1 |  | | | |  |  |  |  |  |  |  |
| 1 | Ath-AT5G55890.1 |  | | | |  |  |  |  |  |  |  |
| 1 | Ath-AT5G55893.2 |  | | | |  |  |  |  |  |  |  |
| 1 | Ath-AT5G55900.1 |  | Vvi-Vitvi19g00704\_t001 |  |  |  |  |  |  |  |
| 2 | Ath-AT5G55910.1 |  | Vvi-Vitvi19g00707\_t001 |  | Vvi-Vitvi19g00789\_t001 |  |  |  |  |  |  |
| 2 | Ath-AT5G55920.1 |  | Vvi-Vitvi19g04281\_t003 |  | | | |  |  |  |  |  |  |
| 2 | Ath-AT5G55930.1 |  | Vvi-Vitvi19g00712\_t001 |  | | | |  |  |  |  |  |  |
| 2 | Ath-AT5G55940.1 |  | Vvi-Vitvi19g00721\_t001 |  | | | |  |  |  |  |  |  |
| 2 | Ath-AT5G55950.1 |  | Vvi-Vitvi19g00724\_t001 |  | | | |  |  |  |  |  |  |
| 2 | Ath-AT5G55960.1 |  | Vvi-Vitvi19g04286\_t001 |  | | | |  |  |  |  |  |  |
| 2 | Ath-AT5G55970.1 |  | Vvi-Vitvi19g00728\_t001 |  | | | |  |  |  |  |  |  |
| 2 | Ath-AT5G55980.1 |  | | | |  | | | |  |  |  |  |  |  |
| 2 | Ath-AT5G55990.1 |  | Vvi-Vitvi19g00731\_t001 |  | | | |  |  |  |  |  |  |
| 2 | Ath-AT5G56000.1 |  | Vvi-Vitvi19g02080\_t001 |  | | | |  |  |  |  |  |  |
| 2 | Ath-AT5G56010.1 |  | | | |  | | | |  |  |  |  |  |  |
| 2 | Ath-AT5G56020.2 |  | Vvi-Vitvi19g00734\_t001 |  | | | |  |  |  |  |  |  |
| 2 | Ath-AT5G56030.2 |  | | | |  | | | |  |  |  |  |  |  |
| 2 | Ath-AT5G56040.2 |  | Vvi-Vitvi19g00735\_t001 |  | | | |  |  |  |  |  |  |
| 2 | Ath-AT5G56050.1 |  | | | |  | | | |  |  |  |  |  |  |
| 2 | Ath-AT5G56060.1 |  | | | |  | | | |  |  |  |  |  |  |
| 2 | Ath-AT5G56070.1 |  | | | |  | | | |  |  |  |  |  |  |
| 2 | Ath-AT5G56075.1 |  | | | |  | Vvi-Vitvi19g02101\_t001 |  |  |  |  |  |  |
| 2 | Ath-AT5G56080.1 |  | | | |  | | | |  |  |  |  |  |  |
| 3 | Ath-AT5G56090.1 |  | | | |  | | | |  | Vvi-Vitvi19g00767\_t001 |  |  |  |  |  |
| 3 | Ath-AT5G56100.1 |  | | | |  | | | |  | | | |  |  |  |  |  |
| 3 | Ath-AT5G56110.1 |  | | | |  | | | |  | Vvi-Vitvi19g00758\_t001 |  |  |  |  |  |
| 3 | Ath-AT5G56120.1 |  | | | |  | | | |  | Vvi-Vitvi19g00750\_t001.1.6037826d |  |  |  |  |  |
| 3 | Ath-AT5G56130.1 |  | | | |  | | | |  | Vvi-Vitvi19g00749\_t001 |  |  |  |  |  |
| 3 | Ath-AT5G56140.1 |  | Vvi-Vitvi19g00747\_t001 |  | | | |  | | | |  |  |  |  |  |
| 2 | Ath-AT5G56150.1 |  |  |  | | | |  | Vvi-Vitvi19g00744\_t002 |  |  |  |  |  |
| 2 | Ath-AT5G56160.1 |  |  |  | | | |  | Vvi-Vitvi19g00740\_t001 |  |  |  |  |  |
| 1 | Ath-AT5G56170.1 |  |  |  | | | |  |  |  |  |  |  |
| 1 | Ath-AT5G56180.1 |  |  |  | Vvi-Vitvi19g00907\_t001 |  |  |  |  |  |  |
| 1 | Ath-AT5G56190.7 |  |  |  | Vvi-Vitvi19g00908\_t001 |  |  |  |  |  |  |
| 1 | Ath-AT5G56200.1 |  |  |  | Vvi-Vitvi19g00912\_t001 |  |  |  |  |  |  |
| 1 | Ath-AT5G56210.1 |  |  |  | Vvi-Vitvi19g00913\_t001 |  |  |  |  |  |  |
| 1 | Ath-AT5G56220.2 |  |  |  | Vvi-Vitvi19g00915\_t001 |  |  |  |  |  |  |
| 1 | Ath-AT5G56230.1 |  |  |  | Vvi-Vitvi19g00916\_t001 |  |  |  |  |  |  |
| 1 | Ath-AT5G56240.3 |  |  |  | Vvi-Vitvi19g00917\_t003 |  |  |  |  |  |  |
| 1 | Ath-AT5G56250.1 |  |  |  | | | |  |  |  |  |  |  |
| 1 | Ath-AT5G56260.3 |  |  |  | | | |  |  |  |  |  |  |
| 1 | Ath-AT5G56270.1 |  |  |  | Vvi-Vitvi19g00927\_t001 |  |  |  |  |  |  |
| 1 | Ath-AT5G56280.1 |  |  |  | | | |  |  |  |  |  |  |
| 1 | Ath-AT5G56290.1 |  |  |  | Vvi-Vitvi19g00933\_t001 |  |  |  |  |  |  |
| 1 | Ath-AT5G56300.1 |  | Vvi-Vitvi06g00008\_t001 |  |  |  |  |  |  |  |
| 1 | Ath-AT5G56310.1 |  | Vvi-Vitvi06g00011\_t001 |  |  |  |  |  |  |  |
| 1 | Ath-AT5G56320.3 |  | Vvi-Vitvi06g00016\_t001 |  |  |  |  |  |  |  |
| 1 | Ath-AT5G56325.1 |  | | | |  |  |  |  |  |  |  |
| 1 | Ath-AT5G56330.1 |  | | | |  |  |  |  |  |  |  |
| 1 | Ath-AT5G56340.1 |  | Vvi-Vitvi06g00020\_t001 |  |  |  |  |  |  |  |
| 1 | Ath-AT5G56350.1 |  | Vvi-Vitvi06g00021\_t001 |  |  |  |  |  |  |  |
| 1 | Ath-AT5G56360.1 |  | Vvi-Vitvi06g00022\_t001 |  |  |  |  |  |  |  |
| 1 | Ath-AT5G56368.1 |  | | | |  |  |  |  |  |  |  |
| 1 | Ath-AT5G56369.1 |  | | | |  |  |  |  |  |  |  |
| 1 | Ath-AT5G56370.1 |  | | | |  |  |  |  |  |  |  |
| 1 | Ath-AT5G56380.2 |  | | | |  |  |  |  |  |  |  |
| 1 | Ath-AT5G56390.1 |  | | | |  |  |  |  |  |  |  |
| 1 | Ath-AT5G56400.1 |  | | | |  |  |  |  |  |  |  |
| 1 | Ath-AT5G56410.1 |  | | | |  |  |  |  |  |  |  |
| 1 | Ath-AT5G56420.1 |  | | | |  |  |  |  |  |  |  |
| 1 | Ath-AT5G56430.1 |  | | | |  |  |  |  |  |  |  |
| 1 | Ath-AT5G56440.1 |  | | | |  |  |  |  |  |  |  |
| 1 | Ath-AT5G56450.1 |  | | | |  |  |  |  |  |  |  |
| 1 | Ath-AT5G56452.1 |  | | | |  |  |  |  |  |  |  |
| 1 | Ath-AT5G56460.1 |  | Vvi-Vitvi06g00031\_t001 |  |  |  |  |  |  |  |
| 1 | Ath-AT5G56470.1 |  | | | |  |  |  |  |  |  |  |
| 1 | Ath-AT5G56480.1 |  | | | |  |  |  |  |  |  |  |
| 1 | Ath-AT5G56490.1 |  | | | |  |  |  |  |  |  |  |
| 1 | Ath-AT5G56500.2 |  | Vvi-Vitvi06g00032\_t001 |  |  |  |  |  |  |  |
| 1 | Ath-AT5G56510.1 |  | Vvi-Vitvi06g01575\_t001 |  |  |  |  |  |  |  |
| 1 | Ath-AT5G56520.1 |  | Vvi-Vitvi06g01582\_t001 |  |  |  |  |  |  |  |
| 1 | Ath-AT5G56530.2 |  | Vvi-Vitvi06g00044\_t001 |  |  |  |  |  |  |  |
| 1 | Ath-AT5G56540.1 |  | Vvi-Vitvi06g04015\_t001 |  |  |  |  |  |  |  |
| 1 | Ath-AT5G56550.1 |  | Vvi-Vitvi11g00251\_t001 |  |  |  |  |  |  |  |
| 1 | Ath-AT5G56555.1 |  | | | |  |  |  |  |  |  |  |
| 1 | Ath-AT5G56560.1 |  | | | |  |  |  |  |  |  |  |
| 1 | Ath-AT5G56570.1 |  | | | |  |  |  |  |  |  |  |
| 1 | Ath-AT5G56580.1 |  | Vvi-Vitvi11g00250\_t001 |  |  |  |  |  |  |  |
| 1 | Ath-AT5G56590.1 |  | Vvi-Vitvi11g00249\_t001 |  |  |  |  |  |  |  |
| 1 | Ath-AT5G56600.1 |  | Vvi-Vitvi11g00247\_t001 |  |  |  |  |  |  |  |
| 1 | Ath-AT5G56610.1 |  | Vvi-Vitvi11g00242\_t001 |  |  |  |  |  |  |  |
| 1 | Ath-AT5G56620.3 |  | Vvi-Vitvi11g00241\_t001 |  |  |  |  |  |  |  |
| 1 | Ath-AT5G56630.1 |  | Vvi-Vitvi11g00237\_t001 |  |  |  |  |  |  |  |
| 1 | Ath-AT5G56640.1 |  | Vvi-Vitvi11g00231\_t001 |  |  |  |  |  |  |  |
| 1 | Ath-AT5G56650.1 |  | Vvi-Vitvi11g00226\_t001 |  |  |  |  |  |  |  |
| 1 | Ath-AT5G56660.1 |  | | | |  |  |  |  |  |  |  |
| 1 | Ath-AT5G56670.1 |  | Vvi-Vitvi11g04055\_t001 |  |  |  |  |  |  |  |
| 1 | Ath-AT5G56680.1 |  | Vvi-Vitvi11g00224\_t001 |  |  |  |  |  |  |  |
| 1 | Ath-AT5G56690.1 |  | | | |  |  |  |  |  |  |  |
| 1 | Ath-AT5G56700.2 |  | | | |  |  |  |  |  |  |  |
| 1 | Ath-AT5G56710.1 |  | Vvi-Vitvi11g00222\_t001 |  |  |  |  |  |  |  |
| 1 | Ath-AT5G56720.1 |  | | | |  |  |  |  |  |  |  |
| 1 | Ath-AT5G56730.1 |  | Vvi-Vitvi11g00221\_t001 |  |  |  |  |  |  |  |
| 1 | Ath-AT5G56740.1 |  | Vvi-Vitvi11g00219\_t001 |  |  |  |  |  |  |  |
| 1 | Ath-AT5G56750.1 |  | Vvi-Vitvi11g00203\_t001 |  |  |  |  |  |  |  |
| 1 | Ath-AT5G56760.1 |  | Vvi-Vitvi11g01377\_t001 |  |  |  |  |  |  |  |
| 1 | Ath-AT5G56770.1 |  | Vvi-Vitvi11g00200\_t001 |  |  |  |  |  |  |  |
| 1 | Ath-AT5G56780.1 |  | | | |  |  |  |  |  |  |  |
| 1 | Ath-AT5G56790.1 |  | Vvi-Vitvi11g00199\_t002 |  |  |  |  |  |  |  |
| 1 | Ath-AT5G56795.1 |  | | | |  |  |  |  |  |  |  |
| 1 | Ath-AT5G56800.1 |  | | | |  |  |  |  |  |  |  |
| 1 | Ath-AT5G56810.1 |  | | | |  |  |  |  |  |  |  |
| 1 | Ath-AT5G56820.1 |  | | | |  |  |  |  |  |  |  |
| 1 | Ath-AT5G56840.1 |  | Vvi-Vitvi11g00197\_t001 |  |  |  |  |  |  |  |
| 1 | Ath-AT5G56850.1 |  | Vvi-Vitvi11g00184\_t001 |  |  |  |  |  |  |  |
| 1 | Ath-AT5G56860.1 |  | Vvi-Vitvi11g00180\_t001 |  |  |  |  |  |  |  |
| 1 | Ath-AT5G56870.1 |  | Vvi-Vitvi11g00178\_t001 |  |  |  |  |  |  |  |
| 1 | Ath-AT5G56880.1 |  | Vvi-Vitvi11g01374\_t001 |  |  |  |  |  |  |  |
| 1 | Ath-AT5G56890.1 |  | Vvi-Vitvi11g01322\_t001 |  |  |  |  |  |  |  |
| 1 | Ath-AT5G56900.2 |  | Vvi-Vitvi11g00170\_t001 |  |  |  |  |  |  |  |
| 1 | Ath-AT5G56910.1 |  | | | |  |  |  |  |  |  |  |
| 1 | Ath-AT5G56920.1 |  | | | |  |  |  |  |  |  |  |
| 2 | Ath-AT5G56930.1 |  | | | |  | Vvi-Vitvi11g01367\_t001 |  |  |  |  |  |  |
| 2 | Ath-AT5G56940.1 |  | | | |  | Vvi-Vitvi11g00162\_t001 |  |  |  |  |  |  |
| 2 | Ath-AT5G56950.1 |  | | | |  | Vvi-Vitvi11g00163\_t001 |  |  |  |  |  |  |
| 2 | Ath-AT5G56960.2 |  | Vvi-Vitvi11g00165\_t001 |  | Vvi-Vitvi11g00165\_t001 |  |  |  |  |  |  |
| 2 | Ath-AT5G56970.1 |  | | | |  | Vvi-Vitvi11g01371\_t001 |  |  |  |  |  |  |
| 2 | Ath-AT5G56980.1 |  | | | |  | Vvi-Vitvi11g01372\_t001 |  |  |  |  |  |  |
| 1 | Ath-AT5G56985.1 |  | | | |  |  |  |  |  |  |  |
| 1 | Ath-AT5G56990.2 |  | | | |  |  |  |  |  |  |  |
| 2 | Ath-AT5G57000.1 |  | Vvi-Vitvi11g00157\_t002 |  | Vvi-Vitvi09g04034\_t001 |  |  |  |  |  |  |
| 2 | Ath-AT5G57010.1 |  | Vvi-Vitvi11g00156\_t001 |  | | | |  |  |  |  |  |  |
| 2 | Ath-AT5G57015.1 |  | Vvi-Vitvi11g00155\_t001 |  | Vvi-Vitvi09g00148\_t001 |  |  |  |  |  |  |
| 2 | Ath-AT5G57020.1 |  | Vvi-Vitvi11g00150\_t001 |  | Vvi-Vitvi09g00150\_t005 |  |  |  |  |  |  |
| 2 | Ath-AT5G57030.1 |  | Vvi-Vitvi11g00148\_t001 |  | | | |  |  |  |  |  |  |
| 2 | Ath-AT5G57035.1 |  | Vvi-Vitvi11g00142\_t001 |  | | | |  |  |  |  |  |  |
| 2 | Ath-AT5G57040.1 |  | Vvi-Vitvi11g00141\_t001 |  | | | |  |  |  |  |  |  |
| 2 | Ath-AT5G57050.1 |  | Vvi-Vitvi11g00137\_t002 |  | Vvi-Vitvi09g00156\_t002 |  |  |  |  |  |  |
| 2 | Ath-AT5G57060.3 |  | Vvi-Vitvi11g00134\_t002 |  | Vvi-Vitvi09g01525\_t002 |  |  |  |  |  |  |
| 2 | Ath-AT5G57070.1 |  | Vvi-Vitvi11g00132\_t001 |  | Vvi-Vitvi09g00159\_t001 |  |  |  |  |  |  |
| 0 | Ath-AT5G57080.1 |  |  |  |  |  |  |  |  |
| 1 | Ath-AT5G57090.1 |  | Vvi-Vitvi11g01186\_t001 |  |  |  |  |  |  |  |
| 2 | Ath-AT5G57100.1 |  | | | |  | Vvi-Vitvi11g01185\_t001 |  |  |  |  |  |  |
| 2 | Ath-AT5G57110.3 |  | | | |  | Vvi-Vitvi11g01176\_t001 |  |  |  |  |  |  |
| 2 | Ath-AT5G57120.1 |  | | | |  | Vvi-Vitvi11g01173\_t001 |  |  |  |  |  |  |
| 2 | Ath-AT5G57123.1 |  | | | |  | Vvi-Vitvi11g01629\_t001 |  |  |  |  |  |  |
| 2 | Ath-AT5G57130.1 |  | | | |  | Vvi-Vitvi11g01162\_t001 |  |  |  |  |  |  |
| 2 | Ath-AT5G57140.1 |  | | | |  | Vvi-Vitvi11g01156\_t001 |  |  |  |  |  |  |
| 2 | Ath-AT5G57150.4 |  | | | |  | Vvi-Vitvi11g01153\_t001 |  |  |  |  |  |  |
| 2 | Ath-AT5G57160.1 |  | | | |  | Vvi-Vitvi11g01148\_t001 |  |  |  |  |  |  |
| 2 | Ath-AT5G57170.2 |  | | | |  | Vvi-Vitvi11g01146\_t001 |  |  |  |  |  |  |
| 2 | Ath-AT5G57180.2 |  | | | |  | Vvi-Vitvi11g01143\_t001 |  |  |  |  |  |  |
| 2 | Ath-AT5G57190.1 |  | | | |  | | | |  |  |  |  |  |  |
| 2 | Ath-AT5G57200.1 |  | | | |  | Vvi-Vitvi11g01139\_t001 |  |  |  |  |  |  |
| 2 | Ath-AT5G57210.1 |  | | | |  | Vvi-Vitvi11g01136\_t001 |  |  |  |  |  |  |
| 2 | Ath-AT5G57220.1 |  | | | |  | | | |  |  |  |  |  |  |
| 2 | Ath-AT5G57230.2 |  | | | |  | Vvi-Vitvi11g01133\_t002 |  |  |  |  |  |  |
| 2 | Ath-AT5G57240.3 |  | | | |  | Vvi-Vitvi11g01132\_t001 |  |  |  |  |  |  |
| 2 | Ath-AT5G57250.1 |  | | | |  | | | |  |  |  |  |  |  |
| 2 | Ath-AT5G57260.1 |  | | | |  | | | |  |  |  |  |  |  |
| 2 | Ath-AT5G57270.2 |  | | | |  | Vvi-Vitvi11g01128\_t001 |  |  |  |  |  |  |
| 2 | Ath-AT5G57280.1 |  | | | |  | Vvi-Vitvi11g01104\_t001 |  |  |  |  |  |  |
| 2 | Ath-AT5G57290.1 |  | | | |  | Vvi-Vitvi11g01101\_t001 |  |  |  |  |  |  |
| 1 | Ath-AT5G57300.1 |  | | | |  |  |  |  |  |  |  |
| 1 | Ath-AT5G57320.2 |  | | | |  |  |  |  |  |  |  |
| 1 | Ath-AT5G57330.1 |  | Vvi-Vitvi11g01211\_t001 |  |  |  |  |  |  |  |
| 1 | Ath-AT5G57340.2 |  | | | |  |  |  |  |  |  |  |
| 1 | Ath-AT5G57345.1 |  | Vvi-Vitvi11g01651\_t001 |  |  |  |  |  |  |  |
| 1 | Ath-AT5G57350.1 |  | | | |  |  |  |  |  |  |  |
| 1 | Ath-AT5G57360.2 |  | Vvi-Vitvi11g01220\_t001 |  |  |  |  |  |  |  |
| 1 | Ath-AT5G57370.1 |  | Vvi-Vitvi11g01656\_t001 |  |  |  |  |  |  |  |
| 1 | Ath-AT5G57380.1 |  | Vvi-Vitvi11g01230\_t001 |  |  |  |  |  |  |  |
| 1 | Ath-AT5G57390.1 |  | Vvi-Vitvi11g01231\_t001 |  |  |  |  |  |  |  |
| 1 | Ath-AT5G57400.2 |  | | | |  |  |  |  |  |  |  |
| 1 | Ath-AT5G57410.3 |  | | | |  |  |  |  |  |  |  |
| 1 | Ath-AT5G57420.1 |  | Vvi-Vitvi11g01235\_t001 |  |  |  |  |  |  |  |
| 1 | Ath-AT5G57440.1 |  | | | |  |  |  |  |  |  |  |
| 1 | Ath-AT5G57450.1 |  | | | |  |  |  |  |  |  |  |
| 1 | Ath-AT5G57460.1 |  | Vvi-Vitvi11g01239\_t001 |  |  |  |  |  |  |  |
| 1 | Ath-AT5G57480.1 |  | Vvi-Vitvi11g01243\_t001 |  |  |  |  |  |  |  |
| 1 | Ath-AT5G57490.1 |  | Vvi-Vitvi11g01662\_t001 |  |  |  |  |  |  |  |
| 1 | Ath-AT5G57500.1 |  | Vvi-Vitvi11g01664\_t001 |  |  |  |  |  |  |  |
| 1 | Ath-AT5G57510.1 |  | Vvi-Vitvi11g01667\_t001 |  |  |  |  |  |  |  |
| 1 | Ath-AT5G57520.1 |  | Vvi-Vitvi11g01259\_t001 |  |  |  |  |  |  |  |
| 1 | Ath-AT5G57530.1 |  | | | |  |  |  |  |  |  |  |
| 1 | Ath-AT5G57535.1 |  | | | |  |  |  |  |  |  |  |
| 1 | Ath-AT5G57540.1 |  | | | |  |  |  |  |  |  |  |
| 1 | Ath-AT5G57550.1 |  | Vvi-Vitvi11g04355\_t001 |  |  |  |  |  |  |  |
| 1 | Ath-AT5G57560.1 |  | Vvi-Vitvi11g01673\_t001 |  |  |  |  |  |  |  |
| 1 | Ath-AT5G57565.4 |  | | | |  |  |  |  |  |  |  |
| 1 | Ath-AT5G57569.1 |  | | | |  |  |  |  |  |  |  |
| 1 | Ath-AT5G57567.2 |  | | | |  |  |  |  |  |  |  |
| 1 | Ath-AT5G57570.1 |  | | | |  |  |  |  |  |  |  |
| 1 | Ath-AT5G57580.1 |  | Vvi-Vitvi11g01272\_t001 |  |  |  |  |  |  |  |
| 1 | Ath-AT5G57590.1 |  | Vvi-Vitvi11g01273\_t001 |  |  |  |  |  |  |  |
| 1 | Ath-AT5G57610.1 |  | Vvi-Vitvi11g01282\_t001 |  |  |  |  |  |  |  |
| 1 | Ath-AT5G57620.1 |  | Vvi-Vitvi11g01283\_t001 |  |  |  |  |  |  |  |
| 1 | Ath-AT5G57625.1 |  | Vvi-Vitvi11g01293\_t001 |  |  |  |  |  |  |  |
| 1 | Ath-AT5G57630.1 |  | Vvi-Vitvi11g01298\_t002 |  |  |  |  |  |  |  |
| 1 | Ath-AT5G57640.1 |  | | | |  |  |  |  |  |  |  |
| 1 | Ath-AT5G57650.1 |  | | | |  |  |  |  |  |  |  |
| 1 | Ath-AT5G57655.2 |  | Vvi-Vitvi11g01300\_t002 |  |  |  |  |  |  |  |
| 1 | Ath-AT5G57660.1 |  | Vvi-Vitvi11g01309\_t001 |  |  |  |  |  |  |  |
| 1 | Ath-AT5G57670.2 |  | Vvi-Vitvi11g01310\_t001 |  |  |  |  |  |  |  |
| 1 | Ath-AT5G57685.1 |  | Vvi-Vitvi11g01702\_t001 |  |  |  |  |  |  |  |
| 1 | Ath-AT5G57690.1 |  | Vvi-Vitvi11g01318\_t001 |  |  |  |  |  |  |  |
| 0 | Ath-AT5G57700.3 |  |  |  |  |  |  |  |  |
| 0 | Ath-AT5G57710.1 |  |  |  |  |  |  |  |  |
| 0 | Ath-AT5G57720.1 |  |  |  |  |  |  |  |  |
| 1 | Ath-AT5G57740.1 |  | Vvi-Vitvi11g00871\_t001 |  |  |  |  |  |  |  |
| 1 | Ath-AT5G57750.1 |  | Vvi-Vitvi11g00869\_t001 |  |  |  |  |  |  |  |
| 1 | Ath-AT5G57760.1 |  | | | |  |  |  |  |  |  |  |
| 1 | Ath-AT5G57770.1 |  | Vvi-Vitvi11g00840\_t001 |  |  |  |  |  |  |  |
| 1 | Ath-AT5G57780.1 |  | Vvi-Vitvi11g00838\_t001 |  |  |  |  |  |  |  |
| 1 | Ath-AT5G57785.1 |  | | | |  |  |  |  |  |  |  |
| 1 | Ath-AT5G57790.1 |  | | | |  |  |  |  |  |  |  |
| 1 | Ath-AT5G57800.1 |  | Vvi-Vitvi11g00835\_t001 |  |  |  |  |  |  |  |
| 1 | Ath-AT5G57810.1 |  | | | |  |  |  |  |  |  |  |
| 1 | Ath-AT5G57815.1 |  | Vvi-Vitvi11g00826\_t001 |  |  |  |  |  |  |  |
| 0 | Ath-AT5G57820.1 |  |  |  |  |  |  |  |  |
| 1 | Ath-AT5G57830.1 |  | Vvi-Vitvi11g00745\_t001 |  |  |  |  |  |  |  |
| 1 | Ath-AT5G57840.1 |  | Vvi-Vitvi11g00730\_t001 |  |  |  |  |  |  |  |
| 1 | Ath-AT5G57850.1 |  | | | |  |  |  |  |  |  |  |
| 1 | Ath-AT5G57860.2 |  | | | |  |  |  |  |  |  |  |
| 1 | Ath-AT5G57870.1 |  | Vvi-Vitvi11g00723\_t001 |  |  |  |  |  |  |  |
| 1 | Ath-AT5G57880.1 |  | Vvi-Vitvi11g00722\_t001 |  |  |  |  |  |  |  |
| 1 | Ath-AT5G57887.1 |  | | | |  |  |  |  |  |  |  |
| 1 | Ath-AT5G57890.1 |  | | | |  |  |  |  |  |  |  |
| 1 | Ath-AT5G57900.1 |  | Vvi-Vitvi11g00719\_t001 |  |  |  |  |  |  |  |
| 1 | Ath-AT5G57910.1 |  | Vvi-Vitvi11g01505\_t001 |  |  |  |  |  |  |  |
| 1 | Ath-AT5G57920.3 |  | Vvi-Vitvi11g00708\_t001 |  |  |  |  |  |  |  |
| 1 | Ath-AT5G57930.2 |  | Vvi-Vitvi11g04175\_t001 |  |  |  |  |  |  |  |
| 1 | Ath-AT5G57940.2 |  | Vvi-Vitvi11g00701\_t002 |  |  |  |  |  |  |  |
| 1 | Ath-AT5G57950.2 |  | Vvi-Vitvi11g00696\_t001 |  |  |  |  |  |  |  |
| 1 | Ath-AT5G57960.1 |  | | | |  |  |  |  |  |  |  |
| 1 | Ath-AT5G57970.1 |  | Vvi-Vitvi11g00695\_t001 |  |  |  |  |  |  |  |
| 1 | Ath-AT5G57980.1 |  | | | |  |  |  |  |  |  |  |
| 2 | Ath-AT5G57990.1 |  | | | |  | Vvi-Vitvi11g01715\_t001 |  |  |  |  |  |  |
| 2 | Ath-AT5G58000.2 |  | | | |  | Vvi-Vitvi11g04169\_t001 |  |  |  |  |  |  |
| 2 | Ath-AT5G58003.1 |  | | | |  | Vvi-Vitvi11g04170\_t001 |  |  |  |  |  |  |
| 2 | Ath-AT5G58005.1 |  | | | |  | Vvi-Vitvi11g00679\_t002 |  |  |  |  |  |  |
| 2 | Ath-AT5G58010.1 |  | Vvi-Vitvi11g00680\_t001 |  | Vvi-Vitvi11g00680\_t001 |  |  |  |  |  |  |
| 2 | Ath-AT5G58020.1 |  | | | |  | Vvi-Vitvi11g00688\_t001 |  |  |  |  |  |  |
| 2 | Ath-AT5G58030.1 |  | | | |  | Vvi-Vitvi11g00690\_t001 |  |  |  |  |  |  |
| 2 | Ath-AT5G58040.1 |  | | | |  | Vvi-Vitvi11g00692\_t001 |  |  |  |  |  |  |
| 1 | Ath-AT5G58050.1 |  | Vvi-Vitvi11g00660\_t001 |  |  |  |  |  |  |  |
| 1 | Ath-AT5G58060.2 |  | Vvi-Vitvi11g00659\_t001 |  |  |  |  |  |  |  |
| 1 | Ath-AT5G58070.1 |  | Vvi-Vitvi11g00658\_t001 |  |  |  |  |  |  |  |
| 1 | Ath-AT5G58080.1 |  | Vvi-Vitvi11g00651\_t001 |  |  |  |  |  |  |  |
| 1 | Ath-AT5G58090.1 |  | | | |  |  |  |  |  |  |  |
| 1 | Ath-AT5G58100.1 |  | | | |  |  |  |  |  |  |  |
| 1 | Ath-AT5G58110.1 |  | | | |  |  |  |  |  |  |  |
| 1 | Ath-AT5G58120.1 |  | | | |  |  |  |  |  |  |  |
| 1 | Ath-AT5G58130.1 |  | | | |  |  |  |  |  |  |  |
| 1 | Ath-AT5G58140.2 |  | | | |  |  |  |  |  |  |  |
| 1 | Ath-AT5G58150.1 |  | | | |  |  |  |  |  |  |  |
| 1 | Ath-AT5G58160.1 |  | | | |  |  |  |  |  |  |  |
| 1 | Ath-AT5G58170.1 |  | | | |  |  |  |  |  |  |  |
| 1 | Ath-AT5G58180.2 |  | | | |  |  |  |  |  |  |  |
| 1 | Ath-AT5G58190.2 |  | | | |  |  |  |  |  |  |  |
| 1 | Ath-AT5G58200.2 |  | Vvi-Vitvi11g04160\_t001 |  |  |  |  |  |  |  |
| 1 | Ath-AT5G58210.4 |  | | | |  |  |  |  |  |  |  |
| 1 | Ath-AT5G58220.1 |  | | | |  |  |  |  |  |  |  |
| 1 | Ath-AT5G58230.1 |  | | | |  |  |  |  |  |  |  |
| 2 | Ath-AT5G58240.1 |  | | | |  | Vvi-Vitvi06g00840\_t001 |  |  |  |  |  |  |
| 2 | Ath-AT5G58250.1 |  | | | |  | Vvi-Vitvi06g00834\_t002 |  |  |  |  |  |  |
| 2 | Ath-AT5G58260.1 |  | | | |  | Vvi-Vitvi06g00832\_t001 |  |  |  |  |  |  |
| 2 | Ath-AT5G58270.1 |  | | | |  | Vvi-Vitvi06g00831\_t001 |  |  |  |  |  |  |
| 2 | Ath-AT5G58280.2 |  | | | |  | Vvi-Vitvi06g00822\_t001 |  |  |  |  |  |  |
| 2 | Ath-AT5G58290.1 |  | | | |  | Vvi-Vitvi06g00813\_t001 |  |  |  |  |  |  |
| 2 | Ath-AT5G58300.1 |  | | | |  | Vvi-Vitvi06g00812\_t001 |  |  |  |  |  |  |
| 2 | Ath-AT5G58310.1 |  | | | |  | | | |  |  |  |  |  |  |
| 2 | Ath-AT5G58320.2 |  | | | |  | Vvi-Vitvi06g00805\_t001 |  |  |  |  |  |  |
| 2 | Ath-AT5G58330.1 |  | | | |  | | | |  |  |  |  |  |  |
| 2 | Ath-AT5G58340.1 |  | | | |  | Vvi-Vitvi06g00798\_t001 |  |  |  |  |  |  |
| 2 | Ath-AT5G58350.1 |  | | | |  | Vvi-Vitvi06g00784\_t001 |  |  |  |  |  |  |
| 2 | Ath-AT5G58360.1 |  | | | |  | Vvi-Vitvi06g00783\_t001 |  |  |  |  |  |  |
| 2 | Ath-AT5G58370.2 |  | | | |  | Vvi-Vitvi06g00777\_t001 |  |  |  |  |  |  |
| 2 | Ath-AT5G58375.1 |  | Vvi-Vitvi11g01486\_t001 |  | Vvi-Vitvi06g01777\_t001 |  |  |  |  |  |  |
| 1 | Ath-AT5G58380.1 |  |  |  | Vvi-Vitvi06g00775\_t003 |  |  |  |  |  |  |
| 1 | Ath-AT5G58390.1 |  |  |  | Vvi-Vitvi06g00768\_t001 |  |  |  |  |  |  |
| 1 | Ath-AT5G58400.1 |  |  |  | | | |  |  |  |  |  |  |
| 1 | Ath-AT5G58410.1 |  |  |  | Vvi-Vitvi06g00746\_t001 |  |  |  |  |  |  |
| 1 | Ath-AT5G58412.1 |  |  |  | | | |  |  |  |  |  |  |
| 2 | Ath-AT5G58420.1 |  | Vvi-Vitvi06g00701\_t001.1.6037826e |  | | | |  |  |  |  |  |  |
| 2 | Ath-AT5G58430.1 |  | | | |  | Vvi-Vitvi06g00733\_t001 |  |  |  |  |  |  |
| 2 | Ath-AT5G58440.1 |  | | | |  | Vvi-Vitvi06g00732\_t002 |  |  |  |  |  |  |
| 2 | Ath-AT5G58450.1 |  | | | |  | Vvi-Vitvi06g00729\_t001 |  |  |  |  |  |  |
| 2 | Ath-AT5G58460.1 |  | | | |  | Vvi-Vitvi06g00726\_t001 |  |  |  |  |  |  |
| 1 | Ath-AT5G58470.2 |  | | | |  |  |  |  |  |  |  |
| 1 | Ath-AT5G58480.1 |  | | | |  |  |  |  |  |  |  |
| 1 | Ath-AT5G58490.1 |  | Vvi-Vitvi06g00699\_t001 |  |  |  |  |  |  |  |
| 1 | Ath-AT5G58500.1 |  | Vvi-Vitvi06g00695\_t001 |  |  |  |  |  |  |  |
| 1 | Ath-AT5G58510.1 |  | Vvi-Vitvi06g00694\_t001 |  |  |  |  |  |  |  |
| 1 | Ath-AT5G58520.1 |  | Vvi-Vitvi06g00693\_t001 |  |  |  |  |  |  |  |
| 1 | Ath-AT5G58530.1 |  | Vvi-Vitvi06g00690\_t001 |  |  |  |  |  |  |  |
| 1 | Ath-AT5G58540.1 |  | Vvi-Vitvi06g00689\_t001 |  |  |  |  |  |  |  |
| 1 | Ath-AT5G58550.1 |  | Vvi-Vitvi06g00688\_t001 |  |  |  |  |  |  |  |
| 1 | Ath-AT5G58560.1 |  | Vvi-Vitvi06g01754\_t001 |  |  |  |  |  |  |  |
| 1 | Ath-AT5G58570.1 |  | | | |  |  |  |  |  |  |  |
| 1 | Ath-AT5G58575.1 |  | Vvi-Vitvi06g00681\_t001 |  |  |  |  |  |  |  |
| 1 | Ath-AT5G58580.1 |  | | | |  |  |  |  |  |  |  |
| 1 | Ath-AT5G58590.1 |  | Vvi-Vitvi06g00671\_t001 |  |  |  |  |  |  |  |
| 1 | Ath-AT5G58600.1 |  | Vvi-Vitvi06g00665\_t001 |  |  |  |  |  |  |  |
| 1 | Ath-AT5G58610.3 |  | Vvi-Vitvi06g00661\_t001 |  |  |  |  |  |  |  |
| 1 | Ath-AT5G58620.1 |  | | | |  |  |  |  |  |  |  |
| 1 | Ath-AT5G58630.1 |  | Vvi-Vitvi06g01749\_t001 |  |  |  |  |  |  |  |
| 1 | Ath-AT5G58640.1 |  | | | |  |  |  |  |  |  |  |
| 1 | Ath-AT5G58650.1 |  | | | |  |  |  |  |  |  |  |
| 1 | Ath-AT5G58660.1 |  | | | |  |  |  |  |  |  |  |
| 1 | Ath-AT5G58670.1 |  | Vvi-Vitvi06g00649\_t001 |  |  |  |  |  |  |  |
| 1 | Ath-AT5G58680.1 |  | | | |  |  |  |  |  |  |  |
| 1 | Ath-AT5G58690.3 |  | Vvi-Vitvi06g00647\_t001 |  |  |  |  |  |  |  |
| 1 | Ath-AT5G58700.3 |  | | | |  |  |  |  |  |  |  |
| 1 | Ath-AT5G58710.1 |  | Vvi-Vitvi06g00643\_t001 |  |  |  |  |  |  |  |
| 1 | Ath-AT5G58720.1 |  | Vvi-Vitvi06g00641\_t001 |  |  |  |  |  |  |  |
| 1 | Ath-AT5G58730.1 |  | Vvi-Vitvi06g00640\_t001 |  |  |  |  |  |  |  |
| 1 | Ath-AT5G58740.1 |  | | | |  |  |  |  |  |  |  |
| 1 | Ath-AT5G58750.1 |  | Vvi-Vitvi06g00638\_t001 |  |  |  |  |  |  |  |
| 1 | Ath-AT5G58760.1 |  | Vvi-Vitvi06g00635\_t001 |  |  |  |  |  |  |  |
| 1 | Ath-AT5G58770.1 |  | Vvi-Vitvi06g00634\_t001 |  |  |  |  |  |  |  |
| 1 | Ath-AT5G58780.1 |  | | | |  |  |  |  |  |  |  |
| 1 | Ath-AT5G58782.2 |  | | | |  |  |  |  |  |  |  |
| 1 | Ath-AT5G58784.1 |  | | | |  |  |  |  |  |  |  |
| 1 | Ath-AT5G58787.1 |  | Vvi-Vitvi06g01748\_t001 |  |  |  |  |  |  |  |
| 1 | Ath-AT5G58790.2 |  | | | |  |  |  |  |  |  |  |
| 1 | Ath-AT5G58800.2 |  | Vvi-Vitvi06g00618\_t001 |  |  |  |  |  |  |  |
| 1 | Ath-AT5G58820.1 |  | | | |  |  |  |  |  |  |  |
| 1 | Ath-AT5G58830.1 |  | | | |  |  |  |  |  |  |  |
| 1 | Ath-AT5G58840.1 |  | | | |  |  |  |  |  |  |  |
| 1 | Ath-AT5G58850.1 |  | Vvi-Vitvi06g00611\_t001 |  |  |  |  |  |  |  |
| 1 | Ath-AT5G58860.1 |  | Vvi-Vitvi06g00605\_t001 |  |  |  |  |  |  |  |
| 1 | Ath-AT5G58870.1 |  | Vvi-Vitvi06g00595\_t001 |  |  |  |  |  |  |  |
| 1 | Ath-AT5G58880.1 |  | Vvi-Vitvi06g00594\_t001 |  |  |  |  |  |  |  |
| 1 | Ath-AT5G58890.1 |  | | | |  |  |  |  |  |  |  |
| 2 | Ath-AT5G58900.1 |  | Vvi-Vitvi06g00592\_t001 |  | Vvi-Vitvi08g01336\_t001 |  |  |  |  |  |  |
| 2 | Ath-AT5G58910.2 |  | Vvi-Vitvi06g00591\_t001 |  | Vvi-Vitvi08g01335\_t001 |  |  |  |  |  |  |
| 2 | Ath-AT5G58920.1 |  | Vvi-Vitvi06g00589\_t001 |  | | | |  |  |  |  |  |  |
| 2 | Ath-AT5G58930.1 |  | Vvi-Vitvi06g00587\_t001 |  | Vvi-Vitvi08g01327\_t001 |  |  |  |  |  |  |
| 2 | Ath-AT5G58940.2 |  | Vvi-Vitvi06g00584\_t001 |  | | | |  |  |  |  |  |  |
| 2 | Ath-AT5G58950.1 |  | | | |  | | | |  |  |  |  |  |  |
| 2 | Ath-AT5G58960.1 |  | Vvi-Vitvi06g00578\_t001 |  | | | |  |  |  |  |  |  |
| 2 | Ath-AT5G58970.1 |  | Vvi-Vitvi06g00577\_t001.1.6037826e |  | Vvi-Vitvi08g01313\_t001 |  |  |  |  |  |  |
| 2 | Ath-AT5G58980.1 |  | Vvi-Vitvi06g00573\_t001 |  | Vvi-Vitvi08g01311\_t001 |  |  |  |  |  |  |
| 2 | Ath-AT5G58990.1 |  | Vvi-Vitvi06g00568\_t001 |  | | | |  |  |  |  |  |  |
| 2 | Ath-AT5G59000.1 |  | Vvi-Vitvi06g00567\_t001 |  | Vvi-Vitvi08g01297\_t001 |  |  |  |  |  |  |
| 2 | Ath-AT5G59010.1 |  | Vvi-Vitvi06g00565\_t001 |  | Vvi-Vitvi08g01296\_t001 |  |  |  |  |  |  |
| 2 | Ath-AT5G59020.1 |  | Vvi-Vitvi06g00563\_t001 |  | Vvi-Vitvi08g01293\_t001 |  |  |  |  |  |  |
| 2 | Ath-AT5G59030.1 |  | Vvi-Vitvi06g01729\_t001 |  | Vvi-Vitvi08g02230\_t001 |  |  |  |  |  |  |
| 2 | Ath-AT5G59040.1 |  | | | |  | | | |  |  |  |  |  |  |
| 2 | Ath-AT5G59050.1 |  | Vvi-Vitvi06g00558\_t001 |  | Vvi-Vitvi08g01287\_t001 |  |  |  |  |  |  |
| 2 | Ath-AT5G59060.1 |  | | | |  | | | |  |  |  |  |  |  |
| 2 | Ath-AT5G59080.1 |  | | | |  | Vvi-Vitvi08g04253\_t006 |  |  |  |  |  |  |
| 1 | Ath-AT5G59070.1 |  | Vvi-Vitvi06g00555\_t001 |  |  |  |  |  |  |  |
| 1 | Ath-AT5G59090.1 |  | Vvi-Vitvi06g00547\_t001 |  |  |  |  |  |  |  |
| 1 | Ath-AT5G59100.1 |  | | | |  |  |  |  |  |  |  |
| 1 | Ath-AT5G59105.1 |  | | | |  |  |  |  |  |  |  |
| 1 | Ath-AT5G59110.1 |  | | | |  |  |  |  |  |  |  |
| 1 | Ath-AT5G59120.1 |  | Vvi-Vitvi06g04180\_t001 |  |  |  |  |  |  |  |
| 1 | Ath-AT5G59130.4 |  | | | |  |  |  |  |  |  |  |
| 1 | Ath-AT5G59140.1 |  | Vvi-Vitvi06g00543\_t001 |  |  |  |  |  |  |  |
| 1 | Ath-AT5G59150.1 |  | Vvi-Vitvi06g00542\_t001 |  |  |  |  |  |  |  |
| 1 | Ath-AT5G59160.1 |  | Vvi-Vitvi06g00540\_t003 |  |  |  |  |  |  |  |
| 1 | Ath-AT5G59170.1 |  | | | |  |  |  |  |  |  |  |
| 1 | Ath-AT5G59180.1 |  | Vvi-Vitvi06g00539\_t001 |  |  |  |  |  |  |  |
| 1 | Ath-AT5G59190.1 |  | | | |  |  |  |  |  |  |  |
| 1 | Ath-AT5G59200.1 |  | | | |  |  |  |  |  |  |  |
| 1 | Ath-AT5G59210.1 |  | Vvi-Vitvi06g00536\_t001 |  |  |  |  |  |  |  |
| 1 | Ath-AT5G59220.1 |  | Vvi-Vitvi06g00533\_t001 |  |  |  |  |  |  |  |
| 1 | Ath-AT5G59230.1 |  | Vvi-Vitvi06g00515\_t001 |  |  |  |  |  |  |  |
| 1 | Ath-AT5G59240.1 |  | | | |  |  |  |  |  |  |  |
| 1 | Ath-AT5G59250.1 |  | Vvi-Vitvi06g00514\_t001 |  |  |  |  |  |  |  |
| 0 | Ath-AT5G59260.1 |  |  |  |  |  |  |  |  |
| 0 | Ath-AT5G59270.2 |  |  |  |  |  |  |  |  |
| 0 | Ath-AT5G59280.1 |  |  |  |  |  |  |  |  |
| 0 | Ath-AT5G59290.2 |  |  |  |  |  |  |  |  |
| 0 | Ath-AT5G59300.1 |  |  |  |  |  |  |  |  |
| 0 | Ath-AT5G59305.1 |  |  |  |  |  |  |  |  |
| 0 | Ath-AT5G59310.1 |  |  |  |  |  |  |  |  |
| 0 | Ath-AT5G59320.1 |  |  |  |  |  |  |  |  |
| 0 | Ath-AT5G59330.1 |  |  |  |  |  |  |  |  |
| 0 | Ath-AT5G59340.1 |  |  |  |  |  |  |  |  |
| 0 | Ath-AT5G59350.1 |  |  |  |  |  |  |  |  |
| 0 | Ath-AT5G59360.1 |  |  |  |  |  |  |  |  |
| 0 | Ath-AT5G59370.1 |  |  |  |  |  |  |  |  |
| 1 | Ath-AT5G59380.1 |  | Vvi-Vitvi06g01703\_t001 |  |  |  |  |  |  |  |
| 1 | Ath-AT5G59390.1 |  | | | |  |  |  |  |  |  |  |
| 1 | Ath-AT5G59400.1 |  | Vvi-Vitvi06g00484\_t001 |  |  |  |  |  |  |  |
| 1 | Ath-AT5G59410.1 |  | Vvi-Vitvi06g00486\_t001 |  |  |  |  |  |  |  |
| 1 | Ath-AT5G59420.1 |  | Vvi-Vitvi06g00487\_t001 |  |  |  |  |  |  |  |
| 1 | Ath-AT5G59430.2 |  | Vvi-Vitvi06g00488\_t001 |  |  |  |  |  |  |  |
| 1 | Ath-AT5G59440.3 |  | | | |  |  |  |  |  |  |  |
| 1 | Ath-AT5G59450.1 |  | Vvi-Vitvi06g00490\_t001 |  |  |  |  |  |  |  |
| 1 | Ath-AT5G59460.1 |  | Vvi-Vitvi06g00493\_t001 |  |  |  |  |  |  |  |
| 1 | Ath-AT5G59470.1 |  | | | |  |  |  |  |  |  |  |
| 1 | Ath-AT5G59480.1 |  | Vvi-Vitvi06g00495\_t001 |  |  |  |  |  |  |  |
| 1 | Ath-AT5G59490.1 |  | | | |  |  |  |  |  |  |  |
| 1 | Ath-AT5G59500.1 |  | Vvi-Vitvi06g00497\_t001 |  |  |  |  |  |  |  |
| 1 | Ath-AT5G59510.1 |  | Vvi-Vitvi06g04166\_t001 |  |  |  |  |  |  |  |
| 1 | Ath-AT5G59520.1 |  | Vvi-Vitvi06g00500\_t001 |  |  |  |  |  |  |  |
| 1 | Ath-AT5G59530.1 |  | | | |  |  |  |  |  |  |  |
| 1 | Ath-AT5G59540.1 |  | | | |  |  |  |  |  |  |  |
| 1 | Ath-AT5G59550.2 |  | Vvi-Vitvi06g00502\_t001 |  |  |  |  |  |  |  |
| 1 | Ath-AT5G59560.1 |  | | | |  |  |  |  |  |  |  |
| 1 | Ath-AT5G59570.1 |  | Vvi-Vitvi06g00505\_t001 |  |  |  |  |  |  |  |
| 1 | Ath-AT5G59580.1 |  | | | |  |  |  |  |  |  |  |
| 1 | Ath-AT5G59590.1 |  | | | |  |  |  |  |  |  |  |
| 1 | Ath-AT5G59610.3 |  | Vvi-Vitvi06g00509\_t001 |  |  |  |  |  |  |  |
| 0 | Ath-AT5G59600.1 |  |  |  |  |  |  |  |  |
| 0 | Ath-AT5G59613.2 |  |  |  |  |  |  |  |  |
| 0 | Ath-AT5G59616.1 |  |  |  |  |  |  |  |  |
| 0 | Ath-AT5G59650.2 |  |  |  |  |  |  |  |  |
| 0 | Ath-AT5G59660.3 |  |  |  |  |  |  |  |  |
| 0 | Ath-AT5G59670.2 |  |  |  |  |  |  |  |  |
| 0 | Ath-AT5G59680.1 |  |  |  |  |  |  |  |  |
| 1 | Ath-AT5G59690.1 |  | Vvi-Vitvi06g04126\_t001 |  |  |  |  |  |  |  |
| 1 | Ath-AT5G59700.1 |  | Vvi-Vitvi06g00388\_t001 |  |  |  |  |  |  |  |
| 1 | Ath-AT5G59710.1 |  | Vvi-Vitvi06g00389\_t001 |  |  |  |  |  |  |  |
| 1 | Ath-AT5G59720.1 |  | | | |  |  |  |  |  |  |  |
| 1 | Ath-AT5G59730.1 |  | Vvi-Vitvi06g00397\_t001 |  |  |  |  |  |  |  |
| 1 | Ath-AT5G59740.1 |  | Vvi-Vitvi06g01681\_t001 |  |  |  |  |  |  |  |
| 1 | Ath-AT5G59750.2 |  | Vvi-Vitvi06g00407\_t001 |  |  |  |  |  |  |  |
| 1 | Ath-AT5G59760.1 |  | Vvi-Vitvi06g00411\_t001 |  |  |  |  |  |  |  |
| 1 | Ath-AT5G59770.1 |  | Vvi-Vitvi06g00413\_t001 |  |  |  |  |  |  |  |
| 1 | Ath-AT5G59780.3 |  | Vvi-Vitvi06g00414\_t002 |  |  |  |  |  |  |  |
| 2 | Ath-AT5G59790.1 |  | Vvi-Vitvi06g00415\_t001 |  | Vvi-Vitvi13g00260\_t002 |  |  |  |  |  |  |
| 2 | Ath-AT5G59800.1 |  | Vvi-Vitvi06g00417\_t001 |  | | | |  |  |  |  |  |  |
| 2 | Ath-AT5G59810.1 |  | | | |  | | | |  |  |  |  |  |  |
| 2 | Ath-AT5G59820.1 |  | Vvi-Vitvi06g01682\_t001 |  | Vvi-Vitvi13g00262\_t001 |  |  |  |  |  |  |
| 2 | Ath-AT5G59830.2 |  | Vvi-Vitvi06g00419\_t001 |  | Vvi-Vitvi13g00263\_t001 |  |  |  |  |  |  |
| 2 | Ath-AT5G59840.1 |  | Vvi-Vitvi06g00420\_t001 |  | Vvi-Vitvi13g00266\_t001 |  |  |  |  |  |  |
| 2 | Ath-AT5G59845.1 |  | | | |  | | | |  |  |  |  |  |  |
| 2 | Ath-AT5G59850.1 |  | | | |  | Vvi-Vitvi13g04080\_t001 |  |  |  |  |  |  |
| 2 | Ath-AT5G59860.1 |  | Vvi-Vitvi06g01688\_t001 |  | | | |  |  |  |  |  |  |
| 2 | Ath-AT5G59865.1 |  | | | |  | | | |  |  |  |  |  |  |
| 2 | Ath-AT5G59870.1 |  | Vvi-Vitvi06g00423\_t001 |  | | | |  |  |  |  |  |  |
| 2 | Ath-AT5G59880.1 |  | Vvi-Vitvi06g00424\_t001 |  | Vvi-Vitvi13g00269\_t003 |  |  |  |  |  |  |
| 2 | Ath-AT5G59890.1 |  | | | |  | | | |  |  |  |  |  |  |
| 2 | Ath-AT5G59895.1 |  | | | |  | | | |  |  |  |  |  |  |
| 2 | Ath-AT5G59900.1 |  | Vvi-Vitvi06g00425\_t001 |  | | | |  |  |  |  |  |  |
| 2 | Ath-AT5G59910.1 |  | Vvi-Vitvi06g00426\_t001 |  | | | |  |  |  |  |  |  |
| 2 | Ath-AT5G59920.1 |  | | | |  | | | |  |  |  |  |  |  |
| 2 | Ath-AT5G59930.1 |  | | | |  | | | |  |  |  |  |  |  |
| 2 | Ath-AT5G59940.1 |  | | | |  | | | |  |  |  |  |  |  |
| 2 | Ath-AT5G59950.5 |  | Vvi-Vitvi06g00429\_t002 |  | Vvi-Vitvi13g00272\_t001 |  |  |  |  |  |  |
| 2 | Ath-AT5G59960.1 |  | Vvi-Vitvi06g00430\_t001 |  | | | |  |  |  |  |  |  |
| 3 | Ath-AT5G59970.2 |  | Vvi-Vitvi06g04151\_t001 |  | Vvi-Vitvi13g04084\_t001 |  | Vvi-Vitvi06g04126\_t001 |  |  |  |  |  |
| 1 | Ath-AT5G59980.2 |  |  |  |  |  | Vvi-Vitvi06g00384\_t001 |  |  |  |  |  |
| 1 | Ath-AT5G59990.1 |  |  |  |  |  | Vvi-Vitvi06g01676\_t001 |  |  |  |  |  |
| 1 | Ath-AT5G60000.1 |  |  |  |  |  | | | |  |  |  |  |  |
| 1 | Ath-AT5G60010.1 |  |  |  |  |  | Vvi-Vitvi06g00381\_t001 |  |  |  |  |  |
| 1 | Ath-AT5G60020.1 |  |  |  |  |  | Vvi-Vitvi06g00378\_t001 |  |  |  |  |  |
| 1 | Ath-AT5G60030.1 |  |  |  |  |  | | | |  |  |  |  |  |
| 1 | Ath-AT5G60040.2 |  |  |  |  |  | Vvi-Vitvi06g00376\_t001 |  |  |  |  |  |
| 1 | Ath-AT5G60050.1 |  |  |  |  |  | Vvi-Vitvi06g00375\_t001 |  |  |  |  |  |
| 1 | Ath-AT5G60060.1 |  |  |  |  |  | | | |  |  |  |  |  |
| 1 | Ath-AT5G60070.1 |  |  |  |  |  | | | |  |  |  |  |  |
| 1 | Ath-AT5G60080.1 |  |  |  |  |  | | | |  |  |  |  |  |
| 1 | Ath-AT5G60090.1 |  |  |  |  |  | | | |  |  |  |  |  |
| 1 | Ath-AT5G60100.2 |  |  |  |  |  | Vvi-Vitvi06g00368\_t001 |  |  |  |  |  |
| 1 | Ath-AT5G60110.1 |  |  |  |  |  | | | |  |  |  |  |  |
| 1 | Ath-AT5G60120.2 |  |  |  |  |  | Vvi-Vitvi06g00360\_t001 |  |  |  |  |  |
| 1 | Ath-AT5G60130.1 |  |  |  |  |  | | | |  |  |  |  |  |
| 1 | Ath-AT5G60140.1 |  |  |  |  |  | | | |  |  |  |  |  |
| 1 | Ath-AT5G60142.1 |  |  |  |  |  | | | |  |  |  |  |  |
| 1 | Ath-AT5G60150.2 |  |  |  |  |  | Vvi-Vitvi06g00350\_t001 |  |  |  |  |  |
| 1 | Ath-AT5G60160.1 |  |  |  |  |  | | | |  |  |  |  |  |
| 1 | Ath-AT5G60170.2 |  |  |  |  |  | | | |  |  |  |  |  |
| 1 | Ath-AT5G60180.1 |  |  |  |  |  | | | |  |  |  |  |  |
| 1 | Ath-AT5G60190.1 |  |  |  |  |  | Vvi-Vitvi06g00347\_t001 |  |  |  |  |  |
| 1 | Ath-AT5G60200.1 |  |  |  |  |  | Vvi-Vitvi06g00345\_t001 |  |  |  |  |  |
| 1 | Ath-AT5G60210.3 |  |  |  |  |  | Vvi-Vitvi06g01665\_t004 |  |  |  |  |  |
| 1 | Ath-AT5G60215.1 |  |  |  |  |  | | | |  |  |  |  |  |
| 1 | Ath-AT5G60220.1 |  |  |  |  |  | Vvi-Vitvi06g00342\_t001 |  |  |  |  |  |
| 1 | Ath-AT5G60230.2 |  |  |  |  |  | Vvi-Vitvi06g00341\_t001 |  |  |  |  |  |
| 1 | Ath-AT5G60240.1 |  |  |  |  |  | | | |  |  |  |  |  |
| 1 | Ath-AT5G60250.1 |  |  |  |  |  | Vvi-Vitvi06g00335\_t001 |  |  |  |  |  |
| 1 | Ath-AT5G60260.1 |  |  |  |  |  | | | |  |  |  |  |  |
| 1 | Ath-AT5G60265.1 |  |  |  |  |  | | | |  |  |  |  |  |
| 1 | Ath-AT5G60270.1 |  |  |  |  |  | | | |  |  |  |  |  |
| 1 | Ath-AT5G60280.1 |  |  |  |  |  | | | |  |  |  |  |  |
| 1 | Ath-AT5G60290.1 |  |  |  |  |  | | | |  |  |  |  |  |
| 1 | Ath-AT5G60300.3 |  |  |  |  |  | | | |  |  |  |  |  |
| 1 | Ath-AT5G60310.1 |  |  |  |  |  | | | |  |  |  |  |  |
| 1 | Ath-AT5G60320.1 |  |  |  |  |  | | | |  |  |  |  |  |
| 1 | Ath-AT5G60335.1 |  |  |  |  |  | Vvi-Vitvi06g00329\_t001 |  |  |  |  |  |
| 1 | Ath-AT5G60340.1 |  |  |  |  |  | Vvi-Vitvi06g00328\_t001 |  |  |  |  |  |
| 1 | Ath-AT5G60350.2 |  |  |  |  |  | | | |  |  |  |  |  |
| 1 | Ath-AT5G60360.3 |  |  |  |  |  | | | |  |  |  |  |  |
| 1 | Ath-AT5G60370.1 |  |  |  |  |  | Vvi-Vitvi06g00325\_t001 |  |  |  |  |  |
| 1 | Ath-AT5G60380.1 |  |  |  |  |  | | | |  |  |  |  |  |
| 2 | Ath-AT5G60390.1 |  | Vvi-Vitvi13g02054\_t001 |  |  |  | Vvi-Vitvi06g04107\_t001 |  |  |  |  |  |
| 2 | Ath-AT5G60400.3 |  | | | |  |  |  | | | |  |  |  |  |  |
| 2 | Ath-AT5G60410.2 |  | | | |  |  |  | Vvi-Vitvi06g00315\_t003 |  |  |  |  |  |
| 2 | Ath-AT5G60430.2 |  | | | |  |  |  | | | |  |  |  |  |  |
| 2 | Ath-AT5G60440.1 |  | | | |  |  |  | | | |  |  |  |  |  |
| 2 | Ath-AT5G60450.1 |  | | | |  |  |  | Vvi-Vitvi06g00311\_t001 |  |  |  |  |  |
| 2 | Ath-AT5G60460.1 |  | Vvi-Vitvi13g00583\_t001 |  |  |  | Vvi-Vitvi06g01661\_t001 |  |  |  |  |  |
| 2 | Ath-AT5G60470.2 |  | | | |  |  |  | Vvi-Vitvi06g00304\_t001 |  |  |  |  |  |
| 2 | Ath-AT5G60480.1 |  | | | |  |  |  | | | |  |  |  |  |  |
| 3 | Ath-AT5G60490.1 |  | | | |  | Vvi-Vitvi08g02147\_t001 |  | Vvi-Vitvi06g00303\_t001 |  |  |  |  |  |
| 3 | Ath-AT5G60500.1 |  | | | |  | | | |  | | | |  |  |  |  |  |
| 3 | Ath-AT5G60510.1 |  | | | |  | | | |  | | | |  |  |  |  |  |
| 3 | Ath-AT5G60520.1 |  | | | |  | | | |  | Vvi-Vitvi06g00299\_t001 |  |  |  |  |  |
| 3 | Ath-AT5G60530.1 |  | | | |  | | | |  | | | |  |  |  |  |  |
| 3 | Ath-AT5G60540.1 |  | | | |  | | | |  | Vvi-Vitvi06g00298\_t001 |  |  |  |  |  |
| 3 | Ath-AT5G60550.1 |  | | | |  | | | |  | Vvi-Vitvi06g00296\_t002 |  |  |  |  |  |
| 3 | Ath-AT5G60553.1 |  | | | |  | | | |  | | | |  |  |  |  |  |
| 3 | Ath-AT5G60560.2 |  | | | |  | | | |  | | | |  |  |  |  |  |
| 3 | Ath-AT5G60570.3 |  | | | |  | | | |  | Vvi-Vitvi06g00295\_t001 |  |  |  |  |  |
| 3 | Ath-AT5G60580.4 |  | | | |  | Vvi-Vitvi08g01043\_t001 |  | Vvi-Vitvi06g00289\_t001 |  |  |  |  |  |
| 3 | Ath-AT5G60590.2 |  | | | |  | | | |  | Vvi-Vitvi06g00288\_t001 |  |  |  |  |  |
| 3 | Ath-AT5G60600.1 |  | | | |  | | | |  | Vvi-Vitvi06g00286\_t001 |  |  |  |  |  |
| 3 | Ath-AT5G60610.1 |  | | | |  | | | |  | | | |  |  |  |  |  |
| 3 | Ath-AT5G60615.1 |  | | | |  | | | |  | | | |  |  |  |  |  |
| 3 | Ath-AT5G60620.1 |  | | | |  | | | |  | | | |  |  |  |  |  |
| 3 | Ath-AT5G60630.1 |  | | | |  | | | |  | | | |  |  |  |  |  |
| 3 | Ath-AT5G60640.1 |  | Vvi-Vitvi13g00602\_t001 |  | | | |  | Vvi-Vitvi06g00284\_t001 |  |  |  |  |  |
| 3 | Ath-AT5G60650.1 |  | | | |  | | | |  | Vvi-Vitvi06g01658\_t001 |  |  |  |  |  |
| 3 | Ath-AT5G60660.1 |  | Vvi-Vitvi13g00605\_t001 |  | Vvi-Vitvi08g01038\_t001 |  | Vvi-Vitvi06g00281\_t001 |  |  |  |  |  |
| 3 | Ath-AT5G60670.1 |  | Vvi-Vitvi13g00606\_t001 |  | Vvi-Vitvi08g01037\_t001 |  | Vvi-Vitvi06g00280\_t001 |  |  |  |  |  |
| 3 | Ath-AT5G60680.1 |  | | | |  | Vvi-Vitvi08g01036\_t001 |  | Vvi-Vitvi06g00279\_t001 |  |  |  |  |  |
| 3 | Ath-AT5G60690.1 |  | Vvi-Vitvi13g00609\_t001 |  | | | |  | Vvi-Vitvi06g00276\_t001 |  |  |  |  |  |
| 3 | Ath-AT5G60700.2 |  | | | |  | | | |  | Vvi-Vitvi06g00266\_t001 |  |  |  |  |  |
| 3 | Ath-AT5G60710.1 |  | Vvi-Vitvi13g00620\_t001 |  | | | |  | Vvi-Vitvi06g00258\_t001 |  |  |  |  |  |
| 3 | Ath-AT5G60720.1 |  | | | |  | | | |  | Vvi-Vitvi06g00244\_t001 |  |  |  |  |  |
| 3 | Ath-AT5G60730.1 |  | Vvi-Vitvi13g00637\_t001.1.6037826b |  | | | |  | Vvi-Vitvi06g00243\_t001 |  |  |  |  |  |
| 3 | Ath-AT5G60740.1 |  | Vvi-Vitvi13g00638\_t001 |  | Vvi-Vitvi08g01013\_t001 |  | Vvi-Vitvi06g00242\_t001 |  |  |  |  |  |
| 1 | Ath-AT5G60750.1 |  |  |  | | | |  |  |  |  |  |  |
| 1 | Ath-AT5G60760.1 |  |  |  | Vvi-Vitvi08g01012\_t002 |  |  |  |  |  |  |
| 1 | Ath-AT5G60770.1 |  |  |  | Vvi-Vitvi08g01004\_t001 |  |  |  |  |  |  |
| 1 | Ath-AT5G60780.1 |  |  |  | | | |  |  |  |  |  |  |
| 1 | Ath-AT5G60790.1 |  |  |  | | | |  |  |  |  |  |  |
| 1 | Ath-AT5G60800.2 |  |  |  | Vvi-Vitvi08g00992\_t001 |  |  |  |  |  |  |
| 0 | Ath-AT5G60805.1 |  |  |  |  |  |  |  |  |
| 0 | Ath-AT5G60810.1 |  |  |  |  |  |  |  |  |
| 1 | Ath-AT5G60820.1 |  | Vvi-Vitvi17g00435\_t001 |  |  |  |  |  |  |  |
| 1 | Ath-AT5G60830.1 |  | | | |  |  |  |  |  |  |  |
| 1 | Ath-AT5G60840.1 |  | | | |  |  |  |  |  |  |  |
| 1 | Ath-AT5G60850.1 |  | Vvi-Vitvi17g00447\_t001 |  |  |  |  |  |  |  |
| 1 | Ath-AT5G60860.1 |  | | | |  |  |  |  |  |  |  |
| 1 | Ath-AT5G60870.2 |  | | | |  |  |  |  |  |  |  |
| 1 | Ath-AT5G60880.1 |  | Vvi-Vitvi17g01436\_t001 |  |  |  |  |  |  |  |
| 1 | Ath-AT5G60890.1 |  | | | |  |  |  |  |  |  |  |
| 2 | Ath-AT5G60910.1 |  | Vvi-Vitvi17g00470\_t001 |  | Vvi-Vitvi01g00008\_t001 |  |  |  |  |  |  |
| 2 | Ath-AT5G60920.1 |  | Vvi-Vitvi17g04133\_t001 |  | | | |  |  |  |  |  |  |
| 2 | Ath-AT5G60930.2 |  | | | |  | | | |  |  |  |  |  |  |
| 2 | Ath-AT5G60940.1 |  | Vvi-Vitvi17g00482\_t001 |  | | | |  |  |  |  |  |  |
| 2 | Ath-AT5G60945.1 |  | | | |  | | | |  |  |  |  |  |  |
| 2 | Ath-AT5G60950.1 |  | | | |  | | | |  |  |  |  |  |  |
| 2 | Ath-AT5G60960.1 |  | Vvi-Vitvi17g00483\_t001 |  | | | |  |  |  |  |  |  |
| 2 | Ath-AT5G60964.1 |  | | | |  | | | |  |  |  |  |  |  |
| 2 | Ath-AT5G60970.1 |  | Vvi-Vitvi17g00495\_t001 |  | | | |  |  |  |  |  |  |
| 2 | Ath-AT5G60980.2 |  | Vvi-Vitvi17g00496\_t002 |  | Vvi-Vitvi01g00036\_t002 |  |  |  |  |  |  |
| 1 | Ath-AT5G60990.1 |  |  |  | | | |  |  |  |  |  |  |
| 1 | Ath-AT5G61000.1 |  |  |  | | | |  |  |  |  |  |  |
| 2 | Ath-AT5G61010.1 |  | Vvi-Vitvi17g00713\_t001 |  | | | |  |  |  |  |  |  |
| 2 | Ath-AT5G61020.1 |  | | | |  | | | |  |  |  |  |  |  |
| 2 | Ath-AT5G61030.1 |  | Vvi-Vitvi17g01512\_t001 |  | | | |  |  |  |  |  |  |
| 2 | Ath-AT5G61040.1 |  | Vvi-Vitvi17g00711\_t001 |  | Vvi-Vitvi01g00041\_t001 |  |  |  |  |  |  |
| 2 | Ath-AT5G61050.1 |  | Vvi-Vitvi17g00705\_t001 |  | | | |  |  |  |  |  |  |
| 2 | Ath-AT5G61060.2 |  | | | |  | | | |  |  |  |  |  |  |
| 2 | Ath-AT5G61070.1 |  | | | |  | | | |  |  |  |  |  |  |
| 2 | Ath-AT5G61090.1 |  | | | |  | | | |  |  |  |  |  |  |
| 2 | Ath-AT5G61100.1 |  | | | |  | | | |  |  |  |  |  |  |
| 2 | Ath-AT5G61110.1 |  | | | |  | | | |  |  |  |  |  |  |
| 2 | Ath-AT5G61120.1 |  | Vvi-Vitvi17g00704\_t002 |  | | | |  |  |  |  |  |  |
| 2 | Ath-AT5G61130.1 |  | Vvi-Vitvi17g01511\_t001 |  | Vvi-Vitvi01g00051\_t001 |  |  |  |  |  |  |
| 2 | Ath-AT5G61140.2 |  | Vvi-Vitvi17g00701\_t001 |  | | | |  |  |  |  |  |  |
| 2 | Ath-AT5G61150.1 |  | Vvi-Vitvi17g00694\_t001 |  | | | |  |  |  |  |  |  |
| 2 | Ath-AT5G61160.1 |  | | | |  | | | |  |  |  |  |  |  |
| 2 | Ath-AT5G61170.1 |  | Vvi-Vitvi17g00684\_t001 |  | | | |  |  |  |  |  |  |
| 2 | Ath-AT5G61180.1 |  | | | |  | | | |  |  |  |  |  |  |
| 2 | Ath-AT5G61190.5 |  | | | |  | | | |  |  |  |  |  |  |
| 2 | Ath-AT5G61200.3 |  | | | |  | | | |  |  |  |  |  |  |
| 2 | Ath-AT5G61210.1 |  | | | |  | Vvi-Vitvi01g00059\_t002 |  |  |  |  |  |  |
| 2 | Ath-AT5G61220.1 |  | | | |  | | | |  |  |  |  |  |  |
| 2 | Ath-AT5G61230.1 |  | | | |  | | | |  |  |  |  |  |  |
| 2 | Ath-AT5G61240.2 |  | | | |  | | | |  |  |  |  |  |  |
| 2 | Ath-AT5G61250.1 |  | Vvi-Vitvi17g00673\_t001 |  | Vvi-Vitvi01g00073\_t001 |  |  |  |  |  |  |
| 2 | Ath-AT5G61260.1 |  | Vvi-Vitvi17g00666\_t001 |  | | | |  |  |  |  |  |  |
| 2 | Ath-AT5G61270.1 |  | | | |  | | | |  |  |  |  |  |  |
| 2 | Ath-AT5G61280.1 |  | | | |  | Vvi-Vitvi01g01845\_t001 |  |  |  |  |  |  |
| 1 | Ath-AT5G61290.1 |  | Vvi-Vitvi17g04186\_t001 |  |  |  |  |  |  |  |
| 1 | Ath-AT5G61300.1 |  | | | |  |  |  |  |  |  |  |
| 1 | Ath-AT5G61310.5 |  | | | |  |  |  |  |  |  |  |
| 1 | Ath-AT5G61320.1 |  | | | |  |  |  |  |  |  |  |
| 1 | Ath-AT5G61330.2 |  | | | |  |  |  |  |  |  |  |
| 1 | Ath-AT5G61340.2 |  | Vvi-Vitvi17g00645\_t001 |  |  |  |  |  |  |  |
| 1 | Ath-AT5G61350.1 |  | Vvi-Vitvi17g00644\_t001 |  |  |  |  |  |  |  |
| 1 | Ath-AT5G61360.1 |  | | | |  |  |  |  |  |  |  |
| 1 | Ath-AT5G61370.1 |  | | | |  |  |  |  |  |  |  |
| 1 | Ath-AT5G61380.1 |  | Vvi-Vitvi17g00636\_t001 |  |  |  |  |  |  |  |
| 1 | Ath-AT5G61390.1 |  | Vvi-Vitvi17g00635\_t001 |  |  |  |  |  |  |  |
| 1 | Ath-AT5G61400.1 |  | Vvi-Vitvi17g00632\_t001 |  |  |  |  |  |  |  |
| 1 | Ath-AT5G61410.2 |  | Vvi-Vitvi17g00629\_t001 |  |  |  |  |  |  |  |
| 1 | Ath-AT5G61412.1 |  | | | |  |  |  |  |  |  |  |
| 1 | Ath-AT5G61420.2 |  | | | |  |  |  |  |  |  |  |
| 1 | Ath-AT5G61430.1 |  | Vvi-Vitvi17g00622\_t001 |  |  |  |  |  |  |  |
| 1 | Ath-AT5G61440.1 |  | Vvi-Vitvi17g00617\_t001 |  |  |  |  |  |  |  |
| 0 | Ath-AT5G61450.1 |  |  |  |  |  |  |  |  |
| 0 | Ath-AT5G61460.1 |  |  |  |  |  |  |  |  |
| 0 | Ath-AT5G61470.1 |  |  |  |  |  |  |  |  |
| 0 | Ath-AT5G61480.1 |  |  |  |  |  |  |  |  |
| 0 | Ath-AT5G61490.1 |  |  |  |  |  |  |  |  |
| 0 | Ath-AT5G61495.1 |  |  |  |  |  |  |  |  |
| 0 | Ath-AT5G61500.1 |  |  |  |  |  |  |  |  |
| 0 | Ath-AT5G61510.1 |  |  |  |  |  |  |  |  |
| 0 | Ath-AT5G61520.1 |  |  |  |  |  |  |  |  |
| 0 | Ath-AT5G61530.1 |  |  |  |  |  |  |  |  |
| 0 | Ath-AT5G61540.1 |  |  |  |  |  |  |  |  |
| 0 | Ath-AT5G61550.2 |  |  |  |  |  |  |  |  |
| 0 | Ath-AT5G61560.3 |  |  |  |  |  |  |  |  |
| 0 | Ath-AT5G61570.1 |  |  |  |  |  |  |  |  |
| 0 | Ath-AT5G61580.1 |  |  |  |  |  |  |  |  |
| 0 | Ath-AT5G61590.1 |  |  |  |  |  |  |  |  |
| 0 | Ath-AT5G61600.1 |  |  |  |  |  |  |  |  |
| 0 | Ath-AT5G61605.1 |  |  |  |  |  |  |  |  |
| 0 | Ath-AT5G61610.1 |  |  |  |  |  |  |  |  |
| 0 | Ath-AT5G61620.1 |  |  |  |  |  |  |  |  |
| 0 | Ath-AT5G61630.1 |  |  |  |  |  |  |  |  |
| 0 | Ath-AT5G61640.2 |  |  |  |  |  |  |  |  |
| 0 | Ath-AT5G61650.1 |  |  |  |  |  |  |  |  |
| 0 | Ath-AT5G61660.1 |  |  |  |  |  |  |  |  |
| 0 | Ath-AT5G61670.1 |  |  |  |  |  |  |  |  |
| 0 | Ath-AT5G61680.1 |  |  |  |  |  |  |  |  |
| 0 | Ath-AT5G61690.2 |  |  |  |  |  |  |  |  |
| 0 | Ath-AT5G61700.1 |  |  |  |  |  |  |  |  |
| 0 | Ath-AT5G61710.1 |  |  |  |  |  |  |  |  |
| 0 | Ath-AT5G61720.1 |  |  |  |  |  |  |  |  |
| 0 | Ath-AT5G61730.2 |  |  |  |  |  |  |  |  |
| 0 | Ath-AT5G61740.1 |  |  |  |  |  |  |  |  |
| 0 | Ath-AT5G61750.1 |  |  |  |  |  |  |  |  |
| 0 | Ath-AT5G61760.1 |  |  |  |  |  |  |  |  |
| 0 | Ath-AT5G61770.2 |  |  |  |  |  |  |  |  |
| 1 | Ath-AT5G61780.1 |  | Vvi-Vitvi17g00002\_t001 |  |  |  |  |  |  |  |
| 2 | Ath-AT5G61790.1 |  | Vvi-Vitvi17g01311\_t001 |  | Vvi-Vitvi01g01800\_t001 |  |  |  |  |  |  |
| 2 | Ath-AT5G61800.1 |  | | | |  | | | |  |  |  |  |  |  |
| 2 | Ath-AT5G61810.1 |  | | | |  | | | |  |  |  |  |  |  |
| 2 | Ath-AT5G61820.1 |  | Vvi-Vitvi17g04002\_t001 |  | | | |  |  |  |  |  |  |
| 2 | Ath-AT5G61830.1 |  | | | |  | | | |  |  |  |  |  |  |
| 2 | Ath-AT5G61840.1 |  | Vvi-Vitvi17g00017\_t001 |  | Vvi-Vitvi01g01778\_t001 |  |  |  |  |  |  |
| 2 | Ath-AT5G61850.2 |  | Vvi-Vitvi17g00021\_t001 |  | | | |  |  |  |  |  |  |
| 2 | Ath-AT5G61865.1 |  | Vvi-Vitvi17g01320\_t001 |  | | | |  |  |  |  |  |  |
| 2 | Ath-AT5G61880.1 |  | Vvi-Vitvi17g00024\_t001 |  | | | |  |  |  |  |  |  |
| 2 | Ath-AT5G61890.1 |  | Vvi-Vitvi17g00025\_t001 |  | Vvi-Vitvi01g01826\_t001 |  |  |  |  |  |  |
| 2 | Ath-AT5G61910.4 |  | Vvi-Vitvi17g00026\_t001 |  | Vvi-Vitvi01g01770\_t001 |  |  |  |  |  |  |
| 2 | Ath-AT5G61900.3 |  | | | |  | | | |  |  |  |  |  |  |
| 2 | Ath-AT5G61920.3 |  | | | |  | | | |  |  |  |  |  |  |
| 2 | Ath-AT5G61930.2 |  | | | |  | | | |  |  |  |  |  |  |
| 2 | Ath-AT5G61940.2 |  | | | |  | | | |  |  |  |  |  |  |
| 2 | Ath-AT5G61950.1 |  | | | |  | | | |  |  |  |  |  |  |
| 2 | Ath-AT5G61960.1 |  | Vvi-Vitvi17g00028\_t001 |  | Vvi-Vitvi01g01768\_t001 |  |  |  |  |  |  |
| 2 | Ath-AT5G61970.1 |  | Vvi-Vitvi17g00032\_t001 |  | | | |  |  |  |  |  |  |
| 2 | Ath-AT5G61980.1 |  | Vvi-Vitvi17g00033\_t001 |  | Vvi-Vitvi01g01762\_t002 |  |  |  |  |  |  |
| 2 | Ath-AT5G61990.1 |  | | | |  | | | |  |  |  |  |  |  |
| 2 | Ath-AT5G61997.1 |  | | | |  | | | |  |  |  |  |  |  |
| 2 | Ath-AT5G62000.2 |  | Vvi-Vitvi17g00036\_t001 |  | Vvi-Vitvi01g01759\_t001 |  |  |  |  |  |  |
| 1 | Ath-AT5G62020.1 |  | Vvi-Vitvi16g00982\_t001 |  |  |  |  |  |  |  |
| 1 | Ath-AT5G62030.1 |  | | | |  |  |  |  |  |  |  |
| 1 | Ath-AT5G62040.1 |  | Vvi-Vitvi16g00977\_t001 |  |  |  |  |  |  |  |
| 1 | Ath-AT5G62050.1 |  | | | |  |  |  |  |  |  |  |
| 1 | Ath-AT5G62060.1 |  | | | |  |  |  |  |  |  |  |
| 1 | Ath-AT5G62065.1 |  | Vvi-Vitvi16g00971\_t001 |  |  |  |  |  |  |  |
| 1 | Ath-AT5G62070.1 |  | Vvi-Vitvi16g00970\_t001 |  |  |  |  |  |  |  |
| 1 | Ath-AT5G62080.1 |  | Vvi-Vitvi16g01864\_t001 |  |  |  |  |  |  |  |
| 1 | Ath-AT5G62090.3 |  | Vvi-Vitvi16g00956\_t001 |  |  |  |  |  |  |  |
| 1 | Ath-AT5G62100.1 |  | Vvi-Vitvi16g00952\_t001 |  |  |  |  |  |  |  |
| 1 | Ath-AT5G62110.1 |  | | | |  |  |  |  |  |  |  |
| 1 | Ath-AT5G62120.1 |  | | | |  |  |  |  |  |  |  |
| 1 | Ath-AT5G62130.2 |  | Vvi-Vitvi16g00937\_t001 |  |  |  |  |  |  |  |
| 1 | Ath-AT5G62140.1 |  | Vvi-Vitvi16g00934\_t001 |  |  |  |  |  |  |  |
| 1 | Ath-AT5G62150.1 |  | Vvi-Vitvi16g00926\_t001 |  |  |  |  |  |  |  |
| 0 | Ath-AT5G62160.1 |  |  |  |  |  |  |  |  |
| 1 | Ath-AT5G62165.2 |  | Vvi-Vitvi16g00898\_t001 |  |  |  |  |  |  |  |
| 1 | Ath-AT5G62170.1 |  | Vvi-Vitvi16g00897\_t001 |  |  |  |  |  |  |  |
| 1 | Ath-AT5G62180.1 |  | Vvi-Vitvi16g00889\_t001 |  |  |  |  |  |  |  |
| 1 | Ath-AT5G62190.1 |  | Vvi-Vitvi16g00869\_t001 |  |  |  |  |  |  |  |
| 1 | Ath-AT5G62200.1 |  | Vvi-Vitvi16g00866\_t001 |  |  |  |  |  |  |  |
| 1 | Ath-AT5G62210.1 |  | | | |  |  |  |  |  |  |  |
| 1 | Ath-AT5G62220.1 |  | Vvi-Vitvi16g00864\_t001 |  |  |  |  |  |  |  |
| 1 | Ath-AT5G62230.1 |  | Vvi-Vitvi16g00856\_t001 |  |  |  |  |  |  |  |
| 2 | Ath-AT5G62240.1 |  | Vvi-Vitvi16g00855\_t001 |  | Vvi-Vitvi02g00454\_t001 |  |  |  |  |  |  |
| 2 | Ath-AT5G62250.1 |  | Vvi-Vitvi16g00836\_t001 |  | | | |  |  |  |  |  |  |
| 2 | Ath-AT5G62260.3 |  | Vvi-Vitvi16g00835\_t001 |  | Vvi-Vitvi02g00468\_t001 |  |  |  |  |  |  |
| 2 | Ath-AT5G62270.2 |  | | | |  | | | |  |  |  |  |  |  |
| 2 | Ath-AT5G62280.1 |  | Vvi-Vitvi16g00809\_t001 |  | Vvi-Vitvi02g00481\_t001 |  |  |  |  |  |  |
| 2 | Ath-AT5G62290.1 |  | Vvi-Vitvi16g00803\_t001 |  | | | |  |  |  |  |  |  |
| 2 | Ath-AT5G62300.1 |  | Vvi-Vitvi16g00798\_t001 |  | | | |  |  |  |  |  |  |
| 2 | Ath-AT5G62310.1 |  | Vvi-Vitvi16g00779\_t001 |  | | | |  |  |  |  |  |  |
| 2 | Ath-AT5G62320.1 |  | Vvi-Vitvi16g00775\_t001 |  | Vvi-Vitvi02g01823\_t001 |  |  |  |  |  |  |
| 1 | Ath-AT5G62330.1 |  |  |  | | | |  |  |  |  |  |  |
| 1 | Ath-AT5G62340.1 |  |  |  | | | |  |  |  |  |  |  |
| 1 | Ath-AT5G62350.1 |  |  |  | Vvi-Vitvi02g00499\_t001 |  |  |  |  |  |  |
| 1 | Ath-AT5G62360.1 |  |  |  | Vvi-Vitvi02g00500\_t001 |  |  |  |  |  |  |
| 1 | Ath-AT5G62370.1 |  |  |  | | | |  |  |  |  |  |  |
| 1 | Ath-AT5G62380.1 |  |  |  | Vvi-Vitvi02g00508\_t001 |  |  |  |  |  |  |
| 0 | Ath-AT5G62390.1 |  |  |  |  |  |  |  |  |
| 0 | Ath-AT5G62400.1 |  |  |  |  |  |  |  |  |
| 0 | Ath-AT5G62410.1 |  |  |  |  |  |  |  |  |
| 0 | Ath-AT5G62420.1 |  |  |  |  |  |  |  |  |
| 1 | Ath-AT5G62430.1 |  | Vvi-Vitvi17g00611\_t001 |  |  |  |  |  |  |  |
| 1 | Ath-AT5G62440.1 |  | Vvi-Vitvi17g00604\_t001 |  |  |  |  |  |  |  |
| 1 | Ath-AT5G62460.3 |  | Vvi-Vitvi17g00603\_t001 |  |  |  |  |  |  |  |
| 1 | Ath-AT5G62470.2 |  | Vvi-Vitvi17g00598\_t001 |  |  |  |  |  |  |  |
| 1 | Ath-AT5G62480.1 |  | Vvi-Vitvi17g01465\_t001 |  |  |  |  |  |  |  |
| 1 | Ath-AT5G62490.1 |  | Vvi-Vitvi17g00577\_t001 |  |  |  |  |  |  |  |
| 1 | Ath-AT5G62500.1 |  | | | |  |  |  |  |  |  |  |
| 1 | Ath-AT5G62510.1 |  | | | |  |  |  |  |  |  |  |
| 1 | Ath-AT5G62520.1 |  | | | |  |  |  |  |  |  |  |
| 1 | Ath-AT5G62530.1 |  | | | |  |  |  |  |  |  |  |
| 1 | Ath-AT5G62540.1 |  | | | |  |  |  |  |  |  |  |
| 1 | Ath-AT5G62550.1 |  | Vvi-Vitvi17g00558\_t001 |  |  |  |  |  |  |  |
| 0 | Ath-AT5G62560.1 |  |  |  |  |  |  |  |  |
| 0 | Ath-AT5G62570.1 |  |  |  |  |  |  |  |  |
| 0 | Ath-AT5G62573.1 |  |  |  |  |  |  |  |  |
| 0 | Ath-AT5G62575.2 |  |  |  |  |  |  |  |  |
| 1 | Ath-AT5G62580.1 |  | Vvi-Vitvi01g00864\_t001 |  |  |  |  |  |  |  |
| 1 | Ath-AT5G62600.1 |  | | | |  |  |  |  |  |  |  |
| 2 | Ath-AT5G62610.1 |  | Vvi-Vitvi01g00876\_t001 |  | Vvi-Vitvi17g00507\_t001 |  |  |  |  |  |  |
| 2 | Ath-AT5G62620.1 |  | Vvi-Vitvi01g00882\_t001 |  | Vvi-Vitvi17g00511\_t001 |  |  |  |  |  |  |
| 2 | Ath-AT5G62623.1 |  | | | |  | | | |  |  |  |  |  |  |
| 2 | Ath-AT5G62627.1 |  | | | |  | | | |  |  |  |  |  |  |
| 2 | Ath-AT5G62630.1 |  | Vvi-Vitvi01g00886\_t001 |  | Vvi-Vitvi17g00514\_t001 |  |  |  |  |  |  |
| 2 | Ath-AT5G62640.3 |  | | | |  | Vvi-Vitvi17g00515\_t001 |  |  |  |  |  |  |
| 2 | Ath-AT5G62650.1 |  | | | |  | | | |  |  |  |  |  |  |
| 2 | Ath-AT5G62660.1 |  | | | |  | | | |  |  |  |  |  |  |
| 2 | Ath-AT5G62670.1 |  | | | |  | Vvi-Vitvi17g00527\_t001 |  |  |  |  |  |  |
| 2 | Ath-AT5G62680.1 |  | Vvi-Vitvi01g00911\_t001 |  | Vvi-Vitvi17g00528\_t001 |  |  |  |  |  |  |
| 2 | Ath-AT5G62690.1 |  | | | |  | | | |  |  |  |  |  |  |
| 2 | Ath-AT5G62700.1 |  | | | |  | | | |  |  |  |  |  |  |
| 2 | Ath-AT5G62710.1 |  | | | |  | Vvi-Vitvi17g00533\_t001 |  |  |  |  |  |  |
| 2 | Ath-AT5G62720.1 |  | | | |  | Vvi-Vitvi17g00539\_t001 |  |  |  |  |  |  |
| 2 | Ath-AT5G62730.1 |  | Vvi-Vitvi01g00921\_t001 |  | Vvi-Vitvi17g00541\_t001 |  |  |  |  |  |  |
| 2 | Ath-AT5G62740.1 |  | Vvi-Vitvi01g00929\_t001 |  | Vvi-Vitvi17g00546\_t002 |  |  |  |  |  |  |
| 1 | Ath-AT5G62750.1 |  |  |  | | | |  |  |  |  |  |  |
| 1 | Ath-AT5G62760.1 |  |  |  | Vvi-Vitvi17g01459\_t001 |  |  |  |  |  |  |
| 1 | Ath-AT5G62770.1 |  | Vvi-Vitvi17g00815\_t001 |  |  |  |  |  |  |  |
| 1 | Ath-AT5G62780.1 |  | | | |  |  |  |  |  |  |  |
| 1 | Ath-AT5G62790.2 |  | Vvi-Vitvi17g00816\_t001 |  |  |  |  |  |  |  |
| 1 | Ath-AT5G62800.1 |  | | | |  |  |  |  |  |  |  |
| 1 | Ath-AT5G62810.1 |  | Vvi-Vitvi17g00817\_t001 |  |  |  |  |  |  |  |
| 2 | Ath-AT5G62820.1 |  | | | |  | Vvi-Vitvi17g00803\_t001 |  |  |  |  |  |  |
| 2 | Ath-AT5G62830.1 |  | | | |  | | | |  |  |  |  |  |  |
| 2 | Ath-AT5G62840.1 |  | | | |  | Vvi-Vitvi17g00799\_t001 |  |  |  |  |  |  |
| 2 | Ath-AT5G62850.1 |  | | | |  | Vvi-Vitvi17g00791\_t001 |  |  |  |  |  |  |
| 2 | Ath-AT5G62860.1 |  | | | |  | | | |  |  |  |  |  |  |
| 2 | Ath-AT5G62865.1 |  | | | |  | Vvi-Vitvi17g01535\_t001 |  |  |  |  |  |  |
| 2 | Ath-AT5G62880.1 |  | | | |  | Vvi-Vitvi17g00762\_t001 |  |  |  |  |  |  |
| 2 | Ath-AT5G62890.2 |  | | | |  | Vvi-Vitvi17g00759\_t001 |  |  |  |  |  |  |
| 2 | Ath-AT5G62900.1 |  | | | |  | Vvi-Vitvi17g00758\_t001 |  |  |  |  |  |  |
| 1 | Ath-AT5G62910.1 |  | | | |  |  |  |  |  |  |  |
| 1 | Ath-AT5G62920.1 |  | | | |  |  |  |  |  |  |  |
| 1 | Ath-AT5G62930.2 |  | Vvi-Vitvi17g00831\_t001 |  |  |  |  |  |  |  |
| 1 | Ath-AT5G62940.1 |  | | | |  |  |  |  |  |  |  |
| 1 | Ath-AT5G62950.4 |  | | | |  |  |  |  |  |  |  |
| 1 | Ath-AT5G62960.1 |  | Vvi-Vitvi17g00837\_t003 |  |  |  |  |  |  |  |
| 1 | Ath-AT5G62970.1 |  | | | |  |  |  |  |  |  |  |
| 1 | Ath-AT5G62980.2 |  | | | |  |  |  |  |  |  |  |
| 1 | Ath-AT5G62990.1 |  | | | |  |  |  |  |  |  |  |
| 1 | Ath-AT5G62998.1 |  | | | |  |  |  |  |  |  |  |
| 1 | Ath-AT5G63000.1 |  | Vvi-Vitvi17g00841\_t001 |  |  |  |  |  |  |  |
| 1 | Ath-AT5G63010.1 |  | | | |  |  |  |  |  |  |  |
| 1 | Ath-AT5G63020.1 |  | | | |  |  |  |  |  |  |  |
| 1 | Ath-AT5G63030.1 |  | Vvi-Vitvi17g00844\_t001 |  |  |  |  |  |  |  |
| 1 | Ath-AT5G63040.2 |  | Vvi-Vitvi17g00864\_t001 |  |  |  |  |  |  |  |
| 1 | Ath-AT5G63050.1 |  | Vvi-Vitvi17g00866\_t001 |  |  |  |  |  |  |  |
| 1 | Ath-AT5G63060.1 |  | Vvi-Vitvi17g00872\_t001 |  |  |  |  |  |  |  |
| 1 | Ath-AT5G63063.1 |  | | | |  |  |  |  |  |  |  |
| 1 | Ath-AT5G63070.1 |  | | | |  |  |  |  |  |  |  |
| 1 | Ath-AT5G63080.1 |  | Vvi-Vitvi17g00875\_t001 |  |  |  |  |  |  |  |
| 1 | Ath-AT5G63085.1 |  | | | |  |  |  |  |  |  |  |
| 1 | Ath-AT5G63087.1 |  | | | |  |  |  |  |  |  |  |
| 1 | Ath-AT5G63090.1 |  | Vvi-Vitvi17g00890\_t002 |  |  |  |  |  |  |  |
| 1 | Ath-AT5G63100.1 |  | Vvi-Vitvi17g04253\_t001 |  |  |  |  |  |  |  |
| 1 | Ath-AT5G63110.1 |  | Vvi-Vitvi17g00894\_t001 |  |  |  |  |  |  |  |
| 1 | Ath-AT5G63120.2 |  | Vvi-Vitvi17g00900\_t001 |  |  |  |  |  |  |  |
| 1 | Ath-AT5G63130.2 |  | Vvi-Vitvi17g04255\_t001 |  |  |  |  |  |  |  |
| 1 | Ath-AT5G63135.1 |  | Vvi-Vitvi17g00907\_t001 |  |  |  |  |  |  |  |
| 0 | Ath-AT5G63140.1 |  |  |  |  |  |  |  |  |
| 0 | Ath-AT5G63150.1 |  |  |  |  |  |  |  |  |
| 1 | Ath-AT5G63160.1 |  | Vvi-Vitvi17g00975\_t001 |  |  |  |  |  |  |  |
| 1 | Ath-AT5G63170.1 |  | | | |  |  |  |  |  |  |  |
| 2 | Ath-AT5G63180.1 |  | Vvi-Vitvi17g00977\_t001 |  | Vvi-Vitvi14g01635\_t001 |  |  |  |  |  |  |
| 2 | Ath-AT5G63190.1 |  | Vvi-Vitvi17g00987\_t001 |  | | | |  |  |  |  |  |  |
| 2 | Ath-AT5G63200.1 |  | Vvi-Vitvi17g00989\_t001 |  | | | |  |  |  |  |  |  |
| 2 | Ath-AT5G63220.1 |  | | | |  | | | |  |  |  |  |  |  |
| 2 | Ath-AT5G63225.1 |  | | | |  | | | |  |  |  |  |  |  |
| 2 | Ath-AT5G63230.1 |  | | | |  | | | |  |  |  |  |  |  |
| 2 | Ath-AT5G63240.1 |  | | | |  | | | |  |  |  |  |  |  |
| 2 | Ath-AT5G63250.1 |  | | | |  | | | |  |  |  |  |  |  |
| 2 | Ath-AT5G63260.2 |  | Vvi-Vitvi17g01002\_t001 |  | Vvi-Vitvi14g01629\_t001 |  |  |  |  |  |  |
| 2 | Ath-AT5G63270.1 |  | Vvi-Vitvi17g01003\_t001 |  | Vvi-Vitvi14g01628\_t001 |  |  |  |  |  |  |
| 2 | Ath-AT5G63280.1 |  | Vvi-Vitvi17g01024\_t001 |  | Vvi-Vitvi14g01617\_t001 |  |  |  |  |  |  |
| 2 | Ath-AT5G63290.1 |  | Vvi-Vitvi17g01025\_t001 |  | | | |  |  |  |  |  |  |
| 2 | Ath-AT5G63300.2 |  | | | |  | | | |  |  |  |  |  |  |
| 2 | Ath-AT5G63310.1 |  | Vvi-Vitvi17g01038\_t002 |  | | | |  |  |  |  |  |  |
| 2 | Ath-AT5G63320.1 |  | Vvi-Vitvi17g00153\_t002 |  | Vvi-Vitvi14g01594\_t002 |  |  |  |  |  |  |
| 2 | Ath-AT5G63340.1 |  | | | |  | | | |  |  |  |  |  |  |
| 2 | Ath-AT5G63350.1 |  | Vvi-Vitvi17g00152\_t001 |  | Vvi-Vitvi14g02990\_t001 |  |  |  |  |  |  |
| 2 | Ath-AT5G63370.1 |  | Vvi-Vitvi17g00150\_t001 |  | | | |  |  |  |  |  |  |
| 2 | Ath-AT5G63375.1 |  | | | |  | | | |  |  |  |  |  |  |
| 2 | Ath-AT5G63380.1 |  | Vvi-Vitvi17g00148\_t001 |  | Vvi-Vitvi14g01588\_t001 |  |  |  |  |  |  |
| 2 | Ath-AT5G63390.1 |  | Vvi-Vitvi17g00145\_t001 |  | | | |  |  |  |  |  |  |
| 2 | Ath-AT5G63400.1 |  | | | |  | | | |  |  |  |  |  |  |
| 2 | Ath-AT5G63410.1 |  | Vvi-Vitvi17g00139\_t001 |  | Vvi-Vitvi14g01576\_t001 |  |  |  |  |  |  |
| 1 | Ath-AT5G63420.1 |  | Vvi-Vitvi17g00132\_t001 |  |  |  |  |  |  |  |
| 1 | Ath-AT5G63440.2 |  | Vvi-Vitvi17g00122\_t001 |  |  |  |  |  |  |  |
| 1 | Ath-AT5G63450.2 |  | Vvi-Vitvi17g00119\_t001 |  |  |  |  |  |  |  |
| 1 | Ath-AT5G63460.4 |  | Vvi-Vitvi17g00118\_t001 |  |  |  |  |  |  |  |
| 1 | Ath-AT5G63470.1 |  | | | |  |  |  |  |  |  |  |
| 1 | Ath-AT5G63480.1 |  | | | |  |  |  |  |  |  |  |
| 1 | Ath-AT5G63490.1 |  | | | |  |  |  |  |  |  |  |
| 1 | Ath-AT5G63500.1 |  | | | |  |  |  |  |  |  |  |
| 1 | Ath-AT5G63510.2 |  | | | |  |  |  |  |  |  |  |
| 1 | Ath-AT5G63520.1 |  | Vvi-Vitvi17g01345\_t001 |  |  |  |  |  |  |  |
| 1 | Ath-AT5G63530.1 |  | Vvi-Vitvi17g00087\_t001 |  |  |  |  |  |  |  |
| 1 | Ath-AT5G63540.2 |  | Vvi-Vitvi17g00083\_t001 |  |  |  |  |  |  |  |
| 1 | Ath-AT5G63550.2 |  | Vvi-Vitvi17g00079\_t001 |  |  |  |  |  |  |  |
| 1 | Ath-AT5G63560.1 |  | Vvi-Vitvi17g00078\_t001 |  |  |  |  |  |  |  |
| 1 | Ath-AT5G63570.1 |  | Vvi-Vitvi17g00074\_t001 |  |  |  |  |  |  |  |
| 0 | Ath-AT5G63580.1 |  |  |  |  |  |  |  |  |
| 0 | Ath-AT5G63590.1 |  |  |  |  |  |  |  |  |
| 0 | Ath-AT5G63595.1 |  |  |  |  |  |  |  |  |
| 0 | Ath-AT5G63600.2 |  |  |  |  |  |  |  |  |
| 0 | Ath-AT5G63610.1 |  |  |  |  |  |  |  |  |
| 1 | Ath-AT5G63620.2 |  | Vvi-Vitvi07g01998\_t001 |  |  |  |  |  |  |  |
| 1 | Ath-AT5G63625.1 |  | | | |  |  |  |  |  |  |  |
| 1 | Ath-AT5G63630.2 |  | | | |  |  |  |  |  |  |  |
| 1 | Ath-AT5G63640.1 |  | | | |  |  |  |  |  |  |  |
| 1 | Ath-AT5G63650.1 |  | | | |  |  |  |  |  |  |  |
| 1 | Ath-AT5G63660.1 |  | | | |  |  |  |  |  |  |  |
| 1 | Ath-AT5G63670.1 |  | | | |  |  |  |  |  |  |  |
| 1 | Ath-AT5G63680.2 |  | | | |  |  |  |  |  |  |  |
| 1 | Ath-AT5G63690.1 |  | | | |  |  |  |  |  |  |  |
| 1 | Ath-AT5G63700.1 |  | | | |  |  |  |  |  |  |  |
| 1 | Ath-AT5G63710.3 |  | Vvi-Vitvi07g01973\_t001 |  |  |  |  |  |  |  |
| 1 | Ath-AT5G63720.1 |  | | | |  |  |  |  |  |  |  |
| 1 | Ath-AT5G63730.1 |  | | | |  |  |  |  |  |  |  |
| 1 | Ath-AT5G63740.1 |  | | | |  |  |  |  |  |  |  |
| 1 | Ath-AT5G63750.1 |  | | | |  |  |  |  |  |  |  |
| 1 | Ath-AT5G63760.2 |  | | | |  |  |  |  |  |  |  |
| 1 | Ath-AT5G63770.1 |  | Vvi-Vitvi07g01961\_t001 |  |  |  |  |  |  |  |
| 1 | Ath-AT5G63780.1 |  | Vvi-Vitvi07g01937\_t002 |  |  |  |  |  |  |  |
| 1 | Ath-AT5G63790.2 |  | Vvi-Vitvi07g01929\_t001 |  |  |  |  |  |  |  |
| 1 | Ath-AT5G63800.1 |  | Vvi-Vitvi07g01927\_t001 |  |  |  |  |  |  |  |
| 1 | Ath-AT5G63810.1 |  | | | |  |  |  |  |  |  |  |
| 1 | Ath-AT5G63820.1 |  | | | |  |  |  |  |  |  |  |
| 1 | Ath-AT5G63830.1 |  | | | |  |  |  |  |  |  |  |
| 1 | Ath-AT5G63840.2 |  | | | |  |  |  |  |  |  |  |
| 1 | Ath-AT5G63850.1 |  | | | |  |  |  |  |  |  |  |
| 1 | Ath-AT5G63860.1 |  | Vvi-Vitvi07g01923\_t001 |  |  |  |  |  |  |  |
| 2 | Ath-AT5G63870.2 |  | | | |  | Vvi-Vitvi04g01742\_t001 |  |  |  |  |  |  |
| 2 | Ath-AT5G63880.2 |  | | | |  | | | |  |  |  |  |  |  |
| 2 | Ath-AT5G63890.2 |  | | | |  | | | |  |  |  |  |  |  |
| 2 | Ath-AT5G63900.1 |  | | | |  | | | |  |  |  |  |  |  |
| 2 | Ath-AT5G63905.2 |  | | | |  | | | |  |  |  |  |  |  |
| 2 | Ath-AT5G63910.1 |  | Vvi-Vitvi07g01917\_t001 |  | | | |  |  |  |  |  |  |
| 2 | Ath-AT5G63920.1 |  | | | |  | | | |  |  |  |  |  |  |
| 2 | Ath-AT5G63930.1 |  | | | |  | | | |  |  |  |  |  |  |
| 2 | Ath-AT5G63940.1 |  | | | |  | | | |  |  |  |  |  |  |
| 2 | Ath-AT5G63950.1 |  | | | |  | Vvi-Vitvi04g01734\_t001 |  |  |  |  |  |  |
| 2 | Ath-AT5G63960.2 |  | | | |  | Vvi-Vitvi04g01729\_t001 |  |  |  |  |  |  |
| 2 | Ath-AT5G63970.2 |  | | | |  | Vvi-Vitvi04g01728\_t001 |  |  |  |  |  |  |
| 2 | Ath-AT5G63980.1 |  | | | |  | Vvi-Vitvi04g01709\_t002 |  |  |  |  |  |  |
| 2 | Ath-AT5G63990.1 |  | | | |  | | | |  |  |  |  |  |  |
| 2 | Ath-AT5G64000.1 |  | | | |  | | | |  |  |  |  |  |  |
| 2 | Ath-AT5G64010.1 |  | | | |  | | | |  |  |  |  |  |  |
| 2 | Ath-AT5G64020.1 |  | Vvi-Vitvi07g04747\_t001 |  | | | |  |  |  |  |  |  |
| 1 | Ath-AT5G64030.1 |  |  |  | | | |  |  |  |  |  |  |
| 1 | Ath-AT5G64040.2 |  |  |  | Vvi-Vitvi04g01700\_t001 |  |  |  |  |  |  |
| 1 | Ath-AT5G64050.1 |  |  |  | | | |  |  |  |  |  |  |
| 1 | Ath-AT5G64060.1 |  |  |  | Vvi-Vitvi04g01684\_t001 |  |  |  |  |  |  |
| 1 | Ath-AT5G64070.1 |  |  |  | Vvi-Vitvi04g01682\_t001 |  |  |  |  |  |  |
| 0 | Ath-AT5G64080.1 |  |  |  |  |  |  |  |  |
| 0 | Ath-AT5G64090.1 |  |  |  |  |  |  |  |  |
| 0 | Ath-AT5G64100.1 |  |  |  |  |  |  |  |  |
| 0 | Ath-AT5G64110.1 |  |  |  |  |  |  |  |  |
| 0 | Ath-AT5G64120.1 |  |  |  |  |  |  |  |  |
| 0 | Ath-AT5G64130.3 |  |  |  |  |  |  |  |  |
| 0 | Ath-AT5G64140.1 |  |  |  |  |  |  |  |  |
| 0 | Ath-AT5G64150.1 |  |  |  |  |  |  |  |  |
| 0 | Ath-AT5G64160.1 |  |  |  |  |  |  |  |  |
| 0 | Ath-AT5G64170.2 |  |  |  |  |  |  |  |  |
| 0 | Ath-AT5G64180.1 |  |  |  |  |  |  |  |  |
| 0 | Ath-AT5G64190.2 |  |  |  |  |  |  |  |  |
| 0 | Ath-AT5G64200.1 |  |  |  |  |  |  |  |  |
| 0 | Ath-AT5G64210.1 |  |  |  |  |  |  |  |  |
| 0 | Ath-AT5G64220.2 |  |  |  |  |  |  |  |  |
| 0 | Ath-AT5G64230.1 |  |  |  |  |  |  |  |  |
| 0 | Ath-AT5G64240.2 |  |  |  |  |  |  |  |  |
| 0 | Ath-AT5G64250.2 |  |  |  |  |  |  |  |  |
| 0 | Ath-AT5G64260.1 |  |  |  |  |  |  |  |  |
| 0 | Ath-AT5G64270.1 |  |  |  |  |  |  |  |  |
| 0 | Ath-AT5G64280.1 |  |  |  |  |  |  |  |  |
| 0 | Ath-AT5G64290.1 |  |  |  |  |  |  |  |  |
| 0 | Ath-AT5G64300.1 |  |  |  |  |  |  |  |  |
| 0 | Ath-AT5G64310.1 |  |  |  |  |  |  |  |  |
| 0 | Ath-AT5G64320.1 |  |  |  |  |  |  |  |  |
| 0 | Ath-AT5G64330.1 |  |  |  |  |  |  |  |  |
| 0 | Ath-AT5G64340.1 |  |  |  |  |  |  |  |  |
| 0 | Ath-AT5G64350.1 |  |  |  |  |  |  |  |  |
| 0 | Ath-AT5G64360.4 |  |  |  |  |  |  |  |  |
| 0 | Ath-AT5G64370.1 |  |  |  |  |  |  |  |  |
| 0 | Ath-AT5G64380.1 |  |  |  |  |  |  |  |  |
| 0 | Ath-AT5G64390.3 |  |  |  |  |  |  |  |  |
| 0 | Ath-AT5G64395.1 |  |  |  |  |  |  |  |  |
| 0 | Ath-AT5G64400.2 |  |  |  |  |  |  |  |  |
| 0 | Ath-AT5G64401.1 |  |  |  |  |  |  |  |  |
| 1 | Ath-AT5G64410.1 |  | Vvi-Vitvi03g00842\_t001 |  |  |  |  |  |  |  |
| 1 | Ath-AT5G64420.1 |  | Vvi-Vitvi03g00846\_t001 |  |  |  |  |  |  |  |
| 1 | Ath-AT5G64430.1 |  | Vvi-Vitvi03g00849\_t001 |  |  |  |  |  |  |  |
| 1 | Ath-AT5G64440.1 |  | Vvi-Vitvi03g00851\_t001 |  |  |  |  |  |  |  |
| 1 | Ath-AT5G64450.1 |  | | | |  |  |  |  |  |  |  |
| 1 | Ath-AT5G64460.3 |  | Vvi-Vitvi03g00852\_t001 |  |  |  |  |  |  |  |
| 1 | Ath-AT5G64470.2 |  | | | |  |  |  |  |  |  |  |
| 1 | Ath-AT5G64480.2 |  | Vvi-Vitvi03g00853\_t001 |  |  |  |  |  |  |  |
| 1 | Ath-AT5G64490.1 |  | | | |  |  |  |  |  |  |  |
| 1 | Ath-AT5G64500.1 |  | Vvi-Vitvi03g04288\_t001 |  |  |  |  |  |  |  |
| 0 | Ath-AT5G64510.1 |  |  |  |  |  |  |  |  |
| 0 | Ath-AT5G64520.4 |  |  |  |  |  |  |  |  |
| 0 | Ath-AT5G64530.1 |  |  |  |  |  |  |  |  |
| 0 | Ath-AT5G64540.1 |  |  |  |  |  |  |  |  |
| 0 | Ath-AT5G64550.1 |  |  |  |  |  |  |  |  |
| 0 | Ath-AT5G64560.1 |  |  |  |  |  |  |  |  |
| 0 | Ath-AT5G64570.1 |  |  |  |  |  |  |  |  |
| 1 | Ath-AT5G64580.1 |  | Vvi-Vitvi04g01677\_t002 |  |  |  |  |  |  |  |
| 1 | Ath-AT5G64590.1 |  | | | |  |  |  |  |  |  |  |
| 1 | Ath-AT5G64600.1 |  | | | |  |  |  |  |  |  |  |
| 1 | Ath-AT5G64610.1 |  | | | |  |  |  |  |  |  |  |
| 1 | Ath-AT5G64620.1 |  | Vvi-Vitvi04g01666\_t001 |  |  |  |  |  |  |  |
| 1 | Ath-AT5G64630.2 |  | Vvi-Vitvi04g01665\_t002 |  |  |  |  |  |  |  |
| 1 | Ath-AT5G64640.1 |  | Vvi-Vitvi04g02246\_t001 |  |  |  |  |  |  |  |
| 1 | Ath-AT5G64650.1 |  | Vvi-Vitvi04g01655\_t001 |  |  |  |  |  |  |  |
| 1 | Ath-AT5G64660.1 |  | Vvi-Vitvi04g02243\_t001 |  |  |  |  |  |  |  |
| 1 | Ath-AT5G64667.1 |  | Vvi-Vitvi04g02242\_t001 |  |  |  |  |  |  |  |
| 1 | Ath-AT5G64670.1 |  | | | |  |  |  |  |  |  |  |
| 1 | Ath-AT5G64680.3 |  | | | |  |  |  |  |  |  |  |
| 1 | Ath-AT5G64687.1 |  | | | |  |  |  |  |  |  |  |
| 1 | Ath-AT5G64690.1 |  | | | |  |  |  |  |  |  |  |
| 1 | Ath-AT5G64700.1 |  | | | |  |  |  |  |  |  |  |
| 1 | Ath-AT5G64710.1 |  | | | |  |  |  |  |  |  |  |
| 2 | Ath-AT5G64720.1 |  | | | |  | Vvi-Vitvi07g01882\_t001 |  |  |  |  |  |  |
| 2 | Ath-AT5G64730.1 |  | | | |  | | | |  |  |  |  |  |  |
| 2 | Ath-AT5G64740.1 |  | Vvi-Vitvi04g02233\_t001 |  | Vvi-Vitvi07g01881\_t001 |  |  |  |  |  |  |
| 1 | Ath-AT5G64750.1 |  |  |  | Vvi-Vitvi07g01874\_t001 |  |  |  |  |  |  |
| 1 | Ath-AT5G64760.2 |  |  |  | Vvi-Vitvi07g01872\_t001 |  |  |  |  |  |  |
| 1 | Ath-AT5G64770.1 |  |  |  | | | |  |  |  |  |  |  |
| 1 | Ath-AT5G64780.1 |  |  |  | Vvi-Vitvi07g02694\_t001 |  |  |  |  |  |  |
| 1 | Ath-AT5G64790.1 |  |  |  | Vvi-Vitvi07g01856\_t001 |  |  |  |  |  |  |
| 1 | Ath-AT5G64800.1 |  |  |  | | | |  |  |  |  |  |  |
| 1 | Ath-AT5G64810.1 |  |  |  | Vvi-Vitvi07g01847\_t001 |  |  |  |  |  |  |
| 1 | Ath-AT5G64813.2 |  |  |  | Vvi-Vitvi07g01846\_t001 |  |  |  |  |  |  |
| 1 | Ath-AT5G64816.3 |  |  |  | | | |  |  |  |  |  |  |
| 1 | Ath-AT5G64820.1 |  |  |  | Vvi-Vitvi07g01845\_t001 |  |  |  |  |  |  |
| 1 | Ath-AT5G64830.1 |  |  |  | | | |  |  |  |  |  |  |
| 1 | Ath-AT5G64840.1 |  |  |  | Vvi-Vitvi07g01840\_t001 |  |  |  |  |  |  |
| 1 | Ath-AT5G64850.1 |  |  |  | Vvi-Vitvi07g02691\_t001 |  |  |  |  |  |  |
| 1 | Ath-AT5G64860.1 |  |  |  | Vvi-Vitvi07g01830\_t001 |  |  |  |  |  |  |
| 1 | Ath-AT5G64870.1 |  |  |  | | | |  |  |  |  |  |  |
| 1 | Ath-AT5G64880.1 |  |  |  | Vvi-Vitvi07g02687\_t001 |  |  |  |  |  |  |
| 1 | Ath-AT5G64890.1 |  |  |  | | | |  |  |  |  |  |  |
| 1 | Ath-AT5G64900.1 |  |  |  | | | |  |  |  |  |  |  |
| 1 | Ath-AT5G64905.1 |  |  |  | | | |  |  |  |  |  |  |
| 1 | Ath-AT5G64910.2 |  |  |  | Vvi-Vitvi07g01825\_t001 |  |  |  |  |  |  |
| 1 | Ath-AT5G64920.1 |  |  |  | | | |  |  |  |  |  |  |
| 1 | Ath-AT5G64930.1 |  |  |  | Vvi-Vitvi07g01824\_t001 |  |  |  |  |  |  |
| 1 | Ath-AT5G64940.1 |  |  |  | | | |  |  |  |  |  |  |
| 1 | Ath-AT5G64950.1 |  |  |  | Vvi-Vitvi07g01822\_t001 |  |  |  |  |  |  |
| 2 | Ath-AT5G64960.1 |  | Vvi-Vitvi07g04551\_t001 |  | | | |  |  |  |  |  |  |
| 2 | Ath-AT5G64970.1 |  | Vvi-Vitvi07g04552\_t001 |  | | | |  |  |  |  |  |  |
| 2 | Ath-AT5G64980.1 |  | | | |  | | | |  |  |  |  |  |  |
| 2 | Ath-AT5G64990.2 |  | Vvi-Vitvi07g04554\_t001 |  | | | |  |  |  |  |  |  |
| 2 | Ath-AT5G65000.1 |  | Vvi-Vitvi07g01381\_t001 |  | | | |  |  |  |  |  |  |
| 2 | Ath-AT5G65005.1 |  | | | |  | | | |  |  |  |  |  |  |
| 2 | Ath-AT5G65010.2 |  | Vvi-Vitvi07g04555\_t001 |  | | | |  |  |  |  |  |  |
| 2 | Ath-AT5G65020.1 |  | | | |  | | | |  |  |  |  |  |  |
| 2 | Ath-AT5G65030.1 |  | Vvi-Vitvi07g04558\_t001 |  | | | |  |  |  |  |  |  |
| 3 | Ath-AT5G65040.1 |  | Vvi-Vitvi07g04543\_t001 |  | | | |  | Vvi-Vitvi18g02569\_t001 |  |  |  |  |  |
| 3 | Ath-AT5G65050.3 |  | | | |  | | | |  | | | |  |  |  |  |  |
| 3 | Ath-AT5G65060.1 |  | | | |  | | | |  | | | |  |  |  |  |  |
| 3 | Ath-AT5G65070.3 |  | | | |  | | | |  | | | |  |  |  |  |  |
| 3 | Ath-AT5G65080.2 |  | | | |  | | | |  | | | |  |  |  |  |  |
| 3 | Ath-AT5G65090.1 |  | Vvi-Vitvi07g01352\_t001 |  | | | |  | | | |  |  |  |  |  |
| 3 | Ath-AT5G65100.1 |  | Vvi-Vitvi07g01353\_t001 |  | | | |  | | | |  |  |  |  |  |
| 3 | Ath-AT5G65110.1 |  | Vvi-Vitvi07g03026\_t001 |  | | | |  | | | |  |  |  |  |  |
| 3 | Ath-AT5G65120.1 |  | Vvi-Vitvi07g03031\_t001 |  | | | |  | | | |  |  |  |  |  |
| 3 | Ath-AT5G65130.2 |  | Vvi-Vitvi07g03030\_t001 |  | | | |  | Vvi-Vitvi18g00381\_t001 |  |  |  |  |  |
| 3 | Ath-AT5G65140.1 |  | Vvi-Vitvi07g01365\_t001 |  | | | |  | Vvi-Vitvi18g00384\_t001 |  |  |  |  |  |
| 3 | Ath-AT5G65158.1 |  | Vvi-Vitvi07g01364\_t001 |  | | | |  | | | |  |  |  |  |  |
| 3 | Ath-AT5G65160.1 |  | Vvi-Vitvi07g01363\_t001 |  | | | |  | Vvi-Vitvi18g00390\_t001 |  |  |  |  |  |
| 3 | Ath-AT5G65165.1 |  | Vvi-Vitvi07g01360\_t001 |  | | | |  | | | |  |  |  |  |  |
| 3 | Ath-AT5G65170.1 |  | Vvi-Vitvi07g01359\_t001 |  | | | |  | Vvi-Vitvi18g00391\_t001 |  |  |  |  |  |
| 3 | Ath-AT5G65180.1 |  | | | |  | Vvi-Vitvi07g01803\_t002 |  | | | |  |  |  |  |  |
| 2 | Ath-AT5G65200.1 |  | | | |  |  |  | | | |  |  |  |  |  |
| 2 | Ath-AT5G65205.1 |  | | | |  |  |  | | | |  |  |  |  |  |
| 2 | Ath-AT5G65207.1 |  | | | |  |  |  | | | |  |  |  |  |  |
| 2 | Ath-AT5G65210.1 |  | | | |  |  |  | | | |  |  |  |  |  |
| 2 | Ath-AT5G65220.1 |  | | | |  |  |  | | | |  |  |  |  |  |
| 2 | Ath-AT5G65225.1 |  | | | |  |  |  | | | |  |  |  |  |  |
| 2 | Ath-AT5G65230.1 |  | | | |  |  |  | Vvi-Vitvi18g00406\_t001 |  |  |  |  |  |
| 1 | Ath-AT5G65240.2 |  | Vvi-Vitvi07g04561\_t001 |  |  |  |  |  |  |  |
| 1 | Ath-AT5G65250.1 |  | Vvi-Vitvi07g04578\_t001 |  |  |  |  |  |  |  |
| 1 | Ath-AT5G65260.1 |  | Vvi-Vitvi07g04579\_t001 |  |  |  |  |  |  |  |
| 1 | Ath-AT5G65270.1 |  | Vvi-Vitvi07g01504\_t001 |  |  |  |  |  |  |  |
| 1 | Ath-AT5G65274.1 |  | | | |  |  |  |  |  |  |  |
| 1 | Ath-AT5G65280.1 |  | Vvi-Vitvi07g01499\_t001 |  |  |  |  |  |  |  |
| 1 | Ath-AT5G65290.1 |  | Vvi-Vitvi07g01489\_t001 |  |  |  |  |  |  |  |
| 1 | Ath-AT5G65300.1 |  | | | |  |  |  |  |  |  |  |
| 2 | Ath-AT5G65310.1 |  | Vvi-Vitvi07g01488\_t001 |  | Vvi-Vitvi18g00448\_t001 |  |  |  |  |  |  |
| 2 | Ath-AT5G65320.1 |  | Vvi-Vitvi07g01475\_t001 |  | Vvi-Vitvi18g00463\_t001 |  |  |  |  |  |  |
| 2 | Ath-AT5G65330.1 |  | | | |  | | | |  |  |  |  |  |  |
| 2 | Ath-AT5G65340.1 |  | Vvi-Vitvi07g01472\_t001 |  | | | |  |  |  |  |  |  |
| 2 | Ath-AT5G65350.1 |  | | | |  | | | |  |  |  |  |  |  |
| 2 | Ath-AT5G65360.1 |  | | | |  | | | |  |  |  |  |  |  |
| 2 | Ath-AT5G65370.1 |  | Vvi-Vitvi07g01471\_t001 |  | Vvi-Vitvi18g00469\_t001 |  |  |  |  |  |  |
| 2 | Ath-AT5G65380.1 |  | Vvi-Vitvi07g04591\_t001 |  | Vvi-Vitvi18g00470\_t001 |  |  |  |  |  |  |
| 2 | Ath-AT5G65390.1 |  | | | |  | | | |  |  |  |  |  |  |
| 2 | Ath-AT5G65400.2 |  | Vvi-Vitvi07g01468\_t001 |  | Vvi-Vitvi18g00489\_t001 |  |  |  |  |  |  |
| 2 | Ath-AT5G65410.1 |  | Vvi-Vitvi07g01465\_t001 |  | Vvi-Vitvi18g00493\_t001 |  |  |  |  |  |  |
| 2 | Ath-AT5G65420.3 |  | Vvi-Vitvi07g01460\_t001 |  | Vvi-Vitvi18g00499\_t001 |  |  |  |  |  |  |
| 2 | Ath-AT5G65430.3 |  | Vvi-Vitvi07g01457\_t001 |  | Vvi-Vitvi18g02600\_t001 |  |  |  |  |  |  |
| 2 | Ath-AT5G65440.3 |  | Vvi-Vitvi07g01451\_t001 |  | Vvi-Vitvi18g00505\_t002 |  |  |  |  |  |  |
| 2 | Ath-AT5G65450.2 |  | Vvi-Vitvi07g01449\_t001 |  | Vvi-Vitvi18g00511\_t001 |  |  |  |  |  |  |
| 2 | Ath-AT5G65460.3 |  | Vvi-Vitvi07g01442\_t001 |  | | | |  |  |  |  |  |  |
| 2 | Ath-AT5G65470.1 |  | Vvi-Vitvi07g01437\_t001 |  | Vvi-Vitvi18g00518\_t002 |  |  |  |  |  |  |
| 2 | Ath-AT5G65480.1 |  | | | |  | Vvi-Vitvi18g00524\_t001 |  |  |  |  |  |  |
| 2 | Ath-AT5G65490.1 |  | Vvi-Vitvi07g01425\_t002 |  | | | |  |  |  |  |  |  |
| 2 | Ath-AT5G65495.1 |  | Vvi-Vitvi07g04610\_t001 |  | | | |  |  |  |  |  |  |
| 2 | Ath-AT5G65500.2 |  | Vvi-Vitvi07g01423\_t001 |  | | | |  |  |  |  |  |  |
| 2 | Ath-AT5G65510.1 |  | Vvi-Vitvi07g01419\_t001 |  | | | |  |  |  |  |  |  |
| 2 | Ath-AT5G65520.1 |  | Vvi-Vitvi07g02572\_t001 |  | Vvi-Vitvi18g00533\_t001 |  |  |  |  |  |  |
| 1 | Ath-AT5G65530.1 |  | Vvi-Vitvi07g04612\_t001 |  |  |  |  |  |  |  |
| 1 | Ath-AT5G65540.1 |  | Vvi-Vitvi07g04615\_t001 |  |  |  |  |  |  |  |
| 1 | Ath-AT5G65550.1 |  | Vvi-Vitvi07g04617\_t001 |  |  |  |  |  |  |  |
| 1 | Ath-AT5G65560.3 |  | | | |  |  |  |  |  |  |  |
| 1 | Ath-AT5G65570.1 |  | Vvi-Vitvi07g01410\_t001 |  |  |  |  |  |  |  |
| 1 | Ath-AT5G65580.1 |  | | | |  |  |  |  |  |  |  |
| 1 | Ath-AT5G65590.1 |  | Vvi-Vitvi07g01401\_t001 |  |  |  |  |  |  |  |
| 1 | Ath-AT5G65600.1 |  | Vvi-Vitvi07g01387\_t001 |  |  |  |  |  |  |  |
| 1 | Ath-AT5G65609.1 |  | | | |  |  |  |  |  |  |  |
| 1 | Ath-AT5G65610.1 |  | | | |  |  |  |  |  |  |  |
| 1 | Ath-AT5G65613.1 |  | | | |  |  |  |  |  |  |  |
| 1 | Ath-AT5G65620.2 |  | Vvi-Vitvi07g01510\_t001 |  |  |  |  |  |  |  |
| 1 | Ath-AT5G65630.1 |  | Vvi-Vitvi07g04637\_t001 |  |  |  |  |  |  |  |
| 1 | Ath-AT5G65640.1 |  | Vvi-Vitvi07g02613\_t001 |  |  |  |  |  |  |  |
| 1 | Ath-AT5G65650.1 |  | | | |  |  |  |  |  |  |  |
| 1 | Ath-AT5G65660.1 |  | Vvi-Vitvi07g01543\_t001 |  |  |  |  |  |  |  |
| 1 | Ath-AT5G65670.1 |  | Vvi-Vitvi07g04642\_t003 |  |  |  |  |  |  |  |
| 1 | Ath-AT5G65683.1 |  | Vvi-Vitvi07g04644\_t001 |  |  |  |  |  |  |  |
| 1 | Ath-AT5G65690.2 |  | Vvi-Vitvi07g04646\_t001 |  |  |  |  |  |  |  |
| 1 | Ath-AT5G65687.1 |  | | | |  |  |  |  |  |  |  |
| 1 | Ath-AT5G65685.5 |  | Vvi-Vitvi07g04648\_t001 |  |  |  |  |  |  |  |
| 1 | Ath-AT5G65700.1 |  | Vvi-Vitvi07g04652\_t001 |  |  |  |  |  |  |  |
| 1 | Ath-AT5G65710.1 |  | Vvi-Vitvi07g04654\_t001 |  |  |  |  |  |  |  |
| 1 | Ath-AT5G65720.1 |  | Vvi-Vitvi07g04660\_t001 |  |  |  |  |  |  |  |
| 1 | Ath-AT5G65730.1 |  | Vvi-Vitvi07g04661\_t001 |  |  |  |  |  |  |  |
| 1 | Ath-AT5G65740.2 |  | Vvi-Vitvi07g04662\_t001 |  |  |  |  |  |  |  |
| 1 | Ath-AT5G65750.1 |  | | | |  |  |  |  |  |  |  |
| 1 | Ath-AT5G65760.1 |  | | | |  |  |  |  |  |  |  |
| 2 | Ath-AT5G65780.2 |  | | | |  | Vvi-Vitvi15g04538\_t001 |  |  |  |  |  |  |
| 2 | Ath-AT5G65770.2 |  | | | |  | | | |  |  |  |  |  |  |
| 3 | Ath-AT5G65790.1 |  | | | |  | | | |  | Vvi-Vitvi18g00605\_t001 |  |  |  |  |  |
| 3 | Ath-AT5G65800.1 |  | Vvi-Vitvi07g03132\_t001 |  | | | |  | Vvi-Vitvi18g00609\_t001 |  |  |  |  |  |
| 2 | Ath-AT5G65810.1 |  |  |  | Vvi-Vitvi15g04535\_t001 |  | Vvi-Vitvi18g00610\_t001 |  |  |  |  |  |
| 2 | Ath-AT5G65820.1 |  |  |  | Vvi-Vitvi15g04529\_t001 |  | | | |  |  |  |  |  |
| 2 | Ath-AT5G65830.1 |  |  |  | Vvi-Vitvi15g04523\_t001 |  | | | |  |  |  |  |  |
| 2 | Ath-AT5G65840.1 |  |  |  | Vvi-Vitvi15g04519\_t003 |  | | | |  |  |  |  |  |
| 2 | Ath-AT5G65850.1 |  |  |  | | | |  | | | |  |  |  |  |  |
| 2 | Ath-AT5G65860.1 |  |  |  | | | |  | | | |  |  |  |  |  |
| 2 | Ath-AT5G65870.1 |  |  |  | Vvi-Vitvi15g04511\_t001 |  | Vvi-Vitvi18g00635\_t001 |  |  |  |  |  |
| 2 | Ath-AT5G65880.1 |  |  |  | Vvi-Vitvi15g04509\_t001 |  | | | |  |  |  |  |  |
| 2 | Ath-AT5G65890.3 |  |  |  | Vvi-Vitvi15g04507\_t001 |  | | | |  |  |  |  |  |
| 2 | Ath-AT5G65900.1 |  |  |  | | | |  | | | |  |  |  |  |  |
| 2 | Ath-AT5G65910.1 |  |  |  | Vvi-Vitvi15g04506\_t001 |  | Vvi-Vitvi18g00637\_t001 |  |  |  |  |  |
| 2 | Ath-AT5G65920.1 |  |  |  | Vvi-Vitvi15g04503\_t001 |  | Vvi-Vitvi18g00638\_t001 |  |  |  |  |  |
| 0 | Ath-AT5G65925.1 |  |  |  |  |  |  |  |  |
| 0 | Ath-AT5G65930.3 |  |  |  |  |  |  |  |  |
| 0 | Ath-AT5G65940.1 |  |  |  |  |  |  |  |  |
| 0 | Ath-AT5G65950.1 |  |  |  |  |  |  |  |  |
| 0 | Ath-AT5G65960.1 |  |  |  |  |  |  |  |  |
| 0 | Ath-AT5G65970.1 |  |  |  |  |  |  |  |  |
| 0 | Ath-AT5G65980.1 |  |  |  |  |  |  |  |  |
| 0 | Ath-AT5G65990.1 |  |  |  |  |  |  |  |  |
| 0 | Ath-AT5G66000.1 |  |  |  |  |  |  |  |  |
| 0 | Ath-AT5G66005.3 |  |  |  |  |  |  |  |  |
| 0 | Ath-AT5G66010.1 |  |  |  |  |  |  |  |  |
| 0 | Ath-AT5G66020.1 |  |  |  |  |  |  |  |  |
| 0 | Ath-AT5G66030.1 |  |  |  |  |  |  |  |  |
| 1 | Ath-AT5G66040.1 |  | Vvi-Vitvi04g01540\_t001 |  |  |  |  |  |  |  |
| 1 | Ath-AT5G66050.1 |  | Vvi-Vitvi04g04407\_t002 |  |  |  |  |  |  |  |
| 1 | Ath-AT5G66052.1 |  | | | |  |  |  |  |  |  |  |
| 1 | Ath-AT5G66053.1 |  | | | |  |  |  |  |  |  |  |
| 1 | Ath-AT5G66055.1 |  | Vvi-Vitvi04g01526\_t001 |  |  |  |  |  |  |  |
| 1 | Ath-AT5G66060.1 |  | Vvi-Vitvi04g02185\_t001 |  |  |  |  |  |  |  |
| 1 | Ath-AT5G66070.2 |  | Vvi-Vitvi04g01536\_t001 |  |  |  |  |  |  |  |
| 1 | Ath-AT5G66080.1 |  | Vvi-Vitvi04g02280\_t001 |  |  |  |  |  |  |  |
| 1 | Ath-AT5G66090.1 |  | Vvi-Vitvi04g01542\_t001 |  |  |  |  |  |  |  |
| 1 | Ath-AT5G66100.1 |  | Vvi-Vitvi04g01506\_t001 |  |  |  |  |  |  |  |
| 1 | Ath-AT5G66110.1 |  | Vvi-Vitvi04g02170\_t001 |  |  |  |  |  |  |  |
| 1 | Ath-AT5G66120.2 |  | Vvi-Vitvi04g01492\_t001 |  |  |  |  |  |  |  |
| 1 | Ath-AT5G66130.1 |  | Vvi-Vitvi04g01490\_t001 |  |  |  |  |  |  |  |
| 1 | Ath-AT5G66140.1 |  | Vvi-Vitvi04g01484\_t001 |  |  |  |  |  |  |  |
| 1 | Ath-AT5G66150.1 |  | Vvi-Vitvi04g01481\_t001 |  |  |  |  |  |  |  |
| 1 | Ath-AT5G66160.1 |  | | | |  |  |  |  |  |  |  |
| 1 | Ath-AT5G66170.2 |  | Vvi-Vitvi04g02158\_t001 |  |  |  |  |  |  |  |
| 1 | Ath-AT5G66180.1 |  | Vvi-Vitvi04g01473\_t001 |  |  |  |  |  |  |  |
| 1 | Ath-AT5G66190.1 |  | Vvi-Vitvi04g01468\_t001 |  |  |  |  |  |  |  |
| 1 | Ath-AT5G66200.1 |  | Vvi-Vitvi04g01467\_t001 |  |  |  |  |  |  |  |
| 1 | Ath-AT5G66210.1 |  | Vvi-Vitvi04g01462\_t001 |  |  |  |  |  |  |  |
| 1 | Ath-AT5G66230.2 |  | Vvi-Vitvi04g01453\_t001 |  |  |  |  |  |  |  |
| 1 | Ath-AT5G66220.1 |  | | | |  |  |  |  |  |  |  |
| 1 | Ath-AT5G66240.2 |  | Vvi-Vitvi04g01450\_t002 |  |  |  |  |  |  |  |
| 1 | Ath-AT5G66250.4 |  | Vvi-Vitvi04g01443\_t001 |  |  |  |  |  |  |  |
| 1 | Ath-AT5G66260.1 |  | | | |  |  |  |  |  |  |  |
| 1 | Ath-AT5G66270.1 |  | Vvi-Vitvi04g01436\_t001 |  |  |  |  |  |  |  |
| 1 | Ath-AT5G66280.1 |  | Vvi-Vitvi04g02147\_t001 |  |  |  |  |  |  |  |
| 1 | Ath-AT5G66290.2 |  | Vvi-Vitvi04g01434\_t001 |  |  |  |  |  |  |  |
| 1 | Ath-AT5G66300.1 |  | Vvi-Vitvi04g01430\_t001 |  |  |  |  |  |  |  |
| 1 | Ath-AT5G66310.1 |  | Vvi-Vitvi04g01425\_t003 |  |  |  |  |  |  |  |
| 1 | Ath-AT5G66320.1 |  | Vvi-Vitvi04g01410\_t001 |  |  |  |  |  |  |  |
| 1 | Ath-AT5G66330.1 |  | Vvi-Vitvi04g01407\_t001 |  |  |  |  |  |  |  |
| 1 | Ath-AT5G66335.1 |  | | | |  |  |  |  |  |  |  |
| 1 | Ath-AT5G66340.1 |  | | | |  |  |  |  |  |  |  |
| 1 | Ath-AT5G66350.2 |  | Vvi-Vitvi04g01399\_t001 |  |  |  |  |  |  |  |
| 1 | Ath-AT5G66360.2 |  | Vvi-Vitvi04g01388\_t001 |  |  |  |  |  |  |  |
| 1 | Ath-AT5G66370.1 |  | | | |  |  |  |  |  |  |  |
| 1 | Ath-AT5G66380.1 |  | Vvi-Vitvi04g01381\_t002 |  |  |  |  |  |  |  |
| 2 | Ath-AT5G66390.1 |  | Vvi-Vitvi04g01378\_t001 |  | Vvi-Vitvi18g01014\_t001 |  |  |  |  |  |  |
| 2 | Ath-AT5G66400.1 |  | Vvi-Vitvi04g01368\_t001 |  | | | |  |  |  |  |  |  |
| 2 | Ath-AT5G66410.1 |  | Vvi-Vitvi04g01359\_t001 |  | | | |  |  |  |  |  |  |
| 2 | Ath-AT5G66420.2 |  | Vvi-Vitvi04g04362\_t001 |  | | | |  |  |  |  |  |  |
| 2 | Ath-AT5G66430.1 |  | Vvi-Vitvi04g01338\_t001 |  | Vvi-Vitvi18g02761\_t002 |  |  |  |  |  |  |
| 2 | Ath-AT5G66440.1 |  | Vvi-Vitvi04g01337\_t001 |  | Vvi-Vitvi18g00992\_t001 |  |  |  |  |  |  |
| 2 | Ath-AT5G66450.3 |  | Vvi-Vitvi04g01336\_t001 |  | | | |  |  |  |  |  |  |
| 2 | Ath-AT5G66460.1 |  | Vvi-Vitvi04g01334\_t001 |  | Vvi-Vitvi18g00991\_t001 |  |  |  |  |  |  |
| 2 | Ath-AT5G66470.1 |  | Vvi-Vitvi04g01332\_t001 |  | | | |  |  |  |  |  |  |
| 2 | Ath-AT5G66480.1 |  | Vvi-Vitvi04g01330\_t001 |  | | | |  |  |  |  |  |  |
| 2 | Ath-AT5G66490.1 |  | Vvi-Vitvi04g04348\_t001 |  | | | |  |  |  |  |  |  |
| 2 | Ath-AT5G66500.1 |  | | | |  | | | |  |  |  |  |  |  |
| 2 | Ath-AT5G66510.2 |  | Vvi-Vitvi04g01325\_t001 |  | Vvi-Vitvi18g00981\_t001 |  |  |  |  |  |  |
| 2 | Ath-AT5G66530.1 |  | Vvi-Vitvi04g01318\_t001 |  | | | |  |  |  |  |  |  |
| 2 | Ath-AT5G66520.1 |  | | | |  | | | |  |  |  |  |  |  |
| 2 | Ath-AT5G66540.1 |  | | | |  | | | |  |  |  |  |  |  |
| 2 | Ath-AT5G66550.2 |  | Vvi-Vitvi04g01296\_t001 |  | Vvi-Vitvi18g00968\_t002 |  |  |  |  |  |  |
| 1 | Ath-AT5G66560.1 |  |  |  | | | |  |  |  |  |  |  |
| 2 | Ath-AT5G66570.1 |  | Vvi-Vitvi18g00894\_t001 |  | | | |  |  |  |  |  |  |
| 2 | Ath-AT5G66580.1 |  | | | |  | | | |  |  |  |  |  |  |
| 2 | Ath-AT5G66590.1 |  | | | |  | | | |  |  |  |  |  |  |
| 2 | Ath-AT5G66600.9 |  | | | |  | | | |  |  |  |  |  |  |
| 2 | Ath-AT5G66595.1 |  | | | |  | | | |  |  |  |  |  |  |
| 2 | Ath-AT5G66607.1 |  | | | |  | | | |  |  |  |  |  |  |
| 3 | Ath-AT5G66610.2 |  | Vvi-Vitvi18g00903\_t001 |  | | | |  | Vvi-Vitvi04g02091\_t001 |  |  |  |  |  |
| 3 | Ath-AT5G66620.1 |  | | | |  | | | |  | | | |  |  |  |  |  |
| 3 | Ath-AT5G66630.1 |  | | | |  | | | |  | | | |  |  |  |  |  |
| 3 | Ath-AT5G66631.1 |  | | | |  | | | |  | Vvi-Vitvi04g01212\_t001 |  |  |  |  |  |
| 3 | Ath-AT5G66640.5 |  | | | |  | | | |  | | | |  |  |  |  |  |
| 3 | Ath-AT5G66650.1 |  | Vvi-Vitvi18g02730\_t001 |  | | | |  | Vvi-Vitvi04g01225\_t001 |  |  |  |  |  |
| 3 | Ath-AT5G66658.1 |  | | | |  | | | |  | | | |  |  |  |  |  |
| 3 | Ath-AT5G66660.1 |  | | | |  | | | |  | | | |  |  |  |  |  |
| 3 | Ath-AT5G66670.2 |  | | | |  | | | |  | Vvi-Vitvi04g01228\_t001 |  |  |  |  |  |
| 3 | Ath-AT5G66675.2 |  | | | |  | | | |  | | | |  |  |  |  |  |
| 3 | Ath-AT5G66680.1 |  | | | |  | | | |  | Vvi-Vitvi04g01231\_t001 |  |  |  |  |  |
| 3 | Ath-AT5G66690.1 |  | Vvi-Vitvi18g04218\_t001 |  | | | |  | Vvi-Vitvi04g01237\_t001 |  |  |  |  |  |
| 3 | Ath-AT5G66700.1 |  | | | |  | | | |  | Vvi-Vitvi04g01244\_t001 |  |  |  |  |  |
| 3 | Ath-AT5G66710.1 |  | | | |  | | | |  | Vvi-Vitvi04g01246\_t001 |  |  |  |  |  |
| 3 | Ath-AT5G66720.1 |  | Vvi-Vitvi18g00933\_t005 |  | | | |  | Vvi-Vitvi04g01249\_t001 |  |  |  |  |  |
| 3 | Ath-AT5G66730.1 |  | Vvi-Vitvi18g00935\_t001 |  | | | |  | Vvi-Vitvi04g01252\_t001 |  |  |  |  |  |
| 3 | Ath-AT5G66740.1 |  | Vvi-Vitvi18g00947\_t001 |  | Vvi-Vitvi18g00947\_t001 |  | Vvi-Vitvi04g01264\_t001 |  |  |  |  |  |
| 1 | Ath-AT5G66750.1 |  |  |  |  |  | Vvi-Vitvi04g01275\_t001 |  |  |  |  |  |
| 1 | Ath-AT5G66760.1 |  |  |  |  |  | Vvi-Vitvi04g01280\_t001 |  |  |  |  |  |
| 1 | Ath-AT5G66770.1 |  |  |  |  |  | Vvi-Vitvi04g01281\_t001 |  |  |  |  |  |
| 1 | Ath-AT5G66780.1 |  |  |  |  |  | Vvi-Vitvi04g01282\_t001 |  |  |  |  |  |
| 0 | Ath-AT5G66790.1 |  |  |  |  |  |  |  |  |
| 1 | Ath-AT5G66800.1 |  | Vvi-Vitvi07g01761\_t001 |  |  |  |  |  |  |  |
| 1 | Ath-AT5G66810.1 |  | | | |  |  |  |  |  |  |  |
| 1 | Ath-AT5G66815.1 |  | | | |  |  |  |  |  |  |  |
[truncated: 7,144 more chars]
