# Supplementary material for: Genome-wide identification, characterization and gene expression of BES1 transcription factor family in grapevine (Vitis vinifera L.)
Source: Sci Rep. 2023 Jan 5;13:240. doi: 10.1038/s41598-022-24407-y (PMC9816167; doi:10.1038/s41598-022-24407-y)
Supplement: Supplementary file 3 — Supplementary Information. [file 41598_2022_24407_MOESM3_ESM.zip › Vvi_Ath/Vitis_vinifera.PN40024.v4.dna_sm.toplevel.fa.vs.Arabidopsis_thaliana.TAIR10.dna_sm.toplevel.fa.html/Ath-Mt.html]

|  |  |  |  |  |  |  |  |  |  |  |  |  |  |  |  |  |  |
| --- | --- | --- | --- | --- | --- | --- | --- | --- | --- | --- | --- | --- | --- | --- | --- | --- | --- |
| Duplication depth | Reference chromosome | Collinear blocks | | | | | | | | | | | | | | | |
| 0 | Ath-ATMG00010.1 |  |  |  |  |  |  |  |  |
| 0 | Ath-ATMG00030.1 |  |  |  |  |  |  |  |  |
| 0 | Ath-ATMG00040.1 |  |  |  |  |  |  |  |  |
| 0 | Ath-ATMG00050.1 |  |  |  |  |  |  |  |  |
| 0 | Ath-ATMG00070.1 |  |  |  |  |  |  |  |  |
| 0 | Ath-ATMG00080.1 |  |  |  |  |  |  |  |  |
| 0 | Ath-ATMG00090.1 |  |  |  |  |  |  |  |  |
| 0 | Ath-ATMG00110.1 |  |  |  |  |  |  |  |  |
| 0 | Ath-ATMG00120.1 |  |  |  |  |  |  |  |  |
| 0 | Ath-ATMG00130.1 |  |  |  |  |  |  |  |  |
| 0 | Ath-ATMG00140.1 |  |  |  |  |  |  |  |  |
| 0 | Ath-ATMG00150.1 |  |  |  |  |  |  |  |  |
| 0 | Ath-ATMG00160.1 |  |  |  |  |  |  |  |  |
| 0 | Ath-ATMG00170.1 |  |  |  |  |  |  |  |  |
| 0 | Ath-ATMG00180.1 |  |  |  |  |  |  |  |  |
| 0 | Ath-ATMG00200.1 |  |  |  |  |  |  |  |  |
| 0 | Ath-ATMG00210.1 |  |  |  |  |  |  |  |  |
| 0 | Ath-ATMG00220.1 |  |  |  |  |  |  |  |  |
| 0 | Ath-ATMG00240.1 |  |  |  |  |  |  |  |  |
| 0 | Ath-ATMG00260.1 |  |  |  |  |  |  |  |  |
| 0 | Ath-ATMG00270.1 |  |  |  |  |  |  |  |  |
| 0 | Ath-ATMG00280.1 |  |  |  |  |  |  |  |  |
| 0 | Ath-ATMG00285.1 |  |  |  |  |  |  |  |  |
| 0 | Ath-ATMG00290.1 |  |  |  |  |  |  |  |  |
| 0 | Ath-ATMG00300.1 |  |  |  |  |  |  |  |  |
| 0 | Ath-ATMG00310.1 |  |  |  |  |  |  |  |  |
| 0 | Ath-ATMG00320.1 |  |  |  |  |  |  |  |  |
| 0 | Ath-ATMG00370.1 |  |  |  |  |  |  |  |  |
| 0 | Ath-ATMG00400.1 |  |  |  |  |  |  |  |  |
| 0 | Ath-ATMG00410.1 |  |  |  |  |  |  |  |  |
| 0 | Ath-ATMG00430.1 |  |  |  |  |  |  |  |  |
| 0 | Ath-ATMG00440.1 |  |  |  |  |  |  |  |  |
| 0 | Ath-ATMG00450.1 |  |  |  |  |  |  |  |  |
| 0 | Ath-ATMG00470.1 |  |  |  |  |  |  |  |  |
| 0 | Ath-ATMG00480.1 |  |  |  |  |  |  |  |  |
| 0 | Ath-ATMG00490.1 |  |  |  |  |  |  |  |  |
| 0 | Ath-ATMG00500.1 |  |  |  |  |  |  |  |  |
| 0 | Ath-ATMG00510.1 |  |  |  |  |  |  |  |  |
| 0 | Ath-ATMG00513.1 |  |  |  |  |  |  |  |  |
| 0 | Ath-ATMG00516.1 |  |  |  |  |  |  |  |  |
| 0 | Ath-ATMG00520.1 |  |  |  |  |  |  |  |  |
| 0 | Ath-ATMG00530.1 |  |  |  |  |  |  |  |  |
| 0 | Ath-ATMG00540.1 |  |  |  |  |  |  |  |  |
| 0 | Ath-ATMG00550.1 |  |  |  |  |  |  |  |  |
| 0 | Ath-ATMG00560.1 |  |  |  |  |  |  |  |  |
| 0 | Ath-ATMG00570.1 |  |  |  |  |  |  |  |  |
| 0 | Ath-ATMG00580.1 |  |  |  |  |  |  |  |  |
| 0 | Ath-ATMG00590.1 |  |  |  |  |  |  |  |  |
| 0 | Ath-ATMG00600.1 |  |  |  |  |  |  |  |  |
| 0 | Ath-ATMG00610.1 |  |  |  |  |  |  |  |  |
| 0 | Ath-ATMG00620.1 |  |  |  |  |  |  |  |  |
| 0 | Ath-ATMG00630.1 |  |  |  |  |  |  |  |  |
| 0 | Ath-ATMG00640.1 |  |  |  |  |  |  |  |  |
| 0 | Ath-ATMG00650.1 |  |  |  |  |  |  |  |  |
| 0 | Ath-ATMG00660.1 |  |  |  |  |  |  |  |  |
| 0 | Ath-ATMG00665.1 |  |  |  |  |  |  |  |  |
| 0 | Ath-ATMG00670.1 |  |  |  |  |  |  |  |  |
| 0 | Ath-ATMG00680.1 |  |  |  |  |  |  |  |  |
| 0 | Ath-ATMG00690.1 |  |  |  |  |  |  |  |  |
| 0 | Ath-ATMG00710.1 |  |  |  |  |  |  |  |  |
| 0 | Ath-ATMG00720.1 |  |  |  |  |  |  |  |  |
| 0 | Ath-ATMG00730.1 |  |  |  |  |  |  |  |  |
| 0 | Ath-ATMG00740.1 |  |  |  |  |  |  |  |  |
| 0 | Ath-ATMG00750.1 |  |  |  |  |  |  |  |  |
| 0 | Ath-ATMG00760.1 |  |  |  |  |  |  |  |  |
| 0 | Ath-ATMG00770.1 |  |  |  |  |  |  |  |  |
| 0 | Ath-ATMG00810.1 |  |  |  |  |  |  |  |  |
| 0 | Ath-ATMG00820.1 |  |  |  |  |  |  |  |  |
| 0 | Ath-ATMG00830.1 |  |  |  |  |  |  |  |  |
| 0 | Ath-ATMG00840.1 |  |  |  |  |  |  |  |  |
| 0 | Ath-ATMG00850.1 |  |  |  |  |  |  |  |  |
| 0 | Ath-ATMG00860.1 |  |  |  |  |  |  |  |  |
| 0 | Ath-ATMG00870.1 |  |  |  |  |  |  |  |  |
| 0 | Ath-ATMG00880.1 |  |  |  |  |  |  |  |  |
| 0 | Ath-ATMG00890.1 |  |  |  |  |  |  |  |  |
| 0 | Ath-ATMG00900.1 |  |  |  |  |  |  |  |  |
| 0 | Ath-ATMG00910.1 |  |  |  |  |  |  |  |  |
| 0 | Ath-ATMG00920.1 |  |  |  |  |  |  |  |  |
| 0 | Ath-ATMG00940.1 |  |  |  |  |  |  |  |  |
| 0 | Ath-ATMG00960.1 |  |  |  |  |  |  |  |  |
| 0 | Ath-ATMG00970.1 |  |  |  |  |  |  |  |  |
| 0 | Ath-ATMG00980.1 |  |  |  |  |  |  |  |  |
| 0 | Ath-ATMG00990.1 |  |  |  |  |  |  |  |  |
| 0 | Ath-ATMG01000.1 |  |  |  |  |  |  |  |  |
| 0 | Ath-ATMG01010.1 |  |  |  |  |  |  |  |  |
| 0 | Ath-ATMG01020.1 |  |  |  |  |  |  |  |  |
| 0 | Ath-ATMG01030.1 |  |  |  |  |  |  |  |  |
| 0 | Ath-ATMG01040.1 |  |  |  |  |  |  |  |  |
| 0 | Ath-ATMG01050.1 |  |  |  |  |  |  |  |  |
| 0 | Ath-ATMG01060.1 |  |  |  |  |  |  |  |  |
| 0 | Ath-ATMG01080.1 |  |  |  |  |  |  |  |  |
| 0 | Ath-ATMG01090.1 |  |  |  |  |  |  |  |  |
| 0 | Ath-ATMG01100.1 |  |  |  |  |  |  |  |  |
| 0 | Ath-ATMG01110.1 |  |  |  |  |  |  |  |  |
| 0 | Ath-ATMG01120.1 |  |  |  |  |  |  |  |  |
| 0 | Ath-ATMG01130.1 |  |  |  |  |  |  |  |  |
| 0 | Ath-ATMG01140.1 |  |  |  |  |  |  |  |  |
| 0 | Ath-ATMG01150.1 |  |  |  |  |  |  |  |  |
| 0 | Ath-ATMG01170.1 |  |  |  |  |  |  |  |  |
| 0 | Ath-ATMG01180.1 |  |  |  |  |  |  |  |  |
| 0 | Ath-ATMG01190.1 |  |  |  |  |  |  |  |  |
| 0 | Ath-ATMG01200.1 |  |  |  |  |  |  |  |  |
| 0 | Ath-ATMG01210.1 |  |  |  |  |  |  |  |  |
| 0 | Ath-ATMG01220.1 |  |  |  |  |  |  |  |  |
| 0 | Ath-ATMG01230.1 |  |  |  |  |  |  |  |  |
| 0 | Ath-ATMG01240.1 |  |  |  |  |  |  |  |  |
| 0 | Ath-ATMG01250.1 |  |  |  |  |  |  |  |  |
| 0 | Ath-ATMG01260.1 |  |  |  |  |  |  |  |  |
| 0 | Ath-ATMG01270.1 |  |  |  |  |  |  |  |  |
| 0 | Ath-ATMG01275.1 |  |  |  |  |  |  |  |  |
| 0 | Ath-ATMG01280.1 |  |  |  |  |  |  |  |  |
| 0 | Ath-ATMG01290.1 |  |  |  |  |  |  |  |  |
| 0 | Ath-ATMG01300.1 |  |  |  |  |  |  |  |  |
| 0 | Ath-ATMG01310.1 |  |  |  |  |  |  |  |  |
| 0 | Ath-ATMG01330.1 |  |  |  |  |  |  |  |  |
| 0 | Ath-ATMG01350.1 |  |  |  |  |  |  |  |  |
| 0 | Ath-ATMG01360.1 |  |  |  |  |  |  |  |  |
| 0 | Ath-ATMG01370.1 |  |  |  |  |  |  |  |  |
| 0 | Ath-ATMG01400.1 |  |  |  |  |  |  |  |  |
| 0 | Ath-ATMG01410.1 |  |  |  |  |  |  |  |  |
