# Supplementary material for: Genome-wide identification, characterization and gene expression of BES1 transcription factor family in grapevine (Vitis vinifera L.)
Source: Sci Rep. 2023 Jan 5;13:240. doi: 10.1038/s41598-022-24407-y (PMC9816167; doi:10.1038/s41598-022-24407-y)
Supplement: Supplementary file 3 — Supplementary Information. [file 41598_2022_24407_MOESM3_ESM.zip › Vvi_Ath/Vitis_vinifera.PN40024.v4.dna_sm.toplevel.fa.vs.Arabidopsis_thaliana.TAIR10.dna_sm.toplevel.fa.html/Ath-Pt.html]

|  |  |  |  |  |  |  |  |  |  |  |  |  |  |  |  |  |  |
| --- | --- | --- | --- | --- | --- | --- | --- | --- | --- | --- | --- | --- | --- | --- | --- | --- | --- |
| Duplication depth | Reference chromosome | Collinear blocks | | | | | | | | | | | | | | | |
| 0 | Ath-ATCG00020.1 |  |  |  |  |  |  |  |  |
| 0 | Ath-ATCG00040.1 |  |  |  |  |  |  |  |  |
| 0 | Ath-ATCG00050.1 |  |  |  |  |  |  |  |  |
| 0 | Ath-ATCG00070.1 |  |  |  |  |  |  |  |  |
| 0 | Ath-ATCG00080.1 |  |  |  |  |  |  |  |  |
| 0 | Ath-ATCG00120.1 |  |  |  |  |  |  |  |  |
| 0 | Ath-ATCG00130.1 |  |  |  |  |  |  |  |  |
| 0 | Ath-ATCG00140.1 |  |  |  |  |  |  |  |  |
| 0 | Ath-ATCG00150.1 |  |  |  |  |  |  |  |  |
| 0 | Ath-ATCG00160.1 |  |  |  |  |  |  |  |  |
| 0 | Ath-ATCG00170.1 |  |  |  |  |  |  |  |  |
| 0 | Ath-ATCG00180.1 |  |  |  |  |  |  |  |  |
| 0 | Ath-ATCG00190.1 |  |  |  |  |  |  |  |  |
| 0 | Ath-ATCG00210.1 |  |  |  |  |  |  |  |  |
| 0 | Ath-ATCG00220.1 |  |  |  |  |  |  |  |  |
| 0 | Ath-ATCG00270.1 |  |  |  |  |  |  |  |  |
| 0 | Ath-ATCG00280.1 |  |  |  |  |  |  |  |  |
| 0 | Ath-ATCG00300.1 |  |  |  |  |  |  |  |  |
| 0 | Ath-ATCG00330.1 |  |  |  |  |  |  |  |  |
| 0 | Ath-ATCG00340.1 |  |  |  |  |  |  |  |  |
| 0 | Ath-ATCG00350.1 |  |  |  |  |  |  |  |  |
| 0 | Ath-ATCG00360.1 |  |  |  |  |  |  |  |  |
| 0 | Ath-ATCG00380.1 |  |  |  |  |  |  |  |  |
| 0 | Ath-ATCG00420.1 |  |  |  |  |  |  |  |  |
| 0 | Ath-ATCG00430.1 |  |  |  |  |  |  |  |  |
| 0 | Ath-ATCG00440.1 |  |  |  |  |  |  |  |  |
| 0 | Ath-ATCG00470.1 |  |  |  |  |  |  |  |  |
| 1 | Ath-ATCG00480.1 |  | Vvi-Vitvi01g02307\_t001 |  |  |  |  |  |  |  |
| 1 | Ath-ATCG00490.1 |  | Vvi-Vitvi00g04191\_t001 |  |  |  |  |  |  |  |
| 1 | Ath-ATCG00500.1 |  | Vvi-Vitvi00g04190\_t001 |  |  |  |  |  |  |  |
| 1 | Ath-ATCG00510.1 |  | | | |  |  |  |  |  |  |  |
| 1 | Ath-ATCG00520.1 |  | Vvi-Vitvi00g04189\_t001 |  |  |  |  |  |  |  |
| 1 | Ath-ATCG00530.1 |  | Vvi-Vitvi00g04188\_t001 |  |  |  |  |  |  |  |
| 1 | Ath-ATCG00540.1 |  | Vvi-Vitvi00g04187\_t001 |  |  |  |  |  |  |  |
| 0 | Ath-ATCG00550.1 |  |  |  |  |  |  |  |  |
| 0 | Ath-ATCG00560.1 |  |  |  |  |  |  |  |  |
| 0 | Ath-ATCG00570.1 |  |  |  |  |  |  |  |  |
| 0 | Ath-ATCG00580.1 |  |  |  |  |  |  |  |  |
| 0 | Ath-ATCG00590.1 |  |  |  |  |  |  |  |  |
| 0 | Ath-ATCG00600.1 |  |  |  |  |  |  |  |  |
| 0 | Ath-ATCG00630.1 |  |  |  |  |  |  |  |  |
| 0 | Ath-ATCG00640.1 |  |  |  |  |  |  |  |  |
| 0 | Ath-ATCG00650.1 |  |  |  |  |  |  |  |  |
| 0 | Ath-ATCG00660.1 |  |  |  |  |  |  |  |  |
| 0 | Ath-ATCG00065.1 |  |  |  |  |  |  |  |  |
| 0 | Ath-ATCG00670.1 |  |  |  |  |  |  |  |  |
| 0 | Ath-ATCG00680.1 |  |  |  |  |  |  |  |  |
| 0 | Ath-ATCG00690.1 |  |  |  |  |  |  |  |  |
| 0 | Ath-ATCG00700.1 |  |  |  |  |  |  |  |  |
| 0 | Ath-ATCG00710.1 |  |  |  |  |  |  |  |  |
| 0 | Ath-ATCG00720.1 |  |  |  |  |  |  |  |  |
| 0 | Ath-ATCG00730.1 |  |  |  |  |  |  |  |  |
| 0 | Ath-ATCG00740.1 |  |  |  |  |  |  |  |  |
| 0 | Ath-ATCG00750.1 |  |  |  |  |  |  |  |  |
| 0 | Ath-ATCG00760.1 |  |  |  |  |  |  |  |  |
| 0 | Ath-ATCG00770.1 |  |  |  |  |  |  |  |  |
| 0 | Ath-ATCG00780.1 |  |  |  |  |  |  |  |  |
| 0 | Ath-ATCG00790.1 |  |  |  |  |  |  |  |  |
| 0 | Ath-ATCG00800.1 |  |  |  |  |  |  |  |  |
| 0 | Ath-ATCG00810.1 |  |  |  |  |  |  |  |  |
| 0 | Ath-ATCG00820.1 |  |  |  |  |  |  |  |  |
| 0 | Ath-ATCG00830.1 |  |  |  |  |  |  |  |  |
| 0 | Ath-ATCG00840.1 |  |  |  |  |  |  |  |  |
| 0 | Ath-ATCG00860.1 |  |  |  |  |  |  |  |  |
| 0 | Ath-ATCG00870.1 |  |  |  |  |  |  |  |  |
| 0 | Ath-ATCG00890.1 |  |  |  |  |  |  |  |  |
| 0 | Ath-ATCG00900.1 |  |  |  |  |  |  |  |  |
| 0 | Ath-ATCG00905.1 |  |  |  |  |  |  |  |  |
| 0 | Ath-ATCG01000.1 |  |  |  |  |  |  |  |  |
| 0 | Ath-ATCG01010.1 |  |  |  |  |  |  |  |  |
| 0 | Ath-ATCG01020.1 |  |  |  |  |  |  |  |  |
| 0 | Ath-ATCG01040.1 |  |  |  |  |  |  |  |  |
| 0 | Ath-ATCG01050.1 |  |  |  |  |  |  |  |  |
| 0 | Ath-ATCG01060.1 |  |  |  |  |  |  |  |  |
| 0 | Ath-ATCG01070.1 |  |  |  |  |  |  |  |  |
| 0 | Ath-ATCG01080.1 |  |  |  |  |  |  |  |  |
| 0 | Ath-ATCG01090.1 |  |  |  |  |  |  |  |  |
| 0 | Ath-ATCG01100.1 |  |  |  |  |  |  |  |  |
| 0 | Ath-ATCG01110.1 |  |  |  |  |  |  |  |  |
| 0 | Ath-ATCG01120.1 |  |  |  |  |  |  |  |  |
| 0 | Ath-ATCG01130.1 |  |  |  |  |  |  |  |  |
| 0 | Ath-ATCG01230.1 |  |  |  |  |  |  |  |  |
| 0 | Ath-ATCG01240.1 |  |  |  |  |  |  |  |  |
| 0 | Ath-ATCG01250.1 |  |  |  |  |  |  |  |  |
| 0 | Ath-ATCG01270.1 |  |  |  |  |  |  |  |  |
| 0 | Ath-ATCG01280.1 |  |  |  |  |  |  |  |  |
| 0 | Ath-ATCG01300.1 |  |  |  |  |  |  |  |  |
| 0 | Ath-ATCG01310.1 |  |  |  |  |  |  |  |  |
