# Supplementary material for: Genome-wide identification, characterization and gene expression of BES1 transcription factor family in grapevine (Vitis vinifera L.)
Source: Sci Rep. 2023 Jan 5;13:240. doi: 10.1038/s41598-022-24407-y (PMC9816167; doi:10.1038/s41598-022-24407-y)
Supplement: Supplementary file 3 — Supplementary Information. [file 41598_2022_24407_MOESM3_ESM.zip › Vvi_Ath/Vitis_vinifera.PN40024.v4.dna_sm.toplevel.fa.vs.Arabidopsis_thaliana.TAIR10.dna_sm.toplevel.fa.html/Vvi-1.html]

|  |  |  |  |  |  |  |  |  |  |  |  |  |  |  |  |  |  |
| --- | --- | --- | --- | --- | --- | --- | --- | --- | --- | --- | --- | --- | --- | --- | --- | --- | --- |
| Duplication depth | Reference chromosome | Collinear blocks | | | | | | | | | | | | | | | |
| 0 | Vvi-Vitvi01g04000\_t001 |  |  |  |  |  |  |  |  |
| 0 | Vvi-Vitvi01g04001\_t001 |  |  |  |  |  |  |  |  |
| 0 | Vvi-Vitvi01g04002\_t001 |  |  |  |  |  |  |  |  |
| 0 | Vvi-Vitvi01g04003\_t001 |  |  |  |  |  |  |  |  |
| 0 | Vvi-Vitvi01g04004\_t001 |  |  |  |  |  |  |  |  |
| 0 | Vvi-Vitvi01g04005\_t001 |  |  |  |  |  |  |  |  |
| 2 | Vvi-Vitvi01g00002\_t001 |  | Ath-AT1G69040.2 |  | Ath-AT2G03730.1 |  |  |  |  |  |  |
| 2 | Vvi-Vitvi01g01833\_t001 |  | | | |  | | | |  |  |  |  |  |  |
| 2 | Vvi-Vitvi01g00005\_t001 |  | Ath-AT1G69060.1 |  | | | |  |  |  |  |  |  |
| 2 | Vvi-Vitvi01g01834\_t001 |  | | | |  | | | |  |  |  |  |  |  |
| 2 | Vvi-Vitvi01g04006\_t001 |  | | | |  | | | |  |  |  |  |  |  |
| 2 | Vvi-Vitvi01g00006\_t001 |  | Ath-AT1G69070.2 |  | | | |  |  |  |  |  |  |
| 2 | Vvi-Vitvi01g01835\_t001 |  | Ath-AT1G69080.1 |  | Ath-AT2G03720.2 |  |  |  |  |  |  |
| 3 | Vvi-Vitvi01g01836\_t001 |  | Ath-AT1G69085.1 |  | | | |  | Ath-AT3G02290.5 |  |  |  |  |  |
| 3 | Vvi-Vitvi01g04007\_t001 |  | | | |  | | | |  | | | |  |  |  |  |  |
| 5 | Vvi-Vitvi01g00008\_t001 |  | Ath-AT1G69120.1 |  | | | |  | | | |  | Ath-AT5G60910.1 |  | Ath-AT1G26310.1 |  |  |  |
| 5 | Vvi-Vitvi01g00011\_t001 |  | | | |  | Ath-AT2G03710.1 |  | | | |  | | | |  | | | |  |  |  |
| 5 | Vvi-Vitvi01g00012\_t001 |  | Ath-AT1G69160.1 |  | | | |  | | | |  | | | |  | | | |  |  |  |
| 5 | Vvi-Vitvi01g01837\_t003 |  | Ath-AT1G69170.1 |  | | | |  | | | |  | | | |  | | | |  |  |  |
| 5 | Vvi-Vitvi01g00013\_t001 |  | Ath-AT1G69180.1 |  | | | |  | | | |  | | | |  | | | |  |  |  |
| 5 | Vvi-Vitvi01g00014\_t001 |  | | | |  | | | |  | | | |  | | | |  | Ath-AT1G26330.2 |  |  |  |
| 5 | Vvi-Vitvi01g01838\_t001 |  | | | |  | | | |  | Ath-AT3G02230.1 |  | | | |  | | | |  |  |  |
| 5 | Vvi-Vitvi01g00015\_t001 |  | | | |  | | | |  | | | |  | | | |  | | | |  |  |  |
| 5 | Vvi-Vitvi01g00019\_t001 |  | | | |  | | | |  | | | |  | | | |  | | | |  |  |  |
| 5 | Vvi-Vitvi01g00023\_t001 |  | | | |  | | | |  | | | |  | | | |  | Ath-AT1G26340.1 |  |  |  |
| 5 | Vvi-Vitvi01g00024\_t001 |  | Ath-AT1G69190.1 |  | | | |  | | | |  | | | |  | | | |  |  |  |
| 5 | Vvi-Vitvi01g00025\_t001 |  | Ath-AT1G69200.1 |  | | | |  | | | |  | | | |  | | | |  |  |  |
| 6 | Vvi-Vitvi01g00026\_t002 |  | | | |  | | | |  | | | |  | | | |  | | | |  | Ath-AT1G13700.1 |  |  |
| 6 | Vvi-Vitvi01g00027\_t001 |  | | | |  | | | |  | | | |  | | | |  | | | |  | | | |  |  |
| 6 | Vvi-Vitvi01g04008\_t001 |  | | | |  | | | |  | | | |  | | | |  | | | |  | | | |  |  |
| 6 | Vvi-Vitvi01g00028\_t001 |  | Ath-AT1G69210.1 |  | | | |  | | | |  | | | |  | | | |  | | | |  |  |
| 6 | Vvi-Vitvi01g00029\_t002 |  | Ath-AT1G69220.1 |  | | | |  | | | |  | | | |  | | | |  | | | |  |  |
| 6 | Vvi-Vitvi01g00030\_t001 |  | | | |  | | | |  | | | |  | | | |  | | | |  | | | |  |  |
| 6 | Vvi-Vitvi01g00031\_t001 |  | | | |  | | | |  | | | |  | | | |  | | | |  | Ath-AT1G13710.1 |  |  |
| 6 | Vvi-Vitvi01g00033\_t002 |  | Ath-AT1G69230.1 |  | Ath-AT2G03680.1 |  | Ath-AT3G02180.1 |  | | | |  | Ath-AT1G26355.1 |  | | | |  |  |
| 6 | Vvi-Vitvi01g00034\_t001 |  | Ath-AT1G69240.1 |  | | | |  | | | |  | | | |  | Ath-AT1G26360.1 |  | | | |  |  |
| 6 | Vvi-Vitvi01g00035\_t001 |  | | | |  | Ath-AT2G03667.1 |  | | | |  | | | |  | | | |  | | | |  |  |
| 6 | Vvi-Vitvi01g00036\_t002 |  | | | |  | Ath-AT2G03640.4 |  | | | |  | Ath-AT5G60980.2 |  | | | |  | Ath-AT1G13730.1 |  |  |
| 6 | Vvi-Vitvi01g00037\_t001 |  | Ath-AT1G69260.1 |  | | | |  | Ath-AT3G02140.1 |  | | | |  | | | |  | Ath-AT1G13740.1 |  |  |
| 6 | Vvi-Vitvi01g00038\_t001 |  | Ath-AT1G69270.1 |  | | | |  | Ath-AT3G02130.1 |  | | | |  | | | |  | | | |  |  |
| 6 | Vvi-Vitvi01g01840\_t001 |  | | | |  | Ath-AT2G03630.1 |  | | | |  | | | |  | | | |  | | | |  |  |
| 6 | Vvi-Vitvi01g00039\_t004 |  | | | |  | Ath-AT2G03620.1 |  | | | |  | | | |  | | | |  | | | |  |  |
| 6 | Vvi-Vitvi01g00041\_t001 |  | | | |  | | | |  | | | |  | Ath-AT5G61040.1 |  | | | |  | | | |  |  |
| 6 | Vvi-Vitvi01g04009\_t001 |  | | | |  | | | |  | | | |  | | | |  | | | |  | | | |  |  |
| 6 | Vvi-Vitvi01g00042\_t001 |  | | | |  | | | |  | | | |  | | | |  | | | |  | Ath-AT1G13750.1 |  |  |
| 6 | Vvi-Vitvi01g00043\_t001 |  | Ath-AT1G69290.1 |  | | | |  | | | |  | | | |  | | | |  | | | |  |  |
| 6 | Vvi-Vitvi01g00045\_t001 |  | | | |  | | | |  | | | |  | | | |  | | | |  | Ath-AT1G13770.1 |  |  |
| 6 | Vvi-Vitvi01g04010\_t001 |  | | | |  | | | |  | | | |  | | | |  | | | |  | | | |  |  |
| 6 | Vvi-Vitvi01g00046\_t001 |  | | | |  | | | |  | | | |  | | | |  | | | |  | | | |  |  |
| 6 | Vvi-Vitvi01g00047\_t001 |  | | | |  | | | |  | | | |  | | | |  | | | |  | | | |  |  |
| 6 | Vvi-Vitvi01g00048\_t001 |  | | | |  | Ath-AT2G03520.1 |  | | | |  | | | |  | Ath-AT1G26440.3 |  | | | |  |  |
| 6 | Vvi-Vitvi01g00049\_t001 |  | | | |  | Ath-AT2G03510.1 |  | | | |  | | | |  | | | |  | | | |  |  |
| 6 | Vvi-Vitvi01g00050\_t001 |  | | | |  | | | |  | | | |  | | | |  | | | |  | Ath-AT1G13820.1 |  |  |
| 6 | Vvi-Vitvi01g00051\_t001 |  | | | |  | Ath-AT2G03505.1 |  | | | |  | Ath-AT5G61130.1 |  | | | |  | | | |  |  |
| 6 | Vvi-Vitvi01g01841\_t001.1.60378269 |  | | | |  | | | |  | | | |  | | | |  | Ath-AT1G26460.1 |  | | | |  |  |
| 6 | Vvi-Vitvi01g00052\_t001 |  | | | |  | | | |  | | | |  | | | |  | | | |  | | | |  |  |
| 6 | Vvi-Vitvi01g00053\_t001 |  | | | |  | | | |  | | | |  | | | |  | | | |  | | | |  |  |
| 6 | Vvi-Vitvi01g00054\_t002 |  | | | |  | | | |  | | | |  | | | |  | Ath-AT1G26470.1 |  | | | |  |  |
| 6 | Vvi-Vitvi01g00055\_t001 |  | | | |  | | | |  | | | |  | | | |  | Ath-AT1G26480.1 |  | | | |  |  |
| 6 | Vvi-Vitvi01g01842\_t001 |  | | | |  | | | |  | | | |  | | | |  | | | |  | | | |  |  |
| 6 | Vvi-Vitvi01g00056\_t001 |  | | | |  | | | |  | | | |  | | | |  | | | |  | Ath-AT1G13910.1 |  |  |
| 6 | Vvi-Vitvi01g00058\_t001 |  | | | |  | | | |  | | | |  | | | |  | | | |  | | | |  |  |
| 6 | Vvi-Vitvi01g01843\_t001 |  | | | |  | | | |  | | | |  | | | |  | | | |  | | | |  |  |
| 6 | Vvi-Vitvi01g00059\_t002 |  | | | |  | | | |  | | | |  | Ath-AT5G61210.1 |  | | | |  | | | |  |  |
| 6 | Vvi-Vitvi01g00060\_t001 |  | | | |  | | | |  | Ath-AT3G02080.1 |  | | | |  | | | |  | | | |  |  |
| 5 | Vvi-Vitvi01g00061\_t001 |  | | | |  | | | |  |  |  | | | |  | Ath-AT1G26570.1 |  | | | |  |  |
| 5 | Vvi-Vitvi01g00062\_t001 |  | | | |  | | | |  |  |  | | | |  | Ath-AT1G26580.1 |  | | | |  |  |
| 5 | Vvi-Vitvi01g04011\_t001 |  | | | |  | | | |  |  |  | | | |  | | | |  | | | |  |  |
| 5 | Vvi-Vitvi01g00063\_t001 |  | | | |  | | | |  |  |  | | | |  | Ath-AT1G26590.2 |  | | | |  |  |
| 5 | Vvi-Vitvi01g01844\_t002 |  | Ath-AT1G69310.1 |  | | | |  |  |  | | | |  | | | |  | | | |  |  |
| 5 | Vvi-Vitvi01g00064\_t001 |  | | | |  | | | |  |  |  | | | |  | | | |  | | | |  |  |
| 5 | Vvi-Vitvi01g00065\_t001 |  | | | |  | | | |  |  |  | | | |  | | | |  | | | |  |  |
| 5 | Vvi-Vitvi01g00066\_t001 |  | | | |  | | | |  |  |  | | | |  | | | |  | | | |  |  |
| 5 | Vvi-Vitvi01g00067\_t001 |  | | | |  | | | |  |  |  | | | |  | | | |  | | | |  |  |
| 5 | Vvi-Vitvi01g04012\_t001 |  | | | |  | | | |  |  |  | | | |  | | | |  | | | |  |  |
| 5 | Vvi-Vitvi01g04013\_t001 |  | | | |  | | | |  |  |  | | | |  | | | |  | | | |  |  |
| 5 | Vvi-Vitvi01g00070\_t001 |  | | | |  | Ath-AT2G03480.1 |  |  |  | | | |  | | | |  | | | |  |  |
| 5 | Vvi-Vitvi01g04014\_t001 |  | | | |  | | | |  |  |  | | | |  | | | |  | | | |  |  |
| 5 | Vvi-Vitvi01g00071\_t001 |  | | | |  | | | |  |  |  | | | |  | | | |  | | | |  |  |
| 5 | Vvi-Vitvi01g00072\_t001 |  | | | |  | | | |  |  |  | | | |  | | | |  | | | |  |  |
| 5 | Vvi-Vitvi01g00073\_t001 |  | | | |  | | | |  |  |  | Ath-AT5G61250.1 |  | | | |  | | | |  |  |
| 5 | Vvi-Vitvi01g01845\_t001 |  | Ath-AT1G69325.1 |  | | | |  |  |  | Ath-AT5G61280.1 |  | | | |  | Ath-AT1G13920.4 |  |  |
| 4 | Vvi-Vitvi01g01846\_t001 |  | | | |  | Ath-AT2G03440.1 |  |  |  |  |  | | | |  | Ath-AT1G13930.1 |  |  |
| 4 | Vvi-Vitvi01g00074\_t001 |  | | | |  | Ath-AT2G03430.1 |  |  |  |  |  | | | |  | | | |  |  |
| 4 | Vvi-Vitvi01g00075\_t001 |  | | | |  | Ath-AT2G03420.1 |  |  |  |  |  | | | |  | | | |  |  |
| 4 | Vvi-Vitvi01g00076\_t002 |  | Ath-AT1G69340.1 |  | | | |  |  |  |  |  | | | |  | | | |  |  |
| 4 | Vvi-Vitvi01g00077\_t001 |  | | | |  | Ath-AT2G03390.4 |  |  |  |  |  | | | |  | | | |  |  |
| 4 | Vvi-Vitvi01g00078\_t001 |  | Ath-AT1G69350.1 |  | | | |  |  |  |  |  | | | |  | | | |  |  |
| 4 | Vvi-Vitvi01g00079\_t001 |  | | | |  | Ath-AT2G03380.1 |  |  |  |  |  | | | |  | | | |  |  |
| 3 | Vvi-Vitvi01g00080\_t001 |  | Ath-AT1G69360.1 |  |  |  |  |  |  |  | Ath-AT1G26620.1 |  | Ath-AT1G13940.1 |  |  |
| 0 | Vvi-Vitvi01g01847\_t001 |  |  |  |  |  |  |  |  |
| 0 | Vvi-Vitvi01g00081\_t001 |  |  |  |  |  |  |  |  |
| 0 | Vvi-Vitvi01g00082\_t001 |  |  |  |  |  |  |  |  |
| 0 | Vvi-Vitvi01g01848\_t001 |  |  |  |  |  |  |  |  |
| 0 | Vvi-Vitvi01g01849\_t001 |  |  |  |  |  |  |  |  |
| 0 | Vvi-Vitvi01g04015\_t001 |  |  |  |  |  |  |  |  |
| 0 | Vvi-Vitvi01g01850\_t001 |  |  |  |  |  |  |  |  |
| 0 | Vvi-Vitvi01g01851\_t001 |  |  |  |  |  |  |  |  |
| 0 | Vvi-Vitvi01g04016\_t001 |  |  |  |  |  |  |  |  |
| 0 | Vvi-Vitvi01g04017\_t001 |  |  |  |  |  |  |  |  |
| 0 | Vvi-Vitvi01g01852\_t001 |  |  |  |  |  |  |  |  |
| 0 | Vvi-Vitvi01g01853\_t001 |  |  |  |  |  |  |  |  |
| 0 | Vvi-Vitvi01g04018\_t001 |  |  |  |  |  |  |  |  |
| 0 | Vvi-Vitvi01g00083\_t001 |  |  |  |  |  |  |  |  |
| 0 | Vvi-Vitvi01g04019\_t001 |  |  |  |  |  |  |  |  |
| 0 | Vvi-Vitvi01g04020\_t001 |  |  |  |  |  |  |  |  |
| 0 | Vvi-Vitvi01g04021\_t001 |  |  |  |  |  |  |  |  |
| 0 | Vvi-Vitvi01g04022\_t001 |  |  |  |  |  |  |  |  |
| 0 | Vvi-Vitvi01g01854\_t001 |  |  |  |  |  |  |  |  |
| 2 | Vvi-Vitvi01g01855\_t001 |  | Ath-AT2G34940.1 |  | Ath-AT1G30900.1 |  |  |  |  |  |  |
| 2 | Vvi-Vitvi01g00085\_t001 |  | | | |  | | | |  |  |  |  |  |  |
| 2 | Vvi-Vitvi01g01856\_t001 |  | | | |  | | | |  |  |  |  |  |  |
| 3 | Vvi-Vitvi01g00086\_t001 |  | | | |  | | | |  | Ath-AT4G20130.1 |  |  |  |  |  |
| 4 | Vvi-Vitvi01g01857\_t001 |  | | | |  | | | |  | | | |  | Ath-AT5G44710.1 |  |  |  |  |
| 4 | Vvi-Vitvi01g01858\_t001 |  | | | |  | | | |  | | | |  | | | |  |  |  |  |
| 4 | Vvi-Vitvi01g00087\_t002 |  | | | |  | Ath-AT1G30910.1 |  | | | |  | Ath-AT5G44720.1 |  |  |  |  |
| 4 | Vvi-Vitvi01g00088\_t001 |  | | | |  | | | |  | | | |  | | | |  |  |  |  |
| 4 | Vvi-Vitvi01g00089\_t001 |  | | | |  | | | |  | Ath-AT4G20090.1 |  | | | |  |  |  |  |
| 4 | Vvi-Vitvi01g01859\_t001 |  | Ath-AT2G34960.1 |  | | | |  | | | |  | | | |  |  |  |  |
| 4 | Vvi-Vitvi01g00090\_t001 |  | | | |  | | | |  | | | |  | | | |  |  |  |  |
| 4 | Vvi-Vitvi01g00091\_t001 |  | | | |  | | | |  | | | |  | Ath-AT5G44730.1 |  |  |  |  |
| 4 | Vvi-Vitvi01g00092\_t001 |  | | | |  | | | |  | | | |  | Ath-AT5G44740.2 |  |  |  |  |
| 4 | Vvi-Vitvi01g00094\_t001 |  | | | |  | | | |  | | | |  | | | |  |  |  |  |
| 4 | Vvi-Vitvi01g00095\_t001 |  | | | |  | | | |  | | | |  | | | |  |  |  |  |
| 4 | Vvi-Vitvi01g00096\_t001 |  | | | |  | Ath-AT1G30950.1 |  | | | |  | | | |  |  |  |  |
| 4 | Vvi-Vitvi01g00097\_t001 |  | | | |  | Ath-AT1G30960.1 |  | | | |  | | | |  |  |  |  |
| 4 | Vvi-Vitvi01g00098\_t002 |  | | | |  | Ath-AT1G30970.3 |  | | | |  | | | |  |  |  |  |
| 4 | Vvi-Vitvi01g04023\_t001 |  | | | |  | | | |  | | | |  | | | |  |  |  |  |
| 4 | Vvi-Vitvi01g04024\_t001 |  | | | |  | | | |  | | | |  | | | |  |  |  |  |
| 4 | Vvi-Vitvi01g00099\_t001 |  | Ath-AT2G34980.1 |  | | | |  | | | |  | | | |  |  |  |  |
| 4 | Vvi-Vitvi01g04025\_t001 |  | | | |  | | | |  | | | |  | | | |  |  |  |  |
| 4 | Vvi-Vitvi01g00101\_t001 |  | | | |  | | | |  | | | |  | | | |  |  |  |  |
| 4 | Vvi-Vitvi01g00102\_t001 |  | | | |  | | | |  | | | |  | | | |  |  |  |  |
| 4 | Vvi-Vitvi01g00103\_t001 |  | | | |  | | | |  | Ath-AT4G20070.1 |  | | | |  |  |  |  |
| 4 | Vvi-Vitvi01g00104\_t001 |  | | | |  | | | |  | Ath-AT4G20040.1 |  | | | |  |  |  |  |
| 4 | Vvi-Vitvi01g04026\_t001 |  | | | |  | | | |  | | | |  | | | |  |  |  |  |
| 4 | Vvi-Vitvi01g00105\_t001 |  | | | |  | | | |  | | | |  | | | |  |  |  |  |
| 4 | Vvi-Vitvi01g01862\_t001 |  | | | |  | | | |  | | | |  | | | |  |  |  |  |
| 4 | Vvi-Vitvi01g00106\_t001 |  | | | |  | | | |  | | | |  | | | |  |  |  |  |
| 4 | Vvi-Vitvi01g04027\_t001 |  | | | |  | | | |  | Ath-AT4G20030.1 |  | | | |  |  |  |  |
| 4 | Vvi-Vitvi01g04028\_t001 |  | | | |  | | | |  | | | |  | | | |  |  |  |  |
| 4 | Vvi-Vitvi01g01865\_t001 |  | | | |  | | | |  | | | |  | | | |  |  |  |  |
| 4 | Vvi-Vitvi01g00108\_t001 |  | | | |  | | | |  | | | |  | Ath-AT5G44790.1 |  |  |  |  |
| 4 | Vvi-Vitvi01g01866\_t001 |  | Ath-AT2G35010.1 |  | Ath-AT1G31020.1 |  | | | |  | | | |  |  |  |  |
| 4 | Vvi-Vitvi01g00110\_t001 |  | | | |  | | | |  | | | |  | | | |  |  |  |  |
| 4 | Vvi-Vitvi01g00111\_t001 |  | | | |  | Ath-AT1G31040.1 |  | | | |  | | | |  |  |  |  |
| 4 | Vvi-Vitvi01g00112\_t001 |  | Ath-AT2G35020.1 |  | Ath-AT1G31070.2 |  | | | |  | | | |  |  |  |  |
| 4 | Vvi-Vitvi01g00113\_t001 |  | Ath-AT2G35035.1 |  | | | |  | | | |  | | | |  |  |  |  |
| 4 | Vvi-Vitvi01g00114\_t001 |  | | | |  | | | |  | | | |  | | | |  |  |  |  |
| 4 | Vvi-Vitvi01g04029\_t001 |  | | | |  | | | |  | | | |  | | | |  |  |  |  |
| 4 | Vvi-Vitvi01g04030\_t001 |  | | | |  | | | |  | | | |  | | | |  |  |  |  |
| 4 | Vvi-Vitvi01g00115\_t001 |  | Ath-AT2G35040.1 |  | | | |  | | | |  | | | |  |  |  |  |
| 4 | Vvi-Vitvi01g00116\_t001 |  | | | |  | | | |  | Ath-AT4G19990.2 |  | | | |  |  |  |  |
| 4 | Vvi-Vitvi01g01870\_t001 |  | | | |  | | | |  | Ath-AT4G19980.1 |  | | | |  |  |  |  |
| 4 | Vvi-Vitvi01g00119\_t001 |  | | | |  | | | |  | | | |  | Ath-AT5G44800.1 |  |  |  |  |
| 4 | Vvi-Vitvi01g04031\_t001 |  | | | |  | | | |  | | | |  | | | |  |  |  |  |
| 4 | Vvi-Vitvi01g00120\_t001 |  | Ath-AT2G35050.1 |  | | | |  | | | |  | | | |  |  |  |  |
| 4 | Vvi-Vitvi01g00121\_t001 |  | Ath-AT2G35060.2 |  | Ath-AT1G31120.1 |  | Ath-AT4G19960.3 |  | | | |  |  |  |  |
| 4 | Vvi-Vitvi01g00122\_t001 |  | | | |  | | | |  | | | |  | | | |  |  |  |  |
| 4 | Vvi-Vitvi01g00124\_t001 |  | | | |  | Ath-AT1G31130.1 |  | Ath-AT4G19950.1 |  | Ath-AT5G44860.2 |  |  |  |  |
| 4 | Vvi-Vitvi01g00126\_t001 |  | | | |  | | | |  | | | |  | | | |  |  |  |  |
| 4 | Vvi-Vitvi01g00128\_t001 |  | | | |  | | | |  | | | |  | | | |  |  |  |  |
| 4 | Vvi-Vitvi01g01871\_t001 |  | | | |  | | | |  | | | |  | | | |  |  |  |  |
| 4 | Vvi-Vitvi01g01872\_t001 |  | | | |  | | | |  | | | |  | | | |  |  |  |  |
| 4 | Vvi-Vitvi01g04032\_t001 |  | | | |  | | | |  | | | |  | | | |  |  |  |  |
| 4 | Vvi-Vitvi01g01873\_t001 |  | | | |  | | | |  | | | |  | | | |  |  |  |  |
| 4 | Vvi-Vitvi01g00129\_t001 |  | | | |  | | | |  | | | |  | | | |  |  |  |  |
| 4 | Vvi-Vitvi01g00130\_t001 |  | | | |  | | | |  | | | |  | | | |  |  |  |  |
| 4 | Vvi-Vitvi01g00131\_t001 |  | Ath-AT2G35100.1 |  | | | |  | | | |  | Ath-AT5G44930.1 |  |  |  |  |
| 4 | Vvi-Vitvi01g00132\_t001 |  | | | |  | Ath-AT1G31160.1 |  | | | |  | | | |  |  |  |  |
| 4 | Vvi-Vitvi01g00133\_t001 |  | | | |  | Ath-AT1G31170.4 |  | | | |  | | | |  |  |  |  |
| 4 | Vvi-Vitvi01g00134\_t001 |  | Ath-AT2G35110.1 |  | | | |  | | | |  | | | |  |  |  |  |
| 4 | Vvi-Vitvi01g00135\_t002 |  | | | |  | | | |  | | | |  | | | |  |  |  |  |
| 5 | Vvi-Vitvi01g01874\_t001 |  | | | |  | | | |  | Ath-AT4G19830.1 |  | | | |  | Ath-AT4G19830.1 |  |  |  |
| 4 | Vvi-Vitvi01g00136\_t001 |  | | | |  | | | |  |  |  | | | |  | | | |  |  |  |
| 4 | Vvi-Vitvi01g01875\_t001 |  | | | |  | Ath-AT1G31240.1 |  |  |  | | | |  | | | |  |  |  |
| 3 | Vvi-Vitvi01g00137\_t001 |  | | | |  |  |  |  |  | | | |  | | | |  |  |  |
| 3 | Vvi-Vitvi01g00138\_t002 |  | | | |  |  |  |  |  | | | |  | | | |  |  |  |
| 3 | Vvi-Vitvi01g00139\_t001 |  | | | |  |  |  |  |  | | | |  | | | |  |  |  |
| 3 | Vvi-Vitvi01g01876\_t001 |  | | | |  |  |  |  |  | | | |  | | | |  |  |  |
| 3 | Vvi-Vitvi01g01877\_t001 |  | | | |  |  |  |  |  | | | |  | | | |  |  |  |
| 3 | Vvi-Vitvi01g01878\_t001 |  | | | |  |  |  |  |  | | | |  | | | |  |  |  |
| 3 | Vvi-Vitvi01g01880\_t001 |  | | | |  |  |  |  |  | | | |  | | | |  |  |  |
| 3 | Vvi-Vitvi01g04033\_t001 |  | | | |  |  |  |  |  | | | |  | | | |  |  |  |
| 3 | Vvi-Vitvi01g01881\_t001 |  | | | |  |  |  |  |  | | | |  | | | |  |  |  |
| 3 | Vvi-Vitvi01g00140\_t001 |  | | | |  |  |  |  |  | | | |  | | | |  |  |  |
| 3 | Vvi-Vitvi01g01883\_t001 |  | | | |  |  |  |  |  | | | |  | | | |  |  |  |
| 3 | Vvi-Vitvi01g01884\_t001 |  | | | |  |  |  |  |  | | | |  | Ath-AT4G19840.1 |  |  |  |
| 3 | Vvi-Vitvi01g04034\_t001 |  | | | |  |  |  |  |  | | | |  | | | |  |  |  |
| 3 | Vvi-Vitvi01g00142\_t001 |  | | | |  |  |  |  |  | | | |  | | | |  |  |  |
| 3 | Vvi-Vitvi01g00143\_t001 |  | | | |  |  |  |  |  | | | |  | Ath-AT4G19860.1 |  |  |  |
| 4 | Vvi-Vitvi01g01885\_t001 |  | | | |  | Ath-AT5G23260.4 |  |  |  | | | |  | | | |  |  |  |
| 4 | Vvi-Vitvi01g04035\_t001 |  | | | |  | | | |  |  |  | | | |  | | | |  |  |  |
| 4 | Vvi-Vitvi01g00145\_t001 |  | | | |  | | | |  |  |  | Ath-AT5G45040.1 |  | | | |  |  |  |
| 3 | Vvi-Vitvi01g04036\_t001 |  | | | |  | | | |  |  |  |  |  | | | |  |  |  |
| 3 | Vvi-Vitvi01g04037\_t001 |  | | | |  | | | |  |  |  |  |  | | | |  |  |  |
| 3 | Vvi-Vitvi01g00146\_t001 |  | Ath-AT2G35155.1 |  | | | |  |  |  |  |  | | | |  |  |  |
| 3 | Vvi-Vitvi01g00147\_t001 |  | | | |  | | | |  |  |  |  |  | | | |  |  |  |
| 3 | Vvi-Vitvi01g00148\_t001 |  | | | |  | | | |  |  |  |  |  | Ath-AT4G19880.2 |  |  |  |
| 3 | Vvi-Vitvi01g00149\_t001 |  | | | |  | | | |  |  |  |  |  | | | |  |  |  |
| 3 | Vvi-Vitvi01g00150\_t001 |  | | | |  | | | |  |  |  |  |  | Ath-AT4G19890.1 |  |  |  |
| 3 | Vvi-Vitvi01g01887\_t001 |  | | | |  | | | |  |  |  |  |  | | | |  |  |  |
| 3 | Vvi-Vitvi01g00151\_t001 |  | | | |  | | | |  |  |  |  |  | Ath-AT4G19900.1 |  |  |  |
| 2 | Vvi-Vitvi01g00152\_t001 |  | | | |  | | | |  |  |  |  |  |  |
| 2 | Vvi-Vitvi01g04038\_t001 |  | | | |  | | | |  |  |  |  |  |  |
| 2 | Vvi-Vitvi01g00155\_t001 |  | | | |  | | | |  |  |  |  |  |  |
| 2 | Vvi-Vitvi01g00156\_t001 |  | | | |  | | | |  |  |  |  |  |  |
| 2 | Vvi-Vitvi01g01888\_t001 |  | | | |  | | | |  |  |  |  |  |  |
| 2 | Vvi-Vitvi01g01889\_t001 |  | | | |  | | | |  |  |  |  |  |  |
| 2 | Vvi-Vitvi01g01890\_t001 |  | | | |  | | | |  |  |  |  |  |  |
| 2 | Vvi-Vitvi01g00157\_t001 |  | Ath-AT2G35370.1 |  | | | |  |  |  |  |  |  |
| 1 | Vvi-Vitvi01g00160\_t001 |  |  |  | Ath-AT5G23210.1 |  |  |  |  |  |  |
| 1 | Vvi-Vitvi01g00159\_t001 |  |  |  | | | |  |  |  |  |  |  |
| 1 | Vvi-Vitvi01g00161\_t001 |  |  |  | | | |  |  |  |  |  |  |
| 1 | Vvi-Vitvi01g04039\_t001 |  |  |  | | | |  |  |  |  |  |  |
| 1 | Vvi-Vitvi01g00162\_t001 |  |  |  | Ath-AT5G23190.1 |  |  |  |  |  |  |
| 1 | Vvi-Vitvi01g01893\_t001.2.6037826a |  |  |  | | | |  |  |  |  |  |  |
| 1 | Vvi-Vitvi01g00163\_t001 |  |  |  | Ath-AT5G23160.1 |  |  |  |  |  |  |
| 1 | Vvi-Vitvi01g04040\_t001 |  |  |  | | | |  |  |  |  |  |  |
| 1 | Vvi-Vitvi01g04041\_t001 |  |  |  | | | |  |  |  |  |  |  |
| 1 | Vvi-Vitvi01g00166\_t002 |  |  |  | Ath-AT5G23140.1 |  |  |  |  |  |  |
| 1 | Vvi-Vitvi01g00167\_t001 |  |  |  | | | |  |  |  |  |  |  |
| 1 | Vvi-Vitvi01g00168\_t002 |  |  |  | | | |  |  |  |  |  |  |
| 1 | Vvi-Vitvi01g00169\_t001 |  |  |  | Ath-AT5G23130.1 |  |  |  |  |  |  |
| 1 | Vvi-Vitvi01g00170\_t001 |  |  |  | Ath-AT5G23120.2 |  |  |  |  |  |  |
| 1 | Vvi-Vitvi01g00171\_t001 |  |  |  | Ath-AT5G23110.1 |  |  |  |  |  |  |
| 0 | Vvi-Vitvi01g00172\_t001 |  |  |  |  |  |  |  |  |
| 0 | Vvi-Vitvi01g00173\_t001 |  |  |  |  |  |  |  |  |
| 0 | Vvi-Vitvi01g00174\_t001 |  |  |  |  |  |  |  |  |
| 0 | Vvi-Vitvi01g04042\_t001 |  |  |  |  |  |  |  |  |
| 0 | Vvi-Vitvi01g00175\_t001 |  |  |  |  |  |  |  |  |
| 0 | Vvi-Vitvi01g00176\_t002 |  |  |  |  |  |  |  |  |
| 0 | Vvi-Vitvi01g00178\_t001 |  |  |  |  |  |  |  |  |
| 0 | Vvi-Vitvi01g00179\_t001 |  |  |  |  |  |  |  |  |
| 0 | Vvi-Vitvi01g00180\_t001 |  |  |  |  |  |  |  |  |
| 0 | Vvi-Vitvi01g04043\_t001 |  |  |  |  |  |  |  |  |
| 0 | Vvi-Vitvi01g00181\_t001 |  |  |  |  |  |  |  |  |
| 0 | Vvi-Vitvi01g00182\_t001 |  |  |  |  |  |  |  |  |
| 0 | Vvi-Vitvi01g00183\_t001 |  |  |  |  |  |  |  |  |
| 0 | Vvi-Vitvi01g04044\_t001 |  |  |  |  |  |  |  |  |
| 0 | Vvi-Vitvi01g00185\_t001 |  |  |  |  |  |  |  |  |
| 0 | Vvi-Vitvi01g01895\_t001 |  |  |  |  |  |  |  |  |
| 1 | Vvi-Vitvi01g00186\_t001 |  | Ath-AT1G26180.1 |  |  |  |  |  |  |  |
| 2 | Vvi-Vitvi01g00187\_t001 |  | | | |  | Ath-AT3G25590.1 |  |  |  |  |  |  |
| 2 | Vvi-Vitvi01g00188\_t001 |  | | | |  | | | |  |  |  |  |  |  |
| 2 | Vvi-Vitvi01g00189\_t001 |  | | | |  | | | |  |  |  |  |  |  |
| 2 | Vvi-Vitvi01g00190\_t001 |  | Ath-AT1G26170.1 |  | | | |  |  |  |  |  |  |
| 2 | Vvi-Vitvi01g00191\_t001 |  | | | |  | | | |  |  |  |  |  |  |
| 2 | Vvi-Vitvi01g04045\_t001 |  | | | |  | | | |  |  |  |  |  |  |
| 2 | Vvi-Vitvi01g00192\_t001 |  | | | |  | | | |  |  |  |  |  |  |
| 2 | Vvi-Vitvi01g01897\_t001 |  | | | |  | | | |  |  |  |  |  |  |
| 2 | Vvi-Vitvi01g01898\_t001 |  | | | |  | | | |  |  |  |  |  |  |
| 2 | Vvi-Vitvi01g01901\_t001 |  | | | |  | | | |  |  |  |  |  |  |
| 2 | Vvi-Vitvi01g04046\_t001 |  | | | |  | | | |  |  |  |  |  |  |
| 2 | Vvi-Vitvi01g01902\_t001 |  | | | |  | | | |  |  |  |  |  |  |
| 2 | Vvi-Vitvi01g00193\_t001 |  | | | |  | | | |  |  |  |  |  |  |
| 2 | Vvi-Vitvi01g01903\_t001 |  | Ath-AT1G26160.1 |  | | | |  |  |  |  |  |  |
| 2 | Vvi-Vitvi01g04047\_t001 |  | | | |  | | | |  |  |  |  |  |  |
| 2 | Vvi-Vitvi01g00196\_t001 |  | Ath-AT1G26150.1 |  | | | |  |  |  |  |  |  |
| 3 | Vvi-Vitvi01g00197\_t001 |  | Ath-AT1G26140.1 |  | Ath-AT3G25597.1 |  | Ath-AT1G68700.1 |  |  |  |  |  |
| 3 | Vvi-Vitvi01g00198\_t001 |  | | | |  | Ath-AT3G25600.1 |  | | | |  |  |  |  |  |
| 3 | Vvi-Vitvi01g00199\_t001 |  | Ath-AT1G26130.3 |  | Ath-AT3G25610.1 |  | Ath-AT1G68710.3 |  |  |  |  |  |
| 3 | Vvi-Vitvi01g00201\_t001 |  | | | |  | | | |  | Ath-AT1G68720.1 |  |  |  |  |  |
| 3 | Vvi-Vitvi01g01907\_t001 |  | | | |  | | | |  | | | |  |  |  |  |  |
| 3 | Vvi-Vitvi01g00202\_t001 |  | | | |  | | | |  | Ath-AT1G68730.1 |  |  |  |  |  |
| 3 | Vvi-Vitvi01g00203\_t001 |  | | | |  | | | |  | Ath-AT1G68740.1 |  |  |  |  |  |
| 3 | Vvi-Vitvi01g00204\_t001 |  | Ath-AT1G26120.1 |  | | | |  | | | |  |  |  |  |  |
| 2 | Vvi-Vitvi01g01908\_t001 |  |  |  | | | |  | | | |  |  |  |  |  |
| 2 | Vvi-Vitvi01g00205\_t001 |  |  |  | | | |  | | | |  |  |  |  |  |
| 2 | Vvi-Vitvi01g00206\_t001 |  |  |  | | | |  | | | |  |  |  |  |  |
| 2 | Vvi-Vitvi01g04048\_t001 |  |  |  | | | |  | | | |  |  |  |  |  |
| 2 | Vvi-Vitvi01g04049\_t001 |  |  |  | | | |  | | | |  |  |  |  |  |
| 2 | Vvi-Vitvi01g04050\_t001 |  |  |  | | | |  | | | |  |  |  |  |  |
| 2 | Vvi-Vitvi01g01912\_t001 |  |  |  | | | |  | | | |  |  |  |  |  |
| 2 | Vvi-Vitvi01g04051\_t001 |  |  |  | | | |  | | | |  |  |  |  |  |
| 2 | Vvi-Vitvi01g01915\_t001 |  |  |  | | | |  | | | |  |  |  |  |  |
| 2 | Vvi-Vitvi01g01916\_t001 |  |  |  | | | |  | | | |  |  |  |  |  |
| 2 | Vvi-Vitvi01g01917\_t001 |  |  |  | | | |  | | | |  |  |  |  |  |
| 2 | Vvi-Vitvi01g01918\_t001 |  |  |  | | | |  | | | |  |  |  |  |  |
| 2 | Vvi-Vitvi01g04052\_t001 |  |  |  | | | |  | | | |  |  |  |  |  |
| 2 | Vvi-Vitvi01g01919\_t001 |  |  |  | | | |  | | | |  |  |  |  |  |
| 2 | Vvi-Vitvi01g04053\_t001 |  |  |  | | | |  | | | |  |  |  |  |  |
| 2 | Vvi-Vitvi01g04054\_t001 |  |  |  | | | |  | | | |  |  |  |  |  |
| 2 | Vvi-Vitvi01g04055\_t001 |  |  |  | | | |  | | | |  |  |  |  |  |
| 2 | Vvi-Vitvi01g04056\_t001 |  |  |  | | | |  | | | |  |  |  |  |  |
| 2 | Vvi-Vitvi01g01922\_t001 |  |  |  | | | |  | | | |  |  |  |  |  |
| 2 | Vvi-Vitvi01g01923\_t001 |  |  |  | | | |  | | | |  |  |  |  |  |
| 2 | Vvi-Vitvi01g00211\_t001 |  |  |  | Ath-AT3G25620.2 |  | | | |  |  |  |  |  |
| 2 | Vvi-Vitvi01g04057\_t001 |  |  |  | | | |  | | | |  |  |  |  |  |
| 2 | Vvi-Vitvi01g00214\_t001 |  |  |  | | | |  | Ath-AT1G68750.1 |  |  |  |  |  |
| 2 | Vvi-Vitvi01g04058\_t001 |  |  |  | | | |  | Ath-AT1G68760.1 |  |  |  |  |  |
| 2 | Vvi-Vitvi01g00217\_t001 |  |  |  | Ath-AT3G25640.2 |  | | | |  |  |  |  |  |
| 2 | Vvi-Vitvi01g01924\_t001 |  |  |  | | | |  | | | |  |  |  |  |  |
| 2 | Vvi-Vitvi01g04059\_t001 |  |  |  | | | |  | | | |  |  |  |  |  |
| 3 | Vvi-Vitvi01g00218\_t001 |  | Ath-AT1G13230.1 |  | Ath-AT3G25670.1 |  | Ath-AT1G68780.1 |  |  |  |  |  |
| 3 | Vvi-Vitvi01g00219\_t001 |  | | | |  | | | |  | Ath-AT1G68790.1 |  |  |  |  |  |
| 4 | Vvi-Vitvi01g00220\_t002 |  | | | |  | | | |  | | | |  | Ath-AT1G26110.1 |  |  |  |  |
| 4 | Vvi-Vitvi01g00221\_t001 |  | | | |  | | | |  | | | |  | | | |  |  |  |  |
| 4 | Vvi-Vitvi01g00222\_t001 |  | | | |  | | | |  | | | |  | Ath-AT1G26100.1 |  |  |  |  |
| 4 | Vvi-Vitvi01g00223\_t001 |  | | | |  | Ath-AT3G25680.1 |  | | | |  | | | |  |  |  |  |
| 4 | Vvi-Vitvi01g00224\_t001 |  | | | |  | Ath-AT3G25690.5 |  | | | |  | | | |  |  |  |  |
| 4 | Vvi-Vitvi01g00225\_t001 |  | | | |  | | | |  | | | |  | Ath-AT1G26090.1 |  |  |  |  |
| 4 | Vvi-Vitvi01g00226\_t001 |  | | | |  | | | |  | | | |  | | | |  |  |  |  |
| 4 | Vvi-Vitvi01g04060\_t001 |  | | | |  | | | |  | | | |  | | | |  |  |  |  |
| 4 | Vvi-Vitvi01g01926\_t001 |  | | | |  | | | |  | Ath-AT1G68795.1 |  | | | |  |  |  |  |
| 4 | Vvi-Vitvi01g00227\_t001 |  | | | |  | | | |  | | | |  | Ath-AT1G25682.1 |  |  |  |  |
| 4 | Vvi-Vitvi01g00228\_t001 |  | | | |  | | | |  | Ath-AT1G68800.2 |  | | | |  |  |  |  |
| 4 | Vvi-Vitvi01g00230\_t001 |  | | | |  | Ath-AT3G25700.1 |  | | | |  | | | |  |  |  |  |
| 4 | Vvi-Vitvi01g00232\_t001 |  | | | |  | Ath-AT3G25710.1 |  | Ath-AT1G68810.1 |  | | | |  |  |  |  |
| 4 | Vvi-Vitvi01g00233\_t001 |  | | | |  | | | |  | Ath-AT1G68820.4 |  | | | |  |  |  |  |
| 4 | Vvi-Vitvi01g00234\_t001 |  | | | |  | | | |  | | | |  | | | |  |  |  |  |
| 4 | Vvi-Vitvi01g04061\_t001 |  | | | |  | | | |  | | | |  | | | |  |  |  |  |
| 4 | Vvi-Vitvi01g04062\_t001 |  | | | |  | | | |  | | | |  | | | |  |  |  |  |
| 4 | Vvi-Vitvi01g00237\_t003 |  | | | |  | | | |  | | | |  | Ath-AT1G25580.1 |  |  |  |  |
| 4 | Vvi-Vitvi01g00238\_t001 |  | | | |  | | | |  | Ath-AT1G68830.1 |  | | | |  |  |  |  |
| 4 | Vvi-Vitvi01g00239\_t001 |  | | | |  | | | |  | | | |  | | | |  |  |  |  |
| 4 | Vvi-Vitvi01g04063\_t001 |  | | | |  | | | |  | | | |  | | | |  |  |  |  |
| 4 | Vvi-Vitvi01g01930\_t001 |  | | | |  | | | |  | | | |  | | | |  |  |  |  |
| 4 | Vvi-Vitvi01g00241\_t001 |  | Ath-AT1G13250.1 |  | | | |  | | | |  | | | |  |  |  |  |
| 4 | Vvi-Vitvi01g00243\_t001 |  | | | |  | | | |  | | | |  | Ath-AT1G25570.1 |  |  |  |  |
| 4 | Vvi-Vitvi01g00244\_t001 |  | Ath-AT1G13260.1 |  | Ath-AT3G25730.1 |  | Ath-AT1G68840.1 |  | Ath-AT1G25560.1 |  |  |  |  |
| 3 | Vvi-Vitvi01g00245\_t001 |  | Ath-AT1G13270.1 |  | Ath-AT3G25740.1 |  |  |  | | | |  |  |  |  |
| 3 | Vvi-Vitvi01g00246\_t001 |  | Ath-AT1G13280.1 |  | Ath-AT3G25760.1 |  |  |  | | | |  |  |  |  |
| 3 | Vvi-Vitvi01g00247\_t001 |  | Ath-AT1G13290.1 |  | | | |  |  |  | | | |  |  |  |  |
| 4 | Vvi-Vitvi01g00249\_t001 |  | Ath-AT1G13300.1 |  | Ath-AT3G25790.1 |  | Ath-AT1G68670.1 |  | Ath-AT1G25550.1 |  |  |  |  |
| 4 | Vvi-Vitvi01g00250\_t001 |  | | | |  | | | |  | | | |  | | | |  |  |  |  |
| 4 | Vvi-Vitvi01g00251\_t001 |  | Ath-AT1G13310.1 |  | | | |  | | | |  | | | |  |  |  |  |
| 4 | Vvi-Vitvi01g00252\_t001 |  | | | |  | | | |  | | | |  | Ath-AT1G25540.1 |  |  |  |  |
| 4 | Vvi-Vitvi01g00253\_t001 |  | | | |  | | | |  | | | |  | Ath-AT1G25530.1 |  |  |  |  |
| 4 | Vvi-Vitvi01g00254\_t001 |  | | | |  | | | |  | Ath-AT1G68660.1 |  | | | |  |  |  |  |
| 4 | Vvi-Vitvi01g00255\_t002 |  | | | |  | | | |  | Ath-AT1G68650.1 |  | Ath-AT1G25520.1 |  |  |  |  |
| 4 | Vvi-Vitvi01g00258\_t001 |  | | | |  | | | |  | | | |  | Ath-AT1G25510.1 |  |  |  |  |
| 4 | Vvi-Vitvi01g00259\_t001 |  | | | |  | | | |  | | | |  | Ath-AT1G25500.2 |  |  |  |  |
| 4 | Vvi-Vitvi01g00260\_t001 |  | | | |  | | | |  | Ath-AT1G68640.1 |  | | | |  |  |  |  |
| 4 | Vvi-Vitvi01g04064\_t001 |  | | | |  | | | |  | | | |  | | | |  |  |  |  |
| 4 | Vvi-Vitvi01g00261\_t001 |  | | | |  | | | |  | Ath-AT1G68630.1 |  | | | |  |  |  |  |
| 4 | Vvi-Vitvi01g00262\_t001 |  | | | |  | | | |  | | | |  | | | |  |  |  |  |
| 4 | Vvi-Vitvi01g00263\_t001 |  | | | |  | | | |  | | | |  | | | |  |  |  |  |
| 4 | Vvi-Vitvi01g00264\_t001 |  | | | |  | | | |  | Ath-AT1G68620.1 |  | | | |  |  |  |  |
| 4 | Vvi-Vitvi01g04065\_t001 |  | | | |  | | | |  | | | |  | | | |  |  |  |  |
| 4 | Vvi-Vitvi01g00265\_t002 |  | Ath-AT1G13320.1 |  | Ath-AT3G25800.1 |  | | | |  | Ath-AT1G25490.1 |  |  |  |  |
| 4 | Vvi-Vitvi01g00266\_t001 |  | | | |  | | | |  | Ath-AT1G68600.1 |  | Ath-AT1G25480.1 |  |  |  |  |
| 4 | Vvi-Vitvi01g00267\_t001 |  | | | |  | | | |  | | | |  | | | |  |  |  |  |
| 4 | Vvi-Vitvi01g04066\_t001 |  | | | |  | | | |  | | | |  | | | |  |  |  |  |
| 4 | Vvi-Vitvi01g04067\_t001 |  | | | |  | | | |  | | | |  | | | |  |  |  |  |
| 4 | Vvi-Vitvi01g00268\_t001 |  | | | |  | | | |  | Ath-AT1G68590.1 |  | | | |  |  |  |  |
| 4 | Vvi-Vitvi01g00269\_t001 |  | | | |  | Ath-AT3G25805.1 |  | | | |  | | | |  |  |  |  |
| 4 | Vvi-Vitvi01g00270\_t001 |  | Ath-AT1G13330.1 |  | | | |  | | | |  | | | |  |  |  |  |
| 4 | Vvi-Vitvi01g00271\_t001 |  | Ath-AT1G13340.1 |  | | | |  | | | |  | | | |  |  |  |  |
| 4 | Vvi-Vitvi01g00272\_t001 |  | Ath-AT1G13350.3 |  | Ath-AT3G25840.1 |  | | | |  | | | |  |  |  |  |
| 4 | Vvi-Vitvi01g01932\_t001 |  | | | |  | | | |  | Ath-AT1G68585.1 |  | | | |  |  |  |  |
| 4 | Vvi-Vitvi01g00273\_t001 |  | | | |  | | | |  | Ath-AT1G68580.2 |  | | | |  |  |  |  |
| 4 | Vvi-Vitvi01g00274\_t001 |  | | | |  | Ath-AT3G25860.1 |  | | | |  | | | |  |  |  |  |
| 4 | Vvi-Vitvi01g00275\_t001 |  | | | |  | | | |  | | | |  | | | |  |  |  |  |
| 4 | Vvi-Vitvi01g00277\_t001 |  | | | |  | | | |  | Ath-AT1G68570.1 |  | | | |  |  |  |  |
| 4 | Vvi-Vitvi01g04068\_t001 |  | | | |  | | | |  | | | |  | | | |  |  |  |  |
| 4 | Vvi-Vitvi01g00278\_t001 |  | | | |  | | | |  | | | |  | | | |  |  |  |  |
| 4 | Vvi-Vitvi01g00279\_t001 |  | Ath-AT1G13360.1 |  | Ath-AT3G25870.1 |  | | | |  | | | |  |  |  |  |
| 4 | Vvi-Vitvi01g01934\_t001 |  | | | |  | | | |  | | | |  | | | |  |  |  |  |
| 4 | Vvi-Vitvi01g01935\_t001 |  | | | |  | | | |  | | | |  | | | |  |  |  |  |
| 4 | Vvi-Vitvi01g00280\_t001 |  | | | |  | | | |  | Ath-AT1G68560.1 |  | | | |  |  |  |  |
| 4 | Vvi-Vitvi01g00281\_t001 |  | | | |  | | | |  | | | |  | | | |  |  |  |  |
| 4 | Vvi-Vitvi01g01827\_t001 |  | | | |  | Ath-AT3G25890.2 |  | Ath-AT1G68550.2 |  | Ath-AT1G25470.2 |  |  |  |  |
| 4 | Vvi-Vitvi01g00282\_t001 |  | | | |  | | | |  | Ath-AT1G68540.1 |  | Ath-AT1G25460.1 |  |  |  |  |
| 4 | Vvi-Vitvi01g00284\_t001 |  | | | |  | | | |  | Ath-AT1G68530.1 |  | Ath-AT1G25450.1 |  |  |  |  |
| 4 | Vvi-Vitvi01g00286\_t001 |  | | | |  | Ath-AT3G25900.1 |  | | | |  | | | |  |  |  |  |
| 5 | Vvi-Vitvi01g00287\_t001 |  | | | |  | | | |  | | | |  | | | |  | Ath-AT1G67170.1 |  |  |  |
| 5 | Vvi-Vitvi01g00288\_t001 |  | | | |  | | | |  | Ath-AT1G68520.1 |  | Ath-AT1G25440.1 |  | | | |  |  |  |
| 5 | Vvi-Vitvi01g00289\_t001 |  | | | |  | | | |  | | | |  | | | |  | | | |  |  |  |
| 5 | Vvi-Vitvi01g00290\_t001 |  | | | |  | | | |  | Ath-AT1G68510.1 |  | | | |  | Ath-AT1G67100.1 |  |  |  |
| 5 | Vvi-Vitvi01g00291\_t001 |  | | | |  | | | |  | | | |  | | | |  | | | |  |  |  |
| 5 | Vvi-Vitvi01g04069\_t001 |  | | | |  | | | |  | | | |  | | | |  | | | |  |  |  |
| 5 | Vvi-Vitvi01g01936\_t001 |  | | | |  | Ath-AT3G25905.1 |  | | | |  | Ath-AT1G25425.1 |  | | | |  |  |  |
| 5 | Vvi-Vitvi01g00292\_t001 |  | Ath-AT1G13380.1 |  | | | |  | | | |  | | | |  | | | |  |  |  |
| 5 | Vvi-Vitvi01g01937\_t001 |  | | | |  | | | |  | Ath-AT1G68500.1 |  | Ath-AT1G25422.1 |  | | | |  |  |  |
| 5 | Vvi-Vitvi01g01938\_t001 |  | Ath-AT1G13390.2 |  | | | |  | Ath-AT1G68490.1 |  | | | |  | | | |  |  |  |
| 5 | Vvi-Vitvi01g00293\_t001 |  | | | |  | Ath-AT3G25920.1 |  | | | |  | | | |  | | | |  |  |  |
| 5 | Vvi-Vitvi01g01939\_t001 |  | Ath-AT1G13400.1 |  | | | |  | Ath-AT1G68480.1 |  | | | |  | | | |  |  |  |
| 5 | Vvi-Vitvi01g00294\_t001 |  | | | |  | | | |  | | | |  | Ath-AT1G25420.1 |  | | | |  |  |  |
| 5 | Vvi-Vitvi01g00295\_t001 |  | | | |  | | | |  | | | |  | | | |  | | | |  |  |  |
| 5 | Vvi-Vitvi01g04070\_t001 |  | | | |  | | | |  | | | |  | | | |  | | | |  |  |  |
| 5 | Vvi-Vitvi01g00296\_t001 |  | Ath-AT1G13410.1 |  | | | |  | | | |  | | | |  | | | |  |  |  |
| 4 | Vvi-Vitvi01g00298\_t001 |  |  |  | | | |  | Ath-AT1G68460.1 |  | Ath-AT1G25410.1 |  | | | |  |  |  |
| 4 | Vvi-Vitvi01g04071\_t001 |  |  |  | | | |  | | | |  | | | |  | | | |  |  |  |
| 5 | Vvi-Vitvi01g01941\_t001 |  | Ath-AT3G18360.1 |  | | | |  | Ath-AT1G68450.1 |  | | | |  | | | |  |  |  |
| 5 | Vvi-Vitvi01g00299\_t001 |  | | | |  | | | |  | Ath-AT1G68440.1 |  | Ath-AT1G25400.1 |  | | | |  |  |  |
| 5 | Vvi-Vitvi01g00300\_t001 |  | | | |  | | | |  | | | |  | Ath-AT1G68290.1 |  | | | |  |  |  |
| 5 | Vvi-Vitvi01g01942\_t001 |  | | | |  | Ath-AT3G25930.1 |  | | | |  | Ath-AT1G68300.1 |  | | | |  |  |  |
| 4 | Vvi-Vitvi01g01943\_t001 |  | | | |  |  |  | | | |  | | | |  | | | |  |  |  |
| 4 | Vvi-Vitvi01g04072\_t001 |  | | | |  |  |  | Ath-AT1G68260.1 |  | | | |  | | | |  |  |  |
| 3 | Vvi-Vitvi01g01944\_t001 |  | | | |  |  |  |  |  | | | |  | | | |  |  |  |
| 3 | Vvi-Vitvi01g04073\_t001 |  | | | |  |  |  |  |  | | | |  | | | |  |  |  |
| 4 | Vvi-Vitvi01g01946\_t001 |  | | | |  | Ath-AT1G25330.1 |  |  |  | | | |  | | | |  |  |  |
| 4 | Vvi-Vitvi01g00302\_t001 |  | | | |  | Ath-AT1G25340.3 |  |  |  | Ath-AT1G68320.1 |  | | | |  |  |  |
| 4 | Vvi-Vitvi01g00303\_t001 |  | | | |  | Ath-AT1G25350.2 |  |  |  | Ath-AT1G68325.1 |  | | | |  |  |  |
| 4 | Vvi-Vitvi01g04074\_t001 |  | | | |  | | | |  |  |  | | | |  | Ath-AT1G67070.1 |  |  |  |
| 4 | Vvi-Vitvi01g04075\_t001 |  | | | |  | | | |  |  |  | | | |  | | | |  |  |  |
| 4 | Vvi-Vitvi01g00305\_t001 |  | Ath-AT3G18300.1 |  | | | |  |  |  | Ath-AT1G68330.1 |  | Ath-AT1G67050.1 |  |  |  |
| 4 | Vvi-Vitvi01g00306\_t002 |  | Ath-AT3G18295.1 |  | Ath-AT1G25370.1 |  |  |  | Ath-AT1G68340.1 |  | | | |  |  |  |
| 4 | Vvi-Vitvi01g04076\_t001 |  | | | |  | | | |  |  |  | Ath-AT1G68350.1 |  | | | |  |  |  |
| 4 | Vvi-Vitvi01g00309\_t001 |  | | | |  | | | |  |  |  | Ath-AT1G68360.1 |  | Ath-AT1G67030.1 |  |  |  |
| 4 | Vvi-Vitvi01g00310\_t001 |  | | | |  | | | |  |  |  | Ath-AT1G68370.1 |  | | | |  |  |  |
| 4 | Vvi-Vitvi01g04077\_t001 |  | | | |  | | | |  |  |  | | | |  | | | |  |  |  |
| 4 | Vvi-Vitvi01g04078\_t001 |  | | | |  | | | |  |  |  | | | |  | | | |  |  |  |
| 4 | Vvi-Vitvi01g00313\_t001 |  | Ath-AT3G18290.1 |  | | | |  |  |  | | | |  | | | |  |  |  |
| 4 | Vvi-Vitvi01g00314\_t002 |  | | | |  | Ath-AT1G25375.1 |  |  |  | | | |  | | | |  |  |  |
| 4 | Vvi-Vitvi01g00315\_t001 |  | | | |  | | | |  |  |  | | | |  | | | |  |  |  |
| 4 | Vvi-Vitvi01g00317\_t001 |  | | | |  | | | |  |  |  | Ath-AT1G68380.1 |  | | | |  |  |  |
| 4 | Vvi-Vitvi01g00318\_t001 |  | | | |  | | | |  |  |  | Ath-AT1G68400.1 |  | | | |  |  |  |
| 4 | Vvi-Vitvi01g00319\_t002 |  | | | |  | | | |  |  |  | | | |  | | | |  |  |  |
| 4 | Vvi-Vitvi01g00320\_t001 |  | | | |  | | | |  |  |  | | | |  | | | |  |  |  |
| 4 | Vvi-Vitvi01g04079\_t001 |  | | | |  | | | |  |  |  | | | |  | | | |  |  |  |
| 4 | Vvi-Vitvi01g04080\_t001 |  | | | |  | | | |  |  |  | | | |  | | | |  |  |  |
| 4 | Vvi-Vitvi01g04081\_t001 |  | | | |  | | | |  |  |  | | | |  | | | |  |  |  |
| 4 | Vvi-Vitvi01g00325\_t001 |  | | | |  | | | |  |  |  | Ath-AT1G68410.1 |  | | | |  |  |  |
| 4 | Vvi-Vitvi01g04082\_t001 |  | | | |  | | | |  |  |  | | | |  | | | |  |  |  |
| 4 | Vvi-Vitvi01g00326\_t001 |  | | | |  | Ath-AT1G25380.1 |  |  |  | | | |  | | | |  |  |  |
| 4 | Vvi-Vitvi01g01949\_t001 |  | | | |  | | | |  |  |  | Ath-AT1G68430.1 |  | Ath-AT1G66890.1 |  |  |  |
| 3 | Vvi-Vitvi01g04083\_t002 |  | | | |  | Ath-AT1G25390.2 |  |  |  |  |  | Ath-AT1G66880.1 |  |  |  |
| 2 | Vvi-Vitvi01g04084\_t001 |  | | | |  |  |  |  |  |  |  | | | |  |  |  |
| 2 | Vvi-Vitvi01g01952\_t001 |  | | | |  |  |  |  |  |  |  | | | |  |  |  |
| 2 | Vvi-Vitvi01g04085\_t001 |  | | | |  |  |  |  |  |  |  | | | |  |  |  |
| 2 | Vvi-Vitvi01g00327\_t001 |  | | | |  |  |  |  |  |  |  | | | |  |  |  |
| 2 | Vvi-Vitvi01g04086\_t001 |  | | | |  |  |  |  |  |  |  | | | |  |  |  |
| 2 | Vvi-Vitvi01g04087\_t001 |  | | | |  |  |  |  |  |  |  | | | |  |  |  |
| 2 | Vvi-Vitvi01g04088\_t001 |  | | | |  |  |  |  |  |  |  | | | |  |  |  |
| 4 | Vvi-Vitvi01g00328\_t001 |  | | | |  | Ath-AT1G15030.1 |  | Ath-AT2G01260.1 |  |  |  | | | |  |  |  |
| 4 | Vvi-Vitvi01g00329\_t001 |  | | | |  | Ath-AT1G15040.1 |  | | | |  |  |  | Ath-AT1G66860.1 |  |  |  |
| 4 | Vvi-Vitvi01g00330\_t001 |  | | | |  | | | |  | | | |  |  |  | | | |  |  |  |
| 4 | Vvi-Vitvi01g00331\_t001 |  | Ath-AT3G18280.2 |  | | | |  | | | |  |  |  | | | |  |  |  |
| 4 | Vvi-Vitvi01g00332\_t001 |  | Ath-AT3G18260.1 |  | | | |  | | | |  |  |  | | | |  |  |  |
| 4 | Vvi-Vitvi01g04089\_t001 |  | | | |  | | | |  | | | |  |  |  | | | |  |  |  |
| 4 | Vvi-Vitvi01g01954\_t001 |  | | | |  | | | |  | | | |  |  |  | | | |  |  |  |
| 4 | Vvi-Vitvi01g00333\_t001 |  | | | |  | | | |  | Ath-AT2G01220.2 |  |  |  | | | |  |  |  |
| 4 | Vvi-Vitvi01g00335\_t001 |  | | | |  | | | |  | Ath-AT2G01210.1 |  |  |  | Ath-AT1G66830.1 |  |  |  |
| 4 | Vvi-Vitvi01g00336\_t001 |  | | | |  | Ath-AT1G15050.1 |  | Ath-AT2G01200.1 |  |  |  | | | |  |  |  |
| 4 | Vvi-Vitvi01g00337\_t001 |  | | | |  | | | |  | | | |  |  |  | | | |  |  |  |
| 4 | Vvi-Vitvi01g00338\_t001 |  | | | |  | Ath-AT1G15060.1 |  | | | |  |  |  | | | |  |  |  |
| 4 | Vvi-Vitvi01g04090\_t001 |  | | | |  | | | |  | | | |  |  |  | | | |  |  |  |
| 4 | Vvi-Vitvi01g00339\_t002 |  | | | |  | | | |  | | | |  |  |  | | | |  |  |  |
| 4 | Vvi-Vitvi01g01955\_t001 |  | Ath-AT3G18230.1 |  | | | |  | Ath-AT2G01190.1 |  |  |  | | | |  |  |  |
| 4 | Vvi-Vitvi01g04091\_t001 |  | | | |  | | | |  | | | |  |  |  | | | |  |  |  |
| 4 | Vvi-Vitvi01g01956\_t001 |  | Ath-AT3G18220.1 |  | Ath-AT1G15080.1 |  | Ath-AT2G01180.3 |  |  |  | | | |  |  |  |
| 4 | Vvi-Vitvi01g00340\_t001 |  | | | |  | | | |  | | | |  |  |  | | | |  |  |  |
| 4 | Vvi-Vitvi01g01958\_t001 |  | | | |  | | | |  | | | |  |  |  | | | |  |  |  |
| 4 | Vvi-Vitvi01g01959\_t001 |  | | | |  | | | |  | | | |  |  |  | | | |  |  |  |
| 4 | Vvi-Vitvi01g00341\_t001 |  | | | |  | | | |  | | | |  |  |  | | | |  |  |  |
| 4 | Vvi-Vitvi01g04092\_t001 |  | | | |  | | | |  | | | |  |  |  | | | |  |  |  |
| 4 | Vvi-Vitvi01g00343\_t001 |  | | | |  | | | |  | Ath-AT2G01170.1 |  |  |  | | | |  |  |  |
| 4 | Vvi-Vitvi01g00344\_t001 |  | | | |  | | | |  | | | |  |  |  | | | |  |  |  |
| 4 | Vvi-Vitvi01g00345\_t001 |  | | | |  | | | |  | | | |  |  |  | | | |  |  |  |
| 4 | Vvi-Vitvi01g00346\_t001 |  | | | |  | | | |  | | | |  |  |  | | | |  |  |  |
| 4 | Vvi-Vitvi01g01960\_t001 |  | | | |  | | | |  | | | |  |  |  | | | |  |  |  |
| 4 | Vvi-Vitvi01g00347\_t001 |  | | | |  | | | |  | | | |  |  |  | Ath-AT1G66810.3 |  |  |  |
| 3 | Vvi-Vitvi01g04093\_t001 |  | | | |  | | | |  | | | |  |  |  |  |  |
| 3 | Vvi-Vitvi01g04094\_t001 |  | | | |  | | | |  | | | |  |  |  |  |  |
| 3 | Vvi-Vitvi01g04095\_t001 |  | | | |  | | | |  | | | |  |  |  |  |  |
| 3 | Vvi-Vitvi01g04096\_t001 |  | | | |  | | | |  | | | |  |  |  |  |  |
| 3 | Vvi-Vitvi01g00353\_t001 |  | | | |  | | | |  | | | |  |  |  |  |  |
| 3 | Vvi-Vitvi01g00354\_t001 |  | | | |  | Ath-AT1G15100.1 |  | Ath-AT2G01150.2 |  |  |  |  |  |
| 3 | Vvi-Vitvi01g00355\_t001 |  | | | |  | | | |  | | | |  |  |  |  |  |
| 3 | Vvi-Vitvi01g04097\_t001 |  | | | |  | | | |  | | | |  |  |  |  |  |
| 3 | Vvi-Vitvi01g00357\_t001 |  | Ath-AT3G18210.1 |  | | | |  | | | |  |  |  |  |  |
| 2 | Vvi-Vitvi01g00358\_t001 |  |  |  | | | |  | | | |  |  |  |  |  |
| 2 | Vvi-Vitvi01g00360\_t001 |  |  |  | | | |  | Ath-AT2G01140.1 |  |  |  |  |  |
| 2 | Vvi-Vitvi01g04098\_t001 |  |  |  | | | |  | Ath-AT2G01130.1 |  |  |  |  |  |
| 2 | Vvi-Vitvi01g00362\_t001.1.60378269 |  |  |  | Ath-AT1G15110.2 |  | | | |  |  |  |  |  |
| 2 | Vvi-Vitvi01g00363\_t001 |  |  |  | | | |  | | | |  |  |  |  |  |
| 2 | Vvi-Vitvi01g00365\_t001 |  |  |  | | | |  | | | |  |  |  |  |  |
| 2 | Vvi-Vitvi01g00366\_t001 |  |  |  | | | |  | Ath-AT2G01120.2 |  |  |  |  |  |
| 2 | Vvi-Vitvi01g00368\_t001 |  |  |  | | | |  | Ath-AT2G01110.1 |  |  |  |  |  |
| 2 | Vvi-Vitvi01g00369\_t001 |  | Ath-AT1G71150.1 |  | | | |  |  |  |  |  |  |
| 2 | Vvi-Vitvi01g00370\_t001 |  | Ath-AT1G71140.1 |  | Ath-AT1G15150.1 |  |  |  |  |  |  |
| 1 | Vvi-Vitvi01g04099\_t001 |  | | | |  |  |  |  |  |  |  |
| 1 | Vvi-Vitvi01g01962\_t001 |  | | | |  |  |  |  |  |  |  |
| 1 | Vvi-Vitvi01g00371\_t001 |  | | | |  |  |  |  |  |  |  |
| 1 | Vvi-Vitvi01g01963\_t001 |  | | | |  |  |  |  |  |  |  |
| 1 | Vvi-Vitvi01g04100\_t001 |  | | | |  |  |  |  |  |  |  |
| 1 | Vvi-Vitvi01g01964\_t001 |  | | | |  |  |  |  |  |  |  |
| 1 | Vvi-Vitvi01g04101\_t001 |  | | | |  |  |  |  |  |  |  |
| 1 | Vvi-Vitvi01g00372\_t001 |  | | | |  |  |  |  |  |  |  |
| 1 | Vvi-Vitvi01g00373\_t001 |  | | | |  |  |  |  |  |  |  |
| 1 | Vvi-Vitvi01g00374\_t001 |  | | | |  |  |  |  |  |  |  |
| 2 | Vvi-Vitvi01g00375\_t001 |  | | | |  | Ath-AT2G01100.2 |  |  |  |  |  |  |
| 2 | Vvi-Vitvi01g00376\_t001 |  | | | |  | | | |  |  |  |  |  |  |
| 2 | Vvi-Vitvi01g00377\_t002 |  | | | |  | | | |  |  |  |  |  |  |
| 2 | Vvi-Vitvi01g00378\_t001 |  | Ath-AT1G71120.1 |  | | | |  |  |  |  |  |  |
| 2 | Vvi-Vitvi01g00379\_t001 |  | | | |  | | | |  |  |  |  |  |  |
| 2 | Vvi-Vitvi01g00380\_t001 |  | | | |  | | | |  |  |  |  |  |  |
| 2 | Vvi-Vitvi01g01965\_t001 |  | | | |  | | | |  |  |  |  |  |  |
| 2 | Vvi-Vitvi01g01966\_t001 |  | | | |  | | | |  |  |  |  |  |  |
| 2 | Vvi-Vitvi01g01967\_t001 |  | | | |  | | | |  |  |  |  |  |  |
| 2 | Vvi-Vitvi01g00381\_t002 |  | | | |  | | | |  |  |  |  |  |  |
| 2 | Vvi-Vitvi01g00382\_t001 |  | | | |  | Ath-AT2G01270.1 |  |  |  |  |  |  |
| 2 | Vvi-Vitvi01g00383\_t001 |  | | | |  | | | |  |  |  |  |  |  |
| 2 | Vvi-Vitvi01g00384\_t001 |  | Ath-AT1G71110.1 |  | | | |  |  |  |  |  |  |
| 2 | Vvi-Vitvi01g00385\_t001 |  | Ath-AT1G71100.1 |  | Ath-AT2G01290.1 |  |  |  |  |  |  |
| 2 | Vvi-Vitvi01g00386\_t001 |  | Ath-AT1G71090.1 |  | | | |  |  |  |  |  |  |
| 2 | Vvi-Vitvi01g04102\_t001 |  | | | |  | | | |  |  |  |  |  |  |
| 2 | Vvi-Vitvi01g04103\_t001 |  | | | |  | | | |  |  |  |  |  |  |
| 2 | Vvi-Vitvi01g00388\_t001 |  | | | |  | Ath-AT2G01300.1 |  |  |  |  |  |  |
| 2 | Vvi-Vitvi01g00389\_t001 |  | | | |  | | | |  |  |  |  |  |  |
| 2 | Vvi-Vitvi01g00390\_t001 |  | | | |  | Ath-AT2G01320.3 |  |  |  |  |  |  |
| 2 | Vvi-Vitvi01g00391\_t001 |  | Ath-AT1G71070.1 |  | | | |  |  |  |  |  |  |
| 2 | Vvi-Vitvi01g04104\_t001 |  | | | |  | | | |  |  |  |  |  |  |
| 2 | Vvi-Vitvi01g00392\_t001 |  | Ath-AT1G71060.1 |  | | | |  |  |  |  |  |  |
| 2 | Vvi-Vitvi01g01968\_t001 |  | | | |  | | | |  |  |  |  |  |  |
| 2 | Vvi-Vitvi01g04105\_t001 |  | | | |  | | | |  |  |  |  |  |  |
| 3 | Vvi-Vitvi01g00393\_t001 |  | Ath-AT1G71050.1 |  | | | |  | Ath-AT1G22990.1 |  |  |  |  |  |
| 3 | Vvi-Vitvi01g00394\_t001 |  | | | |  | | | |  | Ath-AT1G23000.2 |  |  |  |  |  |
| 3 | Vvi-Vitvi01g00397\_t001 |  | Ath-AT1G71040.1 |  | | | |  | Ath-AT1G23010.1 |  |  |  |  |  |
| 3 | Vvi-Vitvi01g04106\_t001 |  | | | |  | | | |  | | | |  |  |  |  |  |
| 3 | Vvi-Vitvi01g04107\_t001 |  | | | |  | | | |  | | | |  |  |  |  |  |
| 3 | Vvi-Vitvi01g00401\_t001 |  | | | |  | | | |  | | | |  |  |  |  |  |
| 3 | Vvi-Vitvi01g00402\_t001 |  | Ath-AT1G71020.1 |  | | | |  | Ath-AT1G23030.1 |  |  |  |  |  |
| 3 | Vvi-Vitvi01g00403\_t001 |  | Ath-AT1G71015.2 |  | Ath-AT2G01340.1 |  | | | |  |  |  |  |  |
| 3 | Vvi-Vitvi01g00404\_t001 |  | Ath-AT1G71010.1 |  | | | |  | | | |  |  |  |  |  |
| 3 | Vvi-Vitvi01g00405\_t006 |  | | | |  | Ath-AT2G01350.1 |  | | | |  |  |  |  |  |
| 3 | Vvi-Vitvi01g04108\_t001 |  | | | |  | Ath-AT2G01379.1 |  | | | |  |  |  |  |  |
| 3 | Vvi-Vitvi01g04109\_t001 |  | | | |  | | | |  | | | |  |  |  |  |  |
| 3 | Vvi-Vitvi01g00407\_t001 |  | | | |  | Ath-AT2G01410.1 |  | | | |  |  |  |  |  |
| 3 | Vvi-Vitvi01g00408\_t001 |  | Ath-AT1G71000.1 |  | | | |  | | | |  |  |  |  |  |
| 3 | Vvi-Vitvi01g01971\_t001 |  | | | |  | | | |  | Ath-AT1G23040.1 |  |  |  |  |  |
| 3 | Vvi-Vitvi01g01972\_t001 |  | | | |  | | | |  | | | |  |  |  |  |  |
| 3 | Vvi-Vitvi01g00409\_t001 |  | Ath-AT1G70950.1 |  | | | |  | Ath-AT1G23060.1 |  |  |  |  |  |
| 3 | Vvi-Vitvi01g04110\_t001 |  | | | |  | | | |  | | | |  |  |  |  |  |
| 3 | Vvi-Vitvi01g00411\_t001 |  | Ath-AT1G70940.1 |  | Ath-AT2G01420.2 |  | Ath-AT1G23080.1 |  |  |  |  |  |
| 3 | Vvi-Vitvi01g00412\_t001 |  | Ath-AT1G70920.1 |  | Ath-AT2G01430.1 |  | | | |  |  |  |  |  |
| 3 | Vvi-Vitvi01g00413\_t001 |  | | | |  | Ath-AT2G01440.1 |  | | | |  |  |  |  |  |
| 3 | Vvi-Vitvi01g04111\_t001 |  | | | |  | | | |  | | | |  |  |  |  |  |
| 3 | Vvi-Vitvi01g00414\_t001 |  | | | |  | | | |  | | | |  |  |  |  |  |
| 4 | Vvi-Vitvi01g00415\_t001 |  | | | |  | | | |  | | | |  | Ath-AT1G15000.1 |  |  |  |  |
| 4 | Vvi-Vitvi01g01973\_t001 |  | | | |  | | | |  | | | |  | | | |  |  |  |  |
| 4 | Vvi-Vitvi01g04112\_t001 |  | | | |  | | | |  | | | |  | | | |  |  |  |  |
| 4 | Vvi-Vitvi01g00417\_t001 |  | | | |  | Ath-AT2G01450.1 |  | | | |  | | | |  |  |  |  |
| 4 | Vvi-Vitvi01g01974\_t001 |  | | | |  | | | |  | | | |  | | | |  |  |  |  |
| 4 | Vvi-Vitvi01g00418\_t001 |  | | | |  | | | |  | | | |  | | | |  |  |  |  |
| 4 | Vvi-Vitvi01g00421\_t001 |  | | | |  | Ath-AT2G01460.1 |  | | | |  | | | |  |  |  |  |
| 4 | Vvi-Vitvi01g00422\_t001 |  | | | |  | Ath-AT2G01470.1 |  | | | |  | | | |  |  |  |  |
| 4 | Vvi-Vitvi01g00423\_t001 |  | | | |  | | | |  | Ath-AT1G23090.1 |  | | | |  |  |  |  |
| 4 | Vvi-Vitvi01g00424\_t001 |  | | | |  | | | |  | Ath-AT1G23100.1 |  | Ath-AT1G14980.1 |  |  |  |  |
| 4 | Vvi-Vitvi01g00425\_t001 |  | | | |  | Ath-AT2G01480.1 |  | | | |  | Ath-AT1G14970.1 |  |  |  |  |
| 4 | Vvi-Vitvi01g00426\_t001 |  | | | |  | Ath-AT2G01490.1 |  | | | |  | | | |  |  |  |  |
| 4 | Vvi-Vitvi01g00427\_t001 |  | | | |  | Ath-AT2G01500.1 |  | | | |  | | | |  |  |  |  |
| 4 | Vvi-Vitvi01g00428\_t001 |  | Ath-AT1G70900.1 |  | | | |  | Ath-AT1G23110.1 |  | | | |  |  |  |  |
| 4 | Vvi-Vitvi01g04113\_t001 |  | | | |  | | | |  | | | |  | | | |  |  |  |  |
| 4 | Vvi-Vitvi01g00430\_t001 |  | | | |  | Ath-AT2G01510.1 |  | | | |  | | | |  |  |  |  |
| 4 | Vvi-Vitvi01g00432\_t001 |  | | | |  | | | |  | | | |  | | | |  |  |  |  |
| 4 | Vvi-Vitvi01g04114\_t001 |  | | | |  | | | |  | | | |  | Ath-AT1G14930.1 |  |  |  |  |
| 4 | Vvi-Vitvi01g00434\_t001 |  | | | |  | | | |  | | | |  | | | |  |  |  |  |
| 4 | Vvi-Vitvi01g00436\_t001 |  | Ath-AT1G70880.1 |  | | | |  | Ath-AT1G23120.2 |  | | | |  |  |  |  |
| 4 | Vvi-Vitvi01g00437\_t001 |  | | | |  | | | |  | | | |  | | | |  |  |  |  |
| 4 | Vvi-Vitvi01g00438\_t001 |  | | | |  | | | |  | | | |  | | | |  |  |  |  |
| 4 | Vvi-Vitvi01g01976\_t001 |  | | | |  | | | |  | | | |  | | | |  |  |  |  |
| 4 | Vvi-Vitvi01g01977\_t001 |  | | | |  | | | |  | | | |  | | | |  |  |  |  |
| 4 | Vvi-Vitvi01g00439\_t001 |  | | | |  | | | |  | | | |  | | | |  |  |  |  |
| 4 | Vvi-Vitvi01g01978\_t001 |  | | | |  | | | |  | | | |  | | | |  |  |  |  |
| 4 | Vvi-Vitvi01g01979\_t001 |  | | | |  | | | |  | | | |  | | | |  |  |  |  |
| 4 | Vvi-Vitvi01g04115\_t001 |  | | | |  | | | |  | | | |  | | | |  |  |  |  |
| 4 | Vvi-Vitvi01g01981\_t001 |  | | | |  | | | |  | | | |  | | | |  |  |  |  |
| 4 | Vvi-Vitvi01g01982\_t001 |  | | | |  | | | |  | | | |  | | | |  |  |  |  |
| 4 | Vvi-Vitvi01g00441\_t002 |  | | | |  | | | |  | | | |  | | | |  |  |  |  |
| 4 | Vvi-Vitvi01g00442\_t001 |  | Ath-AT1G70820.1 |  | | | |  | | | |  | | | |  |  |  |  |
| 4 | Vvi-Vitvi01g00443\_t001 |  | | | |  | | | |  | | | |  | | | |  |  |  |  |
| 4 | Vvi-Vitvi01g00444\_t001 |  | Ath-AT1G70810.1 |  | Ath-AT2G01540.1 |  | | | |  | | | |  |  |  |  |
| 4 | Vvi-Vitvi01g00445\_t001 |  | Ath-AT1G70780.1 |  | | | |  | Ath-AT1G23150.1 |  | | | |  |  |  |  |
| 4 | Vvi-Vitvi01g01983\_t001 |  | | | |  | | | |  | | | |  | | | |  |  |  |  |
| 4 | Vvi-Vitvi01g00446\_t001 |  | | | |  | Ath-AT2G01570.1 |  | | | |  | Ath-AT1G14920.1 |  |  |  |  |
| 4 | Vvi-Vitvi01g00447\_t001 |  | | | |  | | | |  | | | |  | | | |  |  |  |  |
| 4 | Vvi-Vitvi01g00448\_t001 |  | Ath-AT1G70770.2 |  | | | |  | Ath-AT1G23170.2 |  | | | |  |  |  |  |
| 4 | Vvi-Vitvi01g00449\_t001 |  | Ath-AT1G70760.1 |  | | | |  | | | |  | | | |  |  |  |  |
| 4 | Vvi-Vitvi01g00450\_t001 |  | | | |  | | | |  | Ath-AT1G23180.1 |  | | | |  |  |  |  |
| 4 | Vvi-Vitvi01g01984\_t001 |  | | | |  | Ath-AT2G01590.1 |  | | | |  | | | |  |  |  |  |
| 4 | Vvi-Vitvi01g01985\_t001 |  | | | |  | | | |  | | | |  | | | |  |  |  |  |
| 4 | Vvi-Vitvi01g00451\_t002 |  | | | |  | Ath-AT2G01600.1 |  | | | |  | Ath-AT1G14910.1 |  |  |  |  |
| 4 | Vvi-Vitvi01g00452\_t001 |  | Ath-AT1G70750.1 |  | | | |  | | | |  | | | |  |  |  |  |
| 4 | Vvi-Vitvi01g00453\_t001 |  | Ath-AT1G70740.1 |  | | | |  | | | |  | | | |  |  |  |  |
| 4 | Vvi-Vitvi01g00454\_t001 |  | | | |  | | | |  | | | |  | Ath-AT1G14900.1 |  |  |  |  |
| 4 | Vvi-Vitvi01g00455\_t002 |  | Ath-AT1G70730.3 |  | | | |  | Ath-AT1G23190.1 |  | | | |  |  |  |  |
| 4 | Vvi-Vitvi01g00456\_t001 |  | | | |  | | | |  | Ath-AT1G23200.1 |  | | | |  |  |  |  |
| 4 | Vvi-Vitvi01g00457\_t001 |  | Ath-AT1G70720.2 |  | Ath-AT2G01610.1 |  | Ath-AT1G23205.1 |  | Ath-AT1G14890.1 |  |  |  |  |
| 4 | Vvi-Vitvi01g01986\_t002 |  | | | |  | | | |  | | | |  | | | |  |  |  |  |
| 4 | Vvi-Vitvi01g00458\_t001 |  | | | |  | | | |  | | | |  | | | |  |  |  |  |
| 4 | Vvi-Vitvi01g01987\_t001 |  | | | |  | | | |  | | | |  | Ath-AT1G14870.1 |  |  |  |  |
| 4 | Vvi-Vitvi01g00460\_t001 |  | | | |  | | | |  | | | |  | | | |  |  |  |  |
| 4 | Vvi-Vitvi01g00461\_t001 |  | | | |  | | | |  | | | |  | | | |  |  |  |  |
| 4 | Vvi-Vitvi01g01988\_t001 |  | | | |  | | | |  | | | |  | | | |  |  |  |  |
| 4 | Vvi-Vitvi01g04116\_t001 |  | | | |  | | | |  | | | |  | | | |  |  |  |  |
| 4 | Vvi-Vitvi01g01990\_t001 |  | | | |  | | | |  | | | |  | | | |  |  |  |  |
| 4 | Vvi-Vitvi01g00463\_t001 |  | | | |  | | | |  | | | |  | | | |  |  |  |  |
| 4 | Vvi-Vitvi01g00464\_t001 |  | | | |  | | | |  | | | |  | | | |  |  |  |  |
| 4 | Vvi-Vitvi01g00465\_t001 |  | | | |  | | | |  | | | |  | | | |  |  |  |  |
| 4 | Vvi-Vitvi01g04117\_t001 |  | | | |  | | | |  | | | |  | | | |  |  |  |  |
| 4 | Vvi-Vitvi01g00466\_t001 |  | | | |  | | | |  | Ath-AT1G23220.1 |  | | | |  |  |  |  |
| 4 | Vvi-Vitvi01g00467\_t001 |  | | | |  | Ath-AT2G01620.1 |  | | | |  | | | |  |  |  |  |
| 4 | Vvi-Vitvi01g00468\_t001 |  | | | |  | Ath-AT2G01630.1 |  | | | |  | | | |  |  |  |  |
| 4 | Vvi-Vitvi01g04118\_t002 |  | | | |  | | | |  | Ath-AT1G23230.1 |  | | | |  |  |  |  |
| 4 | Vvi-Vitvi01g00473\_t001 |  | Ath-AT1G70700.3 |  | | | |  | | | |  | | | |  |  |  |  |
| 4 | Vvi-Vitvi01g04119\_t001 |  | | | |  | | | |  | | | |  | | | |  |  |  |  |
| 4 | Vvi-Vitvi01g00474\_t001 |  | | | |  | Ath-AT2G01650.1 |  | | | |  | | | |  |  |  |  |
| 4 | Vvi-Vitvi01g00475\_t001 |  | | | |  | | | |  | | | |  | | | |  |  |  |  |
| 4 | Vvi-Vitvi01g04120\_t001 |  | | | |  | | | |  | | | |  | | | |  |  |  |  |
| 4 | Vvi-Vitvi01g00476\_t001 |  | | | |  | | | |  | | | |  | | | |  |  |  |  |
| 4 | Vvi-Vitvi01g00477\_t001 |  | Ath-AT1G70690.1 |  | Ath-AT2G01660.1 |  | | | |  | | | |  |  |  |  |
| 4 | Vvi-Vitvi01g00478\_t001 |  | Ath-AT1G70670.1 |  | | | |  | Ath-AT1G23240.4 |  | | | |  |  |  |  |
| 4 | Vvi-Vitvi01g00479\_t002 |  | Ath-AT1G70650.2 |  | | | |  | Ath-AT1G23260.1 |  | | | |  |  |  |  |
| 2 | Vvi-Vitvi01g00481\_t001 |  |  |  | | | |  |  |  | | | |  |  |  |  |
| 2 | Vvi-Vitvi01g00482\_t001 |  |  |  | | | |  |  |  | | | |  |  |  |  |
| 2 | Vvi-Vitvi01g00483\_t001 |  |  |  | Ath-AT2G01670.1 |  |  |  | Ath-AT1G14860.1 |  |  |  |  |
| 2 | Vvi-Vitvi01g01993\_t001 |  |  |  | | | |  |  |  | | | |  |  |  |  |
| 2 | Vvi-Vitvi01g00484\_t001 |  |  |  | | | |  |  |  | | | |  |  |  |  |
| 2 | Vvi-Vitvi01g00486\_t001 |  |  |  | Ath-AT2G01680.1 |  |  |  | | | |  |  |  |  |
| 2 | Vvi-Vitvi01g00487\_t001 |  |  |  | Ath-AT2G01690.2 |  |  |  | | | |  |  |  |  |
| 2 | Vvi-Vitvi01g00488\_t001 |  |  |  | | | |  |  |  | Ath-AT1G14850.1 |  |  |  |  |
| 2 | Vvi-Vitvi01g00491\_t001 |  |  |  | | | |  |  |  | | | |  |  |  |  |
| 3 | Vvi-Vitvi01g00492\_t001 |  | Ath-AT1G68070.1 |  | Ath-AT2G01735.1 |  |  |  | | | |  |  |  |  |
| 3 | Vvi-Vitvi01g04121\_t001 |  | | | |  | | | |  |  |  | | | |  |  |  |  |
| 3 | Vvi-Vitvi01g00497\_t001 |  | Ath-AT1G68060.1 |  | Ath-AT2G01750.2 |  |  |  | Ath-AT1G14840.1 |  |  |  |  |
| 3 | Vvi-Vitvi01g00498\_t001 |  | | | |  | | | |  |  |  | Ath-AT1G14830.1 |  |  |  |  |
| 3 | Vvi-Vitvi01g00499\_t001 |  | Ath-AT1G68050.1 |  | | | |  |  |  | | | |  |  |  |  |
| 3 | Vvi-Vitvi01g00500\_t001 |  | | | |  | Ath-AT2G01755.2 |  |  |  | | | |  |  |  |  |
| 3 | Vvi-Vitvi01g01995\_t001 |  | | | |  | Ath-AT2G01760.2 |  |  |  | | | |  |  |  |  |
| 3 | Vvi-Vitvi01g00501\_t001 |  | | | |  | | | |  |  |  | Ath-AT1G14820.3 |  |  |  |  |
| 3 | Vvi-Vitvi01g00502\_t001 |  | | | |  | | | |  |  |  | Ath-AT1G14810.1 |  |  |  |  |
| 3 | Vvi-Vitvi01g00503\_t002 |  | | | |  | | | |  |  |  | Ath-AT1G14790.1 |  |  |  |  |
| 3 | Vvi-Vitvi01g00505\_t001 |  | | | |  | | | |  |  |  | | | |  |  |  |  |
| 3 | Vvi-Vitvi01g01997\_t001 |  | Ath-AT1G68040.1 |  | | | |  |  |  | | | |  |  |  |  |
| 3 | Vvi-Vitvi01g01998\_t005 |  | | | |  | | | |  |  |  | | | |  |  |  |  |
| 3 | Vvi-Vitvi01g02001\_t001 |  | | | |  | | | |  |  |  | | | |  |  |  |  |
| 3 | Vvi-Vitvi01g04122\_t001 |  | | | |  | | | |  |  |  | | | |  |  |  |  |
| 3 | Vvi-Vitvi01g04123\_t001 |  | | | |  | | | |  |  |  | | | |  |  |  |  |
| 3 | Vvi-Vitvi01g00508\_t001 |  | | | |  | | | |  |  |  | Ath-AT1G14780.1 |  |  |  |  |
| 3 | Vvi-Vitvi01g00509\_t001 |  | Ath-AT1G68020.2 |  | | | |  |  |  | | | |  |  |  |  |
| 3 | Vvi-Vitvi01g00510\_t001 |  | | | |  | | | |  |  |  | Ath-AT1G14760.1 |  |  |  |  |
| 3 | Vvi-Vitvi01g04124\_t001 |  | | | |  | | | |  |  |  | | | |  |  |  |  |
| 3 | Vvi-Vitvi01g00511\_t001 |  | Ath-AT1G67970.1 |  | | | |  |  |  | | | |  |  |  |  |
| 2 | Vvi-Vitvi01g00512\_t001 |  |  |  | Ath-AT2G01770.1 |  |  |  | | | |  |  |  |  |
| 2 | Vvi-Vitvi01g00513\_t001 |  |  |  | | | |  |  |  | | | |  |  |  |  |
| 2 | Vvi-Vitvi01g04125\_t003 |  |  |  | | | |  |  |  | | | |  |  |  |  |
| 2 | Vvi-Vitvi01g00516\_t002 |  |  |  | | | |  |  |  | | | |  |  |  |  |
| 2 | Vvi-Vitvi01g00517\_t001 |  |  |  | | | |  |  |  | Ath-AT1G14750.3 |  |  |  |  |
| 3 | Vvi-Vitvi01g00518\_t001 |  | Ath-AT1G67950.2 |  | | | |  |  |  | | | |  |  |  |  |
| 3 | Vvi-Vitvi01g00519\_t001 |  | | | |  | Ath-AT2G01818.1 |  |  |  | | | |  |  |  |  |
| 3 | Vvi-Vitvi01g00520\_t001 |  | Ath-AT1G67960.1 |  | | | |  |  |  | | | |  |  |  |  |
| 3 | Vvi-Vitvi01g00521\_t001 |  | | | |  | | | |  |  |  | | | |  |  |  |  |
| 3 | Vvi-Vitvi01g04126\_t001 |  | | | |  | | | |  |  |  | | | |  |  |  |  |
| 3 | Vvi-Vitvi01g00522\_t001 |  | | | |  | | | |  |  |  | | | |  |  |  |  |
| 3 | Vvi-Vitvi01g00523\_t001 |  | | | |  | | | |  |  |  | | | |  |  |  |  |
| 3 | Vvi-Vitvi01g00524\_t001 |  | | | |  | | | |  |  |  | | | |  |  |  |  |
| 3 | Vvi-Vitvi01g00525\_t001 |  | | | |  | | | |  |  |  | | | |  |  |  |  |
| 3 | Vvi-Vitvi01g00526\_t001 |  | | | |  | Ath-AT2G01820.1 |  |  |  | | | |  |  |  |  |
| 3 | Vvi-Vitvi01g00527\_t001 |  | | | |  | | | |  |  |  | | | |  |  |  |  |
| 3 | Vvi-Vitvi01g02003\_t001 |  | | | |  | | | |  |  |  | | | |  |  |  |  |
| 3 | Vvi-Vitvi01g00528\_t002 |  | | | |  | Ath-AT2G01830.2 |  |  |  | | | |  |  |  |  |
| 3 | Vvi-Vitvi01g04127\_t001 |  | | | |  | | | |  |  |  | | | |  |  |  |  |
| 3 | Vvi-Vitvi01g00529\_t001 |  | | | |  | | | |  |  |  | | | |  |  |  |  |
| 3 | Vvi-Vitvi01g00530\_t001 |  | | | |  | | | |  |  |  | Ath-AT1G14740.1 |  |  |  |  |
| 3 | Vvi-Vitvi01g02004\_t001 |  | | | |  | | | |  |  |  | Ath-AT1G14730.1 |  |  |  |  |
| 3 | Vvi-Vitvi01g02005\_t001 |  | | | |  | | | |  |  |  | | | |  |  |  |  |
| 3 | Vvi-Vitvi01g00532\_t001 |  | | | |  | | | |  |  |  | | | |  |  |  |  |
| 3 | Vvi-Vitvi01g00533\_t001 |  | | | |  | Ath-AT2G01850.1 |  |  |  | Ath-AT1G14720.1 |  |  |  |  |
| 3 | Vvi-Vitvi01g00536\_t003 |  | | | |  | | | |  |  |  | | | |  |  |  |  |
| 3 | Vvi-Vitvi01g00537\_t002 |  | | | |  | | | |  |  |  | Ath-AT1G14710.1 |  |  |  |  |
| 3 | Vvi-Vitvi01g00538\_t001 |  | Ath-AT1G68080.1 |  | | | |  |  |  | | | |  |  |  |  |
| 3 | Vvi-Vitvi01g00539\_t001 |  | | | |  | Ath-AT2G01880.1 |  |  |  | Ath-AT1G14700.1 |  |  |  |  |
| 3 | Vvi-Vitvi01g00540\_t001 |  | Ath-AT1G68090.1 |  | | | |  |  |  | | | |  |  |  |  |
| 3 | Vvi-Vitvi01g00541\_t001 |  | | | |  | Ath-AT2G01900.3 |  |  |  | | | |  |  |  |  |
| 3 | Vvi-Vitvi01g00542\_t001 |  | | | |  | Ath-AT2G01905.1 |  |  |  | | | |  |  |  |  |
| 3 | Vvi-Vitvi01g00543\_t001 |  | | | |  | Ath-AT2G01910.1 |  |  |  | Ath-AT1G14690.2 |  |  |  |  |
| 3 | Vvi-Vitvi01g00544\_t001 |  | Ath-AT1G68100.1 |  | | | |  |  |  | | | |  |  |  |  |
| 3 | Vvi-Vitvi01g02006\_t001 |  | | | |  | | | |  |  |  | | | |  |  |  |  |
| 3 | Vvi-Vitvi01g00545\_t001 |  | | | |  | | | |  |  |  | Ath-AT1G14687.1 |  |  |  |  |
| 3 | Vvi-Vitvi01g00546\_t001 |  | Ath-AT1G68110.1 |  | Ath-AT2G01920.1 |  |  |  | Ath-AT1G14686.1 |  |  |  |  |
| 3 | Vvi-Vitvi01g00547\_t001 |  | Ath-AT1G68120.1 |  | Ath-AT2G01930.1 |  |  |  | Ath-AT1G14685.1 |  |  |  |  |
| 3 | Vvi-Vitvi01g00550\_t001 |  | | | |  | | | |  |  |  | | | |  |  |  |  |
| 3 | Vvi-Vitvi01g04128\_t001 |  | | | |  | | | |  |  |  | | | |  |  |  |  |
| 3 | Vvi-Vitvi01g00552\_t001 |  | Ath-AT1G68130.1 |  | Ath-AT2G01940.3 |  |  |  | | | |  |  |  |  |
| 2 | Vvi-Vitvi01g02007\_t001 |  |  |  | | | |  |  |  | | | |  |  |  |  |
| 2 | Vvi-Vitvi01g00553\_t001 |  |  |  | Ath-AT2G01950.1 |  |  |  | | | |  |  |  |  |
| 2 | Vvi-Vitvi01g00554\_t001 |  |  |  | | | |  |  |  | | | |  |  |  |  |
| 2 | Vvi-Vitvi01g04129\_t001 |  |  |  | | | |  |  |  | | | |  |  |  |  |
| 2 | Vvi-Vitvi01g00555\_t001 |  |  |  | | | |  |  |  | | | |  |  |  |  |
| 2 | Vvi-Vitvi01g04130\_t001 |  |  |  | | | |  |  |  | | | |  |  |  |  |
| 2 | Vvi-Vitvi01g00556\_t004 |  |  |  | | | |  |  |  | | | |  |  |  |  |
| 2 | Vvi-Vitvi01g00557\_t001 |  |  |  | | | |  |  |  | | | |  |  |  |  |
| 2 | Vvi-Vitvi01g02011\_t001 |  |  |  | | | |  |  |  | | | |  |  |  |  |
| 2 | Vvi-Vitvi01g00558\_t001 |  |  |  | | | |  |  |  | | | |  |  |  |  |
| 2 | Vvi-Vitvi01g00559\_t001 |  |  |  | | | |  |  |  | | | |  |  |  |  |
| 2 | Vvi-Vitvi01g00560\_t001 |  |  |  | | | |  |  |  | | | |  |  |  |  |
| 2 | Vvi-Vitvi01g00561\_t001 |  |  |  | Ath-AT2G01970.1 |  |  |  | Ath-AT1G14670.1 |  |  |  |  |
| 2 | Vvi-Vitvi01g00562\_t001 |  |  |  | Ath-AT2G01980.1 |  |  |  | Ath-AT1G14660.1 |  |  |  |  |
| 2 | Vvi-Vitvi01g00563\_t003 |  |  |  | | | |  |  |  | | | |  |  |  |  |
| 2 | Vvi-Vitvi01g04131\_t001 |  |  |  | | | |  |  |  | | | |  |  |  |  |
| 2 | Vvi-Vitvi01g00566\_t001 |  |  |  | | | |  |  |  | | | |  |  |  |  |
| 2 | Vvi-Vitvi01g00567\_t001 |  |  |  | Ath-AT2G01990.2 |  |  |  | Ath-AT1G14630.1 |  |  |  |  |
| 2 | Vvi-Vitvi01g02012\_t001 |  |  |  | | | |  |  |  | | | |  |  |  |  |
| 2 | Vvi-Vitvi01g00568\_t001 |  |  |  | Ath-AT2G02000.1 |  |  |  | | | |  |  |  |  |
| 2 | Vvi-Vitvi01g02013\_t001 |  |  |  | | | |  |  |  | | | |  |  |  |  |
| 2 | Vvi-Vitvi01g00571\_t001 |  |  |  | Ath-AT2G02020.1 |  |  |  | | | |  |  |  |  |
| 2 | Vvi-Vitvi01g00572\_t001 |  |  |  | | | |  |  |  | | | |  |  |  |  |
| 2 | Vvi-Vitvi01g00573\_t001 |  |  |  | | | |  |  |  | | | |  |  |  |  |
| 2 | Vvi-Vitvi01g00574\_t001 |  |  |  | | | |  |  |  | | | |  |  |  |  |
| 2 | Vvi-Vitvi01g04132\_t001 |  |  |  | | | |  |  |  | | | |  |  |  |  |
| 2 | Vvi-Vitvi01g04133\_t001 |  |  |  | Ath-AT2G02050.1 |  |  |  | | | |  |  |  |  |
| 2 | Vvi-Vitvi01g00577\_t001 |  |  |  | | | |  |  |  | | | |  |  |  |  |
| 2 | Vvi-Vitvi01g04134\_t001 |  |  |  | | | |  |  |  | | | |  |  |  |  |
| 2 | Vvi-Vitvi01g00578\_t001 |  |  |  | Ath-AT2G02060.2 |  |  |  | Ath-AT1G14600.1 |  |  |  |  |
| 0 | Vvi-Vitvi01g00579\_t001 |  |  |  |  |  |  |  |  |
| 2 | Vvi-Vitvi01g00580\_t001 |  | Ath-AT1G67730.1 |  | Ath-AT1G24470.1 |  |  |  |  |  |  |
| 2 | Vvi-Vitvi01g04135\_t001 |  | | | |  | | | |  |  |  |  |  |  |
| 2 | Vvi-Vitvi01g00583\_t001 |  | | | |  | | | |  |  |  |  |  |  |
| 2 | Vvi-Vitvi01g04136\_t001 |  | | | |  | | | |  |  |  |  |  |  |
| 2 | Vvi-Vitvi01g04137\_t001 |  | | | |  | | | |  |  |  |  |  |  |
| 2 | Vvi-Vitvi01g00585\_t001 |  | | | |  | | | |  |  |  |  |  |  |
| 2 | Vvi-Vitvi01g04138\_t001 |  | | | |  | | | |  |  |  |  |  |  |
| 2 | Vvi-Vitvi01g00586\_t001 |  | | | |  | | | |  |  |  |  |  |  |
| 2 | Vvi-Vitvi01g02020\_t001 |  | | | |  | | | |  |  |  |  |  |  |
| 2 | Vvi-Vitvi01g00587\_t001 |  | | | |  | | | |  |  |  |  |  |  |
| 2 | Vvi-Vitvi01g02021\_t001 |  | | | |  | | | |  |  |  |  |  |  |
| 2 | Vvi-Vitvi01g00588\_t001 |  | | | |  | Ath-AT1G24480.1 |  |  |  |  |  |  |
| 2 | Vvi-Vitvi01g00589\_t001 |  | Ath-AT1G67740.1 |  | | | |  |  |  |  |  |  |
| 2 | Vvi-Vitvi01g00590\_t001 |  | | | |  | Ath-AT1G24490.1 |  |  |  |  |  |  |
| 3 | Vvi-Vitvi01g00591\_t007 |  | | | |  | | | |  | Ath-AT3G26100.2 |  |  |  |  |  |
| 3 | Vvi-Vitvi01g04139\_t001 |  | | | |  | | | |  | | | |  |  |  |  |  |
| 3 | Vvi-Vitvi01g00593\_t001 |  | Ath-AT1G67750.1 |  | | | |  | | | |  |  |  |  |  |
| 3 | Vvi-Vitvi01g00594\_t001 |  | | | |  | Ath-AT1G24510.1 |  | | | |  |  |  |  |  |
| 4 | Vvi-Vitvi01g00595\_t001 |  | | | |  | | | |  | | | |  | Ath-AT1G13190.1 |  |  |  |  |
| 4 | Vvi-Vitvi01g00596\_t001 |  | | | |  | | | |  | | | |  | Ath-AT1G13180.1 |  |  |  |  |
| 4 | Vvi-Vitvi01g04140\_t001 |  | | | |  | | | |  | | | |  | | | |  |  |  |  |
| 4 | Vvi-Vitvi01g00598\_t001 |  | | | |  | | | |  | | | |  | Ath-AT1G13170.2 |  |  |  |  |
| 4 | Vvi-Vitvi01g00599\_t001 |  | | | |  | | | |  | | | |  | Ath-AT1G13160.1 |  |  |  |  |
| 4 | Vvi-Vitvi01g00600\_t001 |  | | | |  | | | |  | Ath-AT3G26115.1 |  | | | |  |  |  |  |
| 4 | Vvi-Vitvi01g04141\_t001 |  | | | |  | | | |  | | | |  | | | |  |  |  |  |
| 4 | Vvi-Vitvi01g00602\_t001 |  | Ath-AT1G67770.1 |  | | | |  | Ath-AT3G26120.1 |  | | | |  |  |  |  |
| 4 | Vvi-Vitvi01g00603\_t001 |  | | | |  | | | |  | | | |  | | | |  |  |  |  |
| 4 | Vvi-Vitvi01g04142\_t001 |  | | | |  | | | |  | | | |  | | | |  |  |  |  |
| 4 | Vvi-Vitvi01g00604\_t001 |  | Ath-AT1G67785.1 |  | | | |  | | | |  | | | |  |  |  |  |
| 4 | Vvi-Vitvi01g04143\_t001 |  | | | |  | | | |  | | | |  | | | |  |  |  |  |
| 4 | Vvi-Vitvi01g00605\_t001 |  | | | |  | | | |  | | | |  | | | |  |  |  |  |
| 4 | Vvi-Vitvi01g00608\_t001 |  | | | |  | Ath-AT1G24530.1 |  | | | |  | | | |  |  |  |  |
| 4 | Vvi-Vitvi01g02022\_t001 |  | | | |  | | | |  | | | |  | | | |  |  |  |  |
| 4 | Vvi-Vitvi01g00609\_t001 |  | Ath-AT1G67790.1 |  | | | |  | | | |  | | | |  |  |  |  |
| 4 | Vvi-Vitvi01g00610\_t001 |  | | | |  | | | |  | | | |  | | | |  |  |  |  |
| 4 | Vvi-Vitvi01g00611\_t001 |  | | | |  | | | |  | | | |  | | | |  |  |  |  |
| 4 | Vvi-Vitvi01g00612\_t001 |  | | | |  | Ath-AT1G24540.1 |  | Ath-AT3G26125.1 |  | Ath-AT1G13140.1 |  |  |  |  |
| 4 | Vvi-Vitvi01g02023\_t002 |  | | | |  | | | |  | | | |  | | | |  |  |  |  |
| 4 | Vvi-Vitvi01g02024\_t001 |  | | | |  | | | |  | | | |  | Ath-AT1G13130.1 |  |  |  |  |
| 4 | Vvi-Vitvi01g00613\_t001 |  | | | |  | | | |  | | | |  | Ath-AT1G13120.1 |  |  |  |  |
| 4 | Vvi-Vitvi01g00614\_t001 |  | Ath-AT1G67800.2 |  | | | |  | | | |  | | | |  |  |  |  |
| 4 | Vvi-Vitvi01g04144\_t001 |  | | | |  | | | |  | | | |  | | | |  |  |  |  |
| 4 | Vvi-Vitvi01g04145\_t001 |  | | | |  | | | |  | | | |  | | | |  |  |  |  |
| 4 | Vvi-Vitvi01g04146\_t001 |  | | | |  | | | |  | | | |  | | | |  |  |  |  |
| 4 | Vvi-Vitvi01g00616\_t001 |  | | | |  | Ath-AT1G24560.1 |  | | | |  | | | |  |  |  |  |
| 3 | Vvi-Vitvi01g00617\_t001 |  | | | |  |  |  | Ath-AT3G26300.1 |  | | | |  |  |  |  |
| 3 | Vvi-Vitvi01g04147\_t001 |  | | | |  |  |  | | | |  | | | |  |  |  |  |
| 3 | Vvi-Vitvi01g00619\_t001 |  | | | |  |  |  | | | |  | | | |  |  |  |  |
| 3 | Vvi-Vitvi01g02026\_t001 |  | | | |  |  |  | | | |  | | | |  |  |  |  |
| 3 | Vvi-Vitvi01g00620\_t001 |  | | | |  |  |  | | | |  | | | |  |  |  |  |
| 3 | Vvi-Vitvi01g00621\_t001 |  | | | |  |  |  | | | |  | | | |  |  |  |  |
| 3 | Vvi-Vitvi01g04148\_t001 |  | | | |  |  |  | | | |  | | | |  |  |  |  |
| 3 | Vvi-Vitvi01g00623\_t001 |  | | | |  |  |  | | | |  | | | |  |  |  |  |
| 3 | Vvi-Vitvi01g04149\_t001 |  | | | |  |  |  | Ath-AT3G26350.1 |  | Ath-AT1G13050.1 |  |  |  |  |
| 3 | Vvi-Vitvi01g00625\_t001 |  | | | |  |  |  | | | |  | Ath-AT1G13040.1 |  |  |  |  |
| 3 | Vvi-Vitvi01g00626\_t002 |  | | | |  |  |  | | | |  | | | |  |  |  |  |
| 3 | Vvi-Vitvi01g00627\_t001 |  | | | |  |  |  | | | |  | Ath-AT1G13030.1 |  |  |  |  |
| 3 | Vvi-Vitvi01g04150\_t001 |  | | | |  |  |  | | | |  | | | |  |  |  |  |
| 3 | Vvi-Vitvi01g00628\_t001 |  | | | |  |  |  | Ath-AT3G26370.1 |  | | | |  |  |  |  |
| 3 | Vvi-Vitvi01g00629\_t001 |  | | | |  |  |  | Ath-AT3G26380.1 |  | | | |  |  |  |  |
| 3 | Vvi-Vitvi01g04151\_t001 |  | | | |  |  |  | | | |  | | | |  |  |  |  |
| 3 | Vvi-Vitvi01g00630\_t001 |  | | | |  |  |  | | | |  | | | |  |  |  |  |
| 3 | Vvi-Vitvi01g00631\_t001 |  | | | |  |  |  | | | |  | | | |  |  |  |  |
| 3 | Vvi-Vitvi01g02028\_t001 |  | | | |  |  |  | | | |  | | | |  |  |  |  |
| 3 | Vvi-Vitvi01g00632\_t001 |  | Ath-AT1G67810.1 |  |  |  | | | |  | | | |  |  |  |  |
| 3 | Vvi-Vitvi01g04152\_t001 |  | | | |  |  |  | | | |  | | | |  |  |  |  |
| 3 | Vvi-Vitvi01g00633\_t001 |  | | | |  |  |  | Ath-AT3G26400.1 |  | Ath-AT1G13020.1 |  |  |  |  |
| 3 | Vvi-Vitvi01g00634\_t001 |  | Ath-AT1G67830.1 |  |  |  | Ath-AT3G26430.1 |  | | | |  |  |  |  |
| 3 | Vvi-Vitvi01g00635\_t001 |  | | | |  |  |  | | | |  | | | |  |  |  |  |
| 3 | Vvi-Vitvi01g00636\_t001 |  | | | |  |  |  | | | |  | | | |  |  |  |  |
| 3 | Vvi-Vitvi01g00637\_t001 |  | | | |  |  |  | | | |  | | | |  |  |  |  |
| 3 | Vvi-Vitvi01g00638\_t001 |  | | | |  |  |  | | | |  | | | |  |  |  |  |
| 3 | Vvi-Vitvi01g00639\_t001 |  | | | |  |  |  | | | |  | | | |  |  |  |  |
| 3 | Vvi-Vitvi01g00640\_t001 |  | Ath-AT1G67840.1 |  |  |  | | | |  | | | |  |  |  |  |
| 3 | Vvi-Vitvi01g00641\_t001 |  | Ath-AT1G67850.1 |  |  |  | Ath-AT3G26440.5 |  | Ath-AT1G13000.1 |  |  |  |  |
| 3 | Vvi-Vitvi01g02030\_t001 |  | Ath-AT1G67856.1 |  |  |  | | | |  | | | |  |  |  |  |
| 3 | Vvi-Vitvi01g04153\_t001 |  | | | |  |  |  | | | |  | | | |  |  |  |  |
| 3 | Vvi-Vitvi01g02031\_t001 |  | | | |  |  |  | | | |  | | | |  |  |  |  |
| 3 | Vvi-Vitvi01g02032\_t001 |  | | | |  |  |  | | | |  | | | |  |  |  |  |
| 3 | Vvi-Vitvi01g04154\_t001 |  | | | |  |  |  | | | |  | | | |  |  |  |  |
| 3 | Vvi-Vitvi01g00642\_t001 |  | Ath-AT1G67880.1 |  |  |  | | | |  | Ath-AT1G12990.1 |  |  |  |  |
| 3 | Vvi-Vitvi01g00644\_t001 |  | Ath-AT1G67900.2 |  |  |  | Ath-AT3G26490.1 |  | | | |  |  |  |  |
| 3 | Vvi-Vitvi01g04155\_t001 |  | Ath-AT1G67910.2 |  |  |  | | | |  | | | |  |  |  |  |
| 2 | Vvi-Vitvi01g00645\_t001 |  |  |  |  |  | | | |  | Ath-AT1G12980.1 |  |  |  |  |
| 2 | Vvi-Vitvi01g04156\_t001 |  |  |  |  |  | | | |  | | | |  |  |  |  |
| 3 | Vvi-Vitvi01g00646\_t002 |  | Ath-AT1G70640.1 |  |  |  | Ath-AT3G26510.7 |  | | | |  |  |  |  |
| 3 | Vvi-Vitvi01g00647\_t001 |  | | | |  |  |  | Ath-AT3G26570.1 |  | | | |  |  |  |  |
| 3 | Vvi-Vitvi01g00648\_t001 |  | Ath-AT1G70630.3 |  |  |  | | | |  | | | |  |  |  |  |
| 3 | Vvi-Vitvi01g00649\_t001 |  | Ath-AT1G70620.3 |  |  |  | | | |  | | | |  |  |  |  |
| 3 | Vvi-Vitvi01g00650\_t001 |  | Ath-AT1G70610.1 |  |  |  | | | |  | | | |  |  |  |  |
| 3 | Vvi-Vitvi01g00652\_t001 |  | | | |  |  |  | Ath-AT3G26580.1 |  | | | |  |  |  |  |
| 4 | Vvi-Vitvi01g04157\_t001 |  | Ath-AT1G70600.1 |  | Ath-AT1G23290.1 |  | | | |  | Ath-AT1G12960.1 |  |  |  |  |
| 4 | Vvi-Vitvi01g02038\_t001 |  | | | |  | | | |  | | | |  | | | |  |  |  |  |
| 4 | Vvi-Vitvi01g00653\_t001 |  | | | |  | Ath-AT1G23300.1 |  | Ath-AT3G26590.1 |  | Ath-AT1G12950.1 |  |  |  |  |
| 3 | Vvi-Vitvi01g00654\_t001 |  | | | |  | | | |  | | | |  |  |  |  |  |
| 3 | Vvi-Vitvi01g00655\_t001 |  | | | |  | | | |  | | | |  |  |  |  |  |
| 3 | Vvi-Vitvi01g00656\_t001 |  | | | |  | | | |  | | | |  |  |  |  |  |
| 3 | Vvi-Vitvi01g00657\_t001 |  | | | |  | | | |  | | | |  |  |  |  |  |
| 3 | Vvi-Vitvi01g00658\_t001 |  | | | |  | | | |  | | | |  |  |  |  |  |
| 3 | Vvi-Vitvi01g00659\_t001 |  | | | |  | | | |  | Ath-AT3G26600.1 |  |  |  |  |  |
| 3 | Vvi-Vitvi01g00660\_t001 |  | Ath-AT1G70590.1 |  | | | |  | | | |  |  |  |  |  |
| 4 | Vvi-Vitvi01g02039\_t001 |  | | | |  | | | |  | | | |  | Ath-AT1G10690.1 |  |  |  |  |
| 4 | Vvi-Vitvi01g00662\_t004 |  | Ath-AT1G70580.2 |  | Ath-AT1G23310.1 |  | | | |  | | | |  |  |  |  |
| 4 | Vvi-Vitvi01g00663\_t001 |  | | | |  | | | |  | | | |  | Ath-AT1G10700.1 |  |  |  |  |
| 4 | Vvi-Vitvi01g00664\_t001 |  | Ath-AT1G70570.2 |  | | | |  | | | |  | | | |  |  |  |  |
| 4 | Vvi-Vitvi01g00665\_t001 |  | | | |  | | | |  | | | |  | Ath-AT1G10710.1 |  |  |  |  |
| 4 | Vvi-Vitvi01g00666\_t001 |  | | | |  | | | |  | | | |  | | | |  |  |  |  |
| 4 | Vvi-Vitvi01g00668\_t001 |  | | | |  | | | |  | | | |  | | | |  |  |  |  |
| 4 | Vvi-Vitvi01g04158\_t001 |  | | | |  | | | |  | | | |  | | | |  |  |  |  |
| 4 | Vvi-Vitvi01g02041\_t001 |  | | | |  | | | |  | | | |  | | | |  |  |  |  |
| 4 | Vvi-Vitvi01g00669\_t001 |  | | | |  | | | |  | | | |  | Ath-AT1G10730.1 |  |  |  |  |
| 4 | Vvi-Vitvi01g04159\_t001 |  | | | |  | | | |  | | | |  | | | |  |  |  |  |
| 4 | Vvi-Vitvi01g04160\_t001 |  | | | |  | | | |  | | | |  | | | |  |  |  |  |
| 4 | Vvi-Vitvi01g00672\_t001 |  | Ath-AT1G70560.1 |  | Ath-AT1G23320.1 |  | | | |  | | | |  |  |  |  |
| 4 | Vvi-Vitvi01g00674\_t001 |  | | | |  | Ath-AT1G23330.1 |  | | | |  | Ath-AT1G10740.4 |  |  |  |  |
| 4 | Vvi-Vitvi01g04161\_t001 |  | | | |  | | | |  | | | |  | | | |  |  |  |  |
| 4 | Vvi-Vitvi01g00675\_t001 |  | | | |  | | | |  | | | |  | | | |  |  |  |  |
| 4 | Vvi-Vitvi01g00676\_t001 |  | Ath-AT1G70550.1 |  | Ath-AT1G23340.2 |  | | | |  | Ath-AT1G10750.1 |  |  |  |  |
| 4 | Vvi-Vitvi01g04162\_t001 |  | | | |  | | | |  | | | |  | | | |  |  |  |  |
| 4 | Vvi-Vitvi01g00680\_t001 |  | | | |  | | | |  | | | |  | | | |  |  |  |  |
| 4 | Vvi-Vitvi01g00681\_t001 |  | | | |  | | | |  | | | |  | Ath-AT1G10760.1 |  |  |  |  |
| 4 | Vvi-Vitvi01g02044\_t001 |  | Ath-AT1G70540.1 |  | Ath-AT1G23350.1 |  | | | |  | Ath-AT1G10770.1 |  |  |  |  |
| 3 | Vvi-Vitvi01g00682\_t001 |  | | | |  | | | |  | | | |  |  |  |  |  |
| 3 | Vvi-Vitvi01g04163\_t001 |  | | | |  | | | |  | | | |  |  |  |  |  |
| 3 | Vvi-Vitvi01g00684\_t001 |  | | | |  | | | |  | Ath-AT3G26610.1 |  |  |  |  |  |
| 2 | Vvi-Vitvi01g00685\_t001 |  | Ath-AT1G70520.1 |  | | | |  |  |  |  |  |  |
| 2 | Vvi-Vitvi01g00687\_t001 |  | | | |  | | | |  |  |  |  |  |  |
| 2 | Vvi-Vitvi01g00688\_t001 |  | | | |  | Ath-AT1G23360.1 |  |  |  |  |  |  |
| 2 | Vvi-Vitvi01g04164\_t001 |  | | | |  | | | |  |  |  |  |  |  |
| 2 | Vvi-Vitvi01g00693\_t001 |  | | | |  | | | |  |  |  |  |  |  |
| 2 | Vvi-Vitvi01g00694\_t001 |  | Ath-AT1G70510.2 |  | Ath-AT1G23380.2 |  |  |  |  |  |  |
| 3 | Vvi-Vitvi01g00695\_t001 |  | | | |  | | | |  | Ath-AT1G60790.1 |  |  |  |  |  |
| 3 | Vvi-Vitvi01g04165\_t001 |  | | | |  | | | |  | | | |  |  |  |  |  |
| 3 | Vvi-Vitvi01g00696\_t001 |  | | | |  | | | |  | | | |  |  |  |  |  |
| 3 | Vvi-Vitvi01g04166\_t001 |  | | | |  | | | |  | | | |  |  |  |  |  |
| 3 | Vvi-Vitvi01g00697\_t001 |  | | | |  | Ath-AT1G23390.1 |  | | | |  |  |  |  |  |
| 4 | Vvi-Vitvi01g00698\_t001 |  | | | |  | | | |  | | | |  | Ath-AT1G10670.3 |  |  |  |  |
| 4 | Vvi-Vitvi01g00700\_t002 |  | | | |  | | | |  | | | |  | | | |  |  |  |  |
| 4 | Vvi-Vitvi01g00701\_t001 |  | Ath-AT1G70505.1 |  | | | |  | | | |  | Ath-AT1G10660.1 |  |  |  |  |
| 4 | Vvi-Vitvi01g04167\_t001 |  | | | |  | | | |  | | | |  | | | |  |  |  |  |
| 4 | Vvi-Vitvi01g04168\_t001 |  | | | |  | | | |  | | | |  | | | |  |  |  |  |
| 4 | Vvi-Vitvi01g02047\_t001 |  | | | |  | Ath-AT1G23400.1 |  | | | |  | | | |  |  |  |  |
| 4 | Vvi-Vitvi01g04169\_t001 |  | | | |  | | | |  | | | |  | | | |  |  |  |  |
| 4 | Vvi-Vitvi01g04170\_t001 |  | | | |  | | | |  | | | |  | | | |  |  |  |  |
| 4 | Vvi-Vitvi01g02049\_t001 |  | | | |  | | | |  | | | |  | | | |  |  |  |  |
| 4 | Vvi-Vitvi01g04171\_t001 |  | | | |  | | | |  | | | |  | | | |  |  |  |  |
| 4 | Vvi-Vitvi01g00703\_t001 |  | | | |  | Ath-AT1G23420.2 |  | | | |  | | | |  |  |  |  |
| 4 | Vvi-Vitvi01g02051\_t001 |  | | | |  | | | |  | | | |  | Ath-AT1G10657.1 |  |  |  |  |
| 4 | Vvi-Vitvi01g00704\_t001 |  | | | |  | | | |  | | | |  | | | |  |  |  |  |
| 4 | Vvi-Vitvi01g04172\_t001 |  | | | |  | | | |  | | | |  | | | |  |  |  |  |
| 4 | Vvi-Vitvi01g00705\_t001 |  | | | |  | | | |  | Ath-AT1G60610.1 |  | Ath-AT1G10650.1 |  |  |  |  |
| 4 | Vvi-Vitvi01g02052\_t001 |  | | | |  | | | |  | | | |  | | | |  |  |  |  |
| 4 | Vvi-Vitvi01g04173\_t001 |  | | | |  | | | |  | | | |  | | | |  |  |  |  |
| 4 | Vvi-Vitvi01g00706\_t001 |  | | | |  | Ath-AT1G23440.1 |  | | | |  | | | |  |  |  |  |
| 4 | Vvi-Vitvi01g04174\_t001 |  | | | |  | Ath-AT1G23450.1 |  | | | |  | | | |  |  |  |  |
| 4 | Vvi-Vitvi01g04175\_t001 |  | Ath-AT1G70500.1 |  | Ath-AT1G23460.1 |  | | | |  | | | |  |  |  |  |
| 4 | Vvi-Vitvi01g00710\_t001 |  | | | |  | | | |  | Ath-AT1G60590.1 |  | Ath-AT1G10640.1 |  |  |  |  |
| 4 | Vvi-Vitvi01g02053\_t001 |  | | | |  | | | |  | | | |  | | | |  |  |  |  |
| 4 | Vvi-Vitvi01g04176\_t001 |  | | | |  | | | |  | | | |  | | | |  |  |  |  |
| 4 | Vvi-Vitvi01g00711\_t001 |  | | | |  | | | |  | Ath-AT1G60550.1 |  | | | |  |  |  |  |
| 4 | Vvi-Vitvi01g00712\_t001 |  | | | |  | | | |  | Ath-AT1G60490.1 |  | | | |  |  |  |  |
| 4 | Vvi-Vitvi01g04177\_t002 |  | Ath-AT1G70490.1 |  | Ath-AT1G23490.1 |  | | | |  | Ath-AT1G10630.1 |  |  |  |  |
| 4 | Vvi-Vitvi01g02056\_t001 |  | | | |  | | | |  | | | |  | | | |  |  |  |  |
| 4 | Vvi-Vitvi01g00714\_t001 |  | | | |  | | | |  | Ath-AT1G60450.1 |  | | | |  |  |  |  |
| 4 | Vvi-Vitvi01g00715\_t001 |  | | | |  | | | |  | | | |  | | | |  |  |  |  |
| 4 | Vvi-Vitvi01g00716\_t001 |  | | | |  | | | |  | Ath-AT1G60440.1 |  | | | |  |  |  |  |
| 4 | Vvi-Vitvi01g00717\_t001 |  | Ath-AT1G70480.2 |  | Ath-AT1G23520.1 |  | | | |  | | | |  |  |  |  |
| 4 | Vvi-Vitvi01g00719\_t001 |  | | | |  | | | |  | Ath-AT1G60420.1 |  | | | |  |  |  |  |
| 3 | Vvi-Vitvi01g04178\_t001 |  | | | |  | | | |  |  |  | | | |  |  |  |  |
| 3 | Vvi-Vitvi01g04179\_t001 |  | | | |  | | | |  |  |  | | | |  |  |  |  |
| 3 | Vvi-Vitvi01g02058\_t001 |  | | | |  | | | |  |  |  | | | |  |  |  |  |
| 3 | Vvi-Vitvi01g04180\_t001 |  | | | |  | | | |  |  |  | | | |  |  |  |  |
| 3 | Vvi-Vitvi01g02060\_t001 |  | | | |  | | | |  |  |  | | | |  |  |  |  |
| 3 | Vvi-Vitvi01g02061\_t001 |  | | | |  | | | |  |  |  | | | |  |  |  |  |
| 3 | Vvi-Vitvi01g00722\_t001 |  | | | |  | | | |  |  |  | | | |  |  |  |  |
| 3 | Vvi-Vitvi01g00723\_t001 |  | | | |  | | | |  |  |  | | | |  |  |  |  |
| 3 | Vvi-Vitvi01g02062\_t001 |  | | | |  | Ath-AT1G23530.1 |  |  |  | | | |  |  |  |  |
| 3 | Vvi-Vitvi01g04181\_t001 |  | | | |  | | | |  |  |  | | | |  |  |  |  |
| 3 | Vvi-Vitvi01g02064\_t001 |  | | | |  | | | |  |  |  | | | |  |  |  |  |
| 3 | Vvi-Vitvi01g04182\_t001 |  | | | |  | | | |  |  |  | | | |  |  |  |  |
| 3 | Vvi-Vitvi01g02065\_t001 |  | | | |  | | | |  |  |  | | | |  |  |  |  |
| 3 | Vvi-Vitvi01g04183\_t001 |  | Ath-AT1G70450.1 |  | Ath-AT1G23540.1 |  |  |  | Ath-AT1G10620.1 |  |  |  |  |
| 3 | Vvi-Vitvi01g00729\_t001 |  | Ath-AT1G70440.1 |  | Ath-AT1G23550.1 |  |  |  | | | |  |  |  |  |
| 3 | Vvi-Vitvi01g00730\_t001 |  | Ath-AT1G70430.3 |  | | | |  |  |  | | | |  |  |  |  |
| 3 | Vvi-Vitvi01g04184\_t001 |  | | | |  | | | |  |  |  | | | |  |  |  |  |
| 3 | Vvi-Vitvi01g00732\_t001 |  | Ath-AT1G70420.1 |  | Ath-AT1G23710.1 |  |  |  | | | |  |  |  |  |
| 3 | Vvi-Vitvi01g00733\_t002 |  | | | |  | | | |  |  |  | Ath-AT1G10600.4 |  |  |  |  |
| 3 | Vvi-Vitvi01g04185\_t001 |  | | | |  | | | |  |  |  | | | |  |  |  |  |
| 3 | Vvi-Vitvi01g00735\_t001 |  | Ath-AT1G70410.2 |  | Ath-AT1G23730.2 |  |  |  | | | |  |  |  |  |
| 3 | Vvi-Vitvi01g00737\_t001 |  | | | |  | | | |  |  |  | | | |  |  |  |  |
| 3 | Vvi-Vitvi01g00738\_t001 |  | | | |  | Ath-AT1G23740.1 |  |  |  | | | |  |  |  |  |
| 3 | Vvi-Vitvi01g00739\_t001 |  | | | |  | | | |  |  |  | | | |  |  |  |  |
| 3 | Vvi-Vitvi01g00740\_t001 |  | | | |  | | | |  |  |  | | | |  |  |  |  |
| 3 | Vvi-Vitvi01g04186\_t001 |  | | | |  | | | |  |  |  | | | |  |  |  |  |
| 3 | Vvi-Vitvi01g00741\_t001 |  | | | |  | | | |  |  |  | | | |  |  |  |  |
| 3 | Vvi-Vitvi01g04187\_t001 |  | | | |  | | | |  |  |  | | | |  |  |  |  |
| 3 | Vvi-Vitvi01g00742\_t001 |  | | | |  | | | |  |  |  | | | |  |  |  |  |
| 3 | Vvi-Vitvi01g02067\_t001 |  | | | |  | | | |  |  |  | | | |  |  |  |  |
| 3 | Vvi-Vitvi01g00744\_t002 |  | | | |  | Ath-AT1G23750.1 |  |  |  | Ath-AT1G10590.3 |  |  |  |  |
| 4 | Vvi-Vitvi01g00745\_t001 |  | Ath-AT1G70370.2 |  | Ath-AT1G23760.1 |  | Ath-AT1G60390.1 |  | | | |  |  |  |  |
| 4 | Vvi-Vitvi01g00746\_t001 |  | | | |  | | | |  | | | |  | | | |  |  |  |  |
| 4 | Vvi-Vitvi01g00747\_t001 |  | | | |  | | | |  | | | |  | | | |  |  |  |  |
| 4 | Vvi-Vitvi01g04188\_t001 |  | | | |  | | | |  | | | |  | | | |  |  |  |  |
| 4 | Vvi-Vitvi01g04189\_t001 |  | | | |  | | | |  | | | |  | | | |  |  |  |  |
| 4 | Vvi-Vitvi01g00752\_t001 |  | | | |  | | | |  | | | |  | Ath-AT1G10585.1 |  |  |  |  |
| 4 | Vvi-Vitvi01g04190\_t001 |  | | | |  | | | |  | | | |  | | | |  |  |  |  |
| 4 | Vvi-Vitvi01g04191\_t001 |  | | | |  | | | |  | | | |  | | | |  |  |  |  |
| 4 | Vvi-Vitvi01g02070\_t001 |  | | | |  | | | |  | | | |  | | | |  |  |  |  |
| 4 | Vvi-Vitvi01g04192\_t001 |  | | | |  | | | |  | | | |  | | | |  |  |  |  |
| 4 | Vvi-Vitvi01g02072\_t001 |  | | | |  | | | |  | | | |  | | | |  |  |  |  |
| 4 | Vvi-Vitvi01g02073\_t001 |  | | | |  | | | |  | | | |  | | | |  |  |  |  |
| 4 | Vvi-Vitvi01g02074\_t001 |  | | | |  | | | |  | | | |  | | | |  |  |  |  |
| 4 | Vvi-Vitvi01g00756\_t001 |  | | | |  | | | |  | | | |  | | | |  |  |  |  |
| 4 | Vvi-Vitvi01g04193\_t001 |  | | | |  | | | |  | | | |  | | | |  |  |  |  |
| 4 | Vvi-Vitvi01g04194\_t001 |  | | | |  | | | |  | | | |  | | | |  |  |  |  |
| 4 | Vvi-Vitvi01g00762\_t001 |  | | | |  | | | |  | | | |  | | | |  |  |  |  |
| 4 | Vvi-Vitvi01g04195\_t001 |  | | | |  | | | |  | | | |  | | | |  |  |  |  |
| 4 | Vvi-Vitvi01g04196\_t001 |  | | | |  | | | |  | | | |  | | | |  |  |  |  |
| 4 | Vvi-Vitvi01g04197\_t001 |  | | | |  | | | |  | | | |  | | | |  |  |  |  |
| 4 | Vvi-Vitvi01g04198\_t001 |  | | | |  | | | |  | | | |  | | | |  |  |  |  |
| 4 | Vvi-Vitvi01g00770\_t001 |  | Ath-AT1G70340.1 |  | Ath-AT1G23790.1 |  | | | |  | | | |  |  |  |  |
| 4 | Vvi-Vitvi01g00772\_t001 |  | | | |  | | | |  | | | |  | Ath-AT1G10580.1 |  |  |  |  |
| 4 | Vvi-Vitvi01g00773\_t001 |  | | | |  | | | |  | Ath-AT1G60230.1 |  | | | |  |  |  |  |
| 4 | Vvi-Vitvi01g00775\_t003 |  | | | |  | | | |  | | | |  | | | |  |  |  |  |
| 4 | Vvi-Vitvi01g00776\_t001 |  | | | |  | | | |  | Ath-AT1G60220.1 |  | Ath-AT1G10570.1 |  |  |  |  |
| 4 | Vvi-Vitvi01g00777\_t005 |  | | | |  | | | |  | Ath-AT1G60200.1 |  | | | |  |  |  |  |
| 4 | Vvi-Vitvi01g00779\_t001 |  | | | |  | | | |  | | | |  | | | |  |  |  |  |
| 4 | Vvi-Vitvi01g00780\_t001 |  | Ath-AT1G70330.1 |  | | | |  | | | |  | | | |  |  |  |  |
| 4 | Vvi-Vitvi01g04199\_t001 |  | | | |  | | | |  | | | |  | | | |  |  |  |  |
| 4 | Vvi-Vitvi01g00781\_t001 |  | | | |  | | | |  | | | |  | | | |  |  |  |  |
| 4 | Vvi-Vitvi01g00782\_t001 |  | | | |  | | | |  | Ath-AT1G60190.1 |  | Ath-AT1G10560.1 |  |  |  |  |
| 4 | Vvi-Vitvi01g00784\_t001 |  | | | |  | | | |  | | | |  | Ath-AT1G10550.1 |  |  |  |  |
| 3 | Vvi-Vitvi01g00785\_t001 |  | | | |  | Ath-AT1G23800.1 |  | | | |  |  |  |  |  |
| 3 | Vvi-Vitvi01g00787\_t001 |  | | | |  | | | |  | | | |  |  |  |  |  |
| 3 | Vvi-Vitvi01g00788\_t001 |  | | | |  | | | |  | Ath-AT1G60170.1 |  |  |  |  |  |
| 3 | Vvi-Vitvi01g00789\_t001 |  | Ath-AT1G70310.1 |  | Ath-AT1G23820.1 |  | | | |  |  |  |  |  |
| 3 | Vvi-Vitvi01g04200\_t001 |  | | | |  | Ath-AT1G23830.1 |  | | | |  |  |  |  |  |
| 3 | Vvi-Vitvi01g04201\_t001 |  | | | |  | | | |  | | | |  |  |  |  |  |
| 3 | Vvi-Vitvi01g04202\_t001 |  | | | |  | | | |  | | | |  |  |  |  |  |
| 3 | Vvi-Vitvi01g00790\_t001 |  | | | |  | | | |  | | | |  |  |  |  |  |
| 3 | Vvi-Vitvi01g02080\_t001 |  | | | |  | Ath-AT1G23860.1 |  | | | |  |  |  |  |  |
| 3 | Vvi-Vitvi01g00791\_t001 |  | | | |  | | | |  | Ath-AT1G60160.1 |  |  |  |  |  |
| 3 | Vvi-Vitvi01g00792\_t002 |  | Ath-AT1G70300.1 |  | | | |  | | | |  |  |  |  |  |
| 3 | Vvi-Vitvi01g04203\_t001 |  | | | |  | | | |  | | | |  |  |  |  |  |
| 3 | Vvi-Vitvi01g00793\_t001 |  | Ath-AT1G70290.1 |  | Ath-AT1G23870.1 |  | Ath-AT1G60140.6 |  |  |  |  |  |
| 3 | Vvi-Vitvi01g04204\_t001 |  | | | |  | Ath-AT1G23965.1 |  | | | |  |  |  |  |  |
| 3 | Vvi-Vitvi01g04205\_t001 |  | | | |  | Ath-AT1G23980.1 |  | | | |  |  |  |  |  |
| 3 | Vvi-Vitvi01g00797\_t001 |  | | | |  | | | |  | Ath-AT1G60070.2 |  |  |  |  |  |
| 3 | Vvi-Vitvi01g00798\_t001 |  | Ath-AT1G70280.2 |  | | | |  | | | |  |  |  |  |  |
| 3 | Vvi-Vitvi01g00799\_t001 |  | | | |  | | | |  | | | |  |  |  |  |  |
| 3 | Vvi-Vitvi01g00800\_t001 |  | | | |  | | | |  | | | |  |  |  |  |  |
| 3 | Vvi-Vitvi01g00801\_t001 |  | | | |  | | | |  | Ath-AT1G60060.1 |  |  |  |  |  |
| 3 | Vvi-Vitvi01g00803\_t002 |  | | | |  | | | |  | | | |  |  |  |  |  |
| 3 | Vvi-Vitvi01g02082\_t001 |  | | | |  | | | |  | | | |  |  |  |  |  |
| 3 | Vvi-Vitvi01g04206\_t001 |  | | | |  | | | |  | | | |  |  |  |  |  |
| 3 | Vvi-Vitvi01g04207\_t001 |  | | | |  | | | |  | | | |  |  |  |  |  |
| 3 | Vvi-Vitvi01g04208\_t001 |  | | | |  | | | |  | | | |  |  |  |  |  |
| 3 | Vvi-Vitvi01g04209\_t001 |  | | | |  | | | |  | | | |  |  |  |  |  |
| 3 | Vvi-Vitvi01g00808\_t001 |  | | | |  | | | |  | | | |  |  |  |  |  |
| 3 | Vvi-Vitvi01g04210\_t001 |  | | | |  | | | |  | | | |  |  |  |  |  |
| 4 | Vvi-Vitvi01g00810\_t001 |  | | | |  | | | |  | | | |  | Ath-AT3G28050.1 |  |  |  |  |
| 4 | Vvi-Vitvi01g00812\_t001 |  | | | |  | | | |  | | | |  | Ath-AT3G28100.1 |  |  |  |  |
| 4 | Vvi-Vitvi01g00813\_t001 |  | | | |  | | | |  | | | |  | | | |  |  |  |  |
| 4 | Vvi-Vitvi01g00814\_t002 |  | | | |  | | | |  | | | |  | Ath-AT3G28130.2 |  |  |  |  |
| 4 | Vvi-Vitvi01g00815\_t001 |  | Ath-AT1G70260.1 |  | | | |  | Ath-AT1G60050.1 |  | | | |  |  |  |  |
| 4 | Vvi-Vitvi01g02090\_t001 |  | | | |  | | | |  | | | |  | | | |  |  |  |  |
| 4 | Vvi-Vitvi01g00816\_t001 |  | | | |  | Ath-AT1G24020.1 |  | | | |  | | | |  |  |  |  |
| 4 | Vvi-Vitvi01g00817\_t001 |  | | | |  | Ath-AT1G24030.1 |  | | | |  | | | |  |  |  |  |
| 4 | Vvi-Vitvi01g00818\_t001 |  | | | |  | Ath-AT1G24040.1 |  | | | |  | | | |  |  |  |  |
| 4 | Vvi-Vitvi01g04211\_t001 |  | | | |  | | | |  | | | |  | | | |  |  |  |  |
| 4 | Vvi-Vitvi01g00819\_t001 |  | | | |  | | | |  | | | |  | | | |  |  |  |  |
| 4 | Vvi-Vitvi01g00821\_t001 |  | | | |  | | | |  | | | |  | | | |  |  |  |  |
| 4 | Vvi-Vitvi01g00822\_t001 |  | | | |  | | | |  | | | |  | | | |  |  |  |  |
| 4 | Vvi-Vitvi01g00823\_t001 |  | | | |  | | | |  | | | |  | | | |  |  |  |  |
| 4 | Vvi-Vitvi01g00824\_t001 |  | | | |  | | | |  | | | |  | | | |  |  |  |  |
| 4 | Vvi-Vitvi01g04212\_t001 |  | | | |  | | | |  | | | |  | | | |  |  |  |  |
| 4 | Vvi-Vitvi01g00825\_t001 |  | | | |  | | | |  | | | |  | | | |  |  |  |  |
| 4 | Vvi-Vitvi01g00826\_t001 |  | | | |  | Ath-AT1G24095.1 |  | | | |  | | | |  |  |  |  |
| 4 | Vvi-Vitvi01g04213\_t001 |  | | | |  | | | |  | | | |  | | | |  |  |  |  |
| 4 | Vvi-Vitvi01g00830\_t002 |  | | | |  | | | |  | | | |  | | | |  |  |  |  |
| 4 | Vvi-Vitvi01g00831\_t001 |  | | | |  | | | |  | | | |  | | | |  |  |  |  |
| 4 | Vvi-Vitvi01g00832\_t001 |  | | | |  | | | |  | | | |  | | | |  |  |  |  |
| 5 | Vvi-Vitvi01g00833\_t001 |  | | | |  | | | |  | Ath-AT1G60030.1 |  | | | |  | Ath-AT1G10540.1 |  |  |  |
| 5 | Vvi-Vitvi01g04214\_t001 |  | | | |  | | | |  | | | |  | | | |  | | | |  |  |  |
| 5 | Vvi-Vitvi01g02091\_t001 |  | Ath-AT1G70220.1 |  | | | |  | | | |  | | | |  | | | |  |  |  |
| 5 | Vvi-Vitvi01g00836\_t001 |  | | | |  | | | |  | | | |  | | | |  | | | |  |  |  |
| 5 | Vvi-Vitvi01g00842\_t001 |  | | | |  | | | |  | | | |  | | | |  | | | |  |  |  |
| 5 | Vvi-Vitvi01g02092\_t001 |  | | | |  | | | |  | | | |  | | | |  | | | |  |  |  |
| 5 | Vvi-Vitvi01g00844\_t001 |  | | | |  | Ath-AT1G24110.1 |  | | | |  | Ath-AT3G28200.1 |  | | | |  |  |  |
| 5 | Vvi-Vitvi01g02093\_t001 |  | | | |  | Ath-AT1G24120.1 |  | Ath-AT1G59980.1 |  | | | |  | | | |  |  |  |
| 5 | Vvi-Vitvi01g00845\_t001 |  | | | |  | | | |  | | | |  | | | |  | Ath-AT1G10480.1 |  |  |  |
| 5 | Vvi-Vitvi01g00846\_t002 |  | Ath-AT1G70180.2 |  | | | |  | | | |  | | | |  | | | |  |  |  |
| 5 | Vvi-Vitvi01g02094\_t001 |  | | | |  | | | |  | | | |  | | | |  | | | |  |  |  |
| 5 | Vvi-Vitvi01g00849\_t001 |  | | | |  | Ath-AT1G24130.1 |  | | | |  | | | |  | | | |  |  |  |
| 5 | Vvi-Vitvi01g00850\_t001 |  | Ath-AT1G70170.1 |  | Ath-AT1G24140.1 |  | Ath-AT1G59970.1 |  | | | |  | | | |  |  |  |
| 5 | Vvi-Vitvi01g02096\_t001 |  | | | |  | | | |  | | | |  | | | |  | | | |  |  |  |
| 5 | Vvi-Vitvi01g00852\_t001 |  | Ath-AT1G70160.1 |  | | | |  | | | |  | | | |  | | | |  |  |  |
| 5 | Vvi-Vitvi01g00853\_t001 |  | Ath-AT1G70150.1 |  | | | |  | | | |  | | | |  | | | |  |  |  |
| 5 | Vvi-Vitvi01g00857\_t001 |  | | | |  | | | |  | Ath-AT1G59940.2 |  | | | |  | Ath-AT1G10470.1 |  |  |  |
| 5 | Vvi-Vitvi01g00858\_t001 |  | Ath-AT1G70140.1 |  | | | |  | Ath-AT1G59910.1 |  | | | |  | | | |  |  |  |
| 5 | Vvi-Vitvi01g00859\_t001 |  | Ath-AT1G70100.3 |  | Ath-AT1G24160.1 |  | | | |  | | | |  | | | |  |  |  |
| 5 | Vvi-Vitvi01g02097\_t001 |  | | | |  | | | |  | | | |  | | | |  | Ath-AT1G10460.1 |  |  |  |
| 5 | Vvi-Vitvi01g00860\_t001 |  | Ath-AT1G70090.1 |  | Ath-AT1G24170.1 |  | | | |  | Ath-AT3G28340.1 |  | | | |  |  |  |
| 5 | Vvi-Vitvi01g00861\_t001 |  | | | |  | Ath-AT1G24180.1 |  | Ath-AT1G59900.1 |  | | | |  | | | |  |  |  |
| 5 | Vvi-Vitvi01g00862\_t001 |  | Ath-AT1G70070.1 |  | | | |  | | | |  | | | |  | | | |  |  |  |
| 5 | Vvi-Vitvi01g02098\_t001 |  | Ath-AT1G70060.1 |  | Ath-AT1G24190.3 |  | Ath-AT1G59890.2 |  | | | |  | | | |  |  |  |
| 4 | Vvi-Vitvi01g04215\_t001 |  | | | |  |  |  | | | |  | | | |  | | | |  |  |  |
| 4 | Vvi-Vitvi01g00863\_t001 |  | | | |  |  |  | | | |  | | | |  | | | |  |  |  |
| 6 | Vvi-Vitvi01g00864\_t001 |  | | | |  | Ath-AT1G27210.1 |  | Ath-AT1G59850.1 |  | | | |  | | | |  | Ath-AT5G62580.1 |  |  |
| 6 | Vvi-Vitvi01g02099\_t001 |  | | | |  | Ath-AT1G27200.1 |  | | | |  | | | |  | | | |  | | | |  |  |
| 6 | Vvi-Vitvi01g00865\_t001 |  | | | |  | | | |  | Ath-AT1G59840.1 |  | | | |  | | | |  | | | |  |  |
| 6 | Vvi-Vitvi01g00867\_t001 |  | Ath-AT1G70000.1 |  | | | |  | | | |  | | | |  | | | |  | | | |  |  |
| 6 | Vvi-Vitvi01g00868\_t001 |  | Ath-AT1G69990.1 |  | Ath-AT1G27190.1 |  | | | |  | Ath-AT3G28450.1 |  | | | |  | | | |  |  |
| 6 | Vvi-Vitvi01g00869\_t001 |  | Ath-AT1G69980.1 |  | | | |  | | | |  | | | |  | | | |  | | | |  |  |
| 6 | Vvi-Vitvi01g00870\_t001 |  | Ath-AT1G69960.1 |  | | | |  | Ath-AT1G59830.1 |  | | | |  | Ath-AT1G10430.1 |  | | | |  |  |
| 5 | Vvi-Vitvi01g02100\_t001 |  | | | |  | | | |  |  |  | Ath-AT3G28455.1 |  | | | |  | | | |  |  |
| 5 | Vvi-Vitvi01g04216\_t001 |  | | | |  | | | |  |  |  | | | |  | | | |  | | | |  |  |
| 5 | Vvi-Vitvi01g00873\_t001 |  | | | |  | Ath-AT1G27170.2 |  |  |  | | | |  | | | |  | | | |  |  |
| 5 | Vvi-Vitvi01g00874\_t001 |  | | | |  | | | |  |  |  | | | |  | | | |  | | | |  |  |
| 5 | Vvi-Vitvi01g00875\_t001 |  | | | |  | | | |  |  |  | | | |  | | | |  | | | |  |  |
| 5 | Vvi-Vitvi01g02101\_t001 |  | Ath-AT1G69935.1 |  | | | |  |  |  | | | |  | | | |  | | | |  |  |
| 6 | Vvi-Vitvi01g00876\_t001 |  | | | |  | | | |  | Ath-AT1G59640.2 |  | | | |  | | | |  | Ath-AT5G62610.1 |  |  |
| 6 | Vvi-Vitvi01g00877\_t001 |  | | | |  | | | |  | | | |  | | | |  | Ath-AT1G10417.4 |  | | | |  |  |
| 6 | Vvi-Vitvi01g00878\_t001 |  | | | |  | | | |  | Ath-AT1G59650.1 |  | | | |  | Ath-AT1G10410.1 |  | | | |  |  |
| 6 | Vvi-Vitvi01g00879\_t001 |  | | | |  | | | |  | | | |  | | | |  | | | |  | | | |  |  |
| 6 | Vvi-Vitvi01g00880\_t001 |  | | | |  | | | |  | | | |  | | | |  | | | |  | | | |  |  |
| 6 | Vvi-Vitvi01g02102\_t001 |  | | | |  | | | |  | | | |  | | | |  | | | |  | | | |  |  |
| 6 | Vvi-Vitvi01g00881\_t001 |  | | | |  | | | |  | | | |  | | | |  | Ath-AT1G10385.1 |  | | | |  |  |
| 6 | Vvi-Vitvi01g00882\_t001 |  | | | |  | | | |  | | | |  | | | |  | | | |  | Ath-AT5G62620.1 |  |  |
| 6 | Vvi-Vitvi01g00883\_t001 |  | | | |  | | | |  | | | |  | | | |  | Ath-AT1G10380.1 |  | | | |  |  |
| 6 | Vvi-Vitvi01g00884\_t001 |  | | | |  | | | |  | | | |  | | | |  | | | |  | | | |  |  |
| 6 | Vvi-Vitvi01g00885\_t001 |  | | | |  | | | |  | | | |  | | | |  | | | |  | | | |  |  |
| 6 | Vvi-Vitvi01g00886\_t001 |  | | | |  | | | |  | | | |  | | | |  | | | |  | Ath-AT5G62630.1 |  |  |
| 6 | Vvi-Vitvi01g00888\_t001 |  | Ath-AT1G69930.1 |  | | | |  | Ath-AT1G59670.1 |  | | | |  | Ath-AT1G10360.1 |  | | | |  |  |
| 6 | Vvi-Vitvi01g00889\_t003 |  | | | |  | | | |  | | | |  | | | |  | | | |  | | | |  |  |
| 6 | Vvi-Vitvi01g00892\_t001 |  | | | |  | Ath-AT1G27130.1 |  | | | |  | | | |  | | | |  | | | |  |  |
| 6 | Vvi-Vitvi01g04217\_t001 |  | | | |  | | | |  | | | |  | | | |  | | | |  | | | |  |  |
| 6 | Vvi-Vitvi01g00893\_t001 |  | | | |  | | | |  | | | |  | | | |  | | | |  | | | |  |  |
| 6 | Vvi-Vitvi01g00898\_t001 |  | | | |  | Ath-AT1G27110.1 |  | | | |  | | | |  | | | |  | | | |  |  |
| 6 | Vvi-Vitvi01g04218\_t001 |  | | | |  | | | |  | | | |  | | | |  | | | |  | | | |  |  |
| 6 | Vvi-Vitvi01g00900\_t001 |  | Ath-AT1G69910.1 |  | | | |  | | | |  | | | |  | | | |  | | | |  |  |
| 6 | Vvi-Vitvi01g00902\_t004 |  | Ath-AT1G69890.1 |  | Ath-AT1G27100.1 |  | Ath-AT1G59710.2 |  | Ath-AT3G28630.1 |  | | | |  | | | |  |  |
| 6 | Vvi-Vitvi01g00903\_t001 |  | | | |  | | | |  | Ath-AT1G59720.1 |  | | | |  | | | |  | | | |  |  |
| 6 | Vvi-Vitvi01g00905\_t001 |  | | | |  | | | |  | | | |  | | | |  | | | |  | | | |  |  |
| 6 | Vvi-Vitvi01g00906\_t001 |  | | | |  | | | |  | Ath-AT1G59725.1 |  | | | |  | Ath-AT1G10350.1 |  | | | |  |  |
| 5 | Vvi-Vitvi01g00907\_t001 |  | | | |  | | | |  | Ath-AT1G59730.1 |  | | | |  |  |  | | | |  |  |
| 5 | Vvi-Vitvi01g00908\_t001 |  | | | |  | Ath-AT1G27090.1 |  | | | |  | | | |  |  |  | | | |  |  |
| 5 | Vvi-Vitvi01g00909\_t001 |  | | | |  | | | |  | | | |  | | | |  |  |  | | | |  |  |
| 5 | Vvi-Vitvi01g00911\_t001 |  | Ath-AT1G69860.1 |  | Ath-AT1G27080.1 |  | | | |  | | | |  |  |  | Ath-AT5G62680.1 |  |  |
| 5 | Vvi-Vitvi01g00914\_t001 |  | | | |  | Ath-AT1G27070.1 |  | | | |  | | | |  |  |  | | | |  |  |
| 5 | Vvi-Vitvi01g00915\_t001 |  | | | |  | Ath-AT1G27060.1 |  | | | |  | | | |  |  |  | | | |  |  |
| 5 | Vvi-Vitvi01g00916\_t001 |  | | | |  | Ath-AT1G27050.1 |  | | | |  | | | |  |  |  | | | |  |  |
| 6 | Vvi-Vitvi01g00917\_t001 |  | | | |  | | | |  | | | |  | | | |  | Ath-AT2G02590.1 |  | | | |  |  |
| 6 | Vvi-Vitvi01g00918\_t001 |  | | | |  | | | |  | | | |  | | | |  | | | |  | | | |  |  |
| 6 | Vvi-Vitvi01g04219\_t001 |  | | | |  | | | |  | | | |  | | | |  | | | |  | | | |  |  |
| 6 | Vvi-Vitvi01g04220\_t001 |  | | | |  | | | |  | | | |  | | | |  | | | |  | | | |  |  |
| 6 | Vvi-Vitvi01g00919\_t001 |  | | | |  | | | |  | | | |  | | | |  | | | |  | | | |  |  |
| 6 | Vvi-Vitvi01g00921\_t001 |  | Ath-AT1G69850.1 |  | Ath-AT1G27040.1 |  | Ath-AT1G59740.1 |  | | | |  | | | |  | Ath-AT5G62730.1 |  |  |
| 6 | Vvi-Vitvi01g00922\_t001 |  | | | |  | | | |  | Ath-AT1G14430.1 |  | | | |  | | | |  | | | |  |  |
| 6 | Vvi-Vitvi01g04221\_t001 |  | | | |  | | | |  | | | |  | | | |  | | | |  | | | |  |  |
| 6 | Vvi-Vitvi01g00924\_t005 |  | | | |  | | | |  | | | |  | | | |  | Ath-AT2G02710.1 |  | | | |  |  |
| 6 | Vvi-Vitvi01g04222\_t001 |  | | | |  | | | |  | | | |  | | | |  | | | |  | | | |  |  |
| 6 | Vvi-Vitvi01g00926\_t002 |  | | | |  | | | |  | | | |  | | | |  | | | |  | | | |  |  |
| 6 | Vvi-Vitvi01g00927\_t001 |  | | | |  | | | |  | | | |  | Ath-AT3G28690.2 |  | | | |  | | | |  |  |
| 5 | Vvi-Vitvi01g00928\_t001 |  | | | |  | Ath-AT1G27020.1 |  | | | |  |  |  | | | |  | | | |  |  |
| 5 | Vvi-Vitvi01g00929\_t001 |  | Ath-AT1G69840.1 |  | | | |  | | | |  |  |  | | | |  | Ath-AT5G62740.1 |  |  |
| 4 | Vvi-Vitvi01g00930\_t001 |  | | | |  | | | |  | | | |  |  |  | | | |  |  |  |
| 4 | Vvi-Vitvi01g00932\_t001 |  | Ath-AT1G69830.1 |  | | | |  | | | |  |  |  | | | |  |  |  |
| 4 | Vvi-Vitvi01g02109\_t001 |  | | | |  | | | |  | Ath-AT1G14420.1 |  |  |  | Ath-AT2G02720.1 |  |  |  |
| 4 | Vvi-Vitvi01g00933\_t001 |  | | | |  | | | |  | | | |  |  |  | | | |  |  |  |
| 4 | Vvi-Vitvi01g00934\_t001 |  | | | |  | | | |  | | | |  |  |  | | | |  |  |  |
| 4 | Vvi-Vitvi01g00935\_t001 |  | | | |  | Ath-AT1G27000.1 |  | | | |  |  |  | Ath-AT2G02730.2 |  |  |  |
| 4 | Vvi-Vitvi01g00936\_t001 |  | | | |  | | | |  | | | |  |  |  | | | |  |  |  |
| 4 | Vvi-Vitvi01g00937\_t001 |  | | | |  | | | |  | | | |  |  |  | | | |  |  |  |
| 4 | Vvi-Vitvi01g00938\_t001 |  | | | |  | | | |  | Ath-AT1G14410.1 |  |  |  | Ath-AT2G02740.1 |  |  |  |
| 4 | Vvi-Vitvi01g00940\_t001 |  | Ath-AT1G69810.1 |  | | | |  | | | |  |  |  | | | |  |  |  |
| 4 | Vvi-Vitvi01g00941\_t001 |  | Ath-AT1G69800.2 |  | | | |  | | | |  |  |  | | | |  |  |  |
| 4 | Vvi-Vitvi01g00942\_t001 |  | | | |  | | | |  | | | |  |  |  | Ath-AT2G02750.1 |  |  |  |
| 4 | Vvi-Vitvi01g00943\_t002 |  | | | |  | | | |  | Ath-AT1G14400.1 |  |  |  | Ath-AT2G02760.2 |  |  |  |
| 4 | Vvi-Vitvi01g00944\_t001 |  | | | |  | | | |  | Ath-AT1G14390.1 |  |  |  | Ath-AT2G02780.1 |  |  |  |
| 4 | Vvi-Vitvi01g00945\_t001 |  | | | |  | | | |  | Ath-AT1G14380.3 |  |  |  | Ath-AT2G02790.1 |  |  |  |
| 4 | Vvi-Vitvi01g02113\_t001 |  | | | |  | | | |  | | | |  |  |  | | | |  |  |  |
| 4 | Vvi-Vitvi01g04223\_t001 |  | | | |  | | | |  | | | |  |  |  | | | |  |  |  |
| 4 | Vvi-Vitvi01g04224\_t001 |  | | | |  | | | |  | | | |  |  |  | | | |  |  |  |
| 4 | Vvi-Vitvi01g02115\_t001 |  | | | |  | | | |  | | | |  |  |  | | | |  |  |  |
| 4 | Vvi-Vitvi01g04225\_t001 |  | | | |  | Ath-AT1G26800.1 |  | | | |  |  |  | | | |  |  |  |
| 3 | Vvi-Vitvi01g04226\_t001 |  | | | |  |  |  | | | |  |  |  | | | |  |  |  |
| 3 | Vvi-Vitvi01g04227\_t001 |  | | | |  |  |  | | | |  |  |  | | | |  |  |  |
| 3 | Vvi-Vitvi01g04228\_t001 |  | | | |  |  |  | | | |  |  |  | | | |  |  |  |
| 3 | Vvi-Vitvi01g04229\_t001 |  | | | |  |  |  | | | |  |  |  | | | |  |  |  |
| 3 | Vvi-Vitvi01g04230\_t001 |  | | | |  |  |  | | | |  |  |  | | | |  |  |  |
| 3 | Vvi-Vitvi01g02117\_t001 |  | | | |  |  |  | | | |  |  |  | | | |  |  |  |
| 3 | Vvi-Vitvi01g02118\_t001 |  | | | |  |  |  | | | |  |  |  | | | |  |  |  |
| 3 | Vvi-Vitvi01g04231\_t001 |  | | | |  |  |  | | | |  |  |  | | | |  |  |  |
| 3 | Vvi-Vitvi01g02119\_t001 |  | | | |  |  |  | | | |  |  |  | | | |  |  |  |
| 3 | Vvi-Vitvi01g02120\_t001 |  | | | |  |  |  | | | |  |  |  | | | |  |  |  |
| 3 | Vvi-Vitvi01g04232\_t001 |  | | | |  |  |  | | | |  |  |  | | | |  |  |  |
| 3 | Vvi-Vitvi01g00950\_t001 |  | | | |  |  |  | | | |  |  |  | | | |  |  |  |
| 3 | Vvi-Vitvi01g02121\_t001 |  | | | |  |  |  | | | |  |  |  | | | |  |  |  |
| 3 | Vvi-Vitvi01g00952\_t001 |  | | | |  |  |  | | | |  |  |  | | | |  |  |  |
| 3 | Vvi-Vitvi01g04233\_t001 |  | | | |  |  |  | | | |  |  |  | | | |  |  |  |
| 3 | Vvi-Vitvi01g00953\_t001 |  | | | |  |  |  | Ath-AT1G14370.1 |  |  |  | Ath-AT2G02800.2 |  |  |  |
| 4 | Vvi-Vitvi01g00954\_t001 |  | Ath-AT1G69790.1 |  | Ath-AT1G26970.1 |  | | | |  |  |  | | | |  |  |  |
| 4 | Vvi-Vitvi01g00955\_t002 |  | | | |  | | | |  | Ath-AT1G14360.1 |  |  |  | Ath-AT2G02810.1 |  |  |  |
| 4 | Vvi-Vitvi01g00956\_t001 |  | | | |  | | | |  | Ath-AT1G14350.2 |  |  |  | Ath-AT2G02820.2 |  |  |  |
| 4 | Vvi-Vitvi01g04234\_t001 |  | | | |  | | | |  | | | |  |  |  | | | |  |  |  |
| 4 | Vvi-Vitvi01g00957\_t001 |  | | | |  | | | |  | Ath-AT1G14345.1 |  |  |  | | | |  |  |  |
| 4 | Vvi-Vitvi01g00958\_t002 |  | Ath-AT1G69780.1 |  | Ath-AT1G26960.1 |  | | | |  |  |  | | | |  |  |  |
| 4 | Vvi-Vitvi01g00959\_t001 |  | | | |  | | | |  | | | |  |  |  | Ath-AT2G02860.1 |  |  |  |
| 4 | Vvi-Vitvi01g00960\_t003 |  | | | |  | | | |  | Ath-AT1G14340.1 |  |  |  | | | |  |  |  |
| 5 | Vvi-Vitvi01g00964\_t001 |  | | | |  | Ath-AT1G26945.1 |  | | | |  | Ath-AT1G74500.1 |  | | | |  |  |  |
| 5 | Vvi-Vitvi01g00968\_t002 |  | | | |  | Ath-AT1G26940.1 |  | | | |  | | | |  | | | |  |  |  |
| 5 | Vvi-Vitvi01g04235\_t001 |  | | | |  | | | |  | | | |  | | | |  | | | |  |  |  |
| 5 | Vvi-Vitvi01g04236\_t001 |  | | | |  | | | |  | | | |  | | | |  | | | |  |  |  |
| 5 | Vvi-Vitvi01g04237\_t001 |  | | | |  | | | |  | | | |  | | | |  | | | |  |  |  |
| 5 | Vvi-Vitvi01g04238\_t001 |  | | | |  | | | |  | | | |  | | | |  | | | |  |  |  |
| 5 | Vvi-Vitvi01g00970\_t001 |  | | | |  | Ath-AT1G26930.1 |  | Ath-AT1G14330.1 |  | Ath-AT1G74510.1 |  | Ath-AT2G02870.3 |  |  |  |
| 5 | Vvi-Vitvi01g02127\_t001 |  | Ath-AT1G69760.1 |  | Ath-AT1G26920.1 |  | | | |  | | | |  | | | |  |  |  |
| 5 | Vvi-Vitvi01g00971\_t001 |  | | | |  | | | |  | | | |  | | | |  | | | |  |  |  |
| 5 | Vvi-Vitvi01g00972\_t001 |  | | | |  | | | |  | | | |  | | | |  | | | |  |  |  |
| 5 | Vvi-Vitvi01g00975\_t001 |  | | | |  | Ath-AT1G26900.1 |  | | | |  | | | |  | | | |  |  |  |
| 5 | Vvi-Vitvi01g00977\_t001 |  | Ath-AT1G69710.1 |  | | | |  | | | |  | | | |  | | | |  |  |  |
| 5 | Vvi-Vitvi01g04239\_t001 |  | | | |  | | | |  | | | |  | | | |  | | | |  |  |  |
| 5 | Vvi-Vitvi01g02128\_t001 |  | | | |  | | | |  | | | |  | | | |  | | | |  |  |  |
| 5 | Vvi-Vitvi01g00979\_t001 |  | Ath-AT1G69700.1 |  | | | |  | | | |  | Ath-AT1G74520.1 |  | | | |  |  |  |
| 5 | Vvi-Vitvi01g00980\_t001 |  | Ath-AT1G69690.1 |  | | | |  | | | |  | | | |  | | | |  |  |  |
| 5 | Vvi-Vitvi01g04240\_t001 |  | Ath-AT1G69680.1 |  | | | |  | | | |  | | | |  | | | |  |  |  |
| 6 | Vvi-Vitvi01g00985\_t001 |  | Ath-AT1G69670.1 |  | | | |  | | | |  | | | |  | | | |  | Ath-AT1G26830.1 |  |  |
| 6 | Vvi-Vitvi01g02129\_t001 |  | | | |  | | | |  | Ath-AT1G14310.1 |  | | | |  | | | |  | | | |  |  |
| 6 | Vvi-Vitvi01g00988\_t001 |  | | | |  | | | |  | | | |  | | | |  | Ath-AT2G02910.1 |  | | | |  |  |
| 6 | Vvi-Vitvi01g04241\_t001 |  | | | |  | | | |  | | | |  | | | |  | | | |  | | | |  |  |
| 6 | Vvi-Vitvi01g00989\_t001 |  | | | |  | | | |  | | | |  | | | |  | | | |  | | | |  |  |
| 6 | Vvi-Vitvi01g00991\_t001 |  | | | |  | | | |  | Ath-AT1G14300.2 |  | | | |  | | | |  | | | |  |  |
| 6 | Vvi-Vitvi01g00992\_t001 |  | | | |  | | | |  | | | |  | | | |  | | | |  | Ath-AT1G26840.1 |  |  |
| 6 | Vvi-Vitvi01g00993\_t001 |  | Ath-AT1G69640.1 |  | | | |  | Ath-AT1G14290.1 |  | | | |  | | | |  | | | |  |  |
| 6 | Vvi-Vitvi01g00994\_t001 |  | | | |  | | | |  | Ath-AT1G14280.1 |  | | | |  | Ath-AT2G02950.1 |  | | | |  |  |
| 6 | Vvi-Vitvi01g00995\_t001 |  | | | |  | | | |  | | | |  | | | |  | | | |  | Ath-AT1G26850.1 |  |  |
| 6 | Vvi-Vitvi01g00997\_t001 |  | | | |  | | | |  | Ath-AT1G14270.1 |  | | | |  | | | |  | | | |  |  |
| 6 | Vvi-Vitvi01g00999\_t001 |  | | | |  | | | |  | | | |  | | | |  | | | |  | Ath-AT1G26870.1 |  |  |
| 6 | Vvi-Vitvi01g04242\_t001 |  | | | |  | | | |  | | | |  | | | |  | | | |  | | | |  |  |
| 6 | Vvi-Vitvi01g01006\_t001 |  | | | |  | | | |  | | | |  | | | |  | | | |  | | | |  |  |
| 6 | Vvi-Vitvi01g01007\_t001 |  | | | |  | | | |  | | | |  | Ath-AT1G74590.1 |  | | | |  | | | |  |  |
| 6 | Vvi-Vitvi01g02131\_t001 |  | | | |  | | | |  | | | |  | | | |  | | | |  | | | |  |  |
| 6 | Vvi-Vitvi01g04244\_t001 |  | | | |  | | | |  | | | |  | | | |  | | | |  | | | |  |  |
| 6 | Vvi-Vitvi01g02132\_t001 |  | | | |  | | | |  | | | |  | | | |  | | | |  | | | |  |  |
| 6 | Vvi-Vitvi01g04245\_t001 |  | | | |  | | | |  | | | |  | | | |  | | | |  | | | |  |  |
| 7 | Vvi-Vitvi01g04246\_t001 |  | Ath-AT1G69620.1 |  | Ath-AT1G26880.1 |  | | | |  | | | |  | | | |  | Ath-AT1G26880.1 |  | Ath-AT3G28900.1 |  |
| 7 | Vvi-Vitvi01g01009\_t001 |  | Ath-AT1G69610.2 |  | | | |  | | | |  | | | |  | | | |  | | | |  | | | |  |
| 7 | Vvi-Vitvi01g01010\_t001 |  | | | |  | | | |  | | | |  | | | |  | Ath-AT2G02955.1 |  | | | |  | | | |  |
| 7 | Vvi-Vitvi01g01012\_t001 |  | | | |  | | | |  | | | |  | Ath-AT1G74660.1 |  | | | |  | | | |  | Ath-AT3G28917.1 |  |
| 7 | Vvi-Vitvi01g01013\_t001 |  | Ath-AT1G69600.1 |  | | | |  | | | |  | | | |  | | | |  | | | |  | Ath-AT3G28920.1 |  |
| 7 | Vvi-Vitvi01g04247\_t001 |  | | | |  | | | |  | | | |  | | | |  | | | |  | | | |  | | | |  |
| 7 | Vvi-Vitvi01g01015\_t001 |  | | | |  | | | |  | Ath-AT1G14260.2 |  | | | |  | Ath-AT2G02960.5 |  | | | |  | | | |  |
| 7 | Vvi-Vitvi01g01016\_t001 |  | | | |  | | | |  | | | |  | | | |  | | | |  | | | |  | | | |  |
| 7 | Vvi-Vitvi01g01017\_t001 |  | | | |  | | | |  | | | |  | | | |  | | | |  | | | |  | | | |  |
| 7 | Vvi-Vitvi01g01018\_t001 |  | | | |  | | | |  | Ath-AT1G14230.1 |  | | | |  | Ath-AT2G02970.1 |  | | | |  | | | |  |
| 7 | Vvi-Vitvi01g01019\_t001 |  | | | |  | | | |  | | | |  | | | |  | | | |  | | | |  | Ath-AT3G28960.3 |  |
| 7 | Vvi-Vitvi01g01020\_t001 |  | | | |  | | | |  | | | |  | | | |  | Ath-AT2G02980.1 |  | | | |  | | | |  |
| 7 | Vvi-Vitvi01g01021\_t001 |  | | | |  | Ath-AT1G26820.1 |  | Ath-AT1G14210.1 |  | | | |  | Ath-AT2G02990.1 |  | | | |  | | | |  |
| 7 | Vvi-Vitvi01g04248\_t001 |  | | | |  | | | |  | | | |  | | | |  | | | |  | | | |  | | | |  |
| 7 | Vvi-Vitvi01g01022\_t001 |  | | | |  | Ath-AT1G26810.2 |  | | | |  | Ath-AT1G74800.1 |  | | | |  | Ath-AT1G27120.1 |  | | | |  |
| 5 | Vvi-Vitvi01g01023\_t001 |  | | | |  | Ath-AT1G26800.1 |  | Ath-AT1G14200.1 |  |  |  | | | |  |  |  | | | |  |
| 5 | Vvi-Vitvi01g01024\_t001 |  | Ath-AT1G69580.2 |  | | | |  | | | |  |  |  | | | |  |  |  | | | |  |
| 5 | Vvi-Vitvi01g01026\_t001 |  | Ath-AT1G69570.1 |  | Ath-AT1G26790.1 |  | | | |  |  |  | | | |  |  |  | | | |  |
| 5 | Vvi-Vitvi01g01027\_t001 |  | | | |  | | | |  | | | |  |  |  | | | |  |  |  | Ath-AT3G29000.1 |  |
| 5 | Vvi-Vitvi01g01028\_t001 |  | Ath-AT1G69560.2 |  | Ath-AT1G26780.2 |  | | | |  |  |  | | | |  |  |  | Ath-AT3G29020.2 |  |
| 4 | Vvi-Vitvi01g04249\_t001 |  | | | |  | | | |  | | | |  |  |  | | | |  |  |  |
| 4 | Vvi-Vitvi01g01030\_t001 |  | Ath-AT1G69530.3 |  | Ath-AT1G26770.2 |  | | | |  |  |  | Ath-AT2G03090.1 |  |  |  |
| 3 | Vvi-Vitvi01g01031\_t001 |  | | | |  |  |  | | | |  |  |  | | | |  |  |  |
| 3 | Vvi-Vitvi01g04250\_t001 |  | | | |  |  |  | | | |  |  |  | | | |  |  |  |
| 3 | Vvi-Vitvi01g01033\_t001 |  | Ath-AT1G69520.2 |  |  |  | | | |  |  |  | | | |  |  |  |
| 3 | Vvi-Vitvi01g01034\_t001 |  | | | |  |  |  | Ath-AT1G14170.3 |  |  |  | | | |  |  |  |
| 3 | Vvi-Vitvi01g04251\_t001 |  | Ath-AT1G69510.1 |  |  |  | | | |  |  |  | | | |  |  |  |
| 3 | Vvi-Vitvi01g01035\_t001 |  | | | |  |  |  | Ath-AT1G14150.1 |  |  |  | | | |  |  |  |
| 3 | Vvi-Vitvi01g01036\_t001 |  | | | |  |  |  | | | |  |  |  | | | |  |  |  |
| 3 | Vvi-Vitvi01g01037\_t001 |  | Ath-AT1G69500.1 |  |  |  | | | |  |  |  | | | |  |  |  |
| 3 | Vvi-Vitvi01g01038\_t001 |  | Ath-AT1G69490.1 |  |  |  | | | |  |  |  | | | |  |  |  |
| 2 | Vvi-Vitvi01g01040\_t001 |  |  |  |  |  | | | |  |  |  | Ath-AT2G03150.1 |  |  |  |
| 2 | Vvi-Vitvi01g04252\_t001 |  |  |  |  |  | | | |  |  |  | | | |  |  |  |
| 2 | Vvi-Vitvi01g04253\_t001 |  |  |  |  |  | | | |  |  |  | | | |  |  |  |
| 2 | Vvi-Vitvi01g04254\_t001 |  |  |  |  |  | | | |  |  |  | | | |  |  |  |
| 2 | Vvi-Vitvi01g01044\_t001 |  |  |  |  |  | | | |  |  |  | | | |  |  |  |
| 2 | Vvi-Vitvi01g01048\_t001 |  |  |  |  |  | | | |  |  |  | | | |  |  |  |
| 2 | Vvi-Vitvi01g01049\_t001 |  |  |  |  |  | Ath-AT1G14140.1 |  |  |  | | | |  |  |  |
| 2 | Vvi-Vitvi01g01052\_t001 |  |  |  |  |  | | | |  |  |  | | | |  |  |  |
| 2 | Vvi-Vitvi01g01053\_t001 |  |  |  |  |  | | | |  |  |  | | | |  |  |  |
| 2 | Vvi-Vitvi01g04255\_t001 |  |  |  |  |  | | | |  |  |  | | | |  |  |  |
| 2 | Vvi-Vitvi01g04256\_t001 |  |  |  |  |  | | | |  |  |  | | | |  |  |  |
| 2 | Vvi-Vitvi01g04257\_t001 |  |  |  |  |  | | | |  |  |  | | | |  |  |  |
| 2 | Vvi-Vitvi01g04258\_t001 |  |  |  |  |  | | | |  |  |  | | | |  |  |  |
| 2 | Vvi-Vitvi01g04259\_t001 |  |  |  |  |  | | | |  |  |  | | | |  |  |  |
| 2 | Vvi-Vitvi01g04260\_t001 |  |  |  |  |  | | | |  |  |  | | | |  |  |  |
| 2 | Vvi-Vitvi01g04261\_t002 |  |  |  |  |  | | | |  |  |  | | | |  |  |  |
| 2 | Vvi-Vitvi01g01089\_t001 |  |  |  |  |  | | | |  |  |  | | | |  |  |  |
| 2 | Vvi-Vitvi01g04262\_t001 |  |  |  |  |  | | | |  |  |  | | | |  |  |  |
| 2 | Vvi-Vitvi01g01087\_t001 |  |  |  |  |  | | | |  |  |  | | | |  |  |  |
| 2 | Vvi-Vitvi01g02140\_t001 |  |  |  |  |  | | | |  |  |  | | | |  |  |  |
| 2 | Vvi-Vitvi01g04263\_t001 |  |  |  |  |  | | | |  |  |  | | | |  |  |  |
| 2 | Vvi-Vitvi01g01085\_t001 |  |  |  |  |  | | | |  |  |  | | | |  |  |  |
| 2 | Vvi-Vitvi01g01083\_t001 |  |  |  |  |  | | | |  |  |  | | | |  |  |  |
| 2 | Vvi-Vitvi01g01082\_t002 |  |  |  |  |  | | | |  |  |  | | | |  |  |  |
| 2 | Vvi-Vitvi01g01079\_t001 |  |  |  |  |  | Ath-AT1G14080.1 |  |  |  | Ath-AT2G03210.2 |  |  |  |
| 2 | Vvi-Vitvi01g01077\_t001 |  |  |  |  |  | Ath-AT1G14040.1 |  |  |  | Ath-AT2G03240.1 |  |  |  |
| 2 | Vvi-Vitvi01g01072\_t001 |  |  |  |  |  | | | |  |  |  | | | |  |  |  |
| 2 | Vvi-Vitvi01g01071\_t001 |  |  |  |  |  | | | |  |  |  | | | |  |  |  |
| 2 | Vvi-Vitvi01g04264\_t001 |  |  |  |  |  | | | |  |  |  | | | |  |  |  |
| 2 | Vvi-Vitvi01g04265\_t001 |  |  |  |  |  | | | |  |  |  | | | |  |  |  |
| 2 | Vvi-Vitvi01g02139\_t001 |  |  |  |  |  | | | |  |  |  | | | |  |  |  |
| 2 | Vvi-Vitvi01g01061\_t001 |  |  |  |  |  | | | |  |  |  | | | |  |  |  |
| 2 | Vvi-Vitvi01g01058\_t001 |  |  |  |  |  | Ath-AT1G14030.1 |  |  |  | | | |  |  |  |
| 2 | Vvi-Vitvi01g01057\_t001 |  |  |  |  |  | Ath-AT1G14020.1 |  |  |  | Ath-AT2G03280.2 |  |  |  |
| 2 | Vvi-Vitvi01g01056\_t001 |  |  |  |  |  | Ath-AT1G14010.1 |  |  |  | Ath-AT2G03290.1 |  |  |  |
| 1 | Vvi-Vitvi01g01055\_t001 |  |  |  |  |  | Ath-AT1G14000.1 |  |  |  |  |  |
| 0 | Vvi-Vitvi01g04266\_t001 |  |  |  |  |  |  |  |  |
| 0 | Vvi-Vitvi01g04267\_t001 |  |  |  |  |  |  |  |  |
| 0 | Vvi-Vitvi01g04268\_t001 |  |  |  |  |  |  |  |  |
| 0 | Vvi-Vitvi01g04269\_t001 |  |  |  |  |  |  |  |  |
| 0 | Vvi-Vitvi01g04270\_t001 |  |  |  |  |  |  |  |  |
| 0 | Vvi-Vitvi01g04271\_t001 |  |  |  |  |  |  |  |  |
| 0 | Vvi-Vitvi01g04272\_t001 |  |  |  |  |  |  |  |  |
| 0 | Vvi-Vitvi01g04273\_t001 |  |  |  |  |  |  |  |  |
| 0 | Vvi-Vitvi01g04274\_t001 |  |  |  |  |  |  |  |  |
| 0 | Vvi-Vitvi01g04275\_t001 |  |  |  |  |  |  |  |  |
| 0 | Vvi-Vitvi01g04276\_t001 |  |  |  |  |  |  |  |  |
| 0 | Vvi-Vitvi01g04277\_t001 |  |  |  |  |  |  |  |  |
| 0 | Vvi-Vitvi01g04278\_t001 |  |  |  |  |  |  |  |  |
| 0 | Vvi-Vitvi01g02141\_t001 |  |  |  |  |  |  |  |  |
| 0 | Vvi-Vitvi01g01116\_t001 |  |  |  |  |  |  |  |  |
| 0 | Vvi-Vitvi01g04280\_t001 |  |  |  |  |  |  |  |  |
| 0 | Vvi-Vitvi01g01121\_t001 |  |  |  |  |  |  |  |  |
| 0 | Vvi-Vitvi01g02150\_t001 |  |  |  |  |  |  |  |  |
| 0 | Vvi-Vitvi01g04281\_t001 |  |  |  |  |  |  |  |  |
| 0 | Vvi-Vitvi01g01125\_t001 |  |  |  |  |  |  |  |  |
| 0 | Vvi-Vitvi01g01126\_t001 |  |  |  |  |  |  |  |  |
| 0 | Vvi-Vitvi01g01127\_t001 |  |  |  |  |  |  |  |  |
| 0 | Vvi-Vitvi01g01129\_t001 |  |  |  |  |  |  |  |  |
| 0 | Vvi-Vitvi01g01130\_t001 |  |  |  |  |  |  |  |  |
| 0 | Vvi-Vitvi01g01132\_t001 |  |  |  |  |  |  |  |  |
| 0 | Vvi-Vitvi01g01133\_t001 |  |  |  |  |  |  |  |  |
| 0 | Vvi-Vitvi01g01134\_t001 |  |  |  |  |  |  |  |  |
| 0 | Vvi-Vitvi01g00978\_t001 |  |  |  |  |  |  |  |  |
| 0 | Vvi-Vitvi01g04282\_t001 |  |  |  |  |  |  |  |  |
| 0 | Vvi-Vitvi01g02154\_t001 |  |  |  |  |  |  |  |  |
| 0 | Vvi-Vitvi01g01137\_t001 |  |  |  |  |  |  |  |  |
| 0 | Vvi-Vitvi01g04283\_t001 |  |  |  |  |  |  |  |  |
| 0 | Vvi-Vitvi01g04284\_t001 |  |  |  |  |  |  |  |  |
| 0 | Vvi-Vitvi01g04285\_t001 |  |  |  |  |  |  |  |  |
| 0 | Vvi-Vitvi01g04286\_t001 |  |  |  |  |  |  |  |  |
| 0 | Vvi-Vitvi01g04287\_t001 |  |  |  |  |  |  |  |  |
| 0 | Vvi-Vitvi01g04288\_t001 |  |  |  |  |  |  |  |  |
| 0 | Vvi-Vitvi01g04289\_t001 |  |  |  |  |  |  |  |  |
| 0 | Vvi-Vitvi01g04290\_t001 |  |  |  |  |  |  |  |  |
| 0 | Vvi-Vitvi01g02157\_t001 |  |  |  |  |  |  |  |  |
| 0 | Vvi-Vitvi01g01153\_t001 |  |  |  |  |  |  |  |  |
| 0 | Vvi-Vitvi01g04291\_t001 |  |  |  |  |  |  |  |  |
| 0 | Vvi-Vitvi01g04292\_t001 |  |  |  |  |  |  |  |  |
| 0 | Vvi-Vitvi01g04293\_t001 |  |  |  |  |  |  |  |  |
| 0 | Vvi-Vitvi01g04294\_t001 |  |  |  |  |  |  |  |  |
| 0 | Vvi-Vitvi01g04295\_t001 |  |  |  |  |  |  |  |  |
| 0 | Vvi-Vitvi01g04296\_t001 |  |  |  |  |  |  |  |  |
| 0 | Vvi-Vitvi01g04297\_t001 |  |  |  |  |  |  |  |  |
| 0 | Vvi-Vitvi01g04298\_t001 |  |  |  |  |  |  |  |  |
| 0 | Vvi-Vitvi01g04299\_t001 |  |  |  |  |  |  |  |  |
| 0 | Vvi-Vitvi01g04300\_t001 |  |  |  |  |  |  |  |  |
| 0 | Vvi-Vitvi01g01160\_t001 |  |  |  |  |  |  |  |  |
| 0 | Vvi-Vitvi01g04301\_t001 |  |  |  |  |  |  |  |  |
| 0 | Vvi-Vitvi01g04302\_t001 |  |  |  |  |  |  |  |  |
| 0 | Vvi-Vitvi01g01163\_t001 |  |  |  |  |  |  |  |  |
| 0 | Vvi-Vitvi01g01164\_t001 |  |  |  |  |  |  |  |  |
| 0 | Vvi-Vitvi01g02160\_t001 |  |  |  |  |  |  |  |  |
| 0 | Vvi-Vitvi01g01165\_t004 |  |  |  |  |  |  |  |  |
| 0 | Vvi-Vitvi01g01167\_t001 |  |  |  |  |  |  |  |  |
| 0 | Vvi-Vitvi01g01169\_t001 |  |  |  |  |  |  |  |  |
| 0 | Vvi-Vitvi01g04303\_t001 |  |  |  |  |  |  |  |  |
| 0 | Vvi-Vitvi01g01173\_t001 |  |  |  |  |  |  |  |  |
| 0 | Vvi-Vitvi01g04304\_t001 |  |  |  |  |  |  |  |  |
| 0 | Vvi-Vitvi01g04305\_t001 |  |  |  |  |  |  |  |  |
| 0 | Vvi-Vitvi01g04306\_t001 |  |  |  |  |  |  |  |  |
| 0 | Vvi-Vitvi01g04307\_t001 |  |  |  |  |  |  |  |  |
| 0 | Vvi-Vitvi01g04308\_t001 |  |  |  |  |  |  |  |  |
| 0 | Vvi-Vitvi01g04309\_t001 |  |  |  |  |  |  |  |  |
| 0 | Vvi-Vitvi01g04310\_t001 |  |  |  |  |  |  |  |  |
| 0 | Vvi-Vitvi01g01236\_t001 |  |  |  |  |  |  |  |  |
| 0 | Vvi-Vitvi01g01253\_t001 |  |  |  |  |  |  |  |  |
| 0 | Vvi-Vitvi01g01258\_t001 |  |  |  |  |  |  |  |  |
| 0 | Vvi-Vitvi01g01259\_t001 |  |  |  |  |  |  |  |  |
| 0 | Vvi-Vitvi01g04311\_t001 |  |  |  |  |  |  |  |  |
| 0 | Vvi-Vitvi01g04312\_t001 |  |  |  |  |  |  |  |  |
| 0 | Vvi-Vitvi01g01265\_t001 |  |  |  |  |  |  |  |  |
| 0 | Vvi-Vitvi01g01267\_t001 |  |  |  |  |  |  |  |  |
| 0 | Vvi-Vitvi01g04313\_t001 |  |  |  |  |  |  |  |  |
| 0 | Vvi-Vitvi01g01268\_t001 |  |  |  |  |  |  |  |  |
| 0 | Vvi-Vitvi01g04314\_t001 |  |  |  |  |  |  |  |  |
| 0 | Vvi-Vitvi01g04315\_t001 |  |  |  |  |  |  |  |  |
| 0 | Vvi-Vitvi01g01271\_t001 |  |  |  |  |  |  |  |  |
| 0 | Vvi-Vitvi01g04316\_t001 |  |  |  |  |  |  |  |  |
| 0 | Vvi-Vitvi01g04317\_t001 |  |  |  |  |  |  |  |  |
| 0 | Vvi-Vitvi01g01278\_t001 |  |  |  |  |  |  |  |  |
| 0 | Vvi-Vitvi01g01280\_t001 |  |  |  |  |  |  |  |  |
| 0 | Vvi-Vitvi01g04318\_t001 |  |  |  |  |  |  |  |  |
| 0 | Vvi-Vitvi01g01282\_t001 |  |  |  |  |  |  |  |  |
| 0 | Vvi-Vitvi01g01283\_t001 |  |  |  |  |  |  |  |  |
| 0 | Vvi-Vitvi01g01284\_t001 |  |  |  |  |  |  |  |  |
| 0 | Vvi-Vitvi01g04319\_t001 |  |  |  |  |  |  |  |  |
| 0 | Vvi-Vitvi01g04320\_t001 |  |  |  |  |  |  |  |  |
| 0 | Vvi-Vitvi01g01287\_t001 |  |  |  |  |  |  |  |  |
| 1 | Vvi-Vitvi01g01289\_t001 |  | Ath-AT1G69020.2 |  |  |  |  |  |  |  |
| 1 | Vvi-Vitvi01g02171\_t001 |  | | | |  |  |  |  |  |  |  |
| 1 | Vvi-Vitvi01g01291\_t001 |  | | | |  |  |  |  |  |  |  |
| 1 | Vvi-Vitvi01g04321\_t001 |  | | | |  |  |  |  |  |  |  |
| 1 | Vvi-Vitvi01g01295\_t001 |  | | | |  |  |  |  |  |  |  |
| 1 | Vvi-Vitvi01g01296\_t001 |  | | | |  |  |  |  |  |  |  |
| 1 | Vvi-Vitvi01g01298\_t001 |  | | | |  |  |  |  |  |  |  |
| 1 | Vvi-Vitvi01g01299\_t001 |  | | | |  |  |  |  |  |  |  |
| 1 | Vvi-Vitvi01g01301\_t001 |  | | | |  |  |  |  |  |  |  |
| 1 | Vvi-Vitvi01g02173\_t001 |  | | | |  |  |  |  |  |  |  |
| 1 | Vvi-Vitvi01g01302\_t001 |  | | | |  |  |  |  |  |  |  |
| 1 | Vvi-Vitvi01g04322\_t001 |  | | | |  |  |  |  |  |  |  |
| 1 | Vvi-Vitvi01g04323\_t001 |  | | | |  |  |  |  |  |  |  |
| 1 | Vvi-Vitvi01g02174\_t001 |  | | | |  |  |  |  |  |  |  |
| 1 | Vvi-Vitvi01g01304\_t001 |  | | | |  |  |  |  |  |  |  |
| 1 | Vvi-Vitvi01g01307\_t001 |  | | | |  |  |  |  |  |  |  |
| 1 | Vvi-Vitvi01g01311\_t001 |  | | | |  |  |  |  |  |  |  |
| 1 | Vvi-Vitvi01g01312\_t001 |  | Ath-AT1G68990.2 |  |  |  |  |  |  |  |
| 1 | Vvi-Vitvi01g01313\_t001 |  | Ath-AT1G68940.2 |  |  |  |  |  |  |  |
| 1 | Vvi-Vitvi01g01314\_t001 |  | | | |  |  |  |  |  |  |  |
| 1 | Vvi-Vitvi01g01315\_t001 |  | Ath-AT1G68930.1 |  |  |  |  |  |  |  |
| 1 | Vvi-Vitvi01g01316\_t002 |  | Ath-AT1G68920.4 |  |  |  |  |  |  |  |
| 1 | Vvi-Vitvi01g01317\_t001 |  | Ath-AT1G68910.1 |  |  |  |  |  |  |  |
| 1 | Vvi-Vitvi01g04324\_t001 |  | | | |  |  |  |  |  |  |  |
| 1 | Vvi-Vitvi01g01320\_t001 |  | | | |  |  |  |  |  |  |  |
| 1 | Vvi-Vitvi01g04325\_t001 |  | | | |  |  |  |  |  |  |  |
| 1 | Vvi-Vitvi01g04326\_t001 |  | | | |  |  |  |  |  |  |  |
| 1 | Vvi-Vitvi01g04327\_t001 |  | | | |  |  |  |  |  |  |  |
| 1 | Vvi-Vitvi01g04328\_t001 |  | | | |  |  |  |  |  |  |  |
| 1 | Vvi-Vitvi01g04329\_t001 |  | | | |  |  |  |  |  |  |  |
| 1 | Vvi-Vitvi01g01328\_t001 |  | | | |  |  |  |  |  |  |  |
| 1 | Vvi-Vitvi01g04330\_t001 |  | | | |  |  |  |  |  |  |  |
| 1 | Vvi-Vitvi01g04331\_t001 |  | | | |  |  |  |  |  |  |  |
| 1 | Vvi-Vitvi01g02178\_t001 |  | | | |  |  |  |  |  |  |  |
| 1 | Vvi-Vitvi01g01331\_t001 |  | Ath-AT1G68890.1 |  |  |  |  |  |  |  |
| 1 | Vvi-Vitvi01g01333\_t001 |  | | | |  |  |  |  |  |  |  |
| 1 | Vvi-Vitvi01g01335\_t001 |  | | | |  |  |  |  |  |  |  |
| 1 | Vvi-Vitvi01g01336\_t001 |  | | | |  |  |  |  |  |  |  |
| 1 | Vvi-Vitvi01g04332\_t001 |  | | | |  |  |  |  |  |  |  |
| 1 | Vvi-Vitvi01g01339\_t001 |  | | | |  |  |  |  |  |  |  |
| 1 | Vvi-Vitvi01g04333\_t001 |  | | | |  |  |  |  |  |  |  |
| 1 | Vvi-Vitvi01g04334\_t001 |  | | | |  |  |  |  |  |  |  |
| 1 | Vvi-Vitvi01g01342\_t001 |  | | | |  |  |  |  |  |  |  |
| 1 | Vvi-Vitvi01g01344\_t001 |  | | | |  |  |  |  |  |  |  |
| 1 | Vvi-Vitvi01g01345\_t001 |  | | | |  |  |  |  |  |  |  |
| 1 | Vvi-Vitvi01g01346\_t001 |  | | | |  |  |  |  |  |  |  |
| 1 | Vvi-Vitvi01g01348\_t001 |  | | | |  |  |  |  |  |  |  |
| 1 | Vvi-Vitvi01g04335\_t001 |  | | | |  |  |  |  |  |  |  |
| 1 | Vvi-Vitvi01g01352\_t001 |  | | | |  |  |  |  |  |  |  |
| 1 | Vvi-Vitvi01g04336\_t001 |  | | | |  |  |  |  |  |  |  |
| 1 | Vvi-Vitvi01g01351\_t001 |  | | | |  |  |  |  |  |  |  |
| 1 | Vvi-Vitvi01g01354\_t001 |  | | | |  |  |  |  |  |  |  |
| 1 | Vvi-Vitvi01g01355\_t001 |  | | | |  |  |  |  |  |  |  |
| 1 | Vvi-Vitvi01g04337\_t001 |  | | | |  |  |  |  |  |  |  |
| 1 | Vvi-Vitvi01g01356\_t001 |  | | | |  |  |  |  |  |  |  |
| 1 | Vvi-Vitvi01g01357\_t001 |  | | | |  |  |  |  |  |  |  |
| 1 | Vvi-Vitvi01g01359\_t001 |  | Ath-AT1G68850.1 |  |  |  |  |  |  |  |
| 0 | Vvi-Vitvi01g01360\_t001 |  |  |  |  |  |  |  |  |
| 0 | Vvi-Vitvi01g01361\_t001 |  |  |  |  |  |  |  |  |
| 0 | Vvi-Vitvi01g02182\_t001 |  |  |  |  |  |  |  |  |
| 0 | Vvi-Vitvi01g01362\_t003 |  |  |  |  |  |  |  |  |
| 0 | Vvi-Vitvi01g04338\_t001 |  |  |  |  |  |  |  |  |
| 0 | Vvi-Vitvi01g04339\_t001 |  |  |  |  |  |  |  |  |
| 0 | Vvi-Vitvi01g01368\_t001 |  |  |  |  |  |  |  |  |
| 0 | Vvi-Vitvi01g01369\_t001 |  |  |  |  |  |  |  |  |
| 0 | Vvi-Vitvi01g04340\_t001 |  |  |  |  |  |  |  |  |
| 0 | Vvi-Vitvi01g01372\_t001 |  |  |  |  |  |  |  |  |
| 0 | Vvi-Vitvi01g01373\_t001 |  |  |  |  |  |  |  |  |
| 0 | Vvi-Vitvi01g01374\_t001 |  |  |  |  |  |  |  |  |
| 0 | Vvi-Vitvi01g04341\_t001 |  |  |  |  |  |  |  |  |
| 0 | Vvi-Vitvi01g01375\_t001 |  |  |  |  |  |  |  |  |
| 0 | Vvi-Vitvi01g01376\_t003 |  |  |  |  |  |  |  |  |
| 0 | Vvi-Vitvi01g01378\_t002 |  |  |  |  |  |  |  |  |
| 0 | Vvi-Vitvi01g01380\_t001 |  |  |  |  |  |  |  |  |
| 0 | Vvi-Vitvi01g04342\_t001 |  |  |  |  |  |  |  |  |
| 0 | Vvi-Vitvi01g01383\_t001 |  |  |  |  |  |  |  |  |
| 0 | Vvi-Vitvi01g01385\_t001 |  |  |  |  |  |  |  |  |
| 0 | Vvi-Vitvi01g02188\_t001 |  |  |  |  |  |  |  |  |
| 0 | Vvi-Vitvi01g04343\_t001 |  |  |  |  |  |  |  |  |
| 0 | Vvi-Vitvi01g04344\_t001 |  |  |  |  |  |  |  |  |
| 0 | Vvi-Vitvi01g04345\_t001 |  |  |  |  |  |  |  |  |
| 0 | Vvi-Vitvi01g01390\_t001 |  |  |  |  |  |  |  |  |
| 0 | Vvi-Vitvi01g01391\_t001 |  |  |  |  |  |  |  |  |
| 0 | Vvi-Vitvi01g04346\_t001 |  |  |  |  |  |  |  |  |
| 0 | Vvi-Vitvi01g01399\_t001 |  |  |  |  |  |  |  |  |
| 0 | Vvi-Vitvi01g04347\_t001 |  |  |  |  |  |  |  |  |
| 0 | Vvi-Vitvi01g01400\_t002 |  |  |  |  |  |  |  |  |
| 0 | Vvi-Vitvi01g04348\_t001 |  |  |  |  |  |  |  |  |
| 0 | Vvi-Vitvi01g04349\_t001 |  |  |  |  |  |  |  |  |
| 0 | Vvi-Vitvi01g04350\_t001 |  |  |  |  |  |  |  |  |
| 0 | Vvi-Vitvi01g04351\_t001 |  |  |  |  |  |  |  |  |
| 0 | Vvi-Vitvi01g01404\_t001 |  |  |  |  |  |  |  |  |
| 0 | Vvi-Vitvi01g01405\_t001 |  |  |  |  |  |  |  |  |
| 0 | Vvi-Vitvi01g04352\_t001 |  |  |  |  |  |  |  |  |
| 0 | Vvi-Vitvi01g01411\_t002 |  |  |  |  |  |  |  |  |
| 0 | Vvi-Vitvi01g01413\_t001 |  |  |  |  |  |  |  |  |
| 0 | Vvi-Vitvi01g01414\_t001 |  |  |  |  |  |  |  |  |
| 0 | Vvi-Vitvi01g01417\_t001 |  |  |  |  |  |  |  |  |
| 0 | Vvi-Vitvi01g01418\_t001 |  |  |  |  |  |  |  |  |
| 0 | Vvi-Vitvi01g01421\_t001 |  |  |  |  |  |  |  |  |
| 0 | Vvi-Vitvi01g04353\_t001 |  |  |  |  |  |  |  |  |
| 0 | Vvi-Vitvi01g01424\_t001 |  |  |  |  |  |  |  |  |
| 0 | Vvi-Vitvi01g01831\_t001 |  |  |  |  |  |  |  |  |
| 0 | Vvi-Vitvi01g01429\_t001 |  |  |  |  |  |  |  |  |
| 0 | Vvi-Vitvi01g04354\_t001 |  |  |  |  |  |  |  |  |
| 0 | Vvi-Vitvi01g01430\_t006 |  |  |  |  |  |  |  |  |
| 0 | Vvi-Vitvi01g01432\_t001 |  |  |  |  |  |  |  |  |
| 0 | Vvi-Vitvi01g02195\_t001 |  |  |  |  |  |  |  |  |
| 0 | Vvi-Vitvi01g01434\_t001 |  |  |  |  |  |  |  |  |
| 0 | Vvi-Vitvi01g01435\_t001 |  |  |  |  |  |  |  |  |
| 0 | Vvi-Vitvi01g01436\_t001 |  |  |  |  |  |  |  |  |
| 0 | Vvi-Vitvi01g01437\_t001 |  |  |  |  |  |  |  |  |
| 0 | Vvi-Vitvi01g01438\_t001 |  |  |  |  |  |  |  |  |
| 1 | Vvi-Vitvi01g01439\_t001 |  | Ath-AT2G02220.1 |  |  |  |  |  |  |  |
| 1 | Vvi-Vitvi01g01440\_t002 |  | | | |  |  |  |  |  |  |  |
| 1 | Vvi-Vitvi01g04355\_t001 |  | | | |  |  |  |  |  |  |  |
| 1 | Vvi-Vitvi01g01443\_t001 |  | Ath-AT2G02180.1 |  |  |  |  |  |  |  |
| 1 | Vvi-Vitvi01g01444\_t001 |  | | | |  |  |  |  |  |  |  |
| 1 | Vvi-Vitvi01g01446\_t001 |  | Ath-AT2G02170.2 |  |  |  |  |  |  |  |
| 1 | Vvi-Vitvi01g01447\_t001 |  | Ath-AT2G02160.1 |  |  |  |  |  |  |  |
| 1 | Vvi-Vitvi01g01449\_t001 |  | Ath-AT2G02150.1 |  |  |  |  |  |  |  |
| 1 | Vvi-Vitvi01g04356\_t001 |  | | | |  |  |  |  |  |  |  |
| 1 | Vvi-Vitvi01g04357\_t001 |  | | | |  |  |  |  |  |  |  |
| 1 | Vvi-Vitvi01g04358\_t001 |  | | | |  |  |  |  |  |  |  |
| 1 | Vvi-Vitvi01g01453\_t001 |  | | | |  |  |  |  |  |  |  |
| 1 | Vvi-Vitvi01g01455\_t001 |  | | | |  |  |  |  |  |  |  |
| 1 | Vvi-Vitvi01g04359\_t001 |  | | | |  |  |  |  |  |  |  |
| 1 | Vvi-Vitvi01g01456\_t001 |  | Ath-AT2G02148.3 |  |  |  |  |  |  |  |
| 1 | Vvi-Vitvi01g01457\_t001 |  | | | |  |  |  |  |  |  |  |
| 1 | Vvi-Vitvi01g04360\_t001 |  | | | |  |  |  |  |  |  |  |
| 1 | Vvi-Vitvi01g04361\_t001 |  | | | |  |  |  |  |  |  |  |
| 1 | Vvi-Vitvi01g04362\_t001 |  | | | |  |  |  |  |  |  |  |
| 1 | Vvi-Vitvi01g01459\_t001 |  | | | |  |  |  |  |  |  |  |
| 1 | Vvi-Vitvi01g04363\_t001 |  | | | |  |  |  |  |  |  |  |
| 1 | Vvi-Vitvi01g04364\_t001 |  | | | |  |  |  |  |  |  |  |
| 1 | Vvi-Vitvi01g02201\_t001 |  | | | |  |  |  |  |  |  |  |
| 1 | Vvi-Vitvi01g01460\_t001 |  | | | |  |  |  |  |  |  |  |
| 1 | Vvi-Vitvi01g04365\_t001 |  | | | |  |  |  |  |  |  |  |
| 1 | Vvi-Vitvi01g04366\_t001 |  | | | |  |  |  |  |  |  |  |
| 1 | Vvi-Vitvi01g01462\_t001 |  | | | |  |  |  |  |  |  |  |
| 1 | Vvi-Vitvi01g01464\_t001 |  | | | |  |  |  |  |  |  |  |
| 1 | Vvi-Vitvi01g01467\_t001 |  | | | |  |  |  |  |  |  |  |
| 1 | Vvi-Vitvi01g01468\_t001 |  | | | |  |  |  |  |  |  |  |
| 1 | Vvi-Vitvi01g01470\_t001 |  | | | |  |  |  |  |  |  |  |
| 1 | Vvi-Vitvi01g02205\_t001 |  | | | |  |  |  |  |  |  |  |
| 1 | Vvi-Vitvi01g02206\_t001 |  | | | |  |  |  |  |  |  |  |
| 1 | Vvi-Vitvi01g04367\_t001 |  | | | |  |  |  |  |  |  |  |
| 1 | Vvi-Vitvi01g04368\_t001 |  | | | |  |  |  |  |  |  |  |
| 1 | Vvi-Vitvi01g04369\_t001 |  | | | |  |  |  |  |  |  |  |
| 1 | Vvi-Vitvi01g02210\_t001 |  | | | |  |  |  |  |  |  |  |
| 1 | Vvi-Vitvi01g04370\_t001 |  | | | |  |  |  |  |  |  |  |
| 1 | Vvi-Vitvi01g04371\_t001 |  | | | |  |  |  |  |  |  |  |
| 1 | Vvi-Vitvi01g01476\_t001 |  | Ath-AT2G02100.1 |  |  |  |  |  |  |  |
| 1 | Vvi-Vitvi01g01477\_t001 |  | | | |  |  |  |  |  |  |  |
| 1 | Vvi-Vitvi01g01478\_t003 |  | | | |  |  |  |  |  |  |  |
| 1 | Vvi-Vitvi01g01480\_t001 |  | | | |  |  |  |  |  |  |  |
| 1 | Vvi-Vitvi01g04372\_t001 |  | | | |  |  |  |  |  |  |  |
| 1 | Vvi-Vitvi01g01482\_t001 |  | | | |  |  |  |  |  |  |  |
| 1 | Vvi-Vitvi01g01488\_t001 |  | Ath-AT2G02090.1 |  |  |  |  |  |  |  |
| 1 | Vvi-Vitvi01g01492\_t001 |  | Ath-AT2G02070.1 |  |  |  |  |  |  |  |
| 1 | Vvi-Vitvi01g01493\_t001 |  | | | |  |  |  |  |  |  |  |
| 1 | Vvi-Vitvi01g04373\_t001 |  | | | |  |  |  |  |  |  |  |
| 1 | Vvi-Vitvi01g04374\_t001 |  | | | |  |  |  |  |  |  |  |
| 1 | Vvi-Vitvi01g01494\_t001 |  | | | |  |  |  |  |  |  |  |
| 1 | Vvi-Vitvi01g04375\_t001 |  | | | |  |  |  |  |  |  |  |
| 1 | Vvi-Vitvi01g04376\_t001 |  | | | |  |  |  |  |  |  |  |
| 1 | Vvi-Vitvi01g01496\_t001 |  | | | |  |  |  |  |  |  |  |
| 1 | Vvi-Vitvi01g01498\_t001 |  | Ath-AT2G02061.1 |  |  |  |  |  |  |  |
| 1 | Vvi-Vitvi01g01499\_t001 |  | Ath-AT3G26090.1 |  |  |  |  |  |  |  |
| 1 | Vvi-Vitvi01g01500\_t001 |  | Ath-AT3G26085.2 |  |  |  |  |  |  |  |
| 1 | Vvi-Vitvi01g01502\_t001 |  | | | |  |  |  |  |  |  |  |
| 1 | Vvi-Vitvi01g04377\_t001 |  | | | |  |  |  |  |  |  |  |
| 1 | Vvi-Vitvi01g01503\_t001 |  | | | |  |  |  |  |  |  |  |
| 2 | Vvi-Vitvi01g01504\_t001 |  | | | |  | Ath-AT1G67720.1 |  |  |  |  |  |  |
| 2 | Vvi-Vitvi01g01505\_t001 |  | | | |  | | | |  |  |  |  |  |  |
| 2 | Vvi-Vitvi01g01506\_t001 |  | | | |  | Ath-AT1G67710.1 |  |  |  |  |  |  |
| 2 | Vvi-Vitvi01g01507\_t001 |  | Ath-AT3G26050.1 |  | | | |  |  |  |  |  |  |
| 2 | Vvi-Vitvi01g01508\_t001 |  | | | |  | | | |  |  |  |  |  |  |
| 2 | Vvi-Vitvi01g01509\_t001 |  | | | |  | | | |  |  |  |  |  |  |
| 2 | Vvi-Vitvi01g01510\_t001 |  | | | |  | Ath-AT1G67700.2 |  |  |  |  |  |  |
| 2 | Vvi-Vitvi01g04378\_t001 |  | | | |  | | | |  |  |  |  |  |  |
| 2 | Vvi-Vitvi01g01512\_t001 |  | | | |  | | | |  |  |  |  |  |  |
| 2 | Vvi-Vitvi01g02213\_t001 |  | Ath-AT3G26040.1 |  | | | |  |  |  |  |  |  |
| 2 | Vvi-Vitvi01g01513\_t001 |  | | | |  | | | |  |  |  |  |  |  |
| 2 | Vvi-Vitvi01g01514\_t001 |  | | | |  | | | |  |  |  |  |  |  |
| 2 | Vvi-Vitvi01g04379\_t001 |  | | | |  | | | |  |  |  |  |  |  |
| 2 | Vvi-Vitvi01g04380\_t001 |  | | | |  | | | |  |  |  |  |  |  |
| 2 | Vvi-Vitvi01g01517\_t001 |  | | | |  | | | |  |  |  |  |  |  |
| 2 | Vvi-Vitvi01g04381\_t001 |  | | | |  | | | |  |  |  |  |  |  |
| 2 | Vvi-Vitvi01g01519\_t001 |  | | | |  | | | |  |  |  |  |  |  |
| 2 | Vvi-Vitvi01g02214\_t001 |  | | | |  | | | |  |  |  |  |  |  |
| 2 | Vvi-Vitvi01g01520\_t001 |  | | | |  | | | |  |  |  |  |  |  |
| 2 | Vvi-Vitvi01g01521\_t001 |  | Ath-AT3G26020.4 |  | | | |  |  |  |  |  |  |
| 2 | Vvi-Vitvi01g01523\_t001 |  | | | |  | Ath-AT1G67690.1 |  |  |  |  |  |  |
| 2 | Vvi-Vitvi01g01528\_t002 |  | | | |  | | | |  |  |  |  |  |  |
| 2 | Vvi-Vitvi01g01529\_t001 |  | Ath-AT3G26000.1 |  | | | |  |  |  |  |  |  |
| 2 | Vvi-Vitvi01g01530\_t001 |  | | | |  | | | |  |  |  |  |  |  |
| 2 | Vvi-Vitvi01g04382\_t001 |  | | | |  | Ath-AT1G67670.1 |  |  |  |  |  |  |
| 2 | Vvi-Vitvi01g01532\_t002 |  | | | |  | Ath-AT1G67660.1 |  |  |  |  |  |  |
| 2 | Vvi-Vitvi01g01533\_t002 |  | Ath-AT3G25990.1 |  | | | |  |  |  |  |  |  |
| 2 | Vvi-Vitvi01g01534\_t001 |  | Ath-AT3G25980.1 |  | | | |  |  |  |  |  |  |
| 1 | Vvi-Vitvi01g01536\_t001 |  |  |  | | | |  |  |  |  |  |  |
| 1 | Vvi-Vitvi01g01537\_t001 |  |  |  | | | |  |  |  |  |  |  |
| 1 | Vvi-Vitvi01g01538\_t001 |  |  |  | | | |  |  |  |  |  |  |
| 1 | Vvi-Vitvi01g01539\_t002 |  |  |  | | | |  |  |  |  |  |  |
| 1 | Vvi-Vitvi01g04383\_t001 |  |  |  | | | |  |  |  |  |  |  |
| 1 | Vvi-Vitvi01g01540\_t001 |  |  |  | | | |  |  |  |  |  |  |
| 1 | Vvi-Vitvi01g01541\_t001 |  |  |  | Ath-AT1G67640.1 |  |  |  |  |  |  |
| 1 | Vvi-Vitvi01g04384\_t001 |  |  |  | | | |  |  |  |  |  |  |
| 1 | Vvi-Vitvi01g04385\_t001 |  |  |  | | | |  |  |  |  |  |  |
| 1 | Vvi-Vitvi01g02218\_t001 |  |  |  | | | |  |  |  |  |  |  |
| 2 | Vvi-Vitvi01g01543\_t001 |  | Ath-AT1G67570.1 |  | | | |  |  |  |  |  |  |
| 2 | Vvi-Vitvi01g04386\_t001 |  | | | |  | | | |  |  |  |  |  |  |
| 2 | Vvi-Vitvi01g01546\_t001 |  | Ath-AT1G67580.1 |  | | | |  |  |  |  |  |  |
| 2 | Vvi-Vitvi01g01547\_t001 |  | Ath-AT1G67590.1 |  | | | |  |  |  |  |  |  |
| 2 | Vvi-Vitvi01g01548\_t001 |  | | | |  | | | |  |  |  |  |  |  |
| 2 | Vvi-Vitvi01g01549\_t001 |  | Ath-AT1G67600.1 |  | | | |  |  |  |  |  |  |
| 2 | Vvi-Vitvi01g01550\_t001 |  | | | |  | | | |  |  |  |  |  |  |
| 2 | Vvi-Vitvi01g01552\_t001 |  | | | |  | | | |  |  |  |  |  |  |
| 2 | Vvi-Vitvi01g04387\_t001 |  | | | |  | | | |  |  |  |  |  |  |
| 2 | Vvi-Vitvi01g01553\_t001 |  | | | |  | | | |  |  |  |  |  |  |
| 2 | Vvi-Vitvi01g01554\_t001 |  | Ath-AT1G67620.1 |  | | | |  |  |  |  |  |  |
| 2 | Vvi-Vitvi01g04388\_t001 |  | Ath-AT1G67623.1 |  | | | |  |  |  |  |  |  |
| 2 | Vvi-Vitvi01g01555\_t001 |  | Ath-AT1G67640.1 |  | | | |  |  |  |  |  |  |
| 1 | Vvi-Vitvi01g02221\_t001 |  |  |  | | | |  |  |  |  |  |  |
| 1 | Vvi-Vitvi01g02222\_t001 |  |  |  | | | |  |  |  |  |  |  |
| 1 | Vvi-Vitvi01g01557\_t001 |  |  |  | | | |  |  |  |  |  |  |
| 1 | Vvi-Vitvi01g01558\_t001 |  |  |  | | | |  |  |  |  |  |  |
| 1 | Vvi-Vitvi01g01559\_t001 |  |  |  | Ath-AT1G67630.1 |  |  |  |  |  |  |
| 1 | Vvi-Vitvi01g02223\_t001 |  |  |  | | | |  |  |  |  |  |  |
| 1 | Vvi-Vitvi01g04389\_t001 |  |  |  | | | |  |  |  |  |  |  |
| 1 | Vvi-Vitvi01g04390\_t001 |  |  |  | | | |  |  |  |  |  |  |
| 1 | Vvi-Vitvi01g04391\_t001 |  |  |  | | | |  |  |  |  |  |  |
| 1 | Vvi-Vitvi01g02224\_t001 |  |  |  | | | |  |  |  |  |  |  |
| 1 | Vvi-Vitvi01g01561\_t001 |  |  |  | | | |  |  |  |  |  |  |
| 1 | Vvi-Vitvi01g01562\_t001 |  |  |  | Ath-AT1G67560.1 |  |  |  |  |  |  |
| 1 | Vvi-Vitvi01g01563\_t001 |  |  |  | Ath-AT1G67550.1 |  |  |  |  |  |  |
| 1 | Vvi-Vitvi01g01564\_t001 |  |  |  | | | |  |  |  |  |  |  |
| 1 | Vvi-Vitvi01g04392\_t001 |  |  |  | | | |  |  |  |  |  |  |
| 1 | Vvi-Vitvi01g01565\_t001 |  |  |  | | | |  |  |  |  |  |  |
| 1 | Vvi-Vitvi01g01566\_t001 |  |  |  | | | |  |  |  |  |  |  |
| 1 | Vvi-Vitvi01g01567\_t001 |  |  |  | | | |  |  |  |  |  |  |
| 1 | Vvi-Vitvi01g01568\_t002 |  |  |  | | | |  |  |  |  |  |  |
| 1 | Vvi-Vitvi01g01569\_t001 |  |  |  | Ath-AT1G67540.2 |  |  |  |  |  |  |
| 1 | Vvi-Vitvi01g04393\_t001 |  |  |  | | | |  |  |  |  |  |  |
| 1 | Vvi-Vitvi01g04394\_t001 |  |  |  | | | |  |  |  |  |  |  |
| 1 | Vvi-Vitvi01g04395\_t001 |  |  |  | | | |  |  |  |  |  |  |
| 1 | Vvi-Vitvi01g04396\_t001 |  |  |  | | | |  |  |  |  |  |  |
| 1 | Vvi-Vitvi01g01572\_t001 |  |  |  | | | |  |  |  |  |  |  |
| 1 | Vvi-Vitvi01g04397\_t001 |  |  |  | | | |  |  |  |  |  |  |
| 1 | Vvi-Vitvi01g04398\_t001 |  |  |  | | | |  |  |  |  |  |  |
| 1 | Vvi-Vitvi01g04399\_t001 |  |  |  | | | |  |  |  |  |  |  |
| 1 | Vvi-Vitvi01g04400\_t001 |  |  |  | | | |  |  |  |  |  |  |
| 1 | Vvi-Vitvi01g01573\_t001 |  |  |  | | | |  |  |  |  |  |  |
| 1 | Vvi-Vitvi01g02227\_t001 |  |  |  | | | |  |  |  |  |  |  |
| 1 | Vvi-Vitvi01g01575\_t001 |  |  |  | | | |  |  |  |  |  |  |
| 1 | Vvi-Vitvi01g01576\_t001 |  |  |  | Ath-AT1G67530.1 |  |  |  |  |  |  |
| 1 | Vvi-Vitvi01g01577\_t002 |  |  |  | | | |  |  |  |  |  |  |
| 1 | Vvi-Vitvi01g01578\_t001 |  |  |  | Ath-AT1G67510.1 |  |  |  |  |  |  |
| 0 | Vvi-Vitvi01g04401\_t001 |  |  |  |  |  |  |  |  |
| 0 | Vvi-Vitvi01g01580\_t001 |  |  |  |  |  |  |  |  |
| 0 | Vvi-Vitvi01g02228\_t001 |  |  |  |  |  |  |  |  |
| 0 | Vvi-Vitvi01g02229\_t001 |  |  |  |  |  |  |  |  |
| 0 | Vvi-Vitvi01g02230\_t001 |  |  |  |  |  |  |  |  |
| 0 | Vvi-Vitvi01g04402\_t001 |  |  |  |  |  |  |  |  |
| 0 | Vvi-Vitvi01g04403\_t001 |  |  |  |  |  |  |  |  |
| 0 | Vvi-Vitvi01g02232\_t001 |  |  |  |  |  |  |  |  |
| 0 | Vvi-Vitvi01g04404\_t001 |  |  |  |  |  |  |  |  |
| 0 | Vvi-Vitvi01g01583\_t001 |  |  |  |  |  |  |  |  |
| 0 | Vvi-Vitvi01g02233\_t001 |  |  |  |  |  |  |  |  |
| 0 | Vvi-Vitvi01g01584\_t001 |  |  |  |  |  |  |  |  |
| 0 | Vvi-Vitvi01g04405\_t001 |  |  |  |  |  |  |  |  |
| 0 | Vvi-Vitvi01g02234\_t001 |  |  |  |  |  |  |  |  |
| 0 | Vvi-Vitvi01g01585\_t001 |  |  |  |  |  |  |  |  |
| 0 | Vvi-Vitvi01g01586\_t001 |  |  |  |  |  |  |  |  |
| 0 | Vvi-Vitvi01g01588\_t001 |  |  |  |  |  |  |  |  |
| 0 | Vvi-Vitvi01g04406\_t001 |  |  |  |  |  |  |  |  |
| 0 | Vvi-Vitvi01g01589\_t001 |  |  |  |  |  |  |  |  |
| 0 | Vvi-Vitvi01g02236\_t001 |  |  |  |  |  |  |  |  |
| 0 | Vvi-Vitvi01g01591\_t001 |  |  |  |  |  |  |  |  |
| 0 | Vvi-Vitvi01g01592\_t001 |  |  |  |  |  |  |  |  |
| 0 | Vvi-Vitvi01g04407\_t001 |  |  |  |  |  |  |  |  |
| 0 | Vvi-Vitvi01g04408\_t001 |  |  |  |  |  |  |  |  |
| 0 | Vvi-Vitvi01g04409\_t001 |  |  |  |  |  |  |  |  |
| 0 | Vvi-Vitvi01g04410\_t001 |  |  |  |  |  |  |  |  |
| 0 | Vvi-Vitvi01g04411\_t001 |  |  |  |  |  |  |  |  |
| 0 | Vvi-Vitvi01g02240\_t001 |  |  |  |  |  |  |  |  |
| 0 | Vvi-Vitvi01g04412\_t001 |  |  |  |  |  |  |  |  |
| 0 | Vvi-Vitvi01g02242\_t001 |  |  |  |  |  |  |  |  |
| 0 | Vvi-Vitvi01g04413\_t001 |  |  |  |  |  |  |  |  |
| 0 | Vvi-Vitvi01g01595\_t001 |  |  |  |  |  |  |  |  |
| 0 | Vvi-Vitvi01g04414\_t001 |  |  |  |  |  |  |  |  |
| 0 | Vvi-Vitvi01g02246\_t001 |  |  |  |  |  |  |  |  |
| 0 | Vvi-Vitvi01g04415\_t001 |  |  |  |  |  |  |  |  |
| 0 | Vvi-Vitvi01g04416\_t001 |  |  |  |  |  |  |  |  |
| 0 | Vvi-Vitvi01g04417\_t001 |  |  |  |  |  |  |  |  |
| 0 | Vvi-Vitvi01g02247\_t001 |  |  |  |  |  |  |  |  |
| 0 | Vvi-Vitvi01g04418\_t001 |  |  |  |  |  |  |  |  |
| 0 | Vvi-Vitvi01g04419\_t001 |  |  |  |  |  |  |  |  |
| 0 | Vvi-Vitvi01g04420\_t001 |  |  |  |  |  |  |  |  |
| 0 | Vvi-Vitvi01g04421\_t001 |  |  |  |  |  |  |  |  |
| 0 | Vvi-Vitvi01g04422\_t001 |  |  |  |  |  |  |  |  |
| 0 | Vvi-Vitvi01g02252\_t001 |  |  |  |  |  |  |  |  |
| 0 | Vvi-Vitvi01g04423\_t001 |  |  |  |  |  |  |  |  |
| 0 | Vvi-Vitvi01g02254\_t001 |  |  |  |  |  |  |  |  |
| 0 | Vvi-Vitvi01g01606\_t001 |  |  |  |  |  |  |  |  |
| 0 | Vvi-Vitvi01g01607\_t001 |  |  |  |  |  |  |  |  |
| 0 | Vvi-Vitvi01g04424\_t001 |  |  |  |  |  |  |  |  |
| 0 | Vvi-Vitvi01g02255\_t001 |  |  |  |  |  |  |  |  |
| 0 | Vvi-Vitvi01g01610\_t001 |  |  |  |  |  |  |  |  |
| 0 | Vvi-Vitvi01g01611\_t001 |  |  |  |  |  |  |  |  |
| 0 | Vvi-Vitvi01g04425\_t001 |  |  |  |  |  |  |  |  |
| 0 | Vvi-Vitvi01g01613\_t001 |  |  |  |  |  |  |  |  |
| 0 | Vvi-Vitvi01g04426\_t001 |  |  |  |  |  |  |  |  |
| 0 | Vvi-Vitvi01g04427\_t001 |  |  |  |  |  |  |  |  |
| 0 | Vvi-Vitvi01g04428\_t001 |  |  |  |  |  |  |  |  |
| 0 | Vvi-Vitvi01g04429\_t001 |  |  |  |  |  |  |  |  |
| 1 | Vvi-Vitvi01g01618\_t001 |  | Ath-AT1G67490.1 |  |  |  |  |  |  |  |
| 1 | Vvi-Vitvi01g01620\_t001 |  | | | |  |  |  |  |  |  |  |
| 1 | Vvi-Vitvi01g01621\_t001 |  | Ath-AT1G67480.2 |  |  |  |  |  |  |  |
| 1 | Vvi-Vitvi01g04430\_t001 |  | | | |  |  |  |  |  |  |  |
| 1 | Vvi-Vitvi01g01623\_t001 |  | | | |  |  |  |  |  |  |  |
| 1 | Vvi-Vitvi01g01624\_t001 |  | Ath-AT1G67440.2 |  |  |  |  |  |  |  |
| 1 | Vvi-Vitvi01g04431\_t001 |  | | | |  |  |  |  |  |  |  |
| 1 | Vvi-Vitvi01g01627\_t001 |  | | | |  |  |  |  |  |  |  |
| 1 | Vvi-Vitvi01g02258\_t004 |  | Ath-AT1G67430.1 |  |  |  |  |  |  |  |
| 1 | Vvi-Vitvi01g01629\_t001 |  | Ath-AT1G67420.4 |  |  |  |  |  |  |  |
| 1 | Vvi-Vitvi01g04432\_t001 |  | Ath-AT1G67410.1 |  |  |  |  |  |  |  |
| 1 | Vvi-Vitvi01g04433\_t001 |  | | | |  |  |  |  |  |  |  |
| 1 | Vvi-Vitvi01g01632\_t001 |  | | | |  |  |  |  |  |  |  |
| 1 | Vvi-Vitvi01g04434\_t001 |  | | | |  |  |  |  |  |  |  |
| 1 | Vvi-Vitvi01g04435\_t001 |  | | | |  |  |  |  |  |  |  |
| 1 | Vvi-Vitvi01g02260\_t001 |  | | | |  |  |  |  |  |  |  |
| 1 | Vvi-Vitvi01g04436\_t001 |  | | | |  |  |  |  |  |  |  |
| 1 | Vvi-Vitvi01g01638\_t001 |  | | | |  |  |  |  |  |  |  |
| 1 | Vvi-Vitvi01g04437\_t001 |  | | | |  |  |  |  |  |  |  |
| 1 | Vvi-Vitvi01g02263\_t001 |  | | | |  |  |  |  |  |  |  |
| 1 | Vvi-Vitvi01g04438\_t001 |  | | | |  |  |  |  |  |  |  |
| 1 | Vvi-Vitvi01g02266\_t001 |  | | | |  |  |  |  |  |  |  |
| 1 | Vvi-Vitvi01g04439\_t001 |  | | | |  |  |  |  |  |  |  |
| 1 | Vvi-Vitvi01g01640\_t001 |  | Ath-AT1G67400.1 |  |  |  |  |  |  |  |
| 2 | Vvi-Vitvi01g01641\_t001 |  | | | |  | Ath-AT1G27920.1 |  |  |  |  |  |  |
| 2 | Vvi-Vitvi01g02267\_t001 |  | | | |  | | | |  |  |  |  |  |  |
| 2 | Vvi-Vitvi01g01642\_t001 |  | | | |  | | | |  |  |  |  |  |  |
| 2 | Vvi-Vitvi01g01644\_t001 |  | | | |  | | | |  |  |  |  |  |  |
| 2 | Vvi-Vitvi01g04440\_t001 |  | | | |  | | | |  |  |  |  |  |  |
| 2 | Vvi-Vitvi01g01645\_t001 |  | | | |  | | | |  |  |  |  |  |  |
| 2 | Vvi-Vitvi01g01646\_t001 |  | Ath-AT1G67370.1 |  | | | |  |  |  |  |  |  |
| 2 | Vvi-Vitvi01g02268\_t001 |  | Ath-AT1G67360.2 |  | | | |  |  |  |  |  |  |
| 2 | Vvi-Vitvi01g02269\_t001 |  | Ath-AT1G67350.1 |  | | | |  |  |  |  |  |  |
| 2 | Vvi-Vitvi01g04441\_t001 |  | | | |  | | | |  |  |  |  |  |  |
| 2 | Vvi-Vitvi01g01648\_t001 |  | | | |  | | | |  |  |  |  |  |  |
| 2 | Vvi-Vitvi01g01650\_t001 |  | Ath-AT1G67340.1 |  | | | |  |  |  |  |  |  |
| 2 | Vvi-Vitvi01g01651\_t001 |  | | | |  | | | |  |  |  |  |  |  |
| 2 | Vvi-Vitvi01g01652\_t001 |  | | | |  | | | |  |  |  |  |  |  |
| 3 | Vvi-Vitvi01g01653\_t002 |  | | | |  | | | |  | Ath-AT1G27385.4 |  |  |  |  |  |
| 3 | Vvi-Vitvi01g01654\_t001 |  | | | |  | | | |  | | | |  |  |  |  |  |
| 3 | Vvi-Vitvi01g01655\_t001 |  | Ath-AT1G67330.1 |  | Ath-AT1G27930.1 |  | | | |  |  |  |  |  |
| 3 | Vvi-Vitvi01g01657\_t001 |  | | | |  | Ath-AT1G27950.1 |  | | | |  |  |  |  |  |
| 3 | Vvi-Vitvi01g01658\_t001 |  | | | |  | Ath-AT1G27960.1 |  | | | |  |  |  |  |  |
| 3 | Vvi-Vitvi01g01659\_t002 |  | Ath-AT1G67325.2 |  | | | |  | | | |  |  |  |  |  |
| 3 | Vvi-Vitvi01g01660\_t001 |  | | | |  | | | |  | Ath-AT1G27360.2 |  |  |  |  |  |
| 3 | Vvi-Vitvi01g01661\_t001 |  | | | |  | | | |  | | | |  |  |  |  |  |
| 3 | Vvi-Vitvi01g01662\_t001 |  | | | |  | | | |  | Ath-AT1G27340.1 |  |  |  |  |  |
| 3 | Vvi-Vitvi01g01663\_t001 |  | | | |  | | | |  | | | |  |  |  |  |  |
| 3 | Vvi-Vitvi01g04442\_t001 |  | | | |  | | | |  | Ath-AT1G27330.1 |  |  |  |  |  |
| 3 | Vvi-Vitvi01g01664\_t001 |  | | | |  | | | |  | | | |  |  |  |  |  |
| 3 | Vvi-Vitvi01g01666\_t001 |  | | | |  | | | |  | Ath-AT1G27320.1 |  |  |  |  |  |
| 3 | Vvi-Vitvi01g04443\_t002 |  | | | |  | Ath-AT1G27970.2 |  | Ath-AT1G27310.1 |  |  |  |  |  |
| 3 | Vvi-Vitvi01g02272\_t001 |  | | | |  | | | |  | Ath-AT1G27300.1 |  |  |  |  |  |
| 3 | Vvi-Vitvi01g01669\_t001 |  | | | |  | | | |  | | | |  |  |  |  |  |
| 3 | Vvi-Vitvi01g01670\_t001 |  | | | |  | | | |  | | | |  |  |  |  |  |
| 3 | Vvi-Vitvi01g01671\_t001 |  | Ath-AT1G67310.1 |  | | | |  | | | |  |  |  |  |  |
| 2 | Vvi-Vitvi01g04444\_t001 |  |  |  | | | |  | | | |  |  |  |  |  |
| 2 | Vvi-Vitvi01g04445\_t001 |  |  |  | | | |  | | | |  |  |  |  |  |
| 2 | Vvi-Vitvi01g02274\_t001 |  |  |  | | | |  | | | |  |  |  |  |  |
| 2 | Vvi-Vitvi01g02275\_t001 |  |  |  | | | |  | | | |  |  |  |  |  |
| 2 | Vvi-Vitvi01g04446\_t003 |  |  |  | | | |  | Ath-AT1G27290.1 |  |  |  |  |  |
| 1 | Vvi-Vitvi01g01673\_t001 |  |  |  | | | |  |  |  |  |  |  |
| 1 | Vvi-Vitvi01g04447\_t001 |  |  |  | | | |  |  |  |  |  |  |
| 1 | Vvi-Vitvi01g04448\_t001 |  |  |  | | | |  |  |  |  |  |  |
| 1 | Vvi-Vitvi01g04449\_t001 |  |  |  | | | |  |  |  |  |  |  |
| 1 | Vvi-Vitvi01g01677\_t001 |  |  |  | | | |  |  |  |  |  |  |
| 1 | Vvi-Vitvi01g04450\_t001 |  |  |  | | | |  |  |  |  |  |  |
| 2 | Vvi-Vitvi01g01678\_t001 |  | Ath-AT5G50670.2 |  | | | |  |  |  |  |  |  |
| 2 | Vvi-Vitvi01g04451\_t001 |  | | | |  | | | |  |  |  |  |  |  |
| 2 | Vvi-Vitvi01g02278\_t001 |  | | | |  | | | |  |  |  |  |  |  |
| 3 | Vvi-Vitvi01g01680\_t001 |  | | | |  | | | |  | Ath-AT5G13080.1 |  |  |  |  |  |
| 3 | Vvi-Vitvi01g01682\_t001 |  | | | |  | Ath-AT1G27980.1 |  | | | |  |  |  |  |  |
| 3 | Vvi-Vitvi01g01683\_t001 |  | | | |  | | | |  | | | |  |  |  |  |  |
| 3 | Vvi-Vitvi01g01684\_t001 |  | | | |  | | | |  | | | |  |  |  |  |  |
| 3 | Vvi-Vitvi01g04452\_t001 |  | | | |  | | | |  | | | |  |  |  |  |  |
| 3 | Vvi-Vitvi01g01686\_t001 |  | | | |  | | | |  | Ath-AT5G13090.1 |  |  |  |  |  |
| 3 | Vvi-Vitvi01g01687\_t001 |  | | | |  | Ath-AT1G27990.1 |  | | | |  |  |  |  |  |
| 2 | Vvi-Vitvi01g01689\_t001 |  | | | |  |  |  | | | |  |  |  |  |  |
| 2 | Vvi-Vitvi01g01690\_t001 |  | | | |  |  |  | | | |  |  |  |  |  |
| 2 | Vvi-Vitvi01g04453\_t001 |  | | | |  |  |  | Ath-AT5G13100.1 |  |  |  |  |  |
| 2 | Vvi-Vitvi01g04454\_t001 |  | | | |  |  |  | | | |  |  |  |  |  |
| 2 | Vvi-Vitvi01g02281\_t001 |  | | | |  |  |  | | | |  |  |  |  |  |
| 2 | Vvi-Vitvi01g04455\_t001 |  | | | |  |  |  | | | |  |  |  |  |  |
| 2 | Vvi-Vitvi01g04456\_t001 |  | | | |  |  |  | | | |  |  |  |  |  |
| 2 | Vvi-Vitvi01g02283\_t003 |  | | | |  |  |  | | | |  |  |  |  |  |
| 2 | Vvi-Vitvi01g01695\_t001 |  | Ath-AT5G50740.3 |  |  |  | | | |  |  |  |  |  |
| 2 | Vvi-Vitvi01g04457\_t001 |  | | | |  |  |  | | | |  |  |  |  |  |
| 2 | Vvi-Vitvi01g04458\_t001 |  | | | |  |  |  | | | |  |  |  |  |  |
| 2 | Vvi-Vitvi01g01700\_t001 |  | | | |  |  |  | Ath-AT5G13110.1 |  |  |  |  |  |
| 2 | Vvi-Vitvi01g01701\_t001 |  | Ath-AT5G50770.1 |  |  |  | | | |  |  |  |  |  |
| 2 | Vvi-Vitvi01g01703\_t001 |  | | | |  |  |  | | | |  |  |  |  |  |
| 2 | Vvi-Vitvi01g01704\_t001 |  | | | |  |  |  | | | |  |  |  |  |  |
| 2 | Vvi-Vitvi01g01705\_t001 |  | | | |  |  |  | Ath-AT5G13120.1 |  |  |  |  |  |
| 2 | Vvi-Vitvi01g01706\_t001 |  | | | |  |  |  | | | |  |  |  |  |  |
| 2 | Vvi-Vitvi01g01707\_t001 |  | | | |  |  |  | | | |  |  |  |  |  |
| 2 | Vvi-Vitvi01g01708\_t001 |  | | | |  |  |  | | | |  |  |  |  |  |
| 2 | Vvi-Vitvi01g01709\_t001 |  | | | |  |  |  | Ath-AT5G13150.1 |  |  |  |  |  |
| 2 | Vvi-Vitvi01g01710\_t001 |  | | | |  |  |  | | | |  |  |  |  |  |
| 2 | Vvi-Vitvi01g01711\_t001 |  | | | |  |  |  | | | |  |  |  |  |  |
| 2 | Vvi-Vitvi01g04459\_t001 |  | | | |  |  |  | | | |  |  |  |  |  |
| 2 | Vvi-Vitvi01g04460\_t001 |  | | | |  |  |  | | | |  |  |  |  |  |
| 2 | Vvi-Vitvi01g04461\_t001 |  | | | |  |  |  | | | |  |  |  |  |  |
| 2 | Vvi-Vitvi01g01713\_t001 |  | | | |  |  |  | | | |  |  |  |  |  |
| 2 | Vvi-Vitvi01g04462\_t001 |  | | | |  |  |  | | | |  |  |  |  |  |
| 2 | Vvi-Vitvi01g02286\_t001 |  | | | |  |  |  | | | |  |  |  |  |  |
| 3 | Vvi-Vitvi01g01714\_t001 |  | | | |  | Ath-AT3G26960.1 |  | | | |  |  |  |  |  |
| 3 | Vvi-Vitvi01g02287\_t001 |  | | | |  | | | |  | | | |  |  |  |  |  |
| 3 | Vvi-Vitvi01g04463\_t001 |  | | | |  | | | |  | | | |  |  |  |  |  |
| 3 | Vvi-Vitvi01g01716\_t001 |  | | | |  | | | |  | | | |  |  |  |  |  |
| 3 | Vvi-Vitvi01g01718\_t001 |  | | | |  | | | |  | Ath-AT5G13160.1 |  |  |  |  |  |
| 3 | Vvi-Vitvi01g01719\_t001 |  | Ath-AT5G50790.1 |  | | | |  | Ath-AT5G13170.1 |  |  |  |  |  |
| 3 | Vvi-Vitvi01g01720\_t001 |  | | | |  | | | |  | | | |  |  |  |  |  |
| 4 | Vvi-Vitvi01g01722\_t001 |  | | | |  | | | |  | Ath-AT5G13180.1 |  | Ath-AT2G33480.1 |  |  |  |  |
| 4 | Vvi-Vitvi01g02289\_t001 |  | | | |  | | | |  | | | |  | | | |  |  |  |  |
| 4 | Vvi-Vitvi01g04464\_t001 |  | | | |  | | | |  | | | |  | | | |  |  |  |  |
| 4 | Vvi-Vitvi01g01723\_t001 |  | | | |  | | | |  | | | |  | | | |  |  |  |  |
| 4 | Vvi-Vitvi01g01724\_t002 |  | Ath-AT5G50850.1 |  | | | |  | | | |  | | | |  |  |  |  |
| 4 | Vvi-Vitvi01g04465\_t001 |  | | | |  | | | |  | | | |  | | | |  |  |  |  |
| 4 | Vvi-Vitvi01g01726\_t002 |  | | | |  | Ath-AT3G26910.3 |  | | | |  | Ath-AT2G33490.1 |  |  |  |  |
| 4 | Vvi-Vitvi01g01727\_t001 |  | | | |  | | | |  | | | |  | | | |  |  |  |  |
| 4 | Vvi-Vitvi01g01728\_t001 |  | | | |  | | | |  | | | |  | | | |  |  |  |  |
| 5 | Vvi-Vitvi01g01729\_t001 |  | | | |  | | | |  | | | |  | Ath-AT2G33500.1 |  | Ath-AT1G28050.1 |  |  |  |
| 5 | Vvi-Vitvi01g01730\_t001 |  | Ath-AT5G50900.1 |  | | | |  | | | |  | | | |  | | | |  |  |  |
| 5 | Vvi-Vitvi01g01731\_t001 |  | | | |  | | | |  | Ath-AT5G13190.2 |  | | | |  | | | |  |  |  |
| 5 | Vvi-Vitvi01g04466\_t001 |  | | | |  | | | |  | | | |  | | | |  | | | |  |  |  |
| 5 | Vvi-Vitvi01g04467\_t001 |  | | | |  | | | |  | | | |  | | | |  | | | |  |  |  |
| 5 | Vvi-Vitvi01g01733\_t001 |  | | | |  | | | |  | | | |  | Ath-AT2G33510.2 |  | Ath-AT1G28070.1 |  |  |  |
| 5 | Vvi-Vitvi01g01734\_t001 |  | | | |  | | | |  | Ath-AT5G13200.1 |  | | | |  | | | |  |  |  |
| 5 | Vvi-Vitvi01g04468\_t001 |  | | | |  | | | |  | | | |  | | | |  | | | |  |  |  |
| 5 | Vvi-Vitvi01g01737\_t001 |  | | | |  | | | |  | Ath-AT5G13210.1 |  | | | |  | | | |  |  |  |
| 5 | Vvi-Vitvi01g01738\_t001 |  | | | |  | Ath-AT3G26810.1 |  | | | |  | | | |  | | | |  |  |  |
| 5 | Vvi-Vitvi01g02291\_t001 |  | | | |  | | | |  | | | |  | | | |  | | | |  |  |  |
| 5 | Vvi-Vitvi01g02293\_t001 |  | | | |  | | | |  | Ath-AT5G13220.7 |  | | | |  | | | |  |  |  |
| 5 | Vvi-Vitvi01g01740\_t001 |  | | | |  | | | |  | Ath-AT5G13230.1 |  | | | |  | | | |  |  |  |
| 5 | Vvi-Vitvi01g04469\_t001 |  | | | |  | | | |  | | | |  | | | |  | | | |  |  |  |
| 5 | Vvi-Vitvi01g02295\_t001 |  | | | |  | | | |  | Ath-AT5G13250.1 |  | | | |  | Ath-AT1G28080.2 |  |  |  |
| 5 | Vvi-Vitvi01g01742\_t001 |  | | | |  | | | |  | | | |  | | | |  | | | |  |  |  |
| 5 | Vvi-Vitvi01g04470\_t001 |  | | | |  | | | |  | | | |  | | | |  | | | |  |  |  |
| 5 | Vvi-Vitvi01g01743\_t001 |  | | | |  | Ath-AT3G26790.1 |  | | | |  | | | |  | | | |  |  |  |
| 5 | Vvi-Vitvi01g04471\_t001 |  | | | |  | | | |  | | | |  | | | |  | | | |  |  |  |
| 5 | Vvi-Vitvi01g01744\_t001 |  | | | |  | | | |  | | | |  | | | |  | | | |  |  |  |
| 5 | Vvi-Vitvi01g01745\_t001 |  | Ath-AT5G50915.2 |  | | | |  | | | |  | | | |  | | | |  |  |  |
| 4 | Vvi-Vitvi01g01747\_t001 |  |  |  | | | |  | | | |  | | | |  | Ath-AT1G28090.1 |  |  |  |
| 4 | Vvi-Vitvi01g01748\_t001 |  |  |  | | | |  | | | |  | | | |  | | | |  |  |  |
| 4 | Vvi-Vitvi01g04472\_t001 |  |  |  | | | |  | | | |  | | | |  | | | |  |  |  |
| 4 | Vvi-Vitvi01g01749\_t001 |  |  |  | | | |  | | | |  | Ath-AT2G33540.1 |  | | | |  |  |  |
| 4 | Vvi-Vitvi01g01750\_t001 |  |  |  | | | |  | Ath-AT5G13260.1 |  | | | |  | | | |  |  |  |
| 4 | Vvi-Vitvi01g01751\_t001 |  |  |  | | | |  | | | |  | | | |  | | | |  |  |  |
| 4 | Vvi-Vitvi01g01752\_t001 |  |  |  | | | |  | | | |  | | | |  | | | |  |  |  |
| 4 | Vvi-Vitvi01g01753\_t001 |  |  |  | | | |  | | | |  | | | |  | Ath-AT1G28110.2 |  |  |  |
| 4 | Vvi-Vitvi01g01754\_t001 |  |  |  | | | |  | | | |  | | | |  | | | |  |  |  |
| 4 | Vvi-Vitvi01g01756\_t001 |  |  |  | | | |  | Ath-AT5G13270.1 |  | | | |  | | | |  |  |  |
| 4 | Vvi-Vitvi01g04473\_t001 |  |  |  | | | |  | | | |  | | | |  | | | |  |  |  |
| 4 | Vvi-Vitvi01g01757\_t001 |  |  |  | Ath-AT3G26744.2 |  | | | |  | | | |  | | | |  |  |  |
| 4 | Vvi-Vitvi01g01758\_t001 |  |  |  | | | |  | Ath-AT5G13280.1 |  | | | |  | | | |  |  |  |
| 5 | Vvi-Vitvi01g01759\_t001 |  | Ath-AT5G62000.2 |  | | | |  | | | |  | | | |  | | | |  |  |  |
| 5 | Vvi-Vitvi01g04474\_t001 |  | | | |  | | | |  | | | |  | | | |  | | | |  |  |  |
| 5 | Vvi-Vitvi01g01760\_t001 |  | | | |  | | | |  | | | |  | | | |  | | | |  |  |  |
| 5 | Vvi-Vitvi01g02296\_t001 |  | | | |  | Ath-AT3G26740.1 |  | | | |  | | | |  | | | |  |  |  |
| 4 | Vvi-Vitvi01g01761\_t001 |  | | | |  |  |  | Ath-AT5G13290.2 |  | | | |  | | | |  |  |  |
| 4 | Vvi-Vitvi01g01762\_t002 |  | Ath-AT5G61980.1 |  |  |  | Ath-AT5G13300.1 |  | | | |  | | | |  |  |  |
| 4 | Vvi-Vitvi01g01763\_t001 |  | | | |  |  |  | Ath-AT5G13310.1 |  | | | |  | | | |  |  |  |
| 4 | Vvi-Vitvi01g02298\_t001 |  | | | |  |  |  | | | |  | Ath-AT2G33550.1 |  | | | |  |  |  |
| 4 | Vvi-Vitvi01g01764\_t001 |  | | | |  |  |  | | | |  | | | |  | | | |  |  |  |
| 4 | Vvi-Vitvi01g01765\_t001 |  | | | |  |  |  | | | |  | | | |  | | | |  |  |  |
| 4 | Vvi-Vitvi01g04475\_t001 |  | | | |  |  |  | | | |  | | | |  | | | |  |  |  |
| 4 | Vvi-Vitvi01g04476\_t001 |  | | | |  |  |  | | | |  | | | |  | | | |  |  |  |
| 4 | Vvi-Vitvi01g01768\_t001 |  | Ath-AT5G61960.1 |  |  |  | | | |  | | | |  | | | |  |  |  |
| 4 | Vvi-Vitvi01g01769\_t001 |  | | | |  |  |  | | | |  | | | |  | Ath-AT1G28120.1 |  |  |  |
| 4 | Vvi-Vitvi01g04477\_t001 |  | | | |  |  |  | | | |  | | | |  | | | |  |  |  |
| 4 | Vvi-Vitvi01g01770\_t001 |  | Ath-AT5G61910.4 |  |  |  | | | |  | | | |  | | | |  |  |  |
| 4 | Vvi-Vitvi01g01826\_t001 |  | Ath-AT5G61890.1 |  |  |  | Ath-AT5G13330.1 |  | Ath-AT2G33710.2 |  | | | |  |  |  |
| 3 | Vvi-Vitvi01g01772\_t001 |  | | | |  |  |  | | | |  |  |  | | | |  |  |  |
| 3 | Vvi-Vitvi01g01773\_t001 |  | | | |  |  |  | | | |  |  |  | | | |  |  |  |
| 3 | Vvi-Vitvi01g01774\_t001 |  | | | |  |  |  | | | |  |  |  | | | |  |  |  |
| 3 | Vvi-Vitvi01g01776\_t001 |  | | | |  |  |  | | | |  |  |  | | | |  |  |  |
| 3 | Vvi-Vitvi01g01777\_t001 |  | | | |  |  |  | | | |  |  |  | | | |  |  |  |
| 3 | Vvi-Vitvi01g01778\_t001 |  | Ath-AT5G61840.1 |  |  |  | | | |  |  |  | | | |  |  |  |
| 3 | Vvi-Vitvi01g01779\_t001 |  | | | |  |  |  | | | |  |  |  | | | |  |  |  |
| 3 | Vvi-Vitvi01g01780\_t001 |  | | | |  |  |  | Ath-AT5G13410.1 |  |  |  | | | |  |  |  |
| 2 | Vvi-Vitvi01g01782\_t002 |  | | | |  |  |  |  |  |  |  | | | |  |  |  |
| 2 | Vvi-Vitvi01g01783\_t001 |  | | | |  |  |  |  |  |  |  | | | |  |  |  |
| 2 | Vvi-Vitvi01g01784\_t001 |  | | | |  |  |  |  |  |  |  | | | |  |  |  |
| 2 | Vvi-Vitvi01g04478\_t001 |  | | | |  |  |  |  |  |  |  | | | |  |  |  |
| 2 | Vvi-Vitvi01g02299\_t003 |  | | | |  |  |  |  |  |  |  | | | |  |  |  |
| 2 | Vvi-Vitvi01g01785\_t001 |  | | | |  |  |  |  |  |  |  | | | |  |  |  |
| 2 | Vvi-Vitvi01g01788\_t001 |  | | | |  |  |  |  |  |  |  | | | |  |  |  |
| 2 | Vvi-Vitvi01g01789\_t001 |  | | | |  |  |  |  |  |  |  | | | |  |  |  |
| 2 | Vvi-Vitvi01g04479\_t001 |  | | | |  |  |  |  |  |  |  | | | |  |  |  |
| 2 | Vvi-Vitvi01g01790\_t001 |  | | | |  |  |  |  |  |  |  | | | |  |  |  |
| 2 | Vvi-Vitvi01g01791\_t001 |  | | | |  |  |  |  |  |  |  | Ath-AT1G28130.1 |  |  |  |
| 3 | Vvi-Vitvi01g01792\_t001 |  | | | |  | Ath-AT5G47930.1 |  |  |  |  |  | | | |  |  |  |
| 3 | Vvi-Vitvi01g01793\_t002 |  | | | |  | | | |  |  |  |  |  | | | |  |  |  |
| 3 | Vvi-Vitvi01g01794\_t001 |  | | | |  | | | |  |  |  |  |  | | | |  |  |  |
| 3 | Vvi-Vitvi01g01795\_t001 |  | | | |  | | | |  |  |  |  |  | | | |  |  |  |
| 3 | Vvi-Vitvi01g01796\_t001 |  | | | |  | | | |  |  |  |  |  | | | |  |  |  |
| 3 | Vvi-Vitvi01g04480\_t001 |  | | | |  | | | |  |  |  |  |  | | | |  |  |  |
| 3 | Vvi-Vitvi01g02300\_t001 |  | | | |  | | | |  |  |  |  |  | | | |  |  |  |
| 3 | Vvi-Vitvi01g01798\_t001 |  | | | |  | Ath-AT5G47920.1 |  |  |  |  |  | | | |  |  |  |
| 3 | Vvi-Vitvi01g01799\_t001 |  | | | |  | | | |  |  |  |  |  | | | |  |  |  |
| 3 | Vvi-Vitvi01g04481\_t001 |  | | | |  | | | |  |  |  |  |  | | | |  |  |  |
| 3 | Vvi-Vitvi01g01800\_t001 |  | Ath-AT5G61790.1 |  | | | |  |  |  |  |  | | | |  |  |  |
| 2 | Vvi-Vitvi01g01801\_t001 |  |  |  | | | |  |  |  |  |  | Ath-AT1G28190.1 |  |  |  |
| 1 | Vvi-Vitvi01g01802\_t001 |  |  |  | | | |  |  |  |  |  |  |
| 1 | Vvi-Vitvi01g01803\_t001 |  |  |  | Ath-AT5G47910.1 |  |  |  |  |  |  |
| 1 | Vvi-Vitvi01g01804\_t001 |  |  |  | | | |  |  |  |  |  |  |
| 1 | Vvi-Vitvi01g01805\_t001 |  |  |  | | | |  |  |  |  |  |  |
| 1 | Vvi-Vitvi01g04482\_t001 |  |  |  | | | |  |  |  |  |  |  |
| 1 | Vvi-Vitvi01g01806\_t001 |  |  |  | | | |  |  |  |  |  |  |
| 1 | Vvi-Vitvi01g01807\_t001 |  |  |  | | | |  |  |  |  |  |  |
| 1 | Vvi-Vitvi01g01808\_t001 |  |  |  | | | |  |  |  |  |  |  |
| 1 | Vvi-Vitvi01g01809\_t001 |  |  |  | | | |  |  |  |  |  |  |
| 1 | Vvi-Vitvi01g01810\_t001 |  |  |  | | | |  |  |  |  |  |  |
| 1 | Vvi-Vitvi01g01811\_t001 |  |  |  | Ath-AT5G47900.9 |  |  |  |  |  |  |
| 1 | Vvi-Vitvi01g01812\_t001 |  |  |  | | | |  |  |  |  |  |  |
| 1 | Vvi-Vitvi01g01813\_t003 |  |  |  | Ath-AT5G47890.1 |  |  |  |  |  |  |
| 1 | Vvi-Vitvi01g01814\_t001 |  |  |  | | | |  |  |  |  |  |  |
| 1 | Vvi-Vitvi01g01815\_t001 |  |  |  | | | |  |  |  |  |  |  |
| 1 | Vvi-Vitvi01g01816\_t001 |  |  |  | | | |  |  |  |  |  |  |
| 1 | Vvi-Vitvi01g01820\_t001 |  |  |  | Ath-AT5G47870.1 |  |  |  |  |  |  |
| 1 | Vvi-Vitvi01g01821\_t001 |  |  |  | Ath-AT5G47860.1 |  |  |  |  |  |  |
| 0 | Vvi-Vitvi01g04483\_t001 |  |  |  |  |  |  |  |  |
| 0 | Vvi-Vitvi01g01822\_t001 |  |  |  |  |  |  |  |  |
| 0 | Vvi-Vitvi01g01824\_t001 |  |  |  |  |  |  |  |  |
