# Supplementary material for: Genome-wide identification, characterization and gene expression of BES1 transcription factor family in grapevine (Vitis vinifera L.)
Source: Sci Rep. 2023 Jan 5;13:240. doi: 10.1038/s41598-022-24407-y (PMC9816167; doi:10.1038/s41598-022-24407-y)
Supplement: Supplementary file 3 — Supplementary Information. [file 41598_2022_24407_MOESM3_ESM.zip › Vvi_Ath/Vitis_vinifera.PN40024.v4.dna_sm.toplevel.fa.vs.Arabidopsis_thaliana.TAIR10.dna_sm.toplevel.fa.html/Vvi-13.html]

|  |  |  |  |  |  |  |  |  |  |  |  |  |  |  |  |  |  |
| --- | --- | --- | --- | --- | --- | --- | --- | --- | --- | --- | --- | --- | --- | --- | --- | --- | --- |
| Duplication depth | Reference chromosome | Collinear blocks | | | | | | | | | | | | | | | |
| 0 | Vvi-Vitvi13g00001\_t002 |  |  |  |  |  |  |  |  |
| 0 | Vvi-Vitvi13g00002\_t001 |  |  |  |  |  |  |  |  |
| 0 | Vvi-Vitvi13g01878\_t001 |  |  |  |  |  |  |  |  |
| 0 | Vvi-Vitvi13g00003\_t001 |  |  |  |  |  |  |  |  |
| 0 | Vvi-Vitvi13g00004\_t001 |  |  |  |  |  |  |  |  |
| 0 | Vvi-Vitvi13g01879\_t001 |  |  |  |  |  |  |  |  |
| 0 | Vvi-Vitvi13g00005\_t001 |  |  |  |  |  |  |  |  |
| 0 | Vvi-Vitvi13g00007\_t001 |  |  |  |  |  |  |  |  |
| 0 | Vvi-Vitvi13g00008\_t001 |  |  |  |  |  |  |  |  |
| 0 | Vvi-Vitvi13g00009\_t001 |  |  |  |  |  |  |  |  |
| 0 | Vvi-Vitvi13g04000\_t001 |  |  |  |  |  |  |  |  |
| 0 | Vvi-Vitvi13g00010\_t001 |  |  |  |  |  |  |  |  |
| 0 | Vvi-Vitvi13g00011\_t001 |  |  |  |  |  |  |  |  |
| 0 | Vvi-Vitvi13g00012\_t001 |  |  |  |  |  |  |  |  |
| 1 | Vvi-Vitvi13g00013\_t001 |  | Ath-AT3G55830.1 |  |  |  |  |  |  |  |
| 1 | Vvi-Vitvi13g00014\_t001 |  | | | |  |  |  |  |  |  |  |
| 2 | Vvi-Vitvi13g00015\_t001 |  | Ath-AT3G55840.1 |  | Ath-AT2G40000.1 |  |  |  |  |  |  |
| 3 | Vvi-Vitvi13g00017\_t001 |  | | | |  | Ath-AT2G40010.1 |  | Ath-AT3G11250.1 |  |  |  |  |  |
| 4 | Vvi-Vitvi13g00018\_t003 |  | | | |  | | | |  | | | |  | Ath-AT5G05760.1 |  |  |  |  |
| 4 | Vvi-Vitvi13g00019\_t001 |  | | | |  | | | |  | | | |  | | | |  |  |  |  |
| 4 | Vvi-Vitvi13g01882\_t001 |  | | | |  | | | |  | | | |  | | | |  |  |  |  |
| 4 | Vvi-Vitvi13g00021\_t001 |  | | | |  | | | |  | | | |  | Ath-AT5G05740.1 |  |  |  |  |
| 4 | Vvi-Vitvi13g04001\_t001 |  | | | |  | | | |  | | | |  | | | |  |  |  |  |
| 4 | Vvi-Vitvi13g00022\_t001 |  | Ath-AT3G55870.3 |  | | | |  | | | |  | Ath-AT5G05730.2 |  |  |  |  |
| 4 | Vvi-Vitvi13g04002\_t001 |  | | | |  | | | |  | | | |  | | | |  |  |  |  |
| 4 | Vvi-Vitvi13g01884\_t001 |  | | | |  | | | |  | | | |  | | | |  |  |  |  |
| 4 | Vvi-Vitvi13g01885\_t001 |  | | | |  | | | |  | | | |  | | | |  |  |  |  |
| 4 | Vvi-Vitvi13g00023\_t001 |  | | | |  | | | |  | | | |  | Ath-AT5G05710.1 |  |  |  |  |
| 4 | Vvi-Vitvi13g00025\_t001 |  | | | |  | | | |  | | | |  | | | |  |  |  |  |
| 4 | Vvi-Vitvi13g00026\_t001 |  | | | |  | | | |  | | | |  | | | |  |  |  |  |
| 4 | Vvi-Vitvi13g04003\_t001 |  | | | |  | | | |  | | | |  | | | |  |  |  |  |
| 4 | Vvi-Vitvi13g04004\_t001 |  | | | |  | | | |  | | | |  | | | |  |  |  |  |
| 4 | Vvi-Vitvi13g00027\_t001 |  | | | |  | Ath-AT2G40060.1 |  | | | |  | | | |  |  |  |  |
| 4 | Vvi-Vitvi13g00028\_t001 |  | | | |  | Ath-AT2G40070.1 |  | | | |  | | | |  |  |  |  |
| 4 | Vvi-Vitvi13g00029\_t002 |  | | | |  | | | |  | Ath-AT3G11240.1 |  | Ath-AT5G05700.1 |  |  |  |  |
| 4 | Vvi-Vitvi13g01886\_t002 |  | Ath-AT3G55890.1 |  | | | |  | Ath-AT3G11230.2 |  | | | |  |  |  |  |
| 4 | Vvi-Vitvi13g00030\_t001 |  | | | |  | | | |  | | | |  | | | |  |  |  |  |
| 4 | Vvi-Vitvi13g00031\_t001 |  | | | |  | | | |  | | | |  | | | |  |  |  |  |
| 4 | Vvi-Vitvi13g00033\_t002 |  | | | |  | | | |  | | | |  | Ath-AT5G05690.1 |  |  |  |  |
| 4 | Vvi-Vitvi13g00034\_t001 |  | | | |  | | | |  | | | |  | Ath-AT5G05680.1 |  |  |  |  |
| 4 | Vvi-Vitvi13g00035\_t001 |  | | | |  | Ath-AT2G40095.2 |  | | | |  | | | |  |  |  |  |
| 4 | Vvi-Vitvi13g00036\_t001 |  | | | |  | | | |  | | | |  | | | |  |  |  |  |
| 4 | Vvi-Vitvi13g00037\_t001 |  | | | |  | | | |  | Ath-AT3G11210.1 |  | | | |  |  |  |  |
| 4 | Vvi-Vitvi13g04005\_t001 |  | | | |  | | | |  | | | |  | | | |  |  |  |  |
| 4 | Vvi-Vitvi13g04006\_t001 |  | | | |  | | | |  | | | |  | | | |  |  |  |  |
| 4 | Vvi-Vitvi13g01889\_t001 |  | | | |  | | | |  | | | |  | | | |  |  |  |  |
| 4 | Vvi-Vitvi13g04007\_t001 |  | | | |  | | | |  | | | |  | | | |  |  |  |  |
| 4 | Vvi-Vitvi13g04008\_t001 |  | | | |  | | | |  | | | |  | | | |  |  |  |  |
| 4 | Vvi-Vitvi13g04009\_t001 |  | | | |  | | | |  | | | |  | | | |  |  |  |  |
| 4 | Vvi-Vitvi13g00041\_t001 |  | | | |  | | | |  | | | |  | | | |  |  |  |  |
| 4 | Vvi-Vitvi13g04010\_t001 |  | | | |  | | | |  | | | |  | | | |  |  |  |  |
| 4 | Vvi-Vitvi13g01890\_t001 |  | | | |  | | | |  | | | |  | | | |  |  |  |  |
| 4 | Vvi-Vitvi13g01891\_t001 |  | | | |  | | | |  | | | |  | | | |  |  |  |  |
| 4 | Vvi-Vitvi13g04011\_t001 |  | | | |  | | | |  | | | |  | | | |  |  |  |  |
| 4 | Vvi-Vitvi13g00042\_t002 |  | | | |  | | | |  | | | |  | | | |  |  |  |  |
| 4 | Vvi-Vitvi13g04012\_t001 |  | | | |  | | | |  | | | |  | | | |  |  |  |  |
| 4 | Vvi-Vitvi13g00043\_t001 |  | | | |  | | | |  | | | |  | | | |  |  |  |  |
| 4 | Vvi-Vitvi13g04013\_t001 |  | | | |  | | | |  | | | |  | | | |  |  |  |  |
| 4 | Vvi-Vitvi13g00045\_t001 |  | | | |  | | | |  | | | |  | | | |  |  |  |  |
| 5 | Vvi-Vitvi13g01892\_t001 |  | | | |  | | | |  | | | |  | | | |  | Ath-AT2G29950.1 |  |  |  |
| 5 | Vvi-Vitvi13g00046\_t001 |  | Ath-AT3G55920.1 |  | | | |  | | | |  | | | |  | Ath-AT2G29960.1 |  |  |  |
| 5 | Vvi-Vitvi13g00047\_t001 |  | Ath-AT3G55940.1 |  | Ath-AT2G40116.1 |  | | | |  | | | |  | | | |  |  |  |
| 5 | Vvi-Vitvi13g00048\_t001 |  | | | |  | | | |  | | | |  | | | |  | | | |  |  |  |
| 5 | Vvi-Vitvi13g00049\_t001 |  | | | |  | | | |  | | | |  | Ath-AT5G05660.1 |  | | | |  |  |  |
| 5 | Vvi-Vitvi13g00050\_t001 |  | Ath-AT3G55950.1 |  | | | |  | | | |  | | | |  | | | |  |  |  |
| 5 | Vvi-Vitvi13g00051\_t001 |  | Ath-AT3G55960.1 |  | | | |  | | | |  | | | |  | | | |  |  |  |
| 5 | Vvi-Vitvi13g00052\_t001 |  | | | |  | Ath-AT2G40120.1 |  | | | |  | | | |  | | | |  |  |  |
| 5 | Vvi-Vitvi13g00053\_t001 |  | | | |  | Ath-AT2G40130.2 |  | | | |  | | | |  | Ath-AT2G29970.1 |  |  |  |
| 5 | Vvi-Vitvi13g00054\_t001 |  | | | |  | | | |  | Ath-AT3G11200.1 |  | Ath-AT5G05610.1 |  | | | |  |  |  |
| 5 | Vvi-Vitvi13g00055\_t001 |  | Ath-AT3G55970.1 |  | | | |  | Ath-AT3G11180.2 |  | Ath-AT5G05600.1 |  | | | |  |  |  |
| 5 | Vvi-Vitvi13g04014\_t001 |  | | | |  | | | |  | | | |  | | | |  | | | |  |  |  |
| 5 | Vvi-Vitvi13g00058\_t001 |  | Ath-AT3G55980.2 |  | Ath-AT2G40140.1 |  | | | |  | | | |  | | | |  |  |  |
| 5 | Vvi-Vitvi13g00059\_t001 |  | | | |  | | | |  | | | |  | Ath-AT5G05590.1 |  | | | |  |  |  |
| 5 | Vvi-Vitvi13g00060\_t001 |  | | | |  | | | |  | Ath-AT3G11170.1 |  | Ath-AT5G05580.1 |  | Ath-AT2G29980.1 |  |  |  |
| 5 | Vvi-Vitvi13g00061\_t001 |  | | | |  | | | |  | | | |  | Ath-AT5G05570.1 |  | | | |  |  |  |
| 5 | Vvi-Vitvi13g00062\_t001 |  | | | |  | | | |  | Ath-AT3G11150.1 |  | | | |  | | | |  |  |  |
| 5 | Vvi-Vitvi13g00063\_t001 |  | | | |  | | | |  | | | |  | | | |  | | | |  |  |  |
| 5 | Vvi-Vitvi13g04015\_t001 |  | | | |  | | | |  | | | |  | | | |  | | | |  |  |  |
| 5 | Vvi-Vitvi13g00064\_t001 |  | | | |  | | | |  | | | |  | | | |  | | | |  |  |  |
| 5 | Vvi-Vitvi13g00065\_t001 |  | | | |  | | | |  | | | |  | | | |  | | | |  |  |  |
| 5 | Vvi-Vitvi13g00066\_t001 |  | Ath-AT3G55990.1 |  | Ath-AT2G40150.1 |  | | | |  | | | |  | | | |  |  |  |
| 5 | Vvi-Vitvi13g00067\_t001 |  | | | |  | | | |  | | | |  | | | |  | | | |  |  |  |
| 5 | Vvi-Vitvi13g00068\_t001 |  | | | |  | | | |  | | | |  | | | |  | | | |  |  |  |
| 5 | Vvi-Vitvi13g00069\_t001 |  | | | |  | | | |  | | | |  | Ath-AT5G05560.2 |  | | | |  |  |  |
| 5 | Vvi-Vitvi13g00070\_t001 |  | | | |  | Ath-AT2G40170.1 |  | | | |  | | | |  | | | |  |  |  |
| 5 | Vvi-Vitvi13g01893\_t001 |  | | | |  | | | |  | | | |  | | | |  | | | |  |  |  |
| 5 | Vvi-Vitvi13g00071\_t001 |  | | | |  | Ath-AT2G40180.1 |  | | | |  | | | |  | Ath-AT2G30020.1 |  |  |  |
| 5 | Vvi-Vitvi13g00072\_t001 |  | | | |  | | | |  | Ath-AT3G11130.1 |  | | | |  | | | |  |  |  |
| 5 | Vvi-Vitvi13g00073\_t001 |  | | | |  | Ath-AT2G40190.1 |  | | | |  | | | |  | | | |  |  |  |
| 5 | Vvi-Vitvi13g00074\_t001 |  | Ath-AT3G56010.1 |  | | | |  | | | |  | | | |  | | | |  |  |  |
| 5 | Vvi-Vitvi13g00075\_t001 |  | | | |  | Ath-AT2G40200.1 |  | | | |  | | | |  | | | |  |  |  |
| 5 | Vvi-Vitvi13g00076\_t001 |  | | | |  | | | |  | | | |  | | | |  | | | |  |  |  |
| 5 | Vvi-Vitvi13g00079\_t001 |  | | | |  | | | |  | Ath-AT3G11110.1 |  | | | |  | | | |  |  |  |
| 5 | Vvi-Vitvi13g01860\_t001 |  | | | |  | | | |  | | | |  | | | |  | Ath-AT2G30080.1 |  |  |  |
| 5 | Vvi-Vitvi13g00080\_t001 |  | | | |  | Ath-AT2G40220.1 |  | | | |  | | | |  | | | |  |  |  |
| 5 | Vvi-Vitvi13g00081\_t001 |  | | | |  | Ath-AT2G40230.1 |  | | | |  | | | |  | | | |  |  |  |
| 5 | Vvi-Vitvi13g00082\_t001 |  | | | |  | | | |  | | | |  | | | |  | | | |  |  |  |
| 5 | Vvi-Vitvi13g00083\_t001 |  | Ath-AT3G56030.1 |  | Ath-AT2G40240.1 |  | | | |  | | | |  | | | |  |  |  |
| 5 | Vvi-Vitvi13g00084\_t001 |  | | | |  | | | |  | Ath-AT3G11100.1 |  | Ath-AT5G05550.2 |  | | | |  |  |  |
| 5 | Vvi-Vitvi13g00085\_t001 |  | | | |  | | | |  | Ath-AT3G11090.1 |  | | | |  | Ath-AT2G30130.1 |  |  |  |
| 4 | Vvi-Vitvi13g00086\_t001 |  | | | |  | | | |  | | | |  | | | |  |  |  |  |
| 4 | Vvi-Vitvi13g00087\_t001 |  | Ath-AT3G56040.1 |  | | | |  | | | |  | | | |  |  |  |  |
| 4 | Vvi-Vitvi13g00088\_t001 |  | | | |  | Ath-AT2G40260.1 |  | | | |  | | | |  |  |  |  |
| 4 | Vvi-Vitvi13g00089\_t001 |  | | | |  | | | |  | Ath-AT3G11070.1 |  | Ath-AT5G05520.1 |  |  |  |  |
| 3 | Vvi-Vitvi13g01894\_t001 |  | | | |  | | | |  |  |  | | | |  |  |  |  |
| 3 | Vvi-Vitvi13g00090\_t001 |  | | | |  | | | |  |  |  | Ath-AT5G05510.1 |  |  |  |  |
| 3 | Vvi-Vitvi13g00091\_t001 |  | | | |  | | | |  |  |  | Ath-AT5G05500.1 |  |  |  |  |
| 3 | Vvi-Vitvi13g00092\_t001 |  | Ath-AT3G56050.1 |  | Ath-AT2G40270.1 |  |  |  | | | |  |  |  |  |
| 3 | Vvi-Vitvi13g04016\_t001 |  | | | |  | | | |  |  |  | | | |  |  |  |  |
| 3 | Vvi-Vitvi13g01895\_t001 |  | | | |  | | | |  |  |  | | | |  |  |  |  |
| 3 | Vvi-Vitvi13g00095\_t001 |  | | | |  | | | |  |  |  | | | |  |  |  |  |
| 3 | Vvi-Vitvi13g04017\_t001 |  | | | |  | | | |  |  |  | | | |  |  |  |  |
| 3 | Vvi-Vitvi13g04018\_t001 |  | | | |  | | | |  |  |  | | | |  |  |  |  |
| 3 | Vvi-Vitvi13g04019\_t001 |  | | | |  | | | |  |  |  | | | |  |  |  |  |
| 3 | Vvi-Vitvi13g00096\_t001 |  | | | |  | | | |  |  |  | | | |  |  |  |  |
| 3 | Vvi-Vitvi13g00097\_t001 |  | | | |  | | | |  |  |  | | | |  |  |  |  |
| 3 | Vvi-Vitvi13g00098\_t001 |  | | | |  | | | |  |  |  | Ath-AT5G05490.1 |  |  |  |  |
| 3 | Vvi-Vitvi13g00099\_t001 |  | | | |  | | | |  |  |  | | | |  |  |  |  |
| 3 | Vvi-Vitvi13g01897\_t001 |  | | | |  | | | |  |  |  | Ath-AT5G05480.1 |  |  |  |  |
| 3 | Vvi-Vitvi13g00100\_t001 |  | Ath-AT3G56080.1 |  | Ath-AT2G40280.1 |  |  |  | | | |  |  |  |  |
| 3 | Vvi-Vitvi13g04020\_t001 |  | | | |  | | | |  |  |  | | | |  |  |  |  |
| 3 | Vvi-Vitvi13g00101\_t001 |  | | | |  | Ath-AT2G40290.1 |  |  |  | Ath-AT5G05470.1 |  |  |  |  |
| 3 | Vvi-Vitvi13g04021\_t001 |  | | | |  | | | |  |  |  | | | |  |  |  |  |
| 3 | Vvi-Vitvi13g00102\_t001 |  | | | |  | | | |  |  |  | | | |  |  |  |  |
| 3 | Vvi-Vitvi13g04022\_t001 |  | | | |  | | | |  |  |  | | | |  |  |  |  |
| 3 | Vvi-Vitvi13g00103\_t001 |  | | | |  | | | |  |  |  | | | |  |  |  |  |
| 3 | Vvi-Vitvi13g00104\_t001 |  | | | |  | | | |  |  |  | | | |  |  |  |  |
| 3 | Vvi-Vitvi13g00105\_t001 |  | | | |  | | | |  |  |  | | | |  |  |  |  |
| 3 | Vvi-Vitvi13g00106\_t001 |  | | | |  | | | |  |  |  | | | |  |  |  |  |
| 3 | Vvi-Vitvi13g04023\_t001 |  | | | |  | | | |  |  |  | | | |  |  |  |  |
| 5 | Vvi-Vitvi13g00107\_t001 |  | Ath-AT3G56090.1 |  | Ath-AT2G40300.1 |  | Ath-AT3G11050.1 |  | | | |  | Ath-AT5G01600.1 |  |  |  |
| 5 | Vvi-Vitvi13g00108\_t001 |  | | | |  | | | |  | | | |  | | | |  | | | |  |  |  |
| 5 | Vvi-Vitvi13g00109\_t001 |  | | | |  | | | |  | | | |  | | | |  | | | |  |  |  |
| 5 | Vvi-Vitvi13g00110\_t001 |  | | | |  | | | |  | Ath-AT3G11040.3 |  | Ath-AT5G05460.1 |  | | | |  |  |  |
| 5 | Vvi-Vitvi13g00111\_t001 |  | | | |  | | | |  | | | |  | Ath-AT5G05450.1 |  | | | |  |  |  |
| 5 | Vvi-Vitvi13g00112\_t001 |  | | | |  | Ath-AT2G40316.1 |  | | | |  | | | |  | | | |  |  |  |
| 5 | Vvi-Vitvi13g00113\_t001 |  | | | |  | Ath-AT2G40320.1 |  | Ath-AT3G11030.1 |  | | | |  | Ath-AT5G01620.3 |  |  |  |
| 5 | Vvi-Vitvi13g00114\_t001 |  | | | |  | Ath-AT2G40330.1 |  | | | |  | Ath-AT5G05440.1 |  | | | |  |  |  |
| 5 | Vvi-Vitvi13g00115\_t001 |  | | | |  | | | |  | | | |  | | | |  | | | |  |  |  |
| 5 | Vvi-Vitvi13g04024\_t001 |  | | | |  | | | |  | | | |  | | | |  | | | |  |  |  |
| 5 | Vvi-Vitvi13g00116\_t001 |  | | | |  | Ath-AT2G40340.6 |  | Ath-AT3G11020.1 |  | Ath-AT5G05410.1 |  | | | |  |  |  |
| 5 | Vvi-Vitvi13g00117\_t001 |  | | | |  | Ath-AT2G40370.1 |  | | | |  | Ath-AT5G05390.1 |  | | | |  |  |  |
| 5 | Vvi-Vitvi13g04025\_t001 |  | | | |  | | | |  | | | |  | | | |  | | | |  |  |  |
| 5 | Vvi-Vitvi13g04026\_t001 |  | | | |  | | | |  | | | |  | | | |  | | | |  |  |  |
| 5 | Vvi-Vitvi13g00118\_t001 |  | | | |  | | | |  | | | |  | | | |  | | | |  |  |  |
| 5 | Vvi-Vitvi13g00120\_t001 |  | Ath-AT3G56100.2 |  | | | |  | | | |  | | | |  | | | |  |  |  |
| 5 | Vvi-Vitvi13g00121\_t001 |  | Ath-AT3G56110.2 |  | Ath-AT2G40380.1 |  | | | |  | Ath-AT5G05380.2 |  | Ath-AT5G01640.1 |  |  |  |
| 5 | Vvi-Vitvi13g00122\_t001 |  | | | |  | Ath-AT2G40390.1 |  | | | |  | | | |  | | | |  |  |  |
| 5 | Vvi-Vitvi13g00123\_t001 |  | Ath-AT3G56130.1 |  | | | |  | | | |  | | | |  | | | |  |  |  |
| 5 | Vvi-Vitvi13g00124\_t001 |  | | | |  | | | |  | Ath-AT3G11000.2 |  | | | |  | Ath-AT5G01660.1 |  |  |  |
| 5 | Vvi-Vitvi13g00126\_t001 |  | Ath-AT3G56140.1 |  | Ath-AT2G40400.1 |  | | | |  | | | |  | | | |  |  |  |
| 5 | Vvi-Vitvi13g01899\_t001 |  | | | |  | | | |  | | | |  | | | |  | | | |  |  |  |
| 5 | Vvi-Vitvi13g01900\_t001 |  | | | |  | | | |  | | | |  | | | |  | | | |  |  |  |
| 5 | Vvi-Vitvi13g01901\_t001 |  | | | |  | | | |  | | | |  | Ath-AT5G05365.1 |  | | | |  |  |  |
| 5 | Vvi-Vitvi13g04027\_t001 |  | | | |  | | | |  | | | |  | | | |  | | | |  |  |  |
| 5 | Vvi-Vitvi13g00128\_t001 |  | Ath-AT3G56170.1 |  | Ath-AT2G40410.2 |  | | | |  | | | |  | | | |  |  |  |
| 5 | Vvi-Vitvi13g00129\_t001.1.6037826b |  | | | |  | | | |  | | | |  | Ath-AT5G05360.1 |  | | | |  |  |  |
| 5 | Vvi-Vitvi13g00131\_t001 |  | | | |  | | | |  | Ath-AT3G10985.1 |  | | | |  | Ath-AT5G01740.1 |  |  |  |
| 5 | Vvi-Vitvi13g00132\_t001 |  | | | |  | | | |  | Ath-AT3G10980.1 |  | Ath-AT5G05350.1 |  | | | |  |  |  |
| 5 | Vvi-Vitvi13g00133\_t001 |  | Ath-AT3G56190.1 |  | | | |  | | | |  | | | |  | | | |  |  |  |
| 5 | Vvi-Vitvi13g00134\_t001 |  | Ath-AT3G56200.1 |  | Ath-AT2G40420.1 |  | | | |  | | | |  | | | |  |  |  |
| 5 | Vvi-Vitvi13g00135\_t001 |  | Ath-AT3G56210.5 |  | | | |  | | | |  | | | |  | | | |  |  |  |
| 5 | Vvi-Vitvi13g00136\_t001 |  | | | |  | Ath-AT2G40430.2 |  | | | |  | | | |  | | | |  |  |  |
| 5 | Vvi-Vitvi13g00137\_t001 |  | | | |  | | | |  | | | |  | | | |  | | | |  |  |  |
| 5 | Vvi-Vitvi13g00138\_t001 |  | | | |  | | | |  | | | |  | | | |  | | | |  |  |  |
| 5 | Vvi-Vitvi13g00139\_t002 |  | Ath-AT3G56220.1 |  | Ath-AT2G40435.1 |  | | | |  | | | |  | | | |  |  |  |
| 5 | Vvi-Vitvi13g00140\_t001 |  | Ath-AT3G56230.1 |  | Ath-AT2G40450.1 |  | | | |  | | | |  | | | |  |  |  |
| 5 | Vvi-Vitvi13g01902\_t001 |  | | | |  | | | |  | | | |  | | | |  | | | |  |  |  |
| 5 | Vvi-Vitvi13g00141\_t001 |  | Ath-AT3G56250.3 |  | | | |  | | | |  | | | |  | | | |  |  |  |
| 5 | Vvi-Vitvi13g00142\_t001 |  | | | |  | Ath-AT2G40460.1 |  | | | |  | | | |  | | | |  |  |  |
| 5 | Vvi-Vitvi13g00143\_t001 |  | | | |  | | | |  | | | |  | Ath-AT5G05340.1 |  | | | |  |  |  |
| 5 | Vvi-Vitvi13g00144\_t001 |  | | | |  | Ath-AT2G40470.1 |  | | | |  | | | |  | | | |  |  |  |
| 5 | Vvi-Vitvi13g01903\_t001 |  | | | |  | Ath-AT2G40475.1 |  | | | |  | | | |  | | | |  |  |  |
| 5 | Vvi-Vitvi13g00145\_t001 |  | Ath-AT3G56270.1 |  | Ath-AT2G40480.1 |  | | | |  | | | |  | | | |  |  |  |
| 5 | Vvi-Vitvi13g01904\_t001 |  | | | |  | | | |  | | | |  | | | |  | | | |  |  |  |
| 5 | Vvi-Vitvi13g00146\_t001 |  | | | |  | | | |  | Ath-AT3G10970.1 |  | | | |  | | | |  |  |  |
| 5 | Vvi-Vitvi13g00147\_t001 |  | | | |  | Ath-AT2G40490.1 |  | | | |  | | | |  | | | |  |  |  |
| 5 | Vvi-Vitvi13g04028\_t001 |  | | | |  | | | |  | | | |  | | | |  | | | |  |  |  |
| 5 | Vvi-Vitvi13g00149\_t001 |  | | | |  | | | |  | | | |  | | | |  | Ath-AT5G01810.1 |  |  |  |
| 4 | Vvi-Vitvi13g00150\_t001 |  | | | |  | | | |  | Ath-AT3G10960.1 |  | | | |  |  |  |  |
| 4 | Vvi-Vitvi13g01905\_t001 |  | | | |  | | | |  | Ath-AT3G10940.1 |  | | | |  |  |  |  |
| 3 | Vvi-Vitvi13g00151\_t001 |  | | | |  | | | |  |  |  | Ath-AT5G05310.7 |  |  |  |  |
| 3 | Vvi-Vitvi13g00153\_t001 |  | | | |  | Ath-AT2G40550.1 |  |  |  | | | |  |  |  |  |
| 3 | Vvi-Vitvi13g00155\_t001 |  | Ath-AT3G56290.1 |  | | | |  |  |  | | | |  |  |  |  |
| 3 | Vvi-Vitvi13g00156\_t001 |  | | | |  | | | |  |  |  | | | |  |  |  |  |
| 3 | Vvi-Vitvi13g01907\_t001 |  | | | |  | | | |  |  |  | | | |  |  |  |  |
| 3 | Vvi-Vitvi13g00157\_t001 |  | Ath-AT3G56310.1 |  | | | |  |  |  | | | |  |  |  |  |
| 3 | Vvi-Vitvi13g00158\_t001 |  | Ath-AT3G56320.1 |  | | | |  |  |  | | | |  |  |  |  |
| 3 | Vvi-Vitvi13g04029\_t001 |  | | | |  | | | |  |  |  | | | |  |  |  |  |
| 3 | Vvi-Vitvi13g00159\_t001 |  | Ath-AT3G56330.1 |  | | | |  |  |  | | | |  |  |  |  |
| 3 | Vvi-Vitvi13g00160\_t001 |  | Ath-AT3G56340.1 |  | Ath-AT2G40590.1 |  |  |  | | | |  |  |  |  |
| 3 | Vvi-Vitvi13g00161\_t001 |  | | | |  | | | |  |  |  | | | |  |  |  |  |
| 3 | Vvi-Vitvi13g04030\_t001 |  | | | |  | | | |  |  |  | | | |  |  |  |  |
| 3 | Vvi-Vitvi13g04031\_t001 |  | | | |  | | | |  |  |  | | | |  |  |  |  |
| 3 | Vvi-Vitvi13g04032\_t001 |  | | | |  | | | |  |  |  | | | |  |  |  |  |
| 3 | Vvi-Vitvi13g04033\_t001 |  | | | |  | | | |  |  |  | | | |  |  |  |  |
| 3 | Vvi-Vitvi13g00164\_t001 |  | | | |  | | | |  |  |  | | | |  |  |  |  |
| 3 | Vvi-Vitvi13g04034\_t001 |  | | | |  | | | |  |  |  | | | |  |  |  |  |
| 3 | Vvi-Vitvi13g04035\_t001 |  | | | |  | | | |  |  |  | | | |  |  |  |  |
| 3 | Vvi-Vitvi13g04036\_t001 |  | | | |  | | | |  |  |  | | | |  |  |  |  |
| 3 | Vvi-Vitvi13g04037\_t001 |  | | | |  | | | |  |  |  | | | |  |  |  |  |
| 3 | Vvi-Vitvi13g00167\_t001 |  | | | |  | | | |  |  |  | | | |  |  |  |  |
| 3 | Vvi-Vitvi13g00168\_t001 |  | | | |  | | | |  |  |  | | | |  |  |  |  |
| 3 | Vvi-Vitvi13g01910\_t001 |  | | | |  | | | |  |  |  | | | |  |  |  |  |
| 3 | Vvi-Vitvi13g04038\_t001 |  | | | |  | | | |  |  |  | | | |  |  |  |  |
| 3 | Vvi-Vitvi13g00170\_t001 |  | | | |  | | | |  |  |  | | | |  |  |  |  |
| 3 | Vvi-Vitvi13g01911\_t002 |  | | | |  | | | |  |  |  | | | |  |  |  |  |
| 3 | Vvi-Vitvi13g01912\_t001 |  | | | |  | | | |  |  |  | Ath-AT5G05280.1 |  |  |  |  |
| 3 | Vvi-Vitvi13g00171\_t001 |  | | | |  | Ath-AT2G40620.1 |  |  |  | | | |  |  |  |  |
| 3 | Vvi-Vitvi13g01913\_t001 |  | | | |  | | | |  |  |  | | | |  |  |  |  |
| 3 | Vvi-Vitvi13g00172\_t001 |  | | | |  | | | |  |  |  | | | |  |  |  |  |
| 3 | Vvi-Vitvi13g00173\_t001 |  | | | |  | | | |  |  |  | | | |  |  |  |  |
| 3 | Vvi-Vitvi13g04039\_t001 |  | | | |  | | | |  |  |  | | | |  |  |  |  |
| 3 | Vvi-Vitvi13g04040\_t001 |  | | | |  | | | |  |  |  | | | |  |  |  |  |
| 3 | Vvi-Vitvi13g00175\_t003 |  | | | |  | | | |  |  |  | | | |  |  |  |  |
| 3 | Vvi-Vitvi13g00176\_t001 |  | | | |  | | | |  |  |  | | | |  |  |  |  |
| 3 | Vvi-Vitvi13g00177\_t001 |  | Ath-AT3G56350.1 |  | | | |  |  |  | | | |  |  |  |  |
| 3 | Vvi-Vitvi13g00178\_t001 |  | Ath-AT3G56360.1 |  | | | |  |  |  | Ath-AT5G05250.1 |  |  |  |  |
| 3 | Vvi-Vitvi13g00179\_t001 |  | | | |  | Ath-AT2G40630.1 |  |  |  | Ath-AT5G05240.1 |  |  |  |  |
| 3 | Vvi-Vitvi13g00180\_t001 |  | | | |  | Ath-AT2G40640.1 |  |  |  | Ath-AT5G05230.1 |  |  |  |  |
| 3 | Vvi-Vitvi13g00181\_t001 |  | | | |  | | | |  |  |  | | | |  |  |  |  |
| 3 | Vvi-Vitvi13g00182\_t001 |  | Ath-AT3G56370.1 |  | | | |  |  |  | | | |  |  |  |  |
| 3 | Vvi-Vitvi13g04041\_t001 |  | | | |  | | | |  |  |  | | | |  |  |  |  |
| 3 | Vvi-Vitvi13g01915\_t001 |  | | | |  | Ath-AT2G40660.1 |  |  |  | | | |  |  |  |  |
| 3 | Vvi-Vitvi13g04042\_t001 |  | | | |  | | | |  |  |  | | | |  |  |  |  |
| 3 | Vvi-Vitvi13g00183\_t001 |  | Ath-AT3G56380.2 |  | Ath-AT2G40670.2 |  |  |  | | | |  |  |  |  |
| 3 | Vvi-Vitvi13g04043\_t001 |  | | | |  | | | |  |  |  | | | |  |  |  |  |
| 3 | Vvi-Vitvi13g00185\_t001 |  | | | |  | Ath-AT2G40690.1 |  |  |  | | | |  |  |  |  |
| 3 | Vvi-Vitvi13g00186\_t001 |  | | | |  | Ath-AT2G40720.1 |  |  |  | | | |  |  |  |  |
| 3 | Vvi-Vitvi13g00187\_t001 |  | | | |  | | | |  |  |  | | | |  |  |  |  |
| 3 | Vvi-Vitvi13g00188\_t001 |  | | | |  | Ath-AT2G40730.1 |  |  |  | | | |  |  |  |  |
| 3 | Vvi-Vitvi13g00189\_t001 |  | Ath-AT3G56400.1 |  | Ath-AT2G40740.3 |  |  |  | | | |  |  |  |  |
| 3 | Vvi-Vitvi13g01916\_t001 |  | | | |  | Ath-AT2G40750.1 |  |  |  | | | |  |  |  |  |
| 3 | Vvi-Vitvi13g00190\_t001 |  | | | |  | Ath-AT2G40760.1 |  |  |  | | | |  |  |  |  |
| 3 | Vvi-Vitvi13g01917\_t001 |  | | | |  | | | |  |  |  | | | |  |  |  |  |
| 3 | Vvi-Vitvi13g00191\_t001 |  | | | |  | | | |  |  |  | | | |  |  |  |  |
| 3 | Vvi-Vitvi13g01918\_t001 |  | | | |  | | | |  |  |  | Ath-AT5G05220.1 |  |  |  |  |
| 3 | Vvi-Vitvi13g00192\_t001 |  | | | |  | Ath-AT2G40770.1 |  |  |  | | | |  |  |  |  |
| 2 | Vvi-Vitvi13g00193\_t001 |  | | | |  |  |  |  |  | Ath-AT5G05200.1 |  |  |  |  |
| 2 | Vvi-Vitvi13g00194\_t001 |  | Ath-AT3G56410.2 |  |  |  |  |  | Ath-AT5G05190.1 |  |  |  |  |
| 1 | Vvi-Vitvi13g04044\_t001 |  |  |  |  |  |  |  | | | |  |  |  |  |
| 1 | Vvi-Vitvi13g00195\_t001 |  |  |  |  |  |  |  | Ath-AT5G05180.1 |  |  |  |  |
| 0 | Vvi-Vitvi13g00196\_t001 |  |  |  |  |  |  |  |  |
| 0 | Vvi-Vitvi13g00197\_t001 |  |  |  |  |  |  |  |  |
| 0 | Vvi-Vitvi13g04045\_t001 |  |  |  |  |  |  |  |  |
| 0 | Vvi-Vitvi13g00198\_t001 |  |  |  |  |  |  |  |  |
| 0 | Vvi-Vitvi13g00199\_t001 |  |  |  |  |  |  |  |  |
| 1 | Vvi-Vitvi13g00200\_t001 |  | Ath-AT3G55000.1 |  |  |  |  |  |  |  |
| 3 | Vvi-Vitvi13g00202\_t001 |  | | | |  | Ath-AT2G37250.1 |  | Ath-AT2G39270.1 |  |  |  |  |  |
| 3 | Vvi-Vitvi13g04046\_t001 |  | | | |  | | | |  | | | |  |  |  |  |  |
| 4 | Vvi-Vitvi13g00203\_t004 |  | | | |  | | | |  | | | |  | Ath-AT5G02810.1 |  |  |  |  |
| 4 | Vvi-Vitvi13g00204\_t001 |  | | | |  | | | |  | | | |  | | | |  |  |  |  |
| 4 | Vvi-Vitvi13g04047\_t001 |  | | | |  | | | |  | | | |  | | | |  |  |  |  |
| 4 | Vvi-Vitvi13g00205\_t001 |  | | | |  | | | |  | | | |  | Ath-AT5G02800.1 |  |  |  |  |
| 4 | Vvi-Vitvi13g00206\_t001 |  | Ath-AT3G55020.1 |  | Ath-AT2G37290.2 |  | Ath-AT2G39280.2 |  | | | |  |  |  |  |
| 4 | Vvi-Vitvi13g00207\_t001 |  | Ath-AT3G55030.1 |  | | | |  | Ath-AT2G39290.1 |  | | | |  |  |  |  |
| 4 | Vvi-Vitvi13g00208\_t001 |  | Ath-AT3G55040.1 |  | | | |  | | | |  | Ath-AT5G02780.1 |  |  |  |  |
| 4 | Vvi-Vitvi13g00210\_t002 |  | Ath-AT3G55050.2 |  | | | |  | | | |  | | | |  |  |  |  |
| 4 | Vvi-Vitvi13g00211\_t001 |  | | | |  | | | |  | | | |  | | | |  |  |  |  |
| 4 | Vvi-Vitvi13g04048\_t001 |  | | | |  | | | |  | | | |  | | | |  |  |  |  |
| 4 | Vvi-Vitvi13g00212\_t001 |  | Ath-AT3G55060.2 |  | | | |  | Ath-AT2G39300.4 |  | | | |  |  |  |  |
| 4 | Vvi-Vitvi13g04049\_t001 |  | | | |  | | | |  | | | |  | | | |  |  |  |  |
| 4 | Vvi-Vitvi13g00213\_t001 |  | Ath-AT3G55070.1 |  | | | |  | | | |  | | | |  |  |  |  |
| 4 | Vvi-Vitvi13g00214\_t001 |  | | | |  | Ath-AT2G37340.1 |  | | | |  | | | |  |  |  |  |
| 4 | Vvi-Vitvi13g00215\_t001 |  | Ath-AT3G55080.1 |  | | | |  | | | |  | | | |  |  |  |  |
| 4 | Vvi-Vitvi13g00218\_t002 |  | | | |  | | | |  | | | |  | | | |  |  |  |  |
| 4 | Vvi-Vitvi13g00219\_t001 |  | | | |  | | | |  | Ath-AT2G39340.1 |  | | | |  |  |  |  |
| 4 | Vvi-Vitvi13g00220\_t001 |  | | | |  | | | |  | | | |  | | | |  |  |  |  |
| 4 | Vvi-Vitvi13g00221\_t001 |  | Ath-AT3G55090.1 |  | Ath-AT2G37360.1 |  | Ath-AT2G39350.1 |  | | | |  |  |  |  |
| 4 | Vvi-Vitvi13g00222\_t001 |  | | | |  | | | |  | | | |  | | | |  |  |  |  |
| 4 | Vvi-Vitvi13g00223\_t001 |  | | | |  | | | |  | Ath-AT2G39360.1 |  | | | |  |  |  |  |
| 4 | Vvi-Vitvi13g01922\_t001 |  | | | |  | | | |  | | | |  | | | |  |  |  |  |
| 4 | Vvi-Vitvi13g04050\_t001 |  | | | |  | | | |  | | | |  | | | |  |  |  |  |
| 4 | Vvi-Vitvi13g01924\_t001 |  | | | |  | | | |  | | | |  | | | |  |  |  |  |
| 4 | Vvi-Vitvi13g01925\_t001 |  | | | |  | | | |  | | | |  | | | |  |  |  |  |
| 4 | Vvi-Vitvi13g01926\_t001 |  | | | |  | | | |  | | | |  | | | |  |  |  |  |
| 4 | Vvi-Vitvi13g01927\_t001 |  | | | |  | | | |  | | | |  | | | |  |  |  |  |
| 4 | Vvi-Vitvi13g01928\_t001 |  | | | |  | | | |  | | | |  | | | |  |  |  |  |
| 4 | Vvi-Vitvi13g00225\_t001 |  | Ath-AT3G55120.1 |  | | | |  | | | |  | | | |  |  |  |  |
| 4 | Vvi-Vitvi13g00226\_t001 |  | | | |  | | | |  | | | |  | Ath-AT5G02630.1 |  |  |  |  |
| 4 | Vvi-Vitvi13g00227\_t002 |  | | | |  | Ath-AT2G37370.2 |  | | | |  | | | |  |  |  |  |
| 4 | Vvi-Vitvi13g01929\_t001 |  | | | |  | Ath-AT2G37380.1 |  | Ath-AT2G39370.1 |  | | | |  |  |  |  |
| 3 | Vvi-Vitvi13g00229\_t001 |  | | | |  |  |  | | | |  | | | |  |  |  |  |
| 3 | Vvi-Vitvi13g00230\_t001 |  | | | |  |  |  | | | |  | | | |  |  |  |  |
| 3 | Vvi-Vitvi13g00231\_t001 |  | | | |  |  |  | | | |  | | | |  |  |  |  |
| 3 | Vvi-Vitvi13g00232\_t001 |  | Ath-AT3G55140.1 |  |  |  | | | |  | | | |  |  |  |  |
| 3 | Vvi-Vitvi13g00233\_t001 |  | Ath-AT3G55150.1 |  |  |  | Ath-AT2G39380.1 |  | | | |  |  |  |  |
| 3 | Vvi-Vitvi13g00234\_t001 |  | Ath-AT3G55170.5 |  |  |  | Ath-AT2G39390.1 |  | Ath-AT5G02610.2 |  |  |  |  |
| 3 | Vvi-Vitvi13g00235\_t001 |  | Ath-AT3G55180.1 |  |  |  | Ath-AT2G39400.1 |  | | | |  |  |  |  |
| 3 | Vvi-Vitvi13g00236\_t001 |  | Ath-AT3G55200.2 |  |  |  | | | |  | | | |  |  |  |  |
| 3 | Vvi-Vitvi13g00237\_t001 |  | Ath-AT3G55230.1 |  |  |  | | | |  | | | |  |  |  |  |
| 3 | Vvi-Vitvi13g00238\_t001 |  | | | |  |  |  | Ath-AT2G39430.1 |  | | | |  |  |  |  |
| 3 | Vvi-Vitvi13g00239\_t001 |  | | | |  |  |  | | | |  | | | |  |  |  |  |
| 3 | Vvi-Vitvi13g00240\_t001 |  | | | |  |  |  | Ath-AT2G39435.2 |  | | | |  |  |  |  |
| 3 | Vvi-Vitvi13g00241\_t001 |  | | | |  |  |  | | | |  | | | |  |  |  |  |
| 3 | Vvi-Vitvi13g04051\_t001 |  | | | |  |  |  | | | |  | | | |  |  |  |  |
| 3 | Vvi-Vitvi13g01932\_t001 |  | Ath-AT3G55240.1 |  |  |  | | | |  | Ath-AT5G02580.1 |  |  |  |  |
| 2 | Vvi-Vitvi13g01933\_t001 |  | | | |  |  |  | | | |  |  |  |  |  |
| 2 | Vvi-Vitvi13g04052\_t001 |  | | | |  |  |  | | | |  |  |  |  |  |
| 2 | Vvi-Vitvi13g04053\_t001 |  | | | |  |  |  | | | |  |  |  |  |  |
| 2 | Vvi-Vitvi13g00242\_t001 |  | | | |  |  |  | | | |  |  |  |  |  |
| 2 | Vvi-Vitvi13g04054\_t001 |  | | | |  |  |  | | | |  |  |  |  |  |
| 2 | Vvi-Vitvi13g04055\_t001 |  | | | |  |  |  | | | |  |  |  |  |  |
| 2 | Vvi-Vitvi13g00244\_t001 |  | | | |  |  |  | Ath-AT2G39450.1 |  |  |  |  |  |
| 2 | Vvi-Vitvi13g00245\_t001 |  | Ath-AT3G55260.1 |  |  |  | | | |  |  |  |  |  |
| 2 | Vvi-Vitvi13g00246\_t002 |  | | | |  |  |  | | | |  |  |  |  |  |
| 2 | Vvi-Vitvi13g01935\_t001 |  | | | |  |  |  | | | |  |  |  |  |  |
| 2 | Vvi-Vitvi13g04056\_t001 |  | | | |  |  |  | | | |  |  |  |  |  |
| 2 | Vvi-Vitvi13g01937\_t005 |  | | | |  |  |  | | | |  |  |  |  |  |
| 2 | Vvi-Vitvi13g04057\_t001 |  | | | |  |  |  | | | |  |  |  |  |  |
| 2 | Vvi-Vitvi13g01941\_t001 |  | | | |  |  |  | | | |  |  |  |  |  |
| 2 | Vvi-Vitvi13g01942\_t001 |  | | | |  |  |  | | | |  |  |  |  |  |
| 2 | Vvi-Vitvi13g04058\_t001 |  | | | |  |  |  | | | |  |  |  |  |  |
| 2 | Vvi-Vitvi13g04059\_t001 |  | | | |  |  |  | | | |  |  |  |  |  |
| 2 | Vvi-Vitvi13g04060\_t001 |  | | | |  |  |  | | | |  |  |  |  |  |
| 2 | Vvi-Vitvi13g01944\_t001 |  | | | |  |  |  | | | |  |  |  |  |  |
| 2 | Vvi-Vitvi13g04061\_t001 |  | | | |  |  |  | | | |  |  |  |  |  |
| 2 | Vvi-Vitvi13g04062\_t001 |  | | | |  |  |  | | | |  |  |  |  |  |
| 2 | Vvi-Vitvi13g04063\_t001 |  | | | |  |  |  | | | |  |  |  |  |  |
| 2 | Vvi-Vitvi13g01946\_t001 |  | | | |  |  |  | | | |  |  |  |  |  |
| 2 | Vvi-Vitvi13g04064\_t001 |  | | | |  |  |  | | | |  |  |  |  |  |
| 2 | Vvi-Vitvi13g04065\_t001 |  | | | |  |  |  | | | |  |  |  |  |  |
| 2 | Vvi-Vitvi13g01947\_t001 |  | | | |  |  |  | | | |  |  |  |  |  |
| 2 | Vvi-Vitvi13g04066\_t001 |  | | | |  |  |  | | | |  |  |  |  |  |
| 2 | Vvi-Vitvi13g01948\_t001 |  | | | |  |  |  | | | |  |  |  |  |  |
| 2 | Vvi-Vitvi13g04067\_t001 |  | | | |  |  |  | | | |  |  |  |  |  |
| 2 | Vvi-Vitvi13g01949\_t001 |  | | | |  |  |  | | | |  |  |  |  |  |
| 2 | Vvi-Vitvi13g04068\_t001 |  | | | |  |  |  | | | |  |  |  |  |  |
| 2 | Vvi-Vitvi13g04069\_t001 |  | | | |  |  |  | | | |  |  |  |  |  |
| 2 | Vvi-Vitvi13g00249\_t001 |  | Ath-AT3G55280.1 |  |  |  | Ath-AT2G39460.2 |  |  |  |  |  |
| 2 | Vvi-Vitvi13g00250\_t001 |  | | | |  |  |  | Ath-AT2G39470.1 |  |  |  |  |  |
| 2 | Vvi-Vitvi13g00252\_t001 |  | Ath-AT3G55320.1 |  |  |  | Ath-AT2G39480.1 |  |  |  |  |  |
| 2 | Vvi-Vitvi13g00254\_t001 |  | | | |  |  |  | | | |  |  |  |  |  |
| 2 | Vvi-Vitvi13g00255\_t001 |  | | | |  |  |  | | | |  |  |  |  |  |
| 2 | Vvi-Vitvi13g01950\_t001 |  | | | |  |  |  | Ath-AT2G39490.1 |  |  |  |  |  |
| 2 | Vvi-Vitvi13g01951\_t001 |  | | | |  |  |  | | | |  |  |  |  |  |
| 2 | Vvi-Vitvi13g04070\_t001 |  | | | |  |  |  | | | |  |  |  |  |  |
| 2 | Vvi-Vitvi13g01952\_t001 |  | | | |  |  |  | | | |  |  |  |  |  |
| 2 | Vvi-Vitvi13g04071\_t001 |  | | | |  |  |  | | | |  |  |  |  |  |
| 2 | Vvi-Vitvi13g01953\_t001 |  | | | |  |  |  | | | |  |  |  |  |  |
| 2 | Vvi-Vitvi13g04072\_t001 |  | | | |  |  |  | | | |  |  |  |  |  |
| 2 | Vvi-Vitvi13g04073\_t001 |  | | | |  |  |  | | | |  |  |  |  |  |
| 2 | Vvi-Vitvi13g04074\_t001 |  | | | |  |  |  | | | |  |  |  |  |  |
| 2 | Vvi-Vitvi13g01955\_t001 |  | | | |  |  |  | | | |  |  |  |  |  |
| 2 | Vvi-Vitvi13g04075\_t001 |  | | | |  |  |  | | | |  |  |  |  |  |
| 2 | Vvi-Vitvi13g00258\_t001 |  | | | |  |  |  | | | |  |  |  |  |  |
| 2 | Vvi-Vitvi13g00259\_t001 |  | Ath-AT3G55340.1 |  |  |  | | | |  |  |  |  |  |
| 4 | Vvi-Vitvi13g00260\_t002 |  | | | |  | Ath-AT5G59790.1 |  | | | |  | Ath-AT3G46110.1 |  |  |  |  |
| 4 | Vvi-Vitvi13g04076\_t001 |  | | | |  | | | |  | | | |  | | | |  |  |  |  |
| 4 | Vvi-Vitvi13g04077\_t001 |  | | | |  | | | |  | | | |  | | | |  |  |  |  |
| 4 | Vvi-Vitvi13g00262\_t001 |  | | | |  | Ath-AT5G59820.1 |  | | | |  | Ath-AT3G46070.1 |  |  |  |  |
| 4 | Vvi-Vitvi13g04078\_t001 |  | | | |  | | | |  | | | |  | | | |  |  |  |  |
| 4 | Vvi-Vitvi13g00263\_t001 |  | | | |  | Ath-AT5G59830.2 |  | | | |  | | | |  |  |  |  |
| 4 | Vvi-Vitvi13g00264\_t001 |  | | | |  | | | |  | Ath-AT2G39510.1 |  | | | |  |  |  |  |
| 4 | Vvi-Vitvi13g04079\_t001 |  | Ath-AT3G55410.1 |  | | | |  | | | |  | | | |  |  |  |  |
| 3 | Vvi-Vitvi13g00266\_t001 |  |  |  | Ath-AT5G59840.1 |  | | | |  | Ath-AT3G46060.1 |  |  |  |  |
| 3 | Vvi-Vitvi13g04080\_t001 |  |  |  | Ath-AT5G59850.1 |  | Ath-AT2G39590.1 |  | Ath-AT3G46040.1 |  |  |  |  |
| 3 | Vvi-Vitvi13g01958\_t001 |  |  |  | | | |  | | | |  | | | |  |  |  |  |
| 3 | Vvi-Vitvi13g00269\_t003 |  |  |  | Ath-AT5G59880.1 |  | | | |  | Ath-AT3G46000.2 |  |  |  |  |
| 3 | Vvi-Vitvi13g00270\_t001 |  |  |  | | | |  | Ath-AT2G39620.1 |  | | | |  |  |  |  |
| 2 | Vvi-Vitvi13g01959\_t001 |  |  |  | | | |  |  |  | | | |  |  |  |  |
| 2 | Vvi-Vitvi13g04081\_t001 |  |  |  | | | |  |  |  | | | |  |  |  |  |
| 2 | Vvi-Vitvi13g00271\_t001 |  |  |  | | | |  |  |  | | | |  |  |  |  |
| 2 | Vvi-Vitvi13g00272\_t001 |  |  |  | Ath-AT5G59950.5 |  |  |  | | | |  |  |  |  |
| 2 | Vvi-Vitvi13g04082\_t001 |  |  |  | | | |  |  |  | | | |  |  |  |  |
| 2 | Vvi-Vitvi13g04083\_t001 |  |  |  | | | |  |  |  | | | |  |  |  |  |
| 2 | Vvi-Vitvi13g00273\_t001 |  |  |  | | | |  |  |  | | | |  |  |  |  |
| 2 | Vvi-Vitvi13g01962\_t001 |  |  |  | | | |  |  |  | | | |  |  |  |  |
| 2 | Vvi-Vitvi13g01963\_t001 |  |  |  | | | |  |  |  | | | |  |  |  |  |
| 2 | Vvi-Vitvi13g00274\_t001 |  |  |  | | | |  |  |  | | | |  |  |  |  |
| 2 | Vvi-Vitvi13g00275\_t001 |  |  |  | | | |  |  |  | | | |  |  |  |  |
| 2 | Vvi-Vitvi13g04084\_t001 |  |  |  | Ath-AT5G59970.2 |  |  |  | Ath-AT3G45930.1 |  |  |  |  |
| 0 | Vvi-Vitvi13g04085\_t001 |  |  |  |  |  |  |  |  |
| 0 | Vvi-Vitvi13g04086\_t001 |  |  |  |  |  |  |  |  |
| 0 | Vvi-Vitvi13g00277\_t001 |  |  |  |  |  |  |  |  |
| 0 | Vvi-Vitvi13g01966\_t003 |  |  |  |  |  |  |  |  |
| 0 | Vvi-Vitvi13g04087\_t001 |  |  |  |  |  |  |  |  |
| 0 | Vvi-Vitvi13g00279\_t001 |  |  |  |  |  |  |  |  |
| 0 | Vvi-Vitvi13g00280\_t001 |  |  |  |  |  |  |  |  |
| 0 | Vvi-Vitvi13g04088\_t001 |  |  |  |  |  |  |  |  |
| 0 | Vvi-Vitvi13g00281\_t001 |  |  |  |  |  |  |  |  |
| 0 | Vvi-Vitvi13g00282\_t002 |  |  |  |  |  |  |  |  |
| 0 | Vvi-Vitvi13g00284\_t001 |  |  |  |  |  |  |  |  |
| 0 | Vvi-Vitvi13g01969\_t001 |  |  |  |  |  |  |  |  |
| 0 | Vvi-Vitvi13g01970\_t001 |  |  |  |  |  |  |  |  |
| 0 | Vvi-Vitvi13g00285\_t001 |  |  |  |  |  |  |  |  |
| 0 | Vvi-Vitvi13g04089\_t001 |  |  |  |  |  |  |  |  |
| 0 | Vvi-Vitvi13g00286\_t001 |  |  |  |  |  |  |  |  |
| 0 | Vvi-Vitvi13g04090\_t001 |  |  |  |  |  |  |  |  |
| 0 | Vvi-Vitvi13g00287\_t001 |  |  |  |  |  |  |  |  |
| 0 | Vvi-Vitvi13g00288\_t001 |  |  |  |  |  |  |  |  |
| 0 | Vvi-Vitvi13g00289\_t001 |  |  |  |  |  |  |  |  |
| 0 | Vvi-Vitvi13g01972\_t001 |  |  |  |  |  |  |  |  |
| 0 | Vvi-Vitvi13g04091\_t001 |  |  |  |  |  |  |  |  |
| 0 | Vvi-Vitvi13g04092\_t001 |  |  |  |  |  |  |  |  |
| 0 | Vvi-Vitvi13g00290\_t001 |  |  |  |  |  |  |  |  |
| 0 | Vvi-Vitvi13g00291\_t001 |  |  |  |  |  |  |  |  |
| 0 | Vvi-Vitvi13g00292\_t001 |  |  |  |  |  |  |  |  |
| 0 | Vvi-Vitvi13g00293\_t001 |  |  |  |  |  |  |  |  |
| 0 | Vvi-Vitvi13g00294\_t001 |  |  |  |  |  |  |  |  |
| 0 | Vvi-Vitvi13g01973\_t002 |  |  |  |  |  |  |  |  |
| 0 | Vvi-Vitvi13g04093\_t001 |  |  |  |  |  |  |  |  |
| 1 | Vvi-Vitvi13g00295\_t001 |  | Ath-AT3G55360.1 |  |  |  |  |  |  |  |
| 1 | Vvi-Vitvi13g00296\_t001 |  | | | |  |  |  |  |  |  |  |
| 1 | Vvi-Vitvi13g04094\_t001 |  | | | |  |  |  |  |  |  |  |
| 1 | Vvi-Vitvi13g04095\_t001 |  | | | |  |  |  |  |  |  |  |
| 1 | Vvi-Vitvi13g01974\_t001 |  | | | |  |  |  |  |  |  |  |
| 1 | Vvi-Vitvi13g04096\_t001 |  | | | |  |  |  |  |  |  |  |
| 1 | Vvi-Vitvi13g04097\_t001 |  | | | |  |  |  |  |  |  |  |
| 1 | Vvi-Vitvi13g01976\_t001 |  | | | |  |  |  |  |  |  |  |
| 1 | Vvi-Vitvi13g01977\_t001 |  | | | |  |  |  |  |  |  |  |
| 1 | Vvi-Vitvi13g01978\_t001 |  | | | |  |  |  |  |  |  |  |
| 1 | Vvi-Vitvi13g04098\_t001 |  | | | |  |  |  |  |  |  |  |
| 1 | Vvi-Vitvi13g01979\_t001 |  | | | |  |  |  |  |  |  |  |
| 1 | Vvi-Vitvi13g01980\_t001 |  | | | |  |  |  |  |  |  |  |
| 1 | Vvi-Vitvi13g01981\_t001 |  | | | |  |  |  |  |  |  |  |
| 1 | Vvi-Vitvi13g00297\_t001 |  | | | |  |  |  |  |  |  |  |
| 2 | Vvi-Vitvi13g01982\_t001 |  | | | |  | Ath-AT2G39518.1 |  |  |  |  |  |  |
| 2 | Vvi-Vitvi13g01983\_t001 |  | | | |  | | | |  |  |  |  |  |  |
| 4 | Vvi-Vitvi13g00298\_t001 |  | Ath-AT3G55370.3 |  | | | |  | Ath-AT2G37590.1 |  | Ath-AT2G28810.1 |  |  |  |  |
| 4 | Vvi-Vitvi13g00300\_t001 |  | | | |  | | | |  | | | |  | | | |  |  |  |  |
| 4 | Vvi-Vitvi13g00301\_t001 |  | | | |  | | | |  | | | |  | | | |  |  |  |  |
| 4 | Vvi-Vitvi13g00302\_t001 |  | | | |  | Ath-AT2G39550.1 |  | | | |  | | | |  |  |  |  |
| 4 | Vvi-Vitvi13g00305\_t001 |  | Ath-AT3G55470.1 |  | | | |  | | | |  | | | |  |  |  |  |
| 4 | Vvi-Vitvi13g00306\_t001 |  | | | |  | Ath-AT2G39690.5 |  | | | |  | | | |  |  |  |  |
| 4 | Vvi-Vitvi13g00307\_t001 |  | Ath-AT3G55480.2 |  | | | |  | | | |  | | | |  |  |  |  |
| 4 | Vvi-Vitvi13g01984\_t001 |  | | | |  | | | |  | | | |  | | | |  |  |  |  |
| 4 | Vvi-Vitvi13g04099\_t001 |  | | | |  | | | |  | | | |  | | | |  |  |  |  |
| 4 | Vvi-Vitvi13g01985\_t001 |  | | | |  | | | |  | | | |  | | | |  |  |  |  |
| 4 | Vvi-Vitvi13g04100\_t001 |  | | | |  | | | |  | | | |  | | | |  |  |  |  |
| 4 | Vvi-Vitvi13g01987\_t001 |  | | | |  | | | |  | | | |  | | | |  |  |  |  |
| 4 | Vvi-Vitvi13g01988\_t001 |  | | | |  | | | |  | | | |  | | | |  |  |  |  |
| 4 | Vvi-Vitvi13g00309\_t001 |  | Ath-AT3G55500.1 |  | Ath-AT2G39700.1 |  | Ath-AT2G37640.1 |  | Ath-AT2G28950.1 |  |  |  |  |
| 4 | Vvi-Vitvi13g00310\_t001 |  | | | |  | | | |  | | | |  | | | |  |  |  |  |
| 4 | Vvi-Vitvi13g00311\_t001 |  | | | |  | | | |  | Ath-AT2G37650.1 |  | Ath-AT2G29060.1 |  |  |  |  |
| 4 | Vvi-Vitvi13g00312\_t001 |  | | | |  | | | |  | | | |  | | | |  |  |  |  |
| 4 | Vvi-Vitvi13g04101\_t001 |  | | | |  | | | |  | | | |  | | | |  |  |  |  |
| 4 | Vvi-Vitvi13g04102\_t001 |  | | | |  | | | |  | | | |  | | | |  |  |  |  |
| 4 | Vvi-Vitvi13g04103\_t001 |  | | | |  | | | |  | | | |  | | | |  |  |  |  |
| 4 | Vvi-Vitvi13g01865\_t001 |  | | | |  | | | |  | | | |  | | | |  |  |  |  |
| 4 | Vvi-Vitvi13g00314\_t002 |  | | | |  | | | |  | | | |  | | | |  |  |  |  |
| 4 | Vvi-Vitvi13g00315\_t001 |  | | | |  | | | |  | | | |  | | | |  |  |  |  |
| 4 | Vvi-Vitvi13g00316\_t001 |  | | | |  | | | |  | | | |  | | | |  |  |  |  |
| 5 | Vvi-Vitvi13g00317\_t001 |  | | | |  | | | |  | | | |  | | | |  | Ath-AT3G53750.2 |  |  |  |
| 5 | Vvi-Vitvi13g01990\_t001 |  | | | |  | | | |  | | | |  | | | |  | | | |  |  |  |
| 5 | Vvi-Vitvi13g01992\_t001 |  | Ath-AT3G55515.1 |  | Ath-AT2G39705.1 |  | | | |  | Ath-AT2G29125.1 |  | | | |  |  |  |
| 5 | Vvi-Vitvi13g00320\_t003 |  | Ath-AT3G55520.3 |  | | | |  | | | |  | | | |  | | | |  |  |  |
| 5 | Vvi-Vitvi13g00321\_t001 |  | | | |  | | | |  | | | |  | Ath-AT2G29130.1 |  | | | |  |  |  |
| 5 | Vvi-Vitvi13g00322\_t001 |  | | | |  | | | |  | | | |  | | | |  | | | |  |  |  |
| 5 | Vvi-Vitvi13g00323\_t001 |  | | | |  | | | |  | | | |  | | | |  | | | |  |  |  |
| 5 | Vvi-Vitvi13g00324\_t001 |  | Ath-AT3G55530.1 |  | | | |  | | | |  | | | |  | | | |  |  |  |
| 5 | Vvi-Vitvi13g04104\_t001 |  | | | |  | | | |  | | | |  | | | |  | | | |  |  |  |
| 5 | Vvi-Vitvi13g00325\_t001 |  | | | |  | Ath-AT2G39710.1 |  | | | |  | | | |  | | | |  |  |  |
| 5 | Vvi-Vitvi13g00326\_t001 |  | | | |  | Ath-AT2G39720.1 |  | | | |  | | | |  | | | |  |  |  |
| 6 | Vvi-Vitvi13g00327\_t001 |  | | | |  | | | |  | | | |  | | | |  | | | |  | Ath-AT5G06090.1 |  |  |
| 6 | Vvi-Vitvi13g00328\_t001 |  | | | |  | | | |  | | | |  | Ath-AT2G29140.2 |  | | | |  | | | |  |  |
| 6 | Vvi-Vitvi13g00329\_t001 |  | | | |  | | | |  | | | |  | | | |  | | | |  | | | |  |  |
| 6 | Vvi-Vitvi13g00330\_t001 |  | | | |  | | | |  | | | |  | | | |  | | | |  | | | |  |  |
| 6 | Vvi-Vitvi13g00331\_t002 |  | | | |  | | | |  | | | |  | | | |  | | | |  | | | |  |  |
| 6 | Vvi-Vitvi13g04105\_t001 |  | | | |  | | | |  | | | |  | | | |  | | | |  | | | |  |  |
| 6 | Vvi-Vitvi13g00332\_t001 |  | Ath-AT3G55550.1 |  | | | |  | Ath-AT2G37710.1 |  | | | |  | Ath-AT3G53810.1 |  | | | |  |  |
| 6 | Vvi-Vitvi13g01994\_t001 |  | | | |  | Ath-AT2G39730.1 |  | | | |  | | | |  | | | |  | | | |  |  |
| 6 | Vvi-Vitvi13g04106\_t001 |  | | | |  | | | |  | | | |  | | | |  | | | |  | | | |  |  |
| 6 | Vvi-Vitvi13g00333\_t001 |  | | | |  | | | |  | | | |  | | | |  | | | |  | Ath-AT5G06080.1 |  |  |
| 6 | Vvi-Vitvi13g04107\_t001 |  | | | |  | | | |  | | | |  | | | |  | | | |  | | | |  |  |
| 6 | Vvi-Vitvi13g00338\_t001 |  | Ath-AT3G55560.1 |  | | | |  | | | |  | | | |  | | | |  | | | |  |  |
| 6 | Vvi-Vitvi13g00339\_t002 |  | | | |  | | | |  | | | |  | | | |  | | | |  | | | |  |  |
| 6 | Vvi-Vitvi13g01996\_t001 |  | | | |  | | | |  | Ath-AT2G37730.1 |  | | | |  | | | |  | | | |  |  |
| 6 | Vvi-Vitvi13g00340\_t001 |  | | | |  | | | |  | Ath-AT2G37740.1 |  | | | |  | | | |  | Ath-AT5G06070.1 |  |  |
| 5 | Vvi-Vitvi13g01997\_t001 |  | | | |  | | | |  |  |  | Ath-AT2G29260.1 |  | | | |  | Ath-AT5G06060.1 |  |  |
| 5 | Vvi-Vitvi13g01998\_t001 |  | | | |  | | | |  |  |  | | | |  | | | |  | | | |  |  |
| 5 | Vvi-Vitvi13g00341\_t001 |  | | | |  | | | |  |  |  | | | |  | | | |  | | | |  |  |
| 5 | Vvi-Vitvi13g00342\_t001 |  | | | |  | | | |  |  |  | | | |  | | | |  | | | |  |  |
| 5 | Vvi-Vitvi13g01999\_t001 |  | | | |  | | | |  |  |  | Ath-AT2G29350.1 |  | | | |  | | | |  |  |
| 5 | Vvi-Vitvi13g04108\_t001 |  | | | |  | | | |  |  |  | | | |  | | | |  | | | |  |  |
| 5 | Vvi-Vitvi13g00343\_t001 |  | | | |  | | | |  |  |  | Ath-AT2G29360.1 |  | | | |  | | | |  |  |
| 5 | Vvi-Vitvi13g00344\_t001 |  | | | |  | | | |  |  |  | Ath-AT2G29380.1 |  | | | |  | | | |  |  |
| 5 | Vvi-Vitvi13g00345\_t001 |  | | | |  | | | |  |  |  | Ath-AT2G29390.1 |  | | | |  | | | |  |  |
| 5 | Vvi-Vitvi13g04109\_t001 |  | | | |  | | | |  |  |  | | | |  | | | |  | | | |  |  |
| 5 | Vvi-Vitvi13g00346\_t001 |  | | | |  | Ath-AT2G39740.2 |  |  |  | | | |  | | | |  | | | |  |  |
| 5 | Vvi-Vitvi13g00347\_t001 |  | | | |  | | | |  |  |  | | | |  | | | |  | | | |  |  |
| 5 | Vvi-Vitvi13g00348\_t002 |  | | | |  | Ath-AT2G39750.1 |  |  |  | | | |  | | | |  | Ath-AT5G06050.1 |  |  |
| 5 | Vvi-Vitvi13g00349\_t001 |  | | | |  | Ath-AT2G39760.1 |  |  |  | | | |  | | | |  | | | |  |  |
| 5 | Vvi-Vitvi13g02000\_t001 |  | Ath-AT3G55570.1 |  | | | |  |  |  | | | |  | | | |  | | | |  |  |
| 5 | Vvi-Vitvi13g04110\_t001 |  | | | |  | | | |  |  |  | | | |  | | | |  | | | |  |  |
| 5 | Vvi-Vitvi13g00350\_t002 |  | Ath-AT3G55580.1 |  | | | |  |  |  | | | |  | Ath-AT3G53830.4 |  | | | |  |  |
| 5 | Vvi-Vitvi13g02001\_t001 |  | | | |  | | | |  |  |  | | | |  | Ath-AT3G53840.1 |  | | | |  |  |
| 5 | Vvi-Vitvi13g04111\_t001 |  | | | |  | | | |  |  |  | | | |  | | | |  | | | |  |  |
| 5 | Vvi-Vitvi13g02002\_t001 |  | | | |  | | | |  |  |  | | | |  | | | |  | | | |  |  |
| 5 | Vvi-Vitvi13g00351\_t001 |  | | | |  | | | |  |  |  | | | |  | | | |  | | | |  |  |
| 5 | Vvi-Vitvi13g02003\_t001 |  | | | |  | | | |  |  |  | | | |  | | | |  | Ath-AT5G06000.1 |  |  |
| 5 | Vvi-Vitvi13g00352\_t001 |  | Ath-AT3G55590.1 |  | Ath-AT2G39770.1 |  |  |  | | | |  | | | |  | | | |  |  |
| 5 | Vvi-Vitvi13g00353\_t001 |  | | | |  | | | |  |  |  | | | |  | Ath-AT3G53850.1 |  | | | |  |  |
| 5 | Vvi-Vitvi13g00354\_t001 |  | Ath-AT3G55605.1 |  | Ath-AT2G39790.1 |  |  |  | | | |  | | | |  | Ath-AT5G05990.1 |  |  |
| 5 | Vvi-Vitvi13g04112\_t001 |  | | | |  | | | |  |  |  | | | |  | | | |  | | | |  |  |
| 5 | Vvi-Vitvi13g00355\_t001 |  | Ath-AT3G55610.1 |  | Ath-AT2G39800.4 |  |  |  | | | |  | | | |  | | | |  |  |
| 5 | Vvi-Vitvi13g00356\_t001 |  | | | |  | Ath-AT2G39805.2 |  |  |  | | | |  | | | |  | | | |  |  |
| 5 | Vvi-Vitvi13g00358\_t001 |  | | | |  | | | |  |  |  | | | |  | | | |  | | | |  |  |
| 5 | Vvi-Vitvi13g00359\_t001 |  | Ath-AT3G55630.3 |  | | | |  |  |  | | | |  | | | |  | Ath-AT5G05980.1 |  |  |
| 5 | Vvi-Vitvi13g00361\_t001 |  | | | |  | | | |  |  |  | | | |  | | | |  | Ath-AT5G05970.2 |  |  |
| 4 | Vvi-Vitvi13g00362\_t003 |  | | | |  | Ath-AT2G39840.1 |  |  |  | Ath-AT2G29400.1 |  | | | |  |  |  |
| 2 | Vvi-Vitvi13g00364\_t001 |  | | | |  |  |  |  |  |  |  | | | |  |  |  |
| 2 | Vvi-Vitvi13g00365\_t001 |  | Ath-AT3G55640.1 |  |  |  |  |  |  |  | Ath-AT3G53940.1 |  |  |  |
| 1 | Vvi-Vitvi13g04113\_t001 |  |  |  |  |  |  |  |  |  | | | |  |  |  |
| 1 | Vvi-Vitvi13g00366\_t001 |  |  |  |  |  |  |  |  |  | | | |  |  |  |
| 1 | Vvi-Vitvi13g00367\_t001 |  |  |  |  |  |  |  |  |  | Ath-AT3G53960.1 |  |  |  |
| 0 | Vvi-Vitvi13g00368\_t001 |  |  |  |  |  |  |  |  |
| 0 | Vvi-Vitvi13g00369\_t001 |  |  |  |  |  |  |  |  |
| 0 | Vvi-Vitvi13g04114\_t001 |  |  |  |  |  |  |  |  |
| 0 | Vvi-Vitvi13g02005\_t001 |  |  |  |  |  |  |  |  |
| 0 | Vvi-Vitvi13g02006\_t001 |  |  |  |  |  |  |  |  |
| 0 | Vvi-Vitvi13g04115\_t001 |  |  |  |  |  |  |  |  |
| 0 | Vvi-Vitvi13g04116\_t001 |  |  |  |  |  |  |  |  |
| 0 | Vvi-Vitvi13g04117\_t001 |  |  |  |  |  |  |  |  |
| 0 | Vvi-Vitvi13g04118\_t001 |  |  |  |  |  |  |  |  |
| 0 | Vvi-Vitvi13g00374\_t001 |  |  |  |  |  |  |  |  |
| 0 | Vvi-Vitvi13g00375\_t001 |  |  |  |  |  |  |  |  |
| 0 | Vvi-Vitvi13g02008\_t001 |  |  |  |  |  |  |  |  |
| 0 | Vvi-Vitvi13g02009\_t001 |  |  |  |  |  |  |  |  |
| 0 | Vvi-Vitvi13g02010\_t001 |  |  |  |  |  |  |  |  |
| 0 | Vvi-Vitvi13g00377\_t001 |  |  |  |  |  |  |  |  |
| 0 | Vvi-Vitvi13g00380\_t001 |  |  |  |  |  |  |  |  |
| 0 | Vvi-Vitvi13g00381\_t001 |  |  |  |  |  |  |  |  |
| 0 | Vvi-Vitvi13g04119\_t001 |  |  |  |  |  |  |  |  |
| 0 | Vvi-Vitvi13g00382\_t001 |  |  |  |  |  |  |  |  |
| 0 | Vvi-Vitvi13g00383\_t001 |  |  |  |  |  |  |  |  |
| 0 | Vvi-Vitvi13g00384\_t001 |  |  |  |  |  |  |  |  |
| 0 | Vvi-Vitvi13g04120\_t001 |  |  |  |  |  |  |  |  |
| 0 | Vvi-Vitvi13g00385\_t001 |  |  |  |  |  |  |  |  |
| 0 | Vvi-Vitvi13g00386\_t001 |  |  |  |  |  |  |  |  |
| 0 | Vvi-Vitvi13g02012\_t001 |  |  |  |  |  |  |  |  |
| 0 | Vvi-Vitvi13g04121\_t001 |  |  |  |  |  |  |  |  |
| 1 | Vvi-Vitvi13g00387\_t001 |  | Ath-AT3G55646.1 |  |  |  |  |  |  |  |
| 2 | Vvi-Vitvi13g00388\_t001 |  | | | |  | Ath-AT5G05960.1 |  |  |  |  |  |  |
| 2 | Vvi-Vitvi13g00389\_t001 |  | | | |  | Ath-AT5G05950.1 |  |  |  |  |  |  |
| 2 | Vvi-Vitvi13g04122\_t001 |  | | | |  | | | |  |  |  |  |  |  |
| 2 | Vvi-Vitvi13g00390\_t001 |  | Ath-AT3G55660.1 |  | Ath-AT5G05940.1 |  |  |  |  |  |  |
| 2 | Vvi-Vitvi13g00391\_t001 |  | | | |  | Ath-AT5G05930.1 |  |  |  |  |  |  |
| 2 | Vvi-Vitvi13g04123\_t001 |  | | | |  | | | |  |  |  |  |  |  |
| 2 | Vvi-Vitvi13g02013\_t001 |  | | | |  | | | |  |  |  |  |  |  |
| 2 | Vvi-Vitvi13g02014\_t001 |  | | | |  | | | |  |  |  |  |  |  |
| 2 | Vvi-Vitvi13g02016\_t001 |  | | | |  | | | |  |  |  |  |  |  |
| 2 | Vvi-Vitvi13g02017\_t001 |  | | | |  | | | |  |  |  |  |  |  |
| 2 | Vvi-Vitvi13g04124\_t001 |  | | | |  | | | |  |  |  |  |  |  |
| 2 | Vvi-Vitvi13g00397\_t001 |  | | | |  | Ath-AT5G05880.1 |  |  |  |  |  |  |
| 2 | Vvi-Vitvi13g00398\_t001 |  | | | |  | | | |  |  |  |  |  |  |
| 2 | Vvi-Vitvi13g00405\_t001 |  | | | |  | | | |  |  |  |  |  |  |
| 2 | Vvi-Vitvi13g00409\_t001 |  | | | |  | | | |  |  |  |  |  |  |
| 2 | Vvi-Vitvi13g00410\_t001 |  | | | |  | | | |  |  |  |  |  |  |
| 2 | Vvi-Vitvi13g00417\_t001 |  | | | |  | | | |  |  |  |  |  |  |
| 2 | Vvi-Vitvi13g00431\_t001 |  | | | |  | | | |  |  |  |  |  |  |
| 2 | Vvi-Vitvi13g04125\_t001 |  | | | |  | | | |  |  |  |  |  |  |
| 2 | Vvi-Vitvi13g02021\_t001 |  | | | |  | | | |  |  |  |  |  |  |
| 2 | Vvi-Vitvi13g02022\_t001 |  | | | |  | | | |  |  |  |  |  |  |
| 2 | Vvi-Vitvi13g02023\_t001 |  | | | |  | | | |  |  |  |  |  |  |
| 2 | Vvi-Vitvi13g02024\_t001 |  | | | |  | | | |  |  |  |  |  |  |
| 2 | Vvi-Vitvi13g00432\_t001 |  | | | |  | | | |  |  |  |  |  |  |
| 2 | Vvi-Vitvi13g04126\_t001 |  | | | |  | Ath-AT5G05860.1 |  |  |  |  |  |  |
| 2 | Vvi-Vitvi13g04127\_t001 |  | Ath-AT3G55700.1 |  | | | |  |  |  |  |  |  |
| 2 | Vvi-Vitvi13g04128\_t001 |  | | | |  | | | |  |  |  |  |  |  |
| 2 | Vvi-Vitvi13g04129\_t001 |  | | | |  | | | |  |  |  |  |  |  |
| 2 | Vvi-Vitvi13g04130\_t001 |  | | | |  | | | |  |  |  |  |  |  |
| 2 | Vvi-Vitvi13g00451\_t001 |  | | | |  | | | |  |  |  |  |  |  |
| 2 | Vvi-Vitvi13g04131\_t001 |  | | | |  | | | |  |  |  |  |  |  |
| 2 | Vvi-Vitvi13g04132\_t001 |  | | | |  | | | |  |  |  |  |  |  |
| 2 | Vvi-Vitvi13g00452\_t001 |  | | | |  | | | |  |  |  |  |  |  |
| 2 | Vvi-Vitvi13g00458\_t001 |  | | | |  | | | |  |  |  |  |  |  |
| 2 | Vvi-Vitvi13g04133\_t001 |  | | | |  | | | |  |  |  |  |  |  |
| 2 | Vvi-Vitvi13g04134\_t001 |  | | | |  | | | |  |  |  |  |  |  |
| 2 | Vvi-Vitvi13g02026\_t001 |  | | | |  | | | |  |  |  |  |  |  |
| 2 | Vvi-Vitvi13g02027\_t001 |  | | | |  | | | |  |  |  |  |  |  |
| 2 | Vvi-Vitvi13g02028\_t001 |  | | | |  | | | |  |  |  |  |  |  |
| 2 | Vvi-Vitvi13g04135\_t001 |  | | | |  | | | |  |  |  |  |  |  |
| 2 | Vvi-Vitvi13g04136\_t001 |  | | | |  | | | |  |  |  |  |  |  |
| 2 | Vvi-Vitvi13g00471\_t001 |  | | | |  | | | |  |  |  |  |  |  |
| 2 | Vvi-Vitvi13g00474\_t001 |  | | | |  | | | |  |  |  |  |  |  |
| 2 | Vvi-Vitvi13g04137\_t001 |  | | | |  | | | |  |  |  |  |  |  |
| 2 | Vvi-Vitvi13g04138\_t001 |  | | | |  | | | |  |  |  |  |  |  |
| 2 | Vvi-Vitvi13g04139\_t001 |  | | | |  | | | |  |  |  |  |  |  |
| 2 | Vvi-Vitvi13g00489\_t001 |  | | | |  | | | |  |  |  |  |  |  |
| 2 | Vvi-Vitvi13g00490\_t001 |  | | | |  | | | |  |  |  |  |  |  |
| 2 | Vvi-Vitvi13g00491\_t001 |  | | | |  | | | |  |  |  |  |  |  |
| 2 | Vvi-Vitvi13g00492\_t001 |  | Ath-AT3G55720.1 |  | Ath-AT5G05840.1 |  |  |  |  |  |  |
| 3 | Vvi-Vitvi13g04140\_t003 |  | | | |  | Ath-AT5G05830.1 |  | Ath-AT5G01070.1 |  |  |  |  |  |
| 4 | Vvi-Vitvi13g00494\_t001 |  | Ath-AT3G55730.1 |  | | | |  | | | |  | Ath-AT2G39880.1 |  |  |  |  |
| 4 | Vvi-Vitvi13g00495\_t001 |  | | | |  | | | |  | Ath-AT5G01090.1 |  | | | |  |  |  |  |
| 4 | Vvi-Vitvi13g00496\_t001 |  | Ath-AT3G55740.1 |  | | | |  | | | |  | Ath-AT2G39890.1 |  |  |  |  |
| 4 | Vvi-Vitvi13g04141\_t001 |  | | | |  | | | |  | | | |  | | | |  |  |  |  |
| 4 | Vvi-Vitvi13g00498\_t001 |  | | | |  | | | |  | | | |  | | | |  |  |  |  |
| 4 | Vvi-Vitvi13g00499\_t001 |  | | | |  | | | |  | Ath-AT5G01100.1 |  | | | |  |  |  |  |
| 4 | Vvi-Vitvi13g04142\_t001 |  | Ath-AT3G55750.1 |  | | | |  | | | |  | | | |  |  |  |  |
| 4 | Vvi-Vitvi13g00501\_t001 |  | | | |  | Ath-AT5G05820.1 |  | | | |  | | | |  |  |  |  |
| 4 | Vvi-Vitvi13g02031\_t001 |  | Ath-AT3G55770.7 |  | | | |  | | | |  | Ath-AT2G39900.1 |  |  |  |  |
| 4 | Vvi-Vitvi13g00502\_t001 |  | | | |  | | | |  | | | |  | | | |  |  |  |  |
| 4 | Vvi-Vitvi13g00503\_t001 |  | | | |  | | | |  | | | |  | Ath-AT2G39910.1 |  |  |  |  |
| 4 | Vvi-Vitvi13g00504\_t001 |  | Ath-AT3G55780.1 |  | | | |  | | | |  | | | |  |  |  |  |
| 4 | Vvi-Vitvi13g02032\_t001 |  | | | |  | Ath-AT5G05800.2 |  | | | |  | | | |  |  |  |  |
| 4 | Vvi-Vitvi13g02033\_t005 |  | | | |  | | | |  | | | |  | Ath-AT2G39920.1 |  |  |  |  |
| 4 | Vvi-Vitvi13g00505\_t002 |  | | | |  | | | |  | | | |  | | | |  |  |  |  |
| 4 | Vvi-Vitvi13g02034\_t001 |  | | | |  | | | |  | | | |  | | | |  |  |  |  |
| 4 | Vvi-Vitvi13g00507\_t001 |  | Ath-AT3G55800.1 |  | | | |  | | | |  | | | |  |  |  |  |
| 4 | Vvi-Vitvi13g02035\_t001 |  | | | |  | | | |  | | | |  | | | |  |  |  |  |
| 4 | Vvi-Vitvi13g00509\_t001 |  | | | |  | | | |  | Ath-AT5G01190.1 |  | | | |  |  |  |  |
| 4 | Vvi-Vitvi13g00510\_t001 |  | | | |  | Ath-AT5G05790.1 |  | Ath-AT5G01200.1 |  | | | |  |  |  |  |
| 4 | Vvi-Vitvi13g00512\_t001 |  | | | |  | | | |  | | | |  | Ath-AT2G39940.1 |  |  |  |  |
| 4 | Vvi-Vitvi13g04143\_t001 |  | | | |  | | | |  | | | |  | | | |  |  |  |  |
| 4 | Vvi-Vitvi13g00513\_t001 |  | | | |  | | | |  | | | |  | Ath-AT2G39950.1 |  |  |  |  |
| 4 | Vvi-Vitvi13g02037\_t001 |  | | | |  | | | |  | | | |  | Ath-AT2G39960.1 |  |  |  |  |
| 4 | Vvi-Vitvi13g00515\_t001 |  | | | |  | | | |  | | | |  | Ath-AT2G39970.1 |  |  |  |  |
| 4 | Vvi-Vitvi13g04144\_t001 |  | | | |  | | | |  | | | |  | | | |  |  |  |  |
| 4 | Vvi-Vitvi13g00516\_t001 |  | | | |  | | | |  | | | |  | | | |  |  |  |  |
| 4 | Vvi-Vitvi13g04145\_t001 |  | | | |  | | | |  | | | |  | | | |  |  |  |  |
| 4 | Vvi-Vitvi13g00517\_t001 |  | | | |  | | | |  | Ath-AT5G01210.1 |  | Ath-AT2G39980.1 |  |  |  |  |
| 3 | Vvi-Vitvi13g00521\_t001 |  | Ath-AT3G55820.1 |  | | | |  |  |  | | | |  |  |  |  |
| 2 | Vvi-Vitvi13g04146\_t001 |  |  |  | | | |  |  |  | | | |  |  |  |  |
| 2 | Vvi-Vitvi13g02039\_t001 |  |  |  | Ath-AT5G05770.1 |  |  |  | | | |  |  |  |  |
| 1 | Vvi-Vitvi13g00522\_t001 |  |  |  |  |  |  |  | Ath-AT2G39990.1 |  |  |  |  |
| 0 | Vvi-Vitvi13g00523\_t001 |  |  |  |  |  |  |  |  |
| 0 | Vvi-Vitvi13g00526\_t001 |  |  |  |  |  |  |  |  |
| 0 | Vvi-Vitvi13g04147\_t001 |  |  |  |  |  |  |  |  |
| 0 | Vvi-Vitvi13g04148\_t001 |  |  |  |  |  |  |  |  |
| 0 | Vvi-Vitvi13g04149\_t001 |  |  |  |  |  |  |  |  |
| 0 | Vvi-Vitvi13g02042\_t001 |  |  |  |  |  |  |  |  |
| 0 | Vvi-Vitvi13g04150\_t001 |  |  |  |  |  |  |  |  |
| 0 | Vvi-Vitvi13g04151\_t001 |  |  |  |  |  |  |  |  |
| 0 | Vvi-Vitvi13g04152\_t001 |  |  |  |  |  |  |  |  |
| 0 | Vvi-Vitvi13g00528\_t001 |  |  |  |  |  |  |  |  |
| 0 | Vvi-Vitvi13g00529\_t001 |  |  |  |  |  |  |  |  |
| 0 | Vvi-Vitvi13g00530\_t001 |  |  |  |  |  |  |  |  |
| 0 | Vvi-Vitvi13g04153\_t001 |  |  |  |  |  |  |  |  |
| 0 | Vvi-Vitvi13g00531\_t001 |  |  |  |  |  |  |  |  |
| 0 | Vvi-Vitvi13g04154\_t001 |  |  |  |  |  |  |  |  |
| 0 | Vvi-Vitvi13g00533\_t001 |  |  |  |  |  |  |  |  |
| 0 | Vvi-Vitvi13g00534\_t001 |  |  |  |  |  |  |  |  |
| 0 | Vvi-Vitvi13g04155\_t001 |  |  |  |  |  |  |  |  |
| 0 | Vvi-Vitvi13g00535\_t001 |  |  |  |  |  |  |  |  |
| 0 | Vvi-Vitvi13g00536\_t001 |  |  |  |  |  |  |  |  |
| 0 | Vvi-Vitvi13g00537\_t004 |  |  |  |  |  |  |  |  |
| 0 | Vvi-Vitvi13g00538\_t001 |  |  |  |  |  |  |  |  |
| 0 | Vvi-Vitvi13g04156\_t001 |  |  |  |  |  |  |  |  |
| 0 | Vvi-Vitvi13g02046\_t001 |  |  |  |  |  |  |  |  |
| 0 | Vvi-Vitvi13g02047\_t001 |  |  |  |  |  |  |  |  |
| 0 | Vvi-Vitvi13g00540\_t001 |  |  |  |  |  |  |  |  |
| 1 | Vvi-Vitvi13g00541\_t001 |  | Ath-AT2G35810.1 |  |  |  |  |  |  |  |
| 1 | Vvi-Vitvi13g00542\_t001 |  | | | |  |  |  |  |  |  |  |
| 1 | Vvi-Vitvi13g00543\_t001 |  | | | |  |  |  |  |  |  |  |
| 1 | Vvi-Vitvi13g00545\_t001 |  | | | |  |  |  |  |  |  |  |
| 1 | Vvi-Vitvi13g00546\_t001 |  | | | |  |  |  |  |  |  |  |
| 1 | Vvi-Vitvi13g04157\_t001 |  | | | |  |  |  |  |  |  |  |
| 1 | Vvi-Vitvi13g04158\_t001 |  | | | |  |  |  |  |  |  |  |
| 1 | Vvi-Vitvi13g00549\_t001 |  | | | |  |  |  |  |  |  |  |
| 1 | Vvi-Vitvi13g00551\_t001 |  | | | |  |  |  |  |  |  |  |
| 1 | Vvi-Vitvi13g02050\_t001 |  | | | |  |  |  |  |  |  |  |
| 1 | Vvi-Vitvi13g00552\_t001 |  | | | |  |  |  |  |  |  |  |
| 1 | Vvi-Vitvi13g01866\_t001 |  | | | |  |  |  |  |  |  |  |
| 1 | Vvi-Vitvi13g01867\_t001 |  | | | |  |  |  |  |  |  |  |
| 1 | Vvi-Vitvi13g04159\_t001 |  | | | |  |  |  |  |  |  |  |
| 1 | Vvi-Vitvi13g00555\_t001 |  | | | |  |  |  |  |  |  |  |
| 1 | Vvi-Vitvi13g00556\_t001 |  | | | |  |  |  |  |  |  |  |
| 1 | Vvi-Vitvi13g04160\_t001 |  | | | |  |  |  |  |  |  |  |
| 2 | Vvi-Vitvi13g00561\_t001 |  | | | |  | Ath-AT3G52300.1 |  |  |  |  |  |  |
| 2 | Vvi-Vitvi13g00562\_t001 |  | Ath-AT2G35800.1 |  | | | |  |  |  |  |  |  |
| 2 | Vvi-Vitvi13g02052\_t001 |  | Ath-AT2G35795.1 |  | | | |  |  |  |  |  |  |
| 2 | Vvi-Vitvi13g00563\_t002 |  | | | |  | Ath-AT3G52290.1 |  |  |  |  |  |  |
| 2 | Vvi-Vitvi13g00564\_t001 |  | | | |  | | | |  |  |  |  |  |  |
| 2 | Vvi-Vitvi13g02053\_t001 |  | | | |  | | | |  |  |  |  |  |  |
| 3 | Vvi-Vitvi13g02054\_t001 |  | | | |  | | | |  | Ath-AT5G60390.1 |  |  |  |  |  |
| 3 | Vvi-Vitvi13g02055\_t001 |  | | | |  | | | |  | | | |  |  |  |  |  |
| 3 | Vvi-Vitvi13g00565\_t001 |  | Ath-AT2G35790.1 |  | | | |  | | | |  |  |  |  |  |
| 3 | Vvi-Vitvi13g04161\_t001 |  | | | |  | | | |  | | | |  |  |  |  |  |
| 3 | Vvi-Vitvi13g00566\_t001 |  | | | |  | Ath-AT3G52280.2 |  | | | |  |  |  |  |  |
| 3 | Vvi-Vitvi13g00567\_t001 |  | | | |  | | | |  | | | |  |  |  |  |  |
| 3 | Vvi-Vitvi13g00568\_t001 |  | | | |  | | | |  | | | |  |  |  |  |  |
| 3 | Vvi-Vitvi13g00569\_t001 |  | | | |  | Ath-AT3G52270.1 |  | | | |  |  |  |  |  |
| 3 | Vvi-Vitvi13g00570\_t002 |  | | | |  | | | |  | | | |  |  |  |  |  |
| 3 | Vvi-Vitvi13g00571\_t001 |  | | | |  | Ath-AT3G52260.3 |  | | | |  |  |  |  |  |
| 3 | Vvi-Vitvi13g00573\_t001 |  | | | |  | Ath-AT3G52250.1 |  | | | |  |  |  |  |  |
| 2 | Vvi-Vitvi13g00574\_t001 |  | | | |  |  |  | | | |  |  |  |  |  |
| 2 | Vvi-Vitvi13g00575\_t001 |  | Ath-AT2G35780.1 |  |  |  | | | |  |  |  |  |  |
| 2 | Vvi-Vitvi13g00576\_t001 |  | Ath-AT2G35770.1 |  |  |  | | | |  |  |  |  |  |
| 1 | Vvi-Vitvi13g00577\_t001 |  |  |  |  |  | | | |  |  |  |  |  |
| 1 | Vvi-Vitvi13g00578\_t001 |  |  |  |  |  | | | |  |  |  |  |  |
| 1 | Vvi-Vitvi13g00579\_t001 |  |  |  |  |  | | | |  |  |  |  |  |
| 1 | Vvi-Vitvi13g00583\_t001 |  |  |  |  |  | Ath-AT5G60460.1 |  |  |  |  |  |
| 1 | Vvi-Vitvi13g00584\_t001 |  |  |  |  |  | | | |  |  |  |  |  |
| 1 | Vvi-Vitvi13g00585\_t001 |  |  |  |  |  | | | |  |  |  |  |  |
| 1 | Vvi-Vitvi13g04162\_t001 |  |  |  |  |  | | | |  |  |  |  |  |
| 1 | Vvi-Vitvi13g00588\_t001 |  |  |  |  |  | | | |  |  |  |  |  |
| 1 | Vvi-Vitvi13g00589\_t001 |  |  |  |  |  | | | |  |  |  |  |  |
| 1 | Vvi-Vitvi13g00590\_t001 |  |  |  |  |  | | | |  |  |  |  |  |
| 1 | Vvi-Vitvi13g00591\_t001 |  |  |  |  |  | | | |  |  |  |  |  |
| 1 | Vvi-Vitvi13g00592\_t001 |  |  |  |  |  | | | |  |  |  |  |  |
| 1 | Vvi-Vitvi13g04163\_t001 |  |  |  |  |  | | | |  |  |  |  |  |
| 1 | Vvi-Vitvi13g04164\_t001 |  |  |  |  |  | | | |  |  |  |  |  |
| 1 | Vvi-Vitvi13g00594\_t001 |  |  |  |  |  | | | |  |  |  |  |  |
| 1 | Vvi-Vitvi13g00596\_t001 |  |  |  |  |  | | | |  |  |  |  |  |
| 1 | Vvi-Vitvi13g00597\_t002 |  |  |  |  |  | | | |  |  |  |  |  |
| 1 | Vvi-Vitvi13g00598\_t001 |  |  |  |  |  | | | |  |  |  |  |  |
| 1 | Vvi-Vitvi13g04165\_t001 |  |  |  |  |  | | | |  |  |  |  |  |
| 1 | Vvi-Vitvi13g04166\_t001 |  |  |  |  |  | | | |  |  |  |  |  |
| 1 | Vvi-Vitvi13g00600\_t001 |  |  |  |  |  | | | |  |  |  |  |  |
| 1 | Vvi-Vitvi13g00601\_t001 |  |  |  |  |  | | | |  |  |  |  |  |
| 1 | Vvi-Vitvi13g00602\_t001 |  |  |  |  |  | Ath-AT5G60640.1 |  |  |  |  |  |
| 1 | Vvi-Vitvi13g00603\_t001 |  |  |  |  |  | | | |  |  |  |  |  |
| 1 | Vvi-Vitvi13g00604\_t001 |  |  |  |  |  | | | |  |  |  |  |  |
| 1 | Vvi-Vitvi13g04167\_t001 |  |  |  |  |  | | | |  |  |  |  |  |
| 1 | Vvi-Vitvi13g00605\_t001 |  |  |  |  |  | Ath-AT5G60660.1 |  |  |  |  |  |
| 1 | Vvi-Vitvi13g04168\_t001 |  |  |  |  |  | | | |  |  |  |  |  |
| 1 | Vvi-Vitvi13g00606\_t001 |  |  |  |  |  | Ath-AT5G60670.1 |  |  |  |  |  |
| 1 | Vvi-Vitvi13g00607\_t001 |  |  |  |  |  | | | |  |  |  |  |  |
| 1 | Vvi-Vitvi13g04169\_t001 |  |  |  |  |  | | | |  |  |  |  |  |
| 1 | Vvi-Vitvi13g00609\_t001 |  |  |  |  |  | Ath-AT5G60690.1 |  |  |  |  |  |
| 1 | Vvi-Vitvi13g00610\_t001 |  |  |  |  |  | | | |  |  |  |  |  |
| 1 | Vvi-Vitvi13g00611\_t001 |  |  |  |  |  | | | |  |  |  |  |  |
| 1 | Vvi-Vitvi13g00612\_t001 |  |  |  |  |  | | | |  |  |  |  |  |
| 1 | Vvi-Vitvi13g00613\_t002 |  |  |  |  |  | | | |  |  |  |  |  |
| 1 | Vvi-Vitvi13g02058\_t001 |  |  |  |  |  | | | |  |  |  |  |  |
| 1 | Vvi-Vitvi13g04170\_t001 |  |  |  |  |  | | | |  |  |  |  |  |
| 1 | Vvi-Vitvi13g00614\_t001 |  |  |  |  |  | | | |  |  |  |  |  |
| 1 | Vvi-Vitvi13g04171\_t001 |  |  |  |  |  | | | |  |  |  |  |  |
| 1 | Vvi-Vitvi13g00615\_t001 |  |  |  |  |  | | | |  |  |  |  |  |
| 1 | Vvi-Vitvi13g00617\_t001 |  |  |  |  |  | | | |  |  |  |  |  |
| 1 | Vvi-Vitvi13g00618\_t001 |  |  |  |  |  | | | |  |  |  |  |  |
| 1 | Vvi-Vitvi13g00619\_t001 |  |  |  |  |  | | | |  |  |  |  |  |
| 3 | Vvi-Vitvi13g00620\_t001 |  | Ath-AT3G54780.4 |  | Ath-AT2G38970.1 |  | Ath-AT5G60710.1 |  |  |  |  |  |
| 4 | Vvi-Vitvi13g00621\_t001 |  | | | |  | | | |  | | | |  | Ath-AT5G04220.2 |  |  |  |  |
| 4 | Vvi-Vitvi13g04172\_t001 |  | | | |  | | | |  | | | |  | | | |  |  |  |  |
| 5 | Vvi-Vitvi13g00622\_t001 |  | | | |  | | | |  | | | |  | Ath-AT5G04230.2 |  | Ath-AT3G10340.1 |  |  |  |
| 5 | Vvi-Vitvi13g04173\_t001 |  | | | |  | | | |  | | | |  | | | |  | | | |  |  |  |
| 5 | Vvi-Vitvi13g04174\_t001 |  | | | |  | | | |  | | | |  | | | |  | | | |  |  |  |
| 5 | Vvi-Vitvi13g04175\_t001 |  | | | |  | | | |  | | | |  | | | |  | | | |  |  |  |
| 5 | Vvi-Vitvi13g04176\_t001 |  | | | |  | | | |  | | | |  | | | |  | | | |  |  |  |
| 5 | Vvi-Vitvi13g00627\_t001 |  | | | |  | | | |  | | | |  | | | |  | | | |  |  |  |
| 5 | Vvi-Vitvi13g04177\_t001 |  | | | |  | | | |  | | | |  | | | |  | | | |  |  |  |
| 5 | Vvi-Vitvi13g04178\_t001 |  | | | |  | | | |  | | | |  | | | |  | | | |  |  |  |
| 5 | Vvi-Vitvi13g02062\_t001 |  | | | |  | | | |  | | | |  | | | |  | | | |  |  |  |
| 5 | Vvi-Vitvi13g04179\_t002 |  | | | |  | | | |  | | | |  | Ath-AT5G04240.1 |  | | | |  |  |  |
| 5 | Vvi-Vitvi13g00631\_t001 |  | | | |  | Ath-AT2G39020.1 |  | | | |  | | | |  | | | |  |  |  |
| 5 | Vvi-Vitvi13g00632\_t001 |  | | | |  | | | |  | | | |  | | | |  | | | |  |  |  |
| 5 | Vvi-Vitvi13g00635\_t001 |  | | | |  | | | |  | | | |  | | | |  | | | |  |  |  |
| 5 | Vvi-Vitvi13g00636\_t001 |  | Ath-AT3G54826.1 |  | | | |  | | | |  | | | |  | | | |  |  |  |
| 5 | Vvi-Vitvi13g00637\_t001.1.6037826b |  | | | |  | | | |  | Ath-AT5G60730.1 |  | | | |  | Ath-AT3G10350.2 |  |  |  |
| 5 | Vvi-Vitvi13g00638\_t001 |  | | | |  | | | |  | Ath-AT5G60740.1 |  | | | |  | | | |  |  |  |
| 4 | Vvi-Vitvi13g00639\_t001 |  | | | |  | Ath-AT2G39110.1 |  |  |  | | | |  | | | |  |  |  |
| 4 | Vvi-Vitvi13g04180\_t001 |  | | | |  | | | |  |  |  | | | |  | | | |  |  |  |
| 4 | Vvi-Vitvi13g00640\_t001 |  | | | |  | | | |  |  |  | Ath-AT5G04250.2 |  | | | |  |  |  |
| 4 | Vvi-Vitvi13g00643\_t001 |  | | | |  | Ath-AT2G39120.1 |  |  |  | | | |  | | | |  |  |  |
| 4 | Vvi-Vitvi13g00644\_t002 |  | | | |  | | | |  |  |  | | | |  | Ath-AT3G10360.1 |  |  |  |
| 4 | Vvi-Vitvi13g04181\_t001 |  | | | |  | | | |  |  |  | Ath-AT5G04260.1 |  | | | |  |  |  |
| 4 | Vvi-Vitvi13g04182\_t001 |  | | | |  | | | |  |  |  | | | |  | | | |  |  |  |
| 4 | Vvi-Vitvi13g00646\_t001 |  | Ath-AT3G54830.1 |  | Ath-AT2G39130.1 |  |  |  | | | |  | | | |  |  |  |
| 4 | Vvi-Vitvi13g00647\_t001 |  | | | |  | | | |  |  |  | Ath-AT5G04270.1 |  | | | |  |  |  |
| 4 | Vvi-Vitvi13g04183\_t001 |  | | | |  | | | |  |  |  | | | |  | | | |  |  |  |
| 4 | Vvi-Vitvi13g04184\_t001 |  | | | |  | | | |  |  |  | | | |  | | | |  |  |  |
| 4 | Vvi-Vitvi13g00650\_t001 |  | Ath-AT3G54840.1 |  | | | |  |  |  | | | |  | | | |  |  |  |
| 4 | Vvi-Vitvi13g00651\_t001 |  | Ath-AT3G54850.1 |  | | | |  |  |  | | | |  | | | |  |  |  |
| 4 | Vvi-Vitvi13g00652\_t001 |  | Ath-AT3G54860.2 |  | | | |  |  |  | | | |  | | | |  |  |  |
| 4 | Vvi-Vitvi13g00654\_t001 |  | | | |  | | | |  |  |  | | | |  | Ath-AT3G10370.1 |  |  |  |
| 4 | Vvi-Vitvi13g00656\_t001 |  | | | |  | Ath-AT2G39140.1 |  |  |  | | | |  | | | |  |  |  |
| 4 | Vvi-Vitvi13g00657\_t001 |  | Ath-AT3G54870.2 |  | | | |  |  |  | | | |  | | | |  |  |  |
| 4 | Vvi-Vitvi13g00658\_t001 |  | | | |  | | | |  |  |  | | | |  | | | |  |  |  |
| 4 | Vvi-Vitvi13g00659\_t001 |  | | | |  | | | |  |  |  | | | |  | Ath-AT3G10380.1 |  |  |  |
| 4 | Vvi-Vitvi13g04185\_t001 |  | | | |  | | | |  |  |  | | | |  | | | |  |  |  |
| 4 | Vvi-Vitvi13g00661\_t001 |  | | | |  | | | |  |  |  | | | |  | Ath-AT3G10390.3 |  |  |  |
| 4 | Vvi-Vitvi13g04186\_t001 |  | | | |  | | | |  |  |  | | | |  | | | |  |  |  |
| 4 | Vvi-Vitvi13g00662\_t003 |  | | | |  | Ath-AT2G39170.1 |  |  |  | | | |  | | | |  |  |  |
| 4 | Vvi-Vitvi13g00663\_t001 |  | | | |  | | | |  |  |  | | | |  | Ath-AT3G10405.1 |  |  |  |
| 4 | Vvi-Vitvi13g00665\_t001 |  | | | |  | | | |  |  |  | | | |  | | | |  |  |  |
| 4 | Vvi-Vitvi13g02064\_t001 |  | | | |  | | | |  |  |  | | | |  | | | |  |  |  |
| 4 | Vvi-Vitvi13g00667\_t001 |  | Ath-AT3G54920.1 |  | | | |  |  |  | | | |  | | | |  |  |  |
| 4 | Vvi-Vitvi13g00669\_t001 |  | | | |  | | | |  |  |  | | | |  | | | |  |  |  |
| 4 | Vvi-Vitvi13g00670\_t001 |  | Ath-AT3G54930.2 |  | | | |  |  |  | | | |  | | | |  |  |  |
| 4 | Vvi-Vitvi13g00673\_t001 |  | | | |  | | | |  |  |  | | | |  | | | |  |  |  |
| 4 | Vvi-Vitvi13g00674\_t001 |  | | | |  | | | |  |  |  | Ath-AT5G04290.1 |  | | | |  |  |  |
| 4 | Vvi-Vitvi13g04187\_t001 |  | | | |  | | | |  |  |  | | | |  | | | |  |  |  |
| 4 | Vvi-Vitvi13g04188\_t001 |  | | | |  | | | |  |  |  | | | |  | | | |  |  |  |
| 4 | Vvi-Vitvi13g00675\_t001 |  | | | |  | Ath-AT2G39210.1 |  |  |  | | | |  | | | |  |  |  |
| 3 | Vvi-Vitvi13g00676\_t001 |  | | | |  |  |  |  |  | | | |  | | | |  |  |  |
| 3 | Vvi-Vitvi13g00677\_t001 |  | | | |  |  |  |  |  | | | |  | | | |  |  |  |
| 3 | Vvi-Vitvi13g00678\_t002 |  | | | |  |  |  |  |  | | | |  | | | |  |  |  |
| 3 | Vvi-Vitvi13g04189\_t001 |  | | | |  |  |  |  |  | | | |  | | | |  |  |  |
| 3 | Vvi-Vitvi13g04190\_t001 |  | | | |  |  |  |  |  | | | |  | | | |  |  |  |
| 3 | Vvi-Vitvi13g04191\_t001 |  | | | |  |  |  |  |  | | | |  | | | |  |  |  |
| 3 | Vvi-Vitvi13g00680\_t001 |  | | | |  |  |  |  |  | | | |  | | | |  |  |  |
| 3 | Vvi-Vitvi13g04192\_t001 |  | | | |  |  |  |  |  | | | |  | | | |  |  |  |
| 3 | Vvi-Vitvi13g04193\_t001 |  | | | |  |  |  |  |  | | | |  | | | |  |  |  |
| 3 | Vvi-Vitvi13g04194\_t001 |  | | | |  |  |  |  |  | | | |  | | | |  |  |  |
| 3 | Vvi-Vitvi13g04195\_t001 |  | | | |  |  |  |  |  | | | |  | | | |  |  |  |
| 3 | Vvi-Vitvi13g00683\_t001 |  | | | |  |  |  |  |  | | | |  | | | |  |  |  |
| 3 | Vvi-Vitvi13g00684\_t001 |  | | | |  |  |  |  |  | | | |  | | | |  |  |  |
| 3 | Vvi-Vitvi13g00685\_t001 |  | | | |  |  |  |  |  | | | |  | Ath-AT3G10420.2 |  |  |  |
| 3 | Vvi-Vitvi13g00686\_t001 |  | | | |  |  |  |  |  | | | |  | | | |  |  |  |
| 3 | Vvi-Vitvi13g00687\_t001 |  | | | |  |  |  |  |  | | | |  | | | |  |  |  |
| 3 | Vvi-Vitvi13g00688\_t001 |  | Ath-AT3G54940.2 |  |  |  |  |  | | | |  | | | |  |  |  |
| 2 | Vvi-Vitvi13g02066\_t001 |  |  |  |  |  |  |  | | | |  | | | |  |  |  |
| 2 | Vvi-Vitvi13g02067\_t001 |  |  |  |  |  |  |  | | | |  | | | |  |  |  |
| 2 | Vvi-Vitvi13g04196\_t001 |  |  |  |  |  |  |  | | | |  | | | |  |  |  |
| 2 | Vvi-Vitvi13g00692\_t001 |  |  |  |  |  |  |  | Ath-AT5G04360.1 |  | | | |  |  |  |
| 2 | Vvi-Vitvi13g00694\_t001 |  |  |  |  |  |  |  | Ath-AT5G04390.1 |  | Ath-AT3G10470.1 |  |  |  |
| 2 | Vvi-Vitvi13g00695\_t001 |  |  |  |  |  |  |  | | | |  | | | |  |  |  |
| 2 | Vvi-Vitvi13g02070\_t003 |  |  |  |  |  |  |  | | | |  | | | |  |  |  |
| 2 | Vvi-Vitvi13g04197\_t001 |  |  |  |  |  |  |  | | | |  | | | |  |  |  |
| 2 | Vvi-Vitvi13g04198\_t001 |  |  |  |  |  |  |  | | | |  | | | |  |  |  |
| 2 | Vvi-Vitvi13g00697\_t001 |  |  |  |  |  |  |  | | | |  | | | |  |  |  |
| 2 | Vvi-Vitvi13g04199\_t001 |  |  |  |  |  |  |  | | | |  | | | |  |  |  |
| 2 | Vvi-Vitvi13g00698\_t001 |  |  |  |  |  |  |  | Ath-AT5G04410.1 |  | Ath-AT3G10480.3 |  |  |  |
| 2 | Vvi-Vitvi13g04200\_t001 |  |  |  |  |  |  |  | | | |  | | | |  |  |  |
| 2 | Vvi-Vitvi13g00699\_t001 |  |  |  |  |  |  |  | | | |  | Ath-AT3G10490.2 |  |  |  |
| 1 | Vvi-Vitvi13g00700\_t002 |  |  |  |  |  |  |  | | | |  |  |  |  |
| 1 | Vvi-Vitvi13g00701\_t001 |  |  |  |  |  |  |  | | | |  |  |  |  |
| 1 | Vvi-Vitvi13g04201\_t001 |  |  |  |  |  |  |  | | | |  |  |  |  |
| 1 | Vvi-Vitvi13g02072\_t001 |  |  |  |  |  |  |  | | | |  |  |  |  |
| 1 | Vvi-Vitvi13g04202\_t001 |  |  |  |  |  |  |  | | | |  |  |  |  |
| 1 | Vvi-Vitvi13g04203\_t001 |  |  |  |  |  |  |  | | | |  |  |  |  |
| 1 | Vvi-Vitvi13g04204\_t001 |  |  |  |  |  |  |  | | | |  |  |  |  |
| 1 | Vvi-Vitvi13g00703\_t001 |  |  |  |  |  |  |  | Ath-AT5G04420.2 |  |  |  |  |
| 1 | Vvi-Vitvi13g04205\_t001 |  |  |  |  |  |  |  | | | |  |  |  |  |
| 1 | Vvi-Vitvi13g02074\_t001 |  |  |  |  |  |  |  | | | |  |  |  |  |
| 1 | Vvi-Vitvi13g02075\_t001 |  |  |  |  |  |  |  | | | |  |  |  |  |
| 1 | Vvi-Vitvi13g02076\_t001 |  |  |  |  |  |  |  | | | |  |  |  |  |
| 1 | Vvi-Vitvi13g04206\_t001 |  |  |  |  |  |  |  | | | |  |  |  |  |
| 1 | Vvi-Vitvi13g00705\_t002 |  |  |  |  |  |  |  | Ath-AT5G04430.2 |  |  |  |  |
| 1 | Vvi-Vitvi13g00706\_t001 |  |  |  |  |  |  |  | | | |  |  |  |  |
| 1 | Vvi-Vitvi13g00707\_t001 |  |  |  |  |  |  |  | Ath-AT5G04440.1 |  |  |  |  |
| 1 | Vvi-Vitvi13g00708\_t001 |  |  |  |  |  |  |  | Ath-AT5G04460.1 |  |  |  |  |
| 1 | Vvi-Vitvi13g00709\_t001 |  |  |  |  |  |  |  | | | |  |  |  |  |
| 1 | Vvi-Vitvi13g01250\_t001 |  |  |  |  |  |  |  | | | |  |  |  |  |
| 1 | Vvi-Vitvi13g04207\_t001 |  |  |  |  |  |  |  | | | |  |  |  |  |
| 1 | Vvi-Vitvi13g04208\_t001 |  |  |  |  |  |  |  | | | |  |  |  |  |
| 1 | Vvi-Vitvi13g04209\_t001 |  |  |  |  |  |  |  | | | |  |  |  |  |
| 1 | Vvi-Vitvi13g04210\_t001 |  |  |  |  |  |  |  | | | |  |  |  |  |
| 1 | Vvi-Vitvi13g04211\_t001 |  |  |  |  |  |  |  | | | |  |  |  |  |
| 1 | Vvi-Vitvi13g01245\_t001 |  |  |  |  |  |  |  | | | |  |  |  |  |
| 1 | Vvi-Vitvi13g02248\_t001 |  |  |  |  |  |  |  | | | |  |  |  |  |
| 2 | Vvi-Vitvi13g01244\_t001 |  | Ath-AT3G52170.2 |  |  |  |  |  | | | |  |  |  |  |
| 2 | Vvi-Vitvi13g01243\_t001 |  | | | |  |  |  |  |  | Ath-AT5G04480.1 |  |  |  |  |
| 2 | Vvi-Vitvi13g01242\_t001 |  | Ath-AT3G52180.1 |  |  |  |  |  | | | |  |  |  |  |
| 2 | Vvi-Vitvi13g01240\_t001 |  | | | |  |  |  |  |  | | | |  |  |  |  |
| 2 | Vvi-Vitvi13g04212\_t001 |  | | | |  |  |  |  |  | | | |  |  |  |  |
| 2 | Vvi-Vitvi13g04213\_t001 |  | | | |  |  |  |  |  | | | |  |  |  |  |
| 2 | Vvi-Vitvi13g04214\_t001 |  | | | |  |  |  |  |  | | | |  |  |  |  |
| 2 | Vvi-Vitvi13g00710\_t001 |  | Ath-AT3G52190.1 |  |  |  |  |  | | | |  |  |  |  |
| 2 | Vvi-Vitvi13g00711\_t001 |  | | | |  |  |  |  |  | | | |  |  |  |  |
| 2 | Vvi-Vitvi13g00712\_t001 |  | | | |  |  |  |  |  | Ath-AT5G04490.1 |  |  |  |  |
| 2 | Vvi-Vitvi13g02078\_t001 |  | | | |  |  |  |  |  | | | |  |  |  |  |
| 2 | Vvi-Vitvi13g04215\_t001 |  | | | |  |  |  |  |  | | | |  |  |  |  |
| 2 | Vvi-Vitvi13g04216\_t001 |  | | | |  |  |  |  |  | | | |  |  |  |  |
| 2 | Vvi-Vitvi13g02081\_t001 |  | | | |  |  |  |  |  | | | |  |  |  |  |
| 2 | Vvi-Vitvi13g02082\_t001 |  | | | |  |  |  |  |  | | | |  |  |  |  |
| 2 | Vvi-Vitvi13g00714\_t001 |  | | | |  |  |  |  |  | Ath-AT5G04500.1 |  |  |  |  |
| 2 | Vvi-Vitvi13g00715\_t001 |  | | | |  |  |  |  |  | Ath-AT5G04510.1 |  |  |  |  |
| 2 | Vvi-Vitvi13g00716\_t001 |  | | | |  |  |  |  |  | | | |  |  |  |  |
| 2 | Vvi-Vitvi13g00717\_t001 |  | | | |  |  |  |  |  | | | |  |  |  |  |
| 2 | Vvi-Vitvi13g00718\_t001 |  | | | |  |  |  |  |  | | | |  |  |  |  |
| 2 | Vvi-Vitvi13g00719\_t001 |  | Ath-AT3G52200.2 |  |  |  |  |  | | | |  |  |  |  |
| 2 | Vvi-Vitvi13g00720\_t001 |  | | | |  |  |  |  |  | Ath-AT5G04520.1 |  |  |  |  |
| 2 | Vvi-Vitvi13g02083\_t001 |  | | | |  |  |  |  |  | | | |  |  |  |  |
| 2 | Vvi-Vitvi13g04217\_t001 |  | | | |  |  |  |  |  | | | |  |  |  |  |
| 2 | Vvi-Vitvi13g04218\_t001 |  | | | |  |  |  |  |  | | | |  |  |  |  |
| 2 | Vvi-Vitvi13g02085\_t001 |  | | | |  |  |  |  |  | | | |  |  |  |  |
| 2 | Vvi-Vitvi13g00722\_t001 |  | Ath-AT3G52210.3 |  |  |  |  |  | | | |  |  |  |  |
| 2 | Vvi-Vitvi13g00723\_t001 |  | | | |  |  |  |  |  | | | |  |  |  |  |
| 2 | Vvi-Vitvi13g00724\_t001 |  | | | |  |  |  |  |  | | | |  |  |  |  |
| 2 | Vvi-Vitvi13g00725\_t001 |  | Ath-AT3G52230.1 |  |  |  |  |  | | | |  |  |  |  |
| 1 | Vvi-Vitvi13g00728\_t001 |  |  |  |  |  |  |  | | | |  |  |  |  |
| 1 | Vvi-Vitvi13g00729\_t001 |  |  |  |  |  |  |  | | | |  |  |  |  |
| 1 | Vvi-Vitvi13g04219\_t001 |  |  |  |  |  |  |  | | | |  |  |  |  |
| 1 | Vvi-Vitvi13g00731\_t001 |  |  |  |  |  |  |  | Ath-AT5G04540.1 |  |  |  |  |
| 1 | Vvi-Vitvi13g00732\_t001 |  |  |  |  |  |  |  | | | |  |  |  |  |
| 1 | Vvi-Vitvi13g02088\_t001 |  |  |  |  |  |  |  | | | |  |  |  |  |
| 1 | Vvi-Vitvi13g04220\_t001 |  |  |  |  |  |  |  | | | |  |  |  |  |
| 1 | Vvi-Vitvi13g04221\_t001 |  |  |  |  |  |  |  | | | |  |  |  |  |
| 1 | Vvi-Vitvi13g04222\_t001 |  |  |  |  |  |  |  | | | |  |  |  |  |
| 1 | Vvi-Vitvi13g02091\_t001 |  |  |  |  |  |  |  | | | |  |  |  |  |
| 1 | Vvi-Vitvi13g04223\_t001 |  |  |  |  |  |  |  | | | |  |  |  |  |
| 1 | Vvi-Vitvi13g04224\_t001 |  |  |  |  |  |  |  | | | |  |  |  |  |
| 1 | Vvi-Vitvi13g00736\_t001 |  |  |  |  |  |  |  | | | |  |  |  |  |
| 1 | Vvi-Vitvi13g00737\_t001 |  |  |  |  |  |  |  | | | |  |  |  |  |
| 1 | Vvi-Vitvi13g00738\_t001 |  |  |  |  |  |  |  | Ath-AT5G04630.1 |  |  |  |  |
| 0 | Vvi-Vitvi13g00740\_t001 |  |  |  |  |  |  |  |  |
| 0 | Vvi-Vitvi13g04225\_t001 |  |  |  |  |  |  |  |  |
| 0 | Vvi-Vitvi13g00743\_t001 |  |  |  |  |  |  |  |  |
| 0 | Vvi-Vitvi13g04226\_t001 |  |  |  |  |  |  |  |  |
| 0 | Vvi-Vitvi13g02093\_t001 |  |  |  |  |  |  |  |  |
| 0 | Vvi-Vitvi13g00744\_t001 |  |  |  |  |  |  |  |  |
| 0 | Vvi-Vitvi13g04227\_t001 |  |  |  |  |  |  |  |  |
| 0 | Vvi-Vitvi13g00745\_t001 |  |  |  |  |  |  |  |  |
| 0 | Vvi-Vitvi13g04228\_t001 |  |  |  |  |  |  |  |  |
| 0 | Vvi-Vitvi13g00747\_t001 |  |  |  |  |  |  |  |  |
| 0 | Vvi-Vitvi13g04229\_t001 |  |  |  |  |  |  |  |  |
| 0 | Vvi-Vitvi13g04230\_t001 |  |  |  |  |  |  |  |  |
| 0 | Vvi-Vitvi13g00749\_t001 |  |  |  |  |  |  |  |  |
| 0 | Vvi-Vitvi13g04231\_t001 |  |  |  |  |  |  |  |  |
| 0 | Vvi-Vitvi13g04232\_t001 |  |  |  |  |  |  |  |  |
| 0 | Vvi-Vitvi13g04233\_t001 |  |  |  |  |  |  |  |  |
| 0 | Vvi-Vitvi13g00751\_t001 |  |  |  |  |  |  |  |  |
| 0 | Vvi-Vitvi13g04234\_t001 |  |  |  |  |  |  |  |  |
| 0 | Vvi-Vitvi13g00754\_t001 |  |  |  |  |  |  |  |  |
| 0 | Vvi-Vitvi13g04235\_t001 |  |  |  |  |  |  |  |  |
| 0 | Vvi-Vitvi13g02096\_t001 |  |  |  |  |  |  |  |  |
| 0 | Vvi-Vitvi13g04236\_t001 |  |  |  |  |  |  |  |  |
| 0 | Vvi-Vitvi13g02097\_t002 |  |  |  |  |  |  |  |  |
| 0 | Vvi-Vitvi13g04237\_t001 |  |  |  |  |  |  |  |  |
| 0 | Vvi-Vitvi13g04238\_t001 |  |  |  |  |  |  |  |  |
| 0 | Vvi-Vitvi13g04239\_t001 |  |  |  |  |  |  |  |  |
| 0 | Vvi-Vitvi13g04240\_t001 |  |  |  |  |  |  |  |  |
| 0 | Vvi-Vitvi13g04241\_t001 |  |  |  |  |  |  |  |  |
| 0 | Vvi-Vitvi13g04242\_t001 |  |  |  |  |  |  |  |  |
| 0 | Vvi-Vitvi13g04243\_t001 |  |  |  |  |  |  |  |  |
| 0 | Vvi-Vitvi13g04244\_t001 |  |  |  |  |  |  |  |  |
| 0 | Vvi-Vitvi13g02099\_t001 |  |  |  |  |  |  |  |  |
| 0 | Vvi-Vitvi13g04245\_t001 |  |  |  |  |  |  |  |  |
| 0 | Vvi-Vitvi13g04246\_t001 |  |  |  |  |  |  |  |  |
| 0 | Vvi-Vitvi13g04247\_t001 |  |  |  |  |  |  |  |  |
| 0 | Vvi-Vitvi13g04248\_t001 |  |  |  |  |  |  |  |  |
| 0 | Vvi-Vitvi13g04249\_t001 |  |  |  |  |  |  |  |  |
| 0 | Vvi-Vitvi13g02101\_t001 |  |  |  |  |  |  |  |  |
| 0 | Vvi-Vitvi13g00764\_t002 |  |  |  |  |  |  |  |  |
| 0 | Vvi-Vitvi13g02102\_t001 |  |  |  |  |  |  |  |  |
| 0 | Vvi-Vitvi13g00766\_t001 |  |  |  |  |  |  |  |  |
| 0 | Vvi-Vitvi13g04250\_t001 |  |  |  |  |  |  |  |  |
| 0 | Vvi-Vitvi13g00767\_t002 |  |  |  |  |  |  |  |  |
| 0 | Vvi-Vitvi13g00768\_t001 |  |  |  |  |  |  |  |  |
| 0 | Vvi-Vitvi13g04251\_t001 |  |  |  |  |  |  |  |  |
| 0 | Vvi-Vitvi13g04252\_t001 |  |  |  |  |  |  |  |  |
| 0 | Vvi-Vitvi13g00772\_t001 |  |  |  |  |  |  |  |  |
| 0 | Vvi-Vitvi13g00773\_t001 |  |  |  |  |  |  |  |  |
| 0 | Vvi-Vitvi13g00774\_t001 |  |  |  |  |  |  |  |  |
| 0 | Vvi-Vitvi13g00777\_t001 |  |  |  |  |  |  |  |  |
| 0 | Vvi-Vitvi13g00778\_t001 |  |  |  |  |  |  |  |  |
| 0 | Vvi-Vitvi13g00779\_t001 |  |  |  |  |  |  |  |  |
| 0 | Vvi-Vitvi13g00786\_t001 |  |  |  |  |  |  |  |  |
| 0 | Vvi-Vitvi13g00790\_t001 |  |  |  |  |  |  |  |  |
| 0 | Vvi-Vitvi13g04253\_t001 |  |  |  |  |  |  |  |  |
| 0 | Vvi-Vitvi13g04254\_t001 |  |  |  |  |  |  |  |  |
| 0 | Vvi-Vitvi13g00792\_t001 |  |  |  |  |  |  |  |  |
| 0 | Vvi-Vitvi13g00793\_t001 |  |  |  |  |  |  |  |  |
| 0 | Vvi-Vitvi13g04255\_t001 |  |  |  |  |  |  |  |  |
| 0 | Vvi-Vitvi13g00794\_t001 |  |  |  |  |  |  |  |  |
| 0 | Vvi-Vitvi13g00796\_t001 |  |  |  |  |  |  |  |  |
| 0 | Vvi-Vitvi13g00797\_t001 |  |  |  |  |  |  |  |  |
| 0 | Vvi-Vitvi13g02104\_t001 |  |  |  |  |  |  |  |  |
| 0 | Vvi-Vitvi13g02105\_t001 |  |  |  |  |  |  |  |  |
| 0 | Vvi-Vitvi13g00802\_t001 |  |  |  |  |  |  |  |  |
| 0 | Vvi-Vitvi13g00804\_t001 |  |  |  |  |  |  |  |  |
| 0 | Vvi-Vitvi13g00805\_t001 |  |  |  |  |  |  |  |  |
| 0 | Vvi-Vitvi13g04256\_t001 |  |  |  |  |  |  |  |  |
| 0 | Vvi-Vitvi13g04257\_t001 |  |  |  |  |  |  |  |  |
| 0 | Vvi-Vitvi13g00807\_t002 |  |  |  |  |  |  |  |  |
| 0 | Vvi-Vitvi13g04258\_t001 |  |  |  |  |  |  |  |  |
| 0 | Vvi-Vitvi13g02109\_t002 |  |  |  |  |  |  |  |  |
| 0 | Vvi-Vitvi13g02110\_t003 |  |  |  |  |  |  |  |  |
| 0 | Vvi-Vitvi13g04259\_t001 |  |  |  |  |  |  |  |  |
| 0 | Vvi-Vitvi13g00808\_t001 |  |  |  |  |  |  |  |  |
| 0 | Vvi-Vitvi13g00810\_t001 |  |  |  |  |  |  |  |  |
| 0 | Vvi-Vitvi13g00811\_t001 |  |  |  |  |  |  |  |  |
| 0 | Vvi-Vitvi13g04260\_t001 |  |  |  |  |  |  |  |  |
| 0 | Vvi-Vitvi13g00813\_t001 |  |  |  |  |  |  |  |  |
| 0 | Vvi-Vitvi13g04261\_t001 |  |  |  |  |  |  |  |  |
| 0 | Vvi-Vitvi13g00815\_t001 |  |  |  |  |  |  |  |  |
| 0 | Vvi-Vitvi13g00816\_t001 |  |  |  |  |  |  |  |  |
| 0 | Vvi-Vitvi13g00818\_t001 |  |  |  |  |  |  |  |  |
| 0 | Vvi-Vitvi13g00819\_t001 |  |  |  |  |  |  |  |  |
| 0 | Vvi-Vitvi13g02119\_t001 |  |  |  |  |  |  |  |  |
| 0 | Vvi-Vitvi13g00824\_t001 |  |  |  |  |  |  |  |  |
| 0 | Vvi-Vitvi13g00829\_t001 |  |  |  |  |  |  |  |  |
| 0 | Vvi-Vitvi13g04262\_t001 |  |  |  |  |  |  |  |  |
| 0 | Vvi-Vitvi13g02120\_t001 |  |  |  |  |  |  |  |  |
| 0 | Vvi-Vitvi13g04263\_t001 |  |  |  |  |  |  |  |  |
| 0 | Vvi-Vitvi13g00832\_t001 |  |  |  |  |  |  |  |  |
| 0 | Vvi-Vitvi13g04264\_t001 |  |  |  |  |  |  |  |  |
| 0 | Vvi-Vitvi13g00835\_t001 |  |  |  |  |  |  |  |  |
| 0 | Vvi-Vitvi13g00837\_t001 |  |  |  |  |  |  |  |  |
| 0 | Vvi-Vitvi13g02126\_t001 |  |  |  |  |  |  |  |  |
| 0 | Vvi-Vitvi13g04265\_t001 |  |  |  |  |  |  |  |  |
| 0 | Vvi-Vitvi13g04266\_t001 |  |  |  |  |  |  |  |  |
| 0 | Vvi-Vitvi13g04267\_t001 |  |  |  |  |  |  |  |  |
| 0 | Vvi-Vitvi13g04268\_t001 |  |  |  |  |  |  |  |  |
| 0 | Vvi-Vitvi13g04269\_t001 |  |  |  |  |  |  |  |  |
| 0 | Vvi-Vitvi13g04270\_t001 |  |  |  |  |  |  |  |  |
| 0 | Vvi-Vitvi13g04271\_t001 |  |  |  |  |  |  |  |  |
| 0 | Vvi-Vitvi13g02130\_t001 |  |  |  |  |  |  |  |  |
| 0 | Vvi-Vitvi13g04272\_t001 |  |  |  |  |  |  |  |  |
| 0 | Vvi-Vitvi13g00843\_t001 |  |  |  |  |  |  |  |  |
| 0 | Vvi-Vitvi13g04273\_t001 |  |  |  |  |  |  |  |  |
| 0 | Vvi-Vitvi13g00850\_t001 |  |  |  |  |  |  |  |  |
| 0 | Vvi-Vitvi13g04274\_t001 |  |  |  |  |  |  |  |  |
| 0 | Vvi-Vitvi13g00851\_t001 |  |  |  |  |  |  |  |  |
| 0 | Vvi-Vitvi13g02132\_t001 |  |  |  |  |  |  |  |  |
| 0 | Vvi-Vitvi13g00852\_t001 |  |  |  |  |  |  |  |  |
| 0 | Vvi-Vitvi13g02133\_t001 |  |  |  |  |  |  |  |  |
| 0 | Vvi-Vitvi13g00856\_t001 |  |  |  |  |  |  |  |  |
| 0 | Vvi-Vitvi13g00858\_t003 |  |  |  |  |  |  |  |  |
| 0 | Vvi-Vitvi13g00859\_t001 |  |  |  |  |  |  |  |  |
| 0 | Vvi-Vitvi13g00860\_t001 |  |  |  |  |  |  |  |  |
| 0 | Vvi-Vitvi13g04275\_t001 |  |  |  |  |  |  |  |  |
| 0 | Vvi-Vitvi13g04276\_t001 |  |  |  |  |  |  |  |  |
| 0 | Vvi-Vitvi13g04277\_t001 |  |  |  |  |  |  |  |  |
| 0 | Vvi-Vitvi13g00864\_t001 |  |  |  |  |  |  |  |  |
| 0 | Vvi-Vitvi13g00865\_t001 |  |  |  |  |  |  |  |  |
| 0 | Vvi-Vitvi13g04278\_t001 |  |  |  |  |  |  |  |  |
| 0 | Vvi-Vitvi13g00867\_t001 |  |  |  |  |  |  |  |  |
| 0 | Vvi-Vitvi13g00870\_t001 |  |  |  |  |  |  |  |  |
| 0 | Vvi-Vitvi13g00871\_t001 |  |  |  |  |  |  |  |  |
| 0 | Vvi-Vitvi13g00872\_t001 |  |  |  |  |  |  |  |  |
| 0 | Vvi-Vitvi13g00873\_t002 |  |  |  |  |  |  |  |  |
| 0 | Vvi-Vitvi13g00877\_t002 |  |  |  |  |  |  |  |  |
| 0 | Vvi-Vitvi13g02139\_t001 |  |  |  |  |  |  |  |  |
| 0 | Vvi-Vitvi13g00878\_t001 |  |  |  |  |  |  |  |  |
| 0 | Vvi-Vitvi13g00879\_t001 |  |  |  |  |  |  |  |  |
| 0 | Vvi-Vitvi13g00880\_t001 |  |  |  |  |  |  |  |  |
| 0 | Vvi-Vitvi13g00883\_t001 |  |  |  |  |  |  |  |  |
| 0 | Vvi-Vitvi13g02140\_t001 |  |  |  |  |  |  |  |  |
| 0 | Vvi-Vitvi13g00886\_t003 |  |  |  |  |  |  |  |  |
| 0 | Vvi-Vitvi13g00887\_t001 |  |  |  |  |  |  |  |  |
| 0 | Vvi-Vitvi13g04279\_t001 |  |  |  |  |  |  |  |  |
| 0 | Vvi-Vitvi13g00890\_t001 |  |  |  |  |  |  |  |  |
| 0 | Vvi-Vitvi13g00891\_t001 |  |  |  |  |  |  |  |  |
| 0 | Vvi-Vitvi13g00925\_t001 |  |  |  |  |  |  |  |  |
| 0 | Vvi-Vitvi13g00924\_t001 |  |  |  |  |  |  |  |  |
| 0 | Vvi-Vitvi13g00923\_t001 |  |  |  |  |  |  |  |  |
| 0 | Vvi-Vitvi13g00922\_t001 |  |  |  |  |  |  |  |  |
| 0 | Vvi-Vitvi13g04280\_t001 |  |  |  |  |  |  |  |  |
| 0 | Vvi-Vitvi13g00921\_t003 |  |  |  |  |  |  |  |  |
| 0 | Vvi-Vitvi13g04281\_t001 |  |  |  |  |  |  |  |  |
| 0 | Vvi-Vitvi13g04282\_t001 |  |  |  |  |  |  |  |  |
| 0 | Vvi-Vitvi13g04283\_t001 |  |  |  |  |  |  |  |  |
| 0 | Vvi-Vitvi13g04284\_t001 |  |  |  |  |  |  |  |  |
| 0 | Vvi-Vitvi13g04285\_t001 |  |  |  |  |  |  |  |  |
| 0 | Vvi-Vitvi13g02144\_t001 |  |  |  |  |  |  |  |  |
| 0 | Vvi-Vitvi13g04286\_t001 |  |  |  |  |  |  |  |  |
| 0 | Vvi-Vitvi13g04287\_t001 |  |  |  |  |  |  |  |  |
| 0 | Vvi-Vitvi13g04288\_t001 |  |  |  |  |  |  |  |  |
| 0 | Vvi-Vitvi13g04289\_t001 |  |  |  |  |  |  |  |  |
| 0 | Vvi-Vitvi13g04290\_t001 |  |  |  |  |  |  |  |  |
| 0 | Vvi-Vitvi13g00918\_t001 |  |  |  |  |  |  |  |  |
| 0 | Vvi-Vitvi13g00917\_t001 |  |  |  |  |  |  |  |  |
| 0 | Vvi-Vitvi13g00914\_t001 |  |  |  |  |  |  |  |  |
| 0 | Vvi-Vitvi13g04291\_t001 |  |  |  |  |  |  |  |  |
| 0 | Vvi-Vitvi13g00907\_t001 |  |  |  |  |  |  |  |  |
| 0 | Vvi-Vitvi13g00905\_t001 |  |  |  |  |  |  |  |  |
| 0 | Vvi-Vitvi13g04292\_t001 |  |  |  |  |  |  |  |  |
| 0 | Vvi-Vitvi13g04293\_t001 |  |  |  |  |  |  |  |  |
| 0 | Vvi-Vitvi13g04294\_t001 |  |  |  |  |  |  |  |  |
| 0 | Vvi-Vitvi13g04295\_t001 |  |  |  |  |  |  |  |  |
| 0 | Vvi-Vitvi13g04296\_t001 |  |  |  |  |  |  |  |  |
| 0 | Vvi-Vitvi13g04297\_t001 |  |  |  |  |  |  |  |  |
| 0 | Vvi-Vitvi13g04298\_t001 |  |  |  |  |  |  |  |  |
| 0 | Vvi-Vitvi13g04299\_t001 |  |  |  |  |  |  |  |  |
| 0 | Vvi-Vitvi13g04300\_t001 |  |  |  |  |  |  |  |  |
| 0 | Vvi-Vitvi13g04301\_t001 |  |  |  |  |  |  |  |  |
| 0 | Vvi-Vitvi13g04302\_t001 |  |  |  |  |  |  |  |  |
| 0 | Vvi-Vitvi13g04303\_t001 |  |  |  |  |  |  |  |  |
| 0 | Vvi-Vitvi13g02589\_t001 |  |  |  |  |  |  |  |  |
| 0 | Vvi-Vitvi13g04304\_t001 |  |  |  |  |  |  |  |  |
| 0 | Vvi-Vitvi13g04305\_t001 |  |  |  |  |  |  |  |  |
| 0 | Vvi-Vitvi13g02586\_t001 |  |  |  |  |  |  |  |  |
| 0 | Vvi-Vitvi13g00981\_t001 |  |  |  |  |  |  |  |  |
| 0 | Vvi-Vitvi13g00979\_t001 |  |  |  |  |  |  |  |  |
| 0 | Vvi-Vitvi13g04306\_t001 |  |  |  |  |  |  |  |  |
| 0 | Vvi-Vitvi13g04307\_t001 |  |  |  |  |  |  |  |  |
| 0 | Vvi-Vitvi13g04308\_t001 |  |  |  |  |  |  |  |  |
| 0 | Vvi-Vitvi13g00975\_t003 |  |  |  |  |  |  |  |  |
| 0 | Vvi-Vitvi13g02173\_t001 |  |  |  |  |  |  |  |  |
| 0 | Vvi-Vitvi13g00974\_t001 |  |  |  |  |  |  |  |  |
| 0 | Vvi-Vitvi13g04309\_t001 |  |  |  |  |  |  |  |  |
| 0 | Vvi-Vitvi13g02169\_t001 |  |  |  |  |  |  |  |  |
| 0 | Vvi-Vitvi13g04310\_t001 |  |  |  |  |  |  |  |  |
| 0 | Vvi-Vitvi13g04311\_t001 |  |  |  |  |  |  |  |  |
| 0 | Vvi-Vitvi13g04312\_t001 |  |  |  |  |  |  |  |  |
| 0 | Vvi-Vitvi13g04313\_t001 |  |  |  |  |  |  |  |  |
| 0 | Vvi-Vitvi13g04314\_t001 |  |  |  |  |  |  |  |  |
| 0 | Vvi-Vitvi13g00954\_t001 |  |  |  |  |  |  |  |  |
| 0 | Vvi-Vitvi13g00926\_t001 |  |  |  |  |  |  |  |  |
| 0 | Vvi-Vitvi13g02148\_t001 |  |  |  |  |  |  |  |  |
| 0 | Vvi-Vitvi13g00927\_t001 |  |  |  |  |  |  |  |  |
| 0 | Vvi-Vitvi13g02149\_t001 |  |  |  |  |  |  |  |  |
| 0 | Vvi-Vitvi13g04315\_t001 |  |  |  |  |  |  |  |  |
| 1 | Vvi-Vitvi13g04316\_t001 |  | Ath-AT5G04750.1 |  |  |  |  |  |  |  |
| 1 | Vvi-Vitvi13g00932\_t001 |  | | | |  |  |  |  |  |  |  |
| 1 | Vvi-Vitvi13g00934\_t001 |  | | | |  |  |  |  |  |  |  |
| 1 | Vvi-Vitvi13g00936\_t001 |  | | | |  |  |  |  |  |  |  |
| 1 | Vvi-Vitvi13g02153\_t001 |  | | | |  |  |  |  |  |  |  |
| 1 | Vvi-Vitvi13g04317\_t001 |  | | | |  |  |  |  |  |  |  |
| 1 | Vvi-Vitvi13g04318\_t001 |  | | | |  |  |  |  |  |  |  |
| 1 | Vvi-Vitvi13g04319\_t001 |  | | | |  |  |  |  |  |  |  |
| 1 | Vvi-Vitvi13g04320\_t001 |  | | | |  |  |  |  |  |  |  |
| 1 | Vvi-Vitvi13g04321\_t001 |  | | | |  |  |  |  |  |  |  |
| 2 | Vvi-Vitvi13g00991\_t001 |  | Ath-AT5G04770.1 |  | Ath-AT3G10600.1 |  |  |  |  |  |  |
| 2 | Vvi-Vitvi13g00994\_t001 |  | Ath-AT5G04780.1 |  | | | |  |  |  |  |  |  |
| 2 | Vvi-Vitvi13g00996\_t001 |  | Ath-AT5G04800.2 |  | Ath-AT3G10610.1 |  |  |  |  |  |  |
| 2 | Vvi-Vitvi13g00998\_t001 |  | Ath-AT5G04810.1 |  | | | |  |  |  |  |  |  |
| 2 | Vvi-Vitvi13g01001\_t001 |  | Ath-AT5G04820.1 |  | | | |  |  |  |  |  |  |
| 2 | Vvi-Vitvi13g01006\_t001 |  | | | |  | | | |  |  |  |  |  |  |
| 2 | Vvi-Vitvi13g01007\_t001 |  | Ath-AT5G04830.2 |  | | | |  |  |  |  |  |  |
| 2 | Vvi-Vitvi13g01008\_t001 |  | | | |  | | | |  |  |  |  |  |  |
| 2 | Vvi-Vitvi13g02178\_t001 |  | | | |  | | | |  |  |  |  |  |  |
| 2 | Vvi-Vitvi13g04322\_t001 |  | | | |  | | | |  |  |  |  |  |  |
| 2 | Vvi-Vitvi13g01009\_t001 |  | | | |  | | | |  |  |  |  |  |  |
| 2 | Vvi-Vitvi13g04323\_t001 |  | | | |  | | | |  |  |  |  |  |  |
| 2 | Vvi-Vitvi13g04324\_t001 |  | | | |  | | | |  |  |  |  |  |  |
| 2 | Vvi-Vitvi13g04325\_t001 |  | | | |  | | | |  |  |  |  |  |  |
| 2 | Vvi-Vitvi13g01012\_t001 |  | | | |  | | | |  |  |  |  |  |  |
| 2 | Vvi-Vitvi13g04326\_t001 |  | | | |  | | | |  |  |  |  |  |  |
| 2 | Vvi-Vitvi13g04327\_t001 |  | | | |  | | | |  |  |  |  |  |  |
| 2 | Vvi-Vitvi13g04328\_t001 |  | | | |  | | | |  |  |  |  |  |  |
| 2 | Vvi-Vitvi13g01017\_t001 |  | | | |  | | | |  |  |  |  |  |  |
| 2 | Vvi-Vitvi13g01020\_t001 |  | | | |  | | | |  |  |  |  |  |  |
| 2 | Vvi-Vitvi13g01021\_t001 |  | | | |  | Ath-AT3G10630.1 |  |  |  |  |  |  |
| 2 | Vvi-Vitvi13g01022\_t001 |  | | | |  | | | |  |  |  |  |  |  |
| 2 | Vvi-Vitvi13g04329\_t001 |  | | | |  | | | |  |  |  |  |  |  |
| 2 | Vvi-Vitvi13g01028\_t001 |  | | | |  | | | |  |  |  |  |  |  |
| 2 | Vvi-Vitvi13g01031\_t001 |  | Ath-AT5G04850.2 |  | Ath-AT3G10640.1 |  |  |  |  |  |  |
| 2 | Vvi-Vitvi13g02182\_t001 |  | | | |  | | | |  |  |  |  |  |  |
| 2 | Vvi-Vitvi13g01036\_t001 |  | | | |  | | | |  |  |  |  |  |  |
| 2 | Vvi-Vitvi13g01037\_t001 |  | | | |  | | | |  |  |  |  |  |  |
| 2 | Vvi-Vitvi13g01038\_t001 |  | | | |  | | | |  |  |  |  |  |  |
| 2 | Vvi-Vitvi13g01043\_t001 |  | | | |  | | | |  |  |  |  |  |  |
| 2 | Vvi-Vitvi13g01044\_t001 |  | | | |  | | | |  |  |  |  |  |  |
| 2 | Vvi-Vitvi13g01048\_t001 |  | | | |  | | | |  |  |  |  |  |  |
| 2 | Vvi-Vitvi13g01049\_t001 |  | | | |  | | | |  |  |  |  |  |  |
| 2 | Vvi-Vitvi13g01050\_t001 |  | Ath-AT5G04885.2 |  | | | |  |  |  |  |  |  |
| 2 | Vvi-Vitvi13g04330\_t001 |  | | | |  | | | |  |  |  |  |  |  |
| 2 | Vvi-Vitvi13g01052\_t001 |  | | | |  | | | |  |  |  |  |  |  |
| 2 | Vvi-Vitvi13g01053\_t001 |  | | | |  | | | |  |  |  |  |  |  |
| 2 | Vvi-Vitvi13g04331\_t001 |  | | | |  | | | |  |  |  |  |  |  |
| 2 | Vvi-Vitvi13g04332\_t001 |  | | | |  | | | |  |  |  |  |  |  |
| 2 | Vvi-Vitvi13g01056\_t001 |  | | | |  | | | |  |  |  |  |  |  |
| 2 | Vvi-Vitvi13g01057\_t001 |  | | | |  | | | |  |  |  |  |  |  |
| 2 | Vvi-Vitvi13g01058\_t001 |  | | | |  | | | |  |  |  |  |  |  |
| 2 | Vvi-Vitvi13g01060\_t001 |  | | | |  | Ath-AT3G10650.1 |  |  |  |  |  |  |
| 2 | Vvi-Vitvi13g01061\_t001 |  | | | |  | | | |  |  |  |  |  |  |
| 2 | Vvi-Vitvi13g01062\_t001 |  | | | |  | | | |  |  |  |  |  |  |
| 2 | Vvi-Vitvi13g01065\_t003 |  | | | |  | | | |  |  |  |  |  |  |
| 2 | Vvi-Vitvi13g04333\_t001 |  | | | |  | | | |  |  |  |  |  |  |
| 2 | Vvi-Vitvi13g01066\_t001 |  | | | |  | | | |  |  |  |  |  |  |
| 2 | Vvi-Vitvi13g01070\_t001 |  | | | |  | | | |  |  |  |  |  |  |
| 2 | Vvi-Vitvi13g01077\_t001 |  | | | |  | | | |  |  |  |  |  |  |
| 2 | Vvi-Vitvi13g04334\_t001 |  | | | |  | | | |  |  |  |  |  |  |
| 2 | Vvi-Vitvi13g04335\_t001 |  | | | |  | Ath-AT3G10660.2 |  |  |  |  |  |  |
| 2 | Vvi-Vitvi13g01074\_t001 |  | Ath-AT5G04890.1 |  | Ath-AT3G10680.1 |  |  |  |  |  |  |
| 1 | Vvi-Vitvi13g01073\_t001 |  | | | |  |  |  |  |  |  |  |
| 1 | Vvi-Vitvi13g02189\_t001 |  | | | |  |  |  |  |  |  |  |
| 1 | Vvi-Vitvi13g04336\_t001 |  | | | |  |  |  |  |  |  |  |
| 1 | Vvi-Vitvi13g04337\_t001 |  | | | |  |  |  |  |  |  |  |
| 1 | Vvi-Vitvi13g04338\_t001 |  | | | |  |  |  |  |  |  |  |
| 1 | Vvi-Vitvi13g01090\_t001 |  | | | |  |  |  |  |  |  |  |
| 1 | Vvi-Vitvi13g01091\_t001 |  | | | |  |  |  |  |  |  |  |
| 1 | Vvi-Vitvi13g04339\_t001 |  | | | |  |  |  |  |  |  |  |
| 1 | Vvi-Vitvi13g01094\_t001 |  | | | |  |  |  |  |  |  |  |
| 1 | Vvi-Vitvi13g01095\_t001 |  | | | |  |  |  |  |  |  |  |
| 1 | Vvi-Vitvi13g01096\_t001 |  | | | |  |  |  |  |  |  |  |
| 1 | Vvi-Vitvi13g04340\_t001 |  | | | |  |  |  |  |  |  |  |
| 1 | Vvi-Vitvi13g01098\_t001 |  | | | |  |  |  |  |  |  |  |
| 1 | Vvi-Vitvi13g01100\_t001 |  | | | |  |  |  |  |  |  |  |
| 1 | Vvi-Vitvi13g04341\_t001 |  | | | |  |  |  |  |  |  |  |
| 1 | Vvi-Vitvi13g01101\_t001 |  | | | |  |  |  |  |  |  |  |
| 1 | Vvi-Vitvi13g01102\_t001 |  | | | |  |  |  |  |  |  |  |
| 1 | Vvi-Vitvi13g04342\_t001 |  | | | |  |  |  |  |  |  |  |
| 1 | Vvi-Vitvi13g04343\_t001 |  | | | |  |  |  |  |  |  |  |
| 1 | Vvi-Vitvi13g04344\_t001 |  | | | |  |  |  |  |  |  |  |
| 1 | Vvi-Vitvi13g01107\_t002 |  | | | |  |  |  |  |  |  |  |
| 1 | Vvi-Vitvi13g01110\_t002 |  | Ath-AT5G04940.2 |  |  |  |  |  |  |  |
| 1 | Vvi-Vitvi13g04345\_t001 |  | | | |  |  |  |  |  |  |  |
| 1 | Vvi-Vitvi13g01111\_t001 |  | | | |  |  |  |  |  |  |  |
| 1 | Vvi-Vitvi13g02198\_t001 |  | | | |  |  |  |  |  |  |  |
| 1 | Vvi-Vitvi13g04346\_t001 |  | | | |  |  |  |  |  |  |  |
| 1 | Vvi-Vitvi13g02199\_t001 |  | | | |  |  |  |  |  |  |  |
| 1 | Vvi-Vitvi13g02200\_t001 |  | | | |  |  |  |  |  |  |  |
| 1 | Vvi-Vitvi13g01113\_t001 |  | | | |  |  |  |  |  |  |  |
| 1 | Vvi-Vitvi13g01114\_t001 |  | | | |  |  |  |  |  |  |  |
| 1 | Vvi-Vitvi13g01119\_t001 |  | | | |  |  |  |  |  |  |  |
| 1 | Vvi-Vitvi13g02201\_t001 |  | | | |  |  |  |  |  |  |  |
| 1 | Vvi-Vitvi13g04347\_t001 |  | | | |  |  |  |  |  |  |  |
| 1 | Vvi-Vitvi13g01123\_t001 |  | Ath-AT5G04970.1 |  |  |  |  |  |  |  |
| 0 | Vvi-Vitvi13g01125\_t001 |  |  |  |  |  |  |  |  |
| 0 | Vvi-Vitvi13g01126\_t001 |  |  |  |  |  |  |  |  |
| 0 | Vvi-Vitvi13g01130\_t001 |  |  |  |  |  |  |  |  |
| 0 | Vvi-Vitvi13g01132\_t001 |  |  |  |  |  |  |  |  |
| 0 | Vvi-Vitvi13g04348\_t001 |  |  |  |  |  |  |  |  |
| 0 | Vvi-Vitvi13g01134\_t001 |  |  |  |  |  |  |  |  |
| 0 | Vvi-Vitvi13g01137\_t001 |  |  |  |  |  |  |  |  |
| 0 | Vvi-Vitvi13g01139\_t001 |  |  |  |  |  |  |  |  |
| 0 | Vvi-Vitvi13g04349\_t001 |  |  |  |  |  |  |  |  |
| 0 | Vvi-Vitvi13g04350\_t001 |  |  |  |  |  |  |  |  |
| 0 | Vvi-Vitvi13g04351\_t001 |  |  |  |  |  |  |  |  |
| 0 | Vvi-Vitvi13g01862\_t001 |  |  |  |  |  |  |  |  |
| 0 | Vvi-Vitvi13g01144\_t001 |  |  |  |  |  |  |  |  |
| 0 | Vvi-Vitvi13g01145\_t001 |  |  |  |  |  |  |  |  |
| 0 | Vvi-Vitvi13g04352\_t001 |  |  |  |  |  |  |  |  |
| 0 | Vvi-Vitvi13g01146\_t001 |  |  |  |  |  |  |  |  |
| 0 | Vvi-Vitvi13g04353\_t001 |  |  |  |  |  |  |  |  |
| 0 | Vvi-Vitvi13g01148\_t001 |  |  |  |  |  |  |  |  |
| 0 | Vvi-Vitvi13g04354\_t001 |  |  |  |  |  |  |  |  |
| 0 | Vvi-Vitvi13g01155\_t001 |  |  |  |  |  |  |  |  |
| 0 | Vvi-Vitvi13g04355\_t001 |  |  |  |  |  |  |  |  |
| 0 | Vvi-Vitvi13g04356\_t001 |  |  |  |  |  |  |  |  |
| 0 | Vvi-Vitvi13g04357\_t001 |  |  |  |  |  |  |  |  |
| 0 | Vvi-Vitvi13g04358\_t001 |  |  |  |  |  |  |  |  |
| 0 | Vvi-Vitvi13g01159\_t001 |  |  |  |  |  |  |  |  |
| 0 | Vvi-Vitvi13g04359\_t001 |  |  |  |  |  |  |  |  |
| 0 | Vvi-Vitvi13g04360\_t001 |  |  |  |  |  |  |  |  |
| 0 | Vvi-Vitvi13g01161\_t001 |  |  |  |  |  |  |  |  |
| 0 | Vvi-Vitvi13g01163\_t001 |  |  |  |  |  |  |  |  |
| 0 | Vvi-Vitvi13g01164\_t001 |  |  |  |  |  |  |  |  |
| 0 | Vvi-Vitvi13g01167\_t001 |  |  |  |  |  |  |  |  |
| 0 | Vvi-Vitvi13g01168\_t001 |  |  |  |  |  |  |  |  |
| 0 | Vvi-Vitvi13g04361\_t001 |  |  |  |  |  |  |  |  |
| 0 | Vvi-Vitvi13g01170\_t001 |  |  |  |  |  |  |  |  |
| 0 | Vvi-Vitvi13g01171\_t001 |  |  |  |  |  |  |  |  |
| 0 | Vvi-Vitvi13g01173\_t001 |  |  |  |  |  |  |  |  |
| 0 | Vvi-Vitvi13g02206\_t001 |  |  |  |  |  |  |  |  |
| 0 | Vvi-Vitvi13g02208\_t001 |  |  |  |  |  |  |  |  |
| 0 | Vvi-Vitvi13g01175\_t001 |  |  |  |  |  |  |  |  |
| 0 | Vvi-Vitvi13g01177\_t001 |  |  |  |  |  |  |  |  |
| 0 | Vvi-Vitvi13g04362\_t001 |  |  |  |  |  |  |  |  |
| 0 | Vvi-Vitvi13g02215\_t001 |  |  |  |  |  |  |  |  |
| 0 | Vvi-Vitvi13g02217\_t001 |  |  |  |  |  |  |  |  |
| 0 | Vvi-Vitvi13g04363\_t001 |  |  |  |  |  |  |  |  |
| 0 | Vvi-Vitvi13g04364\_t001 |  |  |  |  |  |  |  |  |
| 0 | Vvi-Vitvi13g04365\_t001 |  |  |  |  |  |  |  |  |
| 0 | Vvi-Vitvi13g01179\_t001 |  |  |  |  |  |  |  |  |
| 0 | Vvi-Vitvi13g04366\_t001 |  |  |  |  |  |  |  |  |
| 0 | Vvi-Vitvi13g04367\_t001 |  |  |  |  |  |  |  |  |
| 0 | Vvi-Vitvi13g04368\_t001 |  |  |  |  |  |  |  |  |
| 0 | Vvi-Vitvi13g02222\_t001 |  |  |  |  |  |  |  |  |
| 0 | Vvi-Vitvi13g01186\_t001 |  |  |  |  |  |  |  |  |
| 0 | Vvi-Vitvi13g04369\_t001 |  |  |  |  |  |  |  |  |
| 0 | Vvi-Vitvi13g04370\_t001 |  |  |  |  |  |  |  |  |
| 0 | Vvi-Vitvi13g04371\_t001 |  |  |  |  |  |  |  |  |
| 0 | Vvi-Vitvi13g04372\_t001 |  |  |  |  |  |  |  |  |
| 0 | Vvi-Vitvi13g04373\_t001 |  |  |  |  |  |  |  |  |
| 0 | Vvi-Vitvi13g04374\_t001 |  |  |  |  |  |  |  |  |
| 0 | Vvi-Vitvi13g04375\_t001 |  |  |  |  |  |  |  |  |
| 0 | Vvi-Vitvi13g04376\_t001 |  |  |  |  |  |  |  |  |
| 0 | Vvi-Vitvi13g04377\_t001 |  |  |  |  |  |  |  |  |
| 0 | Vvi-Vitvi13g04378\_t001 |  |  |  |  |  |  |  |  |
| 0 | Vvi-Vitvi13g01189\_t001 |  |  |  |  |  |  |  |  |
| 0 | Vvi-Vitvi13g02226\_t001 |  |  |  |  |  |  |  |  |
| 0 | Vvi-Vitvi13g04379\_t001 |  |  |  |  |  |  |  |  |
| 0 | Vvi-Vitvi13g04380\_t001 |  |  |  |  |  |  |  |  |
| 0 | Vvi-Vitvi13g01190\_t001 |  |  |  |  |  |  |  |  |
| 0 | Vvi-Vitvi13g04381\_t001 |  |  |  |  |  |  |  |  |
| 0 | Vvi-Vitvi13g01191\_t001 |  |  |  |  |  |  |  |  |
| 0 | Vvi-Vitvi13g04382\_t001 |  |  |  |  |  |  |  |  |
| 0 | Vvi-Vitvi13g04383\_t001 |  |  |  |  |  |  |  |  |
| 0 | Vvi-Vitvi13g02230\_t001 |  |  |  |  |  |  |  |  |
| 0 | Vvi-Vitvi13g04384\_t001 |  |  |  |  |  |  |  |  |
| 0 | Vvi-Vitvi13g02231\_t001 |  |  |  |  |  |  |  |  |
| 0 | Vvi-Vitvi13g04385\_t001 |  |  |  |  |  |  |  |  |
| 0 | Vvi-Vitvi13g04386\_t001 |  |  |  |  |  |  |  |  |
| 0 | Vvi-Vitvi13g04387\_t001 |  |  |  |  |  |  |  |  |
| 0 | Vvi-Vitvi13g04388\_t001 |  |  |  |  |  |  |  |  |
| 0 | Vvi-Vitvi13g04389\_t001 |  |  |  |  |  |  |  |  |
| 0 | Vvi-Vitvi13g01195\_t001 |  |  |  |  |  |  |  |  |
| 0 | Vvi-Vitvi13g01200\_t001 |  |  |  |  |  |  |  |  |
| 0 | Vvi-Vitvi13g04390\_t001 |  |  |  |  |  |  |  |  |
| 0 | Vvi-Vitvi13g02238\_t001 |  |  |  |  |  |  |  |  |
| 0 | Vvi-Vitvi13g04391\_t001 |  |  |  |  |  |  |  |  |
| 0 | Vvi-Vitvi13g04392\_t001 |  |  |  |  |  |  |  |  |
| 0 | Vvi-Vitvi13g02241\_t001 |  |  |  |  |  |  |  |  |
| 0 | Vvi-Vitvi13g04393\_t001 |  |  |  |  |  |  |  |  |
| 0 | Vvi-Vitvi13g04394\_t001 |  |  |  |  |  |  |  |  |
| 0 | Vvi-Vitvi13g01215\_t001 |  |  |  |  |  |  |  |  |
| 0 | Vvi-Vitvi13g04395\_t001 |  |  |  |  |  |  |  |  |
| 0 | Vvi-Vitvi13g04396\_t001 |  |  |  |  |  |  |  |  |
| 0 | Vvi-Vitvi13g01222\_t002 |  |  |  |  |  |  |  |  |
| 0 | Vvi-Vitvi13g04397\_t001 |  |  |  |  |  |  |  |  |
| 0 | Vvi-Vitvi13g01225\_t001 |  |  |  |  |  |  |  |  |
| 0 | Vvi-Vitvi13g01226\_t001 |  |  |  |  |  |  |  |  |
| 0 | Vvi-Vitvi13g01227\_t001 |  |  |  |  |  |  |  |  |
| 0 | Vvi-Vitvi13g01229\_t001 |  |  |  |  |  |  |  |  |
| 0 | Vvi-Vitvi13g01230\_t001 |  |  |  |  |  |  |  |  |
| 0 | Vvi-Vitvi13g01231\_t001 |  |  |  |  |  |  |  |  |
| 0 | Vvi-Vitvi13g01232\_t001 |  |  |  |  |  |  |  |  |
| 0 | Vvi-Vitvi13g01233\_t001 |  |  |  |  |  |  |  |  |
| 0 | Vvi-Vitvi13g01234\_t001 |  |  |  |  |  |  |  |  |
| 0 | Vvi-Vitvi13g02246\_t001 |  |  |  |  |  |  |  |  |
| 0 | Vvi-Vitvi13g04398\_t001 |  |  |  |  |  |  |  |  |
| 0 | Vvi-Vitvi13g04399\_t001 |  |  |  |  |  |  |  |  |
| 0 | Vvi-Vitvi13g01236\_t001 |  |  |  |  |  |  |  |  |
| 0 | Vvi-Vitvi13g04400\_t001 |  |  |  |  |  |  |  |  |
| 0 | Vvi-Vitvi13g04401\_t005 |  |  |  |  |  |  |  |  |
| 0 | Vvi-Vitvi13g04402\_t001 |  |  |  |  |  |  |  |  |
| 0 | Vvi-Vitvi13g04403\_t001 |  |  |  |  |  |  |  |  |
| 0 | Vvi-Vitvi13g04404\_t001 |  |  |  |  |  |  |  |  |
| 0 | Vvi-Vitvi13g04405\_t001 |  |  |  |  |  |  |  |  |
| 0 | Vvi-Vitvi13g02251\_t001 |  |  |  |  |  |  |  |  |
| 0 | Vvi-Vitvi13g01251\_t001 |  |  |  |  |  |  |  |  |
| 0 | Vvi-Vitvi13g04406\_t001 |  |  |  |  |  |  |  |  |
| 0 | Vvi-Vitvi13g04407\_t001 |  |  |  |  |  |  |  |  |
| 0 | Vvi-Vitvi13g04408\_t001 |  |  |  |  |  |  |  |  |
| 0 | Vvi-Vitvi13g02253\_t001 |  |  |  |  |  |  |  |  |
| 0 | Vvi-Vitvi13g02255\_t001 |  |  |  |  |  |  |  |  |
| 0 | Vvi-Vitvi13g01253\_t001 |  |  |  |  |  |  |  |  |
| 0 | Vvi-Vitvi13g04409\_t001 |  |  |  |  |  |  |  |  |
| 0 | Vvi-Vitvi13g04410\_t001 |  |  |  |  |  |  |  |  |
| 0 | Vvi-Vitvi13g01256\_t001 |  |  |  |  |  |  |  |  |
| 0 | Vvi-Vitvi13g04411\_t001 |  |  |  |  |  |  |  |  |
| 0 | Vvi-Vitvi13g01259\_t001 |  |  |  |  |  |  |  |  |
| 0 | Vvi-Vitvi13g04412\_t001 |  |  |  |  |  |  |  |  |
| 0 | Vvi-Vitvi13g04413\_t001 |  |  |  |  |  |  |  |  |
| 0 | Vvi-Vitvi13g01264\_t001 |  |  |  |  |  |  |  |  |
| 0 | Vvi-Vitvi13g04414\_t001 |  |  |  |  |  |  |  |  |
| 0 | Vvi-Vitvi13g01266\_t001 |  |  |  |  |  |  |  |  |
| 0 | Vvi-Vitvi13g01267\_t001 |  |  |  |  |  |  |  |  |
| 0 | Vvi-Vitvi13g04415\_t001 |  |  |  |  |  |  |  |  |
| 0 | Vvi-Vitvi13g04416\_t001 |  |  |  |  |  |  |  |  |
| 0 | Vvi-Vitvi13g01272\_t001 |  |  |  |  |  |  |  |  |
| 0 | Vvi-Vitvi13g04417\_t001 |  |  |  |  |  |  |  |  |
| 0 | Vvi-Vitvi13g02262\_t001 |  |  |  |  |  |  |  |  |
| 0 | Vvi-Vitvi13g04418\_t001 |  |  |  |  |  |  |  |  |
| 0 | Vvi-Vitvi13g04419\_t001 |  |  |  |  |  |  |  |  |
| 0 | Vvi-Vitvi13g04420\_t001 |  |  |  |  |  |  |  |  |
| 0 | Vvi-Vitvi13g04421\_t001 |  |  |  |  |  |  |  |  |
| 0 | Vvi-Vitvi13g02263\_t001 |  |  |  |  |  |  |  |  |
| 0 | Vvi-Vitvi13g04422\_t001 |  |  |  |  |  |  |  |  |
| 0 | Vvi-Vitvi13g04423\_t001 |  |  |  |  |  |  |  |  |
| 0 | Vvi-Vitvi13g04424\_t001 |  |  |  |  |  |  |  |  |
| 0 | Vvi-Vitvi13g04425\_t001 |  |  |  |  |  |  |  |  |
| 0 | Vvi-Vitvi13g04426\_t001 |  |  |  |  |  |  |  |  |
| 0 | Vvi-Vitvi13g04427\_t001 |  |  |  |  |  |  |  |  |
| 0 | Vvi-Vitvi13g02269\_t001 |  |  |  |  |  |  |  |  |
| 0 | Vvi-Vitvi13g04428\_t001 |  |  |  |  |  |  |  |  |
| 0 | Vvi-Vitvi13g02270\_t001 |  |  |  |  |  |  |  |  |
| 0 | Vvi-Vitvi13g02271\_t001 |  |  |  |  |  |  |  |  |
| 0 | Vvi-Vitvi13g02272\_t001 |  |  |  |  |  |  |  |  |
| 0 | Vvi-Vitvi13g04429\_t001 |  |  |  |  |  |  |  |  |
| 0 | Vvi-Vitvi13g02273\_t001 |  |  |  |  |  |  |  |  |
| 0 | Vvi-Vitvi13g04430\_t001 |  |  |  |  |  |  |  |  |
| 0 | Vvi-Vitvi13g04431\_t001 |  |  |  |  |  |  |  |  |
| 0 | Vvi-Vitvi13g01291\_t002 |  |  |  |  |  |  |  |  |
| 0 | Vvi-Vitvi13g01293\_t001 |  |  |  |  |  |  |  |  |
| 0 | Vvi-Vitvi13g01298\_t001 |  |  |  |  |  |  |  |  |
| 0 | Vvi-Vitvi13g04432\_t001 |  |  |  |  |  |  |  |  |
| 0 | Vvi-Vitvi13g01299\_t001 |  |  |  |  |  |  |  |  |
| 0 | Vvi-Vitvi13g01300\_t001 |  |  |  |  |  |  |  |  |
| 0 | Vvi-Vitvi13g01302\_t001 |  |  |  |  |  |  |  |  |
| 0 | Vvi-Vitvi13g04433\_t001 |  |  |  |  |  |  |  |  |
| 0 | Vvi-Vitvi13g01303\_t001 |  |  |  |  |  |  |  |  |
| 0 | Vvi-Vitvi13g04434\_t001 |  |  |  |  |  |  |  |  |
| 0 | Vvi-Vitvi13g04435\_t001 |  |  |  |  |  |  |  |  |
| 0 | Vvi-Vitvi13g04436\_t001 |  |  |  |  |  |  |  |  |
| 0 | Vvi-Vitvi13g01305\_t001 |  |  |  |  |  |  |  |  |
| 0 | Vvi-Vitvi13g01306\_t001 |  |  |  |  |  |  |  |  |
| 0 | Vvi-Vitvi13g04437\_t001 |  |  |  |  |  |  |  |  |
| 0 | Vvi-Vitvi13g01310\_t001 |  |  |  |  |  |  |  |  |
| 0 | Vvi-Vitvi13g04438\_t001 |  |  |  |  |  |  |  |  |
| 0 | Vvi-Vitvi13g04439\_t001 |  |  |  |  |  |  |  |  |
| 0 | Vvi-Vitvi13g04440\_t001 |  |  |  |  |  |  |  |  |
| 0 | Vvi-Vitvi13g04441\_t001 |  |  |  |  |  |  |  |  |
| 0 | Vvi-Vitvi13g01323\_t001 |  |  |  |  |  |  |  |  |
| 0 | Vvi-Vitvi13g04442\_t001 |  |  |  |  |  |  |  |  |
| 0 | Vvi-Vitvi13g04443\_t001 |  |  |  |  |  |  |  |  |
| 0 | Vvi-Vitvi13g04444\_t001 |  |  |  |  |  |  |  |  |
| 0 | Vvi-Vitvi13g01325\_t001 |  |  |  |  |  |  |  |  |
| 0 | Vvi-Vitvi13g01327\_t001 |  |  |  |  |  |  |  |  |
| 0 | Vvi-Vitvi13g02284\_t001 |  |  |  |  |  |  |  |  |
| 0 | Vvi-Vitvi13g04445\_t001 |  |  |  |  |  |  |  |  |
| 0 | Vvi-Vitvi13g02285\_t001 |  |  |  |  |  |  |  |  |
| 0 | Vvi-Vitvi13g01328\_t001 |  |  |  |  |  |  |  |  |
| 0 | Vvi-Vitvi13g01329\_t001 |  |  |  |  |  |  |  |  |
| 0 | Vvi-Vitvi13g04446\_t001 |  |  |  |  |  |  |  |  |
| 0 | Vvi-Vitvi13g01407\_t001 |  |  |  |  |  |  |  |  |
| 0 | Vvi-Vitvi13g04447\_t001 |  |  |  |  |  |  |  |  |
| 0 | Vvi-Vitvi13g04448\_t001 |  |  |  |  |  |  |  |  |
| 0 | Vvi-Vitvi13g02320\_t001 |  |  |  |  |  |  |  |  |
| 0 | Vvi-Vitvi13g01403\_t001 |  |  |  |  |  |  |  |  |
| 0 | Vvi-Vitvi13g01402\_t001 |  |  |  |  |  |  |  |  |
| 0 | Vvi-Vitvi13g01401\_t002 |  |  |  |  |  |  |  |  |
| 0 | Vvi-Vitvi13g02319\_t001 |  |  |  |  |  |  |  |  |
| 0 | Vvi-Vitvi13g01398\_t001 |  |  |  |  |  |  |  |  |
| 0 | Vvi-Vitvi13g01396\_t001 |  |  |  |  |  |  |  |  |
| 1 | Vvi-Vitvi13g01395\_t001 |  | Ath-AT5G07050.1 |  |  |  |  |  |  |  |
| 1 | Vvi-Vitvi13g01394\_t001 |  | | | |  |  |  |  |  |  |  |
| 1 | Vvi-Vitvi13g01393\_t001 |  | | | |  |  |  |  |  |  |  |
| 1 | Vvi-Vitvi13g01389\_t001 |  | | | |  |  |  |  |  |  |  |
| 1 | Vvi-Vitvi13g04449\_t001 |  | | | |  |  |  |  |  |  |  |
| 1 | Vvi-Vitvi13g01387\_t001 |  | Ath-AT5G07040.1 |  |  |  |  |  |  |  |
| 1 | Vvi-Vitvi13g01386\_t001 |  | | | |  |  |  |  |  |  |  |
| 1 | Vvi-Vitvi13g01385\_t001 |  | Ath-AT5G07020.1 |  |  |  |  |  |  |  |
| 1 | Vvi-Vitvi13g04450\_t001 |  | | | |  |  |  |  |  |  |  |
| 1 | Vvi-Vitvi13g01384\_t001 |  | | | |  |  |  |  |  |  |  |
| 1 | Vvi-Vitvi13g04451\_t001 |  | | | |  |  |  |  |  |  |  |
| 1 | Vvi-Vitvi13g04452\_t001 |  | | | |  |  |  |  |  |  |  |
| 1 | Vvi-Vitvi13g01379\_t001 |  | Ath-AT5G07000.1 |  |  |  |  |  |  |  |
| 1 | Vvi-Vitvi13g04453\_t001 |  | | | |  |  |  |  |  |  |  |
| 1 | Vvi-Vitvi13g04454\_t001 |  | | | |  |  |  |  |  |  |  |
| 1 | Vvi-Vitvi13g01376\_t001 |  | Ath-AT5G06990.1 |  |  |  |  |  |  |  |
| 1 | Vvi-Vitvi13g04455\_t001 |  | | | |  |  |  |  |  |  |  |
| 1 | Vvi-Vitvi13g02316\_t001 |  | Ath-AT5G06980.4 |  |  |  |  |  |  |  |
| 0 | Vvi-Vitvi13g02315\_t001 |  |  |  |  |  |  |  |  |
| 0 | Vvi-Vitvi13g04456\_t001 |  |  |  |  |  |  |  |  |
| 0 | Vvi-Vitvi13g04457\_t001 |  |  |  |  |  |  |  |  |
| 0 | Vvi-Vitvi13g04458\_t001 |  |  |  |  |  |  |  |  |
| 0 | Vvi-Vitvi13g02314\_t001 |  |  |  |  |  |  |  |  |
| 0 | Vvi-Vitvi13g04459\_t001 |  |  |  |  |  |  |  |  |
| 0 | Vvi-Vitvi13g04460\_t001 |  |  |  |  |  |  |  |  |
| 0 | Vvi-Vitvi13g04461\_t001 |  |  |  |  |  |  |  |  |
| 0 | Vvi-Vitvi13g04462\_t001 |  |  |  |  |  |  |  |  |
| 0 | Vvi-Vitvi13g02312\_t001 |  |  |  |  |  |  |  |  |
| 0 | Vvi-Vitvi13g01369\_t002 |  |  |  |  |  |  |  |  |
| 0 | Vvi-Vitvi13g04463\_t001 |  |  |  |  |  |  |  |  |
| 0 | Vvi-Vitvi13g04464\_t001 |  |  |  |  |  |  |  |  |
| 0 | Vvi-Vitvi13g02309\_t001 |  |  |  |  |  |  |  |  |
| 0 | Vvi-Vitvi13g04465\_t001 |  |  |  |  |  |  |  |  |
| 0 | Vvi-Vitvi13g04466\_t001 |  |  |  |  |  |  |  |  |
| 0 | Vvi-Vitvi13g04467\_t001 |  |  |  |  |  |  |  |  |
| 0 | Vvi-Vitvi13g04468\_t001 |  |  |  |  |  |  |  |  |
| 0 | Vvi-Vitvi13g04469\_t001 |  |  |  |  |  |  |  |  |
| 0 | Vvi-Vitvi13g04470\_t001 |  |  |  |  |  |  |  |  |
| 0 | Vvi-Vitvi13g04471\_t001 |  |  |  |  |  |  |  |  |
| 0 | Vvi-Vitvi13g02302\_t001 |  |  |  |  |  |  |  |  |
| 0 | Vvi-Vitvi13g04472\_t001 |  |  |  |  |  |  |  |  |
| 0 | Vvi-Vitvi13g04473\_t001 |  |  |  |  |  |  |  |  |
| 0 | Vvi-Vitvi13g01361\_t001 |  |  |  |  |  |  |  |  |
| 0 | Vvi-Vitvi13g01360\_t001 |  |  |  |  |  |  |  |  |
| 0 | Vvi-Vitvi13g01359\_t001 |  |  |  |  |  |  |  |  |
| 0 | Vvi-Vitvi13g04474\_t001 |  |  |  |  |  |  |  |  |
| 0 | Vvi-Vitvi13g01358\_t001 |  |  |  |  |  |  |  |  |
| 0 | Vvi-Vitvi13g04475\_t001 |  |  |  |  |  |  |  |  |
| 0 | Vvi-Vitvi13g01357\_t001 |  |  |  |  |  |  |  |  |
| 0 | Vvi-Vitvi13g04476\_t001 |  |  |  |  |  |  |  |  |
| 0 | Vvi-Vitvi13g04477\_t001 |  |  |  |  |  |  |  |  |
| 0 | Vvi-Vitvi13g04478\_t001 |  |  |  |  |  |  |  |  |
| 0 | Vvi-Vitvi13g01355\_t001 |  |  |  |  |  |  |  |  |
| 0 | Vvi-Vitvi13g01354\_t001 |  |  |  |  |  |  |  |  |
| 0 | Vvi-Vitvi13g01353\_t001 |  |  |  |  |  |  |  |  |
| 0 | Vvi-Vitvi13g01352\_t001 |  |  |  |  |  |  |  |  |
| 0 | Vvi-Vitvi13g04479\_t001 |  |  |  |  |  |  |  |  |
| 0 | Vvi-Vitvi13g04480\_t001 |  |  |  |  |  |  |  |  |
| 0 | Vvi-Vitvi13g01351\_t001 |  |  |  |  |  |  |  |  |
| 0 | Vvi-Vitvi13g01350\_t001 |  |  |  |  |  |  |  |  |
| 0 | Vvi-Vitvi13g04481\_t001 |  |  |  |  |  |  |  |  |
| 0 | Vvi-Vitvi13g02295\_t001 |  |  |  |  |  |  |  |  |
| 0 | Vvi-Vitvi13g01348\_t002 |  |  |  |  |  |  |  |  |
| 0 | Vvi-Vitvi13g01347\_t001 |  |  |  |  |  |  |  |  |
| 0 | Vvi-Vitvi13g01345\_t001 |  |  |  |  |  |  |  |  |
| 0 | Vvi-Vitvi13g02294\_t001 |  |  |  |  |  |  |  |  |
| 0 | Vvi-Vitvi13g04482\_t001 |  |  |  |  |  |  |  |  |
| 0 | Vvi-Vitvi13g04483\_t001 |  |  |  |  |  |  |  |  |
| 0 | Vvi-Vitvi13g04484\_t001 |  |  |  |  |  |  |  |  |
| 0 | Vvi-Vitvi13g04485\_t001 |  |  |  |  |  |  |  |  |
| 0 | Vvi-Vitvi13g04486\_t001 |  |  |  |  |  |  |  |  |
| 0 | Vvi-Vitvi13g02290\_t001 |  |  |  |  |  |  |  |  |
| 0 | Vvi-Vitvi13g04487\_t001 |  |  |  |  |  |  |  |  |
| 0 | Vvi-Vitvi13g04488\_t001 |  |  |  |  |  |  |  |  |
| 0 | Vvi-Vitvi13g01342\_t001 |  |  |  |  |  |  |  |  |
| 0 | Vvi-Vitvi13g01341\_t001 |  |  |  |  |  |  |  |  |
| 0 | Vvi-Vitvi13g04489\_t001 |  |  |  |  |  |  |  |  |
| 0 | Vvi-Vitvi13g01340\_t001 |  |  |  |  |  |  |  |  |
| 0 | Vvi-Vitvi13g01338\_t001 |  |  |  |  |  |  |  |  |
| 0 | Vvi-Vitvi13g01337\_t001 |  |  |  |  |  |  |  |  |
| 0 | Vvi-Vitvi13g04490\_t001 |  |  |  |  |  |  |  |  |
| 0 | Vvi-Vitvi13g01336\_t001 |  |  |  |  |  |  |  |  |
| 0 | Vvi-Vitvi13g04491\_t001 |  |  |  |  |  |  |  |  |
| 0 | Vvi-Vitvi13g04492\_t001 |  |  |  |  |  |  |  |  |
| 0 | Vvi-Vitvi13g04493\_t001 |  |  |  |  |  |  |  |  |
| 0 | Vvi-Vitvi13g04494\_t001 |  |  |  |  |  |  |  |  |
| 0 | Vvi-Vitvi13g04495\_t001 |  |  |  |  |  |  |  |  |
| 0 | Vvi-Vitvi13g04496\_t001 |  |  |  |  |  |  |  |  |
| 0 | Vvi-Vitvi13g01333\_t001 |  |  |  |  |  |  |  |  |
| 0 | Vvi-Vitvi13g01408\_t001 |  |  |  |  |  |  |  |  |
| 0 | Vvi-Vitvi13g01409\_t001 |  |  |  |  |  |  |  |  |
| 0 | Vvi-Vitvi13g01413\_t002 |  |  |  |  |  |  |  |  |
| 0 | Vvi-Vitvi13g01414\_t001 |  |  |  |  |  |  |  |  |
| 0 | Vvi-Vitvi13g04497\_t001 |  |  |  |  |  |  |  |  |
| 0 | Vvi-Vitvi13g01416\_t002 |  |  |  |  |  |  |  |  |
| 0 | Vvi-Vitvi13g01417\_t001 |  |  |  |  |  |  |  |  |
| 0 | Vvi-Vitvi13g01420\_t001 |  |  |  |  |  |  |  |  |
| 0 | Vvi-Vitvi13g04498\_t001 |  |  |  |  |  |  |  |  |
| 0 | Vvi-Vitvi13g04499\_t001 |  |  |  |  |  |  |  |  |
| 0 | Vvi-Vitvi13g04500\_t001 |  |  |  |  |  |  |  |  |
| 0 | Vvi-Vitvi13g01423\_t001 |  |  |  |  |  |  |  |  |
| 0 | Vvi-Vitvi13g01425\_t001 |  |  |  |  |  |  |  |  |
| 0 | Vvi-Vitvi13g04501\_t001 |  |  |  |  |  |  |  |  |
| 0 | Vvi-Vitvi13g01427\_t001 |  |  |  |  |  |  |  |  |
| 0 | Vvi-Vitvi13g01428\_t001 |  |  |  |  |  |  |  |  |
| 0 | Vvi-Vitvi13g02323\_t001 |  |  |  |  |  |  |  |  |
| 0 | Vvi-Vitvi13g01429\_t002 |  |  |  |  |  |  |  |  |
| 0 | Vvi-Vitvi13g01433\_t001 |  |  |  |  |  |  |  |  |
| 0 | Vvi-Vitvi13g02324\_t001 |  |  |  |  |  |  |  |  |
| 0 | Vvi-Vitvi13g02325\_t001 |  |  |  |  |  |  |  |  |
| 0 | Vvi-Vitvi13g02326\_t001 |  |  |  |  |  |  |  |  |
| 0 | Vvi-Vitvi13g02327\_t001 |  |  |  |  |  |  |  |  |
| 0 | Vvi-Vitvi13g04502\_t001 |  |  |  |  |  |  |  |  |
| 0 | Vvi-Vitvi13g02328\_t001 |  |  |  |  |  |  |  |  |
| 0 | Vvi-Vitvi13g01434\_t001 |  |  |  |  |  |  |  |  |
| 0 | Vvi-Vitvi13g02329\_t001 |  |  |  |  |  |  |  |  |
| 0 | Vvi-Vitvi13g01435\_t001 |  |  |  |  |  |  |  |  |
| 0 | Vvi-Vitvi13g04503\_t001 |  |  |  |  |  |  |  |  |
| 0 | Vvi-Vitvi13g02332\_t001 |  |  |  |  |  |  |  |  |
| 0 | Vvi-Vitvi13g04504\_t001 |  |  |  |  |  |  |  |  |
| 0 | Vvi-Vitvi13g04505\_t001 |  |  |  |  |  |  |  |  |
| 0 | Vvi-Vitvi13g04506\_t001 |  |  |  |  |  |  |  |  |
| 0 | Vvi-Vitvi13g04507\_t001 |  |  |  |  |  |  |  |  |
| 0 | Vvi-Vitvi13g04509\_t001 |  |  |  |  |  |  |  |  |
| 0 | Vvi-Vitvi13g01446\_t001 |  |  |  |  |  |  |  |  |
| 0 | Vvi-Vitvi13g04510\_t001 |  |  |  |  |  |  |  |  |
| 0 | Vvi-Vitvi13g01455\_t001 |  |  |  |  |  |  |  |  |
| 0 | Vvi-Vitvi13g04511\_t001 |  |  |  |  |  |  |  |  |
| 0 | Vvi-Vitvi13g04512\_t001 |  |  |  |  |  |  |  |  |
| 0 | Vvi-Vitvi13g01459\_t001 |  |  |  |  |  |  |  |  |
| 0 | Vvi-Vitvi13g04513\_t001 |  |  |  |  |  |  |  |  |
| 0 | Vvi-Vitvi13g04514\_t001 |  |  |  |  |  |  |  |  |
| 0 | Vvi-Vitvi13g01462\_t001 |  |  |  |  |  |  |  |  |
| 0 | Vvi-Vitvi13g01463\_t001 |  |  |  |  |  |  |  |  |
| 0 | Vvi-Vitvi13g01464\_t001 |  |  |  |  |  |  |  |  |
| 0 | Vvi-Vitvi13g04515\_t001 |  |  |  |  |  |  |  |  |
| 0 | Vvi-Vitvi13g04516\_t001 |  |  |  |  |  |  |  |  |
| 0 | Vvi-Vitvi13g04517\_t001 |  |  |  |  |  |  |  |  |
| 0 | Vvi-Vitvi13g04518\_t001 |  |  |  |  |  |  |  |  |
| 0 | Vvi-Vitvi13g04519\_t001 |  |  |  |  |  |  |  |  |
| 0 | Vvi-Vitvi13g04520\_t001 |  |  |  |  |  |  |  |  |
| 0 | Vvi-Vitvi13g02340\_t001 |  |  |  |  |  |  |  |  |
| 0 | Vvi-Vitvi13g01468\_t001 |  |  |  |  |  |  |  |  |
| 0 | Vvi-Vitvi13g04521\_t001 |  |  |  |  |  |  |  |  |
| 0 | Vvi-Vitvi13g04522\_t001 |  |  |  |  |  |  |  |  |
| 0 | Vvi-Vitvi13g02341\_t001 |  |  |  |  |  |  |  |  |
| 0 | Vvi-Vitvi13g04523\_t001 |  |  |  |  |  |  |  |  |
| 0 | Vvi-Vitvi13g04524\_t001 |  |  |  |  |  |  |  |  |
| 0 | Vvi-Vitvi13g04525\_t001 |  |  |  |  |  |  |  |  |
| 0 | Vvi-Vitvi13g04526\_t001 |  |  |  |  |  |  |  |  |
| 0 | Vvi-Vitvi13g02344\_t001 |  |  |  |  |  |  |  |  |
| 0 | Vvi-Vitvi13g04527\_t001 |  |  |  |  |  |  |  |  |
| 0 | Vvi-Vitvi13g04528\_t001 |  |  |  |  |  |  |  |  |
| 0 | Vvi-Vitvi13g04529\_t001 |  |  |  |  |  |  |  |  |
| 0 | Vvi-Vitvi13g04530\_t001 |  |  |  |  |  |  |  |  |
| 0 | Vvi-Vitvi13g04531\_t001 |  |  |  |  |  |  |  |  |
| 0 | Vvi-Vitvi13g04532\_t001 |  |  |  |  |  |  |  |  |
| 0 | Vvi-Vitvi13g04533\_t001 |  |  |  |  |  |  |  |  |
| 0 | Vvi-Vitvi13g01476\_t001 |  |  |  |  |  |  |  |  |
| 0 | Vvi-Vitvi13g04534\_t001 |  |  |  |  |  |  |  |  |
| 0 | Vvi-Vitvi13g04535\_t001 |  |  |  |  |  |  |  |  |
| 0 | Vvi-Vitvi13g04536\_t001 |  |  |  |  |  |  |  |  |
| 0 | Vvi-Vitvi13g04537\_t001 |  |  |  |  |  |  |  |  |
| 0 | Vvi-Vitvi13g04538\_t001 |  |  |  |  |  |  |  |  |
| 0 | Vvi-Vitvi13g04539\_t001 |  |  |  |  |  |  |  |  |
| 0 | Vvi-Vitvi13g04540\_t001 |  |  |  |  |  |  |  |  |
| 0 | Vvi-Vitvi13g01481\_t001 |  |  |  |  |  |  |  |  |
| 0 | Vvi-Vitvi13g04541\_t001 |  |  |  |  |  |  |  |  |
| 0 | Vvi-Vitvi13g04542\_t001 |  |  |  |  |  |  |  |  |
| 0 | Vvi-Vitvi13g04543\_t001 |  |  |  |  |  |  |  |  |
| 0 | Vvi-Vitvi13g04544\_t001 |  |  |  |  |  |  |  |  |
| 0 | Vvi-Vitvi13g04545\_t001 |  |  |  |  |  |  |  |  |
| 0 | Vvi-Vitvi13g04546\_t001 |  |  |  |  |  |  |  |  |
| 0 | Vvi-Vitvi13g02349\_t001 |  |  |  |  |  |  |  |  |
| 0 | Vvi-Vitvi13g04547\_t001 |  |  |  |  |  |  |  |  |
| 0 | Vvi-Vitvi13g04548\_t001 |  |  |  |  |  |  |  |  |
| 0 | Vvi-Vitvi13g01483\_t002 |  |  |  |  |  |  |  |  |
| 0 | Vvi-Vitvi13g01484\_t001 |  |  |  |  |  |  |  |  |
| 0 | Vvi-Vitvi13g01485\_t001 |  |  |  |  |  |  |  |  |
| 0 | Vvi-Vitvi13g02351\_t001 |  |  |  |  |  |  |  |  |
| 0 | Vvi-Vitvi13g04549\_t001 |  |  |  |  |  |  |  |  |
| 0 | Vvi-Vitvi13g02353\_t001 |  |  |  |  |  |  |  |  |
| 0 | Vvi-Vitvi13g02354\_t001 |  |  |  |  |  |  |  |  |
| 0 | Vvi-Vitvi13g01486\_t001 |  |  |  |  |  |  |  |  |
| 0 | Vvi-Vitvi13g04550\_t001 |  |  |  |  |  |  |  |  |
| 0 | Vvi-Vitvi13g04551\_t001 |  |  |  |  |  |  |  |  |
| 0 | Vvi-Vitvi13g02356\_t001 |  |  |  |  |  |  |  |  |
| 0 | Vvi-Vitvi13g01488\_t001 |  |  |  |  |  |  |  |  |
| 0 | Vvi-Vitvi13g01489\_t001 |  |  |  |  |  |  |  |  |
| 0 | Vvi-Vitvi13g04552\_t001 |  |  |  |  |  |  |  |  |
| 0 | Vvi-Vitvi13g04553\_t001 |  |  |  |  |  |  |  |  |
| 0 | Vvi-Vitvi13g04554\_t001 |  |  |  |  |  |  |  |  |
| 0 | Vvi-Vitvi13g04555\_t001 |  |  |  |  |  |  |  |  |
| 0 | Vvi-Vitvi13g02359\_t001 |  |  |  |  |  |  |  |  |
| 0 | Vvi-Vitvi13g02360\_t001 |  |  |  |  |  |  |  |  |
| 0 | Vvi-Vitvi13g02361\_t001 |  |  |  |  |  |  |  |  |
| 0 | Vvi-Vitvi13g01492\_t001 |  |  |  |  |  |  |  |  |
| 0 | Vvi-Vitvi13g04556\_t001 |  |  |  |  |  |  |  |  |
| 0 | Vvi-Vitvi13g04557\_t001 |  |  |  |  |  |  |  |  |
| 0 | Vvi-Vitvi13g04558\_t001 |  |  |  |  |  |  |  |  |
| 0 | Vvi-Vitvi13g01512\_t001 |  |  |  |  |  |  |  |  |
| 0 | Vvi-Vitvi13g04559\_t001 |  |  |  |  |  |  |  |  |
| 0 | Vvi-Vitvi13g04560\_t001 |  |  |  |  |  |  |  |  |
| 0 | Vvi-Vitvi13g01513\_t001 |  |  |  |  |  |  |  |  |
| 0 | Vvi-Vitvi13g04561\_t001 |  |  |  |  |  |  |  |  |
| 0 | Vvi-Vitvi13g04562\_t001 |  |  |  |  |  |  |  |  |
| 0 | Vvi-Vitvi13g04563\_t001 |  |  |  |  |  |  |  |  |
| 0 | Vvi-Vitvi13g04564\_t001 |  |  |  |  |  |  |  |  |
| 0 | Vvi-Vitvi13g01515\_t001 |  |  |  |  |  |  |  |  |
| 0 | Vvi-Vitvi13g04565\_t001 |  |  |  |  |  |  |  |  |
| 0 | Vvi-Vitvi13g04566\_t001 |  |  |  |  |  |  |  |  |
| 0 | Vvi-Vitvi13g04567\_t001 |  |  |  |  |  |  |  |  |
| 0 | Vvi-Vitvi13g04568\_t001 |  |  |  |  |  |  |  |  |
| 0 | Vvi-Vitvi13g02400\_t001 |  |  |  |  |  |  |  |  |
| 0 | Vvi-Vitvi13g04569\_t001 |  |  |  |  |  |  |  |  |
| 0 | Vvi-Vitvi13g04570\_t001 |  |  |  |  |  |  |  |  |
| 0 | Vvi-Vitvi13g04571\_t001 |  |  |  |  |  |  |  |  |
| 0 | Vvi-Vitvi13g04572\_t001 |  |  |  |  |  |  |  |  |
| 0 | Vvi-Vitvi13g04573\_t001 |  |  |  |  |  |  |  |  |
| 0 | Vvi-Vitvi13g01529\_t001 |  |  |  |  |  |  |  |  |
| 0 | Vvi-Vitvi13g02402\_t001 |  |  |  |  |  |  |  |  |
| 0 | Vvi-Vitvi13g04574\_t001 |  |  |  |  |  |  |  |  |
| 0 | Vvi-Vitvi13g04575\_t001 |  |  |  |  |  |  |  |  |
| 0 | Vvi-Vitvi13g04576\_t001 |  |  |  |  |  |  |  |  |
| 0 | Vvi-Vitvi13g04577\_t001 |  |  |  |  |  |  |  |  |
| 0 | Vvi-Vitvi13g01532\_t001 |  |  |  |  |  |  |  |  |
| 0 | Vvi-Vitvi13g04578\_t001 |  |  |  |  |  |  |  |  |
| 0 | Vvi-Vitvi13g02413\_t002 |  |  |  |  |  |  |  |  |
| 0 | Vvi-Vitvi13g04579\_t001 |  |  |  |  |  |  |  |  |
| 0 | Vvi-Vitvi13g04580\_t001 |  |  |  |  |  |  |  |  |
| 0 | Vvi-Vitvi13g04581\_t001 |  |  |  |  |  |  |  |  |
| 0 | Vvi-Vitvi13g04582\_t001 |  |  |  |  |  |  |  |  |
| 0 | Vvi-Vitvi13g04583\_t001 |  |  |  |  |  |  |  |  |
| 0 | Vvi-Vitvi13g04584\_t001 |  |  |  |  |  |  |  |  |
| 0 | Vvi-Vitvi13g04585\_t001 |  |  |  |  |  |  |  |  |
| 0 | Vvi-Vitvi13g04586\_t001 |  |  |  |  |  |  |  |  |
| 0 | Vvi-Vitvi13g04587\_t001 |  |  |  |  |  |  |  |  |
| 0 | Vvi-Vitvi13g04588\_t001 |  |  |  |  |  |  |  |  |
| 0 | Vvi-Vitvi13g04589\_t001 |  |  |  |  |  |  |  |  |
| 0 | Vvi-Vitvi13g04590\_t001 |  |  |  |  |  |  |  |  |
| 0 | Vvi-Vitvi13g04591\_t001 |  |  |  |  |  |  |  |  |
| 0 | Vvi-Vitvi13g04592\_t001 |  |  |  |  |  |  |  |  |
| 0 | Vvi-Vitvi13g04593\_t001 |  |  |  |  |  |  |  |  |
| 0 | Vvi-Vitvi13g04594\_t001 |  |  |  |  |  |  |  |  |
| 0 | Vvi-Vitvi13g04595\_t001 |  |  |  |  |  |  |  |  |
| 0 | Vvi-Vitvi13g04596\_t001 |  |  |  |  |  |  |  |  |
| 0 | Vvi-Vitvi13g04597\_t001 |  |  |  |  |  |  |  |  |
| 0 | Vvi-Vitvi13g04598\_t001 |  |  |  |  |  |  |  |  |
| 0 | Vvi-Vitvi13g04599\_t001 |  |  |  |  |  |  |  |  |
| 0 | Vvi-Vitvi13g04600\_t001 |  |  |  |  |  |  |  |  |
| 0 | Vvi-Vitvi13g04601\_t001 |  |  |  |  |  |  |  |  |
| 0 | Vvi-Vitvi13g04602\_t001 |  |  |  |  |  |  |  |  |
| 0 | Vvi-Vitvi13g04603\_t001 |  |  |  |  |  |  |  |  |
| 0 | Vvi-Vitvi13g04604\_t001 |  |  |  |  |  |  |  |  |
| 0 | Vvi-Vitvi13g04605\_t001 |  |  |  |  |  |  |  |  |
| 0 | Vvi-Vitvi13g04606\_t001 |  |  |  |  |  |  |  |  |
| 0 | Vvi-Vitvi13g04607\_t001 |  |  |  |  |  |  |  |  |
| 0 | Vvi-Vitvi13g04608\_t001 |  |  |  |  |  |  |  |  |
| 0 | Vvi-Vitvi13g04609\_t001 |  |  |  |  |  |  |  |  |
| 0 | Vvi-Vitvi13g04610\_t001 |  |  |  |  |  |  |  |  |
| 0 | Vvi-Vitvi13g04611\_t001 |  |  |  |  |  |  |  |  |
| 0 | Vvi-Vitvi13g04612\_t001 |  |  |  |  |  |  |  |  |
| 0 | Vvi-Vitvi13g04613\_t001 |  |  |  |  |  |  |  |  |
| 0 | Vvi-Vitvi13g04614\_t001 |  |  |  |  |  |  |  |  |
| 0 | Vvi-Vitvi13g04615\_t001 |  |  |  |  |  |  |  |  |
| 0 | Vvi-Vitvi13g04616\_t001 |  |  |  |  |  |  |  |  |
| 0 | Vvi-Vitvi13g04617\_t001 |  |  |  |  |  |  |  |  |
| 0 | Vvi-Vitvi13g04618\_t001 |  |  |  |  |  |  |  |  |
| 0 | Vvi-Vitvi13g04619\_t001 |  |  |  |  |  |  |  |  |
| 0 | Vvi-Vitvi13g04620\_t001 |  |  |  |  |  |  |  |  |
| 0 | Vvi-Vitvi13g04621\_t001 |  |  |  |  |  |  |  |  |
| 0 | Vvi-Vitvi13g04622\_t001 |  |  |  |  |  |  |  |  |
| 0 | Vvi-Vitvi13g04623\_t001 |  |  |  |  |  |  |  |  |
| 0 | Vvi-Vitvi13g04624\_t001 |  |  |  |  |  |  |  |  |
| 0 | Vvi-Vitvi13g04625\_t001 |  |  |  |  |  |  |  |  |
| 0 | Vvi-Vitvi13g04626\_t001 |  |  |  |  |  |  |  |  |
| 0 | Vvi-Vitvi13g04627\_t001 |  |  |  |  |  |  |  |  |
| 0 | Vvi-Vitvi13g04628\_t001 |  |  |  |  |  |  |  |  |
| 0 | Vvi-Vitvi13g04629\_t001 |  |  |  |  |  |  |  |  |
| 0 | Vvi-Vitvi13g04630\_t001 |  |  |  |  |  |  |  |  |
| 0 | Vvi-Vitvi13g04631\_t001 |  |  |  |  |  |  |  |  |
| 0 | Vvi-Vitvi13g04632\_t001 |  |  |  |  |  |  |  |  |
| 0 | Vvi-Vitvi13g04633\_t001 |  |  |  |  |  |  |  |  |
| 0 | Vvi-Vitvi13g04634\_t001 |  |  |  |  |  |  |  |  |
| 0 | Vvi-Vitvi13g04635\_t001 |  |  |  |  |  |  |  |  |
| 0 | Vvi-Vitvi13g04636\_t001 |  |  |  |  |  |  |  |  |
| 0 | Vvi-Vitvi13g04637\_t001 |  |  |  |  |  |  |  |  |
| 0 | Vvi-Vitvi13g04638\_t001 |  |  |  |  |  |  |  |  |
| 0 | Vvi-Vitvi13g04639\_t001 |  |  |  |  |  |  |  |  |
| 0 | Vvi-Vitvi13g01534\_t001 |  |  |  |  |  |  |  |  |
| 0 | Vvi-Vitvi13g01535\_t001 |  |  |  |  |  |  |  |  |
| 0 | Vvi-Vitvi13g01536\_t001 |  |  |  |  |  |  |  |  |
| 0 | Vvi-Vitvi13g01537\_t001 |  |  |  |  |  |  |  |  |
| 0 | Vvi-Vitvi13g02416\_t001 |  |  |  |  |  |  |  |  |
| 0 | Vvi-Vitvi13g01538\_t001 |  |  |  |  |  |  |  |  |
| 0 | Vvi-Vitvi13g02417\_t001 |  |  |  |  |  |  |  |  |
| 0 | Vvi-Vitvi13g01539\_t001 |  |  |  |  |  |  |  |  |
| 0 | Vvi-Vitvi13g01540\_t002 |  |  |  |  |  |  |  |  |
| 0 | Vvi-Vitvi13g01541\_t001 |  |  |  |  |  |  |  |  |
| 0 | Vvi-Vitvi13g04640\_t001 |  |  |  |  |  |  |  |  |
| 0 | Vvi-Vitvi13g04641\_t001 |  |  |  |  |  |  |  |  |
| 0 | Vvi-Vitvi13g01545\_t001 |  |  |  |  |  |  |  |  |
| 0 | Vvi-Vitvi13g02419\_t001 |  |  |  |  |  |  |  |  |
| 0 | Vvi-Vitvi13g04642\_t001 |  |  |  |  |  |  |  |  |
| 0 | Vvi-Vitvi13g04643\_t001 |  |  |  |  |  |  |  |  |
| 0 | Vvi-Vitvi13g04644\_t001 |  |  |  |  |  |  |  |  |
| 0 | Vvi-Vitvi13g04645\_t001 |  |  |  |  |  |  |  |  |
| 0 | Vvi-Vitvi13g01550\_t001 |  |  |  |  |  |  |  |  |
| 0 | Vvi-Vitvi13g01551\_t001 |  |  |  |  |  |  |  |  |
| 0 | Vvi-Vitvi13g01552\_t001 |  |  |  |  |  |  |  |  |
| 0 | Vvi-Vitvi13g01554\_t001 |  |  |  |  |  |  |  |  |
| 0 | Vvi-Vitvi13g01555\_t001 |  |  |  |  |  |  |  |  |
| 0 | Vvi-Vitvi13g01556\_t002 |  |  |  |  |  |  |  |  |
| 0 | Vvi-Vitvi13g02422\_t001 |  |  |  |  |  |  |  |  |
| 0 | Vvi-Vitvi13g04646\_t001 |  |  |  |  |  |  |  |  |
| 0 | Vvi-Vitvi13g01557\_t001 |  |  |  |  |  |  |  |  |
| 0 | Vvi-Vitvi13g01558\_t001 |  |  |  |  |  |  |  |  |
| 0 | Vvi-Vitvi13g02423\_t001 |  |  |  |  |  |  |  |  |
| 0 | Vvi-Vitvi13g04647\_t001 |  |  |  |  |  |  |  |  |
| 0 | Vvi-Vitvi13g01560\_t001 |  |  |  |  |  |  |  |  |
| 0 | Vvi-Vitvi13g04648\_t001 |  |  |  |  |  |  |  |  |
| 0 | Vvi-Vitvi13g04649\_t001 |  |  |  |  |  |  |  |  |
| 0 | Vvi-Vitvi13g04650\_t001 |  |  |  |  |  |  |  |  |
| 0 | Vvi-Vitvi13g04651\_t001 |  |  |  |  |  |  |  |  |
| 0 | Vvi-Vitvi13g01566\_t001 |  |  |  |  |  |  |  |  |
| 0 | Vvi-Vitvi13g02427\_t001 |  |  |  |  |  |  |  |  |
| 0 | Vvi-Vitvi13g02428\_t001 |  |  |  |  |  |  |  |  |
| 0 | Vvi-Vitvi13g04652\_t001 |  |  |  |  |  |  |  |  |
| 0 | Vvi-Vitvi13g02429\_t001 |  |  |  |  |  |  |  |  |
| 0 | Vvi-Vitvi13g02430\_t001 |  |  |  |  |  |  |  |  |
| 0 | Vvi-Vitvi13g04653\_t001 |  |  |  |  |  |  |  |  |
| 0 | Vvi-Vitvi13g04654\_t001 |  |  |  |  |  |  |  |  |
| 0 | Vvi-Vitvi13g01569\_t002 |  |  |  |  |  |  |  |  |
| 1 | Vvi-Vitvi13g01570\_t001 |  | Ath-AT3G51930.1 |  |  |  |  |  |  |  |
| 1 | Vvi-Vitvi13g01571\_t002 |  | | | |  |  |  |  |  |  |  |
| 1 | Vvi-Vitvi13g01572\_t001 |  | | | |  |  |  |  |  |  |  |
| 1 | Vvi-Vitvi13g01573\_t001 |  | | | |  |  |  |  |  |  |  |
| 1 | Vvi-Vitvi13g01574\_t001 |  | | | |  |  |  |  |  |  |  |
| 1 | Vvi-Vitvi13g01575\_t002 |  | Ath-AT3G51940.2 |  |  |  |  |  |  |  |
| 1 | Vvi-Vitvi13g01576\_t002 |  | | | |  |  |  |  |  |  |  |
| 1 | Vvi-Vitvi13g02435\_t001 |  | | | |  |  |  |  |  |  |  |
| 1 | Vvi-Vitvi13g04655\_t001 |  | | | |  |  |  |  |  |  |  |
| 1 | Vvi-Vitvi13g01579\_t001 |  | | | |  |  |  |  |  |  |  |
| 1 | Vvi-Vitvi13g04656\_t001 |  | | | |  |  |  |  |  |  |  |
| 1 | Vvi-Vitvi13g01601\_t001 |  | | | |  |  |  |  |  |  |  |
| 1 | Vvi-Vitvi13g04657\_t001 |  | | | |  |  |  |  |  |  |  |
| 1 | Vvi-Vitvi13g04658\_t001 |  | | | |  |  |  |  |  |  |  |
| 1 | Vvi-Vitvi13g01603\_t001 |  | | | |  |  |  |  |  |  |  |
| 1 | Vvi-Vitvi13g02445\_t001 |  | | | |  |  |  |  |  |  |  |
| 1 | Vvi-Vitvi13g04659\_t001 |  | | | |  |  |  |  |  |  |  |
| 1 | Vvi-Vitvi13g04660\_t001 |  | | | |  |  |  |  |  |  |  |
| 1 | Vvi-Vitvi13g04661\_t001 |  | | | |  |  |  |  |  |  |  |
| 1 | Vvi-Vitvi13g04662\_t001 |  | | | |  |  |  |  |  |  |  |
| 1 | Vvi-Vitvi13g04663\_t001 |  | | | |  |  |  |  |  |  |  |
| 1 | Vvi-Vitvi13g04664\_t001 |  | | | |  |  |  |  |  |  |  |
| 1 | Vvi-Vitvi13g04665\_t001 |  | | | |  |  |  |  |  |  |  |
| 1 | Vvi-Vitvi13g01608\_t001 |  | | | |  |  |  |  |  |  |  |
| 1 | Vvi-Vitvi13g04666\_t001 |  | | | |  |  |  |  |  |  |  |
| 1 | Vvi-Vitvi13g01609\_t001 |  | Ath-AT3G51960.2 |  |  |  |  |  |  |  |
| 1 | Vvi-Vitvi13g01610\_t002 |  | | | |  |  |  |  |  |  |  |
| 1 | Vvi-Vitvi13g04667\_t001 |  | | | |  |  |  |  |  |  |  |
| 1 | Vvi-Vitvi13g01611\_t002 |  | | | |  |  |  |  |  |  |  |
| 1 | Vvi-Vitvi13g01612\_t001 |  | | | |  |  |  |  |  |  |  |
| 1 | Vvi-Vitvi13g01613\_t001 |  | | | |  |  |  |  |  |  |  |
| 1 | Vvi-Vitvi13g01614\_t001 |  | | | |  |  |  |  |  |  |  |
| 1 | Vvi-Vitvi13g04668\_t001 |  | | | |  |  |  |  |  |  |  |
| 1 | Vvi-Vitvi13g04669\_t001 |  | | | |  |  |  |  |  |  |  |
| 1 | Vvi-Vitvi13g02449\_t001 |  | | | |  |  |  |  |  |  |  |
| 1 | Vvi-Vitvi13g04670\_t001 |  | | | |  |  |  |  |  |  |  |
| 1 | Vvi-Vitvi13g01616\_t001 |  | | | |  |  |  |  |  |  |  |
| 1 | Vvi-Vitvi13g04671\_t001 |  | | | |  |  |  |  |  |  |  |
| 1 | Vvi-Vitvi13g01617\_t001 |  | | | |  |  |  |  |  |  |  |
| 1 | Vvi-Vitvi13g01618\_t001 |  | | | |  |  |  |  |  |  |  |
| 1 | Vvi-Vitvi13g01620\_t001 |  | | | |  |  |  |  |  |  |  |
| 1 | Vvi-Vitvi13g04672\_t001 |  | | | |  |  |  |  |  |  |  |
| 1 | Vvi-Vitvi13g01622\_t001 |  | | | |  |  |  |  |  |  |  |
| 1 | Vvi-Vitvi13g01623\_t001 |  | | | |  |  |  |  |  |  |  |
| 1 | Vvi-Vitvi13g01626\_t001 |  | Ath-AT3G51990.1 |  |  |  |  |  |  |  |
| 1 | Vvi-Vitvi13g01627\_t002 |  | | | |  |  |  |  |  |  |  |
| 1 | Vvi-Vitvi13g01628\_t001 |  | | | |  |  |  |  |  |  |  |
| 1 | Vvi-Vitvi13g01629\_t001 |  | | | |  |  |  |  |  |  |  |
| 1 | Vvi-Vitvi13g04673\_t001 |  | | | |  |  |  |  |  |  |  |
| 1 | Vvi-Vitvi13g04674\_t001 |  | | | |  |  |  |  |  |  |  |
| 1 | Vvi-Vitvi13g01861\_t001 |  | | | |  |  |  |  |  |  |  |
| 1 | Vvi-Vitvi13g02457\_t001 |  | | | |  |  |  |  |  |  |  |
| 1 | Vvi-Vitvi13g01633\_t001 |  | Ath-AT3G52020.1 |  |  |  |  |  |  |  |
| 1 | Vvi-Vitvi13g01634\_t001 |  | | | |  |  |  |  |  |  |  |
| 1 | Vvi-Vitvi13g01636\_t001 |  | | | |  |  |  |  |  |  |  |
| 1 | Vvi-Vitvi13g04675\_t001 |  | | | |  |  |  |  |  |  |  |
| 1 | Vvi-Vitvi13g02459\_t001 |  | | | |  |  |  |  |  |  |  |
| 1 | Vvi-Vitvi13g04676\_t001 |  | | | |  |  |  |  |  |  |  |
| 1 | Vvi-Vitvi13g04677\_t001 |  | | | |  |  |  |  |  |  |  |
| 1 | Vvi-Vitvi13g01638\_t001 |  | | | |  |  |  |  |  |  |  |
| 1 | Vvi-Vitvi13g04678\_t001 |  | | | |  |  |  |  |  |  |  |
| 1 | Vvi-Vitvi13g04679\_t001 |  | | | |  |  |  |  |  |  |  |
| 1 | Vvi-Vitvi13g01640\_t001 |  | Ath-AT3G52030.1 |  |  |  |  |  |  |  |
| 1 | Vvi-Vitvi13g01641\_t001 |  | | | |  |  |  |  |  |  |  |
| 1 | Vvi-Vitvi13g01642\_t001 |  | | | |  |  |  |  |  |  |  |
| 1 | Vvi-Vitvi13g01643\_t001 |  | Ath-AT3G52040.1 |  |  |  |  |  |  |  |
| 0 | Vvi-Vitvi13g04680\_t001 |  |  |  |  |  |  |  |  |
| 0 | Vvi-Vitvi13g01645\_t001 |  |  |  |  |  |  |  |  |
| 0 | Vvi-Vitvi13g04681\_t001 |  |  |  |  |  |  |  |  |
| 0 | Vvi-Vitvi13g04682\_t001 |  |  |  |  |  |  |  |  |
| 0 | Vvi-Vitvi13g04683\_t001 |  |  |  |  |  |  |  |  |
| 0 | Vvi-Vitvi13g04684\_t001 |  |  |  |  |  |  |  |  |
| 0 | Vvi-Vitvi13g04685\_t001 |  |  |  |  |  |  |  |  |
| 0 | Vvi-Vitvi13g04686\_t001 |  |  |  |  |  |  |  |  |
| 0 | Vvi-Vitvi13g04687\_t001 |  |  |  |  |  |  |  |  |
| 0 | Vvi-Vitvi13g04688\_t001 |  |  |  |  |  |  |  |  |
| 0 | Vvi-Vitvi13g04689\_t001 |  |  |  |  |  |  |  |  |
| 0 | Vvi-Vitvi13g04690\_t001 |  |  |  |  |  |  |  |  |
| 0 | Vvi-Vitvi13g02465\_t001 |  |  |  |  |  |  |  |  |
| 0 | Vvi-Vitvi13g04691\_t001 |  |  |  |  |  |  |  |  |
| 0 | Vvi-Vitvi13g04692\_t001 |  |  |  |  |  |  |  |  |
| 0 | Vvi-Vitvi13g04693\_t001 |  |  |  |  |  |  |  |  |
| 0 | Vvi-Vitvi13g02466\_t001 |  |  |  |  |  |  |  |  |
| 0 | Vvi-Vitvi13g04694\_t001 |  |  |  |  |  |  |  |  |
| 0 | Vvi-Vitvi13g04695\_t001 |  |  |  |  |  |  |  |  |
| 0 | Vvi-Vitvi13g04696\_t001 |  |  |  |  |  |  |  |  |
| 0 | Vvi-Vitvi13g04697\_t001 |  |  |  |  |  |  |  |  |
| 0 | Vvi-Vitvi13g01648\_t001 |  |  |  |  |  |  |  |  |
| 0 | Vvi-Vitvi13g01649\_t001 |  |  |  |  |  |  |  |  |
| 0 | Vvi-Vitvi13g01650\_t001 |  |  |  |  |  |  |  |  |
| 0 | Vvi-Vitvi13g01651\_t001 |  |  |  |  |  |  |  |  |
| 0 | Vvi-Vitvi13g01652\_t001 |  |  |  |  |  |  |  |  |
| 0 | Vvi-Vitvi13g01653\_t001 |  |  |  |  |  |  |  |  |
| 0 | Vvi-Vitvi13g01654\_t001 |  |  |  |  |  |  |  |  |
| 0 | Vvi-Vitvi13g04698\_t001 |  |  |  |  |  |  |  |  |
| 0 | Vvi-Vitvi13g04699\_t001 |  |  |  |  |  |  |  |  |
| 0 | Vvi-Vitvi13g04700\_t001 |  |  |  |  |  |  |  |  |
| 0 | Vvi-Vitvi13g04701\_t001 |  |  |  |  |  |  |  |  |
| 0 | Vvi-Vitvi13g04702\_t001 |  |  |  |  |  |  |  |  |
| 0 | Vvi-Vitvi13g04703\_t001 |  |  |  |  |  |  |  |  |
| 0 | Vvi-Vitvi13g04704\_t001 |  |  |  |  |  |  |  |  |
| 0 | Vvi-Vitvi13g01657\_t001 |  |  |  |  |  |  |  |  |
| 0 | Vvi-Vitvi13g04705\_t001 |  |  |  |  |  |  |  |  |
| 0 | Vvi-Vitvi13g04706\_t001 |  |  |  |  |  |  |  |  |
| 0 | Vvi-Vitvi13g04707\_t001 |  |  |  |  |  |  |  |  |
| 0 | Vvi-Vitvi13g04708\_t001 |  |  |  |  |  |  |  |  |
| 0 | Vvi-Vitvi13g02471\_t001 |  |  |  |  |  |  |  |  |
| 0 | Vvi-Vitvi13g04709\_t001 |  |  |  |  |  |  |  |  |
| 0 | Vvi-Vitvi13g04710\_t001 |  |  |  |  |  |  |  |  |
| 0 | Vvi-Vitvi13g04711\_t001 |  |  |  |  |  |  |  |  |
| 0 | Vvi-Vitvi13g04712\_t001 |  |  |  |  |  |  |  |  |
| 0 | Vvi-Vitvi13g04713\_t001 |  |  |  |  |  |  |  |  |
| 0 | Vvi-Vitvi13g04714\_t001 |  |  |  |  |  |  |  |  |
| 0 | Vvi-Vitvi13g04715\_t001 |  |  |  |  |  |  |  |  |
| 0 | Vvi-Vitvi13g04716\_t001 |  |  |  |  |  |  |  |  |
| 0 | Vvi-Vitvi13g04717\_t001 |  |  |  |  |  |  |  |  |
| 0 | Vvi-Vitvi13g01666\_t001 |  |  |  |  |  |  |  |  |
| 0 | Vvi-Vitvi13g01667\_t001 |  |  |  |  |  |  |  |  |
| 0 | Vvi-Vitvi13g02475\_t001 |  |  |  |  |  |  |  |  |
| 0 | Vvi-Vitvi13g04718\_t001 |  |  |  |  |  |  |  |  |
| 0 | Vvi-Vitvi13g01669\_t001 |  |  |  |  |  |  |  |  |
| 0 | Vvi-Vitvi13g01670\_t001 |  |  |  |  |  |  |  |  |
| 0 | Vvi-Vitvi13g01672\_t001 |  |  |  |  |  |  |  |  |
| 0 | Vvi-Vitvi13g01673\_t001 |  |  |  |  |  |  |  |  |
| 0 | Vvi-Vitvi13g01674\_t002 |  |  |  |  |  |  |  |  |
| 0 | Vvi-Vitvi13g02476\_t001 |  |  |  |  |  |  |  |  |
| 0 | Vvi-Vitvi13g04719\_t001 |  |  |  |  |  |  |  |  |
| 0 | Vvi-Vitvi13g04720\_t001 |  |  |  |  |  |  |  |  |
| 0 | Vvi-Vitvi13g01676\_t001 |  |  |  |  |  |  |  |  |
| 0 | Vvi-Vitvi13g04721\_t001 |  |  |  |  |  |  |  |  |
| 0 | Vvi-Vitvi13g04722\_t001 |  |  |  |  |  |  |  |  |
| 0 | Vvi-Vitvi13g04723\_t001 |  |  |  |  |  |  |  |  |
| 0 | Vvi-Vitvi13g04724\_t001 |  |  |  |  |  |  |  |  |
| 0 | Vvi-Vitvi13g04725\_t001 |  |  |  |  |  |  |  |  |
| 0 | Vvi-Vitvi13g04726\_t001 |  |  |  |  |  |  |  |  |
| 0 | Vvi-Vitvi13g04727\_t001 |  |  |  |  |  |  |  |  |
| 0 | Vvi-Vitvi13g01682\_t001 |  |  |  |  |  |  |  |  |
| 0 | Vvi-Vitvi13g01684\_t001 |  |  |  |  |  |  |  |  |
| 0 | Vvi-Vitvi13g01685\_t001 |  |  |  |  |  |  |  |  |
| 0 | Vvi-Vitvi13g01686\_t002 |  |  |  |  |  |  |  |  |
| 0 | Vvi-Vitvi13g04728\_t001 |  |  |  |  |  |  |  |  |
| 0 | Vvi-Vitvi13g04729\_t001 |  |  |  |  |  |  |  |  |
| 0 | Vvi-Vitvi13g04730\_t001 |  |  |  |  |  |  |  |  |
| 0 | Vvi-Vitvi13g04731\_t001 |  |  |  |  |  |  |  |  |
| 0 | Vvi-Vitvi13g01687\_t001 |  |  |  |  |  |  |  |  |
| 0 | Vvi-Vitvi13g02478\_t001 |  |  |  |  |  |  |  |  |
| 0 | Vvi-Vitvi13g04732\_t001 |  |  |  |  |  |  |  |  |
| 0 | Vvi-Vitvi13g01690\_t001 |  |  |  |  |  |  |  |  |
| 0 | Vvi-Vitvi13g01691\_t001 |  |  |  |  |  |  |  |  |
| 0 | Vvi-Vitvi13g01692\_t001 |  |  |  |  |  |  |  |  |
| 0 | Vvi-Vitvi13g04733\_t001 |  |  |  |  |  |  |  |  |
| 0 | Vvi-Vitvi13g01696\_t001 |  |  |  |  |  |  |  |  |
| 0 | Vvi-Vitvi13g01698\_t001 |  |  |  |  |  |  |  |  |
| 0 | Vvi-Vitvi13g04734\_t001 |  |  |  |  |  |  |  |  |
| 0 | Vvi-Vitvi13g04735\_t001 |  |  |  |  |  |  |  |  |
| 0 | Vvi-Vitvi13g04736\_t001 |  |  |  |  |  |  |  |  |
| 0 | Vvi-Vitvi13g01699\_t001 |  |  |  |  |  |  |  |  |
| 0 | Vvi-Vitvi13g01700\_t001 |  |  |  |  |  |  |  |  |
| 0 | Vvi-Vitvi13g02487\_t001 |  |  |  |  |  |  |  |  |
| 0 | Vvi-Vitvi13g01702\_t001 |  |  |  |  |  |  |  |  |
| 0 | Vvi-Vitvi13g01703\_t001 |  |  |  |  |  |  |  |  |
| 0 | Vvi-Vitvi13g01704\_t001 |  |  |  |  |  |  |  |  |
| 0 | Vvi-Vitvi13g01705\_t001 |  |  |  |  |  |  |  |  |
| 0 | Vvi-Vitvi13g01707\_t001 |  |  |  |  |  |  |  |  |
| 0 | Vvi-Vitvi13g01709\_t001 |  |  |  |  |  |  |  |  |
| 0 | Vvi-Vitvi13g01710\_t001 |  |  |  |  |  |  |  |  |
| 0 | Vvi-Vitvi13g01711\_t001 |  |  |  |  |  |  |  |  |
| 0 | Vvi-Vitvi13g02488\_t001 |  |  |  |  |  |  |  |  |
| 0 | Vvi-Vitvi13g01713\_t001 |  |  |  |  |  |  |  |  |
| 0 | Vvi-Vitvi13g04737\_t001 |  |  |  |  |  |  |  |  |
| 0 | Vvi-Vitvi13g04738\_t001 |  |  |  |  |  |  |  |  |
| 0 | Vvi-Vitvi13g01718\_t001 |  |  |  |  |  |  |  |  |
| 0 | Vvi-Vitvi13g04739\_t001 |  |  |  |  |  |  |  |  |
| 0 | Vvi-Vitvi13g01720\_t001 |  |  |  |  |  |  |  |  |
| 0 | Vvi-Vitvi13g02492\_t001 |  |  |  |  |  |  |  |  |
| 0 | Vvi-Vitvi13g01722\_t001 |  |  |  |  |  |  |  |  |
| 0 | Vvi-Vitvi13g02493\_t001 |  |  |  |  |  |  |  |  |
| 0 | Vvi-Vitvi13g02494\_t001 |  |  |  |  |  |  |  |  |
| 0 | Vvi-Vitvi13g04740\_t001 |  |  |  |  |  |  |  |  |
| 0 | Vvi-Vitvi13g02496\_t001 |  |  |  |  |  |  |  |  |
| 0 | Vvi-Vitvi13g02497\_t001 |  |  |  |  |  |  |  |  |
| 0 | Vvi-Vitvi13g04741\_t001 |  |  |  |  |  |  |  |  |
| 0 | Vvi-Vitvi13g04742\_t001 |  |  |  |  |  |  |  |  |
| 0 | Vvi-Vitvi13g04743\_t001 |  |  |  |  |  |  |  |  |
| 0 | Vvi-Vitvi13g01724\_t001 |  |  |  |  |  |  |  |  |
| 0 | Vvi-Vitvi13g04744\_t001 |  |  |  |  |  |  |  |  |
| 0 | Vvi-Vitvi13g04745\_t001 |  |  |  |  |  |  |  |  |
| 0 | Vvi-Vitvi13g02502\_t001 |  |  |  |  |  |  |  |  |
| 0 | Vvi-Vitvi13g04746\_t001 |  |  |  |  |  |  |  |  |
| 0 | Vvi-Vitvi13g04747\_t001 |  |  |  |  |  |  |  |  |
| 0 | Vvi-Vitvi13g01727\_t002 |  |  |  |  |  |  |  |  |
| 0 | Vvi-Vitvi13g04748\_t001 |  |  |  |  |  |  |  |  |
| 0 | Vvi-Vitvi13g01728\_t001 |  |  |  |  |  |  |  |  |
| 0 | Vvi-Vitvi13g01729\_t002 |  |  |  |  |  |  |  |  |
| 0 | Vvi-Vitvi13g02503\_t001 |  |  |  |  |  |  |  |  |
| 0 | Vvi-Vitvi13g04749\_t001 |  |  |  |  |  |  |  |  |
| 0 | Vvi-Vitvi13g04750\_t001 |  |  |  |  |  |  |  |  |
| 0 | Vvi-Vitvi13g01731\_t001 |  |  |  |  |  |  |  |  |
| 0 | Vvi-Vitvi13g04751\_t001 |  |  |  |  |  |  |  |  |
| 0 | Vvi-Vitvi13g02505\_t001 |  |  |  |  |  |  |  |  |
| 0 | Vvi-Vitvi13g01732\_t001 |  |  |  |  |  |  |  |  |
| 0 | Vvi-Vitvi13g01733\_t001 |  |  |  |  |  |  |  |  |
| 0 | Vvi-Vitvi13g01734\_t001 |  |  |  |  |  |  |  |  |
| 0 | Vvi-Vitvi13g01735\_t001 |  |  |  |  |  |  |  |  |
| 0 | Vvi-Vitvi13g04752\_t001 |  |  |  |  |  |  |  |  |
| 0 | Vvi-Vitvi13g01736\_t001 |  |  |  |  |  |  |  |  |
| 0 | Vvi-Vitvi13g01737\_t001 |  |  |  |  |  |  |  |  |
| 0 | Vvi-Vitvi13g01738\_t001 |  |  |  |  |  |  |  |  |
| 0 | Vvi-Vitvi13g01739\_t001 |  |  |  |  |  |  |  |  |
| 0 | Vvi-Vitvi13g01740\_t001 |  |  |  |  |  |  |  |  |
| 0 | Vvi-Vitvi13g01743\_t001 |  |  |  |  |  |  |  |  |
| 0 | Vvi-Vitvi13g01744\_t001 |  |  |  |  |  |  |  |  |
| 0 | Vvi-Vitvi13g02506\_t001 |  |  |  |  |  |  |  |  |
| 0 | Vvi-Vitvi13g01745\_t001 |  |  |  |  |  |  |  |  |
| 0 | Vvi-Vitvi13g04753\_t001 |  |  |  |  |  |  |  |  |
| 0 | Vvi-Vitvi13g01748\_t001 |  |  |  |  |  |  |  |  |
| 0 | Vvi-Vitvi13g02507\_t001 |  |  |  |  |  |  |  |  |
| 0 | Vvi-Vitvi13g01749\_t001 |  |  |  |  |  |  |  |  |
| 0 | Vvi-Vitvi13g01750\_t002 |  |  |  |  |  |  |  |  |
| 0 | Vvi-Vitvi13g02508\_t001 |  |  |  |  |  |  |  |  |
| 0 | Vvi-Vitvi13g04754\_t001 |  |  |  |  |  |  |  |  |
| 0 | Vvi-Vitvi13g02509\_t001 |  |  |  |  |  |  |  |  |
| 0 | Vvi-Vitvi13g02510\_t001 |  |  |  |  |  |  |  |  |
| 0 | Vvi-Vitvi13g04755\_t001 |  |  |  |  |  |  |  |  |
| 0 | Vvi-Vitvi13g04756\_t001 |  |  |  |  |  |  |  |  |
| 0 | Vvi-Vitvi13g04757\_t001 |  |  |  |  |  |  |  |  |
| 0 | Vvi-Vitvi13g04758\_t002 |  |  |  |  |  |  |  |  |
| 0 | Vvi-Vitvi13g02577\_t001 |  |  |  |  |  |  |  |  |
| 0 | Vvi-Vitvi13g04759\_t001 |  |  |  |  |  |  |  |  |
| 0 | Vvi-Vitvi13g04760\_t001 |  |  |  |  |  |  |  |  |
| 0 | Vvi-Vitvi13g01842\_t001 |  |  |  |  |  |  |  |  |
| 0 | Vvi-Vitvi13g01841\_t001 |  |  |  |  |  |  |  |  |
| 1 | Vvi-Vitvi13g01840\_t001 |  | Ath-AT2G44280.2 |  |  |  |  |  |  |  |
| 1 | Vvi-Vitvi13g01839\_t001 |  | Ath-AT2G44290.1 |  |  |  |  |  |  |  |
| 1 | Vvi-Vitvi13g02576\_t001 |  | | | |  |  |  |  |  |  |  |
| 1 | Vvi-Vitvi13g01837\_t001 |  | | | |  |  |  |  |  |  |  |
| 1 | Vvi-Vitvi13g01836\_t001 |  | Ath-AT2G44310.1 |  |  |  |  |  |  |  |
| 1 | Vvi-Vitvi13g01834\_t001 |  | | | |  |  |  |  |  |  |  |
| 1 | Vvi-Vitvi13g02574\_t001 |  | | | |  |  |  |  |  |  |  |
| 1 | Vvi-Vitvi13g02573\_t001 |  | | | |  |  |  |  |  |  |  |
| 1 | Vvi-Vitvi13g04761\_t001 |  | | | |  |  |  |  |  |  |  |
| 1 | Vvi-Vitvi13g04762\_t001 |  | | | |  |  |  |  |  |  |  |
| 1 | Vvi-Vitvi13g02572\_t001 |  | | | |  |  |  |  |  |  |  |
| 1 | Vvi-Vitvi13g02571\_t001 |  | Ath-AT2G44340.1 |  |  |  |  |  |  |  |
| 1 | Vvi-Vitvi13g01833\_t002 |  | | | |  |  |  |  |  |  |  |
| 1 | Vvi-Vitvi13g01832\_t001 |  | Ath-AT2G44350.2 |  |  |  |  |  |  |  |
| 1 | Vvi-Vitvi13g04763\_t001 |  | | | |  |  |  |  |  |  |  |
| 1 | Vvi-Vitvi13g01831\_t001 |  | Ath-AT2G44360.1 |  |  |  |  |  |  |  |
| 1 | Vvi-Vitvi13g04764\_t001 |  | | | |  |  |  |  |  |  |  |
| 1 | Vvi-Vitvi13g01828\_t001 |  | | | |  |  |  |  |  |  |  |
| 1 | Vvi-Vitvi13g01827\_t001 |  | | | |  |  |  |  |  |  |  |
| 1 | Vvi-Vitvi13g04765\_t001 |  | Ath-AT2G44370.1 |  |  |  |  |  |  |  |
| 1 | Vvi-Vitvi13g04766\_t001 |  | | | |  |  |  |  |  |  |  |
| 1 | Vvi-Vitvi13g04767\_t001 |  | | | |  |  |  |  |  |  |  |
| 1 | Vvi-Vitvi13g01825\_t001 |  | Ath-AT2G44400.1 |  |  |  |  |  |  |  |
| 1 | Vvi-Vitvi13g01824\_t001 |  | Ath-AT2G44410.1 |  |  |  |  |  |  |  |
| 1 | Vvi-Vitvi13g01823\_t001 |  | | | |  |  |  |  |  |  |  |
| 1 | Vvi-Vitvi13g01822\_t001 |  | | | |  |  |  |  |  |  |  |
| 1 | Vvi-Vitvi13g01820\_t002 |  | Ath-AT2G44420.2 |  |  |  |  |  |  |  |
| 1 | Vvi-Vitvi13g01819\_t001 |  | | | |  |  |  |  |  |  |  |
| 1 | Vvi-Vitvi13g01818\_t001 |  | | | |  |  |  |  |  |  |  |
| 1 | Vvi-Vitvi13g02569\_t001 |  | Ath-AT2G44430.1 |  |  |  |  |  |  |  |
| 1 | Vvi-Vitvi13g01817\_t001 |  | | | |  |  |  |  |  |  |  |
| 1 | Vvi-Vitvi13g01816\_t001 |  | Ath-AT2G44440.1 |  |  |  |  |  |  |  |
| 0 | Vvi-Vitvi13g02568\_t001 |  |  |  |  |  |  |  |  |
| 0 | Vvi-Vitvi13g04768\_t001 |  |  |  |  |  |  |  |  |
| 0 | Vvi-Vitvi13g04769\_t001 |  |  |  |  |  |  |  |  |
| 0 | Vvi-Vitvi13g02566\_t001 |  |  |  |  |  |  |  |  |
| 0 | Vvi-Vitvi13g04770\_t001 |  |  |  |  |  |  |  |  |
| 0 | Vvi-Vitvi13g04771\_t001 |  |  |  |  |  |  |  |  |
| 0 | Vvi-Vitvi13g04772\_t001 |  |  |  |  |  |  |  |  |
| 0 | Vvi-Vitvi13g04773\_t001 |  |  |  |  |  |  |  |  |
| 0 | Vvi-Vitvi13g04774\_t001 |  |  |  |  |  |  |  |  |
| 0 | Vvi-Vitvi13g02560\_t001 |  |  |  |  |  |  |  |  |
| 0 | Vvi-Vitvi13g04775\_t001 |  |  |  |  |  |  |  |  |
| 0 | Vvi-Vitvi13g04776\_t001 |  |  |  |  |  |  |  |  |
| 0 | Vvi-Vitvi13g04777\_t001 |  |  |  |  |  |  |  |  |
| 0 | Vvi-Vitvi13g01813\_t001 |  |  |  |  |  |  |  |  |
| 0 | Vvi-Vitvi13g04778\_t001 |  |  |  |  |  |  |  |  |
| 0 | Vvi-Vitvi13g01812\_t001 |  |  |  |  |  |  |  |  |
| 0 | Vvi-Vitvi13g01811\_t001 |  |  |  |  |  |  |  |  |
| 0 | Vvi-Vitvi13g04779\_t001 |  |  |  |  |  |  |  |  |
| 0 | Vvi-Vitvi13g04780\_t001 |  |  |  |  |  |  |  |  |
| 0 | Vvi-Vitvi13g04781\_t001 |  |  |  |  |  |  |  |  |
| 0 | Vvi-Vitvi13g04782\_t001 |  |  |  |  |  |  |  |  |
| 0 | Vvi-Vitvi13g04783\_t001 |  |  |  |  |  |  |  |  |
| 0 | Vvi-Vitvi13g04784\_t001 |  |  |  |  |  |  |  |  |
| 0 | Vvi-Vitvi13g04785\_t001 |  |  |  |  |  |  |  |  |
| 0 | Vvi-Vitvi13g04786\_t001 |  |  |  |  |  |  |  |  |
| 0 | Vvi-Vitvi13g04787\_t001 |  |  |  |  |  |  |  |  |
| 0 | Vvi-Vitvi13g04788\_t001 |  |  |  |  |  |  |  |  |
| 0 | Vvi-Vitvi13g02554\_t001 |  |  |  |  |  |  |  |  |
| 0 | Vvi-Vitvi13g02553\_t001 |  |  |  |  |  |  |  |  |
| 0 | Vvi-Vitvi13g02552\_t001 |  |  |  |  |  |  |  |  |
| 0 | Vvi-Vitvi13g04789\_t001 |  |  |  |  |  |  |  |  |
| 0 | Vvi-Vitvi13g02551\_t001 |  |  |  |  |  |  |  |  |
| 0 | Vvi-Vitvi13g04790\_t001 |  |  |  |  |  |  |  |  |
| 0 | Vvi-Vitvi13g02549\_t001 |  |  |  |  |  |  |  |  |
| 0 | Vvi-Vitvi13g02548\_t001 |  |  |  |  |  |  |  |  |
| 0 | Vvi-Vitvi13g04791\_t001 |  |  |  |  |  |  |  |  |
| 0 | Vvi-Vitvi13g04792\_t001 |  |  |  |  |  |  |  |  |
| 0 | Vvi-Vitvi13g01805\_t001 |  |  |  |  |  |  |  |  |
| 0 | Vvi-Vitvi13g01803\_t001 |  |  |  |  |  |  |  |  |
| 0 | Vvi-Vitvi13g01849\_t001 |  |  |  |  |  |  |  |  |
| 0 | Vvi-Vitvi13g01851\_t001 |  |  |  |  |  |  |  |  |
| 0 | Vvi-Vitvi13g01852\_t001 |  |  |  |  |  |  |  |  |
| 0 | Vvi-Vitvi13g01853\_t001 |  |  |  |  |  |  |  |  |
| 0 | Vvi-Vitvi13g04793\_t001 |  |  |  |  |  |  |  |  |
| 0 | Vvi-Vitvi13g01854\_t001 |  |  |  |  |  |  |  |  |
| 0 | Vvi-Vitvi13g01855\_t001 |  |  |  |  |  |  |  |  |
| 0 | Vvi-Vitvi13g01857\_t001 |  |  |  |  |  |  |  |  |
| 0 | Vvi-Vitvi13g04794\_t001 |  |  |  |  |  |  |  |  |
