# Supplementary material for: Genome-wide identification, characterization and gene expression of BES1 transcription factor family in grapevine (Vitis vinifera L.)
Source: Sci Rep. 2023 Jan 5;13:240. doi: 10.1038/s41598-022-24407-y (PMC9816167; doi:10.1038/s41598-022-24407-y)
Supplement: Supplementary file 3 — Supplementary Information. [file 41598_2022_24407_MOESM3_ESM.zip › Vvi_Ath/Vitis_vinifera.PN40024.v4.dna_sm.toplevel.fa.vs.Arabidopsis_thaliana.TAIR10.dna_sm.toplevel.fa.html/Vvi-2.html]

|  |  |  |  |  |  |  |  |  |  |  |  |  |  |  |  |  |  |
| --- | --- | --- | --- | --- | --- | --- | --- | --- | --- | --- | --- | --- | --- | --- | --- | --- | --- |
| Duplication depth | Reference chromosome | Collinear blocks | | | | | | | | | | | | | | | |
| 0 | Vvi-Vitvi02g00001\_t001 |  |  |  |  |  |  |  |  |
| 1 | Vvi-Vitvi02g00002\_t001 |  | Ath-AT4G11400.1 |  |  |  |  |  |  |  |
| 1 | Vvi-Vitvi02g04000\_t001 |  | | | |  |  |  |  |  |  |  |
| 4 | Vvi-Vitvi02g04001\_t001 |  | | | |  | Ath-AT5G41340.1 |  | Ath-AT1G63800.1 |  | Ath-AT2G46030.3 |  |  |  |  |
| 4 | Vvi-Vitvi02g00006\_t001 |  | | | |  | | | |  | | | |  | | | |  |  |  |  |
| 4 | Vvi-Vitvi02g04002\_t001 |  | | | |  | | | |  | | | |  | | | |  |  |  |  |
| 4 | Vvi-Vitvi02g00007\_t001 |  | | | |  | | | |  | | | |  | | | |  |  |  |  |
| 5 | Vvi-Vitvi02g00008\_t001 |  | Ath-AT4G11380.2 |  | | | |  | | | |  | | | |  | Ath-AT4G23460.1 |  |  |  |
| 5 | Vvi-Vitvi02g00009\_t001 |  | | | |  | Ath-AT5G41360.1 |  | | | |  | | | |  | | | |  |  |  |
| 5 | Vvi-Vitvi02g00011\_t001 |  | | | |  | Ath-AT5G41380.1 |  | Ath-AT1G63820.1 |  | | | |  | | | |  |  |  |
| 5 | Vvi-Vitvi02g04003\_t002 |  | | | |  | Ath-AT5G41390.1 |  | Ath-AT1G63830.3 |  | | | |  | Ath-AT4G23470.1 |  |  |  |
| 5 | Vvi-Vitvi02g00013\_t001 |  | Ath-AT4G11360.1 |  | Ath-AT5G41400.1 |  | Ath-AT1G63840.1 |  | | | |  | | | |  |  |  |
| 5 | Vvi-Vitvi02g00014\_t001 |  | | | |  | | | |  | Ath-AT1G63850.1 |  | | | |  | | | |  |  |  |
| 6 | Vvi-Vitvi02g01312\_t001 |  | | | |  | | | |  | | | |  | | | |  | | | |  | Ath-AT5G52530.3 |  |  |
| 6 | Vvi-Vitvi02g04004\_t001 |  | | | |  | | | |  | | | |  | | | |  | | | |  | | | |  |  |
| 6 | Vvi-Vitvi02g00015\_t001 |  | | | |  | | | |  | | | |  | | | |  | | | |  | | | |  |  |
| 6 | Vvi-Vitvi02g04005\_t001 |  | | | |  | | | |  | | | |  | | | |  | | | |  | | | |  |  |
| 6 | Vvi-Vitvi02g00016\_t001 |  | | | |  | Ath-AT5G41410.1 |  | | | |  | | | |  | | | |  | | | |  |  |
| 6 | Vvi-Vitvi02g00017\_t001 |  | Ath-AT4G11350.1 |  | Ath-AT5G41460.1 |  | | | |  | | | |  | Ath-AT4G23490.1 |  | | | |  |  |
| 6 | Vvi-Vitvi02g00018\_t001 |  | | | |  | | | |  | | | |  | | | |  | | | |  | | | |  |  |
| 6 | Vvi-Vitvi02g04006\_t001 |  | | | |  | | | |  | | | |  | | | |  | | | |  | | | |  |  |
| 6 | Vvi-Vitvi02g01313\_t001 |  | | | |  | | | |  | | | |  | | | |  | | | |  | | | |  |  |
| 6 | Vvi-Vitvi02g00020\_t001 |  | | | |  | | | |  | | | |  | | | |  | | | |  | Ath-AT5G52570.1 |  |  |
| 6 | Vvi-Vitvi02g00021\_t001 |  | | | |  | | | |  | | | |  | | | |  | Ath-AT4G23496.1 |  | | | |  |  |
| 6 | Vvi-Vitvi02g00022\_t001 |  | | | |  | | | |  | | | |  | | | |  | Ath-AT4G23500.1 |  | | | |  |  |
| 6 | Vvi-Vitvi02g00023\_t001 |  | Ath-AT4G11330.1 |  | | | |  | | | |  | Ath-AT2G46070.3 |  | | | |  | | | |  |  |
| 6 | Vvi-Vitvi02g00025\_t001 |  | | | |  | | | |  | | | |  | | | |  | | | |  | Ath-AT5G52640.1 |  |  |
| 6 | Vvi-Vitvi02g01314\_t001 |  | | | |  | | | |  | | | |  | | | |  | | | |  | | | |  |  |
| 6 | Vvi-Vitvi02g01315\_t001 |  | | | |  | Ath-AT5G41520.1 |  | | | |  | | | |  | | | |  | Ath-AT5G52650.1 |  |  |
| 6 | Vvi-Vitvi02g00028\_t001 |  | | | |  | | | |  | Ath-AT1G63910.1 |  | | | |  | | | |  | | | |  |  |
| 6 | Vvi-Vitvi02g00029\_t001 |  | Ath-AT4G11300.1 |  | | | |  | Ath-AT1G63930.1 |  | | | |  | Ath-AT4G23530.1 |  | | | |  |  |
| 6 | Vvi-Vitvi02g00030\_t001 |  | | | |  | | | |  | Ath-AT1G63940.2 |  | | | |  | | | |  | | | |  |  |
| 6 | Vvi-Vitvi02g00031\_t001 |  | | | |  | | | |  | Ath-AT1G63950.2 |  | | | |  | | | |  | Ath-AT5G52740.1 |  |  |
| 6 | Vvi-Vitvi02g00032\_t001 |  | Ath-AT4G11280.1 |  | | | |  | | | |  | | | |  | | | |  | | | |  |  |
| 6 | Vvi-Vitvi02g00034\_t001 |  | | | |  | | | |  | Ath-AT1G63970.1 |  | | | |  | | | |  | | | |  |  |
| 6 | Vvi-Vitvi02g00035\_t001 |  | | | |  | Ath-AT5G41560.1 |  | | | |  | | | |  | | | |  | | | |  |  |
| 6 | Vvi-Vitvi02g00037\_t002 |  | | | |  | | | |  | Ath-AT1G63980.1 |  | | | |  | | | |  | | | |  |  |
| 6 | Vvi-Vitvi02g00038\_t001 |  | | | |  | | | |  | Ath-AT1G63990.1 |  | | | |  | | | |  | | | |  |  |
| 6 | Vvi-Vitvi02g00039\_t001 |  | | | |  | | | |  | | | |  | | | |  | Ath-AT4G23550.1 |  | Ath-AT5G52830.1 |  |  |
| 6 | Vvi-Vitvi02g00040\_t001 |  | | | |  | | | |  | | | |  | | | |  | Ath-AT4G23560.1 |  | | | |  |  |
| 6 | Vvi-Vitvi02g00041\_t001 |  | Ath-AT4G11270.1 |  | | | |  | | | |  | | | |  | | | |  | | | |  |  |
| 6 | Vvi-Vitvi02g00042\_t001 |  | Ath-AT4G11260.1 |  | | | |  | | | |  | | | |  | Ath-AT4G23570.3 |  | | | |  |  |
| 6 | Vvi-Vitvi02g00043\_t001 |  | | | |  | Ath-AT5G41580.1 |  | | | |  | | | |  | | | |  | | | |  |  |
| 6 | Vvi-Vitvi02g00044\_t001 |  | Ath-AT4G11240.1 |  | | | |  | Ath-AT1G64040.1 |  | | | |  | | | |  | | | |  |  |
| 6 | Vvi-Vitvi02g00045\_t001 |  | | | |  | | | |  | Ath-AT1G64050.1 |  | | | |  | | | |  | | | |  |  |
| 6 | Vvi-Vitvi02g00046\_t001 |  | | | |  | Ath-AT5G41590.1 |  | | | |  | | | |  | | | |  | | | |  |  |
| 6 | Vvi-Vitvi02g00048\_t001 |  | Ath-AT4G11230.1 |  | | | |  | Ath-AT1G64060.1 |  | | | |  | | | |  | | | |  |  |
| 6 | Vvi-Vitvi02g01317\_t001 |  | | | |  | | | |  | Ath-AT1G64065.1 |  | Ath-AT2G46150.1 |  | | | |  | | | |  |  |
| 6 | Vvi-Vitvi02g01318\_t001 |  | | | |  | | | |  | | | |  | | | |  | | | |  | | | |  |  |
| 6 | Vvi-Vitvi02g01319\_t001 |  | | | |  | | | |  | | | |  | | | |  | | | |  | | | |  |  |
| 6 | Vvi-Vitvi02g04007\_t001 |  | | | |  | | | |  | | | |  | | | |  | | | |  | | | |  |  |
| 6 | Vvi-Vitvi02g00051\_t001 |  | | | |  | | | |  | Ath-AT1G64080.1 |  | | | |  | | | |  | Ath-AT5G52870.1 |  |  |
| 6 | Vvi-Vitvi02g00052\_t001 |  | | | |  | | | |  | | | |  | | | |  | | | |  | | | |  |  |
| 6 | Vvi-Vitvi02g00053\_t001 |  | | | |  | | | |  | | | |  | | | |  | | | |  | | | |  |  |
| 6 | Vvi-Vitvi02g00054\_t001 |  | | | |  | | | |  | | | |  | | | |  | | | |  | | | |  |  |
| 6 | Vvi-Vitvi02g00055\_t001 |  | | | |  | | | |  | | | |  | | | |  | | | |  | | | |  |  |
| 6 | Vvi-Vitvi02g00056\_t001 |  | | | |  | | | |  | | | |  | | | |  | | | |  | | | |  |  |
| 6 | Vvi-Vitvi02g01321\_t001 |  | | | |  | | | |  | | | |  | | | |  | Ath-AT4G23620.2 |  | | | |  |  |
| 6 | Vvi-Vitvi02g00059\_t001 |  | Ath-AT4G11220.1 |  | Ath-AT5G41600.1 |  | Ath-AT1G64090.2 |  | Ath-AT2G46170.1 |  | Ath-AT4G23630.1 |  | | | |  |  |
| 6 | Vvi-Vitvi02g00060\_t001 |  | | | |  | | | |  | Ath-AT1G64110.2 |  | | | |  | | | |  | Ath-AT5G52882.1 |  |  |
| 5 | Vvi-Vitvi02g00062\_t001 |  | | | |  | | | |  | | | |  | | | |  | | | |  |  |  |
| 5 | Vvi-Vitvi02g00063\_t001 |  | | | |  | | | |  | | | |  | | | |  | Ath-AT4G23640.1 |  |  |  |
| 5 | Vvi-Vitvi02g00065\_t002 |  | | | |  | | | |  | | | |  | | | |  | Ath-AT4G23650.1 |  |  |  |
| 5 | Vvi-Vitvi02g00066\_t001 |  | | | |  | | | |  | | | |  | | | |  | | | |  |  |  |
| 5 | Vvi-Vitvi02g00067\_t001 |  | | | |  | | | |  | Ath-AT1G64140.1 |  | | | |  | | | |  |  |  |
| 5 | Vvi-Vitvi02g04008\_t001 |  | | | |  | | | |  | | | |  | | | |  | | | |  |  |  |
| 5 | Vvi-Vitvi02g00068\_t001 |  | | | |  | | | |  | Ath-AT1G64150.1 |  | | | |  | | | |  |  |  |
| 5 | Vvi-Vitvi02g00069\_t001 |  | Ath-AT4G11180.1 |  | | | |  | Ath-AT1G64160.1 |  | | | |  | Ath-AT4G23690.1 |  |  |  |
| 5 | Vvi-Vitvi02g00070\_t001 |  | | | |  | | | |  | | | |  | | | |  | | | |  |  |  |
| 5 | Vvi-Vitvi02g01323\_t001 |  | | | |  | | | |  | | | |  | | | |  | | | |  |  |  |
| 5 | Vvi-Vitvi02g01324\_t001 |  | | | |  | | | |  | | | |  | | | |  | | | |  |  |  |
| 5 | Vvi-Vitvi02g01325\_t001 |  | | | |  | | | |  | | | |  | | | |  | | | |  |  |  |
| 5 | Vvi-Vitvi02g00071\_t001 |  | | | |  | Ath-AT5G41610.1 |  | Ath-AT1G64170.1 |  | | | |  | Ath-AT4G23700.2 |  |  |  |
| 5 | Vvi-Vitvi02g04009\_t001 |  | | | |  | | | |  | | | |  | | | |  | | | |  |  |  |
| 5 | Vvi-Vitvi02g04010\_t001 |  | | | |  | | | |  | | | |  | | | |  | | | |  |  |  |
| 5 | Vvi-Vitvi02g01327\_t001 |  | | | |  | | | |  | | | |  | | | |  | | | |  |  |  |
| 5 | Vvi-Vitvi02g01328\_t001 |  | | | |  | | | |  | | | |  | | | |  | | | |  |  |  |
| 5 | Vvi-Vitvi02g00073\_t001 |  | | | |  | | | |  | | | |  | | | |  | | | |  |  |  |
| 6 | Vvi-Vitvi02g00075\_t001 |  | | | |  | | | |  | | | |  | | | |  | | | |  | Ath-AT3G61590.4 |  |  |
| 6 | Vvi-Vitvi02g04011\_t001 |  | | | |  | | | |  | | | |  | | | |  | | | |  | | | |  |  |
| 6 | Vvi-Vitvi02g01329\_t001 |  | | | |  | Ath-AT5G41620.1 |  | Ath-AT1G64180.1 |  | Ath-AT2G46250.1 |  | | | |  | | | |  |  |
| 6 | Vvi-Vitvi02g00076\_t001 |  | | | |  | Ath-AT5G41650.1 |  | Ath-AT1G64185.1 |  | | | |  | | | |  | | | |  |  |
| 6 | Vvi-Vitvi02g00077\_t001 |  | | | |  | | | |  | | | |  | | | |  | | | |  | | | |  |  |
| 7 | Vvi-Vitvi02g00078\_t001 |  | | | |  | | | |  | | | |  | Ath-AT2G46260.1 |  | | | |  | Ath-AT3G61600.2 |  | Ath-AT4G01160.2 |  |
| 7 | Vvi-Vitvi02g00079\_t001 |  | | | |  | | | |  | | | |  | | | |  | Ath-AT4G23720.1 |  | | | |  | Ath-AT4G01140.1 |  |
| 7 | Vvi-Vitvi02g00080\_t001 |  | | | |  | Ath-AT5G41670.2 |  | Ath-AT1G64190.1 |  | | | |  | | | |  | | | |  | | | |  |
| 7 | Vvi-Vitvi02g00081\_t001 |  | | | |  | | | |  | | | |  | | | |  | | | |  | | | |  | | | |  |
| 7 | Vvi-Vitvi02g01331\_t001 |  | | | |  | | | |  | | | |  | | | |  | | | |  | | | |  | | | |  |
| 7 | Vvi-Vitvi02g00082\_t002 |  | | | |  | | | |  | | | |  | | | |  | | | |  | | | |  | | | |  |
| 7 | Vvi-Vitvi02g01333\_t001 |  | Ath-AT4G11150.1 |  | | | |  | Ath-AT1G64200.2 |  | | | |  | | | |  | | | |  | | | |  |
| 7 | Vvi-Vitvi02g00083\_t001 |  | | | |  | | | |  | | | |  | | | |  | | | |  | | | |  | | | |  |
| 7 | Vvi-Vitvi02g00085\_t001 |  | | | |  | | | |  | | | |  | | | |  | | | |  | | | |  | | | |  |
| 7 | Vvi-Vitvi02g00086\_t001 |  | | | |  | | | |  | | | |  | | | |  | | | |  | | | |  | | | |  |
| 7 | Vvi-Vitvi02g00087\_t001 |  | | | |  | | | |  | | | |  | | | |  | | | |  | | | |  | | | |  |
| 7 | Vvi-Vitvi02g00088\_t001 |  | | | |  | | | |  | | | |  | | | |  | | | |  | | | |  | | | |  |
| 7 | Vvi-Vitvi02g04012\_t001 |  | | | |  | | | |  | | | |  | | | |  | | | |  | | | |  | | | |  |
| 7 | Vvi-Vitvi02g00089\_t001 |  | | | |  | | | |  | | | |  | Ath-AT2G46270.1 |  | | | |  | | | |  | Ath-AT4G01120.1 |  |
| 7 | Vvi-Vitvi02g00090\_t001 |  | | | |  | | | |  | | | |  | | | |  | Ath-AT4G23730.3 |  | Ath-AT3G61610.1 |  | | | |  |
| 7 | Vvi-Vitvi02g00091\_t003 |  | | | |  | | | |  | Ath-AT1G64210.1 |  | | | |  | Ath-AT4G23740.1 |  | | | |  | | | |  |
| 7 | Vvi-Vitvi02g04013\_t001 |  | | | |  | Ath-AT5G41700.4 |  | | | |  | | | |  | | | |  | | | |  | | | |  |
| 7 | Vvi-Vitvi02g00093\_t001 |  | Ath-AT4G11140.1 |  | | | |  | | | |  | | | |  | Ath-AT4G23750.1 |  | Ath-AT3G61630.1 |  | | | |  |
| 7 | Vvi-Vitvi02g01334\_t001 |  | | | |  | | | |  | | | |  | | | |  | | | |  | | | |  | | | |  |
| 7 | Vvi-Vitvi02g00095\_t001 |  | | | |  | | | |  | | | |  | | | |  | Ath-AT4G23760.1 |  | | | |  | | | |  |
| 7 | Vvi-Vitvi02g01335\_t001 |  | | | |  | | | |  | | | |  | Ath-AT2G46330.1 |  | | | |  | Ath-AT3G61640.1 |  | | | |  |
| 7 | Vvi-Vitvi02g04014\_t001 |  | | | |  | | | |  | | | |  | | | |  | | | |  | | | |  | | | |  |
| 7 | Vvi-Vitvi02g00096\_t001 |  | | | |  | Ath-AT5G41761.1 |  | | | |  | | | |  | | | |  | | | |  | | | |  |
| 7 | Vvi-Vitvi02g00097\_t001 |  | | | |  | | | |  | Ath-AT1G64300.2 |  | | | |  | | | |  | | | |  | | | |  |
| 7 | Vvi-Vitvi02g00098\_t001 |  | Ath-AT4G11110.1 |  | | | |  | | | |  | Ath-AT2G46340.1 |  | | | |  | | | |  | | | |  |
| 7 | Vvi-Vitvi02g01336\_t001 |  | | | |  | | | |  | | | |  | | | |  | | | |  | | | |  | | | |  |
| 7 | Vvi-Vitvi02g00099\_t001 |  | | | |  | | | |  | | | |  | | | |  | | | |  | | | |  | | | |  |
| 7 | Vvi-Vitvi02g00100\_t001 |  | | | |  | Ath-AT5G41770.1 |  | | | |  | | | |  | | | |  | | | |  | | | |  |
| 7 | Vvi-Vitvi02g04015\_t001 |  | | | |  | Ath-AT5G41780.2 |  | Ath-AT1G64320.1 |  | | | |  | | | |  | | | |  | | | |  |
| 7 | Vvi-Vitvi02g00101\_t003 |  | | | |  | | | |  | | | |  | | | |  | | | |  | | | |  | | | |  |
| 7 | Vvi-Vitvi02g00103\_t001 |  | | | |  | | | |  | | | |  | | | |  | | | |  | | | |  | Ath-AT4G01100.2 |  |
| 7 | Vvi-Vitvi02g04016\_t001 |  | | | |  | | | |  | | | |  | | | |  | | | |  | | | |  | | | |  |
| 7 | Vvi-Vitvi02g00105\_t001 |  | | | |  | | | |  | | | |  | Ath-AT2G46380.1 |  | | | |  | Ath-AT3G61670.1 |  | | | |  |
| 6 | Vvi-Vitvi02g00106\_t001 |  | Ath-AT4G11090.1 |  | | | |  | | | |  | | | |  | Ath-AT4G23790.1 |  |  |  | Ath-AT4G01080.1 |  |
| 6 | Vvi-Vitvi02g00108\_t001 |  | Ath-AT4G11080.1 |  | | | |  | | | |  | | | |  | Ath-AT4G23800.1 |  |  |  | | | |  |
| 6 | Vvi-Vitvi02g00109\_t001 |  | | | |  | Ath-AT5G41800.1 |  | | | |  | | | |  | | | |  |  |  | | | |  |
| 6 | Vvi-Vitvi02g01337\_t001 |  | | | |  | Ath-AT5G41810.1 |  | Ath-AT1G64340.1 |  | | | |  | | | |  |  |  | | | |  |
| 6 | Vvi-Vitvi02g00110\_t001 |  | | | |  | | | |  | | | |  | | | |  | | | |  |  |  | Ath-AT4G01070.1 |  |
| 6 | Vvi-Vitvi02g00111\_t001 |  | | | |  | | | |  | Ath-AT1G64350.1 |  | | | |  | | | |  |  |  | | | |  |
| 6 | Vvi-Vitvi02g00112\_t001 |  | | | |  | | | |  | | | |  | | | |  | | | |  |  |  | | | |  |
| 6 | Vvi-Vitvi02g00113\_t001 |  | | | |  | | | |  | Ath-AT1G64355.1 |  | | | |  | | | |  |  |  | | | |  |
| 6 | Vvi-Vitvi02g00114\_t001 |  | Ath-AT4G11070.1 |  | | | |  | | | |  | Ath-AT2G46400.1 |  | Ath-AT4G23810.1 |  |  |  | | | |  |
| 6 | Vvi-Vitvi02g04017\_t001 |  | | | |  | | | |  | | | |  | | | |  | | | |  |  |  | | | |  |
| 6 | Vvi-Vitvi02g00115\_t001 |  | | | |  | Ath-AT5G41850.1 |  | | | |  | | | |  | | | |  |  |  | | | |  |
| 6 | Vvi-Vitvi02g00116\_t001 |  | | | |  | | | |  | | | |  | | | |  | | | |  |  |  | | | |  |
| 6 | Vvi-Vitvi02g01338\_t001 |  | | | |  | | | |  | | | |  | | | |  | | | |  |  |  | | | |  |
| 6 | Vvi-Vitvi02g00117\_t001 |  | | | |  | | | |  | | | |  | | | |  | | | |  |  |  | | | |  |
| 6 | Vvi-Vitvi02g00118\_t003 |  | | | |  | Ath-AT5G41870.1 |  | | | |  | | | |  | Ath-AT4G23820.1 |  |  |  | | | |  |
| 6 | Vvi-Vitvi02g00119\_t001 |  | | | |  | | | |  | | | |  | | | |  | | | |  |  |  | Ath-AT4G01026.1 |  |
| 5 | Vvi-Vitvi02g00120\_t001 |  | | | |  | Ath-AT5G41880.1 |  | | | |  | | | |  | | | |  |  |  |
| 4 | Vvi-Vitvi02g00121\_t001 |  | | | |  |  |  | Ath-AT1G64380.1 |  | | | |  | | | |  |  |  |
| 4 | Vvi-Vitvi02g04018\_t001 |  | | | |  |  |  | | | |  | | | |  | | | |  |  |  |
| 4 | Vvi-Vitvi02g00123\_t001 |  | | | |  |  |  | Ath-AT1G64385.1 |  | | | |  | | | |  |  |  |
| 4 | Vvi-Vitvi02g00125\_t001 |  | Ath-AT4G11050.1 |  |  |  | Ath-AT1G64390.1 |  | | | |  | | | |  |  |  |
| 4 | Vvi-Vitvi02g00126\_t001 |  | | | |  |  |  | | | |  | | | |  | | | |  |  |  |
| 4 | Vvi-Vitvi02g00127\_t001 |  | | | |  |  |  | | | |  | | | |  | Ath-AT4G23840.1 |  |  |  |
| 4 | Vvi-Vitvi02g00128\_t001 |  | Ath-AT4G11030.1 |  |  |  | Ath-AT1G64400.1 |  | | | |  | Ath-AT4G23850.1 |  |  |  |
| 4 | Vvi-Vitvi02g00129\_t001 |  | | | |  |  |  | | | |  | Ath-AT2G46470.1 |  | | | |  |  |  |
| 4 | Vvi-Vitvi02g00130\_t001 |  | | | |  |  |  | | | |  | Ath-AT2G46494.1 |  | | | |  |  |  |
| 4 | Vvi-Vitvi02g00131\_t001 |  | | | |  |  |  | | | |  | | | |  | Ath-AT4G23860.3 |  |  |  |
| 4 | Vvi-Vitvi02g01339\_t001 |  | | | |  |  |  | Ath-AT1G64405.1 |  | | | |  | | | |  |  |  |
| 4 | Vvi-Vitvi02g04019\_t001 |  | | | |  |  |  | | | |  | | | |  | | | |  |  |  |
| 4 | Vvi-Vitvi02g00132\_t001 |  | | | |  |  |  | | | |  | | | |  | Ath-AT4G23885.1 |  |  |  |
| 4 | Vvi-Vitvi02g00133\_t001 |  | | | |  |  |  | | | |  | | | |  | Ath-AT4G23890.1 |  |  |  |
| 4 | Vvi-Vitvi02g04020\_t001 |  | | | |  |  |  | | | |  | | | |  | | | |  |  |  |
| 4 | Vvi-Vitvi02g00135\_t001 |  | | | |  |  |  | | | |  | | | |  | | | |  |  |  |
| 4 | Vvi-Vitvi02g04021\_t001 |  | | | |  |  |  | | | |  | | | |  | | | |  |  |  |
| 4 | Vvi-Vitvi02g04022\_t001 |  | | | |  |  |  | | | |  | | | |  | Ath-AT4G23895.3 |  |  |  |
| 4 | Vvi-Vitvi02g04023\_t001 |  | | | |  |  |  | | | |  | | | |  | | | |  |  |  |
| 4 | Vvi-Vitvi02g00136\_t001 |  | | | |  |  |  | | | |  | | | |  | | | |  |  |  |
| 4 | Vvi-Vitvi02g00137\_t001 |  | Ath-AT4G11010.1 |  |  |  | | | |  | | | |  | Ath-AT4G23900.1 |  |  |  |
| 4 | Vvi-Vitvi02g00138\_t001 |  | Ath-AT4G10970.6 |  |  |  | | | |  | | | |  | Ath-AT4G23910.1 |  |  |  |
| 4 | Vvi-Vitvi02g00139\_t001 |  | | | |  |  |  | Ath-AT1G64430.1 |  | | | |  | | | |  |  |  |
| 4 | Vvi-Vitvi02g00140\_t001 |  | | | |  |  |  | | | |  | | | |  | | | |  |  |  |
| 4 | Vvi-Vitvi02g04024\_t001 |  | | | |  |  |  | | | |  | | | |  | | | |  |  |  |
| 4 | Vvi-Vitvi02g00141\_t001 |  | | | |  |  |  | | | |  | | | |  | | | |  |  |  |
| 4 | Vvi-Vitvi02g00143\_t001 |  | Ath-AT4G10960.1 |  |  |  | Ath-AT1G64440.1 |  | | | |  | Ath-AT4G23920.1 |  |  |  |
| 4 | Vvi-Vitvi02g00144\_t001 |  | | | |  |  |  | | | |  | | | |  | | | |  |  |  |
| 4 | Vvi-Vitvi02g00145\_t001 |  | | | |  |  |  | | | |  | | | |  | | | |  |  |  |
| 4 | Vvi-Vitvi02g00146\_t003 |  | Ath-AT4G10955.1 |  |  |  | | | |  | | | |  | | | |  |  |  |
| 4 | Vvi-Vitvi02g00147\_t001 |  | | | |  |  |  | Ath-AT1G64450.1 |  | | | |  | Ath-AT4G23930.1 |  |  |  |
| 4 | Vvi-Vitvi02g00149\_t001 |  | | | |  |  |  | | | |  | Ath-AT2G46500.2 |  | | | |  |  |  |
| 4 | Vvi-Vitvi02g00151\_t001 |  | Ath-AT4G10950.2 |  |  |  | | | |  | | | |  | | | |  |  |  |
| 4 | Vvi-Vitvi02g00152\_t001 |  | | | |  |  |  | Ath-AT1G64480.1 |  | | | |  | | | |  |  |  |
| 4 | Vvi-Vitvi02g01340\_t001 |  | | | |  |  |  | | | |  | | | |  | | | |  |  |  |
| 4 | Vvi-Vitvi02g04025\_t001 |  | | | |  |  |  | | | |  | | | |  | | | |  |  |  |
| 4 | Vvi-Vitvi02g00153\_t001 |  | Ath-AT4G10930.1 |  |  |  | | | |  | | | |  | | | |  |  |  |
| 4 | Vvi-Vitvi02g00155\_t001 |  | | | |  |  |  | | | |  | | | |  | | | |  |  |  |
| 4 | Vvi-Vitvi02g00156\_t001 |  | | | |  |  |  | | | |  | | | |  | Ath-AT4G23950.2 |  |  |  |
| 4 | Vvi-Vitvi02g00157\_t001 |  | Ath-AT4G10925.1 |  |  |  | | | |  | | | |  | Ath-AT4G23960.2 |  |  |  |
| 4 | Vvi-Vitvi02g00158\_t001 |  | | | |  |  |  | | | |  | | | |  | | | |  |  |  |
| 4 | Vvi-Vitvi02g00159\_t001 |  | Ath-AT4G10920.2 |  |  |  | | | |  | | | |  | | | |  |  |  |
| 3 | Vvi-Vitvi02g00160\_t001 |  |  |  |  |  | Ath-AT1G64500.1 |  | | | |  | | | |  |  |  |
| 2 | Vvi-Vitvi02g00161\_t001 |  |  |  |  |  |  |  | | | |  | | | |  |  |  |
| 2 | Vvi-Vitvi02g00162\_t001 |  |  |  |  |  |  |  | | | |  | | | |  |  |  |
| 2 | Vvi-Vitvi02g04026\_t001 |  |  |  |  |  |  |  | | | |  | | | |  |  |  |
| 2 | Vvi-Vitvi02g00163\_t001 |  |  |  |  |  |  |  | Ath-AT2G46530.3 |  | Ath-AT4G23980.1 |  |  |  |
| 1 | Vvi-Vitvi02g04027\_t001 |  |  |  |  |  |  |  |  |  | | | |  |  |  |
| 1 | Vvi-Vitvi02g01341\_t001 |  |  |  |  |  |  |  |  |  | Ath-AT4G23990.1 |  |  |  |
| 1 | Vvi-Vitvi02g04028\_t001 |  |  |  |  |  |  |  |  |  | | | |  |  |  |
| 1 | Vvi-Vitvi02g01343\_t001 |  |  |  |  |  |  |  |  |  | | | |  |  |  |
| 1 | Vvi-Vitvi02g04029\_t001 |  |  |  |  |  |  |  |  |  | | | |  |  |  |
| 1 | Vvi-Vitvi02g01344\_t001 |  |  |  |  |  |  |  |  |  | | | |  |  |  |
| 1 | Vvi-Vitvi02g04030\_t001 |  |  |  |  |  |  |  |  |  | | | |  |  |  |
| 1 | Vvi-Vitvi02g01353\_t001 |  |  |  |  |  |  |  |  |  | | | |  |  |  |
| 1 | Vvi-Vitvi02g04031\_t001 |  |  |  |  |  |  |  |  |  | | | |  |  |  |
| 1 | Vvi-Vitvi02g01347\_t001 |  |  |  |  |  |  |  |  |  | | | |  |  |  |
| 1 | Vvi-Vitvi02g01348\_t002 |  |  |  |  |  |  |  |  |  | | | |  |  |  |
| 1 | Vvi-Vitvi02g04032\_t001 |  |  |  |  |  |  |  |  |  | | | |  |  |  |
| 1 | Vvi-Vitvi02g04033\_t001 |  |  |  |  |  |  |  |  |  | | | |  |  |  |
| 1 | Vvi-Vitvi02g01349\_t001 |  |  |  |  |  |  |  |  |  | | | |  |  |  |
| 1 | Vvi-Vitvi02g01350\_t001 |  |  |  |  |  |  |  |  |  | | | |  |  |  |
| 1 | Vvi-Vitvi02g04034\_t001 |  |  |  |  |  |  |  |  |  | | | |  |  |  |
| 1 | Vvi-Vitvi02g04035\_t001 |  |  |  |  |  |  |  |  |  | | | |  |  |  |
| 1 | Vvi-Vitvi02g04036\_t001 |  |  |  |  |  |  |  |  |  | | | |  |  |  |
| 1 | Vvi-Vitvi02g00167\_t001 |  |  |  |  |  |  |  |  |  | | | |  |  |  |
| 1 | Vvi-Vitvi02g04037\_t001 |  |  |  |  |  |  |  |  |  | | | |  |  |  |
| 1 | Vvi-Vitvi02g04038\_t001 |  |  |  |  |  |  |  |  |  | Ath-AT4G24015.1 |  |  |  |
| 1 | Vvi-Vitvi02g01355\_t004 |  |  |  |  |  |  |  |  |  | | | |  |  |  |
| 1 | Vvi-Vitvi02g04039\_t001 |  |  |  |  |  |  |  |  |  | | | |  |  |  |
| 1 | Vvi-Vitvi02g00174\_t001 |  |  |  |  |  |  |  |  |  | | | |  |  |  |
| 1 | Vvi-Vitvi02g01356\_t001 |  |  |  |  |  |  |  |  |  | | | |  |  |  |
| 2 | Vvi-Vitvi02g00175\_t001 |  | Ath-AT1G64510.2 |  |  |  |  |  |  |  | | | |  |  |  |
| 3 | Vvi-Vitvi02g00176\_t001 |  | Ath-AT1G64520.1 |  | Ath-AT5G42040.1 |  |  |  |  |  | | | |  |  |  |
| 3 | Vvi-Vitvi02g00177\_t001 |  | | | |  | Ath-AT5G42030.2 |  |  |  |  |  | | | |  |  |  |
| 4 | Vvi-Vitvi02g00178\_t001 |  | | | |  | | | |  | Ath-AT4G10890.1 |  |  |  | | | |  |  |  |
| 4 | Vvi-Vitvi02g00179\_t001 |  | Ath-AT1G64530.1 |  | | | |  | | | |  |  |  | Ath-AT4G24020.1 |  |  |  |
| 4 | Vvi-Vitvi02g00180\_t001 |  | | | |  | | | |  | | | |  |  |  | | | |  |  |  |
| 4 | Vvi-Vitvi02g00181\_t001 |  | | | |  | | | |  | Ath-AT4G10850.1 |  |  |  | | | |  |  |  |
| 4 | Vvi-Vitvi02g04040\_t001 |  | | | |  | | | |  | | | |  |  |  | | | |  |  |  |
| 4 | Vvi-Vitvi02g00182\_t001 |  | | | |  | | | |  | Ath-AT4G10840.1 |  |  |  | | | |  |  |  |
| 4 | Vvi-Vitvi02g04041\_t001 |  | | | |  | | | |  | | | |  |  |  | | | |  |  |  |
| 4 | Vvi-Vitvi02g01358\_t001 |  | | | |  | | | |  | | | |  |  |  | | | |  |  |  |
| 4 | Vvi-Vitvi02g01359\_t001 |  | | | |  | | | |  | | | |  |  |  | | | |  |  |  |
| 4 | Vvi-Vitvi02g00184\_t001 |  | | | |  | | | |  | | | |  |  |  | | | |  |  |  |
| 4 | Vvi-Vitvi02g00186\_t001 |  | | | |  | | | |  | | | |  |  |  | | | |  |  |  |
| 4 | Vvi-Vitvi02g00187\_t001 |  | | | |  | Ath-AT5G42020.3 |  | | | |  |  |  | | | |  |  |  |
| 4 | Vvi-Vitvi02g00188\_t001 |  | | | |  | | | |  | | | |  |  |  | | | |  |  |  |
| 4 | Vvi-Vitvi02g04042\_t001 |  | | | |  | | | |  | | | |  |  |  | | | |  |  |  |
| 4 | Vvi-Vitvi02g00189\_t001 |  | Ath-AT1G64550.1 |  | | | |  | | | |  |  |  | | | |  |  |  |
| 4 | Vvi-Vitvi02g04043\_t001 |  | | | |  | | | |  | | | |  |  |  | | | |  |  |  |
| 4 | Vvi-Vitvi02g04044\_t001 |  | | | |  | | | |  | | | |  |  |  | | | |  |  |  |
| 4 | Vvi-Vitvi02g00191\_t001 |  | | | |  | | | |  | | | |  |  |  | Ath-AT4G24040.1 |  |  |  |
| 4 | Vvi-Vitvi02g01360\_t001 |  | | | |  | | | |  | | | |  |  |  | | | |  |  |  |
| 4 | Vvi-Vitvi02g00192\_t001 |  | Ath-AT1G64590.1 |  | | | |  | | | |  |  |  | Ath-AT4G24050.1 |  |  |  |
| 4 | Vvi-Vitvi02g00194\_t001 |  | Ath-AT1G64600.1 |  | | | |  | | | |  |  |  | | | |  |  |  |
| 4 | Vvi-Vitvi02g00195\_t001 |  | | | |  | | | |  | | | |  |  |  | | | |  |  |  |
| 4 | Vvi-Vitvi02g04045\_t001 |  | | | |  | | | |  | | | |  |  |  | | | |  |  |  |
| 4 | Vvi-Vitvi02g00197\_t001 |  | | | |  | | | |  | | | |  |  |  | | | |  |  |  |
| 4 | Vvi-Vitvi02g04046\_t001 |  | | | |  | | | |  | | | |  |  |  | | | |  |  |  |
| 4 | Vvi-Vitvi02g00198\_t001 |  | Ath-AT1G64610.2 |  | Ath-AT5G42010.1 |  | | | |  |  |  | | | |  |  |  |
| 4 | Vvi-Vitvi02g00199\_t001 |  | Ath-AT1G64620.1 |  | | | |  | | | |  |  |  | Ath-AT4G24060.1 |  |  |  |
| 4 | Vvi-Vitvi02g00200\_t001 |  | | | |  | Ath-AT5G42000.1 |  | | | |  |  |  | | | |  |  |  |
| 4 | Vvi-Vitvi02g00201\_t001 |  | | | |  | | | |  | Ath-AT4G10800.1 |  |  |  | | | |  |  |  |
| 4 | Vvi-Vitvi02g01361\_t001 |  | | | |  | | | |  | | | |  |  |  | | | |  |  |  |
| 4 | Vvi-Vitvi02g01363\_t001 |  | | | |  | | | |  | | | |  |  |  | | | |  |  |  |
| 4 | Vvi-Vitvi02g04047\_t001 |  | | | |  | | | |  | | | |  |  |  | | | |  |  |  |
| 4 | Vvi-Vitvi02g00202\_t001 |  | Ath-AT1G64625.1 |  | | | |  | | | |  |  |  | | | |  |  |  |
| 4 | Vvi-Vitvi02g00203\_t001 |  | | | |  | | | |  | | | |  |  |  | | | |  |  |  |
| 4 | Vvi-Vitvi02g00204\_t001 |  | | | |  | | | |  | | | |  |  |  | | | |  |  |  |
| 4 | Vvi-Vitvi02g00205\_t001 |  | | | |  | | | |  | Ath-AT4G10790.1 |  |  |  | | | |  |  |  |
| 4 | Vvi-Vitvi02g00206\_t001 |  | | | |  | | | |  | | | |  |  |  | | | |  |  |  |
| 4 | Vvi-Vitvi02g04048\_t001 |  | | | |  | | | |  | | | |  |  |  | | | |  |  |  |
| 4 | Vvi-Vitvi02g00207\_t001 |  | Ath-AT1G64630.1 |  | Ath-AT5G41990.1 |  | | | |  |  |  | | | |  |  |  |
| 4 | Vvi-Vitvi02g00208\_t002 |  | | | |  | | | |  | | | |  |  |  | | | |  |  |  |
| 4 | Vvi-Vitvi02g00209\_t001 |  | | | |  | | | |  | Ath-AT4G10770.1 |  |  |  | | | |  |  |  |
| 4 | Vvi-Vitvi02g00210\_t001 |  | | | |  | | | |  | Ath-AT4G10760.1 |  |  |  | | | |  |  |  |
| 4 | Vvi-Vitvi02g00211\_t001 |  | Ath-AT1G64640.1 |  | | | |  | | | |  |  |  | | | |  |  |  |
| 4 | Vvi-Vitvi02g00212\_t001 |  | Ath-AT1G64650.1 |  | | | |  | | | |  |  |  | | | |  |  |  |
| 4 | Vvi-Vitvi02g00213\_t001 |  | | | |  | | | |  | Ath-AT4G10750.1 |  |  |  | Ath-AT4G24070.1 |  |  |  |
| 4 | Vvi-Vitvi02g00214\_t001 |  | | | |  | Ath-AT5G41970.1 |  | | | |  |  |  | | | |  |  |  |
| 4 | Vvi-Vitvi02g04049\_t001 |  | | | |  | | | |  | | | |  |  |  | Ath-AT4G24090.1 |  |  |  |
| 4 | Vvi-Vitvi02g01364\_t001 |  | | | |  | Ath-AT5G41960.1 |  | | | |  |  |  | | | |  |  |  |
| 4 | Vvi-Vitvi02g00218\_t003 |  | | | |  | | | |  | Ath-AT4G10730.1 |  |  |  | Ath-AT4G24100.4 |  |  |  |
| 4 | Vvi-Vitvi02g00219\_t001 |  | | | |  | | | |  | | | |  |  |  | Ath-AT4G24110.1 |  |  |  |
| 4 | Vvi-Vitvi02g01365\_t002 |  | | | |  | | | |  | | | |  |  |  | Ath-AT4G24120.1 |  |  |  |
| 4 | Vvi-Vitvi02g04050\_t001 |  | | | |  | | | |  | | | |  |  |  | | | |  |  |  |
| 4 | Vvi-Vitvi02g00221\_t001 |  | | | |  | Ath-AT5G41950.1 |  | | | |  |  |  | | | |  |  |  |
| 4 | Vvi-Vitvi02g00222\_t004 |  | | | |  | Ath-AT5G41940.1 |  | | | |  |  |  | | | |  |  |  |
| 4 | Vvi-Vitvi02g00223\_t001 |  | | | |  | | | |  | | | |  |  |  | Ath-AT4G24130.1 |  |  |  |
| 4 | Vvi-Vitvi02g00224\_t002 |  | | | |  | | | |  | | | |  |  |  | | | |  |  |  |
| 4 | Vvi-Vitvi02g00225\_t001 |  | Ath-AT1G64660.1 |  | | | |  | | | |  |  |  | | | |  |  |  |
| 7 | Vvi-Vitvi02g00226\_t001 |  | | | |  | | | |  | | | |  | Ath-AT3G61880.2 |  | | | |  | Ath-AT1G01190.2 |  | Ath-AT2G46660.1 |  |
| 7 | Vvi-Vitvi02g00227\_t001 |  | | | |  | Ath-AT5G41910.2 |  | | | |  | | | |  | | | |  | | | |  | | | |  |
| 7 | Vvi-Vitvi02g00228\_t001 |  | | | |  | | | |  | | | |  | Ath-AT3G61890.1 |  | | | |  | | | |  | Ath-AT2G46680.1 |  |
| 7 | Vvi-Vitvi02g00230\_t001 |  | | | |  | | | |  | | | |  | | | |  | | | |  | | | |  | | | |  |
| 7 | Vvi-Vitvi02g04051\_t001 |  | | | |  | | | |  | | | |  | | | |  | | | |  | | | |  | | | |  |
| 8 | Vvi-Vitvi02g00231\_t001 |  | | | |  | | | |  | | | |  | | | |  | | | |  | | | |  | | | |  | Ath-AT1G32640.1 |
| 8 | Vvi-Vitvi02g00232\_t001 |  | Ath-AT1G64670.2 |  | Ath-AT5G41900.1 |  | | | |  | | | |  | Ath-AT4G24140.1 |  | | | |  | | | |  | | | |
| 8 | Vvi-Vitvi02g00234\_t001 |  | | | |  | Ath-AT5G41890.1 |  | | | |  | | | |  | | | |  | | | |  | | | |  | | | |
| 7 | Vvi-Vitvi02g00235\_t001 |  | Ath-AT1G64680.1 |  |  |  | | | |  | | | |  | | | |  | | | |  | | | |  | | | |
| 7 | Vvi-Vitvi02g00236\_t001 |  | | | |  |  |  | | | |  | | | |  | | | |  | | | |  | | | |  | | | |
| 7 | Vvi-Vitvi02g00237\_t001 |  | | | |  |  |  | | | |  | | | |  | | | |  | | | |  | | | |  | | | |
| 7 | Vvi-Vitvi02g00238\_t001 |  | Ath-AT1G64690.3 |  |  |  | | | |  | | | |  | | | |  | | | |  | | | |  | | | |
| 7 | Vvi-Vitvi02g04052\_t001 |  | | | |  |  |  | | | |  | | | |  | | | |  | | | |  | | | |  | | | |
| 7 | Vvi-Vitvi02g00239\_t001 |  | | | |  |  |  | | | |  | | | |  | Ath-AT4G24150.1 |  | | | |  | | | |  | | | |
| 6 | Vvi-Vitvi02g00240\_t001 |  | | | |  |  |  | Ath-AT4G10640.1 |  | | | |  |  |  | Ath-AT1G01110.2 |  | | | |  | | | |
| 6 | Vvi-Vitvi02g04053\_t001 |  | | | |  |  |  | | | |  | | | |  |  |  | | | |  | | | |  | | | |
| 6 | Vvi-Vitvi02g00241\_t001 |  | | | |  |  |  | Ath-AT4G10630.1 |  | | | |  |  |  | | | |  | | | |  | Ath-AT1G32760.1 |
| 6 | Vvi-Vitvi02g04054\_t001 |  | | | |  |  |  | | | |  | | | |  |  |  | | | |  | | | |  | | | |
| 6 | Vvi-Vitvi02g00242\_t001 |  | | | |  |  |  | | | |  | Ath-AT3G61910.1 |  |  |  | | | |  | Ath-AT2G46770.1 |  | Ath-AT1G32770.1 |
| 6 | Vvi-Vitvi02g00243\_t001 |  | Ath-AT1G64700.1 |  |  |  | | | |  | Ath-AT3G61920.1 |  |  |  | | | |  | | | |  | | | |
| 6 | Vvi-Vitvi02g04055\_t001 |  | | | |  |  |  | | | |  | | | |  |  |  | | | |  | | | |  | | | |
| 6 | Vvi-Vitvi02g00244\_t001 |  | Ath-AT1G64710.1 |  |  |  | | | |  | | | |  |  |  | | | |  | | | |  | Ath-AT1G32780.1 |
| 6 | Vvi-Vitvi02g00245\_t001 |  | | | |  |  |  | Ath-AT4G10610.1 |  | | | |  |  |  | | | |  | | | |  | Ath-AT1G32790.2 |
| 6 | Vvi-Vitvi02g00246\_t001 |  | | | |  |  |  | Ath-AT4G10600.1 |  | | | |  |  |  | | | |  | | | |  | Ath-AT1G32810.2 |
| 6 | Vvi-Vitvi02g00247\_t001 |  | | | |  |  |  | Ath-AT4G10570.1 |  | | | |  |  |  | | | |  | | | |  | Ath-AT1G32850.1 |
| 6 | Vvi-Vitvi02g00248\_t001 |  | | | |  |  |  | | | |  | | | |  |  |  | | | |  | | | |  | Ath-AT1G32860.1 |
| 6 | Vvi-Vitvi02g00249\_t001 |  | | | |  |  |  | | | |  | | | |  |  |  | Ath-AT1G01100.2 |  | | | |  | | | |
| 6 | Vvi-Vitvi02g00250\_t001 |  | | | |  |  |  | | | |  | | | |  |  |  | | | |  | | | |  | Ath-AT1G32900.1 |
| 6 | Vvi-Vitvi02g00251\_t001 |  | | | |  |  |  | | | |  | | | |  |  |  | | | |  | | | |  | | | |
| 6 | Vvi-Vitvi02g04056\_t001 |  | | | |  |  |  | | | |  | | | |  |  |  | | | |  | | | |  | Ath-AT1G32920.1 |
| 6 | Vvi-Vitvi02g00252\_t001 |  | | | |  |  |  | | | |  | | | |  |  |  | Ath-AT1G01090.1 |  | | | |  | | | |
| 6 | Vvi-Vitvi02g00255\_t001 |  | Ath-AT1G64720.1 |  |  |  | | | |  | | | |  |  |  | | | |  | | | |  | | | |
| 5 | Vvi-Vitvi02g00256\_t001 |  |  |  |  |  | | | |  | | | |  |  |  | | | |  | | | |  | Ath-AT1G32930.1 |
| 5 | Vvi-Vitvi02g04057\_t001 |  |  |  |  |  | | | |  | | | |  |  |  | | | |  | | | |  | | | |
| 5 | Vvi-Vitvi02g00257\_t001 |  |  |  |  |  | | | |  | | | |  |  |  | | | |  | | | |  | | | |
| 5 | Vvi-Vitvi02g00258\_t001 |  |  |  |  |  | | | |  | | | |  |  |  | | | |  | | | |  | Ath-AT1G32940.1 |
| 5 | Vvi-Vitvi02g00259\_t001 |  |  |  |  |  | | | |  | | | |  |  |  | | | |  | | | |  | | | |
| 5 | Vvi-Vitvi02g00260\_t001 |  |  |  |  |  | Ath-AT4G10520.1 |  | | | |  |  |  | | | |  | | | |  | | | |
| 5 | Vvi-Vitvi02g01368\_t001 |  |  |  |  |  | | | |  | | | |  |  |  | | | |  | | | |  | | | |
| 5 | Vvi-Vitvi02g00261\_t001 |  |  |  |  |  | | | |  | | | |  |  |  | | | |  | | | |  | Ath-AT1G32990.1 |
| 5 | Vvi-Vitvi02g01369\_t001 |  |  |  |  |  | | | |  | Ath-AT3G61940.1 |  |  |  | | | |  | Ath-AT2G46800.2 |  | | | |
| 5 | Vvi-Vitvi02g00262\_t001 |  |  |  |  |  | | | |  | | | |  |  |  | | | |  | | | |  | | | |
| 5 | Vvi-Vitvi02g04058\_t001 |  |  |  |  |  | | | |  | | | |  |  |  | | | |  | | | |  | | | |
| 5 | Vvi-Vitvi02g00263\_t001 |  |  |  |  |  | | | |  | | | |  |  |  | | | |  | | | |  | Ath-AT1G33030.1 |
| 5 | Vvi-Vitvi02g04059\_t001 |  |  |  |  |  | Ath-AT4G10490.1 |  | | | |  |  |  | | | |  | | | |  | | | |
| 5 | Vvi-Vitvi02g00265\_t001 |  |  |  |  |  | | | |  | | | |  |  |  | | | |  | | | |  | | | |
| 5 | Vvi-Vitvi02g00267\_t001 |  |  |  |  |  | | | |  | | | |  |  |  | | | |  | | | |  | | | |
| 5 | Vvi-Vitvi02g00268\_t001 |  |  |  |  |  | | | |  | | | |  |  |  | | | |  | | | |  | | | |
| 5 | Vvi-Vitvi02g00270\_t003 |  |  |  |  |  | | | |  | | | |  |  |  | | | |  | | | |  | | | |
| 5 | Vvi-Vitvi02g00272\_t001 |  |  |  |  |  | | | |  | | | |  |  |  | Ath-AT1G01050.1 |  | Ath-AT2G46860.1 |  | | | |
| 5 | Vvi-Vitvi02g00273\_t001 |  |  |  |  |  | | | |  | | | |  |  |  | | | |  | | | |  | | | |
| 5 | Vvi-Vitvi02g00274\_t001 |  |  |  |  |  | | | |  | | | |  |  |  | | | |  | | | |  | | | |
| 5 | Vvi-Vitvi02g04060\_t001 |  |  |  |  |  | | | |  | | | |  |  |  | | | |  | | | |  | | | |
| 5 | Vvi-Vitvi02g00275\_t001 |  |  |  |  |  | | | |  | Ath-AT3G61970.1 |  |  |  | Ath-AT1G01030.1 |  | Ath-AT2G46870.1 |  | | | |
| 2 | Vvi-Vitvi02g04061\_t001 |  |  |  |  |  | | | |  |  |  |  |  |  |  |  |  | | | |
| 2 | Vvi-Vitvi02g00276\_t001 |  |  |  |  |  | Ath-AT4G10465.1 |  |  |  |  |  |  |  |  |  | | | |
| 2 | Vvi-Vitvi02g04062\_t001 |  |  |  |  |  | | | |  |  |  |  |  |  |  |  |  | | | |
| 2 | Vvi-Vitvi02g00277\_t001 |  |  |  |  |  | | | |  |  |  |  |  |  |  |  |  | Ath-AT1G33060.2 |
| 2 | Vvi-Vitvi02g04063\_t001 |  |  |  |  |  | | | |  |  |  |  |  |  |  |  |  | | | |
| 2 | Vvi-Vitvi02g00278\_t001 |  |  |  |  |  | | | |  |  |  |  |  |  |  |  |  | | | |
| 2 | Vvi-Vitvi02g01371\_t001 |  |  |  |  |  | | | |  |  |  |  |  |  |  |  |  | | | |
| 2 | Vvi-Vitvi02g00279\_t001 |  |  |  |  |  | | | |  |  |  |  |  |  |  |  |  | | | |
| 2 | Vvi-Vitvi02g00280\_t001 |  |  |  |  |  | | | |  |  |  |  |  |  |  |  |  | | | |
| 2 | Vvi-Vitvi02g04064\_t001 |  |  |  |  |  | | | |  |  |  |  |  |  |  |  |  | | | |
| 2 | Vvi-Vitvi02g00282\_t001 |  |  |  |  |  | | | |  |  |  |  |  |  |  |  |  | | | |
| 2 | Vvi-Vitvi02g00283\_t001 |  |  |  |  |  | | | |  |  |  |  |  |  |  |  |  | | | |
| 2 | Vvi-Vitvi02g01372\_t001 |  |  |  |  |  | | | |  |  |  |  |  |  |  |  |  | | | |
| 2 | Vvi-Vitvi02g01373\_t001 |  |  |  |  |  | | | |  |  |  |  |  |  |  |  |  | | | |
| 2 | Vvi-Vitvi02g01374\_t001 |  |  |  |  |  | | | |  |  |  |  |  |  |  |  |  | | | |
| 2 | Vvi-Vitvi02g01375\_t002 |  |  |  |  |  | | | |  |  |  |  |  |  |  |  |  | | | |
| 2 | Vvi-Vitvi02g00284\_t001 |  |  |  |  |  | | | |  |  |  |  |  |  |  |  |  | | | |
| 2 | Vvi-Vitvi02g04065\_t001 |  |  |  |  |  | | | |  |  |  |  |  |  |  |  |  | | | |
| 2 | Vvi-Vitvi02g00286\_t001 |  |  |  |  |  | | | |  |  |  |  |  |  |  |  |  | | | |
| 2 | Vvi-Vitvi02g00287\_t001 |  |  |  |  |  | | | |  |  |  |  |  |  |  |  |  | | | |
| 2 | Vvi-Vitvi02g00289\_t001 |  |  |  |  |  | Ath-AT4G10440.1 |  |  |  |  |  |  |  |  |  | Ath-AT1G33170.1 |
| 2 | Vvi-Vitvi02g00290\_t001 |  |  |  |  |  | Ath-AT4G10430.3 |  |  |  |  |  |  |  |  |  | Ath-AT1G33230.2 |
| 2 | Vvi-Vitvi02g00291\_t001 |  |  |  |  |  | | | |  |  |  |  |  |  |  |  |  | Ath-AT1G33240.1 |
| 2 | Vvi-Vitvi02g04066\_t001 |  |  |  |  |  | | | |  |  |  |  |  |  |  |  |  | | | |
| 5 | Vvi-Vitvi02g00293\_t001 |  | Ath-AT4G11350.1 |  | Ath-AT5G41460.1 |  | | | |  | Ath-AT4G23490.1 |  |  |  |  |  |  |  | Ath-AT1G33250.1 |
| 5 | Vvi-Vitvi02g04067\_t001 |  | | | |  | | | |  | Ath-AT4G10390.1 |  | | | |  |  |  |  |  |  |  | Ath-AT1G33260.1 |
| 5 | Vvi-Vitvi02g04068\_t001 |  | | | |  | | | |  | | | |  | | | |  |  |  |  |  |  |  | Ath-AT1G33265.1 |
| 4 | Vvi-Vitvi02g00295\_t001 |  | | | |  | | | |  | Ath-AT4G10380.1 |  | | | |  |  |  |  |
| 4 | Vvi-Vitvi02g00296\_t001 |  | | | |  | | | |  | Ath-AT1G63770.3 |  | | | |  |  |  |  |
| 4 | Vvi-Vitvi02g00297\_t001 |  | | | |  | | | |  | Ath-AT1G63720.1 |  | | | |  |  |  |  |
| 4 | Vvi-Vitvi02g00298\_t001 |  | | | |  | Ath-AT5G41330.1 |  | | | |  | | | |  |  |  |  |
| 4 | Vvi-Vitvi02g00301\_t001 |  | | | |  | | | |  | | | |  | | | |  |  |  |  |
| 5 | Vvi-Vitvi02g00303\_t001 |  | | | |  | | | |  | Ath-AT1G63710.1 |  | | | |  | Ath-AT2G45970.1 |  |  |  |
| 5 | Vvi-Vitvi02g00304\_t001 |  | | | |  | | | |  | | | |  | | | |  | | | |  |  |  |
| 5 | Vvi-Vitvi02g04069\_t001 |  | | | |  | | | |  | | | |  | | | |  | | | |  |  |  |
| 5 | Vvi-Vitvi02g00308\_t001 |  | | | |  | | | |  | Ath-AT1G63700.1 |  | | | |  | | | |  |  |  |
| 5 | Vvi-Vitvi02g00309\_t001 |  | Ath-AT4G11410.1 |  | | | |  | | | |  | Ath-AT4G23420.3 |  | | | |  |  |  |
| 6 | Vvi-Vitvi02g00310\_t001 |  | | | |  | | | |  | | | |  | Ath-AT4G23400.1 |  | Ath-AT2G45960.3 |  | Ath-AT3G61430.1 |  |  |
| 6 | Vvi-Vitvi02g00311\_t001 |  | | | |  | | | |  | Ath-AT1G63690.1 |  | | | |  | | | |  | | | |  |  |
| 6 | Vvi-Vitvi02g04070\_t001 |  | | | |  | | | |  | | | |  | | | |  | | | |  | | | |  |  |
| 6 | Vvi-Vitvi02g01378\_t001 |  | | | |  | | | |  | | | |  | | | |  | | | |  | | | |  |  |
| 6 | Vvi-Vitvi02g00313\_t001 |  | | | |  | | | |  | Ath-AT1G63680.2 |  | | | |  | | | |  | | | |  |  |
| 6 | Vvi-Vitvi02g00314\_t002 |  | | | |  | | | |  | | | |  | | | |  | Ath-AT2G45900.1 |  | | | |  |  |
| 6 | Vvi-Vitvi02g00315\_t001 |  | | | |  | | | |  | Ath-AT1G63660.1 |  | | | |  | | | |  | | | |  |  |
| 6 | Vvi-Vitvi02g00316\_t001 |  | | | |  | | | |  | | | |  | Ath-AT4G23340.1 |  | | | |  | | | |  |  |
| 6 | Vvi-Vitvi02g00317\_t001 |  | | | |  | Ath-AT5G41315.2 |  | Ath-AT1G63650.3 |  | | | |  | | | |  | | | |  |  |
| 6 | Vvi-Vitvi02g01379\_t001 |  | | | |  | | | |  | | | |  | Ath-AT4G23330.1 |  | | | |  | | | |  |  |
| 6 | Vvi-Vitvi02g00318\_t001 |  | | | |  | | | |  | | | |  | | | |  | | | |  | | | |  |  |
| 6 | Vvi-Vitvi02g04071\_t001 |  | | | |  | | | |  | | | |  | | | |  | | | |  | | | |  |  |
| 6 | Vvi-Vitvi02g00319\_t001 |  | | | |  | Ath-AT5G41310.1 |  | Ath-AT1G63640.3 |  | | | |  | | | |  | | | |  |  |
| 6 | Vvi-Vitvi02g00320\_t001 |  | Ath-AT4G11440.1 |  | | | |  | | | |  | | | |  | | | |  | | | |  |  |
| 6 | Vvi-Vitvi02g04072\_t001 |  | Ath-AT4G11450.1 |  | | | |  | Ath-AT1G63520.1 |  | | | |  | | | |  | | | |  |  |
| 6 | Vvi-Vitvi02g04073\_t001 |  | Ath-AT4G11560.1 |  | | | |  | | | |  | Ath-AT4G23120.1 |  | | | |  | | | |  |  |
| 6 | Vvi-Vitvi02g00324\_t001 |  | Ath-AT4G11570.2 |  | | | |  | | | |  | | | |  | | | |  | | | |  |  |
| 6 | Vvi-Vitvi02g00325\_t001 |  | | | |  | | | |  | | | |  | | | |  | | | |  | | | |  |  |
| 6 | Vvi-Vitvi02g00326\_t002 |  | | | |  | | | |  | | | |  | Ath-AT4G23100.3 |  | | | |  | | | |  |  |
| 5 | Vvi-Vitvi02g00327\_t001 |  | | | |  | | | |  | | | |  |  |  | | | |  | | | |  |  |
| 5 | Vvi-Vitvi02g00328\_t003 |  | | | |  | Ath-AT5G41260.1 |  | Ath-AT1G63500.1 |  |  |  | | | |  | | | |  |  |
| 5 | Vvi-Vitvi02g00329\_t001 |  | | | |  | | | |  | Ath-AT1G63490.4 |  |  |  | | | |  | | | |  |  |
| 5 | Vvi-Vitvi02g00330\_t001 |  | | | |  | | | |  | | | |  |  |  | | | |  | | | |  |  |
| 5 | Vvi-Vitvi02g04074\_t001 |  | | | |  | | | |  | | | |  |  |  | | | |  | | | |  |  |
| 5 | Vvi-Vitvi02g01380\_t001 |  | | | |  | | | |  | | | |  |  |  | | | |  | | | |  |  |
| 5 | Vvi-Vitvi02g00331\_t001 |  | | | |  | | | |  | Ath-AT1G63470.1 |  |  |  | Ath-AT2G45850.2 |  | Ath-AT3G61310.1 |  |  |
| 5 | Vvi-Vitvi02g00332\_t001 |  | Ath-AT4G11600.1 |  | | | |  | Ath-AT1G63460.1 |  |  |  | | | |  | | | |  |  |
| 5 | Vvi-Vitvi02g00333\_t001 |  | | | |  | | | |  | | | |  |  |  | | | |  | | | |  |  |
| 5 | Vvi-Vitvi02g00334\_t001 |  | | | |  | Ath-AT5G41250.1 |  | Ath-AT1G63450.1 |  |  |  | | | |  | | | |  |  |
| 5 | Vvi-Vitvi02g01381\_t001 |  | Ath-AT4G11610.1 |  | | | |  | | | |  |  |  | | | |  | | | |  |  |
| 5 | Vvi-Vitvi02g04075\_t001 |  | | | |  | | | |  | | | |  |  |  | | | |  | | | |  |  |
| 5 | Vvi-Vitvi02g00335\_t001 |  | | | |  | Ath-AT5G41210.1 |  | | | |  |  |  | | | |  | | | |  |  |
| 5 | Vvi-Vitvi02g00337\_t001 |  | | | |  | | | |  | Ath-AT1G63440.1 |  |  |  | | | |  | | | |  |  |
| 5 | Vvi-Vitvi02g01382\_t001 |  | | | |  | | | |  | | | |  |  |  | | | |  | | | |  |  |
| 5 | Vvi-Vitvi02g01383\_t001 |  | | | |  | | | |  | | | |  |  |  | | | |  | | | |  |  |
| 5 | Vvi-Vitvi02g00338\_t001 |  | | | |  | | | |  | | | |  |  |  | | | |  | | | |  |  |
| 5 | Vvi-Vitvi02g00339\_t001 |  | Ath-AT4G11640.1 |  | | | |  | | | |  |  |  | | | |  | | | |  |  |
| 4 | Vvi-Vitvi02g00340\_t001 |  |  |  | | | |  | | | |  |  |  | | | |  | | | |  |  |
| 4 | Vvi-Vitvi02g00341\_t001 |  |  |  | Ath-AT5G41190.1 |  | | | |  |  |  | | | |  | | | |  |  |
| 4 | Vvi-Vitvi02g00342\_t001 |  |  |  | Ath-AT5G41180.1 |  | Ath-AT1G63430.2 |  |  |  | | | |  | | | |  |  |
| 4 | Vvi-Vitvi02g01384\_t001 |  |  |  | | | |  | Ath-AT1G63420.1 |  |  |  | Ath-AT2G45830.1 |  | Ath-AT3G61270.1 |  |  |
| 4 | Vvi-Vitvi02g04076\_t001 |  |  |  | | | |  | | | |  |  |  | | | |  | | | |  |  |
| 4 | Vvi-Vitvi02g00344\_t001 |  |  |  | | | |  | Ath-AT1G63410.2 |  |  |  | | | |  | | | |  |  |
| 4 | Vvi-Vitvi02g04077\_t001 |  |  |  | | | |  | | | |  |  |  | | | |  | | | |  |  |
| 4 | Vvi-Vitvi02g01385\_t001 |  |  |  | | | |  | | | |  |  |  | | | |  | | | |  |  |
| 4 | Vvi-Vitvi02g00345\_t001 |  |  |  | | | |  | | | |  |  |  | | | |  | | | |  |  |
| 4 | Vvi-Vitvi02g00346\_t001 |  |  |  | Ath-AT5G41140.1 |  | Ath-AT1G63300.1 |  |  |  | | | |  | | | |  |  |
| 3 | Vvi-Vitvi02g00347\_t001 |  |  |  |  |  | | | |  |  |  | | | |  | | | |  |  |
| 3 | Vvi-Vitvi02g04078\_t001 |  |  |  |  |  | | | |  |  |  | | | |  | | | |  |  |
| 3 | Vvi-Vitvi02g00348\_t001 |  |  |  |  |  | Ath-AT1G63295.2 |  |  |  | Ath-AT2G45820.1 |  | Ath-AT3G61260.1 |  |  |
| 3 | Vvi-Vitvi02g00349\_t001 |  |  |  |  |  | | | |  |  |  | Ath-AT2G45810.1 |  | Ath-AT3G61240.1 |  |  |
| 3 | Vvi-Vitvi02g04079\_t001 |  |  |  |  |  | | | |  |  |  | | | |  | | | |  |  |
| 3 | Vvi-Vitvi02g04080\_t001 |  |  |  |  |  | | | |  |  |  | | | |  | | | |  |  |
| 3 | Vvi-Vitvi02g04081\_t001 |  |  |  |  |  | | | |  |  |  | | | |  | | | |  |  |
| 3 | Vvi-Vitvi02g00350\_t001 |  |  |  |  |  | | | |  |  |  | | | |  | | | |  |  |
| 3 | Vvi-Vitvi02g04082\_t001 |  |  |  |  |  | | | |  |  |  | | | |  | | | |  |  |
| 3 | Vvi-Vitvi02g04083\_t001 |  |  |  |  |  | | | |  |  |  | | | |  | | | |  |  |
| 3 | Vvi-Vitvi02g04084\_t001 |  |  |  |  |  | | | |  |  |  | | | |  | | | |  |  |
| 3 | Vvi-Vitvi02g00358\_t001 |  |  |  |  |  | | | |  |  |  | | | |  | | | |  |  |
| 3 | Vvi-Vitvi02g00360\_t001 |  |  |  |  |  | | | |  |  |  | | | |  | | | |  |  |
| 3 | Vvi-Vitvi02g04085\_t001 |  |  |  |  |  | | | |  |  |  | | | |  | | | |  |  |
| 3 | Vvi-Vitvi02g01392\_t001 |  |  |  |  |  | | | |  |  |  | | | |  | | | |  |  |
| 3 | Vvi-Vitvi02g00363\_t001 |  |  |  |  |  | | | |  |  |  | | | |  | | | |  |  |
| 3 | Vvi-Vitvi02g00364\_t001 |  |  |  |  |  | | | |  |  |  | | | |  | | | |  |  |
| 3 | Vvi-Vitvi02g04086\_t001 |  |  |  |  |  | | | |  |  |  | | | |  | | | |  |  |
| 3 | Vvi-Vitvi02g00365\_t001 |  |  |  |  |  | | | |  |  |  | | | |  | | | |  |  |
| 3 | Vvi-Vitvi02g01393\_t001 |  |  |  |  |  | | | |  |  |  | | | |  | | | |  |  |
| 3 | Vvi-Vitvi02g00366\_t001 |  |  |  |  |  | Ath-AT1G63260.1 |  |  |  | | | |  | | | |  |  |
| 4 | Vvi-Vitvi02g00367\_t001 |  | Ath-AT1G12800.1 |  |  |  | | | |  |  |  | | | |  | | | |  |  |
| 4 | Vvi-Vitvi02g04087\_t001 |  | | | |  |  |  | | | |  |  |  | | | |  | | | |  |  |
| 4 | Vvi-Vitvi02g00368\_t001 |  | | | |  |  |  | | | |  |  |  | Ath-AT2G45800.1 |  | Ath-AT3G61230.1 |  |  |
| 5 | Vvi-Vitvi02g00370\_t001 |  | | | |  | Ath-AT1G63100.2 |  | | | |  |  |  | | | |  | | | |  |  |
| 5 | Vvi-Vitvi02g01397\_t001 |  | | | |  | | | |  | | | |  |  |  | | | |  | | | |  |  |
| 6 | Vvi-Vitvi02g01398\_t001 |  | | | |  | | | |  | | | |  | Ath-AT5G52230.1 |  | | | |  | | | |  |  |
| 6 | Vvi-Vitvi02g04088\_t001 |  | | | |  | | | |  | | | |  | | | |  | | | |  | | | |  |  |
| 7 | Vvi-Vitvi02g00373\_t001 |  | | | |  | | | |  | | | |  | | | |  | | | |  | | | |  | Ath-AT4G11740.1 |  |
| 7 | Vvi-Vitvi02g00374\_t001 |  | | | |  | | | |  | | | |  | | | |  | | | |  | | | |  | | | |  |
| 8 | Vvi-Vitvi02g00375\_t001 |  | | | |  | Ath-AT1G63110.1 |  | | | |  | | | |  | | | |  | | | |  | | | |  | Ath-AT1G12730.1 |
| 8 | Vvi-Vitvi02g00377\_t001 |  | | | |  | | | |  | | | |  | | | |  | | | |  | | | |  | | | |  | Ath-AT1G12740.2 |
| 8 | Vvi-Vitvi02g00378\_t001 |  | | | |  | | | |  | | | |  | | | |  | | | |  | | | |  | Ath-AT4G11720.1 |  | | | |
| 8 | Vvi-Vitvi02g00379\_t001 |  | | | |  | | | |  | | | |  | | | |  | | | |  | | | |  | | | |  | | | |
| 8 | Vvi-Vitvi02g04089\_t001 |  | | | |  | | | |  | | | |  | | | |  | | | |  | | | |  | | | |  | | | |
| 8 | Vvi-Vitvi02g00380\_t001 |  | | | |  | | | |  | | | |  | | | |  | | | |  | | | |  | | | |  | | | |
| 8 | Vvi-Vitvi02g04090\_t001 |  | | | |  | | | |  | | | |  | | | |  | | | |  | | | |  | | | |  | | | |
| 8 | Vvi-Vitvi02g00381\_t001 |  | | | |  | | | |  | | | |  | | | |  | | | |  | | | |  | | | |  | | | |
| 8 | Vvi-Vitvi02g00382\_t001 |  | | | |  | Ath-AT1G63120.1 |  | | | |  | | | |  | | | |  | | | |  | | | |  | Ath-AT1G12750.1 |
| 8 | Vvi-Vitvi02g00383\_t001 |  | | | |  | Ath-AT1G63160.1 |  | | | |  | | | |  | | | |  | | | |  | | | |  | | | |
| 8 | Vvi-Vitvi02g00384\_t001 |  | | | |  | Ath-AT1G63170.1 |  | Ath-AT1G63170.1 |  | | | |  | | | |  | Ath-AT3G61180.1 |  | Ath-AT4G11680.2 |  | Ath-AT1G12760.1 |
| 8 | Vvi-Vitvi02g00385\_t001 |  | | | |  | | | |  | | | |  | | | |  | | | |  | | | |  | Ath-AT4G11670.2 |  | | | |
| 8 | Vvi-Vitvi02g00386\_t001 |  | | | |  | | | |  | | | |  | | | |  | Ath-AT2G45760.1 |  | | | |  | | | |  | | | |
| 8 | Vvi-Vitvi02g00387\_t001 |  | | | |  | | | |  | | | |  | | | |  | | | |  | | | |  | Ath-AT4G11660.1 |  | | | |
| 8 | Vvi-Vitvi02g00388\_t001 |  | | | |  | | | |  | | | |  | Ath-AT5G52200.1 |  | | | |  | | | |  | | | |  | | | |
| 8 | Vvi-Vitvi02g00389\_t001 |  | | | |  | | | |  | | | |  | | | |  | | | |  | | | |  | | | |  | Ath-AT1G12770.1 |
| 8 | Vvi-Vitvi02g01399\_t001 |  | | | |  | | | |  | | | |  | | | |  | | | |  | | | |  | Ath-AT4G11655.1 |  | | | |
| 7 | Vvi-Vitvi02g00390\_t001 |  | Ath-AT1G12780.1 |  | Ath-AT1G63180.1 |  | | | |  | | | |  | | | |  | | | |  |  |  | Ath-AT1G12780.1 |
| 6 | Vvi-Vitvi02g01400\_t001 |  | | | |  | | | |  | | | |  | | | |  | | | |  | | | |  |  |
| 7 | Vvi-Vitvi02g00391\_t001 |  | | | |  | | | |  | | | |  | | | |  | | | |  | | | |  | Ath-AT4G11650.1 |  |
| 7 | Vvi-Vitvi02g00393\_t001 |  | | | |  | | | |  | | | |  | | | |  | | | |  | | | |  | | | |  |
| 7 | Vvi-Vitvi02g01403\_t001 |  | | | |  | | | |  | | | |  | | | |  | | | |  | | | |  | | | |  |
| 7 | Vvi-Vitvi02g01404\_t001 |  | | | |  | | | |  | | | |  | | | |  | | | |  | | | |  | | | |  |
| 7 | Vvi-Vitvi02g01405\_t001 |  | | | |  | | | |  | | | |  | | | |  | | | |  | | | |  | | | |  |
| 7 | Vvi-Vitvi02g04091\_t001 |  | | | |  | | | |  | | | |  | | | |  | | | |  | | | |  | | | |  |
| 7 | Vvi-Vitvi02g01406\_t001 |  | | | |  | | | |  | | | |  | | | |  | | | |  | | | |  | | | |  |
| 7 | Vvi-Vitvi02g04092\_t001 |  | | | |  | | | |  | | | |  | | | |  | | | |  | | | |  | | | |  |
| 7 | Vvi-Vitvi02g01407\_t001 |  | | | |  | | | |  | | | |  | | | |  | | | |  | | | |  | | | |  |
| 7 | Vvi-Vitvi02g01408\_t001 |  | | | |  | | | |  | | | |  | | | |  | | | |  | | | |  | | | |  |
| 7 | Vvi-Vitvi02g01409\_t001 |  | | | |  | | | |  | | | |  | | | |  | | | |  | | | |  | | | |  |
| 7 | Vvi-Vitvi02g00394\_t001 |  | | | |  | Ath-AT1G63220.1 |  | | | |  | | | |  | | | |  | | | |  | | | |  |
| 6 | Vvi-Vitvi02g04093\_t001 |  | | | |  |  |  | | | |  | | | |  | | | |  | | | |  | | | |  |
| 6 | Vvi-Vitvi02g00395\_t001 |  | | | |  |  |  | | | |  | | | |  | Ath-AT2G45550.1 |  | Ath-AT3G61040.1 |  | | | |  |
| 4 | Vvi-Vitvi02g00396\_t001 |  | | | |  |  |  | | | |  | | | |  |  |  |  |  | | | |  |
| 4 | Vvi-Vitvi02g00397\_t001 |  | | | |  |  |  | | | |  | | | |  |  |  |  |  | | | |  |
| 4 | Vvi-Vitvi02g00398\_t001 |  | Ath-AT1G12710.2 |  |  |  | Ath-AT1G63090.1 |  | Ath-AT5G52120.1 |  |  |  |  |  | | | |  |
| 4 | Vvi-Vitvi02g00399\_t001 |  | | | |  |  |  | | | |  | | | |  |  |  |  |  | | | |  |
| 4 | Vvi-Vitvi02g04094\_t001 |  | Ath-AT1G12650.2 |  |  |  | | | |  | | | |  |  |  |  |  | | | |  |
| 4 | Vvi-Vitvi02g00402\_t001 |  | Ath-AT1G12640.1 |  |  |  | Ath-AT1G63050.1 |  | | | |  |  |  |  |  | | | |  |
| 5 | Vvi-Vitvi02g00403\_t001 |  | | | |  | Ath-AT4G23030.1 |  | | | |  | Ath-AT5G52050.1 |  |  |  |  |  | | | |  |
| 5 | Vvi-Vitvi02g00404\_t001 |  | | | |  | | | |  | | | |  | | | |  |  |  |  |  | | | |  |
| 5 | Vvi-Vitvi02g04095\_t001 |  | Ath-AT1G12630.1 |  | | | |  | | | |  | Ath-AT5G51990.1 |  |  |  |  |  | | | |  |
| 5 | Vvi-Vitvi02g00407\_t001 |  | Ath-AT1G12610.1 |  | | | |  | Ath-AT1G63030.2 |  | | | |  |  |  |  |  | | | |  |
| 5 | Vvi-Vitvi02g04096\_t001 |  | | | |  | | | |  | | | |  | | | |  |  |  |  |  | | | |  |
| 5 | Vvi-Vitvi02g04097\_t001 |  | | | |  | | | |  | | | |  | | | |  |  |  |  |  | | | |  |
| 5 | Vvi-Vitvi02g00409\_t001 |  | | | |  | Ath-AT4G23020.2 |  | | | |  | | | |  |  |  |  |  | Ath-AT4G11780.1 |  |
| 5 | Vvi-Vitvi02g04098\_t001 |  | | | |  | | | |  | | | |  | | | |  |  |  |  |  | | | |  |
| 5 | Vvi-Vitvi02g04099\_t001 |  | | | |  | | | |  | | | |  | | | |  |  |  |  |  | | | |  |
| 5 | Vvi-Vitvi02g00410\_t001 |  | Ath-AT1G12600.1 |  | Ath-AT4G23010.3 |  | | | |  | | | |  |  |  |  |  | | | |  |
| 5 | Vvi-Vitvi02g00411\_t001 |  | | | |  | | | |  | | | |  | | | |  |  |  |  |  | Ath-AT4G11790.1 |  |
| 5 | Vvi-Vitvi02g00412\_t001 |  | | | |  | | | |  | | | |  | | | |  |  |  |  |  | | | |  |
| 5 | Vvi-Vitvi02g00413\_t002 |  | Ath-AT1G12580.1 |  | | | |  | | | |  | | | |  |  |  |  |  | | | |  |
| 5 | Vvi-Vitvi02g00414\_t001 |  | | | |  | | | |  | Ath-AT1G63020.2 |  | | | |  |  |  |  |  | | | |  |
| 5 | Vvi-Vitvi02g00415\_t001 |  | | | |  | Ath-AT4G22990.2 |  | Ath-AT1G63010.5 |  | | | |  |  |  |  |  | Ath-AT4G11810.1 |  |
| 5 | Vvi-Vitvi02g00416\_t001 |  | Ath-AT1G12570.1 |  | | | |  | | | |  | Ath-AT5G51930.1 |  |  |  |  |  | | | |  |
| 5 | Vvi-Vitvi02g00417\_t001 |  | | | |  | | | |  | | | |  | | | |  |  |  |  |  | | | |  |
| 5 | Vvi-Vitvi02g00419\_t001 |  | | | |  | Ath-AT4G22980.1 |  | | | |  | Ath-AT5G51920.1 |  |  |  |  |  | | | |  |
| 5 | Vvi-Vitvi02g00420\_t002 |  | | | |  | | | |  | | | |  | | | |  |  |  |  |  | Ath-AT4G11820.2 |  |
| 5 | Vvi-Vitvi02g04100\_t001 |  | | | |  | | | |  | | | |  | | | |  |  |  |  |  | | | |  |
| 5 | Vvi-Vitvi02g00421\_t001 |  | | | |  | | | |  | | | |  | Ath-AT5G51910.1 |  |  |  |  |  | | | |  |
| 5 | Vvi-Vitvi02g00422\_t001 |  | | | |  | | | |  | | | |  | | | |  |  |  |  |  | | | |  |
| 5 | Vvi-Vitvi02g00423\_t001 |  | | | |  | | | |  | Ath-AT1G63000.1 |  | | | |  |  |  |  |  | | | |  |
| 5 | Vvi-Vitvi02g00424\_t001 |  | | | |  | | | |  | | | |  | | | |  |  |  |  |  | Ath-AT4G11830.2 |  |
| 5 | Vvi-Vitvi02g04101\_t001 |  | | | |  | | | |  | | | |  | | | |  |  |  |  |  | | | |  |
| 5 | Vvi-Vitvi02g00425\_t001 |  | | | |  | | | |  | Ath-AT1G62990.1 |  | | | |  |  |  |  |  | | | |  |
| 5 | Vvi-Vitvi02g00426\_t001 |  | | | |  | Ath-AT4G22960.1 |  | | | |  | | | |  |  |  |  |  | Ath-AT4G11860.1 |  |
| 5 | Vvi-Vitvi02g00427\_t001 |  | | | |  | Ath-AT4G22950.1 |  | | | |  | Ath-AT5G51860.1 |  |  |  |  |  | Ath-AT4G11880.1 |  |
| 5 | Vvi-Vitvi02g04102\_t001 |  | | | |  | | | |  | | | |  | | | |  |  |  |  |  | | | |  |
| 5 | Vvi-Vitvi02g00429\_t002 |  | | | |  | Ath-AT4G22920.1 |  | | | |  | | | |  |  |  |  |  | Ath-AT4G11910.1 |  |
| 5 | Vvi-Vitvi02g00431\_t001 |  | | | |  | Ath-AT4G22910.1 |  | | | |  | | | |  |  |  |  |  | Ath-AT4G11920.1 |  |
| 5 | Vvi-Vitvi02g00432\_t001 |  | | | |  | Ath-AT4G22900.1 |  | Ath-AT1G62981.1 |  | | | |  |  |  |  |  | Ath-AT4G11950.1 |  |
| 5 | Vvi-Vitvi02g00433\_t001 |  | Ath-AT1G12560.1 |  | | | |  | Ath-AT1G62980.1 |  | | | |  |  |  |  |  | | | |  |
| 5 | Vvi-Vitvi02g00434\_t001 |  | | | |  | Ath-AT4G22890.3 |  | | | |  | | | |  |  |  |  |  | Ath-AT4G11960.1 |  |
| 5 | Vvi-Vitvi02g04103\_t001 |  | | | |  | | | |  | | | |  | | | |  |  |  |  |  | | | |  |
| 5 | Vvi-Vitvi02g04104\_t001 |  | | | |  | | | |  | | | |  | | | |  |  |  |  |  | | | |  |
| 5 | Vvi-Vitvi02g00435\_t001 |  | | | |  | Ath-AT4G22880.2 |  | | | |  | | | |  |  |  |  |  | | | |  |
| 4 | Vvi-Vitvi02g00436\_t001 |  | Ath-AT1G12550.1 |  |  |  | | | |  | | | |  |  |  |  |  | | | |  |
| 4 | Vvi-Vitvi02g00437\_t002 |  | | | |  |  |  | | | |  | | | |  |  |  |  |  | | | |  |
| 4 | Vvi-Vitvi02g00439\_t001 |  | Ath-AT1G12540.1 |  |  |  | Ath-AT1G62975.1 |  | Ath-AT5G51780.2 |  |  |  |  |  | | | |  |
| 2 | Vvi-Vitvi02g00440\_t002 |  | | | |  |  |  |  |  |  |  |  |  |  |  | Ath-AT4G11970.4 |  |
| 2 | Vvi-Vitvi02g00441\_t002 |  | Ath-AT1G12530.1 |  |  |  |  |  |  |  |  |  |  |  | | | |  |
| 2 | Vvi-Vitvi02g00442\_t001 |  | | | |  |  |  |  |  |  |  |  |  |  |  | | | |  |
| 2 | Vvi-Vitvi02g01411\_t001 |  | | | |  |  |  |  |  |  |  |  |  |  |  | | | |  |
| 2 | Vvi-Vitvi02g01412\_t001 |  | | | |  |  |  |  |  |  |  |  |  |  |  | | | |  |
| 2 | Vvi-Vitvi02g01413\_t001 |  | | | |  |  |  |  |  |  |  |  |  |  |  | | | |  |
| 2 | Vvi-Vitvi02g00443\_t001 |  | | | |  |  |  |  |  |  |  |  |  |  |  | | | |  |
| 2 | Vvi-Vitvi02g00444\_t001 |  | Ath-AT1G12520.1 |  |  |  |  |  |  |  |  |  |  |  | | | |  |
| 2 | Vvi-Vitvi02g04105\_t001 |  | | | |  |  |  |  |  |  |  |  |  |  |  | | | |  |
| 2 | Vvi-Vitvi02g00445\_t001 |  | | | |  |  |  |  |  |  |  |  |  |  |  | Ath-AT4G11980.1 |  |
| 2 | Vvi-Vitvi02g00446\_t001 |  | | | |  |  |  |  |  |  |  |  |  |  |  | | | |  |
| 2 | Vvi-Vitvi02g04106\_t001 |  | | | |  |  |  |  |  |  |  |  |  |  |  | | | |  |
| 2 | Vvi-Vitvi02g04107\_t001 |  | | | |  |  |  |  |  |  |  |  |  |  |  | | | |  |
| 2 | Vvi-Vitvi02g04108\_t001 |  | | | |  |  |  |  |  |  |  |  |  |  |  | | | |  |
| 2 | Vvi-Vitvi02g04109\_t001 |  | | | |  |  |  |  |  |  |  |  |  |  |  | | | |  |
| 2 | Vvi-Vitvi02g04110\_t001 |  | | | |  |  |  |  |  |  |  |  |  |  |  | | | |  |
| 2 | Vvi-Vitvi02g01414\_t001 |  | | | |  |  |  |  |  |  |  |  |  |  |  | | | |  |
| 2 | Vvi-Vitvi02g04111\_t001 |  | | | |  |  |  |  |  |  |  |  |  |  |  | | | |  |
| 2 | Vvi-Vitvi02g04112\_t001 |  | | | |  |  |  |  |  |  |  |  |  |  |  | | | |  |
| 2 | Vvi-Vitvi02g04113\_t001 |  | | | |  |  |  |  |  |  |  |  |  |  |  | | | |  |
| 2 | Vvi-Vitvi02g00448\_t001 |  | | | |  |  |  |  |  |  |  |  |  |  |  | | | |  |
| 2 | Vvi-Vitvi02g00449\_t001 |  | | | |  |  |  |  |  |  |  |  |  |  |  | | | |  |
| 2 | Vvi-Vitvi02g00450\_t001 |  | Ath-AT1G12500.1 |  |  |  |  |  |  |  |  |  |  |  | | | |  |
| 2 | Vvi-Vitvi02g04114\_t001 |  | | | |  |  |  |  |  |  |  |  |  |  |  | | | |  |
| 2 | Vvi-Vitvi02g00451\_t001 |  | Ath-AT1G12480.1 |  |  |  |  |  |  |  |  |  |  |  | | | |  |
| 2 | Vvi-Vitvi02g00452\_t001 |  | | | |  |  |  |  |  |  |  |  |  |  |  | | | |  |
| 2 | Vvi-Vitvi02g01417\_t001 |  | | | |  |  |  |  |  |  |  |  |  |  |  | | | |  |
| 4 | Vvi-Vitvi02g00454\_t001 |  | | | |  | Ath-AT5G62240.1 |  | Ath-AT4G22860.1 |  |  |  |  |  |  |  | Ath-AT4G11990.1 |  |
| 4 | Vvi-Vitvi02g04115\_t001 |  | | | |  | | | |  | | | |  |  |  |  |  |  |  | | | |  |
| 4 | Vvi-Vitvi02g04116\_t001 |  | | | |  | | | |  | | | |  |  |  |  |  |  |  | | | |  |
| 5 | Vvi-Vitvi02g04117\_t001 |  | | | |  | | | |  | | | |  | Ath-AT1G62960.1 |  |  |  |  |  | | | |  |
| 5 | Vvi-Vitvi02g04118\_t001 |  | Ath-AT1G12460.1 |  | | | |  | | | |  | Ath-AT1G62950.1 |  |  |  |  |  | | | |  |
| 5 | Vvi-Vitvi02g04119\_t001 |  | Ath-AT1G12450.1 |  | | | |  | Ath-AT4G22850.1 |  | | | |  |  |  |  |  | Ath-AT4G12000.2 |  |
| 5 | Vvi-Vitvi02g04120\_t001 |  | | | |  | | | |  | Ath-AT4G22840.1 |  | | | |  |  |  |  |  | Ath-AT4G12030.2 |  |
| 5 | Vvi-Vitvi02g00459\_t001 |  | | | |  | | | |  | Ath-AT4G22830.2 |  | | | |  |  |  |  |  | | | |  |
| 5 | Vvi-Vitvi02g00460\_t002 |  | | | |  | | | |  | | | |  | | | |  |  |  |  |  | | | |  |
| 5 | Vvi-Vitvi02g00461\_t002 |  | Ath-AT1G12440.2 |  | | | |  | Ath-AT4G22820.1 |  | | | |  |  |  |  |  | Ath-AT4G12040.1 |  |
| 5 | Vvi-Vitvi02g00462\_t001 |  | Ath-AT1G12430.2 |  | | | |  | | | |  | | | |  |  |  |  |  | | | |  |
| 6 | Vvi-Vitvi02g00465\_t001 |  | | | |  | | | |  | Ath-AT4G22810.1 |  | | | |  | Ath-AT2G45430.1 |  |  |  | Ath-AT4G12050.1 |  |
| 6 | Vvi-Vitvi02g00466\_t001 |  | | | |  | | | |  | Ath-AT4G22790.1 |  | | | |  | | | |  |  |  | | | |  |
| 6 | Vvi-Vitvi02g04121\_t001 |  | | | |  | | | |  | | | |  | | | |  | | | |  |  |  | | | |  |
| 6 | Vvi-Vitvi02g04122\_t001 |  | | | |  | | | |  | | | |  | | | |  | | | |  |  |  | | | |  |
| 6 | Vvi-Vitvi02g00467\_t001 |  | Ath-AT1G12420.1 |  | | | |  | Ath-AT4G22780.1 |  | | | |  | | | |  |  |  | | | |  |
| 6 | Vvi-Vitvi02g00468\_t001 |  | | | |  | Ath-AT5G62260.3 |  | Ath-AT4G22770.1 |  | | | |  | | | |  |  |  | Ath-AT4G12080.1 |  |
| 6 | Vvi-Vitvi02g04123\_t001 |  | | | |  | | | |  | | | |  | | | |  | | | |  |  |  | | | |  |
| 6 | Vvi-Vitvi02g04124\_t001 |  | | | |  | | | |  | | | |  | | | |  | | | |  |  |  | | | |  |
| 6 | Vvi-Vitvi02g00469\_t001 |  | Ath-AT1G12410.1 |  | | | |  | | | |  | | | |  | | | |  |  |  | | | |  |
| 6 | Vvi-Vitvi02g00470\_t001 |  | Ath-AT1G12400.3 |  | | | |  | | | |  | Ath-AT1G62886.1 |  | | | |  |  |  | | | |  |
| 6 | Vvi-Vitvi02g00471\_t001 |  | | | |  | | | |  | | | |  | Ath-AT1G62880.1 |  | | | |  |  |  | Ath-AT4G12090.1 |  |
| 6 | Vvi-Vitvi02g00472\_t001 |  | | | |  | | | |  | | | |  | | | |  | | | |  |  |  | | | |  |
| 6 | Vvi-Vitvi02g04125\_t001 |  | | | |  | | | |  | | | |  | | | |  | | | |  |  |  | | | |  |
| 6 | Vvi-Vitvi02g00474\_t001 |  | | | |  | | | |  | Ath-AT4G22760.1 |  | | | |  | | | |  |  |  | | | |  |
| 6 | Vvi-Vitvi02g01423\_t001 |  | | | |  | | | |  | Ath-AT4G22758.1 |  | | | |  | | | |  |  |  | | | |  |
| 6 | Vvi-Vitvi02g04126\_t001 |  | | | |  | | | |  | Ath-AT4G22755.1 |  | | | |  | | | |  |  |  | Ath-AT4G12110.1 |  |
| 6 | Vvi-Vitvi02g00475\_t001 |  | Ath-AT1G12370.2 |  | | | |  | | | |  | | | |  | | | |  |  |  | | | |  |
| 6 | Vvi-Vitvi02g00476\_t002 |  | | | |  | | | |  | | | |  | Ath-AT1G62870.1 |  | | | |  |  |  | | | |  |
| 6 | Vvi-Vitvi02g04127\_t001 |  | | | |  | | | |  | | | |  | | | |  | | | |  |  |  | | | |  |
| 6 | Vvi-Vitvi02g00477\_t001 |  | Ath-AT1G12330.1 |  | | | |  | | | |  | | | |  | | | |  |  |  | | | |  |
| 6 | Vvi-Vitvi02g04128\_t001 |  | | | |  | | | |  | | | |  | | | |  | | | |  |  |  | | | |  |
| 6 | Vvi-Vitvi02g04129\_t001 |  | | | |  | | | |  | Ath-AT4G22750.1 |  | | | |  | | | |  |  |  | | | |  |
| 6 | Vvi-Vitvi02g00479\_t001 |  | | | |  | | | |  | Ath-AT4G22740.1 |  | | | |  | Ath-AT2G45380.1 |  |  |  | | | |  |
| 6 | Vvi-Vitvi02g00480\_t001 |  | | | |  | | | |  | | | |  | Ath-AT1G62850.2 |  | | | |  |  |  | | | |  |
| 6 | Vvi-Vitvi02g00481\_t001 |  | Ath-AT1G12320.1 |  | Ath-AT5G62280.1 |  | | | |  | Ath-AT1G62840.1 |  | Ath-AT2G45360.1 |  |  |  | | | |  |
| 5 | Vvi-Vitvi02g00482\_t001 |  |  |  | | | |  | | | |  | | | |  | | | |  |  |  | Ath-AT4G12130.1 |  |
| 5 | Vvi-Vitvi02g00483\_t001 |  |  |  | | | |  | Ath-AT4G22730.1 |  | | | |  | Ath-AT2G45340.1 |  |  |  | | | |  |
| 5 | Vvi-Vitvi02g00485\_t001 |  |  |  | | | |  | | | |  | Ath-AT1G62820.1 |  | | | |  |  |  | | | |  |
| 5 | Vvi-Vitvi02g00486\_t001 |  |  |  | | | |  | | | |  | | | |  | | | |  |  |  | Ath-AT4G12230.1 |  |
| 5 | Vvi-Vitvi02g00487\_t001 |  |  |  | | | |  | | | |  | | | |  | | | |  |  |  | | | |  |
| 5 | Vvi-Vitvi02g04130\_t001 |  |  |  | | | |  | | | |  | | | |  | | | |  |  |  | Ath-AT4G12240.1 |  |
| 5 | Vvi-Vitvi02g04131\_t001 |  |  |  | | | |  | | | |  | | | |  | | | |  |  |  | | | |  |
| 5 | Vvi-Vitvi02g04132\_t001 |  |  |  | | | |  | | | |  | | | |  | Ath-AT2G45310.1 |  |  |  | Ath-AT4G12250.1 |  |
| 5 | Vvi-Vitvi02g04133\_t001 |  |  |  | | | |  | | | |  | | | |  | | | |  |  |  | | | |  |
| 5 | Vvi-Vitvi02g04134\_t001 |  |  |  | | | |  | | | |  | | | |  | | | |  |  |  | | | |  |
| 5 | Vvi-Vitvi02g04135\_t001 |  |  |  | | | |  | | | |  | | | |  | | | |  |  |  | | | |  |
| 5 | Vvi-Vitvi02g04136\_t001 |  |  |  | | | |  | | | |  | Ath-AT1G62810.1 |  | | | |  |  |  | Ath-AT4G12290.1 |  |
| 5 | Vvi-Vitvi02g04137\_t001 |  |  |  | | | |  | | | |  | | | |  | | | |  |  |  | | | |  |
| 5 | Vvi-Vitvi02g04138\_t001 |  |  |  | | | |  | | | |  | | | |  | | | |  |  |  | | | |  |
| 5 | Vvi-Vitvi02g01823\_t001 |  |  |  | Ath-AT5G62320.1 |  | Ath-AT4G22680.1 |  | | | |  | | | |  |  |  | | | |  |
| 5 | Vvi-Vitvi02g04139\_t001 |  |  |  | | | |  | | | |  | | | |  | | | |  |  |  | | | |  |
| 5 | Vvi-Vitvi02g00496\_t001 |  |  |  | | | |  | | | |  | | | |  | | | |  |  |  | Ath-AT4G12340.1 |  |
| 5 | Vvi-Vitvi02g00495\_t001 |  |  |  | | | |  | | | |  | | | |  | | | |  |  |  | | | |  |
| 5 | Vvi-Vitvi02g01427\_t001 |  |  |  | | | |  | | | |  | | | |  | | | |  |  |  | | | |  |
| 5 | Vvi-Vitvi02g04140\_t001 |  |  |  | | | |  | | | |  | | | |  | | | |  |  |  | | | |  |
| 5 | Vvi-Vitvi02g04141\_t001 |  |  |  | | | |  | | | |  | | | |  | | | |  |  |  | | | |  |
| 5 | Vvi-Vitvi02g01429\_t003 |  |  |  | | | |  | Ath-AT4G22670.1 |  | | | |  | | | |  |  |  | | | |  |
| 5 | Vvi-Vitvi02g04142\_t001 |  |  |  | | | |  | | | |  | Ath-AT1G62790.1 |  | | | |  |  |  | | | |  |
| 5 | Vvi-Vitvi02g00498\_t001 |  |  |  | | | |  | | | |  | Ath-AT1G62780.1 |  | | | |  |  |  | | | |  |
| 5 | Vvi-Vitvi02g00499\_t001 |  |  |  | Ath-AT5G62350.1 |  | | | |  | Ath-AT1G62770.1 |  | | | |  |  |  | Ath-AT4G12390.1 |  |
| 5 | Vvi-Vitvi02g00500\_t001 |  |  |  | Ath-AT5G62360.1 |  | | | |  | Ath-AT1G62760.1 |  | | | |  |  |  | | | |  |
| 5 | Vvi-Vitvi02g00501\_t001 |  |  |  | | | |  | | | |  | | | |  | | | |  |  |  | | | |  |
| 5 | Vvi-Vitvi02g04143\_t001 |  |  |  | | | |  | | | |  | | | |  | | | |  |  |  | | | |  |
| 5 | Vvi-Vitvi02g04144\_t001 |  |  |  | | | |  | | | |  | | | |  | | | |  |  |  | | | |  |
| 5 | Vvi-Vitvi02g01431\_t001 |  |  |  | | | |  | | | |  | | | |  | | | |  |  |  | | | |  |
| 5 | Vvi-Vitvi02g00502\_t001 |  |  |  | | | |  | | | |  | Ath-AT1G62750.1 |  | | | |  |  |  | | | |  |
| 6 | Vvi-Vitvi02g01852\_t001 |  | Ath-AT1G12270.1 |  | | | |  | | | |  | Ath-AT1G62740.1 |  | | | |  |  |  | Ath-AT4G12400.2 |  |
| 6 | Vvi-Vitvi02g00505\_t001 |  | | | |  | | | |  | | | |  | Ath-AT1G62710.1 |  | | | |  |  |  | | | |  |
| 6 | Vvi-Vitvi02g00507\_t001 |  | | | |  | | | |  | Ath-AT4G22620.1 |  | | | |  | Ath-AT2G45210.1 |  |  |  | Ath-AT4G12410.1 |  |
| 6 | Vvi-Vitvi02g00508\_t001 |  | Ath-AT1G12260.1 |  | Ath-AT5G62380.1 |  | | | |  | Ath-AT1G62700.1 |  | | | |  |  |  | | | |  |
| 5 | Vvi-Vitvi02g01432\_t001 |  | | | |  |  |  | | | |  | | | |  | | | |  |  |  | | | |  |
| 5 | Vvi-Vitvi02g00509\_t004 |  | Ath-AT1G12250.1 |  |  |  | | | |  | | | |  | | | |  |  |  | | | |  |
| 5 | Vvi-Vitvi02g01433\_t001 |  | | | |  |  |  | | | |  | | | |  | | | |  |  |  | | | |  |
| 5 | Vvi-Vitvi02g00510\_t001 |  | | | |  |  |  | | | |  | | | |  | Ath-AT2G45190.1 |  |  |  | | | |  |
| 4 | Vvi-Vitvi02g00511\_t001 |  | | | |  |  |  | | | |  | | | |  |  |  |  |  | Ath-AT4G12420.2 |  |
| 4 | Vvi-Vitvi02g00512\_t001 |  | Ath-AT1G12240.1 |  |  |  | | | |  | Ath-AT1G62660.1 |  |  |  |  |  | | | |  |
| 4 | Vvi-Vitvi02g04145\_t001 |  | Ath-AT1G12230.2 |  |  |  | | | |  | | | |  |  |  |  |  | | | |  |
| 4 | Vvi-Vitvi02g04146\_t001 |  | | | |  |  |  | Ath-AT4G22590.1 |  | | | |  |  |  |  |  | Ath-AT4G12430.1 |  |
| 4 | Vvi-Vitvi02g04147\_t001 |  | | | |  |  |  | | | |  | | | |  |  |  |  |  | | | |  |
| 4 | Vvi-Vitvi02g04148\_t001 |  | | | |  |  |  | | | |  | | | |  |  |  |  |  | | | |  |
| 4 | Vvi-Vitvi02g04149\_t001 |  | | | |  |  |  | | | |  | | | |  |  |  |  |  | | | |  |
| 4 | Vvi-Vitvi02g01697\_t001 |  | | | |  |  |  | | | |  | | | |  |  |  |  |  | | | |  |
| 4 | Vvi-Vitvi02g01696\_t001 |  | | | |  |  |  | Ath-AT4G22580.1 |  | | | |  |  |  |  |  | | | |  |
| 4 | Vvi-Vitvi02g01695\_t001 |  | | | |  |  |  | | | |  | Ath-AT1G62640.2 |  |  |  |  |  | | | |  |
| 4 | Vvi-Vitvi02g01693\_t001 |  | | | |  |  |  | | | |  | | | |  |  |  |  |  | | | |  |
| 4 | Vvi-Vitvi02g01692\_t001 |  | | | |  |  |  | | | |  | | | |  |  |  |  |  | | | |  |
| 4 | Vvi-Vitvi02g01691\_t001 |  | | | |  |  |  | | | |  | | | |  |  |  |  |  | | | |  |
| 4 | Vvi-Vitvi02g04150\_t001 |  | Ath-AT1G12200.1 |  |  |  | | | |  | Ath-AT1G62620.1 |  |  |  |  |  | | | |  |
| 4 | Vvi-Vitvi02g04151\_t001 |  | | | |  |  |  | | | |  | | | |  |  |  |  |  | | | |  |
| 4 | Vvi-Vitvi02g04152\_t001 |  | | | |  |  |  | | | |  | | | |  |  |  |  |  | | | |  |
| 4 | Vvi-Vitvi02g04153\_t001 |  | | | |  |  |  | | | |  | | | |  |  |  |  |  | | | |  |
| 4 | Vvi-Vitvi02g01689\_t001 |  | | | |  |  |  | | | |  | | | |  |  |  |  |  | | | |  |
| 4 | Vvi-Vitvi02g04154\_t001 |  | | | |  |  |  | | | |  | | | |  |  |  |  |  | | | |  |
| 4 | Vvi-Vitvi02g01687\_t001 |  | | | |  |  |  | | | |  | | | |  |  |  |  |  | | | |  |
| 4 | Vvi-Vitvi02g01685\_t001 |  | Ath-AT1G12140.1 |  |  |  | | | |  | Ath-AT1G62600.1 |  |  |  |  |  | | | |  |
| 4 | Vvi-Vitvi02g01683\_t001 |  | | | |  |  |  | | | |  | | | |  |  |  |  |  | | | |  |
| 4 | Vvi-Vitvi02g01682\_t002 |  | Ath-AT1G12120.1 |  |  |  | | | |  | Ath-AT1G62530.2 |  |  |  |  |  | | | |  |
| 4 | Vvi-Vitvi02g01847\_t001 |  | | | |  |  |  | | | |  | | | |  |  |  |  |  | | | |  |
| 4 | Vvi-Vitvi02g04155\_t001 |  | | | |  |  |  | Ath-AT4G22570.1 |  | | | |  |  |  |  |  | Ath-AT4G12440.2 |  |
| 4 | Vvi-Vitvi02g00526\_t001 |  | | | |  |  |  | Ath-AT4G22560.1 |  | Ath-AT1G62520.1 |  |  |  |  |  | Ath-AT4G12450.1 |  |
| 4 | Vvi-Vitvi02g00527\_t001 |  | | | |  |  |  | Ath-AT4G22550.1 |  | | | |  |  |  |  |  | | | |  |
| 4 | Vvi-Vitvi02g04156\_t002 |  | | | |  |  |  | Ath-AT4G22540.1 |  | | | |  |  |  |  |  | Ath-AT4G12460.7 |  |
| 3 | Vvi-Vitvi02g00529\_t001 |  | Ath-AT1G12110.1 |  |  |  |  |  | | | |  |  |  |  |  | | | |  |
| 3 | Vvi-Vitvi02g04157\_t001 |  | | | |  |  |  |  |  | | | |  |  |  |  |  | | | |  |
| 3 | Vvi-Vitvi02g01438\_t001 |  | Ath-AT1G12100.1 |  |  |  |  |  | | | |  |  |  |  |  | Ath-AT4G12490.1 |  |
| 1 | Vvi-Vitvi02g01439\_t001 |  |  |  |  |  |  |  | Ath-AT1G62510.1 |  |  |  |  |
| 2 | Vvi-Vitvi02g01440\_t001 |  | Ath-AT2G45180.1 |  |  |  |  |  | | | |  |  |  |  |
| 2 | Vvi-Vitvi02g00531\_t001 |  | | | |  |  |  |  |  | Ath-AT1G62500.1 |  |  |  |  |
| 1 | Vvi-Vitvi02g00532\_t001 |  | | | |  |  |  |  |  |  |  |
| 1 | Vvi-Vitvi02g04158\_t001 |  | | | |  |  |  |  |  |  |  |
| 1 | Vvi-Vitvi02g01441\_t002 |  | | | |  |  |  |  |  |  |  |
| 1 | Vvi-Vitvi02g01442\_t001 |  | | | |  |  |  |  |  |  |  |
| 1 | Vvi-Vitvi02g00533\_t001 |  | | | |  |  |  |  |  |  |  |
| 2 | Vvi-Vitvi02g00534\_t001 |  | | | |  | Ath-AT4G16510.1 |  |  |  |  |  |  |
| 2 | Vvi-Vitvi02g01443\_t001 |  | | | |  | | | |  |  |  |  |  |  |
| 2 | Vvi-Vitvi02g04159\_t001 |  | | | |  | | | |  |  |  |  |  |  |
| 3 | Vvi-Vitvi02g00535\_t001 |  | Ath-AT2G45170.1 |  | Ath-AT4G16520.1 |  | Ath-AT3G60640.1 |  |  |  |  |  |
| 3 | Vvi-Vitvi02g00536\_t002 |  | Ath-AT2G45160.1 |  | | | |  | Ath-AT3G60630.1 |  |  |  |  |  |
| 3 | Vvi-Vitvi02g04160\_t001 |  | | | |  | | | |  | | | |  |  |  |  |  |
| 5 | Vvi-Vitvi02g00538\_t001 |  | | | |  | | | |  | | | |  | Ath-AT4G17250.1 |  | Ath-AT5G47580.1 |  |  |  |
| 5 | Vvi-Vitvi02g04161\_t001 |  | | | |  | | | |  | | | |  | | | |  | | | |  |  |  |
| 5 | Vvi-Vitvi02g01445\_t001 |  | | | |  | | | |  | | | |  | | | |  | | | |  |  |  |
| 5 | Vvi-Vitvi02g00540\_t001 |  | | | |  | | | |  | | | |  | | | |  | | | |  |  |  |
| 5 | Vvi-Vitvi02g00541\_t001 |  | | | |  | | | |  | | | |  | | | |  | | | |  |  |  |
| 5 | Vvi-Vitvi02g00542\_t001 |  | | | |  | Ath-AT4G16535.1 |  | | | |  | | | |  | | | |  |  |  |
| 5 | Vvi-Vitvi02g00543\_t001 |  | | | |  | | | |  | | | |  | | | |  | | | |  |  |  |
| 5 | Vvi-Vitvi02g01446\_t001 |  | | | |  | | | |  | | | |  | | | |  | | | |  |  |  |
| 5 | Vvi-Vitvi02g01447\_t001 |  | | | |  | | | |  | | | |  | | | |  | | | |  |  |  |
| 5 | Vvi-Vitvi02g00545\_t001 |  | Ath-AT2G45140.1 |  | | | |  | Ath-AT3G60600.1 |  | | | |  | | | |  |  |  |
| 5 | Vvi-Vitvi02g00546\_t001 |  | | | |  | | | |  | | | |  | | | |  | Ath-AT5G47510.4 |  |  |  |
| 5 | Vvi-Vitvi02g00547\_t001 |  | | | |  | Ath-AT4G16563.1 |  | | | |  | | | |  | | | |  |  |  |
| 5 | Vvi-Vitvi02g00548\_t001 |  | | | |  | Ath-AT4G16566.1 |  | | | |  | | | |  | | | |  |  |  |
| 5 | Vvi-Vitvi02g00549\_t001 |  | | | |  | Ath-AT4G16570.1 |  | | | |  | | | |  | | | |  |  |  |
| 5 | Vvi-Vitvi02g00551\_t001 |  | | | |  | | | |  | | | |  | | | |  | | | |  |  |  |
| 5 | Vvi-Vitvi02g04162\_t001 |  | | | |  | | | |  | | | |  | | | |  | | | |  |  |  |
| 5 | Vvi-Vitvi02g04163\_t001 |  | | | |  | | | |  | | | |  | | | |  | | | |  |  |  |
| 5 | Vvi-Vitvi02g04164\_t001 |  | | | |  | Ath-AT4G16600.1 |  | | | |  | | | |  | | | |  |  |  |
| 5 | Vvi-Vitvi02g00553\_t001 |  | | | |  | | | |  | | | |  | | | |  | Ath-AT5G47500.1 |  |  |  |
| 5 | Vvi-Vitvi02g00554\_t001 |  | | | |  | | | |  | | | |  | | | |  | | | |  |  |  |
| 5 | Vvi-Vitvi02g04165\_t001 |  | | | |  | | | |  | | | |  | | | |  | | | |  |  |  |
| 5 | Vvi-Vitvi02g00558\_t001 |  | | | |  | | | |  | | | |  | Ath-AT4G17300.2 |  | | | |  |  |  |
| 5 | Vvi-Vitvi02g04166\_t001 |  | | | |  | | | |  | | | |  | | | |  | | | |  |  |  |
| 5 | Vvi-Vitvi02g04167\_t001 |  | | | |  | | | |  | | | |  | | | |  | Ath-AT5G47480.1 |  |  |  |
| 5 | Vvi-Vitvi02g04168\_t001 |  | | | |  | | | |  | | | |  | | | |  | | | |  |  |  |
| 5 | Vvi-Vitvi02g01452\_t001 |  | | | |  | | | |  | | | |  | | | |  | | | |  |  |  |
| 5 | Vvi-Vitvi02g00560\_t001 |  | Ath-AT2G45120.1 |  | Ath-AT4G16610.1 |  | Ath-AT3G60580.1 |  | | | |  | | | |  |  |  |
| 5 | Vvi-Vitvi02g04169\_t001 |  | | | |  | Ath-AT4G16620.1 |  | | | |  | | | |  | Ath-AT5G47470.2 |  |  |  |
| 5 | Vvi-Vitvi02g04170\_t001 |  | | | |  | | | |  | | | |  | | | |  | | | |  |  |  |
| 5 | Vvi-Vitvi02g00563\_t001 |  | | | |  | | | |  | | | |  | | | |  | Ath-AT5G47460.1 |  |  |  |
| 5 | Vvi-Vitvi02g01706\_t006 |  | | | |  | | | |  | | | |  | Ath-AT4G17310.3 |  | Ath-AT5G47455.4 |  |  |  |
| 5 | Vvi-Vitvi02g00564\_t001 |  | Ath-AT2G45040.1 |  | Ath-AT4G16640.1 |  | | | |  | | | |  | | | |  |  |  |
| 5 | Vvi-Vitvi02g00565\_t001 |  | | | |  | Ath-AT4G16650.1 |  | | | |  | | | |  | | | |  |  |  |
| 5 | Vvi-Vitvi02g00566\_t001 |  | | | |  | | | |  | | | |  | Ath-AT4G17330.1 |  | | | |  |  |  |
| 5 | Vvi-Vitvi02g00567\_t001 |  | | | |  | | | |  | | | |  | | | |  | | | |  |  |  |
| 5 | Vvi-Vitvi02g00568\_t001 |  | | | |  | | | |  | | | |  | Ath-AT4G17340.1 |  | Ath-AT5G47450.1 |  |  |  |
| 5 | Vvi-Vitvi02g00569\_t001 |  | | | |  | | | |  | Ath-AT3G60510.3 |  | | | |  | | | |  |  |  |
| 5 | Vvi-Vitvi02g00570\_t001 |  | | | |  | | | |  | | | |  | | | |  | | | |  |  |  |
| 5 | Vvi-Vitvi02g04171\_t001 |  | | | |  | Ath-AT4G16670.1 |  | | | |  | Ath-AT4G17350.1 |  | Ath-AT5G47440.1 |  |  |  |
| 5 | Vvi-Vitvi02g00572\_t001 |  | | | |  | | | |  | | | |  | Ath-AT4G17360.1 |  | Ath-AT5G47435.1 |  |  |  |
| 5 | Vvi-Vitvi02g04172\_t001 |  | | | |  | Ath-AT4G16695.5 |  | | | |  | | | |  | | | |  |  |  |
| 5 | Vvi-Vitvi02g00573\_t001 |  | | | |  | Ath-AT4G16700.1 |  | | | |  | | | |  | | | |  |  |  |
| 5 | Vvi-Vitvi02g00575\_t001 |  | | | |  | | | |  | | | |  | Ath-AT4G17370.1 |  | | | |  |  |  |
| 5 | Vvi-Vitvi02g04173\_t001 |  | | | |  | | | |  | | | |  | Ath-AT4G17380.1 |  | | | |  |  |  |
| 5 | Vvi-Vitvi02g01712\_t001 |  | | | |  | Ath-AT4G16720.1 |  | | | |  | Ath-AT4G17390.1 |  | | | |  |  |  |
| 5 | Vvi-Vitvi02g01713\_t001 |  | | | |  | | | |  | | | |  | | | |  | | | |  |  |  |
| 5 | Vvi-Vitvi02g01714\_t001 |  | | | |  | | | |  | | | |  | Ath-AT4G17410.2 |  | Ath-AT5G47430.6 |  |  |  |
| 5 | Vvi-Vitvi02g01716\_t001 |  | | | |  | | | |  | | | |  | | | |  | | | |  |  |  |
| 5 | Vvi-Vitvi02g01717\_t001 |  | Ath-AT2G44910.1 |  | Ath-AT4G16780.1 |  | | | |  | Ath-AT4G17460.1 |  | Ath-AT5G47370.1 |  |  |  |
| 3 | Vvi-Vitvi02g04174\_t001 |  | | | |  |  |  | | | |  | | | |  |  |  |  |
| 3 | Vvi-Vitvi02g04175\_t001 |  | | | |  |  |  | | | |  | | | |  |  |  |  |
| 3 | Vvi-Vitvi02g01718\_t001 |  | | | |  |  |  | Ath-AT3G60410.2 |  | | | |  |  |  |  |
| 3 | Vvi-Vitvi02g04176\_t001 |  | | | |  |  |  | | | |  | | | |  |  |  |  |
| 3 | Vvi-Vitvi02g01720\_t001 |  | | | |  |  |  | | | |  | | | |  |  |  |  |
| 3 | Vvi-Vitvi02g01721\_t001 |  | | | |  |  |  | | | |  | | | |  |  |  |  |
| 3 | Vvi-Vitvi02g04177\_t001 |  | | | |  |  |  | | | |  | | | |  |  |  |  |
| 3 | Vvi-Vitvi02g01723\_t001 |  | | | |  |  |  | | | |  | | | |  |  |  |  |
| 3 | Vvi-Vitvi02g01726\_t001 |  | | | |  |  |  | | | |  | | | |  |  |  |  |
| 3 | Vvi-Vitvi02g01727\_t001 |  | | | |  |  |  | | | |  | | | |  |  |  |  |
| 3 | Vvi-Vitvi02g01728\_t001 |  | | | |  |  |  | | | |  | | | |  |  |  |  |
| 3 | Vvi-Vitvi02g04178\_t001 |  | | | |  |  |  | | | |  | | | |  |  |  |  |
| 3 | Vvi-Vitvi02g04179\_t001 |  | | | |  |  |  | | | |  | | | |  |  |  |  |
| 3 | Vvi-Vitvi02g04180\_t001 |  | | | |  |  |  | | | |  | | | |  |  |  |  |
| 3 | Vvi-Vitvi02g04181\_t001 |  | | | |  |  |  | | | |  | | | |  |  |  |  |
| 3 | Vvi-Vitvi02g00590\_t001 |  | | | |  |  |  | | | |  | Ath-AT4G17620.1 |  |  |  |  |
| 4 | Vvi-Vitvi02g00593\_t001 |  | | | |  | Ath-AT5G47090.1 |  | | | |  | | | |  |  |  |  |
| 4 | Vvi-Vitvi02g04182\_t001 |  | | | |  | | | |  | | | |  | | | |  |  |  |  |
| 4 | Vvi-Vitvi02g00595\_t001 |  | | | |  | | | |  | | | |  | | | |  |  |  |  |
| 4 | Vvi-Vitvi02g04183\_t001 |  | | | |  | | | |  | | | |  | | | |  |  |  |  |
| 4 | Vvi-Vitvi02g00597\_t001 |  | Ath-AT2G44680.1 |  | Ath-AT5G47080.1 |  | Ath-AT3G60250.1 |  | Ath-AT4G17640.1 |  |  |  |  |
| 4 | Vvi-Vitvi02g00598\_t003 |  | | | |  | | | |  | | | |  | | | |  |  |  |  |
| 4 | Vvi-Vitvi02g00599\_t001 |  | | | |  | | | |  | Ath-AT3G60245.1 |  | | | |  |  |  |  |
| 3 | Vvi-Vitvi02g00600\_t001 |  | | | |  | | | |  |  |  | | | |  |  |  |  |
| 3 | Vvi-Vitvi02g00603\_t001 |  | | | |  | | | |  |  |  | | | |  |  |  |  |
| 3 | Vvi-Vitvi02g00604\_t001 |  | | | |  | Ath-AT5G47070.1 |  |  |  | Ath-AT4G17660.1 |  |  |  |  |
| 3 | Vvi-Vitvi02g01470\_t001 |  | | | |  | | | |  |  |  | | | |  |  |  |  |
| 3 | Vvi-Vitvi02g00605\_t001 |  | | | |  | | | |  |  |  | | | |  |  |  |  |
| 3 | Vvi-Vitvi02g00606\_t001 |  | | | |  | | | |  |  |  | | | |  |  |  |  |
| 3 | Vvi-Vitvi02g00607\_t001 |  | | | |  | | | |  |  |  | | | |  |  |  |  |
| 3 | Vvi-Vitvi02g00614\_t001 |  | Ath-AT2G44670.1 |  | Ath-AT5G47060.1 |  |  |  | Ath-AT4G17670.1 |  |  |  |  |
| 3 | Vvi-Vitvi02g01471\_t001 |  | | | |  | Ath-AT5G47050.1 |  |  |  | Ath-AT4G17680.1 |  |  |  |  |
| 2 | Vvi-Vitvi02g00615\_t001 |  | | | |  | | | |  |  |  |  |  |  |
| 2 | Vvi-Vitvi02g00617\_t001 |  | | | |  | Ath-AT5G47040.1 |  |  |  |  |  |  |
| 2 | Vvi-Vitvi02g00618\_t001 |  | | | |  | | | |  |  |  |  |  |  |
| 2 | Vvi-Vitvi02g00619\_t001 |  | | | |  | | | |  |  |  |  |  |  |
| 2 | Vvi-Vitvi02g00620\_t001 |  | | | |  | | | |  |  |  |  |  |  |
| 2 | Vvi-Vitvi02g04184\_t001 |  | | | |  | | | |  |  |  |  |  |  |
| 2 | Vvi-Vitvi02g01472\_t001 |  | | | |  | | | |  |  |  |  |  |  |
| 2 | Vvi-Vitvi02g01473\_t001 |  | | | |  | | | |  |  |  |  |  |  |
| 2 | Vvi-Vitvi02g01474\_t001 |  | | | |  | | | |  |  |  |  |  |  |
| 2 | Vvi-Vitvi02g01479\_t001 |  | | | |  | | | |  |  |  |  |  |  |
| 2 | Vvi-Vitvi02g00626\_t001 |  | | | |  | Ath-AT5G47020.1 |  |  |  |  |  |  |
| 2 | Vvi-Vitvi02g04185\_t001 |  | | | |  | | | |  |  |  |  |  |  |
| 2 | Vvi-Vitvi02g01480\_t001 |  | | | |  | | | |  |  |  |  |  |  |
| 2 | Vvi-Vitvi02g00627\_t001 |  | | | |  | Ath-AT5G47010.1 |  |  |  |  |  |  |
| 2 | Vvi-Vitvi02g00628\_t001 |  | | | |  | | | |  |  |  |  |  |  |
| 2 | Vvi-Vitvi02g01481\_t001 |  | | | |  | Ath-AT5G46930.1 |  |  |  |  |  |  |
| 1 | Vvi-Vitvi02g00629\_t001 |  | | | |  |  |  |  |  |  |  |
| 1 | Vvi-Vitvi02g01482\_t001 |  | | | |  |  |  |  |  |  |  |
| 1 | Vvi-Vitvi02g00630\_t001 |  | | | |  |  |  |  |  |  |  |
| 1 | Vvi-Vitvi02g00633\_t001 |  | Ath-AT2G44530.1 |  |  |  |  |  |  |  |
| 0 | Vvi-Vitvi02g00634\_t001 |  |  |  |  |  |  |  |  |
| 0 | Vvi-Vitvi02g01483\_t001 |  |  |  |  |  |  |  |  |
| 0 | Vvi-Vitvi02g00635\_t001 |  |  |  |  |  |  |  |  |
| 0 | Vvi-Vitvi02g04186\_t001 |  |  |  |  |  |  |  |  |
| 0 | Vvi-Vitvi02g00636\_t002 |  |  |  |  |  |  |  |  |
| 0 | Vvi-Vitvi02g04187\_t001 |  |  |  |  |  |  |  |  |
| 0 | Vvi-Vitvi02g00640\_t001 |  |  |  |  |  |  |  |  |
| 0 | Vvi-Vitvi02g00641\_t001 |  |  |  |  |  |  |  |  |
| 0 | Vvi-Vitvi02g00642\_t001 |  |  |  |  |  |  |  |  |
| 0 | Vvi-Vitvi02g00645\_t001 |  |  |  |  |  |  |  |  |
| 0 | Vvi-Vitvi02g01484\_t001 |  |  |  |  |  |  |  |  |
| 0 | Vvi-Vitvi02g01485\_t001 |  |  |  |  |  |  |  |  |
| 0 | Vvi-Vitvi02g01486\_t001 |  |  |  |  |  |  |  |  |
| 0 | Vvi-Vitvi02g04188\_t001 |  |  |  |  |  |  |  |  |
| 0 | Vvi-Vitvi02g04189\_t001 |  |  |  |  |  |  |  |  |
| 0 | Vvi-Vitvi02g01488\_t001 |  |  |  |  |  |  |  |  |
| 0 | Vvi-Vitvi02g04190\_t001 |  |  |  |  |  |  |  |  |
| 0 | Vvi-Vitvi02g04191\_t001 |  |  |  |  |  |  |  |  |
| 0 | Vvi-Vitvi02g01492\_t001 |  |  |  |  |  |  |  |  |
| 0 | Vvi-Vitvi02g00648\_t001 |  |  |  |  |  |  |  |  |
| 0 | Vvi-Vitvi02g00649\_t001 |  |  |  |  |  |  |  |  |
| 0 | Vvi-Vitvi02g00650\_t003 |  |  |  |  |  |  |  |  |
| 0 | Vvi-Vitvi02g04192\_t001 |  |  |  |  |  |  |  |  |
| 0 | Vvi-Vitvi02g00653\_t001 |  |  |  |  |  |  |  |  |
| 0 | Vvi-Vitvi02g00654\_t001 |  |  |  |  |  |  |  |  |
| 0 | Vvi-Vitvi02g00655\_t001 |  |  |  |  |  |  |  |  |
| 0 | Vvi-Vitvi02g04193\_t001 |  |  |  |  |  |  |  |  |
| 0 | Vvi-Vitvi02g00656\_t001 |  |  |  |  |  |  |  |  |
| 1 | Vvi-Vitvi02g00658\_t001 |  | Ath-AT2G35270.1 |  |  |  |  |  |  |  |
| 1 | Vvi-Vitvi02g00659\_t001 |  | Ath-AT2G35290.1 |  |  |  |  |  |  |  |
| 2 | Vvi-Vitvi02g00660\_t001 |  | | | |  | Ath-AT5G46640.1 |  |  |  |  |  |  |
| 2 | Vvi-Vitvi02g00661\_t001 |  | | | |  | Ath-AT5G46630.2 |  |  |  |  |  |  |
| 2 | Vvi-Vitvi02g00662\_t001 |  | | | |  | | | |  |  |  |  |  |  |
| 2 | Vvi-Vitvi02g00663\_t001 |  | | | |  | Ath-AT5G46620.1 |  |  |  |  |  |  |
| 3 | Vvi-Vitvi02g01498\_t001 |  | Ath-AT2G35300.1 |  | | | |  | Ath-AT1G32560.1 |  |  |  |  |  |
| 3 | Vvi-Vitvi02g04194\_t001 |  | | | |  | | | |  | | | |  |  |  |  |  |
| 3 | Vvi-Vitvi02g00664\_t001 |  | | | |  | | | |  | | | |  |  |  |  |  |
| 3 | Vvi-Vitvi02g01499\_t001 |  | | | |  | | | |  | | | |  |  |  |  |  |
| 3 | Vvi-Vitvi02g04195\_t001 |  | | | |  | | | |  | | | |  |  |  |  |  |
| 3 | Vvi-Vitvi02g00665\_t001 |  | | | |  | | | |  | Ath-AT1G32550.2 |  |  |  |  |  |
| 3 | Vvi-Vitvi02g00666\_t003 |  | | | |  | | | |  | Ath-AT1G32540.1 |  |  |  |  |  |
| 3 | Vvi-Vitvi02g00667\_t001 |  | Ath-AT2G35320.1 |  | | | |  | | | |  |  |  |  |  |
| 3 | Vvi-Vitvi02g04196\_t001 |  | | | |  | | | |  | | | |  |  |  |  |  |
| 3 | Vvi-Vitvi02g00669\_t001 |  | | | |  | | | |  | | | |  |  |  |  |  |
| 3 | Vvi-Vitvi02g00670\_t001 |  | Ath-AT2G35330.1 |  | | | |  | Ath-AT1G32530.1 |  |  |  |  |  |
| 3 | Vvi-Vitvi02g04197\_t001 |  | | | |  | | | |  | | | |  |  |  |  |  |
| 3 | Vvi-Vitvi02g04198\_t001 |  | | | |  | | | |  | | | |  |  |  |  |  |
| 3 | Vvi-Vitvi02g04199\_t001 |  | | | |  | | | |  | | | |  |  |  |  |  |
| 3 | Vvi-Vitvi02g00671\_t001 |  | | | |  | Ath-AT5G46600.1 |  | | | |  |  |  |  |  |
| 3 | Vvi-Vitvi02g00672\_t001 |  | | | |  | | | |  | Ath-AT1G32520.1 |  |  |  |  |  |
| 3 | Vvi-Vitvi02g00673\_t001 |  | | | |  | Ath-AT5G46590.1 |  | Ath-AT1G32510.1 |  |  |  |  |  |
| 3 | Vvi-Vitvi02g00674\_t001 |  | | | |  | | | |  | Ath-AT1G32500.1 |  |  |  |  |  |
| 3 | Vvi-Vitvi02g04200\_t001 |  | | | |  | | | |  | | | |  |  |  |  |  |
| 3 | Vvi-Vitvi02g04201\_t001 |  | | | |  | | | |  | | | |  |  |  |  |  |
| 3 | Vvi-Vitvi02g00677\_t001 |  | | | |  | Ath-AT5G46580.1 |  | | | |  |  |  |  |  |
| 3 | Vvi-Vitvi02g00678\_t001 |  | Ath-AT2G35350.1 |  | Ath-AT2G35350.1 |  | | | |  |  |  |  |  |
| 3 | Vvi-Vitvi02g00679\_t002 |  | Ath-AT2G35360.1 |  | | | |  | | | |  |  |  |  |  |
| 3 | Vvi-Vitvi02g00680\_t001 |  | Ath-AT2G35370.1 |  | | | |  | Ath-AT1G32470.1 |  |  |  |  |  |
| 4 | Vvi-Vitvi02g00682\_t001 |  | | | |  | | | |  | | | |  | Ath-AT5G46570.1 |  |  |  |  |
| 4 | Vvi-Vitvi02g00683\_t001 |  | | | |  | | | |  | Ath-AT1G32450.1 |  | | | |  |  |  |  |
| 4 | Vvi-Vitvi02g00684\_t001 |  | | | |  | | | |  | Ath-AT1G32440.1 |  | | | |  |  |  |  |
| 3 | Vvi-Vitvi02g00685\_t001 |  | | | |  | | | |  |  |  | | | |  |  |  |  |
| 4 | Vvi-Vitvi02g00686\_t001 |  | | | |  | | | |  | Ath-AT4G17810.1 |  | | | |  |  |  |  |
| 4 | Vvi-Vitvi02g00688\_t001 |  | | | |  | | | |  | Ath-AT4G17830.2 |  | | | |  |  |  |  |
| 4 | Vvi-Vitvi02g00689\_t001 |  | | | |  | Ath-AT2G35260.1 |  | Ath-AT4G17840.1 |  | | | |  |  |  |  |
| 4 | Vvi-Vitvi02g00691\_t003 |  | | | |  | | | |  | Ath-AT4G17850.1 |  | | | |  |  |  |  |
| 5 | Vvi-Vitvi02g00693\_t001 |  | | | |  | | | |  | | | |  | | | |  | Ath-AT5G46800.1 |  |  |  |
| 5 | Vvi-Vitvi02g00694\_t001 |  | | | |  | | | |  | | | |  | | | |  | Ath-AT5G46795.1 |  |  |  |
| 5 | Vvi-Vitvi02g00695\_t001 |  | | | |  | | | |  | Ath-AT4G17870.1 |  | | | |  | Ath-AT5G46790.1 |  |  |  |
| 5 | Vvi-Vitvi02g00696\_t001 |  | | | |  | Ath-AT2G35230.3 |  | | | |  | | | |  | Ath-AT5G46780.1 |  |  |  |
| 5 | Vvi-Vitvi02g01504\_t001 |  | | | |  | | | |  | | | |  | | | |  | | | |  |  |  |
| 5 | Vvi-Vitvi02g01505\_t001 |  | | | |  | Ath-AT2G35215.1 |  | | | |  | | | |  | | | |  |  |  |
| 5 | Vvi-Vitvi02g00698\_t001 |  | | | |  | | | |  | Ath-AT4G17880.1 |  | | | |  | Ath-AT5G46760.1 |  |  |  |
| 5 | Vvi-Vitvi02g00699\_t001 |  | | | |  | Ath-AT2G35210.1 |  | Ath-AT4G17890.1 |  | | | |  | Ath-AT5G46750.1 |  |  |  |
| 5 | Vvi-Vitvi02g00700\_t001 |  | | | |  | | | |  | Ath-AT4G17895.1 |  | | | |  | Ath-AT5G46740.1 |  |  |  |
| 5 | Vvi-Vitvi02g01506\_t001 |  | | | |  | | | |  | | | |  | | | |  | | | |  |  |  |
| 5 | Vvi-Vitvi02g04202\_t001 |  | | | |  | | | |  | | | |  | | | |  | | | |  |  |  |
| 5 | Vvi-Vitvi02g00701\_t001 |  | | | |  | | | |  | | | |  | | | |  | | | |  |  |  |
| 5 | Vvi-Vitvi02g01507\_t001 |  | | | |  | | | |  | | | |  | | | |  | | | |  |  |  |
| 5 | Vvi-Vitvi02g00702\_t001 |  | | | |  | | | |  | | | |  | | | |  | | | |  |  |  |
| 5 | Vvi-Vitvi02g00703\_t001 |  | | | |  | Ath-AT2G35200.1 |  | | | |  | | | |  | | | |  |  |  |
| 5 | Vvi-Vitvi02g00704\_t001 |  | | | |  | | | |  | Ath-AT4G17900.1 |  | Ath-AT5G46710.1 |  | | | |  |  |  |
| 5 | Vvi-Vitvi02g00705\_t001 |  | | | |  | | | |  | | | |  | | | |  | Ath-AT5G46700.1 |  |  |  |
| 5 | Vvi-Vitvi02g00706\_t001 |  | Ath-AT2G35410.1 |  | | | |  | | | |  | | | |  | | | |  |  |  |
| 5 | Vvi-Vitvi02g00708\_t001 |  | | | |  | | | |  | Ath-AT4G17905.2 |  | | | |  | | | |  |  |  |
| 5 | Vvi-Vitvi02g00709\_t001 |  | | | |  | | | |  | | | |  | | | |  | Ath-AT5G46690.1 |  |  |  |
| 5 | Vvi-Vitvi02g00710\_t001 |  | | | |  | | | |  | Ath-AT4G17910.4 |  | | | |  | | | |  |  |  |
| 5 | Vvi-Vitvi02g04203\_t001 |  | | | |  | | | |  | | | |  | | | |  | | | |  |  |  |
| 5 | Vvi-Vitvi02g04204\_t001 |  | | | |  | | | |  | | | |  | | | |  | | | |  |  |  |
| 5 | Vvi-Vitvi02g00711\_t001 |  | | | |  | | | |  | | | |  | | | |  | | | |  |  |  |
| 5 | Vvi-Vitvi02g04205\_t001 |  | | | |  | | | |  | | | |  | | | |  | | | |  |  |  |
| 5 | Vvi-Vitvi02g04206\_t001 |  | | | |  | | | |  | | | |  | | | |  | | | |  |  |  |
| 5 | Vvi-Vitvi02g00713\_t001 |  | | | |  | | | |  | | | |  | | | |  | | | |  |  |  |
| 6 | Vvi-Vitvi02g00714\_t001 |  | Ath-AT2G35420.1 |  | Ath-AT2G35000.1 |  | Ath-AT4G17920.1 |  | | | |  | Ath-AT5G46650.1 |  | Ath-AT4G17920.1 |  |  |
| 5 | Vvi-Vitvi02g00715\_t001 |  | Ath-AT2G35430.1 |  | Ath-AT1G32360.1 |  | | | |  | | | |  |  |  | | | |  |  |
| 5 | Vvi-Vitvi02g00717\_t002 |  | | | |  | | | |  | | | |  | | | |  |  |  | | | |  |  |
| 5 | Vvi-Vitvi02g00718\_t001 |  | | | |  | | | |  | | | |  | | | |  |  |  | | | |  |  |
| 5 | Vvi-Vitvi02g04207\_t001 |  | | | |  | | | |  | | | |  | | | |  |  |  | | | |  |  |
| 5 | Vvi-Vitvi02g00719\_t001 |  | | | |  | | | |  | Ath-AT4G17940.1 |  | | | |  |  |  | | | |  |  |
| 4 | Vvi-Vitvi02g00721\_t001 |  | | | |  | | | |  |  |  | | | |  |  |  | | | |  |  |
| 4 | Vvi-Vitvi02g00722\_t001 |  | | | |  | | | |  |  |  | Ath-AT5G46840.1 |  |  |  | | | |  |  |
| 4 | Vvi-Vitvi02g00723\_t001 |  | | | |  | | | |  |  |  | | | |  |  |  | Ath-AT4G17790.1 |  |  |
| 4 | Vvi-Vitvi02g00724\_t001 |  | | | |  | | | |  |  |  | | | |  |  |  | | | |  |  |
| 4 | Vvi-Vitvi02g00725\_t001 |  | | | |  | | | |  |  |  | | | |  |  |  | Ath-AT4G17785.1 |  |  |
| 4 | Vvi-Vitvi02g00726\_t001 |  | | | |  | | | |  |  |  | | | |  |  |  | | | |  |  |
| 4 | Vvi-Vitvi02g00728\_t001 |  | | | |  | | | |  |  |  | | | |  |  |  | | | |  |  |
| 4 | Vvi-Vitvi02g00729\_t003 |  | | | |  | | | |  |  |  | | | |  |  |  | Ath-AT4G17770.2 |  |  |
| 4 | Vvi-Vitvi02g04208\_t001 |  | | | |  | | | |  |  |  | | | |  |  |  | | | |  |  |
| 4 | Vvi-Vitvi02g04209\_t001 |  | | | |  | | | |  |  |  | | | |  |  |  | | | |  |  |
| 4 | Vvi-Vitvi02g00732\_t001 |  | | | |  | Ath-AT1G32340.1 |  |  |  | | | |  |  |  | | | |  |  |
| 4 | Vvi-Vitvi02g00733\_t001 |  | | | |  | | | |  |  |  | | | |  |  |  | | | |  |  |
| 4 | Vvi-Vitvi02g04210\_t001 |  | | | |  | | | |  |  |  | | | |  |  |  | | | |  |  |
| 4 | Vvi-Vitvi02g00734\_t001 |  | | | |  | | | |  |  |  | | | |  |  |  | Ath-AT4G17760.1 |  |  |
| 4 | Vvi-Vitvi02g00735\_t001 |  | | | |  | | | |  |  |  | | | |  |  |  | | | |  |  |
| 4 | Vvi-Vitvi02g04211\_t001 |  | | | |  | | | |  |  |  | | | |  |  |  | | | |  |  |
| 4 | Vvi-Vitvi02g00737\_t001 |  | | | |  | | | |  |  |  | | | |  |  |  | | | |  |  |
| 4 | Vvi-Vitvi02g00738\_t001 |  | Ath-AT2G35500.1 |  | | | |  |  |  | | | |  |  |  | | | |  |  |
| 4 | Vvi-Vitvi02g00739\_t001 |  | | | |  | Ath-AT1G32330.1 |  |  |  | | | |  |  |  | Ath-AT4G17750.1 |  |  |
| 4 | Vvi-Vitvi02g04212\_t001 |  | | | |  | | | |  |  |  | | | |  |  |  | | | |  |  |
| 4 | Vvi-Vitvi02g00741\_t001 |  | | | |  | | | |  |  |  | | | |  |  |  | | | |  |  |
| 4 | Vvi-Vitvi02g00744\_t001 |  | | | |  | | | |  |  |  | | | |  |  |  | | | |  |  |
| 4 | Vvi-Vitvi02g00746\_t001 |  | | | |  | | | |  |  |  | Ath-AT5G46850.2 |  |  |  | | | |  |  |
| 4 | Vvi-Vitvi02g00747\_t001 |  | | | |  | Ath-AT1G32270.1 |  |  |  | Ath-AT5G46860.1 |  |  |  | Ath-AT4G17730.2 |  |  |
| 4 | Vvi-Vitvi02g00749\_t001 |  | | | |  | | | |  |  |  | Ath-AT5G46870.1 |  |  |  | Ath-AT4G17720.1 |  |  |
| 4 | Vvi-Vitvi02g01513\_t001 |  | | | |  | | | |  |  |  | | | |  |  |  | | | |  |  |
| 4 | Vvi-Vitvi02g01514\_t002 |  | | | |  | | | |  |  |  | | | |  |  |  | | | |  |  |
| 4 | Vvi-Vitvi02g04213\_t001 |  | | | |  | | | |  |  |  | | | |  |  |  | | | |  |  |
| 4 | Vvi-Vitvi02g00750\_t001 |  | | | |  | | | |  |  |  | | | |  |  |  | | | |  |  |
| 4 | Vvi-Vitvi02g01515\_t001 |  | | | |  | | | |  |  |  | | | |  |  |  | | | |  |  |
| 4 | Vvi-Vitvi02g00751\_t001 |  | Ath-AT2G35510.3 |  | | | |  |  |  | | | |  |  |  | | | |  |  |
| 4 | Vvi-Vitvi02g00752\_t001 |  | | | |  | | | |  |  |  | | | |  |  |  | | | |  |  |
| 4 | Vvi-Vitvi02g00757\_t002 |  | | | |  | Ath-AT1G32240.1 |  |  |  | | | |  |  |  | Ath-AT4G17695.1 |  |  |
| 3 | Vvi-Vitvi02g00758\_t001 |  | | | |  | | | |  |  |  | | | |  |  |  |  |
| 3 | Vvi-Vitvi02g00759\_t001 |  | | | |  | | | |  |  |  | Ath-AT5G46910.1 |  |  |  |  |
| 2 | Vvi-Vitvi02g00762\_t001 |  | | | |  | | | |  |  |  |  |  |  |
| 2 | Vvi-Vitvi02g04214\_t001 |  | | | |  | | | |  |  |  |  |  |  |
| 2 | Vvi-Vitvi02g00763\_t001 |  | | | |  | | | |  |  |  |  |  |  |
| 2 | Vvi-Vitvi02g00768\_t001 |  | | | |  | | | |  |  |  |  |  |  |
| 2 | Vvi-Vitvi02g00771\_t001 |  | | | |  | | | |  |  |  |  |  |  |
| 2 | Vvi-Vitvi02g00772\_t002 |  | | | |  | | | |  |  |  |  |  |  |
| 2 | Vvi-Vitvi02g00773\_t001 |  | | | |  | | | |  |  |  |  |  |  |
| 2 | Vvi-Vitvi02g00774\_t001 |  | | | |  | | | |  |  |  |  |  |  |
| 2 | Vvi-Vitvi02g00775\_t001 |  | | | |  | | | |  |  |  |  |  |  |
| 2 | Vvi-Vitvi02g00777\_t001 |  | | | |  | | | |  |  |  |  |  |  |
| 2 | Vvi-Vitvi02g00780\_t001 |  | Ath-AT2G35520.2 |  | Ath-AT1G32210.1 |  |  |  |  |  |  |
| 2 | Vvi-Vitvi02g04216\_t001 |  | | | |  | | | |  |  |  |  |  |  |
| 2 | Vvi-Vitvi02g00783\_t001 |  | | | |  | | | |  |  |  |  |  |  |
| 2 | Vvi-Vitvi02g00784\_t001 |  | | | |  | | | |  |  |  |  |  |  |
| 2 | Vvi-Vitvi02g00785\_t001 |  | | | |  | Ath-AT1G32200.2 |  |  |  |  |  |  |
| 2 | Vvi-Vitvi02g00787\_t001 |  | | | |  | Ath-AT1G32190.1 |  |  |  |  |  |  |
| 2 | Vvi-Vitvi02g00790\_t001 |  | | | |  | | | |  |  |  |  |  |  |
| 2 | Vvi-Vitvi02g00792\_t001 |  | | | |  | Ath-AT1G32170.1 |  |  |  |  |  |  |
| 2 | Vvi-Vitvi02g00795\_t001 |  | | | |  | Ath-AT1G32160.1 |  |  |  |  |  |  |
| 2 | Vvi-Vitvi02g00796\_t003 |  | Ath-AT2G35530.1 |  | Ath-AT1G32150.1 |  |  |  |  |  |  |
| 1 | Vvi-Vitvi02g00798\_t001 |  | | | |  |  |  |  |  |  |  |
| 1 | Vvi-Vitvi02g00802\_t001 |  | Ath-AT2G35540.1 |  |  |  |  |  |  |  |
| 0 | Vvi-Vitvi02g04217\_t001 |  |  |  |  |  |  |  |  |
| 0 | Vvi-Vitvi02g04218\_t001 |  |  |  |  |  |  |  |  |
| 0 | Vvi-Vitvi02g00805\_t001 |  |  |  |  |  |  |  |  |
| 0 | Vvi-Vitvi02g04219\_t001 |  |  |  |  |  |  |  |  |
| 0 | Vvi-Vitvi02g04220\_t001 |  |  |  |  |  |  |  |  |
| 0 | Vvi-Vitvi02g00808\_t001 |  |  |  |  |  |  |  |  |
| 0 | Vvi-Vitvi02g00809\_t001 |  |  |  |  |  |  |  |  |
| 0 | Vvi-Vitvi02g04221\_t001 |  |  |  |  |  |  |  |  |
| 0 | Vvi-Vitvi02g01526\_t001 |  |  |  |  |  |  |  |  |
| 0 | Vvi-Vitvi02g04222\_t001 |  |  |  |  |  |  |  |  |
| 0 | Vvi-Vitvi02g04223\_t001 |  |  |  |  |  |  |  |  |
| 0 | Vvi-Vitvi02g04224\_t001 |  |  |  |  |  |  |  |  |
| 0 | Vvi-Vitvi02g04225\_t001 |  |  |  |  |  |  |  |  |
| 0 | Vvi-Vitvi02g00818\_t001 |  |  |  |  |  |  |  |  |
| 0 | Vvi-Vitvi02g04226\_t001 |  |  |  |  |  |  |  |  |
| 0 | Vvi-Vitvi02g04227\_t001 |  |  |  |  |  |  |  |  |
| 0 | Vvi-Vitvi02g04228\_t001 |  |  |  |  |  |  |  |  |
| 0 | Vvi-Vitvi02g04229\_t001 |  |  |  |  |  |  |  |  |
| 0 | Vvi-Vitvi02g04230\_t001 |  |  |  |  |  |  |  |  |
| 0 | Vvi-Vitvi02g04231\_t001 |  |  |  |  |  |  |  |  |
| 0 | Vvi-Vitvi02g04232\_t001 |  |  |  |  |  |  |  |  |
| 0 | Vvi-Vitvi02g04233\_t001 |  |  |  |  |  |  |  |  |
| 0 | Vvi-Vitvi02g01528\_t001 |  |  |  |  |  |  |  |  |
| 0 | Vvi-Vitvi02g00828\_t002 |  |  |  |  |  |  |  |  |
| 0 | Vvi-Vitvi02g00831\_t001 |  |  |  |  |  |  |  |  |
| 0 | Vvi-Vitvi02g04234\_t001 |  |  |  |  |  |  |  |  |
| 0 | Vvi-Vitvi02g01529\_t001 |  |  |  |  |  |  |  |  |
| 0 | Vvi-Vitvi02g04235\_t001 |  |  |  |  |  |  |  |  |
| 0 | Vvi-Vitvi02g04237\_t001 |  |  |  |  |  |  |  |  |
| 0 | Vvi-Vitvi02g04238\_t001 |  |  |  |  |  |  |  |  |
| 0 | Vvi-Vitvi02g00836\_t001 |  |  |  |  |  |  |  |  |
| 0 | Vvi-Vitvi02g01532\_t001 |  |  |  |  |  |  |  |  |
| 0 | Vvi-Vitvi02g01533\_t001 |  |  |  |  |  |  |  |  |
| 0 | Vvi-Vitvi02g00839\_t001 |  |  |  |  |  |  |  |  |
| 0 | Vvi-Vitvi02g00840\_t001 |  |  |  |  |  |  |  |  |
| 0 | Vvi-Vitvi02g04239\_t001 |  |  |  |  |  |  |  |  |
| 0 | Vvi-Vitvi02g04240\_t001 |  |  |  |  |  |  |  |  |
| 0 | Vvi-Vitvi02g00842\_t001 |  |  |  |  |  |  |  |  |
| 0 | Vvi-Vitvi02g00843\_t001 |  |  |  |  |  |  |  |  |
| 0 | Vvi-Vitvi02g00847\_t001 |  |  |  |  |  |  |  |  |
| 0 | Vvi-Vitvi02g00848\_t001 |  |  |  |  |  |  |  |  |
| 0 | Vvi-Vitvi02g00851\_t001 |  |  |  |  |  |  |  |  |
| 0 | Vvi-Vitvi02g04241\_t001 |  |  |  |  |  |  |  |  |
| 0 | Vvi-Vitvi02g04242\_t001 |  |  |  |  |  |  |  |  |
| 0 | Vvi-Vitvi02g00853\_t001 |  |  |  |  |  |  |  |  |
| 0 | Vvi-Vitvi02g01536\_t001 |  |  |  |  |  |  |  |  |
| 0 | Vvi-Vitvi02g00855\_t001 |  |  |  |  |  |  |  |  |
| 0 | Vvi-Vitvi02g00857\_t001 |  |  |  |  |  |  |  |  |
| 0 | Vvi-Vitvi02g00859\_t001 |  |  |  |  |  |  |  |  |
| 0 | Vvi-Vitvi02g04243\_t001 |  |  |  |  |  |  |  |  |
| 0 | Vvi-Vitvi02g01537\_t001 |  |  |  |  |  |  |  |  |
| 0 | Vvi-Vitvi02g04244\_t001 |  |  |  |  |  |  |  |  |
| 0 | Vvi-Vitvi02g00867\_t001 |  |  |  |  |  |  |  |  |
| 0 | Vvi-Vitvi02g04245\_t001 |  |  |  |  |  |  |  |  |
| 0 | Vvi-Vitvi02g00868\_t001 |  |  |  |  |  |  |  |  |
| 0 | Vvi-Vitvi02g04246\_t001 |  |  |  |  |  |  |  |  |
| 0 | Vvi-Vitvi02g04247\_t001 |  |  |  |  |  |  |  |  |
| 0 | Vvi-Vitvi02g04248\_t001 |  |  |  |  |  |  |  |  |
| 0 | Vvi-Vitvi02g04249\_t001 |  |  |  |  |  |  |  |  |
| 0 | Vvi-Vitvi02g04250\_t001 |  |  |  |  |  |  |  |  |
| 0 | Vvi-Vitvi02g04251\_t001 |  |  |  |  |  |  |  |  |
| 0 | Vvi-Vitvi02g00893\_t001 |  |  |  |  |  |  |  |  |
| 0 | Vvi-Vitvi02g01542\_t001 |  |  |  |  |  |  |  |  |
| 0 | Vvi-Vitvi02g04252\_t001 |  |  |  |  |  |  |  |  |
| 0 | Vvi-Vitvi02g01543\_t001 |  |  |  |  |  |  |  |  |
| 0 | Vvi-Vitvi02g04253\_t001 |  |  |  |  |  |  |  |  |
| 0 | Vvi-Vitvi02g04254\_t001 |  |  |  |  |  |  |  |  |
| 0 | Vvi-Vitvi02g04255\_t001 |  |  |  |  |  |  |  |  |
| 0 | Vvi-Vitvi02g04256\_t001 |  |  |  |  |  |  |  |  |
| 0 | Vvi-Vitvi02g04257\_t001 |  |  |  |  |  |  |  |  |
| 0 | Vvi-Vitvi02g00896\_t001 |  |  |  |  |  |  |  |  |
| 0 | Vvi-Vitvi02g00897\_t001 |  |  |  |  |  |  |  |  |
| 0 | Vvi-Vitvi02g04258\_t001 |  |  |  |  |  |  |  |  |
| 0 | Vvi-Vitvi02g04259\_t001 |  |  |  |  |  |  |  |  |
| 0 | Vvi-Vitvi02g04260\_t001 |  |  |  |  |  |  |  |  |
| 0 | Vvi-Vitvi02g04261\_t001 |  |  |  |  |  |  |  |  |
| 0 | Vvi-Vitvi02g04262\_t001 |  |  |  |  |  |  |  |  |
| 0 | Vvi-Vitvi02g04263\_t001 |  |  |  |  |  |  |  |  |
| 0 | Vvi-Vitvi02g04264\_t001 |  |  |  |  |  |  |  |  |
| 0 | Vvi-Vitvi02g04265\_t001 |  |  |  |  |  |  |  |  |
| 0 | Vvi-Vitvi02g04266\_t001 |  |  |  |  |  |  |  |  |
| 0 | Vvi-Vitvi02g04267\_t001 |  |  |  |  |  |  |  |  |
| 0 | Vvi-Vitvi02g04268\_t001 |  |  |  |  |  |  |  |  |
| 0 | Vvi-Vitvi02g04269\_t001 |  |  |  |  |  |  |  |  |
| 0 | Vvi-Vitvi02g04270\_t001 |  |  |  |  |  |  |  |  |
| 0 | Vvi-Vitvi02g04271\_t001 |  |  |  |  |  |  |  |  |
| 0 | Vvi-Vitvi02g04272\_t001 |  |  |  |  |  |  |  |  |
| 0 | Vvi-Vitvi02g04273\_t001 |  |  |  |  |  |  |  |  |
| 0 | Vvi-Vitvi02g04274\_t001 |  |  |  |  |  |  |  |  |
| 0 | Vvi-Vitvi02g01842\_t001 |  |  |  |  |  |  |  |  |
| 0 | Vvi-Vitvi02g04276\_t001 |  |  |  |  |  |  |  |  |
| 0 | Vvi-Vitvi02g00915\_t001 |  |  |  |  |  |  |  |  |
| 0 | Vvi-Vitvi02g04277\_t001 |  |  |  |  |  |  |  |  |
| 0 | Vvi-Vitvi02g00919\_t001 |  |  |  |  |  |  |  |  |
| 0 | Vvi-Vitvi02g01556\_t001 |  |  |  |  |  |  |  |  |
| 0 | Vvi-Vitvi02g04278\_t001 |  |  |  |  |  |  |  |  |
| 0 | Vvi-Vitvi02g04279\_t001 |  |  |  |  |  |  |  |  |
| 0 | Vvi-Vitvi02g00936\_t001 |  |  |  |  |  |  |  |  |
| 0 | Vvi-Vitvi02g04280\_t001 |  |  |  |  |  |  |  |  |
| 0 | Vvi-Vitvi02g04281\_t001 |  |  |  |  |  |  |  |  |
| 0 | Vvi-Vitvi02g00938\_t001 |  |  |  |  |  |  |  |  |
| 0 | Vvi-Vitvi02g04282\_t001 |  |  |  |  |  |  |  |  |
| 0 | Vvi-Vitvi02g04283\_t001 |  |  |  |  |  |  |  |  |
| 0 | Vvi-Vitvi02g01557\_t001 |  |  |  |  |  |  |  |  |
| 0 | Vvi-Vitvi02g04284\_t001 |  |  |  |  |  |  |  |  |
| 0 | Vvi-Vitvi02g00942\_t001 |  |  |  |  |  |  |  |  |
| 0 | Vvi-Vitvi02g00945\_t001 |  |  |  |  |  |  |  |  |
| 0 | Vvi-Vitvi02g01559\_t001 |  |  |  |  |  |  |  |  |
| 0 | Vvi-Vitvi02g00950\_t001 |  |  |  |  |  |  |  |  |
| 0 | Vvi-Vitvi02g00952\_t001 |  |  |  |  |  |  |  |  |
| 0 | Vvi-Vitvi02g04285\_t001 |  |  |  |  |  |  |  |  |
| 0 | Vvi-Vitvi02g00953\_t001 |  |  |  |  |  |  |  |  |
| 0 | Vvi-Vitvi02g00954\_t001 |  |  |  |  |  |  |  |  |
| 0 | Vvi-Vitvi02g01306\_t001 |  |  |  |  |  |  |  |  |
| 0 | Vvi-Vitvi02g01303\_t001 |  |  |  |  |  |  |  |  |
| 0 | Vvi-Vitvi02g04286\_t001 |  |  |  |  |  |  |  |  |
| 0 | Vvi-Vitvi02g01567\_t001 |  |  |  |  |  |  |  |  |
| 0 | Vvi-Vitvi02g01568\_t001 |  |  |  |  |  |  |  |  |
| 0 | Vvi-Vitvi02g01569\_t001 |  |  |  |  |  |  |  |  |
| 0 | Vvi-Vitvi02g04287\_t001 |  |  |  |  |  |  |  |  |
| 0 | Vvi-Vitvi02g04288\_t001 |  |  |  |  |  |  |  |  |
| 0 | Vvi-Vitvi02g04289\_t001 |  |  |  |  |  |  |  |  |
| 0 | Vvi-Vitvi02g04290\_t001 |  |  |  |  |  |  |  |  |
| 0 | Vvi-Vitvi02g01571\_t001 |  |  |  |  |  |  |  |  |
| 0 | Vvi-Vitvi02g00963\_t001 |  |  |  |  |  |  |  |  |
| 0 | Vvi-Vitvi02g04291\_t001 |  |  |  |  |  |  |  |  |
| 0 | Vvi-Vitvi02g04292\_t001 |  |  |  |  |  |  |  |  |
| 0 | Vvi-Vitvi02g04293\_t002 |  |  |  |  |  |  |  |  |
| 0 | Vvi-Vitvi02g00972\_t001 |  |  |  |  |  |  |  |  |
| 0 | Vvi-Vitvi02g00974\_t001 |  |  |  |  |  |  |  |  |
| 0 | Vvi-Vitvi02g00975\_t001 |  |  |  |  |  |  |  |  |
| 0 | Vvi-Vitvi02g00978\_t002 |  |  |  |  |  |  |  |  |
| 0 | Vvi-Vitvi02g01575\_t001 |  |  |  |  |  |  |  |  |
| 0 | Vvi-Vitvi02g04294\_t001 |  |  |  |  |  |  |  |  |
| 0 | Vvi-Vitvi02g00982\_t002 |  |  |  |  |  |  |  |  |
| 0 | Vvi-Vitvi02g00983\_t001 |  |  |  |  |  |  |  |  |
| 0 | Vvi-Vitvi02g04295\_t001 |  |  |  |  |  |  |  |  |
| 0 | Vvi-Vitvi02g00985\_t001 |  |  |  |  |  |  |  |  |
| 0 | Vvi-Vitvi02g04296\_t001 |  |  |  |  |  |  |  |  |
| 0 | Vvi-Vitvi02g01576\_t001 |  |  |  |  |  |  |  |  |
| 0 | Vvi-Vitvi02g01577\_t001 |  |  |  |  |  |  |  |  |
| 0 | Vvi-Vitvi02g04297\_t001 |  |  |  |  |  |  |  |  |
| 0 | Vvi-Vitvi02g00991\_t001 |  |  |  |  |  |  |  |  |
| 0 | Vvi-Vitvi02g00992\_t001 |  |  |  |  |  |  |  |  |
| 0 | Vvi-Vitvi02g00994\_t001 |  |  |  |  |  |  |  |  |
| 0 | Vvi-Vitvi02g00995\_t001 |  |  |  |  |  |  |  |  |
| 0 | Vvi-Vitvi02g04298\_t001 |  |  |  |  |  |  |  |  |
| 0 | Vvi-Vitvi02g04299\_t001 |  |  |  |  |  |  |  |  |
| 0 | Vvi-Vitvi02g04300\_t001 |  |  |  |  |  |  |  |  |
| 0 | Vvi-Vitvi02g04301\_t001 |  |  |  |  |  |  |  |  |
| 0 | Vvi-Vitvi02g00998\_t001 |  |  |  |  |  |  |  |  |
| 0 | Vvi-Vitvi02g00999\_t001 |  |  |  |  |  |  |  |  |
| 0 | Vvi-Vitvi02g01001\_t001 |  |  |  |  |  |  |  |  |
| 0 | Vvi-Vitvi02g01003\_t001 |  |  |  |  |  |  |  |  |
| 0 | Vvi-Vitvi02g04302\_t001 |  |  |  |  |  |  |  |  |
| 0 | Vvi-Vitvi02g04303\_t001 |  |  |  |  |  |  |  |  |
| 0 | Vvi-Vitvi02g01004\_t001 |  |  |  |  |  |  |  |  |
| 0 | Vvi-Vitvi02g01005\_t001 |  |  |  |  |  |  |  |  |
| 0 | Vvi-Vitvi02g01011\_t001 |  |  |  |  |  |  |  |  |
| 0 | Vvi-Vitvi02g01012\_t001 |  |  |  |  |  |  |  |  |
| 0 | Vvi-Vitvi02g01584\_t001 |  |  |  |  |  |  |  |  |
| 0 | Vvi-Vitvi02g04304\_t001 |  |  |  |  |  |  |  |  |
| 0 | Vvi-Vitvi02g01015\_t001 |  |  |  |  |  |  |  |  |
| 0 | Vvi-Vitvi02g01016\_t001 |  |  |  |  |  |  |  |  |
| 0 | Vvi-Vitvi02g01307\_t001 |  |  |  |  |  |  |  |  |
| 0 | Vvi-Vitvi02g04305\_t001 |  |  |  |  |  |  |  |  |
| 0 | Vvi-Vitvi02g01019\_t001 |  |  |  |  |  |  |  |  |
| 0 | Vvi-Vitvi02g01585\_t001 |  |  |  |  |  |  |  |  |
| 0 | Vvi-Vitvi02g01308\_t001 |  |  |  |  |  |  |  |  |
| 0 | Vvi-Vitvi02g01022\_t001 |  |  |  |  |  |  |  |  |
| 0 | Vvi-Vitvi02g01024\_t001 |  |  |  |  |  |  |  |  |
| 0 | Vvi-Vitvi02g01309\_t001 |  |  |  |  |  |  |  |  |
| 0 | Vvi-Vitvi02g04306\_t001 |  |  |  |  |  |  |  |  |
| 0 | Vvi-Vitvi02g04307\_t001 |  |  |  |  |  |  |  |  |
| 0 | Vvi-Vitvi02g04308\_t001 |  |  |  |  |  |  |  |  |
| 0 | Vvi-Vitvi02g04309\_t001 |  |  |  |  |  |  |  |  |
| 0 | Vvi-Vitvi02g04310\_t001 |  |  |  |  |  |  |  |  |
| 0 | Vvi-Vitvi02g04311\_t001 |  |  |  |  |  |  |  |  |
| 0 | Vvi-Vitvi02g01594\_t001 |  |  |  |  |  |  |  |  |
| 0 | Vvi-Vitvi02g04312\_t001 |  |  |  |  |  |  |  |  |
| 0 | Vvi-Vitvi02g04313\_t001 |  |  |  |  |  |  |  |  |
| 0 | Vvi-Vitvi02g04314\_t001 |  |  |  |  |  |  |  |  |
| 0 | Vvi-Vitvi02g01038\_t001 |  |  |  |  |  |  |  |  |
| 0 | Vvi-Vitvi02g01040\_t001 |  |  |  |  |  |  |  |  |
| 0 | Vvi-Vitvi02g04315\_t001 |  |  |  |  |  |  |  |  |
| 0 | Vvi-Vitvi02g04316\_t001 |  |  |  |  |  |  |  |  |
| 0 | Vvi-Vitvi02g04317\_t001 |  |  |  |  |  |  |  |  |
| 0 | Vvi-Vitvi02g04318\_t001 |  |  |  |  |  |  |  |  |
| 0 | Vvi-Vitvi02g04319\_t001 |  |  |  |  |  |  |  |  |
| 0 | Vvi-Vitvi02g01045\_t001 |  |  |  |  |  |  |  |  |
| 0 | Vvi-Vitvi02g04320\_t001 |  |  |  |  |  |  |  |  |
| 0 | Vvi-Vitvi02g01047\_t001 |  |  |  |  |  |  |  |  |
| 0 | Vvi-Vitvi02g01050\_t001 |  |  |  |  |  |  |  |  |
| 0 | Vvi-Vitvi02g04321\_t001 |  |  |  |  |  |  |  |  |
| 0 | Vvi-Vitvi02g04322\_t001 |  |  |  |  |  |  |  |  |
| 0 | Vvi-Vitvi02g01057\_t001 |  |  |  |  |  |  |  |  |
| 0 | Vvi-Vitvi02g01602\_t001 |  |  |  |  |  |  |  |  |
| 0 | Vvi-Vitvi02g04323\_t001 |  |  |  |  |  |  |  |  |
| 0 | Vvi-Vitvi02g01605\_t001 |  |  |  |  |  |  |  |  |
| 0 | Vvi-Vitvi02g01606\_t001 |  |  |  |  |  |  |  |  |
| 0 | Vvi-Vitvi02g01607\_t001 |  |  |  |  |  |  |  |  |
| 0 | Vvi-Vitvi02g04325\_t001 |  |  |  |  |  |  |  |  |
| 0 | Vvi-Vitvi02g04326\_t001 |  |  |  |  |  |  |  |  |
| 0 | Vvi-Vitvi02g01609\_t001 |  |  |  |  |  |  |  |  |
| 0 | Vvi-Vitvi02g04327\_t001 |  |  |  |  |  |  |  |  |
| 0 | Vvi-Vitvi02g01066\_t001 |  |  |  |  |  |  |  |  |
| 0 | Vvi-Vitvi02g04328\_t001 |  |  |  |  |  |  |  |  |
| 0 | Vvi-Vitvi02g04329\_t001 |  |  |  |  |  |  |  |  |
| 0 | Vvi-Vitvi02g01067\_t001 |  |  |  |  |  |  |  |  |
| 0 | Vvi-Vitvi02g04330\_t001 |  |  |  |  |  |  |  |  |
| 0 | Vvi-Vitvi02g04331\_t001 |  |  |  |  |  |  |  |  |
| 0 | Vvi-Vitvi02g04332\_t001 |  |  |  |  |  |  |  |  |
| 0 | Vvi-Vitvi02g04333\_t001 |  |  |  |  |  |  |  |  |
| 0 | Vvi-Vitvi02g04334\_t001 |  |  |  |  |  |  |  |  |
| 0 | Vvi-Vitvi02g04335\_t001 |  |  |  |  |  |  |  |  |
| 0 | Vvi-Vitvi02g01611\_t001 |  |  |  |  |  |  |  |  |
| 0 | Vvi-Vitvi02g04336\_t001 |  |  |  |  |  |  |  |  |
| 0 | Vvi-Vitvi02g04337\_t001 |  |  |  |  |  |  |  |  |
| 0 | Vvi-Vitvi02g04338\_t001 |  |  |  |  |  |  |  |  |
| 0 | Vvi-Vitvi02g04339\_t001 |  |  |  |  |  |  |  |  |
| 0 | Vvi-Vitvi02g01613\_t001 |  |  |  |  |  |  |  |  |
| 0 | Vvi-Vitvi02g01075\_t001 |  |  |  |  |  |  |  |  |
| 0 | Vvi-Vitvi02g01614\_t001 |  |  |  |  |  |  |  |  |
| 0 | Vvi-Vitvi02g01615\_t001 |  |  |  |  |  |  |  |  |
| 0 | Vvi-Vitvi02g04340\_t001 |  |  |  |  |  |  |  |  |
| 0 | Vvi-Vitvi02g01081\_t001 |  |  |  |  |  |  |  |  |
| 0 | Vvi-Vitvi02g01082\_t001 |  |  |  |  |  |  |  |  |
| 0 | Vvi-Vitvi02g01618\_t001 |  |  |  |  |  |  |  |  |
| 0 | Vvi-Vitvi02g01621\_t001 |  |  |  |  |  |  |  |  |
| 0 | Vvi-Vitvi02g01084\_t001 |  |  |  |  |  |  |  |  |
| 0 | Vvi-Vitvi02g04341\_t001 |  |  |  |  |  |  |  |  |
| 0 | Vvi-Vitvi02g04342\_t001 |  |  |  |  |  |  |  |  |
| 0 | Vvi-Vitvi02g04343\_t001 |  |  |  |  |  |  |  |  |
| 0 | Vvi-Vitvi02g01091\_t001 |  |  |  |  |  |  |  |  |
| 0 | Vvi-Vitvi02g01092\_t001 |  |  |  |  |  |  |  |  |
| 0 | Vvi-Vitvi02g01094\_t001 |  |  |  |  |  |  |  |  |
| 0 | Vvi-Vitvi02g04344\_t001 |  |  |  |  |  |  |  |  |
| 0 | Vvi-Vitvi02g01098\_t001 |  |  |  |  |  |  |  |  |
| 0 | Vvi-Vitvi02g01100\_t001 |  |  |  |  |  |  |  |  |
| 0 | Vvi-Vitvi02g01101\_t001 |  |  |  |  |  |  |  |  |
| 0 | Vvi-Vitvi02g04345\_t001 |  |  |  |  |  |  |  |  |
| 0 | Vvi-Vitvi02g04346\_t001 |  |  |  |  |  |  |  |  |
| 0 | Vvi-Vitvi02g04347\_t001 |  |  |  |  |  |  |  |  |
| 0 | Vvi-Vitvi02g04348\_t001 |  |  |  |  |  |  |  |  |
| 0 | Vvi-Vitvi02g01113\_t001 |  |  |  |  |  |  |  |  |
| 0 | Vvi-Vitvi02g04349\_t001 |  |  |  |  |  |  |  |  |
| 0 | Vvi-Vitvi02g01118\_t001 |  |  |  |  |  |  |  |  |
| 0 | Vvi-Vitvi02g01121\_t001 |  |  |  |  |  |  |  |  |
| 0 | Vvi-Vitvi02g04350\_t001 |  |  |  |  |  |  |  |  |
| 0 | Vvi-Vitvi02g04351\_t001 |  |  |  |  |  |  |  |  |
| 0 | Vvi-Vitvi02g04352\_t001 |  |  |  |  |  |  |  |  |
| 0 | Vvi-Vitvi02g01126\_t001 |  |  |  |  |  |  |  |  |
| 0 | Vvi-Vitvi02g04353\_t001 |  |  |  |  |  |  |  |  |
| 0 | Vvi-Vitvi02g04354\_t001 |  |  |  |  |  |  |  |  |
| 0 | Vvi-Vitvi02g04355\_t001 |  |  |  |  |  |  |  |  |
| 0 | Vvi-Vitvi02g01133\_t001 |  |  |  |  |  |  |  |  |
| 0 | Vvi-Vitvi02g04356\_t001 |  |  |  |  |  |  |  |  |
| 0 | Vvi-Vitvi02g04357\_t001 |  |  |  |  |  |  |  |  |
| 0 | Vvi-Vitvi02g01138\_t001 |  |  |  |  |  |  |  |  |
| 0 | Vvi-Vitvi02g04358\_t001 |  |  |  |  |  |  |  |  |
| 0 | Vvi-Vitvi02g01139\_t001 |  |  |  |  |  |  |  |  |
| 0 | Vvi-Vitvi02g04359\_t001 |  |  |  |  |  |  |  |  |
| 0 | Vvi-Vitvi02g01140\_t001 |  |  |  |  |  |  |  |  |
| 0 | Vvi-Vitvi02g04360\_t001 |  |  |  |  |  |  |  |  |
| 0 | Vvi-Vitvi02g04361\_t001 |  |  |  |  |  |  |  |  |
| 0 | Vvi-Vitvi02g01143\_t002 |  |  |  |  |  |  |  |  |
| 0 | Vvi-Vitvi02g04362\_t001 |  |  |  |  |  |  |  |  |
| 0 | Vvi-Vitvi02g04363\_t001 |  |  |  |  |  |  |  |  |
| 0 | Vvi-Vitvi02g01149\_t001 |  |  |  |  |  |  |  |  |
| 0 | Vvi-Vitvi02g01150\_t002 |  |  |  |  |  |  |  |  |
| 0 | Vvi-Vitvi02g01152\_t001 |  |  |  |  |  |  |  |  |
| 0 | Vvi-Vitvi02g01153\_t001 |  |  |  |  |  |  |  |  |
| 0 | Vvi-Vitvi02g01154\_t001 |  |  |  |  |  |  |  |  |
| 0 | Vvi-Vitvi02g04364\_t001 |  |  |  |  |  |  |  |  |
| 0 | Vvi-Vitvi02g04365\_t001 |  |  |  |  |  |  |  |  |
| 0 | Vvi-Vitvi02g01158\_t001 |  |  |  |  |  |  |  |  |
| 0 | Vvi-Vitvi02g01160\_t001 |  |  |  |  |  |  |  |  |
| 0 | Vvi-Vitvi02g04366\_t001 |  |  |  |  |  |  |  |  |
| 0 | Vvi-Vitvi02g01161\_t001 |  |  |  |  |  |  |  |  |
| 0 | Vvi-Vitvi02g01162\_t001 |  |  |  |  |  |  |  |  |
| 0 | Vvi-Vitvi02g04367\_t001 |  |  |  |  |  |  |  |  |
| 0 | Vvi-Vitvi02g01163\_t001 |  |  |  |  |  |  |  |  |
| 0 | Vvi-Vitvi02g01164\_t001 |  |  |  |  |  |  |  |  |
| 0 | Vvi-Vitvi02g01166\_t001 |  |  |  |  |  |  |  |  |
| 0 | Vvi-Vitvi02g01167\_t001 |  |  |  |  |  |  |  |  |
| 0 | Vvi-Vitvi02g04368\_t001 |  |  |  |  |  |  |  |  |
| 0 | Vvi-Vitvi02g04369\_t001 |  |  |  |  |  |  |  |  |
| 0 | Vvi-Vitvi02g01171\_t001 |  |  |  |  |  |  |  |  |
| 0 | Vvi-Vitvi02g01172\_t001 |  |  |  |  |  |  |  |  |
| 0 | Vvi-Vitvi02g01173\_t001 |  |  |  |  |  |  |  |  |
| 0 | Vvi-Vitvi02g04370\_t001 |  |  |  |  |  |  |  |  |
| 0 | Vvi-Vitvi02g04371\_t001 |  |  |  |  |  |  |  |  |
| 0 | Vvi-Vitvi02g01177\_t001 |  |  |  |  |  |  |  |  |
| 0 | Vvi-Vitvi02g01179\_t001 |  |  |  |  |  |  |  |  |
| 0 | Vvi-Vitvi02g01180\_t001 |  |  |  |  |  |  |  |  |
| 0 | Vvi-Vitvi02g01182\_t001 |  |  |  |  |  |  |  |  |
| 0 | Vvi-Vitvi02g04372\_t001 |  |  |  |  |  |  |  |  |
| 0 | Vvi-Vitvi02g01640\_t001 |  |  |  |  |  |  |  |  |
| 0 | Vvi-Vitvi02g01183\_t001 |  |  |  |  |  |  |  |  |
| 0 | Vvi-Vitvi02g04373\_t001 |  |  |  |  |  |  |  |  |
| 0 | Vvi-Vitvi02g04374\_t001 |  |  |  |  |  |  |  |  |
| 0 | Vvi-Vitvi02g04375\_t001 |  |  |  |  |  |  |  |  |
| 0 | Vvi-Vitvi02g04376\_t001 |  |  |  |  |  |  |  |  |
| 0 | Vvi-Vitvi02g04377\_t001 |  |  |  |  |  |  |  |  |
| 0 | Vvi-Vitvi02g01642\_t001 |  |  |  |  |  |  |  |  |
| 0 | Vvi-Vitvi02g04378\_t001 |  |  |  |  |  |  |  |  |
| 0 | Vvi-Vitvi02g04379\_t001 |  |  |  |  |  |  |  |  |
| 0 | Vvi-Vitvi02g01187\_t001 |  |  |  |  |  |  |  |  |
| 0 | Vvi-Vitvi02g04380\_t001 |  |  |  |  |  |  |  |  |
| 0 | Vvi-Vitvi02g01189\_t001 |  |  |  |  |  |  |  |  |
| 0 | Vvi-Vitvi02g01190\_t001 |  |  |  |  |  |  |  |  |
| 0 | Vvi-Vitvi02g04381\_t001 |  |  |  |  |  |  |  |  |
| 1 | Vvi-Vitvi02g01191\_t001 |  | Ath-AT1G31360.3 |  |  |  |  |  |  |  |
| 1 | Vvi-Vitvi02g01192\_t001 |  | | | |  |  |  |  |  |  |  |
| 1 | Vvi-Vitvi02g01648\_t001 |  | | | |  |  |  |  |  |  |  |
| 1 | Vvi-Vitvi02g01193\_t001 |  | | | |  |  |  |  |  |  |  |
| 1 | Vvi-Vitvi02g01201\_t001 |  | | | |  |  |  |  |  |  |  |
| 1 | Vvi-Vitvi02g01203\_t001 |  | | | |  |  |  |  |  |  |  |
| 1 | Vvi-Vitvi02g01650\_t001 |  | | | |  |  |  |  |  |  |  |
| 1 | Vvi-Vitvi02g01204\_t001 |  | Ath-AT1G31420.1 |  |  |  |  |  |  |  |
| 1 | Vvi-Vitvi02g01205\_t001 |  | | | |  |  |  |  |  |  |  |
| 1 | Vvi-Vitvi02g04382\_t001 |  | Ath-AT1G31440.1 |  |  |  |  |  |  |  |
| 1 | Vvi-Vitvi02g01207\_t001 |  | Ath-AT1G31450.1 |  |  |  |  |  |  |  |
| 1 | Vvi-Vitvi02g01208\_t001 |  | | | |  |  |  |  |  |  |  |
| 1 | Vvi-Vitvi02g01209\_t001 |  | | | |  |  |  |  |  |  |  |
| 1 | Vvi-Vitvi02g01651\_t001 |  | | | |  |  |  |  |  |  |  |
| 1 | Vvi-Vitvi02g01652\_t001 |  | | | |  |  |  |  |  |  |  |
| 1 | Vvi-Vitvi02g01210\_t001 |  | Ath-AT1G31460.1 |  |  |  |  |  |  |  |
| 1 | Vvi-Vitvi02g01653\_t001 |  | | | |  |  |  |  |  |  |  |
| 1 | Vvi-Vitvi02g01211\_t001 |  | | | |  |  |  |  |  |  |  |
| 1 | Vvi-Vitvi02g04383\_t001 |  | | | |  |  |  |  |  |  |  |
| 1 | Vvi-Vitvi02g01214\_t001 |  | | | |  |  |  |  |  |  |  |
| 1 | Vvi-Vitvi02g01215\_t001 |  | | | |  |  |  |  |  |  |  |
| 1 | Vvi-Vitvi02g04384\_t001 |  | | | |  |  |  |  |  |  |  |
| 1 | Vvi-Vitvi02g01220\_t001 |  | | | |  |  |  |  |  |  |  |
| 1 | Vvi-Vitvi02g01222\_t001 |  | | | |  |  |  |  |  |  |  |
| 2 | Vvi-Vitvi02g01224\_t001 |  | Ath-AT1G31470.1 |  | Ath-AT5G45275.1 |  |  |  |  |  |  |
| 2 | Vvi-Vitvi02g04385\_t001 |  | | | |  | | | |  |  |  |  |  |  |
| 2 | Vvi-Vitvi02g01225\_t001 |  | | | |  | | | |  |  |  |  |  |  |
| 2 | Vvi-Vitvi02g01227\_t001 |  | Ath-AT1G31490.1 |  | | | |  |  |  |  |  |  |
| 2 | Vvi-Vitvi02g01661\_t001 |  | | | |  | | | |  |  |  |  |  |  |
| 2 | Vvi-Vitvi02g01228\_t001 |  | Ath-AT1G31500.7 |  | | | |  |  |  |  |  |  |
| 2 | Vvi-Vitvi02g01230\_t001 |  | | | |  | Ath-AT5G45280.2 |  |  |  |  |  |  |
| 2 | Vvi-Vitvi02g01662\_t001 |  | | | |  | | | |  |  |  |  |  |  |
| 2 | Vvi-Vitvi02g01231\_t002 |  | | | |  | Ath-AT5G45290.2 |  |  |  |  |  |  |
| 2 | Vvi-Vitvi02g01232\_t001 |  | | | |  | Ath-AT5G45300.3 |  |  |  |  |  |  |
| 2 | Vvi-Vitvi02g01233\_t001 |  | | | |  | | | |  |  |  |  |  |  |
| 2 | Vvi-Vitvi02g01235\_t001 |  | Ath-AT1G31600.3 |  | | | |  |  |  |  |  |  |
| 2 | Vvi-Vitvi02g01236\_t005 |  | Ath-AT1G31650.1 |  | | | |  |  |  |  |  |  |
| 1 | Vvi-Vitvi02g04386\_t001 |  |  |  | | | |  |  |  |  |  |  |
| 1 | Vvi-Vitvi02g04387\_t001 |  |  |  | | | |  |  |  |  |  |  |
| 1 | Vvi-Vitvi02g04388\_t001 |  |  |  | | | |  |  |  |  |  |  |
| 1 | Vvi-Vitvi02g01237\_t001 |  |  |  | | | |  |  |  |  |  |  |
| 1 | Vvi-Vitvi02g04389\_t001 |  |  |  | | | |  |  |  |  |  |  |
| 1 | Vvi-Vitvi02g04390\_t001 |  |  |  | | | |  |  |  |  |  |  |
| 1 | Vvi-Vitvi02g01240\_t001 |  |  |  | | | |  |  |  |  |  |  |
| 1 | Vvi-Vitvi02g01241\_t001 |  |  |  | | | |  |  |  |  |  |  |
| 1 | Vvi-Vitvi02g04391\_t001 |  |  |  | | | |  |  |  |  |  |  |
| 1 | Vvi-Vitvi02g04392\_t001 |  |  |  | | | |  |  |  |  |  |  |
| 1 | Vvi-Vitvi02g01243\_t001 |  |  |  | | | |  |  |  |  |  |  |
| 1 | Vvi-Vitvi02g04393\_t001 |  |  |  | | | |  |  |  |  |  |  |
| 1 | Vvi-Vitvi02g04394\_t001 |  |  |  | | | |  |  |  |  |  |  |
| 1 | Vvi-Vitvi02g04395\_t001 |  |  |  | | | |  |  |  |  |  |  |
| 1 | Vvi-Vitvi02g04396\_t001 |  |  |  | | | |  |  |  |  |  |  |
| 1 | Vvi-Vitvi02g04397\_t001 |  |  |  | | | |  |  |  |  |  |  |
| 1 | Vvi-Vitvi02g01245\_t001 |  |  |  | | | |  |  |  |  |  |  |
| 1 | Vvi-Vitvi02g01246\_t001 |  |  |  | | | |  |  |  |  |  |  |
| 1 | Vvi-Vitvi02g04398\_t001 |  |  |  | | | |  |  |  |  |  |  |
| 1 | Vvi-Vitvi02g04399\_t001 |  |  |  | | | |  |  |  |  |  |  |
| 1 | Vvi-Vitvi02g01248\_t001 |  |  |  | Ath-AT5G45310.2 |  |  |  |  |  |  |
| 1 | Vvi-Vitvi02g04400\_t001 |  |  |  | | | |  |  |  |  |  |  |
| 1 | Vvi-Vitvi02g01251\_t001 |  |  |  | | | |  |  |  |  |  |  |
| 1 | Vvi-Vitvi02g01664\_t001 |  |  |  | | | |  |  |  |  |  |  |
| 1 | Vvi-Vitvi02g01252\_t001 |  |  |  | Ath-AT5G45320.1 |  |  |  |  |  |  |
| 1 | Vvi-Vitvi02g04401\_t001 |  |  |  | | | |  |  |  |  |  |  |
| 1 | Vvi-Vitvi02g01253\_t001 |  |  |  | | | |  |  |  |  |  |  |
| 1 | Vvi-Vitvi02g04402\_t001 |  |  |  | | | |  |  |  |  |  |  |
| 1 | Vvi-Vitvi02g04403\_t001 |  |  |  | | | |  |  |  |  |  |  |
| 1 | Vvi-Vitvi02g04404\_t001 |  |  |  | | | |  |  |  |  |  |  |
| 2 | Vvi-Vitvi02g01261\_t001 |  | Ath-AT4G19390.1 |  | | | |  |  |  |  |  |  |
| 2 | Vvi-Vitvi02g01665\_t001 |  | | | |  | | | |  |  |  |  |  |  |
| 2 | Vvi-Vitvi02g04405\_t001 |  | | | |  | | | |  |  |  |  |  |  |
| 2 | Vvi-Vitvi02g01264\_t001 |  | Ath-AT4G19380.2 |  | | | |  |  |  |  |  |  |
| 2 | Vvi-Vitvi02g01266\_t001 |  | Ath-AT4G19370.1 |  | | | |  |  |  |  |  |  |
| 2 | Vvi-Vitvi02g01267\_t001 |  | Ath-AT4G19275.1 |  | Ath-AT5G45330.1 |  |  |  |  |  |  |
| 2 | Vvi-Vitvi02g01268\_t001 |  | | | |  | | | |  |  |  |  |  |  |
| 2 | Vvi-Vitvi02g01666\_t001 |  | | | |  | | | |  |  |  |  |  |  |
| 2 | Vvi-Vitvi02g01269\_t001 |  | Ath-AT4G19230.2 |  | Ath-AT5G45340.1 |  |  |  |  |  |  |
| 2 | Vvi-Vitvi02g01270\_t001 |  | | | |  | | | |  |  |  |  |  |  |
| 2 | Vvi-Vitvi02g04406\_t001 |  | | | |  | | | |  |  |  |  |  |  |
| 2 | Vvi-Vitvi02g01668\_t001 |  | | | |  | | | |  |  |  |  |  |  |
| 2 | Vvi-Vitvi02g01272\_t001 |  | | | |  | | | |  |  |  |  |  |  |
| 2 | Vvi-Vitvi02g01273\_t001 |  | | | |  | | | |  |  |  |  |  |  |
| 2 | Vvi-Vitvi02g01274\_t001 |  | Ath-AT4G19220.1 |  | | | |  |  |  |  |  |  |
| 2 | Vvi-Vitvi02g01669\_t001 |  | Ath-AT4G19210.1 |  | | | |  |  |  |  |  |  |
| 2 | Vvi-Vitvi02g01670\_t001 |  | | | |  | | | |  |  |  |  |  |  |
| 2 | Vvi-Vitvi02g01275\_t001 |  | | | |  | Ath-AT5G45360.1 |  |  |  |  |  |  |
| 2 | Vvi-Vitvi02g01276\_t001 |  | | | |  | | | |  |  |  |  |  |  |
| 2 | Vvi-Vitvi02g04407\_t001 |  | | | |  | | | |  |  |  |  |  |  |
| 2 | Vvi-Vitvi02g01277\_t001 |  | | | |  | | | |  |  |  |  |  |  |
| 2 | Vvi-Vitvi02g01278\_t001 |  | Ath-AT4G19190.1 |  | | | |  |  |  |  |  |  |
| 2 | Vvi-Vitvi02g01279\_t001 |  | | | |  | | | |  |  |  |  |  |  |
| 2 | Vvi-Vitvi02g01280\_t001 |  | | | |  | | | |  |  |  |  |  |  |
| 2 | Vvi-Vitvi02g04408\_t001 |  | | | |  | | | |  |  |  |  |  |  |
| 2 | Vvi-Vitvi02g01283\_t001 |  | | | |  | | | |  |  |  |  |  |  |
| 2 | Vvi-Vitvi02g01284\_t001 |  | Ath-AT4G19185.1 |  | Ath-AT5G45370.4 |  |  |  |  |  |  |
| 1 | Vvi-Vitvi02g01286\_t001 |  | Ath-AT4G19170.1 |  |  |  |  |  |  |  |
| 0 | Vvi-Vitvi02g01288\_t001 |  |  |  |  |  |  |  |  |
| 0 | Vvi-Vitvi02g04409\_t001 |  |  |  |  |  |  |  |  |
| 0 | Vvi-Vitvi02g01290\_t002 |  |  |  |  |  |  |  |  |
