# Supplementary material for: Genome-wide identification, characterization and gene expression of BES1 transcription factor family in grapevine (Vitis vinifera L.)
Source: Sci Rep. 2023 Jan 5;13:240. doi: 10.1038/s41598-022-24407-y (PMC9816167; doi:10.1038/s41598-022-24407-y)
Supplement: Supplementary file 3 — Supplementary Information. [file 41598_2022_24407_MOESM3_ESM.zip › Vvi_Ath/Vitis_vinifera.PN40024.v4.dna_sm.toplevel.fa.vs.Arabidopsis_thaliana.TAIR10.dna_sm.toplevel.fa.html/Vvi-3.html]

|  |  |  |  |  |  |  |  |  |  |  |  |  |  |  |  |  |  |
| --- | --- | --- | --- | --- | --- | --- | --- | --- | --- | --- | --- | --- | --- | --- | --- | --- | --- |
| Duplication depth | Reference chromosome | Collinear blocks | | | | | | | | | | | | | | | |
| 0 | Vvi-Vitvi03g04000\_t002 |  |  |  |  |  |  |  |  |
| 0 | Vvi-Vitvi03g04001\_t001 |  |  |  |  |  |  |  |  |
| 0 | Vvi-Vitvi03g04002\_t001 |  |  |  |  |  |  |  |  |
| 0 | Vvi-Vitvi03g04003\_t001 |  |  |  |  |  |  |  |  |
| 0 | Vvi-Vitvi03g04004\_t001 |  |  |  |  |  |  |  |  |
| 0 | Vvi-Vitvi03g04005\_t001.2.6037826d |  |  |  |  |  |  |  |  |
| 0 | Vvi-Vitvi03g00001\_t001 |  |  |  |  |  |  |  |  |
| 0 | Vvi-Vitvi03g04006\_t001 |  |  |  |  |  |  |  |  |
| 2 | Vvi-Vitvi03g00003\_t001 |  | Ath-AT2G21450.2 |  | Ath-AT2G16390.2 |  |  |  |  |  |  |
| 2 | Vvi-Vitvi03g04007\_t001 |  | | | |  | | | |  |  |  |  |  |  |
| 2 | Vvi-Vitvi03g00004\_t001 |  | | | |  | Ath-AT2G16400.1 |  |  |  |  |  |  |
| 3 | Vvi-Vitvi03g04008\_t001 |  | | | |  | | | |  | Ath-AT3G51000.1 |  |  |  |  |  |
| 3 | Vvi-Vitvi03g04009\_t001 |  | | | |  | | | |  | | | |  |  |  |  |  |
| 3 | Vvi-Vitvi03g00007\_t001 |  | Ath-AT2G21440.1 |  | | | |  | | | |  |  |  |  |  |
| 3 | Vvi-Vitvi03g04010\_t001 |  | | | |  | | | |  | | | |  |  |  |  |  |
| 3 | Vvi-Vitvi03g00009\_t001 |  | | | |  | | | |  | | | |  |  |  |  |  |
| 4 | Vvi-Vitvi03g00011\_t001 |  | | | |  | | | |  | | | |  | Ath-AT4G39100.1 |  |  |  |  |
| 4 | Vvi-Vitvi03g00012\_t001 |  | | | |  | Ath-AT2G16405.1 |  | | | |  | | | |  |  |  |  |
| 4 | Vvi-Vitvi03g04011\_t001 |  | | | |  | | | |  | | | |  | | | |  |  |  |  |
| 4 | Vvi-Vitvi03g04012\_t001 |  | | | |  | | | |  | | | |  | | | |  |  |  |  |
| 4 | Vvi-Vitvi03g01322\_t001 |  | | | |  | | | |  | | | |  | | | |  |  |  |  |
| 4 | Vvi-Vitvi03g04013\_t001 |  | | | |  | | | |  | | | |  | | | |  |  |  |  |
| 4 | Vvi-Vitvi03g04014\_t001 |  | | | |  | | | |  | | | |  | | | |  |  |  |  |
| 4 | Vvi-Vitvi03g04015\_t001 |  | | | |  | | | |  | | | |  | | | |  |  |  |  |
| 4 | Vvi-Vitvi03g00015\_t001 |  | | | |  | Ath-AT2G16430.2 |  | | | |  | | | |  |  |  |  |
| 4 | Vvi-Vitvi03g00016\_t001 |  | | | |  | | | |  | | | |  | | | |  |  |  |  |
| 4 | Vvi-Vitvi03g00017\_t001 |  | | | |  | | | |  | | | |  | | | |  |  |  |  |
| 4 | Vvi-Vitvi03g00021\_t001 |  | Ath-AT2G21430.1 |  | | | |  | | | |  | Ath-AT4G39090.1 |  |  |  |  |
| 4 | Vvi-Vitvi03g00022\_t001 |  | Ath-AT2G21410.1 |  | | | |  | | | |  | Ath-AT4G39080.1 |  |  |  |  |
| 4 | Vvi-Vitvi03g00023\_t001 |  | | | |  | Ath-AT2G16440.1 |  | | | |  | | | |  |  |  |  |
| 4 | Vvi-Vitvi03g00024\_t001 |  | Ath-AT2G21400.2 |  | | | |  | Ath-AT3G51060.1 |  | | | |  |  |  |  |
| 4 | Vvi-Vitvi03g00025\_t001 |  | Ath-AT2G21385.1 |  | | | |  | | | |  | | | |  |  |  |  |
| 4 | Vvi-Vitvi03g00026\_t001 |  | | | |  | | | |  | | | |  | Ath-AT4G39070.1 |  |  |  |  |
| 4 | Vvi-Vitvi03g00028\_t001 |  | Ath-AT2G21380.2 |  | | | |  | | | |  | Ath-AT4G39050.1 |  |  |  |  |
| 4 | Vvi-Vitvi03g00029\_t001 |  | Ath-AT2G21370.1 |  | | | |  | | | |  | | | |  |  |  |  |
| 4 | Vvi-Vitvi03g00030\_t001 |  | | | |  | | | |  | | | |  | | | |  |  |  |  |
| 4 | Vvi-Vitvi03g00031\_t001 |  | Ath-AT2G21350.1 |  | | | |  | | | |  | Ath-AT4G39040.1 |  |  |  |  |
| 4 | Vvi-Vitvi03g00032\_t001 |  | Ath-AT2G21340.1 |  | | | |  | | | |  | Ath-AT4G39030.1 |  |  |  |  |
| 4 | Vvi-Vitvi03g00033\_t001 |  | | | |  | | | |  | | | |  | | | |  |  |  |  |
| 4 | Vvi-Vitvi03g04016\_t001 |  | | | |  | | | |  | | | |  | | | |  |  |  |  |
| 5 | Vvi-Vitvi03g00034\_t004 |  | | | |  | | | |  | | | |  | | | |  | Ath-AT4G34640.1 |  |  |  |
| 5 | Vvi-Vitvi03g00035\_t001 |  | | | |  | | | |  | | | |  | Ath-AT4G39020.1 |  | Ath-AT4G34660.1 |  |  |  |
| 5 | Vvi-Vitvi03g00036\_t001 |  | | | |  | | | |  | | | |  | | | |  | | | |  |  |  |
| 5 | Vvi-Vitvi03g00037\_t001 |  | | | |  | | | |  | Ath-AT3G51080.1 |  | | | |  | Ath-AT4G34680.2 |  |  |  |
| 5 | Vvi-Vitvi03g00038\_t001 |  | | | |  | | | |  | | | |  | | | |  | | | |  |  |  |
| 5 | Vvi-Vitvi03g01326\_t001 |  | | | |  | Ath-AT2G16460.1 |  | Ath-AT3G51090.1 |  | | | |  | | | |  |  |  |
| 5 | Vvi-Vitvi03g04017\_t001 |  | | | |  | | | |  | | | |  | | | |  | | | |  |  |  |
| 5 | Vvi-Vitvi03g04018\_t001 |  | | | |  | | | |  | | | |  | | | |  | | | |  |  |  |
| 5 | Vvi-Vitvi03g01330\_t001 |  | | | |  | | | |  | | | |  | | | |  | | | |  |  |  |
| 5 | Vvi-Vitvi03g00040\_t001 |  | | | |  | | | |  | | | |  | | | |  | | | |  |  |  |
| 5 | Vvi-Vitvi03g04019\_t001 |  | | | |  | | | |  | | | |  | | | |  | | | |  |  |  |
| 5 | Vvi-Vitvi03g00041\_t001 |  | | | |  | | | |  | Ath-AT3G51100.1 |  | | | |  | | | |  |  |  |
| 5 | Vvi-Vitvi03g00042\_t001 |  | | | |  | Ath-AT2G16485.1 |  | Ath-AT3G51120.1 |  | | | |  | | | |  |  |  |
| 5 | Vvi-Vitvi03g00043\_t002 |  | | | |  | | | |  | | | |  | | | |  | | | |  |  |  |
| 5 | Vvi-Vitvi03g00044\_t001 |  | | | |  | | | |  | | | |  | Ath-AT4G39000.1 |  | | | |  |  |  |
| 5 | Vvi-Vitvi03g00045\_t001 |  | | | |  | | | |  | | | |  | | | |  | | | |  |  |  |
| 5 | Vvi-Vitvi03g00046\_t001 |  | | | |  | | | |  | | | |  | | | |  | | | |  |  |  |
| 5 | Vvi-Vitvi03g00047\_t001 |  | | | |  | | | |  | | | |  | | | |  | | | |  |  |  |
| 5 | Vvi-Vitvi03g00048\_t001 |  | Ath-AT2G21330.1 |  | | | |  | | | |  | Ath-AT4G38970.1 |  | | | |  |  |  |
| 5 | Vvi-Vitvi03g00049\_t001 |  | Ath-AT2G21320.1 |  | | | |  | | | |  | Ath-AT4G38960.3 |  | | | |  |  |  |
| 5 | Vvi-Vitvi03g04020\_t001 |  | | | |  | | | |  | | | |  | | | |  | | | |  |  |  |
| 5 | Vvi-Vitvi03g00050\_t001 |  | Ath-AT2G21300.3 |  | | | |  | Ath-AT3G51150.2 |  | Ath-AT4G38950.4 |  | | | |  |  |  |
| 4 | Vvi-Vitvi03g00051\_t001 |  | | | |  | | | |  |  |  | | | |  | Ath-AT4G34700.1 |  |  |  |
| 4 | Vvi-Vitvi03g01333\_t001 |  | | | |  | | | |  |  |  | | | |  | | | |  |  |  |
| 4 | Vvi-Vitvi03g01334\_t001 |  | Ath-AT2G21290.1 |  | | | |  |  |  | | | |  | | | |  |  |  |
| 4 | Vvi-Vitvi03g00052\_t001 |  | Ath-AT2G21280.2 |  | | | |  |  |  | | | |  | | | |  |  |  |
| 4 | Vvi-Vitvi03g00053\_t001 |  | Ath-AT2G21270.3 |  | | | |  |  |  | Ath-AT4G38930.3 |  | | | |  |  |  |
| 4 | Vvi-Vitvi03g04021\_t001 |  | | | |  | | | |  |  |  | | | |  | | | |  |  |  |
| 4 | Vvi-Vitvi03g00054\_t001 |  | | | |  | Ath-AT2G16500.1 |  |  |  | | | |  | Ath-AT4G34710.1 |  |  |  |
| 4 | Vvi-Vitvi03g04022\_t001 |  | | | |  | Ath-AT2G16510.1 |  |  |  | Ath-AT4G38920.1 |  | Ath-AT4G34720.1 |  |  |  |
| 4 | Vvi-Vitvi03g00056\_t001 |  | Ath-AT2G21250.1 |  | | | |  |  |  | | | |  | | | |  |  |  |
| 4 | Vvi-Vitvi03g00057\_t001 |  | | | |  | Ath-AT2G16530.3 |  |  |  | | | |  | | | |  |  |  |
| 4 | Vvi-Vitvi03g04023\_t001 |  | | | |  | | | |  |  |  | | | |  | | | |  |  |  |
| 4 | Vvi-Vitvi03g00058\_t001 |  | Ath-AT2G21240.1 |  | | | |  |  |  | Ath-AT4G38910.2 |  | | | |  |  |  |
| 4 | Vvi-Vitvi03g00059\_t001 |  | Ath-AT2G21230.3 |  | | | |  |  |  | Ath-AT4G38900.1 |  | | | |  |  |  |
| 4 | Vvi-Vitvi03g00060\_t001 |  | | | |  | | | |  |  |  | Ath-AT4G38890.1 |  | | | |  |  |  |
| 4 | Vvi-Vitvi03g00061\_t001 |  | | | |  | Ath-AT2G16570.1 |  |  |  | Ath-AT4G38880.1 |  | Ath-AT4G34740.1 |  |  |  |
| 4 | Vvi-Vitvi03g01335\_t001 |  | | | |  | | | |  |  |  | | | |  | | | |  |  |  |
| 4 | Vvi-Vitvi03g00062\_t002 |  | | | |  | | | |  |  |  | | | |  | | | |  |  |  |
| 4 | Vvi-Vitvi03g00063\_t001 |  | | | |  | | | |  |  |  | | | |  | | | |  |  |  |
| 4 | Vvi-Vitvi03g01336\_t001 |  | | | |  | | | |  |  |  | | | |  | Ath-AT4G34750.2 |  |  |  |
| 4 | Vvi-Vitvi03g01337\_t001 |  | | | |  | | | |  |  |  | | | |  | | | |  |  |  |
| 4 | Vvi-Vitvi03g00064\_t001 |  | Ath-AT2G21220.1 |  | Ath-AT2G16580.1 |  |  |  | Ath-AT4G38860.1 |  | Ath-AT4G34760.1 |  |  |  |
| 3 | Vvi-Vitvi03g00065\_t001 |  | | | |  |  |  |  |  | | | |  | | | |  |  |  |
| 3 | Vvi-Vitvi03g04024\_t001 |  | | | |  |  |  |  |  | | | |  | | | |  |  |  |
| 3 | Vvi-Vitvi03g04025\_t001 |  | | | |  |  |  |  |  | | | |  | | | |  |  |  |
| 3 | Vvi-Vitvi03g00069\_t001 |  | | | |  |  |  |  |  | | | |  | | | |  |  |  |
| 3 | Vvi-Vitvi03g00070\_t001 |  | | | |  |  |  |  |  | | | |  | | | |  |  |  |
| 3 | Vvi-Vitvi03g01338\_t001 |  | | | |  |  |  |  |  | | | |  | | | |  |  |  |
| 3 | Vvi-Vitvi03g01341\_t001 |  | | | |  |  |  |  |  | | | |  | | | |  |  |  |
| 3 | Vvi-Vitvi03g04026\_t001 |  | | | |  |  |  |  |  | | | |  | | | |  |  |  |
| 3 | Vvi-Vitvi03g01344\_t001 |  | | | |  |  |  |  |  | | | |  | | | |  |  |  |
| 3 | Vvi-Vitvi03g04027\_t001 |  | | | |  |  |  |  |  | | | |  | | | |  |  |  |
| 3 | Vvi-Vitvi03g01345\_t001 |  | | | |  |  |  |  |  | | | |  | | | |  |  |  |
| 3 | Vvi-Vitvi03g04028\_t001 |  | | | |  |  |  |  |  | | | |  | | | |  |  |  |
| 3 | Vvi-Vitvi03g00072\_t001 |  | | | |  |  |  |  |  | | | |  | | | |  |  |  |
| 3 | Vvi-Vitvi03g01346\_t001 |  | | | |  |  |  |  |  | Ath-AT4G38840.1 |  | | | |  |  |  |
| 2 | Vvi-Vitvi03g01347\_t001 |  | Ath-AT2G21210.2 |  |  |  |  |  |  |  | Ath-AT4G34770.1 |  |  |  |
| 2 | Vvi-Vitvi03g01348\_t001 |  | | | |  |  |  |  |  |  |  | | | |  |  |  |
| 2 | Vvi-Vitvi03g01349\_t001 |  | | | |  |  |  |  |  |  |  | | | |  |  |  |
| 2 | Vvi-Vitvi03g01350\_t001 |  | | | |  |  |  |  |  |  |  | | | |  |  |  |
| 2 | Vvi-Vitvi03g01351\_t001 |  | | | |  |  |  |  |  |  |  | | | |  |  |  |
| 2 | Vvi-Vitvi03g04029\_t001 |  | | | |  |  |  |  |  |  |  | | | |  |  |  |
| 2 | Vvi-Vitvi03g01353\_t001 |  | | | |  |  |  |  |  |  |  | | | |  |  |  |
| 2 | Vvi-Vitvi03g01354\_t001 |  | | | |  |  |  |  |  |  |  | | | |  |  |  |
| 2 | Vvi-Vitvi03g04030\_t001 |  | | | |  |  |  |  |  |  |  | | | |  |  |  |
| 2 | Vvi-Vitvi03g01355\_t001 |  | | | |  |  |  |  |  |  |  | | | |  |  |  |
| 2 | Vvi-Vitvi03g01356\_t001 |  | | | |  |  |  |  |  |  |  | | | |  |  |  |
| 2 | Vvi-Vitvi03g01357\_t001 |  | | | |  |  |  |  |  |  |  | | | |  |  |  |
| 2 | Vvi-Vitvi03g01358\_t001 |  | | | |  |  |  |  |  |  |  | | | |  |  |  |
| 2 | Vvi-Vitvi03g04031\_t001 |  | | | |  |  |  |  |  |  |  | | | |  |  |  |
| 2 | Vvi-Vitvi03g01359\_t001 |  | | | |  |  |  |  |  |  |  | | | |  |  |  |
| 2 | Vvi-Vitvi03g01360\_t001 |  | | | |  |  |  |  |  |  |  | | | |  |  |  |
| 2 | Vvi-Vitvi03g01361\_t001 |  | | | |  |  |  |  |  |  |  | | | |  |  |  |
| 2 | Vvi-Vitvi03g01362\_t001 |  | | | |  |  |  |  |  |  |  | | | |  |  |  |
| 2 | Vvi-Vitvi03g01363\_t001 |  | | | |  |  |  |  |  |  |  | | | |  |  |  |
| 2 | Vvi-Vitvi03g01364\_t001 |  | | | |  |  |  |  |  |  |  | | | |  |  |  |
| 2 | Vvi-Vitvi03g01365\_t001 |  | | | |  |  |  |  |  |  |  | | | |  |  |  |
| 2 | Vvi-Vitvi03g04032\_t001 |  | | | |  |  |  |  |  |  |  | | | |  |  |  |
| 2 | Vvi-Vitvi03g01367\_t001 |  | | | |  |  |  |  |  |  |  | | | |  |  |  |
| 2 | Vvi-Vitvi03g04033\_t001 |  | | | |  |  |  |  |  |  |  | | | |  |  |  |
| 2 | Vvi-Vitvi03g01369\_t001 |  | Ath-AT2G21200.1 |  |  |  |  |  |  |  | | | |  |  |  |
| 2 | Vvi-Vitvi03g04034\_t001 |  | | | |  |  |  |  |  |  |  | | | |  |  |  |
| 2 | Vvi-Vitvi03g00075\_t001 |  | | | |  |  |  |  |  |  |  | Ath-AT4G34810.1 |  |  |  |
| 2 | Vvi-Vitvi03g00076\_t001 |  | | | |  |  |  |  |  |  |  | Ath-AT4G34830.1 |  |  |  |
| 2 | Vvi-Vitvi03g04035\_t001 |  | | | |  |  |  |  |  |  |  | | | |  |  |  |
| 2 | Vvi-Vitvi03g00077\_t001 |  | | | |  |  |  |  |  |  |  | | | |  |  |  |
| 2 | Vvi-Vitvi03g04036\_t001 |  | | | |  |  |  |  |  |  |  | | | |  |  |  |
| 2 | Vvi-Vitvi03g00078\_t001 |  | | | |  |  |  |  |  |  |  | | | |  |  |  |
| 2 | Vvi-Vitvi03g00079\_t001 |  | | | |  |  |  |  |  |  |  | | | |  |  |  |
| 2 | Vvi-Vitvi03g00080\_t001 |  | | | |  |  |  |  |  |  |  | | | |  |  |  |
| 2 | Vvi-Vitvi03g00081\_t001 |  | | | |  |  |  |  |  |  |  | | | |  |  |  |
| 2 | Vvi-Vitvi03g00082\_t001 |  | | | |  |  |  |  |  |  |  | | | |  |  |  |
| 2 | Vvi-Vitvi03g00083\_t001 |  | | | |  |  |  |  |  |  |  | | | |  |  |  |
| 2 | Vvi-Vitvi03g00084\_t001 |  | | | |  |  |  |  |  |  |  | | | |  |  |  |
| 2 | Vvi-Vitvi03g00085\_t004 |  | | | |  |  |  |  |  |  |  | Ath-AT4G34840.1 |  |  |  |
| 2 | Vvi-Vitvi03g00086\_t001 |  | | | |  |  |  |  |  |  |  | Ath-AT4G34850.1 |  |  |  |
| 2 | Vvi-Vitvi03g00087\_t001 |  | Ath-AT2G21190.1 |  |  |  |  |  |  |  | | | |  |  |  |
| 2 | Vvi-Vitvi03g00088\_t001 |  | | | |  |  |  |  |  |  |  | Ath-AT4G34860.2 |  |  |  |
| 1 | Vvi-Vitvi03g04037\_t001 |  | | | |  |  |  |  |  |  |  |
| 1 | Vvi-Vitvi03g00089\_t001 |  | | | |  |  |  |  |  |  |  |
| 1 | Vvi-Vitvi03g04038\_t001 |  | Ath-AT2G21180.1 |  |  |  |  |  |  |  |
| 0 | Vvi-Vitvi03g01372\_t001 |  |  |  |  |  |  |  |  |
| 0 | Vvi-Vitvi03g01373\_t001 |  |  |  |  |  |  |  |  |
| 0 | Vvi-Vitvi03g04039\_t001 |  |  |  |  |  |  |  |  |
| 0 | Vvi-Vitvi03g01374\_t001 |  |  |  |  |  |  |  |  |
| 0 | Vvi-Vitvi03g01377\_t001 |  |  |  |  |  |  |  |  |
| 0 | Vvi-Vitvi03g04040\_t001 |  |  |  |  |  |  |  |  |
| 0 | Vvi-Vitvi03g04041\_t001 |  |  |  |  |  |  |  |  |
| 0 | Vvi-Vitvi03g00090\_t001 |  |  |  |  |  |  |  |  |
| 0 | Vvi-Vitvi03g04042\_t001 |  |  |  |  |  |  |  |  |
| 0 | Vvi-Vitvi03g01379\_t001 |  |  |  |  |  |  |  |  |
| 0 | Vvi-Vitvi03g04043\_t001 |  |  |  |  |  |  |  |  |
| 0 | Vvi-Vitvi03g01380\_t001 |  |  |  |  |  |  |  |  |
| 0 | Vvi-Vitvi03g00092\_t001 |  |  |  |  |  |  |  |  |
| 0 | Vvi-Vitvi03g00093\_t001 |  |  |  |  |  |  |  |  |
| 0 | Vvi-Vitvi03g04044\_t001 |  |  |  |  |  |  |  |  |
| 0 | Vvi-Vitvi03g04045\_t001 |  |  |  |  |  |  |  |  |
| 0 | Vvi-Vitvi03g04046\_t001 |  |  |  |  |  |  |  |  |
| 0 | Vvi-Vitvi03g04047\_t001 |  |  |  |  |  |  |  |  |
| 0 | Vvi-Vitvi03g04048\_t001 |  |  |  |  |  |  |  |  |
| 0 | Vvi-Vitvi03g04049\_t001 |  |  |  |  |  |  |  |  |
| 0 | Vvi-Vitvi03g04050\_t001 |  |  |  |  |  |  |  |  |
| 0 | Vvi-Vitvi03g01383\_t001 |  |  |  |  |  |  |  |  |
| 0 | Vvi-Vitvi03g01384\_t001 |  |  |  |  |  |  |  |  |
| 0 | Vvi-Vitvi03g04051\_t001 |  |  |  |  |  |  |  |  |
| 0 | Vvi-Vitvi03g04052\_t001 |  |  |  |  |  |  |  |  |
| 0 | Vvi-Vitvi03g04053\_t001 |  |  |  |  |  |  |  |  |
| 1 | Vvi-Vitvi03g00097\_t001 |  | Ath-AT2G21170.1 |  |  |  |  |  |  |  |
| 1 | Vvi-Vitvi03g00098\_t001 |  | | | |  |  |  |  |  |  |  |
| 1 | Vvi-Vitvi03g00099\_t001 |  | | | |  |  |  |  |  |  |  |
| 2 | Vvi-Vitvi03g00100\_t001 |  | Ath-AT2G21160.1 |  | Ath-AT2G16595.1 |  |  |  |  |  |  |
| 2 | Vvi-Vitvi03g04054\_t001 |  | | | |  | | | |  |  |  |  |  |  |
| 3 | Vvi-Vitvi03g01388\_t001 |  | Ath-AT2G21140.1 |  | | | |  | Ath-AT4G38770.1 |  |  |  |  |  |
| 3 | Vvi-Vitvi03g00102\_t001 |  | | | |  | | | |  | | | |  |  |  |  |  |
| 3 | Vvi-Vitvi03g01389\_t001 |  | | | |  | | | |  | | | |  |  |  |  |  |
| 3 | Vvi-Vitvi03g01390\_t001 |  | | | |  | | | |  | | | |  |  |  |  |  |
| 3 | Vvi-Vitvi03g00103\_t001 |  | | | |  | | | |  | Ath-AT4G38760.1 |  |  |  |  |  |
| 4 | Vvi-Vitvi03g04055\_t001 |  | Ath-AT2G21130.1 |  | Ath-AT2G16600.1 |  | Ath-AT4G38740.1 |  | Ath-AT4G34870.1 |  |  |  |  |
| 4 | Vvi-Vitvi03g00106\_t001 |  | Ath-AT2G21120.1 |  | | | |  | Ath-AT4G38730.1 |  | | | |  |  |  |  |
| 4 | Vvi-Vitvi03g00107\_t001 |  | | | |  | | | |  | Ath-AT4G38710.2 |  | | | |  |  |  |  |
| 4 | Vvi-Vitvi03g01391\_t001 |  | | | |  | Ath-AT2G16630.1 |  | | | |  | | | |  |  |  |  |
| 4 | Vvi-Vitvi03g04056\_t001 |  | | | |  | | | |  | | | |  | Ath-AT4G34880.2 |  |  |  |  |
| 4 | Vvi-Vitvi03g04057\_t001 |  | | | |  | | | |  | | | |  | | | |  |  |  |  |
| 4 | Vvi-Vitvi03g04058\_t001 |  | | | |  | | | |  | | | |  | | | |  |  |  |  |
| 4 | Vvi-Vitvi03g00109\_t001 |  | | | |  | | | |  | | | |  | | | |  |  |  |  |
| 4 | Vvi-Vitvi03g00110\_t001 |  | | | |  | | | |  | | | |  | | | |  |  |  |  |
| 4 | Vvi-Vitvi03g00111\_t001 |  | | | |  | | | |  | | | |  | | | |  |  |  |  |
| 4 | Vvi-Vitvi03g01393\_t001 |  | | | |  | | | |  | | | |  | | | |  |  |  |  |
| 4 | Vvi-Vitvi03g00112\_t001 |  | | | |  | | | |  | | | |  | | | |  |  |  |  |
| 4 | Vvi-Vitvi03g01394\_t001 |  | Ath-AT2G21110.1 |  | | | |  | Ath-AT4G38700.1 |  | | | |  |  |  |  |
| 4 | Vvi-Vitvi03g01395\_t001 |  | Ath-AT2G21100.1 |  | | | |  | | | |  | | | |  |  |  |  |
| 4 | Vvi-Vitvi03g00113\_t001 |  | | | |  | | | |  | | | |  | | | |  |  |  |  |
| 4 | Vvi-Vitvi03g00114\_t001 |  | | | |  | Ath-AT2G16640.1 |  | | | |  | | | |  |  |  |  |
| 4 | Vvi-Vitvi03g00116\_t001 |  | Ath-AT2G21090.1 |  | | | |  | | | |  | | | |  |  |  |  |
| 4 | Vvi-Vitvi03g01396\_t001 |  | | | |  | | | |  | Ath-AT4G38690.1 |  | Ath-AT4G34920.1 |  |  |  |  |
| 4 | Vvi-Vitvi03g04059\_t001 |  | | | |  | | | |  | | | |  | | | |  |  |  |  |
| 4 | Vvi-Vitvi03g00118\_t001 |  | Ath-AT2G21070.3 |  | | | |  | | | |  | | | |  |  |  |  |
| 5 | Vvi-Vitvi03g00119\_t001 |  | | | |  | | | |  | | | |  | | | |  | Ath-AT4G36040.1 |  |  |  |
| 5 | Vvi-Vitvi03g00120\_t001 |  | | | |  | | | |  | | | |  | Ath-AT4G34940.1 |  | Ath-AT4G36030.1 |  |  |  |
| 5 | Vvi-Vitvi03g00121\_t001 |  | | | |  | | | |  | Ath-AT4G38680.1 |  | | | |  | Ath-AT4G36020.1 |  |  |  |
| 5 | Vvi-Vitvi03g04060\_t001 |  | | | |  | | | |  | | | |  | | | |  | | | |  |  |  |
| 5 | Vvi-Vitvi03g00122\_t001 |  | Ath-AT2G21050.1 |  | | | |  | | | |  | | | |  | | | |  |  |  |
| 5 | Vvi-Vitvi03g01397\_t001 |  | | | |  | | | |  | | | |  | | | |  | | | |  |  |  |
| 7 | Vvi-Vitvi03g00123\_t001 |  | | | |  | | | |  | Ath-AT4G38670.1 |  | | | |  | Ath-AT4G36010.2 |  | Ath-AT1G75800.1 |  | Ath-AT1G20030.2 |  |
| 7 | Vvi-Vitvi03g00124\_t001 |  | | | |  | | | |  | Ath-AT4G38660.1 |  | | | |  | | | |  | | | |  | | | |  |
| 7 | Vvi-Vitvi03g00125\_t001 |  | | | |  | | | |  | Ath-AT4G38650.1 |  | | | |  | | | |  | | | |  | | | |  |
| 7 | Vvi-Vitvi03g00126\_t001 |  | | | |  | Ath-AT2G16660.1 |  | | | |  | Ath-AT4G34950.1 |  | | | |  | | | |  | | | |  |
| 7 | Vvi-Vitvi03g00127\_t002 |  | | | |  | | | |  | | | |  | Ath-AT4G34960.1 |  | | | |  | | | |  | | | |  |
| 7 | Vvi-Vitvi03g00128\_t001 |  | | | |  | | | |  | Ath-AT4G38640.1 |  | | | |  | | | |  | | | |  | | | |  |
| 7 | Vvi-Vitvi03g00129\_t001 |  | | | |  | Ath-AT2G16700.1 |  | | | |  | Ath-AT4G34970.1 |  | | | |  | | | |  | | | |  |
| 7 | Vvi-Vitvi03g00130\_t001 |  | | | |  | | | |  | | | |  | Ath-AT4G34980.1 |  | | | |  | | | |  | | | |  |
| 7 | Vvi-Vitvi03g04061\_t001 |  | | | |  | | | |  | | | |  | | | |  | | | |  | | | |  | | | |  |
| 7 | Vvi-Vitvi03g00131\_t001 |  | | | |  | | | |  | | | |  | | | |  | | | |  | | | |  | | | |  |
| 7 | Vvi-Vitvi03g04062\_t001 |  | | | |  | | | |  | | | |  | | | |  | | | |  | | | |  | | | |  |
| 7 | Vvi-Vitvi03g00132\_t001 |  | | | |  | | | |  | | | |  | | | |  | | | |  | | | |  | | | |  |
| 7 | Vvi-Vitvi03g00133\_t001 |  | | | |  | | | |  | | | |  | | | |  | Ath-AT4G35985.1 |  | | | |  | | | |  |
| 7 | Vvi-Vitvi03g00134\_t001 |  | | | |  | | | |  | | | |  | | | |  | | | |  | | | |  | | | |  |
| 7 | Vvi-Vitvi03g04063\_t001 |  | | | |  | | | |  | | | |  | | | |  | | | |  | | | |  | | | |  |
| 7 | Vvi-Vitvi03g00135\_t001 |  | | | |  | | | |  | | | |  | | | |  | | | |  | | | |  | | | |  |
| 7 | Vvi-Vitvi03g00136\_t001 |  | | | |  | Ath-AT2G16720.1 |  | Ath-AT4G38620.1 |  | Ath-AT4G34990.1 |  | | | |  | | | |  | | | |  |
| 7 | Vvi-Vitvi03g00137\_t001 |  | | | |  | | | |  | | | |  | Ath-AT4G35000.1 |  | Ath-AT4G35970.1 |  | | | |  | | | |  |
| 6 | Vvi-Vitvi03g00138\_t001 |  | Ath-AT2G20990.3 |  | | | |  | | | |  | | | |  |  |  | | | |  | Ath-AT1G20080.2 |  |
| 6 | Vvi-Vitvi03g00139\_t002 |  | | | |  | | | |  | Ath-AT4G38600.1 |  | | | |  |  |  | | | |  | | | |  |
| 6 | Vvi-Vitvi03g00141\_t001 |  | | | |  | Ath-AT2G16730.1 |  | Ath-AT4G38590.2 |  | Ath-AT4G35010.1 |  |  |  | | | |  | | | |  |
| 6 | Vvi-Vitvi03g04064\_t001 |  | Ath-AT2G20980.1 |  | | | |  | | | |  | | | |  |  |  | | | |  | | | |  |
| 6 | Vvi-Vitvi03g00143\_t001 |  | | | |  | | | |  | | | |  | Ath-AT4G35020.2 |  |  |  | Ath-AT1G75840.1 |  | Ath-AT1G20090.1 |  |
| 6 | Vvi-Vitvi03g01399\_t001 |  | | | |  | | | |  | | | |  | | | |  |  |  | | | |  | | | |  |
| 6 | Vvi-Vitvi03g00144\_t001 |  | | | |  | Ath-AT2G16750.2 |  | | | |  | Ath-AT4G35030.3 |  |  |  | | | |  | | | |  |
| 6 | Vvi-Vitvi03g01400\_t001 |  | | | |  | | | |  | | | |  | | | |  |  |  | | | |  | | | |  |
| 6 | Vvi-Vitvi03g00146\_t003 |  | | | |  | Ath-AT2G16770.1 |  | | | |  | Ath-AT4G35040.2 |  |  |  | | | |  | | | |  |
| 6 | Vvi-Vitvi03g00147\_t001 |  | | | |  | Ath-AT2G16780.1 |  | | | |  | Ath-AT4G35050.1 |  |  |  | | | |  | | | |  |
| 6 | Vvi-Vitvi03g00148\_t001 |  | | | |  | | | |  | Ath-AT4G38580.1 |  | Ath-AT4G35060.1 |  |  |  | | | |  | | | |  |
| 6 | Vvi-Vitvi03g00149\_t001 |  | | | |  | | | |  | | | |  | Ath-AT4G35070.1 |  |  |  | | | |  | | | |  |
| 6 | Vvi-Vitvi03g00150\_t001 |  | | | |  | | | |  | Ath-AT4G38570.1 |  | | | |  |  |  | | | |  | | | |  |
| 6 | Vvi-Vitvi03g00151\_t001 |  | | | |  | Ath-AT2G16800.1 |  | | | |  | Ath-AT4G35080.3 |  |  |  | | | |  | | | |  |
| 6 | Vvi-Vitvi03g00152\_t001 |  | | | |  | | | |  | | | |  | | | |  |  |  | Ath-AT1G75950.1 |  | Ath-AT1G20140.1 |  |
| 6 | Vvi-Vitvi03g04065\_t001 |  | | | |  | | | |  | | | |  | | | |  |  |  | | | |  | | | |  |
| 6 | Vvi-Vitvi03g00153\_t001 |  | | | |  | | | |  | | | |  | | | |  |  |  | | | |  | | | |  |
| 6 | Vvi-Vitvi03g00154\_t001 |  | | | |  | | | |  | | | |  | | | |  |  |  | | | |  | | | |  |
| 6 | Vvi-Vitvi03g00155\_t001 |  | | | |  | Ath-AT2G16850.1 |  | | | |  | Ath-AT4G35100.1 |  |  |  | | | |  | | | |  |
| 5 | Vvi-Vitvi03g00156\_t001 |  | | | |  | Ath-AT2G16860.1 |  | | | |  |  |  |  |  | | | |  | | | |  |
| 5 | Vvi-Vitvi03g01401\_t001 |  | | | |  | | | |  | | | |  |  |  |  |  | | | |  | | | |  |
| 5 | Vvi-Vitvi03g00157\_t001 |  | | | |  | Ath-AT2G16910.1 |  | | | |  |  |  |  |  | | | |  | | | |  |
| 5 | Vvi-Vitvi03g01402\_t001 |  | | | |  | | | |  | | | |  |  |  |  |  | | | |  | | | |  |
| 5 | Vvi-Vitvi03g00158\_t001 |  | Ath-AT2G20940.1 |  | | | |  | | | |  |  |  |  |  | | | |  | | | |  |
| 5 | Vvi-Vitvi03g00159\_t001 |  | | | |  | Ath-AT2G16920.1 |  | | | |  |  |  |  |  | | | |  | | | |  |
| 5 | Vvi-Vitvi03g00160\_t001 |  | Ath-AT2G20930.1 |  | | | |  | | | |  |  |  |  |  | | | |  | | | |  |
| 5 | Vvi-Vitvi03g00161\_t001 |  | | | |  | | | |  | | | |  |  |  |  |  | Ath-AT1G75990.1 |  | Ath-AT1G20200.1 |  |
| 5 | Vvi-Vitvi03g04066\_t001 |  | | | |  | | | |  | Ath-AT4G38540.1 |  |  |  |  |  | | | |  | | | |  |
| 5 | Vvi-Vitvi03g04067\_t001 |  | | | |  | | | |  | | | |  |  |  |  |  | | | |  | | | |  |
| 5 | Vvi-Vitvi03g00164\_t001 |  | | | |  | | | |  | | | |  |  |  |  |  | | | |  | | | |  |
| 5 | Vvi-Vitvi03g04068\_t001 |  | | | |  | Ath-AT2G16940.2 |  | | | |  |  |  |  |  | | | |  | | | |  |
| 5 | Vvi-Vitvi03g04069\_t001 |  | | | |  | | | |  | | | |  |  |  |  |  | | | |  | | | |  |
| 5 | Vvi-Vitvi03g00167\_t001 |  | | | |  | | | |  | Ath-AT4G38520.1 |  |  |  |  |  | | | |  | | | |  |
| 5 | Vvi-Vitvi03g00168\_t001 |  | | | |  | | | |  | | | |  |  |  |  |  | | | |  | | | |  |
| 5 | Vvi-Vitvi03g00169\_t001 |  | Ath-AT2G20920.1 |  | | | |  | | | |  |  |  |  |  | | | |  | | | |  |
| 4 | Vvi-Vitvi03g00170\_t001 |  |  |  | | | |  | | | |  |  |  |  |  | | | |  | | | |  |
| 4 | Vvi-Vitvi03g04070\_t001 |  |  |  | | | |  | | | |  |  |  |  |  | | | |  | | | |  |
| 4 | Vvi-Vitvi03g00172\_t001 |  |  |  | | | |  | | | |  |  |  |  |  | | | |  | | | |  |
| 4 | Vvi-Vitvi03g04071\_t001 |  |  |  | | | |  | | | |  |  |  |  |  | | | |  | | | |  |
| 4 | Vvi-Vitvi03g01406\_t001 |  |  |  | | | |  | | | |  |  |  |  |  | | | |  | | | |  |
| 4 | Vvi-Vitvi03g00173\_t001 |  |  |  | | | |  | Ath-AT4G38510.5 |  |  |  |  |  | Ath-AT1G76030.1 |  | Ath-AT1G20260.1 |  |
| 4 | Vvi-Vitvi03g00174\_t001 |  |  |  | | | |  | | | |  |  |  |  |  | | | |  | | | |  |
| 4 | Vvi-Vitvi03g01408\_t001 |  |  |  | | | |  | | | |  |  |  |  |  | | | |  | | | |  |
| 4 | Vvi-Vitvi03g00175\_t003 |  |  |  | | | |  | | | |  |  |  |  |  | | | |  | | | |  |
| 4 | Vvi-Vitvi03g04072\_t001 |  |  |  | | | |  | | | |  |  |  |  |  | | | |  | | | |  |
| 4 | Vvi-Vitvi03g04073\_t001 |  |  |  | | | |  | | | |  |  |  |  |  | | | |  | | | |  |
| 4 | Vvi-Vitvi03g00178\_t001 |  |  |  | | | |  | | | |  |  |  |  |  | | | |  | | | |  |
| 4 | Vvi-Vitvi03g00179\_t001 |  |  |  | | | |  | Ath-AT4G38500.1 |  |  |  |  |  | | | |  | | | |  |
| 4 | Vvi-Vitvi03g00180\_t001 |  |  |  | Ath-AT2G16950.1 |  | | | |  |  |  |  |  | | | |  | | | |  |
| 4 | Vvi-Vitvi03g01410\_t001 |  |  |  | | | |  | Ath-AT4G38490.1 |  |  |  |  |  | | | |  | | | |  |
| 4 | Vvi-Vitvi03g01411\_t001 |  |  |  | | | |  | | | |  |  |  |  |  | | | |  | | | |  |
| 4 | Vvi-Vitvi03g00181\_t001 |  |  |  | | | |  | | | |  |  |  |  |  | | | |  | | | |  |
| 4 | Vvi-Vitvi03g00182\_t001 |  |  |  | | | |  | | | |  |  |  |  |  | | | |  | | | |  |
| 4 | Vvi-Vitvi03g01412\_t001 |  |  |  | | | |  | | | |  |  |  |  |  | | | |  | | | |  |
| 4 | Vvi-Vitvi03g04074\_t001 |  |  |  | | | |  | | | |  |  |  |  |  | Ath-AT1G76080.1 |  | | | |  |
| 4 | Vvi-Vitvi03g00183\_t001 |  |  |  | | | |  | | | |  |  |  |  |  | | | |  | | | |  |
| 4 | Vvi-Vitvi03g01413\_t001 |  |  |  | | | |  | | | |  |  |  |  |  | | | |  | | | |  |
| 4 | Vvi-Vitvi03g01414\_t001 |  |  |  | | | |  | | | |  |  |  |  |  | | | |  | | | |  |
| 4 | Vvi-Vitvi03g00185\_t001 |  |  |  | | | |  | | | |  |  |  |  |  | Ath-AT1G76090.1 |  | Ath-AT1G20330.1 |  |
| 2 | Vvi-Vitvi03g00186\_t001 |  |  |  | Ath-AT2G16970.2 |  | | | |  |  |  |  |  |
| 2 | Vvi-Vitvi03g00187\_t001 |  |  |  | | | |  | Ath-AT4G38480.1 |  |  |  |  |  |
| 2 | Vvi-Vitvi03g04075\_t001 |  |  |  | | | |  | | | |  |  |  |  |  |
| 2 | Vvi-Vitvi03g00188\_t001 |  |  |  | | | |  | | | |  |  |  |  |  |
| 2 | Vvi-Vitvi03g00189\_t001 |  |  |  | | | |  | | | |  |  |  |  |  |
| 2 | Vvi-Vitvi03g00190\_t001 |  |  |  | | | |  | | | |  |  |  |  |  |
| 2 | Vvi-Vitvi03g04076\_t001 |  |  |  | | | |  | | | |  |  |  |  |  |
| 2 | Vvi-Vitvi03g00191\_t001 |  |  |  | Ath-AT2G17000.1 |  | | | |  |  |  |  |  |
| 2 | Vvi-Vitvi03g01416\_t001 |  |  |  | | | |  | Ath-AT4G38470.1 |  |  |  |  |  |
| 2 | Vvi-Vitvi03g01417\_t001 |  |  |  | | | |  | | | |  |  |  |  |  |
| 2 | Vvi-Vitvi03g01418\_t001 |  |  |  | | | |  | | | |  |  |  |  |  |
| 2 | Vvi-Vitvi03g00192\_t001 |  |  |  | | | |  | | | |  |  |  |  |  |
| 2 | Vvi-Vitvi03g01419\_t001 |  |  |  | | | |  | | | |  |  |  |  |  |
| 2 | Vvi-Vitvi03g04077\_t001 |  |  |  | | | |  | | | |  |  |  |  |  |
| 2 | Vvi-Vitvi03g01420\_t001 |  |  |  | Ath-AT2G17020.1 |  | | | |  |  |  |  |  |
| 2 | Vvi-Vitvi03g01421\_t001 |  |  |  | | | |  | Ath-AT4G38460.1 |  |  |  |  |  |
| 2 | Vvi-Vitvi03g00193\_t001 |  |  |  | | | |  | | | |  |  |  |  |  |
| 2 | Vvi-Vitvi03g04078\_t001 |  |  |  | | | |  | | | |  |  |  |  |  |
| 2 | Vvi-Vitvi03g01424\_t001 |  |  |  | | | |  | | | |  |  |  |  |  |
| 2 | Vvi-Vitvi03g01425\_t001 |  |  |  | | | |  | | | |  |  |  |  |  |
| 2 | Vvi-Vitvi03g00194\_t001 |  |  |  | | | |  | | | |  |  |  |  |  |
| 2 | Vvi-Vitvi03g00195\_t001 |  |  |  | | | |  | Ath-AT4G38440.1 |  |  |  |  |  |
| 2 | Vvi-Vitvi03g00196\_t001 |  |  |  | | | |  | | | |  |  |  |  |  |
| 3 | Vvi-Vitvi03g00197\_t001 |  | Ath-AT4G35260.1 |  | Ath-AT2G17130.1 |  | | | |  |  |  |  |  |
| 2 | Vvi-Vitvi03g01426\_t001 |  | | | |  |  |  | | | |  |  |  |  |  |
| 2 | Vvi-Vitvi03g01427\_t001 |  | | | |  |  |  | | | |  |  |  |  |  |
| 2 | Vvi-Vitvi03g01428\_t001 |  | | | |  |  |  | | | |  |  |  |  |  |
| 2 | Vvi-Vitvi03g01429\_t001 |  | | | |  |  |  | | | |  |  |  |  |  |
| 2 | Vvi-Vitvi03g01431\_t001 |  | | | |  |  |  | | | |  |  |  |  |  |
| 2 | Vvi-Vitvi03g04079\_t001 |  | | | |  |  |  | | | |  |  |  |  |  |
| 2 | Vvi-Vitvi03g00199\_t003 |  | | | |  |  |  | Ath-AT4G38380.2 |  |  |  |  |  |
| 1 | Vvi-Vitvi03g01433\_t001 |  | | | |  |  |  |  |  |  |  |
| 1 | Vvi-Vitvi03g04080\_t001 |  | | | |  |  |  |  |  |  |  |
| 1 | Vvi-Vitvi03g01434\_t001 |  | | | |  |  |  |  |  |  |  |
| 1 | Vvi-Vitvi03g00200\_t002 |  | | | |  |  |  |  |  |  |  |
| 1 | Vvi-Vitvi03g00202\_t001 |  | | | |  |  |  |  |  |  |  |
| 1 | Vvi-Vitvi03g00203\_t001 |  | Ath-AT4G35250.1 |  |  |  |  |  |  |  |
| 1 | Vvi-Vitvi03g00204\_t001 |  | Ath-AT4G35240.1 |  |  |  |  |  |  |  |
| 1 | Vvi-Vitvi03g00205\_t001 |  | Ath-AT4G35230.1 |  |  |  |  |  |  |  |
| 1 | Vvi-Vitvi03g01436\_t001 |  | Ath-AT4G35200.1 |  |  |  |  |  |  |  |
| 1 | Vvi-Vitvi03g04081\_t001 |  | | | |  |  |  |  |  |  |  |
| 1 | Vvi-Vitvi03g01438\_t001 |  | | | |  |  |  |  |  |  |  |
| 1 | Vvi-Vitvi03g01440\_t001 |  | | | |  |  |  |  |  |  |  |
| 1 | Vvi-Vitvi03g01442\_t001 |  | | | |  |  |  |  |  |  |  |
| 1 | Vvi-Vitvi03g01444\_t001 |  | | | |  |  |  |  |  |  |  |
| 1 | Vvi-Vitvi03g04082\_t001 |  | | | |  |  |  |  |  |  |  |
| 1 | Vvi-Vitvi03g04083\_t001 |  | | | |  |  |  |  |  |  |  |
| 1 | Vvi-Vitvi03g01445\_t001 |  | | | |  |  |  |  |  |  |  |
| 1 | Vvi-Vitvi03g01446\_t001 |  | | | |  |  |  |  |  |  |  |
| 1 | Vvi-Vitvi03g01447\_t001 |  | | | |  |  |  |  |  |  |  |
| 1 | Vvi-Vitvi03g01448\_t001 |  | | | |  |  |  |  |  |  |  |
| 1 | Vvi-Vitvi03g00206\_t001 |  | | | |  |  |  |  |  |  |  |
| 1 | Vvi-Vitvi03g00207\_t001 |  | | | |  |  |  |  |  |  |  |
| 1 | Vvi-Vitvi03g00208\_t001 |  | Ath-AT4G35190.1 |  |  |  |  |  |  |  |
| 0 | Vvi-Vitvi03g00209\_t001 |  |  |  |  |  |  |  |  |
| 0 | Vvi-Vitvi03g00210\_t001 |  |  |  |  |  |  |  |  |
| 0 | Vvi-Vitvi03g04084\_t001 |  |  |  |  |  |  |  |  |
| 0 | Vvi-Vitvi03g04085\_t001 |  |  |  |  |  |  |  |  |
| 0 | Vvi-Vitvi03g01449\_t001 |  |  |  |  |  |  |  |  |
| 0 | Vvi-Vitvi03g04086\_t001 |  |  |  |  |  |  |  |  |
| 0 | Vvi-Vitvi03g01450\_t001 |  |  |  |  |  |  |  |  |
| 0 | Vvi-Vitvi03g04087\_t001 |  |  |  |  |  |  |  |  |
| 0 | Vvi-Vitvi03g04088\_t001 |  |  |  |  |  |  |  |  |
| 0 | Vvi-Vitvi03g01451\_t001 |  |  |  |  |  |  |  |  |
| 0 | Vvi-Vitvi03g01452\_t001 |  |  |  |  |  |  |  |  |
| 0 | Vvi-Vitvi03g00211\_t001 |  |  |  |  |  |  |  |  |
| 0 | Vvi-Vitvi03g04089\_t001 |  |  |  |  |  |  |  |  |
| 0 | Vvi-Vitvi03g01453\_t001 |  |  |  |  |  |  |  |  |
| 0 | Vvi-Vitvi03g01454\_t001 |  |  |  |  |  |  |  |  |
| 0 | Vvi-Vitvi03g04090\_t001 |  |  |  |  |  |  |  |  |
| 0 | Vvi-Vitvi03g01455\_t001 |  |  |  |  |  |  |  |  |
| 0 | Vvi-Vitvi03g01456\_t001 |  |  |  |  |  |  |  |  |
| 0 | Vvi-Vitvi03g04091\_t001 |  |  |  |  |  |  |  |  |
| 0 | Vvi-Vitvi03g01457\_t001 |  |  |  |  |  |  |  |  |
| 0 | Vvi-Vitvi03g01458\_t001 |  |  |  |  |  |  |  |  |
| 0 | Vvi-Vitvi03g01459\_t001 |  |  |  |  |  |  |  |  |
| 0 | Vvi-Vitvi03g01460\_t001 |  |  |  |  |  |  |  |  |
| 0 | Vvi-Vitvi03g04092\_t001 |  |  |  |  |  |  |  |  |
| 0 | Vvi-Vitvi03g01461\_t001 |  |  |  |  |  |  |  |  |
| 0 | Vvi-Vitvi03g01462\_t001 |  |  |  |  |  |  |  |  |
| 0 | Vvi-Vitvi03g04093\_t001 |  |  |  |  |  |  |  |  |
| 0 | Vvi-Vitvi03g00213\_t001 |  |  |  |  |  |  |  |  |
| 0 | Vvi-Vitvi03g00214\_t001 |  |  |  |  |  |  |  |  |
| 1 | Vvi-Vitvi03g01463\_t001 |  | Ath-AT1G20440.1 |  |  |  |  |  |  |  |
| 2 | Vvi-Vitvi03g00215\_t001 |  | | | |  | Ath-AT4G35170.1 |  |  |  |  |  |  |
| 2 | Vvi-Vitvi03g00216\_t001 |  | | | |  | | | |  |  |  |  |  |  |
| 4 | Vvi-Vitvi03g00217\_t001 |  | | | |  | | | |  | Ath-AT1G76160.1 |  | Ath-AT4G38420.1 |  |  |  |  |
| 4 | Vvi-Vitvi03g00218\_t001 |  | | | |  | | | |  | | | |  | | | |  |  |  |  |
| 4 | Vvi-Vitvi03g00220\_t001 |  | | | |  | | | |  | | | |  | | | |  |  |  |  |
| 4 | Vvi-Vitvi03g00221\_t001 |  | | | |  | | | |  | | | |  | | | |  |  |  |  |
| 4 | Vvi-Vitvi03g00222\_t001 |  | | | |  | | | |  | | | |  | | | |  |  |  |  |
| 4 | Vvi-Vitvi03g00223\_t001 |  | | | |  | | | |  | | | |  | | | |  |  |  |  |
| 5 | Vvi-Vitvi03g04094\_t001 |  | | | |  | | | |  | | | |  | | | |  | Ath-AT2G17030.1 |  |  |  |
| 5 | Vvi-Vitvi03g00225\_t001 |  | | | |  | | | |  | | | |  | Ath-AT4G38370.1 |  | | | |  |  |  |
| 5 | Vvi-Vitvi03g00226\_t001 |  | Ath-AT1G20580.1 |  | | | |  | Ath-AT1G76300.1 |  | | | |  | | | |  |  |  |
| 5 | Vvi-Vitvi03g00227\_t002 |  | | | |  | | | |  | | | |  | | | |  | | | |  |  |  |
| 5 | Vvi-Vitvi03g00228\_t001 |  | | | |  | | | |  | | | |  | Ath-AT4G38360.2 |  | | | |  |  |  |
| 5 | Vvi-Vitvi03g00229\_t003 |  | | | |  | | | |  | | | |  | | | |  | | | |  |  |  |
| 5 | Vvi-Vitvi03g01464\_t001 |  | | | |  | | | |  | | | |  | | | |  | | | |  |  |  |
| 5 | Vvi-Vitvi03g00230\_t001 |  | | | |  | Ath-AT4G35280.1 |  | | | |  | | | |  | | | |  |  |  |
| 5 | Vvi-Vitvi03g04095\_t001 |  | | | |  | | | |  | | | |  | | | |  | | | |  |  |  |
| 5 | Vvi-Vitvi03g00231\_t001 |  | Ath-AT1G20640.3 |  | | | |  | Ath-AT1G76350.1 |  | | | |  | Ath-AT2G17150.1 |  |  |  |
| 5 | Vvi-Vitvi03g00232\_t001 |  | | | |  | | | |  | | | |  | | | |  | | | |  |  |  |
| 5 | Vvi-Vitvi03g00233\_t001 |  | | | |  | | | |  | | | |  | Ath-AT4G38350.2 |  | | | |  |  |  |
| 5 | Vvi-Vitvi03g00234\_t001 |  | | | |  | | | |  | | | |  | | | |  | Ath-AT2G17210.1 |  |  |  |
| 5 | Vvi-Vitvi03g00235\_t001 |  | | | |  | | | |  | | | |  | Ath-AT4G38270.1 |  | | | |  |  |  |
| 5 | Vvi-Vitvi03g04096\_t001 |  | | | |  | | | |  | | | |  | | | |  | | | |  |  |  |
| 5 | Vvi-Vitvi03g01465\_t001 |  | | | |  | | | |  | | | |  | | | |  | | | |  |  |  |
| 5 | Vvi-Vitvi03g04097\_t001 |  | | | |  | | | |  | | | |  | | | |  | | | |  |  |  |
| 5 | Vvi-Vitvi03g01467\_t001 |  | | | |  | | | |  | | | |  | | | |  | | | |  |  |  |
| 5 | Vvi-Vitvi03g04098\_t001 |  | | | |  | | | |  | | | |  | | | |  | | | |  |  |  |
| 5 | Vvi-Vitvi03g04099\_t001 |  | | | |  | | | |  | | | |  | | | |  | | | |  |  |  |
| 5 | Vvi-Vitvi03g01469\_t001 |  | | | |  | | | |  | | | |  | | | |  | | | |  |  |  |
| 5 | Vvi-Vitvi03g01470\_t001 |  | | | |  | | | |  | | | |  | | | |  | | | |  |  |  |
| 5 | Vvi-Vitvi03g01471\_t001 |  | | | |  | | | |  | | | |  | | | |  | | | |  |  |  |
| 5 | Vvi-Vitvi03g04100\_t001 |  | | | |  | | | |  | | | |  | | | |  | | | |  |  |  |
| 5 | Vvi-Vitvi03g00238\_t001 |  | | | |  | | | |  | Ath-AT1G76360.1 |  | | | |  | Ath-AT2G17220.1 |  |  |  |
| 5 | Vvi-Vitvi03g00239\_t001 |  | Ath-AT1G20650.1 |  | | | |  | Ath-AT1G76370.1 |  | | | |  | | | |  |  |  |
| 5 | Vvi-Vitvi03g00240\_t001 |  | Ath-AT1G20670.1 |  | | | |  | Ath-AT1G76380.2 |  | | | |  | | | |  |  |  |
| 5 | Vvi-Vitvi03g00241\_t001 |  | Ath-AT1G20730.1 |  | | | |  | | | |  | Ath-AT4G38260.1 |  | | | |  |  |  |
| 5 | Vvi-Vitvi03g01472\_t001 |  | | | |  | | | |  | | | |  | | | |  | | | |  |  |  |
| 5 | Vvi-Vitvi03g00242\_t001 |  | | | |  | | | |  | | | |  | | | |  | Ath-AT2G17230.1 |  |  |  |
| 5 | Vvi-Vitvi03g00243\_t001 |  | Ath-AT1G20760.1 |  | | | |  | | | |  | | | |  | | | |  |  |  |
| 5 | Vvi-Vitvi03g00244\_t001 |  | | | |  | | | |  | | | |  | Ath-AT4G38250.1 |  | | | |  |  |  |
| 5 | Vvi-Vitvi03g00245\_t002 |  | | | |  | | | |  | | | |  | | | |  | | | |  |  |  |
| 5 | Vvi-Vitvi03g00246\_t001 |  | | | |  | Ath-AT4G35290.2 |  | | | |  | | | |  | Ath-AT2G17260.1 |  |  |  |
| 5 | Vvi-Vitvi03g01473\_t001 |  | Ath-AT1G20823.1 |  | | | |  | Ath-AT1G76410.1 |  | | | |  | | | |  |  |  |
| 5 | Vvi-Vitvi03g00247\_t001 |  | Ath-AT1G20840.1 |  | Ath-AT4G35300.6 |  | | | |  | | | |  | | | |  |  |  |
| 5 | Vvi-Vitvi03g00248\_t001 |  | | | |  | | | |  | | | |  | | | |  | Ath-AT2G17270.1 |  |  |  |
| 5 | Vvi-Vitvi03g00249\_t001 |  | | | |  | Ath-AT4G35310.1 |  | | | |  | Ath-AT4G38230.2 |  | Ath-AT2G17290.2 |  |  |  |
| 5 | Vvi-Vitvi03g00250\_t001 |  | | | |  | | | |  | | | |  | Ath-AT4G38225.3 |  | | | |  |  |  |
| 5 | Vvi-Vitvi03g00251\_t001 |  | | | |  | Ath-AT4G35320.1 |  | | | |  | | | |  | Ath-AT2G17300.1 |  |  |  |
| 5 | Vvi-Vitvi03g00252\_t001 |  | | | |  | | | |  | | | |  | | | |  | | | |  |  |  |
| 5 | Vvi-Vitvi03g00253\_t001 |  | Ath-AT1G20850.1 |  | Ath-AT4G35350.1 |  | | | |  | | | |  | | | |  |  |  |
| 5 | Vvi-Vitvi03g00254\_t001 |  | | | |  | Ath-AT4G35360.1 |  | | | |  | | | |  | Ath-AT2G17320.1 |  |  |  |
| 5 | Vvi-Vitvi03g00255\_t001 |  | | | |  | | | |  | | | |  | | | |  | | | |  |  |  |
| 5 | Vvi-Vitvi03g00256\_t001 |  | | | |  | | | |  | | | |  | | | |  | | | |  |  |  |
| 5 | Vvi-Vitvi03g00257\_t002 |  | | | |  | | | |  | | | |  | Ath-AT4G38220.2 |  | | | |  |  |  |
| 5 | Vvi-Vitvi03g01474\_t001 |  | | | |  | | | |  | | | |  | Ath-AT4G38213.1 |  | | | |  |  |  |
| 5 | Vvi-Vitvi03g00258\_t001 |  | | | |  | | | |  | | | |  | Ath-AT4G38210.1 |  | | | |  |  |  |
| 5 | Vvi-Vitvi03g00259\_t001 |  | | | |  | | | |  | | | |  | | | |  | | | |  |  |  |
| 5 | Vvi-Vitvi03g00260\_t001 |  | | | |  | | | |  | | | |  | | | |  | | | |  |  |  |
| 5 | Vvi-Vitvi03g00261\_t003 |  | | | |  | Ath-AT4G35380.1 |  | | | |  | Ath-AT4G38200.1 |  | | | |  |  |  |
| 5 | Vvi-Vitvi03g00262\_t001 |  | | | |  | | | |  | Ath-AT1G76490.1 |  | | | |  | Ath-AT2G17370.1 |  |  |  |
| 5 | Vvi-Vitvi03g00263\_t001 |  | Ath-AT1G20900.1 |  | Ath-AT4G35390.1 |  | Ath-AT1G76500.1 |  | | | |  | | | |  |  |  |
| 4 | Vvi-Vitvi03g00264\_t001 |  | | | |  | | | |  |  |  | Ath-AT4G38190.1 |  | | | |  |  |  |
| 4 | Vvi-Vitvi03g00265\_t001 |  | Ath-AT1G20920.1 |  | | | |  |  |  | | | |  | | | |  |  |  |
| 4 | Vvi-Vitvi03g01475\_t003 |  | | | |  | | | |  |  |  | Ath-AT4G38170.1 |  | | | |  |  |  |
| 4 | Vvi-Vitvi03g00266\_t001 |  | | | |  | | | |  |  |  | | | |  | | | |  |  |  |
| 4 | Vvi-Vitvi03g00267\_t001 |  | Ath-AT1G20990.1 |  | | | |  |  |  | | | |  | | | |  |  |  |
| 4 | Vvi-Vitvi03g00268\_t001 |  | | | |  | | | |  |  |  | | | |  | | | |  |  |  |
| 4 | Vvi-Vitvi03g04101\_t001 |  | | | |  | | | |  |  |  | | | |  | | | |  |  |  |
| 4 | Vvi-Vitvi03g00269\_t001 |  | | | |  | | | |  |  |  | Ath-AT4G38160.3 |  | | | |  |  |  |
| 4 | Vvi-Vitvi03g00270\_t001 |  | | | |  | | | |  |  |  | Ath-AT4G38150.1 |  | | | |  |  |  |
| 4 | Vvi-Vitvi03g00271\_t001 |  | | | |  | | | |  |  |  | Ath-AT4G38140.1 |  | | | |  |  |  |
| 4 | Vvi-Vitvi03g00272\_t001.1.6037826d |  | | | |  | | | |  |  |  | | | |  | | | |  |  |  |
| 4 | Vvi-Vitvi03g01477\_t001 |  | | | |  | Ath-AT4G35420.1 |  |  |  | | | |  | | | |  |  |  |
| 4 | Vvi-Vitvi03g01478\_t001 |  | | | |  | | | |  |  |  | | | |  | | | |  |  |  |
| 4 | Vvi-Vitvi03g00273\_t002 |  | | | |  | | | |  |  |  | Ath-AT4G38130.1 |  | | | |  |  |  |
| 3 | Vvi-Vitvi03g00274\_t001 |  | | | |  | | | |  |  |  |  |  | | | |  |  |  |
| 3 | Vvi-Vitvi03g00275\_t001 |  | | | |  | Ath-AT4G35440.2 |  |  |  |  |  | | | |  |  |  |
| 3 | Vvi-Vitvi03g00277\_t003 |  | | | |  | Ath-AT4G35450.5 |  |  |  |  |  | Ath-AT2G17390.1 |  |  |  |
| 1 | Vvi-Vitvi03g00278\_t001 |  | | | |  |  |  |  |  |  |  |
| 3 | Vvi-Vitvi03g04102\_t001 |  | | | |  | Ath-AT2G16390.2 |  | Ath-AT2G21450.2 |  |  |  |  |  |
| 3 | Vvi-Vitvi03g00283\_t001 |  | | | |  | | | |  | Ath-AT2G21470.2 |  |  |  |  |  |
| 3 | Vvi-Vitvi03g01479\_t001 |  | | | |  | | | |  | | | |  |  |  |  |  |
| 3 | Vvi-Vitvi03g04103\_t001 |  | | | |  | | | |  | | | |  |  |  |  |  |
| 4 | Vvi-Vitvi03g00284\_t001 |  | | | |  | | | |  | Ath-AT2G21480.1 |  | Ath-AT4G39110.1 |  |  |  |  |
| 4 | Vvi-Vitvi03g00285\_t001 |  | | | |  | | | |  | | | |  | | | |  |  |  |  |
| 4 | Vvi-Vitvi03g00286\_t001 |  | | | |  | | | |  | | | |  | | | |  |  |  |  |
| 4 | Vvi-Vitvi03g00287\_t001 |  | | | |  | | | |  | | | |  | Ath-AT4G39120.2 |  |  |  |  |
| 4 | Vvi-Vitvi03g01481\_t001 |  | | | |  | | | |  | Ath-AT2G21490.1 |  | Ath-AT4G39130.1 |  |  |  |  |
| 6 | Vvi-Vitvi03g00288\_t001 |  | | | |  | | | |  | Ath-AT2G21500.1 |  | Ath-AT4G39140.4 |  | Ath-AT1G75400.1 |  | Ath-AT1G19680.1 |  |  |
| 6 | Vvi-Vitvi03g04104\_t001 |  | | | |  | | | |  | | | |  | | | |  | | | |  | | | |  |  |
| 7 | Vvi-Vitvi03g01482\_t001 |  | | | |  | Ath-AT2G16385.1 |  | | | |  | | | |  | | | |  | | | |  | Ath-AT4G34600.1 |  |
| 7 | Vvi-Vitvi03g00289\_t001 |  | Ath-AT1G21080.3 |  | | | |  | Ath-AT2G21510.2 |  | Ath-AT4G39150.1 |  | | | |  | | | |  | | | |  |
| 6 | Vvi-Vitvi03g00291\_t001 |  |  |  | | | |  | | | |  | | | |  | | | |  | | | |  | | | |  |
| 7 | Vvi-Vitvi03g00292\_t001 |  | Ath-AT2G18160.1 |  | | | |  | | | |  | | | |  | Ath-AT1G75390.1 |  | | | |  | Ath-AT4G34590.1 |  |
| 7 | Vvi-Vitvi03g04105\_t001 |  | | | |  | | | |  | | | |  | | | |  | | | |  | | | |  | | | |  |
| 7 | Vvi-Vitvi03g04106\_t001 |  | | | |  | | | |  | | | |  | | | |  | | | |  | | | |  | | | |  |
| 7 | Vvi-Vitvi03g00293\_t001 |  | | | |  | | | |  | | | |  | Ath-AT4G39160.2 |  | | | |  | | | |  | | | |  |
| 7 | Vvi-Vitvi03g04107\_t001 |  | | | |  | | | |  | | | |  | | | |  | | | |  | | | |  | | | |  |
| 7 | Vvi-Vitvi03g00294\_t001 |  | | | |  | | | |  | Ath-AT2G21520.2 |  | Ath-AT4G39170.1 |  | Ath-AT1G75370.2 |  | Ath-AT1G19650.1 |  | | | |  |
| 7 | Vvi-Vitvi03g00295\_t001 |  | Ath-AT2G18180.1 |  | | | |  | | | |  | Ath-AT4G39180.1 |  | | | |  | | | |  | Ath-AT4G34580.2 |  |
| 7 | Vvi-Vitvi03g04108\_t001 |  | | | |  | | | |  | | | |  | | | |  | | | |  | | | |  | | | |  |
| 7 | Vvi-Vitvi03g04109\_t001 |  | | | |  | Ath-AT2G16370.1 |  | Ath-AT2G21550.1 |  | | | |  | | | |  | | | |  | Ath-AT4G34570.1 |  |
| 7 | Vvi-Vitvi03g00298\_t001 |  | Ath-AT2G18193.1 |  | | | |  | | | |  | | | |  | | | |  | | | |  | | | |  |
| 7 | Vvi-Vitvi03g00299\_t001 |  | | | |  | | | |  | | | |  | | | |  | | | |  | Ath-AT1G19640.1 |  | | | |  |
| 7 | Vvi-Vitvi03g00300\_t001 |  | | | |  | | | |  | | | |  | | | |  | | | |  | | | |  | | | |  |
| 7 | Vvi-Vitvi03g00301\_t001 |  | | | |  | | | |  | | | |  | | | |  | | | |  | | | |  | | | |  |
| 7 | Vvi-Vitvi03g04110\_t001 |  | | | |  | | | |  | | | |  | | | |  | | | |  | | | |  | | | |  |
| 7 | Vvi-Vitvi03g00302\_t001 |  | | | |  | | | |  | Ath-AT2G21560.1 |  | | | |  | | | |  | | | |  | Ath-AT4G34560.1 |  |
| 7 | Vvi-Vitvi03g00303\_t001 |  | | | |  | Ath-AT2G16360.1 |  | Ath-AT2G21580.1 |  | Ath-AT4G39200.1 |  | | | |  | | | |  | Ath-AT4G34555.1 |  |
| 7 | Vvi-Vitvi03g00304\_t001 |  | | | |  | | | |  | Ath-AT2G21590.1 |  | Ath-AT4G39210.1 |  | | | |  | | | |  | | | |  |
| 7 | Vvi-Vitvi03g01483\_t001 |  | | | |  | | | |  | | | |  | | | |  | | | |  | | | |  | | | |  |
| 7 | Vvi-Vitvi03g00306\_t001 |  | | | |  | Ath-AT2G16365.1 |  | | | |  | | | |  | | | |  | | | |  | Ath-AT4G34550.1 |  |
| 7 | Vvi-Vitvi03g04111\_t001 |  | | | |  | | | |  | | | |  | | | |  | | | |  | | | |  | | | |  |
| 7 | Vvi-Vitvi03g00307\_t003 |  | Ath-AT2G18240.1 |  | | | |  | Ath-AT2G21600.1 |  | Ath-AT4G39220.1 |  | | | |  | | | |  | | | |  |
| 7 | Vvi-Vitvi03g00308\_t001 |  | | | |  | | | |  | | | |  | | | |  | | | |  | | | |  | | | |  |
| 7 | Vvi-Vitvi03g01484\_t001 |  | | | |  | | | |  | | | |  | Ath-AT4G39230.1 |  | Ath-AT1G75280.1 |  | Ath-AT1G19540.1 |  | | | |  |
| 7 | Vvi-Vitvi03g04112\_t001 |  | | | |  | | | |  | | | |  | | | |  | | | |  | | | |  | | | |  |
| 7 | Vvi-Vitvi03g01490\_t005 |  | | | |  | | | |  | | | |  | | | |  | | | |  | | | |  | | | |  |
| 7 | Vvi-Vitvi03g04113\_t001 |  | | | |  | | | |  | | | |  | | | |  | | | |  | | | |  | | | |  |
| 7 | Vvi-Vitvi03g04114\_t001 |  | | | |  | | | |  | | | |  | | | |  | | | |  | | | |  | | | |  |
| 7 | Vvi-Vitvi03g04115\_t001 |  | | | |  | | | |  | | | |  | | | |  | | | |  | | | |  | | | |  |
| 7 | Vvi-Vitvi03g01491\_t001 |  | | | |  | | | |  | | | |  | | | |  | | | |  | | | |  | | | |  |
| 7 | Vvi-Vitvi03g01492\_t001 |  | | | |  | | | |  | | | |  | | | |  | | | |  | | | |  | Ath-AT4G34540.1 |  |
| 7 | Vvi-Vitvi03g04116\_t001 |  | | | |  | | | |  | | | |  | | | |  | | | |  | | | |  | | | |  |
| 7 | Vvi-Vitvi03g00311\_t001 |  | Ath-AT2G18260.1 |  | | | |  | | | |  | | | |  | | | |  | | | |  | | | |  |
| 7 | Vvi-Vitvi03g00312\_t001 |  | | | |  | | | |  | | | |  | | | |  | | | |  | | | |  | | | |  |
| 7 | Vvi-Vitvi03g00313\_t001 |  | | | |  | | | |  | Ath-AT2G21610.2 |  | | | |  | | | |  | | | |  | | | |  |
| 7 | Vvi-Vitvi03g00314\_t001 |  | | | |  | | | |  | Ath-AT2G21620.2 |  | | | |  | | | |  | | | |  | | | |  |
| 7 | Vvi-Vitvi03g00315\_t001 |  | Ath-AT2G18300.3 |  | | | |  | | | |  | | | |  | | | |  | | | |  | Ath-AT4G34530.1 |  |
| 7 | Vvi-Vitvi03g00317\_t001 |  | | | |  | | | |  | | | |  | | | |  | | | |  | | | |  | | | |  |
| 7 | Vvi-Vitvi03g01495\_t001 |  | Ath-AT2G18328.1 |  | | | |  | Ath-AT2G21650.1 |  | Ath-AT4G39250.1 |  | Ath-AT1G75250.1 |  | Ath-AT1G19510.1 |  | | | |  |
| 7 | Vvi-Vitvi03g04117\_t001 |  | | | |  | | | |  | | | |  | | | |  | | | |  | | | |  | | | |  |
| 7 | Vvi-Vitvi03g00318\_t001 |  | | | |  | | | |  | | | |  | | | |  | | | |  | | | |  | | | |  |
| 7 | Vvi-Vitvi03g00319\_t001 |  | | | |  | | | |  | | | |  | | | |  | Ath-AT1G75230.2 |  | Ath-AT1G19480.1 |  | | | |  |
| 7 | Vvi-Vitvi03g00321\_t001 |  | Ath-AT2G18360.1 |  | | | |  | | | |  | | | |  | | | |  | | | |  | | | |  |
| 6 | Vvi-Vitvi03g00322\_t001 |  |  |  | Ath-AT2G16280.1 |  | | | |  | | | |  | | | |  | Ath-AT1G19440.1 |  | Ath-AT4G34510.1 |  |
| 6 | Vvi-Vitvi03g00324\_t003 |  |  |  | | | |  | | | |  | | | |  | | | |  | | | |  | | | |  |
| 6 | Vvi-Vitvi03g00325\_t001 |  |  |  | | | |  | | | |  | | | |  | | | |  | | | |  | | | |  |
| 6 | Vvi-Vitvi03g00327\_t001 |  |  |  | | | |  | Ath-AT2G21660.1 |  | Ath-AT4G39260.1 |  | | | |  | | | |  | | | |  |
| 6 | Vvi-Vitvi03g00328\_t002 |  |  |  | Ath-AT2G16250.1 |  | | | |  | Ath-AT4G39270.1 |  | | | |  | | | |  | | | |  |
| 6 | Vvi-Vitvi03g00329\_t001 |  |  |  | | | |  | Ath-AT2G21710.1 |  | | | |  | | | |  | | | |  | | | |  |
| 5 | Vvi-Vitvi03g00330\_t001 |  |  |  | | | |  |  |  | | | |  | | | |  | | | |  | | | |  |
| 5 | Vvi-Vitvi03g00331\_t001 |  |  |  | | | |  |  |  | Ath-AT4G39280.2 |  | | | |  | | | |  | | | |  |
| 5 | Vvi-Vitvi03g00332\_t001 |  |  |  | | | |  |  |  | | | |  | | | |  | | | |  | | | |  |
| 5 | Vvi-Vitvi03g00333\_t001 |  |  |  | | | |  |  |  | Ath-AT4G39300.2 |  | | | |  | | | |  | | | |  |
| 4 | Vvi-Vitvi03g04118\_t001 |  |  |  | | | |  |  |  |  |  | | | |  | | | |  | | | |  |
| 4 | Vvi-Vitvi03g00334\_t001 |  |  |  | | | |  |  |  |  |  | Ath-AT1G75180.1 |  | Ath-AT1G19400.2 |  | | | |  |
| 2 | Vvi-Vitvi03g00335\_t001 |  |  |  | | | |  |  |  |  |  |  |  |  |  | | | |  |
| 2 | Vvi-Vitvi03g00338\_t001 |  |  |  | | | |  |  |  |  |  |  |  |  |  | Ath-AT4G34500.1 |  |
| 2 | Vvi-Vitvi03g00339\_t001 |  |  |  | | | |  |  |  |  |  |  |  |  |  | Ath-AT4G34490.2 |  |
| 2 | Vvi-Vitvi03g00340\_t001 |  |  |  | Ath-AT2G16230.1 |  |  |  |  |  |  |  |  |  | Ath-AT4G34480.1 |  |
| 2 | Vvi-Vitvi03g00343\_t001 |  |  |  | | | |  |  |  |  |  |  |  |  |  | Ath-AT4G34460.1 |  |
| 2 | Vvi-Vitvi03g04119\_t001 |  |  |  | | | |  |  |  |  |  |  |  |  |  | | | |  |
| 2 | Vvi-Vitvi03g01497\_t001 |  |  |  | | | |  |  |  |  |  |  |  |  |  | | | |  |
| 2 | Vvi-Vitvi03g00344\_t001 |  |  |  | Ath-AT2G16200.2 |  |  |  |  |  |  |  |  |  | Ath-AT4G34450.1 |  |
| 4 | Vvi-Vitvi03g00345\_t001 |  | Ath-AT3G18810.1 |  | | | |  | Ath-AT1G49270.1 |  |  |  |  |  |  |  | Ath-AT4G34440.1 |  |
| 4 | Vvi-Vitvi03g04120\_t001 |  | | | |  | | | |  | | | |  |  |  |  |  |  |  | | | |  |
| 4 | Vvi-Vitvi03g04121\_t001 |  | | | |  | | | |  | | | |  |  |  |  |  |  |  | | | |  |
| 4 | Vvi-Vitvi03g00348\_t001 |  | Ath-AT3G18820.1 |  | | | |  | Ath-AT1G49300.1 |  |  |  |  |  |  |  | | | |  |
| 4 | Vvi-Vitvi03g00349\_t001 |  | | | |  | | | |  | | | |  |  |  |  |  |  |  | Ath-AT4G34430.4 |  |
| 3 | Vvi-Vitvi03g04122\_t001 |  | | | |  | | | |  | | | |  |  |  |  |  |
| 3 | Vvi-Vitvi03g01499\_t001 |  | | | |  | | | |  | | | |  |  |  |  |  |
| 3 | Vvi-Vitvi03g00350\_t001 |  | | | |  | | | |  | | | |  |  |  |  |  |
| 3 | Vvi-Vitvi03g01500\_t001 |  | | | |  | | | |  | | | |  |  |  |  |  |
| 3 | Vvi-Vitvi03g04123\_t001 |  | | | |  | | | |  | | | |  |  |  |  |  |
| 3 | Vvi-Vitvi03g01501\_t001 |  | | | |  | | | |  | | | |  |  |  |  |  |
| 3 | Vvi-Vitvi03g00352\_t003 |  | | | |  | | | |  | Ath-AT1G49320.1 |  |  |  |  |  |
| 3 | Vvi-Vitvi03g01503\_t001 |  | | | |  | | | |  | | | |  |  |  |  |  |
| 3 | Vvi-Vitvi03g00351\_t001 |  | | | |  | | | |  | | | |  |  |  |  |  |
| 3 | Vvi-Vitvi03g00353\_t001 |  | | | |  | | | |  | | | |  |  |  |  |  |
| 3 | Vvi-Vitvi03g00354\_t001 |  | | | |  | | | |  | | | |  |  |  |  |  |
| 3 | Vvi-Vitvi03g01504\_t001 |  | | | |  | | | |  | | | |  |  |  |  |  |
| 3 | Vvi-Vitvi03g00355\_t001 |  | | | |  | | | |  | | | |  |  |  |  |  |
| 3 | Vvi-Vitvi03g00356\_t001 |  | | | |  | | | |  | | | |  |  |  |  |  |
| 3 | Vvi-Vitvi03g01505\_t005 |  | | | |  | | | |  | | | |  |  |  |  |  |
| 3 | Vvi-Vitvi03g04124\_t001 |  | | | |  | | | |  | | | |  |  |  |  |  |
| 3 | Vvi-Vitvi03g01507\_t001 |  | | | |  | Ath-AT2G16190.1 |  | Ath-AT1G49330.1 |  |  |  |  |  |
| 3 | Vvi-Vitvi03g01508\_t001 |  | | | |  | | | |  | | | |  |  |  |  |  |
| 3 | Vvi-Vitvi03g00358\_t001 |  | | | |  | | | |  | Ath-AT1G49340.1 |  |  |  |  |  |
| 3 | Vvi-Vitvi03g00359\_t001 |  | Ath-AT3G18830.1 |  | Ath-AT2G16120.1 |  | | | |  |  |  |  |  |
| 3 | Vvi-Vitvi03g00360\_t001 |  | | | |  | | | |  | | | |  |  |  |  |  |
| 3 | Vvi-Vitvi03g00361\_t001 |  | Ath-AT3G18840.2 |  | | | |  | | | |  |  |  |  |  |
| 3 | Vvi-Vitvi03g01512\_t001 |  | | | |  | | | |  | | | |  |  |  |  |  |
| 3 | Vvi-Vitvi03g04125\_t001 |  | | | |  | | | |  | | | |  |  |  |  |  |
| 3 | Vvi-Vitvi03g04126\_t001 |  | | | |  | | | |  | | | |  |  |  |  |  |
| 3 | Vvi-Vitvi03g04127\_t001 |  | | | |  | | | |  | | | |  |  |  |  |  |
| 3 | Vvi-Vitvi03g00364\_t001 |  | | | |  | | | |  | | | |  |  |  |  |  |
| 3 | Vvi-Vitvi03g00365\_t001 |  | | | |  | | | |  | | | |  |  |  |  |  |
| 3 | Vvi-Vitvi03g04128\_t001 |  | | | |  | | | |  | | | |  |  |  |  |  |
| 3 | Vvi-Vitvi03g04129\_t001 |  | | | |  | | | |  | | | |  |  |  |  |  |
| 3 | Vvi-Vitvi03g04130\_t001 |  | | | |  | | | |  | | | |  |  |  |  |  |
| 3 | Vvi-Vitvi03g04131\_t001 |  | | | |  | | | |  | | | |  |  |  |  |  |
| 3 | Vvi-Vitvi03g04132\_t001 |  | | | |  | | | |  | | | |  |  |  |  |  |
| 3 | Vvi-Vitvi03g00368\_t001 |  | | | |  | | | |  | | | |  |  |  |  |  |
| 4 | Vvi-Vitvi03g00369\_t001 |  | | | |  | | | |  | | | |  | Ath-AT4G34320.2 |  |  |  |  |
| 4 | Vvi-Vitvi03g00370\_t003 |  | Ath-AT3G18850.3 |  | | | |  | | | |  | | | |  |  |  |  |
| 4 | Vvi-Vitvi03g00371\_t003 |  | Ath-AT3G18860.1 |  | | | |  | | | |  | | | |  |  |  |  |
| 4 | Vvi-Vitvi03g00372\_t001 |  | | | |  | | | |  | | | |  | | | |  |  |  |  |
| 4 | Vvi-Vitvi03g00373\_t002 |  | | | |  | | | |  | Ath-AT1G49380.1 |  | | | |  |  |  |  |
| 4 | Vvi-Vitvi03g00374\_t001 |  | | | |  | | | |  | | | |  | Ath-AT4G34350.1 |  |  |  |  |
| 4 | Vvi-Vitvi03g00375\_t002 |  | Ath-AT3G18890.1 |  | | | |  | | | |  | | | |  |  |  |  |
| 4 | Vvi-Vitvi03g00376\_t001 |  | | | |  | | | |  | | | |  | Ath-AT4G34360.1 |  |  |  |  |
| 4 | Vvi-Vitvi03g00377\_t001 |  | | | |  | Ath-AT2G16090.1 |  | | | |  | Ath-AT4G34370.1 |  |  |  |  |
| 4 | Vvi-Vitvi03g00378\_t001 |  | Ath-AT3G18900.4 |  | | | |  | | | |  | | | |  |  |  |  |
| 4 | Vvi-Vitvi03g04133\_t001 |  | | | |  | Ath-AT2G16070.2 |  | | | |  | | | |  |  |  |  |
| 4 | Vvi-Vitvi03g00379\_t002 |  | | | |  | Ath-AT2G16060.1 |  | | | |  | | | |  |  |  |  |
| 4 | Vvi-Vitvi03g01517\_t001 |  | | | |  | | | |  | | | |  | | | |  |  |  |  |
| 4 | Vvi-Vitvi03g00380\_t001 |  | | | |  | | | |  | | | |  | | | |  |  |  |  |
| 4 | Vvi-Vitvi03g01518\_t001 |  | | | |  | | | |  | | | |  | | | |  |  |  |  |
| 4 | Vvi-Vitvi03g00381\_t001 |  | | | |  | Ath-AT2G16050.1 |  | | | |  | | | |  |  |  |  |
| 4 | Vvi-Vitvi03g00383\_t001 |  | | | |  | Ath-AT2G15980.1 |  | | | |  | | | |  |  |  |  |
| 4 | Vvi-Vitvi03g00384\_t001 |  | | | |  | | | |  | Ath-AT1G49405.1 |  | | | |  |  |  |  |
| 4 | Vvi-Vitvi03g00385\_t001 |  | | | |  | | | |  | Ath-AT1G49410.1 |  | | | |  |  |  |  |
| 4 | Vvi-Vitvi03g00386\_t001 |  | Ath-AT3G18930.1 |  | | | |  | | | |  | | | |  |  |  |  |
| 4 | Vvi-Vitvi03g00388\_t001 |  | | | |  | | | |  | Ath-AT1G49430.1 |  | | | |  |  |  |  |
| 4 | Vvi-Vitvi03g00389\_t001 |  | | | |  | Ath-AT2G15970.1 |  | | | |  | | | |  |  |  |  |
| 4 | Vvi-Vitvi03g00391\_t001 |  | | | |  | | | |  | | | |  | | | |  |  |  |  |
| 4 | Vvi-Vitvi03g00392\_t001 |  | | | |  | | | |  | | | |  | | | |  |  |  |  |
| 4 | Vvi-Vitvi03g00393\_t001 |  | | | |  | | | |  | | | |  | | | |  |  |  |  |
| 4 | Vvi-Vitvi03g00394\_t001 |  | | | |  | | | |  | | | |  | | | |  |  |  |  |
| 4 | Vvi-Vitvi03g00395\_t001 |  | | | |  | | | |  | | | |  | | | |  |  |  |  |
| 4 | Vvi-Vitvi03g00396\_t001 |  | Ath-AT3G18940.1 |  | | | |  | | | |  | | | |  |  |  |  |
| 4 | Vvi-Vitvi03g00397\_t001 |  | Ath-AT3G18950.1 |  | | | |  | Ath-AT1G49450.1 |  | Ath-AT4G34380.1 |  |  |  |  |
| 3 | Vvi-Vitvi03g04134\_t001 |  |  |  | | | |  | | | |  | | | |  |  |  |  |
| 3 | Vvi-Vitvi03g00398\_t001 |  |  |  | | | |  | | | |  | Ath-AT4G34390.1 |  |  |  |  |
| 2 | Vvi-Vitvi03g01520\_t001 |  |  |  | | | |  | | | |  |  |  |  |  |
| 2 | Vvi-Vitvi03g01521\_t001 |  |  |  | | | |  | | | |  |  |  |  |  |
| 2 | Vvi-Vitvi03g00400\_t001 |  |  |  | | | |  | | | |  |  |  |  |  |
| 2 | Vvi-Vitvi03g00403\_t001 |  |  |  | Ath-AT2G15910.1 |  | | | |  |  |  |  |  |
| 1 | Vvi-Vitvi03g00405\_t001 |  |  |  |  |  | Ath-AT1G49470.1 |  |  |  |  |  |
| 0 | Vvi-Vitvi03g04135\_t001 |  |  |  |  |  |  |  |  |
| 0 | Vvi-Vitvi03g04136\_t001 |  |  |  |  |  |  |  |  |
| 0 | Vvi-Vitvi03g04137\_t001 |  |  |  |  |  |  |  |  |
| 0 | Vvi-Vitvi03g01524\_t001 |  |  |  |  |  |  |  |  |
| 0 | Vvi-Vitvi03g04138\_t001 |  |  |  |  |  |  |  |  |
| 0 | Vvi-Vitvi03g04139\_t001 |  |  |  |  |  |  |  |  |
| 0 | Vvi-Vitvi03g04140\_t001 |  |  |  |  |  |  |  |  |
| 0 | Vvi-Vitvi03g00407\_t001 |  |  |  |  |  |  |  |  |
| 0 | Vvi-Vitvi03g00408\_t001 |  |  |  |  |  |  |  |  |
| 0 | Vvi-Vitvi03g04141\_t001 |  |  |  |  |  |  |  |  |
| 0 | Vvi-Vitvi03g04142\_t001 |  |  |  |  |  |  |  |  |
| 0 | Vvi-Vitvi03g01528\_t001 |  |  |  |  |  |  |  |  |
| 0 | Vvi-Vitvi03g04143\_t001 |  |  |  |  |  |  |  |  |
| 0 | Vvi-Vitvi03g01531\_t001 |  |  |  |  |  |  |  |  |
| 0 | Vvi-Vitvi03g00409\_t001 |  |  |  |  |  |  |  |  |
| 0 | Vvi-Vitvi03g04144\_t001 |  |  |  |  |  |  |  |  |
| 0 | Vvi-Vitvi03g01532\_t001 |  |  |  |  |  |  |  |  |
| 0 | Vvi-Vitvi03g04145\_t001 |  |  |  |  |  |  |  |  |
| 0 | Vvi-Vitvi03g01536\_t001 |  |  |  |  |  |  |  |  |
| 0 | Vvi-Vitvi03g00413\_t001 |  |  |  |  |  |  |  |  |
| 0 | Vvi-Vitvi03g00414\_t001 |  |  |  |  |  |  |  |  |
| 0 | Vvi-Vitvi03g04146\_t001 |  |  |  |  |  |  |  |  |
| 0 | Vvi-Vitvi03g04147\_t001 |  |  |  |  |  |  |  |  |
| 0 | Vvi-Vitvi03g04148\_t001 |  |  |  |  |  |  |  |  |
| 0 | Vvi-Vitvi03g01537\_t001 |  |  |  |  |  |  |  |  |
| 0 | Vvi-Vitvi03g04149\_t001 |  |  |  |  |  |  |  |  |
| 0 | Vvi-Vitvi03g04150\_t001 |  |  |  |  |  |  |  |  |
| 0 | Vvi-Vitvi03g04151\_t001 |  |  |  |  |  |  |  |  |
| 2 | Vvi-Vitvi03g00416\_t001 |  | Ath-AT3G18960.1 |  | Ath-AT1G49475.1 |  |  |  |  |  |  |
| 2 | Vvi-Vitvi03g00417\_t001 |  | | | |  | | | |  |  |  |  |  |  |
| 2 | Vvi-Vitvi03g00419\_t001 |  | | | |  | | | |  |  |  |  |  |  |
| 2 | Vvi-Vitvi03g00420\_t001 |  | | | |  | | | |  |  |  |  |  |  |
| 2 | Vvi-Vitvi03g04152\_t001 |  | | | |  | | | |  |  |  |  |  |  |
| 2 | Vvi-Vitvi03g00421\_t001 |  | | | |  | | | |  |  |  |  |  |  |
| 2 | Vvi-Vitvi03g00422\_t001 |  | | | |  | | | |  |  |  |  |  |  |
| 2 | Vvi-Vitvi03g00423\_t001 |  | | | |  | | | |  |  |  |  |  |  |
| 2 | Vvi-Vitvi03g00425\_t001 |  | | | |  | | | |  |  |  |  |  |  |
| 2 | Vvi-Vitvi03g00426\_t001 |  | | | |  | | | |  |  |  |  |  |  |
| 2 | Vvi-Vitvi03g00427\_t001 |  | Ath-AT3G19020.1 |  | Ath-AT1G49490.2 |  |  |  |  |  |  |
| 2 | Vvi-Vitvi03g00428\_t001 |  | | | |  | | | |  |  |  |  |  |  |
| 2 | Vvi-Vitvi03g00429\_t001 |  | | | |  | | | |  |  |  |  |  |  |
| 2 | Vvi-Vitvi03g00430\_t001 |  | | | |  | | | |  |  |  |  |  |  |
| 2 | Vvi-Vitvi03g04153\_t001 |  | | | |  | | | |  |  |  |  |  |  |
| 2 | Vvi-Vitvi03g04154\_t001 |  | | | |  | | | |  |  |  |  |  |  |
| 2 | Vvi-Vitvi03g04155\_t001 |  | | | |  | | | |  |  |  |  |  |  |
| 2 | Vvi-Vitvi03g00433\_t001 |  | | | |  | | | |  |  |  |  |  |  |
| 2 | Vvi-Vitvi03g04156\_t001 |  | | | |  | | | |  |  |  |  |  |  |
| 2 | Vvi-Vitvi03g04157\_t001 |  | | | |  | | | |  |  |  |  |  |  |
| 2 | Vvi-Vitvi03g04158\_t001 |  | | | |  | | | |  |  |  |  |  |  |
| 2 | Vvi-Vitvi03g04159\_t001 |  | | | |  | | | |  |  |  |  |  |  |
| 2 | Vvi-Vitvi03g04160\_t001 |  | | | |  | | | |  |  |  |  |  |  |
| 2 | Vvi-Vitvi03g04161\_t001 |  | | | |  | | | |  |  |  |  |  |  |
| 2 | Vvi-Vitvi03g04162\_t001 |  | | | |  | | | |  |  |  |  |  |  |
| 2 | Vvi-Vitvi03g04163\_t001 |  | | | |  | | | |  |  |  |  |  |  |
| 2 | Vvi-Vitvi03g04164\_t001 |  | | | |  | | | |  |  |  |  |  |  |
| 2 | Vvi-Vitvi03g04165\_t001 |  | | | |  | | | |  |  |  |  |  |  |
| 2 | Vvi-Vitvi03g04166\_t001 |  | | | |  | | | |  |  |  |  |  |  |
| 2 | Vvi-Vitvi03g04167\_t001 |  | | | |  | | | |  |  |  |  |  |  |
| 2 | Vvi-Vitvi03g00447\_t001 |  | Ath-AT3G19050.1 |  | | | |  |  |  |  |  |  |
| 2 | Vvi-Vitvi03g01544\_t001 |  | | | |  | | | |  |  |  |  |  |  |
| 2 | Vvi-Vitvi03g00449\_t003 |  | | | |  | Ath-AT1G49510.1 |  |  |  |  |  |  |
| 2 | Vvi-Vitvi03g04168\_t001 |  | | | |  | | | |  |  |  |  |  |  |
| 2 | Vvi-Vitvi03g01545\_t001 |  | | | |  | | | |  |  |  |  |  |  |
| 2 | Vvi-Vitvi03g04169\_t001 |  | | | |  | | | |  |  |  |  |  |  |
| 2 | Vvi-Vitvi03g00452\_t001 |  | | | |  | | | |  |  |  |  |  |  |
| 2 | Vvi-Vitvi03g04170\_t001 |  | Ath-AT3G19080.3 |  | Ath-AT1G49520.1 |  |  |  |  |  |  |
| 2 | Vvi-Vitvi03g00454\_t002 |  | | | |  | Ath-AT1G49560.1 |  |  |  |  |  |  |
| 2 | Vvi-Vitvi03g00455\_t001 |  | Ath-AT3G19090.2 |  | | | |  |  |  |  |  |  |
| 2 | Vvi-Vitvi03g00456\_t001 |  | | | |  | Ath-AT1G49570.1 |  |  |  |  |  |  |
| 2 | Vvi-Vitvi03g04171\_t001 |  | | | |  | | | |  |  |  |  |  |  |
| 3 | Vvi-Vitvi03g00457\_t001 |  | | | |  | | | |  | Ath-AT2G15790.1 |  |  |  |  |  |
| 3 | Vvi-Vitvi03g00458\_t001 |  | | | |  | | | |  | | | |  |  |  |  |  |
| 3 | Vvi-Vitvi03g00459\_t001 |  | | | |  | | | |  | Ath-AT2G15770.1 |  |  |  |  |  |
| 3 | Vvi-Vitvi03g00460\_t001 |  | | | |  | | | |  | | | |  |  |  |  |  |
| 3 | Vvi-Vitvi03g04172\_t001 |  | | | |  | | | |  | | | |  |  |  |  |  |
| 3 | Vvi-Vitvi03g04173\_t001 |  | | | |  | | | |  | | | |  |  |  |  |  |
| 3 | Vvi-Vitvi03g00463\_t001 |  | | | |  | | | |  | | | |  |  |  |  |  |
| 3 | Vvi-Vitvi03g01549\_t001 |  | | | |  | | | |  | | | |  |  |  |  |  |
| 3 | Vvi-Vitvi03g01550\_t001 |  | | | |  | | | |  | | | |  |  |  |  |  |
| 3 | Vvi-Vitvi03g01551\_t001 |  | | | |  | | | |  | | | |  |  |  |  |  |
| 3 | Vvi-Vitvi03g04174\_t001 |  | | | |  | | | |  | | | |  |  |  |  |  |
| 3 | Vvi-Vitvi03g00464\_t001 |  | Ath-AT3G19100.1 |  | Ath-AT1G49580.1 |  | | | |  |  |  |  |  |
| 3 | Vvi-Vitvi03g00467\_t001 |  | | | |  | | | |  | | | |  |  |  |  |  |
| 3 | Vvi-Vitvi03g00469\_t001 |  | Ath-AT3G19120.1 |  | | | |  | | | |  |  |  |  |  |
| 3 | Vvi-Vitvi03g04175\_t001 |  | | | |  | | | |  | | | |  |  |  |  |  |
| 3 | Vvi-Vitvi03g00471\_t001 |  | | | |  | | | |  | | | |  |  |  |  |  |
| 3 | Vvi-Vitvi03g00472\_t002 |  | | | |  | Ath-AT1G49590.1 |  | | | |  |  |  |  |  |
| 3 | Vvi-Vitvi03g00473\_t001 |  | | | |  | | | |  | Ath-AT2G15760.1 |  |  |  |  |  |
| 3 | Vvi-Vitvi03g00474\_t001 |  | | | |  | | | |  | | | |  |  |  |  |  |
| 3 | Vvi-Vitvi03g00475\_t001 |  | | | |  | | | |  | | | |  |  |  |  |  |
| 3 | Vvi-Vitvi03g00476\_t002 |  | Ath-AT3G19130.1 |  | Ath-AT1G49600.2 |  | | | |  |  |  |  |  |
| 3 | Vvi-Vitvi03g00477\_t001 |  | | | |  | | | |  | Ath-AT2G15730.1 |  |  |  |  |  |
| 3 | Vvi-Vitvi03g00479\_t001 |  | | | |  | | | |  | | | |  |  |  |  |  |
| 3 | Vvi-Vitvi03g00480\_t001 |  | | | |  | | | |  | | | |  |  |  |  |  |
| 3 | Vvi-Vitvi03g00481\_t001 |  | | | |  | | | |  | | | |  |  |  |  |  |
| 3 | Vvi-Vitvi03g01553\_t001 |  | | | |  | | | |  | | | |  |  |  |  |  |
| 3 | Vvi-Vitvi03g01554\_t001 |  | | | |  | | | |  | | | |  |  |  |  |  |
| 3 | Vvi-Vitvi03g01555\_t001 |  | | | |  | | | |  | | | |  |  |  |  |  |
| 3 | Vvi-Vitvi03g00482\_t001 |  | | | |  | | | |  | | | |  |  |  |  |  |
| 3 | Vvi-Vitvi03g00483\_t001 |  | | | |  | | | |  | | | |  |  |  |  |  |
| 3 | Vvi-Vitvi03g01557\_t001 |  | | | |  | | | |  | | | |  |  |  |  |  |
| 3 | Vvi-Vitvi03g01558\_t001 |  | | | |  | | | |  | | | |  |  |  |  |  |
| 3 | Vvi-Vitvi03g01561\_t001 |  | | | |  | | | |  | | | |  |  |  |  |  |
| 3 | Vvi-Vitvi03g00485\_t001 |  | Ath-AT3G19170.1 |  | Ath-AT1G49630.2 |  | | | |  |  |  |  |  |
| 3 | Vvi-Vitvi03g01562\_t001 |  | | | |  | | | |  | | | |  |  |  |  |  |
| 3 | Vvi-Vitvi03g00486\_t001 |  | | | |  | | | |  | Ath-AT2G15695.1 |  |  |  |  |  |
| 3 | Vvi-Vitvi03g04176\_t001 |  | Ath-AT3G19184.2 |  | | | |  | | | |  |  |  |  |  |
| 3 | Vvi-Vitvi03g00488\_t001 |  | | | |  | | | |  | | | |  |  |  |  |  |
| 3 | Vvi-Vitvi03g00489\_t001 |  | | | |  | | | |  | | | |  |  |  |  |  |
| 3 | Vvi-Vitvi03g00491\_t001 |  | | | |  | Ath-AT1G49670.2 |  | | | |  |  |  |  |  |
| 3 | Vvi-Vitvi03g00492\_t001 |  | Ath-AT3G19190.2 |  | | | |  | | | |  |  |  |  |  |
| 3 | Vvi-Vitvi03g00493\_t001 |  | Ath-AT3G19210.1 |  | | | |  | | | |  |  |  |  |  |
| 3 | Vvi-Vitvi03g01565\_t001 |  | | | |  | | | |  | | | |  |  |  |  |  |
| 3 | Vvi-Vitvi03g01566\_t001 |  | | | |  | | | |  | | | |  |  |  |  |  |
| 3 | Vvi-Vitvi03g04177\_t001 |  | | | |  | | | |  | | | |  |  |  |  |  |
| 3 | Vvi-Vitvi03g01567\_t001 |  | | | |  | | | |  | | | |  |  |  |  |  |
| 3 | Vvi-Vitvi03g00495\_t001 |  | | | |  | | | |  | Ath-AT2G15690.1 |  |  |  |  |  |
| 3 | Vvi-Vitvi03g00496\_t001 |  | | | |  | | | |  | Ath-AT2G15680.1 |  |  |  |  |  |
| 3 | Vvi-Vitvi03g01568\_t001 |  | Ath-AT3G19220.1 |  | | | |  | | | |  |  |  |  |  |
| 3 | Vvi-Vitvi03g00497\_t001 |  | Ath-AT3G19230.1 |  | | | |  | | | |  |  |  |  |  |
| 3 | Vvi-Vitvi03g00498\_t001 |  | | | |  | | | |  | | | |  |  |  |  |  |
| 3 | Vvi-Vitvi03g04178\_t001 |  | | | |  | | | |  | | | |  |  |  |  |  |
| 3 | Vvi-Vitvi03g00499\_t001 |  | Ath-AT3G19240.1 |  | | | |  | | | |  |  |  |  |  |
| 3 | Vvi-Vitvi03g00500\_t001 |  | | | |  | | | |  | | | |  |  |  |  |  |
| 3 | Vvi-Vitvi03g04179\_t001 |  | | | |  | | | |  | | | |  |  |  |  |  |
| 3 | Vvi-Vitvi03g01571\_t001 |  | | | |  | | | |  | | | |  |  |  |  |  |
| 3 | Vvi-Vitvi03g00501\_t001 |  | | | |  | | | |  | | | |  |  |  |  |  |
| 3 | Vvi-Vitvi03g04180\_t001 |  | | | |  | | | |  | | | |  |  |  |  |  |
| 3 | Vvi-Vitvi03g04181\_t001 |  | | | |  | | | |  | | | |  |  |  |  |  |
| 3 | Vvi-Vitvi03g01572\_t001 |  | | | |  | | | |  | | | |  |  |  |  |  |
| 3 | Vvi-Vitvi03g00505\_t001 |  | Ath-AT3G19260.1 |  | | | |  | | | |  |  |  |  |  |
| 3 | Vvi-Vitvi03g00508\_t001 |  | Ath-AT3G19270.2 |  | | | |  | | | |  |  |  |  |  |
| 3 | Vvi-Vitvi03g00509\_t001 |  | | | |  | | | |  | Ath-AT2G15620.1 |  |  |  |  |  |
| 3 | Vvi-Vitvi03g00510\_t001 |  | Ath-AT3G19280.1 |  | Ath-AT1G49710.1 |  | | | |  |  |  |  |  |
| 4 | Vvi-Vitvi03g01573\_t001 |  | | | |  | | | |  | | | |  | Ath-AT4G33980.2 |  |  |  |  |
| 4 | Vvi-Vitvi03g00511\_t001 |  | | | |  | | | |  | | | |  | Ath-AT4G33990.1 |  |  |  |  |
| 4 | Vvi-Vitvi03g01574\_t003 |  | Ath-AT3G19290.3 |  | Ath-AT1G49720.2 |  | | | |  | Ath-AT4G34000.2 |  |  |  |  |
| 4 | Vvi-Vitvi03g00512\_t001 |  | | | |  | | | |  | | | |  | Ath-AT4G34020.1 |  |  |  |  |
| 4 | Vvi-Vitvi03g00513\_t001 |  | | | |  | | | |  | | | |  | Ath-AT4G34030.1 |  |  |  |  |
| 4 | Vvi-Vitvi03g00514\_t001 |  | | | |  | | | |  | Ath-AT2G15570.2 |  | | | |  |  |  |  |
| 4 | Vvi-Vitvi03g00515\_t003 |  | | | |  | | | |  | Ath-AT2G15560.1 |  | | | |  |  |  |  |
| 4 | Vvi-Vitvi03g01575\_t001 |  | | | |  | | | |  | | | |  | | | |  |  |  |  |
| 4 | Vvi-Vitvi03g04182\_t001 |  | | | |  | | | |  | | | |  | | | |  |  |  |  |
| 4 | Vvi-Vitvi03g01577\_t001 |  | | | |  | | | |  | | | |  | | | |  |  |  |  |
| 4 | Vvi-Vitvi03g01578\_t001 |  | | | |  | | | |  | | | |  | | | |  |  |  |  |
| 4 | Vvi-Vitvi03g00516\_t002 |  | Ath-AT3G19300.1 |  | Ath-AT1G49730.1 |  | | | |  | | | |  |  |  |  |
| 4 | Vvi-Vitvi03g00517\_t001 |  | Ath-AT3G19310.1 |  | Ath-AT1G49740.1 |  | | | |  | | | |  |  |  |  |
| 4 | Vvi-Vitvi03g00519\_t001 |  | | | |  | | | |  | | | |  | | | |  |  |  |  |
| 4 | Vvi-Vitvi03g04183\_t001 |  | | | |  | | | |  | | | |  | | | |  |  |  |  |
| 4 | Vvi-Vitvi03g00520\_t001 |  | | | |  | | | |  | | | |  | | | |  |  |  |  |
| 4 | Vvi-Vitvi03g00521\_t001 |  | | | |  | | | |  | Ath-AT2G15530.4 |  | Ath-AT4G34040.1 |  |  |  |  |
| 4 | Vvi-Vitvi03g04184\_t001 |  | | | |  | | | |  | | | |  | | | |  |  |  |  |
| 4 | Vvi-Vitvi03g00523\_t001 |  | | | |  | | | |  | | | |  | | | |  |  |  |  |
| 4 | Vvi-Vitvi03g00524\_t001 |  | | | |  | | | |  | | | |  | Ath-AT4G34050.3 |  |  |  |  |
| 4 | Vvi-Vitvi03g00525\_t001 |  | | | |  | | | |  | | | |  | | | |  |  |  |  |
| 4 | Vvi-Vitvi03g00527\_t001 |  | | | |  | | | |  | | | |  | | | |  |  |  |  |
| 5 | Vvi-Vitvi03g00528\_t001 |  | | | |  | | | |  | | | |  | Ath-AT4G34090.3 |  | Ath-AT2G23370.1 |  |  |  |
| 5 | Vvi-Vitvi03g00529\_t002 |  | Ath-AT3G19370.1 |  | | | |  | | | |  | | | |  | Ath-AT2G23360.1 |  |  |  |
| 5 | Vvi-Vitvi03g04185\_t001 |  | | | |  | | | |  | | | |  | | | |  | | | |  |  |  |
| 5 | Vvi-Vitvi03g00530\_t002 |  | | | |  | | | |  | | | |  | Ath-AT4G34100.1 |  | | | |  |  |  |
| 5 | Vvi-Vitvi03g00531\_t001 |  | | | |  | Ath-AT1G49760.2 |  | | | |  | Ath-AT4G34110.1 |  | Ath-AT2G23350.1 |  |  |  |
| 5 | Vvi-Vitvi03g00532\_t001 |  | | | |  | | | |  | | | |  | Ath-AT4G34120.1 |  | | | |  |  |  |
| 5 | Vvi-Vitvi03g00533\_t001 |  | | | |  | | | |  | Ath-AT2G15480.2 |  | Ath-AT4G34131.1 |  | | | |  |  |  |
| 5 | Vvi-Vitvi03g00534\_t001 |  | | | |  | | | |  | | | |  | | | |  | | | |  |  |  |
| 5 | Vvi-Vitvi03g00535\_t001 |  | | | |  | | | |  | Ath-AT2G15440.1 |  | | | |  | | | |  |  |  |
| 5 | Vvi-Vitvi03g00536\_t001 |  | | | |  | | | |  | | | |  | | | |  | | | |  |  |  |
| 5 | Vvi-Vitvi03g00537\_t001 |  | | | |  | | | |  | | | |  | | | |  | | | |  |  |  |
| 5 | Vvi-Vitvi03g00539\_t001 |  | Ath-AT3G19390.1 |  | | | |  | | | |  | | | |  | | | |  |  |  |
| 5 | Vvi-Vitvi03g04186\_t001 |  | | | |  | | | |  | | | |  | | | |  | | | |  |  |  |
| 5 | Vvi-Vitvi03g00540\_t001 |  | Ath-AT3G19420.1 |  | | | |  | | | |  | | | |  | | | |  |  |  |
| 5 | Vvi-Vitvi03g00541\_t001 |  | | | |  | | | |  | | | |  | Ath-AT4G34150.1 |  | | | |  |  |  |
| 5 | Vvi-Vitvi03g00542\_t001 |  | | | |  | | | |  | | | |  | Ath-AT4G34160.1 |  | | | |  |  |  |
| 5 | Vvi-Vitvi03g00543\_t001 |  | | | |  | | | |  | Ath-AT2G15400.1 |  | | | |  | | | |  |  |  |
| 5 | Vvi-Vitvi03g04187\_t001 |  | Ath-AT3G19430.2 |  | | | |  | | | |  | | | |  | | | |  |  |  |
| 5 | Vvi-Vitvi03g04188\_t001 |  | | | |  | | | |  | | | |  | | | |  | | | |  |  |  |
| 5 | Vvi-Vitvi03g00545\_t001 |  | | | |  | | | |  | | | |  | Ath-AT4G34180.1 |  | | | |  |  |  |
| 5 | Vvi-Vitvi03g00546\_t001 |  | | | |  | | | |  | | | |  | | | |  | | | |  |  |  |
| 5 | Vvi-Vitvi03g00547\_t001 |  | | | |  | | | |  | | | |  | | | |  | | | |  |  |  |
| 5 | Vvi-Vitvi03g00548\_t001 |  | | | |  | | | |  | Ath-AT2G15320.1 |  | | | |  | | | |  |  |  |
| 5 | Vvi-Vitvi03g01579\_t001 |  | | | |  | | | |  | | | |  | | | |  | | | |  |  |  |
| 5 | Vvi-Vitvi03g04189\_t001 |  | | | |  | | | |  | | | |  | Ath-AT4G34190.1 |  | | | |  |  |  |
| 5 | Vvi-Vitvi03g00550\_t001 |  | | | |  | Ath-AT1G49810.1 |  | | | |  | | | |  | | | |  |  |  |
| 4 | Vvi-Vitvi03g00551\_t001 |  | | | |  |  |  | | | |  | Ath-AT4G34200.1 |  | | | |  |  |  |
| 4 | Vvi-Vitvi03g00552\_t001 |  | | | |  |  |  | | | |  | Ath-AT4G34215.2 |  | | | |  |  |  |
| 4 | Vvi-Vitvi03g00553\_t001 |  | | | |  |  |  | Ath-AT2G15300.1 |  | Ath-AT4G34220.1 |  | Ath-AT2G23300.1 |  |  |  |
| 4 | Vvi-Vitvi03g00556\_t001 |  | | | |  |  |  | Ath-AT2G15290.1 |  | | | |  | | | |  |  |  |
| 4 | Vvi-Vitvi03g01580\_t001 |  | | | |  |  |  | Ath-AT2G15280.1 |  | | | |  | | | |  |  |  |
| 4 | Vvi-Vitvi03g01581\_t001 |  | | | |  |  |  | Ath-AT2G15270.1 |  | | | |  | | | |  |  |  |
| 4 | Vvi-Vitvi03g04190\_t001 |  | | | |  |  |  | | | |  | | | |  | | | |  |  |  |
| 4 | Vvi-Vitvi03g04191\_t001 |  | | | |  |  |  | | | |  | | | |  | | | |  |  |  |
| 4 | Vvi-Vitvi03g00557\_t001 |  | | | |  |  |  | | | |  | | | |  | | | |  |  |  |
| 4 | Vvi-Vitvi03g00559\_t001 |  | | | |  |  |  | | | |  | | | |  | Ath-AT2G23290.1 |  |  |  |
| 4 | Vvi-Vitvi03g00560\_t001 |  | | | |  |  |  | Ath-AT2G15240.1 |  | | | |  | | | |  |  |  |
| 4 | Vvi-Vitvi03g00561\_t001 |  | Ath-AT3G19450.1 |  |  |  | | | |  | Ath-AT4G34230.1 |  | | | |  |  |  |
| 3 | Vvi-Vitvi03g00564\_t001 |  | | | |  |  |  | | | |  |  |  | | | |  |  |  |
| 3 | Vvi-Vitvi03g00565\_t001 |  | | | |  |  |  | | | |  |  |  | | | |  |  |  |
| 3 | Vvi-Vitvi03g04192\_t001 |  | | | |  |  |  | | | |  |  |  | | | |  |  |  |
| 3 | Vvi-Vitvi03g00566\_t001 |  | | | |  |  |  | | | |  |  |  | | | |  |  |  |
| 3 | Vvi-Vitvi03g01584\_t001 |  | | | |  |  |  | | | |  |  |  | | | |  |  |  |
| 3 | Vvi-Vitvi03g00567\_t001 |  | | | |  |  |  | | | |  |  |  | | | |  |  |  |
| 3 | Vvi-Vitvi03g01585\_t001 |  | | | |  |  |  | | | |  |  |  | | | |  |  |  |
| 3 | Vvi-Vitvi03g00568\_t001 |  | | | |  |  |  | | | |  |  |  | | | |  |  |  |
| 3 | Vvi-Vitvi03g04193\_t001 |  | | | |  |  |  | | | |  |  |  | | | |  |  |  |
| 3 | Vvi-Vitvi03g04194\_t001 |  | | | |  |  |  | | | |  |  |  | | | |  |  |  |
| 3 | Vvi-Vitvi03g04195\_t001 |  | | | |  |  |  | | | |  |  |  | | | |  |  |  |
| 3 | Vvi-Vitvi03g00569\_t001 |  | | | |  |  |  | | | |  |  |  | Ath-AT2G23260.1 |  |  |  |
| 3 | Vvi-Vitvi03g00570\_t001 |  | | | |  |  |  | Ath-AT2G15230.1 |  |  |  | | | |  |  |  |
| 3 | Vvi-Vitvi03g04196\_t001 |  | | | |  |  |  | | | |  |  |  | | | |  |  |  |
| 4 | Vvi-Vitvi03g00573\_t001 |  | | | |  | Ath-AT1G49820.1 |  | | | |  |  |  | | | |  |  |  |
| 4 | Vvi-Vitvi03g01592\_t001 |  | | | |  | | | |  | | | |  |  |  | | | |  |  |  |
| 4 | Vvi-Vitvi03g04197\_t001 |  | | | |  | | | |  | | | |  |  |  | | | |  |  |  |
| 4 | Vvi-Vitvi03g04198\_t001 |  | | | |  | | | |  | Ath-AT2G15170.1 |  |  |  | | | |  |  |  |
| 4 | Vvi-Vitvi03g01594\_t001 |  | | | |  | | | |  | | | |  |  |  | | | |  |  |  |
| 4 | Vvi-Vitvi03g04199\_t001 |  | | | |  | | | |  | | | |  |  |  | | | |  |  |  |
| 4 | Vvi-Vitvi03g01597\_t001 |  | | | |  | | | |  | | | |  |  |  | | | |  |  |  |
| 4 | Vvi-Vitvi03g00575\_t001 |  | | | |  | | | |  | | | |  |  |  | | | |  |  |  |
| 4 | Vvi-Vitvi03g04200\_t001 |  | | | |  | | | |  | | | |  |  |  | | | |  |  |  |
| 4 | Vvi-Vitvi03g01598\_t001 |  | | | |  | | | |  | | | |  |  |  | | | |  |  |  |
| 4 | Vvi-Vitvi03g00577\_t001 |  | | | |  | | | |  | | | |  |  |  | | | |  |  |  |
| 4 | Vvi-Vitvi03g04201\_t001 |  | Ath-AT3G19500.1 |  | Ath-AT1G49830.1 |  | | | |  |  |  | | | |  |  |  |
| 4 | Vvi-Vitvi03g00579\_t001 |  | Ath-AT3G19508.1 |  | | | |  | | | |  |  |  | | | |  |  |  |
| 4 | Vvi-Vitvi03g00580\_t001 |  | | | |  | | | |  | Ath-AT2G15020.1 |  |  |  | | | |  |  |  |
| 4 | Vvi-Vitvi03g00581\_t001 |  | | | |  | | | |  | | | |  |  |  | | | |  |  |  |
| 4 | Vvi-Vitvi03g01599\_t001 |  | | | |  | | | |  | Ath-AT2G15000.6 |  |  |  | | | |  |  |  |
| 4 | Vvi-Vitvi03g00582\_t001 |  | | | |  | | | |  | | | |  |  |  | | | |  |  |  |
| 4 | Vvi-Vitvi03g00583\_t001 |  | | | |  | | | |  | | | |  |  |  | | | |  |  |  |
| 4 | Vvi-Vitvi03g00584\_t001 |  | | | |  | | | |  | | | |  |  |  | | | |  |  |  |
| 4 | Vvi-Vitvi03g01600\_t001 |  | Ath-AT3G19510.1 |  | | | |  | | | |  |  |  | | | |  |  |  |
| 4 | Vvi-Vitvi03g00586\_t001 |  | | | |  | | | |  | Ath-AT2G14960.1 |  |  |  | Ath-AT2G23170.1 |  |  |  |
| 4 | Vvi-Vitvi03g00587\_t001 |  | | | |  | | | |  | | | |  |  |  | | | |  |  |  |
| 4 | Vvi-Vitvi03g04202\_t001 |  | | | |  | | | |  | | | |  |  |  | | | |  |  |  |
| 4 | Vvi-Vitvi03g00589\_t001 |  | | | |  | | | |  | Ath-AT2G14910.1 |  |  |  | | | |  |  |  |
| 4 | Vvi-Vitvi03g00591\_t001 |  | | | |  | | | |  | | | |  |  |  | | | |  |  |  |
| 4 | Vvi-Vitvi03g00592\_t001 |  | | | |  | | | |  | | | |  |  |  | | | |  |  |  |
| 4 | Vvi-Vitvi03g00593\_t001 |  | | | |  | | | |  | Ath-AT2G14900.1 |  |  |  | | | |  |  |  |
| 4 | Vvi-Vitvi03g00595\_t002 |  | | | |  | | | |  | | | |  |  |  | | | |  |  |  |
| 4 | Vvi-Vitvi03g04203\_t001 |  | | | |  | | | |  | | | |  |  |  | | | |  |  |  |
| 4 | Vvi-Vitvi03g00597\_t001 |  | Ath-AT3G19540.1 |  | Ath-AT1G49840.1 |  | | | |  |  |  | | | |  |  |  |
| 4 | Vvi-Vitvi03g00598\_t001 |  | | | |  | | | |  | | | |  |  |  | | | |  |  |  |
| 4 | Vvi-Vitvi03g00599\_t001 |  | | | |  | | | |  | Ath-AT2G14880.1 |  |  |  | | | |  |  |  |
| 4 | Vvi-Vitvi03g00600\_t001 |  | | | |  | | | |  | | | |  |  |  | | | |  |  |  |
| 4 | Vvi-Vitvi03g00601\_t001 |  | | | |  | | | |  | | | |  |  |  | | | |  |  |  |
| 5 | Vvi-Vitvi03g00602\_t001 |  | | | |  | | | |  | | | |  | Ath-AT4G33925.1 |  | | | |  |  |  |
| 5 | Vvi-Vitvi03g00603\_t001 |  | | | |  | | | |  | | | |  | | | |  | | | |  |  |  |
| 5 | Vvi-Vitvi03g00604\_t001 |  | | | |  | | | |  | | | |  | | | |  | | | |  |  |  |
| 5 | Vvi-Vitvi03g00605\_t001 |  | | | |  | | | |  | | | |  | Ath-AT4G33920.1 |  | | | |  |  |  |
| 5 | Vvi-Vitvi03g00606\_t001 |  | | | |  | Ath-AT1G49850.1 |  | | | |  | | | |  | | | |  |  |  |
| 5 | Vvi-Vitvi03g04204\_t001 |  | Ath-AT3G19550.1 |  | | | |  | | | |  | | | |  | | | |  |  |  |
| 5 | Vvi-Vitvi03g01603\_t001 |  | | | |  | | | |  | | | |  | | | |  | | | |  |  |  |
| 5 | Vvi-Vitvi03g04205\_t001 |  | | | |  | | | |  | | | |  | | | |  | | | |  |  |  |
| 5 | Vvi-Vitvi03g00609\_t001 |  | | | |  | | | |  | | | |  | Ath-AT4G33910.1 |  | Ath-AT2G23096.1 |  |  |  |
| 5 | Vvi-Vitvi03g00610\_t001 |  | | | |  | | | |  | | | |  | | | |  | | | |  |  |  |
| 5 | Vvi-Vitvi03g00612\_t001 |  | | | |  | | | |  | | | |  | | | |  | | | |  |  |  |
| 5 | Vvi-Vitvi03g00613\_t001 |  | | | |  | Ath-AT1G49870.1 |  | | | |  | | | |  | | | |  |  |  |
| 5 | Vvi-Vitvi03g00614\_t001 |  | | | |  | | | |  | | | |  | | | |  | | | |  |  |  |
| 5 | Vvi-Vitvi03g00615\_t001 |  | | | |  | | | |  | | | |  | | | |  | | | |  |  |  |
| 5 | Vvi-Vitvi03g00617\_t001 |  | | | |  | | | |  | | | |  | | | |  | | | |  |  |  |
| 5 | Vvi-Vitvi03g01605\_t002 |  | | | |  | | | |  | Ath-AT2G14860.1 |  | Ath-AT4G33905.1 |  | | | |  |  |  |
| 6 | Vvi-Vitvi03g00618\_t001 |  | | | |  | | | |  | Ath-AT2G14850.1 |  | Ath-AT4G33890.1 |  | | | |  | Ath-AT5G67410.1 |  |  |
| 6 | Vvi-Vitvi03g00619\_t001 |  | Ath-AT3G19553.1 |  | | | |  | | | |  | | | |  | | | |  | | | |  |  |
| 6 | Vvi-Vitvi03g00621\_t001 |  | | | |  | Ath-AT1G49880.1 |  | | | |  | | | |  | | | |  | | | |  |  |
| 6 | Vvi-Vitvi03g00623\_t001 |  | | | |  | | | |  | Ath-AT2G14825.1 |  | | | |  | | | |  | | | |  |  |
| 6 | Vvi-Vitvi03g04206\_t001 |  | | | |  | | | |  | | | |  | | | |  | | | |  | | | |  |  |
| 6 | Vvi-Vitvi03g00624\_t001 |  | | | |  | | | |  | | | |  | | | |  | | | |  | | | |  |  |
| 6 | Vvi-Vitvi03g00625\_t001 |  | | | |  | | | |  | | | |  | | | |  | | | |  | | | |  |  |
| 6 | Vvi-Vitvi03g01606\_t001 |  | | | |  | | | |  | | | |  | | | |  | | | |  | | | |  |  |
| 6 | Vvi-Vitvi03g00626\_t001 |  | Ath-AT3G19570.2 |  | Ath-AT1G49890.1 |  | | | |  | | | |  | | | |  | | | |  |  |
| 6 | Vvi-Vitvi03g00628\_t001 |  | | | |  | | | |  | | | |  | | | |  | | | |  | Ath-AT5G67420.2 |  |  |
| 6 | Vvi-Vitvi03g00629\_t001 |  | | | |  | | | |  | Ath-AT2G14820.1 |  | | | |  | Ath-AT2G23050.1 |  | Ath-AT5G67440.3 |  |  |
| 5 | Vvi-Vitvi03g00630\_t001 |  | Ath-AT3G19580.1 |  | | | |  | | | |  | | | |  |  |  | Ath-AT5G67450.1 |  |  |
| 5 | Vvi-Vitvi03g00631\_t001 |  | | | |  | | | |  | | | |  | | | |  |  |  | | | |  |  |
| 5 | Vvi-Vitvi03g00633\_t001 |  | Ath-AT3G19590.1 |  | Ath-AT1G49910.1 |  | | | |  | | | |  |  |  | | | |  |  |
| 5 | Vvi-Vitvi03g00634\_t001 |  | | | |  | | | |  | | | |  | | | |  |  |  | | | |  |  |
| 5 | Vvi-Vitvi03g00635\_t001 |  | | | |  | | | |  | Ath-AT2G14760.3 |  | Ath-AT4G33880.1 |  |  |  | | | |  |  |
| 5 | Vvi-Vitvi03g00636\_t001 |  | | | |  | | | |  | | | |  | | | |  |  |  | | | |  |  |
| 5 | Vvi-Vitvi03g04207\_t001 |  | | | |  | | | |  | | | |  | | | |  |  |  | | | |  |  |
| 5 | Vvi-Vitvi03g00638\_t001 |  | Ath-AT3G19620.1 |  | | | |  | | | |  | | | |  |  |  | | | |  |  |
| 5 | Vvi-Vitvi03g00639\_t001 |  | | | |  | | | |  | | | |  | | | |  |  |  | | | |  |  |
| 5 | Vvi-Vitvi03g00640\_t001 |  | | | |  | | | |  | | | |  | | | |  |  |  | | | |  |  |
| 5 | Vvi-Vitvi03g00645\_t001 |  | | | |  | | | |  | | | |  | | | |  |  |  | | | |  |  |
| 5 | Vvi-Vitvi03g00646\_t001 |  | Ath-AT3G19630.1 |  | | | |  | | | |  | | | |  |  |  | | | |  |  |
| 5 | Vvi-Vitvi03g00647\_t001 |  | Ath-AT3G19650.1 |  | | | |  | | | |  | | | |  |  |  | | | |  |  |
| 4 | Vvi-Vitvi03g04208\_t001 |  |  |  | | | |  | | | |  | | | |  |  |  | | | |  |  |
| 4 | Vvi-Vitvi03g00649\_t001 |  |  |  | | | |  | | | |  | | | |  |  |  | | | |  |  |
| 4 | Vvi-Vitvi03g04209\_t001 |  |  |  | | | |  | | | |  | | | |  |  |  | | | |  |  |
| 4 | Vvi-Vitvi03g04210\_t001 |  |  |  | | | |  | | | |  | | | |  |  |  | | | |  |  |
| 4 | Vvi-Vitvi03g00651\_t001 |  |  |  | | | |  | Ath-AT2G14750.1 |  | | | |  |  |  | Ath-AT5G67520.1 |  |  |
| 4 | Vvi-Vitvi03g00653\_t001 |  |  |  | | | |  | | | |  | | | |  |  |  | | | |  |  |
| 4 | Vvi-Vitvi03g00654\_t001 |  |  |  | | | |  | | | |  | Ath-AT4G33820.1 |  |  |  | | | |  |  |
| 4 | Vvi-Vitvi03g00655\_t001 |  |  |  | | | |  | | | |  | | | |  |  |  | | | |  |  |
| 4 | Vvi-Vitvi03g00657\_t001 |  |  |  | | | |  | | | |  | | | |  |  |  | | | |  |  |
| 4 | Vvi-Vitvi03g00660\_t001 |  |  |  | | | |  | | | |  | | | |  |  |  | | | |  |  |
| 4 | Vvi-Vitvi03g00661\_t001 |  |  |  | | | |  | | | |  | | | |  |  |  | | | |  |  |
| 4 | Vvi-Vitvi03g00664\_t001 |  |  |  | | | |  | | | |  | | | |  |  |  | | | |  |  |
| 4 | Vvi-Vitvi03g00665\_t001 |  |  |  | | | |  | | | |  | | | |  |  |  | Ath-AT5G67560.1 |  |  |
| 4 | Vvi-Vitvi03g00666\_t001 |  |  |  | | | |  | | | |  | | | |  |  |  | | | |  |  |
| 4 | Vvi-Vitvi03g00667\_t001 |  |  |  | | | |  | Ath-AT2G14680.2 |  | | | |  |  |  | | | |  |  |
| 3 | Vvi-Vitvi03g00668\_t001 |  |  |  | Ath-AT1G49950.1 |  |  |  | | | |  |  |  | Ath-AT5G67580.2 |  |  |
| 2 | Vvi-Vitvi03g04211\_t001 |  |  |  | | | |  |  |  | | | |  |  |  |  |
| 2 | Vvi-Vitvi03g04212\_t001 |  |  |  | | | |  |  |  | | | |  |  |  |  |
| 2 | Vvi-Vitvi03g00673\_t001 |  |  |  | Ath-AT1G49960.1 |  |  |  | | | |  |  |  |  |
| 2 | Vvi-Vitvi03g00675\_t001 |  |  |  | | | |  |  |  | | | |  |  |  |  |
| 2 | Vvi-Vitvi03g00676\_t001 |  |  |  | | | |  |  |  | | | |  |  |  |  |
| 2 | Vvi-Vitvi03g00677\_t001 |  |  |  | | | |  |  |  | Ath-AT4G33800.1 |  |  |  |  |
| 1 | Vvi-Vitvi03g01612\_t001 |  |  |  | Ath-AT1G49975.1 |  |  |  |  |  |  |
| 0 | Vvi-Vitvi03g00678\_t001 |  |  |  |  |  |  |  |  |
| 0 | Vvi-Vitvi03g01613\_t001 |  |  |  |  |  |  |  |  |
| 0 | Vvi-Vitvi03g01614\_t001 |  |  |  |  |  |  |  |  |
| 0 | Vvi-Vitvi03g01615\_t001 |  |  |  |  |  |  |  |  |
| 0 | Vvi-Vitvi03g04213\_t001 |  |  |  |  |  |  |  |  |
| 0 | Vvi-Vitvi03g04214\_t001 |  |  |  |  |  |  |  |  |
| 0 | Vvi-Vitvi03g04215\_t001 |  |  |  |  |  |  |  |  |
| 0 | Vvi-Vitvi03g00689\_t001 |  |  |  |  |  |  |  |  |
| 0 | Vvi-Vitvi03g04216\_t001 |  |  |  |  |  |  |  |  |
| 0 | Vvi-Vitvi03g00690\_t001 |  |  |  |  |  |  |  |  |
| 0 | Vvi-Vitvi03g00691\_t001 |  |  |  |  |  |  |  |  |
| 0 | Vvi-Vitvi03g04217\_t001 |  |  |  |  |  |  |  |  |
| 0 | Vvi-Vitvi03g01617\_t001 |  |  |  |  |  |  |  |  |
| 0 | Vvi-Vitvi03g01618\_t001 |  |  |  |  |  |  |  |  |
| 0 | Vvi-Vitvi03g00692\_t001 |  |  |  |  |  |  |  |  |
| 0 | Vvi-Vitvi03g00693\_t001 |  |  |  |  |  |  |  |  |
| 0 | Vvi-Vitvi03g01621\_t001 |  |  |  |  |  |  |  |  |
| 0 | Vvi-Vitvi03g00696\_t001 |  |  |  |  |  |  |  |  |
| 0 | Vvi-Vitvi03g01622\_t001 |  |  |  |  |  |  |  |  |
| 0 | Vvi-Vitvi03g04218\_t001 |  |  |  |  |  |  |  |  |
| 0 | Vvi-Vitvi03g00698\_t001 |  |  |  |  |  |  |  |  |
| 0 | Vvi-Vitvi03g04219\_t001 |  |  |  |  |  |  |  |  |
| 0 | Vvi-Vitvi03g00699\_t001 |  |  |  |  |  |  |  |  |
| 0 | Vvi-Vitvi03g00700\_t001 |  |  |  |  |  |  |  |  |
| 0 | Vvi-Vitvi03g01625\_t001 |  |  |  |  |  |  |  |  |
| 0 | Vvi-Vitvi03g00702\_t001 |  |  |  |  |  |  |  |  |
| 0 | Vvi-Vitvi03g04220\_t001 |  |  |  |  |  |  |  |  |
| 0 | Vvi-Vitvi03g00703\_t001 |  |  |  |  |  |  |  |  |
| 0 | Vvi-Vitvi03g04221\_t001 |  |  |  |  |  |  |  |  |
| 0 | Vvi-Vitvi03g00704\_t001 |  |  |  |  |  |  |  |  |
| 0 | Vvi-Vitvi03g04222\_t001 |  |  |  |  |  |  |  |  |
| 0 | Vvi-Vitvi03g01628\_t001 |  |  |  |  |  |  |  |  |
| 0 | Vvi-Vitvi03g01629\_t001 |  |  |  |  |  |  |  |  |
| 0 | Vvi-Vitvi03g04223\_t001 |  |  |  |  |  |  |  |  |
| 0 | Vvi-Vitvi03g04224\_t001 |  |  |  |  |  |  |  |  |
| 0 | Vvi-Vitvi03g04225\_t001 |  |  |  |  |  |  |  |  |
| 0 | Vvi-Vitvi03g04226\_t001 |  |  |  |  |  |  |  |  |
| 0 | Vvi-Vitvi03g00706\_t001 |  |  |  |  |  |  |  |  |
| 0 | Vvi-Vitvi03g04227\_t001 |  |  |  |  |  |  |  |  |
| 0 | Vvi-Vitvi03g04228\_t001 |  |  |  |  |  |  |  |  |
| 0 | Vvi-Vitvi03g04229\_t001 |  |  |  |  |  |  |  |  |
| 0 | Vvi-Vitvi03g00707\_t001 |  |  |  |  |  |  |  |  |
| 0 | Vvi-Vitvi03g00708\_t001 |  |  |  |  |  |  |  |  |
| 0 | Vvi-Vitvi03g01635\_t001 |  |  |  |  |  |  |  |  |
| 0 | Vvi-Vitvi03g04230\_t001 |  |  |  |  |  |  |  |  |
| 0 | Vvi-Vitvi03g04231\_t001 |  |  |  |  |  |  |  |  |
| 1 | Vvi-Vitvi03g00710\_t001 |  | Ath-AT1G50000.1 |  |  |  |  |  |  |  |
| 1 | Vvi-Vitvi03g00711\_t001 |  | | | |  |  |  |  |  |  |  |
| 1 | Vvi-Vitvi03g00712\_t001 |  | | | |  |  |  |  |  |  |  |
| 1 | Vvi-Vitvi03g00713\_t001 |  | | | |  |  |  |  |  |  |  |
| 1 | Vvi-Vitvi03g04232\_t001 |  | | | |  |  |  |  |  |  |  |
| 1 | Vvi-Vitvi03g04233\_t001 |  | | | |  |  |  |  |  |  |  |
| 1 | Vvi-Vitvi03g00717\_t001 |  | | | |  |  |  |  |  |  |  |
| 1 | Vvi-Vitvi03g00718\_t001 |  | | | |  |  |  |  |  |  |  |
| 1 | Vvi-Vitvi03g04234\_t001 |  | | | |  |  |  |  |  |  |  |
| 1 | Vvi-Vitvi03g01638\_t001 |  | | | |  |  |  |  |  |  |  |
| 1 | Vvi-Vitvi03g00721\_t001 |  | Ath-AT1G50010.1 |  |  |  |  |  |  |  |
| 1 | Vvi-Vitvi03g00722\_t002 |  | | | |  |  |  |  |  |  |  |
| 1 | Vvi-Vitvi03g01639\_t001 |  | Ath-AT1G50020.1 |  |  |  |  |  |  |  |
| 1 | Vvi-Vitvi03g00723\_t001 |  | | | |  |  |  |  |  |  |  |
| 1 | Vvi-Vitvi03g04235\_t001 |  | | | |  |  |  |  |  |  |  |
| 1 | Vvi-Vitvi03g04236\_t001 |  | | | |  |  |  |  |  |  |  |
| 1 | Vvi-Vitvi03g00724\_t001 |  | Ath-AT1G50030.1 |  |  |  |  |  |  |  |
| 1 | Vvi-Vitvi03g00725\_t001 |  | | | |  |  |  |  |  |  |  |
| 1 | Vvi-Vitvi03g00726\_t001 |  | | | |  |  |  |  |  |  |  |
| 1 | Vvi-Vitvi03g00728\_t001 |  | | | |  |  |  |  |  |  |  |
| 1 | Vvi-Vitvi03g00729\_t001 |  | | | |  |  |  |  |  |  |  |
| 1 | Vvi-Vitvi03g00730\_t001 |  | | | |  |  |  |  |  |  |  |
| 1 | Vvi-Vitvi03g04237\_t001 |  | | | |  |  |  |  |  |  |  |
| 1 | Vvi-Vitvi03g04238\_t001 |  | | | |  |  |  |  |  |  |  |
| 1 | Vvi-Vitvi03g04239\_t001 |  | | | |  |  |  |  |  |  |  |
| 1 | Vvi-Vitvi03g00732\_t001 |  | | | |  |  |  |  |  |  |  |
| 1 | Vvi-Vitvi03g00733\_t001 |  | | | |  |  |  |  |  |  |  |
| 1 | Vvi-Vitvi03g04240\_t001 |  | | | |  |  |  |  |  |  |  |
| 1 | Vvi-Vitvi03g00734\_t001 |  | | | |  |  |  |  |  |  |  |
| 1 | Vvi-Vitvi03g00735\_t001 |  | Ath-AT1G50040.1 |  |  |  |  |  |  |  |
| 1 | Vvi-Vitvi03g01647\_t001 |  | | | |  |  |  |  |  |  |  |
| 1 | Vvi-Vitvi03g04241\_t001 |  | | | |  |  |  |  |  |  |  |
| 2 | Vvi-Vitvi03g00737\_t001 |  | | | |  | Ath-AT2G14620.1 |  |  |  |  |  |  |
| 2 | Vvi-Vitvi03g01648\_t005 |  | | | |  | | | |  |  |  |  |  |  |
| 2 | Vvi-Vitvi03g00740\_t001 |  | Ath-AT1G50060.1 |  | | | |  |  |  |  |  |  |
| 1 | Vvi-Vitvi03g01649\_t001 |  |  |  | Ath-AT2G14580.1 |  |  |  |  |  |  |
| 1 | Vvi-Vitvi03g01650\_t001 |  |  |  | | | |  |  |  |  |  |  |
| 1 | Vvi-Vitvi03g01651\_t001 |  |  |  | | | |  |  |  |  |  |  |
| 1 | Vvi-Vitvi03g04242\_t001 |  |  |  | | | |  |  |  |  |  |  |
| 1 | Vvi-Vitvi03g01652\_t001 |  |  |  | | | |  |  |  |  |  |  |
| 1 | Vvi-Vitvi03g00743\_t001 |  |  |  | | | |  |  |  |  |  |  |
| 1 | Vvi-Vitvi03g00744\_t001 |  |  |  | | | |  |  |  |  |  |  |
| 1 | Vvi-Vitvi03g00745\_t001 |  |  |  | | | |  |  |  |  |  |  |
| 1 | Vvi-Vitvi03g04243\_t001 |  |  |  | | | |  |  |  |  |  |  |
| 1 | Vvi-Vitvi03g00752\_t001 |  |  |  | | | |  |  |  |  |  |  |
| 1 | Vvi-Vitvi03g01654\_t001 |  |  |  | | | |  |  |  |  |  |  |
| 1 | Vvi-Vitvi03g00754\_t001 |  |  |  | | | |  |  |  |  |  |  |
| 1 | Vvi-Vitvi03g01657\_t001 |  |  |  | | | |  |  |  |  |  |  |
| 1 | Vvi-Vitvi03g04244\_t001 |  |  |  | | | |  |  |  |  |  |  |
| 1 | Vvi-Vitvi03g00755\_t001 |  |  |  | | | |  |  |  |  |  |  |
| 1 | Vvi-Vitvi03g04245\_t001 |  |  |  | | | |  |  |  |  |  |  |
| 1 | Vvi-Vitvi03g04246\_t001 |  |  |  | | | |  |  |  |  |  |  |
| 1 | Vvi-Vitvi03g04247\_t001 |  |  |  | | | |  |  |  |  |  |  |
| 1 | Vvi-Vitvi03g00757\_t001 |  |  |  | | | |  |  |  |  |  |  |
| 1 | Vvi-Vitvi03g04248\_t001 |  |  |  | | | |  |  |  |  |  |  |
| 1 | Vvi-Vitvi03g04249\_t001 |  |  |  | | | |  |  |  |  |  |  |
| 1 | Vvi-Vitvi03g01659\_t001 |  |  |  | Ath-AT2G14530.1 |  |  |  |  |  |  |
| 1 | Vvi-Vitvi03g01660\_t001 |  |  |  | | | |  |  |  |  |  |  |
| 1 | Vvi-Vitvi03g01661\_t001 |  |  |  | | | |  |  |  |  |  |  |
| 1 | Vvi-Vitvi03g04250\_t001 |  |  |  | | | |  |  |  |  |  |  |
| 1 | Vvi-Vitvi03g00763\_t002 |  |  |  | | | |  |  |  |  |  |  |
| 1 | Vvi-Vitvi03g00766\_t001 |  |  |  | Ath-AT2G14520.1 |  |  |  |  |  |  |
| 1 | Vvi-Vitvi03g00767\_t002 |  |  |  | | | |  |  |  |  |  |  |
| 1 | Vvi-Vitvi03g04251\_t001 |  |  |  | | | |  |  |  |  |  |  |
| 1 | Vvi-Vitvi03g04252\_t001 |  |  |  | | | |  |  |  |  |  |  |
| 1 | Vvi-Vitvi03g04253\_t001 |  |  |  | | | |  |  |  |  |  |  |
| 1 | Vvi-Vitvi03g00773\_t006 |  |  |  | | | |  |  |  |  |  |  |
| 1 | Vvi-Vitvi03g04254\_t001 |  |  |  | | | |  |  |  |  |  |  |
| 1 | Vvi-Vitvi03g00775\_t001 |  |  |  | | | |  |  |  |  |  |  |
| 1 | Vvi-Vitvi03g01664\_t001 |  |  |  | | | |  |  |  |  |  |  |
| 1 | Vvi-Vitvi03g01665\_t001 |  |  |  | | | |  |  |  |  |  |  |
| 1 | Vvi-Vitvi03g01666\_t001 |  |  |  | | | |  |  |  |  |  |  |
| 1 | Vvi-Vitvi03g01667\_t001 |  |  |  | | | |  |  |  |  |  |  |
| 1 | Vvi-Vitvi03g01668\_t001 |  |  |  | | | |  |  |  |  |  |  |
| 1 | Vvi-Vitvi03g04255\_t001 |  |  |  | | | |  |  |  |  |  |  |
| 1 | Vvi-Vitvi03g00777\_t001 |  |  |  | | | |  |  |  |  |  |  |
| 1 | Vvi-Vitvi03g00778\_t001 |  |  |  | | | |  |  |  |  |  |  |
| 1 | Vvi-Vitvi03g00779\_t001 |  |  |  | | | |  |  |  |  |  |  |
| 1 | Vvi-Vitvi03g00780\_t001 |  |  |  | | | |  |  |  |  |  |  |
| 1 | Vvi-Vitvi03g00781\_t002 |  |  |  | | | |  |  |  |  |  |  |
| 1 | Vvi-Vitvi03g00782\_t001 |  |  |  | Ath-AT2G14260.1 |  |  |  |  |  |  |
| 1 | Vvi-Vitvi03g00783\_t001 |  |  |  | | | |  |  |  |  |  |  |
| 1 | Vvi-Vitvi03g00785\_t001 |  |  |  | | | |  |  |  |  |  |  |
| 1 | Vvi-Vitvi03g00786\_t001 |  |  |  | | | |  |  |  |  |  |  |
| 1 | Vvi-Vitvi03g04256\_t001 |  |  |  | | | |  |  |  |  |  |  |
| 1 | Vvi-Vitvi03g04257\_t001 |  |  |  | | | |  |  |  |  |  |  |
| 1 | Vvi-Vitvi03g04258\_t001 |  |  |  | | | |  |  |  |  |  |  |
| 1 | Vvi-Vitvi03g04259\_t001 |  |  |  | | | |  |  |  |  |  |  |
| 1 | Vvi-Vitvi03g00790\_t001 |  |  |  | | | |  |  |  |  |  |  |
| 1 | Vvi-Vitvi03g00791\_t001 |  |  |  | | | |  |  |  |  |  |  |
| 1 | Vvi-Vitvi03g00792\_t001 |  |  |  | | | |  |  |  |  |  |  |
| 1 | Vvi-Vitvi03g00793\_t001 |  |  |  | Ath-AT2G14255.1 |  |  |  |  |  |  |
| 1 | Vvi-Vitvi03g00796\_t001 |  |  |  | | | |  |  |  |  |  |  |
| 1 | Vvi-Vitvi03g04260\_t001 |  |  |  | | | |  |  |  |  |  |  |
| 1 | Vvi-Vitvi03g04261\_t001 |  |  |  | | | |  |  |  |  |  |  |
| 1 | Vvi-Vitvi03g00806\_t001 |  |  |  | | | |  |  |  |  |  |  |
| 1 | Vvi-Vitvi03g04262\_t001 |  |  |  | | | |  |  |  |  |  |  |
| 1 | Vvi-Vitvi03g04263\_t001 |  |  |  | | | |  |  |  |  |  |  |
| 1 | Vvi-Vitvi03g01681\_t001 |  |  |  | | | |  |  |  |  |  |  |
| 1 | Vvi-Vitvi03g01682\_t001 |  |  |  | | | |  |  |  |  |  |  |
| 1 | Vvi-Vitvi03g04264\_t001 |  |  |  | | | |  |  |  |  |  |  |
| 1 | Vvi-Vitvi03g00808\_t001 |  |  |  | | | |  |  |  |  |  |  |
| 1 | Vvi-Vitvi03g04265\_t001 |  |  |  | | | |  |  |  |  |  |  |
| 1 | Vvi-Vitvi03g04266\_t001 |  |  |  | | | |  |  |  |  |  |  |
| 1 | Vvi-Vitvi03g04267\_t001 |  |  |  | | | |  |  |  |  |  |  |
| 1 | Vvi-Vitvi03g00813\_t001 |  |  |  | | | |  |  |  |  |  |  |
| 1 | Vvi-Vitvi03g00814\_t001 |  |  |  | | | |  |  |  |  |  |  |
| 1 | Vvi-Vitvi03g04268\_t001 |  |  |  | | | |  |  |  |  |  |  |
| 1 | Vvi-Vitvi03g00816\_t001 |  |  |  | | | |  |  |  |  |  |  |
| 1 | Vvi-Vitvi03g00818\_t001 |  |  |  | | | |  |  |  |  |  |  |
| 1 | Vvi-Vitvi03g00819\_t001 |  |  |  | Ath-AT2G14210.2 |  |  |  |  |  |  |
| 0 | Vvi-Vitvi03g04269\_t001 |  |  |  |  |  |  |  |  |
| 0 | Vvi-Vitvi03g01687\_t001 |  |  |  |  |  |  |  |  |
| 0 | Vvi-Vitvi03g04270\_t001 |  |  |  |  |  |  |  |  |
| 0 | Vvi-Vitvi03g04271\_t001 |  |  |  |  |  |  |  |  |
| 0 | Vvi-Vitvi03g00825\_t001 |  |  |  |  |  |  |  |  |
| 0 | Vvi-Vitvi03g01695\_t001 |  |  |  |  |  |  |  |  |
| 0 | Vvi-Vitvi03g00828\_t001 |  |  |  |  |  |  |  |  |
| 0 | Vvi-Vitvi03g04272\_t001 |  |  |  |  |  |  |  |  |
| 0 | Vvi-Vitvi03g01696\_t001 |  |  |  |  |  |  |  |  |
| 0 | Vvi-Vitvi03g04273\_t001 |  |  |  |  |  |  |  |  |
| 0 | Vvi-Vitvi03g04274\_t001 |  |  |  |  |  |  |  |  |
| 0 | Vvi-Vitvi03g04275\_t001 |  |  |  |  |  |  |  |  |
| 0 | Vvi-Vitvi03g04276\_t001 |  |  |  |  |  |  |  |  |
| 0 | Vvi-Vitvi03g04277\_t001 |  |  |  |  |  |  |  |  |
| 0 | Vvi-Vitvi03g04278\_t001 |  |  |  |  |  |  |  |  |
| 0 | Vvi-Vitvi03g00835\_t001 |  |  |  |  |  |  |  |  |
| 0 | Vvi-Vitvi03g04279\_t001 |  |  |  |  |  |  |  |  |
| 0 | Vvi-Vitvi03g04280\_t001 |  |  |  |  |  |  |  |  |
| 0 | Vvi-Vitvi03g01703\_t001 |  |  |  |  |  |  |  |  |
| 0 | Vvi-Vitvi03g01704\_t001 |  |  |  |  |  |  |  |  |
| 0 | Vvi-Vitvi03g04281\_t001 |  |  |  |  |  |  |  |  |
| 0 | Vvi-Vitvi03g00841\_t001 |  |  |  |  |  |  |  |  |
| 1 | Vvi-Vitvi03g00842\_t001 |  | Ath-AT5G64410.1 |  |  |  |  |  |  |  |
| 1 | Vvi-Vitvi03g04282\_t001 |  | | | |  |  |  |  |  |  |  |
| 1 | Vvi-Vitvi03g00844\_t001 |  | | | |  |  |  |  |  |  |  |
| 1 | Vvi-Vitvi03g00845\_t001 |  | | | |  |  |  |  |  |  |  |
| 1 | Vvi-Vitvi03g00846\_t001 |  | Ath-AT5G64420.1 |  |  |  |  |  |  |  |
| 1 | Vvi-Vitvi03g04283\_t001 |  | | | |  |  |  |  |  |  |  |
| 1 | Vvi-Vitvi03g00847\_t003 |  | | | |  |  |  |  |  |  |  |
| 1 | Vvi-Vitvi03g00848\_t001 |  | | | |  |  |  |  |  |  |  |
| 1 | Vvi-Vitvi03g00849\_t001 |  | Ath-AT5G64430.1 |  |  |  |  |  |  |  |
| 1 | Vvi-Vitvi03g04284\_t001 |  | | | |  |  |  |  |  |  |  |
| 1 | Vvi-Vitvi03g00850\_t001 |  | | | |  |  |  |  |  |  |  |
| 1 | Vvi-Vitvi03g04285\_t001 |  | | | |  |  |  |  |  |  |  |
| 1 | Vvi-Vitvi03g00851\_t001 |  | Ath-AT5G64440.1 |  |  |  |  |  |  |  |
| 1 | Vvi-Vitvi03g00852\_t001 |  | Ath-AT5G64460.3 |  |  |  |  |  |  |  |
| 1 | Vvi-Vitvi03g00853\_t001 |  | Ath-AT5G64480.2 |  |  |  |  |  |  |  |
| 1 | Vvi-Vitvi03g00855\_t004 |  | | | |  |  |  |  |  |  |  |
| 1 | Vvi-Vitvi03g00858\_t001 |  | | | |  |  |  |  |  |  |  |
| 1 | Vvi-Vitvi03g00859\_t001 |  | | | |  |  |  |  |  |  |  |
| 1 | Vvi-Vitvi03g04286\_t001 |  | | | |  |  |  |  |  |  |  |
| 1 | Vvi-Vitvi03g00860\_t001 |  | | | |  |  |  |  |  |  |  |
| 1 | Vvi-Vitvi03g00862\_t001 |  | | | |  |  |  |  |  |  |  |
| 1 | Vvi-Vitvi03g00804\_t001 |  | | | |  |  |  |  |  |  |  |
| 1 | Vvi-Vitvi03g04287\_t001 |  | | | |  |  |  |  |  |  |  |
| 1 | Vvi-Vitvi03g00801\_t001 |  | | | |  |  |  |  |  |  |  |
| 1 | Vvi-Vitvi03g04288\_t001 |  | Ath-AT5G64500.1 |  |  |  |  |  |  |  |
| 0 | Vvi-Vitvi03g04289\_t001 |  |  |  |  |  |  |  |  |
| 0 | Vvi-Vitvi03g04290\_t001 |  |  |  |  |  |  |  |  |
| 0 | Vvi-Vitvi03g04291\_t001 |  |  |  |  |  |  |  |  |
| 0 | Vvi-Vitvi03g04292\_t001 |  |  |  |  |  |  |  |  |
| 0 | Vvi-Vitvi03g00904\_t001 |  |  |  |  |  |  |  |  |
| 0 | Vvi-Vitvi03g04293\_t001 |  |  |  |  |  |  |  |  |
| 0 | Vvi-Vitvi03g01727\_t001 |  |  |  |  |  |  |  |  |
| 0 | Vvi-Vitvi03g04294\_t001 |  |  |  |  |  |  |  |  |
| 0 | Vvi-Vitvi03g04295\_t001 |  |  |  |  |  |  |  |  |
| 0 | Vvi-Vitvi03g04296\_t001 |  |  |  |  |  |  |  |  |
| 0 | Vvi-Vitvi03g04297\_t001 |  |  |  |  |  |  |  |  |
| 0 | Vvi-Vitvi03g01723\_t001 |  |  |  |  |  |  |  |  |
| 0 | Vvi-Vitvi03g04298\_t001 |  |  |  |  |  |  |  |  |
| 0 | Vvi-Vitvi03g00900\_t001 |  |  |  |  |  |  |  |  |
| 0 | Vvi-Vitvi03g04299\_t001 |  |  |  |  |  |  |  |  |
| 0 | Vvi-Vitvi03g00895\_t001 |  |  |  |  |  |  |  |  |
| 0 | Vvi-Vitvi03g00892\_t001 |  |  |  |  |  |  |  |  |
| 0 | Vvi-Vitvi03g04300\_t001 |  |  |  |  |  |  |  |  |
| 0 | Vvi-Vitvi03g04301\_t001 |  |  |  |  |  |  |  |  |
| 0 | Vvi-Vitvi03g04302\_t001 |  |  |  |  |  |  |  |  |
| 0 | Vvi-Vitvi03g00887\_t001 |  |  |  |  |  |  |  |  |
| 0 | Vvi-Vitvi03g01719\_t001 |  |  |  |  |  |  |  |  |
| 0 | Vvi-Vitvi03g04303\_t001 |  |  |  |  |  |  |  |  |
| 0 | Vvi-Vitvi03g00886\_t001 |  |  |  |  |  |  |  |  |
| 0 | Vvi-Vitvi03g04304\_t001 |  |  |  |  |  |  |  |  |
| 0 | Vvi-Vitvi03g04305\_t001 |  |  |  |  |  |  |  |  |
| 0 | Vvi-Vitvi03g00882\_t001 |  |  |  |  |  |  |  |  |
| 0 | Vvi-Vitvi03g00874\_t001 |  |  |  |  |  |  |  |  |
| 0 | Vvi-Vitvi03g04306\_t001 |  |  |  |  |  |  |  |  |
| 0 | Vvi-Vitvi03g00871\_t001 |  |  |  |  |  |  |  |  |
| 0 | Vvi-Vitvi03g04307\_t001 |  |  |  |  |  |  |  |  |
| 0 | Vvi-Vitvi03g04308\_t001 |  |  |  |  |  |  |  |  |
| 0 | Vvi-Vitvi03g04309\_t001 |  |  |  |  |  |  |  |  |
| 0 | Vvi-Vitvi03g04310\_t001 |  |  |  |  |  |  |  |  |
| 0 | Vvi-Vitvi03g04311\_t001 |  |  |  |  |  |  |  |  |
| 0 | Vvi-Vitvi03g00866\_t001 |  |  |  |  |  |  |  |  |
| 0 | Vvi-Vitvi03g04312\_t001 |  |  |  |  |  |  |  |  |
| 0 | Vvi-Vitvi03g04313\_t001 |  |  |  |  |  |  |  |  |
| 0 | Vvi-Vitvi03g04314\_t001 |  |  |  |  |  |  |  |  |
| 0 | Vvi-Vitvi03g04315\_t001 |  |  |  |  |  |  |  |  |
| 0 | Vvi-Vitvi03g01030\_t001 |  |  |  |  |  |  |  |  |
| 0 | Vvi-Vitvi03g01029\_t001 |  |  |  |  |  |  |  |  |
| 0 | Vvi-Vitvi03g01770\_t001 |  |  |  |  |  |  |  |  |
| 0 | Vvi-Vitvi03g01769\_t001 |  |  |  |  |  |  |  |  |
| 0 | Vvi-Vitvi03g04316\_t001 |  |  |  |  |  |  |  |  |
| 0 | Vvi-Vitvi03g01023\_t001 |  |  |  |  |  |  |  |  |
| 0 | Vvi-Vitvi03g01022\_t001 |  |  |  |  |  |  |  |  |
| 0 | Vvi-Vitvi03g01020\_t001 |  |  |  |  |  |  |  |  |
| 0 | Vvi-Vitvi03g01019\_t001 |  |  |  |  |  |  |  |  |
| 0 | Vvi-Vitvi03g01017\_t001 |  |  |  |  |  |  |  |  |
| 0 | Vvi-Vitvi03g04317\_t001 |  |  |  |  |  |  |  |  |
| 0 | Vvi-Vitvi03g01016\_t001 |  |  |  |  |  |  |  |  |
| 0 | Vvi-Vitvi03g01013\_t001 |  |  |  |  |  |  |  |  |
| 0 | Vvi-Vitvi03g01012\_t002 |  |  |  |  |  |  |  |  |
| 0 | Vvi-Vitvi03g01011\_t001 |  |  |  |  |  |  |  |  |
| 0 | Vvi-Vitvi03g01006\_t001 |  |  |  |  |  |  |  |  |
| 0 | Vvi-Vitvi03g01766\_t001 |  |  |  |  |  |  |  |  |
| 0 | Vvi-Vitvi03g04318\_t001 |  |  |  |  |  |  |  |  |
| 0 | Vvi-Vitvi03g01002\_t001 |  |  |  |  |  |  |  |  |
| 0 | Vvi-Vitvi03g01001\_t001 |  |  |  |  |  |  |  |  |
| 0 | Vvi-Vitvi03g00999\_t001 |  |  |  |  |  |  |  |  |
| 0 | Vvi-Vitvi03g00997\_t001 |  |  |  |  |  |  |  |  |
| 0 | Vvi-Vitvi03g04319\_t001 |  |  |  |  |  |  |  |  |
| 0 | Vvi-Vitvi03g00996\_t001 |  |  |  |  |  |  |  |  |
| 0 | Vvi-Vitvi03g00995\_t001 |  |  |  |  |  |  |  |  |
| 0 | Vvi-Vitvi03g00993\_t001 |  |  |  |  |  |  |  |  |
| 0 | Vvi-Vitvi03g00987\_t002 |  |  |  |  |  |  |  |  |
| 0 | Vvi-Vitvi03g04320\_t001 |  |  |  |  |  |  |  |  |
| 0 | Vvi-Vitvi03g04321\_t001 |  |  |  |  |  |  |  |  |
| 0 | Vvi-Vitvi03g04322\_t001 |  |  |  |  |  |  |  |  |
| 0 | Vvi-Vitvi03g04323\_t001 |  |  |  |  |  |  |  |  |
| 0 | Vvi-Vitvi03g04324\_t001 |  |  |  |  |  |  |  |  |
| 0 | Vvi-Vitvi03g00981\_t001 |  |  |  |  |  |  |  |  |
| 0 | Vvi-Vitvi03g04325\_t001 |  |  |  |  |  |  |  |  |
| 0 | Vvi-Vitvi03g04326\_t001 |  |  |  |  |  |  |  |  |
| 0 | Vvi-Vitvi03g01765\_t001 |  |  |  |  |  |  |  |  |
| 0 | Vvi-Vitvi03g04327\_t001 |  |  |  |  |  |  |  |  |
| 0 | Vvi-Vitvi03g04328\_t001 |  |  |  |  |  |  |  |  |
| 0 | Vvi-Vitvi03g04329\_t001 |  |  |  |  |  |  |  |  |
| 0 | Vvi-Vitvi03g04330\_t001 |  |  |  |  |  |  |  |  |
| 0 | Vvi-Vitvi03g04331\_t001 |  |  |  |  |  |  |  |  |
| 0 | Vvi-Vitvi03g04332\_t001 |  |  |  |  |  |  |  |  |
| 0 | Vvi-Vitvi03g04333\_t001 |  |  |  |  |  |  |  |  |
| 0 | Vvi-Vitvi03g04334\_t001 |  |  |  |  |  |  |  |  |
| 0 | Vvi-Vitvi03g04335\_t001 |  |  |  |  |  |  |  |  |
| 0 | Vvi-Vitvi03g04336\_t001 |  |  |  |  |  |  |  |  |
| 0 | Vvi-Vitvi03g04337\_t001 |  |  |  |  |  |  |  |  |
| 0 | Vvi-Vitvi03g04338\_t001 |  |  |  |  |  |  |  |  |
| 0 | Vvi-Vitvi03g04339\_t001 |  |  |  |  |  |  |  |  |
| 0 | Vvi-Vitvi03g04340\_t001 |  |  |  |  |  |  |  |  |
| 0 | Vvi-Vitvi03g04341\_t001 |  |  |  |  |  |  |  |  |
| 0 | Vvi-Vitvi03g04342\_t001 |  |  |  |  |  |  |  |  |
| 0 | Vvi-Vitvi03g04343\_t001 |  |  |  |  |  |  |  |  |
| 0 | Vvi-Vitvi03g04344\_t001 |  |  |  |  |  |  |  |  |
| 0 | Vvi-Vitvi03g04345\_t001 |  |  |  |  |  |  |  |  |
| 0 | Vvi-Vitvi03g04346\_t001 |  |  |  |  |  |  |  |  |
| 0 | Vvi-Vitvi03g04347\_t001 |  |  |  |  |  |  |  |  |
| 0 | Vvi-Vitvi03g04348\_t001 |  |  |  |  |  |  |  |  |
| 0 | Vvi-Vitvi03g04349\_t001 |  |  |  |  |  |  |  |  |
| 0 | Vvi-Vitvi03g04352\_t001 |  |  |  |  |  |  |  |  |
| 0 | Vvi-Vitvi03g04353\_t001 |  |  |  |  |  |  |  |  |
| 0 | Vvi-Vitvi03g04354\_t001 |  |  |  |  |  |  |  |  |
| 0 | Vvi-Vitvi03g00973\_t001 |  |  |  |  |  |  |  |  |
| 0 | Vvi-Vitvi03g04355\_t001 |  |  |  |  |  |  |  |  |
| 0 | Vvi-Vitvi03g04356\_t001 |  |  |  |  |  |  |  |  |
| 0 | Vvi-Vitvi03g01763\_t001 |  |  |  |  |  |  |  |  |
| 0 | Vvi-Vitvi03g04357\_t001 |  |  |  |  |  |  |  |  |
| 0 | Vvi-Vitvi03g04358\_t001 |  |  |  |  |  |  |  |  |
| 0 | Vvi-Vitvi03g04359\_t001 |  |  |  |  |  |  |  |  |
| 0 | Vvi-Vitvi03g04360\_t001 |  |  |  |  |  |  |  |  |
| 0 | Vvi-Vitvi03g04361\_t001 |  |  |  |  |  |  |  |  |
| 0 | Vvi-Vitvi03g01760\_t001 |  |  |  |  |  |  |  |  |
| 0 | Vvi-Vitvi03g01759\_t001 |  |  |  |  |  |  |  |  |
| 0 | Vvi-Vitvi03g00954\_t001 |  |  |  |  |  |  |  |  |
| 0 | Vvi-Vitvi03g04362\_t001 |  |  |  |  |  |  |  |  |
| 0 | Vvi-Vitvi03g00950\_t001 |  |  |  |  |  |  |  |  |
| 0 | Vvi-Vitvi03g01757\_t001 |  |  |  |  |  |  |  |  |
| 0 | Vvi-Vitvi03g04363\_t001 |  |  |  |  |  |  |  |  |
| 0 | Vvi-Vitvi03g04364\_t001 |  |  |  |  |  |  |  |  |
| 0 | Vvi-Vitvi03g01756\_t001 |  |  |  |  |  |  |  |  |
| 0 | Vvi-Vitvi03g01755\_t001 |  |  |  |  |  |  |  |  |
| 0 | Vvi-Vitvi03g04365\_t001 |  |  |  |  |  |  |  |  |
| 0 | Vvi-Vitvi03g04366\_t001 |  |  |  |  |  |  |  |  |
| 0 | Vvi-Vitvi03g01754\_t001 |  |  |  |  |  |  |  |  |
| 0 | Vvi-Vitvi03g04367\_t001 |  |  |  |  |  |  |  |  |
| 0 | Vvi-Vitvi03g00946\_t001 |  |  |  |  |  |  |  |  |
| 0 | Vvi-Vitvi03g04368\_t001 |  |  |  |  |  |  |  |  |
| 0 | Vvi-Vitvi03g00941\_t001 |  |  |  |  |  |  |  |  |
| 0 | Vvi-Vitvi03g04369\_t001 |  |  |  |  |  |  |  |  |
| 0 | Vvi-Vitvi03g01752\_t001 |  |  |  |  |  |  |  |  |
| 0 | Vvi-Vitvi03g04370\_t001 |  |  |  |  |  |  |  |  |
| 0 | Vvi-Vitvi03g00936\_t001 |  |  |  |  |  |  |  |  |
| 0 | Vvi-Vitvi03g04371\_t001 |  |  |  |  |  |  |  |  |
| 0 | Vvi-Vitvi03g04372\_t001 |  |  |  |  |  |  |  |  |
| 0 | Vvi-Vitvi03g04373\_t001 |  |  |  |  |  |  |  |  |
| 0 | Vvi-Vitvi03g01743\_t001 |  |  |  |  |  |  |  |  |
| 0 | Vvi-Vitvi03g01742\_t001 |  |  |  |  |  |  |  |  |
| 0 | Vvi-Vitvi03g00933\_t001 |  |  |  |  |  |  |  |  |
| 0 | Vvi-Vitvi03g00932\_t001 |  |  |  |  |  |  |  |  |
| 0 | Vvi-Vitvi03g01741\_t001 |  |  |  |  |  |  |  |  |
| 0 | Vvi-Vitvi03g04374\_t001 |  |  |  |  |  |  |  |  |
| 0 | Vvi-Vitvi03g04375\_t001 |  |  |  |  |  |  |  |  |
| 0 | Vvi-Vitvi03g04376\_t001 |  |  |  |  |  |  |  |  |
| 0 | Vvi-Vitvi03g04377\_t001 |  |  |  |  |  |  |  |  |
| 0 | Vvi-Vitvi03g04378\_t001 |  |  |  |  |  |  |  |  |
| 0 | Vvi-Vitvi03g01877\_t001 |  |  |  |  |  |  |  |  |
| 0 | Vvi-Vitvi03g01883\_t001 |  |  |  |  |  |  |  |  |
| 0 | Vvi-Vitvi03g04380\_t001 |  |  |  |  |  |  |  |  |
| 0 | Vvi-Vitvi03g04382\_t001 |  |  |  |  |  |  |  |  |
| 0 | Vvi-Vitvi03g04383\_t001 |  |  |  |  |  |  |  |  |
| 0 | Vvi-Vitvi03g04384\_t001 |  |  |  |  |  |  |  |  |
| 0 | Vvi-Vitvi03g01775\_t001 |  |  |  |  |  |  |  |  |
| 0 | Vvi-Vitvi03g04385\_t001 |  |  |  |  |  |  |  |  |
| 0 | Vvi-Vitvi03g01776\_t001 |  |  |  |  |  |  |  |  |
| 0 | Vvi-Vitvi03g01037\_t001 |  |  |  |  |  |  |  |  |
| 0 | Vvi-Vitvi03g04386\_t001 |  |  |  |  |  |  |  |  |
| 0 | Vvi-Vitvi03g01038\_t001 |  |  |  |  |  |  |  |  |
| 0 | Vvi-Vitvi03g04387\_t001 |  |  |  |  |  |  |  |  |
| 0 | Vvi-Vitvi03g04388\_t001 |  |  |  |  |  |  |  |  |
| 0 | Vvi-Vitvi03g01780\_t001 |  |  |  |  |  |  |  |  |
| 0 | Vvi-Vitvi03g01040\_t001 |  |  |  |  |  |  |  |  |
| 0 | Vvi-Vitvi03g04389\_t001 |  |  |  |  |  |  |  |  |
| 0 | Vvi-Vitvi03g04390\_t001 |  |  |  |  |  |  |  |  |
| 0 | Vvi-Vitvi03g01042\_t001 |  |  |  |  |  |  |  |  |
| 0 | Vvi-Vitvi03g01043\_t001 |  |  |  |  |  |  |  |  |
| 0 | Vvi-Vitvi03g04391\_t001 |  |  |  |  |  |  |  |  |
| 0 | Vvi-Vitvi03g04392\_t001 |  |  |  |  |  |  |  |  |
| 0 | Vvi-Vitvi03g04393\_t001 |  |  |  |  |  |  |  |  |
| 0 | Vvi-Vitvi03g04394\_t001 |  |  |  |  |  |  |  |  |
| 0 | Vvi-Vitvi03g04395\_t001 |  |  |  |  |  |  |  |  |
| 0 | Vvi-Vitvi03g01783\_t001 |  |  |  |  |  |  |  |  |
| 0 | Vvi-Vitvi03g04396\_t001 |  |  |  |  |  |  |  |  |
| 0 | Vvi-Vitvi03g01782\_t001 |  |  |  |  |  |  |  |  |
| 0 | Vvi-Vitvi03g01047\_t002 |  |  |  |  |  |  |  |  |
| 0 | Vvi-Vitvi03g04397\_t001 |  |  |  |  |  |  |  |  |
| 0 | Vvi-Vitvi03g01052\_t001 |  |  |  |  |  |  |  |  |
| 0 | Vvi-Vitvi03g04398\_t001 |  |  |  |  |  |  |  |  |
| 0 | Vvi-Vitvi03g04399\_t001 |  |  |  |  |  |  |  |  |
| 0 | Vvi-Vitvi03g01056\_t001 |  |  |  |  |  |  |  |  |
| 0 | Vvi-Vitvi03g01058\_t001 |  |  |  |  |  |  |  |  |
| 0 | Vvi-Vitvi03g01059\_t001 |  |  |  |  |  |  |  |  |
| 0 | Vvi-Vitvi03g04400\_t001 |  |  |  |  |  |  |  |  |
| 0 | Vvi-Vitvi03g04401\_t001 |  |  |  |  |  |  |  |  |
| 0 | Vvi-Vitvi03g04402\_t001 |  |  |  |  |  |  |  |  |
| 0 | Vvi-Vitvi03g01066\_t001 |  |  |  |  |  |  |  |  |
| 0 | Vvi-Vitvi03g04403\_t001 |  |  |  |  |  |  |  |  |
| 0 | Vvi-Vitvi03g04404\_t001 |  |  |  |  |  |  |  |  |
| 0 | Vvi-Vitvi03g04405\_t001 |  |  |  |  |  |  |  |  |
| 0 | Vvi-Vitvi03g04406\_t001 |  |  |  |  |  |  |  |  |
| 0 | Vvi-Vitvi03g04407\_t001 |  |  |  |  |  |  |  |  |
| 0 | Vvi-Vitvi03g01067\_t001 |  |  |  |  |  |  |  |  |
| 0 | Vvi-Vitvi03g01317\_t001 |  |  |  |  |  |  |  |  |
| 0 | Vvi-Vitvi03g01318\_t001 |  |  |  |  |  |  |  |  |
| 0 | Vvi-Vitvi03g01071\_t001 |  |  |  |  |  |  |  |  |
| 0 | Vvi-Vitvi03g01789\_t001 |  |  |  |  |  |  |  |  |
| 0 | Vvi-Vitvi03g01073\_t001 |  |  |  |  |  |  |  |  |
| 0 | Vvi-Vitvi03g04408\_t001 |  |  |  |  |  |  |  |  |
| 0 | Vvi-Vitvi03g01792\_t001 |  |  |  |  |  |  |  |  |
| 0 | Vvi-Vitvi03g01077\_t001 |  |  |  |  |  |  |  |  |
| 0 | Vvi-Vitvi03g01078\_t001 |  |  |  |  |  |  |  |  |
| 0 | Vvi-Vitvi03g04409\_t001 |  |  |  |  |  |  |  |  |
| 0 | Vvi-Vitvi03g04410\_t001 |  |  |  |  |  |  |  |  |
| 0 | Vvi-Vitvi03g01079\_t001 |  |  |  |  |  |  |  |  |
| 0 | Vvi-Vitvi03g01080\_t001 |  |  |  |  |  |  |  |  |
| 0 | Vvi-Vitvi03g01081\_t001 |  |  |  |  |  |  |  |  |
| 0 | Vvi-Vitvi03g04411\_t001 |  |  |  |  |  |  |  |  |
| 0 | Vvi-Vitvi03g04412\_t001 |  |  |  |  |  |  |  |  |
| 0 | Vvi-Vitvi03g04413\_t001 |  |  |  |  |  |  |  |  |
| 0 | Vvi-Vitvi03g04414\_t001 |  |  |  |  |  |  |  |  |
| 0 | Vvi-Vitvi03g01084\_t001 |  |  |  |  |  |  |  |  |
| 0 | Vvi-Vitvi03g04415\_t001 |  |  |  |  |  |  |  |  |
| 0 | Vvi-Vitvi03g01085\_t001 |  |  |  |  |  |  |  |  |
| 0 | Vvi-Vitvi03g04416\_t001 |  |  |  |  |  |  |  |  |
| 0 | Vvi-Vitvi03g01088\_t001 |  |  |  |  |  |  |  |  |
| 0 | Vvi-Vitvi03g04417\_t001 |  |  |  |  |  |  |  |  |
| 0 | Vvi-Vitvi03g04418\_t001 |  |  |  |  |  |  |  |  |
| 0 | Vvi-Vitvi03g01092\_t001 |  |  |  |  |  |  |  |  |
| 0 | Vvi-Vitvi03g04419\_t001 |  |  |  |  |  |  |  |  |
| 0 | Vvi-Vitvi03g04420\_t001 |  |  |  |  |  |  |  |  |
| 0 | Vvi-Vitvi03g01093\_t001 |  |  |  |  |  |  |  |  |
| 0 | Vvi-Vitvi03g04421\_t001 |  |  |  |  |  |  |  |  |
| 0 | Vvi-Vitvi03g01799\_t001 |  |  |  |  |  |  |  |  |
| 0 | Vvi-Vitvi03g01800\_t001 |  |  |  |  |  |  |  |  |
| 0 | Vvi-Vitvi03g01095\_t001 |  |  |  |  |  |  |  |  |
| 0 | Vvi-Vitvi03g01096\_t001 |  |  |  |  |  |  |  |  |
| 0 | Vvi-Vitvi03g01097\_t001 |  |  |  |  |  |  |  |  |
| 0 | Vvi-Vitvi03g01801\_t001 |  |  |  |  |  |  |  |  |
| 0 | Vvi-Vitvi03g01098\_t001 |  |  |  |  |  |  |  |  |
| 0 | Vvi-Vitvi03g04422\_t001 |  |  |  |  |  |  |  |  |
| 0 | Vvi-Vitvi03g04423\_t001 |  |  |  |  |  |  |  |  |
| 0 | Vvi-Vitvi03g01108\_t001 |  |  |  |  |  |  |  |  |
| 0 | Vvi-Vitvi03g01110\_t001 |  |  |  |  |  |  |  |  |
| 0 | Vvi-Vitvi03g01111\_t001 |  |  |  |  |  |  |  |  |
| 0 | Vvi-Vitvi03g01809\_t001 |  |  |  |  |  |  |  |  |
| 0 | Vvi-Vitvi03g04424\_t001 |  |  |  |  |  |  |  |  |
| 0 | Vvi-Vitvi03g04425\_t001 |  |  |  |  |  |  |  |  |
| 0 | Vvi-Vitvi03g01812\_t001 |  |  |  |  |  |  |  |  |
| 0 | Vvi-Vitvi03g04426\_t001 |  |  |  |  |  |  |  |  |
| 0 | Vvi-Vitvi03g01118\_t001 |  |  |  |  |  |  |  |  |
| 0 | Vvi-Vitvi03g04427\_t002 |  |  |  |  |  |  |  |  |
| 0 | Vvi-Vitvi03g01816\_t001 |  |  |  |  |  |  |  |  |
| 0 | Vvi-Vitvi03g04428\_t001 |  |  |  |  |  |  |  |  |
| 0 | Vvi-Vitvi03g04429\_t001 |  |  |  |  |  |  |  |  |
| 0 | Vvi-Vitvi03g04430\_t001 |  |  |  |  |  |  |  |  |
| 0 | Vvi-Vitvi03g04431\_t001 |  |  |  |  |  |  |  |  |
| 0 | Vvi-Vitvi03g04432\_t001 |  |  |  |  |  |  |  |  |
| 0 | Vvi-Vitvi03g04433\_t001 |  |  |  |  |  |  |  |  |
| 0 | Vvi-Vitvi03g04434\_t001 |  |  |  |  |  |  |  |  |
| 0 | Vvi-Vitvi03g04435\_t001 |  |  |  |  |  |  |  |  |
| 0 | Vvi-Vitvi03g01124\_t001 |  |  |  |  |  |  |  |  |
| 0 | Vvi-Vitvi03g01126\_t001 |  |  |  |  |  |  |  |  |
| 0 | Vvi-Vitvi03g01127\_t001 |  |  |  |  |  |  |  |  |
| 0 | Vvi-Vitvi03g04436\_t001 |  |  |  |  |  |  |  |  |
| 0 | Vvi-Vitvi03g01130\_t001 |  |  |  |  |  |  |  |  |
| 0 | Vvi-Vitvi03g04437\_t001 |  |  |  |  |  |  |  |  |
| 0 | Vvi-Vitvi03g04438\_t001 |  |  |  |  |  |  |  |  |
| 0 | Vvi-Vitvi03g04439\_t001 |  |  |  |  |  |  |  |  |
| 0 | Vvi-Vitvi03g01137\_t001 |  |  |  |  |  |  |  |  |
| 0 | Vvi-Vitvi03g01138\_t001 |  |  |  |  |  |  |  |  |
| 0 | Vvi-Vitvi03g01140\_t001 |  |  |  |  |  |  |  |  |
| 0 | Vvi-Vitvi03g04440\_t001 |  |  |  |  |  |  |  |  |
| 0 | Vvi-Vitvi03g04441\_t001 |  |  |  |  |  |  |  |  |
| 0 | Vvi-Vitvi03g01141\_t001 |  |  |  |  |  |  |  |  |
| 0 | Vvi-Vitvi03g01144\_t001 |  |  |  |  |  |  |  |  |
| 0 | Vvi-Vitvi03g01145\_t001 |  |  |  |  |  |  |  |  |
| 0 | Vvi-Vitvi03g04442\_t001 |  |  |  |  |  |  |  |  |
| 0 | Vvi-Vitvi03g04443\_t001 |  |  |  |  |  |  |  |  |
| 0 | Vvi-Vitvi03g04444\_t001 |  |  |  |  |  |  |  |  |
| 0 | Vvi-Vitvi03g01149\_t001 |  |  |  |  |  |  |  |  |
| 0 | Vvi-Vitvi03g04445\_t001 |  |  |  |  |  |  |  |  |
| 0 | Vvi-Vitvi03g04446\_t001 |  |  |  |  |  |  |  |  |
| 0 | Vvi-Vitvi03g04447\_t001 |  |  |  |  |  |  |  |  |
| 0 | Vvi-Vitvi03g01822\_t001 |  |  |  |  |  |  |  |  |
| 0 | Vvi-Vitvi03g04448\_t001 |  |  |  |  |  |  |  |  |
| 0 | Vvi-Vitvi03g04449\_t001 |  |  |  |  |  |  |  |  |
| 0 | Vvi-Vitvi03g01151\_t001 |  |  |  |  |  |  |  |  |
| 0 | Vvi-Vitvi03g01154\_t001 |  |  |  |  |  |  |  |  |
| 0 | Vvi-Vitvi03g01824\_t001 |  |  |  |  |  |  |  |  |
| 0 | Vvi-Vitvi03g04450\_t001 |  |  |  |  |  |  |  |  |
| 0 | Vvi-Vitvi03g04451\_t001 |  |  |  |  |  |  |  |  |
| 0 | Vvi-Vitvi03g04452\_t001 |  |  |  |  |  |  |  |  |
| 0 | Vvi-Vitvi03g01159\_t001 |  |  |  |  |  |  |  |  |
| 0 | Vvi-Vitvi03g04453\_t001 |  |  |  |  |  |  |  |  |
| 0 | Vvi-Vitvi03g01161\_t001 |  |  |  |  |  |  |  |  |
| 0 | Vvi-Vitvi03g01162\_t001 |  |  |  |  |  |  |  |  |
| 0 | Vvi-Vitvi03g01163\_t001 |  |  |  |  |  |  |  |  |
| 0 | Vvi-Vitvi03g01826\_t001 |  |  |  |  |  |  |  |  |
| 0 | Vvi-Vitvi03g04454\_t001 |  |  |  |  |  |  |  |  |
| 0 | Vvi-Vitvi03g01169\_t001 |  |  |  |  |  |  |  |  |
| 0 | Vvi-Vitvi03g01830\_t001 |  |  |  |  |  |  |  |  |
| 0 | Vvi-Vitvi03g04455\_t001 |  |  |  |  |  |  |  |  |
| 0 | Vvi-Vitvi03g04456\_t001 |  |  |  |  |  |  |  |  |
| 0 | Vvi-Vitvi03g01173\_t001 |  |  |  |  |  |  |  |  |
| 0 | Vvi-Vitvi03g04457\_t001 |  |  |  |  |  |  |  |  |
| 0 | Vvi-Vitvi03g01176\_t001 |  |  |  |  |  |  |  |  |
| 0 | Vvi-Vitvi03g01178\_t001 |  |  |  |  |  |  |  |  |
| 0 | Vvi-Vitvi03g04458\_t001 |  |  |  |  |  |  |  |  |
| 0 | Vvi-Vitvi03g01832\_t001 |  |  |  |  |  |  |  |  |
| 0 | Vvi-Vitvi03g01833\_t001 |  |  |  |  |  |  |  |  |
| 0 | Vvi-Vitvi03g01182\_t001 |  |  |  |  |  |  |  |  |
| 0 | Vvi-Vitvi03g01834\_t001 |  |  |  |  |  |  |  |  |
| 0 | Vvi-Vitvi03g01185\_t001 |  |  |  |  |  |  |  |  |
| 0 | Vvi-Vitvi03g04459\_t001 |  |  |  |  |  |  |  |  |
| 0 | Vvi-Vitvi03g04460\_t001 |  |  |  |  |  |  |  |  |
| 0 | Vvi-Vitvi03g01187\_t001 |  |  |  |  |  |  |  |  |
| 0 | Vvi-Vitvi03g01188\_t002 |  |  |  |  |  |  |  |  |
| 0 | Vvi-Vitvi03g01189\_t004 |  |  |  |  |  |  |  |  |
| 0 | Vvi-Vitvi03g01190\_t001 |  |  |  |  |  |  |  |  |
| 0 | Vvi-Vitvi03g04461\_t001 |  |  |  |  |  |  |  |  |
| 0 | Vvi-Vitvi03g01192\_t001 |  |  |  |  |  |  |  |  |
| 0 | Vvi-Vitvi03g01193\_t001 |  |  |  |  |  |  |  |  |
| 0 | Vvi-Vitvi03g01196\_t001 |  |  |  |  |  |  |  |  |
| 0 | Vvi-Vitvi03g01197\_t001 |  |  |  |  |  |  |  |  |
| 0 | Vvi-Vitvi03g01198\_t001 |  |  |  |  |  |  |  |  |
| 0 | Vvi-Vitvi03g04462\_t001 |  |  |  |  |  |  |  |  |
| 0 | Vvi-Vitvi03g01200\_t001 |  |  |  |  |  |  |  |  |
| 0 | Vvi-Vitvi03g01839\_t001 |  |  |  |  |  |  |  |  |
| 0 | Vvi-Vitvi03g04463\_t001 |  |  |  |  |  |  |  |  |
| 0 | Vvi-Vitvi03g01202\_t001 |  |  |  |  |  |  |  |  |
| 0 | Vvi-Vitvi03g04464\_t002 |  |  |  |  |  |  |  |  |
| 0 | Vvi-Vitvi03g04465\_t001 |  |  |  |  |  |  |  |  |
| 0 | Vvi-Vitvi03g04466\_t001 |  |  |  |  |  |  |  |  |
| 0 | Vvi-Vitvi03g01840\_t001 |  |  |  |  |  |  |  |  |
| 0 | Vvi-Vitvi03g01841\_t001 |  |  |  |  |  |  |  |  |
| 0 | Vvi-Vitvi03g04467\_t001 |  |  |  |  |  |  |  |  |
| 0 | Vvi-Vitvi03g04468\_t001 |  |  |  |  |  |  |  |  |
| 0 | Vvi-Vitvi03g04469\_t001 |  |  |  |  |  |  |  |  |
| 0 | Vvi-Vitvi03g04470\_t001 |  |  |  |  |  |  |  |  |
| 0 | Vvi-Vitvi03g04471\_t001 |  |  |  |  |  |  |  |  |
| 0 | Vvi-Vitvi03g01842\_t001 |  |  |  |  |  |  |  |  |
| 0 | Vvi-Vitvi03g01843\_t001 |  |  |  |  |  |  |  |  |
| 0 | Vvi-Vitvi03g04472\_t001 |  |  |  |  |  |  |  |  |
| 0 | Vvi-Vitvi03g04473\_t001 |  |  |  |  |  |  |  |  |
| 0 | Vvi-Vitvi03g01213\_t001 |  |  |  |  |  |  |  |  |
| 0 | Vvi-Vitvi03g01845\_t001 |  |  |  |  |  |  |  |  |
| 0 | Vvi-Vitvi03g01216\_t001 |  |  |  |  |  |  |  |  |
| 0 | Vvi-Vitvi03g04474\_t001 |  |  |  |  |  |  |  |  |
| 0 | Vvi-Vitvi03g04475\_t001 |  |  |  |  |  |  |  |  |
| 0 | Vvi-Vitvi03g01219\_t001 |  |  |  |  |  |  |  |  |
| 0 | Vvi-Vitvi03g04476\_t001 |  |  |  |  |  |  |  |  |
| 0 | Vvi-Vitvi03g01846\_t001 |  |  |  |  |  |  |  |  |
| 0 | Vvi-Vitvi03g04477\_t001 |  |  |  |  |  |  |  |  |
| 0 | Vvi-Vitvi03g04478\_t001 |  |  |  |  |  |  |  |  |
| 0 | Vvi-Vitvi03g04479\_t001 |  |  |  |  |  |  |  |  |
| 0 | Vvi-Vitvi03g01849\_t001 |  |  |  |  |  |  |  |  |
| 0 | Vvi-Vitvi03g04480\_t001 |  |  |  |  |  |  |  |  |
| 0 | Vvi-Vitvi03g01224\_t001 |  |  |  |  |  |  |  |  |
| 0 | Vvi-Vitvi03g01225\_t001 |  |  |  |  |  |  |  |  |
| 0 | Vvi-Vitvi03g01851\_t001 |  |  |  |  |  |  |  |  |
| 0 | Vvi-Vitvi03g04481\_t001 |  |  |  |  |  |  |  |  |
| 0 | Vvi-Vitvi03g01226\_t001 |  |  |  |  |  |  |  |  |
| 0 | Vvi-Vitvi03g04482\_t001 |  |  |  |  |  |  |  |  |
| 0 | Vvi-Vitvi03g04483\_t001 |  |  |  |  |  |  |  |  |
| 0 | Vvi-Vitvi03g04484\_t001 |  |  |  |  |  |  |  |  |
| 0 | Vvi-Vitvi03g04485\_t001 |  |  |  |  |  |  |  |  |
| 0 | Vvi-Vitvi03g04486\_t001 |  |  |  |  |  |  |  |  |
| 0 | Vvi-Vitvi03g01231\_t001 |  |  |  |  |  |  |  |  |
| 0 | Vvi-Vitvi03g01232\_t001 |  |  |  |  |  |  |  |  |
| 0 | Vvi-Vitvi03g01233\_t001 |  |  |  |  |  |  |  |  |
| 0 | Vvi-Vitvi03g01234\_t001 |  |  |  |  |  |  |  |  |
| 0 | Vvi-Vitvi03g01235\_t001 |  |  |  |  |  |  |  |  |
| 0 | Vvi-Vitvi03g01236\_t001 |  |  |  |  |  |  |  |  |
| 0 | Vvi-Vitvi03g01237\_t001 |  |  |  |  |  |  |  |  |
| 0 | Vvi-Vitvi03g01238\_t001 |  |  |  |  |  |  |  |  |
| 0 | Vvi-Vitvi03g04487\_t001 |  |  |  |  |  |  |  |  |
| 0 | Vvi-Vitvi03g01239\_t001 |  |  |  |  |  |  |  |  |
| 0 | Vvi-Vitvi03g04488\_t001 |  |  |  |  |  |  |  |  |
| 0 | Vvi-Vitvi03g01242\_t001 |  |  |  |  |  |  |  |  |
| 0 | Vvi-Vitvi03g01243\_t001 |  |  |  |  |  |  |  |  |
| 0 | Vvi-Vitvi03g04489\_t001 |  |  |  |  |  |  |  |  |
| 0 | Vvi-Vitvi03g01244\_t002 |  |  |  |  |  |  |  |  |
| 0 | Vvi-Vitvi03g04490\_t001 |  |  |  |  |  |  |  |  |
| 0 | Vvi-Vitvi03g04491\_t001 |  |  |  |  |  |  |  |  |
| 0 | Vvi-Vitvi03g01853\_t001 |  |  |  |  |  |  |  |  |
| 0 | Vvi-Vitvi03g04492\_t001 |  |  |  |  |  |  |  |  |
| 0 | Vvi-Vitvi03g01247\_t001 |  |  |  |  |  |  |  |  |
| 0 | Vvi-Vitvi03g04493\_t001 |  |  |  |  |  |  |  |  |
| 0 | Vvi-Vitvi03g01856\_t001 |  |  |  |  |  |  |  |  |
| 0 | Vvi-Vitvi03g04494\_t001 |  |  |  |  |  |  |  |  |
| 0 | Vvi-Vitvi03g01858\_t001 |  |  |  |  |  |  |  |  |
| 0 | Vvi-Vitvi03g01248\_t001 |  |  |  |  |  |  |  |  |
| 0 | Vvi-Vitvi03g01249\_t001 |  |  |  |  |  |  |  |  |
| 0 | Vvi-Vitvi03g04495\_t001 |  |  |  |  |  |  |  |  |
| 0 | Vvi-Vitvi03g04496\_t001 |  |  |  |  |  |  |  |  |
| 0 | Vvi-Vitvi03g04497\_t001 |  |  |  |  |  |  |  |  |
| 0 | Vvi-Vitvi03g01251\_t001 |  |  |  |  |  |  |  |  |
| 0 | Vvi-Vitvi03g01252\_t001 |  |  |  |  |  |  |  |  |
| 0 | Vvi-Vitvi03g01254\_t001 |  |  |  |  |  |  |  |  |
| 0 | Vvi-Vitvi03g01255\_t001 |  |  |  |  |  |  |  |  |
| 0 | Vvi-Vitvi03g01256\_t001 |  |  |  |  |  |  |  |  |
| 0 | Vvi-Vitvi03g01258\_t001 |  |  |  |  |  |  |  |  |
| 0 | Vvi-Vitvi03g01259\_t001 |  |  |  |  |  |  |  |  |
| 0 | Vvi-Vitvi03g04498\_t001 |  |  |  |  |  |  |  |  |
| 0 | Vvi-Vitvi03g04499\_t001 |  |  |  |  |  |  |  |  |
| 0 | Vvi-Vitvi03g04500\_t001 |  |  |  |  |  |  |  |  |
| 0 | Vvi-Vitvi03g04501\_t001 |  |  |  |  |  |  |  |  |
| 0 | Vvi-Vitvi03g01267\_t001 |  |  |  |  |  |  |  |  |
| 0 | Vvi-Vitvi03g04502\_t001 |  |  |  |  |  |  |  |  |
| 0 | Vvi-Vitvi03g04503\_t001 |  |  |  |  |  |  |  |  |
| 0 | Vvi-Vitvi03g01861\_t001 |  |  |  |  |  |  |  |  |
| 0 | Vvi-Vitvi03g01862\_t001 |  |  |  |  |  |  |  |  |
| 0 | Vvi-Vitvi03g04504\_t001 |  |  |  |  |  |  |  |  |
| 0 | Vvi-Vitvi03g04505\_t001 |  |  |  |  |  |  |  |  |
| 0 | Vvi-Vitvi03g04506\_t001 |  |  |  |  |  |  |  |  |
| 0 | Vvi-Vitvi03g01272\_t001 |  |  |  |  |  |  |  |  |
| 0 | Vvi-Vitvi03g04507\_t001 |  |  |  |  |  |  |  |  |
| 0 | Vvi-Vitvi03g04508\_t001 |  |  |  |  |  |  |  |  |
| 0 | Vvi-Vitvi03g04509\_t001 |  |  |  |  |  |  |  |  |
| 0 | Vvi-Vitvi03g04510\_t001 |  |  |  |  |  |  |  |  |
| 0 | Vvi-Vitvi03g04511\_t001 |  |  |  |  |  |  |  |  |
| 0 | Vvi-Vitvi03g01277\_t001 |  |  |  |  |  |  |  |  |
| 0 | Vvi-Vitvi03g04512\_t001 |  |  |  |  |  |  |  |  |
| 0 | Vvi-Vitvi03g04513\_t001 |  |  |  |  |  |  |  |  |
| 0 | Vvi-Vitvi03g04514\_t001 |  |  |  |  |  |  |  |  |
| 0 | Vvi-Vitvi03g01283\_t001 |  |  |  |  |  |  |  |  |
| 0 | Vvi-Vitvi03g04515\_t001 |  |  |  |  |  |  |  |  |
| 0 | Vvi-Vitvi03g01285\_t001 |  |  |  |  |  |  |  |  |
| 0 | Vvi-Vitvi03g04516\_t001 |  |  |  |  |  |  |  |  |
| 0 | Vvi-Vitvi03g04517\_t001 |  |  |  |  |  |  |  |  |
| 0 | Vvi-Vitvi03g01286\_t001 |  |  |  |  |  |  |  |  |
| 0 | Vvi-Vitvi03g01287\_t001 |  |  |  |  |  |  |  |  |
| 0 | Vvi-Vitvi03g01288\_t001 |  |  |  |  |  |  |  |  |
| 0 | Vvi-Vitvi03g01289\_t001 |  |  |  |  |  |  |  |  |
| 0 | Vvi-Vitvi03g01290\_t001 |  |  |  |  |  |  |  |  |
| 0 | Vvi-Vitvi03g01292\_t001 |  |  |  |  |  |  |  |  |
| 0 | Vvi-Vitvi03g01294\_t001 |  |  |  |  |  |  |  |  |
| 0 | Vvi-Vitvi03g01297\_t001 |  |  |  |  |  |  |  |  |
| 0 | Vvi-Vitvi03g04518\_t001 |  |  |  |  |  |  |  |  |
| 0 | Vvi-Vitvi03g01298\_t001 |  |  |  |  |  |  |  |  |
| 0 | Vvi-Vitvi03g04519\_t001 |  |  |  |  |  |  |  |  |
| 0 | Vvi-Vitvi03g01299\_t001 |  |  |  |  |  |  |  |  |
| 0 | Vvi-Vitvi03g01300\_t001 |  |  |  |  |  |  |  |  |
| 0 | Vvi-Vitvi03g01872\_t001 |  |  |  |  |  |  |  |  |
| 0 | Vvi-Vitvi03g04520\_t001 |  |  |  |  |  |  |  |  |
| 0 | Vvi-Vitvi03g01302\_t001 |  |  |  |  |  |  |  |  |
| 0 | Vvi-Vitvi03g01303\_t003 |  |  |  |  |  |  |  |  |
| 0 | Vvi-Vitvi03g01304\_t001 |  |  |  |  |  |  |  |  |
| 0 | Vvi-Vitvi03g01305\_t001 |  |  |  |  |  |  |  |  |
| 0 | Vvi-Vitvi03g01306\_t001 |  |  |  |  |  |  |  |  |
| 0 | Vvi-Vitvi03g01308\_t001 |  |  |  |  |  |  |  |  |
| 0 | Vvi-Vitvi03g01309\_t001 |  |  |  |  |  |  |  |  |
| 0 | Vvi-Vitvi03g01312\_t001 |  |  |  |  |  |  |  |  |
| 0 | Vvi-Vitvi03g01314\_t001 |  |  |  |  |  |  |  |  |
| 0 | Vvi-Vitvi03g04521\_t001 |  |  |  |  |  |  |  |  |
| 0 | Vvi-Vitvi03g04522\_t001 |  |  |  |  |  |  |  |  |
| 0 | Vvi-Vitvi03g01315\_t001 |  |  |  |  |  |  |  |  |
| 0 | Vvi-Vitvi03g01316\_t002 |  |  |  |  |  |  |  |  |
| 0 | Vvi-Vitvi03g04523\_t001 |  |  |  |  |  |  |  |  |
| 0 | Vvi-Vitvi03g04524\_t001 |  |  |  |  |  |  |  |  |
| 0 | Vvi-Vitvi03g04525\_t001 |  |  |  |  |  |  |  |  |
| 0 | Vvi-Vitvi03g04526\_t001 |  |  |  |  |  |  |  |  |
| 0 | Vvi-Vitvi03g04527\_t001 |  |  |  |  |  |  |  |  |
| 0 | Vvi-Vitvi03g04528\_t001 |  |  |  |  |  |  |  |  |
| 0 | Vvi-Vitvi03g04529\_t001 |  |  |  |  |  |  |  |  |
| 0 | Vvi-Vitvi03g04530\_t001 |  |  |  |  |  |  |  |  |
