# Supplementary material for: Genome-wide identification, characterization and gene expression of BES1 transcription factor family in grapevine (Vitis vinifera L.)
Source: Sci Rep. 2023 Jan 5;13:240. doi: 10.1038/s41598-022-24407-y (PMC9816167; doi:10.1038/s41598-022-24407-y)
Supplement: Supplementary file 3 — Supplementary Information. [file 41598_2022_24407_MOESM3_ESM.zip › Vvi_Ath/Vitis_vinifera.PN40024.v4.dna_sm.toplevel.fa.vs.Arabidopsis_thaliana.TAIR10.dna_sm.toplevel.fa.html/Vvi-5.html]

|  |  |  |  |  |  |  |  |  |  |  |  |  |  |  |  |  |  |
| --- | --- | --- | --- | --- | --- | --- | --- | --- | --- | --- | --- | --- | --- | --- | --- | --- | --- |
| Duplication depth | Reference chromosome | Collinear blocks | | | | | | | | | | | | | | | |
| 1 | Vvi-Vitvi05g00001\_t002 |  | Ath-AT5G49060.1 |  |  |  |  |  |  |  |
| 1 | Vvi-Vitvi05g04000\_t001 |  | | | |  |  |  |  |  |  |  |
| 2 | Vvi-Vitvi05g01734\_t001 |  | | | |  | Ath-AT3G06890.1 |  |  |  |  |  |  |
| 2 | Vvi-Vitvi05g00005\_t001 |  | | | |  | | | |  |  |  |  |  |  |
| 2 | Vvi-Vitvi05g00006\_t002 |  | | | |  | Ath-AT3G06880.4 |  |  |  |  |  |  |
| 3 | Vvi-Vitvi05g00007\_t001 |  | | | |  | | | |  | Ath-AT3G05550.1 |  |  |  |  |  |
| 5 | Vvi-Vitvi05g00008\_t001 |  | | | |  | | | |  | | | |  | Ath-AT3G16650.1 |  | Ath-AT4G15900.1 |  |  |  |
| 5 | Vvi-Vitvi05g00009\_t001 |  | | | |  | | | |  | | | |  | | | |  | | | |  |  |  |
| 5 | Vvi-Vitvi05g00010\_t001 |  | | | |  | | | |  | Ath-AT3G05560.1 |  | | | |  | | | |  |  |  |
| 5 | Vvi-Vitvi05g00011\_t001 |  | | | |  | | | |  | | | |  | Ath-AT3G16660.2 |  | | | |  |  |  |
| 5 | Vvi-Vitvi05g00012\_t001 |  | Ath-AT5G49100.1 |  | Ath-AT3G06868.1 |  | | | |  | | | |  | | | |  |  |  |
| 5 | Vvi-Vitvi05g04001\_t001 |  | | | |  | | | |  | | | |  | Ath-AT3G16690.3 |  | Ath-AT4G15920.1 |  |  |  |
| 5 | Vvi-Vitvi05g04002\_t001 |  | | | |  | | | |  | | | |  | | | |  | | | |  |  |  |
| 5 | Vvi-Vitvi05g04003\_t001 |  | | | |  | | | |  | | | |  | | | |  | | | |  |  |  |
| 5 | Vvi-Vitvi05g00015\_t001 |  | | | |  | | | |  | | | |  | | | |  | | | |  |  |  |
| 5 | Vvi-Vitvi05g04004\_t001 |  | | | |  | | | |  | | | |  | | | |  | | | |  |  |  |
| 5 | Vvi-Vitvi05g00016\_t001 |  | | | |  | | | |  | | | |  | | | |  | | | |  |  |  |
| 5 | Vvi-Vitvi05g04005\_t001 |  | | | |  | | | |  | | | |  | | | |  | | | |  |  |  |
| 5 | Vvi-Vitvi05g01740\_t001 |  | | | |  | | | |  | | | |  | | | |  | | | |  |  |  |
| 5 | Vvi-Vitvi05g01741\_t001 |  | | | |  | | | |  | | | |  | | | |  | | | |  |  |  |
| 5 | Vvi-Vitvi05g04006\_t001 |  | | | |  | | | |  | | | |  | | | |  | | | |  |  |  |
| 5 | Vvi-Vitvi05g00018\_t001 |  | | | |  | | | |  | | | |  | | | |  | | | |  |  |  |
| 5 | Vvi-Vitvi05g00020\_t002 |  | | | |  | Ath-AT3G06860.1 |  | | | |  | | | |  | | | |  |  |  |
| 5 | Vvi-Vitvi05g04007\_t001 |  | | | |  | | | |  | | | |  | | | |  | | | |  |  |  |
| 5 | Vvi-Vitvi05g04008\_t001 |  | | | |  | | | |  | | | |  | | | |  | | | |  |  |  |
| 5 | Vvi-Vitvi05g00026\_t001 |  | Ath-AT5G49110.2 |  | | | |  | | | |  | | | |  | | | |  |  |  |
| 5 | Vvi-Vitvi05g00027\_t001 |  | | | |  | | | |  | | | |  | | | |  | Ath-AT4G15930.1 |  |  |  |
| 5 | Vvi-Vitvi05g00028\_t001 |  | Ath-AT5G49120.1 |  | | | |  | | | |  | | | |  | | | |  |  |  |
| 5 | Vvi-Vitvi05g04009\_t001 |  | | | |  | | | |  | | | |  | | | |  | | | |  |  |  |
| 5 | Vvi-Vitvi05g00031\_t001 |  | Ath-AT5G49130.1 |  | | | |  | | | |  | | | |  | | | |  |  |  |
| 5 | Vvi-Vitvi05g00032\_t001 |  | | | |  | Ath-AT3G06850.1 |  | | | |  | | | |  | | | |  |  |  |
| 5 | Vvi-Vitvi05g00033\_t001 |  | | | |  | | | |  | Ath-AT3G05590.1 |  | | | |  | | | |  |  |  |
| 5 | Vvi-Vitvi05g00034\_t001 |  | Ath-AT5G49150.2 |  | | | |  | | | |  | | | |  | | | |  |  |  |
| 5 | Vvi-Vitvi05g00035\_t001 |  | | | |  | | | |  | | | |  | Ath-AT3G16700.1 |  | Ath-AT4G15940.1 |  |  |  |
| 5 | Vvi-Vitvi05g01742\_t002 |  | | | |  | | | |  | Ath-AT3G05600.1 |  | | | |  | | | |  |  |  |
| 5 | Vvi-Vitvi05g00038\_t001 |  | | | |  | | | |  | | | |  | | | |  | | | |  |  |  |
| 5 | Vvi-Vitvi05g00039\_t001 |  | | | |  | | | |  | | | |  | | | |  | Ath-AT4G15955.3 |  |  |  |
| 5 | Vvi-Vitvi05g00040\_t001 |  | | | |  | | | |  | | | |  | | | |  | | | |  |  |  |
| 5 | Vvi-Vitvi05g04010\_t001 |  | | | |  | | | |  | | | |  | | | |  | | | |  |  |  |
| 5 | Vvi-Vitvi05g01743\_t001 |  | Ath-AT5G49170.1 |  | Ath-AT3G06840.1 |  | | | |  | | | |  | | | |  |  |  |
| 5 | Vvi-Vitvi05g00041\_t001 |  | | | |  | | | |  | | | |  | Ath-AT3G16720.1 |  | Ath-AT4G15975.1 |  |  |  |
| 5 | Vvi-Vitvi05g00042\_t001 |  | | | |  | | | |  | | | |  | Ath-AT3G16730.1 |  | | | |  |  |  |
| 5 | Vvi-Vitvi05g00043\_t001 |  | Ath-AT5G49180.1 |  | Ath-AT3G06830.1 |  | Ath-AT3G05610.1 |  | | | |  | | | |  |  |  |
| 5 | Vvi-Vitvi05g00044\_t001 |  | | | |  | Ath-AT3G06810.1 |  | | | |  | | | |  | | | |  |  |  |
| 5 | Vvi-Vitvi05g00045\_t001 |  | Ath-AT5G49190.1 |  | | | |  | | | |  | | | |  | | | |  |  |  |
| 5 | Vvi-Vitvi05g04011\_t001 |  | | | |  | | | |  | | | |  | | | |  | | | |  |  |  |
| 5 | Vvi-Vitvi05g04012\_t001 |  | | | |  | | | |  | | | |  | | | |  | | | |  |  |  |
| 5 | Vvi-Vitvi05g04013\_t001 |  | | | |  | | | |  | | | |  | | | |  | | | |  |  |  |
| 5 | Vvi-Vitvi05g00047\_t001 |  | | | |  | | | |  | | | |  | | | |  | | | |  |  |  |
| 5 | Vvi-Vitvi05g00048\_t001 |  | | | |  | | | |  | | | |  | | | |  | | | |  |  |  |
| 5 | Vvi-Vitvi05g00049\_t001 |  | | | |  | Ath-AT3G06790.1 |  | | | |  | | | |  | | | |  |  |  |
| 5 | Vvi-Vitvi05g00050\_t001 |  | | | |  | | | |  | | | |  | Ath-AT3G16760.1 |  | | | |  |  |  |
| 5 | Vvi-Vitvi05g01724\_t001 |  | | | |  | | | |  | | | |  | Ath-AT3G16770.1 |  | | | |  |  |  |
| 5 | Vvi-Vitvi05g01745\_t001 |  | | | |  | Ath-AT3G06780.1 |  | | | |  | | | |  | | | |  |  |  |
| 5 | Vvi-Vitvi05g00053\_t001 |  | | | |  | | | |  | | | |  | Ath-AT3G16780.1 |  | Ath-AT4G16030.1 |  |  |  |
| 5 | Vvi-Vitvi05g00054\_t001 |  | | | |  | | | |  | Ath-AT3G05630.1 |  | Ath-AT3G16785.4 |  | | | |  |  |  |
| 5 | Vvi-Vitvi05g00055\_t001 |  | | | |  | | | |  | Ath-AT3G05640.1 |  | Ath-AT3G16800.2 |  | | | |  |  |  |
| 4 | Vvi-Vitvi05g00056\_t001 |  | | | |  | | | |  |  |  | | | |  | Ath-AT4G16060.1 |  |  |  |
| 4 | Vvi-Vitvi05g00057\_t001.1.6037826e |  | | | |  | | | |  |  |  | | | |  | | | |  |  |  |
| 4 | Vvi-Vitvi05g01746\_t001 |  | | | |  | | | |  |  |  | | | |  | Ath-AT4G16070.3 |  |  |  |
| 4 | Vvi-Vitvi05g01747\_t001 |  | | | |  | Ath-AT3G06778.1 |  |  |  | | | |  | | | |  |  |  |
| 4 | Vvi-Vitvi05g00060\_t001 |  | | | |  | | | |  |  |  | Ath-AT3G16810.1 |  | | | |  |  |  |
| 4 | Vvi-Vitvi05g00061\_t001 |  | Ath-AT5G49215.1 |  | Ath-AT3G06770.5 |  |  |  | Ath-AT3G16850.1 |  | | | |  |  |  |
| 4 | Vvi-Vitvi05g04014\_t001 |  | | | |  | | | |  |  |  | | | |  | | | |  |  |  |
| 4 | Vvi-Vitvi05g00062\_t003 |  | | | |  | | | |  |  |  | | | |  | | | |  |  |  |
| 4 | Vvi-Vitvi05g00063\_t006 |  | | | |  | | | |  |  |  | | | |  | | | |  |  |  |
| 4 | Vvi-Vitvi05g00064\_t001 |  | | | |  | | | |  |  |  | | | |  | | | |  |  |  |
| 4 | Vvi-Vitvi05g00065\_t001 |  | | | |  | | | |  |  |  | | | |  | | | |  |  |  |
| 4 | Vvi-Vitvi05g00066\_t001 |  | | | |  | | | |  |  |  | | | |  | | | |  |  |  |
| 4 | Vvi-Vitvi05g00067\_t001 |  | | | |  | | | |  |  |  | | | |  | | | |  |  |  |
| 4 | Vvi-Vitvi05g00068\_t001 |  | | | |  | | | |  |  |  | | | |  | | | |  |  |  |
| 4 | Vvi-Vitvi05g01752\_t001 |  | | | |  | | | |  |  |  | | | |  | | | |  |  |  |
| 4 | Vvi-Vitvi05g00069\_t001 |  | | | |  | | | |  |  |  | | | |  | | | |  |  |  |
| 4 | Vvi-Vitvi05g01753\_t001 |  | | | |  | | | |  |  |  | | | |  | | | |  |  |  |
| 4 | Vvi-Vitvi05g01754\_t001 |  | | | |  | | | |  |  |  | | | |  | | | |  |  |  |
| 4 | Vvi-Vitvi05g04015\_t001 |  | | | |  | | | |  |  |  | | | |  | | | |  |  |  |
| 4 | Vvi-Vitvi05g00071\_t001 |  | | | |  | | | |  |  |  | | | |  | | | |  |  |  |
| 4 | Vvi-Vitvi05g01757\_t001 |  | | | |  | | | |  |  |  | | | |  | | | |  |  |  |
| 4 | Vvi-Vitvi05g01759\_t003 |  | | | |  | | | |  |  |  | | | |  | | | |  |  |  |
| 4 | Vvi-Vitvi05g00072\_t001 |  | | | |  | | | |  |  |  | | | |  | | | |  |  |  |
| 4 | Vvi-Vitvi05g01760\_t001 |  | | | |  | | | |  |  |  | | | |  | | | |  |  |  |
| 4 | Vvi-Vitvi05g01761\_t001 |  | | | |  | | | |  |  |  | | | |  | | | |  |  |  |
| 4 | Vvi-Vitvi05g00073\_t002 |  | Ath-AT5G49220.1 |  | | | |  |  |  | | | |  | Ath-AT4G16100.1 |  |  |  |
| 4 | Vvi-Vitvi05g01762\_t001 |  | Ath-AT5G49230.1 |  | Ath-AT3G06760.2 |  |  |  | | | |  | | | |  |  |  |
| 4 | Vvi-Vitvi05g04016\_t001 |  | | | |  | | | |  |  |  | | | |  | | | |  |  |  |
| 4 | Vvi-Vitvi05g00075\_t001 |  | | | |  | | | |  |  |  | Ath-AT3G16857.2 |  | | | |  |  |  |
| 4 | Vvi-Vitvi05g00076\_t001 |  | Ath-AT5G49270.1 |  | | | |  |  |  | Ath-AT3G16860.1 |  | Ath-AT4G16120.1 |  |  |  |
| 4 | Vvi-Vitvi05g01763\_t001 |  | | | |  | | | |  |  |  | | | |  | | | |  |  |  |
| 4 | Vvi-Vitvi05g00077\_t001 |  | Ath-AT5G49300.1 |  | Ath-AT3G06740.1 |  |  |  | Ath-AT3G16870.1 |  | Ath-AT4G16141.1 |  |  |  |
| 4 | Vvi-Vitvi05g04017\_t001 |  | | | |  | | | |  |  |  | | | |  | | | |  |  |  |
| 4 | Vvi-Vitvi05g01765\_t001 |  | | | |  | Ath-AT3G06720.1 |  |  |  | | | |  | Ath-AT4G16143.1 |  |  |  |
| 4 | Vvi-Vitvi05g00079\_t001 |  | Ath-AT5G49320.1 |  | | | |  |  |  | | | |  | | | |  |  |  |
| 4 | Vvi-Vitvi05g00080\_t001 |  | | | |  | | | |  |  |  | | | |  | | | |  |  |  |
| 4 | Vvi-Vitvi05g00081\_t002 |  | | | |  | | | |  |  |  | | | |  | | | |  |  |  |
| 4 | Vvi-Vitvi05g00082\_t001 |  | | | |  | | | |  |  |  | | | |  | | | |  |  |  |
| 5 | Vvi-Vitvi05g01766\_t001 |  | | | |  | | | |  | Ath-AT2G47480.1 |  | | | |  | | | |  |  |  |
| 5 | Vvi-Vitvi05g04018\_t001 |  | | | |  | | | |  | | | |  | | | |  | | | |  |  |  |
| 5 | Vvi-Vitvi05g00083\_t001 |  | | | |  | | | |  | | | |  | | | |  | | | |  |  |  |
| 5 | Vvi-Vitvi05g00084\_t001 |  | Ath-AT5G49330.1 |  | | | |  | Ath-AT2G47460.1 |  | | | |  | | | |  |  |  |
| 5 | Vvi-Vitvi05g00085\_t001 |  | | | |  | | | |  | | | |  | | | |  | Ath-AT4G16144.1 |  |  |  |
| 5 | Vvi-Vitvi05g00086\_t001 |  | Ath-AT5G49340.1 |  | | | |  | | | |  | | | |  | | | |  |  |  |
| 5 | Vvi-Vitvi05g01767\_t001 |  | Ath-AT5G49350.1 |  | | | |  | | | |  | | | |  | | | |  |  |  |
| 5 | Vvi-Vitvi05g00087\_t001 |  | | | |  | | | |  | | | |  | Ath-AT3G16890.1 |  | | | |  |  |  |
| 5 | Vvi-Vitvi05g01768\_t001 |  | | | |  | | | |  | | | |  | | | |  | | | |  |  |  |
| 5 | Vvi-Vitvi05g01769\_t001 |  | | | |  | | | |  | | | |  | | | |  | | | |  |  |  |
| 5 | Vvi-Vitvi05g04019\_t001 |  | | | |  | | | |  | | | |  | | | |  | Ath-AT4G16146.1 |  |  |  |
| 5 | Vvi-Vitvi05g00089\_t001 |  | Ath-AT5G49360.1 |  | | | |  | | | |  | | | |  | | | |  |  |  |
| 5 | Vvi-Vitvi05g00092\_t001 |  | | | |  | | | |  | | | |  | Ath-AT3G16910.1 |  | | | |  |  |  |
| 5 | Vvi-Vitvi05g00094\_t001 |  | | | |  | | | |  | | | |  | Ath-AT3G16920.2 |  | | | |  |  |  |
| 5 | Vvi-Vitvi05g00096\_t001 |  | | | |  | | | |  | | | |  | Ath-AT3G16940.1 |  | | | |  |  |  |
| 5 | Vvi-Vitvi05g00097\_t001 |  | | | |  | | | |  | | | |  | | | |  | | | |  |  |  |
| 5 | Vvi-Vitvi05g00098\_t001 |  | | | |  | Ath-AT3G06680.1 |  | | | |  | | | |  | | | |  |  |  |
| 5 | Vvi-Vitvi05g00100\_t001 |  | | | |  | | | |  | | | |  | Ath-AT3G16950.2 |  | Ath-AT4G16155.1 |  |  |  |
| 5 | Vvi-Vitvi05g00101\_t002 |  | Ath-AT5G49390.1 |  | Ath-AT3G06670.2 |  | | | |  | | | |  | | | |  |  |  |
| 5 | Vvi-Vitvi05g00102\_t001 |  | | | |  | | | |  | | | |  | | | |  | Ath-AT4G16160.2 |  |  |  |
| 5 | Vvi-Vitvi05g00104\_t001 |  | Ath-AT5G49400.1 |  | | | |  | | | |  | | | |  | | | |  |  |  |
| 5 | Vvi-Vitvi05g00105\_t001 |  | | | |  | | | |  | | | |  | Ath-AT3G16980.1 |  | | | |  |  |  |
| 5 | Vvi-Vitvi05g00106\_t001 |  | Ath-AT5G49430.1 |  | | | |  | Ath-AT2G47410.5 |  | | | |  | | | |  |  |  |
| 5 | Vvi-Vitvi05g00107\_t001 |  | | | |  | | | |  | | | |  | | | |  | Ath-AT4G16260.1 |  |  |  |
| 4 | Vvi-Vitvi05g04020\_t001 |  | | | |  | | | |  | | | |  | | | |  |  |  |  |
| 4 | Vvi-Vitvi05g00108\_t001 |  | | | |  | | | |  | | | |  | | | |  |  |  |  |
| 4 | Vvi-Vitvi05g00109\_t001 |  | | | |  | | | |  | | | |  | Ath-AT3G16990.1 |  |  |  |  |
| 4 | Vvi-Vitvi05g00110\_t001 |  | | | |  | | | |  | | | |  | | | |  |  |  |  |
| 4 | Vvi-Vitvi05g04021\_t001 |  | | | |  | | | |  | | | |  | | | |  |  |  |  |
| 4 | Vvi-Vitvi05g04022\_t001 |  | | | |  | | | |  | | | |  | | | |  |  |  |  |
| 4 | Vvi-Vitvi05g04023\_t001 |  | | | |  | | | |  | | | |  | | | |  |  |  |  |
| 4 | Vvi-Vitvi05g00112\_t001 |  | | | |  | | | |  | | | |  | Ath-AT3G17000.1 |  |  |  |  |
| 4 | Vvi-Vitvi05g00113\_t001 |  | | | |  | | | |  | | | |  | | | |  |  |  |  |
| 4 | Vvi-Vitvi05g00114\_t001 |  | | | |  | Ath-AT3G06660.1 |  | Ath-AT2G47350.1 |  | | | |  |  |  |  |
| 4 | Vvi-Vitvi05g01772\_t001 |  | | | |  | Ath-AT3G36659.1 |  | Ath-AT2G47340.1 |  | | | |  |  |  |  |
| 4 | Vvi-Vitvi05g00115\_t001 |  | | | |  | | | |  | | | |  | Ath-AT3G17020.1 |  |  |  |  |
| 4 | Vvi-Vitvi05g00116\_t001 |  | | | |  | | | |  | | | |  | | | |  |  |  |  |
| 4 | Vvi-Vitvi05g00117\_t001 |  | | | |  | Ath-AT3G66658.2 |  | | | |  | | | |  |  |  |  |
| 4 | Vvi-Vitvi05g00118\_t001 |  | | | |  | | | |  | | | |  | Ath-AT3G17030.1 |  |  |  |  |
| 4 | Vvi-Vitvi05g00120\_t001 |  | | | |  | Ath-AT3G66654.3 |  | Ath-AT2G47320.1 |  | | | |  |  |  |  |
| 3 | Vvi-Vitvi05g00122\_t001 |  | | | |  | | | |  |  |  | | | |  |  |  |  |
| 3 | Vvi-Vitvi05g00123\_t001 |  | | | |  | | | |  |  |  | | | |  |  |  |  |
| 3 | Vvi-Vitvi05g00124\_t001 |  | | | |  | | | |  |  |  | Ath-AT3G17040.1 |  |  |  |  |
| 3 | Vvi-Vitvi05g00125\_t001 |  | | | |  | Ath-AT3G66652.2 |  |  |  | | | |  |  |  |  |
| 3 | Vvi-Vitvi05g01773\_t001 |  | | | |  | | | |  |  |  | | | |  |  |  |  |
| 3 | Vvi-Vitvi05g00126\_t001 |  | | | |  | | | |  |  |  | | | |  |  |  |  |
| 3 | Vvi-Vitvi05g00127\_t001 |  | | | |  | | | |  |  |  | | | |  |  |  |  |
| 3 | Vvi-Vitvi05g00128\_t001 |  | | | |  | | | |  |  |  | | | |  |  |  |  |
| 3 | Vvi-Vitvi05g00129\_t002 |  | Ath-AT5G49460.1 |  | Ath-AT3G06650.2 |  |  |  | | | |  |  |  |  |
| 3 | Vvi-Vitvi05g00130\_t001 |  | | | |  | | | |  |  |  | | | |  |  |  |  |
| 3 | Vvi-Vitvi05g00132\_t001 |  | Ath-AT5G49470.2 |  | Ath-AT3G06620.1 |  |  |  | | | |  |  |  |  |
| 3 | Vvi-Vitvi05g01774\_t001 |  | | | |  | Ath-AT3G06610.1 |  |  |  | | | |  |  |  |  |
| 3 | Vvi-Vitvi05g01775\_t001 |  | | | |  | | | |  |  |  | | | |  |  |  |  |
| 3 | Vvi-Vitvi05g00134\_t001 |  | | | |  | | | |  |  |  | Ath-AT3G17060.1 |  |  |  |  |
| 3 | Vvi-Vitvi05g00135\_t001 |  | | | |  | | | |  |  |  | Ath-AT3G17070.1 |  |  |  |  |
| 3 | Vvi-Vitvi05g04024\_t001 |  | | | |  | | | |  |  |  | | | |  |  |  |  |
| 3 | Vvi-Vitvi05g00136\_t001 |  | | | |  | | | |  |  |  | | | |  |  |  |  |
| 3 | Vvi-Vitvi05g00137\_t001 |  | | | |  | | | |  |  |  | | | |  |  |  |  |
| 3 | Vvi-Vitvi05g00138\_t001 |  | | | |  | | | |  |  |  | | | |  |  |  |  |
| 3 | Vvi-Vitvi05g00139\_t001 |  | | | |  | | | |  |  |  | | | |  |  |  |  |
| 3 | Vvi-Vitvi05g00141\_t001 |  | | | |  | | | |  |  |  | Ath-AT3G17090.1 |  |  |  |  |
| 3 | Vvi-Vitvi05g00142\_t001 |  | | | |  | | | |  |  |  | | | |  |  |  |  |
| 3 | Vvi-Vitvi05g01776\_t001 |  | Ath-AT5G49480.1 |  | | | |  |  |  | | | |  |  |  |  |
| 3 | Vvi-Vitvi05g01777\_t001 |  | | | |  | | | |  |  |  | | | |  |  |  |  |
| 3 | Vvi-Vitvi05g01778\_t001 |  | | | |  | | | |  |  |  | | | |  |  |  |  |
| 3 | Vvi-Vitvi05g01780\_t001 |  | | | |  | | | |  |  |  | | | |  |  |  |  |
| 3 | Vvi-Vitvi05g04025\_t001 |  | | | |  | | | |  |  |  | | | |  |  |  |  |
| 3 | Vvi-Vitvi05g01782\_t001 |  | | | |  | | | |  |  |  | | | |  |  |  |  |
| 3 | Vvi-Vitvi05g00143\_t001 |  | | | |  | Ath-AT3G06590.2 |  |  |  | Ath-AT3G17100.2 |  |  |  |  |
| 3 | Vvi-Vitvi05g01783\_t001 |  | | | |  | | | |  |  |  | | | |  |  |  |  |
| 3 | Vvi-Vitvi05g00144\_t001 |  | Ath-AT5G49510.1 |  | | | |  |  |  | | | |  |  |  |  |
| 3 | Vvi-Vitvi05g00145\_t001 |  | Ath-AT5G49520.1 |  | | | |  |  |  | | | |  |  |  |  |
| 3 | Vvi-Vitvi05g00146\_t001 |  | | | |  | | | |  |  |  | | | |  |  |  |  |
| 3 | Vvi-Vitvi05g00147\_t001 |  | | | |  | | | |  |  |  | | | |  |  |  |  |
| 3 | Vvi-Vitvi05g01784\_t001 |  | | | |  | | | |  |  |  | | | |  |  |  |  |
| 3 | Vvi-Vitvi05g00149\_t001 |  | | | |  | Ath-AT3G06580.1 |  |  |  | | | |  |  |  |  |
| 3 | Vvi-Vitvi05g00150\_t001 |  | | | |  | | | |  |  |  | Ath-AT3G17120.3 |  |  |  |  |
| 4 | Vvi-Vitvi05g01786\_t001 |  | | | |  | | | |  | Ath-AT1G47960.1 |  | Ath-AT3G17130.1 |  |  |  |  |
| 4 | Vvi-Vitvi05g01787\_t001 |  | | | |  | | | |  | | | |  | | | |  |  |  |  |
| 4 | Vvi-Vitvi05g01788\_t001 |  | | | |  | | | |  | | | |  | | | |  |  |  |  |
| 4 | Vvi-Vitvi05g01789\_t001 |  | | | |  | | | |  | | | |  | | | |  |  |  |  |
| 4 | Vvi-Vitvi05g04026\_t001 |  | | | |  | | | |  | | | |  | | | |  |  |  |  |
| 4 | Vvi-Vitvi05g00151\_t001 |  | | | |  | Ath-AT3G06560.1 |  | | | |  | | | |  |  |  |  |
| 4 | Vvi-Vitvi05g04027\_t001 |  | | | |  | | | |  | | | |  | | | |  |  |  |  |
| 4 | Vvi-Vitvi05g01790\_t003 |  | | | |  | | | |  | Ath-AT1G47970.1 |  | Ath-AT3G17160.1 |  |  |  |  |
| 4 | Vvi-Vitvi05g00153\_t001 |  | | | |  | Ath-AT3G06550.2 |  | | | |  | | | |  |  |  |  |
| 4 | Vvi-Vitvi05g04028\_t001 |  | Ath-AT5G49540.1 |  | | | |  | Ath-AT1G47980.1 |  | | | |  |  |  |  |
| 4 | Vvi-Vitvi05g00156\_t001 |  | | | |  | | | |  | | | |  | | | |  |  |  |  |
| 4 | Vvi-Vitvi05g00157\_t001 |  | | | |  | | | |  | | | |  | Ath-AT3G17170.1 |  |  |  |  |
| 4 | Vvi-Vitvi05g00158\_t002 |  | Ath-AT5G49580.1 |  | | | |  | | | |  | | | |  |  |  |  |
| 4 | Vvi-Vitvi05g04029\_t001 |  | | | |  | | | |  | | | |  | | | |  |  |  |  |
| 4 | Vvi-Vitvi05g00159\_t001 |  | Ath-AT5G49610.1 |  | | | |  | | | |  | | | |  |  |  |  |
| 4 | Vvi-Vitvi05g00160\_t001 |  | | | |  | | | |  | | | |  | Ath-AT3G17180.1 |  |  |  |  |
| 4 | Vvi-Vitvi05g00161\_t001 |  | | | |  | Ath-AT3G06510.2 |  | | | |  | | | |  |  |  |  |
| 4 | Vvi-Vitvi05g04030\_t001 |  | | | |  | | | |  | | | |  | | | |  |  |  |  |
| 4 | Vvi-Vitvi05g00163\_t001 |  | | | |  | | | |  | Ath-AT1G47990.1 |  | | | |  |  |  |  |
| 4 | Vvi-Vitvi05g00164\_t001 |  | | | |  | Ath-AT3G06500.1 |  | | | |  | | | |  |  |  |  |
| 4 | Vvi-Vitvi05g00166\_t001 |  | Ath-AT5G49620.2 |  | Ath-AT3G06490.1 |  | Ath-AT1G48000.1 |  | | | |  |  |  |  |
| 2 | Vvi-Vitvi05g00167\_t001 |  |  |  |  |  | | | |  | Ath-AT3G17205.1 |  |  |  |  |
| 2 | Vvi-Vitvi05g00168\_t001 |  |  |  |  |  | | | |  | | | |  |  |  |  |
| 2 | Vvi-Vitvi05g00169\_t001 |  |  |  |  |  | | | |  | | | |  |  |  |  |
| 2 | Vvi-Vitvi05g00170\_t001 |  |  |  |  |  | | | |  | | | |  |  |  |  |
| 2 | Vvi-Vitvi05g04031\_t001 |  |  |  |  |  | | | |  | Ath-AT3G17210.1 |  |  |  |  |
| 2 | Vvi-Vitvi05g04032\_t002 |  |  |  |  |  | | | |  | | | |  |  |  |  |
| 2 | Vvi-Vitvi05g04033\_t001 |  |  |  |  |  | | | |  | | | |  |  |  |  |
| 2 | Vvi-Vitvi05g04036\_t001 |  |  |  |  |  | | | |  | | | |  |  |  |  |
| 2 | Vvi-Vitvi05g04037\_t001 |  |  |  |  |  | | | |  | | | |  |  |  |  |
| 2 | Vvi-Vitvi05g04038\_t001 |  |  |  |  |  | | | |  | | | |  |  |  |  |
| 2 | Vvi-Vitvi05g01801\_t001 |  |  |  |  |  | | | |  | | | |  |  |  |  |
| 2 | Vvi-Vitvi05g00177\_t001 |  |  |  |  |  | | | |  | | | |  |  |  |  |
| 2 | Vvi-Vitvi05g01805\_t001 |  |  |  |  |  | | | |  | | | |  |  |  |  |
| 2 | Vvi-Vitvi05g04039\_t001 |  |  |  |  |  | | | |  | | | |  |  |  |  |
| 2 | Vvi-Vitvi05g01808\_t001 |  |  |  |  |  | | | |  | | | |  |  |  |  |
| 2 | Vvi-Vitvi05g00179\_t001 |  |  |  |  |  | | | |  | | | |  |  |  |  |
| 2 | Vvi-Vitvi05g04040\_t001 |  |  |  |  |  | | | |  | | | |  |  |  |  |
| 2 | Vvi-Vitvi05g04041\_t001 |  |  |  |  |  | | | |  | | | |  |  |  |  |
| 2 | Vvi-Vitvi05g00181\_t001 |  |  |  |  |  | Ath-AT1G48130.1 |  | | | |  |  |  |  |
| 2 | Vvi-Vitvi05g04042\_t001 |  |  |  |  |  | | | |  | | | |  |  |  |  |
| 2 | Vvi-Vitvi05g01811\_t001 |  |  |  |  |  | | | |  | | | |  |  |  |  |
| 2 | Vvi-Vitvi05g04043\_t001 |  |  |  |  |  | | | |  | | | |  |  |  |  |
| 2 | Vvi-Vitvi05g04044\_t001 |  |  |  |  |  | | | |  | Ath-AT3G17340.2 |  |  |  |  |
| 1 | Vvi-Vitvi05g04045\_t001 |  |  |  |  |  | | | |  |  |  |  |  |
| 1 | Vvi-Vitvi05g00231\_t001 |  |  |  |  |  | Ath-AT1G48160.1 |  |  |  |  |  |
| 1 | Vvi-Vitvi05g00229\_t001 |  |  |  |  |  | | | |  |  |  |  |  |
| 1 | Vvi-Vitvi05g00186\_t002 |  |  |  |  |  | Ath-AT1G48170.1 |  |  |  |  |  |
| 0 | Vvi-Vitvi05g00227\_t001 |  |  |  |  |  |  |  |  |
| 0 | Vvi-Vitvi05g00225\_t001 |  |  |  |  |  |  |  |  |
| 0 | Vvi-Vitvi05g00223\_t001 |  |  |  |  |  |  |  |  |
| 0 | Vvi-Vitvi05g04046\_t001 |  |  |  |  |  |  |  |  |
| 0 | Vvi-Vitvi05g04047\_t001 |  |  |  |  |  |  |  |  |
| 0 | Vvi-Vitvi05g00218\_t001 |  |  |  |  |  |  |  |  |
| 0 | Vvi-Vitvi05g00217\_t004 |  |  |  |  |  |  |  |  |
| 1 | Vvi-Vitvi05g01830\_t001 |  | Ath-AT3G17330.3 |  |  |  |  |  |  |  |
| 1 | Vvi-Vitvi05g00215\_t001 |  | Ath-AT3G17310.2 |  |  |  |  |  |  |  |
| 1 | Vvi-Vitvi05g00214\_t001 |  | | | |  |  |  |  |  |  |  |
| 1 | Vvi-Vitvi05g00213\_t001 |  | Ath-AT3G17300.1 |  |  |  |  |  |  |  |
| 1 | Vvi-Vitvi05g00212\_t001 |  | | | |  |  |  |  |  |  |  |
| 1 | Vvi-Vitvi05g00210\_t001 |  | | | |  |  |  |  |  |  |  |
| 1 | Vvi-Vitvi05g04048\_t001 |  | | | |  |  |  |  |  |  |  |
| 1 | Vvi-Vitvi05g00209\_t001 |  | | | |  |  |  |  |  |  |  |
| 1 | Vvi-Vitvi05g00208\_t001 |  | Ath-AT3G17250.1 |  |  |  |  |  |  |  |
| 1 | Vvi-Vitvi05g00207\_t001 |  | Ath-AT3G17240.1 |  |  |  |  |  |  |  |
| 1 | Vvi-Vitvi05g00206\_t001 |  | | | |  |  |  |  |  |  |  |
| 1 | Vvi-Vitvi05g00205\_t001 |  | Ath-AT3G17220.1 |  |  |  |  |  |  |  |
| 1 | Vvi-Vitvi05g04049\_t001 |  | | | |  |  |  |  |  |  |  |
| 1 | Vvi-Vitvi05g01828\_t001 |  | Ath-AT3G17210.1 |  |  |  |  |  |  |  |
| 0 | Vvi-Vitvi05g00204\_t001 |  |  |  |  |  |  |  |  |
| 0 | Vvi-Vitvi05g04050\_t001 |  |  |  |  |  |  |  |  |
| 0 | Vvi-Vitvi05g04051\_t001 |  |  |  |  |  |  |  |  |
| 0 | Vvi-Vitvi05g04052\_t001 |  |  |  |  |  |  |  |  |
| 0 | Vvi-Vitvi05g04053\_t001 |  |  |  |  |  |  |  |  |
| 0 | Vvi-Vitvi05g04054\_t001 |  |  |  |  |  |  |  |  |
| 0 | Vvi-Vitvi05g00198\_t001 |  |  |  |  |  |  |  |  |
| 0 | Vvi-Vitvi05g04055\_t001 |  |  |  |  |  |  |  |  |
| 0 | Vvi-Vitvi05g00197\_t001 |  |  |  |  |  |  |  |  |
| 0 | Vvi-Vitvi05g04056\_t001 |  |  |  |  |  |  |  |  |
| 0 | Vvi-Vitvi05g04057\_t001 |  |  |  |  |  |  |  |  |
| 0 | Vvi-Vitvi05g04058\_t001 |  |  |  |  |  |  |  |  |
| 0 | Vvi-Vitvi05g04059\_t001 |  |  |  |  |  |  |  |  |
| 0 | Vvi-Vitvi05g00196\_t001 |  |  |  |  |  |  |  |  |
| 0 | Vvi-Vitvi05g04060\_t001 |  |  |  |  |  |  |  |  |
| 0 | Vvi-Vitvi05g04061\_t001 |  |  |  |  |  |  |  |  |
| 0 | Vvi-Vitvi05g00195\_t001 |  |  |  |  |  |  |  |  |
| 0 | Vvi-Vitvi05g04062\_t001 |  |  |  |  |  |  |  |  |
| 0 | Vvi-Vitvi05g04063\_t001 |  |  |  |  |  |  |  |  |
| 0 | Vvi-Vitvi05g04064\_t001 |  |  |  |  |  |  |  |  |
| 0 | Vvi-Vitvi05g04065\_t001 |  |  |  |  |  |  |  |  |
| 0 | Vvi-Vitvi05g04066\_t001 |  |  |  |  |  |  |  |  |
| 0 | Vvi-Vitvi05g00191\_t001 |  |  |  |  |  |  |  |  |
| 0 | Vvi-Vitvi05g00190\_t001 |  |  |  |  |  |  |  |  |
| 0 | Vvi-Vitvi05g04067\_t001 |  |  |  |  |  |  |  |  |
| 0 | Vvi-Vitvi05g04068\_t001 |  |  |  |  |  |  |  |  |
| 0 | Vvi-Vitvi05g01819\_t001 |  |  |  |  |  |  |  |  |
| 0 | Vvi-Vitvi05g01815\_t001 |  |  |  |  |  |  |  |  |
| 0 | Vvi-Vitvi05g04069\_t001 |  |  |  |  |  |  |  |  |
| 0 | Vvi-Vitvi05g04070\_t001 |  |  |  |  |  |  |  |  |
| 0 | Vvi-Vitvi05g00183\_t001 |  |  |  |  |  |  |  |  |
| 0 | Vvi-Vitvi05g04071\_t001 |  |  |  |  |  |  |  |  |
| 1 | Vvi-Vitvi05g04072\_t001 |  | Ath-AT3G17340.2 |  |  |  |  |  |  |  |
| 1 | Vvi-Vitvi05g04073\_t001 |  | | | |  |  |  |  |  |  |  |
| 2 | Vvi-Vitvi05g00235\_t001 |  | | | |  | Ath-AT1G48130.1 |  |  |  |  |  |  |
| 2 | Vvi-Vitvi05g00236\_t001 |  | Ath-AT3G17350.1 |  | | | |  |  |  |  |  |  |
| 2 | Vvi-Vitvi05g00237\_t001 |  | Ath-AT3G17360.3 |  | | | |  |  |  |  |  |  |
| 2 | Vvi-Vitvi05g00239\_t003 |  | | | |  | | | |  |  |  |  |  |  |
| 2 | Vvi-Vitvi05g00241\_t001 |  | Ath-AT3G17365.2 |  | | | |  |  |  |  |  |  |
| 2 | Vvi-Vitvi05g00242\_t001 |  | Ath-AT3G17390.1 |  | | | |  |  |  |  |  |  |
| 2 | Vvi-Vitvi05g00243\_t002 |  | | | |  | | | |  |  |  |  |  |  |
| 2 | Vvi-Vitvi05g00244\_t001 |  | | | |  | | | |  |  |  |  |  |  |
| 2 | Vvi-Vitvi05g00245\_t001 |  | | | |  | | | |  |  |  |  |  |  |
| 2 | Vvi-Vitvi05g00246\_t003 |  | Ath-AT3G17410.2 |  | Ath-AT1G48210.1 |  |  |  |  |  |  |
| 2 | Vvi-Vitvi05g00247\_t001 |  | | | |  | | | |  |  |  |  |  |  |
| 2 | Vvi-Vitvi05g00248\_t001 |  | Ath-AT3G17430.1 |  | Ath-AT1G48230.1 |  |  |  |  |  |  |
| 2 | Vvi-Vitvi05g00249\_t001 |  | Ath-AT3G17440.1 |  | Ath-AT1G48240.1 |  |  |  |  |  |  |
| 2 | Vvi-Vitvi05g04074\_t001 |  | | | |  | | | |  |  |  |  |  |  |
| 2 | Vvi-Vitvi05g00250\_t001 |  | | | |  | | | |  |  |  |  |  |  |
| 2 | Vvi-Vitvi05g04075\_t001 |  | | | |  | | | |  |  |  |  |  |  |
| 2 | Vvi-Vitvi05g00252\_t001 |  | Ath-AT3G17450.1 |  | | | |  |  |  |  |  |  |
| 2 | Vvi-Vitvi05g00253\_t001 |  | | | |  | | | |  |  |  |  |  |  |
| 2 | Vvi-Vitvi05g01832\_t001 |  | Ath-AT3G17460.1 |  | | | |  |  |  |  |  |  |
| 2 | Vvi-Vitvi05g00254\_t001 |  | Ath-AT3G17465.1 |  | | | |  |  |  |  |  |  |
| 2 | Vvi-Vitvi05g00255\_t001 |  | Ath-AT3G17470.2 |  | | | |  |  |  |  |  |  |
| 2 | Vvi-Vitvi05g00256\_t001 |  | Ath-AT3G17510.1 |  | Ath-AT1G48260.1 |  |  |  |  |  |  |
| 2 | Vvi-Vitvi05g01833\_t001 |  | | | |  | | | |  |  |  |  |  |  |
| 2 | Vvi-Vitvi05g00258\_t001 |  | | | |  | | | |  |  |  |  |  |  |
| 2 | Vvi-Vitvi05g00259\_t001 |  | | | |  | | | |  |  |  |  |  |  |
| 2 | Vvi-Vitvi05g00260\_t001 |  | | | |  | | | |  |  |  |  |  |  |
| 2 | Vvi-Vitvi05g00261\_t001 |  | | | |  | | | |  |  |  |  |  |  |
| 2 | Vvi-Vitvi05g00262\_t001 |  | | | |  | | | |  |  |  |  |  |  |
| 2 | Vvi-Vitvi05g01834\_t001 |  | | | |  | | | |  |  |  |  |  |  |
| 2 | Vvi-Vitvi05g00264\_t001 |  | | | |  | Ath-AT1G48280.1 |  |  |  |  |  |  |
| 2 | Vvi-Vitvi05g01835\_t001 |  | | | |  | Ath-AT1G48300.1 |  |  |  |  |  |  |
| 2 | Vvi-Vitvi05g00265\_t001 |  | | | |  | Ath-AT1G48310.2 |  |  |  |  |  |  |
| 2 | Vvi-Vitvi05g00266\_t001 |  | | | |  | | | |  |  |  |  |  |  |
| 2 | Vvi-Vitvi05g01836\_t001 |  | | | |  | | | |  |  |  |  |  |  |
| 2 | Vvi-Vitvi05g00267\_t001 |  | | | |  | Ath-AT1G48320.1 |  |  |  |  |  |  |
| 2 | Vvi-Vitvi05g01837\_t001 |  | | | |  | | | |  |  |  |  |  |  |
| 2 | Vvi-Vitvi05g04076\_t001 |  | | | |  | | | |  |  |  |  |  |  |
| 2 | Vvi-Vitvi05g04077\_t001 |  | | | |  | | | |  |  |  |  |  |  |
| 2 | Vvi-Vitvi05g04078\_t001 |  | | | |  | | | |  |  |  |  |  |  |
| 2 | Vvi-Vitvi05g04079\_t001 |  | | | |  | | | |  |  |  |  |  |  |
| 2 | Vvi-Vitvi05g01842\_t001 |  | | | |  | | | |  |  |  |  |  |  |
| 2 | Vvi-Vitvi05g01843\_t001 |  | Ath-AT3G17580.1 |  | Ath-AT1G48330.1 |  |  |  |  |  |  |
| 2 | Vvi-Vitvi05g01726\_t001 |  | | | |  | | | |  |  |  |  |  |  |
| 2 | Vvi-Vitvi05g04080\_t001 |  | | | |  | | | |  |  |  |  |  |  |
| 2 | Vvi-Vitvi05g04081\_t001 |  | | | |  | | | |  |  |  |  |  |  |
| 2 | Vvi-Vitvi05g01728\_t001 |  | | | |  | | | |  |  |  |  |  |  |
| 2 | Vvi-Vitvi05g01729\_t001 |  | | | |  | | | |  |  |  |  |  |  |
| 2 | Vvi-Vitvi05g01727\_t001 |  | | | |  | | | |  |  |  |  |  |  |
| 2 | Vvi-Vitvi05g00270\_t001 |  | Ath-AT3G17590.2 |  | | | |  |  |  |  |  |  |
| 2 | Vvi-Vitvi05g00271\_t001 |  | Ath-AT3G17600.1 |  | | | |  |  |  |  |  |  |
| 2 | Vvi-Vitvi05g00272\_t001 |  | | | |  | | | |  |  |  |  |  |  |
| 2 | Vvi-Vitvi05g00273\_t001 |  | | | |  | | | |  |  |  |  |  |  |
| 2 | Vvi-Vitvi05g00274\_t001 |  | Ath-AT3G17609.2 |  | | | |  |  |  |  |  |  |
| 2 | Vvi-Vitvi05g00275\_t001 |  | | | |  | | | |  |  |  |  |  |  |
| 2 | Vvi-Vitvi05g00276\_t001 |  | | | |  | | | |  |  |  |  |  |  |
| 2 | Vvi-Vitvi05g00277\_t001 |  | Ath-AT3G17611.1 |  | | | |  |  |  |  |  |  |
| 2 | Vvi-Vitvi05g00278\_t001 |  | | | |  | | | |  |  |  |  |  |  |
| 2 | Vvi-Vitvi05g00279\_t001 |  | Ath-AT3G17626.1 |  | Ath-AT1G48350.1 |  |  |  |  |  |  |
| 1 | Vvi-Vitvi05g00280\_t001 |  | Ath-AT3G17630.1 |  |  |  |  |  |  |  |
| 1 | Vvi-Vitvi05g00281\_t001 |  | Ath-AT4G15890.1 |  |  |  |  |  |  |  |
| 1 | Vvi-Vitvi05g00282\_t001 |  | | | |  |  |  |  |  |  |  |
| 1 | Vvi-Vitvi05g00284\_t001 |  | | | |  |  |  |  |  |  |  |
| 1 | Vvi-Vitvi05g00285\_t001 |  | | | |  |  |  |  |  |  |  |
| 1 | Vvi-Vitvi05g00286\_t001 |  | | | |  |  |  |  |  |  |  |
| 1 | Vvi-Vitvi05g00287\_t001.1.6037826e |  | Ath-AT4G15885.1 |  |  |  |  |  |  |  |
| 1 | Vvi-Vitvi05g00288\_t001 |  | Ath-AT4G15880.1 |  |  |  |  |  |  |  |
| 1 | Vvi-Vitvi05g00289\_t001 |  | | | |  |  |  |  |  |  |  |
| 1 | Vvi-Vitvi05g00290\_t001 |  | Ath-AT4G15840.1 |  |  |  |  |  |  |  |
| 1 | Vvi-Vitvi05g00291\_t001 |  | | | |  |  |  |  |  |  |  |
| 1 | Vvi-Vitvi05g00292\_t001 |  | Ath-AT4G15830.1 |  |  |  |  |  |  |  |
| 1 | Vvi-Vitvi05g00293\_t001 |  | Ath-AT4G15820.1 |  |  |  |  |  |  |  |
| 1 | Vvi-Vitvi05g00294\_t001 |  | Ath-AT4G15810.1 |  |  |  |  |  |  |  |
| 1 | Vvi-Vitvi05g00295\_t001 |  | | | |  |  |  |  |  |  |  |
| 1 | Vvi-Vitvi05g00296\_t001 |  | | | |  |  |  |  |  |  |  |
| 1 | Vvi-Vitvi05g04082\_t001 |  | | | |  |  |  |  |  |  |  |
| 1 | Vvi-Vitvi05g00299\_t001 |  | | | |  |  |  |  |  |  |  |
| 1 | Vvi-Vitvi05g00300\_t001 |  | | | |  |  |  |  |  |  |  |
| 1 | Vvi-Vitvi05g00301\_t001 |  | | | |  |  |  |  |  |  |  |
| 1 | Vvi-Vitvi05g00302\_t001 |  | | | |  |  |  |  |  |  |  |
| 1 | Vvi-Vitvi05g00303\_t001 |  | | | |  |  |  |  |  |  |  |
| 1 | Vvi-Vitvi05g00305\_t001 |  | | | |  |  |  |  |  |  |  |
| 1 | Vvi-Vitvi05g04083\_t001 |  | | | |  |  |  |  |  |  |  |
| 1 | Vvi-Vitvi05g00309\_t001 |  | | | |  |  |  |  |  |  |  |
| 1 | Vvi-Vitvi05g01845\_t001 |  | | | |  |  |  |  |  |  |  |
| 1 | Vvi-Vitvi05g00310\_t001 |  | | | |  |  |  |  |  |  |  |
| 1 | Vvi-Vitvi05g00311\_t001 |  | | | |  |  |  |  |  |  |  |
| 1 | Vvi-Vitvi05g00312\_t001 |  | Ath-AT4G15790.2 |  |  |  |  |  |  |  |
| 1 | Vvi-Vitvi05g00313\_t001 |  | | | |  |  |  |  |  |  |  |
| 1 | Vvi-Vitvi05g00314\_t001 |  | | | |  |  |  |  |  |  |  |
| 1 | Vvi-Vitvi05g00315\_t001 |  | | | |  |  |  |  |  |  |  |
| 1 | Vvi-Vitvi05g04084\_t001 |  | | | |  |  |  |  |  |  |  |
| 1 | Vvi-Vitvi05g00319\_t001 |  | | | |  |  |  |  |  |  |  |
| 1 | Vvi-Vitvi05g00320\_t001 |  | | | |  |  |  |  |  |  |  |
| 1 | Vvi-Vitvi05g00321\_t001 |  | | | |  |  |  |  |  |  |  |
| 1 | Vvi-Vitvi05g00322\_t001 |  | | | |  |  |  |  |  |  |  |
| 1 | Vvi-Vitvi05g00323\_t001 |  | | | |  |  |  |  |  |  |  |
| 1 | Vvi-Vitvi05g00325\_t001 |  | | | |  |  |  |  |  |  |  |
| 1 | Vvi-Vitvi05g04085\_t001 |  | | | |  |  |  |  |  |  |  |
| 1 | Vvi-Vitvi05g00326\_t001 |  | Ath-AT4G15730.1 |  |  |  |  |  |  |  |
| 1 | Vvi-Vitvi05g04086\_t001 |  | | | |  |  |  |  |  |  |  |
| 1 | Vvi-Vitvi05g00328\_t001 |  | | | |  |  |  |  |  |  |  |
| 1 | Vvi-Vitvi05g01846\_t001 |  | | | |  |  |  |  |  |  |  |
| 2 | Vvi-Vitvi05g00329\_t001 |  | | | |  | Ath-AT5G18540.1 |  |  |  |  |  |  |
| 2 | Vvi-Vitvi05g04087\_t001 |  | | | |  | | | |  |  |  |  |  |  |
| 2 | Vvi-Vitvi05g00330\_t001 |  | | | |  | | | |  |  |  |  |  |  |
| 2 | Vvi-Vitvi05g01847\_t001 |  | | | |  | | | |  |  |  |  |  |  |
| 2 | Vvi-Vitvi05g04088\_t001 |  | | | |  | | | |  |  |  |  |  |  |
| 2 | Vvi-Vitvi05g01849\_t001 |  | | | |  | | | |  |  |  |  |  |  |
| 2 | Vvi-Vitvi05g01850\_t001 |  | | | |  | | | |  |  |  |  |  |  |
| 2 | Vvi-Vitvi05g01851\_t001 |  | | | |  | | | |  |  |  |  |  |  |
| 2 | Vvi-Vitvi05g01852\_t001 |  | | | |  | | | |  |  |  |  |  |  |
| 3 | Vvi-Vitvi05g00333\_t001 |  | | | |  | Ath-AT5G18550.1 |  | Ath-AT3G06410.1 |  |  |  |  |  |
| 3 | Vvi-Vitvi05g00334\_t001 |  | | | |  | Ath-AT5G18560.1 |  | | | |  |  |  |  |  |
| 3 | Vvi-Vitvi05g00335\_t001 |  | | | |  | Ath-AT5G18570.1 |  | | | |  |  |  |  |  |
| 3 | Vvi-Vitvi05g00337\_t001 |  | | | |  | | | |  | | | |  |  |  |  |  |
| 3 | Vvi-Vitvi05g00338\_t001 |  | | | |  | Ath-AT5G18580.1 |  | | | |  |  |  |  |  |
| 3 | Vvi-Vitvi05g04089\_t001 |  | | | |  | | | |  | | | |  |  |  |  |  |
| 3 | Vvi-Vitvi05g00339\_t001 |  | | | |  | | | |  | | | |  |  |  |  |  |
| 3 | Vvi-Vitvi05g00340\_t001 |  | | | |  | | | |  | | | |  |  |  |  |  |
| 3 | Vvi-Vitvi05g00341\_t001 |  | | | |  | Ath-AT5G18590.2 |  | | | |  |  |  |  |  |
| 3 | Vvi-Vitvi05g00342\_t001 |  | Ath-AT4G15670.1 |  | Ath-AT5G18600.1 |  | | | |  |  |  |  |  |
| 3 | Vvi-Vitvi05g00343\_t001 |  | | | |  | | | |  | | | |  |  |  |  |  |
| 3 | Vvi-Vitvi05g00344\_t001 |  | | | |  | Ath-AT5G18610.2 |  | | | |  |  |  |  |  |
| 3 | Vvi-Vitvi05g00345\_t001 |  | | | |  | Ath-AT5G18620.2 |  | Ath-AT3G06400.3 |  |  |  |  |  |
| 3 | Vvi-Vitvi05g00346\_t001 |  | | | |  | Ath-AT5G18630.1 |  | | | |  |  |  |  |  |
| 3 | Vvi-Vitvi05g01856\_t001 |  | | | |  | | | |  | | | |  |  |  |  |  |
| 3 | Vvi-Vitvi05g00347\_t004 |  | | | |  | Ath-AT5G18650.1 |  | | | |  |  |  |  |  |
| 3 | Vvi-Vitvi05g00348\_t001 |  | Ath-AT4G15640.1 |  | | | |  | | | |  |  |  |  |  |
| 3 | Vvi-Vitvi05g01857\_t001 |  | | | |  | | | |  | | | |  |  |  |  |  |
| 3 | Vvi-Vitvi05g00349\_t001 |  | Ath-AT4G15610.1 |  | | | |  | Ath-AT3G06390.1 |  |  |  |  |  |
| 3 | Vvi-Vitvi05g01858\_t001 |  | | | |  | | | |  | | | |  |  |  |  |  |
| 3 | Vvi-Vitvi05g00350\_t001 |  | | | |  | Ath-AT5G18660.1 |  | | | |  |  |  |  |  |
| 3 | Vvi-Vitvi05g00351\_t001 |  | | | |  | | | |  | | | |  |  |  |  |  |
| 3 | Vvi-Vitvi05g00352\_t001 |  | Ath-AT4G15563.7 |  | | | |  | | | |  |  |  |  |  |
| 2 | Vvi-Vitvi05g00353\_t001 |  |  |  | | | |  | | | |  |  |  |  |  |
| 2 | Vvi-Vitvi05g04090\_t001 |  |  |  | | | |  | | | |  |  |  |  |  |
| 2 | Vvi-Vitvi05g04091\_t001 |  |  |  | | | |  | | | |  |  |  |  |  |
| 2 | Vvi-Vitvi05g00354\_t001 |  |  |  | | | |  | | | |  |  |  |  |  |
| 2 | Vvi-Vitvi05g00355\_t001 |  |  |  | | | |  | | | |  |  |  |  |  |
| 2 | Vvi-Vitvi05g01860\_t001 |  |  |  | | | |  | | | |  |  |  |  |  |
| 2 | Vvi-Vitvi05g00357\_t001 |  |  |  | Ath-AT5G18670.1 |  | | | |  |  |  |  |  |
| 2 | Vvi-Vitvi05g01861\_t001 |  |  |  | | | |  | | | |  |  |  |  |  |
| 2 | Vvi-Vitvi05g01862\_t002 |  |  |  | Ath-AT5G18680.1 |  | Ath-AT3G06380.1 |  |  |  |  |  |
| 2 | Vvi-Vitvi05g00358\_t001 |  |  |  | | | |  | | | |  |  |  |  |  |
| 2 | Vvi-Vitvi05g00359\_t001 |  |  |  | | | |  | Ath-AT3G06370.3 |  |  |  |  |  |
| 2 | Vvi-Vitvi05g00360\_t003 |  |  |  | | | |  | | | |  |  |  |  |  |
| 2 | Vvi-Vitvi05g00362\_t001 |  |  |  | | | |  | | | |  |  |  |  |  |
| 2 | Vvi-Vitvi05g04092\_t001 |  |  |  | | | |  | | | |  |  |  |  |  |
| 2 | Vvi-Vitvi05g00363\_t001 |  |  |  | Ath-AT5G18700.1 |  | | | |  |  |  |  |  |
| 2 | Vvi-Vitvi05g01864\_t003 |  |  |  | | | |  | | | |  |  |  |  |  |
| 2 | Vvi-Vitvi05g00364\_t001 |  |  |  | | | |  | Ath-AT3G06350.1 |  |  |  |  |  |
| 2 | Vvi-Vitvi05g00365\_t001 |  |  |  | | | |  | | | |  |  |  |  |  |
| 2 | Vvi-Vitvi05g04093\_t001 |  |  |  | | | |  | | | |  |  |  |  |  |
| 2 | Vvi-Vitvi05g00366\_t001 |  |  |  | Ath-AT5G18750.1 |  | Ath-AT3G06340.3 |  |  |  |  |  |
| 2 | Vvi-Vitvi05g01865\_t001 |  |  |  | | | |  | | | |  |  |  |  |  |
| 3 | Vvi-Vitvi05g00367\_t001 |  | Ath-AT3G21480.1 |  | | | |  | | | |  |  |  |  |  |
| 3 | Vvi-Vitvi05g00368\_t002 |  | | | |  | Ath-AT5G18760.1 |  | Ath-AT3G06330.1 |  |  |  |  |  |
| 3 | Vvi-Vitvi05g00369\_t001 |  | | | |  | | | |  | | | |  |  |  |  |  |
| 3 | Vvi-Vitvi05g04094\_t001 |  | | | |  | | | |  | | | |  |  |  |  |  |
| 3 | Vvi-Vitvi05g00370\_t001 |  | | | |  | | | |  | | | |  |  |  |  |  |
| 3 | Vvi-Vitvi05g00371\_t001 |  | | | |  | | | |  | | | |  |  |  |  |  |
| 3 | Vvi-Vitvi05g01867\_t001 |  | Ath-AT3G21490.1 |  | | | |  | | | |  |  |  |  |  |
| 3 | Vvi-Vitvi05g00372\_t001 |  | Ath-AT3G21500.3 |  | | | |  | | | |  |  |  |  |  |
| 3 | Vvi-Vitvi05g04095\_t001 |  | | | |  | Ath-AT5G18790.1 |  | Ath-AT3G06320.1 |  |  |  |  |  |
| 3 | Vvi-Vitvi05g00374\_t002 |  | | | |  | Ath-AT5G18800.2 |  | Ath-AT3G06310.3 |  |  |  |  |  |
| 3 | Vvi-Vitvi05g00376\_t001 |  | | | |  | Ath-AT5G18830.3 |  | | | |  |  |  |  |  |
| 3 | Vvi-Vitvi05g00377\_t001 |  | | | |  | Ath-AT5G18840.1 |  | | | |  |  |  |  |  |
| 3 | Vvi-Vitvi05g04096\_t001 |  | | | |  | | | |  | | | |  |  |  |  |  |
| 3 | Vvi-Vitvi05g00380\_t001 |  | | | |  | Ath-AT5G18850.1 |  | | | |  |  |  |  |  |
| 3 | Vvi-Vitvi05g00381\_t003 |  | | | |  | Ath-AT5G18860.1 |  | | | |  |  |  |  |  |
| 4 | Vvi-Vitvi05g00382\_t001 |  | Ath-AT3G21510.1 |  | | | |  | | | |  | Ath-AT1G03430.1 |  |  |  |  |
| 4 | Vvi-Vitvi05g00383\_t001 |  | | | |  | Ath-AT5G18900.1 |  | Ath-AT3G06300.1 |  | | | |  |  |  |  |
| 4 | Vvi-Vitvi05g00384\_t001 |  | | | |  | Ath-AT5G18910.1 |  | | | |  | | | |  |  |  |  |
| 4 | Vvi-Vitvi05g01868\_t001 |  | | | |  | | | |  | | | |  | | | |  |  |  |  |
| 4 | Vvi-Vitvi05g01869\_t001 |  | | | |  | | | |  | | | |  | | | |  |  |  |  |
| 4 | Vvi-Vitvi05g01870\_t001 |  | | | |  | | | |  | | | |  | | | |  |  |  |  |
| 4 | Vvi-Vitvi05g01871\_t001 |  | | | |  | | | |  | | | |  | | | |  |  |  |  |
| 4 | Vvi-Vitvi05g00385\_t001 |  | Ath-AT3G21520.1 |  | | | |  | | | |  | | | |  |  |  |  |
| 4 | Vvi-Vitvi05g00386\_t001 |  | Ath-AT3G21530.1 |  | | | |  | | | |  | | | |  |  |  |  |
| 4 | Vvi-Vitvi05g01872\_t001 |  | | | |  | | | |  | | | |  | | | |  |  |  |  |
| 4 | Vvi-Vitvi05g00387\_t001 |  | | | |  | | | |  | | | |  | | | |  |  |  |  |
| 4 | Vvi-Vitvi05g00388\_t002 |  | | | |  | | | |  | | | |  | Ath-AT1G03365.1 |  |  |  |  |
| 4 | Vvi-Vitvi05g00389\_t001 |  | | | |  | Ath-AT5G18920.2 |  | | | |  | | | |  |  |  |  |
| 4 | Vvi-Vitvi05g00390\_t001 |  | | | |  | | | |  | | | |  | | | |  |  |  |  |
| 4 | Vvi-Vitvi05g00391\_t001 |  | | | |  | | | |  | Ath-AT3G06290.1 |  | | | |  |  |  |  |
| 4 | Vvi-Vitvi05g04097\_t001 |  | | | |  | | | |  | | | |  | | | |  |  |  |  |
| 4 | Vvi-Vitvi05g00392\_t001 |  | | | |  | | | |  | Ath-AT3G06270.1 |  | | | |  |  |  |  |
| 4 | Vvi-Vitvi05g04098\_t001 |  | | | |  | | | |  | | | |  | | | |  |  |  |  |
| 4 | Vvi-Vitvi05g00393\_t001 |  | | | |  | Ath-AT5G18930.1 |  | | | |  | | | |  |  |  |  |
| 4 | Vvi-Vitvi05g00394\_t001 |  | | | |  | | | |  | Ath-AT3G06260.1 |  | | | |  |  |  |  |
| 4 | Vvi-Vitvi05g00395\_t001 |  | | | |  | | | |  | | | |  | | | |  |  |  |  |
| 4 | Vvi-Vitvi05g00396\_t001 |  | | | |  | | | |  | | | |  | | | |  |  |  |  |
| 4 | Vvi-Vitvi05g00397\_t001 |  | | | |  | Ath-AT5G18940.1 |  | | | |  | | | |  |  |  |  |
| 4 | Vvi-Vitvi05g00398\_t001 |  | | | |  | | | |  | | | |  | Ath-AT1G03350.1 |  |  |  |  |
| 4 | Vvi-Vitvi05g00399\_t001 |  | | | |  | | | |  | | | |  | | | |  |  |  |  |
| 4 | Vvi-Vitvi05g04099\_t001 |  | | | |  | | | |  | | | |  | | | |  |  |  |  |
| 4 | Vvi-Vitvi05g00400\_t001 |  | | | |  | Ath-AT5G18950.1 |  | | | |  | | | |  |  |  |  |
| 4 | Vvi-Vitvi05g01873\_t001 |  | | | |  | Ath-AT5G18960.2 |  | Ath-AT3G06250.2 |  | | | |  |  |  |  |
| 4 | Vvi-Vitvi05g00401\_t001 |  | Ath-AT3G21580.1 |  | | | |  | | | |  | | | |  |  |  |  |
| 3 | Vvi-Vitvi05g00402\_t001 |  |  |  | Ath-AT5G18970.1 |  | | | |  | | | |  |  |  |  |
| 3 | Vvi-Vitvi05g00403\_t001 |  |  |  | | | |  | | | |  | | | |  |  |  |  |
| 3 | Vvi-Vitvi05g00404\_t003 |  |  |  | | | |  | | | |  | | | |  |  |  |  |
| 3 | Vvi-Vitvi05g00406\_t001 |  |  |  | Ath-AT5G18980.1 |  | Ath-AT3G06210.1 |  | | | |  |  |  |  |
| 3 | Vvi-Vitvi05g00407\_t001 |  |  |  | | | |  | Ath-AT3G06200.1 |  | | | |  |  |  |  |
| 3 | Vvi-Vitvi05g00408\_t001 |  |  |  | Ath-AT5G19000.2 |  | Ath-AT3G06190.1 |  | | | |  |  |  |  |
| 3 | Vvi-Vitvi05g00409\_t001 |  |  |  | Ath-AT5G19010.1 |  | | | |  | | | |  |  |  |  |
| 3 | Vvi-Vitvi05g00410\_t001 |  |  |  | | | |  | | | |  | | | |  |  |  |  |
| 3 | Vvi-Vitvi05g00411\_t001 |  |  |  | | | |  | | | |  | | | |  |  |  |  |
| 3 | Vvi-Vitvi05g00412\_t001 |  |  |  | | | |  | | | |  | | | |  |  |  |  |
| 3 | Vvi-Vitvi05g00413\_t001 |  |  |  | | | |  | | | |  | | | |  |  |  |  |
| 3 | Vvi-Vitvi05g00416\_t001 |  |  |  | Ath-AT5G19020.1 |  | | | |  | | | |  |  |  |  |
| 3 | Vvi-Vitvi05g00418\_t001 |  |  |  | Ath-AT5G19025.2 |  | Ath-AT3G06180.1 |  | | | |  |  |  |  |
| 3 | Vvi-Vitvi05g00419\_t001 |  |  |  | | | |  | Ath-AT3G06170.1 |  | | | |  |  |  |  |
| 3 | Vvi-Vitvi05g00420\_t001 |  |  |  | | | |  | | | |  | Ath-AT1G03270.1 |  |  |  |  |
| 3 | Vvi-Vitvi05g01874\_t001 |  |  |  | Ath-AT5G19030.1 |  | | | |  | | | |  |  |  |  |
| 3 | Vvi-Vitvi05g00421\_t001 |  |  |  | Ath-AT5G19040.1 |  | | | |  | | | |  |  |  |  |
| 3 | Vvi-Vitvi05g04100\_t001 |  |  |  | | | |  | | | |  | | | |  |  |  |  |
| 3 | Vvi-Vitvi05g00422\_t001 |  |  |  | Ath-AT5G19050.1 |  | | | |  | | | |  |  |  |  |
| 3 | Vvi-Vitvi05g00423\_t001 |  |  |  | Ath-AT5G19060.1 |  | Ath-AT3G06150.1 |  | | | |  |  |  |  |
| 3 | Vvi-Vitvi05g01875\_t001 |  |  |  | Ath-AT5G19070.1 |  | | | |  | Ath-AT1G03260.1 |  |  |  |  |
| 3 | Vvi-Vitvi05g00424\_t001 |  |  |  | | | |  | Ath-AT3G06145.1 |  | | | |  |  |  |  |
| 3 | Vvi-Vitvi05g00425\_t002 |  |  |  | Ath-AT5G19080.1 |  | Ath-AT3G06140.1 |  | | | |  |  |  |  |
| 3 | Vvi-Vitvi05g00426\_t001 |  |  |  | Ath-AT5G19090.1 |  | Ath-AT3G06130.1 |  | | | |  |  |  |  |
| 3 | Vvi-Vitvi05g00427\_t001 |  |  |  | | | |  | Ath-AT3G06120.1 |  | | | |  |  |  |  |
| 3 | Vvi-Vitvi05g00429\_t001 |  |  |  | | | |  | Ath-AT3G06110.3 |  | | | |  |  |  |  |
| 3 | Vvi-Vitvi05g04101\_t001 |  |  |  | | | |  | | | |  | | | |  |  |  |  |
| 3 | Vvi-Vitvi05g00430\_t001 |  |  |  | Ath-AT5G19100.1 |  | | | |  | Ath-AT1G03220.1 |  |  |  |  |
| 3 | Vvi-Vitvi05g00431\_t001 |  |  |  | | | |  | | | |  | | | |  |  |  |  |
| 3 | Vvi-Vitvi05g00432\_t001 |  |  |  | | | |  | Ath-AT3G06100.1 |  | | | |  |  |  |  |
| 3 | Vvi-Vitvi05g00433\_t001 |  |  |  | | | |  | | | |  | | | |  |  |  |  |
| 3 | Vvi-Vitvi05g04102\_t001 |  |  |  | | | |  | | | |  | | | |  |  |  |  |
| 3 | Vvi-Vitvi05g00434\_t001 |  |  |  | Ath-AT5G19140.1 |  | | | |  | | | |  |  |  |  |
| 3 | Vvi-Vitvi05g00435\_t001 |  |  |  | | | |  | | | |  | | | |  |  |  |  |
| 3 | Vvi-Vitvi05g00436\_t001 |  |  |  | Ath-AT5G19150.2 |  | | | |  | | | |  |  |  |  |
| 3 | Vvi-Vitvi05g00437\_t001 |  |  |  | Ath-AT5G19160.1 |  | Ath-AT3G06080.2 |  | | | |  |  |  |  |
| 3 | Vvi-Vitvi05g00438\_t001 |  |  |  | Ath-AT5G19180.1 |  | | | |  | | | |  |  |  |  |
| 3 | Vvi-Vitvi05g01877\_t001 |  |  |  | Ath-AT5G19190.1 |  | Ath-AT3G06070.1 |  | | | |  |  |  |  |
| 3 | Vvi-Vitvi05g01878\_t002 |  |  |  | Ath-AT5G19200.1 |  | Ath-AT3G06060.2 |  | | | |  |  |  |  |
| 3 | Vvi-Vitvi05g00439\_t001 |  |  |  | | | |  | Ath-AT3G06050.1 |  | | | |  |  |  |  |
| 3 | Vvi-Vitvi05g00440\_t001 |  |  |  | | | |  | | | |  | | | |  |  |  |  |
| 3 | Vvi-Vitvi05g00441\_t001 |  |  |  | Ath-AT5G19210.2 |  | | | |  | | | |  |  |  |  |
| 3 | Vvi-Vitvi05g00442\_t001 |  |  |  | Ath-AT5G19220.1 |  | | | |  | | | |  |  |  |  |
| 3 | Vvi-Vitvi05g01879\_t001 |  |  |  | Ath-AT5G19230.2 |  | Ath-AT3G06035.1 |  | | | |  |  |  |  |
| 3 | Vvi-Vitvi05g00443\_t001 |  |  |  | | | |  | | | |  | | | |  |  |  |  |
| 3 | Vvi-Vitvi05g00444\_t001 |  |  |  | | | |  | Ath-AT3G06030.1 |  | | | |  |  |  |  |
| 3 | Vvi-Vitvi05g04103\_t001 |  |  |  | | | |  | | | |  | | | |  |  |  |  |
| 3 | Vvi-Vitvi05g00448\_t001 |  |  |  | Ath-AT5G19260.1 |  | Ath-AT3G06020.1 |  | Ath-AT1G03170.1 |  |  |  |  |
| 2 | Vvi-Vitvi05g00449\_t001 |  |  |  | | | |  | | | |  |  |  |  |  |
| 2 | Vvi-Vitvi05g04104\_t001 |  |  |  | | | |  | | | |  |  |  |  |  |
| 2 | Vvi-Vitvi05g00451\_t001 |  |  |  | Ath-AT5G19280.2 |  | | | |  |  |  |  |  |
| 2 | Vvi-Vitvi05g00452\_t001 |  |  |  | Ath-AT5G19300.1 |  | | | |  |  |  |  |  |
| 2 | Vvi-Vitvi05g00453\_t001 |  |  |  | Ath-AT5G19310.1 |  | Ath-AT3G06010.1 |  |  |  |  |  |
| 2 | Vvi-Vitvi05g00454\_t001 |  |  |  | Ath-AT5G19320.1 |  | Ath-AT3G06000.1 |  |  |  |  |  |
| 2 | Vvi-Vitvi05g00455\_t001 |  |  |  | | | |  | | | |  |  |  |  |  |
| 2 | Vvi-Vitvi05g00456\_t001 |  |  |  | | | |  | | | |  |  |  |  |  |
| 2 | Vvi-Vitvi05g00457\_t001 |  |  |  | | | |  | | | |  |  |  |  |  |
| 2 | Vvi-Vitvi05g00458\_t002 |  |  |  | Ath-AT5G19330.1 |  | | | |  |  |  |  |  |
| 2 | Vvi-Vitvi05g04105\_t001 |  |  |  | | | |  | | | |  |  |  |  |  |
| 2 | Vvi-Vitvi05g00459\_t001 |  |  |  | | | |  | Ath-AT3G05990.1 |  |  |  |  |  |
| 2 | Vvi-Vitvi05g00460\_t001 |  |  |  | Ath-AT5G19340.1 |  | Ath-AT3G05980.1 |  |  |  |  |  |
| 2 | Vvi-Vitvi05g00461\_t001 |  |  |  | | | |  | | | |  |  |  |  |  |
| 2 | Vvi-Vitvi05g00462\_t008 |  |  |  | | | |  | | | |  |  |  |  |  |
| 2 | Vvi-Vitvi05g00463\_t001 |  |  |  | | | |  | Ath-AT3G05970.1 |  |  |  |  |  |
| 2 | Vvi-Vitvi05g00464\_t001 |  |  |  | | | |  | | | |  |  |  |  |  |
| 2 | Vvi-Vitvi05g00465\_t001 |  |  |  | | | |  | Ath-AT3G05960.1 |  |  |  |  |  |
| 1 | Vvi-Vitvi05g00466\_t001 |  |  |  | | | |  |  |  |  |  |  |
| 2 | Vvi-Vitvi05g00467\_t001 |  | Ath-AT2G32300.2 |  | | | |  |  |  |  |  |  |
| 2 | Vvi-Vitvi05g00468\_t001 |  | | | |  | | | |  |  |  |  |  |  |
| 2 | Vvi-Vitvi05g04106\_t001 |  | | | |  | | | |  |  |  |  |  |  |
| 3 | Vvi-Vitvi05g00469\_t001 |  | | | |  | | | |  | Ath-AT3G22380.2 |  |  |  |  |  |
| 5 | Vvi-Vitvi05g00470\_t001 |  | | | |  | | | |  | | | |  | Ath-AT4G14950.1 |  | Ath-AT1G05360.1 |  |  |  |
| 5 | Vvi-Vitvi05g04107\_t001 |  | | | |  | | | |  | | | |  | | | |  | | | |  |  |  |
| 5 | Vvi-Vitvi05g00471\_t001 |  | | | |  | | | |  | | | |  | | | |  | | | |  |  |  |
| 5 | Vvi-Vitvi05g00472\_t001 |  | | | |  | | | |  | Ath-AT3G22400.1 |  | | | |  | | | |  |  |  |
| 5 | Vvi-Vitvi05g00474\_t001 |  | | | |  | | | |  | | | |  | | | |  | | | |  |  |  |
| 5 | Vvi-Vitvi05g00475\_t001 |  | Ath-AT2G32150.1 |  | | | |  | | | |  | | | |  | | | |  |  |  |
| 5 | Vvi-Vitvi05g00476\_t001 |  | | | |  | Ath-AT5G19530.1 |  | | | |  | | | |  | | | |  |  |  |
| 4 | Vvi-Vitvi05g01881\_t001 |  | | | |  |  |  | | | |  | | | |  | | | |  |  |  |
| 4 | Vvi-Vitvi05g04108\_t001 |  | | | |  |  |  | | | |  | | | |  | | | |  |  |  |
| 4 | Vvi-Vitvi05g00477\_t001 |  | | | |  |  |  | | | |  | | | |  | | | |  |  |  |
| 4 | Vvi-Vitvi05g00478\_t001 |  | | | |  |  |  | | | |  | | | |  | | | |  |  |  |
| 4 | Vvi-Vitvi05g04109\_t001 |  | | | |  |  |  | | | |  | | | |  | | | |  |  |  |
| 4 | Vvi-Vitvi05g00479\_t001 |  | | | |  |  |  | | | |  | | | |  | | | |  |  |  |
| 4 | Vvi-Vitvi05g00481\_t001 |  | | | |  |  |  | | | |  | Ath-AT4G14940.1 |  | | | |  |  |  |
| 4 | Vvi-Vitvi05g00482\_t001 |  | | | |  |  |  | | | |  | | | |  | | | |  |  |  |
| 4 | Vvi-Vitvi05g00483\_t001 |  | | | |  |  |  | | | |  | | | |  | | | |  |  |  |
| 4 | Vvi-Vitvi05g00484\_t001 |  | | | |  |  |  | | | |  | | | |  | | | |  |  |  |
| 4 | Vvi-Vitvi05g04110\_t001 |  | | | |  |  |  | | | |  | | | |  | | | |  |  |  |
| 4 | Vvi-Vitvi05g04111\_t001 |  | | | |  |  |  | | | |  | | | |  | | | |  |  |  |
| 4 | Vvi-Vitvi05g00485\_t001 |  | | | |  |  |  | | | |  | | | |  | | | |  |  |  |
| 4 | Vvi-Vitvi05g00486\_t001 |  | Ath-AT2G32120.2 |  |  |  | | | |  | | | |  | | | |  |  |  |
| 4 | Vvi-Vitvi05g01885\_t001 |  | | | |  |  |  | | | |  | | | |  | | | |  |  |  |
| 4 | Vvi-Vitvi05g00487\_t001 |  | | | |  |  |  | Ath-AT3G22410.1 |  | | | |  | Ath-AT1G05370.1 |  |  |  |
| 4 | Vvi-Vitvi05g01886\_t001 |  | | | |  |  |  | | | |  | | | |  | | | |  |  |  |
| 4 | Vvi-Vitvi05g00490\_t001 |  | | | |  |  |  | | | |  | Ath-AT4G14930.1 |  | | | |  |  |  |
| 4 | Vvi-Vitvi05g04112\_t001 |  | | | |  |  |  | | | |  | | | |  | | | |  |  |  |
| 4 | Vvi-Vitvi05g00492\_t001 |  | | | |  |  |  | Ath-AT3G22420.3 |  | | | |  | | | |  |  |  |
| 4 | Vvi-Vitvi05g01887\_t001 |  | | | |  |  |  | | | |  | | | |  | | | |  |  |  |
| 4 | Vvi-Vitvi05g04113\_t001 |  | | | |  |  |  | | | |  | | | |  | | | |  |  |  |
| 4 | Vvi-Vitvi05g04114\_t001 |  | | | |  |  |  | | | |  | | | |  | | | |  |  |  |
| 4 | Vvi-Vitvi05g00495\_t001 |  | | | |  |  |  | | | |  | | | |  | | | |  |  |  |
| 4 | Vvi-Vitvi05g04115\_t001 |  | | | |  |  |  | | | |  | | | |  | | | |  |  |  |
| 4 | Vvi-Vitvi05g00496\_t001 |  | | | |  |  |  | | | |  | Ath-AT4G14920.3 |  | Ath-AT1G05380.2 |  |  |  |
| 4 | Vvi-Vitvi05g04116\_t001 |  | | | |  |  |  | | | |  | | | |  | | | |  |  |  |
| 4 | Vvi-Vitvi05g00497\_t001 |  | | | |  |  |  | Ath-AT3G22425.2 |  | Ath-AT4G14910.2 |  | | | |  |  |  |
| 4 | Vvi-Vitvi05g00498\_t001 |  | | | |  |  |  | | | |  | | | |  | Ath-AT1G05385.1 |  |  |  |
| 4 | Vvi-Vitvi05g04117\_t001 |  | | | |  |  |  | | | |  | | | |  | | | |  |  |  |
| 4 | Vvi-Vitvi05g04118\_t001 |  | | | |  |  |  | | | |  | | | |  | | | |  |  |  |
| 4 | Vvi-Vitvi05g04119\_t001 |  | | | |  |  |  | | | |  | | | |  | | | |  |  |  |
| 4 | Vvi-Vitvi05g00500\_t001 |  | | | |  |  |  | Ath-AT3G22430.1 |  | | | |  | | | |  |  |  |
| 4 | Vvi-Vitvi05g00503\_t001 |  | | | |  |  |  | Ath-AT3G22440.1 |  | Ath-AT4G14900.1 |  | | | |  |  |  |
| 4 | Vvi-Vitvi05g00504\_t001 |  | | | |  |  |  | Ath-AT3G22450.1 |  | | | |  | | | |  |  |  |
| 4 | Vvi-Vitvi05g00505\_t001 |  | | | |  |  |  | | | |  | | | |  | | | |  |  |  |
| 4 | Vvi-Vitvi05g00506\_t002 |  | | | |  |  |  | Ath-AT3G22480.1 |  | | | |  | | | |  |  |  |
| 4 | Vvi-Vitvi05g00508\_t001 |  | | | |  |  |  | | | |  | Ath-AT4G14870.1 |  | | | |  |  |  |
| 4 | Vvi-Vitvi05g00509\_t001 |  | Ath-AT2G32100.1 |  |  |  | | | |  | Ath-AT4G14860.1 |  | Ath-AT1G05420.2 |  |  |  |
| 4 | Vvi-Vitvi05g00510\_t001 |  | | | |  |  |  | Ath-AT3G22490.1 |  | | | |  | | | |  |  |  |
| 4 | Vvi-Vitvi05g00512\_t001 |  | Ath-AT2G32090.1 |  |  |  | | | |  | | | |  | | | |  |  |  |
| 4 | Vvi-Vitvi05g00513\_t001 |  | | | |  |  |  | | | |  | Ath-AT4G14850.1 |  | | | |  |  |  |
| 4 | Vvi-Vitvi05g01890\_t001 |  | | | |  |  |  | Ath-AT3G22510.1 |  | | | |  | | | |  |  |  |
| 4 | Vvi-Vitvi05g04120\_t001 |  | | | |  |  |  | Ath-AT3G22520.1 |  | Ath-AT4G14840.1 |  | | | |  |  |  |
| 4 | Vvi-Vitvi05g00517\_t001 |  | | | |  |  |  | Ath-AT3G22530.1 |  | Ath-AT4G14830.1 |  | | | |  |  |  |
| 4 | Vvi-Vitvi05g01891\_t001 |  | | | |  |  |  | | | |  | | | |  | Ath-AT1G05430.1 |  |  |  |
| 4 | Vvi-Vitvi05g00518\_t001 |  | | | |  |  |  | | | |  | Ath-AT4G14820.1 |  | | | |  |  |  |
| 4 | Vvi-Vitvi05g00519\_t001 |  | | | |  |  |  | | | |  | | | |  | Ath-AT1G05440.1 |  |  |  |
| 4 | Vvi-Vitvi05g04121\_t001 |  | | | |  |  |  | | | |  | | | |  | | | |  |  |  |
| 4 | Vvi-Vitvi05g00520\_t001 |  | | | |  |  |  | Ath-AT3G22540.1 |  | Ath-AT4G14819.1 |  | | | |  |  |  |
| 4 | Vvi-Vitvi05g00521\_t001 |  | Ath-AT2G32040.1 |  |  |  | | | |  | | | |  | | | |  |  |  |
| 5 | Vvi-Vitvi05g04122\_t001 |  | | | |  | Ath-AT3G63210.1 |  | Ath-AT3G22550.1 |  | | | |  | | | |  |  |  |
| 5 | Vvi-Vitvi05g00523\_t001 |  | Ath-AT2G32020.1 |  | | | |  | Ath-AT3G22560.1 |  | | | |  | | | |  |  |  |
| 5 | Vvi-Vitvi05g00524\_t001 |  | | | |  | | | |  | | | |  | | | |  | | | |  |  |  |
| 5 | Vvi-Vitvi05g04123\_t001 |  | | | |  | | | |  | | | |  | | | |  | | | |  |  |  |
| 5 | Vvi-Vitvi05g01892\_t001 |  | | | |  | | | |  | | | |  | | | |  | | | |  |  |  |
| 5 | Vvi-Vitvi05g01893\_t001 |  | | | |  | | | |  | | | |  | | | |  | | | |  |  |  |
| 5 | Vvi-Vitvi05g04124\_t001 |  | | | |  | | | |  | | | |  | | | |  | | | |  |  |  |
| 5 | Vvi-Vitvi05g04125\_t001 |  | | | |  | | | |  | | | |  | | | |  | | | |  |  |  |
| 5 | Vvi-Vitvi05g00526\_t001 |  | | | |  | | | |  | Ath-AT3G22590.1 |  | | | |  | | | |  |  |  |
| 5 | Vvi-Vitvi05g00527\_t002 |  | | | |  | | | |  | Ath-AT3G22600.1 |  | Ath-AT4G14815.1 |  | | | |  |  |  |
| 5 | Vvi-Vitvi05g00528\_t001 |  | | | |  | | | |  | | | |  | | | |  | | | |  |  |  |
| 5 | Vvi-Vitvi05g00529\_t001 |  | | | |  | | | |  | Ath-AT3G22620.1 |  | | | |  | Ath-AT1G05450.2 |  |  |  |
| 5 | Vvi-Vitvi05g01894\_t001 |  | | | |  | | | |  | | | |  | Ath-AT4G14805.1 |  | | | |  |  |  |
| 5 | Vvi-Vitvi05g00530\_t001 |  | | | |  | | | |  | | | |  | | | |  | Ath-AT1G05460.1 |  |  |  |
| 5 | Vvi-Vitvi05g04126\_t001 |  | | | |  | | | |  | | | |  | | | |  | | | |  |  |  |
| 5 | Vvi-Vitvi05g04127\_t001 |  | | | |  | | | |  | | | |  | | | |  | | | |  |  |  |
| 5 | Vvi-Vitvi05g00532\_t001 |  | | | |  | | | |  | | | |  | | | |  | | | |  |  |  |
| 5 | Vvi-Vitvi05g04128\_t001 |  | | | |  | | | |  | | | |  | | | |  | | | |  |  |  |
| 5 | Vvi-Vitvi05g00533\_t001 |  | Ath-AT2G32010.1 |  | Ath-AT3G63240.1 |  | | | |  | | | |  | Ath-AT1G05470.1 |  |  |  |
| 3 | Vvi-Vitvi05g00534\_t001 |  |  |  | | | |  | | | |  | Ath-AT4G14790.2 |  |  |  |  |
| 3 | Vvi-Vitvi05g00535\_t001 |  |  |  | | | |  | Ath-AT3G22670.1 |  | | | |  |  |  |  |
| 3 | Vvi-Vitvi05g04129\_t001 |  |  |  | | | |  | | | |  | | | |  |  |  |  |
| 3 | Vvi-Vitvi05g01895\_t001 |  |  |  | | | |  | | | |  | | | |  |  |  |  |
| 3 | Vvi-Vitvi05g04130\_t001 |  |  |  | | | |  | | | |  | | | |  |  |  |  |
| 3 | Vvi-Vitvi05g01896\_t001 |  |  |  | | | |  | | | |  | | | |  |  |  |  |
| 3 | Vvi-Vitvi05g00537\_t002 |  |  |  | | | |  | | | |  | | | |  |  |  |  |
| 3 | Vvi-Vitvi05g00538\_t001 |  |  |  | | | |  | Ath-AT3G22690.1 |  | | | |  |  |  |  |
| 3 | Vvi-Vitvi05g00539\_t003 |  |  |  | Ath-AT3G63250.1 |  | Ath-AT3G22740.1 |  | | | |  |  |  |  |
| 3 | Vvi-Vitvi05g00540\_t001 |  |  |  | Ath-AT3G63260.1 |  | Ath-AT3G22750.1 |  | Ath-AT4G14780.1 |  |  |  |  |
| 3 | Vvi-Vitvi05g04131\_t001 |  |  |  | | | |  | | | |  | | | |  |  |  |  |
| 3 | Vvi-Vitvi05g00541\_t001 |  |  |  | | | |  | Ath-AT3G22760.1 |  | Ath-AT4G14770.1 |  |  |  |  |
| 3 | Vvi-Vitvi05g00542\_t001 |  |  |  | | | |  | Ath-AT3G22790.1 |  | Ath-AT4G14760.2 |  |  |  |  |
| 3 | Vvi-Vitvi05g00543\_t001 |  |  |  | | | |  | | | |  | | | |  |  |  |  |
| 3 | Vvi-Vitvi05g00544\_t001 |  |  |  | | | |  | Ath-AT3G22800.1 |  | | | |  |  |  |  |
| 3 | Vvi-Vitvi05g00545\_t001 |  |  |  | | | |  | | | |  | | | |  |  |  |  |
| 3 | Vvi-Vitvi05g00546\_t001 |  |  |  | | | |  | | | |  | | | |  |  |  |  |
| 3 | Vvi-Vitvi05g04132\_t001 |  |  |  | | | |  | | | |  | | | |  |  |  |  |
| 3 | Vvi-Vitvi05g00548\_t001 |  |  |  | | | |  | | | |  | | | |  |  |  |  |
| 3 | Vvi-Vitvi05g04133\_t001 |  |  |  | | | |  | | | |  | | | |  |  |  |  |
| 3 | Vvi-Vitvi05g00549\_t001 |  |  |  | | | |  | | | |  | Ath-AT4G14750.3 |  |  |  |  |
| 3 | Vvi-Vitvi05g00551\_t001 |  |  |  | | | |  | | | |  | | | |  |  |  |  |
| 3 | Vvi-Vitvi05g00553\_t001 |  |  |  | | | |  | | | |  | Ath-AT4G14746.1 |  |  |  |  |
| 4 | Vvi-Vitvi05g00554\_t001 |  | Ath-AT5G43870.1 |  | Ath-AT3G63300.1 |  | Ath-AT3G22810.1 |  | Ath-AT4G14740.2 |  |  |  |  |
| 4 | Vvi-Vitvi05g00555\_t001 |  | | | |  | Ath-AT3G63310.1 |  | | | |  | Ath-AT4G14730.1 |  |  |  |  |
| 4 | Vvi-Vitvi05g00556\_t001 |  | Ath-AT5G43860.1 |  | | | |  | | | |  | | | |  |  |  |  |
| 4 | Vvi-Vitvi05g00557\_t001 |  | | | |  | | | |  | Ath-AT3G22820.1 |  | Ath-AT4G14723.1 |  |  |  |  |
| 4 | Vvi-Vitvi05g04134\_t001 |  | | | |  | | | |  | | | |  | | | |  |  |  |  |
| 4 | Vvi-Vitvi05g00558\_t002 |  | | | |  | | | |  | | | |  | Ath-AT4G14713.1 |  |  |  |  |
| 4 | Vvi-Vitvi05g00559\_t002 |  | Ath-AT5G43850.1 |  | | | |  | | | |  | Ath-AT4G14710.5 |  |  |  |  |
| 4 | Vvi-Vitvi05g00560\_t001 |  | | | |  | | | |  | | | |  | | | |  |  |  |  |
| 4 | Vvi-Vitvi05g00561\_t001 |  | | | |  | | | |  | Ath-AT3G22830.1 |  | | | |  |  |  |  |
| 4 | Vvi-Vitvi05g04135\_t001 |  | | | |  | | | |  | | | |  | | | |  |  |  |  |
| 4 | Vvi-Vitvi05g00562\_t001 |  | | | |  | | | |  | | | |  | | | |  |  |  |  |
| 4 | Vvi-Vitvi05g00563\_t001 |  | | | |  | | | |  | Ath-AT3G22840.1 |  | Ath-AT4G14690.1 |  |  |  |  |
| 4 | Vvi-Vitvi05g00564\_t002 |  | | | |  | | | |  | | | |  | | | |  |  |  |  |
| 4 | Vvi-Vitvi05g00565\_t001 |  | | | |  | | | |  | | | |  | | | |  |  |  |  |
| 4 | Vvi-Vitvi05g04136\_t001 |  | | | |  | | | |  | | | |  | | | |  |  |  |  |
| 4 | Vvi-Vitvi05g00568\_t001 |  | | | |  | | | |  | Ath-AT3G22845.1 |  | | | |  |  |  |  |
| 4 | Vvi-Vitvi05g00569\_t001 |  | Ath-AT5G43830.1 |  | | | |  | Ath-AT3G22850.1 |  | | | |  |  |  |  |
| 4 | Vvi-Vitvi05g00570\_t001 |  | | | |  | | | |  | Ath-AT3G22880.1 |  | | | |  |  |  |  |
| 4 | Vvi-Vitvi05g00573\_t001 |  | | | |  | | | |  | | | |  | | | |  |  |  |  |
| 4 | Vvi-Vitvi05g00574\_t002 |  | Ath-AT5G43810.1 |  | | | |  | | | |  | | | |  |  |  |  |
| 4 | Vvi-Vitvi05g00575\_t001 |  | Ath-AT5G43790.1 |  | | | |  | | | |  | | | |  |  |  |  |
| 4 | Vvi-Vitvi05g00576\_t001 |  | Ath-AT5G43780.1 |  | | | |  | Ath-AT3G22890.1 |  | Ath-AT4G14680.2 |  |  |  |  |
| 2 | Vvi-Vitvi05g00577\_t001 |  |  |  | Ath-AT3G63380.1 |  | Ath-AT3G22910.1 |  |  |  |  |  |
| 0 | Vvi-Vitvi05g04137\_t001 |  |  |  |  |  |  |  |  |
| 0 | Vvi-Vitvi05g01900\_t001 |  |  |  |  |  |  |  |  |
| 0 | Vvi-Vitvi05g04138\_t001 |  |  |  |  |  |  |  |  |
| 0 | Vvi-Vitvi05g00579\_t001 |  |  |  |  |  |  |  |  |
| 0 | Vvi-Vitvi05g00580\_t001 |  |  |  |  |  |  |  |  |
| 0 | Vvi-Vitvi05g00582\_t001 |  |  |  |  |  |  |  |  |
| 0 | Vvi-Vitvi05g00583\_t001 |  |  |  |  |  |  |  |  |
| 0 | Vvi-Vitvi05g04139\_t001 |  |  |  |  |  |  |  |  |
| 0 | Vvi-Vitvi05g00584\_t001 |  |  |  |  |  |  |  |  |
| 0 | Vvi-Vitvi05g00585\_t001 |  |  |  |  |  |  |  |  |
| 0 | Vvi-Vitvi05g04140\_t001 |  |  |  |  |  |  |  |  |
| 0 | Vvi-Vitvi05g00588\_t001 |  |  |  |  |  |  |  |  |
| 0 | Vvi-Vitvi05g00589\_t001 |  |  |  |  |  |  |  |  |
| 0 | Vvi-Vitvi05g04141\_t001 |  |  |  |  |  |  |  |  |
| 0 | Vvi-Vitvi05g00590\_t001 |  |  |  |  |  |  |  |  |
| 0 | Vvi-Vitvi05g00592\_t001 |  |  |  |  |  |  |  |  |
| 0 | Vvi-Vitvi05g04142\_t001 |  |  |  |  |  |  |  |  |
| 0 | Vvi-Vitvi05g04143\_t001 |  |  |  |  |  |  |  |  |
| 0 | Vvi-Vitvi05g00597\_t001 |  |  |  |  |  |  |  |  |
| 0 | Vvi-Vitvi05g00598\_t001 |  |  |  |  |  |  |  |  |
| 0 | Vvi-Vitvi05g04144\_t001 |  |  |  |  |  |  |  |  |
| 0 | Vvi-Vitvi05g00599\_t001 |  |  |  |  |  |  |  |  |
| 0 | Vvi-Vitvi05g00600\_t001 |  |  |  |  |  |  |  |  |
| 0 | Vvi-Vitvi05g00601\_t001 |  |  |  |  |  |  |  |  |
| 0 | Vvi-Vitvi05g04145\_t001 |  |  |  |  |  |  |  |  |
| 0 | Vvi-Vitvi05g04146\_t001 |  |  |  |  |  |  |  |  |
| 0 | Vvi-Vitvi05g04147\_t001 |  |  |  |  |  |  |  |  |
| 0 | Vvi-Vitvi05g00604\_t002 |  |  |  |  |  |  |  |  |
| 2 | Vvi-Vitvi05g00605\_t001 |  | Ath-AT4G14640.1 |  | Ath-AT3G22930.1 |  |  |  |  |  |  |
| 2 | Vvi-Vitvi05g00606\_t001 |  | | | |  | | | |  |  |  |  |  |  |
| 2 | Vvi-Vitvi05g00607\_t001 |  | | | |  | | | |  |  |  |  |  |  |
| 2 | Vvi-Vitvi05g04148\_t001 |  | | | |  | | | |  |  |  |  |  |  |
| 2 | Vvi-Vitvi05g04149\_t001 |  | | | |  | | | |  |  |  |  |  |  |
| 2 | Vvi-Vitvi05g00609\_t001 |  | | | |  | Ath-AT3G22942.1 |  |  |  |  |  |  |
| 2 | Vvi-Vitvi05g00611\_t001 |  | | | |  | Ath-AT3G22950.2 |  |  |  |  |  |  |
| 2 | Vvi-Vitvi05g00612\_t001 |  | | | |  | Ath-AT3G22960.1 |  |  |  |  |  |  |
| 2 | Vvi-Vitvi05g04150\_t001 |  | Ath-AT4G14620.1 |  | Ath-AT3G22970.1 |  |  |  |  |  |  |
| 2 | Vvi-Vitvi05g00614\_t003 |  | | | |  | | | |  |  |  |  |  |  |
| 2 | Vvi-Vitvi05g00615\_t002 |  | | | |  | Ath-AT3G22990.1 |  |  |  |  |  |  |
| 4 | Vvi-Vitvi05g00616\_t001 |  | | | |  | | | |  | Ath-AT1G04220.1 |  | Ath-AT5G43760.1 |  |  |  |  |
| 4 | Vvi-Vitvi05g01904\_t001 |  | Ath-AT4G14615.1 |  | | | |  | | | |  | | | |  |  |  |  |
| 4 | Vvi-Vitvi05g00617\_t001 |  | | | |  | | | |  | | | |  | | | |  |  |  |  |
| 4 | Vvi-Vitvi05g00618\_t001 |  | Ath-AT4G14580.1 |  | Ath-AT3G23000.1 |  | | | |  | | | |  |  |  |  |
| 4 | Vvi-Vitvi05g00619\_t001 |  | | | |  | Ath-AT3G23020.1 |  | | | |  | | | |  |  |  |  |
| 4 | Vvi-Vitvi05g00620\_t001 |  | Ath-AT4G14570.1 |  | | | |  | | | |  | | | |  |  |  |  |
| 4 | Vvi-Vitvi05g00621\_t001 |  | | | |  | | | |  | | | |  | Ath-AT5G43750.1 |  |  |  |  |
| 4 | Vvi-Vitvi05g00623\_t001 |  | | | |  | | | |  | | | |  | | | |  |  |  |  |
| 4 | Vvi-Vitvi05g00624\_t001 |  | | | |  | | | |  | | | |  | | | |  |  |  |  |
| 4 | Vvi-Vitvi05g00625\_t001 |  | | | |  | | | |  | | | |  | Ath-AT5G43745.1 |  |  |  |  |
| 4 | Vvi-Vitvi05g04151\_t001 |  | | | |  | | | |  | | | |  | | | |  |  |  |  |
| 4 | Vvi-Vitvi05g00626\_t001 |  | | | |  | | | |  | Ath-AT1G04230.1 |  | Ath-AT5G43720.1 |  |  |  |  |
| 4 | Vvi-Vitvi05g00627\_t001 |  | | | |  | | | |  | | | |  | Ath-AT5G43710.1 |  |  |  |  |
| 4 | Vvi-Vitvi05g00628\_t001 |  | | | |  | | | |  | | | |  | | | |  |  |  |  |
| 4 | Vvi-Vitvi05g00630\_t001 |  | Ath-AT4G14550.2 |  | Ath-AT3G23050.1 |  | Ath-AT1G04250.1 |  | | | |  |  |  |  |
| 4 | Vvi-Vitvi05g00631\_t001 |  | | | |  | | | |  | | | |  | Ath-AT5G43680.1 |  |  |  |  |
| 4 | Vvi-Vitvi05g04152\_t001 |  | | | |  | | | |  | | | |  | | | |  |  |  |  |
| 4 | Vvi-Vitvi05g00632\_t001 |  | | | |  | | | |  | | | |  | Ath-AT5G43670.1 |  |  |  |  |
| 4 | Vvi-Vitvi05g01905\_t001 |  | | | |  | | | |  | | | |  | | | |  |  |  |  |
| 4 | Vvi-Vitvi05g00633\_t002 |  | | | |  | | | |  | | | |  | | | |  |  |  |  |
| 4 | Vvi-Vitvi05g00634\_t001 |  | | | |  | Ath-AT3G23060.1 |  | | | |  | | | |  |  |  |  |
| 4 | Vvi-Vitvi05g01906\_t001 |  | | | |  | | | |  | Ath-AT1G04260.1 |  | | | |  |  |  |  |
| 4 | Vvi-Vitvi05g04153\_t001 |  | | | |  | | | |  | | | |  | | | |  |  |  |  |
| 4 | Vvi-Vitvi05g00637\_t001 |  | | | |  | | | |  | | | |  | | | |  |  |  |  |
| 4 | Vvi-Vitvi05g00638\_t001.1.6037826e |  | Ath-AT4G14520.2 |  | | | |  | | | |  | | | |  |  |  |  |
| 4 | Vvi-Vitvi05g04154\_t001 |  | | | |  | | | |  | | | |  | | | |  |  |  |  |
| 4 | Vvi-Vitvi05g00639\_t001 |  | Ath-AT4G14510.1 |  | Ath-AT3G23070.1 |  | | | |  | | | |  |  |  |  |
| 4 | Vvi-Vitvi05g04155\_t001 |  | | | |  | | | |  | | | |  | | | |  |  |  |  |
| 4 | Vvi-Vitvi05g00640\_t001 |  | | | |  | | | |  | | | |  | Ath-AT5G43650.1 |  |  |  |  |
| 4 | Vvi-Vitvi05g00641\_t001 |  | | | |  | | | |  | Ath-AT1G04270.1 |  | Ath-AT5G43640.1 |  |  |  |  |
| 4 | Vvi-Vitvi05g00642\_t001 |  | | | |  | | | |  | | | |  | | | |  |  |  |  |
| 4 | Vvi-Vitvi05g00643\_t001 |  | | | |  | | | |  | | | |  | | | |  |  |  |  |
| 4 | Vvi-Vitvi05g00644\_t001 |  | | | |  | | | |  | | | |  | | | |  |  |  |  |
| 4 | Vvi-Vitvi05g00648\_t001 |  | | | |  | | | |  | Ath-AT1G04280.1 |  | | | |  |  |  |  |
| 4 | Vvi-Vitvi05g00649\_t001 |  | | | |  | | | |  | | | |  | | | |  |  |  |  |
| 4 | Vvi-Vitvi05g00650\_t001 |  | | | |  | | | |  | | | |  | Ath-AT5G43630.2 |  |  |  |  |
| 4 | Vvi-Vitvi05g00651\_t001 |  | Ath-AT4G14500.1 |  | Ath-AT3G23080.1 |  | | | |  | | | |  |  |  |  |
| 4 | Vvi-Vitvi05g00652\_t001 |  | | | |  | Ath-AT3G23090.3 |  | | | |  | | | |  |  |  |  |
| 4 | Vvi-Vitvi05g00653\_t001 |  | | | |  | | | |  | | | |  | | | |  |  |  |  |
| 4 | Vvi-Vitvi05g00654\_t001 |  | | | |  | | | |  | | | |  | Ath-AT5G43600.1 |  |  |  |  |
| 4 | Vvi-Vitvi05g00655\_t001 |  | | | |  | Ath-AT3G23100.1 |  | | | |  | | | |  |  |  |  |
| 4 | Vvi-Vitvi05g00656\_t001 |  | | | |  | | | |  | | | |  | | | |  |  |  |  |
| 4 | Vvi-Vitvi05g04156\_t001 |  | | | |  | | | |  | | | |  | | | |  |  |  |  |
| 4 | Vvi-Vitvi05g04157\_t001 |  | | | |  | | | |  | | | |  | | | |  |  |  |  |
| 4 | Vvi-Vitvi05g00659\_t001 |  | | | |  | | | |  | | | |  | | | |  |  |  |  |
| 4 | Vvi-Vitvi05g01908\_t001 |  | | | |  | | | |  | | | |  | | | |  |  |  |  |
| 4 | Vvi-Vitvi05g04158\_t001 |  | | | |  | | | |  | | | |  | | | |  |  |  |  |
| 4 | Vvi-Vitvi05g00661\_t001 |  | | | |  | | | |  | | | |  | | | |  |  |  |  |
| 4 | Vvi-Vitvi05g00663\_t001 |  | Ath-AT4G14490.1 |  | | | |  | | | |  | | | |  |  |  |  |
| 4 | Vvi-Vitvi05g04159\_t001 |  | | | |  | | | |  | | | |  | | | |  |  |  |  |
| 4 | Vvi-Vitvi05g00664\_t001 |  | | | |  | | | |  | | | |  | | | |  |  |  |  |
| 4 | Vvi-Vitvi05g00666\_t001 |  | | | |  | | | |  | Ath-AT1G04300.3 |  | | | |  |  |  |  |
| 4 | Vvi-Vitvi05g04160\_t001 |  | | | |  | Ath-AT3G23123.1 |  | | | |  | | | |  |  |  |  |
| 4 | Vvi-Vitvi05g01909\_t001 |  | | | |  | | | |  | | | |  | | | |  |  |  |  |
| 4 | Vvi-Vitvi05g04161\_t001 |  | | | |  | | | |  | | | |  | | | |  |  |  |  |
| 4 | Vvi-Vitvi05g04162\_t001 |  | | | |  | | | |  | | | |  | | | |  |  |  |  |
| 4 | Vvi-Vitvi05g04163\_t001 |  | | | |  | | | |  | | | |  | | | |  |  |  |  |
| 4 | Vvi-Vitvi05g01911\_t001 |  | | | |  | | | |  | | | |  | Ath-AT5G43580.1 |  |  |  |  |
| 4 | Vvi-Vitvi05g00669\_t001 |  | | | |  | | | |  | | | |  | Ath-AT5G43570.2 |  |  |  |  |
| 4 | Vvi-Vitvi05g00670\_t001 |  | | | |  | | | |  | | | |  | | | |  |  |  |  |
| 4 | Vvi-Vitvi05g04164\_t001 |  | | | |  | | | |  | | | |  | | | |  |  |  |  |
| 4 | Vvi-Vitvi05g00675\_t001 |  | | | |  | | | |  | | | |  | | | |  |  |  |  |
| 4 | Vvi-Vitvi05g00676\_t001 |  | | | |  | | | |  | | | |  | | | |  |  |  |  |
| 4 | Vvi-Vitvi05g00679\_t001 |  | | | |  | | | |  | | | |  | | | |  |  |  |  |
| 4 | Vvi-Vitvi05g00680\_t001 |  | | | |  | | | |  | | | |  | | | |  |  |  |  |
| 4 | Vvi-Vitvi05g00681\_t001 |  | | | |  | | | |  | | | |  | | | |  |  |  |  |
| 4 | Vvi-Vitvi05g01912\_t001 |  | Ath-AT4G14480.1 |  | | | |  | | | |  | | | |  |  |  |  |
| 4 | Vvi-Vitvi05g01913\_t001 |  | | | |  | Ath-AT3G23130.1 |  | | | |  | | | |  |  |  |  |
| 4 | Vvi-Vitvi05g01914\_t001 |  | | | |  | | | |  | | | |  | | | |  |  |  |  |
| 4 | Vvi-Vitvi05g04165\_t001 |  | | | |  | | | |  | | | |  | | | |  |  |  |  |
| 4 | Vvi-Vitvi05g01915\_t001 |  | | | |  | | | |  | | | |  | Ath-AT5G43540.1 |  |  |  |  |
| 4 | Vvi-Vitvi05g00684\_t001 |  | | | |  | Ath-AT3G23150.1 |  | Ath-AT1G04310.2 |  | | | |  |  |  |  |
| 4 | Vvi-Vitvi05g01916\_t001 |  | Ath-AT4G14465.1 |  | | | |  | | | |  | | | |  |  |  |  |
| 4 | Vvi-Vitvi05g04166\_t001 |  | | | |  | | | |  | | | |  | | | |  |  |  |  |
| 4 | Vvi-Vitvi05g00685\_t001 |  | | | |  | | | |  | | | |  | | | |  |  |  |  |
| 4 | Vvi-Vitvi05g00686\_t001 |  | | | |  | Ath-AT3G23160.1 |  | | | |  | | | |  |  |  |  |
| 4 | Vvi-Vitvi05g01917\_t001 |  | | | |  | | | |  | | | |  | | | |  |  |  |  |
| 4 | Vvi-Vitvi05g01918\_t001 |  | Ath-AT4G14450.1 |  | Ath-AT3G23170.1 |  | Ath-AT1G04330.1 |  | | | |  |  |  |  |
| 4 | Vvi-Vitvi05g00688\_t001 |  | | | |  | | | |  | | | |  | Ath-AT5G43530.1 |  |  |  |  |
| 4 | Vvi-Vitvi05g04167\_t001 |  | | | |  | | | |  | | | |  | | | |  |  |  |  |
| 4 | Vvi-Vitvi05g04168\_t001 |  | | | |  | | | |  | | | |  | | | |  |  |  |  |
| 4 | Vvi-Vitvi05g00690\_t001 |  | Ath-AT4G14430.1 |  | | | |  | | | |  | | | |  |  |  |  |
| 4 | Vvi-Vitvi05g04169\_t001 |  | | | |  | | | |  | | | |  | | | |  |  |  |  |
| 4 | Vvi-Vitvi05g01920\_t001 |  | | | |  | | | |  | | | |  | | | |  |  |  |  |
| 4 | Vvi-Vitvi05g00691\_t001 |  | | | |  | | | |  | | | |  | Ath-AT5G43500.1 |  |  |  |  |
| 4 | Vvi-Vitvi05g00692\_t001 |  | Ath-AT4G14420.1 |  | Ath-AT3G23180.1 |  | Ath-AT1G04340.1 |  | Ath-AT5G43460.2 |  |  |  |  |
| 4 | Vvi-Vitvi05g04170\_t001 |  | | | |  | | | |  | | | |  | | | |  |  |  |  |
| 4 | Vvi-Vitvi05g00694\_t001 |  | | | |  | | | |  | | | |  | | | |  |  |  |  |
| 4 | Vvi-Vitvi05g04171\_t001 |  | | | |  | | | |  | | | |  | | | |  |  |  |  |
| 4 | Vvi-Vitvi05g04172\_t001 |  | | | |  | | | |  | | | |  | Ath-AT5G43440.1 |  |  |  |  |
| 4 | Vvi-Vitvi05g04173\_t001 |  | | | |  | | | |  | | | |  | | | |  |  |  |  |
| 4 | Vvi-Vitvi05g04174\_t001 |  | | | |  | | | |  | | | |  | | | |  |  |  |  |
| 4 | Vvi-Vitvi05g01922\_t001 |  | | | |  | | | |  | | | |  | | | |  |  |  |  |
| 4 | Vvi-Vitvi05g00698\_t001 |  | | | |  | | | |  | | | |  | | | |  |  |  |  |
| 4 | Vvi-Vitvi05g01923\_t001 |  | | | |  | | | |  | | | |  | | | |  |  |  |  |
| 4 | Vvi-Vitvi05g01924\_t001 |  | | | |  | | | |  | | | |  | | | |  |  |  |  |
| 4 | Vvi-Vitvi05g00699\_t001 |  | | | |  | | | |  | | | |  | | | |  |  |  |  |
| 4 | Vvi-Vitvi05g04175\_t001 |  | | | |  | | | |  | | | |  | | | |  |  |  |  |
| 4 | Vvi-Vitvi05g01927\_t001 |  | | | |  | | | |  | | | |  | | | |  |  |  |  |
| 4 | Vvi-Vitvi05g00701\_t001 |  | | | |  | | | |  | | | |  | | | |  |  |  |  |
| 4 | Vvi-Vitvi05g00703\_t001 |  | | | |  | | | |  | | | |  | | | |  |  |  |  |
| 4 | Vvi-Vitvi05g00705\_t001 |  | | | |  | | | |  | | | |  | | | |  |  |  |  |
| 4 | Vvi-Vitvi05g01928\_t001 |  | | | |  | | | |  | | | |  | | | |  |  |  |  |
| 4 | Vvi-Vitvi05g01930\_t004 |  | | | |  | | | |  | | | |  | | | |  |  |  |  |
| 4 | Vvi-Vitvi05g00706\_t001 |  | | | |  | | | |  | | | |  | | | |  |  |  |  |
| 4 | Vvi-Vitvi05g00707\_t001 |  | | | |  | | | |  | Ath-AT1G04350.1 |  | | | |  |  |  |  |
| 4 | Vvi-Vitvi05g00709\_t001 |  | | | |  | Ath-AT3G23200.1 |  | | | |  | | | |  |  |  |  |
| 4 | Vvi-Vitvi05g00710\_t001 |  | | | |  | | | |  | | | |  | | | |  |  |  |  |
| 4 | Vvi-Vitvi05g00711\_t001 |  | Ath-AT4G14410.1 |  | Ath-AT3G23210.1 |  | | | |  | | | |  |  |  |  |
| 3 | Vvi-Vitvi05g00712\_t001 |  |  |  | | | |  | | | |  | Ath-AT5G43430.4 |  |  |  |  |
| 3 | Vvi-Vitvi05g00713\_t001 |  |  |  | | | |  | Ath-AT1G04360.1 |  | Ath-AT5G43420.1 |  |  |  |  |
| 3 | Vvi-Vitvi05g04176\_t001 |  |  |  | | | |  | | | |  | | | |  |  |  |  |
| 3 | Vvi-Vitvi05g01722\_t001 |  |  |  | Ath-AT3G23230.1 |  | Ath-AT1G04370.1 |  | Ath-AT5G43410.1 |  |  |  |  |
| 1 | Vvi-Vitvi05g01723\_t001 |  |  |  |  |  |  |  | | | |  |  |  |  |
| 1 | Vvi-Vitvi05g00715\_t001 |  |  |  |  |  |  |  | | | |  |  |  |  |
| 1 | Vvi-Vitvi05g01931\_t001 |  |  |  |  |  |  |  | | | |  |  |  |  |
| 1 | Vvi-Vitvi05g01933\_t001 |  |  |  |  |  |  |  | | | |  |  |  |  |
| 1 | Vvi-Vitvi05g00718\_t001 |  |  |  |  |  |  |  | Ath-AT5G43390.1 |  |  |  |  |
| 1 | Vvi-Vitvi05g04177\_t001 |  |  |  |  |  |  |  | | | |  |  |  |  |
| 1 | Vvi-Vitvi05g01939\_t001 |  |  |  |  |  |  |  | | | |  |  |  |  |
| 1 | Vvi-Vitvi05g04178\_t001 |  |  |  |  |  |  |  | | | |  |  |  |  |
| 1 | Vvi-Vitvi05g04179\_t001 |  |  |  |  |  |  |  | | | |  |  |  |  |
| 1 | Vvi-Vitvi05g04180\_t001 |  |  |  |  |  |  |  | | | |  |  |  |  |
| 1 | Vvi-Vitvi05g04181\_t001 |  |  |  |  |  |  |  | | | |  |  |  |  |
| 1 | Vvi-Vitvi05g01940\_t001 |  |  |  |  |  |  |  | | | |  |  |  |  |
| 1 | Vvi-Vitvi05g01941\_t001 |  |  |  |  |  |  |  | | | |  |  |  |  |
| 1 | Vvi-Vitvi05g01942\_t001 |  |  |  |  |  |  |  | | | |  |  |  |  |
| 1 | Vvi-Vitvi05g04182\_t001 |  |  |  |  |  |  |  | | | |  |  |  |  |
| 1 | Vvi-Vitvi05g01943\_t001 |  |  |  |  |  |  |  | | | |  |  |  |  |
| 1 | Vvi-Vitvi05g01944\_t001 |  |  |  |  |  |  |  | | | |  |  |  |  |
| 1 | Vvi-Vitvi05g01945\_t001 |  |  |  |  |  |  |  | | | |  |  |  |  |
| 1 | Vvi-Vitvi05g00726\_t001 |  |  |  |  |  |  |  | | | |  |  |  |  |
| 1 | Vvi-Vitvi05g01946\_t001 |  |  |  |  |  |  |  | | | |  |  |  |  |
| 1 | Vvi-Vitvi05g01947\_t001 |  |  |  |  |  |  |  | | | |  |  |  |  |
| 1 | Vvi-Vitvi05g01948\_t001 |  |  |  |  |  |  |  | | | |  |  |  |  |
| 1 | Vvi-Vitvi05g01949\_t001 |  |  |  |  |  |  |  | | | |  |  |  |  |
| 1 | Vvi-Vitvi05g01950\_t001 |  |  |  |  |  |  |  | | | |  |  |  |  |
| 1 | Vvi-Vitvi05g01951\_t001 |  |  |  |  |  |  |  | | | |  |  |  |  |
| 1 | Vvi-Vitvi05g04183\_t001 |  |  |  |  |  |  |  | | | |  |  |  |  |
| 1 | Vvi-Vitvi05g01952\_t001 |  |  |  |  |  |  |  | | | |  |  |  |  |
| 1 | Vvi-Vitvi05g01953\_t001 |  |  |  |  |  |  |  | | | |  |  |  |  |
| 1 | Vvi-Vitvi05g04184\_t001 |  |  |  |  |  |  |  | | | |  |  |  |  |
| 1 | Vvi-Vitvi05g00731\_t002 |  |  |  |  |  |  |  | Ath-AT5G43380.1 |  |  |  |  |
| 1 | Vvi-Vitvi05g00733\_t001 |  |  |  |  |  |  |  | Ath-AT5G43350.1 |  |  |  |  |
| 0 | Vvi-Vitvi05g00734\_t001 |  |  |  |  |  |  |  |  |
| 0 | Vvi-Vitvi05g04185\_t001 |  |  |  |  |  |  |  |  |
| 0 | Vvi-Vitvi05g01956\_t001 |  |  |  |  |  |  |  |  |
| 0 | Vvi-Vitvi05g00736\_t001 |  |  |  |  |  |  |  |  |
| 1 | Vvi-Vitvi05g00737\_t002 |  | Ath-AT1G04400.1 |  |  |  |  |  |  |  |
| 1 | Vvi-Vitvi05g00739\_t001 |  | | | |  |  |  |  |  |  |  |
| 1 | Vvi-Vitvi05g00740\_t001 |  | | | |  |  |  |  |  |  |  |
| 2 | Vvi-Vitvi05g00742\_t001 |  | | | |  | Ath-AT4G14385.1 |  |  |  |  |  |  |
| 3 | Vvi-Vitvi05g01732\_t001 |  | | | |  | | | |  | Ath-AT3G23250.1 |  |  |  |  |  |
| 3 | Vvi-Vitvi05g01733\_t001 |  | | | |  | | | |  | | | |  |  |  |  |  |
| 3 | Vvi-Vitvi05g00746\_t001 |  | | | |  | | | |  | | | |  |  |  |  |  |
| 3 | Vvi-Vitvi05g01958\_t001 |  | | | |  | Ath-AT4G14380.1 |  | | | |  |  |  |  |  |
| 3 | Vvi-Vitvi05g04186\_t001 |  | | | |  | | | |  | | | |  |  |  |  |  |
| 3 | Vvi-Vitvi05g00752\_t002 |  | Ath-AT1G04420.1 |  | | | |  | | | |  |  |  |  |  |
| 3 | Vvi-Vitvi05g00753\_t001 |  | | | |  | Ath-AT4G14368.1 |  | Ath-AT3G23270.2 |  |  |  |  |  |
| 3 | Vvi-Vitvi05g04187\_t001 |  | | | |  | | | |  | | | |  |  |  |  |  |
| 3 | Vvi-Vitvi05g00755\_t002 |  | | | |  | Ath-AT4G14365.1 |  | Ath-AT3G23280.1 |  |  |  |  |  |
| 3 | Vvi-Vitvi05g00757\_t001 |  | | | |  | | | |  | Ath-AT3G23290.2 |  |  |  |  |  |
| 3 | Vvi-Vitvi05g04188\_t001 |  | | | |  | | | |  | | | |  |  |  |  |  |
| 3 | Vvi-Vitvi05g04189\_t001 |  | | | |  | | | |  | | | |  |  |  |  |  |
| 3 | Vvi-Vitvi05g00759\_t001 |  | Ath-AT1G04430.1 |  | Ath-AT4G14360.2 |  | Ath-AT3G23300.1 |  |  |  |  |  |
| 3 | Vvi-Vitvi05g00760\_t001 |  | | | |  | | | |  | | | |  |  |  |  |  |
| 3 | Vvi-Vitvi05g00761\_t001 |  | | | |  | | | |  | | | |  |  |  |  |  |
| 3 | Vvi-Vitvi05g00762\_t001 |  | | | |  | | | |  | | | |  |  |  |  |  |
| 3 | Vvi-Vitvi05g04190\_t001 |  | | | |  | | | |  | | | |  |  |  |  |  |
| 3 | Vvi-Vitvi05g00764\_t001 |  | | | |  | Ath-AT4G14350.1 |  | Ath-AT3G23310.1 |  |  |  |  |  |
| 3 | Vvi-Vitvi05g00765\_t001 |  | | | |  | Ath-AT4G14342.2 |  | Ath-AT3G23325.1 |  |  |  |  |  |
| 3 | Vvi-Vitvi05g00767\_t001 |  | Ath-AT1G04440.1 |  | Ath-AT4G14340.1 |  | Ath-AT3G23340.1 |  |  |  |  |  |
| 3 | Vvi-Vitvi05g04191\_t001 |  | | | |  | | | |  | | | |  |  |  |  |  |
| 3 | Vvi-Vitvi05g04192\_t001 |  | | | |  | | | |  | | | |  |  |  |  |  |
| 3 | Vvi-Vitvi05g04193\_t001 |  | | | |  | | | |  | | | |  |  |  |  |  |
| 3 | Vvi-Vitvi05g04194\_t001 |  | | | |  | | | |  | | | |  |  |  |  |  |
| 3 | Vvi-Vitvi05g04195\_t001 |  | | | |  | | | |  | | | |  |  |  |  |  |
| 3 | Vvi-Vitvi05g00769\_t001 |  | | | |  | | | |  | Ath-AT3G23350.2 |  |  |  |  |  |
| 3 | Vvi-Vitvi05g00770\_t001 |  | | | |  | | | |  | | | |  |  |  |  |  |
| 3 | Vvi-Vitvi05g00771\_t001 |  | | | |  | | | |  | | | |  |  |  |  |  |
| 3 | Vvi-Vitvi05g00772\_t001 |  | | | |  | | | |  | Ath-AT3G23360.1 |  |  |  |  |  |
| 3 | Vvi-Vitvi05g00774\_t001 |  | | | |  | | | |  | Ath-AT3G23370.1 |  |  |  |  |  |
| 3 | Vvi-Vitvi05g00775\_t001 |  | | | |  | | | |  | | | |  |  |  |  |  |
| 3 | Vvi-Vitvi05g00778\_t001 |  | | | |  | | | |  | | | |  |  |  |  |  |
| 4 | Vvi-Vitvi05g00779\_t001 |  | | | |  | | | |  | | | |  | Ath-AT2G33470.1 |  |  |  |  |
| 4 | Vvi-Vitvi05g01965\_t001 |  | Ath-AT1G04450.1 |  | | | |  | Ath-AT3G23380.1 |  | Ath-AT2G33460.1 |  |  |  |  |
| 4 | Vvi-Vitvi05g00780\_t001 |  | | | |  | Ath-AT4G14330.1 |  | | | |  | | | |  |  |  |  |
| 4 | Vvi-Vitvi05g00781\_t001 |  | | | |  | | | |  | Ath-AT3G23400.1 |  | | | |  |  |  |  |
| 4 | Vvi-Vitvi05g00782\_t001 |  | | | |  | | | |  | | | |  | | | |  |  |  |  |
| 4 | Vvi-Vitvi05g00783\_t001 |  | | | |  | Ath-AT4G14310.2 |  | | | |  | | | |  |  |  |  |
| 4 | Vvi-Vitvi05g00784\_t001 |  | | | |  | | | |  | Ath-AT3G23410.1 |  | | | |  |  |  |  |
| 4 | Vvi-Vitvi05g00785\_t001 |  | | | |  | | | |  | Ath-AT3G23430.1 |  | | | |  |  |  |  |
| 4 | Vvi-Vitvi05g04196\_t001 |  | | | |  | | | |  | | | |  | | | |  |  |  |  |
| 4 | Vvi-Vitvi05g00787\_t001 |  | | | |  | Ath-AT4G14305.1 |  | | | |  | | | |  |  |  |  |
| 4 | Vvi-Vitvi05g00788\_t001 |  | | | |  | | | |  | Ath-AT3G23440.1 |  | | | |  |  |  |  |
| 4 | Vvi-Vitvi05g04197\_t001 |  | | | |  | | | |  | | | |  | | | |  |  |  |  |
| 4 | Vvi-Vitvi05g00790\_t002 |  | | | |  | | | |  | | | |  | | | |  |  |  |  |
| 4 | Vvi-Vitvi05g01725\_t001 |  | | | |  | | | |  | | | |  | | | |  |  |  |  |
| 4 | Vvi-Vitvi05g00794\_t002 |  | | | |  | | | |  | | | |  | | | |  |  |  |  |
| 4 | Vvi-Vitvi05g00795\_t001 |  | | | |  | | | |  | | | |  | Ath-AT2G33450.1 |  |  |  |  |
| 4 | Vvi-Vitvi05g00796\_t001 |  | | | |  | | | |  | | | |  | Ath-AT2G33435.1 |  |  |  |  |
| 4 | Vvi-Vitvi05g04198\_t001 |  | | | |  | | | |  | | | |  | | | |  |  |  |  |
| 4 | Vvi-Vitvi05g00799\_t001 |  | | | |  | | | |  | | | |  | | | |  |  |  |  |
| 4 | Vvi-Vitvi05g00800\_t001 |  | | | |  | | | |  | Ath-AT3G23490.1 |  | | | |  |  |  |  |
| 4 | Vvi-Vitvi05g00802\_t001 |  | | | |  | | | |  | | | |  | | | |  |  |  |  |
| 4 | Vvi-Vitvi05g00803\_t001 |  | Ath-AT1G04470.1 |  | | | |  | | | |  | Ath-AT2G33420.1 |  |  |  |  |
| 4 | Vvi-Vitvi05g04199\_t001 |  | | | |  | | | |  | | | |  | | | |  |  |  |  |
| 4 | Vvi-Vitvi05g04200\_t001 |  | | | |  | | | |  | | | |  | | | |  |  |  |  |
| 4 | Vvi-Vitvi05g04201\_t001 |  | | | |  | | | |  | | | |  | | | |  |  |  |  |
| 4 | Vvi-Vitvi05g01972\_t001 |  | | | |  | | | |  | | | |  | | | |  |  |  |  |
| 4 | Vvi-Vitvi05g04202\_t001 |  | | | |  | | | |  | | | |  | | | |  |  |  |  |
| 4 | Vvi-Vitvi05g04203\_t001 |  | | | |  | | | |  | | | |  | | | |  |  |  |  |
| 4 | Vvi-Vitvi05g04204\_t001 |  | | | |  | | | |  | | | |  | | | |  |  |  |  |
| 4 | Vvi-Vitvi05g04205\_t001 |  | | | |  | | | |  | Ath-AT3G23510.1 |  | | | |  |  |  |  |
| 3 | Vvi-Vitvi05g04206\_t001 |  | | | |  | | | |  |  |  | | | |  |  |  |  |
| 3 | Vvi-Vitvi05g04207\_t001 |  | | | |  | | | |  |  |  | | | |  |  |  |  |
| 3 | Vvi-Vitvi05g00810\_t001 |  | | | |  | | | |  |  |  | | | |  |  |  |  |
| 3 | Vvi-Vitvi05g00811\_t001 |  | | | |  | | | |  |  |  | | | |  |  |  |  |
| 3 | Vvi-Vitvi05g00812\_t002 |  | | | |  | Ath-AT4G14300.2 |  |  |  | Ath-AT2G33410.1 |  |  |  |  |
| 2 | Vvi-Vitvi05g04208\_t001 |  | | | |  |  |  |  |  | | | |  |  |  |  |
| 2 | Vvi-Vitvi05g00814\_t001 |  | | | |  |  |  |  |  | | | |  |  |  |  |
| 2 | Vvi-Vitvi05g00815\_t001 |  | | | |  |  |  |  |  | Ath-AT2G33400.1 |  |  |  |  |
| 2 | Vvi-Vitvi05g04209\_t001 |  | | | |  |  |  |  |  | Ath-AT2G33390.1 |  |  |  |  |
| 2 | Vvi-Vitvi05g01981\_t001 |  | | | |  |  |  |  |  | Ath-AT2G33385.2 |  |  |  |  |
| 2 | Vvi-Vitvi05g00816\_t001 |  | | | |  |  |  |  |  | | | |  |  |  |  |
| 2 | Vvi-Vitvi05g00817\_t001 |  | | | |  |  |  |  |  | | | |  |  |  |  |
| 2 | Vvi-Vitvi05g00818\_t001 |  | | | |  |  |  |  |  | | | |  |  |  |  |
| 2 | Vvi-Vitvi05g00819\_t001 |  | | | |  |  |  |  |  | Ath-AT2G33380.1 |  |  |  |  |
| 2 | Vvi-Vitvi05g04210\_t001 |  | Ath-AT1G04480.1 |  |  |  |  |  | Ath-AT2G33370.1 |  |  |  |  |
| 2 | Vvi-Vitvi05g00821\_t001 |  | | | |  |  |  |  |  | | | |  |  |  |  |
| 2 | Vvi-Vitvi05g00822\_t001 |  | Ath-AT1G04490.8 |  |  |  |  |  | Ath-AT2G33360.1 |  |  |  |  |
| 2 | Vvi-Vitvi05g04211\_t001 |  | | | |  |  |  |  |  | | | |  |  |  |  |
| 2 | Vvi-Vitvi05g00823\_t001 |  | Ath-AT1G04500.1 |  |  |  |  |  | Ath-AT2G33350.5 |  |  |  |  |
| 2 | Vvi-Vitvi05g04212\_t001 |  | | | |  |  |  |  |  | | | |  |  |  |  |
| 2 | Vvi-Vitvi05g00825\_t001 |  | Ath-AT1G04510.1 |  |  |  |  |  | Ath-AT2G33340.1 |  |  |  |  |
| 2 | Vvi-Vitvi05g00826\_t001 |  | | | |  |  |  |  |  | | | |  |  |  |  |
| 2 | Vvi-Vitvi05g00828\_t001 |  | Ath-AT1G04520.1 |  |  |  |  |  | Ath-AT2G33330.1 |  |  |  |  |
| 2 | Vvi-Vitvi05g00829\_t001 |  | | | |  |  |  |  |  | | | |  |  |  |  |
| 3 | Vvi-Vitvi05g00830\_t001 |  | | | |  | Ath-AT4G13330.2 |  |  |  | | | |  |  |  |  |
| 3 | Vvi-Vitvi05g00831\_t001 |  | | | |  | | | |  |  |  | | | |  |  |  |  |
| 3 | Vvi-Vitvi05g00832\_t001 |  | | | |  | | | |  |  |  | | | |  |  |  |  |
| 3 | Vvi-Vitvi05g00834\_t001 |  | | | |  | | | |  |  |  | | | |  |  |  |  |
| 3 | Vvi-Vitvi05g00835\_t002 |  | | | |  | | | |  |  |  | | | |  |  |  |  |
| 3 | Vvi-Vitvi05g04213\_t001 |  | | | |  | | | |  |  |  | | | |  |  |  |  |
| 3 | Vvi-Vitvi05g00836\_t002 |  | Ath-AT1G04530.1 |  | | | |  |  |  | | | |  |  |  |  |
| 3 | Vvi-Vitvi05g04214\_t001 |  | | | |  | | | |  |  |  | | | |  |  |  |  |
| 3 | Vvi-Vitvi05g00837\_t001 |  | Ath-AT1G04540.1 |  | | | |  |  |  | Ath-AT2G33320.1 |  |  |  |  |
| 3 | Vvi-Vitvi05g00838\_t001 |  | Ath-AT1G04550.2 |  | | | |  |  |  | Ath-AT2G33310.2 |  |  |  |  |
| 3 | Vvi-Vitvi05g00839\_t001 |  | | | |  | Ath-AT4G13430.1 |  |  |  | | | |  |  |  |  |
| 3 | Vvi-Vitvi05g04215\_t001 |  | | | |  | | | |  |  |  | | | |  |  |  |  |
| 3 | Vvi-Vitvi05g00841\_t001 |  | | | |  | | | |  |  |  | | | |  |  |  |  |
| 3 | Vvi-Vitvi05g04216\_t001 |  | | | |  | | | |  |  |  | | | |  |  |  |  |
| 3 | Vvi-Vitvi05g04217\_t001 |  | | | |  | | | |  |  |  | | | |  |  |  |  |
| 3 | Vvi-Vitvi05g00842\_t001 |  | | | |  | | | |  |  |  | | | |  |  |  |  |
| 3 | Vvi-Vitvi05g00845\_t001 |  | | | |  | Ath-AT4G13440.2 |  |  |  | | | |  |  |  |  |
| 3 | Vvi-Vitvi05g04218\_t001 |  | | | |  | | | |  |  |  | | | |  |  |  |  |
| 3 | Vvi-Vitvi05g00847\_t001 |  | | | |  | | | |  |  |  | | | |  |  |  |  |
| 3 | Vvi-Vitvi05g00848\_t001 |  | | | |  | | | |  |  |  | | | |  |  |  |  |
| 3 | Vvi-Vitvi05g00849\_t001 |  | | | |  | | | |  |  |  | | | |  |  |  |  |
| 3 | Vvi-Vitvi05g01986\_t001 |  | | | |  | | | |  |  |  | | | |  |  |  |  |
| 3 | Vvi-Vitvi05g01987\_t001 |  | | | |  | | | |  |  |  | | | |  |  |  |  |
| 3 | Vvi-Vitvi05g01988\_t001 |  | | | |  | | | |  |  |  | | | |  |  |  |  |
| 3 | Vvi-Vitvi05g01989\_t001 |  | | | |  | | | |  |  |  | | | |  |  |  |  |
| 3 | Vvi-Vitvi05g00851\_t001 |  | | | |  | | | |  |  |  | | | |  |  |  |  |
| 3 | Vvi-Vitvi05g00852\_t001 |  | | | |  | Ath-AT4G13450.1 |  |  |  | | | |  |  |  |  |
| 3 | Vvi-Vitvi05g01990\_t001 |  | | | |  | | | |  |  |  | | | |  |  |  |  |
| 3 | Vvi-Vitvi05g01991\_t001 |  | | | |  | | | |  |  |  | | | |  |  |  |  |
| 3 | Vvi-Vitvi05g04219\_t001 |  | | | |  | | | |  |  |  | | | |  |  |  |  |
| 3 | Vvi-Vitvi05g04220\_t001 |  | Ath-AT1G04555.1 |  | | | |  |  |  | | | |  |  |  |  |
| 3 | Vvi-Vitvi05g01993\_t001 |  | | | |  | | | |  |  |  | | | |  |  |  |  |
| 3 | Vvi-Vitvi05g00855\_t001 |  | | | |  | Ath-AT4G13460.1 |  |  |  | Ath-AT2G33290.2 |  |  |  |  |
| 3 | Vvi-Vitvi05g00857\_t001 |  | Ath-AT1G04560.1 |  | | | |  |  |  | | | |  |  |  |  |
| 3 | Vvi-Vitvi05g00858\_t001 |  | | | |  | | | |  |  |  | | | |  |  |  |  |
| 3 | Vvi-Vitvi05g00861\_t001 |  | | | |  | Ath-AT4G13480.1 |  |  |  | | | |  |  |  |  |
| 3 | Vvi-Vitvi05g04221\_t001 |  | | | |  | | | |  |  |  | | | |  |  |  |  |
| 3 | Vvi-Vitvi05g04222\_t001 |  | | | |  | | | |  |  |  | | | |  |  |  |  |
| 3 | Vvi-Vitvi05g00862\_t001 |  | | | |  | | | |  |  |  | | | |  |  |  |  |
| 3 | Vvi-Vitvi05g00863\_t001 |  | | | |  | | | |  |  |  | | | |  |  |  |  |
| 3 | Vvi-Vitvi05g00864\_t001 |  | | | |  | | | |  |  |  | | | |  |  |  |  |
| 3 | Vvi-Vitvi05g04223\_t001 |  | Ath-AT1G04570.1 |  | | | |  |  |  | Ath-AT2G33280.1 |  |  |  |  |
| 3 | Vvi-Vitvi05g00867\_t001 |  | | | |  | | | |  |  |  | Ath-AT2G33255.1 |  |  |  |  |
| 3 | Vvi-Vitvi05g00871\_t001 |  | | | |  | | | |  |  |  | | | |  |  |  |  |
| 3 | Vvi-Vitvi05g00872\_t001 |  | | | |  | | | |  |  |  | | | |  |  |  |  |
| 3 | Vvi-Vitvi05g00873\_t001 |  | | | |  | Ath-AT4G13510.1 |  |  |  | | | |  |  |  |  |
| 3 | Vvi-Vitvi05g00874\_t001 |  | | | |  | | | |  |  |  | | | |  |  |  |  |
| 3 | Vvi-Vitvi05g00875\_t001 |  | | | |  | | | |  |  |  | Ath-AT2G33250.1 |  |  |  |  |
| 3 | Vvi-Vitvi05g01994\_t001 |  | | | |  | | | |  |  |  | | | |  |  |  |  |
| 3 | Vvi-Vitvi05g00878\_t002 |  | | | |  | | | |  |  |  | | | |  |  |  |  |
| 3 | Vvi-Vitvi05g04224\_t001 |  | | | |  | | | |  |  |  | | | |  |  |  |  |
| 3 | Vvi-Vitvi05g04225\_t001 |  | | | |  | | | |  |  |  | | | |  |  |  |  |
| 3 | Vvi-Vitvi05g04226\_t001 |  | | | |  | | | |  |  |  | | | |  |  |  |  |
| 3 | Vvi-Vitvi05g00881\_t001 |  | | | |  | | | |  |  |  | | | |  |  |  |  |
| 3 | Vvi-Vitvi05g00882\_t001 |  | | | |  | | | |  |  |  | | | |  |  |  |  |
| 4 | Vvi-Vitvi05g00883\_t001 |  | | | |  | | | |  | Ath-AT3G23920.1 |  | | | |  |  |  |  |
| 4 | Vvi-Vitvi05g01996\_t001 |  | | | |  | | | |  | | | |  | | | |  |  |  |  |
| 4 | Vvi-Vitvi05g00884\_t001 |  | | | |  | Ath-AT4G13550.2 |  | | | |  | | | |  |  |  |  |
| 3 | Vvi-Vitvi05g00885\_t001 |  | | | |  |  |  | Ath-AT3G23930.1 |  | | | |  |  |  |  |
| 3 | Vvi-Vitvi05g04227\_t001 |  | | | |  |  |  | | | |  | | | |  |  |  |  |
| 3 | Vvi-Vitvi05g00886\_t001 |  | | | |  |  |  | | | |  | | | |  |  |  |  |
| 3 | Vvi-Vitvi05g04228\_t001 |  | | | |  |  |  | | | |  | | | |  |  |  |  |
| 3 | Vvi-Vitvi05g04229\_t001 |  | | | |  |  |  | | | |  | | | |  |  |  |  |
| 3 | Vvi-Vitvi05g00888\_t001 |  | | | |  |  |  | | | |  | | | |  |  |  |  |
| 3 | Vvi-Vitvi05g01998\_t001 |  | Ath-AT1G04610.1 |  |  |  | | | |  | Ath-AT2G33230.1 |  |  |  |  |
| 3 | Vvi-Vitvi05g00891\_t001 |  | Ath-AT1G04620.1 |  |  |  | | | |  | | | |  |  |  |  |
| 2 | Vvi-Vitvi05g01999\_t001 |  |  |  |  |  | | | |  | | | |  |  |  |  |
| 2 | Vvi-Vitvi05g04230\_t001 |  |  |  |  |  | | | |  | | | |  |  |  |  |
| 2 | Vvi-Vitvi05g04231\_t001 |  |  |  |  |  | | | |  | | | |  |  |  |  |
| 2 | Vvi-Vitvi05g00902\_t001 |  |  |  |  |  | | | |  | | | |  |  |  |  |
| 2 | Vvi-Vitvi05g00903\_t001 |  |  |  |  |  | Ath-AT3G23980.1 |  | | | |  |  |  |  |
| 2 | Vvi-Vitvi05g00905\_t001 |  |  |  |  |  | | | |  | | | |  |  |  |  |
| 2 | Vvi-Vitvi05g04232\_t001 |  |  |  |  |  | | | |  | | | |  |  |  |  |
| 2 | Vvi-Vitvi05g02001\_t001 |  |  |  |  |  | | | |  | | | |  |  |  |  |
| 2 | Vvi-Vitvi05g04233\_t001 |  |  |  |  |  | | | |  | | | |  |  |  |  |
| 2 | Vvi-Vitvi05g00913\_t001 |  |  |  |  |  | | | |  | | | |  |  |  |  |
| 2 | Vvi-Vitvi05g04234\_t001 |  |  |  |  |  | | | |  | | | |  |  |  |  |
| 2 | Vvi-Vitvi05g00910\_t001 |  |  |  |  |  | | | |  | | | |  |  |  |  |
| 2 | Vvi-Vitvi05g04235\_t001 |  |  |  |  |  | | | |  | | | |  |  |  |  |
| 2 | Vvi-Vitvi05g04236\_t001 |  |  |  |  |  | | | |  | | | |  |  |  |  |
| 2 | Vvi-Vitvi05g04237\_t001 |  |  |  |  |  | | | |  | | | |  |  |  |  |
| 2 | Vvi-Vitvi05g02006\_t001 |  |  |  |  |  | | | |  | | | |  |  |  |  |
| 2 | Vvi-Vitvi05g04238\_t001 |  |  |  |  |  | | | |  | | | |  |  |  |  |
| 2 | Vvi-Vitvi05g00918\_t001 |  |  |  |  |  | | | |  | | | |  |  |  |  |
| 2 | Vvi-Vitvi05g00920\_t001 |  |  |  |  |  | | | |  | | | |  |  |  |  |
| 2 | Vvi-Vitvi05g00923\_t001 |  |  |  |  |  | | | |  | | | |  |  |  |  |
| 2 | Vvi-Vitvi05g00924\_t001 |  |  |  |  |  | Ath-AT3G23990.1 |  | Ath-AT2G33210.1 |  |  |  |  |
| 2 | Vvi-Vitvi05g00925\_t001 |  |  |  |  |  | Ath-AT3G24010.1 |  | | | |  |  |  |  |
| 2 | Vvi-Vitvi05g00927\_t001 |  |  |  |  |  | | | |  | | | |  |  |  |  |
| 2 | Vvi-Vitvi05g00928\_t001 |  |  |  |  |  | | | |  | Ath-AT2G33205.5 |  |  |  |  |
| 2 | Vvi-Vitvi05g00929\_t001 |  |  |  |  |  | Ath-AT3G24020.1 |  | | | |  |  |  |  |
| 2 | Vvi-Vitvi05g02009\_t001 |  |  |  |  |  | | | |  | | | |  |  |  |  |
| 2 | Vvi-Vitvi05g00930\_t001 |  |  |  |  |  | Ath-AT3G24030.1 |  | | | |  |  |  |  |
| 2 | Vvi-Vitvi05g00932\_t001 |  |  |  |  |  | Ath-AT3G24040.1 |  | | | |  |  |  |  |
| 2 | Vvi-Vitvi05g00934\_t001 |  |  |  |  |  | | | |  | | | |  |  |  |  |
| 2 | Vvi-Vitvi05g04239\_t001 |  |  |  |  |  | | | |  | | | |  |  |  |  |
| 2 | Vvi-Vitvi05g00936\_t001 |  |  |  |  |  | | | |  | | | |  |  |  |  |
| 2 | Vvi-Vitvi05g00938\_t002 |  |  |  |  |  | Ath-AT3G24050.1 |  | | | |  |  |  |  |
| 2 | Vvi-Vitvi05g04240\_t001 |  |  |  |  |  | | | |  | | | |  |  |  |  |
| 2 | Vvi-Vitvi05g00939\_t001 |  |  |  |  |  | | | |  | Ath-AT2G33170.1 |  |  |  |  |
| 2 | Vvi-Vitvi05g00941\_t001 |  |  |  |  |  | Ath-AT3G24060.1 |  | | | |  |  |  |  |
| 3 | Vvi-Vitvi05g00943\_t001 |  | Ath-AT1G04645.1 |  |  |  | | | |  | | | |  |  |  |  |
| 3 | Vvi-Vitvi05g00944\_t001 |  | Ath-AT1G04650.1 |  |  |  | | | |  | | | |  |  |  |  |
| 3 | Vvi-Vitvi05g04241\_t001 |  | | | |  |  |  | | | |  | | | |  |  |  |  |
| 3 | Vvi-Vitvi05g02012\_t001 |  | | | |  |  |  | | | |  | | | |  |  |  |  |
| 3 | Vvi-Vitvi05g02013\_t001 |  | | | |  |  |  | | | |  | | | |  |  |  |  |
| 3 | Vvi-Vitvi05g00948\_t001 |  | | | |  |  |  | | | |  | | | |  |  |  |  |
| 3 | Vvi-Vitvi05g00952\_t001 |  | | | |  |  |  | | | |  | | | |  |  |  |  |
| 3 | Vvi-Vitvi05g00953\_t001 |  | Ath-AT1G04680.1 |  |  |  | | | |  | | | |  |  |  |  |
| 3 | Vvi-Vitvi05g04242\_t001 |  | | | |  |  |  | | | |  | | | |  |  |  |  |
| 3 | Vvi-Vitvi05g00959\_t001 |  | | | |  |  |  | | | |  | | | |  |  |  |  |
| 3 | Vvi-Vitvi05g00960\_t001 |  | Ath-AT1G04690.1 |  |  |  | | | |  | | | |  |  |  |  |
| 3 | Vvi-Vitvi05g04243\_t001 |  | | | |  |  |  | | | |  | | | |  |  |  |  |
| 3 | Vvi-Vitvi05g00961\_t001 |  | Ath-AT1G04700.1 |  |  |  | | | |  | | | |  |  |  |  |
| 3 | Vvi-Vitvi05g00963\_t001 |  | | | |  |  |  | Ath-AT3G24220.1 |  | | | |  |  |  |  |
| 2 | Vvi-Vitvi05g02017\_t001 |  | | | |  |  |  |  |  | | | |  |  |  |  |
| 2 | Vvi-Vitvi05g02018\_t001 |  | | | |  |  |  |  |  | | | |  |  |  |  |
| 2 | Vvi-Vitvi05g02019\_t001 |  | | | |  |  |  |  |  | | | |  |  |  |  |
| 2 | Vvi-Vitvi05g04244\_t001 |  | | | |  |  |  |  |  | | | |  |  |  |  |
| 2 | Vvi-Vitvi05g00966\_t001 |  | Ath-AT1G04710.1 |  |  |  |  |  | Ath-AT2G33150.1 |  |  |  |  |
| 1 | Vvi-Vitvi05g02021\_t002 |  | Ath-AT1G04790.1 |  |  |  |  |  |  |  |
| 1 | Vvi-Vitvi05g04245\_t001 |  | | | |  |  |  |  |  |  |  |
| 1 | Vvi-Vitvi05g04246\_t001 |  | | | |  |  |  |  |  |  |  |
| 1 | Vvi-Vitvi05g04247\_t002 |  | | | |  |  |  |  |  |  |  |
| 1 | Vvi-Vitvi05g04248\_t001 |  | | | |  |  |  |  |  |  |  |
| 1 | Vvi-Vitvi05g04249\_t001 |  | | | |  |  |  |  |  |  |  |
| 1 | Vvi-Vitvi05g04250\_t001 |  | | | |  |  |  |  |  |  |  |
| 1 | Vvi-Vitvi05g04251\_t001 |  | | | |  |  |  |  |  |  |  |
| 1 | Vvi-Vitvi05g04252\_t001 |  | | | |  |  |  |  |  |  |  |
| 1 | Vvi-Vitvi05g00972\_t001 |  | | | |  |  |  |  |  |  |  |
| 1 | Vvi-Vitvi05g04253\_t001 |  | | | |  |  |  |  |  |  |  |
| 1 | Vvi-Vitvi05g00973\_t001 |  | Ath-AT1G04820.1 |  |  |  |  |  |  |  |
| 0 | Vvi-Vitvi05g00975\_t001 |  |  |  |  |  |  |  |  |
| 0 | Vvi-Vitvi05g00976\_t001 |  |  |  |  |  |  |  |  |
| 0 | Vvi-Vitvi05g02025\_t001 |  |  |  |  |  |  |  |  |
| 0 | Vvi-Vitvi05g00977\_t001 |  |  |  |  |  |  |  |  |
| 0 | Vvi-Vitvi05g00979\_t001 |  |  |  |  |  |  |  |  |
| 0 | Vvi-Vitvi05g00986\_t001 |  |  |  |  |  |  |  |  |
| 0 | Vvi-Vitvi05g02028\_t001 |  |  |  |  |  |  |  |  |
| 0 | Vvi-Vitvi05g00990\_t001 |  |  |  |  |  |  |  |  |
| 0 | Vvi-Vitvi05g00991\_t001 |  |  |  |  |  |  |  |  |
| 0 | Vvi-Vitvi05g04254\_t001 |  |  |  |  |  |  |  |  |
| 0 | Vvi-Vitvi05g00994\_t001 |  |  |  |  |  |  |  |  |
| 0 | Vvi-Vitvi05g04255\_t001 |  |  |  |  |  |  |  |  |
| 0 | Vvi-Vitvi05g04256\_t001 |  |  |  |  |  |  |  |  |
| 0 | Vvi-Vitvi05g04257\_t001 |  |  |  |  |  |  |  |  |
| 0 | Vvi-Vitvi05g04258\_t001 |  |  |  |  |  |  |  |  |
| 0 | Vvi-Vitvi05g04259\_t001 |  |  |  |  |  |  |  |  |
| 0 | Vvi-Vitvi05g04260\_t001 |  |  |  |  |  |  |  |  |
| 0 | Vvi-Vitvi05g04261\_t001 |  |  |  |  |  |  |  |  |
| 0 | Vvi-Vitvi05g01005\_t001 |  |  |  |  |  |  |  |  |
| 0 | Vvi-Vitvi05g04262\_t001 |  |  |  |  |  |  |  |  |
| 0 | Vvi-Vitvi05g04263\_t001 |  |  |  |  |  |  |  |  |
| 0 | Vvi-Vitvi05g04264\_t001 |  |  |  |  |  |  |  |  |
| 0 | Vvi-Vitvi05g04265\_t001 |  |  |  |  |  |  |  |  |
| 0 | Vvi-Vitvi05g01018\_t001 |  |  |  |  |  |  |  |  |
| 0 | Vvi-Vitvi05g01031\_t001 |  |  |  |  |  |  |  |  |
| 0 | Vvi-Vitvi05g01032\_t001 |  |  |  |  |  |  |  |  |
| 0 | Vvi-Vitvi05g04266\_t001 |  |  |  |  |  |  |  |  |
| 0 | Vvi-Vitvi05g04267\_t001 |  |  |  |  |  |  |  |  |
| 1 | Vvi-Vitvi05g01036\_t001 |  | Ath-AT3G24200.2 |  |  |  |  |  |  |  |
| 1 | Vvi-Vitvi05g04268\_t001 |  | | | |  |  |  |  |  |  |  |
| 1 | Vvi-Vitvi05g01038\_t001 |  | Ath-AT3G24190.1 |  |  |  |  |  |  |  |
| 1 | Vvi-Vitvi05g04269\_t001 |  | | | |  |  |  |  |  |  |  |
| 1 | Vvi-Vitvi05g02038\_t001 |  | | | |  |  |  |  |  |  |  |
| 1 | Vvi-Vitvi05g01041\_t001 |  | | | |  |  |  |  |  |  |  |
| 1 | Vvi-Vitvi05g01042\_t001 |  | | | |  |  |  |  |  |  |  |
| 1 | Vvi-Vitvi05g04270\_t001 |  | | | |  |  |  |  |  |  |  |
| 1 | Vvi-Vitvi05g01044\_t001 |  | | | |  |  |  |  |  |  |  |
| 1 | Vvi-Vitvi05g01046\_t001 |  | | | |  |  |  |  |  |  |  |
| 1 | Vvi-Vitvi05g01047\_t001 |  | | | |  |  |  |  |  |  |  |
| 1 | Vvi-Vitvi05g01048\_t001 |  | | | |  |  |  |  |  |  |  |
| 1 | Vvi-Vitvi05g04271\_t001 |  | | | |  |  |  |  |  |  |  |
| 1 | Vvi-Vitvi05g04272\_t001 |  | | | |  |  |  |  |  |  |  |
| 1 | Vvi-Vitvi05g04273\_t001 |  | | | |  |  |  |  |  |  |  |
| 1 | Vvi-Vitvi05g04274\_t001 |  | | | |  |  |  |  |  |  |  |
| 1 | Vvi-Vitvi05g01054\_t002 |  | Ath-AT3G24160.1 |  |  |  |  |  |  |  |
| 2 | Vvi-Vitvi05g01055\_t001 |  | | | |  | Ath-AT4G14050.1 |  |  |  |  |  |  |
| 2 | Vvi-Vitvi05g01057\_t001 |  | | | |  | | | |  |  |  |  |  |  |
| 2 | Vvi-Vitvi05g01059\_t001 |  | | | |  | | | |  |  |  |  |  |  |
| 2 | Vvi-Vitvi05g01061\_t001 |  | | | |  | | | |  |  |  |  |  |  |
| 2 | Vvi-Vitvi05g01063\_t001 |  | Ath-AT3G24140.1 |  | | | |  |  |  |  |  |  |
| 2 | Vvi-Vitvi05g04275\_t001 |  | | | |  | | | |  |  |  |  |  |  |
| 2 | Vvi-Vitvi05g04276\_t001 |  | | | |  | | | |  |  |  |  |  |  |
| 2 | Vvi-Vitvi05g01066\_t002 |  | Ath-AT3G24120.2 |  | | | |  |  |  |  |  |  |
| 2 | Vvi-Vitvi05g01068\_t001 |  | Ath-AT3G24110.1 |  | | | |  |  |  |  |  |  |
| 1 | Vvi-Vitvi05g01069\_t001 |  |  |  | | | |  |  |  |  |  |  |
| 1 | Vvi-Vitvi05g04277\_t001 |  |  |  | | | |  |  |  |  |  |  |
| 1 | Vvi-Vitvi05g04278\_t001 |  |  |  | | | |  |  |  |  |  |  |
| 1 | Vvi-Vitvi05g04279\_t001 |  |  |  | | | |  |  |  |  |  |  |
| 1 | Vvi-Vitvi05g02044\_t001 |  |  |  | | | |  |  |  |  |  |  |
| 1 | Vvi-Vitvi05g01072\_t001 |  |  |  | | | |  |  |  |  |  |  |
| 1 | Vvi-Vitvi05g01073\_t001 |  |  |  | | | |  |  |  |  |  |  |
| 1 | Vvi-Vitvi05g04280\_t001 |  |  |  | | | |  |  |  |  |  |  |
| 1 | Vvi-Vitvi05g01075\_t001 |  |  |  | | | |  |  |  |  |  |  |
| 1 | Vvi-Vitvi05g01076\_t001 |  |  |  | | | |  |  |  |  |  |  |
| 1 | Vvi-Vitvi05g04281\_t001 |  |  |  | | | |  |  |  |  |  |  |
| 1 | Vvi-Vitvi05g04282\_t001 |  |  |  | | | |  |  |  |  |  |  |
| 1 | Vvi-Vitvi05g01080\_t001 |  |  |  | | | |  |  |  |  |  |  |
| 1 | Vvi-Vitvi05g01081\_t001 |  |  |  | | | |  |  |  |  |  |  |
| 1 | Vvi-Vitvi05g01082\_t001 |  |  |  | | | |  |  |  |  |  |  |
| 1 | Vvi-Vitvi05g01087\_t001 |  |  |  | | | |  |  |  |  |  |  |
| 2 | Vvi-Vitvi05g01088\_t001 |  | Ath-AT3G23800.1 |  | Ath-AT4G14030.2 |  |  |  |  |  |  |
| 2 | Vvi-Vitvi05g04283\_t001 |  | | | |  | | | |  |  |  |  |  |  |
| 2 | Vvi-Vitvi05g01091\_t001 |  | | | |  | | | |  |  |  |  |  |  |
| 2 | Vvi-Vitvi05g01094\_t001 |  | Ath-AT3G23810.1 |  | | | |  |  |  |  |  |  |
| 2 | Vvi-Vitvi05g01095\_t001 |  | | | |  | | | |  |  |  |  |  |  |
| 2 | Vvi-Vitvi05g01096\_t001 |  | | | |  | | | |  |  |  |  |  |  |
| 2 | Vvi-Vitvi05g01097\_t001 |  | | | |  | Ath-AT4G13980.1 |  |  |  |  |  |  |
| 2 | Vvi-Vitvi05g02048\_t001 |  | | | |  | Ath-AT4G13950.1 |  |  |  |  |  |  |
| 2 | Vvi-Vitvi05g04284\_t001 |  | | | |  | | | |  |  |  |  |  |  |
| 2 | Vvi-Vitvi05g04285\_t001 |  | | | |  | | | |  |  |  |  |  |  |
| 2 | Vvi-Vitvi05g02049\_t001 |  | | | |  | | | |  |  |  |  |  |  |
| 2 | Vvi-Vitvi05g01103\_t001 |  | | | |  | | | |  |  |  |  |  |  |
| 2 | Vvi-Vitvi05g01104\_t001 |  | | | |  | | | |  |  |  |  |  |  |
| 2 | Vvi-Vitvi05g01106\_t001 |  | | | |  | Ath-AT4G13870.2 |  |  |  |  |  |  |
| 2 | Vvi-Vitvi05g01108\_t001 |  | Ath-AT3G23820.1 |  | | | |  |  |  |  |  |  |
| 2 | Vvi-Vitvi05g01112\_t001 |  | Ath-AT3G23830.1 |  | Ath-AT4G13850.1 |  |  |  |  |  |  |
| 2 | Vvi-Vitvi05g01113\_t001 |  | | | |  | | | |  |  |  |  |  |  |
| 2 | Vvi-Vitvi05g01114\_t001 |  | | | |  | | | |  |  |  |  |  |  |
| 2 | Vvi-Vitvi05g04286\_t001 |  | | | |  | | | |  |  |  |  |  |  |
| 2 | Vvi-Vitvi05g01115\_t001 |  | | | |  | | | |  |  |  |  |  |  |
| 2 | Vvi-Vitvi05g01116\_t001 |  | Ath-AT3G23840.1 |  | Ath-AT4G13840.1 |  |  |  |  |  |  |
| 2 | Vvi-Vitvi05g04287\_t001 |  | | | |  | | | |  |  |  |  |  |  |
| 2 | Vvi-Vitvi05g04288\_t001 |  | | | |  | | | |  |  |  |  |  |  |
| 2 | Vvi-Vitvi05g01117\_t001 |  | | | |  | | | |  |  |  |  |  |  |
| 2 | Vvi-Vitvi05g01118\_t001 |  | | | |  | Ath-AT4G13830.2 |  |  |  |  |  |  |
| 2 | Vvi-Vitvi05g04289\_t001 |  | | | |  | | | |  |  |  |  |  |  |
| 2 | Vvi-Vitvi05g02051\_t001 |  | | | |  | | | |  |  |  |  |  |  |
| 2 | Vvi-Vitvi05g02052\_t001 |  | | | |  | | | |  |  |  |  |  |  |
| 2 | Vvi-Vitvi05g04290\_t001 |  | | | |  | | | |  |  |  |  |  |  |
| 2 | Vvi-Vitvi05g04291\_t001 |  | | | |  | | | |  |  |  |  |  |  |
| 2 | Vvi-Vitvi05g01120\_t001 |  | | | |  | | | |  |  |  |  |  |  |
| 2 | Vvi-Vitvi05g01121\_t001 |  | | | |  | | | |  |  |  |  |  |  |
| 2 | Vvi-Vitvi05g04292\_t001 |  | | | |  | | | |  |  |  |  |  |  |
| 2 | Vvi-Vitvi05g04293\_t001 |  | | | |  | | | |  |  |  |  |  |  |
| 2 | Vvi-Vitvi05g01124\_t001 |  | | | |  | | | |  |  |  |  |  |  |
| 2 | Vvi-Vitvi05g01125\_t001 |  | | | |  | | | |  |  |  |  |  |  |
| 2 | Vvi-Vitvi05g01126\_t001 |  | | | |  | | | |  |  |  |  |  |  |
| 2 | Vvi-Vitvi05g01128\_t001 |  | Ath-AT3G23870.1 |  | Ath-AT4G13800.1 |  |  |  |  |  |  |
| 1 | Vvi-Vitvi05g01129\_t001 |  | | | |  |  |  |  |  |  |  |
| 1 | Vvi-Vitvi05g02056\_t001 |  | Ath-AT3G23910.1 |  |  |  |  |  |  |  |
| 0 | Vvi-Vitvi05g01130\_t002 |  |  |  |  |  |  |  |  |
| 0 | Vvi-Vitvi05g01135\_t001 |  |  |  |  |  |  |  |  |
| 0 | Vvi-Vitvi05g04294\_t001 |  |  |  |  |  |  |  |  |
| 0 | Vvi-Vitvi05g01136\_t001 |  |  |  |  |  |  |  |  |
| 0 | Vvi-Vitvi05g04295\_t001 |  |  |  |  |  |  |  |  |
| 0 | Vvi-Vitvi05g01137\_t001 |  |  |  |  |  |  |  |  |
| 0 | Vvi-Vitvi05g01138\_t001 |  |  |  |  |  |  |  |  |
| 0 | Vvi-Vitvi05g04296\_t001 |  |  |  |  |  |  |  |  |
| 0 | Vvi-Vitvi05g04297\_t001 |  |  |  |  |  |  |  |  |
| 0 | Vvi-Vitvi05g04298\_t001 |  |  |  |  |  |  |  |  |
| 0 | Vvi-Vitvi05g04299\_t001 |  |  |  |  |  |  |  |  |
| 0 | Vvi-Vitvi05g04300\_t001 |  |  |  |  |  |  |  |  |
| 0 | Vvi-Vitvi05g04301\_t001 |  |  |  |  |  |  |  |  |
| 0 | Vvi-Vitvi05g04302\_t001 |  |  |  |  |  |  |  |  |
| 0 | Vvi-Vitvi05g01145\_t001 |  |  |  |  |  |  |  |  |
| 0 | Vvi-Vitvi05g04303\_t001 |  |  |  |  |  |  |  |  |
| 0 | Vvi-Vitvi05g04304\_t001 |  |  |  |  |  |  |  |  |
| 0 | Vvi-Vitvi05g04305\_t001 |  |  |  |  |  |  |  |  |
| 0 | Vvi-Vitvi05g04306\_t001 |  |  |  |  |  |  |  |  |
| 0 | Vvi-Vitvi05g04307\_t001 |  |  |  |  |  |  |  |  |
| 0 | Vvi-Vitvi05g04308\_t001 |  |  |  |  |  |  |  |  |
| 0 | Vvi-Vitvi05g04309\_t001 |  |  |  |  |  |  |  |  |
| 0 | Vvi-Vitvi05g04310\_t001 |  |  |  |  |  |  |  |  |
| 0 | Vvi-Vitvi05g01153\_t001 |  |  |  |  |  |  |  |  |
| 0 | Vvi-Vitvi05g04311\_t001 |  |  |  |  |  |  |  |  |
| 0 | Vvi-Vitvi05g02059\_t001 |  |  |  |  |  |  |  |  |
| 0 | Vvi-Vitvi05g01154\_t001 |  |  |  |  |  |  |  |  |
| 0 | Vvi-Vitvi05g01159\_t001 |  |  |  |  |  |  |  |  |
| 0 | Vvi-Vitvi05g04312\_t001 |  |  |  |  |  |  |  |  |
| 0 | Vvi-Vitvi05g04313\_t001 |  |  |  |  |  |  |  |  |
| 0 | Vvi-Vitvi05g01164\_t001 |  |  |  |  |  |  |  |  |
| 0 | Vvi-Vitvi05g04314\_t001 |  |  |  |  |  |  |  |  |
| 0 | Vvi-Vitvi05g04315\_t001 |  |  |  |  |  |  |  |  |
| 0 | Vvi-Vitvi05g04316\_t001 |  |  |  |  |  |  |  |  |
| 0 | Vvi-Vitvi05g02074\_t001 |  |  |  |  |  |  |  |  |
| 0 | Vvi-Vitvi05g04317\_t001 |  |  |  |  |  |  |  |  |
| 0 | Vvi-Vitvi05g01178\_t001 |  |  |  |  |  |  |  |  |
| 0 | Vvi-Vitvi05g01179\_t001 |  |  |  |  |  |  |  |  |
| 0 | Vvi-Vitvi05g04318\_t001 |  |  |  |  |  |  |  |  |
| 0 | Vvi-Vitvi05g01181\_t001 |  |  |  |  |  |  |  |  |
| 0 | Vvi-Vitvi05g04319\_t001 |  |  |  |  |  |  |  |  |
| 0 | Vvi-Vitvi05g04320\_t001 |  |  |  |  |  |  |  |  |
| 0 | Vvi-Vitvi05g04321\_t001 |  |  |  |  |  |  |  |  |
| 0 | Vvi-Vitvi05g04322\_t001 |  |  |  |  |  |  |  |  |
| 0 | Vvi-Vitvi05g01184\_t001 |  |  |  |  |  |  |  |  |
| 0 | Vvi-Vitvi05g01185\_t004 |  |  |  |  |  |  |  |  |
| 0 | Vvi-Vitvi05g01186\_t001 |  |  |  |  |  |  |  |  |
| 0 | Vvi-Vitvi05g04323\_t001 |  |  |  |  |  |  |  |  |
| 0 | Vvi-Vitvi05g01189\_t001 |  |  |  |  |  |  |  |  |
| 0 | Vvi-Vitvi05g04324\_t001 |  |  |  |  |  |  |  |  |
| 0 | Vvi-Vitvi05g04325\_t001 |  |  |  |  |  |  |  |  |
| 0 | Vvi-Vitvi05g01192\_t001 |  |  |  |  |  |  |  |  |
| 0 | Vvi-Vitvi05g02077\_t001 |  |  |  |  |  |  |  |  |
| 0 | Vvi-Vitvi05g01193\_t003 |  |  |  |  |  |  |  |  |
| 0 | Vvi-Vitvi05g01195\_t001 |  |  |  |  |  |  |  |  |
| 0 | Vvi-Vitvi05g01197\_t001 |  |  |  |  |  |  |  |  |
| 0 | Vvi-Vitvi05g01198\_t001 |  |  |  |  |  |  |  |  |
| 0 | Vvi-Vitvi05g01200\_t001 |  |  |  |  |  |  |  |  |
| 0 | Vvi-Vitvi05g01201\_t001 |  |  |  |  |  |  |  |  |
| 0 | Vvi-Vitvi05g01202\_t001 |  |  |  |  |  |  |  |  |
| 0 | Vvi-Vitvi05g04326\_t001 |  |  |  |  |  |  |  |  |
| 0 | Vvi-Vitvi05g01209\_t001 |  |  |  |  |  |  |  |  |
| 0 | Vvi-Vitvi05g02078\_t001 |  |  |  |  |  |  |  |  |
| 0 | Vvi-Vitvi05g04327\_t001 |  |  |  |  |  |  |  |  |
| 0 | Vvi-Vitvi05g01213\_t001 |  |  |  |  |  |  |  |  |
| 0 | Vvi-Vitvi05g02079\_t001 |  |  |  |  |  |  |  |  |
| 0 | Vvi-Vitvi05g01214\_t001 |  |  |  |  |  |  |  |  |
| 0 | Vvi-Vitvi05g01216\_t001 |  |  |  |  |  |  |  |  |
| 0 | Vvi-Vitvi05g01219\_t001 |  |  |  |  |  |  |  |  |
| 0 | Vvi-Vitvi05g01221\_t002 |  |  |  |  |  |  |  |  |
| 0 | Vvi-Vitvi05g04328\_t001 |  |  |  |  |  |  |  |  |
| 0 | Vvi-Vitvi05g04329\_t001 |  |  |  |  |  |  |  |  |
| 0 | Vvi-Vitvi05g01223\_t001 |  |  |  |  |  |  |  |  |
| 0 | Vvi-Vitvi05g02082\_t001 |  |  |  |  |  |  |  |  |
| 0 | Vvi-Vitvi05g04330\_t001 |  |  |  |  |  |  |  |  |
| 0 | Vvi-Vitvi05g04331\_t001 |  |  |  |  |  |  |  |  |
| 0 | Vvi-Vitvi05g04332\_t001 |  |  |  |  |  |  |  |  |
| 0 | Vvi-Vitvi05g04333\_t001 |  |  |  |  |  |  |  |  |
| 0 | Vvi-Vitvi05g02085\_t004 |  |  |  |  |  |  |  |  |
| 0 | Vvi-Vitvi05g04334\_t001 |  |  |  |  |  |  |  |  |
| 0 | Vvi-Vitvi05g04335\_t001 |  |  |  |  |  |  |  |  |
| 0 | Vvi-Vitvi05g01228\_t001 |  |  |  |  |  |  |  |  |
| 0 | Vvi-Vitvi05g02088\_t002 |  |  |  |  |  |  |  |  |
| 0 | Vvi-Vitvi05g02089\_t001 |  |  |  |  |  |  |  |  |
| 0 | Vvi-Vitvi05g01229\_t001 |  |  |  |  |  |  |  |  |
| 0 | Vvi-Vitvi05g04336\_t001 |  |  |  |  |  |  |  |  |
| 0 | Vvi-Vitvi05g04337\_t001 |  |  |  |  |  |  |  |  |
| 0 | Vvi-Vitvi05g02090\_t001 |  |  |  |  |  |  |  |  |
| 0 | Vvi-Vitvi05g01231\_t001 |  |  |  |  |  |  |  |  |
| 0 | Vvi-Vitvi05g04338\_t001 |  |  |  |  |  |  |  |  |
| 0 | Vvi-Vitvi05g04339\_t001 |  |  |  |  |  |  |  |  |
| 0 | Vvi-Vitvi05g04340\_t001 |  |  |  |  |  |  |  |  |
| 0 | Vvi-Vitvi05g04341\_t001 |  |  |  |  |  |  |  |  |
| 0 | Vvi-Vitvi05g04342\_t001 |  |  |  |  |  |  |  |  |
| 0 | Vvi-Vitvi05g04343\_t001 |  |  |  |  |  |  |  |  |
| 0 | Vvi-Vitvi05g04344\_t001 |  |  |  |  |  |  |  |  |
| 0 | Vvi-Vitvi05g04345\_t001 |  |  |  |  |  |  |  |  |
| 0 | Vvi-Vitvi05g04346\_t001 |  |  |  |  |  |  |  |  |
| 0 | Vvi-Vitvi05g04347\_t001 |  |  |  |  |  |  |  |  |
| 0 | Vvi-Vitvi05g04348\_t001 |  |  |  |  |  |  |  |  |
| 0 | Vvi-Vitvi05g02096\_t001 |  |  |  |  |  |  |  |  |
| 0 | Vvi-Vitvi05g04349\_t001 |  |  |  |  |  |  |  |  |
| 0 | Vvi-Vitvi05g04350\_t001 |  |  |  |  |  |  |  |  |
| 0 | Vvi-Vitvi05g04351\_t001 |  |  |  |  |  |  |  |  |
| 0 | Vvi-Vitvi05g01242\_t001 |  |  |  |  |  |  |  |  |
| 0 | Vvi-Vitvi05g02097\_t001 |  |  |  |  |  |  |  |  |
| 0 | Vvi-Vitvi05g02099\_t003 |  |  |  |  |  |  |  |  |
| 0 | Vvi-Vitvi05g02100\_t001 |  |  |  |  |  |  |  |  |
| 0 | Vvi-Vitvi05g04352\_t001 |  |  |  |  |  |  |  |  |
| 0 | Vvi-Vitvi05g01247\_t001 |  |  |  |  |  |  |  |  |
| 0 | Vvi-Vitvi05g01248\_t001 |  |  |  |  |  |  |  |  |
| 0 | Vvi-Vitvi05g01250\_t001 |  |  |  |  |  |  |  |  |
| 0 | Vvi-Vitvi05g01252\_t001 |  |  |  |  |  |  |  |  |
| 0 | Vvi-Vitvi05g01254\_t001 |  |  |  |  |  |  |  |  |
| 0 | Vvi-Vitvi05g01255\_t001 |  |  |  |  |  |  |  |  |
| 0 | Vvi-Vitvi05g01256\_t001 |  |  |  |  |  |  |  |  |
| 0 | Vvi-Vitvi05g01257\_t001 |  |  |  |  |  |  |  |  |
| 0 | Vvi-Vitvi05g01258\_t002 |  |  |  |  |  |  |  |  |
| 0 | Vvi-Vitvi05g04353\_t001 |  |  |  |  |  |  |  |  |
| 0 | Vvi-Vitvi05g04354\_t001 |  |  |  |  |  |  |  |  |
| 0 | Vvi-Vitvi05g01263\_t001 |  |  |  |  |  |  |  |  |
| 0 | Vvi-Vitvi05g01264\_t001 |  |  |  |  |  |  |  |  |
| 0 | Vvi-Vitvi05g01265\_t001 |  |  |  |  |  |  |  |  |
| 0 | Vvi-Vitvi05g01266\_t001 |  |  |  |  |  |  |  |  |
| 0 | Vvi-Vitvi05g01267\_t001 |  |  |  |  |  |  |  |  |
| 0 | Vvi-Vitvi05g01269\_t001 |  |  |  |  |  |  |  |  |
| 0 | Vvi-Vitvi05g04355\_t001 |  |  |  |  |  |  |  |  |
| 0 | Vvi-Vitvi05g04356\_t001 |  |  |  |  |  |  |  |  |
| 0 | Vvi-Vitvi05g04357\_t001 |  |  |  |  |  |  |  |  |
| 0 | Vvi-Vitvi05g04358\_t001 |  |  |  |  |  |  |  |  |
| 0 | Vvi-Vitvi05g02103\_t001 |  |  |  |  |  |  |  |  |
| 0 | Vvi-Vitvi05g04360\_t001 |  |  |  |  |  |  |  |  |
| 0 | Vvi-Vitvi05g02104\_t001 |  |  |  |  |  |  |  |  |
| 0 | Vvi-Vitvi05g01273\_t001 |  |  |  |  |  |  |  |  |
| 0 | Vvi-Vitvi05g01274\_t001 |  |  |  |  |  |  |  |  |
| 0 | Vvi-Vitvi05g01276\_t001 |  |  |  |  |  |  |  |  |
| 0 | Vvi-Vitvi05g01278\_t001 |  |  |  |  |  |  |  |  |
| 0 | Vvi-Vitvi05g01279\_t001 |  |  |  |  |  |  |  |  |
| 0 | Vvi-Vitvi05g02108\_t001 |  |  |  |  |  |  |  |  |
| 0 | Vvi-Vitvi05g01285\_t001 |  |  |  |  |  |  |  |  |
| 0 | Vvi-Vitvi05g04361\_t001 |  |  |  |  |  |  |  |  |
| 0 | Vvi-Vitvi05g04362\_t001 |  |  |  |  |  |  |  |  |
| 0 | Vvi-Vitvi05g01288\_t001 |  |  |  |  |  |  |  |  |
| 0 | Vvi-Vitvi05g01289\_t001 |  |  |  |  |  |  |  |  |
| 0 | Vvi-Vitvi05g02111\_t001 |  |  |  |  |  |  |  |  |
| 0 | Vvi-Vitvi05g02112\_t001 |  |  |  |  |  |  |  |  |
| 0 | Vvi-Vitvi05g01291\_t001 |  |  |  |  |  |  |  |  |
| 0 | Vvi-Vitvi05g04363\_t001 |  |  |  |  |  |  |  |  |
| 0 | Vvi-Vitvi05g01294\_t001 |  |  |  |  |  |  |  |  |
| 0 | Vvi-Vitvi05g04364\_t001 |  |  |  |  |  |  |  |  |
| 0 | Vvi-Vitvi05g04365\_t001 |  |  |  |  |  |  |  |  |
| 0 | Vvi-Vitvi05g01299\_t001 |  |  |  |  |  |  |  |  |
| 0 | Vvi-Vitvi05g01300\_t001 |  |  |  |  |  |  |  |  |
| 0 | Vvi-Vitvi05g01301\_t001 |  |  |  |  |  |  |  |  |
| 0 | Vvi-Vitvi05g01304\_t001 |  |  |  |  |  |  |  |  |
| 0 | Vvi-Vitvi05g02116\_t001 |  |  |  |  |  |  |  |  |
| 0 | Vvi-Vitvi05g01306\_t001 |  |  |  |  |  |  |  |  |
| 0 | Vvi-Vitvi05g01307\_t001 |  |  |  |  |  |  |  |  |
| 0 | Vvi-Vitvi05g01308\_t002 |  |  |  |  |  |  |  |  |
| 0 | Vvi-Vitvi05g01309\_t001 |  |  |  |  |  |  |  |  |
| 0 | Vvi-Vitvi05g01310\_t001 |  |  |  |  |  |  |  |  |
| 0 | Vvi-Vitvi05g01313\_t001 |  |  |  |  |  |  |  |  |
| 0 | Vvi-Vitvi05g01314\_t001 |  |  |  |  |  |  |  |  |
| 0 | Vvi-Vitvi05g01317\_t001 |  |  |  |  |  |  |  |  |
| 0 | Vvi-Vitvi05g01318\_t001 |  |  |  |  |  |  |  |  |
| 0 | Vvi-Vitvi05g01319\_t001 |  |  |  |  |  |  |  |  |
| 0 | Vvi-Vitvi05g01320\_t001 |  |  |  |  |  |  |  |  |
| 0 | Vvi-Vitvi05g04366\_t001 |  |  |  |  |  |  |  |  |
| 0 | Vvi-Vitvi05g01323\_t001 |  |  |  |  |  |  |  |  |
| 0 | Vvi-Vitvi05g01324\_t002 |  |  |  |  |  |  |  |  |
| 0 | Vvi-Vitvi05g01325\_t001 |  |  |  |  |  |  |  |  |
| 0 | Vvi-Vitvi05g04367\_t001 |  |  |  |  |  |  |  |  |
| 0 | Vvi-Vitvi05g01327\_t002 |  |  |  |  |  |  |  |  |
| 0 | Vvi-Vitvi05g01328\_t001 |  |  |  |  |  |  |  |  |
| 0 | Vvi-Vitvi05g01330\_t001 |  |  |  |  |  |  |  |  |
| 0 | Vvi-Vitvi05g04368\_t001 |  |  |  |  |  |  |  |  |
| 0 | Vvi-Vitvi05g01334\_t001 |  |  |  |  |  |  |  |  |
| 0 | Vvi-Vitvi05g04369\_t001 |  |  |  |  |  |  |  |  |
| 0 | Vvi-Vitvi05g01336\_t001 |  |  |  |  |  |  |  |  |
| 0 | Vvi-Vitvi05g01337\_t001 |  |  |  |  |  |  |  |  |
| 0 | Vvi-Vitvi05g04370\_t001 |  |  |  |  |  |  |  |  |
| 0 | Vvi-Vitvi05g01338\_t001 |  |  |  |  |  |  |  |  |
| 0 | Vvi-Vitvi05g01339\_t001 |  |  |  |  |  |  |  |  |
| 0 | Vvi-Vitvi05g01340\_t001 |  |  |  |  |  |  |  |  |
| 0 | Vvi-Vitvi05g01341\_t001 |  |  |  |  |  |  |  |  |
| 0 | Vvi-Vitvi05g01342\_t004 |  |  |  |  |  |  |  |  |
| 0 | Vvi-Vitvi05g01343\_t001 |  |  |  |  |  |  |  |  |
| 0 | Vvi-Vitvi05g01344\_t001 |  |  |  |  |  |  |  |  |
| 0 | Vvi-Vitvi05g02120\_t001 |  |  |  |  |  |  |  |  |
| 0 | Vvi-Vitvi05g01345\_t001 |  |  |  |  |  |  |  |  |
| 0 | Vvi-Vitvi05g04371\_t001 |  |  |  |  |  |  |  |  |
| 0 | Vvi-Vitvi05g01347\_t001 |  |  |  |  |  |  |  |  |
| 0 | Vvi-Vitvi05g01348\_t001 |  |  |  |  |  |  |  |  |
| 0 | Vvi-Vitvi05g04372\_t001 |  |  |  |  |  |  |  |  |
| 0 | Vvi-Vitvi05g01350\_t001 |  |  |  |  |  |  |  |  |
| 0 | Vvi-Vitvi05g01351\_t001 |  |  |  |  |  |  |  |  |
| 0 | Vvi-Vitvi05g01352\_t001 |  |  |  |  |  |  |  |  |
| 0 | Vvi-Vitvi05g02121\_t001 |  |  |  |  |  |  |  |  |
| 0 | Vvi-Vitvi05g01353\_t001 |  |  |  |  |  |  |  |  |
| 0 | Vvi-Vitvi05g01355\_t001 |  |  |  |  |  |  |  |  |
| 0 | Vvi-Vitvi05g01356\_t001 |  |  |  |  |  |  |  |  |
| 0 | Vvi-Vitvi05g01357\_t001 |  |  |  |  |  |  |  |  |
| 0 | Vvi-Vitvi05g04373\_t001 |  |  |  |  |  |  |  |  |
| 0 | Vvi-Vitvi05g01358\_t001 |  |  |  |  |  |  |  |  |
| 0 | Vvi-Vitvi05g04374\_t001 |  |  |  |  |  |  |  |  |
| 0 | Vvi-Vitvi05g01359\_t001 |  |  |  |  |  |  |  |  |
| 0 | Vvi-Vitvi05g04375\_t001 |  |  |  |  |  |  |  |  |
| 0 | Vvi-Vitvi05g01360\_t001 |  |  |  |  |  |  |  |  |
| 0 | Vvi-Vitvi05g01361\_t001 |  |  |  |  |  |  |  |  |
| 0 | Vvi-Vitvi05g02122\_t001 |  |  |  |  |  |  |  |  |
| 0 | Vvi-Vitvi05g02123\_t001 |  |  |  |  |  |  |  |  |
| 0 | Vvi-Vitvi05g01362\_t001 |  |  |  |  |  |  |  |  |
| 0 | Vvi-Vitvi05g04376\_t001 |  |  |  |  |  |  |  |  |
| 0 | Vvi-Vitvi05g01363\_t001 |  |  |  |  |  |  |  |  |
| 0 | Vvi-Vitvi05g01364\_t001 |  |  |  |  |  |  |  |  |
| 0 | Vvi-Vitvi05g01365\_t001 |  |  |  |  |  |  |  |  |
| 0 | Vvi-Vitvi05g01366\_t001 |  |  |  |  |  |  |  |  |
| 0 | Vvi-Vitvi05g01367\_t001 |  |  |  |  |  |  |  |  |
| 0 | Vvi-Vitvi05g02124\_t001 |  |  |  |  |  |  |  |  |
| 0 | Vvi-Vitvi05g01369\_t001 |  |  |  |  |  |  |  |  |
| 0 | Vvi-Vitvi05g01370\_t001 |  |  |  |  |  |  |  |  |
| 0 | Vvi-Vitvi05g02125\_t001 |  |  |  |  |  |  |  |  |
| 0 | Vvi-Vitvi05g04377\_t001 |  |  |  |  |  |  |  |  |
| 0 | Vvi-Vitvi05g02126\_t001 |  |  |  |  |  |  |  |  |
| 0 | Vvi-Vitvi05g02127\_t001 |  |  |  |  |  |  |  |  |
| 0 | Vvi-Vitvi05g04378\_t001 |  |  |  |  |  |  |  |  |
| 0 | Vvi-Vitvi05g04379\_t001 |  |  |  |  |  |  |  |  |
| 0 | Vvi-Vitvi05g01372\_t001 |  |  |  |  |  |  |  |  |
| 0 | Vvi-Vitvi05g04380\_t001 |  |  |  |  |  |  |  |  |
| 0 | Vvi-Vitvi05g02128\_t001 |  |  |  |  |  |  |  |  |
| 0 | Vvi-Vitvi05g01375\_t001 |  |  |  |  |  |  |  |  |
| 0 | Vvi-Vitvi05g01376\_t001 |  |  |  |  |  |  |  |  |
| 0 | Vvi-Vitvi05g04381\_t001 |  |  |  |  |  |  |  |  |
| 0 | Vvi-Vitvi05g04382\_t001 |  |  |  |  |  |  |  |  |
| 0 | Vvi-Vitvi05g01378\_t001 |  |  |  |  |  |  |  |  |
| 0 | Vvi-Vitvi05g01379\_t001 |  |  |  |  |  |  |  |  |
| 0 | Vvi-Vitvi05g04383\_t001 |  |  |  |  |  |  |  |  |
| 0 | Vvi-Vitvi05g01380\_t001 |  |  |  |  |  |  |  |  |
| 0 | Vvi-Vitvi05g01381\_t002 |  |  |  |  |  |  |  |  |
| 0 | Vvi-Vitvi05g01382\_t001 |  |  |  |  |  |  |  |  |
| 0 | Vvi-Vitvi05g04384\_t001 |  |  |  |  |  |  |  |  |
| 0 | Vvi-Vitvi05g02131\_t001 |  |  |  |  |  |  |  |  |
| 0 | Vvi-Vitvi05g01383\_t001 |  |  |  |  |  |  |  |  |
| 0 | Vvi-Vitvi05g04385\_t001 |  |  |  |  |  |  |  |  |
| 0 | Vvi-Vitvi05g04386\_t001 |  |  |  |  |  |  |  |  |
| 0 | Vvi-Vitvi05g01388\_t001 |  |  |  |  |  |  |  |  |
| 0 | Vvi-Vitvi05g01389\_t001 |  |  |  |  |  |  |  |  |
| 0 | Vvi-Vitvi05g01390\_t001 |  |  |  |  |  |  |  |  |
| 0 | Vvi-Vitvi05g04387\_t001 |  |  |  |  |  |  |  |  |
| 0 | Vvi-Vitvi05g04388\_t001 |  |  |  |  |  |  |  |  |
| 0 | Vvi-Vitvi05g04389\_t001 |  |  |  |  |  |  |  |  |
| 0 | Vvi-Vitvi05g01392\_t001 |  |  |  |  |  |  |  |  |
| 0 | Vvi-Vitvi05g01393\_t002 |  |  |  |  |  |  |  |  |
| 0 | Vvi-Vitvi05g02135\_t001 |  |  |  |  |  |  |  |  |
| 0 | Vvi-Vitvi05g02136\_t001 |  |  |  |  |  |  |  |  |
| 0 | Vvi-Vitvi05g01395\_t001 |  |  |  |  |  |  |  |  |
| 0 | Vvi-Vitvi05g01396\_t001 |  |  |  |  |  |  |  |  |
| 0 | Vvi-Vitvi05g04390\_t001 |  |  |  |  |  |  |  |  |
| 0 | Vvi-Vitvi05g01399\_t001 |  |  |  |  |  |  |  |  |
| 0 | Vvi-Vitvi05g01400\_t001 |  |  |  |  |  |  |  |  |
| 0 | Vvi-Vitvi05g01401\_t001 |  |  |  |  |  |  |  |  |
| 0 | Vvi-Vitvi05g04391\_t001 |  |  |  |  |  |  |  |  |
| 0 | Vvi-Vitvi05g01404\_t001 |  |  |  |  |  |  |  |  |
| 0 | Vvi-Vitvi05g01405\_t001 |  |  |  |  |  |  |  |  |
| 0 | Vvi-Vitvi05g04392\_t001 |  |  |  |  |  |  |  |  |
| 0 | Vvi-Vitvi05g01408\_t001 |  |  |  |  |  |  |  |  |
| 0 | Vvi-Vitvi05g01409\_t003 |  |  |  |  |  |  |  |  |
| 0 | Vvi-Vitvi05g01410\_t001 |  |  |  |  |  |  |  |  |
| 0 | Vvi-Vitvi05g01411\_t001 |  |  |  |  |  |  |  |  |
| 0 | Vvi-Vitvi05g01412\_t001 |  |  |  |  |  |  |  |  |
| 0 | Vvi-Vitvi05g04393\_t001 |  |  |  |  |  |  |  |  |
| 0 | Vvi-Vitvi05g01413\_t001 |  |  |  |  |  |  |  |  |
| 0 | Vvi-Vitvi05g04394\_t001 |  |  |  |  |  |  |  |  |
| 0 | Vvi-Vitvi05g04395\_t001 |  |  |  |  |  |  |  |  |
| 0 | Vvi-Vitvi05g01417\_t001 |  |  |  |  |  |  |  |  |
| 0 | Vvi-Vitvi05g01419\_t001 |  |  |  |  |  |  |  |  |
| 0 | Vvi-Vitvi05g02138\_t004 |  |  |  |  |  |  |  |  |
| 0 | Vvi-Vitvi05g01420\_t001 |  |  |  |  |  |  |  |  |
| 0 | Vvi-Vitvi05g01421\_t001 |  |  |  |  |  |  |  |  |
| 0 | Vvi-Vitvi05g02139\_t001 |  |  |  |  |  |  |  |  |
| 0 | Vvi-Vitvi05g04396\_t001 |  |  |  |  |  |  |  |  |
| 0 | Vvi-Vitvi05g01424\_t001 |  |  |  |  |  |  |  |  |
| 0 | Vvi-Vitvi05g02141\_t001 |  |  |  |  |  |  |  |  |
| 0 | Vvi-Vitvi05g04397\_t001 |  |  |  |  |  |  |  |  |
| 0 | Vvi-Vitvi05g04398\_t001 |  |  |  |  |  |  |  |  |
| 0 | Vvi-Vitvi05g04399\_t001 |  |  |  |  |  |  |  |  |
| 0 | Vvi-Vitvi05g04400\_t001 |  |  |  |  |  |  |  |  |
| 0 | Vvi-Vitvi05g04401\_t001 |  |  |  |  |  |  |  |  |
| 0 | Vvi-Vitvi05g04402\_t001 |  |  |  |  |  |  |  |  |
| 0 | Vvi-Vitvi05g04403\_t001 |  |  |  |  |  |  |  |  |
| 0 | Vvi-Vitvi05g02145\_t001 |  |  |  |  |  |  |  |  |
| 0 | Vvi-Vitvi05g02146\_t001 |  |  |  |  |  |  |  |  |
| 0 | Vvi-Vitvi05g04404\_t001 |  |  |  |  |  |  |  |  |
| 0 | Vvi-Vitvi05g02148\_t001 |  |  |  |  |  |  |  |  |
| 0 | Vvi-Vitvi05g01435\_t001 |  |  |  |  |  |  |  |  |
| 0 | Vvi-Vitvi05g04405\_t001 |  |  |  |  |  |  |  |  |
| 0 | Vvi-Vitvi05g04406\_t001 |  |  |  |  |  |  |  |  |
| 0 | Vvi-Vitvi05g04407\_t001 |  |  |  |  |  |  |  |  |
| 0 | Vvi-Vitvi05g04408\_t002 |  |  |  |  |  |  |  |  |
| 0 | Vvi-Vitvi05g04409\_t001 |  |  |  |  |  |  |  |  |
| 0 | Vvi-Vitvi05g04410\_t001 |  |  |  |  |  |  |  |  |
| 0 | Vvi-Vitvi05g04411\_t001 |  |  |  |  |  |  |  |  |
| 0 | Vvi-Vitvi05g04412\_t001 |  |  |  |  |  |  |  |  |
| 0 | Vvi-Vitvi05g04413\_t001 |  |  |  |  |  |  |  |  |
| 0 | Vvi-Vitvi05g04414\_t001 |  |  |  |  |  |  |  |  |
| 0 | Vvi-Vitvi05g01437\_t001 |  |  |  |  |  |  |  |  |
| 0 | Vvi-Vitvi05g04415\_t001 |  |  |  |  |  |  |  |  |
| 0 | Vvi-Vitvi05g04416\_t001 |  |  |  |  |  |  |  |  |
| 0 | Vvi-Vitvi05g04417\_t001 |  |  |  |  |  |  |  |  |
| 0 | Vvi-Vitvi05g04418\_t001 |  |  |  |  |  |  |  |  |
| 0 | Vvi-Vitvi05g04419\_t001 |  |  |  |  |  |  |  |  |
| 0 | Vvi-Vitvi05g04420\_t001 |  |  |  |  |  |  |  |  |
| 0 | Vvi-Vitvi05g04421\_t001 |  |  |  |  |  |  |  |  |
| 0 | Vvi-Vitvi05g02153\_t001 |  |  |  |  |  |  |  |  |
| 0 | Vvi-Vitvi05g04422\_t001 |  |  |  |  |  |  |  |  |
| 0 | Vvi-Vitvi05g02156\_t001 |  |  |  |  |  |  |  |  |
| 0 | Vvi-Vitvi05g02157\_t001 |  |  |  |  |  |  |  |  |
| 0 | Vvi-Vitvi05g04423\_t001 |  |  |  |  |  |  |  |  |
| 0 | Vvi-Vitvi05g04424\_t001 |  |  |  |  |  |  |  |  |
| 0 | Vvi-Vitvi05g04425\_t001 |  |  |  |  |  |  |  |  |
| 0 | Vvi-Vitvi05g04426\_t001 |  |  |  |  |  |  |  |  |
| 0 | Vvi-Vitvi05g04427\_t001 |  |  |  |  |  |  |  |  |
| 0 | Vvi-Vitvi05g04428\_t001 |  |  |  |  |  |  |  |  |
| 0 | Vvi-Vitvi05g04429\_t001 |  |  |  |  |  |  |  |  |
| 0 | Vvi-Vitvi05g01447\_t001 |  |  |  |  |  |  |  |  |
| 0 | Vvi-Vitvi05g04430\_t001 |  |  |  |  |  |  |  |  |
| 0 | Vvi-Vitvi05g04431\_t001 |  |  |  |  |  |  |  |  |
| 0 | Vvi-Vitvi05g04432\_t001 |  |  |  |  |  |  |  |  |
| 0 | Vvi-Vitvi05g01450\_t001 |  |  |  |  |  |  |  |  |
| 0 | Vvi-Vitvi05g04433\_t001 |  |  |  |  |  |  |  |  |
| 0 | Vvi-Vitvi05g01452\_t001 |  |  |  |  |  |  |  |  |
| 0 | Vvi-Vitvi05g01453\_t001 |  |  |  |  |  |  |  |  |
| 0 | Vvi-Vitvi05g01454\_t001 |  |  |  |  |  |  |  |  |
| 0 | Vvi-Vitvi05g04434\_t001 |  |  |  |  |  |  |  |  |
| 0 | Vvi-Vitvi05g04435\_t001 |  |  |  |  |  |  |  |  |
| 0 | Vvi-Vitvi05g01456\_t001 |  |  |  |  |  |  |  |  |
| 0 | Vvi-Vitvi05g04436\_t001 |  |  |  |  |  |  |  |  |
| 0 | Vvi-Vitvi05g02166\_t001 |  |  |  |  |  |  |  |  |
| 0 | Vvi-Vitvi05g01459\_t001 |  |  |  |  |  |  |  |  |
| 0 | Vvi-Vitvi05g02167\_t001 |  |  |  |  |  |  |  |  |
| 0 | Vvi-Vitvi05g02168\_t001 |  |  |  |  |  |  |  |  |
| 0 | Vvi-Vitvi05g02170\_t001 |  |  |  |  |  |  |  |  |
| 0 | Vvi-Vitvi05g01464\_t001 |  |  |  |  |  |  |  |  |
| 2 | Vvi-Vitvi05g01465\_t001 |  | Ath-AT1G05860.2 |  | Ath-AT2G31600.1 |  |  |  |  |  |  |
| 3 | Vvi-Vitvi05g01466\_t001 |  | | | |  | | | |  | Ath-AT2G43370.1 |  |  |  |  |  |
| 3 | Vvi-Vitvi05g01467\_t001 |  | | | |  | | | |  | Ath-AT2G43360.1 |  |  |  |  |  |
| 3 | Vvi-Vitvi05g01468\_t002 |  | | | |  | | | |  | | | |  |  |  |  |  |
| 3 | Vvi-Vitvi05g01469\_t001 |  | | | |  | Ath-AT2G31570.1 |  | Ath-AT2G43350.2 |  |  |  |  |  |
| 3 | Vvi-Vitvi05g04437\_t001 |  | | | |  | | | |  | | | |  |  |  |  |  |
| 3 | Vvi-Vitvi05g01470\_t001 |  | Ath-AT1G05870.6 |  | Ath-AT2G31560.1 |  | Ath-AT2G43340.1 |  |  |  |  |  |
| 3 | Vvi-Vitvi05g01471\_t001 |  | | | |  | | | |  | Ath-AT2G43330.1 |  |  |  |  |  |
| 3 | Vvi-Vitvi05g04438\_t001 |  | | | |  | | | |  | | | |  |  |  |  |  |
| 3 | Vvi-Vitvi05g01475\_t001 |  | Ath-AT1G05890.2 |  | Ath-AT2G31510.1 |  | | | |  |  |  |  |  |
| 3 | Vvi-Vitvi05g01477\_t001 |  | | | |  | Ath-AT2G31500.1 |  | | | |  |  |  |  |  |
| 3 | Vvi-Vitvi05g01478\_t001 |  | | | |  | | | |  | | | |  |  |  |  |  |
| 3 | Vvi-Vitvi05g01479\_t001 |  | Ath-AT1G05894.1 |  | Ath-AT2G31480.1 |  | | | |  |  |  |  |  |
| 3 | Vvi-Vitvi05g01480\_t001 |  | | | |  | | | |  | Ath-AT2G43320.1 |  |  |  |  |  |
| 3 | Vvi-Vitvi05g01481\_t001 |  | | | |  | | | |  | | | |  |  |  |  |  |
| 4 | Vvi-Vitvi05g01482\_t001 |  | | | |  | | | |  | | | |  | Ath-AT3G59380.1 |  |  |  |  |
| 4 | Vvi-Vitvi05g01484\_t001 |  | Ath-AT1G05900.2 |  | Ath-AT2G31450.1 |  | | | |  | | | |  |  |  |  |
| 3 | Vvi-Vitvi05g01485\_t001 |  | | | |  |  |  | | | |  | | | |  |  |  |  |
| 3 | Vvi-Vitvi05g01486\_t001 |  | Ath-AT1G05910.1 |  |  |  | | | |  | | | |  |  |  |  |
| 3 | Vvi-Vitvi05g04439\_t001 |  | | | |  |  |  | | | |  | | | |  |  |  |  |
| 3 | Vvi-Vitvi05g01488\_t001 |  | Ath-AT1G05960.2 |  |  |  | | | |  | | | |  |  |  |  |
| 3 | Vvi-Vitvi05g04440\_t001 |  | | | |  |  |  | | | |  | | | |  |  |  |  |
| 3 | Vvi-Vitvi05g01489\_t001 |  | | | |  |  |  | Ath-AT2G43310.1 |  | | | |  |  |  |  |
| 3 | Vvi-Vitvi05g01490\_t001 |  | | | |  |  |  | | | |  | Ath-AT3G59390.2 |  |  |  |  |
| 3 | Vvi-Vitvi05g01492\_t001 |  | | | |  |  |  | | | |  | Ath-AT3G59400.1 |  |  |  |  |
| 3 | Vvi-Vitvi05g01493\_t001 |  | | | |  |  |  | | | |  | Ath-AT3G59410.2 |  |  |  |  |
| 3 | Vvi-Vitvi05g04441\_t001 |  | | | |  |  |  | | | |  | | | |  |  |  |  |
| 3 | Vvi-Vitvi05g01494\_t001 |  | | | |  |  |  | | | |  | Ath-AT3G59420.1 |  |  |  |  |
| 3 | Vvi-Vitvi05g01495\_t001 |  | | | |  |  |  | | | |  | Ath-AT3G59430.2 |  |  |  |  |
| 3 | Vvi-Vitvi05g01497\_t001 |  | | | |  |  |  | | | |  | | | |  |  |  |  |
| 3 | Vvi-Vitvi05g01498\_t002 |  | | | |  |  |  | | | |  | | | |  |  |  |  |
| 3 | Vvi-Vitvi05g02178\_t001 |  | | | |  |  |  | | | |  | | | |  |  |  |  |
| 3 | Vvi-Vitvi05g01499\_t001 |  | | | |  |  |  | | | |  | | | |  |  |  |  |
| 3 | Vvi-Vitvi05g04442\_t001 |  | | | |  |  |  | | | |  | | | |  |  |  |  |
| 3 | Vvi-Vitvi05g01500\_t001 |  | Ath-AT1G05990.1 |  |  |  | Ath-AT2G43290.1 |  | Ath-AT3G59440.1 |  |  |  |  |
| 2 | Vvi-Vitvi05g04443\_t001 |  |  |  |  |  | | | |  | | | |  |  |  |  |
| 2 | Vvi-Vitvi05g01501\_t001 |  |  |  |  |  | | | |  | | | |  |  |  |  |
| 2 | Vvi-Vitvi05g04444\_t001 |  |  |  |  |  | | | |  | | | |  |  |  |  |
| 2 | Vvi-Vitvi05g04445\_t001 |  |  |  |  |  | Ath-AT2G43280.1 |  | | | |  |  |  |  |
| 1 | Vvi-Vitvi05g01502\_t001 |  |  |  |  |  |  |  | | | |  |  |  |  |
| 1 | Vvi-Vitvi05g04446\_t001 |  |  |  |  |  |  |  | | | |  |  |  |  |
| 1 | Vvi-Vitvi05g04447\_t001 |  |  |  |  |  |  |  | | | |  |  |  |  |
| 1 | Vvi-Vitvi05g04448\_t001 |  |  |  |  |  |  |  | | | |  |  |  |  |
| 1 | Vvi-Vitvi05g04449\_t001 |  |  |  |  |  |  |  | | | |  |  |  |  |
| 1 | Vvi-Vitvi05g02180\_t001 |  |  |  |  |  |  |  | | | |  |  |  |  |
| 1 | Vvi-Vitvi05g04450\_t001 |  |  |  |  |  |  |  | | | |  |  |  |  |
| 1 | Vvi-Vitvi05g04451\_t001 |  |  |  |  |  |  |  | | | |  |  |  |  |
| 1 | Vvi-Vitvi05g04452\_t001 |  |  |  |  |  |  |  | | | |  |  |  |  |
| 1 | Vvi-Vitvi05g02182\_t001 |  |  |  |  |  |  |  | | | |  |  |  |  |
| 1 | Vvi-Vitvi05g04453\_t001 |  |  |  |  |  |  |  | | | |  |  |  |  |
| 1 | Vvi-Vitvi05g01506\_t001 |  |  |  |  |  |  |  | | | |  |  |  |  |
| 1 | Vvi-Vitvi05g01507\_t001 |  |  |  |  |  |  |  | | | |  |  |  |  |
| 1 | Vvi-Vitvi05g01508\_t001 |  |  |  |  |  |  |  | | | |  |  |  |  |
| 1 | Vvi-Vitvi05g04454\_t001 |  |  |  |  |  |  |  | | | |  |  |  |  |
| 1 | Vvi-Vitvi05g02184\_t001 |  |  |  |  |  |  |  | | | |  |  |  |  |
| 1 | Vvi-Vitvi05g02185\_t001 |  |  |  |  |  |  |  | | | |  |  |  |  |
| 1 | Vvi-Vitvi05g04455\_t001 |  |  |  |  |  |  |  | | | |  |  |  |  |
| 1 | Vvi-Vitvi05g02187\_t001 |  |  |  |  |  |  |  | | | |  |  |  |  |
| 1 | Vvi-Vitvi05g04456\_t001 |  |  |  |  |  |  |  | | | |  |  |  |  |
| 1 | Vvi-Vitvi05g04457\_t001 |  |  |  |  |  |  |  | | | |  |  |  |  |
| 1 | Vvi-Vitvi05g01510\_t001 |  |  |  |  |  |  |  | Ath-AT3G59470.2 |  |  |  |  |
| 1 | Vvi-Vitvi05g01511\_t001 |  |  |  |  |  |  |  | | | |  |  |  |  |
| 1 | Vvi-Vitvi05g02190\_t001 |  |  |  |  |  |  |  | | | |  |  |  |  |
| 1 | Vvi-Vitvi05g04458\_t001 |  |  |  |  |  |  |  | | | |  |  |  |  |
| 1 | Vvi-Vitvi05g02192\_t001 |  |  |  |  |  |  |  | | | |  |  |  |  |
| 1 | Vvi-Vitvi05g01514\_t001 |  |  |  |  |  |  |  | | | |  |  |  |  |
| 1 | Vvi-Vitvi05g01516\_t001 |  |  |  |  |  |  |  | Ath-AT3G59480.1 |  |  |  |  |
| 1 | Vvi-Vitvi05g01517\_t001 |  |  |  |  |  |  |  | Ath-AT3G59490.2 |  |  |  |  |
| 1 | Vvi-Vitvi05g01518\_t001 |  |  |  |  |  |  |  | Ath-AT3G59500.1 |  |  |  |  |
| 1 | Vvi-Vitvi05g04459\_t001 |  |  |  |  |  |  |  | Ath-AT3G59510.1 |  |  |  |  |
| 1 | Vvi-Vitvi05g02193\_t001 |  |  |  |  |  |  |  | | | |  |  |  |  |
| 1 | Vvi-Vitvi05g01519\_t001 |  |  |  |  |  |  |  | | | |  |  |  |  |
| 1 | Vvi-Vitvi05g04460\_t001 |  |  |  |  |  |  |  | | | |  |  |  |  |
| 1 | Vvi-Vitvi05g01520\_t001 |  |  |  |  |  |  |  | Ath-AT3G59520.1 |  |  |  |  |
| 0 | Vvi-Vitvi05g04461\_t001 |  |  |  |  |  |  |  |  |
| 0 | Vvi-Vitvi05g02194\_t001 |  |  |  |  |  |  |  |  |
| 0 | Vvi-Vitvi05g04462\_t001 |  |  |  |  |  |  |  |  |
| 0 | Vvi-Vitvi05g04463\_t001 |  |  |  |  |  |  |  |  |
| 0 | Vvi-Vitvi05g04464\_t001 |  |  |  |  |  |  |  |  |
| 0 | Vvi-Vitvi05g04465\_t001 |  |  |  |  |  |  |  |  |
| 0 | Vvi-Vitvi05g02195\_t001 |  |  |  |  |  |  |  |  |
| 0 | Vvi-Vitvi05g04466\_t001 |  |  |  |  |  |  |  |  |
| 0 | Vvi-Vitvi05g04467\_t001 |  |  |  |  |  |  |  |  |
| 0 | Vvi-Vitvi05g04468\_t001 |  |  |  |  |  |  |  |  |
| 0 | Vvi-Vitvi05g04469\_t001 |  |  |  |  |  |  |  |  |
| 0 | Vvi-Vitvi05g04470\_t001 |  |  |  |  |  |  |  |  |
| 0 | Vvi-Vitvi05g02197\_t001 |  |  |  |  |  |  |  |  |
| 0 | Vvi-Vitvi05g04471\_t001 |  |  |  |  |  |  |  |  |
| 0 | Vvi-Vitvi05g02198\_t001 |  |  |  |  |  |  |  |  |
| 0 | Vvi-Vitvi05g04472\_t001 |  |  |  |  |  |  |  |  |
| 0 | Vvi-Vitvi05g04473\_t001 |  |  |  |  |  |  |  |  |
| 0 | Vvi-Vitvi05g02200\_t001 |  |  |  |  |  |  |  |  |
| 0 | Vvi-Vitvi05g04474\_t001 |  |  |  |  |  |  |  |  |
| 0 | Vvi-Vitvi05g02201\_t001 |  |  |  |  |  |  |  |  |
| 0 | Vvi-Vitvi05g04475\_t001 |  |  |  |  |  |  |  |  |
| 0 | Vvi-Vitvi05g04476\_t001 |  |  |  |  |  |  |  |  |
| 0 | Vvi-Vitvi05g04477\_t001 |  |  |  |  |  |  |  |  |
| 0 | Vvi-Vitvi05g04478\_t001 |  |  |  |  |  |  |  |  |
| 0 | Vvi-Vitvi05g01522\_t001 |  |  |  |  |  |  |  |  |
| 0 | Vvi-Vitvi05g04479\_t001 |  |  |  |  |  |  |  |  |
| 0 | Vvi-Vitvi05g04480\_t001 |  |  |  |  |  |  |  |  |
| 0 | Vvi-Vitvi05g02203\_t001 |  |  |  |  |  |  |  |  |
| 0 | Vvi-Vitvi05g04481\_t001 |  |  |  |  |  |  |  |  |
| 0 | Vvi-Vitvi05g04482\_t001 |  |  |  |  |  |  |  |  |
| 0 | Vvi-Vitvi05g04483\_t001 |  |  |  |  |  |  |  |  |
| 0 | Vvi-Vitvi05g02204\_t001 |  |  |  |  |  |  |  |  |
| 0 | Vvi-Vitvi05g01524\_t001 |  |  |  |  |  |  |  |  |
| 0 | Vvi-Vitvi05g02205\_t001 |  |  |  |  |  |  |  |  |
| 0 | Vvi-Vitvi05g02206\_t001 |  |  |  |  |  |  |  |  |
| 0 | Vvi-Vitvi05g02212\_t001 |  |  |  |  |  |  |  |  |
| 0 | Vvi-Vitvi05g04484\_t001 |  |  |  |  |  |  |  |  |
| 0 | Vvi-Vitvi05g02297\_t001 |  |  |  |  |  |  |  |  |
| 0 | Vvi-Vitvi05g02208\_t001 |  |  |  |  |  |  |  |  |
| 0 | Vvi-Vitvi05g04485\_t001 |  |  |  |  |  |  |  |  |
| 0 | Vvi-Vitvi05g04486\_t001 |  |  |  |  |  |  |  |  |
| 0 | Vvi-Vitvi05g01528\_t001 |  |  |  |  |  |  |  |  |
| 0 | Vvi-Vitvi05g04487\_t001 |  |  |  |  |  |  |  |  |
| 0 | Vvi-Vitvi05g02210\_t001 |  |  |  |  |  |  |  |  |
| 0 | Vvi-Vitvi05g04488\_t001 |  |  |  |  |  |  |  |  |
| 0 | Vvi-Vitvi05g02213\_t001 |  |  |  |  |  |  |  |  |
| 0 | Vvi-Vitvi05g04489\_t001 |  |  |  |  |  |  |  |  |
| 0 | Vvi-Vitvi05g04490\_t001 |  |  |  |  |  |  |  |  |
| 0 | Vvi-Vitvi05g04491\_t001 |  |  |  |  |  |  |  |  |
| 0 | Vvi-Vitvi05g01530\_t001 |  |  |  |  |  |  |  |  |
| 0 | Vvi-Vitvi05g04492\_t001 |  |  |  |  |  |  |  |  |
| 0 | Vvi-Vitvi05g04493\_t001 |  |  |  |  |  |  |  |  |
| 0 | Vvi-Vitvi05g04494\_t001 |  |  |  |  |  |  |  |  |
| 0 | Vvi-Vitvi05g04495\_t001 |  |  |  |  |  |  |  |  |
| 0 | Vvi-Vitvi05g04496\_t001 |  |  |  |  |  |  |  |  |
| 0 | Vvi-Vitvi05g04497\_t001 |  |  |  |  |  |  |  |  |
| 0 | Vvi-Vitvi05g04498\_t001 |  |  |  |  |  |  |  |  |
| 0 | Vvi-Vitvi05g04499\_t001 |  |  |  |  |  |  |  |  |
| 0 | Vvi-Vitvi05g02215\_t001 |  |  |  |  |  |  |  |  |
| 0 | Vvi-Vitvi05g04500\_t001 |  |  |  |  |  |  |  |  |
| 0 | Vvi-Vitvi05g02217\_t001 |  |  |  |  |  |  |  |  |
| 0 | Vvi-Vitvi05g04501\_t001 |  |  |  |  |  |  |  |  |
| 0 | Vvi-Vitvi05g04502\_t001 |  |  |  |  |  |  |  |  |
| 0 | Vvi-Vitvi05g02219\_t001 |  |  |  |  |  |  |  |  |
| 0 | Vvi-Vitvi05g01533\_t001 |  |  |  |  |  |  |  |  |
| 0 | Vvi-Vitvi05g02221\_t001 |  |  |  |  |  |  |  |  |
| 0 | Vvi-Vitvi05g02222\_t001 |  |  |  |  |  |  |  |  |
| 0 | Vvi-Vitvi05g02223\_t001 |  |  |  |  |  |  |  |  |
| 0 | Vvi-Vitvi05g02224\_t001 |  |  |  |  |  |  |  |  |
| 0 | Vvi-Vitvi05g04503\_t001 |  |  |  |  |  |  |  |  |
| 0 | Vvi-Vitvi05g04504\_t001 |  |  |  |  |  |  |  |  |
| 0 | Vvi-Vitvi05g02228\_t001 |  |  |  |  |  |  |  |  |
| 0 | Vvi-Vitvi05g04505\_t001 |  |  |  |  |  |  |  |  |
| 0 | Vvi-Vitvi05g04506\_t001 |  |  |  |  |  |  |  |  |
| 0 | Vvi-Vitvi05g02232\_t001 |  |  |  |  |  |  |  |  |
| 1 | Vvi-Vitvi05g01536\_t001 |  | Ath-AT2G43400.1 |  |  |  |  |  |  |  |
| 1 | Vvi-Vitvi05g04507\_t001 |  | | | |  |  |  |  |  |  |  |
| 1 | Vvi-Vitvi05g01537\_t001 |  | | | |  |  |  |  |  |  |  |
| 1 | Vvi-Vitvi05g04508\_t001 |  | | | |  |  |  |  |  |  |  |
| 1 | Vvi-Vitvi05g02233\_t001 |  | | | |  |  |  |  |  |  |  |
| 1 | Vvi-Vitvi05g02234\_t001 |  | | | |  |  |  |  |  |  |  |
| 1 | Vvi-Vitvi05g01540\_t003 |  | | | |  |  |  |  |  |  |  |
| 1 | Vvi-Vitvi05g01542\_t001 |  | Ath-AT2G43410.2 |  |  |  |  |  |  |  |
| 1 | Vvi-Vitvi05g01543\_t001 |  | Ath-AT2G43420.1 |  |  |  |  |  |  |  |
| 1 | Vvi-Vitvi05g01544\_t001 |  | Ath-AT2G43430.1 |  |  |  |  |  |  |  |
| 1 | Vvi-Vitvi05g04509\_t001 |  | | | |  |  |  |  |  |  |  |
| 1 | Vvi-Vitvi05g01545\_t001 |  | | | |  |  |  |  |  |  |  |
| 1 | Vvi-Vitvi05g01546\_t001 |  | | | |  |  |  |  |  |  |  |
| 1 | Vvi-Vitvi05g01547\_t001 |  | | | |  |  |  |  |  |  |  |
| 1 | Vvi-Vitvi05g01548\_t001 |  | Ath-AT2G43460.1 |  |  |  |  |  |  |  |
| 1 | Vvi-Vitvi05g04510\_t002 |  | | | |  |  |  |  |  |  |  |
| 1 | Vvi-Vitvi05g01550\_t001 |  | | | |  |  |  |  |  |  |  |
| 1 | Vvi-Vitvi05g01551\_t001 |  | | | |  |  |  |  |  |  |  |
| 1 | Vvi-Vitvi05g04511\_t001 |  | | | |  |  |  |  |  |  |  |
| 1 | Vvi-Vitvi05g02235\_t001 |  | | | |  |  |  |  |  |  |  |
| 1 | Vvi-Vitvi05g02236\_t001 |  | | | |  |  |  |  |  |  |  |
| 1 | Vvi-Vitvi05g01553\_t001 |  | Ath-AT2G43465.1 |  |  |  |  |  |  |  |
| 1 | Vvi-Vitvi05g01554\_t001 |  | | | |  |  |  |  |  |  |  |
| 1 | Vvi-Vitvi05g02237\_t001 |  | | | |  |  |  |  |  |  |  |
| 1 | Vvi-Vitvi05g01555\_t001 |  | | | |  |  |  |  |  |  |  |
| 1 | Vvi-Vitvi05g01556\_t001 |  | | | |  |  |  |  |  |  |  |
| 1 | Vvi-Vitvi05g04512\_t001 |  | Ath-AT2G43470.1 |  |  |  |  |  |  |  |
| 1 | Vvi-Vitvi05g01558\_t001 |  | Ath-AT2G43490.7 |  |  |  |  |  |  |  |
| 1 | Vvi-Vitvi05g04513\_t001 |  | | | |  |  |  |  |  |  |  |
| 1 | Vvi-Vitvi05g04514\_t001 |  | | | |  |  |  |  |  |  |  |
| 1 | Vvi-Vitvi05g01560\_t001 |  | Ath-AT2G43500.4 |  |  |  |  |  |  |  |
| 1 | Vvi-Vitvi05g04515\_t001 |  | | | |  |  |  |  |  |  |  |
| 1 | Vvi-Vitvi05g04516\_t001 |  | | | |  |  |  |  |  |  |  |
| 1 | Vvi-Vitvi05g01563\_t001 |  | | | |  |  |  |  |  |  |  |
| 1 | Vvi-Vitvi05g01564\_t001 |  | | | |  |  |  |  |  |  |  |
| 1 | Vvi-Vitvi05g04517\_t001 |  | | | |  |  |  |  |  |  |  |
| 1 | Vvi-Vitvi05g02239\_t001 |  | Ath-AT2G43540.1 |  |  |  |  |  |  |  |
| 1 | Vvi-Vitvi05g01566\_t001 |  | Ath-AT2G43560.1 |  |  |  |  |  |  |  |
| 1 | Vvi-Vitvi05g04518\_t001 |  | | | |  |  |  |  |  |  |  |
| 1 | Vvi-Vitvi05g04519\_t001 |  | | | |  |  |  |  |  |  |  |
| 1 | Vvi-Vitvi05g04520\_t001 |  | | | |  |  |  |  |  |  |  |
| 1 | Vvi-Vitvi05g04521\_t001 |  | | | |  |  |  |  |  |  |  |
| 1 | Vvi-Vitvi05g01568\_t001 |  | | | |  |  |  |  |  |  |  |
| 1 | Vvi-Vitvi05g04522\_t001 |  | | | |  |  |  |  |  |  |  |
| 1 | Vvi-Vitvi05g01569\_t001 |  | | | |  |  |  |  |  |  |  |
| 1 | Vvi-Vitvi05g04523\_t001 |  | | | |  |  |  |  |  |  |  |
| 1 | Vvi-Vitvi05g04524\_t001 |  | | | |  |  |  |  |  |  |  |
| 1 | Vvi-Vitvi05g04525\_t001 |  | | | |  |  |  |  |  |  |  |
| 1 | Vvi-Vitvi05g01572\_t001 |  | | | |  |  |  |  |  |  |  |
| 1 | Vvi-Vitvi05g04526\_t001 |  | | | |  |  |  |  |  |  |  |
| 1 | Vvi-Vitvi05g02241\_t001 |  | | | |  |  |  |  |  |  |  |
| 1 | Vvi-Vitvi05g04527\_t001 |  | | | |  |  |  |  |  |  |  |
| 1 | Vvi-Vitvi05g01573\_t001 |  | Ath-AT2G43570.1 |  |  |  |  |  |  |  |
| 1 | Vvi-Vitvi05g01574\_t001 |  | | | |  |  |  |  |  |  |  |
| 1 | Vvi-Vitvi05g02244\_t001 |  | | | |  |  |  |  |  |  |  |
| 1 | Vvi-Vitvi05g01575\_t001 |  | | | |  |  |  |  |  |  |  |
| 1 | Vvi-Vitvi05g04528\_t001 |  | | | |  |  |  |  |  |  |  |
| 1 | Vvi-Vitvi05g01577\_t001 |  | | | |  |  |  |  |  |  |  |
| 1 | Vvi-Vitvi05g02245\_t001 |  | | | |  |  |  |  |  |  |  |
| 1 | Vvi-Vitvi05g04529\_t001 |  | | | |  |  |  |  |  |  |  |
| 1 | Vvi-Vitvi05g01578\_t001 |  | | | |  |  |  |  |  |  |  |
| 1 | Vvi-Vitvi05g01579\_t001 |  | | | |  |  |  |  |  |  |  |
| 1 | Vvi-Vitvi05g02248\_t001 |  | | | |  |  |  |  |  |  |  |
| 1 | Vvi-Vitvi05g01580\_t001 |  | | | |  |  |  |  |  |  |  |
| 1 | Vvi-Vitvi05g01581\_t001 |  | | | |  |  |  |  |  |  |  |
| 1 | Vvi-Vitvi05g04530\_t001 |  | | | |  |  |  |  |  |  |  |
| 1 | Vvi-Vitvi05g04531\_t001 |  | | | |  |  |  |  |  |  |  |
| 1 | Vvi-Vitvi05g04532\_t001 |  | | | |  |  |  |  |  |  |  |
| 1 | Vvi-Vitvi05g04533\_t001 |  | | | |  |  |  |  |  |  |  |
| 1 | Vvi-Vitvi05g04534\_t001 |  | | | |  |  |  |  |  |  |  |
| 1 | Vvi-Vitvi05g02249\_t001 |  | | | |  |  |  |  |  |  |  |
| 1 | Vvi-Vitvi05g01583\_t001 |  | | | |  |  |  |  |  |  |  |
| 1 | Vvi-Vitvi05g04535\_t001 |  | | | |  |  |  |  |  |  |  |
| 1 | Vvi-Vitvi05g02251\_t001 |  | Ath-AT2G43620.1 |  |  |  |  |  |  |  |
| 2 | Vvi-Vitvi05g01584\_t001 |  | | | |  | Ath-AT3G59630.2 |  |  |  |  |  |  |
| 2 | Vvi-Vitvi05g02252\_t003 |  | | | |  | | | |  |  |  |  |  |  |
| 2 | Vvi-Vitvi05g01585\_t001 |  | Ath-AT2G43630.1 |  | Ath-AT3G59640.1 |  |  |  |  |  |  |
| 2 | Vvi-Vitvi05g01587\_t001 |  | Ath-AT2G43640.2 |  | | | |  |  |  |  |  |  |
| 2 | Vvi-Vitvi05g01588\_t001 |  | | | |  | | | |  |  |  |  |  |  |
| 2 | Vvi-Vitvi05g01589\_t001 |  | | | |  | | | |  |  |  |  |  |  |
| 2 | Vvi-Vitvi05g01590\_t001 |  | | | |  | | | |  |  |  |  |  |  |
| 2 | Vvi-Vitvi05g01592\_t001 |  | | | |  | | | |  |  |  |  |  |  |
| 2 | Vvi-Vitvi05g01593\_t002 |  | Ath-AT2G43650.1 |  | | | |  |  |  |  |  |  |
| 2 | Vvi-Vitvi05g01594\_t002 |  | | | |  | Ath-AT3G59650.2 |  |  |  |  |  |  |
| 2 | Vvi-Vitvi05g01595\_t003 |  | | | |  | | | |  |  |  |  |  |  |
| 2 | Vvi-Vitvi05g01596\_t001 |  | | | |  | | | |  |  |  |  |  |  |
| 2 | Vvi-Vitvi05g01597\_t001 |  | | | |  | | | |  |  |  |  |  |  |
| 2 | Vvi-Vitvi05g01599\_t001 |  | | | |  | | | |  |  |  |  |  |  |
| 2 | Vvi-Vitvi05g01600\_t001 |  | | | |  | Ath-AT3G59660.1 |  |  |  |  |  |  |
| 2 | Vvi-Vitvi05g01601\_t001 |  | | | |  | Ath-AT3G59670.1 |  |  |  |  |  |  |
| 2 | Vvi-Vitvi05g01602\_t001 |  | | | |  | Ath-AT3G59680.2 |  |  |  |  |  |  |
| 2 | Vvi-Vitvi05g01603\_t001 |  | Ath-AT2G43680.1 |  | Ath-AT3G59690.2 |  |  |  |  |  |  |
| 1 | Vvi-Vitvi05g04536\_t001 |  | | | |  |  |  |  |  |  |  |
| 1 | Vvi-Vitvi05g02253\_t001 |  | | | |  |  |  |  |  |  |  |
| 1 | Vvi-Vitvi05g04537\_t001 |  | | | |  |  |  |  |  |  |  |
| 1 | Vvi-Vitvi05g04538\_t001 |  | | | |  |  |  |  |  |  |  |
| 1 | Vvi-Vitvi05g01605\_t001 |  | | | |  |  |  |  |  |  |  |
| 1 | Vvi-Vitvi05g01607\_t001 |  | | | |  |  |  |  |  |  |  |
| 1 | Vvi-Vitvi05g01609\_t001 |  | | | |  |  |  |  |  |  |  |
| 1 | Vvi-Vitvi05g02257\_t001 |  | | | |  |  |  |  |  |  |  |
| 1 | Vvi-Vitvi05g04539\_t001 |  | | | |  |  |  |  |  |  |  |
| 1 | Vvi-Vitvi05g04540\_t001 |  | | | |  |  |  |  |  |  |  |
| 1 | Vvi-Vitvi05g01610\_t001 |  | | | |  |  |  |  |  |  |  |
| 1 | Vvi-Vitvi05g04541\_t001 |  | | | |  |  |  |  |  |  |  |
| 1 | Vvi-Vitvi05g01611\_t001 |  | | | |  |  |  |  |  |  |  |
| 1 | Vvi-Vitvi05g01612\_t001 |  | | | |  |  |  |  |  |  |  |
| 1 | Vvi-Vitvi05g04542\_t001 |  | | | |  |  |  |  |  |  |  |
| 1 | Vvi-Vitvi05g01616\_t001 |  | | | |  |  |  |  |  |  |  |
| 1 | Vvi-Vitvi05g04543\_t001 |  | | | |  |  |  |  |  |  |  |
| 1 | Vvi-Vitvi05g04544\_t001 |  | | | |  |  |  |  |  |  |  |
| 1 | Vvi-Vitvi05g04545\_t001 |  | | | |  |  |  |  |  |  |  |
| 1 | Vvi-Vitvi05g04546\_t001 |  | | | |  |  |  |  |  |  |  |
| 1 | Vvi-Vitvi05g04547\_t001 |  | | | |  |  |  |  |  |  |  |
| 1 | Vvi-Vitvi05g04548\_t001 |  | | | |  |  |  |  |  |  |  |
| 1 | Vvi-Vitvi05g04549\_t001 |  | | | |  |  |  |  |  |  |  |
| 1 | Vvi-Vitvi05g01618\_t001 |  | | | |  |  |  |  |  |  |  |
| 1 | Vvi-Vitvi05g01619\_t001 |  | Ath-AT2G43710.1 |  |  |  |  |  |  |  |
| 1 | Vvi-Vitvi05g01620\_t001 |  | | | |  |  |  |  |  |  |  |
| 1 | Vvi-Vitvi05g01621\_t001 |  | | | |  |  |  |  |  |  |  |
| 1 | Vvi-Vitvi05g01622\_t001 |  | | | |  |  |  |  |  |  |  |
| 2 | Vvi-Vitvi05g01623\_t001 |  | | | |  | Ath-AT3G59710.2 |  |  |  |  |  |  |
| 2 | Vvi-Vitvi05g01624\_t001 |  | Ath-AT2G43750.2 |  | Ath-AT3G59760.1 |  |  |  |  |  |  |
| 2 | Vvi-Vitvi05g01626\_t001 |  | Ath-AT2G43770.1 |  | | | |  |  |  |  |  |  |
| 2 | Vvi-Vitvi05g01627\_t001 |  | | | |  | | | |  |  |  |  |  |  |
| 2 | Vvi-Vitvi05g01629\_t001 |  | | | |  | Ath-AT3G59780.1 |  |  |  |  |  |  |
| 2 | Vvi-Vitvi05g01630\_t001 |  | | | |  | | | |  |  |  |  |  |  |
| 2 | Vvi-Vitvi05g01631\_t001 |  | | | |  | | | |  |  |  |  |  |  |
| 2 | Vvi-Vitvi05g01632\_t001 |  | | | |  | | | |  |  |  |  |  |  |
| 2 | Vvi-Vitvi05g01633\_t001 |  | | | |  | | | |  |  |  |  |  |  |
| 2 | Vvi-Vitvi05g02262\_t001 |  | | | |  | | | |  |  |  |  |  |  |
| 2 | Vvi-Vitvi05g01634\_t001 |  | Ath-AT2G43790.1 |  | Ath-AT3G59790.1 |  |  |  |  |  |  |
| 2 | Vvi-Vitvi05g01636\_t001 |  | Ath-AT2G43795.2 |  | Ath-AT3G59800.1 |  |  |  |  |  |  |
| 2 | Vvi-Vitvi05g01638\_t001 |  | Ath-AT2G43800.1 |  | | | |  |  |  |  |  |  |
| 2 | Vvi-Vitvi05g01639\_t001 |  | | | |  | | | |  |  |  |  |  |  |
| 2 | Vvi-Vitvi05g01640\_t001 |  | Ath-AT2G43810.2 |  | Ath-AT3G59810.1 |  |  |  |  |  |  |
| 2 | Vvi-Vitvi05g01641\_t001 |  | | | |  | | | |  |  |  |  |  |  |
| 2 | Vvi-Vitvi05g04550\_t001 |  | | | |  | | | |  |  |  |  |  |  |
| 2 | Vvi-Vitvi05g01642\_t001 |  | Ath-AT2G43820.1 |  | | | |  |  |  |  |  |  |
| 2 | Vvi-Vitvi05g04551\_t001 |  | | | |  | | | |  |  |  |  |  |  |
| 2 | Vvi-Vitvi05g01643\_t001 |  | | | |  | | | |  |  |  |  |  |  |
| 2 | Vvi-Vitvi05g04552\_t001 |  | | | |  | | | |  |  |  |  |  |  |
| 2 | Vvi-Vitvi05g01647\_t001 |  | | | |  | | | |  |  |  |  |  |  |
| 2 | Vvi-Vitvi05g01648\_t001 |  | | | |  | | | |  |  |  |  |  |  |
| 2 | Vvi-Vitvi05g04553\_t001 |  | | | |  | | | |  |  |  |  |  |  |
| 2 | Vvi-Vitvi05g01649\_t001 |  | | | |  | | | |  |  |  |  |  |  |
| 2 | Vvi-Vitvi05g04554\_t001 |  | | | |  | | | |  |  |  |  |  |  |
| 2 | Vvi-Vitvi05g01650\_t001 |  | | | |  | | | |  |  |  |  |  |  |
| 2 | Vvi-Vitvi05g04555\_t001 |  | | | |  | | | |  |  |  |  |  |  |
| 2 | Vvi-Vitvi05g02266\_t001 |  | | | |  | | | |  |  |  |  |  |  |
| 2 | Vvi-Vitvi05g04556\_t001 |  | | | |  | | | |  |  |  |  |  |  |
| 2 | Vvi-Vitvi05g01652\_t001 |  | | | |  | | | |  |  |  |  |  |  |
| 2 | Vvi-Vitvi05g04557\_t001 |  | | | |  | | | |  |  |  |  |  |  |
| 2 | Vvi-Vitvi05g04558\_t001 |  | | | |  | | | |  |  |  |  |  |  |
| 2 | Vvi-Vitvi05g04559\_t001 |  | | | |  | | | |  |  |  |  |  |  |
| 2 | Vvi-Vitvi05g04560\_t001 |  | | | |  | | | |  |  |  |  |  |  |
| 2 | Vvi-Vitvi05g04561\_t001 |  | | | |  | | | |  |  |  |  |  |  |
| 2 | Vvi-Vitvi05g01654\_t001 |  | | | |  | | | |  |  |  |  |  |  |
| 2 | Vvi-Vitvi05g01655\_t001 |  | | | |  | Ath-AT3G59820.2 |  |  |  |  |  |  |
| 2 | Vvi-Vitvi05g04562\_t001 |  | | | |  | | | |  |  |  |  |  |  |
| 2 | Vvi-Vitvi05g01656\_t001 |  | Ath-AT2G43850.2 |  | Ath-AT3G59830.1 |  |  |  |  |  |  |
| 1 | Vvi-Vitvi05g04563\_t001 |  | | | |  |  |  |  |  |  |  |
| 1 | Vvi-Vitvi05g01658\_t001 |  | | | |  |  |  |  |  |  |  |
| 1 | Vvi-Vitvi05g04564\_t001 |  | | | |  |  |  |  |  |  |  |
| 1 | Vvi-Vitvi05g01659\_t001 |  | Ath-AT2G44065.2 |  |  |  |  |  |  |  |
| 0 | Vvi-Vitvi05g01660\_t001 |  |  |  |  |  |  |  |  |
| 0 | Vvi-Vitvi05g01661\_t001 |  |  |  |  |  |  |  |  |
| 0 | Vvi-Vitvi05g02267\_t001 |  |  |  |  |  |  |  |  |
| 0 | Vvi-Vitvi05g01662\_t001 |  |  |  |  |  |  |  |  |
| 0 | Vvi-Vitvi05g01663\_t001 |  |  |  |  |  |  |  |  |
| 0 | Vvi-Vitvi05g02268\_t001 |  |  |  |  |  |  |  |  |
| 0 | Vvi-Vitvi05g04565\_t001 |  |  |  |  |  |  |  |  |
| 0 | Vvi-Vitvi05g01665\_t001 |  |  |  |  |  |  |  |  |
| 0 | Vvi-Vitvi05g01666\_t001 |  |  |  |  |  |  |  |  |
| 0 | Vvi-Vitvi05g04566\_t001 |  |  |  |  |  |  |  |  |
| 0 | Vvi-Vitvi05g02269\_t001 |  |  |  |  |  |  |  |  |
| 0 | Vvi-Vitvi05g04567\_t001 |  |  |  |  |  |  |  |  |
| 0 | Vvi-Vitvi05g04568\_t001 |  |  |  |  |  |  |  |  |
| 0 | Vvi-Vitvi05g04569\_t001 |  |  |  |  |  |  |  |  |
| 0 | Vvi-Vitvi05g01670\_t001 |  |  |  |  |  |  |  |  |
| 0 | Vvi-Vitvi05g04570\_t001 |  |  |  |  |  |  |  |  |
| 0 | Vvi-Vitvi05g04571\_t001 |  |  |  |  |  |  |  |  |
| 0 | Vvi-Vitvi05g01672\_t001 |  |  |  |  |  |  |  |  |
| 0 | Vvi-Vitvi05g01673\_t003 |  |  |  |  |  |  |  |  |
| 0 | Vvi-Vitvi05g01674\_t001 |  |  |  |  |  |  |  |  |
| 0 | Vvi-Vitvi05g01675\_t001 |  |  |  |  |  |  |  |  |
| 0 | Vvi-Vitvi05g01676\_t001 |  |  |  |  |  |  |  |  |
| 0 | Vvi-Vitvi05g02271\_t001 |  |  |  |  |  |  |  |  |
| 0 | Vvi-Vitvi05g01677\_t001 |  |  |  |  |  |  |  |  |
| 0 | Vvi-Vitvi05g01678\_t001 |  |  |  |  |  |  |  |  |
| 0 | Vvi-Vitvi05g04572\_t001 |  |  |  |  |  |  |  |  |
| 2 | Vvi-Vitvi05g01679\_t001 |  | Ath-AT2G43860.1 |  | Ath-AT3G59850.2 |  |  |  |  |  |  |
| 2 | Vvi-Vitvi05g01681\_t001 |  | Ath-AT2G43880.1 |  | | | |  |  |  |  |  |  |
| 2 | Vvi-Vitvi05g01682\_t001 |  | | | |  | | | |  |  |  |  |  |  |
| 2 | Vvi-Vitvi05g01683\_t001 |  | Ath-AT2G43900.2 |  | | | |  |  |  |  |  |  |
| 2 | Vvi-Vitvi05g02272\_t001 |  | Ath-AT2G43910.1 |  | | | |  |  |  |  |  |  |
| 2 | Vvi-Vitvi05g02273\_t001 |  | | | |  | | | |  |  |  |  |  |  |
| 2 | Vvi-Vitvi05g04573\_t001 |  | | | |  | | | |  |  |  |  |  |  |
| 2 | Vvi-Vitvi05g01684\_t001 |  | | | |  | | | |  |  |  |  |  |  |
| 2 | Vvi-Vitvi05g02275\_t002 |  | Ath-AT2G43945.1 |  | Ath-AT3G59870.1 |  |  |  |  |  |  |
| 2 | Vvi-Vitvi05g04574\_t001 |  | | | |  | | | |  |  |  |  |  |  |
| 2 | Vvi-Vitvi05g01685\_t001 |  | Ath-AT2G43950.1 |  | | | |  |  |  |  |  |  |
| 2 | Vvi-Vitvi05g02276\_t001 |  | Ath-AT2G43970.1 |  | | | |  |  |  |  |  |  |
| 2 | Vvi-Vitvi05g01686\_t001 |  | Ath-AT2G43980.1 |  | | | |  |  |  |  |  |  |
| 2 | Vvi-Vitvi05g01687\_t001 |  | | | |  | | | |  |  |  |  |  |  |
| 2 | Vvi-Vitvi05g01688\_t001 |  | Ath-AT2G43990.1 |  | | | |  |  |  |  |  |  |
| 2 | Vvi-Vitvi05g04575\_t001 |  | Ath-AT2G44010.1 |  | Ath-AT3G59880.1 |  |  |  |  |  |  |
| 2 | Vvi-Vitvi05g02278\_t001 |  | | | |  | | | |  |  |  |  |  |  |
| 2 | Vvi-Vitvi05g01689\_t001 |  | | | |  | | | |  |  |  |  |  |  |
| 2 | Vvi-Vitvi05g01690\_t001 |  | | | |  | | | |  |  |  |  |  |  |
| 2 | Vvi-Vitvi05g01691\_t001 |  | | | |  | | | |  |  |  |  |  |  |
| 2 | Vvi-Vitvi05g01692\_t001 |  | Ath-AT2G44040.1 |  | Ath-AT3G59890.1 |  |  |  |  |  |  |
| 2 | Vvi-Vitvi05g01693\_t001 |  | Ath-AT2G44050.1 |  | | | |  |  |  |  |  |  |
| 2 | Vvi-Vitvi05g01694\_t001 |  | | | |  | | | |  |  |  |  |  |  |
| 2 | Vvi-Vitvi05g01696\_t001 |  | | | |  | | | |  |  |  |  |  |  |
| 2 | Vvi-Vitvi05g01697\_t001 |  | Ath-AT2G44060.1 |  | | | |  |  |  |  |  |  |
| 2 | Vvi-Vitvi05g02279\_t001 |  | Ath-AT2G44080.1 |  | Ath-AT3G59900.1 |  |  |  |  |  |  |
| 2 | Vvi-Vitvi05g04576\_t001 |  | Ath-AT2G44090.1 |  | Ath-AT3G59910.2 |  |  |  |  |  |  |
| 2 | Vvi-Vitvi05g01700\_t001 |  | Ath-AT2G44100.1 |  | Ath-AT3G59920.1 |  |  |  |  |  |  |
| 2 | Vvi-Vitvi05g01701\_t001 |  | | | |  | | | |  |  |  |  |  |  |
| 2 | Vvi-Vitvi05g01702\_t001 |  | Ath-AT2G44110.2 |  | | | |  |  |  |  |  |  |
| 2 | Vvi-Vitvi05g01703\_t001 |  | Ath-AT2G44130.1 |  | Ath-AT3G59940.1 |  |  |  |  |  |  |
| 2 | Vvi-Vitvi05g01704\_t001 |  | Ath-AT2G44160.1 |  | Ath-AT3G59970.3 |  |  |  |  |  |  |
| 1 | Vvi-Vitvi05g04577\_t001 |  |  |  | | | |  |  |  |  |  |  |
| 1 | Vvi-Vitvi05g04578\_t001 |  |  |  | Ath-AT3G59980.1 |  |  |  |  |  |  |
| 0 | Vvi-Vitvi05g04579\_t001 |  |  |  |  |  |  |  |  |
| 0 | Vvi-Vitvi05g01711\_t001 |  |  |  |  |  |  |  |  |
| 0 | Vvi-Vitvi05g01712\_t001 |  |  |  |  |  |  |  |  |
| 0 | Vvi-Vitvi05g01714\_t001 |  |  |  |  |  |  |  |  |
| 0 | Vvi-Vitvi05g04580\_t001 |  |  |  |  |  |  |  |  |
| 0 | Vvi-Vitvi05g04581\_t001 |  |  |  |  |  |  |  |  |
| 0 | Vvi-Vitvi05g01715\_t001 |  |  |  |  |  |  |  |  |
| 0 | Vvi-Vitvi05g04582\_t001 |  |  |  |  |  |  |  |  |
| 0 | Vvi-Vitvi05g01716\_t001 |  |  |  |  |  |  |  |  |
| 0 | Vvi-Vitvi05g02281\_t001 |  |  |  |  |  |  |  |  |
| 0 | Vvi-Vitvi05g04583\_t001 |  |  |  |  |  |  |  |  |
| 0 | Vvi-Vitvi05g04584\_t001 |  |  |  |  |  |  |  |  |
| 0 | Vvi-Vitvi05g04585\_t001 |  |  |  |  |  |  |  |  |
| 0 | Vvi-Vitvi05g04586\_t001 |  |  |  |  |  |  |  |  |
| 0 | Vvi-Vitvi05g04587\_t001 |  |  |  |  |  |  |  |  |
| 0 | Vvi-Vitvi05g04588\_t001 |  |  |  |  |  |  |  |  |
| 0 | Vvi-Vitvi05g04589\_t001 |  |  |  |  |  |  |  |  |
