# Supplementary material for: Genome-wide identification, characterization and gene expression of BES1 transcription factor family in grapevine (Vitis vinifera L.)
Source: Sci Rep. 2023 Jan 5;13:240. doi: 10.1038/s41598-022-24407-y (PMC9816167; doi:10.1038/s41598-022-24407-y)
Supplement: Supplementary file 3 — Supplementary Information. [file 41598_2022_24407_MOESM3_ESM.zip › Vvi_Ath/Vitis_vinifera.PN40024.v4.dna_sm.toplevel.fa.vs.Arabidopsis_thaliana.TAIR10.dna_sm.toplevel.fa.html/Vvi-6.html]

|  |  |  |  |  |  |  |  |  |  |  |  |  |  |  |  |  |  |
| --- | --- | --- | --- | --- | --- | --- | --- | --- | --- | --- | --- | --- | --- | --- | --- | --- | --- |
| Duplication depth | Reference chromosome | Collinear blocks | | | | | | | | | | | | | | | |
| 2 | Vvi-Vitvi06g04002\_t001 |  | Ath-AT3G13390.1 |  | Ath-AT1G55560.1 |  |  |  |  |  |  |
| 2 | Vvi-Vitvi06g04003\_t001 |  | | | |  | | | |  |  |  |  |  |  |
| 2 | Vvi-Vitvi06g04004\_t001 |  | | | |  | | | |  |  |  |  |  |  |
| 2 | Vvi-Vitvi06g00007\_t001 |  | | | |  | | | |  |  |  |  |  |  |
| 4 | Vvi-Vitvi06g00008\_t001 |  | | | |  | | | |  | Ath-AT4G26420.1 |  | Ath-AT5G56300.1 |  |  |  |  |
| 4 | Vvi-Vitvi06g00009\_t001 |  | | | |  | | | |  | | | |  | | | |  |  |  |  |
| 4 | Vvi-Vitvi06g00011\_t001 |  | | | |  | | | |  | | | |  | Ath-AT5G56310.1 |  |  |  |  |
| 4 | Vvi-Vitvi06g00012\_t001 |  | | | |  | Ath-AT1G55550.1 |  | | | |  | | | |  |  |  |  |
| 4 | Vvi-Vitvi06g00013\_t001 |  | Ath-AT3G13410.1 |  | Ath-AT1G55546.1 |  | | | |  | | | |  |  |  |  |
| 4 | Vvi-Vitvi06g00015\_t001 |  | | | |  | | | |  | Ath-AT4G26415.1 |  | | | |  |  |  |  |
| 4 | Vvi-Vitvi06g00016\_t001 |  | | | |  | | | |  | | | |  | Ath-AT5G56320.3 |  |  |  |  |
| 4 | Vvi-Vitvi06g00017\_t001 |  | | | |  | Ath-AT1G55540.2 |  | | | |  | | | |  |  |  |  |
| 4 | Vvi-Vitvi06g00018\_t002 |  | | | |  | | | |  | Ath-AT4G26410.1 |  | | | |  |  |  |  |
| 4 | Vvi-Vitvi06g00019\_t001 |  | Ath-AT3G13420.3 |  | Ath-AT1G55535.1 |  | | | |  | | | |  |  |  |  |
| 4 | Vvi-Vitvi06g04005\_t001 |  | | | |  | | | |  | | | |  | | | |  |  |  |  |
| 4 | Vvi-Vitvi06g04006\_t001 |  | | | |  | | | |  | | | |  | | | |  |  |  |  |
| 4 | Vvi-Vitvi06g00020\_t001 |  | Ath-AT3G13430.3 |  | Ath-AT1G55530.2 |  | Ath-AT4G26400.1 |  | Ath-AT5G56340.1 |  |  |  |  |
| 4 | Vvi-Vitvi06g00021\_t001 |  | | | |  | | | |  | Ath-AT4G26390.1 |  | Ath-AT5G56350.1 |  |  |  |  |
| 4 | Vvi-Vitvi06g00022\_t001 |  | | | |  | | | |  | | | |  | Ath-AT5G56360.1 |  |  |  |  |
| 4 | Vvi-Vitvi06g00024\_t001 |  | | | |  | | | |  | | | |  | | | |  |  |  |  |
| 4 | Vvi-Vitvi06g00026\_t001 |  | | | |  | | | |  | | | |  | | | |  |  |  |  |
| 4 | Vvi-Vitvi06g00027\_t001 |  | | | |  | | | |  | Ath-AT4G26370.1 |  | | | |  |  |  |  |
| 4 | Vvi-Vitvi06g01573\_t001 |  | Ath-AT3G13445.1 |  | Ath-AT1G55520.1 |  | | | |  | | | |  |  |  |  |
| 4 | Vvi-Vitvi06g00028\_t001 |  | Ath-AT3G13450.1 |  | Ath-AT1G55510.1 |  | | | |  | | | |  |  |  |  |
| 4 | Vvi-Vitvi06g00029\_t001 |  | Ath-AT3G13460.1 |  | Ath-AT1G55500.5 |  | | | |  | | | |  |  |  |  |
| 4 | Vvi-Vitvi06g00030\_t001 |  | | | |  | | | |  | Ath-AT4G26330.1 |  | | | |  |  |  |  |
| 3 | Vvi-Vitvi06g00031\_t001 |  | | | |  | | | |  |  |  | Ath-AT5G56460.1 |  |  |  |  |
| 3 | Vvi-Vitvi06g01574\_t001 |  | | | |  | | | |  |  |  | | | |  |  |  |  |
| 3 | Vvi-Vitvi06g00032\_t001 |  | Ath-AT3G13470.1 |  | Ath-AT1G55490.1 |  |  |  | Ath-AT5G56500.2 |  |  |  |  |
| 3 | Vvi-Vitvi06g01575\_t001 |  | | | |  | | | |  |  |  | Ath-AT5G56510.1 |  |  |  |  |
| 3 | Vvi-Vitvi06g04007\_t001 |  | | | |  | Ath-AT1G55480.1 |  |  |  | | | |  |  |  |  |
| 3 | Vvi-Vitvi06g04008\_t001 |  | | | |  | | | |  |  |  | | | |  |  |  |  |
| 3 | Vvi-Vitvi06g00034\_t001 |  | | | |  | | | |  |  |  | | | |  |  |  |  |
| 3 | Vvi-Vitvi06g00035\_t001 |  | | | |  | | | |  |  |  | | | |  |  |  |  |
| 3 | Vvi-Vitvi06g00036\_t001 |  | | | |  | | | |  |  |  | | | |  |  |  |  |
| 3 | Vvi-Vitvi06g04009\_t001 |  | | | |  | | | |  |  |  | | | |  |  |  |  |
| 3 | Vvi-Vitvi06g00037\_t001 |  | | | |  | | | |  |  |  | | | |  |  |  |  |
| 3 | Vvi-Vitvi06g04010\_t001 |  | | | |  | | | |  |  |  | | | |  |  |  |  |
| 3 | Vvi-Vitvi06g04011\_t001 |  | | | |  | | | |  |  |  | | | |  |  |  |  |
| 3 | Vvi-Vitvi06g04012\_t001 |  | | | |  | | | |  |  |  | | | |  |  |  |  |
| 3 | Vvi-Vitvi06g00039\_t001 |  | | | |  | | | |  |  |  | | | |  |  |  |  |
| 3 | Vvi-Vitvi06g00040\_t001 |  | | | |  | | | |  |  |  | | | |  |  |  |  |
| 3 | Vvi-Vitvi06g00041\_t001 |  | | | |  | | | |  |  |  | | | |  |  |  |  |
| 3 | Vvi-Vitvi06g00042\_t001 |  | | | |  | | | |  |  |  | | | |  |  |  |  |
| 3 | Vvi-Vitvi06g01580\_t001 |  | Ath-AT3G13480.2 |  | Ath-AT1G55475.1 |  |  |  | | | |  |  |  |  |
| 3 | Vvi-Vitvi06g01581\_t001 |  | | | |  | | | |  |  |  | | | |  |  |  |  |
| 3 | Vvi-Vitvi06g00043\_t001 |  | Ath-AT3G13490.1 |  | | | |  |  |  | | | |  |  |  |  |
| 3 | Vvi-Vitvi06g04013\_t001 |  | | | |  | | | |  |  |  | | | |  |  |  |  |
| 3 | Vvi-Vitvi06g01582\_t001 |  | | | |  | Ath-AT1G55365.1 |  |  |  | Ath-AT5G56520.1 |  |  |  |  |
| 3 | Vvi-Vitvi06g04014\_t001 |  | | | |  | | | |  |  |  | | | |  |  |  |  |
| 3 | Vvi-Vitvi06g00044\_t001 |  | Ath-AT3G13510.1 |  | Ath-AT1G55360.1 |  |  |  | Ath-AT5G56530.2 |  |  |  |  |
| 3 | Vvi-Vitvi06g00045\_t001 |  | | | |  | Ath-AT1G55350.5 |  |  |  | | | |  |  |  |  |
| 3 | Vvi-Vitvi06g00046\_t002 |  | | | |  | Ath-AT1G55340.1 |  |  |  | | | |  |  |  |  |
| 3 | Vvi-Vitvi06g04015\_t001 |  | Ath-AT3G13520.1 |  | | | |  |  |  | Ath-AT5G56540.1 |  |  |  |  |
| 2 | Vvi-Vitvi06g00048\_t001 |  | | | |  | Ath-AT1G55325.2 |  |  |  |  |  |  |
| 1 | Vvi-Vitvi06g00049\_t001 |  | | | |  |  |  |  |  |  |  |
| 2 | Vvi-Vitvi06g00050\_t001 |  | | | |  | Ath-AT5G19760.1 |  |  |  |  |  |  |
| 2 | Vvi-Vitvi06g00051\_t002 |  | | | |  | Ath-AT5G19770.1 |  |  |  |  |  |  |
| 2 | Vvi-Vitvi06g00052\_t001 |  | | | |  | Ath-AT5G19790.1 |  |  |  |  |  |  |
| 2 | Vvi-Vitvi06g00053\_t001 |  | | | |  | Ath-AT5G19820.1 |  |  |  |  |  |  |
| 2 | Vvi-Vitvi06g01583\_t001 |  | | | |  | | | |  |  |  |  |  |  |
| 2 | Vvi-Vitvi06g00054\_t001 |  | | | |  | | | |  |  |  |  |  |  |
| 2 | Vvi-Vitvi06g00056\_t001 |  | | | |  | | | |  |  |  |  |  |  |
| 2 | Vvi-Vitvi06g00058\_t001 |  | | | |  | Ath-AT5G19830.1 |  |  |  |  |  |  |
| 2 | Vvi-Vitvi06g00059\_t001 |  | Ath-AT3G13540.1 |  | | | |  |  |  |  |  |  |
| 2 | Vvi-Vitvi06g00060\_t001 |  | | | |  | Ath-AT5G19850.1 |  |  |  |  |  |  |
| 1 | Vvi-Vitvi06g00061\_t001 |  | | | |  |  |  |  |  |  |  |
| 1 | Vvi-Vitvi06g00062\_t001 |  | | | |  |  |  |  |  |  |  |
| 1 | Vvi-Vitvi06g00064\_t002 |  | | | |  |  |  |  |  |  |  |
| 1 | Vvi-Vitvi06g04016\_t001 |  | | | |  |  |  |  |  |  |  |
| 1 | Vvi-Vitvi06g04017\_t001 |  | | | |  |  |  |  |  |  |  |
| 1 | Vvi-Vitvi06g04018\_t001 |  | | | |  |  |  |  |  |  |  |
| 1 | Vvi-Vitvi06g04019\_t001 |  | | | |  |  |  |  |  |  |  |
| 1 | Vvi-Vitvi06g04020\_t001 |  | | | |  |  |  |  |  |  |  |
| 1 | Vvi-Vitvi06g00067\_t001 |  | | | |  |  |  |  |  |  |  |
| 1 | Vvi-Vitvi06g04021\_t001 |  | | | |  |  |  |  |  |  |  |
| 1 | Vvi-Vitvi06g04022\_t001 |  | | | |  |  |  |  |  |  |  |
| 1 | Vvi-Vitvi06g00069\_t001 |  | | | |  |  |  |  |  |  |  |
| 1 | Vvi-Vitvi06g04023\_t001 |  | | | |  |  |  |  |  |  |  |
| 1 | Vvi-Vitvi06g00070\_t001 |  | | | |  |  |  |  |  |  |  |
| 1 | Vvi-Vitvi06g00071\_t001 |  | Ath-AT3G13600.1 |  |  |  |  |  |  |  |
| 1 | Vvi-Vitvi06g00072\_t001 |  | | | |  |  |  |  |  |  |  |
| 1 | Vvi-Vitvi06g00073\_t001 |  | | | |  |  |  |  |  |  |  |
| 1 | Vvi-Vitvi06g00074\_t001 |  | | | |  |  |  |  |  |  |  |
| 1 | Vvi-Vitvi06g00075\_t001 |  | | | |  |  |  |  |  |  |  |
| 1 | Vvi-Vitvi06g04024\_t001 |  | | | |  |  |  |  |  |  |  |
| 2 | Vvi-Vitvi06g01588\_t001 |  | | | |  | Ath-AT1G55320.1 |  |  |  |  |  |  |
| 2 | Vvi-Vitvi06g00076\_t001 |  | Ath-AT3G13610.1 |  | Ath-AT1G55290.1 |  |  |  |  |  |  |
| 2 | Vvi-Vitvi06g04025\_t001 |  | | | |  | | | |  |  |  |  |  |  |
| 2 | Vvi-Vitvi06g04026\_t001 |  | | | |  | | | |  |  |  |  |  |  |
| 2 | Vvi-Vitvi06g00077\_t001 |  | | | |  | | | |  |  |  |  |  |  |
| 2 | Vvi-Vitvi06g00078\_t001 |  | | | |  | | | |  |  |  |  |  |  |
| 2 | Vvi-Vitvi06g00079\_t001 |  | Ath-AT3G13620.1 |  | | | |  |  |  |  |  |  |
| 2 | Vvi-Vitvi06g01590\_t001 |  | | | |  | | | |  |  |  |  |  |  |
| 2 | Vvi-Vitvi06g00080\_t001 |  | | | |  | | | |  |  |  |  |  |  |
| 2 | Vvi-Vitvi06g00083\_t001 |  | | | |  | Ath-AT1G55280.2 |  |  |  |  |  |  |
| 2 | Vvi-Vitvi06g00084\_t001 |  | | | |  | | | |  |  |  |  |  |  |
| 2 | Vvi-Vitvi06g00085\_t001 |  | | | |  | | | |  |  |  |  |  |  |
| 2 | Vvi-Vitvi06g00086\_t003 |  | | | |  | | | |  |  |  |  |  |  |
| 2 | Vvi-Vitvi06g00087\_t001 |  | | | |  | Ath-AT1G55270.1 |  |  |  |  |  |  |
| 2 | Vvi-Vitvi06g04027\_t001 |  | | | |  | | | |  |  |  |  |  |  |
| 2 | Vvi-Vitvi06g04028\_t001 |  | | | |  | Ath-AT1G55265.1 |  |  |  |  |  |  |
| 2 | Vvi-Vitvi06g00088\_t001 |  | | | |  | | | |  |  |  |  |  |  |
| 2 | Vvi-Vitvi06g00089\_t001 |  | | | |  | Ath-AT1G55260.1 |  |  |  |  |  |  |
| 2 | Vvi-Vitvi06g00090\_t001 |  | | | |  | | | |  |  |  |  |  |  |
| 2 | Vvi-Vitvi06g00091\_t003 |  | | | |  | | | |  |  |  |  |  |  |
| 2 | Vvi-Vitvi06g01592\_t001 |  | | | |  | | | |  |  |  |  |  |  |
| 2 | Vvi-Vitvi06g00092\_t001 |  | | | |  | | | |  |  |  |  |  |  |
| 2 | Vvi-Vitvi06g00093\_t001 |  | | | |  | | | |  |  |  |  |  |  |
| 2 | Vvi-Vitvi06g00094\_t001 |  | | | |  | | | |  |  |  |  |  |  |
| 2 | Vvi-Vitvi06g00095\_t001 |  | | | |  | | | |  |  |  |  |  |  |
| 2 | Vvi-Vitvi06g00096\_t001 |  | | | |  | | | |  |  |  |  |  |  |
| 2 | Vvi-Vitvi06g00097\_t001 |  | | | |  | Ath-AT1G55250.1 |  |  |  |  |  |  |
| 2 | Vvi-Vitvi06g00098\_t001 |  | | | |  | Ath-AT1G55230.1 |  |  |  |  |  |  |
| 2 | Vvi-Vitvi06g01593\_t001 |  | | | |  | | | |  |  |  |  |  |  |
| 2 | Vvi-Vitvi06g01594\_t001 |  | | | |  | | | |  |  |  |  |  |  |
| 2 | Vvi-Vitvi06g04029\_t001 |  | Ath-AT3G13650.1 |  | Ath-AT1G55210.1 |  |  |  |  |  |  |
| 1 | Vvi-Vitvi06g01596\_t001 |  | Ath-AT3G13662.1 |  |  |  |  |  |  |  |
| 1 | Vvi-Vitvi06g01597\_t001 |  | | | |  |  |  |  |  |  |  |
| 1 | Vvi-Vitvi06g01598\_t001 |  | | | |  |  |  |  |  |  |  |
| 1 | Vvi-Vitvi06g01599\_t001 |  | | | |  |  |  |  |  |  |  |
| 1 | Vvi-Vitvi06g01600\_t001 |  | | | |  |  |  |  |  |  |  |
| 1 | Vvi-Vitvi06g01601\_t001 |  | | | |  |  |  |  |  |  |  |
| 1 | Vvi-Vitvi06g01602\_t001 |  | | | |  |  |  |  |  |  |  |
| 1 | Vvi-Vitvi06g01603\_t001 |  | | | |  |  |  |  |  |  |  |
| 1 | Vvi-Vitvi06g04030\_t001 |  | | | |  |  |  |  |  |  |  |
| 1 | Vvi-Vitvi06g01605\_t001 |  | | | |  |  |  |  |  |  |  |
| 1 | Vvi-Vitvi06g01606\_t001 |  | | | |  |  |  |  |  |  |  |
| 1 | Vvi-Vitvi06g01607\_t001 |  | | | |  |  |  |  |  |  |  |
| 1 | Vvi-Vitvi06g04031\_t001 |  | | | |  |  |  |  |  |  |  |
| 1 | Vvi-Vitvi06g00100\_t001 |  | | | |  |  |  |  |  |  |  |
| 1 | Vvi-Vitvi06g00102\_t001 |  | | | |  |  |  |  |  |  |  |
| 1 | Vvi-Vitvi06g00104\_t001 |  | Ath-AT3G13670.1 |  |  |  |  |  |  |  |
| 0 | Vvi-Vitvi06g00105\_t001 |  |  |  |  |  |  |  |  |
| 0 | Vvi-Vitvi06g00106\_t001 |  |  |  |  |  |  |  |  |
| 0 | Vvi-Vitvi06g00107\_t001 |  |  |  |  |  |  |  |  |
| 0 | Vvi-Vitvi06g00108\_t002 |  |  |  |  |  |  |  |  |
| 0 | Vvi-Vitvi06g00109\_t001 |  |  |  |  |  |  |  |  |
| 0 | Vvi-Vitvi06g01608\_t001 |  |  |  |  |  |  |  |  |
| 0 | Vvi-Vitvi06g04032\_t001 |  |  |  |  |  |  |  |  |
| 0 | Vvi-Vitvi06g00110\_t001 |  |  |  |  |  |  |  |  |
| 0 | Vvi-Vitvi06g00111\_t001 |  |  |  |  |  |  |  |  |
| 0 | Vvi-Vitvi06g00112\_t001 |  |  |  |  |  |  |  |  |
| 0 | Vvi-Vitvi06g00113\_t001 |  |  |  |  |  |  |  |  |
| 0 | Vvi-Vitvi06g00114\_t001.3.6037826e |  |  |  |  |  |  |  |  |
| 0 | Vvi-Vitvi06g00115\_t001 |  |  |  |  |  |  |  |  |
| 0 | Vvi-Vitvi06g00116\_t001 |  |  |  |  |  |  |  |  |
| 0 | Vvi-Vitvi06g00117\_t001 |  |  |  |  |  |  |  |  |
| 0 | Vvi-Vitvi06g00118\_t001 |  |  |  |  |  |  |  |  |
| 0 | Vvi-Vitvi06g04033\_t001 |  |  |  |  |  |  |  |  |
| 0 | Vvi-Vitvi06g00119\_t001 |  |  |  |  |  |  |  |  |
| 0 | Vvi-Vitvi06g04034\_t001 |  |  |  |  |  |  |  |  |
| 0 | Vvi-Vitvi06g00120\_t002 |  |  |  |  |  |  |  |  |
| 0 | Vvi-Vitvi06g00121\_t002 |  |  |  |  |  |  |  |  |
| 0 | Vvi-Vitvi06g04035\_t001 |  |  |  |  |  |  |  |  |
| 0 | Vvi-Vitvi06g04036\_t001 |  |  |  |  |  |  |  |  |
| 0 | Vvi-Vitvi06g01610\_t001 |  |  |  |  |  |  |  |  |
| 0 | Vvi-Vitvi06g00123\_t001 |  |  |  |  |  |  |  |  |
| 0 | Vvi-Vitvi06g00124\_t001 |  |  |  |  |  |  |  |  |
| 0 | Vvi-Vitvi06g00125\_t001 |  |  |  |  |  |  |  |  |
| 0 | Vvi-Vitvi06g00126\_t001 |  |  |  |  |  |  |  |  |
| 0 | Vvi-Vitvi06g00127\_t001 |  |  |  |  |  |  |  |  |
| 0 | Vvi-Vitvi06g00128\_t001 |  |  |  |  |  |  |  |  |
| 0 | Vvi-Vitvi06g04037\_t001 |  |  |  |  |  |  |  |  |
| 0 | Vvi-Vitvi06g00130\_t001 |  |  |  |  |  |  |  |  |
| 0 | Vvi-Vitvi06g04038\_t001 |  |  |  |  |  |  |  |  |
| 0 | Vvi-Vitvi06g00132\_t001 |  |  |  |  |  |  |  |  |
| 0 | Vvi-Vitvi06g04039\_t001 |  |  |  |  |  |  |  |  |
| 0 | Vvi-Vitvi06g00134\_t001 |  |  |  |  |  |  |  |  |
| 0 | Vvi-Vitvi06g01611\_t001 |  |  |  |  |  |  |  |  |
| 0 | Vvi-Vitvi06g00136\_t001 |  |  |  |  |  |  |  |  |
| 0 | Vvi-Vitvi06g00137\_t001 |  |  |  |  |  |  |  |  |
| 0 | Vvi-Vitvi06g01612\_t001 |  |  |  |  |  |  |  |  |
| 0 | Vvi-Vitvi06g00138\_t001 |  |  |  |  |  |  |  |  |
| 0 | Vvi-Vitvi06g01613\_t001 |  |  |  |  |  |  |  |  |
| 0 | Vvi-Vitvi06g04040\_t001 |  |  |  |  |  |  |  |  |
| 0 | Vvi-Vitvi06g04041\_t001 |  |  |  |  |  |  |  |  |
| 0 | Vvi-Vitvi06g00139\_t001 |  |  |  |  |  |  |  |  |
| 0 | Vvi-Vitvi06g00140\_t001 |  |  |  |  |  |  |  |  |
| 0 | Vvi-Vitvi06g01615\_t001 |  |  |  |  |  |  |  |  |
| 0 | Vvi-Vitvi06g00141\_t001 |  |  |  |  |  |  |  |  |
| 0 | Vvi-Vitvi06g04042\_t001 |  |  |  |  |  |  |  |  |
| 0 | Vvi-Vitvi06g04043\_t001 |  |  |  |  |  |  |  |  |
| 0 | Vvi-Vitvi06g04044\_t001 |  |  |  |  |  |  |  |  |
| 0 | Vvi-Vitvi06g04045\_t001 |  |  |  |  |  |  |  |  |
| 0 | Vvi-Vitvi06g00143\_t001 |  |  |  |  |  |  |  |  |
| 0 | Vvi-Vitvi06g00149\_t001 |  |  |  |  |  |  |  |  |
| 0 | Vvi-Vitvi06g04046\_t001 |  |  |  |  |  |  |  |  |
| 0 | Vvi-Vitvi06g00150\_t001 |  |  |  |  |  |  |  |  |
| 0 | Vvi-Vitvi06g00153\_t001 |  |  |  |  |  |  |  |  |
| 0 | Vvi-Vitvi06g00155\_t001 |  |  |  |  |  |  |  |  |
| 0 | Vvi-Vitvi06g00156\_t001 |  |  |  |  |  |  |  |  |
| 0 | Vvi-Vitvi06g00158\_t001 |  |  |  |  |  |  |  |  |
| 0 | Vvi-Vitvi06g00159\_t001 |  |  |  |  |  |  |  |  |
| 1 | Vvi-Vitvi06g00160\_t001 |  | Ath-AT3G20780.1 |  |  |  |  |  |  |  |
| 1 | Vvi-Vitvi06g00161\_t001 |  | Ath-AT3G20790.1 |  |  |  |  |  |  |  |
| 1 | Vvi-Vitvi06g00162\_t001 |  | | | |  |  |  |  |  |  |  |
| 1 | Vvi-Vitvi06g04047\_t001 |  | | | |  |  |  |  |  |  |  |
| 1 | Vvi-Vitvi06g00163\_t001 |  | | | |  |  |  |  |  |  |  |
| 1 | Vvi-Vitvi06g01616\_t001 |  | | | |  |  |  |  |  |  |  |
| 1 | Vvi-Vitvi06g01617\_t001 |  | | | |  |  |  |  |  |  |  |
| 1 | Vvi-Vitvi06g04048\_t001 |  | | | |  |  |  |  |  |  |  |
| 1 | Vvi-Vitvi06g04049\_t001 |  | | | |  |  |  |  |  |  |  |
| 1 | Vvi-Vitvi06g01618\_t001 |  | | | |  |  |  |  |  |  |  |
| 1 | Vvi-Vitvi06g00165\_t001 |  | | | |  |  |  |  |  |  |  |
| 1 | Vvi-Vitvi06g04050\_t001 |  | | | |  |  |  |  |  |  |  |
| 1 | Vvi-Vitvi06g00166\_t001 |  | | | |  |  |  |  |  |  |  |
| 1 | Vvi-Vitvi06g04051\_t001 |  | | | |  |  |  |  |  |  |  |
| 1 | Vvi-Vitvi06g00167\_t001 |  | Ath-AT3G20810.2 |  |  |  |  |  |  |  |
| 2 | Vvi-Vitvi06g00169\_t001 |  | | | |  | Ath-AT5G12890.1 |  |  |  |  |  |  |
| 2 | Vvi-Vitvi06g00170\_t001 |  | | | |  | | | |  |  |  |  |  |  |
| 2 | Vvi-Vitvi06g01620\_t001 |  | | | |  | | | |  |  |  |  |  |  |
| 2 | Vvi-Vitvi06g00172\_t001 |  | | | |  | | | |  |  |  |  |  |  |
| 2 | Vvi-Vitvi06g00173\_t001 |  | | | |  | Ath-AT5G12900.1 |  |  |  |  |  |  |
| 2 | Vvi-Vitvi06g04052\_t001 |  | | | |  | | | |  |  |  |  |  |  |
| 2 | Vvi-Vitvi06g00177\_t001 |  | | | |  | Ath-AT5G12920.2 |  |  |  |  |  |  |
| 2 | Vvi-Vitvi06g04053\_t001 |  | | | |  | | | |  |  |  |  |  |  |
| 2 | Vvi-Vitvi06g00179\_t001 |  | | | |  | | | |  |  |  |  |  |  |
| 2 | Vvi-Vitvi06g00180\_t001 |  | | | |  | Ath-AT5G12930.1 |  |  |  |  |  |  |
| 3 | Vvi-Vitvi06g00181\_t001 |  | | | |  | | | |  | Ath-AT1G51140.1 |  |  |  |  |  |
| 3 | Vvi-Vitvi06g00182\_t001 |  | Ath-AT3G20820.1 |  | Ath-AT5G12940.1 |  | | | |  |  |  |  |  |
| 3 | Vvi-Vitvi06g04054\_t001 |  | | | |  | | | |  | | | |  |  |  |  |  |
| 3 | Vvi-Vitvi06g04055\_t001 |  | | | |  | | | |  | | | |  |  |  |  |  |
| 3 | Vvi-Vitvi06g00184\_t001 |  | | | |  | Ath-AT5G12950.1 |  | | | |  |  |  |  |  |
| 3 | Vvi-Vitvi06g00185\_t001 |  | Ath-AT3G20830.1 |  | | | |  | Ath-AT1G51170.1 |  |  |  |  |  |
| 3 | Vvi-Vitvi06g00186\_t001 |  | | | |  | | | |  | | | |  |  |  |  |  |
| 3 | Vvi-Vitvi06g00187\_t001 |  | Ath-AT3G20840.1 |  | | | |  | Ath-AT1G51190.1 |  |  |  |  |  |
| 3 | Vvi-Vitvi06g00188\_t001 |  | | | |  | Ath-AT5G13070.1 |  | | | |  |  |  |  |  |
| 2 | Vvi-Vitvi06g00189\_t003 |  | | | |  |  |  | Ath-AT1G51200.1 |  |  |  |  |  |
| 2 | Vvi-Vitvi06g00191\_t002 |  | Ath-AT3G20860.1 |  |  |  | | | |  |  |  |  |  |
| 2 | Vvi-Vitvi06g04056\_t001 |  | | | |  |  |  | | | |  |  |  |  |  |
| 2 | Vvi-Vitvi06g04057\_t001 |  | | | |  |  |  | | | |  |  |  |  |  |
| 2 | Vvi-Vitvi06g04058\_t001 |  | | | |  |  |  | | | |  |  |  |  |  |
| 2 | Vvi-Vitvi06g04059\_t001 |  | | | |  |  |  | | | |  |  |  |  |  |
| 2 | Vvi-Vitvi06g00192\_t001 |  | | | |  |  |  | | | |  |  |  |  |  |
| 2 | Vvi-Vitvi06g04060\_t001 |  | | | |  |  |  | | | |  |  |  |  |  |
| 2 | Vvi-Vitvi06g00193\_t001 |  | Ath-AT3G20870.1 |  |  |  | | | |  |  |  |  |  |
| 2 | Vvi-Vitvi06g00195\_t001 |  | Ath-AT3G20880.1 |  |  |  | Ath-AT1G51220.1 |  |  |  |  |  |
| 1 | Vvi-Vitvi06g00198\_t001 |  |  |  |  |  | | | |  |  |  |  |  |
| 1 | Vvi-Vitvi06g00199\_t003 |  |  |  |  |  | | | |  |  |  |  |  |
| 1 | Vvi-Vitvi06g00200\_t001 |  |  |  |  |  | | | |  |  |  |  |  |
| 1 | Vvi-Vitvi06g04061\_t001 |  |  |  |  |  | | | |  |  |  |  |  |
| 1 | Vvi-Vitvi06g00201\_t002 |  |  |  |  |  | | | |  |  |  |  |  |
| 1 | Vvi-Vitvi06g00202\_t001 |  |  |  |  |  | | | |  |  |  |  |  |
| 1 | Vvi-Vitvi06g04062\_t001 |  |  |  |  |  | | | |  |  |  |  |  |
| 1 | Vvi-Vitvi06g01622\_t001 |  |  |  |  |  | | | |  |  |  |  |  |
| 1 | Vvi-Vitvi06g01625\_t001 |  |  |  |  |  | | | |  |  |  |  |  |
| 1 | Vvi-Vitvi06g01626\_t001 |  |  |  |  |  | | | |  |  |  |  |  |
| 1 | Vvi-Vitvi06g04063\_t001 |  |  |  |  |  | | | |  |  |  |  |  |
| 1 | Vvi-Vitvi06g04064\_t001 |  |  |  |  |  | | | |  |  |  |  |  |
| 1 | Vvi-Vitvi06g01628\_t001 |  |  |  |  |  | | | |  |  |  |  |  |
| 1 | Vvi-Vitvi06g01629\_t001 |  |  |  |  |  | | | |  |  |  |  |  |
| 1 | Vvi-Vitvi06g04065\_t001 |  |  |  |  |  | | | |  |  |  |  |  |
| 1 | Vvi-Vitvi06g04066\_t001 |  |  |  |  |  | | | |  |  |  |  |  |
| 1 | Vvi-Vitvi06g04067\_t001 |  |  |  |  |  | | | |  |  |  |  |  |
| 1 | Vvi-Vitvi06g00205\_t001 |  |  |  |  |  | | | |  |  |  |  |  |
| 1 | Vvi-Vitvi06g00206\_t001 |  |  |  |  |  | | | |  |  |  |  |  |
| 1 | Vvi-Vitvi06g00207\_t001 |  |  |  |  |  | | | |  |  |  |  |  |
| 1 | Vvi-Vitvi06g00209\_t001 |  |  |  |  |  | | | |  |  |  |  |  |
| 1 | Vvi-Vitvi06g04068\_t001 |  |  |  |  |  | | | |  |  |  |  |  |
| 1 | Vvi-Vitvi06g00210\_t001 |  |  |  |  |  | | | |  |  |  |  |  |
| 1 | Vvi-Vitvi06g00211\_t001 |  |  |  |  |  | | | |  |  |  |  |  |
| 1 | Vvi-Vitvi06g01631\_t001 |  |  |  |  |  | Ath-AT1G51310.1 |  |  |  |  |  |
| 1 | Vvi-Vitvi06g00212\_t001 |  |  |  |  |  | | | |  |  |  |  |  |
| 1 | Vvi-Vitvi06g00213\_t002 |  |  |  |  |  | | | |  |  |  |  |  |
| 2 | Vvi-Vitvi06g00214\_t001 |  | Ath-AT5G12870.1 |  |  |  | | | |  |  |  |  |  |
| 2 | Vvi-Vitvi06g00215\_t001 |  | Ath-AT5G12860.1 |  |  |  | | | |  |  |  |  |  |
| 2 | Vvi-Vitvi06g04069\_t001 |  | | | |  |  |  | | | |  |  |  |  |  |
| 2 | Vvi-Vitvi06g00216\_t001 |  | | | |  |  |  | Ath-AT1G51340.2 |  |  |  |  |  |
| 2 | Vvi-Vitvi06g00217\_t001 |  | | | |  |  |  | | | |  |  |  |  |  |
| 2 | Vvi-Vitvi06g00218\_t003 |  | Ath-AT5G12850.1 |  |  |  | | | |  |  |  |  |  |
| 2 | Vvi-Vitvi06g04070\_t001 |  | | | |  |  |  | | | |  |  |  |  |  |
| 2 | Vvi-Vitvi06g00220\_t001 |  | | | |  |  |  | | | |  |  |  |  |  |
| 2 | Vvi-Vitvi06g01633\_t001 |  | | | |  |  |  | Ath-AT1G51350.1 |  |  |  |  |  |
| 2 | Vvi-Vitvi06g00222\_t001 |  | | | |  |  |  | | | |  |  |  |  |  |
| 3 | Vvi-Vitvi06g00223\_t001 |  | | | |  | Ath-AT3G20890.1 |  | | | |  |  |  |  |  |
| 3 | Vvi-Vitvi06g01635\_t001 |  | | | |  | Ath-AT3G20898.1 |  | Ath-AT1G51355.1 |  |  |  |  |  |
| 3 | Vvi-Vitvi06g01636\_t001 |  | | | |  | | | |  | | | |  |  |  |  |  |
| 3 | Vvi-Vitvi06g04071\_t001 |  | | | |  | | | |  | | | |  |  |  |  |  |
| 3 | Vvi-Vitvi06g00224\_t001 |  | Ath-AT5G12840.1 |  | Ath-AT3G20910.1 |  | | | |  |  |  |  |  |
| 3 | Vvi-Vitvi06g00225\_t001 |  | | | |  | Ath-AT3G20920.1 |  | | | |  |  |  |  |  |
| 3 | Vvi-Vitvi06g04072\_t001 |  | | | |  | | | |  | | | |  |  |  |  |  |
| 3 | Vvi-Vitvi06g00226\_t001 |  | | | |  | | | |  | | | |  |  |  |  |  |
| 3 | Vvi-Vitvi06g00227\_t001 |  | Ath-AT5G12480.1 |  | | | |  | | | |  |  |  |  |  |
| 3 | Vvi-Vitvi06g04073\_t001 |  | | | |  | | | |  | | | |  |  |  |  |  |
| 3 | Vvi-Vitvi06g00229\_t001 |  | | | |  | | | |  | | | |  |  |  |  |  |
| 3 | Vvi-Vitvi06g00230\_t001 |  | | | |  | | | |  | | | |  |  |  |  |  |
| 3 | Vvi-Vitvi06g01637\_t001 |  | | | |  | Ath-AT3G21055.2 |  | Ath-AT1G51400.1 |  |  |  |  |  |
| 3 | Vvi-Vitvi06g04074\_t001 |  | | | |  | | | |  | | | |  |  |  |  |  |
| 3 | Vvi-Vitvi06g04075\_t001 |  | | | |  | | | |  | | | |  |  |  |  |  |
| 3 | Vvi-Vitvi06g04076\_t001 |  | | | |  | | | |  | | | |  |  |  |  |  |
| 3 | Vvi-Vitvi06g04077\_t001 |  | | | |  | | | |  | | | |  |  |  |  |  |
| 3 | Vvi-Vitvi06g04078\_t001 |  | | | |  | | | |  | | | |  |  |  |  |  |
| 3 | Vvi-Vitvi06g01639\_t001 |  | | | |  | | | |  | | | |  |  |  |  |  |
| 3 | Vvi-Vitvi06g01640\_t001 |  | | | |  | | | |  | | | |  |  |  |  |  |
| 3 | Vvi-Vitvi06g04079\_t001 |  | | | |  | | | |  | | | |  |  |  |  |  |
| 3 | Vvi-Vitvi06g01641\_t001 |  | | | |  | | | |  | | | |  |  |  |  |  |
| 3 | Vvi-Vitvi06g04080\_t001 |  | | | |  | | | |  | | | |  |  |  |  |  |
| 3 | Vvi-Vitvi06g00233\_t001 |  | | | |  | | | |  | Ath-AT1G51410.1 |  |  |  |  |  |
| 3 | Vvi-Vitvi06g01644\_t001 |  | | | |  | | | |  | | | |  |  |  |  |  |
| 3 | Vvi-Vitvi06g00234\_t001 |  | | | |  | | | |  | Ath-AT1G51440.1 |  |  |  |  |  |
| 3 | Vvi-Vitvi06g00235\_t001 |  | Ath-AT5G12470.1 |  | | | |  | | | |  |  |  |  |  |
| 2 | Vvi-Vitvi06g00236\_t001 |  |  |  | Ath-AT3G21070.3 |  | | | |  |  |  |  |  |
| 2 | Vvi-Vitvi06g00237\_t001 |  |  |  | | | |  | Ath-AT1G51450.1 |  |  |  |  |  |
| 2 | Vvi-Vitvi06g04081\_t001 |  |  |  | | | |  | | | |  |  |  |  |  |
| 2 | Vvi-Vitvi06g00238\_t001 |  |  |  | | | |  | | | |  |  |  |  |  |
| 2 | Vvi-Vitvi06g01646\_t001 |  |  |  | | | |  | | | |  |  |  |  |  |
| 2 | Vvi-Vitvi06g00241\_t003 |  |  |  | | | |  | | | |  |  |  |  |  |
| 3 | Vvi-Vitvi06g00242\_t001 |  | Ath-AT5G60740.1 |  | Ath-AT3G21090.1 |  | Ath-AT1G51500.1 |  |  |  |  |  |
| 1 | Vvi-Vitvi06g00243\_t001 |  | Ath-AT5G60730.1 |  |  |  |  |  |  |  |
| 1 | Vvi-Vitvi06g00244\_t001 |  | Ath-AT5G60720.1 |  |  |  |  |  |  |  |
| 1 | Vvi-Vitvi06g00245\_t001 |  | | | |  |  |  |  |  |  |  |
| 1 | Vvi-Vitvi06g00246\_t001 |  | | | |  |  |  |  |  |  |  |
| 1 | Vvi-Vitvi06g01647\_t001 |  | | | |  |  |  |  |  |  |  |
| 1 | Vvi-Vitvi06g01648\_t001 |  | | | |  |  |  |  |  |  |  |
| 1 | Vvi-Vitvi06g04082\_t001 |  | | | |  |  |  |  |  |  |  |
| 1 | Vvi-Vitvi06g01649\_t001 |  | | | |  |  |  |  |  |  |  |
| 1 | Vvi-Vitvi06g04083\_t001 |  | | | |  |  |  |  |  |  |  |
| 1 | Vvi-Vitvi06g04084\_t001 |  | | | |  |  |  |  |  |  |  |
| 1 | Vvi-Vitvi06g04085\_t001 |  | | | |  |  |  |  |  |  |  |
| 2 | Vvi-Vitvi06g00248\_t001 |  | | | |  | Ath-AT2G27990.1 |  |  |  |  |  |  |
| 2 | Vvi-Vitvi06g01651\_t001 |  | | | |  | | | |  |  |  |  |  |  |
| 2 | Vvi-Vitvi06g04086\_t001 |  | | | |  | | | |  |  |  |  |  |  |
| 2 | Vvi-Vitvi06g04087\_t001 |  | | | |  | | | |  |  |  |  |  |  |
| 2 | Vvi-Vitvi06g04088\_t001 |  | | | |  | | | |  |  |  |  |  |  |
| 2 | Vvi-Vitvi06g04089\_t001 |  | | | |  | | | |  |  |  |  |  |  |
| 3 | Vvi-Vitvi06g00250\_t001 |  | | | |  | Ath-AT2G28210.1 |  | Ath-AT1G08065.1 |  |  |  |  |  |
| 3 | Vvi-Vitvi06g00251\_t001 |  | | | |  | | | |  | | | |  |  |  |  |  |
| 3 | Vvi-Vitvi06g04090\_t001 |  | | | |  | | | |  | Ath-AT1G08060.3 |  |  |  |  |  |
| 3 | Vvi-Vitvi06g04091\_t001 |  | | | |  | | | |  | | | |  |  |  |  |  |
| 3 | Vvi-Vitvi06g00258\_t001 |  | Ath-AT5G60710.1 |  | | | |  | Ath-AT1G08050.1 |  |  |  |  |  |
| 3 | Vvi-Vitvi06g04092\_t001 |  | | | |  | | | |  | | | |  |  |  |  |  |
| 3 | Vvi-Vitvi06g00259\_t001 |  | | | |  | Ath-AT2G28250.3 |  | | | |  |  |  |  |  |
| 3 | Vvi-Vitvi06g04093\_t001 |  | | | |  | | | |  | | | |  |  |  |  |  |
| 3 | Vvi-Vitvi06g00261\_t001 |  | | | |  | | | |  | | | |  |  |  |  |  |
| 3 | Vvi-Vitvi06g04094\_t001 |  | | | |  | | | |  | | | |  |  |  |  |  |
| 3 | Vvi-Vitvi06g00262\_t001 |  | | | |  | Ath-AT2G28260.1 |  | | | |  |  |  |  |  |
| 3 | Vvi-Vitvi06g00263\_t001 |  | | | |  | Ath-AT2G28305.1 |  | | | |  |  |  |  |  |
| 3 | Vvi-Vitvi06g00266\_t001 |  | Ath-AT5G60700.2 |  | | | |  | | | |  |  |  |  |  |
| 3 | Vvi-Vitvi06g00267\_t001 |  | | | |  | Ath-AT2G28310.1 |  | Ath-AT1G08040.1 |  |  |  |  |  |
| 3 | Vvi-Vitvi06g00268\_t001 |  | | | |  | Ath-AT2G28315.1 |  | | | |  |  |  |  |  |
| 3 | Vvi-Vitvi06g00269\_t001 |  | | | |  | Ath-AT2G28320.1 |  | | | |  |  |  |  |  |
| 3 | Vvi-Vitvi06g04095\_t001 |  | | | |  | | | |  | Ath-AT1G08000.2 |  |  |  |  |  |
| 3 | Vvi-Vitvi06g00272\_t002 |  | | | |  | Ath-AT2G28350.1 |  | | | |  |  |  |  |  |
| 3 | Vvi-Vitvi06g04096\_t001 |  | | | |  | | | |  | | | |  |  |  |  |  |
| 3 | Vvi-Vitvi06g04097\_t001 |  | | | |  | | | |  | | | |  |  |  |  |  |
| 4 | Vvi-Vitvi06g00274\_t004 |  | | | |  | Ath-AT2G28360.1 |  | Ath-AT1G07990.1 |  | Ath-AT3G45190.1 |  |  |  |  |
| 3 | Vvi-Vitvi06g04098\_t001 |  | | | |  | | | |  |  |  | | | |  |  |  |  |
| 3 | Vvi-Vitvi06g00275\_t001 |  | | | |  | Ath-AT2G28370.1 |  |  |  | | | |  |  |  |  |
| 3 | Vvi-Vitvi06g01656\_t001 |  | | | |  | | | |  |  |  | | | |  |  |  |  |
| 3 | Vvi-Vitvi06g00276\_t001 |  | Ath-AT5G60690.1 |  | | | |  |  |  | | | |  |  |  |  |
| 3 | Vvi-Vitvi06g04099\_t001 |  | | | |  | | | |  |  |  | | | |  |  |  |  |
| 3 | Vvi-Vitvi06g00277\_t001 |  | | | |  | Ath-AT2G28380.1 |  |  |  | | | |  |  |  |  |
| 3 | Vvi-Vitvi06g00278\_t001 |  | | | |  | Ath-AT2G28390.1 |  |  |  | | | |  |  |  |  |
| 3 | Vvi-Vitvi06g04100\_t001 |  | | | |  | | | |  |  |  | | | |  |  |  |  |
| 3 | Vvi-Vitvi06g04101\_t001 |  | | | |  | | | |  |  |  | | | |  |  |  |  |
| 3 | Vvi-Vitvi06g00279\_t001 |  | Ath-AT5G60680.1 |  | Ath-AT2G28400.1 |  |  |  | Ath-AT3G45210.1 |  |  |  |  |
| 3 | Vvi-Vitvi06g00280\_t001 |  | Ath-AT5G60670.1 |  | | | |  |  |  | | | |  |  |  |  |
| 3 | Vvi-Vitvi06g00281\_t001 |  | Ath-AT5G60660.1 |  | | | |  |  |  | | | |  |  |  |  |
| 3 | Vvi-Vitvi06g01658\_t001 |  | Ath-AT5G60650.1 |  | Ath-AT2G28410.1 |  |  |  | | | |  |  |  |  |
| 3 | Vvi-Vitvi06g00282\_t001 |  | | | |  | Ath-AT2G28430.1 |  |  |  | | | |  |  |  |  |
| 3 | Vvi-Vitvi06g00283\_t001 |  | | | |  | | | |  |  |  | | | |  |  |  |  |
| 3 | Vvi-Vitvi06g00284\_t001 |  | Ath-AT5G60640.1 |  | | | |  |  |  | | | |  |  |  |  |
| 3 | Vvi-Vitvi06g00285\_t001 |  | | | |  | Ath-AT2G28440.1 |  |  |  | Ath-AT3G45230.1 |  |  |  |  |
| 3 | Vvi-Vitvi06g00286\_t001 |  | Ath-AT5G60600.1 |  | | | |  |  |  | | | |  |  |  |  |
| 3 | Vvi-Vitvi06g00287\_t001 |  | | | |  | | | |  |  |  | | | |  |  |  |  |
| 3 | Vvi-Vitvi06g00288\_t001 |  | Ath-AT5G60590.2 |  | | | |  |  |  | | | |  |  |  |  |
| 3 | Vvi-Vitvi06g00289\_t001 |  | Ath-AT5G60580.4 |  | | | |  |  |  | | | |  |  |  |  |
| 3 | Vvi-Vitvi06g00290\_t001 |  | | | |  | Ath-AT2G28450.1 |  |  |  | | | |  |  |  |  |
| 3 | Vvi-Vitvi06g04102\_t001 |  | | | |  | | | |  |  |  | | | |  |  |  |  |
| 3 | Vvi-Vitvi06g00292\_t001 |  | | | |  | | | |  |  |  | | | |  |  |  |  |
| 3 | Vvi-Vitvi06g00293\_t001 |  | | | |  | | | |  |  |  | | | |  |  |  |  |
| 3 | Vvi-Vitvi06g00294\_t001 |  | | | |  | | | |  |  |  | | | |  |  |  |  |
| 3 | Vvi-Vitvi06g00295\_t001 |  | Ath-AT5G60570.3 |  | | | |  |  |  | | | |  |  |  |  |
| 3 | Vvi-Vitvi06g04103\_t001 |  | | | |  | | | |  |  |  | | | |  |  |  |  |
| 3 | Vvi-Vitvi06g00296\_t002 |  | Ath-AT5G60550.1 |  | | | |  |  |  | Ath-AT3G45240.1 |  |  |  |  |
| 3 | Vvi-Vitvi06g04104\_t001 |  | | | |  | | | |  |  |  | | | |  |  |  |  |
| 3 | Vvi-Vitvi06g00298\_t001 |  | Ath-AT5G60540.1 |  | | | |  |  |  | | | |  |  |  |  |
| 3 | Vvi-Vitvi06g00299\_t001 |  | Ath-AT5G60520.1 |  | | | |  |  |  | | | |  |  |  |  |
| 3 | Vvi-Vitvi06g00300\_t001 |  | | | |  | Ath-AT2G28470.1 |  |  |  | | | |  |  |  |  |
| 2 | Vvi-Vitvi06g00301\_t001 |  | | | |  |  |  |  |  | | | |  |  |  |  |
| 3 | Vvi-Vitvi06g01659\_t001 |  | | | |  | Ath-AT1G07980.1 |  |  |  | | | |  |  |  |  |
| 3 | Vvi-Vitvi06g00302\_t001 |  | | | |  | | | |  |  |  | | | |  |  |  |  |
| 3 | Vvi-Vitvi06g00303\_t001 |  | Ath-AT5G60490.1 |  | | | |  |  |  | | | |  |  |  |  |
| 3 | Vvi-Vitvi06g00304\_t001 |  | Ath-AT5G60470.2 |  | | | |  |  |  | Ath-AT3G45260.1 |  |  |  |  |
| 3 | Vvi-Vitvi06g00305\_t001 |  | | | |  | | | |  |  |  | | | |  |  |  |  |
| 3 | Vvi-Vitvi06g00306\_t001 |  | | | |  | | | |  |  |  | Ath-AT3G45280.1 |  |  |  |  |
| 3 | Vvi-Vitvi06g04105\_t001 |  | | | |  | | | |  |  |  | | | |  |  |  |  |
| 3 | Vvi-Vitvi06g01661\_t001 |  | Ath-AT5G60460.1 |  | | | |  |  |  | | | |  |  |  |  |
| 3 | Vvi-Vitvi06g00308\_t001 |  | | | |  | | | |  |  |  | | | |  |  |  |  |
| 3 | Vvi-Vitvi06g00309\_t001 |  | | | |  | | | |  |  |  | | | |  |  |  |  |
| 3 | Vvi-Vitvi06g00310\_t001 |  | | | |  | | | |  |  |  | Ath-AT3G45290.1 |  |  |  |  |
| 3 | Vvi-Vitvi06g00311\_t001 |  | Ath-AT5G60450.1 |  | | | |  |  |  | | | |  |  |  |  |
| 3 | Vvi-Vitvi06g00312\_t001 |  | | | |  | | | |  |  |  | Ath-AT3G45300.1 |  |  |  |  |
| 3 | Vvi-Vitvi06g00313\_t001 |  | | | |  | Ath-AT1G07970.1 |  |  |  | | | |  |  |  |  |
| 3 | Vvi-Vitvi06g01662\_t001 |  | | | |  | | | |  |  |  | | | |  |  |  |  |
| 3 | Vvi-Vitvi06g00314\_t001 |  | | | |  | | | |  |  |  | | | |  |  |  |  |
| 3 | Vvi-Vitvi06g00315\_t003 |  | Ath-AT5G60410.2 |  | | | |  |  |  | | | |  |  |  |  |
| 3 | Vvi-Vitvi06g04106\_t001 |  | | | |  | | | |  |  |  | | | |  |  |  |  |
| 3 | Vvi-Vitvi06g00316\_t002 |  | | | |  | | | |  |  |  | | | |  |  |  |  |
| 3 | Vvi-Vitvi06g00318\_t001 |  | | | |  | | | |  |  |  | | | |  |  |  |  |
| 3 | Vvi-Vitvi06g04107\_t001 |  | Ath-AT5G60390.1 |  | | | |  |  |  | | | |  |  |  |  |
| 3 | Vvi-Vitvi06g01664\_t002 |  | | | |  | | | |  |  |  | | | |  |  |  |  |
| 3 | Vvi-Vitvi06g00320\_t001 |  | | | |  | | | |  |  |  | | | |  |  |  |  |
| 3 | Vvi-Vitvi06g04108\_t001 |  | | | |  | | | |  |  |  | | | |  |  |  |  |
| 3 | Vvi-Vitvi06g04109\_t003 |  | | | |  | | | |  |  |  | | | |  |  |  |  |
| 3 | Vvi-Vitvi06g00322\_t001 |  | | | |  | Ath-AT1G07960.3 |  |  |  | | | |  |  |  |  |
| 4 | Vvi-Vitvi06g00323\_t001 |  | | | |  | | | |  | Ath-AT2G28490.1 |  | | | |  |  |  |  |
| 4 | Vvi-Vitvi06g00325\_t001 |  | Ath-AT5G60370.1 |  | | | |  | | | |  | | | |  |  |  |  |
| 4 | Vvi-Vitvi06g00327\_t001 |  | | | |  | | | |  | | | |  | | | |  |  |  |  |
| 4 | Vvi-Vitvi06g00328\_t001 |  | Ath-AT5G60340.1 |  | | | |  | | | |  | | | |  |  |  |  |
| 4 | Vvi-Vitvi06g00329\_t001 |  | Ath-AT5G60335.1 |  | | | |  | | | |  | | | |  |  |  |  |
| 4 | Vvi-Vitvi06g00330\_t001 |  | | | |  | Ath-AT1G07910.2 |  | | | |  | | | |  |  |  |  |
| 4 | Vvi-Vitvi06g04110\_t001 |  | | | |  | | | |  | | | |  | | | |  |  |  |  |
| 4 | Vvi-Vitvi06g00331\_t001 |  | | | |  | | | |  | | | |  | | | |  |  |  |  |
| 4 | Vvi-Vitvi06g00334\_t001 |  | | | |  | | | |  | | | |  | | | |  |  |  |  |
| 4 | Vvi-Vitvi06g00335\_t001 |  | Ath-AT5G60250.1 |  | | | |  | | | |  | Ath-AT3G45580.1 |  |  |  |  |
| 4 | Vvi-Vitvi06g00336\_t001 |  | | | |  | Ath-AT1G07900.1 |  | Ath-AT2G28500.1 |  | | | |  |  |  |  |
| 4 | Vvi-Vitvi06g00337\_t001 |  | | | |  | | | |  | | | |  | | | |  |  |  |  |
| 4 | Vvi-Vitvi06g00338\_t001 |  | | | |  | | | |  | | | |  | | | |  |  |  |  |
| 4 | Vvi-Vitvi06g00339\_t001 |  | | | |  | | | |  | | | |  | | | |  |  |  |  |
| 4 | Vvi-Vitvi06g04111\_t001 |  | | | |  | | | |  | | | |  | | | |  |  |  |  |
| 4 | Vvi-Vitvi06g00340\_t001 |  | | | |  | | | |  | | | |  | | | |  |  |  |  |
| 4 | Vvi-Vitvi06g00341\_t001 |  | Ath-AT5G60230.2 |  | | | |  | | | |  | | | |  |  |  |  |
| 4 | Vvi-Vitvi06g00342\_t001 |  | Ath-AT5G60220.1 |  | | | |  | | | |  | Ath-AT3G45600.1 |  |  |  |  |
| 4 | Vvi-Vitvi06g01665\_t004 |  | Ath-AT5G60210.3 |  | | | |  | | | |  | | | |  |  |  |  |
| 4 | Vvi-Vitvi06g04112\_t001 |  | | | |  | | | |  | | | |  | | | |  |  |  |  |
| 4 | Vvi-Vitvi06g00345\_t001 |  | Ath-AT5G60200.1 |  | | | |  | Ath-AT2G28510.1 |  | Ath-AT3G45610.1 |  |  |  |  |
| 4 | Vvi-Vitvi06g00346\_t001 |  | | | |  | | | |  | | | |  | | | |  |  |  |  |
| 4 | Vvi-Vitvi06g00347\_t001 |  | Ath-AT5G60190.1 |  | | | |  | | | |  | | | |  |  |  |  |
| 4 | Vvi-Vitvi06g04113\_t001 |  | | | |  | | | |  | | | |  | | | |  |  |  |  |
| 4 | Vvi-Vitvi06g00348\_t001 |  | | | |  | | | |  | Ath-AT2G28520.1 |  | | | |  |  |  |  |
| 4 | Vvi-Vitvi06g00349\_t001 |  | | | |  | | | |  | | | |  | Ath-AT3G45620.2 |  |  |  |  |
| 4 | Vvi-Vitvi06g00350\_t001 |  | Ath-AT5G60150.2 |  | | | |  | | | |  | | | |  |  |  |  |
| 4 | Vvi-Vitvi06g00351\_t001 |  | | | |  | | | |  | | | |  | | | |  |  |  |  |
| 4 | Vvi-Vitvi06g00352\_t001 |  | | | |  | | | |  | | | |  | | | |  |  |  |  |
| 4 | Vvi-Vitvi06g04114\_t001 |  | | | |  | | | |  | | | |  | | | |  |  |  |  |
| 4 | Vvi-Vitvi06g00354\_t001 |  | | | |  | | | |  | | | |  | | | |  |  |  |  |
| 4 | Vvi-Vitvi06g01667\_t001 |  | | | |  | | | |  | | | |  | | | |  |  |  |  |
| 4 | Vvi-Vitvi06g00356\_t001 |  | | | |  | | | |  | | | |  | Ath-AT3G45640.1 |  |  |  |  |
| 4 | Vvi-Vitvi06g00358\_t003 |  | | | |  | Ath-AT1G07890.1 |  | | | |  | | | |  |  |  |  |
| 4 | Vvi-Vitvi06g00359\_t002 |  | | | |  | | | |  | Ath-AT2G28540.2 |  | | | |  |  |  |  |
| 4 | Vvi-Vitvi06g00360\_t001 |  | Ath-AT5G60120.2 |  | | | |  | Ath-AT2G28550.3 |  | | | |  |  |  |  |
| 4 | Vvi-Vitvi06g00362\_t001 |  | | | |  | | | |  | Ath-AT2G28560.1 |  | | | |  |  |  |  |
| 4 | Vvi-Vitvi06g04115\_t001 |  | | | |  | | | |  | | | |  | | | |  |  |  |  |
| 4 | Vvi-Vitvi06g04116\_t001 |  | | | |  | | | |  | | | |  | | | |  |  |  |  |
| 4 | Vvi-Vitvi06g04117\_t001 |  | | | |  | | | |  | | | |  | | | |  |  |  |  |
| 4 | Vvi-Vitvi06g04118\_t001 |  | | | |  | | | |  | | | |  | | | |  |  |  |  |
| 4 | Vvi-Vitvi06g04119\_t001 |  | | | |  | | | |  | | | |  | | | |  |  |  |  |
| 4 | Vvi-Vitvi06g04120\_t001 |  | | | |  | | | |  | | | |  | | | |  |  |  |  |
| 4 | Vvi-Vitvi06g04121\_t001 |  | | | |  | | | |  | | | |  | | | |  |  |  |  |
| 4 | Vvi-Vitvi06g04122\_t001 |  | | | |  | | | |  | | | |  | | | |  |  |  |  |
| 4 | Vvi-Vitvi06g00365\_t001 |  | | | |  | Ath-AT1G07880.2 |  | | | |  | | | |  |  |  |  |
| 4 | Vvi-Vitvi06g00366\_t001 |  | | | |  | | | |  | | | |  | Ath-AT3G45740.1 |  |  |  |  |
| 4 | Vvi-Vitvi06g00367\_t001.1.6037826e |  | | | |  | | | |  | | | |  | Ath-AT3G45770.1 |  |  |  |  |
| 5 | Vvi-Vitvi06g00368\_t001 |  | Ath-AT5G60100.2 |  | | | |  | | | |  | | | |  | Ath-AT5G02810.1 |  |  |  |
| 5 | Vvi-Vitvi06g00369\_t001 |  | | | |  | Ath-AT1G07870.2 |  | Ath-AT2G28590.1 |  | | | |  | Ath-AT5G02800.1 |  |  |  |
| 5 | Vvi-Vitvi06g00370\_t001 |  | | | |  | | | |  | | | |  | | | |  | | | |  |  |  |
| 5 | Vvi-Vitvi06g00371\_t001 |  | | | |  | Ath-AT1G07850.1 |  | | | |  | | | |  | | | |  |  |  |
| 5 | Vvi-Vitvi06g00372\_t001 |  | | | |  | | | |  | | | |  | | | |  | Ath-AT5G02780.1 |  |  |  |
| 5 | Vvi-Vitvi06g04123\_t001 |  | | | |  | | | |  | | | |  | | | |  | | | |  |  |  |
| 5 | Vvi-Vitvi06g00374\_t001 |  | | | |  | | | |  | | | |  | Ath-AT3G45780.1 |  | | | |  |  |  |
| 5 | Vvi-Vitvi06g00375\_t001 |  | Ath-AT5G60050.1 |  | | | |  | | | |  | | | |  | | | |  |  |  |
| 5 | Vvi-Vitvi06g04124\_t001 |  | | | |  | | | |  | | | |  | | | |  | | | |  |  |  |
| 5 | Vvi-Vitvi06g00376\_t001 |  | Ath-AT5G60040.2 |  | | | |  | | | |  | | | |  | | | |  |  |  |
| 5 | Vvi-Vitvi06g01670\_t001 |  | | | |  | | | |  | | | |  | | | |  | | | |  |  |  |
| 5 | Vvi-Vitvi06g00378\_t001 |  | Ath-AT5G60020.1 |  | | | |  | | | |  | | | |  | | | |  |  |  |
| 5 | Vvi-Vitvi06g00379\_t001 |  | | | |  | | | |  | | | |  | | | |  | | | |  |  |  |
| 5 | Vvi-Vitvi06g00380\_t001 |  | | | |  | | | |  | Ath-AT2G28610.1 |  | | | |  | | | |  |  |  |
| 5 | Vvi-Vitvi06g00381\_t001 |  | Ath-AT5G60010.1 |  | | | |  | | | |  | Ath-AT3G45810.1 |  | | | |  |  |  |
| 5 | Vvi-Vitvi06g04125\_t001 |  | | | |  | | | |  | | | |  | | | |  | | | |  |  |  |
| 5 | Vvi-Vitvi06g00382\_t001 |  | | | |  | | | |  | | | |  | Ath-AT3G45830.1 |  | | | |  |  |  |
| 5 | Vvi-Vitvi06g00383\_t001 |  | | | |  | | | |  | Ath-AT2G28620.2 |  | Ath-AT3G45850.2 |  | | | |  |  |  |
| 4 | Vvi-Vitvi06g01671\_t001 |  | | | |  | | | |  |  |  | | | |  | | | |  |  |  |
| 4 | Vvi-Vitvi06g01672\_t001 |  | | | |  | | | |  |  |  | | | |  | | | |  |  |  |
| 4 | Vvi-Vitvi06g01673\_t001 |  | | | |  | | | |  |  |  | | | |  | | | |  |  |  |
| 4 | Vvi-Vitvi06g01675\_t001 |  | | | |  | | | |  |  |  | | | |  | | | |  |  |  |
| 4 | Vvi-Vitvi06g01676\_t001 |  | Ath-AT5G59990.1 |  | | | |  |  |  | | | |  | | | |  |  |  |
| 4 | Vvi-Vitvi06g00384\_t001 |  | Ath-AT5G59980.2 |  | | | |  |  |  | | | |  | | | |  |  |  |
| 6 | Vvi-Vitvi06g04126\_t001 |  | Ath-AT5G59970.2 |  | | | |  | Ath-AT5G59690.1 |  | Ath-AT3G45930.1 |  | | | |  | Ath-AT3G46320.1 |  |  |
| 4 | Vvi-Vitvi06g04127\_t001 |  |  |  | | | |  | | | |  |  |  | | | |  | | | |  |  |
| 5 | Vvi-Vitvi06g00386\_t002 |  | Ath-AT1G07670.1 |  | Ath-AT1G07810.1 |  | | | |  |  |  | | | |  | | | |  |  |
| 5 | Vvi-Vitvi06g00387\_t001 |  | | | |  | | | |  | | | |  |  |  | Ath-AT5G02640.1 |  | Ath-AT3G46300.1 |  |  |
| 6 | Vvi-Vitvi06g00388\_t001 |  | | | |  | | | |  | Ath-AT5G59700.1 |  | Ath-AT2G39360.1 |  | | | |  | Ath-AT3G46290.1 |  |  |
| 6 | Vvi-Vitvi06g04128\_t001 |  | | | |  | | | |  | | | |  | | | |  | | | |  | | | |  |  |
| 6 | Vvi-Vitvi06g04129\_t001 |  | | | |  | | | |  | | | |  | | | |  | | | |  | Ath-AT3G46260.1 |  |  |
| 6 | Vvi-Vitvi06g01678\_t001 |  | | | |  | | | |  | | | |  | | | |  | | | |  | | | |  |  |
| 6 | Vvi-Vitvi06g00389\_t001 |  | Ath-AT1G07705.2 |  | | | |  | Ath-AT5G59710.1 |  | | | |  | | | |  | | | |  |  |
| 6 | Vvi-Vitvi06g04130\_t001 |  | | | |  | | | |  | | | |  | | | |  | | | |  | | | |  |  |
| 6 | Vvi-Vitvi06g04131\_t001 |  | | | |  | | | |  | | | |  | | | |  | | | |  | | | |  |  |
| 6 | Vvi-Vitvi06g00391\_t001 |  | | | |  | | | |  | | | |  | | | |  | | | |  | | | |  |  |
| 6 | Vvi-Vitvi06g01680\_t001 |  | | | |  | | | |  | | | |  | | | |  | | | |  | | | |  |  |
| 6 | Vvi-Vitvi06g04132\_t001 |  | | | |  | | | |  | | | |  | | | |  | | | |  | | | |  |  |
| 6 | Vvi-Vitvi06g04133\_t001 |  | | | |  | | | |  | | | |  | | | |  | | | |  | | | |  |  |
| 6 | Vvi-Vitvi06g00392\_t001 |  | | | |  | | | |  | | | |  | | | |  | | | |  | Ath-AT3G46220.2 |  |  |
| 6 | Vvi-Vitvi06g00393\_t001 |  | Ath-AT1G07710.1 |  | | | |  | | | |  | | | |  | Ath-AT5G02620.2 |  | | | |  |  |
| 6 | Vvi-Vitvi06g04134\_t001 |  | | | |  | | | |  | | | |  | | | |  | | | |  | | | |  |  |
| 6 | Vvi-Vitvi06g00394\_t001 |  | | | |  | | | |  | | | |  | | | |  | | | |  | Ath-AT3G46200.1 |  |  |
| 6 | Vvi-Vitvi06g04135\_t001 |  | | | |  | | | |  | | | |  | | | |  | | | |  | | | |  |  |
| 6 | Vvi-Vitvi06g04136\_t001 |  | | | |  | | | |  | | | |  | | | |  | | | |  | | | |  |  |
| 7 | Vvi-Vitvi06g00396\_t001 |  | Ath-AT1G07720.2 |  | | | |  | | | |  | | | |  | | | |  | | | |  | Ath-AT2G28630.1 |  |
| 7 | Vvi-Vitvi06g00397\_t001 |  | | | |  | | | |  | Ath-AT5G59730.1 |  | Ath-AT2G39380.1 |  | | | |  | | | |  | | | |  |
| 7 | Vvi-Vitvi06g00398\_t001 |  | | | |  | | | |  | | | |  | Ath-AT2G39390.1 |  | Ath-AT5G02610.2 |  | | | |  | | | |  |
| 7 | Vvi-Vitvi06g00399\_t001 |  | | | |  | | | |  | | | |  | | | |  | Ath-AT5G02600.2 |  | | | |  | | | |  |
| 6 | Vvi-Vitvi06g04137\_t001 |  | | | |  | | | |  | | | |  | | | |  |  |  | | | |  | | | |  |
| 6 | Vvi-Vitvi06g00400\_t001 |  | Ath-AT1G07730.2 |  | | | |  | | | |  | Ath-AT2G39430.1 |  |  |  | | | |  | Ath-AT2G28670.1 |  |
| 6 | Vvi-Vitvi06g00401\_t001 |  | | | |  | | | |  | | | |  | | | |  |  |  | | | |  | | | |  |
| 6 | Vvi-Vitvi06g00402\_t001 |  | | | |  | Ath-AT1G07740.1 |  | | | |  | | | |  |  |  | | | |  | | | |  |
| 5 | Vvi-Vitvi06g00403\_t001 |  | Ath-AT1G07745.1 |  |  |  | | | |  | | | |  |  |  | | | |  | | | |  |
| 5 | Vvi-Vitvi06g00405\_t001 |  | | | |  |  |  | | | |  | | | |  |  |  | | | |  | | | |  |
| 5 | Vvi-Vitvi06g00406\_t001 |  | Ath-AT1G07750.1 |  |  |  | | | |  | | | |  |  |  | | | |  | Ath-AT2G28680.1 |  |
| 5 | Vvi-Vitvi06g01681\_t001 |  | | | |  |  |  | Ath-AT5G59740.1 |  | | | |  |  |  | Ath-AT3G46180.1 |  | | | |  |
| 5 | Vvi-Vitvi06g04138\_t001 |  | | | |  |  |  | | | |  | | | |  |  |  | | | |  | | | |  |
| 5 | Vvi-Vitvi06g00407\_t001 |  | | | |  |  |  | Ath-AT5G59750.2 |  | | | |  |  |  | | | |  | | | |  |
| 5 | Vvi-Vitvi06g00408\_t001 |  | | | |  |  |  | | | |  | Ath-AT2G39460.2 |  |  |  | | | |  | | | |  |
| 5 | Vvi-Vitvi06g04139\_t001 |  | | | |  |  |  | | | |  | | | |  |  |  | | | |  | | | |  |
| 5 | Vvi-Vitvi06g00409\_t001 |  | | | |  |  |  | | | |  | Ath-AT2G39480.1 |  |  |  | | | |  | | | |  |
| 4 | Vvi-Vitvi06g04140\_t001 |  | | | |  |  |  | | | |  |  |  |  |  | | | |  | | | |  |
| 4 | Vvi-Vitvi06g00410\_t001 |  | | | |  |  |  | | | |  |  |  |  |  | | | |  | | | |  |
| 4 | Vvi-Vitvi06g00411\_t001 |  | | | |  |  |  | Ath-AT5G59760.1 |  |  |  |  |  | | | |  | Ath-AT2G28690.1 |  |
| 4 | Vvi-Vitvi06g00412\_t001 |  | | | |  |  |  | | | |  |  |  |  |  | | | |  | | | |  |
| 4 | Vvi-Vitvi06g00413\_t001 |  | | | |  |  |  | Ath-AT5G59770.1 |  |  |  |  |  | | | |  | | | |  |
| 4 | Vvi-Vitvi06g04141\_t001 |  | | | |  |  |  | | | |  |  |  |  |  | | | |  | | | |  |
| 4 | Vvi-Vitvi06g00414\_t002 |  | | | |  |  |  | Ath-AT5G59780.3 |  |  |  |  |  | Ath-AT3G46130.1 |  | | | |  |
| 4 | Vvi-Vitvi06g04142\_t001 |  | | | |  |  |  | | | |  |  |  |  |  | | | |  | | | |  |
| 4 | Vvi-Vitvi06g00415\_t001 |  | | | |  |  |  | Ath-AT5G59790.1 |  |  |  |  |  | Ath-AT3G46110.1 |  | | | |  |
| 4 | Vvi-Vitvi06g04143\_t001 |  | | | |  |  |  | | | |  |  |  |  |  | | | |  | | | |  |
| 4 | Vvi-Vitvi06g00417\_t001 |  | | | |  |  |  | Ath-AT5G59800.1 |  |  |  |  |  | | | |  | | | |  |
| 4 | Vvi-Vitvi06g01682\_t001 |  | | | |  |  |  | Ath-AT5G59820.1 |  |  |  |  |  | Ath-AT3G46080.1 |  | Ath-AT2G28710.1 |  |
| 4 | Vvi-Vitvi06g00419\_t001 |  | | | |  |  |  | Ath-AT5G59830.2 |  |  |  |  |  | | | |  | | | |  |
| 4 | Vvi-Vitvi06g04144\_t001 |  | | | |  |  |  | | | |  |  |  |  |  | | | |  | | | |  |
| 5 | Vvi-Vitvi06g00420\_t001 |  | | | |  | Ath-AT3G46060.1 |  | Ath-AT5G59840.1 |  |  |  |  |  | | | |  | | | |  |
| 5 | Vvi-Vitvi06g01683\_t001 |  | | | |  | | | |  | | | |  |  |  |  |  | | | |  | | | |  |
| 5 | Vvi-Vitvi06g00421\_t001 |  | | | |  | | | |  | | | |  |  |  |  |  | | | |  | | | |  |
| 5 | Vvi-Vitvi06g00422\_t001 |  | Ath-AT1G07790.1 |  | | | |  | | | |  |  |  |  |  | | | |  | | | |  |
| 4 | Vvi-Vitvi06g01684\_t001 |  |  |  | | | |  | | | |  |  |  |  |  | | | |  | | | |  |
| 4 | Vvi-Vitvi06g01685\_t001 |  |  |  | | | |  | | | |  |  |  |  |  | | | |  | | | |  |
| 4 | Vvi-Vitvi06g01686\_t001 |  |  |  | | | |  | | | |  |  |  |  |  | | | |  | | | |  |
| 4 | Vvi-Vitvi06g04145\_t001 |  |  |  | | | |  | | | |  |  |  |  |  | | | |  | | | |  |
| 4 | Vvi-Vitvi06g01687\_t001 |  |  |  | | | |  | | | |  |  |  |  |  | | | |  | | | |  |
| 4 | Vvi-Vitvi06g04146\_t001 |  |  |  | | | |  | | | |  |  |  |  |  | | | |  | | | |  |
| 4 | Vvi-Vitvi06g01688\_t001 |  |  |  | | | |  | Ath-AT5G59860.1 |  |  |  |  |  | Ath-AT3G46020.1 |  | | | |  |
| 5 | Vvi-Vitvi06g00423\_t001 |  | Ath-AT5G02560.2 |  | | | |  | Ath-AT5G59870.1 |  |  |  |  |  | | | |  | | | |  |
| 5 | Vvi-Vitvi06g00424\_t001 |  | | | |  | | | |  | Ath-AT5G59880.1 |  |  |  |  |  | Ath-AT3G46000.2 |  | | | |  |
| 5 | Vvi-Vitvi06g00425\_t001 |  | | | |  | | | |  | Ath-AT5G59900.1 |  |  |  |  |  | | | |  | | | |  |
| 6 | Vvi-Vitvi06g00426\_t001 |  | | | |  | | | |  | Ath-AT5G59910.1 |  | Ath-AT1G07790.1 |  |  |  | Ath-AT3G45980.1 |  | Ath-AT2G28720.1 |  |
| 6 | Vvi-Vitvi06g04147\_t001 |  | | | |  | | | |  | | | |  | | | |  |  |  | | | |  | | | |  |
| 6 | Vvi-Vitvi06g01689\_t001 |  | | | |  | | | |  | | | |  | | | |  |  |  | | | |  | | | |  |
| 6 | Vvi-Vitvi06g01690\_t001 |  | | | |  | | | |  | | | |  | | | |  |  |  | | | |  | | | |  |
| 6 | Vvi-Vitvi06g01691\_t001 |  | | | |  | | | |  | | | |  | | | |  |  |  | | | |  | | | |  |
| 6 | Vvi-Vitvi06g04148\_t001 |  | | | |  | | | |  | | | |  | | | |  |  |  | | | |  | | | |  |
| 6 | Vvi-Vitvi06g00429\_t002 |  | Ath-AT5G02530.1 |  | | | |  | Ath-AT5G59950.5 |  | | | |  |  |  | | | |  | | | |  |
| 6 | Vvi-Vitvi06g04149\_t001 |  | | | |  | | | |  | | | |  | | | |  |  |  | | | |  | | | |  |
| 6 | Vvi-Vitvi06g04150\_t001 |  | | | |  | | | |  | | | |  | | | |  |  |  | | | |  | | | |  |
| 6 | Vvi-Vitvi06g00430\_t001 |  | | | |  | | | |  | Ath-AT5G59960.1 |  | | | |  |  |  | | | |  | | | |  |
| 6 | Vvi-Vitvi06g00432\_t001 |  | | | |  | | | |  | | | |  | | | |  |  |  | | | |  | | | |  |
| 6 | Vvi-Vitvi06g04151\_t001 |  | | | |  | Ath-AT3G46320.1 |  | Ath-AT5G59970.2 |  | | | |  |  |  | Ath-AT3G45930.1 |  | | | |  |
| 4 | Vvi-Vitvi06g00434\_t001 |  | | | |  | Ath-AT3G46430.1 |  |  |  | | | |  |  |  |  |  | | | |  |
| 4 | Vvi-Vitvi06g00435\_t001 |  | | | |  | Ath-AT3G46440.1 |  |  |  | | | |  |  |  |  |  | Ath-AT2G28760.4 |  |
| 4 | Vvi-Vitvi06g00436\_t001 |  | | | |  | | | |  |  |  | | | |  |  |  |  |  | Ath-AT2G28780.1 |  |
| 4 | Vvi-Vitvi06g00437\_t001 |  | | | |  | Ath-AT3G46450.2 |  |  |  | | | |  |  |  |  |  | | | |  |
| 4 | Vvi-Vitvi06g04152\_t001 |  | | | |  | | | |  |  |  | | | |  |  |  |  |  | | | |  |
| 4 | Vvi-Vitvi06g00438\_t001 |  | | | |  | | | |  |  |  | Ath-AT1G07645.1 |  |  |  |  |  | | | |  |
| 4 | Vvi-Vitvi06g00439\_t003 |  | | | |  | Ath-AT3G46460.1 |  |  |  | | | |  |  |  |  |  | | | |  |
| 4 | Vvi-Vitvi06g00440\_t001 |  | | | |  | | | |  |  |  | | | |  |  |  |  |  | Ath-AT2G28790.2 |  |
| 4 | Vvi-Vitvi06g00441\_t001 |  | | | |  | | | |  |  |  | | | |  |  |  |  |  | | | |  |
| 4 | Vvi-Vitvi06g00442\_t001 |  | | | |  | | | |  |  |  | | | |  |  |  |  |  | Ath-AT2G28800.1 |  |
| 4 | Vvi-Vitvi06g00443\_t001 |  | Ath-AT5G02490.1 |  | | | |  |  |  | | | |  |  |  |  |  | | | |  |
| 4 | Vvi-Vitvi06g00444\_t001 |  | Ath-AT5G02480.1 |  | | | |  |  |  | | | |  |  |  |  |  | | | |  |
| 4 | Vvi-Vitvi06g04153\_t001 |  | | | |  | | | |  |  |  | | | |  |  |  |  |  | | | |  |
| 4 | Vvi-Vitvi06g00445\_t001 |  | | | |  | | | |  |  |  | | | |  |  |  |  |  | | | |  |
| 4 | Vvi-Vitvi06g04154\_t001 |  | | | |  | | | |  |  |  | | | |  |  |  |  |  | | | |  |
| 4 | Vvi-Vitvi06g00447\_t001 |  | | | |  | | | |  |  |  | | | |  |  |  |  |  | | | |  |
| 4 | Vvi-Vitvi06g04155\_t001 |  | | | |  | | | |  |  |  | | | |  |  |  |  |  | | | |  |
| 4 | Vvi-Vitvi06g01693\_t001 |  | | | |  | | | |  |  |  | | | |  |  |  |  |  | | | |  |
| 4 | Vvi-Vitvi06g00449\_t001 |  | Ath-AT5G02460.1 |  | | | |  |  |  | Ath-AT1G07640.3 |  |  |  |  |  | Ath-AT2G28810.1 |  |
| 4 | Vvi-Vitvi06g00450\_t001 |  | | | |  | Ath-AT3G46510.1 |  |  |  | | | |  |  |  |  |  | Ath-AT2G28830.1 |  |
| 4 | Vvi-Vitvi06g01694\_t001 |  | | | |  | | | |  |  |  | | | |  |  |  |  |  | | | |  |
| 4 | Vvi-Vitvi06g00451\_t001 |  | | | |  | | | |  |  |  | | | |  |  |  |  |  | Ath-AT2G28840.1 |  |
| 4 | Vvi-Vitvi06g00452\_t001 |  | Ath-AT5G02440.1 |  | | | |  |  |  | | | |  |  |  |  |  | | | |  |
| 4 | Vvi-Vitvi06g01695\_t001 |  | | | |  | | | |  |  |  | | | |  |  |  |  |  | | | |  |
| 4 | Vvi-Vitvi06g00453\_t003 |  | | | |  | Ath-AT3G46520.1 |  |  |  | | | |  |  |  |  |  | | | |  |
| 4 | Vvi-Vitvi06g00454\_t001 |  | | | |  | | | |  |  |  | | | |  |  |  |  |  | | | |  |
| 4 | Vvi-Vitvi06g00455\_t001.1.6037826f |  | | | |  | Ath-AT3G46540.1 |  |  |  | | | |  |  |  |  |  | | | |  |
| 4 | Vvi-Vitvi06g00456\_t001 |  | Ath-AT5G02400.1 |  | | | |  |  |  | Ath-AT1G07630.1 |  |  |  |  |  | Ath-AT2G28890.1 |  |
| 4 | Vvi-Vitvi06g04156\_t001 |  | | | |  | | | |  |  |  | | | |  |  |  |  |  | | | |  |
| 4 | Vvi-Vitvi06g00459\_t001 |  | | | |  | Ath-AT3G46550.1 |  |  |  | | | |  |  |  |  |  | | | |  |
| 4 | Vvi-Vitvi06g00460\_t001 |  | Ath-AT5G02390.1 |  | | | |  |  |  | Ath-AT1G07620.2 |  |  |  |  |  | | | |  |
| 4 | Vvi-Vitvi06g00462\_t001 |  | | | |  | | | |  |  |  | Ath-AT1G07615.1 |  |  |  |  |  | | | |  |
| 4 | Vvi-Vitvi06g00463\_t001 |  | | | |  | | | |  |  |  | | | |  |  |  |  |  | | | |  |
| 4 | Vvi-Vitvi06g01696\_t003 |  | | | |  | | | |  |  |  | | | |  |  |  |  |  | | | |  |
| 4 | Vvi-Vitvi06g04157\_t001 |  | | | |  | | | |  |  |  | | | |  |  |  |  |  | | | |  |
| 4 | Vvi-Vitvi06g04158\_t001 |  | | | |  | | | |  |  |  | | | |  |  |  |  |  | | | |  |
| 4 | Vvi-Vitvi06g00464\_t001 |  | | | |  | | | |  |  |  | Ath-AT1G07590.1 |  |  |  |  |  | | | |  |
| 4 | Vvi-Vitvi06g00465\_t001 |  | | | |  | | | |  |  |  | | | |  |  |  |  |  | Ath-AT2G28900.1 |  |
| 4 | Vvi-Vitvi06g00466\_t001 |  | | | |  | Ath-AT3G46560.1 |  |  |  | | | |  |  |  |  |  | | | |  |
| 4 | Vvi-Vitvi06g04159\_t001 |  | | | |  | | | |  |  |  | | | |  |  |  |  |  | | | |  |
| 4 | Vvi-Vitvi06g01700\_t001 |  | | | |  | | | |  |  |  | | | |  |  |  |  |  | Ath-AT2G28910.3 |  |
| 4 | Vvi-Vitvi06g01701\_t001 |  | | | |  | | | |  |  |  | | | |  |  |  |  |  | | | |  |
| 4 | Vvi-Vitvi06g01702\_t001 |  | | | |  | | | |  |  |  | | | |  |  |  |  |  | | | |  |
| 4 | Vvi-Vitvi06g04160\_t001 |  | | | |  | | | |  |  |  | | | |  |  |  |  |  | | | |  |
| 4 | Vvi-Vitvi06g00467\_t001 |  | | | |  | | | |  |  |  | | | |  |  |  |  |  | | | |  |
| 5 | Vvi-Vitvi06g00468\_t001 |  | | | |  | | | |  | Ath-AT2G39640.1 |  | | | |  |  |  |  |  | | | |  |
| 5 | Vvi-Vitvi06g04161\_t001 |  | | | |  | | | |  | | | |  | | | |  |  |  |  |  | | | |  |
| 5 | Vvi-Vitvi06g00470\_t001 |  | | | |  | | | |  | | | |  | | | |  |  |  |  |  | | | |  |
| 6 | Vvi-Vitvi06g01703\_t001 |  | | | |  | Ath-AT3G46580.1 |  | | | |  | | | |  | Ath-AT5G59380.1 |  |  |  | | | |  |
| 6 | Vvi-Vitvi06g00471\_t001 |  | | | |  | | | |  | | | |  | | | |  | | | |  |  |  | | | |  |
| 6 | Vvi-Vitvi06g00472\_t001 |  | | | |  | | | |  | | | |  | | | |  | | | |  |  |  | | | |  |
| 6 | Vvi-Vitvi06g00473\_t001 |  | Ath-AT5G02290.1 |  | | | |  | Ath-AT2G39660.1 |  | Ath-AT1G07570.3 |  | | | |  |  |  | Ath-AT2G28930.1 |  |
| 6 | Vvi-Vitvi06g00474\_t001 |  | | | |  | | | |  | | | |  | | | |  | | | |  |  |  | Ath-AT2G28940.2 |  |
| 6 | Vvi-Vitvi06g00475\_t001 |  | | | |  | | | |  | | | |  | | | |  | | | |  |  |  | | | |  |
| 6 | Vvi-Vitvi06g00476\_t001 |  | | | |  | | | |  | | | |  | | | |  | | | |  |  |  | | | |  |
| 6 | Vvi-Vitvi06g04162\_t001 |  | | | |  | | | |  | | | |  | | | |  | | | |  |  |  | | | |  |
| 6 | Vvi-Vitvi06g00477\_t001 |  | | | |  | | | |  | | | |  | | | |  | | | |  |  |  | | | |  |
| 6 | Vvi-Vitvi06g00479\_t001 |  | | | |  | | | |  | | | |  | | | |  | | | |  |  |  | | | |  |
| 6 | Vvi-Vitvi06g00480\_t001 |  | | | |  | | | |  | | | |  | | | |  | | | |  |  |  | | | |  |
| 6 | Vvi-Vitvi06g00481\_t001 |  | Ath-AT5G02260.1 |  | | | |  | Ath-AT2G39700.1 |  | | | |  | | | |  |  |  | Ath-AT2G28950.1 |  |
| 6 | Vvi-Vitvi06g01704\_t001 |  | | | |  | | | |  | | | |  | | | |  | | | |  |  |  | | | |  |
| 6 | Vvi-Vitvi06g04163\_t001 |  | | | |  | | | |  | | | |  | | | |  | | | |  |  |  | | | |  |
| 6 | Vvi-Vitvi06g00483\_t001 |  | | | |  | | | |  | | | |  | | | |  | | | |  |  |  | | | |  |
| 6 | Vvi-Vitvi06g01705\_t001 |  | | | |  | | | |  | | | |  | | | |  | | | |  |  |  | | | |  |
| 6 | Vvi-Vitvi06g00484\_t001 |  | | | |  | | | |  | | | |  | | | |  | Ath-AT5G59400.1 |  |  |  | | | |  |
| 6 | Vvi-Vitvi06g00486\_t001 |  | | | |  | | | |  | | | |  | | | |  | Ath-AT5G59410.1 |  |  |  | Ath-AT2G29020.1 |  |
| 6 | Vvi-Vitvi06g00487\_t001 |  | | | |  | | | |  | | | |  | | | |  | Ath-AT5G59420.1 |  |  |  | | | |  |
| 6 | Vvi-Vitvi06g00488\_t001 |  | | | |  | Ath-AT3G46590.2 |  | | | |  | Ath-AT1G07540.1 |  | Ath-AT5G59430.2 |  |  |  | | | |  |
| 6 | Vvi-Vitvi06g04164\_t001 |  | | | |  | | | |  | | | |  | | | |  | | | |  |  |  | | | |  |
| 6 | Vvi-Vitvi06g01569\_t001 |  | | | |  | | | |  | | | |  | Ath-AT1G07520.3 |  | | | |  |  |  | Ath-AT2G29060.1 |  |
| 6 | Vvi-Vitvi06g00489\_t001 |  | | | |  | | | |  | | | |  | | | |  | | | |  |  |  | | | |  |
| 6 | Vvi-Vitvi06g00490\_t001 |  | | | |  | | | |  | | | |  | | | |  | Ath-AT5G59450.1 |  |  |  | | | |  |
| 6 | Vvi-Vitvi06g00491\_t001 |  | | | |  | | | |  | | | |  | | | |  | | | |  |  |  | | | |  |
| 6 | Vvi-Vitvi06g04165\_t001 |  | | | |  | | | |  | | | |  | | | |  | | | |  |  |  | | | |  |
| 6 | Vvi-Vitvi06g00492\_t001 |  | | | |  | | | |  | | | |  | | | |  | | | |  |  |  | | | |  |
| 6 | Vvi-Vitvi06g00493\_t001 |  | | | |  | | | |  | | | |  | | | |  | Ath-AT5G59460.1 |  |  |  | | | |  |
| 6 | Vvi-Vitvi06g00494\_t001 |  | | | |  | | | |  | | | |  | | | |  | | | |  |  |  | Ath-AT2G29070.2 |  |
| 6 | Vvi-Vitvi06g00495\_t001 |  | Ath-AT5G02230.3 |  | | | |  | | | |  | | | |  | Ath-AT5G59480.1 |  |  |  | | | |  |
| 6 | Vvi-Vitvi06g00496\_t001 |  | | | |  | | | |  | | | |  | Ath-AT1G07510.1 |  | | | |  |  |  | Ath-AT2G29080.1 |  |
| 6 | Vvi-Vitvi06g00497\_t001 |  | | | |  | | | |  | | | |  | | | |  | Ath-AT5G59500.1 |  |  |  | | | |  |
| 6 | Vvi-Vitvi06g00498\_t001 |  | | | |  | | | |  | | | |  | | | |  | | | |  |  |  | Ath-AT2G29090.1 |  |
| 6 | Vvi-Vitvi06g01706\_t001 |  | Ath-AT5G02220.1 |  | | | |  | | | |  | | | |  | | | |  |  |  | | | |  |
| 5 | Vvi-Vitvi06g04166\_t001 |  |  |  | | | |  | Ath-AT2G39705.1 |  | | | |  | Ath-AT5G59510.1 |  |  |  | Ath-AT2G29125.1 |  |
| 5 | Vvi-Vitvi06g00500\_t001 |  |  |  | | | |  | | | |  | | | |  | Ath-AT5G59520.1 |  |  |  | | | |  |
| 5 | Vvi-Vitvi06g00501\_t001 |  |  |  | | | |  | | | |  | | | |  | | | |  |  |  | | | |  |
| 5 | Vvi-Vitvi06g00502\_t001 |  |  |  | Ath-AT3G46620.1 |  | Ath-AT2G39720.1 |  | | | |  | Ath-AT5G59550.2 |  |  |  | | | |  |
| 5 | Vvi-Vitvi06g04167\_t001 |  |  |  | | | |  | | | |  | | | |  | | | |  |  |  | | | |  |
| 5 | Vvi-Vitvi06g00503\_t001 |  |  |  | | | |  | | | |  | | | |  | | | |  |  |  | Ath-AT2G29140.2 |  |
| 5 | Vvi-Vitvi06g01708\_t001 |  |  |  | | | |  | | | |  | | | |  | | | |  |  |  | Ath-AT2G29180.1 |  |
| 5 | Vvi-Vitvi06g00504\_t001 |  |  |  | | | |  | | | |  | | | |  | | | |  |  |  | | | |  |
| 5 | Vvi-Vitvi06g04168\_t001 |  |  |  | | | |  | | | |  | | | |  | | | |  |  |  | | | |  |
| 5 | Vvi-Vitvi06g00505\_t001 |  |  |  | Ath-AT3G46640.3 |  | | | |  | | | |  | Ath-AT5G59570.1 |  |  |  | | | |  |
| 5 | Vvi-Vitvi06g00506\_t001 |  |  |  | | | |  | | | |  | | | |  | | | |  |  |  | | | |  |
| 5 | Vvi-Vitvi06g00508\_t001 |  |  |  | | | |  | | | |  | | | |  | | | |  |  |  | | | |  |
| 5 | Vvi-Vitvi06g00509\_t001 |  |  |  | | | |  | | | |  | | | |  | Ath-AT5G59610.3 |  |  |  | | | |  |
| 4 | Vvi-Vitvi06g00510\_t001 |  |  |  | Ath-AT3G46740.1 |  | | | |  | | | |  |  |  |  |  | | | |  |
| 4 | Vvi-Vitvi06g00511\_t001 |  |  |  | | | |  | | | |  | | | |  |  |  |  |  | | | |  |
| 4 | Vvi-Vitvi06g00512\_t001 |  |  |  | | | |  | | | |  | | | |  |  |  |  |  | | | |  |
| 4 | Vvi-Vitvi06g00513\_t001 |  |  |  | | | |  | Ath-AT2G39730.1 |  | | | |  |  |  |  |  | | | |  |
| 4 | Vvi-Vitvi06g00514\_t001 |  | Ath-AT5G59250.1 |  | | | |  |  |  | | | |  |  |  |  |  | | | |  |
| 4 | Vvi-Vitvi06g04169\_t001 |  | | | |  | | | |  |  |  | | | |  |  |  |  |  | | | |  |
| 4 | Vvi-Vitvi06g00515\_t001 |  | Ath-AT5G59230.1 |  | | | |  |  |  | Ath-AT1G07470.2 |  |  |  |  |  | | | |  |
| 4 | Vvi-Vitvi06g00516\_t001 |  | | | |  | | | |  |  |  | | | |  |  |  |  |  | Ath-AT2G29210.1 |  |
| 4 | Vvi-Vitvi06g00517\_t001 |  | | | |  | Ath-AT3G46780.1 |  |  |  | | | |  |  |  |  |  | | | |  |
| 4 | Vvi-Vitvi06g00518\_t001 |  | | | |  | | | |  |  |  | | | |  |  |  |  |  | | | |  |
| 4 | Vvi-Vitvi06g00520\_t001 |  | | | |  | | | |  |  |  | | | |  |  |  |  |  | | | |  |
| 4 | Vvi-Vitvi06g01710\_t001 |  | | | |  | | | |  |  |  | | | |  |  |  |  |  | | | |  |
| 5 | Vvi-Vitvi06g00521\_t001 |  | | | |  | | | |  | Ath-AT5G06070.1 |  | | | |  |  |  |  |  | | | |  |
| 5 | Vvi-Vitvi06g00522\_t001 |  | | | |  | | | |  | Ath-AT5G06060.1 |  | Ath-AT1G07450.1 |  |  |  |  |  | Ath-AT2G29260.1 |  |
| 5 | Vvi-Vitvi06g04170\_t001 |  | | | |  | | | |  | | | |  | | | |  |  |  |  |  | Ath-AT2G29350.1 |  |
| 5 | Vvi-Vitvi06g01718\_t001 |  | | | |  | | | |  | | | |  | | | |  |  |  |  |  | Ath-AT2G29370.1 |  |
| 5 | Vvi-Vitvi06g04171\_t001 |  | | | |  | | | |  | | | |  | | | |  |  |  |  |  | | | |  |
| 5 | Vvi-Vitvi06g04172\_t001 |  | | | |  | | | |  | | | |  | | | |  |  |  |  |  | | | |  |
| 5 | Vvi-Vitvi06g04173\_t001 |  | | | |  | | | |  | | | |  | | | |  |  |  |  |  | | | |  |
| 5 | Vvi-Vitvi06g00529\_t001 |  | | | |  | | | |  | | | |  | | | |  |  |  |  |  | | | |  |
| 5 | Vvi-Vitvi06g04174\_t001 |  | | | |  | | | |  | | | |  | Ath-AT1G07440.1 |  |  |  |  |  | | | |  |
| 5 | Vvi-Vitvi06g04175\_t001 |  | | | |  | | | |  | | | |  | | | |  |  |  |  |  | | | |  |
| 5 | Vvi-Vitvi06g04176\_t001 |  | | | |  | | | |  | | | |  | | | |  |  |  |  |  | | | |  |
| 5 | Vvi-Vitvi06g00533\_t001 |  | Ath-AT5G59220.1 |  | | | |  | | | |  | Ath-AT1G07430.1 |  |  |  |  |  | Ath-AT2G29380.1 |  |
| 5 | Vvi-Vitvi06g00534\_t001 |  | | | |  | | | |  | | | |  | Ath-AT1G07420.1 |  |  |  |  |  | Ath-AT2G29390.1 |  |
| 5 | Vvi-Vitvi06g04177\_t001 |  | | | |  | | | |  | | | |  | | | |  |  |  |  |  | | | |  |
| 5 | Vvi-Vitvi06g00535\_t001 |  | | | |  | | | |  | Ath-AT5G06000.1 |  | | | |  |  |  |  |  | | | |  |
| 5 | Vvi-Vitvi06g00536\_t001 |  | Ath-AT5G59210.1 |  | | | |  | | | |  | | | |  |  |  |  |  | | | |  |
| 5 | Vvi-Vitvi06g00538\_t001 |  | | | |  | Ath-AT3G46790.1 |  | | | |  | | | |  |  |  |  |  | | | |  |
| 5 | Vvi-Vitvi06g04178\_t001 |  | | | |  | | | |  | | | |  | | | |  |  |  |  |  | | | |  |
| 5 | Vvi-Vitvi06g00539\_t001 |  | Ath-AT5G59180.1 |  | | | |  | | | |  | | | |  |  |  |  |  | | | |  |
| 5 | Vvi-Vitvi06g00540\_t003 |  | Ath-AT5G59160.1 |  | Ath-AT3G46820.1 |  | | | |  | | | |  |  |  |  |  | Ath-AT2G29400.1 |  |
| 5 | Vvi-Vitvi06g00541\_t001 |  | | | |  | | | |  | | | |  | | | |  |  |  |  |  | Ath-AT2G29410.1 |  |
| 5 | Vvi-Vitvi06g00542\_t001 |  | Ath-AT5G59150.1 |  | Ath-AT3G46830.1 |  | | | |  | Ath-AT1G07410.1 |  |  |  |  |  | | | |  |
| 4 | Vvi-Vitvi06g00543\_t001 |  | Ath-AT5G59140.1 |  | | | |  | | | |  |  |  |  |  |  |  | | | |  |
| 4 | Vvi-Vitvi06g04179\_t001 |  | | | |  | | | |  | | | |  |  |  |  |  |  |  | | | |  |
| 4 | Vvi-Vitvi06g04180\_t001 |  | Ath-AT5G59120.1 |  | Ath-AT3G46840.1 |  | | | |  |  |  |  |  |  |  | | | |  |
| 4 | Vvi-Vitvi06g00546\_t001 |  | | | |  | | | |  | | | |  |  |  |  |  |  |  | | | |  |
| 4 | Vvi-Vitvi06g00547\_t001 |  | Ath-AT5G59090.1 |  | Ath-AT3G46850.1 |  | | | |  |  |  |  |  |  |  | | | |  |
| 4 | Vvi-Vitvi06g04181\_t001 |  | | | |  | | | |  | | | |  |  |  |  |  |  |  | | | |  |
| 4 | Vvi-Vitvi06g04182\_t001 |  | | | |  | | | |  | | | |  |  |  |  |  |  |  | | | |  |
| 4 | Vvi-Vitvi06g00548\_t001 |  | | | |  | | | |  | | | |  |  |  |  |  |  |  | | | |  |
| 4 | Vvi-Vitvi06g04183\_t001 |  | | | |  | | | |  | | | |  |  |  |  |  |  |  | | | |  |
| 4 | Vvi-Vitvi06g00549\_t001 |  | | | |  | | | |  | | | |  |  |  |  |  |  |  | | | |  |
| 4 | Vvi-Vitvi06g00550\_t001 |  | | | |  | | | |  | | | |  |  |  |  |  |  |  | | | |  |
| 4 | Vvi-Vitvi06g00552\_t001 |  | | | |  | | | |  | | | |  |  |  |  |  |  |  | | | |  |
| 4 | Vvi-Vitvi06g00553\_t001 |  | | | |  | | | |  | | | |  |  |  |  |  |  |  | | | |  |
| 4 | Vvi-Vitvi06g01722\_t003 |  | | | |  | Ath-AT3G46870.2 |  | | | |  |  |  |  |  |  |  | | | |  |
| 4 | Vvi-Vitvi06g01723\_t001 |  | | | |  | | | |  | | | |  |  |  |  |  |  |  | | | |  |
| 4 | Vvi-Vitvi06g00555\_t001 |  | Ath-AT5G59070.1 |  | Ath-AT3G46890.1 |  | Ath-AT5G05950.1 |  |  |  |  |  |  |  | | | |  |
| 4 | Vvi-Vitvi06g01724\_t001 |  | | | |  | | | |  | | | |  |  |  |  |  |  |  | Ath-AT2G29420.1 |  |
| 4 | Vvi-Vitvi06g01725\_t001 |  | | | |  | | | |  | | | |  |  |  |  |  |  |  | | | |  |
| 4 | Vvi-Vitvi06g04184\_t001 |  | | | |  | | | |  | | | |  |  |  |  |  |  |  | Ath-AT2G29460.1 |  |
| 4 | Vvi-Vitvi06g04185\_t001 |  | | | |  | | | |  | | | |  |  |  |  |  |  |  | | | |  |
| 4 | Vvi-Vitvi06g04186\_t001 |  | | | |  | | | |  | | | |  |  |  |  |  |  |  | Ath-AT2G29470.1 |  |
| 4 | Vvi-Vitvi06g01727\_t001 |  | | | |  | | | |  | | | |  |  |  |  |  |  |  | | | |  |
| 4 | Vvi-Vitvi06g01728\_t001 |  | | | |  | | | |  | | | |  |  |  |  |  |  |  | | | |  |
| 4 | Vvi-Vitvi06g04187\_t001 |  | | | |  | | | |  | | | |  |  |  |  |  |  |  | | | |  |
| 4 | Vvi-Vitvi06g04188\_t001 |  | | | |  | | | |  | | | |  |  |  |  |  |  |  | | | |  |
| 5 | Vvi-Vitvi06g00556\_t001 |  | | | |  | | | |  | | | |  | Ath-AT3G53990.1 |  |  |  |  |  | | | |  |
| 5 | Vvi-Vitvi06g00558\_t001 |  | Ath-AT5G59050.1 |  | | | |  | | | |  | Ath-AT3G54000.1 |  |  |  |  |  | | | |  |
| 5 | Vvi-Vitvi06g04189\_t001 |  | | | |  | | | |  | | | |  | | | |  |  |  |  |  | | | |  |
| 6 | Vvi-Vitvi06g01729\_t001 |  | Ath-AT5G59030.1 |  | Ath-AT3G46900.1 |  | | | |  | | | |  | Ath-AT2G37925.1 |  |  |  | | | |  |
| 5 | Vvi-Vitvi06g01730\_t001 |  | | | |  |  |  | | | |  | | | |  | | | |  |  |  | | | |  |
| 5 | Vvi-Vitvi06g00559\_t001 |  | | | |  |  |  | | | |  | | | |  | | | |  |  |  | | | |  |
| 5 | Vvi-Vitvi06g00560\_t001 |  | | | |  |  |  | | | |  | | | |  | | | |  |  |  | | | |  |
| 6 | Vvi-Vitvi06g00561\_t001 |  | | | |  | Ath-AT1G07400.1 |  | | | |  | | | |  | | | |  |  |  | Ath-AT2G29500.1 |  |
| 6 | Vvi-Vitvi06g00562\_t001 |  | | | |  | | | |  | | | |  | | | |  | | | |  |  |  | | | |  |
| 6 | Vvi-Vitvi06g04190\_t001 |  | | | |  | | | |  | | | |  | | | |  | | | |  |  |  | | | |  |
| 7 | Vvi-Vitvi06g00563\_t001 |  | Ath-AT5G59020.1 |  | | | |  | | | |  | | | |  | Ath-AT2G37930.3 |  | Ath-AT5G01030.2 |  | Ath-AT2G29510.1 |  |
| 7 | Vvi-Vitvi06g00564\_t001 |  | | | |  | | | |  | | | |  | Ath-AT3G54020.1 |  | Ath-AT2G37940.2 |  | | | |  | Ath-AT2G29525.1 |  |
| 7 | Vvi-Vitvi06g04191\_t001 |  | | | |  | | | |  | | | |  | | | |  | | | |  | | | |  | | | |  |
| 7 | Vvi-Vitvi06g00565\_t001 |  | Ath-AT5G59010.1 |  | | | |  | | | |  | Ath-AT3G54030.1 |  | | | |  | | | |  | | | |  |
| 7 | Vvi-Vitvi06g04192\_t001 |  | | | |  | | | |  | | | |  | | | |  | | | |  | | | |  | Ath-AT2G29530.3 |  |
| 7 | Vvi-Vitvi06g01732\_t001 |  | | | |  | | | |  | | | |  | | | |  | | | |  | | | |  | Ath-AT2G29540.3 |  |
| 7 | Vvi-Vitvi06g00567\_t001 |  | Ath-AT5G59000.1 |  | | | |  | Ath-AT5G05830.1 |  | | | |  | Ath-AT2G37950.1 |  | Ath-AT5G01070.1 |  | | | |  |
| 7 | Vvi-Vitvi06g01734\_t001 |  | | | |  | | | |  | | | |  | Ath-AT3G54040.2 |  | | | |  | | | |  | | | |  |
| 7 | Vvi-Vitvi06g00568\_t001 |  | Ath-AT5G58990.1 |  | | | |  | | | |  | | | |  | | | |  | | | |  | | | |  |
| 7 | Vvi-Vitvi06g00569\_t001 |  | | | |  | | | |  | | | |  | | | |  | | | |  | | | |  | Ath-AT2G29550.1 |  |
| 7 | Vvi-Vitvi06g04193\_t001 |  | | | |  | | | |  | | | |  | | | |  | | | |  | | | |  | | | |  |
| 7 | Vvi-Vitvi06g00570\_t001 |  | | | |  | | | |  | | | |  | | | |  | | | |  | | | |  | | | |  |
| 7 | Vvi-Vitvi06g00573\_t001 |  | Ath-AT5G58980.1 |  | Ath-AT1G07380.2 |  | | | |  | | | |  | Ath-AT2G38010.2 |  | | | |  | | | |  |
| 7 | Vvi-Vitvi06g00574\_t001 |  | | | |  | | | |  | | | |  | | | |  | | | |  | | | |  | Ath-AT2G29560.1 |  |
| 7 | Vvi-Vitvi06g04194\_t001 |  | | | |  | | | |  | | | |  | | | |  | | | |  | | | |  | | | |  |
| 7 | Vvi-Vitvi06g00575\_t001 |  | | | |  | | | |  | Ath-AT5G05820.1 |  | | | |  | | | |  | | | |  | | | |  |
| 7 | Vvi-Vitvi06g00576\_t001 |  | | | |  | Ath-AT1G07370.1 |  | | | |  | | | |  | | | |  | | | |  | Ath-AT2G29570.1 |  |
| 7 | Vvi-Vitvi06g01736\_t001 |  | | | |  | | | |  | | | |  | | | |  | | | |  | | | |  | | | |  |
| 7 | Vvi-Vitvi06g00577\_t001.1.6037826e |  | Ath-AT5G58970.1 |  | | | |  | | | |  | Ath-AT3G54110.1 |  | | | |  | | | |  | | | |  |
| 7 | Vvi-Vitvi06g00578\_t001 |  | Ath-AT5G58960.1 |  | | | |  | | | |  | | | |  | | | |  | | | |  | | | |  |
| 7 | Vvi-Vitvi06g04195\_t001 |  | | | |  | | | |  | | | |  | | | |  | | | |  | | | |  | | | |  |
| 7 | Vvi-Vitvi06g00579\_t001 |  | | | |  | | | |  | | | |  | | | |  | | | |  | | | |  | | | |  |
| 7 | Vvi-Vitvi06g01737\_t001 |  | | | |  | | | |  | | | |  | | | |  | | | |  | | | |  | | | |  |
| 7 | Vvi-Vitvi06g04196\_t001 |  | | | |  | | | |  | | | |  | | | |  | | | |  | | | |  | | | |  |
| 8 | Vvi-Vitvi06g00580\_t001 |  | | | |  | | | |  | | | |  | | | |  | | | |  | | | |  | | | |  | Ath-AT3G46960.1 |
| 8 | Vvi-Vitvi06g04197\_t001 |  | | | |  | Ath-AT1G07360.1 |  | | | |  | | | |  | | | |  | | | |  | Ath-AT2G29580.1 |  | | | |
| 8 | Vvi-Vitvi06g04198\_t001 |  | | | |  | Ath-AT1G07350.1 |  | | | |  | | | |  | | | |  | | | |  | | | |  | | | |
| 8 | Vvi-Vitvi06g00583\_t001 |  | | | |  | | | |  | | | |  | | | |  | | | |  | | | |  | | | |  | Ath-AT3G46970.1 |
| 8 | Vvi-Vitvi06g00584\_t001 |  | Ath-AT5G58940.2 |  | | | |  | | | |  | | | |  | | | |  | | | |  | | | |  | | | |
| 8 | Vvi-Vitvi06g00585\_t001 |  | | | |  | | | |  | | | |  | | | |  | Ath-AT2G38060.2 |  | | | |  | | | |  | Ath-AT3G46980.4 |
| 8 | Vvi-Vitvi06g00586\_t001 |  | | | |  | | | |  | | | |  | | | |  | | | |  | | | |  | Ath-AT2G29590.1 |  | | | |
| 8 | Vvi-Vitvi06g00587\_t001 |  | Ath-AT5G58930.1 |  | | | |  | | | |  | | | |  | Ath-AT2G38070.1 |  | Ath-AT5G01170.1 |  | | | |  | Ath-AT3G46990.1 |
| 8 | Vvi-Vitvi06g04199\_t001 |  | | | |  | | | |  | | | |  | | | |  | | | |  | | | |  | | | |  | | | |
| 8 | Vvi-Vitvi06g00588\_t001 |  | | | |  | | | |  | | | |  | | | |  | | | |  | | | |  | | | |  | | | |
| 8 | Vvi-Vitvi06g01738\_t001 |  | | | |  | | | |  | | | |  | | | |  | | | |  | | | |  | | | |  | | | |
| 8 | Vvi-Vitvi06g00589\_t001 |  | Ath-AT5G58920.1 |  | | | |  | | | |  | | | |  | | | |  | | | |  | | | |  | | | |
| 8 | Vvi-Vitvi06g00590\_t001 |  | | | |  | | | |  | | | |  | Ath-AT3G54140.1 |  | | | |  | Ath-AT5G01180.1 |  | | | |  | | | |
| 7 | Vvi-Vitvi06g04200\_t001 |  | | | |  | | | |  | | | |  |  |  | | | |  | | | |  | | | |  | | | |
| 7 | Vvi-Vitvi06g00591\_t001 |  | Ath-AT5G58910.2 |  | | | |  | | | |  |  |  | Ath-AT2G38080.1 |  | Ath-AT5G01190.1 |  | | | |  | | | |
| 7 | Vvi-Vitvi06g00592\_t001 |  | Ath-AT5G58900.1 |  | | | |  | Ath-AT5G05790.1 |  |  |  | Ath-AT2G38090.1 |  | Ath-AT5G01200.1 |  | | | |  | | | |
| 4 | Vvi-Vitvi06g00593\_t001 |  | | | |  | | | |  |  |  |  |  |  |  |  |  | | | |  | Ath-AT3G47000.1 |
| 4 | Vvi-Vitvi06g04201\_t001 |  | | | |  | | | |  |  |  |  |  |  |  |  |  | | | |  | | | |
| 4 | Vvi-Vitvi06g00594\_t001 |  | Ath-AT5G58880.1 |  | Ath-AT1G07330.2 |  |  |  |  |  |  |  |  |  | Ath-AT2G29620.2 |  | | | |
| 4 | Vvi-Vitvi06g00595\_t001 |  | Ath-AT5G58870.1 |  | | | |  |  |  |  |  |  |  |  |  | | | |  | Ath-AT3G47060.1 |
| 4 | Vvi-Vitvi06g01739\_t001 |  | | | |  | | | |  |  |  |  |  |  |  |  |  | Ath-AT2G29630.3 |  | | | |
| 4 | Vvi-Vitvi06g00598\_t001 |  | | | |  | Ath-AT1G07320.1 |  |  |  |  |  |  |  |  |  | | | |  | | | |
| 4 | Vvi-Vitvi06g00599\_t001 |  | | | |  | Ath-AT1G07310.1 |  |  |  |  |  |  |  |  |  | | | |  | | | |
| 4 | Vvi-Vitvi06g00600\_t001 |  | | | |  | | | |  |  |  |  |  |  |  |  |  | Ath-AT2G29640.1 |  | | | |
| 4 | Vvi-Vitvi06g04202\_t001 |  | | | |  | | | |  |  |  |  |  |  |  |  |  | | | |  | | | |
| 4 | Vvi-Vitvi06g04203\_t001 |  | | | |  | | | |  |  |  |  |  |  |  |  |  | | | |  | | | |
| 4 | Vvi-Vitvi06g01740\_t001 |  | | | |  | | | |  |  |  |  |  |  |  |  |  | | | |  | Ath-AT3G47070.1 |
| 4 | Vvi-Vitvi06g04204\_t001 |  | | | |  | | | |  |  |  |  |  |  |  |  |  | | | |  | | | |
| 4 | Vvi-Vitvi06g00601\_t001 |  | | | |  | | | |  |  |  |  |  |  |  |  |  | | | |  | | | |
| 4 | Vvi-Vitvi06g00602\_t001 |  | | | |  | | | |  |  |  |  |  |  |  |  |  | | | |  | | | |
| 4 | Vvi-Vitvi06g00604\_t001 |  | | | |  | | | |  |  |  |  |  |  |  |  |  | | | |  | | | |
| 4 | Vvi-Vitvi06g00605\_t001 |  | Ath-AT5G58860.1 |  | | | |  |  |  |  |  |  |  |  |  | | | |  | | | |
| 4 | Vvi-Vitvi06g00606\_t001 |  | | | |  | | | |  |  |  |  |  |  |  |  |  | Ath-AT2G29650.1 |  | | | |
| 4 | Vvi-Vitvi06g00607\_t001 |  | | | |  | | | |  |  |  |  |  |  |  |  |  | Ath-AT2G29660.1 |  | | | |
| 4 | Vvi-Vitvi06g00608\_t001 |  | | | |  | | | |  |  |  |  |  |  |  |  |  | | | |  | | | |
| 4 | Vvi-Vitvi06g00610\_t001 |  | | | |  | Ath-AT1G07290.1 |  |  |  |  |  |  |  |  |  | | | |  | | | |
| 4 | Vvi-Vitvi06g00611\_t001 |  | Ath-AT5G58850.1 |  | | | |  |  |  |  |  |  |  |  |  | | | |  | | | |
| 4 | Vvi-Vitvi06g00613\_t001 |  | | | |  | Ath-AT1G07280.1 |  |  |  |  |  |  |  |  |  | Ath-AT2G29670.1 |  | Ath-AT3G47080.1 |
| 4 | Vvi-Vitvi06g04205\_t001 |  | | | |  | | | |  |  |  |  |  |  |  |  |  | | | |  | | | |
| 4 | Vvi-Vitvi06g00614\_t001 |  | | | |  | Ath-AT1G07270.1 |  |  |  |  |  |  |  |  |  | Ath-AT2G29680.1 |  | | | |
| 4 | Vvi-Vitvi06g04206\_t001 |  | | | |  | | | |  |  |  |  |  |  |  |  |  | | | |  | | | |
| 4 | Vvi-Vitvi06g00615\_t001 |  | | | |  | | | |  |  |  |  |  |  |  |  |  | | | |  | | | |
| 4 | Vvi-Vitvi06g00617\_t001 |  | | | |  | | | |  |  |  |  |  |  |  |  |  | Ath-AT2G29690.1 |  | | | |
| 4 | Vvi-Vitvi06g00618\_t001 |  | Ath-AT5G58800.2 |  | | | |  |  |  |  |  |  |  |  |  | | | |  | | | |
| 4 | Vvi-Vitvi06g00619\_t001 |  | | | |  | | | |  |  |  |  |  |  |  |  |  | Ath-AT2G29700.1 |  | | | |
| 4 | Vvi-Vitvi06g04207\_t001 |  | | | |  | | | |  |  |  |  |  |  |  |  |  | | | |  | | | |
| 4 | Vvi-Vitvi06g00621\_t001 |  | | | |  | Ath-AT1G07240.1 |  |  |  |  |  |  |  |  |  | Ath-AT2G29710.1 |  | | | |
| 4 | Vvi-Vitvi06g04208\_t001 |  | | | |  | | | |  |  |  |  |  |  |  |  |  | Ath-AT2G29740.1 |  | | | |
| 4 | Vvi-Vitvi06g01743\_t001 |  | | | |  | | | |  |  |  |  |  |  |  |  |  | | | |  | | | |
| 4 | Vvi-Vitvi06g04209\_t001 |  | | | |  | | | |  |  |  |  |  |  |  |  |  | Ath-AT2G29760.1 |  | | | |
| 4 | Vvi-Vitvi06g04210\_t001 |  | | | |  | | | |  |  |  |  |  |  |  |  |  | | | |  | | | |
| 4 | Vvi-Vitvi06g04211\_t001 |  | | | |  | | | |  |  |  |  |  |  |  |  |  | | | |  | | | |
| 4 | Vvi-Vitvi06g04212\_t001 |  | | | |  | | | |  |  |  |  |  |  |  |  |  | | | |  | | | |
| 4 | Vvi-Vitvi06g00625\_t001 |  | | | |  | | | |  |  |  |  |  |  |  |  |  | | | |  | | | |
| 4 | Vvi-Vitvi06g00626\_t001 |  | | | |  | | | |  |  |  |  |  |  |  |  |  | | | |  | | | |
| 4 | Vvi-Vitvi06g04213\_t001 |  | | | |  | | | |  |  |  |  |  |  |  |  |  | | | |  | | | |
| 4 | Vvi-Vitvi06g00627\_t001 |  | | | |  | | | |  |  |  |  |  |  |  |  |  | | | |  | Ath-AT3G47120.1 |
| 4 | Vvi-Vitvi06g01746\_t001 |  | | | |  | | | |  |  |  |  |  |  |  |  |  | | | |  | | | |
| 4 | Vvi-Vitvi06g00629\_t001 |  | | | |  | | | |  |  |  |  |  |  |  |  |  | | | |  | | | |
| 4 | Vvi-Vitvi06g00630\_t001 |  | | | |  | | | |  |  |  |  |  |  |  |  |  | | | |  | | | |
| 4 | Vvi-Vitvi06g00631\_t001 |  | | | |  | | | |  |  |  |  |  |  |  |  |  | Ath-AT2G29890.3 |  | | | |
| 4 | Vvi-Vitvi06g00632\_t001 |  | | | |  | Ath-AT1G07230.1 |  |  |  |  |  |  |  |  |  | | | |  | | | |
| 4 | Vvi-Vitvi06g00633\_t001 |  | | | |  | | | |  |  |  |  |  |  |  |  |  | | | |  | | | |
| 5 | Vvi-Vitvi06g01748\_t001 |  | Ath-AT5G58787.1 |  | | | |  | Ath-AT5G01520.1 |  |  |  |  |  |  |  | | | |  | Ath-AT3G47160.2 |
| 4 | Vvi-Vitvi06g00634\_t001 |  | Ath-AT5G58770.1 |  | | | |  | | | |  |  |  |  |  |  |  | | | |  |
| 4 | Vvi-Vitvi06g00635\_t001 |  | Ath-AT5G58760.1 |  | | | |  | | | |  |  |  |  |  |  |  | | | |  |
| 4 | Vvi-Vitvi06g04214\_t001 |  | | | |  | | | |  | | | |  |  |  |  |  |  |  | Ath-AT2G29940.1 |  |
| 4 | Vvi-Vitvi06g00637\_t001 |  | | | |  | | | |  | | | |  |  |  |  |  |  |  | | | |  |
| 4 | Vvi-Vitvi06g00638\_t001 |  | Ath-AT5G58750.1 |  | | | |  | | | |  |  |  |  |  |  |  | | | |  |
| 4 | Vvi-Vitvi06g00639\_t001 |  | | | |  | | | |  | Ath-AT5G01490.2 |  |  |  |  |  |  |  | | | |  |
| 4 | Vvi-Vitvi06g00640\_t001 |  | Ath-AT5G58730.1 |  | | | |  | | | |  |  |  |  |  |  |  | | | |  |
| 4 | Vvi-Vitvi06g00641\_t001 |  | Ath-AT5G58720.1 |  | | | |  | | | |  |  |  |  |  |  |  | | | |  |
| 5 | Vvi-Vitvi06g00642\_t001 |  | | | |  | | | |  | | | |  | Ath-AT2G40080.1 |  |  |  |  |  | Ath-AT2G29950.1 |  |
| 5 | Vvi-Vitvi06g00643\_t001 |  | Ath-AT5G58710.1 |  | | | |  | | | |  | | | |  |  |  |  |  | Ath-AT2G29960.1 |  |
| 5 | Vvi-Vitvi06g00644\_t001 |  | | | |  | | | |  | Ath-AT5G01450.1 |  | | | |  |  |  |  |  | | | |  |
| 5 | Vvi-Vitvi06g00645\_t001 |  | | | |  | Ath-AT1G07220.1 |  | | | |  | | | |  |  |  |  |  | | | |  |
| 5 | Vvi-Vitvi06g00646\_t001 |  | | | |  | | | |  | Ath-AT5G01410.1 |  | | | |  |  |  |  |  | | | |  |
| 5 | Vvi-Vitvi06g00647\_t001 |  | Ath-AT5G58690.3 |  | | | |  | | | |  | Ath-AT2G40116.1 |  |  |  |  |  | | | |  |
| 5 | Vvi-Vitvi06g00648\_t001 |  | | | |  | | | |  | | | |  | | | |  |  |  |  |  | | | |  |
| 5 | Vvi-Vitvi06g00649\_t001 |  | Ath-AT5G58670.1 |  | | | |  | | | |  | | | |  |  |  |  |  | | | |  |
| 5 | Vvi-Vitvi06g00650\_t001 |  | | | |  | | | |  | | | |  | | | |  |  |  |  |  | | | |  |
| 5 | Vvi-Vitvi06g00651\_t002 |  | | | |  | Ath-AT1G07210.1 |  | | | |  | | | |  |  |  |  |  | | | |  |
| 5 | Vvi-Vitvi06g00652\_t001 |  | | | |  | Ath-AT1G07200.2 |  | | | |  | Ath-AT2G40130.2 |  |  |  |  |  | Ath-AT2G29970.1 |  |
| 5 | Vvi-Vitvi06g00653\_t002 |  | | | |  | | | |  | | | |  | Ath-AT2G40140.1 |  |  |  |  |  | | | |  |
| 5 | Vvi-Vitvi06g01749\_t001 |  | Ath-AT5G58630.1 |  | | | |  | Ath-AT5G01370.1 |  | | | |  |  |  |  |  | | | |  |
| 5 | Vvi-Vitvi06g00654\_t001 |  | | | |  | | | |  | | | |  | | | |  |  |  |  |  | Ath-AT2G29980.1 |  |
| 5 | Vvi-Vitvi06g00656\_t001 |  | | | |  | | | |  | | | |  | | | |  |  |  |  |  | | | |  |
| 5 | Vvi-Vitvi06g04215\_t001 |  | | | |  | | | |  | | | |  | | | |  |  |  |  |  | | | |  |
| 5 | Vvi-Vitvi06g00657\_t001 |  | | | |  | | | |  | | | |  | | | |  |  |  |  |  | | | |  |
| 5 | Vvi-Vitvi06g01751\_t001 |  | | | |  | Ath-AT1G07180.1 |  | | | |  | | | |  |  |  |  |  | Ath-AT2G29990.1 |  |
| 5 | Vvi-Vitvi06g01752\_t001 |  | | | |  | | | |  | | | |  | | | |  |  |  |  |  | | | |  |
| 5 | Vvi-Vitvi06g00658\_t001 |  | | | |  | Ath-AT1G07170.1 |  | | | |  | | | |  |  |  |  |  | Ath-AT2G30000.1 |  |
| 5 | Vvi-Vitvi06g00659\_t001 |  | | | |  | | | |  | | | |  | | | |  |  |  |  |  | | | |  |
| 5 | Vvi-Vitvi06g00661\_t001 |  | Ath-AT5G58610.3 |  | | | |  | | | |  | | | |  |  |  |  |  | | | |  |
| 5 | Vvi-Vitvi06g00662\_t001 |  | | | |  | | | |  | | | |  | | | |  |  |  |  |  | | | |  |
| 5 | Vvi-Vitvi06g00665\_t001 |  | Ath-AT5G58600.1 |  | | | |  | | | |  | | | |  |  |  |  |  | Ath-AT2G30010.2 |  |
| 5 | Vvi-Vitvi06g00666\_t001 |  | | | |  | | | |  | | | |  | | | |  |  |  |  |  | | | |  |
| 5 | Vvi-Vitvi06g00667\_t001 |  | | | |  | Ath-AT1G07160.1 |  | | | |  | Ath-AT2G40180.1 |  |  |  |  |  | Ath-AT2G30020.1 |  |
| 5 | Vvi-Vitvi06g00668\_t001 |  | | | |  | Ath-AT1G07150.1 |  | | | |  | | | |  |  |  |  |  | Ath-AT2G30040.1 |  |
| 5 | Vvi-Vitvi06g00669\_t001 |  | | | |  | | | |  | | | |  | | | |  |  |  |  |  | | | |  |
| 5 | Vvi-Vitvi06g00670\_t001 |  | | | |  | | | |  | | | |  | | | |  |  |  |  |  | | | |  |
| 5 | Vvi-Vitvi06g00671\_t001 |  | Ath-AT5G58590.1 |  | Ath-AT1G07140.1 |  | | | |  | | | |  |  |  |  |  | Ath-AT2G30060.1 |  |
| 5 | Vvi-Vitvi06g04216\_t001 |  | | | |  | | | |  | | | |  | | | |  |  |  |  |  | | | |  |
| 5 | Vvi-Vitvi06g00672\_t001 |  | | | |  | | | |  | | | |  | | | |  |  |  |  |  | Ath-AT2G30070.1 |  |
| 5 | Vvi-Vitvi06g00673\_t001 |  | | | |  | | | |  | Ath-AT5G01320.1 |  | | | |  |  |  |  |  | | | |  |
| 4 | Vvi-Vitvi06g00674\_t001 |  | | | |  | Ath-AT1G07120.1 |  |  |  | | | |  |  |  |  |  | | | |  |
| 4 | Vvi-Vitvi06g04217\_t001 |  | | | |  | | | |  |  |  | | | |  |  |  |  |  | | | |  |
| 4 | Vvi-Vitvi06g00676\_t001 |  | | | |  | Ath-AT1G07110.1 |  |  |  | | | |  |  |  |  |  | | | |  |
| 4 | Vvi-Vitvi06g00677\_t001 |  | | | |  | | | |  |  |  | | | |  |  |  |  |  | | | |  |
| 4 | Vvi-Vitvi06g01753\_t001 |  | | | |  | | | |  |  |  | | | |  |  |  |  |  | | | |  |
| 4 | Vvi-Vitvi06g00678\_t001 |  | | | |  | | | |  |  |  | | | |  |  |  |  |  | Ath-AT2G30080.1 |  |
| 4 | Vvi-Vitvi06g00679\_t001 |  | | | |  | | | |  |  |  | | | |  |  |  |  |  | Ath-AT2G30090.1 |  |
| 4 | Vvi-Vitvi06g00680\_t001 |  | | | |  | | | |  |  |  | | | |  |  |  |  |  | | | |  |
| 4 | Vvi-Vitvi06g00681\_t001 |  | Ath-AT5G58575.1 |  | | | |  |  |  | | | |  |  |  |  |  | | | |  |
| 4 | Vvi-Vitvi06g01754\_t001 |  | Ath-AT5G58560.1 |  | | | |  |  |  | | | |  |  |  |  |  | | | |  |
| 4 | Vvi-Vitvi06g00683\_t001 |  | | | |  | | | |  |  |  | | | |  |  |  |  |  | | | |  |
| 4 | Vvi-Vitvi06g04218\_t001 |  | | | |  | | | |  |  |  | | | |  |  |  |  |  | | | |  |
| 4 | Vvi-Vitvi06g01755\_t001 |  | | | |  | | | |  |  |  | | | |  |  |  |  |  | | | |  |
| 4 | Vvi-Vitvi06g01756\_t001 |  | | | |  | | | |  |  |  | | | |  |  |  |  |  | | | |  |
| 4 | Vvi-Vitvi06g01757\_t001 |  | | | |  | | | |  |  |  | | | |  |  |  |  |  | | | |  |
| 4 | Vvi-Vitvi06g00686\_t001 |  | | | |  | | | |  |  |  | Ath-AT2G40260.1 |  |  |  |  |  | | | |  |
| 4 | Vvi-Vitvi06g00687\_t001 |  | | | |  | | | |  |  |  | | | |  |  |  |  |  | Ath-AT2G30100.1 |  |
| 4 | Vvi-Vitvi06g00688\_t001 |  | Ath-AT5G58550.1 |  | | | |  |  |  | | | |  |  |  |  |  | | | |  |
| 4 | Vvi-Vitvi06g00689\_t001 |  | Ath-AT5G58540.1 |  | | | |  |  |  | Ath-AT2G40270.1 |  |  |  |  |  | | | |  |
| 4 | Vvi-Vitvi06g04219\_t001 |  | | | |  | | | |  |  |  | | | |  |  |  |  |  | | | |  |
| 4 | Vvi-Vitvi06g00690\_t001 |  | Ath-AT5G58530.1 |  | | | |  |  |  | | | |  |  |  |  |  | | | |  |
| 4 | Vvi-Vitvi06g00691\_t001 |  | | | |  | | | |  |  |  | | | |  |  |  |  |  | Ath-AT2G30105.1 |  |
| 4 | Vvi-Vitvi06g00692\_t003 |  | | | |  | | | |  |  |  | | | |  |  |  |  |  | Ath-AT2G30110.1 |  |
| 4 | Vvi-Vitvi06g00693\_t001 |  | Ath-AT5G58520.1 |  | | | |  |  |  | | | |  |  |  |  |  | | | |  |
| 4 | Vvi-Vitvi06g00694\_t001 |  | Ath-AT5G58510.1 |  | | | |  |  |  | | | |  |  |  |  |  | | | |  |
| 4 | Vvi-Vitvi06g04220\_t001 |  | | | |  | | | |  |  |  | | | |  |  |  |  |  | | | |  |
| 4 | Vvi-Vitvi06g04221\_t001 |  | | | |  | | | |  |  |  | | | |  |  |  |  |  | | | |  |
| 4 | Vvi-Vitvi06g00695\_t001 |  | Ath-AT5G58500.1 |  | Ath-AT1G07090.1 |  |  |  | | | |  |  |  |  |  | | | |  |
| 4 | Vvi-Vitvi06g00696\_t001 |  | | | |  | | | |  |  |  | Ath-AT2G40290.1 |  |  |  |  |  | | | |  |
| 4 | Vvi-Vitvi06g04222\_t001 |  | | | |  | | | |  |  |  | | | |  |  |  |  |  | | | |  |
| 4 | Vvi-Vitvi06g04223\_t001 |  | | | |  | | | |  |  |  | | | |  |  |  |  |  | | | |  |
| 4 | Vvi-Vitvi06g01759\_t001 |  | | | |  | Ath-AT1G07080.1 |  |  |  | | | |  |  |  |  |  | | | |  |
| 4 | Vvi-Vitvi06g00699\_t001 |  | Ath-AT5G58490.1 |  | | | |  |  |  | | | |  |  |  |  |  | | | |  |
| 4 | Vvi-Vitvi06g00700\_t001 |  | | | |  | | | |  |  |  | | | |  |  |  |  |  | | | |  |
| 4 | Vvi-Vitvi06g00701\_t001.1.6037826e |  | Ath-AT5G58420.1 |  | | | |  |  |  | | | |  |  |  |  |  | | | |  |
| 3 | Vvi-Vitvi06g01761\_t001 |  |  |  | | | |  |  |  | Ath-AT2G40300.1 |  |  |  |  |  | | | |  |
| 3 | Vvi-Vitvi06g00702\_t001 |  |  |  | | | |  |  |  | | | |  |  |  |  |  | Ath-AT2G30120.5 |  |
| 3 | Vvi-Vitvi06g00704\_t001 |  |  |  | | | |  |  |  | | | |  |  |  |  |  | | | |  |
| 3 | Vvi-Vitvi06g04224\_t001 |  |  |  | | | |  |  |  | | | |  |  |  |  |  | | | |  |
| 3 | Vvi-Vitvi06g00705\_t001 |  |  |  | Ath-AT1G07060.2 |  |  |  | | | |  |  |  |  |  | | | |  |
| 3 | Vvi-Vitvi06g00706\_t001 |  |  |  | | | |  |  |  | | | |  |  |  |  |  | Ath-AT2G30130.1 |  |
| 3 | Vvi-Vitvi06g00707\_t001 |  |  |  | Ath-AT1G07050.2 |  |  |  | | | |  |  |  |  |  | | | |  |
| 3 | Vvi-Vitvi06g00708\_t001 |  |  |  | | | |  |  |  | | | |  |  |  |  |  | Ath-AT2G30140.1 |  |
| 3 | Vvi-Vitvi06g01762\_t001 |  |  |  | | | |  |  |  | | | |  |  |  |  |  | | | |  |
| 3 | Vvi-Vitvi06g00709\_t001 |  |  |  | | | |  |  |  | | | |  |  |  |  |  | | | |  |
| 3 | Vvi-Vitvi06g01763\_t001 |  |  |  | | | |  |  |  | | | |  |  |  |  |  | | | |  |
| 3 | Vvi-Vitvi06g04225\_t001 |  |  |  | | | |  |  |  | | | |  |  |  |  |  | | | |  |
| 3 | Vvi-Vitvi06g04226\_t001 |  |  |  | | | |  |  |  | | | |  |  |  |  |  | | | |  |
| 3 | Vvi-Vitvi06g04227\_t001 |  |  |  | | | |  |  |  | | | |  |  |  |  |  | | | |  |
| 3 | Vvi-Vitvi06g00714\_t001 |  |  |  | | | |  |  |  | | | |  |  |  |  |  | | | |  |
| 3 | Vvi-Vitvi06g00715\_t001 |  |  |  | | | |  |  |  | | | |  |  |  |  |  | | | |  |
| 3 | Vvi-Vitvi06g04228\_t001 |  |  |  | | | |  |  |  | | | |  |  |  |  |  | | | |  |
| 3 | Vvi-Vitvi06g04229\_t001 |  |  |  | | | |  |  |  | | | |  |  |  |  |  | | | |  |
| 3 | Vvi-Vitvi06g04230\_t001 |  |  |  | | | |  |  |  | | | |  |  |  |  |  | | | |  |
| 3 | Vvi-Vitvi06g00718\_t001 |  |  |  | | | |  |  |  | | | |  |  |  |  |  | | | |  |
| 3 | Vvi-Vitvi06g04231\_t001 |  |  |  | | | |  |  |  | | | |  |  |  |  |  | | | |  |
| 3 | Vvi-Vitvi06g00720\_t001 |  |  |  | Ath-AT1G07025.1 |  |  |  | | | |  |  |  |  |  | Ath-AT2G30160.1 |  |
| 3 | Vvi-Vitvi06g00721\_t001 |  |  |  | | | |  |  |  | | | |  |  |  |  |  | Ath-AT2G30170.1 |  |
| 3 | Vvi-Vitvi06g00722\_t001 |  |  |  | Ath-AT1G07020.1 |  |  |  | | | |  |  |  |  |  | | | |  |
| 3 | Vvi-Vitvi06g00723\_t001 |  |  |  | | | |  |  |  | | | |  |  |  |  |  | Ath-AT2G30200.1 |  |
| 3 | Vvi-Vitvi06g00724\_t001 |  |  |  | | | |  |  |  | Ath-AT2G40330.1 |  |  |  |  |  | | | |  |
| 3 | Vvi-Vitvi06g00725\_t001 |  |  |  | Ath-AT1G07010.3 |  |  |  | | | |  |  |  |  |  | | | |  |
| 4 | Vvi-Vitvi06g00726\_t001 |  | Ath-AT5G58460.1 |  | | | |  |  |  | | | |  |  |  |  |  | | | |  |
| 4 | Vvi-Vitvi06g04232\_t001 |  | | | |  | | | |  |  |  | | | |  |  |  |  |  | | | |  |
| 4 | Vvi-Vitvi06g00728\_t001 |  | | | |  | | | |  |  |  | Ath-AT2G40370.1 |  |  |  |  |  | Ath-AT2G30210.1 |  |
| 3 | Vvi-Vitvi06g00729\_t001 |  | Ath-AT5G58450.1 |  | | | |  |  |  |  |  |  |  |  |  | | | |  |
| 3 | Vvi-Vitvi06g04233\_t001 |  | | | |  | | | |  |  |  |  |  |  |  |  |  | | | |  |
| 3 | Vvi-Vitvi06g04234\_t001 |  | | | |  | | | |  |  |  |  |  |  |  |  |  | | | |  |
| 3 | Vvi-Vitvi06g04235\_t001 |  | | | |  | | | |  |  |  |  |  |  |  |  |  | | | |  |
| 3 | Vvi-Vitvi06g04236\_t001 |  | | | |  | | | |  |  |  |  |  |  |  |  |  | | | |  |
| 3 | Vvi-Vitvi06g00731\_t001 |  | | | |  | | | |  |  |  |  |  |  |  |  |  | | | |  |
| 3 | Vvi-Vitvi06g00732\_t002 |  | Ath-AT5G58440.1 |  | | | |  |  |  |  |  |  |  |  |  | | | |  |
| 3 | Vvi-Vitvi06g00733\_t001 |  | Ath-AT5G58430.1 |  | Ath-AT1G07000.1 |  |  |  |  |  |  |  |  |  | | | |  |
| 3 | Vvi-Vitvi06g00734\_t001 |  | | | |  | | | |  |  |  |  |  |  |  |  |  | Ath-AT2G30220.1 |  |
| 3 | Vvi-Vitvi06g00735\_t001 |  | | | |  | | | |  |  |  |  |  |  |  |  |  | | | |  |
| 3 | Vvi-Vitvi06g00736\_t001 |  | | | |  | Ath-AT1G06980.1 |  |  |  |  |  |  |  |  |  | Ath-AT2G30230.1 |  |
| 3 | Vvi-Vitvi06g00737\_t001 |  | | | |  | Ath-AT1G06970.1 |  |  |  |  |  |  |  |  |  | Ath-AT2G30240.1 |  |
| 3 | Vvi-Vitvi06g01768\_t001 |  | | | |  | | | |  |  |  |  |  |  |  |  |  | | | |  |
| 3 | Vvi-Vitvi06g00738\_t001 |  | | | |  | | | |  |  |  |  |  |  |  |  |  | | | |  |
| 3 | Vvi-Vitvi06g00739\_t001 |  | | | |  | | | |  |  |  |  |  |  |  |  |  | | | |  |
| 4 | Vvi-Vitvi06g00740\_t001 |  | | | |  | | | |  | Ath-AT5G01710.1 |  |  |  |  |  |  |  | | | |  |
| 4 | Vvi-Vitvi06g00741\_t001 |  | | | |  | | | |  | | | |  |  |  |  |  |  |  | Ath-AT2G30250.1 |  |
| 4 | Vvi-Vitvi06g00742\_t001 |  | | | |  | | | |  | Ath-AT5G01720.1 |  |  |  |  |  |  |  | | | |  |
| 4 | Vvi-Vitvi06g00743\_t001 |  | | | |  | Ath-AT1G06960.1 |  | | | |  |  |  |  |  |  |  | Ath-AT2G30260.1 |  |
| 4 | Vvi-Vitvi06g00744\_t001 |  | | | |  | | | |  | | | |  |  |  |  |  |  |  | | | |  |
| 4 | Vvi-Vitvi06g00745\_t001 |  | | | |  | | | |  | Ath-AT5G01740.1 |  |  |  |  |  |  |  | | | |  |
| 4 | Vvi-Vitvi06g01769\_t001 |  | | | |  | | | |  | | | |  |  |  |  |  |  |  | | | |  |
| 4 | Vvi-Vitvi06g00746\_t001 |  | Ath-AT5G58410.1 |  | | | |  | | | |  |  |  |  |  |  |  | | | |  |
| 4 | Vvi-Vitvi06g04237\_t001 |  | | | |  | | | |  | | | |  |  |  |  |  |  |  | | | |  |
| 4 | Vvi-Vitvi06g00748\_t001 |  | | | |  | | | |  | | | |  |  |  |  |  |  |  | | | |  |
| 4 | Vvi-Vitvi06g04238\_t001 |  | | | |  | | | |  | | | |  |  |  |  |  |  |  | | | |  |
| 4 | Vvi-Vitvi06g04239\_t001 |  | | | |  | | | |  | | | |  |  |  |  |  |  |  | | | |  |
| 4 | Vvi-Vitvi06g04240\_t001 |  | | | |  | | | |  | | | |  |  |  |  |  |  |  | | | |  |
| 4 | Vvi-Vitvi06g04241\_t001 |  | | | |  | | | |  | | | |  |  |  |  |  |  |  | | | |  |
| 4 | Vvi-Vitvi06g04242\_t001 |  | | | |  | | | |  | | | |  |  |  |  |  |  |  | | | |  |
| 4 | Vvi-Vitvi06g00753\_t001 |  | | | |  | | | |  | | | |  |  |  |  |  |  |  | | | |  |
| 4 | Vvi-Vitvi06g01771\_t001 |  | | | |  | | | |  | | | |  |  |  |  |  |  |  | | | |  |
| 4 | Vvi-Vitvi06g04243\_t001 |  | | | |  | | | |  | | | |  |  |  |  |  |  |  | | | |  |
| 4 | Vvi-Vitvi06g00754\_t001 |  | | | |  | | | |  | | | |  |  |  |  |  |  |  | | | |  |
| 4 | Vvi-Vitvi06g01772\_t001 |  | | | |  | | | |  | Ath-AT5G01750.2 |  |  |  |  |  |  |  | Ath-AT2G30270.1 |  |
| 4 | Vvi-Vitvi06g00757\_t001 |  | | | |  | | | |  | | | |  |  |  |  |  |  |  | | | |  |
| 4 | Vvi-Vitvi06g00758\_t001 |  | | | |  | | | |  | | | |  |  |  |  |  |  |  | | | |  |
| 4 | Vvi-Vitvi06g00759\_t001 |  | | | |  | | | |  | | | |  |  |  |  |  |  |  | | | |  |
| 4 | Vvi-Vitvi06g01773\_t002 |  | | | |  | | | |  | | | |  |  |  |  |  |  |  | Ath-AT2G30280.1 |  |
| 4 | Vvi-Vitvi06g00761\_t001 |  | | | |  | | | |  | | | |  |  |  |  |  |  |  | Ath-AT2G30300.1 |  |
| 4 | Vvi-Vitvi06g00762\_t001 |  | | | |  | | | |  | | | |  |  |  |  |  |  |  | | | |  |
| 5 | Vvi-Vitvi06g00763\_t001 |  | | | |  | | | |  | | | |  | Ath-AT2G40435.1 |  |  |  |  |  | | | |  |
| 5 | Vvi-Vitvi06g00764\_t001 |  | | | |  | | | |  | | | |  | | | |  |  |  |  |  | Ath-AT2G30320.1 |  |
| 5 | Vvi-Vitvi06g04244\_t001 |  | | | |  | | | |  | | | |  | | | |  |  |  |  |  | | | |  |
| 5 | Vvi-Vitvi06g00767\_t001 |  | | | |  | Ath-AT1G06950.1 |  | | | |  | | | |  |  |  |  |  | | | |  |
| 5 | Vvi-Vitvi06g00768\_t001 |  | Ath-AT5G58390.1 |  | | | |  | | | |  | | | |  |  |  |  |  | | | |  |
| 5 | Vvi-Vitvi06g00769\_t001 |  | | | |  | | | |  | | | |  | | | |  |  |  |  |  | | | |  |
| 5 | Vvi-Vitvi06g00770\_t001 |  | | | |  | | | |  | | | |  | | | |  |  |  |  |  | Ath-AT2G30330.1 |  |
| 5 | Vvi-Vitvi06g00771\_t001 |  | | | |  | | | |  | | | |  | | | |  |  |  |  |  | | | |  |
| 5 | Vvi-Vitvi06g04245\_t001 |  | | | |  | | | |  | | | |  | | | |  |  |  |  |  | | | |  |
| 5 | Vvi-Vitvi06g00772\_t001 |  | | | |  | | | |  | | | |  | Ath-AT2G40470.1 |  |  |  |  |  | Ath-AT2G30340.2 |  |
| 5 | Vvi-Vitvi06g00773\_t002 |  | | | |  | | | |  | | | |  | | | |  |  |  |  |  | | | |  |
| 5 | Vvi-Vitvi06g04246\_t001 |  | | | |  | | | |  | | | |  | | | |  |  |  |  |  | | | |  |
| 5 | Vvi-Vitvi06g04247\_t001 |  | | | |  | | | |  | | | |  | | | |  |  |  |  |  | | | |  |
| 5 | Vvi-Vitvi06g01776\_t001 |  | | | |  | | | |  | | | |  | | | |  |  |  |  |  | | | |  |
| 5 | Vvi-Vitvi06g00774\_t001 |  | | | |  | | | |  | | | |  | | | |  |  |  |  |  | | | |  |
| 5 | Vvi-Vitvi06g00775\_t003 |  | Ath-AT5G58380.1 |  | | | |  | Ath-AT5G01810.1 |  | | | |  |  |  |  |  | | | |  |
| 5 | Vvi-Vitvi06g00776\_t001 |  | | | |  | | | |  | | | |  | | | |  |  |  |  |  | Ath-AT2G30350.2 |  |
| 5 | Vvi-Vitvi06g01777\_t001 |  | Ath-AT5G58375.1 |  | | | |  | | | |  | | | |  |  |  |  |  | | | |  |
| 5 | Vvi-Vitvi06g00777\_t001 |  | Ath-AT5G58370.2 |  | | | |  | | | |  | | | |  |  |  |  |  | | | |  |
| 5 | Vvi-Vitvi06g00779\_t001 |  | | | |  | | | |  | Ath-AT5G01820.1 |  | | | |  |  |  |  |  | Ath-AT2G30360.1 |  |
| 5 | Vvi-Vitvi06g00780\_t001 |  | | | |  | | | |  | | | |  | | | |  |  |  |  |  | Ath-AT2G30370.1 |  |
| 5 | Vvi-Vitvi06g00781\_t001 |  | | | |  | | | |  | | | |  | | | |  |  |  |  |  | Ath-AT2G30380.1 |  |
| 5 | Vvi-Vitvi06g00782\_t001 |  | | | |  | Ath-AT1G06923.1 |  | | | |  | | | |  |  |  |  |  | Ath-AT2G30395.1 |  |
| 5 | Vvi-Vitvi06g04248\_t001 |  | | | |  | | | |  | | | |  | | | |  |  |  |  |  | | | |  |
| 5 | Vvi-Vitvi06g00783\_t001 |  | Ath-AT5G58360.1 |  | Ath-AT1G06920.1 |  | Ath-AT5G01840.1 |  | | | |  |  |  |  |  | Ath-AT2G30400.1 |  |
| 5 | Vvi-Vitvi06g00784\_t001 |  | Ath-AT5G58350.1 |  | | | |  | | | |  | | | |  |  |  |  |  | | | |  |
| 5 | Vvi-Vitvi06g00785\_t001 |  | | | |  | | | |  | | | |  | Ath-AT2G40520.2 |  |  |  |  |  | | | |  |
| 5 | Vvi-Vitvi06g00786\_t001 |  | | | |  | | | |  | | | |  | Ath-AT2G40590.1 |  |  |  |  |  | | | |  |
| 5 | Vvi-Vitvi06g00787\_t001 |  | | | |  | | | |  | | | |  | | | |  |  |  |  |  | | | |  |
| 5 | Vvi-Vitvi06g00789\_t002 |  | | | |  | | | |  | | | |  | | | |  |  |  |  |  | Ath-AT2G30460.2 |  |
| 5 | Vvi-Vitvi06g00790\_t001 |  | | | |  | | | |  | | | |  | Ath-AT2G40610.1 |  |  |  |  |  | | | |  |
| 5 | Vvi-Vitvi06g00791\_t001 |  | | | |  | Ath-AT1G06900.1 |  | | | |  | | | |  |  |  |  |  | | | |  |
| 5 | Vvi-Vitvi06g01778\_t001 |  | | | |  | | | |  | | | |  | | | |  |  |  |  |  | | | |  |
| 5 | Vvi-Vitvi06g04249\_t001 |  | | | |  | | | |  | | | |  | | | |  |  |  |  |  | | | |  |
| 5 | Vvi-Vitvi06g04250\_t001 |  | | | |  | | | |  | | | |  | | | |  |  |  |  |  | | | |  |
| 5 | Vvi-Vitvi06g00796\_t001 |  | | | |  | | | |  | | | |  | | | |  |  |  |  |  | | | |  |
| 5 | Vvi-Vitvi06g00798\_t001 |  | Ath-AT5G58340.1 |  | | | |  | | | |  | | | |  |  |  |  |  | | | |  |
| 5 | Vvi-Vitvi06g00799\_t001 |  | | | |  | | | |  | | | |  | | | |  |  |  |  |  | | | |  |
| 5 | Vvi-Vitvi06g00800\_t001 |  | | | |  | Ath-AT1G06850.1 |  | | | |  | Ath-AT2G40620.1 |  |  |  |  |  | | | |  |
| 4 | Vvi-Vitvi06g04251\_t001 |  | | | |  | | | |  | | | |  |  |  |  |  |  |  | | | |  |
| 4 | Vvi-Vitvi06g00801\_t001 |  | | | |  | | | |  | Ath-AT5G01880.1 |  |  |  |  |  |  |  | | | |  |
| 4 | Vvi-Vitvi06g04252\_t001 |  | | | |  | | | |  | | | |  |  |  |  |  |  |  | | | |  |
| 4 | Vvi-Vitvi06g04253\_t001 |  | | | |  | | | |  | | | |  |  |  |  |  |  |  | Ath-AT2G30470.1 |  |
| 4 | Vvi-Vitvi06g04254\_t001 |  | | | |  | | | |  | | | |  |  |  |  |  |  |  | | | |  |
| 4 | Vvi-Vitvi06g01781\_t001 |  | | | |  | | | |  | | | |  |  |  |  |  |  |  | Ath-AT2G30480.5 |  |
| 4 | Vvi-Vitvi06g00803\_t001 |  | | | |  | | | |  | | | |  |  |  |  |  |  |  | Ath-AT2G30490.1 |  |
| 4 | Vvi-Vitvi06g00804\_t001 |  | | | |  | | | |  | | | |  |  |  |  |  |  |  | | | |  |
| 4 | Vvi-Vitvi06g00805\_t001 |  | Ath-AT5G58320.2 |  | | | |  | | | |  |  |  |  |  |  |  | Ath-AT2G30500.1 |  |
| 4 | Vvi-Vitvi06g04255\_t001 |  | | | |  | | | |  | | | |  |  |  |  |  |  |  | | | |  |
| 4 | Vvi-Vitvi06g01782\_t001 |  | | | |  | | | |  | Ath-AT5G01940.3 |  |  |  |  |  |  |  | | | |  |
| 4 | Vvi-Vitvi06g00807\_t001 |  | | | |  | | | |  | | | |  |  |  |  |  |  |  | | | |  |
| 4 | Vvi-Vitvi06g04256\_t001 |  | | | |  | | | |  | | | |  |  |  |  |  |  |  | | | |  |
| 4 | Vvi-Vitvi06g00808\_t001 |  | | | |  | | | |  | | | |  |  |  |  |  |  |  | | | |  |
| 4 | Vvi-Vitvi06g00810\_t001 |  | | | |  | | | |  | | | |  |  |  |  |  |  |  | | | |  |
| 4 | Vvi-Vitvi06g00811\_t001 |  | | | |  | | | |  | | | |  |  |  |  |  |  |  | | | |  |
| 4 | Vvi-Vitvi06g00812\_t001 |  | Ath-AT5G58300.1 |  | | | |  | | | |  |  |  |  |  |  |  | | | |  |
| 4 | Vvi-Vitvi06g00813\_t001 |  | Ath-AT5G58290.1 |  | | | |  | | | |  |  |  |  |  |  |  | | | |  |
| 4 | Vvi-Vitvi06g04257\_t001 |  | | | |  | | | |  | | | |  |  |  |  |  |  |  | | | |  |
| 4 | Vvi-Vitvi06g00814\_t001 |  | | | |  | | | |  | | | |  |  |  |  |  |  |  | Ath-AT2G30520.1 |  |
| 4 | Vvi-Vitvi06g00817\_t001 |  | | | |  | | | |  | | | |  |  |  |  |  |  |  | | | |  |
| 4 | Vvi-Vitvi06g00819\_t002 |  | | | |  | Ath-AT1G06840.1 |  | Ath-AT5G01950.7 |  |  |  |  |  |  |  | | | |  |
| 3 | Vvi-Vitvi06g04258\_t001 |  | | | |  |  |  | | | |  |  |  |  |  |  |  | | | |  |
| 3 | Vvi-Vitvi06g00822\_t001 |  | Ath-AT5G58280.2 |  |  |  | | | |  |  |  |  |  |  |  | | | |  |
| 3 | Vvi-Vitvi06g00823\_t001 |  | | | |  |  |  | | | |  |  |  |  |  |  |  | | | |  |
| 3 | Vvi-Vitvi06g00824\_t001 |  | | | |  |  |  | | | |  |  |  |  |  |  |  | | | |  |
| 3 | Vvi-Vitvi06g00825\_t001 |  | | | |  |  |  | | | |  |  |  |  |  |  |  | | | |  |
| 3 | Vvi-Vitvi06g00826\_t001 |  | | | |  |  |  | Ath-AT5G01960.1 |  |  |  |  |  |  |  | | | |  |
| 3 | Vvi-Vitvi06g00827\_t001 |  | | | |  |  |  | Ath-AT5G01970.1 |  |  |  |  |  |  |  | Ath-AT2G30530.1 |  |
| 1 | Vvi-Vitvi06g00829\_t001 |  | | | |  |  |  |  |  |  |  |
| 1 | Vvi-Vitvi06g00831\_t001 |  | Ath-AT5G58270.1 |  |  |  |  |  |  |  |
| 1 | Vvi-Vitvi06g00832\_t001 |  | Ath-AT5G58260.1 |  |  |  |  |  |  |  |
| 1 | Vvi-Vitvi06g00833\_t001 |  | | | |  |  |  |  |  |  |  |
| 1 | Vvi-Vitvi06g00834\_t002 |  | Ath-AT5G58250.1 |  |  |  |  |  |  |  |
| 1 | Vvi-Vitvi06g00836\_t001 |  | | | |  |  |  |  |  |  |  |
| 1 | Vvi-Vitvi06g00840\_t001 |  | Ath-AT5G58240.1 |  |  |  |  |  |  |  |
| 0 | Vvi-Vitvi06g00841\_t001 |  |  |  |  |  |  |  |  |
| 0 | Vvi-Vitvi06g00843\_t001 |  |  |  |  |  |  |  |  |
| 0 | Vvi-Vitvi06g00844\_t002 |  |  |  |  |  |  |  |  |
| 0 | Vvi-Vitvi06g00845\_t001 |  |  |  |  |  |  |  |  |
| 0 | Vvi-Vitvi06g04259\_t001 |  |  |  |  |  |  |  |  |
| 0 | Vvi-Vitvi06g04260\_t001 |  |  |  |  |  |  |  |  |
| 0 | Vvi-Vitvi06g00846\_t001 |  |  |  |  |  |  |  |  |
| 0 | Vvi-Vitvi06g00848\_t001 |  |  |  |  |  |  |  |  |
| 0 | Vvi-Vitvi06g04261\_t001 |  |  |  |  |  |  |  |  |
| 0 | Vvi-Vitvi06g04262\_t001 |  |  |  |  |  |  |  |  |
| 0 | Vvi-Vitvi06g04263\_t001 |  |  |  |  |  |  |  |  |
| 0 | Vvi-Vitvi06g04264\_t001 |  |  |  |  |  |  |  |  |
| 0 | Vvi-Vitvi06g00876\_t001 |  |  |  |  |  |  |  |  |
| 0 | Vvi-Vitvi06g04265\_t001 |  |  |  |  |  |  |  |  |
| 0 | Vvi-Vitvi06g04266\_t001 |  |  |  |  |  |  |  |  |
| 0 | Vvi-Vitvi06g04267\_t001 |  |  |  |  |  |  |  |  |
| 0 | Vvi-Vitvi06g00893\_t001 |  |  |  |  |  |  |  |  |
| 0 | Vvi-Vitvi06g04268\_t001 |  |  |  |  |  |  |  |  |
| 0 | Vvi-Vitvi06g00894\_t001 |  |  |  |  |  |  |  |  |
| 0 | Vvi-Vitvi06g00895\_t001 |  |  |  |  |  |  |  |  |
| 0 | Vvi-Vitvi06g00896\_t001 |  |  |  |  |  |  |  |  |
| 0 | Vvi-Vitvi06g00897\_t001 |  |  |  |  |  |  |  |  |
| 0 | Vvi-Vitvi06g00898\_t001 |  |  |  |  |  |  |  |  |
| 0 | Vvi-Vitvi06g04269\_t001 |  |  |  |  |  |  |  |  |
| 0 | Vvi-Vitvi06g00899\_t001 |  |  |  |  |  |  |  |  |
| 0 | Vvi-Vitvi06g04270\_t001 |  |  |  |  |  |  |  |  |
| 0 | Vvi-Vitvi06g04271\_t001 |  |  |  |  |  |  |  |  |
| 0 | Vvi-Vitvi06g04272\_t001 |  |  |  |  |  |  |  |  |
| 0 | Vvi-Vitvi06g04273\_t001 |  |  |  |  |  |  |  |  |
| 0 | Vvi-Vitvi06g00903\_t001 |  |  |  |  |  |  |  |  |
| 0 | Vvi-Vitvi06g00906\_t001 |  |  |  |  |  |  |  |  |
| 0 | Vvi-Vitvi06g00910\_t002 |  |  |  |  |  |  |  |  |
| 0 | Vvi-Vitvi06g04274\_t001 |  |  |  |  |  |  |  |  |
| 0 | Vvi-Vitvi06g04275\_t001 |  |  |  |  |  |  |  |  |
| 0 | Vvi-Vitvi06g04276\_t001 |  |  |  |  |  |  |  |  |
| 0 | Vvi-Vitvi06g00915\_t001 |  |  |  |  |  |  |  |  |
| 0 | Vvi-Vitvi06g00916\_t001 |  |  |  |  |  |  |  |  |
| 0 | Vvi-Vitvi06g00917\_t001 |  |  |  |  |  |  |  |  |
| 0 | Vvi-Vitvi06g00918\_t001 |  |  |  |  |  |  |  |  |
| 0 | Vvi-Vitvi06g00920\_t001 |  |  |  |  |  |  |  |  |
| 0 | Vvi-Vitvi06g00922\_t001 |  |  |  |  |  |  |  |  |
| 0 | Vvi-Vitvi06g00928\_t001 |  |  |  |  |  |  |  |  |
| 0 | Vvi-Vitvi06g04277\_t001 |  |  |  |  |  |  |  |  |
| 0 | Vvi-Vitvi06g04278\_t001 |  |  |  |  |  |  |  |  |
| 0 | Vvi-Vitvi06g04279\_t001 |  |  |  |  |  |  |  |  |
| 0 | Vvi-Vitvi06g00931\_t001 |  |  |  |  |  |  |  |  |
| 0 | Vvi-Vitvi06g00932\_t001 |  |  |  |  |  |  |  |  |
| 0 | Vvi-Vitvi06g04280\_t001 |  |  |  |  |  |  |  |  |
| 0 | Vvi-Vitvi06g04281\_t001 |  |  |  |  |  |  |  |  |
| 0 | Vvi-Vitvi06g00937\_t001 |  |  |  |  |  |  |  |  |
| 0 | Vvi-Vitvi06g04282\_t001 |  |  |  |  |  |  |  |  |
| 0 | Vvi-Vitvi06g04283\_t001 |  |  |  |  |  |  |  |  |
| 0 | Vvi-Vitvi06g00943\_t001 |  |  |  |  |  |  |  |  |
| 0 | Vvi-Vitvi06g00944\_t001 |  |  |  |  |  |  |  |  |
| 1 | Vvi-Vitvi06g00946\_t001 |  | Ath-AT5G22140.1 |  |  |  |  |  |  |  |
| 1 | Vvi-Vitvi06g00947\_t001 |  | | | |  |  |  |  |  |  |  |
| 1 | Vvi-Vitvi06g01810\_t001 |  | | | |  |  |  |  |  |  |  |
| 1 | Vvi-Vitvi06g04284\_t001 |  | | | |  |  |  |  |  |  |  |
| 1 | Vvi-Vitvi06g01811\_t001 |  | | | |  |  |  |  |  |  |  |
| 1 | Vvi-Vitvi06g00956\_t001 |  | | | |  |  |  |  |  |  |  |
| 1 | Vvi-Vitvi06g00959\_t001 |  | | | |  |  |  |  |  |  |  |
| 1 | Vvi-Vitvi06g04285\_t001 |  | | | |  |  |  |  |  |  |  |
| 1 | Vvi-Vitvi06g00961\_t001 |  | | | |  |  |  |  |  |  |  |
| 1 | Vvi-Vitvi06g00962\_t003 |  | | | |  |  |  |  |  |  |  |
| 1 | Vvi-Vitvi06g00964\_t001 |  | | | |  |  |  |  |  |  |  |
| 1 | Vvi-Vitvi06g00966\_t001 |  | | | |  |  |  |  |  |  |  |
| 1 | Vvi-Vitvi06g00968\_t001 |  | | | |  |  |  |  |  |  |  |
| 1 | Vvi-Vitvi06g04286\_t001 |  | | | |  |  |  |  |  |  |  |
| 1 | Vvi-Vitvi06g00972\_t001 |  | | | |  |  |  |  |  |  |  |
| 1 | Vvi-Vitvi06g00973\_t001 |  | | | |  |  |  |  |  |  |  |
| 1 | Vvi-Vitvi06g00975\_t001 |  | Ath-AT5G22040.2 |  |  |  |  |  |  |  |
| 1 | Vvi-Vitvi06g04287\_t001 |  | | | |  |  |  |  |  |  |  |
| 1 | Vvi-Vitvi06g00978\_t001 |  | | | |  |  |  |  |  |  |  |
| 1 | Vvi-Vitvi06g00979\_t001 |  | | | |  |  |  |  |  |  |  |
| 1 | Vvi-Vitvi06g00982\_t001 |  | | | |  |  |  |  |  |  |  |
| 1 | Vvi-Vitvi06g04288\_t001 |  | | | |  |  |  |  |  |  |  |
| 1 | Vvi-Vitvi06g01818\_t001 |  | | | |  |  |  |  |  |  |  |
| 1 | Vvi-Vitvi06g00984\_t001 |  | | | |  |  |  |  |  |  |  |
| 1 | Vvi-Vitvi06g00985\_t001 |  | | | |  |  |  |  |  |  |  |
| 1 | Vvi-Vitvi06g00988\_t001 |  | Ath-AT5G22000.1 |  |  |  |  |  |  |  |
| 2 | Vvi-Vitvi06g01819\_t001 |  | | | |  | Ath-AT2G27180.1 |  |  |  |  |  |  |
| 2 | Vvi-Vitvi06g00990\_t001 |  | Ath-AT5G21990.1 |  | | | |  |  |  |  |  |  |
| 2 | Vvi-Vitvi06g00991\_t002 |  | | | |  | | | |  |  |  |  |  |  |
| 2 | Vvi-Vitvi06g00992\_t001 |  | | | |  | | | |  |  |  |  |  |  |
| 3 | Vvi-Vitvi06g00993\_t001 |  | | | |  | | | |  | Ath-AT5G20930.1 |  |  |  |  |  |
| 3 | Vvi-Vitvi06g00994\_t001 |  | | | |  | | | |  | Ath-AT5G20935.1 |  |  |  |  |  |
| 3 | Vvi-Vitvi06g00995\_t001 |  | | | |  | | | |  | | | |  |  |  |  |  |
| 3 | Vvi-Vitvi06g00996\_t001 |  | | | |  | | | |  | | | |  |  |  |  |  |
| 3 | Vvi-Vitvi06g00997\_t001 |  | | | |  | | | |  | | | |  |  |  |  |  |
| 3 | Vvi-Vitvi06g01821\_t001 |  | | | |  | Ath-AT2G27140.1 |  | Ath-AT5G20970.1 |  |  |  |  |  |
| 3 | Vvi-Vitvi06g00998\_t001 |  | | | |  | | | |  | | | |  |  |  |  |  |
| 3 | Vvi-Vitvi06g00999\_t001 |  | | | |  | | | |  | | | |  |  |  |  |  |
| 3 | Vvi-Vitvi06g01000\_t001 |  | | | |  | | | |  | | | |  |  |  |  |  |
| 3 | Vvi-Vitvi06g04289\_t001 |  | | | |  | | | |  | | | |  |  |  |  |  |
| 3 | Vvi-Vitvi06g01004\_t002 |  | | | |  | | | |  | Ath-AT5G20990.1 |  |  |  |  |  |
| 3 | Vvi-Vitvi06g01010\_t001 |  | | | |  | | | |  | | | |  |  |  |  |  |
| 3 | Vvi-Vitvi06g01011\_t001 |  | Ath-AT5G21160.3 |  | | | |  | | | |  |  |  |  |  |
| 3 | Vvi-Vitvi06g04290\_t001 |  | | | |  | | | |  | | | |  |  |  |  |  |
| 3 | Vvi-Vitvi06g04291\_t001 |  | | | |  | | | |  | | | |  |  |  |  |  |
| 3 | Vvi-Vitvi06g04292\_t001 |  | | | |  | | | |  | | | |  |  |  |  |  |
| 3 | Vvi-Vitvi06g01017\_t001 |  | | | |  | | | |  | | | |  |  |  |  |  |
| 3 | Vvi-Vitvi06g04293\_t001 |  | | | |  | | | |  | | | |  |  |  |  |  |
| 3 | Vvi-Vitvi06g01020\_t001 |  | Ath-AT5G21150.1 |  | | | |  | Ath-AT5G21030.2 |  |  |  |  |  |
| 3 | Vvi-Vitvi06g01021\_t001 |  | | | |  | | | |  | | | |  |  |  |  |  |
| 3 | Vvi-Vitvi06g01023\_t001 |  | | | |  | | | |  | | | |  |  |  |  |  |
| 3 | Vvi-Vitvi06g01024\_t001 |  | | | |  | | | |  | | | |  |  |  |  |  |
| 3 | Vvi-Vitvi06g01026\_t001 |  | | | |  | | | |  | | | |  |  |  |  |  |
| 3 | Vvi-Vitvi06g01027\_t001 |  | | | |  | | | |  | | | |  |  |  |  |  |
| 3 | Vvi-Vitvi06g01033\_t001 |  | Ath-AT5G21100.1 |  | | | |  | | | |  |  |  |  |  |
| 2 | Vvi-Vitvi06g01827\_t001 |  |  |  | | | |  | | | |  |  |  |  |  |
| 2 | Vvi-Vitvi06g01034\_t001 |  |  |  | | | |  | | | |  |  |  |  |  |
| 2 | Vvi-Vitvi06g01035\_t001 |  |  |  | | | |  | | | |  |  |  |  |  |
| 2 | Vvi-Vitvi06g01036\_t001 |  |  |  | | | |  | | | |  |  |  |  |  |
| 2 | Vvi-Vitvi06g01829\_t001 |  |  |  | | | |  | Ath-AT5G21050.1 |  |  |  |  |  |
| 2 | Vvi-Vitvi06g01830\_t002 |  |  |  | Ath-AT2G27130.1 |  | | | |  |  |  |  |  |
| 2 | Vvi-Vitvi06g04294\_t001 |  |  |  | | | |  | | | |  |  |  |  |  |
| 2 | Vvi-Vitvi06g04295\_t001 |  |  |  | | | |  | | | |  |  |  |  |  |
| 2 | Vvi-Vitvi06g04296\_t001 |  |  |  | | | |  | | | |  |  |  |  |  |
| 2 | Vvi-Vitvi06g04297\_t001 |  |  |  | | | |  | | | |  |  |  |  |  |
| 2 | Vvi-Vitvi06g04298\_t001 |  |  |  | | | |  | | | |  |  |  |  |  |
| 2 | Vvi-Vitvi06g01043\_t001 |  |  |  | | | |  | | | |  |  |  |  |  |
| 2 | Vvi-Vitvi06g01044\_t001 |  |  |  | | | |  | Ath-AT5G21060.3 |  |  |  |  |  |
| 2 | Vvi-Vitvi06g01045\_t001 |  |  |  | | | |  | | | |  |  |  |  |  |
| 2 | Vvi-Vitvi06g01834\_t001 |  |  |  | | | |  | | | |  |  |  |  |  |
| 2 | Vvi-Vitvi06g01047\_t001 |  |  |  | | | |  | | | |  |  |  |  |  |
| 2 | Vvi-Vitvi06g01835\_t001 |  |  |  | | | |  | | | |  |  |  |  |  |
| 2 | Vvi-Vitvi06g04299\_t001 |  |  |  | | | |  | | | |  |  |  |  |  |
| 2 | Vvi-Vitvi06g01048\_t001 |  |  |  | Ath-AT2G27110.1 |  | | | |  |  |  |  |  |
| 2 | Vvi-Vitvi06g01050\_t001 |  |  |  | Ath-AT2G27100.1 |  | | | |  |  |  |  |  |
| 2 | Vvi-Vitvi06g04300\_t001 |  |  |  | | | |  | | | |  |  |  |  |  |
| 2 | Vvi-Vitvi06g01051\_t001 |  |  |  | | | |  | Ath-AT5G21070.1 |  |  |  |  |  |
| 2 | Vvi-Vitvi06g01052\_t001 |  |  |  | | | |  | | | |  |  |  |  |  |
| 2 | Vvi-Vitvi06g01053\_t001 |  |  |  | Ath-AT2G27090.2 |  | | | |  |  |  |  |  |
| 2 | Vvi-Vitvi06g01055\_t001 |  |  |  | Ath-AT2G27080.2 |  | Ath-AT5G21130.1 |  |  |  |  |  |
| 2 | Vvi-Vitvi06g04301\_t001 |  |  |  | | | |  | | | |  |  |  |  |  |
| 2 | Vvi-Vitvi06g01056\_t001 |  |  |  | | | |  | | | |  |  |  |  |  |
| 2 | Vvi-Vitvi06g01057\_t001 |  |  |  | Ath-AT2G27060.1 |  | | | |  |  |  |  |  |
| 2 | Vvi-Vitvi06g01058\_t001 |  |  |  | | | |  | | | |  |  |  |  |  |
| 2 | Vvi-Vitvi06g01060\_t001 |  |  |  | | | |  | | | |  |  |  |  |  |
| 2 | Vvi-Vitvi06g01061\_t001 |  |  |  | | | |  | | | |  |  |  |  |  |
| 2 | Vvi-Vitvi06g04302\_t001 |  |  |  | | | |  | | | |  |  |  |  |  |
| 2 | Vvi-Vitvi06g04303\_t001 |  |  |  | | | |  | | | |  |  |  |  |  |
| 2 | Vvi-Vitvi06g04304\_t001 |  |  |  | | | |  | | | |  |  |  |  |  |
| 2 | Vvi-Vitvi06g01064\_t001 |  |  |  | | | |  | | | |  |  |  |  |  |
| 2 | Vvi-Vitvi06g04305\_t001 |  |  |  | | | |  | | | |  |  |  |  |  |
| 2 | Vvi-Vitvi06g01069\_t001 |  |  |  | | | |  | | | |  |  |  |  |  |
| 2 | Vvi-Vitvi06g01070\_t001 |  |  |  | | | |  | | | |  |  |  |  |  |
| 2 | Vvi-Vitvi06g01071\_t001 |  |  |  | | | |  | | | |  |  |  |  |  |
| 2 | Vvi-Vitvi06g01839\_t001 |  |  |  | | | |  | | | |  |  |  |  |  |
| 2 | Vvi-Vitvi06g01072\_t001 |  |  |  | | | |  | | | |  |  |  |  |  |
| 2 | Vvi-Vitvi06g01073\_t004 |  |  |  | | | |  | | | |  |  |  |  |  |
| 2 | Vvi-Vitvi06g01074\_t002 |  |  |  | | | |  | | | |  |  |  |  |  |
| 2 | Vvi-Vitvi06g01075\_t001 |  |  |  | | | |  | | | |  |  |  |  |  |
| 2 | Vvi-Vitvi06g01078\_t001 |  |  |  | | | |  | | | |  |  |  |  |  |
| 2 | Vvi-Vitvi06g04306\_t001 |  |  |  | | | |  | | | |  |  |  |  |  |
| 2 | Vvi-Vitvi06g04307\_t001 |  |  |  | | | |  | | | |  |  |  |  |  |
| 2 | Vvi-Vitvi06g01084\_t003 |  |  |  | Ath-AT2G26980.4 |  | Ath-AT5G21326.1 |  |  |  |  |  |
| 1 | Vvi-Vitvi06g04309\_t001 |  |  |  | | | |  |  |  |  |  |  |
| 1 | Vvi-Vitvi06g01842\_t001 |  |  |  | | | |  |  |  |  |  |  |
| 1 | Vvi-Vitvi06g01843\_t001 |  |  |  | | | |  |  |  |  |  |  |
| 1 | Vvi-Vitvi06g01086\_t003 |  |  |  | | | |  |  |  |  |  |  |
| 1 | Vvi-Vitvi06g01087\_t001 |  |  |  | Ath-AT2G26970.1 |  |  |  |  |  |  |
| 1 | Vvi-Vitvi06g01088\_t001 |  |  |  | | | |  |  |  |  |  |  |
| 1 | Vvi-Vitvi06g04310\_t001 |  |  |  | | | |  |  |  |  |  |  |
| 1 | Vvi-Vitvi06g01844\_t001 |  |  |  | | | |  |  |  |  |  |  |
| 1 | Vvi-Vitvi06g01091\_t001 |  |  |  | | | |  |  |  |  |  |  |
| 1 | Vvi-Vitvi06g01092\_t002 |  |  |  | | | |  |  |  |  |  |  |
| 1 | Vvi-Vitvi06g01093\_t001 |  |  |  | | | |  |  |  |  |  |  |
| 1 | Vvi-Vitvi06g01094\_t001 |  |  |  | | | |  |  |  |  |  |  |
| 1 | Vvi-Vitvi06g01095\_t001 |  |  |  | | | |  |  |  |  |  |  |
| 1 | Vvi-Vitvi06g01846\_t001 |  |  |  | | | |  |  |  |  |  |  |
| 1 | Vvi-Vitvi06g01097\_t001 |  |  |  | | | |  |  |  |  |  |  |
| 1 | Vvi-Vitvi06g04311\_t001 |  |  |  | | | |  |  |  |  |  |  |
| 1 | Vvi-Vitvi06g01100\_t001 |  |  |  | | | |  |  |  |  |  |  |
| 1 | Vvi-Vitvi06g01101\_t001 |  |  |  | | | |  |  |  |  |  |  |
| 1 | Vvi-Vitvi06g01847\_t001 |  |  |  | | | |  |  |  |  |  |  |
| 1 | Vvi-Vitvi06g01104\_t001 |  |  |  | Ath-AT2G26770.1 |  |  |  |  |  |  |
| 1 | Vvi-Vitvi06g04312\_t001 |  |  |  | | | |  |  |  |  |  |  |
| 1 | Vvi-Vitvi06g01850\_t001 |  |  |  | | | |  |  |  |  |  |  |
| 1 | Vvi-Vitvi06g01851\_t001 |  |  |  | | | |  |  |  |  |  |  |
| 1 | Vvi-Vitvi06g01106\_t001 |  |  |  | Ath-AT2G26760.1 |  |  |  |  |  |  |
| 0 | Vvi-Vitvi06g01107\_t002 |  |  |  |  |  |  |  |  |
| 0 | Vvi-Vitvi06g01108\_t001 |  |  |  |  |  |  |  |  |
| 0 | Vvi-Vitvi06g01109\_t001 |  |  |  |  |  |  |  |  |
| 0 | Vvi-Vitvi06g01852\_t001 |  |  |  |  |  |  |  |  |
| 0 | Vvi-Vitvi06g04313\_t001 |  |  |  |  |  |  |  |  |
| 0 | Vvi-Vitvi06g01110\_t001 |  |  |  |  |  |  |  |  |
| 0 | Vvi-Vitvi06g04314\_t001 |  |  |  |  |  |  |  |  |
| 0 | Vvi-Vitvi06g01111\_t001 |  |  |  |  |  |  |  |  |
| 0 | Vvi-Vitvi06g04315\_t001 |  |  |  |  |  |  |  |  |
| 0 | Vvi-Vitvi06g01112\_t001 |  |  |  |  |  |  |  |  |
| 0 | Vvi-Vitvi06g01113\_t001 |  |  |  |  |  |  |  |  |
| 0 | Vvi-Vitvi06g01114\_t002 |  |  |  |  |  |  |  |  |
| 0 | Vvi-Vitvi06g01116\_t001 |  |  |  |  |  |  |  |  |
| 0 | Vvi-Vitvi06g01117\_t001 |  |  |  |  |  |  |  |  |
| 0 | Vvi-Vitvi06g04316\_t001 |  |  |  |  |  |  |  |  |
| 0 | Vvi-Vitvi06g01119\_t001 |  |  |  |  |  |  |  |  |
| 0 | Vvi-Vitvi06g01122\_t001 |  |  |  |  |  |  |  |  |
| 0 | Vvi-Vitvi06g04317\_t001 |  |  |  |  |  |  |  |  |
| 0 | Vvi-Vitvi06g04318\_t001 |  |  |  |  |  |  |  |  |
| 0 | Vvi-Vitvi06g01856\_t001 |  |  |  |  |  |  |  |  |
| 0 | Vvi-Vitvi06g01125\_t001 |  |  |  |  |  |  |  |  |
| 0 | Vvi-Vitvi06g01124\_t001 |  |  |  |  |  |  |  |  |
| 0 | Vvi-Vitvi06g04319\_t001 |  |  |  |  |  |  |  |  |
| 0 | Vvi-Vitvi06g01130\_t001 |  |  |  |  |  |  |  |  |
| 0 | Vvi-Vitvi06g01131\_t001 |  |  |  |  |  |  |  |  |
| 0 | Vvi-Vitvi06g01858\_t001 |  |  |  |  |  |  |  |  |
| 0 | Vvi-Vitvi06g04320\_t001 |  |  |  |  |  |  |  |  |
| 0 | Vvi-Vitvi06g01132\_t001 |  |  |  |  |  |  |  |  |
| 0 | Vvi-Vitvi06g01133\_t001 |  |  |  |  |  |  |  |  |
| 0 | Vvi-Vitvi06g01134\_t001 |  |  |  |  |  |  |  |  |
| 0 | Vvi-Vitvi06g04321\_t001 |  |  |  |  |  |  |  |  |
| 0 | Vvi-Vitvi06g01861\_t001 |  |  |  |  |  |  |  |  |
| 0 | Vvi-Vitvi06g04322\_t001 |  |  |  |  |  |  |  |  |
| 0 | Vvi-Vitvi06g04323\_t001 |  |  |  |  |  |  |  |  |
| 0 | Vvi-Vitvi06g01138\_t001 |  |  |  |  |  |  |  |  |
| 0 | Vvi-Vitvi06g04324\_t001 |  |  |  |  |  |  |  |  |
| 0 | Vvi-Vitvi06g04325\_t001 |  |  |  |  |  |  |  |  |
| 0 | Vvi-Vitvi06g01862\_t001 |  |  |  |  |  |  |  |  |
| 0 | Vvi-Vitvi06g01863\_t001 |  |  |  |  |  |  |  |  |
| 0 | Vvi-Vitvi06g01864\_t001 |  |  |  |  |  |  |  |  |
| 0 | Vvi-Vitvi06g01865\_t001 |  |  |  |  |  |  |  |  |
| 0 | Vvi-Vitvi06g01866\_t001 |  |  |  |  |  |  |  |  |
| 0 | Vvi-Vitvi06g04326\_t001 |  |  |  |  |  |  |  |  |
| 0 | Vvi-Vitvi06g01867\_t001 |  |  |  |  |  |  |  |  |
| 0 | Vvi-Vitvi06g01139\_t001 |  |  |  |  |  |  |  |  |
| 0 | Vvi-Vitvi06g04327\_t001 |  |  |  |  |  |  |  |  |
| 0 | Vvi-Vitvi06g01140\_t001 |  |  |  |  |  |  |  |  |
| 0 | Vvi-Vitvi06g01144\_t001 |  |  |  |  |  |  |  |  |
| 0 | Vvi-Vitvi06g01146\_t001 |  |  |  |  |  |  |  |  |
| 0 | Vvi-Vitvi06g01147\_t001 |  |  |  |  |  |  |  |  |
| 0 | Vvi-Vitvi06g01148\_t002 |  |  |  |  |  |  |  |  |
| 0 | Vvi-Vitvi06g01868\_t001 |  |  |  |  |  |  |  |  |
| 0 | Vvi-Vitvi06g01149\_t001 |  |  |  |  |  |  |  |  |
| 0 | Vvi-Vitvi06g04328\_t001 |  |  |  |  |  |  |  |  |
| 0 | Vvi-Vitvi06g01152\_t001 |  |  |  |  |  |  |  |  |
| 0 | Vvi-Vitvi06g04329\_t001 |  |  |  |  |  |  |  |  |
| 0 | Vvi-Vitvi06g04330\_t001 |  |  |  |  |  |  |  |  |
| 0 | Vvi-Vitvi06g04331\_t001 |  |  |  |  |  |  |  |  |
| 0 | Vvi-Vitvi06g04332\_t001 |  |  |  |  |  |  |  |  |
| 0 | Vvi-Vitvi06g01869\_t001 |  |  |  |  |  |  |  |  |
| 0 | Vvi-Vitvi06g01870\_t001 |  |  |  |  |  |  |  |  |
| 0 | Vvi-Vitvi06g01871\_t001 |  |  |  |  |  |  |  |  |
| 0 | Vvi-Vitvi06g01161\_t003 |  |  |  |  |  |  |  |  |
| 0 | Vvi-Vitvi06g01872\_t001 |  |  |  |  |  |  |  |  |
| 0 | Vvi-Vitvi06g01162\_t001 |  |  |  |  |  |  |  |  |
| 0 | Vvi-Vitvi06g01163\_t001 |  |  |  |  |  |  |  |  |
| 0 | Vvi-Vitvi06g01164\_t001 |  |  |  |  |  |  |  |  |
| 0 | Vvi-Vitvi06g01873\_t001 |  |  |  |  |  |  |  |  |
| 0 | Vvi-Vitvi06g01165\_t002 |  |  |  |  |  |  |  |  |
| 0 | Vvi-Vitvi06g01166\_t001 |  |  |  |  |  |  |  |  |
| 0 | Vvi-Vitvi06g01877\_t001 |  |  |  |  |  |  |  |  |
| 0 | Vvi-Vitvi06g01878\_t001 |  |  |  |  |  |  |  |  |
| 0 | Vvi-Vitvi06g01167\_t001 |  |  |  |  |  |  |  |  |
| 0 | Vvi-Vitvi06g04333\_t001 |  |  |  |  |  |  |  |  |
| 0 | Vvi-Vitvi06g04334\_t001 |  |  |  |  |  |  |  |  |
| 0 | Vvi-Vitvi06g01168\_t001 |  |  |  |  |  |  |  |  |
| 0 | Vvi-Vitvi06g01169\_t002 |  |  |  |  |  |  |  |  |
| 0 | Vvi-Vitvi06g01172\_t001 |  |  |  |  |  |  |  |  |
| 0 | Vvi-Vitvi06g01881\_t001 |  |  |  |  |  |  |  |  |
| 0 | Vvi-Vitvi06g04335\_t001 |  |  |  |  |  |  |  |  |
| 0 | Vvi-Vitvi06g04336\_t001 |  |  |  |  |  |  |  |  |
| 0 | Vvi-Vitvi06g01175\_t001 |  |  |  |  |  |  |  |  |
| 0 | Vvi-Vitvi06g04337\_t001 |  |  |  |  |  |  |  |  |
| 0 | Vvi-Vitvi06g04338\_t001 |  |  |  |  |  |  |  |  |
| 0 | Vvi-Vitvi06g04339\_t001 |  |  |  |  |  |  |  |  |
| 0 | Vvi-Vitvi06g04340\_t001 |  |  |  |  |  |  |  |  |
| 0 | Vvi-Vitvi06g04341\_t001 |  |  |  |  |  |  |  |  |
| 0 | Vvi-Vitvi06g04342\_t001 |  |  |  |  |  |  |  |  |
| 0 | Vvi-Vitvi06g01887\_t001 |  |  |  |  |  |  |  |  |
| 0 | Vvi-Vitvi06g01180\_t001 |  |  |  |  |  |  |  |  |
| 0 | Vvi-Vitvi06g01890\_t001 |  |  |  |  |  |  |  |  |
| 0 | Vvi-Vitvi06g04343\_t001 |  |  |  |  |  |  |  |  |
| 0 | Vvi-Vitvi06g04344\_t001 |  |  |  |  |  |  |  |  |
| 0 | Vvi-Vitvi06g04345\_t001 |  |  |  |  |  |  |  |  |
| 0 | Vvi-Vitvi06g01190\_t001 |  |  |  |  |  |  |  |  |
| 0 | Vvi-Vitvi06g04346\_t001 |  |  |  |  |  |  |  |  |
| 0 | Vvi-Vitvi06g04347\_t001 |  |  |  |  |  |  |  |  |
| 0 | Vvi-Vitvi06g01195\_t001 |  |  |  |  |  |  |  |  |
| 0 | Vvi-Vitvi06g04348\_t001 |  |  |  |  |  |  |  |  |
| 0 | Vvi-Vitvi06g01197\_t001 |  |  |  |  |  |  |  |  |
| 0 | Vvi-Vitvi06g04349\_t001 |  |  |  |  |  |  |  |  |
| 0 | Vvi-Vitvi06g01201\_t001 |  |  |  |  |  |  |  |  |
| 0 | Vvi-Vitvi06g04350\_t001 |  |  |  |  |  |  |  |  |
| 0 | Vvi-Vitvi06g01203\_t001 |  |  |  |  |  |  |  |  |
| 0 | Vvi-Vitvi06g01205\_t001 |  |  |  |  |  |  |  |  |
| 1 | Vvi-Vitvi06g01207\_t001 |  | Ath-AT5G19360.1 |  |  |  |  |  |  |  |
| 1 | Vvi-Vitvi06g04351\_t001 |  | | | |  |  |  |  |  |  |  |
| 1 | Vvi-Vitvi06g04352\_t001 |  | | | |  |  |  |  |  |  |  |
| 1 | Vvi-Vitvi06g01211\_t001 |  | Ath-AT5G19370.1 |  |  |  |  |  |  |  |
| 1 | Vvi-Vitvi06g04353\_t001 |  | | | |  |  |  |  |  |  |  |
| 1 | Vvi-Vitvi06g04354\_t001 |  | | | |  |  |  |  |  |  |  |
| 1 | Vvi-Vitvi06g01212\_t001 |  | Ath-AT5G19380.2 |  |  |  |  |  |  |  |
| 1 | Vvi-Vitvi06g01214\_t001 |  | | | |  |  |  |  |  |  |  |
| 1 | Vvi-Vitvi06g01215\_t001 |  | | | |  |  |  |  |  |  |  |
| 1 | Vvi-Vitvi06g04355\_t001 |  | | | |  |  |  |  |  |  |  |
| 1 | Vvi-Vitvi06g01216\_t001 |  | | | |  |  |  |  |  |  |  |
| 2 | Vvi-Vitvi06g01217\_t002 |  | Ath-AT5G19390.2 |  | Ath-AT5G12150.1 |  |  |  |  |  |  |
| 2 | Vvi-Vitvi06g01218\_t001 |  | | | |  | | | |  |  |  |  |  |  |
| 2 | Vvi-Vitvi06g01220\_t001 |  | | | |  | Ath-AT5G12190.1 |  |  |  |  |  |  |
| 2 | Vvi-Vitvi06g04356\_t001 |  | | | |  | | | |  |  |  |  |  |  |
| 2 | Vvi-Vitvi06g01222\_t001 |  | | | |  | | | |  |  |  |  |  |  |
| 2 | Vvi-Vitvi06g04357\_t001 |  | | | |  | | | |  |  |  |  |  |  |
| 2 | Vvi-Vitvi06g04358\_t001 |  | | | |  | | | |  |  |  |  |  |  |
| 2 | Vvi-Vitvi06g04359\_t001 |  | | | |  | | | |  |  |  |  |  |  |
| 2 | Vvi-Vitvi06g01225\_t001 |  | Ath-AT5G19400.3 |  | | | |  |  |  |  |  |  |
| 2 | Vvi-Vitvi06g04360\_t001 |  | | | |  | | | |  |  |  |  |  |  |
| 2 | Vvi-Vitvi06g01226\_t001 |  | | | |  | | | |  |  |  |  |  |  |
| 2 | Vvi-Vitvi06g01227\_t001 |  | | | |  | | | |  |  |  |  |  |  |
| 2 | Vvi-Vitvi06g01231\_t001 |  | Ath-AT5G19410.2 |  | | | |  |  |  |  |  |  |
| 2 | Vvi-Vitvi06g01232\_t001 |  | | | |  | | | |  |  |  |  |  |  |
| 2 | Vvi-Vitvi06g01233\_t001 |  | | | |  | | | |  |  |  |  |  |  |
| 2 | Vvi-Vitvi06g04361\_t001 |  | | | |  | | | |  |  |  |  |  |  |
| 2 | Vvi-Vitvi06g01236\_t001 |  | | | |  | Ath-AT5G12300.1 |  |  |  |  |  |  |
| 2 | Vvi-Vitvi06g01237\_t001 |  | Ath-AT5G19430.5 |  | Ath-AT5G12310.1 |  |  |  |  |  |  |
| 1 | Vvi-Vitvi06g01238\_t001 |  |  |  | Ath-AT5G12320.1 |  |  |  |  |  |  |
| 1 | Vvi-Vitvi06g01239\_t001 |  |  |  | Ath-AT5G12330.4 |  |  |  |  |  |  |
| 1 | Vvi-Vitvi06g01240\_t001 |  |  |  | Ath-AT5G12340.2 |  |  |  |  |  |  |
| 1 | Vvi-Vitvi06g04362\_t001 |  |  |  | | | |  |  |  |  |  |  |
| 1 | Vvi-Vitvi06g04363\_t001 |  |  |  | | | |  |  |  |  |  |  |
| 1 | Vvi-Vitvi06g04364\_t001 |  |  |  | | | |  |  |  |  |  |  |
| 1 | Vvi-Vitvi06g01241\_t001 |  |  |  | | | |  |  |  |  |  |  |
| 1 | Vvi-Vitvi06g04365\_t001 |  |  |  | | | |  |  |  |  |  |  |
| 1 | Vvi-Vitvi06g01242\_t001 |  |  |  | | | |  |  |  |  |  |  |
| 1 | Vvi-Vitvi06g01243\_t001 |  |  |  | | | |  |  |  |  |  |  |
| 1 | Vvi-Vitvi06g01244\_t001 |  |  |  | | | |  |  |  |  |  |  |
| 1 | Vvi-Vitvi06g01907\_t001 |  |  |  | | | |  |  |  |  |  |  |
| 1 | Vvi-Vitvi06g04366\_t001 |  |  |  | | | |  |  |  |  |  |  |
| 1 | Vvi-Vitvi06g01245\_t001 |  |  |  | Ath-AT5G12350.1 |  |  |  |  |  |  |
| 1 | Vvi-Vitvi06g04367\_t001 |  |  |  | | | |  |  |  |  |  |  |
| 1 | Vvi-Vitvi06g01246\_t001 |  |  |  | | | |  |  |  |  |  |  |
| 1 | Vvi-Vitvi06g01248\_t001 |  |  |  | | | |  |  |  |  |  |  |
| 1 | Vvi-Vitvi06g01249\_t001 |  |  |  | | | |  |  |  |  |  |  |
| 1 | Vvi-Vitvi06g01909\_t001 |  |  |  | | | |  |  |  |  |  |  |
| 1 | Vvi-Vitvi06g01910\_t001 |  |  |  | | | |  |  |  |  |  |  |
| 1 | Vvi-Vitvi06g01911\_t001 |  |  |  | Ath-AT5G12360.1 |  |  |  |  |  |  |
| 1 | Vvi-Vitvi06g04368\_t001 |  |  |  | | | |  |  |  |  |  |  |
| 1 | Vvi-Vitvi06g01250\_t001 |  |  |  | | | |  |  |  |  |  |  |
| 1 | Vvi-Vitvi06g04369\_t001 |  |  |  | | | |  |  |  |  |  |  |
| 1 | Vvi-Vitvi06g04370\_t001 |  |  |  | | | |  |  |  |  |  |  |
| 1 | Vvi-Vitvi06g01251\_t001 |  |  |  | | | |  |  |  |  |  |  |
| 1 | Vvi-Vitvi06g01252\_t001 |  |  |  | Ath-AT5G12380.1 |  |  |  |  |  |  |
| 1 | Vvi-Vitvi06g01253\_t001 |  |  |  | | | |  |  |  |  |  |  |
| 1 | Vvi-Vitvi06g01254\_t001 |  |  |  | Ath-AT5G12390.1 |  |  |  |  |  |  |
| 0 | Vvi-Vitvi06g04371\_t001 |  |  |  |  |  |  |  |  |
| 0 | Vvi-Vitvi06g01255\_t001 |  |  |  |  |  |  |  |  |
| 0 | Vvi-Vitvi06g04372\_t001 |  |  |  |  |  |  |  |  |
| 0 | Vvi-Vitvi06g04373\_t001 |  |  |  |  |  |  |  |  |
| 0 | Vvi-Vitvi06g04374\_t001 |  |  |  |  |  |  |  |  |
| 0 | Vvi-Vitvi06g04375\_t001 |  |  |  |  |  |  |  |  |
| 0 | Vvi-Vitvi06g04376\_t001 |  |  |  |  |  |  |  |  |
| 0 | Vvi-Vitvi06g04377\_t001 |  |  |  |  |  |  |  |  |
| 0 | Vvi-Vitvi06g04378\_t001 |  |  |  |  |  |  |  |  |
| 0 | Vvi-Vitvi06g01913\_t001 |  |  |  |  |  |  |  |  |
| 0 | Vvi-Vitvi06g04379\_t001 |  |  |  |  |  |  |  |  |
| 0 | Vvi-Vitvi06g01259\_t001 |  |  |  |  |  |  |  |  |
| 0 | Vvi-Vitvi06g04380\_t001 |  |  |  |  |  |  |  |  |
| 0 | Vvi-Vitvi06g04381\_t001 |  |  |  |  |  |  |  |  |
| 0 | Vvi-Vitvi06g04382\_t001 |  |  |  |  |  |  |  |  |
| 0 | Vvi-Vitvi06g01260\_t001 |  |  |  |  |  |  |  |  |
| 0 | Vvi-Vitvi06g04383\_t001 |  |  |  |  |  |  |  |  |
| 0 | Vvi-Vitvi06g01261\_t002 |  |  |  |  |  |  |  |  |
| 0 | Vvi-Vitvi06g01262\_t002 |  |  |  |  |  |  |  |  |
| 0 | Vvi-Vitvi06g04384\_t001 |  |  |  |  |  |  |  |  |
| 0 | Vvi-Vitvi06g01263\_t001 |  |  |  |  |  |  |  |  |
| 0 | Vvi-Vitvi06g04385\_t001 |  |  |  |  |  |  |  |  |
| 0 | Vvi-Vitvi06g01264\_t001 |  |  |  |  |  |  |  |  |
| 0 | Vvi-Vitvi06g01265\_t001 |  |  |  |  |  |  |  |  |
| 0 | Vvi-Vitvi06g01266\_t002 |  |  |  |  |  |  |  |  |
| 0 | Vvi-Vitvi06g04386\_t001 |  |  |  |  |  |  |  |  |
| 0 | Vvi-Vitvi06g01267\_t001 |  |  |  |  |  |  |  |  |
| 0 | Vvi-Vitvi06g01268\_t001 |  |  |  |  |  |  |  |  |
| 0 | Vvi-Vitvi06g01269\_t001 |  |  |  |  |  |  |  |  |
| 0 | Vvi-Vitvi06g01270\_t001 |  |  |  |  |  |  |  |  |
| 0 | Vvi-Vitvi06g01271\_t001 |  |  |  |  |  |  |  |  |
| 0 | Vvi-Vitvi06g01272\_t001 |  |  |  |  |  |  |  |  |
| 0 | Vvi-Vitvi06g01273\_t001 |  |  |  |  |  |  |  |  |
| 0 | Vvi-Vitvi06g01274\_t001 |  |  |  |  |  |  |  |  |
| 0 | Vvi-Vitvi06g01275\_t001 |  |  |  |  |  |  |  |  |
| 0 | Vvi-Vitvi06g01276\_t001 |  |  |  |  |  |  |  |  |
| 0 | Vvi-Vitvi06g01279\_t001 |  |  |  |  |  |  |  |  |
| 0 | Vvi-Vitvi06g04387\_t001 |  |  |  |  |  |  |  |  |
| 0 | Vvi-Vitvi06g04388\_t001 |  |  |  |  |  |  |  |  |
| 0 | Vvi-Vitvi06g04389\_t001 |  |  |  |  |  |  |  |  |
| 0 | Vvi-Vitvi06g04390\_t001 |  |  |  |  |  |  |  |  |
| 0 | Vvi-Vitvi06g04391\_t001 |  |  |  |  |  |  |  |  |
| 0 | Vvi-Vitvi06g01917\_t001 |  |  |  |  |  |  |  |  |
| 0 | Vvi-Vitvi06g01918\_t001 |  |  |  |  |  |  |  |  |
| 0 | Vvi-Vitvi06g04392\_t001 |  |  |  |  |  |  |  |  |
| 0 | Vvi-Vitvi06g01281\_t001 |  |  |  |  |  |  |  |  |
| 0 | Vvi-Vitvi06g04393\_t001 |  |  |  |  |  |  |  |  |
| 0 | Vvi-Vitvi06g04394\_t001 |  |  |  |  |  |  |  |  |
| 0 | Vvi-Vitvi06g01282\_t001 |  |  |  |  |  |  |  |  |
| 0 | Vvi-Vitvi06g01283\_t001 |  |  |  |  |  |  |  |  |
| 0 | Vvi-Vitvi06g04395\_t001 |  |  |  |  |  |  |  |  |
| 0 | Vvi-Vitvi06g01285\_t001 |  |  |  |  |  |  |  |  |
| 0 | Vvi-Vitvi06g01286\_t001 |  |  |  |  |  |  |  |  |
| 0 | Vvi-Vitvi06g01288\_t001 |  |  |  |  |  |  |  |  |
| 0 | Vvi-Vitvi06g01289\_t001 |  |  |  |  |  |  |  |  |
| 0 | Vvi-Vitvi06g01290\_t001 |  |  |  |  |  |  |  |  |
| 0 | Vvi-Vitvi06g01293\_t001 |  |  |  |  |  |  |  |  |
| 0 | Vvi-Vitvi06g04396\_t001 |  |  |  |  |  |  |  |  |
| 1 | Vvi-Vitvi06g01295\_t001 |  | Ath-AT2G28000.1 |  |  |  |  |  |  |  |
| 2 | Vvi-Vitvi06g01297\_t001 |  | | | |  | Ath-AT3G45090.1 |  |  |  |  |  |  |
| 2 | Vvi-Vitvi06g04397\_t001 |  | | | |  | | | |  |  |  |  |  |  |
| 2 | Vvi-Vitvi06g01298\_t001 |  | Ath-AT2G28060.1 |  | | | |  |  |  |  |  |  |
| 3 | Vvi-Vitvi06g01299\_t001 |  | | | |  | Ath-AT3G45060.1 |  | Ath-AT1G08090.1 |  |  |  |  |  |
| 3 | Vvi-Vitvi06g04398\_t001 |  | | | |  | | | |  | | | |  |  |  |  |  |
| 3 | Vvi-Vitvi06g01301\_t001 |  | | | |  | | | |  | | | |  |  |  |  |  |
| 3 | Vvi-Vitvi06g01302\_t001 |  | | | |  | Ath-AT3G45050.4 |  | | | |  |  |  |  |  |
| 3 | Vvi-Vitvi06g04399\_t001 |  | | | |  | | | |  | | | |  |  |  |  |  |
| 3 | Vvi-Vitvi06g01303\_t001 |  | | | |  | | | |  | | | |  |  |  |  |  |
| 3 | Vvi-Vitvi06g01306\_t001 |  | Ath-AT2G28070.1 |  | | | |  | | | |  |  |  |  |  |
| 4 | Vvi-Vitvi06g01307\_t001 |  | Ath-AT2G28080.1 |  | | | |  | | | |  | Ath-AT2G36970.1 |  |  |  |  |
| 4 | Vvi-Vitvi06g01309\_t001 |  | | | |  | Ath-AT3G45040.1 |  | | | |  | | | |  |  |  |  |
| 4 | Vvi-Vitvi06g01310\_t001 |  | Ath-AT2G28085.1 |  | | | |  | | | |  | | | |  |  |  |  |
| 4 | Vvi-Vitvi06g01312\_t001 |  | | | |  | | | |  | | | |  | | | |  |  |  |  |
| 4 | Vvi-Vitvi06g01313\_t001 |  | | | |  | | | |  | | | |  | | | |  |  |  |  |
| 4 | Vvi-Vitvi06g04400\_t001 |  | | | |  | | | |  | | | |  | | | |  |  |  |  |
| 4 | Vvi-Vitvi06g01314\_t001 |  | | | |  | Ath-AT3G45020.1 |  | | | |  | | | |  |  |  |  |
| 4 | Vvi-Vitvi06g04401\_t001 |  | | | |  | | | |  | | | |  | | | |  |  |  |  |
| 4 | Vvi-Vitvi06g01315\_t001 |  | Ath-AT2G28090.1 |  | | | |  | | | |  | Ath-AT2G36950.1 |  |  |  |  |
| 4 | Vvi-Vitvi06g04402\_t001 |  | | | |  | | | |  | | | |  | | | |  |  |  |  |
| 4 | Vvi-Vitvi06g01318\_t001 |  | | | |  | | | |  | | | |  | | | |  |  |  |  |
| 4 | Vvi-Vitvi06g01320\_t002 |  | | | |  | | | |  | Ath-AT1G08110.4 |  | | | |  |  |  |  |
| 5 | Vvi-Vitvi06g01321\_t001 |  | | | |  | | | |  | | | |  | Ath-AT2G36890.2 |  | Ath-AT5G23000.1 |  |  |  |
| 5 | Vvi-Vitvi06g04403\_t001 |  | | | |  | | | |  | | | |  | | | |  | | | |  |  |  |
| 5 | Vvi-Vitvi06g01322\_t001 |  | | | |  | | | |  | | | |  | | | |  | | | |  |  |  |
| 5 | Vvi-Vitvi06g01323\_t001 |  | Ath-AT2G28100.1 |  | | | |  | | | |  | | | |  | | | |  |  |  |
| 5 | Vvi-Vitvi06g01324\_t002 |  | | | |  | | | |  | Ath-AT1G08125.2 |  | | | |  | | | |  |  |  |
| 5 | Vvi-Vitvi06g04404\_t001 |  | | | |  | | | |  | | | |  | | | |  | | | |  |  |  |
| 5 | Vvi-Vitvi06g01325\_t001 |  | | | |  | | | |  | | | |  | Ath-AT2G36880.1 |  | | | |  |  |  |
| 5 | Vvi-Vitvi06g01327\_t001 |  | Ath-AT2G28105.1 |  | | | |  | | | |  | | | |  | | | |  |  |  |
| 5 | Vvi-Vitvi06g01924\_t001 |  | | | |  | Ath-AT3G45010.1 |  | | | |  | | | |  | Ath-AT5G22960.1 |  |  |  |
| 5 | Vvi-Vitvi06g04405\_t001 |  | | | |  | | | |  | | | |  | | | |  | | | |  |  |  |
| 5 | Vvi-Vitvi06g01328\_t002 |  | | | |  | Ath-AT3G45000.1 |  | | | |  | | | |  | Ath-AT5G22950.1 |  |  |  |
| 5 | Vvi-Vitvi06g01329\_t001 |  | | | |  | Ath-AT3G44990.1 |  | | | |  | Ath-AT2G36870.1 |  | | | |  |  |  |
| 5 | Vvi-Vitvi06g01330\_t001 |  | Ath-AT2G28110.1 |  | | | |  | | | |  | | | |  | Ath-AT5G22940.2 |  |  |  |
| 5 | Vvi-Vitvi06g01331\_t001 |  | | | |  | | | |  | | | |  | | | |  | | | |  |  |  |
| 5 | Vvi-Vitvi06g04406\_t001 |  | | | |  | | | |  | | | |  | | | |  | | | |  |  |  |
| 5 | Vvi-Vitvi06g01334\_t001 |  | Ath-AT2G28120.1 |  | | | |  | | | |  | | | |  | | | |  |  |  |
| 5 | Vvi-Vitvi06g01927\_t001 |  | | | |  | | | |  | | | |  | | | |  | | | |  |  |  |
| 5 | Vvi-Vitvi06g01335\_t001 |  | Ath-AT2G28130.1 |  | | | |  | | | |  | | | |  | | | |  |  |  |
| 5 | Vvi-Vitvi06g01928\_t001 |  | | | |  | Ath-AT3G44960.2 |  | | | |  | | | |  | | | |  |  |  |
| 5 | Vvi-Vitvi06g01929\_t001 |  | | | |  | | | |  | | | |  | | | |  | | | |  |  |  |
| 5 | Vvi-Vitvi06g01930\_t003 |  | | | |  | | | |  | | | |  | | | |  | | | |  |  |  |
| 6 | Vvi-Vitvi06g01340\_t001 |  | Ath-AT2G28140.1 |  | Ath-AT3G44940.1 |  | | | |  | | | |  | Ath-AT5G22930.1 |  | Ath-AT2G28140.1 |  |  |
| 6 | Vvi-Vitvi06g01341\_t001 |  | | | |  | | | |  | Ath-AT1G08130.1 |  | | | |  | | | |  | | | |  |  |
| 6 | Vvi-Vitvi06g01343\_t001 |  | Ath-AT2G28150.1 |  | | | |  | | | |  | | | |  | | | |  | | | |  |  |
| 6 | Vvi-Vitvi06g04407\_t001 |  | | | |  | | | |  | | | |  | | | |  | | | |  | | | |  |  |
| 6 | Vvi-Vitvi06g01344\_t001 |  | | | |  | | | |  | | | |  | | | |  | Ath-AT5G22920.1 |  | | | |  |  |
| 6 | Vvi-Vitvi06g01345\_t001 |  | Ath-AT2G28160.1 |  | | | |  | | | |  | | | |  | | | |  | | | |  |  |
| 6 | Vvi-Vitvi06g01346\_t001 |  | | | |  | | | |  | | | |  | Ath-AT2G36830.1 |  | | | |  | | | |  |  |
| 6 | Vvi-Vitvi06g04408\_t001 |  | | | |  | | | |  | | | |  | | | |  | | | |  | | | |  |  |
| 6 | Vvi-Vitvi06g01348\_t001 |  | | | |  | | | |  | | | |  | | | |  | | | |  | | | |  |  |
| 6 | Vvi-Vitvi06g01349\_t001 |  | Ath-AT2G28190.1 |  | | | |  | | | |  | | | |  | | | |  | | | |  |  |
| 6 | Vvi-Vitvi06g01350\_t001 |  | Ath-AT2G28200.1 |  | | | |  | | | |  | | | |  | | | |  | | | |  |  |
| 5 | Vvi-Vitvi06g04409\_t001 |  |  |  | | | |  | | | |  | | | |  | | | |  | | | |  |  |
| 5 | Vvi-Vitvi06g01351\_t001 |  |  |  | Ath-AT3G44890.1 |  | | | |  | | | |  | | | |  | | | |  |  |
| 5 | Vvi-Vitvi06g01353\_t001 |  |  |  | | | |  | | | |  | | | |  | Ath-AT5G22890.1 |  | | | |  |  |
| 5 | Vvi-Vitvi06g01354\_t001 |  |  |  | Ath-AT3G44880.1 |  | | | |  | | | |  | | | |  | | | |  |  |
| 4 | Vvi-Vitvi06g04410\_t001 |  |  |  |  |  | | | |  | | | |  | Ath-AT5G22875.2 |  | | | |  |  |
| 4 | Vvi-Vitvi06g04411\_t001 |  |  |  |  |  | | | |  | | | |  | | | |  | | | |  |  |
| 4 | Vvi-Vitvi06g01933\_t001 |  |  |  |  |  | | | |  | Ath-AT2G36720.2 |  | | | |  | Ath-AT2G27980.1 |  |  |
| 3 | Vvi-Vitvi06g04412\_t001 |  |  |  |  |  | | | |  |  |  | | | |  | | | |  |  |
| 3 | Vvi-Vitvi06g01934\_t001 |  |  |  |  |  | | | |  |  |  | | | |  | | | |  |  |
| 3 | Vvi-Vitvi06g01357\_t001 |  |  |  |  |  | Ath-AT1G08160.1 |  |  |  | Ath-AT5G22870.1 |  | | | |  |  |
| 3 | Vvi-Vitvi06g04413\_t001 |  |  |  |  |  | | | |  |  |  | | | |  | | | |  |  |
| 3 | Vvi-Vitvi06g01358\_t001 |  |  |  |  |  | | | |  |  |  | | | |  | Ath-AT2G27950.1 |  |  |
| 3 | Vvi-Vitvi06g01359\_t001 |  |  |  |  |  | | | |  |  |  | | | |  | Ath-AT2G27940.1 |  |  |
| 3 | Vvi-Vitvi06g04414\_t001 |  |  |  |  |  | | | |  |  |  | | | |  | | | |  |  |
| 3 | Vvi-Vitvi06g01360\_t001 |  |  |  |  |  | Ath-AT1G08170.1 |  |  |  | | | |  | | | |  |  |
| 3 | Vvi-Vitvi06g04415\_t001 |  |  |  |  |  | | | |  |  |  | | | |  | | | |  |  |
| 3 | Vvi-Vitvi06g01361\_t001 |  |  |  |  |  | | | |  |  |  | | | |  | | | |  |  |
| 4 | Vvi-Vitvi06g01362\_t001 |  | Ath-AT3G45010.1 |  |  |  | | | |  |  |  | | | |  | Ath-AT2G27920.1 |  |  |
| 4 | Vvi-Vitvi06g04416\_t001 |  | | | |  |  |  | | | |  |  |  | | | |  | Ath-AT2G27900.1 |  |  |
| 4 | Vvi-Vitvi06g04417\_t001 |  | | | |  |  |  | | | |  |  |  | | | |  | | | |  |  |
| 4 | Vvi-Vitvi06g04418\_t001 |  | | | |  |  |  | | | |  |  |  | | | |  | | | |  |  |
| 4 | Vvi-Vitvi06g04419\_t001 |  | | | |  |  |  | | | |  |  |  | | | |  | | | |  |  |
| 4 | Vvi-Vitvi06g04420\_t001 |  | | | |  |  |  | | | |  |  |  | | | |  | | | |  |  |
| 4 | Vvi-Vitvi06g01364\_t001 |  | | | |  |  |  | Ath-AT1G08190.1 |  |  |  | | | |  | | | |  |  |
| 4 | Vvi-Vitvi06g01365\_t001 |  | | | |  |  |  | | | |  |  |  | Ath-AT5G22860.1 |  | | | |  |  |
| 4 | Vvi-Vitvi06g01367\_t001 |  | | | |  |  |  | | | |  |  |  | | | |  | | | |  |  |
| 4 | Vvi-Vitvi06g01935\_t001 |  | | | |  |  |  | | | |  |  |  | | | |  | | | |  |  |
| 4 | Vvi-Vitvi06g01936\_t001 |  | | | |  |  |  | | | |  |  |  | | | |  | | | |  |  |
| 4 | Vvi-Vitvi06g04421\_t001 |  | | | |  |  |  | | | |  |  |  | | | |  | | | |  |  |
| 4 | Vvi-Vitvi06g04422\_t001 |  | | | |  |  |  | | | |  |  |  | | | |  | | | |  |  |
| 4 | Vvi-Vitvi06g01374\_t001 |  | | | |  |  |  | | | |  |  |  | | | |  | | | |  |  |
| 4 | Vvi-Vitvi06g01937\_t001 |  | | | |  |  |  | | | |  |  |  | | | |  | | | |  |  |
| 4 | Vvi-Vitvi06g01375\_t001 |  | | | |  |  |  | | | |  |  |  | | | |  | | | |  |  |
| 4 | Vvi-Vitvi06g01376\_t001 |  | | | |  |  |  | Ath-AT1G08220.1 |  |  |  | | | |  | | | |  |  |
| 4 | Vvi-Vitvi06g01377\_t001 |  | | | |  |  |  | | | |  |  |  | Ath-AT5G22850.1 |  | | | |  |  |
| 4 | Vvi-Vitvi06g01378\_t001 |  | | | |  |  |  | | | |  |  |  | | | |  | Ath-AT2G27880.1 |  |  |
| 4 | Vvi-Vitvi06g01380\_t001 |  | Ath-AT3G44850.1 |  |  |  | | | |  |  |  | Ath-AT5G22840.1 |  | | | |  |  |
| 4 | Vvi-Vitvi06g01383\_t003 |  | | | |  |  |  | | | |  |  |  | Ath-AT5G22830.2 |  | | | |  |  |
| 4 | Vvi-Vitvi06g01384\_t001 |  | | | |  |  |  | | | |  |  |  | | | |  | | | |  |  |
| 4 | Vvi-Vitvi06g01385\_t001 |  | | | |  |  |  | | | |  |  |  | | | |  | | | |  |  |
| 4 | Vvi-Vitvi06g04423\_t001 |  | | | |  |  |  | | | |  |  |  | | | |  | | | |  |  |
| 4 | Vvi-Vitvi06g01386\_t001 |  | | | |  |  |  | | | |  |  |  | Ath-AT5G22820.3 |  | | | |  |  |
| 4 | Vvi-Vitvi06g04424\_t001 |  | | | |  |  |  | | | |  |  |  | | | |  | | | |  |  |
| 4 | Vvi-Vitvi06g04425\_t001 |  | | | |  |  |  | | | |  |  |  | | | |  | | | |  |  |
| 4 | Vvi-Vitvi06g01939\_t001 |  | | | |  |  |  | | | |  |  |  | | | |  | | | |  |  |
| 4 | Vvi-Vitvi06g01940\_t001 |  | | | |  |  |  | | | |  |  |  | | | |  | | | |  |  |
| 4 | Vvi-Vitvi06g01387\_t001 |  | | | |  |  |  | | | |  |  |  | Ath-AT5G22810.1 |  | | | |  |  |
| 4 | Vvi-Vitvi06g01388\_t001 |  | Ath-AT3G44830.1 |  |  |  | | | |  |  |  | | | |  | | | |  |  |
| 4 | Vvi-Vitvi06g01389\_t001 |  | | | |  |  |  | | | |  |  |  | | | |  | Ath-AT2G27860.1 |  |  |
| 4 | Vvi-Vitvi06g01942\_t001 |  | Ath-AT3G44820.1 |  |  |  | | | |  |  |  | | | |  | | | |  |  |
| 4 | Vvi-Vitvi06g01943\_t001 |  | | | |  |  |  | | | |  |  |  | | | |  | | | |  |  |
| 4 | Vvi-Vitvi06g01391\_t001 |  | | | |  |  |  | | | |  |  |  | Ath-AT5G22790.1 |  | | | |  |  |
| 4 | Vvi-Vitvi06g01392\_t001 |  | | | |  |  |  | | | |  |  |  | Ath-AT5G22770.4 |  | | | |  |  |
| 4 | Vvi-Vitvi06g01394\_t003 |  | | | |  |  |  | | | |  |  |  | Ath-AT5G22760.1 |  | | | |  |  |
| 4 | Vvi-Vitvi06g01395\_t001 |  | | | |  |  |  | | | |  |  |  | Ath-AT5G22750.1 |  | | | |  |  |
| 4 | Vvi-Vitvi06g01944\_t001 |  | | | |  |  |  | | | |  |  |  | | | |  | | | |  |  |
| 4 | Vvi-Vitvi06g01396\_t001 |  | | | |  |  |  | Ath-AT1G08230.2 |  |  |  | | | |  | | | |  |  |
| 4 | Vvi-Vitvi06g01397\_t001 |  | | | |  |  |  | | | |  |  |  | | | |  | | | |  |  |
| 4 | Vvi-Vitvi06g04426\_t001 |  | | | |  |  |  | | | |  |  |  | | | |  | | | |  |  |
| 4 | Vvi-Vitvi06g04427\_t001 |  | | | |  |  |  | | | |  |  |  | | | |  | | | |  |  |
| 4 | Vvi-Vitvi06g01398\_t001 |  | | | |  |  |  | | | |  |  |  | Ath-AT5G22740.1 |  | | | |  |  |
| 4 | Vvi-Vitvi06g01399\_t001 |  | Ath-AT3G44750.1 |  |  |  | | | |  |  |  | Ath-AT5G22650.1 |  | Ath-AT2G27840.3 |  |  |
| 4 | Vvi-Vitvi06g01400\_t001 |  | | | |  |  |  | | | |  |  |  | | | |  | Ath-AT2G27830.1 |  |  |
| 4 | Vvi-Vitvi06g01401\_t001 |  | | | |  |  |  | | | |  |  |  | Ath-AT5G22640.1 |  | | | |  |  |
| 5 | Vvi-Vitvi06g01402\_t001 |  | | | |  | Ath-AT2G36490.1 |  | | | |  |  |  | | | |  | | | |  |  |
| 5 | Vvi-Vitvi06g01403\_t001 |  | | | |  | | | |  | | | |  |  |  | | | |  | | | |  |  |
| 5 | Vvi-Vitvi06g01945\_t001 |  | Ath-AT3G44735.1 |  | | | |  | | | |  |  |  | | | |  | | | |  |  |
| 5 | Vvi-Vitvi06g01946\_t001 |  | Ath-AT3G44720.1 |  | | | |  | Ath-AT1G08250.1 |  |  |  | Ath-AT5G22630.1 |  | Ath-AT2G27820.1 |  |  |
| 4 | Vvi-Vitvi06g01404\_t001 |  | | | |  | | | |  |  |  |  |  | | | |  | Ath-AT2G27810.4 |  |  |
| 4 | Vvi-Vitvi06g01947\_t001 |  | | | |  | | | |  |  |  |  |  | | | |  | | | |  |  |
| 4 | Vvi-Vitvi06g01406\_t001 |  | | | |  | | | |  |  |  |  |  | | | |  | | | |  |  |
| 4 | Vvi-Vitvi06g01407\_t001 |  | | | |  | | | |  |  |  |  |  | Ath-AT5G22620.5 |  | | | |  |  |
| 4 | Vvi-Vitvi06g01948\_t001 |  | | | |  | | | |  |  |  |  |  | | | |  | | | |  |  |
| 4 | Vvi-Vitvi06g01408\_t001 |  | | | |  | Ath-AT2G36470.1 |  |  |  |  |  | | | |  | Ath-AT2G27770.1 |  |  |
| 4 | Vvi-Vitvi06g01409\_t001 |  | Ath-AT3G44716.1 |  | | | |  |  |  |  |  | | | |  | | | |  |  |
| 4 | Vvi-Vitvi06g01410\_t001 |  | | | |  | | | |  |  |  |  |  | Ath-AT5G22580.1 |  | | | |  |  |
| 4 | Vvi-Vitvi06g01411\_t001 |  | | | |  | | | |  |  |  |  |  | | | |  | | | |  |  |
| 4 | Vvi-Vitvi06g01414\_t001 |  | | | |  | | | |  |  |  |  |  | | | |  | | | |  |  |
| 4 | Vvi-Vitvi06g01415\_t001 |  | | | |  | | | |  |  |  |  |  | | | |  | Ath-AT2G27760.1 |  |  |
| 4 | Vvi-Vitvi06g01416\_t001 |  | | | |  | | | |  |  |  |  |  | | | |  | | | |  |  |
| 4 | Vvi-Vitvi06g01417\_t001 |  | | | |  | | | |  |  |  |  |  | | | |  | | | |  |  |
| 4 | Vvi-Vitvi06g01418\_t001 |  | | | |  | Ath-AT2G36410.1 |  |  |  |  |  | | | |  | Ath-AT2G27740.1 |  |  |
| 4 | Vvi-Vitvi06g01419\_t001 |  | | | |  | | | |  |  |  |  |  | | | |  | | | |  |  |
| 4 | Vvi-Vitvi06g01420\_t001 |  | | | |  | Ath-AT2G36380.1 |  |  |  |  |  | | | |  | | | |  |  |
| 4 | Vvi-Vitvi06g04428\_t001 |  | | | |  | | | |  |  |  |  |  | | | |  | | | |  |  |
| 4 | Vvi-Vitvi06g04429\_t001 |  | | | |  | | | |  |  |  |  |  | | | |  | | | |  |  |
| 4 | Vvi-Vitvi06g04430\_t001 |  | | | |  | | | |  |  |  |  |  | | | |  | | | |  |  |
| 4 | Vvi-Vitvi06g01949\_t001 |  | | | |  | | | |  |  |  |  |  | | | |  | | | |  |  |
| 4 | Vvi-Vitvi06g01950\_t001 |  | | | |  | | | |  |  |  |  |  | | | |  | | | |  |  |
| 4 | Vvi-Vitvi06g04431\_t001 |  | | | |  | | | |  |  |  |  |  | | | |  | | | |  |  |
| 4 | Vvi-Vitvi06g04432\_t001 |  | Ath-AT3G44680.1 |  | | | |  |  |  |  |  | | | |  | | | |  |  |
| 4 | Vvi-Vitvi06g01427\_t001 |  | | | |  | | | |  |  |  |  |  | Ath-AT5G22510.1 |  | | | |  |  |
| 4 | Vvi-Vitvi06g01428\_t001 |  | Ath-AT3G44620.2 |  | | | |  |  |  |  |  | | | |  | | | |  |  |
| 4 | Vvi-Vitvi06g01429\_t001 |  | | | |  | Ath-AT2G36350.1 |  |  |  |  |  | | | |  | | | |  |  |
| 4 | Vvi-Vitvi06g04433\_t001 |  | | | |  | | | |  |  |  |  |  | | | |  | | | |  |  |
| 4 | Vvi-Vitvi06g01430\_t001 |  | Ath-AT3G44610.1 |  | | | |  |  |  |  |  | | | |  | | | |  |  |
| 4 | Vvi-Vitvi06g04434\_t001 |  | Ath-AT3G44600.1 |  | Ath-AT2G36130.1 |  |  |  |  |  | | | |  | | | |  |  |
| 3 | Vvi-Vitvi06g01433\_t001 |  | Ath-AT3G44590.1 |  |  |  |  |  |  |  | | | |  | Ath-AT2G27710.2 |  |  |
| 3 | Vvi-Vitvi06g01435\_t001 |  | | | |  |  |  |  |  |  |  | | | |  | | | |  |  |
| 3 | Vvi-Vitvi06g01442\_t002 |  | | | |  |  |  |  |  |  |  | | | |  | | | |  |  |
| 3 | Vvi-Vitvi06g01443\_t001 |  | | | |  |  |  |  |  |  |  | | | |  | | | |  |  |
| 3 | Vvi-Vitvi06g01444\_t001 |  | | | |  |  |  |  |  |  |  | | | |  | Ath-AT2G27680.1 |  |  |
| 3 | Vvi-Vitvi06g01952\_t001 |  | | | |  |  |  |  |  |  |  | | | |  | | | |  |  |
| 3 | Vvi-Vitvi06g01446\_t001 |  | | | |  |  |  |  |  |  |  | | | |  | | | |  |  |
| 3 | Vvi-Vitvi06g01447\_t001 |  | | | |  |  |  |  |  |  |  | | | |  | | | |  |  |
| 3 | Vvi-Vitvi06g01448\_t001 |  | | | |  |  |  |  |  |  |  | | | |  | | | |  |  |
| 3 | Vvi-Vitvi06g01449\_t001 |  | | | |  |  |  |  |  |  |  | | | |  | | | |  |  |
| 3 | Vvi-Vitvi06g04435\_t001 |  | | | |  |  |  |  |  |  |  | | | |  | | | |  |  |
| 3 | Vvi-Vitvi06g04436\_t001 |  | | | |  |  |  |  |  |  |  | | | |  | | | |  |  |
| 3 | Vvi-Vitvi06g01953\_t001 |  | | | |  |  |  |  |  |  |  | | | |  | | | |  |  |
| 3 | Vvi-Vitvi06g01954\_t001 |  | | | |  |  |  |  |  |  |  | | | |  | | | |  |  |
| 3 | Vvi-Vitvi06g01451\_t001 |  | | | |  |  |  |  |  |  |  | | | |  | | | |  |  |
| 3 | Vvi-Vitvi06g01452\_t001 |  | | | |  |  |  |  |  |  |  | | | |  | | | |  |  |
| 3 | Vvi-Vitvi06g01453\_t001 |  | | | |  |  |  |  |  |  |  | | | |  | | | |  |  |
| 3 | Vvi-Vitvi06g01454\_t001 |  | | | |  |  |  |  |  |  |  | | | |  | | | |  |  |
| 3 | Vvi-Vitvi06g01455\_t001 |  | Ath-AT3G44540.1 |  |  |  |  |  |  |  | Ath-AT5G22500.1 |  | | | |  |  |
| 3 | Vvi-Vitvi06g01456\_t001 |  | | | |  |  |  |  |  |  |  | | | |  | | | |  |  |
| 3 | Vvi-Vitvi06g01457\_t002 |  | Ath-AT3G44530.1 |  |  |  |  |  |  |  | | | |  | | | |  |  |
| 3 | Vvi-Vitvi06g01458\_t001 |  | | | |  |  |  |  |  |  |  | | | |  | Ath-AT2G27610.1 |  |  |
| 3 | Vvi-Vitvi06g01459\_t001 |  | | | |  |  |  |  |  |  |  | | | |  | | | |  |  |
| 3 | Vvi-Vitvi06g01460\_t001 |  | | | |  |  |  |  |  |  |  | | | |  | Ath-AT2G27600.1 |  |  |
| 3 | Vvi-Vitvi06g01462\_t001 |  | | | |  |  |  |  |  |  |  | | | |  | | | |  |  |
| 3 | Vvi-Vitvi06g04437\_t001 |  | | | |  |  |  |  |  |  |  | | | |  | | | |  |  |
| 3 | Vvi-Vitvi06g01464\_t001 |  | | | |  |  |  |  |  |  |  | | | |  | | | |  |  |
| 3 | Vvi-Vitvi06g01465\_t001 |  | | | |  |  |  |  |  |  |  | | | |  | | | |  |  |
| 3 | Vvi-Vitvi06g01958\_t001 |  | | | |  |  |  |  |  |  |  | | | |  | | | |  |  |
| 3 | Vvi-Vitvi06g01467\_t001 |  | | | |  |  |  |  |  |  |  | | | |  | Ath-AT2G27590.1 |  |  |
| 4 | Vvi-Vitvi06g01468\_t001 |  | | | |  | Ath-AT1G08200.1 |  |  |  |  |  | | | |  | | | |  |  |
| 4 | Vvi-Vitvi06g01469\_t001 |  | | | |  | Ath-AT1G08280.1 |  |  |  |  |  | | | |  | | | |  |  |
| 4 | Vvi-Vitvi06g01470\_t001 |  | | | |  | | | |  |  |  |  |  | Ath-AT5G22470.1 |  | | | |  |  |
| 4 | Vvi-Vitvi06g01471\_t001 |  | | | |  | Ath-AT1G08290.1 |  |  |  |  |  | | | |  | | | |  |  |
| 4 | Vvi-Vitvi06g01472\_t001 |  | | | |  | | | |  |  |  |  |  | | | |  | | | |  |  |
| 4 | Vvi-Vitvi06g01473\_t001 |  | | | |  | | | |  |  |  |  |  | | | |  | Ath-AT2G27550.1 |  |  |
| 4 | Vvi-Vitvi06g04438\_t001 |  | | | |  | | | |  |  |  |  |  | Ath-AT5G22460.1 |  | | | |  |  |
| 4 | Vvi-Vitvi06g01476\_t001 |  | Ath-AT3G44510.2 |  | | | |  |  |  |  |  | | | |  | | | |  |  |
| 4 | Vvi-Vitvi06g01477\_t001 |  | | | |  | Ath-AT1G08310.2 |  |  |  |  |  | | | |  | | | |  |  |
| 4 | Vvi-Vitvi06g01478\_t001 |  | Ath-AT3G44460.1 |  | | | |  |  |  |  |  | | | |  | | | |  |  |
| 4 | Vvi-Vitvi06g04439\_t001 |  | | | |  | | | |  |  |  |  |  | | | |  | | | |  |  |
| 4 | Vvi-Vitvi06g01479\_t001 |  | | | |  | | | |  |  |  |  |  | Ath-AT5G22450.2 |  | | | |  |  |
| 4 | Vvi-Vitvi06g01480\_t001 |  | | | |  | Ath-AT1G08320.4 |  |  |  |  |  | | | |  | | | |  |  |
| 4 | Vvi-Vitvi06g01481\_t001 |  | | | |  | | | |  |  |  |  |  | | | |  | | | |  |  |
| 4 | Vvi-Vitvi06g04440\_t001 |  | | | |  | | | |  |  |  |  |  | | | |  | | | |  |  |
| 4 | Vvi-Vitvi06g04441\_t001 |  | | | |  | | | |  |  |  |  |  | | | |  | | | |  |  |
| 4 | Vvi-Vitvi06g01485\_t001 |  | | | |  | Ath-AT1G08350.2 |  |  |  |  |  | | | |  | | | |  |  |
| 4 | Vvi-Vitvi06g01959\_t001 |  | Ath-AT3G44450.1 |  | | | |  |  |  |  |  | | | |  | | | |  |  |
| 4 | Vvi-Vitvi06g01486\_t002 |  | | | |  | Ath-AT1G08360.1 |  |  |  |  |  | Ath-AT5G22440.1 |  | Ath-AT2G27530.2 |  |  |
| 3 | Vvi-Vitvi06g01487\_t002 |  | | | |  |  |  |  |  |  |  | | | |  | Ath-AT2G27510.1 |  |  |
| 3 | Vvi-Vitvi06g01489\_t001 |  | | | |  |  |  |  |  |  |  | | | |  | Ath-AT2G27500.1 |  |  |
| 3 | Vvi-Vitvi06g01491\_t001 |  | | | |  |  |  |  |  |  |  | | | |  | | | |  |  |
| 3 | Vvi-Vitvi06g01961\_t001 |  | | | |  |  |  |  |  |  |  | | | |  | Ath-AT2G27480.1 |  |  |
| 3 | Vvi-Vitvi06g01492\_t001 |  | | | |  |  |  |  |  |  |  | | | |  | Ath-AT2G27470.1 |  |  |
| 3 | Vvi-Vitvi06g01493\_t003 |  | | | |  |  |  |  |  |  |  | | | |  | | | |  |  |
| 3 | Vvi-Vitvi06g01498\_t001 |  | | | |  |  |  |  |  |  |  | | | |  | Ath-AT2G27460.1 |  |  |
| 3 | Vvi-Vitvi06g01499\_t001 |  | | | |  |  |  |  |  |  |  | | | |  | | | |  |  |
| 3 | Vvi-Vitvi06g04442\_t001 |  | | | |  |  |  |  |  |  |  | | | |  | | | |  |  |
| 3 | Vvi-Vitvi06g04443\_t001 |  | | | |  |  |  |  |  |  |  | | | |  | | | |  |  |
| 3 | Vvi-Vitvi06g01962\_t003 |  | | | |  |  |  |  |  |  |  | | | |  | | | |  |  |
| 3 | Vvi-Vitvi06g01501\_t001 |  | | | |  |  |  |  |  |  |  | | | |  | | | |  |  |
| 3 | Vvi-Vitvi06g01502\_t001 |  | | | |  |  |  |  |  |  |  | | | |  | Ath-AT2G27450.2 |  |  |
| 3 | Vvi-Vitvi06g01503\_t001 |  | | | |  |  |  |  |  |  |  | | | |  | | | |  |  |
| 4 | Vvi-Vitvi06g01504\_t001 |  | | | |  | Ath-AT3G11490.1 |  |  |  |  |  | | | |  | | | |  |  |
| 4 | Vvi-Vitvi06g01505\_t001 |  | Ath-AT3G44380.1 |  | | | |  |  |  |  |  | | | |  | | | |  |  |
| 4 | Vvi-Vitvi06g01963\_t001 |  | | | |  | | | |  |  |  |  |  | | | |  | | | |  |  |
| 4 | Vvi-Vitvi06g01506\_t001 |  | | | |  | | | |  |  |  |  |  | | | |  | | | |  |  |
| 4 | Vvi-Vitvi06g04444\_t001 |  | | | |  | | | |  |  |  |  |  | | | |  | | | |  |  |
| 4 | Vvi-Vitvi06g01965\_t001 |  | | | |  | | | |  |  |  |  |  | | | |  | | | |  |  |
| 4 | Vvi-Vitvi06g01966\_t001 |  | | | |  | | | |  |  |  |  |  | | | |  | | | |  |  |
| 4 | Vvi-Vitvi06g01507\_t001 |  | | | |  | | | |  |  |  |  |  | | | |  | | | |  |  |
| 4 | Vvi-Vitvi06g01508\_t001 |  | | | |  | | | |  |  |  |  |  | | | |  | | | |  |  |
| 4 | Vvi-Vitvi06g01509\_t001 |  | | | |  | | | |  |  |  |  |  | | | |  | | | |  |  |
| 4 | Vvi-Vitvi06g01968\_t001 |  | | | |  | | | |  |  |  |  |  | | | |  | | | |  |  |
| 4 | Vvi-Vitvi06g01510\_t001 |  | | | |  | | | |  |  |  |  |  | Ath-AT5G22410.1 |  | | | |  |  |
| 4 | Vvi-Vitvi06g01969\_t001 |  | | | |  | | | |  |  |  |  |  | | | |  | | | |  |  |
| 4 | Vvi-Vitvi06g01970\_t001 |  | | | |  | | | |  |  |  |  |  | | | |  | | | |  |  |
| 4 | Vvi-Vitvi06g01971\_t001 |  | | | |  | | | |  |  |  |  |  | | | |  | | | |  |  |
| 4 | Vvi-Vitvi06g01972\_t001 |  | | | |  | | | |  |  |  |  |  | | | |  | | | |  |  |
| 5 | Vvi-Vitvi06g04445\_t001 |  | | | |  | Ath-AT3G11510.1 |  | Ath-AT2G36160.1 |  |  |  | | | |  | | | |  |  |
| 5 | Vvi-Vitvi06g01512\_t001 |  | | | |  | | | |  | | | |  |  |  | | | |  | | | |  |  |
| 5 | Vvi-Vitvi06g01513\_t001 |  | | | |  | | | |  | | | |  |  |  | | | |  | Ath-AT2G27385.3 |  |  |
| 5 | Vvi-Vitvi16g02097\_t001 |  | | | |  | | | |  | | | |  |  |  | | | |  | | | |  |  |
| 5 | Vvi-Vitvi16g02096\_t001 |  | | | |  | | | |  | | | |  |  |  | | | |  | | | |  |  |
| 5 | Vvi-Vitvi16g02094\_t001 |  | | | |  | | | |  | | | |  |  |  | | | |  | | | |  |  |
| 5 | Vvi-Vitvi16g02093\_t001 |  | | | |  | | | |  | | | |  |  |  | | | |  | | | |  |  |
| 5 | Vvi-Vitvi06g01515\_t001 |  | Ath-AT3G44350.2 |  | | | |  | | | |  |  |  | Ath-AT5G22380.1 |  | | | |  |  |
| 5 | Vvi-Vitvi06g01516\_t001 |  | | | |  | | | |  | | | |  |  |  | | | |  | | | |  |  |
| 5 | Vvi-Vitvi06g01517\_t001 |  | | | |  | | | |  | | | |  |  |  | Ath-AT5G22360.1 |  | | | |  |  |
| 5 | Vvi-Vitvi06g01518\_t001 |  | | | |  | | | |  | | | |  |  |  | | | |  | | | |  |  |
| 5 | Vvi-Vitvi06g01520\_t001 |  | | | |  | | | |  | | | |  |  |  | | | |  | | | |  |  |
| 5 | Vvi-Vitvi06g01521\_t001 |  | | | |  | | | |  | | | |  |  |  | Ath-AT5G22350.1 |  | | | |  |  |
| 5 | Vvi-Vitvi06g01522\_t001 |  | | | |  | Ath-AT3G11550.1 |  | Ath-AT2G36100.1 |  |  |  | | | |  | Ath-AT2G27370.1 |  |  |
| 5 | Vvi-Vitvi06g01523\_t001 |  | | | |  | | | |  | | | |  |  |  | | | |  | Ath-AT2G27350.3 |  |  |
| 5 | Vvi-Vitvi06g01524\_t002 |  | | | |  | | | |  | | | |  |  |  | | | |  | | | |  |  |
| 5 | Vvi-Vitvi06g01974\_t001 |  | | | |  | | | |  | | | |  |  |  | | | |  | Ath-AT2G27330.1 |  |  |
| 5 | Vvi-Vitvi06g01526\_t001 |  | Ath-AT3G44330.1 |  | | | |  | | | |  |  |  | | | |  | | | |  |  |
| 5 | Vvi-Vitvi06g01527\_t001 |  | | | |  | | | |  | | | |  |  |  | Ath-AT5G22340.2 |  | | | |  |  |
| 5 | Vvi-Vitvi06g01529\_t002 |  | | | |  | | | |  | | | |  |  |  | Ath-AT5G22320.1 |  | | | |  |  |
| 5 | Vvi-Vitvi06g01530\_t001 |  | Ath-AT3G44326.1 |  | | | |  | Ath-AT2G36090.1 |  |  |  | | | |  | Ath-AT2G27310.1 |  |  |
| 5 | Vvi-Vitvi06g01531\_t001 |  | | | |  | | | |  | | | |  |  |  | | | |  | | | |  |  |
| 5 | Vvi-Vitvi06g01532\_t001 |  | | | |  | Ath-AT3G11590.1 |  | | | |  |  |  | Ath-AT5G22310.1 |  | | | |  |  |
| 5 | Vvi-Vitvi06g01533\_t001 |  | Ath-AT3G44300.1 |  | | | |  | | | |  |  |  | Ath-AT5G22300.1 |  | | | |  |  |
| 5 | Vvi-Vitvi06g01534\_t001 |  | | | |  | | | |  | | | |  |  |  | | | |  | | | |  |  |
| 5 | Vvi-Vitvi06g01975\_t001 |  | | | |  | | | |  | | | |  |  |  | | | |  | | | |  |  |
| 5 | Vvi-Vitvi06g01536\_t001 |  | Ath-AT3G44290.1 |  | | | |  | | | |  |  |  | Ath-AT5G22290.1 |  | Ath-AT2G27300.1 |  |  |
| 5 | Vvi-Vitvi06g01537\_t001 |  | | | |  | | | |  | | | |  |  |  | | | |  | | | |  |  |
| 5 | Vvi-Vitvi06g01538\_t001 |  | | | |  | | | |  | | | |  |  |  | | | |  | | | |  |  |
| 5 | Vvi-Vitvi06g01539\_t001 |  | | | |  | | | |  | | | |  |  |  | | | |  | | | |  |  |
| 5 | Vvi-Vitvi06g04446\_t001 |  | Ath-AT3G44280.1 |  | | | |  | | | |  |  |  | | | |  | | | |  |  |
| 5 | Vvi-Vitvi06g01542\_t001 |  | | | |  | | | |  | | | |  |  |  | | | |  | | | |  |  |
| 5 | Vvi-Vitvi06g01543\_t001 |  | | | |  | Ath-AT3G11600.1 |  | | | |  |  |  | | | |  | | | |  |  |
| 6 | Vvi-Vitvi06g01545\_t001 |  | | | |  | | | |  | Ath-AT2G36060.2 |  | Ath-AT3G52560.4 |  | | | |  | | | |  |  |
| 6 | Vvi-Vitvi06g01546\_t001 |  | Ath-AT3G44260.1 |  | | | |  | | | |  | | | |  | Ath-AT5G22250.1 |  | | | |  |  |
| 6 | Vvi-Vitvi06g01548\_t001 |  | | | |  | | | |  | Ath-AT2G36050.1 |  | Ath-AT3G52540.1 |  | | | |  | | | |  |  |
| 6 | Vvi-Vitvi06g01549\_t001 |  | | | |  | | | |  | Ath-AT2G36026.1 |  | Ath-AT3G52525.1 |  | | | |  | | | |  |  |
| 6 | Vvi-Vitvi06g01551\_t001 |  | | | |  | | | |  | | | |  | | | |  | | | |  | Ath-AT2G27290.1 |  |  |
| 6 | Vvi-Vitvi06g01552\_t001 |  | | | |  | | | |  | Ath-AT2G36010.2 |  | | | |  | Ath-AT5G22220.2 |  | | | |  |  |
| 6 | Vvi-Vitvi06g01553\_t001 |  | | | |  | | | |  | | | |  | | | |  | Ath-AT5G22210.1 |  | | | |  |  |
| 6 | Vvi-Vitvi06g01555\_t001 |  | | | |  | | | |  | | | |  | | | |  | | | |  | Ath-AT2G27280.1 |  |  |
| 6 | Vvi-Vitvi06g01556\_t001 |  | | | |  | | | |  | | | |  | Ath-AT3G52500.1 |  | | | |  | | | |  |  |
| 6 | Vvi-Vitvi06g04447\_t001 |  | | | |  | | | |  | | | |  | | | |  | | | |  | | | |  |  |
| 6 | Vvi-Vitvi06g01557\_t001 |  | | | |  | | | |  | | | |  | | | |  | | | |  | | | |  |  |
| 6 | Vvi-Vitvi06g01559\_t001 |  | Ath-AT3G44220.1 |  | Ath-AT3G11660.1 |  | Ath-AT2G35960.1 |  | Ath-AT3G52470.1 |  | Ath-AT5G22200.1 |  | | | |  |  |
| 5 | Vvi-Vitvi06g01560\_t001 |  | | | |  | | | |  |  |  | Ath-AT3G52460.1 |  | | | |  | Ath-AT2G27260.1 |  |  |
| 3 | Vvi-Vitvi06g01561\_t001 |  | | | |  | | | |  |  |  |  |  | | | |  |  |  |
| 3 | Vvi-Vitvi06g01562\_t001 |  | Ath-AT3G44110.1 |  | | | |  |  |  |  |  | Ath-AT5G22060.1 |  |  |  |
| 3 | Vvi-Vitvi06g01563\_t001 |  | Ath-AT3G44100.1 |  | Ath-AT3G11780.2 |  |  |  |  |  | | | |  |  |  |
| 1 | Vvi-Vitvi06g01564\_t001 |  |  |  |  |  |  |  |  |  | Ath-AT5G22050.2 |  |  |  |
| 0 | Vvi-Vitvi06g01982\_t001 |  |  |  |  |  |  |  |  |
