# Supplementary material for: Genome-wide identification, characterization and gene expression of BES1 transcription factor family in grapevine (Vitis vinifera L.)
Source: Sci Rep. 2023 Jan 5;13:240. doi: 10.1038/s41598-022-24407-y (PMC9816167; doi:10.1038/s41598-022-24407-y)
Supplement: Supplementary file 3 — Supplementary Information. [file 41598_2022_24407_MOESM3_ESM.zip › Vvi_Ath/Vitis_vinifera.PN40024.v4.dna_sm.toplevel.fa.vs.Arabidopsis_thaliana.TAIR10.dna_sm.toplevel.fa.html/Vvi-9.html]

|  |  |  |  |  |  |  |  |  |  |  |  |  |  |  |  |  |  |
| --- | --- | --- | --- | --- | --- | --- | --- | --- | --- | --- | --- | --- | --- | --- | --- | --- | --- |
| Duplication depth | Reference chromosome | Collinear blocks | | | | | | | | | | | | | | | |
| 0 | Vvi-Vitvi09g04000\_t001 |  |  |  |  |  |  |  |  |
| 0 | Vvi-Vitvi09g04001\_t001 |  |  |  |  |  |  |  |  |
| 0 | Vvi-Vitvi09g04002\_t001 |  |  |  |  |  |  |  |  |
| 0 | Vvi-Vitvi09g04003\_t001 |  |  |  |  |  |  |  |  |
| 0 | Vvi-Vitvi09g04004\_t001 |  |  |  |  |  |  |  |  |
| 0 | Vvi-Vitvi09g04005\_t001 |  |  |  |  |  |  |  |  |
| 0 | Vvi-Vitvi09g00001\_t001 |  |  |  |  |  |  |  |  |
| 0 | Vvi-Vitvi09g04006\_t001 |  |  |  |  |  |  |  |  |
| 0 | Vvi-Vitvi09g00002\_t001 |  |  |  |  |  |  |  |  |
| 0 | Vvi-Vitvi09g04007\_t001 |  |  |  |  |  |  |  |  |
| 2 | Vvi-Vitvi09g00003\_t001 |  | Ath-AT1G72300.1 |  | Ath-AT1G17240.2 |  |  |  |  |  |  |
| 2 | Vvi-Vitvi09g04008\_t001 |  | | | |  | | | |  |  |  |  |  |  |
| 2 | Vvi-Vitvi09g00004\_t001 |  | | | |  | | | |  |  |  |  |  |  |
| 2 | Vvi-Vitvi09g04009\_t001 |  | | | |  | | | |  |  |  |  |  |  |
| 2 | Vvi-Vitvi09g00006\_t001 |  | | | |  | Ath-AT1G17260.1 |  |  |  |  |  |  |
| 3 | Vvi-Vitvi09g00007\_t001 |  | | | |  | | | |  | Ath-AT3G14360.1 |  |  |  |  |  |
| 4 | Vvi-Vitvi09g00008\_t001 |  | | | |  | | | |  | Ath-AT3G14350.1 |  | Ath-AT1G53730.2 |  |  |  |  |
| 4 | Vvi-Vitvi09g00009\_t001 |  | | | |  | | | |  | | | |  | Ath-AT1G53750.1 |  |  |  |  |
| 4 | Vvi-Vitvi09g01491\_t001 |  | | | |  | | | |  | | | |  | Ath-AT1G53760.1 |  |  |  |  |
| 4 | Vvi-Vitvi09g00010\_t001 |  | | | |  | Ath-AT1G17270.2 |  | | | |  | Ath-AT1G53770.2 |  |  |  |  |
| 4 | Vvi-Vitvi09g00011\_t001 |  | | | |  | | | |  | | | |  | Ath-AT1G53800.3 |  |  |  |  |
| 4 | Vvi-Vitvi09g00012\_t001 |  | Ath-AT1G72310.1 |  | | | |  | | | |  | Ath-AT1G53820.1 |  |  |  |  |
| 4 | Vvi-Vitvi09g00013\_t001 |  | | | |  | | | |  | | | |  | | | |  |  |  |  |
| 4 | Vvi-Vitvi09g00014\_t002 |  | | | |  | Ath-AT1G17280.4 |  | | | |  | | | |  |  |  |  |
| 4 | Vvi-Vitvi09g00015\_t001 |  | Ath-AT1G72320.4 |  | | | |  | | | |  | | | |  |  |  |  |
| 4 | Vvi-Vitvi09g00017\_t001 |  | | | |  | | | |  | Ath-AT3G14310.1 |  | Ath-AT1G53830.1 |  |  |  |  |
| 4 | Vvi-Vitvi09g04010\_t001 |  | | | |  | | | |  | | | |  | | | |  |  |  |  |
| 4 | Vvi-Vitvi09g00018\_t001 |  | | | |  | | | |  | Ath-AT3G14300.1 |  | Ath-AT1G53840.1 |  |  |  |  |
| 4 | Vvi-Vitvi09g00019\_t001 |  | Ath-AT1G72330.3 |  | Ath-AT1G17290.1 |  | | | |  | | | |  |  |  |  |
| 4 | Vvi-Vitvi09g00020\_t001 |  | | | |  | | | |  | Ath-AT3G14290.1 |  | Ath-AT1G53850.2 |  |  |  |  |
| 4 | Vvi-Vitvi09g00021\_t001 |  | | | |  | | | |  | | | |  | Ath-AT1G53860.1 |  |  |  |  |
| 4 | Vvi-Vitvi09g00022\_t001 |  | | | |  | | | |  | Ath-AT3G14280.1 |  | | | |  |  |  |  |
| 4 | Vvi-Vitvi09g00023\_t001 |  | | | |  | | | |  | Ath-AT3G14270.2 |  | | | |  |  |  |  |
| 4 | Vvi-Vitvi09g00026\_t001 |  | | | |  | | | |  | Ath-AT3G14260.1 |  | Ath-AT1G53870.1 |  |  |  |  |
| 4 | Vvi-Vitvi09g00027\_t001 |  | Ath-AT1G72340.2 |  | | | |  | | | |  | Ath-AT1G53880.1 |  |  |  |  |
| 4 | Vvi-Vitvi09g04011\_t001 |  | | | |  | | | |  | | | |  | | | |  |  |  |  |
| 4 | Vvi-Vitvi09g00028\_t001 |  | | | |  | | | |  | | | |  | | | |  |  |  |  |
| 4 | Vvi-Vitvi09g01493\_t001 |  | | | |  | | | |  | | | |  | Ath-AT1G53885.1 |  |  |  |  |
| 4 | Vvi-Vitvi09g04012\_t001 |  | | | |  | | | |  | | | |  | | | |  |  |  |  |
| 4 | Vvi-Vitvi09g00029\_t001 |  | | | |  | | | |  | | | |  | | | |  |  |  |  |
| 4 | Vvi-Vitvi09g04013\_t001 |  | | | |  | | | |  | | | |  | | | |  |  |  |  |
| 4 | Vvi-Vitvi09g00030\_t001 |  | | | |  | | | |  | Ath-AT3G14240.1 |  | | | |  |  |  |  |
| 4 | Vvi-Vitvi09g04014\_t001 |  | | | |  | | | |  | | | |  | | | |  |  |  |  |
| 4 | Vvi-Vitvi09g00031\_t002 |  | Ath-AT1G72360.2 |  | | | |  | Ath-AT3G14230.1 |  | Ath-AT1G53910.1 |  |  |  |  |
| 4 | Vvi-Vitvi09g00032\_t001 |  | | | |  | | | |  | | | |  | | | |  |  |  |  |
| 4 | Vvi-Vitvi09g00033\_t001 |  | | | |  | | | |  | Ath-AT3G14225.1 |  | Ath-AT1G53920.1 |  |  |  |  |
| 4 | Vvi-Vitvi09g00034\_t001 |  | | | |  | | | |  | | | |  | | | |  |  |  |  |
| 4 | Vvi-Vitvi09g00035\_t001 |  | | | |  | | | |  | | | |  | | | |  |  |  |  |
| 4 | Vvi-Vitvi09g00036\_t001 |  | | | |  | | | |  | | | |  | | | |  |  |  |  |
| 4 | Vvi-Vitvi09g00038\_t001 |  | | | |  | | | |  | | | |  | | | |  |  |  |  |
| 4 | Vvi-Vitvi09g00039\_t001 |  | | | |  | | | |  | | | |  | | | |  |  |  |  |
| 4 | Vvi-Vitvi09g00040\_t001 |  | | | |  | | | |  | | | |  | | | |  |  |  |  |
| 4 | Vvi-Vitvi09g00041\_t001 |  | | | |  | | | |  | | | |  | | | |  |  |  |  |
| 4 | Vvi-Vitvi09g04015\_t001 |  | | | |  | | | |  | | | |  | | | |  |  |  |  |
| 4 | Vvi-Vitvi09g00042\_t001 |  | | | |  | Ath-AT1G17340.1 |  | Ath-AT3G14205.1 |  | | | |  |  |  |  |
| 4 | Vvi-Vitvi09g00043\_t001 |  | Ath-AT1G72370.1 |  | | | |  | | | |  | | | |  |  |  |  |
| 4 | Vvi-Vitvi09g00044\_t001 |  | | | |  | | | |  | | | |  | | | |  |  |  |  |
| 4 | Vvi-Vitvi09g00045\_t001 |  | | | |  | | | |  | | | |  | Ath-AT1G54050.1 |  |  |  |  |
| 4 | Vvi-Vitvi09g00046\_t001 |  | | | |  | Ath-AT1G17345.1 |  | | | |  | | | |  |  |  |  |
| 4 | Vvi-Vitvi09g01495\_t001 |  | | | |  | | | |  | | | |  | | | |  |  |  |  |
| 4 | Vvi-Vitvi09g00047\_t002 |  | Ath-AT1G72420.2 |  | Ath-AT1G17350.2 |  | | | |  | | | |  |  |  |  |
| 4 | Vvi-Vitvi09g00049\_t001 |  | | | |  | | | |  | Ath-AT3G14200.1 |  | | | |  |  |  |  |
| 4 | Vvi-Vitvi09g04016\_t001 |  | | | |  | | | |  | | | |  | | | |  |  |  |  |
| 4 | Vvi-Vitvi09g00050\_t003 |  | | | |  | | | |  | | | |  | Ath-AT1G54070.1 |  |  |  |  |
| 4 | Vvi-Vitvi09g00051\_t001 |  | | | |  | | | |  | | | |  | | | |  |  |  |  |
| 4 | Vvi-Vitvi09g01496\_t001 |  | | | |  | | | |  | Ath-AT3G14180.1 |  | | | |  |  |  |  |
| 4 | Vvi-Vitvi09g04017\_t001 |  | | | |  | | | |  | | | |  | | | |  |  |  |  |
| 4 | Vvi-Vitvi09g00052\_t001 |  | | | |  | Ath-AT1G17360.1 |  | Ath-AT3G14172.1 |  | | | |  |  |  |  |
| 4 | Vvi-Vitvi09g00053\_t001 |  | | | |  | | | |  | | | |  | | | |  |  |  |  |
| 4 | Vvi-Vitvi09g00055\_t001 |  | | | |  | | | |  | Ath-AT3G14170.2 |  | | | |  |  |  |  |
| 4 | Vvi-Vitvi09g00056\_t001 |  | | | |  | | | |  | Ath-AT3G14140.1 |  | | | |  |  |  |  |
| 4 | Vvi-Vitvi09g00057\_t002 |  | | | |  | | | |  | | | |  | | | |  |  |  |  |
| 4 | Vvi-Vitvi09g00058\_t001 |  | | | |  | | | |  | | | |  | | | |  |  |  |  |
| 4 | Vvi-Vitvi09g00059\_t001 |  | | | |  | | | |  | | | |  | | | |  |  |  |  |
| 4 | Vvi-Vitvi09g01499\_t001 |  | | | |  | | | |  | Ath-AT3G14130.1 |  | | | |  |  |  |  |
| 4 | Vvi-Vitvi09g01500\_t001 |  | | | |  | | | |  | | | |  | | | |  |  |  |  |
| 4 | Vvi-Vitvi09g00060\_t001 |  | | | |  | | | |  | | | |  | | | |  |  |  |  |
| 4 | Vvi-Vitvi09g00061\_t001 |  | | | |  | | | |  | Ath-AT3G14120.1 |  | | | |  |  |  |  |
| 4 | Vvi-Vitvi09g00062\_t001 |  | | | |  | | | |  | Ath-AT3G14110.3 |  | | | |  |  |  |  |
| 4 | Vvi-Vitvi09g00063\_t001 |  | | | |  | Ath-AT1G17370.1 |  | Ath-AT3G14100.1 |  | Ath-AT1G54080.2 |  |  |  |  |
| 4 | Vvi-Vitvi09g00064\_t001 |  | Ath-AT1G72450.1 |  | Ath-AT1G17380.1 |  | | | |  | | | |  |  |  |  |
| 4 | Vvi-Vitvi09g00065\_t001 |  | Ath-AT1G72460.1 |  | | | |  | | | |  | | | |  |  |  |  |
| 4 | Vvi-Vitvi09g00067\_t001 |  | Ath-AT1G72470.1 |  | | | |  | Ath-AT3G14090.1 |  | Ath-AT1G54090.1 |  |  |  |  |
| 4 | Vvi-Vitvi09g00068\_t002 |  | Ath-AT1G72480.1 |  | | | |  | | | |  | | | |  |  |  |  |
| 4 | Vvi-Vitvi09g00069\_t001 |  | | | |  | | | |  | Ath-AT3G14080.2 |  | | | |  |  |  |  |
| 4 | Vvi-Vitvi09g01501\_t001 |  | Ath-AT1G72490.3 |  | Ath-AT1G17400.1 |  | | | |  | | | |  |  |  |  |
| 4 | Vvi-Vitvi09g00070\_t001 |  | | | |  | Ath-AT1G17410.1 |  | | | |  | | | |  |  |  |  |
| 4 | Vvi-Vitvi09g00071\_t001 |  | Ath-AT1G72500.1 |  | | | |  | | | |  | | | |  |  |  |  |
| 4 | Vvi-Vitvi09g04018\_t001 |  | | | |  | | | |  | | | |  | | | |  |  |  |  |
| 4 | Vvi-Vitvi09g00073\_t001 |  | | | |  | | | |  | | | |  | | | |  |  |  |  |
| 4 | Vvi-Vitvi09g00076\_t001 |  | | | |  | | | |  | Ath-AT3G14075.2 |  | | | |  |  |  |  |
| 4 | Vvi-Vitvi09g00077\_t001 |  | Ath-AT1G72510.2 |  | | | |  | | | |  | Ath-AT1G54095.1 |  |  |  |  |
| 4 | Vvi-Vitvi09g00078\_t001 |  | | | |  | | | |  | | | |  | Ath-AT1G54100.2 |  |  |  |  |
| 4 | Vvi-Vitvi09g00079\_t001 |  | | | |  | | | |  | Ath-AT3G14070.1 |  | Ath-AT1G54115.1 |  |  |  |  |
| 4 | Vvi-Vitvi09g00081\_t001 |  | | | |  | | | |  | Ath-AT3G14067.1 |  | | | |  |  |  |  |
| 4 | Vvi-Vitvi09g01502\_t001 |  | | | |  | | | |  | | | |  | | | |  |  |  |  |
| 4 | Vvi-Vitvi09g00082\_t001 |  | | | |  | | | |  | | | |  | | | |  |  |  |  |
| 4 | Vvi-Vitvi09g00083\_t001 |  | | | |  | | | |  | Ath-AT3G14060.1 |  | Ath-AT1G54120.1 |  |  |  |  |
| 4 | Vvi-Vitvi09g04019\_t001 |  | | | |  | | | |  | | | |  | | | |  |  |  |  |
| 4 | Vvi-Vitvi09g04020\_t001 |  | | | |  | | | |  | | | |  | | | |  |  |  |  |
| 4 | Vvi-Vitvi09g00085\_t002 |  | Ath-AT1G72520.1 |  | Ath-AT1G17420.1 |  | | | |  | | | |  |  |  |  |
| 4 | Vvi-Vitvi09g04021\_t001 |  | | | |  | | | |  | | | |  | | | |  |  |  |  |
| 4 | Vvi-Vitvi09g00086\_t001 |  | | | |  | | | |  | | | |  | | | |  |  |  |  |
| 4 | Vvi-Vitvi09g00087\_t001 |  | Ath-AT1G72530.2 |  | | | |  | | | |  | | | |  |  |  |  |
| 4 | Vvi-Vitvi09g00088\_t001 |  | Ath-AT1G72540.1 |  | | | |  | | | |  | | | |  |  |  |  |
| 4 | Vvi-Vitvi09g00089\_t002 |  | Ath-AT1G72560.1 |  | | | |  | | | |  | | | |  |  |  |  |
| 4 | Vvi-Vitvi09g04022\_t001 |  | | | |  | | | |  | | | |  | | | |  |  |  |  |
| 4 | Vvi-Vitvi09g00093\_t001 |  | | | |  | | | |  | Ath-AT3G14050.1 |  | Ath-AT1G54130.1 |  |  |  |  |
| 4 | Vvi-Vitvi09g00094\_t001 |  | | | |  | | | |  | | | |  | Ath-AT1G54140.1 |  |  |  |  |
| 4 | Vvi-Vitvi09g04023\_t001 |  | | | |  | | | |  | | | |  | | | |  |  |  |  |
| 4 | Vvi-Vitvi09g00095\_t001 |  | | | |  | Ath-AT1G17430.1 |  | | | |  | | | |  |  |  |  |
| 3 | Vvi-Vitvi09g00096\_t001 |  | | | |  |  |  | | | |  | Ath-AT1G54150.1 |  |  |  |  |
| 4 | Vvi-Vitvi09g01487\_t001 |  | | | |  | Ath-AT3G13840.1 |  | | | |  | | | |  |  |  |  |
| 4 | Vvi-Vitvi09g01503\_t001 |  | | | |  | | | |  | | | |  | | | |  |  |  |  |
| 4 | Vvi-Vitvi09g04024\_t001 |  | | | |  | | | |  | | | |  | | | |  |  |  |  |
| 4 | Vvi-Vitvi09g01504\_t001 |  | | | |  | | | |  | | | |  | | | |  |  |  |  |
| 4 | Vvi-Vitvi09g01505\_t001 |  | | | |  | | | |  | | | |  | | | |  |  |  |  |
| 4 | Vvi-Vitvi09g00097\_t001 |  | | | |  | | | |  | | | |  | | | |  |  |  |  |
| 4 | Vvi-Vitvi09g04025\_t001 |  | | | |  | | | |  | | | |  | | | |  |  |  |  |
| 4 | Vvi-Vitvi09g00099\_t001 |  | | | |  | | | |  | | | |  | | | |  |  |  |  |
| 4 | Vvi-Vitvi09g04026\_t001 |  | | | |  | | | |  | | | |  | | | |  |  |  |  |
| 4 | Vvi-Vitvi09g04027\_t001 |  | | | |  | | | |  | | | |  | | | |  |  |  |  |
| 4 | Vvi-Vitvi09g04028\_t001 |  | | | |  | | | |  | | | |  | | | |  |  |  |  |
| 4 | Vvi-Vitvi09g04029\_t001 |  | | | |  | | | |  | | | |  | | | |  |  |  |  |
| 4 | Vvi-Vitvi09g00104\_t001 |  | | | |  | | | |  | | | |  | | | |  |  |  |  |
| 4 | Vvi-Vitvi09g04030\_t001 |  | | | |  | | | |  | | | |  | | | |  |  |  |  |
| 4 | Vvi-Vitvi09g01510\_t001 |  | | | |  | | | |  | | | |  | | | |  |  |  |  |
| 4 | Vvi-Vitvi09g01511\_t001 |  | | | |  | | | |  | | | |  | | | |  |  |  |  |
| 4 | Vvi-Vitvi09g01512\_t001 |  | Ath-AT1G72610.1 |  | | | |  | | | |  | | | |  |  |  |  |
| 4 | Vvi-Vitvi09g01513\_t001 |  | | | |  | | | |  | | | |  | | | |  |  |  |  |
| 4 | Vvi-Vitvi09g01514\_t001 |  | | | |  | | | |  | | | |  | | | |  |  |  |  |
| 4 | Vvi-Vitvi09g01515\_t001 |  | | | |  | | | |  | | | |  | | | |  |  |  |  |
| 4 | Vvi-Vitvi09g00107\_t001 |  | | | |  | Ath-AT3G13960.1 |  | Ath-AT3G13960.1 |  | | | |  |  |  |  |
| 3 | Vvi-Vitvi09g01516\_t001 |  | | | |  | | | |  |  |  | | | |  |  |  |  |
| 3 | Vvi-Vitvi09g00108\_t001 |  | | | |  | | | |  |  |  | | | |  |  |  |  |
| 3 | Vvi-Vitvi09g00110\_t001 |  | | | |  | | | |  |  |  | | | |  |  |  |  |
| 3 | Vvi-Vitvi09g04031\_t001 |  | | | |  | | | |  |  |  | | | |  |  |  |  |
| 3 | Vvi-Vitvi09g00111\_t001 |  | | | |  | Ath-AT3G13970.1 |  |  |  | Ath-AT1G54210.1 |  |  |  |  |
| 2 | Vvi-Vitvi09g00112\_t001 |  | | | |  | | | |  |  |  |  |  |  |
| 2 | Vvi-Vitvi09g00113\_t001 |  | | | |  | | | |  |  |  |  |  |  |
| 3 | Vvi-Vitvi09g00115\_t001 |  | | | |  | | | |  | Ath-AT1G17440.1 |  |  |  |  |  |
| 3 | Vvi-Vitvi09g00116\_t001 |  | | | |  | | | |  | Ath-AT1G17450.2 |  |  |  |  |  |
| 3 | Vvi-Vitvi09g00117\_t001 |  | Ath-AT1G72630.1 |  | | | |  | Ath-AT1G17455.1 |  |  |  |  |  |
| 3 | Vvi-Vitvi09g00118\_t001 |  | Ath-AT1G72640.1 |  | | | |  | | | |  |  |  |  |  |
| 3 | Vvi-Vitvi09g00119\_t001 |  | | | |  | | | |  | | | |  |  |  |  |  |
| 3 | Vvi-Vitvi09g00121\_t004 |  | | | |  | | | |  | | | |  |  |  |  |  |
| 3 | Vvi-Vitvi09g04032\_t001 |  | | | |  | | | |  | | | |  |  |  |  |  |
| 3 | Vvi-Vitvi09g00122\_t001 |  | Ath-AT1G72650.2 |  | | | |  | Ath-AT1G17460.2 |  |  |  |  |  |
| 3 | Vvi-Vitvi09g00123\_t001 |  | Ath-AT1G72660.1 |  | | | |  | Ath-AT1G17470.1 |  |  |  |  |  |
| 3 | Vvi-Vitvi09g01517\_t001 |  | | | |  | | | |  | | | |  |  |  |  |  |
| 3 | Vvi-Vitvi09g00126\_t001 |  | | | |  | Ath-AT3G13980.1 |  | | | |  |  |  |  |  |
| 3 | Vvi-Vitvi09g00127\_t001 |  | | | |  | Ath-AT3G13990.1 |  | | | |  |  |  |  |  |
| 3 | Vvi-Vitvi09g00128\_t001 |  | | | |  | Ath-AT3G14000.1 |  | | | |  |  |  |  |  |
| 3 | Vvi-Vitvi09g00129\_t002 |  | | | |  | | | |  | | | |  |  |  |  |  |
| 3 | Vvi-Vitvi09g00130\_t004 |  | | | |  | Ath-AT3G14010.5 |  | | | |  |  |  |  |  |
| 3 | Vvi-Vitvi09g00131\_t001 |  | | | |  | | | |  | | | |  |  |  |  |  |
| 3 | Vvi-Vitvi09g00132\_t001 |  | | | |  | | | |  | | | |  |  |  |  |  |
| 3 | Vvi-Vitvi09g00133\_t001 |  | | | |  | Ath-AT3G14020.1 |  | | | |  |  |  |  |  |
| 2 | Vvi-Vitvi09g01518\_t001 |  | | | |  |  |  | | | |  |  |  |  |  |
| 2 | Vvi-Vitvi09g00134\_t001 |  | | | |  |  |  | | | |  |  |  |  |  |
| 2 | Vvi-Vitvi09g00135\_t001 |  | | | |  |  |  | | | |  |  |  |  |  |
| 2 | Vvi-Vitvi09g00136\_t001 |  | | | |  |  |  | | | |  |  |  |  |  |
| 2 | Vvi-Vitvi09g00137\_t001 |  | | | |  |  |  | | | |  |  |  |  |  |
| 2 | Vvi-Vitvi09g00139\_t001 |  | | | |  |  |  | | | |  |  |  |  |  |
| 2 | Vvi-Vitvi09g04033\_t001 |  | | | |  |  |  | | | |  |  |  |  |  |
| 2 | Vvi-Vitvi09g00140\_t001 |  | | | |  |  |  | | | |  |  |  |  |  |
| 3 | Vvi-Vitvi09g00141\_t001 |  | | | |  | Ath-AT3G13882.2 |  | | | |  |  |  |  |  |
| 3 | Vvi-Vitvi09g00142\_t001 |  | | | |  | Ath-AT3G13890.1 |  | | | |  |  |  |  |  |
| 3 | Vvi-Vitvi09g00143\_t001 |  | Ath-AT1G72670.1 |  | | | |  | Ath-AT1G17480.1 |  |  |  |  |  |
| 3 | Vvi-Vitvi09g00144\_t001 |  | | | |  | | | |  | | | |  |  |  |  |  |
| 4 | Vvi-Vitvi09g04034\_t001 |  | Ath-AT1G72690.1 |  | Ath-AT3G13898.1 |  | Ath-AT1G17490.1 |  | Ath-AT5G57000.1 |  |  |  |  |
| 4 | Vvi-Vitvi09g00146\_t001 |  | | | |  | | | |  | | | |  | | | |  |  |  |  |
| 4 | Vvi-Vitvi09g04035\_t001 |  | | | |  | | | |  | | | |  | | | |  |  |  |  |
| 4 | Vvi-Vitvi09g00147\_t001 |  | Ath-AT1G72700.1 |  | Ath-AT3G13900.1 |  | Ath-AT1G17500.1 |  | | | |  |  |  |  |
| 4 | Vvi-Vitvi09g00148\_t001 |  | Ath-AT1G72710.1 |  | | | |  | | | |  | Ath-AT5G57015.1 |  |  |  |  |
| 4 | Vvi-Vitvi09g01521\_t001 |  | Ath-AT1G72720.1 |  | Ath-AT3G13910.2 |  | | | |  | | | |  |  |  |  |
| 4 | Vvi-Vitvi09g00149\_t001 |  | Ath-AT1G72730.1 |  | Ath-AT3G13920.5 |  | | | |  | | | |  |  |  |  |
| 4 | Vvi-Vitvi09g00150\_t005 |  | | | |  | | | |  | | | |  | Ath-AT5G57020.1 |  |  |  |  |
| 4 | Vvi-Vitvi09g04036\_t001 |  | Ath-AT1G72740.1 |  | | | |  | | | |  | | | |  |  |  |  |
| 4 | Vvi-Vitvi09g00151\_t002 |  | | | |  | Ath-AT3G13930.1 |  | | | |  | | | |  |  |  |  |
| 4 | Vvi-Vitvi09g00152\_t001 |  | Ath-AT1G72750.1 |  | | | |  | Ath-AT1G17530.1 |  | | | |  |  |  |  |
| 4 | Vvi-Vitvi09g00153\_t001 |  | | | |  | | | |  | | | |  | | | |  |  |  |  |
| 4 | Vvi-Vitvi09g00155\_t001 |  | | | |  | | | |  | Ath-AT1G17540.1 |  | | | |  |  |  |  |
| 4 | Vvi-Vitvi09g00156\_t002 |  | Ath-AT1G72770.1 |  | | | |  | Ath-AT1G17550.1 |  | Ath-AT5G57050.1 |  |  |  |  |
| 4 | Vvi-Vitvi09g00157\_t001 |  | | | |  | Ath-AT3G13940.1 |  | | | |  | | | |  |  |  |  |
| 3 | Vvi-Vitvi09g01525\_t002 |  | | | |  |  |  | | | |  | Ath-AT5G57060.3 |  |  |  |  |
| 3 | Vvi-Vitvi09g00159\_t001 |  | Ath-AT1G72790.1 |  |  |  | | | |  | Ath-AT5G57070.1 |  |  |  |  |
| 1 | Vvi-Vitvi09g00161\_t001 |  |  |  |  |  | | | |  |  |  |  |  |
| 1 | Vvi-Vitvi09g00162\_t001 |  |  |  |  |  | Ath-AT1G17580.1 |  |  |  |  |  |
| 1 | Vvi-Vitvi09g00163\_t001 |  |  |  |  |  | | | |  |  |  |  |  |
| 1 | Vvi-Vitvi09g00164\_t001 |  |  |  |  |  | | | |  |  |  |  |  |
| 1 | Vvi-Vitvi09g04037\_t001 |  |  |  |  |  | | | |  |  |  |  |  |
| 1 | Vvi-Vitvi09g01530\_t001 |  |  |  |  |  | | | |  |  |  |  |  |
| 1 | Vvi-Vitvi09g01532\_t001 |  |  |  |  |  | | | |  |  |  |  |  |
| 1 | Vvi-Vitvi09g00166\_t001 |  |  |  |  |  | | | |  |  |  |  |  |
| 1 | Vvi-Vitvi09g04038\_t001 |  |  |  |  |  | | | |  |  |  |  |  |
| 1 | Vvi-Vitvi09g01533\_t001 |  |  |  |  |  | | | |  |  |  |  |  |
| 1 | Vvi-Vitvi09g01534\_t001 |  |  |  |  |  | | | |  |  |  |  |  |
| 1 | Vvi-Vitvi09g04039\_t001 |  |  |  |  |  | | | |  |  |  |  |  |
| 1 | Vvi-Vitvi09g04040\_t001 |  |  |  |  |  | | | |  |  |  |  |  |
| 1 | Vvi-Vitvi09g04041\_t001 |  |  |  |  |  | | | |  |  |  |  |  |
| 1 | Vvi-Vitvi09g00170\_t001 |  |  |  |  |  | | | |  |  |  |  |  |
| 2 | Vvi-Vitvi09g00171\_t001 |  | Ath-AT1G55170.1 |  |  |  | | | |  |  |  |  |  |
| 2 | Vvi-Vitvi09g00172\_t001 |  | | | |  |  |  | | | |  |  |  |  |  |
| 2 | Vvi-Vitvi09g00173\_t001 |  | Ath-AT1G55160.3 |  |  |  | | | |  |  |  |  |  |
| 2 | Vvi-Vitvi09g00175\_t001 |  | Ath-AT1G55150.2 |  |  |  | | | |  |  |  |  |  |
| 2 | Vvi-Vitvi09g00176\_t001 |  | Ath-AT1G55140.1 |  |  |  | | | |  |  |  |  |  |
| 2 | Vvi-Vitvi09g00177\_t001 |  | | | |  |  |  | | | |  |  |  |  |  |
| 2 | Vvi-Vitvi09g00178\_t001 |  | | | |  |  |  | Ath-AT1G17665.1 |  |  |  |  |  |
| 1 | Vvi-Vitvi09g00179\_t001 |  | Ath-AT1G55130.1 |  |  |  |  |  |  |  |
| 1 | Vvi-Vitvi09g00180\_t001 |  | | | |  |  |  |  |  |  |  |
| 1 | Vvi-Vitvi09g04042\_t001 |  | | | |  |  |  |  |  |  |  |
| 1 | Vvi-Vitvi09g01536\_t001 |  | | | |  |  |  |  |  |  |  |
| 1 | Vvi-Vitvi09g04043\_t001 |  | | | |  |  |  |  |  |  |  |
| 1 | Vvi-Vitvi09g04044\_t001 |  | | | |  |  |  |  |  |  |  |
| 1 | Vvi-Vitvi09g04045\_t001 |  | | | |  |  |  |  |  |  |  |
| 1 | Vvi-Vitvi09g00181\_t001 |  | | | |  |  |  |  |  |  |  |
| 3 | Vvi-Vitvi09g00182\_t001 |  | | | |  | Ath-AT1G72960.1 |  | Ath-AT3G13870.1 |  |  |  |  |  |
| 3 | Vvi-Vitvi09g00183\_t001 |  | | | |  | Ath-AT1G72970.1 |  | | | |  |  |  |  |  |
| 3 | Vvi-Vitvi09g04046\_t001 |  | | | |  | | | |  | | | |  |  |  |  |  |
| 3 | Vvi-Vitvi09g00184\_t001 |  | | | |  | | | |  | | | |  |  |  |  |  |
| 4 | Vvi-Vitvi09g00185\_t001 |  | | | |  | | | |  | | | |  | Ath-AT1G17620.1 |  |  |  |  |
| 4 | Vvi-Vitvi09g00186\_t001 |  | | | |  | | | |  | Ath-AT3G13860.1 |  | | | |  |  |  |  |
| 4 | Vvi-Vitvi09g00187\_t001 |  | | | |  | | | |  | | | |  | Ath-AT1G17630.1 |  |  |  |  |
| 4 | Vvi-Vitvi09g00188\_t001 |  | | | |  | Ath-AT1G72980.1 |  | Ath-AT3G13850.1 |  | | | |  |  |  |  |
| 4 | Vvi-Vitvi09g00189\_t001 |  | | | |  | | | |  | | | |  | | | |  |  |  |  |
| 5 | Vvi-Vitvi09g01540\_t001 |  | Ath-AT1G55080.1 |  | | | |  | | | |  | | | |  | Ath-AT1G55080.1 |  |  |  |
| 4 | Vvi-Vitvi09g04047\_t001 |  |  |  | | | |  | | | |  | | | |  | | | |  |  |  |
| 4 | Vvi-Vitvi09g00190\_t001 |  |  |  | Ath-AT1G72990.1 |  | | | |  | | | |  | | | |  |  |  |
| 4 | Vvi-Vitvi09g00191\_t001 |  |  |  | | | |  | Ath-AT3G13810.2 |  | | | |  | Ath-AT1G55110.2 |  |  |  |
| 4 | Vvi-Vitvi09g04048\_t001 |  |  |  | | | |  | | | |  | | | |  | | | |  |  |  |
| 4 | Vvi-Vitvi09g00192\_t001 |  |  |  | | | |  | Ath-AT3G13800.1 |  | | | |  | | | |  |  |  |
| 4 | Vvi-Vitvi09g00193\_t001 |  |  |  | | | |  | Ath-AT3G13784.2 |  | | | |  | Ath-AT1G55120.1 |  |  |  |
| 5 | Vvi-Vitvi09g00194\_t001 |  | Ath-AT2G19480.1 |  | | | |  | Ath-AT3G13782.1 |  | | | |  | | | |  |  |  |
| 5 | Vvi-Vitvi09g00195\_t001 |  | | | |  | | | |  | | | |  | Ath-AT1G17650.1 |  | | | |  |  |  |
| 5 | Vvi-Vitvi09g00196\_t001 |  | | | |  | | | |  | | | |  | | | |  | | | |  |  |  |
| 5 | Vvi-Vitvi09g00197\_t001 |  | | | |  | | | |  | Ath-AT3G13780.1 |  | | | |  | | | |  |  |  |
| 5 | Vvi-Vitvi09g00198\_t001 |  | | | |  | | | |  | | | |  | | | |  | | | |  |  |  |
| 6 | Vvi-Vitvi09g00199\_t002 |  | | | |  | | | |  | | | |  | | | |  | | | |  | Ath-AT4G29670.2 |  |  |
| 6 | Vvi-Vitvi09g00200\_t001 |  | | | |  | | | |  | | | |  | | | |  | Ath-AT1G55180.1 |  | | | |  |  |
| 6 | Vvi-Vitvi09g00201\_t001 |  | | | |  | | | |  | | | |  | Ath-AT1G17700.1 |  | Ath-AT1G55190.1 |  | | | |  |  |
| 7 | Vvi-Vitvi09g00202\_t001 |  | | | |  | | | |  | Ath-AT3G13700.1 |  | | | |  | | | |  | | | |  | Ath-AT3G21215.1 |  |
| 7 | Vvi-Vitvi09g04049\_t001 |  | | | |  | | | |  | | | |  | | | |  | | | |  | | | |  | | | |  |
| 7 | Vvi-Vitvi09g00203\_t001 |  | | | |  | | | |  | Ath-AT3G13690.1 |  | | | |  | Ath-AT1G55200.1 |  | | | |  | | | |  |
| 5 | Vvi-Vitvi09g04050\_t001 |  | | | |  | | | |  |  |  | | | |  |  |  | | | |  | Ath-AT3G21250.4 |  |
| 5 | Vvi-Vitvi09g04051\_t001 |  | | | |  | | | |  |  |  | | | |  |  |  | | | |  | | | |  |
| 5 | Vvi-Vitvi09g00206\_t001 |  | | | |  | | | |  |  |  | | | |  |  |  | | | |  | | | |  |
| 5 | Vvi-Vitvi09g01542\_t002 |  | | | |  | Ath-AT1G73010.1 |  |  |  | Ath-AT1G17710.1 |  |  |  | Ath-AT4G29530.1 |  | | | |  |
| 6 | Vvi-Vitvi09g00208\_t001 |  | | | |  | | | |  | Ath-AT1G51690.5 |  | Ath-AT1G17720.1 |  |  |  | | | |  | | | |  |
| 6 | Vvi-Vitvi09g00209\_t001 |  | Ath-AT2G19680.1 |  | | | |  | | | |  | | | |  |  |  | Ath-AT4G29480.1 |  | | | |  |
| 6 | Vvi-Vitvi09g04052\_t001 |  | | | |  | | | |  | | | |  | | | |  |  |  | | | |  | | | |  |
| 6 | Vvi-Vitvi09g00210\_t001 |  | | | |  | | | |  | Ath-AT1G51700.1 |  | | | |  |  |  | | | |  | Ath-AT3G21270.1 |  |
| 6 | Vvi-Vitvi09g04053\_t001 |  | | | |  | | | |  | | | |  | | | |  |  |  | | | |  | | | |  |
| 6 | Vvi-Vitvi09g00211\_t001 |  | | | |  | | | |  | Ath-AT1G51710.1 |  | | | |  |  |  | | | |  | Ath-AT3G21280.1 |  |
| 6 | Vvi-Vitvi09g00212\_t001 |  | | | |  | | | |  | | | |  | | | |  |  |  | | | |  | Ath-AT3G21290.1 |  |
| 6 | Vvi-Vitvi09g00213\_t002 |  | Ath-AT2G19720.1 |  | | | |  | | | |  | | | |  |  |  | Ath-AT4G29430.1 |  | | | |  |
| 6 | Vvi-Vitvi09g00214\_t003 |  | | | |  | | | |  | Ath-AT1G51720.1 |  | | | |  |  |  | | | |  | | | |  |
| 6 | Vvi-Vitvi09g00215\_t002 |  | | | |  | | | |  | Ath-AT1G51730.1 |  | | | |  |  |  | | | |  | | | |  |
| 6 | Vvi-Vitvi09g04054\_t001 |  | | | |  | | | |  | | | |  | | | |  |  |  | | | |  | | | |  |
| 6 | Vvi-Vitvi09g00216\_t001 |  | | | |  | | | |  | Ath-AT1G51745.1 |  | | | |  |  |  | | | |  | Ath-AT3G21295.1 |  |
| 6 | Vvi-Vitvi09g01545\_t001 |  | Ath-AT2G19730.2 |  | | | |  | | | |  | | | |  |  |  | Ath-AT4G29410.1 |  | | | |  |
| 6 | Vvi-Vitvi09g01546\_t001 |  | | | |  | | | |  | | | |  | | | |  |  |  | | | |  | | | |  |
| 6 | Vvi-Vitvi09g00217\_t001 |  | | | |  | Ath-AT1G73020.1 |  | | | |  | | | |  |  |  | | | |  | | | |  |
| 6 | Vvi-Vitvi09g00218\_t001 |  | | | |  | Ath-AT1G73030.1 |  | | | |  | Ath-AT1G17730.1 |  |  |  | | | |  | | | |  |
| 5 | Vvi-Vitvi09g00219\_t001 |  | | | |  | Ath-AT1G73040.1 |  | | | |  |  |  |  |  | | | |  | | | |  |
| 4 | Vvi-Vitvi09g01548\_t001 |  | | | |  |  |  | | | |  |  |  |  |  | | | |  | | | |  |
| 4 | Vvi-Vitvi09g00220\_t001 |  | | | |  |  |  | Ath-AT1G51760.1 |  |  |  |  |  | | | |  | | | |  |
| 4 | Vvi-Vitvi09g04055\_t001 |  | Ath-AT2G19750.1 |  |  |  | | | |  |  |  |  |  | Ath-AT4G29390.1 |  | | | |  |
| 4 | Vvi-Vitvi09g04056\_t001 |  | | | |  |  |  | | | |  |  |  |  |  | | | |  | | | |  |
| 4 | Vvi-Vitvi09g00223\_t001 |  | | | |  |  |  | Ath-AT1G51770.1 |  |  |  |  |  | | | |  | Ath-AT3G21310.1 |  |
| 4 | Vvi-Vitvi09g00224\_t001 |  | | | |  |  |  | | | |  |  |  |  |  | | | |  | | | |  |
| 4 | Vvi-Vitvi09g00225\_t001 |  | | | |  |  |  | | | |  |  |  |  |  | | | |  | Ath-AT3G21320.1 |  |
| 4 | Vvi-Vitvi09g00226\_t001 |  | | | |  |  |  | | | |  |  |  |  |  | | | |  | | | |  |
| 4 | Vvi-Vitvi09g04057\_t001 |  | | | |  |  |  | | | |  |  |  |  |  | | | |  | | | |  |
| 4 | Vvi-Vitvi09g00227\_t001 |  | | | |  |  |  | | | |  |  |  |  |  | | | |  | Ath-AT3G21330.1 |  |
| 3 | Vvi-Vitvi09g00228\_t001 |  | | | |  |  |  | Ath-AT1G51800.1 |  |  |  |  |  | | | |  |  |
| 3 | Vvi-Vitvi09g00229\_t001 |  | | | |  |  |  | | | |  |  |  |  |  | | | |  |  |
| 3 | Vvi-Vitvi09g00230\_t001 |  | | | |  |  |  | Ath-AT1G51805.1 |  |  |  |  |  | | | |  |  |
| 3 | Vvi-Vitvi09g00231\_t001 |  | | | |  |  |  | | | |  |  |  |  |  | | | |  |  |
| 3 | Vvi-Vitvi09g04058\_t001 |  | | | |  |  |  | | | |  |  |  |  |  | | | |  |  |
| 3 | Vvi-Vitvi09g00232\_t001 |  | | | |  |  |  | | | |  |  |  |  |  | | | |  |  |
| 3 | Vvi-Vitvi09g01550\_t006 |  | Ath-AT2G19830.2 |  |  |  | | | |  |  |  |  |  | Ath-AT4G29160.1 |  |  |
| 1 | Vvi-Vitvi09g04059\_t001 |  |  |  |  |  | | | |  |  |  |  |  |
| 1 | Vvi-Vitvi09g00233\_t001 |  |  |  |  |  | | | |  |  |  |  |  |
| 1 | Vvi-Vitvi09g04060\_t001 |  |  |  |  |  | | | |  |  |  |  |  |
| 1 | Vvi-Vitvi09g04061\_t001 |  |  |  |  |  | | | |  |  |  |  |  |
| 1 | Vvi-Vitvi09g04062\_t001 |  |  |  |  |  | | | |  |  |  |  |  |
| 1 | Vvi-Vitvi09g00235\_t001 |  |  |  |  |  | | | |  |  |  |  |  |
| 1 | Vvi-Vitvi09g01551\_t001 |  |  |  |  |  | | | |  |  |  |  |  |
| 1 | Vvi-Vitvi09g04063\_t001 |  |  |  |  |  | | | |  |  |  |  |  |
| 1 | Vvi-Vitvi09g00239\_t001 |  |  |  |  |  | | | |  |  |  |  |  |
| 1 | Vvi-Vitvi09g00240\_t001 |  |  |  |  |  | | | |  |  |  |  |  |
| 1 | Vvi-Vitvi09g00241\_t001 |  |  |  |  |  | | | |  |  |  |  |  |
| 1 | Vvi-Vitvi09g04064\_t001 |  |  |  |  |  | | | |  |  |  |  |  |
| 1 | Vvi-Vitvi09g01555\_t001 |  |  |  |  |  | | | |  |  |  |  |  |
| 1 | Vvi-Vitvi09g04065\_t001 |  |  |  |  |  | Ath-AT1G51820.1 |  |  |  |  |  |
| 1 | Vvi-Vitvi09g01556\_t001 |  |  |  |  |  | | | |  |  |  |  |  |
| 1 | Vvi-Vitvi09g04066\_t001 |  |  |  |  |  | | | |  |  |  |  |  |
| 1 | Vvi-Vitvi09g00253\_t001 |  |  |  |  |  | | | |  |  |  |  |  |
| 1 | Vvi-Vitvi09g00255\_t001 |  |  |  |  |  | Ath-AT1G51860.1 |  |  |  |  |  |
| 1 | Vvi-Vitvi09g04067\_t001 |  |  |  |  |  | | | |  |  |  |  |  |
| 1 | Vvi-Vitvi09g00257\_t001 |  |  |  |  |  | | | |  |  |  |  |  |
| 1 | Vvi-Vitvi09g00258\_t001 |  |  |  |  |  | | | |  |  |  |  |  |
| 1 | Vvi-Vitvi09g00259\_t001 |  |  |  |  |  | | | |  |  |  |  |  |
| 1 | Vvi-Vitvi09g01558\_t001 |  |  |  |  |  | | | |  |  |  |  |  |
| 1 | Vvi-Vitvi09g00260\_t001 |  |  |  |  |  | | | |  |  |  |  |  |
| 1 | Vvi-Vitvi09g04068\_t001 |  |  |  |  |  | | | |  |  |  |  |  |
| 1 | Vvi-Vitvi09g04069\_t001 |  |  |  |  |  | | | |  |  |  |  |  |
| 1 | Vvi-Vitvi09g04070\_t001 |  |  |  |  |  | | | |  |  |  |  |  |
| 1 | Vvi-Vitvi09g04071\_t001 |  |  |  |  |  | | | |  |  |  |  |  |
| 1 | Vvi-Vitvi09g01560\_t001 |  |  |  |  |  | | | |  |  |  |  |  |
| 1 | Vvi-Vitvi09g00261\_t001 |  |  |  |  |  | | | |  |  |  |  |  |
| 1 | Vvi-Vitvi09g00264\_t001 |  |  |  |  |  | | | |  |  |  |  |  |
| 1 | Vvi-Vitvi09g04072\_t001 |  |  |  |  |  | | | |  |  |  |  |  |
| 1 | Vvi-Vitvi09g00266\_t001 |  |  |  |  |  | | | |  |  |  |  |  |
| 1 | Vvi-Vitvi09g01561\_t002 |  |  |  |  |  | | | |  |  |  |  |  |
| 1 | Vvi-Vitvi09g04073\_t001 |  |  |  |  |  | | | |  |  |  |  |  |
| 1 | Vvi-Vitvi09g04074\_t001 |  |  |  |  |  | | | |  |  |  |  |  |
| 1 | Vvi-Vitvi09g04075\_t001 |  |  |  |  |  | | | |  |  |  |  |  |
| 1 | Vvi-Vitvi09g00270\_t001 |  |  |  |  |  | | | |  |  |  |  |  |
| 1 | Vvi-Vitvi09g00271\_t001 |  |  |  |  |  | Ath-AT1G51980.1 |  |  |  |  |  |
| 0 | Vvi-Vitvi09g00272\_t001 |  |  |  |  |  |  |  |  |
| 0 | Vvi-Vitvi09g01562\_t001 |  |  |  |  |  |  |  |  |
| 0 | Vvi-Vitvi09g01563\_t001 |  |  |  |  |  |  |  |  |
| 0 | Vvi-Vitvi09g01564\_t001 |  |  |  |  |  |  |  |  |
| 0 | Vvi-Vitvi09g00273\_t001 |  |  |  |  |  |  |  |  |
| 0 | Vvi-Vitvi09g01565\_t001 |  |  |  |  |  |  |  |  |
| 0 | Vvi-Vitvi09g04076\_t001 |  |  |  |  |  |  |  |  |
| 0 | Vvi-Vitvi09g04077\_t001 |  |  |  |  |  |  |  |  |
| 0 | Vvi-Vitvi09g01566\_t001 |  |  |  |  |  |  |  |  |
| 0 | Vvi-Vitvi09g01567\_t001 |  |  |  |  |  |  |  |  |
| 0 | Vvi-Vitvi09g01568\_t001 |  |  |  |  |  |  |  |  |
| 0 | Vvi-Vitvi09g04078\_t001 |  |  |  |  |  |  |  |  |
| 0 | Vvi-Vitvi09g00275\_t001 |  |  |  |  |  |  |  |  |
| 0 | Vvi-Vitvi09g01571\_t001 |  |  |  |  |  |  |  |  |
| 1 | Vvi-Vitvi09g00276\_t002 |  | Ath-AT3G16490.1 |  |  |  |  |  |  |  |
| 1 | Vvi-Vitvi09g00277\_t001 |  | | | |  |  |  |  |  |  |  |
| 1 | Vvi-Vitvi09g00279\_t001 |  | | | |  |  |  |  |  |  |  |
| 1 | Vvi-Vitvi09g00281\_t001 |  | | | |  |  |  |  |  |  |  |
| 1 | Vvi-Vitvi09g00282\_t001 |  | | | |  |  |  |  |  |  |  |
| 1 | Vvi-Vitvi09g00283\_t001 |  | | | |  |  |  |  |  |  |  |
| 1 | Vvi-Vitvi09g04079\_t001 |  | | | |  |  |  |  |  |  |  |
| 1 | Vvi-Vitvi09g00284\_t001 |  | | | |  |  |  |  |  |  |  |
| 1 | Vvi-Vitvi09g00285\_t001 |  | | | |  |  |  |  |  |  |  |
| 1 | Vvi-Vitvi09g00286\_t001 |  | | | |  |  |  |  |  |  |  |
| 1 | Vvi-Vitvi09g00287\_t001 |  | | | |  |  |  |  |  |  |  |
| 2 | Vvi-Vitvi09g00288\_t001 |  | | | |  | Ath-AT1G79730.1 |  |  |  |  |  |  |
| 2 | Vvi-Vitvi09g04080\_t001 |  | | | |  | | | |  |  |  |  |  |  |
| 2 | Vvi-Vitvi09g00290\_t001 |  | Ath-AT3G16360.2 |  | | | |  |  |  |  |  |  |
| 2 | Vvi-Vitvi09g04081\_t001 |  | | | |  | | | |  |  |  |  |  |  |
| 2 | Vvi-Vitvi09g04082\_t001 |  | | | |  | | | |  |  |  |  |  |  |
| 2 | Vvi-Vitvi09g04083\_t001 |  | | | |  | | | |  |  |  |  |  |  |
| 2 | Vvi-Vitvi09g00293\_t001 |  | Ath-AT3G16350.1 |  | | | |  |  |  |  |  |  |
| 2 | Vvi-Vitvi09g04084\_t001 |  | Ath-AT3G16340.1 |  | | | |  |  |  |  |  |  |
| 2 | Vvi-Vitvi09g04085\_t001 |  | | | |  | | | |  |  |  |  |  |  |
| 2 | Vvi-Vitvi09g00295\_t001 |  | | | |  | | | |  |  |  |  |  |  |
| 2 | Vvi-Vitvi09g00296\_t001 |  | | | |  | | | |  |  |  |  |  |  |
| 2 | Vvi-Vitvi09g04086\_t001 |  | | | |  | | | |  |  |  |  |  |  |
| 2 | Vvi-Vitvi09g04087\_t001 |  | | | |  | | | |  |  |  |  |  |  |
| 2 | Vvi-Vitvi09g04088\_t001 |  | | | |  | | | |  |  |  |  |  |  |
| 2 | Vvi-Vitvi09g04089\_t001 |  | | | |  | | | |  |  |  |  |  |  |
| 2 | Vvi-Vitvi09g00300\_t001 |  | | | |  | | | |  |  |  |  |  |  |
| 2 | Vvi-Vitvi09g00301\_t001 |  | | | |  | Ath-AT1G79750.1 |  |  |  |  |  |  |
| 2 | Vvi-Vitvi09g00302\_t001 |  | | | |  | | | |  |  |  |  |  |  |
| 2 | Vvi-Vitvi09g00303\_t001 |  | | | |  | | | |  |  |  |  |  |  |
| 2 | Vvi-Vitvi09g00304\_t001 |  | | | |  | Ath-AT1G79760.2 |  |  |  |  |  |  |
| 2 | Vvi-Vitvi09g00305\_t001 |  | | | |  | | | |  |  |  |  |  |  |
| 2 | Vvi-Vitvi09g00306\_t002 |  | | | |  | | | |  |  |  |  |  |  |
| 2 | Vvi-Vitvi09g00307\_t001 |  | Ath-AT3G16330.1 |  | | | |  |  |  |  |  |  |
| 2 | Vvi-Vitvi09g01579\_t001 |  | | | |  | | | |  |  |  |  |  |  |
| 2 | Vvi-Vitvi09g00308\_t001 |  | | | |  | Ath-AT1G79770.1 |  |  |  |  |  |  |
| 2 | Vvi-Vitvi09g01580\_t001 |  | | | |  | | | |  |  |  |  |  |  |
| 2 | Vvi-Vitvi09g00310\_t001 |  | | | |  | | | |  |  |  |  |  |  |
| 2 | Vvi-Vitvi09g04090\_t001 |  | | | |  | | | |  |  |  |  |  |  |
| 2 | Vvi-Vitvi09g00311\_t001 |  | | | |  | | | |  |  |  |  |  |  |
| 2 | Vvi-Vitvi09g01581\_t002 |  | | | |  | | | |  |  |  |  |  |  |
| 2 | Vvi-Vitvi09g00312\_t001 |  | Ath-AT3G16310.1 |  | | | |  |  |  |  |  |  |
| 2 | Vvi-Vitvi09g00314\_t001 |  | Ath-AT3G16300.1 |  | Ath-AT1G79780.1 |  |  |  |  |  |  |
| 2 | Vvi-Vitvi09g04091\_t001 |  | | | |  | | | |  |  |  |  |  |  |
| 2 | Vvi-Vitvi09g04092\_t001 |  | | | |  | | | |  |  |  |  |  |  |
| 2 | Vvi-Vitvi09g01583\_t001 |  | | | |  | | | |  |  |  |  |  |  |
| 2 | Vvi-Vitvi09g04093\_t001 |  | | | |  | | | |  |  |  |  |  |  |
| 2 | Vvi-Vitvi09g04094\_t001 |  | | | |  | | | |  |  |  |  |  |  |
| 2 | Vvi-Vitvi09g04095\_t001 |  | | | |  | | | |  |  |  |  |  |  |
| 2 | Vvi-Vitvi09g04096\_t001 |  | | | |  | | | |  |  |  |  |  |  |
| 2 | Vvi-Vitvi09g04097\_t001 |  | | | |  | | | |  |  |  |  |  |  |
| 2 | Vvi-Vitvi09g00317\_t001 |  | | | |  | | | |  |  |  |  |  |  |
| 2 | Vvi-Vitvi09g01587\_t001 |  | | | |  | | | |  |  |  |  |  |  |
| 2 | Vvi-Vitvi09g04098\_t001 |  | | | |  | | | |  |  |  |  |  |  |
| 2 | Vvi-Vitvi09g04099\_t001 |  | | | |  | | | |  |  |  |  |  |  |
| 2 | Vvi-Vitvi09g00318\_t001 |  | | | |  | | | |  |  |  |  |  |  |
| 2 | Vvi-Vitvi09g01588\_t001 |  | | | |  | | | |  |  |  |  |  |  |
| 2 | Vvi-Vitvi09g01589\_t001 |  | | | |  | | | |  |  |  |  |  |  |
| 2 | Vvi-Vitvi09g00319\_t002 |  | Ath-AT3G16290.1 |  | | | |  |  |  |  |  |  |
| 2 | Vvi-Vitvi09g00320\_t001 |  | | | |  | Ath-AT1G79800.1 |  |  |  |  |  |  |
| 2 | Vvi-Vitvi09g00321\_t001 |  | | | |  | | | |  |  |  |  |  |  |
| 2 | Vvi-Vitvi09g01590\_t001 |  | | | |  | | | |  |  |  |  |  |  |
| 2 | Vvi-Vitvi09g04100\_t001 |  | | | |  | | | |  |  |  |  |  |  |
| 2 | Vvi-Vitvi09g00323\_t001 |  | Ath-AT3G16280.2 |  | | | |  |  |  |  |  |  |
| 3 | Vvi-Vitvi09g00324\_t001 |  | | | |  | | | |  | Ath-AT1G52155.1 |  |  |  |  |  |
| 3 | Vvi-Vitvi09g01591\_t001 |  | | | |  | | | |  | | | |  |  |  |  |  |
| 3 | Vvi-Vitvi09g01592\_t001 |  | | | |  | | | |  | | | |  |  |  |  |  |
| 3 | Vvi-Vitvi09g01593\_t001 |  | | | |  | | | |  | | | |  |  |  |  |  |
| 3 | Vvi-Vitvi09g00326\_t001 |  | Ath-AT3G16270.1 |  | | | |  | | | |  |  |  |  |  |
| 3 | Vvi-Vitvi09g00327\_t001 |  | Ath-AT3G16260.1 |  | | | |  | Ath-AT1G52160.1 |  |  |  |  |  |
| 3 | Vvi-Vitvi09g00328\_t001 |  | Ath-AT3G16250.1 |  | | | |  | | | |  |  |  |  |  |
| 3 | Vvi-Vitvi09g00329\_t001 |  | Ath-AT3G16240.1 |  | | | |  | | | |  |  |  |  |  |
| 3 | Vvi-Vitvi09g00331\_t001 |  | | | |  | | | |  | | | |  |  |  |  |  |
| 3 | Vvi-Vitvi09g00332\_t001 |  | | | |  | | | |  | | | |  |  |  |  |  |
| 3 | Vvi-Vitvi09g00333\_t001 |  | Ath-AT3G16220.1 |  | | | |  | | | |  |  |  |  |  |
| 3 | Vvi-Vitvi09g04101\_t001 |  | | | |  | | | |  | | | |  |  |  |  |  |
| 3 | Vvi-Vitvi09g00334\_t001 |  | | | |  | | | |  | | | |  |  |  |  |  |
| 3 | Vvi-Vitvi09g00335\_t001 |  | Ath-AT3G16200.1 |  | | | |  | | | |  |  |  |  |  |
| 3 | Vvi-Vitvi09g00336\_t002 |  | | | |  | | | |  | | | |  |  |  |  |  |
| 3 | Vvi-Vitvi09g00337\_t001 |  | | | |  | | | |  | | | |  |  |  |  |  |
| 3 | Vvi-Vitvi09g00338\_t001 |  | | | |  | Ath-AT1G79810.1 |  | | | |  |  |  |  |  |
| 3 | Vvi-Vitvi09g00339\_t001 |  | | | |  | | | |  | | | |  |  |  |  |  |
| 3 | Vvi-Vitvi09g00340\_t001 |  | | | |  | | | |  | Ath-AT1G52190.1 |  |  |  |  |  |
| 3 | Vvi-Vitvi09g00341\_t001 |  | Ath-AT3G16190.1 |  | | | |  | | | |  |  |  |  |  |
| 3 | Vvi-Vitvi09g01596\_t001 |  | | | |  | | | |  | | | |  |  |  |  |  |
| 3 | Vvi-Vitvi09g01597\_t001 |  | Ath-AT3G16175.1 |  | | | |  | Ath-AT1G52191.1 |  |  |  |  |  |
| 3 | Vvi-Vitvi09g04102\_t001 |  | | | |  | | | |  | | | |  |  |  |  |  |
| 3 | Vvi-Vitvi09g00342\_t001 |  | Ath-AT3G16170.1 |  | | | |  | | | |  |  |  |  |  |
| 3 | Vvi-Vitvi09g00343\_t001 |  | | | |  | | | |  | Ath-AT1G52200.1 |  |  |  |  |  |
| 4 | Vvi-Vitvi09g00345\_t001 |  | | | |  | | | |  | | | |  | Ath-AT1G15220.2 |  |  |  |  |
| 5 | Vvi-Vitvi09g00347\_t001 |  | | | |  | | | |  | | | |  | | | |  | Ath-AT1G52290.1 |  |  |  |
| 5 | Vvi-Vitvi09g00348\_t001 |  | | | |  | | | |  | | | |  | | | |  | Ath-AT1G52280.1 |  |  |  |
| 5 | Vvi-Vitvi09g01598\_t001 |  | | | |  | | | |  | | | |  | Ath-AT1G15230.1 |  | | | |  |  |  |
| 5 | Vvi-Vitvi09g00350\_t001 |  | | | |  | | | |  | | | |  | | | |  | | | |  |  |  |
| 5 | Vvi-Vitvi09g00351\_t001 |  | | | |  | | | |  | Ath-AT1G52260.1 |  | | | |  | | | |  |  |  |
| 5 | Vvi-Vitvi09g00352\_t001 |  | | | |  | | | |  | | | |  | | | |  | | | |  |  |  |
| 5 | Vvi-Vitvi09g04103\_t001 |  | | | |  | | | |  | | | |  | | | |  | | | |  |  |  |
| 5 | Vvi-Vitvi09g00355\_t001 |  | | | |  | | | |  | | | |  | | | |  | Ath-AT1G52245.1 |  |  |  |
| 5 | Vvi-Vitvi09g00356\_t001 |  | | | |  | Ath-AT1G79870.1 |  | | | |  | | | |  | | | |  |  |  |
| 5 | Vvi-Vitvi09g00358\_t001 |  | | | |  | | | |  | | | |  | | | |  | | | |  |  |  |
| 5 | Vvi-Vitvi09g00359\_t001 |  | | | |  | | | |  | | | |  | | | |  | Ath-AT1G52240.1 |  |  |  |
| 5 | Vvi-Vitvi09g00361\_t001 |  | Ath-AT3G16140.1 |  | | | |  | | | |  | | | |  | Ath-AT1G52230.1 |  |  |  |
| 5 | Vvi-Vitvi09g04104\_t001 |  | | | |  | | | |  | | | |  | | | |  | | | |  |  |  |
| 5 | Vvi-Vitvi09g04105\_t001 |  | | | |  | | | |  | | | |  | | | |  | | | |  |  |  |
| 5 | Vvi-Vitvi09g01600\_t001 |  | | | |  | | | |  | | | |  | | | |  | | | |  |  |  |
| 5 | Vvi-Vitvi09g00363\_t001 |  | | | |  | | | |  | | | |  | | | |  | | | |  |  |  |
| 5 | Vvi-Vitvi09g04106\_t001 |  | | | |  | | | |  | | | |  | | | |  | | | |  |  |  |
| 5 | Vvi-Vitvi09g01601\_t001 |  | | | |  | | | |  | | | |  | | | |  | Ath-AT1G52220.1 |  |  |  |
| 4 | Vvi-Vitvi09g00365\_t001 |  | | | |  | | | |  | | | |  | | | |  |  |  |  |
| 4 | Vvi-Vitvi09g04107\_t001 |  | | | |  | | | |  | | | |  | | | |  |  |  |  |
| 4 | Vvi-Vitvi09g00366\_t001 |  | | | |  | | | |  | | | |  | | | |  |  |  |  |
| 4 | Vvi-Vitvi09g01602\_t001 |  | | | |  | | | |  | | | |  | | | |  |  |  |  |
| 4 | Vvi-Vitvi09g00367\_t001 |  | | | |  | | | |  | | | |  | | | |  |  |  |  |
| 4 | Vvi-Vitvi09g00368\_t001 |  | | | |  | | | |  | | | |  | | | |  |  |  |  |
| 4 | Vvi-Vitvi09g00369\_t001 |  | | | |  | | | |  | | | |  | Ath-AT1G15240.2 |  |  |  |  |
| 4 | Vvi-Vitvi09g00370\_t001 |  | Ath-AT3G16080.1 |  | | | |  | Ath-AT1G52300.1 |  | Ath-AT1G15250.2 |  |  |  |  |
| 4 | Vvi-Vitvi09g00371\_t001 |  | | | |  | Ath-AT1G79890.1 |  | | | |  | | | |  |  |  |  |
| 4 | Vvi-Vitvi09g00372\_t001 |  | Ath-AT3G16070.1 |  | | | |  | | | |  | Ath-AT1G15260.1 |  |  |  |  |
| 4 | Vvi-Vitvi09g01604\_t001 |  | | | |  | | | |  | | | |  | | | |  |  |  |  |
| 4 | Vvi-Vitvi09g00373\_t001 |  | Ath-AT3G16060.1 |  | | | |  | | | |  | | | |  |  |  |  |
| 4 | Vvi-Vitvi09g04108\_t001 |  | | | |  | | | |  | | | |  | | | |  |  |  |  |
| 4 | Vvi-Vitvi09g00375\_t001 |  | Ath-AT3G16050.1 |  | | | |  | | | |  | | | |  |  |  |  |
| 4 | Vvi-Vitvi09g04109\_t001 |  | | | |  | | | |  | | | |  | | | |  |  |  |  |
| 4 | Vvi-Vitvi09g00376\_t001 |  | | | |  | | | |  | | | |  | | | |  |  |  |  |
| 4 | Vvi-Vitvi09g00377\_t001 |  | | | |  | Ath-AT1G79900.1 |  | | | |  | | | |  |  |  |  |
| 4 | Vvi-Vitvi09g00378\_t001 |  | | | |  | | | |  | | | |  | | | |  |  |  |  |
| 4 | Vvi-Vitvi09g01606\_t001 |  | Ath-AT3G16040.1 |  | | | |  | | | |  | Ath-AT1G15270.1 |  |  |  |  |
| 3 | Vvi-Vitvi09g04110\_t001 |  | Ath-AT3G16030.6 |  | | | |  | | | |  |  |  |  |  |
| 3 | Vvi-Vitvi09g01607\_t001 |  | | | |  | | | |  | | | |  |  |  |  |  |
| 3 | Vvi-Vitvi09g00380\_t001 |  | | | |  | | | |  | | | |  |  |  |  |  |
| 3 | Vvi-Vitvi09g00382\_t001 |  | | | |  | | | |  | | | |  |  |  |  |  |
| 3 | Vvi-Vitvi09g00383\_t002 |  | | | |  | | | |  | | | |  |  |  |  |  |
| 3 | Vvi-Vitvi09g00384\_t001 |  | | | |  | Ath-AT1G79915.1 |  | | | |  |  |  |  |  |
| 3 | Vvi-Vitvi09g00385\_t001 |  | | | |  | | | |  | Ath-AT1G52315.1 |  |  |  |  |  |
| 3 | Vvi-Vitvi09g04111\_t001 |  | | | |  | | | |  | | | |  |  |  |  |  |
| 3 | Vvi-Vitvi09g00386\_t001 |  | | | |  | | | |  | Ath-AT1G52320.5 |  |  |  |  |  |
| 3 | Vvi-Vitvi09g00387\_t001 |  | | | |  | | | |  | | | |  |  |  |  |  |
| 3 | Vvi-Vitvi09g00388\_t001 |  | | | |  | | | |  | Ath-AT1G52330.2 |  |  |  |  |  |
| 3 | Vvi-Vitvi09g04112\_t001 |  | | | |  | | | |  | | | |  |  |  |  |  |
| 3 | Vvi-Vitvi09g04113\_t001 |  | | | |  | | | |  | | | |  |  |  |  |  |
| 3 | Vvi-Vitvi09g00389\_t001 |  | | | |  | | | |  | Ath-AT1G52343.1 |  |  |  |  |  |
| 3 | Vvi-Vitvi09g00390\_t001 |  | | | |  | Ath-AT1G79960.1 |  | | | |  |  |  |  |  |
| 3 | Vvi-Vitvi09g00391\_t001 |  | | | |  | | | |  | | | |  |  |  |  |  |
| 3 | Vvi-Vitvi09g00392\_t002 |  | | | |  | | | |  | | | |  |  |  |  |  |
| 3 | Vvi-Vitvi09g01610\_t001 |  | Ath-AT3G16010.1 |  | | | |  | | | |  |  |  |  |  |
| 3 | Vvi-Vitvi09g00393\_t001 |  | | | |  | | | |  | | | |  |  |  |  |  |
| 3 | Vvi-Vitvi09g01611\_t001 |  | | | |  | | | |  | | | |  |  |  |  |  |
| 3 | Vvi-Vitvi09g00394\_t001 |  | | | |  | | | |  | | | |  |  |  |  |  |
| 3 | Vvi-Vitvi09g00395\_t003 |  | Ath-AT3G16000.1 |  | | | |  | | | |  |  |  |  |  |
| 3 | Vvi-Vitvi09g00397\_t001 |  | | | |  | Ath-AT1G79985.1 |  | | | |  |  |  |  |  |
| 3 | Vvi-Vitvi09g00398\_t001 |  | | | |  | | | |  | | | |  |  |  |  |  |
| 3 | Vvi-Vitvi09g00399\_t001 |  | Ath-AT3G15990.1 |  | | | |  | | | |  |  |  |  |  |
| 3 | Vvi-Vitvi09g04114\_t001 |  | | | |  | | | |  | | | |  |  |  |  |  |
| 3 | Vvi-Vitvi09g04115\_t001 |  | | | |  | | | |  | | | |  |  |  |  |  |
| 3 | Vvi-Vitvi09g04116\_t001 |  | | | |  | | | |  | | | |  |  |  |  |  |
| 3 | Vvi-Vitvi09g04117\_t001 |  | | | |  | | | |  | | | |  |  |  |  |  |
| 3 | Vvi-Vitvi09g04118\_t001 |  | | | |  | | | |  | | | |  |  |  |  |  |
| 3 | Vvi-Vitvi09g00400\_t001 |  | Ath-AT3G15980.5 |  | Ath-AT1G79990.1 |  | Ath-AT1G52360.2 |  |  |  |  |  |
| 3 | Vvi-Vitvi09g00401\_t003 |  | | | |  | | | |  | | | |  |  |  |  |  |
| 3 | Vvi-Vitvi09g00402\_t001 |  | Ath-AT3G15970.1 |  | | | |  | Ath-AT1G52380.2 |  |  |  |  |  |
| 3 | Vvi-Vitvi09g01616\_t001 |  | | | |  | | | |  | | | |  |  |  |  |  |
| 3 | Vvi-Vitvi09g01617\_t001 |  | | | |  | | | |  | | | |  |  |  |  |  |
| 3 | Vvi-Vitvi09g01618\_t001 |  | | | |  | | | |  | | | |  |  |  |  |  |
| 3 | Vvi-Vitvi09g00404\_t003 |  | | | |  | | | |  | | | |  |  |  |  |  |
| 3 | Vvi-Vitvi09g00405\_t001 |  | | | |  | Ath-AT1G80000.2 |  | | | |  |  |  |  |  |
| 2 | Vvi-Vitvi09g00406\_t001 |  | Ath-AT3G15940.2 |  |  |  | Ath-AT1G52420.1 |  |  |  |  |  |
| 2 | Vvi-Vitvi09g00409\_t001 |  | | | |  |  |  | | | |  |  |  |  |  |
| 2 | Vvi-Vitvi09g04119\_t001 |  | | | |  |  |  | | | |  |  |  |  |  |
| 2 | Vvi-Vitvi09g04120\_t001 |  | | | |  |  |  | | | |  |  |  |  |  |
| 2 | Vvi-Vitvi09g04121\_t001 |  | | | |  |  |  | | | |  |  |  |  |  |
| 2 | Vvi-Vitvi09g04122\_t001 |  | | | |  |  |  | | | |  |  |  |  |  |
| 2 | Vvi-Vitvi09g01621\_t001 |  | | | |  |  |  | | | |  |  |  |  |  |
| 2 | Vvi-Vitvi09g04123\_t001 |  | | | |  |  |  | | | |  |  |  |  |  |
| 2 | Vvi-Vitvi09g04124\_t001 |  | | | |  |  |  | | | |  |  |  |  |  |
| 2 | Vvi-Vitvi09g04125\_t001 |  | | | |  |  |  | | | |  |  |  |  |  |
| 2 | Vvi-Vitvi09g00412\_t001 |  | | | |  |  |  | | | |  |  |  |  |  |
| 2 | Vvi-Vitvi09g01622\_t001 |  | | | |  |  |  | | | |  |  |  |  |  |
| 2 | Vvi-Vitvi09g04126\_t001 |  | | | |  |  |  | | | |  |  |  |  |  |
| 2 | Vvi-Vitvi09g00416\_t001 |  | | | |  |  |  | | | |  |  |  |  |  |
| 2 | Vvi-Vitvi09g01624\_t001 |  | | | |  |  |  | | | |  |  |  |  |  |
| 2 | Vvi-Vitvi09g04127\_t001 |  | Ath-AT3G15700.1 |  |  |  | | | |  |  |  |  |  |
| 2 | Vvi-Vitvi09g04128\_t001 |  | | | |  |  |  | | | |  |  |  |  |  |
| 2 | Vvi-Vitvi09g00418\_t001 |  | | | |  |  |  | | | |  |  |  |  |  |
| 2 | Vvi-Vitvi09g04129\_t001 |  | | | |  |  |  | | | |  |  |  |  |  |
| 2 | Vvi-Vitvi09g00419\_t001 |  | | | |  |  |  | | | |  |  |  |  |  |
| 2 | Vvi-Vitvi09g00420\_t001 |  | | | |  |  |  | | | |  |  |  |  |  |
| 2 | Vvi-Vitvi09g04130\_t001 |  | | | |  |  |  | Ath-AT1G52660.1 |  |  |  |  |  |
| 2 | Vvi-Vitvi09g04131\_t001 |  | | | |  |  |  | | | |  |  |  |  |  |
| 2 | Vvi-Vitvi09g04132\_t001 |  | | | |  |  |  | | | |  |  |  |  |  |
| 2 | Vvi-Vitvi09g04133\_t001 |  | | | |  |  |  | | | |  |  |  |  |  |
| 2 | Vvi-Vitvi09g04134\_t001 |  | | | |  |  |  | | | |  |  |  |  |  |
| 2 | Vvi-Vitvi09g00426\_t001 |  | | | |  |  |  | | | |  |  |  |  |  |
| 4 | Vvi-Vitvi09g00427\_t001 |  | | | |  | Ath-AT1G80440.1 |  | | | |  | Ath-AT1G15670.1 |  |  |  |  |
| 4 | Vvi-Vitvi09g00428\_t001 |  | | | |  | | | |  | | | |  | Ath-AT1G15660.1 |  |  |  |  |
| 4 | Vvi-Vitvi09g00429\_t002 |  | | | |  | Ath-AT1G80420.1 |  | | | |  | | | |  |  |  |  |
| 4 | Vvi-Vitvi09g00430\_t001 |  | | | |  | Ath-AT1G80410.2 |  | | | |  | | | |  |  |  |  |
| 4 | Vvi-Vitvi09g00431\_t001 |  | | | |  | Ath-AT1G80400.1 |  | | | |  | | | |  |  |  |  |
| 4 | Vvi-Vitvi09g00432\_t001 |  | | | |  | | | |  | | | |  | | | |  |  |  |  |
| 4 | Vvi-Vitvi09g00433\_t001 |  | | | |  | | | |  | | | |  | | | |  |  |  |  |
| 4 | Vvi-Vitvi09g04135\_t001 |  | | | |  | | | |  | | | |  | | | |  |  |  |  |
| 4 | Vvi-Vitvi09g00434\_t001 |  | | | |  | | | |  | | | |  | | | |  |  |  |  |
| 4 | Vvi-Vitvi09g00435\_t001 |  | | | |  | | | |  | | | |  | | | |  |  |  |  |
| 4 | Vvi-Vitvi09g00436\_t001 |  | Ath-AT3G15540.1 |  | | | |  | Ath-AT1G52830.1 |  | Ath-AT1G15580.1 |  |  |  |  |
| 2 | Vvi-Vitvi09g00437\_t001 |  |  |  | | | |  |  |  | | | |  |  |  |  |
| 2 | Vvi-Vitvi09g00438\_t002 |  |  |  | | | |  |  |  | | | |  |  |  |  |
| 2 | Vvi-Vitvi09g04136\_t001 |  |  |  | | | |  |  |  | | | |  |  |  |  |
| 2 | Vvi-Vitvi09g00439\_t001 |  |  |  | Ath-AT1G80380.2 |  |  |  | | | |  |  |  |  |
| 2 | Vvi-Vitvi09g00440\_t001 |  |  |  | | | |  |  |  | | | |  |  |  |  |
| 2 | Vvi-Vitvi09g04137\_t001 |  |  |  | | | |  |  |  | | | |  |  |  |  |
| 2 | Vvi-Vitvi09g04138\_t001 |  |  |  | | | |  |  |  | | | |  |  |  |  |
| 2 | Vvi-Vitvi09g04139\_t001 |  |  |  | | | |  |  |  | | | |  |  |  |  |
| 2 | Vvi-Vitvi09g00442\_t001 |  |  |  | | | |  |  |  | | | |  |  |  |  |
| 2 | Vvi-Vitvi09g00443\_t001 |  |  |  | Ath-AT1G80370.1 |  |  |  | Ath-AT1G15570.1 |  |  |  |  |
| 2 | Vvi-Vitvi09g00444\_t001 |  |  |  | Ath-AT1G80360.1 |  |  |  | | | |  |  |  |  |
| 2 | Vvi-Vitvi09g00446\_t001 |  |  |  | Ath-AT1G80350.1 |  |  |  | | | |  |  |  |  |
| 2 | Vvi-Vitvi09g04140\_t001 |  |  |  | | | |  |  |  | | | |  |  |  |  |
| 2 | Vvi-Vitvi09g04141\_t001 |  |  |  | | | |  |  |  | | | |  |  |  |  |
| 2 | Vvi-Vitvi09g00448\_t001 |  |  |  | Ath-AT1G80330.1 |  |  |  | Ath-AT1G15550.1 |  |  |  |  |
| 2 | Vvi-Vitvi09g04142\_t001 |  |  |  | | | |  |  |  | | | |  |  |  |  |
| 3 | Vvi-Vitvi09g00449\_t001 |  | Ath-AT1G52790.1 |  | | | |  |  |  | | | |  |  |  |  |
| 3 | Vvi-Vitvi09g00450\_t001 |  | | | |  | Ath-AT1G80320.1 |  |  |  | | | |  |  |  |  |
| 3 | Vvi-Vitvi09g00451\_t001 |  | | | |  | | | |  |  |  | Ath-AT1G15540.3 |  |  |  |  |
| 3 | Vvi-Vitvi09g04143\_t001 |  | | | |  | | | |  |  |  | | | |  |  |  |  |
| 3 | Vvi-Vitvi09g04144\_t001 |  | | | |  | | | |  |  |  | | | |  |  |  |  |
| 3 | Vvi-Vitvi09g04145\_t001 |  | | | |  | | | |  |  |  | | | |  |  |  |  |
| 3 | Vvi-Vitvi09g00452\_t001 |  | | | |  | | | |  |  |  | | | |  |  |  |  |
| 3 | Vvi-Vitvi09g00453\_t001 |  | | | |  | | | |  |  |  | | | |  |  |  |  |
| 3 | Vvi-Vitvi09g00454\_t001 |  | | | |  | | | |  |  |  | | | |  |  |  |  |
| 3 | Vvi-Vitvi09g00455\_t001 |  | | | |  | | | |  |  |  | | | |  |  |  |  |
| 3 | Vvi-Vitvi09g01633\_t001 |  | | | |  | | | |  |  |  | | | |  |  |  |  |
| 3 | Vvi-Vitvi09g00457\_t001 |  | | | |  | | | |  |  |  | | | |  |  |  |  |
| 3 | Vvi-Vitvi09g04146\_t001 |  | | | |  | | | |  |  |  | Ath-AT1G15520.1 |  |  |  |  |
| 3 | Vvi-Vitvi09g04147\_t001 |  | | | |  | | | |  |  |  | | | |  |  |  |  |
| 3 | Vvi-Vitvi09g04148\_t001 |  | | | |  | | | |  |  |  | | | |  |  |  |  |
| 3 | Vvi-Vitvi09g00462\_t001 |  | | | |  | | | |  |  |  | | | |  |  |  |  |
| 3 | Vvi-Vitvi09g04149\_t001 |  | | | |  | | | |  |  |  | | | |  |  |  |  |
| 3 | Vvi-Vitvi09g04150\_t001 |  | | | |  | | | |  |  |  | | | |  |  |  |  |
| 3 | Vvi-Vitvi09g04151\_t001 |  | | | |  | | | |  |  |  | | | |  |  |  |  |
| 3 | Vvi-Vitvi09g00476\_t001 |  | | | |  | | | |  |  |  | | | |  |  |  |  |
| 3 | Vvi-Vitvi09g04152\_t001 |  | | | |  | | | |  |  |  | | | |  |  |  |  |
| 3 | Vvi-Vitvi09g04153\_t001 |  | | | |  | | | |  |  |  | | | |  |  |  |  |
| 3 | Vvi-Vitvi09g04154\_t001 |  | | | |  | | | |  |  |  | | | |  |  |  |  |
| 3 | Vvi-Vitvi09g00480\_t001 |  | | | |  | | | |  |  |  | | | |  |  |  |  |
| 3 | Vvi-Vitvi09g00482\_t001 |  | Ath-AT1G52780.1 |  | | | |  |  |  | | | |  |  |  |  |
| 3 | Vvi-Vitvi09g04155\_t001 |  | | | |  | | | |  |  |  | | | |  |  |  |  |
| 3 | Vvi-Vitvi09g04156\_t001 |  | | | |  | | | |  |  |  | | | |  |  |  |  |
| 3 | Vvi-Vitvi09g00484\_t001 |  | | | |  | Ath-AT1G80310.1 |  |  |  | | | |  |  |  |  |
| 3 | Vvi-Vitvi09g04157\_t001 |  | | | |  | | | |  |  |  | | | |  |  |  |  |
| 4 | Vvi-Vitvi09g00486\_t001 |  | | | |  | | | |  | Ath-AT3G15550.1 |  | | | |  |  |  |  |
| 4 | Vvi-Vitvi09g00487\_t001 |  | | | |  | | | |  | | | |  | Ath-AT1G15510.1 |  |  |  |  |
| 4 | Vvi-Vitvi09g00488\_t001 |  | Ath-AT1G52770.1 |  | | | |  | Ath-AT3G15570.1 |  | | | |  |  |  |  |
| 4 | Vvi-Vitvi09g00489\_t001 |  | | | |  | Ath-AT1G80300.1 |  | | | |  | Ath-AT1G15500.1 |  |  |  |  |
| 4 | Vvi-Vitvi09g00490\_t001 |  | | | |  | | | |  | | | |  | | | |  |  |  |  |
| 4 | Vvi-Vitvi09g00491\_t001 |  | Ath-AT1G52760.1 |  | | | |  | | | |  | | | |  |  |  |  |
| 4 | Vvi-Vitvi09g00492\_t001 |  | Ath-AT1G52750.1 |  | Ath-AT1G80280.1 |  | | | |  | Ath-AT1G15490.1 |  |  |  |  |
| 4 | Vvi-Vitvi09g04158\_t001 |  | | | |  | | | |  | | | |  | | | |  |  |  |  |
| 4 | Vvi-Vitvi09g04159\_t001 |  | | | |  | | | |  | | | |  | | | |  |  |  |  |
| 4 | Vvi-Vitvi09g00493\_t001 |  | | | |  | | | |  | Ath-AT3G15605.4 |  | | | |  |  |  |  |
| 4 | Vvi-Vitvi09g04160\_t001 |  | Ath-AT1G52740.1 |  | | | |  | | | |  | | | |  |  |  |  |
| 4 | Vvi-Vitvi09g00496\_t001 |  | Ath-AT1G52730.2 |  | | | |  | Ath-AT3G15610.1 |  | Ath-AT1G15470.1 |  |  |  |  |
| 4 | Vvi-Vitvi09g00499\_t001 |  | | | |  | | | |  | | | |  | | | |  |  |  |  |
| 4 | Vvi-Vitvi09g00500\_t001 |  | | | |  | | | |  | | | |  | Ath-AT1G15460.1 |  |  |  |  |
| 4 | Vvi-Vitvi09g01641\_t001 |  | Ath-AT1G52720.1 |  | | | |  | Ath-AT3G15630.1 |  | | | |  |  |  |  |
| 4 | Vvi-Vitvi09g04161\_t001 |  | | | |  | | | |  | | | |  | | | |  |  |  |  |
| 4 | Vvi-Vitvi09g04162\_t001 |  | | | |  | | | |  | | | |  | | | |  |  |  |  |
| 4 | Vvi-Vitvi09g04163\_t001 |  | | | |  | | | |  | | | |  | | | |  |  |  |  |
| 4 | Vvi-Vitvi09g00502\_t001 |  | | | |  | | | |  | | | |  | | | |  |  |  |  |
| 4 | Vvi-Vitvi09g00503\_t001 |  | | | |  | | | |  | | | |  | | | |  |  |  |  |
| 4 | Vvi-Vitvi09g04164\_t001 |  | | | |  | | | |  | | | |  | | | |  |  |  |  |
| 4 | Vvi-Vitvi09g04165\_t001 |  | | | |  | | | |  | | | |  | | | |  |  |  |  |
| 4 | Vvi-Vitvi09g00504\_t001 |  | | | |  | | | |  | | | |  | | | |  |  |  |  |
| 4 | Vvi-Vitvi09g04166\_t001 |  | | | |  | | | |  | | | |  | | | |  |  |  |  |
| 4 | Vvi-Vitvi09g04167\_t001 |  | | | |  | | | |  | | | |  | | | |  |  |  |  |
| 4 | Vvi-Vitvi09g00505\_t001 |  | | | |  | | | |  | | | |  | | | |  |  |  |  |
| 4 | Vvi-Vitvi09g00508\_t001 |  | | | |  | | | |  | | | |  | | | |  |  |  |  |
| 4 | Vvi-Vitvi09g00510\_t001 |  | | | |  | | | |  | | | |  | | | |  |  |  |  |
| 4 | Vvi-Vitvi09g00512\_t001 |  | | | |  | Ath-AT1G80240.1 |  | | | |  | | | |  |  |  |  |
| 4 | Vvi-Vitvi09g00513\_t001 |  | | | |  | | | |  | | | |  | | | |  |  |  |  |
| 4 | Vvi-Vitvi09g00514\_t001 |  | | | |  | | | |  | | | |  | | | |  |  |  |  |
| 4 | Vvi-Vitvi09g00516\_t001 |  | | | |  | | | |  | | | |  | | | |  |  |  |  |
| 4 | Vvi-Vitvi09g04168\_t001 |  | | | |  | | | |  | | | |  | | | |  |  |  |  |
| 4 | Vvi-Vitvi09g00517\_t001 |  | | | |  | | | |  | | | |  | | | |  |  |  |  |
| 4 | Vvi-Vitvi09g01645\_t001 |  | Ath-AT1G52710.1 |  | Ath-AT1G80230.1 |  | Ath-AT3G15640.1 |  | | | |  |  |  |  |
| 4 | Vvi-Vitvi09g00518\_t001 |  | Ath-AT1G52695.1 |  | | | |  | Ath-AT3G15650.2 |  | | | |  |  |  |  |
| 4 | Vvi-Vitvi09g00519\_t001 |  | | | |  | | | |  | | | |  | | | |  |  |  |  |
| 4 | Vvi-Vitvi09g00520\_t001 |  | | | |  | | | |  | | | |  | Ath-AT1G15440.1 |  |  |  |  |
| 4 | Vvi-Vitvi09g00521\_t002 |  | | | |  | | | |  | Ath-AT3G15660.1 |  | | | |  |  |  |  |
| 4 | Vvi-Vitvi09g00522\_t001 |  | | | |  | | | |  | | | |  | | | |  |  |  |  |
| 4 | Vvi-Vitvi09g00523\_t001 |  | | | |  | | | |  | | | |  | Ath-AT1G15420.1 |  |  |  |  |
| 4 | Vvi-Vitvi09g00524\_t001 |  | Ath-AT1G52690.2 |  | | | |  | Ath-AT3G15670.1 |  | | | |  |  |  |  |
| 4 | Vvi-Vitvi09g00525\_t001 |  | Ath-AT1G52680.1 |  | | | |  | | | |  | Ath-AT1G15415.1 |  |  |  |  |
| 4 | Vvi-Vitvi09g00526\_t002 |  | | | |  | Ath-AT1G80210.1 |  | | | |  | | | |  |  |  |  |
| 4 | Vvi-Vitvi09g04169\_t001 |  | | | |  | | | |  | | | |  | | | |  |  |  |  |
| 4 | Vvi-Vitvi09g04170\_t001 |  | | | |  | | | |  | Ath-AT3G15680.1 |  | | | |  |  |  |  |
| 4 | Vvi-Vitvi09g00529\_t001 |  | Ath-AT1G52670.1 |  | | | |  | Ath-AT3G15690.2 |  | | | |  |  |  |  |
| 2 | Vvi-Vitvi09g00530\_t001 |  |  |  | | | |  |  |  | | | |  |  |  |  |
| 2 | Vvi-Vitvi09g00531\_t001 |  |  |  | | | |  |  |  | | | |  |  |  |  |
| 2 | Vvi-Vitvi09g00532\_t001 |  |  |  | | | |  |  |  | | | |  |  |  |  |
| 2 | Vvi-Vitvi09g04171\_t001 |  |  |  | | | |  |  |  | | | |  |  |  |  |
| 2 | Vvi-Vitvi09g01646\_t001 |  |  |  | | | |  |  |  | | | |  |  |  |  |
| 2 | Vvi-Vitvi09g04172\_t001 |  |  |  | | | |  |  |  | | | |  |  |  |  |
| 2 | Vvi-Vitvi09g00535\_t001 |  |  |  | | | |  |  |  | | | |  |  |  |  |
| 2 | Vvi-Vitvi09g00536\_t001 |  |  |  | | | |  |  |  | | | |  |  |  |  |
| 2 | Vvi-Vitvi09g04173\_t001 |  |  |  | | | |  |  |  | | | |  |  |  |  |
| 2 | Vvi-Vitvi09g00538\_t001 |  |  |  | | | |  |  |  | | | |  |  |  |  |
| 2 | Vvi-Vitvi09g00539\_t001 |  |  |  | | | |  |  |  | Ath-AT1G15410.2 |  |  |  |  |
| 2 | Vvi-Vitvi09g00540\_t001 |  |  |  | | | |  |  |  | | | |  |  |  |  |
| 2 | Vvi-Vitvi09g01650\_t001 |  |  |  | | | |  |  |  | | | |  |  |  |  |
| 2 | Vvi-Vitvi09g04174\_t001 |  |  |  | | | |  |  |  | | | |  |  |  |  |
| 2 | Vvi-Vitvi09g00542\_t001 |  |  |  | | | |  |  |  | | | |  |  |  |  |
| 2 | Vvi-Vitvi09g04175\_t001 |  |  |  | | | |  |  |  | | | |  |  |  |  |
| 2 | Vvi-Vitvi09g01651\_t001 |  |  |  | | | |  |  |  | | | |  |  |  |  |
| 2 | Vvi-Vitvi09g00546\_t001 |  |  |  | | | |  |  |  | | | |  |  |  |  |
| 2 | Vvi-Vitvi09g00549\_t001 |  |  |  | | | |  |  |  | | | |  |  |  |  |
| 2 | Vvi-Vitvi09g00550\_t001 |  |  |  | Ath-AT1G80200.2 |  |  |  | | | |  |  |  |  |
| 2 | Vvi-Vitvi09g00551\_t001 |  |  |  | | | |  |  |  | | | |  |  |  |  |
| 2 | Vvi-Vitvi09g04176\_t001 |  |  |  | | | |  |  |  | | | |  |  |  |  |
| 2 | Vvi-Vitvi09g00553\_t001 |  |  |  | | | |  |  |  | | | |  |  |  |  |
| 2 | Vvi-Vitvi09g00554\_t001 |  |  |  | Ath-AT1G80190.5 |  |  |  | | | |  |  |  |  |
| 2 | Vvi-Vitvi09g01654\_t001 |  |  |  | Ath-AT1G80180.1 |  |  |  | Ath-AT1G15400.3 |  |  |  |  |
| 2 | Vvi-Vitvi09g00556\_t001 |  |  |  | Ath-AT1G80170.1 |  |  |  | | | |  |  |  |  |
| 2 | Vvi-Vitvi09g00557\_t001 |  |  |  | | | |  |  |  | Ath-AT1G15390.1 |  |  |  |  |
| 2 | Vvi-Vitvi09g00558\_t001 |  |  |  | | | |  |  |  | | | |  |  |  |  |
| 2 | Vvi-Vitvi09g00559\_t001 |  |  |  | Ath-AT1G80160.3 |  |  |  | Ath-AT1G15380.1 |  |  |  |  |
| 1 | Vvi-Vitvi09g00560\_t001 |  |  |  | | | |  |  |  |  |  |  |
| 1 | Vvi-Vitvi09g00561\_t001 |  |  |  | | | |  |  |  |  |  |  |
| 1 | Vvi-Vitvi09g00562\_t001 |  |  |  | | | |  |  |  |  |  |  |
| 1 | Vvi-Vitvi09g00563\_t001 |  |  |  | | | |  |  |  |  |  |  |
| 1 | Vvi-Vitvi09g00564\_t001 |  |  |  | | | |  |  |  |  |  |  |
| 1 | Vvi-Vitvi09g04177\_t001 |  |  |  | | | |  |  |  |  |  |  |
| 1 | Vvi-Vitvi09g04178\_t001 |  |  |  | | | |  |  |  |  |  |  |
| 1 | Vvi-Vitvi09g01655\_t001 |  |  |  | | | |  |  |  |  |  |  |
| 1 | Vvi-Vitvi09g00566\_t001 |  |  |  | | | |  |  |  |  |  |  |
| 1 | Vvi-Vitvi09g00567\_t001 |  |  |  | | | |  |  |  |  |  |  |
| 2 | Vvi-Vitvi09g00568\_t001 |  | Ath-AT1G52640.1 |  | | | |  |  |  |  |  |  |
| 2 | Vvi-Vitvi09g04179\_t001 |  | | | |  | | | |  |  |  |  |  |  |
| 2 | Vvi-Vitvi09g00569\_t001 |  | | | |  | | | |  |  |  |  |  |  |
| 2 | Vvi-Vitvi09g00570\_t001 |  | Ath-AT1G52630.1 |  | | | |  |  |  |  |  |  |
| 2 | Vvi-Vitvi09g00573\_t001 |  | | | |  | | | |  |  |  |  |  |  |
| 2 | Vvi-Vitvi09g00574\_t001 |  | Ath-AT1G52620.1 |  | | | |  |  |  |  |  |  |
| 2 | Vvi-Vitvi09g00575\_t001 |  | | | |  | Ath-AT1G80150.1 |  |  |  |  |  |  |
| 3 | Vvi-Vitvi09g00576\_t001 |  | Ath-AT1G52600.1 |  | | | |  | Ath-AT3G15710.1 |  |  |  |  |  |
| 3 | Vvi-Vitvi09g00577\_t001 |  | Ath-AT1G52590.1 |  | | | |  | | | |  |  |  |  |  |
| 3 | Vvi-Vitvi09g00578\_t001 |  | Ath-AT1G52580.1 |  | | | |  | | | |  |  |  |  |  |
| 4 | Vvi-Vitvi09g00579\_t001 |  | | | |  | | | |  | Ath-AT3G15720.1 |  | Ath-AT4G32380.1 |  |  |  |  |
| 4 | Vvi-Vitvi09g00582\_t001 |  | | | |  | | | |  | | | |  | | | |  |  |  |  |
| 4 | Vvi-Vitvi09g04180\_t001 |  | | | |  | | | |  | | | |  | | | |  |  |  |  |
| 4 | Vvi-Vitvi09g00584\_t001 |  | | | |  | | | |  | | | |  | | | |  |  |  |  |
| 4 | Vvi-Vitvi09g00585\_t001 |  | | | |  | | | |  | | | |  | | | |  |  |  |  |
| 4 | Vvi-Vitvi09g00587\_t001 |  | | | |  | | | |  | | | |  | | | |  |  |  |  |
| 4 | Vvi-Vitvi09g00588\_t001 |  | | | |  | | | |  | | | |  | | | |  |  |  |  |
| 4 | Vvi-Vitvi09g00590\_t001 |  | | | |  | | | |  | | | |  | | | |  |  |  |  |
| 4 | Vvi-Vitvi09g01657\_t001 |  | | | |  | | | |  | | | |  | | | |  |  |  |  |
| 4 | Vvi-Vitvi09g04181\_t001 |  | | | |  | | | |  | | | |  | | | |  |  |  |  |
| 4 | Vvi-Vitvi09g00591\_t001 |  | | | |  | | | |  | | | |  | | | |  |  |  |  |
| 5 | Vvi-Vitvi09g00592\_t001 |  | | | |  | | | |  | | | |  | | | |  | Ath-AT1G15370.1 |  |  |  |
| 5 | Vvi-Vitvi09g04182\_t001 |  | | | |  | | | |  | | | |  | | | |  | | | |  |  |  |
| 5 | Vvi-Vitvi09g00593\_t001 |  | | | |  | | | |  | | | |  | | | |  | Ath-AT1G15360.2 |  |  |  |
| 5 | Vvi-Vitvi09g04183\_t001 |  | | | |  | | | |  | | | |  | | | |  | | | |  |  |  |
| 5 | Vvi-Vitvi09g00595\_t001 |  | Ath-AT1G52570.1 |  | | | |  | Ath-AT3G15730.1 |  | | | |  | | | |  |  |  |
| 5 | Vvi-Vitvi09g04184\_t001 |  | Ath-AT1G52565.1 |  | | | |  | Ath-AT3G15760.1 |  | | | |  | | | |  |  |  |
| 5 | Vvi-Vitvi09g04185\_t001 |  | | | |  | | | |  | Ath-AT3G15770.1 |  | Ath-AT4G32342.2 |  | Ath-AT1G15350.2 |  |  |  |
| 5 | Vvi-Vitvi09g00599\_t001 |  | Ath-AT1G52560.1 |  | | | |  | | | |  | | | |  | | | |  |  |  |
| 4 | Vvi-Vitvi09g00600\_t001 |  |  |  | Ath-AT1G80133.1 |  | | | |  | | | |  | | | |  |  |  |
| 4 | Vvi-Vitvi09g01660\_t001 |  |  |  | | | |  | | | |  | | | |  | | | |  |  |  |
| 4 | Vvi-Vitvi09g00601\_t001 |  |  |  | Ath-AT1G80130.1 |  | | | |  | Ath-AT4G32340.1 |  | | | |  |  |  |
| 4 | Vvi-Vitvi09g00603\_t001 |  |  |  | | | |  | | | |  | | | |  | | | |  |  |  |
| 4 | Vvi-Vitvi09g00604\_t001 |  |  |  | | | |  | | | |  | Ath-AT4G32300.1 |  | | | |  |  |  |
| 4 | Vvi-Vitvi09g04186\_t001 |  |  |  | | | |  | | | |  | | | |  | | | |  |  |  |
| 4 | Vvi-Vitvi09g00605\_t001 |  |  |  | | | |  | | | |  | | | |  | | | |  |  |  |
| 4 | Vvi-Vitvi09g01662\_t001 |  |  |  | | | |  | | | |  | | | |  | | | |  |  |  |
| 4 | Vvi-Vitvi09g01663\_t001 |  |  |  | | | |  | | | |  | | | |  | | | |  |  |  |
| 4 | Vvi-Vitvi09g00606\_t001 |  |  |  | | | |  | Ath-AT3G15790.1 |  | | | |  | Ath-AT1G15340.1 |  |  |  |
| 4 | Vvi-Vitvi09g00607\_t002 |  |  |  | | | |  | Ath-AT3G15800.1 |  | | | |  | | | |  |  |  |
| 4 | Vvi-Vitvi09g01664\_t001 |  |  |  | | | |  | | | |  | | | |  | | | |  |  |  |
| 4 | Vvi-Vitvi09g01665\_t001 |  |  |  | | | |  | | | |  | | | |  | | | |  |  |  |
| 4 | Vvi-Vitvi09g01666\_t001 |  |  |  | | | |  | | | |  | | | |  | | | |  |  |  |
| 4 | Vvi-Vitvi09g04187\_t001 |  |  |  | | | |  | | | |  | | | |  | | | |  |  |  |
| 4 | Vvi-Vitvi09g00610\_t001 |  |  |  | Ath-AT1G80120.1 |  | Ath-AT3G15810.1 |  | | | |  | | | |  |  |  |
| 4 | Vvi-Vitvi09g01667\_t001 |  |  |  | | | |  | | | |  | | | |  | | | |  |  |  |
| 4 | Vvi-Vitvi09g04188\_t001 |  |  |  | | | |  | | | |  | | | |  | | | |  |  |  |
| 4 | Vvi-Vitvi09g04189\_t001 |  |  |  | | | |  | | | |  | | | |  | | | |  |  |  |
| 4 | Vvi-Vitvi09g00615\_t001 |  |  |  | Ath-AT1G80100.3 |  | | | |  | | | |  | | | |  |  |  |
| 4 | Vvi-Vitvi09g00616\_t001 |  |  |  | | | |  | Ath-AT3G15850.1 |  | | | |  | | | |  |  |  |
| 4 | Vvi-Vitvi09g00617\_t001 |  |  |  | | | |  | | | |  | | | |  | | | |  |  |  |
| 4 | Vvi-Vitvi09g00618\_t001 |  |  |  | | | |  | | | |  | | | |  | | | |  |  |  |
| 4 | Vvi-Vitvi09g00619\_t001 |  |  |  | Ath-AT1G80090.1 |  | | | |  | | | |  | Ath-AT1G15330.1 |  |  |  |
| 4 | Vvi-Vitvi09g00620\_t001 |  |  |  | | | |  | Ath-AT3G15880.2 |  | | | |  | | | |  |  |  |
| 4 | Vvi-Vitvi09g04190\_t001 |  |  |  | | | |  | | | |  | | | |  | | | |  |  |  |
| 4 | Vvi-Vitvi09g00621\_t001 |  |  |  | Ath-AT1G80080.1 |  | | | |  | | | |  | | | |  |  |  |
| 4 | Vvi-Vitvi09g00622\_t001 |  |  |  | | | |  | Ath-AT3G15890.1 |  | | | |  | | | |  |  |  |
| 4 | Vvi-Vitvi09g00624\_t001 |  |  |  | Ath-AT1G80070.1 |  | | | |  | | | |  | | | |  |  |  |
| 4 | Vvi-Vitvi09g00625\_t001 |  |  |  | Ath-AT1G80060.3 |  | | | |  | Ath-AT4G32270.1 |  | | | |  |  |  |
| 4 | Vvi-Vitvi09g00626\_t001 |  |  |  | | | |  | | | |  | | | |  | | | |  |  |  |
| 4 | Vvi-Vitvi09g04191\_t001 |  |  |  | | | |  | | | |  | | | |  | | | |  |  |  |
| 4 | Vvi-Vitvi09g00627\_t001 |  |  |  | | | |  | | | |  | Ath-AT4G32250.3 |  | Ath-AT1G15320.1 |  |  |  |
| 4 | Vvi-Vitvi09g00629\_t002 |  |  |  | Ath-AT1G80050.1 |  | | | |  | | | |  | | | |  |  |  |
| 4 | Vvi-Vitvi09g00630\_t001 |  |  |  | Ath-AT1G80040.1 |  | | | |  | | | |  | | | |  |  |  |
| 4 | Vvi-Vitvi09g00631\_t001 |  |  |  | | | |  | | | |  | | | |  | | | |  |  |  |
| 4 | Vvi-Vitvi09g00633\_t001 |  |  |  | | | |  | | | |  | | | |  | | | |  |  |  |
| 4 | Vvi-Vitvi09g00634\_t001 |  |  |  | | | |  | | | |  | | | |  | | | |  |  |  |
| 4 | Vvi-Vitvi09g00635\_t001 |  |  |  | Ath-AT1G80030.2 |  | | | |  | | | |  | | | |  |  |  |
| 4 | Vvi-Vitvi09g01670\_t001 |  |  |  | | | |  | Ath-AT3G15900.1 |  | | | |  | | | |  |  |  |
| 4 | Vvi-Vitvi09g01674\_t001 |  |  |  | | | |  | | | |  | | | |  | | | |  |  |  |
| 4 | Vvi-Vitvi09g04192\_t001 |  |  |  | | | |  | | | |  | | | |  | | | |  |  |  |
| 4 | Vvi-Vitvi09g00641\_t001 |  |  |  | | | |  | | | |  | | | |  | | | |  |  |  |
| 4 | Vvi-Vitvi09g00642\_t001 |  |  |  | | | |  | | | |  | | | |  | | | |  |  |  |
| 4 | Vvi-Vitvi09g04193\_t001 |  |  |  | | | |  | | | |  | | | |  | | | |  |  |  |
| 4 | Vvi-Vitvi09g04194\_t001 |  |  |  | | | |  | | | |  | | | |  | | | |  |  |  |
| 4 | Vvi-Vitvi09g04195\_t001 |  |  |  | | | |  | | | |  | | | |  | | | |  |  |  |
| 4 | Vvi-Vitvi09g04196\_t001 |  |  |  | | | |  | | | |  | | | |  | | | |  |  |  |
| 4 | Vvi-Vitvi09g00643\_t001.1.6037826f |  |  |  | | | |  | | | |  | | | |  | Ath-AT1G15310.1 |  |  |  |
| 4 | Vvi-Vitvi09g00644\_t001 |  |  |  | | | |  | Ath-AT3G15920.1 |  | Ath-AT4G32160.1 |  | | | |  |  |  |
| 3 | Vvi-Vitvi09g00647\_t001 |  |  |  | | | |  | Ath-AT3G15930.1 |  |  |  | | | |  |  |  |
| 2 | Vvi-Vitvi09g00648\_t002 |  |  |  | | | |  |  |  |  |  | | | |  |  |  |
| 2 | Vvi-Vitvi09g04197\_t001 |  |  |  | | | |  |  |  |  |  | | | |  |  |  |
| 2 | Vvi-Vitvi09g00649\_t001 |  |  |  | Ath-AT1G80010.1 |  |  |  |  |  | | | |  |  |  |
| 1 | Vvi-Vitvi09g04198\_t001 |  |  |  |  |  |  |  |  |  | | | |  |  |  |
| 1 | Vvi-Vitvi09g04199\_t001 |  |  |  |  |  |  |  |  |  | | | |  |  |  |
| 1 | Vvi-Vitvi09g04200\_t001 |  |  |  |  |  |  |  |  |  | | | |  |  |  |
| 1 | Vvi-Vitvi09g00650\_t002 |  |  |  |  |  |  |  |  |  | | | |  |  |  |
| 1 | Vvi-Vitvi09g04201\_t001 |  |  |  |  |  |  |  |  |  | | | |  |  |  |
| 1 | Vvi-Vitvi09g00653\_t002 |  |  |  |  |  |  |  |  |  | | | |  |  |  |
| 1 | Vvi-Vitvi09g00654\_t001 |  |  |  |  |  |  |  |  |  | | | |  |  |  |
| 1 | Vvi-Vitvi09g00655\_t001 |  |  |  |  |  |  |  |  |  | | | |  |  |  |
| 1 | Vvi-Vitvi09g00658\_t001 |  |  |  |  |  |  |  |  |  | | | |  |  |  |
| 1 | Vvi-Vitvi09g00659\_t001 |  |  |  |  |  |  |  |  |  | Ath-AT1G15290.1 |  |  |  |
| 0 | Vvi-Vitvi09g00660\_t001 |  |  |  |  |  |  |  |  |
| 0 | Vvi-Vitvi09g04202\_t001 |  |  |  |  |  |  |  |  |
| 0 | Vvi-Vitvi09g00664\_t001 |  |  |  |  |  |  |  |  |
| 0 | Vvi-Vitvi09g04203\_t001 |  |  |  |  |  |  |  |  |
| 0 | Vvi-Vitvi09g04204\_t001 |  |  |  |  |  |  |  |  |
| 0 | Vvi-Vitvi09g04205\_t001 |  |  |  |  |  |  |  |  |
| 0 | Vvi-Vitvi09g04206\_t001 |  |  |  |  |  |  |  |  |
| 0 | Vvi-Vitvi09g00665\_t001 |  |  |  |  |  |  |  |  |
| 0 | Vvi-Vitvi09g00666\_t001 |  |  |  |  |  |  |  |  |
| 0 | Vvi-Vitvi09g00667\_t001 |  |  |  |  |  |  |  |  |
| 0 | Vvi-Vitvi09g04207\_t001 |  |  |  |  |  |  |  |  |
| 0 | Vvi-Vitvi09g00669\_t001 |  |  |  |  |  |  |  |  |
| 0 | Vvi-Vitvi09g00671\_t001 |  |  |  |  |  |  |  |  |
| 0 | Vvi-Vitvi09g04208\_t001 |  |  |  |  |  |  |  |  |
| 0 | Vvi-Vitvi09g00673\_t001 |  |  |  |  |  |  |  |  |
| 0 | Vvi-Vitvi09g04209\_t001 |  |  |  |  |  |  |  |  |
| 0 | Vvi-Vitvi09g00674\_t001 |  |  |  |  |  |  |  |  |
| 0 | Vvi-Vitvi09g04210\_t001 |  |  |  |  |  |  |  |  |
| 0 | Vvi-Vitvi09g04211\_t001 |  |  |  |  |  |  |  |  |
| 0 | Vvi-Vitvi09g04212\_t001 |  |  |  |  |  |  |  |  |
| 0 | Vvi-Vitvi09g00677\_t001 |  |  |  |  |  |  |  |  |
| 0 | Vvi-Vitvi09g00678\_t001 |  |  |  |  |  |  |  |  |
| 0 | Vvi-Vitvi09g04213\_t001 |  |  |  |  |  |  |  |  |
| 0 | Vvi-Vitvi09g04214\_t001 |  |  |  |  |  |  |  |  |
| 0 | Vvi-Vitvi09g00680\_t001 |  |  |  |  |  |  |  |  |
| 0 | Vvi-Vitvi09g04215\_t001 |  |  |  |  |  |  |  |  |
| 0 | Vvi-Vitvi09g01693\_t001 |  |  |  |  |  |  |  |  |
| 0 | Vvi-Vitvi09g04216\_t001 |  |  |  |  |  |  |  |  |
| 0 | Vvi-Vitvi09g04217\_t001 |  |  |  |  |  |  |  |  |
| 0 | Vvi-Vitvi09g04218\_t001 |  |  |  |  |  |  |  |  |
| 0 | Vvi-Vitvi09g00682\_t001 |  |  |  |  |  |  |  |  |
| 0 | Vvi-Vitvi09g04219\_t001 |  |  |  |  |  |  |  |  |
| 0 | Vvi-Vitvi09g01694\_t001 |  |  |  |  |  |  |  |  |
| 0 | Vvi-Vitvi09g00686\_t001 |  |  |  |  |  |  |  |  |
| 0 | Vvi-Vitvi09g04220\_t001 |  |  |  |  |  |  |  |  |
| 0 | Vvi-Vitvi09g00689\_t001 |  |  |  |  |  |  |  |  |
| 0 | Vvi-Vitvi09g04221\_t001 |  |  |  |  |  |  |  |  |
| 0 | Vvi-Vitvi09g04222\_t001 |  |  |  |  |  |  |  |  |
| 0 | Vvi-Vitvi09g00691\_t001 |  |  |  |  |  |  |  |  |
| 0 | Vvi-Vitvi09g00692\_t001 |  |  |  |  |  |  |  |  |
| 1 | Vvi-Vitvi09g00693\_t001 |  | Ath-AT1G15690.1 |  |  |  |  |  |  |  |
| 1 | Vvi-Vitvi09g01698\_t001 |  | | | |  |  |  |  |  |  |  |
| 1 | Vvi-Vitvi09g04223\_t001 |  | | | |  |  |  |  |  |  |  |
| 1 | Vvi-Vitvi09g04224\_t001 |  | | | |  |  |  |  |  |  |  |
| 1 | Vvi-Vitvi09g00694\_t001 |  | Ath-AT1G15710.1 |  |  |  |  |  |  |  |
| 1 | Vvi-Vitvi09g04225\_t001 |  | | | |  |  |  |  |  |  |  |
| 1 | Vvi-Vitvi09g04226\_t001 |  | | | |  |  |  |  |  |  |  |
| 1 | Vvi-Vitvi09g00707\_t001 |  | | | |  |  |  |  |  |  |  |
| 1 | Vvi-Vitvi09g00708\_t001 |  | | | |  |  |  |  |  |  |  |
| 1 | Vvi-Vitvi09g04227\_t001 |  | | | |  |  |  |  |  |  |  |
| 1 | Vvi-Vitvi09g04228\_t001 |  | | | |  |  |  |  |  |  |  |
| 1 | Vvi-Vitvi09g00712\_t001 |  | | | |  |  |  |  |  |  |  |
| 1 | Vvi-Vitvi09g01706\_t001 |  | | | |  |  |  |  |  |  |  |
| 1 | Vvi-Vitvi09g04229\_t001 |  | | | |  |  |  |  |  |  |  |
| 1 | Vvi-Vitvi09g00717\_t001 |  | | | |  |  |  |  |  |  |  |
| 1 | Vvi-Vitvi09g00719\_t001 |  | | | |  |  |  |  |  |  |  |
| 1 | Vvi-Vitvi09g00720\_t001 |  | | | |  |  |  |  |  |  |  |
| 1 | Vvi-Vitvi09g00721\_t001 |  | | | |  |  |  |  |  |  |  |
| 1 | Vvi-Vitvi09g01708\_t001 |  | | | |  |  |  |  |  |  |  |
| 1 | Vvi-Vitvi09g00722\_t001 |  | | | |  |  |  |  |  |  |  |
| 1 | Vvi-Vitvi09g01709\_t001 |  | | | |  |  |  |  |  |  |  |
| 1 | Vvi-Vitvi09g00725\_t001 |  | | | |  |  |  |  |  |  |  |
| 1 | Vvi-Vitvi09g04230\_t001 |  | | | |  |  |  |  |  |  |  |
| 1 | Vvi-Vitvi09g04231\_t001 |  | | | |  |  |  |  |  |  |  |
| 1 | Vvi-Vitvi09g04232\_t001 |  | | | |  |  |  |  |  |  |  |
| 1 | Vvi-Vitvi09g04233\_t001 |  | | | |  |  |  |  |  |  |  |
| 1 | Vvi-Vitvi09g04234\_t001 |  | | | |  |  |  |  |  |  |  |
| 1 | Vvi-Vitvi09g04235\_t001 |  | | | |  |  |  |  |  |  |  |
| 2 | Vvi-Vitvi09g00731\_t001 |  | Ath-AT1G15730.1 |  | Ath-AT1G80480.1 |  |  |  |  |  |  |
| 2 | Vvi-Vitvi09g04236\_t001 |  | | | |  | | | |  |  |  |  |  |  |
| 2 | Vvi-Vitvi09g00733\_t002 |  | | | |  | | | |  |  |  |  |  |  |
| 2 | Vvi-Vitvi09g00734\_t001 |  | Ath-AT1G15740.1 |  | | | |  |  |  |  |  |  |
| 2 | Vvi-Vitvi09g04237\_t001 |  | | | |  | | | |  |  |  |  |  |  |
| 2 | Vvi-Vitvi09g04238\_t001 |  | | | |  | | | |  |  |  |  |  |  |
| 2 | Vvi-Vitvi09g04239\_t001 |  | | | |  | | | |  |  |  |  |  |  |
| 2 | Vvi-Vitvi09g04240\_t001 |  | | | |  | | | |  |  |  |  |  |  |
| 2 | Vvi-Vitvi09g01720\_t001 |  | | | |  | | | |  |  |  |  |  |  |
| 2 | Vvi-Vitvi09g04241\_t001 |  | | | |  | | | |  |  |  |  |  |  |
| 2 | Vvi-Vitvi09g04242\_t001 |  | | | |  | | | |  |  |  |  |  |  |
| 2 | Vvi-Vitvi09g01725\_t001 |  | | | |  | | | |  |  |  |  |  |  |
| 2 | Vvi-Vitvi09g04243\_t001 |  | | | |  | | | |  |  |  |  |  |  |
| 2 | Vvi-Vitvi09g04244\_t001 |  | | | |  | | | |  |  |  |  |  |  |
| 2 | Vvi-Vitvi09g04245\_t001 |  | | | |  | | | |  |  |  |  |  |  |
| 2 | Vvi-Vitvi09g04246\_t001 |  | | | |  | | | |  |  |  |  |  |  |
| 2 | Vvi-Vitvi09g00747\_t001 |  | | | |  | | | |  |  |  |  |  |  |
| 2 | Vvi-Vitvi09g00749\_t001 |  | | | |  | | | |  |  |  |  |  |  |
| 2 | Vvi-Vitvi09g00752\_t001 |  | | | |  | | | |  |  |  |  |  |  |
| 2 | Vvi-Vitvi09g00753\_t002 |  | Ath-AT1G15750.3 |  | Ath-AT1G80490.2 |  |  |  |  |  |  |
| 2 | Vvi-Vitvi09g01728\_t001 |  | | | |  | | | |  |  |  |  |  |  |
| 2 | Vvi-Vitvi09g01729\_t001 |  | | | |  | | | |  |  |  |  |  |  |
| 2 | Vvi-Vitvi09g00758\_t001 |  | | | |  | Ath-AT1G80510.1 |  |  |  |  |  |  |
| 2 | Vvi-Vitvi09g00759\_t001 |  | Ath-AT1G15760.1 |  | Ath-AT1G80520.1 |  |  |  |  |  |  |
| 1 | Vvi-Vitvi09g00760\_t001 |  |  |  | Ath-AT1G80530.1 |  |  |  |  |  |  |
| 1 | Vvi-Vitvi09g04247\_t001 |  |  |  | | | |  |  |  |  |  |  |
| 1 | Vvi-Vitvi09g04248\_t001 |  |  |  | | | |  |  |  |  |  |  |
| 1 | Vvi-Vitvi09g00762\_t001 |  |  |  | | | |  |  |  |  |  |  |
| 1 | Vvi-Vitvi09g00763\_t003 |  |  |  | | | |  |  |  |  |  |  |
| 1 | Vvi-Vitvi09g00767\_t001 |  |  |  | | | |  |  |  |  |  |  |
| 1 | Vvi-Vitvi09g00768\_t001 |  |  |  | | | |  |  |  |  |  |  |
| 1 | Vvi-Vitvi09g00772\_t001 |  |  |  | | | |  |  |  |  |  |  |
| 1 | Vvi-Vitvi09g04249\_t001 |  |  |  | | | |  |  |  |  |  |  |
| 1 | Vvi-Vitvi09g04250\_t001 |  |  |  | | | |  |  |  |  |  |  |
| 1 | Vvi-Vitvi09g00773\_t001 |  |  |  | Ath-AT1G80540.1 |  |  |  |  |  |  |
| 1 | Vvi-Vitvi09g00776\_t001 |  |  |  | | | |  |  |  |  |  |  |
| 1 | Vvi-Vitvi09g04251\_t001 |  |  |  | | | |  |  |  |  |  |  |
| 1 | Vvi-Vitvi09g04252\_t001 |  |  |  | | | |  |  |  |  |  |  |
| 1 | Vvi-Vitvi09g04253\_t001 |  |  |  | | | |  |  |  |  |  |  |
| 1 | Vvi-Vitvi09g00780\_t001 |  |  |  | | | |  |  |  |  |  |  |
| 1 | Vvi-Vitvi09g00782\_t001 |  |  |  | | | |  |  |  |  |  |  |
| 1 | Vvi-Vitvi09g01735\_t001 |  |  |  | | | |  |  |  |  |  |  |
| 1 | Vvi-Vitvi09g00784\_t001 |  |  |  | | | |  |  |  |  |  |  |
| 1 | Vvi-Vitvi09g00785\_t001 |  |  |  | | | |  |  |  |  |  |  |
| 1 | Vvi-Vitvi09g04254\_t001 |  |  |  | | | |  |  |  |  |  |  |
| 1 | Vvi-Vitvi09g00790\_t001 |  |  |  | | | |  |  |  |  |  |  |
| 1 | Vvi-Vitvi09g00791\_t001 |  |  |  | | | |  |  |  |  |  |  |
| 1 | Vvi-Vitvi09g00792\_t001 |  |  |  | | | |  |  |  |  |  |  |
| 1 | Vvi-Vitvi09g00793\_t001 |  |  |  | Ath-AT1G80570.2 |  |  |  |  |  |  |
| 1 | Vvi-Vitvi09g04255\_t001 |  |  |  | | | |  |  |  |  |  |  |
| 1 | Vvi-Vitvi09g00794\_t001 |  |  |  | Ath-AT1G80600.1 |  |  |  |  |  |  |
| 1 | Vvi-Vitvi09g04256\_t001 |  |  |  | | | |  |  |  |  |  |  |
| 1 | Vvi-Vitvi09g01736\_t001 |  |  |  | | | |  |  |  |  |  |  |
| 1 | Vvi-Vitvi09g04257\_t001 |  |  |  | | | |  |  |  |  |  |  |
| 1 | Vvi-Vitvi09g04258\_t001 |  |  |  | | | |  |  |  |  |  |  |
| 1 | Vvi-Vitvi09g01739\_t001 |  |  |  | | | |  |  |  |  |  |  |
| 1 | Vvi-Vitvi09g04259\_t001 |  |  |  | | | |  |  |  |  |  |  |
| 1 | Vvi-Vitvi09g04260\_t001 |  |  |  | | | |  |  |  |  |  |  |
| 1 | Vvi-Vitvi09g04261\_t001 |  |  |  | | | |  |  |  |  |  |  |
| 1 | Vvi-Vitvi09g04262\_t001 |  |  |  | | | |  |  |  |  |  |  |
| 1 | Vvi-Vitvi09g01741\_t001 |  |  |  | | | |  |  |  |  |  |  |
| 1 | Vvi-Vitvi09g04263\_t001 |  |  |  | | | |  |  |  |  |  |  |
| 1 | Vvi-Vitvi09g00801\_t001 |  |  |  | | | |  |  |  |  |  |  |
| 1 | Vvi-Vitvi09g01743\_t001 |  |  |  | | | |  |  |  |  |  |  |
| 1 | Vvi-Vitvi09g00802\_t001 |  |  |  | | | |  |  |  |  |  |  |
| 1 | Vvi-Vitvi09g04264\_t001 |  |  |  | | | |  |  |  |  |  |  |
| 1 | Vvi-Vitvi09g00803\_t001 |  |  |  | | | |  |  |  |  |  |  |
| 1 | Vvi-Vitvi09g00805\_t001 |  |  |  | Ath-AT1G80610.1 |  |  |  |  |  |  |
| 1 | Vvi-Vitvi09g04265\_t001 |  |  |  | | | |  |  |  |  |  |  |
| 1 | Vvi-Vitvi09g00806\_t001 |  |  |  | | | |  |  |  |  |  |  |
| 1 | Vvi-Vitvi09g04266\_t001 |  |  |  | | | |  |  |  |  |  |  |
| 1 | Vvi-Vitvi09g04267\_t001 |  |  |  | | | |  |  |  |  |  |  |
| 1 | Vvi-Vitvi09g00809\_t001 |  |  |  | | | |  |  |  |  |  |  |
| 1 | Vvi-Vitvi09g04268\_t001 |  |  |  | | | |  |  |  |  |  |  |
| 1 | Vvi-Vitvi09g04269\_t001 |  |  |  | | | |  |  |  |  |  |  |
| 1 | Vvi-Vitvi09g04270\_t001 |  |  |  | | | |  |  |  |  |  |  |
| 1 | Vvi-Vitvi09g04271\_t001 |  |  |  | | | |  |  |  |  |  |  |
| 1 | Vvi-Vitvi09g04272\_t001 |  |  |  | | | |  |  |  |  |  |  |
| 1 | Vvi-Vitvi09g00815\_t001 |  |  |  | | | |  |  |  |  |  |  |
| 1 | Vvi-Vitvi09g00816\_t001 |  |  |  | Ath-AT1G80680.1 |  |  |  |  |  |  |
| 1 | Vvi-Vitvi09g00817\_t001 |  |  |  | Ath-AT1G80690.2 |  |  |  |  |  |  |
| 1 | Vvi-Vitvi09g01749\_t001 |  |  |  | Ath-AT1G80700.1 |  |  |  |  |  |  |
| 1 | Vvi-Vitvi09g04273\_t001 |  |  |  | | | |  |  |  |  |  |  |
| 1 | Vvi-Vitvi09g00821\_t001 |  |  |  | | | |  |  |  |  |  |  |
| 1 | Vvi-Vitvi09g01750\_t001 |  |  |  | | | |  |  |  |  |  |  |
| 1 | Vvi-Vitvi09g01751\_t001 |  |  |  | | | |  |  |  |  |  |  |
| 1 | Vvi-Vitvi09g00825\_t001 |  |  |  | | | |  |  |  |  |  |  |
| 1 | Vvi-Vitvi09g00826\_t001 |  |  |  | Ath-AT1G80720.2 |  |  |  |  |  |  |
| 1 | Vvi-Vitvi09g00827\_t001 |  |  |  | Ath-AT1G80730.1 |  |  |  |  |  |  |
| 0 | Vvi-Vitvi09g00828\_t001 |  |  |  |  |  |  |  |  |
| 0 | Vvi-Vitvi09g00829\_t001 |  |  |  |  |  |  |  |  |
| 0 | Vvi-Vitvi09g01752\_t001 |  |  |  |  |  |  |  |  |
| 0 | Vvi-Vitvi09g04274\_t001 |  |  |  |  |  |  |  |  |
| 0 | Vvi-Vitvi09g00834\_t001 |  |  |  |  |  |  |  |  |
| 0 | Vvi-Vitvi09g00837\_t001 |  |  |  |  |  |  |  |  |
| 0 | Vvi-Vitvi09g00838\_t001 |  |  |  |  |  |  |  |  |
| 0 | Vvi-Vitvi09g04275\_t001 |  |  |  |  |  |  |  |  |
| 0 | Vvi-Vitvi09g04276\_t001 |  |  |  |  |  |  |  |  |
| 0 | Vvi-Vitvi09g01756\_t001 |  |  |  |  |  |  |  |  |
| 0 | Vvi-Vitvi09g01757\_t001 |  |  |  |  |  |  |  |  |
| 0 | Vvi-Vitvi09g00844\_t001 |  |  |  |  |  |  |  |  |
| 0 | Vvi-Vitvi09g00845\_t001 |  |  |  |  |  |  |  |  |
| 0 | Vvi-Vitvi09g00848\_t001 |  |  |  |  |  |  |  |  |
| 0 | Vvi-Vitvi09g00853\_t001 |  |  |  |  |  |  |  |  |
| 0 | Vvi-Vitvi09g04277\_t001 |  |  |  |  |  |  |  |  |
| 0 | Vvi-Vitvi09g04278\_t001 |  |  |  |  |  |  |  |  |
| 0 | Vvi-Vitvi09g00859\_t001 |  |  |  |  |  |  |  |  |
| 0 | Vvi-Vitvi09g00860\_t001 |  |  |  |  |  |  |  |  |
| 0 | Vvi-Vitvi09g00861\_t002 |  |  |  |  |  |  |  |  |
| 0 | Vvi-Vitvi09g04279\_t001 |  |  |  |  |  |  |  |  |
| 0 | Vvi-Vitvi09g04280\_t001 |  |  |  |  |  |  |  |  |
| 0 | Vvi-Vitvi09g04281\_t001 |  |  |  |  |  |  |  |  |
| 0 | Vvi-Vitvi09g04282\_t001 |  |  |  |  |  |  |  |  |
| 0 | Vvi-Vitvi09g04283\_t001 |  |  |  |  |  |  |  |  |
| 0 | Vvi-Vitvi09g04284\_t001 |  |  |  |  |  |  |  |  |
| 0 | Vvi-Vitvi09g00865\_t001 |  |  |  |  |  |  |  |  |
| 0 | Vvi-Vitvi09g04285\_t001 |  |  |  |  |  |  |  |  |
| 0 | Vvi-Vitvi09g04286\_t001 |  |  |  |  |  |  |  |  |
| 0 | Vvi-Vitvi09g04287\_t001 |  |  |  |  |  |  |  |  |
| 0 | Vvi-Vitvi09g04288\_t001 |  |  |  |  |  |  |  |  |
| 0 | Vvi-Vitvi09g00867\_t001 |  |  |  |  |  |  |  |  |
| 0 | Vvi-Vitvi09g04289\_t001 |  |  |  |  |  |  |  |  |
| 0 | Vvi-Vitvi09g04290\_t001 |  |  |  |  |  |  |  |  |
| 0 | Vvi-Vitvi09g01761\_t001 |  |  |  |  |  |  |  |  |
| 0 | Vvi-Vitvi09g00870\_t001 |  |  |  |  |  |  |  |  |
| 0 | Vvi-Vitvi09g01764\_t001 |  |  |  |  |  |  |  |  |
| 0 | Vvi-Vitvi09g04291\_t001 |  |  |  |  |  |  |  |  |
| 0 | Vvi-Vitvi09g04292\_t001 |  |  |  |  |  |  |  |  |
| 0 | Vvi-Vitvi09g00873\_t001 |  |  |  |  |  |  |  |  |
| 0 | Vvi-Vitvi09g01765\_t001 |  |  |  |  |  |  |  |  |
| 0 | Vvi-Vitvi09g01766\_t001 |  |  |  |  |  |  |  |  |
| 0 | Vvi-Vitvi09g04293\_t001 |  |  |  |  |  |  |  |  |
| 0 | Vvi-Vitvi09g00876\_t002 |  |  |  |  |  |  |  |  |
| 0 | Vvi-Vitvi09g04294\_t001 |  |  |  |  |  |  |  |  |
| 0 | Vvi-Vitvi09g04295\_t001 |  |  |  |  |  |  |  |  |
| 0 | Vvi-Vitvi09g04296\_t001 |  |  |  |  |  |  |  |  |
| 0 | Vvi-Vitvi09g04297\_t001 |  |  |  |  |  |  |  |  |
| 0 | Vvi-Vitvi09g01767\_t001 |  |  |  |  |  |  |  |  |
| 0 | Vvi-Vitvi09g04298\_t001 |  |  |  |  |  |  |  |  |
| 0 | Vvi-Vitvi09g01769\_t001 |  |  |  |  |  |  |  |  |
| 0 | Vvi-Vitvi09g04299\_t001 |  |  |  |  |  |  |  |  |
| 0 | Vvi-Vitvi09g04300\_t001 |  |  |  |  |  |  |  |  |
| 0 | Vvi-Vitvi09g04301\_t001 |  |  |  |  |  |  |  |  |
| 0 | Vvi-Vitvi09g02029\_t001 |  |  |  |  |  |  |  |  |
| 0 | Vvi-Vitvi09g01773\_t001 |  |  |  |  |  |  |  |  |
| 0 | Vvi-Vitvi09g04302\_t001 |  |  |  |  |  |  |  |  |
| 0 | Vvi-Vitvi09g04303\_t001 |  |  |  |  |  |  |  |  |
| 0 | Vvi-Vitvi09g04304\_t001 |  |  |  |  |  |  |  |  |
| 0 | Vvi-Vitvi09g04305\_t001 |  |  |  |  |  |  |  |  |
| 0 | Vvi-Vitvi09g04306\_t001 |  |  |  |  |  |  |  |  |
| 0 | Vvi-Vitvi09g04307\_t001 |  |  |  |  |  |  |  |  |
| 0 | Vvi-Vitvi09g04308\_t001 |  |  |  |  |  |  |  |  |
| 0 | Vvi-Vitvi09g04309\_t001 |  |  |  |  |  |  |  |  |
| 0 | Vvi-Vitvi09g04310\_t001 |  |  |  |  |  |  |  |  |
| 0 | Vvi-Vitvi09g04311\_t001 |  |  |  |  |  |  |  |  |
| 0 | Vvi-Vitvi09g00889\_t001 |  |  |  |  |  |  |  |  |
| 0 | Vvi-Vitvi09g00893\_t001 |  |  |  |  |  |  |  |  |
| 0 | Vvi-Vitvi09g04312\_t001 |  |  |  |  |  |  |  |  |
| 0 | Vvi-Vitvi09g00901\_t001 |  |  |  |  |  |  |  |  |
| 0 | Vvi-Vitvi09g04314\_t001 |  |  |  |  |  |  |  |  |
| 0 | Vvi-Vitvi09g04315\_t001 |  |  |  |  |  |  |  |  |
| 0 | Vvi-Vitvi09g04316\_t001 |  |  |  |  |  |  |  |  |
| 0 | Vvi-Vitvi09g00905\_t001 |  |  |  |  |  |  |  |  |
| 0 | Vvi-Vitvi09g04318\_t001 |  |  |  |  |  |  |  |  |
| 0 | Vvi-Vitvi09g01780\_t001 |  |  |  |  |  |  |  |  |
| 0 | Vvi-Vitvi09g04319\_t001 |  |  |  |  |  |  |  |  |
| 0 | Vvi-Vitvi09g04320\_t001 |  |  |  |  |  |  |  |  |
| 0 | Vvi-Vitvi09g00916\_t001 |  |  |  |  |  |  |  |  |
| 0 | Vvi-Vitvi09g04322\_t001 |  |  |  |  |  |  |  |  |
| 0 | Vvi-Vitvi09g04324\_t001 |  |  |  |  |  |  |  |  |
| 0 | Vvi-Vitvi09g04325\_t001 |  |  |  |  |  |  |  |  |
| 0 | Vvi-Vitvi09g04326\_t001 |  |  |  |  |  |  |  |  |
| 0 | Vvi-Vitvi09g04327\_t001 |  |  |  |  |  |  |  |  |
| 0 | Vvi-Vitvi09g04328\_t001 |  |  |  |  |  |  |  |  |
| 0 | Vvi-Vitvi09g04329\_t001 |  |  |  |  |  |  |  |  |
| 0 | Vvi-Vitvi09g04330\_t001 |  |  |  |  |  |  |  |  |
| 0 | Vvi-Vitvi09g00932\_t001 |  |  |  |  |  |  |  |  |
| 0 | Vvi-Vitvi09g04331\_t001 |  |  |  |  |  |  |  |  |
| 0 | Vvi-Vitvi09g01784\_t001 |  |  |  |  |  |  |  |  |
| 0 | Vvi-Vitvi09g04332\_t001 |  |  |  |  |  |  |  |  |
| 0 | Vvi-Vitvi09g00937\_t001 |  |  |  |  |  |  |  |  |
| 0 | Vvi-Vitvi09g04333\_t001 |  |  |  |  |  |  |  |  |
| 0 | Vvi-Vitvi09g04334\_t001 |  |  |  |  |  |  |  |  |
| 0 | Vvi-Vitvi09g01786\_t001 |  |  |  |  |  |  |  |  |
| 0 | Vvi-Vitvi09g01787\_t001 |  |  |  |  |  |  |  |  |
| 0 | Vvi-Vitvi09g04335\_t001 |  |  |  |  |  |  |  |  |
| 0 | Vvi-Vitvi09g00939\_t001 |  |  |  |  |  |  |  |  |
| 0 | Vvi-Vitvi09g00940\_t001 |  |  |  |  |  |  |  |  |
| 0 | Vvi-Vitvi09g01788\_t001 |  |  |  |  |  |  |  |  |
| 0 | Vvi-Vitvi09g00943\_t001 |  |  |  |  |  |  |  |  |
| 0 | Vvi-Vitvi09g00944\_t001 |  |  |  |  |  |  |  |  |
| 0 | Vvi-Vitvi09g00945\_t001 |  |  |  |  |  |  |  |  |
| 0 | Vvi-Vitvi09g04336\_t001 |  |  |  |  |  |  |  |  |
| 0 | Vvi-Vitvi09g04337\_t001 |  |  |  |  |  |  |  |  |
| 0 | Vvi-Vitvi09g04338\_t001 |  |  |  |  |  |  |  |  |
| 0 | Vvi-Vitvi09g04339\_t001 |  |  |  |  |  |  |  |  |
| 0 | Vvi-Vitvi09g01790\_t002 |  |  |  |  |  |  |  |  |
| 0 | Vvi-Vitvi09g00949\_t001 |  |  |  |  |  |  |  |  |
| 0 | Vvi-Vitvi09g04340\_t001 |  |  |  |  |  |  |  |  |
| 0 | Vvi-Vitvi09g04341\_t001 |  |  |  |  |  |  |  |  |
| 0 | Vvi-Vitvi09g04342\_t001 |  |  |  |  |  |  |  |  |
| 0 | Vvi-Vitvi09g04343\_t001 |  |  |  |  |  |  |  |  |
| 0 | Vvi-Vitvi09g01795\_t001 |  |  |  |  |  |  |  |  |
| 0 | Vvi-Vitvi09g01796\_t001 |  |  |  |  |  |  |  |  |
| 0 | Vvi-Vitvi09g04344\_t001 |  |  |  |  |  |  |  |  |
| 0 | Vvi-Vitvi09g04345\_t001 |  |  |  |  |  |  |  |  |
| 0 | Vvi-Vitvi09g04346\_t001 |  |  |  |  |  |  |  |  |
| 0 | Vvi-Vitvi09g04347\_t001 |  |  |  |  |  |  |  |  |
| 0 | Vvi-Vitvi09g04348\_t001 |  |  |  |  |  |  |  |  |
| 0 | Vvi-Vitvi09g02025\_t001 |  |  |  |  |  |  |  |  |
| 0 | Vvi-Vitvi09g04349\_t001 |  |  |  |  |  |  |  |  |
| 0 | Vvi-Vitvi09g04350\_t001 |  |  |  |  |  |  |  |  |
| 0 | Vvi-Vitvi09g04351\_t001 |  |  |  |  |  |  |  |  |
| 0 | Vvi-Vitvi09g02033\_t001 |  |  |  |  |  |  |  |  |
| 0 | Vvi-Vitvi09g02034\_t001 |  |  |  |  |  |  |  |  |
| 0 | Vvi-Vitvi09g04352\_t001 |  |  |  |  |  |  |  |  |
| 0 | Vvi-Vitvi09g04353\_t001 |  |  |  |  |  |  |  |  |
| 0 | Vvi-Vitvi09g02036\_t001 |  |  |  |  |  |  |  |  |
| 0 | Vvi-Vitvi09g02039\_t001 |  |  |  |  |  |  |  |  |
| 0 | Vvi-Vitvi09g04354\_t001 |  |  |  |  |  |  |  |  |
| 0 | Vvi-Vitvi09g02041\_t001 |  |  |  |  |  |  |  |  |
| 0 | Vvi-Vitvi09g04355\_t001 |  |  |  |  |  |  |  |  |
| 0 | Vvi-Vitvi09g04356\_t001 |  |  |  |  |  |  |  |  |
| 0 | Vvi-Vitvi09g04357\_t001 |  |  |  |  |  |  |  |  |
| 0 | Vvi-Vitvi09g04358\_t001 |  |  |  |  |  |  |  |  |
| 0 | Vvi-Vitvi09g02044\_t001 |  |  |  |  |  |  |  |  |
| 0 | Vvi-Vitvi09g04359\_t001 |  |  |  |  |  |  |  |  |
| 0 | Vvi-Vitvi09g04360\_t001 |  |  |  |  |  |  |  |  |
| 0 | Vvi-Vitvi09g04361\_t001 |  |  |  |  |  |  |  |  |
| 0 | Vvi-Vitvi09g02050\_t001 |  |  |  |  |  |  |  |  |
| 0 | Vvi-Vitvi09g04362\_t001 |  |  |  |  |  |  |  |  |
| 0 | Vvi-Vitvi09g00957\_t001 |  |  |  |  |  |  |  |  |
| 0 | Vvi-Vitvi09g00960\_t001 |  |  |  |  |  |  |  |  |
| 0 | Vvi-Vitvi09g00961\_t001 |  |  |  |  |  |  |  |  |
| 0 | Vvi-Vitvi09g01798\_t001 |  |  |  |  |  |  |  |  |
| 0 | Vvi-Vitvi09g04363\_t001 |  |  |  |  |  |  |  |  |
| 0 | Vvi-Vitvi09g00965\_t001 |  |  |  |  |  |  |  |  |
| 0 | Vvi-Vitvi09g00966\_t001 |  |  |  |  |  |  |  |  |
| 0 | Vvi-Vitvi09g04364\_t001 |  |  |  |  |  |  |  |  |
| 0 | Vvi-Vitvi09g00968\_t001 |  |  |  |  |  |  |  |  |
| 0 | Vvi-Vitvi09g00969\_t001 |  |  |  |  |  |  |  |  |
| 0 | Vvi-Vitvi09g00971\_t001 |  |  |  |  |  |  |  |  |
| 0 | Vvi-Vitvi09g04365\_t001 |  |  |  |  |  |  |  |  |
| 0 | Vvi-Vitvi09g00973\_t001 |  |  |  |  |  |  |  |  |
| 0 | Vvi-Vitvi09g00976\_t001 |  |  |  |  |  |  |  |  |
| 0 | Vvi-Vitvi09g00980\_t001 |  |  |  |  |  |  |  |  |
| 0 | Vvi-Vitvi09g00981\_t001 |  |  |  |  |  |  |  |  |
| 0 | Vvi-Vitvi09g00985\_t001 |  |  |  |  |  |  |  |  |
| 0 | Vvi-Vitvi09g00988\_t001 |  |  |  |  |  |  |  |  |
| 0 | Vvi-Vitvi09g04366\_t001 |  |  |  |  |  |  |  |  |
| 0 | Vvi-Vitvi09g00990\_t001 |  |  |  |  |  |  |  |  |
| 0 | Vvi-Vitvi09g04367\_t001 |  |  |  |  |  |  |  |  |
| 0 | Vvi-Vitvi09g04368\_t001 |  |  |  |  |  |  |  |  |
| 0 | Vvi-Vitvi09g04369\_t001 |  |  |  |  |  |  |  |  |
| 0 | Vvi-Vitvi09g04370\_t001 |  |  |  |  |  |  |  |  |
| 0 | Vvi-Vitvi09g00992\_t001 |  |  |  |  |  |  |  |  |
| 0 | Vvi-Vitvi09g00993\_t001 |  |  |  |  |  |  |  |  |
| 0 | Vvi-Vitvi09g04371\_t001 |  |  |  |  |  |  |  |  |
| 0 | Vvi-Vitvi09g00994\_t001 |  |  |  |  |  |  |  |  |
| 0 | Vvi-Vitvi09g00995\_t001 |  |  |  |  |  |  |  |  |
| 0 | Vvi-Vitvi09g04372\_t001 |  |  |  |  |  |  |  |  |
| 0 | Vvi-Vitvi09g00996\_t001 |  |  |  |  |  |  |  |  |
| 0 | Vvi-Vitvi09g00997\_t001 |  |  |  |  |  |  |  |  |
| 0 | Vvi-Vitvi09g04373\_t001 |  |  |  |  |  |  |  |  |
| 0 | Vvi-Vitvi09g04374\_t001 |  |  |  |  |  |  |  |  |
| 0 | Vvi-Vitvi09g01001\_t001 |  |  |  |  |  |  |  |  |
| 0 | Vvi-Vitvi09g04375\_t001 |  |  |  |  |  |  |  |  |
| 0 | Vvi-Vitvi09g01807\_t001 |  |  |  |  |  |  |  |  |
| 0 | Vvi-Vitvi09g04376\_t001 |  |  |  |  |  |  |  |  |
| 0 | Vvi-Vitvi09g04377\_t001 |  |  |  |  |  |  |  |  |
| 0 | Vvi-Vitvi09g01005\_t001 |  |  |  |  |  |  |  |  |
| 0 | Vvi-Vitvi09g01006\_t001 |  |  |  |  |  |  |  |  |
| 0 | Vvi-Vitvi09g01808\_t001 |  |  |  |  |  |  |  |  |
| 0 | Vvi-Vitvi09g01007\_t001 |  |  |  |  |  |  |  |  |
| 0 | Vvi-Vitvi09g04378\_t001 |  |  |  |  |  |  |  |  |
| 0 | Vvi-Vitvi09g01008\_t001 |  |  |  |  |  |  |  |  |
| 0 | Vvi-Vitvi09g04379\_t001 |  |  |  |  |  |  |  |  |
| 0 | Vvi-Vitvi09g04380\_t001 |  |  |  |  |  |  |  |  |
| 0 | Vvi-Vitvi09g04381\_t001 |  |  |  |  |  |  |  |  |
| 0 | Vvi-Vitvi09g04382\_t001 |  |  |  |  |  |  |  |  |
| 0 | Vvi-Vitvi09g01011\_t001 |  |  |  |  |  |  |  |  |
| 0 | Vvi-Vitvi09g04383\_t001 |  |  |  |  |  |  |  |  |
| 0 | Vvi-Vitvi09g04384\_t001 |  |  |  |  |  |  |  |  |
| 0 | Vvi-Vitvi09g01017\_t001 |  |  |  |  |  |  |  |  |
| 0 | Vvi-Vitvi09g04385\_t001 |  |  |  |  |  |  |  |  |
| 0 | Vvi-Vitvi09g04386\_t001 |  |  |  |  |  |  |  |  |
| 0 | Vvi-Vitvi09g01022\_t001 |  |  |  |  |  |  |  |  |
| 0 | Vvi-Vitvi09g04387\_t001 |  |  |  |  |  |  |  |  |
| 0 | Vvi-Vitvi09g01812\_t001 |  |  |  |  |  |  |  |  |
| 0 | Vvi-Vitvi09g04388\_t001 |  |  |  |  |  |  |  |  |
| 0 | Vvi-Vitvi09g04389\_t001 |  |  |  |  |  |  |  |  |
| 0 | Vvi-Vitvi09g04390\_t001 |  |  |  |  |  |  |  |  |
| 0 | Vvi-Vitvi09g01025\_t001 |  |  |  |  |  |  |  |  |
| 0 | Vvi-Vitvi09g01026\_t001 |  |  |  |  |  |  |  |  |
| 0 | Vvi-Vitvi09g04391\_t001 |  |  |  |  |  |  |  |  |
| 0 | Vvi-Vitvi09g04392\_t001 |  |  |  |  |  |  |  |  |
| 0 | Vvi-Vitvi09g04393\_t001 |  |  |  |  |  |  |  |  |
| 0 | Vvi-Vitvi09g04394\_t001 |  |  |  |  |  |  |  |  |
| 0 | Vvi-Vitvi09g04395\_t001 |  |  |  |  |  |  |  |  |
| 0 | Vvi-Vitvi09g04396\_t001 |  |  |  |  |  |  |  |  |
| 0 | Vvi-Vitvi09g04397\_t001 |  |  |  |  |  |  |  |  |
| 0 | Vvi-Vitvi09g04398\_t001 |  |  |  |  |  |  |  |  |
| 0 | Vvi-Vitvi09g04399\_t001 |  |  |  |  |  |  |  |  |
| 0 | Vvi-Vitvi09g04400\_t001 |  |  |  |  |  |  |  |  |
| 0 | Vvi-Vitvi09g04401\_t001 |  |  |  |  |  |  |  |  |
| 0 | Vvi-Vitvi09g04403\_t001 |  |  |  |  |  |  |  |  |
| 0 | Vvi-Vitvi09g04404\_t001 |  |  |  |  |  |  |  |  |
| 0 | Vvi-Vitvi09g04405\_t001 |  |  |  |  |  |  |  |  |
| 0 | Vvi-Vitvi09g04406\_t001 |  |  |  |  |  |  |  |  |
| 0 | Vvi-Vitvi09g04407\_t001 |  |  |  |  |  |  |  |  |
| 0 | Vvi-Vitvi09g04408\_t001 |  |  |  |  |  |  |  |  |
| 0 | Vvi-Vitvi09g04409\_t001 |  |  |  |  |  |  |  |  |
| 0 | Vvi-Vitvi09g04410\_t001 |  |  |  |  |  |  |  |  |
| 0 | Vvi-Vitvi09g04411\_t001 |  |  |  |  |  |  |  |  |
| 0 | Vvi-Vitvi09g04412\_t001 |  |  |  |  |  |  |  |  |
| 0 | Vvi-Vitvi09g04413\_t001 |  |  |  |  |  |  |  |  |
| 0 | Vvi-Vitvi09g04414\_t001 |  |  |  |  |  |  |  |  |
| 0 | Vvi-Vitvi09g04415\_t001 |  |  |  |  |  |  |  |  |
| 0 | Vvi-Vitvi09g04416\_t001 |  |  |  |  |  |  |  |  |
| 0 | Vvi-Vitvi09g04417\_t001 |  |  |  |  |  |  |  |  |
| 0 | Vvi-Vitvi09g04418\_t001 |  |  |  |  |  |  |  |  |
| 0 | Vvi-Vitvi09g01827\_t001 |  |  |  |  |  |  |  |  |
| 0 | Vvi-Vitvi09g04419\_t001 |  |  |  |  |  |  |  |  |
| 0 | Vvi-Vitvi09g04420\_t001 |  |  |  |  |  |  |  |  |
| 0 | Vvi-Vitvi09g04421\_t001 |  |  |  |  |  |  |  |  |
| 0 | Vvi-Vitvi09g04422\_t001 |  |  |  |  |  |  |  |  |
| 0 | Vvi-Vitvi09g01047\_t001 |  |  |  |  |  |  |  |  |
| 0 | Vvi-Vitvi09g04423\_t001 |  |  |  |  |  |  |  |  |
| 0 | Vvi-Vitvi09g01823\_t001 |  |  |  |  |  |  |  |  |
| 0 | Vvi-Vitvi09g04424\_t001 |  |  |  |  |  |  |  |  |
| 0 | Vvi-Vitvi09g04425\_t001 |  |  |  |  |  |  |  |  |
| 0 | Vvi-Vitvi09g04426\_t001 |  |  |  |  |  |  |  |  |
| 0 | Vvi-Vitvi09g04427\_t001 |  |  |  |  |  |  |  |  |
| 0 | Vvi-Vitvi09g04428\_t001 |  |  |  |  |  |  |  |  |
| 0 | Vvi-Vitvi09g01038\_t001 |  |  |  |  |  |  |  |  |
| 0 | Vvi-Vitvi09g04429\_t001 |  |  |  |  |  |  |  |  |
| 0 | Vvi-Vitvi09g04430\_t001 |  |  |  |  |  |  |  |  |
| 0 | Vvi-Vitvi09g01036\_t001 |  |  |  |  |  |  |  |  |
| 0 | Vvi-Vitvi09g04431\_t001 |  |  |  |  |  |  |  |  |
| 0 | Vvi-Vitvi09g04432\_t001 |  |  |  |  |  |  |  |  |
| 0 | Vvi-Vitvi09g01031\_t001 |  |  |  |  |  |  |  |  |
| 0 | Vvi-Vitvi09g04433\_t001 |  |  |  |  |  |  |  |  |
| 0 | Vvi-Vitvi09g01816\_t001 |  |  |  |  |  |  |  |  |
| 0 | Vvi-Vitvi09g01027\_t001 |  |  |  |  |  |  |  |  |
| 0 | Vvi-Vitvi09g04434\_t001 |  |  |  |  |  |  |  |  |
| 0 | Vvi-Vitvi09g04435\_t001 |  |  |  |  |  |  |  |  |
| 0 | Vvi-Vitvi09g01075\_t001 |  |  |  |  |  |  |  |  |
| 0 | Vvi-Vitvi09g01076\_t001 |  |  |  |  |  |  |  |  |
| 0 | Vvi-Vitvi09g01078\_t001 |  |  |  |  |  |  |  |  |
| 0 | Vvi-Vitvi09g01079\_t001 |  |  |  |  |  |  |  |  |
| 0 | Vvi-Vitvi09g01084\_t001 |  |  |  |  |  |  |  |  |
| 0 | Vvi-Vitvi09g04436\_t001 |  |  |  |  |  |  |  |  |
| 0 | Vvi-Vitvi09g04437\_t001 |  |  |  |  |  |  |  |  |
| 0 | Vvi-Vitvi09g04438\_t001 |  |  |  |  |  |  |  |  |
| 0 | Vvi-Vitvi09g04439\_t001 |  |  |  |  |  |  |  |  |
| 0 | Vvi-Vitvi09g04440\_t001 |  |  |  |  |  |  |  |  |
| 0 | Vvi-Vitvi09g01836\_t001 |  |  |  |  |  |  |  |  |
| 0 | Vvi-Vitvi09g01089\_t001 |  |  |  |  |  |  |  |  |
| 0 | Vvi-Vitvi09g04441\_t001 |  |  |  |  |  |  |  |  |
| 0 | Vvi-Vitvi09g01097\_t001 |  |  |  |  |  |  |  |  |
| 0 | Vvi-Vitvi09g04442\_t001 |  |  |  |  |  |  |  |  |
| 0 | Vvi-Vitvi09g04443\_t001 |  |  |  |  |  |  |  |  |
| 0 | Vvi-Vitvi09g04444\_t001 |  |  |  |  |  |  |  |  |
| 0 | Vvi-Vitvi09g04445\_t001 |  |  |  |  |  |  |  |  |
| 0 | Vvi-Vitvi09g04446\_t001 |  |  |  |  |  |  |  |  |
| 0 | Vvi-Vitvi09g04447\_t001 |  |  |  |  |  |  |  |  |
| 0 | Vvi-Vitvi09g04448\_t001 |  |  |  |  |  |  |  |  |
| 0 | Vvi-Vitvi09g04449\_t001 |  |  |  |  |  |  |  |  |
| 0 | Vvi-Vitvi09g04450\_t001 |  |  |  |  |  |  |  |  |
| 0 | Vvi-Vitvi09g04451\_t001 |  |  |  |  |  |  |  |  |
| 0 | Vvi-Vitvi09g04453\_t001 |  |  |  |  |  |  |  |  |
| 0 | Vvi-Vitvi09g01106\_t001 |  |  |  |  |  |  |  |  |
| 0 | Vvi-Vitvi09g04454\_t001 |  |  |  |  |  |  |  |  |
| 0 | Vvi-Vitvi09g04455\_t001 |  |  |  |  |  |  |  |  |
| 0 | Vvi-Vitvi09g04456\_t001 |  |  |  |  |  |  |  |  |
| 0 | Vvi-Vitvi09g04457\_t001 |  |  |  |  |  |  |  |  |
| 0 | Vvi-Vitvi09g04458\_t001 |  |  |  |  |  |  |  |  |
| 0 | Vvi-Vitvi09g04459\_t001 |  |  |  |  |  |  |  |  |
| 0 | Vvi-Vitvi09g04460\_t001 |  |  |  |  |  |  |  |  |
| 0 | Vvi-Vitvi09g01118\_t001 |  |  |  |  |  |  |  |  |
| 0 | Vvi-Vitvi09g04461\_t001 |  |  |  |  |  |  |  |  |
| 0 | Vvi-Vitvi09g04462\_t001 |  |  |  |  |  |  |  |  |
| 0 | Vvi-Vitvi09g04463\_t001 |  |  |  |  |  |  |  |  |
| 1 | Vvi-Vitvi09g01122\_t001 |  | Ath-AT1G80840.1 |  |  |  |  |  |  |  |
| 1 | Vvi-Vitvi09g01123\_t001 |  | Ath-AT1G80850.1 |  |  |  |  |  |  |  |
| 1 | Vvi-Vitvi09g04464\_t001 |  | Ath-AT1G80860.2 |  |  |  |  |  |  |  |
| 1 | Vvi-Vitvi09g01126\_t001 |  | | | |  |  |  |  |  |  |  |
| 1 | Vvi-Vitvi09g01127\_t001 |  | | | |  |  |  |  |  |  |  |
| 1 | Vvi-Vitvi09g01129\_t001 |  | | | |  |  |  |  |  |  |  |
| 1 | Vvi-Vitvi09g01134\_t001 |  | | | |  |  |  |  |  |  |  |
| 1 | Vvi-Vitvi09g01135\_t001 |  | | | |  |  |  |  |  |  |  |
| 1 | Vvi-Vitvi09g01137\_t001 |  | | | |  |  |  |  |  |  |  |
| 1 | Vvi-Vitvi09g01141\_t001 |  | | | |  |  |  |  |  |  |  |
| 1 | Vvi-Vitvi09g04465\_t001 |  | | | |  |  |  |  |  |  |  |
| 1 | Vvi-Vitvi09g04466\_t001 |  | | | |  |  |  |  |  |  |  |
| 1 | Vvi-Vitvi09g01143\_t001 |  | | | |  |  |  |  |  |  |  |
| 1 | Vvi-Vitvi09g01145\_t001 |  | | | |  |  |  |  |  |  |  |
| 1 | Vvi-Vitvi09g04467\_t001 |  | | | |  |  |  |  |  |  |  |
| 1 | Vvi-Vitvi09g01151\_t001 |  | | | |  |  |  |  |  |  |  |
| 1 | Vvi-Vitvi09g04468\_t001 |  | Ath-AT1G80870.1 |  |  |  |  |  |  |  |
| 1 | Vvi-Vitvi09g04469\_t001 |  | | | |  |  |  |  |  |  |  |
| 1 | Vvi-Vitvi09g01155\_t001 |  | | | |  |  |  |  |  |  |  |
| 1 | Vvi-Vitvi09g01157\_t001 |  | Ath-AT1G80880.1 |  |  |  |  |  |  |  |
| 1 | Vvi-Vitvi09g01158\_t001 |  | Ath-AT1G80890.1 |  |  |  |  |  |  |  |
| 1 | Vvi-Vitvi09g01159\_t001 |  | Ath-AT1G80900.1 |  |  |  |  |  |  |  |
| 1 | Vvi-Vitvi09g01161\_t001 |  | | | |  |  |  |  |  |  |  |
| 1 | Vvi-Vitvi09g04470\_t001 |  | | | |  |  |  |  |  |  |  |
| 1 | Vvi-Vitvi09g01852\_t001 |  | | | |  |  |  |  |  |  |  |
| 1 | Vvi-Vitvi09g01164\_t001 |  | Ath-AT1G80920.1 |  |  |  |  |  |  |  |
| 0 | Vvi-Vitvi09g01168\_t001 |  |  |  |  |  |  |  |  |
| 0 | Vvi-Vitvi09g01169\_t001 |  |  |  |  |  |  |  |  |
| 0 | Vvi-Vitvi09g01853\_t001 |  |  |  |  |  |  |  |  |
| 0 | Vvi-Vitvi09g04472\_t001 |  |  |  |  |  |  |  |  |
| 0 | Vvi-Vitvi09g04473\_t001 |  |  |  |  |  |  |  |  |
| 0 | Vvi-Vitvi09g04474\_t001 |  |  |  |  |  |  |  |  |
| 0 | Vvi-Vitvi09g04475\_t001 |  |  |  |  |  |  |  |  |
| 0 | Vvi-Vitvi09g04476\_t001 |  |  |  |  |  |  |  |  |
| 0 | Vvi-Vitvi09g04477\_t001 |  |  |  |  |  |  |  |  |
| 0 | Vvi-Vitvi09g04478\_t001 |  |  |  |  |  |  |  |  |
| 0 | Vvi-Vitvi09g04479\_t001 |  |  |  |  |  |  |  |  |
| 0 | Vvi-Vitvi09g01181\_t001 |  |  |  |  |  |  |  |  |
| 0 | Vvi-Vitvi09g04480\_t001 |  |  |  |  |  |  |  |  |
| 0 | Vvi-Vitvi09g01868\_t001 |  |  |  |  |  |  |  |  |
| 0 | Vvi-Vitvi09g01186\_t001 |  |  |  |  |  |  |  |  |
| 0 | Vvi-Vitvi09g01187\_t001 |  |  |  |  |  |  |  |  |
| 0 | Vvi-Vitvi09g01869\_t001 |  |  |  |  |  |  |  |  |
| 0 | Vvi-Vitvi09g01194\_t001 |  |  |  |  |  |  |  |  |
| 0 | Vvi-Vitvi09g04481\_t001 |  |  |  |  |  |  |  |  |
| 0 | Vvi-Vitvi09g01870\_t001 |  |  |  |  |  |  |  |  |
| 0 | Vvi-Vitvi09g04482\_t001 |  |  |  |  |  |  |  |  |
| 0 | Vvi-Vitvi09g01195\_t001 |  |  |  |  |  |  |  |  |
| 0 | Vvi-Vitvi09g01196\_t001 |  |  |  |  |  |  |  |  |
| 0 | Vvi-Vitvi09g04483\_t001 |  |  |  |  |  |  |  |  |
| 0 | Vvi-Vitvi09g04484\_t001 |  |  |  |  |  |  |  |  |
| 0 | Vvi-Vitvi09g04485\_t001 |  |  |  |  |  |  |  |  |
| 0 | Vvi-Vitvi09g01201\_t001 |  |  |  |  |  |  |  |  |
| 0 | Vvi-Vitvi09g01875\_t001 |  |  |  |  |  |  |  |  |
| 0 | Vvi-Vitvi09g01202\_t001 |  |  |  |  |  |  |  |  |
| 0 | Vvi-Vitvi09g04486\_t001 |  |  |  |  |  |  |  |  |
| 0 | Vvi-Vitvi09g04487\_t001 |  |  |  |  |  |  |  |  |
| 0 | Vvi-Vitvi09g01214\_t001 |  |  |  |  |  |  |  |  |
| 0 | Vvi-Vitvi09g01215\_t001 |  |  |  |  |  |  |  |  |
| 0 | Vvi-Vitvi09g01216\_t001 |  |  |  |  |  |  |  |  |
| 0 | Vvi-Vitvi09g04488\_t001 |  |  |  |  |  |  |  |  |
| 0 | Vvi-Vitvi09g04489\_t001 |  |  |  |  |  |  |  |  |
| 0 | Vvi-Vitvi09g01217\_t001 |  |  |  |  |  |  |  |  |
| 0 | Vvi-Vitvi09g04490\_t001 |  |  |  |  |  |  |  |  |
| 0 | Vvi-Vitvi09g01220\_t001 |  |  |  |  |  |  |  |  |
| 0 | Vvi-Vitvi09g01882\_t001 |  |  |  |  |  |  |  |  |
| 0 | Vvi-Vitvi09g01225\_t001 |  |  |  |  |  |  |  |  |
| 0 | Vvi-Vitvi09g04491\_t001 |  |  |  |  |  |  |  |  |
| 0 | Vvi-Vitvi09g01884\_t001 |  |  |  |  |  |  |  |  |
| 0 | Vvi-Vitvi09g01227\_t001 |  |  |  |  |  |  |  |  |
| 0 | Vvi-Vitvi09g04492\_t001 |  |  |  |  |  |  |  |  |
| 0 | Vvi-Vitvi09g04493\_t001 |  |  |  |  |  |  |  |  |
| 0 | Vvi-Vitvi09g01229\_t001 |  |  |  |  |  |  |  |  |
| 0 | Vvi-Vitvi09g01231\_t001 |  |  |  |  |  |  |  |  |
| 0 | Vvi-Vitvi09g01233\_t001 |  |  |  |  |  |  |  |  |
| 0 | Vvi-Vitvi09g04494\_t001 |  |  |  |  |  |  |  |  |
| 0 | Vvi-Vitvi09g01236\_t001 |  |  |  |  |  |  |  |  |
| 0 | Vvi-Vitvi09g01885\_t001 |  |  |  |  |  |  |  |  |
| 0 | Vvi-Vitvi09g01237\_t001 |  |  |  |  |  |  |  |  |
| 0 | Vvi-Vitvi09g01238\_t001 |  |  |  |  |  |  |  |  |
| 0 | Vvi-Vitvi09g04495\_t001 |  |  |  |  |  |  |  |  |
| 0 | Vvi-Vitvi09g04496\_t001 |  |  |  |  |  |  |  |  |
| 0 | Vvi-Vitvi09g01243\_t001 |  |  |  |  |  |  |  |  |
| 0 | Vvi-Vitvi09g01244\_t001 |  |  |  |  |  |  |  |  |
| 0 | Vvi-Vitvi09g01889\_t001 |  |  |  |  |  |  |  |  |
| 1 | Vvi-Vitvi09g01246\_t001 |  | Ath-AT1G79640.1 |  |  |  |  |  |  |  |
| 2 | Vvi-Vitvi09g01250\_t001 |  | Ath-AT1G79650.4 |  | Ath-AT1G16190.1 |  |  |  |  |  |  |
| 2 | Vvi-Vitvi09g01251\_t001 |  | | | |  | Ath-AT1G16180.1 |  |  |  |  |  |  |
| 2 | Vvi-Vitvi09g01892\_t001 |  | | | |  | | | |  |  |  |  |  |  |
| 2 | Vvi-Vitvi09g01893\_t001 |  | | | |  | | | |  |  |  |  |  |  |
| 2 | Vvi-Vitvi09g01253\_t001 |  | | | |  | | | |  |  |  |  |  |  |
| 2 | Vvi-Vitvi09g01254\_t001 |  | | | |  | | | |  |  |  |  |  |  |
| 2 | Vvi-Vitvi09g04497\_t001 |  | | | |  | | | |  |  |  |  |  |  |
| 2 | Vvi-Vitvi09g04498\_t001 |  | | | |  | | | |  |  |  |  |  |  |
| 2 | Vvi-Vitvi09g01255\_t001 |  | | | |  | | | |  |  |  |  |  |  |
| 2 | Vvi-Vitvi09g04499\_t001 |  | | | |  | | | |  |  |  |  |  |  |
| 2 | Vvi-Vitvi09g01256\_t001 |  | | | |  | | | |  |  |  |  |  |  |
| 2 | Vvi-Vitvi09g04500\_t001 |  | | | |  | | | |  |  |  |  |  |  |
| 2 | Vvi-Vitvi09g04501\_t001 |  | | | |  | | | |  |  |  |  |  |  |
| 2 | Vvi-Vitvi09g04502\_t001 |  | | | |  | | | |  |  |  |  |  |  |
| 2 | Vvi-Vitvi09g04503\_t001 |  | | | |  | | | |  |  |  |  |  |  |
| 2 | Vvi-Vitvi09g01260\_t001 |  | | | |  | | | |  |  |  |  |  |  |
| 2 | Vvi-Vitvi09g01903\_t001 |  | | | |  | | | |  |  |  |  |  |  |
| 2 | Vvi-Vitvi09g04504\_t001 |  | | | |  | | | |  |  |  |  |  |  |
| 2 | Vvi-Vitvi09g04505\_t001 |  | | | |  | | | |  |  |  |  |  |  |
| 2 | Vvi-Vitvi09g04506\_t001 |  | | | |  | | | |  |  |  |  |  |  |
| 2 | Vvi-Vitvi09g01262\_t001 |  | Ath-AT1G79690.1 |  | | | |  |  |  |  |  |  |
| 2 | Vvi-Vitvi09g01263\_t001 |  | | | |  | Ath-AT1G16080.1 |  |  |  |  |  |  |
| 2 | Vvi-Vitvi09g01265\_t001 |  | | | |  | | | |  |  |  |  |  |  |
| 2 | Vvi-Vitvi09g04507\_t001 |  | | | |  | | | |  |  |  |  |  |  |
| 2 | Vvi-Vitvi09g01266\_t001 |  | | | |  | Ath-AT1G16070.1 |  |  |  |  |  |  |
| 2 | Vvi-Vitvi09g01269\_t001 |  | Ath-AT1G79700.2 |  | Ath-AT1G16060.1 |  |  |  |  |  |  |
| 2 | Vvi-Vitvi09g01271\_t001 |  | Ath-AT1G79710.1 |  | | | |  |  |  |  |  |  |
| 2 | Vvi-Vitvi09g01272\_t001 |  | Ath-AT1G79720.1 |  | | | |  |  |  |  |  |  |
| 1 | Vvi-Vitvi09g04508\_t001 |  |  |  | | | |  |  |  |  |  |  |
| 1 | Vvi-Vitvi09g01273\_t003 |  |  |  | | | |  |  |  |  |  |  |
| 1 | Vvi-Vitvi09g01274\_t001 |  |  |  | | | |  |  |  |  |  |  |
| 1 | Vvi-Vitvi09g04509\_t001 |  |  |  | | | |  |  |  |  |  |  |
| 1 | Vvi-Vitvi09g01275\_t001 |  |  |  | | | |  |  |  |  |  |  |
| 1 | Vvi-Vitvi09g04510\_t001 |  |  |  | | | |  |  |  |  |  |  |
| 1 | Vvi-Vitvi09g01905\_t001 |  |  |  | | | |  |  |  |  |  |  |
| 1 | Vvi-Vitvi09g04511\_t001 |  |  |  | | | |  |  |  |  |  |  |
| 1 | Vvi-Vitvi09g01278\_t001 |  |  |  | Ath-AT1G16040.1 |  |  |  |  |  |  |
| 0 | Vvi-Vitvi09g01279\_t001 |  |  |  |  |  |  |  |  |
| 0 | Vvi-Vitvi09g01280\_t001.1.6037826f |  |  |  |  |  |  |  |  |
| 0 | Vvi-Vitvi09g01281\_t001 |  |  |  |  |  |  |  |  |
| 0 | Vvi-Vitvi09g01282\_t001 |  |  |  |  |  |  |  |  |
| 0 | Vvi-Vitvi09g01285\_t001 |  |  |  |  |  |  |  |  |
| 0 | Vvi-Vitvi09g01287\_t001 |  |  |  |  |  |  |  |  |
| 0 | Vvi-Vitvi09g04512\_t001 |  |  |  |  |  |  |  |  |
| 0 | Vvi-Vitvi09g01289\_t001 |  |  |  |  |  |  |  |  |
| 0 | Vvi-Vitvi09g01291\_t001 |  |  |  |  |  |  |  |  |
| 0 | Vvi-Vitvi09g04513\_t001 |  |  |  |  |  |  |  |  |
| 0 | Vvi-Vitvi09g04514\_t001 |  |  |  |  |  |  |  |  |
| 0 | Vvi-Vitvi09g04515\_t001 |  |  |  |  |  |  |  |  |
| 0 | Vvi-Vitvi09g01297\_t001 |  |  |  |  |  |  |  |  |
| 0 | Vvi-Vitvi09g01298\_t001 |  |  |  |  |  |  |  |  |
| 0 | Vvi-Vitvi09g01299\_t001 |  |  |  |  |  |  |  |  |
| 0 | Vvi-Vitvi09g01912\_t001 |  |  |  |  |  |  |  |  |
| 0 | Vvi-Vitvi09g01300\_t001 |  |  |  |  |  |  |  |  |
| 0 | Vvi-Vitvi09g01913\_t001 |  |  |  |  |  |  |  |  |
| 0 | Vvi-Vitvi09g01301\_t001 |  |  |  |  |  |  |  |  |
| 0 | Vvi-Vitvi09g01302\_t001 |  |  |  |  |  |  |  |  |
| 0 | Vvi-Vitvi09g01303\_t001 |  |  |  |  |  |  |  |  |
| 0 | Vvi-Vitvi09g01304\_t001 |  |  |  |  |  |  |  |  |
| 0 | Vvi-Vitvi09g01914\_t001 |  |  |  |  |  |  |  |  |
| 0 | Vvi-Vitvi09g01915\_t001 |  |  |  |  |  |  |  |  |
| 0 | Vvi-Vitvi09g01916\_t001 |  |  |  |  |  |  |  |  |
| 0 | Vvi-Vitvi09g01305\_t001 |  |  |  |  |  |  |  |  |
| 0 | Vvi-Vitvi09g01306\_t001 |  |  |  |  |  |  |  |  |
| 0 | Vvi-Vitvi09g01307\_t001 |  |  |  |  |  |  |  |  |
| 0 | Vvi-Vitvi09g01309\_t001 |  |  |  |  |  |  |  |  |
| 0 | Vvi-Vitvi09g01310\_t001 |  |  |  |  |  |  |  |  |
| 0 | Vvi-Vitvi09g01311\_t001 |  |  |  |  |  |  |  |  |
| 0 | Vvi-Vitvi09g04516\_t001 |  |  |  |  |  |  |  |  |
| 0 | Vvi-Vitvi09g04517\_t001 |  |  |  |  |  |  |  |  |
| 0 | Vvi-Vitvi09g04518\_t001 |  |  |  |  |  |  |  |  |
| 0 | Vvi-Vitvi09g04519\_t001 |  |  |  |  |  |  |  |  |
| 0 | Vvi-Vitvi09g01313\_t001 |  |  |  |  |  |  |  |  |
| 0 | Vvi-Vitvi09g04520\_t001 |  |  |  |  |  |  |  |  |
| 0 | Vvi-Vitvi09g04521\_t001 |  |  |  |  |  |  |  |  |
| 0 | Vvi-Vitvi09g04522\_t001 |  |  |  |  |  |  |  |  |
| 0 | Vvi-Vitvi09g04523\_t001 |  |  |  |  |  |  |  |  |
| 0 | Vvi-Vitvi09g01918\_t001 |  |  |  |  |  |  |  |  |
| 0 | Vvi-Vitvi09g01919\_t001 |  |  |  |  |  |  |  |  |
| 0 | Vvi-Vitvi09g04524\_t001 |  |  |  |  |  |  |  |  |
| 0 | Vvi-Vitvi09g04525\_t001 |  |  |  |  |  |  |  |  |
| 0 | Vvi-Vitvi09g04526\_t001 |  |  |  |  |  |  |  |  |
| 0 | Vvi-Vitvi09g01921\_t001 |  |  |  |  |  |  |  |  |
| 0 | Vvi-Vitvi09g01314\_t001 |  |  |  |  |  |  |  |  |
| 0 | Vvi-Vitvi09g04527\_t001 |  |  |  |  |  |  |  |  |
| 0 | Vvi-Vitvi09g01316\_t001 |  |  |  |  |  |  |  |  |
| 0 | Vvi-Vitvi09g04528\_t001 |  |  |  |  |  |  |  |  |
| 0 | Vvi-Vitvi09g01924\_t001 |  |  |  |  |  |  |  |  |
| 0 | Vvi-Vitvi09g01317\_t001 |  |  |  |  |  |  |  |  |
| 0 | Vvi-Vitvi09g04529\_t001 |  |  |  |  |  |  |  |  |
| 0 | Vvi-Vitvi09g01320\_t001 |  |  |  |  |  |  |  |  |
| 0 | Vvi-Vitvi09g04530\_t001 |  |  |  |  |  |  |  |  |
| 0 | Vvi-Vitvi09g04531\_t001 |  |  |  |  |  |  |  |  |
| 0 | Vvi-Vitvi09g01927\_t005 |  |  |  |  |  |  |  |  |
| 0 | Vvi-Vitvi09g04532\_t001 |  |  |  |  |  |  |  |  |
| 0 | Vvi-Vitvi09g04533\_t001 |  |  |  |  |  |  |  |  |
| 0 | Vvi-Vitvi09g04534\_t001 |  |  |  |  |  |  |  |  |
| 0 | Vvi-Vitvi09g04535\_t001 |  |  |  |  |  |  |  |  |
| 0 | Vvi-Vitvi09g04536\_t001 |  |  |  |  |  |  |  |  |
| 0 | Vvi-Vitvi09g01324\_t001 |  |  |  |  |  |  |  |  |
| 0 | Vvi-Vitvi09g01327\_t001 |  |  |  |  |  |  |  |  |
| 0 | Vvi-Vitvi09g04537\_t001 |  |  |  |  |  |  |  |  |
| 0 | Vvi-Vitvi09g01936\_t001 |  |  |  |  |  |  |  |  |
| 0 | Vvi-Vitvi09g04538\_t001 |  |  |  |  |  |  |  |  |
| 0 | Vvi-Vitvi09g01938\_t001 |  |  |  |  |  |  |  |  |
| 0 | Vvi-Vitvi09g04539\_t001 |  |  |  |  |  |  |  |  |
| 0 | Vvi-Vitvi09g04540\_t001 |  |  |  |  |  |  |  |  |
| 0 | Vvi-Vitvi09g04541\_t001 |  |  |  |  |  |  |  |  |
| 0 | Vvi-Vitvi09g04542\_t001 |  |  |  |  |  |  |  |  |
| 0 | Vvi-Vitvi09g04543\_t001 |  |  |  |  |  |  |  |  |
| 0 | Vvi-Vitvi09g01329\_t001 |  |  |  |  |  |  |  |  |
| 0 | Vvi-Vitvi09g04544\_t001 |  |  |  |  |  |  |  |  |
| 0 | Vvi-Vitvi09g01943\_t001 |  |  |  |  |  |  |  |  |
| 0 | Vvi-Vitvi09g04545\_t001 |  |  |  |  |  |  |  |  |
| 0 | Vvi-Vitvi09g04546\_t001 |  |  |  |  |  |  |  |  |
| 0 | Vvi-Vitvi09g04547\_t001 |  |  |  |  |  |  |  |  |
| 0 | Vvi-Vitvi09g04548\_t001 |  |  |  |  |  |  |  |  |
| 0 | Vvi-Vitvi09g04549\_t001 |  |  |  |  |  |  |  |  |
| 0 | Vvi-Vitvi09g01946\_t001 |  |  |  |  |  |  |  |  |
| 0 | Vvi-Vitvi09g04550\_t001 |  |  |  |  |  |  |  |  |
| 0 | Vvi-Vitvi09g01947\_t001 |  |  |  |  |  |  |  |  |
| 0 | Vvi-Vitvi09g01332\_t001 |  |  |  |  |  |  |  |  |
| 0 | Vvi-Vitvi09g01949\_t001 |  |  |  |  |  |  |  |  |
| 0 | Vvi-Vitvi09g01335\_t001 |  |  |  |  |  |  |  |  |
| 0 | Vvi-Vitvi09g01951\_t001 |  |  |  |  |  |  |  |  |
| 0 | Vvi-Vitvi09g04551\_t001 |  |  |  |  |  |  |  |  |
| 0 | Vvi-Vitvi09g04552\_t001 |  |  |  |  |  |  |  |  |
| 0 | Vvi-Vitvi09g01338\_t001 |  |  |  |  |  |  |  |  |
| 0 | Vvi-Vitvi09g04553\_t001 |  |  |  |  |  |  |  |  |
| 0 | Vvi-Vitvi09g04554\_t001 |  |  |  |  |  |  |  |  |
| 0 | Vvi-Vitvi09g04555\_t001 |  |  |  |  |  |  |  |  |
| 0 | Vvi-Vitvi09g04556\_t001 |  |  |  |  |  |  |  |  |
| 0 | Vvi-Vitvi09g04557\_t001 |  |  |  |  |  |  |  |  |
| 0 | Vvi-Vitvi09g04558\_t001 |  |  |  |  |  |  |  |  |
| 0 | Vvi-Vitvi09g04559\_t001 |  |  |  |  |  |  |  |  |
| 0 | Vvi-Vitvi09g01341\_t001 |  |  |  |  |  |  |  |  |
| 1 | Vvi-Vitvi09g04560\_t001 |  | Ath-AT1G51610.1 |  |  |  |  |  |  |  |
| 1 | Vvi-Vitvi09g01954\_t001 |  | | | |  |  |  |  |  |  |  |
| 1 | Vvi-Vitvi09g04561\_t001 |  | | | |  |  |  |  |  |  |  |
| 1 | Vvi-Vitvi09g04562\_t001 |  | Ath-AT1G51590.1 |  |  |  |  |  |  |  |
| 1 | Vvi-Vitvi09g04563\_t001 |  | | | |  |  |  |  |  |  |  |
| 1 | Vvi-Vitvi09g01957\_t001 |  | | | |  |  |  |  |  |  |  |
| 1 | Vvi-Vitvi09g04564\_t001 |  | | | |  |  |  |  |  |  |  |
| 1 | Vvi-Vitvi09g04565\_t001 |  | | | |  |  |  |  |  |  |  |
| 1 | Vvi-Vitvi09g01344\_t001 |  | | | |  |  |  |  |  |  |  |
| 1 | Vvi-Vitvi09g01345\_t001 |  | | | |  |  |  |  |  |  |  |
| 1 | Vvi-Vitvi09g01347\_t001 |  | | | |  |  |  |  |  |  |  |
| 1 | Vvi-Vitvi09g01962\_t001 |  | | | |  |  |  |  |  |  |  |
| 1 | Vvi-Vitvi09g01349\_t001 |  | | | |  |  |  |  |  |  |  |
| 1 | Vvi-Vitvi09g01351\_t001 |  | | | |  |  |  |  |  |  |  |
| 1 | Vvi-Vitvi09g04566\_t001 |  | | | |  |  |  |  |  |  |  |
| 1 | Vvi-Vitvi09g01352\_t003 |  | | | |  |  |  |  |  |  |  |
| 1 | Vvi-Vitvi09g01354\_t001 |  | Ath-AT1G51580.1 |  |  |  |  |  |  |  |
| 1 | Vvi-Vitvi09g01355\_t001 |  | | | |  |  |  |  |  |  |  |
| 1 | Vvi-Vitvi09g04567\_t001 |  | | | |  |  |  |  |  |  |  |
| 1 | Vvi-Vitvi09g04568\_t001 |  | | | |  |  |  |  |  |  |  |
| 1 | Vvi-Vitvi09g04569\_t001 |  | | | |  |  |  |  |  |  |  |
| 1 | Vvi-Vitvi09g01966\_t001 |  | | | |  |  |  |  |  |  |  |
| 1 | Vvi-Vitvi09g01970\_t001 |  | | | |  |  |  |  |  |  |  |
| 1 | Vvi-Vitvi09g04570\_t001 |  | | | |  |  |  |  |  |  |  |
| 1 | Vvi-Vitvi09g04571\_t001 |  | | | |  |  |  |  |  |  |  |
| 1 | Vvi-Vitvi09g04572\_t001 |  | | | |  |  |  |  |  |  |  |
| 1 | Vvi-Vitvi09g01357\_t001 |  | | | |  |  |  |  |  |  |  |
| 1 | Vvi-Vitvi09g01972\_t001 |  | | | |  |  |  |  |  |  |  |
| 1 | Vvi-Vitvi09g01359\_t001 |  | | | |  |  |  |  |  |  |  |
| 1 | Vvi-Vitvi09g01361\_t001 |  | | | |  |  |  |  |  |  |  |
| 1 | Vvi-Vitvi09g01362\_t001 |  | | | |  |  |  |  |  |  |  |
| 1 | Vvi-Vitvi09g04573\_t001 |  | | | |  |  |  |  |  |  |  |
| 1 | Vvi-Vitvi09g01975\_t001 |  | | | |  |  |  |  |  |  |  |
| 1 | Vvi-Vitvi09g01364\_t001 |  | | | |  |  |  |  |  |  |  |
| 1 | Vvi-Vitvi09g04574\_t001 |  | | | |  |  |  |  |  |  |  |
| 1 | Vvi-Vitvi09g04575\_t001 |  | | | |  |  |  |  |  |  |  |
| 1 | Vvi-Vitvi09g01980\_t001 |  | | | |  |  |  |  |  |  |  |
| 1 | Vvi-Vitvi09g04576\_t001 |  | | | |  |  |  |  |  |  |  |
| 1 | Vvi-Vitvi09g01372\_t002 |  | Ath-AT1G51560.1 |  |  |  |  |  |  |  |
| 1 | Vvi-Vitvi09g01373\_t001 |  | Ath-AT1G51550.1 |  |  |  |  |  |  |  |
| 1 | Vvi-Vitvi09g01374\_t001 |  | Ath-AT1G51540.1 |  |  |  |  |  |  |  |
| 0 | Vvi-Vitvi09g01376\_t001 |  |  |  |  |  |  |  |  |
| 0 | Vvi-Vitvi09g01982\_t001 |  |  |  |  |  |  |  |  |
| 0 | Vvi-Vitvi09g01983\_t001 |  |  |  |  |  |  |  |  |
| 0 | Vvi-Vitvi09g01379\_t001 |  |  |  |  |  |  |  |  |
| 0 | Vvi-Vitvi09g04577\_t001 |  |  |  |  |  |  |  |  |
| 1 | Vvi-Vitvi09g01380\_t001 |  | Ath-AT3G20210.1 |  |  |  |  |  |  |  |
| 1 | Vvi-Vitvi09g04578\_t001 |  | | | |  |  |  |  |  |  |  |
| 2 | Vvi-Vitvi09g01381\_t001 |  | | | |  | Ath-AT1G16780.1 |  |  |  |  |  |  |
| 2 | Vvi-Vitvi09g01382\_t001 |  | | | |  | Ath-AT1G16790.1 |  |  |  |  |  |  |
| 2 | Vvi-Vitvi09g01383\_t001 |  | | | |  | | | |  |  |  |  |  |  |
| 2 | Vvi-Vitvi09g01384\_t001 |  | | | |  | | | |  |  |  |  |  |  |
| 2 | Vvi-Vitvi09g01385\_t001 |  | Ath-AT3G20220.1 |  | | | |  |  |  |  |  |  |
| 2 | Vvi-Vitvi09g01984\_t001 |  | | | |  | | | |  |  |  |  |  |  |
| 2 | Vvi-Vitvi09g01386\_t001 |  | | | |  | Ath-AT1G16800.2 |  |  |  |  |  |  |
| 2 | Vvi-Vitvi09g01387\_t001 |  | | | |  | | | |  |  |  |  |  |  |
| 2 | Vvi-Vitvi09g01388\_t002 |  | Ath-AT3G20240.1 |  | | | |  |  |  |  |  |  |
| 3 | Vvi-Vitvi09g01985\_t001 |  | | | |  | Ath-AT1G16825.1 |  | Ath-AT1G78895.1 |  |  |  |  |  |
| 3 | Vvi-Vitvi09g01389\_t001 |  | Ath-AT3G20250.2 |  | | | |  | | | |  |  |  |  |  |
| 3 | Vvi-Vitvi09g04579\_t001 |  | | | |  | | | |  | | | |  |  |  |  |  |
| 3 | Vvi-Vitvi09g01390\_t001 |  | Ath-AT3G20260.1 |  | | | |  | | | |  |  |  |  |  |
| 3 | Vvi-Vitvi09g04580\_t001 |  | Ath-AT3G20270.3 |  | | | |  | | | |  |  |  |  |  |
| 2 | Vvi-Vitvi09g01394\_t001 |  |  |  | | | |  | | | |  |  |  |  |  |
| 2 | Vvi-Vitvi09g01395\_t001 |  |  |  | | | |  | | | |  |  |  |  |  |
| 2 | Vvi-Vitvi09g04581\_t001 |  |  |  | | | |  | | | |  |  |  |  |  |
| 2 | Vvi-Vitvi09g04582\_t001 |  |  |  | | | |  | | | |  |  |  |  |  |
| 2 | Vvi-Vitvi09g01986\_t001 |  |  |  | | | |  | | | |  |  |  |  |  |
| 2 | Vvi-Vitvi09g01397\_t001 |  |  |  | | | |  | Ath-AT1G78900.1 |  |  |  |  |  |
| 2 | Vvi-Vitvi09g01398\_t001 |  |  |  | | | |  | | | |  |  |  |  |  |
| 2 | Vvi-Vitvi09g01399\_t001 |  |  |  | | | |  | | | |  |  |  |  |  |
| 2 | Vvi-Vitvi09g01400\_t002 |  |  |  | | | |  | | | |  |  |  |  |  |
| 2 | Vvi-Vitvi09g01401\_t001 |  |  |  | | | |  | | | |  |  |  |  |  |
| 2 | Vvi-Vitvi09g01402\_t001 |  |  |  | Ath-AT1G16830.1 |  | | | |  |  |  |  |  |
| 2 | Vvi-Vitvi09g04583\_t001 |  |  |  | | | |  | | | |  |  |  |  |  |
| 2 | Vvi-Vitvi09g01987\_t001 |  |  |  | | | |  | | | |  |  |  |  |  |
| 2 | Vvi-Vitvi09g01403\_t001 |  |  |  | | | |  | | | |  |  |  |  |  |
| 2 | Vvi-Vitvi09g01404\_t001 |  |  |  | | | |  | | | |  |  |  |  |  |
| 2 | Vvi-Vitvi09g01405\_t001 |  |  |  | Ath-AT1G16840.5 |  | | | |  |  |  |  |  |
| 1 | Vvi-Vitvi09g04584\_t001 |  |  |  |  |  | | | |  |  |  |  |  |
| 1 | Vvi-Vitvi09g01407\_t001 |  |  |  |  |  | | | |  |  |  |  |  |
| 1 | Vvi-Vitvi09g01989\_t001 |  |  |  |  |  | Ath-AT1G78922.1 |  |  |  |  |  |
| 1 | Vvi-Vitvi09g01990\_t001 |  |  |  |  |  | | | |  |  |  |  |  |
| 1 | Vvi-Vitvi09g01408\_t001 |  |  |  |  |  | Ath-AT1G78930.1 |  |  |  |  |  |
| 1 | Vvi-Vitvi09g01409\_t001 |  |  |  |  |  | | | |  |  |  |  |  |
| 1 | Vvi-Vitvi09g01410\_t001 |  |  |  |  |  | Ath-AT1G78940.2 |  |  |  |  |  |
| 1 | Vvi-Vitvi09g01411\_t001 |  |  |  |  |  | | | |  |  |  |  |  |
| 1 | Vvi-Vitvi09g01412\_t001 |  |  |  |  |  | | | |  |  |  |  |  |
| 1 | Vvi-Vitvi09g01992\_t002 |  |  |  |  |  | Ath-AT1G78950.2 |  |  |  |  |  |
| 1 | Vvi-Vitvi09g04585\_t001 |  |  |  |  |  | | | |  |  |  |  |  |
| 1 | Vvi-Vitvi09g04586\_t001 |  |  |  |  |  | | | |  |  |  |  |  |
| 1 | Vvi-Vitvi09g01414\_t001 |  |  |  |  |  | | | |  |  |  |  |  |
| 1 | Vvi-Vitvi09g01416\_t001 |  |  |  |  |  | Ath-AT1G78970.3 |  |  |  |  |  |
| 0 | Vvi-Vitvi09g01995\_t001 |  |  |  |  |  |  |  |  |
| 0 | Vvi-Vitvi09g04587\_t001 |  |  |  |  |  |  |  |  |
| 0 | Vvi-Vitvi09g04588\_t001 |  |  |  |  |  |  |  |  |
| 0 | Vvi-Vitvi09g04589\_t001 |  |  |  |  |  |  |  |  |
| 0 | Vvi-Vitvi09g01420\_t001 |  |  |  |  |  |  |  |  |
| 0 | Vvi-Vitvi09g04590\_t001 |  |  |  |  |  |  |  |  |
| 0 | Vvi-Vitvi09g04591\_t001 |  |  |  |  |  |  |  |  |
| 0 | Vvi-Vitvi09g01425\_t001 |  |  |  |  |  |  |  |  |
| 0 | Vvi-Vitvi09g01426\_t001 |  |  |  |  |  |  |  |  |
| 0 | Vvi-Vitvi09g01427\_t002 |  |  |  |  |  |  |  |  |
| 0 | Vvi-Vitvi09g01431\_t001 |  |  |  |  |  |  |  |  |
| 0 | Vvi-Vitvi09g01432\_t001 |  |  |  |  |  |  |  |  |
| 0 | Vvi-Vitvi09g04592\_t001 |  |  |  |  |  |  |  |  |
| 0 | Vvi-Vitvi09g04593\_t001 |  |  |  |  |  |  |  |  |
| 0 | Vvi-Vitvi09g01441\_t001 |  |  |  |  |  |  |  |  |
| 0 | Vvi-Vitvi09g04594\_t001 |  |  |  |  |  |  |  |  |
| 0 | Vvi-Vitvi09g01998\_t001 |  |  |  |  |  |  |  |  |
| 0 | Vvi-Vitvi09g04595\_t001 |  |  |  |  |  |  |  |  |
| 0 | Vvi-Vitvi09g02001\_t001 |  |  |  |  |  |  |  |  |
| 0 | Vvi-Vitvi09g02002\_t001 |  |  |  |  |  |  |  |  |
| 0 | Vvi-Vitvi09g02006\_t001 |  |  |  |  |  |  |  |  |
| 0 | Vvi-Vitvi09g02008\_t001 |  |  |  |  |  |  |  |  |
| 0 | Vvi-Vitvi09g04596\_t001 |  |  |  |  |  |  |  |  |
| 0 | Vvi-Vitvi09g01447\_t001 |  |  |  |  |  |  |  |  |
| 0 | Vvi-Vitvi09g04597\_t001 |  |  |  |  |  |  |  |  |
| 0 | Vvi-Vitvi09g04598\_t001 |  |  |  |  |  |  |  |  |
| 0 | Vvi-Vitvi09g04599\_t001 |  |  |  |  |  |  |  |  |
| 0 | Vvi-Vitvi09g01453\_t001 |  |  |  |  |  |  |  |  |
| 0 | Vvi-Vitvi09g04600\_t001 |  |  |  |  |  |  |  |  |
| 0 | Vvi-Vitvi09g04601\_t001 |  |  |  |  |  |  |  |  |
| 1 | Vvi-Vitvi09g01455\_t001 |  | Ath-AT3G13065.1 |  |  |  |  |  |  |  |
| 1 | Vvi-Vitvi09g01456\_t001 |  | Ath-AT3G13062.2 |  |  |  |  |  |  |  |
| 1 | Vvi-Vitvi09g01457\_t001 |  | Ath-AT3G13060.2 |  |  |  |  |  |  |  |
| 1 | Vvi-Vitvi09g01458\_t001 |  | Ath-AT3G13050.1 |  |  |  |  |  |  |  |
| 1 | Vvi-Vitvi09g04602\_t001 |  | | | |  |  |  |  |  |  |  |
| 1 | Vvi-Vitvi09g01459\_t001 |  | Ath-AT3G13040.1 |  |  |  |  |  |  |  |
| 1 | Vvi-Vitvi09g04603\_t001 |  | | | |  |  |  |  |  |  |  |
| 1 | Vvi-Vitvi09g04604\_t001 |  | Ath-AT3G13010.1 |  |  |  |  |  |  |  |
| 1 | Vvi-Vitvi09g01460\_t001 |  | | | |  |  |  |  |  |  |  |
| 1 | Vvi-Vitvi09g04605\_t001 |  | | | |  |  |  |  |  |  |  |
| 1 | Vvi-Vitvi09g01462\_t001 |  | | | |  |  |  |  |  |  |  |
| 1 | Vvi-Vitvi09g04606\_t001 |  | | | |  |  |  |  |  |  |  |
| 1 | Vvi-Vitvi09g01464\_t002 |  | Ath-AT3G13000.2 |  |  |  |  |  |  |  |
| 1 | Vvi-Vitvi09g04607\_t001 |  | | | |  |  |  |  |  |  |  |
| 1 | Vvi-Vitvi09g04608\_t001 |  | | | |  |  |  |  |  |  |  |
| 1 | Vvi-Vitvi09g01466\_t001 |  | | | |  |  |  |  |  |  |  |
| 1 | Vvi-Vitvi09g01467\_t001 |  | | | |  |  |  |  |  |  |  |
| 1 | Vvi-Vitvi09g04609\_t001 |  | | | |  |  |  |  |  |  |  |
| 1 | Vvi-Vitvi09g04610\_t001 |  | | | |  |  |  |  |  |  |  |
| 1 | Vvi-Vitvi09g04611\_t001 |  | | | |  |  |  |  |  |  |  |
| 1 | Vvi-Vitvi09g04612\_t001 |  | | | |  |  |  |  |  |  |  |
| 1 | Vvi-Vitvi09g02014\_t001 |  | | | |  |  |  |  |  |  |  |
| 1 | Vvi-Vitvi09g01471\_t001 |  | | | |  |  |  |  |  |  |  |
| 1 | Vvi-Vitvi09g01473\_t001 |  | | | |  |  |  |  |  |  |  |
| 1 | Vvi-Vitvi09g01474\_t001 |  | Ath-AT3G12980.1 |  |  |  |  |  |  |  |
| 0 | Vvi-Vitvi09g04613\_t001 |  |  |  |  |  |  |  |  |
| 0 | Vvi-Vitvi09g04614\_t001 |  |  |  |  |  |  |  |  |
| 0 | Vvi-Vitvi09g04615\_t001 |  |  |  |  |  |  |  |  |
