# Supplementary material for: Genome-wide identification, characterization and gene expression of BES1 transcription factor family in grapevine (Vitis vinifera L.)
Source: Sci Rep. 2023 Jan 5;13:240. doi: 10.1038/s41598-022-24407-y (PMC9816167; doi:10.1038/s41598-022-24407-y)
Supplement: Supplementary file 3 — Supplementary Information. [file 41598_2022_24407_MOESM3_ESM.zip › Vvi_Atr/Vitis_vinifera.PN40024.v4.dna_sm.toplevel.fa.vs.Amborella_trichopoda.AMTR1.0.dna_sm.toplevel.fa.html/Atr-AmTr_v1.0_scaffold00001.html]

|  |  |  |  |  |  |  |  |  |  |  |  |  |  |
| --- | --- | --- | --- | --- | --- | --- | --- | --- | --- | --- | --- | --- | --- |
| Duplication depth | Reference chromosome | Collinear blocks | | | | | | | | | | | |
| 2 | Atr-ERM96124 |  | Vvi-Vitvi03g00231\_t001 |  | Vvi-Vitvi18g02488\_t001 |  |  |  |  |
| 3 | Atr-ERM96125 |  | | | |  | | | |  | Vvi-Vitvi04g01566\_t001 |  |  |  |
| 3 | Atr-ERM96126 |  | | | |  | | | |  | | | |  |  |  |
| 3 | Atr-ERM96127 |  | | | |  | | | |  | | | |  |  |  |
| 3 | Atr-ERM96128 |  | | | |  | | | |  | Vvi-Vitvi04g01565\_t001 |  |  |  |
| 3 | Atr-ERM96129 |  | | | |  | | | |  | | | |  |  |  |
| 3 | Atr-ERM96130 |  | | | |  | Vvi-Vitvi18g00095\_t001 |  | Vvi-Vitvi04g04448\_t001 |  |  |  |
| 3 | Atr-ERM96131 |  | | | |  | | | |  | | | |  |  |  |
| 3 | Atr-ERM96132 |  | Vvi-Vitvi03g00230\_t001 |  | | | |  | Vvi-Vitvi04g02200\_t001 |  |  |  |
| 3 | Atr-ERM96133 |  | Vvi-Vitvi03g01464\_t001 |  | | | |  | Vvi-Vitvi04g02199\_t001 |  |  |  |
| 3 | Atr-ERM96134 |  | Vvi-Vitvi03g00229\_t003 |  | | | |  | Vvi-Vitvi04g04445\_t001 |  |  |  |
| 3 | Atr-ERM96135 |  | | | |  | | | |  | Vvi-Vitvi04g04444\_t001 |  |  |  |
| 3 | Atr-ERM96136 |  | | | |  | | | |  | | | |  |  |  |
| 3 | Atr-ERM96137 |  | | | |  | | | |  | | | |  |  |  |
| 3 | Atr-ERM96138 |  | | | |  | | | |  | Vvi-Vitvi04g04443\_t001 |  |  |  |
| 3 | Atr-ERM96139 |  | | | |  | | | |  | | | |  |  |  |
| 3 | Atr-ERM96140 |  | | | |  | | | |  | | | |  |  |  |
| 3 | Atr-ERM96141 |  | | | |  | | | |  | | | |  |  |  |
| 3 | Atr-ERM96142 |  | | | |  | | | |  | | | |  |  |  |
| 3 | Atr-ERM96143 |  | | | |  | | | |  | | | |  |  |  |
| 3 | Atr-ERM96144 |  | | | |  | | | |  | Vvi-Vitvi04g01559\_t002 |  |  |  |
| 3 | Atr-ERM96145 |  | | | |  | Vvi-Vitvi18g00096\_t001 |  | | | |  |  |  |
| 3 | Atr-ERM96146 |  | | | |  | | | |  | | | |  |  |  |
| 3 | Atr-ERM96147 |  | | | |  | | | |  | | | |  |  |  |
| 3 | Atr-ERM96148 |  | Vvi-Vitvi03g00228\_t001 |  | | | |  | | | |  |  |  |
| 3 | Atr-ERM96149 |  | | | |  | | | |  | | | |  |  |  |
| 3 | Atr-ERM96150 |  | Vvi-Vitvi03g00226\_t001 |  | Vvi-Vitvi18g00098\_t001 |  | | | |  |  |  |
| 3 | Atr-ERM96151 |  | | | |  | | | |  | | | |  |  |  |
| 3 | Atr-ERM96152 |  | Vvi-Vitvi03g00225\_t001 |  | | | |  | | | |  |  |  |
| 2 | Atr-ERM96153 |  |  |  | | | |  | | | |  |  |  |
| 2 | Atr-ERM96154 |  |  |  | | | |  | | | |  |  |  |
| 2 | Atr-ERM96155 |  |  |  | | | |  | | | |  |  |  |
| 2 | Atr-ERM96156 |  |  |  | | | |  | | | |  |  |  |
| 2 | Atr-ERM96157 |  |  |  | | | |  | | | |  |  |  |
| 2 | Atr-ERM96158 |  |  |  | | | |  | Vvi-Vitvi04g01556\_t001 |  |  |  |
| 2 | Atr-ERM96159 |  |  |  | Vvi-Vitvi18g00102\_t001 |  | | | |  |  |  |
| 2 | Atr-ERM96160 |  |  |  | | | |  | | | |  |  |  |
| 2 | Atr-ERM96161 |  |  |  | | | |  | | | |  |  |  |
| 2 | Atr-ERM96162 |  |  |  | | | |  | | | |  |  |  |
| 2 | Atr-ERM96163 |  |  |  | | | |  | | | |  |  |  |
| 2 | Atr-ERM96164 |  |  |  | | | |  | | | |  |  |  |
| 2 | Atr-ERM96165 |  |  |  | Vvi-Vitvi18g00103\_t001 |  | | | |  |  |  |
| 1 | Atr-ERM96166 |  |  |  |  |  | Vvi-Vitvi04g02190\_t001 |  |  |  |
| 1 | Atr-ERM96167 |  |  |  |  |  | | | |  |  |  |
| 1 | Atr-ERM96168 |  |  |  |  |  | | | |  |  |  |
| 1 | Atr-ERM96169 |  |  |  |  |  | | | |  |  |  |
| 1 | Atr-ERM96170 |  |  |  |  |  | | | |  |  |  |
| 1 | Atr-ERM96171 |  |  |  |  |  | | | |  |  |  |
| 1 | Atr-ERM96172 |  |  |  |  |  | | | |  |  |  |
| 1 | Atr-ERM96173 |  |  |  |  |  | | | |  |  |  |
| 1 | Atr-ERM96174 |  |  |  |  |  | | | |  |  |  |
| 1 | Atr-ERM96175 |  |  |  |  |  | | | |  |  |  |
| 1 | Atr-ERM96176 |  |  |  |  |  | | | |  |  |  |
| 1 | Atr-ERM96177 |  |  |  |  |  | Vvi-Vitvi04g04429\_t001 |  |  |  |
| 0 | Atr-ERM96178 |  |  |  |  |  |  |
| 0 | Atr-ERM96179 |  |  |  |  |  |  |
| 0 | Atr-ERM96180 |  |  |  |  |  |  |
| 0 | Atr-ERM96181 |  |  |  |  |  |  |
| 0 | Atr-ERM96182 |  |  |  |  |  |  |
| 0 | Atr-ERM96183 |  |  |  |  |  |  |
| 0 | Atr-ERM96184 |  |  |  |  |  |  |
| 0 | Atr-ERM96185 |  |  |  |  |  |  |
| 0 | Atr-ERM96186 |  |  |  |  |  |  |
| 0 | Atr-ERM96187 |  |  |  |  |  |  |
| 0 | Atr-ERM96188 |  |  |  |  |  |  |
| 0 | Atr-ERM96189 |  |  |  |  |  |  |
| 0 | Atr-ERM96190 |  |  |  |  |  |  |
| 0 | Atr-ERM96191 |  |  |  |  |  |  |
| 0 | Atr-ERM96192 |  |  |  |  |  |  |
| 0 | Atr-ERM96193 |  |  |  |  |  |  |
| 0 | Atr-ERM96194 |  |  |  |  |  |  |
| 0 | Atr-ERM96195 |  |  |  |  |  |  |
| 0 | Atr-ERM96196 |  |  |  |  |  |  |
| 0 | Atr-ERM96197 |  |  |  |  |  |  |
| 0 | Atr-ERM96198 |  |  |  |  |  |  |
| 0 | Atr-ERM96199 |  |  |  |  |  |  |
| 0 | Atr-ERM96200 |  |  |  |  |  |  |
| 0 | Atr-ERM96201 |  |  |  |  |  |  |
| 0 | Atr-ERM96202 |  |  |  |  |  |  |
| 0 | Atr-ERM96203 |  |  |  |  |  |  |
| 0 | Atr-ERM96204 |  |  |  |  |  |  |
| 0 | Atr-ERM96205 |  |  |  |  |  |  |
| 0 | Atr-ERM96206 |  |  |  |  |  |  |
| 0 | Atr-ERM96207 |  |  |  |  |  |  |
| 0 | Atr-ERM96208 |  |  |  |  |  |  |
| 0 | Atr-ERM96209 |  |  |  |  |  |  |
| 0 | Atr-ERM96210 |  |  |  |  |  |  |
| 0 | Atr-ERM96211 |  |  |  |  |  |  |
| 0 | Atr-ERM96212 |  |  |  |  |  |  |
| 0 | Atr-ERM96213 |  |  |  |  |  |  |
| 0 | Atr-ERM96214 |  |  |  |  |  |  |
| 0 | Atr-ERM96215 |  |  |  |  |  |  |
| 0 | Atr-ERM96216 |  |  |  |  |  |  |
| 0 | Atr-ERM96217 |  |  |  |  |  |  |
| 0 | Atr-ERM96218 |  |  |  |  |  |  |
| 0 | Atr-ERM96219 |  |  |  |  |  |  |
| 0 | Atr-ERM96220 |  |  |  |  |  |  |
| 1 | Atr-ERM96221 |  | Vvi-Vitvi04g02179\_t001 |  |  |  |  |  |
| 1 | Atr-ERM96222 |  | | | |  |  |  |  |  |
| 1 | Atr-ERM96223 |  | | | |  |  |  |  |  |
| 2 | Atr-ERM96224 |  | | | |  | Vvi-Vitvi04g04401\_t001 |  |  |  |  |
| 2 | Atr-ERM96225 |  | | | |  | | | |  |  |  |  |
| 2 | Atr-ERM96226 |  | | | |  | | | |  |  |  |  |
| 2 | Atr-ERM96227 |  | | | |  | | | |  |  |  |  |
| 2 | Atr-ERM96228 |  | | | |  | | | |  |  |  |  |
| 2 | Atr-ERM96229 |  | | | |  | | | |  |  |  |  |
| 2 | Atr-ERM96230 |  | | | |  | | | |  |  |  |  |
| 2 | Atr-ERM96231 |  | | | |  | | | |  |  |  |  |
| 2 | Atr-ERM96232 |  | | | |  | | | |  |  |  |  |
| 2 | Atr-ERM96233 |  | | | |  | | | |  |  |  |  |
| 2 | Atr-ERM96234 |  | | | |  | | | |  |  |  |  |
| 2 | Atr-ERM96235 |  | | | |  | | | |  |  |  |  |
| 2 | Atr-ERM96236 |  | | | |  | Vvi-Vitvi04g02280\_t001 |  |  |  |  |
| 2 | Atr-ERM96237 |  | Vvi-Vitvi04g01526\_t001 |  | | | |  |  |  |  |
| 3 | Atr-ERM96238 |  | | | |  | | | |  | Vvi-Vitvi03g00188\_t001 |  |  |  |
| 3 | Atr-ERM96239 |  | | | |  | | | |  | | | |  |  |  |
| 3 | Atr-ERM96240 |  | Vvi-Vitvi04g01527\_t001 |  | | | |  | | | |  |  |  |
| 3 | Atr-ERM96241 |  | | | |  | | | |  | Vvi-Vitvi03g00187\_t001 |  |  |  |
| 3 | Atr-ERM96242 |  | | | |  | | | |  | | | |  |  |  |
| 3 | Atr-ERM96243 |  | | | |  | | | |  | | | |  |  |  |
| 4 | Atr-ERM96244 |  | | | |  | | | |  | | | |  | Vvi-Vitvi18g00157\_t001 |  |  |
| 4 | Atr-ERM96245 |  | | | |  | | | |  | | | |  | Vvi-Vitvi18g00158\_t001 |  |  |
| 5 | Atr-ERM96246 |  | | | |  | | | |  | Vvi-Vitvi03g00185\_t001 |  | | | |  | Vvi-Vitvi18g00163\_t001 |  |
| 5 | Atr-ERM96247 |  | | | |  | | | |  | | | |  | | | |  | | | |  |
| 5 | Atr-ERM96248 |  | | | |  | | | |  | | | |  | | | |  | | | |  |
| 5 | Atr-ERM96249 |  | | | |  | | | |  | | | |  | | | |  | | | |  |
| 5 | Atr-ERM96250 |  | | | |  | | | |  | | | |  | Vvi-Vitvi18g00161\_t001 |  | Vvi-Vitvi18g00161\_t001 |  |
| 5 | Atr-ERM96251 |  | | | |  | | | |  | | | |  | | | |  | | | |  |
| 5 | Atr-ERM96252 |  | Vvi-Vitvi04g01530\_t002 |  | | | |  | | | |  | | | |  | Vvi-Vitvi18g00159\_t001 |  |
| 5 | Atr-ERM96253 |  | | | |  | | | |  | Vvi-Vitvi03g04074\_t001 |  | Vvi-Vitvi18g02503\_t001 |  | | | |  |
| 5 | Atr-ERM96254 |  | | | |  | | | |  | | | |  | | | |  | | | |  |
| 5 | Atr-ERM96255 |  | | | |  | | | |  | | | |  | | | |  | | | |  |
| 5 | Atr-ERM96256 |  | | | |  | | | |  | | | |  | | | |  | | | |  |
| 5 | Atr-ERM96257 |  | | | |  | | | |  | | | |  | | | |  | | | |  |
| 5 | Atr-ERM96258 |  | Vvi-Vitvi04g04405\_t001 |  | | | |  | | | |  | Vvi-Vitvi18g00164\_t001 |  | | | |  |
| 5 | Atr-ERM96259 |  | | | |  | Vvi-Vitvi04g01534\_t001 |  | | | |  | | | |  | | | |  |
| 5 | Atr-ERM96260 |  | | | |  | Vvi-Vitvi04g04403\_t001 |  | | | |  | Vvi-Vitvi18g00167\_t001 |  | | | |  |
| 5 | Atr-ERM96261 |  | | | |  | | | |  | Vvi-Vitvi03g01411\_t001 |  | | | |  | | | |  |
| 5 | Atr-ERM96262 |  | | | |  | | | |  | | | |  | | | |  | | | |  |
| 5 | Atr-ERM96263 |  | | | |  | Vvi-Vitvi04g01529\_t001 |  | | | |  | | | |  | | | |  |
| 5 | Atr-ERM96264 |  | Vvi-Vitvi04g02185\_t001 |  | | | |  | | | |  | Vvi-Vitvi18g00172\_t001.3.6037826c |  | | | |  |
| 5 | Atr-ERM96265 |  | | | |  | | | |  | | | |  | | | |  | | | |  |
| 5 | Atr-ERM96266 |  | Vvi-Vitvi04g01535\_t001 |  | | | |  | | | |  | | | |  | | | |  |
| 5 | Atr-ERM96267 |  | | | |  | | | |  | | | |  | Vvi-Vitvi18g00173\_t001 |  | | | |  |
| 5 | Atr-ERM96268 |  | | | |  | | | |  | | | |  | Vvi-Vitvi18g00174\_t001 |  | | | |  |
| 4 | Atr-ERM96269 |  | | | |  | | | |  | | | |  |  |  | | | |  |
| 4 | Atr-ERM96270 |  | Vvi-Vitvi04g01536\_t001 |  | | | |  | | | |  |  |  | | | |  |
| 3 | Atr-ERM96271 |  |  |  | | | |  | Vvi-Vitvi03g00170\_t001 |  |  |  | | | |  |
| 2 | Atr-ERM96272 |  |  |  | Vvi-Vitvi04g04407\_t002 |  |  |  |  |  | | | |  |
| 2 | Atr-ERM96273 |  |  |  | | | |  |  |  |  |  | | | |  |
| 2 | Atr-ERM96274 |  |  |  | | | |  |  |  |  |  | | | |  |
| 2 | Atr-ERM96275 |  |  |  | | | |  |  |  |  |  | | | |  |
| 2 | Atr-ERM96276 |  |  |  | | | |  |  |  |  |  | | | |  |
| 2 | Atr-ERM96277 |  |  |  | | | |  |  |  |  |  | Vvi-Vitvi18g00156\_t001 |  |
| 2 | Atr-ERM96278 |  |  |  | | | |  |  |  |  |  | | | |  |
| 2 | Atr-ERM96279 |  |  |  | | | |  |  |  |  |  | | | |  |
| 2 | Atr-ERM96280 |  |  |  | | | |  |  |  |  |  | | | |  |
| 2 | Atr-ERM96281 |  |  |  | Vvi-Vitvi04g04409\_t001 |  |  |  |  |  | Vvi-Vitvi18g00154\_t001 |  |
| 2 | Atr-ERM96282 |  |  |  | | | |  |  |  |  |  | Vvi-Vitvi18g00153\_t001 |  |
| 2 | Atr-ERM96283 |  |  |  | | | |  |  |  |  |  | | | |  |
| 2 | Atr-ERM96284 |  |  |  | | | |  |  |  |  |  | | | |  |
| 2 | Atr-ERM96285 |  |  |  | | | |  |  |  |  |  | Vvi-Vitvi18g00152\_t001 |  |
| 2 | Atr-ERM96286 |  |  |  | | | |  |  |  |  |  | | | |  |
| 2 | Atr-ERM96287 |  |  |  | | | |  |  |  |  |  | | | |  |
| 2 | Atr-ERM96288 |  |  |  | | | |  |  |  |  |  | Vvi-Vitvi18g00151\_t001 |  |
| 2 | Atr-ERM96289 |  |  |  | | | |  |  |  |  |  | | | |  |
| 2 | Atr-ERM96290 |  |  |  | | | |  |  |  |  |  | | | |  |
| 2 | Atr-ERM96291 |  |  |  | | | |  |  |  |  |  | | | |  |
| 2 | Atr-ERM96292 |  |  |  | | | |  |  |  |  |  | | | |  |
| 2 | Atr-ERM96293 |  |  |  | | | |  |  |  |  |  | Vvi-Vitvi18g00150\_t001 |  |
| 2 | Atr-ERM96294 |  |  |  | | | |  |  |  |  |  | | | |  |
| 2 | Atr-ERM96295 |  |  |  | | | |  |  |  |  |  | Vvi-Vitvi18g04021\_t001 |  |
| 2 | Atr-ERM96296 |  |  |  | Vvi-Vitvi04g01520\_t001 |  |  |  |  |  | Vvi-Vitvi18g00148\_t001 |  |
| 1 | Atr-ERM96297 |  |  |  |  |  |  |  |  |  | | | |  |
| 1 | Atr-ERM96298 |  |  |  |  |  |  |  |  |  | | | |  |
| 1 | Atr-ERM96299 |  |  |  |  |  |  |  |  |  | | | |  |
| 1 | Atr-ERM96300 |  |  |  |  |  |  |  |  |  | Vvi-Vitvi18g00144\_t001 |  |
| 1 | Atr-ERM96301 |  |  |  |  |  |  |  |  |  | | | |  |
| 1 | Atr-ERM96302 |  |  |  |  |  |  |  |  |  | | | |  |
| 1 | Atr-ERM96303 |  |  |  |  |  |  |  |  |  | Vvi-Vitvi18g00142\_t001 |  |
| 0 | Atr-ERM96304 |  |  |  |  |  |  |
| 0 | Atr-ERM96305 |  |  |  |  |  |  |
| 0 | Atr-ERM96306 |  |  |  |  |  |  |
| 0 | Atr-ERM96307 |  |  |  |  |  |  |
| 0 | Atr-ERM96308 |  |  |  |  |  |  |
| 0 | Atr-ERM96309 |  |  |  |  |  |  |
| 0 | Atr-ERM96310 |  |  |  |  |  |  |
| 0 | Atr-ERM96311 |  |  |  |  |  |  |
| 0 | Atr-ERM96312 |  |  |  |  |  |  |
| 0 | Atr-ERM96313 |  |  |  |  |  |  |
| 0 | Atr-ERM96314 |  |  |  |  |  |  |
| 0 | Atr-ERM96315 |  |  |  |  |  |  |
| 0 | Atr-ERM96316 |  |  |  |  |  |  |
| 0 | Atr-ERM96317 |  |  |  |  |  |  |
| 0 | Atr-ERM96318 |  |  |  |  |  |  |
| 0 | Atr-ERM96319 |  |  |  |  |  |  |
| 0 | Atr-ERM96320 |  |  |  |  |  |  |
| 0 | Atr-ERM96321 |  |  |  |  |  |  |
| 0 | Atr-ERM96322 |  |  |  |  |  |  |
| 1 | Atr-ERM96323 |  | Vvi-Vitvi03g00197\_t001 |  |  |  |  |  |
| 2 | Atr-ERM96324 |  | | | |  | Vvi-Vitvi04g01425\_t003 |  |  |  |  |
| 2 | Atr-ERM96325 |  | | | |  | | | |  |  |  |  |
| 2 | Atr-ERM96326 |  | | | |  | | | |  |  |  |  |
| 2 | Atr-ERM96327 |  | | | |  | | | |  |  |  |  |
| 2 | Atr-ERM96328 |  | | | |  | | | |  |  |  |  |
| 2 | Atr-ERM96329 |  | | | |  | | | |  |  |  |  |
| 2 | Atr-ERM96330 |  | | | |  | | | |  |  |  |  |
| 2 | Atr-ERM96331 |  | | | |  | | | |  |  |  |  |
| 2 | Atr-ERM96332 |  | | | |  | | | |  |  |  |  |
| 3 | Atr-ERM96333 |  | | | |  | Vvi-Vitvi04g02143\_t002 |  | Vvi-Vitvi03g01326\_t001 |  |  |  |
| 3 | Atr-ERM96334 |  | | | |  | | | |  | | | |  |  |  |
| 3 | Atr-ERM96335 |  | | | |  | | | |  | | | |  |  |  |
| 3 | Atr-ERM96336 |  | | | |  | | | |  | | | |  |  |  |
| 3 | Atr-ERM96337 |  | | | |  | | | |  | | | |  |  |  |
| 3 | Atr-ERM96338 |  | | | |  | | | |  | | | |  |  |  |
| 3 | Atr-ERM96339 |  | | | |  | | | |  | | | |  |  |  |
| 3 | Atr-ERM96340 |  | | | |  | Vvi-Vitvi04g01410\_t001 |  | Vvi-Vitvi03g00037\_t001 |  |  |  |
| 3 | Atr-ERM96341 |  | | | |  | | | |  | | | |  |  |  |
| 3 | Atr-ERM96342 |  | | | |  | | | |  | | | |  |  |  |
| 3 | Atr-ERM96343 |  | Vvi-Vitvi03g00199\_t003 |  | | | |  | Vvi-Vitvi03g00032\_t001 |  |  |  |
| 3 | Atr-ERM96344 |  | | | |  | | | |  | | | |  |  |  |
| 3 | Atr-ERM96345 |  | | | |  | | | |  | | | |  |  |  |
| 3 | Atr-ERM96346 |  | | | |  | | | |  | Vvi-Vitvi03g00026\_t001 |  |  |  |
| 3 | Atr-ERM96347 |  | | | |  | | | |  | | | |  |  |  |
| 3 | Atr-ERM96348 |  | | | |  | Vvi-Vitvi04g01404\_t001 |  | | | |  |  |  |
| 3 | Atr-ERM96349 |  | | | |  | | | |  | | | |  |  |  |
| 3 | Atr-ERM96350 |  | | | |  | | | |  | | | |  |  |  |
| 3 | Atr-ERM96351 |  | | | |  | | | |  | | | |  |  |  |
| 3 | Atr-ERM96352 |  | | | |  | | | |  | Vvi-Vitvi03g00025\_t001 |  |  |  |
| 3 | Atr-ERM96353 |  | | | |  | Vvi-Vitvi04g02136\_t001 |  | | | |  |  |  |
| 3 | Atr-ERM96354 |  | | | |  | Vvi-Vitvi04g01401\_t001 |  | | | |  |  |  |
| 3 | Atr-ERM96355 |  | | | |  | | | |  | | | |  |  |  |
| 3 | Atr-ERM96356 |  | | | |  | Vvi-Vitvi04g01399\_t001 |  | Vvi-Vitvi03g00024\_t001 |  |  |  |
| 1 | Atr-ERM96357 |  | | | |  |  |  |  |  |
| 1 | Atr-ERM96358 |  | Vvi-Vitvi03g01433\_t001 |  |  |  |  |  |
| 2 | Atr-ERM96359 |  | | | |  | Vvi-Vitvi18g00113\_t001 |  |  |  |  |
| 2 | Atr-ERM96360 |  | Vvi-Vitvi03g00202\_t001 |  | Vvi-Vitvi18g00114\_t001 |  |  |  |  |
| 2 | Atr-ERM96361 |  | | | |  | | | |  |  |  |  |
| 2 | Atr-ERM96362 |  | Vvi-Vitvi03g00203\_t001 |  | | | |  |  |  |  |
| 2 | Atr-ERM96363 |  | | | |  | | | |  |  |  |  |
| 2 | Atr-ERM96364 |  | | | |  | | | |  |  |  |  |
| 2 | Atr-ERM96365 |  | | | |  | Vvi-Vitvi18g00116\_t002 |  |  |  |  |
| 2 | Atr-ERM96366 |  | | | |  | | | |  |  |  |  |
| 2 | Atr-ERM96367 |  | | | |  | | | |  |  |  |  |
| 2 | Atr-ERM96368 |  | | | |  | | | |  |  |  |  |
| 2 | Atr-ERM96369 |  | Vvi-Vitvi03g00204\_t001 |  | Vvi-Vitvi18g00119\_t001 |  |  |  |  |
| 2 | Atr-ERM96370 |  | | | |  | | | |  |  |  |  |
| 2 | Atr-ERM96371 |  | Vvi-Vitvi03g00205\_t001 |  | Vvi-Vitvi18g00120\_t002 |  |  |  |  |
| 2 | Atr-ERM96372 |  | | | |  | Vvi-Vitvi18g02496\_t001 |  |  |  |  |
| 3 | Atr-ERM96373 |  | Vvi-Vitvi03g01436\_t001 |  | | | |  | Vvi-Vitvi04g02298\_t001 |  |  |  |
| 3 | Atr-ERM96374 |  | | | |  | | | |  | | | |  |  |  |
| 3 | Atr-ERM96375 |  | | | |  | | | |  | Vvi-Vitvi04g02299\_t001 |  |  |  |
| 3 | Atr-ERM96376 |  | | | |  | | | |  | | | |  |  |  |
| 3 | Atr-ERM96377 |  | | | |  | | | |  | | | |  |  |  |
| 3 | Atr-ERM96378 |  | | | |  | | | |  | | | |  |  |  |
| 3 | Atr-ERM96379 |  | | | |  | | | |  | | | |  |  |  |
| 3 | Atr-ERM96380 |  | | | |  | | | |  | Vvi-Vitvi04g04428\_t001 |  |  |  |
| 3 | Atr-ERM96381 |  | | | |  | | | |  | | | |  |  |  |
| 3 | Atr-ERM96382 |  | | | |  | | | |  | | | |  |  |  |
| 3 | Atr-ERM96383 |  | | | |  | Vvi-Vitvi18g00122\_t001 |  | | | |  |  |  |
| 3 | Atr-ERM96384 |  | Vvi-Vitvi03g01438\_t001 |  | | | |  | | | |  |  |  |
| 3 | Atr-ERM96385 |  | | | |  | | | |  | | | |  |  |  |
| 3 | Atr-ERM96386 |  | | | |  | | | |  | | | |  |  |  |
| 3 | Atr-ERM96387 |  | | | |  | | | |  | | | |  |  |  |
| 3 | Atr-ERM96388 |  | | | |  | | | |  | | | |  |  |  |
| 3 | Atr-ERM96389 |  | | | |  | | | |  | | | |  |  |  |
| 3 | Atr-ERM96390 |  | | | |  | | | |  | | | |  |  |  |
| 3 | Atr-ERM96391 |  | | | |  | | | |  | | | |  |  |  |
| 3 | Atr-ERM96392 |  | | | |  | | | |  | | | |  |  |  |
| 3 | Atr-ERM96393 |  | | | |  | | | |  | | | |  |  |  |
| 3 | Atr-ERM96394 |  | | | |  | | | |  | | | |  |  |  |
| 3 | Atr-ERM96395 |  | | | |  | | | |  | Vvi-Vitvi04g01518\_t001 |  |  |  |
| 3 | Atr-ERM96396 |  | | | |  | | | |  | | | |  |  |  |
| 3 | Atr-ERM96397 |  | | | |  | Vvi-Vitvi18g02497\_t001 |  | | | |  |  |  |
| 3 | Atr-ERM96398 |  | | | |  | | | |  | | | |  |  |  |
| 3 | Atr-ERM96399 |  | | | |  | | | |  | | | |  |  |  |
| 3 | Atr-ERM96400 |  | | | |  | | | |  | | | |  |  |  |
| 3 | Atr-ERM96401 |  | | | |  | | | |  | | | |  |  |  |
| 3 | Atr-ERM96402 |  | | | |  | | | |  | | | |  |  |  |
| 3 | Atr-ERM96403 |  | Vvi-Vitvi03g00207\_t001 |  | | | |  | | | |  |  |  |
| 3 | Atr-ERM96404 |  | | | |  | | | |  | | | |  |  |  |
| 3 | Atr-ERM96405 |  | | | |  | | | |  | Vvi-Vitvi04g02300\_t001 |  |  |  |
| 3 | Atr-ERM96406 |  | | | |  | | | |  | | | |  |  |  |
| 3 | Atr-ERM96407 |  | | | |  | | | |  | Vvi-Vitvi04g01510\_t001 |  |  |  |
| 3 | Atr-ERM96408 |  | | | |  | | | |  | | | |  |  |  |
| 3 | Atr-ERM96409 |  | Vvi-Vitvi03g04085\_t001 |  | Vvi-Vitvi18g00127\_t001 |  | | | |  |  |  |
| 3 | Atr-ERM96410 |  | | | |  | | | |  | | | |  |  |  |
| 3 | Atr-ERM96411 |  | | | |  | | | |  | | | |  |  |  |
| 3 | Atr-ERM96412 |  | Vvi-Vitvi03g00213\_t001 |  | Vvi-Vitvi18g00128\_t001 |  | | | |  |  |  |
| 3 | Atr-ERM96413 |  | | | |  | | | |  | | | |  |  |  |
| 3 | Atr-ERM96414 |  | | | |  | | | |  | | | |  |  |  |
| 3 | Atr-ERM96415 |  | | | |  | | | |  | | | |  |  |  |
| 3 | Atr-ERM96416 |  | | | |  | | | |  | | | |  |  |  |
| 3 | Atr-ERM96417 |  | | | |  | | | |  | | | |  |  |  |
| 3 | Atr-ERM96418 |  | | | |  | | | |  | | | |  |  |  |
| 3 | Atr-ERM96419 |  | | | |  | Vvi-Vitvi18g00131\_t001 |  | | | |  |  |  |
| 3 | Atr-ERM96420 |  | | | |  | | | |  | | | |  |  |  |
| 3 | Atr-ERM96421 |  | | | |  | | | |  | | | |  |  |  |
| 3 | Atr-ERM96422 |  | | | |  | | | |  | | | |  |  |  |
| 3 | Atr-ERM96423 |  | Vvi-Vitvi03g00229\_t003 |  | | | |  | Vvi-Vitvi04g04445\_t001 |  |  |  |
| 1 | Atr-ERM96424 |  |  |  | Vvi-Vitvi18g02500\_t001 |  |  |  |  |
| 0 | Atr-ERM96425 |  |  |  |  |  |  |
| 0 | Atr-ERM96426 |  |  |  |  |  |  |
| 0 | Atr-ERM96427 |  |  |  |  |  |  |
| 0 | Atr-ERM96428 |  |  |  |  |  |  |
| 0 | Atr-ERM96429 |  |  |  |  |  |  |
| 0 | Atr-ERM96430 |  |  |  |  |  |  |
| 0 | Atr-ERM96431 |  |  |  |  |  |  |
| 0 | Atr-ERM96432 |  |  |  |  |  |  |
| 0 | Atr-ERM96433 |  |  |  |  |  |  |
| 0 | Atr-ERM96434 |  |  |  |  |  |  |
| 0 | Atr-ERM96435 |  |  |  |  |  |  |
| 0 | Atr-ERM96436 |  |  |  |  |  |  |
| 0 | Atr-ERM96437 |  |  |  |  |  |  |
| 0 | Atr-ERM96438 |  |  |  |  |  |  |
| 0 | Atr-ERM96439 |  |  |  |  |  |  |
| 0 | Atr-ERM96440 |  |  |  |  |  |  |
| 0 | Atr-ERM96441 |  |  |  |  |  |  |
| 0 | Atr-ERM96442 |  |  |  |  |  |  |
| 0 | Atr-ERM96443 |  |  |  |  |  |  |
| 0 | Atr-ERM96444 |  |  |  |  |  |  |
| 0 | Atr-ERM96445 |  |  |  |  |  |  |
| 0 | Atr-ERM96446 |  |  |  |  |  |  |
| 0 | Atr-ERM96447 |  |  |  |  |  |  |
| 0 | Atr-ERM96448 |  |  |  |  |  |  |
| 0 | Atr-ERM96449 |  |  |  |  |  |  |
| 0 | Atr-ERM96450 |  |  |  |  |  |  |
| 0 | Atr-ERM96451 |  |  |  |  |  |  |
| 0 | Atr-ERM96452 |  |  |  |  |  |  |
| 0 | Atr-ERM96453 |  |  |  |  |  |  |
| 0 | Atr-ERM96454 |  |  |  |  |  |  |
| 0 | Atr-ERM96455 |  |  |  |  |  |  |
| 1 | Atr-ERM96456 |  | Vvi-Vitvi16g01090\_t001 |  |  |  |  |  |
| 1 | Atr-ERM96457 |  | | | |  |  |  |  |  |
| 1 | Atr-ERM96458 |  | | | |  |  |  |  |  |
| 1 | Atr-ERM96459 |  | | | |  |  |  |  |  |
| 1 | Atr-ERM96460 |  | Vvi-Vitvi16g01916\_t001 |  |  |  |  |  |
| 1 | Atr-ERM96461 |  | | | |  |  |  |  |  |
| 1 | Atr-ERM96462 |  | | | |  |  |  |  |  |
| 1 | Atr-ERM96463 |  | Vvi-Vitvi16g01095\_t001 |  |  |  |  |  |
| 1 | Atr-ERM96464 |  | | | |  |  |  |  |  |
| 1 | Atr-ERM96465 |  | | | |  |  |  |  |  |
| 1 | Atr-ERM96466 |  | | | |  |  |  |  |  |
| 1 | Atr-ERM96467 |  | | | |  |  |  |  |  |
| 1 | Atr-ERM96468 |  | | | |  |  |  |  |  |
| 1 | Atr-ERM96469 |  | | | |  |  |  |  |  |
| 1 | Atr-ERM96470 |  | | | |  |  |  |  |  |
| 1 | Atr-ERM96471 |  | | | |  |  |  |  |  |
| 1 | Atr-ERM96472 |  | | | |  |  |  |  |  |
| 1 | Atr-ERM96473 |  | | | |  |  |  |  |  |
| 1 | Atr-ERM96474 |  | | | |  |  |  |  |  |
| 1 | Atr-ERM96475 |  | Vvi-Vitvi16g01096\_t001 |  |  |  |  |  |
| 1 | Atr-ERM96476 |  | | | |  |  |  |  |  |
| 1 | Atr-ERM96477 |  | | | |  |  |  |  |  |
| 1 | Atr-ERM96478 |  | | | |  |  |  |  |  |
| 1 | Atr-ERM96479 |  | | | |  |  |  |  |  |
| 1 | Atr-ERM96480 |  | | | |  |  |  |  |  |
| 1 | Atr-ERM96481 |  | | | |  |  |  |  |  |
| 2 | Atr-ERM96482 |  | | | |  | Vvi-Vitvi02g00023\_t001 |  |  |  |  |
| 2 | Atr-ERM96483 |  | | | |  | | | |  |  |  |  |
| 2 | Atr-ERM96484 |  | | | |  | | | |  |  |  |  |
| 2 | Atr-ERM96485 |  | | | |  | | | |  |  |  |  |
| 2 | Atr-ERM96486 |  | | | |  | | | |  |  |  |  |
| 2 | Atr-ERM96487 |  | | | |  | | | |  |  |  |  |
| 2 | Atr-ERM96488 |  | | | |  | | | |  |  |  |  |
| 2 | Atr-ERM96489 |  | Vvi-Vitvi16g01097\_t001 |  | | | |  |  |  |  |
| 2 | Atr-ERM96490 |  | Vvi-Vitvi16g01099\_t001 |  | | | |  |  |  |  |
| 2 | Atr-ERM96491 |  | | | |  | Vvi-Vitvi02g00021\_t001 |  |  |  |  |
| 2 | Atr-ERM96492 |  | | | |  | | | |  |  |  |  |
| 2 | Atr-ERM96493 |  | | | |  | | | |  |  |  |  |
| 2 | Atr-ERM96494 |  | | | |  | | | |  |  |  |  |
| 2 | Atr-ERM96495 |  | | | |  | | | |  |  |  |  |
| 2 | Atr-ERM96496 |  | | | |  | | | |  |  |  |  |
| 2 | Atr-ERM96497 |  | | | |  | | | |  |  |  |  |
| 2 | Atr-ERM96498 |  | | | |  | | | |  |  |  |  |
| 2 | Atr-ERM96499 |  | | | |  | | | |  |  |  |  |
| 2 | Atr-ERM96500 |  | | | |  | | | |  |  |  |  |
| 2 | Atr-ERM96501 |  | | | |  | | | |  |  |  |  |
| 2 | Atr-ERM96502 |  | Vvi-Vitvi16g01105\_t001 |  | | | |  |  |  |  |
| 2 | Atr-ERM96503 |  | Vvi-Vitvi16g01106\_t001 |  | | | |  |  |  |  |
| 2 | Atr-ERM96504 |  | | | |  | | | |  |  |  |  |
| 2 | Atr-ERM96505 |  | | | |  | | | |  |  |  |  |
| 2 | Atr-ERM96506 |  | Vvi-Vitvi16g01127\_t001 |  | | | |  |  |  |  |
| 1 | Atr-ERM96507 |  |  |  | Vvi-Vitvi02g00016\_t001 |  |  |  |  |
| 1 | Atr-ERM96508 |  |  |  | | | |  |  |  |  |
| 1 | Atr-ERM96509 |  |  |  | Vvi-Vitvi02g00015\_t001 |  |  |  |  |
| 1 | Atr-ERM96510 |  |  |  | | | |  |  |  |  |
| 1 | Atr-ERM96511 |  |  |  | Vvi-Vitvi02g00014\_t001 |  |  |  |  |
| 1 | Atr-ERM96512 |  |  |  | | | |  |  |  |  |
| 1 | Atr-ERM96513 |  |  |  | | | |  |  |  |  |
| 1 | Atr-ERM96514 |  |  |  | | | |  |  |  |  |
| 1 | Atr-ERM96515 |  |  |  | Vvi-Vitvi02g00013\_t001 |  |  |  |  |
| 1 | Atr-ERM96516 |  |  |  | | | |  |  |  |  |
| 1 | Atr-ERM96517 |  |  |  | | | |  |  |  |  |
| 1 | Atr-ERM96518 |  |  |  | | | |  |  |  |  |
| 1 | Atr-ERM96519 |  |  |  | | | |  |  |  |  |
| 1 | Atr-ERM96520 |  |  |  | | | |  |  |  |  |
| 1 | Atr-ERM96521 |  |  |  | Vvi-Vitvi02g04003\_t002 |  |  |  |  |
| 1 | Atr-ERM96522 |  |  |  | | | |  |  |  |  |
| 1 | Atr-ERM96523 |  |  |  | | | |  |  |  |  |
| 1 | Atr-ERM96524 |  |  |  | | | |  |  |  |  |
| 1 | Atr-ERM96525 |  |  |  | | | |  |  |  |  |
| 1 | Atr-ERM96526 |  |  |  | Vvi-Vitvi02g00009\_t001 |  |  |  |  |
| 0 | Atr-ERM96527 |  |  |  |  |  |  |
| 0 | Atr-ERM96528 |  |  |  |  |  |  |
| 0 | Atr-ERM96529 |  |  |  |  |  |  |
| 0 | Atr-ERM96530 |  |  |  |  |  |  |
| 0 | Atr-ERM96531 |  |  |  |  |  |  |
| 0 | Atr-ERM96532 |  |  |  |  |  |  |
| 0 | Atr-ERM96533 |  |  |  |  |  |  |
| 0 | Atr-ERM96534 |  |  |  |  |  |  |
| 0 | Atr-ERM96535 |  |  |  |  |  |  |
| 0 | Atr-ERM96536 |  |  |  |  |  |  |
| 0 | Atr-ERM96537 |  |  |  |  |  |  |
| 0 | Atr-ERM96538 |  |  |  |  |  |  |
| 0 | Atr-ERM96539 |  |  |  |  |  |  |
| 0 | Atr-ERM96540 |  |  |  |  |  |  |
| 0 | Atr-ERM96541 |  |  |  |  |  |  |
| 0 | Atr-ERM96542 |  |  |  |  |  |  |
| 0 | Atr-ERM96543 |  |  |  |  |  |  |
| 0 | Atr-ERM96544 |  |  |  |  |  |  |
| 0 | Atr-ERM96545 |  |  |  |  |  |  |
| 0 | Atr-ERM96546 |  |  |  |  |  |  |
| 0 | Atr-ERM96547 |  |  |  |  |  |  |
| 0 | Atr-ERM96548 |  |  |  |  |  |  |
| 0 | Atr-ERM96549 |  |  |  |  |  |  |
| 0 | Atr-ERM96550 |  |  |  |  |  |  |
| 0 | Atr-ERM96551 |  |  |  |  |  |  |
| 0 | Atr-ERM96552 |  |  |  |  |  |  |
| 0 | Atr-ERM96553 |  |  |  |  |  |  |
| 0 | Atr-ERM96554 |  |  |  |  |  |  |
| 0 | Atr-ERM96555 |  |  |  |  |  |  |
| 0 | Atr-ERM96556 |  |  |  |  |  |  |
| 0 | Atr-ERM96557 |  |  |  |  |  |  |
| 0 | Atr-ERM96558 |  |  |  |  |  |  |
| 0 | Atr-ERM96559 |  |  |  |  |  |  |
| 0 | Atr-ERM96560 |  |  |  |  |  |  |
| 0 | Atr-ERM96561 |  |  |  |  |  |  |
| 0 | Atr-ERM96562 |  |  |  |  |  |  |
| 0 | Atr-ERM96563 |  |  |  |  |  |  |
| 0 | Atr-ERM96564 |  |  |  |  |  |  |
| 0 | Atr-ERM96565 |  |  |  |  |  |  |
| 0 | Atr-ERM96566 |  |  |  |  |  |  |
| 0 | Atr-ERM96567 |  |  |  |  |  |  |
| 0 | Atr-ERM96568 |  |  |  |  |  |  |
| 0 | Atr-ERM96569 |  |  |  |  |  |  |
| 0 | Atr-ERM96570 |  |  |  |  |  |  |
| 0 | Atr-ERM96571 |  |  |  |  |  |  |
| 0 | Atr-ERM96572 |  |  |  |  |  |  |
| 0 | Atr-ERM96573 |  |  |  |  |  |  |
| 0 | Atr-ERM96574 |  |  |  |  |  |  |
| 0 | Atr-ERM96575 |  |  |  |  |  |  |
| 0 | Atr-ERM96576 |  |  |  |  |  |  |
| 0 | Atr-ERM96577 |  |  |  |  |  |  |
| 0 | Atr-ERM96578 |  |  |  |  |  |  |
| 0 | Atr-ERM96579 |  |  |  |  |  |  |
| 0 | Atr-ERM96580 |  |  |  |  |  |  |
| 0 | Atr-ERM96581 |  |  |  |  |  |  |
| 0 | Atr-ERM96582 |  |  |  |  |  |  |
| 0 | Atr-ERM96583 |  |  |  |  |  |  |
| 0 | Atr-ERM96584 |  |  |  |  |  |  |
| 0 | Atr-ERM96585 |  |  |  |  |  |  |
| 0 | Atr-ERM96586 |  |  |  |  |  |  |
| 0 | Atr-ERM96587 |  |  |  |  |  |  |
| 0 | Atr-ERM96588 |  |  |  |  |  |  |
| 0 | Atr-ERM96589 |  |  |  |  |  |  |
| 0 | Atr-ERM96590 |  |  |  |  |  |  |
| 0 | Atr-ERM96591 |  |  |  |  |  |  |
| 0 | Atr-ERM96592 |  |  |  |  |  |  |
| 0 | Atr-ERM96593 |  |  |  |  |  |  |
| 0 | Atr-ERM96594 |  |  |  |  |  |  |
| 0 | Atr-ERM96595 |  |  |  |  |  |  |
| 0 | Atr-ERM96596 |  |  |  |  |  |  |
| 0 | Atr-ERM96597 |  |  |  |  |  |  |
| 0 | Atr-ERM96598 |  |  |  |  |  |  |
| 0 | Atr-ERM96599 |  |  |  |  |  |  |
| 0 | Atr-ERM96600 |  |  |  |  |  |  |
| 0 | Atr-ERM96601 |  |  |  |  |  |  |
| 0 | Atr-ERM96602 |  |  |  |  |  |  |
| 0 | Atr-ERM96603 |  |  |  |  |  |  |
| 0 | Atr-ERM96604 |  |  |  |  |  |  |
| 0 | Atr-ERM96605 |  |  |  |  |  |  |
| 0 | Atr-ERM96606 |  |  |  |  |  |  |
| 0 | Atr-ERM96607 |  |  |  |  |  |  |
| 0 | Atr-ERM96608 |  |  |  |  |  |  |
| 0 | Atr-ERM96609 |  |  |  |  |  |  |
| 0 | Atr-ERM96610 |  |  |  |  |  |  |
| 0 | Atr-ERM96611 |  |  |  |  |  |  |
| 0 | Atr-ERM96612 |  |  |  |  |  |  |
| 0 | Atr-ERM96613 |  |  |  |  |  |  |
| 1 | Atr-ERM96614 |  | Vvi-Vitvi16g01340\_t001 |  |  |  |  |  |
| 1 | Atr-ERM96615 |  | | | |  |  |  |  |  |
| 1 | Atr-ERM96616 |  | Vvi-Vitvi16g01339\_t001 |  |  |  |  |  |
| 1 | Atr-ERM96617 |  | | | |  |  |  |  |  |
| 1 | Atr-ERM96618 |  | Vvi-Vitvi16g01338\_t001 |  |  |  |  |  |
| 1 | Atr-ERM96619 |  | | | |  |  |  |  |  |
| 1 | Atr-ERM96620 |  | | | |  |  |  |  |  |
| 1 | Atr-ERM96621 |  | | | |  |  |  |  |  |
| 1 | Atr-ERM96622 |  | Vvi-Vitvi16g02062\_t001 |  |  |  |  |  |
| 1 | Atr-ERM96623 |  | Vvi-Vitvi16g01337\_t001 |  |  |  |  |  |
| 1 | Atr-ERM96624 |  | | | |  |  |  |  |  |
| 1 | Atr-ERM96625 |  | | | |  |  |  |  |  |
| 1 | Atr-ERM96626 |  | | | |  |  |  |  |  |
| 1 | Atr-ERM96627 |  | | | |  |  |  |  |  |
| 1 | Atr-ERM96628 |  | | | |  |  |  |  |  |
| 1 | Atr-ERM96629 |  | | | |  |  |  |  |  |
| 1 | Atr-ERM96630 |  | | | |  |  |  |  |  |
| 1 | Atr-ERM96631 |  | | | |  |  |  |  |  |
| 1 | Atr-ERM96632 |  | Vvi-Vitvi16g01334\_t001 |  |  |  |  |  |
| 0 | Atr-ERM96633 |  |  |  |  |  |  |
| 0 | Atr-ERM96634 |  |  |  |  |  |  |
| 0 | Atr-ERM96635 |  |  |  |  |  |  |
| 0 | Atr-ERM96636 |  |  |  |  |  |  |
| 0 | Atr-ERM96637 |  |  |  |  |  |  |
| 0 | Atr-ERM96638 |  |  |  |  |  |  |
| 0 | Atr-ERM96639 |  |  |  |  |  |  |
| 0 | Atr-ERM96640 |  |  |  |  |  |  |
| 0 | Atr-ERM96641 |  |  |  |  |  |  |
| 0 | Atr-ERM96642 |  |  |  |  |  |  |
| 0 | Atr-ERM96643 |  |  |  |  |  |  |
| 0 | Atr-ERM96644 |  |  |  |  |  |  |
| 1 | Atr-ERM96645 |  | Vvi-Vitvi03g00606\_t001 |  |  |  |  |  |
| 1 | Atr-ERM96646 |  | | | |  |  |  |  |  |
| 1 | Atr-ERM96647 |  | | | |  |  |  |  |  |
| 1 | Atr-ERM96648 |  | | | |  |  |  |  |  |
| 1 | Atr-ERM96649 |  | | | |  |  |  |  |  |
| 1 | Atr-ERM96650 |  | Vvi-Vitvi03g00600\_t001 |  |  |  |  |  |
| 1 | Atr-ERM96651 |  | | | |  |  |  |  |  |
| 1 | Atr-ERM96652 |  | | | |  |  |  |  |  |
| 1 | Atr-ERM96653 |  | | | |  |  |  |  |  |
| 1 | Atr-ERM96654 |  | | | |  |  |  |  |  |
| 1 | Atr-ERM96655 |  | | | |  |  |  |  |  |
| 1 | Atr-ERM96656 |  | | | |  |  |  |  |  |
| 1 | Atr-ERM96657 |  | Vvi-Vitvi03g00599\_t001 |  |  |  |  |  |
| 1 | Atr-ERM96658 |  | | | |  |  |  |  |  |
| 1 | Atr-ERM96659 |  | | | |  |  |  |  |  |
| 1 | Atr-ERM96660 |  | | | |  |  |  |  |  |
| 1 | Atr-ERM96661 |  | | | |  |  |  |  |  |
| 1 | Atr-ERM96662 |  | | | |  |  |  |  |  |
| 1 | Atr-ERM96663 |  | | | |  |  |  |  |  |
| 1 | Atr-ERM96664 |  | | | |  |  |  |  |  |
| 1 | Atr-ERM96665 |  | | | |  |  |  |  |  |
| 1 | Atr-ERM96666 |  | | | |  |  |  |  |  |
| 1 | Atr-ERM96667 |  | | | |  |  |  |  |  |
| 1 | Atr-ERM96668 |  | | | |  |  |  |  |  |
| 1 | Atr-ERM96669 |  | | | |  |  |  |  |  |
| 1 | Atr-ERM96670 |  | | | |  |  |  |  |  |
| 1 | Atr-ERM96671 |  | | | |  |  |  |  |  |
| 1 | Atr-ERM96672 |  | | | |  |  |  |  |  |
| 1 | Atr-ERM96673 |  | | | |  |  |  |  |  |
| 1 | Atr-ERM96674 |  | | | |  |  |  |  |  |
| 1 | Atr-ERM96675 |  | | | |  |  |  |  |  |
| 1 | Atr-ERM96676 |  | | | |  |  |  |  |  |
| 1 | Atr-ERM96677 |  | | | |  |  |  |  |  |
| 1 | Atr-ERM96678 |  | | | |  |  |  |  |  |
| 1 | Atr-ERM96679 |  | Vvi-Vitvi03g00586\_t001 |  |  |  |  |  |
| 1 | Atr-ERM96680 |  | | | |  |  |  |  |  |
| 1 | Atr-ERM96681 |  | | | |  |  |  |  |  |
| 1 | Atr-ERM96682 |  | | | |  |  |  |  |  |
| 1 | Atr-ERM96683 |  | | | |  |  |  |  |  |
| 1 | Atr-ERM96684 |  | | | |  |  |  |  |  |
| 1 | Atr-ERM96685 |  | Vvi-Vitvi03g01600\_t001 |  |  |  |  |  |
| 1 | Atr-ERM96686 |  | Vvi-Vitvi03g00584\_t001 |  |  |  |  |  |
| 1 | Atr-ERM96687 |  | | | |  |  |  |  |  |
| 1 | Atr-ERM96688 |  | | | |  |  |  |  |  |
| 1 | Atr-ERM96689 |  | | | |  |  |  |  |  |
| 1 | Atr-ERM96690 |  | | | |  |  |  |  |  |
| 1 | Atr-ERM96691 |  | Vvi-Vitvi03g00583\_t001 |  |  |  |  |  |
| 1 | Atr-ERM96692 |  | | | |  |  |  |  |  |
| 1 | Atr-ERM96693 |  | | | |  |  |  |  |  |
| 1 | Atr-ERM96694 |  | | | |  |  |  |  |  |
| 1 | Atr-ERM96695 |  | Vvi-Vitvi03g00581\_t001 |  |  |  |  |  |
| 0 | Atr-ERM96696 |  |  |  |  |  |  |
| 1 | Atr-ERM96697 |  | Vvi-Vitvi07g01646\_t001 |  |  |  |  |  |
| 1 | Atr-ERM96698 |  | Vvi-Vitvi07g01645\_t001 |  |  |  |  |  |
| 1 | Atr-ERM96699 |  | | | |  |  |  |  |  |
| 1 | Atr-ERM96700 |  | | | |  |  |  |  |  |
| 1 | Atr-ERM96701 |  | | | |  |  |  |  |  |
| 1 | Atr-ERM96702 |  | | | |  |  |  |  |  |
| 1 | Atr-ERM96703 |  | | | |  |  |  |  |  |
| 1 | Atr-ERM96704 |  | | | |  |  |  |  |  |
| 1 | Atr-ERM96705 |  | | | |  |  |  |  |  |
| 1 | Atr-ERM96706 |  | | | |  |  |  |  |  |
| 1 | Atr-ERM96707 |  | Vvi-Vitvi07g04685\_t001 |  |  |  |  |  |
| 1 | Atr-ERM96708 |  | | | |  |  |  |  |  |
| 1 | Atr-ERM96709 |  | | | |  |  |  |  |  |
| 1 | Atr-ERM96710 |  | | | |  |  |  |  |  |
| 1 | Atr-ERM96711 |  | Vvi-Vitvi07g01642\_t001 |  |  |  |  |  |
| 1 | Atr-ERM96712 |  | | | |  |  |  |  |  |
| 1 | Atr-ERM96713 |  | Vvi-Vitvi07g01641\_t002 |  |  |  |  |  |
| 1 | Atr-ERM96714 |  | Vvi-Vitvi07g01638\_t001 |  |  |  |  |  |
| 0 | Atr-ERM96715 |  |  |  |  |  |  |
| 0 | Atr-ERM96716 |  |  |  |  |  |  |
| 0 | Atr-ERM96717 |  |  |  |  |  |  |
| 0 | Atr-ERM96718 |  |  |  |  |  |  |
| 0 | Atr-ERM96719 |  |  |  |  |  |  |
| 0 | Atr-ERM96720 |  |  |  |  |  |  |
