# Supplementary material for: Genome-wide identification, characterization and gene expression of BES1 transcription factor family in grapevine (Vitis vinifera L.)
Source: Sci Rep. 2023 Jan 5;13:240. doi: 10.1038/s41598-022-24407-y (PMC9816167; doi:10.1038/s41598-022-24407-y)
Supplement: Supplementary file 3 — Supplementary Information. [file 41598_2022_24407_MOESM3_ESM.zip › Vvi_Atr/Vitis_vinifera.PN40024.v4.dna_sm.toplevel.fa.vs.Amborella_trichopoda.AMTR1.0.dna_sm.toplevel.fa.html/Atr-AmTr_v1.0_scaffold00002.html]

|  |  |  |  |  |  |  |  |  |  |  |  |  |  |
| --- | --- | --- | --- | --- | --- | --- | --- | --- | --- | --- | --- | --- | --- |
| Duplication depth | Reference chromosome | Collinear blocks | | | | | | | | | | | |
| 0 | Atr-ERN00925 |  |  |  |  |  |  |
| 0 | Atr-ERN00926 |  |  |  |  |  |  |
| 0 | Atr-ERN00927 |  |  |  |  |  |  |
| 0 | Atr-ERN00928 |  |  |  |  |  |  |
| 0 | Atr-ERN00929 |  |  |  |  |  |  |
| 0 | Atr-ERN00930 |  |  |  |  |  |  |
| 0 | Atr-ERN00931 |  |  |  |  |  |  |
| 0 | Atr-ERN00932 |  |  |  |  |  |  |
| 0 | Atr-ERN00933 |  |  |  |  |  |  |
| 0 | Atr-ERN00934 |  |  |  |  |  |  |
| 0 | Atr-ERN00935 |  |  |  |  |  |  |
| 0 | Atr-ERN00936 |  |  |  |  |  |  |
| 0 | Atr-ERN00937 |  |  |  |  |  |  |
| 0 | Atr-ERN00938 |  |  |  |  |  |  |
| 0 | Atr-ERN00939 |  |  |  |  |  |  |
| 0 | Atr-ERN00940 |  |  |  |  |  |  |
| 0 | Atr-ERN00941 |  |  |  |  |  |  |
| 0 | Atr-ERN00942 |  |  |  |  |  |  |
| 0 | Atr-ERN00943 |  |  |  |  |  |  |
| 0 | Atr-ERN00944 |  |  |  |  |  |  |
| 0 | Atr-ERN00945 |  |  |  |  |  |  |
| 0 | Atr-ERN00946 |  |  |  |  |  |  |
| 0 | Atr-ERN00947 |  |  |  |  |  |  |
| 0 | Atr-ERN00948 |  |  |  |  |  |  |
| 0 | Atr-ERN00949 |  |  |  |  |  |  |
| 0 | Atr-ERN00950 |  |  |  |  |  |  |
| 0 | Atr-ERN00951 |  |  |  |  |  |  |
| 0 | Atr-ERN00952 |  |  |  |  |  |  |
| 0 | Atr-ERN00953 |  |  |  |  |  |  |
| 0 | Atr-ERN00954 |  |  |  |  |  |  |
| 0 | Atr-ERN00955 |  |  |  |  |  |  |
| 0 | Atr-ERN00956 |  |  |  |  |  |  |
| 0 | Atr-ERN00957 |  |  |  |  |  |  |
| 0 | Atr-ERN00958 |  |  |  |  |  |  |
| 0 | Atr-ERN00959 |  |  |  |  |  |  |
| 0 | Atr-ERN00960 |  |  |  |  |  |  |
| 0 | Atr-ERN00961 |  |  |  |  |  |  |
| 0 | Atr-ERN00962 |  |  |  |  |  |  |
| 0 | Atr-ERN00963 |  |  |  |  |  |  |
| 0 | Atr-ERN00964 |  |  |  |  |  |  |
| 0 | Atr-ERN00965 |  |  |  |  |  |  |
| 0 | Atr-ERN00966 |  |  |  |  |  |  |
| 0 | Atr-ERN00967 |  |  |  |  |  |  |
| 0 | Atr-ERN00968 |  |  |  |  |  |  |
| 0 | Atr-ERN00969 |  |  |  |  |  |  |
| 0 | Atr-ERN00970 |  |  |  |  |  |  |
| 0 | Atr-ERN00971 |  |  |  |  |  |  |
| 0 | Atr-ERN00972 |  |  |  |  |  |  |
| 0 | Atr-ERN00973 |  |  |  |  |  |  |
| 0 | Atr-ERN00974 |  |  |  |  |  |  |
| 0 | Atr-ERN00975 |  |  |  |  |  |  |
| 0 | Atr-ERN00976 |  |  |  |  |  |  |
| 0 | Atr-ERN00977 |  |  |  |  |  |  |
| 0 | Atr-ERN00978 |  |  |  |  |  |  |
| 0 | Atr-ERN00979 |  |  |  |  |  |  |
| 0 | Atr-ERN00980 |  |  |  |  |  |  |
| 0 | Atr-ERN00981 |  |  |  |  |  |  |
| 0 | Atr-ERN00982 |  |  |  |  |  |  |
| 0 | Atr-ERN00983 |  |  |  |  |  |  |
| 0 | Atr-ERN00984 |  |  |  |  |  |  |
| 0 | Atr-ERN00985 |  |  |  |  |  |  |
| 0 | Atr-ERN00986 |  |  |  |  |  |  |
| 0 | Atr-ERN00987 |  |  |  |  |  |  |
| 0 | Atr-ERN00988 |  |  |  |  |  |  |
| 0 | Atr-ERN00989 |  |  |  |  |  |  |
| 0 | Atr-ERN00990 |  |  |  |  |  |  |
| 0 | Atr-ERN00991 |  |  |  |  |  |  |
| 0 | Atr-ERN00992 |  |  |  |  |  |  |
| 0 | Atr-ERN00993 |  |  |  |  |  |  |
| 0 | Atr-ERN00994 |  |  |  |  |  |  |
| 0 | Atr-ERN00995 |  |  |  |  |  |  |
| 0 | Atr-ERN00996 |  |  |  |  |  |  |
| 0 | Atr-ERN00997 |  |  |  |  |  |  |
| 0 | Atr-ERN00998 |  |  |  |  |  |  |
| 0 | Atr-ERN00999 |  |  |  |  |  |  |
| 0 | Atr-ERN01000 |  |  |  |  |  |  |
| 0 | Atr-ERN01001 |  |  |  |  |  |  |
| 0 | Atr-ERN01002 |  |  |  |  |  |  |
| 0 | Atr-ERN01003 |  |  |  |  |  |  |
| 0 | Atr-ERN01004 |  |  |  |  |  |  |
| 0 | Atr-ERN01005 |  |  |  |  |  |  |
| 0 | Atr-ERN01006 |  |  |  |  |  |  |
| 1 | Atr-ERN01007 |  | Vvi-Vitvi15g01417\_t001 |  |  |  |  |  |
| 1 | Atr-ERN01008 |  | | | |  |  |  |  |  |
| 1 | Atr-ERN01009 |  | | | |  |  |  |  |  |
| 2 | Atr-ERN01010 |  | | | |  | Vvi-Vitvi15g00492\_t001 |  |  |  |  |
| 2 | Atr-ERN01011 |  | | | |  | | | |  |  |  |  |
| 2 | Atr-ERN01012 |  | | | |  | Vvi-Vitvi15g00500\_t001 |  |  |  |  |
| 2 | Atr-ERN01013 |  | | | |  | | | |  |  |  |  |
| 2 | Atr-ERN01014 |  | | | |  | | | |  |  |  |  |
| 2 | Atr-ERN01015 |  | | | |  | | | |  |  |  |  |
| 2 | Atr-ERN01016 |  | | | |  | | | |  |  |  |  |
| 2 | Atr-ERN01017 |  | | | |  | | | |  |  |  |  |
| 2 | Atr-ERN01018 |  | | | |  | | | |  |  |  |  |
| 2 | Atr-ERN01019 |  | | | |  | | | |  |  |  |  |
| 2 | Atr-ERN01020 |  | Vvi-Vitvi15g00478\_t001 |  | | | |  |  |  |  |
| 2 | Atr-ERN01021 |  | | | |  | | | |  |  |  |  |
| 2 | Atr-ERN01022 |  | | | |  | | | |  |  |  |  |
| 2 | Atr-ERN01023 |  | | | |  | | | |  |  |  |  |
| 2 | Atr-ERN01024 |  | Vvi-Vitvi15g00457\_t001 |  | | | |  |  |  |  |
| 2 | Atr-ERN01025 |  | | | |  | | | |  |  |  |  |
| 2 | Atr-ERN01026 |  | | | |  | | | |  |  |  |  |
| 2 | Atr-ERN01027 |  | | | |  | Vvi-Vitvi15g00513\_t003 |  |  |  |  |
| 2 | Atr-ERN01028 |  | | | |  | | | |  |  |  |  |
| 2 | Atr-ERN01029 |  | | | |  | Vvi-Vitvi15g00514\_t001 |  |  |  |  |
| 2 | Atr-ERN01030 |  | | | |  | | | |  |  |  |  |
| 2 | Atr-ERN01031 |  | | | |  | | | |  |  |  |  |
| 2 | Atr-ERN01032 |  | | | |  | | | |  |  |  |  |
| 2 | Atr-ERN01033 |  | Vvi-Vitvi15g00452\_t001 |  | | | |  |  |  |  |
| 2 | Atr-ERN01034 |  | | | |  | | | |  |  |  |  |
| 3 | Atr-ERN01035 |  | | | |  | | | |  | Vvi-Vitvi13g04637\_t001 |  |  |  |
| 3 | Atr-ERN01036 |  | | | |  | | | |  | | | |  |  |  |
| 3 | Atr-ERN01037 |  | Vvi-Vitvi15g00448\_t001 |  | | | |  | | | |  |  |  |
| 3 | Atr-ERN01038 |  | Vvi-Vitvi15g00447\_t001 |  | | | |  | | | |  |  |  |
| 2 | Atr-ERN01039 |  |  |  | | | |  | | | |  |  |  |
| 2 | Atr-ERN01040 |  |  |  | | | |  | | | |  |  |  |
| 2 | Atr-ERN01041 |  |  |  | | | |  | | | |  |  |  |
| 2 | Atr-ERN01042 |  |  |  | | | |  | | | |  |  |  |
| 2 | Atr-ERN01043 |  |  |  | | | |  | | | |  |  |  |
| 2 | Atr-ERN01044 |  |  |  | Vvi-Vitvi15g00516\_t001 |  | | | |  |  |  |
| 2 | Atr-ERN01045 |  |  |  | | | |  | | | |  |  |  |
| 2 | Atr-ERN01046 |  |  |  | Vvi-Vitvi15g00518\_t001 |  | | | |  |  |  |
| 2 | Atr-ERN01047 |  |  |  | | | |  | | | |  |  |  |
| 2 | Atr-ERN01048 |  |  |  | | | |  | | | |  |  |  |
| 2 | Atr-ERN01049 |  |  |  | | | |  | | | |  |  |  |
| 2 | Atr-ERN01050 |  |  |  | | | |  | | | |  |  |  |
| 2 | Atr-ERN01051 |  |  |  | | | |  | | | |  |  |  |
| 2 | Atr-ERN01052 |  |  |  | | | |  | | | |  |  |  |
| 2 | Atr-ERN01053 |  |  |  | Vvi-Vitvi15g00526\_t001 |  | | | |  |  |  |
| 2 | Atr-ERN01054 |  |  |  | | | |  | | | |  |  |  |
| 2 | Atr-ERN01055 |  |  |  | | | |  | Vvi-Vitvi13g04622\_t001 |  |  |  |
| 2 | Atr-ERN01056 |  |  |  | | | |  | | | |  |  |  |
| 2 | Atr-ERN01057 |  |  |  | | | |  | Vvi-Vitvi13g04621\_t001 |  |  |  |
| 2 | Atr-ERN01058 |  |  |  | | | |  | | | |  |  |  |
| 2 | Atr-ERN01059 |  |  |  | | | |  | | | |  |  |  |
| 2 | Atr-ERN01060 |  |  |  | | | |  | | | |  |  |  |
| 2 | Atr-ERN01061 |  |  |  | | | |  | Vvi-Vitvi13g04618\_t001 |  |  |  |
| 2 | Atr-ERN01062 |  |  |  | | | |  | | | |  |  |  |
| 2 | Atr-ERN01063 |  |  |  | | | |  | | | |  |  |  |
| 2 | Atr-ERN01064 |  |  |  | | | |  | | | |  |  |  |
| 2 | Atr-ERN01065 |  |  |  | | | |  | Vvi-Vitvi13g04616\_t001 |  |  |  |
| 2 | Atr-ERN01066 |  |  |  | | | |  | | | |  |  |  |
| 2 | Atr-ERN01067 |  |  |  | | | |  | | | |  |  |  |
| 2 | Atr-ERN01068 |  |  |  | | | |  | | | |  |  |  |
| 2 | Atr-ERN01069 |  |  |  | | | |  | | | |  |  |  |
| 2 | Atr-ERN01070 |  |  |  | | | |  | | | |  |  |  |
| 3 | Atr-ERN01071 |  | Vvi-Vitvi13g04597\_t001 |  | | | |  | | | |  |  |  |
| 3 | Atr-ERN01072 |  | | | |  | Vvi-Vitvi15g01421\_t001 |  | | | |  |  |  |
| 3 | Atr-ERN01073 |  | | | |  | | | |  | | | |  |  |  |
| 3 | Atr-ERN01074 |  | Vvi-Vitvi13g04602\_t001 |  | | | |  | Vvi-Vitvi13g04602\_t001 |  |  |  |
| 3 | Atr-ERN01075 |  | Vvi-Vitvi13g04614\_t001 |  | Vvi-Vitvi15g00531\_t001 |  | | | |  |  |  |
| 3 | Atr-ERN01076 |  | Vvi-Vitvi13g04615\_t001 |  | | | |  | | | |  |  |  |
| 3 | Atr-ERN01077 |  | | | |  | | | |  | | | |  |  |  |
| 3 | Atr-ERN01078 |  | | | |  | | | |  | | | |  |  |  |
| 3 | Atr-ERN01079 |  | | | |  | | | |  | Vvi-Vitvi13g04591\_t001 |  |  |  |
| 2 | Atr-ERN01080 |  | | | |  | | | |  |  |  |  |
| 2 | Atr-ERN01081 |  | Vvi-Vitvi13g04624\_t001 |  | | | |  |  |  |  |
| 2 | Atr-ERN01082 |  | | | |  | | | |  |  |  |  |
| 2 | Atr-ERN01083 |  | Vvi-Vitvi13g04625\_t001 |  | | | |  |  |  |  |
| 2 | Atr-ERN01084 |  | | | |  | | | |  |  |  |  |
| 2 | Atr-ERN01085 |  | | | |  | | | |  |  |  |  |
| 2 | Atr-ERN01086 |  | | | |  | | | |  |  |  |  |
| 2 | Atr-ERN01087 |  | | | |  | | | |  |  |  |  |
| 2 | Atr-ERN01088 |  | | | |  | | | |  |  |  |  |
| 2 | Atr-ERN01089 |  | | | |  | | | |  |  |  |  |
| 2 | Atr-ERN01090 |  | | | |  | | | |  |  |  |  |
| 2 | Atr-ERN01091 |  | | | |  | | | |  |  |  |  |
| 2 | Atr-ERN01092 |  | | | |  | Vvi-Vitvi15g00537\_t001 |  |  |  |  |
| 2 | Atr-ERN01093 |  | | | |  | | | |  |  |  |  |
| 2 | Atr-ERN01094 |  | | | |  | | | |  |  |  |  |
| 2 | Atr-ERN01095 |  | | | |  | | | |  |  |  |  |
| 2 | Atr-ERN01096 |  | | | |  | | | |  |  |  |  |
| 2 | Atr-ERN01097 |  | | | |  | | | |  |  |  |  |
| 2 | Atr-ERN01098 |  | | | |  | | | |  |  |  |  |
| 2 | Atr-ERN01099 |  | | | |  | | | |  |  |  |  |
| 2 | Atr-ERN01100 |  | Vvi-Vitvi13g04636\_t001 |  | | | |  |  |  |  |
| 2 | Atr-ERN01101 |  | Vvi-Vitvi13g04637\_t001 |  | | | |  |  |  |  |
| 1 | Atr-ERN01102 |  |  |  | | | |  |  |  |  |
| 1 | Atr-ERN01103 |  |  |  | | | |  |  |  |  |
| 1 | Atr-ERN01104 |  |  |  | | | |  |  |  |  |
| 1 | Atr-ERN01105 |  |  |  | | | |  |  |  |  |
| 2 | Atr-ERN01106 |  | Vvi-Vitvi02g00705\_t001 |  | | | |  |  |  |  |
| 2 | Atr-ERN01107 |  | Vvi-Vitvi02g00706\_t001 |  | | | |  |  |  |  |
| 2 | Atr-ERN01108 |  | Vvi-Vitvi02g00708\_t001 |  | | | |  |  |  |  |
| 2 | Atr-ERN01109 |  | | | |  | | | |  |  |  |  |
| 2 | Atr-ERN01110 |  | Vvi-Vitvi02g00710\_t001 |  | | | |  |  |  |  |
| 2 | Atr-ERN01111 |  | | | |  | | | |  |  |  |  |
| 2 | Atr-ERN01112 |  | | | |  | | | |  |  |  |  |
| 2 | Atr-ERN01113 |  | | | |  | | | |  |  |  |  |
| 2 | Atr-ERN01114 |  | | | |  | | | |  |  |  |  |
| 2 | Atr-ERN01115 |  | | | |  | | | |  |  |  |  |
| 2 | Atr-ERN01116 |  | | | |  | | | |  |  |  |  |
| 2 | Atr-ERN01117 |  | | | |  | | | |  |  |  |  |
| 2 | Atr-ERN01118 |  | | | |  | Vvi-Vitvi15g00547\_t001 |  |  |  |  |
| 1 | Atr-ERN01119 |  | | | |  |  |  |  |  |
| 1 | Atr-ERN01120 |  | Vvi-Vitvi02g00711\_t001 |  |  |  |  |  |
| 2 | Atr-ERN01121 |  | Vvi-Vitvi02g00714\_t001 |  | Vvi-Vitvi02g00714\_t001 |  |  |  |  |
| 1 | Atr-ERN01122 |  |  |  | | | |  |  |  |  |
| 1 | Atr-ERN01123 |  |  |  | | | |  |  |  |  |
| 1 | Atr-ERN01124 |  |  |  | | | |  |  |  |  |
| 1 | Atr-ERN01125 |  |  |  | | | |  |  |  |  |
| 1 | Atr-ERN01126 |  |  |  | | | |  |  |  |  |
| 1 | Atr-ERN01127 |  |  |  | | | |  |  |  |  |
| 1 | Atr-ERN01128 |  |  |  | | | |  |  |  |  |
| 1 | Atr-ERN01129 |  |  |  | | | |  |  |  |  |
| 1 | Atr-ERN01130 |  |  |  | | | |  |  |  |  |
| 1 | Atr-ERN01131 |  |  |  | | | |  |  |  |  |
| 1 | Atr-ERN01132 |  |  |  | | | |  |  |  |  |
| 1 | Atr-ERN01133 |  |  |  | | | |  |  |  |  |
| 1 | Atr-ERN01134 |  |  |  | | | |  |  |  |  |
| 1 | Atr-ERN01135 |  |  |  | | | |  |  |  |  |
| 1 | Atr-ERN01136 |  |  |  | | | |  |  |  |  |
| 1 | Atr-ERN01137 |  |  |  | | | |  |  |  |  |
| 1 | Atr-ERN01138 |  |  |  | | | |  |  |  |  |
| 1 | Atr-ERN01139 |  |  |  | | | |  |  |  |  |
| 1 | Atr-ERN01140 |  |  |  | | | |  |  |  |  |
| 1 | Atr-ERN01141 |  |  |  | | | |  |  |  |  |
| 1 | Atr-ERN01142 |  |  |  | | | |  |  |  |  |
| 1 | Atr-ERN01143 |  |  |  | Vvi-Vitvi02g00704\_t001 |  |  |  |  |
| 1 | Atr-ERN01144 |  |  |  | | | |  |  |  |  |
| 1 | Atr-ERN01145 |  |  |  | | | |  |  |  |  |
| 1 | Atr-ERN01146 |  |  |  | | | |  |  |  |  |
| 1 | Atr-ERN01147 |  |  |  | | | |  |  |  |  |
| 1 | Atr-ERN01148 |  |  |  | | | |  |  |  |  |
| 1 | Atr-ERN01149 |  |  |  | | | |  |  |  |  |
| 1 | Atr-ERN01150 |  |  |  | | | |  |  |  |  |
| 1 | Atr-ERN01151 |  |  |  | | | |  |  |  |  |
| 1 | Atr-ERN01152 |  |  |  | | | |  |  |  |  |
| 1 | Atr-ERN01153 |  |  |  | | | |  |  |  |  |
| 1 | Atr-ERN01154 |  |  |  | Vvi-Vitvi02g00703\_t001 |  |  |  |  |
| 1 | Atr-ERN01155 |  |  |  | | | |  |  |  |  |
| 1 | Atr-ERN01156 |  |  |  | | | |  |  |  |  |
| 1 | Atr-ERN01157 |  |  |  | | | |  |  |  |  |
| 1 | Atr-ERN01158 |  |  |  | | | |  |  |  |  |
| 1 | Atr-ERN01159 |  |  |  | | | |  |  |  |  |
| 1 | Atr-ERN01160 |  |  |  | Vvi-Vitvi02g00700\_t001 |  |  |  |  |
| 1 | Atr-ERN01161 |  |  |  | | | |  |  |  |  |
| 1 | Atr-ERN01162 |  |  |  | Vvi-Vitvi02g00699\_t001 |  |  |  |  |
| 1 | Atr-ERN01163 |  |  |  | | | |  |  |  |  |
| 1 | Atr-ERN01164 |  |  |  | | | |  |  |  |  |
| 1 | Atr-ERN01165 |  |  |  | | | |  |  |  |  |
| 1 | Atr-ERN01166 |  |  |  | | | |  |  |  |  |
| 1 | Atr-ERN01167 |  |  |  | | | |  |  |  |  |
| 1 | Atr-ERN01168 |  |  |  | Vvi-Vitvi02g00696\_t001 |  |  |  |  |
| 0 | Atr-ERN01169 |  |  |  |  |  |  |
| 0 | Atr-ERN01170 |  |  |  |  |  |  |
| 0 | Atr-ERN01171 |  |  |  |  |  |  |
| 0 | Atr-ERN01172 |  |  |  |  |  |  |
| 0 | Atr-ERN01173 |  |  |  |  |  |  |
| 0 | Atr-ERN01174 |  |  |  |  |  |  |
| 0 | Atr-ERN01175 |  |  |  |  |  |  |
| 0 | Atr-ERN01176 |  |  |  |  |  |  |
| 0 | Atr-ERN01177 |  |  |  |  |  |  |
| 0 | Atr-ERN01178 |  |  |  |  |  |  |
| 0 | Atr-ERN01179 |  |  |  |  |  |  |
| 0 | Atr-ERN01180 |  |  |  |  |  |  |
| 0 | Atr-ERN01181 |  |  |  |  |  |  |
| 0 | Atr-ERN01182 |  |  |  |  |  |  |
| 0 | Atr-ERN01183 |  |  |  |  |  |  |
| 0 | Atr-ERN01184 |  |  |  |  |  |  |
| 0 | Atr-ERN01185 |  |  |  |  |  |  |
| 0 | Atr-ERN01186 |  |  |  |  |  |  |
| 0 | Atr-ERN01187 |  |  |  |  |  |  |
| 0 | Atr-ERN01188 |  |  |  |  |  |  |
| 0 | Atr-ERN01189 |  |  |  |  |  |  |
| 0 | Atr-ERN01190 |  |  |  |  |  |  |
| 0 | Atr-ERN01191 |  |  |  |  |  |  |
| 0 | Atr-ERN01192 |  |  |  |  |  |  |
| 0 | Atr-ERN01193 |  |  |  |  |  |  |
| 0 | Atr-ERN01194 |  |  |  |  |  |  |
| 0 | Atr-ERN01195 |  |  |  |  |  |  |
| 0 | Atr-ERN01196 |  |  |  |  |  |  |
| 0 | Atr-ERN01197 |  |  |  |  |  |  |
| 0 | Atr-ERN01198 |  |  |  |  |  |  |
| 0 | Atr-ERN01199 |  |  |  |  |  |  |
| 0 | Atr-ERN01200 |  |  |  |  |  |  |
| 0 | Atr-ERN01201 |  |  |  |  |  |  |
| 0 | Atr-ERN01202 |  |  |  |  |  |  |
| 0 | Atr-ERN01203 |  |  |  |  |  |  |
| 0 | Atr-ERN01204 |  |  |  |  |  |  |
| 0 | Atr-ERN01205 |  |  |  |  |  |  |
| 0 | Atr-ERN01206 |  |  |  |  |  |  |
| 0 | Atr-ERN01207 |  |  |  |  |  |  |
| 0 | Atr-ERN01208 |  |  |  |  |  |  |
| 0 | Atr-ERN01209 |  |  |  |  |  |  |
| 0 | Atr-ERN01210 |  |  |  |  |  |  |
| 0 | Atr-ERN01211 |  |  |  |  |  |  |
| 0 | Atr-ERN01212 |  |  |  |  |  |  |
| 0 | Atr-ERN01213 |  |  |  |  |  |  |
| 0 | Atr-ERN01214 |  |  |  |  |  |  |
| 0 | Atr-ERN01215 |  |  |  |  |  |  |
| 0 | Atr-ERN01216 |  |  |  |  |  |  |
| 0 | Atr-ERN01217 |  |  |  |  |  |  |
| 0 | Atr-ERN01218 |  |  |  |  |  |  |
| 0 | Atr-ERN01219 |  |  |  |  |  |  |
| 0 | Atr-ERN01220 |  |  |  |  |  |  |
| 0 | Atr-ERN01221 |  |  |  |  |  |  |
| 0 | Atr-ERN01222 |  |  |  |  |  |  |
| 0 | Atr-ERN01223 |  |  |  |  |  |  |
| 0 | Atr-ERN01224 |  |  |  |  |  |  |
| 0 | Atr-ERN01225 |  |  |  |  |  |  |
| 0 | Atr-ERN01226 |  |  |  |  |  |  |
| 0 | Atr-ERN01227 |  |  |  |  |  |  |
| 0 | Atr-ERN01228 |  |  |  |  |  |  |
| 0 | Atr-ERN01229 |  |  |  |  |  |  |
| 0 | Atr-ERN01230 |  |  |  |  |  |  |
| 0 | Atr-ERN01231 |  |  |  |  |  |  |
| 0 | Atr-ERN01232 |  |  |  |  |  |  |
| 0 | Atr-ERN01233 |  |  |  |  |  |  |
| 0 | Atr-ERN01234 |  |  |  |  |  |  |
| 0 | Atr-ERN01235 |  |  |  |  |  |  |
| 0 | Atr-ERN01236 |  |  |  |  |  |  |
| 0 | Atr-ERN01237 |  |  |  |  |  |  |
| 0 | Atr-ERN01238 |  |  |  |  |  |  |
| 0 | Atr-ERN01239 |  |  |  |  |  |  |
| 0 | Atr-ERN01240 |  |  |  |  |  |  |
| 0 | Atr-ERN01241 |  |  |  |  |  |  |
| 0 | Atr-ERN01242 |  |  |  |  |  |  |
| 0 | Atr-ERN01243 |  |  |  |  |  |  |
| 0 | Atr-ERN01244 |  |  |  |  |  |  |
| 0 | Atr-ERN01245 |  |  |  |  |  |  |
| 0 | Atr-ERN01246 |  |  |  |  |  |  |
| 0 | Atr-ERN01247 |  |  |  |  |  |  |
| 0 | Atr-ERN01248 |  |  |  |  |  |  |
| 0 | Atr-ERN01249 |  |  |  |  |  |  |
| 0 | Atr-ERN01250 |  |  |  |  |  |  |
| 0 | Atr-ERN01251 |  |  |  |  |  |  |
| 0 | Atr-ERN01252 |  |  |  |  |  |  |
| 0 | Atr-ERN01253 |  |  |  |  |  |  |
| 0 | Atr-ERN01254 |  |  |  |  |  |  |
| 0 | Atr-ERN01255 |  |  |  |  |  |  |
| 0 | Atr-ERN01256 |  |  |  |  |  |  |
| 0 | Atr-ERN01257 |  |  |  |  |  |  |
| 0 | Atr-ERN01258 |  |  |  |  |  |  |
| 0 | Atr-ERN01259 |  |  |  |  |  |  |
| 0 | Atr-ERN01260 |  |  |  |  |  |  |
| 0 | Atr-ERN01261 |  |  |  |  |  |  |
| 0 | Atr-ERN01262 |  |  |  |  |  |  |
| 0 | Atr-ERN01263 |  |  |  |  |  |  |
| 0 | Atr-ERN01264 |  |  |  |  |  |  |
| 0 | Atr-ERN01265 |  |  |  |  |  |  |
| 0 | Atr-ERN01266 |  |  |  |  |  |  |
| 0 | Atr-ERN01267 |  |  |  |  |  |  |
| 0 | Atr-ERN01268 |  |  |  |  |  |  |
| 0 | Atr-ERN01269 |  |  |  |  |  |  |
| 0 | Atr-ERN01270 |  |  |  |  |  |  |
| 0 | Atr-ERN01271 |  |  |  |  |  |  |
| 0 | Atr-ERN01272 |  |  |  |  |  |  |
| 0 | Atr-ERN01273 |  |  |  |  |  |  |
| 0 | Atr-ERN01274 |  |  |  |  |  |  |
| 0 | Atr-ERN01275 |  |  |  |  |  |  |
| 0 | Atr-ERN01276 |  |  |  |  |  |  |
| 0 | Atr-ERN01277 |  |  |  |  |  |  |
| 0 | Atr-ERN01278 |  |  |  |  |  |  |
| 0 | Atr-ERN01279 |  |  |  |  |  |  |
| 0 | Atr-ERN01280 |  |  |  |  |  |  |
| 0 | Atr-ERN01281 |  |  |  |  |  |  |
| 0 | Atr-ERN01282 |  |  |  |  |  |  |
| 0 | Atr-ERN01283 |  |  |  |  |  |  |
| 1 | Atr-ERN01284 |  | Vvi-Vitvi06g01376\_t001 |  |  |  |  |  |
| 1 | Atr-ERN01285 |  | | | |  |  |  |  |  |
| 1 | Atr-ERN01286 |  | | | |  |  |  |  |  |
| 1 | Atr-ERN01287 |  | | | |  |  |  |  |  |
| 1 | Atr-ERN01288 |  | | | |  |  |  |  |  |
| 1 | Atr-ERN01289 |  | | | |  |  |  |  |  |
| 1 | Atr-ERN01290 |  | | | |  |  |  |  |  |
| 1 | Atr-ERN01291 |  | | | |  |  |  |  |  |
| 1 | Atr-ERN01292 |  | | | |  |  |  |  |  |
| 1 | Atr-ERN01293 |  | | | |  |  |  |  |  |
| 1 | Atr-ERN01294 |  | | | |  |  |  |  |  |
| 1 | Atr-ERN01295 |  | | | |  |  |  |  |  |
| 1 | Atr-ERN01296 |  | | | |  |  |  |  |  |
| 1 | Atr-ERN01297 |  | | | |  |  |  |  |  |
| 1 | Atr-ERN01298 |  | | | |  |  |  |  |  |
| 1 | Atr-ERN01299 |  | | | |  |  |  |  |  |
| 2 | Atr-ERN01300 |  | Vvi-Vitvi06g01380\_t001 |  | Vvi-Vitvi08g01545\_t001 |  |  |  |  |
| 2 | Atr-ERN01301 |  | | | |  | | | |  |  |  |  |
| 2 | Atr-ERN01302 |  | | | |  | | | |  |  |  |  |
| 2 | Atr-ERN01303 |  | | | |  | | | |  |  |  |  |
| 2 | Atr-ERN01304 |  | | | |  | | | |  |  |  |  |
| 2 | Atr-ERN01305 |  | | | |  | | | |  |  |  |  |
| 2 | Atr-ERN01306 |  | | | |  | | | |  |  |  |  |
| 2 | Atr-ERN01307 |  | | | |  | | | |  |  |  |  |
| 2 | Atr-ERN01308 |  | | | |  | Vvi-Vitvi08g01546\_t001 |  |  |  |  |
| 2 | Atr-ERN01309 |  | | | |  | | | |  |  |  |  |
| 2 | Atr-ERN01310 |  | | | |  | | | |  |  |  |  |
| 2 | Atr-ERN01311 |  | Vvi-Vitvi06g01383\_t003 |  | | | |  |  |  |  |
| 2 | Atr-ERN01312 |  | | | |  | Vvi-Vitvi08g01547\_t001 |  |  |  |  |
| 2 | Atr-ERN01313 |  | | | |  | | | |  |  |  |  |
| 2 | Atr-ERN01314 |  | | | |  | | | |  |  |  |  |
| 2 | Atr-ERN01315 |  | | | |  | | | |  |  |  |  |
| 2 | Atr-ERN01316 |  | | | |  | | | |  |  |  |  |
| 2 | Atr-ERN01317 |  | | | |  | | | |  |  |  |  |
| 2 | Atr-ERN01318 |  | Vvi-Vitvi06g01386\_t001 |  | | | |  |  |  |  |
| 2 | Atr-ERN01319 |  | Vvi-Vitvi06g01940\_t001 |  | | | |  |  |  |  |
| 2 | Atr-ERN01320 |  | | | |  | Vvi-Vitvi08g01551\_t001.1.6037826f |  |  |  |  |
| 2 | Atr-ERN01321 |  | Vvi-Vitvi06g01387\_t001 |  | Vvi-Vitvi08g01553\_t001 |  |  |  |  |
| 1 | Atr-ERN01322 |  |  |  | | | |  |  |  |  |
| 1 | Atr-ERN01323 |  |  |  | Vvi-Vitvi08g01555\_t001 |  |  |  |  |
| 1 | Atr-ERN01324 |  |  |  | | | |  |  |  |  |
| 1 | Atr-ERN01325 |  |  |  | | | |  |  |  |  |
| 1 | Atr-ERN01326 |  |  |  | | | |  |  |  |  |
| 1 | Atr-ERN01327 |  |  |  | | | |  |  |  |  |
| 1 | Atr-ERN01328 |  |  |  | | | |  |  |  |  |
| 1 | Atr-ERN01329 |  |  |  | | | |  |  |  |  |
| 1 | Atr-ERN01330 |  |  |  | | | |  |  |  |  |
| 1 | Atr-ERN01331 |  |  |  | | | |  |  |  |  |
| 1 | Atr-ERN01332 |  |  |  | Vvi-Vitvi08g01556\_t001 |  |  |  |  |
| 1 | Atr-ERN01333 |  |  |  | | | |  |  |  |  |
| 1 | Atr-ERN01334 |  |  |  | | | |  |  |  |  |
| 1 | Atr-ERN01335 |  |  |  | | | |  |  |  |  |
| 1 | Atr-ERN01336 |  |  |  | | | |  |  |  |  |
| 1 | Atr-ERN01337 |  |  |  | | | |  |  |  |  |
| 1 | Atr-ERN01338 |  |  |  | | | |  |  |  |  |
| 1 | Atr-ERN01339 |  |  |  | | | |  |  |  |  |
| 1 | Atr-ERN01340 |  |  |  | | | |  |  |  |  |
| 1 | Atr-ERN01341 |  |  |  | | | |  |  |  |  |
| 1 | Atr-ERN01342 |  |  |  | | | |  |  |  |  |
| 1 | Atr-ERN01343 |  |  |  | Vvi-Vitvi08g01558\_t001 |  |  |  |  |
| 1 | Atr-ERN01344 |  |  |  | Vvi-Vitvi08g01561\_t001 |  |  |  |  |
| 1 | Atr-ERN01345 |  |  |  | | | |  |  |  |  |
| 1 | Atr-ERN01346 |  |  |  | | | |  |  |  |  |
| 1 | Atr-ERN01347 |  |  |  | Vvi-Vitvi08g01562\_t001 |  |  |  |  |
| 0 | Atr-ERN01348 |  |  |  |  |  |  |
| 0 | Atr-ERN01349 |  |  |  |  |  |  |
| 0 | Atr-ERN01350 |  |  |  |  |  |  |
| 1 | Atr-ERN01351 |  | Vvi-Vitvi01g00915\_t001 |  |  |  |  |  |
| 1 | Atr-ERN01352 |  | Vvi-Vitvi01g00914\_t001 |  |  |  |  |  |
| 1 | Atr-ERN01353 |  | | | |  |  |  |  |  |
| 1 | Atr-ERN01354 |  | | | |  |  |  |  |  |
| 1 | Atr-ERN01355 |  | | | |  |  |  |  |  |
| 1 | Atr-ERN01356 |  | Vvi-Vitvi01g00911\_t001 |  |  |  |  |  |
| 1 | Atr-ERN01357 |  | | | |  |  |  |  |  |
| 1 | Atr-ERN01358 |  | | | |  |  |  |  |  |
| 1 | Atr-ERN01359 |  | | | |  |  |  |  |  |
| 1 | Atr-ERN01360 |  | | | |  |  |  |  |  |
| 1 | Atr-ERN01361 |  | Vvi-Vitvi01g00909\_t001 |  |  |  |  |  |
| 1 | Atr-ERN01362 |  | | | |  |  |  |  |  |
| 1 | Atr-ERN01363 |  | | | |  |  |  |  |  |
| 1 | Atr-ERN01364 |  | | | |  |  |  |  |  |
| 1 | Atr-ERN01365 |  | | | |  |  |  |  |  |
| 1 | Atr-ERN01366 |  | | | |  |  |  |  |  |
| 1 | Atr-ERN01367 |  | Vvi-Vitvi01g00908\_t001 |  |  |  |  |  |
| 1 | Atr-ERN01368 |  | Vvi-Vitvi01g00906\_t001 |  |  |  |  |  |
| 0 | Atr-ERN01369 |  |  |  |  |  |  |
| 0 | Atr-ERN01370 |  |  |  |  |  |  |
| 1 | Atr-ERN01371 |  | Vvi-Vitvi05g04160\_t001 |  |  |  |  |  |
| 1 | Atr-ERN01372 |  | | | |  |  |  |  |  |
| 1 | Atr-ERN01373 |  | | | |  |  |  |  |  |
| 1 | Atr-ERN01374 |  | | | |  |  |  |  |  |
| 1 | Atr-ERN01375 |  | | | |  |  |  |  |  |
| 1 | Atr-ERN01376 |  | | | |  |  |  |  |  |
| 1 | Atr-ERN01377 |  | | | |  |  |  |  |  |
| 1 | Atr-ERN01378 |  | | | |  |  |  |  |  |
| 1 | Atr-ERN01379 |  | | | |  |  |  |  |  |
| 1 | Atr-ERN01380 |  | | | |  |  |  |  |  |
| 1 | Atr-ERN01381 |  | | | |  |  |  |  |  |
| 1 | Atr-ERN01382 |  | | | |  |  |  |  |  |
| 3 | Atr-ERN01383 |  | | | |  | Vvi-Vitvi14g00508\_t001 |  | Vvi-Vitvi07g00013\_t001 |  |  |  |
| 3 | Atr-ERN01384 |  | Vvi-Vitvi05g00661\_t001 |  | | | |  | | | |  |  |  |
| 3 | Atr-ERN01385 |  | | | |  | | | |  | Vvi-Vitvi07g00015\_t001 |  |  |  |
| 3 | Atr-ERN01386 |  | | | |  | Vvi-Vitvi14g00509\_t001 |  | | | |  |  |  |
| 3 | Atr-ERN01387 |  | | | |  | | | |  | Vvi-Vitvi07g00016\_t001 |  |  |  |
| 3 | Atr-ERN01388 |  | | | |  | | | |  | | | |  |  |  |
| 3 | Atr-ERN01389 |  | | | |  | | | |  | | | |  |  |  |
| 3 | Atr-ERN01390 |  | | | |  | | | |  | | | |  |  |  |
| 3 | Atr-ERN01391 |  | | | |  | | | |  | | | |  |  |  |
| 3 | Atr-ERN01392 |  | | | |  | | | |  | | | |  |  |  |
| 3 | Atr-ERN01393 |  | | | |  | | | |  | | | |  |  |  |
| 3 | Atr-ERN01394 |  | | | |  | | | |  | | | |  |  |  |
| 3 | Atr-ERN01395 |  | | | |  | | | |  | | | |  |  |  |
| 3 | Atr-ERN01396 |  | | | |  | | | |  | | | |  |  |  |
| 3 | Atr-ERN01397 |  | | | |  | | | |  | | | |  |  |  |
| 3 | Atr-ERN01398 |  | | | |  | | | |  | | | |  |  |  |
| 3 | Atr-ERN01399 |  | | | |  | | | |  | | | |  |  |  |
| 4 | Atr-ERN01400 |  | | | |  | | | |  | | | |  | Vvi-Vitvi14g00535\_t001 |  |  |
| 4 | Atr-ERN01401 |  | | | |  | | | |  | | | |  | | | |  |  |
| 4 | Atr-ERN01402 |  | Vvi-Vitvi05g00654\_t001 |  | | | |  | | | |  | | | |  |  |
| 4 | Atr-ERN01403 |  | | | |  | Vvi-Vitvi14g00521\_t001 |  | Vvi-Vitvi07g00018\_t001 |  | Vvi-Vitvi14g00521\_t001 |  |  |
| 4 | Atr-ERN01404 |  | | | |  | | | |  | Vvi-Vitvi07g00020\_t001 |  | Vvi-Vitvi14g02679\_t001 |  |  |
| 4 | Atr-ERN01405 |  | | | |  | Vvi-Vitvi14g00536\_t001 |  | | | |  | | | |  |  |
| 4 | Atr-ERN01406 |  | | | |  | | | |  | | | |  | | | |  |  |
| 4 | Atr-ERN01407 |  | | | |  | | | |  | | | |  | | | |  |  |
| 4 | Atr-ERN01408 |  | Vvi-Vitvi05g00651\_t001 |  | | | |  | | | |  | | | |  |  |
| 4 | Atr-ERN01409 |  | Vvi-Vitvi05g00650\_t001 |  | | | |  | | | |  | | | |  |  |
| 4 | Atr-ERN01410 |  | | | |  | | | |  | | | |  | | | |  |  |
| 4 | Atr-ERN01411 |  | Vvi-Vitvi05g00642\_t001 |  | | | |  | Vvi-Vitvi07g00024\_t001 |  | | | |  |  |
| 4 | Atr-ERN01412 |  | | | |  | | | |  | | | |  | | | |  |  |
| 4 | Atr-ERN01413 |  | | | |  | Vvi-Vitvi14g02049\_t001 |  | Vvi-Vitvi07g02066\_t001 |  | | | |  |  |
| 4 | Atr-ERN01414 |  | | | |  | Vvi-Vitvi14g00540\_t001 |  | Vvi-Vitvi07g00026\_t001 |  | | | |  |  |
| 3 | Atr-ERN01415 |  | | | |  |  |  | Vvi-Vitvi07g00027\_t001 |  | | | |  |  |
| 3 | Atr-ERN01416 |  | Vvi-Vitvi05g00640\_t001 |  |  |  | | | |  | | | |  |  |
| 3 | Atr-ERN01417 |  | | | |  |  |  | Vvi-Vitvi07g00029\_t001 |  | | | |  |  |
| 3 | Atr-ERN01418 |  | Vvi-Vitvi05g00639\_t001 |  |  |  | | | |  | | | |  |  |
| 3 | Atr-ERN01419 |  | | | |  |  |  | | | |  | Vvi-Vitvi14g00497\_t001 |  |  |
| 3 | Atr-ERN01420 |  | Vvi-Vitvi05g00638\_t001.1.6037826e |  |  |  | | | |  | | | |  |  |
| 3 | Atr-ERN01421 |  | Vvi-Vitvi05g01906\_t001 |  |  |  | Vvi-Vitvi07g00030\_t001 |  | Vvi-Vitvi14g02667\_t001 |  |  |
| 3 | Atr-ERN01422 |  | | | |  |  |  | | | |  | Vvi-Vitvi14g00491\_t001 |  |  |
| 3 | Atr-ERN01423 |  | | | |  |  |  | | | |  | | | |  |  |
| 3 | Atr-ERN01424 |  | | | |  |  |  | | | |  | | | |  |  |
| 3 | Atr-ERN01425 |  | | | |  |  |  | | | |  | | | |  |  |
| 3 | Atr-ERN01426 |  | Vvi-Vitvi05g00634\_t001 |  |  |  | Vvi-Vitvi07g00031\_t001 |  | Vvi-Vitvi14g00490\_t001 |  |  |
| 3 | Atr-ERN01427 |  | Vvi-Vitvi05g00633\_t002 |  |  |  | Vvi-Vitvi07g00032\_t001 |  | | | |  |  |
| 3 | Atr-ERN01428 |  | | | |  |  |  | Vvi-Vitvi07g00034\_t001 |  | | | |  |  |
| 3 | Atr-ERN01429 |  | | | |  |  |  | Vvi-Vitvi07g00035\_t001 |  | Vvi-Vitvi14g00489\_t001 |  |  |
| 3 | Atr-ERN01430 |  | Vvi-Vitvi05g00632\_t001 |  |  |  | | | |  | | | |  |  |
| 3 | Atr-ERN01431 |  | | | |  |  |  | | | |  | | | |  |  |
| 3 | Atr-ERN01432 |  | | | |  |  |  | Vvi-Vitvi07g04010\_t001 |  | | | |  |  |
| 3 | Atr-ERN01433 |  | | | |  |  |  | Vvi-Vitvi07g00040\_t001 |  | | | |  |  |
| 3 | Atr-ERN01434 |  | | | |  |  |  | Vvi-Vitvi07g00041\_t001 |  | | | |  |  |
| 3 | Atr-ERN01435 |  | | | |  |  |  | | | |  | Vvi-Vitvi14g00487\_t001 |  |  |
| 3 | Atr-ERN01436 |  | Vvi-Vitvi05g00630\_t001 |  |  |  | Vvi-Vitvi07g00042\_t001 |  | Vvi-Vitvi14g00483\_t001 |  |  |
| 3 | Atr-ERN01437 |  | | | |  |  |  | | | |  | | | |  |  |
| 3 | Atr-ERN01438 |  | | | |  |  |  | | | |  | | | |  |  |
| 3 | Atr-ERN01439 |  | Vvi-Vitvi05g00628\_t001 |  |  |  | | | |  | | | |  |  |
| 3 | Atr-ERN01440 |  | | | |  |  |  | | | |  | Vvi-Vitvi14g00482\_t001 |  |  |
| 3 | Atr-ERN01441 |  | | | |  |  |  | | | |  | | | |  |  |
| 3 | Atr-ERN01442 |  | | | |  |  |  | | | |  | | | |  |  |
| 3 | Atr-ERN01443 |  | | | |  |  |  | Vvi-Vitvi07g00044\_t001 |  | | | |  |  |
| 3 | Atr-ERN01444 |  | Vvi-Vitvi05g00627\_t001 |  |  |  | | | |  | | | |  |  |
| 3 | Atr-ERN01445 |  | Vvi-Vitvi05g00626\_t001 |  |  |  | | | |  | | | |  |  |
| 3 | Atr-ERN01446 |  | Vvi-Vitvi05g00625\_t001 |  |  |  | | | |  | | | |  |  |
| 3 | Atr-ERN01447 |  | | | |  |  |  | Vvi-Vitvi07g00045\_t001 |  | | | |  |  |
| 3 | Atr-ERN01448 |  | | | |  |  |  | | | |  | | | |  |  |
| 3 | Atr-ERN01449 |  | | | |  |  |  | | | |  | | | |  |  |
| 3 | Atr-ERN01450 |  | Vvi-Vitvi05g00624\_t001 |  |  |  | Vvi-Vitvi07g00046\_t001 |  | Vvi-Vitvi14g00477\_t001 |  |  |
| 3 | Atr-ERN01451 |  | | | |  |  |  | | | |  | | | |  |  |
| 3 | Atr-ERN01452 |  | | | |  |  |  | | | |  | | | |  |  |
| 3 | Atr-ERN01453 |  | | | |  |  |  | | | |  | Vvi-Vitvi14g00476\_t001 |  |  |
| 3 | Atr-ERN01454 |  | Vvi-Vitvi05g00621\_t001 |  |  |  | | | |  | | | |  |  |
| 3 | Atr-ERN01455 |  | | | |  |  |  | Vvi-Vitvi07g00047\_t001 |  | | | |  |  |
| 3 | Atr-ERN01456 |  | | | |  |  |  | | | |  | | | |  |  |
| 3 | Atr-ERN01457 |  | | | |  |  |  | | | |  | | | |  |  |
| 3 | Atr-ERN01458 |  | | | |  |  |  | Vvi-Vitvi07g00049\_t001 |  | Vvi-Vitvi14g00473\_t001 |  |  |
| 3 | Atr-ERN01459 |  | Vvi-Vitvi05g00620\_t001 |  |  |  | | | |  | | | |  |  |
| 3 | Atr-ERN01460 |  | | | |  |  |  | Vvi-Vitvi07g00050\_t001 |  | | | |  |  |
| 3 | Atr-ERN01461 |  | Vvi-Vitvi05g00619\_t001 |  |  |  | | | |  | | | |  |  |
| 3 | Atr-ERN01462 |  | | | |  |  |  | | | |  | Vvi-Vitvi14g00471\_t002 |  |  |
| 3 | Atr-ERN01463 |  | | | |  |  |  | | | |  | | | |  |  |
| 3 | Atr-ERN01464 |  | | | |  |  |  | Vvi-Vitvi07g00054\_t001 |  | | | |  |  |
| 3 | Atr-ERN01465 |  | | | |  |  |  | | | |  | | | |  |  |
| 3 | Atr-ERN01466 |  | | | |  |  |  | | | |  | | | |  |  |
| 3 | Atr-ERN01467 |  | Vvi-Vitvi05g00617\_t001 |  |  |  | | | |  | | | |  |  |
| 3 | Atr-ERN01468 |  | Vvi-Vitvi05g01904\_t001 |  |  |  | | | |  | | | |  |  |
| 3 | Atr-ERN01469 |  | | | |  |  |  | | | |  | | | |  |  |
| 3 | Atr-ERN01470 |  | | | |  |  |  | Vvi-Vitvi07g00060\_t001 |  | | | |  |  |
| 3 | Atr-ERN01471 |  | Vvi-Vitvi05g04150\_t001 |  |  |  | Vvi-Vitvi07g00061\_t002 |  | | | |  |  |
| 3 | Atr-ERN01472 |  | Vvi-Vitvi05g00612\_t001 |  |  |  | Vvi-Vitvi07g00062\_t001 |  | | | |  |  |
| 3 | Atr-ERN01473 |  | | | |  |  |  | Vvi-Vitvi07g00064\_t001 |  | | | |  |  |
| 3 | Atr-ERN01474 |  | | | |  |  |  | | | |  | | | |  |  |
| 3 | Atr-ERN01475 |  | | | |  |  |  | | | |  | | | |  |  |
| 3 | Atr-ERN01476 |  | Vvi-Vitvi05g00611\_t001 |  |  |  | | | |  | | | |  |  |
| 3 | Atr-ERN01477 |  | Vvi-Vitvi05g00609\_t001 |  |  |  | | | |  | | | |  |  |
| 3 | Atr-ERN01478 |  | | | |  |  |  | | | |  | | | |  |  |
| 3 | Atr-ERN01479 |  | | | |  |  |  | Vvi-Vitvi07g00065\_t001 |  | Vvi-Vitvi14g00469\_t001 |  |  |
| 3 | Atr-ERN01480 |  | | | |  |  |  | | | |  | | | |  |  |
| 3 | Atr-ERN01481 |  | Vvi-Vitvi05g00605\_t001 |  |  |  | | | |  | Vvi-Vitvi14g00468\_t001 |  |  |
| 2 | Atr-ERN01482 |  |  |  |  |  | | | |  | | | |  |  |
| 2 | Atr-ERN01483 |  |  |  |  |  | Vvi-Vitvi07g00066\_t001 |  | | | |  |  |
| 2 | Atr-ERN01484 |  |  |  |  |  | | | |  | | | |  |  |
| 2 | Atr-ERN01485 |  |  |  |  |  | | | |  | | | |  |  |
| 2 | Atr-ERN01486 |  |  |  |  |  | | | |  | | | |  |  |
| 2 | Atr-ERN01487 |  |  |  |  |  | | | |  | | | |  |  |
| 2 | Atr-ERN01488 |  |  |  |  |  | | | |  | | | |  |  |
| 3 | Atr-ERN01489 |  | Vvi-Vitvi05g00576\_t001 |  |  |  | | | |  | | | |  |  |
| 3 | Atr-ERN01490 |  | Vvi-Vitvi05g00574\_t002 |  |  |  | | | |  | | | |  |  |
| 3 | Atr-ERN01491 |  | Vvi-Vitvi05g00573\_t001 |  |  |  | | | |  | | | |  |  |
| 3 | Atr-ERN01492 |  | Vvi-Vitvi05g00570\_t001 |  |  |  | | | |  | | | |  |  |
| 3 | Atr-ERN01493 |  | | | |  |  |  | | | |  | | | |  |  |
| 3 | Atr-ERN01494 |  | Vvi-Vitvi05g00569\_t001 |  |  |  | | | |  | | | |  |  |
| 3 | Atr-ERN01495 |  | | | |  |  |  | Vvi-Vitvi07g00073\_t002 |  | | | |  |  |
| 3 | Atr-ERN01496 |  | | | |  |  |  | | | |  | | | |  |  |
| 3 | Atr-ERN01497 |  | | | |  |  |  | | | |  | | | |  |  |
| 3 | Atr-ERN01498 |  | | | |  |  |  | | | |  | | | |  |  |
| 3 | Atr-ERN01499 |  | | | |  |  |  | | | |  | | | |  |  |
| 3 | Atr-ERN01500 |  | | | |  |  |  | | | |  | | | |  |  |
| 3 | Atr-ERN01501 |  | | | |  |  |  | | | |  | | | |  |  |
| 3 | Atr-ERN01502 |  | | | |  |  |  | | | |  | | | |  |  |
| 3 | Atr-ERN01503 |  | | | |  |  |  | | | |  | | | |  |  |
| 3 | Atr-ERN01504 |  | Vvi-Vitvi05g00568\_t001 |  |  |  | Vvi-Vitvi07g00074\_t001 |  | | | |  |  |
| 3 | Atr-ERN01505 |  | | | |  |  |  | | | |  | | | |  |  |
| 3 | Atr-ERN01506 |  | Vvi-Vitvi05g00565\_t001 |  |  |  | | | |  | Vvi-Vitvi14g00446\_t001 |  |  |
| 3 | Atr-ERN01507 |  | | | |  |  |  | | | |  | | | |  |  |
| 3 | Atr-ERN01508 |  | | | |  |  |  | | | |  | | | |  |  |
| 3 | Atr-ERN01509 |  | Vvi-Vitvi05g00563\_t001 |  |  |  | | | |  | | | |  |  |
| 3 | Atr-ERN01510 |  | | | |  |  |  | Vvi-Vitvi07g00075\_t001 |  | | | |  |  |
| 3 | Atr-ERN01511 |  | | | |  |  |  | Vvi-Vitvi07g00077\_t001 |  | | | |  |  |
| 3 | Atr-ERN01512 |  | Vvi-Vitvi05g00562\_t001 |  |  |  | | | |  | | | |  |  |
| 3 | Atr-ERN01513 |  | Vvi-Vitvi05g00561\_t001 |  |  |  | Vvi-Vitvi07g00078\_t001 |  | | | |  |  |
| 3 | Atr-ERN01514 |  | | | |  |  |  | | | |  | | | |  |  |
| 3 | Atr-ERN01515 |  | | | |  |  |  | Vvi-Vitvi07g00079\_t001 |  | | | |  |  |
| 3 | Atr-ERN01516 |  | | | |  |  |  | | | |  | | | |  |  |
| 3 | Atr-ERN01517 |  | Vvi-Vitvi05g00559\_t002 |  |  |  | | | |  | | | |  |  |
| 3 | Atr-ERN01518 |  | | | |  |  |  | | | |  | | | |  |  |
| 3 | Atr-ERN01519 |  | | | |  |  |  | Vvi-Vitvi07g00081\_t001 |  | | | |  |  |
| 3 | Atr-ERN01520 |  | | | |  |  |  | | | |  | | | |  |  |
| 3 | Atr-ERN01521 |  | Vvi-Vitvi05g00558\_t002 |  |  |  | Vvi-Vitvi07g00082\_t001 |  | | | |  |  |
| 3 | Atr-ERN01522 |  | | | |  |  |  | | | |  | Vvi-Vitvi14g00445\_t001 |  |  |
| 3 | Atr-ERN01523 |  | | | |  |  |  | | | |  | | | |  |  |
| 3 | Atr-ERN01524 |  | Vvi-Vitvi05g00557\_t001 |  |  |  | | | |  | | | |  |  |
| 3 | Atr-ERN01525 |  | | | |  |  |  | Vvi-Vitvi07g02102\_t001 |  | | | |  |  |
| 3 | Atr-ERN01526 |  | | | |  |  |  | | | |  | | | |  |  |
| 3 | Atr-ERN01527 |  | Vvi-Vitvi05g00556\_t001 |  |  |  | Vvi-Vitvi07g02114\_t001 |  | | | |  |  |
| 3 | Atr-ERN01528 |  | | | |  |  |  | | | |  | | | |  |  |
| 3 | Atr-ERN01529 |  | Vvi-Vitvi05g00555\_t001 |  |  |  | Vvi-Vitvi07g00087\_t001 |  | | | |  |  |
| 3 | Atr-ERN01530 |  | Vvi-Vitvi05g00554\_t001 |  |  |  | Vvi-Vitvi07g00088\_t001 |  | | | |  |  |
| 3 | Atr-ERN01531 |  | | | |  |  |  | | | |  | | | |  |  |
| 3 | Atr-ERN01532 |  | | | |  |  |  | | | |  | | | |  |  |
| 3 | Atr-ERN01533 |  | | | |  |  |  | | | |  | | | |  |  |
| 3 | Atr-ERN01534 |  | | | |  |  |  | | | |  | | | |  |  |
| 3 | Atr-ERN01535 |  | | | |  |  |  | | | |  | | | |  |  |
| 3 | Atr-ERN01536 |  | | | |  |  |  | | | |  | Vvi-Vitvi14g00440\_t002 |  |  |
| 3 | Atr-ERN01537 |  | | | |  |  |  | | | |  | Vvi-Vitvi14g00439\_t001 |  |  |
| 3 | Atr-ERN01538 |  | Vvi-Vitvi05g00553\_t001 |  |  |  | | | |  | Vvi-Vitvi14g00438\_t001 |  |  |
| 3 | Atr-ERN01539 |  | | | |  |  |  | | | |  | | | |  |  |
| 3 | Atr-ERN01540 |  | Vvi-Vitvi05g00551\_t001 |  |  |  | | | |  | | | |  |  |
| 3 | Atr-ERN01541 |  | | | |  |  |  | | | |  | | | |  |  |
| 3 | Atr-ERN01542 |  | | | |  |  |  | | | |  | | | |  |  |
| 3 | Atr-ERN01543 |  | | | |  |  |  | | | |  | | | |  |  |
| 3 | Atr-ERN01544 |  | Vvi-Vitvi05g00549\_t001 |  |  |  | | | |  | Vvi-Vitvi14g00437\_t001 |  |  |
| 3 | Atr-ERN01545 |  | | | |  |  |  | Vvi-Vitvi07g04029\_t001 |  | | | |  |  |
| 3 | Atr-ERN01546 |  | | | |  |  |  | | | |  | | | |  |  |
| 3 | Atr-ERN01547 |  | | | |  |  |  | | | |  | | | |  |  |
| 3 | Atr-ERN01548 |  | | | |  |  |  | Vvi-Vitvi07g00090\_t001 |  | Vvi-Vitvi14g00434\_t001 |  |  |
| 3 | Atr-ERN01549 |  | | | |  |  |  | | | |  | | | |  |  |
| 3 | Atr-ERN01550 |  | | | |  |  |  | | | |  | | | |  |  |
| 3 | Atr-ERN01551 |  | | | |  |  |  | | | |  | Vvi-Vitvi14g00433\_t001 |  |  |
| 3 | Atr-ERN01552 |  | | | |  |  |  | | | |  | | | |  |  |
| 3 | Atr-ERN01553 |  | | | |  |  |  | | | |  | | | |  |  |
| 3 | Atr-ERN01554 |  | Vvi-Vitvi05g00546\_t001 |  |  |  | Vvi-Vitvi07g00091\_t001 |  | | | |  |  |
| 3 | Atr-ERN01555 |  | Vvi-Vitvi05g00545\_t001 |  |  |  | | | |  | | | |  |  |
| 3 | Atr-ERN01556 |  | Vvi-Vitvi05g00544\_t001 |  |  |  | | | |  | | | |  |  |
| 3 | Atr-ERN01557 |  | | | |  |  |  | | | |  | Vvi-Vitvi14g00432\_t002 |  |  |
| 3 | Atr-ERN01558 |  | | | |  |  |  | | | |  | Vvi-Vitvi14g00430\_t001 |  |  |
| 3 | Atr-ERN01559 |  | Vvi-Vitvi05g00543\_t001 |  |  |  | | | |  | Vvi-Vitvi14g00429\_t001 |  |  |
| 3 | Atr-ERN01560 |  | Vvi-Vitvi05g00542\_t001 |  |  |  | Vvi-Vitvi07g00097\_t002 |  | | | |  |  |
| 3 | Atr-ERN01561 |  | Vvi-Vitvi05g00541\_t001 |  |  |  | | | |  | Vvi-Vitvi14g00428\_t001 |  |  |
| 2 | Atr-ERN01562 |  | | | |  |  |  | | | |  |  |  |
| 2 | Atr-ERN01563 |  | | | |  |  |  | | | |  |  |  |
| 2 | Atr-ERN01564 |  | | | |  |  |  | | | |  |  |  |
| 2 | Atr-ERN01565 |  | | | |  |  |  | | | |  |  |  |
| 2 | Atr-ERN01566 |  | | | |  |  |  | Vvi-Vitvi07g00099\_t001 |  |  |  |
| 2 | Atr-ERN01567 |  | Vvi-Vitvi05g00540\_t001 |  |  |  | Vvi-Vitvi07g04032\_t001 |  |  |  |
| 2 | Atr-ERN01568 |  | Vvi-Vitvi05g00539\_t003 |  |  |  | | | |  |  |  |
| 2 | Atr-ERN01569 |  | | | |  |  |  | Vvi-Vitvi07g00102\_t001 |  |  |  |
| 2 | Atr-ERN01570 |  | Vvi-Vitvi05g00537\_t002 |  |  |  | | | |  |  |  |
| 2 | Atr-ERN01571 |  | Vvi-Vitvi05g00535\_t001 |  |  |  | | | |  |  |  |
| 2 | Atr-ERN01572 |  | | | |  |  |  | Vvi-Vitvi07g00103\_t001 |  |  |  |
| 2 | Atr-ERN01573 |  | Vvi-Vitvi05g00534\_t001 |  |  |  | | | |  |  |  |
| 2 | Atr-ERN01574 |  | | | |  |  |  | | | |  |  |  |
| 2 | Atr-ERN01575 |  | Vvi-Vitvi05g00533\_t001 |  |  |  | Vvi-Vitvi07g00105\_t001 |  |  |  |
| 2 | Atr-ERN01576 |  | | | |  |  |  | | | |  |  |  |
| 2 | Atr-ERN01577 |  | | | |  |  |  | | | |  |  |  |
| 2 | Atr-ERN01578 |  | | | |  |  |  | Vvi-Vitvi07g00106\_t001 |  |  |  |
| 2 | Atr-ERN01579 |  | | | |  |  |  | Vvi-Vitvi07g00107\_t001 |  |  |  |
| 2 | Atr-ERN01580 |  | | | |  |  |  | | | |  |  |  |
| 2 | Atr-ERN01581 |  | | | |  |  |  | | | |  |  |  |
| 2 | Atr-ERN01582 |  | | | |  |  |  | Vvi-Vitvi07g00109\_t001 |  |  |  |
| 2 | Atr-ERN01583 |  | | | |  |  |  | | | |  |  |  |
| 2 | Atr-ERN01584 |  | | | |  |  |  | | | |  |  |  |
| 2 | Atr-ERN01585 |  | Vvi-Vitvi05g00530\_t001 |  |  |  | | | |  |  |  |
| 2 | Atr-ERN01586 |  | | | |  |  |  | | | |  |  |  |
| 2 | Atr-ERN01587 |  | Vvi-Vitvi05g00528\_t001 |  |  |  | Vvi-Vitvi07g00110\_t001 |  |  |  |
| 2 | Atr-ERN01588 |  | Vvi-Vitvi05g00527\_t002 |  |  |  | Vvi-Vitvi07g02131\_t001 |  |  |  |
| 2 | Atr-ERN01589 |  | Vvi-Vitvi05g00526\_t001 |  |  |  | | | |  |  |  |
| 1 | Atr-ERN01590 |  |  |  |  |  | Vvi-Vitvi07g00112\_t001 |  |  |  |
| 1 | Atr-ERN01591 |  |  |  |  |  | | | |  |  |  |
| 1 | Atr-ERN01592 |  |  |  |  |  | | | |  |  |  |
| 1 | Atr-ERN01593 |  |  |  |  |  | | | |  |  |  |
| 1 | Atr-ERN01594 |  |  |  |  |  | | | |  |  |  |
| 1 | Atr-ERN01595 |  |  |  |  |  | | | |  |  |  |
| 1 | Atr-ERN01596 |  |  |  |  |  | Vvi-Vitvi07g00113\_t001 |  |  |  |
