# Supplementary material for: Genome-wide identification, characterization and gene expression of BES1 transcription factor family in grapevine (Vitis vinifera L.)
Source: Sci Rep. 2023 Jan 5;13:240. doi: 10.1038/s41598-022-24407-y (PMC9816167; doi:10.1038/s41598-022-24407-y)
Supplement: Supplementary file 3 — Supplementary Information. [file 41598_2022_24407_MOESM3_ESM.zip › Vvi_Atr/Vitis_vinifera.PN40024.v4.dna_sm.toplevel.fa.vs.Amborella_trichopoda.AMTR1.0.dna_sm.toplevel.fa.html/Atr-AmTr_v1.0_scaffold00003.html]

|  |  |  |  |  |  |  |  |  |  |  |  |  |  |
| --- | --- | --- | --- | --- | --- | --- | --- | --- | --- | --- | --- | --- | --- |
| Duplication depth | Reference chromosome | Collinear blocks | | | | | | | | | | | |
| 0 | Atr-ERN03079 |  |  |  |  |  |  |
| 0 | Atr-ERN03080 |  |  |  |  |  |  |
| 0 | Atr-ERN03081 |  |  |  |  |  |  |
| 0 | Atr-ERN03082 |  |  |  |  |  |  |
| 1 | Atr-ERN03083 |  | Vvi-Vitvi05g00101\_t002 |  |  |  |  |  |
| 1 | Atr-ERN03084 |  | | | |  |  |  |  |  |
| 1 | Atr-ERN03085 |  | | | |  |  |  |  |  |
| 1 | Atr-ERN03086 |  | | | |  |  |  |  |  |
| 1 | Atr-ERN03087 |  | | | |  |  |  |  |  |
| 1 | Atr-ERN03088 |  | | | |  |  |  |  |  |
| 1 | Atr-ERN03089 |  | | | |  |  |  |  |  |
| 1 | Atr-ERN03090 |  | Vvi-Vitvi05g00115\_t001 |  |  |  |  |  |
| 1 | Atr-ERN03091 |  | | | |  |  |  |  |  |
| 1 | Atr-ERN03092 |  | | | |  |  |  |  |  |
| 1 | Atr-ERN03093 |  | | | |  |  |  |  |  |
| 1 | Atr-ERN03094 |  | | | |  |  |  |  |  |
| 1 | Atr-ERN03095 |  | | | |  |  |  |  |  |
| 1 | Atr-ERN03096 |  | | | |  |  |  |  |  |
| 1 | Atr-ERN03097 |  | | | |  |  |  |  |  |
| 1 | Atr-ERN03098 |  | | | |  |  |  |  |  |
| 1 | Atr-ERN03099 |  | | | |  |  |  |  |  |
| 1 | Atr-ERN03100 |  | | | |  |  |  |  |  |
| 1 | Atr-ERN03101 |  | | | |  |  |  |  |  |
| 1 | Atr-ERN03102 |  | | | |  |  |  |  |  |
| 1 | Atr-ERN03103 |  | | | |  |  |  |  |  |
| 1 | Atr-ERN03104 |  | | | |  |  |  |  |  |
| 1 | Atr-ERN03105 |  | | | |  |  |  |  |  |
| 1 | Atr-ERN03106 |  | | | |  |  |  |  |  |
| 1 | Atr-ERN03107 |  | Vvi-Vitvi05g00124\_t001 |  |  |  |  |  |
| 1 | Atr-ERN03108 |  | Vvi-Vitvi05g00125\_t001 |  |  |  |  |  |
| 1 | Atr-ERN03109 |  | | | |  |  |  |  |  |
| 1 | Atr-ERN03110 |  | | | |  |  |  |  |  |
| 1 | Atr-ERN03111 |  | | | |  |  |  |  |  |
| 1 | Atr-ERN03112 |  | | | |  |  |  |  |  |
| 1 | Atr-ERN03113 |  | Vvi-Vitvi05g00126\_t001 |  |  |  |  |  |
| 1 | Atr-ERN03114 |  | | | |  |  |  |  |  |
| 1 | Atr-ERN03115 |  | | | |  |  |  |  |  |
| 1 | Atr-ERN03116 |  | | | |  |  |  |  |  |
| 1 | Atr-ERN03117 |  | | | |  |  |  |  |  |
| 1 | Atr-ERN03118 |  | | | |  |  |  |  |  |
| 1 | Atr-ERN03119 |  | | | |  |  |  |  |  |
| 1 | Atr-ERN03120 |  | | | |  |  |  |  |  |
| 1 | Atr-ERN03121 |  | | | |  |  |  |  |  |
| 1 | Atr-ERN03122 |  | Vvi-Vitvi05g00129\_t002 |  |  |  |  |  |
| 1 | Atr-ERN03123 |  | Vvi-Vitvi05g00130\_t001 |  |  |  |  |  |
| 1 | Atr-ERN03124 |  | | | |  |  |  |  |  |
| 1 | Atr-ERN03125 |  | Vvi-Vitvi05g00132\_t001 |  |  |  |  |  |
| 1 | Atr-ERN03126 |  | Vvi-Vitvi05g01774\_t001 |  |  |  |  |  |
| 1 | Atr-ERN03127 |  | | | |  |  |  |  |  |
| 1 | Atr-ERN03128 |  | | | |  |  |  |  |  |
| 1 | Atr-ERN03129 |  | | | |  |  |  |  |  |
| 1 | Atr-ERN03130 |  | | | |  |  |  |  |  |
| 1 | Atr-ERN03131 |  | | | |  |  |  |  |  |
| 1 | Atr-ERN03132 |  | | | |  |  |  |  |  |
| 1 | Atr-ERN03133 |  | | | |  |  |  |  |  |
| 1 | Atr-ERN03134 |  | | | |  |  |  |  |  |
| 1 | Atr-ERN03135 |  | Vvi-Vitvi05g00137\_t001 |  |  |  |  |  |
| 1 | Atr-ERN03136 |  | | | |  |  |  |  |  |
| 1 | Atr-ERN03137 |  | | | |  |  |  |  |  |
| 1 | Atr-ERN03138 |  | | | |  |  |  |  |  |
| 1 | Atr-ERN03139 |  | | | |  |  |  |  |  |
| 1 | Atr-ERN03140 |  | | | |  |  |  |  |  |
| 1 | Atr-ERN03141 |  | Vvi-Vitvi05g00141\_t001 |  |  |  |  |  |
| 1 | Atr-ERN03142 |  | | | |  |  |  |  |  |
| 1 | Atr-ERN03143 |  | Vvi-Vitvi05g00142\_t001 |  |  |  |  |  |
| 0 | Atr-ERN03144 |  |  |  |  |  |  |
| 0 | Atr-ERN03145 |  |  |  |  |  |  |
| 0 | Atr-ERN03146 |  |  |  |  |  |  |
| 0 | Atr-ERN03147 |  |  |  |  |  |  |
| 0 | Atr-ERN03148 |  |  |  |  |  |  |
| 0 | Atr-ERN03149 |  |  |  |  |  |  |
| 0 | Atr-ERN03150 |  |  |  |  |  |  |
| 0 | Atr-ERN03151 |  |  |  |  |  |  |
| 0 | Atr-ERN03152 |  |  |  |  |  |  |
| 0 | Atr-ERN03153 |  |  |  |  |  |  |
| 0 | Atr-ERN03154 |  |  |  |  |  |  |
| 0 | Atr-ERN03155 |  |  |  |  |  |  |
| 0 | Atr-ERN03156 |  |  |  |  |  |  |
| 0 | Atr-ERN03157 |  |  |  |  |  |  |
| 0 | Atr-ERN03158 |  |  |  |  |  |  |
| 0 | Atr-ERN03159 |  |  |  |  |  |  |
| 0 | Atr-ERN03160 |  |  |  |  |  |  |
| 1 | Atr-ERN03161 |  | Vvi-Vitvi07g00350\_t001 |  |  |  |  |  |
| 1 | Atr-ERN03162 |  | | | |  |  |  |  |  |
| 1 | Atr-ERN03163 |  | | | |  |  |  |  |  |
| 1 | Atr-ERN03164 |  | | | |  |  |  |  |  |
| 1 | Atr-ERN03165 |  | | | |  |  |  |  |  |
| 2 | Atr-ERN03166 |  | | | |  | Vvi-Vitvi05g00044\_t001 |  |  |  |  |
| 2 | Atr-ERN03167 |  | | | |  | | | |  |  |  |  |
| 2 | Atr-ERN03168 |  | | | |  | | | |  |  |  |  |
| 2 | Atr-ERN03169 |  | | | |  | | | |  |  |  |  |
| 2 | Atr-ERN03170 |  | | | |  | | | |  |  |  |  |
| 2 | Atr-ERN03171 |  | | | |  | | | |  |  |  |  |
| 2 | Atr-ERN03172 |  | | | |  | | | |  |  |  |  |
| 2 | Atr-ERN03173 |  | | | |  | | | |  |  |  |  |
| 2 | Atr-ERN03174 |  | Vvi-Vitvi07g00355\_t001 |  | | | |  |  |  |  |
| 2 | Atr-ERN03175 |  | | | |  | | | |  |  |  |  |
| 2 | Atr-ERN03176 |  | | | |  | | | |  |  |  |  |
| 2 | Atr-ERN03177 |  | | | |  | | | |  |  |  |  |
| 2 | Atr-ERN03178 |  | Vvi-Vitvi07g02208\_t001 |  | | | |  |  |  |  |
| 2 | Atr-ERN03179 |  | Vvi-Vitvi07g00357\_t001 |  | Vvi-Vitvi05g01724\_t001 |  |  |  |  |
| 2 | Atr-ERN03180 |  | | | |  | | | |  |  |  |  |
| 2 | Atr-ERN03181 |  | | | |  | | | |  |  |  |  |
| 2 | Atr-ERN03182 |  | | | |  | | | |  |  |  |  |
| 2 | Atr-ERN03183 |  | | | |  | | | |  |  |  |  |
| 2 | Atr-ERN03184 |  | | | |  | | | |  |  |  |  |
| 2 | Atr-ERN03185 |  | Vvi-Vitvi07g00358\_t001 |  | | | |  |  |  |  |
| 2 | Atr-ERN03186 |  | | | |  | Vvi-Vitvi05g00060\_t001 |  |  |  |  |
| 2 | Atr-ERN03187 |  | Vvi-Vitvi07g00363\_t001 |  | Vvi-Vitvi05g00061\_t001 |  |  |  |  |
| 2 | Atr-ERN03188 |  | | | |  | | | |  |  |  |  |
| 2 | Atr-ERN03189 |  | | | |  | | | |  |  |  |  |
| 2 | Atr-ERN03190 |  | | | |  | | | |  |  |  |  |
| 2 | Atr-ERN03191 |  | | | |  | | | |  |  |  |  |
| 2 | Atr-ERN03192 |  | | | |  | | | |  |  |  |  |
| 2 | Atr-ERN03193 |  | | | |  | | | |  |  |  |  |
| 2 | Atr-ERN03194 |  | | | |  | Vvi-Vitvi05g00063\_t006 |  |  |  |  |
| 2 | Atr-ERN03195 |  | | | |  | Vvi-Vitvi05g00064\_t001 |  |  |  |  |
| 2 | Atr-ERN03196 |  | Vvi-Vitvi07g00365\_t001 |  | Vvi-Vitvi05g00065\_t001 |  |  |  |  |
| 0 | Atr-ERN03197 |  |  |  |  |  |  |
| 0 | Atr-ERN03198 |  |  |  |  |  |  |
| 0 | Atr-ERN03199 |  |  |  |  |  |  |
| 0 | Atr-ERN03200 |  |  |  |  |  |  |
| 0 | Atr-ERN03201 |  |  |  |  |  |  |
| 0 | Atr-ERN03202 |  |  |  |  |  |  |
| 0 | Atr-ERN03203 |  |  |  |  |  |  |
| 0 | Atr-ERN03204 |  |  |  |  |  |  |
| 0 | Atr-ERN03205 |  |  |  |  |  |  |
| 0 | Atr-ERN03206 |  |  |  |  |  |  |
| 0 | Atr-ERN03207 |  |  |  |  |  |  |
| 0 | Atr-ERN03208 |  |  |  |  |  |  |
| 0 | Atr-ERN03209 |  |  |  |  |  |  |
| 0 | Atr-ERN03210 |  |  |  |  |  |  |
| 0 | Atr-ERN03211 |  |  |  |  |  |  |
| 0 | Atr-ERN03212 |  |  |  |  |  |  |
| 0 | Atr-ERN03213 |  |  |  |  |  |  |
| 0 | Atr-ERN03214 |  |  |  |  |  |  |
| 0 | Atr-ERN03215 |  |  |  |  |  |  |
| 0 | Atr-ERN03216 |  |  |  |  |  |  |
| 0 | Atr-ERN03217 |  |  |  |  |  |  |
| 1 | Atr-ERN03218 |  | Vvi-Vitvi05g00254\_t001 |  |  |  |  |  |
| 1 | Atr-ERN03219 |  | | | |  |  |  |  |  |
| 1 | Atr-ERN03220 |  | | | |  |  |  |  |  |
| 1 | Atr-ERN03221 |  | | | |  |  |  |  |  |
| 1 | Atr-ERN03222 |  | | | |  |  |  |  |  |
| 1 | Atr-ERN03223 |  | | | |  |  |  |  |  |
| 1 | Atr-ERN03224 |  | | | |  |  |  |  |  |
| 1 | Atr-ERN03225 |  | | | |  |  |  |  |  |
| 1 | Atr-ERN03226 |  | | | |  |  |  |  |  |
| 1 | Atr-ERN03227 |  | | | |  |  |  |  |  |
| 1 | Atr-ERN03228 |  | | | |  |  |  |  |  |
| 1 | Atr-ERN03229 |  | | | |  |  |  |  |  |
| 1 | Atr-ERN03230 |  | | | |  |  |  |  |  |
| 1 | Atr-ERN03231 |  | | | |  |  |  |  |  |
| 1 | Atr-ERN03232 |  | | | |  |  |  |  |  |
| 1 | Atr-ERN03233 |  | | | |  |  |  |  |  |
| 1 | Atr-ERN03234 |  | | | |  |  |  |  |  |
| 1 | Atr-ERN03235 |  | | | |  |  |  |  |  |
| 1 | Atr-ERN03236 |  | | | |  |  |  |  |  |
| 1 | Atr-ERN03237 |  | Vvi-Vitvi05g04070\_t001 |  |  |  |  |  |
| 1 | Atr-ERN03238 |  | | | |  |  |  |  |  |
| 1 | Atr-ERN03239 |  | | | |  |  |  |  |  |
| 2 | Atr-ERN03240 |  | | | |  | Vvi-Vitvi07g00485\_t001 |  |  |  |  |
| 2 | Atr-ERN03241 |  | | | |  | | | |  |  |  |  |
| 2 | Atr-ERN03242 |  | | | |  | | | |  |  |  |  |
| 2 | Atr-ERN03243 |  | | | |  | Vvi-Vitvi07g02245\_t001 |  |  |  |  |
| 2 | Atr-ERN03244 |  | | | |  | | | |  |  |  |  |
| 2 | Atr-ERN03245 |  | | | |  | | | |  |  |  |  |
| 2 | Atr-ERN03246 |  | | | |  | | | |  |  |  |  |
| 2 | Atr-ERN03247 |  | | | |  | | | |  |  |  |  |
| 2 | Atr-ERN03248 |  | | | |  | | | |  |  |  |  |
| 2 | Atr-ERN03249 |  | | | |  | | | |  |  |  |  |
| 2 | Atr-ERN03250 |  | | | |  | Vvi-Vitvi07g00475\_t001 |  |  |  |  |
| 2 | Atr-ERN03251 |  | Vvi-Vitvi05g04059\_t001 |  | | | |  |  |  |  |
| 2 | Atr-ERN03252 |  | | | |  | | | |  |  |  |  |
| 3 | Atr-ERN03253 |  | | | |  | | | |  | Vvi-Vitvi14g00052\_t003 |  |  |  |
| 3 | Atr-ERN03254 |  | Vvi-Vitvi05g00208\_t001 |  | Vvi-Vitvi07g00474\_t001 |  | | | |  |  |  |
| 3 | Atr-ERN03255 |  | | | |  | | | |  | | | |  |  |  |
| 3 | Atr-ERN03256 |  | Vvi-Vitvi05g00209\_t001 |  | | | |  | | | |  |  |  |
| 3 | Atr-ERN03257 |  | | | |  | Vvi-Vitvi07g00472\_t002 |  | Vvi-Vitvi14g00053\_t003 |  |  |  |
| 3 | Atr-ERN03258 |  | Vvi-Vitvi05g00212\_t001 |  | Vvi-Vitvi07g00471\_t001 |  | | | |  |  |  |
| 3 | Atr-ERN03259 |  | | | |  | | | |  | | | |  |  |  |
| 3 | Atr-ERN03260 |  | | | |  | | | |  | Vvi-Vitvi14g02452\_t001 |  |  |  |
| 3 | Atr-ERN03261 |  | | | |  | | | |  | | | |  |  |  |
| 3 | Atr-ERN03262 |  | | | |  | | | |  | | | |  |  |  |
| 3 | Atr-ERN03263 |  | | | |  | | | |  | | | |  |  |  |
| 3 | Atr-ERN03264 |  | | | |  | | | |  | Vvi-Vitvi14g04006\_t001 |  |  |  |
| 3 | Atr-ERN03265 |  | | | |  | | | |  | | | |  |  |  |
| 3 | Atr-ERN03266 |  | | | |  | | | |  | | | |  |  |  |
| 3 | Atr-ERN03267 |  | | | |  | | | |  | Vvi-Vitvi14g00056\_t001 |  |  |  |
| 3 | Atr-ERN03268 |  | | | |  | | | |  | Vvi-Vitvi14g00057\_t002 |  |  |  |
| 3 | Atr-ERN03269 |  | Vvi-Vitvi05g00214\_t001 |  | Vvi-Vitvi07g00470\_t001 |  | | | |  |  |  |
| 3 | Atr-ERN03270 |  | Vvi-Vitvi05g00215\_t001 |  | | | |  | | | |  |  |  |
| 3 | Atr-ERN03271 |  | | | |  | Vvi-Vitvi07g04113\_t001 |  | Vvi-Vitvi14g00059\_t001 |  |  |  |
| 3 | Atr-ERN03272 |  | | | |  | Vvi-Vitvi07g00465\_t001 |  | | | |  |  |  |
| 3 | Atr-ERN03273 |  | Vvi-Vitvi05g00218\_t001 |  | | | |  | | | |  |  |  |
| 3 | Atr-ERN03274 |  | | | |  | | | |  | | | |  |  |  |
| 3 | Atr-ERN03275 |  | Vvi-Vitvi05g04047\_t001 |  | Vvi-Vitvi07g00464\_t001 |  | Vvi-Vitvi14g04009\_t001 |  |  |  |
| 2 | Atr-ERN03276 |  |  |  | Vvi-Vitvi07g00463\_t001 |  | | | |  |  |  |
| 2 | Atr-ERN03277 |  |  |  | | | |  | Vvi-Vitvi14g00062\_t001 |  |  |  |
| 3 | Atr-ERN03278 |  | Vvi-Vitvi05g04031\_t001 |  | | | |  | | | |  |  |  |
| 3 | Atr-ERN03279 |  | Vvi-Vitvi05g00170\_t001 |  | Vvi-Vitvi07g00457\_t001 |  | Vvi-Vitvi14g02457\_t001 |  |  |  |
| 3 | Atr-ERN03280 |  | | | |  | | | |  | Vvi-Vitvi14g00066\_t001 |  |  |  |
| 3 | Atr-ERN03281 |  | Vvi-Vitvi05g00169\_t001 |  | | | |  | | | |  |  |  |
| 3 | Atr-ERN03282 |  | Vvi-Vitvi05g00168\_t001 |  | | | |  | | | |  |  |  |
| 3 | Atr-ERN03283 |  | | | |  | Vvi-Vitvi07g00456\_t001 |  | Vvi-Vitvi14g00067\_t001 |  |  |  |
| 3 | Atr-ERN03284 |  | | | |  | | | |  | Vvi-Vitvi14g00068\_t001 |  |  |  |
| 3 | Atr-ERN03285 |  | Vvi-Vitvi05g00167\_t001 |  | | | |  | | | |  |  |  |
| 3 | Atr-ERN03286 |  | | | |  | | | |  | | | |  |  |  |
| 3 | Atr-ERN03287 |  | | | |  | | | |  | | | |  |  |  |
| 3 | Atr-ERN03288 |  | Vvi-Vitvi05g00166\_t001 |  | Vvi-Vitvi07g00455\_t001 |  | | | |  |  |  |
| 3 | Atr-ERN03289 |  | | | |  | | | |  | | | |  |  |  |
| 3 | Atr-ERN03290 |  | Vvi-Vitvi05g00164\_t001 |  | | | |  | Vvi-Vitvi14g00070\_t001 |  |  |  |
| 3 | Atr-ERN03291 |  | | | |  | | | |  | | | |  |  |  |
| 3 | Atr-ERN03292 |  | Vvi-Vitvi05g00163\_t001 |  | Vvi-Vitvi07g00453\_t001 |  | | | |  |  |  |
| 3 | Atr-ERN03293 |  | | | |  | | | |  | Vvi-Vitvi14g00071\_t001 |  |  |  |
| 3 | Atr-ERN03294 |  | Vvi-Vitvi05g00161\_t001 |  | | | |  | | | |  |  |  |
| 3 | Atr-ERN03295 |  | | | |  | | | |  | | | |  |  |  |
| 3 | Atr-ERN03296 |  | | | |  | Vvi-Vitvi07g00450\_t001 |  | | | |  |  |  |
| 4 | Atr-ERN03297 |  | | | |  | | | |  | | | |  | Vvi-Vitvi05g01776\_t001 |  |  |
| 4 | Atr-ERN03298 |  | | | |  | | | |  | | | |  | | | |  |  |
| 4 | Atr-ERN03299 |  | | | |  | | | |  | | | |  | | | |  |  |
| 4 | Atr-ERN03300 |  | | | |  | | | |  | | | |  | | | |  |  |
| 4 | Atr-ERN03301 |  | | | |  | | | |  | | | |  | Vvi-Vitvi05g00143\_t001 |  |  |
| 5 | Atr-ERN03302 |  | | | |  | | | |  | | | |  | Vvi-Vitvi05g01783\_t001 |  | Vvi-Vitvi07g02230\_t001 |  |
| 5 | Atr-ERN03303 |  | | | |  | | | |  | | | |  | Vvi-Vitvi05g00144\_t001 |  | | | |  |
| 5 | Atr-ERN03304 |  | | | |  | | | |  | | | |  | Vvi-Vitvi05g00145\_t001 |  | Vvi-Vitvi07g00434\_t001 |  |
| 5 | Atr-ERN03305 |  | | | |  | | | |  | | | |  | | | |  | | | |  |
| 5 | Atr-ERN03306 |  | | | |  | | | |  | | | |  | | | |  | Vvi-Vitvi07g00437\_t001 |  |
| 5 | Atr-ERN03307 |  | | | |  | | | |  | | | |  | | | |  | | | |  |
| 5 | Atr-ERN03308 |  | | | |  | | | |  | Vvi-Vitvi14g00079\_t001 |  | | | |  | | | |  |
| 5 | Atr-ERN03309 |  | | | |  | Vvi-Vitvi07g00439\_t001 |  | Vvi-Vitvi14g02465\_t001 |  | Vvi-Vitvi05g00150\_t001 |  | Vvi-Vitvi07g00439\_t001 |  |
| 4 | Atr-ERN03310 |  | | | |  | | | |  |  |  | | | |  | | | |  |
| 4 | Atr-ERN03311 |  | | | |  | | | |  |  |  | | | |  | | | |  |
| 4 | Atr-ERN03312 |  | Vvi-Vitvi05g00149\_t001 |  | | | |  |  |  | | | |  | | | |  |
| 4 | Atr-ERN03313 |  | | | |  | | | |  |  |  | | | |  | | | |  |
| 4 | Atr-ERN03314 |  | | | |  | | | |  |  |  | | | |  | | | |  |
| 4 | Atr-ERN03315 |  | Vvi-Vitvi05g01784\_t001 |  | | | |  |  |  | | | |  | | | |  |
| 3 | Atr-ERN03316 |  |  |  | Vvi-Vitvi07g00436\_t001 |  |  |  | | | |  | | | |  |
| 2 | Atr-ERN03317 |  |  |  |  |  |  |  | | | |  | Vvi-Vitvi07g00441\_t001 |  |
| 2 | Atr-ERN03318 |  |  |  |  |  |  |  | | | |  | | | |  |
| 2 | Atr-ERN03319 |  |  |  |  |  |  |  | | | |  | | | |  |
| 2 | Atr-ERN03320 |  |  |  |  |  |  |  | Vvi-Vitvi05g00153\_t001 |  | | | |  |
| 1 | Atr-ERN03321 |  |  |  |  |  |  |  |  |  | | | |  |
| 1 | Atr-ERN03322 |  |  |  |  |  |  |  |  |  | | | |  |
| 1 | Atr-ERN03323 |  |  |  |  |  |  |  |  |  | | | |  |
| 1 | Atr-ERN03324 |  |  |  |  |  |  |  |  |  | | | |  |
| 1 | Atr-ERN03325 |  |  |  |  |  |  |  |  |  | Vvi-Vitvi07g00458\_t001 |  |
| 0 | Atr-ERN03326 |  |  |  |  |  |  |
| 0 | Atr-ERN03327 |  |  |  |  |  |  |
| 0 | Atr-ERN03328 |  |  |  |  |  |  |
| 0 | Atr-ERN03329 |  |  |  |  |  |  |
| 0 | Atr-ERN03330 |  |  |  |  |  |  |
| 0 | Atr-ERN03331 |  |  |  |  |  |  |
| 0 | Atr-ERN03332 |  |  |  |  |  |  |
| 0 | Atr-ERN03333 |  |  |  |  |  |  |
| 0 | Atr-ERN03334 |  |  |  |  |  |  |
| 0 | Atr-ERN03335 |  |  |  |  |  |  |
| 0 | Atr-ERN03336 |  |  |  |  |  |  |
| 0 | Atr-ERN03337 |  |  |  |  |  |  |
| 0 | Atr-ERN03338 |  |  |  |  |  |  |
| 0 | Atr-ERN03339 |  |  |  |  |  |  |
| 0 | Atr-ERN03340 |  |  |  |  |  |  |
| 0 | Atr-ERN03341 |  |  |  |  |  |  |
| 0 | Atr-ERN03342 |  |  |  |  |  |  |
| 0 | Atr-ERN03343 |  |  |  |  |  |  |
| 0 | Atr-ERN03344 |  |  |  |  |  |  |
| 0 | Atr-ERN03345 |  |  |  |  |  |  |
| 1 | Atr-ERN03346 |  | Vvi-Vitvi12g00468\_t001 |  |  |  |  |  |
| 1 | Atr-ERN03347 |  | | | |  |  |  |  |  |
| 1 | Atr-ERN03348 |  | Vvi-Vitvi12g00469\_t001 |  |  |  |  |  |
| 1 | Atr-ERN03349 |  | | | |  |  |  |  |  |
| 3 | Atr-ERN03350 |  | | | |  | Vvi-Vitvi19g00262\_t001 |  | Vvi-Vitvi10g04278\_t001 |  |  |  |
| 3 | Atr-ERN03351 |  | | | |  | | | |  | | | |  |  |  |
| 3 | Atr-ERN03352 |  | | | |  | | | |  | | | |  |  |  |
| 3 | Atr-ERN03353 |  | | | |  | | | |  | | | |  |  |  |
| 3 | Atr-ERN03354 |  | | | |  | | | |  | | | |  |  |  |
| 3 | Atr-ERN03355 |  | Vvi-Vitvi12g00472\_t001 |  | Vvi-Vitvi19g01896\_t001 |  | | | |  |  |  |
| 3 | Atr-ERN03356 |  | | | |  | | | |  | | | |  |  |  |
| 3 | Atr-ERN03357 |  | | | |  | | | |  | | | |  |  |  |
| 3 | Atr-ERN03358 |  | Vvi-Vitvi12g00473\_t001 |  | | | |  | | | |  |  |  |
| 3 | Atr-ERN03359 |  | | | |  | | | |  | | | |  |  |  |
| 3 | Atr-ERN03360 |  | | | |  | | | |  | Vvi-Vitvi10g00436\_t001 |  |  |  |
| 3 | Atr-ERN03361 |  | | | |  | | | |  | | | |  |  |  |
| 3 | Atr-ERN03362 |  | | | |  | | | |  | | | |  |  |  |
| 3 | Atr-ERN03363 |  | | | |  | | | |  | | | |  |  |  |
| 3 | Atr-ERN03364 |  | | | |  | | | |  | | | |  |  |  |
| 3 | Atr-ERN03365 |  | Vvi-Vitvi12g02389\_t001 |  | | | |  | | | |  |  |  |
| 3 | Atr-ERN03366 |  | Vvi-Vitvi12g04142\_t001 |  | | | |  | | | |  |  |  |
| 3 | Atr-ERN03367 |  | | | |  | | | |  | | | |  |  |  |
| 3 | Atr-ERN03368 |  | | | |  | | | |  | | | |  |  |  |
| 3 | Atr-ERN03369 |  | | | |  | | | |  | | | |  |  |  |
| 3 | Atr-ERN03370 |  | | | |  | | | |  | | | |  |  |  |
| 3 | Atr-ERN03371 |  | | | |  | | | |  | | | |  |  |  |
| 3 | Atr-ERN03372 |  | | | |  | | | |  | | | |  |  |  |
| 3 | Atr-ERN03373 |  | | | |  | | | |  | | | |  |  |  |
| 3 | Atr-ERN03374 |  | | | |  | | | |  | | | |  |  |  |
| 3 | Atr-ERN03375 |  | | | |  | | | |  | | | |  |  |  |
| 3 | Atr-ERN03376 |  | | | |  | | | |  | | | |  |  |  |
| 3 | Atr-ERN03377 |  | | | |  | | | |  | | | |  |  |  |
| 3 | Atr-ERN03378 |  | Vvi-Vitvi12g00475\_t001 |  | | | |  | | | |  |  |  |
| 3 | Atr-ERN03379 |  | | | |  | | | |  | | | |  |  |  |
| 3 | Atr-ERN03380 |  | | | |  | Vvi-Vitvi19g00270\_t001 |  | Vvi-Vitvi10g00437\_t001 |  |  |  |
| 3 | Atr-ERN03381 |  | Vvi-Vitvi12g02394\_t001 |  | | | |  | | | |  |  |  |
| 3 | Atr-ERN03382 |  | | | |  | | | |  | | | |  |  |  |
| 3 | Atr-ERN03383 |  | | | |  | | | |  | | | |  |  |  |
| 3 | Atr-ERN03384 |  | | | |  | | | |  | | | |  |  |  |
| 3 | Atr-ERN03385 |  | | | |  | | | |  | | | |  |  |  |
| 3 | Atr-ERN03386 |  | | | |  | | | |  | | | |  |  |  |
| 3 | Atr-ERN03387 |  | Vvi-Vitvi12g00478\_t001 |  | Vvi-Vitvi19g00272\_t001 |  | | | |  |  |  |
| 3 | Atr-ERN03388 |  | | | |  | | | |  | | | |  |  |  |
| 3 | Atr-ERN03389 |  | | | |  | | | |  | | | |  |  |  |
| 3 | Atr-ERN03390 |  | | | |  | | | |  | | | |  |  |  |
| 4 | Atr-ERN03391 |  | | | |  | | | |  | Vvi-Vitvi10g04280\_t001 |  | Vvi-Vitvi10g04280\_t001 |  |  |
| 4 | Atr-ERN03392 |  | | | |  | | | |  | | | |  | | | |  |  |
| 4 | Atr-ERN03393 |  | | | |  | | | |  | Vvi-Vitvi10g04281\_t001 |  | Vvi-Vitvi10g01600\_t001 |  |  |
| 4 | Atr-ERN03394 |  | | | |  | | | |  | | | |  | | | |  |  |
| 4 | Atr-ERN03395 |  | | | |  | | | |  | | | |  | Vvi-Vitvi10g00427\_t001 |  |  |
| 4 | Atr-ERN03396 |  | | | |  | | | |  | | | |  | Vvi-Vitvi10g00426\_t001 |  |  |
| 4 | Atr-ERN03397 |  | | | |  | | | |  | | | |  | | | |  |  |
| 4 | Atr-ERN03398 |  | | | |  | Vvi-Vitvi19g04107\_t001 |  | | | |  | | | |  |  |
| 4 | Atr-ERN03399 |  | | | |  | | | |  | | | |  | | | |  |  |
| 4 | Atr-ERN03400 |  | | | |  | Vvi-Vitvi19g00274\_t001 |  | | | |  | | | |  |  |
| 4 | Atr-ERN03401 |  | | | |  | | | |  | | | |  | | | |  |  |
| 4 | Atr-ERN03402 |  | | | |  | | | |  | | | |  | | | |  |  |
| 4 | Atr-ERN03403 |  | | | |  | | | |  | | | |  | | | |  |  |
| 4 | Atr-ERN03404 |  | Vvi-Vitvi12g00480\_t001 |  | | | |  | | | |  | | | |  |  |
| 4 | Atr-ERN03405 |  | | | |  | | | |  | | | |  | Vvi-Vitvi10g00425\_t001 |  |  |
| 4 | Atr-ERN03406 |  | | | |  | | | |  | | | |  | | | |  |  |
| 4 | Atr-ERN03407 |  | | | |  | | | |  | | | |  | | | |  |  |
| 4 | Atr-ERN03408 |  | | | |  | | | |  | Vvi-Vitvi10g04283\_t001 |  | Vvi-Vitvi10g04277\_t001 |  |  |
| 4 | Atr-ERN03409 |  | | | |  | | | |  | Vvi-Vitvi10g04284\_t001 |  | Vvi-Vitvi10g04276\_t001 |  |  |
| 4 | Atr-ERN03410 |  | Vvi-Vitvi12g00481\_t001 |  | | | |  | Vvi-Vitvi10g00420\_t001 |  | Vvi-Vitvi10g00423\_t001 |  |  |
| 3 | Atr-ERN03411 |  | | | |  | | | |  |  |  | | | |  |  |
| 3 | Atr-ERN03412 |  | Vvi-Vitvi12g00482\_t001 |  | | | |  |  |  | Vvi-Vitvi10g00422\_t001 |  |  |
| 3 | Atr-ERN03413 |  | | | |  | | | |  |  |  | | | |  |  |
| 3 | Atr-ERN03414 |  | | | |  | Vvi-Vitvi19g00275\_t001 |  |  |  | | | |  |  |
| 3 | Atr-ERN03415 |  | | | |  | | | |  |  |  | | | |  |  |
| 3 | Atr-ERN03416 |  | | | |  | | | |  |  |  | | | |  |  |
| 3 | Atr-ERN03417 |  | Vvi-Vitvi12g00483\_t003 |  | | | |  |  |  | Vvi-Vitvi10g00417\_t002 |  |  |
| 3 | Atr-ERN03418 |  | | | |  | | | |  |  |  | | | |  |  |
| 3 | Atr-ERN03419 |  | | | |  | | | |  |  |  | | | |  |  |
| 3 | Atr-ERN03420 |  | | | |  | | | |  |  |  | | | |  |  |
| 3 | Atr-ERN03421 |  | | | |  | | | |  |  |  | Vvi-Vitvi10g04272\_t001 |  |  |
| 3 | Atr-ERN03422 |  | | | |  | | | |  |  |  | Vvi-Vitvi10g04271\_t001 |  |  |
| 3 | Atr-ERN03423 |  | Vvi-Vitvi12g00484\_t001 |  | | | |  |  |  | Vvi-Vitvi10g00397\_t001 |  |  |
| 3 | Atr-ERN03424 |  | | | |  | | | |  |  |  | | | |  |  |
| 3 | Atr-ERN03425 |  | Vvi-Vitvi12g00485\_t001 |  | | | |  |  |  | | | |  |  |
| 3 | Atr-ERN03426 |  | | | |  | | | |  |  |  | | | |  |  |
| 3 | Atr-ERN03427 |  | | | |  | | | |  |  |  | Vvi-Vitvi10g01753\_t001 |  |  |
| 3 | Atr-ERN03428 |  | | | |  | Vvi-Vitvi19g00276\_t001 |  |  |  | Vvi-Vitvi10g00398\_t001 |  |  |
| 3 | Atr-ERN03429 |  | | | |  | | | |  |  |  | Vvi-Vitvi10g00399\_t001 |  |  |
| 3 | Atr-ERN03430 |  | Vvi-Vitvi12g00486\_t001 |  | | | |  |  |  | | | |  |  |
| 3 | Atr-ERN03431 |  | Vvi-Vitvi12g00489\_t001 |  | | | |  |  |  | | | |  |  |
| 3 | Atr-ERN03432 |  | | | |  | | | |  |  |  | Vvi-Vitvi10g00401\_t001 |  |  |
| 3 | Atr-ERN03433 |  | | | |  | Vvi-Vitvi19g00280\_t001 |  |  |  | | | |  |  |
| 3 | Atr-ERN03434 |  | | | |  | | | |  |  |  | | | |  |  |
| 3 | Atr-ERN03435 |  | | | |  | | | |  |  |  | | | |  |  |
| 3 | Atr-ERN03436 |  | | | |  | Vvi-Vitvi19g00282\_t001 |  |  |  | Vvi-Vitvi10g00406\_t001 |  |  |
| 3 | Atr-ERN03437 |  | | | |  | | | |  |  |  | | | |  |  |
| 3 | Atr-ERN03438 |  | | | |  | | | |  |  |  | Vvi-Vitvi10g01757\_t001 |  |  |
| 3 | Atr-ERN03439 |  | | | |  | | | |  |  |  | | | |  |  |
| 3 | Atr-ERN03440 |  | | | |  | Vvi-Vitvi19g01899\_t001 |  |  |  | | | |  |  |
| 3 | Atr-ERN03441 |  | Vvi-Vitvi12g00490\_t001 |  | Vvi-Vitvi19g01900\_t001 |  |  |  | | | |  |  |
| 3 | Atr-ERN03442 |  | | | |  | Vvi-Vitvi19g00284\_t001 |  |  |  | | | |  |  |
| 3 | Atr-ERN03443 |  | | | |  | | | |  |  |  | | | |  |  |
| 3 | Atr-ERN03444 |  | Vvi-Vitvi12g00491\_t001 |  | | | |  |  |  | | | |  |  |
| 3 | Atr-ERN03445 |  | Vvi-Vitvi12g00493\_t002 |  | | | |  |  |  | | | |  |  |
| 3 | Atr-ERN03446 |  | | | |  | Vvi-Vitvi19g00286\_t001 |  |  |  | Vvi-Vitvi10g00394\_t001 |  |  |
| 3 | Atr-ERN03447 |  | | | |  | Vvi-Vitvi19g01901\_t001.2.6037826c |  |  |  | | | |  |  |
| 3 | Atr-ERN03448 |  | | | |  | | | |  |  |  | | | |  |  |
| 3 | Atr-ERN03449 |  | | | |  | | | |  |  |  | | | |  |  |
| 3 | Atr-ERN03450 |  | | | |  | | | |  |  |  | | | |  |  |
| 3 | Atr-ERN03451 |  | | | |  | | | |  |  |  | | | |  |  |
| 3 | Atr-ERN03452 |  | Vvi-Vitvi12g02402\_t001 |  | Vvi-Vitvi19g01902\_t001 |  |  |  | | | |  |  |
| 3 | Atr-ERN03453 |  | | | |  | | | |  |  |  | | | |  |  |
| 3 | Atr-ERN03454 |  | | | |  | | | |  |  |  | | | |  |  |
| 3 | Atr-ERN03455 |  | | | |  | | | |  |  |  | | | |  |  |
| 3 | Atr-ERN03456 |  | Vvi-Vitvi12g00501\_t001 |  | | | |  |  |  | | | |  |  |
| 3 | Atr-ERN03457 |  | | | |  | | | |  |  |  | | | |  |  |
| 3 | Atr-ERN03458 |  | | | |  | | | |  |  |  | Vvi-Vitvi10g04259\_t002 |  |  |
| 3 | Atr-ERN03459 |  | | | |  | | | |  |  |  | | | |  |  |
| 3 | Atr-ERN03460 |  | | | |  | | | |  |  |  | Vvi-Vitvi10g00383\_t001 |  |  |
| 3 | Atr-ERN03461 |  | | | |  | | | |  |  |  | | | |  |  |
| 3 | Atr-ERN03462 |  | | | |  | | | |  |  |  | | | |  |  |
| 3 | Atr-ERN03463 |  | | | |  | | | |  |  |  | | | |  |  |
| 3 | Atr-ERN03464 |  | | | |  | | | |  |  |  | | | |  |  |
| 3 | Atr-ERN03465 |  | | | |  | | | |  |  |  | | | |  |  |
| 3 | Atr-ERN03466 |  | | | |  | | | |  |  |  | | | |  |  |
| 3 | Atr-ERN03467 |  | | | |  | | | |  |  |  | | | |  |  |
| 3 | Atr-ERN03468 |  | | | |  | | | |  |  |  | | | |  |  |
| 3 | Atr-ERN03469 |  | | | |  | | | |  |  |  | | | |  |  |
| 3 | Atr-ERN03470 |  | | | |  | | | |  |  |  | | | |  |  |
| 3 | Atr-ERN03471 |  | | | |  | | | |  |  |  | | | |  |  |
| 3 | Atr-ERN03472 |  | | | |  | | | |  |  |  | | | |  |  |
| 3 | Atr-ERN03473 |  | | | |  | | | |  |  |  | | | |  |  |
| 3 | Atr-ERN03474 |  | | | |  | | | |  |  |  | | | |  |  |
| 3 | Atr-ERN03475 |  | | | |  | | | |  |  |  | | | |  |  |
| 3 | Atr-ERN03476 |  | | | |  | | | |  |  |  | | | |  |  |
| 3 | Atr-ERN03477 |  | | | |  | | | |  |  |  | | | |  |  |
| 3 | Atr-ERN03478 |  | | | |  | Vvi-Vitvi19g00290\_t001 |  |  |  | | | |  |  |
| 3 | Atr-ERN03479 |  | | | |  | | | |  |  |  | Vvi-Vitvi10g00365\_t001 |  |  |
| 3 | Atr-ERN03480 |  | Vvi-Vitvi12g00502\_t001 |  | | | |  |  |  | | | |  |  |
| 2 | Atr-ERN03481 |  |  |  | | | |  |  |  | | | |  |  |
| 2 | Atr-ERN03482 |  |  |  | | | |  |  |  | | | |  |  |
| 2 | Atr-ERN03483 |  |  |  | | | |  |  |  | | | |  |  |
| 2 | Atr-ERN03484 |  |  |  | | | |  |  |  | Vvi-Vitvi10g01748\_t001 |  |  |
| 2 | Atr-ERN03485 |  |  |  | | | |  |  |  | | | |  |  |
| 2 | Atr-ERN03486 |  |  |  | | | |  |  |  | Vvi-Vitvi10g00363\_t001 |  |  |
| 2 | Atr-ERN03487 |  |  |  | | | |  |  |  | | | |  |  |
| 2 | Atr-ERN03488 |  |  |  | | | |  |  |  | Vvi-Vitvi10g01746\_t001 |  |  |
| 2 | Atr-ERN03489 |  |  |  | | | |  |  |  | | | |  |  |
| 2 | Atr-ERN03490 |  |  |  | | | |  |  |  | | | |  |  |
| 2 | Atr-ERN03491 |  |  |  | | | |  |  |  | | | |  |  |
| 2 | Atr-ERN03492 |  |  |  | Vvi-Vitvi19g01910\_t001 |  |  |  | | | |  |  |
| 2 | Atr-ERN03493 |  |  |  | | | |  |  |  | | | |  |  |
| 2 | Atr-ERN03494 |  |  |  | | | |  |  |  | | | |  |  |
| 3 | Atr-ERN03495 |  | Vvi-Vitvi12g00643\_t001 |  | | | |  |  |  | | | |  |  |
| 3 | Atr-ERN03496 |  | | | |  | | | |  |  |  | | | |  |  |
| 3 | Atr-ERN03497 |  | | | |  | | | |  |  |  | | | |  |  |
| 3 | Atr-ERN03498 |  | | | |  | | | |  |  |  | | | |  |  |
| 3 | Atr-ERN03499 |  | Vvi-Vitvi12g00641\_t001 |  | | | |  |  |  | Vvi-Vitvi10g04257\_t001 |  |  |
| 3 | Atr-ERN03500 |  | | | |  | | | |  |  |  | | | |  |  |
| 3 | Atr-ERN03501 |  | | | |  | | | |  |  |  | | | |  |  |
| 3 | Atr-ERN03502 |  | | | |  | | | |  |  |  | | | |  |  |
| 3 | Atr-ERN03503 |  | | | |  | Vvi-Vitvi19g00300\_t001 |  |  |  | | | |  |  |
| 3 | Atr-ERN03504 |  | | | |  | | | |  |  |  | Vvi-Vitvi10g01740\_t001 |  |  |
| 3 | Atr-ERN03505 |  | | | |  | Vvi-Vitvi19g00302\_t001 |  |  |  | | | |  |  |
| 3 | Atr-ERN03506 |  | | | |  | | | |  |  |  | | | |  |  |
| 3 | Atr-ERN03507 |  | | | |  | | | |  |  |  | Vvi-Vitvi10g00359\_t001 |  |  |
| 3 | Atr-ERN03508 |  | | | |  | | | |  |  |  | | | |  |  |
| 3 | Atr-ERN03509 |  | | | |  | | | |  |  |  | | | |  |  |
| 3 | Atr-ERN03510 |  | | | |  | | | |  |  |  | | | |  |  |
| 3 | Atr-ERN03511 |  | Vvi-Vitvi12g00640\_t001 |  | | | |  |  |  | | | |  |  |
| 3 | Atr-ERN03512 |  | | | |  | | | |  |  |  | | | |  |  |
| 3 | Atr-ERN03513 |  | | | |  | | | |  |  |  | | | |  |  |
| 3 | Atr-ERN03514 |  | | | |  | | | |  |  |  | | | |  |  |
| 3 | Atr-ERN03515 |  | | | |  | | | |  |  |  | | | |  |  |
| 3 | Atr-ERN03516 |  | | | |  | | | |  |  |  | | | |  |  |
| 3 | Atr-ERN03517 |  | | | |  | | | |  |  |  | | | |  |  |
| 3 | Atr-ERN03518 |  | | | |  | | | |  |  |  | | | |  |  |
| 3 | Atr-ERN03519 |  | | | |  | | | |  |  |  | | | |  |  |
| 3 | Atr-ERN03520 |  | | | |  | | | |  |  |  | | | |  |  |
| 3 | Atr-ERN03521 |  | | | |  | Vvi-Vitvi19g00303\_t001 |  |  |  | | | |  |  |
| 3 | Atr-ERN03522 |  | | | |  | | | |  |  |  | | | |  |  |
| 3 | Atr-ERN03523 |  | Vvi-Vitvi12g00638\_t001 |  | Vvi-Vitvi19g00304\_t001 |  |  |  | Vvi-Vitvi10g04245\_t001 |  |  |
| 3 | Atr-ERN03524 |  | | | |  | | | |  |  |  | | | |  |  |
| 3 | Atr-ERN03525 |  | | | |  | | | |  |  |  | | | |  |  |
| 3 | Atr-ERN03526 |  | | | |  | | | |  |  |  | | | |  |  |
| 3 | Atr-ERN03527 |  | | | |  | | | |  |  |  | | | |  |  |
| 3 | Atr-ERN03528 |  | Vvi-Vitvi12g02457\_t001 |  | | | |  |  |  | | | |  |  |
| 3 | Atr-ERN03529 |  | | | |  | | | |  |  |  | | | |  |  |
| 3 | Atr-ERN03530 |  | Vvi-Vitvi12g00634\_t001 |  | | | |  |  |  | | | |  |  |
| 3 | Atr-ERN03531 |  | Vvi-Vitvi12g00633\_t003 |  | Vvi-Vitvi19g00305\_t001 |  |  |  | | | |  |  |
| 3 | Atr-ERN03532 |  | | | |  | | | |  |  |  | | | |  |  |
| 3 | Atr-ERN03533 |  | | | |  | | | |  |  |  | | | |  |  |
| 3 | Atr-ERN03534 |  | | | |  | | | |  |  |  | | | |  |  |
| 3 | Atr-ERN03535 |  | | | |  | | | |  |  |  | | | |  |  |
| 3 | Atr-ERN03536 |  | Vvi-Vitvi12g00632\_t001 |  | Vvi-Vitvi19g00306\_t001 |  |  |  | Vvi-Vitvi10g00345\_t001 |  |  |
| 1 | Atr-ERN03537 |  | | | |  |  |  |  |  |
| 1 | Atr-ERN03538 |  | | | |  |  |  |  |  |
| 1 | Atr-ERN03539 |  | | | |  |  |  |  |  |
| 1 | Atr-ERN03540 |  | | | |  |  |  |  |  |
| 1 | Atr-ERN03541 |  | | | |  |  |  |  |  |
| 1 | Atr-ERN03542 |  | Vvi-Vitvi12g00631\_t001 |  |  |  |  |  |
| 0 | Atr-ERN03543 |  |  |  |  |  |  |
| 0 | Atr-ERN03544 |  |  |  |  |  |  |
| 0 | Atr-ERN03545 |  |  |  |  |  |  |
| 0 | Atr-ERN03546 |  |  |  |  |  |  |
| 0 | Atr-ERN03547 |  |  |  |  |  |  |
| 0 | Atr-ERN03548 |  |  |  |  |  |  |
