# Supplementary material for: Genome-wide identification, characterization and gene expression of BES1 transcription factor family in grapevine (Vitis vinifera L.)
Source: Sci Rep. 2023 Jan 5;13:240. doi: 10.1038/s41598-022-24407-y (PMC9816167; doi:10.1038/s41598-022-24407-y)
Supplement: Supplementary file 3 — Supplementary Information. [file 41598_2022_24407_MOESM3_ESM.zip › Vvi_Atr/Vitis_vinifera.PN40024.v4.dna_sm.toplevel.fa.vs.Amborella_trichopoda.AMTR1.0.dna_sm.toplevel.fa.html/Atr-AmTr_v1.0_scaffold00004.html]

|  |  |  |  |  |  |  |  |  |  |  |  |  |  |
| --- | --- | --- | --- | --- | --- | --- | --- | --- | --- | --- | --- | --- | --- |
| Duplication depth | Reference chromosome | Collinear blocks | | | | | | | | | | | |
| 1 | Atr-ERM93485 |  | Vvi-Vitvi05g04122\_t001 |  |  |  |  |  |
| 1 | Atr-ERM93486 |  | Vvi-Vitvi05g00521\_t001 |  |  |  |  |  |
| 1 | Atr-ERM93487 |  | Vvi-Vitvi05g00520\_t001 |  |  |  |  |  |
| 1 | Atr-ERM93488 |  | Vvi-Vitvi05g00519\_t001 |  |  |  |  |  |
| 1 | Atr-ERM93489 |  | | | |  |  |  |  |  |
| 1 | Atr-ERM93490 |  | | | |  |  |  |  |  |
| 1 | Atr-ERM93491 |  | Vvi-Vitvi05g01891\_t001 |  |  |  |  |  |
| 1 | Atr-ERM93492 |  | | | |  |  |  |  |  |
| 1 | Atr-ERM93493 |  | | | |  |  |  |  |  |
| 1 | Atr-ERM93494 |  | | | |  |  |  |  |  |
| 1 | Atr-ERM93495 |  | Vvi-Vitvi05g00517\_t001 |  |  |  |  |  |
| 1 | Atr-ERM93496 |  | Vvi-Vitvi05g04120\_t001 |  |  |  |  |  |
| 1 | Atr-ERM93497 |  | Vvi-Vitvi05g01890\_t001 |  |  |  |  |  |
| 1 | Atr-ERM93498 |  | | | |  |  |  |  |  |
| 1 | Atr-ERM93499 |  | | | |  |  |  |  |  |
| 1 | Atr-ERM93500 |  | | | |  |  |  |  |  |
| 1 | Atr-ERM93501 |  | | | |  |  |  |  |  |
| 1 | Atr-ERM93502 |  | | | |  |  |  |  |  |
| 1 | Atr-ERM93503 |  | | | |  |  |  |  |  |
| 1 | Atr-ERM93504 |  | | | |  |  |  |  |  |
| 1 | Atr-ERM93505 |  | | | |  |  |  |  |  |
| 1 | Atr-ERM93506 |  | Vvi-Vitvi05g00510\_t001 |  |  |  |  |  |
| 1 | Atr-ERM93507 |  | | | |  |  |  |  |  |
| 1 | Atr-ERM93508 |  | | | |  |  |  |  |  |
| 1 | Atr-ERM93509 |  | | | |  |  |  |  |  |
| 1 | Atr-ERM93510 |  | | | |  |  |  |  |  |
| 1 | Atr-ERM93511 |  | Vvi-Vitvi05g00505\_t001 |  |  |  |  |  |
| 1 | Atr-ERM93512 |  | Vvi-Vitvi05g00503\_t001 |  |  |  |  |  |
| 1 | Atr-ERM93513 |  | | | |  |  |  |  |  |
| 1 | Atr-ERM93514 |  | | | |  |  |  |  |  |
| 1 | Atr-ERM93515 |  | Vvi-Vitvi05g00497\_t001 |  |  |  |  |  |
| 1 | Atr-ERM93516 |  | | | |  |  |  |  |  |
| 1 | Atr-ERM93517 |  | | | |  |  |  |  |  |
| 1 | Atr-ERM93518 |  | | | |  |  |  |  |  |
| 1 | Atr-ERM93519 |  | Vvi-Vitvi05g00496\_t001 |  |  |  |  |  |
| 0 | Atr-ERM93520 |  |  |  |  |  |  |
| 0 | Atr-ERM93521 |  |  |  |  |  |  |
| 0 | Atr-ERM93522 |  |  |  |  |  |  |
| 0 | Atr-ERM93523 |  |  |  |  |  |  |
| 1 | Atr-ERM93524 |  | Vvi-Vitvi08g01812\_t002 |  |  |  |  |  |
| 1 | Atr-ERM93525 |  | | | |  |  |  |  |  |
| 1 | Atr-ERM93526 |  | Vvi-Vitvi08g01813\_t001 |  |  |  |  |  |
| 2 | Atr-ERM93527 |  | | | |  | Vvi-Vitvi06g01581\_t001 |  |  |  |  |
| 2 | Atr-ERM93528 |  | | | |  | | | |  |  |  |  |
| 2 | Atr-ERM93529 |  | | | |  | | | |  |  |  |  |
| 2 | Atr-ERM93530 |  | | | |  | Vvi-Vitvi06g00035\_t001 |  |  |  |  |
| 2 | Atr-ERM93531 |  | Vvi-Vitvi08g01815\_t001 |  | | | |  |  |  |  |
| 2 | Atr-ERM93532 |  | | | |  | Vvi-Vitvi06g04007\_t001 |  |  |  |  |
| 2 | Atr-ERM93533 |  | Vvi-Vitvi08g01816\_t001 |  | Vvi-Vitvi06g01575\_t001 |  |  |  |  |
| 2 | Atr-ERM93534 |  | | | |  | | | |  |  |  |  |
| 2 | Atr-ERM93535 |  | | | |  | Vvi-Vitvi06g01574\_t001 |  |  |  |  |
| 2 | Atr-ERM93536 |  | | | |  | Vvi-Vitvi06g00031\_t001 |  |  |  |  |
| 2 | Atr-ERM93537 |  | | | |  | Vvi-Vitvi06g00030\_t001 |  |  |  |  |
| 2 | Atr-ERM93538 |  | Vvi-Vitvi08g01817\_t003 |  | Vvi-Vitvi06g00029\_t001 |  |  |  |  |
| 3 | Atr-ERM93539 |  | | | |  | | | |  | Vvi-Vitvi08g01831\_t001 |  |  |  |
| 3 | Atr-ERM93540 |  | | | |  | | | |  | Vvi-Vitvi08g01829\_t001 |  |  |  |
| 3 | Atr-ERM93541 |  | | | |  | Vvi-Vitvi06g00028\_t001 |  | | | |  |  |  |
| 3 | Atr-ERM93542 |  | | | |  | | | |  | Vvi-Vitvi08g01826\_t001 |  |  |  |
| 3 | Atr-ERM93543 |  | | | |  | | | |  | Vvi-Vitvi08g01825\_t001 |  |  |  |
| 3 | Atr-ERM93544 |  | | | |  | Vvi-Vitvi06g00027\_t001 |  | | | |  |  |  |
| 2 | Atr-ERM93545 |  | Vvi-Vitvi08g01824\_t001 |  |  |  | | | |  |  |  |
| 2 | Atr-ERM93546 |  | | | |  |  |  | Vvi-Vitvi08g01822\_t001 |  |  |  |
| 2 | Atr-ERM93547 |  | | | |  |  |  | Vvi-Vitvi08g01820\_t001 |  |  |  |
| 2 | Atr-ERM93548 |  | | | |  |  |  | | | |  |  |  |
| 2 | Atr-ERM93549 |  | | | |  |  |  | Vvi-Vitvi08g01818\_t001 |  |  |  |
| 1 | Atr-ERM93550 |  | Vvi-Vitvi08g01832\_t001 |  |  |  |  |  |
| 1 | Atr-ERM93551 |  | | | |  |  |  |  |  |
| 2 | Atr-ERM93552 |  | | | |  | Vvi-Vitvi08g01999\_t001 |  |  |  |  |
| 2 | Atr-ERM93553 |  | | | |  | Vvi-Vitvi08g02000\_t001 |  |  |  |  |
| 2 | Atr-ERM93554 |  | | | |  | | | |  |  |  |  |
| 2 | Atr-ERM93555 |  | | | |  | Vvi-Vitvi08g00130\_t001 |  |  |  |  |
| 2 | Atr-ERM93556 |  | | | |  | | | |  |  |  |  |
| 2 | Atr-ERM93557 |  | | | |  | Vvi-Vitvi08g00132\_t001 |  |  |  |  |
| 2 | Atr-ERM93558 |  | | | |  | Vvi-Vitvi08g00133\_t001 |  |  |  |  |
| 3 | Atr-ERM93559 |  | | | |  | | | |  | Vvi-Vitvi08g00112\_t001 |  |  |  |
| 3 | Atr-ERM93560 |  | Vvi-Vitvi08g01833\_t004 |  | | | |  | | | |  |  |  |
| 2 | Atr-ERM93561 |  |  |  | | | |  | | | |  |  |  |
| 2 | Atr-ERM93562 |  |  |  | | | |  | | | |  |  |  |
| 3 | Atr-ERM93563 |  | Vvi-Vitvi08g00160\_t001 |  | | | |  | | | |  |  |  |
| 3 | Atr-ERM93564 |  | | | |  | | | |  | | | |  |  |  |
| 3 | Atr-ERM93565 |  | | | |  | | | |  | | | |  |  |  |
| 3 | Atr-ERM93566 |  | | | |  | | | |  | | | |  |  |  |
| 3 | Atr-ERM93567 |  | Vvi-Vitvi08g00152\_t001 |  | | | |  | | | |  |  |  |
| 3 | Atr-ERM93568 |  | | | |  | | | |  | | | |  |  |  |
| 3 | Atr-ERM93569 |  | | | |  | Vvi-Vitvi08g00146\_t001 |  | | | |  |  |  |
| 3 | Atr-ERM93570 |  | | | |  | | | |  | Vvi-Vitvi08g00113\_t001 |  |  |  |
| 3 | Atr-ERM93571 |  | | | |  | | | |  | | | |  |  |  |
| 3 | Atr-ERM93572 |  | | | |  | | | |  | | | |  |  |  |
| 3 | Atr-ERM93573 |  | | | |  | | | |  | Vvi-Vitvi08g00115\_t001 |  |  |  |
| 3 | Atr-ERM93574 |  | | | |  | | | |  | | | |  |  |  |
| 3 | Atr-ERM93575 |  | | | |  | | | |  | Vvi-Vitvi08g00120\_t001 |  |  |  |
| 3 | Atr-ERM93576 |  | | | |  | | | |  | | | |  |  |  |
| 3 | Atr-ERM93577 |  | | | |  | | | |  | Vvi-Vitvi08g01995\_t001 |  |  |  |
| 3 | Atr-ERM93578 |  | | | |  | | | |  | | | |  |  |  |
| 3 | Atr-ERM93579 |  | Vvi-Vitvi08g00140\_t001 |  | | | |  | | | |  |  |  |
| 3 | Atr-ERM93580 |  | Vvi-Vitvi08g00137\_t001 |  | | | |  | | | |  |  |  |
| 3 | Atr-ERM93581 |  | | | |  | | | |  | | | |  |  |  |
| 3 | Atr-ERM93582 |  | | | |  | | | |  | | | |  |  |  |
| 3 | Atr-ERM93583 |  | Vvi-Vitvi08g00135\_t003 |  | | | |  | | | |  |  |  |
| 3 | Atr-ERM93584 |  | | | |  | | | |  | Vvi-Vitvi08g00134\_t001 |  |  |  |
| 3 | Atr-ERM93585 |  | Vvi-Vitvi08g00114\_t001 |  | | | |  | | | |  |  |  |
| 2 | Atr-ERM93586 |  |  |  | | | |  | | | |  |  |  |
| 2 | Atr-ERM93587 |  |  |  | | | |  | | | |  |  |  |
| 2 | Atr-ERM93588 |  |  |  | | | |  | | | |  |  |  |
| 2 | Atr-ERM93589 |  |  |  | | | |  | | | |  |  |  |
| 2 | Atr-ERM93590 |  |  |  | | | |  | | | |  |  |  |
| 2 | Atr-ERM93591 |  |  |  | Vvi-Vitvi08g00163\_t001 |  | | | |  |  |  |
| 2 | Atr-ERM93592 |  |  |  | | | |  | | | |  |  |  |
| 2 | Atr-ERM93593 |  |  |  | | | |  | Vvi-Vitvi08g00161\_t001 |  |  |  |
| 2 | Atr-ERM93594 |  |  |  | | | |  | | | |  |  |  |
| 2 | Atr-ERM93595 |  |  |  | | | |  | | | |  |  |  |
| 2 | Atr-ERM93596 |  |  |  | | | |  | Vvi-Vitvi08g00174\_t002 |  |  |  |
| 2 | Atr-ERM93597 |  |  |  | Vvi-Vitvi08g00173\_t001 |  | | | |  |  |  |
| 2 | Atr-ERM93598 |  |  |  | | | |  | | | |  |  |  |
| 2 | Atr-ERM93599 |  |  |  | | | |  | | | |  |  |  |
| 2 | Atr-ERM93600 |  |  |  | | | |  | | | |  |  |  |
| 3 | Atr-ERM93601 |  | Vvi-Vitvi06g00956\_t001 |  | | | |  | | | |  |  |  |
| 3 | Atr-ERM93602 |  | | | |  | | | |  | | | |  |  |  |
| 3 | Atr-ERM93603 |  | | | |  | | | |  | | | |  |  |  |
| 3 | Atr-ERM93604 |  | Vvi-Vitvi06g00959\_t001 |  | | | |  | | | |  |  |  |
| 3 | Atr-ERM93605 |  | | | |  | | | |  | | | |  |  |  |
| 3 | Atr-ERM93606 |  | | | |  | | | |  | | | |  |  |  |
| 3 | Atr-ERM93607 |  | | | |  | | | |  | | | |  |  |  |
| 3 | Atr-ERM93608 |  | | | |  | | | |  | | | |  |  |  |
| 3 | Atr-ERM93609 |  | | | |  | | | |  | | | |  |  |  |
| 3 | Atr-ERM93610 |  | | | |  | Vvi-Vitvi08g00186\_t001 |  | | | |  |  |  |
| 3 | Atr-ERM93611 |  | | | |  | | | |  | | | |  |  |  |
| 3 | Atr-ERM93612 |  | | | |  | | | |  | | | |  |  |  |
| 3 | Atr-ERM93613 |  | | | |  | Vvi-Vitvi08g00198\_t001 |  | Vvi-Vitvi08g00180\_t001 |  |  |  |
| 3 | Atr-ERM93614 |  | | | |  | Vvi-Vitvi08g02021\_t001 |  | | | |  |  |  |
| 3 | Atr-ERM93615 |  | | | |  | | | |  | | | |  |  |  |
| 3 | Atr-ERM93616 |  | | | |  | | | |  | | | |  |  |  |
| 3 | Atr-ERM93617 |  | | | |  | | | |  | | | |  |  |  |
| 3 | Atr-ERM93618 |  | | | |  | | | |  | | | |  |  |  |
| 3 | Atr-ERM93619 |  | | | |  | | | |  | | | |  |  |  |
| 3 | Atr-ERM93620 |  | | | |  | | | |  | | | |  |  |  |
| 3 | Atr-ERM93621 |  | Vvi-Vitvi06g00979\_t001 |  | Vvi-Vitvi08g00200\_t001 |  | | | |  |  |  |
| 3 | Atr-ERM93622 |  | | | |  | Vvi-Vitvi08g00202\_t001 |  | | | |  |  |  |
| 3 | Atr-ERM93623 |  | | | |  | Vvi-Vitvi08g00205\_t001 |  | | | |  |  |  |
| 3 | Atr-ERM93624 |  | | | |  | | | |  | | | |  |  |  |
| 3 | Atr-ERM93625 |  | | | |  | | | |  | | | |  |  |  |
| 3 | Atr-ERM93626 |  | Vvi-Vitvi06g01818\_t001 |  | | | |  | | | |  |  |  |
| 3 | Atr-ERM93627 |  | | | |  | Vvi-Vitvi08g00209\_t001 |  | | | |  |  |  |
| 3 | Atr-ERM93628 |  | Vvi-Vitvi06g00984\_t001 |  | Vvi-Vitvi08g00212\_t001 |  | Vvi-Vitvi08g00195\_t001 |  |  |  |
| 2 | Atr-ERM93629 |  | | | |  | | | |  |  |  |  |
| 2 | Atr-ERM93630 |  | | | |  | | | |  |  |  |  |
| 2 | Atr-ERM93631 |  | | | |  | Vvi-Vitvi08g00215\_t001 |  |  |  |  |
| 2 | Atr-ERM93632 |  | | | |  | | | |  |  |  |  |
| 2 | Atr-ERM93633 |  | | | |  | | | |  |  |  |  |
| 2 | Atr-ERM93634 |  | | | |  | | | |  |  |  |  |
| 2 | Atr-ERM93635 |  | | | |  | | | |  |  |  |  |
| 2 | Atr-ERM93636 |  | | | |  | | | |  |  |  |  |
| 2 | Atr-ERM93637 |  | | | |  | Vvi-Vitvi08g00216\_t001 |  |  |  |  |
| 2 | Atr-ERM93638 |  | | | |  | | | |  |  |  |  |
| 2 | Atr-ERM93639 |  | | | |  | | | |  |  |  |  |
| 2 | Atr-ERM93640 |  | | | |  | | | |  |  |  |  |
| 2 | Atr-ERM93641 |  | | | |  | | | |  |  |  |  |
| 2 | Atr-ERM93642 |  | | | |  | | | |  |  |  |  |
| 2 | Atr-ERM93643 |  | | | |  | Vvi-Vitvi08g02028\_t001 |  |  |  |  |
| 2 | Atr-ERM93644 |  | | | |  | Vvi-Vitvi08g00222\_t001 |  |  |  |  |
| 2 | Atr-ERM93645 |  | | | |  | Vvi-Vitvi08g00223\_t001 |  |  |  |  |
| 2 | Atr-ERM93646 |  | | | |  | | | |  |  |  |  |
| 2 | Atr-ERM93647 |  | Vvi-Vitvi06g00990\_t001 |  | | | |  |  |  |  |
| 1 | Atr-ERM93648 |  |  |  | | | |  |  |  |  |
| 1 | Atr-ERM93649 |  |  |  | Vvi-Vitvi08g00225\_t001 |  |  |  |  |
| 1 | Atr-ERM93650 |  |  |  | | | |  |  |  |  |
| 1 | Atr-ERM93651 |  |  |  | | | |  |  |  |  |
| 1 | Atr-ERM93652 |  |  |  | | | |  |  |  |  |
| 1 | Atr-ERM93653 |  |  |  | | | |  |  |  |  |
| 1 | Atr-ERM93654 |  |  |  | Vvi-Vitvi08g00235\_t003 |  |  |  |  |
| 1 | Atr-ERM93655 |  |  |  | | | |  |  |  |  |
| 1 | Atr-ERM93656 |  |  |  | | | |  |  |  |  |
| 1 | Atr-ERM93657 |  |  |  | | | |  |  |  |  |
| 2 | Atr-ERM93658 |  | Vvi-Vitvi08g00298\_t001 |  | | | |  |  |  |  |
| 2 | Atr-ERM93659 |  | | | |  | | | |  |  |  |  |
| 2 | Atr-ERM93660 |  | Vvi-Vitvi08g00297\_t001 |  | | | |  |  |  |  |
| 2 | Atr-ERM93661 |  | | | |  | | | |  |  |  |  |
| 2 | Atr-ERM93662 |  | | | |  | Vvi-Vitvi08g02036\_t001 |  |  |  |  |
| 2 | Atr-ERM93663 |  | | | |  | | | |  |  |  |  |
| 2 | Atr-ERM93664 |  | | | |  | | | |  |  |  |  |
| 2 | Atr-ERM93665 |  | | | |  | | | |  |  |  |  |
| 2 | Atr-ERM93666 |  | Vvi-Vitvi08g00274\_t001 |  | | | |  |  |  |  |
| 2 | Atr-ERM93667 |  | Vvi-Vitvi08g00271\_t002 |  | | | |  |  |  |  |
| 2 | Atr-ERM93668 |  | | | |  | | | |  |  |  |  |
| 2 | Atr-ERM93669 |  | Vvi-Vitvi08g00262\_t001 |  | | | |  |  |  |  |
| 2 | Atr-ERM93670 |  | | | |  | | | |  |  |  |  |
| 2 | Atr-ERM93671 |  | | | |  | | | |  |  |  |  |
| 2 | Atr-ERM93672 |  | | | |  | | | |  |  |  |  |
| 2 | Atr-ERM93673 |  | | | |  | | | |  |  |  |  |
| 2 | Atr-ERM93674 |  | | | |  | Vvi-Vitvi08g00261\_t001 |  |  |  |  |
| 2 | Atr-ERM93675 |  | Vvi-Vitvi08g02039\_t001 |  | | | |  |  |  |  |
| 2 | Atr-ERM93676 |  | | | |  | | | |  |  |  |  |
| 2 | Atr-ERM93677 |  | | | |  | | | |  |  |  |  |
| 2 | Atr-ERM93678 |  | | | |  | | | |  |  |  |  |
| 2 | Atr-ERM93679 |  | | | |  | | | |  |  |  |  |
| 2 | Atr-ERM93680 |  | Vvi-Vitvi08g00258\_t001 |  | | | |  |  |  |  |
| 2 | Atr-ERM93681 |  | Vvi-Vitvi08g00241\_t001 |  | | | |  |  |  |  |
| 1 | Atr-ERM93682 |  |  |  | | | |  |  |  |  |
| 1 | Atr-ERM93683 |  |  |  | | | |  |  |  |  |
| 1 | Atr-ERM93684 |  |  |  | | | |  |  |  |  |
| 1 | Atr-ERM93685 |  |  |  | | | |  |  |  |  |
| 1 | Atr-ERM93686 |  |  |  | Vvi-Vitvi08g00289\_t002 |  |  |  |  |
| 0 | Atr-ERM93687 |  |  |  |  |  |  |
| 0 | Atr-ERM93688 |  |  |  |  |  |  |
| 0 | Atr-ERM93689 |  |  |  |  |  |  |
| 0 | Atr-ERM93690 |  |  |  |  |  |  |
| 0 | Atr-ERM93691 |  |  |  |  |  |  |
| 0 | Atr-ERM93692 |  |  |  |  |  |  |
| 0 | Atr-ERM93693 |  |  |  |  |  |  |
| 0 | Atr-ERM93694 |  |  |  |  |  |  |
| 0 | Atr-ERM93695 |  |  |  |  |  |  |
| 0 | Atr-ERM93696 |  |  |  |  |  |  |
| 0 | Atr-ERM93697 |  |  |  |  |  |  |
| 0 | Atr-ERM93698 |  |  |  |  |  |  |
| 0 | Atr-ERM93699 |  |  |  |  |  |  |
| 0 | Atr-ERM93700 |  |  |  |  |  |  |
| 0 | Atr-ERM93701 |  |  |  |  |  |  |
| 0 | Atr-ERM93702 |  |  |  |  |  |  |
| 0 | Atr-ERM93703 |  |  |  |  |  |  |
| 0 | Atr-ERM93704 |  |  |  |  |  |  |
| 0 | Atr-ERM93705 |  |  |  |  |  |  |
| 0 | Atr-ERM93706 |  |  |  |  |  |  |
| 0 | Atr-ERM93707 |  |  |  |  |  |  |
| 0 | Atr-ERM93708 |  |  |  |  |  |  |
| 0 | Atr-ERM93709 |  |  |  |  |  |  |
| 0 | Atr-ERM93710 |  |  |  |  |  |  |
| 0 | Atr-ERM93711 |  |  |  |  |  |  |
| 0 | Atr-ERM93712 |  |  |  |  |  |  |
| 0 | Atr-ERM93713 |  |  |  |  |  |  |
| 0 | Atr-ERM93714 |  |  |  |  |  |  |
| 0 | Atr-ERM93715 |  |  |  |  |  |  |
| 0 | Atr-ERM93716 |  |  |  |  |  |  |
| 0 | Atr-ERM93717 |  |  |  |  |  |  |
| 0 | Atr-ERM93718 |  |  |  |  |  |  |
| 0 | Atr-ERM93719 |  |  |  |  |  |  |
| 0 | Atr-ERM93720 |  |  |  |  |  |  |
| 0 | Atr-ERM93721 |  |  |  |  |  |  |
| 0 | Atr-ERM93722 |  |  |  |  |  |  |
| 0 | Atr-ERM93723 |  |  |  |  |  |  |
| 0 | Atr-ERM93724 |  |  |  |  |  |  |
| 0 | Atr-ERM93725 |  |  |  |  |  |  |
| 0 | Atr-ERM93726 |  |  |  |  |  |  |
| 0 | Atr-ERM93727 |  |  |  |  |  |  |
| 0 | Atr-ERM93728 |  |  |  |  |  |  |
| 0 | Atr-ERM93729 |  |  |  |  |  |  |
| 0 | Atr-ERM93730 |  |  |  |  |  |  |
| 0 | Atr-ERM93731 |  |  |  |  |  |  |
| 0 | Atr-ERM93732 |  |  |  |  |  |  |
| 0 | Atr-ERM93733 |  |  |  |  |  |  |
| 0 | Atr-ERM93734 |  |  |  |  |  |  |
| 0 | Atr-ERM93735 |  |  |  |  |  |  |
| 0 | Atr-ERM93736 |  |  |  |  |  |  |
| 0 | Atr-ERM93737 |  |  |  |  |  |  |
| 0 | Atr-ERM93738 |  |  |  |  |  |  |
| 0 | Atr-ERM93739 |  |  |  |  |  |  |
| 0 | Atr-ERM93740 |  |  |  |  |  |  |
| 0 | Atr-ERM93741 |  |  |  |  |  |  |
| 0 | Atr-ERM93742 |  |  |  |  |  |  |
| 0 | Atr-ERM93743 |  |  |  |  |  |  |
| 0 | Atr-ERM93744 |  |  |  |  |  |  |
| 0 | Atr-ERM93745 |  |  |  |  |  |  |
| 0 | Atr-ERM93746 |  |  |  |  |  |  |
| 0 | Atr-ERM93747 |  |  |  |  |  |  |
| 0 | Atr-ERM93748 |  |  |  |  |  |  |
| 0 | Atr-ERM93749 |  |  |  |  |  |  |
| 0 | Atr-ERM93750 |  |  |  |  |  |  |
| 0 | Atr-ERM93751 |  |  |  |  |  |  |
| 0 | Atr-ERM93752 |  |  |  |  |  |  |
| 0 | Atr-ERM93753 |  |  |  |  |  |  |
| 0 | Atr-ERM93754 |  |  |  |  |  |  |
| 0 | Atr-ERM93755 |  |  |  |  |  |  |
| 0 | Atr-ERM93756 |  |  |  |  |  |  |
| 0 | Atr-ERM93757 |  |  |  |  |  |  |
| 0 | Atr-ERM93758 |  |  |  |  |  |  |
| 0 | Atr-ERM93759 |  |  |  |  |  |  |
| 0 | Atr-ERM93760 |  |  |  |  |  |  |
| 0 | Atr-ERM93761 |  |  |  |  |  |  |
| 0 | Atr-ERM93762 |  |  |  |  |  |  |
| 0 | Atr-ERM93763 |  |  |  |  |  |  |
| 0 | Atr-ERM93764 |  |  |  |  |  |  |
| 0 | Atr-ERM93765 |  |  |  |  |  |  |
| 0 | Atr-ERM93766 |  |  |  |  |  |  |
| 0 | Atr-ERM93767 |  |  |  |  |  |  |
| 0 | Atr-ERM93768 |  |  |  |  |  |  |
| 0 | Atr-ERM93769 |  |  |  |  |  |  |
| 0 | Atr-ERM93770 |  |  |  |  |  |  |
| 0 | Atr-ERM93771 |  |  |  |  |  |  |
| 0 | Atr-ERM93772 |  |  |  |  |  |  |
| 0 | Atr-ERM93773 |  |  |  |  |  |  |
| 0 | Atr-ERM93774 |  |  |  |  |  |  |
| 0 | Atr-ERM93775 |  |  |  |  |  |  |
| 0 | Atr-ERM93776 |  |  |  |  |  |  |
| 0 | Atr-ERM93777 |  |  |  |  |  |  |
| 0 | Atr-ERM93778 |  |  |  |  |  |  |
| 0 | Atr-ERM93779 |  |  |  |  |  |  |
| 0 | Atr-ERM93780 |  |  |  |  |  |  |
| 0 | Atr-ERM93781 |  |  |  |  |  |  |
| 0 | Atr-ERM93782 |  |  |  |  |  |  |
| 0 | Atr-ERM93783 |  |  |  |  |  |  |
| 0 | Atr-ERM93784 |  |  |  |  |  |  |
| 0 | Atr-ERM93785 |  |  |  |  |  |  |
| 0 | Atr-ERM93786 |  |  |  |  |  |  |
| 0 | Atr-ERM93787 |  |  |  |  |  |  |
| 0 | Atr-ERM93788 |  |  |  |  |  |  |
