# Supplementary material for: Genome-wide identification, characterization and gene expression of BES1 transcription factor family in grapevine (Vitis vinifera L.)
Source: Sci Rep. 2023 Jan 5;13:240. doi: 10.1038/s41598-022-24407-y (PMC9816167; doi:10.1038/s41598-022-24407-y)
Supplement: Supplementary file 3 — Supplementary Information. [file 41598_2022_24407_MOESM3_ESM.zip › Vvi_Atr/Vitis_vinifera.PN40024.v4.dna_sm.toplevel.fa.vs.Amborella_trichopoda.AMTR1.0.dna_sm.toplevel.fa.html/Atr-AmTr_v1.0_scaffold00005.html]

|  |  |  |  |  |  |  |  |  |  |  |  |  |  |
| --- | --- | --- | --- | --- | --- | --- | --- | --- | --- | --- | --- | --- | --- |
| Duplication depth | Reference chromosome | Collinear blocks | | | | | | | | | | | |
| 0 | Atr-ERN06727 |  |  |  |  |  |  |
| 0 | Atr-ERN06728 |  |  |  |  |  |  |
| 0 | Atr-ERN06729 |  |  |  |  |  |  |
| 0 | Atr-ERN06730 |  |  |  |  |  |  |
| 0 | Atr-ERN06731 |  |  |  |  |  |  |
| 0 | Atr-ERN06732 |  |  |  |  |  |  |
| 0 | Atr-ERN06733 |  |  |  |  |  |  |
| 0 | Atr-ERN06734 |  |  |  |  |  |  |
| 0 | Atr-ERN06735 |  |  |  |  |  |  |
| 0 | Atr-ERN06736 |  |  |  |  |  |  |
| 0 | Atr-ERN06737 |  |  |  |  |  |  |
| 0 | Atr-ERN06738 |  |  |  |  |  |  |
| 0 | Atr-ERN06739 |  |  |  |  |  |  |
| 0 | Atr-ERN06740 |  |  |  |  |  |  |
| 0 | Atr-ERN06741 |  |  |  |  |  |  |
| 0 | Atr-ERN06742 |  |  |  |  |  |  |
| 0 | Atr-ERN06743 |  |  |  |  |  |  |
| 0 | Atr-ERN06744 |  |  |  |  |  |  |
| 0 | Atr-ERN06745 |  |  |  |  |  |  |
| 0 | Atr-ERN06746 |  |  |  |  |  |  |
| 0 | Atr-ERN06747 |  |  |  |  |  |  |
| 0 | Atr-ERN06748 |  |  |  |  |  |  |
| 0 | Atr-ERN06749 |  |  |  |  |  |  |
| 0 | Atr-ERN06750 |  |  |  |  |  |  |
| 0 | Atr-ERN06751 |  |  |  |  |  |  |
| 0 | Atr-ERN06752 |  |  |  |  |  |  |
| 0 | Atr-ERN06753 |  |  |  |  |  |  |
| 0 | Atr-ERN06754 |  |  |  |  |  |  |
| 0 | Atr-ERN06755 |  |  |  |  |  |  |
| 0 | Atr-ERN06756 |  |  |  |  |  |  |
| 0 | Atr-ERN06757 |  |  |  |  |  |  |
| 0 | Atr-ERN06758 |  |  |  |  |  |  |
| 0 | Atr-ERN06759 |  |  |  |  |  |  |
| 0 | Atr-ERN06760 |  |  |  |  |  |  |
| 0 | Atr-ERN06761 |  |  |  |  |  |  |
| 0 | Atr-ERN06762 |  |  |  |  |  |  |
| 0 | Atr-ERN06763 |  |  |  |  |  |  |
| 0 | Atr-ERN06764 |  |  |  |  |  |  |
| 0 | Atr-ERN06765 |  |  |  |  |  |  |
| 0 | Atr-ERN06766 |  |  |  |  |  |  |
| 0 | Atr-ERN06767 |  |  |  |  |  |  |
| 0 | Atr-ERN06768 |  |  |  |  |  |  |
| 0 | Atr-ERN06769 |  |  |  |  |  |  |
| 0 | Atr-ERN06770 |  |  |  |  |  |  |
| 0 | Atr-ERN06771 |  |  |  |  |  |  |
| 0 | Atr-ERN06772 |  |  |  |  |  |  |
| 0 | Atr-ERN06773 |  |  |  |  |  |  |
| 0 | Atr-ERN06774 |  |  |  |  |  |  |
| 0 | Atr-ERN06775 |  |  |  |  |  |  |
| 0 | Atr-ERN06776 |  |  |  |  |  |  |
| 0 | Atr-ERN06777 |  |  |  |  |  |  |
| 0 | Atr-ERN06778 |  |  |  |  |  |  |
| 0 | Atr-ERN06779 |  |  |  |  |  |  |
| 0 | Atr-ERN06780 |  |  |  |  |  |  |
| 0 | Atr-ERN06781 |  |  |  |  |  |  |
| 0 | Atr-ERN06782 |  |  |  |  |  |  |
| 0 | Atr-ERN06783 |  |  |  |  |  |  |
| 0 | Atr-ERN06784 |  |  |  |  |  |  |
| 0 | Atr-ERN06785 |  |  |  |  |  |  |
| 0 | Atr-ERN06786 |  |  |  |  |  |  |
| 0 | Atr-ERN06787 |  |  |  |  |  |  |
| 0 | Atr-ERN06788 |  |  |  |  |  |  |
| 0 | Atr-ERN06789 |  |  |  |  |  |  |
| 0 | Atr-ERN06790 |  |  |  |  |  |  |
| 0 | Atr-ERN06791 |  |  |  |  |  |  |
| 2 | Atr-ERN06792 |  | Vvi-Vitvi01g01533\_t002 |  | Vvi-Vitvi14g01602\_t001 |  |  |  |  |
| 2 | Atr-ERN06793 |  | | | |  | | | |  |  |  |  |
| 2 | Atr-ERN06794 |  | | | |  | | | |  |  |  |  |
| 2 | Atr-ERN06795 |  | Vvi-Vitvi01g01534\_t001 |  | | | |  |  |  |  |
| 2 | Atr-ERN06796 |  | Vvi-Vitvi01g01536\_t001 |  | | | |  |  |  |  |
| 2 | Atr-ERN06797 |  | Vvi-Vitvi01g01537\_t001 |  | | | |  |  |  |  |
| 2 | Atr-ERN06798 |  | Vvi-Vitvi01g01540\_t001 |  | Vvi-Vitvi14g01597\_t001 |  |  |  |  |
| 2 | Atr-ERN06799 |  | | | |  | | | |  |  |  |  |
| 2 | Atr-ERN06800 |  | | | |  | | | |  |  |  |  |
| 2 | Atr-ERN06801 |  | | | |  | | | |  |  |  |  |
| 2 | Atr-ERN06802 |  | Vvi-Vitvi01g01555\_t001 |  | Vvi-Vitvi14g01596\_t001 |  |  |  |  |
| 2 | Atr-ERN06803 |  | | | |  | | | |  |  |  |  |
| 2 | Atr-ERN06804 |  | | | |  | | | |  |  |  |  |
| 2 | Atr-ERN06805 |  | Vvi-Vitvi01g01557\_t001 |  | | | |  |  |  |  |
| 1 | Atr-ERN06806 |  |  |  | | | |  |  |  |  |
| 1 | Atr-ERN06807 |  |  |  | Vvi-Vitvi14g01594\_t002 |  |  |  |  |
| 1 | Atr-ERN06808 |  |  |  | Vvi-Vitvi14g02987\_t001 |  |  |  |  |
| 1 | Atr-ERN06809 |  |  |  | | | |  |  |  |  |
| 1 | Atr-ERN06810 |  |  |  | | | |  |  |  |  |
| 1 | Atr-ERN06811 |  |  |  | | | |  |  |  |  |
| 1 | Atr-ERN06812 |  |  |  | | | |  |  |  |  |
| 1 | Atr-ERN06813 |  |  |  | | | |  |  |  |  |
| 1 | Atr-ERN06814 |  |  |  | | | |  |  |  |  |
| 1 | Atr-ERN06815 |  |  |  | | | |  |  |  |  |
| 1 | Atr-ERN06816 |  |  |  | | | |  |  |  |  |
| 1 | Atr-ERN06817 |  |  |  | | | |  |  |  |  |
| 1 | Atr-ERN06818 |  |  |  | | | |  |  |  |  |
| 1 | Atr-ERN06819 |  |  |  | | | |  |  |  |  |
| 1 | Atr-ERN06820 |  |  |  | | | |  |  |  |  |
| 1 | Atr-ERN06821 |  |  |  | Vvi-Vitvi14g01579\_t001 |  |  |  |  |
| 1 | Atr-ERN06822 |  |  |  | | | |  |  |  |  |
| 1 | Atr-ERN06823 |  |  |  | | | |  |  |  |  |
| 1 | Atr-ERN06824 |  |  |  | | | |  |  |  |  |
| 1 | Atr-ERN06825 |  |  |  | | | |  |  |  |  |
| 1 | Atr-ERN06826 |  |  |  | | | |  |  |  |  |
| 1 | Atr-ERN06827 |  |  |  | | | |  |  |  |  |
| 1 | Atr-ERN06828 |  |  |  | | | |  |  |  |  |
| 1 | Atr-ERN06829 |  |  |  | | | |  |  |  |  |
| 1 | Atr-ERN06830 |  |  |  | | | |  |  |  |  |
| 1 | Atr-ERN06831 |  |  |  | | | |  |  |  |  |
| 1 | Atr-ERN06832 |  |  |  | | | |  |  |  |  |
| 1 | Atr-ERN06833 |  |  |  | | | |  |  |  |  |
| 1 | Atr-ERN06834 |  |  |  | | | |  |  |  |  |
| 1 | Atr-ERN06835 |  |  |  | | | |  |  |  |  |
| 1 | Atr-ERN06836 |  |  |  | | | |  |  |  |  |
| 1 | Atr-ERN06837 |  |  |  | | | |  |  |  |  |
| 1 | Atr-ERN06838 |  |  |  | | | |  |  |  |  |
| 1 | Atr-ERN06839 |  |  |  | | | |  |  |  |  |
| 1 | Atr-ERN06840 |  |  |  | | | |  |  |  |  |
| 1 | Atr-ERN06841 |  |  |  | | | |  |  |  |  |
| 1 | Atr-ERN06842 |  |  |  | | | |  |  |  |  |
| 1 | Atr-ERN06843 |  |  |  | | | |  |  |  |  |
| 1 | Atr-ERN06844 |  |  |  | | | |  |  |  |  |
| 1 | Atr-ERN06845 |  |  |  | | | |  |  |  |  |
| 1 | Atr-ERN06846 |  |  |  | | | |  |  |  |  |
| 1 | Atr-ERN06847 |  |  |  | Vvi-Vitvi14g01574\_t001 |  |  |  |  |
| 0 | Atr-ERN06848 |  |  |  |  |  |  |
| 0 | Atr-ERN06849 |  |  |  |  |  |  |
| 0 | Atr-ERN06850 |  |  |  |  |  |  |
| 0 | Atr-ERN06851 |  |  |  |  |  |  |
| 0 | Atr-ERN06852 |  |  |  |  |  |  |
| 0 | Atr-ERN06853 |  |  |  |  |  |  |
| 0 | Atr-ERN06854 |  |  |  |  |  |  |
| 0 | Atr-ERN06855 |  |  |  |  |  |  |
| 0 | Atr-ERN06856 |  |  |  |  |  |  |
| 0 | Atr-ERN06857 |  |  |  |  |  |  |
| 0 | Atr-ERN06858 |  |  |  |  |  |  |
| 0 | Atr-ERN06859 |  |  |  |  |  |  |
| 0 | Atr-ERN06860 |  |  |  |  |  |  |
| 0 | Atr-ERN06861 |  |  |  |  |  |  |
| 0 | Atr-ERN06862 |  |  |  |  |  |  |
| 0 | Atr-ERN06863 |  |  |  |  |  |  |
| 0 | Atr-ERN06864 |  |  |  |  |  |  |
| 0 | Atr-ERN06865 |  |  |  |  |  |  |
| 0 | Atr-ERN06866 |  |  |  |  |  |  |
| 0 | Atr-ERN06867 |  |  |  |  |  |  |
| 0 | Atr-ERN06868 |  |  |  |  |  |  |
| 0 | Atr-ERN06869 |  |  |  |  |  |  |
| 0 | Atr-ERN06870 |  |  |  |  |  |  |
| 0 | Atr-ERN06871 |  |  |  |  |  |  |
| 0 | Atr-ERN06872 |  |  |  |  |  |  |
| 0 | Atr-ERN06873 |  |  |  |  |  |  |
| 0 | Atr-ERN06874 |  |  |  |  |  |  |
| 0 | Atr-ERN06875 |  |  |  |  |  |  |
| 0 | Atr-ERN06876 |  |  |  |  |  |  |
| 0 | Atr-ERN06877 |  |  |  |  |  |  |
| 0 | Atr-ERN06878 |  |  |  |  |  |  |
| 0 | Atr-ERN06879 |  |  |  |  |  |  |
| 0 | Atr-ERN06880 |  |  |  |  |  |  |
| 0 | Atr-ERN06881 |  |  |  |  |  |  |
| 0 | Atr-ERN06882 |  |  |  |  |  |  |
| 0 | Atr-ERN06883 |  |  |  |  |  |  |
| 0 | Atr-ERN06884 |  |  |  |  |  |  |
| 0 | Atr-ERN06885 |  |  |  |  |  |  |
| 0 | Atr-ERN06886 |  |  |  |  |  |  |
| 0 | Atr-ERN06887 |  |  |  |  |  |  |
| 0 | Atr-ERN06888 |  |  |  |  |  |  |
| 0 | Atr-ERN06889 |  |  |  |  |  |  |
| 0 | Atr-ERN06890 |  |  |  |  |  |  |
| 0 | Atr-ERN06891 |  |  |  |  |  |  |
| 0 | Atr-ERN06892 |  |  |  |  |  |  |
| 0 | Atr-ERN06893 |  |  |  |  |  |  |
| 0 | Atr-ERN06894 |  |  |  |  |  |  |
| 0 | Atr-ERN06895 |  |  |  |  |  |  |
| 0 | Atr-ERN06896 |  |  |  |  |  |  |
| 0 | Atr-ERN06897 |  |  |  |  |  |  |
| 0 | Atr-ERN06898 |  |  |  |  |  |  |
| 0 | Atr-ERN06899 |  |  |  |  |  |  |
| 0 | Atr-ERN06900 |  |  |  |  |  |  |
| 0 | Atr-ERN06901 |  |  |  |  |  |  |
| 0 | Atr-ERN06902 |  |  |  |  |  |  |
| 0 | Atr-ERN06903 |  |  |  |  |  |  |
| 0 | Atr-ERN06904 |  |  |  |  |  |  |
| 0 | Atr-ERN06905 |  |  |  |  |  |  |
| 0 | Atr-ERN06906 |  |  |  |  |  |  |
| 0 | Atr-ERN06907 |  |  |  |  |  |  |
| 0 | Atr-ERN06908 |  |  |  |  |  |  |
| 0 | Atr-ERN06909 |  |  |  |  |  |  |
| 0 | Atr-ERN06910 |  |  |  |  |  |  |
| 0 | Atr-ERN06911 |  |  |  |  |  |  |
| 0 | Atr-ERN06912 |  |  |  |  |  |  |
| 0 | Atr-ERN06913 |  |  |  |  |  |  |
| 0 | Atr-ERN06914 |  |  |  |  |  |  |
| 0 | Atr-ERN06915 |  |  |  |  |  |  |
| 0 | Atr-ERN06916 |  |  |  |  |  |  |
| 0 | Atr-ERN06917 |  |  |  |  |  |  |
| 0 | Atr-ERN06918 |  |  |  |  |  |  |
| 0 | Atr-ERN06919 |  |  |  |  |  |  |
| 0 | Atr-ERN06920 |  |  |  |  |  |  |
| 0 | Atr-ERN06921 |  |  |  |  |  |  |
| 0 | Atr-ERN06922 |  |  |  |  |  |  |
| 0 | Atr-ERN06923 |  |  |  |  |  |  |
| 0 | Atr-ERN06924 |  |  |  |  |  |  |
| 0 | Atr-ERN06925 |  |  |  |  |  |  |
| 0 | Atr-ERN06926 |  |  |  |  |  |  |
| 0 | Atr-ERN06927 |  |  |  |  |  |  |
| 0 | Atr-ERN06928 |  |  |  |  |  |  |
| 0 | Atr-ERN06929 |  |  |  |  |  |  |
| 0 | Atr-ERN06930 |  |  |  |  |  |  |
| 0 | Atr-ERN06931 |  |  |  |  |  |  |
| 0 | Atr-ERN06932 |  |  |  |  |  |  |
| 0 | Atr-ERN06933 |  |  |  |  |  |  |
| 0 | Atr-ERN06934 |  |  |  |  |  |  |
| 0 | Atr-ERN06935 |  |  |  |  |  |  |
| 0 | Atr-ERN06936 |  |  |  |  |  |  |
| 0 | Atr-ERN06937 |  |  |  |  |  |  |
| 0 | Atr-ERN06938 |  |  |  |  |  |  |
| 0 | Atr-ERN06939 |  |  |  |  |  |  |
| 0 | Atr-ERN06940 |  |  |  |  |  |  |
| 0 | Atr-ERN06941 |  |  |  |  |  |  |
| 0 | Atr-ERN06942 |  |  |  |  |  |  |
| 0 | Atr-ERN06943 |  |  |  |  |  |  |
| 0 | Atr-ERN06944 |  |  |  |  |  |  |
| 0 | Atr-ERN06945 |  |  |  |  |  |  |
| 0 | Atr-ERN06946 |  |  |  |  |  |  |
| 0 | Atr-ERN06947 |  |  |  |  |  |  |
| 1 | Atr-ERN06948 |  | Vvi-Vitvi14g01003\_t002 |  |  |  |  |  |
| 1 | Atr-ERN06949 |  | Vvi-Vitvi14g00999\_t001 |  |  |  |  |  |
| 1 | Atr-ERN06950 |  | | | |  |  |  |  |  |
| 1 | Atr-ERN06951 |  | Vvi-Vitvi14g00997\_t001 |  |  |  |  |  |
| 1 | Atr-ERN06952 |  | Vvi-Vitvi14g00996\_t001 |  |  |  |  |  |
| 1 | Atr-ERN06953 |  | | | |  |  |  |  |  |
| 1 | Atr-ERN06954 |  | | | |  |  |  |  |  |
| 1 | Atr-ERN06955 |  | | | |  |  |  |  |  |
| 1 | Atr-ERN06956 |  | | | |  |  |  |  |  |
| 1 | Atr-ERN06957 |  | | | |  |  |  |  |  |
| 1 | Atr-ERN06958 |  | | | |  |  |  |  |  |
| 1 | Atr-ERN06959 |  | | | |  |  |  |  |  |
| 1 | Atr-ERN06960 |  | | | |  |  |  |  |  |
| 1 | Atr-ERN06961 |  | | | |  |  |  |  |  |
| 1 | Atr-ERN06962 |  | | | |  |  |  |  |  |
| 1 | Atr-ERN06963 |  | | | |  |  |  |  |  |
| 1 | Atr-ERN06964 |  | | | |  |  |  |  |  |
| 1 | Atr-ERN06965 |  | Vvi-Vitvi14g00992\_t001 |  |  |  |  |  |
| 1 | Atr-ERN06966 |  | | | |  |  |  |  |  |
| 1 | Atr-ERN06967 |  | | | |  |  |  |  |  |
| 1 | Atr-ERN06968 |  | | | |  |  |  |  |  |
| 1 | Atr-ERN06969 |  | | | |  |  |  |  |  |
| 1 | Atr-ERN06970 |  | | | |  |  |  |  |  |
| 1 | Atr-ERN06971 |  | | | |  |  |  |  |  |
| 1 | Atr-ERN06972 |  | | | |  |  |  |  |  |
| 1 | Atr-ERN06973 |  | | | |  |  |  |  |  |
| 1 | Atr-ERN06974 |  | | | |  |  |  |  |  |
| 1 | Atr-ERN06975 |  | Vvi-Vitvi14g00974\_t001 |  |  |  |  |  |
| 0 | Atr-ERN06976 |  |  |  |  |  |  |
| 0 | Atr-ERN06977 |  |  |  |  |  |  |
| 0 | Atr-ERN06978 |  |  |  |  |  |  |
| 0 | Atr-ERN06979 |  |  |  |  |  |  |
| 0 | Atr-ERN06980 |  |  |  |  |  |  |
| 0 | Atr-ERN06981 |  |  |  |  |  |  |
