# Supplementary material for: Genome-wide identification, characterization and gene expression of BES1 transcription factor family in grapevine (Vitis vinifera L.)
Source: Sci Rep. 2023 Jan 5;13:240. doi: 10.1038/s41598-022-24407-y (PMC9816167; doi:10.1038/s41598-022-24407-y)
Supplement: Supplementary file 3 — Supplementary Information. [file 41598_2022_24407_MOESM3_ESM.zip › Vvi_Atr/Vitis_vinifera.PN40024.v4.dna_sm.toplevel.fa.vs.Amborella_trichopoda.AMTR1.0.dna_sm.toplevel.fa.html/Atr-AmTr_v1.0_scaffold00006.html]

|  |  |  |  |  |  |  |  |  |  |  |  |  |  |
| --- | --- | --- | --- | --- | --- | --- | --- | --- | --- | --- | --- | --- | --- |
| Duplication depth | Reference chromosome | Collinear blocks | | | | | | | | | | | |
| 0 | Atr-ERN05603 |  |  |  |  |  |  |
| 0 | Atr-ERN05604 |  |  |  |  |  |  |
| 0 | Atr-ERN05605 |  |  |  |  |  |  |
| 0 | Atr-ERN05606 |  |  |  |  |  |  |
| 0 | Atr-ERN05607 |  |  |  |  |  |  |
| 0 | Atr-ERN05608 |  |  |  |  |  |  |
| 0 | Atr-ERN05609 |  |  |  |  |  |  |
| 0 | Atr-ERN05610 |  |  |  |  |  |  |
| 0 | Atr-ERN05611 |  |  |  |  |  |  |
| 0 | Atr-ERN05612 |  |  |  |  |  |  |
| 0 | Atr-ERN05613 |  |  |  |  |  |  |
| 0 | Atr-ERN05614 |  |  |  |  |  |  |
| 0 | Atr-ERN05615 |  |  |  |  |  |  |
| 0 | Atr-ERN05616 |  |  |  |  |  |  |
| 0 | Atr-ERN05617 |  |  |  |  |  |  |
| 1 | Atr-ERN05618 |  | Vvi-Vitvi01g00374\_t001 |  |  |  |  |  |
| 1 | Atr-ERN05619 |  | Vvi-Vitvi01g00373\_t001 |  |  |  |  |  |
| 1 | Atr-ERN05620 |  | | | |  |  |  |  |  |
| 1 | Atr-ERN05621 |  | | | |  |  |  |  |  |
| 1 | Atr-ERN05622 |  | | | |  |  |  |  |  |
| 1 | Atr-ERN05623 |  | | | |  |  |  |  |  |
| 1 | Atr-ERN05624 |  | | | |  |  |  |  |  |
| 1 | Atr-ERN05625 |  | | | |  |  |  |  |  |
| 1 | Atr-ERN05626 |  | | | |  |  |  |  |  |
| 1 | Atr-ERN05627 |  | | | |  |  |  |  |  |
| 1 | Atr-ERN05628 |  | | | |  |  |  |  |  |
| 1 | Atr-ERN05629 |  | | | |  |  |  |  |  |
| 1 | Atr-ERN05630 |  | | | |  |  |  |  |  |
| 1 | Atr-ERN05631 |  | Vvi-Vitvi01g00372\_t001 |  |  |  |  |  |
| 1 | Atr-ERN05632 |  | | | |  |  |  |  |  |
| 1 | Atr-ERN05633 |  | | | |  |  |  |  |  |
| 1 | Atr-ERN05634 |  | | | |  |  |  |  |  |
| 1 | Atr-ERN05635 |  | | | |  |  |  |  |  |
| 1 | Atr-ERN05636 |  | | | |  |  |  |  |  |
| 1 | Atr-ERN05637 |  | | | |  |  |  |  |  |
| 1 | Atr-ERN05638 |  | | | |  |  |  |  |  |
| 1 | Atr-ERN05639 |  | | | |  |  |  |  |  |
| 1 | Atr-ERN05640 |  | | | |  |  |  |  |  |
| 1 | Atr-ERN05641 |  | | | |  |  |  |  |  |
| 1 | Atr-ERN05642 |  | | | |  |  |  |  |  |
| 1 | Atr-ERN05643 |  | | | |  |  |  |  |  |
| 1 | Atr-ERN05644 |  | | | |  |  |  |  |  |
| 1 | Atr-ERN05645 |  | | | |  |  |  |  |  |
| 1 | Atr-ERN05646 |  | Vvi-Vitvi01g00370\_t001 |  |  |  |  |  |
| 1 | Atr-ERN05647 |  | | | |  |  |  |  |  |
| 1 | Atr-ERN05648 |  | | | |  |  |  |  |  |
| 1 | Atr-ERN05649 |  | | | |  |  |  |  |  |
| 1 | Atr-ERN05650 |  | | | |  |  |  |  |  |
| 1 | Atr-ERN05651 |  | | | |  |  |  |  |  |
| 1 | Atr-ERN05652 |  | | | |  |  |  |  |  |
| 1 | Atr-ERN05653 |  | | | |  |  |  |  |  |
| 1 | Atr-ERN05654 |  | | | |  |  |  |  |  |
| 1 | Atr-ERN05655 |  | | | |  |  |  |  |  |
| 1 | Atr-ERN05656 |  | | | |  |  |  |  |  |
| 1 | Atr-ERN05657 |  | | | |  |  |  |  |  |
| 1 | Atr-ERN05658 |  | | | |  |  |  |  |  |
| 1 | Atr-ERN05659 |  | Vvi-Vitvi01g00366\_t001 |  |  |  |  |  |
| 1 | Atr-ERN05660 |  | | | |  |  |  |  |  |
| 1 | Atr-ERN05661 |  | | | |  |  |  |  |  |
| 1 | Atr-ERN05662 |  | | | |  |  |  |  |  |
| 1 | Atr-ERN05663 |  | | | |  |  |  |  |  |
| 1 | Atr-ERN05664 |  | | | |  |  |  |  |  |
| 1 | Atr-ERN05665 |  | | | |  |  |  |  |  |
| 1 | Atr-ERN05666 |  | | | |  |  |  |  |  |
| 1 | Atr-ERN05667 |  | | | |  |  |  |  |  |
| 1 | Atr-ERN05668 |  | | | |  |  |  |  |  |
| 1 | Atr-ERN05669 |  | | | |  |  |  |  |  |
| 1 | Atr-ERN05670 |  | Vvi-Vitvi01g00363\_t001 |  |  |  |  |  |
| 1 | Atr-ERN05671 |  | | | |  |  |  |  |  |
| 1 | Atr-ERN05672 |  | | | |  |  |  |  |  |
| 1 | Atr-ERN05673 |  | | | |  |  |  |  |  |
| 1 | Atr-ERN05674 |  | | | |  |  |  |  |  |
| 1 | Atr-ERN05675 |  | | | |  |  |  |  |  |
| 1 | Atr-ERN05676 |  | | | |  |  |  |  |  |
| 1 | Atr-ERN05677 |  | | | |  |  |  |  |  |
| 1 | Atr-ERN05678 |  | | | |  |  |  |  |  |
| 1 | Atr-ERN05679 |  | | | |  |  |  |  |  |
| 1 | Atr-ERN05680 |  | | | |  |  |  |  |  |
| 1 | Atr-ERN05681 |  | Vvi-Vitvi01g04098\_t001 |  |  |  |  |  |
| 0 | Atr-ERN05682 |  |  |  |  |  |  |
| 0 | Atr-ERN05683 |  |  |  |  |  |  |
| 0 | Atr-ERN05684 |  |  |  |  |  |  |
| 1 | Atr-ERN05685 |  | Vvi-Vitvi03g01579\_t001 |  |  |  |  |  |
| 1 | Atr-ERN05686 |  | | | |  |  |  |  |  |
| 1 | Atr-ERN05687 |  | Vvi-Vitvi03g00550\_t001 |  |  |  |  |  |
| 1 | Atr-ERN05688 |  | | | |  |  |  |  |  |
| 1 | Atr-ERN05689 |  | | | |  |  |  |  |  |
| 1 | Atr-ERN05690 |  | | | |  |  |  |  |  |
| 1 | Atr-ERN05691 |  | | | |  |  |  |  |  |
| 1 | Atr-ERN05692 |  | | | |  |  |  |  |  |
| 1 | Atr-ERN05693 |  | Vvi-Vitvi03g00553\_t001 |  |  |  |  |  |
| 1 | Atr-ERN05694 |  | | | |  |  |  |  |  |
| 1 | Atr-ERN05695 |  | | | |  |  |  |  |  |
| 1 | Atr-ERN05696 |  | | | |  |  |  |  |  |
| 1 | Atr-ERN05697 |  | | | |  |  |  |  |  |
| 1 | Atr-ERN05698 |  | | | |  |  |  |  |  |
| 1 | Atr-ERN05699 |  | | | |  |  |  |  |  |
| 1 | Atr-ERN05700 |  | | | |  |  |  |  |  |
| 1 | Atr-ERN05701 |  | | | |  |  |  |  |  |
| 1 | Atr-ERN05702 |  | | | |  |  |  |  |  |
| 1 | Atr-ERN05703 |  | Vvi-Vitvi03g00556\_t001 |  |  |  |  |  |
| 1 | Atr-ERN05704 |  | Vvi-Vitvi03g01580\_t001 |  |  |  |  |  |
| 1 | Atr-ERN05705 |  | | | |  |  |  |  |  |
| 1 | Atr-ERN05706 |  | | | |  |  |  |  |  |
| 1 | Atr-ERN05707 |  | | | |  |  |  |  |  |
| 1 | Atr-ERN05708 |  | | | |  |  |  |  |  |
| 1 | Atr-ERN05709 |  | | | |  |  |  |  |  |
| 1 | Atr-ERN05710 |  | | | |  |  |  |  |  |
| 1 | Atr-ERN05711 |  | | | |  |  |  |  |  |
| 1 | Atr-ERN05712 |  | | | |  |  |  |  |  |
| 1 | Atr-ERN05713 |  | | | |  |  |  |  |  |
| 1 | Atr-ERN05714 |  | | | |  |  |  |  |  |
| 1 | Atr-ERN05715 |  | Vvi-Vitvi03g00560\_t001 |  |  |  |  |  |
| 0 | Atr-ERN05716 |  |  |  |  |  |  |
| 0 | Atr-ERN05717 |  |  |  |  |  |  |
| 0 | Atr-ERN05718 |  |  |  |  |  |  |
| 0 | Atr-ERN05719 |  |  |  |  |  |  |
| 0 | Atr-ERN05720 |  |  |  |  |  |  |
| 0 | Atr-ERN05721 |  |  |  |  |  |  |
| 0 | Atr-ERN05722 |  |  |  |  |  |  |
| 0 | Atr-ERN05723 |  |  |  |  |  |  |
| 0 | Atr-ERN05724 |  |  |  |  |  |  |
| 0 | Atr-ERN05725 |  |  |  |  |  |  |
| 0 | Atr-ERN05726 |  |  |  |  |  |  |
| 0 | Atr-ERN05727 |  |  |  |  |  |  |
| 0 | Atr-ERN05728 |  |  |  |  |  |  |
| 0 | Atr-ERN05729 |  |  |  |  |  |  |
| 0 | Atr-ERN05730 |  |  |  |  |  |  |
| 0 | Atr-ERN05731 |  |  |  |  |  |  |
| 0 | Atr-ERN05732 |  |  |  |  |  |  |
| 0 | Atr-ERN05733 |  |  |  |  |  |  |
| 1 | Atr-ERN05734 |  | Vvi-Vitvi17g00451\_t001 |  |  |  |  |  |
| 1 | Atr-ERN05735 |  | Vvi-Vitvi17g01435\_t001 |  |  |  |  |  |
| 1 | Atr-ERN05736 |  | | | |  |  |  |  |  |
| 1 | Atr-ERN05737 |  | Vvi-Vitvi17g01434\_t001 |  |  |  |  |  |
| 1 | Atr-ERN05738 |  | | | |  |  |  |  |  |
| 1 | Atr-ERN05739 |  | | | |  |  |  |  |  |
| 2 | Atr-ERN05740 |  | | | |  | Vvi-Vitvi14g01330\_t001 |  |  |  |  |
| 2 | Atr-ERN05741 |  | | | |  | | | |  |  |  |  |
| 2 | Atr-ERN05742 |  | | | |  | | | |  |  |  |  |
| 2 | Atr-ERN05743 |  | Vvi-Vitvi17g00447\_t001 |  | | | |  |  |  |  |
| 2 | Atr-ERN05744 |  | | | |  | | | |  |  |  |  |
| 2 | Atr-ERN05745 |  | | | |  | | | |  |  |  |  |
| 2 | Atr-ERN05746 |  | Vvi-Vitvi17g00444\_t004 |  | | | |  |  |  |  |
| 2 | Atr-ERN05747 |  | | | |  | | | |  |  |  |  |
| 3 | Atr-ERN05748 |  | | | |  | | | |  | Vvi-Vitvi01g01289\_t001 |  |  |  |
| 3 | Atr-ERN05749 |  | | | |  | | | |  | | | |  |  |  |
| 3 | Atr-ERN05750 |  | Vvi-Vitvi17g00443\_t001 |  | Vvi-Vitvi14g01327\_t001 |  | | | |  |  |  |
| 3 | Atr-ERN05751 |  | | | |  | | | |  | | | |  |  |  |
| 3 | Atr-ERN05752 |  | Vvi-Vitvi17g00442\_t001 |  | Vvi-Vitvi14g01326\_t001 |  | | | |  |  |  |
| 3 | Atr-ERN05753 |  | | | |  | | | |  | | | |  |  |  |
| 3 | Atr-ERN05754 |  | | | |  | Vvi-Vitvi14g01325\_t001 |  | | | |  |  |  |
| 3 | Atr-ERN05755 |  | Vvi-Vitvi17g00441\_t001 |  | Vvi-Vitvi14g01321\_t001 |  | Vvi-Vitvi01g01299\_t001 |  |  |  |
| 3 | Atr-ERN05756 |  | | | |  | | | |  | | | |  |  |  |
| 3 | Atr-ERN05757 |  | | | |  | | | |  | | | |  |  |  |
| 3 | Atr-ERN05758 |  | | | |  | Vvi-Vitvi14g01319\_t001 |  | | | |  |  |  |
| 3 | Atr-ERN05759 |  | | | |  | | | |  | | | |  |  |  |
| 3 | Atr-ERN05760 |  | | | |  | | | |  | Vvi-Vitvi01g01301\_t001 |  |  |  |
| 3 | Atr-ERN05761 |  | | | |  | Vvi-Vitvi14g01316\_t001 |  | Vvi-Vitvi01g01304\_t001 |  |  |  |
| 3 | Atr-ERN05762 |  | Vvi-Vitvi17g00440\_t001 |  | | | |  | | | |  |  |  |
| 2 | Atr-ERN05763 |  |  |  | | | |  | | | |  |  |  |
| 2 | Atr-ERN05764 |  |  |  | | | |  | | | |  |  |  |
| 2 | Atr-ERN05765 |  |  |  | | | |  | | | |  |  |  |
| 2 | Atr-ERN05766 |  |  |  | | | |  | Vvi-Vitvi01g01307\_t001 |  |  |  |
| 2 | Atr-ERN05767 |  |  |  | | | |  | Vvi-Vitvi01g01311\_t001 |  |  |  |
| 2 | Atr-ERN05768 |  |  |  | | | |  | | | |  |  |  |
| 2 | Atr-ERN05769 |  |  |  | Vvi-Vitvi14g01314\_t001 |  | | | |  |  |  |
| 2 | Atr-ERN05770 |  |  |  | | | |  | | | |  |  |  |
| 2 | Atr-ERN05771 |  |  |  | | | |  | | | |  |  |  |
| 2 | Atr-ERN05772 |  |  |  | | | |  | | | |  |  |  |
| 2 | Atr-ERN05773 |  |  |  | | | |  | | | |  |  |  |
| 2 | Atr-ERN05774 |  |  |  | | | |  | | | |  |  |  |
| 2 | Atr-ERN05775 |  |  |  | | | |  | | | |  |  |  |
| 2 | Atr-ERN05776 |  |  |  | | | |  | | | |  |  |  |
| 2 | Atr-ERN05777 |  |  |  | | | |  | | | |  |  |  |
| 2 | Atr-ERN05778 |  |  |  | Vvi-Vitvi14g01311\_t001 |  | Vvi-Vitvi01g01312\_t001 |  |  |  |
| 2 | Atr-ERN05779 |  |  |  | Vvi-Vitvi14g01310\_t001 |  | | | |  |  |  |
| 2 | Atr-ERN05780 |  |  |  | Vvi-Vitvi14g01309\_t001 |  | | | |  |  |  |
| 2 | Atr-ERN05781 |  |  |  | | | |  | | | |  |  |  |
| 2 | Atr-ERN05782 |  |  |  | | | |  | | | |  |  |  |
| 2 | Atr-ERN05783 |  |  |  | | | |  | | | |  |  |  |
| 2 | Atr-ERN05784 |  |  |  | | | |  | | | |  |  |  |
| 2 | Atr-ERN05785 |  |  |  | | | |  | | | |  |  |  |
| 2 | Atr-ERN05786 |  |  |  | | | |  | | | |  |  |  |
| 2 | Atr-ERN05787 |  |  |  | | | |  | | | |  |  |  |
| 2 | Atr-ERN05788 |  |  |  | Vvi-Vitvi14g01307\_t001 |  | | | |  |  |  |
| 2 | Atr-ERN05789 |  |  |  | | | |  | | | |  |  |  |
| 2 | Atr-ERN05790 |  |  |  | | | |  | | | |  |  |  |
| 2 | Atr-ERN05791 |  |  |  | | | |  | | | |  |  |  |
| 2 | Atr-ERN05792 |  |  |  | | | |  | Vvi-Vitvi01g01313\_t001 |  |  |  |
| 2 | Atr-ERN05793 |  |  |  | | | |  | | | |  |  |  |
| 2 | Atr-ERN05794 |  |  |  | | | |  | | | |  |  |  |
| 2 | Atr-ERN05795 |  |  |  | | | |  | Vvi-Vitvi01g01314\_t001 |  |  |  |
| 2 | Atr-ERN05796 |  |  |  | | | |  | | | |  |  |  |
| 2 | Atr-ERN05797 |  |  |  | | | |  | | | |  |  |  |
| 2 | Atr-ERN05798 |  |  |  | | | |  | Vvi-Vitvi01g01315\_t001 |  |  |  |
| 2 | Atr-ERN05799 |  |  |  | Vvi-Vitvi14g01306\_t003 |  | Vvi-Vitvi01g01317\_t001 |  |  |  |
| 2 | Atr-ERN05800 |  |  |  | Vvi-Vitvi14g01305\_t001 |  | | | |  |  |  |
| 3 | Atr-ERN05801 |  | Vvi-Vitvi17g00436\_t001 |  | | | |  | | | |  |  |  |
| 3 | Atr-ERN05802 |  | | | |  | | | |  | | | |  |  |  |
| 3 | Atr-ERN05803 |  | | | |  | | | |  | | | |  |  |  |
| 3 | Atr-ERN05804 |  | | | |  | | | |  | | | |  |  |  |
| 3 | Atr-ERN05805 |  | | | |  | | | |  | | | |  |  |  |
| 3 | Atr-ERN05806 |  | | | |  | | | |  | | | |  |  |  |
| 3 | Atr-ERN05807 |  | Vvi-Vitvi17g00435\_t001 |  | Vvi-Vitvi14g01303\_t001 |  | | | |  |  |  |
| 3 | Atr-ERN05808 |  | | | |  | | | |  | Vvi-Vitvi01g01331\_t001 |  |  |  |
| 3 | Atr-ERN05809 |  | | | |  | | | |  | | | |  |  |  |
| 3 | Atr-ERN05810 |  | | | |  | | | |  | | | |  |  |  |
| 3 | Atr-ERN05811 |  | | | |  | | | |  | | | |  |  |  |
| 3 | Atr-ERN05812 |  | | | |  | | | |  | | | |  |  |  |
| 3 | Atr-ERN05813 |  | | | |  | | | |  | | | |  |  |  |
| 3 | Atr-ERN05814 |  | | | |  | | | |  | | | |  |  |  |
| 3 | Atr-ERN05815 |  | | | |  | Vvi-Vitvi14g01302\_t001 |  | | | |  |  |  |
| 3 | Atr-ERN05816 |  | | | |  | | | |  | | | |  |  |  |
| 3 | Atr-ERN05817 |  | Vvi-Vitvi17g04120\_t001 |  | | | |  | | | |  |  |  |
| 3 | Atr-ERN05818 |  | | | |  | | | |  | | | |  |  |  |
| 3 | Atr-ERN05819 |  | | | |  | | | |  | | | |  |  |  |
| 3 | Atr-ERN05820 |  | | | |  | | | |  | | | |  |  |  |
| 3 | Atr-ERN05821 |  | | | |  | | | |  | | | |  |  |  |
| 3 | Atr-ERN05822 |  | | | |  | | | |  | | | |  |  |  |
| 3 | Atr-ERN05823 |  | | | |  | | | |  | | | |  |  |  |
| 3 | Atr-ERN05824 |  | | | |  | | | |  | Vvi-Vitvi01g01339\_t001 |  |  |  |
| 3 | Atr-ERN05825 |  | Vvi-Vitvi17g00430\_t001 |  | | | |  | | | |  |  |  |
| 3 | Atr-ERN05826 |  | | | |  | | | |  | | | |  |  |  |
| 3 | Atr-ERN05827 |  | | | |  | | | |  | | | |  |  |  |
| 3 | Atr-ERN05828 |  | | | |  | | | |  | | | |  |  |  |
| 3 | Atr-ERN05829 |  | | | |  | | | |  | | | |  |  |  |
| 3 | Atr-ERN05830 |  | | | |  | | | |  | | | |  |  |  |
| 3 | Atr-ERN05831 |  | | | |  | | | |  | | | |  |  |  |
| 3 | Atr-ERN05832 |  | | | |  | | | |  | | | |  |  |  |
| 3 | Atr-ERN05833 |  | | | |  | | | |  | | | |  |  |  |
| 3 | Atr-ERN05834 |  | | | |  | | | |  | | | |  |  |  |
| 3 | Atr-ERN05835 |  | | | |  | | | |  | | | |  |  |  |
| 3 | Atr-ERN05836 |  | | | |  | Vvi-Vitvi14g01294\_t001 |  | Vvi-Vitvi01g01351\_t001 |  |  |  |
| 3 | Atr-ERN05837 |  | Vvi-Vitvi17g00426\_t001 |  | | | |  | Vvi-Vitvi01g01357\_t001 |  |  |  |
| 3 | Atr-ERN05838 |  | | | |  | | | |  | | | |  |  |  |
| 3 | Atr-ERN05839 |  | | | |  | | | |  | | | |  |  |  |
| 3 | Atr-ERN05840 |  | | | |  | | | |  | Vvi-Vitvi01g01359\_t001 |  |  |  |
| 3 | Atr-ERN05841 |  | | | |  | | | |  | | | |  |  |  |
| 3 | Atr-ERN05842 |  | | | |  | | | |  | | | |  |  |  |
| 3 | Atr-ERN05843 |  | | | |  | | | |  | | | |  |  |  |
| 3 | Atr-ERN05844 |  | | | |  | | | |  | | | |  |  |  |
| 3 | Atr-ERN05845 |  | | | |  | | | |  | | | |  |  |  |
| 3 | Atr-ERN05846 |  | | | |  | | | |  | Vvi-Vitvi01g01361\_t001 |  |  |  |
| 2 | Atr-ERN05847 |  | | | |  | | | |  |  |  |  |
| 2 | Atr-ERN05848 |  | | | |  | | | |  |  |  |  |
| 2 | Atr-ERN05849 |  | | | |  | | | |  |  |  |  |
| 2 | Atr-ERN05850 |  | | | |  | | | |  |  |  |  |
| 2 | Atr-ERN05851 |  | | | |  | | | |  |  |  |  |
| 2 | Atr-ERN05852 |  | | | |  | | | |  |  |  |  |
| 2 | Atr-ERN05853 |  | | | |  | Vvi-Vitvi14g01283\_t001 |  |  |  |  |
| 2 | Atr-ERN05854 |  | | | |  | | | |  |  |  |  |
| 2 | Atr-ERN05855 |  | Vvi-Vitvi17g00414\_t001 |  | | | |  |  |  |  |
| 2 | Atr-ERN05856 |  | | | |  | | | |  |  |  |  |
| 2 | Atr-ERN05857 |  | | | |  | | | |  |  |  |  |
| 2 | Atr-ERN05858 |  | | | |  | | | |  |  |  |  |
| 2 | Atr-ERN05859 |  | | | |  | | | |  |  |  |  |
| 2 | Atr-ERN05860 |  | | | |  | | | |  |  |  |  |
| 2 | Atr-ERN05861 |  | | | |  | | | |  |  |  |  |
| 2 | Atr-ERN05862 |  | | | |  | | | |  |  |  |  |
| 2 | Atr-ERN05863 |  | | | |  | | | |  |  |  |  |
| 2 | Atr-ERN05864 |  | | | |  | | | |  |  |  |  |
| 2 | Atr-ERN05865 |  | | | |  | | | |  |  |  |  |
| 2 | Atr-ERN05866 |  | Vvi-Vitvi17g00413\_t001 |  | | | |  |  |  |  |
| 2 | Atr-ERN05867 |  | | | |  | | | |  |  |  |  |
| 2 | Atr-ERN05868 |  | | | |  | | | |  |  |  |  |
| 2 | Atr-ERN05869 |  | | | |  | | | |  |  |  |  |
| 2 | Atr-ERN05870 |  | | | |  | | | |  |  |  |  |
| 2 | Atr-ERN05871 |  | | | |  | | | |  |  |  |  |
| 2 | Atr-ERN05872 |  | | | |  | | | |  |  |  |  |
| 2 | Atr-ERN05873 |  | | | |  | Vvi-Vitvi14g01281\_t001 |  |  |  |  |
| 2 | Atr-ERN05874 |  | | | |  | Vvi-Vitvi14g01280\_t001 |  |  |  |  |
| 2 | Atr-ERN05875 |  | | | |  | | | |  |  |  |  |
| 2 | Atr-ERN05876 |  | | | |  | Vvi-Vitvi14g01279\_t001 |  |  |  |  |
| 2 | Atr-ERN05877 |  | | | |  | | | |  |  |  |  |
| 2 | Atr-ERN05878 |  | | | |  | | | |  |  |  |  |
| 2 | Atr-ERN05879 |  | | | |  | | | |  |  |  |  |
| 2 | Atr-ERN05880 |  | | | |  | | | |  |  |  |  |
| 2 | Atr-ERN05881 |  | Vvi-Vitvi17g00411\_t001 |  | | | |  |  |  |  |
| 2 | Atr-ERN05882 |  | | | |  | | | |  |  |  |  |
| 2 | Atr-ERN05883 |  | | | |  | | | |  |  |  |  |
| 2 | Atr-ERN05884 |  | | | |  | | | |  |  |  |  |
| 2 | Atr-ERN05885 |  | | | |  | Vvi-Vitvi14g01268\_t001 |  |  |  |  |
| 2 | Atr-ERN05886 |  | | | |  | | | |  |  |  |  |
| 2 | Atr-ERN05887 |  | | | |  | | | |  |  |  |  |
| 2 | Atr-ERN05888 |  | | | |  | | | |  |  |  |  |
| 2 | Atr-ERN05889 |  | | | |  | | | |  |  |  |  |
| 2 | Atr-ERN05890 |  | | | |  | | | |  |  |  |  |
| 2 | Atr-ERN05891 |  | Vvi-Vitvi17g00409\_t001 |  | | | |  |  |  |  |
| 2 | Atr-ERN05892 |  | | | |  | | | |  |  |  |  |
| 2 | Atr-ERN05893 |  | | | |  | | | |  |  |  |  |
| 2 | Atr-ERN05894 |  | | | |  | | | |  |  |  |  |
| 2 | Atr-ERN05895 |  | | | |  | | | |  |  |  |  |
| 2 | Atr-ERN05896 |  | Vvi-Vitvi17g00406\_t001 |  | | | |  |  |  |  |
| 1 | Atr-ERN05897 |  |  |  | | | |  |  |  |  |
| 2 | Atr-ERN05898 |  | Vvi-Vitvi01g00777\_t005 |  | | | |  |  |  |  |
| 2 | Atr-ERN05899 |  | | | |  | | | |  |  |  |  |
| 2 | Atr-ERN05900 |  | | | |  | | | |  |  |  |  |
| 2 | Atr-ERN05901 |  | | | |  | Vvi-Vitvi14g02897\_t001 |  |  |  |  |
| 1 | Atr-ERN05902 |  | | | |  |  |  |  |  |
| 1 | Atr-ERN05903 |  | | | |  |  |  |  |  |
| 2 | Atr-ERN05904 |  | | | |  | Vvi-Vitvi14g01786\_t001 |  |  |  |  |
| 2 | Atr-ERN05905 |  | Vvi-Vitvi01g00779\_t001 |  | Vvi-Vitvi14g01787\_t001 |  |  |  |  |
| 2 | Atr-ERN05906 |  | | | |  | Vvi-Vitvi14g04630\_t001 |  |  |  |  |
| 3 | Atr-ERN05907 |  | | | |  | Vvi-Vitvi14g01789\_t001 |  | Vvi-Vitvi17g00790\_t001 |  |  |  |
| 3 | Atr-ERN05908 |  | | | |  | | | |  | | | |  |  |  |
| 3 | Atr-ERN05909 |  | Vvi-Vitvi01g00781\_t001 |  | Vvi-Vitvi14g01795\_t001 |  | | | |  |  |  |
| 3 | Atr-ERN05910 |  | Vvi-Vitvi01g00782\_t001 |  | | | |  | Vvi-Vitvi17g00787\_t001 |  |  |  |
| 3 | Atr-ERN05911 |  | Vvi-Vitvi01g00785\_t001 |  | Vvi-Vitvi14g01796\_t001 |  | Vvi-Vitvi17g00786\_t001 |  |  |  |
| 3 | Atr-ERN05912 |  | | | |  | | | |  | | | |  |  |  |
| 3 | Atr-ERN05913 |  | | | |  | | | |  | | | |  |  |  |
| 3 | Atr-ERN05914 |  | | | |  | Vvi-Vitvi14g01797\_t001 |  | Vvi-Vitvi17g04228\_t001 |  |  |  |
| 3 | Atr-ERN05915 |  | | | |  | | | |  | | | |  |  |  |
| 3 | Atr-ERN05916 |  | | | |  | | | |  | | | |  |  |  |
| 3 | Atr-ERN05917 |  | | | |  | | | |  | Vvi-Vitvi17g00781\_t001 |  |  |  |
| 3 | Atr-ERN05918 |  | Vvi-Vitvi01g00789\_t001 |  | | | |  | Vvi-Vitvi17g00780\_t001 |  |  |  |
| 3 | Atr-ERN05919 |  | | | |  | | | |  | | | |  |  |  |
| 3 | Atr-ERN05920 |  | | | |  | | | |  | | | |  |  |  |
| 3 | Atr-ERN05921 |  | | | |  | | | |  | | | |  |  |  |
| 3 | Atr-ERN05922 |  | | | |  | | | |  | | | |  |  |  |
| 3 | Atr-ERN05923 |  | | | |  | | | |  | Vvi-Vitvi17g00779\_t001 |  |  |  |
| 3 | Atr-ERN05924 |  | | | |  | | | |  | | | |  |  |  |
| 3 | Atr-ERN05925 |  | | | |  | | | |  | Vvi-Vitvi17g00778\_t001 |  |  |  |
| 2 | Atr-ERN05926 |  | Vvi-Vitvi01g00792\_t002 |  | Vvi-Vitvi14g01798\_t001 |  |  |  |  |
