# Supplementary material for: Genome-wide identification, characterization and gene expression of BES1 transcription factor family in grapevine (Vitis vinifera L.)
Source: Sci Rep. 2023 Jan 5;13:240. doi: 10.1038/s41598-022-24407-y (PMC9816167; doi:10.1038/s41598-022-24407-y)
Supplement: Supplementary file 3 — Supplementary Information. [file 41598_2022_24407_MOESM3_ESM.zip › Vvi_Atr/Vitis_vinifera.PN40024.v4.dna_sm.toplevel.fa.vs.Amborella_trichopoda.AMTR1.0.dna_sm.toplevel.fa.html/Atr-AmTr_v1.0_scaffold00007.html]

|  |  |  |  |  |  |  |  |  |  |  |  |  |  |
| --- | --- | --- | --- | --- | --- | --- | --- | --- | --- | --- | --- | --- | --- |
| Duplication depth | Reference chromosome | Collinear blocks | | | | | | | | | | | |
| 0 | Atr-ERN05190 |  |  |  |  |  |  |
| 0 | Atr-ERN05191 |  |  |  |  |  |  |
| 0 | Atr-ERN05192 |  |  |  |  |  |  |
| 0 | Atr-ERN05193 |  |  |  |  |  |  |
| 0 | Atr-ERN05194 |  |  |  |  |  |  |
| 0 | Atr-ERN05195 |  |  |  |  |  |  |
| 0 | Atr-ERN05196 |  |  |  |  |  |  |
| 0 | Atr-ERN05197 |  |  |  |  |  |  |
| 0 | Atr-ERN05198 |  |  |  |  |  |  |
| 0 | Atr-ERN05199 |  |  |  |  |  |  |
| 0 | Atr-ERN05200 |  |  |  |  |  |  |
| 0 | Atr-ERN05201 |  |  |  |  |  |  |
| 0 | Atr-ERN05202 |  |  |  |  |  |  |
| 0 | Atr-ERN05203 |  |  |  |  |  |  |
| 0 | Atr-ERN05204 |  |  |  |  |  |  |
| 0 | Atr-ERN05205 |  |  |  |  |  |  |
| 0 | Atr-ERN05206 |  |  |  |  |  |  |
| 0 | Atr-ERN05207 |  |  |  |  |  |  |
| 0 | Atr-ERN05208 |  |  |  |  |  |  |
| 0 | Atr-ERN05209 |  |  |  |  |  |  |
| 0 | Atr-ERN05210 |  |  |  |  |  |  |
| 0 | Atr-ERN05211 |  |  |  |  |  |  |
| 0 | Atr-ERN05212 |  |  |  |  |  |  |
| 0 | Atr-ERN05213 |  |  |  |  |  |  |
| 0 | Atr-ERN05214 |  |  |  |  |  |  |
| 0 | Atr-ERN05215 |  |  |  |  |  |  |
| 0 | Atr-ERN05216 |  |  |  |  |  |  |
| 0 | Atr-ERN05217 |  |  |  |  |  |  |
| 0 | Atr-ERN05218 |  |  |  |  |  |  |
| 0 | Atr-ERN05219 |  |  |  |  |  |  |
| 0 | Atr-ERN05220 |  |  |  |  |  |  |
| 0 | Atr-ERN05221 |  |  |  |  |  |  |
| 0 | Atr-ERN05222 |  |  |  |  |  |  |
| 3 | Atr-ERN05223 |  | Vvi-Vitvi13g00047\_t001 |  | Vvi-Vitvi06g00647\_t001 |  | Vvi-Vitvi08g00642\_t001 |  |  |  |
| 3 | Atr-ERN05224 |  | | | |  | | | |  | | | |  |  |  |
| 3 | Atr-ERN05225 |  | Vvi-Vitvi13g00049\_t001 |  | | | |  | | | |  |  |  |
| 3 | Atr-ERN05226 |  | | | |  | | | |  | | | |  |  |  |
| 3 | Atr-ERN05227 |  | Vvi-Vitvi13g00050\_t001 |  | Vvi-Vitvi06g00650\_t001 |  | | | |  |  |  |
| 3 | Atr-ERN05228 |  | | | |  | | | |  | | | |  |  |  |
| 3 | Atr-ERN05229 |  | | | |  | | | |  | | | |  |  |  |
| 3 | Atr-ERN05230 |  | | | |  | | | |  | Vvi-Vitvi08g00643\_t001 |  |  |  |
| 3 | Atr-ERN05231 |  | | | |  | | | |  | | | |  |  |  |
| 3 | Atr-ERN05232 |  | | | |  | Vvi-Vitvi06g00651\_t002 |  | | | |  |  |  |
| 3 | Atr-ERN05233 |  | | | |  | | | |  | | | |  |  |  |
| 3 | Atr-ERN05234 |  | | | |  | | | |  | | | |  |  |  |
| 3 | Atr-ERN05235 |  | | | |  | | | |  | | | |  |  |  |
| 3 | Atr-ERN05236 |  | | | |  | | | |  | | | |  |  |  |
| 3 | Atr-ERN05237 |  | | | |  | | | |  | | | |  |  |  |
| 3 | Atr-ERN05238 |  | | | |  | | | |  | | | |  |  |  |
| 3 | Atr-ERN05239 |  | | | |  | | | |  | | | |  |  |  |
| 3 | Atr-ERN05240 |  | Vvi-Vitvi13g00051\_t001 |  | | | |  | | | |  |  |  |
| 3 | Atr-ERN05241 |  | | | |  | | | |  | Vvi-Vitvi08g00644\_t001 |  |  |  |
| 3 | Atr-ERN05242 |  | Vvi-Vitvi13g00053\_t001 |  | Vvi-Vitvi06g00652\_t001 |  | | | |  |  |  |
| 3 | Atr-ERN05243 |  | | | |  | | | |  | | | |  |  |  |
| 3 | Atr-ERN05244 |  | | | |  | | | |  | | | |  |  |  |
| 3 | Atr-ERN05245 |  | | | |  | | | |  | | | |  |  |  |
| 3 | Atr-ERN05246 |  | | | |  | | | |  | | | |  |  |  |
| 3 | Atr-ERN05247 |  | | | |  | | | |  | | | |  |  |  |
| 3 | Atr-ERN05248 |  | | | |  | | | |  | | | |  |  |  |
| 3 | Atr-ERN05249 |  | | | |  | | | |  | | | |  |  |  |
| 3 | Atr-ERN05250 |  | | | |  | Vvi-Vitvi06g00653\_t002 |  | | | |  |  |  |
| 3 | Atr-ERN05251 |  | | | |  | | | |  | | | |  |  |  |
| 3 | Atr-ERN05252 |  | | | |  | | | |  | | | |  |  |  |
| 3 | Atr-ERN05253 |  | Vvi-Vitvi13g00055\_t001 |  | | | |  | Vvi-Vitvi08g00656\_t001 |  |  |  |
| 3 | Atr-ERN05254 |  | | | |  | | | |  | | | |  |  |  |
| 3 | Atr-ERN05255 |  | | | |  | | | |  | | | |  |  |  |
| 3 | Atr-ERN05256 |  | | | |  | | | |  | | | |  |  |  |
| 3 | Atr-ERN05257 |  | | | |  | | | |  | | | |  |  |  |
| 3 | Atr-ERN05258 |  | | | |  | | | |  | | | |  |  |  |
| 3 | Atr-ERN05259 |  | | | |  | | | |  | | | |  |  |  |
| 3 | Atr-ERN05260 |  | | | |  | | | |  | | | |  |  |  |
| 3 | Atr-ERN05261 |  | | | |  | | | |  | | | |  |  |  |
| 3 | Atr-ERN05262 |  | Vvi-Vitvi13g00060\_t001 |  | Vvi-Vitvi06g00654\_t001 |  | Vvi-Vitvi08g00662\_t001 |  |  |  |
| 3 | Atr-ERN05263 |  | | | |  | Vvi-Vitvi06g00657\_t001 |  | | | |  |  |  |
| 3 | Atr-ERN05264 |  | | | |  | | | |  | Vvi-Vitvi08g00665\_t001 |  |  |  |
| 3 | Atr-ERN05265 |  | Vvi-Vitvi13g00061\_t001 |  | | | |  | Vvi-Vitvi08g00666\_t001 |  |  |  |
| 3 | Atr-ERN05266 |  | | | |  | Vvi-Vitvi06g01751\_t001 |  | | | |  |  |  |
| 3 | Atr-ERN05267 |  | Vvi-Vitvi13g00062\_t001 |  | | | |  | | | |  |  |  |
| 3 | Atr-ERN05268 |  | | | |  | | | |  | | | |  |  |  |
| 3 | Atr-ERN05269 |  | | | |  | | | |  | Vvi-Vitvi08g00667\_t001 |  |  |  |
| 3 | Atr-ERN05270 |  | | | |  | | | |  | | | |  |  |  |
| 3 | Atr-ERN05271 |  | | | |  | | | |  | | | |  |  |  |
| 3 | Atr-ERN05272 |  | Vvi-Vitvi13g00066\_t001 |  | | | |  | | | |  |  |  |
| 2 | Atr-ERN05273 |  |  |  | | | |  | | | |  |  |  |
| 2 | Atr-ERN05274 |  |  |  | | | |  | | | |  |  |  |
| 2 | Atr-ERN05275 |  |  |  | Vvi-Vitvi06g00666\_t001 |  | | | |  |  |  |
| 1 | Atr-ERN05276 |  |  |  |  |  | | | |  |  |  |
| 1 | Atr-ERN05277 |  |  |  |  |  | | | |  |  |  |
| 1 | Atr-ERN05278 |  |  |  |  |  | | | |  |  |  |
| 1 | Atr-ERN05279 |  |  |  |  |  | | | |  |  |  |
| 1 | Atr-ERN05280 |  |  |  |  |  | | | |  |  |  |
| 1 | Atr-ERN05281 |  |  |  |  |  | Vvi-Vitvi08g00671\_t001 |  |  |  |
| 1 | Atr-ERN05282 |  |  |  |  |  | Vvi-Vitvi08g00672\_t001 |  |  |  |
| 1 | Atr-ERN05283 |  |  |  |  |  | | | |  |  |  |
| 1 | Atr-ERN05284 |  |  |  |  |  | | | |  |  |  |
| 1 | Atr-ERN05285 |  |  |  |  |  | | | |  |  |  |
| 1 | Atr-ERN05286 |  |  |  |  |  | | | |  |  |  |
| 1 | Atr-ERN05287 |  |  |  |  |  | | | |  |  |  |
| 1 | Atr-ERN05288 |  |  |  |  |  | | | |  |  |  |
| 1 | Atr-ERN05289 |  |  |  |  |  | | | |  |  |  |
| 1 | Atr-ERN05290 |  |  |  |  |  | | | |  |  |  |
| 1 | Atr-ERN05291 |  |  |  |  |  | | | |  |  |  |
| 1 | Atr-ERN05292 |  |  |  |  |  | | | |  |  |  |
| 1 | Atr-ERN05293 |  |  |  |  |  | | | |  |  |  |
| 1 | Atr-ERN05294 |  |  |  |  |  | | | |  |  |  |
| 1 | Atr-ERN05295 |  |  |  |  |  | | | |  |  |  |
| 1 | Atr-ERN05296 |  |  |  |  |  | | | |  |  |  |
| 1 | Atr-ERN05297 |  |  |  |  |  | | | |  |  |  |
| 1 | Atr-ERN05298 |  |  |  |  |  | | | |  |  |  |
| 1 | Atr-ERN05299 |  |  |  |  |  | Vvi-Vitvi08g00674\_t002 |  |  |  |
| 1 | Atr-ERN05300 |  |  |  |  |  | | | |  |  |  |
| 1 | Atr-ERN05301 |  |  |  |  |  | Vvi-Vitvi08g00676\_t002 |  |  |  |
| 1 | Atr-ERN05302 |  |  |  |  |  | | | |  |  |  |
| 1 | Atr-ERN05303 |  |  |  |  |  | | | |  |  |  |
| 1 | Atr-ERN05304 |  |  |  |  |  | | | |  |  |  |
| 1 | Atr-ERN05305 |  |  |  |  |  | | | |  |  |  |
| 1 | Atr-ERN05306 |  |  |  |  |  | Vvi-Vitvi08g00678\_t001 |  |  |  |
| 1 | Atr-ERN05307 |  |  |  |  |  | | | |  |  |  |
| 1 | Atr-ERN05308 |  |  |  |  |  | | | |  |  |  |
| 1 | Atr-ERN05309 |  |  |  |  |  | | | |  |  |  |
| 2 | Atr-ERN05310 |  | Vvi-Vitvi06g00667\_t001 |  |  |  | | | |  |  |  |
| 2 | Atr-ERN05311 |  | | | |  |  |  | | | |  |  |  |
| 2 | Atr-ERN05312 |  | | | |  |  |  | | | |  |  |  |
| 2 | Atr-ERN05313 |  | | | |  |  |  | Vvi-Vitvi08g00679\_t001 |  |  |  |
| 2 | Atr-ERN05314 |  | | | |  |  |  | | | |  |  |  |
| 2 | Atr-ERN05315 |  | | | |  |  |  | | | |  |  |  |
| 2 | Atr-ERN05316 |  | Vvi-Vitvi06g00668\_t001 |  |  |  | | | |  |  |  |
| 2 | Atr-ERN05317 |  | | | |  |  |  | | | |  |  |  |
| 2 | Atr-ERN05318 |  | | | |  |  |  | | | |  |  |  |
| 2 | Atr-ERN05319 |  | | | |  |  |  | Vvi-Vitvi08g00685\_t001 |  |  |  |
| 2 | Atr-ERN05320 |  | Vvi-Vitvi06g00669\_t001 |  |  |  | | | |  |  |  |
| 2 | Atr-ERN05321 |  | | | |  |  |  | | | |  |  |  |
| 2 | Atr-ERN05322 |  | | | |  |  |  | | | |  |  |  |
| 2 | Atr-ERN05323 |  | | | |  |  |  | | | |  |  |  |
| 2 | Atr-ERN05324 |  | Vvi-Vitvi06g00672\_t001 |  |  |  | | | |  |  |  |
| 2 | Atr-ERN05325 |  | | | |  |  |  | Vvi-Vitvi08g00687\_t001 |  |  |  |
| 2 | Atr-ERN05326 |  | Vvi-Vitvi06g00679\_t001 |  |  |  | | | |  |  |  |
| 2 | Atr-ERN05327 |  | | | |  |  |  | | | |  |  |  |
| 2 | Atr-ERN05328 |  | Vvi-Vitvi06g00680\_t001 |  |  |  | | | |  |  |  |
| 1 | Atr-ERN05329 |  |  |  |  |  | | | |  |  |  |
| 1 | Atr-ERN05330 |  |  |  |  |  | | | |  |  |  |
| 1 | Atr-ERN05331 |  |  |  |  |  | Vvi-Vitvi08g00690\_t001 |  |  |  |
| 0 | Atr-ERN05332 |  |  |  |  |  |  |
| 0 | Atr-ERN05333 |  |  |  |  |  |  |
| 0 | Atr-ERN05334 |  |  |  |  |  |  |
| 0 | Atr-ERN05335 |  |  |  |  |  |  |
| 0 | Atr-ERN05336 |  |  |  |  |  |  |
| 0 | Atr-ERN05337 |  |  |  |  |  |  |
| 0 | Atr-ERN05338 |  |  |  |  |  |  |
| 0 | Atr-ERN05339 |  |  |  |  |  |  |
| 0 | Atr-ERN05340 |  |  |  |  |  |  |
| 0 | Atr-ERN05341 |  |  |  |  |  |  |
| 0 | Atr-ERN05342 |  |  |  |  |  |  |
| 0 | Atr-ERN05343 |  |  |  |  |  |  |
| 0 | Atr-ERN05344 |  |  |  |  |  |  |
| 0 | Atr-ERN05345 |  |  |  |  |  |  |
| 0 | Atr-ERN05346 |  |  |  |  |  |  |
| 0 | Atr-ERN05347 |  |  |  |  |  |  |
| 0 | Atr-ERN05348 |  |  |  |  |  |  |
| 0 | Atr-ERN05349 |  |  |  |  |  |  |
| 0 | Atr-ERN05350 |  |  |  |  |  |  |
| 0 | Atr-ERN05351 |  |  |  |  |  |  |
| 0 | Atr-ERN05352 |  |  |  |  |  |  |
| 0 | Atr-ERN05353 |  |  |  |  |  |  |
| 0 | Atr-ERN05354 |  |  |  |  |  |  |
| 0 | Atr-ERN05355 |  |  |  |  |  |  |
| 0 | Atr-ERN05356 |  |  |  |  |  |  |
| 0 | Atr-ERN05357 |  |  |  |  |  |  |
| 0 | Atr-ERN05358 |  |  |  |  |  |  |
| 2 | Atr-ERN05359 |  | Vvi-Vitvi13g00132\_t001 |  | Vvi-Vitvi08g00803\_t001 |  |  |  |  |
| 2 | Atr-ERN05360 |  | | | |  | Vvi-Vitvi08g00805\_t001 |  |  |  |  |
| 3 | Atr-ERN05361 |  | | | |  | | | |  | Vvi-Vitvi06g01769\_t001 |  |  |  |
| 3 | Atr-ERN05362 |  | Vvi-Vitvi13g00133\_t001 |  | Vvi-Vitvi08g00806\_t001 |  | | | |  |  |  |
| 3 | Atr-ERN05363 |  | | | |  | | | |  | | | |  |  |  |
| 3 | Atr-ERN05364 |  | Vvi-Vitvi13g00134\_t001 |  | | | |  | | | |  |  |  |
| 3 | Atr-ERN05365 |  | | | |  | | | |  | | | |  |  |  |
| 3 | Atr-ERN05366 |  | | | |  | | | |  | Vvi-Vitvi06g00746\_t001 |  |  |  |
| 3 | Atr-ERN05367 |  | | | |  | | | |  | | | |  |  |  |
| 3 | Atr-ERN05368 |  | | | |  | | | |  | | | |  |  |  |
| 3 | Atr-ERN05369 |  | | | |  | Vvi-Vitvi08g02086\_t001 |  | Vvi-Vitvi06g01772\_t001 |  |  |  |
| 3 | Atr-ERN05370 |  | | | |  | Vvi-Vitvi08g00812\_t001 |  | | | |  |  |  |
| 3 | Atr-ERN05371 |  | | | |  | | | |  | | | |  |  |  |
| 3 | Atr-ERN05372 |  | | | |  | Vvi-Vitvi08g02087\_t001 |  | | | |  |  |  |
| 3 | Atr-ERN05373 |  | | | |  | | | |  | | | |  |  |  |
| 3 | Atr-ERN05374 |  | Vvi-Vitvi13g00135\_t001 |  | Vvi-Vitvi08g00816\_t001 |  | Vvi-Vitvi06g00759\_t001 |  |  |  |
| 3 | Atr-ERN05375 |  | | | |  | Vvi-Vitvi08g00817\_t001 |  | | | |  |  |  |
| 3 | Atr-ERN05376 |  | | | |  | | | |  | | | |  |  |  |
| 3 | Atr-ERN05377 |  | | | |  | Vvi-Vitvi08g00819\_t001 |  | | | |  |  |  |
| 4 | Atr-ERN05378 |  | | | |  | | | |  | Vvi-Vitvi06g01773\_t002 |  | Vvi-Vitvi08g00845\_t001 |  |  |
| 6 | Atr-ERN05379 |  | | | |  | | | |  | | | |  | Vvi-Vitvi08g00844\_t001 |  | Vvi-Vitvi13g00158\_t001 |  | Vvi-Vitvi06g00785\_t001 |
| 6 | Atr-ERN05380 |  | | | |  | | | |  | | | |  | | | |  | Vvi-Vitvi13g00157\_t001 |  | | | |
| 6 | Atr-ERN05381 |  | | | |  | | | |  | | | |  | Vvi-Vitvi08g00843\_t001 |  | | | |  | Vvi-Vitvi06g00784\_t001 |
| 6 | Atr-ERN05382 |  | | | |  | | | |  | | | |  | Vvi-Vitvi08g00841\_t001 |  | | | |  | | | |
| 6 | Atr-ERN05383 |  | | | |  | | | |  | | | |  | Vvi-Vitvi08g00840\_t001 |  | | | |  | Vvi-Vitvi06g00783\_t001 |
| 6 | Atr-ERN05384 |  | | | |  | | | |  | | | |  | | | |  | | | |  | | | |
| 6 | Atr-ERN05385 |  | | | |  | | | |  | | | |  | | | |  | | | |  | | | |
| 6 | Atr-ERN05386 |  | | | |  | | | |  | | | |  | | | |  | | | |  | | | |
| 6 | Atr-ERN05387 |  | | | |  | | | |  | | | |  | | | |  | | | |  | Vvi-Vitvi06g00782\_t001 |
| 6 | Atr-ERN05388 |  | | | |  | | | |  | | | |  | Vvi-Vitvi08g00839\_t001 |  | | | |  | | | |
| 6 | Atr-ERN05389 |  | | | |  | | | |  | | | |  | | | |  | Vvi-Vitvi13g00156\_t001 |  | | | |
| 6 | Atr-ERN05390 |  | | | |  | | | |  | | | |  | | | |  | Vvi-Vitvi13g00155\_t001 |  | | | |
| 6 | Atr-ERN05391 |  | | | |  | | | |  | | | |  | | | |  | | | |  | Vvi-Vitvi06g00781\_t001 |
| 6 | Atr-ERN05392 |  | | | |  | | | |  | | | |  | Vvi-Vitvi08g00836\_t001 |  | | | |  | | | |
| 6 | Atr-ERN05393 |  | | | |  | | | |  | | | |  | | | |  | Vvi-Vitvi13g00153\_t001 |  | | | |
| 6 | Atr-ERN05394 |  | | | |  | | | |  | | | |  | | | |  | | | |  | | | |
| 6 | Atr-ERN05395 |  | Vvi-Vitvi13g00146\_t001 |  | | | |  | | | |  | | | |  | | | |  | | | |
| 6 | Atr-ERN05396 |  | Vvi-Vitvi13g00147\_t001 |  | | | |  | | | |  | | | |  | | | |  | | | |
| 6 | Atr-ERN05397 |  | | | |  | | | |  | | | |  | | | |  | | | |  | | | |
| 6 | Atr-ERN05398 |  | Vvi-Vitvi13g00149\_t001 |  | Vvi-Vitvi08g00828\_t001 |  | Vvi-Vitvi06g00775\_t003 |  | Vvi-Vitvi08g00835\_t001 |  | | | |  | | | |
| 6 | Atr-ERN05399 |  | | | |  | Vvi-Vitvi08g00829\_t001 |  | | | |  | | | |  | | | |  | | | |
| 6 | Atr-ERN05400 |  | | | |  | | | |  | | | |  | | | |  | | | |  | | | |
| 6 | Atr-ERN05401 |  | | | |  | | | |  | | | |  | | | |  | | | |  | | | |
| 6 | Atr-ERN05402 |  | | | |  | | | |  | | | |  | | | |  | | | |  | | | |
| 6 | Atr-ERN05403 |  | | | |  | | | |  | | | |  | | | |  | | | |  | | | |
| 6 | Atr-ERN05404 |  | | | |  | Vvi-Vitvi08g00835\_t001 |  | Vvi-Vitvi06g00779\_t001 |  | | | |  | | | |  | | | |
| 4 | Atr-ERN05405 |  | | | |  |  |  |  |  | | | |  | Vvi-Vitvi13g01905\_t001 |  | | | |
| 4 | Atr-ERN05406 |  | | | |  |  |  |  |  | | | |  | | | |  | | | |
| 4 | Atr-ERN05407 |  | Vvi-Vitvi13g00150\_t001 |  |  |  |  |  | Vvi-Vitvi08g00834\_t001 |  | | | |  | | | |
| 4 | Atr-ERN05408 |  | | | |  |  |  |  |  | | | |  | | | |  | Vvi-Vitvi06g00777\_t001 |
| 4 | Atr-ERN05409 |  | | | |  |  |  |  |  | | | |  | | | |  | Vvi-Vitvi06g00776\_t001 |
| 4 | Atr-ERN05410 |  | Vvi-Vitvi13g00151\_t001 |  |  |  |  |  | | | |  | | | |  | | | |
| 3 | Atr-ERN05411 |  |  |  |  |  |  |  | Vvi-Vitvi08g00826\_t002 |  | | | |  | | | |
| 3 | Atr-ERN05412 |  |  |  |  |  |  |  | Vvi-Vitvi08g00825\_t002 |  | Vvi-Vitvi13g00145\_t001 |  | | | |
| 3 | Atr-ERN05413 |  |  |  |  |  |  |  | Vvi-Vitvi08g02089\_t001 |  | Vvi-Vitvi13g01903\_t001 |  | Vvi-Vitvi06g01776\_t001 |
| 3 | Atr-ERN05414 |  |  |  |  |  |  |  | | | |  | | | |  | | | |
| 3 | Atr-ERN05415 |  |  |  |  |  |  |  | | | |  | | | |  | | | |
| 3 | Atr-ERN05416 |  |  |  |  |  |  |  | | | |  | Vvi-Vitvi13g00144\_t001 |  | Vvi-Vitvi06g00772\_t001 |
| 3 | Atr-ERN05417 |  |  |  |  |  |  |  | | | |  | | | |  | | | |
| 3 | Atr-ERN05418 |  |  |  |  |  |  |  | | | |  | | | |  | | | |
| 3 | Atr-ERN05419 |  |  |  |  |  |  |  | Vvi-Vitvi08g00821\_t001 |  | Vvi-Vitvi13g00143\_t001 |  | Vvi-Vitvi06g00768\_t001 |
| 0 | Atr-ERN05420 |  |  |  |  |  |  |
| 0 | Atr-ERN05421 |  |  |  |  |  |  |
| 0 | Atr-ERN05422 |  |  |  |  |  |  |
| 0 | Atr-ERN05423 |  |  |  |  |  |  |
| 0 | Atr-ERN05424 |  |  |  |  |  |  |
| 0 | Atr-ERN05425 |  |  |  |  |  |  |
| 0 | Atr-ERN05426 |  |  |  |  |  |  |
| 0 | Atr-ERN05427 |  |  |  |  |  |  |
| 0 | Atr-ERN05428 |  |  |  |  |  |  |
| 0 | Atr-ERN05429 |  |  |  |  |  |  |
| 0 | Atr-ERN05430 |  |  |  |  |  |  |
| 0 | Atr-ERN05431 |  |  |  |  |  |  |
| 0 | Atr-ERN05432 |  |  |  |  |  |  |
| 0 | Atr-ERN05433 |  |  |  |  |  |  |
| 0 | Atr-ERN05434 |  |  |  |  |  |  |
| 0 | Atr-ERN05435 |  |  |  |  |  |  |
| 0 | Atr-ERN05436 |  |  |  |  |  |  |
| 0 | Atr-ERN05437 |  |  |  |  |  |  |
| 0 | Atr-ERN05438 |  |  |  |  |  |  |
| 0 | Atr-ERN05439 |  |  |  |  |  |  |
| 0 | Atr-ERN05440 |  |  |  |  |  |  |
| 0 | Atr-ERN05441 |  |  |  |  |  |  |
| 0 | Atr-ERN05442 |  |  |  |  |  |  |
| 0 | Atr-ERN05443 |  |  |  |  |  |  |
| 0 | Atr-ERN05444 |  |  |  |  |  |  |
| 0 | Atr-ERN05445 |  |  |  |  |  |  |
| 0 | Atr-ERN05446 |  |  |  |  |  |  |
| 0 | Atr-ERN05447 |  |  |  |  |  |  |
| 0 | Atr-ERN05448 |  |  |  |  |  |  |
| 0 | Atr-ERN05449 |  |  |  |  |  |  |
| 0 | Atr-ERN05450 |  |  |  |  |  |  |
| 0 | Atr-ERN05451 |  |  |  |  |  |  |
| 0 | Atr-ERN05452 |  |  |  |  |  |  |
| 0 | Atr-ERN05453 |  |  |  |  |  |  |
| 0 | Atr-ERN05454 |  |  |  |  |  |  |
| 0 | Atr-ERN05455 |  |  |  |  |  |  |
| 0 | Atr-ERN05456 |  |  |  |  |  |  |
| 0 | Atr-ERN05457 |  |  |  |  |  |  |
| 0 | Atr-ERN05458 |  |  |  |  |  |  |
| 0 | Atr-ERN05459 |  |  |  |  |  |  |
| 0 | Atr-ERN05460 |  |  |  |  |  |  |
| 0 | Atr-ERN05461 |  |  |  |  |  |  |
| 0 | Atr-ERN05462 |  |  |  |  |  |  |
| 0 | Atr-ERN05463 |  |  |  |  |  |  |
| 0 | Atr-ERN05464 |  |  |  |  |  |  |
| 0 | Atr-ERN05465 |  |  |  |  |  |  |
| 0 | Atr-ERN05466 |  |  |  |  |  |  |
| 0 | Atr-ERN05467 |  |  |  |  |  |  |
| 0 | Atr-ERN05468 |  |  |  |  |  |  |
| 0 | Atr-ERN05469 |  |  |  |  |  |  |
| 0 | Atr-ERN05470 |  |  |  |  |  |  |
| 0 | Atr-ERN05471 |  |  |  |  |  |  |
| 0 | Atr-ERN05472 |  |  |  |  |  |  |
| 0 | Atr-ERN05473 |  |  |  |  |  |  |
| 0 | Atr-ERN05474 |  |  |  |  |  |  |
| 0 | Atr-ERN05475 |  |  |  |  |  |  |
| 0 | Atr-ERN05476 |  |  |  |  |  |  |
| 0 | Atr-ERN05477 |  |  |  |  |  |  |
| 0 | Atr-ERN05478 |  |  |  |  |  |  |
| 0 | Atr-ERN05479 |  |  |  |  |  |  |
| 0 | Atr-ERN05480 |  |  |  |  |  |  |
| 0 | Atr-ERN05481 |  |  |  |  |  |  |
| 0 | Atr-ERN05482 |  |  |  |  |  |  |
| 0 | Atr-ERN05483 |  |  |  |  |  |  |
| 0 | Atr-ERN05484 |  |  |  |  |  |  |
| 0 | Atr-ERN05485 |  |  |  |  |  |  |
| 0 | Atr-ERN05486 |  |  |  |  |  |  |
| 0 | Atr-ERN05487 |  |  |  |  |  |  |
| 0 | Atr-ERN05488 |  |  |  |  |  |  |
| 0 | Atr-ERN05489 |  |  |  |  |  |  |
| 0 | Atr-ERN05490 |  |  |  |  |  |  |
| 0 | Atr-ERN05491 |  |  |  |  |  |  |
| 0 | Atr-ERN05492 |  |  |  |  |  |  |
| 0 | Atr-ERN05493 |  |  |  |  |  |  |
| 0 | Atr-ERN05494 |  |  |  |  |  |  |
| 0 | Atr-ERN05495 |  |  |  |  |  |  |
| 0 | Atr-ERN05496 |  |  |  |  |  |  |
| 0 | Atr-ERN05497 |  |  |  |  |  |  |
| 0 | Atr-ERN05498 |  |  |  |  |  |  |
| 0 | Atr-ERN05499 |  |  |  |  |  |  |
| 0 | Atr-ERN05500 |  |  |  |  |  |  |
| 0 | Atr-ERN05501 |  |  |  |  |  |  |
| 0 | Atr-ERN05502 |  |  |  |  |  |  |
| 2 | Atr-ERN05503 |  | Vvi-Vitvi09g00033\_t001 |  | Vvi-Vitvi11g00025\_t001 |  |  |  |  |
| 2 | Atr-ERN05504 |  | | | |  | | | |  |  |  |  |
| 2 | Atr-ERN05505 |  | | | |  | | | |  |  |  |  |
| 2 | Atr-ERN05506 |  | | | |  | | | |  |  |  |  |
| 2 | Atr-ERN05507 |  | | | |  | | | |  |  |  |  |
| 2 | Atr-ERN05508 |  | | | |  | Vvi-Vitvi11g00032\_t001 |  |  |  |  |
| 2 | Atr-ERN05509 |  | | | |  | | | |  |  |  |  |
| 2 | Atr-ERN05510 |  | | | |  | | | |  |  |  |  |
| 2 | Atr-ERN05511 |  | | | |  | | | |  |  |  |  |
| 2 | Atr-ERN05512 |  | | | |  | | | |  |  |  |  |
| 2 | Atr-ERN05513 |  | | | |  | | | |  |  |  |  |
| 2 | Atr-ERN05514 |  | | | |  | | | |  |  |  |  |
| 2 | Atr-ERN05515 |  | | | |  | | | |  |  |  |  |
| 2 | Atr-ERN05516 |  | | | |  | | | |  |  |  |  |
| 2 | Atr-ERN05517 |  | | | |  | Vvi-Vitvi11g00035\_t001 |  |  |  |  |
| 2 | Atr-ERN05518 |  | Vvi-Vitvi09g00049\_t001 |  | | | |  |  |  |  |
| 2 | Atr-ERN05519 |  | | | |  | | | |  |  |  |  |
| 2 | Atr-ERN05520 |  | Vvi-Vitvi09g00050\_t003 |  | | | |  |  |  |  |
| 2 | Atr-ERN05521 |  | | | |  | | | |  |  |  |  |
| 2 | Atr-ERN05522 |  | | | |  | | | |  |  |  |  |
| 2 | Atr-ERN05523 |  | | | |  | | | |  |  |  |  |
| 2 | Atr-ERN05524 |  | | | |  | | | |  |  |  |  |
| 2 | Atr-ERN05525 |  | | | |  | Vvi-Vitvi11g00037\_t001 |  |  |  |  |
| 2 | Atr-ERN05526 |  | | | |  | | | |  |  |  |  |
| 2 | Atr-ERN05527 |  | Vvi-Vitvi09g01496\_t001 |  | | | |  |  |  |  |
| 2 | Atr-ERN05528 |  | | | |  | | | |  |  |  |  |
| 2 | Atr-ERN05529 |  | Vvi-Vitvi09g00052\_t001 |  | | | |  |  |  |  |
| 2 | Atr-ERN05530 |  | Vvi-Vitvi09g00053\_t001 |  | | | |  |  |  |  |
| 2 | Atr-ERN05531 |  | Vvi-Vitvi09g00055\_t001 |  | | | |  |  |  |  |
| 2 | Atr-ERN05532 |  | | | |  | Vvi-Vitvi11g00038\_t001 |  |  |  |  |
| 2 | Atr-ERN05533 |  | | | |  | | | |  |  |  |  |
| 2 | Atr-ERN05534 |  | | | |  | | | |  |  |  |  |
| 2 | Atr-ERN05535 |  | | | |  | | | |  |  |  |  |
| 2 | Atr-ERN05536 |  | | | |  | | | |  |  |  |  |
| 2 | Atr-ERN05537 |  | | | |  | | | |  |  |  |  |
| 2 | Atr-ERN05538 |  | | | |  | | | |  |  |  |  |
| 2 | Atr-ERN05539 |  | | | |  | | | |  |  |  |  |
| 2 | Atr-ERN05540 |  | | | |  | | | |  |  |  |  |
| 2 | Atr-ERN05541 |  | | | |  | | | |  |  |  |  |
| 2 | Atr-ERN05542 |  | | | |  | | | |  |  |  |  |
| 2 | Atr-ERN05543 |  | | | |  | | | |  |  |  |  |
| 2 | Atr-ERN05544 |  | | | |  | | | |  |  |  |  |
| 2 | Atr-ERN05545 |  | Vvi-Vitvi09g00058\_t001 |  | Vvi-Vitvi11g00041\_t002 |  |  |  |  |
| 2 | Atr-ERN05546 |  | | | |  | | | |  |  |  |  |
| 2 | Atr-ERN05547 |  | | | |  | | | |  |  |  |  |
| 2 | Atr-ERN05548 |  | Vvi-Vitvi09g00059\_t001 |  | | | |  |  |  |  |
| 2 | Atr-ERN05549 |  | | | |  | | | |  |  |  |  |
| 2 | Atr-ERN05550 |  | | | |  | | | |  |  |  |  |
| 2 | Atr-ERN05551 |  | | | |  | | | |  |  |  |  |
| 2 | Atr-ERN05552 |  | | | |  | | | |  |  |  |  |
| 2 | Atr-ERN05553 |  | | | |  | | | |  |  |  |  |
| 2 | Atr-ERN05554 |  | | | |  | | | |  |  |  |  |
| 2 | Atr-ERN05555 |  | | | |  | | | |  |  |  |  |
| 2 | Atr-ERN05556 |  | | | |  | | | |  |  |  |  |
| 2 | Atr-ERN05557 |  | | | |  | | | |  |  |  |  |
| 2 | Atr-ERN05558 |  | | | |  | | | |  |  |  |  |
| 2 | Atr-ERN05559 |  | | | |  | | | |  |  |  |  |
| 2 | Atr-ERN05560 |  | | | |  | | | |  |  |  |  |
| 2 | Atr-ERN05561 |  | | | |  | | | |  |  |  |  |
| 2 | Atr-ERN05562 |  | | | |  | | | |  |  |  |  |
| 2 | Atr-ERN05563 |  | | | |  | | | |  |  |  |  |
| 2 | Atr-ERN05564 |  | Vvi-Vitvi09g01499\_t001 |  | | | |  |  |  |  |
| 2 | Atr-ERN05565 |  | | | |  | | | |  |  |  |  |
| 2 | Atr-ERN05566 |  | | | |  | Vvi-Vitvi11g01326\_t001 |  |  |  |  |
| 2 | Atr-ERN05567 |  | | | |  | Vvi-Vitvi11g00042\_t002 |  |  |  |  |
| 2 | Atr-ERN05568 |  | Vvi-Vitvi09g00060\_t001 |  | | | |  |  |  |  |
| 2 | Atr-ERN05569 |  | Vvi-Vitvi09g00061\_t001 |  | | | |  |  |  |  |
| 2 | Atr-ERN05570 |  | | | |  | | | |  |  |  |  |
| 2 | Atr-ERN05571 |  | | | |  | Vvi-Vitvi11g00043\_t001 |  |  |  |  |
| 2 | Atr-ERN05572 |  | | | |  | Vvi-Vitvi11g00044\_t002 |  |  |  |  |
| 2 | Atr-ERN05573 |  | | | |  | | | |  |  |  |  |
| 2 | Atr-ERN05574 |  | | | |  | | | |  |  |  |  |
| 2 | Atr-ERN05575 |  | | | |  | | | |  |  |  |  |
| 2 | Atr-ERN05576 |  | | | |  | | | |  |  |  |  |
| 2 | Atr-ERN05577 |  | | | |  | | | |  |  |  |  |
| 2 | Atr-ERN05578 |  | | | |  | | | |  |  |  |  |
| 2 | Atr-ERN05579 |  | | | |  | | | |  |  |  |  |
| 2 | Atr-ERN05580 |  | | | |  | Vvi-Vitvi11g00045\_t001 |  |  |  |  |
| 2 | Atr-ERN05581 |  | | | |  | | | |  |  |  |  |
| 2 | Atr-ERN05582 |  | | | |  | | | |  |  |  |  |
| 2 | Atr-ERN05583 |  | | | |  | | | |  |  |  |  |
| 2 | Atr-ERN05584 |  | | | |  | | | |  |  |  |  |
| 2 | Atr-ERN05585 |  | | | |  | | | |  |  |  |  |
| 2 | Atr-ERN05586 |  | | | |  | | | |  |  |  |  |
| 2 | Atr-ERN05587 |  | | | |  | | | |  |  |  |  |
| 2 | Atr-ERN05588 |  | | | |  | Vvi-Vitvi11g00047\_t001 |  |  |  |  |
| 2 | Atr-ERN05589 |  | | | |  | | | |  |  |  |  |
| 2 | Atr-ERN05590 |  | | | |  | | | |  |  |  |  |
| 2 | Atr-ERN05591 |  | | | |  | Vvi-Vitvi11g00048\_t001 |  |  |  |  |
| 2 | Atr-ERN05592 |  | Vvi-Vitvi09g00063\_t001 |  | Vvi-Vitvi11g00049\_t001 |  |  |  |  |
| 0 | Atr-ERN05593 |  |  |  |  |  |  |
| 0 | Atr-ERN05594 |  |  |  |  |  |  |
| 0 | Atr-ERN05595 |  |  |  |  |  |  |
