# Supplementary material for: Genome-wide identification, characterization and gene expression of BES1 transcription factor family in grapevine (Vitis vinifera L.)
Source: Sci Rep. 2023 Jan 5;13:240. doi: 10.1038/s41598-022-24407-y (PMC9816167; doi:10.1038/s41598-022-24407-y)
Supplement: Supplementary file 3 — Supplementary Information. [file 41598_2022_24407_MOESM3_ESM.zip › Vvi_Atr/Vitis_vinifera.PN40024.v4.dna_sm.toplevel.fa.vs.Amborella_trichopoda.AMTR1.0.dna_sm.toplevel.fa.html/Atr-AmTr_v1.0_scaffold00008.html]

|  |  |  |  |  |  |  |  |  |  |  |  |  |  |
| --- | --- | --- | --- | --- | --- | --- | --- | --- | --- | --- | --- | --- | --- |
| Duplication depth | Reference chromosome | Collinear blocks | | | | | | | | | | | |
| 0 | Atr-ERM95252 |  |  |  |  |  |  |
| 0 | Atr-ERM95253 |  |  |  |  |  |  |
| 0 | Atr-ERM95254 |  |  |  |  |  |  |
| 0 | Atr-ERM95255 |  |  |  |  |  |  |
| 0 | Atr-ERM95256 |  |  |  |  |  |  |
| 0 | Atr-ERM95257 |  |  |  |  |  |  |
| 0 | Atr-ERM95258 |  |  |  |  |  |  |
| 0 | Atr-ERM95259 |  |  |  |  |  |  |
| 0 | Atr-ERM95260 |  |  |  |  |  |  |
| 0 | Atr-ERM95261 |  |  |  |  |  |  |
| 0 | Atr-ERM95262 |  |  |  |  |  |  |
| 0 | Atr-ERM95263 |  |  |  |  |  |  |
| 0 | Atr-ERM95264 |  |  |  |  |  |  |
| 1 | Atr-ERM95265 |  | Vvi-Vitvi18g00328\_t001 |  |  |  |  |  |
| 1 | Atr-ERM95266 |  | | | |  |  |  |  |  |
| 1 | Atr-ERM95267 |  | | | |  |  |  |  |  |
| 1 | Atr-ERM95268 |  | | | |  |  |  |  |  |
| 1 | Atr-ERM95269 |  | | | |  |  |  |  |  |
| 1 | Atr-ERM95270 |  | Vvi-Vitvi18g00326\_t001 |  |  |  |  |  |
| 1 | Atr-ERM95271 |  | | | |  |  |  |  |  |
| 1 | Atr-ERM95272 |  | | | |  |  |  |  |  |
| 1 | Atr-ERM95273 |  | | | |  |  |  |  |  |
| 1 | Atr-ERM95274 |  | Vvi-Vitvi18g02548\_t001 |  |  |  |  |  |
| 1 | Atr-ERM95275 |  | | | |  |  |  |  |  |
| 1 | Atr-ERM95276 |  | | | |  |  |  |  |  |
| 1 | Atr-ERM95277 |  | | | |  |  |  |  |  |
| 1 | Atr-ERM95278 |  | | | |  |  |  |  |  |
| 1 | Atr-ERM95279 |  | | | |  |  |  |  |  |
| 1 | Atr-ERM95280 |  | | | |  |  |  |  |  |
| 1 | Atr-ERM95281 |  | | | |  |  |  |  |  |
| 1 | Atr-ERM95282 |  | | | |  |  |  |  |  |
| 1 | Atr-ERM95283 |  | Vvi-Vitvi18g00325\_t001 |  |  |  |  |  |
| 1 | Atr-ERM95284 |  | | | |  |  |  |  |  |
| 1 | Atr-ERM95285 |  | | | |  |  |  |  |  |
| 1 | Atr-ERM95286 |  | | | |  |  |  |  |  |
| 1 | Atr-ERM95287 |  | | | |  |  |  |  |  |
| 1 | Atr-ERM95288 |  | | | |  |  |  |  |  |
| 1 | Atr-ERM95289 |  | | | |  |  |  |  |  |
| 1 | Atr-ERM95290 |  | | | |  |  |  |  |  |
| 1 | Atr-ERM95291 |  | | | |  |  |  |  |  |
| 1 | Atr-ERM95292 |  | | | |  |  |  |  |  |
| 1 | Atr-ERM95293 |  | | | |  |  |  |  |  |
| 1 | Atr-ERM95294 |  | | | |  |  |  |  |  |
| 1 | Atr-ERM95295 |  | | | |  |  |  |  |  |
| 1 | Atr-ERM95296 |  | | | |  |  |  |  |  |
| 1 | Atr-ERM95297 |  | | | |  |  |  |  |  |
| 1 | Atr-ERM95298 |  | | | |  |  |  |  |  |
| 1 | Atr-ERM95299 |  | | | |  |  |  |  |  |
| 1 | Atr-ERM95300 |  | | | |  |  |  |  |  |
| 1 | Atr-ERM95301 |  | | | |  |  |  |  |  |
| 1 | Atr-ERM95302 |  | | | |  |  |  |  |  |
| 1 | Atr-ERM95303 |  | | | |  |  |  |  |  |
| 1 | Atr-ERM95304 |  | Vvi-Vitvi18g02546\_t001 |  |  |  |  |  |
| 1 | Atr-ERM95305 |  | | | |  |  |  |  |  |
| 1 | Atr-ERM95306 |  | | | |  |  |  |  |  |
| 1 | Atr-ERM95307 |  | | | |  |  |  |  |  |
| 1 | Atr-ERM95308 |  | | | |  |  |  |  |  |
| 1 | Atr-ERM95309 |  | | | |  |  |  |  |  |
| 1 | Atr-ERM95310 |  | | | |  |  |  |  |  |
| 1 | Atr-ERM95311 |  | Vvi-Vitvi18g00317\_t001 |  |  |  |  |  |
| 0 | Atr-ERM95312 |  |  |  |  |  |  |
| 0 | Atr-ERM95313 |  |  |  |  |  |  |
| 0 | Atr-ERM95314 |  |  |  |  |  |  |
| 0 | Atr-ERM95315 |  |  |  |  |  |  |
| 0 | Atr-ERM95316 |  |  |  |  |  |  |
| 0 | Atr-ERM95317 |  |  |  |  |  |  |
| 0 | Atr-ERM95318 |  |  |  |  |  |  |
| 0 | Atr-ERM95319 |  |  |  |  |  |  |
| 0 | Atr-ERM95320 |  |  |  |  |  |  |
| 0 | Atr-ERM95321 |  |  |  |  |  |  |
| 0 | Atr-ERM95322 |  |  |  |  |  |  |
| 0 | Atr-ERM95323 |  |  |  |  |  |  |
| 0 | Atr-ERM95324 |  |  |  |  |  |  |
| 0 | Atr-ERM95325 |  |  |  |  |  |  |
| 0 | Atr-ERM95326 |  |  |  |  |  |  |
| 0 | Atr-ERM95327 |  |  |  |  |  |  |
| 0 | Atr-ERM95328 |  |  |  |  |  |  |
| 0 | Atr-ERM95329 |  |  |  |  |  |  |
| 0 | Atr-ERM95330 |  |  |  |  |  |  |
| 0 | Atr-ERM95331 |  |  |  |  |  |  |
| 0 | Atr-ERM95332 |  |  |  |  |  |  |
| 0 | Atr-ERM95333 |  |  |  |  |  |  |
| 0 | Atr-ERM95334 |  |  |  |  |  |  |
| 0 | Atr-ERM95335 |  |  |  |  |  |  |
| 0 | Atr-ERM95336 |  |  |  |  |  |  |
| 0 | Atr-ERM95337 |  |  |  |  |  |  |
| 0 | Atr-ERM95338 |  |  |  |  |  |  |
| 0 | Atr-ERM95339 |  |  |  |  |  |  |
| 0 | Atr-ERM95340 |  |  |  |  |  |  |
| 0 | Atr-ERM95341 |  |  |  |  |  |  |
| 0 | Atr-ERM95342 |  |  |  |  |  |  |
| 0 | Atr-ERM95343 |  |  |  |  |  |  |
| 0 | Atr-ERM95344 |  |  |  |  |  |  |
| 0 | Atr-ERM95345 |  |  |  |  |  |  |
| 0 | Atr-ERM95346 |  |  |  |  |  |  |
| 2 | Atr-ERM95347 |  | Vvi-Vitvi18g00470\_t001 |  | Vvi-Vitvi07g04591\_t001 |  |  |  |  |
| 2 | Atr-ERM95348 |  | | | |  | | | |  |  |  |  |
| 2 | Atr-ERM95349 |  | | | |  | | | |  |  |  |  |
| 2 | Atr-ERM95350 |  | | | |  | | | |  |  |  |  |
| 2 | Atr-ERM95351 |  | | | |  | | | |  |  |  |  |
| 2 | Atr-ERM95352 |  | Vvi-Vitvi18g00466\_t001 |  | | | |  |  |  |  |
| 2 | Atr-ERM95353 |  | | | |  | | | |  |  |  |  |
| 2 | Atr-ERM95354 |  | | | |  | | | |  |  |  |  |
| 2 | Atr-ERM95355 |  | Vvi-Vitvi18g00464\_t001 |  | Vvi-Vitvi07g04590\_t001 |  |  |  |  |
| 2 | Atr-ERM95356 |  | | | |  | | | |  |  |  |  |
| 2 | Atr-ERM95357 |  | | | |  | | | |  |  |  |  |
| 2 | Atr-ERM95358 |  | | | |  | | | |  |  |  |  |
| 2 | Atr-ERM95359 |  | | | |  | | | |  |  |  |  |
| 2 | Atr-ERM95360 |  | | | |  | | | |  |  |  |  |
| 2 | Atr-ERM95361 |  | | | |  | | | |  |  |  |  |
| 2 | Atr-ERM95362 |  | | | |  | | | |  |  |  |  |
| 2 | Atr-ERM95363 |  | | | |  | Vvi-Vitvi07g02600\_t001 |  |  |  |  |
| 2 | Atr-ERM95364 |  | | | |  | Vvi-Vitvi07g01473\_t001 |  |  |  |  |
| 2 | Atr-ERM95365 |  | | | |  | | | |  |  |  |  |
| 2 | Atr-ERM95366 |  | Vvi-Vitvi18g00463\_t001 |  | Vvi-Vitvi07g01475\_t001 |  |  |  |  |
| 2 | Atr-ERM95367 |  | | | |  | | | |  |  |  |  |
| 2 | Atr-ERM95368 |  | | | |  | | | |  |  |  |  |
| 2 | Atr-ERM95369 |  | Vvi-Vitvi18g00462\_t001 |  | Vvi-Vitvi07g01476\_t001 |  |  |  |  |
| 2 | Atr-ERM95370 |  | | | |  | | | |  |  |  |  |
| 2 | Atr-ERM95371 |  | Vvi-Vitvi18g00456\_t001 |  | | | |  |  |  |  |
| 2 | Atr-ERM95372 |  | | | |  | | | |  |  |  |  |
| 2 | Atr-ERM95373 |  | | | |  | | | |  |  |  |  |
| 2 | Atr-ERM95374 |  | | | |  | | | |  |  |  |  |
| 2 | Atr-ERM95375 |  | | | |  | | | |  |  |  |  |
| 2 | Atr-ERM95376 |  | | | |  | | | |  |  |  |  |
| 2 | Atr-ERM95377 |  | | | |  | | | |  |  |  |  |
| 2 | Atr-ERM95378 |  | | | |  | | | |  |  |  |  |
| 2 | Atr-ERM95379 |  | | | |  | | | |  |  |  |  |
| 2 | Atr-ERM95380 |  | | | |  | | | |  |  |  |  |
| 2 | Atr-ERM95381 |  | | | |  | | | |  |  |  |  |
| 2 | Atr-ERM95382 |  | | | |  | | | |  |  |  |  |
| 2 | Atr-ERM95383 |  | | | |  | | | |  |  |  |  |
| 2 | Atr-ERM95384 |  | | | |  | Vvi-Vitvi07g01482\_t001 |  |  |  |  |
| 2 | Atr-ERM95385 |  | | | |  | | | |  |  |  |  |
| 2 | Atr-ERM95386 |  | Vvi-Vitvi18g00448\_t001 |  | Vvi-Vitvi07g01488\_t001 |  |  |  |  |
| 2 | Atr-ERM95387 |  | | | |  | | | |  |  |  |  |
| 2 | Atr-ERM95388 |  | | | |  | | | |  |  |  |  |
| 2 | Atr-ERM95389 |  | | | |  | | | |  |  |  |  |
| 2 | Atr-ERM95390 |  | | | |  | | | |  |  |  |  |
| 2 | Atr-ERM95391 |  | | | |  | | | |  |  |  |  |
| 2 | Atr-ERM95392 |  | | | |  | | | |  |  |  |  |
| 2 | Atr-ERM95393 |  | | | |  | | | |  |  |  |  |
| 2 | Atr-ERM95394 |  | | | |  | | | |  |  |  |  |
| 2 | Atr-ERM95395 |  | | | |  | | | |  |  |  |  |
| 2 | Atr-ERM95396 |  | | | |  | Vvi-Vitvi07g01489\_t001 |  |  |  |  |
| 2 | Atr-ERM95397 |  | | | |  | | | |  |  |  |  |
| 2 | Atr-ERM95398 |  | | | |  | | | |  |  |  |  |
| 2 | Atr-ERM95399 |  | | | |  | | | |  |  |  |  |
| 2 | Atr-ERM95400 |  | | | |  | | | |  |  |  |  |
| 2 | Atr-ERM95401 |  | | | |  | | | |  |  |  |  |
| 2 | Atr-ERM95402 |  | | | |  | Vvi-Vitvi07g01492\_t001 |  |  |  |  |
| 2 | Atr-ERM95403 |  | | | |  | | | |  |  |  |  |
| 2 | Atr-ERM95404 |  | | | |  | | | |  |  |  |  |
| 2 | Atr-ERM95405 |  | | | |  | | | |  |  |  |  |
| 2 | Atr-ERM95406 |  | | | |  | | | |  |  |  |  |
| 2 | Atr-ERM95407 |  | | | |  | | | |  |  |  |  |
| 2 | Atr-ERM95408 |  | | | |  | | | |  |  |  |  |
| 2 | Atr-ERM95409 |  | | | |  | | | |  |  |  |  |
| 2 | Atr-ERM95410 |  | | | |  | | | |  |  |  |  |
| 2 | Atr-ERM95411 |  | | | |  | | | |  |  |  |  |
| 2 | Atr-ERM95412 |  | Vvi-Vitvi18g00446\_t001 |  | | | |  |  |  |  |
| 2 | Atr-ERM95413 |  | | | |  | | | |  |  |  |  |
| 2 | Atr-ERM95414 |  | | | |  | | | |  |  |  |  |
| 2 | Atr-ERM95415 |  | | | |  | | | |  |  |  |  |
| 2 | Atr-ERM95416 |  | | | |  | | | |  |  |  |  |
| 2 | Atr-ERM95417 |  | | | |  | | | |  |  |  |  |
| 2 | Atr-ERM95418 |  | | | |  | | | |  |  |  |  |
| 2 | Atr-ERM95419 |  | | | |  | | | |  |  |  |  |
| 2 | Atr-ERM95420 |  | | | |  | Vvi-Vitvi07g01494\_t001 |  |  |  |  |
| 2 | Atr-ERM95421 |  | Vvi-Vitvi18g00444\_t001 |  | | | |  |  |  |  |
| 1 | Atr-ERM95422 |  |  |  | | | |  |  |  |  |
| 1 | Atr-ERM95423 |  |  |  | | | |  |  |  |  |
| 1 | Atr-ERM95424 |  |  |  | | | |  |  |  |  |
| 1 | Atr-ERM95425 |  |  |  | | | |  |  |  |  |
| 1 | Atr-ERM95426 |  |  |  | | | |  |  |  |  |
| 1 | Atr-ERM95427 |  |  |  | | | |  |  |  |  |
| 1 | Atr-ERM95428 |  |  |  | | | |  |  |  |  |
| 1 | Atr-ERM95429 |  |  |  | | | |  |  |  |  |
| 1 | Atr-ERM95430 |  |  |  | | | |  |  |  |  |
| 1 | Atr-ERM95431 |  |  |  | Vvi-Vitvi07g04587\_t001 |  |  |  |  |
| 1 | Atr-ERM95432 |  |  |  | | | |  |  |  |  |
| 1 | Atr-ERM95433 |  |  |  | | | |  |  |  |  |
| 1 | Atr-ERM95434 |  |  |  | | | |  |  |  |  |
| 1 | Atr-ERM95435 |  |  |  | | | |  |  |  |  |
| 1 | Atr-ERM95436 |  |  |  | | | |  |  |  |  |
| 1 | Atr-ERM95437 |  |  |  | | | |  |  |  |  |
| 1 | Atr-ERM95438 |  |  |  | | | |  |  |  |  |
| 1 | Atr-ERM95439 |  |  |  | | | |  |  |  |  |
| 1 | Atr-ERM95440 |  |  |  | | | |  |  |  |  |
| 1 | Atr-ERM95441 |  |  |  | | | |  |  |  |  |
| 1 | Atr-ERM95442 |  |  |  | | | |  |  |  |  |
| 1 | Atr-ERM95443 |  |  |  | | | |  |  |  |  |
| 1 | Atr-ERM95444 |  |  |  | | | |  |  |  |  |
| 1 | Atr-ERM95445 |  |  |  | | | |  |  |  |  |
| 1 | Atr-ERM95446 |  |  |  | | | |  |  |  |  |
| 1 | Atr-ERM95447 |  |  |  | | | |  |  |  |  |
| 1 | Atr-ERM95448 |  |  |  | | | |  |  |  |  |
| 1 | Atr-ERM95449 |  |  |  | | | |  |  |  |  |
| 1 | Atr-ERM95450 |  |  |  | | | |  |  |  |  |
| 1 | Atr-ERM95451 |  |  |  | | | |  |  |  |  |
| 1 | Atr-ERM95452 |  |  |  | | | |  |  |  |  |
| 1 | Atr-ERM95453 |  |  |  | | | |  |  |  |  |
| 1 | Atr-ERM95454 |  |  |  | | | |  |  |  |  |
| 1 | Atr-ERM95455 |  |  |  | Vvi-Vitvi07g01498\_t001 |  |  |  |  |
| 1 | Atr-ERM95456 |  |  |  | | | |  |  |  |  |
| 1 | Atr-ERM95457 |  |  |  | | | |  |  |  |  |
| 1 | Atr-ERM95458 |  |  |  | | | |  |  |  |  |
| 1 | Atr-ERM95459 |  |  |  | | | |  |  |  |  |
| 1 | Atr-ERM95460 |  |  |  | | | |  |  |  |  |
| 1 | Atr-ERM95461 |  |  |  | | | |  |  |  |  |
| 1 | Atr-ERM95462 |  |  |  | | | |  |  |  |  |
| 1 | Atr-ERM95463 |  |  |  | | | |  |  |  |  |
| 1 | Atr-ERM95464 |  |  |  | | | |  |  |  |  |
| 1 | Atr-ERM95465 |  |  |  | | | |  |  |  |  |
| 1 | Atr-ERM95466 |  |  |  | | | |  |  |  |  |
| 1 | Atr-ERM95467 |  |  |  | | | |  |  |  |  |
| 1 | Atr-ERM95468 |  |  |  | | | |  |  |  |  |
| 1 | Atr-ERM95469 |  |  |  | | | |  |  |  |  |
| 1 | Atr-ERM95470 |  |  |  | Vvi-Vitvi07g03049\_t001 |  |  |  |  |
| 1 | Atr-ERM95471 |  |  |  | | | |  |  |  |  |
| 1 | Atr-ERM95472 |  |  |  | | | |  |  |  |  |
| 1 | Atr-ERM95473 |  |  |  | | | |  |  |  |  |
| 1 | Atr-ERM95474 |  |  |  | | | |  |  |  |  |
| 1 | Atr-ERM95475 |  |  |  | | | |  |  |  |  |
| 1 | Atr-ERM95476 |  |  |  | | | |  |  |  |  |
| 1 | Atr-ERM95477 |  |  |  | | | |  |  |  |  |
| 1 | Atr-ERM95478 |  |  |  | Vvi-Vitvi07g04577\_t001 |  |  |  |  |
| 0 | Atr-ERM95479 |  |  |  |  |  |  |
| 0 | Atr-ERM95480 |  |  |  |  |  |  |
| 0 | Atr-ERM95481 |  |  |  |  |  |  |
| 0 | Atr-ERM95482 |  |  |  |  |  |  |
| 0 | Atr-ERM95483 |  |  |  |  |  |  |
| 0 | Atr-ERM95484 |  |  |  |  |  |  |
| 0 | Atr-ERM95485 |  |  |  |  |  |  |
| 0 | Atr-ERM95486 |  |  |  |  |  |  |
| 0 | Atr-ERM95487 |  |  |  |  |  |  |
| 0 | Atr-ERM95488 |  |  |  |  |  |  |
| 0 | Atr-ERM95489 |  |  |  |  |  |  |
| 0 | Atr-ERM95490 |  |  |  |  |  |  |
| 0 | Atr-ERM95491 |  |  |  |  |  |  |
| 0 | Atr-ERM95492 |  |  |  |  |  |  |
