# Supplementary material for: Genome-wide identification, characterization and gene expression of BES1 transcription factor family in grapevine (Vitis vinifera L.)
Source: Sci Rep. 2023 Jan 5;13:240. doi: 10.1038/s41598-022-24407-y (PMC9816167; doi:10.1038/s41598-022-24407-y)
Supplement: Supplementary file 3 — Supplementary Information. [file 41598_2022_24407_MOESM3_ESM.zip › Vvi_Atr/Vitis_vinifera.PN40024.v4.dna_sm.toplevel.fa.vs.Amborella_trichopoda.AMTR1.0.dna_sm.toplevel.fa.html/Atr-AmTr_v1.0_scaffold00009.html]

|  |  |  |  |  |  |  |  |  |  |  |  |  |  |
| --- | --- | --- | --- | --- | --- | --- | --- | --- | --- | --- | --- | --- | --- |
| Duplication depth | Reference chromosome | Collinear blocks | | | | | | | | | | | |
| 0 | Atr-ERM94814 |  |  |  |  |  |  |
| 0 | Atr-ERM94815 |  |  |  |  |  |  |
| 0 | Atr-ERM94816 |  |  |  |  |  |  |
| 0 | Atr-ERM94817 |  |  |  |  |  |  |
| 0 | Atr-ERM94818 |  |  |  |  |  |  |
| 0 | Atr-ERM94819 |  |  |  |  |  |  |
| 0 | Atr-ERM94820 |  |  |  |  |  |  |
| 0 | Atr-ERM94821 |  |  |  |  |  |  |
| 0 | Atr-ERM94822 |  |  |  |  |  |  |
| 0 | Atr-ERM94823 |  |  |  |  |  |  |
| 0 | Atr-ERM94824 |  |  |  |  |  |  |
| 0 | Atr-ERM94825 |  |  |  |  |  |  |
| 0 | Atr-ERM94826 |  |  |  |  |  |  |
| 1 | Atr-ERM94827 |  | Vvi-Vitvi13g00647\_t001 |  |  |  |  |  |
| 1 | Atr-ERM94828 |  | | | |  |  |  |  |  |
| 1 | Atr-ERM94829 |  | Vvi-Vitvi13g00646\_t001 |  |  |  |  |  |
| 1 | Atr-ERM94830 |  | | | |  |  |  |  |  |
| 1 | Atr-ERM94831 |  | | | |  |  |  |  |  |
| 1 | Atr-ERM94832 |  | | | |  |  |  |  |  |
| 1 | Atr-ERM94833 |  | | | |  |  |  |  |  |
| 1 | Atr-ERM94834 |  | | | |  |  |  |  |  |
| 1 | Atr-ERM94835 |  | | | |  |  |  |  |  |
| 1 | Atr-ERM94836 |  | | | |  |  |  |  |  |
| 2 | Atr-ERM94837 |  | | | |  | Vvi-Vitvi08g02139\_t001 |  |  |  |  |
| 2 | Atr-ERM94838 |  | Vvi-Vitvi13g04181\_t001 |  | | | |  |  |  |  |
| 2 | Atr-ERM94839 |  | | | |  | | | |  |  |  |  |
| 2 | Atr-ERM94840 |  | | | |  | | | |  |  |  |  |
| 3 | Atr-ERM94841 |  | | | |  | | | |  | Vvi-Vitvi06g01646\_t001 |  |  |  |
| 3 | Atr-ERM94842 |  | Vvi-Vitvi13g00640\_t001 |  | Vvi-Vitvi08g01010\_t005 |  | Vvi-Vitvi06g00241\_t003 |  |  |  |
| 3 | Atr-ERM94843 |  | | | |  | | | |  | | | |  |  |  |
| 3 | Atr-ERM94844 |  | | | |  | | | |  | | | |  |  |  |
| 3 | Atr-ERM94845 |  | | | |  | | | |  | | | |  |  |  |
| 3 | Atr-ERM94846 |  | | | |  | | | |  | | | |  |  |  |
| 3 | Atr-ERM94847 |  | | | |  | | | |  | | | |  |  |  |
| 3 | Atr-ERM94848 |  | Vvi-Vitvi13g00639\_t001 |  | | | |  | | | |  |  |  |
| 3 | Atr-ERM94849 |  | Vvi-Vitvi13g00638\_t001 |  | Vvi-Vitvi08g01013\_t001 |  | Vvi-Vitvi06g00242\_t001 |  |  |  |
| 3 | Atr-ERM94850 |  | | | |  | Vvi-Vitvi08g01014\_t001 |  | | | |  |  |  |
| 3 | Atr-ERM94851 |  | | | |  | | | |  | | | |  |  |  |
| 3 | Atr-ERM94852 |  | | | |  | | | |  | | | |  |  |  |
| 3 | Atr-ERM94853 |  | | | |  | | | |  | | | |  |  |  |
| 3 | Atr-ERM94854 |  | | | |  | | | |  | | | |  |  |  |
| 3 | Atr-ERM94855 |  | | | |  | | | |  | | | |  |  |  |
| 3 | Atr-ERM94856 |  | Vvi-Vitvi13g00637\_t001.1.6037826b |  | | | |  | Vvi-Vitvi06g00243\_t001 |  |  |  |
| 3 | Atr-ERM94857 |  | | | |  | | | |  | | | |  |  |  |
| 3 | Atr-ERM94858 |  | | | |  | | | |  | | | |  |  |  |
| 3 | Atr-ERM94859 |  | | | |  | | | |  | | | |  |  |  |
| 3 | Atr-ERM94860 |  | | | |  | | | |  | | | |  |  |  |
| 3 | Atr-ERM94861 |  | | | |  | Vvi-Vitvi08g02140\_t003 |  | Vvi-Vitvi06g00245\_t001 |  |  |  |
| 3 | Atr-ERM94862 |  | Vvi-Vitvi13g00636\_t001 |  | | | |  | | | |  |  |  |
| 3 | Atr-ERM94863 |  | | | |  | | | |  | | | |  |  |  |
| 3 | Atr-ERM94864 |  | | | |  | | | |  | | | |  |  |  |
| 3 | Atr-ERM94865 |  | | | |  | | | |  | | | |  |  |  |
| 3 | Atr-ERM94866 |  | Vvi-Vitvi13g00632\_t001 |  | Vvi-Vitvi08g01015\_t001 |  | Vvi-Vitvi06g00246\_t001 |  |  |  |
| 1 | Atr-ERM94867 |  | | | |  |  |  |  |  |
| 1 | Atr-ERM94868 |  | Vvi-Vitvi13g00631\_t001 |  |  |  |  |  |
| 1 | Atr-ERM94869 |  | Vvi-Vitvi13g04179\_t002 |  |  |  |  |  |
| 1 | Atr-ERM94870 |  | | | |  |  |  |  |  |
| 1 | Atr-ERM94871 |  | | | |  |  |  |  |  |
| 1 | Atr-ERM94872 |  | | | |  |  |  |  |  |
| 1 | Atr-ERM94873 |  | | | |  |  |  |  |  |
| 1 | Atr-ERM94874 |  | | | |  |  |  |  |  |
| 1 | Atr-ERM94875 |  | | | |  |  |  |  |  |
| 1 | Atr-ERM94876 |  | | | |  |  |  |  |  |
| 1 | Atr-ERM94877 |  | | | |  |  |  |  |  |
| 1 | Atr-ERM94878 |  | | | |  |  |  |  |  |
| 1 | Atr-ERM94879 |  | Vvi-Vitvi13g04177\_t001 |  |  |  |  |  |
| 0 | Atr-ERM94880 |  |  |  |  |  |  |
| 0 | Atr-ERM94881 |  |  |  |  |  |  |
| 0 | Atr-ERM94882 |  |  |  |  |  |  |
| 0 | Atr-ERM94883 |  |  |  |  |  |  |
| 1 | Atr-ERM94884 |  | Vvi-Vitvi07g02266\_t001 |  |  |  |  |  |
| 1 | Atr-ERM94885 |  | | | |  |  |  |  |  |
| 1 | Atr-ERM94886 |  | Vvi-Vitvi07g00594\_t001 |  |  |  |  |  |
| 1 | Atr-ERM94887 |  | | | |  |  |  |  |  |
| 1 | Atr-ERM94888 |  | | | |  |  |  |  |  |
| 1 | Atr-ERM94889 |  | | | |  |  |  |  |  |
| 1 | Atr-ERM94890 |  | | | |  |  |  |  |  |
| 1 | Atr-ERM94891 |  | | | |  |  |  |  |  |
| 1 | Atr-ERM94892 |  | | | |  |  |  |  |  |
| 1 | Atr-ERM94893 |  | | | |  |  |  |  |  |
| 1 | Atr-ERM94894 |  | | | |  |  |  |  |  |
| 1 | Atr-ERM94895 |  | | | |  |  |  |  |  |
| 3 | Atr-ERM94896 |  | Vvi-Vitvi07g02067\_t001 |  | Vvi-Vitvi14g00564\_t001 |  | Vvi-Vitvi05g00715\_t001 |  |  |  |
| 3 | Atr-ERM94897 |  | | | |  | | | |  | | | |  |  |  |
| 3 | Atr-ERM94898 |  | Vvi-Vitvi07g02062\_t001 |  | | | |  | Vvi-Vitvi05g01722\_t001 |  |  |  |
| 3 | Atr-ERM94899 |  | | | |  | | | |  | | | |  |  |  |
| 3 | Atr-ERM94900 |  | | | |  | | | |  | | | |  |  |  |
| 3 | Atr-ERM94901 |  | | | |  | | | |  | | | |  |  |  |
| 3 | Atr-ERM94902 |  | | | |  | | | |  | | | |  |  |  |
| 3 | Atr-ERM94903 |  | | | |  | | | |  | | | |  |  |  |
| 3 | Atr-ERM94904 |  | | | |  | | | |  | | | |  |  |  |
| 3 | Atr-ERM94905 |  | | | |  | | | |  | | | |  |  |  |
| 3 | Atr-ERM94906 |  | | | |  | | | |  | | | |  |  |  |
| 3 | Atr-ERM94907 |  | | | |  | | | |  | | | |  |  |  |
| 3 | Atr-ERM94908 |  | | | |  | | | |  | | | |  |  |  |
| 3 | Atr-ERM94909 |  | | | |  | | | |  | | | |  |  |  |
| 3 | Atr-ERM94910 |  | | | |  | | | |  | | | |  |  |  |
| 3 | Atr-ERM94911 |  | | | |  | | | |  | | | |  |  |  |
| 3 | Atr-ERM94912 |  | | | |  | | | |  | | | |  |  |  |
| 3 | Atr-ERM94913 |  | | | |  | | | |  | | | |  |  |  |
| 3 | Atr-ERM94914 |  | Vvi-Vitvi07g00581\_t001 |  | | | |  | | | |  |  |  |
| 3 | Atr-ERM94915 |  | | | |  | | | |  | Vvi-Vitvi05g00713\_t001 |  |  |  |
| 3 | Atr-ERM94916 |  | | | |  | | | |  | | | |  |  |  |
| 3 | Atr-ERM94917 |  | Vvi-Vitvi07g00578\_t001 |  | | | |  | Vvi-Vitvi05g00710\_t001 |  |  |  |
| 3 | Atr-ERM94918 |  | | | |  | Vvi-Vitvi14g00562\_t001 |  | | | |  |  |  |
| 3 | Atr-ERM94919 |  | | | |  | | | |  | | | |  |  |  |
| 3 | Atr-ERM94920 |  | | | |  | | | |  | Vvi-Vitvi05g00691\_t001 |  |  |  |
| 3 | Atr-ERM94921 |  | | | |  | | | |  | | | |  |  |  |
| 3 | Atr-ERM94922 |  | | | |  | Vvi-Vitvi14g00560\_t001 |  | | | |  |  |  |
| 3 | Atr-ERM94923 |  | | | |  | | | |  | Vvi-Vitvi05g00688\_t001 |  |  |  |
| 3 | Atr-ERM94924 |  | | | |  | | | |  | Vvi-Vitvi05g01918\_t001 |  |  |  |
| 3 | Atr-ERM94925 |  | | | |  | | | |  | | | |  |  |  |
| 3 | Atr-ERM94926 |  | | | |  | | | |  | | | |  |  |  |
| 3 | Atr-ERM94927 |  | Vvi-Vitvi07g00574\_t001 |  | | | |  | | | |  |  |  |
| 3 | Atr-ERM94928 |  | | | |  | | | |  | | | |  |  |  |
| 3 | Atr-ERM94929 |  | | | |  | | | |  | | | |  |  |  |
| 3 | Atr-ERM94930 |  | | | |  | | | |  | | | |  |  |  |
| 3 | Atr-ERM94931 |  | | | |  | Vvi-Vitvi14g00557\_t001 |  | | | |  |  |  |
| 3 | Atr-ERM94932 |  | | | |  | | | |  | Vvi-Vitvi05g00686\_t001 |  |  |  |
| 3 | Atr-ERM94933 |  | | | |  | Vvi-Vitvi14g00555\_t001 |  | Vvi-Vitvi05g01916\_t001 |  |  |  |
| 3 | Atr-ERM94934 |  | | | |  | Vvi-Vitvi14g00551\_t001 |  | | | |  |  |  |
| 3 | Atr-ERM94935 |  | | | |  | Vvi-Vitvi14g00547\_t001 |  | Vvi-Vitvi05g00684\_t001 |  |  |  |
| 2 | Atr-ERM94936 |  | | | |  |  |  | | | |  |  |  |
| 2 | Atr-ERM94937 |  | Vvi-Vitvi07g02262\_t001 |  |  |  | Vvi-Vitvi05g01913\_t001 |  |  |  |
| 1 | Atr-ERM94938 |  | Vvi-Vitvi07g02261\_t001 |  |  |  |  |  |
| 0 | Atr-ERM94939 |  |  |  |  |  |  |
| 0 | Atr-ERM94940 |  |  |  |  |  |  |
| 0 | Atr-ERM94941 |  |  |  |  |  |  |
| 0 | Atr-ERM94942 |  |  |  |  |  |  |
| 1 | Atr-ERM94943 |  | Vvi-Vitvi06g04198\_t001 |  |  |  |  |  |
| 1 | Atr-ERM94944 |  | Vvi-Vitvi06g00584\_t001 |  |  |  |  |  |
| 1 | Atr-ERM94945 |  | | | |  |  |  |  |  |
| 1 | Atr-ERM94946 |  | | | |  |  |  |  |  |
| 1 | Atr-ERM94947 |  | | | |  |  |  |  |  |
| 1 | Atr-ERM94948 |  | | | |  |  |  |  |  |
| 1 | Atr-ERM94949 |  | | | |  |  |  |  |  |
| 2 | Atr-ERM94950 |  | | | |  | Vvi-Vitvi16g04377\_t001 |  |  |  |  |
| 2 | Atr-ERM94951 |  | | | |  | Vvi-Vitvi16g01912\_t001 |  |  |  |  |
| 2 | Atr-ERM94952 |  | | | |  | | | |  |  |  |  |
| 2 | Atr-ERM94953 |  | | | |  | | | |  |  |  |  |
| 2 | Atr-ERM94954 |  | | | |  | | | |  |  |  |  |
| 2 | Atr-ERM94955 |  | | | |  | | | |  |  |  |  |
| 2 | Atr-ERM94956 |  | | | |  | | | |  |  |  |  |
| 2 | Atr-ERM94957 |  | Vvi-Vitvi06g00588\_t001 |  | | | |  |  |  |  |
| 2 | Atr-ERM94958 |  | | | |  | | | |  |  |  |  |
| 2 | Atr-ERM94959 |  | | | |  | | | |  |  |  |  |
| 2 | Atr-ERM94960 |  | | | |  | | | |  |  |  |  |
| 3 | Atr-ERM94961 |  | | | |  | | | |  | Vvi-Vitvi15g01099\_t003 |  |  |  |
| 3 | Atr-ERM94962 |  | | | |  | | | |  | | | |  |  |  |
| 3 | Atr-ERM94963 |  | | | |  | | | |  | | | |  |  |  |
| 3 | Atr-ERM94964 |  | | | |  | | | |  | | | |  |  |  |
| 3 | Atr-ERM94965 |  | | | |  | | | |  | | | |  |  |  |
| 3 | Atr-ERM94966 |  | | | |  | | | |  | Vvi-Vitvi15g01100\_t001 |  |  |  |
| 3 | Atr-ERM94967 |  | | | |  | | | |  | | | |  |  |  |
| 3 | Atr-ERM94968 |  | | | |  | Vvi-Vitvi16g01083\_t001 |  | | | |  |  |  |
| 3 | Atr-ERM94969 |  | | | |  | | | |  | | | |  |  |  |
| 3 | Atr-ERM94970 |  | | | |  | | | |  | Vvi-Vitvi15g01101\_t002 |  |  |  |
| 4 | Atr-ERM94971 |  | | | |  | | | |  | | | |  | Vvi-Vitvi02g00296\_t001 |  |  |
| 4 | Atr-ERM94972 |  | Vvi-Vitvi06g00599\_t001 |  | Vvi-Vitvi16g01080\_t001 |  | | | |  | | | |  |  |
| 4 | Atr-ERM94973 |  | | | |  | | | |  | | | |  | | | |  |  |
| 4 | Atr-ERM94974 |  | | | |  | | | |  | | | |  | | | |  |  |
| 4 | Atr-ERM94975 |  | | | |  | | | |  | | | |  | | | |  |  |
| 4 | Atr-ERM94976 |  | | | |  | | | |  | | | |  | | | |  |  |
| 4 | Atr-ERM94977 |  | | | |  | | | |  | | | |  | | | |  |  |
| 4 | Atr-ERM94978 |  | | | |  | | | |  | | | |  | | | |  |  |
| 4 | Atr-ERM94979 |  | | | |  | | | |  | Vvi-Vitvi15g01103\_t001 |  | | | |  |  |
| 4 | Atr-ERM94980 |  | | | |  | | | |  | Vvi-Vitvi15g01104\_t001 |  | | | |  |  |
| 4 | Atr-ERM94981 |  | | | |  | Vvi-Vitvi16g01079\_t001 |  | | | |  | | | |  |  |
| 4 | Atr-ERM94982 |  | | | |  | Vvi-Vitvi16g01078\_t001 |  | | | |  | | | |  |  |
| 4 | Atr-ERM94983 |  | | | |  | | | |  | Vvi-Vitvi15g04613\_t001 |  | | | |  |  |
| 4 | Atr-ERM94984 |  | | | |  | Vvi-Vitvi16g01909\_t001 |  | | | |  | | | |  |  |
| 4 | Atr-ERM94985 |  | | | |  | | | |  | | | |  | | | |  |  |
| 4 | Atr-ERM94986 |  | | | |  | Vvi-Vitvi16g01074\_t001 |  | | | |  | Vvi-Vitvi02g00297\_t001 |  |  |
| 4 | Atr-ERM94987 |  | | | |  | | | |  | | | |  | Vvi-Vitvi02g00298\_t001 |  |  |
| 4 | Atr-ERM94988 |  | | | |  | Vvi-Vitvi16g01073\_t001 |  | | | |  | Vvi-Vitvi02g00301\_t001 |  |  |
| 4 | Atr-ERM94989 |  | Vvi-Vitvi06g00605\_t001 |  | | | |  | Vvi-Vitvi15g01106\_t001 |  | Vvi-Vitvi02g00303\_t001 |  |  |
| 4 | Atr-ERM94990 |  | Vvi-Vitvi06g00606\_t001 |  | | | |  | Vvi-Vitvi15g01107\_t001 |  | | | |  |  |
| 3 | Atr-ERM94991 |  |  |  | Vvi-Vitvi16g01071\_t001 |  | | | |  | Vvi-Vitvi02g00308\_t001 |  |  |
| 3 | Atr-ERM94992 |  |  |  | | | |  | | | |  | | | |  |  |
| 3 | Atr-ERM94993 |  |  |  | Vvi-Vitvi16g04376\_t001 |  | | | |  | Vvi-Vitvi02g00309\_t001 |  |  |
| 3 | Atr-ERM94994 |  |  |  | | | |  | | | |  | | | |  |  |
| 3 | Atr-ERM94995 |  |  |  | | | |  | Vvi-Vitvi15g01108\_t001 |  | | | |  |  |
| 3 | Atr-ERM94996 |  |  |  | | | |  | Vvi-Vitvi15g01110\_t002 |  | Vvi-Vitvi02g00310\_t001 |  |  |
| 3 | Atr-ERM94997 |  |  |  | Vvi-Vitvi16g01068\_t002 |  | Vvi-Vitvi15g01113\_t001 |  | | | |  |  |
| 3 | Atr-ERM94998 |  |  |  | | | |  | Vvi-Vitvi15g01114\_t001 |  | Vvi-Vitvi02g00311\_t001 |  |  |
| 3 | Atr-ERM94999 |  |  |  | Vvi-Vitvi16g01904\_t001 |  | | | |  | | | |  |  |
| 3 | Atr-ERM95000 |  |  |  | | | |  | | | |  | | | |  |  |
| 3 | Atr-ERM95001 |  |  |  | | | |  | | | |  | | | |  |  |
| 3 | Atr-ERM95002 |  |  |  | Vvi-Vitvi16g01067\_t001 |  | | | |  | | | |  |  |
| 3 | Atr-ERM95003 |  |  |  | | | |  | | | |  | | | |  |  |
| 3 | Atr-ERM95004 |  |  |  | | | |  | | | |  | | | |  |  |
| 3 | Atr-ERM95005 |  |  |  | | | |  | | | |  | | | |  |  |
| 3 | Atr-ERM95006 |  |  |  | | | |  | | | |  | | | |  |  |
| 3 | Atr-ERM95007 |  |  |  | | | |  | | | |  | | | |  |  |
| 3 | Atr-ERM95008 |  |  |  | Vvi-Vitvi16g01066\_t001 |  | Vvi-Vitvi15g01115\_t002 |  | | | |  |  |
| 3 | Atr-ERM95009 |  |  |  | | | |  | | | |  | Vvi-Vitvi02g00313\_t001 |  |  |
| 3 | Atr-ERM95010 |  |  |  | Vvi-Vitvi16g01065\_t001 |  | | | |  | | | |  |  |
| 3 | Atr-ERM95011 |  |  |  | | | |  | Vvi-Vitvi15g01116\_t001 |  | | | |  |  |
| 3 | Atr-ERM95012 |  |  |  | | | |  | | | |  | | | |  |  |
| 3 | Atr-ERM95013 |  |  |  | | | |  | Vvi-Vitvi15g01117\_t002 |  | Vvi-Vitvi02g00314\_t002 |  |  |
| 3 | Atr-ERM95014 |  |  |  | | | |  | | | |  | | | |  |  |
| 3 | Atr-ERM95015 |  |  |  | | | |  | | | |  | | | |  |  |
| 3 | Atr-ERM95016 |  |  |  | | | |  | | | |  | | | |  |  |
| 3 | Atr-ERM95017 |  |  |  | | | |  | | | |  | | | |  |  |
| 3 | Atr-ERM95018 |  |  |  | | | |  | Vvi-Vitvi15g01118\_t005 |  | | | |  |  |
| 3 | Atr-ERM95019 |  |  |  | Vvi-Vitvi16g01063\_t002 |  | | | |  | | | |  |  |
| 3 | Atr-ERM95020 |  |  |  | | | |  | | | |  | | | |  |  |
| 3 | Atr-ERM95021 |  |  |  | | | |  | | | |  | | | |  |  |
| 3 | Atr-ERM95022 |  |  |  | | | |  | | | |  | | | |  |  |
| 3 | Atr-ERM95023 |  |  |  | | | |  | | | |  | | | |  |  |
| 3 | Atr-ERM95024 |  |  |  | | | |  | | | |  | | | |  |  |
| 3 | Atr-ERM95025 |  |  |  | | | |  | | | |  | | | |  |  |
| 3 | Atr-ERM95026 |  |  |  | | | |  | | | |  | | | |  |  |
| 3 | Atr-ERM95027 |  |  |  | | | |  | | | |  | | | |  |  |
| 3 | Atr-ERM95028 |  |  |  | | | |  | | | |  | | | |  |  |
| 3 | Atr-ERM95029 |  |  |  | | | |  | | | |  | | | |  |  |
| 3 | Atr-ERM95030 |  |  |  | | | |  | | | |  | | | |  |  |
| 3 | Atr-ERM95031 |  |  |  | | | |  | | | |  | | | |  |  |
| 3 | Atr-ERM95032 |  |  |  | | | |  | | | |  | | | |  |  |
| 3 | Atr-ERM95033 |  |  |  | | | |  | | | |  | | | |  |  |
| 3 | Atr-ERM95034 |  |  |  | | | |  | | | |  | Vvi-Vitvi02g00315\_t001 |  |  |
| 3 | Atr-ERM95035 |  |  |  | | | |  | | | |  | | | |  |  |
| 3 | Atr-ERM95036 |  |  |  | Vvi-Vitvi16g01058\_t001 |  | Vvi-Vitvi15g01123\_t001 |  | Vvi-Vitvi02g00316\_t001 |  |  |
| 3 | Atr-ERM95037 |  |  |  | | | |  | Vvi-Vitvi15g01124\_t001 |  | Vvi-Vitvi02g00317\_t001 |  |  |
| 3 | Atr-ERM95038 |  |  |  | | | |  | | | |  | | | |  |  |
| 3 | Atr-ERM95039 |  |  |  | | | |  | | | |  | | | |  |  |
| 3 | Atr-ERM95040 |  |  |  | | | |  | | | |  | Vvi-Vitvi02g01379\_t001 |  |  |
| 3 | Atr-ERM95041 |  |  |  | | | |  | | | |  | | | |  |  |
| 3 | Atr-ERM95042 |  |  |  | | | |  | | | |  | | | |  |  |
| 3 | Atr-ERM95043 |  |  |  | | | |  | Vvi-Vitvi15g01127\_t001 |  | | | |  |  |
| 3 | Atr-ERM95044 |  |  |  | Vvi-Vitvi16g01053\_t001 |  | | | |  | | | |  |  |
| 3 | Atr-ERM95045 |  |  |  | | | |  | | | |  | | | |  |  |
| 3 | Atr-ERM95046 |  |  |  | | | |  | | | |  | | | |  |  |
| 3 | Atr-ERM95047 |  |  |  | | | |  | | | |  | | | |  |  |
| 3 | Atr-ERM95048 |  |  |  | | | |  | | | |  | | | |  |  |
| 3 | Atr-ERM95049 |  |  |  | | | |  | | | |  | | | |  |  |
| 3 | Atr-ERM95050 |  |  |  | | | |  | | | |  | | | |  |  |
| 3 | Atr-ERM95051 |  |  |  | | | |  | | | |  | | | |  |  |
| 3 | Atr-ERM95052 |  |  |  | | | |  | | | |  | | | |  |  |
| 3 | Atr-ERM95053 |  |  |  | | | |  | | | |  | Vvi-Vitvi02g00320\_t001 |  |  |
| 3 | Atr-ERM95054 |  |  |  | | | |  | | | |  | | | |  |  |
| 3 | Atr-ERM95055 |  |  |  | | | |  | | | |  | | | |  |  |
| 3 | Atr-ERM95056 |  |  |  | | | |  | | | |  | | | |  |  |
| 3 | Atr-ERM95057 |  |  |  | Vvi-Vitvi16g01052\_t001 |  | | | |  | | | |  |  |
| 3 | Atr-ERM95058 |  |  |  | | | |  | Vvi-Vitvi15g01129\_t002 |  | | | |  |  |
| 3 | Atr-ERM95059 |  |  |  | | | |  | Vvi-Vitvi15g01134\_t001 |  | | | |  |  |
| 3 | Atr-ERM95060 |  |  |  | | | |  | | | |  | | | |  |  |
| 3 | Atr-ERM95061 |  |  |  | Vvi-Vitvi16g01051\_t001 |  | | | |  | | | |  |  |
| 3 | Atr-ERM95062 |  |  |  | | | |  | | | |  | | | |  |  |
| 3 | Atr-ERM95063 |  |  |  | | | |  | | | |  | | | |  |  |
| 3 | Atr-ERM95064 |  |  |  | | | |  | | | |  | | | |  |  |
| 3 | Atr-ERM95065 |  |  |  | | | |  | | | |  | | | |  |  |
| 3 | Atr-ERM95066 |  |  |  | | | |  | | | |  | | | |  |  |
| 3 | Atr-ERM95067 |  |  |  | | | |  | | | |  | | | |  |  |
| 3 | Atr-ERM95068 |  |  |  | | | |  | | | |  | | | |  |  |
| 3 | Atr-ERM95069 |  |  |  | | | |  | | | |  | | | |  |  |
| 3 | Atr-ERM95070 |  |  |  | | | |  | | | |  | Vvi-Vitvi02g04073\_t001 |  |  |
| 3 | Atr-ERM95071 |  |  |  | Vvi-Vitvi16g01897\_t002 |  | | | |  | | | |  |  |
| 3 | Atr-ERM95072 |  |  |  | | | |  | | | |  | | | |  |  |
| 3 | Atr-ERM95073 |  |  |  | | | |  | Vvi-Vitvi15g01142\_t001 |  | | | |  |  |
| 5 | Atr-ERM95074 |  | Vvi-Vitvi06g00563\_t001 |  | | | |  | | | |  | | | |  | Vvi-Vitvi08g01293\_t001 |  |
| 5 | Atr-ERM95075 |  | | | |  | | | |  | | | |  | Vvi-Vitvi02g00327\_t001 |  | | | |  |
| 5 | Atr-ERM95076 |  | Vvi-Vitvi06g00564\_t001 |  | | | |  | | | |  | | | |  | Vvi-Vitvi08g01295\_t001 |  |
| 5 | Atr-ERM95077 |  | | | |  | | | |  | | | |  | | | |  | | | |  |
| 5 | Atr-ERM95078 |  | Vvi-Vitvi06g00565\_t001 |  | | | |  | Vvi-Vitvi15g01144\_t001 |  | Vvi-Vitvi02g00328\_t003 |  | Vvi-Vitvi08g01296\_t001 |  |
| 5 | Atr-ERM95079 |  | | | |  | | | |  | | | |  | Vvi-Vitvi02g00329\_t001 |  | | | |  |
| 5 | Atr-ERM95080 |  | Vvi-Vitvi06g00567\_t001 |  | | | |  | | | |  | | | |  | Vvi-Vitvi08g01297\_t001 |  |
| 5 | Atr-ERM95081 |  | | | |  | | | |  | | | |  | | | |  | | | |  |
| 5 | Atr-ERM95082 |  | | | |  | | | |  | | | |  | | | |  | | | |  |
| 5 | Atr-ERM95083 |  | Vvi-Vitvi06g01734\_t001 |  | Vvi-Vitvi16g04365\_t001 |  | | | |  | | | |  | Vvi-Vitvi08g01300\_t001 |  |
| 5 | Atr-ERM95084 |  | | | |  | | | |  | | | |  | | | |  | | | |  |
| 5 | Atr-ERM95085 |  | | | |  | | | |  | Vvi-Vitvi15g01674\_t001 |  | | | |  | | | |  |
| 5 | Atr-ERM95086 |  | | | |  | | | |  | Vvi-Vitvi15g01145\_t001 |  | Vvi-Vitvi02g00331\_t001 |  | | | |  |
| 5 | Atr-ERM95087 |  | | | |  | | | |  | | | |  | | | |  | | | |  |
| 5 | Atr-ERM95088 |  | | | |  | | | |  | | | |  | | | |  | | | |  |
| 5 | Atr-ERM95089 |  | | | |  | | | |  | | | |  | Vvi-Vitvi02g00332\_t001 |  | | | |  |
| 5 | Atr-ERM95090 |  | | | |  | | | |  | | | |  | | | |  | | | |  |
| 5 | Atr-ERM95091 |  | | | |  | | | |  | | | |  | | | |  | | | |  |
| 5 | Atr-ERM95092 |  | | | |  | | | |  | Vvi-Vitvi15g01147\_t001 |  | Vvi-Vitvi02g01381\_t001 |  | | | |  |
| 5 | Atr-ERM95093 |  | | | |  | | | |  | | | |  | | | |  | | | |  |
| 5 | Atr-ERM95094 |  | | | |  | Vvi-Vitvi16g01036\_t001 |  | | | |  | Vvi-Vitvi02g00338\_t001 |  | | | |  |
| 5 | Atr-ERM95095 |  | | | |  | | | |  | | | |  | Vvi-Vitvi02g00339\_t001 |  | | | |  |
| 5 | Atr-ERM95096 |  | Vvi-Vitvi06g00569\_t001 |  | Vvi-Vitvi16g01029\_t001 |  | | | |  | Vvi-Vitvi02g00340\_t001 |  | Vvi-Vitvi08g02232\_t002 |  |
| 5 | Atr-ERM95097 |  | | | |  | Vvi-Vitvi16g01027\_t001 |  | | | |  | | | |  | | | |  |
| 5 | Atr-ERM95098 |  | | | |  | | | |  | | | |  | | | |  | | | |  |
| 5 | Atr-ERM95099 |  | | | |  | | | |  | | | |  | | | |  | | | |  |
| 5 | Atr-ERM95100 |  | | | |  | | | |  | | | |  | | | |  | | | |  |
| 5 | Atr-ERM95101 |  | | | |  | | | |  | | | |  | | | |  | | | |  |
| 5 | Atr-ERM95102 |  | | | |  | | | |  | | | |  | | | |  | | | |  |
| 5 | Atr-ERM95103 |  | | | |  | Vvi-Vitvi16g01022\_t001 |  | | | |  | | | |  | | | |  |
| 5 | Atr-ERM95104 |  | | | |  | Vvi-Vitvi16g01021\_t001 |  | | | |  | | | |  | | | |  |
| 5 | Atr-ERM95105 |  | | | |  | Vvi-Vitvi16g01874\_t001 |  | | | |  | Vvi-Vitvi02g00346\_t001 |  | | | |  |
| 5 | Atr-ERM95106 |  | | | |  | | | |  | | | |  | Vvi-Vitvi02g00347\_t001 |  | | | |  |
| 5 | Atr-ERM95107 |  | | | |  | Vvi-Vitvi16g01019\_t001 |  | | | |  | | | |  | | | |  |
| 5 | Atr-ERM95108 |  | | | |  | | | |  | | | |  | | | |  | | | |  |
| 5 | Atr-ERM95109 |  | | | |  | Vvi-Vitvi16g01018\_t001 |  | Vvi-Vitvi15g01160\_t001 |  | Vvi-Vitvi02g00348\_t001 |  | | | |  |
| 5 | Atr-ERM95110 |  | | | |  | | | |  | | | |  | | | |  | | | |  |
| 5 | Atr-ERM95111 |  | | | |  | | | |  | | | |  | | | |  | | | |  |
| 5 | Atr-ERM95112 |  | | | |  | | | |  | Vvi-Vitvi15g01162\_t002 |  | Vvi-Vitvi02g00349\_t001 |  | | | |  |
| 5 | Atr-ERM95113 |  | | | |  | | | |  | Vvi-Vitvi15g01168\_t001 |  | | | |  | Vvi-Vitvi08g01319\_t001 |  |
| 4 | Atr-ERM95114 |  | | | |  | | | |  | | | |  | | | |  |  |
| 4 | Atr-ERM95115 |  | | | |  | | | |  | | | |  | | | |  |  |
| 4 | Atr-ERM95116 |  | | | |  | | | |  | | | |  | | | |  |  |
| 4 | Atr-ERM95117 |  | | | |  | | | |  | | | |  | | | |  |  |
| 4 | Atr-ERM95118 |  | | | |  | | | |  | | | |  | | | |  |  |
| 4 | Atr-ERM95119 |  | | | |  | Vvi-Vitvi16g01012\_t001 |  | Vvi-Vitvi15g01169\_t001 |  | | | |  |  |
| 4 | Atr-ERM95120 |  | | | |  | | | |  | Vvi-Vitvi15g01170\_t001 |  | | | |  |  |
| 4 | Atr-ERM95121 |  | | | |  | | | |  | | | |  | | | |  |  |
| 4 | Atr-ERM95122 |  | Vvi-Vitvi06g01738\_t001 |  | | | |  | | | |  | | | |  |  |
| 3 | Atr-ERM95123 |  |  |  | | | |  | | | |  | | | |  |  |
| 3 | Atr-ERM95124 |  |  |  | | | |  | Vvi-Vitvi15g01171\_t001 |  | | | |  |  |
| 3 | Atr-ERM95125 |  |  |  | | | |  | | | |  | | | |  |  |
| 3 | Atr-ERM95126 |  |  |  | Vvi-Vitvi16g01011\_t001 |  | | | |  | | | |  |  |
| 3 | Atr-ERM95127 |  |  |  | Vvi-Vitvi16g01009\_t001 |  | | | |  | | | |  |  |
| 3 | Atr-ERM95128 |  |  |  | | | |  | | | |  | | | |  |  |
| 3 | Atr-ERM95129 |  |  |  | | | |  | | | |  | | | |  |  |
| 3 | Atr-ERM95130 |  |  |  | | | |  | | | |  | | | |  |  |
| 3 | Atr-ERM95131 |  |  |  | | | |  | Vvi-Vitvi15g01172\_t002 |  | | | |  |  |
| 3 | Atr-ERM95132 |  |  |  | | | |  | | | |  | | | |  |  |
| 3 | Atr-ERM95133 |  |  |  | | | |  | | | |  | Vvi-Vitvi02g00366\_t001 |  |  |
| 3 | Atr-ERM95134 |  |  |  | | | |  | | | |  | | | |  |  |
| 3 | Atr-ERM95135 |  |  |  | | | |  | | | |  | | | |  |  |
| 3 | Atr-ERM95136 |  |  |  | | | |  | | | |  | | | |  |  |
| 3 | Atr-ERM95137 |  |  |  | | | |  | | | |  | | | |  |  |
| 3 | Atr-ERM95138 |  |  |  | | | |  | | | |  | | | |  |  |
| 3 | Atr-ERM95139 |  |  |  | | | |  | | | |  | | | |  |  |
| 3 | Atr-ERM95140 |  |  |  | | | |  | | | |  | | | |  |  |
| 3 | Atr-ERM95141 |  |  |  | | | |  | | | |  | | | |  |  |
| 3 | Atr-ERM95142 |  |  |  | | | |  | | | |  | | | |  |  |
| 3 | Atr-ERM95143 |  |  |  | | | |  | | | |  | | | |  |  |
| 3 | Atr-ERM95144 |  |  |  | | | |  | | | |  | | | |  |  |
| 3 | Atr-ERM95145 |  |  |  | Vvi-Vitvi16g01872\_t001 |  | | | |  | Vvi-Vitvi02g01398\_t001 |  |  |
| 2 | Atr-ERM95146 |  |  |  |  |  | | | |  | | | |  |  |
| 2 | Atr-ERM95147 |  |  |  |  |  | | | |  | | | |  |  |
| 2 | Atr-ERM95148 |  |  |  |  |  | | | |  | | | |  |  |
| 2 | Atr-ERM95149 |  |  |  |  |  | | | |  | | | |  |  |
| 2 | Atr-ERM95150 |  |  |  |  |  | | | |  | | | |  |  |
| 2 | Atr-ERM95151 |  |  |  |  |  | | | |  | | | |  |  |
| 2 | Atr-ERM95152 |  |  |  |  |  | | | |  | | | |  |  |
| 2 | Atr-ERM95153 |  |  |  |  |  | | | |  | | | |  |  |
| 2 | Atr-ERM95154 |  |  |  |  |  | | | |  | | | |  |  |
| 2 | Atr-ERM95155 |  |  |  |  |  | | | |  | | | |  |  |
| 2 | Atr-ERM95156 |  |  |  |  |  | Vvi-Vitvi15g01191\_t002 |  | | | |  |  |
| 1 | Atr-ERM95157 |  |  |  |  |  |  |  | | | |  |  |
| 1 | Atr-ERM95158 |  |  |  |  |  |  |  | | | |  |  |
| 1 | Atr-ERM95159 |  |  |  |  |  |  |  | | | |  |  |
| 1 | Atr-ERM95160 |  |  |  |  |  |  |  | | | |  |  |
| 1 | Atr-ERM95161 |  |  |  |  |  |  |  | | | |  |  |
| 1 | Atr-ERM95162 |  |  |  |  |  |  |  | | | |  |  |
| 1 | Atr-ERM95163 |  |  |  |  |  |  |  | | | |  |  |
| 1 | Atr-ERM95164 |  |  |  |  |  |  |  | | | |  |  |
| 1 | Atr-ERM95165 |  |  |  |  |  |  |  | | | |  |  |
| 1 | Atr-ERM95166 |  |  |  |  |  |  |  | | | |  |  |
| 1 | Atr-ERM95167 |  |  |  |  |  |  |  | | | |  |  |
| 1 | Atr-ERM95168 |  |  |  |  |  |  |  | | | |  |  |
| 1 | Atr-ERM95169 |  |  |  |  |  |  |  | | | |  |  |
| 1 | Atr-ERM95170 |  |  |  |  |  |  |  | Vvi-Vitvi02g01400\_t001 |  |  |
| 0 | Atr-ERM95171 |  |  |  |  |  |  |
| 0 | Atr-ERM95172 |  |  |  |  |  |  |
| 0 | Atr-ERM95173 |  |  |  |  |  |  |
| 0 | Atr-ERM95174 |  |  |  |  |  |  |
| 0 | Atr-ERM95175 |  |  |  |  |  |  |
| 0 | Atr-ERM95176 |  |  |  |  |  |  |
| 0 | Atr-ERM95177 |  |  |  |  |  |  |
| 0 | Atr-ERM95178 |  |  |  |  |  |  |
| 0 | Atr-ERM95179 |  |  |  |  |  |  |
| 0 | Atr-ERM95180 |  |  |  |  |  |  |
| 0 | Atr-ERM95181 |  |  |  |  |  |  |
| 0 | Atr-ERM95182 |  |  |  |  |  |  |
| 0 | Atr-ERM95183 |  |  |  |  |  |  |
| 0 | Atr-ERM95184 |  |  |  |  |  |  |
| 2 | Atr-ERM95185 |  | Vvi-Vitvi10g00452\_t002 |  | Vvi-Vitvi19g00239\_t001 |  |  |  |  |
| 3 | Atr-ERM95186 |  | | | |  | Vvi-Vitvi19g00241\_t001 |  | Vvi-Vitvi10g00470\_t001 |  |  |  |
| 3 | Atr-ERM95187 |  | | | |  | | | |  | | | |  |  |  |
| 3 | Atr-ERM95188 |  | | | |  | Vvi-Vitvi19g01881\_t001 |  | | | |  |  |  |
| 3 | Atr-ERM95189 |  | Vvi-Vitvi10g00469\_t001 |  | | | |  | | | |  |  |  |
| 3 | Atr-ERM95190 |  | Vvi-Vitvi10g00471\_t001 |  | | | |  | | | |  |  |  |
| 3 | Atr-ERM95191 |  | | | |  | Vvi-Vitvi19g00242\_t001 |  | | | |  |  |  |
| 3 | Atr-ERM95192 |  | Vvi-Vitvi10g00473\_t003 |  | | | |  | | | |  |  |  |
| 3 | Atr-ERM95193 |  | | | |  | | | |  | | | |  |  |  |
| 3 | Atr-ERM95194 |  | | | |  | | | |  | | | |  |  |  |
| 3 | Atr-ERM95195 |  | | | |  | | | |  | | | |  |  |  |
| 3 | Atr-ERM95196 |  | Vvi-Vitvi10g04308\_t001 |  | | | |  | | | |  |  |  |
| 3 | Atr-ERM95197 |  | Vvi-Vitvi10g04307\_t001 |  | | | |  | | | |  |  |  |
| 3 | Atr-ERM95198 |  | | | |  | Vvi-Vitvi19g00245\_t001 |  | | | |  |  |  |
| 4 | Atr-ERM95199 |  | | | |  | | | |  | | | |  | Vvi-Vitvi12g00340\_t001 |  |  |
| 4 | Atr-ERM95200 |  | Vvi-Vitvi10g04304\_t001 |  | | | |  | | | |  | | | |  |  |
| 4 | Atr-ERM95201 |  | | | |  | | | |  | | | |  | | | |  |  |
| 4 | Atr-ERM95202 |  | | | |  | | | |  | | | |  | | | |  |  |
| 4 | Atr-ERM95203 |  | | | |  | | | |  | Vvi-Vitvi10g04306\_t001 |  | | | |  |  |
| 4 | Atr-ERM95204 |  | Vvi-Vitvi10g00413\_t001 |  | | | |  | | | |  | Vvi-Vitvi12g00341\_t001 |  |  |
| 4 | Atr-ERM95205 |  | Vvi-Vitvi10g00414\_t001 |  | | | |  | | | |  | | | |  |  |
| 4 | Atr-ERM95206 |  | Vvi-Vitvi10g00415\_t001 |  | | | |  | | | |  | | | |  |  |
| 4 | Atr-ERM95207 |  | | | |  | | | |  | | | |  | Vvi-Vitvi12g00342\_t001 |  |  |
| 4 | Atr-ERM95208 |  | | | |  | | | |  | | | |  | | | |  |  |
| 4 | Atr-ERM95209 |  | | | |  | Vvi-Vitvi19g00246\_t001 |  | | | |  | | | |  |  |
| 4 | Atr-ERM95210 |  | Vvi-Vitvi10g04303\_t001 |  | | | |  | | | |  | | | |  |  |
| 4 | Atr-ERM95211 |  | | | |  | | | |  | | | |  | | | |  |  |
| 4 | Atr-ERM95212 |  | | | |  | Vvi-Vitvi19g00251\_t001 |  | | | |  | | | |  |  |
| 4 | Atr-ERM95213 |  | Vvi-Vitvi10g04302\_t001 |  | Vvi-Vitvi19g00252\_t001 |  | | | |  | | | |  |  |
| 4 | Atr-ERM95214 |  | | | |  | Vvi-Vitvi19g00255\_t001 |  | Vvi-Vitvi10g00451\_t001 |  | | | |  |  |
| 4 | Atr-ERM95215 |  | Vvi-Vitvi10g04301\_t001 |  | | | |  | | | |  | | | |  |  |
| 4 | Atr-ERM95216 |  | | | |  | | | |  | | | |  | | | |  |  |
| 4 | Atr-ERM95217 |  | Vvi-Vitvi10g00450\_t001 |  | | | |  | | | |  | | | |  |  |
| 4 | Atr-ERM95218 |  | Vvi-Vitvi10g04300\_t001 |  | | | |  | | | |  | | | |  |  |
| 4 | Atr-ERM95219 |  | Vvi-Vitvi10g02342\_t001 |  | | | |  | | | |  | | | |  |  |
| 4 | Atr-ERM95220 |  | Vvi-Vitvi10g02343\_t001 |  | | | |  | | | |  | | | |  |  |
| 4 | Atr-ERM95221 |  | | | |  | | | |  | | | |  | | | |  |  |
| 4 | Atr-ERM95222 |  | Vvi-Vitvi10g02344\_t001 |  | | | |  | | | |  | | | |  |  |
| 4 | Atr-ERM95223 |  | | | |  | Vvi-Vitvi19g00256\_t001 |  | | | |  | Vvi-Vitvi12g00343\_t001 |  |  |
| 4 | Atr-ERM95224 |  | | | |  | | | |  | | | |  | | | |  |  |
| 4 | Atr-ERM95225 |  | | | |  | | | |  | | | |  | | | |  |  |
| 4 | Atr-ERM95226 |  | Vvi-Vitvi10g04285\_t001 |  | | | |  | Vvi-Vitvi10g02340\_t001 |  | | | |  |  |
| 4 | Atr-ERM95227 |  | Vvi-Vitvi10g02346\_t001 |  | | | |  | | | |  | Vvi-Vitvi12g02332\_t001 |  |  |
| 3 | Atr-ERM95228 |  |  |  | | | |  | | | |  | | | |  |  |
| 3 | Atr-ERM95229 |  |  |  | | | |  | | | |  | | | |  |  |
| 3 | Atr-ERM95230 |  |  |  | | | |  | | | |  | | | |  |  |
| 3 | Atr-ERM95231 |  |  |  | | | |  | Vvi-Vitvi10g04299\_t001 |  | Vvi-Vitvi12g00344\_t001 |  |  |
| 3 | Atr-ERM95232 |  |  |  | | | |  | | | |  | | | |  |  |
| 3 | Atr-ERM95233 |  |  |  | | | |  | | | |  | | | |  |  |
| 3 | Atr-ERM95234 |  |  |  | | | |  | | | |  | | | |  |  |
| 3 | Atr-ERM95235 |  |  |  | | | |  | | | |  | | | |  |  |
| 3 | Atr-ERM95236 |  |  |  | | | |  | | | |  | | | |  |  |
| 3 | Atr-ERM95237 |  |  |  | | | |  | Vvi-Vitvi10g01762\_t001 |  | | | |  |  |
| 2 | Atr-ERM95238 |  |  |  | | | |  |  |  | | | |  |  |
| 2 | Atr-ERM95239 |  |  |  | Vvi-Vitvi19g00258\_t001 |  |  |  | | | |  |  |
| 2 | Atr-ERM95240 |  |  |  | Vvi-Vitvi19g00260\_t001 |  |  |  | Vvi-Vitvi12g00348\_t001 |  |  |
| 0 | Atr-ERM95241 |  |  |  |  |  |  |
| 0 | Atr-ERM95242 |  |  |  |  |  |  |
| 0 | Atr-ERM95243 |  |  |  |  |  |  |
| 0 | Atr-ERM95244 |  |  |  |  |  |  |
| 0 | Atr-ERM95245 |  |  |  |  |  |  |
| 0 | Atr-ERM95246 |  |  |  |  |  |  |
| 0 | Atr-ERM95247 |  |  |  |  |  |  |
| 0 | Atr-ERM95248 |  |  |  |  |  |  |
| 0 | Atr-ERM95249 |  |  |  |  |  |  |
