# Supplementary material for: Genome-wide identification, characterization and gene expression of BES1 transcription factor family in grapevine (Vitis vinifera L.)
Source: Sci Rep. 2023 Jan 5;13:240. doi: 10.1038/s41598-022-24407-y (PMC9816167; doi:10.1038/s41598-022-24407-y)
Supplement: Supplementary file 3 — Supplementary Information. [file 41598_2022_24407_MOESM3_ESM.zip › Vvi_Atr/Vitis_vinifera.PN40024.v4.dna_sm.toplevel.fa.vs.Amborella_trichopoda.AMTR1.0.dna_sm.toplevel.fa.html/Atr-AmTr_v1.0_scaffold00010.html]

|  |  |  |  |  |  |  |  |  |  |  |  |  |  |
| --- | --- | --- | --- | --- | --- | --- | --- | --- | --- | --- | --- | --- | --- |
| Duplication depth | Reference chromosome | Collinear blocks | | | | | | | | | | | |
| 0 | Atr-ERM94025 |  |  |  |  |  |  |
| 0 | Atr-ERM94026 |  |  |  |  |  |  |
| 0 | Atr-ERM94027 |  |  |  |  |  |  |
| 0 | Atr-ERM94028 |  |  |  |  |  |  |
| 0 | Atr-ERM94029 |  |  |  |  |  |  |
| 0 | Atr-ERM94030 |  |  |  |  |  |  |
| 0 | Atr-ERM94031 |  |  |  |  |  |  |
| 0 | Atr-ERM94032 |  |  |  |  |  |  |
| 0 | Atr-ERM94033 |  |  |  |  |  |  |
| 0 | Atr-ERM94034 |  |  |  |  |  |  |
| 0 | Atr-ERM94035 |  |  |  |  |  |  |
| 0 | Atr-ERM94036 |  |  |  |  |  |  |
| 0 | Atr-ERM94037 |  |  |  |  |  |  |
| 0 | Atr-ERM94038 |  |  |  |  |  |  |
| 0 | Atr-ERM94039 |  |  |  |  |  |  |
| 0 | Atr-ERM94040 |  |  |  |  |  |  |
| 0 | Atr-ERM94041 |  |  |  |  |  |  |
| 0 | Atr-ERM94042 |  |  |  |  |  |  |
| 0 | Atr-ERM94043 |  |  |  |  |  |  |
| 0 | Atr-ERM94044 |  |  |  |  |  |  |
| 0 | Atr-ERM94045 |  |  |  |  |  |  |
| 0 | Atr-ERM94046 |  |  |  |  |  |  |
| 0 | Atr-ERM94047 |  |  |  |  |  |  |
| 0 | Atr-ERM94048 |  |  |  |  |  |  |
| 0 | Atr-ERM94049 |  |  |  |  |  |  |
| 0 | Atr-ERM94050 |  |  |  |  |  |  |
| 0 | Atr-ERM94051 |  |  |  |  |  |  |
| 0 | Atr-ERM94052 |  |  |  |  |  |  |
| 0 | Atr-ERM94053 |  |  |  |  |  |  |
| 0 | Atr-ERM94054 |  |  |  |  |  |  |
| 0 | Atr-ERM94055 |  |  |  |  |  |  |
| 0 | Atr-ERM94056 |  |  |  |  |  |  |
| 0 | Atr-ERM94057 |  |  |  |  |  |  |
| 0 | Atr-ERM94058 |  |  |  |  |  |  |
| 0 | Atr-ERM94059 |  |  |  |  |  |  |
| 0 | Atr-ERM94060 |  |  |  |  |  |  |
| 0 | Atr-ERM94061 |  |  |  |  |  |  |
| 0 | Atr-ERM94062 |  |  |  |  |  |  |
| 0 | Atr-ERM94063 |  |  |  |  |  |  |
| 0 | Atr-ERM94064 |  |  |  |  |  |  |
| 0 | Atr-ERM94065 |  |  |  |  |  |  |
| 0 | Atr-ERM94066 |  |  |  |  |  |  |
| 0 | Atr-ERM94067 |  |  |  |  |  |  |
| 0 | Atr-ERM94068 |  |  |  |  |  |  |
| 0 | Atr-ERM94069 |  |  |  |  |  |  |
| 0 | Atr-ERM94070 |  |  |  |  |  |  |
| 0 | Atr-ERM94071 |  |  |  |  |  |  |
| 0 | Atr-ERM94072 |  |  |  |  |  |  |
| 0 | Atr-ERM94073 |  |  |  |  |  |  |
| 0 | Atr-ERM94074 |  |  |  |  |  |  |
| 0 | Atr-ERM94075 |  |  |  |  |  |  |
| 0 | Atr-ERM94076 |  |  |  |  |  |  |
| 0 | Atr-ERM94077 |  |  |  |  |  |  |
| 0 | Atr-ERM94078 |  |  |  |  |  |  |
| 0 | Atr-ERM94079 |  |  |  |  |  |  |
| 0 | Atr-ERM94080 |  |  |  |  |  |  |
| 0 | Atr-ERM94081 |  |  |  |  |  |  |
| 0 | Atr-ERM94082 |  |  |  |  |  |  |
| 0 | Atr-ERM94083 |  |  |  |  |  |  |
| 0 | Atr-ERM94084 |  |  |  |  |  |  |
| 1 | Atr-ERM94085 |  | Vvi-Vitvi01g00423\_t001 |  |  |  |  |  |
| 1 | Atr-ERM94086 |  | | | |  |  |  |  |  |
| 1 | Atr-ERM94087 |  | | | |  |  |  |  |  |
| 1 | Atr-ERM94088 |  | | | |  |  |  |  |  |
| 1 | Atr-ERM94089 |  | | | |  |  |  |  |  |
| 1 | Atr-ERM94090 |  | | | |  |  |  |  |  |
| 1 | Atr-ERM94091 |  | | | |  |  |  |  |  |
| 1 | Atr-ERM94092 |  | | | |  |  |  |  |  |
| 1 | Atr-ERM94093 |  | | | |  |  |  |  |  |
| 1 | Atr-ERM94094 |  | | | |  |  |  |  |  |
| 1 | Atr-ERM94095 |  | | | |  |  |  |  |  |
| 1 | Atr-ERM94096 |  | | | |  |  |  |  |  |
| 1 | Atr-ERM94097 |  | | | |  |  |  |  |  |
| 1 | Atr-ERM94098 |  | | | |  |  |  |  |  |
| 1 | Atr-ERM94099 |  | | | |  |  |  |  |  |
| 1 | Atr-ERM94100 |  | | | |  |  |  |  |  |
| 1 | Atr-ERM94101 |  | | | |  |  |  |  |  |
| 1 | Atr-ERM94102 |  | | | |  |  |  |  |  |
| 1 | Atr-ERM94103 |  | Vvi-Vitvi01g00424\_t001 |  |  |  |  |  |
| 1 | Atr-ERM94104 |  | Vvi-Vitvi01g00426\_t001 |  |  |  |  |  |
| 1 | Atr-ERM94105 |  | | | |  |  |  |  |  |
| 1 | Atr-ERM94106 |  | | | |  |  |  |  |  |
| 1 | Atr-ERM94107 |  | | | |  |  |  |  |  |
| 1 | Atr-ERM94108 |  | Vvi-Vitvi01g00428\_t001 |  |  |  |  |  |
| 1 | Atr-ERM94109 |  | | | |  |  |  |  |  |
| 1 | Atr-ERM94110 |  | | | |  |  |  |  |  |
| 1 | Atr-ERM94111 |  | | | |  |  |  |  |  |
| 1 | Atr-ERM94112 |  | | | |  |  |  |  |  |
| 1 | Atr-ERM94113 |  | | | |  |  |  |  |  |
| 1 | Atr-ERM94114 |  | | | |  |  |  |  |  |
| 1 | Atr-ERM94115 |  | | | |  |  |  |  |  |
| 1 | Atr-ERM94116 |  | | | |  |  |  |  |  |
| 1 | Atr-ERM94117 |  | | | |  |  |  |  |  |
| 1 | Atr-ERM94118 |  | | | |  |  |  |  |  |
| 1 | Atr-ERM94119 |  | | | |  |  |  |  |  |
| 1 | Atr-ERM94120 |  | | | |  |  |  |  |  |
| 1 | Atr-ERM94121 |  | | | |  |  |  |  |  |
| 1 | Atr-ERM94122 |  | | | |  |  |  |  |  |
| 1 | Atr-ERM94123 |  | | | |  |  |  |  |  |
| 1 | Atr-ERM94124 |  | | | |  |  |  |  |  |
| 1 | Atr-ERM94125 |  | | | |  |  |  |  |  |
| 1 | Atr-ERM94126 |  | | | |  |  |  |  |  |
| 1 | Atr-ERM94127 |  | | | |  |  |  |  |  |
| 1 | Atr-ERM94128 |  | | | |  |  |  |  |  |
| 1 | Atr-ERM94129 |  | | | |  |  |  |  |  |
| 1 | Atr-ERM94130 |  | Vvi-Vitvi01g00446\_t001 |  |  |  |  |  |
| 1 | Atr-ERM94131 |  | | | |  |  |  |  |  |
| 2 | Atr-ERM94132 |  | | | |  | Vvi-Vitvi01g00479\_t002 |  |  |  |  |
| 2 | Atr-ERM94133 |  | | | |  | | | |  |  |  |  |
| 2 | Atr-ERM94134 |  | | | |  | | | |  |  |  |  |
| 2 | Atr-ERM94135 |  | | | |  | | | |  |  |  |  |
| 2 | Atr-ERM94136 |  | | | |  | Vvi-Vitvi01g00478\_t001 |  |  |  |  |
| 2 | Atr-ERM94137 |  | | | |  | | | |  |  |  |  |
| 2 | Atr-ERM94138 |  | | | |  | Vvi-Vitvi01g00477\_t001 |  |  |  |  |
| 2 | Atr-ERM94139 |  | | | |  | | | |  |  |  |  |
| 2 | Atr-ERM94140 |  | | | |  | | | |  |  |  |  |
| 2 | Atr-ERM94141 |  | | | |  | | | |  |  |  |  |
| 2 | Atr-ERM94142 |  | | | |  | | | |  |  |  |  |
| 2 | Atr-ERM94143 |  | | | |  | | | |  |  |  |  |
| 2 | Atr-ERM94144 |  | | | |  | Vvi-Vitvi01g00476\_t001 |  |  |  |  |
| 2 | Atr-ERM94145 |  | | | |  | | | |  |  |  |  |
| 2 | Atr-ERM94146 |  | | | |  | Vvi-Vitvi01g00475\_t001 |  |  |  |  |
| 2 | Atr-ERM94147 |  | Vvi-Vitvi01g00455\_t002 |  | | | |  |  |  |  |
| 2 | Atr-ERM94148 |  | Vvi-Vitvi01g00456\_t001 |  | | | |  |  |  |  |
| 2 | Atr-ERM94149 |  | | | |  | | | |  |  |  |  |
| 2 | Atr-ERM94150 |  | | | |  | | | |  |  |  |  |
| 2 | Atr-ERM94151 |  | | | |  | | | |  |  |  |  |
| 2 | Atr-ERM94152 |  | | | |  | | | |  |  |  |  |
| 2 | Atr-ERM94153 |  | Vvi-Vitvi01g00457\_t001 |  | | | |  |  |  |  |
| 2 | Atr-ERM94154 |  | | | |  | | | |  |  |  |  |
| 2 | Atr-ERM94155 |  | | | |  | | | |  |  |  |  |
| 2 | Atr-ERM94156 |  | | | |  | | | |  |  |  |  |
| 2 | Atr-ERM94157 |  | | | |  | | | |  |  |  |  |
| 2 | Atr-ERM94158 |  | | | |  | | | |  |  |  |  |
| 2 | Atr-ERM94159 |  | Vvi-Vitvi01g00466\_t001 |  | Vvi-Vitvi01g00466\_t001 |  |  |  |  |
| 1 | Atr-ERM94160 |  | Vvi-Vitvi07g04551\_t001 |  |  |  |  |  |
| 1 | Atr-ERM94161 |  | | | |  |  |  |  |  |
| 1 | Atr-ERM94162 |  | | | |  |  |  |  |  |
| 2 | Atr-ERM94163 |  | | | |  | Vvi-Vitvi18g00393\_t001 |  |  |  |  |
| 2 | Atr-ERM94164 |  | Vvi-Vitvi07g01358\_t001 |  | Vvi-Vitvi18g00392\_t001 |  |  |  |  |
| 2 | Atr-ERM94165 |  | | | |  | | | |  |  |  |  |
| 2 | Atr-ERM94166 |  | Vvi-Vitvi07g01359\_t001 |  | Vvi-Vitvi18g00391\_t001 |  |  |  |  |
| 2 | Atr-ERM94167 |  | | | |  | | | |  |  |  |  |
| 2 | Atr-ERM94168 |  | Vvi-Vitvi07g01360\_t001 |  | | | |  |  |  |  |
| 2 | Atr-ERM94169 |  | Vvi-Vitvi07g01361\_t001 |  | | | |  |  |  |  |
| 2 | Atr-ERM94170 |  | | | |  | | | |  |  |  |  |
| 2 | Atr-ERM94171 |  | | | |  | | | |  |  |  |  |
| 2 | Atr-ERM94172 |  | Vvi-Vitvi07g01363\_t001 |  | | | |  |  |  |  |
| 2 | Atr-ERM94173 |  | | | |  | Vvi-Vitvi18g00390\_t001 |  |  |  |  |
| 2 | Atr-ERM94174 |  | | | |  | | | |  |  |  |  |
| 2 | Atr-ERM94175 |  | | | |  | Vvi-Vitvi18g00388\_t001 |  |  |  |  |
| 2 | Atr-ERM94176 |  | Vvi-Vitvi07g01364\_t001 |  | | | |  |  |  |  |
| 2 | Atr-ERM94177 |  | | | |  | Vvi-Vitvi18g00387\_t001 |  |  |  |  |
| 2 | Atr-ERM94178 |  | | | |  | | | |  |  |  |  |
| 2 | Atr-ERM94179 |  | | | |  | | | |  |  |  |  |
| 2 | Atr-ERM94180 |  | | | |  | | | |  |  |  |  |
| 2 | Atr-ERM94181 |  | | | |  | | | |  |  |  |  |
| 2 | Atr-ERM94182 |  | | | |  | | | |  |  |  |  |
| 2 | Atr-ERM94183 |  | | | |  | | | |  |  |  |  |
| 2 | Atr-ERM94184 |  | Vvi-Vitvi07g02540\_t001 |  | Vvi-Vitvi18g00385\_t001 |  |  |  |  |
| 2 | Atr-ERM94185 |  | Vvi-Vitvi07g01365\_t001 |  | Vvi-Vitvi18g00384\_t001 |  |  |  |  |
| 2 | Atr-ERM94186 |  | | | |  | | | |  |  |  |  |
| 2 | Atr-ERM94187 |  | | | |  | | | |  |  |  |  |
| 2 | Atr-ERM94188 |  | | | |  | Vvi-Vitvi18g00382\_t001 |  |  |  |  |
| 2 | Atr-ERM94189 |  | | | |  | | | |  |  |  |  |
| 2 | Atr-ERM94190 |  | | | |  | | | |  |  |  |  |
| 2 | Atr-ERM94191 |  | | | |  | | | |  |  |  |  |
| 2 | Atr-ERM94192 |  | | | |  | | | |  |  |  |  |
| 2 | Atr-ERM94193 |  | | | |  | | | |  |  |  |  |
| 2 | Atr-ERM94194 |  | | | |  | | | |  |  |  |  |
| 2 | Atr-ERM94195 |  | | | |  | | | |  |  |  |  |
| 2 | Atr-ERM94196 |  | Vvi-Vitvi07g03031\_t001 |  | | | |  |  |  |  |
| 2 | Atr-ERM94197 |  | | | |  | | | |  |  |  |  |
| 2 | Atr-ERM94198 |  | | | |  | | | |  |  |  |  |
| 2 | Atr-ERM94199 |  | | | |  | Vvi-Vitvi18g00378\_t001 |  |  |  |  |
| 2 | Atr-ERM94200 |  | | | |  | | | |  |  |  |  |
| 2 | Atr-ERM94201 |  | Vvi-Vitvi07g01354\_t001 |  | | | |  |  |  |  |
| 2 | Atr-ERM94202 |  | | | |  | Vvi-Vitvi18g00374\_t001 |  |  |  |  |
| 3 | Atr-ERM94203 |  | | | |  | | | |  | Vvi-Vitvi12g01603\_t001 |  |  |  |
| 3 | Atr-ERM94204 |  | | | |  | Vvi-Vitvi18g00373\_t001 |  | | | |  |  |  |
| 3 | Atr-ERM94205 |  | | | |  | | | |  | | | |  |  |  |
| 3 | Atr-ERM94206 |  | | | |  | | | |  | | | |  |  |  |
| 3 | Atr-ERM94207 |  | | | |  | | | |  | | | |  |  |  |
| 3 | Atr-ERM94208 |  | | | |  | Vvi-Vitvi18g00372\_t001 |  | | | |  |  |  |
| 3 | Atr-ERM94209 |  | | | |  | | | |  | Vvi-Vitvi12g04347\_t001 |  |  |  |
| 3 | Atr-ERM94210 |  | | | |  | | | |  | | | |  |  |  |
| 3 | Atr-ERM94211 |  | | | |  | | | |  | | | |  |  |  |
| 3 | Atr-ERM94212 |  | Vvi-Vitvi07g01352\_t001 |  | | | |  | | | |  |  |  |
| 3 | Atr-ERM94213 |  | | | |  | Vvi-Vitvi18g02571\_t001 |  | | | |  |  |  |
| 3 | Atr-ERM94214 |  | Vvi-Vitvi07g01350\_t001 |  | | | |  | | | |  |  |  |
| 3 | Atr-ERM94215 |  | Vvi-Vitvi07g02536\_t001 |  | Vvi-Vitvi18g02570\_t001 |  | | | |  |  |  |
| 3 | Atr-ERM94216 |  | | | |  | | | |  | | | |  |  |  |
| 3 | Atr-ERM94217 |  | Vvi-Vitvi07g02535\_t001 |  | | | |  | Vvi-Vitvi12g01555\_t001 |  |  |  |
| 3 | Atr-ERM94218 |  | Vvi-Vitvi07g04543\_t001 |  | Vvi-Vitvi18g02569\_t001 |  | | | |  |  |  |
| 2 | Atr-ERM94219 |  |  |  | | | |  | Vvi-Vitvi12g01529\_t001 |  |  |  |
| 2 | Atr-ERM94220 |  |  |  | | | |  | Vvi-Vitvi12g01497\_t001 |  |  |  |
| 3 | Atr-ERM94221 |  | Vvi-Vitvi07g01777\_t001 |  | | | |  | | | |  |  |  |
| 3 | Atr-ERM94222 |  | | | |  | Vvi-Vitvi18g00368\_t003 |  | | | |  |  |  |
| 3 | Atr-ERM94223 |  | Vvi-Vitvi07g01778\_t001 |  | | | |  | Vvi-Vitvi12g04345\_t001 |  |  |  |
| 2 | Atr-ERM94224 |  | | | |  | Vvi-Vitvi18g00367\_t001 |  |  |  |  |
| 2 | Atr-ERM94225 |  | | | |  | | | |  |  |  |  |
| 2 | Atr-ERM94226 |  | Vvi-Vitvi07g01779\_t001 |  | Vvi-Vitvi18g00363\_t001 |  |  |  |  |
| 2 | Atr-ERM94227 |  | | | |  | | | |  |  |  |  |
| 2 | Atr-ERM94228 |  | | | |  | Vvi-Vitvi18g02565\_t002 |  |  |  |  |
| 2 | Atr-ERM94229 |  | Vvi-Vitvi07g01783\_t002 |  | Vvi-Vitvi18g00362\_t001 |  |  |  |  |
| 2 | Atr-ERM94230 |  | | | |  | | | |  |  |  |  |
| 2 | Atr-ERM94231 |  | | | |  | | | |  |  |  |  |
| 2 | Atr-ERM94232 |  | | | |  | | | |  |  |  |  |
| 2 | Atr-ERM94233 |  | | | |  | | | |  |  |  |  |
| 2 | Atr-ERM94234 |  | | | |  | | | |  |  |  |  |
| 2 | Atr-ERM94235 |  | | | |  | | | |  |  |  |  |
| 2 | Atr-ERM94236 |  | Vvi-Vitvi07g02674\_t001 |  | | | |  |  |  |  |
| 2 | Atr-ERM94237 |  | | | |  | | | |  |  |  |  |
| 2 | Atr-ERM94238 |  | | | |  | | | |  |  |  |  |
| 2 | Atr-ERM94239 |  | | | |  | | | |  |  |  |  |
| 2 | Atr-ERM94240 |  | | | |  | | | |  |  |  |  |
| 2 | Atr-ERM94241 |  | Vvi-Vitvi07g01792\_t001 |  | Vvi-Vitvi18g00361\_t001 |  |  |  |  |
| 2 | Atr-ERM94242 |  | Vvi-Vitvi07g04732\_t003 |  | | | |  |  |  |  |
| 2 | Atr-ERM94243 |  | Vvi-Vitvi07g01794\_t001 |  | Vvi-Vitvi18g02559\_t001 |  |  |  |  |
| 2 | Atr-ERM94244 |  | Vvi-Vitvi07g01795\_t001 |  | Vvi-Vitvi18g00360\_t001 |  |  |  |  |
| 2 | Atr-ERM94245 |  | | | |  | | | |  |  |  |  |
| 2 | Atr-ERM94246 |  | Vvi-Vitvi07g01796\_t001 |  | | | |  |  |  |  |
| 2 | Atr-ERM94247 |  | Vvi-Vitvi07g01798\_t001 |  | | | |  |  |  |  |
| 2 | Atr-ERM94248 |  | Vvi-Vitvi07g01800\_t001 |  | Vvi-Vitvi18g00359\_t001 |  |  |  |  |
| 2 | Atr-ERM94249 |  | | | |  | | | |  |  |  |  |
| 2 | Atr-ERM94250 |  | Vvi-Vitvi07g01802\_t001 |  | Vvi-Vitvi18g00358\_t002 |  |  |  |  |
| 2 | Atr-ERM94251 |  | Vvi-Vitvi07g01803\_t002 |  | | | |  |  |  |  |
| 2 | Atr-ERM94252 |  | | | |  | | | |  |  |  |  |
| 2 | Atr-ERM94253 |  | Vvi-Vitvi07g01804\_t001 |  | | | |  |  |  |  |
| 2 | Atr-ERM94254 |  | | | |  | Vvi-Vitvi18g02557\_t001 |  |  |  |  |
| 2 | Atr-ERM94255 |  | | | |  | | | |  |  |  |  |
| 2 | Atr-ERM94256 |  | | | |  | | | |  |  |  |  |
| 2 | Atr-ERM94257 |  | | | |  | Vvi-Vitvi18g00356\_t001 |  |  |  |  |
| 3 | Atr-ERM94258 |  | | | |  | | | |  | Vvi-Vitvi18g00350\_t001 |  |  |  |
| 3 | Atr-ERM94259 |  | | | |  | | | |  | Vvi-Vitvi18g00351\_t001 |  |  |  |
| 3 | Atr-ERM94260 |  | | | |  | | | |  | | | |  |  |  |
| 3 | Atr-ERM94261 |  | | | |  | | | |  | | | |  |  |  |
| 3 | Atr-ERM94262 |  | | | |  | | | |  | Vvi-Vitvi18g00352\_t002 |  |  |  |
| 3 | Atr-ERM94263 |  | | | |  | | | |  | Vvi-Vitvi18g00353\_t001 |  |  |  |
| 3 | Atr-ERM94264 |  | | | |  | | | |  | | | |  |  |  |
| 3 | Atr-ERM94265 |  | | | |  | | | |  | | | |  |  |  |
| 3 | Atr-ERM94266 |  | Vvi-Vitvi07g02679\_t001 |  | | | |  | | | |  |  |  |
| 3 | Atr-ERM94267 |  | | | |  | | | |  | Vvi-Vitvi18g00355\_t001 |  |  |  |
| 3 | Atr-ERM94268 |  | | | |  | | | |  | Vvi-Vitvi18g02556\_t001 |  |  |  |
| 2 | Atr-ERM94269 |  | | | |  | Vvi-Vitvi18g00349\_t001 |  |  |  |  |
| 2 | Atr-ERM94270 |  | | | |  | Vvi-Vitvi18g00348\_t001 |  |  |  |  |
| 2 | Atr-ERM94271 |  | | | |  | | | |  |  |  |  |
| 2 | Atr-ERM94272 |  | | | |  | | | |  |  |  |  |
| 2 | Atr-ERM94273 |  | | | |  | | | |  |  |  |  |
| 2 | Atr-ERM94274 |  | | | |  | | | |  |  |  |  |
| 2 | Atr-ERM94275 |  | Vvi-Vitvi07g01806\_t001 |  | | | |  |  |  |  |
| 2 | Atr-ERM94276 |  | | | |  | | | |  |  |  |  |
| 2 | Atr-ERM94277 |  | | | |  | | | |  |  |  |  |
| 2 | Atr-ERM94278 |  | | | |  | | | |  |  |  |  |
| 2 | Atr-ERM94279 |  | | | |  | | | |  |  |  |  |
| 2 | Atr-ERM94280 |  | Vvi-Vitvi07g02683\_t001 |  | | | |  |  |  |  |
| 2 | Atr-ERM94281 |  | | | |  | | | |  |  |  |  |
| 2 | Atr-ERM94282 |  | | | |  | | | |  |  |  |  |
| 2 | Atr-ERM94283 |  | | | |  | | | |  |  |  |  |
| 2 | Atr-ERM94284 |  | | | |  | Vvi-Vitvi18g00347\_t001 |  |  |  |  |
| 2 | Atr-ERM94285 |  | | | |  | | | |  |  |  |  |
| 2 | Atr-ERM94286 |  | | | |  | | | |  |  |  |  |
| 2 | Atr-ERM94287 |  | | | |  | | | |  |  |  |  |
| 2 | Atr-ERM94288 |  | | | |  | | | |  |  |  |  |
| 2 | Atr-ERM94289 |  | | | |  | | | |  |  |  |  |
| 2 | Atr-ERM94290 |  | | | |  | | | |  |  |  |  |
| 2 | Atr-ERM94291 |  | Vvi-Vitvi07g01807\_t001 |  | Vvi-Vitvi18g00346\_t001 |  |  |  |  |
| 2 | Atr-ERM94292 |  | | | |  | | | |  |  |  |  |
| 2 | Atr-ERM94293 |  | Vvi-Vitvi07g01808\_t001 |  | | | |  |  |  |  |
| 2 | Atr-ERM94294 |  | | | |  | | | |  |  |  |  |
| 2 | Atr-ERM94295 |  | | | |  | | | |  |  |  |  |
| 2 | Atr-ERM94296 |  | | | |  | | | |  |  |  |  |
| 2 | Atr-ERM94297 |  | | | |  | | | |  |  |  |  |
| 2 | Atr-ERM94298 |  | | | |  | | | |  |  |  |  |
| 2 | Atr-ERM94299 |  | | | |  | | | |  |  |  |  |
| 2 | Atr-ERM94300 |  | | | |  | Vvi-Vitvi18g00345\_t001 |  |  |  |  |
| 2 | Atr-ERM94301 |  | | | |  | Vvi-Vitvi18g00344\_t001 |  |  |  |  |
| 2 | Atr-ERM94302 |  | | | |  | | | |  |  |  |  |
| 2 | Atr-ERM94303 |  | | | |  | | | |  |  |  |  |
| 2 | Atr-ERM94304 |  | | | |  | | | |  |  |  |  |
| 2 | Atr-ERM94305 |  | | | |  | | | |  |  |  |  |
| 2 | Atr-ERM94306 |  | | | |  | | | |  |  |  |  |
| 2 | Atr-ERM94307 |  | | | |  | | | |  |  |  |  |
| 2 | Atr-ERM94308 |  | | | |  | | | |  |  |  |  |
| 2 | Atr-ERM94309 |  | | | |  | | | |  |  |  |  |
| 2 | Atr-ERM94310 |  | | | |  | | | |  |  |  |  |
| 2 | Atr-ERM94311 |  | | | |  | | | |  |  |  |  |
| 2 | Atr-ERM94312 |  | | | |  | | | |  |  |  |  |
| 2 | Atr-ERM94313 |  | | | |  | | | |  |  |  |  |
| 2 | Atr-ERM94314 |  | | | |  | | | |  |  |  |  |
| 2 | Atr-ERM94315 |  | | | |  | | | |  |  |  |  |
| 2 | Atr-ERM94316 |  | | | |  | | | |  |  |  |  |
| 2 | Atr-ERM94317 |  | Vvi-Vitvi07g01818\_t001 |  | | | |  |  |  |  |
| 2 | Atr-ERM94318 |  | | | |  | | | |  |  |  |  |
| 3 | Atr-ERM94319 |  | | | |  | | | |  | Vvi-Vitvi18g01436\_t001 |  |  |  |
| 3 | Atr-ERM94320 |  | | | |  | | | |  | | | |  |  |  |
| 3 | Atr-ERM94321 |  | | | |  | Vvi-Vitvi18g00333\_t001 |  | | | |  |  |  |
| 2 | Atr-ERM94322 |  | Vvi-Vitvi07g01823\_t001 |  |  |  | | | |  |  |  |
| 2 | Atr-ERM94323 |  | | | |  |  |  | | | |  |  |  |
| 2 | Atr-ERM94324 |  | | | |  |  |  | | | |  |  |  |
| 2 | Atr-ERM94325 |  | | | |  |  |  | | | |  |  |  |
| 2 | Atr-ERM94326 |  | Vvi-Vitvi07g01825\_t001 |  |  |  | | | |  |  |  |
| 2 | Atr-ERM94327 |  | | | |  |  |  | | | |  |  |  |
| 2 | Atr-ERM94328 |  | | | |  |  |  | | | |  |  |  |
| 2 | Atr-ERM94329 |  | Vvi-Vitvi07g01826\_t001 |  |  |  | | | |  |  |  |
| 2 | Atr-ERM94330 |  | Vvi-Vitvi07g01827\_t001 |  |  |  | | | |  |  |  |
| 2 | Atr-ERM94331 |  | | | |  |  |  | | | |  |  |  |
| 2 | Atr-ERM94332 |  | Vvi-Vitvi07g01828\_t001 |  |  |  | | | |  |  |  |
| 2 | Atr-ERM94333 |  | | | |  |  |  | | | |  |  |  |
| 2 | Atr-ERM94334 |  | Vvi-Vitvi07g01829\_t001 |  |  |  | | | |  |  |  |
| 2 | Atr-ERM94335 |  | Vvi-Vitvi07g01830\_t001 |  |  |  | | | |  |  |  |
| 2 | Atr-ERM94336 |  | Vvi-Vitvi07g01831\_t002 |  |  |  | Vvi-Vitvi18g01488\_t001 |  |  |  |
| 2 | Atr-ERM94337 |  | | | |  |  |  | Vvi-Vitvi18g04366\_t001 |  |  |  |
| 2 | Atr-ERM94338 |  | Vvi-Vitvi07g01832\_t001 |  |  |  | Vvi-Vitvi18g01579\_t001 |  |  |  |
| 2 | Atr-ERM94339 |  | | | |  |  |  | | | |  |  |  |
| 2 | Atr-ERM94340 |  | | | |  |  |  | | | |  |  |  |
| 2 | Atr-ERM94341 |  | | | |  |  |  | | | |  |  |  |
| 2 | Atr-ERM94342 |  | | | |  |  |  | | | |  |  |  |
| 2 | Atr-ERM94343 |  | | | |  |  |  | | | |  |  |  |
| 2 | Atr-ERM94344 |  | | | |  |  |  | | | |  |  |  |
| 2 | Atr-ERM94345 |  | Vvi-Vitvi07g02689\_t001 |  |  |  | | | |  |  |  |
| 2 | Atr-ERM94346 |  | | | |  |  |  | | | |  |  |  |
| 2 | Atr-ERM94347 |  | | | |  |  |  | Vvi-Vitvi18g01501\_t001 |  |  |  |
| 2 | Atr-ERM94348 |  | | | |  |  |  | Vvi-Vitvi18g01510\_t001 |  |  |  |
| 2 | Atr-ERM94349 |  | | | |  |  |  | | | |  |  |  |
| 2 | Atr-ERM94350 |  | | | |  |  |  | | | |  |  |  |
| 2 | Atr-ERM94351 |  | | | |  |  |  | | | |  |  |  |
| 2 | Atr-ERM94352 |  | | | |  |  |  | | | |  |  |  |
| 2 | Atr-ERM94353 |  | Vvi-Vitvi07g01835\_t001 |  |  |  | | | |  |  |  |
| 2 | Atr-ERM94354 |  | | | |  |  |  | | | |  |  |  |
| 2 | Atr-ERM94355 |  | | | |  |  |  | | | |  |  |  |
| 2 | Atr-ERM94356 |  | | | |  |  |  | Vvi-Vitvi18g01512\_t001 |  |  |  |
| 2 | Atr-ERM94357 |  | | | |  |  |  | | | |  |  |  |
| 2 | Atr-ERM94358 |  | | | |  |  |  | | | |  |  |  |
| 2 | Atr-ERM94359 |  | Vvi-Vitvi07g01836\_t001 |  |  |  | | | |  |  |  |
| 2 | Atr-ERM94360 |  | Vvi-Vitvi07g01838\_t001 |  |  |  | | | |  |  |  |
| 2 | Atr-ERM94361 |  | Vvi-Vitvi07g02691\_t001 |  |  |  | | | |  |  |  |
| 2 | Atr-ERM94362 |  | | | |  |  |  | | | |  |  |  |
| 2 | Atr-ERM94363 |  | Vvi-Vitvi07g01840\_t001 |  |  |  | | | |  |  |  |
| 1 | Atr-ERM94364 |  |  |  |  |  | | | |  |  |  |
| 1 | Atr-ERM94365 |  |  |  |  |  | | | |  |  |  |
| 1 | Atr-ERM94366 |  |  |  |  |  | | | |  |  |  |
| 1 | Atr-ERM94367 |  |  |  |  |  | Vvi-Vitvi18g01522\_t002 |  |  |  |
| 1 | Atr-ERM94368 |  |  |  |  |  | | | |  |  |  |
| 1 | Atr-ERM94369 |  |  |  |  |  | | | |  |  |  |
| 1 | Atr-ERM94370 |  |  |  |  |  | | | |  |  |  |
| 1 | Atr-ERM94371 |  |  |  |  |  | Vvi-Vitvi18g01527\_t001 |  |  |  |
| 1 | Atr-ERM94372 |  |  |  |  |  | | | |  |  |  |
| 1 | Atr-ERM94373 |  |  |  |  |  | | | |  |  |  |
| 1 | Atr-ERM94374 |  |  |  |  |  | Vvi-Vitvi18g02917\_t001 |  |  |  |
| 0 | Atr-ERM94375 |  |  |  |  |  |  |
| 0 | Atr-ERM94376 |  |  |  |  |  |  |
| 0 | Atr-ERM94377 |  |  |  |  |  |  |
| 2 | Atr-ERM94378 |  | Vvi-Vitvi17g00565\_t001 |  | Vvi-Vitvi14g01916\_t001 |  |  |  |  |
| 2 | Atr-ERM94379 |  | | | |  | | | |  |  |  |  |
| 2 | Atr-ERM94380 |  | | | |  | | | |  |  |  |  |
| 2 | Atr-ERM94381 |  | | | |  | Vvi-Vitvi14g01919\_t001 |  |  |  |  |
| 2 | Atr-ERM94382 |  | | | |  | | | |  |  |  |  |
| 3 | Atr-ERM94383 |  | Vvi-Vitvi17g00567\_t001 |  | | | |  | Vvi-Vitvi01g00952\_t001 |  |  |  |
| 3 | Atr-ERM94384 |  | Vvi-Vitvi17g00568\_t001 |  | Vvi-Vitvi14g01921\_t001 |  | Vvi-Vitvi01g00953\_t001 |  |  |  |
| 3 | Atr-ERM94385 |  | | | |  | | | |  | | | |  |  |  |
| 3 | Atr-ERM94386 |  | | | |  | | | |  | | | |  |  |  |
| 3 | Atr-ERM94387 |  | | | |  | | | |  | | | |  |  |  |
| 3 | Atr-ERM94388 |  | | | |  | | | |  | | | |  |  |  |
| 3 | Atr-ERM94389 |  | | | |  | | | |  | | | |  |  |  |
| 3 | Atr-ERM94390 |  | | | |  | | | |  | | | |  |  |  |
| 3 | Atr-ERM94391 |  | | | |  | | | |  | | | |  |  |  |
| 3 | Atr-ERM94392 |  | | | |  | | | |  | | | |  |  |  |
| 3 | Atr-ERM94393 |  | | | |  | | | |  | Vvi-Vitvi01g00955\_t002 |  |  |  |
| 3 | Atr-ERM94394 |  | | | |  | | | |  | Vvi-Vitvi01g00956\_t001 |  |  |  |
| 3 | Atr-ERM94395 |  | | | |  | Vvi-Vitvi14g01922\_t001 |  | Vvi-Vitvi01g00958\_t002 |  |  |  |
| 3 | Atr-ERM94396 |  | | | |  | | | |  | | | |  |  |  |
| 3 | Atr-ERM94397 |  | | | |  | Vvi-Vitvi14g01923\_t001 |  | | | |  |  |  |
| 3 | Atr-ERM94398 |  | | | |  | | | |  | | | |  |  |  |
| 3 | Atr-ERM94399 |  | | | |  | | | |  | Vvi-Vitvi01g00959\_t001 |  |  |  |
| 3 | Atr-ERM94400 |  | | | |  | | | |  | | | |  |  |  |
| 3 | Atr-ERM94401 |  | | | |  | | | |  | | | |  |  |  |
| 3 | Atr-ERM94402 |  | | | |  | | | |  | | | |  |  |  |
| 3 | Atr-ERM94403 |  | | | |  | Vvi-Vitvi14g03076\_t001 |  | Vvi-Vitvi01g00960\_t003 |  |  |  |
| 3 | Atr-ERM94404 |  | Vvi-Vitvi17g00569\_t001 |  | Vvi-Vitvi14g01926\_t001 |  | Vvi-Vitvi01g00964\_t001 |  |  |  |
| 3 | Atr-ERM94405 |  | | | |  | Vvi-Vitvi14g01928\_t001 |  | | | |  |  |  |
| 3 | Atr-ERM94406 |  | | | |  | Vvi-Vitvi14g01929\_t001 |  | | | |  |  |  |
| 3 | Atr-ERM94407 |  | Vvi-Vitvi17g00570\_t001 |  | | | |  | Vvi-Vitvi01g00970\_t001 |  |  |  |
| 3 | Atr-ERM94408 |  | Vvi-Vitvi17g00571\_t001 |  | | | |  | | | |  |  |  |
| 3 | Atr-ERM94409 |  | | | |  | | | |  | | | |  |  |  |
| 3 | Atr-ERM94410 |  | | | |  | | | |  | Vvi-Vitvi01g02127\_t001 |  |  |  |
| 3 | Atr-ERM94411 |  | | | |  | | | |  | | | |  |  |  |
| 3 | Atr-ERM94412 |  | | | |  | | | |  | | | |  |  |  |
| 3 | Atr-ERM94413 |  | Vvi-Vitvi17g04155\_t001 |  | Vvi-Vitvi14g04680\_t001 |  | Vvi-Vitvi01g00971\_t001 |  |  |  |
| 3 | Atr-ERM94414 |  | | | |  | | | |  | Vvi-Vitvi01g00972\_t001 |  |  |  |
| 3 | Atr-ERM94415 |  | | | |  | | | |  | | | |  |  |  |
| 3 | Atr-ERM94416 |  | | | |  | | | |  | | | |  |  |  |
| 3 | Atr-ERM94417 |  | | | |  | Vvi-Vitvi14g01932\_t001 |  | | | |  |  |  |
| 3 | Atr-ERM94418 |  | | | |  | | | |  | | | |  |  |  |
| 3 | Atr-ERM94419 |  | Vvi-Vitvi17g04156\_t001 |  | | | |  | Vvi-Vitvi01g00977\_t001 |  |  |  |
| 3 | Atr-ERM94420 |  | | | |  | | | |  | | | |  |  |  |
| 3 | Atr-ERM94421 |  | | | |  | | | |  | | | |  |  |  |
| 3 | Atr-ERM94422 |  | | | |  | | | |  | | | |  |  |  |
| 3 | Atr-ERM94423 |  | | | |  | | | |  | | | |  |  |  |
| 3 | Atr-ERM94424 |  | | | |  | | | |  | | | |  |  |  |
| 3 | Atr-ERM94425 |  | | | |  | | | |  | | | |  |  |  |
| 3 | Atr-ERM94426 |  | Vvi-Vitvi17g00577\_t001 |  | | | |  | Vvi-Vitvi01g00979\_t001 |  |  |  |
| 3 | Atr-ERM94427 |  | | | |  | | | |  | | | |  |  |  |
| 3 | Atr-ERM94428 |  | Vvi-Vitvi17g00578\_t001 |  | Vvi-Vitvi14g03079\_t001 |  | | | |  |  |  |
| 3 | Atr-ERM94429 |  | Vvi-Vitvi17g00579\_t001 |  | | | |  | | | |  |  |  |
| 3 | Atr-ERM94430 |  | | | |  | | | |  | | | |  |  |  |
| 3 | Atr-ERM94431 |  | Vvi-Vitvi17g00581\_t001 |  | | | |  | Vvi-Vitvi01g00980\_t001 |  |  |  |
| 3 | Atr-ERM94432 |  | | | |  | | | |  | Vvi-Vitvi01g04240\_t001 |  |  |  |
| 3 | Atr-ERM94433 |  | | | |  | | | |  | Vvi-Vitvi01g02129\_t001 |  |  |  |
| 3 | Atr-ERM94434 |  | | | |  | | | |  | Vvi-Vitvi01g00988\_t001 |  |  |  |
| 3 | Atr-ERM94435 |  | | | |  | | | |  | | | |  |  |  |
| 3 | Atr-ERM94436 |  | | | |  | | | |  | | | |  |  |  |
| 3 | Atr-ERM94437 |  | | | |  | | | |  | | | |  |  |  |
| 3 | Atr-ERM94438 |  | | | |  | Vvi-Vitvi14g01935\_t001 |  | | | |  |  |  |
| 3 | Atr-ERM94439 |  | | | |  | | | |  | Vvi-Vitvi01g00989\_t001 |  |  |  |
| 3 | Atr-ERM94440 |  | | | |  | | | |  | | | |  |  |  |
| 3 | Atr-ERM94441 |  | | | |  | | | |  | Vvi-Vitvi01g00991\_t001 |  |  |  |
| 3 | Atr-ERM94442 |  | | | |  | | | |  | | | |  |  |  |
| 3 | Atr-ERM94443 |  | Vvi-Vitvi17g00582\_t001 |  | Vvi-Vitvi14g01936\_t001 |  | | | |  |  |  |
| 3 | Atr-ERM94444 |  | | | |  | | | |  | Vvi-Vitvi01g00993\_t001 |  |  |  |
| 3 | Atr-ERM94445 |  | Vvi-Vitvi17g00583\_t001 |  | | | |  | Vvi-Vitvi01g00994\_t001 |  |  |  |
| 3 | Atr-ERM94446 |  | | | |  | Vvi-Vitvi14g01938\_t001 |  | | | |  |  |  |
| 3 | Atr-ERM94447 |  | | | |  | | | |  | | | |  |  |  |
| 3 | Atr-ERM94448 |  | | | |  | Vvi-Vitvi14g01940\_t001 |  | | | |  |  |  |
| 3 | Atr-ERM94449 |  | | | |  | | | |  | | | |  |  |  |
| 3 | Atr-ERM94450 |  | Vvi-Vitvi17g00584\_t001 |  | | | |  | Vvi-Vitvi01g00995\_t001 |  |  |  |
| 3 | Atr-ERM94451 |  | | | |  | Vvi-Vitvi14g01941\_t002 |  | | | |  |  |  |
| 3 | Atr-ERM94452 |  | | | |  | | | |  | | | |  |  |  |
| 3 | Atr-ERM94453 |  | | | |  | | | |  | | | |  |  |  |
| 3 | Atr-ERM94454 |  | | | |  | | | |  | Vvi-Vitvi01g00997\_t001 |  |  |  |
| 3 | Atr-ERM94455 |  | | | |  | | | |  | | | |  |  |  |
| 3 | Atr-ERM94456 |  | | | |  | | | |  | Vvi-Vitvi01g00999\_t001 |  |  |  |
| 3 | Atr-ERM94457 |  | Vvi-Vitvi17g00586\_t001 |  | | | |  | | | |  |  |  |
| 3 | Atr-ERM94458 |  | | | |  | | | |  | | | |  |  |  |
| 3 | Atr-ERM94459 |  | | | |  | | | |  | | | |  |  |  |
| 3 | Atr-ERM94460 |  | | | |  | | | |  | | | |  |  |  |
| 3 | Atr-ERM94461 |  | | | |  | | | |  | | | |  |  |  |
| 3 | Atr-ERM94462 |  | | | |  | | | |  | | | |  |  |  |
| 3 | Atr-ERM94463 |  | Vvi-Vitvi17g01465\_t001 |  | | | |  | Vvi-Vitvi01g01007\_t001 |  |  |  |
| 3 | Atr-ERM94464 |  | Vvi-Vitvi17g00592\_t001 |  | | | |  | | | |  |  |  |
| 3 | Atr-ERM94465 |  | | | |  | | | |  | | | |  |  |  |
| 3 | Atr-ERM94466 |  | | | |  | | | |  | Vvi-Vitvi01g04246\_t001 |  |  |  |
| 3 | Atr-ERM94467 |  | | | |  | | | |  | Vvi-Vitvi01g01009\_t001 |  |  |  |
| 3 | Atr-ERM94468 |  | | | |  | | | |  | | | |  |  |  |
| 3 | Atr-ERM94469 |  | | | |  | | | |  | | | |  |  |  |
| 3 | Atr-ERM94470 |  | | | |  | | | |  | | | |  |  |  |
| 3 | Atr-ERM94471 |  | Vvi-Vitvi17g00597\_t001 |  | | | |  | | | |  |  |  |
| 3 | Atr-ERM94472 |  | | | |  | | | |  | | | |  |  |  |
| 3 | Atr-ERM94473 |  | | | |  | Vvi-Vitvi14g01952\_t001 |  | | | |  |  |  |
| 3 | Atr-ERM94474 |  | | | |  | | | |  | | | |  |  |  |
| 3 | Atr-ERM94475 |  | | | |  | | | |  | | | |  |  |  |
| 3 | Atr-ERM94476 |  | | | |  | | | |  | | | |  |  |  |
| 3 | Atr-ERM94477 |  | | | |  | Vvi-Vitvi14g03084\_t001 |  | | | |  |  |  |
| 3 | Atr-ERM94478 |  | | | |  | Vvi-Vitvi14g01955\_t001 |  | Vvi-Vitvi01g01013\_t001 |  |  |  |
| 3 | Atr-ERM94479 |  | | | |  | Vvi-Vitvi14g01956\_t001 |  | | | |  |  |  |
| 3 | Atr-ERM94480 |  | | | |  | | | |  | | | |  |  |  |
| 3 | Atr-ERM94481 |  | Vvi-Vitvi17g00598\_t001 |  | Vvi-Vitvi14g01960\_t002 |  | | | |  |  |  |
| 3 | Atr-ERM94482 |  | | | |  | | | |  | | | |  |  |  |
| 3 | Atr-ERM94483 |  | | | |  | | | |  | | | |  |  |  |
| 3 | Atr-ERM94484 |  | | | |  | | | |  | Vvi-Vitvi01g01018\_t001 |  |  |  |
| 3 | Atr-ERM94485 |  | | | |  | | | |  | | | |  |  |  |
| 3 | Atr-ERM94486 |  | | | |  | | | |  | | | |  |  |  |
| 3 | Atr-ERM94487 |  | | | |  | | | |  | | | |  |  |  |
| 3 | Atr-ERM94488 |  | | | |  | | | |  | | | |  |  |  |
| 3 | Atr-ERM94489 |  | | | |  | | | |  | | | |  |  |  |
| 3 | Atr-ERM94490 |  | | | |  | | | |  | | | |  |  |  |
| 3 | Atr-ERM94491 |  | | | |  | | | |  | | | |  |  |  |
| 3 | Atr-ERM94492 |  | | | |  | | | |  | | | |  |  |  |
| 3 | Atr-ERM94493 |  | | | |  | | | |  | | | |  |  |  |
| 3 | Atr-ERM94494 |  | | | |  | | | |  | | | |  |  |  |
| 3 | Atr-ERM94495 |  | | | |  | | | |  | Vvi-Vitvi01g01021\_t001 |  |  |  |
| 3 | Atr-ERM94496 |  | Vvi-Vitvi17g00607\_t001 |  | Vvi-Vitvi14g01964\_t001 |  | | | |  |  |  |
| 3 | Atr-ERM94497 |  | | | |  | | | |  | | | |  |  |  |
| 3 | Atr-ERM94498 |  | Vvi-Vitvi17g00608\_t001 |  | | | |  | | | |  |  |  |
| 3 | Atr-ERM94499 |  | Vvi-Vitvi17g00609\_t001 |  | | | |  | | | |  |  |  |
| 3 | Atr-ERM94500 |  | | | |  | Vvi-Vitvi14g01965\_t001 |  | Vvi-Vitvi01g01022\_t001 |  |  |  |
| 3 | Atr-ERM94501 |  | | | |  | Vvi-Vitvi14g01966\_t001 |  | | | |  |  |  |
| 3 | Atr-ERM94502 |  | | | |  | | | |  | | | |  |  |  |
| 3 | Atr-ERM94503 |  | | | |  | | | |  | | | |  |  |  |
| 3 | Atr-ERM94504 |  | | | |  | | | |  | | | |  |  |  |
| 3 | Atr-ERM94505 |  | | | |  | Vvi-Vitvi14g01967\_t001 |  | | | |  |  |  |
| 3 | Atr-ERM94506 |  | | | |  | | | |  | | | |  |  |  |
| 3 | Atr-ERM94507 |  | | | |  | Vvi-Vitvi14g01968\_t001 |  | | | |  |  |  |
| 3 | Atr-ERM94508 |  | | | |  | | | |  | | | |  |  |  |
| 3 | Atr-ERM94509 |  | | | |  | | | |  | | | |  |  |  |
| 3 | Atr-ERM94510 |  | | | |  | | | |  | | | |  |  |  |
| 3 | Atr-ERM94511 |  | | | |  | | | |  | | | |  |  |  |
| 3 | Atr-ERM94512 |  | | | |  | Vvi-Vitvi14g01971\_t001 |  | Vvi-Vitvi01g01023\_t001 |  |  |  |
| 3 | Atr-ERM94513 |  | | | |  | | | |  | | | |  |  |  |
| 3 | Atr-ERM94514 |  | | | |  | | | |  | | | |  |  |  |
| 3 | Atr-ERM94515 |  | | | |  | | | |  | | | |  |  |  |
| 3 | Atr-ERM94516 |  | | | |  | | | |  | | | |  |  |  |
| 3 | Atr-ERM94517 |  | | | |  | Vvi-Vitvi14g01972\_t001 |  | | | |  |  |  |
| 3 | Atr-ERM94518 |  | | | |  | | | |  | | | |  |  |  |
| 3 | Atr-ERM94519 |  | | | |  | | | |  | | | |  |  |  |
| 3 | Atr-ERM94520 |  | Vvi-Vitvi17g00611\_t001 |  | Vvi-Vitvi14g01973\_t001 |  | Vvi-Vitvi01g01026\_t001 |  |  |  |
| 3 | Atr-ERM94521 |  | | | |  | | | |  | | | |  |  |  |
| 3 | Atr-ERM94522 |  | Vvi-Vitvi17g00612\_t001 |  | | | |  | | | |  |  |  |
| 3 | Atr-ERM94523 |  | | | |  | | | |  | | | |  |  |  |
| 3 | Atr-ERM94524 |  | Vvi-Vitvi17g01471\_t001 |  | Vvi-Vitvi14g01975\_t001 |  | Vvi-Vitvi01g01027\_t001 |  |  |  |
| 3 | Atr-ERM94525 |  | | | |  | | | |  | | | |  |  |  |
| 3 | Atr-ERM94526 |  | | | |  | | | |  | | | |  |  |  |
| 3 | Atr-ERM94527 |  | | | |  | Vvi-Vitvi14g01976\_t001 |  | Vvi-Vitvi01g01028\_t001 |  |  |  |
| 3 | Atr-ERM94528 |  | Vvi-Vitvi17g00614\_t001 |  | | | |  | | | |  |  |  |
| 3 | Atr-ERM94529 |  | Vvi-Vitvi17g00615\_t001 |  | | | |  | | | |  |  |  |
| 3 | Atr-ERM94530 |  | | | |  | | | |  | | | |  |  |  |
| 3 | Atr-ERM94531 |  | | | |  | | | |  | | | |  |  |  |
| 3 | Atr-ERM94532 |  | | | |  | | | |  | | | |  |  |  |
| 3 | Atr-ERM94533 |  | | | |  | | | |  | | | |  |  |  |
| 3 | Atr-ERM94534 |  | | | |  | Vvi-Vitvi14g01978\_t001 |  | | | |  |  |  |
| 3 | Atr-ERM94535 |  | | | |  | | | |  | Vvi-Vitvi01g01033\_t001 |  |  |  |
| 3 | Atr-ERM94536 |  | | | |  | | | |  | | | |  |  |  |
| 3 | Atr-ERM94537 |  | | | |  | Vvi-Vitvi14g01981\_t002 |  | Vvi-Vitvi01g01034\_t001 |  |  |  |
| 3 | Atr-ERM94538 |  | Vvi-Vitvi17g00617\_t001 |  | | | |  | | | |  |  |  |
| 3 | Atr-ERM94539 |  | Vvi-Vitvi17g04171\_t001 |  | | | |  | | | |  |  |  |
| 3 | Atr-ERM94540 |  | | | |  | | | |  | | | |  |  |  |
| 3 | Atr-ERM94541 |  | | | |  | Vvi-Vitvi14g03091\_t001 |  | | | |  |  |  |
| 3 | Atr-ERM94542 |  | | | |  | | | |  | Vvi-Vitvi01g01035\_t001 |  |  |  |
| 3 | Atr-ERM94543 |  | | | |  | | | |  | | | |  |  |  |
| 3 | Atr-ERM94544 |  | | | |  | | | |  | | | |  |  |  |
| 3 | Atr-ERM94545 |  | | | |  | | | |  | | | |  |  |  |
| 3 | Atr-ERM94546 |  | | | |  | | | |  | Vvi-Vitvi01g01037\_t001 |  |  |  |
| 3 | Atr-ERM94547 |  | Vvi-Vitvi17g00622\_t001 |  | Vvi-Vitvi14g01985\_t001 |  | | | |  |  |  |
| 3 | Atr-ERM94548 |  | | | |  | | | |  | | | |  |  |  |
| 3 | Atr-ERM94549 |  | | | |  | | | |  | | | |  |  |  |
| 3 | Atr-ERM94550 |  | | | |  | | | |  | | | |  |  |  |
| 3 | Atr-ERM94551 |  | | | |  | | | |  | Vvi-Vitvi01g01040\_t001 |  |  |  |
| 3 | Atr-ERM94552 |  | | | |  | | | |  | | | |  |  |  |
| 3 | Atr-ERM94553 |  | | | |  | | | |  | | | |  |  |  |
| 3 | Atr-ERM94554 |  | | | |  | | | |  | | | |  |  |  |
| 3 | Atr-ERM94555 |  | | | |  | | | |  | Vvi-Vitvi01g01044\_t001 |  |  |  |
| 3 | Atr-ERM94556 |  | | | |  | | | |  | Vvi-Vitvi01g01049\_t001 |  |  |  |
| 3 | Atr-ERM94557 |  | Vvi-Vitvi17g00623\_t001 |  | Vvi-Vitvi14g01987\_t001 |  | Vvi-Vitvi01g01052\_t001 |  |  |  |
| 3 | Atr-ERM94558 |  | Vvi-Vitvi17g00624\_t001 |  | | | |  | | | |  |  |  |
| 3 | Atr-ERM94559 |  | | | |  | | | |  | Vvi-Vitvi01g01085\_t001 |  |  |  |
| 3 | Atr-ERM94560 |  | Vvi-Vitvi17g04173\_t001 |  | | | |  | | | |  |  |  |
| 3 | Atr-ERM94561 |  | Vvi-Vitvi17g00627\_t001 |  | | | |  | Vvi-Vitvi01g01079\_t001 |  |  |  |
| 3 | Atr-ERM94562 |  | | | |  | Vvi-Vitvi14g01992\_t001 |  | Vvi-Vitvi01g01077\_t001 |  |  |  |
| 3 | Atr-ERM94563 |  | | | |  | | | |  | | | |  |  |  |
| 3 | Atr-ERM94564 |  | | | |  | | | |  | Vvi-Vitvi01g01058\_t001 |  |  |  |
| 3 | Atr-ERM94565 |  | Vvi-Vitvi17g00630\_t001 |  | | | |  | Vvi-Vitvi01g01057\_t001 |  |  |  |
| 3 | Atr-ERM94566 |  | | | |  | Vvi-Vitvi14g03093\_t001 |  | Vvi-Vitvi01g01056\_t001 |  |  |  |
| 3 | Atr-ERM94567 |  | | | |  | Vvi-Vitvi14g01993\_t001 |  | | | |  |  |  |
| 3 | Atr-ERM94568 |  | | | |  | | | |  | | | |  |  |  |
| 3 | Atr-ERM94569 |  | | | |  | Vvi-Vitvi14g01995\_t001 |  | | | |  |  |  |
| 3 | Atr-ERM94570 |  | | | |  | | | |  | Vvi-Vitvi01g01055\_t001 |  |  |  |
| 2 | Atr-ERM94571 |  | Vvi-Vitvi17g00635\_t001 |  | | | |  |  |  |  |
| 1 | Atr-ERM94572 |  |  |  | Vvi-Vitvi14g01996\_t001 |  |  |  |  |
| 0 | Atr-ERM94573 |  |  |  |  |  |  |
| 0 | Atr-ERM94574 |  |  |  |  |  |  |
| 0 | Atr-ERM94575 |  |  |  |  |  |  |
