# Supplementary material for: Genome-wide identification, characterization and gene expression of BES1 transcription factor family in grapevine (Vitis vinifera L.)
Source: Sci Rep. 2023 Jan 5;13:240. doi: 10.1038/s41598-022-24407-y (PMC9816167; doi:10.1038/s41598-022-24407-y)
Supplement: Supplementary file 3 — Supplementary Information. [file 41598_2022_24407_MOESM3_ESM.zip › Vvi_Atr/Vitis_vinifera.PN40024.v4.dna_sm.toplevel.fa.vs.Amborella_trichopoda.AMTR1.0.dna_sm.toplevel.fa.html/Atr-AmTr_v1.0_scaffold00011.html]

|  |  |  |  |  |  |  |  |  |  |  |  |  |  |
| --- | --- | --- | --- | --- | --- | --- | --- | --- | --- | --- | --- | --- | --- |
| Duplication depth | Reference chromosome | Collinear blocks | | | | | | | | | | | |
| 0 | Atr-ERM94577 |  |  |  |  |  |  |
| 0 | Atr-ERM94578 |  |  |  |  |  |  |
| 0 | Atr-ERM94579 |  |  |  |  |  |  |
| 0 | Atr-ERM94580 |  |  |  |  |  |  |
| 0 | Atr-ERM94581 |  |  |  |  |  |  |
| 0 | Atr-ERM94582 |  |  |  |  |  |  |
| 0 | Atr-ERM94583 |  |  |  |  |  |  |
| 0 | Atr-ERM94584 |  |  |  |  |  |  |
| 0 | Atr-ERM94585 |  |  |  |  |  |  |
| 0 | Atr-ERM94586 |  |  |  |  |  |  |
| 0 | Atr-ERM94587 |  |  |  |  |  |  |
| 0 | Atr-ERM94588 |  |  |  |  |  |  |
| 0 | Atr-ERM94589 |  |  |  |  |  |  |
| 0 | Atr-ERM94590 |  |  |  |  |  |  |
| 0 | Atr-ERM94591 |  |  |  |  |  |  |
| 0 | Atr-ERM94592 |  |  |  |  |  |  |
| 0 | Atr-ERM94593 |  |  |  |  |  |  |
| 0 | Atr-ERM94594 |  |  |  |  |  |  |
| 0 | Atr-ERM94595 |  |  |  |  |  |  |
| 0 | Atr-ERM94596 |  |  |  |  |  |  |
| 0 | Atr-ERM94597 |  |  |  |  |  |  |
| 0 | Atr-ERM94598 |  |  |  |  |  |  |
| 0 | Atr-ERM94599 |  |  |  |  |  |  |
| 0 | Atr-ERM94600 |  |  |  |  |  |  |
| 0 | Atr-ERM94601 |  |  |  |  |  |  |
| 0 | Atr-ERM94602 |  |  |  |  |  |  |
| 0 | Atr-ERM94603 |  |  |  |  |  |  |
| 0 | Atr-ERM94604 |  |  |  |  |  |  |
| 0 | Atr-ERM94605 |  |  |  |  |  |  |
| 0 | Atr-ERM94606 |  |  |  |  |  |  |
| 0 | Atr-ERM94607 |  |  |  |  |  |  |
| 0 | Atr-ERM94608 |  |  |  |  |  |  |
| 0 | Atr-ERM94609 |  |  |  |  |  |  |
| 0 | Atr-ERM94610 |  |  |  |  |  |  |
| 0 | Atr-ERM94611 |  |  |  |  |  |  |
| 0 | Atr-ERM94612 |  |  |  |  |  |  |
| 0 | Atr-ERM94613 |  |  |  |  |  |  |
| 0 | Atr-ERM94614 |  |  |  |  |  |  |
| 0 | Atr-ERM94615 |  |  |  |  |  |  |
| 0 | Atr-ERM94616 |  |  |  |  |  |  |
| 0 | Atr-ERM94617 |  |  |  |  |  |  |
| 0 | Atr-ERM94618 |  |  |  |  |  |  |
| 0 | Atr-ERM94619 |  |  |  |  |  |  |
| 0 | Atr-ERM94620 |  |  |  |  |  |  |
| 0 | Atr-ERM94621 |  |  |  |  |  |  |
| 0 | Atr-ERM94622 |  |  |  |  |  |  |
| 0 | Atr-ERM94623 |  |  |  |  |  |  |
| 0 | Atr-ERM94624 |  |  |  |  |  |  |
| 0 | Atr-ERM94625 |  |  |  |  |  |  |
| 1 | Atr-ERM94626 |  | Vvi-Vitvi04g04362\_t001 |  |  |  |  |  |
| 2 | Atr-ERM94627 |  | | | |  | Vvi-Vitvi18g00994\_t001 |  |  |  |  |
| 2 | Atr-ERM94628 |  | | | |  | | | |  |  |  |  |
| 3 | Atr-ERM94629 |  | Vvi-Vitvi04g01338\_t001 |  | Vvi-Vitvi18g02761\_t002 |  | Vvi-Vitvi03g00299\_t001 |  |  |  |
| 3 | Atr-ERM94630 |  | | | |  | | | |  | | | |  |  |  |
| 3 | Atr-ERM94631 |  | Vvi-Vitvi04g01337\_t001 |  | Vvi-Vitvi18g00992\_t001 |  | Vvi-Vitvi03g00302\_t001 |  |  |  |
| 3 | Atr-ERM94632 |  | | | |  | | | |  | | | |  |  |  |
| 3 | Atr-ERM94633 |  | | | |  | | | |  | | | |  |  |  |
| 3 | Atr-ERM94634 |  | | | |  | | | |  | | | |  |  |  |
| 3 | Atr-ERM94635 |  | | | |  | Vvi-Vitvi18g02759\_t001 |  | | | |  |  |  |
| 3 | Atr-ERM94636 |  | Vvi-Vitvi04g01336\_t001 |  | | | |  | | | |  |  |  |
| 3 | Atr-ERM94637 |  | | | |  | | | |  | | | |  |  |  |
| 3 | Atr-ERM94638 |  | | | |  | | | |  | | | |  |  |  |
| 3 | Atr-ERM94639 |  | | | |  | | | |  | | | |  |  |  |
| 3 | Atr-ERM94640 |  | | | |  | | | |  | | | |  |  |  |
| 3 | Atr-ERM94641 |  | | | |  | | | |  | Vvi-Vitvi03g00303\_t001 |  |  |  |
| 3 | Atr-ERM94642 |  | | | |  | | | |  | | | |  |  |  |
| 3 | Atr-ERM94643 |  | | | |  | | | |  | | | |  |  |  |
| 3 | Atr-ERM94644 |  | | | |  | | | |  | | | |  |  |  |
| 3 | Atr-ERM94645 |  | | | |  | Vvi-Vitvi18g02758\_t002 |  | Vvi-Vitvi03g00304\_t001 |  |  |  |
| 3 | Atr-ERM94646 |  | Vvi-Vitvi04g01334\_t001 |  | Vvi-Vitvi18g00991\_t001 |  | | | |  |  |  |
| 3 | Atr-ERM94647 |  | Vvi-Vitvi04g01332\_t001 |  | | | |  | | | |  |  |  |
| 3 | Atr-ERM94648 |  | | | |  | | | |  | | | |  |  |  |
| 3 | Atr-ERM94649 |  | | | |  | | | |  | | | |  |  |  |
| 3 | Atr-ERM94650 |  | Vvi-Vitvi04g01330\_t001 |  | | | |  | | | |  |  |  |
| 3 | Atr-ERM94651 |  | | | |  | | | |  | Vvi-Vitvi03g01483\_t001 |  |  |  |
| 3 | Atr-ERM94652 |  | | | |  | | | |  | | | |  |  |  |
| 3 | Atr-ERM94653 |  | | | |  | | | |  | | | |  |  |  |
| 3 | Atr-ERM94654 |  | | | |  | | | |  | | | |  |  |  |
| 3 | Atr-ERM94655 |  | | | |  | | | |  | Vvi-Vitvi03g00306\_t001 |  |  |  |
| 3 | Atr-ERM94656 |  | | | |  | | | |  | | | |  |  |  |
| 3 | Atr-ERM94657 |  | | | |  | | | |  | | | |  |  |  |
| 3 | Atr-ERM94658 |  | Vvi-Vitvi04g04348\_t001 |  | | | |  | | | |  |  |  |
| 3 | Atr-ERM94659 |  | | | |  | | | |  | | | |  |  |  |
| 3 | Atr-ERM94660 |  | | | |  | | | |  | | | |  |  |  |
| 3 | Atr-ERM94661 |  | | | |  | | | |  | | | |  |  |  |
| 3 | Atr-ERM94662 |  | | | |  | | | |  | | | |  |  |  |
| 3 | Atr-ERM94663 |  | | | |  | | | |  | | | |  |  |  |
| 3 | Atr-ERM94664 |  | | | |  | | | |  | | | |  |  |  |
| 3 | Atr-ERM94665 |  | | | |  | | | |  | | | |  |  |  |
| 3 | Atr-ERM94666 |  | | | |  | | | |  | | | |  |  |  |
| 3 | Atr-ERM94667 |  | | | |  | | | |  | | | |  |  |  |
| 3 | Atr-ERM94668 |  | | | |  | | | |  | | | |  |  |  |
| 3 | Atr-ERM94669 |  | | | |  | | | |  | | | |  |  |  |
| 3 | Atr-ERM94670 |  | | | |  | | | |  | | | |  |  |  |
| 3 | Atr-ERM94671 |  | Vvi-Vitvi04g04347\_t001 |  | Vvi-Vitvi18g04232\_t001 |  | | | |  |  |  |
| 3 | Atr-ERM94672 |  | | | |  | | | |  | | | |  |  |  |
| 3 | Atr-ERM94673 |  | | | |  | Vvi-Vitvi18g00986\_t001 |  | | | |  |  |  |
| 3 | Atr-ERM94674 |  | Vvi-Vitvi04g01327\_t002 |  | Vvi-Vitvi18g00983\_t001 |  | | | |  |  |  |
| 3 | Atr-ERM94675 |  | | | |  | | | |  | | | |  |  |  |
| 3 | Atr-ERM94676 |  | | | |  | Vvi-Vitvi18g02757\_t001 |  | | | |  |  |  |
| 3 | Atr-ERM94677 |  | Vvi-Vitvi04g01325\_t001 |  | Vvi-Vitvi18g00981\_t001 |  | | | |  |  |  |
| 3 | Atr-ERM94678 |  | | | |  | | | |  | | | |  |  |  |
| 3 | Atr-ERM94679 |  | | | |  | | | |  | | | |  |  |  |
| 3 | Atr-ERM94680 |  | Vvi-Vitvi04g01324\_t002 |  | | | |  | | | |  |  |  |
| 3 | Atr-ERM94681 |  | Vvi-Vitvi04g01323\_t002 |  | Vvi-Vitvi18g00980\_t001 |  | Vvi-Vitvi03g00307\_t003 |  |  |  |
| 3 | Atr-ERM94682 |  | Vvi-Vitvi04g02109\_t001 |  | | | |  | | | |  |  |  |
| 3 | Atr-ERM94683 |  | | | |  | | | |  | | | |  |  |  |
| 3 | Atr-ERM94684 |  | | | |  | | | |  | | | |  |  |  |
| 3 | Atr-ERM94685 |  | | | |  | | | |  | Vvi-Vitvi03g00308\_t001 |  |  |  |
| 3 | Atr-ERM94686 |  | | | |  | Vvi-Vitvi18g00979\_t001 |  | Vvi-Vitvi03g01484\_t001 |  |  |  |
| 3 | Atr-ERM94687 |  | | | |  | | | |  | | | |  |  |  |
| 3 | Atr-ERM94688 |  | Vvi-Vitvi04g01322\_t001 |  | | | |  | | | |  |  |  |
| 3 | Atr-ERM94689 |  | | | |  | | | |  | | | |  |  |  |
| 3 | Atr-ERM94690 |  | | | |  | | | |  | | | |  |  |  |
| 3 | Atr-ERM94691 |  | | | |  | | | |  | | | |  |  |  |
| 3 | Atr-ERM94692 |  | | | |  | | | |  | | | |  |  |  |
| 3 | Atr-ERM94693 |  | | | |  | | | |  | | | |  |  |  |
| 3 | Atr-ERM94694 |  | | | |  | | | |  | | | |  |  |  |
| 3 | Atr-ERM94695 |  | | | |  | | | |  | | | |  |  |  |
| 3 | Atr-ERM94696 |  | | | |  | | | |  | | | |  |  |  |
| 3 | Atr-ERM94697 |  | | | |  | Vvi-Vitvi18g02754\_t002 |  | | | |  |  |  |
| 3 | Atr-ERM94698 |  | Vvi-Vitvi04g01321\_t001 |  | | | |  | | | |  |  |  |
| 3 | Atr-ERM94699 |  | Vvi-Vitvi04g01319\_t001 |  | | | |  | Vvi-Vitvi03g00311\_t001 |  |  |  |
| 3 | Atr-ERM94700 |  | Vvi-Vitvi04g01318\_t001 |  | | | |  | | | |  |  |  |
| 3 | Atr-ERM94701 |  | Vvi-Vitvi04g01317\_t001 |  | | | |  | | | |  |  |  |
| 3 | Atr-ERM94702 |  | | | |  | | | |  | | | |  |  |  |
| 3 | Atr-ERM94703 |  | | | |  | | | |  | | | |  |  |  |
| 3 | Atr-ERM94704 |  | | | |  | | | |  | | | |  |  |  |
| 3 | Atr-ERM94705 |  | | | |  | | | |  | | | |  |  |  |
| 3 | Atr-ERM94706 |  | Vvi-Vitvi04g01315\_t001 |  | Vvi-Vitvi18g00976\_t001 |  | | | |  |  |  |
| 3 | Atr-ERM94707 |  | | | |  | | | |  | | | |  |  |  |
| 3 | Atr-ERM94708 |  | | | |  | Vvi-Vitvi18g02752\_t001 |  | | | |  |  |  |
| 3 | Atr-ERM94709 |  | | | |  | | | |  | | | |  |  |  |
| 3 | Atr-ERM94710 |  | | | |  | | | |  | Vvi-Vitvi03g00314\_t001 |  |  |  |
| 3 | Atr-ERM94711 |  | | | |  | Vvi-Vitvi18g00975\_t001 |  | | | |  |  |  |
| 3 | Atr-ERM94712 |  | | | |  | | | |  | | | |  |  |  |
| 3 | Atr-ERM94713 |  | | | |  | | | |  | | | |  |  |  |
| 3 | Atr-ERM94714 |  | Vvi-Vitvi04g01312\_t001 |  | | | |  | Vvi-Vitvi03g00315\_t001 |  |  |  |
| 3 | Atr-ERM94715 |  | | | |  | | | |  | | | |  |  |  |
| 3 | Atr-ERM94716 |  | | | |  | | | |  | | | |  |  |  |
| 3 | Atr-ERM94717 |  | Vvi-Vitvi04g01311\_t001 |  | Vvi-Vitvi18g00974\_t001 |  | | | |  |  |  |
| 3 | Atr-ERM94718 |  | | | |  | | | |  | | | |  |  |  |
| 3 | Atr-ERM94719 |  | | | |  | | | |  | | | |  |  |  |
| 3 | Atr-ERM94720 |  | | | |  | | | |  | | | |  |  |  |
| 3 | Atr-ERM94721 |  | | | |  | | | |  | | | |  |  |  |
| 3 | Atr-ERM94722 |  | | | |  | | | |  | | | |  |  |  |
| 3 | Atr-ERM94723 |  | | | |  | | | |  | | | |  |  |  |
| 3 | Atr-ERM94724 |  | Vvi-Vitvi04g01309\_t001 |  | Vvi-Vitvi18g00973\_t001 |  | Vvi-Vitvi03g01495\_t001 |  |  |  |
| 3 | Atr-ERM94725 |  | | | |  | | | |  | | | |  |  |  |
| 3 | Atr-ERM94726 |  | | | |  | | | |  | | | |  |  |  |
| 3 | Atr-ERM94727 |  | | | |  | | | |  | | | |  |  |  |
| 3 | Atr-ERM94728 |  | | | |  | | | |  | | | |  |  |  |
| 3 | Atr-ERM94729 |  | | | |  | | | |  | | | |  |  |  |
| 3 | Atr-ERM94730 |  | | | |  | | | |  | | | |  |  |  |
| 3 | Atr-ERM94731 |  | | | |  | | | |  | | | |  |  |  |
| 3 | Atr-ERM94732 |  | | | |  | | | |  | | | |  |  |  |
| 3 | Atr-ERM94733 |  | | | |  | | | |  | | | |  |  |  |
| 3 | Atr-ERM94734 |  | | | |  | | | |  | | | |  |  |  |
| 3 | Atr-ERM94735 |  | | | |  | | | |  | | | |  |  |  |
| 3 | Atr-ERM94736 |  | | | |  | | | |  | | | |  |  |  |
| 3 | Atr-ERM94737 |  | | | |  | | | |  | | | |  |  |  |
| 3 | Atr-ERM94738 |  | | | |  | | | |  | | | |  |  |  |
| 3 | Atr-ERM94739 |  | | | |  | | | |  | | | |  |  |  |
| 3 | Atr-ERM94740 |  | Vvi-Vitvi04g01299\_t001 |  | | | |  | | | |  |  |  |
| 2 | Atr-ERM94741 |  |  |  | | | |  | Vvi-Vitvi03g00321\_t001 |  |  |  |
| 1 | Atr-ERM94742 |  |  |  | | | |  |  |  |  |
| 1 | Atr-ERM94743 |  |  |  | Vvi-Vitvi18g00970\_t002 |  |  |  |  |
| 0 | Atr-ERM94744 |  |  |  |  |  |  |
| 0 | Atr-ERM94745 |  |  |  |  |  |  |
| 0 | Atr-ERM94746 |  |  |  |  |  |  |
| 0 | Atr-ERM94747 |  |  |  |  |  |  |
| 1 | Atr-ERM94748 |  | Vvi-Vitvi02g01286\_t001 |  |  |  |  |  |
| 1 | Atr-ERM94749 |  | | | |  |  |  |  |  |
| 1 | Atr-ERM94750 |  | | | |  |  |  |  |  |
| 1 | Atr-ERM94751 |  | | | |  |  |  |  |  |
| 1 | Atr-ERM94752 |  | | | |  |  |  |  |  |
| 1 | Atr-ERM94753 |  | | | |  |  |  |  |  |
| 1 | Atr-ERM94754 |  | | | |  |  |  |  |  |
| 1 | Atr-ERM94755 |  | | | |  |  |  |  |  |
| 1 | Atr-ERM94756 |  | | | |  |  |  |  |  |
| 1 | Atr-ERM94757 |  | Vvi-Vitvi02g01284\_t001 |  |  |  |  |  |
| 1 | Atr-ERM94758 |  | | | |  |  |  |  |  |
| 1 | Atr-ERM94759 |  | | | |  |  |  |  |  |
| 1 | Atr-ERM94760 |  | Vvi-Vitvi02g01279\_t001 |  |  |  |  |  |
| 1 | Atr-ERM94761 |  | | | |  |  |  |  |  |
| 1 | Atr-ERM94762 |  | | | |  |  |  |  |  |
| 1 | Atr-ERM94763 |  | Vvi-Vitvi02g01278\_t001 |  |  |  |  |  |
| 1 | Atr-ERM94764 |  | Vvi-Vitvi02g01275\_t001 |  |  |  |  |  |
| 1 | Atr-ERM94765 |  | | | |  |  |  |  |  |
| 1 | Atr-ERM94766 |  | | | |  |  |  |  |  |
| 1 | Atr-ERM94767 |  | | | |  |  |  |  |  |
| 1 | Atr-ERM94768 |  | | | |  |  |  |  |  |
| 1 | Atr-ERM94769 |  | Vvi-Vitvi02g01272\_t001 |  |  |  |  |  |
| 1 | Atr-ERM94770 |  | | | |  |  |  |  |  |
| 1 | Atr-ERM94771 |  | Vvi-Vitvi02g01269\_t001 |  |  |  |  |  |
| 1 | Atr-ERM94772 |  | | | |  |  |  |  |  |
| 1 | Atr-ERM94773 |  | Vvi-Vitvi02g01267\_t001 |  |  |  |  |  |
| 1 | Atr-ERM94774 |  | Vvi-Vitvi02g01264\_t001 |  |  |  |  |  |
| 1 | Atr-ERM94775 |  | | | |  |  |  |  |  |
| 1 | Atr-ERM94776 |  | | | |  |  |  |  |  |
| 1 | Atr-ERM94777 |  | | | |  |  |  |  |  |
| 1 | Atr-ERM94778 |  | Vvi-Vitvi02g01261\_t001 |  |  |  |  |  |
| 1 | Atr-ERM94779 |  | Vvi-Vitvi02g01253\_t001 |  |  |  |  |  |
| 1 | Atr-ERM94780 |  | Vvi-Vitvi02g01252\_t001 |  |  |  |  |  |
| 1 | Atr-ERM94781 |  | Vvi-Vitvi02g01248\_t001 |  |  |  |  |  |
| 1 | Atr-ERM94782 |  | | | |  |  |  |  |  |
| 1 | Atr-ERM94783 |  | | | |  |  |  |  |  |
| 1 | Atr-ERM94784 |  | | | |  |  |  |  |  |
| 1 | Atr-ERM94785 |  | | | |  |  |  |  |  |
| 1 | Atr-ERM94786 |  | Vvi-Vitvi02g04388\_t001 |  |  |  |  |  |
| 1 | Atr-ERM94787 |  | | | |  |  |  |  |  |
| 1 | Atr-ERM94788 |  | | | |  |  |  |  |  |
| 1 | Atr-ERM94789 |  | | | |  |  |  |  |  |
| 1 | Atr-ERM94790 |  | Vvi-Vitvi02g04386\_t001 |  |  |  |  |  |
| 1 | Atr-ERM94791 |  | Vvi-Vitvi02g01236\_t005 |  |  |  |  |  |
| 1 | Atr-ERM94792 |  | | | |  |  |  |  |  |
| 1 | Atr-ERM94793 |  | | | |  |  |  |  |  |
| 1 | Atr-ERM94794 |  | | | |  |  |  |  |  |
| 1 | Atr-ERM94795 |  | | | |  |  |  |  |  |
| 1 | Atr-ERM94796 |  | Vvi-Vitvi02g01235\_t001 |  |  |  |  |  |
| 1 | Atr-ERM94797 |  | Vvi-Vitvi02g01232\_t001 |  |  |  |  |  |
| 1 | Atr-ERM94798 |  | | | |  |  |  |  |  |
| 1 | Atr-ERM94799 |  | | | |  |  |  |  |  |
| 1 | Atr-ERM94800 |  | | | |  |  |  |  |  |
| 1 | Atr-ERM94801 |  | | | |  |  |  |  |  |
| 1 | Atr-ERM94802 |  | | | |  |  |  |  |  |
| 1 | Atr-ERM94803 |  | | | |  |  |  |  |  |
| 1 | Atr-ERM94804 |  | | | |  |  |  |  |  |
| 1 | Atr-ERM94805 |  | | | |  |  |  |  |  |
| 1 | Atr-ERM94806 |  | Vvi-Vitvi02g01231\_t002 |  |  |  |  |  |
| 1 | Atr-ERM94807 |  | | | |  |  |  |  |  |
| 1 | Atr-ERM94808 |  | | | |  |  |  |  |  |
| 1 | Atr-ERM94809 |  | Vvi-Vitvi02g01230\_t001 |  |  |  |  |  |
| 1 | Atr-ERM94810 |  | | | |  |  |  |  |  |
| 1 | Atr-ERM94811 |  | Vvi-Vitvi02g01228\_t001 |  |  |  |  |  |
| 0 | Atr-ERM94812 |  |  |  |  |  |  |
