# Supplementary material for: Genome-wide identification, characterization and gene expression of BES1 transcription factor family in grapevine (Vitis vinifera L.)
Source: Sci Rep. 2023 Jan 5;13:240. doi: 10.1038/s41598-022-24407-y (PMC9816167; doi:10.1038/s41598-022-24407-y)
Supplement: Supplementary file 3 — Supplementary Information. [file 41598_2022_24407_MOESM3_ESM.zip › Vvi_Atr/Vitis_vinifera.PN40024.v4.dna_sm.toplevel.fa.vs.Amborella_trichopoda.AMTR1.0.dna_sm.toplevel.fa.html/Atr-AmTr_v1.0_scaffold00012.html]

|  |  |  |  |  |  |  |  |  |  |  |  |  |  |
| --- | --- | --- | --- | --- | --- | --- | --- | --- | --- | --- | --- | --- | --- |
| Duplication depth | Reference chromosome | Collinear blocks | | | | | | | | | | | |
| 0 | Atr-ERN07713 |  |  |  |  |  |  |
| 0 | Atr-ERN07714 |  |  |  |  |  |  |
| 0 | Atr-ERN07715 |  |  |  |  |  |  |
| 0 | Atr-ERN07716 |  |  |  |  |  |  |
| 0 | Atr-ERN07717 |  |  |  |  |  |  |
| 0 | Atr-ERN07718 |  |  |  |  |  |  |
| 0 | Atr-ERN07719 |  |  |  |  |  |  |
| 0 | Atr-ERN07720 |  |  |  |  |  |  |
| 0 | Atr-ERN07721 |  |  |  |  |  |  |
| 0 | Atr-ERN07722 |  |  |  |  |  |  |
| 0 | Atr-ERN07723 |  |  |  |  |  |  |
| 0 | Atr-ERN07724 |  |  |  |  |  |  |
| 0 | Atr-ERN07725 |  |  |  |  |  |  |
| 0 | Atr-ERN07726 |  |  |  |  |  |  |
| 0 | Atr-ERN07727 |  |  |  |  |  |  |
| 0 | Atr-ERN07728 |  |  |  |  |  |  |
| 0 | Atr-ERN07729 |  |  |  |  |  |  |
| 0 | Atr-ERN07730 |  |  |  |  |  |  |
| 0 | Atr-ERN07731 |  |  |  |  |  |  |
| 0 | Atr-ERN07732 |  |  |  |  |  |  |
| 0 | Atr-ERN07733 |  |  |  |  |  |  |
| 1 | Atr-ERN07734 |  | Vvi-Vitvi08g01177\_t001 |  |  |  |  |  |
| 1 | Atr-ERN07735 |  | | | |  |  |  |  |  |
| 2 | Atr-ERN07736 |  | | | |  | Vvi-Vitvi06g00405\_t001 |  |  |  |  |
| 2 | Atr-ERN07737 |  | | | |  | | | |  |  |  |  |
| 2 | Atr-ERN07738 |  | | | |  | | | |  |  |  |  |
| 2 | Atr-ERN07739 |  | | | |  | | | |  |  |  |  |
| 2 | Atr-ERN07740 |  | | | |  | | | |  |  |  |  |
| 2 | Atr-ERN07741 |  | | | |  | | | |  |  |  |  |
| 2 | Atr-ERN07742 |  | | | |  | | | |  |  |  |  |
| 2 | Atr-ERN07743 |  | | | |  | | | |  |  |  |  |
| 2 | Atr-ERN07744 |  | | | |  | | | |  |  |  |  |
| 2 | Atr-ERN07745 |  | | | |  | | | |  |  |  |  |
| 2 | Atr-ERN07746 |  | Vvi-Vitvi08g01168\_t001 |  | | | |  |  |  |  |
| 2 | Atr-ERN07747 |  | | | |  | | | |  |  |  |  |
| 2 | Atr-ERN07748 |  | | | |  | | | |  |  |  |  |
| 2 | Atr-ERN07749 |  | | | |  | | | |  |  |  |  |
| 2 | Atr-ERN07750 |  | | | |  | Vvi-Vitvi06g00422\_t001 |  |  |  |  |
| 2 | Atr-ERN07751 |  | | | |  | | | |  |  |  |  |
| 2 | Atr-ERN07752 |  | | | |  | | | |  |  |  |  |
| 2 | Atr-ERN07753 |  | Vvi-Vitvi08g01166\_t001 |  | | | |  |  |  |  |
| 2 | Atr-ERN07754 |  | | | |  | | | |  |  |  |  |
| 2 | Atr-ERN07755 |  | Vvi-Vitvi08g01165\_t001 |  | Vvi-Vitvi06g00424\_t001 |  |  |  |  |
| 2 | Atr-ERN07756 |  | | | |  | | | |  |  |  |  |
| 2 | Atr-ERN07757 |  | | | |  | | | |  |  |  |  |
| 2 | Atr-ERN07758 |  | | | |  | | | |  |  |  |  |
| 2 | Atr-ERN07759 |  | | | |  | | | |  |  |  |  |
| 2 | Atr-ERN07760 |  | | | |  | | | |  |  |  |  |
| 2 | Atr-ERN07761 |  | | | |  | | | |  |  |  |  |
| 2 | Atr-ERN07762 |  | | | |  | | | |  |  |  |  |
| 2 | Atr-ERN07763 |  | | | |  | | | |  |  |  |  |
| 2 | Atr-ERN07764 |  | | | |  | | | |  |  |  |  |
| 2 | Atr-ERN07765 |  | | | |  | | | |  |  |  |  |
| 2 | Atr-ERN07766 |  | | | |  | | | |  |  |  |  |
| 2 | Atr-ERN07767 |  | Vvi-Vitvi08g01164\_t001 |  | | | |  |  |  |  |
| 2 | Atr-ERN07768 |  | Vvi-Vitvi08g01163\_t002 |  | | | |  |  |  |  |
| 2 | Atr-ERN07769 |  | | | |  | | | |  |  |  |  |
| 2 | Atr-ERN07770 |  | | | |  | | | |  |  |  |  |
| 2 | Atr-ERN07771 |  | Vvi-Vitvi08g01162\_t001 |  | | | |  |  |  |  |
| 2 | Atr-ERN07772 |  | Vvi-Vitvi08g01161\_t003 |  | | | |  |  |  |  |
| 1 | Atr-ERN07773 |  |  |  | | | |  |  |  |  |
| 1 | Atr-ERN07774 |  |  |  | Vvi-Vitvi06g00432\_t001 |  |  |  |  |
| 1 | Atr-ERN07775 |  |  |  | | | |  |  |  |  |
| 1 | Atr-ERN07776 |  |  |  | | | |  |  |  |  |
| 1 | Atr-ERN07777 |  |  |  | | | |  |  |  |  |
| 1 | Atr-ERN07778 |  |  |  | | | |  |  |  |  |
| 1 | Atr-ERN07779 |  |  |  | | | |  |  |  |  |
| 1 | Atr-ERN07780 |  |  |  | | | |  |  |  |  |
| 1 | Atr-ERN07781 |  |  |  | | | |  |  |  |  |
| 1 | Atr-ERN07782 |  |  |  | | | |  |  |  |  |
| 1 | Atr-ERN07783 |  |  |  | | | |  |  |  |  |
| 1 | Atr-ERN07784 |  |  |  | | | |  |  |  |  |
| 1 | Atr-ERN07785 |  |  |  | | | |  |  |  |  |
| 1 | Atr-ERN07786 |  |  |  | | | |  |  |  |  |
| 1 | Atr-ERN07787 |  |  |  | | | |  |  |  |  |
| 1 | Atr-ERN07788 |  |  |  | | | |  |  |  |  |
| 1 | Atr-ERN07789 |  |  |  | | | |  |  |  |  |
| 1 | Atr-ERN07790 |  |  |  | | | |  |  |  |  |
| 1 | Atr-ERN07791 |  |  |  | | | |  |  |  |  |
| 1 | Atr-ERN07792 |  |  |  | | | |  |  |  |  |
| 1 | Atr-ERN07793 |  |  |  | | | |  |  |  |  |
| 2 | Atr-ERN07794 |  | Vvi-Vitvi06g00447\_t001 |  | | | |  |  |  |  |
| 2 | Atr-ERN07795 |  | | | |  | | | |  |  |  |  |
| 2 | Atr-ERN07796 |  | | | |  | | | |  |  |  |  |
| 2 | Atr-ERN07797 |  | | | |  | Vvi-Vitvi06g00445\_t001 |  |  |  |  |
| 2 | Atr-ERN07798 |  | | | |  | | | |  |  |  |  |
| 2 | Atr-ERN07799 |  | | | |  | | | |  |  |  |  |
| 2 | Atr-ERN07800 |  | | | |  | | | |  |  |  |  |
| 2 | Atr-ERN07801 |  | Vvi-Vitvi06g00444\_t001 |  | | | |  |  |  |  |
| 2 | Atr-ERN07802 |  | | | |  | | | |  |  |  |  |
| 2 | Atr-ERN07803 |  | | | |  | | | |  |  |  |  |
| 2 | Atr-ERN07804 |  | Vvi-Vitvi06g00442\_t001 |  | | | |  |  |  |  |
| 2 | Atr-ERN07805 |  | | | |  | | | |  |  |  |  |
| 2 | Atr-ERN07806 |  | Vvi-Vitvi06g00440\_t001 |  | | | |  |  |  |  |
| 2 | Atr-ERN07807 |  | | | |  | | | |  |  |  |  |
| 2 | Atr-ERN07808 |  | Vvi-Vitvi06g00439\_t003 |  | | | |  |  |  |  |
| 2 | Atr-ERN07809 |  | Vvi-Vitvi06g00438\_t001 |  | | | |  |  |  |  |
| 2 | Atr-ERN07810 |  | | | |  | | | |  |  |  |  |
| 2 | Atr-ERN07811 |  | | | |  | | | |  |  |  |  |
| 2 | Atr-ERN07812 |  | Vvi-Vitvi06g00437\_t001 |  | | | |  |  |  |  |
| 2 | Atr-ERN07813 |  | | | |  | | | |  |  |  |  |
| 3 | Atr-ERN07814 |  | Vvi-Vitvi06g00436\_t001 |  | | | |  | Vvi-Vitvi08g01179\_t001 |  |  |  |
| 2 | Atr-ERN07815 |  |  |  | | | |  | | | |  |  |  |
| 3 | Atr-ERN07816 |  | Vvi-Vitvi13g00302\_t001 |  | | | |  | | | |  |  |  |
| 3 | Atr-ERN07817 |  | Vvi-Vitvi13g00301\_t001 |  | | | |  | | | |  |  |  |
| 3 | Atr-ERN07818 |  | | | |  | | | |  | | | |  |  |  |
| 3 | Atr-ERN07819 |  | | | |  | | | |  | | | |  |  |  |
| 3 | Atr-ERN07820 |  | | | |  | | | |  | | | |  |  |  |
| 3 | Atr-ERN07821 |  | Vvi-Vitvi13g00298\_t001 |  | Vvi-Vitvi06g00449\_t001 |  | Vvi-Vitvi08g01186\_t001 |  |  |  |
| 3 | Atr-ERN07822 |  | | | |  | | | |  | | | |  |  |  |
| 3 | Atr-ERN07823 |  | | | |  | | | |  | | | |  |  |  |
| 3 | Atr-ERN07824 |  | | | |  | | | |  | | | |  |  |  |
| 3 | Atr-ERN07825 |  | | | |  | Vvi-Vitvi06g00451\_t001 |  | Vvi-Vitvi08g01188\_t001 |  |  |  |
| 3 | Atr-ERN07826 |  | | | |  | | | |  | | | |  |  |  |
| 3 | Atr-ERN07827 |  | | | |  | | | |  | | | |  |  |  |
| 3 | Atr-ERN07828 |  | | | |  | | | |  | | | |  |  |  |
| 3 | Atr-ERN07829 |  | | | |  | Vvi-Vitvi06g00452\_t001 |  | Vvi-Vitvi08g02191\_t001 |  |  |  |
| 3 | Atr-ERN07830 |  | | | |  | | | |  | Vvi-Vitvi08g01189\_t001 |  |  |  |
| 3 | Atr-ERN07831 |  | | | |  | | | |  | | | |  |  |  |
| 3 | Atr-ERN07832 |  | | | |  | | | |  | | | |  |  |  |
| 3 | Atr-ERN07833 |  | Vvi-Vitvi13g00295\_t001 |  | | | |  | | | |  |  |  |
| 3 | Atr-ERN07834 |  | | | |  | | | |  | Vvi-Vitvi08g01193\_t001 |  |  |  |
| 3 | Atr-ERN07835 |  | | | |  | | | |  | | | |  |  |  |
| 3 | Atr-ERN07836 |  | | | |  | Vvi-Vitvi06g00455\_t001.1.6037826f |  | | | |  |  |  |
| 3 | Atr-ERN07837 |  | | | |  | | | |  | | | |  |  |  |
| 3 | Atr-ERN07838 |  | | | |  | Vvi-Vitvi06g00456\_t001 |  | Vvi-Vitvi08g01194\_t001 |  |  |  |
| 3 | Atr-ERN07839 |  | | | |  | Vvi-Vitvi06g00459\_t001 |  | | | |  |  |  |
| 3 | Atr-ERN07840 |  | | | |  | | | |  | | | |  |  |  |
| 3 | Atr-ERN07841 |  | | | |  | Vvi-Vitvi06g00460\_t001 |  | Vvi-Vitvi08g01195\_t001 |  |  |  |
| 3 | Atr-ERN07842 |  | Vvi-Vitvi13g00294\_t001 |  | | | |  | | | |  |  |  |
| 3 | Atr-ERN07843 |  | | | |  | | | |  | | | |  |  |  |
| 3 | Atr-ERN07844 |  | | | |  | | | |  | | | |  |  |  |
| 3 | Atr-ERN07845 |  | | | |  | | | |  | | | |  |  |  |
| 3 | Atr-ERN07846 |  | | | |  | | | |  | | | |  |  |  |
| 3 | Atr-ERN07847 |  | | | |  | | | |  | Vvi-Vitvi08g01197\_t001 |  |  |  |
| 3 | Atr-ERN07848 |  | | | |  | Vvi-Vitvi06g00465\_t001 |  | | | |  |  |  |
| 3 | Atr-ERN07849 |  | | | |  | Vvi-Vitvi06g00466\_t001 |  | Vvi-Vitvi08g02195\_t001 |  |  |  |
| 3 | Atr-ERN07850 |  | | | |  | | | |  | | | |  |  |  |
| 3 | Atr-ERN07851 |  | | | |  | | | |  | | | |  |  |  |
| 3 | Atr-ERN07852 |  | Vvi-Vitvi13g00293\_t001 |  | | | |  | | | |  |  |  |
| 3 | Atr-ERN07853 |  | | | |  | Vvi-Vitvi06g00467\_t001 |  | | | |  |  |  |
| 3 | Atr-ERN07854 |  | Vvi-Vitvi13g00292\_t001 |  | Vvi-Vitvi06g00468\_t001 |  | | | |  |  |  |
| 3 | Atr-ERN07855 |  | | | |  | Vvi-Vitvi06g00470\_t001 |  | | | |  |  |  |
| 3 | Atr-ERN07856 |  | | | |  | | | |  | | | |  |  |  |
| 3 | Atr-ERN07857 |  | | | |  | | | |  | | | |  |  |  |
| 3 | Atr-ERN07858 |  | | | |  | | | |  | Vvi-Vitvi08g02196\_t001 |  |  |  |
| 3 | Atr-ERN07859 |  | | | |  | | | |  | | | |  |  |  |
| 3 | Atr-ERN07860 |  | | | |  | | | |  | Vvi-Vitvi08g01201\_t003 |  |  |  |
| 3 | Atr-ERN07861 |  | | | |  | Vvi-Vitvi06g01703\_t001 |  | | | |  |  |  |
| 3 | Atr-ERN07862 |  | Vvi-Vitvi13g00289\_t001 |  | | | |  | | | |  |  |  |
| 3 | Atr-ERN07863 |  | | | |  | | | |  | | | |  |  |  |
| 3 | Atr-ERN07864 |  | | | |  | | | |  | | | |  |  |  |
| 3 | Atr-ERN07865 |  | | | |  | | | |  | | | |  |  |  |
| 3 | Atr-ERN07866 |  | | | |  | | | |  | | | |  |  |  |
| 3 | Atr-ERN07867 |  | | | |  | | | |  | | | |  |  |  |
| 3 | Atr-ERN07868 |  | | | |  | | | |  | | | |  |  |  |
| 3 | Atr-ERN07869 |  | | | |  | | | |  | | | |  |  |  |
| 3 | Atr-ERN07870 |  | | | |  | | | |  | | | |  |  |  |
| 3 | Atr-ERN07871 |  | | | |  | | | |  | | | |  |  |  |
| 3 | Atr-ERN07872 |  | | | |  | | | |  | | | |  |  |  |
| 3 | Atr-ERN07873 |  | | | |  | | | |  | | | |  |  |  |
| 3 | Atr-ERN07874 |  | | | |  | | | |  | | | |  |  |  |
| 3 | Atr-ERN07875 |  | | | |  | | | |  | | | |  |  |  |
| 3 | Atr-ERN07876 |  | Vvi-Vitvi13g00288\_t001 |  | Vvi-Vitvi06g00472\_t001 |  | | | |  |  |  |
| 3 | Atr-ERN07877 |  | Vvi-Vitvi13g00287\_t001 |  | Vvi-Vitvi06g00473\_t001 |  | Vvi-Vitvi08g01204\_t004 |  |  |  |
| 3 | Atr-ERN07878 |  | Vvi-Vitvi13g00286\_t001 |  | | | |  | | | |  |  |  |
| 3 | Atr-ERN07879 |  | | | |  | | | |  | | | |  |  |  |
| 3 | Atr-ERN07880 |  | | | |  | | | |  | | | |  |  |  |
| 3 | Atr-ERN07881 |  | | | |  | | | |  | | | |  |  |  |
| 3 | Atr-ERN07882 |  | Vvi-Vitvi13g04089\_t001 |  | | | |  | | | |  |  |  |
| 2 | Atr-ERN07883 |  |  |  | | | |  | | | |  |  |  |
| 2 | Atr-ERN07884 |  |  |  | Vvi-Vitvi06g00474\_t001 |  | | | |  |  |  |
| 2 | Atr-ERN07885 |  |  |  | Vvi-Vitvi06g00475\_t001 |  | | | |  |  |  |
| 2 | Atr-ERN07886 |  |  |  | | | |  | | | |  |  |  |
| 3 | Atr-ERN07887 |  | Vvi-Vitvi13g00306\_t001 |  | | | |  | | | |  |  |  |
| 3 | Atr-ERN07888 |  | Vvi-Vitvi13g00307\_t001 |  | | | |  | | | |  |  |  |
| 3 | Atr-ERN07889 |  | Vvi-Vitvi13g01987\_t001 |  | Vvi-Vitvi06g00477\_t001 |  | | | |  |  |  |
| 3 | Atr-ERN07890 |  | | | |  | | | |  | | | |  |  |  |
| 3 | Atr-ERN07891 |  | | | |  | | | |  | | | |  |  |  |
| 3 | Atr-ERN07892 |  | | | |  | | | |  | | | |  |  |  |
| 3 | Atr-ERN07893 |  | | | |  | | | |  | | | |  |  |  |
| 3 | Atr-ERN07894 |  | | | |  | | | |  | | | |  |  |  |
| 3 | Atr-ERN07895 |  | | | |  | | | |  | | | |  |  |  |
| 3 | Atr-ERN07896 |  | | | |  | | | |  | | | |  |  |  |
| 3 | Atr-ERN07897 |  | | | |  | | | |  | | | |  |  |  |
| 3 | Atr-ERN07898 |  | Vvi-Vitvi13g00309\_t001 |  | Vvi-Vitvi06g00481\_t001 |  | Vvi-Vitvi08g01206\_t001 |  |  |  |
| 3 | Atr-ERN07899 |  | | | |  | Vvi-Vitvi06g01704\_t001 |  | | | |  |  |  |
| 3 | Atr-ERN07900 |  | | | |  | | | |  | Vvi-Vitvi08g01208\_t001 |  |  |  |
| 3 | Atr-ERN07901 |  | | | |  | | | |  | | | |  |  |  |
| 3 | Atr-ERN07902 |  | | | |  | Vvi-Vitvi06g00483\_t001 |  | Vvi-Vitvi08g01209\_t001 |  |  |  |
| 3 | Atr-ERN07903 |  | | | |  | | | |  | | | |  |  |  |
| 3 | Atr-ERN07904 |  | | | |  | | | |  | | | |  |  |  |
| 3 | Atr-ERN07905 |  | | | |  | | | |  | | | |  |  |  |
| 3 | Atr-ERN07906 |  | | | |  | | | |  | | | |  |  |  |
| 3 | Atr-ERN07907 |  | | | |  | | | |  | | | |  |  |  |
| 3 | Atr-ERN07908 |  | | | |  | | | |  | | | |  |  |  |
| 3 | Atr-ERN07909 |  | | | |  | | | |  | | | |  |  |  |
| 3 | Atr-ERN07910 |  | | | |  | | | |  | | | |  |  |  |
| 3 | Atr-ERN07911 |  | | | |  | | | |  | | | |  |  |  |
| 3 | Atr-ERN07912 |  | | | |  | | | |  | | | |  |  |  |
| 3 | Atr-ERN07913 |  | | | |  | | | |  | | | |  |  |  |
| 3 | Atr-ERN07914 |  | | | |  | Vvi-Vitvi06g01705\_t001 |  | | | |  |  |  |
| 3 | Atr-ERN07915 |  | | | |  | | | |  | | | |  |  |  |
| 3 | Atr-ERN07916 |  | | | |  | Vvi-Vitvi06g00484\_t001 |  | | | |  |  |  |
| 3 | Atr-ERN07917 |  | | | |  | Vvi-Vitvi06g00486\_t001 |  | | | |  |  |  |
| 3 | Atr-ERN07918 |  | | | |  | | | |  | | | |  |  |  |
| 3 | Atr-ERN07919 |  | | | |  | Vvi-Vitvi06g00487\_t001 |  | Vvi-Vitvi08g01211\_t001 |  |  |  |
| 3 | Atr-ERN07920 |  | Vvi-Vitvi13g00310\_t001 |  | Vvi-Vitvi06g00488\_t001 |  | Vvi-Vitvi08g01213\_t002 |  |  |  |
| 3 | Atr-ERN07921 |  | Vvi-Vitvi13g00311\_t001 |  | Vvi-Vitvi06g01569\_t001 |  | Vvi-Vitvi08g01214\_t001 |  |  |  |
| 3 | Atr-ERN07922 |  | | | |  | | | |  | | | |  |  |  |
| 3 | Atr-ERN07923 |  | | | |  | | | |  | Vvi-Vitvi08g01215\_t001 |  |  |  |
| 3 | Atr-ERN07924 |  | | | |  | | | |  | | | |  |  |  |
| 3 | Atr-ERN07925 |  | | | |  | Vvi-Vitvi06g00493\_t001 |  | | | |  |  |  |
| 3 | Atr-ERN07926 |  | | | |  | | | |  | | | |  |  |  |
| 3 | Atr-ERN07927 |  | | | |  | | | |  | | | |  |  |  |
| 3 | Atr-ERN07928 |  | | | |  | | | |  | Vvi-Vitvi08g01216\_t001 |  |  |  |
| 3 | Atr-ERN07929 |  | | | |  | | | |  | | | |  |  |  |
| 3 | Atr-ERN07930 |  | | | |  | Vvi-Vitvi06g00494\_t001 |  | | | |  |  |  |
| 3 | Atr-ERN07931 |  | | | |  | Vvi-Vitvi06g00495\_t001 |  | Vvi-Vitvi08g01217\_t001 |  |  |  |
| 3 | Atr-ERN07932 |  | Vvi-Vitvi13g00315\_t001 |  | | | |  | | | |  |  |  |
| 3 | Atr-ERN07933 |  | | | |  | | | |  | | | |  |  |  |
| 3 | Atr-ERN07934 |  | Vvi-Vitvi13g00316\_t001 |  | | | |  | | | |  |  |  |
| 3 | Atr-ERN07935 |  | | | |  | | | |  | Vvi-Vitvi08g01221\_t001 |  |  |  |
| 3 | Atr-ERN07936 |  | | | |  | Vvi-Vitvi06g00498\_t001 |  | | | |  |  |  |
| 3 | Atr-ERN07937 |  | Vvi-Vitvi13g00317\_t001 |  | | | |  | | | |  |  |  |
| 3 | Atr-ERN07938 |  | | | |  | | | |  | | | |  |  |  |
| 3 | Atr-ERN07939 |  | Vvi-Vitvi13g01990\_t001 |  | Vvi-Vitvi06g01706\_t001 |  | | | |  |  |  |
| 3 | Atr-ERN07940 |  | | | |  | | | |  | Vvi-Vitvi08g01223\_t001 |  |  |  |
| 3 | Atr-ERN07941 |  | | | |  | | | |  | | | |  |  |  |
| 3 | Atr-ERN07942 |  | | | |  | | | |  | | | |  |  |  |
| 3 | Atr-ERN07943 |  | | | |  | | | |  | | | |  |  |  |
| 3 | Atr-ERN07944 |  | | | |  | | | |  | | | |  |  |  |
| 3 | Atr-ERN07945 |  | Vvi-Vitvi13g00320\_t003 |  | | | |  | | | |  |  |  |
| 3 | Atr-ERN07946 |  | | | |  | | | |  | | | |  |  |  |
| 3 | Atr-ERN07947 |  | | | |  | | | |  | | | |  |  |  |
| 3 | Atr-ERN07948 |  | | | |  | | | |  | Vvi-Vitvi08g01224\_t001 |  |  |  |
| 3 | Atr-ERN07949 |  | | | |  | | | |  | | | |  |  |  |
| 3 | Atr-ERN07950 |  | Vvi-Vitvi13g00321\_t001 |  | | | |  | Vvi-Vitvi08g04233\_t001 |  |  |  |
| 3 | Atr-ERN07951 |  | | | |  | Vvi-Vitvi06g00500\_t001 |  | | | |  |  |  |
| 3 | Atr-ERN07952 |  | | | |  | | | |  | | | |  |  |  |
| 3 | Atr-ERN07953 |  | Vvi-Vitvi13g00324\_t001 |  | Vvi-Vitvi06g00501\_t001 |  | | | |  |  |  |
| 3 | Atr-ERN07954 |  | | | |  | | | |  | | | |  |  |  |
| 3 | Atr-ERN07955 |  | Vvi-Vitvi13g00325\_t001 |  | | | |  | Vvi-Vitvi08g01230\_t001 |  |  |  |
| 3 | Atr-ERN07956 |  | | | |  | | | |  | | | |  |  |  |
| 3 | Atr-ERN07957 |  | Vvi-Vitvi13g00326\_t001 |  | Vvi-Vitvi06g00502\_t001 |  | | | |  |  |  |
| 3 | Atr-ERN07958 |  | | | |  | | | |  | Vvi-Vitvi08g01231\_t001 |  |  |  |
| 3 | Atr-ERN07959 |  | | | |  | | | |  | | | |  |  |  |
| 3 | Atr-ERN07960 |  | Vvi-Vitvi13g00327\_t001 |  | | | |  | | | |  |  |  |
| 3 | Atr-ERN07961 |  | | | |  | | | |  | | | |  |  |  |
| 3 | Atr-ERN07962 |  | | | |  | | | |  | | | |  |  |  |
| 3 | Atr-ERN07963 |  | Vvi-Vitvi13g00328\_t001 |  | Vvi-Vitvi06g00503\_t001 |  | | | |  |  |  |
| 3 | Atr-ERN07964 |  | | | |  | Vvi-Vitvi06g01708\_t001 |  | | | |  |  |  |
| 3 | Atr-ERN07965 |  | | | |  | | | |  | Vvi-Vitvi08g01234\_t001 |  |  |  |
| 3 | Atr-ERN07966 |  | | | |  | Vvi-Vitvi06g00505\_t001 |  | | | |  |  |  |
| 3 | Atr-ERN07967 |  | Vvi-Vitvi13g00329\_t001 |  | | | |  | Vvi-Vitvi08g01235\_t001 |  |  |  |
| 3 | Atr-ERN07968 |  | | | |  | | | |  | | | |  |  |  |
| 3 | Atr-ERN07969 |  | | | |  | | | |  | | | |  |  |  |
| 3 | Atr-ERN07970 |  | Vvi-Vitvi13g00330\_t001 |  | | | |  | Vvi-Vitvi08g01237\_t001 |  |  |  |
| 3 | Atr-ERN07971 |  | | | |  | Vvi-Vitvi06g00509\_t001 |  | | | |  |  |  |
| 3 | Atr-ERN07972 |  | | | |  | Vvi-Vitvi06g00510\_t001 |  | | | |  |  |  |
| 3 | Atr-ERN07973 |  | | | |  | | | |  | Vvi-Vitvi08g01238\_t001 |  |  |  |
| 3 | Atr-ERN07974 |  | | | |  | Vvi-Vitvi06g00511\_t001 |  | | | |  |  |  |
| 3 | Atr-ERN07975 |  | | | |  | | | |  | Vvi-Vitvi08g01239\_t001 |  |  |  |
| 3 | Atr-ERN07976 |  | | | |  | | | |  | | | |  |  |  |
| 3 | Atr-ERN07977 |  | | | |  | | | |  | | | |  |  |  |
| 3 | Atr-ERN07978 |  | | | |  | | | |  | Vvi-Vitvi08g02202\_t001 |  |  |  |
| 3 | Atr-ERN07979 |  | | | |  | | | |  | Vvi-Vitvi08g01240\_t001 |  |  |  |
| 3 | Atr-ERN07980 |  | | | |  | | | |  | | | |  |  |  |
| 3 | Atr-ERN07981 |  | | | |  | | | |  | | | |  |  |  |
| 3 | Atr-ERN07982 |  | | | |  | | | |  | Vvi-Vitvi08g01241\_t001 |  |  |  |
| 3 | Atr-ERN07983 |  | | | |  | | | |  | | | |  |  |  |
| 3 | Atr-ERN07984 |  | | | |  | | | |  | | | |  |  |  |
| 3 | Atr-ERN07985 |  | | | |  | | | |  | Vvi-Vitvi08g01243\_t001 |  |  |  |
| 3 | Atr-ERN07986 |  | | | |  | | | |  | | | |  |  |  |
| 3 | Atr-ERN07987 |  | | | |  | | | |  | | | |  |  |  |
| 3 | Atr-ERN07988 |  | | | |  | | | |  | | | |  |  |  |
| 3 | Atr-ERN07989 |  | | | |  | | | |  | | | |  |  |  |
| 3 | Atr-ERN07990 |  | | | |  | | | |  | | | |  |  |  |
| 3 | Atr-ERN07991 |  | | | |  | | | |  | | | |  |  |  |
| 3 | Atr-ERN07992 |  | | | |  | | | |  | | | |  |  |  |
| 3 | Atr-ERN07993 |  | | | |  | Vvi-Vitvi06g00512\_t001 |  | | | |  |  |  |
| 3 | Atr-ERN07994 |  | | | |  | | | |  | | | |  |  |  |
| 3 | Atr-ERN07995 |  | Vvi-Vitvi13g00332\_t001 |  | | | |  | | | |  |  |  |
| 3 | Atr-ERN07996 |  | | | |  | | | |  | | | |  |  |  |
| 3 | Atr-ERN07997 |  | | | |  | | | |  | | | |  |  |  |
| 3 | Atr-ERN07998 |  | | | |  | | | |  | | | |  |  |  |
| 3 | Atr-ERN07999 |  | | | |  | | | |  | | | |  |  |  |
| 3 | Atr-ERN08000 |  | | | |  | | | |  | | | |  |  |  |
| 3 | Atr-ERN08001 |  | | | |  | | | |  | | | |  |  |  |
| 3 | Atr-ERN08002 |  | | | |  | | | |  | | | |  |  |  |
| 3 | Atr-ERN08003 |  | | | |  | | | |  | | | |  |  |  |
| 3 | Atr-ERN08004 |  | Vvi-Vitvi13g01994\_t001 |  | Vvi-Vitvi06g00513\_t001 |  | Vvi-Vitvi08g01245\_t001 |  |  |  |
| 3 | Atr-ERN08005 |  | | | |  | Vvi-Vitvi06g00514\_t001 |  | | | |  |  |  |
| 3 | Atr-ERN08006 |  | | | |  | | | |  | | | |  |  |  |
| 3 | Atr-ERN08007 |  | | | |  | | | |  | | | |  |  |  |
| 3 | Atr-ERN08008 |  | | | |  | | | |  | Vvi-Vitvi08g01247\_t001 |  |  |  |
| 3 | Atr-ERN08009 |  | | | |  | | | |  | | | |  |  |  |
| 3 | Atr-ERN08010 |  | | | |  | Vvi-Vitvi06g00515\_t001 |  | | | |  |  |  |
| 3 | Atr-ERN08011 |  | | | |  | | | |  | | | |  |  |  |
| 3 | Atr-ERN08012 |  | | | |  | Vvi-Vitvi06g00516\_t001 |  | | | |  |  |  |
| 3 | Atr-ERN08013 |  | | | |  | Vvi-Vitvi06g00517\_t001 |  | | | |  |  |  |
| 3 | Atr-ERN08014 |  | Vvi-Vitvi13g00338\_t001 |  | | | |  | | | |  |  |  |
| 3 | Atr-ERN08015 |  | | | |  | | | |  | | | |  |  |  |
| 3 | Atr-ERN08016 |  | Vvi-Vitvi13g00339\_t002 |  | | | |  | | | |  |  |  |
| 3 | Atr-ERN08017 |  | | | |  | | | |  | | | |  |  |  |
| 3 | Atr-ERN08018 |  | Vvi-Vitvi13g01996\_t001 |  | Vvi-Vitvi06g00520\_t001 |  | Vvi-Vitvi08g01248\_t001 |  |  |  |
| 3 | Atr-ERN08019 |  | | | |  | | | |  | | | |  |  |  |
| 3 | Atr-ERN08020 |  | Vvi-Vitvi13g00340\_t001 |  | Vvi-Vitvi06g01710\_t001 |  | Vvi-Vitvi08g02203\_t001 |  |  |  |
| 3 | Atr-ERN08021 |  | | | |  | | | |  | | | |  |  |  |
| 3 | Atr-ERN08022 |  | | | |  | | | |  | | | |  |  |  |
| 3 | Atr-ERN08023 |  | | | |  | | | |  | | | |  |  |  |
| 3 | Atr-ERN08024 |  | | | |  | | | |  | | | |  |  |  |
| 3 | Atr-ERN08025 |  | | | |  | | | |  | | | |  |  |  |
| 3 | Atr-ERN08026 |  | Vvi-Vitvi13g01997\_t001 |  | Vvi-Vitvi06g00522\_t001 |  | Vvi-Vitvi08g01250\_t001 |  |  |  |
| 3 | Atr-ERN08027 |  | | | |  | | | |  | | | |  |  |  |
| 3 | Atr-ERN08028 |  | | | |  | Vvi-Vitvi06g04175\_t001 |  | Vvi-Vitvi08g04238\_t001 |  |  |  |
| 2 | Atr-ERN08029 |  | | | |  |  |  | | | |  |  |  |
| 2 | Atr-ERN08030 |  | | | |  |  |  | | | |  |  |  |
| 2 | Atr-ERN08031 |  | | | |  |  |  | | | |  |  |  |
| 2 | Atr-ERN08032 |  | | | |  |  |  | | | |  |  |  |
| 2 | Atr-ERN08033 |  | | | |  |  |  | Vvi-Vitvi08g02204\_t001 |  |  |  |
| 2 | Atr-ERN08034 |  | | | |  |  |  | | | |  |  |  |
| 2 | Atr-ERN08035 |  | Vvi-Vitvi13g00346\_t001 |  |  |  | | | |  |  |  |
| 2 | Atr-ERN08036 |  | Vvi-Vitvi13g00348\_t002 |  |  |  | | | |  |  |  |
| 2 | Atr-ERN08037 |  | Vvi-Vitvi13g00349\_t001 |  |  |  | | | |  |  |  |
| 2 | Atr-ERN08038 |  | Vvi-Vitvi13g00350\_t002 |  |  |  | Vvi-Vitvi08g02205\_t001 |  |  |  |
| 2 | Atr-ERN08039 |  | | | |  |  |  | Vvi-Vitvi08g01254\_t002 |  |  |  |
| 2 | Atr-ERN08040 |  | | | |  |  |  | | | |  |  |  |
| 2 | Atr-ERN08041 |  | | | |  |  |  | | | |  |  |  |
| 2 | Atr-ERN08042 |  | | | |  |  |  | | | |  |  |  |
| 2 | Atr-ERN08043 |  | | | |  |  |  | | | |  |  |  |
| 2 | Atr-ERN08044 |  | Vvi-Vitvi13g00352\_t001 |  |  |  | | | |  |  |  |
| 2 | Atr-ERN08045 |  | | | |  |  |  | | | |  |  |  |
| 2 | Atr-ERN08046 |  | | | |  |  |  | | | |  |  |  |
| 2 | Atr-ERN08047 |  | | | |  |  |  | | | |  |  |  |
| 2 | Atr-ERN08048 |  | Vvi-Vitvi13g00353\_t001 |  |  |  | Vvi-Vitvi08g01257\_t001 |  |  |  |
| 2 | Atr-ERN08049 |  | | | |  |  |  | | | |  |  |  |
| 2 | Atr-ERN08050 |  | | | |  |  |  | Vvi-Vitvi08g04241\_t001 |  |  |  |
| 2 | Atr-ERN08051 |  | | | |  |  |  | | | |  |  |  |
| 2 | Atr-ERN08052 |  | | | |  |  |  | | | |  |  |  |
| 2 | Atr-ERN08053 |  | | | |  |  |  | Vvi-Vitvi08g01261\_t001 |  |  |  |
| 2 | Atr-ERN08054 |  | | | |  |  |  | | | |  |  |  |
| 2 | Atr-ERN08055 |  | Vvi-Vitvi13g00354\_t001 |  |  |  | | | |  |  |  |
| 2 | Atr-ERN08056 |  | | | |  |  |  | | | |  |  |  |
| 2 | Atr-ERN08057 |  | | | |  |  |  | Vvi-Vitvi08g01262\_t001 |  |  |  |
| 2 | Atr-ERN08058 |  | | | |  |  |  | | | |  |  |  |
| 2 | Atr-ERN08059 |  | | | |  |  |  | Vvi-Vitvi08g01265\_t001 |  |  |  |
| 2 | Atr-ERN08060 |  | | | |  |  |  | | | |  |  |  |
| 2 | Atr-ERN08061 |  | | | |  |  |  | | | |  |  |  |
| 2 | Atr-ERN08062 |  | | | |  |  |  | | | |  |  |  |
| 2 | Atr-ERN08063 |  | | | |  |  |  | | | |  |  |  |
| 2 | Atr-ERN08064 |  | | | |  |  |  | | | |  |  |  |
| 2 | Atr-ERN08065 |  | | | |  |  |  | | | |  |  |  |
| 2 | Atr-ERN08066 |  | | | |  |  |  | | | |  |  |  |
| 2 | Atr-ERN08067 |  | | | |  |  |  | | | |  |  |  |
| 2 | Atr-ERN08068 |  | | | |  |  |  | | | |  |  |  |
| 2 | Atr-ERN08069 |  | | | |  |  |  | | | |  |  |  |
| 2 | Atr-ERN08070 |  | | | |  |  |  | | | |  |  |  |
| 2 | Atr-ERN08071 |  | | | |  |  |  | Vvi-Vitvi08g01266\_t002 |  |  |  |
| 2 | Atr-ERN08072 |  | Vvi-Vitvi13g00358\_t001 |  |  |  | | | |  |  |  |
| 2 | Atr-ERN08073 |  | Vvi-Vitvi13g00359\_t001 |  |  |  | | | |  |  |  |
| 2 | Atr-ERN08074 |  | | | |  |  |  | Vvi-Vitvi08g01267\_t001 |  |  |  |
| 2 | Atr-ERN08075 |  | Vvi-Vitvi13g00361\_t001 |  |  |  | | | |  |  |  |
| 1 | Atr-ERN08076 |  |  |  |  |  | | | |  |  |  |
| 1 | Atr-ERN08077 |  |  |  |  |  | | | |  |  |  |
| 1 | Atr-ERN08078 |  |  |  |  |  | Vvi-Vitvi08g02214\_t001 |  |  |  |
| 1 | Atr-ERN08079 |  |  |  |  |  | | | |  |  |  |
| 1 | Atr-ERN08080 |  |  |  |  |  | Vvi-Vitvi08g01270\_t001 |  |  |  |
| 1 | Atr-ERN08081 |  |  |  |  |  | | | |  |  |  |
| 1 | Atr-ERN08082 |  |  |  |  |  | | | |  |  |  |
| 1 | Atr-ERN08083 |  |  |  |  |  | | | |  |  |  |
| 1 | Atr-ERN08084 |  |  |  |  |  | | | |  |  |  |
| 1 | Atr-ERN08085 |  |  |  |  |  | Vvi-Vitvi08g01272\_t001 |  |  |  |
| 0 | Atr-ERN08086 |  |  |  |  |  |  |
| 0 | Atr-ERN08087 |  |  |  |  |  |  |
| 0 | Atr-ERN08088 |  |  |  |  |  |  |
