# Supplementary material for: Genome-wide identification, characterization and gene expression of BES1 transcription factor family in grapevine (Vitis vinifera L.)
Source: Sci Rep. 2023 Jan 5;13:240. doi: 10.1038/s41598-022-24407-y (PMC9816167; doi:10.1038/s41598-022-24407-y)
Supplement: Supplementary file 3 — Supplementary Information. [file 41598_2022_24407_MOESM3_ESM.zip › Vvi_Atr/Vitis_vinifera.PN40024.v4.dna_sm.toplevel.fa.vs.Amborella_trichopoda.AMTR1.0.dna_sm.toplevel.fa.html/Atr-AmTr_v1.0_scaffold00013.html]

|  |  |  |  |  |  |  |  |  |  |  |  |  |  |
| --- | --- | --- | --- | --- | --- | --- | --- | --- | --- | --- | --- | --- | --- |
| Duplication depth | Reference chromosome | Collinear blocks | | | | | | | | | | | |
| 0 | Atr-ERN09823 |  |  |  |  |  |  |
| 0 | Atr-ERN09824 |  |  |  |  |  |  |
| 0 | Atr-ERN09825 |  |  |  |  |  |  |
| 1 | Atr-ERN09826 |  | Vvi-Vitvi17g00087\_t001 |  |  |  |  |  |
| 1 | Atr-ERN09827 |  | | | |  |  |  |  |  |
| 2 | Atr-ERN09828 |  | | | |  | Vvi-Vitvi01g01657\_t001 |  |  |  |  |
| 3 | Atr-ERN09829 |  | | | |  | | | |  | Vvi-Vitvi14g01535\_t001 |  |  |  |
| 3 | Atr-ERN09830 |  | | | |  | | | |  | | | |  |  |  |
| 3 | Atr-ERN09831 |  | | | |  | Vvi-Vitvi01g01662\_t001 |  | | | |  |  |  |
| 3 | Atr-ERN09832 |  | | | |  | | | |  | Vvi-Vitvi14g01534\_t001 |  |  |  |
| 3 | Atr-ERN09833 |  | | | |  | | | |  | Vvi-Vitvi14g01533\_t001 |  |  |  |
| 3 | Atr-ERN09834 |  | | | |  | | | |  | | | |  |  |  |
| 3 | Atr-ERN09835 |  | | | |  | | | |  | | | |  |  |  |
| 3 | Atr-ERN09836 |  | | | |  | | | |  | | | |  |  |  |
| 3 | Atr-ERN09837 |  | | | |  | | | |  | Vvi-Vitvi14g04567\_t001 |  |  |  |
| 3 | Atr-ERN09838 |  | | | |  | Vvi-Vitvi01g04442\_t001 |  | | | |  |  |  |
| 3 | Atr-ERN09839 |  | Vvi-Vitvi17g00088\_t001 |  | | | |  | | | |  |  |  |
| 3 | Atr-ERN09840 |  | Vvi-Vitvi17g04018\_t001 |  | Vvi-Vitvi01g01664\_t001 |  | | | |  |  |  |
| 3 | Atr-ERN09841 |  | | | |  | | | |  | | | |  |  |  |
| 3 | Atr-ERN09842 |  | | | |  | | | |  | | | |  |  |  |
| 3 | Atr-ERN09843 |  | Vvi-Vitvi17g00091\_t001 |  | | | |  | Vvi-Vitvi14g01530\_t004 |  |  |  |
| 3 | Atr-ERN09844 |  | | | |  | | | |  | | | |  |  |  |
| 3 | Atr-ERN09845 |  | | | |  | | | |  | | | |  |  |  |
| 3 | Atr-ERN09846 |  | | | |  | | | |  | | | |  |  |  |
| 3 | Atr-ERN09847 |  | | | |  | | | |  | | | |  |  |  |
| 3 | Atr-ERN09848 |  | | | |  | | | |  | | | |  |  |  |
| 3 | Atr-ERN09849 |  | | | |  | | | |  | | | |  |  |  |
| 3 | Atr-ERN09850 |  | | | |  | | | |  | | | |  |  |  |
| 3 | Atr-ERN09851 |  | | | |  | | | |  | | | |  |  |  |
| 3 | Atr-ERN09852 |  | | | |  | | | |  | | | |  |  |  |
| 3 | Atr-ERN09853 |  | | | |  | | | |  | | | |  |  |  |
| 3 | Atr-ERN09854 |  | | | |  | | | |  | | | |  |  |  |
| 3 | Atr-ERN09855 |  | | | |  | | | |  | | | |  |  |  |
| 3 | Atr-ERN09856 |  | | | |  | Vvi-Vitvi01g01666\_t001 |  | | | |  |  |  |
| 3 | Atr-ERN09857 |  | | | |  | Vvi-Vitvi01g04443\_t002 |  | | | |  |  |  |
| 3 | Atr-ERN09858 |  | | | |  | | | |  | | | |  |  |  |
| 3 | Atr-ERN09859 |  | Vvi-Vitvi17g00095\_t001 |  | | | |  | | | |  |  |  |
| 3 | Atr-ERN09860 |  | | | |  | Vvi-Vitvi01g02272\_t001 |  | | | |  |  |  |
| 3 | Atr-ERN09861 |  | | | |  | Vvi-Vitvi01g01671\_t001 |  | | | |  |  |  |
| 3 | Atr-ERN09862 |  | | | |  | | | |  | | | |  |  |  |
| 3 | Atr-ERN09863 |  | Vvi-Vitvi17g00096\_t002 |  | | | |  | | | |  |  |  |
| 3 | Atr-ERN09864 |  | | | |  | | | |  | | | |  |  |  |
| 3 | Atr-ERN09865 |  | | | |  | | | |  | | | |  |  |  |
| 3 | Atr-ERN09866 |  | | | |  | | | |  | | | |  |  |  |
| 3 | Atr-ERN09867 |  | | | |  | Vvi-Vitvi01g04445\_t001 |  | Vvi-Vitvi14g02964\_t001 |  |  |  |
| 3 | Atr-ERN09868 |  | | | |  | | | |  | | | |  |  |  |
| 3 | Atr-ERN09869 |  | | | |  | | | |  | | | |  |  |  |
| 3 | Atr-ERN09870 |  | | | |  | | | |  | | | |  |  |  |
| 3 | Atr-ERN09871 |  | | | |  | | | |  | | | |  |  |  |
| 3 | Atr-ERN09872 |  | | | |  | | | |  | | | |  |  |  |
| 3 | Atr-ERN09873 |  | | | |  | | | |  | | | |  |  |  |
| 3 | Atr-ERN09874 |  | | | |  | Vvi-Vitvi01g04446\_t003 |  | | | |  |  |  |
| 3 | Atr-ERN09875 |  | | | |  | Vvi-Vitvi01g01677\_t001 |  | Vvi-Vitvi14g01526\_t001 |  |  |  |
| 3 | Atr-ERN09876 |  | | | |  | | | |  | | | |  |  |  |
| 3 | Atr-ERN09877 |  | | | |  | Vvi-Vitvi01g04450\_t001 |  | Vvi-Vitvi14g01525\_t001 |  |  |  |
| 3 | Atr-ERN09878 |  | | | |  | | | |  | | | |  |  |  |
| 4 | Atr-ERN09879 |  | | | |  | | | |  | | | |  | Vvi-Vitvi01g04448\_t001 |  |  |
| 4 | Atr-ERN09880 |  | | | |  | | | |  | | | |  | | | |  |  |
| 4 | Atr-ERN09881 |  | | | |  | | | |  | | | |  | | | |  |  |
| 4 | Atr-ERN09882 |  | Vvi-Vitvi17g00098\_t004 |  | | | |  | | | |  | | | |  |  |
| 4 | Atr-ERN09883 |  | | | |  | | | |  | | | |  | | | |  |  |
| 4 | Atr-ERN09884 |  | | | |  | | | |  | | | |  | | | |  |  |
| 4 | Atr-ERN09885 |  | | | |  | | | |  | | | |  | | | |  |  |
| 4 | Atr-ERN09886 |  | | | |  | | | |  | | | |  | | | |  |  |
| 4 | Atr-ERN09887 |  | | | |  | | | |  | | | |  | | | |  |  |
| 4 | Atr-ERN09888 |  | | | |  | | | |  | | | |  | | | |  |  |
| 4 | Atr-ERN09889 |  | | | |  | | | |  | | | |  | | | |  |  |
| 4 | Atr-ERN09890 |  | Vvi-Vitvi17g01345\_t001 |  | | | |  | | | |  | | | |  |  |
| 4 | Atr-ERN09891 |  | | | |  | | | |  | | | |  | | | |  |  |
| 4 | Atr-ERN09892 |  | | | |  | | | |  | | | |  | | | |  |  |
| 4 | Atr-ERN09893 |  | | | |  | | | |  | | | |  | | | |  |  |
| 4 | Atr-ERN09894 |  | | | |  | | | |  | | | |  | | | |  |  |
| 4 | Atr-ERN09895 |  | | | |  | | | |  | | | |  | | | |  |  |
| 4 | Atr-ERN09896 |  | | | |  | | | |  | | | |  | | | |  |  |
| 4 | Atr-ERN09897 |  | Vvi-Vitvi17g00100\_t001 |  | Vvi-Vitvi01g01678\_t001 |  | Vvi-Vitvi14g01524\_t001 |  | Vvi-Vitvi01g01660\_t001 |  |  |
| 4 | Atr-ERN09898 |  | | | |  | | | |  | | | |  | | | |  |  |
| 4 | Atr-ERN09899 |  | | | |  | | | |  | | | |  | | | |  |  |
| 4 | Atr-ERN09900 |  | | | |  | | | |  | | | |  | | | |  |  |
| 4 | Atr-ERN09901 |  | | | |  | | | |  | | | |  | | | |  |  |
| 4 | Atr-ERN09902 |  | Vvi-Vitvi17g00101\_t001 |  | | | |  | | | |  | | | |  |  |
| 4 | Atr-ERN09903 |  | | | |  | Vvi-Vitvi01g02278\_t001 |  | | | |  | | | |  |  |
| 4 | Atr-ERN09904 |  | | | |  | | | |  | | | |  | | | |  |  |
| 4 | Atr-ERN09905 |  | | | |  | | | |  | | | |  | | | |  |  |
| 4 | Atr-ERN09906 |  | | | |  | | | |  | | | |  | | | |  |  |
| 4 | Atr-ERN09907 |  | | | |  | | | |  | | | |  | | | |  |  |
| 4 | Atr-ERN09908 |  | Vvi-Vitvi17g00103\_t002 |  | | | |  | | | |  | | | |  |  |
| 5 | Atr-ERN09909 |  | | | |  | | | |  | | | |  | | | |  | Vvi-Vitvi14g01522\_t001 |  |
| 5 | Atr-ERN09910 |  | | | |  | | | |  | | | |  | | | |  | | | |  |
| 5 | Atr-ERN09911 |  | | | |  | Vvi-Vitvi01g01682\_t001 |  | | | |  | | | |  | | | |  |
| 5 | Atr-ERN09912 |  | | | |  | | | |  | | | |  | | | |  | | | |  |
| 5 | Atr-ERN09913 |  | | | |  | | | |  | | | |  | | | |  | | | |  |
| 5 | Atr-ERN09914 |  | | | |  | | | |  | | | |  | | | |  | | | |  |
| 5 | Atr-ERN09915 |  | | | |  | Vvi-Vitvi01g01684\_t001 |  | Vvi-Vitvi14g02961\_t001 |  | | | |  | | | |  |
| 4 | Atr-ERN09916 |  | | | |  | Vvi-Vitvi01g01686\_t001 |  |  |  | | | |  | | | |  |
| 3 | Atr-ERN09917 |  | | | |  |  |  |  |  | | | |  | | | |  |
| 3 | Atr-ERN09918 |  | | | |  |  |  |  |  | Vvi-Vitvi01g01655\_t001 |  | Vvi-Vitvi14g01540\_t001 |  |
| 3 | Atr-ERN09919 |  | Vvi-Vitvi17g00105\_t001 |  |  |  |  |  | Vvi-Vitvi01g01654\_t001 |  | | | |  |
| 3 | Atr-ERN09920 |  | | | |  |  |  |  |  | | | |  | | | |  |
| 3 | Atr-ERN09921 |  | | | |  |  |  |  |  | | | |  | | | |  |
| 3 | Atr-ERN09922 |  | | | |  |  |  |  |  | Vvi-Vitvi01g01652\_t001 |  | | | |  |
| 3 | Atr-ERN09923 |  | Vvi-Vitvi17g01347\_t001 |  |  |  |  |  | | | |  | | | |  |
| 3 | Atr-ERN09924 |  | | | |  |  |  |  |  | Vvi-Vitvi01g01651\_t001 |  | | | |  |
| 3 | Atr-ERN09925 |  | | | |  |  |  |  |  | | | |  | | | |  |
| 3 | Atr-ERN09926 |  | | | |  |  |  |  |  | | | |  | | | |  |
| 3 | Atr-ERN09927 |  | | | |  |  |  |  |  | | | |  | Vvi-Vitvi14g02971\_t001 |  |
| 3 | Atr-ERN09928 |  | Vvi-Vitvi17g00109\_t001 |  |  |  |  |  | | | |  | | | |  |
| 3 | Atr-ERN09929 |  | | | |  |  |  |  |  | Vvi-Vitvi01g01648\_t001 |  | | | |  |
| 3 | Atr-ERN09930 |  | | | |  |  |  |  |  | | | |  | | | |  |
| 3 | Atr-ERN09931 |  | | | |  |  |  |  |  | | | |  | | | |  |
| 3 | Atr-ERN09932 |  | | | |  |  |  |  |  | | | |  | | | |  |
| 3 | Atr-ERN09933 |  | Vvi-Vitvi17g00110\_t001 |  |  |  |  |  | | | |  | | | |  |
| 3 | Atr-ERN09934 |  | | | |  |  |  |  |  | | | |  | | | |  |
| 3 | Atr-ERN09935 |  | | | |  |  |  |  |  | | | |  | Vvi-Vitvi14g01544\_t001 |  |
| 3 | Atr-ERN09936 |  | | | |  |  |  |  |  | | | |  | | | |  |
| 3 | Atr-ERN09937 |  | | | |  |  |  |  |  | Vvi-Vitvi01g02268\_t001 |  | | | |  |
| 3 | Atr-ERN09938 |  | | | |  |  |  |  |  | Vvi-Vitvi01g01646\_t001 |  | | | |  |
| 3 | Atr-ERN09939 |  | | | |  |  |  |  |  | | | |  | | | |  |
| 3 | Atr-ERN09940 |  | | | |  |  |  |  |  | Vvi-Vitvi01g01645\_t001 |  | | | |  |
| 3 | Atr-ERN09941 |  | | | |  |  |  |  |  | | | |  | Vvi-Vitvi14g01545\_t001 |  |
| 3 | Atr-ERN09942 |  | | | |  |  |  |  |  | | | |  | | | |  |
| 3 | Atr-ERN09943 |  | | | |  |  |  |  |  | Vvi-Vitvi01g01644\_t001 |  | | | |  |
| 3 | Atr-ERN09944 |  | | | |  |  |  |  |  | | | |  | | | |  |
| 3 | Atr-ERN09945 |  | | | |  |  |  |  |  | | | |  | | | |  |
| 3 | Atr-ERN09946 |  | | | |  |  |  |  |  | | | |  | | | |  |
| 3 | Atr-ERN09947 |  | Vvi-Vitvi17g00112\_t001 |  |  |  |  |  | Vvi-Vitvi01g02267\_t001 |  | | | |  |
| 3 | Atr-ERN09948 |  | | | |  |  |  |  |  | Vvi-Vitvi01g01641\_t001 |  | | | |  |
| 3 | Atr-ERN09949 |  | | | |  |  |  |  |  | | | |  | Vvi-Vitvi14g01548\_t001 |  |
| 3 | Atr-ERN09950 |  | | | |  |  |  |  |  | | | |  | | | |  |
| 3 | Atr-ERN09951 |  | Vvi-Vitvi17g01350\_t001 |  |  |  |  |  | | | |  | | | |  |
| 3 | Atr-ERN09952 |  | | | |  |  |  |  |  | | | |  | | | |  |
| 3 | Atr-ERN09953 |  | | | |  |  |  |  |  | | | |  | | | |  |
| 3 | Atr-ERN09954 |  | | | |  |  |  |  |  | | | |  | | | |  |
| 3 | Atr-ERN09955 |  | | | |  |  |  |  |  | Vvi-Vitvi01g01640\_t001 |  | | | |  |
| 3 | Atr-ERN09956 |  | | | |  |  |  |  |  | | | |  | Vvi-Vitvi14g01549\_t001 |  |
| 3 | Atr-ERN09957 |  | Vvi-Vitvi17g01351\_t001 |  |  |  |  |  | | | |  | | | |  |
| 3 | Atr-ERN09958 |  | | | |  |  |  |  |  | | | |  | | | |  |
| 3 | Atr-ERN09959 |  | | | |  |  |  |  |  | | | |  | | | |  |
| 3 | Atr-ERN09960 |  | | | |  |  |  |  |  | | | |  | | | |  |
| 3 | Atr-ERN09961 |  | | | |  |  |  |  |  | | | |  | | | |  |
| 3 | Atr-ERN09962 |  | Vvi-Vitvi17g00116\_t001 |  |  |  |  |  | | | |  | | | |  |
| 3 | Atr-ERN09963 |  | | | |  |  |  |  |  | | | |  | | | |  |
| 3 | Atr-ERN09964 |  | | | |  |  |  |  |  | | | |  | Vvi-Vitvi14g01551\_t001 |  |
| 3 | Atr-ERN09965 |  | Vvi-Vitvi17g00118\_t001 |  |  |  |  |  | | | |  | | | |  |
| 3 | Atr-ERN09966 |  | | | |  |  |  |  |  | | | |  | | | |  |
| 3 | Atr-ERN09967 |  | | | |  |  |  |  |  | | | |  | | | |  |
| 3 | Atr-ERN09968 |  | | | |  |  |  |  |  | | | |  | | | |  |
| 3 | Atr-ERN09969 |  | | | |  |  |  |  |  | | | |  | | | |  |
| 3 | Atr-ERN09970 |  | | | |  |  |  |  |  | | | |  | | | |  |
| 3 | Atr-ERN09971 |  | | | |  |  |  |  |  | | | |  | | | |  |
| 3 | Atr-ERN09972 |  | Vvi-Vitvi17g00119\_t001 |  |  |  |  |  | | | |  | Vvi-Vitvi14g01552\_t001 |  |
| 3 | Atr-ERN09973 |  | | | |  |  |  |  |  | | | |  | | | |  |
| 3 | Atr-ERN09974 |  | | | |  |  |  |  |  | | | |  | | | |  |
| 3 | Atr-ERN09975 |  | | | |  |  |  |  |  | | | |  | Vvi-Vitvi14g01553\_t001 |  |
| 3 | Atr-ERN09976 |  | | | |  |  |  |  |  | Vvi-Vitvi01g04432\_t001 |  | | | |  |
| 3 | Atr-ERN09977 |  | | | |  |  |  |  |  | | | |  | | | |  |
| 3 | Atr-ERN09978 |  | | | |  |  |  |  |  | | | |  | | | |  |
| 3 | Atr-ERN09979 |  | Vvi-Vitvi17g00120\_t001 |  |  |  |  |  | | | |  | Vvi-Vitvi14g01554\_t001 |  |
| 3 | Atr-ERN09980 |  | | | |  |  |  |  |  | Vvi-Vitvi01g01629\_t001 |  | | | |  |
| 3 | Atr-ERN09981 |  | Vvi-Vitvi17g00121\_t001 |  |  |  |  |  | Vvi-Vitvi01g02258\_t004 |  | | | |  |
| 3 | Atr-ERN09982 |  | | | |  |  |  |  |  | | | |  | | | |  |
| 3 | Atr-ERN09983 |  | | | |  |  |  |  |  | | | |  | Vvi-Vitvi14g01555\_t002 |  |
| 3 | Atr-ERN09984 |  | | | |  |  |  |  |  | | | |  | | | |  |
| 3 | Atr-ERN09985 |  | | | |  |  |  |  |  | | | |  | | | |  |
| 3 | Atr-ERN09986 |  | | | |  |  |  |  |  | | | |  | | | |  |
| 3 | Atr-ERN09987 |  | | | |  |  |  |  |  | | | |  | | | |  |
| 3 | Atr-ERN09988 |  | | | |  |  |  |  |  | | | |  | | | |  |
| 3 | Atr-ERN09989 |  | | | |  |  |  |  |  | | | |  | | | |  |
| 3 | Atr-ERN09990 |  | | | |  |  |  |  |  | | | |  | | | |  |
| 3 | Atr-ERN09991 |  | Vvi-Vitvi17g00122\_t001 |  |  |  |  |  | | | |  | | | |  |
| 3 | Atr-ERN09992 |  | | | |  |  |  |  |  | | | |  | | | |  |
| 3 | Atr-ERN09993 |  | | | |  |  |  |  |  | | | |  | | | |  |
| 3 | Atr-ERN09994 |  | | | |  |  |  |  |  | | | |  | | | |  |
| 3 | Atr-ERN09995 |  | | | |  |  |  |  |  | | | |  | | | |  |
| 3 | Atr-ERN09996 |  | | | |  |  |  |  |  | | | |  | | | |  |
| 3 | Atr-ERN09997 |  | | | |  |  |  |  |  | | | |  | | | |  |
| 3 | Atr-ERN09998 |  | | | |  |  |  |  |  | Vvi-Vitvi01g01627\_t001 |  | | | |  |
| 3 | Atr-ERN09999 |  | | | |  |  |  |  |  | | | |  | Vvi-Vitvi14g01558\_t001 |  |
| 3 | Atr-ERN10000 |  | | | |  |  |  |  |  | Vvi-Vitvi01g01624\_t001 |  | | | |  |
| 3 | Atr-ERN10001 |  | | | |  |  |  |  |  | | | |  | | | |  |
| 3 | Atr-ERN10002 |  | | | |  |  |  |  |  | | | |  | | | |  |
| 3 | Atr-ERN10003 |  | | | |  |  |  |  |  | Vvi-Vitvi01g01621\_t001 |  | | | |  |
| 3 | Atr-ERN10004 |  | Vvi-Vitvi17g00124\_t002 |  |  |  |  |  | | | |  | | | |  |
| 3 | Atr-ERN10005 |  | | | |  |  |  |  |  | | | |  | Vvi-Vitvi14g01560\_t001 |  |
| 3 | Atr-ERN10006 |  | | | |  |  |  |  |  | | | |  | | | |  |
| 3 | Atr-ERN10007 |  | | | |  |  |  |  |  | | | |  | Vvi-Vitvi14g04581\_t001 |  |
| 3 | Atr-ERN10008 |  | Vvi-Vitvi17g00125\_t001 |  |  |  |  |  | | | |  | | | |  |
| 3 | Atr-ERN10009 |  | | | |  |  |  |  |  | | | |  | | | |  |
| 3 | Atr-ERN10010 |  | | | |  |  |  |  |  | Vvi-Vitvi01g01620\_t001 |  | | | |  |
| 3 | Atr-ERN10011 |  | | | |  |  |  |  |  | Vvi-Vitvi01g01618\_t001 |  | | | |  |
| 2 | Atr-ERN10012 |  | | | |  |  |  |  |  |  |  | | | |  |
| 2 | Atr-ERN10013 |  | | | |  |  |  |  |  |  |  | | | |  |
| 2 | Atr-ERN10014 |  | | | |  |  |  |  |  |  |  | | | |  |
| 2 | Atr-ERN10015 |  | Vvi-Vitvi17g00127\_t001 |  |  |  |  |  |  |  | Vvi-Vitvi14g02984\_t001 |  |
| 1 | Atr-ERN10016 |  |  |  |  |  |  |  |  |  | | | |  |
| 1 | Atr-ERN10017 |  |  |  |  |  |  |  |  |  | Vvi-Vitvi14g01568\_t001 |  |
| 1 | Atr-ERN10018 |  |  |  |  |  |  |  |  |  | Vvi-Vitvi14g02985\_t001 |  |
| 1 | Atr-ERN10019 |  |  |  |  |  |  |  |  |  | Vvi-Vitvi14g01571\_t001 |  |
| 0 | Atr-ERN10020 |  |  |  |  |  |  |
| 0 | Atr-ERN10021 |  |  |  |  |  |  |
| 0 | Atr-ERN10022 |  |  |  |  |  |  |
| 0 | Atr-ERN10023 |  |  |  |  |  |  |
| 0 | Atr-ERN10024 |  |  |  |  |  |  |
| 0 | Atr-ERN10025 |  |  |  |  |  |  |
| 0 | Atr-ERN10026 |  |  |  |  |  |  |
| 0 | Atr-ERN10027 |  |  |  |  |  |  |
| 0 | Atr-ERN10028 |  |  |  |  |  |  |
| 0 | Atr-ERN10029 |  |  |  |  |  |  |
| 0 | Atr-ERN10030 |  |  |  |  |  |  |
| 0 | Atr-ERN10031 |  |  |  |  |  |  |
| 0 | Atr-ERN10032 |  |  |  |  |  |  |
| 0 | Atr-ERN10033 |  |  |  |  |  |  |
| 0 | Atr-ERN10034 |  |  |  |  |  |  |
| 0 | Atr-ERN10035 |  |  |  |  |  |  |
| 0 | Atr-ERN10036 |  |  |  |  |  |  |
| 0 | Atr-ERN10037 |  |  |  |  |  |  |
| 0 | Atr-ERN10038 |  |  |  |  |  |  |
| 0 | Atr-ERN10039 |  |  |  |  |  |  |
| 0 | Atr-ERN10040 |  |  |  |  |  |  |
| 0 | Atr-ERN10041 |  |  |  |  |  |  |
| 0 | Atr-ERN10042 |  |  |  |  |  |  |
| 0 | Atr-ERN10043 |  |  |  |  |  |  |
| 0 | Atr-ERN10044 |  |  |  |  |  |  |
| 0 | Atr-ERN10045 |  |  |  |  |  |  |
| 0 | Atr-ERN10046 |  |  |  |  |  |  |
| 0 | Atr-ERN10047 |  |  |  |  |  |  |
| 0 | Atr-ERN10048 |  |  |  |  |  |  |
| 0 | Atr-ERN10049 |  |  |  |  |  |  |
| 0 | Atr-ERN10050 |  |  |  |  |  |  |
| 0 | Atr-ERN10051 |  |  |  |  |  |  |
| 0 | Atr-ERN10052 |  |  |  |  |  |  |
| 1 | Atr-ERN10053 |  | Vvi-Vitvi05g01129\_t001 |  |  |  |  |  |
| 1 | Atr-ERN10054 |  | | | |  |  |  |  |  |
| 1 | Atr-ERN10055 |  | | | |  |  |  |  |  |
| 1 | Atr-ERN10056 |  | | | |  |  |  |  |  |
| 1 | Atr-ERN10057 |  | | | |  |  |  |  |  |
| 2 | Atr-ERN10058 |  | | | |  | Vvi-Vitvi07g01078\_t001 |  |  |  |  |
| 2 | Atr-ERN10059 |  | | | |  | | | |  |  |  |  |
| 2 | Atr-ERN10060 |  | Vvi-Vitvi05g01128\_t001 |  | | | |  |  |  |  |
| 2 | Atr-ERN10061 |  | | | |  | | | |  |  |  |  |
| 2 | Atr-ERN10062 |  | Vvi-Vitvi05g01124\_t001 |  | | | |  |  |  |  |
| 2 | Atr-ERN10063 |  | | | |  | | | |  |  |  |  |
| 2 | Atr-ERN10064 |  | | | |  | | | |  |  |  |  |
| 2 | Atr-ERN10065 |  | | | |  | Vvi-Vitvi07g01114\_t001 |  |  |  |  |
| 2 | Atr-ERN10066 |  | | | |  | Vvi-Vitvi07g01120\_t001 |  |  |  |  |
| 2 | Atr-ERN10067 |  | | | |  | | | |  |  |  |  |
| 2 | Atr-ERN10068 |  | | | |  | | | |  |  |  |  |
| 2 | Atr-ERN10069 |  | | | |  | | | |  |  |  |  |
| 2 | Atr-ERN10070 |  | | | |  | | | |  |  |  |  |
| 2 | Atr-ERN10071 |  | Vvi-Vitvi05g01115\_t001 |  | | | |  |  |  |  |
| 2 | Atr-ERN10072 |  | | | |  | | | |  |  |  |  |
| 2 | Atr-ERN10073 |  | Vvi-Vitvi05g01112\_t001 |  | | | |  |  |  |  |
| 2 | Atr-ERN10074 |  | | | |  | | | |  |  |  |  |
| 2 | Atr-ERN10075 |  | Vvi-Vitvi05g01108\_t001 |  | | | |  |  |  |  |
| 2 | Atr-ERN10076 |  | | | |  | | | |  |  |  |  |
| 2 | Atr-ERN10077 |  | | | |  | | | |  |  |  |  |
| 2 | Atr-ERN10078 |  | | | |  | | | |  |  |  |  |
| 2 | Atr-ERN10079 |  | | | |  | | | |  |  |  |  |
| 2 | Atr-ERN10080 |  | | | |  | | | |  |  |  |  |
| 2 | Atr-ERN10081 |  | | | |  | | | |  |  |  |  |
| 2 | Atr-ERN10082 |  | | | |  | | | |  |  |  |  |
| 2 | Atr-ERN10083 |  | | | |  | | | |  |  |  |  |
| 2 | Atr-ERN10084 |  | | | |  | | | |  |  |  |  |
| 2 | Atr-ERN10085 |  | | | |  | Vvi-Vitvi07g01110\_t003 |  |  |  |  |
| 2 | Atr-ERN10086 |  | | | |  | Vvi-Vitvi07g01108\_t001 |  |  |  |  |
| 2 | Atr-ERN10087 |  | | | |  | | | |  |  |  |  |
| 2 | Atr-ERN10088 |  | | | |  | | | |  |  |  |  |
| 2 | Atr-ERN10089 |  | | | |  | Vvi-Vitvi07g04329\_t001 |  |  |  |  |
| 1 | Atr-ERN10090 |  | | | |  |  |  |  |  |
| 1 | Atr-ERN10091 |  | | | |  |  |  |  |  |
| 1 | Atr-ERN10092 |  | | | |  |  |  |  |  |
| 1 | Atr-ERN10093 |  | | | |  |  |  |  |  |
| 1 | Atr-ERN10094 |  | | | |  |  |  |  |  |
| 1 | Atr-ERN10095 |  | | | |  |  |  |  |  |
| 1 | Atr-ERN10096 |  | | | |  |  |  |  |  |
| 1 | Atr-ERN10097 |  | | | |  |  |  |  |  |
| 1 | Atr-ERN10098 |  | | | |  |  |  |  |  |
| 1 | Atr-ERN10099 |  | Vvi-Vitvi05g01094\_t001 |  |  |  |  |  |
| 1 | Atr-ERN10100 |  | | | |  |  |  |  |  |
| 1 | Atr-ERN10101 |  | Vvi-Vitvi05g01091\_t001 |  |  |  |  |  |
| 0 | Atr-ERN10102 |  |  |  |  |  |  |
| 0 | Atr-ERN10103 |  |  |  |  |  |  |
| 0 | Atr-ERN10104 |  |  |  |  |  |  |
| 0 | Atr-ERN10105 |  |  |  |  |  |  |
