# Supplementary material for: Genome-wide identification, characterization and gene expression of BES1 transcription factor family in grapevine (Vitis vinifera L.)
Source: Sci Rep. 2023 Jan 5;13:240. doi: 10.1038/s41598-022-24407-y (PMC9816167; doi:10.1038/s41598-022-24407-y)
Supplement: Supplementary file 3 — Supplementary Information. [file 41598_2022_24407_MOESM3_ESM.zip › Vvi_Atr/Vitis_vinifera.PN40024.v4.dna_sm.toplevel.fa.vs.Amborella_trichopoda.AMTR1.0.dna_sm.toplevel.fa.html/Atr-AmTr_v1.0_scaffold00014.html]

|  |  |  |  |  |  |  |  |  |  |  |  |  |  |
| --- | --- | --- | --- | --- | --- | --- | --- | --- | --- | --- | --- | --- | --- |
| Duplication depth | Reference chromosome | Collinear blocks | | | | | | | | | | | |
| 0 | Atr-ERN09076 |  |  |  |  |  |  |
| 0 | Atr-ERN09077 |  |  |  |  |  |  |
| 0 | Atr-ERN09078 |  |  |  |  |  |  |
| 0 | Atr-ERN09079 |  |  |  |  |  |  |
| 0 | Atr-ERN09080 |  |  |  |  |  |  |
| 0 | Atr-ERN09081 |  |  |  |  |  |  |
| 0 | Atr-ERN09082 |  |  |  |  |  |  |
| 0 | Atr-ERN09083 |  |  |  |  |  |  |
| 0 | Atr-ERN09084 |  |  |  |  |  |  |
| 0 | Atr-ERN09085 |  |  |  |  |  |  |
| 0 | Atr-ERN09086 |  |  |  |  |  |  |
| 0 | Atr-ERN09087 |  |  |  |  |  |  |
| 0 | Atr-ERN09088 |  |  |  |  |  |  |
| 0 | Atr-ERN09089 |  |  |  |  |  |  |
| 0 | Atr-ERN09090 |  |  |  |  |  |  |
| 0 | Atr-ERN09091 |  |  |  |  |  |  |
| 0 | Atr-ERN09092 |  |  |  |  |  |  |
| 0 | Atr-ERN09093 |  |  |  |  |  |  |
| 0 | Atr-ERN09094 |  |  |  |  |  |  |
| 0 | Atr-ERN09095 |  |  |  |  |  |  |
| 0 | Atr-ERN09096 |  |  |  |  |  |  |
| 0 | Atr-ERN09097 |  |  |  |  |  |  |
| 0 | Atr-ERN09098 |  |  |  |  |  |  |
| 0 | Atr-ERN09099 |  |  |  |  |  |  |
| 0 | Atr-ERN09100 |  |  |  |  |  |  |
| 0 | Atr-ERN09101 |  |  |  |  |  |  |
| 0 | Atr-ERN09102 |  |  |  |  |  |  |
| 0 | Atr-ERN09103 |  |  |  |  |  |  |
| 0 | Atr-ERN09104 |  |  |  |  |  |  |
| 0 | Atr-ERN09105 |  |  |  |  |  |  |
| 0 | Atr-ERN09106 |  |  |  |  |  |  |
| 0 | Atr-ERN09107 |  |  |  |  |  |  |
| 0 | Atr-ERN09108 |  |  |  |  |  |  |
| 0 | Atr-ERN09109 |  |  |  |  |  |  |
| 0 | Atr-ERN09110 |  |  |  |  |  |  |
| 0 | Atr-ERN09111 |  |  |  |  |  |  |
| 0 | Atr-ERN09112 |  |  |  |  |  |  |
| 0 | Atr-ERN09113 |  |  |  |  |  |  |
| 0 | Atr-ERN09114 |  |  |  |  |  |  |
| 0 | Atr-ERN09115 |  |  |  |  |  |  |
| 0 | Atr-ERN09116 |  |  |  |  |  |  |
| 0 | Atr-ERN09117 |  |  |  |  |  |  |
| 0 | Atr-ERN09118 |  |  |  |  |  |  |
| 0 | Atr-ERN09119 |  |  |  |  |  |  |
| 0 | Atr-ERN09120 |  |  |  |  |  |  |
| 0 | Atr-ERN09121 |  |  |  |  |  |  |
| 0 | Atr-ERN09122 |  |  |  |  |  |  |
| 0 | Atr-ERN09123 |  |  |  |  |  |  |
| 0 | Atr-ERN09124 |  |  |  |  |  |  |
| 0 | Atr-ERN09125 |  |  |  |  |  |  |
| 0 | Atr-ERN09126 |  |  |  |  |  |  |
| 0 | Atr-ERN09127 |  |  |  |  |  |  |
| 0 | Atr-ERN09128 |  |  |  |  |  |  |
| 0 | Atr-ERN09129 |  |  |  |  |  |  |
| 0 | Atr-ERN09130 |  |  |  |  |  |  |
| 0 | Atr-ERN09131 |  |  |  |  |  |  |
| 0 | Atr-ERN09132 |  |  |  |  |  |  |
| 0 | Atr-ERN09133 |  |  |  |  |  |  |
| 0 | Atr-ERN09134 |  |  |  |  |  |  |
| 0 | Atr-ERN09135 |  |  |  |  |  |  |
| 0 | Atr-ERN09136 |  |  |  |  |  |  |
| 0 | Atr-ERN09137 |  |  |  |  |  |  |
| 0 | Atr-ERN09138 |  |  |  |  |  |  |
| 0 | Atr-ERN09139 |  |  |  |  |  |  |
| 0 | Atr-ERN09140 |  |  |  |  |  |  |
| 0 | Atr-ERN09141 |  |  |  |  |  |  |
| 0 | Atr-ERN09142 |  |  |  |  |  |  |
| 0 | Atr-ERN09143 |  |  |  |  |  |  |
| 0 | Atr-ERN09144 |  |  |  |  |  |  |
| 0 | Atr-ERN09145 |  |  |  |  |  |  |
| 0 | Atr-ERN09146 |  |  |  |  |  |  |
| 0 | Atr-ERN09147 |  |  |  |  |  |  |
| 0 | Atr-ERN09148 |  |  |  |  |  |  |
| 0 | Atr-ERN09149 |  |  |  |  |  |  |
| 0 | Atr-ERN09150 |  |  |  |  |  |  |
| 0 | Atr-ERN09151 |  |  |  |  |  |  |
| 0 | Atr-ERN09152 |  |  |  |  |  |  |
| 0 | Atr-ERN09153 |  |  |  |  |  |  |
| 0 | Atr-ERN09154 |  |  |  |  |  |  |
| 0 | Atr-ERN09155 |  |  |  |  |  |  |
| 0 | Atr-ERN09156 |  |  |  |  |  |  |
| 0 | Atr-ERN09157 |  |  |  |  |  |  |
| 0 | Atr-ERN09158 |  |  |  |  |  |  |
| 0 | Atr-ERN09159 |  |  |  |  |  |  |
| 0 | Atr-ERN09160 |  |  |  |  |  |  |
| 0 | Atr-ERN09161 |  |  |  |  |  |  |
| 0 | Atr-ERN09162 |  |  |  |  |  |  |
| 0 | Atr-ERN09163 |  |  |  |  |  |  |
| 0 | Atr-ERN09164 |  |  |  |  |  |  |
| 0 | Atr-ERN09165 |  |  |  |  |  |  |
| 0 | Atr-ERN09166 |  |  |  |  |  |  |
| 0 | Atr-ERN09167 |  |  |  |  |  |  |
| 0 | Atr-ERN09168 |  |  |  |  |  |  |
| 0 | Atr-ERN09169 |  |  |  |  |  |  |
| 0 | Atr-ERN09170 |  |  |  |  |  |  |
| 0 | Atr-ERN09171 |  |  |  |  |  |  |
| 0 | Atr-ERN09172 |  |  |  |  |  |  |
| 0 | Atr-ERN09173 |  |  |  |  |  |  |
| 0 | Atr-ERN09174 |  |  |  |  |  |  |
| 0 | Atr-ERN09175 |  |  |  |  |  |  |
| 0 | Atr-ERN09176 |  |  |  |  |  |  |
| 0 | Atr-ERN09177 |  |  |  |  |  |  |
| 0 | Atr-ERN09178 |  |  |  |  |  |  |
| 0 | Atr-ERN09179 |  |  |  |  |  |  |
| 0 | Atr-ERN09180 |  |  |  |  |  |  |
| 0 | Atr-ERN09181 |  |  |  |  |  |  |
| 0 | Atr-ERN09182 |  |  |  |  |  |  |
| 0 | Atr-ERN09183 |  |  |  |  |  |  |
| 0 | Atr-ERN09184 |  |  |  |  |  |  |
| 0 | Atr-ERN09185 |  |  |  |  |  |  |
| 0 | Atr-ERN09186 |  |  |  |  |  |  |
| 0 | Atr-ERN09187 |  |  |  |  |  |  |
| 0 | Atr-ERN09188 |  |  |  |  |  |  |
| 0 | Atr-ERN09189 |  |  |  |  |  |  |
| 0 | Atr-ERN09190 |  |  |  |  |  |  |
| 0 | Atr-ERN09191 |  |  |  |  |  |  |
| 0 | Atr-ERN09192 |  |  |  |  |  |  |
| 0 | Atr-ERN09193 |  |  |  |  |  |  |
| 0 | Atr-ERN09194 |  |  |  |  |  |  |
| 0 | Atr-ERN09195 |  |  |  |  |  |  |
| 0 | Atr-ERN09196 |  |  |  |  |  |  |
| 0 | Atr-ERN09197 |  |  |  |  |  |  |
| 0 | Atr-ERN09198 |  |  |  |  |  |  |
| 0 | Atr-ERN09199 |  |  |  |  |  |  |
| 0 | Atr-ERN09200 |  |  |  |  |  |  |
| 0 | Atr-ERN09201 |  |  |  |  |  |  |
| 0 | Atr-ERN09202 |  |  |  |  |  |  |
| 0 | Atr-ERN09203 |  |  |  |  |  |  |
| 0 | Atr-ERN09204 |  |  |  |  |  |  |
| 0 | Atr-ERN09205 |  |  |  |  |  |  |
| 0 | Atr-ERN09206 |  |  |  |  |  |  |
| 0 | Atr-ERN09207 |  |  |  |  |  |  |
| 0 | Atr-ERN09208 |  |  |  |  |  |  |
| 0 | Atr-ERN09209 |  |  |  |  |  |  |
| 0 | Atr-ERN09210 |  |  |  |  |  |  |
| 0 | Atr-ERN09211 |  |  |  |  |  |  |
| 0 | Atr-ERN09212 |  |  |  |  |  |  |
| 0 | Atr-ERN09213 |  |  |  |  |  |  |
| 0 | Atr-ERN09214 |  |  |  |  |  |  |
| 0 | Atr-ERN09215 |  |  |  |  |  |  |
| 0 | Atr-ERN09216 |  |  |  |  |  |  |
| 0 | Atr-ERN09217 |  |  |  |  |  |  |
| 0 | Atr-ERN09218 |  |  |  |  |  |  |
| 0 | Atr-ERN09219 |  |  |  |  |  |  |
| 0 | Atr-ERN09220 |  |  |  |  |  |  |
| 0 | Atr-ERN09221 |  |  |  |  |  |  |
| 0 | Atr-ERN09222 |  |  |  |  |  |  |
| 0 | Atr-ERN09223 |  |  |  |  |  |  |
| 0 | Atr-ERN09224 |  |  |  |  |  |  |
| 0 | Atr-ERN09225 |  |  |  |  |  |  |
| 0 | Atr-ERN09226 |  |  |  |  |  |  |
| 0 | Atr-ERN09227 |  |  |  |  |  |  |
| 0 | Atr-ERN09228 |  |  |  |  |  |  |
