# Supplementary material for: Genome-wide identification, characterization and gene expression of BES1 transcription factor family in grapevine (Vitis vinifera L.)
Source: Sci Rep. 2023 Jan 5;13:240. doi: 10.1038/s41598-022-24407-y (PMC9816167; doi:10.1038/s41598-022-24407-y)
Supplement: Supplementary file 3 — Supplementary Information. [file 41598_2022_24407_MOESM3_ESM.zip › Vvi_Atr/Vitis_vinifera.PN40024.v4.dna_sm.toplevel.fa.vs.Amborella_trichopoda.AMTR1.0.dna_sm.toplevel.fa.html/Atr-AmTr_v1.0_scaffold00015.html]

|  |  |  |  |  |  |  |  |  |  |  |  |  |  |
| --- | --- | --- | --- | --- | --- | --- | --- | --- | --- | --- | --- | --- | --- |
| Duplication depth | Reference chromosome | Collinear blocks | | | | | | | | | | | |
| 0 | Atr-ERN08825 |  |  |  |  |  |  |
| 0 | Atr-ERN08826 |  |  |  |  |  |  |
| 0 | Atr-ERN08827 |  |  |  |  |  |  |
| 0 | Atr-ERN08828 |  |  |  |  |  |  |
| 0 | Atr-ERN08829 |  |  |  |  |  |  |
| 0 | Atr-ERN08830 |  |  |  |  |  |  |
| 0 | Atr-ERN08831 |  |  |  |  |  |  |
| 0 | Atr-ERN08832 |  |  |  |  |  |  |
| 0 | Atr-ERN08833 |  |  |  |  |  |  |
| 0 | Atr-ERN08834 |  |  |  |  |  |  |
| 0 | Atr-ERN08835 |  |  |  |  |  |  |
| 0 | Atr-ERN08836 |  |  |  |  |  |  |
| 0 | Atr-ERN08837 |  |  |  |  |  |  |
| 0 | Atr-ERN08838 |  |  |  |  |  |  |
| 0 | Atr-ERN08839 |  |  |  |  |  |  |
| 0 | Atr-ERN08840 |  |  |  |  |  |  |
| 0 | Atr-ERN08841 |  |  |  |  |  |  |
| 0 | Atr-ERN08842 |  |  |  |  |  |  |
| 0 | Atr-ERN08843 |  |  |  |  |  |  |
| 0 | Atr-ERN08844 |  |  |  |  |  |  |
| 0 | Atr-ERN08845 |  |  |  |  |  |  |
| 0 | Atr-ERN08846 |  |  |  |  |  |  |
| 0 | Atr-ERN08847 |  |  |  |  |  |  |
| 0 | Atr-ERN08848 |  |  |  |  |  |  |
| 0 | Atr-ERN08849 |  |  |  |  |  |  |
| 0 | Atr-ERN08850 |  |  |  |  |  |  |
| 0 | Atr-ERN08851 |  |  |  |  |  |  |
| 0 | Atr-ERN08852 |  |  |  |  |  |  |
| 0 | Atr-ERN08853 |  |  |  |  |  |  |
| 0 | Atr-ERN08854 |  |  |  |  |  |  |
| 0 | Atr-ERN08855 |  |  |  |  |  |  |
| 0 | Atr-ERN08856 |  |  |  |  |  |  |
| 0 | Atr-ERN08857 |  |  |  |  |  |  |
| 0 | Atr-ERN08858 |  |  |  |  |  |  |
| 0 | Atr-ERN08859 |  |  |  |  |  |  |
| 0 | Atr-ERN08860 |  |  |  |  |  |  |
| 0 | Atr-ERN08861 |  |  |  |  |  |  |
| 0 | Atr-ERN08862 |  |  |  |  |  |  |
| 0 | Atr-ERN08863 |  |  |  |  |  |  |
| 0 | Atr-ERN08864 |  |  |  |  |  |  |
| 0 | Atr-ERN08865 |  |  |  |  |  |  |
| 0 | Atr-ERN08866 |  |  |  |  |  |  |
| 0 | Atr-ERN08867 |  |  |  |  |  |  |
| 0 | Atr-ERN08868 |  |  |  |  |  |  |
| 0 | Atr-ERN08869 |  |  |  |  |  |  |
| 0 | Atr-ERN08870 |  |  |  |  |  |  |
| 0 | Atr-ERN08871 |  |  |  |  |  |  |
| 0 | Atr-ERN08872 |  |  |  |  |  |  |
| 0 | Atr-ERN08873 |  |  |  |  |  |  |
| 0 | Atr-ERN08874 |  |  |  |  |  |  |
| 0 | Atr-ERN08875 |  |  |  |  |  |  |
| 0 | Atr-ERN08876 |  |  |  |  |  |  |
| 0 | Atr-ERN08877 |  |  |  |  |  |  |
| 0 | Atr-ERN08878 |  |  |  |  |  |  |
| 0 | Atr-ERN08879 |  |  |  |  |  |  |
| 0 | Atr-ERN08880 |  |  |  |  |  |  |
| 0 | Atr-ERN08881 |  |  |  |  |  |  |
| 0 | Atr-ERN08882 |  |  |  |  |  |  |
| 0 | Atr-ERN08883 |  |  |  |  |  |  |
| 0 | Atr-ERN08884 |  |  |  |  |  |  |
| 0 | Atr-ERN08885 |  |  |  |  |  |  |
| 0 | Atr-ERN08886 |  |  |  |  |  |  |
| 0 | Atr-ERN08887 |  |  |  |  |  |  |
| 0 | Atr-ERN08888 |  |  |  |  |  |  |
| 0 | Atr-ERN08889 |  |  |  |  |  |  |
| 0 | Atr-ERN08890 |  |  |  |  |  |  |
| 0 | Atr-ERN08891 |  |  |  |  |  |  |
| 0 | Atr-ERN08892 |  |  |  |  |  |  |
| 0 | Atr-ERN08893 |  |  |  |  |  |  |
| 0 | Atr-ERN08894 |  |  |  |  |  |  |
| 0 | Atr-ERN08895 |  |  |  |  |  |  |
| 0 | Atr-ERN08896 |  |  |  |  |  |  |
| 0 | Atr-ERN08897 |  |  |  |  |  |  |
| 0 | Atr-ERN08898 |  |  |  |  |  |  |
| 0 | Atr-ERN08899 |  |  |  |  |  |  |
| 0 | Atr-ERN08900 |  |  |  |  |  |  |
| 0 | Atr-ERN08901 |  |  |  |  |  |  |
| 0 | Atr-ERN08902 |  |  |  |  |  |  |
| 0 | Atr-ERN08903 |  |  |  |  |  |  |
| 0 | Atr-ERN08904 |  |  |  |  |  |  |
| 0 | Atr-ERN08905 |  |  |  |  |  |  |
| 0 | Atr-ERN08906 |  |  |  |  |  |  |
| 0 | Atr-ERN08907 |  |  |  |  |  |  |
| 0 | Atr-ERN08908 |  |  |  |  |  |  |
| 0 | Atr-ERN08909 |  |  |  |  |  |  |
| 0 | Atr-ERN08910 |  |  |  |  |  |  |
| 0 | Atr-ERN08911 |  |  |  |  |  |  |
| 0 | Atr-ERN08912 |  |  |  |  |  |  |
| 0 | Atr-ERN08913 |  |  |  |  |  |  |
| 0 | Atr-ERN08914 |  |  |  |  |  |  |
| 0 | Atr-ERN08915 |  |  |  |  |  |  |
| 0 | Atr-ERN08916 |  |  |  |  |  |  |
| 0 | Atr-ERN08917 |  |  |  |  |  |  |
| 0 | Atr-ERN08918 |  |  |  |  |  |  |
| 0 | Atr-ERN08919 |  |  |  |  |  |  |
| 0 | Atr-ERN08920 |  |  |  |  |  |  |
| 0 | Atr-ERN08921 |  |  |  |  |  |  |
| 0 | Atr-ERN08922 |  |  |  |  |  |  |
| 0 | Atr-ERN08923 |  |  |  |  |  |  |
| 0 | Atr-ERN08924 |  |  |  |  |  |  |
| 0 | Atr-ERN08925 |  |  |  |  |  |  |
| 0 | Atr-ERN08926 |  |  |  |  |  |  |
| 0 | Atr-ERN08927 |  |  |  |  |  |  |
| 0 | Atr-ERN08928 |  |  |  |  |  |  |
| 0 | Atr-ERN08929 |  |  |  |  |  |  |
| 0 | Atr-ERN08930 |  |  |  |  |  |  |
| 0 | Atr-ERN08931 |  |  |  |  |  |  |
| 0 | Atr-ERN08932 |  |  |  |  |  |  |
| 0 | Atr-ERN08933 |  |  |  |  |  |  |
| 0 | Atr-ERN08934 |  |  |  |  |  |  |
| 0 | Atr-ERN08935 |  |  |  |  |  |  |
| 0 | Atr-ERN08936 |  |  |  |  |  |  |
| 0 | Atr-ERN08937 |  |  |  |  |  |  |
| 0 | Atr-ERN08938 |  |  |  |  |  |  |
| 0 | Atr-ERN08939 |  |  |  |  |  |  |
| 0 | Atr-ERN08940 |  |  |  |  |  |  |
| 0 | Atr-ERN08941 |  |  |  |  |  |  |
| 0 | Atr-ERN08942 |  |  |  |  |  |  |
| 0 | Atr-ERN08943 |  |  |  |  |  |  |
| 0 | Atr-ERN08944 |  |  |  |  |  |  |
| 0 | Atr-ERN08945 |  |  |  |  |  |  |
| 0 | Atr-ERN08946 |  |  |  |  |  |  |
| 0 | Atr-ERN08947 |  |  |  |  |  |  |
| 0 | Atr-ERN08948 |  |  |  |  |  |  |
| 0 | Atr-ERN08949 |  |  |  |  |  |  |
| 0 | Atr-ERN08950 |  |  |  |  |  |  |
| 0 | Atr-ERN08951 |  |  |  |  |  |  |
| 0 | Atr-ERN08952 |  |  |  |  |  |  |
