# Supplementary material for: Genome-wide identification, characterization and gene expression of BES1 transcription factor family in grapevine (Vitis vinifera L.)
Source: Sci Rep. 2023 Jan 5;13:240. doi: 10.1038/s41598-022-24407-y (PMC9816167; doi:10.1038/s41598-022-24407-y)
Supplement: Supplementary file 3 — Supplementary Information. [file 41598_2022_24407_MOESM3_ESM.zip › Vvi_Atr/Vitis_vinifera.PN40024.v4.dna_sm.toplevel.fa.vs.Amborella_trichopoda.AMTR1.0.dna_sm.toplevel.fa.html/Atr-AmTr_v1.0_scaffold00016.html]

|  |  |  |  |  |  |  |  |  |  |  |  |  |  |
| --- | --- | --- | --- | --- | --- | --- | --- | --- | --- | --- | --- | --- | --- |
| Duplication depth | Reference chromosome | Collinear blocks | | | | | | | | | | | |
| 0 | Atr-ERN06088 |  |  |  |  |  |  |
| 0 | Atr-ERN06089 |  |  |  |  |  |  |
| 0 | Atr-ERN06090 |  |  |  |  |  |  |
| 1 | Atr-ERN06091 |  | Vvi-Vitvi01g01788\_t001 |  |  |  |  |  |
| 1 | Atr-ERN06092 |  | | | |  |  |  |  |  |
| 1 | Atr-ERN06093 |  | | | |  |  |  |  |  |
| 1 | Atr-ERN06094 |  | Vvi-Vitvi01g01785\_t001 |  |  |  |  |  |
| 1 | Atr-ERN06095 |  | Vvi-Vitvi01g02299\_t003 |  |  |  |  |  |
| 1 | Atr-ERN06096 |  | | | |  |  |  |  |  |
| 1 | Atr-ERN06097 |  | | | |  |  |  |  |  |
| 1 | Atr-ERN06098 |  | | | |  |  |  |  |  |
| 1 | Atr-ERN06099 |  | | | |  |  |  |  |  |
| 1 | Atr-ERN06100 |  | | | |  |  |  |  |  |
| 1 | Atr-ERN06101 |  | | | |  |  |  |  |  |
| 1 | Atr-ERN06102 |  | | | |  |  |  |  |  |
| 1 | Atr-ERN06103 |  | | | |  |  |  |  |  |
| 1 | Atr-ERN06104 |  | | | |  |  |  |  |  |
| 1 | Atr-ERN06105 |  | | | |  |  |  |  |  |
| 1 | Atr-ERN06106 |  | | | |  |  |  |  |  |
| 1 | Atr-ERN06107 |  | | | |  |  |  |  |  |
| 1 | Atr-ERN06108 |  | | | |  |  |  |  |  |
| 1 | Atr-ERN06109 |  | | | |  |  |  |  |  |
| 1 | Atr-ERN06110 |  | | | |  |  |  |  |  |
| 1 | Atr-ERN06111 |  | | | |  |  |  |  |  |
| 1 | Atr-ERN06112 |  | | | |  |  |  |  |  |
| 1 | Atr-ERN06113 |  | | | |  |  |  |  |  |
| 1 | Atr-ERN06114 |  | | | |  |  |  |  |  |
| 1 | Atr-ERN06115 |  | | | |  |  |  |  |  |
| 1 | Atr-ERN06116 |  | | | |  |  |  |  |  |
| 1 | Atr-ERN06117 |  | | | |  |  |  |  |  |
| 1 | Atr-ERN06118 |  | Vvi-Vitvi01g01783\_t001 |  |  |  |  |  |
| 1 | Atr-ERN06119 |  | Vvi-Vitvi01g01782\_t002 |  |  |  |  |  |
| 1 | Atr-ERN06120 |  | | | |  |  |  |  |  |
| 1 | Atr-ERN06121 |  | | | |  |  |  |  |  |
| 1 | Atr-ERN06122 |  | | | |  |  |  |  |  |
| 1 | Atr-ERN06123 |  | | | |  |  |  |  |  |
| 2 | Atr-ERN06124 |  | | | |  | Vvi-Vitvi17g00016\_t001 |  |  |  |  |
| 3 | Atr-ERN06125 |  | | | |  | | | |  | Vvi-Vitvi14g01456\_t001 |  |  |  |
| 3 | Atr-ERN06126 |  | Vvi-Vitvi01g01780\_t001 |  | | | |  | | | |  |  |  |
| 3 | Atr-ERN06127 |  | Vvi-Vitvi01g01779\_t001 |  | | | |  | | | |  |  |  |
| 3 | Atr-ERN06128 |  | Vvi-Vitvi01g01778\_t001 |  | Vvi-Vitvi17g00017\_t001 |  | | | |  |  |  |
| 3 | Atr-ERN06129 |  | | | |  | | | |  | | | |  |  |  |
| 3 | Atr-ERN06130 |  | Vvi-Vitvi01g01777\_t001 |  | Vvi-Vitvi17g00018\_t001 |  | | | |  |  |  |
| 3 | Atr-ERN06131 |  | | | |  | | | |  | | | |  |  |  |
| 3 | Atr-ERN06132 |  | | | |  | Vvi-Vitvi17g00021\_t001 |  | | | |  |  |  |
| 3 | Atr-ERN06133 |  | | | |  | | | |  | | | |  |  |  |
| 3 | Atr-ERN06134 |  | | | |  | | | |  | | | |  |  |  |
| 3 | Atr-ERN06135 |  | Vvi-Vitvi01g01773\_t001 |  | Vvi-Vitvi17g04007\_t001 |  | | | |  |  |  |
| 3 | Atr-ERN06136 |  | Vvi-Vitvi01g01772\_t001 |  | | | |  | | | |  |  |  |
| 3 | Atr-ERN06137 |  | | | |  | | | |  | | | |  |  |  |
| 3 | Atr-ERN06138 |  | | | |  | | | |  | | | |  |  |  |
| 3 | Atr-ERN06139 |  | | | |  | | | |  | | | |  |  |  |
| 3 | Atr-ERN06140 |  | Vvi-Vitvi01g01826\_t001 |  | Vvi-Vitvi17g00025\_t001 |  | Vvi-Vitvi14g01458\_t001 |  |  |  |
| 3 | Atr-ERN06141 |  | | | |  | | | |  | Vvi-Vitvi14g01459\_t001 |  |  |  |
| 3 | Atr-ERN06142 |  | Vvi-Vitvi01g01770\_t001 |  | | | |  | Vvi-Vitvi14g01460\_t001 |  |  |  |
| 3 | Atr-ERN06143 |  | Vvi-Vitvi01g01769\_t001 |  | | | |  | | | |  |  |  |
| 3 | Atr-ERN06144 |  | | | |  | | | |  | | | |  |  |  |
| 3 | Atr-ERN06145 |  | | | |  | | | |  | | | |  |  |  |
| 3 | Atr-ERN06146 |  | | | |  | | | |  | | | |  |  |  |
| 3 | Atr-ERN06147 |  | | | |  | | | |  | | | |  |  |  |
| 3 | Atr-ERN06148 |  | | | |  | | | |  | | | |  |  |  |
| 3 | Atr-ERN06149 |  | Vvi-Vitvi01g01768\_t001 |  | Vvi-Vitvi17g00028\_t001 |  | | | |  |  |  |
| 3 | Atr-ERN06150 |  | Vvi-Vitvi01g01764\_t001 |  | | | |  | | | |  |  |  |
| 3 | Atr-ERN06151 |  | | | |  | | | |  | | | |  |  |  |
| 3 | Atr-ERN06152 |  | Vvi-Vitvi01g02298\_t001 |  | Vvi-Vitvi17g00029\_t001 |  | | | |  |  |  |
| 3 | Atr-ERN06153 |  | Vvi-Vitvi01g01763\_t001 |  | | | |  | Vvi-Vitvi14g01463\_t001 |  |  |  |
| 3 | Atr-ERN06154 |  | | | |  | | | |  | | | |  |  |  |
| 3 | Atr-ERN06155 |  | | | |  | Vvi-Vitvi17g00031\_t001 |  | Vvi-Vitvi14g01465\_t002 |  |  |  |
| 2 | Atr-ERN06156 |  | | | |  |  |  | | | |  |  |  |
| 2 | Atr-ERN06157 |  | | | |  |  |  | | | |  |  |  |
| 2 | Atr-ERN06158 |  | | | |  |  |  | Vvi-Vitvi14g01466\_t001 |  |  |  |
| 2 | Atr-ERN06159 |  | | | |  |  |  | Vvi-Vitvi14g01467\_t001 |  |  |  |
| 2 | Atr-ERN06160 |  | | | |  |  |  | | | |  |  |  |
| 2 | Atr-ERN06161 |  | | | |  |  |  | | | |  |  |  |
| 2 | Atr-ERN06162 |  | | | |  |  |  | Vvi-Vitvi14g01474\_t001 |  |  |  |
| 2 | Atr-ERN06163 |  | | | |  |  |  | | | |  |  |  |
| 3 | Atr-ERN06164 |  | | | |  | Vvi-Vitvi01g01731\_t001 |  | | | |  |  |  |
| 3 | Atr-ERN06165 |  | | | |  | Vvi-Vitvi01g01733\_t001 |  | | | |  |  |  |
| 3 | Atr-ERN06166 |  | | | |  | | | |  | | | |  |  |  |
| 4 | Atr-ERN06167 |  | | | |  | Vvi-Vitvi01g01734\_t001 |  | | | |  | Vvi-Vitvi14g01485\_t001 |  |  |
| 4 | Atr-ERN06168 |  | | | |  | | | |  | | | |  | | | |  |  |
| 4 | Atr-ERN06169 |  | | | |  | | | |  | | | |  | | | |  |  |
| 4 | Atr-ERN06170 |  | | | |  | | | |  | | | |  | | | |  |  |
| 4 | Atr-ERN06171 |  | | | |  | | | |  | | | |  | | | |  |  |
| 4 | Atr-ERN06172 |  | | | |  | Vvi-Vitvi01g01738\_t001 |  | Vvi-Vitvi14g01482\_t001 |  | | | |  |  |
| 3 | Atr-ERN06173 |  | | | |  | | | |  |  |  | | | |  |  |
| 3 | Atr-ERN06174 |  | | | |  | | | |  |  |  | | | |  |  |
| 3 | Atr-ERN06175 |  | | | |  | | | |  |  |  | | | |  |  |
| 3 | Atr-ERN06176 |  | | | |  | | | |  |  |  | | | |  |  |
| 3 | Atr-ERN06177 |  | | | |  | Vvi-Vitvi01g02293\_t001 |  |  |  | | | |  |  |
| 3 | Atr-ERN06178 |  | Vvi-Vitvi01g01747\_t001 |  | | | |  |  |  | | | |  |  |
| 3 | Atr-ERN06179 |  | | | |  | | | |  |  |  | | | |  |  |
| 3 | Atr-ERN06180 |  | | | |  | | | |  |  |  | | | |  |  |
| 3 | Atr-ERN06181 |  | | | |  | | | |  |  |  | | | |  |  |
| 3 | Atr-ERN06182 |  | | | |  | | | |  |  |  | | | |  |  |
| 3 | Atr-ERN06183 |  | Vvi-Vitvi01g01745\_t001 |  | | | |  |  |  | | | |  |  |
| 3 | Atr-ERN06184 |  | | | |  | | | |  |  |  | Vvi-Vitvi14g01479\_t001 |  |  |
| 3 | Atr-ERN06185 |  | Vvi-Vitvi01g01742\_t001 |  | | | |  |  |  | | | |  |  |
| 2 | Atr-ERN06186 |  |  |  | Vvi-Vitvi01g02295\_t001 |  |  |  | | | |  |  |
| 2 | Atr-ERN06187 |  |  |  | | | |  |  |  | | | |  |  |
| 2 | Atr-ERN06188 |  |  |  | | | |  |  |  | | | |  |  |
| 2 | Atr-ERN06189 |  |  |  | | | |  |  |  | | | |  |  |
| 2 | Atr-ERN06190 |  |  |  | | | |  |  |  | | | |  |  |
| 2 | Atr-ERN06191 |  |  |  | | | |  |  |  | | | |  |  |
| 2 | Atr-ERN06192 |  |  |  | | | |  |  |  | | | |  |  |
| 2 | Atr-ERN06193 |  |  |  | | | |  |  |  | | | |  |  |
| 2 | Atr-ERN06194 |  |  |  | | | |  |  |  | Vvi-Vitvi14g01478\_t002 |  |  |
| 2 | Atr-ERN06195 |  |  |  | Vvi-Vitvi01g01748\_t001 |  |  |  | | | |  |  |
| 2 | Atr-ERN06196 |  |  |  | | | |  |  |  | | | |  |  |
| 2 | Atr-ERN06197 |  |  |  | Vvi-Vitvi01g01749\_t001 |  |  |  | | | |  |  |
| 2 | Atr-ERN06198 |  |  |  | | | |  |  |  | | | |  |  |
| 2 | Atr-ERN06199 |  |  |  | | | |  |  |  | | | |  |  |
| 2 | Atr-ERN06200 |  |  |  | | | |  |  |  | Vvi-Vitvi14g02951\_t001 |  |  |
| 2 | Atr-ERN06201 |  |  |  | | | |  |  |  | | | |  |  |
| 2 | Atr-ERN06202 |  |  |  | | | |  |  |  | | | |  |  |
| 2 | Atr-ERN06203 |  |  |  | | | |  |  |  | Vvi-Vitvi14g01476\_t001 |  |  |
| 2 | Atr-ERN06204 |  |  |  | Vvi-Vitvi01g01750\_t001 |  |  |  | | | |  |  |
| 2 | Atr-ERN06205 |  |  |  | Vvi-Vitvi01g01753\_t001 |  |  |  | | | |  |  |
| 2 | Atr-ERN06206 |  |  |  | | | |  |  |  | | | |  |  |
| 2 | Atr-ERN06207 |  |  |  | | | |  |  |  | Vvi-Vitvi14g01475\_t001 |  |  |
| 1 | Atr-ERN06208 |  |  |  | | | |  |  |  |  |
| 1 | Atr-ERN06209 |  |  |  | | | |  |  |  |  |
| 2 | Atr-ERN06210 |  | Vvi-Vitvi17g00033\_t001 |  | | | |  |  |  |  |
| 2 | Atr-ERN06211 |  | | | |  | | | |  |  |  |  |
| 3 | Atr-ERN06212 |  | | | |  | Vvi-Vitvi01g02296\_t001 |  | Vvi-Vitvi14g01469\_t001 |  |  |  |
| 3 | Atr-ERN06213 |  | | | |  | Vvi-Vitvi01g01761\_t001 |  | | | |  |  |  |
| 2 | Atr-ERN06214 |  | | | |  |  |  | | | |  |  |  |
| 2 | Atr-ERN06215 |  | Vvi-Vitvi17g00036\_t001 |  |  |  | | | |  |  |  |
| 2 | Atr-ERN06216 |  | | | |  |  |  | Vvi-Vitvi14g01472\_t001 |  |  |  |
| 2 | Atr-ERN06217 |  | Vvi-Vitvi17g00037\_t001 |  |  |  | Vvi-Vitvi14g01473\_t001 |  |  |  |
| 2 | Atr-ERN06218 |  | | | |  |  |  | Vvi-Vitvi14g04554\_t002 |  |  |  |
| 3 | Atr-ERN06219 |  | Vvi-Vitvi17g00052\_t001 |  | Vvi-Vitvi01g01730\_t001 |  | | | |  |  |  |
| 3 | Atr-ERN06220 |  | | | |  | Vvi-Vitvi01g01729\_t001 |  | Vvi-Vitvi14g01487\_t001 |  |  |  |
| 3 | Atr-ERN06221 |  | | | |  | | | |  | | | |  |  |  |
| 3 | Atr-ERN06222 |  | | | |  | | | |  | Vvi-Vitvi14g01488\_t001 |  |  |  |
| 3 | Atr-ERN06223 |  | Vvi-Vitvi17g00053\_t001 |  | | | |  | Vvi-Vitvi14g01489\_t001 |  |  |  |
| 3 | Atr-ERN06224 |  | | | |  | | | |  | | | |  |  |  |
| 3 | Atr-ERN06225 |  | | | |  | | | |  | | | |  |  |  |
| 3 | Atr-ERN06226 |  | | | |  | | | |  | | | |  |  |  |
| 3 | Atr-ERN06227 |  | | | |  | | | |  | | | |  |  |  |
| 3 | Atr-ERN06228 |  | | | |  | | | |  | Vvi-Vitvi14g01491\_t001 |  |  |  |
| 3 | Atr-ERN06229 |  | | | |  | | | |  | | | |  |  |  |
| 3 | Atr-ERN06230 |  | | | |  | | | |  | | | |  |  |  |
| 3 | Atr-ERN06231 |  | | | |  | | | |  | Vvi-Vitvi14g02957\_t001 |  |  |  |
| 3 | Atr-ERN06232 |  | | | |  | | | |  | | | |  |  |  |
| 3 | Atr-ERN06233 |  | | | |  | Vvi-Vitvi01g01727\_t001 |  | | | |  |  |  |
| 3 | Atr-ERN06234 |  | Vvi-Vitvi17g00054\_t001 |  | | | |  | Vvi-Vitvi14g01496\_t003 |  |  |  |
| 3 | Atr-ERN06235 |  | Vvi-Vitvi17g00056\_t001 |  | | | |  | | | |  |  |  |
| 3 | Atr-ERN06236 |  | | | |  | | | |  | Vvi-Vitvi14g01497\_t001 |  |  |  |
| 3 | Atr-ERN06237 |  | Vvi-Vitvi17g00057\_t001 |  | | | |  | | | |  |  |  |
| 3 | Atr-ERN06238 |  | | | |  | Vvi-Vitvi01g01726\_t002 |  | Vvi-Vitvi14g01498\_t002 |  |  |  |
| 3 | Atr-ERN06239 |  | Vvi-Vitvi17g00060\_t001 |  | Vvi-Vitvi01g01724\_t002 |  | | | |  |  |  |
| 3 | Atr-ERN06240 |  | Vvi-Vitvi17g00061\_t001 |  | | | |  | | | |  |  |  |
| 3 | Atr-ERN06241 |  | | | |  | | | |  | | | |  |  |  |
| 3 | Atr-ERN06242 |  | | | |  | | | |  | | | |  |  |  |
| 3 | Atr-ERN06243 |  | | | |  | | | |  | | | |  |  |  |
| 3 | Atr-ERN06244 |  | | | |  | | | |  | | | |  |  |  |
| 3 | Atr-ERN06245 |  | | | |  | | | |  | | | |  |  |  |
| 3 | Atr-ERN06246 |  | | | |  | | | |  | | | |  |  |  |
| 3 | Atr-ERN06247 |  | | | |  | | | |  | | | |  |  |  |
| 3 | Atr-ERN06248 |  | | | |  | Vvi-Vitvi01g02289\_t001 |  | | | |  |  |  |
| 3 | Atr-ERN06249 |  | Vvi-Vitvi17g00066\_t001 |  | Vvi-Vitvi01g01722\_t001 |  | Vvi-Vitvi14g01499\_t001 |  |  |  |
| 3 | Atr-ERN06250 |  | | | |  | | | |  | Vvi-Vitvi14g01500\_t001 |  |  |  |
| 3 | Atr-ERN06251 |  | Vvi-Vitvi17g00067\_t001 |  | | | |  | Vvi-Vitvi14g01501\_t001 |  |  |  |
| 3 | Atr-ERN06252 |  | | | |  | | | |  | Vvi-Vitvi14g01502\_t001 |  |  |  |
| 3 | Atr-ERN06253 |  | | | |  | | | |  | | | |  |  |  |
| 3 | Atr-ERN06254 |  | Vvi-Vitvi17g00068\_t001 |  | | | |  | Vvi-Vitvi14g01503\_t001 |  |  |  |
| 3 | Atr-ERN06255 |  | | | |  | Vvi-Vitvi01g01720\_t001 |  | | | |  |  |  |
| 3 | Atr-ERN06256 |  | Vvi-Vitvi17g00069\_t001 |  | Vvi-Vitvi01g01719\_t001 |  | | | |  |  |  |
| 3 | Atr-ERN06257 |  | | | |  | Vvi-Vitvi01g01718\_t001 |  | Vvi-Vitvi14g01504\_t001 |  |  |  |
| 3 | Atr-ERN06258 |  | Vvi-Vitvi17g00071\_t001 |  | | | |  | Vvi-Vitvi14g01505\_t001 |  |  |  |
| 3 | Atr-ERN06259 |  | Vvi-Vitvi17g00072\_t001 |  | | | |  | Vvi-Vitvi14g01506\_t002 |  |  |  |
| 3 | Atr-ERN06260 |  | | | |  | | | |  | | | |  |  |  |
| 3 | Atr-ERN06261 |  | | | |  | Vvi-Vitvi01g02287\_t001 |  | Vvi-Vitvi14g01507\_t001 |  |  |  |
| 3 | Atr-ERN06262 |  | Vvi-Vitvi17g00073\_t001 |  | | | |  | | | |  |  |  |
| 3 | Atr-ERN06263 |  | Vvi-Vitvi17g01331\_t001 |  | | | |  | Vvi-Vitvi14g01509\_t001 |  |  |  |
| 3 | Atr-ERN06264 |  | | | |  | | | |  | Vvi-Vitvi14g04561\_t001 |  |  |  |
| 3 | Atr-ERN06265 |  | | | |  | | | |  | | | |  |  |  |
| 3 | Atr-ERN06266 |  | | | |  | | | |  | | | |  |  |  |
| 3 | Atr-ERN06267 |  | Vvi-Vitvi17g00074\_t001 |  | | | |  | | | |  |  |  |
| 3 | Atr-ERN06268 |  | Vvi-Vitvi17g00075\_t001 |  | Vvi-Vitvi01g04459\_t001 |  | | | |  |  |  |
| 3 | Atr-ERN06269 |  | Vvi-Vitvi17g00076\_t001 |  | | | |  | | | |  |  |  |
| 3 | Atr-ERN06270 |  | | | |  | | | |  | | | |  |  |  |
| 3 | Atr-ERN06271 |  | | | |  | | | |  | | | |  |  |  |
| 3 | Atr-ERN06272 |  | Vvi-Vitvi17g01333\_t001 |  | Vvi-Vitvi01g01711\_t001 |  | | | |  |  |  |
| 3 | Atr-ERN06273 |  | | | |  | | | |  | | | |  |  |  |
| 3 | Atr-ERN06274 |  | Vvi-Vitvi17g00078\_t001 |  | | | |  | Vvi-Vitvi14g04562\_t001 |  |  |  |
| 3 | Atr-ERN06275 |  | | | |  | | | |  | | | |  |  |  |
| 3 | Atr-ERN06276 |  | | | |  | | | |  | | | |  |  |  |
| 3 | Atr-ERN06277 |  | | | |  | | | |  | | | |  |  |  |
| 3 | Atr-ERN06278 |  | | | |  | Vvi-Vitvi01g01710\_t001 |  | | | |  |  |  |
| 3 | Atr-ERN06279 |  | | | |  | Vvi-Vitvi01g01708\_t001 |  | | | |  |  |  |
| 3 | Atr-ERN06280 |  | | | |  | | | |  | | | |  |  |  |
| 3 | Atr-ERN06281 |  | | | |  | | | |  | Vvi-Vitvi14g01513\_t001 |  |  |  |
| 3 | Atr-ERN06282 |  | Vvi-Vitvi17g00080\_t001 |  | | | |  | Vvi-Vitvi14g01514\_t001 |  |  |  |
| 3 | Atr-ERN06283 |  | | | |  | | | |  | | | |  |  |  |
| 3 | Atr-ERN06284 |  | | | |  | Vvi-Vitvi01g01707\_t001 |  | Vvi-Vitvi14g01515\_t002 |  |  |  |
| 3 | Atr-ERN06285 |  | | | |  | | | |  | | | |  |  |  |
| 3 | Atr-ERN06286 |  | | | |  | Vvi-Vitvi01g01706\_t001 |  | | | |  |  |  |
| 3 | Atr-ERN06287 |  | | | |  | | | |  | | | |  |  |  |
| 3 | Atr-ERN06288 |  | | | |  | | | |  | | | |  |  |  |
| 3 | Atr-ERN06289 |  | | | |  | | | |  | | | |  |  |  |
| 3 | Atr-ERN06290 |  | | | |  | | | |  | | | |  |  |  |
| 3 | Atr-ERN06291 |  | | | |  | | | |  | | | |  |  |  |
| 3 | Atr-ERN06292 |  | | | |  | | | |  | | | |  |  |  |
| 3 | Atr-ERN06293 |  | | | |  | | | |  | | | |  |  |  |
| 3 | Atr-ERN06294 |  | Vvi-Vitvi17g00081\_t002 |  | | | |  | | | |  |  |  |
| 3 | Atr-ERN06295 |  | Vvi-Vitvi17g00082\_t001 |  | | | |  | Vvi-Vitvi14g01517\_t001 |  |  |  |
| 3 | Atr-ERN06296 |  | | | |  | | | |  | | | |  |  |  |
| 3 | Atr-ERN06297 |  | | | |  | Vvi-Vitvi01g01705\_t001 |  | | | |  |  |  |
| 3 | Atr-ERN06298 |  | | | |  | | | |  | | | |  |  |  |
| 3 | Atr-ERN06299 |  | | | |  | | | |  | Vvi-Vitvi14g01519\_t001 |  |  |  |
| 3 | Atr-ERN06300 |  | | | |  | | | |  | | | |  |  |  |
| 3 | Atr-ERN06301 |  | Vvi-Vitvi17g00083\_t001 |  | | | |  | | | |  |  |  |
| 3 | Atr-ERN06302 |  | Vvi-Vitvi17g04015\_t001 |  | Vvi-Vitvi01g01701\_t001 |  | | | |  |  |  |
| 3 | Atr-ERN06303 |  | | | |  | | | |  | | | |  |  |  |
| 3 | Atr-ERN06304 |  | Vvi-Vitvi17g00084\_t001 |  | | | |  | | | |  |  |  |
| 3 | Atr-ERN06305 |  | | | |  | | | |  | | | |  |  |  |
| 3 | Atr-ERN06306 |  | | | |  | | | |  | Vvi-Vitvi14g01520\_t001 |  |  |  |
| 2 | Atr-ERN06307 |  | | | |  | | | |  |  |  |  |
| 2 | Atr-ERN06308 |  | | | |  | | | |  |  |  |  |
| 2 | Atr-ERN06309 |  | | | |  | | | |  |  |  |  |
| 2 | Atr-ERN06310 |  | | | |  | | | |  |  |  |  |
| 2 | Atr-ERN06311 |  | | | |  | | | |  |  |  |  |
| 2 | Atr-ERN06312 |  | | | |  | Vvi-Vitvi01g01700\_t001 |  |  |  |  |
| 2 | Atr-ERN06313 |  | | | |  | Vvi-Vitvi01g04457\_t001 |  |  |  |  |
| 1 | Atr-ERN06314 |  | Vvi-Vitvi17g00085\_t001 |  |  |  |  |  |
| 1 | Atr-ERN06315 |  | Vvi-Vitvi14g02950\_t001 |  |  |  |  |  |
| 1 | Atr-ERN06316 |  | | | |  |  |  |  |  |
| 1 | Atr-ERN06317 |  | | | |  |  |  |  |  |
| 1 | Atr-ERN06318 |  | | | |  |  |  |  |  |
| 2 | Atr-ERN06319 |  | | | |  | Vvi-Vitvi01g01790\_t001 |  |  |  |  |
| 2 | Atr-ERN06320 |  | | | |  | | | |  |  |  |  |
| 2 | Atr-ERN06321 |  | | | |  | Vvi-Vitvi01g01791\_t001 |  |  |  |  |
| 2 | Atr-ERN06322 |  | Vvi-Vitvi14g01450\_t001 |  | | | |  |  |  |  |
| 2 | Atr-ERN06323 |  | | | |  | | | |  |  |  |  |
| 2 | Atr-ERN06324 |  | | | |  | | | |  |  |  |  |
| 2 | Atr-ERN06325 |  | | | |  | | | |  |  |  |  |
| 2 | Atr-ERN06326 |  | | | |  | | | |  |  |  |  |
| 2 | Atr-ERN06327 |  | | | |  | | | |  |  |  |  |
| 2 | Atr-ERN06328 |  | | | |  | | | |  |  |  |  |
| 2 | Atr-ERN06329 |  | Vvi-Vitvi14g01445\_t001 |  | | | |  |  |  |  |
| 2 | Atr-ERN06330 |  | | | |  | | | |  |  |  |  |
| 2 | Atr-ERN06331 |  | | | |  | | | |  |  |  |  |
| 2 | Atr-ERN06332 |  | | | |  | | | |  |  |  |  |
| 2 | Atr-ERN06333 |  | Vvi-Vitvi14g01442\_t001 |  | Vvi-Vitvi01g01793\_t002 |  |  |  |  |
| 2 | Atr-ERN06334 |  | Vvi-Vitvi14g01441\_t001 |  | | | |  |  |  |  |
| 2 | Atr-ERN06335 |  | Vvi-Vitvi14g01440\_t001 |  | | | |  |  |  |  |
| 2 | Atr-ERN06336 |  | Vvi-Vitvi14g01439\_t001 |  | | | |  |  |  |  |
| 2 | Atr-ERN06337 |  | | | |  | | | |  |  |  |  |
| 2 | Atr-ERN06338 |  | | | |  | | | |  |  |  |  |
| 2 | Atr-ERN06339 |  | | | |  | Vvi-Vitvi01g01794\_t001 |  |  |  |  |
| 2 | Atr-ERN06340 |  | Vvi-Vitvi14g04536\_t001 |  | | | |  |  |  |  |
| 2 | Atr-ERN06341 |  | | | |  | | | |  |  |  |  |
| 2 | Atr-ERN06342 |  | | | |  | | | |  |  |  |  |
| 2 | Atr-ERN06343 |  | | | |  | | | |  |  |  |  |
| 2 | Atr-ERN06344 |  | | | |  | | | |  |  |  |  |
| 2 | Atr-ERN06345 |  | Vvi-Vitvi14g01436\_t001 |  | | | |  |  |  |  |
| 2 | Atr-ERN06346 |  | Vvi-Vitvi14g01435\_t001 |  | | | |  |  |  |  |
| 2 | Atr-ERN06347 |  | | | |  | | | |  |  |  |  |
| 2 | Atr-ERN06348 |  | | | |  | | | |  |  |  |  |
| 2 | Atr-ERN06349 |  | | | |  | | | |  |  |  |  |
| 2 | Atr-ERN06350 |  | | | |  | | | |  |  |  |  |
| 2 | Atr-ERN06351 |  | | | |  | | | |  |  |  |  |
| 2 | Atr-ERN06352 |  | | | |  | | | |  |  |  |  |
| 2 | Atr-ERN06353 |  | | | |  | | | |  |  |  |  |
| 2 | Atr-ERN06354 |  | | | |  | | | |  |  |  |  |
| 2 | Atr-ERN06355 |  | | | |  | | | |  |  |  |  |
| 2 | Atr-ERN06356 |  | | | |  | | | |  |  |  |  |
| 2 | Atr-ERN06357 |  | Vvi-Vitvi14g01433\_t001 |  | | | |  |  |  |  |
| 2 | Atr-ERN06358 |  | | | |  | | | |  |  |  |  |
| 2 | Atr-ERN06359 |  | | | |  | Vvi-Vitvi01g01796\_t001 |  |  |  |  |
| 2 | Atr-ERN06360 |  | | | |  | | | |  |  |  |  |
| 2 | Atr-ERN06361 |  | | | |  | | | |  |  |  |  |
| 2 | Atr-ERN06362 |  | | | |  | | | |  |  |  |  |
| 2 | Atr-ERN06363 |  | | | |  | | | |  |  |  |  |
| 2 | Atr-ERN06364 |  | | | |  | | | |  |  |  |  |
| 2 | Atr-ERN06365 |  | | | |  | | | |  |  |  |  |
| 2 | Atr-ERN06366 |  | | | |  | Vvi-Vitvi01g01800\_t001 |  |  |  |  |
| 2 | Atr-ERN06367 |  | | | |  | Vvi-Vitvi01g01801\_t001 |  |  |  |  |
| 2 | Atr-ERN06368 |  | Vvi-Vitvi14g01428\_t001 |  | | | |  |  |  |  |
| 2 | Atr-ERN06369 |  | | | |  | | | |  |  |  |  |
| 2 | Atr-ERN06370 |  | | | |  | | | |  |  |  |  |
| 2 | Atr-ERN06371 |  | | | |  | | | |  |  |  |  |
| 2 | Atr-ERN06372 |  | | | |  | | | |  |  |  |  |
| 2 | Atr-ERN06373 |  | | | |  | | | |  |  |  |  |
| 2 | Atr-ERN06374 |  | | | |  | | | |  |  |  |  |
| 2 | Atr-ERN06375 |  | | | |  | | | |  |  |  |  |
| 2 | Atr-ERN06376 |  | | | |  | | | |  |  |  |  |
| 2 | Atr-ERN06377 |  | | | |  | | | |  |  |  |  |
| 2 | Atr-ERN06378 |  | | | |  | | | |  |  |  |  |
| 2 | Atr-ERN06379 |  | | | |  | | | |  |  |  |  |
| 2 | Atr-ERN06380 |  | | | |  | | | |  |  |  |  |
| 2 | Atr-ERN06381 |  | | | |  | Vvi-Vitvi01g01802\_t001 |  |  |  |  |
| 2 | Atr-ERN06382 |  | | | |  | | | |  |  |  |  |
| 2 | Atr-ERN06383 |  | | | |  | | | |  |  |  |  |
| 2 | Atr-ERN06384 |  | | | |  | | | |  |  |  |  |
| 2 | Atr-ERN06385 |  | | | |  | Vvi-Vitvi01g01805\_t001 |  |  |  |  |
| 2 | Atr-ERN06386 |  | Vvi-Vitvi14g01425\_t001 |  | | | |  |  |  |  |
| 2 | Atr-ERN06387 |  | | | |  | | | |  |  |  |  |
| 2 | Atr-ERN06388 |  | | | |  | Vvi-Vitvi01g01806\_t001 |  |  |  |  |
| 2 | Atr-ERN06389 |  | Vvi-Vitvi14g01424\_t001 |  | Vvi-Vitvi01g01807\_t001 |  |  |  |  |
| 2 | Atr-ERN06390 |  | Vvi-Vitvi14g01423\_t001 |  | | | |  |  |  |  |
| 2 | Atr-ERN06391 |  | | | |  | | | |  |  |  |  |
| 2 | Atr-ERN06392 |  | | | |  | | | |  |  |  |  |
| 2 | Atr-ERN06393 |  | Vvi-Vitvi14g01422\_t001 |  | | | |  |  |  |  |
| 2 | Atr-ERN06394 |  | | | |  | | | |  |  |  |  |
| 2 | Atr-ERN06395 |  | | | |  | Vvi-Vitvi01g01808\_t001 |  |  |  |  |
| 2 | Atr-ERN06396 |  | | | |  | | | |  |  |  |  |
| 2 | Atr-ERN06397 |  | | | |  | Vvi-Vitvi01g01809\_t001 |  |  |  |  |
| 2 | Atr-ERN06398 |  | | | |  | | | |  |  |  |  |
| 2 | Atr-ERN06399 |  | | | |  | | | |  |  |  |  |
| 2 | Atr-ERN06400 |  | | | |  | | | |  |  |  |  |
| 2 | Atr-ERN06401 |  | | | |  | | | |  |  |  |  |
| 2 | Atr-ERN06402 |  | | | |  | | | |  |  |  |  |
| 2 | Atr-ERN06403 |  | | | |  | | | |  |  |  |  |
| 2 | Atr-ERN06404 |  | | | |  | | | |  |  |  |  |
| 2 | Atr-ERN06405 |  | | | |  | | | |  |  |  |  |
| 2 | Atr-ERN06406 |  | Vvi-Vitvi14g01419\_t001 |  | | | |  |  |  |  |
| 2 | Atr-ERN06407 |  | | | |  | | | |  |  |  |  |
| 2 | Atr-ERN06408 |  | | | |  | | | |  |  |  |  |
| 2 | Atr-ERN06409 |  | | | |  | | | |  |  |  |  |
| 2 | Atr-ERN06410 |  | | | |  | | | |  |  |  |  |
| 2 | Atr-ERN06411 |  | | | |  | | | |  |  |  |  |
| 2 | Atr-ERN06412 |  | | | |  | | | |  |  |  |  |
| 2 | Atr-ERN06413 |  | | | |  | | | |  |  |  |  |
| 2 | Atr-ERN06414 |  | | | |  | Vvi-Vitvi01g01810\_t001 |  |  |  |  |
| 2 | Atr-ERN06415 |  | | | |  | | | |  |  |  |  |
| 2 | Atr-ERN06416 |  | | | |  | | | |  |  |  |  |
| 2 | Atr-ERN06417 |  | | | |  | | | |  |  |  |  |
| 2 | Atr-ERN06418 |  | | | |  | Vvi-Vitvi01g01811\_t001 |  |  |  |  |
| 2 | Atr-ERN06419 |  | Vvi-Vitvi14g01418\_t001 |  | | | |  |  |  |  |
| 2 | Atr-ERN06420 |  | | | |  | | | |  |  |  |  |
| 2 | Atr-ERN06421 |  | | | |  | Vvi-Vitvi01g01812\_t001 |  |  |  |  |
| 2 | Atr-ERN06422 |  | | | |  | | | |  |  |  |  |
| 2 | Atr-ERN06423 |  | | | |  | | | |  |  |  |  |
| 2 | Atr-ERN06424 |  | | | |  | | | |  |  |  |  |
| 2 | Atr-ERN06425 |  | | | |  | | | |  |  |  |  |
| 2 | Atr-ERN06426 |  | | | |  | | | |  |  |  |  |
| 2 | Atr-ERN06427 |  | | | |  | | | |  |  |  |  |
| 2 | Atr-ERN06428 |  | | | |  | | | |  |  |  |  |
| 2 | Atr-ERN06429 |  | | | |  | | | |  |  |  |  |
| 2 | Atr-ERN06430 |  | | | |  | | | |  |  |  |  |
| 2 | Atr-ERN06431 |  | | | |  | | | |  |  |  |  |
| 2 | Atr-ERN06432 |  | | | |  | | | |  |  |  |  |
| 2 | Atr-ERN06433 |  | | | |  | | | |  |  |  |  |
| 2 | Atr-ERN06434 |  | | | |  | Vvi-Vitvi01g01813\_t003 |  |  |  |  |
| 2 | Atr-ERN06435 |  | | | |  | | | |  |  |  |  |
| 2 | Atr-ERN06436 |  | Vvi-Vitvi14g01417\_t001 |  | | | |  |  |  |  |
| 1 | Atr-ERN06437 |  |  |  | | | |  |  |  |  |
| 1 | Atr-ERN06438 |  |  |  | | | |  |  |  |  |
| 1 | Atr-ERN06439 |  |  |  | | | |  |  |  |  |
| 1 | Atr-ERN06440 |  |  |  | | | |  |  |  |  |
| 1 | Atr-ERN06441 |  |  |  | | | |  |  |  |  |
| 1 | Atr-ERN06442 |  |  |  | | | |  |  |  |  |
| 1 | Atr-ERN06443 |  |  |  | | | |  |  |  |  |
| 1 | Atr-ERN06444 |  |  |  | | | |  |  |  |  |
| 1 | Atr-ERN06445 |  |  |  | | | |  |  |  |  |
| 1 | Atr-ERN06446 |  |  |  | Vvi-Vitvi01g01815\_t001 |  |  |  |  |
| 1 | Atr-ERN06447 |  |  |  | Vvi-Vitvi01g01816\_t001 |  |  |  |  |
| 1 | Atr-ERN06448 |  |  |  | | | |  |  |  |  |
| 1 | Atr-ERN06449 |  |  |  | | | |  |  |  |  |
| 1 | Atr-ERN06450 |  |  |  | | | |  |  |  |  |
| 1 | Atr-ERN06451 |  |  |  | | | |  |  |  |  |
| 1 | Atr-ERN06452 |  |  |  | | | |  |  |  |  |
| 1 | Atr-ERN06453 |  |  |  | Vvi-Vitvi01g01820\_t001 |  |  |  |  |
| 1 | Atr-ERN06454 |  |  |  | Vvi-Vitvi01g01821\_t001 |  |  |  |  |
| 1 | Atr-ERN06455 |  |  |  | | | |  |  |  |  |
| 1 | Atr-ERN06456 |  |  |  | Vvi-Vitvi01g01822\_t001 |  |  |  |  |
| 1 | Atr-ERN06457 |  |  |  | Vvi-Vitvi01g01824\_t001 |  |  |  |  |
| 0 | Atr-ERN06458 |  |  |  |  |  |  |
| 0 | Atr-ERN06459 |  |  |  |  |  |  |
| 0 | Atr-ERN06460 |  |  |  |  |  |  |
| 0 | Atr-ERN06461 |  |  |  |  |  |  |
| 0 | Atr-ERN06462 |  |  |  |  |  |  |
| 0 | Atr-ERN06463 |  |  |  |  |  |  |
| 0 | Atr-ERN06464 |  |  |  |  |  |  |
| 0 | Atr-ERN06465 |  |  |  |  |  |  |
| 0 | Atr-ERN06466 |  |  |  |  |  |  |
| 0 | Atr-ERN06467 |  |  |  |  |  |  |
| 0 | Atr-ERN06468 |  |  |  |  |  |  |
| 0 | Atr-ERN06469 |  |  |  |  |  |  |
| 0 | Atr-ERN06470 |  |  |  |  |  |  |
