# Supplementary material for: Genome-wide identification, characterization and gene expression of BES1 transcription factor family in grapevine (Vitis vinifera L.)
Source: Sci Rep. 2023 Jan 5;13:240. doi: 10.1038/s41598-022-24407-y (PMC9816167; doi:10.1038/s41598-022-24407-y)
Supplement: Supplementary file 3 — Supplementary Information. [file 41598_2022_24407_MOESM3_ESM.zip › Vvi_Atr/Vitis_vinifera.PN40024.v4.dna_sm.toplevel.fa.vs.Amborella_trichopoda.AMTR1.0.dna_sm.toplevel.fa.html/Atr-AmTr_v1.0_scaffold00017.html]

|  |  |  |  |  |  |  |  |  |  |  |  |  |  |
| --- | --- | --- | --- | --- | --- | --- | --- | --- | --- | --- | --- | --- | --- |
| Duplication depth | Reference chromosome | Collinear blocks | | | | | | | | | | | |
| 0 | Atr-ERN08523 |  |  |  |  |  |  |
| 0 | Atr-ERN08524 |  |  |  |  |  |  |
| 0 | Atr-ERN08525 |  |  |  |  |  |  |
| 0 | Atr-ERN08526 |  |  |  |  |  |  |
| 0 | Atr-ERN08527 |  |  |  |  |  |  |
| 0 | Atr-ERN08528 |  |  |  |  |  |  |
| 0 | Atr-ERN08529 |  |  |  |  |  |  |
| 0 | Atr-ERN08530 |  |  |  |  |  |  |
| 0 | Atr-ERN08531 |  |  |  |  |  |  |
| 0 | Atr-ERN08532 |  |  |  |  |  |  |
| 0 | Atr-ERN08533 |  |  |  |  |  |  |
| 0 | Atr-ERN08534 |  |  |  |  |  |  |
| 0 | Atr-ERN08535 |  |  |  |  |  |  |
| 0 | Atr-ERN08536 |  |  |  |  |  |  |
| 0 | Atr-ERN08537 |  |  |  |  |  |  |
| 0 | Atr-ERN08538 |  |  |  |  |  |  |
| 0 | Atr-ERN08539 |  |  |  |  |  |  |
| 0 | Atr-ERN08540 |  |  |  |  |  |  |
| 1 | Atr-ERN08541 |  | Vvi-Vitvi18g01719\_t001 |  |  |  |  |  |
| 1 | Atr-ERN08542 |  | Vvi-Vitvi18g03004\_t001 |  |  |  |  |  |
| 1 | Atr-ERN08543 |  | | | |  |  |  |  |  |
| 1 | Atr-ERN08544 |  | | | |  |  |  |  |  |
| 1 | Atr-ERN08545 |  | | | |  |  |  |  |  |
| 1 | Atr-ERN08546 |  | Vvi-Vitvi18g01701\_t001 |  |  |  |  |  |
| 1 | Atr-ERN08547 |  | | | |  |  |  |  |  |
| 1 | Atr-ERN08548 |  | | | |  |  |  |  |  |
| 1 | Atr-ERN08549 |  | | | |  |  |  |  |  |
| 1 | Atr-ERN08550 |  | | | |  |  |  |  |  |
| 1 | Atr-ERN08551 |  | | | |  |  |  |  |  |
| 1 | Atr-ERN08552 |  | | | |  |  |  |  |  |
| 1 | Atr-ERN08553 |  | | | |  |  |  |  |  |
| 1 | Atr-ERN08554 |  | | | |  |  |  |  |  |
| 1 | Atr-ERN08555 |  | | | |  |  |  |  |  |
| 1 | Atr-ERN08556 |  | | | |  |  |  |  |  |
| 1 | Atr-ERN08557 |  | | | |  |  |  |  |  |
| 1 | Atr-ERN08558 |  | | | |  |  |  |  |  |
| 1 | Atr-ERN08559 |  | | | |  |  |  |  |  |
| 1 | Atr-ERN08560 |  | | | |  |  |  |  |  |
| 1 | Atr-ERN08561 |  | | | |  |  |  |  |  |
| 1 | Atr-ERN08562 |  | | | |  |  |  |  |  |
| 1 | Atr-ERN08563 |  | | | |  |  |  |  |  |
| 1 | Atr-ERN08564 |  | | | |  |  |  |  |  |
| 1 | Atr-ERN08565 |  | | | |  |  |  |  |  |
| 1 | Atr-ERN08566 |  | | | |  |  |  |  |  |
| 1 | Atr-ERN08567 |  | Vvi-Vitvi18g01684\_t001 |  |  |  |  |  |
| 1 | Atr-ERN08568 |  | | | |  |  |  |  |  |
| 1 | Atr-ERN08569 |  | | | |  |  |  |  |  |
| 1 | Atr-ERN08570 |  | | | |  |  |  |  |  |
| 1 | Atr-ERN08571 |  | | | |  |  |  |  |  |
| 1 | Atr-ERN08572 |  | Vvi-Vitvi18g01683\_t001 |  |  |  |  |  |
| 1 | Atr-ERN08573 |  | | | |  |  |  |  |  |
| 1 | Atr-ERN08574 |  | Vvi-Vitvi18g01681\_t001 |  |  |  |  |  |
| 0 | Atr-ERN08575 |  |  |  |  |  |  |
| 0 | Atr-ERN08576 |  |  |  |  |  |  |
| 0 | Atr-ERN08577 |  |  |  |  |  |  |
| 0 | Atr-ERN08578 |  |  |  |  |  |  |
| 0 | Atr-ERN08579 |  |  |  |  |  |  |
| 0 | Atr-ERN08580 |  |  |  |  |  |  |
| 0 | Atr-ERN08581 |  |  |  |  |  |  |
| 0 | Atr-ERN08582 |  |  |  |  |  |  |
| 0 | Atr-ERN08583 |  |  |  |  |  |  |
| 0 | Atr-ERN08584 |  |  |  |  |  |  |
| 0 | Atr-ERN08585 |  |  |  |  |  |  |
| 0 | Atr-ERN08586 |  |  |  |  |  |  |
| 0 | Atr-ERN08587 |  |  |  |  |  |  |
| 0 | Atr-ERN08588 |  |  |  |  |  |  |
| 0 | Atr-ERN08589 |  |  |  |  |  |  |
| 0 | Atr-ERN08590 |  |  |  |  |  |  |
| 0 | Atr-ERN08591 |  |  |  |  |  |  |
| 0 | Atr-ERN08592 |  |  |  |  |  |  |
| 1 | Atr-ERN08593 |  | Vvi-Vitvi18g00272\_t001 |  |  |  |  |  |
| 1 | Atr-ERN08594 |  | | | |  |  |  |  |  |
| 1 | Atr-ERN08595 |  | | | |  |  |  |  |  |
| 1 | Atr-ERN08596 |  | Vvi-Vitvi18g00271\_t001 |  |  |  |  |  |
| 1 | Atr-ERN08597 |  | Vvi-Vitvi18g00270\_t001 |  |  |  |  |  |
| 1 | Atr-ERN08598 |  | Vvi-Vitvi18g00269\_t001 |  |  |  |  |  |
| 1 | Atr-ERN08599 |  | Vvi-Vitvi18g00268\_t001 |  |  |  |  |  |
| 1 | Atr-ERN08600 |  | | | |  |  |  |  |  |
| 1 | Atr-ERN08601 |  | | | |  |  |  |  |  |
| 1 | Atr-ERN08602 |  | | | |  |  |  |  |  |
| 1 | Atr-ERN08603 |  | | | |  |  |  |  |  |
| 1 | Atr-ERN08604 |  | | | |  |  |  |  |  |
| 1 | Atr-ERN08605 |  | | | |  |  |  |  |  |
| 1 | Atr-ERN08606 |  | Vvi-Vitvi18g00267\_t002 |  |  |  |  |  |
| 1 | Atr-ERN08607 |  | | | |  |  |  |  |  |
| 1 | Atr-ERN08608 |  | Vvi-Vitvi18g04041\_t001 |  |  |  |  |  |
| 1 | Atr-ERN08609 |  | Vvi-Vitvi18g00265\_t001 |  |  |  |  |  |
| 1 | Atr-ERN08610 |  | | | |  |  |  |  |  |
| 1 | Atr-ERN08611 |  | | | |  |  |  |  |  |
| 1 | Atr-ERN08612 |  | | | |  |  |  |  |  |
| 1 | Atr-ERN08613 |  | | | |  |  |  |  |  |
| 1 | Atr-ERN08614 |  | | | |  |  |  |  |  |
| 1 | Atr-ERN08615 |  | | | |  |  |  |  |  |
| 1 | Atr-ERN08616 |  | | | |  |  |  |  |  |
| 1 | Atr-ERN08617 |  | | | |  |  |  |  |  |
| 1 | Atr-ERN08618 |  | | | |  |  |  |  |  |
| 1 | Atr-ERN08619 |  | | | |  |  |  |  |  |
| 1 | Atr-ERN08620 |  | Vvi-Vitvi18g00264\_t001 |  |  |  |  |  |
| 0 | Atr-ERN08621 |  |  |  |  |  |  |
| 0 | Atr-ERN08622 |  |  |  |  |  |  |
| 0 | Atr-ERN08623 |  |  |  |  |  |  |
| 0 | Atr-ERN08624 |  |  |  |  |  |  |
| 0 | Atr-ERN08625 |  |  |  |  |  |  |
| 0 | Atr-ERN08626 |  |  |  |  |  |  |
| 0 | Atr-ERN08627 |  |  |  |  |  |  |
| 0 | Atr-ERN08628 |  |  |  |  |  |  |
| 0 | Atr-ERN08629 |  |  |  |  |  |  |
| 0 | Atr-ERN08630 |  |  |  |  |  |  |
| 0 | Atr-ERN08631 |  |  |  |  |  |  |
| 0 | Atr-ERN08632 |  |  |  |  |  |  |
| 0 | Atr-ERN08633 |  |  |  |  |  |  |
| 0 | Atr-ERN08634 |  |  |  |  |  |  |
| 0 | Atr-ERN08635 |  |  |  |  |  |  |
| 0 | Atr-ERN08636 |  |  |  |  |  |  |
| 0 | Atr-ERN08637 |  |  |  |  |  |  |
| 0 | Atr-ERN08638 |  |  |  |  |  |  |
| 1 | Atr-ERN08639 |  | Vvi-Vitvi18g00316\_t001 |  |  |  |  |  |
| 1 | Atr-ERN08640 |  | | | |  |  |  |  |  |
| 1 | Atr-ERN08641 |  | Vvi-Vitvi18g00315\_t001 |  |  |  |  |  |
| 1 | Atr-ERN08642 |  | Vvi-Vitvi18g00313\_t002 |  |  |  |  |  |
| 1 | Atr-ERN08643 |  | Vvi-Vitvi18g00311\_t001 |  |  |  |  |  |
| 1 | Atr-ERN08644 |  | | | |  |  |  |  |  |
| 1 | Atr-ERN08645 |  | | | |  |  |  |  |  |
| 1 | Atr-ERN08646 |  | Vvi-Vitvi18g00310\_t001 |  |  |  |  |  |
| 1 | Atr-ERN08647 |  | | | |  |  |  |  |  |
| 1 | Atr-ERN08648 |  | Vvi-Vitvi18g00309\_t001 |  |  |  |  |  |
| 0 | Atr-ERN08649 |  |  |  |  |  |  |
| 0 | Atr-ERN08650 |  |  |  |  |  |  |
| 0 | Atr-ERN08651 |  |  |  |  |  |  |
| 0 | Atr-ERN08652 |  |  |  |  |  |  |
| 0 | Atr-ERN08653 |  |  |  |  |  |  |
| 0 | Atr-ERN08654 |  |  |  |  |  |  |
| 0 | Atr-ERN08655 |  |  |  |  |  |  |
| 1 | Atr-ERN08656 |  | Vvi-Vitvi03g00514\_t001 |  |  |  |  |  |
| 1 | Atr-ERN08657 |  | | | |  |  |  |  |  |
| 1 | Atr-ERN08658 |  | | | |  |  |  |  |  |
| 2 | Atr-ERN08659 |  | | | |  | Vvi-Vitvi07g01739\_t001 |  |  |  |  |
| 2 | Atr-ERN08660 |  | | | |  | | | |  |  |  |  |
| 2 | Atr-ERN08661 |  | | | |  | | | |  |  |  |  |
| 2 | Atr-ERN08662 |  | | | |  | | | |  |  |  |  |
| 2 | Atr-ERN08663 |  | | | |  | | | |  |  |  |  |
| 2 | Atr-ERN08664 |  | | | |  | | | |  |  |  |  |
| 2 | Atr-ERN08665 |  | | | |  | | | |  |  |  |  |
| 2 | Atr-ERN08666 |  | | | |  | | | |  |  |  |  |
| 2 | Atr-ERN08667 |  | | | |  | | | |  |  |  |  |
| 2 | Atr-ERN08668 |  | | | |  | | | |  |  |  |  |
| 2 | Atr-ERN08669 |  | | | |  | | | |  |  |  |  |
| 2 | Atr-ERN08670 |  | | | |  | Vvi-Vitvi07g01736\_t001 |  |  |  |  |
| 2 | Atr-ERN08671 |  | | | |  | | | |  |  |  |  |
| 2 | Atr-ERN08672 |  | | | |  | | | |  |  |  |  |
| 2 | Atr-ERN08673 |  | | | |  | | | |  |  |  |  |
| 2 | Atr-ERN08674 |  | | | |  | | | |  |  |  |  |
| 2 | Atr-ERN08675 |  | | | |  | | | |  |  |  |  |
| 2 | Atr-ERN08676 |  | | | |  | | | |  |  |  |  |
| 2 | Atr-ERN08677 |  | | | |  | | | |  |  |  |  |
| 2 | Atr-ERN08678 |  | Vvi-Vitvi03g01575\_t001 |  | | | |  |  |  |  |
| 2 | Atr-ERN08679 |  | | | |  | | | |  |  |  |  |
| 2 | Atr-ERN08680 |  | | | |  | | | |  |  |  |  |
| 2 | Atr-ERN08681 |  | | | |  | | | |  |  |  |  |
| 2 | Atr-ERN08682 |  | | | |  | | | |  |  |  |  |
| 2 | Atr-ERN08683 |  | | | |  | | | |  |  |  |  |
| 2 | Atr-ERN08684 |  | | | |  | | | |  |  |  |  |
| 2 | Atr-ERN08685 |  | | | |  | Vvi-Vitvi07g01734\_t001 |  |  |  |  |
| 2 | Atr-ERN08686 |  | | | |  | | | |  |  |  |  |
| 2 | Atr-ERN08687 |  | | | |  | | | |  |  |  |  |
| 2 | Atr-ERN08688 |  | | | |  | | | |  |  |  |  |
| 2 | Atr-ERN08689 |  | Vvi-Vitvi03g00516\_t002 |  | | | |  |  |  |  |
| 2 | Atr-ERN08690 |  | | | |  | | | |  |  |  |  |
| 2 | Atr-ERN08691 |  | Vvi-Vitvi03g00517\_t001 |  | Vvi-Vitvi07g01733\_t001 |  |  |  |  |
| 2 | Atr-ERN08692 |  | | | |  | | | |  |  |  |  |
| 2 | Atr-ERN08693 |  | | | |  | Vvi-Vitvi07g01731\_t001 |  |  |  |  |
| 2 | Atr-ERN08694 |  | | | |  | Vvi-Vitvi07g01730\_t002 |  |  |  |  |
| 1 | Atr-ERN08695 |  | Vvi-Vitvi03g00519\_t001 |  |  |  |  |  |
| 1 | Atr-ERN08696 |  | | | |  |  |  |  |  |
| 1 | Atr-ERN08697 |  | | | |  |  |  |  |  |
| 1 | Atr-ERN08698 |  | Vvi-Vitvi03g00520\_t001 |  |  |  |  |  |
| 1 | Atr-ERN08699 |  | Vvi-Vitvi07g01865\_t001 |  |  |  |  |  |
| 1 | Atr-ERN08700 |  | | | |  |  |  |  |  |
| 2 | Atr-ERN08701 |  | | | |  | Vvi-Vitvi18g01610\_t001 |  |  |  |  |
| 2 | Atr-ERN08702 |  | | | |  | | | |  |  |  |  |
| 2 | Atr-ERN08703 |  | | | |  | | | |  |  |  |  |
| 2 | Atr-ERN08704 |  | | | |  | | | |  |  |  |  |
| 2 | Atr-ERN08705 |  | Vvi-Vitvi07g01871\_t001 |  | Vvi-Vitvi18g01614\_t001 |  |  |  |  |
| 2 | Atr-ERN08706 |  | | | |  | | | |  |  |  |  |
| 2 | Atr-ERN08707 |  | | | |  | | | |  |  |  |  |
| 2 | Atr-ERN08708 |  | | | |  | | | |  |  |  |  |
| 2 | Atr-ERN08709 |  | | | |  | | | |  |  |  |  |
| 2 | Atr-ERN08710 |  | Vvi-Vitvi07g01875\_t001 |  | | | |  |  |  |  |
| 2 | Atr-ERN08711 |  | Vvi-Vitvi07g01876\_t001 |  | | | |  |  |  |  |
| 2 | Atr-ERN08712 |  | | | |  | | | |  |  |  |  |
| 2 | Atr-ERN08713 |  | Vvi-Vitvi07g01877\_t001 |  | | | |  |  |  |  |
| 2 | Atr-ERN08714 |  | | | |  | | | |  |  |  |  |
| 2 | Atr-ERN08715 |  | | | |  | | | |  |  |  |  |
| 2 | Atr-ERN08716 |  | | | |  | | | |  |  |  |  |
| 2 | Atr-ERN08717 |  | | | |  | Vvi-Vitvi18g01620\_t001 |  |  |  |  |
| 3 | Atr-ERN08718 |  | Vvi-Vitvi07g01879\_t001 |  | Vvi-Vitvi18g01624\_t001 |  | Vvi-Vitvi18g00184\_t001 |  |  |  |
| 3 | Atr-ERN08719 |  | | | |  | | | |  | | | |  |  |  |
| 3 | Atr-ERN08720 |  | | | |  | | | |  | | | |  |  |  |
| 3 | Atr-ERN08721 |  | | | |  | | | |  | | | |  |  |  |
| 3 | Atr-ERN08722 |  | | | |  | | | |  | | | |  |  |  |
| 3 | Atr-ERN08723 |  | | | |  | | | |  | | | |  |  |  |
| 3 | Atr-ERN08724 |  | | | |  | | | |  | | | |  |  |  |
| 3 | Atr-ERN08725 |  | | | |  | | | |  | | | |  |  |  |
| 3 | Atr-ERN08726 |  | | | |  | | | |  | | | |  |  |  |
| 3 | Atr-ERN08727 |  | | | |  | | | |  | | | |  |  |  |
| 3 | Atr-ERN08728 |  | | | |  | | | |  | | | |  |  |  |
| 3 | Atr-ERN08729 |  | | | |  | | | |  | | | |  |  |  |
| 3 | Atr-ERN08730 |  | | | |  | | | |  | | | |  |  |  |
| 3 | Atr-ERN08731 |  | | | |  | | | |  | | | |  |  |  |
| 3 | Atr-ERN08732 |  | | | |  | | | |  | | | |  |  |  |
| 3 | Atr-ERN08733 |  | Vvi-Vitvi07g01881\_t001 |  | Vvi-Vitvi18g01628\_t001 |  | | | |  |  |  |
| 3 | Atr-ERN08734 |  | Vvi-Vitvi07g01882\_t001 |  | Vvi-Vitvi18g02972\_t001 |  | | | |  |  |  |
| 1 | Atr-ERN08735 |  |  |  |  |  | Vvi-Vitvi18g00196\_t003 |  |  |  |
| 1 | Atr-ERN08736 |  |  |  |  |  | | | |  |  |  |
| 1 | Atr-ERN08737 |  |  |  |  |  | | | |  |  |  |
| 1 | Atr-ERN08738 |  |  |  |  |  | | | |  |  |  |
| 1 | Atr-ERN08739 |  |  |  |  |  | | | |  |  |  |
| 1 | Atr-ERN08740 |  |  |  |  |  | Vvi-Vitvi18g00209\_t001 |  |  |  |
| 1 | Atr-ERN08741 |  |  |  |  |  | | | |  |  |  |
| 1 | Atr-ERN08742 |  |  |  |  |  | | | |  |  |  |
| 1 | Atr-ERN08743 |  |  |  |  |  | | | |  |  |  |
| 1 | Atr-ERN08744 |  |  |  |  |  | | | |  |  |  |
| 1 | Atr-ERN08745 |  |  |  |  |  | | | |  |  |  |
| 1 | Atr-ERN08746 |  |  |  |  |  | Vvi-Vitvi18g00219\_t001 |  |  |  |
| 1 | Atr-ERN08747 |  |  |  |  |  | Vvi-Vitvi18g00220\_t001 |  |  |  |
| 1 | Atr-ERN08748 |  |  |  |  |  | | | |  |  |  |
| 1 | Atr-ERN08749 |  |  |  |  |  | | | |  |  |  |
| 1 | Atr-ERN08750 |  |  |  |  |  | | | |  |  |  |
| 1 | Atr-ERN08751 |  |  |  |  |  | | | |  |  |  |
| 1 | Atr-ERN08752 |  |  |  |  |  | | | |  |  |  |
| 1 | Atr-ERN08753 |  |  |  |  |  | | | |  |  |  |
| 1 | Atr-ERN08754 |  |  |  |  |  | Vvi-Vitvi18g00223\_t004 |  |  |  |
| 1 | Atr-ERN08755 |  |  |  |  |  | | | |  |  |  |
| 1 | Atr-ERN08756 |  |  |  |  |  | Vvi-Vitvi18g00228\_t001 |  |  |  |
| 1 | Atr-ERN08757 |  |  |  |  |  | Vvi-Vitvi18g00230\_t001.1.6037826c |  |  |  |
| 1 | Atr-ERN08758 |  |  |  |  |  | | | |  |  |  |
| 1 | Atr-ERN08759 |  |  |  |  |  | | | |  |  |  |
| 1 | Atr-ERN08760 |  |  |  |  |  | | | |  |  |  |
| 1 | Atr-ERN08761 |  |  |  |  |  | | | |  |  |  |
| 1 | Atr-ERN08762 |  |  |  |  |  | | | |  |  |  |
| 1 | Atr-ERN08763 |  |  |  |  |  | | | |  |  |  |
| 1 | Atr-ERN08764 |  |  |  |  |  | | | |  |  |  |
| 1 | Atr-ERN08765 |  |  |  |  |  | | | |  |  |  |
| 1 | Atr-ERN08766 |  |  |  |  |  | | | |  |  |  |
| 1 | Atr-ERN08767 |  |  |  |  |  | | | |  |  |  |
| 1 | Atr-ERN08768 |  |  |  |  |  | | | |  |  |  |
| 1 | Atr-ERN08769 |  |  |  |  |  | | | |  |  |  |
| 1 | Atr-ERN08770 |  |  |  |  |  | | | |  |  |  |
| 1 | Atr-ERN08771 |  |  |  |  |  | | | |  |  |  |
| 1 | Atr-ERN08772 |  |  |  |  |  | | | |  |  |  |
| 1 | Atr-ERN08773 |  |  |  |  |  | | | |  |  |  |
| 1 | Atr-ERN08774 |  |  |  |  |  | | | |  |  |  |
| 1 | Atr-ERN08775 |  |  |  |  |  | | | |  |  |  |
| 1 | Atr-ERN08776 |  |  |  |  |  | Vvi-Vitvi18g02526\_t001 |  |  |  |
| 1 | Atr-ERN08777 |  |  |  |  |  | | | |  |  |  |
| 1 | Atr-ERN08778 |  |  |  |  |  | | | |  |  |  |
| 1 | Atr-ERN08779 |  |  |  |  |  | | | |  |  |  |
| 1 | Atr-ERN08780 |  |  |  |  |  | | | |  |  |  |
| 1 | Atr-ERN08781 |  |  |  |  |  | | | |  |  |  |
| 1 | Atr-ERN08782 |  |  |  |  |  | | | |  |  |  |
| 1 | Atr-ERN08783 |  |  |  |  |  | | | |  |  |  |
| 1 | Atr-ERN08784 |  |  |  |  |  | | | |  |  |  |
| 1 | Atr-ERN08785 |  |  |  |  |  | | | |  |  |  |
| 1 | Atr-ERN08786 |  |  |  |  |  | | | |  |  |  |
| 1 | Atr-ERN08787 |  |  |  |  |  | | | |  |  |  |
| 1 | Atr-ERN08788 |  |  |  |  |  | | | |  |  |  |
| 1 | Atr-ERN08789 |  |  |  |  |  | | | |  |  |  |
| 1 | Atr-ERN08790 |  |  |  |  |  | | | |  |  |  |
| 1 | Atr-ERN08791 |  |  |  |  |  | Vvi-Vitvi18g00251\_t001 |  |  |  |
| 0 | Atr-ERN08792 |  |  |  |  |  |  |
| 0 | Atr-ERN08793 |  |  |  |  |  |  |
| 0 | Atr-ERN08794 |  |  |  |  |  |  |
| 0 | Atr-ERN08795 |  |  |  |  |  |  |
| 0 | Atr-ERN08796 |  |  |  |  |  |  |
| 1 | Atr-ERN08797 |  | Vvi-Vitvi04g01720\_t001 |  |  |  |  |  |
| 1 | Atr-ERN08798 |  | | | |  |  |  |  |  |
| 1 | Atr-ERN08799 |  | Vvi-Vitvi04g01714\_t001 |  |  |  |  |  |
| 1 | Atr-ERN08800 |  | | | |  |  |  |  |  |
| 1 | Atr-ERN08801 |  | | | |  |  |  |  |  |
| 2 | Atr-ERN08802 |  | Vvi-Vitvi04g01711\_t001 |  | Vvi-Vitvi18g01174\_t001 |  |  |  |  |
| 2 | Atr-ERN08803 |  | Vvi-Vitvi04g01709\_t002 |  | | | |  |  |  |  |
| 2 | Atr-ERN08804 |  | | | |  | Vvi-Vitvi18g01177\_t001 |  |  |  |  |
| 2 | Atr-ERN08805 |  | | | |  | Vvi-Vitvi18g01180\_t001 |  |  |  |  |
| 2 | Atr-ERN08806 |  | | | |  | | | |  |  |  |  |
| 2 | Atr-ERN08807 |  | Vvi-Vitvi04g01706\_t001 |  | | | |  |  |  |  |
| 2 | Atr-ERN08808 |  | | | |  | Vvi-Vitvi18g01182\_t001 |  |  |  |  |
| 2 | Atr-ERN08809 |  | | | |  | | | |  |  |  |  |
| 2 | Atr-ERN08810 |  | | | |  | | | |  |  |  |  |
| 2 | Atr-ERN08811 |  | | | |  | | | |  |  |  |  |
| 2 | Atr-ERN08812 |  | | | |  | | | |  |  |  |  |
| 2 | Atr-ERN08813 |  | | | |  | Vvi-Vitvi18g01183\_t002 |  |  |  |  |
| 2 | Atr-ERN08814 |  | Vvi-Vitvi04g01705\_t001 |  | | | |  |  |  |  |
| 1 | Atr-ERN08815 |  |  |  | Vvi-Vitvi18g01187\_t001 |  |  |  |  |
| 1 | Atr-ERN08816 |  |  |  | Vvi-Vitvi18g01188\_t001 |  |  |  |  |
