# Supplementary material for: Genome-wide identification, characterization and gene expression of BES1 transcription factor family in grapevine (Vitis vinifera L.)
Source: Sci Rep. 2023 Jan 5;13:240. doi: 10.1038/s41598-022-24407-y (PMC9816167; doi:10.1038/s41598-022-24407-y)
Supplement: Supplementary file 3 — Supplementary Information. [file 41598_2022_24407_MOESM3_ESM.zip › Vvi_Atr/Vitis_vinifera.PN40024.v4.dna_sm.toplevel.fa.vs.Amborella_trichopoda.AMTR1.0.dna_sm.toplevel.fa.html/Atr-AmTr_v1.0_scaffold00018.html]

|  |  |  |  |  |  |  |  |  |  |  |  |  |  |
| --- | --- | --- | --- | --- | --- | --- | --- | --- | --- | --- | --- | --- | --- |
| Duplication depth | Reference chromosome | Collinear blocks | | | | | | | | | | | |
| 0 | Atr-ERN08095 |  |  |  |  |  |  |
| 0 | Atr-ERN08096 |  |  |  |  |  |  |
| 0 | Atr-ERN08097 |  |  |  |  |  |  |
| 1 | Atr-ERN08098 |  | Vvi-Vitvi16g01333\_t001 |  |  |  |  |  |
| 1 | Atr-ERN08099 |  | Vvi-Vitvi16g01331\_t001 |  |  |  |  |  |
| 1 | Atr-ERN08100 |  | | | |  |  |  |  |  |
| 1 | Atr-ERN08101 |  | | | |  |  |  |  |  |
| 1 | Atr-ERN08102 |  | | | |  |  |  |  |  |
| 1 | Atr-ERN08103 |  | | | |  |  |  |  |  |
| 1 | Atr-ERN08104 |  | | | |  |  |  |  |  |
| 1 | Atr-ERN08105 |  | Vvi-Vitvi16g01330\_t002 |  |  |  |  |  |
| 1 | Atr-ERN08106 |  | | | |  |  |  |  |  |
| 1 | Atr-ERN08107 |  | | | |  |  |  |  |  |
| 1 | Atr-ERN08108 |  | | | |  |  |  |  |  |
| 1 | Atr-ERN08109 |  | | | |  |  |  |  |  |
| 1 | Atr-ERN08110 |  | | | |  |  |  |  |  |
| 1 | Atr-ERN08111 |  | | | |  |  |  |  |  |
| 1 | Atr-ERN08112 |  | Vvi-Vitvi16g01328\_t001 |  |  |  |  |  |
| 1 | Atr-ERN08113 |  | | | |  |  |  |  |  |
| 1 | Atr-ERN08114 |  | | | |  |  |  |  |  |
| 1 | Atr-ERN08115 |  | Vvi-Vitvi16g02059\_t001 |  |  |  |  |  |
| 1 | Atr-ERN08116 |  | Vvi-Vitvi16g02057\_t001 |  |  |  |  |  |
| 1 | Atr-ERN08117 |  | | | |  |  |  |  |  |
| 1 | Atr-ERN08118 |  | Vvi-Vitvi16g01327\_t002 |  |  |  |  |  |
| 1 | Atr-ERN08119 |  | | | |  |  |  |  |  |
| 1 | Atr-ERN08120 |  | | | |  |  |  |  |  |
| 1 | Atr-ERN08121 |  | | | |  |  |  |  |  |
| 1 | Atr-ERN08122 |  | | | |  |  |  |  |  |
| 1 | Atr-ERN08123 |  | | | |  |  |  |  |  |
| 1 | Atr-ERN08124 |  | | | |  |  |  |  |  |
| 1 | Atr-ERN08125 |  | | | |  |  |  |  |  |
| 1 | Atr-ERN08126 |  | | | |  |  |  |  |  |
| 1 | Atr-ERN08127 |  | | | |  |  |  |  |  |
| 1 | Atr-ERN08128 |  | | | |  |  |  |  |  |
| 1 | Atr-ERN08129 |  | | | |  |  |  |  |  |
| 1 | Atr-ERN08130 |  | | | |  |  |  |  |  |
| 1 | Atr-ERN08131 |  | | | |  |  |  |  |  |
| 1 | Atr-ERN08132 |  | | | |  |  |  |  |  |
| 1 | Atr-ERN08133 |  | | | |  |  |  |  |  |
| 1 | Atr-ERN08134 |  | | | |  |  |  |  |  |
| 1 | Atr-ERN08135 |  | | | |  |  |  |  |  |
| 1 | Atr-ERN08136 |  | | | |  |  |  |  |  |
| 1 | Atr-ERN08137 |  | | | |  |  |  |  |  |
| 1 | Atr-ERN08138 |  | | | |  |  |  |  |  |
| 1 | Atr-ERN08139 |  | | | |  |  |  |  |  |
| 1 | Atr-ERN08140 |  | | | |  |  |  |  |  |
| 1 | Atr-ERN08141 |  | | | |  |  |  |  |  |
| 1 | Atr-ERN08142 |  | | | |  |  |  |  |  |
| 1 | Atr-ERN08143 |  | | | |  |  |  |  |  |
| 1 | Atr-ERN08144 |  | Vvi-Vitvi16g01326\_t001 |  |  |  |  |  |
| 1 | Atr-ERN08145 |  | | | |  |  |  |  |  |
| 1 | Atr-ERN08146 |  | | | |  |  |  |  |  |
| 1 | Atr-ERN08147 |  | | | |  |  |  |  |  |
| 1 | Atr-ERN08148 |  | | | |  |  |  |  |  |
| 1 | Atr-ERN08149 |  | | | |  |  |  |  |  |
| 1 | Atr-ERN08150 |  | | | |  |  |  |  |  |
| 1 | Atr-ERN08151 |  | | | |  |  |  |  |  |
| 1 | Atr-ERN08152 |  | | | |  |  |  |  |  |
| 1 | Atr-ERN08153 |  | | | |  |  |  |  |  |
| 2 | Atr-ERN08154 |  | | | |  | Vvi-Vitvi02g00287\_t001 |  |  |  |  |
| 2 | Atr-ERN08155 |  | | | |  | | | |  |  |  |  |
| 2 | Atr-ERN08156 |  | Vvi-Vitvi16g01324\_t001 |  | | | |  |  |  |  |
| 2 | Atr-ERN08157 |  | | | |  | | | |  |  |  |  |
| 2 | Atr-ERN08158 |  | | | |  | | | |  |  |  |  |
| 2 | Atr-ERN08159 |  | | | |  | | | |  |  |  |  |
| 2 | Atr-ERN08160 |  | | | |  | Vvi-Vitvi02g01373\_t001 |  |  |  |  |
| 2 | Atr-ERN08161 |  | | | |  | | | |  |  |  |  |
| 2 | Atr-ERN08162 |  | | | |  | | | |  |  |  |  |
| 2 | Atr-ERN08163 |  | | | |  | | | |  |  |  |  |
| 2 | Atr-ERN08164 |  | | | |  | | | |  |  |  |  |
| 2 | Atr-ERN08165 |  | | | |  | | | |  |  |  |  |
| 2 | Atr-ERN08166 |  | | | |  | | | |  |  |  |  |
| 2 | Atr-ERN08167 |  | | | |  | | | |  |  |  |  |
| 2 | Atr-ERN08168 |  | | | |  | | | |  |  |  |  |
| 2 | Atr-ERN08169 |  | | | |  | | | |  |  |  |  |
| 2 | Atr-ERN08170 |  | | | |  | | | |  |  |  |  |
| 2 | Atr-ERN08171 |  | | | |  | | | |  |  |  |  |
| 2 | Atr-ERN08172 |  | | | |  | | | |  |  |  |  |
| 2 | Atr-ERN08173 |  | | | |  | | | |  |  |  |  |
| 2 | Atr-ERN08174 |  | | | |  | Vvi-Vitvi02g00283\_t001 |  |  |  |  |
| 2 | Atr-ERN08175 |  | | | |  | | | |  |  |  |  |
| 3 | Atr-ERN08176 |  | | | |  | | | |  | Vvi-Vitvi15g01538\_t004 |  |  |  |
| 3 | Atr-ERN08177 |  | | | |  | | | |  | Vvi-Vitvi15g00857\_t001 |  |  |  |
| 3 | Atr-ERN08178 |  | | | |  | | | |  | | | |  |  |  |
| 3 | Atr-ERN08179 |  | | | |  | | | |  | | | |  |  |  |
| 3 | Atr-ERN08180 |  | | | |  | | | |  | | | |  |  |  |
| 3 | Atr-ERN08181 |  | | | |  | Vvi-Vitvi02g00282\_t001 |  | | | |  |  |  |
| 3 | Atr-ERN08182 |  | Vvi-Vitvi16g01320\_t001 |  | | | |  | | | |  |  |  |
| 3 | Atr-ERN08183 |  | | | |  | | | |  | | | |  |  |  |
| 3 | Atr-ERN08184 |  | | | |  | | | |  | | | |  |  |  |
| 3 | Atr-ERN08185 |  | | | |  | | | |  | Vvi-Vitvi15g00855\_t001 |  |  |  |
| 3 | Atr-ERN08186 |  | | | |  | | | |  | | | |  |  |  |
| 3 | Atr-ERN08187 |  | | | |  | | | |  | | | |  |  |  |
| 3 | Atr-ERN08188 |  | Vvi-Vitvi16g01316\_t001 |  | | | |  | | | |  |  |  |
| 3 | Atr-ERN08189 |  | Vvi-Vitvi16g02055\_t001.1.6037826c |  | | | |  | | | |  |  |  |
| 3 | Atr-ERN08190 |  | | | |  | | | |  | Vvi-Vitvi15g00854\_t002 |  |  |  |
| 3 | Atr-ERN08191 |  | | | |  | | | |  | | | |  |  |  |
| 3 | Atr-ERN08192 |  | | | |  | | | |  | Vvi-Vitvi15g00853\_t001 |  |  |  |
| 3 | Atr-ERN08193 |  | | | |  | | | |  | | | |  |  |  |
| 3 | Atr-ERN08194 |  | Vvi-Vitvi16g01310\_t001 |  | | | |  | | | |  |  |  |
| 3 | Atr-ERN08195 |  | | | |  | | | |  | | | |  |  |  |
| 3 | Atr-ERN08196 |  | | | |  | | | |  | | | |  |  |  |
| 3 | Atr-ERN08197 |  | | | |  | | | |  | | | |  |  |  |
| 3 | Atr-ERN08198 |  | | | |  | | | |  | | | |  |  |  |
| 3 | Atr-ERN08199 |  | Vvi-Vitvi16g01309\_t001 |  | | | |  | | | |  |  |  |
| 3 | Atr-ERN08200 |  | | | |  | | | |  | | | |  |  |  |
| 3 | Atr-ERN08201 |  | | | |  | | | |  | | | |  |  |  |
| 3 | Atr-ERN08202 |  | | | |  | Vvi-Vitvi02g00280\_t001 |  | | | |  |  |  |
| 3 | Atr-ERN08203 |  | | | |  | | | |  | | | |  |  |  |
| 3 | Atr-ERN08204 |  | Vvi-Vitvi16g01308\_t001 |  | | | |  | | | |  |  |  |
| 3 | Atr-ERN08205 |  | Vvi-Vitvi16g01307\_t001 |  | | | |  | | | |  |  |  |
| 2 | Atr-ERN08206 |  |  |  | | | |  | | | |  |  |  |
| 2 | Atr-ERN08207 |  |  |  | | | |  | | | |  |  |  |
| 2 | Atr-ERN08208 |  |  |  | | | |  | | | |  |  |  |
| 2 | Atr-ERN08209 |  |  |  | Vvi-Vitvi02g00279\_t001 |  | | | |  |  |  |
| 1 | Atr-ERN08210 |  |  |  |  |  | | | |  |  |  |
| 1 | Atr-ERN08211 |  |  |  |  |  | | | |  |  |  |
| 1 | Atr-ERN08212 |  |  |  |  |  | Vvi-Vitvi15g00833\_t001 |  |  |  |
| 0 | Atr-ERN08213 |  |  |  |  |  |  |
| 0 | Atr-ERN08214 |  |  |  |  |  |  |
| 0 | Atr-ERN08215 |  |  |  |  |  |  |
| 0 | Atr-ERN08216 |  |  |  |  |  |  |
| 0 | Atr-ERN08217 |  |  |  |  |  |  |
| 0 | Atr-ERN08218 |  |  |  |  |  |  |
| 0 | Atr-ERN08219 |  |  |  |  |  |  |
| 0 | Atr-ERN08220 |  |  |  |  |  |  |
| 0 | Atr-ERN08221 |  |  |  |  |  |  |
| 0 | Atr-ERN08222 |  |  |  |  |  |  |
| 0 | Atr-ERN08223 |  |  |  |  |  |  |
| 0 | Atr-ERN08224 |  |  |  |  |  |  |
| 0 | Atr-ERN08225 |  |  |  |  |  |  |
| 0 | Atr-ERN08226 |  |  |  |  |  |  |
| 0 | Atr-ERN08227 |  |  |  |  |  |  |
| 0 | Atr-ERN08228 |  |  |  |  |  |  |
| 0 | Atr-ERN08229 |  |  |  |  |  |  |
| 0 | Atr-ERN08230 |  |  |  |  |  |  |
| 0 | Atr-ERN08231 |  |  |  |  |  |  |
| 0 | Atr-ERN08232 |  |  |  |  |  |  |
| 0 | Atr-ERN08233 |  |  |  |  |  |  |
| 0 | Atr-ERN08234 |  |  |  |  |  |  |
| 0 | Atr-ERN08235 |  |  |  |  |  |  |
| 0 | Atr-ERN08236 |  |  |  |  |  |  |
| 0 | Atr-ERN08237 |  |  |  |  |  |  |
| 0 | Atr-ERN08238 |  |  |  |  |  |  |
| 0 | Atr-ERN08239 |  |  |  |  |  |  |
| 0 | Atr-ERN08240 |  |  |  |  |  |  |
| 0 | Atr-ERN08241 |  |  |  |  |  |  |
| 0 | Atr-ERN08242 |  |  |  |  |  |  |
| 0 | Atr-ERN08243 |  |  |  |  |  |  |
| 0 | Atr-ERN08244 |  |  |  |  |  |  |
| 0 | Atr-ERN08245 |  |  |  |  |  |  |
| 0 | Atr-ERN08246 |  |  |  |  |  |  |
| 0 | Atr-ERN08247 |  |  |  |  |  |  |
| 0 | Atr-ERN08248 |  |  |  |  |  |  |
| 0 | Atr-ERN08249 |  |  |  |  |  |  |
| 0 | Atr-ERN08250 |  |  |  |  |  |  |
| 1 | Atr-ERN08251 |  | Vvi-Vitvi02g00240\_t001 |  |  |  |  |  |
| 1 | Atr-ERN08252 |  | | | |  |  |  |  |  |
| 1 | Atr-ERN08253 |  | | | |  |  |  |  |  |
| 1 | Atr-ERN08254 |  | | | |  |  |  |  |  |
| 1 | Atr-ERN08255 |  | Vvi-Vitvi02g00239\_t001 |  |  |  |  |  |
| 1 | Atr-ERN08256 |  | | | |  |  |  |  |  |
| 1 | Atr-ERN08257 |  | Vvi-Vitvi02g00238\_t001 |  |  |  |  |  |
| 1 | Atr-ERN08258 |  | | | |  |  |  |  |  |
| 1 | Atr-ERN08259 |  | Vvi-Vitvi02g00237\_t001 |  |  |  |  |  |
| 1 | Atr-ERN08260 |  | | | |  |  |  |  |  |
| 1 | Atr-ERN08261 |  | | | |  |  |  |  |  |
| 1 | Atr-ERN08262 |  | | | |  |  |  |  |  |
| 1 | Atr-ERN08263 |  | | | |  |  |  |  |  |
| 1 | Atr-ERN08264 |  | Vvi-Vitvi02g00236\_t001 |  |  |  |  |  |
| 1 | Atr-ERN08265 |  | Vvi-Vitvi02g00235\_t001 |  |  |  |  |  |
| 0 | Atr-ERN08266 |  |  |  |  |  |  |
| 0 | Atr-ERN08267 |  |  |  |  |  |  |
| 0 | Atr-ERN08268 |  |  |  |  |  |  |
| 0 | Atr-ERN08269 |  |  |  |  |  |  |
| 0 | Atr-ERN08270 |  |  |  |  |  |  |
| 0 | Atr-ERN08271 |  |  |  |  |  |  |
