# Supplementary material for: Genome-wide identification, characterization and gene expression of BES1 transcription factor family in grapevine (Vitis vinifera L.)
Source: Sci Rep. 2023 Jan 5;13:240. doi: 10.1038/s41598-022-24407-y (PMC9816167; doi:10.1038/s41598-022-24407-y)
Supplement: Supplementary file 3 — Supplementary Information. [file 41598_2022_24407_MOESM3_ESM.zip › Vvi_Atr/Vitis_vinifera.PN40024.v4.dna_sm.toplevel.fa.vs.Amborella_trichopoda.AMTR1.0.dna_sm.toplevel.fa.html/Atr-AmTr_v1.0_scaffold00019.html]

|  |  |  |  |  |  |  |  |  |  |  |  |  |  |
| --- | --- | --- | --- | --- | --- | --- | --- | --- | --- | --- | --- | --- | --- |
| Duplication depth | Reference chromosome | Collinear blocks | | | | | | | | | | | |
| 0 | Atr-ERN07033 |  |  |  |  |  |  |
| 0 | Atr-ERN07034 |  |  |  |  |  |  |
| 1 | Atr-ERN07035 |  | Vvi-Vitvi09g04468\_t001 |  |  |  |  |  |
| 1 | Atr-ERN07036 |  | Vvi-Vitvi09g01155\_t001 |  |  |  |  |  |
| 1 | Atr-ERN07037 |  | | | |  |  |  |  |  |
| 1 | Atr-ERN07038 |  | | | |  |  |  |  |  |
| 2 | Atr-ERN07039 |  | | | |  | Vvi-Vitvi04g00602\_t001 |  |  |  |  |
| 2 | Atr-ERN07040 |  | | | |  | | | |  |  |  |  |
| 2 | Atr-ERN07041 |  | Vvi-Vitvi09g01157\_t001 |  | | | |  |  |  |  |
| 2 | Atr-ERN07042 |  | | | |  | | | |  |  |  |  |
| 2 | Atr-ERN07043 |  | | | |  | | | |  |  |  |  |
| 2 | Atr-ERN07044 |  | | | |  | | | |  |  |  |  |
| 2 | Atr-ERN07045 |  | | | |  | | | |  |  |  |  |
| 2 | Atr-ERN07046 |  | | | |  | | | |  |  |  |  |
| 2 | Atr-ERN07047 |  | | | |  | | | |  |  |  |  |
| 2 | Atr-ERN07048 |  | Vvi-Vitvi09g01158\_t001 |  | | | |  |  |  |  |
| 2 | Atr-ERN07049 |  | | | |  | | | |  |  |  |  |
| 2 | Atr-ERN07050 |  | Vvi-Vitvi09g01161\_t001 |  | | | |  |  |  |  |
| 2 | Atr-ERN07051 |  | | | |  | | | |  |  |  |  |
| 2 | Atr-ERN07052 |  | | | |  | | | |  |  |  |  |
| 2 | Atr-ERN07053 |  | | | |  | Vvi-Vitvi04g00603\_t001 |  |  |  |  |
| 2 | Atr-ERN07054 |  | | | |  | | | |  |  |  |  |
| 2 | Atr-ERN07055 |  | | | |  | | | |  |  |  |  |
| 2 | Atr-ERN07056 |  | | | |  | | | |  |  |  |  |
| 2 | Atr-ERN07057 |  | | | |  | | | |  |  |  |  |
| 2 | Atr-ERN07058 |  | Vvi-Vitvi09g01164\_t001 |  | | | |  |  |  |  |
| 1 | Atr-ERN07059 |  |  |  | | | |  |  |  |  |
| 1 | Atr-ERN07060 |  |  |  | | | |  |  |  |  |
| 1 | Atr-ERN07061 |  |  |  | | | |  |  |  |  |
| 1 | Atr-ERN07062 |  |  |  | | | |  |  |  |  |
| 2 | Atr-ERN07063 |  | Vvi-Vitvi11g00778\_t001 |  | | | |  |  |  |  |
| 2 | Atr-ERN07064 |  | | | |  | | | |  |  |  |  |
| 2 | Atr-ERN07065 |  | | | |  | | | |  |  |  |  |
| 2 | Atr-ERN07066 |  | | | |  | | | |  |  |  |  |
| 2 | Atr-ERN07067 |  | | | |  | | | |  |  |  |  |
| 2 | Atr-ERN07068 |  | | | |  | | | |  |  |  |  |
| 2 | Atr-ERN07069 |  | | | |  | | | |  |  |  |  |
| 2 | Atr-ERN07070 |  | | | |  | Vvi-Vitvi04g00611\_t001 |  |  |  |  |
| 2 | Atr-ERN07071 |  | | | |  | | | |  |  |  |  |
| 2 | Atr-ERN07072 |  | Vvi-Vitvi11g00786\_t001 |  | | | |  |  |  |  |
| 2 | Atr-ERN07073 |  | | | |  | | | |  |  |  |  |
| 2 | Atr-ERN07074 |  | | | |  | | | |  |  |  |  |
| 2 | Atr-ERN07075 |  | | | |  | | | |  |  |  |  |
| 2 | Atr-ERN07076 |  | | | |  | Vvi-Vitvi04g00617\_t001 |  |  |  |  |
| 2 | Atr-ERN07077 |  | | | |  | Vvi-Vitvi04g00618\_t001 |  |  |  |  |
| 2 | Atr-ERN07078 |  | | | |  | | | |  |  |  |  |
| 2 | Atr-ERN07079 |  | | | |  | Vvi-Vitvi04g00619\_t001 |  |  |  |  |
| 2 | Atr-ERN07080 |  | | | |  | | | |  |  |  |  |
| 2 | Atr-ERN07081 |  | | | |  | Vvi-Vitvi04g00620\_t001 |  |  |  |  |
| 2 | Atr-ERN07082 |  | | | |  | Vvi-Vitvi04g00623\_t001 |  |  |  |  |
| 2 | Atr-ERN07083 |  | | | |  | Vvi-Vitvi04g00625\_t001 |  |  |  |  |
| 2 | Atr-ERN07084 |  | | | |  | Vvi-Vitvi04g00626\_t001 |  |  |  |  |
| 2 | Atr-ERN07085 |  | Vvi-Vitvi11g00787\_t001 |  | | | |  |  |  |  |
| 2 | Atr-ERN07086 |  | | | |  | | | |  |  |  |  |
| 2 | Atr-ERN07087 |  | | | |  | Vvi-Vitvi04g00629\_t001 |  |  |  |  |
| 1 | Atr-ERN07088 |  | | | |  |  |  |  |  |
| 1 | Atr-ERN07089 |  | | | |  |  |  |  |  |
| 1 | Atr-ERN07090 |  | | | |  |  |  |  |  |
| 1 | Atr-ERN07091 |  | | | |  |  |  |  |  |
| 1 | Atr-ERN07092 |  | | | |  |  |  |  |  |
| 1 | Atr-ERN07093 |  | | | |  |  |  |  |  |
| 1 | Atr-ERN07094 |  | Vvi-Vitvi11g00796\_t001 |  |  |  |  |  |
| 1 | Atr-ERN07095 |  | | | |  |  |  |  |  |
| 1 | Atr-ERN07096 |  | Vvi-Vitvi11g00798\_t001 |  |  |  |  |  |
| 1 | Atr-ERN07097 |  | | | |  |  |  |  |  |
| 1 | Atr-ERN07098 |  | | | |  |  |  |  |  |
| 1 | Atr-ERN07099 |  | | | |  |  |  |  |  |
| 1 | Atr-ERN07100 |  | | | |  |  |  |  |  |
| 1 | Atr-ERN07101 |  | | | |  |  |  |  |  |
| 2 | Atr-ERN07102 |  | | | |  | Vvi-Vitvi11g00815\_t001 |  |  |  |  |
| 2 | Atr-ERN07103 |  | | | |  | | | |  |  |  |  |
| 2 | Atr-ERN07104 |  | | | |  | | | |  |  |  |  |
| 2 | Atr-ERN07105 |  | | | |  | Vvi-Vitvi11g00814\_t001 |  |  |  |  |
| 2 | Atr-ERN07106 |  | | | |  | | | |  |  |  |  |
| 2 | Atr-ERN07107 |  | | | |  | | | |  |  |  |  |
| 2 | Atr-ERN07108 |  | | | |  | Vvi-Vitvi11g00813\_t001 |  |  |  |  |
| 2 | Atr-ERN07109 |  | | | |  | | | |  |  |  |  |
| 2 | Atr-ERN07110 |  | | | |  | | | |  |  |  |  |
| 2 | Atr-ERN07111 |  | | | |  | | | |  |  |  |  |
| 2 | Atr-ERN07112 |  | | | |  | | | |  |  |  |  |
| 2 | Atr-ERN07113 |  | | | |  | Vvi-Vitvi11g00812\_t001 |  |  |  |  |
| 2 | Atr-ERN07114 |  | | | |  | | | |  |  |  |  |
| 2 | Atr-ERN07115 |  | | | |  | | | |  |  |  |  |
| 2 | Atr-ERN07116 |  | Vvi-Vitvi11g04209\_t001 |  | | | |  |  |  |  |
| 2 | Atr-ERN07117 |  | Vvi-Vitvi11g00811\_t001 |  | | | |  |  |  |  |
| 2 | Atr-ERN07118 |  | | | |  | | | |  |  |  |  |
| 2 | Atr-ERN07119 |  | | | |  | | | |  |  |  |  |
| 2 | Atr-ERN07120 |  | | | |  | | | |  |  |  |  |
| 2 | Atr-ERN07121 |  | | | |  | | | |  |  |  |  |
| 2 | Atr-ERN07122 |  | | | |  | Vvi-Vitvi11g00805\_t001 |  |  |  |  |
| 2 | Atr-ERN07123 |  | | | |  | | | |  |  |  |  |
| 2 | Atr-ERN07124 |  | | | |  | | | |  |  |  |  |
| 2 | Atr-ERN07125 |  | | | |  | | | |  |  |  |  |
| 2 | Atr-ERN07126 |  | | | |  | | | |  |  |  |  |
| 2 | Atr-ERN07127 |  | | | |  | | | |  |  |  |  |
| 2 | Atr-ERN07128 |  | | | |  | Vvi-Vitvi11g00800\_t001 |  |  |  |  |
| 1 | Atr-ERN07129 |  | | | |  |  |  |  |  |
| 1 | Atr-ERN07130 |  | | | |  |  |  |  |  |
| 1 | Atr-ERN07131 |  | | | |  |  |  |  |  |
| 1 | Atr-ERN07132 |  | | | |  |  |  |  |  |
| 1 | Atr-ERN07133 |  | | | |  |  |  |  |  |
| 1 | Atr-ERN07134 |  | | | |  |  |  |  |  |
| 1 | Atr-ERN07135 |  | | | |  |  |  |  |  |
| 1 | Atr-ERN07136 |  | | | |  |  |  |  |  |
| 1 | Atr-ERN07137 |  | Vvi-Vitvi11g00822\_t001 |  |  |  |  |  |
| 1 | Atr-ERN07138 |  | | | |  |  |  |  |  |
| 1 | Atr-ERN07139 |  | | | |  |  |  |  |  |
| 1 | Atr-ERN07140 |  | Vvi-Vitvi11g00823\_t001 |  |  |  |  |  |
| 1 | Atr-ERN07141 |  | | | |  |  |  |  |  |
| 1 | Atr-ERN07142 |  | | | |  |  |  |  |  |
| 1 | Atr-ERN07143 |  | | | |  |  |  |  |  |
| 1 | Atr-ERN07144 |  | | | |  |  |  |  |  |
| 1 | Atr-ERN07145 |  | | | |  |  |  |  |  |
| 1 | Atr-ERN07146 |  | | | |  |  |  |  |  |
| 1 | Atr-ERN07147 |  | | | |  |  |  |  |  |
| 1 | Atr-ERN07148 |  | | | |  |  |  |  |  |
| 1 | Atr-ERN07149 |  | | | |  |  |  |  |  |
| 1 | Atr-ERN07150 |  | Vvi-Vitvi11g00826\_t001 |  |  |  |  |  |
| 1 | Atr-ERN07151 |  | Vvi-Vitvi11g00828\_t001 |  |  |  |  |  |
| 1 | Atr-ERN07152 |  | | | |  |  |  |  |  |
| 1 | Atr-ERN07153 |  | | | |  |  |  |  |  |
| 1 | Atr-ERN07154 |  | | | |  |  |  |  |  |
| 1 | Atr-ERN07155 |  | | | |  |  |  |  |  |
| 1 | Atr-ERN07156 |  | | | |  |  |  |  |  |
| 1 | Atr-ERN07157 |  | | | |  |  |  |  |  |
| 1 | Atr-ERN07158 |  | Vvi-Vitvi11g04210\_t001 |  |  |  |  |  |
| 1 | Atr-ERN07159 |  | Vvi-Vitvi11g00835\_t001 |  |  |  |  |  |
| 1 | Atr-ERN07160 |  | | | |  |  |  |  |  |
| 1 | Atr-ERN07161 |  | Vvi-Vitvi11g00838\_t001 |  |  |  |  |  |
| 1 | Atr-ERN07162 |  | Vvi-Vitvi11g00840\_t001 |  |  |  |  |  |
| 1 | Atr-ERN07163 |  | Vvi-Vitvi11g00847\_t001 |  |  |  |  |  |
| 1 | Atr-ERN07164 |  | | | |  |  |  |  |  |
| 1 | Atr-ERN07165 |  | | | |  |  |  |  |  |
| 1 | Atr-ERN07166 |  | | | |  |  |  |  |  |
| 1 | Atr-ERN07167 |  | | | |  |  |  |  |  |
| 1 | Atr-ERN07168 |  | | | |  |  |  |  |  |
| 1 | Atr-ERN07169 |  | Vvi-Vitvi11g01536\_t001 |  |  |  |  |  |
| 1 | Atr-ERN07170 |  | Vvi-Vitvi04g00741\_t001 |  |  |  |  |  |
| 1 | Atr-ERN07171 |  | Vvi-Vitvi04g04188\_t003 |  |  |  |  |  |
| 1 | Atr-ERN07172 |  | | | |  |  |  |  |  |
| 1 | Atr-ERN07173 |  | Vvi-Vitvi04g00736\_t001 |  |  |  |  |  |
| 1 | Atr-ERN07174 |  | | | |  |  |  |  |  |
| 1 | Atr-ERN07175 |  | | | |  |  |  |  |  |
| 1 | Atr-ERN07176 |  | | | |  |  |  |  |  |
| 1 | Atr-ERN07177 |  | | | |  |  |  |  |  |
| 1 | Atr-ERN07178 |  | | | |  |  |  |  |  |
| 1 | Atr-ERN07179 |  | | | |  |  |  |  |  |
| 1 | Atr-ERN07180 |  | | | |  |  |  |  |  |
| 1 | Atr-ERN07181 |  | | | |  |  |  |  |  |
| 1 | Atr-ERN07182 |  | | | |  |  |  |  |  |
| 1 | Atr-ERN07183 |  | | | |  |  |  |  |  |
| 1 | Atr-ERN07184 |  | | | |  |  |  |  |  |
| 1 | Atr-ERN07185 |  | | | |  |  |  |  |  |
| 1 | Atr-ERN07186 |  | | | |  |  |  |  |  |
| 1 | Atr-ERN07187 |  | | | |  |  |  |  |  |
| 1 | Atr-ERN07188 |  | | | |  |  |  |  |  |
| 1 | Atr-ERN07189 |  | | | |  |  |  |  |  |
| 1 | Atr-ERN07190 |  | | | |  |  |  |  |  |
| 1 | Atr-ERN07191 |  | | | |  |  |  |  |  |
| 1 | Atr-ERN07192 |  | | | |  |  |  |  |  |
| 1 | Atr-ERN07193 |  | Vvi-Vitvi04g00732\_t001 |  |  |  |  |  |
| 1 | Atr-ERN07194 |  | | | |  |  |  |  |  |
| 1 | Atr-ERN07195 |  | | | |  |  |  |  |  |
| 1 | Atr-ERN07196 |  | | | |  |  |  |  |  |
| 1 | Atr-ERN07197 |  | | | |  |  |  |  |  |
| 1 | Atr-ERN07198 |  | | | |  |  |  |  |  |
| 1 | Atr-ERN07199 |  | | | |  |  |  |  |  |
| 1 | Atr-ERN07200 |  | | | |  |  |  |  |  |
| 1 | Atr-ERN07201 |  | | | |  |  |  |  |  |
| 1 | Atr-ERN07202 |  | | | |  |  |  |  |  |
| 1 | Atr-ERN07203 |  | | | |  |  |  |  |  |
| 1 | Atr-ERN07204 |  | | | |  |  |  |  |  |
| 1 | Atr-ERN07205 |  | | | |  |  |  |  |  |
| 1 | Atr-ERN07206 |  | | | |  |  |  |  |  |
| 1 | Atr-ERN07207 |  | | | |  |  |  |  |  |
| 1 | Atr-ERN07208 |  | | | |  |  |  |  |  |
| 1 | Atr-ERN07209 |  | | | |  |  |  |  |  |
| 1 | Atr-ERN07210 |  | | | |  |  |  |  |  |
| 1 | Atr-ERN07211 |  | Vvi-Vitvi04g00728\_t001 |  |  |  |  |  |
| 1 | Atr-ERN07212 |  | | | |  |  |  |  |  |
| 1 | Atr-ERN07213 |  | | | |  |  |  |  |  |
| 1 | Atr-ERN07214 |  | Vvi-Vitvi04g00716\_t001 |  |  |  |  |  |
| 2 | Atr-ERN07215 |  | Vvi-Vitvi04g00715\_t001 |  | Vvi-Vitvi04g00680\_t001 |  |  |  |  |
| 2 | Atr-ERN07216 |  | | | |  | | | |  |  |  |  |
| 2 | Atr-ERN07217 |  | | | |  | | | |  |  |  |  |
| 2 | Atr-ERN07218 |  | | | |  | | | |  |  |  |  |
| 2 | Atr-ERN07219 |  | | | |  | | | |  |  |  |  |
| 2 | Atr-ERN07220 |  | | | |  | | | |  |  |  |  |
| 2 | Atr-ERN07221 |  | Vvi-Vitvi04g04171\_t001 |  | Vvi-Vitvi04g00681\_t001 |  |  |  |  |
| 2 | Atr-ERN07222 |  | | | |  | | | |  |  |  |  |
| 2 | Atr-ERN07223 |  | Vvi-Vitvi04g04167\_t001 |  | | | |  |  |  |  |
| 2 | Atr-ERN07224 |  | Vvi-Vitvi04g00696\_t001 |  | | | |  |  |  |  |
| 2 | Atr-ERN07225 |  | Vvi-Vitvi04g04161\_t001 |  | Vvi-Vitvi04g04161\_t001 |  |  |  |  |
| 2 | Atr-ERN07226 |  | | | |  | | | |  |  |  |  |
| 2 | Atr-ERN07227 |  | | | |  | | | |  |  |  |  |
| 2 | Atr-ERN07228 |  | | | |  | | | |  |  |  |  |
| 2 | Atr-ERN07229 |  | | | |  | | | |  |  |  |  |
| 2 | Atr-ERN07230 |  | Vvi-Vitvi04g00681\_t001 |  | | | |  |  |  |  |
| 2 | Atr-ERN07231 |  | | | |  | Vvi-Vitvi04g04163\_t001 |  |  |  |  |
| 2 | Atr-ERN07232 |  | | | |  | Vvi-Vitvi04g04168\_t001 |  |  |  |  |
| 2 | Atr-ERN07233 |  | | | |  | | | |  |  |  |  |
| 2 | Atr-ERN07234 |  | | | |  | | | |  |  |  |  |
| 2 | Atr-ERN07235 |  | | | |  | | | |  |  |  |  |
| 2 | Atr-ERN07236 |  | | | |  | Vvi-Vitvi04g04181\_t001 |  |  |  |  |
| 1 | Atr-ERN07237 |  | | | |  |  |  |  |  |
| 1 | Atr-ERN07238 |  | | | |  |  |  |  |  |
| 1 | Atr-ERN07239 |  | | | |  |  |  |  |  |
| 1 | Atr-ERN07240 |  | | | |  |  |  |  |  |
| 1 | Atr-ERN07241 |  | | | |  |  |  |  |  |
| 1 | Atr-ERN07242 |  | | | |  |  |  |  |  |
| 2 | Atr-ERN07243 |  | Vvi-Vitvi04g00670\_t001 |  | Vvi-Vitvi11g01702\_t001 |  |  |  |  |
| 2 | Atr-ERN07244 |  | | | |  | Vvi-Vitvi11g01311\_t001 |  |  |  |  |
| 2 | Atr-ERN07245 |  | | | |  | | | |  |  |  |  |
| 2 | Atr-ERN07246 |  | Vvi-Vitvi04g00665\_t001 |  | Vvi-Vitvi11g01309\_t001 |  |  |  |  |
| 1 | Atr-ERN07247 |  |  |  | Vvi-Vitvi11g01303\_t001 |  |  |  |  |
| 1 | Atr-ERN07248 |  |  |  | | | |  |  |  |  |
| 1 | Atr-ERN07249 |  |  |  | Vvi-Vitvi11g01301\_t001 |  |  |  |  |
| 1 | Atr-ERN07250 |  |  |  | | | |  |  |  |  |
| 1 | Atr-ERN07251 |  |  |  | | | |  |  |  |  |
| 1 | Atr-ERN07252 |  |  |  | | | |  |  |  |  |
| 1 | Atr-ERN07253 |  |  |  | | | |  |  |  |  |
| 1 | Atr-ERN07254 |  |  |  | | | |  |  |  |  |
| 1 | Atr-ERN07255 |  |  |  | | | |  |  |  |  |
| 1 | Atr-ERN07256 |  |  |  | | | |  |  |  |  |
| 1 | Atr-ERN07257 |  |  |  | | | |  |  |  |  |
| 1 | Atr-ERN07258 |  |  |  | Vvi-Vitvi11g01300\_t002 |  |  |  |  |
| 1 | Atr-ERN07259 |  |  |  | Vvi-Vitvi11g01298\_t002 |  |  |  |  |
| 1 | Atr-ERN07260 |  |  |  | Vvi-Vitvi11g01297\_t001.1.6037826a |  |  |  |  |
| 1 | Atr-ERN07261 |  |  |  | | | |  |  |  |  |
| 1 | Atr-ERN07262 |  |  |  | Vvi-Vitvi11g01293\_t001 |  |  |  |  |
| 1 | Atr-ERN07263 |  |  |  | | | |  |  |  |  |
| 1 | Atr-ERN07264 |  |  |  | | | |  |  |  |  |
| 1 | Atr-ERN07265 |  |  |  | | | |  |  |  |  |
| 1 | Atr-ERN07266 |  |  |  | | | |  |  |  |  |
| 1 | Atr-ERN07267 |  |  |  | Vvi-Vitvi11g01283\_t001 |  |  |  |  |
| 1 | Atr-ERN07268 |  |  |  | Vvi-Vitvi11g01282\_t001 |  |  |  |  |
| 1 | Atr-ERN07269 |  |  |  | | | |  |  |  |  |
| 1 | Atr-ERN07270 |  |  |  | | | |  |  |  |  |
| 1 | Atr-ERN07271 |  |  |  | | | |  |  |  |  |
| 1 | Atr-ERN07272 |  |  |  | | | |  |  |  |  |
| 1 | Atr-ERN07273 |  |  |  | | | |  |  |  |  |
| 1 | Atr-ERN07274 |  |  |  | | | |  |  |  |  |
| 1 | Atr-ERN07275 |  |  |  | Vvi-Vitvi11g01277\_t001 |  |  |  |  |
| 1 | Atr-ERN07276 |  |  |  | | | |  |  |  |  |
| 1 | Atr-ERN07277 |  |  |  | Vvi-Vitvi11g01273\_t001 |  |  |  |  |
| 1 | Atr-ERN07278 |  |  |  | Vvi-Vitvi11g01272\_t001 |  |  |  |  |
| 1 | Atr-ERN07279 |  |  |  | | | |  |  |  |  |
| 1 | Atr-ERN07280 |  |  |  | | | |  |  |  |  |
| 1 | Atr-ERN07281 |  |  |  | | | |  |  |  |  |
| 1 | Atr-ERN07282 |  |  |  | Vvi-Vitvi11g04354\_t001 |  |  |  |  |
| 1 | Atr-ERN07283 |  |  |  | | | |  |  |  |  |
| 1 | Atr-ERN07284 |  |  |  | | | |  |  |  |  |
| 1 | Atr-ERN07285 |  |  |  | Vvi-Vitvi11g01671\_t001 |  |  |  |  |
| 1 | Atr-ERN07286 |  |  |  | | | |  |  |  |  |
| 1 | Atr-ERN07287 |  |  |  | | | |  |  |  |  |
| 1 | Atr-ERN07288 |  |  |  | | | |  |  |  |  |
| 1 | Atr-ERN07289 |  |  |  | | | |  |  |  |  |
| 1 | Atr-ERN07290 |  |  |  | | | |  |  |  |  |
| 1 | Atr-ERN07291 |  |  |  | Vvi-Vitvi11g01259\_t001 |  |  |  |  |
| 1 | Atr-ERN07292 |  |  |  | | | |  |  |  |  |
| 1 | Atr-ERN07293 |  |  |  | Vvi-Vitvi11g01257\_t001 |  |  |  |  |
| 1 | Atr-ERN07294 |  |  |  | Vvi-Vitvi11g01667\_t001 |  |  |  |  |
| 1 | Atr-ERN07295 |  |  |  | | | |  |  |  |  |
| 1 | Atr-ERN07296 |  |  |  | | | |  |  |  |  |
| 1 | Atr-ERN07297 |  |  |  | | | |  |  |  |  |
| 1 | Atr-ERN07298 |  |  |  | | | |  |  |  |  |
| 1 | Atr-ERN07299 |  |  |  | | | |  |  |  |  |
| 1 | Atr-ERN07300 |  |  |  | Vvi-Vitvi11g01662\_t001 |  |  |  |  |
| 1 | Atr-ERN07301 |  |  |  | | | |  |  |  |  |
| 1 | Atr-ERN07302 |  |  |  | | | |  |  |  |  |
| 1 | Atr-ERN07303 |  |  |  | | | |  |  |  |  |
| 1 | Atr-ERN07304 |  |  |  | | | |  |  |  |  |
| 1 | Atr-ERN07305 |  |  |  | | | |  |  |  |  |
| 1 | Atr-ERN07306 |  |  |  | Vvi-Vitvi11g04343\_t001 |  |  |  |  |
| 1 | Atr-ERN07307 |  |  |  | Vvi-Vitvi11g01241\_t001 |  |  |  |  |
| 1 | Atr-ERN07308 |  |  |  | | | |  |  |  |  |
| 1 | Atr-ERN07309 |  |  |  | Vvi-Vitvi11g01658\_t001 |  |  |  |  |
| 1 | Atr-ERN07310 |  |  |  | | | |  |  |  |  |
| 1 | Atr-ERN07311 |  |  |  | | | |  |  |  |  |
| 1 | Atr-ERN07312 |  |  |  | | | |  |  |  |  |
| 1 | Atr-ERN07313 |  |  |  | | | |  |  |  |  |
| 1 | Atr-ERN07314 |  |  |  | | | |  |  |  |  |
| 1 | Atr-ERN07315 |  |  |  | Vvi-Vitvi11g01236\_t001 |  |  |  |  |
| 2 | Atr-ERN07316 |  | Vvi-Vitvi04g00225\_t001 |  | | | |  |  |  |  |
| 2 | Atr-ERN07317 |  | | | |  | | | |  |  |  |  |
| 2 | Atr-ERN07318 |  | | | |  | | | |  |  |  |  |
| 2 | Atr-ERN07319 |  | | | |  | | | |  |  |  |  |
| 2 | Atr-ERN07320 |  | | | |  | | | |  |  |  |  |
| 2 | Atr-ERN07321 |  | | | |  | | | |  |  |  |  |
| 2 | Atr-ERN07322 |  | | | |  | | | |  |  |  |  |
| 2 | Atr-ERN07323 |  | | | |  | | | |  |  |  |  |
| 2 | Atr-ERN07324 |  | | | |  | Vvi-Vitvi11g01231\_t001 |  |  |  |  |
| 2 | Atr-ERN07325 |  | | | |  | Vvi-Vitvi11g01230\_t001 |  |  |  |  |
| 2 | Atr-ERN07326 |  | Vvi-Vitvi04g00219\_t001 |  | | | |  |  |  |  |
| 2 | Atr-ERN07327 |  | | | |  | | | |  |  |  |  |
| 2 | Atr-ERN07328 |  | | | |  | | | |  |  |  |  |
| 2 | Atr-ERN07329 |  | | | |  | | | |  |  |  |  |
| 2 | Atr-ERN07330 |  | | | |  | | | |  |  |  |  |
| 2 | Atr-ERN07331 |  | Vvi-Vitvi04g00216\_t001 |  | Vvi-Vitvi11g04337\_t001 |  |  |  |  |
| 2 | Atr-ERN07332 |  | | | |  | | | |  |  |  |  |
| 2 | Atr-ERN07333 |  | | | |  | | | |  |  |  |  |
| 2 | Atr-ERN07334 |  | | | |  | | | |  |  |  |  |
| 2 | Atr-ERN07335 |  | | | |  | | | |  |  |  |  |
| 2 | Atr-ERN07336 |  | | | |  | | | |  |  |  |  |
| 2 | Atr-ERN07337 |  | | | |  | | | |  |  |  |  |
| 2 | Atr-ERN07338 |  | | | |  | | | |  |  |  |  |
| 2 | Atr-ERN07339 |  | | | |  | | | |  |  |  |  |
| 2 | Atr-ERN07340 |  | | | |  | | | |  |  |  |  |
| 2 | Atr-ERN07341 |  | | | |  | | | |  |  |  |  |
| 2 | Atr-ERN07342 |  | | | |  | | | |  |  |  |  |
| 2 | Atr-ERN07343 |  | | | |  | | | |  |  |  |  |
| 2 | Atr-ERN07344 |  | | | |  | | | |  |  |  |  |
| 2 | Atr-ERN07345 |  | Vvi-Vitvi04g01824\_t001 |  | | | |  |  |  |  |
| 2 | Atr-ERN07346 |  | Vvi-Vitvi04g00214\_t001 |  | Vvi-Vitvi11g01220\_t001 |  |  |  |  |
| 2 | Atr-ERN07347 |  | | | |  | | | |  |  |  |  |
| 2 | Atr-ERN07348 |  | | | |  | | | |  |  |  |  |
| 2 | Atr-ERN07349 |  | | | |  | | | |  |  |  |  |
| 2 | Atr-ERN07350 |  | | | |  | | | |  |  |  |  |
| 2 | Atr-ERN07351 |  | | | |  | | | |  |  |  |  |
| 2 | Atr-ERN07352 |  | | | |  | | | |  |  |  |  |
| 2 | Atr-ERN07353 |  | | | |  | | | |  |  |  |  |
| 2 | Atr-ERN07354 |  | | | |  | | | |  |  |  |  |
| 3 | Atr-ERN07355 |  | | | |  | | | |  | Vvi-Vitvi11g01205\_t001 |  |  |  |
| 3 | Atr-ERN07356 |  | | | |  | | | |  | | | |  |  |  |
| 3 | Atr-ERN07357 |  | | | |  | | | |  | | | |  |  |  |
| 3 | Atr-ERN07358 |  | Vvi-Vitvi04g00211\_t001 |  | | | |  | Vvi-Vitvi11g01208\_t001 |  |  |  |
| 3 | Atr-ERN07359 |  | | | |  | | | |  | Vvi-Vitvi11g01650\_t002 |  |  |  |
| 3 | Atr-ERN07360 |  | | | |  | | | |  | | | |  |  |  |
| 3 | Atr-ERN07361 |  | | | |  | | | |  | | | |  |  |  |
| 3 | Atr-ERN07362 |  | | | |  | Vvi-Vitvi11g01651\_t001 |  | | | |  |  |  |
| 3 | Atr-ERN07363 |  | | | |  | | | |  | | | |  |  |  |
| 3 | Atr-ERN07364 |  | | | |  | | | |  | Vvi-Vitvi11g01209\_t001 |  |  |  |
| 3 | Atr-ERN07365 |  | | | |  | | | |  | | | |  |  |  |
| 3 | Atr-ERN07366 |  | | | |  | | | |  | | | |  |  |  |
| 3 | Atr-ERN07367 |  | | | |  | Vvi-Vitvi11g01210\_t001 |  | Vvi-Vitvi11g01210\_t001 |  |  |  |
| 3 | Atr-ERN07368 |  | Vvi-Vitvi04g00209\_t002 |  | | | |  | | | |  |  |  |
| 3 | Atr-ERN07369 |  | | | |  | | | |  | | | |  |  |  |
| 3 | Atr-ERN07370 |  | | | |  | | | |  | | | |  |  |  |
| 3 | Atr-ERN07371 |  | | | |  | | | |  | Vvi-Vitvi11g01211\_t001 |  |  |  |
| 3 | Atr-ERN07372 |  | | | |  | | | |  | | | |  |  |  |
| 3 | Atr-ERN07373 |  | | | |  | | | |  | | | |  |  |  |
| 3 | Atr-ERN07374 |  | | | |  | | | |  | | | |  |  |  |
| 3 | Atr-ERN07375 |  | | | |  | | | |  | | | |  |  |  |
| 3 | Atr-ERN07376 |  | | | |  | | | |  | | | |  |  |  |
| 3 | Atr-ERN07377 |  | | | |  | | | |  | | | |  |  |  |
| 3 | Atr-ERN07378 |  | Vvi-Vitvi04g00206\_t001 |  | | | |  | | | |  |  |  |
| 3 | Atr-ERN07379 |  | | | |  | | | |  | | | |  |  |  |
| 3 | Atr-ERN07380 |  | | | |  | | | |  | | | |  |  |  |
| 3 | Atr-ERN07381 |  | Vvi-Vitvi04g00205\_t002 |  | | | |  | | | |  |  |  |
| 2 | Atr-ERN07382 |  |  |  | | | |  | | | |  |  |  |
| 2 | Atr-ERN07383 |  |  |  | Vvi-Vitvi11g01199\_t001 |  | | | |  |  |  |
| 2 | Atr-ERN07384 |  |  |  | | | |  | | | |  |  |  |
| 2 | Atr-ERN07385 |  |  |  | | | |  | Vvi-Vitvi11g01212\_t001 |  |  |  |
| 2 | Atr-ERN07386 |  |  |  | Vvi-Vitvi11g01198\_t001 |  | | | |  |  |  |
| 2 | Atr-ERN07387 |  |  |  | Vvi-Vitvi11g01197\_t001 |  | | | |  |  |  |
| 2 | Atr-ERN07388 |  |  |  | | | |  | | | |  |  |  |
| 2 | Atr-ERN07389 |  |  |  | | | |  | | | |  |  |  |
| 2 | Atr-ERN07390 |  |  |  | Vvi-Vitvi11g01195\_t001 |  | | | |  |  |  |
| 2 | Atr-ERN07391 |  |  |  | Vvi-Vitvi11g01192\_t001 |  | | | |  |  |  |
| 2 | Atr-ERN07392 |  |  |  | | | |  | | | |  |  |  |
| 2 | Atr-ERN07393 |  |  |  | | | |  | | | |  |  |  |
| 2 | Atr-ERN07394 |  |  |  | | | |  | | | |  |  |  |
| 2 | Atr-ERN07395 |  |  |  | | | |  | | | |  |  |  |
| 2 | Atr-ERN07396 |  |  |  | | | |  | | | |  |  |  |
| 2 | Atr-ERN07397 |  |  |  | | | |  | | | |  |  |  |
| 2 | Atr-ERN07398 |  |  |  | | | |  | | | |  |  |  |
| 2 | Atr-ERN07399 |  |  |  | | | |  | | | |  |  |  |
| 2 | Atr-ERN07400 |  |  |  | | | |  | | | |  |  |  |
| 2 | Atr-ERN07401 |  |  |  | | | |  | | | |  |  |  |
| 2 | Atr-ERN07402 |  |  |  | | | |  | | | |  |  |  |
| 2 | Atr-ERN07403 |  |  |  | | | |  | | | |  |  |  |
| 2 | Atr-ERN07404 |  |  |  | | | |  | | | |  |  |  |
| 2 | Atr-ERN07405 |  |  |  | | | |  | | | |  |  |  |
| 2 | Atr-ERN07406 |  |  |  | | | |  | | | |  |  |  |
| 2 | Atr-ERN07407 |  |  |  | | | |  | | | |  |  |  |
| 2 | Atr-ERN07408 |  |  |  | | | |  | Vvi-Vitvi11g01213\_t001 |  |  |  |
| 2 | Atr-ERN07409 |  |  |  | | | |  | Vvi-Vitvi11g04336\_t001 |  |  |  |
| 1 | Atr-ERN07410 |  |  |  | | | |  |  |  |  |
| 1 | Atr-ERN07411 |  |  |  | | | |  |  |  |  |
| 1 | Atr-ERN07412 |  |  |  | | | |  |  |  |  |
| 1 | Atr-ERN07413 |  |  |  | | | |  |  |  |  |
| 1 | Atr-ERN07414 |  |  |  | | | |  |  |  |  |
| 1 | Atr-ERN07415 |  |  |  | Vvi-Vitvi11g04330\_t001 |  |  |  |  |
| 1 | Atr-ERN07416 |  |  |  | | | |  |  |  |  |
| 1 | Atr-ERN07417 |  |  |  | | | |  |  |  |  |
| 1 | Atr-ERN07418 |  |  |  | | | |  |  |  |  |
| 1 | Atr-ERN07419 |  |  |  | | | |  |  |  |  |
| 1 | Atr-ERN07420 |  |  |  | | | |  |  |  |  |
| 1 | Atr-ERN07421 |  |  |  | | | |  |  |  |  |
| 1 | Atr-ERN07422 |  |  |  | | | |  |  |  |  |
| 1 | Atr-ERN07423 |  |  |  | | | |  |  |  |  |
| 1 | Atr-ERN07424 |  |  |  | | | |  |  |  |  |
| 1 | Atr-ERN07425 |  |  |  | | | |  |  |  |  |
| 1 | Atr-ERN07426 |  |  |  | | | |  |  |  |  |
| 1 | Atr-ERN07427 |  |  |  | | | |  |  |  |  |
| 1 | Atr-ERN07428 |  |  |  | | | |  |  |  |  |
| 1 | Atr-ERN07429 |  |  |  | | | |  |  |  |  |
| 1 | Atr-ERN07430 |  |  |  | Vvi-Vitvi11g01644\_t001 |  |  |  |  |
| 1 | Atr-ERN07431 |  |  |  | Vvi-Vitvi11g01188\_t001 |  |  |  |  |
| 1 | Atr-ERN07432 |  |  |  | | | |  |  |  |  |
| 1 | Atr-ERN07433 |  |  |  | | | |  |  |  |  |
| 1 | Atr-ERN07434 |  |  |  | | | |  |  |  |  |
| 1 | Atr-ERN07435 |  |  |  | | | |  |  |  |  |
| 1 | Atr-ERN07436 |  |  |  | | | |  |  |  |  |
| 1 | Atr-ERN07437 |  |  |  | | | |  |  |  |  |
| 1 | Atr-ERN07438 |  |  |  | | | |  |  |  |  |
| 1 | Atr-ERN07439 |  |  |  | | | |  |  |  |  |
| 1 | Atr-ERN07440 |  |  |  | Vvi-Vitvi11g01186\_t001 |  |  |  |  |
| 1 | Atr-ERN07441 |  |  |  | | | |  |  |  |  |
| 1 | Atr-ERN07442 |  |  |  | | | |  |  |  |  |
| 1 | Atr-ERN07443 |  |  |  | | | |  |  |  |  |
| 1 | Atr-ERN07444 |  |  |  | | | |  |  |  |  |
| 1 | Atr-ERN07445 |  |  |  | | | |  |  |  |  |
| 1 | Atr-ERN07446 |  |  |  | | | |  |  |  |  |
| 1 | Atr-ERN07447 |  |  |  | Vvi-Vitvi11g01183\_t001 |  |  |  |  |
| 1 | Atr-ERN07448 |  |  |  | Vvi-Vitvi11g01182\_t001 |  |  |  |  |
| 1 | Atr-ERN07449 |  |  |  | Vvi-Vitvi11g01180\_t001 |  |  |  |  |
| 1 | Atr-ERN07450 |  |  |  | Vvi-Vitvi11g01177\_t001 |  |  |  |  |
| 1 | Atr-ERN07451 |  |  |  | Vvi-Vitvi11g01176\_t001 |  |  |  |  |
| 1 | Atr-ERN07452 |  |  |  | Vvi-Vitvi11g01629\_t001 |  |  |  |  |
| 1 | Atr-ERN07453 |  |  |  | | | |  |  |  |  |
| 1 | Atr-ERN07454 |  |  |  | | | |  |  |  |  |
| 1 | Atr-ERN07455 |  |  |  | | | |  |  |  |  |
| 1 | Atr-ERN07456 |  |  |  | Vvi-Vitvi11g01163\_t002 |  |  |  |  |
| 1 | Atr-ERN07457 |  |  |  | Vvi-Vitvi11g01162\_t001 |  |  |  |  |
| 1 | Atr-ERN07458 |  |  |  | Vvi-Vitvi11g01160\_t001 |  |  |  |  |
| 1 | Atr-ERN07459 |  |  |  | | | |  |  |  |  |
| 1 | Atr-ERN07460 |  |  |  | Vvi-Vitvi11g01156\_t001 |  |  |  |  |
| 1 | Atr-ERN07461 |  |  |  | | | |  |  |  |  |
| 1 | Atr-ERN07462 |  |  |  | | | |  |  |  |  |
| 1 | Atr-ERN07463 |  |  |  | | | |  |  |  |  |
| 1 | Atr-ERN07464 |  |  |  | | | |  |  |  |  |
| 1 | Atr-ERN07465 |  |  |  | Vvi-Vitvi11g01153\_t001 |  |  |  |  |
| 0 | Atr-ERN07466 |  |  |  |  |  |  |
| 0 | Atr-ERN07467 |  |  |  |  |  |  |
| 1 | Atr-ERN07468 |  | Vvi-Vitvi05g01872\_t001 |  |  |  |  |  |
| 2 | Atr-ERN07469 |  | | | |  | Vvi-Vitvi07g00201\_t001 |  |  |  |  |
| 2 | Atr-ERN07470 |  | | | |  | | | |  |  |  |  |
| 2 | Atr-ERN07471 |  | Vvi-Vitvi05g00386\_t001 |  | Vvi-Vitvi07g00202\_t001 |  |  |  |  |
| 2 | Atr-ERN07472 |  | | | |  | | | |  |  |  |  |
| 2 | Atr-ERN07473 |  | | | |  | | | |  |  |  |  |
| 2 | Atr-ERN07474 |  | | | |  | | | |  |  |  |  |
| 2 | Atr-ERN07475 |  | Vvi-Vitvi05g00385\_t001 |  | | | |  |  |  |  |
| 2 | Atr-ERN07476 |  | | | |  | | | |  |  |  |  |
| 2 | Atr-ERN07477 |  | | | |  | | | |  |  |  |  |
| 2 | Atr-ERN07478 |  | | | |  | | | |  |  |  |  |
| 2 | Atr-ERN07479 |  | Vvi-Vitvi05g01871\_t001 |  | | | |  |  |  |  |
| 2 | Atr-ERN07480 |  | | | |  | | | |  |  |  |  |
| 2 | Atr-ERN07481 |  | Vvi-Vitvi05g01869\_t001 |  | Vvi-Vitvi07g00207\_t001 |  |  |  |  |
| 2 | Atr-ERN07482 |  | | | |  | | | |  |  |  |  |
| 2 | Atr-ERN07483 |  | | | |  | | | |  |  |  |  |
| 2 | Atr-ERN07484 |  | | | |  | | | |  |  |  |  |
| 2 | Atr-ERN07485 |  | | | |  | | | |  |  |  |  |
| 2 | Atr-ERN07486 |  | | | |  | | | |  |  |  |  |
| 2 | Atr-ERN07487 |  | | | |  | Vvi-Vitvi07g00208\_t001 |  |  |  |  |
| 2 | Atr-ERN07488 |  | Vvi-Vitvi05g01868\_t001 |  | | | |  |  |  |  |
| 2 | Atr-ERN07489 |  | | | |  | | | |  |  |  |  |
| 2 | Atr-ERN07490 |  | | | |  | | | |  |  |  |  |
| 2 | Atr-ERN07491 |  | | | |  | | | |  |  |  |  |
| 2 | Atr-ERN07492 |  | Vvi-Vitvi05g00384\_t001 |  | Vvi-Vitvi07g00209\_t001 |  |  |  |  |
| 2 | Atr-ERN07493 |  | | | |  | Vvi-Vitvi07g00210\_t001 |  |  |  |  |
| 1 | Atr-ERN07494 |  | Vvi-Vitvi05g00383\_t001 |  |  |  |  |  |
