# Supplementary material for: Genome-wide identification, characterization and gene expression of BES1 transcription factor family in grapevine (Vitis vinifera L.)
Source: Sci Rep. 2023 Jan 5;13:240. doi: 10.1038/s41598-022-24407-y (PMC9816167; doi:10.1038/s41598-022-24407-y)
Supplement: Supplementary file 3 — Supplementary Information. [file 41598_2022_24407_MOESM3_ESM.zip › Vvi_Atr/Vitis_vinifera.PN40024.v4.dna_sm.toplevel.fa.vs.Amborella_trichopoda.AMTR1.0.dna_sm.toplevel.fa.html/Atr-AmTr_v1.0_scaffold00020.html]

|  |  |  |  |  |  |  |  |  |  |  |  |  |  |
| --- | --- | --- | --- | --- | --- | --- | --- | --- | --- | --- | --- | --- | --- |
| Duplication depth | Reference chromosome | Collinear blocks | | | | | | | | | | | |
| 0 | Atr-ERN11813 |  |  |  |  |  |  |
| 0 | Atr-ERN11814 |  |  |  |  |  |  |
| 0 | Atr-ERN11815 |  |  |  |  |  |  |
| 0 | Atr-ERN11816 |  |  |  |  |  |  |
| 0 | Atr-ERN11817 |  |  |  |  |  |  |
| 0 | Atr-ERN11818 |  |  |  |  |  |  |
| 0 | Atr-ERN11819 |  |  |  |  |  |  |
| 0 | Atr-ERN11820 |  |  |  |  |  |  |
| 0 | Atr-ERN11821 |  |  |  |  |  |  |
| 0 | Atr-ERN11822 |  |  |  |  |  |  |
| 0 | Atr-ERN11823 |  |  |  |  |  |  |
| 0 | Atr-ERN11824 |  |  |  |  |  |  |
| 0 | Atr-ERN11825 |  |  |  |  |  |  |
| 0 | Atr-ERN11826 |  |  |  |  |  |  |
| 0 | Atr-ERN11827 |  |  |  |  |  |  |
| 0 | Atr-ERN11828 |  |  |  |  |  |  |
| 0 | Atr-ERN11829 |  |  |  |  |  |  |
| 0 | Atr-ERN11830 |  |  |  |  |  |  |
| 0 | Atr-ERN11831 |  |  |  |  |  |  |
| 0 | Atr-ERN11832 |  |  |  |  |  |  |
| 0 | Atr-ERN11833 |  |  |  |  |  |  |
| 0 | Atr-ERN11834 |  |  |  |  |  |  |
| 0 | Atr-ERN11835 |  |  |  |  |  |  |
| 0 | Atr-ERN11836 |  |  |  |  |  |  |
| 0 | Atr-ERN11837 |  |  |  |  |  |  |
| 0 | Atr-ERN11838 |  |  |  |  |  |  |
| 0 | Atr-ERN11839 |  |  |  |  |  |  |
| 0 | Atr-ERN11840 |  |  |  |  |  |  |
| 0 | Atr-ERN11841 |  |  |  |  |  |  |
| 0 | Atr-ERN11842 |  |  |  |  |  |  |
| 0 | Atr-ERN11843 |  |  |  |  |  |  |
| 0 | Atr-ERN11844 |  |  |  |  |  |  |
| 0 | Atr-ERN11845 |  |  |  |  |  |  |
| 0 | Atr-ERN11846 |  |  |  |  |  |  |
| 0 | Atr-ERN11847 |  |  |  |  |  |  |
| 0 | Atr-ERN11848 |  |  |  |  |  |  |
| 0 | Atr-ERN11849 |  |  |  |  |  |  |
| 0 | Atr-ERN11850 |  |  |  |  |  |  |
| 0 | Atr-ERN11851 |  |  |  |  |  |  |
| 0 | Atr-ERN11852 |  |  |  |  |  |  |
| 0 | Atr-ERN11853 |  |  |  |  |  |  |
| 0 | Atr-ERN11854 |  |  |  |  |  |  |
| 0 | Atr-ERN11855 |  |  |  |  |  |  |
| 0 | Atr-ERN11856 |  |  |  |  |  |  |
| 0 | Atr-ERN11857 |  |  |  |  |  |  |
| 0 | Atr-ERN11858 |  |  |  |  |  |  |
| 0 | Atr-ERN11859 |  |  |  |  |  |  |
| 0 | Atr-ERN11860 |  |  |  |  |  |  |
| 0 | Atr-ERN11861 |  |  |  |  |  |  |
| 0 | Atr-ERN11862 |  |  |  |  |  |  |
| 0 | Atr-ERN11863 |  |  |  |  |  |  |
| 0 | Atr-ERN11864 |  |  |  |  |  |  |
| 0 | Atr-ERN11865 |  |  |  |  |  |  |
| 0 | Atr-ERN11866 |  |  |  |  |  |  |
| 0 | Atr-ERN11867 |  |  |  |  |  |  |
| 0 | Atr-ERN11868 |  |  |  |  |  |  |
| 0 | Atr-ERN11869 |  |  |  |  |  |  |
| 0 | Atr-ERN11870 |  |  |  |  |  |  |
| 0 | Atr-ERN11871 |  |  |  |  |  |  |
| 0 | Atr-ERN11872 |  |  |  |  |  |  |
| 0 | Atr-ERN11873 |  |  |  |  |  |  |
| 0 | Atr-ERN11874 |  |  |  |  |  |  |
| 0 | Atr-ERN11875 |  |  |  |  |  |  |
| 0 | Atr-ERN11876 |  |  |  |  |  |  |
| 0 | Atr-ERN11877 |  |  |  |  |  |  |
| 0 | Atr-ERN11878 |  |  |  |  |  |  |
| 0 | Atr-ERN11879 |  |  |  |  |  |  |
| 0 | Atr-ERN11880 |  |  |  |  |  |  |
| 0 | Atr-ERN11881 |  |  |  |  |  |  |
| 0 | Atr-ERN11882 |  |  |  |  |  |  |
| 0 | Atr-ERN11883 |  |  |  |  |  |  |
| 0 | Atr-ERN11884 |  |  |  |  |  |  |
| 0 | Atr-ERN11885 |  |  |  |  |  |  |
| 0 | Atr-ERN11886 |  |  |  |  |  |  |
| 0 | Atr-ERN11887 |  |  |  |  |  |  |
| 0 | Atr-ERN11888 |  |  |  |  |  |  |
| 0 | Atr-ERN11889 |  |  |  |  |  |  |
| 0 | Atr-ERN11890 |  |  |  |  |  |  |
| 0 | Atr-ERN11891 |  |  |  |  |  |  |
| 0 | Atr-ERN11892 |  |  |  |  |  |  |
| 0 | Atr-ERN11893 |  |  |  |  |  |  |
| 0 | Atr-ERN11894 |  |  |  |  |  |  |
| 0 | Atr-ERN11895 |  |  |  |  |  |  |
| 0 | Atr-ERN11896 |  |  |  |  |  |  |
| 0 | Atr-ERN11897 |  |  |  |  |  |  |
| 0 | Atr-ERN11898 |  |  |  |  |  |  |
| 0 | Atr-ERN11899 |  |  |  |  |  |  |
| 0 | Atr-ERN11900 |  |  |  |  |  |  |
| 0 | Atr-ERN11901 |  |  |  |  |  |  |
| 0 | Atr-ERN11902 |  |  |  |  |  |  |
| 0 | Atr-ERN11903 |  |  |  |  |  |  |
| 0 | Atr-ERN11904 |  |  |  |  |  |  |
| 0 | Atr-ERN11905 |  |  |  |  |  |  |
| 0 | Atr-ERN11906 |  |  |  |  |  |  |
| 0 | Atr-ERN11907 |  |  |  |  |  |  |
| 0 | Atr-ERN11908 |  |  |  |  |  |  |
| 0 | Atr-ERN11909 |  |  |  |  |  |  |
| 0 | Atr-ERN11910 |  |  |  |  |  |  |
| 0 | Atr-ERN11911 |  |  |  |  |  |  |
| 0 | Atr-ERN11912 |  |  |  |  |  |  |
| 0 | Atr-ERN11913 |  |  |  |  |  |  |
| 0 | Atr-ERN11914 |  |  |  |  |  |  |
| 0 | Atr-ERN11915 |  |  |  |  |  |  |
| 0 | Atr-ERN11916 |  |  |  |  |  |  |
| 0 | Atr-ERN11917 |  |  |  |  |  |  |
| 0 | Atr-ERN11918 |  |  |  |  |  |  |
| 0 | Atr-ERN11919 |  |  |  |  |  |  |
| 0 | Atr-ERN11920 |  |  |  |  |  |  |
| 0 | Atr-ERN11921 |  |  |  |  |  |  |
| 0 | Atr-ERN11922 |  |  |  |  |  |  |
| 0 | Atr-ERN11923 |  |  |  |  |  |  |
| 0 | Atr-ERN11924 |  |  |  |  |  |  |
| 0 | Atr-ERN11925 |  |  |  |  |  |  |
| 0 | Atr-ERN11926 |  |  |  |  |  |  |
| 0 | Atr-ERN11927 |  |  |  |  |  |  |
| 0 | Atr-ERN11928 |  |  |  |  |  |  |
| 0 | Atr-ERN11929 |  |  |  |  |  |  |
| 0 | Atr-ERN11930 |  |  |  |  |  |  |
| 0 | Atr-ERN11931 |  |  |  |  |  |  |
| 0 | Atr-ERN11932 |  |  |  |  |  |  |
| 0 | Atr-ERN11933 |  |  |  |  |  |  |
| 0 | Atr-ERN11934 |  |  |  |  |  |  |
| 0 | Atr-ERN11935 |  |  |  |  |  |  |
| 0 | Atr-ERN11936 |  |  |  |  |  |  |
| 0 | Atr-ERN11937 |  |  |  |  |  |  |
| 0 | Atr-ERN11938 |  |  |  |  |  |  |
| 0 | Atr-ERN11939 |  |  |  |  |  |  |
| 0 | Atr-ERN11940 |  |  |  |  |  |  |
| 0 | Atr-ERN11941 |  |  |  |  |  |  |
| 0 | Atr-ERN11942 |  |  |  |  |  |  |
| 0 | Atr-ERN11943 |  |  |  |  |  |  |
