# Supplementary material for: Genome-wide identification, characterization and gene expression of BES1 transcription factor family in grapevine (Vitis vinifera L.)
Source: Sci Rep. 2023 Jan 5;13:240. doi: 10.1038/s41598-022-24407-y (PMC9816167; doi:10.1038/s41598-022-24407-y)
Supplement: Supplementary file 3 — Supplementary Information. [file 41598_2022_24407_MOESM3_ESM.zip › Vvi_Atr/Vitis_vinifera.PN40024.v4.dna_sm.toplevel.fa.vs.Amborella_trichopoda.AMTR1.0.dna_sm.toplevel.fa.html/Atr-AmTr_v1.0_scaffold00021.html]

|  |  |  |  |  |  |  |  |  |  |  |  |  |  |
| --- | --- | --- | --- | --- | --- | --- | --- | --- | --- | --- | --- | --- | --- |
| Duplication depth | Reference chromosome | Collinear blocks | | | | | | | | | | | |
| 0 | Atr-ERN13862 |  |  |  |  |  |  |
| 0 | Atr-ERN13863 |  |  |  |  |  |  |
| 0 | Atr-ERN13864 |  |  |  |  |  |  |
| 1 | Atr-ERN13865 |  | Vvi-Vitvi19g00105\_t001 |  |  |  |  |  |
| 3 | Atr-ERN13866 |  | | | |  | Vvi-Vitvi12g00175\_t001 |  | Vvi-Vitvi10g00593\_t001 |  |  |  |
| 3 | Atr-ERN13867 |  | | | |  | | | |  | | | |  |  |  |
| 3 | Atr-ERN13868 |  | | | |  | Vvi-Vitvi12g00179\_t001 |  | Vvi-Vitvi10g00592\_t001 |  |  |  |
| 3 | Atr-ERN13869 |  | | | |  | | | |  | | | |  |  |  |
| 3 | Atr-ERN13870 |  | Vvi-Vitvi19g00106\_t001 |  | | | |  | | | |  |  |  |
| 3 | Atr-ERN13871 |  | | | |  | | | |  | Vvi-Vitvi10g00591\_t001 |  |  |  |
| 3 | Atr-ERN13872 |  | | | |  | Vvi-Vitvi12g00181\_t002 |  | | | |  |  |  |
| 3 | Atr-ERN13873 |  | | | |  | | | |  | | | |  |  |  |
| 3 | Atr-ERN13874 |  | | | |  | | | |  | | | |  |  |  |
| 3 | Atr-ERN13875 |  | | | |  | | | |  | | | |  |  |  |
| 3 | Atr-ERN13876 |  | | | |  | Vvi-Vitvi12g02250\_t001 |  | | | |  |  |  |
| 3 | Atr-ERN13877 |  | Vvi-Vitvi19g00114\_t001 |  | | | |  | | | |  |  |  |
| 3 | Atr-ERN13878 |  | | | |  | | | |  | | | |  |  |  |
| 3 | Atr-ERN13879 |  | Vvi-Vitvi19g00121\_t003 |  | | | |  | | | |  |  |  |
| 3 | Atr-ERN13880 |  | | | |  | | | |  | | | |  |  |  |
| 3 | Atr-ERN13881 |  | | | |  | | | |  | | | |  |  |  |
| 3 | Atr-ERN13882 |  | | | |  | | | |  | | | |  |  |  |
| 3 | Atr-ERN13883 |  | | | |  | | | |  | | | |  |  |  |
| 3 | Atr-ERN13884 |  | | | |  | | | |  | | | |  |  |  |
| 3 | Atr-ERN13885 |  | | | |  | | | |  | Vvi-Vitvi10g00585\_t001 |  |  |  |
| 3 | Atr-ERN13886 |  | | | |  | | | |  | | | |  |  |  |
| 3 | Atr-ERN13887 |  | | | |  | Vvi-Vitvi12g00184\_t001 |  | | | |  |  |  |
| 3 | Atr-ERN13888 |  | | | |  | | | |  | Vvi-Vitvi10g00581\_t001 |  |  |  |
| 3 | Atr-ERN13889 |  | | | |  | Vvi-Vitvi12g00185\_t001 |  | | | |  |  |  |
| 3 | Atr-ERN13890 |  | | | |  | | | |  | | | |  |  |  |
| 3 | Atr-ERN13891 |  | | | |  | | | |  | | | |  |  |  |
| 3 | Atr-ERN13892 |  | | | |  | | | |  | Vvi-Vitvi10g00577\_t001 |  |  |  |
| 3 | Atr-ERN13893 |  | | | |  | | | |  | | | |  |  |  |
| 3 | Atr-ERN13894 |  | | | |  | Vvi-Vitvi12g02253\_t001 |  | Vvi-Vitvi10g00575\_t001 |  |  |  |
| 3 | Atr-ERN13895 |  | Vvi-Vitvi19g00122\_t001 |  | Vvi-Vitvi12g00193\_t001 |  | | | |  |  |  |
| 3 | Atr-ERN13896 |  | | | |  | | | |  | | | |  |  |  |
| 3 | Atr-ERN13897 |  | | | |  | | | |  | | | |  |  |  |
| 3 | Atr-ERN13898 |  | | | |  | | | |  | | | |  |  |  |
| 3 | Atr-ERN13899 |  | | | |  | | | |  | | | |  |  |  |
| 3 | Atr-ERN13900 |  | Vvi-Vitvi19g00123\_t001 |  | | | |  | | | |  |  |  |
| 3 | Atr-ERN13901 |  | | | |  | | | |  | | | |  |  |  |
| 3 | Atr-ERN13902 |  | | | |  | | | |  | | | |  |  |  |
| 3 | Atr-ERN13903 |  | | | |  | Vvi-Vitvi12g00198\_t001 |  | Vvi-Vitvi10g00573\_t001 |  |  |  |
| 3 | Atr-ERN13904 |  | Vvi-Vitvi19g00125\_t001 |  | | | |  | | | |  |  |  |
| 3 | Atr-ERN13905 |  | | | |  | | | |  | | | |  |  |  |
| 3 | Atr-ERN13906 |  | | | |  | | | |  | | | |  |  |  |
| 3 | Atr-ERN13907 |  | | | |  | | | |  | | | |  |  |  |
| 3 | Atr-ERN13908 |  | | | |  | | | |  | | | |  |  |  |
| 3 | Atr-ERN13909 |  | | | |  | | | |  | | | |  |  |  |
| 3 | Atr-ERN13910 |  | | | |  | | | |  | | | |  |  |  |
| 3 | Atr-ERN13911 |  | | | |  | | | |  | | | |  |  |  |
| 3 | Atr-ERN13912 |  | | | |  | Vvi-Vitvi12g00200\_t001 |  | Vvi-Vitvi10g00572\_t001 |  |  |  |
| 3 | Atr-ERN13913 |  | Vvi-Vitvi19g00128\_t001 |  | Vvi-Vitvi12g00201\_t001 |  | | | |  |  |  |
| 3 | Atr-ERN13914 |  | Vvi-Vitvi19g00129\_t001 |  | | | |  | | | |  |  |  |
| 3 | Atr-ERN13915 |  | | | |  | Vvi-Vitvi12g00204\_t001 |  | | | |  |  |  |
| 3 | Atr-ERN13916 |  | | | |  | | | |  | | | |  |  |  |
| 3 | Atr-ERN13917 |  | | | |  | | | |  | | | |  |  |  |
| 3 | Atr-ERN13918 |  | | | |  | | | |  | | | |  |  |  |
| 3 | Atr-ERN13919 |  | | | |  | | | |  | Vvi-Vitvi10g00570\_t001 |  |  |  |
| 3 | Atr-ERN13920 |  | Vvi-Vitvi19g00131\_t001 |  | | | |  | Vvi-Vitvi10g04360\_t001 |  |  |  |
| 3 | Atr-ERN13921 |  | | | |  | | | |  | | | |  |  |  |
| 3 | Atr-ERN13922 |  | | | |  | | | |  | | | |  |  |  |
| 3 | Atr-ERN13923 |  | | | |  | | | |  | | | |  |  |  |
| 3 | Atr-ERN13924 |  | | | |  | Vvi-Vitvi12g00206\_t001 |  | | | |  |  |  |
| 3 | Atr-ERN13925 |  | | | |  | | | |  | Vvi-Vitvi10g00568\_t001 |  |  |  |
| 3 | Atr-ERN13926 |  | | | |  | | | |  | | | |  |  |  |
| 3 | Atr-ERN13927 |  | | | |  | | | |  | Vvi-Vitvi10g00565\_t001 |  |  |  |
| 3 | Atr-ERN13928 |  | | | |  | | | |  | | | |  |  |  |
| 3 | Atr-ERN13929 |  | | | |  | Vvi-Vitvi12g00209\_t001 |  | Vvi-Vitvi10g00564\_t001 |  |  |  |
| 3 | Atr-ERN13930 |  | | | |  | | | |  | | | |  |  |  |
| 3 | Atr-ERN13931 |  | | | |  | | | |  | | | |  |  |  |
| 3 | Atr-ERN13932 |  | | | |  | | | |  | | | |  |  |  |
| 3 | Atr-ERN13933 |  | | | |  | | | |  | | | |  |  |  |
| 3 | Atr-ERN13934 |  | | | |  | | | |  | | | |  |  |  |
| 3 | Atr-ERN13935 |  | | | |  | | | |  | | | |  |  |  |
| 3 | Atr-ERN13936 |  | Vvi-Vitvi19g00134\_t001 |  | | | |  | | | |  |  |  |
| 3 | Atr-ERN13937 |  | | | |  | | | |  | | | |  |  |  |
| 3 | Atr-ERN13938 |  | | | |  | | | |  | Vvi-Vitvi10g00563\_t001 |  |  |  |
| 3 | Atr-ERN13939 |  | | | |  | | | |  | Vvi-Vitvi10g00562\_t001 |  |  |  |
| 3 | Atr-ERN13940 |  | | | |  | | | |  | | | |  |  |  |
| 3 | Atr-ERN13941 |  | | | |  | | | |  | | | |  |  |  |
| 3 | Atr-ERN13942 |  | | | |  | | | |  | | | |  |  |  |
| 3 | Atr-ERN13943 |  | | | |  | Vvi-Vitvi12g00212\_t001 |  | Vvi-Vitvi10g00561\_t001 |  |  |  |
| 3 | Atr-ERN13944 |  | | | |  | | | |  | Vvi-Vitvi10g04358\_t001 |  |  |  |
| 3 | Atr-ERN13945 |  | | | |  | | | |  | | | |  |  |  |
| 3 | Atr-ERN13946 |  | Vvi-Vitvi19g00137\_t001 |  | | | |  | | | |  |  |  |
| 3 | Atr-ERN13947 |  | | | |  | | | |  | | | |  |  |  |
| 3 | Atr-ERN13948 |  | Vvi-Vitvi19g00138\_t001 |  | | | |  | Vvi-Vitvi10g00554\_t001 |  |  |  |
| 3 | Atr-ERN13949 |  | | | |  | | | |  | | | |  |  |  |
| 3 | Atr-ERN13950 |  | | | |  | Vvi-Vitvi12g02262\_t001 |  | Vvi-Vitvi10g01790\_t001 |  |  |  |
| 3 | Atr-ERN13951 |  | | | |  | Vvi-Vitvi12g00214\_t001 |  | | | |  |  |  |
| 3 | Atr-ERN13952 |  | | | |  | | | |  | | | |  |  |  |
| 3 | Atr-ERN13953 |  | Vvi-Vitvi19g01842\_t001 |  | Vvi-Vitvi12g00215\_t003 |  | | | |  |  |  |
| 3 | Atr-ERN13954 |  | | | |  | | | |  | | | |  |  |  |
| 3 | Atr-ERN13955 |  | | | |  | | | |  | | | |  |  |  |
| 3 | Atr-ERN13956 |  | | | |  | | | |  | | | |  |  |  |
| 3 | Atr-ERN13957 |  | | | |  | | | |  | | | |  |  |  |
| 3 | Atr-ERN13958 |  | | | |  | | | |  | | | |  |  |  |
| 3 | Atr-ERN13959 |  | | | |  | | | |  | | | |  |  |  |
| 3 | Atr-ERN13960 |  | | | |  | | | |  | | | |  |  |  |
| 3 | Atr-ERN13961 |  | | | |  | | | |  | | | |  |  |  |
| 3 | Atr-ERN13962 |  | | | |  | | | |  | | | |  |  |  |
| 3 | Atr-ERN13963 |  | | | |  | | | |  | Vvi-Vitvi10g00551\_t002 |  |  |  |
| 3 | Atr-ERN13964 |  | | | |  | | | |  | | | |  |  |  |
| 3 | Atr-ERN13965 |  | | | |  | | | |  | | | |  |  |  |
| 3 | Atr-ERN13966 |  | | | |  | Vvi-Vitvi12g00217\_t001 |  | | | |  |  |  |
| 3 | Atr-ERN13967 |  | | | |  | | | |  | | | |  |  |  |
| 3 | Atr-ERN13968 |  | Vvi-Vitvi19g00139\_t001 |  | | | |  | | | |  |  |  |
| 3 | Atr-ERN13969 |  | | | |  | | | |  | | | |  |  |  |
| 3 | Atr-ERN13970 |  | | | |  | | | |  | Vvi-Vitvi10g01789\_t001 |  |  |  |
| 3 | Atr-ERN13971 |  | | | |  | | | |  | Vvi-Vitvi10g00541\_t001 |  |  |  |
| 3 | Atr-ERN13972 |  | Vvi-Vitvi19g00140\_t001 |  | Vvi-Vitvi12g00219\_t001 |  | Vvi-Vitvi10g00540\_t001 |  |  |  |
| 3 | Atr-ERN13973 |  | Vvi-Vitvi19g00141\_t001 |  | | | |  | Vvi-Vitvi10g00539\_t001 |  |  |  |
| 3 | Atr-ERN13974 |  | | | |  | | | |  | | | |  |  |  |
| 3 | Atr-ERN13975 |  | | | |  | | | |  | | | |  |  |  |
| 3 | Atr-ERN13976 |  | | | |  | Vvi-Vitvi12g00220\_t001 |  | Vvi-Vitvi10g01788\_t001 |  |  |  |
| 3 | Atr-ERN13977 |  | | | |  | | | |  | | | |  |  |  |
| 3 | Atr-ERN13978 |  | | | |  | Vvi-Vitvi12g00221\_t001 |  | | | |  |  |  |
| 3 | Atr-ERN13979 |  | | | |  | | | |  | | | |  |  |  |
| 3 | Atr-ERN13980 |  | Vvi-Vitvi19g00143\_t001 |  | Vvi-Vitvi12g04057\_t001 |  | Vvi-Vitvi10g00537\_t001 |  |  |  |
| 3 | Atr-ERN13981 |  | | | |  | | | |  | Vvi-Vitvi10g00536\_t001 |  |  |  |
| 3 | Atr-ERN13982 |  | | | |  | Vvi-Vitvi12g00225\_t002 |  | Vvi-Vitvi10g00535\_t001 |  |  |  |
| 3 | Atr-ERN13983 |  | Vvi-Vitvi19g00147\_t001 |  | Vvi-Vitvi12g00226\_t001 |  | | | |  |  |  |
| 3 | Atr-ERN13984 |  | | | |  | | | |  | | | |  |  |  |
| 3 | Atr-ERN13985 |  | | | |  | | | |  | | | |  |  |  |
| 3 | Atr-ERN13986 |  | | | |  | | | |  | Vvi-Vitvi10g04338\_t001 |  |  |  |
| 3 | Atr-ERN13987 |  | Vvi-Vitvi19g00148\_t001 |  | | | |  | Vvi-Vitvi10g00529\_t001 |  |  |  |
| 3 | Atr-ERN13988 |  | | | |  | | | |  | | | |  |  |  |
| 3 | Atr-ERN13989 |  | | | |  | | | |  | | | |  |  |  |
| 3 | Atr-ERN13990 |  | | | |  | | | |  | | | |  |  |  |
| 3 | Atr-ERN13991 |  | | | |  | | | |  | | | |  |  |  |
| 3 | Atr-ERN13992 |  | | | |  | | | |  | | | |  |  |  |
| 3 | Atr-ERN13993 |  | | | |  | | | |  | | | |  |  |  |
| 3 | Atr-ERN13994 |  | | | |  | | | |  | | | |  |  |  |
| 3 | Atr-ERN13995 |  | | | |  | | | |  | | | |  |  |  |
| 3 | Atr-ERN13996 |  | | | |  | | | |  | | | |  |  |  |
| 3 | Atr-ERN13997 |  | | | |  | | | |  | | | |  |  |  |
| 3 | Atr-ERN13998 |  | | | |  | | | |  | Vvi-Vitvi10g00527\_t001 |  |  |  |
| 3 | Atr-ERN13999 |  | | | |  | | | |  | Vvi-Vitvi10g00525\_t001 |  |  |  |
| 3 | Atr-ERN14000 |  | | | |  | | | |  | | | |  |  |  |
| 3 | Atr-ERN14001 |  | | | |  | | | |  | Vvi-Vitvi10g00523\_t001 |  |  |  |
| 3 | Atr-ERN14002 |  | | | |  | | | |  | | | |  |  |  |
| 3 | Atr-ERN14003 |  | | | |  | | | |  | Vvi-Vitvi10g00521\_t001 |  |  |  |
| 3 | Atr-ERN14004 |  | | | |  | Vvi-Vitvi12g00229\_t001 |  | | | |  |  |  |
| 3 | Atr-ERN14005 |  | | | |  | | | |  | | | |  |  |  |
| 3 | Atr-ERN14006 |  | | | |  | | | |  | | | |  |  |  |
| 3 | Atr-ERN14007 |  | | | |  | Vvi-Vitvi12g00231\_t001 |  | Vvi-Vitvi10g00520\_t001 |  |  |  |
| 3 | Atr-ERN14008 |  | | | |  | | | |  | | | |  |  |  |
| 3 | Atr-ERN14009 |  | | | |  | | | |  | | | |  |  |  |
| 3 | Atr-ERN14010 |  | | | |  | | | |  | Vvi-Vitvi10g00519\_t001 |  |  |  |
| 3 | Atr-ERN14011 |  | Vvi-Vitvi19g00153\_t001 |  | Vvi-Vitvi12g00233\_t001 |  | Vvi-Vitvi10g01781\_t001 |  |  |  |
| 3 | Atr-ERN14012 |  | Vvi-Vitvi19g00155\_t003 |  | | | |  | | | |  |  |  |
| 3 | Atr-ERN14013 |  | | | |  | | | |  | | | |  |  |  |
| 3 | Atr-ERN14014 |  | | | |  | | | |  | Vvi-Vitvi10g00518\_t001 |  |  |  |
| 3 | Atr-ERN14015 |  | | | |  | Vvi-Vitvi12g00234\_t001 |  | | | |  |  |  |
| 3 | Atr-ERN14016 |  | | | |  | | | |  | | | |  |  |  |
| 3 | Atr-ERN14017 |  | Vvi-Vitvi19g00156\_t001 |  | | | |  | | | |  |  |  |
| 3 | Atr-ERN14018 |  | | | |  | | | |  | | | |  |  |  |
| 3 | Atr-ERN14019 |  | | | |  | | | |  | | | |  |  |  |
| 3 | Atr-ERN14020 |  | | | |  | | | |  | Vvi-Vitvi10g00517\_t002 |  |  |  |
| 3 | Atr-ERN14021 |  | | | |  | Vvi-Vitvi12g00235\_t001 |  | Vvi-Vitvi10g00515\_t001 |  |  |  |
| 3 | Atr-ERN14022 |  | | | |  | | | |  | | | |  |  |  |
| 3 | Atr-ERN14023 |  | Vvi-Vitvi19g00158\_t001 |  | | | |  | | | |  |  |  |
| 3 | Atr-ERN14024 |  | | | |  | Vvi-Vitvi12g00236\_t001 |  | Vvi-Vitvi10g00514\_t001 |  |  |  |
| 3 | Atr-ERN14025 |  | Vvi-Vitvi19g00159\_t001 |  | | | |  | | | |  |  |  |
| 3 | Atr-ERN14026 |  | Vvi-Vitvi19g00161\_t001 |  | | | |  | Vvi-Vitvi10g00511\_t001 |  |  |  |
| 3 | Atr-ERN14027 |  | Vvi-Vitvi19g00162\_t001 |  | | | |  | | | |  |  |  |
| 3 | Atr-ERN14028 |  | | | |  | | | |  | | | |  |  |  |
| 3 | Atr-ERN14029 |  | | | |  | | | |  | Vvi-Vitvi10g00510\_t002 |  |  |  |
| 3 | Atr-ERN14030 |  | Vvi-Vitvi19g00178\_t001 |  | | | |  | | | |  |  |  |
| 3 | Atr-ERN14031 |  | | | |  | | | |  | | | |  |  |  |
| 3 | Atr-ERN14032 |  | Vvi-Vitvi19g00179\_t001 |  | | | |  | | | |  |  |  |
| 3 | Atr-ERN14033 |  | | | |  | | | |  | | | |  |  |  |
| 3 | Atr-ERN14034 |  | Vvi-Vitvi19g00180\_t001 |  | | | |  | | | |  |  |  |
| 2 | Atr-ERN14035 |  |  |  | | | |  | | | |  |  |  |
| 2 | Atr-ERN14036 |  |  |  | Vvi-Vitvi12g00251\_t001 |  | Vvi-Vitvi10g00508\_t001 |  |  |  |
| 2 | Atr-ERN14037 |  |  |  | Vvi-Vitvi12g02295\_t001 |  | | | |  |  |  |
| 2 | Atr-ERN14038 |  |  |  | | | |  | | | |  |  |  |
| 2 | Atr-ERN14039 |  |  |  | | | |  | | | |  |  |  |
| 2 | Atr-ERN14040 |  |  |  | Vvi-Vitvi12g02296\_t001 |  | | | |  |  |  |
| 2 | Atr-ERN14041 |  |  |  | | | |  | Vvi-Vitvi10g00506\_t001 |  |  |  |
| 2 | Atr-ERN14042 |  |  |  | Vvi-Vitvi12g00255\_t001 |  | Vvi-Vitvi10g00505\_t001 |  |  |  |
| 2 | Atr-ERN14043 |  |  |  | | | |  | | | |  |  |  |
| 2 | Atr-ERN14044 |  |  |  | | | |  | | | |  |  |  |
| 2 | Atr-ERN14045 |  |  |  | | | |  | Vvi-Vitvi10g00504\_t001 |  |  |  |
| 2 | Atr-ERN14046 |  |  |  | | | |  | | | |  |  |  |
| 2 | Atr-ERN14047 |  |  |  | Vvi-Vitvi12g00256\_t001 |  | Vvi-Vitvi10g00503\_t001 |  |  |  |
| 2 | Atr-ERN14048 |  |  |  | | | |  | | | |  |  |  |
| 2 | Atr-ERN14049 |  |  |  | | | |  | Vvi-Vitvi10g00499\_t003 |  |  |  |
| 1 | Atr-ERN14050 |  |  |  | Vvi-Vitvi12g00269\_t001 |  |  |  |  |
| 0 | Atr-ERN14051 |  |  |  |  |  |  |
| 0 | Atr-ERN14052 |  |  |  |  |  |  |
| 1 | Atr-ERN14053 |  | Vvi-Vitvi02g00178\_t001 |  |  |  |  |  |
| 1 | Atr-ERN14054 |  | | | |  |  |  |  |  |
| 3 | Atr-ERN14055 |  | | | |  | Vvi-Vitvi16g02085\_t001 |  | Vvi-Vitvi15g00945\_t008 |  |  |  |
| 3 | Atr-ERN14056 |  | | | |  | Vvi-Vitvi16g01399\_t002 |  | | | |  |  |  |
| 3 | Atr-ERN14057 |  | | | |  | Vvi-Vitvi16g04519\_t002 |  | | | |  |  |  |
| 3 | Atr-ERN14058 |  | Vvi-Vitvi02g00177\_t001 |  | Vvi-Vitvi16g01401\_t001.2.6037826c |  | | | |  |  |  |
| 3 | Atr-ERN14059 |  | | | |  | | | |  | Vvi-Vitvi15g01569\_t001 |  |  |  |
| 3 | Atr-ERN14060 |  | | | |  | | | |  | Vvi-Vitvi15g01570\_t001 |  |  |  |
| 3 | Atr-ERN14061 |  | | | |  | Vvi-Vitvi16g01402\_t001 |  | | | |  |  |  |
| 3 | Atr-ERN14062 |  | Vvi-Vitvi02g00176\_t001 |  | | | |  | | | |  |  |  |
| 3 | Atr-ERN14063 |  | | | |  | Vvi-Vitvi16g01403\_t002 |  | | | |  |  |  |
| 3 | Atr-ERN14064 |  | | | |  | | | |  | | | |  |  |  |
| 3 | Atr-ERN14065 |  | | | |  | | | |  | | | |  |  |  |
| 3 | Atr-ERN14066 |  | | | |  | | | |  | | | |  |  |  |
| 3 | Atr-ERN14067 |  | Vvi-Vitvi02g00175\_t001 |  | | | |  | | | |  |  |  |
| 3 | Atr-ERN14068 |  | | | |  | | | |  | | | |  |  |  |
| 3 | Atr-ERN14069 |  | | | |  | | | |  | | | |  |  |  |
| 3 | Atr-ERN14070 |  | | | |  | | | |  | | | |  |  |  |
| 3 | Atr-ERN14071 |  | | | |  | | | |  | Vvi-Vitvi15g00946\_t001 |  |  |  |
| 3 | Atr-ERN14072 |  | | | |  | Vvi-Vitvi16g01404\_t001 |  | | | |  |  |  |
| 3 | Atr-ERN14073 |  | | | |  | | | |  | | | |  |  |  |
| 3 | Atr-ERN14074 |  | Vvi-Vitvi02g01341\_t001 |  | | | |  | | | |  |  |  |
| 3 | Atr-ERN14075 |  | | | |  | | | |  | Vvi-Vitvi15g00947\_t001 |  |  |  |
| 3 | Atr-ERN14076 |  | Vvi-Vitvi02g00161\_t001 |  | | | |  | | | |  |  |  |
| 3 | Atr-ERN14077 |  | | | |  | Vvi-Vitvi16g01405\_t001 |  | | | |  |  |  |
| 3 | Atr-ERN14078 |  | | | |  | | | |  | | | |  |  |  |
| 3 | Atr-ERN14079 |  | Vvi-Vitvi02g00160\_t001 |  | | | |  | | | |  |  |  |
| 3 | Atr-ERN14080 |  | | | |  | Vvi-Vitvi16g01406\_t001 |  | | | |  |  |  |
| 3 | Atr-ERN14081 |  | | | |  | | | |  | Vvi-Vitvi15g00948\_t001 |  |  |  |
| 3 | Atr-ERN14082 |  | | | |  | | | |  | | | |  |  |  |
| 3 | Atr-ERN14083 |  | | | |  | Vvi-Vitvi16g01407\_t001 |  | | | |  |  |  |
| 3 | Atr-ERN14084 |  | | | |  | | | |  | | | |  |  |  |
| 3 | Atr-ERN14085 |  | | | |  | | | |  | | | |  |  |  |
| 3 | Atr-ERN14086 |  | Vvi-Vitvi02g00159\_t001 |  | | | |  | | | |  |  |  |
| 3 | Atr-ERN14087 |  | Vvi-Vitvi02g00158\_t001 |  | | | |  | | | |  |  |  |
| 3 | Atr-ERN14088 |  | Vvi-Vitvi02g00157\_t001 |  | | | |  | | | |  |  |  |
| 3 | Atr-ERN14089 |  | | | |  | | | |  | | | |  |  |  |
| 3 | Atr-ERN14090 |  | | | |  | | | |  | | | |  |  |  |
| 3 | Atr-ERN14091 |  | | | |  | Vvi-Vitvi16g01410\_t001 |  | Vvi-Vitvi15g00950\_t001 |  |  |  |
| 3 | Atr-ERN14092 |  | | | |  | | | |  | | | |  |  |  |
| 3 | Atr-ERN14093 |  | | | |  | | | |  | Vvi-Vitvi15g00951\_t001 |  |  |  |
| 3 | Atr-ERN14094 |  | Vvi-Vitvi02g00156\_t001 |  | | | |  | | | |  |  |  |
| 3 | Atr-ERN14095 |  | | | |  | | | |  | Vvi-Vitvi15g00952\_t001 |  |  |  |
| 3 | Atr-ERN14096 |  | | | |  | | | |  | Vvi-Vitvi15g00953\_t001 |  |  |  |
| 3 | Atr-ERN14097 |  | | | |  | | | |  | | | |  |  |  |
| 3 | Atr-ERN14098 |  | Vvi-Vitvi02g00155\_t001 |  | | | |  | Vvi-Vitvi15g04556\_t001 |  |  |  |
| 3 | Atr-ERN14099 |  | | | |  | | | |  | Vvi-Vitvi15g00954\_t002 |  |  |  |
| 3 | Atr-ERN14100 |  | | | |  | | | |  | | | |  |  |  |
| 3 | Atr-ERN14101 |  | | | |  | | | |  | | | |  |  |  |
| 3 | Atr-ERN14102 |  | | | |  | | | |  | | | |  |  |  |
| 3 | Atr-ERN14103 |  | | | |  | Vvi-Vitvi16g01411\_t001 |  | | | |  |  |  |
| 3 | Atr-ERN14104 |  | Vvi-Vitvi02g00153\_t001 |  | | | |  | | | |  |  |  |
| 3 | Atr-ERN14105 |  | | | |  | | | |  | | | |  |  |  |
| 3 | Atr-ERN14106 |  | Vvi-Vitvi02g00152\_t001 |  | Vvi-Vitvi16g01415\_t001 |  | | | |  |  |  |
| 3 | Atr-ERN14107 |  | | | |  | | | |  | | | |  |  |  |
| 3 | Atr-ERN14108 |  | | | |  | | | |  | | | |  |  |  |
| 3 | Atr-ERN14109 |  | Vvi-Vitvi02g00151\_t001 |  | | | |  | | | |  |  |  |
| 3 | Atr-ERN14110 |  | | | |  | Vvi-Vitvi16g01416\_t002 |  | | | |  |  |  |
| 3 | Atr-ERN14111 |  | Vvi-Vitvi02g00149\_t001 |  | Vvi-Vitvi16g01417\_t003 |  | | | |  |  |  |
| 3 | Atr-ERN14112 |  | Vvi-Vitvi02g00147\_t001 |  | | | |  | Vvi-Vitvi15g00955\_t001 |  |  |  |
| 3 | Atr-ERN14113 |  | | | |  | | | |  | | | |  |  |  |
| 3 | Atr-ERN14114 |  | | | |  | | | |  | | | |  |  |  |
| 3 | Atr-ERN14115 |  | | | |  | | | |  | | | |  |  |  |
| 3 | Atr-ERN14116 |  | | | |  | Vvi-Vitvi16g01418\_t001 |  | | | |  |  |  |
| 2 | Atr-ERN14117 |  | Vvi-Vitvi02g00144\_t001 |  |  |  | Vvi-Vitvi15g00958\_t001 |  |  |  |
| 3 | Atr-ERN14118 |  | | | |  | Vvi-Vitvi16g01297\_t001 |  | | | |  |  |  |
| 3 | Atr-ERN14119 |  | | | |  | | | |  | | | |  |  |  |
| 3 | Atr-ERN14120 |  | | | |  | Vvi-Vitvi16g01291\_t001 |  | Vvi-Vitvi15g00960\_t002 |  |  |  |
| 3 | Atr-ERN14121 |  | | | |  | | | |  | | | |  |  |  |
| 3 | Atr-ERN14122 |  | | | |  | | | |  | Vvi-Vitvi15g00972\_t001 |  |  |  |
| 3 | Atr-ERN14123 |  | Vvi-Vitvi02g00140\_t001 |  | | | |  | Vvi-Vitvi15g00973\_t001 |  |  |  |
| 3 | Atr-ERN14124 |  | | | |  | Vvi-Vitvi16g01289\_t001 |  | | | |  |  |  |
| 3 | Atr-ERN14125 |  | Vvi-Vitvi02g00138\_t001 |  | | | |  | | | |  |  |  |
| 3 | Atr-ERN14126 |  | | | |  | | | |  | Vvi-Vitvi15g00974\_t001 |  |  |  |
| 3 | Atr-ERN14127 |  | | | |  | | | |  | | | |  |  |  |
| 3 | Atr-ERN14128 |  | Vvi-Vitvi02g00137\_t001 |  | | | |  | | | |  |  |  |
| 3 | Atr-ERN14129 |  | Vvi-Vitvi02g04022\_t001 |  | | | |  | | | |  |  |  |
| 3 | Atr-ERN14130 |  | | | |  | Vvi-Vitvi16g01288\_t001 |  | Vvi-Vitvi15g00976\_t001 |  |  |  |
| 3 | Atr-ERN14131 |  | Vvi-Vitvi02g00135\_t001 |  | Vvi-Vitvi16g01286\_t001 |  | | | |  |  |  |
| 3 | Atr-ERN14132 |  | | | |  | | | |  | | | |  |  |  |
| 3 | Atr-ERN14133 |  | | | |  | | | |  | | | |  |  |  |
| 3 | Atr-ERN14134 |  | | | |  | Vvi-Vitvi16g01285\_t002 |  | | | |  |  |  |
| 3 | Atr-ERN14135 |  | | | |  | Vvi-Vitvi16g01284\_t001 |  | | | |  |  |  |
| 3 | Atr-ERN14136 |  | | | |  | | | |  | | | |  |  |  |
| 3 | Atr-ERN14137 |  | | | |  | Vvi-Vitvi16g01283\_t001 |  | | | |  |  |  |
| 3 | Atr-ERN14138 |  | | | |  | Vvi-Vitvi16g01282\_t003 |  | | | |  |  |  |
| 3 | Atr-ERN14139 |  | | | |  | | | |  | Vvi-Vitvi15g01595\_t001 |  |  |  |
| 2 | Atr-ERN14140 |  | | | |  | | | |  |  |  |  |
| 2 | Atr-ERN14141 |  | | | |  | Vvi-Vitvi16g01281\_t001 |  |  |  |  |
| 1 | Atr-ERN14142 |  | | | |  |  |  |  |  |
| 1 | Atr-ERN14143 |  | | | |  |  |  |  |  |
| 1 | Atr-ERN14144 |  | | | |  |  |  |  |  |
| 1 | Atr-ERN14145 |  | | | |  |  |  |  |  |
| 1 | Atr-ERN14146 |  | | | |  |  |  |  |  |
| 1 | Atr-ERN14147 |  | Vvi-Vitvi02g00131\_t001 |  |  |  |  |  |
| 0 | Atr-ERN14148 |  |  |  |  |  |  |
| 0 | Atr-ERN14149 |  |  |  |  |  |  |
| 0 | Atr-ERN14150 |  |  |  |  |  |  |
| 0 | Atr-ERN14151 |  |  |  |  |  |  |
| 0 | Atr-ERN14152 |  |  |  |  |  |  |
| 0 | Atr-ERN14153 |  |  |  |  |  |  |
| 0 | Atr-ERN14154 |  |  |  |  |  |  |
| 0 | Atr-ERN14155 |  |  |  |  |  |  |
| 0 | Atr-ERN14156 |  |  |  |  |  |  |
| 0 | Atr-ERN14157 |  |  |  |  |  |  |
| 0 | Atr-ERN14158 |  |  |  |  |  |  |
| 0 | Atr-ERN14159 |  |  |  |  |  |  |
| 0 | Atr-ERN14160 |  |  |  |  |  |  |
