# Supplementary material for: Genome-wide identification, characterization and gene expression of BES1 transcription factor family in grapevine (Vitis vinifera L.)
Source: Sci Rep. 2023 Jan 5;13:240. doi: 10.1038/s41598-022-24407-y (PMC9816167; doi:10.1038/s41598-022-24407-y)
Supplement: Supplementary file 3 — Supplementary Information. [file 41598_2022_24407_MOESM3_ESM.zip › Vvi_Atr/Vitis_vinifera.PN40024.v4.dna_sm.toplevel.fa.vs.Amborella_trichopoda.AMTR1.0.dna_sm.toplevel.fa.html/Atr-AmTr_v1.0_scaffold00022.html]

|  |  |  |  |  |  |  |  |  |  |  |  |  |  |
| --- | --- | --- | --- | --- | --- | --- | --- | --- | --- | --- | --- | --- | --- |
| Duplication depth | Reference chromosome | Collinear blocks | | | | | | | | | | | |
| 0 | Atr-ERN11402 |  |  |  |  |  |  |
| 0 | Atr-ERN11403 |  |  |  |  |  |  |
| 0 | Atr-ERN11404 |  |  |  |  |  |  |
| 0 | Atr-ERN11405 |  |  |  |  |  |  |
| 0 | Atr-ERN11406 |  |  |  |  |  |  |
| 0 | Atr-ERN11407 |  |  |  |  |  |  |
| 0 | Atr-ERN11408 |  |  |  |  |  |  |
| 0 | Atr-ERN11409 |  |  |  |  |  |  |
| 0 | Atr-ERN11410 |  |  |  |  |  |  |
| 0 | Atr-ERN11411 |  |  |  |  |  |  |
| 0 | Atr-ERN11412 |  |  |  |  |  |  |
| 0 | Atr-ERN11413 |  |  |  |  |  |  |
| 0 | Atr-ERN11414 |  |  |  |  |  |  |
| 1 | Atr-ERN11415 |  | Vvi-Vitvi18g04003\_t001 |  |  |  |  |  |
| 1 | Atr-ERN11416 |  | | | |  |  |  |  |  |
| 1 | Atr-ERN11417 |  | | | |  |  |  |  |  |
| 1 | Atr-ERN11418 |  | | | |  |  |  |  |  |
| 2 | Atr-ERN11419 |  | Vvi-Vitvi18g00023\_t001 |  | Vvi-Vitvi03g00267\_t001 |  |  |  |  |
| 2 | Atr-ERN11420 |  | | | |  | | | |  |  |  |  |
| 2 | Atr-ERN11421 |  | | | |  | Vvi-Vitvi03g00268\_t001 |  |  |  |  |
| 2 | Atr-ERN11422 |  | Vvi-Vitvi18g02472\_t001 |  | | | |  |  |  |  |
| 2 | Atr-ERN11423 |  | Vvi-Vitvi18g00020\_t001 |  | | | |  |  |  |  |
| 2 | Atr-ERN11424 |  | | | |  | | | |  |  |  |  |
| 2 | Atr-ERN11425 |  | Vvi-Vitvi18g02471\_t001 |  | | | |  |  |  |  |
| 2 | Atr-ERN11426 |  | | | |  | | | |  |  |  |  |
| 2 | Atr-ERN11427 |  | Vvi-Vitvi18g00018\_t001 |  | | | |  |  |  |  |
| 2 | Atr-ERN11428 |  | | | |  | | | |  |  |  |  |
| 2 | Atr-ERN11429 |  | | | |  | | | |  |  |  |  |
| 2 | Atr-ERN11430 |  | | | |  | Vvi-Vitvi03g00269\_t001 |  |  |  |  |
| 2 | Atr-ERN11431 |  | | | |  | Vvi-Vitvi03g00270\_t001 |  |  |  |  |
| 2 | Atr-ERN11432 |  | | | |  | Vvi-Vitvi03g00271\_t001 |  |  |  |  |
| 2 | Atr-ERN11433 |  | | | |  | Vvi-Vitvi03g00272\_t001.1.6037826d |  |  |  |  |
| 2 | Atr-ERN11434 |  | | | |  | | | |  |  |  |  |
| 2 | Atr-ERN11435 |  | | | |  | | | |  |  |  |  |
| 2 | Atr-ERN11436 |  | | | |  | Vvi-Vitvi03g01477\_t001 |  |  |  |  |
| 1 | Atr-ERN11437 |  | | | |  |  |  |  |  |
| 1 | Atr-ERN11438 |  | | | |  |  |  |  |  |
| 1 | Atr-ERN11439 |  | | | |  |  |  |  |  |
| 1 | Atr-ERN11440 |  | Vvi-Vitvi18g00015\_t001 |  |  |  |  |  |
| 1 | Atr-ERN11441 |  | | | |  |  |  |  |  |
| 1 | Atr-ERN11442 |  | | | |  |  |  |  |  |
| 1 | Atr-ERN11443 |  | Vvi-Vitvi18g00013\_t001 |  |  |  |  |  |
| 1 | Atr-ERN11444 |  | | | |  |  |  |  |  |
| 1 | Atr-ERN11445 |  | | | |  |  |  |  |  |
| 1 | Atr-ERN11446 |  | | | |  |  |  |  |  |
| 1 | Atr-ERN11447 |  | Vvi-Vitvi18g00011\_t001 |  |  |  |  |  |
| 1 | Atr-ERN11448 |  | Vvi-Vitvi18g02468\_t001 |  |  |  |  |  |
| 1 | Atr-ERN11449 |  | | | |  |  |  |  |  |
| 1 | Atr-ERN11450 |  | | | |  |  |  |  |  |
| 1 | Atr-ERN11451 |  | | | |  |  |  |  |  |
| 1 | Atr-ERN11452 |  | Vvi-Vitvi18g00010\_t001 |  |  |  |  |  |
| 1 | Atr-ERN11453 |  | | | |  |  |  |  |  |
| 1 | Atr-ERN11454 |  | | | |  |  |  |  |  |
| 1 | Atr-ERN11455 |  | | | |  |  |  |  |  |
| 1 | Atr-ERN11456 |  | | | |  |  |  |  |  |
| 1 | Atr-ERN11457 |  | | | |  |  |  |  |  |
| 1 | Atr-ERN11458 |  | | | |  |  |  |  |  |
| 1 | Atr-ERN11459 |  | Vvi-Vitvi18g00009\_t002 |  |  |  |  |  |
| 1 | Atr-ERN11460 |  | | | |  |  |  |  |  |
| 1 | Atr-ERN11461 |  | | | |  |  |  |  |  |
| 1 | Atr-ERN11462 |  | | | |  |  |  |  |  |
| 1 | Atr-ERN11463 |  | | | |  |  |  |  |  |
| 1 | Atr-ERN11464 |  | | | |  |  |  |  |  |
| 1 | Atr-ERN11465 |  | | | |  |  |  |  |  |
| 1 | Atr-ERN11466 |  | | | |  |  |  |  |  |
| 1 | Atr-ERN11467 |  | Vvi-Vitvi18g00008\_t001 |  |  |  |  |  |
| 1 | Atr-ERN11468 |  | | | |  |  |  |  |  |
| 1 | Atr-ERN11469 |  | | | |  |  |  |  |  |
| 1 | Atr-ERN11470 |  | Vvi-Vitvi18g00006\_t001 |  |  |  |  |  |
| 1 | Atr-ERN11471 |  | | | |  |  |  |  |  |
| 1 | Atr-ERN11472 |  | | | |  |  |  |  |  |
| 2 | Atr-ERN11473 |  | | | |  | Vvi-Vitvi18g00004\_t001 |  |  |  |  |
| 2 | Atr-ERN11474 |  | | | |  | | | |  |  |  |  |
| 2 | Atr-ERN11475 |  | | | |  | | | |  |  |  |  |
| 2 | Atr-ERN11476 |  | | | |  | | | |  |  |  |  |
| 2 | Atr-ERN11477 |  | Vvi-Vitvi18g00003\_t001 |  | | | |  |  |  |  |
| 2 | Atr-ERN11478 |  | | | |  | | | |  |  |  |  |
| 2 | Atr-ERN11479 |  | | | |  | | | |  |  |  |  |
| 2 | Atr-ERN11480 |  | | | |  | | | |  |  |  |  |
| 2 | Atr-ERN11481 |  | Vvi-Vitvi18g00002\_t003 |  | | | |  |  |  |  |
| 2 | Atr-ERN11482 |  | Vvi-Vitvi18g04000\_t002 |  | | | |  |  |  |  |
| 1 | Atr-ERN11483 |  |  |  | | | |  |  |  |  |
| 1 | Atr-ERN11484 |  |  |  | | | |  |  |  |  |
| 1 | Atr-ERN11485 |  |  |  | | | |  |  |  |  |
| 1 | Atr-ERN11486 |  |  |  | | | |  |  |  |  |
| 1 | Atr-ERN11487 |  |  |  | | | |  |  |  |  |
| 1 | Atr-ERN11488 |  |  |  | | | |  |  |  |  |
| 1 | Atr-ERN11489 |  |  |  | | | |  |  |  |  |
| 1 | Atr-ERN11490 |  |  |  | Vvi-Vitvi18g04003\_t001 |  |  |  |  |
| 1 | Atr-ERN11491 |  |  |  | | | |  |  |  |  |
| 1 | Atr-ERN11492 |  |  |  | | | |  |  |  |  |
| 1 | Atr-ERN11493 |  |  |  | | | |  |  |  |  |
| 1 | Atr-ERN11494 |  |  |  | | | |  |  |  |  |
| 1 | Atr-ERN11495 |  |  |  | | | |  |  |  |  |
| 1 | Atr-ERN11496 |  |  |  | Vvi-Vitvi18g00027\_t001 |  |  |  |  |
| 1 | Atr-ERN11497 |  |  |  | | | |  |  |  |  |
| 2 | Atr-ERN11498 |  | Vvi-Vitvi04g02260\_t001 |  | | | |  |  |  |  |
| 2 | Atr-ERN11499 |  | Vvi-Vitvi04g01754\_t001 |  | | | |  |  |  |  |
| 2 | Atr-ERN11500 |  | | | |  | Vvi-Vitvi18g00030\_t001 |  |  |  |  |
| 2 | Atr-ERN11501 |  | | | |  | Vvi-Vitvi18g00031\_t001 |  |  |  |  |
| 2 | Atr-ERN11502 |  | | | |  | | | |  |  |  |  |
| 2 | Atr-ERN11503 |  | | | |  | | | |  |  |  |  |
| 2 | Atr-ERN11504 |  | | | |  | | | |  |  |  |  |
| 2 | Atr-ERN11505 |  | | | |  | | | |  |  |  |  |
| 2 | Atr-ERN11506 |  | | | |  | | | |  |  |  |  |
| 2 | Atr-ERN11507 |  | Vvi-Vitvi04g02259\_t001 |  | | | |  |  |  |  |
| 2 | Atr-ERN11508 |  | | | |  | Vvi-Vitvi18g02474\_t001 |  |  |  |  |
| 2 | Atr-ERN11509 |  | | | |  | Vvi-Vitvi18g00034\_t001 |  |  |  |  |
| 2 | Atr-ERN11510 |  | | | |  | | | |  |  |  |  |
| 2 | Atr-ERN11511 |  | | | |  | Vvi-Vitvi18g00035\_t003 |  |  |  |  |
| 2 | Atr-ERN11512 |  | | | |  | | | |  |  |  |  |
| 2 | Atr-ERN11513 |  | | | |  | Vvi-Vitvi18g00036\_t001 |  |  |  |  |
| 2 | Atr-ERN11514 |  | | | |  | | | |  |  |  |  |
| 2 | Atr-ERN11515 |  | | | |  | Vvi-Vitvi18g00037\_t001 |  |  |  |  |
| 2 | Atr-ERN11516 |  | Vvi-Vitvi04g01751\_t001 |  | | | |  |  |  |  |
| 2 | Atr-ERN11517 |  | | | |  | | | |  |  |  |  |
| 2 | Atr-ERN11518 |  | Vvi-Vitvi04g01746\_t003 |  | | | |  |  |  |  |
| 2 | Atr-ERN11519 |  | | | |  | | | |  |  |  |  |
| 2 | Atr-ERN11520 |  | | | |  | | | |  |  |  |  |
| 2 | Atr-ERN11521 |  | | | |  | | | |  |  |  |  |
| 2 | Atr-ERN11522 |  | Vvi-Vitvi04g01745\_t002 |  | Vvi-Vitvi18g00041\_t001 |  |  |  |  |
| 2 | Atr-ERN11523 |  | | | |  | | | |  |  |  |  |
| 3 | Atr-ERN11524 |  | | | |  | | | |  | Vvi-Vitvi03g00264\_t001 |  |  |  |
| 3 | Atr-ERN11525 |  | | | |  | | | |  | | | |  |  |  |
| 3 | Atr-ERN11526 |  | | | |  | | | |  | Vvi-Vitvi03g00262\_t001 |  |  |  |
| 3 | Atr-ERN11527 |  | | | |  | | | |  | | | |  |  |  |
| 3 | Atr-ERN11528 |  | | | |  | | | |  | | | |  |  |  |
| 3 | Atr-ERN11529 |  | | | |  | Vvi-Vitvi18g04010\_t001 |  | | | |  |  |  |
| 3 | Atr-ERN11530 |  | | | |  | | | |  | | | |  |  |  |
| 3 | Atr-ERN11531 |  | | | |  | | | |  | | | |  |  |  |
| 3 | Atr-ERN11532 |  | Vvi-Vitvi04g01742\_t001 |  | | | |  | | | |  |  |  |
| 3 | Atr-ERN11533 |  | | | |  | | | |  | | | |  |  |  |
| 3 | Atr-ERN11534 |  | | | |  | Vvi-Vitvi18g02476\_t001 |  | | | |  |  |  |
| 3 | Atr-ERN11535 |  | | | |  | | | |  | | | |  |  |  |
| 3 | Atr-ERN11536 |  | | | |  | | | |  | | | |  |  |  |
| 3 | Atr-ERN11537 |  | | | |  | | | |  | | | |  |  |  |
| 3 | Atr-ERN11538 |  | | | |  | | | |  | | | |  |  |  |
| 3 | Atr-ERN11539 |  | | | |  | Vvi-Vitvi18g00044\_t001 |  | | | |  |  |  |
| 3 | Atr-ERN11540 |  | | | |  | | | |  | Vvi-Vitvi03g00261\_t003 |  |  |  |
| 3 | Atr-ERN11541 |  | | | |  | | | |  | | | |  |  |  |
| 3 | Atr-ERN11542 |  | | | |  | | | |  | Vvi-Vitvi03g00260\_t001 |  |  |  |
| 3 | Atr-ERN11543 |  | Vvi-Vitvi04g01738\_t001 |  | | | |  | | | |  |  |  |
| 3 | Atr-ERN11544 |  | | | |  | Vvi-Vitvi18g00045\_t001 |  | | | |  |  |  |
| 3 | Atr-ERN11545 |  | | | |  | | | |  | | | |  |  |  |
| 3 | Atr-ERN11546 |  | Vvi-Vitvi04g01737\_t001 |  | | | |  | | | |  |  |  |
| 3 | Atr-ERN11547 |  | | | |  | Vvi-Vitvi18g00047\_t004 |  | | | |  |  |  |
| 3 | Atr-ERN11548 |  | | | |  | | | |  | | | |  |  |  |
| 3 | Atr-ERN11549 |  | | | |  | | | |  | | | |  |  |  |
| 3 | Atr-ERN11550 |  | | | |  | | | |  | | | |  |  |  |
| 3 | Atr-ERN11551 |  | | | |  | | | |  | | | |  |  |  |
| 3 | Atr-ERN11552 |  | | | |  | | | |  | | | |  |  |  |
| 3 | Atr-ERN11553 |  | | | |  | | | |  | | | |  |  |  |
| 3 | Atr-ERN11554 |  | | | |  | | | |  | Vvi-Vitvi03g00259\_t001 |  |  |  |
| 3 | Atr-ERN11555 |  | | | |  | | | |  | | | |  |  |  |
| 3 | Atr-ERN11556 |  | | | |  | Vvi-Vitvi18g00048\_t001 |  | | | |  |  |  |
| 3 | Atr-ERN11557 |  | Vvi-Vitvi04g01736\_t001 |  | | | |  | | | |  |  |  |
| 2 | Atr-ERN11558 |  |  |  | | | |  | Vvi-Vitvi03g00257\_t002 |  |  |  |
| 2 | Atr-ERN11559 |  |  |  | | | |  | | | |  |  |  |
| 2 | Atr-ERN11560 |  |  |  | | | |  | | | |  |  |  |
| 2 | Atr-ERN11561 |  |  |  | Vvi-Vitvi18g00049\_t001 |  | | | |  |  |  |
| 2 | Atr-ERN11562 |  |  |  | | | |  | Vvi-Vitvi03g00254\_t001 |  |  |  |
| 2 | Atr-ERN11563 |  |  |  | | | |  | | | |  |  |  |
| 2 | Atr-ERN11564 |  |  |  | Vvi-Vitvi18g00052\_t001 |  | | | |  |  |  |
| 1 | Atr-ERN11565 |  |  |  |  |  | Vvi-Vitvi03g00251\_t001 |  |  |  |
| 0 | Atr-ERN11566 |  |  |  |  |  |  |
| 0 | Atr-ERN11567 |  |  |  |  |  |  |
| 0 | Atr-ERN11568 |  |  |  |  |  |  |
| 0 | Atr-ERN11569 |  |  |  |  |  |  |
| 0 | Atr-ERN11570 |  |  |  |  |  |  |
| 0 | Atr-ERN11571 |  |  |  |  |  |  |
| 0 | Atr-ERN11572 |  |  |  |  |  |  |
| 0 | Atr-ERN11573 |  |  |  |  |  |  |
| 0 | Atr-ERN11574 |  |  |  |  |  |  |
| 0 | Atr-ERN11575 |  |  |  |  |  |  |
| 1 | Atr-ERN11576 |  | Vvi-Vitvi08g02346\_t001 |  |  |  |  |  |
| 2 | Atr-ERN11577 |  | | | |  | Vvi-Vitvi13g01833\_t002 |  |  |  |  |
| 2 | Atr-ERN11578 |  | | | |  | | | |  |  |  |  |
| 2 | Atr-ERN11579 |  | | | |  | | | |  |  |  |  |
| 2 | Atr-ERN11580 |  | | | |  | | | |  |  |  |  |
| 2 | Atr-ERN11581 |  | | | |  | | | |  |  |  |  |
| 3 | Atr-ERN11582 |  | Vvi-Vitvi08g02345\_t001 |  | | | |  | Vvi-Vitvi06g00119\_t001 |  |  |  |
| 3 | Atr-ERN11583 |  | Vvi-Vitvi08g02343\_t001 |  | | | |  | | | |  |  |  |
| 3 | Atr-ERN11584 |  | | | |  | | | |  | | | |  |  |  |
| 3 | Atr-ERN11585 |  | | | |  | | | |  | | | |  |  |  |
| 3 | Atr-ERN11586 |  | | | |  | Vvi-Vitvi13g01820\_t002 |  | | | |  |  |  |
| 3 | Atr-ERN11587 |  | | | |  | | | |  | | | |  |  |  |
| 3 | Atr-ERN11588 |  | Vvi-Vitvi08g01732\_t001 |  | Vvi-Vitvi13g01819\_t001 |  | Vvi-Vitvi06g00123\_t001 |  |  |  |
| 3 | Atr-ERN11589 |  | | | |  | | | |  | | | |  |  |  |
| 3 | Atr-ERN11590 |  | | | |  | | | |  | Vvi-Vitvi06g00124\_t001 |  |  |  |
| 3 | Atr-ERN11591 |  | Vvi-Vitvi08g01731\_t001 |  | | | |  | Vvi-Vitvi06g00125\_t001 |  |  |  |
| 3 | Atr-ERN11592 |  | | | |  | Vvi-Vitvi13g02569\_t001 |  | | | |  |  |  |
| 3 | Atr-ERN11593 |  | | | |  | | | |  | | | |  |  |  |
| 3 | Atr-ERN11594 |  | Vvi-Vitvi08g01728\_t001 |  | | | |  | | | |  |  |  |
| 3 | Atr-ERN11595 |  | | | |  | Vvi-Vitvi13g01817\_t001 |  | | | |  |  |  |
| 3 | Atr-ERN11596 |  | | | |  | | | |  | Vvi-Vitvi06g04037\_t001 |  |  |  |
| 3 | Atr-ERN11597 |  | | | |  | | | |  | | | |  |  |  |
| 3 | Atr-ERN11598 |  | | | |  | | | |  | | | |  |  |  |
| 3 | Atr-ERN11599 |  | Vvi-Vitvi08g01725\_t001 |  | Vvi-Vitvi13g01816\_t001 |  | Vvi-Vitvi06g00130\_t001 |  |  |  |
| 3 | Atr-ERN11600 |  | | | |  | | | |  | Vvi-Vitvi06g00132\_t001 |  |  |  |
| 3 | Atr-ERN11601 |  | | | |  | | | |  | | | |  |  |  |
| 3 | Atr-ERN11602 |  | Vvi-Vitvi08g01724\_t001 |  | | | |  | | | |  |  |  |
| 3 | Atr-ERN11603 |  | | | |  | | | |  | | | |  |  |  |
| 3 | Atr-ERN11604 |  | | | |  | | | |  | | | |  |  |  |
| 3 | Atr-ERN11605 |  | | | |  | | | |  | | | |  |  |  |
| 3 | Atr-ERN11606 |  | | | |  | | | |  | | | |  |  |  |
| 3 | Atr-ERN11607 |  | | | |  | | | |  | | | |  |  |  |
| 3 | Atr-ERN11608 |  | | | |  | | | |  | | | |  |  |  |
| 3 | Atr-ERN11609 |  | | | |  | | | |  | | | |  |  |  |
| 3 | Atr-ERN11610 |  | Vvi-Vitvi08g01723\_t002 |  | Vvi-Vitvi13g02568\_t001 |  | | | |  |  |  |
| 3 | Atr-ERN11611 |  | | | |  | | | |  | | | |  |  |  |
| 3 | Atr-ERN11612 |  | | | |  | | | |  | | | |  |  |  |
| 3 | Atr-ERN11613 |  | | | |  | | | |  | | | |  |  |  |
| 3 | Atr-ERN11614 |  | | | |  | | | |  | | | |  |  |  |
| 3 | Atr-ERN11615 |  | | | |  | | | |  | | | |  |  |  |
| 3 | Atr-ERN11616 |  | | | |  | | | |  | | | |  |  |  |
| 3 | Atr-ERN11617 |  | | | |  | Vvi-Vitvi13g04787\_t001 |  | Vvi-Vitvi06g00134\_t001 |  |  |  |
| 3 | Atr-ERN11618 |  | | | |  | | | |  | | | |  |  |  |
| 3 | Atr-ERN11619 |  | | | |  | Vvi-Vitvi13g02548\_t001 |  | Vvi-Vitvi06g01613\_t001 |  |  |  |
| 2 | Atr-ERN11620 |  | | | |  |  |  | | | |  |  |  |
| 2 | Atr-ERN11621 |  | | | |  |  |  | | | |  |  |  |
| 2 | Atr-ERN11622 |  | | | |  |  |  | | | |  |  |  |
| 3 | Atr-ERN11623 |  | | | |  | Vvi-Vitvi14g04497\_t001 |  | | | |  |  |  |
| 3 | Atr-ERN11624 |  | | | |  | | | |  | | | |  |  |  |
| 3 | Atr-ERN11625 |  | | | |  | | | |  | | | |  |  |  |
| 3 | Atr-ERN11626 |  | | | |  | | | |  | | | |  |  |  |
| 3 | Atr-ERN11627 |  | Vvi-Vitvi08g02342\_t001 |  | | | |  | Vvi-Vitvi06g01615\_t001 |  |  |  |
| 3 | Atr-ERN11628 |  | | | |  | | | |  | | | |  |  |  |
| 3 | Atr-ERN11629 |  | Vvi-Vitvi08g01720\_t001 |  | | | |  | | | |  |  |  |
| 3 | Atr-ERN11630 |  | Vvi-Vitvi08g01719\_t001 |  | | | |  | | | |  |  |  |
| 3 | Atr-ERN11631 |  | Vvi-Vitvi08g01718\_t001 |  | | | |  | | | |  |  |  |
| 3 | Atr-ERN11632 |  | | | |  | Vvi-Vitvi14g04491\_t001 |  | | | |  |  |  |
| 3 | Atr-ERN11633 |  | | | |  | | | |  | | | |  |  |  |
| 3 | Atr-ERN11634 |  | | | |  | | | |  | Vvi-Vitvi06g00141\_t001 |  |  |  |
| 3 | Atr-ERN11635 |  | | | |  | | | |  | | | |  |  |  |
| 3 | Atr-ERN11636 |  | | | |  | | | |  | | | |  |  |  |
| 3 | Atr-ERN11637 |  | | | |  | | | |  | | | |  |  |  |
| 3 | Atr-ERN11638 |  | Vvi-Vitvi08g01717\_t001 |  | | | |  | | | |  |  |  |
| 3 | Atr-ERN11639 |  | | | |  | | | |  | | | |  |  |  |
| 3 | Atr-ERN11640 |  | | | |  | | | |  | | | |  |  |  |
| 3 | Atr-ERN11641 |  | | | |  | Vvi-Vitvi13g01783\_t001 |  | | | |  |  |  |
| 3 | Atr-ERN11642 |  | | | |  | | | |  | | | |  |  |  |
| 3 | Atr-ERN11643 |  | | | |  | | | |  | Vvi-Vitvi06g00149\_t001 |  |  |  |
| 3 | Atr-ERN11644 |  | | | |  | Vvi-Vitvi13g01782\_t001 |  | | | |  |  |  |
| 3 | Atr-ERN11645 |  | | | |  | Vvi-Vitvi14g04490\_t001 |  | | | |  |  |  |
| 3 | Atr-ERN11646 |  | | | |  | | | |  | | | |  |  |  |
| 3 | Atr-ERN11647 |  | | | |  | | | |  | | | |  |  |  |
| 3 | Atr-ERN11648 |  | | | |  | | | |  | | | |  |  |  |
| 3 | Atr-ERN11649 |  | Vvi-Vitvi08g01716\_t002 |  | | | |  | | | |  |  |  |
| 3 | Atr-ERN11650 |  | | | |  | | | |  | | | |  |  |  |
| 3 | Atr-ERN11651 |  | | | |  | Vvi-Vitvi13g02522\_t002 |  | | | |  |  |  |
| 3 | Atr-ERN11652 |  | | | |  | | | |  | Vvi-Vitvi06g00160\_t001 |  |  |  |
| 3 | Atr-ERN11653 |  | | | |  | | | |  | Vvi-Vitvi06g00161\_t001 |  |  |  |
| 3 | Atr-ERN11654 |  | Vvi-Vitvi08g01713\_t001 |  | | | |  | | | |  |  |  |
| 3 | Atr-ERN11655 |  | | | |  | | | |  | Vvi-Vitvi06g00162\_t001 |  |  |  |
| 3 | Atr-ERN11656 |  | | | |  | | | |  | | | |  |  |  |
| 3 | Atr-ERN11657 |  | | | |  | | | |  | | | |  |  |  |
| 3 | Atr-ERN11658 |  | | | |  | | | |  | | | |  |  |  |
| 3 | Atr-ERN11659 |  | | | |  | | | |  | | | |  |  |  |
| 3 | Atr-ERN11660 |  | | | |  | | | |  | Vvi-Vitvi06g00163\_t001 |  |  |  |
| 3 | Atr-ERN11661 |  | | | |  | | | |  | Vvi-Vitvi06g00166\_t001 |  |  |  |
| 3 | Atr-ERN11662 |  | Vvi-Vitvi08g01710\_t001 |  | | | |  | | | |  |  |  |
| 3 | Atr-ERN11663 |  | | | |  | | | |  | | | |  |  |  |
| 3 | Atr-ERN11664 |  | Vvi-Vitvi08g01709\_t001 |  | Vvi-Vitvi13g01774\_t001 |  | | | |  |  |  |
| 3 | Atr-ERN11665 |  | | | |  | | | |  | | | |  |  |  |
| 3 | Atr-ERN11666 |  | Vvi-Vitvi08g01708\_t001 |  | Vvi-Vitvi13g01773\_t001 |  | | | |  |  |  |
| 2 | Atr-ERN11667 |  |  |  | | | |  | | | |  |  |  |
| 2 | Atr-ERN11668 |  |  |  | | | |  | | | |  |  |  |
| 2 | Atr-ERN11669 |  |  |  | | | |  | | | |  |  |  |
| 3 | Atr-ERN11670 |  | Vvi-Vitvi08g01844\_t001 |  | Vvi-Vitvi13g01771\_t001 |  | | | |  |  |  |
| 3 | Atr-ERN11671 |  | | | |  | | | |  | | | |  |  |  |
| 3 | Atr-ERN11672 |  | | | |  | | | |  | | | |  |  |  |
| 3 | Atr-ERN11673 |  | | | |  | | | |  | | | |  |  |  |
| 3 | Atr-ERN11674 |  | | | |  | | | |  | | | |  |  |  |
| 3 | Atr-ERN11675 |  | | | |  | | | |  | | | |  |  |  |
| 3 | Atr-ERN11676 |  | | | |  | | | |  | | | |  |  |  |
| 3 | Atr-ERN11677 |  | | | |  | | | |  | | | |  |  |  |
| 3 | Atr-ERN11678 |  | | | |  | | | |  | | | |  |  |  |
| 3 | Atr-ERN11679 |  | | | |  | Vvi-Vitvi13g01769\_t002 |  | | | |  |  |  |
| 3 | Atr-ERN11680 |  | | | |  | | | |  | Vvi-Vitvi06g00167\_t001 |  |  |  |
| 3 | Atr-ERN11681 |  | Vvi-Vitvi08g01845\_t001 |  | | | |  | | | |  |  |  |
| 3 | Atr-ERN11682 |  | | | |  | | | |  | Vvi-Vitvi06g00173\_t001 |  |  |  |
| 3 | Atr-ERN11683 |  | | | |  | | | |  | Vvi-Vitvi06g00177\_t001 |  |  |  |
| 3 | Atr-ERN11684 |  | | | |  | | | |  | | | |  |  |  |
| 3 | Atr-ERN11685 |  | | | |  | | | |  | | | |  |  |  |
| 3 | Atr-ERN11686 |  | | | |  | Vvi-Vitvi13g01767\_t001 |  | | | |  |  |  |
| 3 | Atr-ERN11687 |  | | | |  | | | |  | | | |  |  |  |
| 3 | Atr-ERN11688 |  | Vvi-Vitvi08g02372\_t001 |  | Vvi-Vitvi13g01766\_t001 |  | | | |  |  |  |
| 3 | Atr-ERN11689 |  | | | |  | | | |  | | | |  |  |  |
| 3 | Atr-ERN11690 |  | | | |  | | | |  | | | |  |  |  |
| 3 | Atr-ERN11691 |  | | | |  | | | |  | | | |  |  |  |
| 3 | Atr-ERN11692 |  | | | |  | | | |  | | | |  |  |  |
| 3 | Atr-ERN11693 |  | | | |  | | | |  | | | |  |  |  |
| 3 | Atr-ERN11694 |  | | | |  | | | |  | | | |  |  |  |
| 3 | Atr-ERN11695 |  | | | |  | | | |  | | | |  |  |  |
| 3 | Atr-ERN11696 |  | | | |  | | | |  | | | |  |  |  |
| 3 | Atr-ERN11697 |  | | | |  | | | |  | Vvi-Vitvi06g00179\_t001 |  |  |  |
| 3 | Atr-ERN11698 |  | | | |  | Vvi-Vitvi14g04480\_t001 |  | | | |  |  |  |
| 3 | Atr-ERN11699 |  | | | |  | | | |  | | | |  |  |  |
| 3 | Atr-ERN11700 |  | Vvi-Vitvi08g02377\_t001 |  | Vvi-Vitvi13g01765\_t001 |  | | | |  |  |  |
| 3 | Atr-ERN11701 |  | | | |  | | | |  | | | |  |  |  |
| 3 | Atr-ERN11702 |  | | | |  | | | |  | | | |  |  |  |
| 3 | Atr-ERN11703 |  | | | |  | | | |  | | | |  |  |  |
| 3 | Atr-ERN11704 |  | Vvi-Vitvi08g01852\_t001 |  | Vvi-Vitvi13g01764\_t001 |  | Vvi-Vitvi06g00181\_t001 |  |  |  |
| 3 | Atr-ERN11705 |  | | | |  | Vvi-Vitvi13g01761\_t001 |  | | | |  |  |  |
| 3 | Atr-ERN11706 |  | Vvi-Vitvi08g01853\_t001 |  | | | |  | | | |  |  |  |
| 3 | Atr-ERN11707 |  | Vvi-Vitvi08g01854\_t001 |  | | | |  | | | |  |  |  |
| 3 | Atr-ERN11708 |  | | | |  | | | |  | | | |  |  |  |
| 3 | Atr-ERN11709 |  | | | |  | | | |  | | | |  |  |  |
| 3 | Atr-ERN11710 |  | Vvi-Vitvi08g02378\_t004 |  | | | |  | | | |  |  |  |
| 3 | Atr-ERN11711 |  | | | |  | | | |  | | | |  |  |  |
| 3 | Atr-ERN11712 |  | | | |  | Vvi-Vitvi13g01760\_t001 |  | Vvi-Vitvi06g00182\_t001 |  |  |  |
| 3 | Atr-ERN11713 |  | Vvi-Vitvi08g01856\_t001 |  | | | |  | | | |  |  |  |
| 3 | Atr-ERN11714 |  | | | |  | | | |  | | | |  |  |  |
| 3 | Atr-ERN11715 |  | Vvi-Vitvi08g01857\_t001 |  | | | |  | | | |  |  |  |
| 3 | Atr-ERN11716 |  | | | |  | | | |  | | | |  |  |  |
| 3 | Atr-ERN11717 |  | | | |  | | | |  | | | |  |  |  |
| 3 | Atr-ERN11718 |  | Vvi-Vitvi08g01859\_t001 |  | | | |  | | | |  |  |  |
| 3 | Atr-ERN11719 |  | Vvi-Vitvi08g01860\_t003 |  | | | |  | | | |  |  |  |
| 3 | Atr-ERN11720 |  | | | |  | | | |  | | | |  |  |  |
| 3 | Atr-ERN11721 |  | | | |  | Vvi-Vitvi13g01758\_t001 |  | | | |  |  |  |
| 3 | Atr-ERN11722 |  | | | |  | | | |  | Vvi-Vitvi06g00184\_t001 |  |  |  |
| 3 | Atr-ERN11723 |  | | | |  | | | |  | | | |  |  |  |
| 3 | Atr-ERN11724 |  | | | |  | | | |  | | | |  |  |  |
| 3 | Atr-ERN11725 |  | | | |  | | | |  | | | |  |  |  |
| 3 | Atr-ERN11726 |  | | | |  | | | |  | | | |  |  |  |
| 3 | Atr-ERN11727 |  | | | |  | Vvi-Vitvi13g01757\_t003 |  | | | |  |  |  |
| 3 | Atr-ERN11728 |  | | | |  | | | |  | Vvi-Vitvi06g00185\_t001 |  |  |  |
| 3 | Atr-ERN11729 |  | | | |  | | | |  | | | |  |  |  |
| 3 | Atr-ERN11730 |  | | | |  | Vvi-Vitvi13g01755\_t001 |  | | | |  |  |  |
| 3 | Atr-ERN11731 |  | | | |  | | | |  | | | |  |  |  |
| 3 | Atr-ERN11732 |  | | | |  | | | |  | | | |  |  |  |
| 3 | Atr-ERN11733 |  | | | |  | | | |  | | | |  |  |  |
| 3 | Atr-ERN11734 |  | | | |  | | | |  | | | |  |  |  |
| 3 | Atr-ERN11735 |  | | | |  | | | |  | | | |  |  |  |
| 3 | Atr-ERN11736 |  | Vvi-Vitvi08g01863\_t001 |  | | | |  | | | |  |  |  |
| 3 | Atr-ERN11737 |  | | | |  | Vvi-Vitvi13g01754\_t001 |  | | | |  |  |  |
| 3 | Atr-ERN11738 |  | Vvi-Vitvi08g01864\_t001 |  | | | |  | Vvi-Vitvi06g00186\_t001 |  |  |  |
| 3 | Atr-ERN11739 |  | | | |  | | | |  | | | |  |  |  |
| 3 | Atr-ERN11740 |  | | | |  | | | |  | | | |  |  |  |
| 3 | Atr-ERN11741 |  | | | |  | | | |  | | | |  |  |  |
| 3 | Atr-ERN11742 |  | | | |  | | | |  | Vvi-Vitvi06g00188\_t001 |  |  |  |
| 3 | Atr-ERN11743 |  | | | |  | | | |  | | | |  |  |  |
| 3 | Atr-ERN11744 |  | Vvi-Vitvi08g01866\_t002 |  | | | |  | Vvi-Vitvi06g00191\_t002 |  |  |  |
| 3 | Atr-ERN11745 |  | | | |  | Vvi-Vitvi13g02518\_t001 |  | | | |  |  |  |
| 2 | Atr-ERN11746 |  | | | |  |  |  | | | |  |  |  |
| 2 | Atr-ERN11747 |  | | | |  |  |  | | | |  |  |  |
| 2 | Atr-ERN11748 |  | | | |  |  |  | | | |  |  |  |
| 2 | Atr-ERN11749 |  | | | |  |  |  | | | |  |  |  |
| 2 | Atr-ERN11750 |  | | | |  |  |  | Vvi-Vitvi06g00192\_t001 |  |  |  |
| 3 | Atr-ERN11751 |  | | | |  | Vvi-Vitvi13g01750\_t002 |  | | | |  |  |  |
| 3 | Atr-ERN11752 |  | Vvi-Vitvi08g01867\_t001 |  | | | |  | | | |  |  |  |
| 3 | Atr-ERN11753 |  | | | |  | Vvi-Vitvi13g01749\_t001 |  | | | |  |  |  |
| 3 | Atr-ERN11754 |  | | | |  | | | |  | | | |  |  |  |
| 3 | Atr-ERN11755 |  | | | |  | Vvi-Vitvi13g02507\_t001 |  | | | |  |  |  |
| 3 | Atr-ERN11756 |  | | | |  | | | |  | Vvi-Vitvi06g00193\_t001 |  |  |  |
| 3 | Atr-ERN11757 |  | Vvi-Vitvi08g01869\_t001 |  | Vvi-Vitvi13g01748\_t001 |  | Vvi-Vitvi06g00195\_t001 |  |  |  |
| 3 | Atr-ERN11758 |  | | | |  | | | |  | | | |  |  |  |
| 3 | Atr-ERN11759 |  | | | |  | | | |  | Vvi-Vitvi06g00198\_t001 |  |  |  |
| 3 | Atr-ERN11760 |  | | | |  | | | |  | Vvi-Vitvi06g00199\_t003 |  |  |  |
| 3 | Atr-ERN11761 |  | | | |  | | | |  | Vvi-Vitvi06g00200\_t001 |  |  |  |
| 3 | Atr-ERN11762 |  | Vvi-Vitvi08g01871\_t001 |  | | | |  | | | |  |  |  |
| 3 | Atr-ERN11763 |  | | | |  | | | |  | | | |  |  |  |
| 3 | Atr-ERN11764 |  | | | |  | | | |  | | | |  |  |  |
| 3 | Atr-ERN11765 |  | | | |  | | | |  | | | |  |  |  |
| 3 | Atr-ERN11766 |  | | | |  | Vvi-Vitvi13g01744\_t001 |  | Vvi-Vitvi06g04062\_t001 |  |  |  |
| 3 | Atr-ERN11767 |  | | | |  | | | |  | | | |  |  |  |
| 3 | Atr-ERN11768 |  | | | |  | | | |  | | | |  |  |  |
| 3 | Atr-ERN11769 |  | | | |  | | | |  | | | |  |  |  |
| 3 | Atr-ERN11770 |  | | | |  | | | |  | Vvi-Vitvi06g00205\_t001 |  |  |  |
| 3 | Atr-ERN11771 |  | | | |  | | | |  | Vvi-Vitvi06g00206\_t001 |  |  |  |
| 3 | Atr-ERN11772 |  | | | |  | Vvi-Vitvi13g01743\_t001 |  | | | |  |  |  |
| 3 | Atr-ERN11773 |  | | | |  | | | |  | | | |  |  |  |
| 3 | Atr-ERN11774 |  | | | |  | | | |  | | | |  |  |  |
| 3 | Atr-ERN11775 |  | | | |  | | | |  | | | |  |  |  |
| 3 | Atr-ERN11776 |  | Vvi-Vitvi08g01873\_t001 |  | | | |  | | | |  |  |  |
| 3 | Atr-ERN11777 |  | | | |  | | | |  | | | |  |  |  |
| 3 | Atr-ERN11778 |  | | | |  | | | |  | | | |  |  |  |
| 3 | Atr-ERN11779 |  | | | |  | | | |  | Vvi-Vitvi06g00207\_t001 |  |  |  |
| 3 | Atr-ERN11780 |  | | | |  | | | |  | | | |  |  |  |
| 3 | Atr-ERN11781 |  | | | |  | | | |  | | | |  |  |  |
| 3 | Atr-ERN11782 |  | | | |  | | | |  | Vvi-Vitvi06g00211\_t001 |  |  |  |
| 3 | Atr-ERN11783 |  | | | |  | | | |  | | | |  |  |  |
| 3 | Atr-ERN11784 |  | Vvi-Vitvi08g02390\_t001 |  | | | |  | | | |  |  |  |
| 3 | Atr-ERN11785 |  | | | |  | | | |  | | | |  |  |  |
| 3 | Atr-ERN11786 |  | | | |  | | | |  | | | |  |  |  |
| 3 | Atr-ERN11787 |  | | | |  | | | |  | | | |  |  |  |
| 3 | Atr-ERN11788 |  | | | |  | | | |  | | | |  |  |  |
| 3 | Atr-ERN11789 |  | | | |  | | | |  | Vvi-Vitvi06g01631\_t001 |  |  |  |
| 3 | Atr-ERN11790 |  | Vvi-Vitvi08g01876\_t002 |  | | | |  | | | |  |  |  |
| 3 | Atr-ERN11791 |  | | | |  | | | |  | | | |  |  |  |
| 3 | Atr-ERN11792 |  | | | |  | | | |  | | | |  |  |  |
| 3 | Atr-ERN11793 |  | | | |  | Vvi-Vitvi13g01738\_t001 |  | | | |  |  |  |
| 3 | Atr-ERN11794 |  | | | |  | Vvi-Vitvi13g01737\_t001 |  | Vvi-Vitvi06g00214\_t001 |  |  |  |
| 3 | Atr-ERN11795 |  | Vvi-Vitvi08g01878\_t001 |  | | | |  | | | |  |  |  |
| 3 | Atr-ERN11796 |  | | | |  | Vvi-Vitvi13g01736\_t001 |  | | | |  |  |  |
| 3 | Atr-ERN11797 |  | Vvi-Vitvi08g01879\_t001 |  | Vvi-Vitvi13g01734\_t001 |  | Vvi-Vitvi06g00216\_t001 |  |  |  |
| 1 | Atr-ERN11798 |  |  |  | | | |  |  |  |  |
| 1 | Atr-ERN11799 |  |  |  | | | |  |  |  |  |
| 1 | Atr-ERN11800 |  |  |  | | | |  |  |  |  |
| 1 | Atr-ERN11801 |  |  |  | Vvi-Vitvi13g01727\_t002 |  |  |  |  |
| 0 | Atr-ERN11802 |  |  |  |  |  |  |
