# Supplementary material for: Genome-wide identification, characterization and gene expression of BES1 transcription factor family in grapevine (Vitis vinifera L.)
Source: Sci Rep. 2023 Jan 5;13:240. doi: 10.1038/s41598-022-24407-y (PMC9816167; doi:10.1038/s41598-022-24407-y)
Supplement: Supplementary file 3 — Supplementary Information. [file 41598_2022_24407_MOESM3_ESM.zip › Vvi_Atr/Vitis_vinifera.PN40024.v4.dna_sm.toplevel.fa.vs.Amborella_trichopoda.AMTR1.0.dna_sm.toplevel.fa.html/Atr-AmTr_v1.0_scaffold00023.html]

|  |  |  |  |  |  |  |  |  |  |  |  |  |  |
| --- | --- | --- | --- | --- | --- | --- | --- | --- | --- | --- | --- | --- | --- |
| Duplication depth | Reference chromosome | Collinear blocks | | | | | | | | | | | |
| 1 | Atr-ERM95529 |  | Vvi-Vitvi06g00723\_t001 |  |  |  |  |  |
| 1 | Atr-ERM95530 |  | | | |  |  |  |  |  |
| 1 | Atr-ERM95531 |  | | | |  |  |  |  |  |
| 1 | Atr-ERM95532 |  | | | |  |  |  |  |  |
| 1 | Atr-ERM95533 |  | | | |  |  |  |  |  |
| 1 | Atr-ERM95534 |  | | | |  |  |  |  |  |
| 1 | Atr-ERM95535 |  | | | |  |  |  |  |  |
| 1 | Atr-ERM95536 |  | | | |  |  |  |  |  |
| 1 | Atr-ERM95537 |  | | | |  |  |  |  |  |
| 1 | Atr-ERM95538 |  | | | |  |  |  |  |  |
| 3 | Atr-ERM95539 |  | | | |  | Vvi-Vitvi13g00113\_t001 |  | Vvi-Vitvi08g00769\_t001 |  |  |  |
| 3 | Atr-ERM95540 |  | Vvi-Vitvi06g00724\_t001 |  | Vvi-Vitvi13g00114\_t001 |  | | | |  |  |  |
| 3 | Atr-ERM95541 |  | | | |  | | | |  | | | |  |  |  |
| 3 | Atr-ERM95542 |  | | | |  | Vvi-Vitvi13g00116\_t001 |  | Vvi-Vitvi08g00778\_t001 |  |  |  |
| 3 | Atr-ERM95543 |  | | | |  | | | |  | | | |  |  |  |
| 3 | Atr-ERM95544 |  | | | |  | | | |  | | | |  |  |  |
| 3 | Atr-ERM95545 |  | | | |  | | | |  | | | |  |  |  |
| 3 | Atr-ERM95546 |  | | | |  | | | |  | Vvi-Vitvi08g00780\_t001 |  |  |  |
| 3 | Atr-ERM95547 |  | Vvi-Vitvi06g00728\_t001 |  | Vvi-Vitvi13g00117\_t001 |  | | | |  |  |  |
| 3 | Atr-ERM95548 |  | | | |  | | | |  | | | |  |  |  |
| 3 | Atr-ERM95549 |  | | | |  | | | |  | | | |  |  |  |
| 3 | Atr-ERM95550 |  | | | |  | | | |  | | | |  |  |  |
| 3 | Atr-ERM95551 |  | | | |  | | | |  | Vvi-Vitvi08g00781\_t001 |  |  |  |
| 3 | Atr-ERM95552 |  | | | |  | | | |  | | | |  |  |  |
| 3 | Atr-ERM95553 |  | | | |  | | | |  | | | |  |  |  |
| 3 | Atr-ERM95554 |  | | | |  | | | |  | | | |  |  |  |
| 3 | Atr-ERM95555 |  | | | |  | Vvi-Vitvi13g00118\_t001 |  | | | |  |  |  |
| 3 | Atr-ERM95556 |  | Vvi-Vitvi06g00729\_t001 |  | | | |  | | | |  |  |  |
| 3 | Atr-ERM95557 |  | | | |  | | | |  | | | |  |  |  |
| 3 | Atr-ERM95558 |  | | | |  | | | |  | | | |  |  |  |
| 3 | Atr-ERM95559 |  | | | |  | Vvi-Vitvi13g00122\_t001 |  | | | |  |  |  |
| 3 | Atr-ERM95560 |  | | | |  | | | |  | | | |  |  |  |
| 3 | Atr-ERM95561 |  | | | |  | | | |  | Vvi-Vitvi08g00783\_t003 |  |  |  |
| 3 | Atr-ERM95562 |  | | | |  | | | |  | | | |  |  |  |
| 3 | Atr-ERM95563 |  | Vvi-Vitvi06g00733\_t001 |  | | | |  | | | |  |  |  |
| 3 | Atr-ERM95564 |  | Vvi-Vitvi06g00734\_t001 |  | | | |  | | | |  |  |  |
| 3 | Atr-ERM95565 |  | | | |  | Vvi-Vitvi13g00123\_t001 |  | | | |  |  |  |
| 3 | Atr-ERM95566 |  | | | |  | | | |  | | | |  |  |  |
| 3 | Atr-ERM95567 |  | | | |  | | | |  | | | |  |  |  |
| 3 | Atr-ERM95568 |  | | | |  | Vvi-Vitvi13g00124\_t001 |  | Vvi-Vitvi08g00786\_t002 |  |  |  |
| 3 | Atr-ERM95569 |  | | | |  | | | |  | Vvi-Vitvi08g00788\_t002 |  |  |  |
| 3 | Atr-ERM95570 |  | | | |  | | | |  | | | |  |  |  |
| 3 | Atr-ERM95571 |  | | | |  | Vvi-Vitvi13g00126\_t001 |  | | | |  |  |  |
| 3 | Atr-ERM95572 |  | | | |  | Vvi-Vitvi13g01899\_t001 |  | | | |  |  |  |
| 3 | Atr-ERM95573 |  | | | |  | | | |  | | | |  |  |  |
| 3 | Atr-ERM95574 |  | Vvi-Vitvi06g00740\_t001 |  | | | |  | | | |  |  |  |
| 3 | Atr-ERM95575 |  | | | |  | | | |  | Vvi-Vitvi08g02080\_t001 |  |  |  |
| 3 | Atr-ERM95576 |  | | | |  | | | |  | | | |  |  |  |
| 3 | Atr-ERM95577 |  | | | |  | | | |  | | | |  |  |  |
| 3 | Atr-ERM95578 |  | | | |  | Vvi-Vitvi13g01900\_t001 |  | | | |  |  |  |
| 3 | Atr-ERM95579 |  | Vvi-Vitvi06g00741\_t001 |  | | | |  | Vvi-Vitvi08g00793\_t001 |  |  |  |
| 3 | Atr-ERM95580 |  | Vvi-Vitvi06g00742\_t001 |  | | | |  | Vvi-Vitvi08g00794\_t001 |  |  |  |
| 3 | Atr-ERM95581 |  | | | |  | Vvi-Vitvi13g01901\_t001 |  | Vvi-Vitvi08g00795\_t002 |  |  |  |
| 3 | Atr-ERM95582 |  | | | |  | Vvi-Vitvi13g00128\_t001 |  | | | |  |  |  |
| 3 | Atr-ERM95583 |  | | | |  | Vvi-Vitvi13g00129\_t001.1.6037826b |  | Vvi-Vitvi08g00797\_t002 |  |  |  |
| 3 | Atr-ERM95584 |  | | | |  | | | |  | | | |  |  |  |
| 3 | Atr-ERM95585 |  | | | |  | | | |  | Vvi-Vitvi08g00799\_t001 |  |  |  |
| 3 | Atr-ERM95586 |  | Vvi-Vitvi06g00743\_t001 |  | | | |  | | | |  |  |  |
| 3 | Atr-ERM95587 |  | Vvi-Vitvi06g00744\_t001 |  | | | |  | Vvi-Vitvi08g00801\_t001 |  |  |  |
| 3 | Atr-ERM95588 |  | | | |  | | | |  | | | |  |  |  |
| 3 | Atr-ERM95589 |  | | | |  | | | |  | | | |  |  |  |
| 3 | Atr-ERM95590 |  | | | |  | | | |  | | | |  |  |  |
| 3 | Atr-ERM95591 |  | | | |  | | | |  | | | |  |  |  |
| 3 | Atr-ERM95592 |  | | | |  | | | |  | | | |  |  |  |
| 3 | Atr-ERM95593 |  | | | |  | | | |  | | | |  |  |  |
| 3 | Atr-ERM95594 |  | | | |  | | | |  | | | |  |  |  |
| 3 | Atr-ERM95595 |  | | | |  | | | |  | | | |  |  |  |
| 3 | Atr-ERM95596 |  | | | |  | | | |  | | | |  |  |  |
| 3 | Atr-ERM95597 |  | | | |  | | | |  | | | |  |  |  |
| 3 | Atr-ERM95598 |  | | | |  | | | |  | | | |  |  |  |
| 3 | Atr-ERM95599 |  | | | |  | | | |  | | | |  |  |  |
| 3 | Atr-ERM95600 |  | | | |  | | | |  | | | |  |  |  |
| 3 | Atr-ERM95601 |  | | | |  | | | |  | | | |  |  |  |
| 3 | Atr-ERM95602 |  | | | |  | | | |  | | | |  |  |  |
| 3 | Atr-ERM95603 |  | | | |  | | | |  | | | |  |  |  |
| 3 | Atr-ERM95604 |  | | | |  | | | |  | | | |  |  |  |
| 3 | Atr-ERM95605 |  | | | |  | | | |  | | | |  |  |  |
| 3 | Atr-ERM95606 |  | Vvi-Vitvi06g00745\_t001 |  | Vvi-Vitvi13g00131\_t001 |  | Vvi-Vitvi08g00802\_t001 |  |  |  |
| 0 | Atr-ERM95607 |  |  |  |  |  |  |
| 0 | Atr-ERM95608 |  |  |  |  |  |  |
| 0 | Atr-ERM95609 |  |  |  |  |  |  |
| 0 | Atr-ERM95610 |  |  |  |  |  |  |
| 0 | Atr-ERM95611 |  |  |  |  |  |  |
| 0 | Atr-ERM95612 |  |  |  |  |  |  |
| 0 | Atr-ERM95613 |  |  |  |  |  |  |
| 0 | Atr-ERM95614 |  |  |  |  |  |  |
| 0 | Atr-ERM95615 |  |  |  |  |  |  |
| 0 | Atr-ERM95616 |  |  |  |  |  |  |
| 0 | Atr-ERM95617 |  |  |  |  |  |  |
| 0 | Atr-ERM95618 |  |  |  |  |  |  |
| 0 | Atr-ERM95619 |  |  |  |  |  |  |
| 0 | Atr-ERM95620 |  |  |  |  |  |  |
| 0 | Atr-ERM95621 |  |  |  |  |  |  |
| 0 | Atr-ERM95622 |  |  |  |  |  |  |
| 0 | Atr-ERM95623 |  |  |  |  |  |  |
| 0 | Atr-ERM95624 |  |  |  |  |  |  |
| 0 | Atr-ERM95625 |  |  |  |  |  |  |
| 0 | Atr-ERM95626 |  |  |  |  |  |  |
| 0 | Atr-ERM95627 |  |  |  |  |  |  |
| 0 | Atr-ERM95628 |  |  |  |  |  |  |
| 0 | Atr-ERM95629 |  |  |  |  |  |  |
| 0 | Atr-ERM95630 |  |  |  |  |  |  |
| 0 | Atr-ERM95631 |  |  |  |  |  |  |
| 0 | Atr-ERM95632 |  |  |  |  |  |  |
| 0 | Atr-ERM95633 |  |  |  |  |  |  |
| 0 | Atr-ERM95634 |  |  |  |  |  |  |
| 1 | Atr-ERM95635 |  | Vvi-Vitvi08g04200\_t001 |  |  |  |  |  |
| 1 | Atr-ERM95636 |  | Vvi-Vitvi08g01117\_t001 |  |  |  |  |  |
| 1 | Atr-ERM95637 |  | | | |  |  |  |  |  |
| 1 | Atr-ERM95638 |  | | | |  |  |  |  |  |
| 1 | Atr-ERM95639 |  | Vvi-Vitvi08g01118\_t001 |  |  |  |  |  |
| 1 | Atr-ERM95640 |  | | | |  |  |  |  |  |
| 1 | Atr-ERM95641 |  | | | |  |  |  |  |  |
| 1 | Atr-ERM95642 |  | | | |  |  |  |  |  |
| 1 | Atr-ERM95643 |  | | | |  |  |  |  |  |
| 2 | Atr-ERM95644 |  | | | |  | Vvi-Vitvi13g04048\_t001 |  |  |  |  |
| 2 | Atr-ERM95645 |  | | | |  | | | |  |  |  |  |
| 2 | Atr-ERM95646 |  | | | |  | | | |  |  |  |  |
| 2 | Atr-ERM95647 |  | | | |  | | | |  |  |  |  |
| 2 | Atr-ERM95648 |  | | | |  | | | |  |  |  |  |
| 2 | Atr-ERM95649 |  | Vvi-Vitvi08g01120\_t001 |  | | | |  |  |  |  |
| 2 | Atr-ERM95650 |  | | | |  | | | |  |  |  |  |
| 2 | Atr-ERM95651 |  | | | |  | | | |  |  |  |  |
| 2 | Atr-ERM95652 |  | | | |  | | | |  |  |  |  |
| 2 | Atr-ERM95653 |  | | | |  | | | |  |  |  |  |
| 2 | Atr-ERM95654 |  | | | |  | | | |  |  |  |  |
| 2 | Atr-ERM95655 |  | | | |  | | | |  |  |  |  |
| 2 | Atr-ERM95656 |  | | | |  | | | |  |  |  |  |
| 2 | Atr-ERM95657 |  | | | |  | | | |  |  |  |  |
| 3 | Atr-ERM95658 |  | | | |  | | | |  | Vvi-Vitvi06g01671\_t001 |  |  |  |
| 3 | Atr-ERM95659 |  | | | |  | | | |  | | | |  |  |  |
| 3 | Atr-ERM95660 |  | | | |  | | | |  | | | |  |  |  |
| 3 | Atr-ERM95661 |  | | | |  | | | |  | | | |  |  |  |
| 3 | Atr-ERM95662 |  | | | |  | | | |  | Vvi-Vitvi06g00383\_t001 |  |  |  |
| 3 | Atr-ERM95663 |  | Vvi-Vitvi08g01124\_t001 |  | | | |  | | | |  |  |  |
| 3 | Atr-ERM95664 |  | Vvi-Vitvi08g01125\_t001 |  | | | |  | | | |  |  |  |
| 3 | Atr-ERM95665 |  | | | |  | | | |  | Vvi-Vitvi06g00376\_t001 |  |  |  |
| 3 | Atr-ERM95666 |  | | | |  | Vvi-Vitvi13g00211\_t001 |  | | | |  |  |  |
| 3 | Atr-ERM95667 |  | | | |  | | | |  | | | |  |  |  |
| 3 | Atr-ERM95668 |  | | | |  | | | |  | | | |  |  |  |
| 3 | Atr-ERM95669 |  | | | |  | | | |  | | | |  |  |  |
| 3 | Atr-ERM95670 |  | | | |  | | | |  | | | |  |  |  |
| 3 | Atr-ERM95671 |  | | | |  | | | |  | | | |  |  |  |
| 3 | Atr-ERM95672 |  | | | |  | | | |  | | | |  |  |  |
| 3 | Atr-ERM95673 |  | | | |  | | | |  | | | |  |  |  |
| 3 | Atr-ERM95674 |  | | | |  | | | |  | | | |  |  |  |
| 3 | Atr-ERM95675 |  | | | |  | | | |  | | | |  |  |  |
| 3 | Atr-ERM95676 |  | | | |  | | | |  | | | |  |  |  |
| 3 | Atr-ERM95677 |  | | | |  | | | |  | | | |  |  |  |
| 3 | Atr-ERM95678 |  | | | |  | Vvi-Vitvi13g00207\_t001 |  | | | |  |  |  |
| 3 | Atr-ERM95679 |  | | | |  | | | |  | | | |  |  |  |
| 3 | Atr-ERM95680 |  | | | |  | | | |  | | | |  |  |  |
| 3 | Atr-ERM95681 |  | | | |  | | | |  | Vvi-Vitvi06g00369\_t001 |  |  |  |
| 3 | Atr-ERM95682 |  | | | |  | | | |  | | | |  |  |  |
| 3 | Atr-ERM95683 |  | Vvi-Vitvi08g01130\_t001 |  | Vvi-Vitvi13g00206\_t001 |  | | | |  |  |  |
| 3 | Atr-ERM95684 |  | | | |  | | | |  | | | |  |  |  |
| 3 | Atr-ERM95685 |  | | | |  | | | |  | | | |  |  |  |
| 3 | Atr-ERM95686 |  | | | |  | | | |  | | | |  |  |  |
| 3 | Atr-ERM95687 |  | | | |  | | | |  | | | |  |  |  |
| 3 | Atr-ERM95688 |  | | | |  | | | |  | | | |  |  |  |
| 3 | Atr-ERM95689 |  | | | |  | | | |  | | | |  |  |  |
| 3 | Atr-ERM95690 |  | | | |  | | | |  | | | |  |  |  |
| 3 | Atr-ERM95691 |  | | | |  | | | |  | | | |  |  |  |
| 3 | Atr-ERM95692 |  | | | |  | | | |  | | | |  |  |  |
| 3 | Atr-ERM95693 |  | Vvi-Vitvi08g01139\_t001 |  | Vvi-Vitvi13g00203\_t004 |  | Vvi-Vitvi06g00368\_t001 |  |  |  |
| 3 | Atr-ERM95694 |  | | | |  | Vvi-Vitvi13g00200\_t001 |  | | | |  |  |  |
| 2 | Atr-ERM95695 |  | | | |  |  |  | | | |  |  |  |
| 2 | Atr-ERM95696 |  | | | |  |  |  | | | |  |  |  |
| 2 | Atr-ERM95697 |  | | | |  |  |  | Vvi-Vitvi06g00367\_t001.1.6037826e |  |  |  |
| 2 | Atr-ERM95698 |  | | | |  |  |  | | | |  |  |  |
| 2 | Atr-ERM95699 |  | | | |  |  |  | | | |  |  |  |
| 2 | Atr-ERM95700 |  | | | |  |  |  | | | |  |  |  |
| 2 | Atr-ERM95701 |  | | | |  |  |  | | | |  |  |  |
| 2 | Atr-ERM95702 |  | | | |  |  |  | | | |  |  |  |
| 2 | Atr-ERM95703 |  | | | |  |  |  | | | |  |  |  |
| 2 | Atr-ERM95704 |  | | | |  |  |  | | | |  |  |  |
| 2 | Atr-ERM95705 |  | | | |  |  |  | | | |  |  |  |
| 2 | Atr-ERM95706 |  | | | |  |  |  | | | |  |  |  |
| 2 | Atr-ERM95707 |  | | | |  |  |  | | | |  |  |  |
| 2 | Atr-ERM95708 |  | | | |  |  |  | | | |  |  |  |
| 2 | Atr-ERM95709 |  | | | |  |  |  | | | |  |  |  |
| 2 | Atr-ERM95710 |  | | | |  |  |  | | | |  |  |  |
| 2 | Atr-ERM95711 |  | Vvi-Vitvi08g01153\_t001 |  |  |  | | | |  |  |  |
| 1 | Atr-ERM95712 |  |  |  |  |  | | | |  |  |  |
| 1 | Atr-ERM95713 |  |  |  |  |  | | | |  |  |  |
| 1 | Atr-ERM95714 |  |  |  |  |  | | | |  |  |  |
| 1 | Atr-ERM95715 |  |  |  |  |  | | | |  |  |  |
| 1 | Atr-ERM95716 |  |  |  |  |  | | | |  |  |  |
| 1 | Atr-ERM95717 |  |  |  |  |  | | | |  |  |  |
| 1 | Atr-ERM95718 |  |  |  |  |  | | | |  |  |  |
| 1 | Atr-ERM95719 |  |  |  |  |  | Vvi-Vitvi06g00365\_t001 |  |  |  |
| 1 | Atr-ERM95720 |  |  |  |  |  | | | |  |  |  |
| 1 | Atr-ERM95721 |  |  |  |  |  | | | |  |  |  |
| 1 | Atr-ERM95722 |  |  |  |  |  | | | |  |  |  |
| 1 | Atr-ERM95723 |  |  |  |  |  | | | |  |  |  |
| 1 | Atr-ERM95724 |  |  |  |  |  | | | |  |  |  |
| 1 | Atr-ERM95725 |  |  |  |  |  | | | |  |  |  |
| 1 | Atr-ERM95726 |  |  |  |  |  | | | |  |  |  |
| 1 | Atr-ERM95727 |  |  |  |  |  | | | |  |  |  |
| 1 | Atr-ERM95728 |  |  |  |  |  | | | |  |  |  |
| 1 | Atr-ERM95729 |  |  |  |  |  | | | |  |  |  |
| 1 | Atr-ERM95730 |  |  |  |  |  | | | |  |  |  |
| 1 | Atr-ERM95731 |  |  |  |  |  | | | |  |  |  |
| 1 | Atr-ERM95732 |  |  |  |  |  | | | |  |  |  |
| 1 | Atr-ERM95733 |  |  |  |  |  | | | |  |  |  |
| 1 | Atr-ERM95734 |  |  |  |  |  | | | |  |  |  |
| 1 | Atr-ERM95735 |  |  |  |  |  | | | |  |  |  |
| 1 | Atr-ERM95736 |  |  |  |  |  | | | |  |  |  |
| 1 | Atr-ERM95737 |  |  |  |  |  | | | |  |  |  |
| 1 | Atr-ERM95738 |  |  |  |  |  | | | |  |  |  |
| 1 | Atr-ERM95739 |  |  |  |  |  | | | |  |  |  |
| 1 | Atr-ERM95740 |  |  |  |  |  | | | |  |  |  |
| 1 | Atr-ERM95741 |  |  |  |  |  | | | |  |  |  |
| 1 | Atr-ERM95742 |  |  |  |  |  | Vvi-Vitvi06g00362\_t001 |  |  |  |
| 1 | Atr-ERM95743 |  |  |  |  |  | | | |  |  |  |
| 1 | Atr-ERM95744 |  |  |  |  |  | | | |  |  |  |
| 1 | Atr-ERM95745 |  |  |  |  |  | | | |  |  |  |
| 1 | Atr-ERM95746 |  |  |  |  |  | | | |  |  |  |
| 1 | Atr-ERM95747 |  |  |  |  |  | Vvi-Vitvi06g00359\_t002 |  |  |  |
| 1 | Atr-ERM95748 |  |  |  |  |  | | | |  |  |  |
| 1 | Atr-ERM95749 |  |  |  |  |  | | | |  |  |  |
| 1 | Atr-ERM95750 |  |  |  |  |  | | | |  |  |  |
| 1 | Atr-ERM95751 |  |  |  |  |  | | | |  |  |  |
| 1 | Atr-ERM95752 |  |  |  |  |  | | | |  |  |  |
| 1 | Atr-ERM95753 |  |  |  |  |  | | | |  |  |  |
| 1 | Atr-ERM95754 |  |  |  |  |  | | | |  |  |  |
| 1 | Atr-ERM95755 |  |  |  |  |  | | | |  |  |  |
| 1 | Atr-ERM95756 |  |  |  |  |  | | | |  |  |  |
| 1 | Atr-ERM95757 |  |  |  |  |  | | | |  |  |  |
| 1 | Atr-ERM95758 |  |  |  |  |  | | | |  |  |  |
| 1 | Atr-ERM95759 |  |  |  |  |  | | | |  |  |  |
| 1 | Atr-ERM95760 |  |  |  |  |  | | | |  |  |  |
| 1 | Atr-ERM95761 |  |  |  |  |  | | | |  |  |  |
| 1 | Atr-ERM95762 |  |  |  |  |  | | | |  |  |  |
| 1 | Atr-ERM95763 |  |  |  |  |  | | | |  |  |  |
| 1 | Atr-ERM95764 |  |  |  |  |  | Vvi-Vitvi06g00358\_t003 |  |  |  |
| 1 | Atr-ERM95765 |  |  |  |  |  | Vvi-Vitvi06g00356\_t001 |  |  |  |
| 1 | Atr-ERM95766 |  |  |  |  |  | | | |  |  |  |
| 1 | Atr-ERM95767 |  |  |  |  |  | | | |  |  |  |
| 1 | Atr-ERM95768 |  |  |  |  |  | | | |  |  |  |
| 1 | Atr-ERM95769 |  |  |  |  |  | | | |  |  |  |
| 1 | Atr-ERM95770 |  |  |  |  |  | Vvi-Vitvi06g00352\_t001 |  |  |  |
| 0 | Atr-ERM95771 |  |  |  |  |  |  |
| 0 | Atr-ERM95772 |  |  |  |  |  |  |
| 0 | Atr-ERM95773 |  |  |  |  |  |  |
| 0 | Atr-ERM95774 |  |  |  |  |  |  |
| 0 | Atr-ERM95775 |  |  |  |  |  |  |
| 0 | Atr-ERM95776 |  |  |  |  |  |  |
| 0 | Atr-ERM95777 |  |  |  |  |  |  |
