# Supplementary material for: Genome-wide identification, characterization and gene expression of BES1 transcription factor family in grapevine (Vitis vinifera L.)
Source: Sci Rep. 2023 Jan 5;13:240. doi: 10.1038/s41598-022-24407-y (PMC9816167; doi:10.1038/s41598-022-24407-y)
Supplement: Supplementary file 3 — Supplementary Information. [file 41598_2022_24407_MOESM3_ESM.zip › Vvi_Atr/Vitis_vinifera.PN40024.v4.dna_sm.toplevel.fa.vs.Amborella_trichopoda.AMTR1.0.dna_sm.toplevel.fa.html/Atr-AmTr_v1.0_scaffold00024.html]

|  |  |  |  |  |  |  |  |  |  |  |  |  |  |
| --- | --- | --- | --- | --- | --- | --- | --- | --- | --- | --- | --- | --- | --- |
| Duplication depth | Reference chromosome | Collinear blocks | | | | | | | | | | | |
| 1 | Atr-ERN11002 |  | Vvi-Vitvi01g00530\_t001 |  |  |  |  |  |
| 1 | Atr-ERN11003 |  | | | |  |  |  |  |  |
| 1 | Atr-ERN11004 |  | | | |  |  |  |  |  |
| 1 | Atr-ERN11005 |  | | | |  |  |  |  |  |
| 1 | Atr-ERN11006 |  | | | |  |  |  |  |  |
| 1 | Atr-ERN11007 |  | | | |  |  |  |  |  |
| 1 | Atr-ERN11008 |  | | | |  |  |  |  |  |
| 1 | Atr-ERN11009 |  | Vvi-Vitvi01g00533\_t001 |  |  |  |  |  |
| 1 | Atr-ERN11010 |  | Vvi-Vitvi01g00536\_t003 |  |  |  |  |  |
| 1 | Atr-ERN11011 |  | | | |  |  |  |  |  |
| 1 | Atr-ERN11012 |  | | | |  |  |  |  |  |
| 1 | Atr-ERN11013 |  | | | |  |  |  |  |  |
| 1 | Atr-ERN11014 |  | | | |  |  |  |  |  |
| 1 | Atr-ERN11015 |  | Vvi-Vitvi01g00537\_t002 |  |  |  |  |  |
| 1 | Atr-ERN11016 |  | Vvi-Vitvi01g00538\_t001 |  |  |  |  |  |
| 1 | Atr-ERN11017 |  | Vvi-Vitvi01g00539\_t001 |  |  |  |  |  |
| 1 | Atr-ERN11018 |  | | | |  |  |  |  |  |
| 1 | Atr-ERN11019 |  | | | |  |  |  |  |  |
| 1 | Atr-ERN11020 |  | | | |  |  |  |  |  |
| 1 | Atr-ERN11021 |  | | | |  |  |  |  |  |
| 1 | Atr-ERN11022 |  | | | |  |  |  |  |  |
| 1 | Atr-ERN11023 |  | | | |  |  |  |  |  |
| 1 | Atr-ERN11024 |  | | | |  |  |  |  |  |
| 1 | Atr-ERN11025 |  | | | |  |  |  |  |  |
| 1 | Atr-ERN11026 |  | Vvi-Vitvi01g00540\_t001 |  |  |  |  |  |
| 1 | Atr-ERN11027 |  | | | |  |  |  |  |  |
| 1 | Atr-ERN11028 |  | Vvi-Vitvi01g00541\_t001 |  |  |  |  |  |
| 1 | Atr-ERN11029 |  | | | |  |  |  |  |  |
| 1 | Atr-ERN11030 |  | | | |  |  |  |  |  |
| 1 | Atr-ERN11031 |  | | | |  |  |  |  |  |
| 1 | Atr-ERN11032 |  | | | |  |  |  |  |  |
| 1 | Atr-ERN11033 |  | | | |  |  |  |  |  |
| 1 | Atr-ERN11034 |  | | | |  |  |  |  |  |
| 1 | Atr-ERN11035 |  | | | |  |  |  |  |  |
| 1 | Atr-ERN11036 |  | Vvi-Vitvi01g00544\_t001 |  |  |  |  |  |
| 1 | Atr-ERN11037 |  | | | |  |  |  |  |  |
| 1 | Atr-ERN11038 |  | | | |  |  |  |  |  |
| 1 | Atr-ERN11039 |  | | | |  |  |  |  |  |
| 1 | Atr-ERN11040 |  | | | |  |  |  |  |  |
| 1 | Atr-ERN11041 |  | Vvi-Vitvi01g00545\_t001 |  |  |  |  |  |
| 1 | Atr-ERN11042 |  | Vvi-Vitvi01g00546\_t001 |  |  |  |  |  |
| 1 | Atr-ERN11043 |  | | | |  |  |  |  |  |
| 1 | Atr-ERN11044 |  | | | |  |  |  |  |  |
| 1 | Atr-ERN11045 |  | | | |  |  |  |  |  |
| 1 | Atr-ERN11046 |  | | | |  |  |  |  |  |
| 1 | Atr-ERN11047 |  | | | |  |  |  |  |  |
| 1 | Atr-ERN11048 |  | Vvi-Vitvi01g00547\_t001 |  |  |  |  |  |
| 1 | Atr-ERN11049 |  | | | |  |  |  |  |  |
| 1 | Atr-ERN11050 |  | | | |  |  |  |  |  |
| 1 | Atr-ERN11051 |  | Vvi-Vitvi01g00550\_t001 |  |  |  |  |  |
| 1 | Atr-ERN11052 |  | | | |  |  |  |  |  |
| 1 | Atr-ERN11053 |  | | | |  |  |  |  |  |
| 1 | Atr-ERN11054 |  | | | |  |  |  |  |  |
| 1 | Atr-ERN11055 |  | | | |  |  |  |  |  |
| 1 | Atr-ERN11056 |  | Vvi-Vitvi01g02007\_t001 |  |  |  |  |  |
| 1 | Atr-ERN11057 |  | Vvi-Vitvi01g00553\_t001 |  |  |  |  |  |
| 1 | Atr-ERN11058 |  | Vvi-Vitvi01g00554\_t001 |  |  |  |  |  |
| 1 | Atr-ERN11059 |  | | | |  |  |  |  |  |
| 1 | Atr-ERN11060 |  | | | |  |  |  |  |  |
| 1 | Atr-ERN11061 |  | | | |  |  |  |  |  |
| 1 | Atr-ERN11062 |  | | | |  |  |  |  |  |
| 1 | Atr-ERN11063 |  | Vvi-Vitvi01g02011\_t001 |  |  |  |  |  |
| 1 | Atr-ERN11064 |  | | | |  |  |  |  |  |
| 1 | Atr-ERN11065 |  | Vvi-Vitvi01g00561\_t001 |  |  |  |  |  |
| 1 | Atr-ERN11066 |  | | | |  |  |  |  |  |
| 1 | Atr-ERN11067 |  | | | |  |  |  |  |  |
| 1 | Atr-ERN11068 |  | | | |  |  |  |  |  |
| 1 | Atr-ERN11069 |  | | | |  |  |  |  |  |
| 1 | Atr-ERN11070 |  | | | |  |  |  |  |  |
| 1 | Atr-ERN11071 |  | | | |  |  |  |  |  |
| 1 | Atr-ERN11072 |  | | | |  |  |  |  |  |
| 1 | Atr-ERN11073 |  | Vvi-Vitvi01g00562\_t001 |  |  |  |  |  |
| 1 | Atr-ERN11074 |  | | | |  |  |  |  |  |
| 1 | Atr-ERN11075 |  | | | |  |  |  |  |  |
| 1 | Atr-ERN11076 |  | | | |  |  |  |  |  |
| 1 | Atr-ERN11077 |  | | | |  |  |  |  |  |
| 1 | Atr-ERN11078 |  | | | |  |  |  |  |  |
| 1 | Atr-ERN11079 |  | | | |  |  |  |  |  |
| 1 | Atr-ERN11080 |  | | | |  |  |  |  |  |
| 1 | Atr-ERN11081 |  | | | |  |  |  |  |  |
| 1 | Atr-ERN11082 |  | Vvi-Vitvi01g00563\_t003 |  |  |  |  |  |
| 1 | Atr-ERN11083 |  | | | |  |  |  |  |  |
| 1 | Atr-ERN11084 |  | | | |  |  |  |  |  |
| 1 | Atr-ERN11085 |  | | | |  |  |  |  |  |
| 1 | Atr-ERN11086 |  | | | |  |  |  |  |  |
| 1 | Atr-ERN11087 |  | | | |  |  |  |  |  |
| 1 | Atr-ERN11088 |  | | | |  |  |  |  |  |
| 1 | Atr-ERN11089 |  | Vvi-Vitvi01g02012\_t001 |  |  |  |  |  |
| 1 | Atr-ERN11090 |  | | | |  |  |  |  |  |
| 1 | Atr-ERN11091 |  | | | |  |  |  |  |  |
| 1 | Atr-ERN11092 |  | | | |  |  |  |  |  |
| 1 | Atr-ERN11093 |  | Vvi-Vitvi01g00568\_t001 |  |  |  |  |  |
| 1 | Atr-ERN11094 |  | | | |  |  |  |  |  |
| 1 | Atr-ERN11095 |  | | | |  |  |  |  |  |
| 1 | Atr-ERN11096 |  | | | |  |  |  |  |  |
| 1 | Atr-ERN11097 |  | | | |  |  |  |  |  |
| 2 | Atr-ERN11098 |  | Vvi-Vitvi01g00571\_t001 |  | Vvi-Vitvi06g00124\_t001 |  |  |  |  |
| 1 | Atr-ERN11099 |  |  |  | | | |  |  |  |  |
| 1 | Atr-ERN11100 |  |  |  | | | |  |  |  |  |
| 1 | Atr-ERN11101 |  |  |  | | | |  |  |  |  |
| 1 | Atr-ERN11102 |  |  |  | | | |  |  |  |  |
| 1 | Atr-ERN11103 |  |  |  | | | |  |  |  |  |
| 1 | Atr-ERN11104 |  |  |  | | | |  |  |  |  |
| 1 | Atr-ERN11105 |  |  |  | | | |  |  |  |  |
| 1 | Atr-ERN11106 |  |  |  | | | |  |  |  |  |
| 1 | Atr-ERN11107 |  |  |  | | | |  |  |  |  |
| 1 | Atr-ERN11108 |  |  |  | | | |  |  |  |  |
| 1 | Atr-ERN11109 |  |  |  | | | |  |  |  |  |
| 1 | Atr-ERN11110 |  |  |  | | | |  |  |  |  |
| 1 | Atr-ERN11111 |  |  |  | | | |  |  |  |  |
| 1 | Atr-ERN11112 |  |  |  | | | |  |  |  |  |
| 1 | Atr-ERN11113 |  |  |  | | | |  |  |  |  |
| 1 | Atr-ERN11114 |  |  |  | | | |  |  |  |  |
| 2 | Atr-ERN11115 |  | Vvi-Vitvi08g01749\_t001 |  | | | |  |  |  |  |
| 2 | Atr-ERN11116 |  | | | |  | | | |  |  |  |  |
| 2 | Atr-ERN11117 |  | | | |  | | | |  |  |  |  |
| 2 | Atr-ERN11118 |  | | | |  | | | |  |  |  |  |
| 2 | Atr-ERN11119 |  | | | |  | | | |  |  |  |  |
| 2 | Atr-ERN11120 |  | | | |  | | | |  |  |  |  |
| 2 | Atr-ERN11121 |  | Vvi-Vitvi08g01751\_t001 |  | | | |  |  |  |  |
| 2 | Atr-ERN11122 |  | | | |  | Vvi-Vitvi06g00111\_t001 |  |  |  |  |
| 2 | Atr-ERN11123 |  | | | |  | | | |  |  |  |  |
| 2 | Atr-ERN11124 |  | | | |  | Vvi-Vitvi06g00110\_t001 |  |  |  |  |
| 2 | Atr-ERN11125 |  | | | |  | Vvi-Vitvi06g00109\_t001 |  |  |  |  |
| 2 | Atr-ERN11126 |  | Vvi-Vitvi08g01756\_t001 |  | | | |  |  |  |  |
| 2 | Atr-ERN11127 |  | | | |  | | | |  |  |  |  |
| 2 | Atr-ERN11128 |  | Vvi-Vitvi08g01757\_t001 |  | | | |  |  |  |  |
| 2 | Atr-ERN11129 |  | Vvi-Vitvi08g01758\_t001 |  | | | |  |  |  |  |
| 2 | Atr-ERN11130 |  | Vvi-Vitvi08g01759\_t001 |  | | | |  |  |  |  |
| 2 | Atr-ERN11131 |  | | | |  | | | |  |  |  |  |
| 2 | Atr-ERN11132 |  | | | |  | | | |  |  |  |  |
| 2 | Atr-ERN11133 |  | Vvi-Vitvi08g01762\_t001 |  | Vvi-Vitvi06g00106\_t001 |  |  |  |  |
| 2 | Atr-ERN11134 |  | | | |  | | | |  |  |  |  |
| 2 | Atr-ERN11135 |  | | | |  | | | |  |  |  |  |
| 2 | Atr-ERN11136 |  | | | |  | Vvi-Vitvi06g00104\_t001 |  |  |  |  |
| 2 | Atr-ERN11137 |  | Vvi-Vitvi08g01764\_t001 |  | | | |  |  |  |  |
| 2 | Atr-ERN11138 |  | | | |  | | | |  |  |  |  |
| 3 | Atr-ERN11139 |  | | | |  | | | |  | Vvi-Vitvi06g00094\_t001 |  |  |  |
| 3 | Atr-ERN11140 |  | | | |  | | | |  | | | |  |  |  |
| 3 | Atr-ERN11141 |  | | | |  | | | |  | | | |  |  |  |
| 3 | Atr-ERN11142 |  | | | |  | | | |  | Vvi-Vitvi06g00095\_t001 |  |  |  |
| 3 | Atr-ERN11143 |  | | | |  | | | |  | Vvi-Vitvi06g00096\_t001 |  |  |  |
| 3 | Atr-ERN11144 |  | | | |  | | | |  | Vvi-Vitvi06g00097\_t001 |  |  |  |
| 3 | Atr-ERN11145 |  | | | |  | | | |  | | | |  |  |  |
| 3 | Atr-ERN11146 |  | | | |  | | | |  | Vvi-Vitvi06g00098\_t001 |  |  |  |
| 3 | Atr-ERN11147 |  | | | |  | | | |  | | | |  |  |  |
| 3 | Atr-ERN11148 |  | | | |  | | | |  | | | |  |  |  |
| 3 | Atr-ERN11149 |  | | | |  | | | |  | | | |  |  |  |
| 3 | Atr-ERN11150 |  | | | |  | | | |  | | | |  |  |  |
| 3 | Atr-ERN11151 |  | Vvi-Vitvi08g01769\_t001 |  | | | |  | | | |  |  |  |
| 3 | Atr-ERN11152 |  | Vvi-Vitvi08g01771\_t001 |  | | | |  | | | |  |  |  |
| 2 | Atr-ERN11153 |  |  |  | | | |  | | | |  |  |  |
| 2 | Atr-ERN11154 |  |  |  | Vvi-Vitvi06g01606\_t001 |  | Vvi-Vitvi06g01606\_t001 |  |  |  |
| 1 | Atr-ERN11155 |  |  |  |  |  | | | |  |  |  |
| 1 | Atr-ERN11156 |  |  |  |  |  | | | |  |  |  |
| 1 | Atr-ERN11157 |  |  |  |  |  | | | |  |  |  |
| 1 | Atr-ERN11158 |  |  |  |  |  | | | |  |  |  |
| 1 | Atr-ERN11159 |  |  |  |  |  | Vvi-Vitvi06g00100\_t001 |  |  |  |
| 1 | Atr-ERN11160 |  |  |  |  |  | | | |  |  |  |
| 1 | Atr-ERN11161 |  |  |  |  |  | | | |  |  |  |
| 1 | Atr-ERN11162 |  |  |  |  |  | | | |  |  |  |
| 1 | Atr-ERN11163 |  |  |  |  |  | | | |  |  |  |
| 1 | Atr-ERN11164 |  |  |  |  |  | Vvi-Vitvi06g00102\_t001 |  |  |  |
| 0 | Atr-ERN11165 |  |  |  |  |  |  |
| 0 | Atr-ERN11166 |  |  |  |  |  |  |
| 0 | Atr-ERN11167 |  |  |  |  |  |  |
| 0 | Atr-ERN11168 |  |  |  |  |  |  |
| 0 | Atr-ERN11169 |  |  |  |  |  |  |
| 0 | Atr-ERN11170 |  |  |  |  |  |  |
| 0 | Atr-ERN11171 |  |  |  |  |  |  |
| 0 | Atr-ERN11172 |  |  |  |  |  |  |
| 0 | Atr-ERN11173 |  |  |  |  |  |  |
| 0 | Atr-ERN11174 |  |  |  |  |  |  |
| 0 | Atr-ERN11175 |  |  |  |  |  |  |
| 0 | Atr-ERN11176 |  |  |  |  |  |  |
| 1 | Atr-ERN11177 |  | Vvi-Vitvi11g01359\_t001 |  |  |  |  |  |
| 1 | Atr-ERN11178 |  | Vvi-Vitvi11g00115\_t001 |  |  |  |  |  |
| 1 | Atr-ERN11179 |  | | | |  |  |  |  |  |
| 1 | Atr-ERN11180 |  | | | |  |  |  |  |  |
| 1 | Atr-ERN11181 |  | | | |  |  |  |  |  |
| 1 | Atr-ERN11182 |  | | | |  |  |  |  |  |
| 1 | Atr-ERN11183 |  | | | |  |  |  |  |  |
| 1 | Atr-ERN11184 |  | | | |  |  |  |  |  |
| 3 | Atr-ERN11185 |  | | | |  | Vvi-Vitvi09g00134\_t001 |  | Vvi-Vitvi04g01805\_t001 |  |  |  |
| 3 | Atr-ERN11186 |  | | | |  | | | |  | | | |  |  |  |
| 3 | Atr-ERN11187 |  | | | |  | | | |  | | | |  |  |  |
| 3 | Atr-ERN11188 |  | | | |  | | | |  | Vvi-Vitvi04g00141\_t001 |  |  |  |
| 3 | Atr-ERN11189 |  | | | |  | | | |  | | | |  |  |  |
| 3 | Atr-ERN11190 |  | | | |  | | | |  | | | |  |  |  |
| 3 | Atr-ERN11191 |  | | | |  | | | |  | | | |  |  |  |
| 3 | Atr-ERN11192 |  | Vvi-Vitvi11g00108\_t001 |  | Vvi-Vitvi09g00129\_t002 |  | | | |  |  |  |
| 3 | Atr-ERN11193 |  | | | |  | | | |  | Vvi-Vitvi04g00142\_t001 |  |  |  |
| 3 | Atr-ERN11194 |  | | | |  | | | |  | | | |  |  |  |
| 3 | Atr-ERN11195 |  | | | |  | | | |  | | | |  |  |  |
| 3 | Atr-ERN11196 |  | | | |  | | | |  | Vvi-Vitvi04g00144\_t001 |  |  |  |
| 3 | Atr-ERN11197 |  | Vvi-Vitvi11g00106\_t001 |  | Vvi-Vitvi09g00126\_t001 |  | Vvi-Vitvi04g00145\_t001 |  |  |  |
| 3 | Atr-ERN11198 |  | | | |  | | | |  | | | |  |  |  |
| 3 | Atr-ERN11199 |  | | | |  | | | |  | | | |  |  |  |
| 3 | Atr-ERN11200 |  | | | |  | | | |  | Vvi-Vitvi04g00146\_t001 |  |  |  |
| 3 | Atr-ERN11201 |  | | | |  | | | |  | | | |  |  |  |
| 3 | Atr-ERN11202 |  | | | |  | | | |  | | | |  |  |  |
| 3 | Atr-ERN11203 |  | | | |  | | | |  | | | |  |  |  |
| 3 | Atr-ERN11204 |  | | | |  | Vvi-Vitvi09g00122\_t001 |  | | | |  |  |  |
| 3 | Atr-ERN11205 |  | | | |  | | | |  | | | |  |  |  |
| 3 | Atr-ERN11206 |  | | | |  | | | |  | | | |  |  |  |
| 3 | Atr-ERN11207 |  | Vvi-Vitvi11g01346\_t001 |  | | | |  | Vvi-Vitvi04g00147\_t001 |  |  |  |
| 3 | Atr-ERN11208 |  | | | |  | | | |  | | | |  |  |  |
| 3 | Atr-ERN11209 |  | Vvi-Vitvi11g01345\_t001 |  | | | |  | Vvi-Vitvi04g01806\_t001 |  |  |  |
| 3 | Atr-ERN11210 |  | Vvi-Vitvi11g00104\_t001 |  | | | |  | | | |  |  |  |
| 3 | Atr-ERN11211 |  | | | |  | | | |  | Vvi-Vitvi04g00148\_t001 |  |  |  |
| 3 | Atr-ERN11212 |  | Vvi-Vitvi11g00103\_t001 |  | Vvi-Vitvi09g00119\_t001 |  | Vvi-Vitvi04g00149\_t001 |  |  |  |
| 3 | Atr-ERN11213 |  | | | |  | | | |  | | | |  |  |  |
| 3 | Atr-ERN11214 |  | | | |  | | | |  | | | |  |  |  |
| 3 | Atr-ERN11215 |  | | | |  | | | |  | | | |  |  |  |
| 3 | Atr-ERN11216 |  | | | |  | | | |  | | | |  |  |  |
| 3 | Atr-ERN11217 |  | | | |  | | | |  | Vvi-Vitvi04g00150\_t001 |  |  |  |
| 3 | Atr-ERN11218 |  | | | |  | Vvi-Vitvi09g00118\_t001 |  | | | |  |  |  |
| 3 | Atr-ERN11219 |  | | | |  | | | |  | | | |  |  |  |
| 3 | Atr-ERN11220 |  | Vvi-Vitvi11g00102\_t001 |  | Vvi-Vitvi09g00117\_t001 |  | | | |  |  |  |
| 3 | Atr-ERN11221 |  | Vvi-Vitvi11g00101\_t001 |  | | | |  | | | |  |  |  |
| 3 | Atr-ERN11222 |  | Vvi-Vitvi11g00097\_t001 |  | | | |  | Vvi-Vitvi04g00153\_t001 |  |  |  |
| 3 | Atr-ERN11223 |  | | | |  | | | |  | | | |  |  |  |
| 3 | Atr-ERN11224 |  | | | |  | | | |  | | | |  |  |  |
| 3 | Atr-ERN11225 |  | | | |  | | | |  | | | |  |  |  |
| 3 | Atr-ERN11226 |  | | | |  | | | |  | Vvi-Vitvi04g00158\_t001 |  |  |  |
| 3 | Atr-ERN11227 |  | | | |  | Vvi-Vitvi09g00111\_t001 |  | | | |  |  |  |
| 3 | Atr-ERN11228 |  | Vvi-Vitvi11g00094\_t002 |  | | | |  | | | |  |  |  |
| 3 | Atr-ERN11229 |  | | | |  | | | |  | Vvi-Vitvi04g00161\_t001 |  |  |  |
| 3 | Atr-ERN11230 |  | | | |  | Vvi-Vitvi09g00108\_t001 |  | | | |  |  |  |
| 3 | Atr-ERN11231 |  | Vvi-Vitvi11g00093\_t001 |  | | | |  | | | |  |  |  |
| 3 | Atr-ERN11232 |  | | | |  | Vvi-Vitvi09g01516\_t001 |  | | | |  |  |  |
| 3 | Atr-ERN11233 |  | | | |  | | | |  | | | |  |  |  |
| 3 | Atr-ERN11234 |  | Vvi-Vitvi11g00092\_t001 |  | Vvi-Vitvi09g00107\_t001 |  | | | |  |  |  |
| 3 | Atr-ERN11235 |  | Vvi-Vitvi11g00091\_t002 |  | | | |  | | | |  |  |  |
| 3 | Atr-ERN11236 |  | | | |  | | | |  | | | |  |  |  |
| 3 | Atr-ERN11237 |  | | | |  | | | |  | | | |  |  |  |
| 3 | Atr-ERN11238 |  | | | |  | | | |  | | | |  |  |  |
| 3 | Atr-ERN11239 |  | | | |  | | | |  | | | |  |  |  |
| 3 | Atr-ERN11240 |  | Vvi-Vitvi11g00090\_t001 |  | | | |  | | | |  |  |  |
| 3 | Atr-ERN11241 |  | | | |  | | | |  | | | |  |  |  |
| 3 | Atr-ERN11242 |  | | | |  | | | |  | | | |  |  |  |
| 3 | Atr-ERN11243 |  | | | |  | | | |  | | | |  |  |  |
| 3 | Atr-ERN11244 |  | | | |  | | | |  | | | |  |  |  |
| 3 | Atr-ERN11245 |  | | | |  | | | |  | Vvi-Vitvi04g00162\_t001 |  |  |  |
| 3 | Atr-ERN11246 |  | Vvi-Vitvi11g00089\_t001 |  | Vvi-Vitvi09g01512\_t001 |  | | | |  |  |  |
| 3 | Atr-ERN11247 |  | Vvi-Vitvi11g00087\_t001 |  | | | |  | | | |  |  |  |
| 3 | Atr-ERN11248 |  | Vvi-Vitvi11g00086\_t001 |  | | | |  | | | |  |  |  |
| 3 | Atr-ERN11249 |  | | | |  | | | |  | | | |  |  |  |
| 3 | Atr-ERN11250 |  | | | |  | | | |  | Vvi-Vitvi04g00163\_t001 |  |  |  |
| 3 | Atr-ERN11251 |  | | | |  | | | |  | Vvi-Vitvi04g00164\_t001 |  |  |  |
| 3 | Atr-ERN11252 |  | Vvi-Vitvi11g00085\_t001 |  | | | |  | Vvi-Vitvi04g00165\_t001 |  |  |  |
| 3 | Atr-ERN11253 |  | | | |  | | | |  | Vvi-Vitvi04g01809\_t001 |  |  |  |
| 3 | Atr-ERN11254 |  | Vvi-Vitvi11g00083\_t001 |  | | | |  | | | |  |  |  |
| 3 | Atr-ERN11255 |  | | | |  | | | |  | | | |  |  |  |
| 4 | Atr-ERN11256 |  | | | |  | | | |  | | | |  | Vvi-Vitvi04g00178\_t001 |  |  |
| 4 | Atr-ERN11257 |  | | | |  | | | |  | | | |  | Vvi-Vitvi04g00176\_t002 |  |  |
| 4 | Atr-ERN11258 |  | | | |  | | | |  | | | |  | Vvi-Vitvi04g00175\_t002 |  |  |
| 4 | Atr-ERN11259 |  | | | |  | | | |  | | | |  | Vvi-Vitvi04g00174\_t001 |  |  |
| 4 | Atr-ERN11260 |  | | | |  | | | |  | | | |  | Vvi-Vitvi04g00173\_t001 |  |  |
| 4 | Atr-ERN11261 |  | | | |  | | | |  | | | |  | Vvi-Vitvi04g00172\_t001 |  |  |
| 4 | Atr-ERN11262 |  | | | |  | | | |  | | | |  | | | |  |  |
| 4 | Atr-ERN11263 |  | | | |  | Vvi-Vitvi09g01487\_t001 |  | | | |  | | | |  |  |
| 4 | Atr-ERN11264 |  | | | |  | | | |  | | | |  | | | |  |  |
| 4 | Atr-ERN11265 |  | | | |  | | | |  | | | |  | | | |  |  |
| 4 | Atr-ERN11266 |  | | | |  | | | |  | | | |  | | | |  |  |
| 4 | Atr-ERN11267 |  | | | |  | | | |  | | | |  | | | |  |  |
| 4 | Atr-ERN11268 |  | | | |  | | | |  | | | |  | Vvi-Vitvi04g00168\_t001 |  |  |
| 4 | Atr-ERN11269 |  | Vvi-Vitvi11g01341\_t001 |  | | | |  | | | |  | | | |  |  |
| 4 | Atr-ERN11270 |  | | | |  | | | |  | | | |  | Vvi-Vitvi04g00167\_t001 |  |  |
| 3 | Atr-ERN11271 |  | | | |  | | | |  | Vvi-Vitvi04g00166\_t001 |  |  |  |
| 3 | Atr-ERN11272 |  | | | |  | | | |  | | | |  |  |  |
| 3 | Atr-ERN11273 |  | | | |  | | | |  | | | |  |  |  |
| 3 | Atr-ERN11274 |  | Vvi-Vitvi11g00079\_t001 |  | | | |  | | | |  |  |  |
| 3 | Atr-ERN11275 |  | | | |  | | | |  | | | |  |  |  |
| 3 | Atr-ERN11276 |  | | | |  | | | |  | | | |  |  |  |
| 3 | Atr-ERN11277 |  | | | |  | | | |  | | | |  |  |  |
| 3 | Atr-ERN11278 |  | | | |  | | | |  | | | |  |  |  |
| 3 | Atr-ERN11279 |  | Vvi-Vitvi11g00078\_t001 |  | | | |  | | | |  |  |  |
| 3 | Atr-ERN11280 |  | Vvi-Vitvi11g00077\_t001 |  | | | |  | | | |  |  |  |
| 3 | Atr-ERN11281 |  | | | |  | Vvi-Vitvi09g00094\_t001 |  | | | |  |  |  |
| 3 | Atr-ERN11282 |  | | | |  | Vvi-Vitvi09g00093\_t001 |  | | | |  |  |  |
| 3 | Atr-ERN11283 |  | | | |  | | | |  | | | |  |  |  |
| 3 | Atr-ERN11284 |  | | | |  | | | |  | | | |  |  |  |
| 3 | Atr-ERN11285 |  | | | |  | | | |  | | | |  |  |  |
| 3 | Atr-ERN11286 |  | Vvi-Vitvi11g00076\_t001 |  | | | |  | | | |  |  |  |
| 3 | Atr-ERN11287 |  | | | |  | Vvi-Vitvi09g00089\_t002 |  | | | |  |  |  |
| 3 | Atr-ERN11288 |  | Vvi-Vitvi11g01336\_t001 |  | Vvi-Vitvi09g00088\_t001 |  | Vvi-Vitvi04g00179\_t001 |  |  |  |
| 3 | Atr-ERN11289 |  | | | |  | | | |  | | | |  |  |  |
| 3 | Atr-ERN11290 |  | | | |  | Vvi-Vitvi09g00086\_t001 |  | | | |  |  |  |
| 3 | Atr-ERN11291 |  | Vvi-Vitvi11g01335\_t001 |  | | | |  | | | |  |  |  |
| 3 | Atr-ERN11292 |  | | | |  | | | |  | | | |  |  |  |
| 3 | Atr-ERN11293 |  | | | |  | | | |  | | | |  |  |  |
| 3 | Atr-ERN11294 |  | | | |  | | | |  | | | |  |  |  |
| 3 | Atr-ERN11295 |  | | | |  | | | |  | | | |  |  |  |
| 3 | Atr-ERN11296 |  | | | |  | | | |  | | | |  |  |  |
| 3 | Atr-ERN11297 |  | | | |  | | | |  | | | |  |  |  |
| 3 | Atr-ERN11298 |  | | | |  | | | |  | | | |  |  |  |
| 3 | Atr-ERN11299 |  | | | |  | | | |  | Vvi-Vitvi04g00181\_t001 |  |  |  |
| 3 | Atr-ERN11300 |  | Vvi-Vitvi11g00072\_t001 |  | Vvi-Vitvi09g00082\_t001 |  | | | |  |  |  |
| 3 | Atr-ERN11301 |  | | | |  | | | |  | | | |  |  |  |
| 3 | Atr-ERN11302 |  | | | |  | | | |  | | | |  |  |  |
| 3 | Atr-ERN11303 |  | | | |  | | | |  | | | |  |  |  |
| 3 | Atr-ERN11304 |  | Vvi-Vitvi11g00068\_t001 |  | | | |  | Vvi-Vitvi04g01813\_t001 |  |  |  |
| 3 | Atr-ERN11305 |  | | | |  | | | |  | | | |  |  |  |
| 3 | Atr-ERN11306 |  | Vvi-Vitvi11g00067\_t001 |  | Vvi-Vitvi09g00079\_t001 |  | | | |  |  |  |
| 3 | Atr-ERN11307 |  | Vvi-Vitvi11g00066\_t001 |  | Vvi-Vitvi09g00078\_t001 |  | | | |  |  |  |
| 3 | Atr-ERN11308 |  | Vvi-Vitvi11g01331\_t001 |  | Vvi-Vitvi09g00077\_t001 |  | Vvi-Vitvi04g00182\_t001 |  |  |  |
| 1 | Atr-ERN11309 |  |  |  |  |  | | | |  |  |  |
| 1 | Atr-ERN11310 |  |  |  |  |  | | | |  |  |  |
| 1 | Atr-ERN11311 |  |  |  |  |  | Vvi-Vitvi04g00183\_t001 |  |  |  |
| 0 | Atr-ERN11312 |  |  |  |  |  |  |
| 0 | Atr-ERN11313 |  |  |  |  |  |  |
| 0 | Atr-ERN11314 |  |  |  |  |  |  |
| 0 | Atr-ERN11315 |  |  |  |  |  |  |
| 0 | Atr-ERN11316 |  |  |  |  |  |  |
| 0 | Atr-ERN11317 |  |  |  |  |  |  |
| 0 | Atr-ERN11318 |  |  |  |  |  |  |
| 0 | Atr-ERN11319 |  |  |  |  |  |  |
| 0 | Atr-ERN11320 |  |  |  |  |  |  |
| 0 | Atr-ERN11321 |  |  |  |  |  |  |
| 0 | Atr-ERN11322 |  |  |  |  |  |  |
| 0 | Atr-ERN11323 |  |  |  |  |  |  |
| 0 | Atr-ERN11324 |  |  |  |  |  |  |
| 0 | Atr-ERN11325 |  |  |  |  |  |  |
| 0 | Atr-ERN11326 |  |  |  |  |  |  |
| 0 | Atr-ERN11327 |  |  |  |  |  |  |
| 0 | Atr-ERN11328 |  |  |  |  |  |  |
| 0 | Atr-ERN11329 |  |  |  |  |  |  |
| 0 | Atr-ERN11330 |  |  |  |  |  |  |
| 0 | Atr-ERN11331 |  |  |  |  |  |  |
| 0 | Atr-ERN11332 |  |  |  |  |  |  |
| 0 | Atr-ERN11333 |  |  |  |  |  |  |
| 0 | Atr-ERN11334 |  |  |  |  |  |  |
| 0 | Atr-ERN11335 |  |  |  |  |  |  |
| 0 | Atr-ERN11336 |  |  |  |  |  |  |
| 0 | Atr-ERN11337 |  |  |  |  |  |  |
| 0 | Atr-ERN11338 |  |  |  |  |  |  |
| 0 | Atr-ERN11339 |  |  |  |  |  |  |
| 0 | Atr-ERN11340 |  |  |  |  |  |  |
| 0 | Atr-ERN11341 |  |  |  |  |  |  |
| 0 | Atr-ERN11342 |  |  |  |  |  |  |
| 0 | Atr-ERN11343 |  |  |  |  |  |  |
| 0 | Atr-ERN11344 |  |  |  |  |  |  |
| 0 | Atr-ERN11345 |  |  |  |  |  |  |
| 0 | Atr-ERN11346 |  |  |  |  |  |  |
| 0 | Atr-ERN11347 |  |  |  |  |  |  |
| 0 | Atr-ERN11348 |  |  |  |  |  |  |
| 0 | Atr-ERN11349 |  |  |  |  |  |  |
| 0 | Atr-ERN11350 |  |  |  |  |  |  |
| 0 | Atr-ERN11351 |  |  |  |  |  |  |
| 0 | Atr-ERN11352 |  |  |  |  |  |  |
| 0 | Atr-ERN11353 |  |  |  |  |  |  |
| 0 | Atr-ERN11354 |  |  |  |  |  |  |
| 0 | Atr-ERN11355 |  |  |  |  |  |  |
| 0 | Atr-ERN11356 |  |  |  |  |  |  |
| 0 | Atr-ERN11357 |  |  |  |  |  |  |
| 0 | Atr-ERN11358 |  |  |  |  |  |  |
| 0 | Atr-ERN11359 |  |  |  |  |  |  |
| 0 | Atr-ERN11360 |  |  |  |  |  |  |
