# Supplementary material for: Genome-wide identification, characterization and gene expression of BES1 transcription factor family in grapevine (Vitis vinifera L.)
Source: Sci Rep. 2023 Jan 5;13:240. doi: 10.1038/s41598-022-24407-y (PMC9816167; doi:10.1038/s41598-022-24407-y)
Supplement: Supplementary file 3 — Supplementary Information. [file 41598_2022_24407_MOESM3_ESM.zip › Vvi_Atr/Vitis_vinifera.PN40024.v4.dna_sm.toplevel.fa.vs.Amborella_trichopoda.AMTR1.0.dna_sm.toplevel.fa.html/Atr-AmTr_v1.0_scaffold00025.html]

|  |  |  |  |  |  |  |  |  |  |  |  |  |  |
| --- | --- | --- | --- | --- | --- | --- | --- | --- | --- | --- | --- | --- | --- |
| Duplication depth | Reference chromosome | Collinear blocks | | | | | | | | | | | |
| 0 | Atr-ERN12287 |  |  |  |  |  |  |
| 0 | Atr-ERN12288 |  |  |  |  |  |  |
| 0 | Atr-ERN12289 |  |  |  |  |  |  |
| 0 | Atr-ERN12290 |  |  |  |  |  |  |
| 0 | Atr-ERN12291 |  |  |  |  |  |  |
| 0 | Atr-ERN12292 |  |  |  |  |  |  |
| 0 | Atr-ERN12293 |  |  |  |  |  |  |
| 0 | Atr-ERN12294 |  |  |  |  |  |  |
| 0 | Atr-ERN12295 |  |  |  |  |  |  |
| 0 | Atr-ERN12296 |  |  |  |  |  |  |
| 0 | Atr-ERN12297 |  |  |  |  |  |  |
| 0 | Atr-ERN12298 |  |  |  |  |  |  |
| 0 | Atr-ERN12299 |  |  |  |  |  |  |
| 1 | Atr-ERN12300 |  | Vvi-Vitvi07g00136\_t001 |  |  |  |  |  |
| 3 | Atr-ERN12301 |  | | | |  | Vvi-Vitvi14g02560\_t001 |  | Vvi-Vitvi05g01879\_t001 |  |  |  |
| 3 | Atr-ERN12302 |  | Vvi-Vitvi07g00137\_t001 |  | | | |  | | | |  |  |  |
| 3 | Atr-ERN12303 |  | | | |  | | | |  | | | |  |  |  |
| 3 | Atr-ERN12304 |  | | | |  | | | |  | | | |  |  |  |
| 3 | Atr-ERN12305 |  | | | |  | | | |  | | | |  |  |  |
| 3 | Atr-ERN12306 |  | | | |  | | | |  | Vvi-Vitvi05g00442\_t001 |  |  |  |
| 3 | Atr-ERN12307 |  | | | |  | | | |  | | | |  |  |  |
| 3 | Atr-ERN12308 |  | | | |  | | | |  | | | |  |  |  |
| 3 | Atr-ERN12309 |  | | | |  | | | |  | | | |  |  |  |
| 3 | Atr-ERN12310 |  | | | |  | | | |  | | | |  |  |  |
| 3 | Atr-ERN12311 |  | | | |  | | | |  | Vvi-Vitvi05g00439\_t001 |  |  |  |
| 3 | Atr-ERN12312 |  | Vvi-Vitvi07g00139\_t001 |  | | | |  | | | |  |  |  |
| 3 | Atr-ERN12313 |  | | | |  | Vvi-Vitvi14g00257\_t001 |  | | | |  |  |  |
| 3 | Atr-ERN12314 |  | | | |  | | | |  | | | |  |  |  |
| 3 | Atr-ERN12315 |  | | | |  | Vvi-Vitvi14g00259\_t001 |  | | | |  |  |  |
| 3 | Atr-ERN12316 |  | | | |  | Vvi-Vitvi14g00262\_t001 |  | | | |  |  |  |
| 3 | Atr-ERN12317 |  | | | |  | | | |  | | | |  |  |  |
| 3 | Atr-ERN12318 |  | | | |  | | | |  | | | |  |  |  |
| 3 | Atr-ERN12319 |  | | | |  | | | |  | | | |  |  |  |
| 3 | Atr-ERN12320 |  | | | |  | | | |  | | | |  |  |  |
| 3 | Atr-ERN12321 |  | | | |  | | | |  | | | |  |  |  |
| 3 | Atr-ERN12322 |  | | | |  | | | |  | | | |  |  |  |
| 3 | Atr-ERN12323 |  | | | |  | | | |  | | | |  |  |  |
| 3 | Atr-ERN12324 |  | | | |  | | | |  | | | |  |  |  |
| 3 | Atr-ERN12325 |  | | | |  | | | |  | Vvi-Vitvi05g00438\_t001 |  |  |  |
| 3 | Atr-ERN12326 |  | Vvi-Vitvi07g00141\_t001 |  | | | |  | | | |  |  |  |
| 3 | Atr-ERN12327 |  | | | |  | | | |  | | | |  |  |  |
| 3 | Atr-ERN12328 |  | | | |  | | | |  | | | |  |  |  |
| 3 | Atr-ERN12329 |  | | | |  | | | |  | | | |  |  |  |
| 3 | Atr-ERN12330 |  | | | |  | | | |  | | | |  |  |  |
| 3 | Atr-ERN12331 |  | | | |  | | | |  | | | |  |  |  |
| 3 | Atr-ERN12332 |  | | | |  | | | |  | Vvi-Vitvi05g00434\_t001 |  |  |  |
| 3 | Atr-ERN12333 |  | | | |  | | | |  | | | |  |  |  |
| 3 | Atr-ERN12334 |  | | | |  | Vvi-Vitvi14g00267\_t001 |  | Vvi-Vitvi05g00433\_t001 |  |  |  |
| 3 | Atr-ERN12335 |  | | | |  | | | |  | | | |  |  |  |
| 3 | Atr-ERN12336 |  | | | |  | | | |  | | | |  |  |  |
| 3 | Atr-ERN12337 |  | | | |  | | | |  | | | |  |  |  |
| 3 | Atr-ERN12338 |  | | | |  | | | |  | | | |  |  |  |
| 3 | Atr-ERN12339 |  | | | |  | | | |  | | | |  |  |  |
| 3 | Atr-ERN12340 |  | | | |  | | | |  | | | |  |  |  |
| 3 | Atr-ERN12341 |  | | | |  | | | |  | | | |  |  |  |
| 3 | Atr-ERN12342 |  | | | |  | | | |  | | | |  |  |  |
| 3 | Atr-ERN12343 |  | | | |  | | | |  | | | |  |  |  |
| 3 | Atr-ERN12344 |  | Vvi-Vitvi07g00146\_t001 |  | Vvi-Vitvi14g00268\_t001 |  | Vvi-Vitvi05g00430\_t001 |  |  |  |
| 3 | Atr-ERN12345 |  | | | |  | | | |  | | | |  |  |  |
| 3 | Atr-ERN12346 |  | | | |  | | | |  | | | |  |  |  |
| 3 | Atr-ERN12347 |  | | | |  | | | |  | | | |  |  |  |
| 3 | Atr-ERN12348 |  | | | |  | | | |  | Vvi-Vitvi05g00429\_t001 |  |  |  |
| 3 | Atr-ERN12349 |  | | | |  | | | |  | | | |  |  |  |
| 3 | Atr-ERN12350 |  | | | |  | | | |  | Vvi-Vitvi05g00427\_t001 |  |  |  |
| 3 | Atr-ERN12351 |  | | | |  | Vvi-Vitvi14g00270\_t001 |  | Vvi-Vitvi05g00426\_t001 |  |  |  |
| 3 | Atr-ERN12352 |  | | | |  | Vvi-Vitvi14g00271\_t001 |  | Vvi-Vitvi05g00425\_t002 |  |  |  |
| 3 | Atr-ERN12353 |  | Vvi-Vitvi07g02140\_t002 |  | | | |  | | | |  |  |  |
| 3 | Atr-ERN12354 |  | Vvi-Vitvi07g00150\_t002 |  | | | |  | | | |  |  |  |
| 3 | Atr-ERN12355 |  | Vvi-Vitvi07g00151\_t001 |  | | | |  | | | |  |  |  |
| 3 | Atr-ERN12356 |  | | | |  | | | |  | Vvi-Vitvi05g00424\_t001 |  |  |  |
| 3 | Atr-ERN12357 |  | Vvi-Vitvi07g00152\_t001 |  | | | |  | Vvi-Vitvi05g01875\_t001 |  |  |  |
| 3 | Atr-ERN12358 |  | | | |  | | | |  | | | |  |  |  |
| 3 | Atr-ERN12359 |  | | | |  | | | |  | Vvi-Vitvi05g00423\_t001 |  |  |  |
| 3 | Atr-ERN12360 |  | | | |  | | | |  | | | |  |  |  |
| 3 | Atr-ERN12361 |  | Vvi-Vitvi07g00154\_t001 |  | | | |  | Vvi-Vitvi05g00421\_t001 |  |  |  |
| 3 | Atr-ERN12362 |  | | | |  | | | |  | | | |  |  |  |
| 3 | Atr-ERN12363 |  | | | |  | | | |  | Vvi-Vitvi05g01874\_t001 |  |  |  |
| 3 | Atr-ERN12364 |  | | | |  | | | |  | | | |  |  |  |
| 3 | Atr-ERN12365 |  | Vvi-Vitvi07g00158\_t001 |  | | | |  | Vvi-Vitvi05g00420\_t001 |  |  |  |
| 3 | Atr-ERN12366 |  | | | |  | | | |  | | | |  |  |  |
| 3 | Atr-ERN12367 |  | Vvi-Vitvi07g00159\_t005 |  | | | |  | | | |  |  |  |
| 3 | Atr-ERN12368 |  | | | |  | | | |  | | | |  |  |  |
| 3 | Atr-ERN12369 |  | Vvi-Vitvi07g00162\_t001 |  | | | |  | | | |  |  |  |
| 3 | Atr-ERN12370 |  | | | |  | | | |  | | | |  |  |  |
| 3 | Atr-ERN12371 |  | Vvi-Vitvi07g00166\_t001 |  | | | |  | | | |  |  |  |
| 3 | Atr-ERN12372 |  | | | |  | | | |  | Vvi-Vitvi05g00419\_t001 |  |  |  |
| 3 | Atr-ERN12373 |  | Vvi-Vitvi07g00168\_t001 |  | | | |  | | | |  |  |  |
| 3 | Atr-ERN12374 |  | | | |  | | | |  | Vvi-Vitvi05g00418\_t001 |  |  |  |
| 3 | Atr-ERN12375 |  | | | |  | Vvi-Vitvi14g00273\_t001 |  | | | |  |  |  |
| 3 | Atr-ERN12376 |  | | | |  | | | |  | | | |  |  |  |
| 3 | Atr-ERN12377 |  | | | |  | | | |  | | | |  |  |  |
| 3 | Atr-ERN12378 |  | Vvi-Vitvi07g00169\_t001 |  | | | |  | Vvi-Vitvi05g00413\_t001 |  |  |  |
| 3 | Atr-ERN12379 |  | | | |  | | | |  | | | |  |  |  |
| 3 | Atr-ERN12380 |  | Vvi-Vitvi07g00171\_t003 |  | | | |  | | | |  |  |  |
| 3 | Atr-ERN12381 |  | | | |  | | | |  | Vvi-Vitvi05g00412\_t001 |  |  |  |
| 3 | Atr-ERN12382 |  | | | |  | | | |  | | | |  |  |  |
| 3 | Atr-ERN12383 |  | | | |  | | | |  | | | |  |  |  |
| 3 | Atr-ERN12384 |  | | | |  | | | |  | | | |  |  |  |
| 3 | Atr-ERN12385 |  | Vvi-Vitvi07g02143\_t001 |  | | | |  | Vvi-Vitvi05g00410\_t001 |  |  |  |
| 3 | Atr-ERN12386 |  | | | |  | | | |  | | | |  |  |  |
| 3 | Atr-ERN12387 |  | Vvi-Vitvi07g04046\_t001 |  | | | |  | | | |  |  |  |
| 3 | Atr-ERN12388 |  | | | |  | | | |  | Vvi-Vitvi05g00409\_t001 |  |  |  |
| 3 | Atr-ERN12389 |  | Vvi-Vitvi07g00184\_t001 |  | | | |  | Vvi-Vitvi05g00408\_t001 |  |  |  |
| 3 | Atr-ERN12390 |  | | | |  | | | |  | | | |  |  |  |
| 3 | Atr-ERN12391 |  | Vvi-Vitvi07g00186\_t001 |  | | | |  | | | |  |  |  |
| 3 | Atr-ERN12392 |  | | | |  | | | |  | Vvi-Vitvi05g00406\_t001 |  |  |  |
| 3 | Atr-ERN12393 |  | | | |  | | | |  | | | |  |  |  |
| 3 | Atr-ERN12394 |  | | | |  | | | |  | | | |  |  |  |
| 3 | Atr-ERN12395 |  | | | |  | | | |  | Vvi-Vitvi05g00404\_t003 |  |  |  |
| 3 | Atr-ERN12396 |  | Vvi-Vitvi07g00187\_t001 |  | | | |  | Vvi-Vitvi05g00403\_t001 |  |  |  |
| 3 | Atr-ERN12397 |  | | | |  | | | |  | | | |  |  |  |
| 3 | Atr-ERN12398 |  | | | |  | | | |  | Vvi-Vitvi05g00402\_t001 |  |  |  |
| 3 | Atr-ERN12399 |  | | | |  | | | |  | Vvi-Vitvi05g00401\_t001 |  |  |  |
| 3 | Atr-ERN12400 |  | | | |  | | | |  | | | |  |  |  |
| 3 | Atr-ERN12401 |  | | | |  | Vvi-Vitvi14g00281\_t001 |  | | | |  |  |  |
| 3 | Atr-ERN12402 |  | | | |  | | | |  | | | |  |  |  |
| 3 | Atr-ERN12403 |  | Vvi-Vitvi07g00188\_t001 |  | Vvi-Vitvi14g00282\_t001 |  | | | |  |  |  |
| 3 | Atr-ERN12404 |  | | | |  | Vvi-Vitvi14g00284\_t001 |  | | | |  |  |  |
| 3 | Atr-ERN12405 |  | | | |  | Vvi-Vitvi14g00286\_t001 |  | | | |  |  |  |
| 3 | Atr-ERN12406 |  | Vvi-Vitvi07g00189\_t001 |  | | | |  | | | |  |  |  |
| 3 | Atr-ERN12407 |  | | | |  | | | |  | | | |  |  |  |
| 3 | Atr-ERN12408 |  | | | |  | | | |  | | | |  |  |  |
| 3 | Atr-ERN12409 |  | | | |  | | | |  | | | |  |  |  |
| 3 | Atr-ERN12410 |  | | | |  | | | |  | | | |  |  |  |
| 3 | Atr-ERN12411 |  | | | |  | | | |  | | | |  |  |  |
| 3 | Atr-ERN12412 |  | | | |  | | | |  | | | |  |  |  |
| 3 | Atr-ERN12413 |  | | | |  | | | |  | | | |  |  |  |
| 3 | Atr-ERN12414 |  | | | |  | | | |  | | | |  |  |  |
| 3 | Atr-ERN12415 |  | | | |  | | | |  | | | |  |  |  |
| 3 | Atr-ERN12416 |  | | | |  | | | |  | | | |  |  |  |
| 3 | Atr-ERN12417 |  | | | |  | Vvi-Vitvi14g00289\_t001 |  | | | |  |  |  |
| 3 | Atr-ERN12418 |  | | | |  | | | |  | Vvi-Vitvi05g00398\_t001 |  |  |  |
| 3 | Atr-ERN12419 |  | Vvi-Vitvi07g00190\_t001 |  | | | |  | | | |  |  |  |
| 3 | Atr-ERN12420 |  | | | |  | | | |  | | | |  |  |  |
| 3 | Atr-ERN12421 |  | | | |  | | | |  | | | |  |  |  |
| 3 | Atr-ERN12422 |  | | | |  | | | |  | | | |  |  |  |
| 3 | Atr-ERN12423 |  | | | |  | | | |  | | | |  |  |  |
| 3 | Atr-ERN12424 |  | | | |  | | | |  | Vvi-Vitvi05g00395\_t001 |  |  |  |
| 3 | Atr-ERN12425 |  | | | |  | Vvi-Vitvi14g00291\_t001 |  | | | |  |  |  |
| 3 | Atr-ERN12426 |  | | | |  | | | |  | | | |  |  |  |
| 3 | Atr-ERN12427 |  | Vvi-Vitvi07g00193\_t001 |  | | | |  | Vvi-Vitvi05g00394\_t001 |  |  |  |
| 3 | Atr-ERN12428 |  | Vvi-Vitvi07g00195\_t001 |  | | | |  | | | |  |  |  |
| 3 | Atr-ERN12429 |  | | | |  | | | |  | | | |  |  |  |
| 3 | Atr-ERN12430 |  | Vvi-Vitvi07g00196\_t001 |  | | | |  | | | |  |  |  |
| 3 | Atr-ERN12431 |  | | | |  | | | |  | Vvi-Vitvi05g00393\_t001 |  |  |  |
| 3 | Atr-ERN12432 |  | | | |  | | | |  | | | |  |  |  |
| 3 | Atr-ERN12433 |  | | | |  | | | |  | Vvi-Vitvi05g00392\_t001 |  |  |  |
| 3 | Atr-ERN12434 |  | | | |  | | | |  | Vvi-Vitvi05g00391\_t001 |  |  |  |
| 3 | Atr-ERN12435 |  | | | |  | | | |  | | | |  |  |  |
| 3 | Atr-ERN12436 |  | | | |  | | | |  | | | |  |  |  |
| 3 | Atr-ERN12437 |  | | | |  | | | |  | | | |  |  |  |
| 3 | Atr-ERN12438 |  | | | |  | | | |  | Vvi-Vitvi05g00390\_t001 |  |  |  |
| 3 | Atr-ERN12439 |  | | | |  | Vvi-Vitvi14g00300\_t001 |  | | | |  |  |  |
| 3 | Atr-ERN12440 |  | | | |  | | | |  | Vvi-Vitvi05g00389\_t001 |  |  |  |
| 3 | Atr-ERN12441 |  | | | |  | | | |  | | | |  |  |  |
| 3 | Atr-ERN12442 |  | | | |  | | | |  | | | |  |  |  |
| 3 | Atr-ERN12443 |  | | | |  | | | |  | | | |  |  |  |
| 3 | Atr-ERN12444 |  | Vvi-Vitvi07g00198\_t001 |  | | | |  | | | |  |  |  |
| 3 | Atr-ERN12445 |  | Vvi-Vitvi07g00199\_t001 |  | Vvi-Vitvi14g00301\_t001 |  | | | |  |  |  |
| 2 | Atr-ERN12446 |  | | | |  |  |  | | | |  |  |  |
| 2 | Atr-ERN12447 |  | Vvi-Vitvi07g00200\_t001 |  |  |  | | | |  |  |  |
| 1 | Atr-ERN12448 |  |  |  |  |  | Vvi-Vitvi05g00388\_t002 |  |  |  |
| 1 | Atr-ERN12449 |  |  |  |  |  | Vvi-Vitvi05g00387\_t001 |  |  |  |
| 1 | Atr-ERN12450 |  | Vvi-Vitvi11g01150\_t001 |  |  |  |  |  |
| 1 | Atr-ERN12451 |  | | | |  |  |  |  |  |
| 1 | Atr-ERN12452 |  | | | |  |  |  |  |  |
| 1 | Atr-ERN12453 |  | | | |  |  |  |  |  |
| 1 | Atr-ERN12454 |  | | | |  |  |  |  |  |
| 1 | Atr-ERN12455 |  | Vvi-Vitvi11g01148\_t001 |  |  |  |  |  |
| 1 | Atr-ERN12456 |  | | | |  |  |  |  |  |
| 1 | Atr-ERN12457 |  | | | |  |  |  |  |  |
| 2 | Atr-ERN12458 |  | | | |  | Vvi-Vitvi04g00214\_t001 |  |  |  |  |
| 2 | Atr-ERN12459 |  | Vvi-Vitvi11g01146\_t001 |  | | | |  |  |  |  |
| 2 | Atr-ERN12460 |  | Vvi-Vitvi11g01144\_t001 |  | | | |  |  |  |  |
| 2 | Atr-ERN12461 |  | | | |  | | | |  |  |  |  |
| 2 | Atr-ERN12462 |  | Vvi-Vitvi11g01143\_t001 |  | | | |  |  |  |  |
| 2 | Atr-ERN12463 |  | | | |  | | | |  |  |  |  |
| 2 | Atr-ERN12464 |  | | | |  | | | |  |  |  |  |
| 2 | Atr-ERN12465 |  | | | |  | | | |  |  |  |  |
| 2 | Atr-ERN12466 |  | Vvi-Vitvi11g01141\_t001 |  | | | |  |  |  |  |
| 2 | Atr-ERN12467 |  | Vvi-Vitvi11g01140\_t001 |  | | | |  |  |  |  |
| 2 | Atr-ERN12468 |  | | | |  | | | |  |  |  |  |
| 2 | Atr-ERN12469 |  | Vvi-Vitvi11g04316\_t001 |  | | | |  |  |  |  |
| 2 | Atr-ERN12470 |  | Vvi-Vitvi11g01139\_t001 |  | | | |  |  |  |  |
| 3 | Atr-ERN12471 |  | Vvi-Vitvi11g01138\_t002 |  | | | |  | Vvi-Vitvi09g01405\_t001 |  |  |  |
| 3 | Atr-ERN12472 |  | | | |  | | | |  | Vvi-Vitvi09g01404\_t001 |  |  |  |
| 3 | Atr-ERN12473 |  | | | |  | | | |  | Vvi-Vitvi09g01403\_t001 |  |  |  |
| 3 | Atr-ERN12474 |  | | | |  | | | |  | | | |  |  |  |
| 3 | Atr-ERN12475 |  | Vvi-Vitvi11g01137\_t001 |  | | | |  | | | |  |  |  |
| 3 | Atr-ERN12476 |  | | | |  | | | |  | Vvi-Vitvi09g01401\_t001 |  |  |  |
| 3 | Atr-ERN12477 |  | | | |  | | | |  | | | |  |  |  |
| 3 | Atr-ERN12478 |  | | | |  | | | |  | | | |  |  |  |
| 3 | Atr-ERN12479 |  | Vvi-Vitvi11g01136\_t001 |  | | | |  | | | |  |  |  |
| 3 | Atr-ERN12480 |  | | | |  | | | |  | | | |  |  |  |
| 3 | Atr-ERN12481 |  | | | |  | | | |  | | | |  |  |  |
| 3 | Atr-ERN12482 |  | | | |  | | | |  | | | |  |  |  |
| 3 | Atr-ERN12483 |  | | | |  | | | |  | | | |  |  |  |
| 3 | Atr-ERN12484 |  | | | |  | Vvi-Vitvi04g00232\_t001 |  | | | |  |  |  |
| 3 | Atr-ERN12485 |  | | | |  | | | |  | | | |  |  |  |
| 3 | Atr-ERN12486 |  | | | |  | | | |  | | | |  |  |  |
| 3 | Atr-ERN12487 |  | Vvi-Vitvi11g01134\_t006 |  | | | |  | | | |  |  |  |
| 3 | Atr-ERN12488 |  | | | |  | | | |  | | | |  |  |  |
| 3 | Atr-ERN12489 |  | Vvi-Vitvi11g01132\_t001 |  | | | |  | | | |  |  |  |
| 3 | Atr-ERN12490 |  | | | |  | | | |  | | | |  |  |  |
| 3 | Atr-ERN12491 |  | Vvi-Vitvi11g01623\_t001 |  | | | |  | | | |  |  |  |
| 3 | Atr-ERN12492 |  | | | |  | | | |  | | | |  |  |  |
| 3 | Atr-ERN12493 |  | Vvi-Vitvi11g01128\_t001 |  | | | |  | | | |  |  |  |
| 3 | Atr-ERN12494 |  | | | |  | | | |  | Vvi-Vitvi09g01397\_t001 |  |  |  |
| 3 | Atr-ERN12495 |  | | | |  | Vvi-Vitvi04g00236\_t001 |  | | | |  |  |  |
| 3 | Atr-ERN12496 |  | | | |  | Vvi-Vitvi04g00237\_t001 |  | | | |  |  |  |
| 3 | Atr-ERN12497 |  | | | |  | | | |  | | | |  |  |  |
| 3 | Atr-ERN12498 |  | | | |  | | | |  | | | |  |  |  |
| 3 | Atr-ERN12499 |  | Vvi-Vitvi11g04307\_t002 |  | | | |  | | | |  |  |  |
| 3 | Atr-ERN12500 |  | | | |  | | | |  | Vvi-Vitvi09g04580\_t001 |  |  |  |
| 3 | Atr-ERN12501 |  | | | |  | | | |  | Vvi-Vitvi09g01390\_t001 |  |  |  |
| 3 | Atr-ERN12502 |  | Vvi-Vitvi11g01107\_t002 |  | | | |  | | | |  |  |  |
| 3 | Atr-ERN12503 |  | Vvi-Vitvi11g01106\_t001 |  | | | |  | | | |  |  |  |
| 3 | Atr-ERN12504 |  | | | |  | | | |  | | | |  |  |  |
| 3 | Atr-ERN12505 |  | | | |  | | | |  | Vvi-Vitvi09g01389\_t001 |  |  |  |
| 3 | Atr-ERN12506 |  | | | |  | Vvi-Vitvi04g00239\_t001 |  | | | |  |  |  |
| 3 | Atr-ERN12507 |  | Vvi-Vitvi11g01101\_t001 |  | | | |  | | | |  |  |  |
| 3 | Atr-ERN12508 |  | | | |  | | | |  | | | |  |  |  |
| 3 | Atr-ERN12509 |  | | | |  | | | |  | | | |  |  |  |
| 3 | Atr-ERN12510 |  | | | |  | | | |  | | | |  |  |  |
| 3 | Atr-ERN12511 |  | | | |  | Vvi-Vitvi04g01830\_t001 |  | | | |  |  |  |
| 3 | Atr-ERN12512 |  | | | |  | | | |  | Vvi-Vitvi09g01985\_t001 |  |  |  |
| 3 | Atr-ERN12513 |  | | | |  | Vvi-Vitvi04g00240\_t001 |  | | | |  |  |  |
| 3 | Atr-ERN12514 |  | | | |  | | | |  | Vvi-Vitvi09g01388\_t002 |  |  |  |
| 3 | Atr-ERN12515 |  | | | |  | | | |  | | | |  |  |  |
| 3 | Atr-ERN12516 |  | | | |  | | | |  | Vvi-Vitvi09g01386\_t001 |  |  |  |
| 3 | Atr-ERN12517 |  | Vvi-Vitvi11g01072\_t001 |  | | | |  | | | |  |  |  |
| 3 | Atr-ERN12518 |  | Vvi-Vitvi11g01055\_t001 |  | | | |  | | | |  |  |  |
| 3 | Atr-ERN12519 |  | | | |  | | | |  | | | |  |  |  |
| 3 | Atr-ERN12520 |  | | | |  | Vvi-Vitvi04g00241\_t001 |  | | | |  |  |  |
| 3 | Atr-ERN12521 |  | | | |  | | | |  | | | |  |  |  |
| 3 | Atr-ERN12522 |  | | | |  | | | |  | | | |  |  |  |
| 3 | Atr-ERN12523 |  | | | |  | | | |  | | | |  |  |  |
| 3 | Atr-ERN12524 |  | Vvi-Vitvi11g01053\_t001 |  | Vvi-Vitvi04g01831\_t001 |  | Vvi-Vitvi09g01384\_t001 |  |  |  |
| 3 | Atr-ERN12525 |  | | | |  | | | |  | | | |  |  |  |
| 3 | Atr-ERN12526 |  | | | |  | | | |  | | | |  |  |  |
| 3 | Atr-ERN12527 |  | Vvi-Vitvi11g01048\_t001 |  | | | |  | | | |  |  |  |
| 3 | Atr-ERN12528 |  | | | |  | Vvi-Vitvi04g00248\_t001 |  | | | |  |  |  |
| 3 | Atr-ERN12529 |  | Vvi-Vitvi11g01041\_t001 |  | | | |  | | | |  |  |  |
| 3 | Atr-ERN12530 |  | Vvi-Vitvi11g01039\_t001 |  | | | |  | | | |  |  |  |
| 2 | Atr-ERN12531 |  |  |  | | | |  | Vvi-Vitvi09g01381\_t001 |  |  |  |
| 1 | Atr-ERN12532 |  |  |  | | | |  |  |  |  |
| 1 | Atr-ERN12533 |  |  |  | | | |  |  |  |  |
| 1 | Atr-ERN12534 |  |  |  | | | |  |  |  |  |
| 1 | Atr-ERN12535 |  |  |  | | | |  |  |  |  |
| 2 | Atr-ERN12536 |  | Vvi-Vitvi11g00919\_t001 |  | Vvi-Vitvi04g00249\_t001 |  |  |  |  |
| 2 | Atr-ERN12537 |  | Vvi-Vitvi11g00920\_t001 |  | | | |  |  |  |  |
| 2 | Atr-ERN12538 |  | | | |  | Vvi-Vitvi04g00250\_t001 |  |  |  |  |
| 2 | Atr-ERN12539 |  | Vvi-Vitvi11g00921\_t001 |  | Vvi-Vitvi04g00251\_t001 |  |  |  |  |
| 2 | Atr-ERN12540 |  | Vvi-Vitvi11g00923\_t001 |  | | | |  |  |  |  |
| 2 | Atr-ERN12541 |  | | | |  | Vvi-Vitvi04g00252\_t001 |  |  |  |  |
| 2 | Atr-ERN12542 |  | | | |  | | | |  |  |  |  |
| 2 | Atr-ERN12543 |  | | | |  | | | |  |  |  |  |
| 2 | Atr-ERN12544 |  | Vvi-Vitvi11g00924\_t001 |  | | | |  |  |  |  |
| 2 | Atr-ERN12545 |  | | | |  | | | |  |  |  |  |
| 2 | Atr-ERN12546 |  | | | |  | | | |  |  |  |  |
| 2 | Atr-ERN12547 |  | Vvi-Vitvi11g00926\_t001 |  | | | |  |  |  |  |
| 2 | Atr-ERN12548 |  | | | |  | | | |  |  |  |  |
| 2 | Atr-ERN12549 |  | | | |  | | | |  |  |  |  |
| 2 | Atr-ERN12550 |  | Vvi-Vitvi11g00927\_t002 |  | | | |  |  |  |  |
| 2 | Atr-ERN12551 |  | Vvi-Vitvi11g00928\_t001.1.6037826a |  | | | |  |  |  |  |
| 2 | Atr-ERN12552 |  | Vvi-Vitvi11g00929\_t001 |  | | | |  |  |  |  |
| 2 | Atr-ERN12553 |  | Vvi-Vitvi11g00931\_t001 |  | Vvi-Vitvi04g00255\_t001 |  |  |  |  |
| 2 | Atr-ERN12554 |  | | | |  | | | |  |  |  |  |
| 2 | Atr-ERN12555 |  | | | |  | | | |  |  |  |  |
| 2 | Atr-ERN12556 |  | | | |  | | | |  |  |  |  |
| 2 | Atr-ERN12557 |  | | | |  | | | |  |  |  |  |
| 2 | Atr-ERN12558 |  | Vvi-Vitvi11g00935\_t001 |  | | | |  |  |  |  |
| 2 | Atr-ERN12559 |  | | | |  | | | |  |  |  |  |
| 2 | Atr-ERN12560 |  | | | |  | | | |  |  |  |  |
| 2 | Atr-ERN12561 |  | | | |  | | | |  |  |  |  |
| 2 | Atr-ERN12562 |  | | | |  | | | |  |  |  |  |
| 2 | Atr-ERN12563 |  | | | |  | | | |  |  |  |  |
| 2 | Atr-ERN12564 |  | | | |  | | | |  |  |  |  |
| 2 | Atr-ERN12565 |  | | | |  | Vvi-Vitvi04g01832\_t001 |  |  |  |  |
| 2 | Atr-ERN12566 |  | | | |  | | | |  |  |  |  |
| 2 | Atr-ERN12567 |  | | | |  | | | |  |  |  |  |
| 2 | Atr-ERN12568 |  | Vvi-Vitvi11g00936\_t001 |  | | | |  |  |  |  |
| 2 | Atr-ERN12569 |  | Vvi-Vitvi11g00937\_t001 |  | | | |  |  |  |  |
| 1 | Atr-ERN12570 |  |  |  | Vvi-Vitvi04g00265\_t002 |  |  |  |  |
| 1 | Atr-ERN12571 |  |  |  | | | |  |  |  |  |
| 1 | Atr-ERN12572 |  |  |  | | | |  |  |  |  |
| 1 | Atr-ERN12573 |  |  |  | | | |  |  |  |  |
| 1 | Atr-ERN12574 |  |  |  | | | |  |  |  |  |
| 1 | Atr-ERN12575 |  |  |  | | | |  |  |  |  |
| 1 | Atr-ERN12576 |  |  |  | | | |  |  |  |  |
| 1 | Atr-ERN12577 |  |  |  | | | |  |  |  |  |
| 1 | Atr-ERN12578 |  |  |  | Vvi-Vitvi04g00266\_t001 |  |  |  |  |
| 1 | Atr-ERN12579 |  |  |  | | | |  |  |  |  |
| 2 | Atr-ERN12580 |  | Vvi-Vitvi09g01455\_t001 |  | | | |  |  |  |  |
| 2 | Atr-ERN12581 |  | | | |  | | | |  |  |  |  |
| 2 | Atr-ERN12582 |  | | | |  | | | |  |  |  |  |
| 2 | Atr-ERN12583 |  | | | |  | | | |  |  |  |  |
| 2 | Atr-ERN12584 |  | | | |  | | | |  |  |  |  |
| 3 | Atr-ERN12585 |  | | | |  | | | |  | Vvi-Vitvi09g00994\_t001 |  |  |  |
| 3 | Atr-ERN12586 |  | | | |  | | | |  | | | |  |  |  |
| 3 | Atr-ERN12587 |  | | | |  | | | |  | | | |  |  |  |
| 3 | Atr-ERN12588 |  | | | |  | | | |  | | | |  |  |  |
| 3 | Atr-ERN12589 |  | | | |  | | | |  | | | |  |  |  |
| 3 | Atr-ERN12590 |  | | | |  | | | |  | | | |  |  |  |
| 3 | Atr-ERN12591 |  | | | |  | Vvi-Vitvi04g00270\_t001 |  | | | |  |  |  |
| 3 | Atr-ERN12592 |  | | | |  | | | |  | | | |  |  |  |
| 3 | Atr-ERN12593 |  | | | |  | | | |  | Vvi-Vitvi09g00997\_t001 |  |  |  |
| 3 | Atr-ERN12594 |  | | | |  | | | |  | | | |  |  |  |
| 3 | Atr-ERN12595 |  | | | |  | | | |  | | | |  |  |  |
| 3 | Atr-ERN12596 |  | | | |  | | | |  | Vvi-Vitvi09g01006\_t001 |  |  |  |
| 3 | Atr-ERN12597 |  | | | |  | Vvi-Vitvi04g00275\_t001 |  | | | |  |  |  |
| 3 | Atr-ERN12598 |  | | | |  | Vvi-Vitvi04g00277\_t001.1.6037826d |  | | | |  |  |  |
| 2 | Atr-ERN12599 |  | | | |  |  |  | Vvi-Vitvi09g01808\_t001 |  |  |  |
| 2 | Atr-ERN12600 |  | Vvi-Vitvi09g01459\_t001 |  |  |  | Vvi-Vitvi09g01008\_t001 |  |  |  |
| 2 | Atr-ERN12601 |  | | | |  |  |  | | | |  |  |  |
| 2 | Atr-ERN12602 |  | | | |  |  |  | | | |  |  |  |
| 2 | Atr-ERN12603 |  | Vvi-Vitvi09g01462\_t001 |  |  |  | | | |  |  |  |
| 2 | Atr-ERN12604 |  | | | |  |  |  | | | |  |  |  |
| 2 | Atr-ERN12605 |  | | | |  |  |  | | | |  |  |  |
| 2 | Atr-ERN12606 |  | Vvi-Vitvi09g01464\_t002 |  |  |  | | | |  |  |  |
| 2 | Atr-ERN12607 |  | | | |  |  |  | | | |  |  |  |
| 2 | Atr-ERN12608 |  | | | |  |  |  | | | |  |  |  |
| 2 | Atr-ERN12609 |  | | | |  |  |  | | | |  |  |  |
| 2 | Atr-ERN12610 |  | Vvi-Vitvi09g04607\_t001 |  |  |  | | | |  |  |  |
| 2 | Atr-ERN12611 |  | Vvi-Vitvi09g04611\_t001 |  |  |  | | | |  |  |  |
| 2 | Atr-ERN12612 |  | | | |  |  |  | | | |  |  |  |
| 2 | Atr-ERN12613 |  | | | |  |  |  | | | |  |  |  |
| 2 | Atr-ERN12614 |  | | | |  |  |  | | | |  |  |  |
| 2 | Atr-ERN12615 |  | | | |  |  |  | | | |  |  |  |
| 2 | Atr-ERN12616 |  | | | |  |  |  | | | |  |  |  |
| 2 | Atr-ERN12617 |  | | | |  |  |  | | | |  |  |  |
| 2 | Atr-ERN12618 |  | | | |  |  |  | | | |  |  |  |
| 2 | Atr-ERN12619 |  | | | |  |  |  | | | |  |  |  |
| 2 | Atr-ERN12620 |  | | | |  |  |  | | | |  |  |  |
| 2 | Atr-ERN12621 |  | Vvi-Vitvi09g02014\_t001 |  |  |  | | | |  |  |  |
| 2 | Atr-ERN12622 |  | Vvi-Vitvi09g01471\_t001 |  |  |  | | | |  |  |  |
| 2 | Atr-ERN12623 |  | | | |  |  |  | Vvi-Vitvi09g01011\_t001 |  |  |  |
| 2 | Atr-ERN12624 |  | | | |  |  |  | | | |  |  |  |
| 2 | Atr-ERN12625 |  | | | |  |  |  | | | |  |  |  |
| 2 | Atr-ERN12626 |  | | | |  |  |  | Vvi-Vitvi09g01017\_t001 |  |  |  |
| 1 | Atr-ERN12627 |  | | | |  |  |  |  |  |
| 1 | Atr-ERN12628 |  | Vvi-Vitvi09g01473\_t001 |  |  |  |  |  |
| 0 | Atr-ERN12629 |  |  |  |  |  |  |
| 0 | Atr-ERN12630 |  |  |  |  |  |  |
| 0 | Atr-ERN12631 |  |  |  |  |  |  |
| 0 | Atr-ERN12632 |  |  |  |  |  |  |
| 0 | Atr-ERN12633 |  |  |  |  |  |  |
| 0 | Atr-ERN12634 |  |  |  |  |  |  |
| 0 | Atr-ERN12635 |  |  |  |  |  |  |
| 0 | Atr-ERN12636 |  |  |  |  |  |  |
| 0 | Atr-ERN12637 |  |  |  |  |  |  |
| 0 | Atr-ERN12638 |  |  |  |  |  |  |
| 0 | Atr-ERN12639 |  |  |  |  |  |  |
| 0 | Atr-ERN12640 |  |  |  |  |  |  |
| 0 | Atr-ERN12641 |  |  |  |  |  |  |
| 0 | Atr-ERN12642 |  |  |  |  |  |  |
| 0 | Atr-ERN12643 |  |  |  |  |  |  |
| 0 | Atr-ERN12644 |  |  |  |  |  |  |
| 0 | Atr-ERN12645 |  |  |  |  |  |  |
| 0 | Atr-ERN12646 |  |  |  |  |  |  |
| 0 | Atr-ERN12647 |  |  |  |  |  |  |
| 0 | Atr-ERN12648 |  |  |  |  |  |  |
| 0 | Atr-ERN12649 |  |  |  |  |  |  |
| 0 | Atr-ERN12650 |  |  |  |  |  |  |
| 0 | Atr-ERN12651 |  |  |  |  |  |  |
| 0 | Atr-ERN12652 |  |  |  |  |  |  |
| 0 | Atr-ERN12653 |  |  |  |  |  |  |
| 0 | Atr-ERN12654 |  |  |  |  |  |  |
| 0 | Atr-ERN12655 |  |  |  |  |  |  |
| 0 | Atr-ERN12656 |  |  |  |  |  |  |
| 0 | Atr-ERN12657 |  |  |  |  |  |  |
| 0 | Atr-ERN12658 |  |  |  |  |  |  |
| 0 | Atr-ERN12659 |  |  |  |  |  |  |
| 0 | Atr-ERN12660 |  |  |  |  |  |  |
| 0 | Atr-ERN12661 |  |  |  |  |  |  |
| 1 | Atr-ERN12662 |  | Vvi-Vitvi05g00463\_t001 |  |  |  |  |  |
| 1 | Atr-ERN12663 |  | | | |  |  |  |  |  |
| 1 | Atr-ERN12664 |  | | | |  |  |  |  |  |
| 1 | Atr-ERN12665 |  | Vvi-Vitvi05g00462\_t008 |  |  |  |  |  |
| 1 | Atr-ERN12666 |  | Vvi-Vitvi05g00461\_t001 |  |  |  |  |  |
| 1 | Atr-ERN12667 |  | | | |  |  |  |  |  |
| 1 | Atr-ERN12668 |  | Vvi-Vitvi05g00460\_t001 |  |  |  |  |  |
| 1 | Atr-ERN12669 |  | Vvi-Vitvi05g00459\_t001 |  |  |  |  |  |
| 2 | Atr-ERN12670 |  | Vvi-Vitvi05g00458\_t002 |  | Vvi-Vitvi07g00184\_t001 |  |  |  |  |
| 2 | Atr-ERN12671 |  | | | |  | | | |  |  |  |  |
| 2 | Atr-ERN12672 |  | | | |  | | | |  |  |  |  |
| 2 | Atr-ERN12673 |  | | | |  | Vvi-Vitvi07g00181\_t001 |  |  |  |  |
| 2 | Atr-ERN12674 |  | | | |  | | | |  |  |  |  |
| 2 | Atr-ERN12675 |  | Vvi-Vitvi05g00453\_t001 |  | | | |  |  |  |  |
| 2 | Atr-ERN12676 |  | | | |  | | | |  |  |  |  |
| 2 | Atr-ERN12677 |  | | | |  | | | |  |  |  |  |
| 2 | Atr-ERN12678 |  | | | |  | | | |  |  |  |  |
| 2 | Atr-ERN12679 |  | | | |  | | | |  |  |  |  |
| 2 | Atr-ERN12680 |  | | | |  | Vvi-Vitvi07g00178\_t001 |  |  |  |  |
| 2 | Atr-ERN12681 |  | | | |  | | | |  |  |  |  |
| 2 | Atr-ERN12682 |  | | | |  | | | |  |  |  |  |
| 2 | Atr-ERN12683 |  | Vvi-Vitvi05g00452\_t001 |  | | | |  |  |  |  |
| 2 | Atr-ERN12684 |  | | | |  | Vvi-Vitvi07g00176\_t001 |  |  |  |  |
| 2 | Atr-ERN12685 |  | | | |  | | | |  |  |  |  |
| 2 | Atr-ERN12686 |  | | | |  | | | |  |  |  |  |
| 2 | Atr-ERN12687 |  | | | |  | | | |  |  |  |  |
| 2 | Atr-ERN12688 |  | | | |  | Vvi-Vitvi07g00175\_t001 |  |  |  |  |
| 2 | Atr-ERN12689 |  | | | |  | | | |  |  |  |  |
| 2 | Atr-ERN12690 |  | | | |  | | | |  |  |  |  |
| 2 | Atr-ERN12691 |  | | | |  | | | |  |  |  |  |
| 2 | Atr-ERN12692 |  | | | |  | | | |  |  |  |  |
| 2 | Atr-ERN12693 |  | | | |  | | | |  |  |  |  |
| 2 | Atr-ERN12694 |  | | | |  | | | |  |  |  |  |
| 2 | Atr-ERN12695 |  | | | |  | | | |  |  |  |  |
| 2 | Atr-ERN12696 |  | | | |  | | | |  |  |  |  |
| 2 | Atr-ERN12697 |  | Vvi-Vitvi05g00448\_t001 |  | Vvi-Vitvi07g00174\_t001 |  |  |  |  |
| 0 | Atr-ERN12698 |  |  |  |  |  |  |
| 0 | Atr-ERN12699 |  |  |  |  |  |  |
