# Supplementary material for: Genome-wide identification, characterization and gene expression of BES1 transcription factor family in grapevine (Vitis vinifera L.)
Source: Sci Rep. 2023 Jan 5;13:240. doi: 10.1038/s41598-022-24407-y (PMC9816167; doi:10.1038/s41598-022-24407-y)
Supplement: Supplementary file 3 — Supplementary Information. [file 41598_2022_24407_MOESM3_ESM.zip › Vvi_Atr/Vitis_vinifera.PN40024.v4.dna_sm.toplevel.fa.vs.Amborella_trichopoda.AMTR1.0.dna_sm.toplevel.fa.html/Atr-AmTr_v1.0_scaffold00026.html]

|  |  |  |  |  |  |  |  |  |  |  |  |  |  |
| --- | --- | --- | --- | --- | --- | --- | --- | --- | --- | --- | --- | --- | --- |
| Duplication depth | Reference chromosome | Collinear blocks | | | | | | | | | | | |
| 0 | Atr-ERN10326 |  |  |  |  |  |  |
| 0 | Atr-ERN10327 |  |  |  |  |  |  |
| 1 | Atr-ERN10328 |  | Vvi-Vitvi11g01483\_t001 |  |  |  |  |  |
| 1 | Atr-ERN10329 |  | Vvi-Vitvi11g00622\_t001 |  |  |  |  |  |
| 1 | Atr-ERN10330 |  | | | |  |  |  |  |  |
| 1 | Atr-ERN10331 |  | | | |  |  |  |  |  |
| 1 | Atr-ERN10332 |  | | | |  |  |  |  |  |
| 1 | Atr-ERN10333 |  | | | |  |  |  |  |  |
| 1 | Atr-ERN10334 |  | | | |  |  |  |  |  |
| 1 | Atr-ERN10335 |  | | | |  |  |  |  |  |
| 1 | Atr-ERN10336 |  | | | |  |  |  |  |  |
| 1 | Atr-ERN10337 |  | | | |  |  |  |  |  |
| 1 | Atr-ERN10338 |  | Vvi-Vitvi11g00620\_t001 |  |  |  |  |  |
| 1 | Atr-ERN10339 |  | | | |  |  |  |  |  |
| 1 | Atr-ERN10340 |  | Vvi-Vitvi11g00619\_t001 |  |  |  |  |  |
| 1 | Atr-ERN10341 |  | Vvi-Vitvi11g00618\_t001 |  |  |  |  |  |
| 1 | Atr-ERN10342 |  | | | |  |  |  |  |  |
| 1 | Atr-ERN10343 |  | | | |  |  |  |  |  |
| 1 | Atr-ERN10344 |  | | | |  |  |  |  |  |
| 1 | Atr-ERN10345 |  | | | |  |  |  |  |  |
| 1 | Atr-ERN10346 |  | | | |  |  |  |  |  |
| 1 | Atr-ERN10347 |  | Vvi-Vitvi11g01479\_t001 |  |  |  |  |  |
| 1 | Atr-ERN10348 |  | | | |  |  |  |  |  |
| 1 | Atr-ERN10349 |  | | | |  |  |  |  |  |
| 1 | Atr-ERN10350 |  | | | |  |  |  |  |  |
| 1 | Atr-ERN10351 |  | | | |  |  |  |  |  |
| 1 | Atr-ERN10352 |  | | | |  |  |  |  |  |
| 1 | Atr-ERN10353 |  | | | |  |  |  |  |  |
| 1 | Atr-ERN10354 |  | Vvi-Vitvi11g00615\_t001 |  |  |  |  |  |
| 1 | Atr-ERN10355 |  | | | |  |  |  |  |  |
| 1 | Atr-ERN10356 |  | | | |  |  |  |  |  |
| 1 | Atr-ERN10357 |  | | | |  |  |  |  |  |
| 1 | Atr-ERN10358 |  | | | |  |  |  |  |  |
| 1 | Atr-ERN10359 |  | | | |  |  |  |  |  |
| 1 | Atr-ERN10360 |  | | | |  |  |  |  |  |
| 1 | Atr-ERN10361 |  | | | |  |  |  |  |  |
| 1 | Atr-ERN10362 |  | | | |  |  |  |  |  |
| 1 | Atr-ERN10363 |  | | | |  |  |  |  |  |
| 1 | Atr-ERN10364 |  | | | |  |  |  |  |  |
| 1 | Atr-ERN10365 |  | | | |  |  |  |  |  |
| 1 | Atr-ERN10366 |  | | | |  |  |  |  |  |
| 1 | Atr-ERN10367 |  | | | |  |  |  |  |  |
| 1 | Atr-ERN10368 |  | Vvi-Vitvi11g04151\_t001 |  |  |  |  |  |
| 1 | Atr-ERN10369 |  | | | |  |  |  |  |  |
| 1 | Atr-ERN10370 |  | | | |  |  |  |  |  |
| 1 | Atr-ERN10371 |  | | | |  |  |  |  |  |
| 1 | Atr-ERN10372 |  | | | |  |  |  |  |  |
| 1 | Atr-ERN10373 |  | | | |  |  |  |  |  |
| 1 | Atr-ERN10374 |  | | | |  |  |  |  |  |
| 1 | Atr-ERN10375 |  | | | |  |  |  |  |  |
| 1 | Atr-ERN10376 |  | | | |  |  |  |  |  |
| 1 | Atr-ERN10377 |  | | | |  |  |  |  |  |
| 1 | Atr-ERN10378 |  | | | |  |  |  |  |  |
| 1 | Atr-ERN10379 |  | | | |  |  |  |  |  |
| 1 | Atr-ERN10380 |  | | | |  |  |  |  |  |
| 1 | Atr-ERN10381 |  | Vvi-Vitvi11g00606\_t001 |  |  |  |  |  |
| 1 | Atr-ERN10382 |  | | | |  |  |  |  |  |
| 1 | Atr-ERN10383 |  | | | |  |  |  |  |  |
| 1 | Atr-ERN10384 |  | | | |  |  |  |  |  |
| 1 | Atr-ERN10385 |  | | | |  |  |  |  |  |
| 1 | Atr-ERN10386 |  | | | |  |  |  |  |  |
| 1 | Atr-ERN10387 |  | | | |  |  |  |  |  |
| 1 | Atr-ERN10388 |  | | | |  |  |  |  |  |
| 1 | Atr-ERN10389 |  | | | |  |  |  |  |  |
| 1 | Atr-ERN10390 |  | | | |  |  |  |  |  |
| 1 | Atr-ERN10391 |  | | | |  |  |  |  |  |
| 1 | Atr-ERN10392 |  | | | |  |  |  |  |  |
| 1 | Atr-ERN10393 |  | | | |  |  |  |  |  |
| 1 | Atr-ERN10394 |  | | | |  |  |  |  |  |
| 1 | Atr-ERN10395 |  | Vvi-Vitvi11g00604\_t001 |  |  |  |  |  |
| 1 | Atr-ERN10396 |  | | | |  |  |  |  |  |
| 1 | Atr-ERN10397 |  | | | |  |  |  |  |  |
| 1 | Atr-ERN10398 |  | | | |  |  |  |  |  |
| 1 | Atr-ERN10399 |  | | | |  |  |  |  |  |
| 1 | Atr-ERN10400 |  | | | |  |  |  |  |  |
| 1 | Atr-ERN10401 |  | | | |  |  |  |  |  |
| 1 | Atr-ERN10402 |  | | | |  |  |  |  |  |
| 1 | Atr-ERN10403 |  | | | |  |  |  |  |  |
| 1 | Atr-ERN10404 |  | Vvi-Vitvi11g00603\_t001 |  |  |  |  |  |
| 1 | Atr-ERN10405 |  | Vvi-Vitvi11g00601\_t002 |  |  |  |  |  |
| 1 | Atr-ERN10406 |  | Vvi-Vitvi11g01475\_t001 |  |  |  |  |  |
| 1 | Atr-ERN10407 |  | | | |  |  |  |  |  |
| 1 | Atr-ERN10408 |  | | | |  |  |  |  |  |
| 1 | Atr-ERN10409 |  | | | |  |  |  |  |  |
| 1 | Atr-ERN10410 |  | | | |  |  |  |  |  |
| 1 | Atr-ERN10411 |  | | | |  |  |  |  |  |
| 1 | Atr-ERN10412 |  | | | |  |  |  |  |  |
| 1 | Atr-ERN10413 |  | | | |  |  |  |  |  |
| 1 | Atr-ERN10414 |  | | | |  |  |  |  |  |
| 1 | Atr-ERN10415 |  | Vvi-Vitvi11g00600\_t001 |  |  |  |  |  |
| 1 | Atr-ERN10416 |  | | | |  |  |  |  |  |
| 1 | Atr-ERN10417 |  | | | |  |  |  |  |  |
| 1 | Atr-ERN10418 |  | | | |  |  |  |  |  |
| 1 | Atr-ERN10419 |  | | | |  |  |  |  |  |
| 1 | Atr-ERN10420 |  | | | |  |  |  |  |  |
| 1 | Atr-ERN10421 |  | Vvi-Vitvi11g00599\_t002 |  |  |  |  |  |
| 1 | Atr-ERN10422 |  | | | |  |  |  |  |  |
| 1 | Atr-ERN10423 |  | | | |  |  |  |  |  |
| 1 | Atr-ERN10424 |  | | | |  |  |  |  |  |
| 1 | Atr-ERN10425 |  | Vvi-Vitvi11g00598\_t001 |  |  |  |  |  |
| 0 | Atr-ERN10426 |  |  |  |  |  |  |
| 0 | Atr-ERN10427 |  |  |  |  |  |  |
| 0 | Atr-ERN10428 |  |  |  |  |  |  |
| 0 | Atr-ERN10429 |  |  |  |  |  |  |
| 0 | Atr-ERN10430 |  |  |  |  |  |  |
| 0 | Atr-ERN10431 |  |  |  |  |  |  |
| 0 | Atr-ERN10432 |  |  |  |  |  |  |
| 0 | Atr-ERN10433 |  |  |  |  |  |  |
| 0 | Atr-ERN10434 |  |  |  |  |  |  |
| 0 | Atr-ERN10435 |  |  |  |  |  |  |
| 0 | Atr-ERN10436 |  |  |  |  |  |  |
| 0 | Atr-ERN10437 |  |  |  |  |  |  |
| 0 | Atr-ERN10438 |  |  |  |  |  |  |
| 0 | Atr-ERN10439 |  |  |  |  |  |  |
| 0 | Atr-ERN10440 |  |  |  |  |  |  |
| 0 | Atr-ERN10441 |  |  |  |  |  |  |
| 0 | Atr-ERN10442 |  |  |  |  |  |  |
| 0 | Atr-ERN10443 |  |  |  |  |  |  |
| 0 | Atr-ERN10444 |  |  |  |  |  |  |
| 0 | Atr-ERN10445 |  |  |  |  |  |  |
| 0 | Atr-ERN10446 |  |  |  |  |  |  |
| 0 | Atr-ERN10447 |  |  |  |  |  |  |
| 0 | Atr-ERN10448 |  |  |  |  |  |  |
| 0 | Atr-ERN10449 |  |  |  |  |  |  |
| 0 | Atr-ERN10450 |  |  |  |  |  |  |
| 0 | Atr-ERN10451 |  |  |  |  |  |  |
| 0 | Atr-ERN10452 |  |  |  |  |  |  |
| 0 | Atr-ERN10453 |  |  |  |  |  |  |
| 0 | Atr-ERN10454 |  |  |  |  |  |  |
| 0 | Atr-ERN10455 |  |  |  |  |  |  |
| 0 | Atr-ERN10456 |  |  |  |  |  |  |
| 0 | Atr-ERN10457 |  |  |  |  |  |  |
| 0 | Atr-ERN10458 |  |  |  |  |  |  |
| 0 | Atr-ERN10459 |  |  |  |  |  |  |
| 0 | Atr-ERN10460 |  |  |  |  |  |  |
| 0 | Atr-ERN10461 |  |  |  |  |  |  |
| 0 | Atr-ERN10462 |  |  |  |  |  |  |
| 0 | Atr-ERN10463 |  |  |  |  |  |  |
