# Supplementary material for: Genome-wide identification, characterization and gene expression of BES1 transcription factor family in grapevine (Vitis vinifera L.)
Source: Sci Rep. 2023 Jan 5;13:240. doi: 10.1038/s41598-022-24407-y (PMC9816167; doi:10.1038/s41598-022-24407-y)
Supplement: Supplementary file 3 — Supplementary Information. [file 41598_2022_24407_MOESM3_ESM.zip › Vvi_Atr/Vitis_vinifera.PN40024.v4.dna_sm.toplevel.fa.vs.Amborella_trichopoda.AMTR1.0.dna_sm.toplevel.fa.html/Atr-AmTr_v1.0_scaffold00027.html]

|  |  |  |  |  |  |  |  |  |  |  |  |  |  |
| --- | --- | --- | --- | --- | --- | --- | --- | --- | --- | --- | --- | --- | --- |
| Duplication depth | Reference chromosome | Collinear blocks | | | | | | | | | | | |
| 0 | Atr-ERN10697 |  |  |  |  |  |  |
| 0 | Atr-ERN10698 |  |  |  |  |  |  |
| 0 | Atr-ERN10699 |  |  |  |  |  |  |
| 0 | Atr-ERN10700 |  |  |  |  |  |  |
| 0 | Atr-ERN10701 |  |  |  |  |  |  |
| 1 | Atr-ERN10702 |  | Vvi-Vitvi16g01371\_t001 |  |  |  |  |  |
| 2 | Atr-ERN10703 |  | | | |  | Vvi-Vitvi02g00219\_t001 |  |  |  |  |
| 2 | Atr-ERN10704 |  | | | |  | | | |  |  |  |  |
| 2 | Atr-ERN10705 |  | | | |  | | | |  |  |  |  |
| 2 | Atr-ERN10706 |  | | | |  | | | |  |  |  |  |
| 2 | Atr-ERN10707 |  | | | |  | | | |  |  |  |  |
| 2 | Atr-ERN10708 |  | | | |  | Vvi-Vitvi02g00221\_t001 |  |  |  |  |
| 2 | Atr-ERN10709 |  | | | |  | | | |  |  |  |  |
| 2 | Atr-ERN10710 |  | | | |  | | | |  |  |  |  |
| 2 | Atr-ERN10711 |  | | | |  | | | |  |  |  |  |
| 2 | Atr-ERN10712 |  | Vvi-Vitvi16g01367\_t001 |  | | | |  |  |  |  |
| 2 | Atr-ERN10713 |  | | | |  | | | |  |  |  |  |
| 2 | Atr-ERN10714 |  | | | |  | | | |  |  |  |  |
| 2 | Atr-ERN10715 |  | | | |  | | | |  |  |  |  |
| 2 | Atr-ERN10716 |  | | | |  | | | |  |  |  |  |
| 2 | Atr-ERN10717 |  | | | |  | | | |  |  |  |  |
| 2 | Atr-ERN10718 |  | | | |  | | | |  |  |  |  |
| 2 | Atr-ERN10719 |  | | | |  | | | |  |  |  |  |
| 2 | Atr-ERN10720 |  | | | |  | | | |  |  |  |  |
| 2 | Atr-ERN10721 |  | Vvi-Vitvi16g01366\_t002 |  | Vvi-Vitvi02g00222\_t004 |  |  |  |  |
| 2 | Atr-ERN10722 |  | | | |  | Vvi-Vitvi02g00223\_t001 |  |  |  |  |
| 2 | Atr-ERN10723 |  | Vvi-Vitvi16g01365\_t001 |  | | | |  |  |  |  |
| 2 | Atr-ERN10724 |  | | | |  | | | |  |  |  |  |
| 2 | Atr-ERN10725 |  | | | |  | Vvi-Vitvi02g00224\_t002 |  |  |  |  |
| 2 | Atr-ERN10726 |  | | | |  | | | |  |  |  |  |
| 2 | Atr-ERN10727 |  | | | |  | Vvi-Vitvi02g00225\_t001 |  |  |  |  |
| 2 | Atr-ERN10728 |  | | | |  | | | |  |  |  |  |
| 2 | Atr-ERN10729 |  | | | |  | | | |  |  |  |  |
| 2 | Atr-ERN10730 |  | | | |  | | | |  |  |  |  |
| 2 | Atr-ERN10731 |  | | | |  | | | |  |  |  |  |
| 2 | Atr-ERN10732 |  | | | |  | Vvi-Vitvi02g00226\_t001 |  |  |  |  |
| 2 | Atr-ERN10733 |  | | | |  | | | |  |  |  |  |
| 2 | Atr-ERN10734 |  | | | |  | | | |  |  |  |  |
| 2 | Atr-ERN10735 |  | | | |  | | | |  |  |  |  |
| 2 | Atr-ERN10736 |  | Vvi-Vitvi16g01364\_t001 |  | | | |  |  |  |  |
| 2 | Atr-ERN10737 |  | | | |  | | | |  |  |  |  |
| 2 | Atr-ERN10738 |  | | | |  | | | |  |  |  |  |
| 2 | Atr-ERN10739 |  | Vvi-Vitvi16g01363\_t001 |  | | | |  |  |  |  |
| 2 | Atr-ERN10740 |  | | | |  | | | |  |  |  |  |
| 2 | Atr-ERN10741 |  | | | |  | Vvi-Vitvi02g00227\_t001 |  |  |  |  |
| 2 | Atr-ERN10742 |  | Vvi-Vitvi16g01362\_t001 |  | Vvi-Vitvi02g00228\_t001 |  |  |  |  |
| 2 | Atr-ERN10743 |  | | | |  | | | |  |  |  |  |
| 2 | Atr-ERN10744 |  | | | |  | | | |  |  |  |  |
| 2 | Atr-ERN10745 |  | | | |  | | | |  |  |  |  |
| 2 | Atr-ERN10746 |  | | | |  | | | |  |  |  |  |
| 2 | Atr-ERN10747 |  | | | |  | | | |  |  |  |  |
| 2 | Atr-ERN10748 |  | | | |  | | | |  |  |  |  |
| 2 | Atr-ERN10749 |  | | | |  | | | |  |  |  |  |
| 2 | Atr-ERN10750 |  | | | |  | | | |  |  |  |  |
| 3 | Atr-ERN10751 |  | | | |  | Vvi-Vitvi02g00230\_t001 |  | Vvi-Vitvi02g00230\_t001 |  |  |  |
| 2 | Atr-ERN10752 |  | | | |  |  |  | | | |  |  |  |
| 2 | Atr-ERN10753 |  | | | |  |  |  | | | |  |  |  |
| 2 | Atr-ERN10754 |  | | | |  |  |  | | | |  |  |  |
| 3 | Atr-ERN10755 |  | Vvi-Vitvi16g01360\_t001 |  | Vvi-Vitvi16g01360\_t001 |  | | | |  |  |  |
| 3 | Atr-ERN10756 |  | | | |  | | | |  | | | |  |  |  |
| 3 | Atr-ERN10757 |  | | | |  | | | |  | | | |  |  |  |
| 3 | Atr-ERN10758 |  | | | |  | | | |  | | | |  |  |  |
| 3 | Atr-ERN10759 |  | | | |  | | | |  | | | |  |  |  |
| 3 | Atr-ERN10760 |  | | | |  | | | |  | | | |  |  |  |
| 3 | Atr-ERN10761 |  | | | |  | | | |  | | | |  |  |  |
| 3 | Atr-ERN10762 |  | Vvi-Vitvi16g01359\_t001 |  | | | |  | | | |  |  |  |
| 2 | Atr-ERN10763 |  |  |  | | | |  | | | |  |  |  |
| 2 | Atr-ERN10764 |  |  |  | | | |  | | | |  |  |  |
| 2 | Atr-ERN10765 |  |  |  | Vvi-Vitvi16g01373\_t001 |  | | | |  |  |  |
| 2 | Atr-ERN10766 |  |  |  | | | |  | | | |  |  |  |
| 2 | Atr-ERN10767 |  |  |  | | | |  | | | |  |  |  |
| 2 | Atr-ERN10768 |  |  |  | | | |  | | | |  |  |  |
| 2 | Atr-ERN10769 |  |  |  | | | |  | Vvi-Vitvi02g00218\_t003 |  |  |  |
| 2 | Atr-ERN10770 |  |  |  | | | |  | | | |  |  |  |
| 2 | Atr-ERN10771 |  |  |  | Vvi-Vitvi16g01374\_t001 |  | | | |  |  |  |
| 2 | Atr-ERN10772 |  |  |  | | | |  | | | |  |  |  |
| 2 | Atr-ERN10773 |  |  |  | | | |  | | | |  |  |  |
| 2 | Atr-ERN10774 |  |  |  | | | |  | | | |  |  |  |
| 2 | Atr-ERN10775 |  |  |  | | | |  | Vvi-Vitvi02g04049\_t001 |  |  |  |
| 2 | Atr-ERN10776 |  |  |  | | | |  | Vvi-Vitvi02g00214\_t001 |  |  |  |
| 2 | Atr-ERN10777 |  |  |  | Vvi-Vitvi16g02073\_t001 |  | | | |  |  |  |
| 2 | Atr-ERN10778 |  |  |  | | | |  | Vvi-Vitvi02g00213\_t001 |  |  |  |
| 2 | Atr-ERN10779 |  |  |  | | | |  | | | |  |  |  |
| 2 | Atr-ERN10780 |  |  |  | Vvi-Vitvi16g02074\_t001 |  | Vvi-Vitvi02g00212\_t001 |  |  |  |
| 1 | Atr-ERN10781 |  |  |  | | | |  |  |  |  |
| 1 | Atr-ERN10782 |  |  |  | | | |  |  |  |  |
| 1 | Atr-ERN10783 |  |  |  | | | |  |  |  |  |
| 1 | Atr-ERN10784 |  |  |  | | | |  |  |  |  |
| 1 | Atr-ERN10785 |  |  |  | Vvi-Vitvi16g02075\_t001 |  |  |  |  |
| 0 | Atr-ERN10786 |  |  |  |  |  |  |
| 0 | Atr-ERN10787 |  |  |  |  |  |  |
| 0 | Atr-ERN10788 |  |  |  |  |  |  |
| 0 | Atr-ERN10789 |  |  |  |  |  |  |
| 0 | Atr-ERN10790 |  |  |  |  |  |  |
| 0 | Atr-ERN10791 |  |  |  |  |  |  |
| 0 | Atr-ERN10792 |  |  |  |  |  |  |
| 0 | Atr-ERN10793 |  |  |  |  |  |  |
| 0 | Atr-ERN10794 |  |  |  |  |  |  |
| 0 | Atr-ERN10795 |  |  |  |  |  |  |
| 0 | Atr-ERN10796 |  |  |  |  |  |  |
| 0 | Atr-ERN10797 |  |  |  |  |  |  |
| 0 | Atr-ERN10798 |  |  |  |  |  |  |
| 0 | Atr-ERN10799 |  |  |  |  |  |  |
| 0 | Atr-ERN10800 |  |  |  |  |  |  |
| 0 | Atr-ERN10801 |  |  |  |  |  |  |
| 0 | Atr-ERN10802 |  |  |  |  |  |  |
| 0 | Atr-ERN10803 |  |  |  |  |  |  |
| 0 | Atr-ERN10804 |  |  |  |  |  |  |
| 0 | Atr-ERN10805 |  |  |  |  |  |  |
| 0 | Atr-ERN10806 |  |  |  |  |  |  |
| 0 | Atr-ERN10807 |  |  |  |  |  |  |
| 0 | Atr-ERN10808 |  |  |  |  |  |  |
| 0 | Atr-ERN10809 |  |  |  |  |  |  |
| 0 | Atr-ERN10810 |  |  |  |  |  |  |
| 0 | Atr-ERN10811 |  |  |  |  |  |  |
| 0 | Atr-ERN10812 |  |  |  |  |  |  |
| 0 | Atr-ERN10813 |  |  |  |  |  |  |
| 0 | Atr-ERN10814 |  |  |  |  |  |  |
| 0 | Atr-ERN10815 |  |  |  |  |  |  |
| 0 | Atr-ERN10816 |  |  |  |  |  |  |
| 0 | Atr-ERN10817 |  |  |  |  |  |  |
| 0 | Atr-ERN10818 |  |  |  |  |  |  |
| 0 | Atr-ERN10819 |  |  |  |  |  |  |
| 0 | Atr-ERN10820 |  |  |  |  |  |  |
| 0 | Atr-ERN10821 |  |  |  |  |  |  |
| 0 | Atr-ERN10822 |  |  |  |  |  |  |
| 0 | Atr-ERN10823 |  |  |  |  |  |  |
| 0 | Atr-ERN10824 |  |  |  |  |  |  |
| 0 | Atr-ERN10825 |  |  |  |  |  |  |
| 0 | Atr-ERN10826 |  |  |  |  |  |  |
| 0 | Atr-ERN10827 |  |  |  |  |  |  |
| 0 | Atr-ERN10828 |  |  |  |  |  |  |
| 0 | Atr-ERN10829 |  |  |  |  |  |  |
| 0 | Atr-ERN10830 |  |  |  |  |  |  |
| 0 | Atr-ERN10831 |  |  |  |  |  |  |
| 0 | Atr-ERN10832 |  |  |  |  |  |  |
| 0 | Atr-ERN10833 |  |  |  |  |  |  |
| 0 | Atr-ERN10834 |  |  |  |  |  |  |
| 0 | Atr-ERN10835 |  |  |  |  |  |  |
| 0 | Atr-ERN10836 |  |  |  |  |  |  |
| 0 | Atr-ERN10837 |  |  |  |  |  |  |
| 0 | Atr-ERN10838 |  |  |  |  |  |  |
| 0 | Atr-ERN10839 |  |  |  |  |  |  |
| 0 | Atr-ERN10840 |  |  |  |  |  |  |
| 0 | Atr-ERN10841 |  |  |  |  |  |  |
| 0 | Atr-ERN10842 |  |  |  |  |  |  |
| 0 | Atr-ERN10843 |  |  |  |  |  |  |
| 0 | Atr-ERN10844 |  |  |  |  |  |  |
| 0 | Atr-ERN10845 |  |  |  |  |  |  |
| 0 | Atr-ERN10846 |  |  |  |  |  |  |
| 0 | Atr-ERN10847 |  |  |  |  |  |  |
| 0 | Atr-ERN10848 |  |  |  |  |  |  |
| 0 | Atr-ERN10849 |  |  |  |  |  |  |
| 0 | Atr-ERN10850 |  |  |  |  |  |  |
| 0 | Atr-ERN10851 |  |  |  |  |  |  |
| 0 | Atr-ERN10852 |  |  |  |  |  |  |
| 0 | Atr-ERN10853 |  |  |  |  |  |  |
| 0 | Atr-ERN10854 |  |  |  |  |  |  |
| 0 | Atr-ERN10855 |  |  |  |  |  |  |
| 0 | Atr-ERN10856 |  |  |  |  |  |  |
| 0 | Atr-ERN10857 |  |  |  |  |  |  |
| 0 | Atr-ERN10858 |  |  |  |  |  |  |
| 0 | Atr-ERN10859 |  |  |  |  |  |  |
| 0 | Atr-ERN10860 |  |  |  |  |  |  |
