# Supplementary material for: Genome-wide identification, characterization and gene expression of BES1 transcription factor family in grapevine (Vitis vinifera L.)
Source: Sci Rep. 2023 Jan 5;13:240. doi: 10.1038/s41598-022-24407-y (PMC9816167; doi:10.1038/s41598-022-24407-y)
Supplement: Supplementary file 3 — Supplementary Information. [file 41598_2022_24407_MOESM3_ESM.zip › Vvi_Atr/Vitis_vinifera.PN40024.v4.dna_sm.toplevel.fa.vs.Amborella_trichopoda.AMTR1.0.dna_sm.toplevel.fa.html/Atr-AmTr_v1.0_scaffold00028.html]

|  |  |  |  |  |  |  |  |  |  |  |  |  |  |
| --- | --- | --- | --- | --- | --- | --- | --- | --- | --- | --- | --- | --- | --- |
| Duplication depth | Reference chromosome | Collinear blocks | | | | | | | | | | | |
| 0 | Atr-ERN10552 |  |  |  |  |  |  |
| 0 | Atr-ERN10553 |  |  |  |  |  |  |
| 0 | Atr-ERN10554 |  |  |  |  |  |  |
| 0 | Atr-ERN10555 |  |  |  |  |  |  |
| 0 | Atr-ERN10556 |  |  |  |  |  |  |
| 0 | Atr-ERN10557 |  |  |  |  |  |  |
| 0 | Atr-ERN10558 |  |  |  |  |  |  |
| 0 | Atr-ERN10559 |  |  |  |  |  |  |
| 0 | Atr-ERN10560 |  |  |  |  |  |  |
| 0 | Atr-ERN10561 |  |  |  |  |  |  |
| 0 | Atr-ERN10562 |  |  |  |  |  |  |
| 0 | Atr-ERN10563 |  |  |  |  |  |  |
| 0 | Atr-ERN10564 |  |  |  |  |  |  |
| 0 | Atr-ERN10565 |  |  |  |  |  |  |
| 0 | Atr-ERN10566 |  |  |  |  |  |  |
| 0 | Atr-ERN10567 |  |  |  |  |  |  |
| 0 | Atr-ERN10568 |  |  |  |  |  |  |
| 0 | Atr-ERN10569 |  |  |  |  |  |  |
| 0 | Atr-ERN10570 |  |  |  |  |  |  |
| 0 | Atr-ERN10571 |  |  |  |  |  |  |
| 0 | Atr-ERN10572 |  |  |  |  |  |  |
| 0 | Atr-ERN10573 |  |  |  |  |  |  |
| 0 | Atr-ERN10574 |  |  |  |  |  |  |
| 0 | Atr-ERN10575 |  |  |  |  |  |  |
| 0 | Atr-ERN10576 |  |  |  |  |  |  |
| 0 | Atr-ERN10577 |  |  |  |  |  |  |
| 0 | Atr-ERN10578 |  |  |  |  |  |  |
| 0 | Atr-ERN10579 |  |  |  |  |  |  |
| 0 | Atr-ERN10580 |  |  |  |  |  |  |
| 0 | Atr-ERN10581 |  |  |  |  |  |  |
| 0 | Atr-ERN10582 |  |  |  |  |  |  |
| 0 | Atr-ERN10583 |  |  |  |  |  |  |
| 0 | Atr-ERN10584 |  |  |  |  |  |  |
| 0 | Atr-ERN10585 |  |  |  |  |  |  |
| 0 | Atr-ERN10586 |  |  |  |  |  |  |
| 0 | Atr-ERN10587 |  |  |  |  |  |  |
| 0 | Atr-ERN10588 |  |  |  |  |  |  |
| 0 | Atr-ERN10589 |  |  |  |  |  |  |
| 0 | Atr-ERN10590 |  |  |  |  |  |  |
| 0 | Atr-ERN10591 |  |  |  |  |  |  |
| 0 | Atr-ERN10592 |  |  |  |  |  |  |
| 0 | Atr-ERN10593 |  |  |  |  |  |  |
| 0 | Atr-ERN10594 |  |  |  |  |  |  |
| 0 | Atr-ERN10595 |  |  |  |  |  |  |
| 0 | Atr-ERN10596 |  |  |  |  |  |  |
| 0 | Atr-ERN10597 |  |  |  |  |  |  |
| 0 | Atr-ERN10598 |  |  |  |  |  |  |
| 0 | Atr-ERN10599 |  |  |  |  |  |  |
| 0 | Atr-ERN10600 |  |  |  |  |  |  |
| 0 | Atr-ERN10601 |  |  |  |  |  |  |
| 0 | Atr-ERN10602 |  |  |  |  |  |  |
| 0 | Atr-ERN10603 |  |  |  |  |  |  |
| 0 | Atr-ERN10604 |  |  |  |  |  |  |
| 0 | Atr-ERN10605 |  |  |  |  |  |  |
| 0 | Atr-ERN10606 |  |  |  |  |  |  |
| 0 | Atr-ERN10607 |  |  |  |  |  |  |
| 0 | Atr-ERN10608 |  |  |  |  |  |  |
| 0 | Atr-ERN10609 |  |  |  |  |  |  |
| 0 | Atr-ERN10610 |  |  |  |  |  |  |
| 0 | Atr-ERN10611 |  |  |  |  |  |  |
| 0 | Atr-ERN10612 |  |  |  |  |  |  |
| 0 | Atr-ERN10613 |  |  |  |  |  |  |
| 0 | Atr-ERN10614 |  |  |  |  |  |  |
| 0 | Atr-ERN10615 |  |  |  |  |  |  |
| 0 | Atr-ERN10616 |  |  |  |  |  |  |
| 0 | Atr-ERN10617 |  |  |  |  |  |  |
| 0 | Atr-ERN10618 |  |  |  |  |  |  |
| 0 | Atr-ERN10619 |  |  |  |  |  |  |
| 0 | Atr-ERN10620 |  |  |  |  |  |  |
| 0 | Atr-ERN10621 |  |  |  |  |  |  |
| 0 | Atr-ERN10622 |  |  |  |  |  |  |
| 0 | Atr-ERN10623 |  |  |  |  |  |  |
| 0 | Atr-ERN10624 |  |  |  |  |  |  |
| 0 | Atr-ERN10625 |  |  |  |  |  |  |
| 0 | Atr-ERN10626 |  |  |  |  |  |  |
| 0 | Atr-ERN10627 |  |  |  |  |  |  |
| 0 | Atr-ERN10628 |  |  |  |  |  |  |
| 0 | Atr-ERN10629 |  |  |  |  |  |  |
| 0 | Atr-ERN10630 |  |  |  |  |  |  |
| 0 | Atr-ERN10631 |  |  |  |  |  |  |
| 0 | Atr-ERN10632 |  |  |  |  |  |  |
| 0 | Atr-ERN10633 |  |  |  |  |  |  |
| 0 | Atr-ERN10634 |  |  |  |  |  |  |
| 0 | Atr-ERN10635 |  |  |  |  |  |  |
| 0 | Atr-ERN10636 |  |  |  |  |  |  |
| 0 | Atr-ERN10637 |  |  |  |  |  |  |
| 0 | Atr-ERN10638 |  |  |  |  |  |  |
| 0 | Atr-ERN10639 |  |  |  |  |  |  |
| 0 | Atr-ERN10640 |  |  |  |  |  |  |
| 0 | Atr-ERN10641 |  |  |  |  |  |  |
| 0 | Atr-ERN10642 |  |  |  |  |  |  |
| 0 | Atr-ERN10643 |  |  |  |  |  |  |
| 0 | Atr-ERN10644 |  |  |  |  |  |  |
| 0 | Atr-ERN10645 |  |  |  |  |  |  |
| 0 | Atr-ERN10646 |  |  |  |  |  |  |
| 0 | Atr-ERN10647 |  |  |  |  |  |  |
| 0 | Atr-ERN10648 |  |  |  |  |  |  |
| 0 | Atr-ERN10649 |  |  |  |  |  |  |
| 0 | Atr-ERN10650 |  |  |  |  |  |  |
| 0 | Atr-ERN10651 |  |  |  |  |  |  |
| 0 | Atr-ERN10652 |  |  |  |  |  |  |
| 0 | Atr-ERN10653 |  |  |  |  |  |  |
| 0 | Atr-ERN10654 |  |  |  |  |  |  |
| 0 | Atr-ERN10655 |  |  |  |  |  |  |
| 0 | Atr-ERN10656 |  |  |  |  |  |  |
| 0 | Atr-ERN10657 |  |  |  |  |  |  |
| 0 | Atr-ERN10658 |  |  |  |  |  |  |
| 0 | Atr-ERN10659 |  |  |  |  |  |  |
| 0 | Atr-ERN10660 |  |  |  |  |  |  |
| 0 | Atr-ERN10661 |  |  |  |  |  |  |
| 0 | Atr-ERN10662 |  |  |  |  |  |  |
| 0 | Atr-ERN10663 |  |  |  |  |  |  |
| 0 | Atr-ERN10664 |  |  |  |  |  |  |
| 0 | Atr-ERN10665 |  |  |  |  |  |  |
| 0 | Atr-ERN10666 |  |  |  |  |  |  |
| 0 | Atr-ERN10667 |  |  |  |  |  |  |
| 0 | Atr-ERN10668 |  |  |  |  |  |  |
| 0 | Atr-ERN10669 |  |  |  |  |  |  |
| 0 | Atr-ERN10670 |  |  |  |  |  |  |
| 0 | Atr-ERN10671 |  |  |  |  |  |  |
| 0 | Atr-ERN10672 |  |  |  |  |  |  |
| 0 | Atr-ERN10673 |  |  |  |  |  |  |
| 0 | Atr-ERN10674 |  |  |  |  |  |  |
| 0 | Atr-ERN10675 |  |  |  |  |  |  |
| 0 | Atr-ERN10676 |  |  |  |  |  |  |
| 0 | Atr-ERN10677 |  |  |  |  |  |  |
| 0 | Atr-ERN10678 |  |  |  |  |  |  |
| 0 | Atr-ERN10679 |  |  |  |  |  |  |
| 0 | Atr-ERN10680 |  |  |  |  |  |  |
| 0 | Atr-ERN10681 |  |  |  |  |  |  |
| 0 | Atr-ERN10682 |  |  |  |  |  |  |
| 0 | Atr-ERN10683 |  |  |  |  |  |  |
| 0 | Atr-ERN10684 |  |  |  |  |  |  |
| 0 | Atr-ERN10685 |  |  |  |  |  |  |
| 0 | Atr-ERN10686 |  |  |  |  |  |  |
| 0 | Atr-ERN10687 |  |  |  |  |  |  |
| 0 | Atr-ERN10688 |  |  |  |  |  |  |
| 0 | Atr-ERN10689 |  |  |  |  |  |  |
| 0 | Atr-ERN10690 |  |  |  |  |  |  |
| 0 | Atr-ERN10691 |  |  |  |  |  |  |
| 0 | Atr-ERN10692 |  |  |  |  |  |  |
| 0 | Atr-ERN10693 |  |  |  |  |  |  |
| 0 | Atr-ERN10694 |  |  |  |  |  |  |
| 0 | Atr-ERN10695 |  |  |  |  |  |  |
| 0 | Atr-ERN10696 |  |  |  |  |  |  |
