# Supplementary material for: Genome-wide identification, characterization and gene expression of BES1 transcription factor family in grapevine (Vitis vinifera L.)
Source: Sci Rep. 2023 Jan 5;13:240. doi: 10.1038/s41598-022-24407-y (PMC9816167; doi:10.1038/s41598-022-24407-y)
Supplement: Supplementary file 3 — Supplementary Information. [file 41598_2022_24407_MOESM3_ESM.zip › Vvi_Atr/Vitis_vinifera.PN40024.v4.dna_sm.toplevel.fa.vs.Amborella_trichopoda.AMTR1.0.dna_sm.toplevel.fa.html/Atr-AmTr_v1.0_scaffold00029.html]

|  |  |  |  |  |  |  |  |  |  |  |  |  |  |
| --- | --- | --- | --- | --- | --- | --- | --- | --- | --- | --- | --- | --- | --- |
| Duplication depth | Reference chromosome | Collinear blocks | | | | | | | | | | | |
| 0 | Atr-ERN09370 |  |  |  |  |  |  |
| 0 | Atr-ERN09371 |  |  |  |  |  |  |
| 0 | Atr-ERN09372 |  |  |  |  |  |  |
| 0 | Atr-ERN09373 |  |  |  |  |  |  |
| 0 | Atr-ERN09374 |  |  |  |  |  |  |
| 0 | Atr-ERN09375 |  |  |  |  |  |  |
| 0 | Atr-ERN09376 |  |  |  |  |  |  |
| 0 | Atr-ERN09377 |  |  |  |  |  |  |
| 0 | Atr-ERN09378 |  |  |  |  |  |  |
| 0 | Atr-ERN09379 |  |  |  |  |  |  |
| 0 | Atr-ERN09380 |  |  |  |  |  |  |
| 1 | Atr-ERN09381 |  | Vvi-Vitvi10g01478\_t001 |  |  |  |  |  |
| 2 | Atr-ERN09382 |  | | | |  | Vvi-Vitvi17g01647\_t001 |  |  |  |  |
| 2 | Atr-ERN09383 |  | | | |  | Vvi-Vitvi17g01190\_t001 |  |  |  |  |
| 2 | Atr-ERN09384 |  | | | |  | | | |  |  |  |  |
| 2 | Atr-ERN09385 |  | | | |  | | | |  |  |  |  |
| 2 | Atr-ERN09386 |  | | | |  | | | |  |  |  |  |
| 2 | Atr-ERN09387 |  | Vvi-Vitvi10g01471\_t001 |  | | | |  |  |  |  |
| 2 | Atr-ERN09388 |  | Vvi-Vitvi10g01470\_t001 |  | | | |  |  |  |  |
| 2 | Atr-ERN09389 |  | | | |  | | | |  |  |  |  |
| 2 | Atr-ERN09390 |  | | | |  | | | |  |  |  |  |
| 2 | Atr-ERN09391 |  | | | |  | | | |  |  |  |  |
| 2 | Atr-ERN09392 |  | Vvi-Vitvi10g01468\_t001 |  | | | |  |  |  |  |
| 2 | Atr-ERN09393 |  | | | |  | Vvi-Vitvi17g01644\_t001 |  |  |  |  |
| 2 | Atr-ERN09394 |  | | | |  | | | |  |  |  |  |
| 2 | Atr-ERN09395 |  | | | |  | | | |  |  |  |  |
| 2 | Atr-ERN09396 |  | | | |  | | | |  |  |  |  |
| 2 | Atr-ERN09397 |  | Vvi-Vitvi10g01466\_t001 |  | | | |  |  |  |  |
| 2 | Atr-ERN09398 |  | Vvi-Vitvi10g01465\_t001 |  | | | |  |  |  |  |
| 2 | Atr-ERN09399 |  | | | |  | | | |  |  |  |  |
| 2 | Atr-ERN09400 |  | Vvi-Vitvi10g01458\_t001 |  | | | |  |  |  |  |
| 2 | Atr-ERN09401 |  | | | |  | Vvi-Vitvi17g01176\_t001 |  |  |  |  |
| 2 | Atr-ERN09402 |  | | | |  | | | |  |  |  |  |
| 2 | Atr-ERN09403 |  | | | |  | | | |  |  |  |  |
| 2 | Atr-ERN09404 |  | | | |  | | | |  |  |  |  |
| 2 | Atr-ERN09405 |  | | | |  | | | |  |  |  |  |
| 2 | Atr-ERN09406 |  | | | |  | | | |  |  |  |  |
| 2 | Atr-ERN09407 |  | | | |  | | | |  |  |  |  |
| 2 | Atr-ERN09408 |  | | | |  | | | |  |  |  |  |
| 2 | Atr-ERN09409 |  | | | |  | | | |  |  |  |  |
| 2 | Atr-ERN09410 |  | | | |  | | | |  |  |  |  |
| 2 | Atr-ERN09411 |  | Vvi-Vitvi10g04669\_t001 |  | | | |  |  |  |  |
| 1 | Atr-ERN09412 |  |  |  | | | |  |  |  |  |
| 1 | Atr-ERN09413 |  |  |  | | | |  |  |  |  |
| 1 | Atr-ERN09414 |  |  |  | | | |  |  |  |  |
| 1 | Atr-ERN09415 |  |  |  | Vvi-Vitvi17g01162\_t001 |  |  |  |  |
| 1 | Atr-ERN09416 |  |  |  | | | |  |  |  |  |
| 1 | Atr-ERN09417 |  |  |  | | | |  |  |  |  |
| 1 | Atr-ERN09418 |  |  |  | | | |  |  |  |  |
| 1 | Atr-ERN09419 |  |  |  | | | |  |  |  |  |
| 2 | Atr-ERN09420 |  | Vvi-Vitvi01g00155\_t001 |  | | | |  |  |  |  |
| 2 | Atr-ERN09421 |  | | | |  | | | |  |  |  |  |
| 2 | Atr-ERN09422 |  | | | |  | | | |  |  |  |  |
| 2 | Atr-ERN09423 |  | | | |  | Vvi-Vitvi17g01635\_t001 |  |  |  |  |
| 2 | Atr-ERN09424 |  | | | |  | | | |  |  |  |  |
| 2 | Atr-ERN09425 |  | | | |  | | | |  |  |  |  |
| 2 | Atr-ERN09426 |  | | | |  | | | |  |  |  |  |
| 2 | Atr-ERN09427 |  | | | |  | | | |  |  |  |  |
| 2 | Atr-ERN09428 |  | | | |  | | | |  |  |  |  |
| 2 | Atr-ERN09429 |  | | | |  | | | |  |  |  |  |
| 2 | Atr-ERN09430 |  | Vvi-Vitvi01g00160\_t001 |  | | | |  |  |  |  |
| 2 | Atr-ERN09431 |  | | | |  | | | |  |  |  |  |
| 2 | Atr-ERN09432 |  | | | |  | | | |  |  |  |  |
| 2 | Atr-ERN09433 |  | | | |  | | | |  |  |  |  |
| 2 | Atr-ERN09434 |  | Vvi-Vitvi01g00162\_t001 |  | | | |  |  |  |  |
| 2 | Atr-ERN09435 |  | Vvi-Vitvi01g01893\_t001.2.6037826a |  | | | |  |  |  |  |
| 2 | Atr-ERN09436 |  | Vvi-Vitvi01g00163\_t001 |  | | | |  |  |  |  |
| 2 | Atr-ERN09437 |  | | | |  | | | |  |  |  |  |
| 2 | Atr-ERN09438 |  | | | |  | | | |  |  |  |  |
| 2 | Atr-ERN09439 |  | Vvi-Vitvi01g00166\_t002 |  | | | |  |  |  |  |
| 2 | Atr-ERN09440 |  | | | |  | | | |  |  |  |  |
| 2 | Atr-ERN09441 |  | | | |  | | | |  |  |  |  |
| 2 | Atr-ERN09442 |  | | | |  | | | |  |  |  |  |
| 2 | Atr-ERN09443 |  | Vvi-Vitvi01g00168\_t002 |  | | | |  |  |  |  |
| 2 | Atr-ERN09444 |  | Vvi-Vitvi01g00169\_t001 |  | | | |  |  |  |  |
| 2 | Atr-ERN09445 |  | Vvi-Vitvi01g00170\_t001 |  | | | |  |  |  |  |
| 2 | Atr-ERN09446 |  | Vvi-Vitvi01g00171\_t001 |  | | | |  |  |  |  |
| 2 | Atr-ERN09447 |  | | | |  | Vvi-Vitvi17g04311\_t001 |  |  |  |  |
| 1 | Atr-ERN09448 |  | Vvi-Vitvi01g00172\_t001 |  |  |  |  |  |
| 1 | Atr-ERN09449 |  | | | |  |  |  |  |  |
| 1 | Atr-ERN09450 |  | Vvi-Vitvi01g00179\_t001 |  |  |  |  |  |
| 0 | Atr-ERN09451 |  |  |  |  |  |  |
| 0 | Atr-ERN09452 |  |  |  |  |  |  |
| 0 | Atr-ERN09453 |  |  |  |  |  |  |
| 0 | Atr-ERN09454 |  |  |  |  |  |  |
| 0 | Atr-ERN09455 |  |  |  |  |  |  |
| 0 | Atr-ERN09456 |  |  |  |  |  |  |
| 1 | Atr-ERN09457 |  | Vvi-Vitvi01g01345\_t001 |  |  |  |  |  |
| 1 | Atr-ERN09458 |  | | | |  |  |  |  |  |
| 1 | Atr-ERN09459 |  | | | |  |  |  |  |  |
| 1 | Atr-ERN09460 |  | Vvi-Vitvi01g01373\_t001 |  |  |  |  |  |
| 1 | Atr-ERN09461 |  | Vvi-Vitvi01g01374\_t001 |  |  |  |  |  |
| 1 | Atr-ERN09462 |  | Vvi-Vitvi01g01376\_t003 |  |  |  |  |  |
| 1 | Atr-ERN09463 |  | | | |  |  |  |  |  |
| 1 | Atr-ERN09464 |  | | | |  |  |  |  |  |
| 1 | Atr-ERN09465 |  | | | |  |  |  |  |  |
| 1 | Atr-ERN09466 |  | | | |  |  |  |  |  |
| 1 | Atr-ERN09467 |  | | | |  |  |  |  |  |
| 1 | Atr-ERN09468 |  | | | |  |  |  |  |  |
| 1 | Atr-ERN09469 |  | | | |  |  |  |  |  |
| 1 | Atr-ERN09470 |  | | | |  |  |  |  |  |
| 1 | Atr-ERN09471 |  | | | |  |  |  |  |  |
| 1 | Atr-ERN09472 |  | | | |  |  |  |  |  |
| 1 | Atr-ERN09473 |  | | | |  |  |  |  |  |
| 1 | Atr-ERN09474 |  | | | |  |  |  |  |  |
| 1 | Atr-ERN09475 |  | | | |  |  |  |  |  |
| 1 | Atr-ERN09476 |  | | | |  |  |  |  |  |
| 1 | Atr-ERN09477 |  | | | |  |  |  |  |  |
| 1 | Atr-ERN09478 |  | | | |  |  |  |  |  |
| 1 | Atr-ERN09479 |  | | | |  |  |  |  |  |
| 1 | Atr-ERN09480 |  | | | |  |  |  |  |  |
| 1 | Atr-ERN09481 |  | | | |  |  |  |  |  |
| 1 | Atr-ERN09482 |  | | | |  |  |  |  |  |
| 1 | Atr-ERN09483 |  | | | |  |  |  |  |  |
| 1 | Atr-ERN09484 |  | Vvi-Vitvi01g01404\_t001 |  |  |  |  |  |
| 1 | Atr-ERN09485 |  | | | |  |  |  |  |  |
| 1 | Atr-ERN09486 |  | | | |  |  |  |  |  |
| 1 | Atr-ERN09487 |  | | | |  |  |  |  |  |
| 1 | Atr-ERN09488 |  | | | |  |  |  |  |  |
| 1 | Atr-ERN09489 |  | | | |  |  |  |  |  |
| 1 | Atr-ERN09490 |  | | | |  |  |  |  |  |
| 1 | Atr-ERN09491 |  | | | |  |  |  |  |  |
| 1 | Atr-ERN09492 |  | | | |  |  |  |  |  |
| 1 | Atr-ERN09493 |  | | | |  |  |  |  |  |
| 1 | Atr-ERN09494 |  | | | |  |  |  |  |  |
| 1 | Atr-ERN09495 |  | | | |  |  |  |  |  |
| 1 | Atr-ERN09496 |  | | | |  |  |  |  |  |
| 1 | Atr-ERN09497 |  | | | |  |  |  |  |  |
| 1 | Atr-ERN09498 |  | | | |  |  |  |  |  |
| 1 | Atr-ERN09499 |  | | | |  |  |  |  |  |
| 1 | Atr-ERN09500 |  | | | |  |  |  |  |  |
| 1 | Atr-ERN09501 |  | | | |  |  |  |  |  |
| 1 | Atr-ERN09502 |  | | | |  |  |  |  |  |
| 1 | Atr-ERN09503 |  | | | |  |  |  |  |  |
| 1 | Atr-ERN09504 |  | Vvi-Vitvi01g01418\_t001 |  |  |  |  |  |
| 1 | Atr-ERN09505 |  | | | |  |  |  |  |  |
| 1 | Atr-ERN09506 |  | | | |  |  |  |  |  |
| 1 | Atr-ERN09507 |  | | | |  |  |  |  |  |
| 1 | Atr-ERN09508 |  | | | |  |  |  |  |  |
| 1 | Atr-ERN09509 |  | | | |  |  |  |  |  |
| 1 | Atr-ERN09510 |  | | | |  |  |  |  |  |
| 1 | Atr-ERN09511 |  | | | |  |  |  |  |  |
| 1 | Atr-ERN09512 |  | | | |  |  |  |  |  |
| 1 | Atr-ERN09513 |  | | | |  |  |  |  |  |
| 1 | Atr-ERN09514 |  | | | |  |  |  |  |  |
| 1 | Atr-ERN09515 |  | | | |  |  |  |  |  |
| 1 | Atr-ERN09516 |  | | | |  |  |  |  |  |
| 1 | Atr-ERN09517 |  | | | |  |  |  |  |  |
| 1 | Atr-ERN09518 |  | | | |  |  |  |  |  |
| 1 | Atr-ERN09519 |  | | | |  |  |  |  |  |
| 1 | Atr-ERN09520 |  | | | |  |  |  |  |  |
| 1 | Atr-ERN09521 |  | Vvi-Vitvi01g01430\_t006 |  |  |  |  |  |
| 1 | Atr-ERN09522 |  | | | |  |  |  |  |  |
| 1 | Atr-ERN09523 |  | | | |  |  |  |  |  |
| 1 | Atr-ERN09524 |  | | | |  |  |  |  |  |
| 1 | Atr-ERN09525 |  | | | |  |  |  |  |  |
| 1 | Atr-ERN09526 |  | | | |  |  |  |  |  |
| 1 | Atr-ERN09527 |  | | | |  |  |  |  |  |
| 1 | Atr-ERN09528 |  | | | |  |  |  |  |  |
| 1 | Atr-ERN09529 |  | | | |  |  |  |  |  |
| 1 | Atr-ERN09530 |  | | | |  |  |  |  |  |
| 1 | Atr-ERN09531 |  | | | |  |  |  |  |  |
| 1 | Atr-ERN09532 |  | Vvi-Vitvi01g02195\_t001 |  |  |  |  |  |
| 1 | Atr-ERN09533 |  | | | |  |  |  |  |  |
| 1 | Atr-ERN09534 |  | | | |  |  |  |  |  |
| 1 | Atr-ERN09535 |  | | | |  |  |  |  |  |
| 1 | Atr-ERN09536 |  | | | |  |  |  |  |  |
| 1 | Atr-ERN09537 |  | | | |  |  |  |  |  |
| 1 | Atr-ERN09538 |  | | | |  |  |  |  |  |
| 1 | Atr-ERN09539 |  | Vvi-Vitvi01g01434\_t001 |  |  |  |  |  |
| 1 | Atr-ERN09540 |  | Vvi-Vitvi01g01435\_t001 |  |  |  |  |  |
| 1 | Atr-ERN09541 |  | | | |  |  |  |  |  |
| 1 | Atr-ERN09542 |  | | | |  |  |  |  |  |
| 1 | Atr-ERN09543 |  | Vvi-Vitvi01g01436\_t001 |  |  |  |  |  |
| 1 | Atr-ERN09544 |  | | | |  |  |  |  |  |
| 1 | Atr-ERN09545 |  | | | |  |  |  |  |  |
| 1 | Atr-ERN09546 |  | Vvi-Vitvi01g01439\_t001 |  |  |  |  |  |
| 1 | Atr-ERN09547 |  | Vvi-Vitvi01g01440\_t002 |  |  |  |  |  |
| 1 | Atr-ERN09548 |  | | | |  |  |  |  |  |
| 1 | Atr-ERN09549 |  | | | |  |  |  |  |  |
| 1 | Atr-ERN09550 |  | | | |  |  |  |  |  |
| 1 | Atr-ERN09551 |  | | | |  |  |  |  |  |
| 1 | Atr-ERN09552 |  | | | |  |  |  |  |  |
| 1 | Atr-ERN09553 |  | Vvi-Vitvi01g01443\_t001 |  |  |  |  |  |
| 1 | Atr-ERN09554 |  | Vvi-Vitvi01g01446\_t001 |  |  |  |  |  |
| 1 | Atr-ERN09555 |  | | | |  |  |  |  |  |
| 1 | Atr-ERN09556 |  | | | |  |  |  |  |  |
| 1 | Atr-ERN09557 |  | Vvi-Vitvi01g01447\_t001 |  |  |  |  |  |
| 1 | Atr-ERN09558 |  | | | |  |  |  |  |  |
| 1 | Atr-ERN09559 |  | | | |  |  |  |  |  |
| 1 | Atr-ERN09560 |  | | | |  |  |  |  |  |
| 1 | Atr-ERN09561 |  | | | |  |  |  |  |  |
| 1 | Atr-ERN09562 |  | | | |  |  |  |  |  |
| 1 | Atr-ERN09563 |  | | | |  |  |  |  |  |
| 1 | Atr-ERN09564 |  | | | |  |  |  |  |  |
| 1 | Atr-ERN09565 |  | | | |  |  |  |  |  |
| 1 | Atr-ERN09566 |  | | | |  |  |  |  |  |
| 1 | Atr-ERN09567 |  | | | |  |  |  |  |  |
| 1 | Atr-ERN09568 |  | | | |  |  |  |  |  |
| 1 | Atr-ERN09569 |  | | | |  |  |  |  |  |
| 1 | Atr-ERN09570 |  | | | |  |  |  |  |  |
| 1 | Atr-ERN09571 |  | | | |  |  |  |  |  |
| 1 | Atr-ERN09572 |  | | | |  |  |  |  |  |
| 1 | Atr-ERN09573 |  | Vvi-Vitvi01g01456\_t001 |  |  |  |  |  |
| 1 | Atr-ERN09574 |  | Vvi-Vitvi01g01457\_t001 |  |  |  |  |  |
| 1 | Atr-ERN09575 |  | | | |  |  |  |  |  |
| 1 | Atr-ERN09576 |  | Vvi-Vitvi01g04362\_t001 |  |  |  |  |  |
| 1 | Atr-ERN09577 |  | | | |  |  |  |  |  |
| 1 | Atr-ERN09578 |  | | | |  |  |  |  |  |
| 1 | Atr-ERN09579 |  | | | |  |  |  |  |  |
| 1 | Atr-ERN09580 |  | | | |  |  |  |  |  |
| 1 | Atr-ERN09581 |  | | | |  |  |  |  |  |
| 1 | Atr-ERN09582 |  | | | |  |  |  |  |  |
| 1 | Atr-ERN09583 |  | | | |  |  |  |  |  |
| 1 | Atr-ERN09584 |  | | | |  |  |  |  |  |
| 1 | Atr-ERN09585 |  | | | |  |  |  |  |  |
| 1 | Atr-ERN09586 |  | | | |  |  |  |  |  |
| 1 | Atr-ERN09587 |  | | | |  |  |  |  |  |
| 1 | Atr-ERN09588 |  | | | |  |  |  |  |  |
| 1 | Atr-ERN09589 |  | Vvi-Vitvi01g01467\_t001 |  |  |  |  |  |
| 1 | Atr-ERN09590 |  | | | |  |  |  |  |  |
| 1 | Atr-ERN09591 |  | | | |  |  |  |  |  |
| 1 | Atr-ERN09592 |  | | | |  |  |  |  |  |
| 1 | Atr-ERN09593 |  | Vvi-Vitvi01g01470\_t001 |  |  |  |  |  |
| 0 | Atr-ERN09594 |  |  |  |  |  |  |
| 0 | Atr-ERN09595 |  |  |  |  |  |  |
| 0 | Atr-ERN09596 |  |  |  |  |  |  |
| 0 | Atr-ERN09597 |  |  |  |  |  |  |
| 0 | Atr-ERN09598 |  |  |  |  |  |  |
| 0 | Atr-ERN09599 |  |  |  |  |  |  |
| 0 | Atr-ERN09600 |  |  |  |  |  |  |
| 0 | Atr-ERN09601 |  |  |  |  |  |  |
| 0 | Atr-ERN09602 |  |  |  |  |  |  |
| 0 | Atr-ERN09603 |  |  |  |  |  |  |
| 0 | Atr-ERN09604 |  |  |  |  |  |  |
| 0 | Atr-ERN09605 |  |  |  |  |  |  |
| 0 | Atr-ERN09606 |  |  |  |  |  |  |
| 0 | Atr-ERN09607 |  |  |  |  |  |  |
| 0 | Atr-ERN09608 |  |  |  |  |  |  |
| 0 | Atr-ERN09609 |  |  |  |  |  |  |
| 1 | Atr-ERN09610 |  | Vvi-Vitvi13g00492\_t001 |  |  |  |  |  |
| 3 | Atr-ERN09611 |  | | | |  | Vvi-Vitvi06g00564\_t001 |  | Vvi-Vitvi08g01295\_t001 |  |  |  |
| 3 | Atr-ERN09612 |  | | | |  | Vvi-Vitvi06g00565\_t001 |  | Vvi-Vitvi08g01296\_t001 |  |  |  |
| 3 | Atr-ERN09613 |  | Vvi-Vitvi13g04140\_t003 |  | Vvi-Vitvi06g00567\_t001 |  | Vvi-Vitvi08g01297\_t001 |  |  |  |
| 3 | Atr-ERN09614 |  | Vvi-Vitvi13g00494\_t001 |  | | | |  | Vvi-Vitvi08g01298\_t001 |  |  |  |
| 3 | Atr-ERN09615 |  | | | |  | | | |  | Vvi-Vitvi08g01299\_t001 |  |  |  |
| 3 | Atr-ERN09616 |  | | | |  | Vvi-Vitvi06g01734\_t001 |  | Vvi-Vitvi08g01300\_t001 |  |  |  |
| 3 | Atr-ERN09617 |  | | | |  | | | |  | | | |  |  |  |
| 3 | Atr-ERN09618 |  | | | |  | | | |  | Vvi-Vitvi08g01301\_t001 |  |  |  |
| 3 | Atr-ERN09619 |  | | | |  | | | |  | Vvi-Vitvi08g01303\_t001 |  |  |  |
| 3 | Atr-ERN09620 |  | | | |  | | | |  | | | |  |  |  |
| 3 | Atr-ERN09621 |  | | | |  | | | |  | | | |  |  |  |
| 3 | Atr-ERN09622 |  | | | |  | | | |  | Vvi-Vitvi08g01304\_t001 |  |  |  |
| 3 | Atr-ERN09623 |  | | | |  | Vvi-Vitvi06g00568\_t001 |  | | | |  |  |  |
| 3 | Atr-ERN09624 |  | Vvi-Vitvi13g00495\_t001 |  | | | |  | Vvi-Vitvi08g01305\_t001 |  |  |  |
| 3 | Atr-ERN09625 |  | | | |  | Vvi-Vitvi06g00569\_t001 |  | Vvi-Vitvi08g02232\_t002 |  |  |  |
| 3 | Atr-ERN09626 |  | | | |  | | | |  | | | |  |  |  |
| 3 | Atr-ERN09627 |  | | | |  | Vvi-Vitvi06g00570\_t001 |  | | | |  |  |  |
| 3 | Atr-ERN09628 |  | Vvi-Vitvi13g00496\_t001 |  | | | |  | | | |  |  |  |
| 3 | Atr-ERN09629 |  | | | |  | | | |  | | | |  |  |  |
| 3 | Atr-ERN09630 |  | | | |  | | | |  | Vvi-Vitvi08g02233\_t001 |  |  |  |
| 3 | Atr-ERN09631 |  | | | |  | | | |  | | | |  |  |  |
| 3 | Atr-ERN09632 |  | Vvi-Vitvi13g00499\_t001 |  | | | |  | Vvi-Vitvi08g01308\_t001 |  |  |  |
| 3 | Atr-ERN09633 |  | | | |  | | | |  | | | |  |  |  |
| 3 | Atr-ERN09634 |  | | | |  | | | |  | Vvi-Vitvi08g01309\_t002 |  |  |  |
| 3 | Atr-ERN09635 |  | Vvi-Vitvi13g04142\_t001 |  | | | |  | Vvi-Vitvi08g04263\_t001 |  |  |  |
| 3 | Atr-ERN09636 |  | | | |  | | | |  | | | |  |  |  |
| 3 | Atr-ERN09637 |  | | | |  | Vvi-Vitvi06g00573\_t001 |  | Vvi-Vitvi08g01311\_t001 |  |  |  |
| 3 | Atr-ERN09638 |  | | | |  | | | |  | | | |  |  |  |
| 3 | Atr-ERN09639 |  | | | |  | | | |  | | | |  |  |  |
| 3 | Atr-ERN09640 |  | | | |  | Vvi-Vitvi06g00574\_t001 |  | | | |  |  |  |
| 3 | Atr-ERN09641 |  | | | |  | Vvi-Vitvi06g00576\_t001 |  | | | |  |  |  |
| 3 | Atr-ERN09642 |  | | | |  | | | |  | | | |  |  |  |
| 3 | Atr-ERN09643 |  | | | |  | | | |  | | | |  |  |  |
| 3 | Atr-ERN09644 |  | | | |  | | | |  | | | |  |  |  |
| 3 | Atr-ERN09645 |  | Vvi-Vitvi13g02031\_t001 |  | | | |  | | | |  |  |  |
| 3 | Atr-ERN09646 |  | | | |  | | | |  | | | |  |  |  |
| 3 | Atr-ERN09647 |  | | | |  | | | |  | | | |  |  |  |
| 3 | Atr-ERN09648 |  | | | |  | | | |  | | | |  |  |  |
| 3 | Atr-ERN09649 |  | | | |  | | | |  | | | |  |  |  |
| 3 | Atr-ERN09650 |  | | | |  | | | |  | | | |  |  |  |
| 3 | Atr-ERN09651 |  | | | |  | Vvi-Vitvi06g00577\_t001.1.6037826e |  | Vvi-Vitvi08g01313\_t001 |  |  |  |
| 3 | Atr-ERN09652 |  | | | |  | | | |  | | | |  |  |  |
| 3 | Atr-ERN09653 |  | | | |  | | | |  | Vvi-Vitvi08g01315\_t001 |  |  |  |
| 3 | Atr-ERN09654 |  | | | |  | | | |  | Vvi-Vitvi08g01316\_t001 |  |  |  |
| 3 | Atr-ERN09655 |  | | | |  | | | |  | | | |  |  |  |
| 3 | Atr-ERN09656 |  | | | |  | | | |  | | | |  |  |  |
| 3 | Atr-ERN09657 |  | | | |  | | | |  | Vvi-Vitvi08g01318\_t001 |  |  |  |
| 3 | Atr-ERN09658 |  | | | |  | Vvi-Vitvi06g00578\_t001 |  | | | |  |  |  |
| 3 | Atr-ERN09659 |  | | | |  | | | |  | | | |  |  |  |
| 3 | Atr-ERN09660 |  | | | |  | | | |  | | | |  |  |  |
| 3 | Atr-ERN09661 |  | | | |  | | | |  | Vvi-Vitvi08g01319\_t001 |  |  |  |
| 3 | Atr-ERN09662 |  | | | |  | | | |  | | | |  |  |  |
| 3 | Atr-ERN09663 |  | | | |  | Vvi-Vitvi06g00579\_t001 |  | | | |  |  |  |
| 3 | Atr-ERN09664 |  | | | |  | | | |  | Vvi-Vitvi08g01320\_t001 |  |  |  |
| 3 | Atr-ERN09665 |  | | | |  | | | |  | | | |  |  |  |
| 3 | Atr-ERN09666 |  | Vvi-Vitvi13g02033\_t005 |  | | | |  | | | |  |  |  |
| 3 | Atr-ERN09667 |  | | | |  | Vvi-Vitvi06g00580\_t001 |  | | | |  |  |  |
| 3 | Atr-ERN09668 |  | | | |  | | | |  | | | |  |  |  |
| 3 | Atr-ERN09669 |  | | | |  | | | |  | Vvi-Vitvi08g01323\_t001 |  |  |  |
| 3 | Atr-ERN09670 |  | | | |  | | | |  | Vvi-Vitvi08g04267\_t001 |  |  |  |
| 3 | Atr-ERN09671 |  | | | |  | Vvi-Vitvi06g04198\_t001 |  | | | |  |  |  |
| 3 | Atr-ERN09672 |  | | | |  | Vvi-Vitvi06g00583\_t001 |  | | | |  |  |  |
| 3 | Atr-ERN09673 |  | | | |  | Vvi-Vitvi06g00585\_t001 |  | Vvi-Vitvi08g01325\_t001 |  |  |  |
| 3 | Atr-ERN09674 |  | | | |  | | | |  | | | |  |  |  |
| 3 | Atr-ERN09675 |  | | | |  | | | |  | | | |  |  |  |
| 3 | Atr-ERN09676 |  | | | |  | | | |  | | | |  |  |  |
| 3 | Atr-ERN09677 |  | | | |  | | | |  | | | |  |  |  |
| 3 | Atr-ERN09678 |  | | | |  | | | |  | | | |  |  |  |
| 3 | Atr-ERN09679 |  | | | |  | | | |  | Vvi-Vitvi08g01326\_t001 |  |  |  |
| 3 | Atr-ERN09680 |  | | | |  | Vvi-Vitvi06g00586\_t001 |  | | | |  |  |  |
| 3 | Atr-ERN09681 |  | | | |  | | | |  | | | |  |  |  |
| 3 | Atr-ERN09682 |  | Vvi-Vitvi13g02035\_t001 |  | | | |  | | | |  |  |  |
| 3 | Atr-ERN09683 |  | | | |  | Vvi-Vitvi06g00587\_t001 |  | Vvi-Vitvi08g01327\_t001 |  |  |  |
| 3 | Atr-ERN09684 |  | | | |  | Vvi-Vitvi06g00588\_t001 |  | Vvi-Vitvi08g01329\_t001 |  |  |  |
| 3 | Atr-ERN09685 |  | | | |  | | | |  | Vvi-Vitvi08g04271\_t001 |  |  |  |
| 3 | Atr-ERN09686 |  | | | |  | | | |  | Vvi-Vitvi08g01332\_t001 |  |  |  |
| 3 | Atr-ERN09687 |  | | | |  | | | |  | | | |  |  |  |
| 3 | Atr-ERN09688 |  | | | |  | Vvi-Vitvi06g00590\_t001 |  | Vvi-Vitvi08g01333\_t001 |  |  |  |
| 3 | Atr-ERN09689 |  | | | |  | | | |  | | | |  |  |  |
| 3 | Atr-ERN09690 |  | | | |  | | | |  | | | |  |  |  |
| 3 | Atr-ERN09691 |  | | | |  | | | |  | | | |  |  |  |
| 3 | Atr-ERN09692 |  | Vvi-Vitvi13g00510\_t001 |  | Vvi-Vitvi06g00592\_t001 |  | Vvi-Vitvi08g01336\_t001 |  |  |  |
| 3 | Atr-ERN09693 |  | | | |  | Vvi-Vitvi06g00593\_t001 |  | | | |  |  |  |
| 3 | Atr-ERN09694 |  | | | |  | | | |  | | | |  |  |  |
| 3 | Atr-ERN09695 |  | | | |  | | | |  | | | |  |  |  |
| 3 | Atr-ERN09696 |  | Vvi-Vitvi13g00512\_t001 |  | | | |  | | | |  |  |  |
| 3 | Atr-ERN09697 |  | Vvi-Vitvi13g00513\_t001 |  | | | |  | | | |  |  |  |
| 3 | Atr-ERN09698 |  | | | |  | | | |  | | | |  |  |  |
| 3 | Atr-ERN09699 |  | | | |  | | | |  | | | |  |  |  |
| 3 | Atr-ERN09700 |  | | | |  | Vvi-Vitvi06g00595\_t001 |  | | | |  |  |  |
| 3 | Atr-ERN09701 |  | Vvi-Vitvi13g02037\_t001 |  | | | |  | | | |  |  |  |
| 3 | Atr-ERN09702 |  | | | |  | | | |  | | | |  |  |  |
| 3 | Atr-ERN09703 |  | | | |  | | | |  | | | |  |  |  |
| 3 | Atr-ERN09704 |  | | | |  | | | |  | | | |  |  |  |
| 3 | Atr-ERN09705 |  | | | |  | | | |  | | | |  |  |  |
| 3 | Atr-ERN09706 |  | | | |  | | | |  | | | |  |  |  |
| 3 | Atr-ERN09707 |  | | | |  | Vvi-Vitvi06g00598\_t001 |  | | | |  |  |  |
| 3 | Atr-ERN09708 |  | Vvi-Vitvi13g00515\_t001 |  | | | |  | | | |  |  |  |
| 3 | Atr-ERN09709 |  | Vvi-Vitvi13g00517\_t001 |  | | | |  | Vvi-Vitvi08g01337\_t001 |  |  |  |
| 3 | Atr-ERN09710 |  | Vvi-Vitvi13g00521\_t001 |  | Vvi-Vitvi06g00600\_t001 |  | | | |  |  |  |
| 3 | Atr-ERN09711 |  | | | |  | | | |  | Vvi-Vitvi08g01338\_t001 |  |  |  |
| 3 | Atr-ERN09712 |  | | | |  | | | |  | | | |  |  |  |
| 3 | Atr-ERN09713 |  | | | |  | | | |  | Vvi-Vitvi08g01339\_t001 |  |  |  |
| 3 | Atr-ERN09714 |  | | | |  | | | |  | | | |  |  |  |
| 3 | Atr-ERN09715 |  | Vvi-Vitvi13g00522\_t001 |  | | | |  | Vvi-Vitvi08g01340\_t001 |  |  |  |
| 2 | Atr-ERN09716 |  |  |  | | | |  | | | |  |  |  |
| 2 | Atr-ERN09717 |  |  |  | Vvi-Vitvi06g01740\_t001 |  | | | |  |  |  |
| 2 | Atr-ERN09718 |  |  |  | | | |  | | | |  |  |  |
| 2 | Atr-ERN09719 |  |  |  | | | |  | Vvi-Vitvi08g01341\_t001 |  |  |  |
| 2 | Atr-ERN09720 |  |  |  | Vvi-Vitvi06g00605\_t001 |  | | | |  |  |  |
| 2 | Atr-ERN09721 |  |  |  | Vvi-Vitvi06g00606\_t001 |  | | | |  |  |  |
| 2 | Atr-ERN09722 |  |  |  | | | |  | | | |  |  |  |
| 2 | Atr-ERN09723 |  |  |  | | | |  | | | |  |  |  |
| 2 | Atr-ERN09724 |  |  |  | Vvi-Vitvi06g00607\_t001 |  | | | |  |  |  |
| 2 | Atr-ERN09725 |  |  |  | | | |  | Vvi-Vitvi08g01342\_t001 |  |  |  |
| 3 | Atr-ERN09726 |  | Vvi-Vitvi13g00012\_t001 |  | | | |  | | | |  |  |  |
| 3 | Atr-ERN09727 |  | | | |  | | | |  | | | |  |  |  |
| 3 | Atr-ERN09728 |  | | | |  | | | |  | | | |  |  |  |
| 3 | Atr-ERN09729 |  | Vvi-Vitvi13g00013\_t001 |  | | | |  | | | |  |  |  |
| 3 | Atr-ERN09730 |  | | | |  | | | |  | | | |  |  |  |
| 3 | Atr-ERN09731 |  | | | |  | Vvi-Vitvi06g00610\_t001 |  | | | |  |  |  |
| 3 | Atr-ERN09732 |  | Vvi-Vitvi13g00014\_t001 |  | Vvi-Vitvi06g00613\_t001 |  | | | |  |  |  |
| 3 | Atr-ERN09733 |  | | | |  | | | |  | | | |  |  |  |
| 3 | Atr-ERN09734 |  | Vvi-Vitvi13g00015\_t001 |  | | | |  | | | |  |  |  |
| 3 | Atr-ERN09735 |  | | | |  | | | |  | | | |  |  |  |
| 3 | Atr-ERN09736 |  | | | |  | Vvi-Vitvi06g00614\_t001 |  | | | |  |  |  |
| 3 | Atr-ERN09737 |  | Vvi-Vitvi13g00017\_t001 |  | Vvi-Vitvi06g00615\_t001 |  | | | |  |  |  |
| 3 | Atr-ERN09738 |  | | | |  | | | |  | | | |  |  |  |
| 3 | Atr-ERN09739 |  | | | |  | | | |  | Vvi-Vitvi08g01346\_t001 |  |  |  |
| 3 | Atr-ERN09740 |  | | | |  | | | |  | | | |  |  |  |
| 3 | Atr-ERN09741 |  | Vvi-Vitvi13g00018\_t003 |  | | | |  | | | |  |  |  |
| 3 | Atr-ERN09742 |  | | | |  | | | |  | | | |  |  |  |
| 3 | Atr-ERN09743 |  | | | |  | | | |  | | | |  |  |  |
| 3 | Atr-ERN09744 |  | Vvi-Vitvi13g01882\_t001 |  | | | |  | | | |  |  |  |
| 3 | Atr-ERN09745 |  | Vvi-Vitvi13g00021\_t001 |  | | | |  | | | |  |  |  |
| 3 | Atr-ERN09746 |  | | | |  | | | |  | | | |  |  |  |
| 3 | Atr-ERN09747 |  | Vvi-Vitvi13g00022\_t001 |  | Vvi-Vitvi06g00617\_t001 |  | | | |  |  |  |
| 3 | Atr-ERN09748 |  | | | |  | | | |  | | | |  |  |  |
| 3 | Atr-ERN09749 |  | | | |  | | | |  | | | |  |  |  |
| 3 | Atr-ERN09750 |  | | | |  | | | |  | | | |  |  |  |
| 3 | Atr-ERN09751 |  | | | |  | | | |  | | | |  |  |  |
| 3 | Atr-ERN09752 |  | | | |  | | | |  | | | |  |  |  |
| 3 | Atr-ERN09753 |  | | | |  | | | |  | | | |  |  |  |
| 3 | Atr-ERN09754 |  | | | |  | | | |  | | | |  |  |  |
| 3 | Atr-ERN09755 |  | | | |  | | | |  | Vvi-Vitvi08g02243\_t002 |  |  |  |
| 3 | Atr-ERN09756 |  | | | |  | | | |  | | | |  |  |  |
| 3 | Atr-ERN09757 |  | | | |  | | | |  | Vvi-Vitvi08g01349\_t001 |  |  |  |
| 3 | Atr-ERN09758 |  | Vvi-Vitvi13g04002\_t001 |  | | | |  | Vvi-Vitvi08g01352\_t001 |  |  |  |
| 3 | Atr-ERN09759 |  | | | |  | | | |  | | | |  |  |  |
| 3 | Atr-ERN09760 |  | Vvi-Vitvi13g01884\_t001 |  | | | |  | | | |  |  |  |
| 3 | Atr-ERN09761 |  | Vvi-Vitvi13g01885\_t001 |  | | | |  | | | |  |  |  |
| 3 | Atr-ERN09762 |  | | | |  | | | |  | | | |  |  |  |
| 3 | Atr-ERN09763 |  | | | |  | | | |  | Vvi-Vitvi08g01353\_t001 |  |  |  |
| 3 | Atr-ERN09764 |  | | | |  | | | |  | Vvi-Vitvi08g01354\_t001 |  |  |  |
| 3 | Atr-ERN09765 |  | | | |  | | | |  | | | |  |  |  |
| 3 | Atr-ERN09766 |  | Vvi-Vitvi13g00027\_t001 |  | | | |  | Vvi-Vitvi08g01355\_t001 |  |  |  |
| 3 | Atr-ERN09767 |  | | | |  | Vvi-Vitvi06g00626\_t001 |  | Vvi-Vitvi08g01356\_t001 |  |  |  |
| 3 | Atr-ERN09768 |  | | | |  | Vvi-Vitvi06g00631\_t001 |  | | | |  |  |  |
| 3 | Atr-ERN09769 |  | Vvi-Vitvi13g00028\_t001 |  | | | |  | Vvi-Vitvi08g01357\_t001 |  |  |  |
| 3 | Atr-ERN09770 |  | | | |  | Vvi-Vitvi06g00632\_t001 |  | | | |  |  |  |
| 3 | Atr-ERN09771 |  | Vvi-Vitvi13g00029\_t002 |  | | | |  | | | |  |  |  |
| 3 | Atr-ERN09772 |  | Vvi-Vitvi13g01886\_t002 |  | | | |  | Vvi-Vitvi08g01358\_t001 |  |  |  |
| 3 | Atr-ERN09773 |  | | | |  | | | |  | Vvi-Vitvi08g01359\_t003 |  |  |  |
| 3 | Atr-ERN09774 |  | | | |  | | | |  | Vvi-Vitvi08g01360\_t001 |  |  |  |
| 3 | Atr-ERN09775 |  | | | |  | | | |  | | | |  |  |  |
| 3 | Atr-ERN09776 |  | | | |  | | | |  | | | |  |  |  |
| 3 | Atr-ERN09777 |  | | | |  | | | |  | | | |  |  |  |
| 3 | Atr-ERN09778 |  | | | |  | | | |  | | | |  |  |  |
| 3 | Atr-ERN09779 |  | | | |  | | | |  | | | |  |  |  |
| 3 | Atr-ERN09780 |  | | | |  | Vvi-Vitvi06g00633\_t001 |  | | | |  |  |  |
| 3 | Atr-ERN09781 |  | Vvi-Vitvi13g00031\_t001 |  | | | |  | Vvi-Vitvi08g02244\_t003 |  |  |  |
| 3 | Atr-ERN09782 |  | Vvi-Vitvi13g00033\_t002 |  | | | |  | | | |  |  |  |
| 3 | Atr-ERN09783 |  | | | |  | Vvi-Vitvi06g01748\_t001 |  | Vvi-Vitvi08g01361\_t006 |  |  |  |
| 3 | Atr-ERN09784 |  | | | |  | | | |  | | | |  |  |  |
| 3 | Atr-ERN09785 |  | | | |  | | | |  | | | |  |  |  |
| 3 | Atr-ERN09786 |  | | | |  | Vvi-Vitvi06g00634\_t001 |  | | | |  |  |  |
| 3 | Atr-ERN09787 |  | | | |  | | | |  | | | |  |  |  |
| 3 | Atr-ERN09788 |  | | | |  | Vvi-Vitvi06g00635\_t001 |  | | | |  |  |  |
| 3 | Atr-ERN09789 |  | | | |  | Vvi-Vitvi06g04214\_t001 |  | | | |  |  |  |
| 3 | Atr-ERN09790 |  | | | |  | | | |  | | | |  |  |  |
| 3 | Atr-ERN09791 |  | | | |  | | | |  | | | |  |  |  |
| 3 | Atr-ERN09792 |  | | | |  | | | |  | | | |  |  |  |
| 3 | Atr-ERN09793 |  | | | |  | | | |  | Vvi-Vitvi08g01363\_t001 |  |  |  |
| 3 | Atr-ERN09794 |  | | | |  | Vvi-Vitvi06g00638\_t001 |  | | | |  |  |  |
| 3 | Atr-ERN09795 |  | | | |  | | | |  | Vvi-Vitvi08g01364\_t001 |  |  |  |
| 3 | Atr-ERN09796 |  | | | |  | Vvi-Vitvi06g00639\_t001 |  | Vvi-Vitvi08g02245\_t002 |  |  |  |
| 3 | Atr-ERN09797 |  | | | |  | | | |  | | | |  |  |  |
| 3 | Atr-ERN09798 |  | Vvi-Vitvi13g00036\_t001 |  | | | |  | | | |  |  |  |
| 3 | Atr-ERN09799 |  | | | |  | | | |  | Vvi-Vitvi08g01365\_t001 |  |  |  |
| 3 | Atr-ERN09800 |  | | | |  | Vvi-Vitvi06g00640\_t001 |  | | | |  |  |  |
| 3 | Atr-ERN09801 |  | Vvi-Vitvi13g00037\_t001 |  | | | |  | Vvi-Vitvi08g01366\_t001 |  |  |  |
| 3 | Atr-ERN09802 |  | | | |  | | | |  | Vvi-Vitvi08g01367\_t001 |  |  |  |
| 2 | Atr-ERN09803 |  | | | |  | | | |  |  |  |  |
| 2 | Atr-ERN09804 |  | Vvi-Vitvi13g01892\_t001 |  | | | |  |  |  |  |
| 2 | Atr-ERN09805 |  | | | |  | Vvi-Vitvi06g00641\_t001 |  |  |  |  |
| 2 | Atr-ERN09806 |  | | | |  | | | |  |  |  |  |
| 2 | Atr-ERN09807 |  | Vvi-Vitvi13g00046\_t001 |  | Vvi-Vitvi06g00643\_t001 |  |  |  |  |
| 1 | Atr-ERN09808 |  |  |  | | | |  |  |  |  |
| 1 | Atr-ERN09809 |  |  |  | | | |  |  |  |  |
| 1 | Atr-ERN09810 |  |  |  | Vvi-Vitvi06g00644\_t001 |  |  |  |  |
| 1 | Atr-ERN09811 |  |  |  | Vvi-Vitvi06g00645\_t001 |  |  |  |  |
| 0 | Atr-ERN09812 |  |  |  |  |  |  |
| 0 | Atr-ERN09813 |  |  |  |  |  |  |
| 0 | Atr-ERN09814 |  |  |  |  |  |  |
| 0 | Atr-ERN09815 |  |  |  |  |  |  |
| 0 | Atr-ERN09816 |  |  |  |  |  |  |
| 0 | Atr-ERN09817 |  |  |  |  |  |  |
| 0 | Atr-ERN09818 |  |  |  |  |  |  |
| 0 | Atr-ERN09819 |  |  |  |  |  |  |
| 0 | Atr-ERN09820 |  |  |  |  |  |  |
| 0 | Atr-ERN09821 |  |  |  |  |  |  |
| 0 | Atr-ERN09822 |  |  |  |  |  |  |
