# Supplementary material for: Genome-wide identification, characterization and gene expression of BES1 transcription factor family in grapevine (Vitis vinifera L.)
Source: Sci Rep. 2023 Jan 5;13:240. doi: 10.1038/s41598-022-24407-y (PMC9816167; doi:10.1038/s41598-022-24407-y)
Supplement: Supplementary file 3 — Supplementary Information. [file 41598_2022_24407_MOESM3_ESM.zip › Vvi_Atr/Vitis_vinifera.PN40024.v4.dna_sm.toplevel.fa.vs.Amborella_trichopoda.AMTR1.0.dna_sm.toplevel.fa.html/Atr-AmTr_v1.0_scaffold00030.html]

|  |  |  |  |  |  |  |  |  |  |  |  |  |  |
| --- | --- | --- | --- | --- | --- | --- | --- | --- | --- | --- | --- | --- | --- |
| Duplication depth | Reference chromosome | Collinear blocks | | | | | | | | | | | |
| 0 | Atr-ERN15987 |  |  |  |  |  |  |
| 0 | Atr-ERN15988 |  |  |  |  |  |  |
| 0 | Atr-ERN15989 |  |  |  |  |  |  |
| 0 | Atr-ERN15990 |  |  |  |  |  |  |
| 0 | Atr-ERN15991 |  |  |  |  |  |  |
| 0 | Atr-ERN15992 |  |  |  |  |  |  |
| 0 | Atr-ERN15993 |  |  |  |  |  |  |
| 0 | Atr-ERN15994 |  |  |  |  |  |  |
| 0 | Atr-ERN15995 |  |  |  |  |  |  |
| 0 | Atr-ERN15996 |  |  |  |  |  |  |
| 1 | Atr-ERN15997 |  | Vvi-Vitvi18g04428\_t001 |  |  |  |  |  |
| 1 | Atr-ERN15998 |  | | | |  |  |  |  |  |
| 1 | Atr-ERN15999 |  | | | |  |  |  |  |  |
| 1 | Atr-ERN16000 |  | | | |  |  |  |  |  |
| 1 | Atr-ERN16001 |  | | | |  |  |  |  |  |
| 2 | Atr-ERN16002 |  | Vvi-Vitvi18g04427\_t001 |  | Vvi-Vitvi04g04474\_t001 |  |  |  |  |
| 2 | Atr-ERN16003 |  | | | |  | Vvi-Vitvi04g01613\_t001 |  |  |  |  |
| 2 | Atr-ERN16004 |  | Vvi-Vitvi18g01659\_t001 |  | | | |  |  |  |  |
| 2 | Atr-ERN16005 |  | | | |  | | | |  |  |  |  |
| 2 | Atr-ERN16006 |  | | | |  | | | |  |  |  |  |
| 2 | Atr-ERN16007 |  | Vvi-Vitvi18g01657\_t001 |  | | | |  |  |  |  |
| 2 | Atr-ERN16008 |  | | | |  | | | |  |  |  |  |
| 2 | Atr-ERN16009 |  | | | |  | | | |  |  |  |  |
| 2 | Atr-ERN16010 |  | | | |  | | | |  |  |  |  |
| 2 | Atr-ERN16011 |  | | | |  | | | |  |  |  |  |
| 2 | Atr-ERN16012 |  | | | |  | | | |  |  |  |  |
| 2 | Atr-ERN16013 |  | Vvi-Vitvi18g01655\_t001 |  | | | |  |  |  |  |
| 2 | Atr-ERN16014 |  | | | |  | | | |  |  |  |  |
| 2 | Atr-ERN16015 |  | | | |  | | | |  |  |  |  |
| 2 | Atr-ERN16016 |  | | | |  | | | |  |  |  |  |
| 2 | Atr-ERN16017 |  | | | |  | | | |  |  |  |  |
| 2 | Atr-ERN16018 |  | | | |  | | | |  |  |  |  |
| 2 | Atr-ERN16019 |  | | | |  | | | |  |  |  |  |
| 2 | Atr-ERN16020 |  | Vvi-Vitvi18g01649\_t001 |  | | | |  |  |  |  |
| 1 | Atr-ERN16021 |  |  |  | | | |  |  |  |  |
| 1 | Atr-ERN16022 |  |  |  | | | |  |  |  |  |
| 1 | Atr-ERN16023 |  |  |  | | | |  |  |  |  |
| 1 | Atr-ERN16024 |  |  |  | | | |  |  |  |  |
| 1 | Atr-ERN16025 |  |  |  | | | |  |  |  |  |
| 1 | Atr-ERN16026 |  |  |  | | | |  |  |  |  |
| 2 | Atr-ERN16027 |  | Vvi-Vitvi18g01301\_t001 |  | | | |  |  |  |  |
| 2 | Atr-ERN16028 |  | | | |  | | | |  |  |  |  |
| 2 | Atr-ERN16029 |  | | | |  | Vvi-Vitvi04g01622\_t001 |  |  |  |  |
| 2 | Atr-ERN16030 |  | Vvi-Vitvi18g01290\_t002 |  | | | |  |  |  |  |
| 2 | Atr-ERN16031 |  | | | |  | | | |  |  |  |  |
| 2 | Atr-ERN16032 |  | | | |  | | | |  |  |  |  |
| 2 | Atr-ERN16033 |  | | | |  | | | |  |  |  |  |
| 2 | Atr-ERN16034 |  | | | |  | | | |  |  |  |  |
| 2 | Atr-ERN16035 |  | | | |  | | | |  |  |  |  |
| 2 | Atr-ERN16036 |  | | | |  | | | |  |  |  |  |
| 2 | Atr-ERN16037 |  | | | |  | | | |  |  |  |  |
| 2 | Atr-ERN16038 |  | | | |  | | | |  |  |  |  |
| 2 | Atr-ERN16039 |  | | | |  | Vvi-Vitvi04g01635\_t001 |  |  |  |  |
| 2 | Atr-ERN16040 |  | | | |  | | | |  |  |  |  |
| 2 | Atr-ERN16041 |  | | | |  | | | |  |  |  |  |
| 2 | Atr-ERN16042 |  | | | |  | | | |  |  |  |  |
| 2 | Atr-ERN16043 |  | | | |  | | | |  |  |  |  |
| 2 | Atr-ERN16044 |  | | | |  | | | |  |  |  |  |
| 2 | Atr-ERN16045 |  | | | |  | | | |  |  |  |  |
| 2 | Atr-ERN16046 |  | | | |  | | | |  |  |  |  |
| 2 | Atr-ERN16047 |  | | | |  | | | |  |  |  |  |
| 2 | Atr-ERN16048 |  | Vvi-Vitvi18g01281\_t001 |  | Vvi-Vitvi04g02237\_t001 |  |  |  |  |
| 2 | Atr-ERN16049 |  | | | |  | | | |  |  |  |  |
| 2 | Atr-ERN16050 |  | | | |  | | | |  |  |  |  |
| 2 | Atr-ERN16051 |  | | | |  | | | |  |  |  |  |
| 2 | Atr-ERN16052 |  | | | |  | | | |  |  |  |  |
| 2 | Atr-ERN16053 |  | | | |  | | | |  |  |  |  |
| 2 | Atr-ERN16054 |  | | | |  | | | |  |  |  |  |
| 2 | Atr-ERN16055 |  | Vvi-Vitvi18g01277\_t001 |  | | | |  |  |  |  |
| 2 | Atr-ERN16056 |  | | | |  | | | |  |  |  |  |
| 2 | Atr-ERN16057 |  | | | |  | | | |  |  |  |  |
| 2 | Atr-ERN16058 |  | | | |  | | | |  |  |  |  |
| 2 | Atr-ERN16059 |  | | | |  | Vvi-Vitvi04g01640\_t001 |  |  |  |  |
| 2 | Atr-ERN16060 |  | | | |  | | | |  |  |  |  |
| 2 | Atr-ERN16061 |  | | | |  | Vvi-Vitvi04g01641\_t001 |  |  |  |  |
| 2 | Atr-ERN16062 |  | | | |  | | | |  |  |  |  |
| 2 | Atr-ERN16063 |  | | | |  | | | |  |  |  |  |
| 2 | Atr-ERN16064 |  | Vvi-Vitvi18g01276\_t001 |  | Vvi-Vitvi04g02243\_t001 |  |  |  |  |
| 2 | Atr-ERN16065 |  | Vvi-Vitvi18g01275\_t002 |  | | | |  |  |  |  |
| 2 | Atr-ERN16066 |  | | | |  | | | |  |  |  |  |
| 2 | Atr-ERN16067 |  | | | |  | | | |  |  |  |  |
| 2 | Atr-ERN16068 |  | | | |  | | | |  |  |  |  |
| 2 | Atr-ERN16069 |  | | | |  | | | |  |  |  |  |
| 2 | Atr-ERN16070 |  | | | |  | | | |  |  |  |  |
| 2 | Atr-ERN16071 |  | | | |  | | | |  |  |  |  |
| 2 | Atr-ERN16072 |  | | | |  | | | |  |  |  |  |
| 2 | Atr-ERN16073 |  | | | |  | Vvi-Vitvi04g01643\_t001 |  |  |  |  |
| 2 | Atr-ERN16074 |  | Vvi-Vitvi18g01268\_t001 |  | | | |  |  |  |  |
| 2 | Atr-ERN16075 |  | Vvi-Vitvi18g01266\_t001 |  | | | |  |  |  |  |
| 2 | Atr-ERN16076 |  | | | |  | Vvi-Vitvi04g01645\_t001 |  |  |  |  |
| 2 | Atr-ERN16077 |  | Vvi-Vitvi18g01265\_t001 |  | Vvi-Vitvi04g01646\_t001 |  |  |  |  |
| 1 | Atr-ERN16078 |  | Vvi-Vitvi18g01264\_t001 |  |  |  |  |  |
| 2 | Atr-ERN16079 |  | | | |  | Vvi-Vitvi02g00195\_t001 |  |  |  |  |
| 2 | Atr-ERN16080 |  | | | |  | | | |  |  |  |  |
| 2 | Atr-ERN16081 |  | Vvi-Vitvi18g01254\_t001 |  | | | |  |  |  |  |
| 1 | Atr-ERN16082 |  |  |  | Vvi-Vitvi02g00199\_t001 |  |  |  |  |
| 1 | Atr-ERN16083 |  |  |  | | | |  |  |  |  |
| 1 | Atr-ERN16084 |  |  |  | | | |  |  |  |  |
| 3 | Atr-ERN16085 |  | Vvi-Vitvi06g00906\_t001 |  | | | |  | Vvi-Vitvi08g02051\_t001 |  |  |  |
| 3 | Atr-ERN16086 |  | | | |  | | | |  | | | |  |  |  |
| 3 | Atr-ERN16087 |  | | | |  | | | |  | | | |  |  |  |
| 3 | Atr-ERN16088 |  | | | |  | Vvi-Vitvi02g00201\_t001 |  | Vvi-Vitvi08g00632\_t001 |  |  |  |
| 3 | Atr-ERN16089 |  | | | |  | | | |  | | | |  |  |  |
| 3 | Atr-ERN16090 |  | | | |  | | | |  | | | |  |  |  |
| 3 | Atr-ERN16091 |  | Vvi-Vitvi06g00910\_t002 |  | | | |  | | | |  |  |  |
| 3 | Atr-ERN16092 |  | | | |  | | | |  | | | |  |  |  |
| 3 | Atr-ERN16093 |  | | | |  | | | |  | | | |  |  |  |
| 3 | Atr-ERN16094 |  | Vvi-Vitvi06g00918\_t001 |  | | | |  | Vvi-Vitvi08g00633\_t001 |  |  |  |
| 3 | Atr-ERN16095 |  | Vvi-Vitvi06g00920\_t001 |  | Vvi-Vitvi02g00202\_t001 |  | | | |  |  |  |
| 3 | Atr-ERN16096 |  | Vvi-Vitvi06g00922\_t001 |  | Vvi-Vitvi02g00203\_t001 |  | Vvi-Vitvi08g00636\_t001 |  |  |  |
| 3 | Atr-ERN16097 |  | | | |  | | | |  | Vvi-Vitvi08g00637\_t001 |  |  |  |
| 3 | Atr-ERN16098 |  | | | |  | | | |  | | | |  |  |  |
| 3 | Atr-ERN16099 |  | | | |  | | | |  | | | |  |  |  |
| 3 | Atr-ERN16100 |  | | | |  | | | |  | Vvi-Vitvi08g00639\_t001 |  |  |  |
| 3 | Atr-ERN16101 |  | | | |  | | | |  | | | |  |  |  |
| 3 | Atr-ERN16102 |  | | | |  | | | |  | | | |  |  |  |
| 3 | Atr-ERN16103 |  | | | |  | Vvi-Vitvi02g00205\_t001 |  | | | |  |  |  |
| 2 | Atr-ERN16104 |  | | | |  |  |  | Vvi-Vitvi08g00640\_t001 |  |  |  |
| 2 | Atr-ERN16105 |  | | | |  | Vvi-Vitvi13g01043\_t001 |  |  |  |  |
| 3 | Atr-ERN16106 |  | Vvi-Vitvi06g00937\_t001 |  | | | |  | Vvi-Vitvi08g01370\_t001 |  |  |  |
| 3 | Atr-ERN16107 |  | Vvi-Vitvi06g00946\_t001 |  | | | |  | | | |  |  |  |
| 2 | Atr-ERN16108 |  |  |  | | | |  | | | |  |  |  |
| 2 | Atr-ERN16109 |  |  |  | | | |  | | | |  |  |  |
| 2 | Atr-ERN16110 |  |  |  | | | |  | | | |  |  |  |
| 2 | Atr-ERN16111 |  |  |  | Vvi-Vitvi13g01031\_t001 |  | Vvi-Vitvi08g01371\_t001 |  |  |  |
| 2 | Atr-ERN16112 |  |  |  | | | |  | | | |  |  |  |
| 2 | Atr-ERN16113 |  |  |  | | | |  | | | |  |  |  |
| 2 | Atr-ERN16114 |  |  |  | | | |  | | | |  |  |  |
| 2 | Atr-ERN16115 |  |  |  | | | |  | | | |  |  |  |
| 2 | Atr-ERN16116 |  |  |  | | | |  | | | |  |  |  |
| 2 | Atr-ERN16117 |  |  |  | | | |  | | | |  |  |  |
| 2 | Atr-ERN16118 |  |  |  | | | |  | | | |  |  |  |
| 2 | Atr-ERN16119 |  |  |  | | | |  | | | |  |  |  |
| 2 | Atr-ERN16120 |  |  |  | | | |  | | | |  |  |  |
| 2 | Atr-ERN16121 |  |  |  | | | |  | | | |  |  |  |
| 2 | Atr-ERN16122 |  |  |  | | | |  | | | |  |  |  |
| 2 | Atr-ERN16123 |  |  |  | | | |  | | | |  |  |  |
| 2 | Atr-ERN16124 |  |  |  | | | |  | | | |  |  |  |
| 2 | Atr-ERN16125 |  |  |  | Vvi-Vitvi13g01022\_t001 |  | | | |  |  |  |
| 2 | Atr-ERN16126 |  |  |  | | | |  | | | |  |  |  |
| 2 | Atr-ERN16127 |  |  |  | Vvi-Vitvi13g01021\_t001 |  | | | |  |  |  |
| 2 | Atr-ERN16128 |  |  |  | | | |  | | | |  |  |  |
| 2 | Atr-ERN16129 |  |  |  | | | |  | | | |  |  |  |
| 2 | Atr-ERN16130 |  |  |  | | | |  | | | |  |  |  |
| 2 | Atr-ERN16131 |  |  |  | | | |  | | | |  |  |  |
| 2 | Atr-ERN16132 |  |  |  | | | |  | | | |  |  |  |
| 2 | Atr-ERN16133 |  |  |  | | | |  | | | |  |  |  |
| 2 | Atr-ERN16134 |  |  |  | | | |  | | | |  |  |  |
| 2 | Atr-ERN16135 |  |  |  | | | |  | | | |  |  |  |
| 2 | Atr-ERN16136 |  |  |  | | | |  | Vvi-Vitvi08g01373\_t001 |  |  |  |
| 2 | Atr-ERN16137 |  |  |  | | | |  | Vvi-Vitvi08g01374\_t001 |  |  |  |
| 2 | Atr-ERN16138 |  |  |  | | | |  | | | |  |  |  |
| 2 | Atr-ERN16139 |  |  |  | | | |  | | | |  |  |  |
| 2 | Atr-ERN16140 |  |  |  | | | |  | | | |  |  |  |
| 2 | Atr-ERN16141 |  |  |  | Vvi-Vitvi13g01008\_t001 |  | Vvi-Vitvi08g01375\_t001 |  |  |  |
| 3 | Atr-ERN16142 |  | Vvi-Vitvi06g01560\_t001 |  | | | |  | | | |  |  |  |
| 3 | Atr-ERN16143 |  | Vvi-Vitvi06g01559\_t001 |  | | | |  | Vvi-Vitvi08g01376\_t001 |  |  |  |
| 3 | Atr-ERN16144 |  | | | |  | | | |  | | | |  |  |  |
| 3 | Atr-ERN16145 |  | | | |  | | | |  | | | |  |  |  |
| 3 | Atr-ERN16146 |  | | | |  | | | |  | Vvi-Vitvi08g01377\_t001 |  |  |  |
| 3 | Atr-ERN16147 |  | | | |  | | | |  | | | |  |  |  |
| 3 | Atr-ERN16148 |  | | | |  | | | |  | | | |  |  |  |
| 3 | Atr-ERN16149 |  | | | |  | | | |  | | | |  |  |  |
| 3 | Atr-ERN16150 |  | | | |  | Vvi-Vitvi13g01007\_t001 |  | | | |  |  |  |
| 3 | Atr-ERN16151 |  | | | |  | | | |  | Vvi-Vitvi08g02246\_t001 |  |  |  |
| 3 | Atr-ERN16152 |  | | | |  | | | |  | | | |  |  |  |
| 3 | Atr-ERN16153 |  | | | |  | | | |  | | | |  |  |  |
| 3 | Atr-ERN16154 |  | | | |  | | | |  | | | |  |  |  |
| 3 | Atr-ERN16155 |  | | | |  | Vvi-Vitvi13g01006\_t001 |  | Vvi-Vitvi08g01378\_t001 |  |  |  |
| 2 | Atr-ERN16156 |  | | | |  |  |  | | | |  |  |  |
| 2 | Atr-ERN16157 |  | | | |  |  |  | Vvi-Vitvi08g01379\_t001 |  |  |  |
| 2 | Atr-ERN16158 |  | | | |  |  |  | | | |  |  |  |
| 2 | Atr-ERN16159 |  | Vvi-Vitvi06g01556\_t001 |  |  |  | Vvi-Vitvi08g04284\_t001 |  |  |  |
| 2 | Atr-ERN16160 |  | | | |  |  |  | Vvi-Vitvi08g01385\_t001 |  |  |  |
| 2 | Atr-ERN16161 |  | | | |  |  |  | | | |  |  |  |
| 2 | Atr-ERN16162 |  | | | |  |  |  | | | |  |  |  |
| 2 | Atr-ERN16163 |  | | | |  |  |  | | | |  |  |  |
| 2 | Atr-ERN16164 |  | | | |  |  |  | | | |  |  |  |
| 2 | Atr-ERN16165 |  | | | |  |  |  | | | |  |  |  |
| 2 | Atr-ERN16166 |  | | | |  |  |  | Vvi-Vitvi08g01386\_t001 |  |  |  |
| 2 | Atr-ERN16167 |  | Vvi-Vitvi06g01553\_t001 |  |  |  | | | |  |  |  |
| 2 | Atr-ERN16168 |  | | | |  |  |  | Vvi-Vitvi08g01387\_t001 |  |  |  |
| 2 | Atr-ERN16169 |  | | | |  |  |  | | | |  |  |  |
| 2 | Atr-ERN16170 |  | | | |  |  |  | | | |  |  |  |
| 2 | Atr-ERN16171 |  | | | |  |  |  | | | |  |  |  |
| 2 | Atr-ERN16172 |  | Vvi-Vitvi06g01552\_t001 |  |  |  | Vvi-Vitvi08g01388\_t001 |  |  |  |
| 2 | Atr-ERN16173 |  | Vvi-Vitvi06g01551\_t001 |  |  |  | | | |  |  |  |
| 2 | Atr-ERN16174 |  | | | |  |  |  | | | |  |  |  |
| 2 | Atr-ERN16175 |  | | | |  |  |  | | | |  |  |  |
| 2 | Atr-ERN16176 |  | | | |  |  |  | Vvi-Vitvi08g01389\_t001 |  |  |  |
| 2 | Atr-ERN16177 |  | | | |  |  |  | Vvi-Vitvi08g02251\_t001.1.6037826f |  |  |  |
| 2 | Atr-ERN16178 |  | | | |  |  |  | | | |  |  |  |
| 2 | Atr-ERN16179 |  | | | |  |  |  | Vvi-Vitvi08g01390\_t001 |  |  |  |
| 2 | Atr-ERN16180 |  | | | |  |  |  | | | |  |  |  |
| 2 | Atr-ERN16181 |  | Vvi-Vitvi06g01549\_t001 |  |  |  | Vvi-Vitvi08g01391\_t001 |  |  |  |
| 2 | Atr-ERN16182 |  | | | |  |  |  | | | |  |  |  |
| 2 | Atr-ERN16183 |  | Vvi-Vitvi06g01548\_t001 |  |  |  | Vvi-Vitvi08g01392\_t001 |  |  |  |
| 2 | Atr-ERN16184 |  | | | |  |  |  | | | |  |  |  |
| 2 | Atr-ERN16185 |  | | | |  |  |  | | | |  |  |  |
| 2 | Atr-ERN16186 |  | Vvi-Vitvi06g01546\_t001 |  |  |  | Vvi-Vitvi08g01393\_t001 |  |  |  |
| 2 | Atr-ERN16187 |  | | | |  |  |  | | | |  |  |  |
| 2 | Atr-ERN16188 |  | | | |  |  |  | | | |  |  |  |
| 2 | Atr-ERN16189 |  | Vvi-Vitvi06g01545\_t001 |  |  |  | Vvi-Vitvi08g01394\_t001 |  |  |  |
| 2 | Atr-ERN16190 |  | | | |  |  |  | Vvi-Vitvi08g02252\_t001 |  |  |  |
| 2 | Atr-ERN16191 |  | Vvi-Vitvi06g01543\_t001 |  |  |  | Vvi-Vitvi08g01395\_t001 |  |  |  |
| 2 | Atr-ERN16192 |  | | | |  |  |  | | | |  |  |  |
| 2 | Atr-ERN16193 |  | | | |  |  |  | | | |  |  |  |
| 2 | Atr-ERN16194 |  | | | |  |  |  | | | |  |  |  |
| 2 | Atr-ERN16195 |  | | | |  |  |  | | | |  |  |  |
| 2 | Atr-ERN16196 |  | | | |  |  |  | Vvi-Vitvi08g01396\_t001 |  |  |  |
| 2 | Atr-ERN16197 |  | | | |  |  |  | | | |  |  |  |
| 2 | Atr-ERN16198 |  | | | |  |  |  | | | |  |  |  |
| 2 | Atr-ERN16199 |  | | | |  |  |  | | | |  |  |  |
| 2 | Atr-ERN16200 |  | | | |  |  |  | | | |  |  |  |
| 2 | Atr-ERN16201 |  | | | |  |  |  | | | |  |  |  |
| 2 | Atr-ERN16202 |  | | | |  |  |  | Vvi-Vitvi08g01397\_t001 |  |  |  |
| 2 | Atr-ERN16203 |  | | | |  |  |  | | | |  |  |  |
| 2 | Atr-ERN16204 |  | Vvi-Vitvi06g01537\_t001 |  |  |  | Vvi-Vitvi08g02253\_t001 |  |  |  |
| 2 | Atr-ERN16205 |  | | | |  |  |  | | | |  |  |  |
| 2 | Atr-ERN16206 |  | | | |  |  |  | | | |  |  |  |
| 2 | Atr-ERN16207 |  | | | |  |  |  | | | |  |  |  |
| 2 | Atr-ERN16208 |  | | | |  |  |  | Vvi-Vitvi08g01406\_t002 |  |  |  |
| 2 | Atr-ERN16209 |  | | | |  |  |  | | | |  |  |  |
| 2 | Atr-ERN16210 |  | Vvi-Vitvi06g01533\_t001 |  |  |  | | | |  |  |  |
| 2 | Atr-ERN16211 |  | | | |  |  |  | | | |  |  |  |
| 2 | Atr-ERN16212 |  | | | |  |  |  | | | |  |  |  |
| 2 | Atr-ERN16213 |  | | | |  |  |  | | | |  |  |  |
| 2 | Atr-ERN16214 |  | | | |  |  |  | Vvi-Vitvi08g01411\_t001 |  |  |  |
| 1 | Atr-ERN16215 |  | Vvi-Vitvi06g01531\_t001 |  |  |  |  |  |
