# Supplementary material for: Genome-wide identification, characterization and gene expression of BES1 transcription factor family in grapevine (Vitis vinifera L.)
Source: Sci Rep. 2023 Jan 5;13:240. doi: 10.1038/s41598-022-24407-y (PMC9816167; doi:10.1038/s41598-022-24407-y)
Supplement: Supplementary file 3 — Supplementary Information. [file 41598_2022_24407_MOESM3_ESM.zip › Vvi_Atr/Vitis_vinifera.PN40024.v4.dna_sm.toplevel.fa.vs.Amborella_trichopoda.AMTR1.0.dna_sm.toplevel.fa.html/Atr-AmTr_v1.0_scaffold00031.html]

|  |  |  |  |  |  |  |  |  |  |  |  |  |  |
| --- | --- | --- | --- | --- | --- | --- | --- | --- | --- | --- | --- | --- | --- |
| Duplication depth | Reference chromosome | Collinear blocks | | | | | | | | | | | |
| 0 | Atr-ERN16482 |  |  |  |  |  |  |
| 0 | Atr-ERN16483 |  |  |  |  |  |  |
| 1 | Atr-ERN16484 |  | Vvi-Vitvi08g01469\_t001 |  |  |  |  |  |
| 1 | Atr-ERN16485 |  | Vvi-Vitvi08g01467\_t001 |  |  |  |  |  |
| 1 | Atr-ERN16486 |  | Vvi-Vitvi08g01464\_t001 |  |  |  |  |  |
| 1 | Atr-ERN16487 |  | | | |  |  |  |  |  |
| 1 | Atr-ERN16488 |  | | | |  |  |  |  |  |
| 1 | Atr-ERN16489 |  | | | |  |  |  |  |  |
| 1 | Atr-ERN16490 |  | | | |  |  |  |  |  |
| 1 | Atr-ERN16491 |  | Vvi-Vitvi08g02265\_t001 |  |  |  |  |  |
| 1 | Atr-ERN16492 |  | | | |  |  |  |  |  |
| 1 | Atr-ERN16493 |  | | | |  |  |  |  |  |
| 1 | Atr-ERN16494 |  | | | |  |  |  |  |  |
| 1 | Atr-ERN16495 |  | | | |  |  |  |  |  |
| 1 | Atr-ERN16496 |  | | | |  |  |  |  |  |
| 1 | Atr-ERN16497 |  | | | |  |  |  |  |  |
| 1 | Atr-ERN16498 |  | | | |  |  |  |  |  |
| 1 | Atr-ERN16499 |  | | | |  |  |  |  |  |
| 1 | Atr-ERN16500 |  | | | |  |  |  |  |  |
| 1 | Atr-ERN16501 |  | | | |  |  |  |  |  |
| 1 | Atr-ERN16502 |  | | | |  |  |  |  |  |
| 1 | Atr-ERN16503 |  | | | |  |  |  |  |  |
| 1 | Atr-ERN16504 |  | | | |  |  |  |  |  |
| 1 | Atr-ERN16505 |  | | | |  |  |  |  |  |
| 1 | Atr-ERN16506 |  | | | |  |  |  |  |  |
| 1 | Atr-ERN16507 |  | Vvi-Vitvi08g01459\_t001 |  |  |  |  |  |
| 1 | Atr-ERN16508 |  | | | |  |  |  |  |  |
| 1 | Atr-ERN16509 |  | | | |  |  |  |  |  |
| 1 | Atr-ERN16510 |  | | | |  |  |  |  |  |
| 1 | Atr-ERN16511 |  | | | |  |  |  |  |  |
| 1 | Atr-ERN16512 |  | Vvi-Vitvi08g01456\_t001 |  |  |  |  |  |
| 1 | Atr-ERN16513 |  | | | |  |  |  |  |  |
| 1 | Atr-ERN16514 |  | | | |  |  |  |  |  |
| 1 | Atr-ERN16515 |  | | | |  |  |  |  |  |
| 1 | Atr-ERN16516 |  | | | |  |  |  |  |  |
| 1 | Atr-ERN16517 |  | | | |  |  |  |  |  |
| 1 | Atr-ERN16518 |  | | | |  |  |  |  |  |
| 1 | Atr-ERN16519 |  | | | |  |  |  |  |  |
| 1 | Atr-ERN16520 |  | | | |  |  |  |  |  |
| 1 | Atr-ERN16521 |  | | | |  |  |  |  |  |
| 1 | Atr-ERN16522 |  | | | |  |  |  |  |  |
| 1 | Atr-ERN16523 |  | | | |  |  |  |  |  |
| 1 | Atr-ERN16524 |  | | | |  |  |  |  |  |
| 1 | Atr-ERN16525 |  | | | |  |  |  |  |  |
| 1 | Atr-ERN16526 |  | | | |  |  |  |  |  |
| 1 | Atr-ERN16527 |  | | | |  |  |  |  |  |
| 1 | Atr-ERN16528 |  | | | |  |  |  |  |  |
| 1 | Atr-ERN16529 |  | | | |  |  |  |  |  |
| 1 | Atr-ERN16530 |  | | | |  |  |  |  |  |
| 1 | Atr-ERN16531 |  | | | |  |  |  |  |  |
| 1 | Atr-ERN16532 |  | Vvi-Vitvi08g01455\_t001 |  |  |  |  |  |
| 1 | Atr-ERN16533 |  | | | |  |  |  |  |  |
| 1 | Atr-ERN16534 |  | | | |  |  |  |  |  |
| 1 | Atr-ERN16535 |  | | | |  |  |  |  |  |
| 1 | Atr-ERN16536 |  | | | |  |  |  |  |  |
| 1 | Atr-ERN16537 |  | Vvi-Vitvi08g01451\_t001 |  |  |  |  |  |
| 1 | Atr-ERN16538 |  | | | |  |  |  |  |  |
| 1 | Atr-ERN16539 |  | | | |  |  |  |  |  |
| 1 | Atr-ERN16540 |  | | | |  |  |  |  |  |
| 1 | Atr-ERN16541 |  | | | |  |  |  |  |  |
| 1 | Atr-ERN16542 |  | | | |  |  |  |  |  |
| 1 | Atr-ERN16543 |  | | | |  |  |  |  |  |
| 1 | Atr-ERN16544 |  | | | |  |  |  |  |  |
| 1 | Atr-ERN16545 |  | | | |  |  |  |  |  |
| 1 | Atr-ERN16546 |  | | | |  |  |  |  |  |
| 1 | Atr-ERN16547 |  | | | |  |  |  |  |  |
| 1 | Atr-ERN16548 |  | | | |  |  |  |  |  |
| 1 | Atr-ERN16549 |  | | | |  |  |  |  |  |
| 1 | Atr-ERN16550 |  | | | |  |  |  |  |  |
| 1 | Atr-ERN16551 |  | Vvi-Vitvi08g01450\_t002 |  |  |  |  |  |
| 1 | Atr-ERN16552 |  | | | |  |  |  |  |  |
| 1 | Atr-ERN16553 |  | | | |  |  |  |  |  |
| 1 | Atr-ERN16554 |  | | | |  |  |  |  |  |
| 1 | Atr-ERN16555 |  | Vvi-Vitvi08g01448\_t001 |  |  |  |  |  |
| 1 | Atr-ERN16556 |  | | | |  |  |  |  |  |
| 1 | Atr-ERN16557 |  | | | |  |  |  |  |  |
| 1 | Atr-ERN16558 |  | | | |  |  |  |  |  |
| 1 | Atr-ERN16559 |  | | | |  |  |  |  |  |
| 1 | Atr-ERN16560 |  | | | |  |  |  |  |  |
| 1 | Atr-ERN16561 |  | | | |  |  |  |  |  |
| 1 | Atr-ERN16562 |  | | | |  |  |  |  |  |
| 1 | Atr-ERN16563 |  | | | |  |  |  |  |  |
| 1 | Atr-ERN16564 |  | | | |  |  |  |  |  |
| 1 | Atr-ERN16565 |  | | | |  |  |  |  |  |
| 1 | Atr-ERN16566 |  | | | |  |  |  |  |  |
| 1 | Atr-ERN16567 |  | | | |  |  |  |  |  |
| 1 | Atr-ERN16568 |  | | | |  |  |  |  |  |
| 1 | Atr-ERN16569 |  | | | |  |  |  |  |  |
| 1 | Atr-ERN16570 |  | | | |  |  |  |  |  |
| 1 | Atr-ERN16571 |  | | | |  |  |  |  |  |
| 1 | Atr-ERN16572 |  | | | |  |  |  |  |  |
| 1 | Atr-ERN16573 |  | | | |  |  |  |  |  |
| 1 | Atr-ERN16574 |  | | | |  |  |  |  |  |
| 1 | Atr-ERN16575 |  | | | |  |  |  |  |  |
| 1 | Atr-ERN16576 |  | | | |  |  |  |  |  |
| 1 | Atr-ERN16577 |  | | | |  |  |  |  |  |
| 1 | Atr-ERN16578 |  | | | |  |  |  |  |  |
| 1 | Atr-ERN16579 |  | | | |  |  |  |  |  |
| 1 | Atr-ERN16580 |  | Vvi-Vitvi08g01444\_t001 |  |  |  |  |  |
| 0 | Atr-ERN16581 |  |  |  |  |  |  |
| 0 | Atr-ERN16582 |  |  |  |  |  |  |
| 0 | Atr-ERN16583 |  |  |  |  |  |  |
| 0 | Atr-ERN16584 |  |  |  |  |  |  |
| 0 | Atr-ERN16585 |  |  |  |  |  |  |
| 0 | Atr-ERN16586 |  |  |  |  |  |  |
| 0 | Atr-ERN16587 |  |  |  |  |  |  |
| 0 | Atr-ERN16588 |  |  |  |  |  |  |
| 0 | Atr-ERN16589 |  |  |  |  |  |  |
| 0 | Atr-ERN16590 |  |  |  |  |  |  |
| 0 | Atr-ERN16591 |  |  |  |  |  |  |
| 0 | Atr-ERN16592 |  |  |  |  |  |  |
| 0 | Atr-ERN16593 |  |  |  |  |  |  |
| 0 | Atr-ERN16594 |  |  |  |  |  |  |
| 0 | Atr-ERN16595 |  |  |  |  |  |  |
| 0 | Atr-ERN16596 |  |  |  |  |  |  |
