# Supplementary material for: Genome-wide identification, characterization and gene expression of BES1 transcription factor family in grapevine (Vitis vinifera L.)
Source: Sci Rep. 2023 Jan 5;13:240. doi: 10.1038/s41598-022-24407-y (PMC9816167; doi:10.1038/s41598-022-24407-y)
Supplement: Supplementary file 3 — Supplementary Information. [file 41598_2022_24407_MOESM3_ESM.zip › Vvi_Atr/Vitis_vinifera.PN40024.v4.dna_sm.toplevel.fa.vs.Amborella_trichopoda.AMTR1.0.dna_sm.toplevel.fa.html/Atr-AmTr_v1.0_scaffold00032.html]

|  |  |  |  |  |  |  |  |  |  |  |  |  |  |
| --- | --- | --- | --- | --- | --- | --- | --- | --- | --- | --- | --- | --- | --- |
| Duplication depth | Reference chromosome | Collinear blocks | | | | | | | | | | | |
| 1 | Atr-ERN14754 |  | Vvi-Vitvi10g01077\_t001 |  |  |  |  |  |
| 1 | Atr-ERN14755 |  | Vvi-Vitvi10g01078\_t001 |  |  |  |  |  |
| 1 | Atr-ERN14756 |  | | | |  |  |  |  |  |
| 1 | Atr-ERN14757 |  | | | |  |  |  |  |  |
| 1 | Atr-ERN14758 |  | Vvi-Vitvi10g01940\_t001 |  |  |  |  |  |
| 1 | Atr-ERN14759 |  | Vvi-Vitvi10g01084\_t001 |  |  |  |  |  |
| 1 | Atr-ERN14760 |  | Vvi-Vitvi10g01942\_t001 |  |  |  |  |  |
| 1 | Atr-ERN14761 |  | | | |  |  |  |  |  |
| 1 | Atr-ERN14762 |  | | | |  |  |  |  |  |
| 1 | Atr-ERN14763 |  | Vvi-Vitvi10g01086\_t001 |  |  |  |  |  |
| 1 | Atr-ERN14764 |  | Vvi-Vitvi10g04519\_t002 |  |  |  |  |  |
| 1 | Atr-ERN14765 |  | | | |  |  |  |  |  |
| 1 | Atr-ERN14766 |  | | | |  |  |  |  |  |
| 1 | Atr-ERN14767 |  | | | |  |  |  |  |  |
| 1 | Atr-ERN14768 |  | Vvi-Vitvi10g01092\_t001 |  |  |  |  |  |
| 0 | Atr-ERN14769 |  |  |  |  |  |  |
| 0 | Atr-ERN14770 |  |  |  |  |  |  |
| 0 | Atr-ERN14771 |  |  |  |  |  |  |
| 0 | Atr-ERN14772 |  |  |  |  |  |  |
| 0 | Atr-ERN14773 |  |  |  |  |  |  |
| 0 | Atr-ERN14774 |  |  |  |  |  |  |
| 0 | Atr-ERN14775 |  |  |  |  |  |  |
| 0 | Atr-ERN14776 |  |  |  |  |  |  |
| 0 | Atr-ERN14777 |  |  |  |  |  |  |
| 0 | Atr-ERN14778 |  |  |  |  |  |  |
| 0 | Atr-ERN14779 |  |  |  |  |  |  |
| 0 | Atr-ERN14780 |  |  |  |  |  |  |
| 0 | Atr-ERN14781 |  |  |  |  |  |  |
| 0 | Atr-ERN14782 |  |  |  |  |  |  |
| 0 | Atr-ERN14783 |  |  |  |  |  |  |
| 0 | Atr-ERN14784 |  |  |  |  |  |  |
| 0 | Atr-ERN14785 |  |  |  |  |  |  |
| 0 | Atr-ERN14786 |  |  |  |  |  |  |
| 0 | Atr-ERN14787 |  |  |  |  |  |  |
| 0 | Atr-ERN14788 |  |  |  |  |  |  |
| 0 | Atr-ERN14789 |  |  |  |  |  |  |
| 0 | Atr-ERN14790 |  |  |  |  |  |  |
| 0 | Atr-ERN14791 |  |  |  |  |  |  |
| 0 | Atr-ERN14792 |  |  |  |  |  |  |
| 0 | Atr-ERN14793 |  |  |  |  |  |  |
| 0 | Atr-ERN14794 |  |  |  |  |  |  |
| 0 | Atr-ERN14795 |  |  |  |  |  |  |
| 0 | Atr-ERN14796 |  |  |  |  |  |  |
| 0 | Atr-ERN14797 |  |  |  |  |  |  |
| 0 | Atr-ERN14798 |  |  |  |  |  |  |
| 0 | Atr-ERN14799 |  |  |  |  |  |  |
| 0 | Atr-ERN14800 |  |  |  |  |  |  |
| 0 | Atr-ERN14801 |  |  |  |  |  |  |
| 0 | Atr-ERN14802 |  |  |  |  |  |  |
| 0 | Atr-ERN14803 |  |  |  |  |  |  |
| 0 | Atr-ERN14804 |  |  |  |  |  |  |
| 0 | Atr-ERN14805 |  |  |  |  |  |  |
| 0 | Atr-ERN14806 |  |  |  |  |  |  |
| 0 | Atr-ERN14807 |  |  |  |  |  |  |
| 0 | Atr-ERN14808 |  |  |  |  |  |  |
| 0 | Atr-ERN14809 |  |  |  |  |  |  |
| 0 | Atr-ERN14810 |  |  |  |  |  |  |
| 0 | Atr-ERN14811 |  |  |  |  |  |  |
| 0 | Atr-ERN14812 |  |  |  |  |  |  |
| 0 | Atr-ERN14813 |  |  |  |  |  |  |
| 0 | Atr-ERN14814 |  |  |  |  |  |  |
| 1 | Atr-ERN14815 |  | Vvi-Vitvi10g01359\_t001 |  |  |  |  |  |
| 1 | Atr-ERN14816 |  | | | |  |  |  |  |  |
| 1 | Atr-ERN14817 |  | Vvi-Vitvi10g01351\_t001 |  |  |  |  |  |
| 1 | Atr-ERN14818 |  | Vvi-Vitvi10g01350\_t001 |  |  |  |  |  |
| 1 | Atr-ERN14819 |  | | | |  |  |  |  |  |
| 1 | Atr-ERN14820 |  | | | |  |  |  |  |  |
| 1 | Atr-ERN14821 |  | | | |  |  |  |  |  |
| 1 | Atr-ERN14822 |  | | | |  |  |  |  |  |
| 1 | Atr-ERN14823 |  | | | |  |  |  |  |  |
| 1 | Atr-ERN14824 |  | Vvi-Vitvi10g01336\_t001 |  |  |  |  |  |
| 1 | Atr-ERN14825 |  | Vvi-Vitvi10g01335\_t001 |  |  |  |  |  |
| 1 | Atr-ERN14826 |  | | | |  |  |  |  |  |
| 1 | Atr-ERN14827 |  | | | |  |  |  |  |  |
| 1 | Atr-ERN14828 |  | Vvi-Vitvi10g01325\_t001 |  |  |  |  |  |
| 1 | Atr-ERN14829 |  | | | |  |  |  |  |  |
| 1 | Atr-ERN14830 |  | Vvi-Vitvi10g01319\_t001 |  |  |  |  |  |
| 0 | Atr-ERN14831 |  |  |  |  |  |  |
| 0 | Atr-ERN14832 |  |  |  |  |  |  |
| 0 | Atr-ERN14833 |  |  |  |  |  |  |
| 0 | Atr-ERN14834 |  |  |  |  |  |  |
| 0 | Atr-ERN14835 |  |  |  |  |  |  |
| 0 | Atr-ERN14836 |  |  |  |  |  |  |
| 0 | Atr-ERN14837 |  |  |  |  |  |  |
| 0 | Atr-ERN14838 |  |  |  |  |  |  |
| 0 | Atr-ERN14839 |  |  |  |  |  |  |
| 0 | Atr-ERN14840 |  |  |  |  |  |  |
| 0 | Atr-ERN14841 |  |  |  |  |  |  |
| 1 | Atr-ERN14842 |  | Vvi-Vitvi10g01248\_t001 |  |  |  |  |  |
| 1 | Atr-ERN14843 |  | | | |  |  |  |  |  |
| 1 | Atr-ERN14844 |  | | | |  |  |  |  |  |
| 1 | Atr-ERN14845 |  | | | |  |  |  |  |  |
| 1 | Atr-ERN14846 |  | | | |  |  |  |  |  |
| 1 | Atr-ERN14847 |  | Vvi-Vitvi10g01978\_t001 |  |  |  |  |  |
| 1 | Atr-ERN14848 |  | | | |  |  |  |  |  |
| 1 | Atr-ERN14849 |  | Vvi-Vitvi10g01245\_t001 |  |  |  |  |  |
| 1 | Atr-ERN14850 |  | | | |  |  |  |  |  |
| 1 | Atr-ERN14851 |  | | | |  |  |  |  |  |
| 1 | Atr-ERN14852 |  | | | |  |  |  |  |  |
| 1 | Atr-ERN14853 |  | | | |  |  |  |  |  |
| 1 | Atr-ERN14854 |  | Vvi-Vitvi10g01972\_t001 |  |  |  |  |  |
| 1 | Atr-ERN14855 |  | | | |  |  |  |  |  |
| 1 | Atr-ERN14856 |  | | | |  |  |  |  |  |
| 1 | Atr-ERN14857 |  | | | |  |  |  |  |  |
| 1 | Atr-ERN14858 |  | | | |  |  |  |  |  |
| 1 | Atr-ERN14859 |  | Vvi-Vitvi10g01236\_t001 |  |  |  |  |  |
| 1 | Atr-ERN14860 |  | | | |  |  |  |  |  |
| 1 | Atr-ERN14861 |  | | | |  |  |  |  |  |
| 1 | Atr-ERN14862 |  | | | |  |  |  |  |  |
| 1 | Atr-ERN14863 |  | | | |  |  |  |  |  |
| 1 | Atr-ERN14864 |  | | | |  |  |  |  |  |
| 1 | Atr-ERN14865 |  | | | |  |  |  |  |  |
| 1 | Atr-ERN14866 |  | | | |  |  |  |  |  |
| 1 | Atr-ERN14867 |  | | | |  |  |  |  |  |
| 1 | Atr-ERN14868 |  | | | |  |  |  |  |  |
| 1 | Atr-ERN14869 |  | | | |  |  |  |  |  |
| 1 | Atr-ERN14870 |  | | | |  |  |  |  |  |
| 1 | Atr-ERN14871 |  | | | |  |  |  |  |  |
| 1 | Atr-ERN14872 |  | Vvi-Vitvi10g01216\_t001 |  |  |  |  |  |
| 1 | Atr-ERN14873 |  | | | |  |  |  |  |  |
| 1 | Atr-ERN14874 |  | | | |  |  |  |  |  |
| 1 | Atr-ERN14875 |  | | | |  |  |  |  |  |
| 1 | Atr-ERN14876 |  | | | |  |  |  |  |  |
| 1 | Atr-ERN14877 |  | | | |  |  |  |  |  |
| 1 | Atr-ERN14878 |  | | | |  |  |  |  |  |
| 1 | Atr-ERN14879 |  | | | |  |  |  |  |  |
| 1 | Atr-ERN14880 |  | | | |  |  |  |  |  |
| 1 | Atr-ERN14881 |  | | | |  |  |  |  |  |
| 1 | Atr-ERN14882 |  | | | |  |  |  |  |  |
| 1 | Atr-ERN14883 |  | | | |  |  |  |  |  |
| 1 | Atr-ERN14884 |  | | | |  |  |  |  |  |
| 1 | Atr-ERN14885 |  | | | |  |  |  |  |  |
| 1 | Atr-ERN14886 |  | | | |  |  |  |  |  |
| 1 | Atr-ERN14887 |  | | | |  |  |  |  |  |
| 1 | Atr-ERN14888 |  | Vvi-Vitvi10g01207\_t001 |  |  |  |  |  |
| 0 | Atr-ERN14889 |  |  |  |  |  |  |
| 0 | Atr-ERN14890 |  |  |  |  |  |  |
| 0 | Atr-ERN14891 |  |  |  |  |  |  |
| 0 | Atr-ERN14892 |  |  |  |  |  |  |
| 0 | Atr-ERN14893 |  |  |  |  |  |  |
| 0 | Atr-ERN14894 |  |  |  |  |  |  |
| 0 | Atr-ERN14895 |  |  |  |  |  |  |
| 0 | Atr-ERN14896 |  |  |  |  |  |  |
| 0 | Atr-ERN14897 |  |  |  |  |  |  |
| 0 | Atr-ERN14898 |  |  |  |  |  |  |
| 0 | Atr-ERN14899 |  |  |  |  |  |  |
| 0 | Atr-ERN14900 |  |  |  |  |  |  |
| 0 | Atr-ERN14901 |  |  |  |  |  |  |
| 0 | Atr-ERN14902 |  |  |  |  |  |  |
| 0 | Atr-ERN14903 |  |  |  |  |  |  |
| 0 | Atr-ERN14904 |  |  |  |  |  |  |
| 0 | Atr-ERN14905 |  |  |  |  |  |  |
| 0 | Atr-ERN14906 |  |  |  |  |  |  |
| 0 | Atr-ERN14907 |  |  |  |  |  |  |
| 0 | Atr-ERN14908 |  |  |  |  |  |  |
| 0 | Atr-ERN14909 |  |  |  |  |  |  |
| 0 | Atr-ERN14910 |  |  |  |  |  |  |
| 0 | Atr-ERN14911 |  |  |  |  |  |  |
| 0 | Atr-ERN14912 |  |  |  |  |  |  |
| 0 | Atr-ERN14913 |  |  |  |  |  |  |
| 0 | Atr-ERN14914 |  |  |  |  |  |  |
| 0 | Atr-ERN14915 |  |  |  |  |  |  |
| 0 | Atr-ERN14916 |  |  |  |  |  |  |
| 0 | Atr-ERN14917 |  |  |  |  |  |  |
| 0 | Atr-ERN14918 |  |  |  |  |  |  |
| 0 | Atr-ERN14919 |  |  |  |  |  |  |
| 0 | Atr-ERN14920 |  |  |  |  |  |  |
| 0 | Atr-ERN14921 |  |  |  |  |  |  |
| 0 | Atr-ERN14922 |  |  |  |  |  |  |
| 0 | Atr-ERN14923 |  |  |  |  |  |  |
| 0 | Atr-ERN14924 |  |  |  |  |  |  |
| 0 | Atr-ERN14925 |  |  |  |  |  |  |
| 0 | Atr-ERN14926 |  |  |  |  |  |  |
| 0 | Atr-ERN14927 |  |  |  |  |  |  |
| 0 | Atr-ERN14928 |  |  |  |  |  |  |
| 0 | Atr-ERN14929 |  |  |  |  |  |  |
| 0 | Atr-ERN14930 |  |  |  |  |  |  |
| 0 | Atr-ERN14931 |  |  |  |  |  |  |
| 0 | Atr-ERN14932 |  |  |  |  |  |  |
| 0 | Atr-ERN14933 |  |  |  |  |  |  |
| 0 | Atr-ERN14934 |  |  |  |  |  |  |
| 0 | Atr-ERN14935 |  |  |  |  |  |  |
| 0 | Atr-ERN14936 |  |  |  |  |  |  |
| 0 | Atr-ERN14937 |  |  |  |  |  |  |
| 0 | Atr-ERN14938 |  |  |  |  |  |  |
| 0 | Atr-ERN14939 |  |  |  |  |  |  |
| 0 | Atr-ERN14940 |  |  |  |  |  |  |
| 0 | Atr-ERN14941 |  |  |  |  |  |  |
| 0 | Atr-ERN14942 |  |  |  |  |  |  |
| 0 | Atr-ERN14943 |  |  |  |  |  |  |
| 0 | Atr-ERN14944 |  |  |  |  |  |  |
| 0 | Atr-ERN14945 |  |  |  |  |  |  |
| 0 | Atr-ERN14946 |  |  |  |  |  |  |
| 0 | Atr-ERN14947 |  |  |  |  |  |  |
| 0 | Atr-ERN14948 |  |  |  |  |  |  |
| 0 | Atr-ERN14949 |  |  |  |  |  |  |
| 0 | Atr-ERN14950 |  |  |  |  |  |  |
| 0 | Atr-ERN14951 |  |  |  |  |  |  |
| 0 | Atr-ERN14952 |  |  |  |  |  |  |
| 0 | Atr-ERN14953 |  |  |  |  |  |  |
| 0 | Atr-ERN14954 |  |  |  |  |  |  |
| 0 | Atr-ERN14955 |  |  |  |  |  |  |
| 0 | Atr-ERN14956 |  |  |  |  |  |  |
| 0 | Atr-ERN14957 |  |  |  |  |  |  |
| 0 | Atr-ERN14958 |  |  |  |  |  |  |
| 0 | Atr-ERN14959 |  |  |  |  |  |  |
| 0 | Atr-ERN14960 |  |  |  |  |  |  |
| 0 | Atr-ERN14961 |  |  |  |  |  |  |
| 0 | Atr-ERN14962 |  |  |  |  |  |  |
| 0 | Atr-ERN14963 |  |  |  |  |  |  |
| 0 | Atr-ERN14964 |  |  |  |  |  |  |
| 0 | Atr-ERN14965 |  |  |  |  |  |  |
| 0 | Atr-ERN14966 |  |  |  |  |  |  |
| 0 | Atr-ERN14967 |  |  |  |  |  |  |
| 0 | Atr-ERN14968 |  |  |  |  |  |  |
| 0 | Atr-ERN14969 |  |  |  |  |  |  |
| 0 | Atr-ERN14970 |  |  |  |  |  |  |
| 0 | Atr-ERN14971 |  |  |  |  |  |  |
| 0 | Atr-ERN14972 |  |  |  |  |  |  |
| 0 | Atr-ERN14973 |  |  |  |  |  |  |
| 0 | Atr-ERN14974 |  |  |  |  |  |  |
| 0 | Atr-ERN14975 |  |  |  |  |  |  |
| 0 | Atr-ERN14976 |  |  |  |  |  |  |
| 0 | Atr-ERN14977 |  |  |  |  |  |  |
| 0 | Atr-ERN14978 |  |  |  |  |  |  |
| 0 | Atr-ERN14979 |  |  |  |  |  |  |
| 0 | Atr-ERN14980 |  |  |  |  |  |  |
| 0 | Atr-ERN14981 |  |  |  |  |  |  |
| 0 | Atr-ERN14982 |  |  |  |  |  |  |
| 0 | Atr-ERN14983 |  |  |  |  |  |  |
| 0 | Atr-ERN14984 |  |  |  |  |  |  |
| 0 | Atr-ERN14985 |  |  |  |  |  |  |
| 0 | Atr-ERN14986 |  |  |  |  |  |  |
| 0 | Atr-ERN14987 |  |  |  |  |  |  |
| 0 | Atr-ERN14988 |  |  |  |  |  |  |
| 0 | Atr-ERN14989 |  |  |  |  |  |  |
| 0 | Atr-ERN14990 |  |  |  |  |  |  |
| 0 | Atr-ERN14991 |  |  |  |  |  |  |
| 0 | Atr-ERN14992 |  |  |  |  |  |  |
| 0 | Atr-ERN14993 |  |  |  |  |  |  |
| 0 | Atr-ERN14994 |  |  |  |  |  |  |
| 0 | Atr-ERN14995 |  |  |  |  |  |  |
| 0 | Atr-ERN14996 |  |  |  |  |  |  |
| 0 | Atr-ERN14997 |  |  |  |  |  |  |
| 0 | Atr-ERN14998 |  |  |  |  |  |  |
| 0 | Atr-ERN14999 |  |  |  |  |  |  |
| 0 | Atr-ERN15000 |  |  |  |  |  |  |
| 0 | Atr-ERN15001 |  |  |  |  |  |  |
| 0 | Atr-ERN15002 |  |  |  |  |  |  |
| 0 | Atr-ERN15003 |  |  |  |  |  |  |
| 0 | Atr-ERN15004 |  |  |  |  |  |  |
| 0 | Atr-ERN15005 |  |  |  |  |  |  |
| 0 | Atr-ERN15006 |  |  |  |  |  |  |
| 0 | Atr-ERN15007 |  |  |  |  |  |  |
| 0 | Atr-ERN15008 |  |  |  |  |  |  |
| 0 | Atr-ERN15009 |  |  |  |  |  |  |
| 0 | Atr-ERN15010 |  |  |  |  |  |  |
| 0 | Atr-ERN15011 |  |  |  |  |  |  |
| 0 | Atr-ERN15012 |  |  |  |  |  |  |
| 0 | Atr-ERN15013 |  |  |  |  |  |  |
| 0 | Atr-ERN15014 |  |  |  |  |  |  |
| 0 | Atr-ERN15015 |  |  |  |  |  |  |
| 0 | Atr-ERN15016 |  |  |  |  |  |  |
| 0 | Atr-ERN15017 |  |  |  |  |  |  |
| 0 | Atr-ERN15018 |  |  |  |  |  |  |
| 0 | Atr-ERN15019 |  |  |  |  |  |  |
| 0 | Atr-ERN15020 |  |  |  |  |  |  |
| 0 | Atr-ERN15021 |  |  |  |  |  |  |
| 0 | Atr-ERN15022 |  |  |  |  |  |  |
| 0 | Atr-ERN15023 |  |  |  |  |  |  |
| 0 | Atr-ERN15024 |  |  |  |  |  |  |
| 0 | Atr-ERN15025 |  |  |  |  |  |  |
| 0 | Atr-ERN15026 |  |  |  |  |  |  |
| 0 | Atr-ERN15027 |  |  |  |  |  |  |
| 0 | Atr-ERN15028 |  |  |  |  |  |  |
| 0 | Atr-ERN15029 |  |  |  |  |  |  |
| 0 | Atr-ERN15030 |  |  |  |  |  |  |
| 0 | Atr-ERN15031 |  |  |  |  |  |  |
| 0 | Atr-ERN15032 |  |  |  |  |  |  |
| 0 | Atr-ERN15033 |  |  |  |  |  |  |
| 0 | Atr-ERN15034 |  |  |  |  |  |  |
| 0 | Atr-ERN15035 |  |  |  |  |  |  |
| 0 | Atr-ERN15036 |  |  |  |  |  |  |
| 0 | Atr-ERN15037 |  |  |  |  |  |  |
| 0 | Atr-ERN15038 |  |  |  |  |  |  |
| 0 | Atr-ERN15039 |  |  |  |  |  |  |
| 0 | Atr-ERN15040 |  |  |  |  |  |  |
| 0 | Atr-ERN15041 |  |  |  |  |  |  |
| 0 | Atr-ERN15042 |  |  |  |  |  |  |
| 0 | Atr-ERN15043 |  |  |  |  |  |  |
