# Supplementary material for: Genome-wide identification, characterization and gene expression of BES1 transcription factor family in grapevine (Vitis vinifera L.)
Source: Sci Rep. 2023 Jan 5;13:240. doi: 10.1038/s41598-022-24407-y (PMC9816167; doi:10.1038/s41598-022-24407-y)
Supplement: Supplementary file 3 — Supplementary Information. [file 41598_2022_24407_MOESM3_ESM.zip › Vvi_Atr/Vitis_vinifera.PN40024.v4.dna_sm.toplevel.fa.vs.Amborella_trichopoda.AMTR1.0.dna_sm.toplevel.fa.html/Atr-AmTr_v1.0_scaffold00033.html]

|  |  |  |  |  |  |  |  |  |  |  |  |  |  |
| --- | --- | --- | --- | --- | --- | --- | --- | --- | --- | --- | --- | --- | --- |
| Duplication depth | Reference chromosome | Collinear blocks | | | | | | | | | | | |
| 0 | Atr-ERN14162 |  |  |  |  |  |  |
| 0 | Atr-ERN14163 |  |  |  |  |  |  |
| 0 | Atr-ERN14164 |  |  |  |  |  |  |
| 0 | Atr-ERN14165 |  |  |  |  |  |  |
| 0 | Atr-ERN14166 |  |  |  |  |  |  |
| 2 | Atr-ERN14167 |  | Vvi-Vitvi18g00572\_t001 |  | Vvi-Vitvi07g04644\_t001 |  |  |  |  |
| 2 | Atr-ERN14168 |  | | | |  | | | |  |  |  |  |
| 2 | Atr-ERN14169 |  | | | |  | | | |  |  |  |  |
| 2 | Atr-ERN14170 |  | | | |  | | | |  |  |  |  |
| 2 | Atr-ERN14171 |  | | | |  | | | |  |  |  |  |
| 2 | Atr-ERN14172 |  | | | |  | | | |  |  |  |  |
| 2 | Atr-ERN14173 |  | Vvi-Vitvi18g00574\_t001 |  | | | |  |  |  |  |
| 2 | Atr-ERN14174 |  | | | |  | | | |  |  |  |  |
| 2 | Atr-ERN14175 |  | Vvi-Vitvi18g00576\_t001 |  | | | |  |  |  |  |
| 2 | Atr-ERN14176 |  | Vvi-Vitvi18g00577\_t001 |  | | | |  |  |  |  |
| 2 | Atr-ERN14177 |  | | | |  | Vvi-Vitvi07g04648\_t001 |  |  |  |  |
| 2 | Atr-ERN14178 |  | | | |  | Vvi-Vitvi07g01536\_t001 |  |  |  |  |
| 3 | Atr-ERN14179 |  | Vvi-Vitvi18g04128\_t001 |  | | | |  | Vvi-Vitvi03g04261\_t001 |  |  |  |
| 3 | Atr-ERN14180 |  | | | |  | | | |  | | | |  |  |  |
| 3 | Atr-ERN14181 |  | | | |  | | | |  | | | |  |  |  |
| 3 | Atr-ERN14182 |  | | | |  | | | |  | | | |  |  |  |
| 3 | Atr-ERN14183 |  | | | |  | Vvi-Vitvi07g01535\_t001 |  | | | |  |  |  |
| 3 | Atr-ERN14184 |  | | | |  | | | |  | | | |  |  |  |
| 3 | Atr-ERN14185 |  | | | |  | | | |  | | | |  |  |  |
| 3 | Atr-ERN14186 |  | | | |  | | | |  | | | |  |  |  |
| 3 | Atr-ERN14187 |  | | | |  | | | |  | Vvi-Vitvi03g00793\_t001 |  |  |  |
| 3 | Atr-ERN14188 |  | | | |  | | | |  | Vvi-Vitvi03g00792\_t001 |  |  |  |
| 3 | Atr-ERN14189 |  | Vvi-Vitvi18g00580\_t001 |  | | | |  | | | |  |  |  |
| 3 | Atr-ERN14190 |  | Vvi-Vitvi18g00583\_t001 |  | | | |  | | | |  |  |  |
| 3 | Atr-ERN14191 |  | Vvi-Vitvi18g04132\_t001 |  | | | |  | | | |  |  |  |
| 3 | Atr-ERN14192 |  | | | |  | | | |  | | | |  |  |  |
| 3 | Atr-ERN14193 |  | | | |  | | | |  | | | |  |  |  |
| 3 | Atr-ERN14194 |  | | | |  | | | |  | | | |  |  |  |
| 3 | Atr-ERN14195 |  | | | |  | | | |  | Vvi-Vitvi03g00786\_t001 |  |  |  |
| 3 | Atr-ERN14196 |  | | | |  | Vvi-Vitvi07g01532\_t001 |  | | | |  |  |  |
| 3 | Atr-ERN14197 |  | | | |  | Vvi-Vitvi07g04652\_t001 |  | | | |  |  |  |
| 3 | Atr-ERN14198 |  | | | |  | | | |  | | | |  |  |  |
| 3 | Atr-ERN14199 |  | | | |  | Vvi-Vitvi07g03070\_t001 |  | Vvi-Vitvi03g00785\_t001 |  |  |  |
| 3 | Atr-ERN14200 |  | | | |  | | | |  | | | |  |  |  |
| 3 | Atr-ERN14201 |  | | | |  | | | |  | | | |  |  |  |
| 3 | Atr-ERN14202 |  | | | |  | | | |  | | | |  |  |  |
| 3 | Atr-ERN14203 |  | | | |  | | | |  | | | |  |  |  |
| 3 | Atr-ERN14204 |  | | | |  | | | |  | | | |  |  |  |
| 3 | Atr-ERN14205 |  | | | |  | | | |  | | | |  |  |  |
| 3 | Atr-ERN14206 |  | | | |  | | | |  | | | |  |  |  |
| 3 | Atr-ERN14207 |  | | | |  | | | |  | | | |  |  |  |
| 3 | Atr-ERN14208 |  | Vvi-Vitvi18g00587\_t001 |  | Vvi-Vitvi07g04654\_t001 |  | Vvi-Vitvi03g00783\_t001 |  |  |  |
| 2 | Atr-ERN14209 |  | | | |  | | | |  |  |  |  |
| 2 | Atr-ERN14210 |  | | | |  | | | |  |  |  |  |
| 2 | Atr-ERN14211 |  | | | |  | | | |  |  |  |  |
| 2 | Atr-ERN14212 |  | | | |  | | | |  |  |  |  |
| 3 | Atr-ERN14213 |  | Vvi-Vitvi18g00590\_t001 |  | Vvi-Vitvi07g04656\_t001 |  | Vvi-Vitvi03g00721\_t001 |  |  |  |
| 3 | Atr-ERN14214 |  | Vvi-Vitvi18g00593\_t001 |  | | | |  | | | |  |  |  |
| 3 | Atr-ERN14215 |  | | | |  | | | |  | | | |  |  |  |
| 3 | Atr-ERN14216 |  | | | |  | Vvi-Vitvi07g04657\_t001 |  | | | |  |  |  |
| 3 | Atr-ERN14217 |  | | | |  | | | |  | | | |  |  |  |
| 3 | Atr-ERN14218 |  | Vvi-Vitvi18g00594\_t002 |  | | | |  | | | |  |  |  |
| 3 | Atr-ERN14219 |  | Vvi-Vitvi18g00595\_t006 |  | | | |  | | | |  |  |  |
| 3 | Atr-ERN14220 |  | Vvi-Vitvi18g00596\_t001 |  | | | |  | Vvi-Vitvi03g01639\_t001 |  |  |  |
| 3 | Atr-ERN14221 |  | | | |  | | | |  | | | |  |  |  |
| 3 | Atr-ERN14222 |  | | | |  | | | |  | | | |  |  |  |
| 3 | Atr-ERN14223 |  | | | |  | | | |  | | | |  |  |  |
| 3 | Atr-ERN14224 |  | Vvi-Vitvi18g00597\_t002 |  | | | |  | Vvi-Vitvi03g00723\_t001 |  |  |  |
| 3 | Atr-ERN14225 |  | | | |  | | | |  | | | |  |  |  |
| 3 | Atr-ERN14226 |  | | | |  | | | |  | | | |  |  |  |
| 3 | Atr-ERN14227 |  | | | |  | | | |  | | | |  |  |  |
| 3 | Atr-ERN14228 |  | | | |  | | | |  | Vvi-Vitvi03g00726\_t001 |  |  |  |
| 3 | Atr-ERN14229 |  | | | |  | | | |  | | | |  |  |  |
| 3 | Atr-ERN14230 |  | | | |  | | | |  | | | |  |  |  |
| 3 | Atr-ERN14231 |  | | | |  | Vvi-Vitvi07g04658\_t001 |  | | | |  |  |  |
| 3 | Atr-ERN14232 |  | | | |  | | | |  | Vvi-Vitvi03g00728\_t001 |  |  |  |
| 3 | Atr-ERN14233 |  | | | |  | | | |  | | | |  |  |  |
| 3 | Atr-ERN14234 |  | Vvi-Vitvi18g00598\_t001 |  | | | |  | Vvi-Vitvi03g00735\_t001 |  |  |  |
| 3 | Atr-ERN14235 |  | | | |  | Vvi-Vitvi07g03064\_t001 |  | | | |  |  |  |
| 3 | Atr-ERN14236 |  | Vvi-Vitvi18g02621\_t001 |  | Vvi-Vitvi07g03063\_t002 |  | Vvi-Vitvi03g01647\_t001 |  |  |  |
| 0 | Atr-ERN14237 |  |  |  |  |  |  |
| 0 | Atr-ERN14238 |  |  |  |  |  |  |
| 0 | Atr-ERN14239 |  |  |  |  |  |  |
| 0 | Atr-ERN14240 |  |  |  |  |  |  |
| 0 | Atr-ERN14241 |  |  |  |  |  |  |
| 0 | Atr-ERN14242 |  |  |  |  |  |  |
| 0 | Atr-ERN14243 |  |  |  |  |  |  |
| 0 | Atr-ERN14244 |  |  |  |  |  |  |
| 0 | Atr-ERN14245 |  |  |  |  |  |  |
| 1 | Atr-ERN14246 |  | Vvi-Vitvi04g00548\_t002 |  |  |  |  |  |
| 1 | Atr-ERN14247 |  | | | |  |  |  |  |  |
| 2 | Atr-ERN14248 |  | | | |  | Vvi-Vitvi09g00793\_t001 |  |  |  |  |
| 2 | Atr-ERN14249 |  | | | |  | | | |  |  |  |  |
| 2 | Atr-ERN14250 |  | | | |  | | | |  |  |  |  |
| 2 | Atr-ERN14251 |  | | | |  | | | |  |  |  |  |
| 2 | Atr-ERN14252 |  | | | |  | | | |  |  |  |  |
| 2 | Atr-ERN14253 |  | | | |  | | | |  |  |  |  |
| 2 | Atr-ERN14254 |  | | | |  | | | |  |  |  |  |
| 2 | Atr-ERN14255 |  | | | |  | | | |  |  |  |  |
| 2 | Atr-ERN14256 |  | Vvi-Vitvi04g00549\_t001 |  | | | |  |  |  |  |
| 2 | Atr-ERN14257 |  | | | |  | | | |  |  |  |  |
| 2 | Atr-ERN14258 |  | Vvi-Vitvi04g00550\_t001 |  | | | |  |  |  |  |
| 2 | Atr-ERN14259 |  | | | |  | | | |  |  |  |  |
| 3 | Atr-ERN14260 |  | | | |  | | | |  | Vvi-Vitvi11g00593\_t001 |  |  |  |
| 3 | Atr-ERN14261 |  | | | |  | | | |  | | | |  |  |  |
| 3 | Atr-ERN14262 |  | | | |  | | | |  | | | |  |  |  |
| 3 | Atr-ERN14263 |  | | | |  | | | |  | Vvi-Vitvi11g00588\_t001 |  |  |  |
| 3 | Atr-ERN14264 |  | Vvi-Vitvi04g00554\_t001 |  | | | |  | | | |  |  |  |
| 3 | Atr-ERN14265 |  | | | |  | | | |  | | | |  |  |  |
| 3 | Atr-ERN14266 |  | | | |  | | | |  | Vvi-Vitvi11g01466\_t001 |  |  |  |
| 3 | Atr-ERN14267 |  | | | |  | | | |  | | | |  |  |  |
| 3 | Atr-ERN14268 |  | | | |  | | | |  | | | |  |  |  |
| 3 | Atr-ERN14269 |  | | | |  | | | |  | | | |  |  |  |
| 3 | Atr-ERN14270 |  | | | |  | | | |  | | | |  |  |  |
| 3 | Atr-ERN14271 |  | | | |  | | | |  | | | |  |  |  |
| 3 | Atr-ERN14272 |  | | | |  | | | |  | Vvi-Vitvi11g00585\_t001 |  |  |  |
| 3 | Atr-ERN14273 |  | Vvi-Vitvi04g00556\_t001 |  | Vvi-Vitvi09g00773\_t001 |  | Vvi-Vitvi11g00584\_t001 |  |  |  |
| 3 | Atr-ERN14274 |  | | | |  | | | |  | | | |  |  |  |
| 3 | Atr-ERN14275 |  | | | |  | | | |  | | | |  |  |  |
| 3 | Atr-ERN14276 |  | | | |  | | | |  | | | |  |  |  |
| 3 | Atr-ERN14277 |  | | | |  | | | |  | | | |  |  |  |
| 3 | Atr-ERN14278 |  | | | |  | | | |  | | | |  |  |  |
| 3 | Atr-ERN14279 |  | | | |  | | | |  | | | |  |  |  |
| 3 | Atr-ERN14280 |  | | | |  | | | |  | | | |  |  |  |
| 3 | Atr-ERN14281 |  | | | |  | | | |  | | | |  |  |  |
| 3 | Atr-ERN14282 |  | | | |  | | | |  | | | |  |  |  |
| 3 | Atr-ERN14283 |  | Vvi-Vitvi04g00557\_t001 |  | Vvi-Vitvi09g04250\_t001 |  | | | |  |  |  |
| 3 | Atr-ERN14284 |  | | | |  | Vvi-Vitvi09g00772\_t001 |  | Vvi-Vitvi11g00582\_t001 |  |  |  |
| 3 | Atr-ERN14285 |  | | | |  | Vvi-Vitvi09g00760\_t001 |  | Vvi-Vitvi11g00581\_t001 |  |  |  |
| 3 | Atr-ERN14286 |  | | | |  | Vvi-Vitvi09g00759\_t001 |  | Vvi-Vitvi11g00580\_t001 |  |  |  |
| 3 | Atr-ERN14287 |  | Vvi-Vitvi04g00559\_t001 |  | | | |  | Vvi-Vitvi11g00579\_t001 |  |  |  |
| 3 | Atr-ERN14288 |  | | | |  | Vvi-Vitvi09g00758\_t001 |  | | | |  |  |  |
| 3 | Atr-ERN14289 |  | | | |  | | | |  | Vvi-Vitvi11g00578\_t001 |  |  |  |
| 3 | Atr-ERN14290 |  | | | |  | | | |  | Vvi-Vitvi11g00577\_t001 |  |  |  |
| 3 | Atr-ERN14291 |  | | | |  | | | |  | Vvi-Vitvi11g00576\_t001 |  |  |  |
| 3 | Atr-ERN14292 |  | Vvi-Vitvi04g04136\_t002 |  | | | |  | | | |  |  |  |
| 3 | Atr-ERN14293 |  | | | |  | Vvi-Vitvi09g00734\_t001 |  | Vvi-Vitvi11g00574\_t001 |  |  |  |
| 3 | Atr-ERN14294 |  | | | |  | | | |  | | | |  |  |  |
| 3 | Atr-ERN14295 |  | | | |  | | | |  | Vvi-Vitvi11g00573\_t001 |  |  |  |
| 3 | Atr-ERN14296 |  | | | |  | Vvi-Vitvi09g00731\_t001 |  | | | |  |  |  |
| 3 | Atr-ERN14297 |  | | | |  | | | |  | | | |  |  |  |
| 3 | Atr-ERN14298 |  | | | |  | | | |  | | | |  |  |  |
| 3 | Atr-ERN14299 |  | | | |  | | | |  | Vvi-Vitvi11g00571\_t002 |  |  |  |
| 3 | Atr-ERN14300 |  | | | |  | | | |  | | | |  |  |  |
| 3 | Atr-ERN14301 |  | | | |  | | | |  | | | |  |  |  |
| 3 | Atr-ERN14302 |  | | | |  | | | |  | | | |  |  |  |
| 3 | Atr-ERN14303 |  | Vvi-Vitvi04g01925\_t001 |  | | | |  | Vvi-Vitvi11g01463\_t001 |  |  |  |
| 3 | Atr-ERN14304 |  | | | |  | | | |  | Vvi-Vitvi11g00567\_t001 |  |  |  |
| 3 | Atr-ERN14305 |  | | | |  | | | |  | | | |  |  |  |
| 3 | Atr-ERN14306 |  | | | |  | Vvi-Vitvi09g00721\_t001 |  | | | |  |  |  |
| 3 | Atr-ERN14307 |  | | | |  | | | |  | | | |  |  |  |
| 3 | Atr-ERN14308 |  | | | |  | | | |  | | | |  |  |  |
| 3 | Atr-ERN14309 |  | | | |  | | | |  | | | |  |  |  |
| 3 | Atr-ERN14310 |  | | | |  | | | |  | | | |  |  |  |
| 3 | Atr-ERN14311 |  | | | |  | | | |  | | | |  |  |  |
| 3 | Atr-ERN14312 |  | | | |  | Vvi-Vitvi09g00720\_t001 |  | Vvi-Vitvi11g00561\_t002 |  |  |  |
| 3 | Atr-ERN14313 |  | | | |  | | | |  | | | |  |  |  |
| 3 | Atr-ERN14314 |  | | | |  | | | |  | | | |  |  |  |
| 3 | Atr-ERN14315 |  | | | |  | | | |  | | | |  |  |  |
| 3 | Atr-ERN14316 |  | Vvi-Vitvi04g00580\_t001 |  | | | |  | | | |  |  |  |
| 3 | Atr-ERN14317 |  | | | |  | | | |  | | | |  |  |  |
| 3 | Atr-ERN14318 |  | | | |  | Vvi-Vitvi09g00693\_t001 |  | Vvi-Vitvi11g00560\_t001 |  |  |  |
| 3 | Atr-ERN14319 |  | | | |  | | | |  | | | |  |  |  |
| 3 | Atr-ERN14320 |  | | | |  | Vvi-Vitvi09g00691\_t001 |  | | | |  |  |  |
| 2 | Atr-ERN14321 |  | Vvi-Vitvi04g00581\_t001 |  |  |  | | | |  |  |  |
| 2 | Atr-ERN14322 |  | | | |  |  |  | | | |  |  |  |
| 2 | Atr-ERN14323 |  | | | |  |  |  | | | |  |  |  |
| 2 | Atr-ERN14324 |  | | | |  |  |  | | | |  |  |  |
| 2 | Atr-ERN14325 |  | | | |  |  |  | | | |  |  |  |
| 2 | Atr-ERN14326 |  | | | |  |  |  | | | |  |  |  |
| 2 | Atr-ERN14327 |  | | | |  |  |  | | | |  |  |  |
| 2 | Atr-ERN14328 |  | | | |  |  |  | | | |  |  |  |
| 2 | Atr-ERN14329 |  | | | |  |  |  | | | |  |  |  |
| 2 | Atr-ERN14330 |  | | | |  |  |  | | | |  |  |  |
| 2 | Atr-ERN14331 |  | | | |  |  |  | | | |  |  |  |
| 2 | Atr-ERN14332 |  | | | |  |  |  | Vvi-Vitvi11g00555\_t001 |  |  |  |
| 2 | Atr-ERN14333 |  | Vvi-Vitvi04g00582\_t001 |  |  |  | | | |  |  |  |
| 2 | Atr-ERN14334 |  | Vvi-Vitvi04g00583\_t001 |  |  |  | | | |  |  |  |
| 3 | Atr-ERN14335 |  | | | |  | Vvi-Vitvi11g00539\_t001 |  | | | |  |  |  |
| 3 | Atr-ERN14336 |  | | | |  | | | |  | | | |  |  |  |
| 3 | Atr-ERN14337 |  | | | |  | Vvi-Vitvi11g00542\_t001 |  | | | |  |  |  |
| 3 | Atr-ERN14338 |  | | | |  | Vvi-Vitvi11g00543\_t001 |  | | | |  |  |  |
| 3 | Atr-ERN14339 |  | | | |  | Vvi-Vitvi11g00545\_t001 |  | Vvi-Vitvi11g00545\_t001 |  |  |  |
| 2 | Atr-ERN14340 |  | | | |  | Vvi-Vitvi11g00547\_t001 |  |  |  |  |
| 2 | Atr-ERN14341 |  | | | |  | | | |  |  |  |  |
| 2 | Atr-ERN14342 |  | Vvi-Vitvi04g00585\_t001 |  | Vvi-Vitvi11g00548\_t001 |  |  |  |  |
| 1 | Atr-ERN14343 |  | Vvi-Vitvi18g00548\_t001 |  |  |  |  |  |
| 1 | Atr-ERN14344 |  | | | |  |  |  |  |  |
| 1 | Atr-ERN14345 |  | | | |  |  |  |  |  |
| 1 | Atr-ERN14346 |  | Vvi-Vitvi18g00546\_t001 |  |  |  |  |  |
| 2 | Atr-ERN14347 |  | | | |  | Vvi-Vitvi07g01401\_t001 |  |  |  |  |
| 2 | Atr-ERN14348 |  | Vvi-Vitvi18g00544\_t001 |  | Vvi-Vitvi07g01402\_t001 |  |  |  |  |
| 2 | Atr-ERN14349 |  | | | |  | Vvi-Vitvi07g01403\_t001 |  |  |  |  |
| 2 | Atr-ERN14350 |  | Vvi-Vitvi18g02615\_t001 |  | | | |  |  |  |  |
| 2 | Atr-ERN14351 |  | | | |  | | | |  |  |  |  |
| 2 | Atr-ERN14352 |  | | | |  | | | |  |  |  |  |
| 2 | Atr-ERN14353 |  | | | |  | | | |  |  |  |  |
| 2 | Atr-ERN14354 |  | | | |  | | | |  |  |  |  |
| 2 | Atr-ERN14355 |  | | | |  | | | |  |  |  |  |
| 3 | Atr-ERN14356 |  | Vvi-Vitvi18g00542\_t001 |  | | | |  | Vvi-Vitvi03g01019\_t001 |  |  |  |
| 3 | Atr-ERN14357 |  | | | |  | | | |  | | | |  |  |  |
| 3 | Atr-ERN14358 |  | | | |  | | | |  | | | |  |  |  |
| 3 | Atr-ERN14359 |  | Vvi-Vitvi18g00539\_t001 |  | | | |  | | | |  |  |  |
| 3 | Atr-ERN14360 |  | | | |  | Vvi-Vitvi07g01408\_t001 |  | | | |  |  |  |
| 3 | Atr-ERN14361 |  | Vvi-Vitvi18g00537\_t001 |  | | | |  | Vvi-Vitvi03g01766\_t001 |  |  |  |
| 3 | Atr-ERN14362 |  | | | |  | | | |  | Vvi-Vitvi03g00999\_t001 |  |  |  |
| 3 | Atr-ERN14363 |  | | | |  | | | |  | Vvi-Vitvi03g00997\_t001 |  |  |  |
| 3 | Atr-ERN14364 |  | Vvi-Vitvi18g02614\_t001 |  | | | |  | | | |  |  |  |
| 3 | Atr-ERN14365 |  | | | |  | Vvi-Vitvi07g04617\_t001 |  | | | |  |  |  |
| 3 | Atr-ERN14366 |  | | | |  | | | |  | | | |  |  |  |
| 3 | Atr-ERN14367 |  | | | |  | | | |  | | | |  |  |  |
| 3 | Atr-ERN14368 |  | | | |  | | | |  | | | |  |  |  |
| 3 | Atr-ERN14369 |  | | | |  | Vvi-Vitvi07g04615\_t001 |  | | | |  |  |  |
| 3 | Atr-ERN14370 |  | | | |  | | | |  | | | |  |  |  |
| 3 | Atr-ERN14371 |  | | | |  | | | |  | | | |  |  |  |
| 3 | Atr-ERN14372 |  | Vvi-Vitvi18g00535\_t001 |  | | | |  | | | |  |  |  |
| 3 | Atr-ERN14373 |  | | | |  | | | |  | | | |  |  |  |
| 3 | Atr-ERN14374 |  | | | |  | | | |  | | | |  |  |  |
| 3 | Atr-ERN14375 |  | | | |  | | | |  | | | |  |  |  |
| 3 | Atr-ERN14376 |  | | | |  | | | |  | | | |  |  |  |
| 3 | Atr-ERN14377 |  | | | |  | | | |  | | | |  |  |  |
| 3 | Atr-ERN14378 |  | | | |  | | | |  | | | |  |  |  |
| 3 | Atr-ERN14379 |  | Vvi-Vitvi18g00534\_t001 |  | | | |  | | | |  |  |  |
| 3 | Atr-ERN14380 |  | Vvi-Vitvi18g00533\_t001 |  | Vvi-Vitvi07g02572\_t001 |  | | | |  |  |  |
| 3 | Atr-ERN14381 |  | Vvi-Vitvi18g00532\_t001 |  | | | |  | | | |  |  |  |
| 3 | Atr-ERN14382 |  | | | |  | | | |  | | | |  |  |  |
| 3 | Atr-ERN14383 |  | | | |  | | | |  | | | |  |  |  |
| 3 | Atr-ERN14384 |  | | | |  | | | |  | | | |  |  |  |
| 3 | Atr-ERN14385 |  | Vvi-Vitvi18g00531\_t001 |  | | | |  | | | |  |  |  |
| 3 | Atr-ERN14386 |  | Vvi-Vitvi18g02613\_t003 |  | | | |  | | | |  |  |  |
| 3 | Atr-ERN14387 |  | Vvi-Vitvi18g00529\_t001 |  | | | |  | | | |  |  |  |
| 3 | Atr-ERN14388 |  | | | |  | | | |  | Vvi-Vitvi03g00996\_t001 |  |  |  |
| 3 | Atr-ERN14389 |  | | | |  | Vvi-Vitvi07g04611\_t002 |  | | | |  |  |  |
| 3 | Atr-ERN14390 |  | | | |  | | | |  | | | |  |  |  |
| 3 | Atr-ERN14391 |  | | | |  | | | |  | | | |  |  |  |
| 3 | Atr-ERN14392 |  | | | |  | | | |  | | | |  |  |  |
| 3 | Atr-ERN14393 |  | | | |  | | | |  | | | |  |  |  |
| 3 | Atr-ERN14394 |  | | | |  | Vvi-Vitvi07g01428\_t001 |  | Vvi-Vitvi03g00995\_t001 |  |  |  |
| 3 | Atr-ERN14395 |  | | | |  | | | |  | | | |  |  |  |
| 3 | Atr-ERN14396 |  | Vvi-Vitvi18g00525\_t001 |  | Vvi-Vitvi07g04608\_t001 |  | | | |  |  |  |
| 3 | Atr-ERN14397 |  | | | |  | | | |  | Vvi-Vitvi03g00993\_t001 |  |  |  |
| 2 | Atr-ERN14398 |  | | | |  | | | |  |  |  |  |
| 2 | Atr-ERN14399 |  | Vvi-Vitvi18g00523\_t001 |  | | | |  |  |  |  |
| 2 | Atr-ERN14400 |  | | | |  | | | |  |  |  |  |
| 2 | Atr-ERN14401 |  | | | |  | | | |  |  |  |  |
| 2 | Atr-ERN14402 |  | Vvi-Vitvi18g00521\_t001 |  | | | |  |  |  |  |
| 2 | Atr-ERN14403 |  | Vvi-Vitvi18g00520\_t001 |  | | | |  |  |  |  |
| 2 | Atr-ERN14404 |  | | | |  | | | |  |  |  |  |
| 2 | Atr-ERN14405 |  | | | |  | Vvi-Vitvi07g01433\_t001 |  |  |  |  |
| 2 | Atr-ERN14406 |  | | | |  | | | |  |  |  |  |
| 2 | Atr-ERN14407 |  | | | |  | | | |  |  |  |  |
| 2 | Atr-ERN14408 |  | Vvi-Vitvi18g02610\_t001.1.6037826d |  | Vvi-Vitvi07g04607\_t001 |  |  |  |  |
| 2 | Atr-ERN14409 |  | | | |  | | | |  |  |  |  |
| 2 | Atr-ERN14410 |  | Vvi-Vitvi18g00518\_t002 |  | Vvi-Vitvi07g01437\_t001 |  |  |  |  |
| 2 | Atr-ERN14411 |  | Vvi-Vitvi18g02609\_t003 |  | | | |  |  |  |  |
| 1 | Atr-ERN14412 |  |  |  | Vvi-Vitvi07g01439\_t001 |  |  |  |  |
| 1 | Atr-ERN14413 |  |  |  | Vvi-Vitvi07g04606\_t001 |  |  |  |  |
| 0 | Atr-ERN14414 |  |  |  |  |  |  |
| 0 | Atr-ERN14415 |  |  |  |  |  |  |
| 0 | Atr-ERN14416 |  |  |  |  |  |  |
| 0 | Atr-ERN14417 |  |  |  |  |  |  |
| 0 | Atr-ERN14418 |  |  |  |  |  |  |
| 1 | Atr-ERN14419 |  | Vvi-Vitvi07g02529\_t001.1.6037826f |  |  |  |  |  |
| 1 | Atr-ERN14420 |  | Vvi-Vitvi07g01325\_t001 |  |  |  |  |  |
| 1 | Atr-ERN14421 |  | Vvi-Vitvi07g01324\_t001 |  |  |  |  |  |
| 1 | Atr-ERN14422 |  | Vvi-Vitvi07g01323\_t001 |  |  |  |  |  |
| 1 | Atr-ERN14423 |  | | | |  |  |  |  |  |
| 1 | Atr-ERN14424 |  | Vvi-Vitvi07g01313\_t001 |  |  |  |  |  |
| 1 | Atr-ERN14425 |  | | | |  |  |  |  |  |
| 1 | Atr-ERN14426 |  | Vvi-Vitvi07g01309\_t001 |  |  |  |  |  |
| 0 | Atr-ERN14427 |  |  |  |  |  |  |
| 0 | Atr-ERN14428 |  |  |  |  |  |  |
| 0 | Atr-ERN14429 |  |  |  |  |  |  |
| 0 | Atr-ERN14430 |  |  |  |  |  |  |
| 0 | Atr-ERN14431 |  |  |  |  |  |  |
