# Supplementary material for: Genome-wide identification, characterization and gene expression of BES1 transcription factor family in grapevine (Vitis vinifera L.)
Source: Sci Rep. 2023 Jan 5;13:240. doi: 10.1038/s41598-022-24407-y (PMC9816167; doi:10.1038/s41598-022-24407-y)
Supplement: Supplementary file 3 — Supplementary Information. [file 41598_2022_24407_MOESM3_ESM.zip › Vvi_Atr/Vitis_vinifera.PN40024.v4.dna_sm.toplevel.fa.vs.Amborella_trichopoda.AMTR1.0.dna_sm.toplevel.fa.html/Atr-AmTr_v1.0_scaffold00034.html]

|  |  |  |  |  |  |  |  |  |  |  |  |  |  |
| --- | --- | --- | --- | --- | --- | --- | --- | --- | --- | --- | --- | --- | --- |
| Duplication depth | Reference chromosome | Collinear blocks | | | | | | | | | | | |
| 0 | Atr-ERN12151 |  |  |  |  |  |  |
| 0 | Atr-ERN12152 |  |  |  |  |  |  |
| 0 | Atr-ERN12153 |  |  |  |  |  |  |
| 0 | Atr-ERN12154 |  |  |  |  |  |  |
| 0 | Atr-ERN12155 |  |  |  |  |  |  |
| 0 | Atr-ERN12156 |  |  |  |  |  |  |
| 0 | Atr-ERN12157 |  |  |  |  |  |  |
| 0 | Atr-ERN12158 |  |  |  |  |  |  |
| 0 | Atr-ERN12159 |  |  |  |  |  |  |
| 0 | Atr-ERN12160 |  |  |  |  |  |  |
| 0 | Atr-ERN12161 |  |  |  |  |  |  |
| 0 | Atr-ERN12162 |  |  |  |  |  |  |
| 0 | Atr-ERN12163 |  |  |  |  |  |  |
| 0 | Atr-ERN12164 |  |  |  |  |  |  |
| 0 | Atr-ERN12165 |  |  |  |  |  |  |
| 0 | Atr-ERN12166 |  |  |  |  |  |  |
| 0 | Atr-ERN12167 |  |  |  |  |  |  |
| 0 | Atr-ERN12168 |  |  |  |  |  |  |
| 0 | Atr-ERN12169 |  |  |  |  |  |  |
| 0 | Atr-ERN12170 |  |  |  |  |  |  |
| 0 | Atr-ERN12171 |  |  |  |  |  |  |
| 0 | Atr-ERN12172 |  |  |  |  |  |  |
| 0 | Atr-ERN12173 |  |  |  |  |  |  |
| 0 | Atr-ERN12174 |  |  |  |  |  |  |
| 0 | Atr-ERN12175 |  |  |  |  |  |  |
| 0 | Atr-ERN12176 |  |  |  |  |  |  |
| 0 | Atr-ERN12177 |  |  |  |  |  |  |
| 0 | Atr-ERN12178 |  |  |  |  |  |  |
| 0 | Atr-ERN12179 |  |  |  |  |  |  |
| 0 | Atr-ERN12180 |  |  |  |  |  |  |
| 0 | Atr-ERN12181 |  |  |  |  |  |  |
| 0 | Atr-ERN12182 |  |  |  |  |  |  |
| 0 | Atr-ERN12183 |  |  |  |  |  |  |
| 0 | Atr-ERN12184 |  |  |  |  |  |  |
| 0 | Atr-ERN12185 |  |  |  |  |  |  |
| 0 | Atr-ERN12186 |  |  |  |  |  |  |
| 0 | Atr-ERN12187 |  |  |  |  |  |  |
| 0 | Atr-ERN12188 |  |  |  |  |  |  |
| 0 | Atr-ERN12189 |  |  |  |  |  |  |
| 0 | Atr-ERN12190 |  |  |  |  |  |  |
| 0 | Atr-ERN12191 |  |  |  |  |  |  |
| 0 | Atr-ERN12192 |  |  |  |  |  |  |
| 0 | Atr-ERN12193 |  |  |  |  |  |  |
| 0 | Atr-ERN12194 |  |  |  |  |  |  |
| 0 | Atr-ERN12195 |  |  |  |  |  |  |
| 0 | Atr-ERN12196 |  |  |  |  |  |  |
| 0 | Atr-ERN12197 |  |  |  |  |  |  |
| 0 | Atr-ERN12198 |  |  |  |  |  |  |
| 0 | Atr-ERN12199 |  |  |  |  |  |  |
| 0 | Atr-ERN12200 |  |  |  |  |  |  |
| 0 | Atr-ERN12201 |  |  |  |  |  |  |
| 0 | Atr-ERN12202 |  |  |  |  |  |  |
| 0 | Atr-ERN12203 |  |  |  |  |  |  |
| 0 | Atr-ERN12204 |  |  |  |  |  |  |
| 0 | Atr-ERN12205 |  |  |  |  |  |  |
| 0 | Atr-ERN12206 |  |  |  |  |  |  |
| 0 | Atr-ERN12207 |  |  |  |  |  |  |
| 0 | Atr-ERN12208 |  |  |  |  |  |  |
| 0 | Atr-ERN12209 |  |  |  |  |  |  |
| 0 | Atr-ERN12210 |  |  |  |  |  |  |
| 0 | Atr-ERN12211 |  |  |  |  |  |  |
| 0 | Atr-ERN12212 |  |  |  |  |  |  |
| 0 | Atr-ERN12213 |  |  |  |  |  |  |
| 0 | Atr-ERN12214 |  |  |  |  |  |  |
| 0 | Atr-ERN12215 |  |  |  |  |  |  |
| 0 | Atr-ERN12216 |  |  |  |  |  |  |
| 0 | Atr-ERN12217 |  |  |  |  |  |  |
| 0 | Atr-ERN12218 |  |  |  |  |  |  |
| 0 | Atr-ERN12219 |  |  |  |  |  |  |
| 0 | Atr-ERN12220 |  |  |  |  |  |  |
| 0 | Atr-ERN12221 |  |  |  |  |  |  |
| 0 | Atr-ERN12222 |  |  |  |  |  |  |
| 0 | Atr-ERN12223 |  |  |  |  |  |  |
| 0 | Atr-ERN12224 |  |  |  |  |  |  |
| 0 | Atr-ERN12225 |  |  |  |  |  |  |
| 0 | Atr-ERN12226 |  |  |  |  |  |  |
| 0 | Atr-ERN12227 |  |  |  |  |  |  |
| 0 | Atr-ERN12228 |  |  |  |  |  |  |
| 0 | Atr-ERN12229 |  |  |  |  |  |  |
| 0 | Atr-ERN12230 |  |  |  |  |  |  |
| 0 | Atr-ERN12231 |  |  |  |  |  |  |
| 0 | Atr-ERN12232 |  |  |  |  |  |  |
| 0 | Atr-ERN12233 |  |  |  |  |  |  |
| 0 | Atr-ERN12234 |  |  |  |  |  |  |
| 0 | Atr-ERN12235 |  |  |  |  |  |  |
| 0 | Atr-ERN12236 |  |  |  |  |  |  |
| 0 | Atr-ERN12237 |  |  |  |  |  |  |
| 0 | Atr-ERN12238 |  |  |  |  |  |  |
| 0 | Atr-ERN12239 |  |  |  |  |  |  |
| 0 | Atr-ERN12240 |  |  |  |  |  |  |
| 0 | Atr-ERN12241 |  |  |  |  |  |  |
| 0 | Atr-ERN12242 |  |  |  |  |  |  |
| 0 | Atr-ERN12243 |  |  |  |  |  |  |
| 0 | Atr-ERN12244 |  |  |  |  |  |  |
| 0 | Atr-ERN12245 |  |  |  |  |  |  |
| 0 | Atr-ERN12246 |  |  |  |  |  |  |
| 0 | Atr-ERN12247 |  |  |  |  |  |  |
| 0 | Atr-ERN12248 |  |  |  |  |  |  |
| 0 | Atr-ERN12249 |  |  |  |  |  |  |
| 0 | Atr-ERN12250 |  |  |  |  |  |  |
| 0 | Atr-ERN12251 |  |  |  |  |  |  |
| 0 | Atr-ERN12252 |  |  |  |  |  |  |
| 0 | Atr-ERN12253 |  |  |  |  |  |  |
| 0 | Atr-ERN12254 |  |  |  |  |  |  |
| 0 | Atr-ERN12255 |  |  |  |  |  |  |
| 0 | Atr-ERN12256 |  |  |  |  |  |  |
| 0 | Atr-ERN12257 |  |  |  |  |  |  |
| 0 | Atr-ERN12258 |  |  |  |  |  |  |
| 0 | Atr-ERN12259 |  |  |  |  |  |  |
| 0 | Atr-ERN12260 |  |  |  |  |  |  |
| 0 | Atr-ERN12261 |  |  |  |  |  |  |
| 0 | Atr-ERN12262 |  |  |  |  |  |  |
| 0 | Atr-ERN12263 |  |  |  |  |  |  |
| 0 | Atr-ERN12264 |  |  |  |  |  |  |
| 0 | Atr-ERN12265 |  |  |  |  |  |  |
| 0 | Atr-ERN12266 |  |  |  |  |  |  |
| 0 | Atr-ERN12267 |  |  |  |  |  |  |
| 0 | Atr-ERN12268 |  |  |  |  |  |  |
| 0 | Atr-ERN12269 |  |  |  |  |  |  |
| 0 | Atr-ERN12270 |  |  |  |  |  |  |
| 0 | Atr-ERN12271 |  |  |  |  |  |  |
| 0 | Atr-ERN12272 |  |  |  |  |  |  |
| 0 | Atr-ERN12273 |  |  |  |  |  |  |
| 0 | Atr-ERN12274 |  |  |  |  |  |  |
| 0 | Atr-ERN12275 |  |  |  |  |  |  |
| 0 | Atr-ERN12276 |  |  |  |  |  |  |
| 0 | Atr-ERN12277 |  |  |  |  |  |  |
| 0 | Atr-ERN12278 |  |  |  |  |  |  |
| 0 | Atr-ERN12279 |  |  |  |  |  |  |
| 0 | Atr-ERN12280 |  |  |  |  |  |  |
| 0 | Atr-ERN12281 |  |  |  |  |  |  |
| 0 | Atr-ERN12282 |  |  |  |  |  |  |
| 0 | Atr-ERN12283 |  |  |  |  |  |  |
| 0 | Atr-ERN12284 |  |  |  |  |  |  |
| 0 | Atr-ERN12285 |  |  |  |  |  |  |
| 0 | Atr-ERN12286 |  |  |  |  |  |  |
