# Supplementary material for: Genome-wide identification, characterization and gene expression of BES1 transcription factor family in grapevine (Vitis vinifera L.)
Source: Sci Rep. 2023 Jan 5;13:240. doi: 10.1038/s41598-022-24407-y (PMC9816167; doi:10.1038/s41598-022-24407-y)
Supplement: Supplementary file 3 — Supplementary Information. [file 41598_2022_24407_MOESM3_ESM.zip › Vvi_Atr/Vitis_vinifera.PN40024.v4.dna_sm.toplevel.fa.vs.Amborella_trichopoda.AMTR1.0.dna_sm.toplevel.fa.html/Atr-AmTr_v1.0_scaffold00035.html]

|  |  |  |  |  |  |  |  |  |  |  |  |  |  |
| --- | --- | --- | --- | --- | --- | --- | --- | --- | --- | --- | --- | --- | --- |
| Duplication depth | Reference chromosome | Collinear blocks | | | | | | | | | | | |
| 0 | Atr-ERN12033 |  |  |  |  |  |  |
| 0 | Atr-ERN12034 |  |  |  |  |  |  |
| 0 | Atr-ERN12035 |  |  |  |  |  |  |
| 0 | Atr-ERN12036 |  |  |  |  |  |  |
| 0 | Atr-ERN12037 |  |  |  |  |  |  |
| 0 | Atr-ERN12038 |  |  |  |  |  |  |
| 0 | Atr-ERN12039 |  |  |  |  |  |  |
| 0 | Atr-ERN12040 |  |  |  |  |  |  |
| 0 | Atr-ERN12041 |  |  |  |  |  |  |
| 0 | Atr-ERN12042 |  |  |  |  |  |  |
| 0 | Atr-ERN12043 |  |  |  |  |  |  |
| 0 | Atr-ERN12044 |  |  |  |  |  |  |
| 0 | Atr-ERN12045 |  |  |  |  |  |  |
| 0 | Atr-ERN12046 |  |  |  |  |  |  |
| 0 | Atr-ERN12047 |  |  |  |  |  |  |
| 0 | Atr-ERN12048 |  |  |  |  |  |  |
| 0 | Atr-ERN12049 |  |  |  |  |  |  |
| 0 | Atr-ERN12050 |  |  |  |  |  |  |
| 0 | Atr-ERN12051 |  |  |  |  |  |  |
| 0 | Atr-ERN12052 |  |  |  |  |  |  |
| 0 | Atr-ERN12053 |  |  |  |  |  |  |
| 0 | Atr-ERN12054 |  |  |  |  |  |  |
| 0 | Atr-ERN12055 |  |  |  |  |  |  |
| 0 | Atr-ERN12056 |  |  |  |  |  |  |
| 0 | Atr-ERN12057 |  |  |  |  |  |  |
| 0 | Atr-ERN12058 |  |  |  |  |  |  |
| 0 | Atr-ERN12059 |  |  |  |  |  |  |
| 0 | Atr-ERN12060 |  |  |  |  |  |  |
| 0 | Atr-ERN12061 |  |  |  |  |  |  |
| 0 | Atr-ERN12062 |  |  |  |  |  |  |
| 0 | Atr-ERN12063 |  |  |  |  |  |  |
| 0 | Atr-ERN12064 |  |  |  |  |  |  |
| 0 | Atr-ERN12065 |  |  |  |  |  |  |
| 0 | Atr-ERN12066 |  |  |  |  |  |  |
| 0 | Atr-ERN12067 |  |  |  |  |  |  |
| 0 | Atr-ERN12068 |  |  |  |  |  |  |
| 0 | Atr-ERN12069 |  |  |  |  |  |  |
| 0 | Atr-ERN12070 |  |  |  |  |  |  |
| 0 | Atr-ERN12071 |  |  |  |  |  |  |
| 0 | Atr-ERN12072 |  |  |  |  |  |  |
| 0 | Atr-ERN12073 |  |  |  |  |  |  |
| 0 | Atr-ERN12074 |  |  |  |  |  |  |
| 0 | Atr-ERN12075 |  |  |  |  |  |  |
| 0 | Atr-ERN12076 |  |  |  |  |  |  |
| 0 | Atr-ERN12077 |  |  |  |  |  |  |
| 0 | Atr-ERN12078 |  |  |  |  |  |  |
| 0 | Atr-ERN12079 |  |  |  |  |  |  |
| 0 | Atr-ERN12080 |  |  |  |  |  |  |
| 0 | Atr-ERN12081 |  |  |  |  |  |  |
| 0 | Atr-ERN12082 |  |  |  |  |  |  |
| 0 | Atr-ERN12083 |  |  |  |  |  |  |
| 0 | Atr-ERN12084 |  |  |  |  |  |  |
| 0 | Atr-ERN12085 |  |  |  |  |  |  |
| 0 | Atr-ERN12086 |  |  |  |  |  |  |
| 0 | Atr-ERN12087 |  |  |  |  |  |  |
| 0 | Atr-ERN12088 |  |  |  |  |  |  |
| 0 | Atr-ERN12089 |  |  |  |  |  |  |
| 0 | Atr-ERN12090 |  |  |  |  |  |  |
| 0 | Atr-ERN12091 |  |  |  |  |  |  |
| 0 | Atr-ERN12092 |  |  |  |  |  |  |
| 0 | Atr-ERN12093 |  |  |  |  |  |  |
| 0 | Atr-ERN12094 |  |  |  |  |  |  |
| 0 | Atr-ERN12095 |  |  |  |  |  |  |
| 0 | Atr-ERN12096 |  |  |  |  |  |  |
| 0 | Atr-ERN12097 |  |  |  |  |  |  |
| 0 | Atr-ERN12098 |  |  |  |  |  |  |
