# Supplementary material for: Genome-wide identification, characterization and gene expression of BES1 transcription factor family in grapevine (Vitis vinifera L.)
Source: Sci Rep. 2023 Jan 5;13:240. doi: 10.1038/s41598-022-24407-y (PMC9816167; doi:10.1038/s41598-022-24407-y)
Supplement: Supplementary file 3 — Supplementary Information. [file 41598_2022_24407_MOESM3_ESM.zip › Vvi_Atr/Vitis_vinifera.PN40024.v4.dna_sm.toplevel.fa.vs.Amborella_trichopoda.AMTR1.0.dna_sm.toplevel.fa.html/Atr-AmTr_v1.0_scaffold00036.html]

|  |  |  |  |  |  |  |  |  |  |  |  |  |  |
| --- | --- | --- | --- | --- | --- | --- | --- | --- | --- | --- | --- | --- | --- |
| Duplication depth | Reference chromosome | Collinear blocks | | | | | | | | | | | |
| 0 | Atr-ERN15285 |  |  |  |  |  |  |
| 0 | Atr-ERN15286 |  |  |  |  |  |  |
| 0 | Atr-ERN15287 |  |  |  |  |  |  |
| 0 | Atr-ERN15288 |  |  |  |  |  |  |
| 0 | Atr-ERN15289 |  |  |  |  |  |  |
| 0 | Atr-ERN15290 |  |  |  |  |  |  |
| 0 | Atr-ERN15291 |  |  |  |  |  |  |
| 0 | Atr-ERN15292 |  |  |  |  |  |  |
| 0 | Atr-ERN15293 |  |  |  |  |  |  |
| 0 | Atr-ERN15294 |  |  |  |  |  |  |
| 0 | Atr-ERN15295 |  |  |  |  |  |  |
| 0 | Atr-ERN15296 |  |  |  |  |  |  |
| 0 | Atr-ERN15297 |  |  |  |  |  |  |
| 1 | Atr-ERN15298 |  | Vvi-Vitvi04g00109\_t001 |  |  |  |  |  |
| 2 | Atr-ERN15299 |  | | | |  | Vvi-Vitvi11g01322\_t001 |  |  |  |  |
| 2 | Atr-ERN15300 |  | | | |  | | | |  |  |  |  |
| 2 | Atr-ERN15301 |  | | | |  | | | |  |  |  |  |
| 2 | Atr-ERN15302 |  | | | |  | | | |  |  |  |  |
| 2 | Atr-ERN15303 |  | | | |  | | | |  |  |  |  |
| 2 | Atr-ERN15304 |  | | | |  | | | |  |  |  |  |
| 2 | Atr-ERN15305 |  | | | |  | | | |  |  |  |  |
| 2 | Atr-ERN15306 |  | | | |  | | | |  |  |  |  |
| 2 | Atr-ERN15307 |  | | | |  | | | |  |  |  |  |
| 3 | Atr-ERN15308 |  | | | |  | | | |  | Vvi-Vitvi09g00123\_t001 |  |  |  |
| 3 | Atr-ERN15309 |  | | | |  | | | |  | | | |  |  |  |
| 3 | Atr-ERN15310 |  | | | |  | | | |  | | | |  |  |  |
| 3 | Atr-ERN15311 |  | | | |  | | | |  | | | |  |  |  |
| 3 | Atr-ERN15312 |  | | | |  | | | |  | | | |  |  |  |
| 3 | Atr-ERN15313 |  | Vvi-Vitvi04g00096\_t001 |  | | | |  | | | |  |  |  |
| 3 | Atr-ERN15314 |  | | | |  | | | |  | | | |  |  |  |
| 3 | Atr-ERN15315 |  | | | |  | | | |  | | | |  |  |  |
| 3 | Atr-ERN15316 |  | | | |  | | | |  | | | |  |  |  |
| 3 | Atr-ERN15317 |  | | | |  | | | |  | | | |  |  |  |
| 3 | Atr-ERN15318 |  | | | |  | | | |  | | | |  |  |  |
| 3 | Atr-ERN15319 |  | | | |  | | | |  | | | |  |  |  |
| 3 | Atr-ERN15320 |  | | | |  | | | |  | | | |  |  |  |
| 3 | Atr-ERN15321 |  | | | |  | | | |  | | | |  |  |  |
| 3 | Atr-ERN15322 |  | | | |  | | | |  | | | |  |  |  |
| 3 | Atr-ERN15323 |  | | | |  | | | |  | | | |  |  |  |
| 3 | Atr-ERN15324 |  | | | |  | | | |  | | | |  |  |  |
| 3 | Atr-ERN15325 |  | | | |  | Vvi-Vitvi11g00160\_t001 |  | Vvi-Vitvi09g00140\_t001 |  |  |  |
| 3 | Atr-ERN15326 |  | | | |  | | | |  | | | |  |  |  |
| 3 | Atr-ERN15327 |  | | | |  | | | |  | | | |  |  |  |
| 3 | Atr-ERN15328 |  | | | |  | | | |  | | | |  |  |  |
| 3 | Atr-ERN15329 |  | Vvi-Vitvi04g01788\_t001 |  | | | |  | | | |  |  |  |
| 3 | Atr-ERN15330 |  | | | |  | | | |  | | | |  |  |  |
| 3 | Atr-ERN15331 |  | | | |  | | | |  | | | |  |  |  |
| 3 | Atr-ERN15332 |  | Vvi-Vitvi04g00092\_t001 |  | Vvi-Vitvi11g00159\_t001 |  | | | |  |  |  |
| 3 | Atr-ERN15333 |  | | | |  | | | |  | Vvi-Vitvi09g00141\_t001 |  |  |  |
| 3 | Atr-ERN15334 |  | | | |  | | | |  | | | |  |  |  |
| 3 | Atr-ERN15335 |  | | | |  | Vvi-Vitvi11g00157\_t002 |  | | | |  |  |  |
| 3 | Atr-ERN15336 |  | | | |  | | | |  | | | |  |  |  |
| 3 | Atr-ERN15337 |  | | | |  | | | |  | | | |  |  |  |
| 3 | Atr-ERN15338 |  | | | |  | | | |  | | | |  |  |  |
| 3 | Atr-ERN15339 |  | | | |  | | | |  | | | |  |  |  |
| 3 | Atr-ERN15340 |  | | | |  | | | |  | | | |  |  |  |
| 3 | Atr-ERN15341 |  | | | |  | | | |  | | | |  |  |  |
| 3 | Atr-ERN15342 |  | | | |  | Vvi-Vitvi11g00156\_t001 |  | Vvi-Vitvi09g00144\_t001 |  |  |  |
| 3 | Atr-ERN15343 |  | Vvi-Vitvi04g00090\_t001 |  | | | |  | | | |  |  |  |
| 3 | Atr-ERN15344 |  | | | |  | | | |  | | | |  |  |  |
| 3 | Atr-ERN15345 |  | | | |  | | | |  | | | |  |  |  |
| 3 | Atr-ERN15346 |  | | | |  | | | |  | | | |  |  |  |
| 3 | Atr-ERN15347 |  | | | |  | | | |  | | | |  |  |  |
| 3 | Atr-ERN15348 |  | | | |  | | | |  | | | |  |  |  |
| 3 | Atr-ERN15349 |  | | | |  | | | |  | | | |  |  |  |
| 3 | Atr-ERN15350 |  | | | |  | | | |  | | | |  |  |  |
| 3 | Atr-ERN15351 |  | Vvi-Vitvi04g00086\_t001 |  | | | |  | | | |  |  |  |
| 3 | Atr-ERN15352 |  | | | |  | | | |  | | | |  |  |  |
| 3 | Atr-ERN15353 |  | | | |  | | | |  | | | |  |  |  |
| 3 | Atr-ERN15354 |  | | | |  | | | |  | | | |  |  |  |
| 3 | Atr-ERN15355 |  | | | |  | Vvi-Vitvi11g00155\_t001 |  | Vvi-Vitvi09g00148\_t001 |  |  |  |
| 3 | Atr-ERN15356 |  | Vvi-Vitvi04g00084\_t001 |  | | | |  | | | |  |  |  |
| 3 | Atr-ERN15357 |  | | | |  | | | |  | | | |  |  |  |
| 3 | Atr-ERN15358 |  | Vvi-Vitvi04g01786\_t001 |  | Vvi-Vitvi11g00154\_t001 |  | Vvi-Vitvi09g01521\_t001 |  |  |  |
| 3 | Atr-ERN15359 |  | | | |  | | | |  | | | |  |  |  |
| 3 | Atr-ERN15360 |  | | | |  | | | |  | | | |  |  |  |
| 3 | Atr-ERN15361 |  | | | |  | Vvi-Vitvi11g00153\_t001 |  | | | |  |  |  |
| 3 | Atr-ERN15362 |  | | | |  | | | |  | | | |  |  |  |
| 3 | Atr-ERN15363 |  | | | |  | Vvi-Vitvi11g00150\_t001 |  | Vvi-Vitvi09g00150\_t005 |  |  |  |
| 3 | Atr-ERN15364 |  | | | |  | | | |  | | | |  |  |  |
| 3 | Atr-ERN15365 |  | | | |  | | | |  | | | |  |  |  |
| 3 | Atr-ERN15366 |  | | | |  | Vvi-Vitvi11g00149\_t001 |  | Vvi-Vitvi09g04036\_t001 |  |  |  |
| 2 | Atr-ERN15367 |  | | | |  |  |  | | | |  |  |  |
| 2 | Atr-ERN15368 |  | | | |  |  |  | | | |  |  |  |
| 2 | Atr-ERN15369 |  | | | |  |  |  | | | |  |  |  |
| 2 | Atr-ERN15370 |  | | | |  |  |  | | | |  |  |  |
| 2 | Atr-ERN15371 |  | | | |  |  |  | | | |  |  |  |
| 3 | Atr-ERN15372 |  | | | |  | Vvi-Vitvi09g00227\_t001 |  | | | |  |  |  |
| 4 | Atr-ERN15373 |  | | | |  | Vvi-Vitvi09g00226\_t001 |  | | | |  | Vvi-Vitvi04g00051\_t001 |  |  |
| 4 | Atr-ERN15374 |  | | | |  | | | |  | | | |  | | | |  |  |
| 4 | Atr-ERN15375 |  | Vvi-Vitvi04g04031\_t001 |  | | | |  | | | |  | | | |  |  |
| 4 | Atr-ERN15376 |  | | | |  | | | |  | | | |  | | | |  |  |
| 4 | Atr-ERN15377 |  | | | |  | | | |  | | | |  | | | |  |  |
| 4 | Atr-ERN15378 |  | | | |  | | | |  | | | |  | | | |  |  |
| 4 | Atr-ERN15379 |  | | | |  | | | |  | | | |  | | | |  |  |
| 4 | Atr-ERN15380 |  | | | |  | | | |  | | | |  | | | |  |  |
| 4 | Atr-ERN15381 |  | | | |  | | | |  | | | |  | | | |  |  |
| 4 | Atr-ERN15382 |  | | | |  | | | |  | | | |  | | | |  |  |
| 4 | Atr-ERN15383 |  | | | |  | | | |  | | | |  | | | |  |  |
| 4 | Atr-ERN15384 |  | | | |  | | | |  | | | |  | | | |  |  |
| 4 | Atr-ERN15385 |  | | | |  | | | |  | | | |  | | | |  |  |
| 4 | Atr-ERN15386 |  | | | |  | | | |  | | | |  | | | |  |  |
| 4 | Atr-ERN15387 |  | | | |  | Vvi-Vitvi09g00223\_t001 |  | | | |  | | | |  |  |
| 4 | Atr-ERN15388 |  | | | |  | Vvi-Vitvi09g04055\_t001 |  | | | |  | | | |  |  |
| 4 | Atr-ERN15389 |  | | | |  | | | |  | | | |  | | | |  |  |
| 4 | Atr-ERN15390 |  | | | |  | | | |  | Vvi-Vitvi09g01533\_t001 |  | Vvi-Vitvi04g00055\_t001 |  |  |
| 3 | Atr-ERN15391 |  | | | |  | | | |  |  |  | | | |  |  |
| 3 | Atr-ERN15392 |  | | | |  | | | |  |  |  | | | |  |  |
| 3 | Atr-ERN15393 |  | | | |  | | | |  |  |  | | | |  |  |
| 3 | Atr-ERN15394 |  | | | |  | | | |  |  |  | | | |  |  |
| 3 | Atr-ERN15395 |  | | | |  | | | |  |  |  | | | |  |  |
| 3 | Atr-ERN15396 |  | | | |  | | | |  |  |  | | | |  |  |
| 3 | Atr-ERN15397 |  | | | |  | | | |  |  |  | | | |  |  |
| 3 | Atr-ERN15398 |  | | | |  | | | |  |  |  | | | |  |  |
| 3 | Atr-ERN15399 |  | | | |  | | | |  |  |  | | | |  |  |
| 3 | Atr-ERN15400 |  | Vvi-Vitvi04g00060\_t001 |  | | | |  |  |  | Vvi-Vitvi04g00060\_t001 |  |  |
| 2 | Atr-ERN15401 |  |  |  | | | |  |  |  | Vvi-Vitvi04g04025\_t001 |  |  |
| 2 | Atr-ERN15402 |  |  |  | | | |  |  |  | | | |  |  |
| 2 | Atr-ERN15403 |  |  |  | | | |  |  |  | Vvi-Vitvi04g00062\_t001 |  |  |
| 2 | Atr-ERN15404 |  |  |  | | | |  |  |  | | | |  |  |
| 2 | Atr-ERN15405 |  |  |  | | | |  |  |  | | | |  |  |
| 2 | Atr-ERN15406 |  |  |  | | | |  |  |  | | | |  |  |
| 2 | Atr-ERN15407 |  |  |  | | | |  |  |  | | | |  |  |
| 2 | Atr-ERN15408 |  |  |  | Vvi-Vitvi09g00218\_t001 |  |  |  | | | |  |  |
| 2 | Atr-ERN15409 |  |  |  | Vvi-Vitvi09g00217\_t001 |  |  |  | | | |  |  |
| 2 | Atr-ERN15410 |  |  |  | | | |  |  |  | | | |  |  |
| 2 | Atr-ERN15411 |  |  |  | | | |  |  |  | | | |  |  |
| 2 | Atr-ERN15412 |  |  |  | | | |  |  |  | | | |  |  |
| 2 | Atr-ERN15413 |  |  |  | | | |  |  |  | | | |  |  |
| 2 | Atr-ERN15414 |  |  |  | | | |  |  |  | | | |  |  |
| 2 | Atr-ERN15415 |  |  |  | Vvi-Vitvi09g01545\_t001 |  |  |  | Vvi-Vitvi04g00064\_t001 |  |  |
| 1 | Atr-ERN15416 |  |  |  | | | |  |  |  |  |
| 1 | Atr-ERN15417 |  |  |  | | | |  |  |  |  |
| 1 | Atr-ERN15418 |  |  |  | | | |  |  |  |  |
| 1 | Atr-ERN15419 |  |  |  | | | |  |  |  |  |
| 1 | Atr-ERN15420 |  |  |  | | | |  |  |  |  |
| 1 | Atr-ERN15421 |  |  |  | | | |  |  |  |  |
| 1 | Atr-ERN15422 |  |  |  | | | |  |  |  |  |
| 1 | Atr-ERN15423 |  |  |  | | | |  |  |  |  |
| 1 | Atr-ERN15424 |  |  |  | | | |  |  |  |  |
| 1 | Atr-ERN15425 |  |  |  | | | |  |  |  |  |
| 1 | Atr-ERN15426 |  |  |  | Vvi-Vitvi09g00216\_t001 |  |  |  |  |
| 1 | Atr-ERN15427 |  |  |  | | | |  |  |  |  |
| 1 | Atr-ERN15428 |  |  |  | | | |  |  |  |  |
| 1 | Atr-ERN15429 |  |  |  | | | |  |  |  |  |
| 1 | Atr-ERN15430 |  |  |  | | | |  |  |  |  |
| 1 | Atr-ERN15431 |  |  |  | | | |  |  |  |  |
| 1 | Atr-ERN15432 |  |  |  | | | |  |  |  |  |
| 1 | Atr-ERN15433 |  |  |  | | | |  |  |  |  |
| 1 | Atr-ERN15434 |  |  |  | | | |  |  |  |  |
| 1 | Atr-ERN15435 |  |  |  | | | |  |  |  |  |
| 1 | Atr-ERN15436 |  |  |  | | | |  |  |  |  |
| 1 | Atr-ERN15437 |  |  |  | Vvi-Vitvi09g00215\_t002 |  |  |  |  |
| 1 | Atr-ERN15438 |  |  |  | Vvi-Vitvi09g00214\_t003 |  |  |  |  |
| 1 | Atr-ERN15439 |  |  |  | | | |  |  |  |  |
| 1 | Atr-ERN15440 |  |  |  | Vvi-Vitvi09g00213\_t002 |  |  |  |  |
| 1 | Atr-ERN15441 |  |  |  | | | |  |  |  |  |
| 1 | Atr-ERN15442 |  |  |  | | | |  |  |  |  |
| 1 | Atr-ERN15443 |  |  |  | | | |  |  |  |  |
| 1 | Atr-ERN15444 |  |  |  | | | |  |  |  |  |
| 1 | Atr-ERN15445 |  |  |  | | | |  |  |  |  |
| 1 | Atr-ERN15446 |  |  |  | | | |  |  |  |  |
| 1 | Atr-ERN15447 |  |  |  | | | |  |  |  |  |
| 1 | Atr-ERN15448 |  |  |  | | | |  |  |  |  |
| 1 | Atr-ERN15449 |  |  |  | | | |  |  |  |  |
| 1 | Atr-ERN15450 |  |  |  | Vvi-Vitvi09g00211\_t001 |  |  |  |  |
| 1 | Atr-ERN15451 |  |  |  | | | |  |  |  |  |
| 2 | Atr-ERN15452 |  | Vvi-Vitvi11g04050\_t001 |  | | | |  |  |  |  |
| 2 | Atr-ERN15453 |  | | | |  | | | |  |  |  |  |
| 2 | Atr-ERN15454 |  | | | |  | | | |  |  |  |  |
| 2 | Atr-ERN15455 |  | | | |  | | | |  |  |  |  |
| 2 | Atr-ERN15456 |  | | | |  | | | |  |  |  |  |
| 2 | Atr-ERN15457 |  | | | |  | | | |  |  |  |  |
| 2 | Atr-ERN15458 |  | | | |  | | | |  |  |  |  |
| 2 | Atr-ERN15459 |  | | | |  | Vvi-Vitvi09g00210\_t001 |  |  |  |  |
| 2 | Atr-ERN15460 |  | Vvi-Vitvi11g00213\_t001 |  | | | |  |  |  |  |
| 2 | Atr-ERN15461 |  | Vvi-Vitvi11g01379\_t001 |  | | | |  |  |  |  |
| 3 | Atr-ERN15462 |  | | | |  | | | |  | Vvi-Vitvi04g00065\_t001 |  |  |  |
| 3 | Atr-ERN15463 |  | Vvi-Vitvi11g00212\_t001 |  | | | |  | | | |  |  |  |
| 3 | Atr-ERN15464 |  | | | |  | Vvi-Vitvi09g00209\_t001 |  | Vvi-Vitvi04g04026\_t001 |  |  |  |
| 3 | Atr-ERN15465 |  | | | |  | | | |  | Vvi-Vitvi04g00066\_t001 |  |  |  |
| 3 | Atr-ERN15466 |  | | | |  | | | |  | | | |  |  |  |
| 3 | Atr-ERN15467 |  | Vvi-Vitvi11g00210\_t001 |  | Vvi-Vitvi09g00208\_t001 |  | Vvi-Vitvi04g00068\_t001 |  |  |  |
| 3 | Atr-ERN15468 |  | | | |  | | | |  | | | |  |  |  |
| 3 | Atr-ERN15469 |  | | | |  | | | |  | | | |  |  |  |
| 3 | Atr-ERN15470 |  | | | |  | | | |  | | | |  |  |  |
| 3 | Atr-ERN15471 |  | Vvi-Vitvi11g00209\_t001 |  | | | |  | | | |  |  |  |
| 3 | Atr-ERN15472 |  | Vvi-Vitvi11g00208\_t001 |  | | | |  | | | |  |  |  |
| 3 | Atr-ERN15473 |  | Vvi-Vitvi11g00207\_t001 |  | | | |  | | | |  |  |  |
| 3 | Atr-ERN15474 |  | Vvi-Vitvi11g00206\_t001 |  | | | |  | Vvi-Vitvi04g00069\_t001 |  |  |  |
| 3 | Atr-ERN15475 |  | | | |  | | | |  | | | |  |  |  |
| 3 | Atr-ERN15476 |  | | | |  | | | |  | | | |  |  |  |
| 3 | Atr-ERN15477 |  | | | |  | | | |  | | | |  |  |  |
| 3 | Atr-ERN15478 |  | Vvi-Vitvi11g00203\_t001 |  | | | |  | Vvi-Vitvi04g00070\_t001 |  |  |  |
| 3 | Atr-ERN15479 |  | Vvi-Vitvi11g00202\_t001 |  | Vvi-Vitvi09g01542\_t002 |  | Vvi-Vitvi04g00071\_t001 |  |  |  |
| 0 | Atr-ERN15480 |  |  |  |  |  |  |
| 0 | Atr-ERN15481 |  |  |  |  |  |  |
