# Supplementary material for: Genome-wide identification, characterization and gene expression of BES1 transcription factor family in grapevine (Vitis vinifera L.)
Source: Sci Rep. 2023 Jan 5;13:240. doi: 10.1038/s41598-022-24407-y (PMC9816167; doi:10.1038/s41598-022-24407-y)
Supplement: Supplementary file 3 — Supplementary Information. [file 41598_2022_24407_MOESM3_ESM.zip › Vvi_Atr/Vitis_vinifera.PN40024.v4.dna_sm.toplevel.fa.vs.Amborella_trichopoda.AMTR1.0.dna_sm.toplevel.fa.html/Atr-AmTr_v1.0_scaffold00037.html]

|  |  |  |  |  |  |  |  |  |  |  |  |  |  |
| --- | --- | --- | --- | --- | --- | --- | --- | --- | --- | --- | --- | --- | --- |
| Duplication depth | Reference chromosome | Collinear blocks | | | | | | | | | | | |
| 0 | Atr-ERN17289 |  |  |  |  |  |  |
| 0 | Atr-ERN17290 |  |  |  |  |  |  |
| 0 | Atr-ERN17291 |  |  |  |  |  |  |
| 0 | Atr-ERN17292 |  |  |  |  |  |  |
| 0 | Atr-ERN17293 |  |  |  |  |  |  |
| 0 | Atr-ERN17294 |  |  |  |  |  |  |
| 0 | Atr-ERN17295 |  |  |  |  |  |  |
| 0 | Atr-ERN17296 |  |  |  |  |  |  |
| 0 | Atr-ERN17297 |  |  |  |  |  |  |
| 0 | Atr-ERN17298 |  |  |  |  |  |  |
| 0 | Atr-ERN17299 |  |  |  |  |  |  |
| 0 | Atr-ERN17300 |  |  |  |  |  |  |
| 0 | Atr-ERN17301 |  |  |  |  |  |  |
| 0 | Atr-ERN17302 |  |  |  |  |  |  |
| 0 | Atr-ERN17303 |  |  |  |  |  |  |
| 0 | Atr-ERN17304 |  |  |  |  |  |  |
| 0 | Atr-ERN17305 |  |  |  |  |  |  |
| 0 | Atr-ERN17306 |  |  |  |  |  |  |
| 0 | Atr-ERN17307 |  |  |  |  |  |  |
| 0 | Atr-ERN17308 |  |  |  |  |  |  |
| 0 | Atr-ERN17309 |  |  |  |  |  |  |
| 0 | Atr-ERN17310 |  |  |  |  |  |  |
| 0 | Atr-ERN17311 |  |  |  |  |  |  |
| 0 | Atr-ERN17312 |  |  |  |  |  |  |
| 0 | Atr-ERN17313 |  |  |  |  |  |  |
| 0 | Atr-ERN17314 |  |  |  |  |  |  |
| 0 | Atr-ERN17315 |  |  |  |  |  |  |
| 0 | Atr-ERN17316 |  |  |  |  |  |  |
| 0 | Atr-ERN17317 |  |  |  |  |  |  |
| 0 | Atr-ERN17318 |  |  |  |  |  |  |
| 0 | Atr-ERN17319 |  |  |  |  |  |  |
| 0 | Atr-ERN17320 |  |  |  |  |  |  |
| 0 | Atr-ERN17321 |  |  |  |  |  |  |
| 0 | Atr-ERN17322 |  |  |  |  |  |  |
| 1 | Atr-ERN17323 |  | Vvi-Vitvi10g01642\_t001 |  |  |  |  |  |
| 1 | Atr-ERN17324 |  | | | |  |  |  |  |  |
| 1 | Atr-ERN17325 |  | | | |  |  |  |  |  |
| 1 | Atr-ERN17326 |  | | | |  |  |  |  |  |
| 1 | Atr-ERN17327 |  | | | |  |  |  |  |  |
| 1 | Atr-ERN17328 |  | | | |  |  |  |  |  |
| 1 | Atr-ERN17329 |  | | | |  |  |  |  |  |
| 1 | Atr-ERN17330 |  | | | |  |  |  |  |  |
| 1 | Atr-ERN17331 |  | | | |  |  |  |  |  |
| 1 | Atr-ERN17332 |  | | | |  |  |  |  |  |
| 1 | Atr-ERN17333 |  | | | |  |  |  |  |  |
| 1 | Atr-ERN17334 |  | | | |  |  |  |  |  |
| 1 | Atr-ERN17335 |  | | | |  |  |  |  |  |
| 1 | Atr-ERN17336 |  | | | |  |  |  |  |  |
| 1 | Atr-ERN17337 |  | | | |  |  |  |  |  |
| 1 | Atr-ERN17338 |  | | | |  |  |  |  |  |
| 1 | Atr-ERN17339 |  | | | |  |  |  |  |  |
| 1 | Atr-ERN17340 |  | | | |  |  |  |  |  |
| 1 | Atr-ERN17341 |  | | | |  |  |  |  |  |
| 1 | Atr-ERN17342 |  | | | |  |  |  |  |  |
| 1 | Atr-ERN17343 |  | | | |  |  |  |  |  |
| 1 | Atr-ERN17344 |  | Vvi-Vitvi10g00101\_t001 |  |  |  |  |  |
| 1 | Atr-ERN17345 |  | | | |  |  |  |  |  |
| 1 | Atr-ERN17346 |  | Vvi-Vitvi10g00098\_t001 |  |  |  |  |  |
| 1 | Atr-ERN17347 |  | | | |  |  |  |  |  |
| 1 | Atr-ERN17348 |  | | | |  |  |  |  |  |
| 1 | Atr-ERN17349 |  | | | |  |  |  |  |  |
| 1 | Atr-ERN17350 |  | | | |  |  |  |  |  |
| 1 | Atr-ERN17351 |  | | | |  |  |  |  |  |
| 1 | Atr-ERN17352 |  | | | |  |  |  |  |  |
| 1 | Atr-ERN17353 |  | | | |  |  |  |  |  |
| 1 | Atr-ERN17354 |  | | | |  |  |  |  |  |
| 1 | Atr-ERN17355 |  | | | |  |  |  |  |  |
| 1 | Atr-ERN17356 |  | | | |  |  |  |  |  |
| 1 | Atr-ERN17357 |  | | | |  |  |  |  |  |
| 2 | Atr-ERN17358 |  | Vvi-Vitvi10g00093\_t001 |  | Vvi-Vitvi19g04237\_t001 |  |  |  |  |
| 2 | Atr-ERN17359 |  | Vvi-Vitvi10g00092\_t001 |  | Vvi-Vitvi19g02020\_t001 |  |  |  |  |
| 2 | Atr-ERN17360 |  | | | |  | Vvi-Vitvi19g00507\_t001 |  |  |  |  |
| 2 | Atr-ERN17361 |  | | | |  | | | |  |  |  |  |
| 3 | Atr-ERN17362 |  | | | |  | | | |  | Vvi-Vitvi12g00368\_t001 |  |  |  |
| 3 | Atr-ERN17363 |  | | | |  | | | |  | | | |  |  |  |
| 3 | Atr-ERN17364 |  | | | |  | | | |  | | | |  |  |  |
| 3 | Atr-ERN17365 |  | | | |  | | | |  | | | |  |  |  |
| 3 | Atr-ERN17366 |  | | | |  | | | |  | | | |  |  |  |
| 3 | Atr-ERN17367 |  | | | |  | | | |  | | | |  |  |  |
| 3 | Atr-ERN17368 |  | | | |  | | | |  | | | |  |  |  |
| 3 | Atr-ERN17369 |  | | | |  | | | |  | | | |  |  |  |
| 3 | Atr-ERN17370 |  | | | |  | | | |  | | | |  |  |  |
| 3 | Atr-ERN17371 |  | Vvi-Vitvi10g00090\_t001 |  | | | |  | | | |  |  |  |
| 3 | Atr-ERN17372 |  | | | |  | | | |  | | | |  |  |  |
| 3 | Atr-ERN17373 |  | | | |  | Vvi-Vitvi19g00508\_t001 |  | Vvi-Vitvi12g00376\_t001 |  |  |  |
| 3 | Atr-ERN17374 |  | | | |  | | | |  | | | |  |  |  |
| 3 | Atr-ERN17375 |  | | | |  | | | |  | | | |  |  |  |
| 3 | Atr-ERN17376 |  | | | |  | | | |  | | | |  |  |  |
| 3 | Atr-ERN17377 |  | | | |  | | | |  | | | |  |  |  |
| 3 | Atr-ERN17378 |  | | | |  | | | |  | | | |  |  |  |
| 3 | Atr-ERN17379 |  | | | |  | Vvi-Vitvi19g00509\_t001 |  | | | |  |  |  |
| 3 | Atr-ERN17380 |  | | | |  | | | |  | | | |  |  |  |
| 3 | Atr-ERN17381 |  | | | |  | | | |  | | | |  |  |  |
| 3 | Atr-ERN17382 |  | | | |  | | | |  | | | |  |  |  |
| 3 | Atr-ERN17383 |  | | | |  | | | |  | | | |  |  |  |
| 3 | Atr-ERN17384 |  | | | |  | | | |  | | | |  |  |  |
| 3 | Atr-ERN17385 |  | | | |  | | | |  | Vvi-Vitvi12g00377\_t001 |  |  |  |
| 3 | Atr-ERN17386 |  | | | |  | | | |  | | | |  |  |  |
| 3 | Atr-ERN17387 |  | | | |  | | | |  | | | |  |  |  |
| 3 | Atr-ERN17388 |  | | | |  | | | |  | | | |  |  |  |
| 3 | Atr-ERN17389 |  | | | |  | Vvi-Vitvi19g00512\_t001 |  | | | |  |  |  |
| 3 | Atr-ERN17390 |  | | | |  | | | |  | | | |  |  |  |
| 3 | Atr-ERN17391 |  | Vvi-Vitvi10g00088\_t001 |  | | | |  | Vvi-Vitvi12g00380\_t001 |  |  |  |
| 3 | Atr-ERN17392 |  | | | |  | | | |  | | | |  |  |  |
| 3 | Atr-ERN17393 |  | | | |  | | | |  | | | |  |  |  |
| 3 | Atr-ERN17394 |  | Vvi-Vitvi10g00087\_t001 |  | Vvi-Vitvi19g02023\_t001 |  | | | |  |  |  |
| 3 | Atr-ERN17395 |  | Vvi-Vitvi10g00086\_t001 |  | | | |  | Vvi-Vitvi12g00381\_t001 |  |  |  |
| 3 | Atr-ERN17396 |  | | | |  | | | |  | | | |  |  |  |
| 3 | Atr-ERN17397 |  | | | |  | | | |  | | | |  |  |  |
| 3 | Atr-ERN17398 |  | Vvi-Vitvi10g00085\_t001 |  | | | |  | | | |  |  |  |
| 3 | Atr-ERN17399 |  | | | |  | | | |  | | | |  |  |  |
| 3 | Atr-ERN17400 |  | Vvi-Vitvi10g00084\_t001 |  | | | |  | | | |  |  |  |
| 3 | Atr-ERN17401 |  | | | |  | | | |  | | | |  |  |  |
| 3 | Atr-ERN17402 |  | Vvi-Vitvi10g00083\_t001 |  | | | |  | | | |  |  |  |
| 3 | Atr-ERN17403 |  | | | |  | | | |  | | | |  |  |  |
| 3 | Atr-ERN17404 |  | | | |  | Vvi-Vitvi19g00514\_t001 |  | | | |  |  |  |
| 3 | Atr-ERN17405 |  | | | |  | Vvi-Vitvi19g00515\_t001 |  | | | |  |  |  |
| 3 | Atr-ERN17406 |  | Vvi-Vitvi10g00082\_t001 |  | | | |  | Vvi-Vitvi12g00382\_t001 |  |  |  |
| 2 | Atr-ERN17407 |  | | | |  | | | |  |  |  |  |
| 2 | Atr-ERN17408 |  | | | |  | | | |  |  |  |  |
| 2 | Atr-ERN17409 |  | | | |  | | | |  |  |  |  |
| 2 | Atr-ERN17410 |  | | | |  | | | |  |  |  |  |
| 2 | Atr-ERN17411 |  | | | |  | | | |  |  |  |  |
| 2 | Atr-ERN17412 |  | | | |  | | | |  |  |  |  |
| 2 | Atr-ERN17413 |  | Vvi-Vitvi10g04022\_t001 |  | | | |  |  |  |  |
| 2 | Atr-ERN17414 |  | | | |  | | | |  |  |  |  |
| 2 | Atr-ERN17415 |  | | | |  | Vvi-Vitvi19g00518\_t001 |  |  |  |  |
| 1 | Atr-ERN17416 |  | | | |  |  |  |  |  |
| 1 | Atr-ERN17417 |  | | | |  |  |  |  |  |
| 1 | Atr-ERN17418 |  | | | |  |  |  |  |  |
| 1 | Atr-ERN17419 |  | | | |  |  |  |  |  |
| 1 | Atr-ERN17420 |  | | | |  |  |  |  |  |
| 1 | Atr-ERN17421 |  | | | |  |  |  |  |  |
| 1 | Atr-ERN17422 |  | | | |  |  |  |  |  |
| 1 | Atr-ERN17423 |  | | | |  |  |  |  |  |
| 1 | Atr-ERN17424 |  | | | |  |  |  |  |  |
| 1 | Atr-ERN17425 |  | | | |  |  |  |  |  |
| 1 | Atr-ERN17426 |  | | | |  |  |  |  |  |
| 1 | Atr-ERN17427 |  | | | |  |  |  |  |  |
| 1 | Atr-ERN17428 |  | | | |  |  |  |  |  |
| 1 | Atr-ERN17429 |  | | | |  |  |  |  |  |
| 1 | Atr-ERN17430 |  | | | |  |  |  |  |  |
| 1 | Atr-ERN17431 |  | | | |  |  |  |  |  |
| 1 | Atr-ERN17432 |  | | | |  |  |  |  |  |
| 1 | Atr-ERN17433 |  | Vvi-Vitvi10g00079\_t001 |  |  |  |  |  |
| 1 | Atr-ERN17434 |  | | | |  |  |  |  |  |
| 1 | Atr-ERN17435 |  | | | |  |  |  |  |  |
| 1 | Atr-ERN17436 |  | | | |  |  |  |  |  |
| 1 | Atr-ERN17437 |  | | | |  |  |  |  |  |
| 1 | Atr-ERN17438 |  | | | |  |  |  |  |  |
| 1 | Atr-ERN17439 |  | | | |  |  |  |  |  |
| 1 | Atr-ERN17440 |  | Vvi-Vitvi10g00078\_t001.1.6037826a |  |  |  |  |  |
| 1 | Atr-ERN17441 |  | | | |  |  |  |  |  |
| 1 | Atr-ERN17442 |  | Vvi-Vitvi10g04018\_t001 |  |  |  |  |  |
| 1 | Atr-ERN17443 |  | | | |  |  |  |  |  |
| 1 | Atr-ERN17444 |  | | | |  |  |  |  |  |
| 1 | Atr-ERN17445 |  | Vvi-Vitvi10g00070\_t002 |  |  |  |  |  |
| 0 | Atr-ERN17446 |  |  |  |  |  |  |
| 0 | Atr-ERN17447 |  |  |  |  |  |  |
| 0 | Atr-ERN17448 |  |  |  |  |  |  |
| 0 | Atr-ERN17449 |  |  |  |  |  |  |
| 0 | Atr-ERN17450 |  |  |  |  |  |  |
| 0 | Atr-ERN17451 |  |  |  |  |  |  |
