# Supplementary material for: Genome-wide identification, characterization and gene expression of BES1 transcription factor family in grapevine (Vitis vinifera L.)
Source: Sci Rep. 2023 Jan 5;13:240. doi: 10.1038/s41598-022-24407-y (PMC9816167; doi:10.1038/s41598-022-24407-y)
Supplement: Supplementary file 3 — Supplementary Information. [file 41598_2022_24407_MOESM3_ESM.zip › Vvi_Atr/Vitis_vinifera.PN40024.v4.dna_sm.toplevel.fa.vs.Amborella_trichopoda.AMTR1.0.dna_sm.toplevel.fa.html/Atr-AmTr_v1.0_scaffold00038.html]

|  |  |  |  |  |  |  |  |  |  |  |  |  |  |
| --- | --- | --- | --- | --- | --- | --- | --- | --- | --- | --- | --- | --- | --- |
| Duplication depth | Reference chromosome | Collinear blocks | | | | | | | | | | | |
| 0 | Atr-ERN14501 |  |  |  |  |  |  |
| 0 | Atr-ERN14502 |  |  |  |  |  |  |
| 0 | Atr-ERN14503 |  |  |  |  |  |  |
| 0 | Atr-ERN14504 |  |  |  |  |  |  |
| 0 | Atr-ERN14505 |  |  |  |  |  |  |
| 0 | Atr-ERN14506 |  |  |  |  |  |  |
| 0 | Atr-ERN14507 |  |  |  |  |  |  |
| 0 | Atr-ERN14508 |  |  |  |  |  |  |
| 0 | Atr-ERN14509 |  |  |  |  |  |  |
| 0 | Atr-ERN14510 |  |  |  |  |  |  |
| 0 | Atr-ERN14511 |  |  |  |  |  |  |
| 0 | Atr-ERN14512 |  |  |  |  |  |  |
| 0 | Atr-ERN14513 |  |  |  |  |  |  |
| 0 | Atr-ERN14514 |  |  |  |  |  |  |
| 0 | Atr-ERN14515 |  |  |  |  |  |  |
| 0 | Atr-ERN14516 |  |  |  |  |  |  |
| 0 | Atr-ERN14517 |  |  |  |  |  |  |
| 0 | Atr-ERN14518 |  |  |  |  |  |  |
| 0 | Atr-ERN14519 |  |  |  |  |  |  |
| 0 | Atr-ERN14520 |  |  |  |  |  |  |
| 0 | Atr-ERN14521 |  |  |  |  |  |  |
| 0 | Atr-ERN14522 |  |  |  |  |  |  |
| 0 | Atr-ERN14523 |  |  |  |  |  |  |
| 0 | Atr-ERN14524 |  |  |  |  |  |  |
| 0 | Atr-ERN14525 |  |  |  |  |  |  |
| 0 | Atr-ERN14526 |  |  |  |  |  |  |
| 0 | Atr-ERN14527 |  |  |  |  |  |  |
| 0 | Atr-ERN14528 |  |  |  |  |  |  |
| 0 | Atr-ERN14529 |  |  |  |  |  |  |
| 0 | Atr-ERN14530 |  |  |  |  |  |  |
| 0 | Atr-ERN14531 |  |  |  |  |  |  |
| 0 | Atr-ERN14532 |  |  |  |  |  |  |
| 0 | Atr-ERN14533 |  |  |  |  |  |  |
| 0 | Atr-ERN14534 |  |  |  |  |  |  |
| 0 | Atr-ERN14535 |  |  |  |  |  |  |
| 0 | Atr-ERN14536 |  |  |  |  |  |  |
| 0 | Atr-ERN14537 |  |  |  |  |  |  |
| 0 | Atr-ERN14538 |  |  |  |  |  |  |
| 0 | Atr-ERN14539 |  |  |  |  |  |  |
| 0 | Atr-ERN14540 |  |  |  |  |  |  |
| 0 | Atr-ERN14541 |  |  |  |  |  |  |
| 0 | Atr-ERN14542 |  |  |  |  |  |  |
| 0 | Atr-ERN14543 |  |  |  |  |  |  |
| 0 | Atr-ERN14544 |  |  |  |  |  |  |
| 0 | Atr-ERN14545 |  |  |  |  |  |  |
| 0 | Atr-ERN14546 |  |  |  |  |  |  |
| 0 | Atr-ERN14547 |  |  |  |  |  |  |
| 0 | Atr-ERN14548 |  |  |  |  |  |  |
| 0 | Atr-ERN14549 |  |  |  |  |  |  |
| 0 | Atr-ERN14550 |  |  |  |  |  |  |
| 0 | Atr-ERN14551 |  |  |  |  |  |  |
| 0 | Atr-ERN14552 |  |  |  |  |  |  |
| 0 | Atr-ERN14553 |  |  |  |  |  |  |
| 0 | Atr-ERN14554 |  |  |  |  |  |  |
| 0 | Atr-ERN14555 |  |  |  |  |  |  |
| 0 | Atr-ERN14556 |  |  |  |  |  |  |
| 0 | Atr-ERN14557 |  |  |  |  |  |  |
| 0 | Atr-ERN14558 |  |  |  |  |  |  |
| 0 | Atr-ERN14559 |  |  |  |  |  |  |
| 0 | Atr-ERN14560 |  |  |  |  |  |  |
| 0 | Atr-ERN14561 |  |  |  |  |  |  |
| 0 | Atr-ERN14562 |  |  |  |  |  |  |
| 0 | Atr-ERN14563 |  |  |  |  |  |  |
| 0 | Atr-ERN14564 |  |  |  |  |  |  |
| 0 | Atr-ERN14565 |  |  |  |  |  |  |
| 0 | Atr-ERN14566 |  |  |  |  |  |  |
| 0 | Atr-ERN14567 |  |  |  |  |  |  |
| 0 | Atr-ERN14568 |  |  |  |  |  |  |
| 0 | Atr-ERN14569 |  |  |  |  |  |  |
| 0 | Atr-ERN14570 |  |  |  |  |  |  |
| 0 | Atr-ERN14571 |  |  |  |  |  |  |
| 0 | Atr-ERN14572 |  |  |  |  |  |  |
| 0 | Atr-ERN14573 |  |  |  |  |  |  |
| 0 | Atr-ERN14574 |  |  |  |  |  |  |
| 0 | Atr-ERN14575 |  |  |  |  |  |  |
| 0 | Atr-ERN14576 |  |  |  |  |  |  |
| 0 | Atr-ERN14577 |  |  |  |  |  |  |
| 0 | Atr-ERN14578 |  |  |  |  |  |  |
| 0 | Atr-ERN14579 |  |  |  |  |  |  |
| 0 | Atr-ERN14580 |  |  |  |  |  |  |
| 0 | Atr-ERN14581 |  |  |  |  |  |  |
| 0 | Atr-ERN14582 |  |  |  |  |  |  |
| 0 | Atr-ERN14583 |  |  |  |  |  |  |
| 0 | Atr-ERN14584 |  |  |  |  |  |  |
| 0 | Atr-ERN14585 |  |  |  |  |  |  |
| 0 | Atr-ERN14586 |  |  |  |  |  |  |
| 0 | Atr-ERN14587 |  |  |  |  |  |  |
| 0 | Atr-ERN14588 |  |  |  |  |  |  |
| 0 | Atr-ERN14589 |  |  |  |  |  |  |
| 0 | Atr-ERN14590 |  |  |  |  |  |  |
| 0 | Atr-ERN14591 |  |  |  |  |  |  |
| 0 | Atr-ERN14592 |  |  |  |  |  |  |
| 0 | Atr-ERN14593 |  |  |  |  |  |  |
| 0 | Atr-ERN14594 |  |  |  |  |  |  |
| 1 | Atr-ERN14595 |  | Vvi-Vitvi18g00397\_t001 |  |  |  |  |  |
| 1 | Atr-ERN14596 |  | | | |  |  |  |  |  |
| 1 | Atr-ERN14597 |  | | | |  |  |  |  |  |
| 1 | Atr-ERN14598 |  | | | |  |  |  |  |  |
| 1 | Atr-ERN14599 |  | Vvi-Vitvi18g00398\_t001 |  |  |  |  |  |
| 1 | Atr-ERN14600 |  | | | |  |  |  |  |  |
| 1 | Atr-ERN14601 |  | | | |  |  |  |  |  |
| 1 | Atr-ERN14602 |  | | | |  |  |  |  |  |
| 1 | Atr-ERN14603 |  | | | |  |  |  |  |  |
| 1 | Atr-ERN14604 |  | | | |  |  |  |  |  |
| 1 | Atr-ERN14605 |  | | | |  |  |  |  |  |
| 1 | Atr-ERN14606 |  | | | |  |  |  |  |  |
| 1 | Atr-ERN14607 |  | | | |  |  |  |  |  |
| 1 | Atr-ERN14608 |  | | | |  |  |  |  |  |
| 2 | Atr-ERN14609 |  | | | |  | Vvi-Vitvi12g02554\_t001 |  |  |  |  |
| 2 | Atr-ERN14610 |  | | | |  | Vvi-Vitvi12g04300\_t002 |  |  |  |  |
| 2 | Atr-ERN14611 |  | | | |  | | | |  |  |  |  |
| 2 | Atr-ERN14612 |  | | | |  | | | |  |  |  |  |
| 2 | Atr-ERN14613 |  | | | |  | Vvi-Vitvi12g04299\_t002 |  |  |  |  |
| 2 | Atr-ERN14614 |  | | | |  | | | |  |  |  |  |
| 2 | Atr-ERN14615 |  | | | |  | | | |  |  |  |  |
| 2 | Atr-ERN14616 |  | | | |  | | | |  |  |  |  |
| 2 | Atr-ERN14617 |  | | | |  | | | |  |  |  |  |
| 2 | Atr-ERN14618 |  | | | |  | | | |  |  |  |  |
| 2 | Atr-ERN14619 |  | | | |  | Vvi-Vitvi12g00905\_t001 |  |  |  |  |
| 2 | Atr-ERN14620 |  | Vvi-Vitvi18g00401\_t001 |  | | | |  |  |  |  |
| 2 | Atr-ERN14621 |  | | | |  | | | |  |  |  |  |
| 2 | Atr-ERN14622 |  | | | |  | | | |  |  |  |  |
| 2 | Atr-ERN14623 |  | | | |  | | | |  |  |  |  |
| 2 | Atr-ERN14624 |  | | | |  | | | |  |  |  |  |
| 2 | Atr-ERN14625 |  | Vvi-Vitvi18g00406\_t001 |  | | | |  |  |  |  |
| 2 | Atr-ERN14626 |  | | | |  | | | |  |  |  |  |
| 2 | Atr-ERN14627 |  | | | |  | | | |  |  |  |  |
| 2 | Atr-ERN14628 |  | | | |  | | | |  |  |  |  |
| 2 | Atr-ERN14629 |  | | | |  | | | |  |  |  |  |
| 2 | Atr-ERN14630 |  | | | |  | | | |  |  |  |  |
| 2 | Atr-ERN14631 |  | Vvi-Vitvi18g00408\_t001 |  | | | |  |  |  |  |
| 2 | Atr-ERN14632 |  | | | |  | | | |  |  |  |  |
| 2 | Atr-ERN14633 |  | | | |  | Vvi-Vitvi12g00866\_t001 |  |  |  |  |
| 2 | Atr-ERN14634 |  | | | |  | | | |  |  |  |  |
| 2 | Atr-ERN14635 |  | | | |  | | | |  |  |  |  |
| 2 | Atr-ERN14636 |  | | | |  | | | |  |  |  |  |
| 2 | Atr-ERN14637 |  | | | |  | | | |  |  |  |  |
| 2 | Atr-ERN14638 |  | | | |  | | | |  |  |  |  |
| 2 | Atr-ERN14639 |  | | | |  | | | |  |  |  |  |
| 2 | Atr-ERN14640 |  | | | |  | | | |  |  |  |  |
| 2 | Atr-ERN14641 |  | | | |  | | | |  |  |  |  |
| 2 | Atr-ERN14642 |  | | | |  | | | |  |  |  |  |
| 2 | Atr-ERN14643 |  | | | |  | | | |  |  |  |  |
| 2 | Atr-ERN14644 |  | | | |  | | | |  |  |  |  |
| 2 | Atr-ERN14645 |  | | | |  | | | |  |  |  |  |
| 2 | Atr-ERN14646 |  | | | |  | | | |  |  |  |  |
| 2 | Atr-ERN14647 |  | | | |  | Vvi-Vitvi12g00857\_t001 |  |  |  |  |
| 1 | Atr-ERN14648 |  | | | |  |  |  |  |  |
| 1 | Atr-ERN14649 |  | Vvi-Vitvi18g00409\_t001 |  |  |  |  |  |
| 1 | Atr-ERN14650 |  | | | |  |  |  |  |  |
| 1 | Atr-ERN14651 |  | | | |  |  |  |  |  |
| 1 | Atr-ERN14652 |  | Vvi-Vitvi18g00410\_t001 |  |  |  |  |  |
| 1 | Atr-ERN14653 |  | | | |  |  |  |  |  |
| 1 | Atr-ERN14654 |  | Vvi-Vitvi18g00411\_t001 |  |  |  |  |  |
| 1 | Atr-ERN14655 |  | | | |  |  |  |  |  |
| 1 | Atr-ERN14656 |  | | | |  |  |  |  |  |
| 1 | Atr-ERN14657 |  | | | |  |  |  |  |  |
| 1 | Atr-ERN14658 |  | | | |  |  |  |  |  |
| 1 | Atr-ERN14659 |  | | | |  |  |  |  |  |
| 1 | Atr-ERN14660 |  | | | |  |  |  |  |  |
| 1 | Atr-ERN14661 |  | | | |  |  |  |  |  |
| 1 | Atr-ERN14662 |  | | | |  |  |  |  |  |
| 1 | Atr-ERN14663 |  | | | |  |  |  |  |  |
| 1 | Atr-ERN14664 |  | | | |  |  |  |  |  |
| 1 | Atr-ERN14665 |  | Vvi-Vitvi18g00412\_t002 |  |  |  |  |  |
| 1 | Atr-ERN14666 |  | | | |  |  |  |  |  |
| 1 | Atr-ERN14667 |  | | | |  |  |  |  |  |
| 1 | Atr-ERN14668 |  | | | |  |  |  |  |  |
| 1 | Atr-ERN14669 |  | | | |  |  |  |  |  |
| 1 | Atr-ERN14670 |  | | | |  |  |  |  |  |
| 1 | Atr-ERN14671 |  | Vvi-Vitvi18g00416\_t001 |  |  |  |  |  |
| 1 | Atr-ERN14672 |  | | | |  |  |  |  |  |
| 1 | Atr-ERN14673 |  | | | |  |  |  |  |  |
| 1 | Atr-ERN14674 |  | | | |  |  |  |  |  |
| 1 | Atr-ERN14675 |  | | | |  |  |  |  |  |
| 1 | Atr-ERN14676 |  | | | |  |  |  |  |  |
| 1 | Atr-ERN14677 |  | | | |  |  |  |  |  |
| 1 | Atr-ERN14678 |  | | | |  |  |  |  |  |
| 1 | Atr-ERN14679 |  | | | |  |  |  |  |  |
| 1 | Atr-ERN14680 |  | | | |  |  |  |  |  |
| 1 | Atr-ERN14681 |  | | | |  |  |  |  |  |
| 1 | Atr-ERN14682 |  | | | |  |  |  |  |  |
| 1 | Atr-ERN14683 |  | | | |  |  |  |  |  |
| 1 | Atr-ERN14684 |  | | | |  |  |  |  |  |
| 1 | Atr-ERN14685 |  | | | |  |  |  |  |  |
| 1 | Atr-ERN14686 |  | | | |  |  |  |  |  |
| 1 | Atr-ERN14687 |  | | | |  |  |  |  |  |
| 1 | Atr-ERN14688 |  | | | |  |  |  |  |  |
| 1 | Atr-ERN14689 |  | | | |  |  |  |  |  |
| 1 | Atr-ERN14690 |  | Vvi-Vitvi18g04082\_t001 |  |  |  |  |  |
| 1 | Atr-ERN14691 |  | | | |  |  |  |  |  |
| 1 | Atr-ERN14692 |  | | | |  |  |  |  |  |
| 1 | Atr-ERN14693 |  | | | |  |  |  |  |  |
| 1 | Atr-ERN14694 |  | | | |  |  |  |  |  |
| 1 | Atr-ERN14695 |  | | | |  |  |  |  |  |
| 1 | Atr-ERN14696 |  | | | |  |  |  |  |  |
| 1 | Atr-ERN14697 |  | | | |  |  |  |  |  |
| 1 | Atr-ERN14698 |  | | | |  |  |  |  |  |
| 1 | Atr-ERN14699 |  | | | |  |  |  |  |  |
| 1 | Atr-ERN14700 |  | | | |  |  |  |  |  |
| 1 | Atr-ERN14701 |  | | | |  |  |  |  |  |
| 1 | Atr-ERN14702 |  | | | |  |  |  |  |  |
| 1 | Atr-ERN14703 |  | | | |  |  |  |  |  |
| 1 | Atr-ERN14704 |  | | | |  |  |  |  |  |
| 1 | Atr-ERN14705 |  | | | |  |  |  |  |  |
| 1 | Atr-ERN14706 |  | | | |  |  |  |  |  |
| 1 | Atr-ERN14707 |  | | | |  |  |  |  |  |
| 1 | Atr-ERN14708 |  | | | |  |  |  |  |  |
| 1 | Atr-ERN14709 |  | Vvi-Vitvi18g00420\_t001 |  |  |  |  |  |
| 1 | Atr-ERN14710 |  | | | |  |  |  |  |  |
| 1 | Atr-ERN14711 |  | | | |  |  |  |  |  |
| 1 | Atr-ERN14712 |  | | | |  |  |  |  |  |
| 1 | Atr-ERN14713 |  | | | |  |  |  |  |  |
| 1 | Atr-ERN14714 |  | | | |  |  |  |  |  |
| 1 | Atr-ERN14715 |  | | | |  |  |  |  |  |
| 1 | Atr-ERN14716 |  | | | |  |  |  |  |  |
| 1 | Atr-ERN14717 |  | | | |  |  |  |  |  |
| 1 | Atr-ERN14718 |  | | | |  |  |  |  |  |
| 1 | Atr-ERN14719 |  | | | |  |  |  |  |  |
| 1 | Atr-ERN14720 |  | | | |  |  |  |  |  |
| 1 | Atr-ERN14721 |  | | | |  |  |  |  |  |
| 1 | Atr-ERN14722 |  | | | |  |  |  |  |  |
| 1 | Atr-ERN14723 |  | | | |  |  |  |  |  |
| 1 | Atr-ERN14724 |  | | | |  |  |  |  |  |
| 1 | Atr-ERN14725 |  | | | |  |  |  |  |  |
| 1 | Atr-ERN14726 |  | Vvi-Vitvi18g00421\_t001 |  |  |  |  |  |
| 1 | Atr-ERN14727 |  | | | |  |  |  |  |  |
| 1 | Atr-ERN14728 |  | | | |  |  |  |  |  |
| 1 | Atr-ERN14729 |  | | | |  |  |  |  |  |
| 1 | Atr-ERN14730 |  | | | |  |  |  |  |  |
| 1 | Atr-ERN14731 |  | | | |  |  |  |  |  |
| 1 | Atr-ERN14732 |  | | | |  |  |  |  |  |
| 1 | Atr-ERN14733 |  | | | |  |  |  |  |  |
| 1 | Atr-ERN14734 |  | | | |  |  |  |  |  |
| 1 | Atr-ERN14735 |  | | | |  |  |  |  |  |
| 1 | Atr-ERN14736 |  | | | |  |  |  |  |  |
| 1 | Atr-ERN14737 |  | Vvi-Vitvi18g00431\_t001 |  |  |  |  |  |
| 1 | Atr-ERN14738 |  | | | |  |  |  |  |  |
| 1 | Atr-ERN14739 |  | | | |  |  |  |  |  |
| 1 | Atr-ERN14740 |  | | | |  |  |  |  |  |
| 1 | Atr-ERN14741 |  | | | |  |  |  |  |  |
| 1 | Atr-ERN14742 |  | | | |  |  |  |  |  |
| 1 | Atr-ERN14743 |  | | | |  |  |  |  |  |
| 1 | Atr-ERN14744 |  | | | |  |  |  |  |  |
| 1 | Atr-ERN14745 |  | Vvi-Vitvi18g00437\_t001 |  |  |  |  |  |
| 1 | Atr-ERN14746 |  | Vvi-Vitvi18g00438\_t001 |  |  |  |  |  |
| 0 | Atr-ERN14747 |  |  |  |  |  |  |
| 0 | Atr-ERN14748 |  |  |  |  |  |  |
| 0 | Atr-ERN14749 |  |  |  |  |  |  |
