# Supplementary material for: Genome-wide identification, characterization and gene expression of BES1 transcription factor family in grapevine (Vitis vinifera L.)
Source: Sci Rep. 2023 Jan 5;13:240. doi: 10.1038/s41598-022-24407-y (PMC9816167; doi:10.1038/s41598-022-24407-y)
Supplement: Supplementary file 3 — Supplementary Information. [file 41598_2022_24407_MOESM3_ESM.zip › Vvi_Atr/Vitis_vinifera.PN40024.v4.dna_sm.toplevel.fa.vs.Amborella_trichopoda.AMTR1.0.dna_sm.toplevel.fa.html/Atr-AmTr_v1.0_scaffold00039.html]

|  |  |  |  |  |  |  |  |  |  |  |  |  |  |
| --- | --- | --- | --- | --- | --- | --- | --- | --- | --- | --- | --- | --- | --- |
| Duplication depth | Reference chromosome | Collinear blocks | | | | | | | | | | | |
| 0 | Atr-ERN15715 |  |  |  |  |  |  |
| 0 | Atr-ERN15716 |  |  |  |  |  |  |
| 0 | Atr-ERN15717 |  |  |  |  |  |  |
| 0 | Atr-ERN15718 |  |  |  |  |  |  |
| 0 | Atr-ERN15719 |  |  |  |  |  |  |
| 0 | Atr-ERN15720 |  |  |  |  |  |  |
| 0 | Atr-ERN15721 |  |  |  |  |  |  |
| 0 | Atr-ERN15722 |  |  |  |  |  |  |
| 0 | Atr-ERN15723 |  |  |  |  |  |  |
| 0 | Atr-ERN15724 |  |  |  |  |  |  |
| 0 | Atr-ERN15725 |  |  |  |  |  |  |
| 0 | Atr-ERN15726 |  |  |  |  |  |  |
| 0 | Atr-ERN15727 |  |  |  |  |  |  |
| 0 | Atr-ERN15728 |  |  |  |  |  |  |
| 0 | Atr-ERN15729 |  |  |  |  |  |  |
| 0 | Atr-ERN15730 |  |  |  |  |  |  |
| 0 | Atr-ERN15731 |  |  |  |  |  |  |
| 0 | Atr-ERN15732 |  |  |  |  |  |  |
| 0 | Atr-ERN15733 |  |  |  |  |  |  |
| 0 | Atr-ERN15734 |  |  |  |  |  |  |
| 0 | Atr-ERN15735 |  |  |  |  |  |  |
| 0 | Atr-ERN15736 |  |  |  |  |  |  |
| 0 | Atr-ERN15737 |  |  |  |  |  |  |
| 0 | Atr-ERN15738 |  |  |  |  |  |  |
| 0 | Atr-ERN15739 |  |  |  |  |  |  |
| 0 | Atr-ERN15740 |  |  |  |  |  |  |
| 0 | Atr-ERN15741 |  |  |  |  |  |  |
| 0 | Atr-ERN15742 |  |  |  |  |  |  |
| 0 | Atr-ERN15743 |  |  |  |  |  |  |
| 0 | Atr-ERN15744 |  |  |  |  |  |  |
| 0 | Atr-ERN15745 |  |  |  |  |  |  |
| 0 | Atr-ERN15746 |  |  |  |  |  |  |
| 0 | Atr-ERN15747 |  |  |  |  |  |  |
| 0 | Atr-ERN15748 |  |  |  |  |  |  |
| 0 | Atr-ERN15749 |  |  |  |  |  |  |
| 0 | Atr-ERN15750 |  |  |  |  |  |  |
| 0 | Atr-ERN15751 |  |  |  |  |  |  |
| 0 | Atr-ERN15752 |  |  |  |  |  |  |
| 0 | Atr-ERN15753 |  |  |  |  |  |  |
| 0 | Atr-ERN15754 |  |  |  |  |  |  |
| 0 | Atr-ERN15755 |  |  |  |  |  |  |
| 0 | Atr-ERN15756 |  |  |  |  |  |  |
| 0 | Atr-ERN15757 |  |  |  |  |  |  |
| 0 | Atr-ERN15758 |  |  |  |  |  |  |
| 0 | Atr-ERN15759 |  |  |  |  |  |  |
| 0 | Atr-ERN15760 |  |  |  |  |  |  |
| 0 | Atr-ERN15761 |  |  |  |  |  |  |
| 0 | Atr-ERN15762 |  |  |  |  |  |  |
| 0 | Atr-ERN15763 |  |  |  |  |  |  |
| 0 | Atr-ERN15764 |  |  |  |  |  |  |
| 0 | Atr-ERN15765 |  |  |  |  |  |  |
| 0 | Atr-ERN15766 |  |  |  |  |  |  |
| 0 | Atr-ERN15767 |  |  |  |  |  |  |
| 0 | Atr-ERN15768 |  |  |  |  |  |  |
| 0 | Atr-ERN15769 |  |  |  |  |  |  |
| 0 | Atr-ERN15770 |  |  |  |  |  |  |
| 0 | Atr-ERN15771 |  |  |  |  |  |  |
| 0 | Atr-ERN15772 |  |  |  |  |  |  |
| 0 | Atr-ERN15773 |  |  |  |  |  |  |
| 0 | Atr-ERN15774 |  |  |  |  |  |  |
| 0 | Atr-ERN15775 |  |  |  |  |  |  |
| 0 | Atr-ERN15776 |  |  |  |  |  |  |
| 0 | Atr-ERN15777 |  |  |  |  |  |  |
| 0 | Atr-ERN15778 |  |  |  |  |  |  |
| 0 | Atr-ERN15779 |  |  |  |  |  |  |
| 0 | Atr-ERN15780 |  |  |  |  |  |  |
| 0 | Atr-ERN15781 |  |  |  |  |  |  |
| 0 | Atr-ERN15782 |  |  |  |  |  |  |
| 0 | Atr-ERN15783 |  |  |  |  |  |  |
| 0 | Atr-ERN15784 |  |  |  |  |  |  |
| 0 | Atr-ERN15785 |  |  |  |  |  |  |
| 1 | Atr-ERN15786 |  | Vvi-Vitvi07g02313\_t001 |  |  |  |  |  |
| 1 | Atr-ERN15787 |  | Vvi-Vitvi07g00740\_t001 |  |  |  |  |  |
| 1 | Atr-ERN15788 |  | Vvi-Vitvi07g00739\_t001 |  |  |  |  |  |
| 1 | Atr-ERN15789 |  | | | |  |  |  |  |  |
| 2 | Atr-ERN15790 |  | | | |  | Vvi-Vitvi07g00733\_t001 |  |  |  |  |
| 2 | Atr-ERN15791 |  | | | |  | | | |  |  |  |  |
| 2 | Atr-ERN15792 |  | | | |  | | | |  |  |  |  |
| 2 | Atr-ERN15793 |  | | | |  | | | |  |  |  |  |
| 2 | Atr-ERN15794 |  | | | |  | | | |  |  |  |  |
| 2 | Atr-ERN15795 |  | | | |  | | | |  |  |  |  |
| 2 | Atr-ERN15796 |  | | | |  | Vvi-Vitvi07g00737\_t001 |  |  |  |  |
| 2 | Atr-ERN15797 |  | | | |  | | | |  |  |  |  |
| 2 | Atr-ERN15798 |  | | | |  | | | |  |  |  |  |
| 2 | Atr-ERN15799 |  | | | |  | | | |  |  |  |  |
| 2 | Atr-ERN15800 |  | Vvi-Vitvi07g02309\_t001 |  | Vvi-Vitvi07g02309\_t001 |  |  |  |  |
| 2 | Atr-ERN15801 |  | | | |  | | | |  |  |  |  |
| 2 | Atr-ERN15802 |  | | | |  | | | |  |  |  |  |
| 2 | Atr-ERN15803 |  | | | |  | | | |  |  |  |  |
| 2 | Atr-ERN15804 |  | | | |  | | | |  |  |  |  |
| 2 | Atr-ERN15805 |  | | | |  | | | |  |  |  |  |
| 2 | Atr-ERN15806 |  | | | |  | | | |  |  |  |  |
| 2 | Atr-ERN15807 |  | | | |  | | | |  |  |  |  |
| 2 | Atr-ERN15808 |  | | | |  | | | |  |  |  |  |
| 2 | Atr-ERN15809 |  | | | |  | | | |  |  |  |  |
| 2 | Atr-ERN15810 |  | | | |  | Vvi-Vitvi07g00741\_t001 |  |  |  |  |
| 2 | Atr-ERN15811 |  | | | |  | | | |  |  |  |  |
| 2 | Atr-ERN15812 |  | | | |  | Vvi-Vitvi07g00743\_t001 |  |  |  |  |
| 2 | Atr-ERN15813 |  | Vvi-Vitvi07g00730\_t001 |  | Vvi-Vitvi07g00744\_t001 |  |  |  |  |
| 1 | Atr-ERN15814 |  | | | |  |  |  |  |  |
| 1 | Atr-ERN15815 |  | | | |  |  |  |  |  |
| 1 | Atr-ERN15816 |  | | | |  |  |  |  |  |
| 1 | Atr-ERN15817 |  | | | |  |  |  |  |  |
| 1 | Atr-ERN15818 |  | | | |  |  |  |  |  |
| 1 | Atr-ERN15819 |  | | | |  |  |  |  |  |
| 1 | Atr-ERN15820 |  | | | |  |  |  |  |  |
| 1 | Atr-ERN15821 |  | | | |  |  |  |  |  |
| 1 | Atr-ERN15822 |  | | | |  |  |  |  |  |
| 1 | Atr-ERN15823 |  | | | |  |  |  |  |  |
| 1 | Atr-ERN15824 |  | | | |  |  |  |  |  |
| 1 | Atr-ERN15825 |  | | | |  |  |  |  |  |
| 1 | Atr-ERN15826 |  | | | |  |  |  |  |  |
| 1 | Atr-ERN15827 |  | | | |  |  |  |  |  |
| 1 | Atr-ERN15828 |  | | | |  |  |  |  |  |
| 1 | Atr-ERN15829 |  | | | |  |  |  |  |  |
| 1 | Atr-ERN15830 |  | | | |  |  |  |  |  |
| 1 | Atr-ERN15831 |  | | | |  |  |  |  |  |
| 1 | Atr-ERN15832 |  | | | |  |  |  |  |  |
| 1 | Atr-ERN15833 |  | | | |  |  |  |  |  |
| 1 | Atr-ERN15834 |  | | | |  |  |  |  |  |
| 1 | Atr-ERN15835 |  | | | |  |  |  |  |  |
| 1 | Atr-ERN15836 |  | Vvi-Vitvi07g02304\_t001 |  |  |  |  |  |
| 1 | Atr-ERN15837 |  | | | |  |  |  |  |  |
| 1 | Atr-ERN15838 |  | | | |  |  |  |  |  |
| 1 | Atr-ERN15839 |  | | | |  |  |  |  |  |
| 1 | Atr-ERN15840 |  | | | |  |  |  |  |  |
| 1 | Atr-ERN15841 |  | | | |  |  |  |  |  |
| 1 | Atr-ERN15842 |  | | | |  |  |  |  |  |
| 1 | Atr-ERN15843 |  | | | |  |  |  |  |  |
| 1 | Atr-ERN15844 |  | | | |  |  |  |  |  |
| 1 | Atr-ERN15845 |  | | | |  |  |  |  |  |
| 1 | Atr-ERN15846 |  | | | |  |  |  |  |  |
| 1 | Atr-ERN15847 |  | | | |  |  |  |  |  |
| 1 | Atr-ERN15848 |  | | | |  |  |  |  |  |
| 1 | Atr-ERN15849 |  | | | |  |  |  |  |  |
| 1 | Atr-ERN15850 |  | | | |  |  |  |  |  |
| 1 | Atr-ERN15851 |  | | | |  |  |  |  |  |
| 1 | Atr-ERN15852 |  | | | |  |  |  |  |  |
| 1 | Atr-ERN15853 |  | | | |  |  |  |  |  |
| 1 | Atr-ERN15854 |  | Vvi-Vitvi07g00696\_t001 |  |  |  |  |  |
| 1 | Atr-ERN15855 |  | | | |  |  |  |  |  |
| 1 | Atr-ERN15856 |  | | | |  |  |  |  |  |
| 1 | Atr-ERN15857 |  | | | |  |  |  |  |  |
| 1 | Atr-ERN15858 |  | | | |  |  |  |  |  |
| 1 | Atr-ERN15859 |  | | | |  |  |  |  |  |
| 1 | Atr-ERN15860 |  | | | |  |  |  |  |  |
| 1 | Atr-ERN15861 |  | | | |  |  |  |  |  |
| 1 | Atr-ERN15862 |  | | | |  |  |  |  |  |
| 1 | Atr-ERN15863 |  | | | |  |  |  |  |  |
| 1 | Atr-ERN15864 |  | | | |  |  |  |  |  |
| 1 | Atr-ERN15865 |  | | | |  |  |  |  |  |
| 1 | Atr-ERN15866 |  | | | |  |  |  |  |  |
| 1 | Atr-ERN15867 |  | | | |  |  |  |  |  |
| 1 | Atr-ERN15868 |  | | | |  |  |  |  |  |
| 1 | Atr-ERN15869 |  | | | |  |  |  |  |  |
| 1 | Atr-ERN15870 |  | | | |  |  |  |  |  |
| 1 | Atr-ERN15871 |  | | | |  |  |  |  |  |
| 1 | Atr-ERN15872 |  | | | |  |  |  |  |  |
| 1 | Atr-ERN15873 |  | | | |  |  |  |  |  |
| 1 | Atr-ERN15874 |  | Vvi-Vitvi07g00687\_t001 |  |  |  |  |  |
| 1 | Atr-ERN15875 |  | | | |  |  |  |  |  |
| 1 | Atr-ERN15876 |  | | | |  |  |  |  |  |
| 1 | Atr-ERN15877 |  | | | |  |  |  |  |  |
| 1 | Atr-ERN15878 |  | | | |  |  |  |  |  |
| 1 | Atr-ERN15879 |  | | | |  |  |  |  |  |
| 1 | Atr-ERN15880 |  | Vvi-Vitvi07g00686\_t001 |  |  |  |  |  |
| 0 | Atr-ERN15881 |  |  |  |  |  |  |
| 0 | Atr-ERN15882 |  |  |  |  |  |  |
| 0 | Atr-ERN15883 |  |  |  |  |  |  |
| 0 | Atr-ERN15884 |  |  |  |  |  |  |
| 0 | Atr-ERN15885 |  |  |  |  |  |  |
| 0 | Atr-ERN15886 |  |  |  |  |  |  |
| 0 | Atr-ERN15887 |  |  |  |  |  |  |
| 0 | Atr-ERN15888 |  |  |  |  |  |  |
| 0 | Atr-ERN15889 |  |  |  |  |  |  |
| 0 | Atr-ERN15890 |  |  |  |  |  |  |
| 0 | Atr-ERN15891 |  |  |  |  |  |  |
| 0 | Atr-ERN15892 |  |  |  |  |  |  |
| 1 | Atr-ERN15893 |  | Vvi-Vitvi05g00864\_t001 |  |  |  |  |  |
| 1 | Atr-ERN15894 |  | | | |  |  |  |  |  |
| 1 | Atr-ERN15895 |  | | | |  |  |  |  |  |
| 1 | Atr-ERN15896 |  | | | |  |  |  |  |  |
| 1 | Atr-ERN15897 |  | | | |  |  |  |  |  |
| 1 | Atr-ERN15898 |  | | | |  |  |  |  |  |
| 1 | Atr-ERN15899 |  | | | |  |  |  |  |  |
| 1 | Atr-ERN15900 |  | | | |  |  |  |  |  |
| 1 | Atr-ERN15901 |  | | | |  |  |  |  |  |
| 1 | Atr-ERN15902 |  | | | |  |  |  |  |  |
| 1 | Atr-ERN15903 |  | | | |  |  |  |  |  |
| 1 | Atr-ERN15904 |  | | | |  |  |  |  |  |
| 1 | Atr-ERN15905 |  | | | |  |  |  |  |  |
| 1 | Atr-ERN15906 |  | | | |  |  |  |  |  |
| 1 | Atr-ERN15907 |  | | | |  |  |  |  |  |
| 1 | Atr-ERN15908 |  | | | |  |  |  |  |  |
| 1 | Atr-ERN15909 |  | | | |  |  |  |  |  |
| 1 | Atr-ERN15910 |  | | | |  |  |  |  |  |
| 1 | Atr-ERN15911 |  | | | |  |  |  |  |  |
| 1 | Atr-ERN15912 |  | | | |  |  |  |  |  |
| 1 | Atr-ERN15913 |  | | | |  |  |  |  |  |
| 1 | Atr-ERN15914 |  | | | |  |  |  |  |  |
| 1 | Atr-ERN15915 |  | | | |  |  |  |  |  |
| 1 | Atr-ERN15916 |  | Vvi-Vitvi05g04220\_t001 |  |  |  |  |  |
| 1 | Atr-ERN15917 |  | | | |  |  |  |  |  |
| 1 | Atr-ERN15918 |  | | | |  |  |  |  |  |
| 1 | Atr-ERN15919 |  | | | |  |  |  |  |  |
| 1 | Atr-ERN15920 |  | | | |  |  |  |  |  |
| 1 | Atr-ERN15921 |  | | | |  |  |  |  |  |
| 1 | Atr-ERN15922 |  | | | |  |  |  |  |  |
| 1 | Atr-ERN15923 |  | | | |  |  |  |  |  |
| 1 | Atr-ERN15924 |  | | | |  |  |  |  |  |
| 1 | Atr-ERN15925 |  | | | |  |  |  |  |  |
| 1 | Atr-ERN15926 |  | | | |  |  |  |  |  |
| 1 | Atr-ERN15927 |  | | | |  |  |  |  |  |
| 1 | Atr-ERN15928 |  | | | |  |  |  |  |  |
| 1 | Atr-ERN15929 |  | | | |  |  |  |  |  |
| 1 | Atr-ERN15930 |  | Vvi-Vitvi05g00836\_t002 |  |  |  |  |  |
| 1 | Atr-ERN15931 |  | | | |  |  |  |  |  |
| 1 | Atr-ERN15932 |  | | | |  |  |  |  |  |
| 1 | Atr-ERN15933 |  | | | |  |  |  |  |  |
| 1 | Atr-ERN15934 |  | | | |  |  |  |  |  |
| 1 | Atr-ERN15935 |  | | | |  |  |  |  |  |
| 1 | Atr-ERN15936 |  | Vvi-Vitvi05g00830\_t001 |  |  |  |  |  |
| 1 | Atr-ERN15937 |  | | | |  |  |  |  |  |
| 1 | Atr-ERN15938 |  | | | |  |  |  |  |  |
| 1 | Atr-ERN15939 |  | | | |  |  |  |  |  |
| 1 | Atr-ERN15940 |  | | | |  |  |  |  |  |
| 1 | Atr-ERN15941 |  | Vvi-Vitvi05g00823\_t001 |  |  |  |  |  |
| 1 | Atr-ERN15942 |  | | | |  |  |  |  |  |
| 1 | Atr-ERN15943 |  | Vvi-Vitvi05g00822\_t001 |  |  |  |  |  |
| 1 | Atr-ERN15944 |  | Vvi-Vitvi05g00821\_t001 |  |  |  |  |  |
| 0 | Atr-ERN15945 |  |  |  |  |  |  |
