# Supplementary material for: Genome-wide identification, characterization and gene expression of BES1 transcription factor family in grapevine (Vitis vinifera L.)
Source: Sci Rep. 2023 Jan 5;13:240. doi: 10.1038/s41598-022-24407-y (PMC9816167; doi:10.1038/s41598-022-24407-y)
Supplement: Supplementary file 3 — Supplementary Information. [file 41598_2022_24407_MOESM3_ESM.zip › Vvi_Atr/Vitis_vinifera.PN40024.v4.dna_sm.toplevel.fa.vs.Amborella_trichopoda.AMTR1.0.dna_sm.toplevel.fa.html/Atr-AmTr_v1.0_scaffold00040.html]

|  |  |  |  |  |  |  |  |  |  |  |  |  |  |
| --- | --- | --- | --- | --- | --- | --- | --- | --- | --- | --- | --- | --- | --- |
| Duplication depth | Reference chromosome | Collinear blocks | | | | | | | | | | | |
| 0 | Atr-ERN12958 |  |  |  |  |  |  |
| 1 | Atr-ERN12959 |  | Vvi-Vitvi04g01602\_t001 |  |  |  |  |  |
| 1 | Atr-ERN12960 |  | | | |  |  |  |  |  |
| 1 | Atr-ERN12961 |  | | | |  |  |  |  |  |
| 1 | Atr-ERN12962 |  | | | |  |  |  |  |  |
| 2 | Atr-ERN12963 |  | | | |  | Vvi-Vitvi18g01648\_t001 |  |  |  |  |
| 2 | Atr-ERN12964 |  | | | |  | | | |  |  |  |  |
| 2 | Atr-ERN12965 |  | Vvi-Vitvi04g02214\_t001 |  | | | |  |  |  |  |
| 2 | Atr-ERN12966 |  | | | |  | | | |  |  |  |  |
| 2 | Atr-ERN12967 |  | | | |  | | | |  |  |  |  |
| 2 | Atr-ERN12968 |  | Vvi-Vitvi04g01598\_t001 |  | | | |  |  |  |  |
| 2 | Atr-ERN12969 |  | | | |  | | | |  |  |  |  |
| 2 | Atr-ERN12970 |  | | | |  | | | |  |  |  |  |
| 2 | Atr-ERN12971 |  | | | |  | | | |  |  |  |  |
| 2 | Atr-ERN12972 |  | | | |  | | | |  |  |  |  |
| 2 | Atr-ERN12973 |  | | | |  | | | |  |  |  |  |
| 2 | Atr-ERN12974 |  | | | |  | | | |  |  |  |  |
| 2 | Atr-ERN12975 |  | | | |  | Vvi-Vitvi18g01647\_t004 |  |  |  |  |
| 2 | Atr-ERN12976 |  | | | |  | | | |  |  |  |  |
| 2 | Atr-ERN12977 |  | Vvi-Vitvi04g01592\_t001 |  | | | |  |  |  |  |
| 2 | Atr-ERN12978 |  | | | |  | | | |  |  |  |  |
| 2 | Atr-ERN12979 |  | | | |  | | | |  |  |  |  |
| 2 | Atr-ERN12980 |  | | | |  | | | |  |  |  |  |
| 2 | Atr-ERN12981 |  | | | |  | | | |  |  |  |  |
| 2 | Atr-ERN12982 |  | | | |  | Vvi-Vitvi18g01639\_t001 |  |  |  |  |
| 2 | Atr-ERN12983 |  | | | |  | Vvi-Vitvi18g01637\_t001 |  |  |  |  |
| 3 | Atr-ERN12984 |  | | | |  | Vvi-Vitvi18g02978\_t001 |  | Vvi-Vitvi06g01478\_t001 |  |  |  |
| 3 | Atr-ERN12985 |  | Vvi-Vitvi04g01591\_t005 |  | Vvi-Vitvi18g01634\_t002 |  | | | |  |  |  |
| 3 | Atr-ERN12986 |  | | | |  | | | |  | | | |  |  |  |
| 3 | Atr-ERN12987 |  | Vvi-Vitvi04g02210\_t001 |  | | | |  | | | |  |  |  |
| 3 | Atr-ERN12988 |  | | | |  | | | |  | | | |  |  |  |
| 3 | Atr-ERN12989 |  | | | |  | | | |  | | | |  |  |  |
| 3 | Atr-ERN12990 |  | | | |  | Vvi-Vitvi18g02977\_t001 |  | | | |  |  |  |
| 3 | Atr-ERN12991 |  | | | |  | | | |  | | | |  |  |  |
| 3 | Atr-ERN12992 |  | Vvi-Vitvi04g01585\_t001 |  | Vvi-Vitvi18g01632\_t003 |  | | | |  |  |  |
| 3 | Atr-ERN12993 |  | | | |  | | | |  | | | |  |  |  |
| 3 | Atr-ERN12994 |  | | | |  | | | |  | | | |  |  |  |
| 3 | Atr-ERN12995 |  | | | |  | | | |  | | | |  |  |  |
| 3 | Atr-ERN12996 |  | | | |  | | | |  | | | |  |  |  |
| 3 | Atr-ERN12997 |  | | | |  | | | |  | | | |  |  |  |
| 3 | Atr-ERN12998 |  | Vvi-Vitvi04g01583\_t001 |  | Vvi-Vitvi18g01631\_t001 |  | | | |  |  |  |
| 2 | Atr-ERN12999 |  |  |  | | | |  | | | |  |  |  |
| 2 | Atr-ERN13000 |  |  |  | | | |  | | | |  |  |  |
| 2 | Atr-ERN13001 |  |  |  | Vvi-Vitvi18g02975\_t001 |  | | | |  |  |  |
| 2 | Atr-ERN13002 |  |  |  | | | |  | | | |  |  |  |
| 2 | Atr-ERN13003 |  |  |  | Vvi-Vitvi18g01629\_t001 |  | | | |  |  |  |
| 2 | Atr-ERN13004 |  |  |  | | | |  | | | |  |  |  |
| 2 | Atr-ERN13005 |  |  |  | Vvi-Vitvi18g02973\_t001 |  | | | |  |  |  |
| 1 | Atr-ERN13006 |  |  |  |  |  | | | |  |  |  |
| 1 | Atr-ERN13007 |  |  |  |  |  | Vvi-Vitvi06g01502\_t001 |  |  |  |
| 1 | Atr-ERN13008 |  |  |  |  |  | Vvi-Vitvi06g01503\_t001 |  |  |  |
| 2 | Atr-ERN13009 |  | Vvi-Vitvi08g01442\_t001 |  |  |  | Vvi-Vitvi06g01504\_t001 |  |  |  |
| 2 | Atr-ERN13010 |  | Vvi-Vitvi08g01441\_t001 |  |  |  | | | |  |  |  |
| 2 | Atr-ERN13011 |  | | | |  |  |  | | | |  |  |  |
| 2 | Atr-ERN13012 |  | | | |  |  |  | | | |  |  |  |
| 2 | Atr-ERN13013 |  | Vvi-Vitvi08g01439\_t001 |  |  |  | | | |  |  |  |
| 2 | Atr-ERN13014 |  | | | |  |  |  | | | |  |  |  |
| 2 | Atr-ERN13015 |  | | | |  |  |  | | | |  |  |  |
| 2 | Atr-ERN13016 |  | | | |  |  |  | | | |  |  |  |
| 2 | Atr-ERN13017 |  | | | |  |  |  | | | |  |  |  |
| 2 | Atr-ERN13018 |  | | | |  |  |  | | | |  |  |  |
| 2 | Atr-ERN13019 |  | | | |  |  |  | | | |  |  |  |
| 2 | Atr-ERN13020 |  | Vvi-Vitvi08g01437\_t001 |  |  |  | | | |  |  |  |
| 2 | Atr-ERN13021 |  | | | |  |  |  | | | |  |  |  |
| 2 | Atr-ERN13022 |  | | | |  |  |  | Vvi-Vitvi06g01505\_t001 |  |  |  |
| 2 | Atr-ERN13023 |  | | | |  |  |  | | | |  |  |  |
| 2 | Atr-ERN13024 |  | | | |  |  |  | | | |  |  |  |
| 2 | Atr-ERN13025 |  | | | |  |  |  | | | |  |  |  |
| 2 | Atr-ERN13026 |  | | | |  |  |  | Vvi-Vitvi06g01969\_t001 |  |  |  |
| 2 | Atr-ERN13027 |  | | | |  |  |  | | | |  |  |  |
| 2 | Atr-ERN13028 |  | Vvi-Vitvi08g01432\_t001 |  |  |  | | | |  |  |  |
| 2 | Atr-ERN13029 |  | Vvi-Vitvi08g04305\_t001 |  |  |  | Vvi-Vitvi06g04445\_t001 |  |  |  |
| 2 | Atr-ERN13030 |  | | | |  |  |  | | | |  |  |  |
| 2 | Atr-ERN13031 |  | | | |  |  |  | | | |  |  |  |
| 2 | Atr-ERN13032 |  | | | |  |  |  | | | |  |  |  |
| 2 | Atr-ERN13033 |  | | | |  |  |  | | | |  |  |  |
| 2 | Atr-ERN13034 |  | | | |  |  |  | | | |  |  |  |
| 2 | Atr-ERN13035 |  | | | |  |  |  | | | |  |  |  |
| 2 | Atr-ERN13036 |  | | | |  |  |  | | | |  |  |  |
| 2 | Atr-ERN13037 |  | Vvi-Vitvi08g01429\_t001 |  |  |  | | | |  |  |  |
| 2 | Atr-ERN13038 |  | Vvi-Vitvi08g02259\_t001 |  |  |  | | | |  |  |  |
| 2 | Atr-ERN13039 |  | | | |  |  |  | | | |  |  |  |
| 2 | Atr-ERN13040 |  | Vvi-Vitvi08g02258\_t001 |  |  |  | | | |  |  |  |
| 2 | Atr-ERN13041 |  | Vvi-Vitvi08g01428\_t001 |  |  |  | | | |  |  |  |
| 2 | Atr-ERN13042 |  | | | |  |  |  | | | |  |  |  |
| 2 | Atr-ERN13043 |  | Vvi-Vitvi08g01426\_t001 |  |  |  | Vvi-Vitvi06g01515\_t001 |  |  |  |
| 2 | Atr-ERN13044 |  | | | |  |  |  | | | |  |  |  |
| 2 | Atr-ERN13045 |  | Vvi-Vitvi08g01425\_t001 |  |  |  | | | |  |  |  |
| 2 | Atr-ERN13046 |  | | | |  |  |  | | | |  |  |  |
| 2 | Atr-ERN13047 |  | | | |  |  |  | | | |  |  |  |
| 2 | Atr-ERN13048 |  | | | |  |  |  | | | |  |  |  |
| 2 | Atr-ERN13049 |  | | | |  |  |  | | | |  |  |  |
| 2 | Atr-ERN13050 |  | | | |  |  |  | | | |  |  |  |
| 2 | Atr-ERN13051 |  | | | |  |  |  | | | |  |  |  |
| 2 | Atr-ERN13052 |  | | | |  |  |  | | | |  |  |  |
| 2 | Atr-ERN13053 |  | | | |  |  |  | | | |  |  |  |
| 2 | Atr-ERN13054 |  | | | |  |  |  | | | |  |  |  |
| 2 | Atr-ERN13055 |  | Vvi-Vitvi08g01423\_t001 |  |  |  | | | |  |  |  |
| 2 | Atr-ERN13056 |  | Vvi-Vitvi08g01422\_t001 |  |  |  | | | |  |  |  |
| 2 | Atr-ERN13057 |  | Vvi-Vitvi08g01421\_t001 |  |  |  | | | |  |  |  |
| 2 | Atr-ERN13058 |  | | | |  |  |  | Vvi-Vitvi06g01517\_t001 |  |  |  |
| 2 | Atr-ERN13059 |  | | | |  |  |  | Vvi-Vitvi06g01518\_t001 |  |  |  |
| 2 | Atr-ERN13060 |  | Vvi-Vitvi08g01419\_t001 |  |  |  | Vvi-Vitvi06g01521\_t001 |  |  |  |
| 2 | Atr-ERN13061 |  | Vvi-Vitvi08g01418\_t001 |  |  |  | Vvi-Vitvi06g01522\_t001 |  |  |  |
| 1 | Atr-ERN13062 |  |  |  |  |  | Vvi-Vitvi06g01523\_t001 |  |  |  |
| 0 | Atr-ERN13063 |  |  |  |  |  |  |
| 0 | Atr-ERN13064 |  |  |  |  |  |  |
| 0 | Atr-ERN13065 |  |  |  |  |  |  |
| 1 | Atr-ERN13066 |  | Vvi-Vitvi06g01469\_t001 |  |  |  |  |  |
| 1 | Atr-ERN13067 |  | | | |  |  |  |  |  |
| 1 | Atr-ERN13068 |  | | | |  |  |  |  |  |
| 1 | Atr-ERN13069 |  | | | |  |  |  |  |  |
| 1 | Atr-ERN13070 |  | | | |  |  |  |  |  |
| 1 | Atr-ERN13071 |  | | | |  |  |  |  |  |
| 1 | Atr-ERN13072 |  | | | |  |  |  |  |  |
| 1 | Atr-ERN13073 |  | | | |  |  |  |  |  |
| 1 | Atr-ERN13074 |  | | | |  |  |  |  |  |
| 1 | Atr-ERN13075 |  | | | |  |  |  |  |  |
| 1 | Atr-ERN13076 |  | | | |  |  |  |  |  |
| 1 | Atr-ERN13077 |  | | | |  |  |  |  |  |
| 1 | Atr-ERN13078 |  | | | |  |  |  |  |  |
| 1 | Atr-ERN13079 |  | Vvi-Vitvi06g01467\_t001 |  |  |  |  |  |
| 1 | Atr-ERN13080 |  | Vvi-Vitvi06g01459\_t001 |  |  |  |  |  |
| 2 | Atr-ERN13081 |  | | | |  | Vvi-Vitvi08g01492\_t001 |  |  |  |  |
| 2 | Atr-ERN13082 |  | | | |  | | | |  |  |  |  |
| 2 | Atr-ERN13083 |  | | | |  | Vvi-Vitvi08g01490\_t001 |  |  |  |  |
| 2 | Atr-ERN13084 |  | | | |  | | | |  |  |  |  |
| 2 | Atr-ERN13085 |  | Vvi-Vitvi06g01457\_t002 |  | | | |  |  |  |  |
| 2 | Atr-ERN13086 |  | | | |  | | | |  |  |  |  |
| 2 | Atr-ERN13087 |  | | | |  | | | |  |  |  |  |
| 2 | Atr-ERN13088 |  | | | |  | Vvi-Vitvi08g01489\_t001 |  |  |  |  |
| 3 | Atr-ERN13089 |  | | | |  | Vvi-Vitvi08g01488\_t001 |  | Vvi-Vitvi13g00829\_t001 |  |  |  |
| 3 | Atr-ERN13090 |  | Vvi-Vitvi06g01455\_t001 |  | | | |  | | | |  |  |  |
| 3 | Atr-ERN13091 |  | | | |  | | | |  | | | |  |  |  |
| 3 | Atr-ERN13092 |  | | | |  | | | |  | | | |  |  |  |
| 3 | Atr-ERN13093 |  | | | |  | | | |  | | | |  |  |  |
| 3 | Atr-ERN13094 |  | | | |  | | | |  | | | |  |  |  |
| 3 | Atr-ERN13095 |  | | | |  | | | |  | | | |  |  |  |
| 3 | Atr-ERN13096 |  | | | |  | | | |  | | | |  |  |  |
| 3 | Atr-ERN13097 |  | | | |  | | | |  | | | |  |  |  |
| 3 | Atr-ERN13098 |  | Vvi-Vitvi06g01454\_t001 |  | Vvi-Vitvi08g01487\_t001 |  | | | |  |  |  |
| 3 | Atr-ERN13099 |  | | | |  | | | |  | | | |  |  |  |
| 3 | Atr-ERN13100 |  | | | |  | | | |  | | | |  |  |  |
| 3 | Atr-ERN13101 |  | | | |  | | | |  | | | |  |  |  |
| 3 | Atr-ERN13102 |  | Vvi-Vitvi06g01453\_t001 |  | | | |  | | | |  |  |  |
| 3 | Atr-ERN13103 |  | Vvi-Vitvi06g01452\_t001 |  | | | |  | | | |  |  |  |
| 3 | Atr-ERN13104 |  | Vvi-Vitvi06g01451\_t001 |  | | | |  | Vvi-Vitvi13g00802\_t001 |  |  |  |
| 3 | Atr-ERN13105 |  | | | |  | | | |  | | | |  |  |  |
| 3 | Atr-ERN13106 |  | | | |  | | | |  | | | |  |  |  |
| 3 | Atr-ERN13107 |  | | | |  | | | |  | | | |  |  |  |
| 3 | Atr-ERN13108 |  | | | |  | | | |  | | | |  |  |  |
| 3 | Atr-ERN13109 |  | | | |  | | | |  | | | |  |  |  |
| 3 | Atr-ERN13110 |  | | | |  | | | |  | | | |  |  |  |
| 3 | Atr-ERN13111 |  | | | |  | | | |  | | | |  |  |  |
| 3 | Atr-ERN13112 |  | | | |  | | | |  | | | |  |  |  |
| 3 | Atr-ERN13113 |  | | | |  | | | |  | | | |  |  |  |
| 3 | Atr-ERN13114 |  | | | |  | | | |  | | | |  |  |  |
| 3 | Atr-ERN13115 |  | | | |  | Vvi-Vitvi08g02272\_t004 |  | | | |  |  |  |
| 3 | Atr-ERN13116 |  | | | |  | | | |  | | | |  |  |  |
| 3 | Atr-ERN13117 |  | | | |  | | | |  | Vvi-Vitvi13g02105\_t001 |  |  |  |
| 3 | Atr-ERN13118 |  | | | |  | | | |  | | | |  |  |  |
| 3 | Atr-ERN13119 |  | | | |  | | | |  | | | |  |  |  |
| 3 | Atr-ERN13120 |  | | | |  | | | |  | | | |  |  |  |
| 3 | Atr-ERN13121 |  | Vvi-Vitvi06g01444\_t001 |  | | | |  | | | |  |  |  |
| 3 | Atr-ERN13122 |  | | | |  | | | |  | | | |  |  |  |
| 3 | Atr-ERN13123 |  | | | |  | | | |  | | | |  |  |  |
| 3 | Atr-ERN13124 |  | Vvi-Vitvi06g01435\_t001 |  | | | |  | | | |  |  |  |
| 3 | Atr-ERN13125 |  | | | |  | | | |  | | | |  |  |  |
| 4 | Atr-ERN13126 |  | | | |  | | | |  | Vvi-Vitvi13g02104\_t001 |  | Vvi-Vitvi08g01484\_t001 |  |  |
| 4 | Atr-ERN13127 |  | | | |  | Vvi-Vitvi08g01483\_t001 |  | | | |  | | | |  |  |
| 4 | Atr-ERN13128 |  | Vvi-Vitvi06g04434\_t001 |  | | | |  | | | |  | | | |  |  |
| 4 | Atr-ERN13129 |  | | | |  | | | |  | | | |  | | | |  |  |
| 4 | Atr-ERN13130 |  | Vvi-Vitvi06g01429\_t001 |  | Vvi-Vitvi08g01482\_t001 |  | | | |  | | | |  |  |
| 3 | Atr-ERN13131 |  | Vvi-Vitvi06g01428\_t001 |  |  |  | | | |  | | | |  |  |
| 3 | Atr-ERN13132 |  | Vvi-Vitvi06g01427\_t001 |  |  |  | Vvi-Vitvi13g00792\_t001 |  | | | |  |  |
| 3 | Atr-ERN13133 |  | Vvi-Vitvi06g04432\_t001 |  |  |  | | | |  | | | |  |  |
| 3 | Atr-ERN13134 |  | | | |  |  |  | | | |  | | | |  |  |
| 3 | Atr-ERN13135 |  | | | |  |  |  | Vvi-Vitvi13g00790\_t001 |  | | | |  |  |
| 3 | Atr-ERN13136 |  | Vvi-Vitvi06g01420\_t001 |  |  |  | Vvi-Vitvi13g00779\_t001 |  | Vvi-Vitvi08g01493\_t001 |  |  |
| 3 | Atr-ERN13137 |  | | | |  |  |  | | | |  | | | |  |  |
| 3 | Atr-ERN13138 |  | | | |  |  |  | | | |  | | | |  |  |
| 3 | Atr-ERN13139 |  | | | |  |  |  | | | |  | Vvi-Vitvi08g01494\_t001 |  |  |
| 3 | Atr-ERN13140 |  | | | |  |  |  | | | |  | | | |  |  |
| 3 | Atr-ERN13141 |  | | | |  |  |  | | | |  | Vvi-Vitvi08g01497\_t001 |  |  |
| 3 | Atr-ERN13142 |  | | | |  |  |  | | | |  | Vvi-Vitvi08g01498\_t001 |  |  |
| 3 | Atr-ERN13143 |  | | | |  |  |  | | | |  | | | |  |  |
| 3 | Atr-ERN13144 |  | Vvi-Vitvi06g01419\_t001 |  |  |  | Vvi-Vitvi13g00778\_t001 |  | | | |  |  |
| 3 | Atr-ERN13145 |  | Vvi-Vitvi06g01418\_t001 |  |  |  | Vvi-Vitvi13g00777\_t001 |  | Vvi-Vitvi08g01499\_t001 |  |  |
| 3 | Atr-ERN13146 |  | | | |  |  |  | Vvi-Vitvi13g00774\_t001 |  | | | |  |  |
| 3 | Atr-ERN13147 |  | Vvi-Vitvi06g01417\_t001 |  |  |  | Vvi-Vitvi13g04252\_t001 |  | | | |  |  |
| 3 | Atr-ERN13148 |  | Vvi-Vitvi06g01416\_t001 |  |  |  | Vvi-Vitvi13g00768\_t001 |  | | | |  |  |
| 2 | Atr-ERN13149 |  | | | |  |  |  |  |  | | | |  |  |
| 2 | Atr-ERN13150 |  | | | |  |  |  |  |  | | | |  |  |
| 2 | Atr-ERN13151 |  | Vvi-Vitvi06g01411\_t001 |  |  |  |  |  | Vvi-Vitvi08g01501\_t001 |  |  |
| 2 | Atr-ERN13152 |  | | | |  |  |  |  |  | | | |  |  |
| 2 | Atr-ERN13153 |  | | | |  |  |  |  |  | | | |  |  |
| 2 | Atr-ERN13154 |  | | | |  |  |  |  |  | | | |  |  |
| 3 | Atr-ERN13155 |  | | | |  | Vvi-Vitvi13g00736\_t001 |  |  |  | | | |  |  |
| 3 | Atr-ERN13156 |  | | | |  | | | |  |  |  | | | |  |  |
| 3 | Atr-ERN13157 |  | Vvi-Vitvi06g01410\_t001 |  | | | |  |  |  | | | |  |  |
| 3 | Atr-ERN13158 |  | Vvi-Vitvi06g01409\_t001 |  | | | |  |  |  | | | |  |  |
| 3 | Atr-ERN13159 |  | | | |  | Vvi-Vitvi13g00738\_t001 |  |  |  | | | |  |  |
| 3 | Atr-ERN13160 |  | | | |  | Vvi-Vitvi13g00740\_t001 |  |  |  | Vvi-Vitvi08g01505\_t001 |  |  |
| 3 | Atr-ERN13161 |  | | | |  | | | |  |  |  | Vvi-Vitvi08g01506\_t001 |  |  |
| 3 | Atr-ERN13162 |  | Vvi-Vitvi06g01408\_t001 |  | | | |  |  |  | Vvi-Vitvi08g01507\_t001 |  |  |
| 3 | Atr-ERN13163 |  | Vvi-Vitvi06g01407\_t001 |  | | | |  |  |  | | | |  |  |
| 3 | Atr-ERN13164 |  | | | |  | | | |  |  |  | Vvi-Vitvi08g01509\_t001 |  |  |
| 3 | Atr-ERN13165 |  | | | |  | Vvi-Vitvi13g02093\_t001 |  |  |  | | | |  |  |
| 3 | Atr-ERN13166 |  | | | |  | | | |  |  |  | | | |  |  |
| 3 | Atr-ERN13167 |  | | | |  | | | |  |  |  | | | |  |  |
| 3 | Atr-ERN13168 |  | | | |  | Vvi-Vitvi13g00745\_t001 |  |  |  | | | |  |  |
| 3 | Atr-ERN13169 |  | | | |  | | | |  |  |  | | | |  |  |
| 3 | Atr-ERN13170 |  | | | |  | | | |  |  |  | | | |  |  |
| 3 | Atr-ERN13171 |  | | | |  | | | |  |  |  | | | |  |  |
| 3 | Atr-ERN13172 |  | | | |  | | | |  |  |  | | | |  |  |
| 3 | Atr-ERN13173 |  | | | |  | | | |  |  |  | | | |  |  |
| 3 | Atr-ERN13174 |  | | | |  | | | |  |  |  | | | |  |  |
| 3 | Atr-ERN13175 |  | | | |  | | | |  |  |  | | | |  |  |
| 3 | Atr-ERN13176 |  | | | |  | | | |  |  |  | | | |  |  |
| 3 | Atr-ERN13177 |  | | | |  | | | |  |  |  | | | |  |  |
| 3 | Atr-ERN13178 |  | | | |  | | | |  |  |  | | | |  |  |
| 3 | Atr-ERN13179 |  | | | |  | | | |  |  |  | | | |  |  |
| 3 | Atr-ERN13180 |  | | | |  | | | |  |  |  | Vvi-Vitvi08g01510\_t001 |  |  |
| 3 | Atr-ERN13181 |  | | | |  | Vvi-Vitvi13g00749\_t001 |  |  |  | | | |  |  |
| 2 | Atr-ERN13182 |  | | | |  |  |  |  |  | | | |  |  |
| 2 | Atr-ERN13183 |  | | | |  |  |  |  |  | | | |  |  |
| 2 | Atr-ERN13184 |  | | | |  |  |  |  |  | | | |  |  |
| 2 | Atr-ERN13185 |  | | | |  |  |  |  |  | | | |  |  |
| 2 | Atr-ERN13186 |  | | | |  |  |  |  |  | | | |  |  |
| 2 | Atr-ERN13187 |  | | | |  |  |  |  |  | Vvi-Vitvi08g01515\_t001 |  |  |
| 2 | Atr-ERN13188 |  | | | |  |  |  |  |  | | | |  |  |
| 2 | Atr-ERN13189 |  | Vvi-Vitvi06g01404\_t001 |  |  |  |  |  | | | |  |  |
| 2 | Atr-ERN13190 |  | Vvi-Vitvi06g01946\_t001 |  |  |  |  |  | | | |  |  |
| 2 | Atr-ERN13191 |  | | | |  |  |  |  |  | | | |  |  |
| 2 | Atr-ERN13192 |  | | | |  |  |  |  |  | Vvi-Vitvi08g01519\_t003 |  |  |
| 2 | Atr-ERN13193 |  | | | |  |  |  |  |  | | | |  |  |
| 2 | Atr-ERN13194 |  | | | |  |  |  |  |  | | | |  |  |
| 2 | Atr-ERN13195 |  | | | |  |  |  |  |  | | | |  |  |
| 2 | Atr-ERN13196 |  | | | |  |  |  |  |  | | | |  |  |
| 2 | Atr-ERN13197 |  | | | |  |  |  |  |  | | | |  |  |
| 2 | Atr-ERN13198 |  | | | |  |  |  |  |  | | | |  |  |
| 2 | Atr-ERN13199 |  | | | |  |  |  |  |  | | | |  |  |
| 2 | Atr-ERN13200 |  | | | |  |  |  |  |  | | | |  |  |
| 2 | Atr-ERN13201 |  | | | |  |  |  |  |  | | | |  |  |
| 2 | Atr-ERN13202 |  | | | |  |  |  |  |  | | | |  |  |
| 2 | Atr-ERN13203 |  | | | |  |  |  |  |  | | | |  |  |
| 2 | Atr-ERN13204 |  | | | |  |  |  |  |  | | | |  |  |
| 2 | Atr-ERN13205 |  | | | |  |  |  |  |  | | | |  |  |
| 2 | Atr-ERN13206 |  | | | |  |  |  |  |  | | | |  |  |
| 2 | Atr-ERN13207 |  | | | |  |  |  |  |  | | | |  |  |
| 2 | Atr-ERN13208 |  | | | |  |  |  |  |  | | | |  |  |
| 2 | Atr-ERN13209 |  | | | |  |  |  |  |  | | | |  |  |
| 2 | Atr-ERN13210 |  | | | |  |  |  |  |  | | | |  |  |
| 2 | Atr-ERN13211 |  | | | |  |  |  |  |  | | | |  |  |
| 2 | Atr-ERN13212 |  | | | |  |  |  |  |  | | | |  |  |
| 2 | Atr-ERN13213 |  | | | |  |  |  |  |  | Vvi-Vitvi08g01521\_t001 |  |  |
| 2 | Atr-ERN13214 |  | | | |  |  |  |  |  | | | |  |  |
| 2 | Atr-ERN13215 |  | | | |  |  |  |  |  | | | |  |  |
| 2 | Atr-ERN13216 |  | Vvi-Vitvi06g01398\_t001 |  |  |  |  |  | Vvi-Vitvi08g01523\_t002 |  |  |
| 2 | Atr-ERN13217 |  | | | |  |  |  |  |  | | | |  |  |
| 2 | Atr-ERN13218 |  | Vvi-Vitvi06g01396\_t001 |  |  |  |  |  | | | |  |  |
| 2 | Atr-ERN13219 |  | | | |  |  |  |  |  | | | |  |  |
| 2 | Atr-ERN13220 |  | | | |  |  |  |  |  | | | |  |  |
| 2 | Atr-ERN13221 |  | Vvi-Vitvi06g01944\_t001 |  |  |  |  |  | Vvi-Vitvi08g01525\_t001 |  |  |
| 2 | Atr-ERN13222 |  | | | |  |  |  |  |  | | | |  |  |
| 2 | Atr-ERN13223 |  | | | |  |  |  |  |  | | | |  |  |
| 2 | Atr-ERN13224 |  | Vvi-Vitvi06g01395\_t001 |  |  |  |  |  | | | |  |  |
| 2 | Atr-ERN13225 |  | | | |  |  |  |  |  | Vvi-Vitvi08g01527\_t001 |  |  |
| 2 | Atr-ERN13226 |  | Vvi-Vitvi06g01394\_t003 |  |  |  |  |  | | | |  |  |
| 2 | Atr-ERN13227 |  | | | |  |  |  |  |  | Vvi-Vitvi08g01536\_t001 |  |  |
| 2 | Atr-ERN13228 |  | | | |  |  |  |  |  | | | |  |  |
| 2 | Atr-ERN13229 |  | Vvi-Vitvi06g01392\_t001 |  |  |  |  |  | | | |  |  |
| 2 | Atr-ERN13230 |  | | | |  |  |  |  |  | | | |  |  |
| 2 | Atr-ERN13231 |  | | | |  |  |  |  |  | | | |  |  |
| 2 | Atr-ERN13232 |  | | | |  |  |  |  |  | | | |  |  |
| 2 | Atr-ERN13233 |  | | | |  |  |  |  |  | Vvi-Vitvi08g02281\_t001 |  |  |
| 2 | Atr-ERN13234 |  | | | |  |  |  |  |  | | | |  |  |
| 2 | Atr-ERN13235 |  | | | |  |  |  |  |  | Vvi-Vitvi08g01537\_t001 |  |  |
| 2 | Atr-ERN13236 |  | | | |  |  |  |  |  | | | |  |  |
| 2 | Atr-ERN13237 |  | | | |  |  |  |  |  | | | |  |  |
| 2 | Atr-ERN13238 |  | Vvi-Vitvi06g01942\_t001 |  |  |  |  |  | | | |  |  |
| 2 | Atr-ERN13239 |  | | | |  |  |  |  |  | | | |  |  |
| 2 | Atr-ERN13240 |  | | | |  |  |  |  |  | | | |  |  |
| 2 | Atr-ERN13241 |  | | | |  |  |  |  |  | | | |  |  |
| 2 | Atr-ERN13242 |  | | | |  |  |  |  |  | Vvi-Vitvi08g01539\_t001 |  |  |
| 2 | Atr-ERN13243 |  | | | |  |  |  |  |  | | | |  |  |
| 2 | Atr-ERN13244 |  | | | |  |  |  |  |  | | | |  |  |
| 2 | Atr-ERN13245 |  | | | |  |  |  |  |  | | | |  |  |
| 2 | Atr-ERN13246 |  | | | |  |  |  |  |  | | | |  |  |
| 2 | Atr-ERN13247 |  | | | |  |  |  |  |  | Vvi-Vitvi08g01540\_t001 |  |  |
| 2 | Atr-ERN13248 |  | | | |  |  |  |  |  | | | |  |  |
| 2 | Atr-ERN13249 |  | | | |  |  |  |  |  | | | |  |  |
| 2 | Atr-ERN13250 |  | | | |  |  |  |  |  | | | |  |  |
| 2 | Atr-ERN13251 |  | | | |  |  |  |  |  | | | |  |  |
| 2 | Atr-ERN13252 |  | | | |  |  |  |  |  | | | |  |  |
| 2 | Atr-ERN13253 |  | | | |  |  |  |  |  | | | |  |  |
| 2 | Atr-ERN13254 |  | | | |  |  |  |  |  | | | |  |  |
| 2 | Atr-ERN13255 |  | Vvi-Vitvi06g01389\_t001 |  |  |  |  |  | | | |  |  |
| 2 | Atr-ERN13256 |  | | | |  |  |  |  |  | | | |  |  |
| 2 | Atr-ERN13257 |  | | | |  |  |  |  |  | | | |  |  |
| 2 | Atr-ERN13258 |  | Vvi-Vitvi06g01388\_t001 |  |  |  |  |  | | | |  |  |
| 1 | Atr-ERN13259 |  |  |  |  |  |  |  | Vvi-Vitvi08g01542\_t001 |  |  |
| 1 | Atr-ERN13260 |  |  |  |  |  |  |  | Vvi-Vitvi08g01543\_t001 |  |  |
| 0 | Atr-ERN13261 |  |  |  |  |  |  |
| 0 | Atr-ERN13262 |  |  |  |  |  |  |
| 0 | Atr-ERN13263 |  |  |  |  |  |  |
