# Supplementary material for: Genome-wide identification, characterization and gene expression of BES1 transcription factor family in grapevine (Vitis vinifera L.)
Source: Sci Rep. 2023 Jan 5;13:240. doi: 10.1038/s41598-022-24407-y (PMC9816167; doi:10.1038/s41598-022-24407-y)
Supplement: Supplementary file 3 — Supplementary Information. [file 41598_2022_24407_MOESM3_ESM.zip › Vvi_Atr/Vitis_vinifera.PN40024.v4.dna_sm.toplevel.fa.vs.Amborella_trichopoda.AMTR1.0.dna_sm.toplevel.fa.html/Atr-AmTr_v1.0_scaffold00041.html]

|  |  |  |  |  |  |  |  |  |  |  |  |  |  |
| --- | --- | --- | --- | --- | --- | --- | --- | --- | --- | --- | --- | --- | --- |
| Duplication depth | Reference chromosome | Collinear blocks | | | | | | | | | | | |
| 1 | Atr-ERN13265 |  | Vvi-Vitvi02g04120\_t001 |  |  |  |  |  |
| 1 | Atr-ERN13266 |  | Vvi-Vitvi02g00459\_t001 |  |  |  |  |  |
| 2 | Atr-ERN13267 |  | Vvi-Vitvi02g00460\_t002 |  | Vvi-Vitvi15g00727\_t001 |  |  |  |  |
| 2 | Atr-ERN13268 |  | | | |  | | | |  |  |  |  |
| 2 | Atr-ERN13269 |  | | | |  | | | |  |  |  |  |
| 2 | Atr-ERN13270 |  | Vvi-Vitvi02g00462\_t001 |  | Vvi-Vitvi15g00731\_t001 |  |  |  |  |
| 2 | Atr-ERN13271 |  | | | |  | | | |  |  |  |  |
| 2 | Atr-ERN13272 |  | | | |  | | | |  |  |  |  |
| 2 | Atr-ERN13273 |  | Vvi-Vitvi02g00465\_t001 |  | Vvi-Vitvi15g00732\_t001 |  |  |  |  |
| 2 | Atr-ERN13274 |  | | | |  | | | |  |  |  |  |
| 2 | Atr-ERN13275 |  | | | |  | | | |  |  |  |  |
| 2 | Atr-ERN13276 |  | | | |  | Vvi-Vitvi15g01499\_t001 |  |  |  |  |
| 2 | Atr-ERN13277 |  | Vvi-Vitvi02g00466\_t001 |  | | | |  |  |  |  |
| 2 | Atr-ERN13278 |  | | | |  | Vvi-Vitvi15g00733\_t001 |  |  |  |  |
| 2 | Atr-ERN13279 |  | | | |  | | | |  |  |  |  |
| 3 | Atr-ERN13280 |  | | | |  | | | |  | Vvi-Vitvi16g00843\_t001 |  |  |  |
| 3 | Atr-ERN13281 |  | | | |  | | | |  | Vvi-Vitvi16g00841\_t001 |  |  |  |
| 3 | Atr-ERN13282 |  | | | |  | Vvi-Vitvi15g00735\_t001 |  | | | |  |  |  |
| 3 | Atr-ERN13283 |  | | | |  | | | |  | | | |  |  |  |
| 3 | Atr-ERN13284 |  | | | |  | Vvi-Vitvi15g00737\_t001 |  | | | |  |  |  |
| 3 | Atr-ERN13285 |  | | | |  | | | |  | | | |  |  |  |
| 3 | Atr-ERN13286 |  | Vvi-Vitvi02g00467\_t001 |  | | | |  | | | |  |  |  |
| 3 | Atr-ERN13287 |  | | | |  | | | |  | | | |  |  |  |
| 3 | Atr-ERN13288 |  | | | |  | Vvi-Vitvi15g00739\_t001 |  | Vvi-Vitvi16g00836\_t001 |  |  |  |
| 3 | Atr-ERN13289 |  | Vvi-Vitvi02g00468\_t001 |  | Vvi-Vitvi15g00740\_t001 |  | Vvi-Vitvi16g00835\_t001 |  |  |  |
| 3 | Atr-ERN13290 |  | | | |  | | | |  | | | |  |  |  |
| 3 | Atr-ERN13291 |  | Vvi-Vitvi02g00469\_t001 |  | | | |  | | | |  |  |  |
| 3 | Atr-ERN13292 |  | | | |  | | | |  | | | |  |  |  |
| 3 | Atr-ERN13293 |  | | | |  | | | |  | | | |  |  |  |
| 3 | Atr-ERN13294 |  | | | |  | | | |  | | | |  |  |  |
| 3 | Atr-ERN13295 |  | | | |  | | | |  | | | |  |  |  |
| 3 | Atr-ERN13296 |  | | | |  | | | |  | | | |  |  |  |
| 3 | Atr-ERN13297 |  | | | |  | | | |  | | | |  |  |  |
| 3 | Atr-ERN13298 |  | | | |  | | | |  | Vvi-Vitvi16g00831\_t001 |  |  |  |
| 3 | Atr-ERN13299 |  | Vvi-Vitvi02g00471\_t001 |  | Vvi-Vitvi15g00741\_t001 |  | | | |  |  |  |
| 3 | Atr-ERN13300 |  | Vvi-Vitvi02g00472\_t001 |  | | | |  | | | |  |  |  |
| 3 | Atr-ERN13301 |  | | | |  | Vvi-Vitvi15g00743\_t001 |  | | | |  |  |  |
| 3 | Atr-ERN13302 |  | | | |  | | | |  | Vvi-Vitvi16g00830\_t001 |  |  |  |
| 3 | Atr-ERN13303 |  | | | |  | | | |  | | | |  |  |  |
| 3 | Atr-ERN13304 |  | | | |  | Vvi-Vitvi15g00746\_t001 |  | | | |  |  |  |
| 3 | Atr-ERN13305 |  | | | |  | | | |  | | | |  |  |  |
| 3 | Atr-ERN13306 |  | Vvi-Vitvi02g00474\_t001 |  | | | |  | | | |  |  |  |
| 3 | Atr-ERN13307 |  | | | |  | | | |  | | | |  |  |  |
| 3 | Atr-ERN13308 |  | Vvi-Vitvi02g04126\_t001 |  | | | |  | | | |  |  |  |
| 3 | Atr-ERN13309 |  | | | |  | | | |  | | | |  |  |  |
| 3 | Atr-ERN13310 |  | | | |  | | | |  | | | |  |  |  |
| 3 | Atr-ERN13311 |  | | | |  | | | |  | Vvi-Vitvi16g00826\_t001 |  |  |  |
| 3 | Atr-ERN13312 |  | | | |  | Vvi-Vitvi15g00747\_t001 |  | | | |  |  |  |
| 3 | Atr-ERN13313 |  | | | |  | | | |  | | | |  |  |  |
| 3 | Atr-ERN13314 |  | | | |  | | | |  | | | |  |  |  |
| 3 | Atr-ERN13315 |  | | | |  | | | |  | Vvi-Vitvi16g00817\_t002 |  |  |  |
| 3 | Atr-ERN13316 |  | Vvi-Vitvi02g00475\_t001 |  | | | |  | | | |  |  |  |
| 3 | Atr-ERN13317 |  | | | |  | Vvi-Vitvi15g00748\_t001 |  | | | |  |  |  |
| 2 | Atr-ERN13318 |  | | | |  |  |  | | | |  |  |  |
| 2 | Atr-ERN13319 |  | | | |  |  |  | | | |  |  |  |
| 2 | Atr-ERN13320 |  | | | |  |  |  | | | |  |  |  |
| 2 | Atr-ERN13321 |  | | | |  |  |  | | | |  |  |  |
| 2 | Atr-ERN13322 |  | | | |  |  |  | | | |  |  |  |
| 2 | Atr-ERN13323 |  | | | |  |  |  | | | |  |  |  |
| 2 | Atr-ERN13324 |  | | | |  |  |  | | | |  |  |  |
| 2 | Atr-ERN13325 |  | | | |  |  |  | | | |  |  |  |
| 2 | Atr-ERN13326 |  | | | |  |  |  | | | |  |  |  |
| 2 | Atr-ERN13327 |  | Vvi-Vitvi02g00477\_t001 |  |  |  | | | |  |  |  |
| 2 | Atr-ERN13328 |  | Vvi-Vitvi02g04129\_t001 |  |  |  | | | |  |  |  |
| 2 | Atr-ERN13329 |  | Vvi-Vitvi02g00479\_t001 |  |  |  | | | |  |  |  |
| 2 | Atr-ERN13330 |  | | | |  |  |  | | | |  |  |  |
| 2 | Atr-ERN13331 |  | | | |  |  |  | | | |  |  |  |
| 2 | Atr-ERN13332 |  | | | |  |  |  | | | |  |  |  |
| 2 | Atr-ERN13333 |  | | | |  |  |  | | | |  |  |  |
| 2 | Atr-ERN13334 |  | | | |  |  |  | | | |  |  |  |
| 2 | Atr-ERN13335 |  | Vvi-Vitvi02g00481\_t001 |  |  |  | Vvi-Vitvi16g00809\_t001 |  |  |  |
| 0 | Atr-ERN13336 |  |  |  |  |  |  |
| 0 | Atr-ERN13337 |  |  |  |  |  |  |
| 0 | Atr-ERN13338 |  |  |  |  |  |  |
| 0 | Atr-ERN13339 |  |  |  |  |  |  |
| 2 | Atr-ERN13340 |  | Vvi-Vitvi15g00931\_t002 |  | Vvi-Vitvi02g00207\_t001 |  |  |  |  |
| 2 | Atr-ERN13341 |  | Vvi-Vitvi15g00929\_t001 |  | Vvi-Vitvi02g00208\_t002 |  |  |  |  |
| 2 | Atr-ERN13342 |  | Vvi-Vitvi15g00928\_t001 |  | | | |  |  |  |  |
| 2 | Atr-ERN13343 |  | Vvi-Vitvi15g00926\_t003 |  | | | |  |  |  |  |
| 2 | Atr-ERN13344 |  | Vvi-Vitvi15g00925\_t001 |  | | | |  |  |  |  |
| 2 | Atr-ERN13345 |  | | | |  | | | |  |  |  |  |
| 2 | Atr-ERN13346 |  | Vvi-Vitvi15g00923\_t001 |  | | | |  |  |  |  |
| 1 | Atr-ERN13347 |  |  |  | | | |  |  |  |  |
| 1 | Atr-ERN13348 |  |  |  | | | |  |  |  |  |
| 1 | Atr-ERN13349 |  |  |  | | | |  |  |  |  |
| 1 | Atr-ERN13350 |  |  |  | | | |  |  |  |  |
| 1 | Atr-ERN13351 |  |  |  | | | |  |  |  |  |
| 1 | Atr-ERN13352 |  |  |  | | | |  |  |  |  |
| 1 | Atr-ERN13353 |  |  |  | | | |  |  |  |  |
| 1 | Atr-ERN13354 |  |  |  | | | |  |  |  |  |
| 1 | Atr-ERN13355 |  |  |  | | | |  |  |  |  |
| 1 | Atr-ERN13356 |  |  |  | | | |  |  |  |  |
| 1 | Atr-ERN13357 |  |  |  | | | |  |  |  |  |
| 1 | Atr-ERN13358 |  |  |  | Vvi-Vitvi02g00209\_t001 |  |  |  |  |
| 1 | Atr-ERN13359 |  |  |  | Vvi-Vitvi02g00210\_t001 |  |  |  |  |
| 1 | Atr-ERN13360 |  |  |  | Vvi-Vitvi02g00211\_t001 |  |  |  |  |
| 1 | Atr-ERN13361 |  |  |  | | | |  |  |  |  |
| 1 | Atr-ERN13362 |  |  |  | | | |  |  |  |  |
| 1 | Atr-ERN13363 |  |  |  | | | |  |  |  |  |
| 1 | Atr-ERN13364 |  |  |  | | | |  |  |  |  |
| 1 | Atr-ERN13365 |  |  |  | | | |  |  |  |  |
| 1 | Atr-ERN13366 |  |  |  | | | |  |  |  |  |
| 1 | Atr-ERN13367 |  |  |  | | | |  |  |  |  |
| 1 | Atr-ERN13368 |  |  |  | | | |  |  |  |  |
| 1 | Atr-ERN13369 |  |  |  | | | |  |  |  |  |
| 1 | Atr-ERN13370 |  |  |  | | | |  |  |  |  |
| 1 | Atr-ERN13371 |  |  |  | | | |  |  |  |  |
| 2 | Atr-ERN13372 |  | Vvi-Vitvi03g00126\_t001 |  | | | |  |  |  |  |
| 2 | Atr-ERN13373 |  | | | |  | Vvi-Vitvi02g00212\_t001 |  |  |  |  |
| 1 | Atr-ERN13374 |  | | | |  |  |  |  |  |
| 1 | Atr-ERN13375 |  | | | |  |  |  |  |  |
| 1 | Atr-ERN13376 |  | | | |  |  |  |  |  |
| 1 | Atr-ERN13377 |  | Vvi-Vitvi03g00125\_t001 |  |  |  |  |  |
| 1 | Atr-ERN13378 |  | | | |  |  |  |  |  |
| 1 | Atr-ERN13379 |  | | | |  |  |  |  |  |
| 1 | Atr-ERN13380 |  | Vvi-Vitvi03g00123\_t001 |  |  |  |  |  |
| 1 | Atr-ERN13381 |  | | | |  |  |  |  |  |
| 1 | Atr-ERN13382 |  | | | |  |  |  |  |  |
| 1 | Atr-ERN13383 |  | Vvi-Vitvi03g00122\_t001 |  |  |  |  |  |
| 1 | Atr-ERN13384 |  | | | |  |  |  |  |  |
| 1 | Atr-ERN13385 |  | Vvi-Vitvi03g00121\_t001 |  |  |  |  |  |
| 1 | Atr-ERN13386 |  | Vvi-Vitvi03g00119\_t001 |  |  |  |  |  |
| 1 | Atr-ERN13387 |  | | | |  |  |  |  |  |
| 1 | Atr-ERN13388 |  | | | |  |  |  |  |  |
| 1 | Atr-ERN13389 |  | Vvi-Vitvi03g01396\_t001 |  |  |  |  |  |
| 1 | Atr-ERN13390 |  | | | |  |  |  |  |  |
| 1 | Atr-ERN13391 |  | | | |  |  |  |  |  |
| 1 | Atr-ERN13392 |  | | | |  |  |  |  |  |
| 1 | Atr-ERN13393 |  | | | |  |  |  |  |  |
| 1 | Atr-ERN13394 |  | | | |  |  |  |  |  |
| 1 | Atr-ERN13395 |  | Vvi-Vitvi03g00114\_t001 |  |  |  |  |  |
| 1 | Atr-ERN13396 |  | | | |  |  |  |  |  |
| 1 | Atr-ERN13397 |  | | | |  |  |  |  |  |
| 1 | Atr-ERN13398 |  | | | |  |  |  |  |  |
| 1 | Atr-ERN13399 |  | | | |  |  |  |  |  |
| 1 | Atr-ERN13400 |  | | | |  |  |  |  |  |
| 1 | Atr-ERN13401 |  | | | |  |  |  |  |  |
| 1 | Atr-ERN13402 |  | | | |  |  |  |  |  |
| 1 | Atr-ERN13403 |  | | | |  |  |  |  |  |
| 1 | Atr-ERN13404 |  | | | |  |  |  |  |  |
| 1 | Atr-ERN13405 |  | | | |  |  |  |  |  |
| 1 | Atr-ERN13406 |  | | | |  |  |  |  |  |
| 1 | Atr-ERN13407 |  | | | |  |  |  |  |  |
| 1 | Atr-ERN13408 |  | | | |  |  |  |  |  |
| 1 | Atr-ERN13409 |  | | | |  |  |  |  |  |
| 1 | Atr-ERN13410 |  | | | |  |  |  |  |  |
| 1 | Atr-ERN13411 |  | | | |  |  |  |  |  |
| 1 | Atr-ERN13412 |  | | | |  |  |  |  |  |
| 1 | Atr-ERN13413 |  | | | |  |  |  |  |  |
| 1 | Atr-ERN13414 |  | | | |  |  |  |  |  |
| 1 | Atr-ERN13415 |  | Vvi-Vitvi03g01394\_t001 |  |  |  |  |  |
| 1 | Atr-ERN13416 |  | | | |  |  |  |  |  |
| 1 | Atr-ERN13417 |  | | | |  |  |  |  |  |
| 1 | Atr-ERN13418 |  | | | |  |  |  |  |  |
| 1 | Atr-ERN13419 |  | | | |  |  |  |  |  |
| 1 | Atr-ERN13420 |  | | | |  |  |  |  |  |
| 1 | Atr-ERN13421 |  | | | |  |  |  |  |  |
| 1 | Atr-ERN13422 |  | | | |  |  |  |  |  |
| 1 | Atr-ERN13423 |  | | | |  |  |  |  |  |
| 1 | Atr-ERN13424 |  | | | |  |  |  |  |  |
| 1 | Atr-ERN13425 |  | Vvi-Vitvi03g01391\_t001 |  |  |  |  |  |
| 1 | Atr-ERN13426 |  | Vvi-Vitvi03g00107\_t001 |  |  |  |  |  |
| 1 | Atr-ERN13427 |  | Vvi-Vitvi03g00106\_t001 |  |  |  |  |  |
| 1 | Atr-ERN13428 |  | | | |  |  |  |  |  |
| 1 | Atr-ERN13429 |  | | | |  |  |  |  |  |
| 1 | Atr-ERN13430 |  | | | |  |  |  |  |  |
| 2 | Atr-ERN13431 |  | | | |  | Vvi-Vitvi03g00098\_t001 |  |  |  |  |
| 2 | Atr-ERN13432 |  | | | |  | | | |  |  |  |  |
| 2 | Atr-ERN13433 |  | | | |  | Vvi-Vitvi03g01388\_t001 |  |  |  |  |
| 2 | Atr-ERN13434 |  | | | |  | | | |  |  |  |  |
| 2 | Atr-ERN13435 |  | | | |  | | | |  |  |  |  |
| 2 | Atr-ERN13436 |  | | | |  | | | |  |  |  |  |
| 2 | Atr-ERN13437 |  | | | |  | Vvi-Vitvi03g00103\_t001 |  |  |  |  |
| 2 | Atr-ERN13438 |  | | | |  | | | |  |  |  |  |
| 2 | Atr-ERN13439 |  | | | |  | | | |  |  |  |  |
| 2 | Atr-ERN13440 |  | | | |  | | | |  |  |  |  |
| 2 | Atr-ERN13441 |  | | | |  | | | |  |  |  |  |
| 2 | Atr-ERN13442 |  | | | |  | | | |  |  |  |  |
| 2 | Atr-ERN13443 |  | | | |  | Vvi-Vitvi03g04055\_t001 |  |  |  |  |
| 2 | Atr-ERN13444 |  | | | |  | | | |  |  |  |  |
| 2 | Atr-ERN13445 |  | | | |  | | | |  |  |  |  |
| 2 | Atr-ERN13446 |  | | | |  | | | |  |  |  |  |
| 2 | Atr-ERN13447 |  | | | |  | | | |  |  |  |  |
| 2 | Atr-ERN13448 |  | | | |  | | | |  |  |  |  |
| 2 | Atr-ERN13449 |  | | | |  | | | |  |  |  |  |
| 2 | Atr-ERN13450 |  | Vvi-Vitvi03g00099\_t001 |  | | | |  |  |  |  |
| 1 | Atr-ERN13451 |  |  |  | | | |  |  |  |  |
| 1 | Atr-ERN13452 |  |  |  | | | |  |  |  |  |
| 1 | Atr-ERN13453 |  |  |  | | | |  |  |  |  |
| 1 | Atr-ERN13454 |  |  |  | | | |  |  |  |  |
| 1 | Atr-ERN13455 |  |  |  | | | |  |  |  |  |
| 1 | Atr-ERN13456 |  |  |  | | | |  |  |  |  |
| 1 | Atr-ERN13457 |  |  |  | | | |  |  |  |  |
| 1 | Atr-ERN13458 |  |  |  | | | |  |  |  |  |
| 1 | Atr-ERN13459 |  |  |  | | | |  |  |  |  |
| 1 | Atr-ERN13460 |  |  |  | | | |  |  |  |  |
| 1 | Atr-ERN13461 |  |  |  | | | |  |  |  |  |
| 1 | Atr-ERN13462 |  |  |  | Vvi-Vitvi03g04056\_t001 |  |  |  |  |
| 1 | Atr-ERN13463 |  |  |  | | | |  |  |  |  |
| 1 | Atr-ERN13464 |  |  |  | | | |  |  |  |  |
| 1 | Atr-ERN13465 |  |  |  | | | |  |  |  |  |
| 1 | Atr-ERN13466 |  |  |  | | | |  |  |  |  |
| 1 | Atr-ERN13467 |  |  |  | | | |  |  |  |  |
| 1 | Atr-ERN13468 |  |  |  | | | |  |  |  |  |
| 1 | Atr-ERN13469 |  |  |  | | | |  |  |  |  |
| 1 | Atr-ERN13470 |  |  |  | | | |  |  |  |  |
| 1 | Atr-ERN13471 |  |  |  | | | |  |  |  |  |
| 1 | Atr-ERN13472 |  |  |  | | | |  |  |  |  |
| 1 | Atr-ERN13473 |  |  |  | | | |  |  |  |  |
| 1 | Atr-ERN13474 |  |  |  | | | |  |  |  |  |
| 1 | Atr-ERN13475 |  |  |  | | | |  |  |  |  |
| 1 | Atr-ERN13476 |  |  |  | | | |  |  |  |  |
| 1 | Atr-ERN13477 |  |  |  | | | |  |  |  |  |
| 1 | Atr-ERN13478 |  |  |  | Vvi-Vitvi03g00111\_t001 |  |  |  |  |
| 1 | Atr-ERN13479 |  |  |  | | | |  |  |  |  |
| 1 | Atr-ERN13480 |  |  |  | | | |  |  |  |  |
| 1 | Atr-ERN13481 |  |  |  | | | |  |  |  |  |
| 1 | Atr-ERN13482 |  |  |  | | | |  |  |  |  |
| 1 | Atr-ERN13483 |  |  |  | Vvi-Vitvi03g00112\_t001 |  |  |  |  |
| 0 | Atr-ERN13484 |  |  |  |  |  |  |
| 0 | Atr-ERN13485 |  |  |  |  |  |  |
| 0 | Atr-ERN13486 |  |  |  |  |  |  |
| 0 | Atr-ERN13487 |  |  |  |  |  |  |
| 0 | Atr-ERN13488 |  |  |  |  |  |  |
| 0 | Atr-ERN13489 |  |  |  |  |  |  |
| 0 | Atr-ERN13490 |  |  |  |  |  |  |
| 0 | Atr-ERN13491 |  |  |  |  |  |  |
| 0 | Atr-ERN13492 |  |  |  |  |  |  |
| 0 | Atr-ERN13493 |  |  |  |  |  |  |
| 0 | Atr-ERN13494 |  |  |  |  |  |  |
| 0 | Atr-ERN13495 |  |  |  |  |  |  |
| 0 | Atr-ERN13496 |  |  |  |  |  |  |
| 0 | Atr-ERN13497 |  |  |  |  |  |  |
| 0 | Atr-ERN13498 |  |  |  |  |  |  |
| 0 | Atr-ERN13499 |  |  |  |  |  |  |
| 0 | Atr-ERN13500 |  |  |  |  |  |  |
| 0 | Atr-ERN13501 |  |  |  |  |  |  |
| 0 | Atr-ERN13502 |  |  |  |  |  |  |
| 0 | Atr-ERN13503 |  |  |  |  |  |  |
| 0 | Atr-ERN13504 |  |  |  |  |  |  |
| 0 | Atr-ERN13505 |  |  |  |  |  |  |
| 0 | Atr-ERN13506 |  |  |  |  |  |  |
| 0 | Atr-ERN13507 |  |  |  |  |  |  |
| 0 | Atr-ERN13508 |  |  |  |  |  |  |
| 0 | Atr-ERN13509 |  |  |  |  |  |  |
| 0 | Atr-ERN13510 |  |  |  |  |  |  |
| 0 | Atr-ERN13511 |  |  |  |  |  |  |
| 0 | Atr-ERN13512 |  |  |  |  |  |  |
| 0 | Atr-ERN13513 |  |  |  |  |  |  |
| 0 | Atr-ERN13514 |  |  |  |  |  |  |
| 0 | Atr-ERN13515 |  |  |  |  |  |  |
| 0 | Atr-ERN13516 |  |  |  |  |  |  |
| 0 | Atr-ERN13517 |  |  |  |  |  |  |
| 0 | Atr-ERN13518 |  |  |  |  |  |  |
| 0 | Atr-ERN13519 |  |  |  |  |  |  |
| 0 | Atr-ERN13520 |  |  |  |  |  |  |
| 0 | Atr-ERN13521 |  |  |  |  |  |  |
| 0 | Atr-ERN13522 |  |  |  |  |  |  |
| 0 | Atr-ERN13523 |  |  |  |  |  |  |
| 0 | Atr-ERN13524 |  |  |  |  |  |  |
| 0 | Atr-ERN13525 |  |  |  |  |  |  |
| 0 | Atr-ERN13526 |  |  |  |  |  |  |
| 0 | Atr-ERN13527 |  |  |  |  |  |  |
| 0 | Atr-ERN13528 |  |  |  |  |  |  |
| 0 | Atr-ERN13529 |  |  |  |  |  |  |
| 0 | Atr-ERN13530 |  |  |  |  |  |  |
| 0 | Atr-ERN13531 |  |  |  |  |  |  |
| 0 | Atr-ERN13532 |  |  |  |  |  |  |
| 0 | Atr-ERN13533 |  |  |  |  |  |  |
| 0 | Atr-ERN13534 |  |  |  |  |  |  |
| 0 | Atr-ERN13535 |  |  |  |  |  |  |
| 0 | Atr-ERN13536 |  |  |  |  |  |  |
| 0 | Atr-ERN13537 |  |  |  |  |  |  |
| 0 | Atr-ERN13538 |  |  |  |  |  |  |
| 0 | Atr-ERN13539 |  |  |  |  |  |  |
| 0 | Atr-ERN13540 |  |  |  |  |  |  |
| 0 | Atr-ERN13541 |  |  |  |  |  |  |
| 0 | Atr-ERN13542 |  |  |  |  |  |  |
| 0 | Atr-ERN13543 |  |  |  |  |  |  |
| 0 | Atr-ERN13544 |  |  |  |  |  |  |
| 0 | Atr-ERN13545 |  |  |  |  |  |  |
| 0 | Atr-ERN13546 |  |  |  |  |  |  |
| 0 | Atr-ERN13547 |  |  |  |  |  |  |
| 0 | Atr-ERN13548 |  |  |  |  |  |  |
| 0 | Atr-ERN13549 |  |  |  |  |  |  |
| 0 | Atr-ERN13550 |  |  |  |  |  |  |
| 0 | Atr-ERN13551 |  |  |  |  |  |  |
| 0 | Atr-ERN13552 |  |  |  |  |  |  |
| 0 | Atr-ERN13553 |  |  |  |  |  |  |
| 0 | Atr-ERN13554 |  |  |  |  |  |  |
| 0 | Atr-ERN13555 |  |  |  |  |  |  |
| 0 | Atr-ERN13556 |  |  |  |  |  |  |
| 0 | Atr-ERN13557 |  |  |  |  |  |  |
| 0 | Atr-ERN13558 |  |  |  |  |  |  |
| 0 | Atr-ERN13559 |  |  |  |  |  |  |
| 0 | Atr-ERN13560 |  |  |  |  |  |  |
| 0 | Atr-ERN13561 |  |  |  |  |  |  |
| 0 | Atr-ERN13562 |  |  |  |  |  |  |
| 0 | Atr-ERN13563 |  |  |  |  |  |  |
| 0 | Atr-ERN13564 |  |  |  |  |  |  |
| 0 | Atr-ERN13565 |  |  |  |  |  |  |
| 0 | Atr-ERN13566 |  |  |  |  |  |  |
