# Supplementary material for: Genome-wide identification, characterization and gene expression of BES1 transcription factor family in grapevine (Vitis vinifera L.)
Source: Sci Rep. 2023 Jan 5;13:240. doi: 10.1038/s41598-022-24407-y (PMC9816167; doi:10.1038/s41598-022-24407-y)
Supplement: Supplementary file 3 — Supplementary Information. [file 41598_2022_24407_MOESM3_ESM.zip › Vvi_Atr/Vitis_vinifera.PN40024.v4.dna_sm.toplevel.fa.vs.Amborella_trichopoda.AMTR1.0.dna_sm.toplevel.fa.html/Atr-AmTr_v1.0_scaffold00042.html]

|  |  |  |  |  |  |  |  |  |  |  |  |  |  |
| --- | --- | --- | --- | --- | --- | --- | --- | --- | --- | --- | --- | --- | --- |
| Duplication depth | Reference chromosome | Collinear blocks | | | | | | | | | | | |
| 0 | Atr-ERN03551 |  |  |  |  |  |  |
| 0 | Atr-ERN03552 |  |  |  |  |  |  |
| 0 | Atr-ERN03553 |  |  |  |  |  |  |
| 0 | Atr-ERN03554 |  |  |  |  |  |  |
| 0 | Atr-ERN03555 |  |  |  |  |  |  |
| 0 | Atr-ERN03556 |  |  |  |  |  |  |
| 0 | Atr-ERN03557 |  |  |  |  |  |  |
| 0 | Atr-ERN03558 |  |  |  |  |  |  |
| 0 | Atr-ERN03559 |  |  |  |  |  |  |
| 0 | Atr-ERN03560 |  |  |  |  |  |  |
| 0 | Atr-ERN03561 |  |  |  |  |  |  |
| 0 | Atr-ERN03562 |  |  |  |  |  |  |
| 0 | Atr-ERN03563 |  |  |  |  |  |  |
| 0 | Atr-ERN03564 |  |  |  |  |  |  |
| 0 | Atr-ERN03565 |  |  |  |  |  |  |
| 0 | Atr-ERN03566 |  |  |  |  |  |  |
| 0 | Atr-ERN03567 |  |  |  |  |  |  |
| 0 | Atr-ERN03568 |  |  |  |  |  |  |
| 0 | Atr-ERN03569 |  |  |  |  |  |  |
| 0 | Atr-ERN03570 |  |  |  |  |  |  |
| 0 | Atr-ERN03571 |  |  |  |  |  |  |
| 0 | Atr-ERN03572 |  |  |  |  |  |  |
| 0 | Atr-ERN03573 |  |  |  |  |  |  |
| 0 | Atr-ERN03574 |  |  |  |  |  |  |
| 0 | Atr-ERN03575 |  |  |  |  |  |  |
| 0 | Atr-ERN03576 |  |  |  |  |  |  |
| 0 | Atr-ERN03577 |  |  |  |  |  |  |
| 0 | Atr-ERN03578 |  |  |  |  |  |  |
| 0 | Atr-ERN03579 |  |  |  |  |  |  |
| 0 | Atr-ERN03580 |  |  |  |  |  |  |
| 0 | Atr-ERN03581 |  |  |  |  |  |  |
| 0 | Atr-ERN03582 |  |  |  |  |  |  |
| 0 | Atr-ERN03583 |  |  |  |  |  |  |
| 0 | Atr-ERN03584 |  |  |  |  |  |  |
| 0 | Atr-ERN03585 |  |  |  |  |  |  |
| 0 | Atr-ERN03586 |  |  |  |  |  |  |
| 0 | Atr-ERN03587 |  |  |  |  |  |  |
| 0 | Atr-ERN03588 |  |  |  |  |  |  |
| 0 | Atr-ERN03589 |  |  |  |  |  |  |
| 0 | Atr-ERN03590 |  |  |  |  |  |  |
| 0 | Atr-ERN03591 |  |  |  |  |  |  |
| 0 | Atr-ERN03592 |  |  |  |  |  |  |
| 0 | Atr-ERN03593 |  |  |  |  |  |  |
| 0 | Atr-ERN03594 |  |  |  |  |  |  |
| 0 | Atr-ERN03595 |  |  |  |  |  |  |
| 0 | Atr-ERN03596 |  |  |  |  |  |  |
| 0 | Atr-ERN03597 |  |  |  |  |  |  |
| 0 | Atr-ERN03598 |  |  |  |  |  |  |
| 0 | Atr-ERN03599 |  |  |  |  |  |  |
| 0 | Atr-ERN03600 |  |  |  |  |  |  |
| 0 | Atr-ERN03601 |  |  |  |  |  |  |
| 0 | Atr-ERN03602 |  |  |  |  |  |  |
| 0 | Atr-ERN03603 |  |  |  |  |  |  |
| 0 | Atr-ERN03604 |  |  |  |  |  |  |
| 0 | Atr-ERN03605 |  |  |  |  |  |  |
| 0 | Atr-ERN03606 |  |  |  |  |  |  |
| 0 | Atr-ERN03607 |  |  |  |  |  |  |
| 0 | Atr-ERN03608 |  |  |  |  |  |  |
| 0 | Atr-ERN03609 |  |  |  |  |  |  |
| 0 | Atr-ERN03610 |  |  |  |  |  |  |
| 0 | Atr-ERN03611 |  |  |  |  |  |  |
| 0 | Atr-ERN03612 |  |  |  |  |  |  |
| 0 | Atr-ERN03613 |  |  |  |  |  |  |
| 0 | Atr-ERN03614 |  |  |  |  |  |  |
| 0 | Atr-ERN03615 |  |  |  |  |  |  |
| 0 | Atr-ERN03616 |  |  |  |  |  |  |
| 0 | Atr-ERN03617 |  |  |  |  |  |  |
| 0 | Atr-ERN03618 |  |  |  |  |  |  |
| 0 | Atr-ERN03619 |  |  |  |  |  |  |
| 0 | Atr-ERN03620 |  |  |  |  |  |  |
| 0 | Atr-ERN03621 |  |  |  |  |  |  |
| 0 | Atr-ERN03622 |  |  |  |  |  |  |
| 0 | Atr-ERN03623 |  |  |  |  |  |  |
| 0 | Atr-ERN03624 |  |  |  |  |  |  |
| 0 | Atr-ERN03625 |  |  |  |  |  |  |
| 0 | Atr-ERN03626 |  |  |  |  |  |  |
| 0 | Atr-ERN03627 |  |  |  |  |  |  |
| 0 | Atr-ERN03628 |  |  |  |  |  |  |
| 0 | Atr-ERN03629 |  |  |  |  |  |  |
