# Supplementary material for: Genome-wide identification, characterization and gene expression of BES1 transcription factor family in grapevine (Vitis vinifera L.)
Source: Sci Rep. 2023 Jan 5;13:240. doi: 10.1038/s41598-022-24407-y (PMC9816167; doi:10.1038/s41598-022-24407-y)
Supplement: Supplementary file 3 — Supplementary Information. [file 41598_2022_24407_MOESM3_ESM.zip › Vvi_Atr/Vitis_vinifera.PN40024.v4.dna_sm.toplevel.fa.vs.Amborella_trichopoda.AMTR1.0.dna_sm.toplevel.fa.html/Atr-AmTr_v1.0_scaffold00043.html]

|  |  |  |  |  |  |  |  |  |  |  |  |  |  |
| --- | --- | --- | --- | --- | --- | --- | --- | --- | --- | --- | --- | --- | --- |
| Duplication depth | Reference chromosome | Collinear blocks | | | | | | | | | | | |
| 0 | Atr-ERN12715 |  |  |  |  |  |  |
| 0 | Atr-ERN12716 |  |  |  |  |  |  |
| 0 | Atr-ERN12717 |  |  |  |  |  |  |
| 0 | Atr-ERN12718 |  |  |  |  |  |  |
| 0 | Atr-ERN12719 |  |  |  |  |  |  |
| 0 | Atr-ERN12720 |  |  |  |  |  |  |
| 0 | Atr-ERN12721 |  |  |  |  |  |  |
| 0 | Atr-ERN12722 |  |  |  |  |  |  |
| 0 | Atr-ERN12723 |  |  |  |  |  |  |
| 0 | Atr-ERN12724 |  |  |  |  |  |  |
| 0 | Atr-ERN12725 |  |  |  |  |  |  |
| 0 | Atr-ERN12726 |  |  |  |  |  |  |
| 0 | Atr-ERN12727 |  |  |  |  |  |  |
| 1 | Atr-ERN12728 |  | Vvi-Vitvi12g00546\_t001 |  |  |  |  |  |
| 1 | Atr-ERN12729 |  | | | |  |  |  |  |  |
| 1 | Atr-ERN12730 |  | | | |  |  |  |  |  |
| 1 | Atr-ERN12731 |  | | | |  |  |  |  |  |
| 1 | Atr-ERN12732 |  | | | |  |  |  |  |  |
| 1 | Atr-ERN12733 |  | | | |  |  |  |  |  |
| 1 | Atr-ERN12734 |  | | | |  |  |  |  |  |
| 1 | Atr-ERN12735 |  | | | |  |  |  |  |  |
| 1 | Atr-ERN12736 |  | Vvi-Vitvi12g00567\_t001 |  |  |  |  |  |
| 1 | Atr-ERN12737 |  | | | |  |  |  |  |  |
| 2 | Atr-ERN12738 |  | | | |  | Vvi-Vitvi19g00392\_t001 |  |  |  |  |
| 2 | Atr-ERN12739 |  | | | |  | | | |  |  |  |  |
| 2 | Atr-ERN12740 |  | | | |  | | | |  |  |  |  |
| 2 | Atr-ERN12741 |  | | | |  | | | |  |  |  |  |
| 2 | Atr-ERN12742 |  | Vvi-Vitvi12g00568\_t001 |  | | | |  |  |  |  |
| 2 | Atr-ERN12743 |  | | | |  | | | |  |  |  |  |
| 2 | Atr-ERN12744 |  | Vvi-Vitvi12g00569\_t002 |  | | | |  |  |  |  |
| 2 | Atr-ERN12745 |  | | | |  | | | |  |  |  |  |
| 2 | Atr-ERN12746 |  | | | |  | | | |  |  |  |  |
| 2 | Atr-ERN12747 |  | Vvi-Vitvi12g00571\_t001 |  | | | |  |  |  |  |
| 2 | Atr-ERN12748 |  | | | |  | | | |  |  |  |  |
| 2 | Atr-ERN12749 |  | | | |  | | | |  |  |  |  |
| 2 | Atr-ERN12750 |  | | | |  | | | |  |  |  |  |
| 2 | Atr-ERN12751 |  | Vvi-Vitvi12g00572\_t001 |  | | | |  |  |  |  |
| 2 | Atr-ERN12752 |  | | | |  | | | |  |  |  |  |
| 2 | Atr-ERN12753 |  | | | |  | | | |  |  |  |  |
| 2 | Atr-ERN12754 |  | | | |  | | | |  |  |  |  |
| 2 | Atr-ERN12755 |  | | | |  | | | |  |  |  |  |
| 2 | Atr-ERN12756 |  | | | |  | | | |  |  |  |  |
| 2 | Atr-ERN12757 |  | | | |  | | | |  |  |  |  |
| 3 | Atr-ERN12758 |  | | | |  | | | |  | Vvi-Vitvi10g04219\_t001 |  |  |  |
| 3 | Atr-ERN12759 |  | Vvi-Vitvi12g02177\_t001 |  | Vvi-Vitvi19g01791\_t003 |  | | | |  |  |  |
| 3 | Atr-ERN12760 |  | | | |  | | | |  | | | |  |  |  |
| 3 | Atr-ERN12761 |  | | | |  | | | |  | | | |  |  |  |
| 3 | Atr-ERN12762 |  | | | |  | | | |  | | | |  |  |  |
| 3 | Atr-ERN12763 |  | | | |  | | | |  | Vvi-Vitvi10g04224\_t001 |  |  |  |
| 3 | Atr-ERN12764 |  | | | |  | | | |  | | | |  |  |  |
| 3 | Atr-ERN12765 |  | | | |  | | | |  | | | |  |  |  |
| 3 | Atr-ERN12766 |  | | | |  | Vvi-Vitvi19g00373\_t001 |  | | | |  |  |  |
| 3 | Atr-ERN12767 |  | | | |  | | | |  | | | |  |  |  |
| 3 | Atr-ERN12768 |  | | | |  | | | |  | | | |  |  |  |
| 3 | Atr-ERN12769 |  | | | |  | | | |  | | | |  |  |  |
| 3 | Atr-ERN12770 |  | | | |  | | | |  | | | |  |  |  |
| 3 | Atr-ERN12771 |  | | | |  | Vvi-Vitvi19g00371\_t001 |  | | | |  |  |  |
| 3 | Atr-ERN12772 |  | Vvi-Vitvi12g04177\_t001 |  | | | |  | | | |  |  |  |
| 3 | Atr-ERN12773 |  | | | |  | | | |  | | | |  |  |  |
| 3 | Atr-ERN12774 |  | | | |  | | | |  | | | |  |  |  |
| 3 | Atr-ERN12775 |  | | | |  | Vvi-Vitvi19g01972\_t001 |  | Vvi-Vitvi10g00321\_t001 |  |  |  |
| 3 | Atr-ERN12776 |  | | | |  | | | |  | | | |  |  |  |
| 3 | Atr-ERN12777 |  | | | |  | | | |  | | | |  |  |  |
| 3 | Atr-ERN12778 |  | | | |  | Vvi-Vitvi19g00370\_t003 |  | | | |  |  |  |
| 3 | Atr-ERN12779 |  | | | |  | | | |  | | | |  |  |  |
| 3 | Atr-ERN12780 |  | Vvi-Vitvi12g00590\_t001 |  | | | |  | | | |  |  |  |
| 3 | Atr-ERN12781 |  | | | |  | | | |  | | | |  |  |  |
| 3 | Atr-ERN12782 |  | | | |  | | | |  | | | |  |  |  |
| 3 | Atr-ERN12783 |  | | | |  | | | |  | | | |  |  |  |
| 3 | Atr-ERN12784 |  | | | |  | | | |  | | | |  |  |  |
| 3 | Atr-ERN12785 |  | | | |  | | | |  | | | |  |  |  |
| 3 | Atr-ERN12786 |  | Vvi-Vitvi12g00593\_t001 |  | Vvi-Vitvi19g00369\_t001 |  | | | |  |  |  |
| 3 | Atr-ERN12787 |  | | | |  | | | |  | | | |  |  |  |
| 3 | Atr-ERN12788 |  | | | |  | Vvi-Vitvi19g00368\_t001 |  | | | |  |  |  |
| 3 | Atr-ERN12789 |  | | | |  | | | |  | | | |  |  |  |
| 3 | Atr-ERN12790 |  | | | |  | | | |  | | | |  |  |  |
| 3 | Atr-ERN12791 |  | | | |  | Vvi-Vitvi19g00366\_t001 |  | Vvi-Vitvi10g01734\_t001 |  |  |  |
| 3 | Atr-ERN12792 |  | | | |  | | | |  | | | |  |  |  |
| 3 | Atr-ERN12793 |  | | | |  | | | |  | Vvi-Vitvi10g04226\_t001 |  |  |  |
| 3 | Atr-ERN12794 |  | | | |  | | | |  | | | |  |  |  |
| 3 | Atr-ERN12795 |  | | | |  | | | |  | | | |  |  |  |
| 3 | Atr-ERN12796 |  | | | |  | | | |  | | | |  |  |  |
| 3 | Atr-ERN12797 |  | | | |  | | | |  | | | |  |  |  |
| 3 | Atr-ERN12798 |  | | | |  | | | |  | | | |  |  |  |
| 3 | Atr-ERN12799 |  | | | |  | | | |  | | | |  |  |  |
| 3 | Atr-ERN12800 |  | | | |  | | | |  | | | |  |  |  |
| 3 | Atr-ERN12801 |  | | | |  | | | |  | | | |  |  |  |
| 3 | Atr-ERN12802 |  | Vvi-Vitvi12g04185\_t001 |  | Vvi-Vitvi19g04192\_t001 |  | Vvi-Vitvi10g04227\_t001 |  |  |  |
| 0 | Atr-ERN12803 |  |  |  |  |  |  |
| 0 | Atr-ERN12804 |  |  |  |  |  |  |
| 0 | Atr-ERN12805 |  |  |  |  |  |  |
| 0 | Atr-ERN12806 |  |  |  |  |  |  |
| 0 | Atr-ERN12807 |  |  |  |  |  |  |
| 0 | Atr-ERN12808 |  |  |  |  |  |  |
