# Supplementary material for: Genome-wide identification, characterization and gene expression of BES1 transcription factor family in grapevine (Vitis vinifera L.)
Source: Sci Rep. 2023 Jan 5;13:240. doi: 10.1038/s41598-022-24407-y (PMC9816167; doi:10.1038/s41598-022-24407-y)
Supplement: Supplementary file 3 — Supplementary Information. [file 41598_2022_24407_MOESM3_ESM.zip › Vvi_Atr/Vitis_vinifera.PN40024.v4.dna_sm.toplevel.fa.vs.Amborella_trichopoda.AMTR1.0.dna_sm.toplevel.fa.html/Atr-AmTr_v1.0_scaffold00044.html]

|  |  |  |  |  |  |  |  |  |  |  |  |  |  |
| --- | --- | --- | --- | --- | --- | --- | --- | --- | --- | --- | --- | --- | --- |
| Duplication depth | Reference chromosome | Collinear blocks | | | | | | | | | | | |
| 0 | Atr-ERN17032 |  |  |  |  |  |  |
| 0 | Atr-ERN17033 |  |  |  |  |  |  |
| 1 | Atr-ERN17034 |  | Vvi-Vitvi01g01477\_t001 |  |  |  |  |  |
| 1 | Atr-ERN17035 |  | Vvi-Vitvi01g01478\_t003 |  |  |  |  |  |
| 1 | Atr-ERN17036 |  | | | |  |  |  |  |  |
| 1 | Atr-ERN17037 |  | | | |  |  |  |  |  |
| 1 | Atr-ERN17038 |  | | | |  |  |  |  |  |
| 2 | Atr-ERN17039 |  | | | |  | Vvi-Vitvi04g00890\_t001 |  |  |  |  |
| 2 | Atr-ERN17040 |  | | | |  | | | |  |  |  |  |
| 2 | Atr-ERN17041 |  | Vvi-Vitvi01g01488\_t001 |  | | | |  |  |  |  |
| 2 | Atr-ERN17042 |  | Vvi-Vitvi01g01492\_t001 |  | | | |  |  |  |  |
| 2 | Atr-ERN17043 |  | Vvi-Vitvi01g01496\_t001 |  | | | |  |  |  |  |
| 2 | Atr-ERN17044 |  | Vvi-Vitvi01g01498\_t001 |  | | | |  |  |  |  |
| 1 | Atr-ERN17045 |  |  |  | Vvi-Vitvi04g02011\_t001 |  |  |  |  |
| 1 | Atr-ERN17046 |  |  |  | | | |  |  |  |  |
| 1 | Atr-ERN17047 |  |  |  | | | |  |  |  |  |
| 1 | Atr-ERN17048 |  |  |  | | | |  |  |  |  |
| 1 | Atr-ERN17049 |  |  |  | | | |  |  |  |  |
| 1 | Atr-ERN17050 |  |  |  | Vvi-Vitvi04g00878\_t001 |  |  |  |  |
| 1 | Atr-ERN17051 |  |  |  | | | |  |  |  |  |
| 1 | Atr-ERN17052 |  |  |  | | | |  |  |  |  |
| 1 | Atr-ERN17053 |  |  |  | | | |  |  |  |  |
| 1 | Atr-ERN17054 |  |  |  | | | |  |  |  |  |
| 1 | Atr-ERN17055 |  |  |  | | | |  |  |  |  |
| 1 | Atr-ERN17056 |  |  |  | | | |  |  |  |  |
| 1 | Atr-ERN17057 |  |  |  | | | |  |  |  |  |
| 1 | Atr-ERN17058 |  |  |  | | | |  |  |  |  |
| 1 | Atr-ERN17059 |  |  |  | | | |  |  |  |  |
| 1 | Atr-ERN17060 |  |  |  | | | |  |  |  |  |
| 1 | Atr-ERN17061 |  |  |  | | | |  |  |  |  |
| 1 | Atr-ERN17062 |  |  |  | | | |  |  |  |  |
| 1 | Atr-ERN17063 |  |  |  | Vvi-Vitvi04g00846\_t001 |  |  |  |  |
| 1 | Atr-ERN17064 |  |  |  | | | |  |  |  |  |
| 1 | Atr-ERN17065 |  |  |  | | | |  |  |  |  |
| 1 | Atr-ERN17066 |  |  |  | | | |  |  |  |  |
| 1 | Atr-ERN17067 |  |  |  | | | |  |  |  |  |
| 1 | Atr-ERN17068 |  |  |  | Vvi-Vitvi04g00841\_t001 |  |  |  |  |
| 1 | Atr-ERN17069 |  |  |  | | | |  |  |  |  |
| 1 | Atr-ERN17070 |  |  |  | | | |  |  |  |  |
| 2 | Atr-ERN17071 |  | Vvi-Vitvi08g02349\_t001 |  | | | |  |  |  |  |
| 2 | Atr-ERN17072 |  | | | |  | | | |  |  |  |  |
| 2 | Atr-ERN17073 |  | | | |  | | | |  |  |  |  |
| 2 | Atr-ERN17074 |  | | | |  | | | |  |  |  |  |
| 2 | Atr-ERN17075 |  | | | |  | | | |  |  |  |  |
| 2 | Atr-ERN17076 |  | | | |  | | | |  |  |  |  |
| 2 | Atr-ERN17077 |  | | | |  | | | |  |  |  |  |
| 2 | Atr-ERN17078 |  | | | |  | Vvi-Vitvi04g00838\_t001 |  |  |  |  |
| 1 | Atr-ERN17079 |  | | | |  |  |  |  |  |
| 1 | Atr-ERN17080 |  | | | |  |  |  |  |  |
| 1 | Atr-ERN17081 |  | | | |  |  |  |  |  |
| 1 | Atr-ERN17082 |  | | | |  |  |  |  |  |
| 1 | Atr-ERN17083 |  | | | |  |  |  |  |  |
| 1 | Atr-ERN17084 |  | | | |  |  |  |  |  |
| 1 | Atr-ERN17085 |  | Vvi-Vitvi08g01742\_t001 |  |  |  |  |  |
| 1 | Atr-ERN17086 |  | | | |  |  |  |  |  |
| 1 | Atr-ERN17087 |  | | | |  |  |  |  |  |
| 1 | Atr-ERN17088 |  | | | |  |  |  |  |  |
| 1 | Atr-ERN17089 |  | Vvi-Vitvi08g01741\_t001 |  |  |  |  |  |
| 1 | Atr-ERN17090 |  | | | |  |  |  |  |  |
| 1 | Atr-ERN17091 |  | | | |  |  |  |  |  |
| 1 | Atr-ERN17092 |  | Vvi-Vitvi08g01740\_t001 |  |  |  |  |  |
| 1 | Atr-ERN17093 |  | | | |  |  |  |  |  |
| 1 | Atr-ERN17094 |  | | | |  |  |  |  |  |
| 1 | Atr-ERN17095 |  | | | |  |  |  |  |  |
| 1 | Atr-ERN17096 |  | | | |  |  |  |  |  |
| 1 | Atr-ERN17097 |  | | | |  |  |  |  |  |
| 1 | Atr-ERN17098 |  | Vvi-Vitvi08g01739\_t002 |  |  |  |  |  |
| 1 | Atr-ERN17099 |  | | | |  |  |  |  |  |
| 1 | Atr-ERN17100 |  | | | |  |  |  |  |  |
| 1 | Atr-ERN17101 |  | Vvi-Vitvi08g01738\_t001 |  |  |  |  |  |
| 1 | Atr-ERN17102 |  | | | |  |  |  |  |  |
| 1 | Atr-ERN17103 |  | | | |  |  |  |  |  |
| 1 | Atr-ERN17104 |  | Vvi-Vitvi08g01737\_t002 |  |  |  |  |  |
| 0 | Atr-ERN17105 |  |  |  |  |  |  |
| 0 | Atr-ERN17106 |  |  |  |  |  |  |
| 0 | Atr-ERN17107 |  |  |  |  |  |  |
| 0 | Atr-ERN17108 |  |  |  |  |  |  |
| 1 | Atr-ERN17109 |  | Vvi-Vitvi18g00054\_t001 |  |  |  |  |  |
| 1 | Atr-ERN17110 |  | | | |  |  |  |  |  |
| 2 | Atr-ERN17111 |  | | | |  | Vvi-Vitvi07g01901\_t001 |  |  |  |  |
| 2 | Atr-ERN17112 |  | | | |  | Vvi-Vitvi07g01900\_t001 |  |  |  |  |
| 2 | Atr-ERN17113 |  | Vvi-Vitvi18g00056\_t002 |  | Vvi-Vitvi07g01898\_t001 |  |  |  |  |
| 2 | Atr-ERN17114 |  | | | |  | Vvi-Vitvi07g01897\_t001 |  |  |  |  |
| 2 | Atr-ERN17115 |  | Vvi-Vitvi18g00057\_t001 |  | | | |  |  |  |  |
| 2 | Atr-ERN17116 |  | Vvi-Vitvi18g00058\_t001 |  | Vvi-Vitvi07g02702\_t001 |  |  |  |  |
| 2 | Atr-ERN17117 |  | Vvi-Vitvi18g00059\_t001 |  | | | |  |  |  |  |
| 2 | Atr-ERN17118 |  | | | |  | | | |  |  |  |  |
| 2 | Atr-ERN17119 |  | | | |  | | | |  |  |  |  |
| 2 | Atr-ERN17120 |  | Vvi-Vitvi18g00060\_t001 |  | | | |  |  |  |  |
| 2 | Atr-ERN17121 |  | Vvi-Vitvi18g02483\_t001 |  | | | |  |  |  |  |
| 2 | Atr-ERN17122 |  | | | |  | | | |  |  |  |  |
| 2 | Atr-ERN17123 |  | | | |  | | | |  |  |  |  |
| 2 | Atr-ERN17124 |  | | | |  | | | |  |  |  |  |
| 2 | Atr-ERN17125 |  | | | |  | | | |  |  |  |  |
| 2 | Atr-ERN17126 |  | | | |  | | | |  |  |  |  |
| 2 | Atr-ERN17127 |  | | | |  | | | |  |  |  |  |
| 2 | Atr-ERN17128 |  | | | |  | | | |  |  |  |  |
| 2 | Atr-ERN17129 |  | | | |  | | | |  |  |  |  |
| 2 | Atr-ERN17130 |  | | | |  | | | |  |  |  |  |
| 2 | Atr-ERN17131 |  | | | |  | | | |  |  |  |  |
| 2 | Atr-ERN17132 |  | | | |  | | | |  |  |  |  |
| 2 | Atr-ERN17133 |  | | | |  | | | |  |  |  |  |
| 2 | Atr-ERN17134 |  | | | |  | Vvi-Vitvi07g04745\_t001 |  |  |  |  |
| 2 | Atr-ERN17135 |  | | | |  | | | |  |  |  |  |
| 2 | Atr-ERN17136 |  | | | |  | | | |  |  |  |  |
| 2 | Atr-ERN17137 |  | Vvi-Vitvi18g00061\_t001 |  | | | |  |  |  |  |
| 2 | Atr-ERN17138 |  | | | |  | | | |  |  |  |  |
| 2 | Atr-ERN17139 |  | | | |  | | | |  |  |  |  |
| 2 | Atr-ERN17140 |  | | | |  | | | |  |  |  |  |
| 2 | Atr-ERN17141 |  | | | |  | Vvi-Vitvi07g01893\_t001 |  |  |  |  |
| 2 | Atr-ERN17142 |  | | | |  | | | |  |  |  |  |
| 2 | Atr-ERN17143 |  | | | |  | | | |  |  |  |  |
| 2 | Atr-ERN17144 |  | | | |  | | | |  |  |  |  |
| 2 | Atr-ERN17145 |  | | | |  | | | |  |  |  |  |
| 2 | Atr-ERN17146 |  | | | |  | | | |  |  |  |  |
| 2 | Atr-ERN17147 |  | | | |  | | | |  |  |  |  |
| 2 | Atr-ERN17148 |  | | | |  | Vvi-Vitvi07g01892\_t001 |  |  |  |  |
| 2 | Atr-ERN17149 |  | | | |  | | | |  |  |  |  |
| 2 | Atr-ERN17150 |  | Vvi-Vitvi18g00068\_t001 |  | | | |  |  |  |  |
| 2 | Atr-ERN17151 |  | Vvi-Vitvi18g00069\_t001 |  | Vvi-Vitvi07g01891\_t001 |  |  |  |  |
| 2 | Atr-ERN17152 |  | | | |  | | | |  |  |  |  |
| 2 | Atr-ERN17153 |  | | | |  | | | |  |  |  |  |
| 2 | Atr-ERN17154 |  | | | |  | | | |  |  |  |  |
| 2 | Atr-ERN17155 |  | | | |  | | | |  |  |  |  |
| 2 | Atr-ERN17156 |  | | | |  | | | |  |  |  |  |
| 2 | Atr-ERN17157 |  | | | |  | | | |  |  |  |  |
| 2 | Atr-ERN17158 |  | | | |  | | | |  |  |  |  |
| 3 | Atr-ERN17159 |  | Vvi-Vitvi18g00071\_t001 |  | Vvi-Vitvi07g01890\_t001 |  | Vvi-Vitvi03g00246\_t001 |  |  |  |
| 3 | Atr-ERN17160 |  | | | |  | | | |  | | | |  |  |  |
| 3 | Atr-ERN17161 |  | | | |  | | | |  | | | |  |  |  |
| 3 | Atr-ERN17162 |  | | | |  | | | |  | | | |  |  |  |
| 3 | Atr-ERN17163 |  | | | |  | | | |  | | | |  |  |  |
| 3 | Atr-ERN17164 |  | | | |  | | | |  | | | |  |  |  |
| 3 | Atr-ERN17165 |  | | | |  | | | |  | | | |  |  |  |
| 3 | Atr-ERN17166 |  | Vvi-Vitvi18g00072\_t005 |  | Vvi-Vitvi07g01889\_t001 |  | | | |  |  |  |
| 3 | Atr-ERN17167 |  | Vvi-Vitvi18g00073\_t001 |  | | | |  | | | |  |  |  |
| 3 | Atr-ERN17168 |  | | | |  | | | |  | | | |  |  |  |
| 3 | Atr-ERN17169 |  | | | |  | | | |  | | | |  |  |  |
| 3 | Atr-ERN17170 |  | | | |  | | | |  | | | |  |  |  |
| 3 | Atr-ERN17171 |  | Vvi-Vitvi18g00075\_t001 |  | | | |  | | | |  |  |  |
| 3 | Atr-ERN17172 |  | | | |  | | | |  | | | |  |  |  |
| 3 | Atr-ERN17173 |  | | | |  | | | |  | | | |  |  |  |
| 3 | Atr-ERN17174 |  | | | |  | | | |  | | | |  |  |  |
| 3 | Atr-ERN17175 |  | | | |  | | | |  | | | |  |  |  |
| 3 | Atr-ERN17176 |  | | | |  | | | |  | | | |  |  |  |
| 3 | Atr-ERN17177 |  | | | |  | | | |  | | | |  |  |  |
| 3 | Atr-ERN17178 |  | Vvi-Vitvi18g00076\_t001 |  | | | |  | | | |  |  |  |
| 3 | Atr-ERN17179 |  | | | |  | | | |  | | | |  |  |  |
| 3 | Atr-ERN17180 |  | | | |  | | | |  | | | |  |  |  |
| 3 | Atr-ERN17181 |  | Vvi-Vitvi18g00077\_t001 |  | | | |  | | | |  |  |  |
| 3 | Atr-ERN17182 |  | | | |  | Vvi-Vitvi07g01887\_t001 |  | Vvi-Vitvi03g00244\_t001 |  |  |  |
| 3 | Atr-ERN17183 |  | Vvi-Vitvi18g00078\_t001 |  | | | |  | | | |  |  |  |
| 3 | Atr-ERN17184 |  | Vvi-Vitvi18g00079\_t001 |  | | | |  | Vvi-Vitvi03g00243\_t001 |  |  |  |
| 3 | Atr-ERN17185 |  | | | |  | Vvi-Vitvi07g01886\_t002 |  | | | |  |  |  |
| 2 | Atr-ERN17186 |  | | | |  |  |  | Vvi-Vitvi03g00242\_t001 |  |  |  |
| 2 | Atr-ERN17187 |  | | | |  |  |  | | | |  |  |  |
| 2 | Atr-ERN17188 |  | | | |  |  |  | | | |  |  |  |
| 3 | Atr-ERN17189 |  | | | |  | Vvi-Vitvi04g01580\_t001 |  | | | |  |  |  |
| 3 | Atr-ERN17190 |  | | | |  | | | |  | | | |  |  |  |
| 3 | Atr-ERN17191 |  | Vvi-Vitvi18g00080\_t001 |  | | | |  | | | |  |  |  |
| 3 | Atr-ERN17192 |  | Vvi-Vitvi18g00081\_t001 |  | | | |  | | | |  |  |  |
| 3 | Atr-ERN17193 |  | Vvi-Vitvi18g00082\_t001 |  | | | |  | | | |  |  |  |
| 3 | Atr-ERN17194 |  | | | |  | | | |  | | | |  |  |  |
| 3 | Atr-ERN17195 |  | Vvi-Vitvi18g00084\_t001 |  | Vvi-Vitvi04g01578\_t001 |  | | | |  |  |  |
| 3 | Atr-ERN17196 |  | | | |  | | | |  | | | |  |  |  |
| 3 | Atr-ERN17197 |  | | | |  | Vvi-Vitvi04g01577\_t001 |  | | | |  |  |  |
| 3 | Atr-ERN17198 |  | | | |  | | | |  | | | |  |  |  |
| 3 | Atr-ERN17199 |  | | | |  | Vvi-Vitvi04g01576\_t001 |  | | | |  |  |  |
| 3 | Atr-ERN17200 |  | | | |  | Vvi-Vitvi04g01575\_t001 |  | | | |  |  |  |
| 3 | Atr-ERN17201 |  | Vvi-Vitvi18g02486\_t001 |  | Vvi-Vitvi04g02203\_t004 |  | | | |  |  |  |
| 3 | Atr-ERN17202 |  | | | |  | | | |  | | | |  |  |  |
| 3 | Atr-ERN17203 |  | | | |  | | | |  | | | |  |  |  |
| 3 | Atr-ERN17204 |  | | | |  | Vvi-Vitvi04g01573\_t001 |  | | | |  |  |  |
| 3 | Atr-ERN17205 |  | | | |  | | | |  | | | |  |  |  |
| 3 | Atr-ERN17206 |  | | | |  | | | |  | | | |  |  |  |
| 3 | Atr-ERN17207 |  | Vvi-Vitvi18g00086\_t001 |  | | | |  | | | |  |  |  |
| 3 | Atr-ERN17208 |  | Vvi-Vitvi18g00087\_t001 |  | | | |  | Vvi-Vitvi03g00241\_t001 |  |  |  |
| 3 | Atr-ERN17209 |  | | | |  | | | |  | | | |  |  |  |
| 3 | Atr-ERN17210 |  | | | |  | | | |  | | | |  |  |  |
| 3 | Atr-ERN17211 |  | Vvi-Vitvi18g00088\_t001 |  | | | |  | Vvi-Vitvi03g00240\_t001 |  |  |  |
| 3 | Atr-ERN17212 |  | | | |  | Vvi-Vitvi04g04452\_t001 |  | | | |  |  |  |
| 3 | Atr-ERN17213 |  | Vvi-Vitvi18g00089\_t001 |  | | | |  | | | |  |  |  |
| 3 | Atr-ERN17214 |  | Vvi-Vitvi18g00090\_t001 |  | | | |  | Vvi-Vitvi03g00239\_t001 |  |  |  |
| 3 | Atr-ERN17215 |  | | | |  | | | |  | | | |  |  |  |
| 3 | Atr-ERN17216 |  | Vvi-Vitvi18g00091\_t002 |  | Vvi-Vitvi04g01568\_t001 |  | Vvi-Vitvi03g00238\_t001 |  |  |  |
| 3 | Atr-ERN17217 |  | | | |  | Vvi-Vitvi04g04451\_t001 |  | | | |  |  |  |
| 2 | Atr-ERN17218 |  | | | |  |  |  | Vvi-Vitvi03g00234\_t001 |  |  |  |
| 2 | Atr-ERN17219 |  | Vvi-Vitvi18g00093\_t001 |  |  |  | Vvi-Vitvi03g00233\_t001 |  |  |  |
| 2 | Atr-ERN17220 |  | | | |  |  |  | | | |  |  |  |
| 2 | Atr-ERN17221 |  | Vvi-Vitvi18g02488\_t001 |  |  |  | Vvi-Vitvi03g00231\_t001 |  |  |  |
| 0 | Atr-ERN17222 |  |  |  |  |  |  |
| 0 | Atr-ERN17223 |  |  |  |  |  |  |
| 0 | Atr-ERN17224 |  |  |  |  |  |  |
| 0 | Atr-ERN17225 |  |  |  |  |  |  |
| 0 | Atr-ERN17226 |  |  |  |  |  |  |
| 0 | Atr-ERN17227 |  |  |  |  |  |  |
| 0 | Atr-ERN17228 |  |  |  |  |  |  |
| 0 | Atr-ERN17229 |  |  |  |  |  |  |
| 0 | Atr-ERN17230 |  |  |  |  |  |  |
| 0 | Atr-ERN17231 |  |  |  |  |  |  |
| 0 | Atr-ERN17232 |  |  |  |  |  |  |
| 0 | Atr-ERN17233 |  |  |  |  |  |  |
| 0 | Atr-ERN17234 |  |  |  |  |  |  |
| 0 | Atr-ERN17235 |  |  |  |  |  |  |
| 0 | Atr-ERN17236 |  |  |  |  |  |  |
| 0 | Atr-ERN17237 |  |  |  |  |  |  |
| 0 | Atr-ERN17238 |  |  |  |  |  |  |
| 0 | Atr-ERN17239 |  |  |  |  |  |  |
| 0 | Atr-ERN17240 |  |  |  |  |  |  |
| 0 | Atr-ERN17241 |  |  |  |  |  |  |
| 0 | Atr-ERN17242 |  |  |  |  |  |  |
| 0 | Atr-ERN17243 |  |  |  |  |  |  |
| 0 | Atr-ERN17244 |  |  |  |  |  |  |
| 0 | Atr-ERN17245 |  |  |  |  |  |  |
| 0 | Atr-ERN17246 |  |  |  |  |  |  |
| 0 | Atr-ERN17247 |  |  |  |  |  |  |
| 0 | Atr-ERN17248 |  |  |  |  |  |  |
| 0 | Atr-ERN17249 |  |  |  |  |  |  |
| 0 | Atr-ERN17250 |  |  |  |  |  |  |
| 0 | Atr-ERN17251 |  |  |  |  |  |  |
| 0 | Atr-ERN17252 |  |  |  |  |  |  |
| 0 | Atr-ERN17253 |  |  |  |  |  |  |
| 0 | Atr-ERN17254 |  |  |  |  |  |  |
| 0 | Atr-ERN17255 |  |  |  |  |  |  |
| 0 | Atr-ERN17256 |  |  |  |  |  |  |
| 0 | Atr-ERN17257 |  |  |  |  |  |  |
| 0 | Atr-ERN17258 |  |  |  |  |  |  |
| 0 | Atr-ERN17259 |  |  |  |  |  |  |
| 0 | Atr-ERN17260 |  |  |  |  |  |  |
| 0 | Atr-ERN17261 |  |  |  |  |  |  |
| 0 | Atr-ERN17262 |  |  |  |  |  |  |
| 0 | Atr-ERN17263 |  |  |  |  |  |  |
| 0 | Atr-ERN17264 |  |  |  |  |  |  |
| 0 | Atr-ERN17265 |  |  |  |  |  |  |
| 0 | Atr-ERN17266 |  |  |  |  |  |  |
| 0 | Atr-ERN17267 |  |  |  |  |  |  |
| 0 | Atr-ERN17268 |  |  |  |  |  |  |
| 0 | Atr-ERN17269 |  |  |  |  |  |  |
| 0 | Atr-ERN17270 |  |  |  |  |  |  |
| 0 | Atr-ERN17271 |  |  |  |  |  |  |
| 0 | Atr-ERN17272 |  |  |  |  |  |  |
| 0 | Atr-ERN17273 |  |  |  |  |  |  |
| 0 | Atr-ERN17274 |  |  |  |  |  |  |
| 0 | Atr-ERN17275 |  |  |  |  |  |  |
| 0 | Atr-ERN17276 |  |  |  |  |  |  |
| 0 | Atr-ERN17277 |  |  |  |  |  |  |
| 0 | Atr-ERN17278 |  |  |  |  |  |  |
| 0 | Atr-ERN17279 |  |  |  |  |  |  |
| 0 | Atr-ERN17280 |  |  |  |  |  |  |
| 0 | Atr-ERN17281 |  |  |  |  |  |  |
| 0 | Atr-ERN17282 |  |  |  |  |  |  |
| 0 | Atr-ERN17283 |  |  |  |  |  |  |
| 0 | Atr-ERN17284 |  |  |  |  |  |  |
| 0 | Atr-ERN17285 |  |  |  |  |  |  |
| 0 | Atr-ERN17286 |  |  |  |  |  |  |
