# Supplementary material for: Genome-wide identification, characterization and gene expression of BES1 transcription factor family in grapevine (Vitis vinifera L.)
Source: Sci Rep. 2023 Jan 5;13:240. doi: 10.1038/s41598-022-24407-y (PMC9816167; doi:10.1038/s41598-022-24407-y)
Supplement: Supplementary file 3 — Supplementary Information. [file 41598_2022_24407_MOESM3_ESM.zip › Vvi_Atr/Vitis_vinifera.PN40024.v4.dna_sm.toplevel.fa.vs.Amborella_trichopoda.AMTR1.0.dna_sm.toplevel.fa.html/Atr-AmTr_v1.0_scaffold00045.html]

|  |  |  |  |  |  |  |  |  |  |  |  |  |  |
| --- | --- | --- | --- | --- | --- | --- | --- | --- | --- | --- | --- | --- | --- |
| Duplication depth | Reference chromosome | Collinear blocks | | | | | | | | | | | |
| 0 | Atr-ERN01922 |  |  |  |  |  |  |
| 0 | Atr-ERN01923 |  |  |  |  |  |  |
| 0 | Atr-ERN01924 |  |  |  |  |  |  |
| 0 | Atr-ERN01925 |  |  |  |  |  |  |
| 0 | Atr-ERN01926 |  |  |  |  |  |  |
| 0 | Atr-ERN01927 |  |  |  |  |  |  |
| 0 | Atr-ERN01928 |  |  |  |  |  |  |
| 0 | Atr-ERN01929 |  |  |  |  |  |  |
| 0 | Atr-ERN01930 |  |  |  |  |  |  |
| 0 | Atr-ERN01931 |  |  |  |  |  |  |
| 0 | Atr-ERN01932 |  |  |  |  |  |  |
| 0 | Atr-ERN01933 |  |  |  |  |  |  |
| 0 | Atr-ERN01934 |  |  |  |  |  |  |
| 0 | Atr-ERN01935 |  |  |  |  |  |  |
| 0 | Atr-ERN01936 |  |  |  |  |  |  |
| 0 | Atr-ERN01937 |  |  |  |  |  |  |
| 0 | Atr-ERN01938 |  |  |  |  |  |  |
| 0 | Atr-ERN01939 |  |  |  |  |  |  |
| 0 | Atr-ERN01940 |  |  |  |  |  |  |
| 0 | Atr-ERN01941 |  |  |  |  |  |  |
| 0 | Atr-ERN01942 |  |  |  |  |  |  |
| 0 | Atr-ERN01943 |  |  |  |  |  |  |
| 0 | Atr-ERN01944 |  |  |  |  |  |  |
| 0 | Atr-ERN01945 |  |  |  |  |  |  |
| 0 | Atr-ERN01946 |  |  |  |  |  |  |
| 0 | Atr-ERN01947 |  |  |  |  |  |  |
| 0 | Atr-ERN01948 |  |  |  |  |  |  |
| 0 | Atr-ERN01949 |  |  |  |  |  |  |
| 0 | Atr-ERN01950 |  |  |  |  |  |  |
| 0 | Atr-ERN01951 |  |  |  |  |  |  |
| 0 | Atr-ERN01952 |  |  |  |  |  |  |
| 0 | Atr-ERN01953 |  |  |  |  |  |  |
| 1 | Atr-ERN01954 |  | Vvi-Vitvi05g00241\_t001 |  |  |  |  |  |
| 1 | Atr-ERN01955 |  | Vvi-Vitvi05g00239\_t003 |  |  |  |  |  |
| 1 | Atr-ERN01956 |  | Vvi-Vitvi05g00237\_t001 |  |  |  |  |  |
| 1 | Atr-ERN01957 |  | Vvi-Vitvi05g00236\_t001 |  |  |  |  |  |
| 1 | Atr-ERN01958 |  | Vvi-Vitvi05g00235\_t001 |  |  |  |  |  |
| 0 | Atr-ERN01959 |  |  |  |  |  |  |
| 0 | Atr-ERN01960 |  |  |  |  |  |  |
| 0 | Atr-ERN01961 |  |  |  |  |  |  |
| 1 | Atr-ERN01962 |  | Vvi-Vitvi07g00476\_t001 |  |  |  |  |  |
| 1 | Atr-ERN01963 |  | | | |  |  |  |  |  |
| 1 | Atr-ERN01964 |  | | | |  |  |  |  |  |
| 2 | Atr-ERN01965 |  | | | |  | Vvi-Vitvi05g04072\_t001 |  |  |  |  |
| 2 | Atr-ERN01966 |  | Vvi-Vitvi07g00477\_t001 |  | | | |  |  |  |  |
| 2 | Atr-ERN01967 |  | Vvi-Vitvi07g00478\_t001 |  | | | |  |  |  |  |
| 2 | Atr-ERN01968 |  | | | |  | | | |  |  |  |  |
| 2 | Atr-ERN01969 |  | | | |  | | | |  |  |  |  |
| 2 | Atr-ERN01970 |  | | | |  | | | |  |  |  |  |
| 2 | Atr-ERN01971 |  | | | |  | | | |  |  |  |  |
| 2 | Atr-ERN01972 |  | | | |  | | | |  |  |  |  |
| 2 | Atr-ERN01973 |  | | | |  | | | |  |  |  |  |
| 3 | Atr-ERN01974 |  | | | |  | | | |  | Vvi-Vitvi14g02447\_t001 |  |  |  |
| 3 | Atr-ERN01975 |  | | | |  | | | |  | | | |  |  |  |
| 3 | Atr-ERN01976 |  | Vvi-Vitvi07g00486\_t001 |  | | | |  | | | |  |  |  |
| 3 | Atr-ERN01977 |  | | | |  | | | |  | | | |  |  |  |
| 3 | Atr-ERN01978 |  | | | |  | | | |  | | | |  |  |  |
| 3 | Atr-ERN01979 |  | Vvi-Vitvi07g02246\_t001 |  | | | |  | | | |  |  |  |
| 3 | Atr-ERN01980 |  | | | |  | | | |  | | | |  |  |  |
| 3 | Atr-ERN01981 |  | | | |  | | | |  | | | |  |  |  |
| 3 | Atr-ERN01982 |  | | | |  | | | |  | | | |  |  |  |
| 3 | Atr-ERN01983 |  | Vvi-Vitvi07g02247\_t001 |  | Vvi-Vitvi05g00242\_t001 |  | Vvi-Vitvi14g02444\_t001 |  |  |  |
| 3 | Atr-ERN01984 |  | | | |  | | | |  | | | |  |  |  |
| 3 | Atr-ERN01985 |  | | | |  | | | |  | | | |  |  |  |
| 3 | Atr-ERN01986 |  | | | |  | Vvi-Vitvi05g00244\_t001 |  | | | |  |  |  |
| 3 | Atr-ERN01987 |  | Vvi-Vitvi07g00491\_t001 |  | Vvi-Vitvi05g00245\_t001 |  | | | |  |  |  |
| 3 | Atr-ERN01988 |  | | | |  | | | |  | | | |  |  |  |
| 3 | Atr-ERN01989 |  | | | |  | | | |  | | | |  |  |  |
| 3 | Atr-ERN01990 |  | Vvi-Vitvi07g00492\_t001 |  | Vvi-Vitvi05g00246\_t003 |  | | | |  |  |  |
| 3 | Atr-ERN01991 |  | | | |  | Vvi-Vitvi05g00247\_t001 |  | Vvi-Vitvi14g00042\_t001 |  |  |  |
| 3 | Atr-ERN01992 |  | | | |  | | | |  | Vvi-Vitvi14g00041\_t001 |  |  |  |
| 3 | Atr-ERN01993 |  | | | |  | | | |  | Vvi-Vitvi14g00040\_t001 |  |  |  |
| 3 | Atr-ERN01994 |  | Vvi-Vitvi07g00494\_t001 |  | | | |  | Vvi-Vitvi14g00039\_t001 |  |  |  |
| 3 | Atr-ERN01995 |  | | | |  | Vvi-Vitvi05g00249\_t001 |  | | | |  |  |  |
| 3 | Atr-ERN01996 |  | | | |  | | | |  | | | |  |  |  |
| 3 | Atr-ERN01997 |  | | | |  | Vvi-Vitvi05g00250\_t001 |  | Vvi-Vitvi14g00038\_t001 |  |  |  |
| 3 | Atr-ERN01998 |  | | | |  | | | |  | | | |  |  |  |
| 3 | Atr-ERN01999 |  | Vvi-Vitvi07g00495\_t001 |  | | | |  | Vvi-Vitvi14g00037\_t001 |  |  |  |
| 3 | Atr-ERN02000 |  | Vvi-Vitvi07g00540\_t001 |  | | | |  | | | |  |  |  |
| 3 | Atr-ERN02001 |  | | | |  | | | |  | | | |  |  |  |
| 3 | Atr-ERN02002 |  | | | |  | | | |  | | | |  |  |  |
| 3 | Atr-ERN02003 |  | | | |  | | | |  | | | |  |  |  |
| 3 | Atr-ERN02004 |  | | | |  | | | |  | | | |  |  |  |
| 3 | Atr-ERN02005 |  | | | |  | | | |  | | | |  |  |  |
| 3 | Atr-ERN02006 |  | | | |  | | | |  | Vvi-Vitvi14g00036\_t001 |  |  |  |
| 3 | Atr-ERN02007 |  | | | |  | | | |  | | | |  |  |  |
| 3 | Atr-ERN02008 |  | | | |  | | | |  | | | |  |  |  |
| 3 | Atr-ERN02009 |  | Vvi-Vitvi07g00539\_t001 |  | | | |  | | | |  |  |  |
| 3 | Atr-ERN02010 |  | | | |  | Vvi-Vitvi05g01832\_t001 |  | Vvi-Vitvi14g00034\_t001 |  |  |  |
| 3 | Atr-ERN02011 |  | | | |  | Vvi-Vitvi05g00254\_t001 |  | | | |  |  |  |
| 3 | Atr-ERN02012 |  | | | |  | Vvi-Vitvi05g00255\_t001 |  | | | |  |  |  |
| 3 | Atr-ERN02013 |  | Vvi-Vitvi07g02253\_t002 |  | | | |  | | | |  |  |  |
| 3 | Atr-ERN02014 |  | | | |  | Vvi-Vitvi05g00256\_t001 |  | | | |  |  |  |
| 3 | Atr-ERN02015 |  | | | |  | Vvi-Vitvi05g00258\_t001 |  | | | |  |  |  |
| 3 | Atr-ERN02016 |  | Vvi-Vitvi07g00538\_t001 |  | | | |  | | | |  |  |  |
| 3 | Atr-ERN02017 |  | Vvi-Vitvi07g00537\_t001 |  | | | |  | | | |  |  |  |
| 3 | Atr-ERN02018 |  | | | |  | Vvi-Vitvi05g00259\_t001 |  | | | |  |  |  |
| 3 | Atr-ERN02019 |  | | | |  | | | |  | | | |  |  |  |
| 3 | Atr-ERN02020 |  | Vvi-Vitvi07g00535\_t001 |  | | | |  | | | |  |  |  |
| 3 | Atr-ERN02021 |  | | | |  | | | |  | | | |  |  |  |
| 3 | Atr-ERN02022 |  | | | |  | Vvi-Vitvi05g00260\_t001 |  | | | |  |  |  |
| 3 | Atr-ERN02023 |  | | | |  | | | |  | | | |  |  |  |
| 3 | Atr-ERN02024 |  | Vvi-Vitvi07g00534\_t001 |  | Vvi-Vitvi05g00261\_t001 |  | | | |  |  |  |
| 3 | Atr-ERN02025 |  | | | |  | | | |  | | | |  |  |  |
| 3 | Atr-ERN02026 |  | | | |  | | | |  | | | |  |  |  |
| 3 | Atr-ERN02027 |  | | | |  | | | |  | | | |  |  |  |
| 3 | Atr-ERN02028 |  | | | |  | | | |  | | | |  |  |  |
| 3 | Atr-ERN02029 |  | Vvi-Vitvi07g00533\_t001 |  | | | |  | | | |  |  |  |
| 3 | Atr-ERN02030 |  | | | |  | Vvi-Vitvi05g00264\_t001 |  | | | |  |  |  |
| 3 | Atr-ERN02031 |  | | | |  | | | |  | | | |  |  |  |
| 3 | Atr-ERN02032 |  | Vvi-Vitvi07g00532\_t001 |  | Vvi-Vitvi05g01835\_t001 |  | | | |  |  |  |
| 3 | Atr-ERN02033 |  | Vvi-Vitvi07g00531\_t001 |  | | | |  | Vvi-Vitvi14g00033\_t001 |  |  |  |
| 3 | Atr-ERN02034 |  | Vvi-Vitvi07g00527\_t001 |  | | | |  | | | |  |  |  |
| 3 | Atr-ERN02035 |  | Vvi-Vitvi07g00526\_t001 |  | | | |  | | | |  |  |  |
| 3 | Atr-ERN02036 |  | Vvi-Vitvi07g00525\_t001 |  | | | |  | | | |  |  |  |
| 3 | Atr-ERN02037 |  | | | |  | | | |  | Vvi-Vitvi14g00030\_t002 |  |  |  |
| 3 | Atr-ERN02038 |  | | | |  | | | |  | | | |  |  |  |
| 3 | Atr-ERN02039 |  | | | |  | Vvi-Vitvi05g00265\_t001 |  | | | |  |  |  |
| 3 | Atr-ERN02040 |  | | | |  | Vvi-Vitvi05g00266\_t001 |  | Vvi-Vitvi14g00029\_t001 |  |  |  |
| 3 | Atr-ERN02041 |  | | | |  | | | |  | | | |  |  |  |
| 3 | Atr-ERN02042 |  | Vvi-Vitvi07g00524\_t001 |  | | | |  | | | |  |  |  |
| 3 | Atr-ERN02043 |  | | | |  | | | |  | | | |  |  |  |
| 3 | Atr-ERN02044 |  | | | |  | | | |  | | | |  |  |  |
| 3 | Atr-ERN02045 |  | | | |  | | | |  | | | |  |  |  |
| 3 | Atr-ERN02046 |  | | | |  | | | |  | Vvi-Vitvi14g00028\_t001 |  |  |  |
| 2 | Atr-ERN02047 |  | Vvi-Vitvi07g00523\_t001 |  | | | |  |  |  |  |
| 2 | Atr-ERN02048 |  | | | |  | | | |  |  |  |  |
| 2 | Atr-ERN02049 |  | | | |  | | | |  |  |  |  |
| 2 | Atr-ERN02050 |  | | | |  | Vvi-Vitvi05g01836\_t001 |  |  |  |  |
| 2 | Atr-ERN02051 |  | | | |  | Vvi-Vitvi05g00267\_t001 |  |  |  |  |
| 2 | Atr-ERN02052 |  | | | |  | | | |  |  |  |  |
| 2 | Atr-ERN02053 |  | | | |  | | | |  |  |  |  |
| 2 | Atr-ERN02054 |  | | | |  | Vvi-Vitvi05g01843\_t001 |  |  |  |  |
| 2 | Atr-ERN02055 |  | | | |  | | | |  |  |  |  |
| 2 | Atr-ERN02056 |  | Vvi-Vitvi07g00522\_t001 |  | | | |  |  |  |  |
| 2 | Atr-ERN02057 |  | | | |  | | | |  |  |  |  |
| 2 | Atr-ERN02058 |  | | | |  | | | |  |  |  |  |
| 2 | Atr-ERN02059 |  | | | |  | | | |  |  |  |  |
| 2 | Atr-ERN02060 |  | | | |  | | | |  |  |  |  |
| 2 | Atr-ERN02061 |  | | | |  | | | |  |  |  |  |
| 2 | Atr-ERN02062 |  | Vvi-Vitvi07g00521\_t001 |  | | | |  |  |  |  |
| 2 | Atr-ERN02063 |  | | | |  | Vvi-Vitvi05g00270\_t001 |  |  |  |  |
| 2 | Atr-ERN02064 |  | | | |  | | | |  |  |  |  |
| 2 | Atr-ERN02065 |  | | | |  | | | |  |  |  |  |
| 2 | Atr-ERN02066 |  | Vvi-Vitvi07g02251\_t002 |  | | | |  |  |  |  |
| 2 | Atr-ERN02067 |  | Vvi-Vitvi07g00517\_t001 |  | | | |  |  |  |  |
| 2 | Atr-ERN02068 |  | | | |  | | | |  |  |  |  |
| 2 | Atr-ERN02069 |  | | | |  | | | |  |  |  |  |
| 2 | Atr-ERN02070 |  | | | |  | | | |  |  |  |  |
| 2 | Atr-ERN02071 |  | | | |  | | | |  |  |  |  |
| 2 | Atr-ERN02072 |  | | | |  | | | |  |  |  |  |
| 2 | Atr-ERN02073 |  | | | |  | | | |  |  |  |  |
| 2 | Atr-ERN02074 |  | | | |  | Vvi-Vitvi05g00274\_t001 |  |  |  |  |
| 2 | Atr-ERN02075 |  | | | |  | | | |  |  |  |  |
| 2 | Atr-ERN02076 |  | Vvi-Vitvi07g00515\_t001 |  | Vvi-Vitvi05g00275\_t001 |  |  |  |  |
| 2 | Atr-ERN02077 |  | Vvi-Vitvi07g00513\_t001 |  | | | |  |  |  |  |
| 2 | Atr-ERN02078 |  | | | |  | | | |  |  |  |  |
| 2 | Atr-ERN02079 |  | Vvi-Vitvi07g02074\_t001 |  | | | |  |  |  |  |
| 2 | Atr-ERN02080 |  | Vvi-Vitvi07g02249\_t003 |  | Vvi-Vitvi05g00276\_t001 |  |  |  |  |
| 2 | Atr-ERN02081 |  | | | |  | | | |  |  |  |  |
| 2 | Atr-ERN02082 |  | | | |  | Vvi-Vitvi05g00278\_t001 |  |  |  |  |
| 1 | Atr-ERN02083 |  | | | |  |  |  |  |  |
| 1 | Atr-ERN02084 |  | | | |  |  |  |  |  |
| 1 | Atr-ERN02085 |  | | | |  |  |  |  |  |
| 2 | Atr-ERN02086 |  | | | |  | Vvi-Vitvi05g00130\_t001 |  |  |  |  |
| 2 | Atr-ERN02087 |  | | | |  | | | |  |  |  |  |
| 2 | Atr-ERN02088 |  | | | |  | | | |  |  |  |  |
| 2 | Atr-ERN02089 |  | | | |  | | | |  |  |  |  |
| 2 | Atr-ERN02090 |  | Vvi-Vitvi07g00502\_t001 |  | | | |  |  |  |  |
| 2 | Atr-ERN02091 |  | | | |  | | | |  |  |  |  |
| 2 | Atr-ERN02092 |  | | | |  | | | |  |  |  |  |
| 2 | Atr-ERN02093 |  | | | |  | | | |  |  |  |  |
| 2 | Atr-ERN02094 |  | | | |  | | | |  |  |  |  |
| 2 | Atr-ERN02095 |  | | | |  | | | |  |  |  |  |
| 2 | Atr-ERN02096 |  | | | |  | | | |  |  |  |  |
| 2 | Atr-ERN02097 |  | | | |  | | | |  |  |  |  |
| 2 | Atr-ERN02098 |  | | | |  | | | |  |  |  |  |
| 2 | Atr-ERN02099 |  | | | |  | | | |  |  |  |  |
| 3 | Atr-ERN02100 |  | Vvi-Vitvi07g00492\_t001 |  | | | |  | Vvi-Vitvi07g00424\_t001 |  |  |  |
| 3 | Atr-ERN02101 |  | | | |  | Vvi-Vitvi05g00115\_t001 |  | | | |  |  |  |
| 3 | Atr-ERN02102 |  | | | |  | Vvi-Vitvi05g01772\_t001 |  | Vvi-Vitvi07g02228\_t001 |  |  |  |
| 3 | Atr-ERN02103 |  | | | |  | | | |  | | | |  |  |  |
| 3 | Atr-ERN02104 |  | | | |  | | | |  | | | |  |  |  |
| 3 | Atr-ERN02105 |  | Vvi-Vitvi07g00471\_t001 |  | | | |  | | | |  |  |  |
| 2 | Atr-ERN02106 |  |  |  | | | |  | | | |  |  |  |
| 3 | Atr-ERN02107 |  | Vvi-Vitvi14g00090\_t001 |  | Vvi-Vitvi05g00114\_t001 |  | Vvi-Vitvi07g00423\_t001.3.6037826e |  |  |  |
| 3 | Atr-ERN02108 |  | | | |  | Vvi-Vitvi05g00113\_t001 |  | | | |  |  |  |
| 3 | Atr-ERN02109 |  | Vvi-Vitvi14g00091\_t001 |  | | | |  | | | |  |  |  |
| 3 | Atr-ERN02110 |  | Vvi-Vitvi14g00092\_t002 |  | | | |  | | | |  |  |  |
| 3 | Atr-ERN02111 |  | | | |  | | | |  | Vvi-Vitvi07g00422\_t001 |  |  |  |
| 3 | Atr-ERN02112 |  | | | |  | | | |  | Vvi-Vitvi07g00421\_t003 |  |  |  |
| 3 | Atr-ERN02113 |  | | | |  | Vvi-Vitvi05g00112\_t001 |  | Vvi-Vitvi07g04099\_t001 |  |  |  |
| 3 | Atr-ERN02114 |  | | | |  | Vvi-Vitvi05g00110\_t001 |  | Vvi-Vitvi07g00418\_t001 |  |  |  |
| 3 | Atr-ERN02115 |  | | | |  | | | |  | | | |  |  |  |
| 3 | Atr-ERN02116 |  | | | |  | | | |  | | | |  |  |  |
| 3 | Atr-ERN02117 |  | | | |  | | | |  | | | |  |  |  |
| 3 | Atr-ERN02118 |  | | | |  | | | |  | | | |  |  |  |
| 3 | Atr-ERN02119 |  | | | |  | | | |  | | | |  |  |  |
| 3 | Atr-ERN02120 |  | | | |  | | | |  | | | |  |  |  |
| 3 | Atr-ERN02121 |  | | | |  | | | |  | Vvi-Vitvi07g00417\_t001 |  |  |  |
| 3 | Atr-ERN02122 |  | | | |  | | | |  | Vvi-Vitvi07g00416\_t001 |  |  |  |
| 3 | Atr-ERN02123 |  | | | |  | | | |  | | | |  |  |  |
| 3 | Atr-ERN02124 |  | | | |  | | | |  | | | |  |  |  |
| 3 | Atr-ERN02125 |  | | | |  | | | |  | | | |  |  |  |
| 3 | Atr-ERN02126 |  | Vvi-Vitvi14g02473\_t001 |  | | | |  | | | |  |  |  |
| 3 | Atr-ERN02127 |  | | | |  | | | |  | Vvi-Vitvi07g00415\_t001 |  |  |  |
| 3 | Atr-ERN02128 |  | | | |  | | | |  | | | |  |  |  |
| 3 | Atr-ERN02129 |  | | | |  | Vvi-Vitvi05g00108\_t001 |  | | | |  |  |  |
| 2 | Atr-ERN02130 |  | | | |  |  |  | | | |  |  |  |
| 2 | Atr-ERN02131 |  | | | |  |  |  | | | |  |  |  |
| 2 | Atr-ERN02132 |  | Vvi-Vitvi14g02475\_t001 |  |  |  | | | |  |  |  |
| 2 | Atr-ERN02133 |  | | | |  |  |  | Vvi-Vitvi07g00414\_t003 |  |  |  |
| 2 | Atr-ERN02134 |  | | | |  |  |  | | | |  |  |  |
| 2 | Atr-ERN02135 |  | | | |  |  |  | | | |  |  |  |
| 2 | Atr-ERN02136 |  | | | |  |  |  | | | |  |  |  |
| 2 | Atr-ERN02137 |  | | | |  |  |  | Vvi-Vitvi07g00412\_t001 |  |  |  |
| 1 | Atr-ERN02138 |  | | | |  |  |  |  |  |
| 1 | Atr-ERN02139 |  | Vvi-Vitvi14g00097\_t001 |  |  |  |  |  |
| 0 | Atr-ERN02140 |  |  |  |  |  |  |
| 0 | Atr-ERN02141 |  |  |  |  |  |  |
| 0 | Atr-ERN02142 |  |  |  |  |  |  |
| 0 | Atr-ERN02143 |  |  |  |  |  |  |
| 0 | Atr-ERN02144 |  |  |  |  |  |  |
| 0 | Atr-ERN02145 |  |  |  |  |  |  |
| 0 | Atr-ERN02146 |  |  |  |  |  |  |
| 0 | Atr-ERN02147 |  |  |  |  |  |  |
| 0 | Atr-ERN02148 |  |  |  |  |  |  |
| 0 | Atr-ERN02149 |  |  |  |  |  |  |
| 1 | Atr-ERN02150 |  | Vvi-Vitvi14g00119\_t001 |  |  |  |  |  |
| 1 | Atr-ERN02151 |  | Vvi-Vitvi14g00129\_t001 |  |  |  |  |  |
| 1 | Atr-ERN02152 |  | | | |  |  |  |  |  |
| 1 | Atr-ERN02153 |  | Vvi-Vitvi14g02487\_t001 |  |  |  |  |  |
| 1 | Atr-ERN02154 |  | Vvi-Vitvi14g00147\_t001 |  |  |  |  |  |
| 1 | Atr-ERN02155 |  | | | |  |  |  |  |  |
| 1 | Atr-ERN02156 |  | | | |  |  |  |  |  |
| 1 | Atr-ERN02157 |  | | | |  |  |  |  |  |
| 1 | Atr-ERN02158 |  | | | |  |  |  |  |  |
| 1 | Atr-ERN02159 |  | | | |  |  |  |  |  |
| 1 | Atr-ERN02160 |  | | | |  |  |  |  |  |
| 1 | Atr-ERN02161 |  | | | |  |  |  |  |  |
| 1 | Atr-ERN02162 |  | | | |  |  |  |  |  |
| 1 | Atr-ERN02163 |  | Vvi-Vitvi14g00150\_t002 |  |  |  |  |  |
| 1 | Atr-ERN02164 |  | | | |  |  |  |  |  |
| 1 | Atr-ERN02165 |  | | | |  |  |  |  |  |
| 1 | Atr-ERN02166 |  | | | |  |  |  |  |  |
| 1 | Atr-ERN02167 |  | | | |  |  |  |  |  |
| 1 | Atr-ERN02168 |  | | | |  |  |  |  |  |
| 1 | Atr-ERN02169 |  | | | |  |  |  |  |  |
| 1 | Atr-ERN02170 |  | | | |  |  |  |  |  |
| 1 | Atr-ERN02171 |  | | | |  |  |  |  |  |
| 1 | Atr-ERN02172 |  | | | |  |  |  |  |  |
| 3 | Atr-ERN02173 |  | Vvi-Vitvi14g00163\_t001 |  | Vvi-Vitvi07g00290\_t001 |  | Vvi-Vitvi05g00313\_t001 |  |  |  |
| 3 | Atr-ERN02174 |  | | | |  | | | |  | Vvi-Vitvi05g00314\_t001 |  |  |  |
| 3 | Atr-ERN02175 |  | | | |  | | | |  | Vvi-Vitvi05g00315\_t001 |  |  |  |
| 3 | Atr-ERN02176 |  | | | |  | Vvi-Vitvi07g00291\_t001 |  | | | |  |  |  |
| 3 | Atr-ERN02177 |  | | | |  | | | |  | | | |  |  |  |
| 3 | Atr-ERN02178 |  | | | |  | | | |  | | | |  |  |  |
| 3 | Atr-ERN02179 |  | | | |  | Vvi-Vitvi07g00293\_t001 |  | | | |  |  |  |
| 3 | Atr-ERN02180 |  | Vvi-Vitvi14g00165\_t001 |  | | | |  | Vvi-Vitvi05g04084\_t001 |  |  |  |
| 3 | Atr-ERN02181 |  | | | |  | Vvi-Vitvi07g00294\_t001 |  | | | |  |  |  |
| 3 | Atr-ERN02182 |  | | | |  | | | |  | | | |  |  |  |
| 3 | Atr-ERN02183 |  | Vvi-Vitvi14g00166\_t001 |  | | | |  | | | |  |  |  |
| 3 | Atr-ERN02184 |  | | | |  | Vvi-Vitvi07g00295\_t001 |  | | | |  |  |  |
| 3 | Atr-ERN02185 |  | | | |  | Vvi-Vitvi07g00296\_t001 |  | | | |  |  |  |
| 3 | Atr-ERN02186 |  | | | |  | | | |  | | | |  |  |  |
| 4 | Atr-ERN02187 |  | | | |  | Vvi-Vitvi07g00298\_t001 |  | Vvi-Vitvi05g00319\_t001 |  | Vvi-Vitvi05g00319\_t001 |  |  |
| 4 | Atr-ERN02188 |  | | | |  | | | |  | | | |  | | | |  |  |
| 4 | Atr-ERN02189 |  | | | |  | Vvi-Vitvi07g00300\_t001 |  | | | |  | | | |  |  |
| 4 | Atr-ERN02190 |  | | | |  | | | |  | Vvi-Vitvi05g00322\_t001 |  | | | |  |  |
| 4 | Atr-ERN02191 |  | | | |  | | | |  | | | |  | | | |  |  |
| 4 | Atr-ERN02192 |  | | | |  | | | |  | | | |  | | | |  |  |
| 4 | Atr-ERN02193 |  | | | |  | | | |  | Vvi-Vitvi05g00323\_t001 |  | | | |  |  |
| 3 | Atr-ERN02194 |  | | | |  | | | |  |  |  | | | |  |  |
| 3 | Atr-ERN02195 |  | Vvi-Vitvi14g00167\_t001 |  | Vvi-Vitvi07g00301\_t001 |  |  |  | | | |  |  |
| 3 | Atr-ERN02196 |  | Vvi-Vitvi14g02506\_t002 |  | | | |  |  |  | | | |  |  |
| 3 | Atr-ERN02197 |  | | | |  | | | |  |  |  | | | |  |  |
| 3 | Atr-ERN02198 |  | | | |  | | | |  |  |  | | | |  |  |
| 3 | Atr-ERN02199 |  | | | |  | | | |  |  |  | | | |  |  |
| 3 | Atr-ERN02200 |  | | | |  | | | |  |  |  | | | |  |  |
| 3 | Atr-ERN02201 |  | | | |  | | | |  |  |  | | | |  |  |
| 3 | Atr-ERN02202 |  | | | |  | | | |  |  |  | | | |  |  |
| 3 | Atr-ERN02203 |  | | | |  | | | |  |  |  | | | |  |  |
| 3 | Atr-ERN02204 |  | | | |  | | | |  |  |  | | | |  |  |
| 3 | Atr-ERN02205 |  | | | |  | | | |  |  |  | | | |  |  |
| 3 | Atr-ERN02206 |  | | | |  | | | |  |  |  | Vvi-Vitvi05g00294\_t001 |  |  |
| 3 | Atr-ERN02207 |  | | | |  | | | |  |  |  | | | |  |  |
| 3 | Atr-ERN02208 |  | | | |  | | | |  |  |  | Vvi-Vitvi05g00293\_t001 |  |  |
| 3 | Atr-ERN02209 |  | | | |  | | | |  |  |  | | | |  |  |
| 3 | Atr-ERN02210 |  | Vvi-Vitvi14g00171\_t001 |  | | | |  |  |  | | | |  |  |
| 3 | Atr-ERN02211 |  | | | |  | | | |  |  |  | | | |  |  |
| 3 | Atr-ERN02212 |  | | | |  | | | |  |  |  | | | |  |  |
| 3 | Atr-ERN02213 |  | | | |  | | | |  |  |  | | | |  |  |
| 3 | Atr-ERN02214 |  | Vvi-Vitvi14g00172\_t001 |  | | | |  |  |  | | | |  |  |
| 3 | Atr-ERN02215 |  | Vvi-Vitvi14g00174\_t001 |  | | | |  |  |  | | | |  |  |
| 3 | Atr-ERN02216 |  | | | |  | | | |  |  |  | Vvi-Vitvi05g00292\_t001 |  |  |
| 3 | Atr-ERN02217 |  | | | |  | Vvi-Vitvi07g00307\_t001 |  |  |  | | | |  |  |
| 3 | Atr-ERN02218 |  | | | |  | | | |  |  |  | | | |  |  |
| 3 | Atr-ERN02219 |  | Vvi-Vitvi14g00175\_t001 |  | Vvi-Vitvi07g00308\_t002 |  |  |  | | | |  |  |
| 3 | Atr-ERN02220 |  | | | |  | | | |  |  |  | | | |  |  |
| 3 | Atr-ERN02221 |  | | | |  | Vvi-Vitvi07g00309\_t001 |  |  |  | | | |  |  |
| 3 | Atr-ERN02222 |  | | | |  | Vvi-Vitvi07g00310\_t003 |  |  |  | | | |  |  |
| 3 | Atr-ERN02223 |  | | | |  | | | |  |  |  | | | |  |  |
| 3 | Atr-ERN02224 |  | | | |  | | | |  |  |  | | | |  |  |
| 3 | Atr-ERN02225 |  | | | |  | | | |  |  |  | | | |  |  |
| 3 | Atr-ERN02226 |  | | | |  | | | |  |  |  | | | |  |  |
| 3 | Atr-ERN02227 |  | Vvi-Vitvi14g00176\_t001 |  | | | |  |  |  | | | |  |  |
| 3 | Atr-ERN02228 |  | Vvi-Vitvi14g00178\_t001 |  | | | |  |  |  | | | |  |  |
| 3 | Atr-ERN02229 |  | | | |  | | | |  |  |  | | | |  |  |
| 3 | Atr-ERN02230 |  | | | |  | Vvi-Vitvi07g00311\_t001 |  |  |  | | | |  |  |
| 3 | Atr-ERN02231 |  | | | |  | | | |  |  |  | | | |  |  |
| 3 | Atr-ERN02232 |  | | | |  | | | |  |  |  | | | |  |  |
| 3 | Atr-ERN02233 |  | | | |  | | | |  |  |  | | | |  |  |
| 3 | Atr-ERN02234 |  | | | |  | | | |  |  |  | Vvi-Vitvi05g00288\_t001 |  |  |
| 3 | Atr-ERN02235 |  | | | |  | Vvi-Vitvi07g00312\_t001 |  |  |  | | | |  |  |
| 3 | Atr-ERN02236 |  | | | |  | | | |  |  |  | Vvi-Vitvi05g00287\_t001.1.6037826e |  |  |
| 3 | Atr-ERN02237 |  | | | |  | | | |  |  |  | | | |  |  |
| 3 | Atr-ERN02238 |  | | | |  | Vvi-Vitvi07g00313\_t001 |  |  |  | | | |  |  |
| 3 | Atr-ERN02239 |  | | | |  | Vvi-Vitvi07g00314\_t001 |  |  |  | Vvi-Vitvi05g00286\_t001 |  |  |
| 3 | Atr-ERN02240 |  | Vvi-Vitvi14g00179\_t001 |  | | | |  |  |  | Vvi-Vitvi05g00284\_t001 |  |  |
| 3 | Atr-ERN02241 |  | Vvi-Vitvi14g00180\_t003 |  | | | |  |  |  | Vvi-Vitvi05g00282\_t001 |  |  |
| 3 | Atr-ERN02242 |  | | | |  | | | |  |  |  | | | |  |  |
| 3 | Atr-ERN02243 |  | | | |  | | | |  |  |  | | | |  |  |
| 3 | Atr-ERN02244 |  | | | |  | | | |  |  |  | | | |  |  |
| 3 | Atr-ERN02245 |  | | | |  | | | |  |  |  | | | |  |  |
| 3 | Atr-ERN02246 |  | | | |  | Vvi-Vitvi07g02198\_t001 |  |  |  | | | |  |  |
| 3 | Atr-ERN02247 |  | | | |  | | | |  |  |  | | | |  |  |
| 3 | Atr-ERN02248 |  | | | |  | | | |  |  |  | | | |  |  |
| 3 | Atr-ERN02249 |  | | | |  | | | |  |  |  | Vvi-Vitvi05g00281\_t001 |  |  |
| 2 | Atr-ERN02250 |  | | | |  | | | |  |  |  |  |
| 3 | Atr-ERN02251 |  | | | |  | | | |  | Vvi-Vitvi05g00001\_t002 |  |  |  |
| 3 | Atr-ERN02252 |  | | | |  | | | |  | Vvi-Vitvi05g01734\_t001 |  |  |  |
| 3 | Atr-ERN02253 |  | | | |  | | | |  | Vvi-Vitvi05g00005\_t001 |  |  |  |
| 3 | Atr-ERN02254 |  | | | |  | | | |  | | | |  |  |  |
| 3 | Atr-ERN02255 |  | | | |  | | | |  | | | |  |  |  |
| 3 | Atr-ERN02256 |  | | | |  | Vvi-Vitvi07g02203\_t001 |  | | | |  |  |  |
| 3 | Atr-ERN02257 |  | | | |  | Vvi-Vitvi07g00322\_t001 |  | | | |  |  |  |
| 2 | Atr-ERN02258 |  | | | |  |  |  | Vvi-Vitvi05g00006\_t002 |  |  |  |
| 2 | Atr-ERN02259 |  | | | |  |  |  | | | |  |  |  |
| 2 | Atr-ERN02260 |  | | | |  |  |  | | | |  |  |  |
| 2 | Atr-ERN02261 |  | | | |  |  |  | | | |  |  |  |
| 2 | Atr-ERN02262 |  | | | |  |  |  | | | |  |  |  |
| 2 | Atr-ERN02263 |  | | | |  |  |  | | | |  |  |  |
| 2 | Atr-ERN02264 |  | | | |  |  |  | | | |  |  |  |
| 2 | Atr-ERN02265 |  | Vvi-Vitvi14g02510\_t001 |  |  |  | Vvi-Vitvi05g00007\_t001 |  |  |  |
| 1 | Atr-ERN02266 |  |  |  |  |  | Vvi-Vitvi05g00008\_t001 |  |  |  |
