# Supplementary material for: Genome-wide identification, characterization and gene expression of BES1 transcription factor family in grapevine (Vitis vinifera L.)
Source: Sci Rep. 2023 Jan 5;13:240. doi: 10.1038/s41598-022-24407-y (PMC9816167; doi:10.1038/s41598-022-24407-y)
Supplement: Supplementary file 3 — Supplementary Information. [file 41598_2022_24407_MOESM3_ESM.zip › Vvi_Atr/Vitis_vinifera.PN40024.v4.dna_sm.toplevel.fa.vs.Amborella_trichopoda.AMTR1.0.dna_sm.toplevel.fa.html/Atr-AmTr_v1.0_scaffold00046.html]

|  |  |  |  |  |  |  |  |  |  |  |  |  |  |
| --- | --- | --- | --- | --- | --- | --- | --- | --- | --- | --- | --- | --- | --- |
| Duplication depth | Reference chromosome | Collinear blocks | | | | | | | | | | | |
| 0 | Atr-ERN17925 |  |  |  |  |  |  |
| 0 | Atr-ERN17926 |  |  |  |  |  |  |
| 0 | Atr-ERN17927 |  |  |  |  |  |  |
| 0 | Atr-ERN17928 |  |  |  |  |  |  |
| 0 | Atr-ERN17929 |  |  |  |  |  |  |
| 0 | Atr-ERN17930 |  |  |  |  |  |  |
| 0 | Atr-ERN17931 |  |  |  |  |  |  |
| 0 | Atr-ERN17932 |  |  |  |  |  |  |
| 0 | Atr-ERN17933 |  |  |  |  |  |  |
| 0 | Atr-ERN17934 |  |  |  |  |  |  |
| 0 | Atr-ERN17935 |  |  |  |  |  |  |
| 0 | Atr-ERN17936 |  |  |  |  |  |  |
| 0 | Atr-ERN17937 |  |  |  |  |  |  |
| 0 | Atr-ERN17938 |  |  |  |  |  |  |
| 0 | Atr-ERN17939 |  |  |  |  |  |  |
| 0 | Atr-ERN17940 |  |  |  |  |  |  |
| 0 | Atr-ERN17941 |  |  |  |  |  |  |
| 0 | Atr-ERN17942 |  |  |  |  |  |  |
| 0 | Atr-ERN17943 |  |  |  |  |  |  |
| 0 | Atr-ERN17944 |  |  |  |  |  |  |
| 0 | Atr-ERN17945 |  |  |  |  |  |  |
| 0 | Atr-ERN17946 |  |  |  |  |  |  |
| 0 | Atr-ERN17947 |  |  |  |  |  |  |
| 0 | Atr-ERN17948 |  |  |  |  |  |  |
| 0 | Atr-ERN17949 |  |  |  |  |  |  |
| 0 | Atr-ERN17950 |  |  |  |  |  |  |
| 0 | Atr-ERN17951 |  |  |  |  |  |  |
| 0 | Atr-ERN17952 |  |  |  |  |  |  |
| 0 | Atr-ERN17953 |  |  |  |  |  |  |
| 0 | Atr-ERN17954 |  |  |  |  |  |  |
| 0 | Atr-ERN17955 |  |  |  |  |  |  |
| 0 | Atr-ERN17956 |  |  |  |  |  |  |
| 0 | Atr-ERN17957 |  |  |  |  |  |  |
| 0 | Atr-ERN17958 |  |  |  |  |  |  |
| 1 | Atr-ERN17959 |  | Vvi-Vitvi11g00493\_t001 |  |  |  |  |  |
| 1 | Atr-ERN17960 |  | | | |  |  |  |  |  |
| 1 | Atr-ERN17961 |  | | | |  |  |  |  |  |
| 1 | Atr-ERN17962 |  | | | |  |  |  |  |  |
| 1 | Atr-ERN17963 |  | | | |  |  |  |  |  |
| 1 | Atr-ERN17964 |  | | | |  |  |  |  |  |
| 1 | Atr-ERN17965 |  | | | |  |  |  |  |  |
| 1 | Atr-ERN17966 |  | | | |  |  |  |  |  |
| 1 | Atr-ERN17967 |  | | | |  |  |  |  |  |
| 1 | Atr-ERN17968 |  | | | |  |  |  |  |  |
| 1 | Atr-ERN17969 |  | | | |  |  |  |  |  |
| 1 | Atr-ERN17970 |  | | | |  |  |  |  |  |
| 2 | Atr-ERN17971 |  | Vvi-Vitvi11g00505\_t001 |  | Vvi-Vitvi04g00494\_t001 |  |  |  |  |
| 2 | Atr-ERN17972 |  | | | |  | | | |  |  |  |  |
| 2 | Atr-ERN17973 |  | | | |  | | | |  |  |  |  |
| 2 | Atr-ERN17974 |  | | | |  | | | |  |  |  |  |
| 2 | Atr-ERN17975 |  | | | |  | | | |  |  |  |  |
| 2 | Atr-ERN17976 |  | | | |  | | | |  |  |  |  |
| 2 | Atr-ERN17977 |  | | | |  | | | |  |  |  |  |
| 2 | Atr-ERN17978 |  | | | |  | | | |  |  |  |  |
| 2 | Atr-ERN17979 |  | | | |  | | | |  |  |  |  |
| 2 | Atr-ERN17980 |  | Vvi-Vitvi11g01447\_t001 |  | Vvi-Vitvi04g00495\_t001 |  |  |  |  |
| 2 | Atr-ERN17981 |  | | | |  | | | |  |  |  |  |
| 2 | Atr-ERN17982 |  | | | |  | | | |  |  |  |  |
| 2 | Atr-ERN17983 |  | | | |  | | | |  |  |  |  |
| 2 | Atr-ERN17984 |  | | | |  | | | |  |  |  |  |
| 2 | Atr-ERN17985 |  | | | |  | Vvi-Vitvi04g00497\_t001 |  |  |  |  |
| 2 | Atr-ERN17986 |  | | | |  | Vvi-Vitvi04g00498\_t001 |  |  |  |  |
| 2 | Atr-ERN17987 |  | Vvi-Vitvi11g00513\_t001 |  | | | |  |  |  |  |
| 2 | Atr-ERN17988 |  | | | |  | | | |  |  |  |  |
| 2 | Atr-ERN17989 |  | Vvi-Vitvi11g00514\_t001 |  | | | |  |  |  |  |
| 2 | Atr-ERN17990 |  | | | |  | | | |  |  |  |  |
| 2 | Atr-ERN17991 |  | Vvi-Vitvi11g00517\_t001 |  | | | |  |  |  |  |
| 2 | Atr-ERN17992 |  | | | |  | | | |  |  |  |  |
| 2 | Atr-ERN17993 |  | | | |  | | | |  |  |  |  |
| 2 | Atr-ERN17994 |  | Vvi-Vitvi11g00518\_t001 |  | | | |  |  |  |  |
| 2 | Atr-ERN17995 |  | | | |  | Vvi-Vitvi04g00501\_t001 |  |  |  |  |
| 2 | Atr-ERN17996 |  | | | |  | | | |  |  |  |  |
| 2 | Atr-ERN17997 |  | | | |  | | | |  |  |  |  |
| 2 | Atr-ERN17998 |  | | | |  | | | |  |  |  |  |
| 2 | Atr-ERN17999 |  | | | |  | | | |  |  |  |  |
| 2 | Atr-ERN18000 |  | | | |  | | | |  |  |  |  |
| 2 | Atr-ERN18001 |  | Vvi-Vitvi11g04113\_t001 |  | Vvi-Vitvi04g00503\_t001 |  |  |  |  |
| 2 | Atr-ERN18002 |  | | | |  | | | |  |  |  |  |
| 2 | Atr-ERN18003 |  | | | |  | | | |  |  |  |  |
| 2 | Atr-ERN18004 |  | | | |  | | | |  |  |  |  |
| 2 | Atr-ERN18005 |  | Vvi-Vitvi11g04116\_t001 |  | | | |  |  |  |  |
| 2 | Atr-ERN18006 |  | | | |  | | | |  |  |  |  |
| 2 | Atr-ERN18007 |  | | | |  | | | |  |  |  |  |
| 2 | Atr-ERN18008 |  | | | |  | | | |  |  |  |  |
| 2 | Atr-ERN18009 |  | Vvi-Vitvi11g01454\_t001 |  | | | |  |  |  |  |
| 2 | Atr-ERN18010 |  | | | |  | | | |  |  |  |  |
| 2 | Atr-ERN18011 |  | Vvi-Vitvi11g00524\_t001 |  | | | |  |  |  |  |
| 2 | Atr-ERN18012 |  | | | |  | | | |  |  |  |  |
| 2 | Atr-ERN18013 |  | | | |  | | | |  |  |  |  |
| 2 | Atr-ERN18014 |  | | | |  | | | |  |  |  |  |
| 2 | Atr-ERN18015 |  | | | |  | | | |  |  |  |  |
| 2 | Atr-ERN18016 |  | | | |  | | | |  |  |  |  |
| 2 | Atr-ERN18017 |  | | | |  | | | |  |  |  |  |
| 2 | Atr-ERN18018 |  | | | |  | | | |  |  |  |  |
| 2 | Atr-ERN18019 |  | | | |  | | | |  |  |  |  |
| 2 | Atr-ERN18020 |  | Vvi-Vitvi11g00525\_t001 |  | Vvi-Vitvi04g00507\_t001 |  |  |  |  |
| 1 | Atr-ERN18021 |  | | | |  |  |  |  |  |
| 2 | Atr-ERN18022 |  | | | |  | Vvi-Vitvi07g01406\_t001 |  |  |  |  |
| 2 | Atr-ERN18023 |  | | | |  | | | |  |  |  |  |
| 2 | Atr-ERN18024 |  | | | |  | | | |  |  |  |  |
| 2 | Atr-ERN18025 |  | | | |  | | | |  |  |  |  |
| 2 | Atr-ERN18026 |  | | | |  | | | |  |  |  |  |
| 2 | Atr-ERN18027 |  | | | |  | | | |  |  |  |  |
| 2 | Atr-ERN18028 |  | | | |  | | | |  |  |  |  |
| 2 | Atr-ERN18029 |  | | | |  | | | |  |  |  |  |
| 2 | Atr-ERN18030 |  | | | |  | | | |  |  |  |  |
| 2 | Atr-ERN18031 |  | Vvi-Vitvi11g00538\_t001 |  | | | |  |  |  |  |
| 2 | Atr-ERN18032 |  | Vvi-Vitvi11g00549\_t001 |  | | | |  |  |  |  |
| 1 | Atr-ERN18033 |  |  |  | | | |  |  |  |  |
| 1 | Atr-ERN18034 |  |  |  | | | |  |  |  |  |
| 1 | Atr-ERN18035 |  |  |  | | | |  |  |  |  |
| 1 | Atr-ERN18036 |  |  |  | | | |  |  |  |  |
| 2 | Atr-ERN18037 |  | Vvi-Vitvi18g00546\_t001 |  | | | |  |  |  |  |
| 2 | Atr-ERN18038 |  | | | |  | | | |  |  |  |  |
| 2 | Atr-ERN18039 |  | | | |  | | | |  |  |  |  |
| 2 | Atr-ERN18040 |  | Vvi-Vitvi18g00549\_t001 |  | Vvi-Vitvi07g01388\_t001 |  |  |  |  |
| 2 | Atr-ERN18041 |  | | | |  | | | |  |  |  |  |
| 2 | Atr-ERN18042 |  | | | |  | | | |  |  |  |  |
| 2 | Atr-ERN18043 |  | | | |  | | | |  |  |  |  |
| 2 | Atr-ERN18044 |  | | | |  | | | |  |  |  |  |
| 2 | Atr-ERN18045 |  | | | |  | | | |  |  |  |  |
| 3 | Atr-ERN18046 |  | | | |  | Vvi-Vitvi07g01387\_t001 |  | Vvi-Vitvi03g00806\_t001 |  |  |  |
| 3 | Atr-ERN18047 |  | | | |  | | | |  | | | |  |  |  |
| 3 | Atr-ERN18048 |  | | | |  | | | |  | | | |  |  |  |
| 3 | Atr-ERN18049 |  | | | |  | | | |  | | | |  |  |  |
| 3 | Atr-ERN18050 |  | | | |  | | | |  | | | |  |  |  |
| 3 | Atr-ERN18051 |  | Vvi-Vitvi18g00550\_t001 |  | | | |  | | | |  |  |  |
| 3 | Atr-ERN18052 |  | | | |  | | | |  | | | |  |  |  |
| 3 | Atr-ERN18053 |  | | | |  | | | |  | | | |  |  |  |
| 3 | Atr-ERN18054 |  | | | |  | | | |  | | | |  |  |  |
| 3 | Atr-ERN18055 |  | | | |  | | | |  | | | |  |  |  |
| 3 | Atr-ERN18056 |  | | | |  | | | |  | | | |  |  |  |
| 3 | Atr-ERN18057 |  | | | |  | | | |  | | | |  |  |  |
| 3 | Atr-ERN18058 |  | Vvi-Vitvi18g00553\_t001 |  | Vvi-Vitvi07g01520\_t001 |  | Vvi-Vitvi03g00819\_t001 |  |  |  |
| 3 | Atr-ERN18059 |  | | | |  | | | |  | | | |  |  |  |
| 3 | Atr-ERN18060 |  | | | |  | | | |  | | | |  |  |  |
| 3 | Atr-ERN18061 |  | | | |  | | | |  | | | |  |  |  |
| 3 | Atr-ERN18062 |  | | | |  | | | |  | | | |  |  |  |
| 3 | Atr-ERN18063 |  | Vvi-Vitvi18g00557\_t001 |  | | | |  | Vvi-Vitvi03g00842\_t001 |  |  |  |
| 3 | Atr-ERN18064 |  | | | |  | | | |  | | | |  |  |  |
| 3 | Atr-ERN18065 |  | | | |  | | | |  | | | |  |  |  |
| 3 | Atr-ERN18066 |  | | | |  | | | |  | | | |  |  |  |
| 3 | Atr-ERN18067 |  | | | |  | | | |  | | | |  |  |  |
| 3 | Atr-ERN18068 |  | | | |  | | | |  | | | |  |  |  |
| 3 | Atr-ERN18069 |  | | | |  | | | |  | | | |  |  |  |
| 3 | Atr-ERN18070 |  | | | |  | | | |  | | | |  |  |  |
| 3 | Atr-ERN18071 |  | | | |  | | | |  | | | |  |  |  |
| 3 | Atr-ERN18072 |  | | | |  | | | |  | | | |  |  |  |
| 3 | Atr-ERN18073 |  | | | |  | | | |  | | | |  |  |  |
| 3 | Atr-ERN18074 |  | | | |  | | | |  | | | |  |  |  |
| 3 | Atr-ERN18075 |  | Vvi-Vitvi18g00560\_t002 |  | Vvi-Vitvi07g02610\_t001 |  | | | |  |  |  |
| 3 | Atr-ERN18076 |  | | | |  | | | |  | | | |  |  |  |
| 3 | Atr-ERN18077 |  | | | |  | | | |  | Vvi-Vitvi03g00844\_t001 |  |  |  |
| 3 | Atr-ERN18078 |  | | | |  | Vvi-Vitvi07g01510\_t001 |  | | | |  |  |  |
| 3 | Atr-ERN18079 |  | | | |  | | | |  | | | |  |  |  |
| 3 | Atr-ERN18080 |  | | | |  | | | |  | | | |  |  |  |
| 3 | Atr-ERN18081 |  | | | |  | | | |  | | | |  |  |  |
| 3 | Atr-ERN18082 |  | | | |  | | | |  | | | |  |  |  |
| 3 | Atr-ERN18083 |  | | | |  | | | |  | | | |  |  |  |
| 3 | Atr-ERN18084 |  | | | |  | | | |  | | | |  |  |  |
| 3 | Atr-ERN18085 |  | | | |  | Vvi-Vitvi07g04636\_t002 |  | | | |  |  |  |
| 3 | Atr-ERN18086 |  | | | |  | Vvi-Vitvi07g01508\_t001 |  | | | |  |  |  |
| 3 | Atr-ERN18087 |  | | | |  | | | |  | | | |  |  |  |
| 3 | Atr-ERN18088 |  | | | |  | | | |  | | | |  |  |  |
| 3 | Atr-ERN18089 |  | | | |  | | | |  | | | |  |  |  |
| 3 | Atr-ERN18090 |  | | | |  | Vvi-Vitvi07g01507\_t001 |  | | | |  |  |  |
| 3 | Atr-ERN18091 |  | | | |  | Vvi-Vitvi07g01506\_t001 |  | | | |  |  |  |
| 3 | Atr-ERN18092 |  | | | |  | | | |  | | | |  |  |  |
| 3 | Atr-ERN18093 |  | | | |  | | | |  | | | |  |  |  |
| 3 | Atr-ERN18094 |  | | | |  | Vvi-Vitvi07g01524\_t001 |  | Vvi-Vitvi03g00845\_t001 |  |  |  |
| 3 | Atr-ERN18095 |  | | | |  | | | |  | Vvi-Vitvi03g00846\_t001 |  |  |  |
| 3 | Atr-ERN18096 |  | | | |  | | | |  | | | |  |  |  |
| 3 | Atr-ERN18097 |  | Vvi-Vitvi18g00561\_t001 |  | | | |  | | | |  |  |  |
| 3 | Atr-ERN18098 |  | Vvi-Vitvi18g00562\_t001 |  | Vvi-Vitvi07g01523\_t001 |  | | | |  |  |  |
| 3 | Atr-ERN18099 |  | | | |  | Vvi-Vitvi07g04637\_t001 |  | Vvi-Vitvi03g00847\_t003 |  |  |  |
| 2 | Atr-ERN18100 |  | | | |  |  |  | | | |  |  |  |
| 2 | Atr-ERN18101 |  | | | |  |  |  | | | |  |  |  |
| 2 | Atr-ERN18102 |  | Vvi-Vitvi18g00563\_t001 |  |  |  | Vvi-Vitvi03g00849\_t001 |  |  |  |
| 2 | Atr-ERN18103 |  | | | |  |  |  | Vvi-Vitvi03g00851\_t001 |  |  |  |
| 1 | Atr-ERN18104 |  | Vvi-Vitvi18g00565\_t003 |  |  |  |  |  |
