# Supplementary material for: Genome-wide identification, characterization and gene expression of BES1 transcription factor family in grapevine (Vitis vinifera L.)
Source: Sci Rep. 2023 Jan 5;13:240. doi: 10.1038/s41598-022-24407-y (PMC9816167; doi:10.1038/s41598-022-24407-y)
Supplement: Supplementary file 3 — Supplementary Information. [file 41598_2022_24407_MOESM3_ESM.zip › Vvi_Atr/Vitis_vinifera.PN40024.v4.dna_sm.toplevel.fa.vs.Amborella_trichopoda.AMTR1.0.dna_sm.toplevel.fa.html/Atr-AmTr_v1.0_scaffold00047.html]

|  |  |  |  |  |  |  |  |  |  |  |  |  |  |
| --- | --- | --- | --- | --- | --- | --- | --- | --- | --- | --- | --- | --- | --- |
| Duplication depth | Reference chromosome | Collinear blocks | | | | | | | | | | | |
| 0 | Atr-ERN17719 |  |  |  |  |  |  |
| 0 | Atr-ERN17720 |  |  |  |  |  |  |
| 0 | Atr-ERN17721 |  |  |  |  |  |  |
| 0 | Atr-ERN17722 |  |  |  |  |  |  |
| 0 | Atr-ERN17723 |  |  |  |  |  |  |
| 0 | Atr-ERN17724 |  |  |  |  |  |  |
| 0 | Atr-ERN17725 |  |  |  |  |  |  |
| 0 | Atr-ERN17726 |  |  |  |  |  |  |
| 0 | Atr-ERN17727 |  |  |  |  |  |  |
| 0 | Atr-ERN17728 |  |  |  |  |  |  |
| 0 | Atr-ERN17729 |  |  |  |  |  |  |
| 1 | Atr-ERN17730 |  | Vvi-Vitvi10g00259\_t001 |  |  |  |  |  |
| 1 | Atr-ERN17731 |  | | | |  |  |  |  |  |
| 1 | Atr-ERN17732 |  | | | |  |  |  |  |  |
| 1 | Atr-ERN17733 |  | | | |  |  |  |  |  |
| 1 | Atr-ERN17734 |  | | | |  |  |  |  |  |
| 1 | Atr-ERN17735 |  | | | |  |  |  |  |  |
| 1 | Atr-ERN17736 |  | Vvi-Vitvi10g00232\_t001 |  |  |  |  |  |
| 1 | Atr-ERN17737 |  | | | |  |  |  |  |  |
| 1 | Atr-ERN17738 |  | | | |  |  |  |  |  |
| 1 | Atr-ERN17739 |  | | | |  |  |  |  |  |
| 1 | Atr-ERN17740 |  | | | |  |  |  |  |  |
| 1 | Atr-ERN17741 |  | | | |  |  |  |  |  |
| 1 | Atr-ERN17742 |  | | | |  |  |  |  |  |
| 1 | Atr-ERN17743 |  | | | |  |  |  |  |  |
| 1 | Atr-ERN17744 |  | | | |  |  |  |  |  |
| 1 | Atr-ERN17745 |  | Vvi-Vitvi10g00231\_t001 |  |  |  |  |  |
| 1 | Atr-ERN17746 |  | | | |  |  |  |  |  |
| 1 | Atr-ERN17747 |  | | | |  |  |  |  |  |
| 1 | Atr-ERN17748 |  | Vvi-Vitvi10g04101\_t001 |  |  |  |  |  |
| 1 | Atr-ERN17749 |  | | | |  |  |  |  |  |
| 1 | Atr-ERN17750 |  | | | |  |  |  |  |  |
| 1 | Atr-ERN17751 |  | Vvi-Vitvi10g04100\_t001 |  |  |  |  |  |
| 1 | Atr-ERN17752 |  | | | |  |  |  |  |  |
| 1 | Atr-ERN17753 |  | Vvi-Vitvi10g00229\_t001 |  |  |  |  |  |
| 1 | Atr-ERN17754 |  | | | |  |  |  |  |  |
| 1 | Atr-ERN17755 |  | | | |  |  |  |  |  |
| 1 | Atr-ERN17756 |  | | | |  |  |  |  |  |
| 1 | Atr-ERN17757 |  | | | |  |  |  |  |  |
| 1 | Atr-ERN17758 |  | | | |  |  |  |  |  |
| 1 | Atr-ERN17759 |  | Vvi-Vitvi10g00228\_t001 |  |  |  |  |  |
| 0 | Atr-ERN17760 |  |  |  |  |  |  |
| 0 | Atr-ERN17761 |  |  |  |  |  |  |
| 0 | Atr-ERN17762 |  |  |  |  |  |  |
| 0 | Atr-ERN17763 |  |  |  |  |  |  |
| 0 | Atr-ERN17764 |  |  |  |  |  |  |
| 0 | Atr-ERN17765 |  |  |  |  |  |  |
| 0 | Atr-ERN17766 |  |  |  |  |  |  |
| 0 | Atr-ERN17767 |  |  |  |  |  |  |
| 0 | Atr-ERN17768 |  |  |  |  |  |  |
| 0 | Atr-ERN17769 |  |  |  |  |  |  |
| 0 | Atr-ERN17770 |  |  |  |  |  |  |
| 0 | Atr-ERN17771 |  |  |  |  |  |  |
| 0 | Atr-ERN17772 |  |  |  |  |  |  |
| 1 | Atr-ERN17773 |  | Vvi-Vitvi17g00452\_t001 |  |  |  |  |  |
| 1 | Atr-ERN17774 |  | | | |  |  |  |  |  |
| 1 | Atr-ERN17775 |  | | | |  |  |  |  |  |
| 1 | Atr-ERN17776 |  | | | |  |  |  |  |  |
| 1 | Atr-ERN17777 |  | | | |  |  |  |  |  |
| 1 | Atr-ERN17778 |  | | | |  |  |  |  |  |
| 1 | Atr-ERN17779 |  | | | |  |  |  |  |  |
| 1 | Atr-ERN17780 |  | | | |  |  |  |  |  |
| 1 | Atr-ERN17781 |  | Vvi-Vitvi17g00457\_t001 |  |  |  |  |  |
| 1 | Atr-ERN17782 |  | | | |  |  |  |  |  |
| 1 | Atr-ERN17783 |  | | | |  |  |  |  |  |
| 1 | Atr-ERN17784 |  | | | |  |  |  |  |  |
| 1 | Atr-ERN17785 |  | | | |  |  |  |  |  |
| 1 | Atr-ERN17786 |  | | | |  |  |  |  |  |
| 1 | Atr-ERN17787 |  | | | |  |  |  |  |  |
| 1 | Atr-ERN17788 |  | | | |  |  |  |  |  |
| 1 | Atr-ERN17789 |  | | | |  |  |  |  |  |
| 1 | Atr-ERN17790 |  | | | |  |  |  |  |  |
| 1 | Atr-ERN17791 |  | | | |  |  |  |  |  |
| 1 | Atr-ERN17792 |  | | | |  |  |  |  |  |
| 1 | Atr-ERN17793 |  | Vvi-Vitvi17g04130\_t001 |  |  |  |  |  |
| 1 | Atr-ERN17794 |  | | | |  |  |  |  |  |
| 1 | Atr-ERN17795 |  | | | |  |  |  |  |  |
| 1 | Atr-ERN17796 |  | | | |  |  |  |  |  |
| 1 | Atr-ERN17797 |  | | | |  |  |  |  |  |
| 1 | Atr-ERN17798 |  | | | |  |  |  |  |  |
| 1 | Atr-ERN17799 |  | | | |  |  |  |  |  |
| 1 | Atr-ERN17800 |  | | | |  |  |  |  |  |
| 1 | Atr-ERN17801 |  | | | |  |  |  |  |  |
| 1 | Atr-ERN17802 |  | | | |  |  |  |  |  |
| 1 | Atr-ERN17803 |  | | | |  |  |  |  |  |
| 1 | Atr-ERN17804 |  | | | |  |  |  |  |  |
| 1 | Atr-ERN17805 |  | | | |  |  |  |  |  |
| 1 | Atr-ERN17806 |  | | | |  |  |  |  |  |
| 1 | Atr-ERN17807 |  | | | |  |  |  |  |  |
| 1 | Atr-ERN17808 |  | | | |  |  |  |  |  |
| 1 | Atr-ERN17809 |  | | | |  |  |  |  |  |
| 1 | Atr-ERN17810 |  | | | |  |  |  |  |  |
| 1 | Atr-ERN17811 |  | | | |  |  |  |  |  |
| 1 | Atr-ERN17812 |  | | | |  |  |  |  |  |
| 1 | Atr-ERN17813 |  | | | |  |  |  |  |  |
| 1 | Atr-ERN17814 |  | | | |  |  |  |  |  |
| 1 | Atr-ERN17815 |  | | | |  |  |  |  |  |
| 1 | Atr-ERN17816 |  | | | |  |  |  |  |  |
| 2 | Atr-ERN17817 |  | | | |  | Vvi-Vitvi01g00006\_t001 |  |  |  |  |
| 2 | Atr-ERN17818 |  | Vvi-Vitvi17g00469\_t001 |  | Vvi-Vitvi01g01835\_t001 |  |  |  |  |
| 3 | Atr-ERN17819 |  | | | |  | Vvi-Vitvi01g01836\_t001 |  | Vvi-Vitvi14g01338\_t001 |  |  |  |
| 3 | Atr-ERN17820 |  | | | |  | | | |  | | | |  |  |  |
| 3 | Atr-ERN17821 |  | | | |  | | | |  | Vvi-Vitvi14g01339\_t001 |  |  |  |
| 3 | Atr-ERN17822 |  | | | |  | | | |  | Vvi-Vitvi14g01340\_t002 |  |  |  |
| 3 | Atr-ERN17823 |  | Vvi-Vitvi17g00470\_t001 |  | | | |  | | | |  |  |  |
| 3 | Atr-ERN17824 |  | | | |  | | | |  | | | |  |  |  |
| 3 | Atr-ERN17825 |  | | | |  | | | |  | | | |  |  |  |
| 3 | Atr-ERN17826 |  | | | |  | | | |  | | | |  |  |  |
| 3 | Atr-ERN17827 |  | | | |  | | | |  | | | |  |  |  |
| 3 | Atr-ERN17828 |  | | | |  | | | |  | | | |  |  |  |
| 3 | Atr-ERN17829 |  | | | |  | | | |  | | | |  |  |  |
| 3 | Atr-ERN17830 |  | | | |  | | | |  | | | |  |  |  |
| 3 | Atr-ERN17831 |  | | | |  | | | |  | | | |  |  |  |
| 3 | Atr-ERN17832 |  | | | |  | | | |  | | | |  |  |  |
| 3 | Atr-ERN17833 |  | | | |  | | | |  | | | |  |  |  |
| 3 | Atr-ERN17834 |  | | | |  | | | |  | | | |  |  |  |
| 3 | Atr-ERN17835 |  | | | |  | | | |  | | | |  |  |  |
| 3 | Atr-ERN17836 |  | | | |  | | | |  | | | |  |  |  |
| 3 | Atr-ERN17837 |  | | | |  | | | |  | | | |  |  |  |
| 3 | Atr-ERN17838 |  | | | |  | | | |  | | | |  |  |  |
| 3 | Atr-ERN17839 |  | | | |  | Vvi-Vitvi01g00008\_t001 |  | Vvi-Vitvi14g01341\_t001 |  |  |  |
| 3 | Atr-ERN17840 |  | | | |  | | | |  | | | |  |  |  |
| 3 | Atr-ERN17841 |  | | | |  | | | |  | | | |  |  |  |
| 3 | Atr-ERN17842 |  | | | |  | Vvi-Vitvi01g00012\_t001 |  | | | |  |  |  |
| 3 | Atr-ERN17843 |  | Vvi-Vitvi17g00473\_t001 |  | Vvi-Vitvi01g01837\_t003 |  | | | |  |  |  |
| 3 | Atr-ERN17844 |  | | | |  | Vvi-Vitvi01g00013\_t001 |  | | | |  |  |  |
| 3 | Atr-ERN17845 |  | | | |  | | | |  | | | |  |  |  |
| 3 | Atr-ERN17846 |  | | | |  | | | |  | | | |  |  |  |
| 3 | Atr-ERN17847 |  | | | |  | | | |  | | | |  |  |  |
| 3 | Atr-ERN17848 |  | | | |  | | | |  | | | |  |  |  |
| 3 | Atr-ERN17849 |  | | | |  | | | |  | | | |  |  |  |
| 3 | Atr-ERN17850 |  | | | |  | | | |  | | | |  |  |  |
| 3 | Atr-ERN17851 |  | | | |  | | | |  | | | |  |  |  |
| 3 | Atr-ERN17852 |  | | | |  | | | |  | | | |  |  |  |
| 3 | Atr-ERN17853 |  | | | |  | | | |  | | | |  |  |  |
| 3 | Atr-ERN17854 |  | | | |  | | | |  | | | |  |  |  |
| 3 | Atr-ERN17855 |  | | | |  | | | |  | | | |  |  |  |
| 3 | Atr-ERN17856 |  | | | |  | | | |  | | | |  |  |  |
| 3 | Atr-ERN17857 |  | | | |  | | | |  | | | |  |  |  |
| 3 | Atr-ERN17858 |  | | | |  | | | |  | | | |  |  |  |
| 3 | Atr-ERN17859 |  | | | |  | | | |  | | | |  |  |  |
| 3 | Atr-ERN17860 |  | | | |  | | | |  | | | |  |  |  |
| 3 | Atr-ERN17861 |  | | | |  | | | |  | | | |  |  |  |
| 3 | Atr-ERN17862 |  | | | |  | | | |  | | | |  |  |  |
| 3 | Atr-ERN17863 |  | | | |  | | | |  | Vvi-Vitvi14g01349\_t001 |  |  |  |
| 3 | Atr-ERN17864 |  | | | |  | Vvi-Vitvi01g00014\_t001 |  | | | |  |  |  |
| 3 | Atr-ERN17865 |  | | | |  | | | |  | | | |  |  |  |
| 3 | Atr-ERN17866 |  | | | |  | | | |  | | | |  |  |  |
| 3 | Atr-ERN17867 |  | Vvi-Vitvi17g01440\_t001 |  | Vvi-Vitvi01g01838\_t001 |  | Vvi-Vitvi14g01350\_t001 |  |  |  |
| 3 | Atr-ERN17868 |  | | | |  | | | |  | | | |  |  |  |
| 3 | Atr-ERN17869 |  | | | |  | Vvi-Vitvi01g00015\_t001 |  | Vvi-Vitvi14g01351\_t001 |  |  |  |
| 3 | Atr-ERN17870 |  | | | |  | | | |  | | | |  |  |  |
| 3 | Atr-ERN17871 |  | | | |  | | | |  | Vvi-Vitvi14g01352\_t001 |  |  |  |
| 3 | Atr-ERN17872 |  | | | |  | | | |  | | | |  |  |  |
| 3 | Atr-ERN17873 |  | | | |  | Vvi-Vitvi01g00023\_t001 |  | | | |  |  |  |
| 3 | Atr-ERN17874 |  | | | |  | | | |  | Vvi-Vitvi14g01354\_t001 |  |  |  |
| 3 | Atr-ERN17875 |  | | | |  | | | |  | | | |  |  |  |
| 3 | Atr-ERN17876 |  | | | |  | | | |  | | | |  |  |  |
| 3 | Atr-ERN17877 |  | | | |  | | | |  | | | |  |  |  |
| 3 | Atr-ERN17878 |  | | | |  | | | |  | | | |  |  |  |
| 3 | Atr-ERN17879 |  | | | |  | | | |  | | | |  |  |  |
| 3 | Atr-ERN17880 |  | Vvi-Vitvi17g04133\_t001 |  | | | |  | Vvi-Vitvi14g01357\_t001 |  |  |  |
| 3 | Atr-ERN17881 |  | | | |  | | | |  | | | |  |  |  |
| 3 | Atr-ERN17882 |  | | | |  | | | |  | | | |  |  |  |
| 3 | Atr-ERN17883 |  | | | |  | | | |  | | | |  |  |  |
| 3 | Atr-ERN17884 |  | | | |  | Vvi-Vitvi01g00024\_t001 |  | | | |  |  |  |
| 3 | Atr-ERN17885 |  | | | |  | Vvi-Vitvi01g00025\_t001 |  | | | |  |  |  |
| 3 | Atr-ERN17886 |  | | | |  | Vvi-Vitvi01g00026\_t002 |  | | | |  |  |  |
| 3 | Atr-ERN17887 |  | | | |  | | | |  | Vvi-Vitvi14g01360\_t001 |  |  |  |
| 3 | Atr-ERN17888 |  | | | |  | | | |  | | | |  |  |  |
| 3 | Atr-ERN17889 |  | Vvi-Vitvi17g00482\_t001 |  | | | |  | | | |  |  |  |
| 3 | Atr-ERN17890 |  | | | |  | | | |  | Vvi-Vitvi14g01361\_t001 |  |  |  |
| 2 | Atr-ERN17891 |  | Vvi-Vitvi17g00484\_t001 |  | Vvi-Vitvi01g00031\_t001 |  |  |  |  |
| 2 | Atr-ERN17892 |  | | | |  | | | |  |  |  |  |
| 2 | Atr-ERN17893 |  | | | |  | | | |  |  |  |  |
| 2 | Atr-ERN17894 |  | | | |  | | | |  |  |  |  |
| 2 | Atr-ERN17895 |  | | | |  | | | |  |  |  |  |
| 2 | Atr-ERN17896 |  | | | |  | | | |  |  |  |  |
| 2 | Atr-ERN17897 |  | | | |  | | | |  |  |  |  |
| 2 | Atr-ERN17898 |  | | | |  | | | |  |  |  |  |
| 2 | Atr-ERN17899 |  | | | |  | | | |  |  |  |  |
| 2 | Atr-ERN17900 |  | | | |  | | | |  |  |  |  |
| 2 | Atr-ERN17901 |  | | | |  | | | |  |  |  |  |
| 2 | Atr-ERN17902 |  | | | |  | | | |  |  |  |  |
| 2 | Atr-ERN17903 |  | | | |  | | | |  |  |  |  |
| 2 | Atr-ERN17904 |  | | | |  | | | |  |  |  |  |
| 2 | Atr-ERN17905 |  | | | |  | | | |  |  |  |  |
| 2 | Atr-ERN17906 |  | Vvi-Vitvi17g00485\_t001 |  | | | |  |  |  |  |
| 2 | Atr-ERN17907 |  | | | |  | | | |  |  |  |  |
| 2 | Atr-ERN17908 |  | Vvi-Vitvi17g00487\_t001 |  | | | |  |  |  |  |
| 2 | Atr-ERN17909 |  | | | |  | | | |  |  |  |  |
| 2 | Atr-ERN17910 |  | | | |  | | | |  |  |  |  |
| 2 | Atr-ERN17911 |  | | | |  | | | |  |  |  |  |
| 2 | Atr-ERN17912 |  | | | |  | | | |  |  |  |  |
| 2 | Atr-ERN17913 |  | | | |  | | | |  |  |  |  |
| 2 | Atr-ERN17914 |  | | | |  | | | |  |  |  |  |
| 2 | Atr-ERN17915 |  | Vvi-Vitvi17g00488\_t001 |  | | | |  |  |  |  |
| 2 | Atr-ERN17916 |  | Vvi-Vitvi17g00489\_t001 |  | Vvi-Vitvi01g00033\_t002 |  |  |  |  |
