# Supplementary material for: Genome-wide identification, characterization and gene expression of BES1 transcription factor family in grapevine (Vitis vinifera L.)
Source: Sci Rep. 2023 Jan 5;13:240. doi: 10.1038/s41598-022-24407-y (PMC9816167; doi:10.1038/s41598-022-24407-y)
Supplement: Supplementary file 3 — Supplementary Information. [file 41598_2022_24407_MOESM3_ESM.zip › Vvi_Atr/Vitis_vinifera.PN40024.v4.dna_sm.toplevel.fa.vs.Amborella_trichopoda.AMTR1.0.dna_sm.toplevel.fa.html/Atr-AmTr_v1.0_scaffold00048.html]

|  |  |  |  |  |  |  |  |  |  |  |  |  |  |
| --- | --- | --- | --- | --- | --- | --- | --- | --- | --- | --- | --- | --- | --- |
| Duplication depth | Reference chromosome | Collinear blocks | | | | | | | | | | | |
| 1 | Atr-ERN15482 |  | Vvi-Vitvi02g00617\_t001 |  |  |  |  |  |
| 1 | Atr-ERN15483 |  | Vvi-Vitvi02g00615\_t001 |  |  |  |  |  |
| 1 | Atr-ERN15484 |  | | | |  |  |  |  |  |
| 1 | Atr-ERN15485 |  | | | |  |  |  |  |  |
| 1 | Atr-ERN15486 |  | Vvi-Vitvi02g01471\_t001 |  |  |  |  |  |
| 1 | Atr-ERN15487 |  | | | |  |  |  |  |  |
| 1 | Atr-ERN15488 |  | Vvi-Vitvi02g00614\_t001 |  |  |  |  |  |
| 1 | Atr-ERN15489 |  | Vvi-Vitvi02g00607\_t001 |  |  |  |  |  |
| 1 | Atr-ERN15490 |  | | | |  |  |  |  |  |
| 1 | Atr-ERN15491 |  | Vvi-Vitvi02g00606\_t001 |  |  |  |  |  |
| 0 | Atr-ERN15492 |  |  |  |  |  |  |
| 1 | Atr-ERN15493 |  | Vvi-Vitvi16g01526\_t001 |  |  |  |  |  |
| 1 | Atr-ERN15494 |  | | | |  |  |  |  |  |
| 1 | Atr-ERN15495 |  | | | |  |  |  |  |  |
| 1 | Atr-ERN15496 |  | | | |  |  |  |  |  |
| 1 | Atr-ERN15497 |  | | | |  |  |  |  |  |
| 2 | Atr-ERN15498 |  | | | |  | Vvi-Vitvi02g00724\_t001 |  |  |  |  |
| 2 | Atr-ERN15499 |  | | | |  | | | |  |  |  |  |
| 2 | Atr-ERN15500 |  | | | |  | | | |  |  |  |  |
| 2 | Atr-ERN15501 |  | | | |  | | | |  |  |  |  |
| 2 | Atr-ERN15502 |  | | | |  | | | |  |  |  |  |
| 2 | Atr-ERN15503 |  | | | |  | | | |  |  |  |  |
| 2 | Atr-ERN15504 |  | | | |  | | | |  |  |  |  |
| 2 | Atr-ERN15505 |  | | | |  | | | |  |  |  |  |
| 2 | Atr-ERN15506 |  | | | |  | | | |  |  |  |  |
| 2 | Atr-ERN15507 |  | | | |  | Vvi-Vitvi02g00732\_t001 |  |  |  |  |
| 2 | Atr-ERN15508 |  | | | |  | | | |  |  |  |  |
| 2 | Atr-ERN15509 |  | | | |  | | | |  |  |  |  |
| 2 | Atr-ERN15510 |  | | | |  | | | |  |  |  |  |
| 2 | Atr-ERN15511 |  | Vvi-Vitvi16g00111\_t001 |  | | | |  |  |  |  |
| 2 | Atr-ERN15512 |  | | | |  | | | |  |  |  |  |
| 2 | Atr-ERN15513 |  | | | |  | | | |  |  |  |  |
| 2 | Atr-ERN15514 |  | Vvi-Vitvi16g00112\_t001 |  | | | |  |  |  |  |
| 2 | Atr-ERN15515 |  | Vvi-Vitvi16g00114\_t001 |  | Vvi-Vitvi02g00739\_t001 |  |  |  |  |
| 2 | Atr-ERN15516 |  | | | |  | | | |  |  |  |  |
| 2 | Atr-ERN15517 |  | Vvi-Vitvi16g00116\_t001 |  | Vvi-Vitvi02g00749\_t001 |  |  |  |  |
| 2 | Atr-ERN15518 |  | | | |  | Vvi-Vitvi02g01513\_t001 |  |  |  |  |
| 3 | Atr-ERN15519 |  | | | |  | | | |  | Vvi-Vitvi02g00777\_t001 |  |  |  |
| 3 | Atr-ERN15520 |  | Vvi-Vitvi16g00119\_t001 |  | | | |  | Vvi-Vitvi02g00774\_t001 |  |  |  |
| 3 | Atr-ERN15521 |  | | | |  | | | |  | | | |  |  |  |
| 3 | Atr-ERN15522 |  | | | |  | | | |  | Vvi-Vitvi02g00773\_t001 |  |  |  |
| 3 | Atr-ERN15523 |  | | | |  | | | |  | Vvi-Vitvi02g00772\_t002 |  |  |  |
| 3 | Atr-ERN15524 |  | | | |  | Vvi-Vitvi02g00750\_t001 |  | | | |  |  |  |
| 3 | Atr-ERN15525 |  | | | |  | | | |  | | | |  |  |  |
| 3 | Atr-ERN15526 |  | | | |  | | | |  | | | |  |  |  |
| 3 | Atr-ERN15527 |  | | | |  | | | |  | | | |  |  |  |
| 3 | Atr-ERN15528 |  | Vvi-Vitvi16g00120\_t001 |  | | | |  | Vvi-Vitvi02g00759\_t001 |  |  |  |
| 3 | Atr-ERN15529 |  | | | |  | | | |  | | | |  |  |  |
| 3 | Atr-ERN15530 |  | | | |  | | | |  | | | |  |  |  |
| 3 | Atr-ERN15531 |  | | | |  | | | |  | | | |  |  |  |
| 3 | Atr-ERN15532 |  | | | |  | | | |  | | | |  |  |  |
| 4 | Atr-ERN15533 |  | | | |  | | | |  | | | |  | Vvi-Vitvi02g00802\_t001 |  |  |
| 4 | Atr-ERN15534 |  | | | |  | | | |  | | | |  | Vvi-Vitvi02g00796\_t003 |  |  |
| 4 | Atr-ERN15535 |  | | | |  | | | |  | | | |  | | | |  |  |
| 4 | Atr-ERN15536 |  | | | |  | | | |  | | | |  | | | |  |  |
| 4 | Atr-ERN15537 |  | | | |  | | | |  | | | |  | | | |  |  |
| 4 | Atr-ERN15538 |  | | | |  | | | |  | | | |  | Vvi-Vitvi02g00795\_t001 |  |  |
| 4 | Atr-ERN15539 |  | | | |  | | | |  | | | |  | Vvi-Vitvi02g00792\_t001 |  |  |
| 4 | Atr-ERN15540 |  | | | |  | | | |  | | | |  | Vvi-Vitvi02g00790\_t001 |  |  |
| 4 | Atr-ERN15541 |  | | | |  | | | |  | | | |  | | | |  |  |
| 4 | Atr-ERN15542 |  | Vvi-Vitvi16g00136\_t001 |  | | | |  | | | |  | | | |  |  |
| 4 | Atr-ERN15543 |  | | | |  | | | |  | | | |  | Vvi-Vitvi02g00785\_t001 |  |  |
| 3 | Atr-ERN15544 |  | | | |  | | | |  | | | |  |  |  |
| 3 | Atr-ERN15545 |  | | | |  | | | |  | | | |  |  |  |
| 3 | Atr-ERN15546 |  | | | |  | | | |  | | | |  |  |  |
| 3 | Atr-ERN15547 |  | | | |  | Vvi-Vitvi02g00751\_t001 |  | | | |  |  |  |
| 3 | Atr-ERN15548 |  | | | |  | | | |  | Vvi-Vitvi02g00738\_t001 |  |  |  |
| 3 | Atr-ERN15549 |  | | | |  | Vvi-Vitvi02g00757\_t002 |  | | | |  |  |  |
| 3 | Atr-ERN15550 |  | | | |  | | | |  | | | |  |  |  |
| 3 | Atr-ERN15551 |  | | | |  | Vvi-Vitvi02g00774\_t001 |  | | | |  |  |  |
| 2 | Atr-ERN15552 |  | | | |  |  |  | | | |  |  |  |
| 2 | Atr-ERN15553 |  | | | |  |  |  | | | |  |  |  |
| 2 | Atr-ERN15554 |  | | | |  |  |  | | | |  |  |  |
| 2 | Atr-ERN15555 |  | | | |  |  |  | | | |  |  |  |
| 2 | Atr-ERN15556 |  | | | |  |  |  | | | |  |  |  |
| 2 | Atr-ERN15557 |  | | | |  |  |  | | | |  |  |  |
| 2 | Atr-ERN15558 |  | | | |  |  |  | | | |  |  |  |
| 2 | Atr-ERN15559 |  | | | |  |  |  | | | |  |  |  |
| 2 | Atr-ERN15560 |  | Vvi-Vitvi16g00147\_t001 |  |  |  | | | |  |  |  |
| 2 | Atr-ERN15561 |  | | | |  |  |  | | | |  |  |  |
| 2 | Atr-ERN15562 |  | | | |  |  |  | | | |  |  |  |
| 2 | Atr-ERN15563 |  | | | |  |  |  | | | |  |  |  |
| 2 | Atr-ERN15564 |  | | | |  |  |  | | | |  |  |  |
| 2 | Atr-ERN15565 |  | | | |  |  |  | | | |  |  |  |
| 2 | Atr-ERN15566 |  | | | |  |  |  | | | |  |  |  |
| 2 | Atr-ERN15567 |  | | | |  |  |  | | | |  |  |  |
| 2 | Atr-ERN15568 |  | Vvi-Vitvi16g00149\_t001 |  |  |  | | | |  |  |  |
| 2 | Atr-ERN15569 |  | | | |  |  |  | | | |  |  |  |
| 2 | Atr-ERN15570 |  | | | |  |  |  | Vvi-Vitvi02g00733\_t001 |  |  |  |
| 1 | Atr-ERN15571 |  | | | |  |  |  |  |  |
| 1 | Atr-ERN15572 |  | | | |  |  |  |  |  |
| 1 | Atr-ERN15573 |  | | | |  |  |  |  |  |
| 1 | Atr-ERN15574 |  | Vvi-Vitvi16g00150\_t001 |  |  |  |  |  |
| 1 | Atr-ERN15575 |  | | | |  |  |  |  |  |
| 1 | Atr-ERN15576 |  | Vvi-Vitvi16g01537\_t001 |  |  |  |  |  |
| 1 | Atr-ERN15577 |  | | | |  |  |  |  |  |
| 1 | Atr-ERN15578 |  | Vvi-Vitvi16g01538\_t001 |  |  |  |  |  |
| 1 | Atr-ERN15579 |  | | | |  |  |  |  |  |
| 1 | Atr-ERN15580 |  | | | |  |  |  |  |  |
| 1 | Atr-ERN15581 |  | Vvi-Vitvi16g00151\_t001 |  |  |  |  |  |
| 1 | Atr-ERN15582 |  | | | |  |  |  |  |  |
| 1 | Atr-ERN15583 |  | | | |  |  |  |  |  |
| 1 | Atr-ERN15584 |  | Vvi-Vitvi16g00154\_t001 |  |  |  |  |  |
| 1 | Atr-ERN15585 |  | | | |  |  |  |  |  |
| 1 | Atr-ERN15586 |  | | | |  |  |  |  |  |
| 1 | Atr-ERN15587 |  | | | |  |  |  |  |  |
| 1 | Atr-ERN15588 |  | | | |  |  |  |  |  |
| 1 | Atr-ERN15589 |  | | | |  |  |  |  |  |
| 1 | Atr-ERN15590 |  | | | |  |  |  |  |  |
| 1 | Atr-ERN15591 |  | Vvi-Vitvi16g01539\_t001 |  |  |  |  |  |
| 1 | Atr-ERN15592 |  | Vvi-Vitvi16g00159\_t001 |  |  |  |  |  |
| 1 | Atr-ERN15593 |  | | | |  |  |  |  |  |
| 1 | Atr-ERN15594 |  | Vvi-Vitvi16g00168\_t002 |  |  |  |  |  |
| 1 | Atr-ERN15595 |  | | | |  |  |  |  |  |
| 1 | Atr-ERN15596 |  | | | |  |  |  |  |  |
| 1 | Atr-ERN15597 |  | | | |  |  |  |  |  |
| 1 | Atr-ERN15598 |  | | | |  |  |  |  |  |
| 1 | Atr-ERN15599 |  | | | |  |  |  |  |  |
| 1 | Atr-ERN15600 |  | | | |  |  |  |  |  |
| 1 | Atr-ERN15601 |  | | | |  |  |  |  |  |
| 1 | Atr-ERN15602 |  | | | |  |  |  |  |  |
| 1 | Atr-ERN15603 |  | | | |  |  |  |  |  |
| 1 | Atr-ERN15604 |  | | | |  |  |  |  |  |
| 1 | Atr-ERN15605 |  | | | |  |  |  |  |  |
| 1 | Atr-ERN15606 |  | | | |  |  |  |  |  |
| 1 | Atr-ERN15607 |  | | | |  |  |  |  |  |
| 1 | Atr-ERN15608 |  | | | |  |  |  |  |  |
| 1 | Atr-ERN15609 |  | | | |  |  |  |  |  |
| 1 | Atr-ERN15610 |  | | | |  |  |  |  |  |
| 1 | Atr-ERN15611 |  | | | |  |  |  |  |  |
| 1 | Atr-ERN15612 |  | | | |  |  |  |  |  |
| 1 | Atr-ERN15613 |  | | | |  |  |  |  |  |
| 1 | Atr-ERN15614 |  | | | |  |  |  |  |  |
| 1 | Atr-ERN15615 |  | | | |  |  |  |  |  |
| 1 | Atr-ERN15616 |  | Vvi-Vitvi16g00174\_t001 |  |  |  |  |  |
| 1 | Atr-ERN15617 |  | | | |  |  |  |  |  |
| 1 | Atr-ERN15618 |  | | | |  |  |  |  |  |
| 1 | Atr-ERN15619 |  | | | |  |  |  |  |  |
| 1 | Atr-ERN15620 |  | | | |  |  |  |  |  |
| 1 | Atr-ERN15621 |  | | | |  |  |  |  |  |
| 1 | Atr-ERN15622 |  | | | |  |  |  |  |  |
| 1 | Atr-ERN15623 |  | | | |  |  |  |  |  |
| 1 | Atr-ERN15624 |  | | | |  |  |  |  |  |
| 1 | Atr-ERN15625 |  | Vvi-Vitvi16g00177\_t001 |  |  |  |  |  |
| 1 | Atr-ERN15626 |  | | | |  |  |  |  |  |
| 1 | Atr-ERN15627 |  | Vvi-Vitvi16g00178\_t001 |  |  |  |  |  |
| 1 | Atr-ERN15628 |  | Vvi-Vitvi16g00179\_t001 |  |  |  |  |  |
| 1 | Atr-ERN15629 |  | Vvi-Vitvi16g00180\_t001 |  |  |  |  |  |
| 1 | Atr-ERN15630 |  | | | |  |  |  |  |  |
| 1 | Atr-ERN15631 |  | | | |  |  |  |  |  |
| 1 | Atr-ERN15632 |  | | | |  |  |  |  |  |
| 1 | Atr-ERN15633 |  | Vvi-Vitvi16g00181\_t001 |  |  |  |  |  |
| 0 | Atr-ERN15634 |  |  |  |  |  |  |
| 0 | Atr-ERN15635 |  |  |  |  |  |  |
| 0 | Atr-ERN15636 |  |  |  |  |  |  |
| 0 | Atr-ERN15637 |  |  |  |  |  |  |
| 0 | Atr-ERN15638 |  |  |  |  |  |  |
| 1 | Atr-ERN15639 |  | Vvi-Vitvi02g01182\_t001 |  |  |  |  |  |
| 1 | Atr-ERN15640 |  | Vvi-Vitvi02g01179\_t001 |  |  |  |  |  |
| 1 | Atr-ERN15641 |  | | | |  |  |  |  |  |
| 1 | Atr-ERN15642 |  | Vvi-Vitvi02g01172\_t001 |  |  |  |  |  |
| 1 | Atr-ERN15643 |  | Vvi-Vitvi02g01164\_t001 |  |  |  |  |  |
| 1 | Atr-ERN15644 |  | | | |  |  |  |  |  |
| 1 | Atr-ERN15645 |  | Vvi-Vitvi02g01163\_t001 |  |  |  |  |  |
| 1 | Atr-ERN15646 |  | | | |  |  |  |  |  |
| 1 | Atr-ERN15647 |  | | | |  |  |  |  |  |
| 1 | Atr-ERN15648 |  | Vvi-Vitvi02g01158\_t001 |  |  |  |  |  |
| 1 | Atr-ERN15649 |  | Vvi-Vitvi02g01154\_t001 |  |  |  |  |  |
| 1 | Atr-ERN15650 |  | | | |  |  |  |  |  |
| 1 | Atr-ERN15651 |  | | | |  |  |  |  |  |
| 1 | Atr-ERN15652 |  | Vvi-Vitvi02g01153\_t001 |  |  |  |  |  |
| 1 | Atr-ERN15653 |  | | | |  |  |  |  |  |
| 1 | Atr-ERN15654 |  | Vvi-Vitvi02g01152\_t001 |  |  |  |  |  |
| 1 | Atr-ERN15655 |  | | | |  |  |  |  |  |
| 1 | Atr-ERN15656 |  | | | |  |  |  |  |  |
| 1 | Atr-ERN15657 |  | Vvi-Vitvi02g01143\_t002 |  |  |  |  |  |
| 1 | Atr-ERN15658 |  | | | |  |  |  |  |  |
| 1 | Atr-ERN15659 |  | | | |  |  |  |  |  |
| 2 | Atr-ERN15660 |  | | | |  | Vvi-Vitvi16g00079\_t001 |  |  |  |  |
| 2 | Atr-ERN15661 |  | | | |  | | | |  |  |  |  |
| 2 | Atr-ERN15662 |  | | | |  | | | |  |  |  |  |
| 2 | Atr-ERN15663 |  | | | |  | | | |  |  |  |  |
| 2 | Atr-ERN15664 |  | | | |  | | | |  |  |  |  |
| 2 | Atr-ERN15665 |  | Vvi-Vitvi02g01139\_t001 |  | | | |  |  |  |  |
| 1 | Atr-ERN15666 |  |  |  | | | |  |  |  |  |
| 1 | Atr-ERN15667 |  |  |  | | | |  |  |  |  |
| 1 | Atr-ERN15668 |  |  |  | | | |  |  |  |  |
| 1 | Atr-ERN15669 |  |  |  | | | |  |  |  |  |
| 1 | Atr-ERN15670 |  |  |  | | | |  |  |  |  |
| 1 | Atr-ERN15671 |  |  |  | | | |  |  |  |  |
| 1 | Atr-ERN15672 |  |  |  | | | |  |  |  |  |
| 1 | Atr-ERN15673 |  |  |  | | | |  |  |  |  |
| 1 | Atr-ERN15674 |  |  |  | | | |  |  |  |  |
| 1 | Atr-ERN15675 |  |  |  | | | |  |  |  |  |
| 1 | Atr-ERN15676 |  |  |  | | | |  |  |  |  |
| 1 | Atr-ERN15677 |  |  |  | Vvi-Vitvi16g00073\_t001 |  |  |  |  |
| 1 | Atr-ERN15678 |  |  |  | | | |  |  |  |  |
| 1 | Atr-ERN15679 |  |  |  | | | |  |  |  |  |
| 1 | Atr-ERN15680 |  |  |  | | | |  |  |  |  |
| 1 | Atr-ERN15681 |  |  |  | Vvi-Vitvi16g04016\_t001 |  |  |  |  |
| 1 | Atr-ERN15682 |  |  |  | | | |  |  |  |  |
| 1 | Atr-ERN15683 |  |  |  | Vvi-Vitvi16g00071\_t004 |  |  |  |  |
| 1 | Atr-ERN15684 |  |  |  | | | |  |  |  |  |
| 1 | Atr-ERN15685 |  |  |  | | | |  |  |  |  |
| 1 | Atr-ERN15686 |  |  |  | Vvi-Vitvi16g01509\_t001 |  |  |  |  |
| 1 | Atr-ERN15687 |  |  |  | | | |  |  |  |  |
| 1 | Atr-ERN15688 |  |  |  | Vvi-Vitvi16g00070\_t001 |  |  |  |  |
| 0 | Atr-ERN15689 |  |  |  |  |  |  |
| 0 | Atr-ERN15690 |  |  |  |  |  |  |
| 0 | Atr-ERN15691 |  |  |  |  |  |  |
| 0 | Atr-ERN15692 |  |  |  |  |  |  |
| 1 | Atr-ERN15693 |  | Vvi-Vitvi02g01191\_t001 |  |  |  |  |  |
| 1 | Atr-ERN15694 |  | | | |  |  |  |  |  |
| 1 | Atr-ERN15695 |  | | | |  |  |  |  |  |
| 1 | Atr-ERN15696 |  | Vvi-Vitvi02g01193\_t001 |  |  |  |  |  |
| 1 | Atr-ERN15697 |  | | | |  |  |  |  |  |
| 1 | Atr-ERN15698 |  | | | |  |  |  |  |  |
| 1 | Atr-ERN15699 |  | Vvi-Vitvi02g01203\_t001 |  |  |  |  |  |
| 1 | Atr-ERN15700 |  | | | |  |  |  |  |  |
| 1 | Atr-ERN15701 |  | | | |  |  |  |  |  |
| 1 | Atr-ERN15702 |  | Vvi-Vitvi02g01205\_t001 |  |  |  |  |  |
| 1 | Atr-ERN15703 |  | | | |  |  |  |  |  |
| 1 | Atr-ERN15704 |  | Vvi-Vitvi02g04382\_t001 |  |  |  |  |  |
| 1 | Atr-ERN15705 |  | | | |  |  |  |  |  |
| 1 | Atr-ERN15706 |  | | | |  |  |  |  |  |
| 1 | Atr-ERN15707 |  | | | |  |  |  |  |  |
| 1 | Atr-ERN15708 |  | | | |  |  |  |  |  |
| 1 | Atr-ERN15709 |  | Vvi-Vitvi02g01214\_t001 |  |  |  |  |  |
| 1 | Atr-ERN15710 |  | | | |  |  |  |  |  |
| 1 | Atr-ERN15711 |  | | | |  |  |  |  |  |
| 1 | Atr-ERN15712 |  | Vvi-Vitvi02g01224\_t001 |  |  |  |  |  |
| 0 | Atr-ERN15713 |  |  |  |  |  |  |
| 0 | Atr-ERN15714 |  |  |  |  |  |  |
