# Supplementary material for: Genome-wide identification, characterization and gene expression of BES1 transcription factor family in grapevine (Vitis vinifera L.)
Source: Sci Rep. 2023 Jan 5;13:240. doi: 10.1038/s41598-022-24407-y (PMC9816167; doi:10.1038/s41598-022-24407-y)
Supplement: Supplementary file 3 — Supplementary Information. [file 41598_2022_24407_MOESM3_ESM.zip › Vvi_Atr/Vitis_vinifera.PN40024.v4.dna_sm.toplevel.fa.vs.Amborella_trichopoda.AMTR1.0.dna_sm.toplevel.fa.html/Atr-AmTr_v1.0_scaffold00049.html]

|  |  |  |  |  |  |  |  |  |  |  |  |  |  |
| --- | --- | --- | --- | --- | --- | --- | --- | --- | --- | --- | --- | --- | --- |
| Duplication depth | Reference chromosome | Collinear blocks | | | | | | | | | | | |
| 0 | Atr-ERN13571 |  |  |  |  |  |  |
| 0 | Atr-ERN13572 |  |  |  |  |  |  |
| 0 | Atr-ERN13573 |  |  |  |  |  |  |
| 0 | Atr-ERN13574 |  |  |  |  |  |  |
| 0 | Atr-ERN13575 |  |  |  |  |  |  |
| 0 | Atr-ERN13576 |  |  |  |  |  |  |
| 0 | Atr-ERN13577 |  |  |  |  |  |  |
| 0 | Atr-ERN13578 |  |  |  |  |  |  |
| 0 | Atr-ERN13579 |  |  |  |  |  |  |
| 0 | Atr-ERN13580 |  |  |  |  |  |  |
| 0 | Atr-ERN13581 |  |  |  |  |  |  |
| 0 | Atr-ERN13582 |  |  |  |  |  |  |
| 0 | Atr-ERN13583 |  |  |  |  |  |  |
| 0 | Atr-ERN13584 |  |  |  |  |  |  |
| 1 | Atr-ERN13585 |  | Vvi-Vitvi14g01624\_t003 |  |  |  |  |  |
| 1 | Atr-ERN13586 |  | | | |  |  |  |  |  |
| 1 | Atr-ERN13587 |  | | | |  |  |  |  |  |
| 1 | Atr-ERN13588 |  | | | |  |  |  |  |  |
| 1 | Atr-ERN13589 |  | | | |  |  |  |  |  |
| 1 | Atr-ERN13590 |  | | | |  |  |  |  |  |
| 1 | Atr-ERN13591 |  | | | |  |  |  |  |  |
| 1 | Atr-ERN13592 |  | | | |  |  |  |  |  |
| 1 | Atr-ERN13593 |  | | | |  |  |  |  |  |
| 1 | Atr-ERN13594 |  | | | |  |  |  |  |  |
| 1 | Atr-ERN13595 |  | | | |  |  |  |  |  |
| 1 | Atr-ERN13596 |  | | | |  |  |  |  |  |
| 1 | Atr-ERN13597 |  | | | |  |  |  |  |  |
| 1 | Atr-ERN13598 |  | | | |  |  |  |  |  |
| 1 | Atr-ERN13599 |  | | | |  |  |  |  |  |
| 1 | Atr-ERN13600 |  | | | |  |  |  |  |  |
| 2 | Atr-ERN13601 |  | | | |  | Vvi-Vitvi01g04377\_t001 |  |  |  |  |
| 2 | Atr-ERN13602 |  | | | |  | | | |  |  |  |  |
| 3 | Atr-ERN13603 |  | | | |  | | | |  | Vvi-Vitvi17g01040\_t001 |  |  |  |
| 3 | Atr-ERN13604 |  | | | |  | | | |  | | | |  |  |  |
| 3 | Atr-ERN13605 |  | | | |  | | | |  | Vvi-Vitvi17g01038\_t002 |  |  |  |
| 3 | Atr-ERN13606 |  | Vvi-Vitvi14g01611\_t001 |  | | | |  | | | |  |  |  |
| 3 | Atr-ERN13607 |  | | | |  | | | |  | Vvi-Vitvi17g01592\_t001 |  |  |  |
| 3 | Atr-ERN13608 |  | | | |  | | | |  | | | |  |  |  |
| 3 | Atr-ERN13609 |  | | | |  | | | |  | | | |  |  |  |
| 3 | Atr-ERN13610 |  | Vvi-Vitvi14g04601\_t001 |  | | | |  | | | |  |  |  |
| 3 | Atr-ERN13611 |  | | | |  | | | |  | | | |  |  |  |
| 3 | Atr-ERN13612 |  | Vvi-Vitvi14g01607\_t002 |  | | | |  | | | |  |  |  |
| 3 | Atr-ERN13613 |  | | | |  | | | |  | | | |  |  |  |
| 3 | Atr-ERN13614 |  | | | |  | | | |  | | | |  |  |  |
| 3 | Atr-ERN13615 |  | | | |  | | | |  | | | |  |  |  |
| 3 | Atr-ERN13616 |  | | | |  | | | |  | | | |  |  |  |
| 3 | Atr-ERN13617 |  | | | |  | | | |  | | | |  |  |  |
| 3 | Atr-ERN13618 |  | | | |  | | | |  | | | |  |  |  |
| 3 | Atr-ERN13619 |  | | | |  | | | |  | Vvi-Vitvi17g01037\_t002 |  |  |  |
| 3 | Atr-ERN13620 |  | | | |  | | | |  | | | |  |  |  |
| 3 | Atr-ERN13621 |  | | | |  | Vvi-Vitvi01g01506\_t001 |  | | | |  |  |  |
| 3 | Atr-ERN13622 |  | | | |  | Vvi-Vitvi01g01507\_t001 |  | | | |  |  |  |
| 3 | Atr-ERN13623 |  | | | |  | | | |  | | | |  |  |  |
| 3 | Atr-ERN13624 |  | | | |  | | | |  | Vvi-Vitvi17g01021\_t001 |  |  |  |
| 3 | Atr-ERN13625 |  | | | |  | | | |  | | | |  |  |  |
| 3 | Atr-ERN13626 |  | | | |  | | | |  | | | |  |  |  |
| 3 | Atr-ERN13627 |  | | | |  | | | |  | | | |  |  |  |
| 3 | Atr-ERN13628 |  | | | |  | | | |  | | | |  |  |  |
| 3 | Atr-ERN13629 |  | | | |  | | | |  | Vvi-Vitvi17g01013\_t002 |  |  |  |
| 2 | Atr-ERN13630 |  | | | |  | | | |  |  |  |  |
| 2 | Atr-ERN13631 |  | | | |  | Vvi-Vitvi01g01512\_t001 |  |  |  |  |
| 2 | Atr-ERN13632 |  | | | |  | | | |  |  |  |  |
| 2 | Atr-ERN13633 |  | | | |  | | | |  |  |  |  |
| 2 | Atr-ERN13634 |  | | | |  | | | |  |  |  |  |
| 2 | Atr-ERN13635 |  | | | |  | | | |  |  |  |  |
| 2 | Atr-ERN13636 |  | | | |  | | | |  |  |  |  |
| 2 | Atr-ERN13637 |  | | | |  | | | |  |  |  |  |
| 2 | Atr-ERN13638 |  | Vvi-Vitvi14g01605\_t005 |  | | | |  |  |  |  |
| 2 | Atr-ERN13639 |  | | | |  | | | |  |  |  |  |
| 2 | Atr-ERN13640 |  | Vvi-Vitvi14g01604\_t001 |  | Vvi-Vitvi01g01529\_t001 |  |  |  |  |
| 2 | Atr-ERN13641 |  | | | |  | | | |  |  |  |  |
| 2 | Atr-ERN13642 |  | | | |  | | | |  |  |  |  |
| 2 | Atr-ERN13643 |  | | | |  | | | |  |  |  |  |
| 2 | Atr-ERN13644 |  | | | |  | | | |  |  |  |  |
| 2 | Atr-ERN13645 |  | | | |  | | | |  |  |  |  |
| 2 | Atr-ERN13646 |  | | | |  | | | |  |  |  |  |
| 2 | Atr-ERN13647 |  | | | |  | | | |  |  |  |  |
| 2 | Atr-ERN13648 |  | | | |  | | | |  |  |  |  |
| 2 | Atr-ERN13649 |  | | | |  | | | |  |  |  |  |
| 2 | Atr-ERN13650 |  | | | |  | Vvi-Vitvi01g01543\_t001 |  |  |  |  |
| 2 | Atr-ERN13651 |  | | | |  | | | |  |  |  |  |
| 2 | Atr-ERN13652 |  | Vvi-Vitvi14g01591\_t001 |  | | | |  |  |  |  |
| 1 | Atr-ERN13653 |  |  |  | Vvi-Vitvi01g01547\_t001 |  |  |  |  |
| 1 | Atr-ERN13654 |  |  |  | Vvi-Vitvi01g01548\_t001 |  |  |  |  |
| 1 | Atr-ERN13655 |  |  |  | | | |  |  |  |  |
| 1 | Atr-ERN13656 |  |  |  | | | |  |  |  |  |
| 1 | Atr-ERN13657 |  |  |  | Vvi-Vitvi01g01549\_t001 |  |  |  |  |
| 1 | Atr-ERN13658 |  |  |  | Vvi-Vitvi01g01550\_t001 |  |  |  |  |
| 0 | Atr-ERN13659 |  |  |  |  |  |  |
| 0 | Atr-ERN13660 |  |  |  |  |  |  |
| 0 | Atr-ERN13661 |  |  |  |  |  |  |
| 0 | Atr-ERN13662 |  |  |  |  |  |  |
| 0 | Atr-ERN13663 |  |  |  |  |  |  |
| 0 | Atr-ERN13664 |  |  |  |  |  |  |
| 0 | Atr-ERN13665 |  |  |  |  |  |  |
| 0 | Atr-ERN13666 |  |  |  |  |  |  |
| 0 | Atr-ERN13667 |  |  |  |  |  |  |
| 0 | Atr-ERN13668 |  |  |  |  |  |  |
| 0 | Atr-ERN13669 |  |  |  |  |  |  |
| 0 | Atr-ERN13670 |  |  |  |  |  |  |
| 2 | Atr-ERN13671 |  | Vvi-Vitvi01g00608\_t001 |  | Vvi-Vitvi17g00968\_t001 |  |  |  |  |
| 3 | Atr-ERN13672 |  | | | |  | Vvi-Vitvi17g00969\_t001 |  | Vvi-Vitvi14g01640\_t001 |  |  |  |
| 3 | Atr-ERN13673 |  | | | |  | | | |  | | | |  |  |  |
| 3 | Atr-ERN13674 |  | Vvi-Vitvi01g04143\_t001 |  | | | |  | | | |  |  |  |
| 3 | Atr-ERN13675 |  | | | |  | | | |  | | | |  |  |  |
| 3 | Atr-ERN13676 |  | | | |  | | | |  | | | |  |  |  |
| 3 | Atr-ERN13677 |  | Vvi-Vitvi01g00604\_t001 |  | | | |  | | | |  |  |  |
| 3 | Atr-ERN13678 |  | | | |  | | | |  | | | |  |  |  |
| 3 | Atr-ERN13679 |  | | | |  | | | |  | | | |  |  |  |
| 3 | Atr-ERN13680 |  | | | |  | | | |  | | | |  |  |  |
| 3 | Atr-ERN13681 |  | | | |  | | | |  | Vvi-Vitvi14g01639\_t001 |  |  |  |
| 3 | Atr-ERN13682 |  | | | |  | | | |  | | | |  |  |  |
| 3 | Atr-ERN13683 |  | Vvi-Vitvi01g00603\_t001 |  | Vvi-Vitvi17g00973\_t001 |  | | | |  |  |  |
| 3 | Atr-ERN13684 |  | | | |  | | | |  | | | |  |  |  |
| 3 | Atr-ERN13685 |  | Vvi-Vitvi01g00602\_t001 |  | | | |  | Vvi-Vitvi14g01637\_t001 |  |  |  |
| 3 | Atr-ERN13686 |  | | | |  | | | |  | | | |  |  |  |
| 3 | Atr-ERN13687 |  | Vvi-Vitvi01g00599\_t001 |  | | | |  | | | |  |  |  |
| 3 | Atr-ERN13688 |  | | | |  | | | |  | | | |  |  |  |
| 3 | Atr-ERN13689 |  | | | |  | | | |  | | | |  |  |  |
| 3 | Atr-ERN13690 |  | | | |  | Vvi-Vitvi17g00975\_t001 |  | | | |  |  |  |
| 3 | Atr-ERN13691 |  | | | |  | | | |  | | | |  |  |  |
| 3 | Atr-ERN13692 |  | Vvi-Vitvi01g00598\_t001 |  | | | |  | | | |  |  |  |
| 3 | Atr-ERN13693 |  | | | |  | | | |  | | | |  |  |  |
| 3 | Atr-ERN13694 |  | | | |  | | | |  | | | |  |  |  |
| 3 | Atr-ERN13695 |  | Vvi-Vitvi01g00596\_t001 |  | | | |  | | | |  |  |  |
| 3 | Atr-ERN13696 |  | | | |  | | | |  | Vvi-Vitvi14g01636\_t001 |  |  |  |
| 3 | Atr-ERN13697 |  | Vvi-Vitvi01g00595\_t001 |  | | | |  | | | |  |  |  |
| 4 | Atr-ERN13698 |  | | | |  | | | |  | | | |  | Vvi-Vitvi01g02021\_t001 |  |  |
| 4 | Atr-ERN13699 |  | | | |  | | | |  | | | |  | | | |  |  |
| 4 | Atr-ERN13700 |  | | | |  | | | |  | Vvi-Vitvi14g01633\_t001 |  | | | |  |  |
| 4 | Atr-ERN13701 |  | | | |  | | | |  | Vvi-Vitvi14g01632\_t001 |  | | | |  |  |
| 3 | Atr-ERN13702 |  | | | |  | | | |  |  |  | Vvi-Vitvi01g00588\_t001 |  |  |
| 3 | Atr-ERN13703 |  | | | |  | | | |  |  |  | | | |  |  |
| 3 | Atr-ERN13704 |  | | | |  | | | |  |  |  | | | |  |  |
| 3 | Atr-ERN13705 |  | | | |  | | | |  |  |  | | | |  |  |
| 3 | Atr-ERN13706 |  | | | |  | | | |  |  |  | | | |  |  |
| 3 | Atr-ERN13707 |  | | | |  | | | |  |  |  | | | |  |  |
| 3 | Atr-ERN13708 |  | | | |  | | | |  |  |  | | | |  |  |
| 3 | Atr-ERN13709 |  | | | |  | | | |  |  |  | | | |  |  |
| 3 | Atr-ERN13710 |  | | | |  | | | |  |  |  | | | |  |  |
| 3 | Atr-ERN13711 |  | | | |  | | | |  |  |  | | | |  |  |
| 3 | Atr-ERN13712 |  | | | |  | | | |  |  |  | Vvi-Vitvi01g00589\_t001 |  |  |
| 3 | Atr-ERN13713 |  | | | |  | Vvi-Vitvi17g00989\_t001 |  |  |  | | | |  |  |
| 3 | Atr-ERN13714 |  | | | |  | | | |  |  |  | | | |  |  |
| 3 | Atr-ERN13715 |  | | | |  | | | |  |  |  | | | |  |  |
| 3 | Atr-ERN13716 |  | | | |  | | | |  |  |  | | | |  |  |
| 3 | Atr-ERN13717 |  | | | |  | | | |  |  |  | | | |  |  |
| 3 | Atr-ERN13718 |  | | | |  | | | |  |  |  | | | |  |  |
| 3 | Atr-ERN13719 |  | Vvi-Vitvi01g00590\_t001 |  | | | |  |  |  | Vvi-Vitvi01g00590\_t001 |  |  |
| 3 | Atr-ERN13720 |  | | | |  | | | |  |  |  | | | |  |  |
| 3 | Atr-ERN13721 |  | | | |  | | | |  |  |  | | | |  |  |
| 3 | Atr-ERN13722 |  | | | |  | | | |  |  |  | | | |  |  |
| 3 | Atr-ERN13723 |  | | | |  | | | |  |  |  | | | |  |  |
| 3 | Atr-ERN13724 |  | | | |  | | | |  |  |  | Vvi-Vitvi01g00591\_t007 |  |  |
| 3 | Atr-ERN13725 |  | | | |  | | | |  |  |  | | | |  |  |
| 3 | Atr-ERN13726 |  | | | |  | | | |  |  |  | | | |  |  |
| 3 | Atr-ERN13727 |  | | | |  | | | |  |  |  | | | |  |  |
| 3 | Atr-ERN13728 |  | | | |  | | | |  |  |  | | | |  |  |
| 3 | Atr-ERN13729 |  | | | |  | | | |  |  |  | | | |  |  |
| 3 | Atr-ERN13730 |  | | | |  | | | |  |  |  | | | |  |  |
| 3 | Atr-ERN13731 |  | | | |  | | | |  |  |  | Vvi-Vitvi01g00593\_t001 |  |  |
| 3 | Atr-ERN13732 |  | | | |  | | | |  |  |  | Vvi-Vitvi01g00594\_t001 |  |  |
| 2 | Atr-ERN13733 |  | | | |  | | | |  |  |  |  |
| 2 | Atr-ERN13734 |  | Vvi-Vitvi01g00580\_t001 |  | | | |  |  |  |  |
| 1 | Atr-ERN13735 |  |  |  | | | |  |  |  |  |
| 1 | Atr-ERN13736 |  |  |  | | | |  |  |  |  |
| 1 | Atr-ERN13737 |  |  |  | | | |  |  |  |  |
| 1 | Atr-ERN13738 |  |  |  | Vvi-Vitvi17g01585\_t001 |  |  |  |  |
| 1 | Atr-ERN13739 |  |  |  | | | |  |  |  |  |
| 1 | Atr-ERN13740 |  |  |  | | | |  |  |  |  |
| 1 | Atr-ERN13741 |  |  |  | | | |  |  |  |  |
| 1 | Atr-ERN13742 |  |  |  | | | |  |  |  |  |
| 1 | Atr-ERN13743 |  |  |  | | | |  |  |  |  |
| 1 | Atr-ERN13744 |  |  |  | | | |  |  |  |  |
| 1 | Atr-ERN13745 |  |  |  | Vvi-Vitvi17g01001\_t001 |  |  |  |  |
| 0 | Atr-ERN13746 |  |  |  |  |  |  |
| 0 | Atr-ERN13747 |  |  |  |  |  |  |
| 0 | Atr-ERN13748 |  |  |  |  |  |  |
| 0 | Atr-ERN13749 |  |  |  |  |  |  |
| 0 | Atr-ERN13750 |  |  |  |  |  |  |
| 0 | Atr-ERN13751 |  |  |  |  |  |  |
| 0 | Atr-ERN13752 |  |  |  |  |  |  |
| 0 | Atr-ERN13753 |  |  |  |  |  |  |
| 0 | Atr-ERN13754 |  |  |  |  |  |  |
| 0 | Atr-ERN13755 |  |  |  |  |  |  |
| 0 | Atr-ERN13756 |  |  |  |  |  |  |
| 0 | Atr-ERN13757 |  |  |  |  |  |  |
| 0 | Atr-ERN13758 |  |  |  |  |  |  |
| 0 | Atr-ERN13759 |  |  |  |  |  |  |
| 0 | Atr-ERN13760 |  |  |  |  |  |  |
| 0 | Atr-ERN13761 |  |  |  |  |  |  |
| 1 | Atr-ERN13762 |  | Vvi-Vitvi01g00654\_t001 |  |  |  |  |  |
| 1 | Atr-ERN13763 |  | | | |  |  |  |  |  |
| 1 | Atr-ERN13764 |  | | | |  |  |  |  |  |
| 1 | Atr-ERN13765 |  | | | |  |  |  |  |  |
| 1 | Atr-ERN13766 |  | | | |  |  |  |  |  |
| 1 | Atr-ERN13767 |  | | | |  |  |  |  |  |
| 1 | Atr-ERN13768 |  | | | |  |  |  |  |  |
| 1 | Atr-ERN13769 |  | | | |  |  |  |  |  |
| 1 | Atr-ERN13770 |  | | | |  |  |  |  |  |
| 1 | Atr-ERN13771 |  | | | |  |  |  |  |  |
| 2 | Atr-ERN13772 |  | | | |  | Vvi-Vitvi14g01695\_t001 |  |  |  |  |
| 2 | Atr-ERN13773 |  | | | |  | | | |  |  |  |  |
| 2 | Atr-ERN13774 |  | | | |  | | | |  |  |  |  |
| 2 | Atr-ERN13775 |  | | | |  | | | |  |  |  |  |
| 2 | Atr-ERN13776 |  | | | |  | | | |  |  |  |  |
| 2 | Atr-ERN13777 |  | | | |  | | | |  |  |  |  |
| 3 | Atr-ERN13778 |  | | | |  | | | |  | Vvi-Vitvi17g00903\_t001 |  |  |  |
| 3 | Atr-ERN13779 |  | | | |  | | | |  | | | |  |  |  |
| 3 | Atr-ERN13780 |  | | | |  | | | |  | | | |  |  |  |
| 3 | Atr-ERN13781 |  | | | |  | | | |  | | | |  |  |  |
| 3 | Atr-ERN13782 |  | | | |  | Vvi-Vitvi14g01694\_t001 |  | | | |  |  |  |
| 3 | Atr-ERN13783 |  | | | |  | | | |  | | | |  |  |  |
| 3 | Atr-ERN13784 |  | | | |  | | | |  | | | |  |  |  |
| 3 | Atr-ERN13785 |  | | | |  | | | |  | | | |  |  |  |
| 3 | Atr-ERN13786 |  | Vvi-Vitvi01g00649\_t001 |  | | | |  | | | |  |  |  |
| 3 | Atr-ERN13787 |  | Vvi-Vitvi01g00648\_t001 |  | | | |  | | | |  |  |  |
| 3 | Atr-ERN13788 |  | | | |  | | | |  | | | |  |  |  |
| 3 | Atr-ERN13789 |  | | | |  | Vvi-Vitvi14g01690\_t001 |  | | | |  |  |  |
| 3 | Atr-ERN13790 |  | | | |  | | | |  | | | |  |  |  |
| 3 | Atr-ERN13791 |  | Vvi-Vitvi01g00646\_t002 |  | | | |  | | | |  |  |  |
| 3 | Atr-ERN13792 |  | | | |  | | | |  | Vvi-Vitvi17g00905\_t001 |  |  |  |
| 3 | Atr-ERN13793 |  | | | |  | | | |  | | | |  |  |  |
| 3 | Atr-ERN13794 |  | | | |  | | | |  | | | |  |  |  |
| 3 | Atr-ERN13795 |  | | | |  | | | |  | | | |  |  |  |
| 3 | Atr-ERN13796 |  | | | |  | | | |  | | | |  |  |  |
| 3 | Atr-ERN13797 |  | | | |  | | | |  | | | |  |  |  |
| 3 | Atr-ERN13798 |  | | | |  | | | |  | Vvi-Vitvi17g00907\_t001 |  |  |  |
| 3 | Atr-ERN13799 |  | | | |  | | | |  | Vvi-Vitvi17g00909\_t001 |  |  |  |
| 3 | Atr-ERN13800 |  | | | |  | | | |  | | | |  |  |  |
| 3 | Atr-ERN13801 |  | | | |  | | | |  | Vvi-Vitvi17g00910\_t001 |  |  |  |
| 3 | Atr-ERN13802 |  | | | |  | | | |  | Vvi-Vitvi17g00911\_t001 |  |  |  |
| 3 | Atr-ERN13803 |  | | | |  | | | |  | | | |  |  |  |
| 3 | Atr-ERN13804 |  | | | |  | Vvi-Vitvi14g01685\_t001 |  | | | |  |  |  |
| 3 | Atr-ERN13805 |  | | | |  | | | |  | | | |  |  |  |
| 3 | Atr-ERN13806 |  | | | |  | | | |  | | | |  |  |  |
| 3 | Atr-ERN13807 |  | | | |  | | | |  | | | |  |  |  |
| 3 | Atr-ERN13808 |  | | | |  | | | |  | Vvi-Vitvi17g00916\_t003 |  |  |  |
| 3 | Atr-ERN13809 |  | | | |  | | | |  | | | |  |  |  |
| 3 | Atr-ERN13810 |  | | | |  | Vvi-Vitvi14g01684\_t001.2.6037826b |  | | | |  |  |  |
| 3 | Atr-ERN13811 |  | | | |  | Vvi-Vitvi14g01683\_t001 |  | | | |  |  |  |
| 3 | Atr-ERN13812 |  | | | |  | | | |  | | | |  |  |  |
| 3 | Atr-ERN13813 |  | | | |  | | | |  | | | |  |  |  |
| 3 | Atr-ERN13814 |  | | | |  | Vvi-Vitvi14g01682\_t001 |  | | | |  |  |  |
| 3 | Atr-ERN13815 |  | | | |  | | | |  | | | |  |  |  |
| 3 | Atr-ERN13816 |  | | | |  | Vvi-Vitvi14g01679\_t001 |  | Vvi-Vitvi17g00917\_t001 |  |  |  |
| 3 | Atr-ERN13817 |  | Vvi-Vitvi01g00645\_t001 |  | | | |  | | | |  |  |  |
| 3 | Atr-ERN13818 |  | Vvi-Vitvi01g04155\_t001 |  | | | |  | | | |  |  |  |
| 3 | Atr-ERN13819 |  | Vvi-Vitvi01g00644\_t001 |  | | | |  | | | |  |  |  |
| 3 | Atr-ERN13820 |  | | | |  | | | |  | | | |  |  |  |
| 3 | Atr-ERN13821 |  | | | |  | Vvi-Vitvi14g01678\_t001 |  | | | |  |  |  |
| 3 | Atr-ERN13822 |  | | | |  | | | |  | | | |  |  |  |
| 3 | Atr-ERN13823 |  | | | |  | Vvi-Vitvi14g01677\_t001 |  | | | |  |  |  |
| 3 | Atr-ERN13824 |  | Vvi-Vitvi01g00642\_t001 |  | Vvi-Vitvi14g01676\_t002 |  | | | |  |  |  |
| 3 | Atr-ERN13825 |  | | | |  | | | |  | | | |  |  |  |
| 3 | Atr-ERN13826 |  | | | |  | | | |  | | | |  |  |  |
| 3 | Atr-ERN13827 |  | | | |  | | | |  | | | |  |  |  |
| 3 | Atr-ERN13828 |  | | | |  | | | |  | | | |  |  |  |
| 3 | Atr-ERN13829 |  | | | |  | | | |  | | | |  |  |  |
| 3 | Atr-ERN13830 |  | | | |  | | | |  | | | |  |  |  |
| 3 | Atr-ERN13831 |  | | | |  | | | |  | | | |  |  |  |
| 3 | Atr-ERN13832 |  | | | |  | | | |  | Vvi-Vitvi17g04259\_t001 |  |  |  |
| 3 | Atr-ERN13833 |  | | | |  | | | |  | Vvi-Vitvi17g00920\_t001 |  |  |  |
| 3 | Atr-ERN13834 |  | | | |  | | | |  | | | |  |  |  |
| 3 | Atr-ERN13835 |  | Vvi-Vitvi01g02030\_t001 |  | | | |  | | | |  |  |  |
| 3 | Atr-ERN13836 |  | | | |  | | | |  | Vvi-Vitvi17g00921\_t001 |  |  |  |
| 3 | Atr-ERN13837 |  | | | |  | | | |  | | | |  |  |  |
| 3 | Atr-ERN13838 |  | | | |  | | | |  | Vvi-Vitvi17g00927\_t001 |  |  |  |
| 3 | Atr-ERN13839 |  | | | |  | Vvi-Vitvi14g01674\_t001 |  | | | |  |  |  |
| 3 | Atr-ERN13840 |  | | | |  | Vvi-Vitvi14g01673\_t001 |  | | | |  |  |  |
| 3 | Atr-ERN13841 |  | Vvi-Vitvi01g00641\_t001 |  | Vvi-Vitvi14g01672\_t001 |  | | | |  |  |  |
| 3 | Atr-ERN13842 |  | Vvi-Vitvi01g00640\_t001 |  | | | |  | | | |  |  |  |
| 3 | Atr-ERN13843 |  | | | |  | | | |  | | | |  |  |  |
| 3 | Atr-ERN13844 |  | | | |  | | | |  | | | |  |  |  |
| 3 | Atr-ERN13845 |  | | | |  | | | |  | Vvi-Vitvi17g00928\_t001 |  |  |  |
| 3 | Atr-ERN13846 |  | | | |  | | | |  | Vvi-Vitvi17g00929\_t002 |  |  |  |
| 3 | Atr-ERN13847 |  | Vvi-Vitvi01g00634\_t001 |  | Vvi-Vitvi14g04610\_t001 |  | | | |  |  |  |
| 2 | Atr-ERN13848 |  | | | |  |  |  | Vvi-Vitvi17g00932\_t001 |  |  |  |
| 2 | Atr-ERN13849 |  | | | |  |  |  | | | |  |  |  |
| 2 | Atr-ERN13850 |  | | | |  |  |  | | | |  |  |  |
| 2 | Atr-ERN13851 |  | | | |  |  |  | | | |  |  |  |
| 2 | Atr-ERN13852 |  | | | |  |  |  | | | |  |  |  |
| 2 | Atr-ERN13853 |  | | | |  |  |  | | | |  |  |  |
| 2 | Atr-ERN13854 |  | | | |  |  |  | | | |  |  |  |
| 2 | Atr-ERN13855 |  | | | |  |  |  | | | |  |  |  |
| 2 | Atr-ERN13856 |  | Vvi-Vitvi01g00629\_t001 |  |  |  | | | |  |  |  |
| 1 | Atr-ERN13857 |  |  |  |  |  | | | |  |  |  |
| 1 | Atr-ERN13858 |  |  |  |  |  | Vvi-Vitvi17g00941\_t001 |  |  |  |
