# Supplementary material for: Genome-wide identification, characterization and gene expression of BES1 transcription factor family in grapevine (Vitis vinifera L.)
Source: Sci Rep. 2023 Jan 5;13:240. doi: 10.1038/s41598-022-24407-y (PMC9816167; doi:10.1038/s41598-022-24407-y)
Supplement: Supplementary file 3 — Supplementary Information. [file 41598_2022_24407_MOESM3_ESM.zip › Vvi_Atr/Vitis_vinifera.PN40024.v4.dna_sm.toplevel.fa.vs.Amborella_trichopoda.AMTR1.0.dna_sm.toplevel.fa.html/Atr-AmTr_v1.0_scaffold00050.html]

|  |  |  |  |  |  |  |  |  |  |  |  |  |  |
| --- | --- | --- | --- | --- | --- | --- | --- | --- | --- | --- | --- | --- | --- |
| Duplication depth | Reference chromosome | Collinear blocks | | | | | | | | | | | |
| 0 | Atr-ERN12853 |  |  |  |  |  |  |
| 0 | Atr-ERN12854 |  |  |  |  |  |  |
| 0 | Atr-ERN12855 |  |  |  |  |  |  |
| 0 | Atr-ERN12856 |  |  |  |  |  |  |
| 0 | Atr-ERN12857 |  |  |  |  |  |  |
| 0 | Atr-ERN12858 |  |  |  |  |  |  |
| 0 | Atr-ERN12859 |  |  |  |  |  |  |
| 0 | Atr-ERN12860 |  |  |  |  |  |  |
| 0 | Atr-ERN12861 |  |  |  |  |  |  |
| 0 | Atr-ERN12862 |  |  |  |  |  |  |
| 0 | Atr-ERN12863 |  |  |  |  |  |  |
| 0 | Atr-ERN12864 |  |  |  |  |  |  |
| 0 | Atr-ERN12865 |  |  |  |  |  |  |
| 0 | Atr-ERN12866 |  |  |  |  |  |  |
| 0 | Atr-ERN12867 |  |  |  |  |  |  |
| 0 | Atr-ERN12868 |  |  |  |  |  |  |
| 0 | Atr-ERN12869 |  |  |  |  |  |  |
| 0 | Atr-ERN12870 |  |  |  |  |  |  |
| 0 | Atr-ERN12871 |  |  |  |  |  |  |
| 0 | Atr-ERN12872 |  |  |  |  |  |  |
| 0 | Atr-ERN12873 |  |  |  |  |  |  |
| 0 | Atr-ERN12874 |  |  |  |  |  |  |
| 0 | Atr-ERN12875 |  |  |  |  |  |  |
| 0 | Atr-ERN12876 |  |  |  |  |  |  |
| 0 | Atr-ERN12877 |  |  |  |  |  |  |
| 0 | Atr-ERN12878 |  |  |  |  |  |  |
| 0 | Atr-ERN12879 |  |  |  |  |  |  |
| 0 | Atr-ERN12880 |  |  |  |  |  |  |
| 0 | Atr-ERN12881 |  |  |  |  |  |  |
| 0 | Atr-ERN12882 |  |  |  |  |  |  |
| 0 | Atr-ERN12883 |  |  |  |  |  |  |
| 0 | Atr-ERN12884 |  |  |  |  |  |  |
| 0 | Atr-ERN12885 |  |  |  |  |  |  |
| 0 | Atr-ERN12886 |  |  |  |  |  |  |
| 0 | Atr-ERN12887 |  |  |  |  |  |  |
| 0 | Atr-ERN12888 |  |  |  |  |  |  |
| 0 | Atr-ERN12889 |  |  |  |  |  |  |
| 0 | Atr-ERN12890 |  |  |  |  |  |  |
| 0 | Atr-ERN12891 |  |  |  |  |  |  |
| 0 | Atr-ERN12892 |  |  |  |  |  |  |
| 0 | Atr-ERN12893 |  |  |  |  |  |  |
| 0 | Atr-ERN12894 |  |  |  |  |  |  |
| 0 | Atr-ERN12895 |  |  |  |  |  |  |
| 0 | Atr-ERN12896 |  |  |  |  |  |  |
| 0 | Atr-ERN12897 |  |  |  |  |  |  |
| 0 | Atr-ERN12898 |  |  |  |  |  |  |
| 0 | Atr-ERN12899 |  |  |  |  |  |  |
| 0 | Atr-ERN12900 |  |  |  |  |  |  |
| 0 | Atr-ERN12901 |  |  |  |  |  |  |
| 0 | Atr-ERN12902 |  |  |  |  |  |  |
| 0 | Atr-ERN12903 |  |  |  |  |  |  |
| 0 | Atr-ERN12904 |  |  |  |  |  |  |
| 0 | Atr-ERN12905 |  |  |  |  |  |  |
| 0 | Atr-ERN12906 |  |  |  |  |  |  |
| 0 | Atr-ERN12907 |  |  |  |  |  |  |
| 0 | Atr-ERN12908 |  |  |  |  |  |  |
| 0 | Atr-ERN12909 |  |  |  |  |  |  |
| 0 | Atr-ERN12910 |  |  |  |  |  |  |
| 0 | Atr-ERN12911 |  |  |  |  |  |  |
| 0 | Atr-ERN12912 |  |  |  |  |  |  |
| 0 | Atr-ERN12913 |  |  |  |  |  |  |
| 0 | Atr-ERN12914 |  |  |  |  |  |  |
| 0 | Atr-ERN12915 |  |  |  |  |  |  |
| 0 | Atr-ERN12916 |  |  |  |  |  |  |
| 0 | Atr-ERN12917 |  |  |  |  |  |  |
| 0 | Atr-ERN12918 |  |  |  |  |  |  |
| 0 | Atr-ERN12919 |  |  |  |  |  |  |
| 0 | Atr-ERN12920 |  |  |  |  |  |  |
| 0 | Atr-ERN12921 |  |  |  |  |  |  |
| 0 | Atr-ERN12922 |  |  |  |  |  |  |
| 0 | Atr-ERN12923 |  |  |  |  |  |  |
| 0 | Atr-ERN12924 |  |  |  |  |  |  |
| 0 | Atr-ERN12925 |  |  |  |  |  |  |
| 0 | Atr-ERN12926 |  |  |  |  |  |  |
| 0 | Atr-ERN12927 |  |  |  |  |  |  |
| 0 | Atr-ERN12928 |  |  |  |  |  |  |
| 0 | Atr-ERN12929 |  |  |  |  |  |  |
| 0 | Atr-ERN12930 |  |  |  |  |  |  |
| 0 | Atr-ERN12931 |  |  |  |  |  |  |
| 0 | Atr-ERN12932 |  |  |  |  |  |  |
| 0 | Atr-ERN12933 |  |  |  |  |  |  |
| 0 | Atr-ERN12934 |  |  |  |  |  |  |
| 0 | Atr-ERN12935 |  |  |  |  |  |  |
| 0 | Atr-ERN12936 |  |  |  |  |  |  |
| 0 | Atr-ERN12937 |  |  |  |  |  |  |
| 0 | Atr-ERN12938 |  |  |  |  |  |  |
| 0 | Atr-ERN12939 |  |  |  |  |  |  |
| 0 | Atr-ERN12940 |  |  |  |  |  |  |
| 0 | Atr-ERN12941 |  |  |  |  |  |  |
| 0 | Atr-ERN12942 |  |  |  |  |  |  |
| 0 | Atr-ERN12943 |  |  |  |  |  |  |
| 0 | Atr-ERN12944 |  |  |  |  |  |  |
| 0 | Atr-ERN12945 |  |  |  |  |  |  |
| 0 | Atr-ERN12946 |  |  |  |  |  |  |
| 0 | Atr-ERN12947 |  |  |  |  |  |  |
| 0 | Atr-ERN12948 |  |  |  |  |  |  |
| 0 | Atr-ERN12949 |  |  |  |  |  |  |
| 0 | Atr-ERN12950 |  |  |  |  |  |  |
| 0 | Atr-ERN12951 |  |  |  |  |  |  |
| 0 | Atr-ERN12952 |  |  |  |  |  |  |
| 0 | Atr-ERN12953 |  |  |  |  |  |  |
| 0 | Atr-ERN12954 |  |  |  |  |  |  |
| 0 | Atr-ERN12955 |  |  |  |  |  |  |
| 0 | Atr-ERN12956 |  |  |  |  |  |  |
