# Supplementary material for: Genome-wide identification, characterization and gene expression of BES1 transcription factor family in grapevine (Vitis vinifera L.)
Source: Sci Rep. 2023 Jan 5;13:240. doi: 10.1038/s41598-022-24407-y (PMC9816167; doi:10.1038/s41598-022-24407-y)
Supplement: Supplementary file 3 — Supplementary Information. [file 41598_2022_24407_MOESM3_ESM.zip › Vvi_Atr/Vitis_vinifera.PN40024.v4.dna_sm.toplevel.fa.vs.Amborella_trichopoda.AMTR1.0.dna_sm.toplevel.fa.html/Atr-AmTr_v1.0_scaffold00051.html]

|  |  |  |  |  |  |  |  |  |  |  |  |  |  |
| --- | --- | --- | --- | --- | --- | --- | --- | --- | --- | --- | --- | --- | --- |
| Duplication depth | Reference chromosome | Collinear blocks | | | | | | | | | | | |
| 0 | Atr-ERN16602 |  |  |  |  |  |  |
| 0 | Atr-ERN16603 |  |  |  |  |  |  |
| 0 | Atr-ERN16604 |  |  |  |  |  |  |
| 0 | Atr-ERN16605 |  |  |  |  |  |  |
| 0 | Atr-ERN16606 |  |  |  |  |  |  |
| 0 | Atr-ERN16607 |  |  |  |  |  |  |
| 0 | Atr-ERN16608 |  |  |  |  |  |  |
| 0 | Atr-ERN16609 |  |  |  |  |  |  |
| 0 | Atr-ERN16610 |  |  |  |  |  |  |
| 0 | Atr-ERN16611 |  |  |  |  |  |  |
| 0 | Atr-ERN16612 |  |  |  |  |  |  |
| 0 | Atr-ERN16613 |  |  |  |  |  |  |
| 0 | Atr-ERN16614 |  |  |  |  |  |  |
| 0 | Atr-ERN16615 |  |  |  |  |  |  |
| 0 | Atr-ERN16616 |  |  |  |  |  |  |
| 0 | Atr-ERN16617 |  |  |  |  |  |  |
| 0 | Atr-ERN16618 |  |  |  |  |  |  |
| 0 | Atr-ERN16619 |  |  |  |  |  |  |
| 0 | Atr-ERN16620 |  |  |  |  |  |  |
| 0 | Atr-ERN16621 |  |  |  |  |  |  |
| 0 | Atr-ERN16622 |  |  |  |  |  |  |
| 0 | Atr-ERN16623 |  |  |  |  |  |  |
| 0 | Atr-ERN16624 |  |  |  |  |  |  |
| 0 | Atr-ERN16625 |  |  |  |  |  |  |
| 0 | Atr-ERN16626 |  |  |  |  |  |  |
| 0 | Atr-ERN16627 |  |  |  |  |  |  |
| 0 | Atr-ERN16628 |  |  |  |  |  |  |
| 0 | Atr-ERN16629 |  |  |  |  |  |  |
| 0 | Atr-ERN16630 |  |  |  |  |  |  |
| 0 | Atr-ERN16631 |  |  |  |  |  |  |
| 0 | Atr-ERN16632 |  |  |  |  |  |  |
| 0 | Atr-ERN16633 |  |  |  |  |  |  |
| 0 | Atr-ERN16634 |  |  |  |  |  |  |
| 0 | Atr-ERN16635 |  |  |  |  |  |  |
| 0 | Atr-ERN16636 |  |  |  |  |  |  |
| 0 | Atr-ERN16637 |  |  |  |  |  |  |
| 0 | Atr-ERN16638 |  |  |  |  |  |  |
| 0 | Atr-ERN16639 |  |  |  |  |  |  |
| 0 | Atr-ERN16640 |  |  |  |  |  |  |
| 0 | Atr-ERN16641 |  |  |  |  |  |  |
| 0 | Atr-ERN16642 |  |  |  |  |  |  |
| 0 | Atr-ERN16643 |  |  |  |  |  |  |
| 0 | Atr-ERN16644 |  |  |  |  |  |  |
| 0 | Atr-ERN16645 |  |  |  |  |  |  |
| 0 | Atr-ERN16646 |  |  |  |  |  |  |
| 0 | Atr-ERN16647 |  |  |  |  |  |  |
| 0 | Atr-ERN16648 |  |  |  |  |  |  |
| 0 | Atr-ERN16649 |  |  |  |  |  |  |
| 0 | Atr-ERN16650 |  |  |  |  |  |  |
| 0 | Atr-ERN16651 |  |  |  |  |  |  |
| 0 | Atr-ERN16652 |  |  |  |  |  |  |
| 0 | Atr-ERN16653 |  |  |  |  |  |  |
| 0 | Atr-ERN16654 |  |  |  |  |  |  |
| 0 | Atr-ERN16655 |  |  |  |  |  |  |
| 0 | Atr-ERN16656 |  |  |  |  |  |  |
| 0 | Atr-ERN16657 |  |  |  |  |  |  |
| 0 | Atr-ERN16658 |  |  |  |  |  |  |
| 0 | Atr-ERN16659 |  |  |  |  |  |  |
| 0 | Atr-ERN16660 |  |  |  |  |  |  |
| 0 | Atr-ERN16661 |  |  |  |  |  |  |
| 0 | Atr-ERN16662 |  |  |  |  |  |  |
| 0 | Atr-ERN16663 |  |  |  |  |  |  |
| 0 | Atr-ERN16664 |  |  |  |  |  |  |
| 0 | Atr-ERN16665 |  |  |  |  |  |  |
| 0 | Atr-ERN16666 |  |  |  |  |  |  |
| 0 | Atr-ERN16667 |  |  |  |  |  |  |
| 0 | Atr-ERN16668 |  |  |  |  |  |  |
| 0 | Atr-ERN16669 |  |  |  |  |  |  |
| 0 | Atr-ERN16670 |  |  |  |  |  |  |
| 0 | Atr-ERN16671 |  |  |  |  |  |  |
| 0 | Atr-ERN16672 |  |  |  |  |  |  |
| 0 | Atr-ERN16673 |  |  |  |  |  |  |
| 0 | Atr-ERN16674 |  |  |  |  |  |  |
| 0 | Atr-ERN16675 |  |  |  |  |  |  |
| 0 | Atr-ERN16676 |  |  |  |  |  |  |
| 0 | Atr-ERN16677 |  |  |  |  |  |  |
| 0 | Atr-ERN16678 |  |  |  |  |  |  |
| 0 | Atr-ERN16679 |  |  |  |  |  |  |
| 0 | Atr-ERN16680 |  |  |  |  |  |  |
| 0 | Atr-ERN16681 |  |  |  |  |  |  |
| 0 | Atr-ERN16682 |  |  |  |  |  |  |
| 0 | Atr-ERN16683 |  |  |  |  |  |  |
| 0 | Atr-ERN16684 |  |  |  |  |  |  |
| 0 | Atr-ERN16685 |  |  |  |  |  |  |
| 0 | Atr-ERN16686 |  |  |  |  |  |  |
| 0 | Atr-ERN16687 |  |  |  |  |  |  |
| 0 | Atr-ERN16688 |  |  |  |  |  |  |
| 0 | Atr-ERN16689 |  |  |  |  |  |  |
| 0 | Atr-ERN16690 |  |  |  |  |  |  |
| 0 | Atr-ERN16691 |  |  |  |  |  |  |
| 0 | Atr-ERN16692 |  |  |  |  |  |  |
| 0 | Atr-ERN16693 |  |  |  |  |  |  |
| 0 | Atr-ERN16694 |  |  |  |  |  |  |
| 0 | Atr-ERN16695 |  |  |  |  |  |  |
| 0 | Atr-ERN16696 |  |  |  |  |  |  |
| 0 | Atr-ERN16697 |  |  |  |  |  |  |
| 0 | Atr-ERN16698 |  |  |  |  |  |  |
| 0 | Atr-ERN16699 |  |  |  |  |  |  |
| 0 | Atr-ERN16700 |  |  |  |  |  |  |
| 0 | Atr-ERN16701 |  |  |  |  |  |  |
| 0 | Atr-ERN16702 |  |  |  |  |  |  |
| 0 | Atr-ERN16703 |  |  |  |  |  |  |
| 0 | Atr-ERN16704 |  |  |  |  |  |  |
