# Supplementary material for: Genome-wide identification, characterization and gene expression of BES1 transcription factor family in grapevine (Vitis vinifera L.)
Source: Sci Rep. 2023 Jan 5;13:240. doi: 10.1038/s41598-022-24407-y (PMC9816167; doi:10.1038/s41598-022-24407-y)
Supplement: Supplementary file 3 — Supplementary Information. [file 41598_2022_24407_MOESM3_ESM.zip › Vvi_Atr/Vitis_vinifera.PN40024.v4.dna_sm.toplevel.fa.vs.Amborella_trichopoda.AMTR1.0.dna_sm.toplevel.fa.html/Atr-AmTr_v1.0_scaffold00052.html]

|  |  |  |  |  |  |  |  |  |  |  |  |  |  |
| --- | --- | --- | --- | --- | --- | --- | --- | --- | --- | --- | --- | --- | --- |
| Duplication depth | Reference chromosome | Collinear blocks | | | | | | | | | | | |
| 0 | Atr-ERN16358 |  |  |  |  |  |  |
| 0 | Atr-ERN16359 |  |  |  |  |  |  |
| 0 | Atr-ERN16360 |  |  |  |  |  |  |
| 0 | Atr-ERN16361 |  |  |  |  |  |  |
| 0 | Atr-ERN16362 |  |  |  |  |  |  |
| 0 | Atr-ERN16363 |  |  |  |  |  |  |
| 0 | Atr-ERN16364 |  |  |  |  |  |  |
| 0 | Atr-ERN16365 |  |  |  |  |  |  |
| 0 | Atr-ERN16366 |  |  |  |  |  |  |
| 0 | Atr-ERN16367 |  |  |  |  |  |  |
| 0 | Atr-ERN16368 |  |  |  |  |  |  |
| 0 | Atr-ERN16369 |  |  |  |  |  |  |
| 0 | Atr-ERN16370 |  |  |  |  |  |  |
| 0 | Atr-ERN16371 |  |  |  |  |  |  |
| 0 | Atr-ERN16372 |  |  |  |  |  |  |
| 0 | Atr-ERN16373 |  |  |  |  |  |  |
| 0 | Atr-ERN16374 |  |  |  |  |  |  |
| 0 | Atr-ERN16375 |  |  |  |  |  |  |
| 0 | Atr-ERN16376 |  |  |  |  |  |  |
| 0 | Atr-ERN16377 |  |  |  |  |  |  |
| 0 | Atr-ERN16378 |  |  |  |  |  |  |
| 0 | Atr-ERN16379 |  |  |  |  |  |  |
| 0 | Atr-ERN16380 |  |  |  |  |  |  |
| 0 | Atr-ERN16381 |  |  |  |  |  |  |
| 0 | Atr-ERN16382 |  |  |  |  |  |  |
| 0 | Atr-ERN16383 |  |  |  |  |  |  |
| 0 | Atr-ERN16384 |  |  |  |  |  |  |
| 0 | Atr-ERN16385 |  |  |  |  |  |  |
| 0 | Atr-ERN16386 |  |  |  |  |  |  |
| 0 | Atr-ERN16387 |  |  |  |  |  |  |
| 0 | Atr-ERN16388 |  |  |  |  |  |  |
| 0 | Atr-ERN16389 |  |  |  |  |  |  |
| 0 | Atr-ERN16390 |  |  |  |  |  |  |
| 0 | Atr-ERN16391 |  |  |  |  |  |  |
| 0 | Atr-ERN16392 |  |  |  |  |  |  |
| 0 | Atr-ERN16393 |  |  |  |  |  |  |
| 0 | Atr-ERN16394 |  |  |  |  |  |  |
| 0 | Atr-ERN16395 |  |  |  |  |  |  |
| 0 | Atr-ERN16396 |  |  |  |  |  |  |
| 0 | Atr-ERN16397 |  |  |  |  |  |  |
| 0 | Atr-ERN16398 |  |  |  |  |  |  |
| 0 | Atr-ERN16399 |  |  |  |  |  |  |
| 0 | Atr-ERN16400 |  |  |  |  |  |  |
| 0 | Atr-ERN16401 |  |  |  |  |  |  |
| 0 | Atr-ERN16402 |  |  |  |  |  |  |
| 0 | Atr-ERN16403 |  |  |  |  |  |  |
| 0 | Atr-ERN16404 |  |  |  |  |  |  |
| 0 | Atr-ERN16405 |  |  |  |  |  |  |
| 0 | Atr-ERN16406 |  |  |  |  |  |  |
| 0 | Atr-ERN16407 |  |  |  |  |  |  |
| 0 | Atr-ERN16408 |  |  |  |  |  |  |
| 0 | Atr-ERN16409 |  |  |  |  |  |  |
| 0 | Atr-ERN16410 |  |  |  |  |  |  |
| 0 | Atr-ERN16411 |  |  |  |  |  |  |
| 0 | Atr-ERN16412 |  |  |  |  |  |  |
| 0 | Atr-ERN16413 |  |  |  |  |  |  |
| 0 | Atr-ERN16414 |  |  |  |  |  |  |
| 0 | Atr-ERN16415 |  |  |  |  |  |  |
| 0 | Atr-ERN16416 |  |  |  |  |  |  |
| 0 | Atr-ERN16417 |  |  |  |  |  |  |
| 0 | Atr-ERN16418 |  |  |  |  |  |  |
| 0 | Atr-ERN16419 |  |  |  |  |  |  |
| 0 | Atr-ERN16420 |  |  |  |  |  |  |
| 0 | Atr-ERN16421 |  |  |  |  |  |  |
| 0 | Atr-ERN16422 |  |  |  |  |  |  |
| 0 | Atr-ERN16423 |  |  |  |  |  |  |
| 0 | Atr-ERN16424 |  |  |  |  |  |  |
| 0 | Atr-ERN16425 |  |  |  |  |  |  |
| 0 | Atr-ERN16426 |  |  |  |  |  |  |
| 0 | Atr-ERN16427 |  |  |  |  |  |  |
| 0 | Atr-ERN16428 |  |  |  |  |  |  |
| 0 | Atr-ERN16429 |  |  |  |  |  |  |
| 0 | Atr-ERN16430 |  |  |  |  |  |  |
| 0 | Atr-ERN16431 |  |  |  |  |  |  |
| 0 | Atr-ERN16432 |  |  |  |  |  |  |
| 0 | Atr-ERN16433 |  |  |  |  |  |  |
| 0 | Atr-ERN16434 |  |  |  |  |  |  |
| 0 | Atr-ERN16435 |  |  |  |  |  |  |
| 0 | Atr-ERN16436 |  |  |  |  |  |  |
| 0 | Atr-ERN16437 |  |  |  |  |  |  |
| 0 | Atr-ERN16438 |  |  |  |  |  |  |
| 0 | Atr-ERN16439 |  |  |  |  |  |  |
| 0 | Atr-ERN16440 |  |  |  |  |  |  |
| 0 | Atr-ERN16441 |  |  |  |  |  |  |
| 0 | Atr-ERN16442 |  |  |  |  |  |  |
| 0 | Atr-ERN16443 |  |  |  |  |  |  |
| 0 | Atr-ERN16444 |  |  |  |  |  |  |
| 0 | Atr-ERN16445 |  |  |  |  |  |  |
| 0 | Atr-ERN16446 |  |  |  |  |  |  |
| 0 | Atr-ERN16447 |  |  |  |  |  |  |
| 0 | Atr-ERN16448 |  |  |  |  |  |  |
| 0 | Atr-ERN16449 |  |  |  |  |  |  |
| 0 | Atr-ERN16450 |  |  |  |  |  |  |
| 0 | Atr-ERN16451 |  |  |  |  |  |  |
| 0 | Atr-ERN16452 |  |  |  |  |  |  |
| 0 | Atr-ERN16453 |  |  |  |  |  |  |
| 0 | Atr-ERN16454 |  |  |  |  |  |  |
| 0 | Atr-ERN16455 |  |  |  |  |  |  |
| 0 | Atr-ERN16456 |  |  |  |  |  |  |
| 0 | Atr-ERN16457 |  |  |  |  |  |  |
| 0 | Atr-ERN16458 |  |  |  |  |  |  |
| 0 | Atr-ERN16459 |  |  |  |  |  |  |
| 0 | Atr-ERN16460 |  |  |  |  |  |  |
| 0 | Atr-ERN16461 |  |  |  |  |  |  |
| 0 | Atr-ERN16462 |  |  |  |  |  |  |
| 0 | Atr-ERN16463 |  |  |  |  |  |  |
| 0 | Atr-ERN16464 |  |  |  |  |  |  |
| 0 | Atr-ERN16465 |  |  |  |  |  |  |
| 0 | Atr-ERN16466 |  |  |  |  |  |  |
| 0 | Atr-ERN16467 |  |  |  |  |  |  |
| 0 | Atr-ERN16468 |  |  |  |  |  |  |
| 0 | Atr-ERN16469 |  |  |  |  |  |  |
| 0 | Atr-ERN16470 |  |  |  |  |  |  |
| 0 | Atr-ERN16471 |  |  |  |  |  |  |
| 0 | Atr-ERN16472 |  |  |  |  |  |  |
| 0 | Atr-ERN16473 |  |  |  |  |  |  |
| 0 | Atr-ERN16474 |  |  |  |  |  |  |
| 0 | Atr-ERN16475 |  |  |  |  |  |  |
| 0 | Atr-ERN16476 |  |  |  |  |  |  |
| 0 | Atr-ERN16477 |  |  |  |  |  |  |
| 0 | Atr-ERN16478 |  |  |  |  |  |  |
| 0 | Atr-ERN16479 |  |  |  |  |  |  |
| 0 | Atr-ERN16480 |  |  |  |  |  |  |
| 0 | Atr-ERN16481 |  |  |  |  |  |  |
