# Supplementary material for: Genome-wide identification, characterization and gene expression of BES1 transcription factor family in grapevine (Vitis vinifera L.)
Source: Sci Rep. 2023 Jan 5;13:240. doi: 10.1038/s41598-022-24407-y (PMC9816167; doi:10.1038/s41598-022-24407-y)
Supplement: Supplementary file 3 — Supplementary Information. [file 41598_2022_24407_MOESM3_ESM.zip › Vvi_Atr/Vitis_vinifera.PN40024.v4.dna_sm.toplevel.fa.vs.Amborella_trichopoda.AMTR1.0.dna_sm.toplevel.fa.html/Atr-AmTr_v1.0_scaffold00053.html]

|  |  |  |  |  |  |  |  |  |  |  |  |  |  |
| --- | --- | --- | --- | --- | --- | --- | --- | --- | --- | --- | --- | --- | --- |
| Duplication depth | Reference chromosome | Collinear blocks | | | | | | | | | | | |
| 0 | Atr-ERN05004 |  |  |  |  |  |  |
| 0 | Atr-ERN05005 |  |  |  |  |  |  |
| 0 | Atr-ERN05006 |  |  |  |  |  |  |
| 0 | Atr-ERN05007 |  |  |  |  |  |  |
| 0 | Atr-ERN05008 |  |  |  |  |  |  |
| 0 | Atr-ERN05009 |  |  |  |  |  |  |
| 0 | Atr-ERN05010 |  |  |  |  |  |  |
| 0 | Atr-ERN05011 |  |  |  |  |  |  |
| 0 | Atr-ERN05012 |  |  |  |  |  |  |
| 0 | Atr-ERN05013 |  |  |  |  |  |  |
| 0 | Atr-ERN05014 |  |  |  |  |  |  |
| 0 | Atr-ERN05015 |  |  |  |  |  |  |
| 0 | Atr-ERN05016 |  |  |  |  |  |  |
| 0 | Atr-ERN05017 |  |  |  |  |  |  |
| 0 | Atr-ERN05018 |  |  |  |  |  |  |
| 0 | Atr-ERN05019 |  |  |  |  |  |  |
| 0 | Atr-ERN05020 |  |  |  |  |  |  |
| 0 | Atr-ERN05021 |  |  |  |  |  |  |
| 0 | Atr-ERN05022 |  |  |  |  |  |  |
| 0 | Atr-ERN05023 |  |  |  |  |  |  |
| 0 | Atr-ERN05024 |  |  |  |  |  |  |
| 0 | Atr-ERN05025 |  |  |  |  |  |  |
| 0 | Atr-ERN05026 |  |  |  |  |  |  |
| 1 | Atr-ERN05027 |  | Vvi-Vitvi06g01253\_t001 |  |  |  |  |  |
| 1 | Atr-ERN05028 |  | | | |  |  |  |  |  |
| 1 | Atr-ERN05029 |  | | | |  |  |  |  |  |
| 1 | Atr-ERN05030 |  | | | |  |  |  |  |  |
| 1 | Atr-ERN05031 |  | | | |  |  |  |  |  |
| 1 | Atr-ERN05032 |  | | | |  |  |  |  |  |
| 1 | Atr-ERN05033 |  | | | |  |  |  |  |  |
| 1 | Atr-ERN05034 |  | | | |  |  |  |  |  |
| 1 | Atr-ERN05035 |  | | | |  |  |  |  |  |
| 1 | Atr-ERN05036 |  | | | |  |  |  |  |  |
| 1 | Atr-ERN05037 |  | | | |  |  |  |  |  |
| 1 | Atr-ERN05038 |  | | | |  |  |  |  |  |
| 1 | Atr-ERN05039 |  | | | |  |  |  |  |  |
| 1 | Atr-ERN05040 |  | | | |  |  |  |  |  |
| 1 | Atr-ERN05041 |  | | | |  |  |  |  |  |
| 1 | Atr-ERN05042 |  | | | |  |  |  |  |  |
| 1 | Atr-ERN05043 |  | | | |  |  |  |  |  |
| 1 | Atr-ERN05044 |  | | | |  |  |  |  |  |
| 1 | Atr-ERN05045 |  | | | |  |  |  |  |  |
| 1 | Atr-ERN05046 |  | | | |  |  |  |  |  |
| 1 | Atr-ERN05047 |  | | | |  |  |  |  |  |
| 1 | Atr-ERN05048 |  | | | |  |  |  |  |  |
| 1 | Atr-ERN05049 |  | | | |  |  |  |  |  |
| 1 | Atr-ERN05050 |  | | | |  |  |  |  |  |
| 1 | Atr-ERN05051 |  | | | |  |  |  |  |  |
| 1 | Atr-ERN05052 |  | Vvi-Vitvi06g01261\_t002 |  |  |  |  |  |
| 1 | Atr-ERN05053 |  | | | |  |  |  |  |  |
| 1 | Atr-ERN05054 |  | | | |  |  |  |  |  |
| 1 | Atr-ERN05055 |  | | | |  |  |  |  |  |
| 1 | Atr-ERN05056 |  | | | |  |  |  |  |  |
| 1 | Atr-ERN05057 |  | | | |  |  |  |  |  |
| 1 | Atr-ERN05058 |  | Vvi-Vitvi06g01262\_t002 |  |  |  |  |  |
| 1 | Atr-ERN05059 |  | | | |  |  |  |  |  |
| 1 | Atr-ERN05060 |  | | | |  |  |  |  |  |
| 1 | Atr-ERN05061 |  | | | |  |  |  |  |  |
| 1 | Atr-ERN05062 |  | | | |  |  |  |  |  |
| 1 | Atr-ERN05063 |  | | | |  |  |  |  |  |
| 1 | Atr-ERN05064 |  | | | |  |  |  |  |  |
| 1 | Atr-ERN05065 |  | | | |  |  |  |  |  |
| 1 | Atr-ERN05066 |  | | | |  |  |  |  |  |
| 1 | Atr-ERN05067 |  | | | |  |  |  |  |  |
| 2 | Atr-ERN05068 |  | | | |  | Vvi-Vitvi08g01678\_t001 |  |  |  |  |
| 2 | Atr-ERN05069 |  | | | |  | | | |  |  |  |  |
| 2 | Atr-ERN05070 |  | | | |  | | | |  |  |  |  |
| 2 | Atr-ERN05071 |  | | | |  | | | |  |  |  |  |
| 2 | Atr-ERN05072 |  | | | |  | Vvi-Vitvi08g01683\_t001 |  |  |  |  |
| 2 | Atr-ERN05073 |  | | | |  | | | |  |  |  |  |
| 3 | Atr-ERN05074 |  | | | |  | | | |  | Vvi-Vitvi13g01545\_t001 |  |  |  |
| 3 | Atr-ERN05075 |  | | | |  | | | |  | | | |  |  |  |
| 3 | Atr-ERN05076 |  | Vvi-Vitvi06g01265\_t001 |  | | | |  | | | |  |  |  |
| 3 | Atr-ERN05077 |  | | | |  | | | |  | | | |  |  |  |
| 3 | Atr-ERN05078 |  | | | |  | Vvi-Vitvi08g01685\_t001 |  | | | |  |  |  |
| 3 | Atr-ERN05079 |  | | | |  | | | |  | | | |  |  |  |
| 3 | Atr-ERN05080 |  | | | |  | | | |  | Vvi-Vitvi13g01556\_t002 |  |  |  |
| 3 | Atr-ERN05081 |  | | | |  | | | |  | Vvi-Vitvi13g02422\_t001 |  |  |  |
| 3 | Atr-ERN05082 |  | | | |  | | | |  | | | |  |  |  |
| 3 | Atr-ERN05083 |  | | | |  | | | |  | | | |  |  |  |
| 3 | Atr-ERN05084 |  | | | |  | Vvi-Vitvi08g01686\_t001 |  | | | |  |  |  |
| 3 | Atr-ERN05085 |  | | | |  | | | |  | | | |  |  |  |
| 3 | Atr-ERN05086 |  | | | |  | | | |  | Vvi-Vitvi13g01558\_t001 |  |  |  |
| 3 | Atr-ERN05087 |  | | | |  | | | |  | | | |  |  |  |
| 3 | Atr-ERN05088 |  | | | |  | | | |  | | | |  |  |  |
| 3 | Atr-ERN05089 |  | Vvi-Vitvi06g01266\_t002 |  | | | |  | | | |  |  |  |
| 3 | Atr-ERN05090 |  | Vvi-Vitvi06g01267\_t001 |  | | | |  | | | |  |  |  |
| 3 | Atr-ERN05091 |  | | | |  | | | |  | | | |  |  |  |
| 3 | Atr-ERN05092 |  | | | |  | | | |  | | | |  |  |  |
| 3 | Atr-ERN05093 |  | | | |  | | | |  | | | |  |  |  |
| 3 | Atr-ERN05094 |  | | | |  | | | |  | | | |  |  |  |
| 3 | Atr-ERN05095 |  | | | |  | | | |  | | | |  |  |  |
| 3 | Atr-ERN05096 |  | | | |  | | | |  | | | |  |  |  |
| 3 | Atr-ERN05097 |  | | | |  | | | |  | | | |  |  |  |
| 3 | Atr-ERN05098 |  | | | |  | | | |  | Vvi-Vitvi13g02428\_t001 |  |  |  |
| 3 | Atr-ERN05099 |  | | | |  | | | |  | Vvi-Vitvi13g02429\_t001 |  |  |  |
| 3 | Atr-ERN05100 |  | | | |  | | | |  | | | |  |  |  |
| 3 | Atr-ERN05101 |  | | | |  | | | |  | | | |  |  |  |
| 3 | Atr-ERN05102 |  | Vvi-Vitvi06g01269\_t001 |  | | | |  | | | |  |  |  |
| 3 | Atr-ERN05103 |  | | | |  | | | |  | | | |  |  |  |
| 3 | Atr-ERN05104 |  | | | |  | Vvi-Vitvi08g01691\_t001 |  | | | |  |  |  |
| 3 | Atr-ERN05105 |  | | | |  | | | |  | | | |  |  |  |
| 3 | Atr-ERN05106 |  | | | |  | Vvi-Vitvi08g01692\_t001 |  | | | |  |  |  |
| 3 | Atr-ERN05107 |  | | | |  | | | |  | | | |  |  |  |
| 3 | Atr-ERN05108 |  | | | |  | | | |  | | | |  |  |  |
| 3 | Atr-ERN05109 |  | | | |  | | | |  | | | |  |  |  |
| 3 | Atr-ERN05110 |  | | | |  | Vvi-Vitvi08g01693\_t001 |  | | | |  |  |  |
| 3 | Atr-ERN05111 |  | | | |  | | | |  | Vvi-Vitvi13g01570\_t001 |  |  |  |
| 3 | Atr-ERN05112 |  | | | |  | | | |  | | | |  |  |  |
| 3 | Atr-ERN05113 |  | | | |  | | | |  | | | |  |  |  |
| 3 | Atr-ERN05114 |  | | | |  | | | |  | | | |  |  |  |
| 3 | Atr-ERN05115 |  | | | |  | | | |  | | | |  |  |  |
| 3 | Atr-ERN05116 |  | Vvi-Vitvi06g01274\_t001 |  | | | |  | | | |  |  |  |
| 3 | Atr-ERN05117 |  | | | |  | | | |  | | | |  |  |  |
| 3 | Atr-ERN05118 |  | | | |  | | | |  | | | |  |  |  |
| 3 | Atr-ERN05119 |  | | | |  | | | |  | | | |  |  |  |
| 3 | Atr-ERN05120 |  | | | |  | | | |  | Vvi-Vitvi13g01571\_t002 |  |  |  |
| 3 | Atr-ERN05121 |  | | | |  | | | |  | | | |  |  |  |
| 3 | Atr-ERN05122 |  | | | |  | | | |  | | | |  |  |  |
| 3 | Atr-ERN05123 |  | | | |  | | | |  | | | |  |  |  |
| 3 | Atr-ERN05124 |  | Vvi-Vitvi06g01275\_t001 |  | | | |  | | | |  |  |  |
| 3 | Atr-ERN05125 |  | | | |  | | | |  | | | |  |  |  |
| 3 | Atr-ERN05126 |  | | | |  | Vvi-Vitvi08g01697\_t001 |  | Vvi-Vitvi13g01575\_t002 |  |  |  |
| 3 | Atr-ERN05127 |  | | | |  | | | |  | | | |  |  |  |
| 3 | Atr-ERN05128 |  | | | |  | Vvi-Vitvi08g01698\_t001 |  | | | |  |  |  |
| 3 | Atr-ERN05129 |  | | | |  | | | |  | | | |  |  |  |
| 3 | Atr-ERN05130 |  | | | |  | Vvi-Vitvi08g02330\_t001 |  | Vvi-Vitvi13g01576\_t002 |  |  |  |
| 3 | Atr-ERN05131 |  | | | |  | | | |  | | | |  |  |  |
| 3 | Atr-ERN05132 |  | | | |  | Vvi-Vitvi08g01699\_t001 |  | | | |  |  |  |
| 3 | Atr-ERN05133 |  | | | |  | | | |  | | | |  |  |  |
| 3 | Atr-ERN05134 |  | | | |  | Vvi-Vitvi08g02334\_t001 |  | | | |  |  |  |
| 2 | Atr-ERN05135 |  | | | |  |  |  | | | |  |  |  |
| 2 | Atr-ERN05136 |  | | | |  |  |  | | | |  |  |  |
| 2 | Atr-ERN05137 |  | | | |  |  |  | | | |  |  |  |
| 2 | Atr-ERN05138 |  | | | |  |  |  | | | |  |  |  |
| 2 | Atr-ERN05139 |  | | | |  |  |  | Vvi-Vitvi13g01613\_t001 |  |  |  |
| 2 | Atr-ERN05140 |  | Vvi-Vitvi06g01276\_t001 |  |  |  | | | |  |  |  |
| 2 | Atr-ERN05141 |  | | | |  |  |  | | | |  |  |  |
| 2 | Atr-ERN05142 |  | | | |  |  |  | | | |  |  |  |
| 2 | Atr-ERN05143 |  | | | |  |  |  | | | |  |  |  |
| 2 | Atr-ERN05144 |  | | | |  |  |  | | | |  |  |  |
| 2 | Atr-ERN05145 |  | | | |  |  |  | | | |  |  |  |
| 2 | Atr-ERN05146 |  | | | |  |  |  | | | |  |  |  |
| 2 | Atr-ERN05147 |  | | | |  |  |  | | | |  |  |  |
| 2 | Atr-ERN05148 |  | | | |  |  |  | | | |  |  |  |
| 3 | Atr-ERN05149 |  | | | |  | Vvi-Vitvi08g01955\_t001 |  | | | |  |  |  |
| 3 | Atr-ERN05150 |  | | | |  | Vvi-Vitvi08g01957\_t001 |  | | | |  |  |  |
| 3 | Atr-ERN05151 |  | | | |  | | | |  | | | |  |  |  |
| 3 | Atr-ERN05152 |  | | | |  | | | |  | | | |  |  |  |
| 3 | Atr-ERN05153 |  | | | |  | | | |  | | | |  |  |  |
| 3 | Atr-ERN05154 |  | | | |  | | | |  | | | |  |  |  |
| 3 | Atr-ERN05155 |  | | | |  | | | |  | | | |  |  |  |
| 3 | Atr-ERN05156 |  | | | |  | | | |  | | | |  |  |  |
| 3 | Atr-ERN05157 |  | | | |  | | | |  | | | |  |  |  |
| 3 | Atr-ERN05158 |  | Vvi-Vitvi06g01279\_t001 |  | Vvi-Vitvi08g01959\_t001 |  | | | |  |  |  |
| 2 | Atr-ERN05159 |  |  |  | | | |  | | | |  |  |  |
| 2 | Atr-ERN05160 |  |  |  | | | |  | | | |  |  |  |
| 2 | Atr-ERN05161 |  |  |  | Vvi-Vitvi08g01962\_t001 |  | Vvi-Vitvi13g01614\_t001 |  |  |  |
| 1 | Atr-ERN05162 |  |  |  | | | |  |  |  |  |
| 1 | Atr-ERN05163 |  |  |  | | | |  |  |  |  |
| 1 | Atr-ERN05164 |  |  |  | | | |  |  |  |  |
| 1 | Atr-ERN05165 |  |  |  | Vvi-Vitvi08g02415\_t001 |  |  |  |  |
| 1 | Atr-ERN05166 |  |  |  | Vvi-Vitvi08g01949\_t001 |  |  |  |  |
| 1 | Atr-ERN05167 |  |  |  | | | |  |  |  |  |
| 1 | Atr-ERN05168 |  |  |  | Vvi-Vitvi08g02413\_t001 |  |  |  |  |
| 1 | Atr-ERN05169 |  |  |  | | | |  |  |  |  |
| 1 | Atr-ERN05170 |  |  |  | | | |  |  |  |  |
| 1 | Atr-ERN05171 |  |  |  | | | |  |  |  |  |
| 1 | Atr-ERN05172 |  |  |  | | | |  |  |  |  |
| 1 | Atr-ERN05173 |  |  |  | | | |  |  |  |  |
| 1 | Atr-ERN05174 |  |  |  | | | |  |  |  |  |
| 1 | Atr-ERN05175 |  |  |  | | | |  |  |  |  |
| 1 | Atr-ERN05176 |  |  |  | | | |  |  |  |  |
| 1 | Atr-ERN05177 |  |  |  | | | |  |  |  |  |
| 1 | Atr-ERN05178 |  |  |  | | | |  |  |  |  |
| 1 | Atr-ERN05179 |  |  |  | | | |  |  |  |  |
| 1 | Atr-ERN05180 |  |  |  | | | |  |  |  |  |
| 1 | Atr-ERN05181 |  |  |  | | | |  |  |  |  |
| 1 | Atr-ERN05182 |  |  |  | | | |  |  |  |  |
| 1 | Atr-ERN05183 |  |  |  | Vvi-Vitvi08g01934\_t001 |  |  |  |  |
| 0 | Atr-ERN05184 |  |  |  |  |  |  |
| 0 | Atr-ERN05185 |  |  |  |  |  |  |
| 0 | Atr-ERN05186 |  |  |  |  |  |  |
| 0 | Atr-ERN05187 |  |  |  |  |  |  |
| 0 | Atr-ERN05188 |  |  |  |  |  |  |
| 0 | Atr-ERN05189 |  |  |  |  |  |  |
