# Supplementary material for: Genome-wide identification, characterization and gene expression of BES1 transcription factor family in grapevine (Vitis vinifera L.)
Source: Sci Rep. 2023 Jan 5;13:240. doi: 10.1038/s41598-022-24407-y (PMC9816167; doi:10.1038/s41598-022-24407-y)
Supplement: Supplementary file 3 — Supplementary Information. [file 41598_2022_24407_MOESM3_ESM.zip › Vvi_Atr/Vitis_vinifera.PN40024.v4.dna_sm.toplevel.fa.vs.Amborella_trichopoda.AMTR1.0.dna_sm.toplevel.fa.html/Atr-AmTr_v1.0_scaffold00054.html]

|  |  |  |  |  |  |  |  |  |  |  |  |  |  |
| --- | --- | --- | --- | --- | --- | --- | --- | --- | --- | --- | --- | --- | --- |
| Duplication depth | Reference chromosome | Collinear blocks | | | | | | | | | | | |
| 0 | Atr-ERN18113 |  |  |  |  |  |  |
| 0 | Atr-ERN18114 |  |  |  |  |  |  |
| 0 | Atr-ERN18115 |  |  |  |  |  |  |
| 0 | Atr-ERN18116 |  |  |  |  |  |  |
| 0 | Atr-ERN18117 |  |  |  |  |  |  |
| 0 | Atr-ERN18118 |  |  |  |  |  |  |
| 0 | Atr-ERN18119 |  |  |  |  |  |  |
| 0 | Atr-ERN18120 |  |  |  |  |  |  |
| 0 | Atr-ERN18121 |  |  |  |  |  |  |
| 0 | Atr-ERN18122 |  |  |  |  |  |  |
| 0 | Atr-ERN18123 |  |  |  |  |  |  |
| 0 | Atr-ERN18124 |  |  |  |  |  |  |
| 0 | Atr-ERN18125 |  |  |  |  |  |  |
| 0 | Atr-ERN18126 |  |  |  |  |  |  |
| 0 | Atr-ERN18127 |  |  |  |  |  |  |
| 0 | Atr-ERN18128 |  |  |  |  |  |  |
| 0 | Atr-ERN18129 |  |  |  |  |  |  |
| 0 | Atr-ERN18130 |  |  |  |  |  |  |
| 0 | Atr-ERN18131 |  |  |  |  |  |  |
| 0 | Atr-ERN18132 |  |  |  |  |  |  |
| 0 | Atr-ERN18133 |  |  |  |  |  |  |
| 0 | Atr-ERN18134 |  |  |  |  |  |  |
| 0 | Atr-ERN18135 |  |  |  |  |  |  |
| 0 | Atr-ERN18136 |  |  |  |  |  |  |
| 0 | Atr-ERN18137 |  |  |  |  |  |  |
| 0 | Atr-ERN18138 |  |  |  |  |  |  |
| 0 | Atr-ERN18139 |  |  |  |  |  |  |
| 0 | Atr-ERN18140 |  |  |  |  |  |  |
| 0 | Atr-ERN18141 |  |  |  |  |  |  |
| 0 | Atr-ERN18142 |  |  |  |  |  |  |
| 0 | Atr-ERN18143 |  |  |  |  |  |  |
| 0 | Atr-ERN18144 |  |  |  |  |  |  |
| 0 | Atr-ERN18145 |  |  |  |  |  |  |
| 0 | Atr-ERN18146 |  |  |  |  |  |  |
| 0 | Atr-ERN18147 |  |  |  |  |  |  |
| 0 | Atr-ERN18148 |  |  |  |  |  |  |
| 0 | Atr-ERN18149 |  |  |  |  |  |  |
| 0 | Atr-ERN18150 |  |  |  |  |  |  |
| 0 | Atr-ERN18151 |  |  |  |  |  |  |
| 0 | Atr-ERN18152 |  |  |  |  |  |  |
| 0 | Atr-ERN18153 |  |  |  |  |  |  |
| 0 | Atr-ERN18154 |  |  |  |  |  |  |
| 0 | Atr-ERN18155 |  |  |  |  |  |  |
| 0 | Atr-ERN18156 |  |  |  |  |  |  |
| 0 | Atr-ERN18157 |  |  |  |  |  |  |
| 0 | Atr-ERN18158 |  |  |  |  |  |  |
| 0 | Atr-ERN18159 |  |  |  |  |  |  |
| 0 | Atr-ERN18160 |  |  |  |  |  |  |
| 0 | Atr-ERN18161 |  |  |  |  |  |  |
| 0 | Atr-ERN18162 |  |  |  |  |  |  |
| 0 | Atr-ERN18163 |  |  |  |  |  |  |
| 0 | Atr-ERN18164 |  |  |  |  |  |  |
| 0 | Atr-ERN18165 |  |  |  |  |  |  |
| 0 | Atr-ERN18166 |  |  |  |  |  |  |
| 0 | Atr-ERN18167 |  |  |  |  |  |  |
| 0 | Atr-ERN18168 |  |  |  |  |  |  |
| 0 | Atr-ERN18169 |  |  |  |  |  |  |
| 0 | Atr-ERN18170 |  |  |  |  |  |  |
| 0 | Atr-ERN18171 |  |  |  |  |  |  |
| 0 | Atr-ERN18172 |  |  |  |  |  |  |
| 0 | Atr-ERN18173 |  |  |  |  |  |  |
| 0 | Atr-ERN18174 |  |  |  |  |  |  |
| 0 | Atr-ERN18175 |  |  |  |  |  |  |
| 0 | Atr-ERN18176 |  |  |  |  |  |  |
| 0 | Atr-ERN18177 |  |  |  |  |  |  |
| 0 | Atr-ERN18178 |  |  |  |  |  |  |
| 0 | Atr-ERN18179 |  |  |  |  |  |  |
| 0 | Atr-ERN18180 |  |  |  |  |  |  |
| 0 | Atr-ERN18181 |  |  |  |  |  |  |
| 0 | Atr-ERN18182 |  |  |  |  |  |  |
| 0 | Atr-ERN18183 |  |  |  |  |  |  |
| 0 | Atr-ERN18184 |  |  |  |  |  |  |
| 0 | Atr-ERN18185 |  |  |  |  |  |  |
| 0 | Atr-ERN18186 |  |  |  |  |  |  |
| 0 | Atr-ERN18187 |  |  |  |  |  |  |
| 0 | Atr-ERN18188 |  |  |  |  |  |  |
| 0 | Atr-ERN18189 |  |  |  |  |  |  |
| 0 | Atr-ERN18190 |  |  |  |  |  |  |
| 0 | Atr-ERN18191 |  |  |  |  |  |  |
| 0 | Atr-ERN18192 |  |  |  |  |  |  |
| 0 | Atr-ERN18193 |  |  |  |  |  |  |
| 0 | Atr-ERN18194 |  |  |  |  |  |  |
| 0 | Atr-ERN18195 |  |  |  |  |  |  |
| 0 | Atr-ERN18196 |  |  |  |  |  |  |
| 0 | Atr-ERN18197 |  |  |  |  |  |  |
| 0 | Atr-ERN18198 |  |  |  |  |  |  |
| 0 | Atr-ERN18199 |  |  |  |  |  |  |
| 0 | Atr-ERN18200 |  |  |  |  |  |  |
| 0 | Atr-ERN18201 |  |  |  |  |  |  |
| 0 | Atr-ERN18202 |  |  |  |  |  |  |
| 0 | Atr-ERN18203 |  |  |  |  |  |  |
