# Supplementary material for: Genome-wide identification, characterization and gene expression of BES1 transcription factor family in grapevine (Vitis vinifera L.)
Source: Sci Rep. 2023 Jan 5;13:240. doi: 10.1038/s41598-022-24407-y (PMC9816167; doi:10.1038/s41598-022-24407-y)
Supplement: Supplementary file 3 — Supplementary Information. [file 41598_2022_24407_MOESM3_ESM.zip › Vvi_Atr/Vitis_vinifera.PN40024.v4.dna_sm.toplevel.fa.vs.Amborella_trichopoda.AMTR1.0.dna_sm.toplevel.fa.html/Atr-AmTr_v1.0_scaffold00055.html]

|  |  |  |  |  |  |  |  |  |  |  |  |  |  |
| --- | --- | --- | --- | --- | --- | --- | --- | --- | --- | --- | --- | --- | --- |
| Duplication depth | Reference chromosome | Collinear blocks | | | | | | | | | | | |
| 0 | Atr-ERN18211 |  |  |  |  |  |  |
| 0 | Atr-ERN18212 |  |  |  |  |  |  |
| 1 | Atr-ERN18213 |  | Vvi-Vitvi04g01482\_t001 |  |  |  |  |  |
| 1 | Atr-ERN18214 |  | | | |  |  |  |  |  |
| 1 | Atr-ERN18215 |  | | | |  |  |  |  |  |
| 1 | Atr-ERN18216 |  | | | |  |  |  |  |  |
| 1 | Atr-ERN18217 |  | | | |  |  |  |  |  |
| 1 | Atr-ERN18218 |  | | | |  |  |  |  |  |
| 1 | Atr-ERN18219 |  | | | |  |  |  |  |  |
| 1 | Atr-ERN18220 |  | | | |  |  |  |  |  |
| 1 | Atr-ERN18221 |  | | | |  |  |  |  |  |
| 1 | Atr-ERN18222 |  | Vvi-Vitvi04g01483\_t001 |  |  |  |  |  |
| 1 | Atr-ERN18223 |  | | | |  |  |  |  |  |
| 1 | Atr-ERN18224 |  | | | |  |  |  |  |  |
| 1 | Atr-ERN18225 |  | | | |  |  |  |  |  |
| 1 | Atr-ERN18226 |  | | | |  |  |  |  |  |
| 1 | Atr-ERN18227 |  | | | |  |  |  |  |  |
| 1 | Atr-ERN18228 |  | | | |  |  |  |  |  |
| 1 | Atr-ERN18229 |  | Vvi-Vitvi04g01486\_t001 |  |  |  |  |  |
| 1 | Atr-ERN18230 |  | | | |  |  |  |  |  |
| 1 | Atr-ERN18231 |  | | | |  |  |  |  |  |
| 1 | Atr-ERN18232 |  | Vvi-Vitvi04g02163\_t001 |  |  |  |  |  |
| 1 | Atr-ERN18233 |  | Vvi-Vitvi04g04391\_t001 |  |  |  |  |  |
| 1 | Atr-ERN18234 |  | | | |  |  |  |  |  |
| 1 | Atr-ERN18235 |  | | | |  |  |  |  |  |
| 1 | Atr-ERN18236 |  | | | |  |  |  |  |  |
| 1 | Atr-ERN18237 |  | Vvi-Vitvi04g04392\_t001 |  |  |  |  |  |
| 1 | Atr-ERN18238 |  | | | |  |  |  |  |  |
| 1 | Atr-ERN18239 |  | Vvi-Vitvi04g01490\_t001 |  |  |  |  |  |
| 1 | Atr-ERN18240 |  | Vvi-Vitvi04g01492\_t001 |  |  |  |  |  |
| 0 | Atr-ERN18241 |  |  |  |  |  |  |
| 0 | Atr-ERN18242 |  |  |  |  |  |  |
| 0 | Atr-ERN18243 |  |  |  |  |  |  |
| 0 | Atr-ERN18244 |  |  |  |  |  |  |
| 0 | Atr-ERN18245 |  |  |  |  |  |  |
| 0 | Atr-ERN18246 |  |  |  |  |  |  |
| 0 | Atr-ERN18247 |  |  |  |  |  |  |
| 0 | Atr-ERN18248 |  |  |  |  |  |  |
| 0 | Atr-ERN18249 |  |  |  |  |  |  |
| 0 | Atr-ERN18250 |  |  |  |  |  |  |
| 0 | Atr-ERN18251 |  |  |  |  |  |  |
| 0 | Atr-ERN18252 |  |  |  |  |  |  |
| 0 | Atr-ERN18253 |  |  |  |  |  |  |
| 0 | Atr-ERN18254 |  |  |  |  |  |  |
| 0 | Atr-ERN18255 |  |  |  |  |  |  |
| 0 | Atr-ERN18256 |  |  |  |  |  |  |
| 0 | Atr-ERN18257 |  |  |  |  |  |  |
| 0 | Atr-ERN18258 |  |  |  |  |  |  |
| 0 | Atr-ERN18259 |  |  |  |  |  |  |
| 0 | Atr-ERN18260 |  |  |  |  |  |  |
| 0 | Atr-ERN18261 |  |  |  |  |  |  |
| 0 | Atr-ERN18262 |  |  |  |  |  |  |
| 0 | Atr-ERN18263 |  |  |  |  |  |  |
| 0 | Atr-ERN18264 |  |  |  |  |  |  |
| 0 | Atr-ERN18265 |  |  |  |  |  |  |
| 0 | Atr-ERN18266 |  |  |  |  |  |  |
| 0 | Atr-ERN18267 |  |  |  |  |  |  |
| 0 | Atr-ERN18268 |  |  |  |  |  |  |
| 0 | Atr-ERN18269 |  |  |  |  |  |  |
| 1 | Atr-ERN18270 |  | Vvi-Vitvi19g00611\_t001 |  |  |  |  |  |
| 1 | Atr-ERN18271 |  | | | |  |  |  |  |  |
| 1 | Atr-ERN18272 |  | Vvi-Vitvi19g00610\_t001 |  |  |  |  |  |
| 1 | Atr-ERN18273 |  | | | |  |  |  |  |  |
| 1 | Atr-ERN18274 |  | | | |  |  |  |  |  |
| 1 | Atr-ERN18275 |  | | | |  |  |  |  |  |
| 1 | Atr-ERN18276 |  | Vvi-Vitvi19g00609\_t001 |  |  |  |  |  |
| 1 | Atr-ERN18277 |  | Vvi-Vitvi19g00608\_t001 |  |  |  |  |  |
| 1 | Atr-ERN18278 |  | Vvi-Vitvi19g00607\_t001 |  |  |  |  |  |
| 1 | Atr-ERN18279 |  | | | |  |  |  |  |  |
| 1 | Atr-ERN18280 |  | | | |  |  |  |  |  |
| 1 | Atr-ERN18281 |  | | | |  |  |  |  |  |
| 1 | Atr-ERN18282 |  | Vvi-Vitvi19g00606\_t001 |  |  |  |  |  |
| 1 | Atr-ERN18283 |  | | | |  |  |  |  |  |
| 1 | Atr-ERN18284 |  | | | |  |  |  |  |  |
| 1 | Atr-ERN18285 |  | | | |  |  |  |  |  |
| 1 | Atr-ERN18286 |  | | | |  |  |  |  |  |
| 1 | Atr-ERN18287 |  | Vvi-Vitvi19g00604\_t001 |  |  |  |  |  |
| 1 | Atr-ERN18288 |  | | | |  |  |  |  |  |
| 1 | Atr-ERN18289 |  | | | |  |  |  |  |  |
| 1 | Atr-ERN18290 |  | | | |  |  |  |  |  |
| 1 | Atr-ERN18291 |  | | | |  |  |  |  |  |
| 1 | Atr-ERN18292 |  | | | |  |  |  |  |  |
| 1 | Atr-ERN18293 |  | | | |  |  |  |  |  |
| 1 | Atr-ERN18294 |  | | | |  |  |  |  |  |
| 1 | Atr-ERN18295 |  | Vvi-Vitvi19g00602\_t001 |  |  |  |  |  |
| 1 | Atr-ERN18296 |  | | | |  |  |  |  |  |
| 1 | Atr-ERN18297 |  | | | |  |  |  |  |  |
| 1 | Atr-ERN18298 |  | | | |  |  |  |  |  |
| 1 | Atr-ERN18299 |  | | | |  |  |  |  |  |
| 1 | Atr-ERN18300 |  | | | |  |  |  |  |  |
| 1 | Atr-ERN18301 |  | Vvi-Vitvi19g00596\_t001 |  |  |  |  |  |
| 1 | Atr-ERN18302 |  | | | |  |  |  |  |  |
| 1 | Atr-ERN18303 |  | | | |  |  |  |  |  |
| 1 | Atr-ERN18304 |  | | | |  |  |  |  |  |
| 1 | Atr-ERN18305 |  | | | |  |  |  |  |  |
| 1 | Atr-ERN18306 |  | | | |  |  |  |  |  |
| 1 | Atr-ERN18307 |  | | | |  |  |  |  |  |
| 1 | Atr-ERN18308 |  | | | |  |  |  |  |  |
| 1 | Atr-ERN18309 |  | | | |  |  |  |  |  |
| 1 | Atr-ERN18310 |  | | | |  |  |  |  |  |
| 1 | Atr-ERN18311 |  | | | |  |  |  |  |  |
| 1 | Atr-ERN18312 |  | | | |  |  |  |  |  |
| 1 | Atr-ERN18313 |  | | | |  |  |  |  |  |
| 1 | Atr-ERN18314 |  | | | |  |  |  |  |  |
| 1 | Atr-ERN18315 |  | Vvi-Vitvi19g00592\_t001 |  |  |  |  |  |
| 1 | Atr-ERN18316 |  | | | |  |  |  |  |  |
| 1 | Atr-ERN18317 |  | | | |  |  |  |  |  |
| 1 | Atr-ERN18318 |  | | | |  |  |  |  |  |
| 1 | Atr-ERN18319 |  | | | |  |  |  |  |  |
| 1 | Atr-ERN18320 |  | | | |  |  |  |  |  |
| 1 | Atr-ERN18321 |  | Vvi-Vitvi19g00588\_t001 |  |  |  |  |  |
| 0 | Atr-ERN18322 |  |  |  |  |  |  |
| 0 | Atr-ERN18323 |  |  |  |  |  |  |
| 0 | Atr-ERN18324 |  |  |  |  |  |  |
| 0 | Atr-ERN18325 |  |  |  |  |  |  |
| 0 | Atr-ERN18326 |  |  |  |  |  |  |
| 0 | Atr-ERN18327 |  |  |  |  |  |  |
| 0 | Atr-ERN18328 |  |  |  |  |  |  |
| 0 | Atr-ERN18329 |  |  |  |  |  |  |
| 0 | Atr-ERN18330 |  |  |  |  |  |  |
| 0 | Atr-ERN18331 |  |  |  |  |  |  |
| 0 | Atr-ERN18332 |  |  |  |  |  |  |
| 0 | Atr-ERN18333 |  |  |  |  |  |  |
| 0 | Atr-ERN18334 |  |  |  |  |  |  |
| 0 | Atr-ERN18335 |  |  |  |  |  |  |
| 0 | Atr-ERN18336 |  |  |  |  |  |  |
| 0 | Atr-ERN18337 |  |  |  |  |  |  |
| 0 | Atr-ERN18338 |  |  |  |  |  |  |
| 0 | Atr-ERN18339 |  |  |  |  |  |  |
| 0 | Atr-ERN18340 |  |  |  |  |  |  |
| 0 | Atr-ERN18341 |  |  |  |  |  |  |
| 0 | Atr-ERN18342 |  |  |  |  |  |  |
| 0 | Atr-ERN18343 |  |  |  |  |  |  |
| 0 | Atr-ERN18344 |  |  |  |  |  |  |
| 0 | Atr-ERN18345 |  |  |  |  |  |  |
| 0 | Atr-ERN18346 |  |  |  |  |  |  |
| 0 | Atr-ERN18347 |  |  |  |  |  |  |
| 0 | Atr-ERN18348 |  |  |  |  |  |  |
| 0 | Atr-ERN18349 |  |  |  |  |  |  |
| 1 | Atr-ERN18350 |  | Vvi-Vitvi01g00850\_t001 |  |  |  |  |  |
| 1 | Atr-ERN18351 |  | | | |  |  |  |  |  |
| 2 | Atr-ERN18352 |  | | | |  | Vvi-Vitvi17g00739\_t001 |  |  |  |  |
| 2 | Atr-ERN18353 |  | Vvi-Vitvi01g02096\_t001 |  | Vvi-Vitvi17g01527\_t001 |  |  |  |  |
| 2 | Atr-ERN18354 |  | | | |  | | | |  |  |  |  |
| 2 | Atr-ERN18355 |  | | | |  | Vvi-Vitvi17g01525\_t001 |  |  |  |  |
| 2 | Atr-ERN18356 |  | | | |  | | | |  |  |  |  |
| 2 | Atr-ERN18357 |  | | | |  | | | |  |  |  |  |
| 2 | Atr-ERN18358 |  | | | |  | | | |  |  |  |  |
| 2 | Atr-ERN18359 |  | | | |  | | | |  |  |  |  |
| 2 | Atr-ERN18360 |  | | | |  | | | |  |  |  |  |
| 2 | Atr-ERN18361 |  | | | |  | | | |  |  |  |  |
| 2 | Atr-ERN18362 |  | | | |  | | | |  |  |  |  |
| 2 | Atr-ERN18363 |  | | | |  | | | |  |  |  |  |
| 2 | Atr-ERN18364 |  | Vvi-Vitvi01g00852\_t001 |  | | | |  |  |  |  |
| 2 | Atr-ERN18365 |  | | | |  | | | |  |  |  |  |
| 2 | Atr-ERN18366 |  | | | |  | | | |  |  |  |  |
| 2 | Atr-ERN18367 |  | | | |  | | | |  |  |  |  |
| 2 | Atr-ERN18368 |  | | | |  | | | |  |  |  |  |
| 2 | Atr-ERN18369 |  | | | |  | | | |  |  |  |  |
| 2 | Atr-ERN18370 |  | | | |  | | | |  |  |  |  |
| 2 | Atr-ERN18371 |  | | | |  | | | |  |  |  |  |
| 2 | Atr-ERN18372 |  | Vvi-Vitvi01g00853\_t001 |  | | | |  |  |  |  |
| 2 | Atr-ERN18373 |  | | | |  | | | |  |  |  |  |
| 2 | Atr-ERN18374 |  | | | |  | | | |  |  |  |  |
| 2 | Atr-ERN18375 |  | | | |  | | | |  |  |  |  |
| 2 | Atr-ERN18376 |  | | | |  | Vvi-Vitvi17g00736\_t002 |  |  |  |  |
| 2 | Atr-ERN18377 |  | | | |  | | | |  |  |  |  |
| 2 | Atr-ERN18378 |  | | | |  | | | |  |  |  |  |
| 2 | Atr-ERN18379 |  | | | |  | Vvi-Vitvi17g00734\_t001 |  |  |  |  |
| 2 | Atr-ERN18380 |  | | | |  | Vvi-Vitvi17g00733\_t002 |  |  |  |  |
| 2 | Atr-ERN18381 |  | | | |  | | | |  |  |  |  |
| 2 | Atr-ERN18382 |  | Vvi-Vitvi01g00857\_t001 |  | Vvi-Vitvi17g00732\_t001 |  |  |  |  |
| 2 | Atr-ERN18383 |  | | | |  | | | |  |  |  |  |
| 2 | Atr-ERN18384 |  | Vvi-Vitvi01g00858\_t001 |  | Vvi-Vitvi17g00731\_t001 |  |  |  |  |
| 0 | Atr-ERN18385 |  |  |  |  |  |  |
| 0 | Atr-ERN18386 |  |  |  |  |  |  |
| 0 | Atr-ERN18387 |  |  |  |  |  |  |
| 0 | Atr-ERN18388 |  |  |  |  |  |  |
