# Supplementary material for: Genome-wide identification, characterization and gene expression of BES1 transcription factor family in grapevine (Vitis vinifera L.)
Source: Sci Rep. 2023 Jan 5;13:240. doi: 10.1038/s41598-022-24407-y (PMC9816167; doi:10.1038/s41598-022-24407-y)
Supplement: Supplementary file 3 — Supplementary Information. [file 41598_2022_24407_MOESM3_ESM.zip › Vvi_Atr/Vitis_vinifera.PN40024.v4.dna_sm.toplevel.fa.vs.Amborella_trichopoda.AMTR1.0.dna_sm.toplevel.fa.html/Atr-AmTr_v1.0_scaffold00056.html]

|  |  |  |  |  |  |  |  |  |  |  |  |  |  |
| --- | --- | --- | --- | --- | --- | --- | --- | --- | --- | --- | --- | --- | --- |
| Duplication depth | Reference chromosome | Collinear blocks | | | | | | | | | | | |
| 0 | Atr-ERN15051 |  |  |  |  |  |  |
| 0 | Atr-ERN15052 |  |  |  |  |  |  |
| 0 | Atr-ERN15053 |  |  |  |  |  |  |
| 0 | Atr-ERN15054 |  |  |  |  |  |  |
| 0 | Atr-ERN15055 |  |  |  |  |  |  |
| 0 | Atr-ERN15056 |  |  |  |  |  |  |
| 0 | Atr-ERN15057 |  |  |  |  |  |  |
| 0 | Atr-ERN15058 |  |  |  |  |  |  |
| 1 | Atr-ERN15059 |  | Vvi-Vitvi04g01845\_t001 |  |  |  |  |  |
| 1 | Atr-ERN15060 |  | | | |  |  |  |  |  |
| 1 | Atr-ERN15061 |  | | | |  |  |  |  |  |
| 1 | Atr-ERN15062 |  | | | |  |  |  |  |  |
| 2 | Atr-ERN15063 |  | | | |  | Vvi-Vitvi11g00293\_t001 |  |  |  |  |
| 2 | Atr-ERN15064 |  | | | |  | | | |  |  |  |  |
| 2 | Atr-ERN15065 |  | Vvi-Vitvi04g00303\_t001 |  | | | |  |  |  |  |
| 2 | Atr-ERN15066 |  | | | |  | | | |  |  |  |  |
| 2 | Atr-ERN15067 |  | | | |  | | | |  |  |  |  |
| 2 | Atr-ERN15068 |  | | | |  | | | |  |  |  |  |
| 2 | Atr-ERN15069 |  | | | |  | | | |  |  |  |  |
| 2 | Atr-ERN15070 |  | | | |  | Vvi-Vitvi11g00292\_t001 |  |  |  |  |
| 3 | Atr-ERN15071 |  | | | |  | | | |  | Vvi-Vitvi09g00327\_t001 |  |  |  |
| 3 | Atr-ERN15072 |  | | | |  | | | |  | Vvi-Vitvi09g00326\_t001 |  |  |  |
| 3 | Atr-ERN15073 |  | | | |  | | | |  | Vvi-Vitvi09g01592\_t001 |  |  |  |
| 3 | Atr-ERN15074 |  | | | |  | | | |  | | | |  |  |  |
| 3 | Atr-ERN15075 |  | | | |  | Vvi-Vitvi11g00290\_t001 |  | | | |  |  |  |
| 3 | Atr-ERN15076 |  | | | |  | Vvi-Vitvi11g00288\_t001 |  | | | |  |  |  |
| 3 | Atr-ERN15077 |  | Vvi-Vitvi04g00302\_t002 |  | | | |  | | | |  |  |  |
| 4 | Atr-ERN15078 |  | | | |  | | | |  | | | |  | Vvi-Vitvi11g00280\_t001 |  |  |
| 4 | Atr-ERN15079 |  | | | |  | | | |  | | | |  | | | |  |  |
| 4 | Atr-ERN15080 |  | | | |  | | | |  | Vvi-Vitvi09g00320\_t001 |  | | | |  |  |
| 4 | Atr-ERN15081 |  | | | |  | | | |  | | | |  | | | |  |  |
| 5 | Atr-ERN15082 |  | | | |  | | | |  | | | |  | | | |  | Vvi-Vitvi04g00293\_t001 |  |
| 5 | Atr-ERN15083 |  | | | |  | | | |  | | | |  | Vvi-Vitvi11g00282\_t001 |  | | | |  |
| 5 | Atr-ERN15084 |  | | | |  | | | |  | | | |  | Vvi-Vitvi11g00283\_t001 |  | Vvi-Vitvi04g00294\_t002 |  |
| 5 | Atr-ERN15085 |  | | | |  | | | |  | | | |  | | | |  | | | |  |
| 5 | Atr-ERN15086 |  | | | |  | | | |  | | | |  | | | |  | Vvi-Vitvi04g00295\_t001 |  |
| 5 | Atr-ERN15087 |  | | | |  | | | |  | | | |  | | | |  | Vvi-Vitvi04g00297\_t001 |  |
| 5 | Atr-ERN15088 |  | Vvi-Vitvi04g00298\_t001 |  | | | |  | | | |  | | | |  | Vvi-Vitvi04g00298\_t001 |  |
| 5 | Atr-ERN15089 |  | | | |  | | | |  | | | |  | | | |  | | | |  |
| 5 | Atr-ERN15090 |  | | | |  | | | |  | | | |  | | | |  | | | |  |
| 5 | Atr-ERN15091 |  | | | |  | | | |  | | | |  | Vvi-Vitvi11g00285\_t001 |  | Vvi-Vitvi04g00300\_t001 |  |
| 5 | Atr-ERN15092 |  | | | |  | | | |  | | | |  | | | |  | | | |  |
| 5 | Atr-ERN15093 |  | | | |  | | | |  | | | |  | | | |  | Vvi-Vitvi04g01840\_t001 |  |
| 5 | Atr-ERN15094 |  | | | |  | | | |  | | | |  | | | |  | | | |  |
| 5 | Atr-ERN15095 |  | | | |  | Vvi-Vitvi11g00286\_t001 |  | | | |  | Vvi-Vitvi11g00286\_t001 |  | | | |  |
| 5 | Atr-ERN15096 |  | | | |  | | | |  | | | |  | | | |  | | | |  |
| 5 | Atr-ERN15097 |  | | | |  | | | |  | | | |  | | | |  | | | |  |
| 5 | Atr-ERN15098 |  | | | |  | | | |  | | | |  | Vvi-Vitvi11g01394\_t001 |  | Vvi-Vitvi04g01841\_t001 |  |
| 3 | Atr-ERN15099 |  | Vvi-Vitvi04g00292\_t001 |  | | | |  | | | |  |  |  |
| 3 | Atr-ERN15100 |  | | | |  | | | |  | Vvi-Vitvi09g00314\_t001 |  |  |  |
| 3 | Atr-ERN15101 |  | | | |  | | | |  | | | |  |  |  |
| 3 | Atr-ERN15102 |  | | | |  | | | |  | | | |  |  |  |
| 3 | Atr-ERN15103 |  | | | |  | Vvi-Vitvi11g00279\_t001 |  | | | |  |  |  |
| 3 | Atr-ERN15104 |  | | | |  | | | |  | Vvi-Vitvi09g00312\_t001 |  |  |  |
| 3 | Atr-ERN15105 |  | Vvi-Vitvi04g00290\_t001 |  | | | |  | | | |  |  |  |
| 3 | Atr-ERN15106 |  | | | |  | | | |  | | | |  |  |  |
| 3 | Atr-ERN15107 |  | Vvi-Vitvi04g00289\_t001 |  | | | |  | Vvi-Vitvi09g00311\_t001 |  |  |  |
| 3 | Atr-ERN15108 |  | | | |  | | | |  | | | |  |  |  |
| 3 | Atr-ERN15109 |  | Vvi-Vitvi04g00288\_t001 |  | | | |  | | | |  |  |  |
| 3 | Atr-ERN15110 |  | Vvi-Vitvi04g00287\_t001 |  | | | |  | Vvi-Vitvi09g00310\_t001 |  |  |  |
| 3 | Atr-ERN15111 |  | | | |  | | | |  | | | |  |  |  |
| 3 | Atr-ERN15112 |  | | | |  | | | |  | | | |  |  |  |
| 3 | Atr-ERN15113 |  | | | |  | | | |  | | | |  |  |  |
| 3 | Atr-ERN15114 |  | | | |  | | | |  | | | |  |  |  |
| 3 | Atr-ERN15115 |  | Vvi-Vitvi04g00286\_t001 |  | | | |  | | | |  |  |  |
| 3 | Atr-ERN15116 |  | Vvi-Vitvi04g00284\_t001 |  | | | |  | Vvi-Vitvi09g00308\_t001 |  |  |  |
| 3 | Atr-ERN15117 |  | | | |  | | | |  | | | |  |  |  |
| 3 | Atr-ERN15118 |  | Vvi-Vitvi04g00283\_t001 |  | | | |  | | | |  |  |  |
| 3 | Atr-ERN15119 |  | | | |  | | | |  | | | |  |  |  |
| 4 | Atr-ERN15120 |  | Vvi-Vitvi04g00282\_t001 |  | Vvi-Vitvi11g00276\_t001 |  | | | |  | Vvi-Vitvi04g00001\_t001 |  |  |
| 3 | Atr-ERN15121 |  |  |  | | | |  | | | |  | | | |  |  |
| 3 | Atr-ERN15122 |  |  |  | | | |  | | | |  | | | |  |  |
| 3 | Atr-ERN15123 |  |  |  | Vvi-Vitvi11g00275\_t001 |  | Vvi-Vitvi09g00307\_t001 |  | Vvi-Vitvi04g00005\_t001 |  |  |
| 3 | Atr-ERN15124 |  |  |  | | | |  | | | |  | | | |  |  |
| 3 | Atr-ERN15125 |  |  |  | Vvi-Vitvi11g00274\_t001 |  | | | |  | | | |  |  |
| 3 | Atr-ERN15126 |  |  |  | | | |  | Vvi-Vitvi09g00306\_t002 |  | Vvi-Vitvi04g00006\_t001 |  |  |
| 3 | Atr-ERN15127 |  |  |  | | | |  | Vvi-Vitvi09g00305\_t001 |  | Vvi-Vitvi04g00007\_t001 |  |  |
| 3 | Atr-ERN15128 |  |  |  | | | |  | | | |  | | | |  |  |
| 3 | Atr-ERN15129 |  |  |  | | | |  | | | |  | | | |  |  |
| 3 | Atr-ERN15130 |  |  |  | | | |  | | | |  | | | |  |  |
| 3 | Atr-ERN15131 |  |  |  | | | |  | | | |  | | | |  |  |
| 3 | Atr-ERN15132 |  |  |  | Vvi-Vitvi11g00273\_t002 |  | | | |  | | | |  |  |
| 3 | Atr-ERN15133 |  |  |  | | | |  | Vvi-Vitvi09g00304\_t001 |  | | | |  |  |
| 3 | Atr-ERN15134 |  |  |  | | | |  | Vvi-Vitvi09g00302\_t001 |  | | | |  |  |
| 3 | Atr-ERN15135 |  |  |  | | | |  | | | |  | | | |  |  |
| 3 | Atr-ERN15136 |  |  |  | | | |  | | | |  | | | |  |  |
| 3 | Atr-ERN15137 |  |  |  | Vvi-Vitvi11g00272\_t001 |  | Vvi-Vitvi09g00301\_t001 |  | Vvi-Vitvi04g00009\_t001 |  |  |
| 3 | Atr-ERN15138 |  |  |  | Vvi-Vitvi11g00271\_t002 |  | | | |  | | | |  |  |
| 3 | Atr-ERN15139 |  |  |  | | | |  | | | |  | | | |  |  |
| 3 | Atr-ERN15140 |  |  |  | | | |  | | | |  | | | |  |  |
| 3 | Atr-ERN15141 |  |  |  | Vvi-Vitvi11g01393\_t001 |  | | | |  | | | |  |  |
| 3 | Atr-ERN15142 |  |  |  | | | |  | | | |  | | | |  |  |
| 3 | Atr-ERN15143 |  |  |  | | | |  | | | |  | | | |  |  |
| 3 | Atr-ERN15144 |  |  |  | | | |  | | | |  | | | |  |  |
| 3 | Atr-ERN15145 |  |  |  | | | |  | | | |  | Vvi-Vitvi04g00010\_t001 |  |  |
| 3 | Atr-ERN15146 |  |  |  | | | |  | Vvi-Vitvi09g00300\_t001 |  | | | |  |  |
| 3 | Atr-ERN15147 |  |  |  | | | |  | | | |  | | | |  |  |
| 3 | Atr-ERN15148 |  |  |  | Vvi-Vitvi11g00270\_t001 |  | Vvi-Vitvi09g04087\_t001 |  | | | |  |  |
| 3 | Atr-ERN15149 |  |  |  | | | |  | | | |  | Vvi-Vitvi04g00011\_t001 |  |  |
| 3 | Atr-ERN15150 |  |  |  | Vvi-Vitvi11g01392\_t001 |  | Vvi-Vitvi09g00290\_t001 |  | Vvi-Vitvi04g00012\_t001 |  |  |
| 3 | Atr-ERN15151 |  |  |  | | | |  | | | |  | | | |  |  |
| 3 | Atr-ERN15152 |  |  |  | | | |  | | | |  | | | |  |  |
| 3 | Atr-ERN15153 |  |  |  | | | |  | | | |  | | | |  |  |
| 3 | Atr-ERN15154 |  |  |  | Vvi-Vitvi11g00269\_t001 |  | | | |  | | | |  |  |
| 3 | Atr-ERN15155 |  |  |  | | | |  | | | |  | | | |  |  |
| 3 | Atr-ERN15156 |  |  |  | | | |  | | | |  | | | |  |  |
| 3 | Atr-ERN15157 |  |  |  | | | |  | | | |  | | | |  |  |
| 3 | Atr-ERN15158 |  |  |  | Vvi-Vitvi11g00268\_t001 |  | | | |  | | | |  |  |
| 3 | Atr-ERN15159 |  |  |  | | | |  | Vvi-Vitvi09g00287\_t001 |  | | | |  |  |
| 3 | Atr-ERN15160 |  |  |  | | | |  | Vvi-Vitvi09g00286\_t001 |  | | | |  |  |
| 3 | Atr-ERN15161 |  |  |  | | | |  | Vvi-Vitvi09g00284\_t001 |  | | | |  |  |
| 3 | Atr-ERN15162 |  |  |  | Vvi-Vitvi11g00267\_t001 |  | | | |  | | | |  |  |
| 3 | Atr-ERN15163 |  |  |  | | | |  | | | |  | | | |  |  |
| 3 | Atr-ERN15164 |  |  |  | | | |  | | | |  | | | |  |  |
| 3 | Atr-ERN15165 |  |  |  | | | |  | Vvi-Vitvi09g00283\_t001 |  | | | |  |  |
| 3 | Atr-ERN15166 |  |  |  | | | |  | | | |  | | | |  |  |
| 3 | Atr-ERN15167 |  |  |  | | | |  | | | |  | | | |  |  |
| 3 | Atr-ERN15168 |  |  |  | | | |  | Vvi-Vitvi09g00281\_t001 |  | Vvi-Vitvi04g00013\_t002 |  |  |
| 3 | Atr-ERN15169 |  |  |  | | | |  | | | |  | | | |  |  |
| 3 | Atr-ERN15170 |  |  |  | | | |  | | | |  | | | |  |  |
| 3 | Atr-ERN15171 |  |  |  | | | |  | | | |  | | | |  |  |
| 3 | Atr-ERN15172 |  |  |  | Vvi-Vitvi11g00261\_t001 |  | | | |  | Vvi-Vitvi04g04007\_t001 |  |  |
| 3 | Atr-ERN15173 |  |  |  | | | |  | | | |  | | | |  |  |
| 3 | Atr-ERN15174 |  |  |  | | | |  | | | |  | | | |  |  |
| 3 | Atr-ERN15175 |  |  |  | | | |  | | | |  | | | |  |  |
| 3 | Atr-ERN15176 |  |  |  | | | |  | | | |  | Vvi-Vitvi04g04010\_t001 |  |  |
| 3 | Atr-ERN15177 |  |  |  | | | |  | | | |  | | | |  |  |
| 3 | Atr-ERN15178 |  |  |  | Vvi-Vitvi11g00260\_t001 |  | Vvi-Vitvi09g00279\_t001 |  | | | |  |  |
| 3 | Atr-ERN15179 |  |  |  | Vvi-Vitvi11g01390\_t001 |  | | | |  | | | |  |  |
| 3 | Atr-ERN15180 |  |  |  | Vvi-Vitvi11g00251\_t001 |  | Vvi-Vitvi09g00275\_t001 |  | | | |  |  |
| 3 | Atr-ERN15181 |  |  |  | Vvi-Vitvi11g00250\_t001 |  | | | |  | | | |  |  |
| 3 | Atr-ERN15182 |  |  |  | | | |  | | | |  | | | |  |  |
| 3 | Atr-ERN15183 |  |  |  | | | |  | | | |  | | | |  |  |
| 3 | Atr-ERN15184 |  |  |  | Vvi-Vitvi11g00249\_t001 |  | | | |  | | | |  |  |
| 3 | Atr-ERN15185 |  |  |  | | | |  | | | |  | | | |  |  |
| 3 | Atr-ERN15186 |  |  |  | Vvi-Vitvi11g00247\_t001 |  | Vvi-Vitvi09g00273\_t001 |  | | | |  |  |
| 3 | Atr-ERN15187 |  |  |  | | | |  | | | |  | | | |  |  |
| 3 | Atr-ERN15188 |  |  |  | | | |  | | | |  | | | |  |  |
| 3 | Atr-ERN15189 |  |  |  | Vvi-Vitvi11g00245\_t001 |  | | | |  | | | |  |  |
| 3 | Atr-ERN15190 |  |  |  | | | |  | | | |  | | | |  |  |
| 3 | Atr-ERN15191 |  |  |  | | | |  | | | |  | | | |  |  |
| 3 | Atr-ERN15192 |  |  |  | | | |  | | | |  | | | |  |  |
| 3 | Atr-ERN15193 |  |  |  | | | |  | | | |  | | | |  |  |
| 3 | Atr-ERN15194 |  |  |  | | | |  | | | |  | | | |  |  |
| 3 | Atr-ERN15195 |  |  |  | | | |  | | | |  | | | |  |  |
| 3 | Atr-ERN15196 |  |  |  | | | |  | | | |  | | | |  |  |
| 3 | Atr-ERN15197 |  |  |  | | | |  | | | |  | | | |  |  |
| 3 | Atr-ERN15198 |  |  |  | | | |  | | | |  | | | |  |  |
| 3 | Atr-ERN15199 |  |  |  | | | |  | | | |  | | | |  |  |
| 3 | Atr-ERN15200 |  |  |  | | | |  | | | |  | Vvi-Vitvi04g00035\_t001 |  |  |
| 3 | Atr-ERN15201 |  |  |  | | | |  | | | |  | | | |  |  |
| 3 | Atr-ERN15202 |  |  |  | | | |  | | | |  | | | |  |  |
| 3 | Atr-ERN15203 |  |  |  | | | |  | | | |  | | | |  |  |
| 3 | Atr-ERN15204 |  |  |  | | | |  | | | |  | | | |  |  |
| 3 | Atr-ERN15205 |  |  |  | | | |  | | | |  | | | |  |  |
| 3 | Atr-ERN15206 |  |  |  | Vvi-Vitvi11g00243\_t001 |  | | | |  | | | |  |  |
| 3 | Atr-ERN15207 |  |  |  | | | |  | Vvi-Vitvi09g01562\_t001 |  | | | |  |  |
| 3 | Atr-ERN15208 |  |  |  | | | |  | | | |  | | | |  |  |
| 4 | Atr-ERN15209 |  | Vvi-Vitvi11g00219\_t001 |  | | | |  | | | |  | | | |  |  |
| 4 | Atr-ERN15210 |  | | | |  | Vvi-Vitvi11g00240\_t001 |  | Vvi-Vitvi09g00271\_t001 |  | | | |  |  |
| 4 | Atr-ERN15211 |  | | | |  | | | |  | | | |  | | | |  |  |
| 4 | Atr-ERN15212 |  | | | |  | Vvi-Vitvi11g00238\_t001 |  | | | |  | | | |  |  |
| 4 | Atr-ERN15213 |  | | | |  | Vvi-Vitvi11g00237\_t001 |  | | | |  | Vvi-Vitvi04g00040\_t003 |  |  |
| 4 | Atr-ERN15214 |  | | | |  | | | |  | Vvi-Vitvi09g04074\_t001 |  | | | |  |  |
| 4 | Atr-ERN15215 |  | | | |  | | | |  | | | |  | | | |  |  |
| 4 | Atr-ERN15216 |  | | | |  | | | |  | | | |  | Vvi-Vitvi04g00041\_t001 |  |  |
| 4 | Atr-ERN15217 |  | | | |  | Vvi-Vitvi11g00235\_t001 |  | | | |  | | | |  |  |
| 4 | Atr-ERN15218 |  | | | |  | | | |  | | | |  | | | |  |  |
| 4 | Atr-ERN15219 |  | | | |  | | | |  | | | |  | Vvi-Vitvi04g00042\_t001 |  |  |
| 4 | Atr-ERN15220 |  | | | |  | | | |  | | | |  | | | |  |  |
| 4 | Atr-ERN15221 |  | | | |  | | | |  | | | |  | | | |  |  |
| 4 | Atr-ERN15222 |  | | | |  | | | |  | | | |  | | | |  |  |
| 4 | Atr-ERN15223 |  | | | |  | | | |  | | | |  | | | |  |  |
| 4 | Atr-ERN15224 |  | Vvi-Vitvi11g01381\_t001 |  | | | |  | | | |  | Vvi-Vitvi04g01778\_t003 |  |  |
| 3 | Atr-ERN15225 |  | | | |  | | | |  | | | |  |  |  |
| 3 | Atr-ERN15226 |  | Vvi-Vitvi11g00227\_t001 |  | Vvi-Vitvi11g00227\_t001 |  | | | |  |  |  |
| 2 | Atr-ERN15227 |  | | | |  |  |  | | | |  |  |  |
| 2 | Atr-ERN15228 |  | | | |  |  |  | | | |  |  |  |
| 2 | Atr-ERN15229 |  | | | |  |  |  | | | |  |  |  |
| 2 | Atr-ERN15230 |  | | | |  |  |  | Vvi-Vitvi09g04068\_t001 |  |  |  |
| 2 | Atr-ERN15231 |  | | | |  |  |  | Vvi-Vitvi09g00260\_t001 |  |  |  |
| 2 | Atr-ERN15232 |  | | | |  |  |  | | | |  |  |  |
| 2 | Atr-ERN15233 |  | | | |  |  |  | Vvi-Vitvi09g01558\_t001 |  |  |  |
| 2 | Atr-ERN15234 |  | | | |  |  |  | | | |  |  |  |
| 2 | Atr-ERN15235 |  | | | |  |  |  | | | |  |  |  |
| 2 | Atr-ERN15236 |  | | | |  |  |  | | | |  |  |  |
| 2 | Atr-ERN15237 |  | | | |  |  |  | Vvi-Vitvi09g00257\_t001 |  |  |  |
| 2 | Atr-ERN15238 |  | | | |  |  |  | | | |  |  |  |
| 2 | Atr-ERN15239 |  | | | |  |  |  | Vvi-Vitvi09g00229\_t001 |  |  |  |
| 2 | Atr-ERN15240 |  | | | |  |  |  | | | |  |  |  |
| 2 | Atr-ERN15241 |  | | | |  |  |  | Vvi-Vitvi09g00228\_t001 |  |  |  |
| 1 | Atr-ERN15242 |  | | | |  |  |  |  |  |
| 1 | Atr-ERN15243 |  | | | |  |  |  |  |  |
| 1 | Atr-ERN15244 |  | | | |  |  |  |  |  |
| 1 | Atr-ERN15245 |  | Vvi-Vitvi11g00228\_t001 |  |  |  |  |  |
| 1 | Atr-ERN15246 |  | | | |  |  |  |  |  |
| 1 | Atr-ERN15247 |  | Vvi-Vitvi11g00229\_t001 |  |  |  |  |  |
| 1 | Atr-ERN15248 |  | | | |  |  |  |  |  |
| 1 | Atr-ERN15249 |  | Vvi-Vitvi11g00231\_t001 |  |  |  |  |  |
| 0 | Atr-ERN15250 |  |  |  |  |  |  |
| 0 | Atr-ERN15251 |  |  |  |  |  |  |
| 0 | Atr-ERN15252 |  |  |  |  |  |  |
| 0 | Atr-ERN15253 |  |  |  |  |  |  |
| 0 | Atr-ERN15254 |  |  |  |  |  |  |
| 0 | Atr-ERN15255 |  |  |  |  |  |  |
| 0 | Atr-ERN15256 |  |  |  |  |  |  |
| 0 | Atr-ERN15257 |  |  |  |  |  |  |
| 0 | Atr-ERN15258 |  |  |  |  |  |  |
| 0 | Atr-ERN15259 |  |  |  |  |  |  |
| 0 | Atr-ERN15260 |  |  |  |  |  |  |
| 0 | Atr-ERN15261 |  |  |  |  |  |  |
| 0 | Atr-ERN15262 |  |  |  |  |  |  |
| 0 | Atr-ERN15263 |  |  |  |  |  |  |
| 0 | Atr-ERN15264 |  |  |  |  |  |  |
| 0 | Atr-ERN15265 |  |  |  |  |  |  |
| 0 | Atr-ERN15266 |  |  |  |  |  |  |
| 0 | Atr-ERN15267 |  |  |  |  |  |  |
| 0 | Atr-ERN15268 |  |  |  |  |  |  |
| 0 | Atr-ERN15269 |  |  |  |  |  |  |
| 0 | Atr-ERN15270 |  |  |  |  |  |  |
| 0 | Atr-ERN15271 |  |  |  |  |  |  |
| 0 | Atr-ERN15272 |  |  |  |  |  |  |
| 0 | Atr-ERN15273 |  |  |  |  |  |  |
| 0 | Atr-ERN15274 |  |  |  |  |  |  |
| 0 | Atr-ERN15275 |  |  |  |  |  |  |
| 0 | Atr-ERN15276 |  |  |  |  |  |  |
| 0 | Atr-ERN15277 |  |  |  |  |  |  |
| 0 | Atr-ERN15278 |  |  |  |  |  |  |
| 0 | Atr-ERN15279 |  |  |  |  |  |  |
| 0 | Atr-ERN15280 |  |  |  |  |  |  |
| 0 | Atr-ERN15281 |  |  |  |  |  |  |
| 0 | Atr-ERN15282 |  |  |  |  |  |  |
| 0 | Atr-ERN15283 |  |  |  |  |  |  |
