# Supplementary material for: Genome-wide identification, characterization and gene expression of BES1 transcription factor family in grapevine (Vitis vinifera L.)
Source: Sci Rep. 2023 Jan 5;13:240. doi: 10.1038/s41598-022-24407-y (PMC9816167; doi:10.1038/s41598-022-24407-y)
Supplement: Supplementary file 3 — Supplementary Information. [file 41598_2022_24407_MOESM3_ESM.zip › Vvi_Atr/Vitis_vinifera.PN40024.v4.dna_sm.toplevel.fa.vs.Amborella_trichopoda.AMTR1.0.dna_sm.toplevel.fa.html/Atr-AmTr_v1.0_scaffold00057.html]

|  |  |  |  |  |  |  |  |  |  |  |  |  |  |
| --- | --- | --- | --- | --- | --- | --- | --- | --- | --- | --- | --- | --- | --- |
| Duplication depth | Reference chromosome | Collinear blocks | | | | | | | | | | | |
| 0 | Atr-ERN16736 |  |  |  |  |  |  |
| 0 | Atr-ERN16737 |  |  |  |  |  |  |
| 0 | Atr-ERN16738 |  |  |  |  |  |  |
| 0 | Atr-ERN16739 |  |  |  |  |  |  |
| 0 | Atr-ERN16740 |  |  |  |  |  |  |
| 0 | Atr-ERN16741 |  |  |  |  |  |  |
| 0 | Atr-ERN16742 |  |  |  |  |  |  |
| 0 | Atr-ERN16743 |  |  |  |  |  |  |
| 0 | Atr-ERN16744 |  |  |  |  |  |  |
| 2 | Atr-ERN16745 |  | Vvi-Vitvi07g00410\_t002 |  | Vvi-Vitvi05g00106\_t001 |  |  |  |  |
| 2 | Atr-ERN16746 |  | | | |  | | | |  |  |  |  |
| 2 | Atr-ERN16747 |  | | | |  | | | |  |  |  |  |
| 3 | Atr-ERN16748 |  | | | |  | | | |  | Vvi-Vitvi14g00097\_t001 |  |  |  |
| 3 | Atr-ERN16749 |  | | | |  | | | |  | | | |  |  |  |
| 3 | Atr-ERN16750 |  | | | |  | | | |  | | | |  |  |  |
| 3 | Atr-ERN16751 |  | | | |  | | | |  | | | |  |  |  |
| 3 | Atr-ERN16752 |  | | | |  | | | |  | | | |  |  |  |
| 3 | Atr-ERN16753 |  | | | |  | | | |  | | | |  |  |  |
| 3 | Atr-ERN16754 |  | | | |  | | | |  | | | |  |  |  |
| 3 | Atr-ERN16755 |  | Vvi-Vitvi07g02221\_t001 |  | | | |  | | | |  |  |  |
| 3 | Atr-ERN16756 |  | | | |  | | | |  | | | |  |  |  |
| 3 | Atr-ERN16757 |  | Vvi-Vitvi07g02220\_t001 |  | | | |  | | | |  |  |  |
| 3 | Atr-ERN16758 |  | | | |  | Vvi-Vitvi05g00096\_t001 |  | | | |  |  |  |
| 3 | Atr-ERN16759 |  | | | |  | | | |  | | | |  |  |  |
| 3 | Atr-ERN16760 |  | | | |  | Vvi-Vitvi05g00092\_t001 |  | | | |  |  |  |
| 3 | Atr-ERN16761 |  | Vvi-Vitvi07g00401\_t001 |  | Vvi-Vitvi05g00089\_t001 |  | | | |  |  |  |
| 3 | Atr-ERN16762 |  | | | |  | Vvi-Vitvi05g04019\_t001 |  | | | |  |  |  |
| 3 | Atr-ERN16763 |  | | | |  | | | |  | | | |  |  |  |
| 3 | Atr-ERN16764 |  | Vvi-Vitvi07g00400\_t001 |  | | | |  | | | |  |  |  |
| 3 | Atr-ERN16765 |  | | | |  | Vvi-Vitvi05g01768\_t001 |  | | | |  |  |  |
| 3 | Atr-ERN16766 |  | | | |  | | | |  | | | |  |  |  |
| 3 | Atr-ERN16767 |  | | | |  | | | |  | | | |  |  |  |
| 3 | Atr-ERN16768 |  | Vvi-Vitvi07g00399\_t001 |  | | | |  | | | |  |  |  |
| 3 | Atr-ERN16769 |  | Vvi-Vitvi07g00396\_t001 |  | | | |  | | | |  |  |  |
| 3 | Atr-ERN16770 |  | | | |  | | | |  | | | |  |  |  |
| 3 | Atr-ERN16771 |  | | | |  | | | |  | | | |  |  |  |
| 3 | Atr-ERN16772 |  | | | |  | | | |  | | | |  |  |  |
| 3 | Atr-ERN16773 |  | Vvi-Vitvi07g00395\_t001 |  | | | |  | | | |  |  |  |
| 3 | Atr-ERN16774 |  | | | |  | Vvi-Vitvi05g01767\_t001 |  | Vvi-Vitvi14g02477\_t001 |  |  |  |
| 3 | Atr-ERN16775 |  | Vvi-Vitvi07g00394\_t001 |  | | | |  | | | |  |  |  |
| 3 | Atr-ERN16776 |  | | | |  | Vvi-Vitvi05g00085\_t001 |  | | | |  |  |  |
| 3 | Atr-ERN16777 |  | | | |  | | | |  | | | |  |  |  |
| 3 | Atr-ERN16778 |  | Vvi-Vitvi07g00392\_t001 |  | | | |  | | | |  |  |  |
| 3 | Atr-ERN16779 |  | | | |  | | | |  | Vvi-Vitvi14g00111\_t001 |  |  |  |
| 3 | Atr-ERN16780 |  | | | |  | | | |  | | | |  |  |  |
| 3 | Atr-ERN16781 |  | Vvi-Vitvi07g00391\_t001 |  | | | |  | | | |  |  |  |
| 3 | Atr-ERN16782 |  | Vvi-Vitvi07g00390\_t001 |  | | | |  | Vvi-Vitvi14g00113\_t001 |  |  |  |
| 3 | Atr-ERN16783 |  | | | |  | Vvi-Vitvi05g00083\_t001 |  | Vvi-Vitvi14g00114\_t001 |  |  |  |
| 3 | Atr-ERN16784 |  | | | |  | | | |  | | | |  |  |  |
| 3 | Atr-ERN16785 |  | | | |  | | | |  | | | |  |  |  |
| 3 | Atr-ERN16786 |  | Vvi-Vitvi07g00387\_t001 |  | | | |  | | | |  |  |  |
| 3 | Atr-ERN16787 |  | Vvi-Vitvi07g00386\_t001 |  | | | |  | | | |  |  |  |
| 3 | Atr-ERN16788 |  | Vvi-Vitvi07g02217\_t001 |  | | | |  | | | |  |  |  |
| 3 | Atr-ERN16789 |  | | | |  | | | |  | | | |  |  |  |
| 3 | Atr-ERN16790 |  | | | |  | | | |  | Vvi-Vitvi14g00115\_t001 |  |  |  |
| 3 | Atr-ERN16791 |  | Vvi-Vitvi07g02216\_t001 |  | Vvi-Vitvi05g01766\_t001 |  | Vvi-Vitvi14g02479\_t001 |  |  |  |
| 3 | Atr-ERN16792 |  | | | |  | | | |  | Vvi-Vitvi14g00116\_t001 |  |  |  |
| 3 | Atr-ERN16793 |  | | | |  | | | |  | | | |  |  |  |
| 4 | Atr-ERN16794 |  | | | |  | Vvi-Vitvi05g00082\_t001 |  | | | |  | Vvi-Vitvi01g00309\_t001 |  |  |
| 4 | Atr-ERN16795 |  | | | |  | | | |  | | | |  | | | |  |  |
| 4 | Atr-ERN16796 |  | | | |  | | | |  | | | |  | | | |  |  |
| 4 | Atr-ERN16797 |  | | | |  | | | |  | | | |  | Vvi-Vitvi01g00310\_t001 |  |  |
| 4 | Atr-ERN16798 |  | | | |  | | | |  | | | |  | | | |  |  |
| 4 | Atr-ERN16799 |  | | | |  | | | |  | | | |  | | | |  |  |
| 4 | Atr-ERN16800 |  | | | |  | | | |  | | | |  | | | |  |  |
| 4 | Atr-ERN16801 |  | | | |  | | | |  | Vvi-Vitvi14g00117\_t001 |  | | | |  |  |
| 4 | Atr-ERN16802 |  | | | |  | | | |  | | | |  | | | |  |  |
| 4 | Atr-ERN16803 |  | Vvi-Vitvi07g00383\_t001 |  | | | |  | | | |  | | | |  |  |
| 4 | Atr-ERN16804 |  | | | |  | | | |  | Vvi-Vitvi14g00118\_t001 |  | | | |  |  |
| 4 | Atr-ERN16805 |  | | | |  | Vvi-Vitvi05g00079\_t001 |  | | | |  | | | |  |  |
| 4 | Atr-ERN16806 |  | | | |  | | | |  | | | |  | Vvi-Vitvi01g00325\_t001 |  |  |
| 4 | Atr-ERN16807 |  | | | |  | | | |  | | | |  | | | |  |  |
| 5 | Atr-ERN16808 |  | | | |  | | | |  | | | |  | | | |  | Vvi-Vitvi07g00368\_t001 |  |
| 5 | Atr-ERN16809 |  | | | |  | | | |  | | | |  | | | |  | | | |  |
| 5 | Atr-ERN16810 |  | | | |  | | | |  | | | |  | | | |  | | | |  |
| 5 | Atr-ERN16811 |  | | | |  | | | |  | | | |  | | | |  | | | |  |
| 5 | Atr-ERN16812 |  | | | |  | | | |  | | | |  | Vvi-Vitvi01g00326\_t001 |  | Vvi-Vitvi07g00369\_t001 |  |
| 5 | Atr-ERN16813 |  | | | |  | | | |  | | | |  | | | |  | | | |  |
| 5 | Atr-ERN16814 |  | | | |  | | | |  | | | |  | | | |  | | | |  |
| 5 | Atr-ERN16815 |  | | | |  | | | |  | | | |  | | | |  | | | |  |
| 5 | Atr-ERN16816 |  | | | |  | | | |  | | | |  | Vvi-Vitvi01g00328\_t001 |  | Vvi-Vitvi07g00370\_t001 |  |
| 5 | Atr-ERN16817 |  | | | |  | | | |  | | | |  | | | |  | | | |  |
| 5 | Atr-ERN16818 |  | | | |  | | | |  | | | |  | | | |  | | | |  |
| 5 | Atr-ERN16819 |  | | | |  | | | |  | | | |  | | | |  | | | |  |
| 5 | Atr-ERN16820 |  | Vvi-Vitvi07g00374\_t001 |  | Vvi-Vitvi05g00075\_t001 |  | | | |  | Vvi-Vitvi01g01959\_t001 |  | | | |  |
| 5 | Atr-ERN16821 |  | | | |  | Vvi-Vitvi05g04016\_t001 |  | Vvi-Vitvi14g00124\_t001 |  | | | |  | | | |  |
| 5 | Atr-ERN16822 |  | | | |  | | | |  | Vvi-Vitvi14g00125\_t001 |  | | | |  | Vvi-Vitvi07g00373\_t001 |  |
| 5 | Atr-ERN16823 |  | Vvi-Vitvi07g04089\_t001 |  | Vvi-Vitvi05g01762\_t001 |  | Vvi-Vitvi14g00126\_t001 |  | | | |  | | | |  |
| 2 | Atr-ERN16824 |  |  |  |  |  |  |  | | | |  | Vvi-Vitvi07g00375\_t001 |  |
| 2 | Atr-ERN16825 |  |  |  |  |  |  |  | | | |  | | | |  |
| 2 | Atr-ERN16826 |  |  |  |  |  |  |  | Vvi-Vitvi01g04092\_t001 |  | | | |  |
| 1 | Atr-ERN16827 |  |  |  |  |  |  |  |  |  | | | |  |
| 1 | Atr-ERN16828 |  |  |  |  |  |  |  |  |  | | | |  |
| 1 | Atr-ERN16829 |  |  |  |  |  |  |  |  |  | Vvi-Vitvi07g00376\_t001 |  |
| 2 | Atr-ERN16830 |  | Vvi-Vitvi05g00394\_t001 |  |  |  |  |  |  |  | Vvi-Vitvi07g00378\_t001 |  |
| 2 | Atr-ERN16831 |  | Vvi-Vitvi05g00392\_t001 |  |  |  |  |  |  |  | | | |  |
| 2 | Atr-ERN16832 |  | | | |  |  |  |  |  |  |  | | | |  |
| 2 | Atr-ERN16833 |  | | | |  |  |  |  |  |  |  | | | |  |
| 2 | Atr-ERN16834 |  | | | |  |  |  |  |  |  |  | Vvi-Vitvi07g02214\_t001 |  |
| 1 | Atr-ERN16835 |  | | | |  |  |  |  |  |
| 2 | Atr-ERN16836 |  | | | |  | Vvi-Vitvi07g00227\_t001 |  |  |  |  |
| 2 | Atr-ERN16837 |  | Vvi-Vitvi05g00372\_t001 |  | | | |  |  |  |  |
| 2 | Atr-ERN16838 |  | | | |  | Vvi-Vitvi07g00228\_t001 |  |  |  |  |
| 2 | Atr-ERN16839 |  | | | |  | | | |  |  |  |  |
| 2 | Atr-ERN16840 |  | | | |  | | | |  |  |  |  |
| 2 | Atr-ERN16841 |  | | | |  | | | |  |  |  |  |
| 2 | Atr-ERN16842 |  | | | |  | | | |  |  |  |  |
| 2 | Atr-ERN16843 |  | | | |  | | | |  |  |  |  |
| 2 | Atr-ERN16844 |  | Vvi-Vitvi05g00370\_t001 |  | Vvi-Vitvi07g00230\_t001 |  |  |  |  |
| 2 | Atr-ERN16845 |  | | | |  | Vvi-Vitvi07g00231\_t001 |  |  |  |  |
| 2 | Atr-ERN16846 |  | | | |  | | | |  |  |  |  |
| 2 | Atr-ERN16847 |  | | | |  | Vvi-Vitvi07g00232\_t001 |  |  |  |  |
| 2 | Atr-ERN16848 |  | | | |  | Vvi-Vitvi07g00233\_t001 |  |  |  |  |
| 2 | Atr-ERN16849 |  | | | |  | | | |  |  |  |  |
| 2 | Atr-ERN16850 |  | | | |  | Vvi-Vitvi07g00234\_t001 |  |  |  |  |
| 3 | Atr-ERN16851 |  | | | |  | | | |  | Vvi-Vitvi14g01460\_t001 |  |  |  |
| 3 | Atr-ERN16852 |  | Vvi-Vitvi05g00367\_t001 |  | | | |  | | | |  |  |  |
| 3 | Atr-ERN16853 |  | Vvi-Vitvi05g00366\_t001 |  | | | |  | Vvi-Vitvi14g01467\_t001 |  |  |  |
| 3 | Atr-ERN16854 |  | | | |  | | | |  | | | |  |  |  |
| 3 | Atr-ERN16855 |  | Vvi-Vitvi05g00365\_t001 |  | | | |  | | | |  |  |  |
| 3 | Atr-ERN16856 |  | | | |  | | | |  | Vvi-Vitvi14g01474\_t001 |  |  |  |
| 3 | Atr-ERN16857 |  | Vvi-Vitvi05g00364\_t001 |  | | | |  | | | |  |  |  |
| 3 | Atr-ERN16858 |  | | | |  | | | |  | | | |  |  |  |
| 3 | Atr-ERN16859 |  | | | |  | Vvi-Vitvi07g00237\_t001 |  | | | |  |  |  |
| 3 | Atr-ERN16860 |  | | | |  | | | |  | | | |  |  |  |
| 3 | Atr-ERN16861 |  | | | |  | Vvi-Vitvi07g00238\_t001 |  | | | |  |  |  |
| 3 | Atr-ERN16862 |  | | | |  | | | |  | | | |  |  |  |
| 3 | Atr-ERN16863 |  | | | |  | Vvi-Vitvi07g00240\_t001 |  | | | |  |  |  |
| 3 | Atr-ERN16864 |  | | | |  | | | |  | | | |  |  |  |
| 3 | Atr-ERN16865 |  | | | |  | | | |  | | | |  |  |  |
| 3 | Atr-ERN16866 |  | | | |  | | | |  | | | |  |  |  |
| 3 | Atr-ERN16867 |  | Vvi-Vitvi05g00363\_t001 |  | | | |  | | | |  |  |  |
| 3 | Atr-ERN16868 |  | | | |  | Vvi-Vitvi07g00243\_t001 |  | Vvi-Vitvi14g02951\_t001 |  |  |  |
| 3 | Atr-ERN16869 |  | Vvi-Vitvi05g00362\_t001 |  | | | |  | | | |  |  |  |
| 3 | Atr-ERN16870 |  | Vvi-Vitvi05g00360\_t003 |  | | | |  | | | |  |  |  |
| 3 | Atr-ERN16871 |  | | | |  | | | |  | | | |  |  |  |
| 3 | Atr-ERN16872 |  | Vvi-Vitvi05g00359\_t001 |  | Vvi-Vitvi07g00244\_t001 |  | | | |  |  |  |
| 3 | Atr-ERN16873 |  | | | |  | | | |  | | | |  |  |  |
| 3 | Atr-ERN16874 |  | | | |  | | | |  | | | |  |  |  |
| 3 | Atr-ERN16875 |  | Vvi-Vitvi05g00358\_t001 |  | | | |  | | | |  |  |  |
| 3 | Atr-ERN16876 |  | Vvi-Vitvi05g01862\_t002 |  | Vvi-Vitvi07g02159\_t002 |  | | | |  |  |  |
| 3 | Atr-ERN16877 |  | Vvi-Vitvi05g00357\_t001 |  | | | |  | | | |  |  |  |
| 3 | Atr-ERN16878 |  | | | |  | | | |  | | | |  |  |  |
| 3 | Atr-ERN16879 |  | | | |  | | | |  | | | |  |  |  |
| 3 | Atr-ERN16880 |  | | | |  | | | |  | | | |  |  |  |
| 4 | Atr-ERN16881 |  | | | |  | | | |  | | | |  | Vvi-Vitvi05g01857\_t001 |  |  |
| 4 | Atr-ERN16882 |  | | | |  | | | |  | | | |  | | | |  |  |
| 4 | Atr-ERN16883 |  | | | |  | Vvi-Vitvi07g00251\_t001 |  | Vvi-Vitvi14g01489\_t001 |  | Vvi-Vitvi05g00349\_t001 |  |  |
| 4 | Atr-ERN16884 |  | | | |  | | | |  | Vvi-Vitvi14g01491\_t001 |  | | | |  |  |
| 4 | Atr-ERN16885 |  | | | |  | | | |  | | | |  | | | |  |  |
| 4 | Atr-ERN16886 |  | | | |  | | | |  | Vvi-Vitvi14g01493\_t001 |  | | | |  |  |
| 4 | Atr-ERN16887 |  | | | |  | | | |  | | | |  | | | |  |  |
| 4 | Atr-ERN16888 |  | | | |  | | | |  | | | |  | | | |  |  |
| 4 | Atr-ERN16889 |  | | | |  | | | |  | | | |  | | | |  |  |
| 4 | Atr-ERN16890 |  | | | |  | | | |  | | | |  | | | |  |  |
| 4 | Atr-ERN16891 |  | | | |  | | | |  | | | |  | | | |  |  |
| 4 | Atr-ERN16892 |  | | | |  | | | |  | | | |  | | | |  |  |
| 4 | Atr-ERN16893 |  | | | |  | | | |  | | | |  | | | |  |  |
| 4 | Atr-ERN16894 |  | | | |  | | | |  | | | |  | | | |  |  |
| 4 | Atr-ERN16895 |  | | | |  | | | |  | | | |  | | | |  |  |
| 4 | Atr-ERN16896 |  | | | |  | | | |  | | | |  | | | |  |  |
| 4 | Atr-ERN16897 |  | | | |  | | | |  | | | |  | | | |  |  |
| 4 | Atr-ERN16898 |  | | | |  | | | |  | | | |  | | | |  |  |
| 4 | Atr-ERN16899 |  | | | |  | | | |  | | | |  | | | |  |  |
| 4 | Atr-ERN16900 |  | | | |  | | | |  | | | |  | | | |  |  |
| 4 | Atr-ERN16901 |  | | | |  | | | |  | | | |  | | | |  |  |
| 4 | Atr-ERN16902 |  | Vvi-Vitvi05g00350\_t001 |  | | | |  | | | |  | Vvi-Vitvi05g00350\_t001 |  |  |
| 4 | Atr-ERN16903 |  | | | |  | | | |  | | | |  | | | |  |  |
| 4 | Atr-ERN16904 |  | | | |  | | | |  | | | |  | | | |  |  |
| 4 | Atr-ERN16905 |  | | | |  | | | |  | | | |  | | | |  |  |
| 4 | Atr-ERN16906 |  | | | |  | | | |  | | | |  | | | |  |  |
| 4 | Atr-ERN16907 |  | | | |  | Vvi-Vitvi07g00253\_t001 |  | | | |  | | | |  |  |
| 4 | Atr-ERN16908 |  | | | |  | | | |  | | | |  | | | |  |  |
| 4 | Atr-ERN16909 |  | Vvi-Vitvi05g00348\_t001 |  | | | |  | | | |  | | | |  |  |
| 4 | Atr-ERN16910 |  | | | |  | | | |  | Vvi-Vitvi14g01498\_t002 |  | | | |  |  |
| 4 | Atr-ERN16911 |  | | | |  | | | |  | | | |  | | | |  |  |
| 4 | Atr-ERN16912 |  | | | |  | | | |  | | | |  | | | |  |  |
| 4 | Atr-ERN16913 |  | | | |  | | | |  | Vvi-Vitvi14g01502\_t001 |  | Vvi-Vitvi05g00351\_t001 |  |  |
| 4 | Atr-ERN16914 |  | | | |  | | | |  | | | |  | | | |  |  |
| 4 | Atr-ERN16915 |  | | | |  | | | |  | | | |  | | | |  |  |
| 4 | Atr-ERN16916 |  | | | |  | | | |  | | | |  | | | |  |  |
| 4 | Atr-ERN16917 |  | | | |  | | | |  | | | |  | | | |  |  |
| 4 | Atr-ERN16918 |  | Vvi-Vitvi05g00346\_t001 |  | | | |  | | | |  | | | |  |  |
| 4 | Atr-ERN16919 |  | | | |  | | | |  | | | |  | | | |  |  |
| 4 | Atr-ERN16920 |  | | | |  | | | |  | | | |  | | | |  |  |
| 4 | Atr-ERN16921 |  | | | |  | Vvi-Vitvi07g00255\_t001 |  | Vvi-Vitvi14g01507\_t001 |  | | | |  |  |
| 4 | Atr-ERN16922 |  | | | |  | | | |  | | | |  | | | |  |  |
| 4 | Atr-ERN16923 |  | | | |  | | | |  | | | |  | | | |  |  |
| 4 | Atr-ERN16924 |  | Vvi-Vitvi05g00343\_t001 |  | | | |  | | | |  | | | |  |  |
| 4 | Atr-ERN16925 |  | | | |  | | | |  | | | |  | | | |  |  |
| 4 | Atr-ERN16926 |  | | | |  | | | |  | | | |  | | | |  |  |
| 4 | Atr-ERN16927 |  | | | |  | | | |  | Vvi-Vitvi14g04563\_t001 |  | | | |  |  |
| 3 | Atr-ERN16928 |  | Vvi-Vitvi05g00341\_t001 |  | | | |  |  |  | | | |  |  |
| 3 | Atr-ERN16929 |  | | | |  | | | |  |  |  | Vvi-Vitvi05g00352\_t001 |  |  |
| 3 | Atr-ERN16930 |  | | | |  | | | |  |  |  | Vvi-Vitvi05g00354\_t001 |  |  |
| 3 | Atr-ERN16931 |  | | | |  | | | |  |  |  | Vvi-Vitvi05g00355\_t001 |  |  |
| 2 | Atr-ERN16932 |  | | | |  | | | |  |  |  |  |
| 2 | Atr-ERN16933 |  | Vvi-Vitvi05g00339\_t001 |  | | | |  |  |  |  |
| 2 | Atr-ERN16934 |  | Vvi-Vitvi05g00338\_t001 |  | | | |  |  |  |  |
| 2 | Atr-ERN16935 |  | | | |  | | | |  |  |  |  |
| 3 | Atr-ERN16936 |  | | | |  | | | |  | Vvi-Vitvi14g00232\_t001 |  |  |  |
| 3 | Atr-ERN16937 |  | Vvi-Vitvi05g00337\_t001 |  | | | |  | | | |  |  |  |
| 3 | Atr-ERN16938 |  | | | |  | | | |  | | | |  |  |  |
| 3 | Atr-ERN16939 |  | Vvi-Vitvi05g00335\_t001 |  | | | |  | | | |  |  |  |
| 3 | Atr-ERN16940 |  | | | |  | | | |  | Vvi-Vitvi14g00231\_t001 |  |  |  |
| 3 | Atr-ERN16941 |  | | | |  | Vvi-Vitvi07g00260\_t001 |  | | | |  |  |  |
| 3 | Atr-ERN16942 |  | | | |  | | | |  | | | |  |  |  |
| 3 | Atr-ERN16943 |  | | | |  | | | |  | | | |  |  |  |
| 3 | Atr-ERN16944 |  | | | |  | | | |  | | | |  |  |  |
| 3 | Atr-ERN16945 |  | | | |  | | | |  | | | |  |  |  |
| 3 | Atr-ERN16946 |  | | | |  | | | |  | | | |  |  |  |
| 3 | Atr-ERN16947 |  | Vvi-Vitvi05g00334\_t001 |  | | | |  | | | |  |  |  |
| 3 | Atr-ERN16948 |  | | | |  | | | |  | | | |  |  |  |
| 3 | Atr-ERN16949 |  | | | |  | | | |  | | | |  |  |  |
| 3 | Atr-ERN16950 |  | Vvi-Vitvi05g00329\_t001 |  | | | |  | | | |  |  |  |
| 3 | Atr-ERN16951 |  | | | |  | Vvi-Vitvi07g00265\_t001 |  | | | |  |  |  |
| 3 | Atr-ERN16952 |  | | | |  | | | |  | | | |  |  |  |
| 3 | Atr-ERN16953 |  | | | |  | | | |  | | | |  |  |  |
| 3 | Atr-ERN16954 |  | Vvi-Vitvi05g00328\_t001 |  | | | |  | Vvi-Vitvi14g00229\_t001 |  |  |  |
| 3 | Atr-ERN16955 |  | Vvi-Vitvi05g00326\_t001 |  | Vvi-Vitvi07g00269\_t001 |  | Vvi-Vitvi14g04081\_t001 |  |  |  |
| 3 | Atr-ERN16956 |  | | | |  | | | |  | | | |  |  |  |
| 3 | Atr-ERN16957 |  | | | |  | | | |  | | | |  |  |  |
| 4 | Atr-ERN16958 |  | | | |  | | | |  | | | |  | Vvi-Vitvi05g00295\_t001 |  |  |
| 4 | Atr-ERN16959 |  | | | |  | | | |  | | | |  | | | |  |  |
| 4 | Atr-ERN16960 |  | | | |  | | | |  | | | |  | | | |  |  |
| 4 | Atr-ERN16961 |  | | | |  | | | |  | | | |  | | | |  |  |
| 4 | Atr-ERN16962 |  | | | |  | | | |  | | | |  | | | |  |  |
| 4 | Atr-ERN16963 |  | | | |  | | | |  | | | |  | | | |  |  |
| 4 | Atr-ERN16964 |  | Vvi-Vitvi05g00325\_t001 |  | | | |  | | | |  | | | |  |  |
| 4 | Atr-ERN16965 |  | | | |  | | | |  | | | |  | | | |  |  |
| 4 | Atr-ERN16966 |  | | | |  | | | |  | | | |  | | | |  |  |
| 4 | Atr-ERN16967 |  | | | |  | | | |  | | | |  | | | |  |  |
| 4 | Atr-ERN16968 |  | | | |  | | | |  | | | |  | | | |  |  |
| 4 | Atr-ERN16969 |  | | | |  | | | |  | | | |  | | | |  |  |
| 4 | Atr-ERN16970 |  | | | |  | Vvi-Vitvi07g00273\_t001 |  | Vvi-Vitvi14g00217\_t001 |  | Vvi-Vitvi05g00299\_t001 |  |  |
| 4 | Atr-ERN16971 |  | | | |  | | | |  | | | |  | | | |  |  |
| 4 | Atr-ERN16972 |  | | | |  | | | |  | | | |  | | | |  |  |
| 4 | Atr-ERN16973 |  | | | |  | | | |  | | | |  | | | |  |  |
| 4 | Atr-ERN16974 |  | | | |  | | | |  | Vvi-Vitvi14g00216\_t001 |  | | | |  |  |
| 4 | Atr-ERN16975 |  | | | |  | | | |  | Vvi-Vitvi14g00215\_t002 |  | Vvi-Vitvi05g00301\_t001 |  |  |
| 4 | Atr-ERN16976 |  | | | |  | | | |  | Vvi-Vitvi14g00197\_t001 |  | | | |  |  |
| 4 | Atr-ERN16977 |  | | | |  | | | |  | | | |  | | | |  |  |
| 4 | Atr-ERN16978 |  | | | |  | | | |  | | | |  | | | |  |  |
| 4 | Atr-ERN16979 |  | Vvi-Vitvi05g00302\_t001 |  | | | |  | | | |  | Vvi-Vitvi05g00302\_t001 |  |  |
| 3 | Atr-ERN16980 |  |  |  | | | |  | Vvi-Vitvi14g02511\_t001 |  | | | |  |  |
| 3 | Atr-ERN16981 |  |  |  | | | |  | | | |  | | | |  |  |
| 3 | Atr-ERN16982 |  |  |  | | | |  | | | |  | | | |  |  |
| 3 | Atr-ERN16983 |  |  |  | | | |  | Vvi-Vitvi14g00196\_t001 |  | | | |  |  |
| 3 | Atr-ERN16984 |  |  |  | | | |  | Vvi-Vitvi14g00195\_t001 |  | | | |  |  |
| 3 | Atr-ERN16985 |  |  |  | | | |  | | | |  | Vvi-Vitvi05g00303\_t001 |  |  |
| 3 | Atr-ERN16986 |  |  |  | | | |  | Vvi-Vitvi14g00194\_t001 |  | Vvi-Vitvi05g00305\_t001 |  |  |
| 3 | Atr-ERN16987 |  |  |  | | | |  | | | |  | | | |  |  |
| 3 | Atr-ERN16988 |  |  |  | | | |  | | | |  | | | |  |  |
| 3 | Atr-ERN16989 |  |  |  | | | |  | | | |  | | | |  |  |
| 3 | Atr-ERN16990 |  |  |  | Vvi-Vitvi07g00278\_t001 |  | Vvi-Vitvi14g00193\_t001 |  | Vvi-Vitvi05g00309\_t001 |  |  |
| 3 | Atr-ERN16991 |  |  |  | | | |  | | | |  | | | |  |  |
| 3 | Atr-ERN16992 |  |  |  | | | |  | | | |  | | | |  |  |
| 3 | Atr-ERN16993 |  |  |  | | | |  | | | |  | | | |  |  |
| 3 | Atr-ERN16994 |  |  |  | Vvi-Vitvi07g00280\_t001 |  | Vvi-Vitvi14g00191\_t001 |  | | | |  |  |
| 3 | Atr-ERN16995 |  |  |  | Vvi-Vitvi07g02188\_t003 |  | | | |  | | | |  |  |
| 3 | Atr-ERN16996 |  |  |  | | | |  | | | |  | | | |  |  |
| 3 | Atr-ERN16997 |  |  |  | | | |  | | | |  | | | |  |  |
| 3 | Atr-ERN16998 |  |  |  | | | |  | | | |  | | | |  |  |
| 3 | Atr-ERN16999 |  |  |  | | | |  | | | |  | | | |  |  |
| 3 | Atr-ERN17000 |  |  |  | Vvi-Vitvi07g02190\_t001 |  | | | |  | | | |  |  |
| 3 | Atr-ERN17001 |  |  |  | | | |  | | | |  | | | |  |  |
| 3 | Atr-ERN17002 |  |  |  | | | |  | | | |  | | | |  |  |
| 3 | Atr-ERN17003 |  |  |  | | | |  | | | |  | | | |  |  |
| 3 | Atr-ERN17004 |  |  |  | | | |  | | | |  | | | |  |  |
| 3 | Atr-ERN17005 |  |  |  | | | |  | Vvi-Vitvi14g04053\_t001 |  | | | |  |  |
| 2 | Atr-ERN17006 |  |  |  | | | |  |  |  | Vvi-Vitvi05g01845\_t001 |  |  |
| 2 | Atr-ERN17007 |  |  |  | | | |  |  |  | | | |  |  |
| 2 | Atr-ERN17008 |  |  |  | | | |  |  |  | | | |  |  |
| 2 | Atr-ERN17009 |  |  |  | | | |  |  |  | Vvi-Vitvi05g00310\_t001 |  |  |
| 2 | Atr-ERN17010 |  |  |  | Vvi-Vitvi07g00289\_t001 |  |  |  | | | |  |  |
| 1 | Atr-ERN17011 |  |  |  |  |  |  |  | Vvi-Vitvi05g00311\_t001 |  |  |
| 1 | Atr-ERN17012 |  |  |  |  |  |  |  | | | |  |  |
| 1 | Atr-ERN17013 |  |  |  |  |  |  |  | Vvi-Vitvi05g00312\_t001 |  |  |
| 0 | Atr-ERN17014 |  |  |  |  |  |  |
| 0 | Atr-ERN17015 |  |  |  |  |  |  |
| 0 | Atr-ERN17016 |  |  |  |  |  |  |
| 0 | Atr-ERN17017 |  |  |  |  |  |  |
| 0 | Atr-ERN17018 |  |  |  |  |  |  |
| 0 | Atr-ERN17019 |  |  |  |  |  |  |
| 0 | Atr-ERN17020 |  |  |  |  |  |  |
| 0 | Atr-ERN17021 |  |  |  |  |  |  |
| 0 | Atr-ERN17022 |  |  |  |  |  |  |
| 0 | Atr-ERN17023 |  |  |  |  |  |  |
| 0 | Atr-ERN17024 |  |  |  |  |  |  |
| 0 | Atr-ERN17025 |  |  |  |  |  |  |
| 0 | Atr-ERN17026 |  |  |  |  |  |  |
| 0 | Atr-ERN17027 |  |  |  |  |  |  |
| 0 | Atr-ERN17028 |  |  |  |  |  |  |
