# Supplementary material for: Genome-wide identification, characterization and gene expression of BES1 transcription factor family in grapevine (Vitis vinifera L.)
Source: Sci Rep. 2023 Jan 5;13:240. doi: 10.1038/s41598-022-24407-y (PMC9816167; doi:10.1038/s41598-022-24407-y)
Supplement: Supplementary file 3 — Supplementary Information. [file 41598_2022_24407_MOESM3_ESM.zip › Vvi_Atr/Vitis_vinifera.PN40024.v4.dna_sm.toplevel.fa.vs.Amborella_trichopoda.AMTR1.0.dna_sm.toplevel.fa.html/Atr-AmTr_v1.0_scaffold00058.html]

|  |  |  |  |  |  |  |  |  |  |  |  |  |  |
| --- | --- | --- | --- | --- | --- | --- | --- | --- | --- | --- | --- | --- | --- |
| Duplication depth | Reference chromosome | Collinear blocks | | | | | | | | | | | |
| 0 | Atr-ERN06474 |  |  |  |  |  |  |
| 0 | Atr-ERN06475 |  |  |  |  |  |  |
| 0 | Atr-ERN06476 |  |  |  |  |  |  |
| 0 | Atr-ERN06477 |  |  |  |  |  |  |
| 0 | Atr-ERN06478 |  |  |  |  |  |  |
| 0 | Atr-ERN06479 |  |  |  |  |  |  |
| 0 | Atr-ERN06480 |  |  |  |  |  |  |
| 0 | Atr-ERN06481 |  |  |  |  |  |  |
| 0 | Atr-ERN06482 |  |  |  |  |  |  |
| 0 | Atr-ERN06483 |  |  |  |  |  |  |
| 0 | Atr-ERN06484 |  |  |  |  |  |  |
| 0 | Atr-ERN06485 |  |  |  |  |  |  |
| 0 | Atr-ERN06486 |  |  |  |  |  |  |
| 0 | Atr-ERN06487 |  |  |  |  |  |  |
| 0 | Atr-ERN06488 |  |  |  |  |  |  |
| 0 | Atr-ERN06489 |  |  |  |  |  |  |
| 0 | Atr-ERN06490 |  |  |  |  |  |  |
| 0 | Atr-ERN06491 |  |  |  |  |  |  |
| 0 | Atr-ERN06492 |  |  |  |  |  |  |
| 0 | Atr-ERN06493 |  |  |  |  |  |  |
| 0 | Atr-ERN06494 |  |  |  |  |  |  |
| 0 | Atr-ERN06495 |  |  |  |  |  |  |
| 0 | Atr-ERN06496 |  |  |  |  |  |  |
| 0 | Atr-ERN06497 |  |  |  |  |  |  |
| 1 | Atr-ERN06498 |  | Vvi-Vitvi17g00643\_t001 |  |  |  |  |  |
| 1 | Atr-ERN06499 |  | | | |  |  |  |  |  |
| 2 | Atr-ERN06500 |  | | | |  | Vvi-Vitvi14g02000\_t001 |  |  |  |  |
| 2 | Atr-ERN06501 |  | | | |  | | | |  |  |  |  |
| 2 | Atr-ERN06502 |  | | | |  | Vvi-Vitvi14g02001\_t001 |  |  |  |  |
| 2 | Atr-ERN06503 |  | | | |  | | | |  |  |  |  |
| 2 | Atr-ERN06504 |  | | | |  | | | |  |  |  |  |
| 2 | Atr-ERN06505 |  | | | |  | | | |  |  |  |  |
| 2 | Atr-ERN06506 |  | | | |  | | | |  |  |  |  |
| 2 | Atr-ERN06507 |  | | | |  | | | |  |  |  |  |
| 2 | Atr-ERN06508 |  | | | |  | | | |  |  |  |  |
| 2 | Atr-ERN06509 |  | Vvi-Vitvi17g00645\_t001 |  | | | |  |  |  |  |
| 2 | Atr-ERN06510 |  | | | |  | | | |  |  |  |  |
| 2 | Atr-ERN06511 |  | | | |  | Vvi-Vitvi14g02004\_t001 |  |  |  |  |
| 2 | Atr-ERN06512 |  | | | |  | | | |  |  |  |  |
| 2 | Atr-ERN06513 |  | Vvi-Vitvi17g00646\_t001 |  | | | |  |  |  |  |
| 2 | Atr-ERN06514 |  | | | |  | | | |  |  |  |  |
| 2 | Atr-ERN06515 |  | | | |  | | | |  |  |  |  |
| 2 | Atr-ERN06516 |  | | | |  | | | |  |  |  |  |
| 2 | Atr-ERN06517 |  | | | |  | Vvi-Vitvi14g02005\_t001 |  |  |  |  |
| 2 | Atr-ERN06518 |  | | | |  | | | |  |  |  |  |
| 2 | Atr-ERN06519 |  | | | |  | | | |  |  |  |  |
| 2 | Atr-ERN06520 |  | Vvi-Vitvi17g00648\_t001 |  | Vvi-Vitvi14g02006\_t001 |  |  |  |  |
| 2 | Atr-ERN06521 |  | | | |  | Vvi-Vitvi14g02007\_t001 |  |  |  |  |
| 2 | Atr-ERN06522 |  | Vvi-Vitvi17g00649\_t001 |  | | | |  |  |  |  |
| 2 | Atr-ERN06523 |  | | | |  | Vvi-Vitvi14g02008\_t001 |  |  |  |  |
| 2 | Atr-ERN06524 |  | | | |  | | | |  |  |  |  |
| 2 | Atr-ERN06525 |  | | | |  | | | |  |  |  |  |
| 2 | Atr-ERN06526 |  | | | |  | | | |  |  |  |  |
| 2 | Atr-ERN06527 |  | Vvi-Vitvi17g00650\_t001 |  | | | |  |  |  |  |
| 2 | Atr-ERN06528 |  | | | |  | | | |  |  |  |  |
| 2 | Atr-ERN06529 |  | | | |  | | | |  |  |  |  |
| 2 | Atr-ERN06530 |  | | | |  | | | |  |  |  |  |
| 2 | Atr-ERN06531 |  | | | |  | Vvi-Vitvi14g02009\_t001 |  |  |  |  |
| 2 | Atr-ERN06532 |  | | | |  | | | |  |  |  |  |
| 2 | Atr-ERN06533 |  | | | |  | | | |  |  |  |  |
| 2 | Atr-ERN06534 |  | | | |  | | | |  |  |  |  |
| 2 | Atr-ERN06535 |  | Vvi-Vitvi17g00651\_t001 |  | | | |  |  |  |  |
| 2 | Atr-ERN06536 |  | | | |  | | | |  |  |  |  |
| 2 | Atr-ERN06537 |  | | | |  | | | |  |  |  |  |
| 2 | Atr-ERN06538 |  | | | |  | | | |  |  |  |  |
| 2 | Atr-ERN06539 |  | | | |  | | | |  |  |  |  |
| 2 | Atr-ERN06540 |  | | | |  | | | |  |  |  |  |
| 2 | Atr-ERN06541 |  | | | |  | Vvi-Vitvi14g02011\_t001 |  |  |  |  |
| 1 | Atr-ERN06542 |  | | | |  |  |  |  |  |
| 1 | Atr-ERN06543 |  | | | |  |  |  |  |  |
| 1 | Atr-ERN06544 |  | | | |  |  |  |  |  |
| 1 | Atr-ERN06545 |  | | | |  |  |  |  |  |
| 1 | Atr-ERN06546 |  | Vvi-Vitvi17g04183\_t001 |  |  |  |  |  |
| 0 | Atr-ERN06547 |  |  |  |  |  |  |
| 0 | Atr-ERN06548 |  |  |  |  |  |  |
| 0 | Atr-ERN06549 |  |  |  |  |  |  |
| 0 | Atr-ERN06550 |  |  |  |  |  |  |
| 0 | Atr-ERN06551 |  |  |  |  |  |  |
| 0 | Atr-ERN06552 |  |  |  |  |  |  |
| 0 | Atr-ERN06553 |  |  |  |  |  |  |
| 0 | Atr-ERN06554 |  |  |  |  |  |  |
| 0 | Atr-ERN06555 |  |  |  |  |  |  |
| 0 | Atr-ERN06556 |  |  |  |  |  |  |
| 0 | Atr-ERN06557 |  |  |  |  |  |  |
| 0 | Atr-ERN06558 |  |  |  |  |  |  |
| 0 | Atr-ERN06559 |  |  |  |  |  |  |
| 0 | Atr-ERN06560 |  |  |  |  |  |  |
| 0 | Atr-ERN06561 |  |  |  |  |  |  |
| 0 | Atr-ERN06562 |  |  |  |  |  |  |
| 0 | Atr-ERN06563 |  |  |  |  |  |  |
| 0 | Atr-ERN06564 |  |  |  |  |  |  |
| 0 | Atr-ERN06565 |  |  |  |  |  |  |
| 0 | Atr-ERN06566 |  |  |  |  |  |  |
| 0 | Atr-ERN06567 |  |  |  |  |  |  |
| 0 | Atr-ERN06568 |  |  |  |  |  |  |
| 0 | Atr-ERN06569 |  |  |  |  |  |  |
| 0 | Atr-ERN06570 |  |  |  |  |  |  |
| 0 | Atr-ERN06571 |  |  |  |  |  |  |
| 0 | Atr-ERN06572 |  |  |  |  |  |  |
| 0 | Atr-ERN06573 |  |  |  |  |  |  |
| 0 | Atr-ERN06574 |  |  |  |  |  |  |
| 0 | Atr-ERN06575 |  |  |  |  |  |  |
| 0 | Atr-ERN06576 |  |  |  |  |  |  |
| 0 | Atr-ERN06577 |  |  |  |  |  |  |
| 0 | Atr-ERN06578 |  |  |  |  |  |  |
| 0 | Atr-ERN06579 |  |  |  |  |  |  |
| 0 | Atr-ERN06580 |  |  |  |  |  |  |
| 0 | Atr-ERN06581 |  |  |  |  |  |  |
| 0 | Atr-ERN06582 |  |  |  |  |  |  |
| 0 | Atr-ERN06583 |  |  |  |  |  |  |
| 0 | Atr-ERN06584 |  |  |  |  |  |  |
| 0 | Atr-ERN06585 |  |  |  |  |  |  |
| 0 | Atr-ERN06586 |  |  |  |  |  |  |
| 0 | Atr-ERN06587 |  |  |  |  |  |  |
| 0 | Atr-ERN06588 |  |  |  |  |  |  |
| 0 | Atr-ERN06589 |  |  |  |  |  |  |
| 0 | Atr-ERN06590 |  |  |  |  |  |  |
| 0 | Atr-ERN06591 |  |  |  |  |  |  |
| 0 | Atr-ERN06592 |  |  |  |  |  |  |
| 0 | Atr-ERN06593 |  |  |  |  |  |  |
| 0 | Atr-ERN06594 |  |  |  |  |  |  |
| 0 | Atr-ERN06595 |  |  |  |  |  |  |
| 0 | Atr-ERN06596 |  |  |  |  |  |  |
| 0 | Atr-ERN06597 |  |  |  |  |  |  |
| 0 | Atr-ERN06598 |  |  |  |  |  |  |
| 0 | Atr-ERN06599 |  |  |  |  |  |  |
| 0 | Atr-ERN06600 |  |  |  |  |  |  |
| 0 | Atr-ERN06601 |  |  |  |  |  |  |
| 0 | Atr-ERN06602 |  |  |  |  |  |  |
| 0 | Atr-ERN06603 |  |  |  |  |  |  |
| 0 | Atr-ERN06604 |  |  |  |  |  |  |
| 0 | Atr-ERN06605 |  |  |  |  |  |  |
| 0 | Atr-ERN06606 |  |  |  |  |  |  |
| 0 | Atr-ERN06607 |  |  |  |  |  |  |
| 0 | Atr-ERN06608 |  |  |  |  |  |  |
| 0 | Atr-ERN06609 |  |  |  |  |  |  |
| 0 | Atr-ERN06610 |  |  |  |  |  |  |
| 0 | Atr-ERN06611 |  |  |  |  |  |  |
| 0 | Atr-ERN06612 |  |  |  |  |  |  |
| 0 | Atr-ERN06613 |  |  |  |  |  |  |
| 0 | Atr-ERN06614 |  |  |  |  |  |  |
| 0 | Atr-ERN06615 |  |  |  |  |  |  |
| 0 | Atr-ERN06616 |  |  |  |  |  |  |
| 0 | Atr-ERN06617 |  |  |  |  |  |  |
| 0 | Atr-ERN06618 |  |  |  |  |  |  |
| 0 | Atr-ERN06619 |  |  |  |  |  |  |
| 0 | Atr-ERN06620 |  |  |  |  |  |  |
| 0 | Atr-ERN06621 |  |  |  |  |  |  |
| 0 | Atr-ERN06622 |  |  |  |  |  |  |
| 0 | Atr-ERN06623 |  |  |  |  |  |  |
| 2 | Atr-ERN06624 |  | Vvi-Vitvi17g00791\_t001 |  | Vvi-Vitvi14g01783\_t001 |  |  |  |  |
| 2 | Atr-ERN06625 |  | | | |  | Vvi-Vitvi14g01782\_t001 |  |  |  |  |
| 3 | Atr-ERN06626 |  | | | |  | | | |  | Vvi-Vitvi01g00776\_t001 |  |  |  |
| 3 | Atr-ERN06627 |  | Vvi-Vitvi17g00794\_t001 |  | Vvi-Vitvi14g01781\_t001 |  | | | |  |  |  |
| 3 | Atr-ERN06628 |  | | | |  | Vvi-Vitvi14g01780\_t001 |  | Vvi-Vitvi01g00775\_t003 |  |  |  |
| 3 | Atr-ERN06629 |  | | | |  | | | |  | Vvi-Vitvi01g00773\_t001 |  |  |  |
| 3 | Atr-ERN06630 |  | | | |  | Vvi-Vitvi14g01777\_t001 |  | | | |  |  |  |
| 3 | Atr-ERN06631 |  | | | |  | | | |  | Vvi-Vitvi01g00770\_t001 |  |  |  |
| 3 | Atr-ERN06632 |  | | | |  | | | |  | | | |  |  |  |
| 3 | Atr-ERN06633 |  | | | |  | | | |  | | | |  |  |  |
| 3 | Atr-ERN06634 |  | | | |  | | | |  | | | |  |  |  |
| 3 | Atr-ERN06635 |  | Vvi-Vitvi17g00796\_t001 |  | | | |  | Vvi-Vitvi01g00745\_t001 |  |  |  |
| 3 | Atr-ERN06636 |  | | | |  | Vvi-Vitvi14g01775\_t001 |  | Vvi-Vitvi01g00744\_t002 |  |  |  |
| 3 | Atr-ERN06637 |  | | | |  | Vvi-Vitvi14g01773\_t001 |  | | | |  |  |  |
| 3 | Atr-ERN06638 |  | | | |  | Vvi-Vitvi14g01772\_t001 |  | | | |  |  |  |
| 3 | Atr-ERN06639 |  | Vvi-Vitvi17g00797\_t001 |  | | | |  | | | |  |  |  |
| 3 | Atr-ERN06640 |  | Vvi-Vitvi17g00799\_t001 |  | | | |  | | | |  |  |  |
| 3 | Atr-ERN06641 |  | | | |  | | | |  | | | |  |  |  |
| 3 | Atr-ERN06642 |  | | | |  | | | |  | | | |  |  |  |
| 3 | Atr-ERN06643 |  | | | |  | | | |  | | | |  |  |  |
| 3 | Atr-ERN06644 |  | Vvi-Vitvi17g00801\_t001 |  | | | |  | | | |  |  |  |
| 3 | Atr-ERN06645 |  | Vvi-Vitvi17g00802\_t001 |  | | | |  | | | |  |  |  |
| 3 | Atr-ERN06646 |  | Vvi-Vitvi17g00803\_t001 |  | Vvi-Vitvi14g01771\_t001 |  | | | |  |  |  |
| 3 | Atr-ERN06647 |  | | | |  | | | |  | Vvi-Vitvi01g00738\_t001 |  |  |  |
| 3 | Atr-ERN06648 |  | | | |  | | | |  | | | |  |  |  |
| 3 | Atr-ERN06649 |  | | | |  | Vvi-Vitvi14g01770\_t001 |  | | | |  |  |  |
| 3 | Atr-ERN06650 |  | | | |  | | | |  | | | |  |  |  |
| 3 | Atr-ERN06651 |  | Vvi-Vitvi17g00804\_t001 |  | | | |  | | | |  |  |  |
| 3 | Atr-ERN06652 |  | | | |  | | | |  | | | |  |  |  |
| 3 | Atr-ERN06653 |  | | | |  | | | |  | | | |  |  |  |
| 3 | Atr-ERN06654 |  | | | |  | | | |  | | | |  |  |  |
| 3 | Atr-ERN06655 |  | Vvi-Vitvi17g00807\_t002 |  | Vvi-Vitvi14g01769\_t001.1.6037826b |  | | | |  |  |  |
| 3 | Atr-ERN06656 |  | Vvi-Vitvi17g00818\_t001 |  | | | |  | | | |  |  |  |
| 3 | Atr-ERN06657 |  | | | |  | Vvi-Vitvi14g01765\_t001 |  | | | |  |  |  |
| 3 | Atr-ERN06658 |  | | | |  | Vvi-Vitvi14g01764\_t001 |  | | | |  |  |  |
| 3 | Atr-ERN06659 |  | | | |  | | | |  | | | |  |  |  |
| 3 | Atr-ERN06660 |  | | | |  | | | |  | | | |  |  |  |
| 3 | Atr-ERN06661 |  | | | |  | | | |  | | | |  |  |  |
| 3 | Atr-ERN06662 |  | | | |  | | | |  | | | |  |  |  |
| 3 | Atr-ERN06663 |  | Vvi-Vitvi17g00819\_t001 |  | Vvi-Vitvi14g01763\_t001 |  | Vvi-Vitvi01g00735\_t001 |  |  |  |
| 3 | Atr-ERN06664 |  | Vvi-Vitvi17g00817\_t001 |  | | | |  | | | |  |  |  |
| 3 | Atr-ERN06665 |  | | | |  | | | |  | Vvi-Vitvi01g00733\_t002 |  |  |  |
| 3 | Atr-ERN06666 |  | | | |  | Vvi-Vitvi14g01762\_t002 |  | | | |  |  |  |
| 3 | Atr-ERN06667 |  | Vvi-Vitvi17g00816\_t001 |  | | | |  | | | |  |  |  |
| 3 | Atr-ERN06668 |  | Vvi-Vitvi17g00815\_t001 |  | Vvi-Vitvi14g01761\_t001 |  | Vvi-Vitvi01g00732\_t001 |  |  |  |
| 3 | Atr-ERN06669 |  | | | |  | | | |  | | | |  |  |  |
| 3 | Atr-ERN06670 |  | | | |  | | | |  | | | |  |  |  |
| 3 | Atr-ERN06671 |  | | | |  | | | |  | | | |  |  |  |
| 3 | Atr-ERN06672 |  | | | |  | | | |  | | | |  |  |  |
| 3 | Atr-ERN06673 |  | | | |  | Vvi-Vitvi14g01758\_t003 |  | Vvi-Vitvi01g00730\_t001 |  |  |  |
| 3 | Atr-ERN06674 |  | Vvi-Vitvi17g00814\_t001 |  | | | |  | Vvi-Vitvi01g00729\_t001 |  |  |  |
| 3 | Atr-ERN06675 |  | | | |  | Vvi-Vitvi14g03019\_t001 |  | | | |  |  |  |
| 3 | Atr-ERN06676 |  | | | |  | | | |  | Vvi-Vitvi01g04183\_t001 |  |  |  |
| 3 | Atr-ERN06677 |  | | | |  | Vvi-Vitvi14g01757\_t001 |  | | | |  |  |  |
| 3 | Atr-ERN06678 |  | | | |  | Vvi-Vitvi14g03018\_t001 |  | | | |  |  |  |
| 3 | Atr-ERN06679 |  | | | |  | Vvi-Vitvi14g03017\_t003 |  | | | |  |  |  |
| 3 | Atr-ERN06680 |  | | | |  | | | |  | Vvi-Vitvi01g02062\_t001 |  |  |  |
| 2 | Atr-ERN06681 |  | | | |  | | | |  |  |  |  |
| 2 | Atr-ERN06682 |  | | | |  | | | |  |  |  |  |
| 2 | Atr-ERN06683 |  | | | |  | | | |  |  |  |  |
| 2 | Atr-ERN06684 |  | | | |  | | | |  |  |  |  |
| 2 | Atr-ERN06685 |  | | | |  | | | |  |  |  |  |
| 2 | Atr-ERN06686 |  | | | |  | | | |  |  |  |  |
| 2 | Atr-ERN06687 |  | | | |  | | | |  |  |  |  |
| 2 | Atr-ERN06688 |  | | | |  | | | |  |  |  |  |
| 2 | Atr-ERN06689 |  | Vvi-Vitvi17g00813\_t001 |  | | | |  |  |  |  |
| 2 | Atr-ERN06690 |  | | | |  | | | |  |  |  |  |
| 2 | Atr-ERN06691 |  | | | |  | | | |  |  |  |  |
| 2 | Atr-ERN06692 |  | | | |  | | | |  |  |  |  |
| 2 | Atr-ERN06693 |  | | | |  | | | |  |  |  |  |
| 2 | Atr-ERN06694 |  | Vvi-Vitvi17g00809\_t001 |  | | | |  |  |  |  |
| 1 | Atr-ERN06695 |  |  |  | Vvi-Vitvi14g01756\_t001 |  |  |  |  |
| 0 | Atr-ERN06696 |  |  |  |  |  |  |
| 0 | Atr-ERN06697 |  |  |  |  |  |  |
| 0 | Atr-ERN06698 |  |  |  |  |  |  |
| 0 | Atr-ERN06699 |  |  |  |  |  |  |
| 0 | Atr-ERN06700 |  |  |  |  |  |  |
| 0 | Atr-ERN06701 |  |  |  |  |  |  |
| 0 | Atr-ERN06702 |  |  |  |  |  |  |
| 0 | Atr-ERN06703 |  |  |  |  |  |  |
| 0 | Atr-ERN06704 |  |  |  |  |  |  |
| 0 | Atr-ERN06705 |  |  |  |  |  |  |
| 0 | Atr-ERN06706 |  |  |  |  |  |  |
| 0 | Atr-ERN06707 |  |  |  |  |  |  |
| 0 | Atr-ERN06708 |  |  |  |  |  |  |
| 0 | Atr-ERN06709 |  |  |  |  |  |  |
| 0 | Atr-ERN06710 |  |  |  |  |  |  |
| 0 | Atr-ERN06711 |  |  |  |  |  |  |
| 0 | Atr-ERN06712 |  |  |  |  |  |  |
| 0 | Atr-ERN06713 |  |  |  |  |  |  |
| 0 | Atr-ERN06714 |  |  |  |  |  |  |
| 0 | Atr-ERN06715 |  |  |  |  |  |  |
| 0 | Atr-ERN06716 |  |  |  |  |  |  |
