# Supplementary material for: Genome-wide identification, characterization and gene expression of BES1 transcription factor family in grapevine (Vitis vinifera L.)
Source: Sci Rep. 2023 Jan 5;13:240. doi: 10.1038/s41598-022-24407-y (PMC9816167; doi:10.1038/s41598-022-24407-y)
Supplement: Supplementary file 3 — Supplementary Information. [file 41598_2022_24407_MOESM3_ESM.zip › Vvi_Atr/Vitis_vinifera.PN40024.v4.dna_sm.toplevel.fa.vs.Amborella_trichopoda.AMTR1.0.dna_sm.toplevel.fa.html/Atr-AmTr_v1.0_scaffold00059.html]

|  |  |  |  |  |  |  |  |  |  |  |  |  |  |
| --- | --- | --- | --- | --- | --- | --- | --- | --- | --- | --- | --- | --- | --- |
| Duplication depth | Reference chromosome | Collinear blocks | | | | | | | | | | | |
| 0 | Atr-ERN17456 |  |  |  |  |  |  |
| 0 | Atr-ERN17457 |  |  |  |  |  |  |
| 0 | Atr-ERN17458 |  |  |  |  |  |  |
| 0 | Atr-ERN17459 |  |  |  |  |  |  |
| 1 | Atr-ERN17460 |  | Vvi-Vitvi03g00328\_t002 |  |  |  |  |  |
| 1 | Atr-ERN17461 |  | | | |  |  |  |  |  |
| 1 | Atr-ERN17462 |  | | | |  |  |  |  |  |
| 1 | Atr-ERN17463 |  | | | |  |  |  |  |  |
| 3 | Atr-ERN17464 |  | | | |  | Vvi-Vitvi18g00968\_t002 |  | Vvi-Vitvi04g01296\_t001 |  |  |  |
| 3 | Atr-ERN17465 |  | | | |  | | | |  | Vvi-Vitvi04g01291\_t001 |  |  |  |
| 3 | Atr-ERN17466 |  | Vvi-Vitvi03g00332\_t001 |  | | | |  | | | |  |  |  |
| 3 | Atr-ERN17467 |  | | | |  | Vvi-Vitvi18g00964\_t001 |  | | | |  |  |  |
| 3 | Atr-ERN17468 |  | | | |  | | | |  | | | |  |  |  |
| 3 | Atr-ERN17469 |  | | | |  | | | |  | | | |  |  |  |
| 3 | Atr-ERN17470 |  | | | |  | | | |  | | | |  |  |  |
| 3 | Atr-ERN17471 |  | | | |  | | | |  | | | |  |  |  |
| 3 | Atr-ERN17472 |  | | | |  | Vvi-Vitvi18g00961\_t001 |  | | | |  |  |  |
| 3 | Atr-ERN17473 |  | | | |  | Vvi-Vitvi18g00960\_t001 |  | | | |  |  |  |
| 3 | Atr-ERN17474 |  | | | |  | | | |  | | | |  |  |  |
| 3 | Atr-ERN17475 |  | | | |  | Vvi-Vitvi18g00959\_t001 |  | | | |  |  |  |
| 3 | Atr-ERN17476 |  | | | |  | | | |  | | | |  |  |  |
| 3 | Atr-ERN17477 |  | | | |  | | | |  | Vvi-Vitvi04g04343\_t001 |  |  |  |
| 3 | Atr-ERN17478 |  | | | |  | | | |  | | | |  |  |  |
| 3 | Atr-ERN17479 |  | Vvi-Vitvi03g00334\_t001 |  | Vvi-Vitvi18g00958\_t001 |  | | | |  |  |  |
| 3 | Atr-ERN17480 |  | | | |  | Vvi-Vitvi18g00957\_t003 |  | Vvi-Vitvi04g01287\_t001 |  |  |  |
| 3 | Atr-ERN17481 |  | | | |  | | | |  | Vvi-Vitvi04g04342\_t001 |  |  |  |
| 3 | Atr-ERN17482 |  | Vvi-Vitvi03g00335\_t001 |  | | | |  | | | |  |  |  |
| 3 | Atr-ERN17483 |  | | | |  | | | |  | | | |  |  |  |
| 3 | Atr-ERN17484 |  | | | |  | | | |  | | | |  |  |  |
| 3 | Atr-ERN17485 |  | Vvi-Vitvi03g00338\_t001 |  | | | |  | | | |  |  |  |
| 3 | Atr-ERN17486 |  | | | |  | | | |  | Vvi-Vitvi04g01282\_t001 |  |  |  |
| 3 | Atr-ERN17487 |  | | | |  | | | |  | Vvi-Vitvi04g01281\_t001 |  |  |  |
| 3 | Atr-ERN17488 |  | | | |  | Vvi-Vitvi18g00956\_t001 |  | | | |  |  |  |
| 3 | Atr-ERN17489 |  | | | |  | | | |  | Vvi-Vitvi04g01280\_t001 |  |  |  |
| 3 | Atr-ERN17490 |  | | | |  | | | |  | Vvi-Vitvi04g01278\_t001 |  |  |  |
| 3 | Atr-ERN17491 |  | | | |  | | | |  | Vvi-Vitvi04g01276\_t003 |  |  |  |
| 3 | Atr-ERN17492 |  | Vvi-Vitvi03g00339\_t001 |  | | | |  | | | |  |  |  |
| 3 | Atr-ERN17493 |  | Vvi-Vitvi03g00340\_t001 |  | Vvi-Vitvi18g00955\_t001 |  | | | |  |  |  |
| 3 | Atr-ERN17494 |  | | | |  | | | |  | | | |  |  |  |
| 3 | Atr-ERN17495 |  | | | |  | Vvi-Vitvi18g00954\_t001 |  | | | |  |  |  |
| 3 | Atr-ERN17496 |  | | | |  | | | |  | Vvi-Vitvi04g01275\_t001 |  |  |  |
| 3 | Atr-ERN17497 |  | Vvi-Vitvi03g00343\_t001 |  | | | |  | | | |  |  |  |
| 3 | Atr-ERN17498 |  | | | |  | | | |  | | | |  |  |  |
| 3 | Atr-ERN17499 |  | Vvi-Vitvi03g00344\_t001 |  | | | |  | | | |  |  |  |
| 3 | Atr-ERN17500 |  | Vvi-Vitvi03g00345\_t001 |  | | | |  | Vvi-Vitvi04g01269\_t001 |  |  |  |
| 3 | Atr-ERN17501 |  | Vvi-Vitvi03g00348\_t001 |  | | | |  | | | |  |  |  |
| 3 | Atr-ERN17502 |  | Vvi-Vitvi03g00349\_t001 |  | | | |  | | | |  |  |  |
| 3 | Atr-ERN17503 |  | | | |  | | | |  | | | |  |  |  |
| 3 | Atr-ERN17504 |  | | | |  | Vvi-Vitvi18g00949\_t001 |  | | | |  |  |  |
| 3 | Atr-ERN17505 |  | | | |  | | | |  | | | |  |  |  |
| 3 | Atr-ERN17506 |  | | | |  | | | |  | | | |  |  |  |
| 3 | Atr-ERN17507 |  | | | |  | | | |  | | | |  |  |  |
| 3 | Atr-ERN17508 |  | | | |  | | | |  | | | |  |  |  |
| 3 | Atr-ERN17509 |  | | | |  | | | |  | Vvi-Vitvi04g01267\_t001 |  |  |  |
| 3 | Atr-ERN17510 |  | | | |  | | | |  | Vvi-Vitvi04g02101\_t001 |  |  |  |
| 3 | Atr-ERN17511 |  | | | |  | | | |  | | | |  |  |  |
| 3 | Atr-ERN17512 |  | | | |  | | | |  | | | |  |  |  |
| 3 | Atr-ERN17513 |  | | | |  | | | |  | | | |  |  |  |
| 3 | Atr-ERN17514 |  | | | |  | | | |  | | | |  |  |  |
| 3 | Atr-ERN17515 |  | | | |  | | | |  | | | |  |  |  |
| 3 | Atr-ERN17516 |  | | | |  | | | |  | Vvi-Vitvi04g01266\_t001 |  |  |  |
| 3 | Atr-ERN17517 |  | | | |  | | | |  | | | |  |  |  |
| 3 | Atr-ERN17518 |  | | | |  | | | |  | | | |  |  |  |
| 3 | Atr-ERN17519 |  | | | |  | | | |  | | | |  |  |  |
| 3 | Atr-ERN17520 |  | | | |  | | | |  | | | |  |  |  |
| 3 | Atr-ERN17521 |  | | | |  | | | |  | | | |  |  |  |
| 3 | Atr-ERN17522 |  | | | |  | Vvi-Vitvi18g00931\_t001 |  | | | |  |  |  |
| 3 | Atr-ERN17523 |  | | | |  | | | |  | | | |  |  |  |
| 5 | Atr-ERN17524 |  | | | |  | Vvi-Vitvi18g00930\_t003 |  | | | |  | Vvi-Vitvi04g02098\_t001 |  | Vvi-Vitvi18g00930\_t003 |  |
| 4 | Atr-ERN17525 |  | Vvi-Vitvi03g00352\_t003 |  |  |  | | | |  | | | |  | | | |  |
| 4 | Atr-ERN17526 |  | | | |  |  |  | | | |  | | | |  | | | |  |
| 4 | Atr-ERN17527 |  | | | |  |  |  | | | |  | | | |  | | | |  |
| 4 | Atr-ERN17528 |  | | | |  |  |  | | | |  | | | |  | | | |  |
| 4 | Atr-ERN17529 |  | | | |  |  |  | | | |  | | | |  | | | |  |
| 4 | Atr-ERN17530 |  | | | |  |  |  | | | |  | | | |  | | | |  |
| 4 | Atr-ERN17531 |  | | | |  |  |  | | | |  | | | |  | | | |  |
| 4 | Atr-ERN17532 |  | | | |  |  |  | Vvi-Vitvi04g01257\_t001 |  | | | |  | | | |  |
| 5 | Atr-ERN17533 |  | | | |  | Vvi-Vitvi18g00938\_t001 |  | | | |  | | | |  | | | |  |
| 5 | Atr-ERN17534 |  | | | |  | | | |  | Vvi-Vitvi04g04338\_t001 |  | | | |  | | | |  |
| 5 | Atr-ERN17535 |  | | | |  | Vvi-Vitvi18g00936\_t001 |  | | | |  | | | |  | | | |  |
| 5 | Atr-ERN17536 |  | | | |  | Vvi-Vitvi18g00935\_t001 |  | Vvi-Vitvi04g01252\_t001 |  | Vvi-Vitvi04g01252\_t001 |  | | | |  |
| 5 | Atr-ERN17537 |  | | | |  | | | |  | | | |  | | | |  | Vvi-Vitvi18g02738\_t001 |  |
| 5 | Atr-ERN17538 |  | | | |  | Vvi-Vitvi18g02739\_t001 |  | | | |  | | | |  | Vvi-Vitvi18g02739\_t001 |  |
| 5 | Atr-ERN17539 |  | | | |  | | | |  | | | |  | | | |  | | | |  |
| 5 | Atr-ERN17540 |  | | | |  | | | |  | | | |  | | | |  | | | |  |
| 5 | Atr-ERN17541 |  | | | |  | Vvi-Vitvi18g00933\_t005 |  | Vvi-Vitvi04g01249\_t001 |  | | | |  | | | |  |
| 5 | Atr-ERN17542 |  | | | |  | | | |  | | | |  | | | |  | Vvi-Vitvi18g00940\_t001 |  |
| 5 | Atr-ERN17543 |  | | | |  | | | |  | | | |  | | | |  | | | |  |
| 5 | Atr-ERN17544 |  | | | |  | | | |  | | | |  | | | |  | Vvi-Vitvi18g00942\_t001 |  |
| 5 | Atr-ERN17545 |  | | | |  | | | |  | | | |  | | | |  | | | |  |
| 5 | Atr-ERN17546 |  | | | |  | | | |  | | | |  | Vvi-Vitvi04g01260\_t001 |  | | | |  |
| 5 | Atr-ERN17547 |  | | | |  | | | |  | | | |  | | | |  | | | |  |
| 5 | Atr-ERN17548 |  | | | |  | | | |  | | | |  | | | |  | Vvi-Vitvi18g00943\_t001 |  |
| 5 | Atr-ERN17549 |  | | | |  | | | |  | | | |  | Vvi-Vitvi04g01262\_t001 |  | Vvi-Vitvi18g00946\_t001 |  |
| 5 | Atr-ERN17550 |  | | | |  | | | |  | | | |  | | | |  | | | |  |
| 5 | Atr-ERN17551 |  | Vvi-Vitvi03g00359\_t001 |  | | | |  | | | |  | Vvi-Vitvi04g01263\_t001 |  | | | |  |
| 5 | Atr-ERN17552 |  | | | |  | | | |  | | | |  | | | |  | | | |  |
| 5 | Atr-ERN17553 |  | | | |  | | | |  | | | |  | | | |  | | | |  |
| 5 | Atr-ERN17554 |  | | | |  | | | |  | | | |  | | | |  | | | |  |
| 5 | Atr-ERN17555 |  | | | |  | | | |  | | | |  | | | |  | | | |  |
| 5 | Atr-ERN17556 |  | | | |  | | | |  | | | |  | | | |  | | | |  |
| 5 | Atr-ERN17557 |  | | | |  | | | |  | | | |  | Vvi-Vitvi04g01265\_t001 |  | Vvi-Vitvi18g00948\_t001 |  |
| 3 | Atr-ERN17558 |  | | | |  | Vvi-Vitvi18g00927\_t002 |  | | | |  |  |  |
| 3 | Atr-ERN17559 |  | | | |  | | | |  | | | |  |  |  |
| 3 | Atr-ERN17560 |  | | | |  | | | |  | | | |  |  |  |
| 3 | Atr-ERN17561 |  | | | |  | | | |  | | | |  |  |  |
| 3 | Atr-ERN17562 |  | | | |  | | | |  | | | |  |  |  |
| 3 | Atr-ERN17563 |  | | | |  | | | |  | | | |  |  |  |
| 3 | Atr-ERN17564 |  | | | |  | | | |  | | | |  |  |  |
| 3 | Atr-ERN17565 |  | | | |  | | | |  | | | |  |  |  |
| 3 | Atr-ERN17566 |  | | | |  | | | |  | | | |  |  |  |
| 3 | Atr-ERN17567 |  | | | |  | | | |  | Vvi-Vitvi04g01231\_t001 |  |  |  |
| 3 | Atr-ERN17568 |  | Vvi-Vitvi03g04128\_t001 |  | | | |  | | | |  |  |  |
| 3 | Atr-ERN17569 |  | | | |  | | | |  | | | |  |  |  |
| 3 | Atr-ERN17570 |  | | | |  | | | |  | | | |  |  |  |
| 3 | Atr-ERN17571 |  | | | |  | Vvi-Vitvi18g00924\_t001 |  | | | |  |  |  |
| 3 | Atr-ERN17572 |  | | | |  | | | |  | | | |  |  |  |
| 3 | Atr-ERN17573 |  | | | |  | | | |  | | | |  |  |  |
| 3 | Atr-ERN17574 |  | | | |  | | | |  | | | |  |  |  |
| 3 | Atr-ERN17575 |  | | | |  | | | |  | | | |  |  |  |
| 3 | Atr-ERN17576 |  | | | |  | | | |  | | | |  |  |  |
| 3 | Atr-ERN17577 |  | | | |  | | | |  | | | |  |  |  |
| 3 | Atr-ERN17578 |  | | | |  | | | |  | | | |  |  |  |
| 3 | Atr-ERN17579 |  | | | |  | | | |  | | | |  |  |  |
| 3 | Atr-ERN17580 |  | | | |  | | | |  | | | |  |  |  |
| 3 | Atr-ERN17581 |  | Vvi-Vitvi03g00369\_t001 |  | | | |  | Vvi-Vitvi04g01228\_t001 |  |  |  |
| 3 | Atr-ERN17582 |  | | | |  | Vvi-Vitvi18g00919\_t001 |  | Vvi-Vitvi04g01227\_t001 |  |  |  |
| 3 | Atr-ERN17583 |  | | | |  | Vvi-Vitvi18g02730\_t001 |  | Vvi-Vitvi04g01225\_t001 |  |  |  |
| 3 | Atr-ERN17584 |  | | | |  | Vvi-Vitvi18g00918\_t001 |  | | | |  |  |  |
| 3 | Atr-ERN17585 |  | | | |  | | | |  | | | |  |  |  |
| 3 | Atr-ERN17586 |  | | | |  | | | |  | | | |  |  |  |
| 3 | Atr-ERN17587 |  | | | |  | Vvi-Vitvi18g00917\_t001 |  | | | |  |  |  |
| 3 | Atr-ERN17588 |  | | | |  | | | |  | | | |  |  |  |
| 3 | Atr-ERN17589 |  | | | |  | | | |  | | | |  |  |  |
| 3 | Atr-ERN17590 |  | | | |  | | | |  | | | |  |  |  |
| 3 | Atr-ERN17591 |  | Vvi-Vitvi03g00370\_t003 |  | Vvi-Vitvi18g00915\_t001 |  | | | |  |  |  |
| 3 | Atr-ERN17592 |  | Vvi-Vitvi03g00371\_t003 |  | | | |  | | | |  |  |  |
| 3 | Atr-ERN17593 |  | | | |  | Vvi-Vitvi18g00913\_t001 |  | Vvi-Vitvi04g01224\_t001 |  |  |  |
| 3 | Atr-ERN17594 |  | Vvi-Vitvi03g00372\_t001 |  | Vvi-Vitvi18g00912\_t001 |  | Vvi-Vitvi04g01223\_t001 |  |  |  |
| 3 | Atr-ERN17595 |  | | | |  | Vvi-Vitvi18g00909\_t001 |  | Vvi-Vitvi04g01222\_t001 |  |  |  |
| 3 | Atr-ERN17596 |  | | | |  | | | |  | | | |  |  |  |
| 3 | Atr-ERN17597 |  | Vvi-Vitvi03g00373\_t002 |  | | | |  | | | |  |  |  |
| 3 | Atr-ERN17598 |  | | | |  | Vvi-Vitvi18g00908\_t001 |  | | | |  |  |  |
| 3 | Atr-ERN17599 |  | | | |  | | | |  | Vvi-Vitvi04g01221\_t002 |  |  |  |
| 3 | Atr-ERN17600 |  | | | |  | | | |  | | | |  |  |  |
| 3 | Atr-ERN17601 |  | Vvi-Vitvi03g00374\_t001 |  | | | |  | | | |  |  |  |
| 3 | Atr-ERN17602 |  | | | |  | | | |  | Vvi-Vitvi04g02094\_t001 |  |  |  |
| 3 | Atr-ERN17603 |  | | | |  | Vvi-Vitvi18g00904\_t001 |  | Vvi-Vitvi04g01216\_t001 |  |  |  |
| 3 | Atr-ERN17604 |  | | | |  | | | |  | | | |  |  |  |
| 3 | Atr-ERN17605 |  | | | |  | | | |  | Vvi-Vitvi04g01214\_t001 |  |  |  |
| 3 | Atr-ERN17606 |  | | | |  | Vvi-Vitvi18g00903\_t001 |  | Vvi-Vitvi04g02091\_t001 |  |  |  |
| 3 | Atr-ERN17607 |  | | | |  | | | |  | Vvi-Vitvi04g04327\_t001 |  |  |  |
| 3 | Atr-ERN17608 |  | | | |  | | | |  | | | |  |  |  |
| 3 | Atr-ERN17609 |  | | | |  | Vvi-Vitvi18g00902\_t001 |  | Vvi-Vitvi04g01210\_t001 |  |  |  |
| 3 | Atr-ERN17610 |  | | | |  | Vvi-Vitvi18g00901\_t001 |  | | | |  |  |  |
| 3 | Atr-ERN17611 |  | | | |  | | | |  | Vvi-Vitvi04g01209\_t001 |  |  |  |
| 3 | Atr-ERN17612 |  | Vvi-Vitvi03g00375\_t002 |  | | | |  | | | |  |  |  |
| 3 | Atr-ERN17613 |  | Vvi-Vitvi03g00376\_t001 |  | | | |  | | | |  |  |  |
| 3 | Atr-ERN17614 |  | | | |  | Vvi-Vitvi18g00899\_t001 |  | | | |  |  |  |
| 3 | Atr-ERN17615 |  | | | |  | | | |  | | | |  |  |  |
| 3 | Atr-ERN17616 |  | | | |  | | | |  | | | |  |  |  |
| 3 | Atr-ERN17617 |  | | | |  | | | |  | | | |  |  |  |
| 3 | Atr-ERN17618 |  | | | |  | | | |  | | | |  |  |  |
| 3 | Atr-ERN17619 |  | | | |  | | | |  | | | |  |  |  |
| 3 | Atr-ERN17620 |  | | | |  | | | |  | | | |  |  |  |
| 3 | Atr-ERN17621 |  | | | |  | Vvi-Vitvi18g00898\_t001 |  | | | |  |  |  |
| 4 | Atr-ERN17622 |  | Vvi-Vitvi03g00377\_t001 |  | Vvi-Vitvi18g00897\_t001 |  | | | |  | Vvi-Vitvi07g01261\_t001 |  |  |
| 4 | Atr-ERN17623 |  | | | |  | | | |  | Vvi-Vitvi04g01208\_t002 |  | | | |  |  |
| 3 | Atr-ERN17624 |  | | | |  | Vvi-Vitvi18g00896\_t001 |  |  |  | | | |  |  |
| 3 | Atr-ERN17625 |  | | | |  | | | |  |  |  | | | |  |  |
| 3 | Atr-ERN17626 |  | Vvi-Vitvi03g00378\_t001 |  | | | |  |  |  | Vvi-Vitvi07g01264\_t001 |  |  |
| 3 | Atr-ERN17627 |  | | | |  | | | |  |  |  | Vvi-Vitvi07g01265\_t001 |  |  |
| 3 | Atr-ERN17628 |  | Vvi-Vitvi03g04133\_t001 |  | | | |  |  |  | | | |  |  |
| 3 | Atr-ERN17629 |  | Vvi-Vitvi03g00379\_t002 |  | | | |  |  |  | | | |  |  |
| 3 | Atr-ERN17630 |  | | | |  | | | |  |  |  | Vvi-Vitvi07g01266\_t001 |  |  |
| 3 | Atr-ERN17631 |  | Vvi-Vitvi03g00380\_t001 |  | | | |  |  |  | Vvi-Vitvi07g02502\_t001 |  |  |
| 3 | Atr-ERN17632 |  | Vvi-Vitvi03g00381\_t001 |  | | | |  |  |  | | | |  |  |
| 3 | Atr-ERN17633 |  | | | |  | | | |  |  |  | | | |  |  |
| 3 | Atr-ERN17634 |  | | | |  | Vvi-Vitvi18g00895\_t001 |  |  |  | | | |  |  |
| 3 | Atr-ERN17635 |  | Vvi-Vitvi03g00383\_t001 |  | | | |  |  |  | | | |  |  |
| 3 | Atr-ERN17636 |  | | | |  | Vvi-Vitvi18g00894\_t001 |  |  |  | | | |  |  |
| 3 | Atr-ERN17637 |  | | | |  | | | |  |  |  | | | |  |  |
| 3 | Atr-ERN17638 |  | | | |  | | | |  |  |  | | | |  |  |
| 3 | Atr-ERN17639 |  | | | |  | | | |  |  |  | | | |  |  |
| 3 | Atr-ERN17640 |  | | | |  | | | |  |  |  | | | |  |  |
| 3 | Atr-ERN17641 |  | Vvi-Vitvi03g00384\_t001 |  | | | |  |  |  | | | |  |  |
| 3 | Atr-ERN17642 |  | | | |  | | | |  |  |  | Vvi-Vitvi07g01274\_t001 |  |  |
| 3 | Atr-ERN17643 |  | | | |  | | | |  |  |  | | | |  |  |
| 3 | Atr-ERN17644 |  | | | |  | Vvi-Vitvi18g00893\_t001 |  |  |  | | | |  |  |
| 3 | Atr-ERN17645 |  | Vvi-Vitvi03g00388\_t001 |  | | | |  |  |  | | | |  |  |
| 3 | Atr-ERN17646 |  | | | |  | Vvi-Vitvi18g02727\_t001 |  |  |  | | | |  |  |
| 3 | Atr-ERN17647 |  | Vvi-Vitvi03g00389\_t001 |  | | | |  |  |  | Vvi-Vitvi07g01276\_t001 |  |  |
| 3 | Atr-ERN17648 |  | | | |  | | | |  |  |  | | | |  |  |
| 3 | Atr-ERN17649 |  | | | |  | | | |  |  |  | | | |  |  |
| 3 | Atr-ERN17650 |  | | | |  | | | |  |  |  | | | |  |  |
| 3 | Atr-ERN17651 |  | | | |  | | | |  |  |  | Vvi-Vitvi07g01277\_t001 |  |  |
| 3 | Atr-ERN17652 |  | | | |  | Vvi-Vitvi18g04213\_t001 |  |  |  | Vvi-Vitvi07g01278\_t002 |  |  |
| 3 | Atr-ERN17653 |  | | | |  | | | |  |  |  | Vvi-Vitvi07g01279\_t001 |  |  |
| 3 | Atr-ERN17654 |  | | | |  | | | |  |  |  | | | |  |  |
| 3 | Atr-ERN17655 |  | | | |  | | | |  |  |  | | | |  |  |
| 3 | Atr-ERN17656 |  | | | |  | | | |  |  |  | | | |  |  |
| 3 | Atr-ERN17657 |  | | | |  | | | |  |  |  | | | |  |  |
| 3 | Atr-ERN17658 |  | | | |  | | | |  |  |  | | | |  |  |
| 3 | Atr-ERN17659 |  | Vvi-Vitvi03g00391\_t001 |  | | | |  |  |  | | | |  |  |
| 3 | Atr-ERN17660 |  | | | |  | Vvi-Vitvi18g00887\_t001 |  |  |  | | | |  |  |
| 3 | Atr-ERN17661 |  | | | |  | | | |  |  |  | | | |  |  |
| 3 | Atr-ERN17662 |  | | | |  | | | |  |  |  | | | |  |  |
| 3 | Atr-ERN17663 |  | | | |  | | | |  |  |  | | | |  |  |
| 3 | Atr-ERN17664 |  | | | |  | | | |  |  |  | | | |  |  |
| 3 | Atr-ERN17665 |  | | | |  | | | |  |  |  | | | |  |  |
| 3 | Atr-ERN17666 |  | | | |  | | | |  |  |  | | | |  |  |
| 3 | Atr-ERN17667 |  | | | |  | Vvi-Vitvi18g00885\_t001 |  |  |  | Vvi-Vitvi07g01280\_t001 |  |  |
| 3 | Atr-ERN17668 |  | Vvi-Vitvi03g00396\_t001 |  | | | |  |  |  | | | |  |  |
| 3 | Atr-ERN17669 |  | | | |  | | | |  |  |  | | | |  |  |
| 3 | Atr-ERN17670 |  | | | |  | | | |  |  |  | | | |  |  |
| 3 | Atr-ERN17671 |  | | | |  | | | |  |  |  | Vvi-Vitvi07g04496\_t001 |  |  |
| 3 | Atr-ERN17672 |  | | | |  | | | |  |  |  | | | |  |  |
| 3 | Atr-ERN17673 |  | Vvi-Vitvi03g00397\_t001 |  | Vvi-Vitvi18g00882\_t001 |  |  |  | Vvi-Vitvi07g01286\_t001 |  |  |
| 3 | Atr-ERN17674 |  | Vvi-Vitvi03g00398\_t001 |  | | | |  |  |  | Vvi-Vitvi07g01287\_t001 |  |  |
| 3 | Atr-ERN17675 |  | | | |  | Vvi-Vitvi18g00881\_t001 |  |  |  | | | |  |  |
| 3 | Atr-ERN17676 |  | | | |  | | | |  |  |  | | | |  |  |
| 3 | Atr-ERN17677 |  | Vvi-Vitvi03g00400\_t001 |  | | | |  |  |  | | | |  |  |
| 3 | Atr-ERN17678 |  | | | |  | | | |  |  |  | | | |  |  |
| 3 | Atr-ERN17679 |  | | | |  | | | |  |  |  | | | |  |  |
| 3 | Atr-ERN17680 |  | | | |  | | | |  |  |  | | | |  |  |
| 3 | Atr-ERN17681 |  | Vvi-Vitvi03g04137\_t001 |  | | | |  |  |  | Vvi-Vitvi07g01288\_t001 |  |  |
| 3 | Atr-ERN17682 |  | | | |  | | | |  |  |  | | | |  |  |
| 3 | Atr-ERN17683 |  | | | |  | | | |  |  |  | | | |  |  |
| 3 | Atr-ERN17684 |  | | | |  | | | |  |  |  | | | |  |  |
| 3 | Atr-ERN17685 |  | | | |  | | | |  |  |  | | | |  |  |
| 3 | Atr-ERN17686 |  | | | |  | | | |  |  |  | | | |  |  |
| 3 | Atr-ERN17687 |  | | | |  | | | |  |  |  | | | |  |  |
| 3 | Atr-ERN17688 |  | Vvi-Vitvi03g04144\_t001 |  | Vvi-Vitvi18g04204\_t001 |  |  |  | | | |  |  |
| 3 | Atr-ERN17689 |  | Vvi-Vitvi03g00414\_t001 |  | | | |  |  |  | | | |  |  |
| 3 | Atr-ERN17690 |  | | | |  | Vvi-Vitvi18g00869\_t001 |  |  |  | | | |  |  |
| 3 | Atr-ERN17691 |  | | | |  | Vvi-Vitvi18g00868\_t001 |  |  |  | | | |  |  |
| 3 | Atr-ERN17692 |  | | | |  | | | |  |  |  | | | |  |  |
| 3 | Atr-ERN17693 |  | | | |  | | | |  |  |  | | | |  |  |
| 3 | Atr-ERN17694 |  | | | |  | | | |  |  |  | | | |  |  |
| 3 | Atr-ERN17695 |  | Vvi-Vitvi03g04146\_t001 |  | Vvi-Vitvi18g00867\_t002 |  |  |  | | | |  |  |
| 3 | Atr-ERN17696 |  | | | |  | | | |  |  |  | Vvi-Vitvi07g01294\_t001 |  |  |
| 3 | Atr-ERN17697 |  | | | |  | Vvi-Vitvi18g00863\_t001 |  |  |  | Vvi-Vitvi07g01295\_t001 |  |  |
| 3 | Atr-ERN17698 |  | Vvi-Vitvi03g00423\_t001 |  | | | |  |  |  | Vvi-Vitvi07g01296\_t001 |  |  |
| 3 | Atr-ERN17699 |  | Vvi-Vitvi03g00425\_t001 |  | | | |  |  |  | | | |  |  |
| 3 | Atr-ERN17700 |  | Vvi-Vitvi03g00426\_t001 |  | | | |  |  |  | | | |  |  |
| 3 | Atr-ERN17701 |  | Vvi-Vitvi03g00428\_t001 |  | | | |  |  |  | | | |  |  |
| 3 | Atr-ERN17702 |  | | | |  | | | |  |  |  | Vvi-Vitvi07g01297\_t001 |  |  |
| 3 | Atr-ERN17703 |  | Vvi-Vitvi03g00429\_t001 |  | | | |  |  |  | | | |  |  |
| 3 | Atr-ERN17704 |  | Vvi-Vitvi03g00430\_t001 |  | Vvi-Vitvi18g00858\_t001 |  |  |  | Vvi-Vitvi07g01298\_t001 |  |  |
| 3 | Atr-ERN17705 |  | | | |  | | | |  |  |  | | | |  |  |
| 3 | Atr-ERN17706 |  | | | |  | Vvi-Vitvi18g00857\_t001 |  |  |  | | | |  |  |
| 3 | Atr-ERN17707 |  | | | |  | | | |  |  |  | | | |  |  |
| 3 | Atr-ERN17708 |  | | | |  | Vvi-Vitvi18g00856\_t001 |  |  |  | | | |  |  |
| 2 | Atr-ERN17709 |  | | | |  |  |  |  |  | Vvi-Vitvi07g04505\_t001 |  |  |
| 2 | Atr-ERN17710 |  | | | |  |  |  |  |  | | | |  |  |
| 2 | Atr-ERN17711 |  | | | |  |  |  |  |  | | | |  |  |
| 2 | Atr-ERN17712 |  | | | |  |  |  |  |  | Vvi-Vitvi07g04507\_t001 |  |  |
| 1 | Atr-ERN17713 |  | Vvi-Vitvi03g04153\_t001 |  |  |  |  |  |
| 1 | Atr-ERN17714 |  | | | |  |  |  |  |  |
| 1 | Atr-ERN17715 |  | | | |  |  |  |  |  |
| 1 | Atr-ERN17716 |  | | | |  |  |  |  |  |
| 1 | Atr-ERN17717 |  | | | |  |  |  |  |  |
| 1 | Atr-ERN17718 |  | Vvi-Vitvi03g04158\_t001 |  |  |  |  |  |
