# Supplementary material for: Genome-wide identification, characterization and gene expression of BES1 transcription factor family in grapevine (Vitis vinifera L.)
Source: Sci Rep. 2023 Jan 5;13:240. doi: 10.1038/s41598-022-24407-y (PMC9816167; doi:10.1038/s41598-022-24407-y)
Supplement: Supplementary file 3 — Supplementary Information. [file 41598_2022_24407_MOESM3_ESM.zip › Vvi_Atr/Vitis_vinifera.PN40024.v4.dna_sm.toplevel.fa.vs.Amborella_trichopoda.AMTR1.0.dna_sm.toplevel.fa.html/Atr-AmTr_v1.0_scaffold00060.html]

|  |  |  |  |  |  |  |  |  |  |  |  |  |  |
| --- | --- | --- | --- | --- | --- | --- | --- | --- | --- | --- | --- | --- | --- |
| Duplication depth | Reference chromosome | Collinear blocks | | | | | | | | | | | |
| 0 | Atr-ERM95803 |  |  |  |  |  |  |
| 0 | Atr-ERM95804 |  |  |  |  |  |  |
| 0 | Atr-ERM95805 |  |  |  |  |  |  |
| 0 | Atr-ERM95806 |  |  |  |  |  |  |
| 0 | Atr-ERM95807 |  |  |  |  |  |  |
| 0 | Atr-ERM95808 |  |  |  |  |  |  |
| 0 | Atr-ERM95809 |  |  |  |  |  |  |
| 0 | Atr-ERM95810 |  |  |  |  |  |  |
| 0 | Atr-ERM95811 |  |  |  |  |  |  |
| 0 | Atr-ERM95812 |  |  |  |  |  |  |
| 0 | Atr-ERM95813 |  |  |  |  |  |  |
| 0 | Atr-ERM95814 |  |  |  |  |  |  |
| 0 | Atr-ERM95815 |  |  |  |  |  |  |
| 0 | Atr-ERM95816 |  |  |  |  |  |  |
| 0 | Atr-ERM95817 |  |  |  |  |  |  |
| 0 | Atr-ERM95818 |  |  |  |  |  |  |
| 0 | Atr-ERM95819 |  |  |  |  |  |  |
| 0 | Atr-ERM95820 |  |  |  |  |  |  |
| 0 | Atr-ERM95821 |  |  |  |  |  |  |
| 0 | Atr-ERM95822 |  |  |  |  |  |  |
| 0 | Atr-ERM95823 |  |  |  |  |  |  |
| 0 | Atr-ERM95824 |  |  |  |  |  |  |
| 0 | Atr-ERM95825 |  |  |  |  |  |  |
| 0 | Atr-ERM95826 |  |  |  |  |  |  |
| 0 | Atr-ERM95827 |  |  |  |  |  |  |
| 0 | Atr-ERM95828 |  |  |  |  |  |  |
| 0 | Atr-ERM95829 |  |  |  |  |  |  |
| 0 | Atr-ERM95830 |  |  |  |  |  |  |
| 0 | Atr-ERM95831 |  |  |  |  |  |  |
| 0 | Atr-ERM95832 |  |  |  |  |  |  |
| 0 | Atr-ERM95833 |  |  |  |  |  |  |
| 0 | Atr-ERM95834 |  |  |  |  |  |  |
| 0 | Atr-ERM95835 |  |  |  |  |  |  |
| 1 | Atr-ERM95836 |  | Vvi-Vitvi01g00482\_t001 |  |  |  |  |  |
| 1 | Atr-ERM95837 |  | | | |  |  |  |  |  |
| 1 | Atr-ERM95838 |  | | | |  |  |  |  |  |
| 1 | Atr-ERM95839 |  | | | |  |  |  |  |  |
| 1 | Atr-ERM95840 |  | | | |  |  |  |  |  |
| 1 | Atr-ERM95841 |  | | | |  |  |  |  |  |
| 1 | Atr-ERM95842 |  | | | |  |  |  |  |  |
| 1 | Atr-ERM95843 |  | Vvi-Vitvi01g00483\_t001 |  |  |  |  |  |
| 1 | Atr-ERM95844 |  | | | |  |  |  |  |  |
| 1 | Atr-ERM95845 |  | | | |  |  |  |  |  |
| 1 | Atr-ERM95846 |  | | | |  |  |  |  |  |
| 1 | Atr-ERM95847 |  | | | |  |  |  |  |  |
| 1 | Atr-ERM95848 |  | Vvi-Vitvi01g00484\_t001 |  |  |  |  |  |
| 1 | Atr-ERM95849 |  | | | |  |  |  |  |  |
| 1 | Atr-ERM95850 |  | | | |  |  |  |  |  |
| 1 | Atr-ERM95851 |  | | | |  |  |  |  |  |
| 1 | Atr-ERM95852 |  | | | |  |  |  |  |  |
| 1 | Atr-ERM95853 |  | | | |  |  |  |  |  |
| 1 | Atr-ERM95854 |  | | | |  |  |  |  |  |
| 1 | Atr-ERM95855 |  | Vvi-Vitvi01g00486\_t001 |  |  |  |  |  |
| 1 | Atr-ERM95856 |  | | | |  |  |  |  |  |
| 1 | Atr-ERM95857 |  | | | |  |  |  |  |  |
| 1 | Atr-ERM95858 |  | Vvi-Vitvi01g00487\_t001 |  |  |  |  |  |
| 1 | Atr-ERM95859 |  | | | |  |  |  |  |  |
| 1 | Atr-ERM95860 |  | | | |  |  |  |  |  |
| 1 | Atr-ERM95861 |  | | | |  |  |  |  |  |
| 1 | Atr-ERM95862 |  | | | |  |  |  |  |  |
| 1 | Atr-ERM95863 |  | | | |  |  |  |  |  |
| 1 | Atr-ERM95864 |  | | | |  |  |  |  |  |
| 1 | Atr-ERM95865 |  | | | |  |  |  |  |  |
| 1 | Atr-ERM95866 |  | | | |  |  |  |  |  |
| 1 | Atr-ERM95867 |  | | | |  |  |  |  |  |
| 1 | Atr-ERM95868 |  | | | |  |  |  |  |  |
| 1 | Atr-ERM95869 |  | | | |  |  |  |  |  |
| 1 | Atr-ERM95870 |  | | | |  |  |  |  |  |
| 1 | Atr-ERM95871 |  | | | |  |  |  |  |  |
| 1 | Atr-ERM95872 |  | | | |  |  |  |  |  |
| 1 | Atr-ERM95873 |  | Vvi-Vitvi01g00488\_t001 |  |  |  |  |  |
| 1 | Atr-ERM95874 |  | | | |  |  |  |  |  |
| 1 | Atr-ERM95875 |  | Vvi-Vitvi01g00491\_t001 |  |  |  |  |  |
| 1 | Atr-ERM95876 |  | | | |  |  |  |  |  |
| 1 | Atr-ERM95877 |  | | | |  |  |  |  |  |
| 1 | Atr-ERM95878 |  | Vvi-Vitvi01g00492\_t001 |  |  |  |  |  |
| 1 | Atr-ERM95879 |  | | | |  |  |  |  |  |
| 1 | Atr-ERM95880 |  | | | |  |  |  |  |  |
| 1 | Atr-ERM95881 |  | | | |  |  |  |  |  |
| 1 | Atr-ERM95882 |  | | | |  |  |  |  |  |
| 1 | Atr-ERM95883 |  | | | |  |  |  |  |  |
| 1 | Atr-ERM95884 |  | | | |  |  |  |  |  |
| 1 | Atr-ERM95885 |  | | | |  |  |  |  |  |
| 1 | Atr-ERM95886 |  | Vvi-Vitvi01g04121\_t001 |  |  |  |  |  |
| 1 | Atr-ERM95887 |  | | | |  |  |  |  |  |
| 1 | Atr-ERM95888 |  | | | |  |  |  |  |  |
| 1 | Atr-ERM95889 |  | | | |  |  |  |  |  |
| 1 | Atr-ERM95890 |  | | | |  |  |  |  |  |
| 1 | Atr-ERM95891 |  | Vvi-Vitvi01g00497\_t001 |  |  |  |  |  |
| 0 | Atr-ERM95892 |  |  |  |  |  |  |
| 0 | Atr-ERM95893 |  |  |  |  |  |  |
| 0 | Atr-ERM95894 |  |  |  |  |  |  |
| 0 | Atr-ERM95895 |  |  |  |  |  |  |
| 0 | Atr-ERM95896 |  |  |  |  |  |  |
| 0 | Atr-ERM95897 |  |  |  |  |  |  |
| 0 | Atr-ERM95898 |  |  |  |  |  |  |
| 0 | Atr-ERM95899 |  |  |  |  |  |  |
| 0 | Atr-ERM95900 |  |  |  |  |  |  |
| 0 | Atr-ERM95901 |  |  |  |  |  |  |
| 0 | Atr-ERM95902 |  |  |  |  |  |  |
| 0 | Atr-ERM95903 |  |  |  |  |  |  |
| 0 | Atr-ERM95904 |  |  |  |  |  |  |
| 0 | Atr-ERM95905 |  |  |  |  |  |  |
| 0 | Atr-ERM95906 |  |  |  |  |  |  |
| 0 | Atr-ERM95907 |  |  |  |  |  |  |
| 0 | Atr-ERM95908 |  |  |  |  |  |  |
| 0 | Atr-ERM95909 |  |  |  |  |  |  |
| 0 | Atr-ERM95910 |  |  |  |  |  |  |
| 0 | Atr-ERM95911 |  |  |  |  |  |  |
| 0 | Atr-ERM95912 |  |  |  |  |  |  |
| 0 | Atr-ERM95913 |  |  |  |  |  |  |
| 0 | Atr-ERM95914 |  |  |  |  |  |  |
| 0 | Atr-ERM95915 |  |  |  |  |  |  |
| 0 | Atr-ERM95916 |  |  |  |  |  |  |
| 0 | Atr-ERM95917 |  |  |  |  |  |  |
| 0 | Atr-ERM95918 |  |  |  |  |  |  |
| 0 | Atr-ERM95919 |  |  |  |  |  |  |
| 0 | Atr-ERM95920 |  |  |  |  |  |  |
| 0 | Atr-ERM95921 |  |  |  |  |  |  |
| 0 | Atr-ERM95922 |  |  |  |  |  |  |
| 0 | Atr-ERM95923 |  |  |  |  |  |  |
| 0 | Atr-ERM95924 |  |  |  |  |  |  |
| 0 | Atr-ERM95925 |  |  |  |  |  |  |
| 0 | Atr-ERM95926 |  |  |  |  |  |  |
| 0 | Atr-ERM95927 |  |  |  |  |  |  |
| 0 | Atr-ERM95928 |  |  |  |  |  |  |
| 0 | Atr-ERM95929 |  |  |  |  |  |  |
| 0 | Atr-ERM95930 |  |  |  |  |  |  |
| 0 | Atr-ERM95931 |  |  |  |  |  |  |
| 0 | Atr-ERM95932 |  |  |  |  |  |  |
| 0 | Atr-ERM95933 |  |  |  |  |  |  |
| 0 | Atr-ERM95934 |  |  |  |  |  |  |
| 0 | Atr-ERM95935 |  |  |  |  |  |  |
| 0 | Atr-ERM95936 |  |  |  |  |  |  |
| 0 | Atr-ERM95937 |  |  |  |  |  |  |
| 0 | Atr-ERM95938 |  |  |  |  |  |  |
| 0 | Atr-ERM95939 |  |  |  |  |  |  |
| 0 | Atr-ERM95940 |  |  |  |  |  |  |
| 0 | Atr-ERM95941 |  |  |  |  |  |  |
| 0 | Atr-ERM95942 |  |  |  |  |  |  |
| 0 | Atr-ERM95943 |  |  |  |  |  |  |
| 0 | Atr-ERM95944 |  |  |  |  |  |  |
| 0 | Atr-ERM95945 |  |  |  |  |  |  |
| 0 | Atr-ERM95946 |  |  |  |  |  |  |
| 0 | Atr-ERM95947 |  |  |  |  |  |  |
| 0 | Atr-ERM95948 |  |  |  |  |  |  |
| 0 | Atr-ERM95949 |  |  |  |  |  |  |
| 0 | Atr-ERM95950 |  |  |  |  |  |  |
| 0 | Atr-ERM95951 |  |  |  |  |  |  |
| 0 | Atr-ERM95952 |  |  |  |  |  |  |
| 0 | Atr-ERM95953 |  |  |  |  |  |  |
| 0 | Atr-ERM95954 |  |  |  |  |  |  |
| 0 | Atr-ERM95955 |  |  |  |  |  |  |
| 0 | Atr-ERM95956 |  |  |  |  |  |  |
| 0 | Atr-ERM95957 |  |  |  |  |  |  |
| 0 | Atr-ERM95958 |  |  |  |  |  |  |
| 0 | Atr-ERM95959 |  |  |  |  |  |  |
| 0 | Atr-ERM95960 |  |  |  |  |  |  |
| 0 | Atr-ERM95961 |  |  |  |  |  |  |
| 0 | Atr-ERM95962 |  |  |  |  |  |  |
| 0 | Atr-ERM95963 |  |  |  |  |  |  |
| 0 | Atr-ERM95964 |  |  |  |  |  |  |
| 0 | Atr-ERM95965 |  |  |  |  |  |  |
| 0 | Atr-ERM95966 |  |  |  |  |  |  |
| 0 | Atr-ERM95967 |  |  |  |  |  |  |
| 0 | Atr-ERM95968 |  |  |  |  |  |  |
| 0 | Atr-ERM95969 |  |  |  |  |  |  |
| 0 | Atr-ERM95970 |  |  |  |  |  |  |
| 0 | Atr-ERM95971 |  |  |  |  |  |  |
| 0 | Atr-ERM95972 |  |  |  |  |  |  |
| 0 | Atr-ERM95973 |  |  |  |  |  |  |
| 0 | Atr-ERM95974 |  |  |  |  |  |  |
