# Supplementary material for: Genome-wide identification, characterization and gene expression of BES1 transcription factor family in grapevine (Vitis vinifera L.)
Source: Sci Rep. 2023 Jan 5;13:240. doi: 10.1038/s41598-022-24407-y (PMC9816167; doi:10.1038/s41598-022-24407-y)
Supplement: Supplementary file 3 — Supplementary Information. [file 41598_2022_24407_MOESM3_ESM.zip › Vvi_Atr/Vitis_vinifera.PN40024.v4.dna_sm.toplevel.fa.vs.Amborella_trichopoda.AMTR1.0.dna_sm.toplevel.fa.html/Atr-AmTr_v1.0_scaffold00061.html]

|  |  |  |  |  |  |  |  |  |  |  |  |  |  |
| --- | --- | --- | --- | --- | --- | --- | --- | --- | --- | --- | --- | --- | --- |
| Duplication depth | Reference chromosome | Collinear blocks | | | | | | | | | | | |
| 0 | Atr-ERN18992 |  |  |  |  |  |  |
| 0 | Atr-ERN18993 |  |  |  |  |  |  |
| 0 | Atr-ERN18994 |  |  |  |  |  |  |
| 0 | Atr-ERN18995 |  |  |  |  |  |  |
| 0 | Atr-ERN18996 |  |  |  |  |  |  |
| 0 | Atr-ERN18997 |  |  |  |  |  |  |
| 0 | Atr-ERN18998 |  |  |  |  |  |  |
| 0 | Atr-ERN18999 |  |  |  |  |  |  |
| 0 | Atr-ERN19000 |  |  |  |  |  |  |
| 0 | Atr-ERN19001 |  |  |  |  |  |  |
| 1 | Atr-ERN19002 |  | Vvi-Vitvi19g00906\_t001 |  |  |  |  |  |
| 1 | Atr-ERN19003 |  | | | |  |  |  |  |  |
| 1 | Atr-ERN19004 |  | | | |  |  |  |  |  |
| 1 | Atr-ERN19005 |  | | | |  |  |  |  |  |
| 1 | Atr-ERN19006 |  | | | |  |  |  |  |  |
| 1 | Atr-ERN19007 |  | | | |  |  |  |  |  |
| 1 | Atr-ERN19008 |  | | | |  |  |  |  |  |
| 1 | Atr-ERN19009 |  | | | |  |  |  |  |  |
| 1 | Atr-ERN19010 |  | | | |  |  |  |  |  |
| 2 | Atr-ERN19011 |  | | | |  | Vvi-Vitvi19g00933\_t001 |  |  |  |  |
| 2 | Atr-ERN19012 |  | | | |  | Vvi-Vitvi19g00932\_t001 |  |  |  |  |
| 2 | Atr-ERN19013 |  | | | |  | | | |  |  |  |  |
| 2 | Atr-ERN19014 |  | | | |  | | | |  |  |  |  |
| 2 | Atr-ERN19015 |  | | | |  | | | |  |  |  |  |
| 2 | Atr-ERN19016 |  | | | |  | Vvi-Vitvi19g00929\_t001 |  |  |  |  |
| 2 | Atr-ERN19017 |  | | | |  | | | |  |  |  |  |
| 2 | Atr-ERN19018 |  | | | |  | | | |  |  |  |  |
| 2 | Atr-ERN19019 |  | | | |  | Vvi-Vitvi19g00927\_t001 |  |  |  |  |
| 2 | Atr-ERN19020 |  | | | |  | | | |  |  |  |  |
| 2 | Atr-ERN19021 |  | | | |  | | | |  |  |  |  |
| 2 | Atr-ERN19022 |  | | | |  | | | |  |  |  |  |
| 2 | Atr-ERN19023 |  | | | |  | | | |  |  |  |  |
| 2 | Atr-ERN19024 |  | | | |  | Vvi-Vitvi19g00917\_t003 |  |  |  |  |
| 2 | Atr-ERN19025 |  | | | |  | | | |  |  |  |  |
| 2 | Atr-ERN19026 |  | | | |  | | | |  |  |  |  |
| 2 | Atr-ERN19027 |  | | | |  | | | |  |  |  |  |
| 2 | Atr-ERN19028 |  | Vvi-Vitvi19g00907\_t001 |  | | | |  |  |  |  |
| 2 | Atr-ERN19029 |  | | | |  | | | |  |  |  |  |
| 2 | Atr-ERN19030 |  | Vvi-Vitvi19g00908\_t001 |  | | | |  |  |  |  |
| 2 | Atr-ERN19031 |  | Vvi-Vitvi19g00909\_t001 |  | | | |  |  |  |  |
| 2 | Atr-ERN19032 |  | | | |  | | | |  |  |  |  |
| 2 | Atr-ERN19033 |  | Vvi-Vitvi19g00911\_t001 |  | | | |  |  |  |  |
| 2 | Atr-ERN19034 |  | | | |  | | | |  |  |  |  |
| 2 | Atr-ERN19035 |  | | | |  | Vvi-Vitvi19g00913\_t001 |  |  |  |  |
| 1 | Atr-ERN19036 |  | | | |  |  |  |  |  |
| 1 | Atr-ERN19037 |  | Vvi-Vitvi19g00912\_t001 |  |  |  |  |  |
| 1 | Atr-ERN19038 |  | | | |  |  |  |  |  |
| 1 | Atr-ERN19039 |  | | | |  |  |  |  |  |
| 1 | Atr-ERN19040 |  | Vvi-Vitvi19g00915\_t001 |  |  |  |  |  |
| 1 | Atr-ERN19041 |  | Vvi-Vitvi19g00916\_t001 |  |  |  |  |  |
| 0 | Atr-ERN19042 |  |  |  |  |  |  |
| 0 | Atr-ERN19043 |  |  |  |  |  |  |
| 0 | Atr-ERN19044 |  |  |  |  |  |  |
| 0 | Atr-ERN19045 |  |  |  |  |  |  |
| 0 | Atr-ERN19046 |  |  |  |  |  |  |
| 0 | Atr-ERN19047 |  |  |  |  |  |  |
| 0 | Atr-ERN19048 |  |  |  |  |  |  |
| 0 | Atr-ERN19049 |  |  |  |  |  |  |
| 1 | Atr-ERN19050 |  | Vvi-Vitvi19g00740\_t001 |  |  |  |  |  |
| 1 | Atr-ERN19051 |  | | | |  |  |  |  |  |
| 1 | Atr-ERN19052 |  | | | |  |  |  |  |  |
| 1 | Atr-ERN19053 |  | | | |  |  |  |  |  |
| 1 | Atr-ERN19054 |  | | | |  |  |  |  |  |
| 1 | Atr-ERN19055 |  | | | |  |  |  |  |  |
| 1 | Atr-ERN19056 |  | | | |  |  |  |  |  |
| 1 | Atr-ERN19057 |  | | | |  |  |  |  |  |
| 1 | Atr-ERN19058 |  | | | |  |  |  |  |  |
| 2 | Atr-ERN19059 |  | | | |  | Vvi-Vitvi12g00777\_t001 |  |  |  |  |
| 2 | Atr-ERN19060 |  | | | |  | | | |  |  |  |  |
| 3 | Atr-ERN19061 |  | | | |  | | | |  | Vvi-Vitvi10g04155\_t001 |  |  |  |
| 3 | Atr-ERN19062 |  | Vvi-Vitvi19g00745\_t001 |  | | | |  | | | |  |  |  |
| 3 | Atr-ERN19063 |  | | | |  | | | |  | Vvi-Vitvi10g00302\_t001 |  |  |  |
| 3 | Atr-ERN19064 |  | | | |  | Vvi-Vitvi12g02510\_t001 |  | Vvi-Vitvi10g04153\_t001 |  |  |  |
| 3 | Atr-ERN19065 |  | | | |  | | | |  | | | |  |  |  |
| 3 | Atr-ERN19066 |  | | | |  | | | |  | | | |  |  |  |
| 3 | Atr-ERN19067 |  | | | |  | | | |  | | | |  |  |  |
| 3 | Atr-ERN19068 |  | Vvi-Vitvi19g00747\_t001 |  | | | |  | | | |  |  |  |
| 3 | Atr-ERN19069 |  | Vvi-Vitvi19g00749\_t001 |  | | | |  | | | |  |  |  |
| 3 | Atr-ERN19070 |  | | | |  | | | |  | Vvi-Vitvi10g04151\_t001 |  |  |  |
| 3 | Atr-ERN19071 |  | | | |  | Vvi-Vitvi12g00774\_t001 |  | | | |  |  |  |
| 3 | Atr-ERN19072 |  | | | |  | Vvi-Vitvi12g00773\_t001 |  | | | |  |  |  |
| 3 | Atr-ERN19073 |  | Vvi-Vitvi19g02084\_t002 |  | | | |  | | | |  |  |  |
| 3 | Atr-ERN19074 |  | | | |  | | | |  | | | |  |  |  |
| 3 | Atr-ERN19075 |  | Vvi-Vitvi19g00757\_t001 |  | | | |  | | | |  |  |  |
| 3 | Atr-ERN19076 |  | Vvi-Vitvi19g00758\_t001 |  | | | |  | | | |  |  |  |
| 3 | Atr-ERN19077 |  | | | |  | | | |  | | | |  |  |  |
| 3 | Atr-ERN19078 |  | | | |  | | | |  | | | |  |  |  |
| 3 | Atr-ERN19079 |  | | | |  | | | |  | | | |  |  |  |
| 3 | Atr-ERN19080 |  | Vvi-Vitvi19g00759\_t001 |  | | | |  | | | |  |  |  |
| 3 | Atr-ERN19081 |  | | | |  | | | |  | | | |  |  |  |
| 3 | Atr-ERN19082 |  | | | |  | | | |  | | | |  |  |  |
| 3 | Atr-ERN19083 |  | | | |  | | | |  | | | |  |  |  |
| 3 | Atr-ERN19084 |  | | | |  | | | |  | Vvi-Vitvi10g04150\_t001 |  |  |  |
| 3 | Atr-ERN19085 |  | | | |  | | | |  | | | |  |  |  |
| 3 | Atr-ERN19086 |  | | | |  | | | |  | | | |  |  |  |
| 3 | Atr-ERN19087 |  | | | |  | Vvi-Vitvi12g00770\_t001 |  | | | |  |  |  |
| 3 | Atr-ERN19088 |  | | | |  | | | |  | | | |  |  |  |
| 3 | Atr-ERN19089 |  | | | |  | | | |  | | | |  |  |  |
| 3 | Atr-ERN19090 |  | | | |  | | | |  | | | |  |  |  |
| 3 | Atr-ERN19091 |  | | | |  | | | |  | | | |  |  |  |
| 3 | Atr-ERN19092 |  | | | |  | | | |  | | | |  |  |  |
| 3 | Atr-ERN19093 |  | | | |  | | | |  | Vvi-Vitvi10g00307\_t003 |  |  |  |
| 3 | Atr-ERN19094 |  | | | |  | | | |  | | | |  |  |  |
| 3 | Atr-ERN19095 |  | Vvi-Vitvi19g00766\_t001 |  | | | |  | | | |  |  |  |
| 3 | Atr-ERN19096 |  | | | |  | | | |  | | | |  |  |  |
| 3 | Atr-ERN19097 |  | Vvi-Vitvi19g00767\_t001 |  | | | |  | | | |  |  |  |
| 3 | Atr-ERN19098 |  | | | |  | | | |  | | | |  |  |  |
| 3 | Atr-ERN19099 |  | Vvi-Vitvi19g00768\_t001 |  | | | |  | | | |  |  |  |
| 3 | Atr-ERN19100 |  | Vvi-Vitvi19g00775\_t001 |  | | | |  | | | |  |  |  |
| 3 | Atr-ERN19101 |  | Vvi-Vitvi19g00776\_t001 |  | | | |  | Vvi-Vitvi10g00304\_t001 |  |  |  |
| 3 | Atr-ERN19102 |  | | | |  | | | |  | | | |  |  |  |
| 3 | Atr-ERN19103 |  | | | |  | | | |  | | | |  |  |  |
| 3 | Atr-ERN19104 |  | Vvi-Vitvi19g00778\_t001 |  | | | |  | | | |  |  |  |
| 3 | Atr-ERN19105 |  | | | |  | | | |  | | | |  |  |  |
| 3 | Atr-ERN19106 |  | | | |  | Vvi-Vitvi12g00769\_t001 |  | Vvi-Vitvi10g04148\_t001 |  |  |  |
| 3 | Atr-ERN19107 |  | | | |  | Vvi-Vitvi12g00767\_t001 |  | | | |  |  |  |
| 3 | Atr-ERN19108 |  | | | |  | | | |  | Vvi-Vitvi10g02295\_t001 |  |  |  |
| 3 | Atr-ERN19109 |  | | | |  | | | |  | | | |  |  |  |
| 3 | Atr-ERN19110 |  | | | |  | | | |  | | | |  |  |  |
| 3 | Atr-ERN19111 |  | | | |  | | | |  | | | |  |  |  |
| 3 | Atr-ERN19112 |  | Vvi-Vitvi19g00781\_t001 |  | Vvi-Vitvi12g00766\_t001 |  | Vvi-Vitvi10g04145\_t001 |  |  |  |
| 2 | Atr-ERN19113 |  | | | |  | | | |  |  |  |  |
| 3 | Atr-ERN19114 |  | | | |  | | | |  | Vvi-Vitvi17g00958\_t001 |  |  |  |
| 3 | Atr-ERN19115 |  | Vvi-Vitvi19g00782\_t001 |  | | | |  | | | |  |  |  |
| 3 | Atr-ERN19116 |  | | | |  | | | |  | | | |  |  |  |
| 3 | Atr-ERN19117 |  | | | |  | | | |  | | | |  |  |  |
| 3 | Atr-ERN19118 |  | | | |  | | | |  | | | |  |  |  |
| 3 | Atr-ERN19119 |  | | | |  | | | |  | | | |  |  |  |
| 3 | Atr-ERN19120 |  | | | |  | | | |  | | | |  |  |  |
| 3 | Atr-ERN19121 |  | | | |  | | | |  | | | |  |  |  |
| 4 | Atr-ERN19122 |  | Vvi-Vitvi19g04298\_t001 |  | Vvi-Vitvi12g02508\_t001 |  | | | |  | Vvi-Vitvi10g01684\_t001 |  |  |
| 4 | Atr-ERN19123 |  | | | |  | | | |  | | | |  | Vvi-Vitvi10g04093\_t001 |  |  |
| 4 | Atr-ERN19124 |  | | | |  | | | |  | | | |  | | | |  |  |
| 4 | Atr-ERN19125 |  | | | |  | | | |  | | | |  | Vvi-Vitvi10g00212\_t001 |  |  |
| 4 | Atr-ERN19126 |  | | | |  | | | |  | | | |  | Vvi-Vitvi10g00213\_t001 |  |  |
| 4 | Atr-ERN19127 |  | Vvi-Vitvi19g04300\_t001 |  | | | |  | | | |  | | | |  |  |
| 4 | Atr-ERN19128 |  | | | |  | | | |  | Vvi-Vitvi17g00954\_t001 |  | Vvi-Vitvi10g00214\_t002 |  |  |
| 4 | Atr-ERN19129 |  | | | |  | | | |  | | | |  | | | |  |  |
| 4 | Atr-ERN19130 |  | | | |  | Vvi-Vitvi12g02507\_t001 |  | | | |  | Vvi-Vitvi10g00215\_t001 |  |  |
| 3 | Atr-ERN19131 |  | Vvi-Vitvi19g04305\_t001 |  | | | |  | | | |  |  |  |
| 3 | Atr-ERN19132 |  | | | |  | | | |  | | | |  |  |  |
| 3 | Atr-ERN19133 |  | | | |  | | | |  | | | |  |  |  |
| 3 | Atr-ERN19134 |  | | | |  | | | |  | | | |  |  |  |
| 3 | Atr-ERN19135 |  | | | |  | | | |  | | | |  |  |  |
| 3 | Atr-ERN19136 |  | | | |  | | | |  | | | |  |  |  |
| 3 | Atr-ERN19137 |  | Vvi-Vitvi19g00899\_t001 |  | | | |  | | | |  |  |  |
| 3 | Atr-ERN19138 |  | | | |  | | | |  | | | |  |  |  |
| 3 | Atr-ERN19139 |  | | | |  | | | |  | | | |  |  |  |
| 3 | Atr-ERN19140 |  | | | |  | | | |  | Vvi-Vitvi17g00942\_t001 |  |  |  |
| 3 | Atr-ERN19141 |  | | | |  | | | |  | | | |  |  |  |
| 3 | Atr-ERN19142 |  | | | |  | | | |  | | | |  |  |  |
| 3 | Atr-ERN19143 |  | | | |  | | | |  | | | |  |  |  |
| 3 | Atr-ERN19144 |  | | | |  | | | |  | | | |  |  |  |
| 3 | Atr-ERN19145 |  | | | |  | | | |  | | | |  |  |  |
| 3 | Atr-ERN19146 |  | | | |  | | | |  | | | |  |  |  |
| 3 | Atr-ERN19147 |  | | | |  | Vvi-Vitvi12g00763\_t001 |  | | | |  |  |  |
| 3 | Atr-ERN19148 |  | Vvi-Vitvi19g00900\_t001 |  | | | |  | Vvi-Vitvi17g00938\_t001 |  |  |  |
| 3 | Atr-ERN19149 |  | | | |  | | | |  | | | |  |  |  |
| 3 | Atr-ERN19150 |  | Vvi-Vitvi19g00904\_t001 |  | | | |  | | | |  |  |  |
| 3 | Atr-ERN19151 |  | | | |  | | | |  | | | |  |  |  |
| 3 | Atr-ERN19152 |  | | | |  | | | |  | | | |  |  |  |
| 3 | Atr-ERN19153 |  | | | |  | | | |  | | | |  |  |  |
| 3 | Atr-ERN19154 |  | Vvi-Vitvi19g00905\_t001 |  | | | |  | | | |  |  |  |
| 2 | Atr-ERN19155 |  |  |  | | | |  | | | |  |  |  |
| 2 | Atr-ERN19156 |  |  |  | | | |  | | | |  |  |  |
| 2 | Atr-ERN19157 |  |  |  | | | |  | | | |  |  |  |
| 2 | Atr-ERN19158 |  |  |  | | | |  | | | |  |  |  |
| 2 | Atr-ERN19159 |  |  |  | | | |  | | | |  |  |  |
| 3 | Atr-ERN19160 |  | Vvi-Vitvi19g00739\_t001 |  | | | |  | | | |  |  |  |
| 3 | Atr-ERN19161 |  | Vvi-Vitvi19g00736\_t001 |  | | | |  | | | |  |  |  |
| 3 | Atr-ERN19162 |  | | | |  | | | |  | | | |  |  |  |
| 3 | Atr-ERN19163 |  | | | |  | | | |  | | | |  |  |  |
| 3 | Atr-ERN19164 |  | | | |  | | | |  | | | |  |  |  |
| 3 | Atr-ERN19165 |  | | | |  | Vvi-Vitvi12g00762\_t001 |  | Vvi-Vitvi17g00931\_t001 |  |  |  |
| 3 | Atr-ERN19166 |  | Vvi-Vitvi19g00734\_t001 |  | Vvi-Vitvi12g00761\_t001 |  | | | |  |  |  |
| 4 | Atr-ERN19167 |  | | | |  | | | |  | | | |  | Vvi-Vitvi10g01687\_t001 |  |  |
| 4 | Atr-ERN19168 |  | Vvi-Vitvi19g00732\_t001 |  | | | |  | | | |  | | | |  |  |
| 4 | Atr-ERN19169 |  | | | |  | Vvi-Vitvi12g00760\_t001 |  | | | |  | | | |  |  |
| 4 | Atr-ERN19170 |  | | | |  | | | |  | | | |  | | | |  |  |
| 4 | Atr-ERN19171 |  | | | |  | | | |  | | | |  | | | |  |  |
| 4 | Atr-ERN19172 |  | | | |  | | | |  | | | |  | | | |  |  |
| 4 | Atr-ERN19173 |  | Vvi-Vitvi19g00730\_t001 |  | | | |  | | | |  | | | |  |  |
| 4 | Atr-ERN19174 |  | | | |  | Vvi-Vitvi12g00759\_t001 |  | | | |  | | | |  |  |
| 4 | Atr-ERN19175 |  | | | |  | Vvi-Vitvi12g00757\_t001 |  | | | |  | Vvi-Vitvi10g00219\_t001 |  |  |
| 4 | Atr-ERN19176 |  | | | |  | | | |  | Vvi-Vitvi17g00921\_t001 |  | | | |  |  |
| 4 | Atr-ERN19177 |  | | | |  | Vvi-Vitvi12g00755\_t001 |  | | | |  | | | |  |  |
| 4 | Atr-ERN19178 |  | | | |  | | | |  | | | |  | | | |  |  |
| 4 | Atr-ERN19179 |  | | | |  | | | |  | | | |  | | | |  |  |
| 4 | Atr-ERN19180 |  | | | |  | | | |  | | | |  | | | |  |  |
| 4 | Atr-ERN19181 |  | Vvi-Vitvi19g02077\_t001 |  | | | |  | | | |  | | | |  |  |
| 4 | Atr-ERN19182 |  | | | |  | | | |  | | | |  | | | |  |  |
| 4 | Atr-ERN19183 |  | | | |  | | | |  | | | |  | | | |  |  |
| 4 | Atr-ERN19184 |  | | | |  | | | |  | | | |  | | | |  |  |
| 4 | Atr-ERN19185 |  | | | |  | | | |  | | | |  | | | |  |  |
| 4 | Atr-ERN19186 |  | | | |  | | | |  | | | |  | | | |  |  |
| 4 | Atr-ERN19187 |  | | | |  | | | |  | | | |  | | | |  |  |
| 4 | Atr-ERN19188 |  | | | |  | | | |  | | | |  | | | |  |  |
| 4 | Atr-ERN19189 |  | | | |  | | | |  | | | |  | | | |  |  |
| 4 | Atr-ERN19190 |  | | | |  | | | |  | | | |  | Vvi-Vitvi10g00221\_t001 |  |  |
| 4 | Atr-ERN19191 |  | | | |  | | | |  | | | |  | | | |  |  |
| 4 | Atr-ERN19192 |  | | | |  | | | |  | Vvi-Vitvi17g00909\_t001 |  | Vvi-Vitvi10g00222\_t001 |  |  |
| 3 | Atr-ERN19193 |  | Vvi-Vitvi19g00729\_t001 |  | | | |  |  |  | | | |  |  |
| 3 | Atr-ERN19194 |  | | | |  | | | |  |  |  | | | |  |  |
| 3 | Atr-ERN19195 |  | | | |  | Vvi-Vitvi12g00754\_t001 |  |  |  | | | |  |  |
| 3 | Atr-ERN19196 |  | Vvi-Vitvi19g00728\_t001 |  | | | |  |  |  | | | |  |  |
| 3 | Atr-ERN19197 |  | | | |  | Vvi-Vitvi12g00753\_t001 |  |  |  | | | |  |  |
| 3 | Atr-ERN19198 |  | | | |  | | | |  |  |  | | | |  |  |
| 3 | Atr-ERN19199 |  | Vvi-Vitvi19g04286\_t001 |  | | | |  |  |  | | | |  |  |
| 3 | Atr-ERN19200 |  | | | |  | Vvi-Vitvi12g00752\_t001 |  |  |  | | | |  |  |
| 3 | Atr-ERN19201 |  | | | |  | | | |  |  |  | | | |  |  |
| 3 | Atr-ERN19202 |  | | | |  | | | |  |  |  | | | |  |  |
| 3 | Atr-ERN19203 |  | | | |  | | | |  |  |  | | | |  |  |
| 3 | Atr-ERN19204 |  | Vvi-Vitvi19g00726\_t001 |  | | | |  |  |  | | | |  |  |
| 3 | Atr-ERN19205 |  | Vvi-Vitvi19g00725\_t001 |  | | | |  |  |  | | | |  |  |
| 3 | Atr-ERN19206 |  | Vvi-Vitvi19g00723\_t001 |  | Vvi-Vitvi12g00751\_t001 |  |  |  | | | |  |  |
| 4 | Atr-ERN19207 |  | Vvi-Vitvi19g00722\_t001 |  | Vvi-Vitvi12g00750\_t001 |  | Vvi-Vitvi10g04141\_t001 |  | | | |  |  |
| 4 | Atr-ERN19208 |  | | | |  | | | |  | | | |  | Vvi-Vitvi10g00223\_t001 |  |  |
| 4 | Atr-ERN19209 |  | | | |  | | | |  | | | |  | | | |  |  |
| 4 | Atr-ERN19210 |  | Vvi-Vitvi19g00712\_t001 |  | | | |  | | | |  | | | |  |  |
| 4 | Atr-ERN19211 |  | | | |  | | | |  | | | |  | Vvi-Vitvi10g04108\_t001 |  |  |
| 4 | Atr-ERN19212 |  | Vvi-Vitvi19g00708\_t001 |  | | | |  | | | |  | | | |  |  |
| 4 | Atr-ERN19213 |  | | | |  | | | |  | | | |  | | | |  |  |
| 4 | Atr-ERN19214 |  | Vvi-Vitvi19g00706\_t001 |  | | | |  | | | |  | | | |  |  |
| 4 | Atr-ERN19215 |  | | | |  | | | |  | | | |  | | | |  |  |
| 4 | Atr-ERN19216 |  | | | |  | | | |  | | | |  | | | |  |  |
| 4 | Atr-ERN19217 |  | Vvi-Vitvi19g00704\_t001 |  | | | |  | | | |  | | | |  |  |
| 4 | Atr-ERN19218 |  | Vvi-Vitvi19g00703\_t001 |  | | | |  | | | |  | Vvi-Vitvi10g04117\_t001 |  |  |
| 3 | Atr-ERN19219 |  | | | |  | | | |  | | | |  |  |  |
| 3 | Atr-ERN19220 |  | | | |  | | | |  | | | |  |  |  |
| 3 | Atr-ERN19221 |  | | | |  | Vvi-Vitvi12g02505\_t001 |  | | | |  |  |  |
| 3 | Atr-ERN19222 |  | | | |  | Vvi-Vitvi12g00747\_t001 |  | | | |  |  |  |
| 3 | Atr-ERN19223 |  | | | |  | Vvi-Vitvi12g00746\_t001 |  | | | |  |  |  |
| 3 | Atr-ERN19224 |  | | | |  | Vvi-Vitvi12g00745\_t001 |  | | | |  |  |  |
| 3 | Atr-ERN19225 |  | | | |  | | | |  | | | |  |  |  |
| 3 | Atr-ERN19226 |  | | | |  | Vvi-Vitvi12g00742\_t001 |  | | | |  |  |  |
| 3 | Atr-ERN19227 |  | | | |  | | | |  | | | |  |  |  |
| 3 | Atr-ERN19228 |  | | | |  | | | |  | | | |  |  |  |
| 3 | Atr-ERN19229 |  | | | |  | | | |  | | | |  |  |  |
| 3 | Atr-ERN19230 |  | | | |  | | | |  | | | |  |  |  |
| 3 | Atr-ERN19231 |  | Vvi-Vitvi19g00697\_t001 |  | | | |  | Vvi-Vitvi10g04129\_t001 |  |  |  |
| 3 | Atr-ERN19232 |  | Vvi-Vitvi19g00696\_t001 |  | Vvi-Vitvi12g00741\_t001 |  | | | |  |  |  |
| 3 | Atr-ERN19233 |  | | | |  | | | |  | | | |  |  |  |
| 3 | Atr-ERN19234 |  | Vvi-Vitvi19g00695\_t001 |  | Vvi-Vitvi12g04252\_t001 |  | | | |  |  |  |
| 3 | Atr-ERN19235 |  | | | |  | | | |  | | | |  |  |  |
| 3 | Atr-ERN19236 |  | | | |  | Vvi-Vitvi12g00736\_t001 |  | | | |  |  |  |
| 3 | Atr-ERN19237 |  | | | |  | | | |  | | | |  |  |  |
| 3 | Atr-ERN19238 |  | | | |  | | | |  | | | |  |  |  |
| 3 | Atr-ERN19239 |  | | | |  | | | |  | Vvi-Vitvi10g02269\_t001 |  |  |  |
| 3 | Atr-ERN19240 |  | Vvi-Vitvi19g00694\_t002 |  | | | |  | | | |  |  |  |
| 3 | Atr-ERN19241 |  | | | |  | | | |  | | | |  |  |  |
| 3 | Atr-ERN19242 |  | | | |  | | | |  | | | |  |  |  |
| 3 | Atr-ERN19243 |  | | | |  | | | |  | | | |  |  |  |
| 3 | Atr-ERN19244 |  | | | |  | | | |  | | | |  |  |  |
| 3 | Atr-ERN19245 |  | | | |  | | | |  | | | |  |  |  |
| 3 | Atr-ERN19246 |  | | | |  | | | |  | | | |  |  |  |
| 3 | Atr-ERN19247 |  | | | |  | | | |  | Vvi-Vitvi10g02268\_t001 |  |  |  |
| 3 | Atr-ERN19248 |  | | | |  | | | |  | | | |  |  |  |
| 3 | Atr-ERN19249 |  | | | |  | | | |  | | | |  |  |  |
| 3 | Atr-ERN19250 |  | Vvi-Vitvi19g02068\_t001 |  | | | |  | | | |  |  |  |
| 3 | Atr-ERN19251 |  | | | |  | Vvi-Vitvi12g00725\_t001 |  | | | |  |  |  |
| 3 | Atr-ERN19252 |  | | | |  | | | |  | | | |  |  |  |
| 3 | Atr-ERN19253 |  | | | |  | | | |  | | | |  |  |  |
| 3 | Atr-ERN19254 |  | | | |  | | | |  | | | |  |  |  |
| 3 | Atr-ERN19255 |  | | | |  | | | |  | Vvi-Vitvi10g02267\_t001 |  |  |  |
| 3 | Atr-ERN19256 |  | | | |  | | | |  | Vvi-Vitvi10g04127\_t001 |  |  |  |
| 3 | Atr-ERN19257 |  | | | |  | | | |  | Vvi-Vitvi10g04126\_t001 |  |  |  |
| 3 | Atr-ERN19258 |  | Vvi-Vitvi19g00690\_t001 |  | | | |  | | | |  |  |  |
| 3 | Atr-ERN19259 |  | | | |  | | | |  | | | |  |  |  |
| 3 | Atr-ERN19260 |  | | | |  | | | |  | | | |  |  |  |
| 3 | Atr-ERN19261 |  | | | |  | | | |  | | | |  |  |  |
| 3 | Atr-ERN19262 |  | | | |  | | | |  | | | |  |  |  |
| 3 | Atr-ERN19263 |  | | | |  | | | |  | | | |  |  |  |
| 3 | Atr-ERN19264 |  | | | |  | | | |  | | | |  |  |  |
| 3 | Atr-ERN19265 |  | | | |  | | | |  | | | |  |  |  |
| 3 | Atr-ERN19266 |  | | | |  | | | |  | | | |  |  |  |
| 3 | Atr-ERN19267 |  | | | |  | | | |  | | | |  |  |  |
| 3 | Atr-ERN19268 |  | | | |  | | | |  | | | |  |  |  |
| 3 | Atr-ERN19269 |  | | | |  | | | |  | | | |  |  |  |
| 3 | Atr-ERN19270 |  | Vvi-Vitvi19g00688\_t001 |  | | | |  | | | |  |  |  |
| 3 | Atr-ERN19271 |  | | | |  | | | |  | | | |  |  |  |
| 3 | Atr-ERN19272 |  | | | |  | | | |  | | | |  |  |  |
| 3 | Atr-ERN19273 |  | Vvi-Vitvi19g00686\_t001 |  | Vvi-Vitvi12g00719\_t001 |  | Vvi-Vitvi10g02273\_t001 |  |  |  |
