# Supplementary material for: Genome-wide identification, characterization and gene expression of BES1 transcription factor family in grapevine (Vitis vinifera L.)
Source: Sci Rep. 2023 Jan 5;13:240. doi: 10.1038/s41598-022-24407-y (PMC9816167; doi:10.1038/s41598-022-24407-y)
Supplement: Supplementary file 3 — Supplementary Information. [file 41598_2022_24407_MOESM3_ESM.zip › Vvi_Atr/Vitis_vinifera.PN40024.v4.dna_sm.toplevel.fa.vs.Amborella_trichopoda.AMTR1.0.dna_sm.toplevel.fa.html/Atr-AmTr_v1.0_scaffold00062.html]

|  |  |  |  |  |  |  |  |  |  |  |  |  |  |
| --- | --- | --- | --- | --- | --- | --- | --- | --- | --- | --- | --- | --- | --- |
| Duplication depth | Reference chromosome | Collinear blocks | | | | | | | | | | | |
| 0 | Atr-ERN19502 |  |  |  |  |  |  |
| 0 | Atr-ERN19503 |  |  |  |  |  |  |
| 0 | Atr-ERN19504 |  |  |  |  |  |  |
| 0 | Atr-ERN19505 |  |  |  |  |  |  |
| 0 | Atr-ERN19506 |  |  |  |  |  |  |
| 0 | Atr-ERN19507 |  |  |  |  |  |  |
| 0 | Atr-ERN19508 |  |  |  |  |  |  |
| 0 | Atr-ERN19509 |  |  |  |  |  |  |
| 0 | Atr-ERN19510 |  |  |  |  |  |  |
| 0 | Atr-ERN19511 |  |  |  |  |  |  |
| 0 | Atr-ERN19512 |  |  |  |  |  |  |
| 0 | Atr-ERN19513 |  |  |  |  |  |  |
| 0 | Atr-ERN19514 |  |  |  |  |  |  |
| 0 | Atr-ERN19515 |  |  |  |  |  |  |
| 0 | Atr-ERN19516 |  |  |  |  |  |  |
| 0 | Atr-ERN19517 |  |  |  |  |  |  |
| 1 | Atr-ERN19518 |  | Vvi-Vitvi10g00035\_t001 |  |  |  |  |  |
| 2 | Atr-ERN19519 |  | Vvi-Vitvi10g00037\_t001 |  | Vvi-Vitvi19g00457\_t001 |  |  |  |  |
| 2 | Atr-ERN19520 |  | | | |  | Vvi-Vitvi19g00458\_t001 |  |  |  |  |
| 2 | Atr-ERN19521 |  | | | |  | | | |  |  |  |  |
| 2 | Atr-ERN19522 |  | | | |  | | | |  |  |  |  |
| 2 | Atr-ERN19523 |  | | | |  | | | |  |  |  |  |
| 2 | Atr-ERN19524 |  | Vvi-Vitvi10g00039\_t001 |  | | | |  |  |  |  |
| 2 | Atr-ERN19525 |  | Vvi-Vitvi10g00040\_t001 |  | | | |  |  |  |  |
| 2 | Atr-ERN19526 |  | | | |  | | | |  |  |  |  |
| 2 | Atr-ERN19527 |  | | | |  | | | |  |  |  |  |
| 2 | Atr-ERN19528 |  | Vvi-Vitvi10g00041\_t001 |  | | | |  |  |  |  |
| 2 | Atr-ERN19529 |  | | | |  | | | |  |  |  |  |
| 2 | Atr-ERN19530 |  | | | |  | | | |  |  |  |  |
| 2 | Atr-ERN19531 |  | | | |  | | | |  |  |  |  |
| 2 | Atr-ERN19532 |  | | | |  | | | |  |  |  |  |
| 2 | Atr-ERN19533 |  | Vvi-Vitvi10g00044\_t001 |  | | | |  |  |  |  |
| 2 | Atr-ERN19534 |  | | | |  | | | |  |  |  |  |
| 2 | Atr-ERN19535 |  | | | |  | | | |  |  |  |  |
| 2 | Atr-ERN19536 |  | | | |  | | | |  |  |  |  |
| 2 | Atr-ERN19537 |  | | | |  | | | |  |  |  |  |
| 2 | Atr-ERN19538 |  | | | |  | | | |  |  |  |  |
| 2 | Atr-ERN19539 |  | | | |  | Vvi-Vitvi19g00460\_t001 |  |  |  |  |
| 2 | Atr-ERN19540 |  | | | |  | | | |  |  |  |  |
| 2 | Atr-ERN19541 |  | | | |  | | | |  |  |  |  |
| 2 | Atr-ERN19542 |  | | | |  | | | |  |  |  |  |
| 2 | Atr-ERN19543 |  | | | |  | | | |  |  |  |  |
| 2 | Atr-ERN19544 |  | | | |  | | | |  |  |  |  |
| 2 | Atr-ERN19545 |  | | | |  | | | |  |  |  |  |
| 2 | Atr-ERN19546 |  | | | |  | | | |  |  |  |  |
| 2 | Atr-ERN19547 |  | Vvi-Vitvi10g00045\_t001 |  | | | |  |  |  |  |
| 2 | Atr-ERN19548 |  | | | |  | | | |  |  |  |  |
| 2 | Atr-ERN19549 |  | | | |  | | | |  |  |  |  |
| 2 | Atr-ERN19550 |  | | | |  | Vvi-Vitvi19g02003\_t001 |  |  |  |  |
| 2 | Atr-ERN19551 |  | | | |  | | | |  |  |  |  |
| 2 | Atr-ERN19552 |  | | | |  | | | |  |  |  |  |
| 2 | Atr-ERN19553 |  | | | |  | | | |  |  |  |  |
| 2 | Atr-ERN19554 |  | | | |  | | | |  |  |  |  |
| 2 | Atr-ERN19555 |  | | | |  | | | |  |  |  |  |
| 2 | Atr-ERN19556 |  | | | |  | | | |  |  |  |  |
| 2 | Atr-ERN19557 |  | | | |  | | | |  |  |  |  |
| 2 | Atr-ERN19558 |  | | | |  | | | |  |  |  |  |
| 2 | Atr-ERN19559 |  | | | |  | | | |  |  |  |  |
| 2 | Atr-ERN19560 |  | | | |  | | | |  |  |  |  |
| 2 | Atr-ERN19561 |  | | | |  | | | |  |  |  |  |
| 2 | Atr-ERN19562 |  | Vvi-Vitvi10g01612\_t001 |  | | | |  |  |  |  |
| 2 | Atr-ERN19563 |  | | | |  | Vvi-Vitvi19g00463\_t001 |  |  |  |  |
| 2 | Atr-ERN19564 |  | | | |  | | | |  |  |  |  |
| 2 | Atr-ERN19565 |  | | | |  | | | |  |  |  |  |
| 2 | Atr-ERN19566 |  | | | |  | | | |  |  |  |  |
| 2 | Atr-ERN19567 |  | | | |  | | | |  |  |  |  |
| 2 | Atr-ERN19568 |  | | | |  | | | |  |  |  |  |
| 2 | Atr-ERN19569 |  | | | |  | | | |  |  |  |  |
| 2 | Atr-ERN19570 |  | | | |  | | | |  |  |  |  |
| 2 | Atr-ERN19571 |  | | | |  | | | |  |  |  |  |
| 2 | Atr-ERN19572 |  | | | |  | | | |  |  |  |  |
| 2 | Atr-ERN19573 |  | | | |  | | | |  |  |  |  |
| 2 | Atr-ERN19574 |  | | | |  | | | |  |  |  |  |
| 2 | Atr-ERN19575 |  | | | |  | Vvi-Vitvi19g00469\_t001 |  |  |  |  |
| 2 | Atr-ERN19576 |  | | | |  | | | |  |  |  |  |
| 2 | Atr-ERN19577 |  | | | |  | | | |  |  |  |  |
| 2 | Atr-ERN19578 |  | | | |  | | | |  |  |  |  |
| 2 | Atr-ERN19579 |  | | | |  | | | |  |  |  |  |
| 2 | Atr-ERN19580 |  | | | |  | | | |  |  |  |  |
| 2 | Atr-ERN19581 |  | | | |  | | | |  |  |  |  |
| 2 | Atr-ERN19582 |  | | | |  | | | |  |  |  |  |
| 2 | Atr-ERN19583 |  | | | |  | | | |  |  |  |  |
| 2 | Atr-ERN19584 |  | Vvi-Vitvi10g00046\_t001 |  | Vvi-Vitvi19g00470\_t001 |  |  |  |  |
| 2 | Atr-ERN19585 |  | | | |  | | | |  |  |  |  |
| 2 | Atr-ERN19586 |  | Vvi-Vitvi10g00047\_t001 |  | | | |  |  |  |  |
| 1 | Atr-ERN19587 |  |  |  | | | |  |  |  |  |
| 1 | Atr-ERN19588 |  |  |  | | | |  |  |  |  |
| 1 | Atr-ERN19589 |  |  |  | Vvi-Vitvi19g00472\_t001 |  |  |  |  |
| 1 | Atr-ERN19590 |  |  |  | | | |  |  |  |  |
| 1 | Atr-ERN19591 |  |  |  | | | |  |  |  |  |
| 1 | Atr-ERN19592 |  |  |  | | | |  |  |  |  |
| 1 | Atr-ERN19593 |  |  |  | | | |  |  |  |  |
| 1 | Atr-ERN19594 |  |  |  | | | |  |  |  |  |
| 1 | Atr-ERN19595 |  |  |  | Vvi-Vitvi19g00473\_t001 |  |  |  |  |
| 1 | Atr-ERN19596 |  |  |  | | | |  |  |  |  |
| 1 | Atr-ERN19597 |  |  |  | | | |  |  |  |  |
| 1 | Atr-ERN19598 |  |  |  | | | |  |  |  |  |
| 1 | Atr-ERN19599 |  |  |  | | | |  |  |  |  |
| 1 | Atr-ERN19600 |  |  |  | | | |  |  |  |  |
| 1 | Atr-ERN19601 |  |  |  | | | |  |  |  |  |
| 1 | Atr-ERN19602 |  |  |  | | | |  |  |  |  |
| 1 | Atr-ERN19603 |  |  |  | | | |  |  |  |  |
| 1 | Atr-ERN19604 |  |  |  | | | |  |  |  |  |
| 1 | Atr-ERN19605 |  |  |  | | | |  |  |  |  |
| 1 | Atr-ERN19606 |  |  |  | | | |  |  |  |  |
| 1 | Atr-ERN19607 |  |  |  | | | |  |  |  |  |
| 1 | Atr-ERN19608 |  |  |  | | | |  |  |  |  |
| 1 | Atr-ERN19609 |  |  |  | | | |  |  |  |  |
| 1 | Atr-ERN19610 |  |  |  | | | |  |  |  |  |
| 1 | Atr-ERN19611 |  |  |  | | | |  |  |  |  |
| 1 | Atr-ERN19612 |  |  |  | | | |  |  |  |  |
| 1 | Atr-ERN19613 |  |  |  | | | |  |  |  |  |
| 1 | Atr-ERN19614 |  |  |  | | | |  |  |  |  |
| 1 | Atr-ERN19615 |  |  |  | | | |  |  |  |  |
| 1 | Atr-ERN19616 |  |  |  | | | |  |  |  |  |
| 1 | Atr-ERN19617 |  |  |  | | | |  |  |  |  |
| 1 | Atr-ERN19618 |  |  |  | | | |  |  |  |  |
| 1 | Atr-ERN19619 |  |  |  | | | |  |  |  |  |
| 1 | Atr-ERN19620 |  |  |  | | | |  |  |  |  |
| 1 | Atr-ERN19621 |  |  |  | Vvi-Vitvi19g04230\_t001 |  |  |  |  |
| 1 | Atr-ERN19622 |  |  |  | Vvi-Vitvi19g00476\_t001 |  |  |  |  |
| 1 | Atr-ERN19623 |  |  |  | Vvi-Vitvi19g00478\_t001 |  |  |  |  |
| 1 | Atr-ERN19624 |  |  |  | Vvi-Vitvi19g02009\_t001 |  |  |  |  |
| 1 | Atr-ERN19625 |  |  |  | Vvi-Vitvi19g00480\_t001 |  |  |  |  |
| 1 | Atr-ERN19626 |  |  |  | | | |  |  |  |  |
| 1 | Atr-ERN19627 |  |  |  | Vvi-Vitvi19g00481\_t001 |  |  |  |  |
| 1 | Atr-ERN19628 |  |  |  | | | |  |  |  |  |
| 1 | Atr-ERN19629 |  |  |  | | | |  |  |  |  |
| 1 | Atr-ERN19630 |  |  |  | | | |  |  |  |  |
| 1 | Atr-ERN19631 |  |  |  | | | |  |  |  |  |
| 1 | Atr-ERN19632 |  |  |  | | | |  |  |  |  |
| 1 | Atr-ERN19633 |  |  |  | Vvi-Vitvi19g00482\_t001 |  |  |  |  |
| 1 | Atr-ERN19634 |  |  |  | | | |  |  |  |  |
| 1 | Atr-ERN19635 |  |  |  | Vvi-Vitvi19g00483\_t001 |  |  |  |  |
| 1 | Atr-ERN19636 |  |  |  | | | |  |  |  |  |
| 1 | Atr-ERN19637 |  |  |  | | | |  |  |  |  |
| 1 | Atr-ERN19638 |  |  |  | | | |  |  |  |  |
| 1 | Atr-ERN19639 |  |  |  | | | |  |  |  |  |
| 1 | Atr-ERN19640 |  |  |  | | | |  |  |  |  |
| 1 | Atr-ERN19641 |  |  |  | | | |  |  |  |  |
| 1 | Atr-ERN19642 |  |  |  | | | |  |  |  |  |
| 1 | Atr-ERN19643 |  |  |  | | | |  |  |  |  |
| 1 | Atr-ERN19644 |  |  |  | | | |  |  |  |  |
| 1 | Atr-ERN19645 |  |  |  | | | |  |  |  |  |
| 1 | Atr-ERN19646 |  |  |  | | | |  |  |  |  |
| 1 | Atr-ERN19647 |  |  |  | | | |  |  |  |  |
| 1 | Atr-ERN19648 |  |  |  | | | |  |  |  |  |
| 1 | Atr-ERN19649 |  |  |  | | | |  |  |  |  |
| 1 | Atr-ERN19650 |  |  |  | | | |  |  |  |  |
| 1 | Atr-ERN19651 |  |  |  | | | |  |  |  |  |
| 1 | Atr-ERN19652 |  |  |  | Vvi-Vitvi19g00486\_t001 |  |  |  |  |
| 1 | Atr-ERN19653 |  |  |  | | | |  |  |  |  |
| 1 | Atr-ERN19654 |  |  |  | | | |  |  |  |  |
| 1 | Atr-ERN19655 |  |  |  | | | |  |  |  |  |
| 1 | Atr-ERN19656 |  |  |  | | | |  |  |  |  |
| 1 | Atr-ERN19657 |  |  |  | | | |  |  |  |  |
| 1 | Atr-ERN19658 |  |  |  | | | |  |  |  |  |
| 1 | Atr-ERN19659 |  |  |  | | | |  |  |  |  |
| 1 | Atr-ERN19660 |  |  |  | | | |  |  |  |  |
| 1 | Atr-ERN19661 |  |  |  | | | |  |  |  |  |
| 1 | Atr-ERN19662 |  |  |  | Vvi-Vitvi19g02011\_t001 |  |  |  |  |
| 1 | Atr-ERN19663 |  |  |  | | | |  |  |  |  |
| 1 | Atr-ERN19664 |  |  |  | Vvi-Vitvi19g00490\_t001 |  |  |  |  |
| 1 | Atr-ERN19665 |  |  |  | | | |  |  |  |  |
| 1 | Atr-ERN19666 |  |  |  | | | |  |  |  |  |
| 1 | Atr-ERN19667 |  |  |  | | | |  |  |  |  |
| 1 | Atr-ERN19668 |  |  |  | | | |  |  |  |  |
| 1 | Atr-ERN19669 |  |  |  | | | |  |  |  |  |
| 1 | Atr-ERN19670 |  |  |  | Vvi-Vitvi19g00491\_t002 |  |  |  |  |
| 1 | Atr-ERN19671 |  |  |  | | | |  |  |  |  |
| 1 | Atr-ERN19672 |  |  |  | | | |  |  |  |  |
| 1 | Atr-ERN19673 |  |  |  | | | |  |  |  |  |
| 1 | Atr-ERN19674 |  |  |  | Vvi-Vitvi19g02012\_t001 |  |  |  |  |
| 1 | Atr-ERN19675 |  |  |  | Vvi-Vitvi19g00492\_t001 |  |  |  |  |
| 0 | Atr-ERN19676 |  |  |  |  |  |  |
| 0 | Atr-ERN19677 |  |  |  |  |  |  |
| 0 | Atr-ERN19678 |  |  |  |  |  |  |
| 0 | Atr-ERN19679 |  |  |  |  |  |  |
| 0 | Atr-ERN19680 |  |  |  |  |  |  |
| 0 | Atr-ERN19681 |  |  |  |  |  |  |
| 0 | Atr-ERN19682 |  |  |  |  |  |  |
| 0 | Atr-ERN19683 |  |  |  |  |  |  |
| 0 | Atr-ERN19684 |  |  |  |  |  |  |
| 0 | Atr-ERN19685 |  |  |  |  |  |  |
| 0 | Atr-ERN19686 |  |  |  |  |  |  |
| 0 | Atr-ERN19687 |  |  |  |  |  |  |
| 0 | Atr-ERN19688 |  |  |  |  |  |  |
| 0 | Atr-ERN19689 |  |  |  |  |  |  |
| 0 | Atr-ERN19690 |  |  |  |  |  |  |
| 0 | Atr-ERN19691 |  |  |  |  |  |  |
| 0 | Atr-ERN19692 |  |  |  |  |  |  |
| 0 | Atr-ERN19693 |  |  |  |  |  |  |
| 0 | Atr-ERN19694 |  |  |  |  |  |  |
| 0 | Atr-ERN19695 |  |  |  |  |  |  |
| 0 | Atr-ERN19696 |  |  |  |  |  |  |
| 0 | Atr-ERN19697 |  |  |  |  |  |  |
| 0 | Atr-ERN19698 |  |  |  |  |  |  |
| 0 | Atr-ERN19699 |  |  |  |  |  |  |
| 0 | Atr-ERN19700 |  |  |  |  |  |  |
| 0 | Atr-ERN19701 |  |  |  |  |  |  |
| 0 | Atr-ERN19702 |  |  |  |  |  |  |
| 0 | Atr-ERN19703 |  |  |  |  |  |  |
| 0 | Atr-ERN19704 |  |  |  |  |  |  |
| 0 | Atr-ERN19705 |  |  |  |  |  |  |
| 0 | Atr-ERN19706 |  |  |  |  |  |  |
| 0 | Atr-ERN19707 |  |  |  |  |  |  |
| 0 | Atr-ERN19708 |  |  |  |  |  |  |
| 0 | Atr-ERN19709 |  |  |  |  |  |  |
| 0 | Atr-ERN19710 |  |  |  |  |  |  |
| 0 | Atr-ERN19711 |  |  |  |  |  |  |
| 0 | Atr-ERN19712 |  |  |  |  |  |  |
| 0 | Atr-ERN19713 |  |  |  |  |  |  |
| 0 | Atr-ERN19714 |  |  |  |  |  |  |
| 0 | Atr-ERN19715 |  |  |  |  |  |  |
| 0 | Atr-ERN19716 |  |  |  |  |  |  |
| 0 | Atr-ERN19717 |  |  |  |  |  |  |
| 0 | Atr-ERN19718 |  |  |  |  |  |  |
| 0 | Atr-ERN19719 |  |  |  |  |  |  |
| 0 | Atr-ERN19720 |  |  |  |  |  |  |
| 0 | Atr-ERN19721 |  |  |  |  |  |  |
| 0 | Atr-ERN19722 |  |  |  |  |  |  |
| 0 | Atr-ERN19723 |  |  |  |  |  |  |
| 0 | Atr-ERN19724 |  |  |  |  |  |  |
| 0 | Atr-ERN19725 |  |  |  |  |  |  |
| 0 | Atr-ERN19726 |  |  |  |  |  |  |
| 0 | Atr-ERN19727 |  |  |  |  |  |  |
| 0 | Atr-ERN19728 |  |  |  |  |  |  |
| 0 | Atr-ERN19729 |  |  |  |  |  |  |
| 0 | Atr-ERN19730 |  |  |  |  |  |  |
| 0 | Atr-ERN19731 |  |  |  |  |  |  |
| 0 | Atr-ERN19732 |  |  |  |  |  |  |
| 0 | Atr-ERN19733 |  |  |  |  |  |  |
| 0 | Atr-ERN19734 |  |  |  |  |  |  |
| 0 | Atr-ERN19735 |  |  |  |  |  |  |
| 0 | Atr-ERN19736 |  |  |  |  |  |  |
| 0 | Atr-ERN19737 |  |  |  |  |  |  |
| 0 | Atr-ERN19738 |  |  |  |  |  |  |
| 0 | Atr-ERN19739 |  |  |  |  |  |  |
| 0 | Atr-ERN19740 |  |  |  |  |  |  |
| 0 | Atr-ERN19741 |  |  |  |  |  |  |
