# Supplementary material for: Genome-wide identification, characterization and gene expression of BES1 transcription factor family in grapevine (Vitis vinifera L.)
Source: Sci Rep. 2023 Jan 5;13:240. doi: 10.1038/s41598-022-24407-y (PMC9816167; doi:10.1038/s41598-022-24407-y)
Supplement: Supplementary file 3 — Supplementary Information. [file 41598_2022_24407_MOESM3_ESM.zip › Vvi_Atr/Vitis_vinifera.PN40024.v4.dna_sm.toplevel.fa.vs.Amborella_trichopoda.AMTR1.0.dna_sm.toplevel.fa.html/Atr-AmTr_v1.0_scaffold00063.html]

|  |  |  |  |  |  |  |  |  |  |  |  |  |  |
| --- | --- | --- | --- | --- | --- | --- | --- | --- | --- | --- | --- | --- | --- |
| Duplication depth | Reference chromosome | Collinear blocks | | | | | | | | | | | |
| 1 | Atr-ERN16219 |  | Vvi-Vitvi13g01463\_t001 |  |  |  |  |  |
| 2 | Atr-ERN16220 |  | Vvi-Vitvi13g01462\_t001 |  | Vvi-Vitvi08g01668\_t001 |  |  |  |  |
| 2 | Atr-ERN16221 |  | | | |  | Vvi-Vitvi08g01666\_t001 |  |  |  |  |
| 2 | Atr-ERN16222 |  | | | |  | | | |  |  |  |  |
| 2 | Atr-ERN16223 |  | | | |  | | | |  |  |  |  |
| 2 | Atr-ERN16224 |  | Vvi-Vitvi13g01459\_t001 |  | | | |  |  |  |  |
| 2 | Atr-ERN16225 |  | | | |  | | | |  |  |  |  |
| 2 | Atr-ERN16226 |  | | | |  | | | |  |  |  |  |
| 2 | Atr-ERN16227 |  | | | |  | | | |  |  |  |  |
| 2 | Atr-ERN16228 |  | | | |  | | | |  |  |  |  |
| 2 | Atr-ERN16229 |  | | | |  | | | |  |  |  |  |
| 2 | Atr-ERN16230 |  | | | |  | | | |  |  |  |  |
| 2 | Atr-ERN16231 |  | | | |  | | | |  |  |  |  |
| 2 | Atr-ERN16232 |  | | | |  | | | |  |  |  |  |
| 3 | Atr-ERN16233 |  | | | |  | | | |  | Vvi-Vitvi06g01909\_t001 |  |  |  |
| 3 | Atr-ERN16234 |  | | | |  | Vvi-Vitvi08g01659\_t001 |  | | | |  |  |  |
| 3 | Atr-ERN16235 |  | Vvi-Vitvi13g01429\_t002 |  | | | |  | Vvi-Vitvi06g01245\_t001 |  |  |  |
| 3 | Atr-ERN16236 |  | | | |  | | | |  | | | |  |  |  |
| 3 | Atr-ERN16237 |  | | | |  | | | |  | | | |  |  |  |
| 3 | Atr-ERN16238 |  | | | |  | | | |  | | | |  |  |  |
| 3 | Atr-ERN16239 |  | | | |  | | | |  | | | |  |  |  |
| 3 | Atr-ERN16240 |  | | | |  | | | |  | | | |  |  |  |
| 3 | Atr-ERN16241 |  | Vvi-Vitvi13g01417\_t001 |  | | | |  | | | |  |  |  |
| 3 | Atr-ERN16242 |  | | | |  | | | |  | | | |  |  |  |
| 3 | Atr-ERN16243 |  | | | |  | Vvi-Vitvi08g02307\_t001 |  | | | |  |  |  |
| 3 | Atr-ERN16244 |  | | | |  | | | |  | | | |  |  |  |
| 3 | Atr-ERN16245 |  | | | |  | | | |  | | | |  |  |  |
| 3 | Atr-ERN16246 |  | | | |  | | | |  | | | |  |  |  |
| 3 | Atr-ERN16247 |  | | | |  | | | |  | | | |  |  |  |
| 3 | Atr-ERN16248 |  | | | |  | | | |  | | | |  |  |  |
| 3 | Atr-ERN16249 |  | | | |  | | | |  | Vvi-Vitvi06g01244\_t001 |  |  |  |
| 3 | Atr-ERN16250 |  | | | |  | | | |  | | | |  |  |  |
| 3 | Atr-ERN16251 |  | | | |  | | | |  | | | |  |  |  |
| 3 | Atr-ERN16252 |  | | | |  | | | |  | | | |  |  |  |
| 3 | Atr-ERN16253 |  | | | |  | | | |  | | | |  |  |  |
| 3 | Atr-ERN16254 |  | | | |  | | | |  | | | |  |  |  |
| 3 | Atr-ERN16255 |  | | | |  | Vvi-Vitvi08g01655\_t002 |  | | | |  |  |  |
| 3 | Atr-ERN16256 |  | | | |  | | | |  | | | |  |  |  |
| 3 | Atr-ERN16257 |  | | | |  | | | |  | | | |  |  |  |
| 3 | Atr-ERN16258 |  | Vvi-Vitvi13g01416\_t002 |  | | | |  | | | |  |  |  |
| 3 | Atr-ERN16259 |  | | | |  | | | |  | | | |  |  |  |
| 3 | Atr-ERN16260 |  | | | |  | | | |  | Vvi-Vitvi06g01240\_t001 |  |  |  |
| 3 | Atr-ERN16261 |  | | | |  | | | |  | | | |  |  |  |
| 3 | Atr-ERN16262 |  | | | |  | | | |  | | | |  |  |  |
| 3 | Atr-ERN16263 |  | | | |  | Vvi-Vitvi08g01649\_t001 |  | | | |  |  |  |
| 2 | Atr-ERN16264 |  | | | |  |  |  | | | |  |  |  |
| 2 | Atr-ERN16265 |  | | | |  |  |  | | | |  |  |  |
| 2 | Atr-ERN16266 |  | | | |  |  |  | Vvi-Vitvi06g01239\_t001 |  |  |  |
| 2 | Atr-ERN16267 |  | | | |  |  |  | | | |  |  |  |
| 2 | Atr-ERN16268 |  | | | |  |  |  | | | |  |  |  |
| 2 | Atr-ERN16269 |  | Vvi-Vitvi13g01414\_t001 |  |  |  | | | |  |  |  |
| 2 | Atr-ERN16270 |  | | | |  |  |  | | | |  |  |  |
| 2 | Atr-ERN16271 |  | | | |  |  |  | | | |  |  |  |
| 2 | Atr-ERN16272 |  | | | |  |  |  | | | |  |  |  |
| 2 | Atr-ERN16273 |  | | | |  |  |  | Vvi-Vitvi06g01236\_t001 |  |  |  |
| 2 | Atr-ERN16274 |  | | | |  |  |  | | | |  |  |  |
| 2 | Atr-ERN16275 |  | | | |  |  |  | | | |  |  |  |
| 2 | Atr-ERN16276 |  | | | |  |  |  | Vvi-Vitvi06g01231\_t001 |  |  |  |
| 2 | Atr-ERN16277 |  | | | |  |  |  | | | |  |  |  |
| 2 | Atr-ERN16278 |  | Vvi-Vitvi13g04492\_t001 |  |  |  | | | |  |  |  |
| 2 | Atr-ERN16279 |  | | | |  |  |  | | | |  |  |  |
| 2 | Atr-ERN16280 |  | | | |  |  |  | | | |  |  |  |
| 2 | Atr-ERN16281 |  | Vvi-Vitvi13g01336\_t001 |  |  |  | Vvi-Vitvi06g01227\_t001 |  |  |  |
| 2 | Atr-ERN16282 |  | | | |  |  |  | | | |  |  |  |
| 2 | Atr-ERN16283 |  | | | |  |  |  | | | |  |  |  |
| 2 | Atr-ERN16284 |  | | | |  |  |  | | | |  |  |  |
| 2 | Atr-ERN16285 |  | | | |  |  |  | | | |  |  |  |
| 2 | Atr-ERN16286 |  | | | |  |  |  | | | |  |  |  |
| 2 | Atr-ERN16287 |  | Vvi-Vitvi13g01337\_t001 |  |  |  | | | |  |  |  |
| 2 | Atr-ERN16288 |  | | | |  |  |  | | | |  |  |  |
| 2 | Atr-ERN16289 |  | | | |  |  |  | | | |  |  |  |
| 2 | Atr-ERN16290 |  | | | |  |  |  | Vvi-Vitvi06g01226\_t001 |  |  |  |
| 2 | Atr-ERN16291 |  | | | |  |  |  | | | |  |  |  |
| 2 | Atr-ERN16292 |  | | | |  |  |  | Vvi-Vitvi06g01218\_t001 |  |  |  |
| 2 | Atr-ERN16293 |  | | | |  |  |  | | | |  |  |  |
| 2 | Atr-ERN16294 |  | | | |  |  |  | | | |  |  |  |
| 2 | Atr-ERN16295 |  | | | |  |  |  | | | |  |  |  |
| 2 | Atr-ERN16296 |  | | | |  |  |  | Vvi-Vitvi06g01217\_t002 |  |  |  |
| 1 | Atr-ERN16297 |  | Vvi-Vitvi13g01338\_t001 |  |  |  |  |  |
| 1 | Atr-ERN16298 |  | | | |  |  |  |  |  |
| 1 | Atr-ERN16299 |  | | | |  |  |  |  |  |
| 1 | Atr-ERN16300 |  | | | |  |  |  |  |  |
| 1 | Atr-ERN16301 |  | | | |  |  |  |  |  |
| 1 | Atr-ERN16302 |  | | | |  |  |  |  |  |
| 1 | Atr-ERN16303 |  | | | |  |  |  |  |  |
| 1 | Atr-ERN16304 |  | | | |  |  |  |  |  |
| 1 | Atr-ERN16305 |  | | | |  |  |  |  |  |
| 1 | Atr-ERN16306 |  | | | |  |  |  |  |  |
| 1 | Atr-ERN16307 |  | | | |  |  |  |  |  |
| 1 | Atr-ERN16308 |  | | | |  |  |  |  |  |
| 1 | Atr-ERN16309 |  | | | |  |  |  |  |  |
| 1 | Atr-ERN16310 |  | | | |  |  |  |  |  |
| 1 | Atr-ERN16311 |  | | | |  |  |  |  |  |
| 1 | Atr-ERN16312 |  | | | |  |  |  |  |  |
| 1 | Atr-ERN16313 |  | | | |  |  |  |  |  |
| 1 | Atr-ERN16314 |  | | | |  |  |  |  |  |
| 1 | Atr-ERN16315 |  | | | |  |  |  |  |  |
| 1 | Atr-ERN16316 |  | Vvi-Vitvi13g01340\_t001 |  |  |  |  |  |
