# Supplementary material for: Genome-wide identification, characterization and gene expression of BES1 transcription factor family in grapevine (Vitis vinifera L.)
Source: Sci Rep. 2023 Jan 5;13:240. doi: 10.1038/s41598-022-24407-y (PMC9816167; doi:10.1038/s41598-022-24407-y)
Supplement: Supplementary file 3 — Supplementary Information. [file 41598_2022_24407_MOESM3_ESM.zip › Vvi_Atr/Vitis_vinifera.PN40024.v4.dna_sm.toplevel.fa.vs.Amborella_trichopoda.AMTR1.0.dna_sm.toplevel.fa.html/Atr-AmTr_v1.0_scaffold00064.html]

|  |  |  |  |  |  |  |  |  |  |  |  |  |  |
| --- | --- | --- | --- | --- | --- | --- | --- | --- | --- | --- | --- | --- | --- |
| Duplication depth | Reference chromosome | Collinear blocks | | | | | | | | | | | |
| 0 | Atr-ERN19743 |  |  |  |  |  |  |
| 0 | Atr-ERN19744 |  |  |  |  |  |  |
| 0 | Atr-ERN19745 |  |  |  |  |  |  |
| 0 | Atr-ERN19746 |  |  |  |  |  |  |
| 0 | Atr-ERN19747 |  |  |  |  |  |  |
| 0 | Atr-ERN19748 |  |  |  |  |  |  |
| 0 | Atr-ERN19749 |  |  |  |  |  |  |
| 0 | Atr-ERN19750 |  |  |  |  |  |  |
| 0 | Atr-ERN19751 |  |  |  |  |  |  |
| 0 | Atr-ERN19752 |  |  |  |  |  |  |
| 0 | Atr-ERN19753 |  |  |  |  |  |  |
| 0 | Atr-ERN19754 |  |  |  |  |  |  |
| 0 | Atr-ERN19755 |  |  |  |  |  |  |
| 0 | Atr-ERN19756 |  |  |  |  |  |  |
| 0 | Atr-ERN19757 |  |  |  |  |  |  |
| 0 | Atr-ERN19758 |  |  |  |  |  |  |
| 0 | Atr-ERN19759 |  |  |  |  |  |  |
| 0 | Atr-ERN19760 |  |  |  |  |  |  |
| 0 | Atr-ERN19761 |  |  |  |  |  |  |
| 0 | Atr-ERN19762 |  |  |  |  |  |  |
| 0 | Atr-ERN19763 |  |  |  |  |  |  |
| 0 | Atr-ERN19764 |  |  |  |  |  |  |
| 0 | Atr-ERN19765 |  |  |  |  |  |  |
| 0 | Atr-ERN19766 |  |  |  |  |  |  |
| 0 | Atr-ERN19767 |  |  |  |  |  |  |
| 0 | Atr-ERN19768 |  |  |  |  |  |  |
| 0 | Atr-ERN19769 |  |  |  |  |  |  |
| 0 | Atr-ERN19770 |  |  |  |  |  |  |
| 0 | Atr-ERN19771 |  |  |  |  |  |  |
| 0 | Atr-ERN19772 |  |  |  |  |  |  |
| 0 | Atr-ERN19773 |  |  |  |  |  |  |
| 0 | Atr-ERN19774 |  |  |  |  |  |  |
| 0 | Atr-ERN19775 |  |  |  |  |  |  |
| 0 | Atr-ERN19776 |  |  |  |  |  |  |
| 0 | Atr-ERN19777 |  |  |  |  |  |  |
| 0 | Atr-ERN19778 |  |  |  |  |  |  |
| 0 | Atr-ERN19779 |  |  |  |  |  |  |
| 0 | Atr-ERN19780 |  |  |  |  |  |  |
| 0 | Atr-ERN19781 |  |  |  |  |  |  |
| 0 | Atr-ERN19782 |  |  |  |  |  |  |
| 0 | Atr-ERN19783 |  |  |  |  |  |  |
| 0 | Atr-ERN19784 |  |  |  |  |  |  |
| 0 | Atr-ERN19785 |  |  |  |  |  |  |
| 0 | Atr-ERN19786 |  |  |  |  |  |  |
| 0 | Atr-ERN19787 |  |  |  |  |  |  |
| 0 | Atr-ERN19788 |  |  |  |  |  |  |
| 0 | Atr-ERN19789 |  |  |  |  |  |  |
| 0 | Atr-ERN19790 |  |  |  |  |  |  |
| 0 | Atr-ERN19791 |  |  |  |  |  |  |
| 0 | Atr-ERN19792 |  |  |  |  |  |  |
| 0 | Atr-ERN19793 |  |  |  |  |  |  |
| 0 | Atr-ERN19794 |  |  |  |  |  |  |
| 0 | Atr-ERN19795 |  |  |  |  |  |  |
| 0 | Atr-ERN19796 |  |  |  |  |  |  |
| 0 | Atr-ERN19797 |  |  |  |  |  |  |
| 0 | Atr-ERN19798 |  |  |  |  |  |  |
| 0 | Atr-ERN19799 |  |  |  |  |  |  |
| 0 | Atr-ERN19800 |  |  |  |  |  |  |
| 0 | Atr-ERN19801 |  |  |  |  |  |  |
| 0 | Atr-ERN19802 |  |  |  |  |  |  |
| 0 | Atr-ERN19803 |  |  |  |  |  |  |
| 0 | Atr-ERN19804 |  |  |  |  |  |  |
| 0 | Atr-ERN19805 |  |  |  |  |  |  |
| 0 | Atr-ERN19806 |  |  |  |  |  |  |
| 0 | Atr-ERN19807 |  |  |  |  |  |  |
| 0 | Atr-ERN19808 |  |  |  |  |  |  |
| 0 | Atr-ERN19809 |  |  |  |  |  |  |
| 0 | Atr-ERN19810 |  |  |  |  |  |  |
| 0 | Atr-ERN19811 |  |  |  |  |  |  |
| 0 | Atr-ERN19812 |  |  |  |  |  |  |
| 0 | Atr-ERN19813 |  |  |  |  |  |  |
| 0 | Atr-ERN19814 |  |  |  |  |  |  |
| 0 | Atr-ERN19815 |  |  |  |  |  |  |
| 0 | Atr-ERN19816 |  |  |  |  |  |  |
| 0 | Atr-ERN19817 |  |  |  |  |  |  |
| 0 | Atr-ERN19818 |  |  |  |  |  |  |
| 0 | Atr-ERN19819 |  |  |  |  |  |  |
| 0 | Atr-ERN19820 |  |  |  |  |  |  |
| 0 | Atr-ERN19821 |  |  |  |  |  |  |
| 0 | Atr-ERN19822 |  |  |  |  |  |  |
| 0 | Atr-ERN19823 |  |  |  |  |  |  |
| 0 | Atr-ERN19824 |  |  |  |  |  |  |
| 0 | Atr-ERN19825 |  |  |  |  |  |  |
| 0 | Atr-ERN19826 |  |  |  |  |  |  |
| 0 | Atr-ERN19827 |  |  |  |  |  |  |
| 0 | Atr-ERN19828 |  |  |  |  |  |  |
| 0 | Atr-ERN19829 |  |  |  |  |  |  |
| 0 | Atr-ERN19830 |  |  |  |  |  |  |
| 0 | Atr-ERN19831 |  |  |  |  |  |  |
| 0 | Atr-ERN19832 |  |  |  |  |  |  |
| 0 | Atr-ERN19833 |  |  |  |  |  |  |
| 0 | Atr-ERN19834 |  |  |  |  |  |  |
| 0 | Atr-ERN19835 |  |  |  |  |  |  |
| 0 | Atr-ERN19836 |  |  |  |  |  |  |
| 0 | Atr-ERN19837 |  |  |  |  |  |  |
| 0 | Atr-ERN19838 |  |  |  |  |  |  |
| 0 | Atr-ERN19839 |  |  |  |  |  |  |
| 0 | Atr-ERN19840 |  |  |  |  |  |  |
| 0 | Atr-ERN19841 |  |  |  |  |  |  |
| 0 | Atr-ERN19842 |  |  |  |  |  |  |
| 0 | Atr-ERN19843 |  |  |  |  |  |  |
| 0 | Atr-ERN19844 |  |  |  |  |  |  |
| 0 | Atr-ERN19845 |  |  |  |  |  |  |
| 0 | Atr-ERN19846 |  |  |  |  |  |  |
| 0 | Atr-ERN19847 |  |  |  |  |  |  |
| 0 | Atr-ERN19848 |  |  |  |  |  |  |
| 0 | Atr-ERN19849 |  |  |  |  |  |  |
| 0 | Atr-ERN19850 |  |  |  |  |  |  |
| 0 | Atr-ERN19851 |  |  |  |  |  |  |
| 0 | Atr-ERN19852 |  |  |  |  |  |  |
| 0 | Atr-ERN19853 |  |  |  |  |  |  |
