# Supplementary material for: Genome-wide identification, characterization and gene expression of BES1 transcription factor family in grapevine (Vitis vinifera L.)
Source: Sci Rep. 2023 Jan 5;13:240. doi: 10.1038/s41598-022-24407-y (PMC9816167; doi:10.1038/s41598-022-24407-y)
Supplement: Supplementary file 3 — Supplementary Information. [file 41598_2022_24407_MOESM3_ESM.zip › Vvi_Atr/Vitis_vinifera.PN40024.v4.dna_sm.toplevel.fa.vs.Amborella_trichopoda.AMTR1.0.dna_sm.toplevel.fa.html/Atr-AmTr_v1.0_scaffold00065.html]

|  |  |  |  |  |  |  |  |  |  |  |  |  |  |
| --- | --- | --- | --- | --- | --- | --- | --- | --- | --- | --- | --- | --- | --- |
| Duplication depth | Reference chromosome | Collinear blocks | | | | | | | | | | | |
| 0 | Atr-ERN18497 |  |  |  |  |  |  |
| 0 | Atr-ERN18498 |  |  |  |  |  |  |
| 0 | Atr-ERN18499 |  |  |  |  |  |  |
| 0 | Atr-ERN18500 |  |  |  |  |  |  |
| 0 | Atr-ERN18501 |  |  |  |  |  |  |
| 0 | Atr-ERN18502 |  |  |  |  |  |  |
| 0 | Atr-ERN18503 |  |  |  |  |  |  |
| 0 | Atr-ERN18504 |  |  |  |  |  |  |
| 0 | Atr-ERN18505 |  |  |  |  |  |  |
| 0 | Atr-ERN18506 |  |  |  |  |  |  |
| 0 | Atr-ERN18507 |  |  |  |  |  |  |
| 0 | Atr-ERN18508 |  |  |  |  |  |  |
| 0 | Atr-ERN18509 |  |  |  |  |  |  |
| 1 | Atr-ERN18510 |  | Vvi-Vitvi18g00612\_t001 |  |  |  |  |  |
| 2 | Atr-ERN18511 |  | | | |  | Vvi-Vitvi15g04528\_t001 |  |  |  |  |
| 2 | Atr-ERN18512 |  | | | |  | | | |  |  |  |  |
| 2 | Atr-ERN18513 |  | | | |  | | | |  |  |  |  |
| 2 | Atr-ERN18514 |  | | | |  | | | |  |  |  |  |
| 2 | Atr-ERN18515 |  | | | |  | | | |  |  |  |  |
| 2 | Atr-ERN18516 |  | Vvi-Vitvi18g00614\_t001 |  | | | |  |  |  |  |
| 2 | Atr-ERN18517 |  | | | |  | | | |  |  |  |  |
| 2 | Atr-ERN18518 |  | Vvi-Vitvi18g00615\_t001 |  | | | |  |  |  |  |
| 2 | Atr-ERN18519 |  | | | |  | Vvi-Vitvi15g04525\_t001 |  |  |  |  |
| 2 | Atr-ERN18520 |  | | | |  | | | |  |  |  |  |
| 2 | Atr-ERN18521 |  | | | |  | | | |  |  |  |  |
| 2 | Atr-ERN18522 |  | | | |  | | | |  |  |  |  |
| 2 | Atr-ERN18523 |  | Vvi-Vitvi18g00616\_t001 |  | | | |  |  |  |  |
| 2 | Atr-ERN18524 |  | | | |  | | | |  |  |  |  |
| 2 | Atr-ERN18525 |  | | | |  | | | |  |  |  |  |
| 2 | Atr-ERN18526 |  | | | |  | Vvi-Vitvi15g04523\_t001 |  |  |  |  |
| 2 | Atr-ERN18527 |  | | | |  | | | |  |  |  |  |
| 2 | Atr-ERN18528 |  | | | |  | | | |  |  |  |  |
| 2 | Atr-ERN18529 |  | | | |  | | | |  |  |  |  |
| 2 | Atr-ERN18530 |  | | | |  | | | |  |  |  |  |
| 3 | Atr-ERN18531 |  | | | |  | | | |  | Vvi-Vitvi03g01638\_t001 |  |  |  |
| 3 | Atr-ERN18532 |  | Vvi-Vitvi18g00617\_t001 |  | Vvi-Vitvi15g04522\_t001 |  | | | |  |  |  |
| 3 | Atr-ERN18533 |  | | | |  | | | |  | Vvi-Vitvi03g00718\_t001 |  |  |  |
| 3 | Atr-ERN18534 |  | | | |  | | | |  | Vvi-Vitvi03g00717\_t001 |  |  |  |
| 3 | Atr-ERN18535 |  | | | |  | | | |  | | | |  |  |  |
| 3 | Atr-ERN18536 |  | | | |  | | | |  | | | |  |  |  |
| 3 | Atr-ERN18537 |  | | | |  | | | |  | | | |  |  |  |
| 3 | Atr-ERN18538 |  | Vvi-Vitvi18g00618\_t001 |  | Vvi-Vitvi15g04521\_t001 |  | | | |  |  |  |
| 3 | Atr-ERN18539 |  | | | |  | Vvi-Vitvi15g04520\_t001 |  | | | |  |  |  |
| 3 | Atr-ERN18540 |  | Vvi-Vitvi18g00621\_t001 |  | | | |  | | | |  |  |  |
| 3 | Atr-ERN18541 |  | Vvi-Vitvi18g00622\_t001 |  | | | |  | | | |  |  |  |
| 3 | Atr-ERN18542 |  | | | |  | | | |  | | | |  |  |  |
| 3 | Atr-ERN18543 |  | | | |  | | | |  | | | |  |  |  |
| 3 | Atr-ERN18544 |  | | | |  | | | |  | | | |  |  |  |
| 3 | Atr-ERN18545 |  | | | |  | | | |  | | | |  |  |  |
| 3 | Atr-ERN18546 |  | | | |  | | | |  | | | |  |  |  |
| 3 | Atr-ERN18547 |  | Vvi-Vitvi18g00623\_t001 |  | Vvi-Vitvi15g04517\_t001 |  | | | |  |  |  |
| 3 | Atr-ERN18548 |  | | | |  | | | |  | | | |  |  |  |
| 3 | Atr-ERN18549 |  | Vvi-Vitvi18g02631\_t001 |  | Vvi-Vitvi15g04515\_t001 |  | | | |  |  |  |
| 3 | Atr-ERN18550 |  | | | |  | | | |  | | | |  |  |  |
| 3 | Atr-ERN18551 |  | | | |  | | | |  | | | |  |  |  |
| 3 | Atr-ERN18552 |  | | | |  | | | |  | | | |  |  |  |
| 3 | Atr-ERN18553 |  | Vvi-Vitvi18g02632\_t001 |  | | | |  | Vvi-Vitvi03g00713\_t001 |  |  |  |
| 3 | Atr-ERN18554 |  | Vvi-Vitvi18g00626\_t001 |  | | | |  | | | |  |  |  |
| 3 | Atr-ERN18555 |  | Vvi-Vitvi18g00628\_t001 |  | Vvi-Vitvi15g04514\_t001 |  | | | |  |  |  |
| 3 | Atr-ERN18556 |  | Vvi-Vitvi18g00632\_t001 |  | | | |  | | | |  |  |  |
| 3 | Atr-ERN18557 |  | | | |  | | | |  | | | |  |  |  |
| 3 | Atr-ERN18558 |  | | | |  | | | |  | | | |  |  |  |
| 3 | Atr-ERN18559 |  | | | |  | | | |  | | | |  |  |  |
| 3 | Atr-ERN18560 |  | | | |  | | | |  | | | |  |  |  |
| 3 | Atr-ERN18561 |  | Vvi-Vitvi18g00635\_t001 |  | Vvi-Vitvi15g04511\_t001 |  | Vvi-Vitvi03g00712\_t001 |  |  |  |
| 3 | Atr-ERN18562 |  | | | |  | | | |  | Vvi-Vitvi03g00711\_t001 |  |  |  |
| 3 | Atr-ERN18563 |  | | | |  | | | |  | | | |  |  |  |
| 3 | Atr-ERN18564 |  | Vvi-Vitvi18g00636\_t001 |  | Vvi-Vitvi15g04508\_t001 |  | | | |  |  |  |
| 3 | Atr-ERN18565 |  | | | |  | | | |  | Vvi-Vitvi03g00710\_t001 |  |  |  |
| 3 | Atr-ERN18566 |  | | | |  | | | |  | | | |  |  |  |
| 3 | Atr-ERN18567 |  | | | |  | | | |  | | | |  |  |  |
| 4 | Atr-ERN18568 |  | | | |  | | | |  | | | |  | Vvi-Vitvi03g00678\_t001 |  |  |
| 4 | Atr-ERN18569 |  | | | |  | Vvi-Vitvi15g04507\_t001 |  | | | |  | | | |  |  |
| 4 | Atr-ERN18570 |  | Vvi-Vitvi18g00637\_t001 |  | Vvi-Vitvi15g04506\_t001 |  | | | |  | | | |  |  |
| 4 | Atr-ERN18571 |  | | | |  | Vvi-Vitvi15g04505\_t001 |  | | | |  | | | |  |  |
| 4 | Atr-ERN18572 |  | | | |  | | | |  | | | |  | | | |  |  |
| 4 | Atr-ERN18573 |  | | | |  | | | |  | | | |  | | | |  |  |
| 4 | Atr-ERN18574 |  | | | |  | | | |  | | | |  | | | |  |  |
| 4 | Atr-ERN18575 |  | | | |  | | | |  | | | |  | | | |  |  |
| 4 | Atr-ERN18576 |  | | | |  | | | |  | | | |  | | | |  |  |
| 4 | Atr-ERN18577 |  | | | |  | | | |  | | | |  | | | |  |  |
| 4 | Atr-ERN18578 |  | | | |  | | | |  | | | |  | | | |  |  |
| 4 | Atr-ERN18579 |  | Vvi-Vitvi18g00638\_t001 |  | Vvi-Vitvi15g04503\_t001 |  | | | |  | | | |  |  |
| 4 | Atr-ERN18580 |  | Vvi-Vitvi18g00639\_t001 |  | | | |  | | | |  | | | |  |  |
| 4 | Atr-ERN18581 |  | | | |  | | | |  | | | |  | | | |  |  |
| 4 | Atr-ERN18582 |  | Vvi-Vitvi18g02636\_t001 |  | Vvi-Vitvi15g04501\_t001 |  | Vvi-Vitvi03g01635\_t001 |  | | | |  |  |
| 3 | Atr-ERN18583 |  | Vvi-Vitvi18g00641\_t001 |  | | | |  |  |  | | | |  |  |
| 3 | Atr-ERN18584 |  | | | |  | | | |  |  |  | | | |  |  |
| 3 | Atr-ERN18585 |  | | | |  | | | |  |  |  | | | |  |  |
| 3 | Atr-ERN18586 |  | | | |  | | | |  |  |  | | | |  |  |
| 3 | Atr-ERN18587 |  | Vvi-Vitvi18g00643\_t001 |  | | | |  |  |  | | | |  |  |
| 3 | Atr-ERN18588 |  | | | |  | | | |  |  |  | | | |  |  |
| 3 | Atr-ERN18589 |  | | | |  | | | |  |  |  | | | |  |  |
| 3 | Atr-ERN18590 |  | | | |  | | | |  |  |  | Vvi-Vitvi03g01612\_t001 |  |  |
| 3 | Atr-ERN18591 |  | | | |  | | | |  |  |  | | | |  |  |
| 3 | Atr-ERN18592 |  | Vvi-Vitvi18g00644\_t001 |  | | | |  |  |  | | | |  |  |
| 3 | Atr-ERN18593 |  | Vvi-Vitvi18g02638\_t001 |  | | | |  |  |  | Vvi-Vitvi03g00677\_t001 |  |  |
| 3 | Atr-ERN18594 |  | | | |  | Vvi-Vitvi15g04497\_t001 |  |  |  | | | |  |  |
| 3 | Atr-ERN18595 |  | Vvi-Vitvi18g00645\_t003 |  | Vvi-Vitvi15g04494\_t001 |  |  |  | | | |  |  |
| 3 | Atr-ERN18596 |  | | | |  | | | |  |  |  | | | |  |  |
| 3 | Atr-ERN18597 |  | | | |  | | | |  |  |  | | | |  |  |
| 3 | Atr-ERN18598 |  | | | |  | | | |  |  |  | | | |  |  |
| 3 | Atr-ERN18599 |  | | | |  | | | |  |  |  | | | |  |  |
| 3 | Atr-ERN18600 |  | | | |  | | | |  |  |  | | | |  |  |
| 3 | Atr-ERN18601 |  | | | |  | Vvi-Vitvi15g04493\_t001 |  |  |  | Vvi-Vitvi03g00675\_t001 |  |  |
| 3 | Atr-ERN18602 |  | | | |  | Vvi-Vitvi15g04491\_t001 |  |  |  | | | |  |  |
| 3 | Atr-ERN18603 |  | Vvi-Vitvi18g02644\_t001 |  | | | |  |  |  | Vvi-Vitvi03g00673\_t001 |  |  |
| 3 | Atr-ERN18604 |  | | | |  | | | |  |  |  | | | |  |  |
| 3 | Atr-ERN18605 |  | | | |  | Vvi-Vitvi15g04490\_t001 |  |  |  | | | |  |  |
| 2 | Atr-ERN18606 |  | Vvi-Vitvi18g00647\_t001 |  |  |  |  |  | | | |  |  |
| 2 | Atr-ERN18607 |  | | | |  |  |  |  |  | | | |  |  |
| 2 | Atr-ERN18608 |  | | | |  |  |  |  |  | | | |  |  |
| 2 | Atr-ERN18609 |  | | | |  |  |  |  |  | | | |  |  |
| 2 | Atr-ERN18610 |  | | | |  |  |  |  |  | | | |  |  |
| 2 | Atr-ERN18611 |  | | | |  |  |  |  |  | | | |  |  |
| 2 | Atr-ERN18612 |  | | | |  |  |  |  |  | | | |  |  |
| 2 | Atr-ERN18613 |  | | | |  |  |  |  |  | | | |  |  |
| 2 | Atr-ERN18614 |  | | | |  |  |  |  |  | | | |  |  |
| 2 | Atr-ERN18615 |  | | | |  |  |  |  |  | | | |  |  |
| 2 | Atr-ERN18616 |  | | | |  |  |  |  |  | | | |  |  |
| 2 | Atr-ERN18617 |  | | | |  |  |  |  |  | | | |  |  |
| 2 | Atr-ERN18618 |  | | | |  |  |  |  |  | | | |  |  |
| 2 | Atr-ERN18619 |  | | | |  |  |  |  |  | | | |  |  |
| 2 | Atr-ERN18620 |  | | | |  |  |  |  |  | | | |  |  |
| 2 | Atr-ERN18621 |  | | | |  |  |  |  |  | | | |  |  |
| 2 | Atr-ERN18622 |  | | | |  |  |  |  |  | | | |  |  |
| 2 | Atr-ERN18623 |  | | | |  |  |  |  |  | | | |  |  |
| 2 | Atr-ERN18624 |  | Vvi-Vitvi18g00651\_t001 |  |  |  |  |  | | | |  |  |
| 2 | Atr-ERN18625 |  | | | |  |  |  |  |  | | | |  |  |
| 2 | Atr-ERN18626 |  | Vvi-Vitvi18g00652\_t001 |  |  |  |  |  | | | |  |  |
| 2 | Atr-ERN18627 |  | | | |  |  |  |  |  | | | |  |  |
| 2 | Atr-ERN18628 |  | | | |  |  |  |  |  | Vvi-Vitvi03g00667\_t001 |  |  |
| 2 | Atr-ERN18629 |  | | | |  |  |  |  |  | | | |  |  |
| 2 | Atr-ERN18630 |  | Vvi-Vitvi18g00653\_t001 |  |  |  |  |  | | | |  |  |
| 2 | Atr-ERN18631 |  | Vvi-Vitvi18g00654\_t001 |  |  |  |  |  | Vvi-Vitvi03g00666\_t001 |  |  |
| 2 | Atr-ERN18632 |  | | | |  |  |  |  |  | | | |  |  |
| 2 | Atr-ERN18633 |  | | | |  |  |  |  |  | | | |  |  |
| 2 | Atr-ERN18634 |  | Vvi-Vitvi18g00656\_t001 |  |  |  |  |  | | | |  |  |
| 2 | Atr-ERN18635 |  | Vvi-Vitvi18g00657\_t001 |  |  |  |  |  | | | |  |  |
| 3 | Atr-ERN18636 |  | | | |  | Vvi-Vitvi11g00692\_t001 |  |  |  | | | |  |  |
| 3 | Atr-ERN18637 |  | | | |  | | | |  |  |  | | | |  |  |
| 3 | Atr-ERN18638 |  | | | |  | | | |  |  |  | | | |  |  |
| 3 | Atr-ERN18639 |  | | | |  | | | |  |  |  | | | |  |  |
| 3 | Atr-ERN18640 |  | | | |  | | | |  |  |  | | | |  |  |
| 3 | Atr-ERN18641 |  | | | |  | | | |  |  |  | | | |  |  |
| 3 | Atr-ERN18642 |  | | | |  | Vvi-Vitvi11g00686\_t001 |  |  |  | | | |  |  |
| 3 | Atr-ERN18643 |  | | | |  | | | |  |  |  | | | |  |  |
| 3 | Atr-ERN18644 |  | | | |  | | | |  |  |  | | | |  |  |
| 3 | Atr-ERN18645 |  | | | |  | | | |  |  |  | | | |  |  |
| 3 | Atr-ERN18646 |  | | | |  | | | |  |  |  | | | |  |  |
| 3 | Atr-ERN18647 |  | | | |  | | | |  |  |  | | | |  |  |
| 3 | Atr-ERN18648 |  | | | |  | | | |  |  |  | | | |  |  |
| 3 | Atr-ERN18649 |  | | | |  | | | |  |  |  | | | |  |  |
| 3 | Atr-ERN18650 |  | | | |  | | | |  |  |  | | | |  |  |
| 3 | Atr-ERN18651 |  | | | |  | | | |  |  |  | | | |  |  |
| 3 | Atr-ERN18652 |  | | | |  | | | |  |  |  | | | |  |  |
| 3 | Atr-ERN18653 |  | Vvi-Vitvi18g00673\_t001 |  | Vvi-Vitvi11g00680\_t001 |  |  |  | Vvi-Vitvi03g00635\_t001 |  |  |
| 1 | Atr-ERN18654 |  |  |  | | | |  |  |  |  |
| 1 | Atr-ERN18655 |  |  |  | | | |  |  |  |  |
| 1 | Atr-ERN18656 |  |  |  | Vvi-Vitvi11g00679\_t002 |  |  |  |  |
| 1 | Atr-ERN18657 |  |  |  | | | |  |  |  |  |
| 1 | Atr-ERN18658 |  |  |  | | | |  |  |  |  |
| 1 | Atr-ERN18659 |  |  |  | | | |  |  |  |  |
| 1 | Atr-ERN18660 |  |  |  | | | |  |  |  |  |
| 1 | Atr-ERN18661 |  |  |  | | | |  |  |  |  |
| 1 | Atr-ERN18662 |  |  |  | | | |  |  |  |  |
| 1 | Atr-ERN18663 |  |  |  | | | |  |  |  |  |
| 1 | Atr-ERN18664 |  |  |  | | | |  |  |  |  |
| 1 | Atr-ERN18665 |  |  |  | | | |  |  |  |  |
| 1 | Atr-ERN18666 |  |  |  | | | |  |  |  |  |
| 1 | Atr-ERN18667 |  |  |  | | | |  |  |  |  |
| 1 | Atr-ERN18668 |  |  |  | | | |  |  |  |  |
| 1 | Atr-ERN18669 |  |  |  | | | |  |  |  |  |
| 1 | Atr-ERN18670 |  |  |  | Vvi-Vitvi11g04172\_t001 |  |  |  |  |
| 1 | Atr-ERN18671 |  |  |  | | | |  |  |  |  |
| 1 | Atr-ERN18672 |  |  |  | | | |  |  |  |  |
| 1 | Atr-ERN18673 |  |  |  | | | |  |  |  |  |
| 1 | Atr-ERN18674 |  |  |  | | | |  |  |  |  |
| 1 | Atr-ERN18675 |  |  |  | | | |  |  |  |  |
| 1 | Atr-ERN18676 |  |  |  | | | |  |  |  |  |
| 1 | Atr-ERN18677 |  |  |  | | | |  |  |  |  |
| 1 | Atr-ERN18678 |  |  |  | | | |  |  |  |  |
| 1 | Atr-ERN18679 |  |  |  | | | |  |  |  |  |
| 1 | Atr-ERN18680 |  |  |  | | | |  |  |  |  |
| 1 | Atr-ERN18681 |  |  |  | | | |  |  |  |  |
| 1 | Atr-ERN18682 |  |  |  | | | |  |  |  |  |
| 2 | Atr-ERN18683 |  | Vvi-Vitvi11g04169\_t001 |  | Vvi-Vitvi11g04169\_t001 |  |  |  |  |
| 3 | Atr-ERN18684 |  | | | |  | Vvi-Vitvi11g00673\_t001 |  | Vvi-Vitvi04g00586\_t001 |  |  |  |
| 3 | Atr-ERN18685 |  | | | |  | | | |  | | | |  |  |  |
| 3 | Atr-ERN18686 |  | | | |  | | | |  | | | |  |  |  |
| 3 | Atr-ERN18687 |  | | | |  | Vvi-Vitvi11g00672\_t001 |  | | | |  |  |  |
| 3 | Atr-ERN18688 |  | | | |  | Vvi-Vitvi11g00671\_t001 |  | | | |  |  |  |
| 3 | Atr-ERN18689 |  | | | |  | | | |  | | | |  |  |  |
| 3 | Atr-ERN18690 |  | | | |  | Vvi-Vitvi11g01715\_t001 |  | | | |  |  |  |
| 3 | Atr-ERN18691 |  | | | |  | | | |  | Vvi-Vitvi04g00590\_t001 |  |  |  |
| 3 | Atr-ERN18692 |  | | | |  | Vvi-Vitvi11g00668\_t001 |  | | | |  |  |  |
| 3 | Atr-ERN18693 |  | | | |  | Vvi-Vitvi11g00667\_t001 |  | Vvi-Vitvi04g00591\_t001 |  |  |  |
| 3 | Atr-ERN18694 |  | | | |  | | | |  | Vvi-Vitvi04g00594\_t001 |  |  |  |
| 3 | Atr-ERN18695 |  | | | |  | Vvi-Vitvi11g00666\_t001 |  | | | |  |  |  |
| 2 | Atr-ERN18696 |  | | | |  |  |  | | | |  |  |  |
| 2 | Atr-ERN18697 |  | Vvi-Vitvi11g00695\_t001 |  |  |  | | | |  |  |  |
| 2 | Atr-ERN18698 |  | | | |  |  |  | | | |  |  |  |
| 2 | Atr-ERN18699 |  | Vvi-Vitvi11g00696\_t001 |  |  |  | | | |  |  |  |
| 2 | Atr-ERN18700 |  | Vvi-Vitvi11g00698\_t001 |  |  |  | | | |  |  |  |
| 2 | Atr-ERN18701 |  | | | |  |  |  | | | |  |  |  |
| 2 | Atr-ERN18702 |  | Vvi-Vitvi11g00699\_t001 |  |  |  | | | |  |  |  |
| 2 | Atr-ERN18703 |  | | | |  |  |  | Vvi-Vitvi04g00598\_t001 |  |  |  |
| 2 | Atr-ERN18704 |  | | | |  |  |  | | | |  |  |  |
| 2 | Atr-ERN18705 |  | | | |  |  |  | | | |  |  |  |
| 2 | Atr-ERN18706 |  | | | |  |  |  | | | |  |  |  |
| 2 | Atr-ERN18707 |  | | | |  |  |  | Vvi-Vitvi04g00599\_t001 |  |  |  |
| 2 | Atr-ERN18708 |  | Vvi-Vitvi11g00700\_t001 |  |  |  | Vvi-Vitvi04g00600\_t001 |  |  |  |
| 0 | Atr-ERN18709 |  |  |  |  |  |  |
