# Supplementary material for: Genome-wide identification, characterization and gene expression of BES1 transcription factor family in grapevine (Vitis vinifera L.)
Source: Sci Rep. 2023 Jan 5;13:240. doi: 10.1038/s41598-022-24407-y (PMC9816167; doi:10.1038/s41598-022-24407-y)
Supplement: Supplementary file 3 — Supplementary Information. [file 41598_2022_24407_MOESM3_ESM.zip › Vvi_Atr/Vitis_vinifera.PN40024.v4.dna_sm.toplevel.fa.vs.Amborella_trichopoda.AMTR1.0.dna_sm.toplevel.fa.html/Atr-AmTr_v1.0_scaffold00066.html]

|  |  |  |  |  |  |  |  |  |  |  |  |  |  |
| --- | --- | --- | --- | --- | --- | --- | --- | --- | --- | --- | --- | --- | --- |
| Duplication depth | Reference chromosome | Collinear blocks | | | | | | | | | | | |
| 0 | Atr-ERN20085 |  |  |  |  |  |  |
| 0 | Atr-ERN20086 |  |  |  |  |  |  |
| 0 | Atr-ERN20087 |  |  |  |  |  |  |
| 0 | Atr-ERN20088 |  |  |  |  |  |  |
| 0 | Atr-ERN20089 |  |  |  |  |  |  |
| 0 | Atr-ERN20090 |  |  |  |  |  |  |
| 0 | Atr-ERN20091 |  |  |  |  |  |  |
| 0 | Atr-ERN20092 |  |  |  |  |  |  |
| 1 | Atr-ERN20093 |  | Vvi-Vitvi05g04385\_t001 |  |  |  |  |  |
| 1 | Atr-ERN20094 |  | | | |  |  |  |  |  |
| 1 | Atr-ERN20095 |  | | | |  |  |  |  |  |
| 1 | Atr-ERN20096 |  | | | |  |  |  |  |  |
| 1 | Atr-ERN20097 |  | | | |  |  |  |  |  |
| 1 | Atr-ERN20098 |  | | | |  |  |  |  |  |
| 1 | Atr-ERN20099 |  | Vvi-Vitvi05g01395\_t001 |  |  |  |  |  |
| 1 | Atr-ERN20100 |  | Vvi-Vitvi05g01396\_t001 |  |  |  |  |  |
| 2 | Atr-ERN20101 |  | | | |  | Vvi-Vitvi05g02138\_t004 |  |  |  |  |
| 2 | Atr-ERN20102 |  | | | |  | Vvi-Vitvi05g01413\_t001 |  |  |  |  |
| 2 | Atr-ERN20103 |  | | | |  | | | |  |  |  |  |
| 2 | Atr-ERN20104 |  | | | |  | Vvi-Vitvi05g01409\_t003 |  |  |  |  |
| 2 | Atr-ERN20105 |  | Vvi-Vitvi05g01408\_t001 |  | Vvi-Vitvi05g01408\_t001 |  |  |  |  |
| 2 | Atr-ERN20106 |  | | | |  | | | |  |  |  |  |
| 2 | Atr-ERN20107 |  | | | |  | | | |  |  |  |  |
| 2 | Atr-ERN20108 |  | | | |  | | | |  |  |  |  |
| 2 | Atr-ERN20109 |  | | | |  | Vvi-Vitvi05g01405\_t001 |  |  |  |  |
| 2 | Atr-ERN20110 |  | | | |  | Vvi-Vitvi05g01401\_t001 |  |  |  |  |
| 1 | Atr-ERN20111 |  | Vvi-Vitvi05g01421\_t001 |  |  |  |  |  |
| 1 | Atr-ERN20112 |  | | | |  |  |  |  |  |
| 1 | Atr-ERN20113 |  | Vvi-Vitvi05g02139\_t001 |  |  |  |  |  |
| 1 | Atr-ERN20114 |  | | | |  |  |  |  |  |
| 1 | Atr-ERN20115 |  | Vvi-Vitvi05g01424\_t001 |  |  |  |  |  |
| 0 | Atr-ERN20116 |  |  |  |  |  |  |
| 0 | Atr-ERN20117 |  |  |  |  |  |  |
| 1 | Atr-ERN20118 |  | Vvi-Vitvi05g01465\_t001 |  |  |  |  |  |
| 1 | Atr-ERN20119 |  | Vvi-Vitvi05g01467\_t001 |  |  |  |  |  |
| 1 | Atr-ERN20120 |  | | | |  |  |  |  |  |
| 1 | Atr-ERN20121 |  | | | |  |  |  |  |  |
| 1 | Atr-ERN20122 |  | Vvi-Vitvi05g01469\_t001 |  |  |  |  |  |
| 1 | Atr-ERN20123 |  | | | |  |  |  |  |  |
| 1 | Atr-ERN20124 |  | | | |  |  |  |  |  |
| 1 | Atr-ERN20125 |  | | | |  |  |  |  |  |
| 1 | Atr-ERN20126 |  | Vvi-Vitvi05g01470\_t001 |  |  |  |  |  |
| 1 | Atr-ERN20127 |  | | | |  |  |  |  |  |
| 1 | Atr-ERN20128 |  | | | |  |  |  |  |  |
| 1 | Atr-ERN20129 |  | | | |  |  |  |  |  |
| 1 | Atr-ERN20130 |  | | | |  |  |  |  |  |
| 1 | Atr-ERN20131 |  | | | |  |  |  |  |  |
| 1 | Atr-ERN20132 |  | | | |  |  |  |  |  |
| 1 | Atr-ERN20133 |  | Vvi-Vitvi05g01471\_t001 |  |  |  |  |  |
| 1 | Atr-ERN20134 |  | | | |  |  |  |  |  |
| 1 | Atr-ERN20135 |  | | | |  |  |  |  |  |
| 1 | Atr-ERN20136 |  | Vvi-Vitvi05g01475\_t001 |  |  |  |  |  |
| 1 | Atr-ERN20137 |  | | | |  |  |  |  |  |
| 1 | Atr-ERN20138 |  | | | |  |  |  |  |  |
| 1 | Atr-ERN20139 |  | | | |  |  |  |  |  |
| 1 | Atr-ERN20140 |  | | | |  |  |  |  |  |
| 1 | Atr-ERN20141 |  | | | |  |  |  |  |  |
| 1 | Atr-ERN20142 |  | Vvi-Vitvi05g01480\_t001 |  |  |  |  |  |
| 1 | Atr-ERN20143 |  | | | |  |  |  |  |  |
| 1 | Atr-ERN20144 |  | | | |  |  |  |  |  |
| 1 | Atr-ERN20145 |  | | | |  |  |  |  |  |
| 1 | Atr-ERN20146 |  | | | |  |  |  |  |  |
| 1 | Atr-ERN20147 |  | | | |  |  |  |  |  |
| 1 | Atr-ERN20148 |  | Vvi-Vitvi05g01482\_t001 |  |  |  |  |  |
| 1 | Atr-ERN20149 |  | | | |  |  |  |  |  |
| 1 | Atr-ERN20150 |  | Vvi-Vitvi05g01486\_t001 |  |  |  |  |  |
| 1 | Atr-ERN20151 |  | Vvi-Vitvi05g01488\_t001 |  |  |  |  |  |
| 1 | Atr-ERN20152 |  | | | |  |  |  |  |  |
| 1 | Atr-ERN20153 |  | | | |  |  |  |  |  |
| 1 | Atr-ERN20154 |  | | | |  |  |  |  |  |
| 1 | Atr-ERN20155 |  | | | |  |  |  |  |  |
| 2 | Atr-ERN20156 |  | | | |  | Vvi-Vitvi05g01702\_t001 |  |  |  |  |
| 2 | Atr-ERN20157 |  | | | |  | | | |  |  |  |  |
| 2 | Atr-ERN20158 |  | | | |  | Vvi-Vitvi05g01701\_t001 |  |  |  |  |
| 2 | Atr-ERN20159 |  | | | |  | | | |  |  |  |  |
| 2 | Atr-ERN20160 |  | | | |  | | | |  |  |  |  |
| 2 | Atr-ERN20161 |  | | | |  | Vvi-Vitvi05g01700\_t001 |  |  |  |  |
| 2 | Atr-ERN20162 |  | | | |  | | | |  |  |  |  |
| 2 | Atr-ERN20163 |  | | | |  | Vvi-Vitvi05g04576\_t001 |  |  |  |  |
| 2 | Atr-ERN20164 |  | | | |  | Vvi-Vitvi05g01697\_t001 |  |  |  |  |
| 3 | Atr-ERN20165 |  | | | |  | | | |  | Vvi-Vitvi07g01215\_t001 |  |  |  |
| 3 | Atr-ERN20166 |  | | | |  | | | |  | | | |  |  |  |
| 3 | Atr-ERN20167 |  | | | |  | Vvi-Vitvi05g01696\_t001 |  | | | |  |  |  |
| 3 | Atr-ERN20168 |  | | | |  | | | |  | | | |  |  |  |
| 3 | Atr-ERN20169 |  | | | |  | | | |  | | | |  |  |  |
| 3 | Atr-ERN20170 |  | | | |  | | | |  | Vvi-Vitvi07g01219\_t001 |  |  |  |
| 3 | Atr-ERN20171 |  | | | |  | Vvi-Vitvi05g01694\_t001 |  | | | |  |  |  |
| 3 | Atr-ERN20172 |  | | | |  | Vvi-Vitvi05g01693\_t001 |  | | | |  |  |  |
| 3 | Atr-ERN20173 |  | | | |  | Vvi-Vitvi05g01692\_t001 |  | | | |  |  |  |
| 3 | Atr-ERN20174 |  | | | |  | | | |  | | | |  |  |  |
| 3 | Atr-ERN20175 |  | Vvi-Vitvi05g04445\_t001 |  | | | |  | | | |  |  |  |
| 2 | Atr-ERN20176 |  |  |  | Vvi-Vitvi05g01691\_t001 |  | | | |  |  |  |
| 2 | Atr-ERN20177 |  |  |  | | | |  | | | |  |  |  |
| 2 | Atr-ERN20178 |  |  |  | Vvi-Vitvi05g01690\_t001 |  | | | |  |  |  |
| 2 | Atr-ERN20179 |  |  |  | | | |  | | | |  |  |  |
| 2 | Atr-ERN20180 |  |  |  | Vvi-Vitvi05g01689\_t001 |  | | | |  |  |  |
| 2 | Atr-ERN20181 |  |  |  | | | |  | | | |  |  |  |
| 2 | Atr-ERN20182 |  |  |  | | | |  | | | |  |  |  |
| 2 | Atr-ERN20183 |  |  |  | | | |  | | | |  |  |  |
| 2 | Atr-ERN20184 |  |  |  | Vvi-Vitvi05g02278\_t001 |  | | | |  |  |  |
| 2 | Atr-ERN20185 |  |  |  | | | |  | | | |  |  |  |
| 2 | Atr-ERN20186 |  |  |  | Vvi-Vitvi05g04575\_t001 |  | | | |  |  |  |
| 2 | Atr-ERN20187 |  |  |  | | | |  | | | |  |  |  |
| 2 | Atr-ERN20188 |  |  |  | | | |  | | | |  |  |  |
| 2 | Atr-ERN20189 |  |  |  | Vvi-Vitvi05g01688\_t001 |  | | | |  |  |  |
| 2 | Atr-ERN20190 |  |  |  | Vvi-Vitvi05g01687\_t001 |  | | | |  |  |  |
| 2 | Atr-ERN20191 |  |  |  | | | |  | | | |  |  |  |
| 2 | Atr-ERN20192 |  |  |  | | | |  | | | |  |  |  |
| 2 | Atr-ERN20193 |  |  |  | | | |  | | | |  |  |  |
| 2 | Atr-ERN20194 |  |  |  | | | |  | | | |  |  |  |
| 2 | Atr-ERN20195 |  |  |  | Vvi-Vitvi05g01686\_t001 |  | | | |  |  |  |
| 2 | Atr-ERN20196 |  |  |  | | | |  | Vvi-Vitvi07g02992\_t001 |  |  |  |
| 2 | Atr-ERN20197 |  |  |  | | | |  | | | |  |  |  |
| 2 | Atr-ERN20198 |  |  |  | | | |  | | | |  |  |  |
| 2 | Atr-ERN20199 |  |  |  | | | |  | | | |  |  |  |
| 2 | Atr-ERN20200 |  |  |  | | | |  | | | |  |  |  |
| 2 | Atr-ERN20201 |  |  |  | | | |  | | | |  |  |  |
| 2 | Atr-ERN20202 |  |  |  | Vvi-Vitvi05g02276\_t001 |  | | | |  |  |  |
| 2 | Atr-ERN20203 |  |  |  | | | |  | | | |  |  |  |
| 2 | Atr-ERN20204 |  |  |  | Vvi-Vitvi05g01685\_t001 |  | | | |  |  |  |
| 2 | Atr-ERN20205 |  |  |  | | | |  | Vvi-Vitvi07g02990\_t001 |  |  |  |
| 2 | Atr-ERN20206 |  |  |  | | | |  | | | |  |  |  |
| 2 | Atr-ERN20207 |  |  |  | | | |  | | | |  |  |  |
| 2 | Atr-ERN20208 |  |  |  | Vvi-Vitvi05g02272\_t001 |  | | | |  |  |  |
| 2 | Atr-ERN20209 |  |  |  | | | |  | | | |  |  |  |
| 2 | Atr-ERN20210 |  |  |  | | | |  | | | |  |  |  |
| 2 | Atr-ERN20211 |  |  |  | | | |  | | | |  |  |  |
| 2 | Atr-ERN20212 |  |  |  | | | |  | Vvi-Vitvi07g04480\_t001 |  |  |  |
| 2 | Atr-ERN20213 |  |  |  | | | |  | | | |  |  |  |
| 2 | Atr-ERN20214 |  |  |  | Vvi-Vitvi05g01679\_t001 |  | Vvi-Vitvi07g02498\_t001 |  |  |  |
| 2 | Atr-ERN20215 |  |  |  | | | |  | | | |  |  |  |
| 2 | Atr-ERN20216 |  |  |  | Vvi-Vitvi05g01672\_t001 |  | Vvi-Vitvi07g01244\_t001 |  |  |  |
| 2 | Atr-ERN20217 |  |  |  | | | |  | | | |  |  |  |
| 2 | Atr-ERN20218 |  |  |  | Vvi-Vitvi05g04565\_t001 |  | | | |  |  |  |
| 2 | Atr-ERN20219 |  |  |  | | | |  | | | |  |  |  |
| 2 | Atr-ERN20220 |  |  |  | | | |  | | | |  |  |  |
| 2 | Atr-ERN20221 |  |  |  | | | |  | | | |  |  |  |
| 2 | Atr-ERN20222 |  |  |  | | | |  | | | |  |  |  |
| 2 | Atr-ERN20223 |  |  |  | | | |  | | | |  |  |  |
| 2 | Atr-ERN20224 |  |  |  | | | |  | | | |  |  |  |
| 2 | Atr-ERN20225 |  |  |  | Vvi-Vitvi05g01660\_t001 |  | Vvi-Vitvi07g01247\_t001 |  |  |  |
| 2 | Atr-ERN20226 |  |  |  | | | |  | | | |  |  |  |
| 2 | Atr-ERN20227 |  |  |  | Vvi-Vitvi05g01656\_t001 |  | | | |  |  |  |
| 2 | Atr-ERN20228 |  |  |  | | | |  | | | |  |  |  |
| 2 | Atr-ERN20229 |  |  |  | | | |  | | | |  |  |  |
| 2 | Atr-ERN20230 |  |  |  | | | |  | | | |  |  |  |
| 2 | Atr-ERN20231 |  |  |  | Vvi-Vitvi05g01655\_t001 |  | Vvi-Vitvi07g04485\_t002 |  |  |  |
| 1 | Atr-ERN20232 |  |  |  |  |  | | | |  |  |  |
| 1 | Atr-ERN20233 |  |  |  |  |  | | | |  |  |  |
| 1 | Atr-ERN20234 |  |  |  |  |  | | | |  |  |  |
| 1 | Atr-ERN20235 |  |  |  |  |  | | | |  |  |  |
| 1 | Atr-ERN20236 |  |  |  |  |  | | | |  |  |  |
| 1 | Atr-ERN20237 |  |  |  |  |  | | | |  |  |  |
| 1 | Atr-ERN20238 |  |  |  |  |  | | | |  |  |  |
| 1 | Atr-ERN20239 |  |  |  |  |  | | | |  |  |  |
| 1 | Atr-ERN20240 |  |  |  |  |  | | | |  |  |  |
| 1 | Atr-ERN20241 |  |  |  |  |  | Vvi-Vitvi07g04486\_t001 |  |  |  |
| 0 | Atr-ERN20242 |  |  |  |  |  |  |
| 0 | Atr-ERN20243 |  |  |  |  |  |  |
| 0 | Atr-ERN20244 |  |  |  |  |  |  |
| 0 | Atr-ERN20245 |  |  |  |  |  |  |
| 0 | Atr-ERN20246 |  |  |  |  |  |  |
| 0 | Atr-ERN20247 |  |  |  |  |  |  |
| 0 | Atr-ERN20248 |  |  |  |  |  |  |
| 0 | Atr-ERN20249 |  |  |  |  |  |  |
| 1 | Atr-ERN20250 |  | Vvi-Vitvi05g01622\_t001 |  |  |  |  |  |
| 1 | Atr-ERN20251 |  | Vvi-Vitvi05g01623\_t001 |  |  |  |  |  |
| 1 | Atr-ERN20252 |  | Vvi-Vitvi05g01624\_t001 |  |  |  |  |  |
| 1 | Atr-ERN20253 |  | | | |  |  |  |  |  |
| 1 | Atr-ERN20254 |  | | | |  |  |  |  |  |
| 1 | Atr-ERN20255 |  | Vvi-Vitvi05g01626\_t001 |  |  |  |  |  |
| 1 | Atr-ERN20256 |  | Vvi-Vitvi05g01627\_t001 |  |  |  |  |  |
| 1 | Atr-ERN20257 |  | Vvi-Vitvi05g01629\_t001 |  |  |  |  |  |
| 1 | Atr-ERN20258 |  | | | |  |  |  |  |  |
| 1 | Atr-ERN20259 |  | Vvi-Vitvi05g01632\_t001 |  |  |  |  |  |
| 1 | Atr-ERN20260 |  | | | |  |  |  |  |  |
| 1 | Atr-ERN20261 |  | | | |  |  |  |  |  |
| 1 | Atr-ERN20262 |  | | | |  |  |  |  |  |
| 1 | Atr-ERN20263 |  | | | |  |  |  |  |  |
| 1 | Atr-ERN20264 |  | | | |  |  |  |  |  |
| 1 | Atr-ERN20265 |  | | | |  |  |  |  |  |
| 1 | Atr-ERN20266 |  | | | |  |  |  |  |  |
| 1 | Atr-ERN20267 |  | | | |  |  |  |  |  |
| 1 | Atr-ERN20268 |  | | | |  |  |  |  |  |
| 1 | Atr-ERN20269 |  | | | |  |  |  |  |  |
| 1 | Atr-ERN20270 |  | | | |  |  |  |  |  |
| 1 | Atr-ERN20271 |  | | | |  |  |  |  |  |
| 1 | Atr-ERN20272 |  | | | |  |  |  |  |  |
| 1 | Atr-ERN20273 |  | | | |  |  |  |  |  |
| 1 | Atr-ERN20274 |  | | | |  |  |  |  |  |
| 1 | Atr-ERN20275 |  | Vvi-Vitvi05g02262\_t001 |  |  |  |  |  |
| 1 | Atr-ERN20276 |  | | | |  |  |  |  |  |
| 1 | Atr-ERN20277 |  | | | |  |  |  |  |  |
| 1 | Atr-ERN20278 |  | | | |  |  |  |  |  |
| 1 | Atr-ERN20279 |  | | | |  |  |  |  |  |
| 1 | Atr-ERN20280 |  | Vvi-Vitvi05g01636\_t001 |  |  |  |  |  |
| 1 | Atr-ERN20281 |  | | | |  |  |  |  |  |
| 1 | Atr-ERN20282 |  | | | |  |  |  |  |  |
| 1 | Atr-ERN20283 |  | | | |  |  |  |  |  |
| 1 | Atr-ERN20284 |  | | | |  |  |  |  |  |
| 1 | Atr-ERN20285 |  | | | |  |  |  |  |  |
| 1 | Atr-ERN20286 |  | | | |  |  |  |  |  |
| 1 | Atr-ERN20287 |  | Vvi-Vitvi05g01638\_t001 |  |  |  |  |  |
| 1 | Atr-ERN20288 |  | Vvi-Vitvi05g01639\_t001 |  |  |  |  |  |
| 1 | Atr-ERN20289 |  | | | |  |  |  |  |  |
| 1 | Atr-ERN20290 |  | | | |  |  |  |  |  |
| 1 | Atr-ERN20291 |  | Vvi-Vitvi05g01641\_t001 |  |  |  |  |  |
| 1 | Atr-ERN20292 |  | | | |  |  |  |  |  |
| 1 | Atr-ERN20293 |  | Vvi-Vitvi05g01642\_t001 |  |  |  |  |  |
| 1 | Atr-ERN20294 |  | | | |  |  |  |  |  |
| 1 | Atr-ERN20295 |  | Vvi-Vitvi05g01648\_t001 |  |  |  |  |  |
| 1 | Atr-ERN20296 |  | | | |  |  |  |  |  |
| 1 | Atr-ERN20297 |  | | | |  |  |  |  |  |
| 1 | Atr-ERN20298 |  | Vvi-Vitvi05g04553\_t001 |  |  |  |  |  |
| 1 | Atr-ERN20299 |  | | | |  |  |  |  |  |
| 1 | Atr-ERN20300 |  | Vvi-Vitvi05g04554\_t001 |  |  |  |  |  |
| 1 | Atr-ERN20301 |  | | | |  |  |  |  |  |
| 1 | Atr-ERN20302 |  | | | |  |  |  |  |  |
| 1 | Atr-ERN20303 |  | | | |  |  |  |  |  |
| 1 | Atr-ERN20304 |  | Vvi-Vitvi05g02266\_t001 |  |  |  |  |  |
| 0 | Atr-ERN20305 |  |  |  |  |  |  |
| 2 | Atr-ERN20306 |  | Vvi-Vitvi05g01551\_t001 |  | Vvi-Vitvi05g04512\_t001 |  |  |  |  |
| 2 | Atr-ERN20307 |  | | | |  | Vvi-Vitvi05g01556\_t001 |  |  |  |  |
| 2 | Atr-ERN20308 |  | | | |  | Vvi-Vitvi05g01555\_t001 |  |  |  |  |
| 2 | Atr-ERN20309 |  | | | |  | | | |  |  |  |  |
| 2 | Atr-ERN20310 |  | Vvi-Vitvi05g01553\_t001 |  | | | |  |  |  |  |
| 2 | Atr-ERN20311 |  | | | |  | | | |  |  |  |  |
| 2 | Atr-ERN20312 |  | | | |  | | | |  |  |  |  |
| 2 | Atr-ERN20313 |  | Vvi-Vitvi05g01554\_t001 |  | | | |  |  |  |  |
| 2 | Atr-ERN20314 |  | | | |  | | | |  |  |  |  |
| 2 | Atr-ERN20315 |  | | | |  | | | |  |  |  |  |
| 3 | Atr-ERN20316 |  | | | |  | | | |  | Vvi-Vitvi04g01201\_t001 |  |  |  |
| 3 | Atr-ERN20317 |  | | | |  | | | |  | Vvi-Vitvi04g01200\_t001 |  |  |  |
| 3 | Atr-ERN20318 |  | | | |  | Vvi-Vitvi05g01546\_t001 |  | | | |  |  |  |
| 3 | Atr-ERN20319 |  | | | |  | Vvi-Vitvi05g01545\_t001 |  | Vvi-Vitvi04g01198\_t001 |  |  |  |
| 3 | Atr-ERN20320 |  | | | |  | Vvi-Vitvi05g01544\_t001 |  | | | |  |  |  |
| 3 | Atr-ERN20321 |  | | | |  | Vvi-Vitvi05g01543\_t001 |  | | | |  |  |  |
| 3 | Atr-ERN20322 |  | | | |  | Vvi-Vitvi05g01542\_t001 |  | | | |  |  |  |
| 3 | Atr-ERN20323 |  | | | |  | Vvi-Vitvi05g01540\_t003 |  | | | |  |  |  |
| 3 | Atr-ERN20324 |  | | | |  | | | |  | | | |  |  |  |
| 3 | Atr-ERN20325 |  | | | |  | | | |  | | | |  |  |  |
| 3 | Atr-ERN20326 |  | | | |  | Vvi-Vitvi05g01537\_t001 |  | | | |  |  |  |
| 3 | Atr-ERN20327 |  | | | |  | | | |  | Vvi-Vitvi04g02083\_t001 |  |  |  |
| 3 | Atr-ERN20328 |  | | | |  | Vvi-Vitvi05g01536\_t001 |  | | | |  |  |  |
| 2 | Atr-ERN20329 |  | | | |  |  |  | | | |  |  |  |
| 2 | Atr-ERN20330 |  | | | |  |  |  | | | |  |  |  |
| 2 | Atr-ERN20331 |  | | | |  |  |  | Vvi-Vitvi04g01197\_t001 |  |  |  |
| 2 | Atr-ERN20332 |  | | | |  |  |  | | | |  |  |  |
| 2 | Atr-ERN20333 |  | | | |  |  |  | | | |  |  |  |
| 2 | Atr-ERN20334 |  | | | |  |  |  | | | |  |  |  |
| 2 | Atr-ERN20335 |  | | | |  |  |  | | | |  |  |  |
| 2 | Atr-ERN20336 |  | Vvi-Vitvi05g04516\_t001 |  |  |  | | | |  |  |  |
| 2 | Atr-ERN20337 |  | | | |  |  |  | | | |  |  |  |
| 2 | Atr-ERN20338 |  | | | |  |  |  | | | |  |  |  |
| 2 | Atr-ERN20339 |  | | | |  |  |  | | | |  |  |  |
| 2 | Atr-ERN20340 |  | | | |  |  |  | | | |  |  |  |
| 2 | Atr-ERN20341 |  | | | |  |  |  | | | |  |  |  |
| 2 | Atr-ERN20342 |  | | | |  |  |  | | | |  |  |  |
| 2 | Atr-ERN20343 |  | Vvi-Vitvi05g01564\_t001 |  |  |  | | | |  |  |  |
| 2 | Atr-ERN20344 |  | Vvi-Vitvi05g04517\_t001 |  |  |  | | | |  |  |  |
| 2 | Atr-ERN20345 |  | Vvi-Vitvi05g02239\_t001 |  |  |  | | | |  |  |  |
| 2 | Atr-ERN20346 |  | Vvi-Vitvi05g01566\_t001 |  |  |  | | | |  |  |  |
| 2 | Atr-ERN20347 |  | | | |  |  |  | Vvi-Vitvi04g01196\_t001 |  |  |  |
| 2 | Atr-ERN20348 |  | Vvi-Vitvi05g01572\_t001 |  |  |  | Vvi-Vitvi04g01195\_t001 |  |  |  |
| 1 | Atr-ERN20349 |  | | | |  |  |  |  |  |
| 1 | Atr-ERN20350 |  | Vvi-Vitvi05g02249\_t001 |  |  |  |  |  |
| 1 | Atr-ERN20351 |  | | | |  |  |  |  |  |
| 1 | Atr-ERN20352 |  | Vvi-Vitvi05g01584\_t001 |  |  |  |  |  |
| 1 | Atr-ERN20353 |  | | | |  |  |  |  |  |
| 1 | Atr-ERN20354 |  | Vvi-Vitvi05g01585\_t001 |  |  |  |  |  |
| 1 | Atr-ERN20355 |  | | | |  |  |  |  |  |
| 1 | Atr-ERN20356 |  | | | |  |  |  |  |  |
| 1 | Atr-ERN20357 |  | Vvi-Vitvi05g01592\_t001 |  |  |  |  |  |
| 1 | Atr-ERN20358 |  | Vvi-Vitvi05g01596\_t001 |  |  |  |  |  |
| 1 | Atr-ERN20359 |  | Vvi-Vitvi05g01599\_t001 |  |  |  |  |  |
| 0 | Atr-ERN20360 |  |  |  |  |  |  |
| 0 | Atr-ERN20361 |  |  |  |  |  |  |
| 0 | Atr-ERN20362 |  |  |  |  |  |  |
| 0 | Atr-ERN20363 |  |  |  |  |  |  |
| 0 | Atr-ERN20364 |  |  |  |  |  |  |
| 0 | Atr-ERN20365 |  |  |  |  |  |  |
| 0 | Atr-ERN20366 |  |  |  |  |  |  |
