# Supplementary material for: Genome-wide identification, characterization and gene expression of BES1 transcription factor family in grapevine (Vitis vinifera L.)
Source: Sci Rep. 2023 Jan 5;13:240. doi: 10.1038/s41598-022-24407-y (PMC9816167; doi:10.1038/s41598-022-24407-y)
Supplement: Supplementary file 3 — Supplementary Information. [file 41598_2022_24407_MOESM3_ESM.zip › Vvi_Atr/Vitis_vinifera.PN40024.v4.dna_sm.toplevel.fa.vs.Amborella_trichopoda.AMTR1.0.dna_sm.toplevel.fa.html/Atr-AmTr_v1.0_scaffold00067.html]

|  |  |  |  |  |  |  |  |  |  |  |  |  |  |
| --- | --- | --- | --- | --- | --- | --- | --- | --- | --- | --- | --- | --- | --- |
| Duplication depth | Reference chromosome | Collinear blocks | | | | | | | | | | | |
| 0 | Atr-ERN18736 |  |  |  |  |  |  |
| 0 | Atr-ERN18737 |  |  |  |  |  |  |
| 0 | Atr-ERN18738 |  |  |  |  |  |  |
| 0 | Atr-ERN18739 |  |  |  |  |  |  |
| 1 | Atr-ERN18740 |  | Vvi-Vitvi07g00597\_t001 |  |  |  |  |  |
| 1 | Atr-ERN18741 |  | Vvi-Vitvi07g02267\_t001 |  |  |  |  |  |
| 1 | Atr-ERN18742 |  | | | |  |  |  |  |  |
| 2 | Atr-ERN18743 |  | | | |  | Vvi-Vitvi05g00746\_t001 |  |  |  |  |
| 2 | Atr-ERN18744 |  | | | |  | | | |  |  |  |  |
| 2 | Atr-ERN18745 |  | | | |  | | | |  |  |  |  |
| 2 | Atr-ERN18746 |  | | | |  | | | |  |  |  |  |
| 2 | Atr-ERN18747 |  | | | |  | | | |  |  |  |  |
| 2 | Atr-ERN18748 |  | Vvi-Vitvi07g00603\_t001 |  | | | |  |  |  |  |
| 2 | Atr-ERN18749 |  | Vvi-Vitvi07g00604\_t001 |  | | | |  |  |  |  |
| 2 | Atr-ERN18750 |  | | | |  | | | |  |  |  |  |
| 2 | Atr-ERN18751 |  | | | |  | | | |  |  |  |  |
| 2 | Atr-ERN18752 |  | Vvi-Vitvi07g00606\_t001 |  | | | |  |  |  |  |
| 2 | Atr-ERN18753 |  | | | |  | | | |  |  |  |  |
| 2 | Atr-ERN18754 |  | | | |  | | | |  |  |  |  |
| 2 | Atr-ERN18755 |  | | | |  | Vvi-Vitvi05g00752\_t002 |  |  |  |  |
| 3 | Atr-ERN18756 |  | | | |  | | | |  | Vvi-Vitvi14g00614\_t001 |  |  |  |
| 3 | Atr-ERN18757 |  | | | |  | | | |  | | | |  |  |  |
| 3 | Atr-ERN18758 |  | Vvi-Vitvi07g00612\_t001 |  | | | |  | | | |  |  |  |
| 3 | Atr-ERN18759 |  | | | |  | | | |  | | | |  |  |  |
| 3 | Atr-ERN18760 |  | | | |  | | | |  | | | |  |  |  |
| 3 | Atr-ERN18761 |  | | | |  | | | |  | | | |  |  |  |
| 3 | Atr-ERN18762 |  | Vvi-Vitvi07g00613\_t001 |  | | | |  | | | |  |  |  |
| 3 | Atr-ERN18763 |  | | | |  | Vvi-Vitvi05g00755\_t002 |  | | | |  |  |  |
| 3 | Atr-ERN18764 |  | Vvi-Vitvi07g00614\_t001 |  | | | |  | | | |  |  |  |
| 3 | Atr-ERN18765 |  | | | |  | | | |  | | | |  |  |  |
| 3 | Atr-ERN18766 |  | Vvi-Vitvi07g00615\_t001 |  | Vvi-Vitvi05g00757\_t001 |  | | | |  |  |  |
| 3 | Atr-ERN18767 |  | | | |  | | | |  | Vvi-Vitvi14g00619\_t003 |  |  |  |
| 3 | Atr-ERN18768 |  | | | |  | | | |  | | | |  |  |  |
| 3 | Atr-ERN18769 |  | | | |  | | | |  | | | |  |  |  |
| 3 | Atr-ERN18770 |  | | | |  | | | |  | | | |  |  |  |
| 3 | Atr-ERN18771 |  | | | |  | | | |  | | | |  |  |  |
| 3 | Atr-ERN18772 |  | | | |  | | | |  | | | |  |  |  |
| 3 | Atr-ERN18773 |  | Vvi-Vitvi07g00618\_t002 |  | | | |  | Vvi-Vitvi14g00627\_t001 |  |  |  |
| 3 | Atr-ERN18774 |  | | | |  | | | |  | | | |  |  |  |
| 3 | Atr-ERN18775 |  | Vvi-Vitvi07g00619\_t001 |  | | | |  | | | |  |  |  |
| 3 | Atr-ERN18776 |  | | | |  | | | |  | | | |  |  |  |
| 3 | Atr-ERN18777 |  | | | |  | | | |  | | | |  |  |  |
| 3 | Atr-ERN18778 |  | | | |  | | | |  | | | |  |  |  |
| 3 | Atr-ERN18779 |  | Vvi-Vitvi07g00620\_t001 |  | | | |  | | | |  |  |  |
| 3 | Atr-ERN18780 |  | | | |  | | | |  | | | |  |  |  |
| 3 | Atr-ERN18781 |  | | | |  | | | |  | | | |  |  |  |
| 3 | Atr-ERN18782 |  | | | |  | | | |  | Vvi-Vitvi14g04267\_t001 |  |  |  |
| 3 | Atr-ERN18783 |  | Vvi-Vitvi07g00621\_t002 |  | Vvi-Vitvi05g00759\_t001 |  | | | |  |  |  |
| 3 | Atr-ERN18784 |  | | | |  | | | |  | | | |  |  |  |
| 3 | Atr-ERN18785 |  | | | |  | | | |  | Vvi-Vitvi14g00632\_t001 |  |  |  |
| 3 | Atr-ERN18786 |  | | | |  | | | |  | Vvi-Vitvi14g00637\_t001 |  |  |  |
| 3 | Atr-ERN18787 |  | | | |  | | | |  | | | |  |  |  |
| 3 | Atr-ERN18788 |  | Vvi-Vitvi07g00622\_t002 |  | | | |  | | | |  |  |  |
| 3 | Atr-ERN18789 |  | | | |  | | | |  | Vvi-Vitvi14g00640\_t001 |  |  |  |
| 3 | Atr-ERN18790 |  | | | |  | | | |  | | | |  |  |  |
| 3 | Atr-ERN18791 |  | Vvi-Vitvi07g00623\_t001 |  | | | |  | | | |  |  |  |
| 3 | Atr-ERN18792 |  | | | |  | | | |  | | | |  |  |  |
| 3 | Atr-ERN18793 |  | Vvi-Vitvi07g00624\_t001 |  | | | |  | | | |  |  |  |
| 3 | Atr-ERN18794 |  | Vvi-Vitvi07g00625\_t001 |  | | | |  | | | |  |  |  |
| 3 | Atr-ERN18795 |  | Vvi-Vitvi07g00626\_t001 |  | Vvi-Vitvi05g00764\_t001 |  | | | |  |  |  |
| 3 | Atr-ERN18796 |  | | | |  | | | |  | | | |  |  |  |
| 3 | Atr-ERN18797 |  | | | |  | | | |  | | | |  |  |  |
| 3 | Atr-ERN18798 |  | Vvi-Vitvi07g00628\_t001 |  | Vvi-Vitvi05g00767\_t001 |  | | | |  |  |  |
| 3 | Atr-ERN18799 |  | Vvi-Vitvi07g00630\_t002 |  | | | |  | Vvi-Vitvi14g00643\_t001 |  |  |  |
| 3 | Atr-ERN18800 |  | | | |  | Vvi-Vitvi05g00769\_t001 |  | | | |  |  |  |
| 3 | Atr-ERN18801 |  | | | |  | Vvi-Vitvi05g00771\_t001 |  | | | |  |  |  |
| 3 | Atr-ERN18802 |  | | | |  | Vvi-Vitvi05g00772\_t001 |  | | | |  |  |  |
| 3 | Atr-ERN18803 |  | | | |  | | | |  | | | |  |  |  |
| 3 | Atr-ERN18804 |  | | | |  | | | |  | Vvi-Vitvi14g00648\_t001 |  |  |  |
| 3 | Atr-ERN18805 |  | | | |  | | | |  | | | |  |  |  |
| 3 | Atr-ERN18806 |  | | | |  | | | |  | | | |  |  |  |
| 3 | Atr-ERN18807 |  | | | |  | | | |  | | | |  |  |  |
| 3 | Atr-ERN18808 |  | | | |  | Vvi-Vitvi05g00774\_t001 |  | | | |  |  |  |
| 3 | Atr-ERN18809 |  | | | |  | Vvi-Vitvi05g00775\_t001 |  | | | |  |  |  |
| 3 | Atr-ERN18810 |  | | | |  | | | |  | | | |  |  |  |
| 3 | Atr-ERN18811 |  | | | |  | Vvi-Vitvi05g00778\_t001 |  | | | |  |  |  |
| 3 | Atr-ERN18812 |  | Vvi-Vitvi07g00633\_t002 |  | | | |  | | | |  |  |  |
| 3 | Atr-ERN18813 |  | | | |  | | | |  | | | |  |  |  |
| 3 | Atr-ERN18814 |  | | | |  | Vvi-Vitvi05g00779\_t001 |  | | | |  |  |  |
| 3 | Atr-ERN18815 |  | | | |  | Vvi-Vitvi05g01965\_t001 |  | | | |  |  |  |
| 3 | Atr-ERN18816 |  | Vvi-Vitvi07g00634\_t001 |  | | | |  | | | |  |  |  |
| 3 | Atr-ERN18817 |  | Vvi-Vitvi07g00635\_t001 |  | | | |  | | | |  |  |  |
| 3 | Atr-ERN18818 |  | | | |  | | | |  | Vvi-Vitvi14g00674\_t001 |  |  |  |
| 3 | Atr-ERN18819 |  | | | |  | | | |  | | | |  |  |  |
| 3 | Atr-ERN18820 |  | | | |  | | | |  | | | |  |  |  |
| 3 | Atr-ERN18821 |  | | | |  | | | |  | | | |  |  |  |
| 3 | Atr-ERN18822 |  | | | |  | Vvi-Vitvi05g00780\_t001 |  | | | |  |  |  |
| 3 | Atr-ERN18823 |  | | | |  | Vvi-Vitvi05g00783\_t001 |  | | | |  |  |  |
| 3 | Atr-ERN18824 |  | | | |  | | | |  | | | |  |  |  |
| 3 | Atr-ERN18825 |  | Vvi-Vitvi07g00636\_t001 |  | Vvi-Vitvi05g00784\_t001 |  | | | |  |  |  |
| 3 | Atr-ERN18826 |  | | | |  | | | |  | | | |  |  |  |
| 3 | Atr-ERN18827 |  | | | |  | | | |  | Vvi-Vitvi14g00679\_t001 |  |  |  |
| 2 | Atr-ERN18828 |  | | | |  | | | |  |  |  |  |
| 2 | Atr-ERN18829 |  | | | |  | Vvi-Vitvi05g00785\_t001 |  |  |  |  |
| 1 | Atr-ERN18830 |  | | | |  |  |  |  |  |
| 1 | Atr-ERN18831 |  | | | |  |  |  |  |  |
| 1 | Atr-ERN18832 |  | | | |  |  |  |  |  |
| 1 | Atr-ERN18833 |  | Vvi-Vitvi07g00637\_t001 |  |  |  |  |  |
| 1 | Atr-ERN18834 |  | | | |  |  |  |  |  |
| 1 | Atr-ERN18835 |  | | | |  |  |  |  |  |
| 1 | Atr-ERN18836 |  | | | |  |  |  |  |  |
| 1 | Atr-ERN18837 |  | | | |  |  |  |  |  |
| 1 | Atr-ERN18838 |  | Vvi-Vitvi07g00654\_t001 |  |  |  |  |  |
| 0 | Atr-ERN18839 |  |  |  |  |  |  |
| 0 | Atr-ERN18840 |  |  |  |  |  |  |
| 0 | Atr-ERN18841 |  |  |  |  |  |  |
| 0 | Atr-ERN18842 |  |  |  |  |  |  |
| 0 | Atr-ERN18843 |  |  |  |  |  |  |
| 0 | Atr-ERN18844 |  |  |  |  |  |  |
| 0 | Atr-ERN18845 |  |  |  |  |  |  |
| 0 | Atr-ERN18846 |  |  |  |  |  |  |
| 0 | Atr-ERN18847 |  |  |  |  |  |  |
| 0 | Atr-ERN18848 |  |  |  |  |  |  |
| 0 | Atr-ERN18849 |  |  |  |  |  |  |
| 0 | Atr-ERN18850 |  |  |  |  |  |  |
| 0 | Atr-ERN18851 |  |  |  |  |  |  |
| 0 | Atr-ERN18852 |  |  |  |  |  |  |
| 0 | Atr-ERN18853 |  |  |  |  |  |  |
| 0 | Atr-ERN18854 |  |  |  |  |  |  |
| 0 | Atr-ERN18855 |  |  |  |  |  |  |
| 0 | Atr-ERN18856 |  |  |  |  |  |  |
| 0 | Atr-ERN18857 |  |  |  |  |  |  |
| 0 | Atr-ERN18858 |  |  |  |  |  |  |
| 0 | Atr-ERN18859 |  |  |  |  |  |  |
| 0 | Atr-ERN18860 |  |  |  |  |  |  |
| 0 | Atr-ERN18861 |  |  |  |  |  |  |
| 0 | Atr-ERN18862 |  |  |  |  |  |  |
| 0 | Atr-ERN18863 |  |  |  |  |  |  |
| 1 | Atr-ERN18864 |  | Vvi-Vitvi07g00830\_t001 |  |  |  |  |  |
| 1 | Atr-ERN18865 |  | | | |  |  |  |  |  |
| 1 | Atr-ERN18866 |  | | | |  |  |  |  |  |
| 1 | Atr-ERN18867 |  | | | |  |  |  |  |  |
| 1 | Atr-ERN18868 |  | | | |  |  |  |  |  |
| 1 | Atr-ERN18869 |  | | | |  |  |  |  |  |
| 1 | Atr-ERN18870 |  | | | |  |  |  |  |  |
| 1 | Atr-ERN18871 |  | | | |  |  |  |  |  |
| 1 | Atr-ERN18872 |  | | | |  |  |  |  |  |
| 1 | Atr-ERN18873 |  | | | |  |  |  |  |  |
| 1 | Atr-ERN18874 |  | | | |  |  |  |  |  |
| 1 | Atr-ERN18875 |  | Vvi-Vitvi07g00825\_t001 |  |  |  |  |  |
| 1 | Atr-ERN18876 |  | Vvi-Vitvi07g00824\_t001 |  |  |  |  |  |
| 1 | Atr-ERN18877 |  | Vvi-Vitvi07g00807\_t001 |  |  |  |  |  |
| 1 | Atr-ERN18878 |  | Vvi-Vitvi07g00806\_t001 |  |  |  |  |  |
| 1 | Atr-ERN18879 |  | | | |  |  |  |  |  |
| 1 | Atr-ERN18880 |  | | | |  |  |  |  |  |
| 1 | Atr-ERN18881 |  | | | |  |  |  |  |  |
| 1 | Atr-ERN18882 |  | | | |  |  |  |  |  |
| 1 | Atr-ERN18883 |  | | | |  |  |  |  |  |
| 1 | Atr-ERN18884 |  | | | |  |  |  |  |  |
| 1 | Atr-ERN18885 |  | | | |  |  |  |  |  |
| 1 | Atr-ERN18886 |  | | | |  |  |  |  |  |
| 1 | Atr-ERN18887 |  | | | |  |  |  |  |  |
| 1 | Atr-ERN18888 |  | | | |  |  |  |  |  |
| 1 | Atr-ERN18889 |  | | | |  |  |  |  |  |
| 1 | Atr-ERN18890 |  | | | |  |  |  |  |  |
| 1 | Atr-ERN18891 |  | | | |  |  |  |  |  |
| 1 | Atr-ERN18892 |  | | | |  |  |  |  |  |
| 1 | Atr-ERN18893 |  | | | |  |  |  |  |  |
| 1 | Atr-ERN18894 |  | | | |  |  |  |  |  |
| 1 | Atr-ERN18895 |  | | | |  |  |  |  |  |
| 1 | Atr-ERN18896 |  | | | |  |  |  |  |  |
| 1 | Atr-ERN18897 |  | | | |  |  |  |  |  |
| 2 | Atr-ERN18898 |  | | | |  | Vvi-Vitvi07g00639\_t001 |  |  |  |  |
| 2 | Atr-ERN18899 |  | | | |  | | | |  |  |  |  |
| 2 | Atr-ERN18900 |  | | | |  | | | |  |  |  |  |
| 2 | Atr-ERN18901 |  | | | |  | | | |  |  |  |  |
| 2 | Atr-ERN18902 |  | | | |  | | | |  |  |  |  |
| 3 | Atr-ERN18903 |  | | | |  | | | |  | Vvi-Vitvi05g00787\_t001 |  |  |  |
| 3 | Atr-ERN18904 |  | Vvi-Vitvi07g02326\_t001 |  | | | |  | | | |  |  |  |
| 2 | Atr-ERN18905 |  |  |  | | | |  | | | |  |  |  |
| 2 | Atr-ERN18906 |  |  |  | | | |  | | | |  |  |  |
| 2 | Atr-ERN18907 |  |  |  | | | |  | | | |  |  |  |
| 2 | Atr-ERN18908 |  |  |  | | | |  | | | |  |  |  |
| 2 | Atr-ERN18909 |  |  |  | | | |  | | | |  |  |  |
| 2 | Atr-ERN18910 |  |  |  | | | |  | | | |  |  |  |
| 2 | Atr-ERN18911 |  |  |  | Vvi-Vitvi07g00643\_t001 |  | Vvi-Vitvi05g00790\_t002 |  |  |  |
| 2 | Atr-ERN18912 |  |  |  | Vvi-Vitvi07g00647\_t001 |  | | | |  |  |  |
| 2 | Atr-ERN18913 |  |  |  | | | |  | Vvi-Vitvi05g00795\_t001 |  |  |  |
| 2 | Atr-ERN18914 |  |  |  | Vvi-Vitvi07g00649\_t001 |  | | | |  |  |  |
| 2 | Atr-ERN18915 |  |  |  | | | |  | Vvi-Vitvi05g00796\_t001 |  |  |  |
| 2 | Atr-ERN18916 |  |  |  | | | |  | | | |  |  |  |
| 2 | Atr-ERN18917 |  |  |  | | | |  | | | |  |  |  |
| 2 | Atr-ERN18918 |  |  |  | | | |  | | | |  |  |  |
| 2 | Atr-ERN18919 |  |  |  | Vvi-Vitvi07g00650\_t001 |  | | | |  |  |  |
| 2 | Atr-ERN18920 |  |  |  | | | |  | | | |  |  |  |
| 2 | Atr-ERN18921 |  |  |  | Vvi-Vitvi07g00652\_t001 |  | Vvi-Vitvi05g00799\_t001 |  |  |  |
| 2 | Atr-ERN18922 |  |  |  | | | |  | Vvi-Vitvi05g00800\_t001 |  |  |  |
| 2 | Atr-ERN18923 |  |  |  | Vvi-Vitvi07g00653\_t001 |  | | | |  |  |  |
| 2 | Atr-ERN18924 |  |  |  | | | |  | | | |  |  |  |
| 2 | Atr-ERN18925 |  |  |  | Vvi-Vitvi07g00656\_t001 |  | Vvi-Vitvi05g00802\_t001 |  |  |  |
| 2 | Atr-ERN18926 |  |  |  | | | |  | | | |  |  |  |
| 2 | Atr-ERN18927 |  |  |  | | | |  | | | |  |  |  |
| 2 | Atr-ERN18928 |  |  |  | | | |  | | | |  |  |  |
| 2 | Atr-ERN18929 |  |  |  | | | |  | | | |  |  |  |
| 2 | Atr-ERN18930 |  |  |  | | | |  | | | |  |  |  |
| 2 | Atr-ERN18931 |  |  |  | | | |  | | | |  |  |  |
| 2 | Atr-ERN18932 |  |  |  | Vvi-Vitvi07g00658\_t002 |  | | | |  |  |  |
| 2 | Atr-ERN18933 |  |  |  | | | |  | Vvi-Vitvi05g04205\_t001 |  |  |  |
| 2 | Atr-ERN18934 |  |  |  | | | |  | | | |  |  |  |
| 2 | Atr-ERN18935 |  |  |  | | | |  | | | |  |  |  |
| 2 | Atr-ERN18936 |  |  |  | | | |  | | | |  |  |  |
| 2 | Atr-ERN18937 |  |  |  | | | |  | | | |  |  |  |
| 2 | Atr-ERN18938 |  |  |  | Vvi-Vitvi07g00659\_t001 |  | Vvi-Vitvi05g00815\_t001 |  |  |  |
| 2 | Atr-ERN18939 |  |  |  | | | |  | | | |  |  |  |
| 2 | Atr-ERN18940 |  |  |  | | | |  | | | |  |  |  |
| 2 | Atr-ERN18941 |  |  |  | | | |  | | | |  |  |  |
| 2 | Atr-ERN18942 |  |  |  | | | |  | | | |  |  |  |
| 2 | Atr-ERN18943 |  |  |  | | | |  | | | |  |  |  |
| 2 | Atr-ERN18944 |  |  |  | | | |  | | | |  |  |  |
| 2 | Atr-ERN18945 |  |  |  | | | |  | | | |  |  |  |
| 2 | Atr-ERN18946 |  |  |  | | | |  | | | |  |  |  |
| 2 | Atr-ERN18947 |  |  |  | | | |  | | | |  |  |  |
| 2 | Atr-ERN18948 |  |  |  | | | |  | | | |  |  |  |
| 2 | Atr-ERN18949 |  |  |  | Vvi-Vitvi07g02289\_t001 |  | | | |  |  |  |
| 2 | Atr-ERN18950 |  |  |  | | | |  | | | |  |  |  |
| 2 | Atr-ERN18951 |  |  |  | | | |  | | | |  |  |  |
| 2 | Atr-ERN18952 |  |  |  | | | |  | | | |  |  |  |
| 2 | Atr-ERN18953 |  |  |  | | | |  | Vvi-Vitvi05g00816\_t001 |  |  |  |
| 2 | Atr-ERN18954 |  |  |  | | | |  | | | |  |  |  |
| 2 | Atr-ERN18955 |  |  |  | | | |  | | | |  |  |  |
| 2 | Atr-ERN18956 |  |  |  | | | |  | Vvi-Vitvi05g00818\_t001 |  |  |  |
| 2 | Atr-ERN18957 |  |  |  | | | |  | | | |  |  |  |
| 2 | Atr-ERN18958 |  |  |  | | | |  | | | |  |  |  |
| 2 | Atr-ERN18959 |  |  |  | | | |  | | | |  |  |  |
| 2 | Atr-ERN18960 |  |  |  | | | |  | | | |  |  |  |
| 2 | Atr-ERN18961 |  |  |  | Vvi-Vitvi07g00667\_t001 |  | | | |  |  |  |
| 2 | Atr-ERN18962 |  |  |  | | | |  | | | |  |  |  |
| 2 | Atr-ERN18963 |  |  |  | Vvi-Vitvi07g00668\_t001 |  | | | |  |  |  |
| 2 | Atr-ERN18964 |  |  |  | Vvi-Vitvi07g04166\_t001 |  | Vvi-Vitvi05g04210\_t001 |  |  |  |
| 2 | Atr-ERN18965 |  |  |  | Vvi-Vitvi07g00674\_t001 |  | | | |  |  |  |
| 2 | Atr-ERN18966 |  |  |  | Vvi-Vitvi07g00675\_t001 |  | Vvi-Vitvi05g00821\_t001 |  |  |  |
| 0 | Atr-ERN18967 |  |  |  |  |  |  |
