# Supplementary material for: Genome-wide identification, characterization and gene expression of BES1 transcription factor family in grapevine (Vitis vinifera L.)
Source: Sci Rep. 2023 Jan 5;13:240. doi: 10.1038/s41598-022-24407-y (PMC9816167; doi:10.1038/s41598-022-24407-y)
Supplement: Supplementary file 3 — Supplementary Information. [file 41598_2022_24407_MOESM3_ESM.zip › Vvi_Atr/Vitis_vinifera.PN40024.v4.dna_sm.toplevel.fa.vs.Amborella_trichopoda.AMTR1.0.dna_sm.toplevel.fa.html/Atr-AmTr_v1.0_scaffold00068.html]

|  |  |  |  |  |  |  |  |  |  |  |  |  |  |
| --- | --- | --- | --- | --- | --- | --- | --- | --- | --- | --- | --- | --- | --- |
| Duplication depth | Reference chromosome | Collinear blocks | | | | | | | | | | | |
| 0 | Atr-ERN20367 |  |  |  |  |  |  |
| 0 | Atr-ERN20368 |  |  |  |  |  |  |
| 0 | Atr-ERN20369 |  |  |  |  |  |  |
| 0 | Atr-ERN20370 |  |  |  |  |  |  |
| 0 | Atr-ERN20371 |  |  |  |  |  |  |
| 0 | Atr-ERN20372 |  |  |  |  |  |  |
| 0 | Atr-ERN20373 |  |  |  |  |  |  |
| 0 | Atr-ERN20374 |  |  |  |  |  |  |
| 0 | Atr-ERN20375 |  |  |  |  |  |  |
| 0 | Atr-ERN20376 |  |  |  |  |  |  |
| 0 | Atr-ERN20377 |  |  |  |  |  |  |
| 0 | Atr-ERN20378 |  |  |  |  |  |  |
| 0 | Atr-ERN20379 |  |  |  |  |  |  |
| 0 | Atr-ERN20380 |  |  |  |  |  |  |
| 0 | Atr-ERN20381 |  |  |  |  |  |  |
| 0 | Atr-ERN20382 |  |  |  |  |  |  |
| 0 | Atr-ERN20383 |  |  |  |  |  |  |
| 1 | Atr-ERN20384 |  | Vvi-Vitvi08g01811\_t001 |  |  |  |  |  |
| 1 | Atr-ERN20385 |  | Vvi-Vitvi08g01810\_t001 |  |  |  |  |  |
| 1 | Atr-ERN20386 |  | | | |  |  |  |  |  |
| 1 | Atr-ERN20387 |  | | | |  |  |  |  |  |
| 1 | Atr-ERN20388 |  | | | |  |  |  |  |  |
| 2 | Atr-ERN20389 |  | Vvi-Vitvi08g02369\_t001 |  | Vvi-Vitvi06g01582\_t001 |  |  |  |  |
| 2 | Atr-ERN20390 |  | | | |  | | | |  |  |  |  |
| 2 | Atr-ERN20391 |  | | | |  | Vvi-Vitvi06g00044\_t001 |  |  |  |  |
| 2 | Atr-ERN20392 |  | | | |  | | | |  |  |  |  |
| 2 | Atr-ERN20393 |  | Vvi-Vitvi08g01808\_t002 |  | | | |  |  |  |  |
| 2 | Atr-ERN20394 |  | Vvi-Vitvi08g01807\_t001 |  | Vvi-Vitvi06g00046\_t002 |  |  |  |  |
| 2 | Atr-ERN20395 |  | | | |  | | | |  |  |  |  |
| 2 | Atr-ERN20396 |  | Vvi-Vitvi08g01805\_t001 |  | | | |  |  |  |  |
| 2 | Atr-ERN20397 |  | | | |  | | | |  |  |  |  |
| 2 | Atr-ERN20398 |  | Vvi-Vitvi08g01804\_t001 |  | | | |  |  |  |  |
| 2 | Atr-ERN20399 |  | | | |  | | | |  |  |  |  |
| 2 | Atr-ERN20400 |  | | | |  | | | |  |  |  |  |
| 2 | Atr-ERN20401 |  | | | |  | | | |  |  |  |  |
| 2 | Atr-ERN20402 |  | | | |  | | | |  |  |  |  |
| 2 | Atr-ERN20403 |  | | | |  | Vvi-Vitvi06g00049\_t001 |  |  |  |  |
| 2 | Atr-ERN20404 |  | Vvi-Vitvi08g01803\_t001 |  | | | |  |  |  |  |
| 2 | Atr-ERN20405 |  | Vvi-Vitvi08g01802\_t001 |  | | | |  |  |  |  |
| 2 | Atr-ERN20406 |  | | | |  | | | |  |  |  |  |
| 2 | Atr-ERN20407 |  | | | |  | Vvi-Vitvi06g00050\_t001 |  |  |  |  |
| 2 | Atr-ERN20408 |  | | | |  | | | |  |  |  |  |
| 2 | Atr-ERN20409 |  | | | |  | | | |  |  |  |  |
| 2 | Atr-ERN20410 |  | | | |  | | | |  |  |  |  |
| 2 | Atr-ERN20411 |  | Vvi-Vitvi08g01800\_t001 |  | Vvi-Vitvi06g00051\_t002 |  |  |  |  |
| 2 | Atr-ERN20412 |  | | | |  | | | |  |  |  |  |
| 2 | Atr-ERN20413 |  | | | |  | | | |  |  |  |  |
| 2 | Atr-ERN20414 |  | | | |  | | | |  |  |  |  |
| 2 | Atr-ERN20415 |  | | | |  | | | |  |  |  |  |
| 2 | Atr-ERN20416 |  | Vvi-Vitvi08g01965\_t001 |  | Vvi-Vitvi06g00052\_t001 |  |  |  |  |
| 2 | Atr-ERN20417 |  | | | |  | | | |  |  |  |  |
| 2 | Atr-ERN20418 |  | Vvi-Vitvi08g01798\_t001 |  | Vvi-Vitvi06g00053\_t001 |  |  |  |  |
| 2 | Atr-ERN20419 |  | | | |  | | | |  |  |  |  |
| 2 | Atr-ERN20420 |  | | | |  | | | |  |  |  |  |
| 2 | Atr-ERN20421 |  | | | |  | | | |  |  |  |  |
| 2 | Atr-ERN20422 |  | | | |  | | | |  |  |  |  |
| 2 | Atr-ERN20423 |  | | | |  | | | |  |  |  |  |
| 2 | Atr-ERN20424 |  | | | |  | | | |  |  |  |  |
| 2 | Atr-ERN20425 |  | | | |  | | | |  |  |  |  |
| 2 | Atr-ERN20426 |  | | | |  | | | |  |  |  |  |
| 2 | Atr-ERN20427 |  | | | |  | | | |  |  |  |  |
| 2 | Atr-ERN20428 |  | | | |  | | | |  |  |  |  |
| 2 | Atr-ERN20429 |  | | | |  | | | |  |  |  |  |
| 2 | Atr-ERN20430 |  | | | |  | | | |  |  |  |  |
| 2 | Atr-ERN20431 |  | | | |  | | | |  |  |  |  |
| 2 | Atr-ERN20432 |  | | | |  | | | |  |  |  |  |
| 2 | Atr-ERN20433 |  | | | |  | | | |  |  |  |  |
| 2 | Atr-ERN20434 |  | | | |  | | | |  |  |  |  |
| 2 | Atr-ERN20435 |  | | | |  | Vvi-Vitvi06g00058\_t001 |  |  |  |  |
| 2 | Atr-ERN20436 |  | | | |  | | | |  |  |  |  |
| 2 | Atr-ERN20437 |  | | | |  | | | |  |  |  |  |
| 2 | Atr-ERN20438 |  | | | |  | | | |  |  |  |  |
| 2 | Atr-ERN20439 |  | | | |  | | | |  |  |  |  |
| 2 | Atr-ERN20440 |  | Vvi-Vitvi08g01797\_t001 |  | Vvi-Vitvi06g00059\_t001 |  |  |  |  |
| 2 | Atr-ERN20441 |  | Vvi-Vitvi08g01796\_t002 |  | | | |  |  |  |  |
| 2 | Atr-ERN20442 |  | | | |  | | | |  |  |  |  |
| 2 | Atr-ERN20443 |  | | | |  | | | |  |  |  |  |
| 2 | Atr-ERN20444 |  | | | |  | | | |  |  |  |  |
| 2 | Atr-ERN20445 |  | | | |  | | | |  |  |  |  |
| 2 | Atr-ERN20446 |  | | | |  | | | |  |  |  |  |
| 2 | Atr-ERN20447 |  | | | |  | Vvi-Vitvi06g00060\_t001 |  |  |  |  |
| 2 | Atr-ERN20448 |  | | | |  | | | |  |  |  |  |
| 2 | Atr-ERN20449 |  | | | |  | | | |  |  |  |  |
| 2 | Atr-ERN20450 |  | | | |  | | | |  |  |  |  |
| 2 | Atr-ERN20451 |  | | | |  | | | |  |  |  |  |
| 2 | Atr-ERN20452 |  | | | |  | Vvi-Vitvi06g00062\_t001 |  |  |  |  |
| 2 | Atr-ERN20453 |  | | | |  | Vvi-Vitvi06g00064\_t002 |  |  |  |  |
| 2 | Atr-ERN20454 |  | | | |  | | | |  |  |  |  |
| 2 | Atr-ERN20455 |  | | | |  | | | |  |  |  |  |
| 2 | Atr-ERN20456 |  | | | |  | Vvi-Vitvi06g04016\_t001 |  |  |  |  |
| 2 | Atr-ERN20457 |  | | | |  | | | |  |  |  |  |
| 2 | Atr-ERN20458 |  | | | |  | | | |  |  |  |  |
| 2 | Atr-ERN20459 |  | | | |  | | | |  |  |  |  |
| 2 | Atr-ERN20460 |  | | | |  | | | |  |  |  |  |
| 2 | Atr-ERN20461 |  | | | |  | | | |  |  |  |  |
| 2 | Atr-ERN20462 |  | Vvi-Vitvi08g01788\_t001 |  | | | |  |  |  |  |
| 1 | Atr-ERN20463 |  |  |  | Vvi-Vitvi06g00071\_t001 |  |  |  |  |
| 0 | Atr-ERN20464 |  |  |  |  |  |  |
| 0 | Atr-ERN20465 |  |  |  |  |  |  |
| 0 | Atr-ERN20466 |  |  |  |  |  |  |
| 0 | Atr-ERN20467 |  |  |  |  |  |  |
| 0 | Atr-ERN20468 |  |  |  |  |  |  |
| 0 | Atr-ERN20469 |  |  |  |  |  |  |
| 0 | Atr-ERN20470 |  |  |  |  |  |  |
| 0 | Atr-ERN20471 |  |  |  |  |  |  |
| 0 | Atr-ERN20472 |  |  |  |  |  |  |
| 0 | Atr-ERN20473 |  |  |  |  |  |  |
| 0 | Atr-ERN20474 |  |  |  |  |  |  |
| 0 | Atr-ERN20475 |  |  |  |  |  |  |
| 0 | Atr-ERN20476 |  |  |  |  |  |  |
| 0 | Atr-ERN20477 |  |  |  |  |  |  |
| 1 | Atr-ERN20478 |  | Vvi-Vitvi19g00207\_t004 |  |  |  |  |  |
| 1 | Atr-ERN20479 |  | | | |  |  |  |  |  |
| 1 | Atr-ERN20480 |  | | | |  |  |  |  |  |
| 1 | Atr-ERN20481 |  | | | |  |  |  |  |  |
| 2 | Atr-ERN20482 |  | Vvi-Vitvi19g00208\_t001 |  | Vvi-Vitvi12g04066\_t001 |  |  |  |  |
| 2 | Atr-ERN20483 |  | Vvi-Vitvi19g00210\_t001 |  | | | |  |  |  |  |
| 2 | Atr-ERN20484 |  | | | |  | | | |  |  |  |  |
| 2 | Atr-ERN20485 |  | Vvi-Vitvi19g00217\_t001 |  | | | |  |  |  |  |
| 2 | Atr-ERN20486 |  | | | |  | | | |  |  |  |  |
| 2 | Atr-ERN20487 |  | Vvi-Vitvi19g04086\_t001 |  | | | |  |  |  |  |
| 2 | Atr-ERN20488 |  | | | |  | | | |  |  |  |  |
| 2 | Atr-ERN20489 |  | | | |  | Vvi-Vitvi12g00294\_t001 |  |  |  |  |
| 2 | Atr-ERN20490 |  | | | |  | | | |  |  |  |  |
| 2 | Atr-ERN20491 |  | | | |  | | | |  |  |  |  |
| 2 | Atr-ERN20492 |  | Vvi-Vitvi19g01869\_t001.1.6037826c |  | | | |  |  |  |  |
| 2 | Atr-ERN20493 |  | | | |  | Vvi-Vitvi12g00298\_t001 |  |  |  |  |
| 2 | Atr-ERN20494 |  | Vvi-Vitvi19g00220\_t001 |  | Vvi-Vitvi12g00299\_t002 |  |  |  |  |
| 2 | Atr-ERN20495 |  | | | |  | | | |  |  |  |  |
| 2 | Atr-ERN20496 |  | | | |  | Vvi-Vitvi12g00300\_t001 |  |  |  |  |
| 2 | Atr-ERN20497 |  | | | |  | | | |  |  |  |  |
| 2 | Atr-ERN20498 |  | | | |  | | | |  |  |  |  |
| 2 | Atr-ERN20499 |  | Vvi-Vitvi19g00221\_t001 |  | Vvi-Vitvi12g00301\_t001 |  |  |  |  |
| 2 | Atr-ERN20500 |  | | | |  | | | |  |  |  |  |
| 2 | Atr-ERN20501 |  | | | |  | | | |  |  |  |  |
| 2 | Atr-ERN20502 |  | | | |  | | | |  |  |  |  |
| 2 | Atr-ERN20503 |  | | | |  | | | |  |  |  |  |
| 2 | Atr-ERN20504 |  | | | |  | | | |  |  |  |  |
| 2 | Atr-ERN20505 |  | | | |  | | | |  |  |  |  |
| 2 | Atr-ERN20506 |  | | | |  | Vvi-Vitvi12g00303\_t004 |  |  |  |  |
| 2 | Atr-ERN20507 |  | Vvi-Vitvi19g01871\_t001 |  | | | |  |  |  |  |
| 2 | Atr-ERN20508 |  | Vvi-Vitvi19g00227\_t001 |  | | | |  |  |  |  |
| 2 | Atr-ERN20509 |  | | | |  | | | |  |  |  |  |
| 2 | Atr-ERN20510 |  | Vvi-Vitvi19g00228\_t001 |  | Vvi-Vitvi12g00304\_t001 |  |  |  |  |
| 1 | Atr-ERN20511 |  |  |  | | | |  |  |  |  |
| 1 | Atr-ERN20512 |  |  |  | | | |  |  |  |  |
| 1 | Atr-ERN20513 |  |  |  | Vvi-Vitvi12g00305\_t001 |  |  |  |  |
| 1 | Atr-ERN20514 |  |  |  | Vvi-Vitvi12g00306\_t001 |  |  |  |  |
| 0 | Atr-ERN20515 |  |  |  |  |  |  |
| 0 | Atr-ERN20516 |  |  |  |  |  |  |
| 0 | Atr-ERN20517 |  |  |  |  |  |  |
| 0 | Atr-ERN20518 |  |  |  |  |  |  |
| 0 | Atr-ERN20519 |  |  |  |  |  |  |
| 0 | Atr-ERN20520 |  |  |  |  |  |  |
| 0 | Atr-ERN20521 |  |  |  |  |  |  |
| 0 | Atr-ERN20522 |  |  |  |  |  |  |
| 0 | Atr-ERN20523 |  |  |  |  |  |  |
| 0 | Atr-ERN20524 |  |  |  |  |  |  |
| 0 | Atr-ERN20525 |  |  |  |  |  |  |
| 0 | Atr-ERN20526 |  |  |  |  |  |  |
| 0 | Atr-ERN20527 |  |  |  |  |  |  |
| 0 | Atr-ERN20528 |  |  |  |  |  |  |
| 0 | Atr-ERN20529 |  |  |  |  |  |  |
| 0 | Atr-ERN20530 |  |  |  |  |  |  |
| 0 | Atr-ERN20531 |  |  |  |  |  |  |
| 0 | Atr-ERN20532 |  |  |  |  |  |  |
| 0 | Atr-ERN20533 |  |  |  |  |  |  |
| 0 | Atr-ERN20534 |  |  |  |  |  |  |
| 0 | Atr-ERN20535 |  |  |  |  |  |  |
| 0 | Atr-ERN20536 |  |  |  |  |  |  |
| 0 | Atr-ERN20537 |  |  |  |  |  |  |
| 0 | Atr-ERN20538 |  |  |  |  |  |  |
| 0 | Atr-ERN20539 |  |  |  |  |  |  |
| 0 | Atr-ERN20540 |  |  |  |  |  |  |
| 0 | Atr-ERN20541 |  |  |  |  |  |  |
| 0 | Atr-ERN20542 |  |  |  |  |  |  |
| 0 | Atr-ERN20543 |  |  |  |  |  |  |
| 0 | Atr-ERN20544 |  |  |  |  |  |  |
| 0 | Atr-ERN20545 |  |  |  |  |  |  |
| 0 | Atr-ERN20546 |  |  |  |  |  |  |
| 0 | Atr-ERN20547 |  |  |  |  |  |  |
| 0 | Atr-ERN20548 |  |  |  |  |  |  |
| 0 | Atr-ERN20549 |  |  |  |  |  |  |
| 0 | Atr-ERN20550 |  |  |  |  |  |  |
| 0 | Atr-ERN20551 |  |  |  |  |  |  |
| 0 | Atr-ERN20552 |  |  |  |  |  |  |
| 0 | Atr-ERN20553 |  |  |  |  |  |  |
