# Supplementary material for: Genome-wide identification, characterization and gene expression of BES1 transcription factor family in grapevine (Vitis vinifera L.)
Source: Sci Rep. 2023 Jan 5;13:240. doi: 10.1038/s41598-022-24407-y (PMC9816167; doi:10.1038/s41598-022-24407-y)
Supplement: Supplementary file 3 — Supplementary Information. [file 41598_2022_24407_MOESM3_ESM.zip › Vvi_Atr/Vitis_vinifera.PN40024.v4.dna_sm.toplevel.fa.vs.Amborella_trichopoda.AMTR1.0.dna_sm.toplevel.fa.html/Atr-AmTr_v1.0_scaffold00069.html]

|  |  |  |  |  |  |  |  |  |  |  |  |  |  |
| --- | --- | --- | --- | --- | --- | --- | --- | --- | --- | --- | --- | --- | --- |
| Duplication depth | Reference chromosome | Collinear blocks | | | | | | | | | | | |
| 0 | Atr-ERN19278 |  |  |  |  |  |  |
| 0 | Atr-ERN19279 |  |  |  |  |  |  |
| 0 | Atr-ERN19280 |  |  |  |  |  |  |
| 0 | Atr-ERN19281 |  |  |  |  |  |  |
| 0 | Atr-ERN19282 |  |  |  |  |  |  |
| 0 | Atr-ERN19283 |  |  |  |  |  |  |
| 0 | Atr-ERN19284 |  |  |  |  |  |  |
| 0 | Atr-ERN19285 |  |  |  |  |  |  |
| 0 | Atr-ERN19286 |  |  |  |  |  |  |
| 0 | Atr-ERN19287 |  |  |  |  |  |  |
| 0 | Atr-ERN19288 |  |  |  |  |  |  |
| 0 | Atr-ERN19289 |  |  |  |  |  |  |
| 0 | Atr-ERN19290 |  |  |  |  |  |  |
| 0 | Atr-ERN19291 |  |  |  |  |  |  |
| 0 | Atr-ERN19292 |  |  |  |  |  |  |
| 0 | Atr-ERN19293 |  |  |  |  |  |  |
| 0 | Atr-ERN19294 |  |  |  |  |  |  |
| 0 | Atr-ERN19295 |  |  |  |  |  |  |
| 1 | Atr-ERN19296 |  | Vvi-Vitvi15g00932\_t001 |  |  |  |  |  |
| 1 | Atr-ERN19297 |  | Vvi-Vitvi15g00933\_t001 |  |  |  |  |  |
| 1 | Atr-ERN19298 |  | | | |  |  |  |  |  |
| 1 | Atr-ERN19299 |  | | | |  |  |  |  |  |
| 1 | Atr-ERN19300 |  | | | |  |  |  |  |  |
| 1 | Atr-ERN19301 |  | | | |  |  |  |  |  |
| 1 | Atr-ERN19302 |  | | | |  |  |  |  |  |
| 1 | Atr-ERN19303 |  | Vvi-Vitvi15g00934\_t001 |  |  |  |  |  |
| 1 | Atr-ERN19304 |  | | | |  |  |  |  |  |
| 1 | Atr-ERN19305 |  | | | |  |  |  |  |  |
| 1 | Atr-ERN19306 |  | | | |  |  |  |  |  |
| 1 | Atr-ERN19307 |  | Vvi-Vitvi15g00935\_t001 |  |  |  |  |  |
| 1 | Atr-ERN19308 |  | | | |  |  |  |  |  |
| 1 | Atr-ERN19309 |  | | | |  |  |  |  |  |
| 1 | Atr-ERN19310 |  | Vvi-Vitvi15g00937\_t001 |  |  |  |  |  |
| 1 | Atr-ERN19311 |  | | | |  |  |  |  |  |
| 1 | Atr-ERN19312 |  | | | |  |  |  |  |  |
| 1 | Atr-ERN19313 |  | | | |  |  |  |  |  |
| 1 | Atr-ERN19314 |  | | | |  |  |  |  |  |
| 2 | Atr-ERN19315 |  | | | |  | Vvi-Vitvi02g00383\_t001 |  |  |  |  |
| 2 | Atr-ERN19316 |  | | | |  | Vvi-Vitvi02g00384\_t001 |  |  |  |  |
| 2 | Atr-ERN19317 |  | | | |  | Vvi-Vitvi02g00385\_t001 |  |  |  |  |
| 2 | Atr-ERN19318 |  | | | |  | Vvi-Vitvi02g00387\_t001 |  |  |  |  |
| 3 | Atr-ERN19319 |  | | | |  | | | |  | Vvi-Vitvi16g00984\_t001 |  |  |  |
| 3 | Atr-ERN19320 |  | | | |  | | | |  | | | |  |  |  |
| 3 | Atr-ERN19321 |  | | | |  | | | |  | | | |  |  |  |
| 3 | Atr-ERN19322 |  | | | |  | Vvi-Vitvi02g00388\_t001 |  | | | |  |  |  |
| 4 | Atr-ERN19323 |  | | | |  | | | |  | | | |  | Vvi-Vitvi15g00846\_t001 |  |  |
| 4 | Atr-ERN19324 |  | | | |  | | | |  | | | |  | | | |  |  |
| 4 | Atr-ERN19325 |  | | | |  | | | |  | | | |  | | | |  |  |
| 5 | Atr-ERN19326 |  | | | |  | Vvi-Vitvi02g00390\_t001 |  | | | |  | | | |  | Vvi-Vitvi02g00390\_t001 |  |
| 4 | Atr-ERN19327 |  | | | |  |  |  | | | |  | | | |  | | | |  |
| 4 | Atr-ERN19328 |  | | | |  |  |  | | | |  | | | |  | | | |  |
| 4 | Atr-ERN19329 |  | | | |  |  |  | | | |  | | | |  | | | |  |
| 4 | Atr-ERN19330 |  | | | |  |  |  | | | |  | | | |  | | | |  |
| 4 | Atr-ERN19331 |  | Vvi-Vitvi15g00941\_t001 |  |  |  | | | |  | | | |  | | | |  |
| 3 | Atr-ERN19332 |  |  |  |  |  | | | |  | | | |  | | | |  |
| 3 | Atr-ERN19333 |  |  |  |  |  | | | |  | Vvi-Vitvi15g00839\_t001 |  | | | |  |
| 3 | Atr-ERN19334 |  |  |  |  |  | Vvi-Vitvi16g00975\_t001 |  | | | |  | | | |  |
| 3 | Atr-ERN19335 |  |  |  |  |  | | | |  | | | |  | | | |  |
| 3 | Atr-ERN19336 |  |  |  |  |  | Vvi-Vitvi16g00973\_t001 |  | | | |  | | | |  |
| 3 | Atr-ERN19337 |  |  |  |  |  | | | |  | | | |  | | | |  |
| 3 | Atr-ERN19338 |  |  |  |  |  | Vvi-Vitvi16g00972\_t001 |  | | | |  | Vvi-Vitvi02g00382\_t001 |  |
| 3 | Atr-ERN19339 |  |  |  |  |  | | | |  | Vvi-Vitvi15g00838\_t003 |  | | | |  |
| 3 | Atr-ERN19340 |  |  |  |  |  | | | |  | | | |  | Vvi-Vitvi02g00381\_t001 |  |
| 3 | Atr-ERN19341 |  |  |  |  |  | Vvi-Vitvi16g00971\_t001 |  | | | |  | | | |  |
| 3 | Atr-ERN19342 |  |  |  |  |  | | | |  | | | |  | Vvi-Vitvi02g00380\_t001 |  |
| 3 | Atr-ERN19343 |  |  |  |  |  | | | |  | Vvi-Vitvi15g00837\_t001 |  | | | |  |
| 3 | Atr-ERN19344 |  |  |  |  |  | | | |  | | | |  | | | |  |
| 3 | Atr-ERN19345 |  |  |  |  |  | | | |  | | | |  | Vvi-Vitvi02g00378\_t001 |  |
| 3 | Atr-ERN19346 |  |  |  |  |  | | | |  | | | |  | | | |  |
| 3 | Atr-ERN19347 |  |  |  |  |  | Vvi-Vitvi16g00966\_t001 |  | Vvi-Vitvi15g00835\_t001 |  | Vvi-Vitvi02g00377\_t001 |  |
| 3 | Atr-ERN19348 |  |  |  |  |  | | | |  | | | |  | Vvi-Vitvi02g00373\_t001 |  |
| 3 | Atr-ERN19349 |  |  |  |  |  | Vvi-Vitvi16g01864\_t001 |  | | | |  | | | |  |
| 3 | Atr-ERN19350 |  |  |  |  |  | Vvi-Vitvi16g00964\_t001 |  | Vvi-Vitvi15g00833\_t001 |  | | | |  |
| 3 | Atr-ERN19351 |  |  |  |  |  | | | |  | | | |  | | | |  |
| 3 | Atr-ERN19352 |  |  |  |  |  | | | |  | | | |  | Vvi-Vitvi02g00370\_t001 |  |
| 2 | Atr-ERN19353 |  |  |  |  |  | Vvi-Vitvi16g00959\_t002 |  | | | |  |  |
| 2 | Atr-ERN19354 |  |  |  |  |  | | | |  | | | |  |  |
| 3 | Atr-ERN19355 |  | Vvi-Vitvi02g00398\_t001 |  |  |  | Vvi-Vitvi16g00957\_t001 |  | Vvi-Vitvi15g00832\_t001 |  |  |
| 3 | Atr-ERN19356 |  | | | |  |  |  | Vvi-Vitvi16g01861\_t001 |  | Vvi-Vitvi15g00831\_t002 |  |  |
| 3 | Atr-ERN19357 |  | Vvi-Vitvi02g00399\_t001 |  |  |  | Vvi-Vitvi16g00956\_t001 |  | Vvi-Vitvi15g00830\_t001 |  |  |
| 3 | Atr-ERN19358 |  | Vvi-Vitvi02g04094\_t001 |  |  |  | | | |  | | | |  |  |
| 3 | Atr-ERN19359 |  | | | |  |  |  | | | |  | | | |  |  |
| 3 | Atr-ERN19360 |  | Vvi-Vitvi02g00402\_t001 |  |  |  | | | |  | | | |  |  |
| 3 | Atr-ERN19361 |  | | | |  |  |  | Vvi-Vitvi16g00955\_t001 |  | | | |  |  |
| 3 | Atr-ERN19362 |  | | | |  |  |  | Vvi-Vitvi16g00953\_t001 |  | | | |  |  |
| 3 | Atr-ERN19363 |  | | | |  |  |  | | | |  | | | |  |  |
| 3 | Atr-ERN19364 |  | | | |  |  |  | Vvi-Vitvi16g00952\_t001 |  | | | |  |  |
| 3 | Atr-ERN19365 |  | | | |  |  |  | | | |  | | | |  |  |
| 3 | Atr-ERN19366 |  | | | |  |  |  | | | |  | | | |  |  |
| 3 | Atr-ERN19367 |  | Vvi-Vitvi02g00403\_t001 |  |  |  | Vvi-Vitvi16g00951\_t001 |  | | | |  |  |
| 3 | Atr-ERN19368 |  | | | |  |  |  | | | |  | | | |  |  |
| 3 | Atr-ERN19369 |  | | | |  |  |  | | | |  | | | |  |  |
| 3 | Atr-ERN19370 |  | | | |  |  |  | | | |  | Vvi-Vitvi15g00828\_t008 |  |  |
| 3 | Atr-ERN19371 |  | | | |  |  |  | Vvi-Vitvi16g00949\_t004 |  | Vvi-Vitvi15g00827\_t001 |  |  |
| 3 | Atr-ERN19372 |  | | | |  |  |  | | | |  | Vvi-Vitvi15g00826\_t001 |  |  |
| 3 | Atr-ERN19373 |  | | | |  |  |  | | | |  | Vvi-Vitvi15g00825\_t001.2.6037826c |  |  |
| 3 | Atr-ERN19374 |  | | | |  |  |  | | | |  | | | |  |  |
| 3 | Atr-ERN19375 |  | | | |  |  |  | | | |  | | | |  |  |
| 3 | Atr-ERN19376 |  | | | |  |  |  | | | |  | Vvi-Vitvi15g00821\_t001 |  |  |
| 3 | Atr-ERN19377 |  | | | |  |  |  | | | |  | | | |  |  |
| 3 | Atr-ERN19378 |  | | | |  |  |  | | | |  | Vvi-Vitvi15g00820\_t001 |  |  |
| 2 | Atr-ERN19379 |  | | | |  |  |  | Vvi-Vitvi16g00944\_t001 |  |  |  |
| 2 | Atr-ERN19380 |  | | | |  |  |  | | | |  |  |  |
| 2 | Atr-ERN19381 |  | | | |  |  |  | | | |  |  |  |
| 2 | Atr-ERN19382 |  | Vvi-Vitvi02g04095\_t001 |  |  |  | Vvi-Vitvi16g00942\_t001 |  |  |  |
| 2 | Atr-ERN19383 |  | Vvi-Vitvi02g00407\_t001 |  |  |  | Vvi-Vitvi16g04340\_t001 |  |  |  |
| 3 | Atr-ERN19384 |  | | | |  | Vvi-Vitvi15g00752\_t001 |  | | | |  |  |  |
| 3 | Atr-ERN19385 |  | | | |  | | | |  | | | |  |  |  |
| 3 | Atr-ERN19386 |  | | | |  | Vvi-Vitvi15g00754\_t001 |  | | | |  |  |  |
| 3 | Atr-ERN19387 |  | | | |  | | | |  | | | |  |  |  |
| 3 | Atr-ERN19388 |  | Vvi-Vitvi02g00409\_t001 |  | | | |  | | | |  |  |  |
| 3 | Atr-ERN19389 |  | | | |  | | | |  | Vvi-Vitvi16g00937\_t001 |  |  |  |
| 3 | Atr-ERN19390 |  | | | |  | | | |  | | | |  |  |  |
| 3 | Atr-ERN19391 |  | | | |  | | | |  | Vvi-Vitvi16g00936\_t003 |  |  |  |
| 3 | Atr-ERN19392 |  | Vvi-Vitvi02g00411\_t001 |  | | | |  | | | |  |  |  |
| 3 | Atr-ERN19393 |  | | | |  | | | |  | Vvi-Vitvi16g00934\_t001 |  |  |  |
| 3 | Atr-ERN19394 |  | | | |  | | | |  | | | |  |  |  |
| 3 | Atr-ERN19395 |  | Vvi-Vitvi02g00413\_t002 |  | | | |  | | | |  |  |  |
| 3 | Atr-ERN19396 |  | Vvi-Vitvi02g00414\_t001 |  | | | |  | | | |  |  |  |
| 3 | Atr-ERN19397 |  | | | |  | | | |  | | | |  |  |  |
| 3 | Atr-ERN19398 |  | | | |  | | | |  | | | |  |  |  |
| 3 | Atr-ERN19399 |  | | | |  | | | |  | | | |  |  |  |
| 3 | Atr-ERN19400 |  | | | |  | | | |  | Vvi-Vitvi16g01857\_t006 |  |  |  |
| 3 | Atr-ERN19401 |  | | | |  | | | |  | | | |  |  |  |
| 3 | Atr-ERN19402 |  | | | |  | | | |  | | | |  |  |  |
| 3 | Atr-ERN19403 |  | | | |  | | | |  | | | |  |  |  |
| 3 | Atr-ERN19404 |  | | | |  | | | |  | | | |  |  |  |
| 3 | Atr-ERN19405 |  | | | |  | Vvi-Vitvi15g01505\_t001 |  | Vvi-Vitvi16g00926\_t001 |  |  |  |
| 3 | Atr-ERN19406 |  | | | |  | | | |  | | | |  |  |  |
| 3 | Atr-ERN19407 |  | | | |  | | | |  | | | |  |  |  |
| 3 | Atr-ERN19408 |  | | | |  | | | |  | | | |  |  |  |
| 3 | Atr-ERN19409 |  | | | |  | | | |  | | | |  |  |  |
| 3 | Atr-ERN19410 |  | | | |  | | | |  | | | |  |  |  |
| 3 | Atr-ERN19411 |  | Vvi-Vitvi02g00415\_t001 |  | Vvi-Vitvi15g00759\_t001 |  | | | |  |  |  |
| 3 | Atr-ERN19412 |  | Vvi-Vitvi02g00416\_t001 |  | | | |  | Vvi-Vitvi16g01854\_t001 |  |  |  |
| 3 | Atr-ERN19413 |  | | | |  | Vvi-Vitvi15g00760\_t001 |  | | | |  |  |  |
| 3 | Atr-ERN19414 |  | | | |  | | | |  | | | |  |  |  |
| 3 | Atr-ERN19415 |  | | | |  | | | |  | | | |  |  |  |
| 3 | Atr-ERN19416 |  | | | |  | | | |  | | | |  |  |  |
| 3 | Atr-ERN19417 |  | | | |  | | | |  | | | |  |  |  |
| 3 | Atr-ERN19418 |  | Vvi-Vitvi02g00419\_t001 |  | | | |  | Vvi-Vitvi16g00922\_t001 |  |  |  |
| 3 | Atr-ERN19419 |  | | | |  | Vvi-Vitvi15g00761\_t001 |  | | | |  |  |  |
| 3 | Atr-ERN19420 |  | | | |  | | | |  | | | |  |  |  |
| 3 | Atr-ERN19421 |  | Vvi-Vitvi02g00420\_t002 |  | | | |  | Vvi-Vitvi16g00919\_t001 |  |  |  |
| 3 | Atr-ERN19422 |  | | | |  | Vvi-Vitvi15g00764\_t001 |  | | | |  |  |  |
| 3 | Atr-ERN19423 |  | | | |  | | | |  | | | |  |  |  |
| 3 | Atr-ERN19424 |  | Vvi-Vitvi02g00421\_t001 |  | Vvi-Vitvi15g00765\_t001 |  | Vvi-Vitvi16g01846\_t001 |  |  |  |
| 3 | Atr-ERN19425 |  | | | |  | | | |  | | | |  |  |  |
| 3 | Atr-ERN19426 |  | | | |  | | | |  | | | |  |  |  |
| 3 | Atr-ERN19427 |  | | | |  | | | |  | | | |  |  |  |
| 3 | Atr-ERN19428 |  | Vvi-Vitvi02g00423\_t001 |  | | | |  | | | |  |  |  |
| 3 | Atr-ERN19429 |  | | | |  | | | |  | Vvi-Vitvi16g01844\_t001 |  |  |  |
| 3 | Atr-ERN19430 |  | | | |  | | | |  | | | |  |  |  |
| 3 | Atr-ERN19431 |  | Vvi-Vitvi02g00424\_t001 |  | Vvi-Vitvi15g00766\_t001 |  | | | |  |  |  |
| 3 | Atr-ERN19432 |  | | | |  | | | |  | | | |  |  |  |
| 3 | Atr-ERN19433 |  | | | |  | Vvi-Vitvi15g00770\_t001 |  | Vvi-Vitvi16g00899\_t001 |  |  |  |
| 2 | Atr-ERN19434 |  | | | |  |  |  | | | |  |  |  |
| 2 | Atr-ERN19435 |  | | | |  |  |  | | | |  |  |  |
| 2 | Atr-ERN19436 |  | | | |  |  |  | | | |  |  |  |
| 2 | Atr-ERN19437 |  | | | |  |  |  | | | |  |  |  |
| 2 | Atr-ERN19438 |  | | | |  |  |  | | | |  |  |  |
| 2 | Atr-ERN19439 |  | | | |  |  |  | | | |  |  |  |
| 2 | Atr-ERN19440 |  | | | |  |  |  | | | |  |  |  |
| 2 | Atr-ERN19441 |  | | | |  |  |  | | | |  |  |  |
| 2 | Atr-ERN19442 |  | | | |  |  |  | | | |  |  |  |
| 2 | Atr-ERN19443 |  | | | |  |  |  | | | |  |  |  |
| 2 | Atr-ERN19444 |  | Vvi-Vitvi02g00442\_t001 |  |  |  | | | |  |  |  |
| 2 | Atr-ERN19445 |  | | | |  |  |  | | | |  |  |  |
| 2 | Atr-ERN19446 |  | | | |  |  |  | | | |  |  |  |
| 2 | Atr-ERN19447 |  | | | |  |  |  | | | |  |  |  |
| 2 | Atr-ERN19448 |  | | | |  |  |  | | | |  |  |  |
| 2 | Atr-ERN19449 |  | | | |  |  |  | | | |  |  |  |
| 2 | Atr-ERN19450 |  | | | |  |  |  | | | |  |  |  |
| 2 | Atr-ERN19451 |  | | | |  |  |  | | | |  |  |  |
| 2 | Atr-ERN19452 |  | | | |  |  |  | Vvi-Vitvi16g00878\_t001 |  |  |  |
| 2 | Atr-ERN19453 |  | Vvi-Vitvi02g00444\_t001 |  |  |  | | | |  |  |  |
| 2 | Atr-ERN19454 |  | | | |  |  |  | | | |  |  |  |
| 2 | Atr-ERN19455 |  | | | |  |  |  | Vvi-Vitvi16g00877\_t001 |  |  |  |
| 2 | Atr-ERN19456 |  | | | |  |  |  | | | |  |  |  |
| 2 | Atr-ERN19457 |  | | | |  |  |  | Vvi-Vitvi16g00875\_t001 |  |  |  |
| 2 | Atr-ERN19458 |  | | | |  |  |  | | | |  |  |  |
| 3 | Atr-ERN19459 |  | | | |  | Vvi-Vitvi15g00810\_t001 |  | | | |  |  |  |
| 3 | Atr-ERN19460 |  | | | |  | | | |  | | | |  |  |  |
| 3 | Atr-ERN19461 |  | | | |  | | | |  | | | |  |  |  |
| 3 | Atr-ERN19462 |  | | | |  | Vvi-Vitvi15g04449\_t001 |  | | | |  |  |  |
| 3 | Atr-ERN19463 |  | | | |  | Vvi-Vitvi15g00812\_t001 |  | | | |  |  |  |
| 3 | Atr-ERN19464 |  | | | |  | | | |  | | | |  |  |  |
| 3 | Atr-ERN19465 |  | | | |  | Vvi-Vitvi15g00813\_t003 |  | | | |  |  |  |
| 3 | Atr-ERN19466 |  | | | |  | | | |  | Vvi-Vitvi16g00870\_t001 |  |  |  |
| 3 | Atr-ERN19467 |  | | | |  | | | |  | | | |  |  |  |
| 3 | Atr-ERN19468 |  | | | |  | | | |  | | | |  |  |  |
| 3 | Atr-ERN19469 |  | | | |  | | | |  | | | |  |  |  |
| 3 | Atr-ERN19470 |  | | | |  | | | |  | | | |  |  |  |
| 3 | Atr-ERN19471 |  | | | |  | Vvi-Vitvi15g00814\_t001 |  | | | |  |  |  |
| 3 | Atr-ERN19472 |  | Vvi-Vitvi02g00448\_t001 |  | | | |  | | | |  |  |  |
| 3 | Atr-ERN19473 |  | | | |  | | | |  | Vvi-Vitvi16g00869\_t001 |  |  |  |
| 3 | Atr-ERN19474 |  | | | |  | | | |  | | | |  |  |  |
| 3 | Atr-ERN19475 |  | | | |  | | | |  | | | |  |  |  |
| 3 | Atr-ERN19476 |  | | | |  | | | |  | | | |  |  |  |
| 3 | Atr-ERN19477 |  | | | |  | | | |  | | | |  |  |  |
| 3 | Atr-ERN19478 |  | | | |  | | | |  | | | |  |  |  |
| 3 | Atr-ERN19479 |  | | | |  | | | |  | | | |  |  |  |
| 3 | Atr-ERN19480 |  | | | |  | | | |  | Vvi-Vitvi16g00864\_t001 |  |  |  |
| 3 | Atr-ERN19481 |  | | | |  | | | |  | Vvi-Vitvi16g00863\_t001 |  |  |  |
| 3 | Atr-ERN19482 |  | | | |  | | | |  | | | |  |  |  |
| 3 | Atr-ERN19483 |  | | | |  | Vvi-Vitvi15g00816\_t001 |  | | | |  |  |  |
| 3 | Atr-ERN19484 |  | | | |  | | | |  | Vvi-Vitvi16g00860\_t003 |  |  |  |
| 3 | Atr-ERN19485 |  | | | |  | | | |  | | | |  |  |  |
| 3 | Atr-ERN19486 |  | Vvi-Vitvi02g00449\_t001 |  | | | |  | | | |  |  |  |
| 3 | Atr-ERN19487 |  | | | |  | Vvi-Vitvi15g00817\_t001 |  | | | |  |  |  |
| 3 | Atr-ERN19488 |  | | | |  | | | |  | | | |  |  |  |
| 3 | Atr-ERN19489 |  | Vvi-Vitvi02g00450\_t001 |  | | | |  | | | |  |  |  |
| 3 | Atr-ERN19490 |  | | | |  | | | |  | | | |  |  |  |
| 3 | Atr-ERN19491 |  | | | |  | | | |  | Vvi-Vitvi16g00856\_t001 |  |  |  |
| 3 | Atr-ERN19492 |  | Vvi-Vitvi02g00451\_t001 |  | | | |  | | | |  |  |  |
| 3 | Atr-ERN19493 |  | Vvi-Vitvi02g00454\_t001 |  | | | |  | Vvi-Vitvi16g00855\_t001 |  |  |  |
| 3 | Atr-ERN19494 |  | Vvi-Vitvi02g04117\_t001 |  | | | |  | Vvi-Vitvi16g00853\_t001 |  |  |  |
| 2 | Atr-ERN19495 |  | | | |  | | | |  |  |  |  |
| 2 | Atr-ERN19496 |  | Vvi-Vitvi02g04118\_t001 |  | | | |  |  |  |  |
| 2 | Atr-ERN19497 |  | Vvi-Vitvi02g04119\_t001 |  | Vvi-Vitvi15g00818\_t001 |  |  |  |  |
| 2 | Atr-ERN19498 |  | | | |  | | | |  |  |  |  |
| 2 | Atr-ERN19499 |  | | | |  | Vvi-Vitvi15g00819\_t001 |  |  |  |  |
| 1 | Atr-ERN19500 |  | | | |  |  |  |  |  |
| 1 | Atr-ERN19501 |  | Vvi-Vitvi02g04120\_t001 |  |  |  |  |  |
