# Supplementary material for: Genome-wide identification, characterization and gene expression of BES1 transcription factor family in grapevine (Vitis vinifera L.)
Source: Sci Rep. 2023 Jan 5;13:240. doi: 10.1038/s41598-022-24407-y (PMC9816167; doi:10.1038/s41598-022-24407-y)
Supplement: Supplementary file 3 — Supplementary Information. [file 41598_2022_24407_MOESM3_ESM.zip › Vvi_Atr/Vitis_vinifera.PN40024.v4.dna_sm.toplevel.fa.vs.Amborella_trichopoda.AMTR1.0.dna_sm.toplevel.fa.html/Atr-AmTr_v1.0_scaffold00070.html]

|  |  |  |  |  |  |  |  |  |  |  |  |  |  |
| --- | --- | --- | --- | --- | --- | --- | --- | --- | --- | --- | --- | --- | --- |
| Duplication depth | Reference chromosome | Collinear blocks | | | | | | | | | | | |
| 0 | Atr-ERN20554 |  |  |  |  |  |  |
| 0 | Atr-ERN20555 |  |  |  |  |  |  |
| 0 | Atr-ERN20556 |  |  |  |  |  |  |
| 0 | Atr-ERN20557 |  |  |  |  |  |  |
| 0 | Atr-ERN20558 |  |  |  |  |  |  |
| 0 | Atr-ERN20559 |  |  |  |  |  |  |
| 0 | Atr-ERN20560 |  |  |  |  |  |  |
| 0 | Atr-ERN20561 |  |  |  |  |  |  |
| 0 | Atr-ERN20562 |  |  |  |  |  |  |
| 0 | Atr-ERN20563 |  |  |  |  |  |  |
| 0 | Atr-ERN20564 |  |  |  |  |  |  |
| 0 | Atr-ERN20565 |  |  |  |  |  |  |
| 0 | Atr-ERN20566 |  |  |  |  |  |  |
| 0 | Atr-ERN20567 |  |  |  |  |  |  |
| 0 | Atr-ERN20568 |  |  |  |  |  |  |
| 0 | Atr-ERN20569 |  |  |  |  |  |  |
| 0 | Atr-ERN20570 |  |  |  |  |  |  |
| 0 | Atr-ERN20571 |  |  |  |  |  |  |
| 0 | Atr-ERN20572 |  |  |  |  |  |  |
| 0 | Atr-ERN20573 |  |  |  |  |  |  |
| 0 | Atr-ERN20574 |  |  |  |  |  |  |
| 0 | Atr-ERN20575 |  |  |  |  |  |  |
| 0 | Atr-ERN20576 |  |  |  |  |  |  |
| 0 | Atr-ERN20577 |  |  |  |  |  |  |
| 0 | Atr-ERN20578 |  |  |  |  |  |  |
| 0 | Atr-ERN20579 |  |  |  |  |  |  |
| 0 | Atr-ERN20580 |  |  |  |  |  |  |
| 0 | Atr-ERN20581 |  |  |  |  |  |  |
| 0 | Atr-ERN20582 |  |  |  |  |  |  |
| 0 | Atr-ERN20583 |  |  |  |  |  |  |
| 0 | Atr-ERN20584 |  |  |  |  |  |  |
| 0 | Atr-ERN20585 |  |  |  |  |  |  |
| 0 | Atr-ERN20586 |  |  |  |  |  |  |
| 0 | Atr-ERN20587 |  |  |  |  |  |  |
| 0 | Atr-ERN20588 |  |  |  |  |  |  |
| 0 | Atr-ERN20589 |  |  |  |  |  |  |
| 0 | Atr-ERN20590 |  |  |  |  |  |  |
| 0 | Atr-ERN20591 |  |  |  |  |  |  |
| 0 | Atr-ERN20592 |  |  |  |  |  |  |
| 0 | Atr-ERN20593 |  |  |  |  |  |  |
| 0 | Atr-ERN20594 |  |  |  |  |  |  |
| 0 | Atr-ERN20595 |  |  |  |  |  |  |
| 0 | Atr-ERN20596 |  |  |  |  |  |  |
| 0 | Atr-ERN20597 |  |  |  |  |  |  |
| 0 | Atr-ERN20598 |  |  |  |  |  |  |
| 0 | Atr-ERN20599 |  |  |  |  |  |  |
| 0 | Atr-ERN20600 |  |  |  |  |  |  |
| 0 | Atr-ERN20601 |  |  |  |  |  |  |
| 0 | Atr-ERN20602 |  |  |  |  |  |  |
| 0 | Atr-ERN20603 |  |  |  |  |  |  |
| 0 | Atr-ERN20604 |  |  |  |  |  |  |
| 0 | Atr-ERN20605 |  |  |  |  |  |  |
| 0 | Atr-ERN20606 |  |  |  |  |  |  |
| 0 | Atr-ERN20607 |  |  |  |  |  |  |
| 0 | Atr-ERN20608 |  |  |  |  |  |  |
| 0 | Atr-ERN20609 |  |  |  |  |  |  |
| 0 | Atr-ERN20610 |  |  |  |  |  |  |
| 0 | Atr-ERN20611 |  |  |  |  |  |  |
| 0 | Atr-ERN20612 |  |  |  |  |  |  |
| 0 | Atr-ERN20613 |  |  |  |  |  |  |
| 0 | Atr-ERN20614 |  |  |  |  |  |  |
| 0 | Atr-ERN20615 |  |  |  |  |  |  |
| 0 | Atr-ERN20616 |  |  |  |  |  |  |
| 0 | Atr-ERN20617 |  |  |  |  |  |  |
| 0 | Atr-ERN20618 |  |  |  |  |  |  |
| 0 | Atr-ERN20619 |  |  |  |  |  |  |
| 0 | Atr-ERN20620 |  |  |  |  |  |  |
| 0 | Atr-ERN20621 |  |  |  |  |  |  |
| 0 | Atr-ERN20622 |  |  |  |  |  |  |
| 0 | Atr-ERN20623 |  |  |  |  |  |  |
| 0 | Atr-ERN20624 |  |  |  |  |  |  |
| 0 | Atr-ERN20625 |  |  |  |  |  |  |
| 0 | Atr-ERN20626 |  |  |  |  |  |  |
| 0 | Atr-ERN20627 |  |  |  |  |  |  |
| 0 | Atr-ERN20628 |  |  |  |  |  |  |
| 0 | Atr-ERN20629 |  |  |  |  |  |  |
| 0 | Atr-ERN20630 |  |  |  |  |  |  |
| 0 | Atr-ERN20631 |  |  |  |  |  |  |
| 0 | Atr-ERN20632 |  |  |  |  |  |  |
| 0 | Atr-ERN20633 |  |  |  |  |  |  |
| 0 | Atr-ERN20634 |  |  |  |  |  |  |
| 0 | Atr-ERN20635 |  |  |  |  |  |  |
| 0 | Atr-ERN20636 |  |  |  |  |  |  |
| 0 | Atr-ERN20637 |  |  |  |  |  |  |
| 0 | Atr-ERN20638 |  |  |  |  |  |  |
| 0 | Atr-ERN20639 |  |  |  |  |  |  |
| 0 | Atr-ERN20640 |  |  |  |  |  |  |
| 0 | Atr-ERN20641 |  |  |  |  |  |  |
| 0 | Atr-ERN20642 |  |  |  |  |  |  |
| 0 | Atr-ERN20643 |  |  |  |  |  |  |
| 0 | Atr-ERN20644 |  |  |  |  |  |  |
| 0 | Atr-ERN20645 |  |  |  |  |  |  |
| 0 | Atr-ERN20646 |  |  |  |  |  |  |
| 0 | Atr-ERN20647 |  |  |  |  |  |  |
| 0 | Atr-ERN20648 |  |  |  |  |  |  |
| 0 | Atr-ERN20649 |  |  |  |  |  |  |
| 0 | Atr-ERN20650 |  |  |  |  |  |  |
| 0 | Atr-ERN20651 |  |  |  |  |  |  |
| 0 | Atr-ERN20652 |  |  |  |  |  |  |
| 0 | Atr-ERN20653 |  |  |  |  |  |  |
| 0 | Atr-ERN20654 |  |  |  |  |  |  |
| 0 | Atr-ERN20655 |  |  |  |  |  |  |
| 0 | Atr-ERN20656 |  |  |  |  |  |  |
| 0 | Atr-ERN20657 |  |  |  |  |  |  |
| 0 | Atr-ERN20658 |  |  |  |  |  |  |
| 0 | Atr-ERN20659 |  |  |  |  |  |  |
| 0 | Atr-ERN20660 |  |  |  |  |  |  |
| 0 | Atr-ERN20661 |  |  |  |  |  |  |
| 0 | Atr-ERN20662 |  |  |  |  |  |  |
| 0 | Atr-ERN20663 |  |  |  |  |  |  |
| 0 | Atr-ERN20664 |  |  |  |  |  |  |
| 0 | Atr-ERN20665 |  |  |  |  |  |  |
| 0 | Atr-ERN20666 |  |  |  |  |  |  |
| 0 | Atr-ERN20667 |  |  |  |  |  |  |
| 0 | Atr-ERN20668 |  |  |  |  |  |  |
| 0 | Atr-ERN20669 |  |  |  |  |  |  |
| 0 | Atr-ERN20670 |  |  |  |  |  |  |
| 0 | Atr-ERN20671 |  |  |  |  |  |  |
| 0 | Atr-ERN20672 |  |  |  |  |  |  |
| 0 | Atr-ERN20673 |  |  |  |  |  |  |
| 0 | Atr-ERN20674 |  |  |  |  |  |  |
| 0 | Atr-ERN20675 |  |  |  |  |  |  |
| 0 | Atr-ERN20676 |  |  |  |  |  |  |
| 0 | Atr-ERN20677 |  |  |  |  |  |  |
| 0 | Atr-ERN20678 |  |  |  |  |  |  |
| 0 | Atr-ERN20679 |  |  |  |  |  |  |
| 0 | Atr-ERN20680 |  |  |  |  |  |  |
| 0 | Atr-ERN20681 |  |  |  |  |  |  |
| 0 | Atr-ERN20682 |  |  |  |  |  |  |
| 0 | Atr-ERN20683 |  |  |  |  |  |  |
| 0 | Atr-ERN20684 |  |  |  |  |  |  |
| 0 | Atr-ERN20685 |  |  |  |  |  |  |
| 0 | Atr-ERN20686 |  |  |  |  |  |  |
| 0 | Atr-ERN20687 |  |  |  |  |  |  |
| 0 | Atr-ERN20688 |  |  |  |  |  |  |
| 0 | Atr-ERN20689 |  |  |  |  |  |  |
| 0 | Atr-ERN20690 |  |  |  |  |  |  |
| 0 | Atr-ERN20691 |  |  |  |  |  |  |
