# Supplementary material for: Genome-wide identification, characterization and gene expression of BES1 transcription factor family in grapevine (Vitis vinifera L.)
Source: Sci Rep. 2023 Jan 5;13:240. doi: 10.1038/s41598-022-24407-y (PMC9816167; doi:10.1038/s41598-022-24407-y)
Supplement: Supplementary file 3 — Supplementary Information. [file 41598_2022_24407_MOESM3_ESM.zip › Vvi_Atr/Vitis_vinifera.PN40024.v4.dna_sm.toplevel.fa.vs.Amborella_trichopoda.AMTR1.0.dna_sm.toplevel.fa.html/Atr-AmTr_v1.0_scaffold00071.html]

|  |  |  |  |  |  |  |  |  |  |  |  |  |  |
| --- | --- | --- | --- | --- | --- | --- | --- | --- | --- | --- | --- | --- | --- |
| Duplication depth | Reference chromosome | Collinear blocks | | | | | | | | | | | |
| 0 | Atr-ERN19854 |  |  |  |  |  |  |
| 0 | Atr-ERN19855 |  |  |  |  |  |  |
| 0 | Atr-ERN19856 |  |  |  |  |  |  |
| 0 | Atr-ERN19857 |  |  |  |  |  |  |
| 0 | Atr-ERN19858 |  |  |  |  |  |  |
| 0 | Atr-ERN19859 |  |  |  |  |  |  |
| 0 | Atr-ERN19860 |  |  |  |  |  |  |
| 0 | Atr-ERN19861 |  |  |  |  |  |  |
| 0 | Atr-ERN19862 |  |  |  |  |  |  |
| 1 | Atr-ERN19863 |  | Vvi-Vitvi18g01212\_t001 |  |  |  |  |  |
| 2 | Atr-ERN19864 |  | | | |  | Vvi-Vitvi04g01695\_t001 |  |  |  |  |
| 2 | Atr-ERN19865 |  | | | |  | Vvi-Vitvi04g01693\_t002 |  |  |  |  |
| 2 | Atr-ERN19866 |  | | | |  | | | |  |  |  |  |
| 2 | Atr-ERN19867 |  | | | |  | | | |  |  |  |  |
| 2 | Atr-ERN19868 |  | | | |  | | | |  |  |  |  |
| 2 | Atr-ERN19869 |  | | | |  | | | |  |  |  |  |
| 2 | Atr-ERN19870 |  | | | |  | | | |  |  |  |  |
| 2 | Atr-ERN19871 |  | | | |  | | | |  |  |  |  |
| 2 | Atr-ERN19872 |  | | | |  | | | |  |  |  |  |
| 2 | Atr-ERN19873 |  | | | |  | | | |  |  |  |  |
| 2 | Atr-ERN19874 |  | Vvi-Vitvi18g01214\_t001 |  | Vvi-Vitvi04g04505\_t001 |  |  |  |  |
| 2 | Atr-ERN19875 |  | | | |  | | | |  |  |  |  |
| 2 | Atr-ERN19876 |  | | | |  | | | |  |  |  |  |
| 2 | Atr-ERN19877 |  | | | |  | | | |  |  |  |  |
| 2 | Atr-ERN19878 |  | | | |  | | | |  |  |  |  |
| 2 | Atr-ERN19879 |  | | | |  | | | |  |  |  |  |
| 2 | Atr-ERN19880 |  | | | |  | Vvi-Vitvi04g01685\_t001 |  |  |  |  |
| 2 | Atr-ERN19881 |  | | | |  | | | |  |  |  |  |
| 2 | Atr-ERN19882 |  | Vvi-Vitvi18g01215\_t001 |  | | | |  |  |  |  |
| 2 | Atr-ERN19883 |  | | | |  | Vvi-Vitvi04g01684\_t001 |  |  |  |  |
| 2 | Atr-ERN19884 |  | | | |  | | | |  |  |  |  |
| 2 | Atr-ERN19885 |  | | | |  | | | |  |  |  |  |
| 2 | Atr-ERN19886 |  | | | |  | | | |  |  |  |  |
| 2 | Atr-ERN19887 |  | | | |  | | | |  |  |  |  |
| 2 | Atr-ERN19888 |  | | | |  | Vvi-Vitvi04g01681\_t001 |  |  |  |  |
| 2 | Atr-ERN19889 |  | | | |  | | | |  |  |  |  |
| 2 | Atr-ERN19890 |  | | | |  | | | |  |  |  |  |
| 2 | Atr-ERN19891 |  | | | |  | Vvi-Vitvi04g01679\_t001 |  |  |  |  |
| 2 | Atr-ERN19892 |  | | | |  | | | |  |  |  |  |
| 2 | Atr-ERN19893 |  | | | |  | | | |  |  |  |  |
| 2 | Atr-ERN19894 |  | | | |  | | | |  |  |  |  |
| 2 | Atr-ERN19895 |  | | | |  | Vvi-Vitvi04g01677\_t002 |  |  |  |  |
| 2 | Atr-ERN19896 |  | | | |  | | | |  |  |  |  |
| 2 | Atr-ERN19897 |  | | | |  | | | |  |  |  |  |
| 2 | Atr-ERN19898 |  | | | |  | | | |  |  |  |  |
| 2 | Atr-ERN19899 |  | | | |  | | | |  |  |  |  |
| 2 | Atr-ERN19900 |  | | | |  | | | |  |  |  |  |
| 2 | Atr-ERN19901 |  | | | |  | | | |  |  |  |  |
| 2 | Atr-ERN19902 |  | | | |  | | | |  |  |  |  |
| 2 | Atr-ERN19903 |  | | | |  | | | |  |  |  |  |
| 2 | Atr-ERN19904 |  | Vvi-Vitvi18g01218\_t001 |  | | | |  |  |  |  |
| 2 | Atr-ERN19905 |  | | | |  | | | |  |  |  |  |
| 2 | Atr-ERN19906 |  | | | |  | | | |  |  |  |  |
| 2 | Atr-ERN19907 |  | | | |  | | | |  |  |  |  |
| 2 | Atr-ERN19908 |  | | | |  | Vvi-Vitvi04g04502\_t001 |  |  |  |  |
| 2 | Atr-ERN19909 |  | | | |  | | | |  |  |  |  |
| 2 | Atr-ERN19910 |  | | | |  | | | |  |  |  |  |
| 2 | Atr-ERN19911 |  | | | |  | | | |  |  |  |  |
| 2 | Atr-ERN19912 |  | | | |  | | | |  |  |  |  |
| 2 | Atr-ERN19913 |  | Vvi-Vitvi18g01219\_t001 |  | | | |  |  |  |  |
| 2 | Atr-ERN19914 |  | Vvi-Vitvi18g01221\_t001 |  | | | |  |  |  |  |
| 2 | Atr-ERN19915 |  | | | |  | | | |  |  |  |  |
| 2 | Atr-ERN19916 |  | | | |  | | | |  |  |  |  |
| 2 | Atr-ERN19917 |  | Vvi-Vitvi18g01223\_t001 |  | Vvi-Vitvi04g01670\_t001 |  |  |  |  |
| 2 | Atr-ERN19918 |  | | | |  | | | |  |  |  |  |
| 3 | Atr-ERN19919 |  | | | |  | | | |  | Vvi-Vitvi18g01253\_t001 |  |  |  |
| 3 | Atr-ERN19920 |  | | | |  | | | |  | Vvi-Vitvi18g01252\_t001 |  |  |  |
| 3 | Atr-ERN19921 |  | | | |  | | | |  | Vvi-Vitvi18g01251\_t001 |  |  |  |
| 3 | Atr-ERN19922 |  | | | |  | | | |  | | | |  |  |  |
| 3 | Atr-ERN19923 |  | | | |  | | | |  | Vvi-Vitvi18g01250\_t001 |  |  |  |
| 3 | Atr-ERN19924 |  | | | |  | | | |  | Vvi-Vitvi18g01249\_t002 |  |  |  |
| 3 | Atr-ERN19925 |  | | | |  | | | |  | | | |  |  |  |
| 3 | Atr-ERN19926 |  | | | |  | | | |  | Vvi-Vitvi18g02836\_t001 |  |  |  |
| 3 | Atr-ERN19927 |  | | | |  | | | |  | | | |  |  |  |
| 3 | Atr-ERN19928 |  | | | |  | | | |  | | | |  |  |  |
| 3 | Atr-ERN19929 |  | | | |  | | | |  | | | |  |  |  |
| 3 | Atr-ERN19930 |  | | | |  | | | |  | | | |  |  |  |
| 3 | Atr-ERN19931 |  | | | |  | | | |  | Vvi-Vitvi18g01247\_t001 |  |  |  |
| 4 | Atr-ERN19932 |  | | | |  | Vvi-Vitvi04g01654\_t001 |  | Vvi-Vitvi18g01244\_t001 |  | Vvi-Vitvi04g01654\_t001 |  |  |
| 3 | Atr-ERN19933 |  | | | |  |  |  | | | |  | | | |  |  |
| 3 | Atr-ERN19934 |  | | | |  |  |  | | | |  | | | |  |  |
| 3 | Atr-ERN19935 |  | Vvi-Vitvi18g01242\_t002 |  |  |  | | | |  | Vvi-Vitvi04g01657\_t001 |  |  |
| 2 | Atr-ERN19936 |  |  |  |  |  | | | |  | | | |  |  |
| 2 | Atr-ERN19937 |  |  |  |  |  | | | |  | Vvi-Vitvi04g02246\_t001 |  |  |
| 2 | Atr-ERN19938 |  |  |  |  |  | Vvi-Vitvi18g01240\_t003 |  | Vvi-Vitvi04g01661\_t001 |  |  |
| 2 | Atr-ERN19939 |  |  |  |  |  | | | |  | Vvi-Vitvi04g01662\_t001 |  |  |
| 2 | Atr-ERN19940 |  |  |  |  |  | Vvi-Vitvi18g01239\_t001 |  | | | |  |  |
| 2 | Atr-ERN19941 |  |  |  |  |  | Vvi-Vitvi18g01238\_t001 |  | | | |  |  |
| 2 | Atr-ERN19942 |  |  |  |  |  | Vvi-Vitvi18g01235\_t004 |  | Vvi-Vitvi04g01663\_t001 |  |  |
| 1 | Atr-ERN19943 |  |  |  |  |  |  |  | | | |  |  |
| 1 | Atr-ERN19944 |  |  |  |  |  |  |  | Vvi-Vitvi04g01665\_t002 |  |  |
| 0 | Atr-ERN19945 |  |  |  |  |  |  |
| 0 | Atr-ERN19946 |  |  |  |  |  |  |
| 0 | Atr-ERN19947 |  |  |  |  |  |  |
| 0 | Atr-ERN19948 |  |  |  |  |  |  |
| 0 | Atr-ERN19949 |  |  |  |  |  |  |
| 0 | Atr-ERN19950 |  |  |  |  |  |  |
| 0 | Atr-ERN19951 |  |  |  |  |  |  |
| 0 | Atr-ERN19952 |  |  |  |  |  |  |
| 0 | Atr-ERN19953 |  |  |  |  |  |  |
| 0 | Atr-ERN19954 |  |  |  |  |  |  |
| 0 | Atr-ERN19955 |  |  |  |  |  |  |
| 0 | Atr-ERN19956 |  |  |  |  |  |  |
| 0 | Atr-ERN19957 |  |  |  |  |  |  |
| 0 | Atr-ERN19958 |  |  |  |  |  |  |
| 0 | Atr-ERN19959 |  |  |  |  |  |  |
| 0 | Atr-ERN19960 |  |  |  |  |  |  |
| 0 | Atr-ERN19961 |  |  |  |  |  |  |
| 0 | Atr-ERN19962 |  |  |  |  |  |  |
| 0 | Atr-ERN19963 |  |  |  |  |  |  |
| 0 | Atr-ERN19964 |  |  |  |  |  |  |
| 0 | Atr-ERN19965 |  |  |  |  |  |  |
| 0 | Atr-ERN19966 |  |  |  |  |  |  |
| 0 | Atr-ERN19967 |  |  |  |  |  |  |
| 0 | Atr-ERN19968 |  |  |  |  |  |  |
| 0 | Atr-ERN19969 |  |  |  |  |  |  |
| 0 | Atr-ERN19970 |  |  |  |  |  |  |
| 0 | Atr-ERN19971 |  |  |  |  |  |  |
| 0 | Atr-ERN19972 |  |  |  |  |  |  |
| 0 | Atr-ERN19973 |  |  |  |  |  |  |
| 0 | Atr-ERN19974 |  |  |  |  |  |  |
| 0 | Atr-ERN19975 |  |  |  |  |  |  |
| 0 | Atr-ERN19976 |  |  |  |  |  |  |
| 0 | Atr-ERN19977 |  |  |  |  |  |  |
| 0 | Atr-ERN19978 |  |  |  |  |  |  |
| 0 | Atr-ERN19979 |  |  |  |  |  |  |
| 0 | Atr-ERN19980 |  |  |  |  |  |  |
| 0 | Atr-ERN19981 |  |  |  |  |  |  |
| 0 | Atr-ERN19982 |  |  |  |  |  |  |
| 0 | Atr-ERN19983 |  |  |  |  |  |  |
| 0 | Atr-ERN19984 |  |  |  |  |  |  |
| 0 | Atr-ERN19985 |  |  |  |  |  |  |
| 0 | Atr-ERN19986 |  |  |  |  |  |  |
| 0 | Atr-ERN19987 |  |  |  |  |  |  |
| 0 | Atr-ERN19988 |  |  |  |  |  |  |
| 0 | Atr-ERN19989 |  |  |  |  |  |  |
| 0 | Atr-ERN19990 |  |  |  |  |  |  |
| 0 | Atr-ERN19991 |  |  |  |  |  |  |
| 0 | Atr-ERN19992 |  |  |  |  |  |  |
| 0 | Atr-ERN19993 |  |  |  |  |  |  |
| 0 | Atr-ERN19994 |  |  |  |  |  |  |
| 0 | Atr-ERN19995 |  |  |  |  |  |  |
| 0 | Atr-ERN19996 |  |  |  |  |  |  |
| 0 | Atr-ERN19997 |  |  |  |  |  |  |
| 0 | Atr-ERN19998 |  |  |  |  |  |  |
| 0 | Atr-ERN19999 |  |  |  |  |  |  |
| 0 | Atr-ERN20000 |  |  |  |  |  |  |
| 0 | Atr-ERN20001 |  |  |  |  |  |  |
| 0 | Atr-ERN20002 |  |  |  |  |  |  |
| 0 | Atr-ERN20003 |  |  |  |  |  |  |
| 0 | Atr-ERN20004 |  |  |  |  |  |  |
| 0 | Atr-ERN20005 |  |  |  |  |  |  |
| 0 | Atr-ERN20006 |  |  |  |  |  |  |
| 0 | Atr-ERN20007 |  |  |  |  |  |  |
| 0 | Atr-ERN20008 |  |  |  |  |  |  |
| 0 | Atr-ERN20009 |  |  |  |  |  |  |
| 0 | Atr-ERN20010 |  |  |  |  |  |  |
| 0 | Atr-ERN20011 |  |  |  |  |  |  |
| 0 | Atr-ERN20012 |  |  |  |  |  |  |
| 0 | Atr-ERN20013 |  |  |  |  |  |  |
| 0 | Atr-ERN20014 |  |  |  |  |  |  |
| 0 | Atr-ERN20015 |  |  |  |  |  |  |
| 0 | Atr-ERN20016 |  |  |  |  |  |  |
| 0 | Atr-ERN20017 |  |  |  |  |  |  |
| 0 | Atr-ERN20018 |  |  |  |  |  |  |
| 0 | Atr-ERN20019 |  |  |  |  |  |  |
| 0 | Atr-ERN20020 |  |  |  |  |  |  |
| 1 | Atr-ERN20021 |  | Vvi-Vitvi12g04585\_t001 |  |  |  |  |  |
| 1 | Atr-ERN20022 |  | | | |  |  |  |  |  |
| 1 | Atr-ERN20023 |  | Vvi-Vitvi12g02033\_t001 |  |  |  |  |  |
| 1 | Atr-ERN20024 |  | | | |  |  |  |  |  |
| 1 | Atr-ERN20025 |  | | | |  |  |  |  |  |
| 1 | Atr-ERN20026 |  | | | |  |  |  |  |  |
| 1 | Atr-ERN20027 |  | | | |  |  |  |  |  |
| 1 | Atr-ERN20028 |  | Vvi-Vitvi12g02034\_t001 |  |  |  |  |  |
| 1 | Atr-ERN20029 |  | | | |  |  |  |  |  |
| 1 | Atr-ERN20030 |  | | | |  |  |  |  |  |
| 1 | Atr-ERN20031 |  | | | |  |  |  |  |  |
| 1 | Atr-ERN20032 |  | Vvi-Vitvi12g02037\_t001 |  |  |  |  |  |
| 1 | Atr-ERN20033 |  | | | |  |  |  |  |  |
| 1 | Atr-ERN20034 |  | | | |  |  |  |  |  |
| 1 | Atr-ERN20035 |  | | | |  |  |  |  |  |
| 1 | Atr-ERN20036 |  | Vvi-Vitvi12g02041\_t001 |  |  |  |  |  |
| 1 | Atr-ERN20037 |  | Vvi-Vitvi12g02042\_t001 |  |  |  |  |  |
| 1 | Atr-ERN20038 |  | | | |  |  |  |  |  |
| 2 | Atr-ERN20039 |  | | | |  | Vvi-Vitvi18g02128\_t001 |  |  |  |  |
| 2 | Atr-ERN20040 |  | | | |  | | | |  |  |  |  |
| 2 | Atr-ERN20041 |  | Vvi-Vitvi12g02721\_t001 |  | | | |  |  |  |  |
| 2 | Atr-ERN20042 |  | | | |  | | | |  |  |  |  |
| 2 | Atr-ERN20043 |  | Vvi-Vitvi12g02044\_t001 |  | | | |  |  |  |  |
| 2 | Atr-ERN20044 |  | | | |  | | | |  |  |  |  |
| 2 | Atr-ERN20045 |  | | | |  | | | |  |  |  |  |
| 2 | Atr-ERN20046 |  | | | |  | | | |  |  |  |  |
| 2 | Atr-ERN20047 |  | | | |  | | | |  |  |  |  |
| 2 | Atr-ERN20048 |  | Vvi-Vitvi12g02045\_t003 |  | | | |  |  |  |  |
| 2 | Atr-ERN20049 |  | Vvi-Vitvi12g02046\_t001 |  | | | |  |  |  |  |
| 2 | Atr-ERN20050 |  | | | |  | Vvi-Vitvi18g02130\_t001 |  |  |  |  |
| 2 | Atr-ERN20051 |  | Vvi-Vitvi12g02047\_t003 |  | | | |  |  |  |  |
| 2 | Atr-ERN20052 |  | | | |  | | | |  |  |  |  |
| 2 | Atr-ERN20053 |  | | | |  | Vvi-Vitvi18g02131\_t001 |  |  |  |  |
| 2 | Atr-ERN20054 |  | | | |  | | | |  |  |  |  |
| 2 | Atr-ERN20055 |  | Vvi-Vitvi12g02050\_t001 |  | | | |  |  |  |  |
| 2 | Atr-ERN20056 |  | | | |  | Vvi-Vitvi18g02133\_t001 |  |  |  |  |
| 2 | Atr-ERN20057 |  | Vvi-Vitvi12g02723\_t002 |  | Vvi-Vitvi18g04611\_t001 |  |  |  |  |
| 2 | Atr-ERN20058 |  | | | |  | | | |  |  |  |  |
| 2 | Atr-ERN20059 |  | | | |  | Vvi-Vitvi18g04620\_t001 |  |  |  |  |
| 2 | Atr-ERN20060 |  | | | |  | | | |  |  |  |  |
| 2 | Atr-ERN20061 |  | | | |  | | | |  |  |  |  |
| 2 | Atr-ERN20062 |  | | | |  | | | |  |  |  |  |
| 2 | Atr-ERN20063 |  | | | |  | | | |  |  |  |  |
| 2 | Atr-ERN20064 |  | | | |  | | | |  |  |  |  |
| 2 | Atr-ERN20065 |  | | | |  | | | |  |  |  |  |
| 2 | Atr-ERN20066 |  | | | |  | Vvi-Vitvi18g02144\_t002 |  |  |  |  |
| 2 | Atr-ERN20067 |  | | | |  | | | |  |  |  |  |
| 2 | Atr-ERN20068 |  | | | |  | | | |  |  |  |  |
| 2 | Atr-ERN20069 |  | | | |  | Vvi-Vitvi18g02145\_t001 |  |  |  |  |
| 2 | Atr-ERN20070 |  | | | |  | | | |  |  |  |  |
| 2 | Atr-ERN20071 |  | | | |  | Vvi-Vitvi18g02146\_t001 |  |  |  |  |
| 2 | Atr-ERN20072 |  | | | |  | | | |  |  |  |  |
| 2 | Atr-ERN20073 |  | Vvi-Vitvi12g02056\_t001 |  | | | |  |  |  |  |
| 2 | Atr-ERN20074 |  | Vvi-Vitvi12g02057\_t001 |  | | | |  |  |  |  |
| 1 | Atr-ERN20075 |  |  |  | Vvi-Vitvi18g02148\_t001 |  |  |  |  |
| 0 | Atr-ERN20076 |  |  |  |  |  |  |
