# Supplementary material for: Genome-wide identification, characterization and gene expression of BES1 transcription factor family in grapevine (Vitis vinifera L.)
Source: Sci Rep. 2023 Jan 5;13:240. doi: 10.1038/s41598-022-24407-y (PMC9816167; doi:10.1038/s41598-022-24407-y)
Supplement: Supplementary file 3 — Supplementary Information. [file 41598_2022_24407_MOESM3_ESM.zip › Vvi_Atr/Vitis_vinifera.PN40024.v4.dna_sm.toplevel.fa.vs.Amborella_trichopoda.AMTR1.0.dna_sm.toplevel.fa.html/Atr-AmTr_v1.0_scaffold00072.html]

|  |  |  |  |  |  |  |  |  |  |  |  |  |  |
| --- | --- | --- | --- | --- | --- | --- | --- | --- | --- | --- | --- | --- | --- |
| Duplication depth | Reference chromosome | Collinear blocks | | | | | | | | | | | |
| 2 | Atr-ERM98369 |  | Vvi-Vitvi10g02328\_t001 |  | Vvi-Vitvi12g02441\_t001 |  |  |  |  |
| 2 | Atr-ERM98370 |  | | | |  | | | |  |  |  |  |
| 2 | Atr-ERM98371 |  | | | |  | | | |  |  |  |  |
| 2 | Atr-ERM98372 |  | | | |  | Vvi-Vitvi12g02442\_t001 |  |  |  |  |
| 2 | Atr-ERM98373 |  | | | |  | Vvi-Vitvi12g00619\_t001 |  |  |  |  |
| 2 | Atr-ERM98374 |  | | | |  | | | |  |  |  |  |
| 2 | Atr-ERM98375 |  | Vvi-Vitvi10g00352\_t001 |  | Vvi-Vitvi12g00620\_t002 |  |  |  |  |
| 2 | Atr-ERM98376 |  | | | |  | Vvi-Vitvi12g00621\_t001 |  |  |  |  |
| 2 | Atr-ERM98377 |  | | | |  | | | |  |  |  |  |
| 2 | Atr-ERM98378 |  | | | |  | | | |  |  |  |  |
| 2 | Atr-ERM98379 |  | | | |  | | | |  |  |  |  |
| 2 | Atr-ERM98380 |  | | | |  | | | |  |  |  |  |
| 2 | Atr-ERM98381 |  | | | |  | | | |  |  |  |  |
| 2 | Atr-ERM98382 |  | | | |  | | | |  |  |  |  |
| 2 | Atr-ERM98383 |  | | | |  | Vvi-Vitvi12g00622\_t001 |  |  |  |  |
| 2 | Atr-ERM98384 |  | | | |  | | | |  |  |  |  |
| 2 | Atr-ERM98385 |  | Vvi-Vitvi10g00350\_t001 |  | | | |  |  |  |  |
| 2 | Atr-ERM98386 |  | | | |  | | | |  |  |  |  |
| 2 | Atr-ERM98387 |  | | | |  | | | |  |  |  |  |
| 2 | Atr-ERM98388 |  | | | |  | | | |  |  |  |  |
| 2 | Atr-ERM98389 |  | | | |  | | | |  |  |  |  |
| 2 | Atr-ERM98390 |  | Vvi-Vitvi10g04243\_t001 |  | | | |  |  |  |  |
| 2 | Atr-ERM98391 |  | | | |  | | | |  |  |  |  |
| 2 | Atr-ERM98392 |  | | | |  | | | |  |  |  |  |
| 2 | Atr-ERM98393 |  | | | |  | | | |  |  |  |  |
| 2 | Atr-ERM98394 |  | | | |  | | | |  |  |  |  |
| 2 | Atr-ERM98395 |  | | | |  | | | |  |  |  |  |
| 2 | Atr-ERM98396 |  | | | |  | | | |  |  |  |  |
| 2 | Atr-ERM98397 |  | | | |  | | | |  |  |  |  |
| 2 | Atr-ERM98398 |  | | | |  | | | |  |  |  |  |
| 2 | Atr-ERM98399 |  | Vvi-Vitvi10g00344\_t001 |  | Vvi-Vitvi12g00627\_t002 |  |  |  |  |
| 2 | Atr-ERM98400 |  | | | |  | | | |  |  |  |  |
| 2 | Atr-ERM98401 |  | | | |  | | | |  |  |  |  |
| 2 | Atr-ERM98402 |  | | | |  | | | |  |  |  |  |
| 2 | Atr-ERM98403 |  | | | |  | | | |  |  |  |  |
| 2 | Atr-ERM98404 |  | | | |  | | | |  |  |  |  |
| 2 | Atr-ERM98405 |  | | | |  | Vvi-Vitvi12g00628\_t002 |  |  |  |  |
| 2 | Atr-ERM98406 |  | | | |  | | | |  |  |  |  |
| 2 | Atr-ERM98407 |  | | | |  | | | |  |  |  |  |
| 2 | Atr-ERM98408 |  | | | |  | | | |  |  |  |  |
| 2 | Atr-ERM98409 |  | Vvi-Vitvi10g00342\_t001 |  | Vvi-Vitvi12g00630\_t001 |  |  |  |  |
| 1 | Atr-ERM98410 |  | | | |  |  |  |  |  |
| 1 | Atr-ERM98411 |  | | | |  |  |  |  |  |
| 1 | Atr-ERM98412 |  | | | |  |  |  |  |  |
| 1 | Atr-ERM98413 |  | | | |  |  |  |  |  |
| 1 | Atr-ERM98414 |  | | | |  |  |  |  |  |
| 1 | Atr-ERM98415 |  | Vvi-Vitvi10g00339\_t001 |  |  |  |  |  |
| 0 | Atr-ERM98416 |  |  |  |  |  |  |
| 0 | Atr-ERM98417 |  |  |  |  |  |  |
| 0 | Atr-ERM98418 |  |  |  |  |  |  |
| 0 | Atr-ERM98419 |  |  |  |  |  |  |
| 0 | Atr-ERM98420 |  |  |  |  |  |  |
| 0 | Atr-ERM98421 |  |  |  |  |  |  |
| 0 | Atr-ERM98422 |  |  |  |  |  |  |
| 0 | Atr-ERM98423 |  |  |  |  |  |  |
| 0 | Atr-ERM98424 |  |  |  |  |  |  |
| 0 | Atr-ERM98425 |  |  |  |  |  |  |
| 0 | Atr-ERM98426 |  |  |  |  |  |  |
| 0 | Atr-ERM98427 |  |  |  |  |  |  |
| 0 | Atr-ERM98428 |  |  |  |  |  |  |
| 0 | Atr-ERM98429 |  |  |  |  |  |  |
| 0 | Atr-ERM98430 |  |  |  |  |  |  |
| 0 | Atr-ERM98431 |  |  |  |  |  |  |
| 0 | Atr-ERM98432 |  |  |  |  |  |  |
| 0 | Atr-ERM98433 |  |  |  |  |  |  |
| 0 | Atr-ERM98434 |  |  |  |  |  |  |
| 0 | Atr-ERM98435 |  |  |  |  |  |  |
| 0 | Atr-ERM98436 |  |  |  |  |  |  |
| 0 | Atr-ERM98437 |  |  |  |  |  |  |
| 0 | Atr-ERM98438 |  |  |  |  |  |  |
| 0 | Atr-ERM98439 |  |  |  |  |  |  |
| 0 | Atr-ERM98440 |  |  |  |  |  |  |
| 0 | Atr-ERM98441 |  |  |  |  |  |  |
| 0 | Atr-ERM98442 |  |  |  |  |  |  |
| 0 | Atr-ERM98443 |  |  |  |  |  |  |
| 0 | Atr-ERM98444 |  |  |  |  |  |  |
| 0 | Atr-ERM98445 |  |  |  |  |  |  |
| 0 | Atr-ERM98446 |  |  |  |  |  |  |
| 0 | Atr-ERM98447 |  |  |  |  |  |  |
| 0 | Atr-ERM98448 |  |  |  |  |  |  |
| 0 | Atr-ERM98449 |  |  |  |  |  |  |
| 0 | Atr-ERM98450 |  |  |  |  |  |  |
| 0 | Atr-ERM98451 |  |  |  |  |  |  |
| 0 | Atr-ERM98452 |  |  |  |  |  |  |
| 0 | Atr-ERM98453 |  |  |  |  |  |  |
| 0 | Atr-ERM98454 |  |  |  |  |  |  |
| 0 | Atr-ERM98455 |  |  |  |  |  |  |
| 0 | Atr-ERM98456 |  |  |  |  |  |  |
| 0 | Atr-ERM98457 |  |  |  |  |  |  |
| 0 | Atr-ERM98458 |  |  |  |  |  |  |
| 0 | Atr-ERM98459 |  |  |  |  |  |  |
| 0 | Atr-ERM98460 |  |  |  |  |  |  |
| 0 | Atr-ERM98461 |  |  |  |  |  |  |
| 0 | Atr-ERM98462 |  |  |  |  |  |  |
| 0 | Atr-ERM98463 |  |  |  |  |  |  |
| 0 | Atr-ERM98464 |  |  |  |  |  |  |
| 0 | Atr-ERM98465 |  |  |  |  |  |  |
| 0 | Atr-ERM98466 |  |  |  |  |  |  |
| 0 | Atr-ERM98467 |  |  |  |  |  |  |
| 0 | Atr-ERM98468 |  |  |  |  |  |  |
| 0 | Atr-ERM98469 |  |  |  |  |  |  |
| 0 | Atr-ERM98470 |  |  |  |  |  |  |
| 0 | Atr-ERM98471 |  |  |  |  |  |  |
| 0 | Atr-ERM98472 |  |  |  |  |  |  |
| 0 | Atr-ERM98473 |  |  |  |  |  |  |
| 0 | Atr-ERM98474 |  |  |  |  |  |  |
| 0 | Atr-ERM98475 |  |  |  |  |  |  |
| 0 | Atr-ERM98476 |  |  |  |  |  |  |
| 0 | Atr-ERM98477 |  |  |  |  |  |  |
| 0 | Atr-ERM98478 |  |  |  |  |  |  |
| 0 | Atr-ERM98479 |  |  |  |  |  |  |
| 0 | Atr-ERM98480 |  |  |  |  |  |  |
| 0 | Atr-ERM98481 |  |  |  |  |  |  |
| 0 | Atr-ERM98482 |  |  |  |  |  |  |
| 0 | Atr-ERM98483 |  |  |  |  |  |  |
| 0 | Atr-ERM98484 |  |  |  |  |  |  |
| 0 | Atr-ERM98485 |  |  |  |  |  |  |
| 0 | Atr-ERM98486 |  |  |  |  |  |  |
| 0 | Atr-ERM98487 |  |  |  |  |  |  |
| 0 | Atr-ERM98488 |  |  |  |  |  |  |
| 0 | Atr-ERM98489 |  |  |  |  |  |  |
| 0 | Atr-ERM98490 |  |  |  |  |  |  |
| 0 | Atr-ERM98491 |  |  |  |  |  |  |
| 0 | Atr-ERM98492 |  |  |  |  |  |  |
| 0 | Atr-ERM98493 |  |  |  |  |  |  |
| 0 | Atr-ERM98494 |  |  |  |  |  |  |
| 0 | Atr-ERM98495 |  |  |  |  |  |  |
| 0 | Atr-ERM98496 |  |  |  |  |  |  |
| 0 | Atr-ERM98497 |  |  |  |  |  |  |
| 0 | Atr-ERM98498 |  |  |  |  |  |  |
| 0 | Atr-ERM98499 |  |  |  |  |  |  |
| 0 | Atr-ERM98500 |  |  |  |  |  |  |
| 0 | Atr-ERM98501 |  |  |  |  |  |  |
| 0 | Atr-ERM98502 |  |  |  |  |  |  |
| 0 | Atr-ERM98503 |  |  |  |  |  |  |
| 0 | Atr-ERM98504 |  |  |  |  |  |  |
| 0 | Atr-ERM98505 |  |  |  |  |  |  |
| 0 | Atr-ERM98506 |  |  |  |  |  |  |
| 0 | Atr-ERM98507 |  |  |  |  |  |  |
| 0 | Atr-ERM98508 |  |  |  |  |  |  |
