# Supplementary material for: Genome-wide identification, characterization and gene expression of BES1 transcription factor family in grapevine (Vitis vinifera L.)
Source: Sci Rep. 2023 Jan 5;13:240. doi: 10.1038/s41598-022-24407-y (PMC9816167; doi:10.1038/s41598-022-24407-y)
Supplement: Supplementary file 3 — Supplementary Information. [file 41598_2022_24407_MOESM3_ESM.zip › Vvi_Atr/Vitis_vinifera.PN40024.v4.dna_sm.toplevel.fa.vs.Amborella_trichopoda.AMTR1.0.dna_sm.toplevel.fa.html/Atr-AmTr_v1.0_scaffold00073.html]

|  |  |  |  |  |  |  |  |  |  |  |  |  |  |
| --- | --- | --- | --- | --- | --- | --- | --- | --- | --- | --- | --- | --- | --- |
| Duplication depth | Reference chromosome | Collinear blocks | | | | | | | | | | | |
| 0 | Atr-ERM97302 |  |  |  |  |  |  |
| 0 | Atr-ERM97303 |  |  |  |  |  |  |
| 0 | Atr-ERM97304 |  |  |  |  |  |  |
| 0 | Atr-ERM97305 |  |  |  |  |  |  |
| 0 | Atr-ERM97306 |  |  |  |  |  |  |
| 0 | Atr-ERM97307 |  |  |  |  |  |  |
| 0 | Atr-ERM97308 |  |  |  |  |  |  |
| 0 | Atr-ERM97309 |  |  |  |  |  |  |
| 0 | Atr-ERM97310 |  |  |  |  |  |  |
| 0 | Atr-ERM97311 |  |  |  |  |  |  |
| 0 | Atr-ERM97312 |  |  |  |  |  |  |
| 0 | Atr-ERM97313 |  |  |  |  |  |  |
| 0 | Atr-ERM97314 |  |  |  |  |  |  |
| 0 | Atr-ERM97315 |  |  |  |  |  |  |
| 0 | Atr-ERM97316 |  |  |  |  |  |  |
| 0 | Atr-ERM97317 |  |  |  |  |  |  |
| 0 | Atr-ERM97318 |  |  |  |  |  |  |
| 0 | Atr-ERM97319 |  |  |  |  |  |  |
| 0 | Atr-ERM97320 |  |  |  |  |  |  |
| 0 | Atr-ERM97321 |  |  |  |  |  |  |
| 0 | Atr-ERM97322 |  |  |  |  |  |  |
| 0 | Atr-ERM97323 |  |  |  |  |  |  |
| 0 | Atr-ERM97324 |  |  |  |  |  |  |
| 0 | Atr-ERM97325 |  |  |  |  |  |  |
| 0 | Atr-ERM97326 |  |  |  |  |  |  |
| 0 | Atr-ERM97327 |  |  |  |  |  |  |
| 0 | Atr-ERM97328 |  |  |  |  |  |  |
| 0 | Atr-ERM97329 |  |  |  |  |  |  |
| 0 | Atr-ERM97330 |  |  |  |  |  |  |
| 0 | Atr-ERM97331 |  |  |  |  |  |  |
| 0 | Atr-ERM97332 |  |  |  |  |  |  |
| 0 | Atr-ERM97333 |  |  |  |  |  |  |
| 0 | Atr-ERM97334 |  |  |  |  |  |  |
| 0 | Atr-ERM97335 |  |  |  |  |  |  |
| 0 | Atr-ERM97336 |  |  |  |  |  |  |
| 0 | Atr-ERM97337 |  |  |  |  |  |  |
| 0 | Atr-ERM97338 |  |  |  |  |  |  |
| 0 | Atr-ERM97339 |  |  |  |  |  |  |
| 0 | Atr-ERM97340 |  |  |  |  |  |  |
| 0 | Atr-ERM97341 |  |  |  |  |  |  |
| 0 | Atr-ERM97342 |  |  |  |  |  |  |
| 0 | Atr-ERM97343 |  |  |  |  |  |  |
| 0 | Atr-ERM97344 |  |  |  |  |  |  |
| 0 | Atr-ERM97345 |  |  |  |  |  |  |
| 0 | Atr-ERM97346 |  |  |  |  |  |  |
| 0 | Atr-ERM97347 |  |  |  |  |  |  |
| 0 | Atr-ERM97348 |  |  |  |  |  |  |
| 0 | Atr-ERM97349 |  |  |  |  |  |  |
| 0 | Atr-ERM97350 |  |  |  |  |  |  |
| 0 | Atr-ERM97351 |  |  |  |  |  |  |
| 0 | Atr-ERM97352 |  |  |  |  |  |  |
| 0 | Atr-ERM97353 |  |  |  |  |  |  |
| 0 | Atr-ERM97354 |  |  |  |  |  |  |
| 0 | Atr-ERM97355 |  |  |  |  |  |  |
| 0 | Atr-ERM97356 |  |  |  |  |  |  |
| 0 | Atr-ERM97357 |  |  |  |  |  |  |
| 0 | Atr-ERM97358 |  |  |  |  |  |  |
| 0 | Atr-ERM97359 |  |  |  |  |  |  |
| 0 | Atr-ERM97360 |  |  |  |  |  |  |
| 0 | Atr-ERM97361 |  |  |  |  |  |  |
| 0 | Atr-ERM97362 |  |  |  |  |  |  |
| 0 | Atr-ERM97363 |  |  |  |  |  |  |
| 0 | Atr-ERM97364 |  |  |  |  |  |  |
| 0 | Atr-ERM97365 |  |  |  |  |  |  |
| 0 | Atr-ERM97366 |  |  |  |  |  |  |
| 0 | Atr-ERM97367 |  |  |  |  |  |  |
