# Supplementary material for: Genome-wide identification, characterization and gene expression of BES1 transcription factor family in grapevine (Vitis vinifera L.)
Source: Sci Rep. 2023 Jan 5;13:240. doi: 10.1038/s41598-022-24407-y (PMC9816167; doi:10.1038/s41598-022-24407-y)
Supplement: Supplementary file 3 — Supplementary Information. [file 41598_2022_24407_MOESM3_ESM.zip › Vvi_Atr/Vitis_vinifera.PN40024.v4.dna_sm.toplevel.fa.vs.Amborella_trichopoda.AMTR1.0.dna_sm.toplevel.fa.html/Atr-AmTr_v1.0_scaffold00074.html]

|  |  |  |  |  |  |  |  |  |  |  |  |  |  |
| --- | --- | --- | --- | --- | --- | --- | --- | --- | --- | --- | --- | --- | --- |
| Duplication depth | Reference chromosome | Collinear blocks | | | | | | | | | | | |
| 0 | Atr-ERM96883 |  |  |  |  |  |  |
| 0 | Atr-ERM96884 |  |  |  |  |  |  |
| 0 | Atr-ERM96885 |  |  |  |  |  |  |
| 0 | Atr-ERM96886 |  |  |  |  |  |  |
| 0 | Atr-ERM96887 |  |  |  |  |  |  |
| 0 | Atr-ERM96888 |  |  |  |  |  |  |
| 0 | Atr-ERM96889 |  |  |  |  |  |  |
| 0 | Atr-ERM96890 |  |  |  |  |  |  |
| 0 | Atr-ERM96891 |  |  |  |  |  |  |
| 0 | Atr-ERM96892 |  |  |  |  |  |  |
| 0 | Atr-ERM96893 |  |  |  |  |  |  |
| 0 | Atr-ERM96894 |  |  |  |  |  |  |
| 0 | Atr-ERM96895 |  |  |  |  |  |  |
| 0 | Atr-ERM96896 |  |  |  |  |  |  |
| 0 | Atr-ERM96897 |  |  |  |  |  |  |
| 0 | Atr-ERM96898 |  |  |  |  |  |  |
| 0 | Atr-ERM96899 |  |  |  |  |  |  |
| 0 | Atr-ERM96900 |  |  |  |  |  |  |
| 0 | Atr-ERM96901 |  |  |  |  |  |  |
| 0 | Atr-ERM96902 |  |  |  |  |  |  |
| 0 | Atr-ERM96903 |  |  |  |  |  |  |
| 0 | Atr-ERM96904 |  |  |  |  |  |  |
| 0 | Atr-ERM96905 |  |  |  |  |  |  |
| 0 | Atr-ERM96906 |  |  |  |  |  |  |
| 0 | Atr-ERM96907 |  |  |  |  |  |  |
| 0 | Atr-ERM96908 |  |  |  |  |  |  |
| 0 | Atr-ERM96909 |  |  |  |  |  |  |
| 0 | Atr-ERM96910 |  |  |  |  |  |  |
| 1 | Atr-ERM96911 |  | Vvi-Vitvi12g00446\_t001 |  |  |  |  |  |
| 1 | Atr-ERM96912 |  | | | |  |  |  |  |  |
| 1 | Atr-ERM96913 |  | | | |  |  |  |  |  |
| 1 | Atr-ERM96914 |  | | | |  |  |  |  |  |
| 1 | Atr-ERM96915 |  | Vvi-Vitvi12g00449\_t001 |  |  |  |  |  |
| 1 | Atr-ERM96916 |  | Vvi-Vitvi12g02381\_t001 |  |  |  |  |  |
| 1 | Atr-ERM96917 |  | | | |  |  |  |  |  |
| 1 | Atr-ERM96918 |  | Vvi-Vitvi12g02382\_t001 |  |  |  |  |  |
| 1 | Atr-ERM96919 |  | | | |  |  |  |  |  |
| 1 | Atr-ERM96920 |  | | | |  |  |  |  |  |
| 1 | Atr-ERM96921 |  | Vvi-Vitvi12g00453\_t001 |  |  |  |  |  |
| 1 | Atr-ERM96922 |  | | | |  |  |  |  |  |
| 1 | Atr-ERM96923 |  | | | |  |  |  |  |  |
| 1 | Atr-ERM96924 |  | | | |  |  |  |  |  |
| 1 | Atr-ERM96925 |  | | | |  |  |  |  |  |
| 1 | Atr-ERM96926 |  | | | |  |  |  |  |  |
| 1 | Atr-ERM96927 |  | | | |  |  |  |  |  |
| 1 | Atr-ERM96928 |  | | | |  |  |  |  |  |
| 1 | Atr-ERM96929 |  | | | |  |  |  |  |  |
| 1 | Atr-ERM96930 |  | Vvi-Vitvi12g00454\_t001 |  |  |  |  |  |
| 2 | Atr-ERM96931 |  | | | |  | Vvi-Vitvi10g04071\_t001 |  |  |  |  |
| 2 | Atr-ERM96932 |  | | | |  | Vvi-Vitvi10g04070\_t001 |  |  |  |  |
| 2 | Atr-ERM96933 |  | | | |  | Vvi-Vitvi10g00164\_t001 |  |  |  |  |
| 2 | Atr-ERM96934 |  | | | |  | | | |  |  |  |  |
| 2 | Atr-ERM96935 |  | | | |  | | | |  |  |  |  |
| 2 | Atr-ERM96936 |  | | | |  | Vvi-Vitvi10g04069\_t001 |  |  |  |  |
| 2 | Atr-ERM96937 |  | | | |  | Vvi-Vitvi10g04068\_t001 |  |  |  |  |
| 2 | Atr-ERM96938 |  | | | |  | | | |  |  |  |  |
| 2 | Atr-ERM96939 |  | | | |  | | | |  |  |  |  |
| 2 | Atr-ERM96940 |  | | | |  | Vvi-Vitvi10g04067\_t001 |  |  |  |  |
| 2 | Atr-ERM96941 |  | Vvi-Vitvi12g00455\_t001 |  | Vvi-Vitvi10g00159\_t001 |  |  |  |  |
| 2 | Atr-ERM96942 |  | | | |  | | | |  |  |  |  |
| 2 | Atr-ERM96943 |  | | | |  | | | |  |  |  |  |
| 2 | Atr-ERM96944 |  | | | |  | | | |  |  |  |  |
| 2 | Atr-ERM96945 |  | | | |  | | | |  |  |  |  |
| 2 | Atr-ERM96946 |  | | | |  | | | |  |  |  |  |
| 2 | Atr-ERM96947 |  | | | |  | | | |  |  |  |  |
| 2 | Atr-ERM96948 |  | | | |  | | | |  |  |  |  |
| 2 | Atr-ERM96949 |  | | | |  | | | |  |  |  |  |
| 2 | Atr-ERM96950 |  | | | |  | | | |  |  |  |  |
| 2 | Atr-ERM96951 |  | Vvi-Vitvi12g02387\_t001 |  | | | |  |  |  |  |
| 1 | Atr-ERM96952 |  |  |  | | | |  |  |  |  |
| 1 | Atr-ERM96953 |  |  |  | | | |  |  |  |  |
| 1 | Atr-ERM96954 |  |  |  | | | |  |  |  |  |
| 1 | Atr-ERM96955 |  |  |  | | | |  |  |  |  |
| 1 | Atr-ERM96956 |  |  |  | | | |  |  |  |  |
| 1 | Atr-ERM96957 |  |  |  | | | |  |  |  |  |
| 1 | Atr-ERM96958 |  |  |  | Vvi-Vitvi10g04063\_t001 |  |  |  |  |
| 0 | Atr-ERM96959 |  |  |  |  |  |  |
| 0 | Atr-ERM96960 |  |  |  |  |  |  |
| 0 | Atr-ERM96961 |  |  |  |  |  |  |
| 0 | Atr-ERM96962 |  |  |  |  |  |  |
| 0 | Atr-ERM96963 |  |  |  |  |  |  |
| 0 | Atr-ERM96964 |  |  |  |  |  |  |
| 0 | Atr-ERM96965 |  |  |  |  |  |  |
| 0 | Atr-ERM96966 |  |  |  |  |  |  |
| 0 | Atr-ERM96967 |  |  |  |  |  |  |
| 0 | Atr-ERM96968 |  |  |  |  |  |  |
| 0 | Atr-ERM96969 |  |  |  |  |  |  |
| 0 | Atr-ERM96970 |  |  |  |  |  |  |
| 0 | Atr-ERM96971 |  |  |  |  |  |  |
| 0 | Atr-ERM96972 |  |  |  |  |  |  |
| 0 | Atr-ERM96973 |  |  |  |  |  |  |
| 0 | Atr-ERM96974 |  |  |  |  |  |  |
| 0 | Atr-ERM96975 |  |  |  |  |  |  |
| 0 | Atr-ERM96976 |  |  |  |  |  |  |
| 0 | Atr-ERM96977 |  |  |  |  |  |  |
| 0 | Atr-ERM96978 |  |  |  |  |  |  |
| 0 | Atr-ERM96979 |  |  |  |  |  |  |
| 0 | Atr-ERM96980 |  |  |  |  |  |  |
| 0 | Atr-ERM96981 |  |  |  |  |  |  |
| 0 | Atr-ERM96982 |  |  |  |  |  |  |
| 0 | Atr-ERM96983 |  |  |  |  |  |  |
| 0 | Atr-ERM96984 |  |  |  |  |  |  |
| 0 | Atr-ERM96985 |  |  |  |  |  |  |
| 0 | Atr-ERM96986 |  |  |  |  |  |  |
| 0 | Atr-ERM96987 |  |  |  |  |  |  |
| 0 | Atr-ERM96988 |  |  |  |  |  |  |
| 0 | Atr-ERM96989 |  |  |  |  |  |  |
| 0 | Atr-ERM96990 |  |  |  |  |  |  |
| 0 | Atr-ERM96991 |  |  |  |  |  |  |
| 0 | Atr-ERM96992 |  |  |  |  |  |  |
| 0 | Atr-ERM96993 |  |  |  |  |  |  |
| 0 | Atr-ERM96994 |  |  |  |  |  |  |
| 0 | Atr-ERM96995 |  |  |  |  |  |  |
| 0 | Atr-ERM96996 |  |  |  |  |  |  |
| 0 | Atr-ERM96997 |  |  |  |  |  |  |
| 0 | Atr-ERM96998 |  |  |  |  |  |  |
| 0 | Atr-ERM96999 |  |  |  |  |  |  |
| 0 | Atr-ERM97000 |  |  |  |  |  |  |
| 0 | Atr-ERM97001 |  |  |  |  |  |  |
| 0 | Atr-ERM97002 |  |  |  |  |  |  |
| 0 | Atr-ERM97003 |  |  |  |  |  |  |
| 0 | Atr-ERM97004 |  |  |  |  |  |  |
| 0 | Atr-ERM97005 |  |  |  |  |  |  |
| 0 | Atr-ERM97006 |  |  |  |  |  |  |
| 0 | Atr-ERM97007 |  |  |  |  |  |  |
| 0 | Atr-ERM97008 |  |  |  |  |  |  |
| 0 | Atr-ERM97009 |  |  |  |  |  |  |
| 0 | Atr-ERM97010 |  |  |  |  |  |  |
| 0 | Atr-ERM97011 |  |  |  |  |  |  |
| 0 | Atr-ERM97012 |  |  |  |  |  |  |
