# Supplementary material for: Genome-wide identification, characterization and gene expression of BES1 transcription factor family in grapevine (Vitis vinifera L.)
Source: Sci Rep. 2023 Jan 5;13:240. doi: 10.1038/s41598-022-24407-y (PMC9816167; doi:10.1038/s41598-022-24407-y)
Supplement: Supplementary file 3 — Supplementary Information. [file 41598_2022_24407_MOESM3_ESM.zip › Vvi_Atr/Vitis_vinifera.PN40024.v4.dna_sm.toplevel.fa.vs.Amborella_trichopoda.AMTR1.0.dna_sm.toplevel.fa.html/Atr-AmTr_v1.0_scaffold00075.html]

|  |  |  |  |  |  |  |  |  |  |  |  |  |  |
| --- | --- | --- | --- | --- | --- | --- | --- | --- | --- | --- | --- | --- | --- |
| Duplication depth | Reference chromosome | Collinear blocks | | | | | | | | | | | |
| 0 | Atr-ERN04560 |  |  |  |  |  |  |
| 0 | Atr-ERN04561 |  |  |  |  |  |  |
| 0 | Atr-ERN04562 |  |  |  |  |  |  |
| 0 | Atr-ERN04563 |  |  |  |  |  |  |
| 0 | Atr-ERN04564 |  |  |  |  |  |  |
| 0 | Atr-ERN04565 |  |  |  |  |  |  |
| 0 | Atr-ERN04566 |  |  |  |  |  |  |
| 0 | Atr-ERN04567 |  |  |  |  |  |  |
| 0 | Atr-ERN04568 |  |  |  |  |  |  |
| 0 | Atr-ERN04569 |  |  |  |  |  |  |
| 0 | Atr-ERN04570 |  |  |  |  |  |  |
| 0 | Atr-ERN04571 |  |  |  |  |  |  |
| 0 | Atr-ERN04572 |  |  |  |  |  |  |
| 0 | Atr-ERN04573 |  |  |  |  |  |  |
| 0 | Atr-ERN04574 |  |  |  |  |  |  |
| 0 | Atr-ERN04575 |  |  |  |  |  |  |
| 0 | Atr-ERN04576 |  |  |  |  |  |  |
| 0 | Atr-ERN04577 |  |  |  |  |  |  |
| 0 | Atr-ERN04578 |  |  |  |  |  |  |
| 0 | Atr-ERN04579 |  |  |  |  |  |  |
| 0 | Atr-ERN04580 |  |  |  |  |  |  |
| 0 | Atr-ERN04581 |  |  |  |  |  |  |
| 0 | Atr-ERN04582 |  |  |  |  |  |  |
| 0 | Atr-ERN04583 |  |  |  |  |  |  |
| 0 | Atr-ERN04584 |  |  |  |  |  |  |
| 0 | Atr-ERN04585 |  |  |  |  |  |  |
| 0 | Atr-ERN04586 |  |  |  |  |  |  |
| 0 | Atr-ERN04587 |  |  |  |  |  |  |
| 0 | Atr-ERN04588 |  |  |  |  |  |  |
| 0 | Atr-ERN04589 |  |  |  |  |  |  |
| 0 | Atr-ERN04590 |  |  |  |  |  |  |
| 0 | Atr-ERN04591 |  |  |  |  |  |  |
| 0 | Atr-ERN04592 |  |  |  |  |  |  |
| 0 | Atr-ERN04593 |  |  |  |  |  |  |
| 0 | Atr-ERN04594 |  |  |  |  |  |  |
| 0 | Atr-ERN04595 |  |  |  |  |  |  |
| 0 | Atr-ERN04596 |  |  |  |  |  |  |
| 0 | Atr-ERN04597 |  |  |  |  |  |  |
| 0 | Atr-ERN04598 |  |  |  |  |  |  |
| 0 | Atr-ERN04599 |  |  |  |  |  |  |
| 0 | Atr-ERN04600 |  |  |  |  |  |  |
| 0 | Atr-ERN04601 |  |  |  |  |  |  |
| 0 | Atr-ERN04602 |  |  |  |  |  |  |
| 0 | Atr-ERN04603 |  |  |  |  |  |  |
| 0 | Atr-ERN04604 |  |  |  |  |  |  |
| 0 | Atr-ERN04605 |  |  |  |  |  |  |
| 0 | Atr-ERN04606 |  |  |  |  |  |  |
| 0 | Atr-ERN04607 |  |  |  |  |  |  |
| 0 | Atr-ERN04608 |  |  |  |  |  |  |
| 0 | Atr-ERN04609 |  |  |  |  |  |  |
| 0 | Atr-ERN04610 |  |  |  |  |  |  |
| 0 | Atr-ERN04611 |  |  |  |  |  |  |
| 0 | Atr-ERN04612 |  |  |  |  |  |  |
| 0 | Atr-ERN04613 |  |  |  |  |  |  |
| 0 | Atr-ERN04614 |  |  |  |  |  |  |
| 0 | Atr-ERN04615 |  |  |  |  |  |  |
| 0 | Atr-ERN04616 |  |  |  |  |  |  |
| 0 | Atr-ERN04617 |  |  |  |  |  |  |
| 0 | Atr-ERN04618 |  |  |  |  |  |  |
| 0 | Atr-ERN04619 |  |  |  |  |  |  |
| 0 | Atr-ERN04620 |  |  |  |  |  |  |
| 0 | Atr-ERN04621 |  |  |  |  |  |  |
| 0 | Atr-ERN04622 |  |  |  |  |  |  |
| 0 | Atr-ERN04623 |  |  |  |  |  |  |
| 0 | Atr-ERN04624 |  |  |  |  |  |  |
| 0 | Atr-ERN04625 |  |  |  |  |  |  |
| 0 | Atr-ERN04626 |  |  |  |  |  |  |
| 0 | Atr-ERN04627 |  |  |  |  |  |  |
| 0 | Atr-ERN04628 |  |  |  |  |  |  |
