# Supplementary material for: Genome-wide identification, characterization and gene expression of BES1 transcription factor family in grapevine (Vitis vinifera L.)
Source: Sci Rep. 2023 Jan 5;13:240. doi: 10.1038/s41598-022-24407-y (PMC9816167; doi:10.1038/s41598-022-24407-y)
Supplement: Supplementary file 3 — Supplementary Information. [file 41598_2022_24407_MOESM3_ESM.zip › Vvi_Atr/Vitis_vinifera.PN40024.v4.dna_sm.toplevel.fa.vs.Amborella_trichopoda.AMTR1.0.dna_sm.toplevel.fa.html/Atr-AmTr_v1.0_scaffold00076.html]

|  |  |  |  |  |  |  |  |  |  |  |  |  |  |
| --- | --- | --- | --- | --- | --- | --- | --- | --- | --- | --- | --- | --- | --- |
| Duplication depth | Reference chromosome | Collinear blocks | | | | | | | | | | | |
| 0 | Atr-ERN04636 |  |  |  |  |  |  |
| 0 | Atr-ERN04637 |  |  |  |  |  |  |
| 0 | Atr-ERN04638 |  |  |  |  |  |  |
| 0 | Atr-ERN04639 |  |  |  |  |  |  |
| 0 | Atr-ERN04640 |  |  |  |  |  |  |
| 0 | Atr-ERN04641 |  |  |  |  |  |  |
| 0 | Atr-ERN04642 |  |  |  |  |  |  |
| 0 | Atr-ERN04643 |  |  |  |  |  |  |
| 0 | Atr-ERN04644 |  |  |  |  |  |  |
| 0 | Atr-ERN04645 |  |  |  |  |  |  |
| 0 | Atr-ERN04646 |  |  |  |  |  |  |
| 0 | Atr-ERN04647 |  |  |  |  |  |  |
| 0 | Atr-ERN04648 |  |  |  |  |  |  |
| 0 | Atr-ERN04649 |  |  |  |  |  |  |
| 0 | Atr-ERN04650 |  |  |  |  |  |  |
| 0 | Atr-ERN04651 |  |  |  |  |  |  |
| 0 | Atr-ERN04652 |  |  |  |  |  |  |
| 0 | Atr-ERN04653 |  |  |  |  |  |  |
| 0 | Atr-ERN04654 |  |  |  |  |  |  |
| 0 | Atr-ERN04655 |  |  |  |  |  |  |
| 0 | Atr-ERN04656 |  |  |  |  |  |  |
| 0 | Atr-ERN04657 |  |  |  |  |  |  |
| 0 | Atr-ERN04658 |  |  |  |  |  |  |
| 0 | Atr-ERN04659 |  |  |  |  |  |  |
| 0 | Atr-ERN04660 |  |  |  |  |  |  |
| 0 | Atr-ERN04661 |  |  |  |  |  |  |
| 0 | Atr-ERN04662 |  |  |  |  |  |  |
| 0 | Atr-ERN04663 |  |  |  |  |  |  |
| 0 | Atr-ERN04664 |  |  |  |  |  |  |
| 0 | Atr-ERN04665 |  |  |  |  |  |  |
| 0 | Atr-ERN04666 |  |  |  |  |  |  |
| 0 | Atr-ERN04667 |  |  |  |  |  |  |
| 0 | Atr-ERN04668 |  |  |  |  |  |  |
| 0 | Atr-ERN04669 |  |  |  |  |  |  |
| 0 | Atr-ERN04670 |  |  |  |  |  |  |
| 0 | Atr-ERN04671 |  |  |  |  |  |  |
| 0 | Atr-ERN04672 |  |  |  |  |  |  |
| 0 | Atr-ERN04673 |  |  |  |  |  |  |
| 0 | Atr-ERN04674 |  |  |  |  |  |  |
| 0 | Atr-ERN04675 |  |  |  |  |  |  |
| 0 | Atr-ERN04676 |  |  |  |  |  |  |
| 0 | Atr-ERN04677 |  |  |  |  |  |  |
| 0 | Atr-ERN04678 |  |  |  |  |  |  |
| 0 | Atr-ERN04679 |  |  |  |  |  |  |
| 0 | Atr-ERN04680 |  |  |  |  |  |  |
| 0 | Atr-ERN04681 |  |  |  |  |  |  |
| 0 | Atr-ERN04682 |  |  |  |  |  |  |
| 0 | Atr-ERN04683 |  |  |  |  |  |  |
| 0 | Atr-ERN04684 |  |  |  |  |  |  |
| 0 | Atr-ERN04685 |  |  |  |  |  |  |
| 0 | Atr-ERN04686 |  |  |  |  |  |  |
| 0 | Atr-ERN04687 |  |  |  |  |  |  |
| 0 | Atr-ERN04688 |  |  |  |  |  |  |
| 0 | Atr-ERN04689 |  |  |  |  |  |  |
| 0 | Atr-ERN04690 |  |  |  |  |  |  |
| 0 | Atr-ERN04691 |  |  |  |  |  |  |
| 0 | Atr-ERN04692 |  |  |  |  |  |  |
| 0 | Atr-ERN04693 |  |  |  |  |  |  |
| 0 | Atr-ERN04694 |  |  |  |  |  |  |
| 0 | Atr-ERN04695 |  |  |  |  |  |  |
| 0 | Atr-ERN04696 |  |  |  |  |  |  |
| 0 | Atr-ERN04697 |  |  |  |  |  |  |
| 0 | Atr-ERN04698 |  |  |  |  |  |  |
| 0 | Atr-ERN04699 |  |  |  |  |  |  |
| 0 | Atr-ERN04700 |  |  |  |  |  |  |
| 0 | Atr-ERN04701 |  |  |  |  |  |  |
| 0 | Atr-ERN04702 |  |  |  |  |  |  |
| 0 | Atr-ERN04703 |  |  |  |  |  |  |
| 0 | Atr-ERN04704 |  |  |  |  |  |  |
| 0 | Atr-ERN04705 |  |  |  |  |  |  |
| 0 | Atr-ERN04706 |  |  |  |  |  |  |
| 0 | Atr-ERN04707 |  |  |  |  |  |  |
| 0 | Atr-ERN04708 |  |  |  |  |  |  |
| 0 | Atr-ERN04709 |  |  |  |  |  |  |
| 0 | Atr-ERN04710 |  |  |  |  |  |  |
| 0 | Atr-ERN04711 |  |  |  |  |  |  |
| 0 | Atr-ERN04712 |  |  |  |  |  |  |
| 0 | Atr-ERN04713 |  |  |  |  |  |  |
| 0 | Atr-ERN04714 |  |  |  |  |  |  |
| 0 | Atr-ERN04715 |  |  |  |  |  |  |
| 0 | Atr-ERN04716 |  |  |  |  |  |  |
| 0 | Atr-ERN04717 |  |  |  |  |  |  |
| 0 | Atr-ERN04718 |  |  |  |  |  |  |
| 0 | Atr-ERN04719 |  |  |  |  |  |  |
| 0 | Atr-ERN04720 |  |  |  |  |  |  |
| 0 | Atr-ERN04721 |  |  |  |  |  |  |
