# Supplementary material for: Genome-wide identification, characterization and gene expression of BES1 transcription factor family in grapevine (Vitis vinifera L.)
Source: Sci Rep. 2023 Jan 5;13:240. doi: 10.1038/s41598-022-24407-y (PMC9816167; doi:10.1038/s41598-022-24407-y)
Supplement: Supplementary file 3 — Supplementary Information. [file 41598_2022_24407_MOESM3_ESM.zip › Vvi_Atr/Vitis_vinifera.PN40024.v4.dna_sm.toplevel.fa.vs.Amborella_trichopoda.AMTR1.0.dna_sm.toplevel.fa.html/Atr-AmTr_v1.0_scaffold00077.html]

|  |  |  |  |  |  |  |  |  |  |  |  |  |  |
| --- | --- | --- | --- | --- | --- | --- | --- | --- | --- | --- | --- | --- | --- |
| Duplication depth | Reference chromosome | Collinear blocks | | | | | | | | | | | |
| 0 | Atr-ERN04094 |  |  |  |  |  |  |
| 1 | Atr-ERN04095 |  | Vvi-Vitvi06g00768\_t001 |  |  |  |  |  |
| 1 | Atr-ERN04096 |  | | | |  |  |  |  |  |
| 1 | Atr-ERN04097 |  | | | |  |  |  |  |  |
| 1 | Atr-ERN04098 |  | | | |  |  |  |  |  |
| 1 | Atr-ERN04099 |  | | | |  |  |  |  |  |
| 1 | Atr-ERN04100 |  | | | |  |  |  |  |  |
| 1 | Atr-ERN04101 |  | | | |  |  |  |  |  |
| 1 | Atr-ERN04102 |  | | | |  |  |  |  |  |
| 1 | Atr-ERN04103 |  | | | |  |  |  |  |  |
| 1 | Atr-ERN04104 |  | | | |  |  |  |  |  |
| 1 | Atr-ERN04105 |  | | | |  |  |  |  |  |
| 1 | Atr-ERN04106 |  | | | |  |  |  |  |  |
| 2 | Atr-ERN04107 |  | | | |  | Vvi-Vitvi08g00820\_t001 |  |  |  |  |
| 3 | Atr-ERN04108 |  | | | |  | | | |  | Vvi-Vitvi13g00137\_t001 |  |  |  |
| 3 | Atr-ERN04109 |  | | | |  | | | |  | Vvi-Vitvi13g00138\_t001 |  |  |  |
| 3 | Atr-ERN04110 |  | | | |  | | | |  | | | |  |  |  |
| 3 | Atr-ERN04111 |  | | | |  | | | |  | | | |  |  |  |
| 3 | Atr-ERN04112 |  | | | |  | | | |  | | | |  |  |  |
| 3 | Atr-ERN04113 |  | | | |  | | | |  | | | |  |  |  |
| 3 | Atr-ERN04114 |  | | | |  | | | |  | | | |  |  |  |
| 3 | Atr-ERN04115 |  | | | |  | | | |  | | | |  |  |  |
| 3 | Atr-ERN04116 |  | | | |  | | | |  | | | |  |  |  |
| 3 | Atr-ERN04117 |  | | | |  | | | |  | Vvi-Vitvi13g00159\_t001 |  |  |  |
| 3 | Atr-ERN04118 |  | Vvi-Vitvi06g00786\_t001 |  | | | |  | Vvi-Vitvi13g00160\_t001 |  |  |  |
| 4 | Atr-ERN04119 |  | Vvi-Vitvi06g00787\_t001 |  | | | |  | | | |  | Vvi-Vitvi13g00177\_t001 |  |  |
| 4 | Atr-ERN04120 |  | | | |  | Vvi-Vitvi08g00847\_t001 |  | | | |  | Vvi-Vitvi13g00176\_t001 |  |  |
| 4 | Atr-ERN04121 |  | Vvi-Vitvi06g00789\_t002 |  | | | |  | | | |  | Vvi-Vitvi13g00175\_t003 |  |  |
| 4 | Atr-ERN04122 |  | | | |  | Vvi-Vitvi08g00850\_t001 |  | | | |  | | | |  |  |
| 4 | Atr-ERN04123 |  | | | |  | | | |  | | | |  | Vvi-Vitvi13g04040\_t001 |  |  |
| 4 | Atr-ERN04124 |  | | | |  | | | |  | | | |  | | | |  |  |
| 4 | Atr-ERN04125 |  | | | |  | | | |  | | | |  | | | |  |  |
| 4 | Atr-ERN04126 |  | | | |  | | | |  | | | |  | | | |  |  |
| 4 | Atr-ERN04127 |  | | | |  | | | |  | | | |  | | | |  |  |
| 4 | Atr-ERN04128 |  | | | |  | | | |  | | | |  | | | |  |  |
| 4 | Atr-ERN04129 |  | | | |  | | | |  | | | |  | Vvi-Vitvi13g00173\_t001 |  |  |
| 4 | Atr-ERN04130 |  | | | |  | | | |  | | | |  | | | |  |  |
| 4 | Atr-ERN04131 |  | | | |  | | | |  | | | |  | | | |  |  |
| 4 | Atr-ERN04132 |  | Vvi-Vitvi06g00791\_t001 |  | | | |  | | | |  | | | |  |  |
| 4 | Atr-ERN04133 |  | | | |  | Vvi-Vitvi08g00852\_t001 |  | | | |  | Vvi-Vitvi13g00172\_t001 |  |  |
| 4 | Atr-ERN04134 |  | | | |  | | | |  | | | |  | | | |  |  |
| 4 | Atr-ERN04135 |  | | | |  | | | |  | | | |  | | | |  |  |
| 4 | Atr-ERN04136 |  | | | |  | | | |  | | | |  | | | |  |  |
| 4 | Atr-ERN04137 |  | | | |  | | | |  | | | |  | | | |  |  |
| 4 | Atr-ERN04138 |  | Vvi-Vitvi06g00799\_t001 |  | Vvi-Vitvi08g00853\_t001 |  | | | |  | | | |  |  |
| 4 | Atr-ERN04139 |  | | | |  | | | |  | | | |  | | | |  |  |
| 4 | Atr-ERN04140 |  | | | |  | | | |  | | | |  | | | |  |  |
| 4 | Atr-ERN04141 |  | | | |  | | | |  | | | |  | Vvi-Vitvi13g01913\_t001 |  |  |
| 4 | Atr-ERN04142 |  | | | |  | | | |  | | | |  | | | |  |  |
| 4 | Atr-ERN04143 |  | | | |  | | | |  | | | |  | | | |  |  |
| 4 | Atr-ERN04144 |  | Vvi-Vitvi06g00800\_t001 |  | | | |  | Vvi-Vitvi13g00171\_t001 |  | | | |  |  |
| 4 | Atr-ERN04145 |  | | | |  | | | |  | | | |  | | | |  |  |
| 4 | Atr-ERN04146 |  | Vvi-Vitvi06g00801\_t001 |  | Vvi-Vitvi08g00857\_t001 |  | | | |  | Vvi-Vitvi13g01912\_t001 |  |  |
| 4 | Atr-ERN04147 |  | | | |  | | | |  | | | |  | Vvi-Vitvi13g01911\_t002 |  |  |
| 4 | Atr-ERN04148 |  | | | |  | | | |  | | | |  | | | |  |  |
| 4 | Atr-ERN04149 |  | | | |  | | | |  | | | |  | | | |  |  |
| 4 | Atr-ERN04150 |  | | | |  | Vvi-Vitvi08g00858\_t001 |  | | | |  | | | |  |  |
| 4 | Atr-ERN04151 |  | | | |  | | | |  | | | |  | | | |  |  |
| 4 | Atr-ERN04152 |  | | | |  | | | |  | | | |  | | | |  |  |
| 4 | Atr-ERN04153 |  | | | |  | | | |  | | | |  | | | |  |  |
| 4 | Atr-ERN04154 |  | | | |  | | | |  | | | |  | | | |  |  |
| 4 | Atr-ERN04155 |  | | | |  | | | |  | | | |  | | | |  |  |
| 4 | Atr-ERN04156 |  | | | |  | | | |  | | | |  | | | |  |  |
| 4 | Atr-ERN04157 |  | | | |  | | | |  | | | |  | Vvi-Vitvi13g00170\_t001 |  |  |
| 3 | Atr-ERN04158 |  | Vvi-Vitvi06g04252\_t001 |  | | | |  | | | |  |  |  |
| 3 | Atr-ERN04159 |  | Vvi-Vitvi06g04253\_t001 |  | Vvi-Vitvi08g00859\_t001 |  | | | |  |  |  |
| 3 | Atr-ERN04160 |  | | | |  | | | |  | Vvi-Vitvi13g00179\_t001 |  |  |  |
| 3 | Atr-ERN04161 |  | | | |  | | | |  | | | |  |  |  |
| 3 | Atr-ERN04162 |  | | | |  | | | |  | Vvi-Vitvi13g00180\_t001 |  |  |  |
| 3 | Atr-ERN04163 |  | | | |  | Vvi-Vitvi08g00860\_t001 |  | | | |  |  |  |
| 3 | Atr-ERN04164 |  | | | |  | Vvi-Vitvi08g00861\_t001 |  | | | |  |  |  |
| 3 | Atr-ERN04165 |  | | | |  | | | |  | | | |  |  |  |
| 4 | Atr-ERN04166 |  | | | |  | Vvi-Vitvi08g00862\_t001 |  | Vvi-Vitvi13g00181\_t001 |  | Vvi-Vitvi02g00123\_t001 |  |  |
| 4 | Atr-ERN04167 |  | | | |  | | | |  | | | |  | | | |  |  |
| 4 | Atr-ERN04168 |  | | | |  | Vvi-Vitvi08g00863\_t001 |  | Vvi-Vitvi13g00182\_t001 |  | | | |  |  |
| 4 | Atr-ERN04169 |  | | | |  | | | |  | Vvi-Vitvi13g01915\_t001 |  | | | |  |  |
| 4 | Atr-ERN04170 |  | | | |  | Vvi-Vitvi08g00864\_t001 |  | | | |  | | | |  |  |
| 4 | Atr-ERN04171 |  | | | |  | Vvi-Vitvi08g00865\_t001 |  | | | |  | | | |  |  |
| 4 | Atr-ERN04172 |  | | | |  | | | |  | Vvi-Vitvi13g00183\_t001 |  | | | |  |  |
| 4 | Atr-ERN04173 |  | | | |  | | | |  | | | |  | | | |  |  |
| 4 | Atr-ERN04174 |  | Vvi-Vitvi06g01781\_t001 |  | | | |  | | | |  | | | |  |  |
| 4 | Atr-ERN04175 |  | | | |  | | | |  | Vvi-Vitvi13g00185\_t001 |  | | | |  |  |
| 4 | Atr-ERN04176 |  | | | |  | | | |  | | | |  | | | |  |  |
| 4 | Atr-ERN04177 |  | | | |  | | | |  | | | |  | | | |  |  |
| 4 | Atr-ERN04178 |  | | | |  | | | |  | | | |  | | | |  |  |
| 4 | Atr-ERN04179 |  | | | |  | | | |  | | | |  | | | |  |  |
| 4 | Atr-ERN04180 |  | | | |  | | | |  | | | |  | Vvi-Vitvi02g00114\_t001 |  |  |
| 4 | Atr-ERN04181 |  | | | |  | | | |  | | | |  | | | |  |  |
| 4 | Atr-ERN04182 |  | | | |  | | | |  | | | |  | | | |  |  |
| 4 | Atr-ERN04183 |  | Vvi-Vitvi06g00804\_t001 |  | | | |  | | | |  | | | |  |  |
| 3 | Atr-ERN04184 |  |  |  | | | |  | | | |  | | | |  |  |
| 3 | Atr-ERN04185 |  |  |  | | | |  | | | |  | | | |  |  |
| 4 | Atr-ERN04186 |  | Vvi-Vitvi15g00994\_t001 |  | | | |  | | | |  | | | |  |  |
| 4 | Atr-ERN04187 |  | | | |  | | | |  | Vvi-Vitvi13g00188\_t001 |  | | | |  |  |
| 4 | Atr-ERN04188 |  | | | |  | | | |  | | | |  | | | |  |  |
| 4 | Atr-ERN04189 |  | | | |  | | | |  | | | |  | | | |  |  |
| 4 | Atr-ERN04190 |  | | | |  | Vvi-Vitvi08g00866\_t001 |  | | | |  | | | |  |  |
| 4 | Atr-ERN04191 |  | | | |  | | | |  | | | |  | | | |  |  |
| 4 | Atr-ERN04192 |  | | | |  | | | |  | | | |  | | | |  |  |
| 4 | Atr-ERN04193 |  | | | |  | Vvi-Vitvi08g00869\_t001 |  | | | |  | | | |  |  |
| 4 | Atr-ERN04194 |  | | | |  | | | |  | | | |  | | | |  |  |
| 4 | Atr-ERN04195 |  | | | |  | Vvi-Vitvi08g00870\_t001 |  | Vvi-Vitvi13g00191\_t001 |  | | | |  |  |
| 4 | Atr-ERN04196 |  | | | |  | Vvi-Vitvi08g00871\_t001 |  | | | |  | Vvi-Vitvi02g00108\_t001 |  |  |
| 4 | Atr-ERN04197 |  | | | |  | | | |  | Vvi-Vitvi13g01918\_t001 |  | | | |  |  |
| 4 | Atr-ERN04198 |  | | | |  | | | |  | Vvi-Vitvi13g00192\_t001 |  | | | |  |  |
| 4 | Atr-ERN04199 |  | | | |  | | | |  | | | |  | | | |  |  |
| 4 | Atr-ERN04200 |  | | | |  | | | |  | | | |  | | | |  |  |
| 4 | Atr-ERN04201 |  | | | |  | | | |  | | | |  | | | |  |  |
| 4 | Atr-ERN04202 |  | | | |  | | | |  | | | |  | | | |  |  |
| 4 | Atr-ERN04203 |  | | | |  | | | |  | | | |  | | | |  |  |
| 4 | Atr-ERN04204 |  | | | |  | | | |  | | | |  | | | |  |  |
| 4 | Atr-ERN04205 |  | | | |  | | | |  | | | |  | | | |  |  |
| 4 | Atr-ERN04206 |  | | | |  | | | |  | | | |  | | | |  |  |
| 4 | Atr-ERN04207 |  | | | |  | | | |  | | | |  | | | |  |  |
| 4 | Atr-ERN04208 |  | | | |  | | | |  | Vvi-Vitvi13g00193\_t001 |  | | | |  |  |
| 4 | Atr-ERN04209 |  | Vvi-Vitvi15g01013\_t001 |  | | | |  | Vvi-Vitvi13g00194\_t001 |  | Vvi-Vitvi02g00105\_t001 |  |  |
| 4 | Atr-ERN04210 |  | | | |  | | | |  | | | |  | | | |  |  |
| 4 | Atr-ERN04211 |  | | | |  | | | |  | | | |  | | | |  |  |
| 5 | Atr-ERN04212 |  | | | |  | | | |  | | | |  | | | |  | Vvi-Vitvi06g00805\_t001 |  |
| 5 | Atr-ERN04213 |  | | | |  | | | |  | | | |  | | | |  | | | |  |
| 5 | Atr-ERN04214 |  | | | |  | | | |  | | | |  | | | |  | | | |  |
| 5 | Atr-ERN04215 |  | | | |  | | | |  | | | |  | | | |  | | | |  |
| 5 | Atr-ERN04216 |  | | | |  | | | |  | Vvi-Vitvi13g00196\_t001 |  | | | |  | | | |  |
| 5 | Atr-ERN04217 |  | | | |  | | | |  | | | |  | | | |  | | | |  |
| 5 | Atr-ERN04218 |  | | | |  | | | |  | | | |  | | | |  | | | |  |
| 5 | Atr-ERN04219 |  | | | |  | | | |  | | | |  | | | |  | | | |  |
| 5 | Atr-ERN04220 |  | | | |  | Vvi-Vitvi08g00872\_t001 |  | | | |  | | | |  | | | |  |
| 5 | Atr-ERN04221 |  | | | |  | Vvi-Vitvi08g00874\_t001 |  | | | |  | | | |  | | | |  |
| 5 | Atr-ERN04222 |  | | | |  | Vvi-Vitvi08g02097\_t001 |  | | | |  | | | |  | | | |  |
| 5 | Atr-ERN04223 |  | | | |  | | | |  | Vvi-Vitvi13g00197\_t001 |  | | | |  | | | |  |
| 5 | Atr-ERN04224 |  | | | |  | | | |  | Vvi-Vitvi13g04045\_t001 |  | | | |  | | | |  |
| 4 | Atr-ERN04225 |  | | | |  | | | |  |  |  | | | |  | | | |  |
| 4 | Atr-ERN04226 |  | | | |  | Vvi-Vitvi08g00877\_t001 |  |  |  | | | |  | | | |  |
| 4 | Atr-ERN04227 |  | | | |  | | | |  |  |  | | | |  | | | |  |
| 4 | Atr-ERN04228 |  | | | |  | | | |  |  |  | | | |  | | | |  |
| 4 | Atr-ERN04229 |  | | | |  | Vvi-Vitvi08g04140\_t001 |  |  |  | | | |  | | | |  |
| 4 | Atr-ERN04230 |  | | | |  | Vvi-Vitvi08g04141\_t001 |  |  |  | | | |  | | | |  |
| 4 | Atr-ERN04231 |  | | | |  | | | |  |  |  | | | |  | | | |  |
| 4 | Atr-ERN04232 |  | Vvi-Vitvi15g01021\_t001 |  | | | |  |  |  | Vvi-Vitvi02g00093\_t001 |  | Vvi-Vitvi06g00810\_t001 |  |
| 4 | Atr-ERN04233 |  | Vvi-Vitvi15g04595\_t001 |  | Vvi-Vitvi08g04142\_t002 |  |  |  | Vvi-Vitvi02g04013\_t001 |  | Vvi-Vitvi06g00811\_t001 |  |
| 4 | Atr-ERN04234 |  | Vvi-Vitvi15g01024\_t001 |  | | | |  |  |  | | | |  | | | |  |
| 4 | Atr-ERN04235 |  | | | |  | Vvi-Vitvi08g00881\_t001 |  |  |  | | | |  | | | |  |
| 4 | Atr-ERN04236 |  | | | |  | | | |  |  |  | | | |  | | | |  |
| 4 | Atr-ERN04237 |  | | | |  | | | |  |  |  | | | |  | | | |  |
| 4 | Atr-ERN04238 |  | | | |  | Vvi-Vitvi08g00883\_t004 |  |  |  | | | |  | Vvi-Vitvi06g00812\_t001 |  |
| 3 | Atr-ERN04239 |  | | | |  |  |  |  |  | | | |  | | | |  |
| 5 | Atr-ERN04240 |  | | | |  | Vvi-Vitvi13g00028\_t001 |  | Vvi-Vitvi08g00055\_t001 |  | | | |  | | | |  |
| 5 | Atr-ERN04241 |  | | | |  | | | |  | | | |  | | | |  | | | |  |
| 6 | Atr-ERN04242 |  | Vvi-Vitvi15g01029\_t001 |  | | | |  | | | |  | | | |  | | | |  | Vvi-Vitvi08g00041\_t001 |
| 6 | Atr-ERN04243 |  | | | |  | | | |  | | | |  | | | |  | | | |  | | | |
| 6 | Atr-ERN04244 |  | | | |  | | | |  | | | |  | | | |  | | | |  | Vvi-Vitvi08g00042\_t001 |
| 6 | Atr-ERN04245 |  | | | |  | | | |  | | | |  | | | |  | | | |  | | | |
| 6 | Atr-ERN04246 |  | | | |  | | | |  | | | |  | | | |  | | | |  | Vvi-Vitvi08g00046\_t002 |
| 6 | Atr-ERN04247 |  | | | |  | | | |  | | | |  | | | |  | | | |  | | | |
| 6 | Atr-ERN04248 |  | | | |  | | | |  | | | |  | | | |  | | | |  | | | |
| 6 | Atr-ERN04249 |  | | | |  | | | |  | Vvi-Vitvi08g00047\_t001 |  | | | |  | Vvi-Vitvi06g00819\_t002 |  | Vvi-Vitvi08g00047\_t001 |
| 6 | Atr-ERN04250 |  | | | |  | | | |  | | | |  | | | |  | | | |  | | | |
| 6 | Atr-ERN04251 |  | | | |  | | | |  | | | |  | | | |  | | | |  | | | |
| 6 | Atr-ERN04252 |  | | | |  | | | |  | | | |  | | | |  | | | |  | | | |
| 6 | Atr-ERN04253 |  | | | |  | | | |  | | | |  | | | |  | | | |  | Vvi-Vitvi08g00051\_t002 |
| 6 | Atr-ERN04254 |  | | | |  | | | |  | | | |  | | | |  | | | |  | Vvi-Vitvi08g00052\_t001 |
| 6 | Atr-ERN04255 |  | | | |  | Vvi-Vitvi13g00011\_t001 |  | | | |  | | | |  | | | |  | | | |
| 6 | Atr-ERN04256 |  | | | |  | | | |  | | | |  | Vvi-Vitvi02g01333\_t001 |  | | | |  | Vvi-Vitvi08g00053\_t001 |
| 6 | Atr-ERN04257 |  | Vvi-Vitvi15g01047\_t001 |  | | | |  | | | |  | | | |  | | | |  | | | |
| 6 | Atr-ERN04258 |  | | | |  | | | |  | | | |  | | | |  | | | |  | | | |
| 6 | Atr-ERN04259 |  | | | |  | | | |  | | | |  | | | |  | | | |  | Vvi-Vitvi08g00054\_t001 |
| 5 | Atr-ERN04260 |  | | | |  | | | |  | | | |  | | | |  | | | |  |
| 5 | Atr-ERN04261 |  | | | |  | | | |  | | | |  | | | |  | | | |  |
| 5 | Atr-ERN04262 |  | | | |  | | | |  | | | |  | | | |  | | | |  |
| 5 | Atr-ERN04263 |  | | | |  | | | |  | Vvi-Vitvi08g00041\_t001 |  | Vvi-Vitvi02g00079\_t001 |  | | | |  |
| 5 | Atr-ERN04264 |  | | | |  | | | |  | | | |  | | | |  | | | |  |
| 5 | Atr-ERN04265 |  | | | |  | | | |  | Vvi-Vitvi08g00038\_t001 |  | | | |  | Vvi-Vitvi06g00826\_t001 |  |
| 5 | Atr-ERN04266 |  | | | |  | | | |  | | | |  | | | |  | | | |  |
| 5 | Atr-ERN04267 |  | | | |  | | | |  | Vvi-Vitvi08g00037\_t001 |  | | | |  | Vvi-Vitvi06g00827\_t001 |  |
| 5 | Atr-ERN04268 |  | | | |  | | | |  | | | |  | | | |  | | | |  |
| 5 | Atr-ERN04269 |  | | | |  | Vvi-Vitvi13g00009\_t001 |  | | | |  | | | |  | | | |  |
| 5 | Atr-ERN04270 |  | Vvi-Vitvi15g01061\_t001 |  | | | |  | | | |  | | | |  | | | |  |
| 4 | Atr-ERN04271 |  |  |  | Vvi-Vitvi13g00008\_t001 |  | | | |  | | | |  | | | |  |
| 4 | Atr-ERN04272 |  |  |  | Vvi-Vitvi13g00007\_t001 |  | | | |  | | | |  | | | |  |
| 4 | Atr-ERN04273 |  |  |  | | | |  | | | |  | | | |  | | | |  |
| 4 | Atr-ERN04274 |  |  |  | | | |  | | | |  | | | |  | | | |  |
| 4 | Atr-ERN04275 |  |  |  | Vvi-Vitvi13g00005\_t001 |  | | | |  | | | |  | | | |  |
| 4 | Atr-ERN04276 |  |  |  | Vvi-Vitvi13g01879\_t001 |  | | | |  | | | |  | | | |  |
| 4 | Atr-ERN04277 |  |  |  | | | |  | Vvi-Vitvi08g00030\_t001 |  | | | |  | Vvi-Vitvi06g00829\_t001 |  |
| 4 | Atr-ERN04278 |  |  |  | | | |  | Vvi-Vitvi08g00028\_t002 |  | | | |  | | | |  |
| 4 | Atr-ERN04279 |  |  |  | | | |  | | | |  | | | |  | | | |  |
| 4 | Atr-ERN04280 |  |  |  | | | |  | | | |  | | | |  | Vvi-Vitvi06g00831\_t001 |  |
| 4 | Atr-ERN04281 |  |  |  | | | |  | | | |  | | | |  | | | |  |
| 4 | Atr-ERN04282 |  |  |  | | | |  | Vvi-Vitvi08g00027\_t001 |  | | | |  | | | |  |
| 4 | Atr-ERN04283 |  |  |  | | | |  | | | |  | | | |  | | | |  |
| 4 | Atr-ERN04284 |  |  |  | | | |  | | | |  | | | |  | Vvi-Vitvi06g00832\_t001 |  |
| 4 | Atr-ERN04285 |  |  |  | | | |  | | | |  | | | |  | Vvi-Vitvi06g00833\_t001 |  |
| 4 | Atr-ERN04286 |  |  |  | | | |  | | | |  | Vvi-Vitvi02g00060\_t001 |  | | | |  |
| 4 | Atr-ERN04287 |  |  |  | | | |  | | | |  | | | |  | | | |  |
| 4 | Atr-ERN04288 |  |  |  | | | |  | | | |  | | | |  | | | |  |
| 4 | Atr-ERN04289 |  |  |  | | | |  | | | |  | | | |  | | | |  |
| 4 | Atr-ERN04290 |  |  |  | | | |  | Vvi-Vitvi08g00026\_t001 |  | | | |  | | | |  |
| 4 | Atr-ERN04291 |  |  |  | | | |  | | | |  | | | |  | | | |  |
| 4 | Atr-ERN04292 |  |  |  | | | |  | | | |  | Vvi-Vitvi02g00059\_t001 |  | | | |  |
| 4 | Atr-ERN04293 |  |  |  | | | |  | | | |  | | | |  | | | |  |
| 4 | Atr-ERN04294 |  |  |  | | | |  | | | |  | | | |  | Vvi-Vitvi06g00834\_t002 |  |
| 4 | Atr-ERN04295 |  |  |  | | | |  | | | |  | | | |  | | | |  |
| 4 | Atr-ERN04296 |  |  |  | | | |  | Vvi-Vitvi08g01973\_t001 |  | | | |  | | | |  |
| 4 | Atr-ERN04297 |  |  |  | | | |  | Vvi-Vitvi08g00023\_t001 |  | Vvi-Vitvi02g01317\_t001 |  | | | |  |
| 4 | Atr-ERN04298 |  |  |  | | | |  | Vvi-Vitvi08g00022\_t001 |  | | | |  | | | |  |
| 4 | Atr-ERN04299 |  |  |  | Vvi-Vitvi13g00003\_t001 |  | Vvi-Vitvi08g00021\_t001 |  | | | |  | | | |  |
| 4 | Atr-ERN04300 |  |  |  | Vvi-Vitvi13g01878\_t001 |  | | | |  | | | |  | | | |  |
| 3 | Atr-ERN04301 |  |  |  |  |  | Vvi-Vitvi08g00020\_t001 |  | Vvi-Vitvi02g00044\_t001 |  | | | |  |
| 2 | Atr-ERN04302 |  |  |  |  |  | Vvi-Vitvi08g00019\_t001 |  |  |  | | | |  |
| 2 | Atr-ERN04303 |  |  |  |  |  | | | |  |  |  | | | |  |
| 2 | Atr-ERN04304 |  |  |  |  |  | Vvi-Vitvi08g00018\_t001 |  |  |  | | | |  |
| 2 | Atr-ERN04305 |  |  |  |  |  | | | |  |  |  | | | |  |
| 2 | Atr-ERN04306 |  |  |  |  |  | | | |  |  |  | | | |  |
| 2 | Atr-ERN04307 |  |  |  |  |  | | | |  |  |  | | | |  |
| 2 | Atr-ERN04308 |  |  |  |  |  | Vvi-Vitvi08g01970\_t001 |  |  |  | | | |  |
| 2 | Atr-ERN04309 |  |  |  |  |  | | | |  |  |  | | | |  |
| 2 | Atr-ERN04310 |  |  |  |  |  | | | |  |  |  | Vvi-Vitvi06g00841\_t001 |  |
| 2 | Atr-ERN04311 |  |  |  |  |  | | | |  |  |  | | | |  |
| 2 | Atr-ERN04312 |  |  |  |  |  | | | |  |  |  | | | |  |
| 2 | Atr-ERN04313 |  |  |  |  |  | Vvi-Vitvi08g00013\_t002 |  |  |  | | | |  |
| 2 | Atr-ERN04314 |  |  |  |  |  | Vvi-Vitvi08g00012\_t001 |  |  |  | | | |  |
| 2 | Atr-ERN04315 |  |  |  |  |  | Vvi-Vitvi08g00008\_t001 |  |  |  | | | |  |
| 2 | Atr-ERN04316 |  |  |  |  |  | | | |  |  |  | | | |  |
| 2 | Atr-ERN04317 |  |  |  |  |  | | | |  |  |  | | | |  |
| 2 | Atr-ERN04318 |  |  |  |  |  | | | |  |  |  | | | |  |
| 2 | Atr-ERN04319 |  |  |  |  |  | Vvi-Vitvi08g04002\_t001 |  |  |  | | | |  |
| 1 | Atr-ERN04320 |  |  |  |  |  |  |  |  |  | Vvi-Vitvi06g00844\_t002 |  |
| 0 | Atr-ERN04321 |  |  |  |  |  |  |
| 0 | Atr-ERN04322 |  |  |  |  |  |  |
| 0 | Atr-ERN04323 |  |  |  |  |  |  |
| 0 | Atr-ERN04324 |  |  |  |  |  |  |
| 0 | Atr-ERN04325 |  |  |  |  |  |  |
| 0 | Atr-ERN04326 |  |  |  |  |  |  |
| 0 | Atr-ERN04327 |  |  |  |  |  |  |
| 0 | Atr-ERN04328 |  |  |  |  |  |  |
| 0 | Atr-ERN04329 |  |  |  |  |  |  |
| 0 | Atr-ERN04330 |  |  |  |  |  |  |
| 0 | Atr-ERN04331 |  |  |  |  |  |  |
| 0 | Atr-ERN04332 |  |  |  |  |  |  |
| 0 | Atr-ERN04333 |  |  |  |  |  |  |
| 0 | Atr-ERN04334 |  |  |  |  |  |  |
