# Supplementary material for: Genome-wide identification, characterization and gene expression of BES1 transcription factor family in grapevine (Vitis vinifera L.)
Source: Sci Rep. 2023 Jan 5;13:240. doi: 10.1038/s41598-022-24407-y (PMC9816167; doi:10.1038/s41598-022-24407-y)
Supplement: Supplementary file 3 — Supplementary Information. [file 41598_2022_24407_MOESM3_ESM.zip › Vvi_Atr/Vitis_vinifera.PN40024.v4.dna_sm.toplevel.fa.vs.Amborella_trichopoda.AMTR1.0.dna_sm.toplevel.fa.html/Atr-AmTr_v1.0_scaffold00078.html]

|  |  |  |  |  |  |  |  |  |  |  |  |  |  |
| --- | --- | --- | --- | --- | --- | --- | --- | --- | --- | --- | --- | --- | --- |
| Duplication depth | Reference chromosome | Collinear blocks | | | | | | | | | | | |
| 0 | Atr-ERN03720 |  |  |  |  |  |  |
| 1 | Atr-ERN03721 |  | Vvi-Vitvi09g00615\_t001 |  |  |  |  |  |
| 2 | Atr-ERN03722 |  | | | |  | Vvi-Vitvi04g00487\_t001 |  |  |  |  |
| 3 | Atr-ERN03723 |  | Vvi-Vitvi09g01666\_t001 |  | | | |  | Vvi-Vitvi11g01445\_t001 |  |  |  |
| 3 | Atr-ERN03724 |  | Vvi-Vitvi09g01664\_t001 |  | | | |  | | | |  |  |  |
| 3 | Atr-ERN03725 |  | Vvi-Vitvi09g00607\_t002 |  | | | |  | | | |  |  |  |
| 3 | Atr-ERN03726 |  | | | |  | | | |  | | | |  |  |  |
| 3 | Atr-ERN03727 |  | | | |  | | | |  | Vvi-Vitvi11g00481\_t001 |  |  |  |
| 3 | Atr-ERN03728 |  | | | |  | | | |  | | | |  |  |  |
| 3 | Atr-ERN03729 |  | | | |  | | | |  | | | |  |  |  |
| 3 | Atr-ERN03730 |  | | | |  | | | |  | | | |  |  |  |
| 3 | Atr-ERN03731 |  | Vvi-Vitvi09g00604\_t001 |  | Vvi-Vitvi04g00485\_t001 |  | | | |  |  |  |
| 3 | Atr-ERN03732 |  | Vvi-Vitvi09g00603\_t001 |  | | | |  | Vvi-Vitvi11g00479\_t001 |  |  |  |
| 3 | Atr-ERN03733 |  | | | |  | Vvi-Vitvi04g00484\_t001 |  | | | |  |  |  |
| 3 | Atr-ERN03734 |  | | | |  | Vvi-Vitvi04g00483\_t002 |  | | | |  |  |  |
| 3 | Atr-ERN03735 |  | Vvi-Vitvi09g00601\_t001 |  | | | |  | Vvi-Vitvi11g00477\_t001 |  |  |  |
| 3 | Atr-ERN03736 |  | | | |  | | | |  | | | |  |  |  |
| 3 | Atr-ERN03737 |  | | | |  | | | |  | | | |  |  |  |
| 3 | Atr-ERN03738 |  | | | |  | | | |  | Vvi-Vitvi11g00476\_t003 |  |  |  |
| 3 | Atr-ERN03739 |  | | | |  | | | |  | | | |  |  |  |
| 3 | Atr-ERN03740 |  | | | |  | Vvi-Vitvi04g00482\_t001 |  | Vvi-Vitvi11g00475\_t001 |  |  |  |
| 3 | Atr-ERN03741 |  | | | |  | | | |  | | | |  |  |  |
| 3 | Atr-ERN03742 |  | Vvi-Vitvi09g00599\_t001 |  | | | |  | | | |  |  |  |
| 3 | Atr-ERN03743 |  | Vvi-Vitvi09g04185\_t001 |  | Vvi-Vitvi04g00481\_t003 |  | Vvi-Vitvi11g00474\_t001 |  |  |  |
| 3 | Atr-ERN03744 |  | Vvi-Vitvi09g00595\_t001 |  | Vvi-Vitvi04g00480\_t001 |  | Vvi-Vitvi11g00469\_t001 |  |  |  |
| 3 | Atr-ERN03745 |  | | | |  | | | |  | Vvi-Vitvi11g00468\_t001 |  |  |  |
| 3 | Atr-ERN03746 |  | Vvi-Vitvi09g00592\_t001 |  | | | |  | | | |  |  |  |
| 3 | Atr-ERN03747 |  | | | |  | Vvi-Vitvi04g00477\_t001 |  | | | |  |  |  |
| 3 | Atr-ERN03748 |  | | | |  | | | |  | | | |  |  |  |
| 3 | Atr-ERN03749 |  | | | |  | | | |  | | | |  |  |  |
| 3 | Atr-ERN03750 |  | Vvi-Vitvi09g00591\_t001 |  | Vvi-Vitvi04g00476\_t001 |  | Vvi-Vitvi11g00466\_t001 |  |  |  |
| 3 | Atr-ERN03751 |  | | | |  | | | |  | | | |  |  |  |
| 3 | Atr-ERN03752 |  | | | |  | | | |  | Vvi-Vitvi11g04106\_t001 |  |  |  |
| 3 | Atr-ERN03753 |  | | | |  | | | |  | | | |  |  |  |
| 3 | Atr-ERN03754 |  | Vvi-Vitvi09g00590\_t001 |  | | | |  | | | |  |  |  |
| 2 | Atr-ERN03755 |  |  |  | Vvi-Vitvi04g01903\_t001 |  | | | |  |  |  |
| 2 | Atr-ERN03756 |  |  |  | Vvi-Vitvi04g01902\_t001 |  | Vvi-Vitvi11g04105\_t003 |  |  |  |
| 1 | Atr-ERN03757 |  |  |  |  |  | | | |  |  |  |
| 1 | Atr-ERN03758 |  |  |  |  |  | | | |  |  |  |
| 2 | Atr-ERN03759 |  | Vvi-Vitvi14g03051\_t001 |  |  |  | | | |  |  |  |
| 2 | Atr-ERN03760 |  | | | |  |  |  | | | |  |  |  |
| 2 | Atr-ERN03761 |  | | | |  |  |  | | | |  |  |  |
| 2 | Atr-ERN03762 |  | | | |  |  |  | | | |  |  |  |
| 2 | Atr-ERN03763 |  | | | |  |  |  | Vvi-Vitvi11g00464\_t001 |  |  |  |
| 1 | Atr-ERN03764 |  | | | |  |  |  |  |  |
| 1 | Atr-ERN03765 |  | | | |  |  |  |  |  |
| 1 | Atr-ERN03766 |  | | | |  |  |  |  |  |
| 1 | Atr-ERN03767 |  | | | |  |  |  |  |  |
| 1 | Atr-ERN03768 |  | | | |  |  |  |  |  |
| 1 | Atr-ERN03769 |  | | | |  |  |  |  |  |
| 1 | Atr-ERN03770 |  | | | |  |  |  |  |  |
| 1 | Atr-ERN03771 |  | | | |  |  |  |  |  |
| 1 | Atr-ERN03772 |  | | | |  |  |  |  |  |
| 1 | Atr-ERN03773 |  | Vvi-Vitvi14g01894\_t001 |  |  |  |  |  |
| 3 | Atr-ERN03774 |  | | | |  | Vvi-Vitvi01g00921\_t001 |  | Vvi-Vitvi17g00541\_t001 |  |  |  |
| 3 | Atr-ERN03775 |  | | | |  | | | |  | | | |  |  |  |
| 3 | Atr-ERN03776 |  | | | |  | | | |  | Vvi-Vitvi17g00543\_t001 |  |  |  |
| 3 | Atr-ERN03777 |  | | | |  | | | |  | | | |  |  |  |
| 3 | Atr-ERN03778 |  | | | |  | Vvi-Vitvi01g00927\_t001 |  | | | |  |  |  |
| 3 | Atr-ERN03779 |  | | | |  | Vvi-Vitvi01g00928\_t001 |  | | | |  |  |  |
| 3 | Atr-ERN03780 |  | | | |  | | | |  | | | |  |  |  |
| 3 | Atr-ERN03781 |  | | | |  | | | |  | | | |  |  |  |
| 3 | Atr-ERN03782 |  | | | |  | | | |  | | | |  |  |  |
| 3 | Atr-ERN03783 |  | | | |  | | | |  | | | |  |  |  |
| 3 | Atr-ERN03784 |  | | | |  | | | |  | | | |  |  |  |
| 3 | Atr-ERN03785 |  | | | |  | | | |  | | | |  |  |  |
| 3 | Atr-ERN03786 |  | | | |  | | | |  | | | |  |  |  |
| 3 | Atr-ERN03787 |  | | | |  | | | |  | | | |  |  |  |
| 3 | Atr-ERN03788 |  | | | |  | Vvi-Vitvi01g00929\_t001 |  | Vvi-Vitvi17g00546\_t002 |  |  |  |
| 3 | Atr-ERN03789 |  | | | |  | | | |  | | | |  |  |  |
| 3 | Atr-ERN03790 |  | Vvi-Vitvi14g01898\_t001 |  | Vvi-Vitvi01g00930\_t001 |  | Vvi-Vitvi17g00547\_t001 |  |  |  |
| 3 | Atr-ERN03791 |  | | | |  | | | |  | | | |  |  |  |
| 3 | Atr-ERN03792 |  | | | |  | | | |  | Vvi-Vitvi17g00548\_t001 |  |  |  |
| 3 | Atr-ERN03793 |  | | | |  | | | |  | | | |  |  |  |
| 3 | Atr-ERN03794 |  | | | |  | | | |  | | | |  |  |  |
| 3 | Atr-ERN03795 |  | | | |  | Vvi-Vitvi01g00932\_t001 |  | | | |  |  |  |
| 3 | Atr-ERN03796 |  | Vvi-Vitvi14g01900\_t001 |  | Vvi-Vitvi01g02109\_t001 |  | Vvi-Vitvi17g00550\_t001 |  |  |  |
| 3 | Atr-ERN03797 |  | | | |  | | | |  | Vvi-Vitvi17g00551\_t002 |  |  |  |
| 3 | Atr-ERN03798 |  | Vvi-Vitvi14g01901\_t001 |  | | | |  | | | |  |  |  |
| 3 | Atr-ERN03799 |  | | | |  | | | |  | | | |  |  |  |
| 3 | Atr-ERN03800 |  | | | |  | | | |  | Vvi-Vitvi17g01459\_t001 |  |  |  |
| 3 | Atr-ERN03801 |  | | | |  | | | |  | | | |  |  |  |
| 3 | Atr-ERN03802 |  | | | |  | Vvi-Vitvi01g00935\_t001 |  | | | |  |  |  |
| 3 | Atr-ERN03803 |  | | | |  | | | |  | Vvi-Vitvi17g00552\_t001 |  |  |  |
| 3 | Atr-ERN03804 |  | | | |  | | | |  | | | |  |  |  |
| 3 | Atr-ERN03805 |  | | | |  | | | |  | | | |  |  |  |
| 3 | Atr-ERN03806 |  | | | |  | Vvi-Vitvi01g00937\_t001 |  | | | |  |  |  |
| 3 | Atr-ERN03807 |  | | | |  | | | |  | | | |  |  |  |
| 3 | Atr-ERN03808 |  | | | |  | | | |  | | | |  |  |  |
| 3 | Atr-ERN03809 |  | | | |  | | | |  | Vvi-Vitvi17g00553\_t001 |  |  |  |
| 3 | Atr-ERN03810 |  | | | |  | | | |  | | | |  |  |  |
| 3 | Atr-ERN03811 |  | | | |  | | | |  | | | |  |  |  |
| 3 | Atr-ERN03812 |  | Vvi-Vitvi14g01903\_t001 |  | | | |  | | | |  |  |  |
| 3 | Atr-ERN03813 |  | Vvi-Vitvi14g01906\_t001 |  | | | |  | Vvi-Vitvi17g00554\_t001 |  |  |  |
| 3 | Atr-ERN03814 |  | | | |  | | | |  | | | |  |  |  |
| 3 | Atr-ERN03815 |  | | | |  | | | |  | Vvi-Vitvi17g00555\_t001 |  |  |  |
| 3 | Atr-ERN03816 |  | Vvi-Vitvi14g01907\_t001 |  | Vvi-Vitvi01g00940\_t001 |  | Vvi-Vitvi17g00556\_t001 |  |  |  |
| 3 | Atr-ERN03817 |  | | | |  | Vvi-Vitvi01g00941\_t001 |  | | | |  |  |  |
| 3 | Atr-ERN03818 |  | | | |  | Vvi-Vitvi01g00942\_t001 |  | | | |  |  |  |
| 3 | Atr-ERN03819 |  | Vvi-Vitvi14g01908\_t001 |  | | | |  | | | |  |  |  |
| 3 | Atr-ERN03820 |  | | | |  | | | |  | | | |  |  |  |
| 3 | Atr-ERN03821 |  | | | |  | | | |  | | | |  |  |  |
| 3 | Atr-ERN03822 |  | Vvi-Vitvi14g01909\_t003 |  | Vvi-Vitvi01g00943\_t002 |  | Vvi-Vitvi17g04153\_t001 |  |  |  |
| 3 | Atr-ERN03823 |  | | | |  | Vvi-Vitvi01g00944\_t001 |  | | | |  |  |  |
| 3 | Atr-ERN03824 |  | | | |  | | | |  | | | |  |  |  |
| 3 | Atr-ERN03825 |  | | | |  | | | |  | Vvi-Vitvi17g00558\_t001 |  |  |  |
| 3 | Atr-ERN03826 |  | | | |  | | | |  | | | |  |  |  |
| 3 | Atr-ERN03827 |  | | | |  | | | |  | | | |  |  |  |
| 3 | Atr-ERN03828 |  | Vvi-Vitvi14g01910\_t001 |  | Vvi-Vitvi01g00945\_t001 |  | Vvi-Vitvi17g00560\_t001 |  |  |  |
| 1 | Atr-ERN03829 |  |  |  |  |  | | | |  |  |  |
| 1 | Atr-ERN03830 |  |  |  |  |  | | | |  |  |  |
| 1 | Atr-ERN03831 |  |  |  |  |  | Vvi-Vitvi17g00561\_t001 |  |  |  |
| 0 | Atr-ERN03832 |  |  |  |  |  |  |
| 0 | Atr-ERN03833 |  |  |  |  |  |  |
| 0 | Atr-ERN03834 |  |  |  |  |  |  |
| 0 | Atr-ERN03835 |  |  |  |  |  |  |
| 0 | Atr-ERN03836 |  |  |  |  |  |  |
| 0 | Atr-ERN03837 |  |  |  |  |  |  |
| 2 | Atr-ERN03838 |  | Vvi-Vitvi18g01531\_t001 |  | Vvi-Vitvi07g01846\_t001 |  |  |  |  |
| 2 | Atr-ERN03839 |  | | | |  | Vvi-Vitvi07g01847\_t001 |  |  |  |  |
| 2 | Atr-ERN03840 |  | Vvi-Vitvi18g01535\_t001 |  | Vvi-Vitvi07g01848\_t001 |  |  |  |  |
| 2 | Atr-ERN03841 |  | Vvi-Vitvi18g01539\_t003 |  | | | |  |  |  |  |
| 2 | Atr-ERN03842 |  | | | |  | Vvi-Vitvi07g01849\_t001 |  |  |  |  |
| 2 | Atr-ERN03843 |  | | | |  | | | |  |  |  |  |
| 2 | Atr-ERN03844 |  | | | |  | Vvi-Vitvi07g01851\_t001 |  |  |  |  |
| 2 | Atr-ERN03845 |  | Vvi-Vitvi18g04407\_t001 |  | | | |  |  |  |  |
| 2 | Atr-ERN03846 |  | | | |  | | | |  |  |  |  |
| 2 | Atr-ERN03847 |  | Vvi-Vitvi18g01541\_t001 |  | | | |  |  |  |  |
| 2 | Atr-ERN03848 |  | | | |  | | | |  |  |  |  |
| 2 | Atr-ERN03849 |  | | | |  | | | |  |  |  |  |
| 2 | Atr-ERN03850 |  | | | |  | | | |  |  |  |  |
| 2 | Atr-ERN03851 |  | | | |  | | | |  |  |  |  |
| 2 | Atr-ERN03852 |  | Vvi-Vitvi18g01542\_t001 |  | | | |  |  |  |  |
| 2 | Atr-ERN03853 |  | | | |  | | | |  |  |  |  |
| 2 | Atr-ERN03854 |  | | | |  | | | |  |  |  |  |
| 2 | Atr-ERN03855 |  | | | |  | | | |  |  |  |  |
| 2 | Atr-ERN03856 |  | | | |  | | | |  |  |  |  |
| 2 | Atr-ERN03857 |  | | | |  | | | |  |  |  |  |
| 2 | Atr-ERN03858 |  | | | |  | | | |  |  |  |  |
| 2 | Atr-ERN03859 |  | | | |  | | | |  |  |  |  |
| 2 | Atr-ERN03860 |  | | | |  | | | |  |  |  |  |
| 2 | Atr-ERN03861 |  | | | |  | | | |  |  |  |  |
| 2 | Atr-ERN03862 |  | | | |  | | | |  |  |  |  |
| 2 | Atr-ERN03863 |  | Vvi-Vitvi18g01544\_t001 |  | | | |  |  |  |  |
| 2 | Atr-ERN03864 |  | | | |  | | | |  |  |  |  |
| 2 | Atr-ERN03865 |  | | | |  | Vvi-Vitvi07g01852\_t001 |  |  |  |  |
| 2 | Atr-ERN03866 |  | | | |  | | | |  |  |  |  |
| 2 | Atr-ERN03867 |  | | | |  | | | |  |  |  |  |
| 2 | Atr-ERN03868 |  | | | |  | | | |  |  |  |  |
| 2 | Atr-ERN03869 |  | Vvi-Vitvi18g01574\_t005 |  | | | |  |  |  |  |
| 2 | Atr-ERN03870 |  | | | |  | | | |  |  |  |  |
| 2 | Atr-ERN03871 |  | | | |  | | | |  |  |  |  |
| 2 | Atr-ERN03872 |  | | | |  | | | |  |  |  |  |
| 2 | Atr-ERN03873 |  | Vvi-Vitvi18g01571\_t001 |  | Vvi-Vitvi07g01857\_t001 |  |  |  |  |
| 2 | Atr-ERN03874 |  | | | |  | | | |  |  |  |  |
| 2 | Atr-ERN03875 |  | Vvi-Vitvi18g01557\_t001 |  | Vvi-Vitvi07g01858\_t002 |  |  |  |  |
| 2 | Atr-ERN03876 |  | | | |  | | | |  |  |  |  |
| 2 | Atr-ERN03877 |  | | | |  | | | |  |  |  |  |
| 2 | Atr-ERN03878 |  | | | |  | Vvi-Vitvi07g01859\_t001 |  |  |  |  |
| 2 | Atr-ERN03879 |  | | | |  | | | |  |  |  |  |
| 2 | Atr-ERN03880 |  | | | |  | | | |  |  |  |  |
| 2 | Atr-ERN03881 |  | | | |  | Vvi-Vitvi07g01860\_t001 |  |  |  |  |
| 2 | Atr-ERN03882 |  | | | |  | | | |  |  |  |  |
| 2 | Atr-ERN03883 |  | | | |  | | | |  |  |  |  |
| 2 | Atr-ERN03884 |  | Vvi-Vitvi18g01553\_t001 |  | | | |  |  |  |  |
| 2 | Atr-ERN03885 |  | | | |  | | | |  |  |  |  |
| 2 | Atr-ERN03886 |  | | | |  | Vvi-Vitvi07g02694\_t001 |  |  |  |  |
| 2 | Atr-ERN03887 |  | | | |  | | | |  |  |  |  |
| 2 | Atr-ERN03888 |  | | | |  | | | |  |  |  |  |
| 2 | Atr-ERN03889 |  | | | |  | | | |  |  |  |  |
| 2 | Atr-ERN03890 |  | | | |  | | | |  |  |  |  |
| 2 | Atr-ERN03891 |  | | | |  | | | |  |  |  |  |
| 2 | Atr-ERN03892 |  | | | |  | | | |  |  |  |  |
| 2 | Atr-ERN03893 |  | | | |  | | | |  |  |  |  |
| 2 | Atr-ERN03894 |  | Vvi-Vitvi18g04412\_t001 |  | | | |  |  |  |  |
| 2 | Atr-ERN03895 |  | | | |  | | | |  |  |  |  |
| 2 | Atr-ERN03896 |  | Vvi-Vitvi18g01604\_t001 |  | | | |  |  |  |  |
| 1 | Atr-ERN03897 |  |  |  | | | |  |  |  |  |
| 2 | Atr-ERN03898 |  | Vvi-Vitvi11g00349\_t002 |  | | | |  |  |  |  |
| 2 | Atr-ERN03899 |  | | | |  | | | |  |  |  |  |
| 2 | Atr-ERN03900 |  | Vvi-Vitvi11g01409\_t001 |  | | | |  |  |  |  |
| 2 | Atr-ERN03901 |  | | | |  | | | |  |  |  |  |
| 2 | Atr-ERN03902 |  | | | |  | | | |  |  |  |  |
| 2 | Atr-ERN03903 |  | | | |  | | | |  |  |  |  |
| 2 | Atr-ERN03904 |  | | | |  | | | |  |  |  |  |
| 2 | Atr-ERN03905 |  | | | |  | | | |  |  |  |  |
| 2 | Atr-ERN03906 |  | | | |  | | | |  |  |  |  |
| 2 | Atr-ERN03907 |  | | | |  | | | |  |  |  |  |
| 2 | Atr-ERN03908 |  | | | |  | | | |  |  |  |  |
| 2 | Atr-ERN03909 |  | | | |  | Vvi-Vitvi07g01886\_t002 |  |  |  |  |
| 1 | Atr-ERN03910 |  | | | |  |  |  |  |  |
| 1 | Atr-ERN03911 |  | Vvi-Vitvi11g00350\_t001 |  |  |  |  |  |
| 1 | Atr-ERN03912 |  | | | |  |  |  |  |  |
| 1 | Atr-ERN03913 |  | | | |  |  |  |  |  |
| 1 | Atr-ERN03914 |  | | | |  |  |  |  |  |
| 1 | Atr-ERN03915 |  | | | |  |  |  |  |  |
| 1 | Atr-ERN03916 |  | | | |  |  |  |  |  |
| 1 | Atr-ERN03917 |  | | | |  |  |  |  |  |
| 1 | Atr-ERN03918 |  | Vvi-Vitvi11g00351\_t001 |  |  |  |  |  |
| 1 | Atr-ERN03919 |  | Vvi-Vitvi11g00352\_t001 |  |  |  |  |  |
| 1 | Atr-ERN03920 |  | | | |  |  |  |  |  |
| 1 | Atr-ERN03921 |  | | | |  |  |  |  |  |
| 1 | Atr-ERN03922 |  | | | |  |  |  |  |  |
| 1 | Atr-ERN03923 |  | | | |  |  |  |  |  |
| 1 | Atr-ERN03924 |  | | | |  |  |  |  |  |
| 1 | Atr-ERN03925 |  | | | |  |  |  |  |  |
| 1 | Atr-ERN03926 |  | | | |  |  |  |  |  |
| 1 | Atr-ERN03927 |  | Vvi-Vitvi11g00360\_t001 |  |  |  |  |  |
