# Supplementary material for: Genome-wide identification, characterization and gene expression of BES1 transcription factor family in grapevine (Vitis vinifera L.)
Source: Sci Rep. 2023 Jan 5;13:240. doi: 10.1038/s41598-022-24407-y (PMC9816167; doi:10.1038/s41598-022-24407-y)
Supplement: Supplementary file 3 — Supplementary Information. [file 41598_2022_24407_MOESM3_ESM.zip › Vvi_Atr/Vitis_vinifera.PN40024.v4.dna_sm.toplevel.fa.vs.Amborella_trichopoda.AMTR1.0.dna_sm.toplevel.fa.html/Atr-AmTr_v1.0_scaffold00079.html]

|  |  |  |  |  |  |  |  |  |  |  |  |  |  |
| --- | --- | --- | --- | --- | --- | --- | --- | --- | --- | --- | --- | --- | --- |
| Duplication depth | Reference chromosome | Collinear blocks | | | | | | | | | | | |
| 0 | Atr-ERN03934 |  |  |  |  |  |  |
| 0 | Atr-ERN03935 |  |  |  |  |  |  |
| 0 | Atr-ERN03936 |  |  |  |  |  |  |
| 0 | Atr-ERN03937 |  |  |  |  |  |  |
| 2 | Atr-ERN03938 |  | Vvi-Vitvi01g00279\_t001 |  | Vvi-Vitvi17g00331\_t001 |  |  |  |  |
| 2 | Atr-ERN03939 |  | | | |  | | | |  |  |  |  |
| 2 | Atr-ERN03940 |  | | | |  | | | |  |  |  |  |
| 2 | Atr-ERN03941 |  | Vvi-Vitvi01g00280\_t001 |  | | | |  |  |  |  |
| 2 | Atr-ERN03942 |  | | | |  | | | |  |  |  |  |
| 2 | Atr-ERN03943 |  | | | |  | | | |  |  |  |  |
| 2 | Atr-ERN03944 |  | | | |  | | | |  |  |  |  |
| 2 | Atr-ERN03945 |  | | | |  | | | |  |  |  |  |
| 2 | Atr-ERN03946 |  | | | |  | | | |  |  |  |  |
| 2 | Atr-ERN03947 |  | | | |  | | | |  |  |  |  |
| 2 | Atr-ERN03948 |  | | | |  | | | |  |  |  |  |
| 2 | Atr-ERN03949 |  | | | |  | | | |  |  |  |  |
| 2 | Atr-ERN03950 |  | | | |  | | | |  |  |  |  |
| 2 | Atr-ERN03951 |  | | | |  | | | |  |  |  |  |
| 2 | Atr-ERN03952 |  | | | |  | Vvi-Vitvi17g00320\_t001 |  |  |  |  |
| 2 | Atr-ERN03953 |  | | | |  | | | |  |  |  |  |
| 2 | Atr-ERN03954 |  | | | |  | | | |  |  |  |  |
| 2 | Atr-ERN03955 |  | | | |  | | | |  |  |  |  |
| 2 | Atr-ERN03956 |  | | | |  | | | |  |  |  |  |
| 2 | Atr-ERN03957 |  | | | |  | | | |  |  |  |  |
| 2 | Atr-ERN03958 |  | | | |  | | | |  |  |  |  |
| 3 | Atr-ERN03959 |  | | | |  | | | |  | Vvi-Vitvi14g01230\_t001 |  |  |  |
| 3 | Atr-ERN03960 |  | | | |  | | | |  | | | |  |  |  |
| 3 | Atr-ERN03961 |  | | | |  | | | |  | | | |  |  |  |
| 3 | Atr-ERN03962 |  | | | |  | | | |  | | | |  |  |  |
| 3 | Atr-ERN03963 |  | Vvi-Vitvi01g00290\_t001 |  | | | |  | | | |  |  |  |
| 3 | Atr-ERN03964 |  | | | |  | | | |  | | | |  |  |  |
| 3 | Atr-ERN03965 |  | | | |  | | | |  | | | |  |  |  |
| 3 | Atr-ERN03966 |  | | | |  | | | |  | | | |  |  |  |
| 3 | Atr-ERN03967 |  | | | |  | Vvi-Vitvi17g00318\_t001 |  | | | |  |  |  |
| 3 | Atr-ERN03968 |  | | | |  | | | |  | | | |  |  |  |
| 3 | Atr-ERN03969 |  | | | |  | | | |  | Vvi-Vitvi14g01211\_t001 |  |  |  |
| 3 | Atr-ERN03970 |  | | | |  | | | |  | | | |  |  |  |
| 3 | Atr-ERN03971 |  | | | |  | | | |  | | | |  |  |  |
| 3 | Atr-ERN03972 |  | | | |  | | | |  | | | |  |  |  |
| 3 | Atr-ERN03973 |  | Vvi-Vitvi01g01939\_t001 |  | | | |  | | | |  |  |  |
| 3 | Atr-ERN03974 |  | | | |  | Vvi-Vitvi17g00315\_t001 |  | | | |  |  |  |
| 3 | Atr-ERN03975 |  | | | |  | | | |  | | | |  |  |  |
| 3 | Atr-ERN03976 |  | Vvi-Vitvi01g00295\_t001 |  | | | |  | Vvi-Vitvi14g01187\_t001 |  |  |  |
| 3 | Atr-ERN03977 |  | | | |  | Vvi-Vitvi17g00313\_t001 |  | Vvi-Vitvi14g01186\_t001 |  |  |  |
| 3 | Atr-ERN03978 |  | | | |  | | | |  | | | |  |  |  |
| 3 | Atr-ERN03979 |  | Vvi-Vitvi01g00298\_t001 |  | | | |  | | | |  |  |  |
| 3 | Atr-ERN03980 |  | | | |  | | | |  | | | |  |  |  |
| 3 | Atr-ERN03981 |  | Vvi-Vitvi01g01941\_t001 |  | Vvi-Vitvi17g01402\_t001 |  | | | |  |  |  |
| 3 | Atr-ERN03982 |  | | | |  | | | |  | | | |  |  |  |
| 3 | Atr-ERN03983 |  | | | |  | Vvi-Vitvi17g00312\_t002 |  | | | |  |  |  |
| 3 | Atr-ERN03984 |  | | | |  | | | |  | Vvi-Vitvi14g01185\_t001 |  |  |  |
| 3 | Atr-ERN03985 |  | Vvi-Vitvi01g00300\_t001 |  | | | |  | | | |  |  |  |
| 3 | Atr-ERN03986 |  | Vvi-Vitvi01g01942\_t001 |  | | | |  | | | |  |  |  |
| 3 | Atr-ERN03987 |  | Vvi-Vitvi01g04072\_t001 |  | | | |  | | | |  |  |  |
| 3 | Atr-ERN03988 |  | | | |  | | | |  | Vvi-Vitvi14g02875\_t001 |  |  |  |
| 3 | Atr-ERN03989 |  | Vvi-Vitvi01g01946\_t001 |  | Vvi-Vitvi17g00311\_t001 |  | Vvi-Vitvi14g01181\_t001 |  |  |  |
| 3 | Atr-ERN03990 |  | | | |  | | | |  | Vvi-Vitvi14g01180\_t001 |  |  |  |
| 3 | Atr-ERN03991 |  | Vvi-Vitvi01g00302\_t001 |  | Vvi-Vitvi17g00309\_t001 |  | | | |  |  |  |
| 3 | Atr-ERN03992 |  | Vvi-Vitvi01g04074\_t001 |  | | | |  | Vvi-Vitvi14g01178\_t001 |  |  |  |
| 3 | Atr-ERN03993 |  | | | |  | | | |  | | | |  |  |  |
| 3 | Atr-ERN03994 |  | | | |  | | | |  | | | |  |  |  |
| 3 | Atr-ERN03995 |  | | | |  | | | |  | Vvi-Vitvi14g01177\_t001 |  |  |  |
| 3 | Atr-ERN03996 |  | | | |  | Vvi-Vitvi17g00304\_t001 |  | Vvi-Vitvi14g01176\_t001 |  |  |  |
| 3 | Atr-ERN03997 |  | | | |  | | | |  | | | |  |  |  |
| 3 | Atr-ERN03998 |  | | | |  | Vvi-Vitvi17g00303\_t001 |  | Vvi-Vitvi14g01166\_t001 |  |  |  |
| 3 | Atr-ERN03999 |  | | | |  | | | |  | | | |  |  |  |
| 3 | Atr-ERN04000 |  | | | |  | Vvi-Vitvi17g00301\_t001 |  | | | |  |  |  |
| 3 | Atr-ERN04001 |  | Vvi-Vitvi01g00305\_t001 |  | Vvi-Vitvi17g00300\_t001 |  | Vvi-Vitvi14g01164\_t001 |  |  |  |
| 3 | Atr-ERN04002 |  | | | |  | | | |  | | | |  |  |  |
| 3 | Atr-ERN04003 |  | | | |  | | | |  | | | |  |  |  |
| 3 | Atr-ERN04004 |  | | | |  | | | |  | | | |  |  |  |
| 3 | Atr-ERN04005 |  | | | |  | | | |  | | | |  |  |  |
| 3 | Atr-ERN04006 |  | Vvi-Vitvi01g00306\_t002 |  | Vvi-Vitvi17g04082\_t001 |  | | | |  |  |  |
| 3 | Atr-ERN04007 |  | | | |  | | | |  | | | |  |  |  |
| 3 | Atr-ERN04008 |  | | | |  | | | |  | | | |  |  |  |
| 3 | Atr-ERN04009 |  | | | |  | | | |  | | | |  |  |  |
| 3 | Atr-ERN04010 |  | | | |  | | | |  | | | |  |  |  |
| 3 | Atr-ERN04011 |  | | | |  | | | |  | Vvi-Vitvi14g01162\_t001 |  |  |  |
| 3 | Atr-ERN04012 |  | | | |  | | | |  | Vvi-Vitvi14g01160\_t001 |  |  |  |
| 3 | Atr-ERN04013 |  | | | |  | | | |  | | | |  |  |  |
| 3 | Atr-ERN04014 |  | Vvi-Vitvi01g00309\_t001 |  | | | |  | Vvi-Vitvi14g02872\_t001 |  |  |  |
| 3 | Atr-ERN04015 |  | Vvi-Vitvi01g00310\_t001 |  | | | |  | | | |  |  |  |
| 3 | Atr-ERN04016 |  | | | |  | | | |  | | | |  |  |  |
| 3 | Atr-ERN04017 |  | | | |  | | | |  | | | |  |  |  |
| 3 | Atr-ERN04018 |  | | | |  | | | |  | | | |  |  |  |
| 3 | Atr-ERN04019 |  | | | |  | | | |  | | | |  |  |  |
| 3 | Atr-ERN04020 |  | | | |  | | | |  | | | |  |  |  |
| 3 | Atr-ERN04021 |  | | | |  | | | |  | Vvi-Vitvi14g01157\_t001 |  |  |  |
| 3 | Atr-ERN04022 |  | Vvi-Vitvi01g00313\_t001 |  | Vvi-Vitvi17g00299\_t001 |  | | | |  |  |  |
| 3 | Atr-ERN04023 |  | | | |  | | | |  | | | |  |  |  |
| 3 | Atr-ERN04024 |  | | | |  | | | |  | | | |  |  |  |
| 3 | Atr-ERN04025 |  | | | |  | | | |  | | | |  |  |  |
| 3 | Atr-ERN04026 |  | | | |  | | | |  | | | |  |  |  |
| 3 | Atr-ERN04027 |  | | | |  | Vvi-Vitvi17g00298\_t001 |  | | | |  |  |  |
| 3 | Atr-ERN04028 |  | | | |  | | | |  | | | |  |  |  |
| 3 | Atr-ERN04029 |  | | | |  | | | |  | | | |  |  |  |
| 3 | Atr-ERN04030 |  | Vvi-Vitvi01g00314\_t002 |  | | | |  | | | |  |  |  |
| 3 | Atr-ERN04031 |  | Vvi-Vitvi01g00315\_t001 |  | Vvi-Vitvi17g00294\_t001 |  | Vvi-Vitvi14g01156\_t001 |  |  |  |
| 3 | Atr-ERN04032 |  | | | |  | | | |  | | | |  |  |  |
| 3 | Atr-ERN04033 |  | | | |  | | | |  | | | |  |  |  |
| 3 | Atr-ERN04034 |  | Vvi-Vitvi01g00318\_t001 |  | | | |  | | | |  |  |  |
| 3 | Atr-ERN04035 |  | Vvi-Vitvi01g00319\_t002 |  | | | |  | Vvi-Vitvi14g01152\_t001 |  |  |  |
| 3 | Atr-ERN04036 |  | | | |  | | | |  | | | |  |  |  |
| 3 | Atr-ERN04037 |  | | | |  | | | |  | | | |  |  |  |
| 3 | Atr-ERN04038 |  | | | |  | | | |  | | | |  |  |  |
| 3 | Atr-ERN04039 |  | | | |  | | | |  | Vvi-Vitvi14g01151\_t001 |  |  |  |
| 3 | Atr-ERN04040 |  | | | |  | | | |  | | | |  |  |  |
| 3 | Atr-ERN04041 |  | | | |  | | | |  | | | |  |  |  |
| 3 | Atr-ERN04042 |  | Vvi-Vitvi01g00325\_t001 |  | | | |  | | | |  |  |  |
| 3 | Atr-ERN04043 |  | Vvi-Vitvi01g00326\_t001 |  | | | |  | | | |  |  |  |
| 3 | Atr-ERN04044 |  | Vvi-Vitvi01g01949\_t001 |  | | | |  | Vvi-Vitvi14g01139\_t001 |  |  |  |
| 3 | Atr-ERN04045 |  | Vvi-Vitvi01g04083\_t002 |  | Vvi-Vitvi17g00289\_t001 |  | Vvi-Vitvi14g04419\_t001 |  |  |  |
| 2 | Atr-ERN04046 |  | Vvi-Vitvi01g04084\_t001 |  | Vvi-Vitvi17g01392\_t001 |  |  |  |  |
| 1 | Atr-ERN04047 |  |  |  | | | |  |  |  |  |
| 1 | Atr-ERN04048 |  |  |  | | | |  |  |  |  |
| 1 | Atr-ERN04049 |  |  |  | | | |  |  |  |  |
| 1 | Atr-ERN04050 |  |  |  | Vvi-Vitvi17g00284\_t001 |  |  |  |  |
| 0 | Atr-ERN04051 |  |  |  |  |  |  |
| 0 | Atr-ERN04052 |  |  |  |  |  |  |
| 0 | Atr-ERN04053 |  |  |  |  |  |  |
| 0 | Atr-ERN04054 |  |  |  |  |  |  |
| 0 | Atr-ERN04055 |  |  |  |  |  |  |
| 0 | Atr-ERN04056 |  |  |  |  |  |  |
| 0 | Atr-ERN04057 |  |  |  |  |  |  |
| 0 | Atr-ERN04058 |  |  |  |  |  |  |
| 0 | Atr-ERN04059 |  |  |  |  |  |  |
| 0 | Atr-ERN04060 |  |  |  |  |  |  |
| 0 | Atr-ERN04061 |  |  |  |  |  |  |
| 0 | Atr-ERN04062 |  |  |  |  |  |  |
