# Supplementary material for: Genome-wide identification, characterization and gene expression of BES1 transcription factor family in grapevine (Vitis vinifera L.)
Source: Sci Rep. 2023 Jan 5;13:240. doi: 10.1038/s41598-022-24407-y (PMC9816167; doi:10.1038/s41598-022-24407-y)
Supplement: Supplementary file 3 — Supplementary Information. [file 41598_2022_24407_MOESM3_ESM.zip › Vvi_Atr/Vitis_vinifera.PN40024.v4.dna_sm.toplevel.fa.vs.Amborella_trichopoda.AMTR1.0.dna_sm.toplevel.fa.html/Atr-AmTr_v1.0_scaffold00080.html]

|  |  |  |  |  |  |  |  |  |  |  |  |  |  |
| --- | --- | --- | --- | --- | --- | --- | --- | --- | --- | --- | --- | --- | --- |
| Duplication depth | Reference chromosome | Collinear blocks | | | | | | | | | | | |
| 0 | Atr-ERN04888 |  |  |  |  |  |  |
| 0 | Atr-ERN04889 |  |  |  |  |  |  |
| 2 | Atr-ERN04890 |  | Vvi-Vitvi13g00535\_t001 |  | Vvi-Vitvi06g00351\_t001 |  |  |  |  |
| 2 | Atr-ERN04891 |  | | | |  | | | |  |  |  |  |
| 2 | Atr-ERN04892 |  | | | |  | | | |  |  |  |  |
| 2 | Atr-ERN04893 |  | | | |  | | | |  |  |  |  |
| 2 | Atr-ERN04894 |  | | | |  | | | |  |  |  |  |
| 2 | Atr-ERN04895 |  | | | |  | | | |  |  |  |  |
| 2 | Atr-ERN04896 |  | | | |  | | | |  |  |  |  |
| 2 | Atr-ERN04897 |  | | | |  | | | |  |  |  |  |
| 2 | Atr-ERN04898 |  | | | |  | | | |  |  |  |  |
| 2 | Atr-ERN04899 |  | | | |  | | | |  |  |  |  |
| 2 | Atr-ERN04900 |  | | | |  | | | |  |  |  |  |
| 2 | Atr-ERN04901 |  | Vvi-Vitvi13g00536\_t001 |  | | | |  |  |  |  |
| 2 | Atr-ERN04902 |  | | | |  | | | |  |  |  |  |
| 2 | Atr-ERN04903 |  | Vvi-Vitvi13g00538\_t001 |  | Vvi-Vitvi06g00350\_t001 |  |  |  |  |
| 2 | Atr-ERN04904 |  | | | |  | | | |  |  |  |  |
| 2 | Atr-ERN04905 |  | | | |  | | | |  |  |  |  |
| 2 | Atr-ERN04906 |  | | | |  | | | |  |  |  |  |
| 2 | Atr-ERN04907 |  | | | |  | | | |  |  |  |  |
| 2 | Atr-ERN04908 |  | | | |  | | | |  |  |  |  |
| 2 | Atr-ERN04909 |  | | | |  | | | |  |  |  |  |
| 2 | Atr-ERN04910 |  | | | |  | | | |  |  |  |  |
| 2 | Atr-ERN04911 |  | | | |  | | | |  |  |  |  |
| 2 | Atr-ERN04912 |  | | | |  | | | |  |  |  |  |
| 2 | Atr-ERN04913 |  | Vvi-Vitvi13g04156\_t001 |  | | | |  |  |  |  |
| 2 | Atr-ERN04914 |  | | | |  | | | |  |  |  |  |
| 2 | Atr-ERN04915 |  | | | |  | | | |  |  |  |  |
| 2 | Atr-ERN04916 |  | | | |  | | | |  |  |  |  |
| 2 | Atr-ERN04917 |  | | | |  | | | |  |  |  |  |
| 2 | Atr-ERN04918 |  | | | |  | | | |  |  |  |  |
| 2 | Atr-ERN04919 |  | | | |  | | | |  |  |  |  |
| 2 | Atr-ERN04920 |  | | | |  | | | |  |  |  |  |
| 2 | Atr-ERN04921 |  | | | |  | | | |  |  |  |  |
| 2 | Atr-ERN04922 |  | | | |  | | | |  |  |  |  |
| 2 | Atr-ERN04923 |  | | | |  | | | |  |  |  |  |
| 2 | Atr-ERN04924 |  | | | |  | | | |  |  |  |  |
| 2 | Atr-ERN04925 |  | | | |  | Vvi-Vitvi06g00349\_t001 |  |  |  |  |
| 2 | Atr-ERN04926 |  | | | |  | | | |  |  |  |  |
| 2 | Atr-ERN04927 |  | | | |  | | | |  |  |  |  |
| 2 | Atr-ERN04928 |  | | | |  | | | |  |  |  |  |
| 2 | Atr-ERN04929 |  | | | |  | | | |  |  |  |  |
| 2 | Atr-ERN04930 |  | | | |  | | | |  |  |  |  |
| 2 | Atr-ERN04931 |  | | | |  | | | |  |  |  |  |
| 2 | Atr-ERN04932 |  | | | |  | | | |  |  |  |  |
| 2 | Atr-ERN04933 |  | | | |  | Vvi-Vitvi06g01665\_t004 |  |  |  |  |
| 2 | Atr-ERN04934 |  | Vvi-Vitvi13g02046\_t001 |  | | | |  |  |  |  |
| 2 | Atr-ERN04935 |  | | | |  | Vvi-Vitvi06g00342\_t001 |  |  |  |  |
| 2 | Atr-ERN04936 |  | | | |  | | | |  |  |  |  |
| 2 | Atr-ERN04937 |  | | | |  | | | |  |  |  |  |
| 2 | Atr-ERN04938 |  | | | |  | Vvi-Vitvi06g00331\_t001 |  |  |  |  |
| 2 | Atr-ERN04939 |  | | | |  | | | |  |  |  |  |
| 2 | Atr-ERN04940 |  | | | |  | | | |  |  |  |  |
| 2 | Atr-ERN04941 |  | | | |  | | | |  |  |  |  |
| 2 | Atr-ERN04942 |  | | | |  | | | |  |  |  |  |
| 2 | Atr-ERN04943 |  | | | |  | | | |  |  |  |  |
| 2 | Atr-ERN04944 |  | | | |  | | | |  |  |  |  |
| 2 | Atr-ERN04945 |  | | | |  | | | |  |  |  |  |
| 2 | Atr-ERN04946 |  | | | |  | | | |  |  |  |  |
| 2 | Atr-ERN04947 |  | | | |  | | | |  |  |  |  |
| 2 | Atr-ERN04948 |  | | | |  | | | |  |  |  |  |
| 2 | Atr-ERN04949 |  | | | |  | | | |  |  |  |  |
| 2 | Atr-ERN04950 |  | | | |  | | | |  |  |  |  |
| 2 | Atr-ERN04951 |  | | | |  | Vvi-Vitvi06g00330\_t001 |  |  |  |  |
| 2 | Atr-ERN04952 |  | | | |  | | | |  |  |  |  |
| 2 | Atr-ERN04953 |  | | | |  | | | |  |  |  |  |
| 2 | Atr-ERN04954 |  | | | |  | | | |  |  |  |  |
| 2 | Atr-ERN04955 |  | | | |  | | | |  |  |  |  |
| 2 | Atr-ERN04956 |  | Vvi-Vitvi13g00543\_t001 |  | | | |  |  |  |  |
| 2 | Atr-ERN04957 |  | | | |  | | | |  |  |  |  |
| 2 | Atr-ERN04958 |  | | | |  | | | |  |  |  |  |
| 2 | Atr-ERN04959 |  | | | |  | | | |  |  |  |  |
| 2 | Atr-ERN04960 |  | | | |  | | | |  |  |  |  |
| 2 | Atr-ERN04961 |  | | | |  | | | |  |  |  |  |
| 2 | Atr-ERN04962 |  | | | |  | | | |  |  |  |  |
| 2 | Atr-ERN04963 |  | | | |  | | | |  |  |  |  |
| 2 | Atr-ERN04964 |  | Vvi-Vitvi13g00562\_t001 |  | | | |  |  |  |  |
| 2 | Atr-ERN04965 |  | | | |  | | | |  |  |  |  |
| 2 | Atr-ERN04966 |  | | | |  | | | |  |  |  |  |
| 2 | Atr-ERN04967 |  | | | |  | | | |  |  |  |  |
| 2 | Atr-ERN04968 |  | | | |  | | | |  |  |  |  |
| 2 | Atr-ERN04969 |  | | | |  | Vvi-Vitvi06g00323\_t001 |  |  |  |  |
| 2 | Atr-ERN04970 |  | | | |  | | | |  |  |  |  |
| 2 | Atr-ERN04971 |  | | | |  | | | |  |  |  |  |
| 2 | Atr-ERN04972 |  | | | |  | | | |  |  |  |  |
| 2 | Atr-ERN04973 |  | | | |  | Vvi-Vitvi06g00322\_t001 |  |  |  |  |
| 1 | Atr-ERN04974 |  | | | |  |  |  |  |  |
| 1 | Atr-ERN04975 |  | | | |  |  |  |  |  |
| 1 | Atr-ERN04976 |  | Vvi-Vitvi13g00565\_t001 |  |  |  |  |  |
| 0 | Atr-ERN04977 |  |  |  |  |  |  |
| 0 | Atr-ERN04978 |  |  |  |  |  |  |
| 0 | Atr-ERN04979 |  |  |  |  |  |  |
| 0 | Atr-ERN04980 |  |  |  |  |  |  |
| 0 | Atr-ERN04981 |  |  |  |  |  |  |
| 0 | Atr-ERN04982 |  |  |  |  |  |  |
| 0 | Atr-ERN04983 |  |  |  |  |  |  |
| 0 | Atr-ERN04984 |  |  |  |  |  |  |
| 0 | Atr-ERN04985 |  |  |  |  |  |  |
| 0 | Atr-ERN04986 |  |  |  |  |  |  |
| 0 | Atr-ERN04987 |  |  |  |  |  |  |
