# Supplementary material for: Genome-wide identification, characterization and gene expression of BES1 transcription factor family in grapevine (Vitis vinifera L.)
Source: Sci Rep. 2023 Jan 5;13:240. doi: 10.1038/s41598-022-24407-y (PMC9816167; doi:10.1038/s41598-022-24407-y)
Supplement: Supplementary file 3 — Supplementary Information. [file 41598_2022_24407_MOESM3_ESM.zip › Vvi_Atr/Vitis_vinifera.PN40024.v4.dna_sm.toplevel.fa.vs.Amborella_trichopoda.AMTR1.0.dna_sm.toplevel.fa.html/Atr-AmTr_v1.0_scaffold00081.html]

|  |  |  |  |  |  |  |  |  |  |  |  |  |  |
| --- | --- | --- | --- | --- | --- | --- | --- | --- | --- | --- | --- | --- | --- |
| Duplication depth | Reference chromosome | Collinear blocks | | | | | | | | | | | |
| 2 | Atr-ERN04472 |  | Vvi-Vitvi09g00522\_t001 |  | Vvi-Vitvi11g00430\_t001 |  |  |  |  |
| 2 | Atr-ERN04473 |  | | | |  | | | |  |  |  |  |
| 2 | Atr-ERN04474 |  | | | |  | | | |  |  |  |  |
| 2 | Atr-ERN04475 |  | | | |  | | | |  |  |  |  |
| 2 | Atr-ERN04476 |  | | | |  | | | |  |  |  |  |
| 2 | Atr-ERN04477 |  | | | |  | | | |  |  |  |  |
| 2 | Atr-ERN04478 |  | | | |  | | | |  |  |  |  |
| 2 | Atr-ERN04479 |  | | | |  | | | |  |  |  |  |
| 2 | Atr-ERN04480 |  | | | |  | | | |  |  |  |  |
| 2 | Atr-ERN04481 |  | | | |  | | | |  |  |  |  |
| 2 | Atr-ERN04482 |  | | | |  | | | |  |  |  |  |
| 2 | Atr-ERN04483 |  | | | |  | | | |  |  |  |  |
| 2 | Atr-ERN04484 |  | | | |  | | | |  |  |  |  |
| 2 | Atr-ERN04485 |  | | | |  | | | |  |  |  |  |
| 2 | Atr-ERN04486 |  | | | |  | | | |  |  |  |  |
| 2 | Atr-ERN04487 |  | | | |  | | | |  |  |  |  |
| 2 | Atr-ERN04488 |  | | | |  | | | |  |  |  |  |
| 2 | Atr-ERN04489 |  | | | |  | | | |  |  |  |  |
| 2 | Atr-ERN04490 |  | | | |  | | | |  |  |  |  |
| 2 | Atr-ERN04491 |  | | | |  | Vvi-Vitvi11g00435\_t001 |  |  |  |  |
| 2 | Atr-ERN04492 |  | | | |  | | | |  |  |  |  |
| 2 | Atr-ERN04493 |  | Vvi-Vitvi09g00526\_t002 |  | | | |  |  |  |  |
| 2 | Atr-ERN04494 |  | | | |  | | | |  |  |  |  |
| 2 | Atr-ERN04495 |  | | | |  | | | |  |  |  |  |
| 2 | Atr-ERN04496 |  | | | |  | | | |  |  |  |  |
| 2 | Atr-ERN04497 |  | | | |  | | | |  |  |  |  |
| 2 | Atr-ERN04498 |  | | | |  | Vvi-Vitvi11g00437\_t001 |  |  |  |  |
| 2 | Atr-ERN04499 |  | | | |  | | | |  |  |  |  |
| 2 | Atr-ERN04500 |  | | | |  | | | |  |  |  |  |
| 2 | Atr-ERN04501 |  | | | |  | | | |  |  |  |  |
| 2 | Atr-ERN04502 |  | | | |  | | | |  |  |  |  |
| 2 | Atr-ERN04503 |  | | | |  | | | |  |  |  |  |
| 2 | Atr-ERN04504 |  | | | |  | Vvi-Vitvi11g00439\_t001 |  |  |  |  |
| 2 | Atr-ERN04505 |  | | | |  | | | |  |  |  |  |
| 2 | Atr-ERN04506 |  | | | |  | | | |  |  |  |  |
| 2 | Atr-ERN04507 |  | Vvi-Vitvi09g00529\_t001 |  | | | |  |  |  |  |
| 2 | Atr-ERN04508 |  | | | |  | | | |  |  |  |  |
| 2 | Atr-ERN04509 |  | Vvi-Vitvi09g00531\_t001 |  | | | |  |  |  |  |
| 2 | Atr-ERN04510 |  | | | |  | | | |  |  |  |  |
| 2 | Atr-ERN04511 |  | | | |  | | | |  |  |  |  |
| 2 | Atr-ERN04512 |  | | | |  | | | |  |  |  |  |
| 2 | Atr-ERN04513 |  | Vvi-Vitvi09g00551\_t001 |  | | | |  |  |  |  |
| 2 | Atr-ERN04514 |  | | | |  | | | |  |  |  |  |
| 2 | Atr-ERN04515 |  | | | |  | | | |  |  |  |  |
| 2 | Atr-ERN04516 |  | Vvi-Vitvi09g00553\_t001 |  | | | |  |  |  |  |
| 2 | Atr-ERN04517 |  | | | |  | Vvi-Vitvi11g01429\_t001 |  |  |  |  |
| 2 | Atr-ERN04518 |  | | | |  | | | |  |  |  |  |
| 2 | Atr-ERN04519 |  | | | |  | | | |  |  |  |  |
| 2 | Atr-ERN04520 |  | | | |  | | | |  |  |  |  |
| 2 | Atr-ERN04521 |  | | | |  | | | |  |  |  |  |
| 2 | Atr-ERN04522 |  | | | |  | | | |  |  |  |  |
| 2 | Atr-ERN04523 |  | | | |  | | | |  |  |  |  |
| 2 | Atr-ERN04524 |  | | | |  | | | |  |  |  |  |
| 2 | Atr-ERN04525 |  | | | |  | | | |  |  |  |  |
| 2 | Atr-ERN04526 |  | Vvi-Vitvi09g00556\_t001 |  | | | |  |  |  |  |
| 2 | Atr-ERN04527 |  | | | |  | Vvi-Vitvi11g00442\_t001 |  |  |  |  |
| 2 | Atr-ERN04528 |  | | | |  | | | |  |  |  |  |
| 2 | Atr-ERN04529 |  | | | |  | | | |  |  |  |  |
| 2 | Atr-ERN04530 |  | | | |  | | | |  |  |  |  |
| 2 | Atr-ERN04531 |  | | | |  | | | |  |  |  |  |
| 2 | Atr-ERN04532 |  | | | |  | | | |  |  |  |  |
| 2 | Atr-ERN04533 |  | | | |  | | | |  |  |  |  |
| 2 | Atr-ERN04534 |  | | | |  | | | |  |  |  |  |
| 2 | Atr-ERN04535 |  | | | |  | | | |  |  |  |  |
| 2 | Atr-ERN04536 |  | | | |  | Vvi-Vitvi11g00447\_t001 |  |  |  |  |
| 1 | Atr-ERN04537 |  | | | |  |  |  |  |  |
| 1 | Atr-ERN04538 |  | | | |  |  |  |  |  |
| 1 | Atr-ERN04539 |  | | | |  |  |  |  |  |
| 1 | Atr-ERN04540 |  | | | |  |  |  |  |  |
| 1 | Atr-ERN04541 |  | | | |  |  |  |  |  |
| 1 | Atr-ERN04542 |  | | | |  |  |  |  |  |
| 1 | Atr-ERN04543 |  | | | |  |  |  |  |  |
| 1 | Atr-ERN04544 |  | | | |  |  |  |  |  |
| 1 | Atr-ERN04545 |  | Vvi-Vitvi09g00559\_t001 |  |  |  |  |  |
| 0 | Atr-ERN04546 |  |  |  |  |  |  |
| 0 | Atr-ERN04547 |  |  |  |  |  |  |
| 0 | Atr-ERN04548 |  |  |  |  |  |  |
| 0 | Atr-ERN04549 |  |  |  |  |  |  |
| 0 | Atr-ERN04550 |  |  |  |  |  |  |
| 0 | Atr-ERN04551 |  |  |  |  |  |  |
| 0 | Atr-ERN04552 |  |  |  |  |  |  |
| 0 | Atr-ERN04553 |  |  |  |  |  |  |
| 0 | Atr-ERN04554 |  |  |  |  |  |  |
| 0 | Atr-ERN04555 |  |  |  |  |  |  |
