# Supplementary material for: Genome-wide identification, characterization and gene expression of BES1 transcription factor family in grapevine (Vitis vinifera L.)
Source: Sci Rep. 2023 Jan 5;13:240. doi: 10.1038/s41598-022-24407-y (PMC9816167; doi:10.1038/s41598-022-24407-y)
Supplement: Supplementary file 3 — Supplementary Information. [file 41598_2022_24407_MOESM3_ESM.zip › Vvi_Atr/Vitis_vinifera.PN40024.v4.dna_sm.toplevel.fa.vs.Amborella_trichopoda.AMTR1.0.dna_sm.toplevel.fa.html/Atr-AmTr_v1.0_scaffold00082.html]

|  |  |  |  |  |  |  |  |  |  |  |  |  |  |
| --- | --- | --- | --- | --- | --- | --- | --- | --- | --- | --- | --- | --- | --- |
| Duplication depth | Reference chromosome | Collinear blocks | | | | | | | | | | | |
| 0 | Atr-ERM98730 |  |  |  |  |  |  |
| 0 | Atr-ERM98731 |  |  |  |  |  |  |
| 0 | Atr-ERM98732 |  |  |  |  |  |  |
| 0 | Atr-ERM98733 |  |  |  |  |  |  |
| 0 | Atr-ERM98734 |  |  |  |  |  |  |
| 0 | Atr-ERM98735 |  |  |  |  |  |  |
| 0 | Atr-ERM98736 |  |  |  |  |  |  |
| 0 | Atr-ERM98737 |  |  |  |  |  |  |
| 0 | Atr-ERM98738 |  |  |  |  |  |  |
| 0 | Atr-ERM98739 |  |  |  |  |  |  |
| 0 | Atr-ERM98740 |  |  |  |  |  |  |
| 0 | Atr-ERM98741 |  |  |  |  |  |  |
| 0 | Atr-ERM98742 |  |  |  |  |  |  |
| 0 | Atr-ERM98743 |  |  |  |  |  |  |
| 0 | Atr-ERM98744 |  |  |  |  |  |  |
| 0 | Atr-ERM98745 |  |  |  |  |  |  |
| 0 | Atr-ERM98746 |  |  |  |  |  |  |
| 0 | Atr-ERM98747 |  |  |  |  |  |  |
| 0 | Atr-ERM98748 |  |  |  |  |  |  |
| 0 | Atr-ERM98749 |  |  |  |  |  |  |
| 0 | Atr-ERM98750 |  |  |  |  |  |  |
| 0 | Atr-ERM98751 |  |  |  |  |  |  |
| 0 | Atr-ERM98752 |  |  |  |  |  |  |
| 0 | Atr-ERM98753 |  |  |  |  |  |  |
| 0 | Atr-ERM98754 |  |  |  |  |  |  |
| 0 | Atr-ERM98755 |  |  |  |  |  |  |
| 0 | Atr-ERM98756 |  |  |  |  |  |  |
| 0 | Atr-ERM98757 |  |  |  |  |  |  |
| 0 | Atr-ERM98758 |  |  |  |  |  |  |
| 0 | Atr-ERM98759 |  |  |  |  |  |  |
| 0 | Atr-ERM98760 |  |  |  |  |  |  |
| 0 | Atr-ERM98761 |  |  |  |  |  |  |
| 0 | Atr-ERM98762 |  |  |  |  |  |  |
| 0 | Atr-ERM98763 |  |  |  |  |  |  |
| 0 | Atr-ERM98764 |  |  |  |  |  |  |
| 0 | Atr-ERM98765 |  |  |  |  |  |  |
| 0 | Atr-ERM98766 |  |  |  |  |  |  |
| 0 | Atr-ERM98767 |  |  |  |  |  |  |
| 0 | Atr-ERM98768 |  |  |  |  |  |  |
| 0 | Atr-ERM98769 |  |  |  |  |  |  |
| 0 | Atr-ERM98770 |  |  |  |  |  |  |
| 0 | Atr-ERM98771 |  |  |  |  |  |  |
| 0 | Atr-ERM98772 |  |  |  |  |  |  |
