# Supplementary material for: Genome-wide identification, characterization and gene expression of BES1 transcription factor family in grapevine (Vitis vinifera L.)
Source: Sci Rep. 2023 Jan 5;13:240. doi: 10.1038/s41598-022-24407-y (PMC9816167; doi:10.1038/s41598-022-24407-y)
Supplement: Supplementary file 3 — Supplementary Information. [file 41598_2022_24407_MOESM3_ESM.zip › Vvi_Atr/Vitis_vinifera.PN40024.v4.dna_sm.toplevel.fa.vs.Amborella_trichopoda.AMTR1.0.dna_sm.toplevel.fa.html/Atr-AmTr_v1.0_scaffold00083.html]

|  |  |  |  |  |  |  |  |  |  |  |  |  |  |
| --- | --- | --- | --- | --- | --- | --- | --- | --- | --- | --- | --- | --- | --- |
| Duplication depth | Reference chromosome | Collinear blocks | | | | | | | | | | | |
| 0 | Atr-ERN02497 |  |  |  |  |  |  |
| 0 | Atr-ERN02498 |  |  |  |  |  |  |
| 0 | Atr-ERN02499 |  |  |  |  |  |  |
| 0 | Atr-ERN02500 |  |  |  |  |  |  |
| 0 | Atr-ERN02501 |  |  |  |  |  |  |
| 0 | Atr-ERN02502 |  |  |  |  |  |  |
| 0 | Atr-ERN02503 |  |  |  |  |  |  |
| 0 | Atr-ERN02504 |  |  |  |  |  |  |
| 0 | Atr-ERN02505 |  |  |  |  |  |  |
| 0 | Atr-ERN02506 |  |  |  |  |  |  |
| 0 | Atr-ERN02507 |  |  |  |  |  |  |
| 0 | Atr-ERN02508 |  |  |  |  |  |  |
| 0 | Atr-ERN02509 |  |  |  |  |  |  |
| 0 | Atr-ERN02510 |  |  |  |  |  |  |
| 0 | Atr-ERN02511 |  |  |  |  |  |  |
| 0 | Atr-ERN02512 |  |  |  |  |  |  |
| 0 | Atr-ERN02513 |  |  |  |  |  |  |
| 0 | Atr-ERN02514 |  |  |  |  |  |  |
| 0 | Atr-ERN02515 |  |  |  |  |  |  |
| 0 | Atr-ERN02516 |  |  |  |  |  |  |
| 0 | Atr-ERN02517 |  |  |  |  |  |  |
| 0 | Atr-ERN02518 |  |  |  |  |  |  |
| 0 | Atr-ERN02519 |  |  |  |  |  |  |
| 0 | Atr-ERN02520 |  |  |  |  |  |  |
| 0 | Atr-ERN02521 |  |  |  |  |  |  |
| 0 | Atr-ERN02522 |  |  |  |  |  |  |
| 0 | Atr-ERN02523 |  |  |  |  |  |  |
| 0 | Atr-ERN02524 |  |  |  |  |  |  |
| 0 | Atr-ERN02525 |  |  |  |  |  |  |
| 0 | Atr-ERN02526 |  |  |  |  |  |  |
| 0 | Atr-ERN02527 |  |  |  |  |  |  |
| 0 | Atr-ERN02528 |  |  |  |  |  |  |
| 0 | Atr-ERN02529 |  |  |  |  |  |  |
| 0 | Atr-ERN02530 |  |  |  |  |  |  |
| 0 | Atr-ERN02531 |  |  |  |  |  |  |
| 0 | Atr-ERN02532 |  |  |  |  |  |  |
| 0 | Atr-ERN02533 |  |  |  |  |  |  |
| 0 | Atr-ERN02534 |  |  |  |  |  |  |
| 0 | Atr-ERN02535 |  |  |  |  |  |  |
| 0 | Atr-ERN02536 |  |  |  |  |  |  |
| 0 | Atr-ERN02537 |  |  |  |  |  |  |
| 0 | Atr-ERN02538 |  |  |  |  |  |  |
| 0 | Atr-ERN02539 |  |  |  |  |  |  |
| 0 | Atr-ERN02540 |  |  |  |  |  |  |
| 0 | Atr-ERN02541 |  |  |  |  |  |  |
| 0 | Atr-ERN02542 |  |  |  |  |  |  |
| 0 | Atr-ERN02543 |  |  |  |  |  |  |
| 0 | Atr-ERN02544 |  |  |  |  |  |  |
| 0 | Atr-ERN02545 |  |  |  |  |  |  |
| 0 | Atr-ERN02546 |  |  |  |  |  |  |
| 0 | Atr-ERN02547 |  |  |  |  |  |  |
| 0 | Atr-ERN02548 |  |  |  |  |  |  |
| 0 | Atr-ERN02549 |  |  |  |  |  |  |
| 0 | Atr-ERN02550 |  |  |  |  |  |  |
| 0 | Atr-ERN02551 |  |  |  |  |  |  |
| 0 | Atr-ERN02552 |  |  |  |  |  |  |
