# Supplementary material for: Genome-wide identification, characterization and gene expression of BES1 transcription factor family in grapevine (Vitis vinifera L.)
Source: Sci Rep. 2023 Jan 5;13:240. doi: 10.1038/s41598-022-24407-y (PMC9816167; doi:10.1038/s41598-022-24407-y)
Supplement: Supplementary file 3 — Supplementary Information. [file 41598_2022_24407_MOESM3_ESM.zip › Vvi_Atr/Vitis_vinifera.PN40024.v4.dna_sm.toplevel.fa.vs.Amborella_trichopoda.AMTR1.0.dna_sm.toplevel.fa.html/Atr-AmTr_v1.0_scaffold00084.html]

|  |  |  |  |  |  |  |  |  |  |  |  |  |  |
| --- | --- | --- | --- | --- | --- | --- | --- | --- | --- | --- | --- | --- | --- |
| Duplication depth | Reference chromosome | Collinear blocks | | | | | | | | | | | |
| 0 | Atr-ERN02274 |  |  |  |  |  |  |
| 0 | Atr-ERN02275 |  |  |  |  |  |  |
| 0 | Atr-ERN02276 |  |  |  |  |  |  |
| 0 | Atr-ERN02277 |  |  |  |  |  |  |
| 0 | Atr-ERN02278 |  |  |  |  |  |  |
| 0 | Atr-ERN02279 |  |  |  |  |  |  |
| 0 | Atr-ERN02280 |  |  |  |  |  |  |
| 0 | Atr-ERN02281 |  |  |  |  |  |  |
| 0 | Atr-ERN02282 |  |  |  |  |  |  |
| 0 | Atr-ERN02283 |  |  |  |  |  |  |
| 0 | Atr-ERN02284 |  |  |  |  |  |  |
| 0 | Atr-ERN02285 |  |  |  |  |  |  |
| 0 | Atr-ERN02286 |  |  |  |  |  |  |
| 0 | Atr-ERN02287 |  |  |  |  |  |  |
| 0 | Atr-ERN02288 |  |  |  |  |  |  |
| 0 | Atr-ERN02289 |  |  |  |  |  |  |
| 0 | Atr-ERN02290 |  |  |  |  |  |  |
| 0 | Atr-ERN02291 |  |  |  |  |  |  |
| 0 | Atr-ERN02292 |  |  |  |  |  |  |
| 0 | Atr-ERN02293 |  |  |  |  |  |  |
| 0 | Atr-ERN02294 |  |  |  |  |  |  |
| 0 | Atr-ERN02295 |  |  |  |  |  |  |
| 0 | Atr-ERN02296 |  |  |  |  |  |  |
| 0 | Atr-ERN02297 |  |  |  |  |  |  |
| 0 | Atr-ERN02298 |  |  |  |  |  |  |
| 0 | Atr-ERN02299 |  |  |  |  |  |  |
| 0 | Atr-ERN02300 |  |  |  |  |  |  |
| 0 | Atr-ERN02301 |  |  |  |  |  |  |
| 0 | Atr-ERN02302 |  |  |  |  |  |  |
| 0 | Atr-ERN02303 |  |  |  |  |  |  |
| 0 | Atr-ERN02304 |  |  |  |  |  |  |
| 0 | Atr-ERN02305 |  |  |  |  |  |  |
| 0 | Atr-ERN02306 |  |  |  |  |  |  |
| 0 | Atr-ERN02307 |  |  |  |  |  |  |
| 0 | Atr-ERN02308 |  |  |  |  |  |  |
| 0 | Atr-ERN02309 |  |  |  |  |  |  |
| 0 | Atr-ERN02310 |  |  |  |  |  |  |
| 0 | Atr-ERN02311 |  |  |  |  |  |  |
| 0 | Atr-ERN02312 |  |  |  |  |  |  |
| 0 | Atr-ERN02313 |  |  |  |  |  |  |
| 0 | Atr-ERN02314 |  |  |  |  |  |  |
| 0 | Atr-ERN02315 |  |  |  |  |  |  |
| 0 | Atr-ERN02316 |  |  |  |  |  |  |
| 0 | Atr-ERN02317 |  |  |  |  |  |  |
| 0 | Atr-ERN02318 |  |  |  |  |  |  |
| 0 | Atr-ERN02319 |  |  |  |  |  |  |
| 0 | Atr-ERN02320 |  |  |  |  |  |  |
| 0 | Atr-ERN02321 |  |  |  |  |  |  |
| 0 | Atr-ERN02322 |  |  |  |  |  |  |
| 0 | Atr-ERN02323 |  |  |  |  |  |  |
