# Supplementary material for: Genome-wide identification, characterization and gene expression of BES1 transcription factor family in grapevine (Vitis vinifera L.)
Source: Sci Rep. 2023 Jan 5;13:240. doi: 10.1038/s41598-022-24407-y (PMC9816167; doi:10.1038/s41598-022-24407-y)
Supplement: Supplementary file 3 — Supplementary Information. [file 41598_2022_24407_MOESM3_ESM.zip › Vvi_Atr/Vitis_vinifera.PN40024.v4.dna_sm.toplevel.fa.vs.Amborella_trichopoda.AMTR1.0.dna_sm.toplevel.fa.html/Atr-AmTr_v1.0_scaffold00085.html]

|  |  |  |  |  |  |  |  |  |  |  |  |  |  |
| --- | --- | --- | --- | --- | --- | --- | --- | --- | --- | --- | --- | --- | --- |
| Duplication depth | Reference chromosome | Collinear blocks | | | | | | | | | | | |
| 0 | Atr-ERN02629 |  |  |  |  |  |  |
| 0 | Atr-ERN02630 |  |  |  |  |  |  |
| 0 | Atr-ERN02631 |  |  |  |  |  |  |
| 0 | Atr-ERN02632 |  |  |  |  |  |  |
| 0 | Atr-ERN02633 |  |  |  |  |  |  |
| 0 | Atr-ERN02634 |  |  |  |  |  |  |
| 0 | Atr-ERN02635 |  |  |  |  |  |  |
| 0 | Atr-ERN02636 |  |  |  |  |  |  |
| 0 | Atr-ERN02637 |  |  |  |  |  |  |
| 0 | Atr-ERN02638 |  |  |  |  |  |  |
| 0 | Atr-ERN02639 |  |  |  |  |  |  |
| 0 | Atr-ERN02640 |  |  |  |  |  |  |
| 0 | Atr-ERN02641 |  |  |  |  |  |  |
| 1 | Atr-ERN02642 |  | Vvi-Vitvi17g00885\_t001 |  |  |  |  |  |
| 1 | Atr-ERN02643 |  | Vvi-Vitvi17g00884\_t001 |  |  |  |  |  |
| 1 | Atr-ERN02644 |  | | | |  |  |  |  |  |
| 1 | Atr-ERN02645 |  | | | |  |  |  |  |  |
| 1 | Atr-ERN02646 |  | | | |  |  |  |  |  |
| 1 | Atr-ERN02647 |  | | | |  |  |  |  |  |
| 1 | Atr-ERN02648 |  | Vvi-Vitvi17g00883\_t001 |  |  |  |  |  |
| 1 | Atr-ERN02649 |  | | | |  |  |  |  |  |
| 1 | Atr-ERN02650 |  | | | |  |  |  |  |  |
| 1 | Atr-ERN02651 |  | Vvi-Vitvi17g00881\_t001 |  |  |  |  |  |
| 1 | Atr-ERN02652 |  | | | |  |  |  |  |  |
| 1 | Atr-ERN02653 |  | Vvi-Vitvi17g00880\_t001 |  |  |  |  |  |
| 2 | Atr-ERN02654 |  | | | |  | Vvi-Vitvi01g00675\_t001 |  |  |  |  |
| 2 | Atr-ERN02655 |  | Vvi-Vitvi17g00875\_t001 |  | | | |  |  |  |  |
| 2 | Atr-ERN02656 |  | | | |  | | | |  |  |  |  |
| 2 | Atr-ERN02657 |  | Vvi-Vitvi17g00874\_t001 |  | Vvi-Vitvi01g00676\_t001 |  |  |  |  |
| 2 | Atr-ERN02658 |  | | | |  | | | |  |  |  |  |
| 2 | Atr-ERN02659 |  | | | |  | | | |  |  |  |  |
| 2 | Atr-ERN02660 |  | | | |  | | | |  |  |  |  |
| 2 | Atr-ERN02661 |  | | | |  | | | |  |  |  |  |
| 2 | Atr-ERN02662 |  | | | |  | | | |  |  |  |  |
| 2 | Atr-ERN02663 |  | | | |  | | | |  |  |  |  |
| 2 | Atr-ERN02664 |  | | | |  | | | |  |  |  |  |
| 2 | Atr-ERN02665 |  | | | |  | | | |  |  |  |  |
| 2 | Atr-ERN02666 |  | Vvi-Vitvi17g00872\_t001 |  | | | |  |  |  |  |
| 2 | Atr-ERN02667 |  | | | |  | | | |  |  |  |  |
| 2 | Atr-ERN02668 |  | | | |  | | | |  |  |  |  |
| 2 | Atr-ERN02669 |  | | | |  | | | |  |  |  |  |
| 2 | Atr-ERN02670 |  | Vvi-Vitvi17g01553\_t001 |  | | | |  |  |  |  |
| 2 | Atr-ERN02671 |  | | | |  | | | |  |  |  |  |
| 2 | Atr-ERN02672 |  | | | |  | | | |  |  |  |  |
| 2 | Atr-ERN02673 |  | Vvi-Vitvi17g00870\_t001 |  | Vvi-Vitvi01g00680\_t001 |  |  |  |  |
| 2 | Atr-ERN02674 |  | | | |  | | | |  |  |  |  |
| 2 | Atr-ERN02675 |  | | | |  | | | |  |  |  |  |
| 2 | Atr-ERN02676 |  | | | |  | | | |  |  |  |  |
| 2 | Atr-ERN02677 |  | | | |  | | | |  |  |  |  |
| 2 | Atr-ERN02678 |  | | | |  | | | |  |  |  |  |
| 2 | Atr-ERN02679 |  | | | |  | | | |  |  |  |  |
| 2 | Atr-ERN02680 |  | | | |  | | | |  |  |  |  |
| 2 | Atr-ERN02681 |  | | | |  | | | |  |  |  |  |
| 2 | Atr-ERN02682 |  | | | |  | | | |  |  |  |  |
| 2 | Atr-ERN02683 |  | | | |  | | | |  |  |  |  |
| 2 | Atr-ERN02684 |  | | | |  | | | |  |  |  |  |
| 2 | Atr-ERN02685 |  | | | |  | | | |  |  |  |  |
| 2 | Atr-ERN02686 |  | Vvi-Vitvi17g00869\_t001 |  | | | |  |  |  |  |
| 2 | Atr-ERN02687 |  | | | |  | | | |  |  |  |  |
| 2 | Atr-ERN02688 |  | | | |  | | | |  |  |  |  |
| 2 | Atr-ERN02689 |  | | | |  | | | |  |  |  |  |
| 2 | Atr-ERN02690 |  | | | |  | | | |  |  |  |  |
| 2 | Atr-ERN02691 |  | | | |  | | | |  |  |  |  |
| 2 | Atr-ERN02692 |  | | | |  | | | |  |  |  |  |
| 2 | Atr-ERN02693 |  | | | |  | Vvi-Vitvi01g00681\_t001 |  |  |  |  |
| 2 | Atr-ERN02694 |  | | | |  | | | |  |  |  |  |
| 2 | Atr-ERN02695 |  | | | |  | | | |  |  |  |  |
| 2 | Atr-ERN02696 |  | Vvi-Vitvi17g00864\_t001 |  | | | |  |  |  |  |
| 2 | Atr-ERN02697 |  | | | |  | Vvi-Vitvi01g00684\_t001 |  |  |  |  |
| 2 | Atr-ERN02698 |  | Vvi-Vitvi17g00863\_t001 |  | | | |  |  |  |  |
| 2 | Atr-ERN02699 |  | Vvi-Vitvi17g00862\_t001 |  | | | |  |  |  |  |
| 2 | Atr-ERN02700 |  | | | |  | | | |  |  |  |  |
| 2 | Atr-ERN02701 |  | | | |  | | | |  |  |  |  |
| 2 | Atr-ERN02702 |  | | | |  | | | |  |  |  |  |
| 2 | Atr-ERN02703 |  | | | |  | | | |  |  |  |  |
| 2 | Atr-ERN02704 |  | | | |  | | | |  |  |  |  |
| 2 | Atr-ERN02705 |  | | | |  | | | |  |  |  |  |
| 2 | Atr-ERN02706 |  | | | |  | | | |  |  |  |  |
| 2 | Atr-ERN02707 |  | | | |  | | | |  |  |  |  |
| 2 | Atr-ERN02708 |  | | | |  | | | |  |  |  |  |
| 2 | Atr-ERN02709 |  | | | |  | | | |  |  |  |  |
| 2 | Atr-ERN02710 |  | | | |  | | | |  |  |  |  |
| 2 | Atr-ERN02711 |  | | | |  | | | |  |  |  |  |
| 2 | Atr-ERN02712 |  | | | |  | | | |  |  |  |  |
| 2 | Atr-ERN02713 |  | | | |  | | | |  |  |  |  |
| 2 | Atr-ERN02714 |  | | | |  | | | |  |  |  |  |
| 2 | Atr-ERN02715 |  | | | |  | | | |  |  |  |  |
| 2 | Atr-ERN02716 |  | | | |  | | | |  |  |  |  |
| 2 | Atr-ERN02717 |  | Vvi-Vitvi17g00861\_t002 |  | | | |  |  |  |  |
| 2 | Atr-ERN02718 |  | | | |  | | | |  |  |  |  |
| 2 | Atr-ERN02719 |  | | | |  | | | |  |  |  |  |
| 2 | Atr-ERN02720 |  | | | |  | | | |  |  |  |  |
| 2 | Atr-ERN02721 |  | | | |  | | | |  |  |  |  |
| 2 | Atr-ERN02722 |  | Vvi-Vitvi17g00856\_t002 |  | | | |  |  |  |  |
| 2 | Atr-ERN02723 |  | Vvi-Vitvi17g00854\_t001 |  | Vvi-Vitvi01g00685\_t001 |  |  |  |  |
| 2 | Atr-ERN02724 |  | | | |  | | | |  |  |  |  |
| 2 | Atr-ERN02725 |  | | | |  | Vvi-Vitvi01g00687\_t001 |  |  |  |  |
| 2 | Atr-ERN02726 |  | | | |  | | | |  |  |  |  |
| 2 | Atr-ERN02727 |  | | | |  | | | |  |  |  |  |
| 2 | Atr-ERN02728 |  | | | |  | | | |  |  |  |  |
| 2 | Atr-ERN02729 |  | Vvi-Vitvi17g00850\_t001 |  | Vvi-Vitvi01g00694\_t001 |  |  |  |  |
| 1 | Atr-ERN02730 |  |  |  | | | |  |  |  |  |
| 1 | Atr-ERN02731 |  |  |  | | | |  |  |  |  |
| 1 | Atr-ERN02732 |  |  |  | | | |  |  |  |  |
| 1 | Atr-ERN02733 |  |  |  | | | |  |  |  |  |
| 1 | Atr-ERN02734 |  |  |  | | | |  |  |  |  |
| 1 | Atr-ERN02735 |  |  |  | | | |  |  |  |  |
| 1 | Atr-ERN02736 |  |  |  | Vvi-Vitvi01g00695\_t001 |  |  |  |  |
| 1 | Atr-ERN02737 |  |  |  | Vvi-Vitvi01g00696\_t001 |  |  |  |  |
| 0 | Atr-ERN02738 |  |  |  |  |  |  |
| 0 | Atr-ERN02739 |  |  |  |  |  |  |
| 0 | Atr-ERN02740 |  |  |  |  |  |  |
| 0 | Atr-ERN02741 |  |  |  |  |  |  |
| 0 | Atr-ERN02742 |  |  |  |  |  |  |
| 0 | Atr-ERN02743 |  |  |  |  |  |  |
