# Supplementary material for: Genome-wide identification, characterization and gene expression of BES1 transcription factor family in grapevine (Vitis vinifera L.)
Source: Sci Rep. 2023 Jan 5;13:240. doi: 10.1038/s41598-022-24407-y (PMC9816167; doi:10.1038/s41598-022-24407-y)
Supplement: Supplementary file 3 — Supplementary Information. [file 41598_2022_24407_MOESM3_ESM.zip › Vvi_Atr/Vitis_vinifera.PN40024.v4.dna_sm.toplevel.fa.vs.Amborella_trichopoda.AMTR1.0.dna_sm.toplevel.fa.html/Atr-AmTr_v1.0_scaffold00086.html]

|  |  |  |  |  |  |  |  |  |  |  |  |  |  |
| --- | --- | --- | --- | --- | --- | --- | --- | --- | --- | --- | --- | --- | --- |
| Duplication depth | Reference chromosome | Collinear blocks | | | | | | | | | | | |
| 0 | Atr-ERN02745 |  |  |  |  |  |  |
| 0 | Atr-ERN02746 |  |  |  |  |  |  |
| 0 | Atr-ERN02747 |  |  |  |  |  |  |
| 0 | Atr-ERN02748 |  |  |  |  |  |  |
| 0 | Atr-ERN02749 |  |  |  |  |  |  |
| 0 | Atr-ERN02750 |  |  |  |  |  |  |
| 1 | Atr-ERN02751 |  | Vvi-Vitvi03g00646\_t001 |  |  |  |  |  |
| 1 | Atr-ERN02752 |  | | | |  |  |  |  |  |
| 1 | Atr-ERN02753 |  | | | |  |  |  |  |  |
| 1 | Atr-ERN02754 |  | | | |  |  |  |  |  |
| 1 | Atr-ERN02755 |  | Vvi-Vitvi03g00647\_t001 |  |  |  |  |  |
| 1 | Atr-ERN02756 |  | | | |  |  |  |  |  |
| 2 | Atr-ERN02757 |  | | | |  | Vvi-Vitvi07g01593\_t001 |  |  |  |  |
| 2 | Atr-ERN02758 |  | | | |  | | | |  |  |  |  |
| 2 | Atr-ERN02759 |  | | | |  | Vvi-Vitvi07g01591\_t001 |  |  |  |  |
| 2 | Atr-ERN02760 |  | | | |  | | | |  |  |  |  |
| 2 | Atr-ERN02761 |  | | | |  | | | |  |  |  |  |
| 2 | Atr-ERN02762 |  | | | |  | | | |  |  |  |  |
| 2 | Atr-ERN02763 |  | Vvi-Vitvi03g04210\_t001 |  | | | |  |  |  |  |
| 2 | Atr-ERN02764 |  | Vvi-Vitvi03g00651\_t001 |  | Vvi-Vitvi07g01590\_t001 |  |  |  |  |
| 2 | Atr-ERN02765 |  | | | |  | | | |  |  |  |  |
| 2 | Atr-ERN02766 |  | Vvi-Vitvi03g00653\_t001 |  | | | |  |  |  |  |
| 2 | Atr-ERN02767 |  | | | |  | Vvi-Vitvi07g02633\_t001 |  |  |  |  |
| 2 | Atr-ERN02768 |  | | | |  | | | |  |  |  |  |
| 2 | Atr-ERN02769 |  | | | |  | | | |  |  |  |  |
| 2 | Atr-ERN02770 |  | | | |  | | | |  |  |  |  |
| 2 | Atr-ERN02771 |  | | | |  | Vvi-Vitvi07g01581\_t001 |  |  |  |  |
| 2 | Atr-ERN02772 |  | | | |  | | | |  |  |  |  |
| 2 | Atr-ERN02773 |  | | | |  | Vvi-Vitvi07g02073\_t001 |  |  |  |  |
| 2 | Atr-ERN02774 |  | | | |  | Vvi-Vitvi07g01580\_t002 |  |  |  |  |
| 2 | Atr-ERN02775 |  | | | |  | | | |  |  |  |  |
| 2 | Atr-ERN02776 |  | | | |  | | | |  |  |  |  |
| 2 | Atr-ERN02777 |  | | | |  | | | |  |  |  |  |
| 2 | Atr-ERN02778 |  | | | |  | | | |  |  |  |  |
| 2 | Atr-ERN02779 |  | Vvi-Vitvi03g00665\_t001 |  | Vvi-Vitvi07g01577\_t001 |  |  |  |  |
| 1 | Atr-ERN02780 |  |  |  | | | |  |  |  |  |
| 1 | Atr-ERN02781 |  |  |  | | | |  |  |  |  |
| 1 | Atr-ERN02782 |  |  |  | | | |  |  |  |  |
| 1 | Atr-ERN02783 |  |  |  | | | |  |  |  |  |
| 1 | Atr-ERN02784 |  |  |  | | | |  |  |  |  |
| 1 | Atr-ERN02785 |  |  |  | | | |  |  |  |  |
| 1 | Atr-ERN02786 |  |  |  | | | |  |  |  |  |
| 1 | Atr-ERN02787 |  |  |  | | | |  |  |  |  |
| 1 | Atr-ERN02788 |  |  |  | | | |  |  |  |  |
| 1 | Atr-ERN02789 |  |  |  | | | |  |  |  |  |
| 1 | Atr-ERN02790 |  |  |  | | | |  |  |  |  |
| 1 | Atr-ERN02791 |  |  |  | Vvi-Vitvi07g04671\_t001 |  |  |  |  |
| 0 | Atr-ERN02792 |  |  |  |  |  |  |
| 0 | Atr-ERN02793 |  |  |  |  |  |  |
| 0 | Atr-ERN02794 |  |  |  |  |  |  |
| 0 | Atr-ERN02795 |  |  |  |  |  |  |
| 0 | Atr-ERN02796 |  |  |  |  |  |  |
| 0 | Atr-ERN02797 |  |  |  |  |  |  |
| 0 | Atr-ERN02798 |  |  |  |  |  |  |
| 0 | Atr-ERN02799 |  |  |  |  |  |  |
| 0 | Atr-ERN02800 |  |  |  |  |  |  |
| 0 | Atr-ERN02801 |  |  |  |  |  |  |
| 0 | Atr-ERN02802 |  |  |  |  |  |  |
| 0 | Atr-ERN02803 |  |  |  |  |  |  |
| 0 | Atr-ERN02804 |  |  |  |  |  |  |
| 0 | Atr-ERN02805 |  |  |  |  |  |  |
| 0 | Atr-ERN02806 |  |  |  |  |  |  |
| 0 | Atr-ERN02807 |  |  |  |  |  |  |
| 0 | Atr-ERN02808 |  |  |  |  |  |  |
| 0 | Atr-ERN02809 |  |  |  |  |  |  |
| 0 | Atr-ERN02810 |  |  |  |  |  |  |
| 0 | Atr-ERN02811 |  |  |  |  |  |  |
| 0 | Atr-ERN02812 |  |  |  |  |  |  |
| 0 | Atr-ERN02813 |  |  |  |  |  |  |
| 0 | Atr-ERN02814 |  |  |  |  |  |  |
| 0 | Atr-ERN02815 |  |  |  |  |  |  |
| 0 | Atr-ERN02816 |  |  |  |  |  |  |
| 0 | Atr-ERN02817 |  |  |  |  |  |  |
| 0 | Atr-ERN02818 |  |  |  |  |  |  |
| 0 | Atr-ERN02819 |  |  |  |  |  |  |
| 0 | Atr-ERN02820 |  |  |  |  |  |  |
| 0 | Atr-ERN02821 |  |  |  |  |  |  |
| 0 | Atr-ERN02822 |  |  |  |  |  |  |
| 1 | Atr-ERN02823 |  | Vvi-Vitvi02g04155\_t001 |  |  |  |  |  |
| 1 | Atr-ERN02824 |  | | | |  |  |  |  |  |
| 1 | Atr-ERN02825 |  | | | |  |  |  |  |  |
| 1 | Atr-ERN02826 |  | | | |  |  |  |  |  |
| 1 | Atr-ERN02827 |  | | | |  |  |  |  |  |
| 1 | Atr-ERN02828 |  | Vvi-Vitvi02g00526\_t001 |  |  |  |  |  |
| 1 | Atr-ERN02829 |  | | | |  |  |  |  |  |
| 1 | Atr-ERN02830 |  | | | |  |  |  |  |  |
| 1 | Atr-ERN02831 |  | | | |  |  |  |  |  |
| 1 | Atr-ERN02832 |  | | | |  |  |  |  |  |
| 1 | Atr-ERN02833 |  | Vvi-Vitvi02g04156\_t002 |  |  |  |  |  |
| 1 | Atr-ERN02834 |  | | | |  |  |  |  |  |
| 1 | Atr-ERN02835 |  | Vvi-Vitvi02g00531\_t001 |  |  |  |  |  |
| 1 | Atr-ERN02836 |  | | | |  |  |  |  |  |
| 1 | Atr-ERN02837 |  | | | |  |  |  |  |  |
| 1 | Atr-ERN02838 |  | | | |  |  |  |  |  |
| 1 | Atr-ERN02839 |  | | | |  |  |  |  |  |
| 1 | Atr-ERN02840 |  | | | |  |  |  |  |  |
| 1 | Atr-ERN02841 |  | | | |  |  |  |  |  |
| 1 | Atr-ERN02842 |  | Vvi-Vitvi02g00541\_t001 |  |  |  |  |  |
| 1 | Atr-ERN02843 |  | | | |  |  |  |  |  |
| 1 | Atr-ERN02844 |  | Vvi-Vitvi02g00545\_t001 |  |  |  |  |  |
| 0 | Atr-ERN02845 |  |  |  |  |  |  |
| 0 | Atr-ERN02846 |  |  |  |  |  |  |
| 0 | Atr-ERN02847 |  |  |  |  |  |  |
| 0 | Atr-ERN02848 |  |  |  |  |  |  |
| 0 | Atr-ERN02849 |  |  |  |  |  |  |
| 0 | Atr-ERN02850 |  |  |  |  |  |  |
| 0 | Atr-ERN02851 |  |  |  |  |  |  |
| 0 | Atr-ERN02852 |  |  |  |  |  |  |
| 0 | Atr-ERN02853 |  |  |  |  |  |  |
| 0 | Atr-ERN02854 |  |  |  |  |  |  |
| 0 | Atr-ERN02855 |  |  |  |  |  |  |
| 0 | Atr-ERN02856 |  |  |  |  |  |  |
| 0 | Atr-ERN02857 |  |  |  |  |  |  |
| 0 | Atr-ERN02858 |  |  |  |  |  |  |
| 0 | Atr-ERN02859 |  |  |  |  |  |  |
| 0 | Atr-ERN02860 |  |  |  |  |  |  |
| 0 | Atr-ERN02861 |  |  |  |  |  |  |
| 0 | Atr-ERN02862 |  |  |  |  |  |  |
| 0 | Atr-ERN02863 |  |  |  |  |  |  |
| 0 | Atr-ERN02864 |  |  |  |  |  |  |
