# Supplementary material for: Genome-wide identification, characterization and gene expression of BES1 transcription factor family in grapevine (Vitis vinifera L.)
Source: Sci Rep. 2023 Jan 5;13:240. doi: 10.1038/s41598-022-24407-y (PMC9816167; doi:10.1038/s41598-022-24407-y)
Supplement: Supplementary file 3 — Supplementary Information. [file 41598_2022_24407_MOESM3_ESM.zip › Vvi_Atr/Vitis_vinifera.PN40024.v4.dna_sm.toplevel.fa.vs.Amborella_trichopoda.AMTR1.0.dna_sm.toplevel.fa.html/Atr-AmTr_v1.0_scaffold00087.html]

|  |  |  |  |  |  |  |  |  |  |  |  |  |  |
| --- | --- | --- | --- | --- | --- | --- | --- | --- | --- | --- | --- | --- | --- |
| Duplication depth | Reference chromosome | Collinear blocks | | | | | | | | | | | |
| 0 | Atr-ERN02553 |  |  |  |  |  |  |
| 0 | Atr-ERN02554 |  |  |  |  |  |  |
| 0 | Atr-ERN02555 |  |  |  |  |  |  |
| 0 | Atr-ERN02556 |  |  |  |  |  |  |
| 0 | Atr-ERN02557 |  |  |  |  |  |  |
| 0 | Atr-ERN02558 |  |  |  |  |  |  |
| 0 | Atr-ERN02559 |  |  |  |  |  |  |
| 0 | Atr-ERN02560 |  |  |  |  |  |  |
| 0 | Atr-ERN02561 |  |  |  |  |  |  |
| 0 | Atr-ERN02562 |  |  |  |  |  |  |
| 0 | Atr-ERN02563 |  |  |  |  |  |  |
| 0 | Atr-ERN02564 |  |  |  |  |  |  |
| 0 | Atr-ERN02565 |  |  |  |  |  |  |
| 0 | Atr-ERN02566 |  |  |  |  |  |  |
| 0 | Atr-ERN02567 |  |  |  |  |  |  |
| 0 | Atr-ERN02568 |  |  |  |  |  |  |
| 0 | Atr-ERN02569 |  |  |  |  |  |  |
| 0 | Atr-ERN02570 |  |  |  |  |  |  |
| 0 | Atr-ERN02571 |  |  |  |  |  |  |
| 0 | Atr-ERN02572 |  |  |  |  |  |  |
| 0 | Atr-ERN02573 |  |  |  |  |  |  |
| 0 | Atr-ERN02574 |  |  |  |  |  |  |
| 0 | Atr-ERN02575 |  |  |  |  |  |  |
| 0 | Atr-ERN02576 |  |  |  |  |  |  |
| 0 | Atr-ERN02577 |  |  |  |  |  |  |
| 0 | Atr-ERN02578 |  |  |  |  |  |  |
| 0 | Atr-ERN02579 |  |  |  |  |  |  |
| 0 | Atr-ERN02580 |  |  |  |  |  |  |
| 0 | Atr-ERN02581 |  |  |  |  |  |  |
| 0 | Atr-ERN02582 |  |  |  |  |  |  |
| 0 | Atr-ERN02583 |  |  |  |  |  |  |
| 0 | Atr-ERN02584 |  |  |  |  |  |  |
| 0 | Atr-ERN02585 |  |  |  |  |  |  |
| 0 | Atr-ERN02586 |  |  |  |  |  |  |
| 0 | Atr-ERN02587 |  |  |  |  |  |  |
| 0 | Atr-ERN02588 |  |  |  |  |  |  |
| 0 | Atr-ERN02589 |  |  |  |  |  |  |
| 0 | Atr-ERN02590 |  |  |  |  |  |  |
| 0 | Atr-ERN02591 |  |  |  |  |  |  |
| 0 | Atr-ERN02592 |  |  |  |  |  |  |
| 0 | Atr-ERN02593 |  |  |  |  |  |  |
| 0 | Atr-ERN02594 |  |  |  |  |  |  |
| 0 | Atr-ERN02595 |  |  |  |  |  |  |
| 0 | Atr-ERN02596 |  |  |  |  |  |  |
| 0 | Atr-ERN02597 |  |  |  |  |  |  |
| 0 | Atr-ERN02598 |  |  |  |  |  |  |
| 0 | Atr-ERN02599 |  |  |  |  |  |  |
| 0 | Atr-ERN02600 |  |  |  |  |  |  |
| 0 | Atr-ERN02601 |  |  |  |  |  |  |
| 0 | Atr-ERN02602 |  |  |  |  |  |  |
| 0 | Atr-ERN02603 |  |  |  |  |  |  |
| 0 | Atr-ERN02604 |  |  |  |  |  |  |
| 0 | Atr-ERN02605 |  |  |  |  |  |  |
| 0 | Atr-ERN02606 |  |  |  |  |  |  |
| 0 | Atr-ERN02607 |  |  |  |  |  |  |
| 0 | Atr-ERN02608 |  |  |  |  |  |  |
| 0 | Atr-ERN02609 |  |  |  |  |  |  |
| 0 | Atr-ERN02610 |  |  |  |  |  |  |
