# Supplementary material for: Genome-wide identification, characterization and gene expression of BES1 transcription factor family in grapevine (Vitis vinifera L.)
Source: Sci Rep. 2023 Jan 5;13:240. doi: 10.1038/s41598-022-24407-y (PMC9816167; doi:10.1038/s41598-022-24407-y)
Supplement: Supplementary file 3 — Supplementary Information. [file 41598_2022_24407_MOESM3_ESM.zip › Vvi_Atr/Vitis_vinifera.PN40024.v4.dna_sm.toplevel.fa.vs.Amborella_trichopoda.AMTR1.0.dna_sm.toplevel.fa.html/Atr-AmTr_v1.0_scaffold00088.html]

|  |  |  |  |  |  |  |  |  |  |  |  |  |  |
| --- | --- | --- | --- | --- | --- | --- | --- | --- | --- | --- | --- | --- | --- |
| Duplication depth | Reference chromosome | Collinear blocks | | | | | | | | | | | |
| 0 | Atr-ERM99486 |  |  |  |  |  |  |
| 0 | Atr-ERM99487 |  |  |  |  |  |  |
| 0 | Atr-ERM99488 |  |  |  |  |  |  |
| 0 | Atr-ERM99489 |  |  |  |  |  |  |
| 0 | Atr-ERM99490 |  |  |  |  |  |  |
| 1 | Atr-ERM99491 |  | Vvi-Vitvi01g02080\_t001 |  |  |  |  |  |
| 2 | Atr-ERM99492 |  | | | |  | Vvi-Vitvi14g01799\_t001 |  |  |  |  |
| 2 | Atr-ERM99493 |  | | | |  | | | |  |  |  |  |
| 3 | Atr-ERM99494 |  | | | |  | | | |  | Vvi-Vitvi17g00777\_t001 |  |  |  |
| 3 | Atr-ERM99495 |  | | | |  | | | |  | | | |  |  |  |
| 3 | Atr-ERM99496 |  | | | |  | | | |  | Vvi-Vitvi17g00776\_t001 |  |  |  |
| 3 | Atr-ERM99497 |  | | | |  | | | |  | | | |  |  |  |
| 3 | Atr-ERM99498 |  | | | |  | | | |  | | | |  |  |  |
| 3 | Atr-ERM99499 |  | | | |  | | | |  | | | |  |  |  |
| 3 | Atr-ERM99500 |  | | | |  | | | |  | | | |  |  |  |
| 3 | Atr-ERM99501 |  | | | |  | | | |  | | | |  |  |  |
| 3 | Atr-ERM99502 |  | | | |  | | | |  | | | |  |  |  |
| 3 | Atr-ERM99503 |  | | | |  | | | |  | | | |  |  |  |
| 3 | Atr-ERM99504 |  | | | |  | Vvi-Vitvi14g01801\_t001 |  | Vvi-Vitvi17g00775\_t001 |  |  |  |
| 3 | Atr-ERM99505 |  | | | |  | Vvi-Vitvi14g01802\_t001 |  | Vvi-Vitvi17g00773\_t001 |  |  |  |
| 3 | Atr-ERM99506 |  | | | |  | | | |  | | | |  |  |  |
| 3 | Atr-ERM99507 |  | Vvi-Vitvi01g04204\_t001 |  | | | |  | Vvi-Vitvi17g04225\_t001 |  |  |  |
| 3 | Atr-ERM99508 |  | | | |  | | | |  | | | |  |  |  |
| 3 | Atr-ERM99509 |  | | | |  | | | |  | | | |  |  |  |
| 3 | Atr-ERM99510 |  | | | |  | | | |  | | | |  |  |  |
| 3 | Atr-ERM99511 |  | | | |  | | | |  | | | |  |  |  |
| 3 | Atr-ERM99512 |  | | | |  | | | |  | | | |  |  |  |
| 3 | Atr-ERM99513 |  | | | |  | | | |  | | | |  |  |  |
| 3 | Atr-ERM99514 |  | | | |  | | | |  | | | |  |  |  |
| 3 | Atr-ERM99515 |  | | | |  | | | |  | | | |  |  |  |
| 3 | Atr-ERM99516 |  | | | |  | | | |  | | | |  |  |  |
| 3 | Atr-ERM99517 |  | | | |  | | | |  | | | |  |  |  |
| 3 | Atr-ERM99518 |  | | | |  | | | |  | | | |  |  |  |
| 3 | Atr-ERM99519 |  | | | |  | | | |  | | | |  |  |  |
| 3 | Atr-ERM99520 |  | | | |  | | | |  | | | |  |  |  |
| 3 | Atr-ERM99521 |  | | | |  | | | |  | | | |  |  |  |
| 3 | Atr-ERM99522 |  | | | |  | | | |  | | | |  |  |  |
| 3 | Atr-ERM99523 |  | | | |  | | | |  | | | |  |  |  |
| 3 | Atr-ERM99524 |  | | | |  | | | |  | | | |  |  |  |
| 3 | Atr-ERM99525 |  | | | |  | | | |  | | | |  |  |  |
| 3 | Atr-ERM99526 |  | Vvi-Vitvi01g00800\_t001 |  | | | |  | | | |  |  |  |
| 3 | Atr-ERM99527 |  | Vvi-Vitvi01g00801\_t001 |  | | | |  | Vvi-Vitvi17g00770\_t001 |  |  |  |
| 3 | Atr-ERM99528 |  | | | |  | | | |  | | | |  |  |  |
| 3 | Atr-ERM99529 |  | | | |  | | | |  | | | |  |  |  |
| 3 | Atr-ERM99530 |  | | | |  | Vvi-Vitvi14g01803\_t001 |  | Vvi-Vitvi17g00767\_t001 |  |  |  |
| 3 | Atr-ERM99531 |  | | | |  | | | |  | | | |  |  |  |
| 3 | Atr-ERM99532 |  | | | |  | Vvi-Vitvi14g01807\_t001 |  | Vvi-Vitvi17g00764\_t001 |  |  |  |
| 3 | Atr-ERM99533 |  | Vvi-Vitvi01g00816\_t001 |  | | | |  | | | |  |  |  |
| 3 | Atr-ERM99534 |  | | | |  | Vvi-Vitvi14g01808\_t001 |  | | | |  |  |  |
| 3 | Atr-ERM99535 |  | | | |  | | | |  | | | |  |  |  |
| 3 | Atr-ERM99536 |  | | | |  | | | |  | | | |  |  |  |
| 3 | Atr-ERM99537 |  | | | |  | | | |  | | | |  |  |  |
| 3 | Atr-ERM99538 |  | | | |  | | | |  | | | |  |  |  |
| 3 | Atr-ERM99539 |  | | | |  | | | |  | | | |  |  |  |
| 3 | Atr-ERM99540 |  | | | |  | | | |  | | | |  |  |  |
| 3 | Atr-ERM99541 |  | | | |  | | | |  | | | |  |  |  |
| 3 | Atr-ERM99542 |  | | | |  | | | |  | | | |  |  |  |
| 3 | Atr-ERM99543 |  | | | |  | | | |  | | | |  |  |  |
| 3 | Atr-ERM99544 |  | | | |  | | | |  | | | |  |  |  |
| 3 | Atr-ERM99545 |  | | | |  | | | |  | | | |  |  |  |
| 3 | Atr-ERM99546 |  | | | |  | | | |  | | | |  |  |  |
| 3 | Atr-ERM99547 |  | | | |  | | | |  | | | |  |  |  |
| 3 | Atr-ERM99548 |  | | | |  | | | |  | | | |  |  |  |
| 3 | Atr-ERM99549 |  | | | |  | | | |  | | | |  |  |  |
| 3 | Atr-ERM99550 |  | | | |  | | | |  | Vvi-Vitvi17g00759\_t001 |  |  |  |
| 3 | Atr-ERM99551 |  | | | |  | | | |  | | | |  |  |  |
| 3 | Atr-ERM99552 |  | | | |  | | | |  | | | |  |  |  |
| 3 | Atr-ERM99553 |  | | | |  | | | |  | | | |  |  |  |
| 3 | Atr-ERM99554 |  | | | |  | | | |  | | | |  |  |  |
| 3 | Atr-ERM99555 |  | | | |  | | | |  | | | |  |  |  |
| 3 | Atr-ERM99556 |  | Vvi-Vitvi01g00817\_t001 |  | | | |  | | | |  |  |  |
| 3 | Atr-ERM99557 |  | Vvi-Vitvi01g00818\_t001 |  | | | |  | | | |  |  |  |
| 3 | Atr-ERM99558 |  | | | |  | Vvi-Vitvi14g04639\_t001 |  | | | |  |  |  |
| 3 | Atr-ERM99559 |  | | | |  | | | |  | | | |  |  |  |
| 3 | Atr-ERM99560 |  | | | |  | Vvi-Vitvi14g01812\_t002 |  | | | |  |  |  |
| 2 | Atr-ERM99561 |  | | | |  |  |  | | | |  |  |  |
| 2 | Atr-ERM99562 |  | | | |  |  |  | | | |  |  |  |
| 2 | Atr-ERM99563 |  | | | |  |  |  | | | |  |  |  |
| 2 | Atr-ERM99564 |  | | | |  |  |  | | | |  |  |  |
| 2 | Atr-ERM99565 |  | | | |  |  |  | | | |  |  |  |
| 2 | Atr-ERM99566 |  | | | |  |  |  | | | |  |  |  |
| 2 | Atr-ERM99567 |  | | | |  |  |  | | | |  |  |  |
| 2 | Atr-ERM99568 |  | | | |  |  |  | | | |  |  |  |
| 2 | Atr-ERM99569 |  | | | |  |  |  | | | |  |  |  |
| 2 | Atr-ERM99570 |  | | | |  |  |  | | | |  |  |  |
| 2 | Atr-ERM99571 |  | | | |  |  |  | | | |  |  |  |
| 2 | Atr-ERM99572 |  | | | |  |  |  | Vvi-Vitvi17g00755\_t001 |  |  |  |
| 2 | Atr-ERM99573 |  | Vvi-Vitvi01g00824\_t001 |  |  |  | | | |  |  |  |
| 2 | Atr-ERM99574 |  | Vvi-Vitvi01g00825\_t001 |  |  |  | | | |  |  |  |
| 2 | Atr-ERM99575 |  | | | |  |  |  | | | |  |  |  |
| 2 | Atr-ERM99576 |  | | | |  |  |  | | | |  |  |  |
| 2 | Atr-ERM99577 |  | | | |  |  |  | | | |  |  |  |
| 2 | Atr-ERM99578 |  | | | |  |  |  | | | |  |  |  |
| 2 | Atr-ERM99579 |  | | | |  |  |  | | | |  |  |  |
| 2 | Atr-ERM99580 |  | | | |  |  |  | | | |  |  |  |
| 2 | Atr-ERM99581 |  | | | |  |  |  | | | |  |  |  |
| 2 | Atr-ERM99582 |  | | | |  |  |  | | | |  |  |  |
| 2 | Atr-ERM99583 |  | | | |  |  |  | | | |  |  |  |
| 2 | Atr-ERM99584 |  | Vvi-Vitvi01g04213\_t001 |  |  |  | | | |  |  |  |
| 2 | Atr-ERM99585 |  | | | |  |  |  | | | |  |  |  |
| 2 | Atr-ERM99586 |  | | | |  |  |  | | | |  |  |  |
| 2 | Atr-ERM99587 |  | | | |  |  |  | | | |  |  |  |
| 2 | Atr-ERM99588 |  | | | |  |  |  | Vvi-Vitvi17g01531\_t001 |  |  |  |
| 2 | Atr-ERM99589 |  | | | |  |  |  | | | |  |  |  |
| 2 | Atr-ERM99590 |  | | | |  |  |  | | | |  |  |  |
| 2 | Atr-ERM99591 |  | | | |  |  |  | Vvi-Vitvi17g00754\_t001 |  |  |  |
| 2 | Atr-ERM99592 |  | | | |  |  |  | | | |  |  |  |
| 2 | Atr-ERM99593 |  | | | |  |  |  | | | |  |  |  |
| 2 | Atr-ERM99594 |  | Vvi-Vitvi01g02093\_t001 |  |  |  | | | |  |  |  |
| 2 | Atr-ERM99595 |  | | | |  |  |  | | | |  |  |  |
| 2 | Atr-ERM99596 |  | Vvi-Vitvi01g00845\_t001 |  |  |  | Vvi-Vitvi17g01530\_t001 |  |  |  |
| 1 | Atr-ERM99597 |  |  |  |  |  | Vvi-Vitvi17g00744\_t001 |  |  |  |
| 1 | Atr-ERM99598 |  |  |  |  |  | Vvi-Vitvi17g01523\_t001 |  |  |  |
| 0 | Atr-ERM99599 |  |  |  |  |  |  |
| 0 | Atr-ERM99600 |  |  |  |  |  |  |
| 0 | Atr-ERM99601 |  |  |  |  |  |  |
| 0 | Atr-ERM99602 |  |  |  |  |  |  |
| 0 | Atr-ERM99603 |  |  |  |  |  |  |
| 0 | Atr-ERM99604 |  |  |  |  |  |  |
| 0 | Atr-ERM99605 |  |  |  |  |  |  |
| 0 | Atr-ERM99606 |  |  |  |  |  |  |
| 1 | Atr-ERM99607 |  | Vvi-Vitvi12g02485\_t001 |  |  |  |  |  |
| 1 | Atr-ERM99608 |  | Vvi-Vitvi12g00671\_t001 |  |  |  |  |  |
| 1 | Atr-ERM99609 |  | Vvi-Vitvi12g00670\_t001 |  |  |  |  |  |
| 2 | Atr-ERM99610 |  | | | |  | Vvi-Vitvi19g00625\_t001 |  |  |  |  |
| 2 | Atr-ERM99611 |  | | | |  | | | |  |  |  |  |
| 2 | Atr-ERM99612 |  | | | |  | | | |  |  |  |  |
| 2 | Atr-ERM99613 |  | Vvi-Vitvi12g00667\_t001 |  | Vvi-Vitvi19g00623\_t003 |  |  |  |  |
| 2 | Atr-ERM99614 |  | | | |  | Vvi-Vitvi19g00621\_t001 |  |  |  |  |
| 2 | Atr-ERM99615 |  | | | |  | | | |  |  |  |  |
| 2 | Atr-ERM99616 |  | | | |  | Vvi-Vitvi19g04255\_t001 |  |  |  |  |
| 2 | Atr-ERM99617 |  | Vvi-Vitvi12g00665\_t002 |  | Vvi-Vitvi19g00619\_t001 |  |  |  |  |
| 2 | Atr-ERM99618 |  | | | |  | | | |  |  |  |  |
| 2 | Atr-ERM99619 |  | | | |  | Vvi-Vitvi19g00618\_t001 |  |  |  |  |
| 2 | Atr-ERM99620 |  | Vvi-Vitvi12g00664\_t003 |  | Vvi-Vitvi19g00617\_t001 |  |  |  |  |
| 2 | Atr-ERM99621 |  | | | |  | | | |  |  |  |  |
| 2 | Atr-ERM99622 |  | | | |  | | | |  |  |  |  |
| 2 | Atr-ERM99623 |  | | | |  | | | |  |  |  |  |
| 2 | Atr-ERM99624 |  | | | |  | | | |  |  |  |  |
| 2 | Atr-ERM99625 |  | | | |  | | | |  |  |  |  |
| 2 | Atr-ERM99626 |  | | | |  | | | |  |  |  |  |
| 2 | Atr-ERM99627 |  | | | |  | | | |  |  |  |  |
| 2 | Atr-ERM99628 |  | | | |  | | | |  |  |  |  |
| 2 | Atr-ERM99629 |  | | | |  | | | |  |  |  |  |
| 2 | Atr-ERM99630 |  | | | |  | Vvi-Vitvi19g00615\_t001 |  |  |  |  |
| 2 | Atr-ERM99631 |  | Vvi-Vitvi12g04217\_t001 |  | Vvi-Vitvi19g00614\_t001 |  |  |  |  |
| 2 | Atr-ERM99632 |  | | | |  | | | |  |  |  |  |
| 2 | Atr-ERM99633 |  | | | |  | | | |  |  |  |  |
| 2 | Atr-ERM99634 |  | | | |  | | | |  |  |  |  |
| 2 | Atr-ERM99635 |  | Vvi-Vitvi12g00656\_t001 |  | Vvi-Vitvi19g00612\_t001 |  |  |  |  |
| 0 | Atr-ERM99636 |  |  |  |  |  |  |
| 0 | Atr-ERM99637 |  |  |  |  |  |  |
| 0 | Atr-ERM99638 |  |  |  |  |  |  |
| 0 | Atr-ERM99639 |  |  |  |  |  |  |
| 0 | Atr-ERM99640 |  |  |  |  |  |  |
| 0 | Atr-ERM99641 |  |  |  |  |  |  |
| 0 | Atr-ERM99642 |  |  |  |  |  |  |
| 0 | Atr-ERM99643 |  |  |  |  |  |  |
| 0 | Atr-ERM99644 |  |  |  |  |  |  |
| 0 | Atr-ERM99645 |  |  |  |  |  |  |
