# Supplementary material for: Genome-wide identification, characterization and gene expression of BES1 transcription factor family in grapevine (Vitis vinifera L.)
Source: Sci Rep. 2023 Jan 5;13:240. doi: 10.1038/s41598-022-24407-y (PMC9816167; doi:10.1038/s41598-022-24407-y)
Supplement: Supplementary file 3 — Supplementary Information. [file 41598_2022_24407_MOESM3_ESM.zip › Vvi_Atr/Vitis_vinifera.PN40024.v4.dna_sm.toplevel.fa.vs.Amborella_trichopoda.AMTR1.0.dna_sm.toplevel.fa.html/Atr-AmTr_v1.0_scaffold00089.html]

|  |  |  |  |  |  |  |  |  |  |  |  |  |  |
| --- | --- | --- | --- | --- | --- | --- | --- | --- | --- | --- | --- | --- | --- |
| Duplication depth | Reference chromosome | Collinear blocks | | | | | | | | | | | |
| 0 | Atr-ERN01804 |  |  |  |  |  |  |
| 0 | Atr-ERN01805 |  |  |  |  |  |  |
| 0 | Atr-ERN01806 |  |  |  |  |  |  |
| 0 | Atr-ERN01807 |  |  |  |  |  |  |
| 0 | Atr-ERN01808 |  |  |  |  |  |  |
| 0 | Atr-ERN01809 |  |  |  |  |  |  |
| 0 | Atr-ERN01810 |  |  |  |  |  |  |
| 0 | Atr-ERN01811 |  |  |  |  |  |  |
| 0 | Atr-ERN01812 |  |  |  |  |  |  |
| 0 | Atr-ERN01813 |  |  |  |  |  |  |
| 1 | Atr-ERN01814 |  | Vvi-Vitvi05g01057\_t001 |  |  |  |  |  |
| 1 | Atr-ERN01815 |  | Vvi-Vitvi05g01063\_t001 |  |  |  |  |  |
| 1 | Atr-ERN01816 |  | | | |  |  |  |  |  |
| 1 | Atr-ERN01817 |  | | | |  |  |  |  |  |
| 1 | Atr-ERN01818 |  | | | |  |  |  |  |  |
| 1 | Atr-ERN01819 |  | | | |  |  |  |  |  |
| 1 | Atr-ERN01820 |  | Vvi-Vitvi05g01066\_t002 |  |  |  |  |  |
| 1 | Atr-ERN01821 |  | Vvi-Vitvi05g01068\_t001 |  |  |  |  |  |
| 1 | Atr-ERN01822 |  | | | |  |  |  |  |  |
| 1 | Atr-ERN01823 |  | | | |  |  |  |  |  |
| 1 | Atr-ERN01824 |  | | | |  |  |  |  |  |
| 1 | Atr-ERN01825 |  | Vvi-Vitvi05g01069\_t001 |  |  |  |  |  |
| 1 | Atr-ERN01826 |  | | | |  |  |  |  |  |
| 1 | Atr-ERN01827 |  | Vvi-Vitvi05g02044\_t001 |  |  |  |  |  |
| 0 | Atr-ERN01828 |  |  |  |  |  |  |
| 0 | Atr-ERN01829 |  |  |  |  |  |  |
| 0 | Atr-ERN01830 |  |  |  |  |  |  |
| 0 | Atr-ERN01831 |  |  |  |  |  |  |
| 0 | Atr-ERN01832 |  |  |  |  |  |  |
| 0 | Atr-ERN01833 |  |  |  |  |  |  |
| 0 | Atr-ERN01834 |  |  |  |  |  |  |
| 0 | Atr-ERN01835 |  |  |  |  |  |  |
| 0 | Atr-ERN01836 |  |  |  |  |  |  |
| 0 | Atr-ERN01837 |  |  |  |  |  |  |
| 0 | Atr-ERN01838 |  |  |  |  |  |  |
| 0 | Atr-ERN01839 |  |  |  |  |  |  |
| 0 | Atr-ERN01840 |  |  |  |  |  |  |
| 0 | Atr-ERN01841 |  |  |  |  |  |  |
| 0 | Atr-ERN01842 |  |  |  |  |  |  |
| 0 | Atr-ERN01843 |  |  |  |  |  |  |
| 0 | Atr-ERN01844 |  |  |  |  |  |  |
| 0 | Atr-ERN01845 |  |  |  |  |  |  |
| 0 | Atr-ERN01846 |  |  |  |  |  |  |
| 0 | Atr-ERN01847 |  |  |  |  |  |  |
| 0 | Atr-ERN01848 |  |  |  |  |  |  |
| 0 | Atr-ERN01849 |  |  |  |  |  |  |
| 0 | Atr-ERN01850 |  |  |  |  |  |  |
| 0 | Atr-ERN01851 |  |  |  |  |  |  |
| 0 | Atr-ERN01852 |  |  |  |  |  |  |
| 0 | Atr-ERN01853 |  |  |  |  |  |  |
| 0 | Atr-ERN01854 |  |  |  |  |  |  |
| 0 | Atr-ERN01855 |  |  |  |  |  |  |
| 0 | Atr-ERN01856 |  |  |  |  |  |  |
| 0 | Atr-ERN01857 |  |  |  |  |  |  |
| 0 | Atr-ERN01858 |  |  |  |  |  |  |
| 0 | Atr-ERN01859 |  |  |  |  |  |  |
| 0 | Atr-ERN01860 |  |  |  |  |  |  |
| 0 | Atr-ERN01861 |  |  |  |  |  |  |
| 0 | Atr-ERN01862 |  |  |  |  |  |  |
| 0 | Atr-ERN01863 |  |  |  |  |  |  |
| 0 | Atr-ERN01864 |  |  |  |  |  |  |
| 0 | Atr-ERN01865 |  |  |  |  |  |  |
| 0 | Atr-ERN01866 |  |  |  |  |  |  |
| 0 | Atr-ERN01867 |  |  |  |  |  |  |
| 0 | Atr-ERN01868 |  |  |  |  |  |  |
| 0 | Atr-ERN01869 |  |  |  |  |  |  |
| 0 | Atr-ERN01870 |  |  |  |  |  |  |
| 0 | Atr-ERN01871 |  |  |  |  |  |  |
| 0 | Atr-ERN01872 |  |  |  |  |  |  |
| 0 | Atr-ERN01873 |  |  |  |  |  |  |
| 0 | Atr-ERN01874 |  |  |  |  |  |  |
| 0 | Atr-ERN01875 |  |  |  |  |  |  |
| 0 | Atr-ERN01876 |  |  |  |  |  |  |
| 0 | Atr-ERN01877 |  |  |  |  |  |  |
| 0 | Atr-ERN01878 |  |  |  |  |  |  |
| 0 | Atr-ERN01879 |  |  |  |  |  |  |
| 0 | Atr-ERN01880 |  |  |  |  |  |  |
| 0 | Atr-ERN01881 |  |  |  |  |  |  |
| 0 | Atr-ERN01882 |  |  |  |  |  |  |
| 0 | Atr-ERN01883 |  |  |  |  |  |  |
| 0 | Atr-ERN01884 |  |  |  |  |  |  |
| 0 | Atr-ERN01885 |  |  |  |  |  |  |
| 0 | Atr-ERN01886 |  |  |  |  |  |  |
| 0 | Atr-ERN01887 |  |  |  |  |  |  |
| 0 | Atr-ERN01888 |  |  |  |  |  |  |
| 0 | Atr-ERN01889 |  |  |  |  |  |  |
| 0 | Atr-ERN01890 |  |  |  |  |  |  |
| 0 | Atr-ERN01891 |  |  |  |  |  |  |
| 0 | Atr-ERN01892 |  |  |  |  |  |  |
| 0 | Atr-ERN01893 |  |  |  |  |  |  |
| 0 | Atr-ERN01894 |  |  |  |  |  |  |
| 0 | Atr-ERN01895 |  |  |  |  |  |  |
| 0 | Atr-ERN01896 |  |  |  |  |  |  |
| 0 | Atr-ERN01897 |  |  |  |  |  |  |
| 0 | Atr-ERN01898 |  |  |  |  |  |  |
| 0 | Atr-ERN01899 |  |  |  |  |  |  |
| 0 | Atr-ERN01900 |  |  |  |  |  |  |
| 0 | Atr-ERN01901 |  |  |  |  |  |  |
| 0 | Atr-ERN01902 |  |  |  |  |  |  |
| 0 | Atr-ERN01903 |  |  |  |  |  |  |
| 0 | Atr-ERN01904 |  |  |  |  |  |  |
| 0 | Atr-ERN01905 |  |  |  |  |  |  |
| 0 | Atr-ERN01906 |  |  |  |  |  |  |
| 0 | Atr-ERN01907 |  |  |  |  |  |  |
| 0 | Atr-ERN01908 |  |  |  |  |  |  |
| 0 | Atr-ERN01909 |  |  |  |  |  |  |
| 0 | Atr-ERN01910 |  |  |  |  |  |  |
| 0 | Atr-ERN01911 |  |  |  |  |  |  |
| 0 | Atr-ERN01912 |  |  |  |  |  |  |
| 0 | Atr-ERN01913 |  |  |  |  |  |  |
| 0 | Atr-ERN01914 |  |  |  |  |  |  |
