# Supplementary material for: Genome-wide identification, characterization and gene expression of BES1 transcription factor family in grapevine (Vitis vinifera L.)
Source: Sci Rep. 2023 Jan 5;13:240. doi: 10.1038/s41598-022-24407-y (PMC9816167; doi:10.1038/s41598-022-24407-y)
Supplement: Supplementary file 3 — Supplementary Information. [file 41598_2022_24407_MOESM3_ESM.zip › Vvi_Atr/Vitis_vinifera.PN40024.v4.dna_sm.toplevel.fa.vs.Amborella_trichopoda.AMTR1.0.dna_sm.toplevel.fa.html/Atr-AmTr_v1.0_scaffold00090.html]

|  |  |  |  |  |  |  |  |  |  |  |  |  |  |
| --- | --- | --- | --- | --- | --- | --- | --- | --- | --- | --- | --- | --- | --- |
| Duplication depth | Reference chromosome | Collinear blocks | | | | | | | | | | | |
| 0 | Atr-ERN01601 |  |  |  |  |  |  |
| 0 | Atr-ERN01602 |  |  |  |  |  |  |
| 0 | Atr-ERN01603 |  |  |  |  |  |  |
| 0 | Atr-ERN01604 |  |  |  |  |  |  |
| 0 | Atr-ERN01605 |  |  |  |  |  |  |
| 0 | Atr-ERN01606 |  |  |  |  |  |  |
| 0 | Atr-ERN01607 |  |  |  |  |  |  |
| 0 | Atr-ERN01608 |  |  |  |  |  |  |
| 0 | Atr-ERN01609 |  |  |  |  |  |  |
| 0 | Atr-ERN01610 |  |  |  |  |  |  |
| 0 | Atr-ERN01611 |  |  |  |  |  |  |
| 0 | Atr-ERN01612 |  |  |  |  |  |  |
| 0 | Atr-ERN01613 |  |  |  |  |  |  |
| 0 | Atr-ERN01614 |  |  |  |  |  |  |
| 0 | Atr-ERN01615 |  |  |  |  |  |  |
| 0 | Atr-ERN01616 |  |  |  |  |  |  |
| 0 | Atr-ERN01617 |  |  |  |  |  |  |
| 0 | Atr-ERN01618 |  |  |  |  |  |  |
| 0 | Atr-ERN01619 |  |  |  |  |  |  |
| 0 | Atr-ERN01620 |  |  |  |  |  |  |
| 0 | Atr-ERN01621 |  |  |  |  |  |  |
| 0 | Atr-ERN01622 |  |  |  |  |  |  |
| 0 | Atr-ERN01623 |  |  |  |  |  |  |
| 0 | Atr-ERN01624 |  |  |  |  |  |  |
| 0 | Atr-ERN01625 |  |  |  |  |  |  |
| 0 | Atr-ERN01626 |  |  |  |  |  |  |
| 0 | Atr-ERN01627 |  |  |  |  |  |  |
| 0 | Atr-ERN01628 |  |  |  |  |  |  |
| 0 | Atr-ERN01629 |  |  |  |  |  |  |
| 0 | Atr-ERN01630 |  |  |  |  |  |  |
| 0 | Atr-ERN01631 |  |  |  |  |  |  |
| 0 | Atr-ERN01632 |  |  |  |  |  |  |
| 0 | Atr-ERN01633 |  |  |  |  |  |  |
| 0 | Atr-ERN01634 |  |  |  |  |  |  |
| 0 | Atr-ERN01635 |  |  |  |  |  |  |
| 0 | Atr-ERN01636 |  |  |  |  |  |  |
| 0 | Atr-ERN01637 |  |  |  |  |  |  |
| 0 | Atr-ERN01638 |  |  |  |  |  |  |
| 0 | Atr-ERN01639 |  |  |  |  |  |  |
| 0 | Atr-ERN01640 |  |  |  |  |  |  |
| 0 | Atr-ERN01641 |  |  |  |  |  |  |
| 0 | Atr-ERN01642 |  |  |  |  |  |  |
| 0 | Atr-ERN01643 |  |  |  |  |  |  |
| 0 | Atr-ERN01644 |  |  |  |  |  |  |
| 0 | Atr-ERN01645 |  |  |  |  |  |  |
| 0 | Atr-ERN01646 |  |  |  |  |  |  |
| 0 | Atr-ERN01647 |  |  |  |  |  |  |
| 0 | Atr-ERN01648 |  |  |  |  |  |  |
| 0 | Atr-ERN01649 |  |  |  |  |  |  |
| 0 | Atr-ERN01650 |  |  |  |  |  |  |
| 0 | Atr-ERN01651 |  |  |  |  |  |  |
| 0 | Atr-ERN01652 |  |  |  |  |  |  |
| 0 | Atr-ERN01653 |  |  |  |  |  |  |
| 0 | Atr-ERN01654 |  |  |  |  |  |  |
| 0 | Atr-ERN01655 |  |  |  |  |  |  |
| 0 | Atr-ERN01656 |  |  |  |  |  |  |
| 0 | Atr-ERN01657 |  |  |  |  |  |  |
| 0 | Atr-ERN01658 |  |  |  |  |  |  |
| 0 | Atr-ERN01659 |  |  |  |  |  |  |
| 0 | Atr-ERN01660 |  |  |  |  |  |  |
| 0 | Atr-ERN01661 |  |  |  |  |  |  |
| 0 | Atr-ERN01662 |  |  |  |  |  |  |
| 0 | Atr-ERN01663 |  |  |  |  |  |  |
| 0 | Atr-ERN01664 |  |  |  |  |  |  |
| 0 | Atr-ERN01665 |  |  |  |  |  |  |
| 0 | Atr-ERN01666 |  |  |  |  |  |  |
| 0 | Atr-ERN01667 |  |  |  |  |  |  |
| 0 | Atr-ERN01668 |  |  |  |  |  |  |
| 0 | Atr-ERN01669 |  |  |  |  |  |  |
| 0 | Atr-ERN01670 |  |  |  |  |  |  |
| 0 | Atr-ERN01671 |  |  |  |  |  |  |
| 0 | Atr-ERN01672 |  |  |  |  |  |  |
| 0 | Atr-ERN01673 |  |  |  |  |  |  |
| 0 | Atr-ERN01674 |  |  |  |  |  |  |
| 0 | Atr-ERN01675 |  |  |  |  |  |  |
| 0 | Atr-ERN01676 |  |  |  |  |  |  |
| 0 | Atr-ERN01677 |  |  |  |  |  |  |
| 0 | Atr-ERN01678 |  |  |  |  |  |  |
| 0 | Atr-ERN01679 |  |  |  |  |  |  |
| 0 | Atr-ERN01680 |  |  |  |  |  |  |
| 0 | Atr-ERN01681 |  |  |  |  |  |  |
| 0 | Atr-ERN01682 |  |  |  |  |  |  |
| 0 | Atr-ERN01683 |  |  |  |  |  |  |
| 0 | Atr-ERN01684 |  |  |  |  |  |  |
| 0 | Atr-ERN01685 |  |  |  |  |  |  |
| 0 | Atr-ERN01686 |  |  |  |  |  |  |
| 0 | Atr-ERN01687 |  |  |  |  |  |  |
| 0 | Atr-ERN01688 |  |  |  |  |  |  |
| 0 | Atr-ERN01689 |  |  |  |  |  |  |
| 0 | Atr-ERN01690 |  |  |  |  |  |  |
| 0 | Atr-ERN01691 |  |  |  |  |  |  |
| 0 | Atr-ERN01692 |  |  |  |  |  |  |
| 0 | Atr-ERN01693 |  |  |  |  |  |  |
| 0 | Atr-ERN01694 |  |  |  |  |  |  |
| 0 | Atr-ERN01695 |  |  |  |  |  |  |
| 0 | Atr-ERN01696 |  |  |  |  |  |  |
| 0 | Atr-ERN01697 |  |  |  |  |  |  |
| 0 | Atr-ERN01698 |  |  |  |  |  |  |
| 0 | Atr-ERN01699 |  |  |  |  |  |  |
| 0 | Atr-ERN01700 |  |  |  |  |  |  |
| 0 | Atr-ERN01701 |  |  |  |  |  |  |
| 0 | Atr-ERN01702 |  |  |  |  |  |  |
| 0 | Atr-ERN01703 |  |  |  |  |  |  |
| 0 | Atr-ERN01704 |  |  |  |  |  |  |
| 0 | Atr-ERN01705 |  |  |  |  |  |  |
| 0 | Atr-ERN01706 |  |  |  |  |  |  |
| 0 | Atr-ERN01707 |  |  |  |  |  |  |
| 0 | Atr-ERN01708 |  |  |  |  |  |  |
| 0 | Atr-ERN01709 |  |  |  |  |  |  |
| 0 | Atr-ERN01710 |  |  |  |  |  |  |
| 0 | Atr-ERN01711 |  |  |  |  |  |  |
| 0 | Atr-ERN01712 |  |  |  |  |  |  |
| 0 | Atr-ERN01713 |  |  |  |  |  |  |
