# Supplementary material for: Genome-wide identification, characterization and gene expression of BES1 transcription factor family in grapevine (Vitis vinifera L.)
Source: Sci Rep. 2023 Jan 5;13:240. doi: 10.1038/s41598-022-24407-y (PMC9816167; doi:10.1038/s41598-022-24407-y)
Supplement: Supplementary file 3 — Supplementary Information. [file 41598_2022_24407_MOESM3_ESM.zip › Vvi_Atr/Vitis_vinifera.PN40024.v4.dna_sm.toplevel.fa.vs.Amborella_trichopoda.AMTR1.0.dna_sm.toplevel.fa.html/Atr-AmTr_v1.0_scaffold00091.html]

|  |  |  |  |  |  |  |  |  |  |  |  |  |  |
| --- | --- | --- | --- | --- | --- | --- | --- | --- | --- | --- | --- | --- | --- |
| Duplication depth | Reference chromosome | Collinear blocks | | | | | | | | | | | |
| 0 | Atr-ERN00576 |  |  |  |  |  |  |
| 0 | Atr-ERN00577 |  |  |  |  |  |  |
| 0 | Atr-ERN00578 |  |  |  |  |  |  |
| 0 | Atr-ERN00579 |  |  |  |  |  |  |
| 0 | Atr-ERN00580 |  |  |  |  |  |  |
| 0 | Atr-ERN00581 |  |  |  |  |  |  |
| 0 | Atr-ERN00582 |  |  |  |  |  |  |
| 0 | Atr-ERN00583 |  |  |  |  |  |  |
| 0 | Atr-ERN00584 |  |  |  |  |  |  |
| 0 | Atr-ERN00585 |  |  |  |  |  |  |
| 0 | Atr-ERN00586 |  |  |  |  |  |  |
| 0 | Atr-ERN00587 |  |  |  |  |  |  |
| 0 | Atr-ERN00588 |  |  |  |  |  |  |
| 0 | Atr-ERN00589 |  |  |  |  |  |  |
| 0 | Atr-ERN00590 |  |  |  |  |  |  |
| 0 | Atr-ERN00591 |  |  |  |  |  |  |
| 0 | Atr-ERN00592 |  |  |  |  |  |  |
| 0 | Atr-ERN00593 |  |  |  |  |  |  |
| 0 | Atr-ERN00594 |  |  |  |  |  |  |
| 0 | Atr-ERN00595 |  |  |  |  |  |  |
| 0 | Atr-ERN00596 |  |  |  |  |  |  |
| 0 | Atr-ERN00597 |  |  |  |  |  |  |
| 0 | Atr-ERN00598 |  |  |  |  |  |  |
| 0 | Atr-ERN00599 |  |  |  |  |  |  |
| 0 | Atr-ERN00600 |  |  |  |  |  |  |
| 0 | Atr-ERN00601 |  |  |  |  |  |  |
| 0 | Atr-ERN00602 |  |  |  |  |  |  |
| 0 | Atr-ERN00603 |  |  |  |  |  |  |
| 0 | Atr-ERN00604 |  |  |  |  |  |  |
| 0 | Atr-ERN00605 |  |  |  |  |  |  |
| 0 | Atr-ERN00606 |  |  |  |  |  |  |
| 0 | Atr-ERN00607 |  |  |  |  |  |  |
| 0 | Atr-ERN00608 |  |  |  |  |  |  |
| 0 | Atr-ERN00609 |  |  |  |  |  |  |
| 0 | Atr-ERN00610 |  |  |  |  |  |  |
| 0 | Atr-ERN00611 |  |  |  |  |  |  |
| 0 | Atr-ERN00612 |  |  |  |  |  |  |
| 0 | Atr-ERN00613 |  |  |  |  |  |  |
| 0 | Atr-ERN00614 |  |  |  |  |  |  |
| 0 | Atr-ERN00615 |  |  |  |  |  |  |
| 0 | Atr-ERN00616 |  |  |  |  |  |  |
| 0 | Atr-ERN00617 |  |  |  |  |  |  |
| 0 | Atr-ERN00618 |  |  |  |  |  |  |
| 0 | Atr-ERN00619 |  |  |  |  |  |  |
| 0 | Atr-ERN00620 |  |  |  |  |  |  |
| 0 | Atr-ERN00621 |  |  |  |  |  |  |
| 0 | Atr-ERN00622 |  |  |  |  |  |  |
| 0 | Atr-ERN00623 |  |  |  |  |  |  |
| 0 | Atr-ERN00624 |  |  |  |  |  |  |
| 0 | Atr-ERN00625 |  |  |  |  |  |  |
| 0 | Atr-ERN00626 |  |  |  |  |  |  |
| 0 | Atr-ERN00627 |  |  |  |  |  |  |
| 0 | Atr-ERN00628 |  |  |  |  |  |  |
| 0 | Atr-ERN00629 |  |  |  |  |  |  |
| 0 | Atr-ERN00630 |  |  |  |  |  |  |
| 0 | Atr-ERN00631 |  |  |  |  |  |  |
| 0 | Atr-ERN00632 |  |  |  |  |  |  |
| 0 | Atr-ERN00633 |  |  |  |  |  |  |
| 0 | Atr-ERN00634 |  |  |  |  |  |  |
| 0 | Atr-ERN00635 |  |  |  |  |  |  |
| 0 | Atr-ERN00636 |  |  |  |  |  |  |
| 0 | Atr-ERN00637 |  |  |  |  |  |  |
| 0 | Atr-ERN00638 |  |  |  |  |  |  |
| 0 | Atr-ERN00639 |  |  |  |  |  |  |
| 0 | Atr-ERN00640 |  |  |  |  |  |  |
| 0 | Atr-ERN00641 |  |  |  |  |  |  |
