# Supplementary material for: Genome-wide identification, characterization and gene expression of BES1 transcription factor family in grapevine (Vitis vinifera L.)
Source: Sci Rep. 2023 Jan 5;13:240. doi: 10.1038/s41598-022-24407-y (PMC9816167; doi:10.1038/s41598-022-24407-y)
Supplement: Supplementary file 3 — Supplementary Information. [file 41598_2022_24407_MOESM3_ESM.zip › Vvi_Atr/Vitis_vinifera.PN40024.v4.dna_sm.toplevel.fa.vs.Amborella_trichopoda.AMTR1.0.dna_sm.toplevel.fa.html/Atr-AmTr_v1.0_scaffold00092.html]

|  |  |  |  |  |  |  |  |  |  |  |  |  |  |
| --- | --- | --- | --- | --- | --- | --- | --- | --- | --- | --- | --- | --- | --- |
| Duplication depth | Reference chromosome | Collinear blocks | | | | | | | | | | | |
| 0 | Atr-ERM99145 |  |  |  |  |  |  |
| 0 | Atr-ERM99146 |  |  |  |  |  |  |
| 0 | Atr-ERM99147 |  |  |  |  |  |  |
| 0 | Atr-ERM99148 |  |  |  |  |  |  |
| 0 | Atr-ERM99149 |  |  |  |  |  |  |
| 0 | Atr-ERM99150 |  |  |  |  |  |  |
| 0 | Atr-ERM99151 |  |  |  |  |  |  |
| 0 | Atr-ERM99152 |  |  |  |  |  |  |
| 0 | Atr-ERM99153 |  |  |  |  |  |  |
| 0 | Atr-ERM99154 |  |  |  |  |  |  |
| 2 | Atr-ERM99155 |  | Vvi-Vitvi10g00878\_t001 |  | Vvi-Vitvi10g00878\_t001 |  |  |  |  |
| 2 | Atr-ERM99156 |  | | | |  | Vvi-Vitvi10g00879\_t001 |  |  |  |  |
| 2 | Atr-ERM99157 |  | | | |  | | | |  |  |  |  |
| 2 | Atr-ERM99158 |  | | | |  | Vvi-Vitvi10g00881\_t001 |  |  |  |  |
| 2 | Atr-ERM99159 |  | | | |  | Vvi-Vitvi10g00886\_t001 |  |  |  |  |
| 2 | Atr-ERM99160 |  | | | |  | Vvi-Vitvi10g00889\_t001 |  |  |  |  |
| 2 | Atr-ERM99161 |  | | | |  | Vvi-Vitvi10g00892\_t001 |  |  |  |  |
| 2 | Atr-ERM99162 |  | | | |  | Vvi-Vitvi10g00899\_t001 |  |  |  |  |
| 2 | Atr-ERM99163 |  | | | |  | | | |  |  |  |  |
| 2 | Atr-ERM99164 |  | | | |  | Vvi-Vitvi10g00902\_t003 |  |  |  |  |
| 1 | Atr-ERM99165 |  | | | |  |  |  |  |  |
| 1 | Atr-ERM99166 |  | | | |  |  |  |  |  |
| 1 | Atr-ERM99167 |  | | | |  |  |  |  |  |
| 1 | Atr-ERM99168 |  | Vvi-Vitvi10g00870\_t001 |  |  |  |  |  |
| 1 | Atr-ERM99169 |  | | | |  |  |  |  |  |
| 1 | Atr-ERM99170 |  | | | |  |  |  |  |  |
| 1 | Atr-ERM99171 |  | | | |  |  |  |  |  |
| 1 | Atr-ERM99172 |  | | | |  |  |  |  |  |
| 1 | Atr-ERM99173 |  | | | |  |  |  |  |  |
| 1 | Atr-ERM99174 |  | | | |  |  |  |  |  |
| 1 | Atr-ERM99175 |  | | | |  |  |  |  |  |
| 1 | Atr-ERM99176 |  | | | |  |  |  |  |  |
| 1 | Atr-ERM99177 |  | | | |  |  |  |  |  |
| 1 | Atr-ERM99178 |  | Vvi-Vitvi10g00858\_t001 |  |  |  |  |  |
| 1 | Atr-ERM99179 |  | Vvi-Vitvi10g00855\_t001 |  |  |  |  |  |
| 1 | Atr-ERM99180 |  | Vvi-Vitvi10g00854\_t001 |  |  |  |  |  |
| 1 | Atr-ERM99181 |  | Vvi-Vitvi10g00853\_t001 |  |  |  |  |  |
| 1 | Atr-ERM99182 |  | | | |  |  |  |  |  |
| 1 | Atr-ERM99183 |  | | | |  |  |  |  |  |
| 2 | Atr-ERM99184 |  | | | |  | Vvi-Vitvi10g00843\_t001 |  |  |  |  |
| 2 | Atr-ERM99185 |  | | | |  | Vvi-Vitvi10g00844\_t001 |  |  |  |  |
| 2 | Atr-ERM99186 |  | | | |  | | | |  |  |  |  |
| 2 | Atr-ERM99187 |  | | | |  | | | |  |  |  |  |
| 2 | Atr-ERM99188 |  | | | |  | | | |  |  |  |  |
| 2 | Atr-ERM99189 |  | | | |  | | | |  |  |  |  |
| 2 | Atr-ERM99190 |  | | | |  | | | |  |  |  |  |
| 2 | Atr-ERM99191 |  | | | |  | | | |  |  |  |  |
| 2 | Atr-ERM99192 |  | | | |  | | | |  |  |  |  |
| 2 | Atr-ERM99193 |  | | | |  | | | |  |  |  |  |
| 2 | Atr-ERM99194 |  | | | |  | | | |  |  |  |  |
| 2 | Atr-ERM99195 |  | | | |  | | | |  |  |  |  |
| 2 | Atr-ERM99196 |  | | | |  | | | |  |  |  |  |
| 2 | Atr-ERM99197 |  | | | |  | Vvi-Vitvi10g01887\_t001 |  |  |  |  |
| 2 | Atr-ERM99198 |  | | | |  | | | |  |  |  |  |
| 2 | Atr-ERM99199 |  | Vvi-Vitvi10g00848\_t001 |  | Vvi-Vitvi10g00848\_t001 |  |  |  |  |
| 2 | Atr-ERM99200 |  | | | |  | Vvi-Vitvi10g00849\_t001 |  |  |  |  |
| 2 | Atr-ERM99201 |  | | | |  | | | |  |  |  |  |
| 2 | Atr-ERM99202 |  | | | |  | | | |  |  |  |  |
| 2 | Atr-ERM99203 |  | | | |  | Vvi-Vitvi10g01888\_t001 |  |  |  |  |
| 2 | Atr-ERM99204 |  | | | |  | Vvi-Vitvi10g00850\_t001 |  |  |  |  |
| 2 | Atr-ERM99205 |  | | | |  | Vvi-Vitvi10g00851\_t002 |  |  |  |  |
| 2 | Atr-ERM99206 |  | | | |  | Vvi-Vitvi10g00852\_t001 |  |  |  |  |
| 2 | Atr-ERM99207 |  | Vvi-Vitvi10g01885\_t001 |  | | | |  |  |  |  |
| 2 | Atr-ERM99208 |  | | | |  | | | |  |  |  |  |
| 2 | Atr-ERM99209 |  | | | |  | | | |  |  |  |  |
| 2 | Atr-ERM99210 |  | Vvi-Vitvi10g04445\_t001 |  | | | |  |  |  |  |
| 2 | Atr-ERM99211 |  | Vvi-Vitvi10g01884\_t001 |  | | | |  |  |  |  |
| 2 | Atr-ERM99212 |  | | | |  | | | |  |  |  |  |
| 2 | Atr-ERM99213 |  | Vvi-Vitvi10g00839\_t001 |  | | | |  |  |  |  |
| 2 | Atr-ERM99214 |  | Vvi-Vitvi10g01883\_t001 |  | | | |  |  |  |  |
| 2 | Atr-ERM99215 |  | | | |  | | | |  |  |  |  |
| 2 | Atr-ERM99216 |  | Vvi-Vitvi10g00838\_t001 |  | | | |  |  |  |  |
| 2 | Atr-ERM99217 |  | | | |  | | | |  |  |  |  |
| 2 | Atr-ERM99218 |  | Vvi-Vitvi10g00837\_t001 |  | | | |  |  |  |  |
| 2 | Atr-ERM99219 |  | Vvi-Vitvi10g00835\_t001 |  | | | |  |  |  |  |
| 2 | Atr-ERM99220 |  | | | |  | | | |  |  |  |  |
| 2 | Atr-ERM99221 |  | Vvi-Vitvi10g00834\_t001 |  | | | |  |  |  |  |
| 2 | Atr-ERM99222 |  | | | |  | | | |  |  |  |  |
| 2 | Atr-ERM99223 |  | | | |  | | | |  |  |  |  |
| 2 | Atr-ERM99224 |  | | | |  | | | |  |  |  |  |
| 2 | Atr-ERM99225 |  | | | |  | Vvi-Vitvi10g04454\_t001 |  |  |  |  |
| 1 | Atr-ERM99226 |  | Vvi-Vitvi10g00832\_t001 |  |  |  |  |  |
| 0 | Atr-ERM99227 |  |  |  |  |  |  |
| 0 | Atr-ERM99228 |  |  |  |  |  |  |
| 0 | Atr-ERM99229 |  |  |  |  |  |  |
| 0 | Atr-ERM99230 |  |  |  |  |  |  |
| 0 | Atr-ERM99231 |  |  |  |  |  |  |
| 0 | Atr-ERM99232 |  |  |  |  |  |  |
| 0 | Atr-ERM99233 |  |  |  |  |  |  |
| 0 | Atr-ERM99234 |  |  |  |  |  |  |
| 0 | Atr-ERM99235 |  |  |  |  |  |  |
| 1 | Atr-ERM99236 |  | Vvi-Vitvi10g01156\_t001 |  |  |  |  |  |
| 1 | Atr-ERM99237 |  | | | |  |  |  |  |  |
| 1 | Atr-ERM99238 |  | | | |  |  |  |  |  |
| 1 | Atr-ERM99239 |  | Vvi-Vitvi10g04533\_t001 |  |  |  |  |  |
| 1 | Atr-ERM99240 |  | | | |  |  |  |  |  |
| 1 | Atr-ERM99241 |  | | | |  |  |  |  |  |
| 1 | Atr-ERM99242 |  | | | |  |  |  |  |  |
| 1 | Atr-ERM99243 |  | | | |  |  |  |  |  |
| 1 | Atr-ERM99244 |  | Vvi-Vitvi10g01123\_t002 |  |  |  |  |  |
| 1 | Atr-ERM99245 |  | | | |  |  |  |  |  |
| 1 | Atr-ERM99246 |  | | | |  |  |  |  |  |
| 1 | Atr-ERM99247 |  | | | |  |  |  |  |  |
| 1 | Atr-ERM99248 |  | | | |  |  |  |  |  |
| 1 | Atr-ERM99249 |  | | | |  |  |  |  |  |
| 1 | Atr-ERM99250 |  | | | |  |  |  |  |  |
| 1 | Atr-ERM99251 |  | Vvi-Vitvi10g04530\_t001 |  |  |  |  |  |
| 1 | Atr-ERM99252 |  | | | |  |  |  |  |  |
| 1 | Atr-ERM99253 |  | | | |  |  |  |  |  |
| 1 | Atr-ERM99254 |  | Vvi-Vitvi10g01121\_t001 |  |  |  |  |  |
| 1 | Atr-ERM99255 |  | | | |  |  |  |  |  |
| 1 | Atr-ERM99256 |  | | | |  |  |  |  |  |
| 1 | Atr-ERM99257 |  | Vvi-Vitvi10g01120\_t001 |  |  |  |  |  |
| 1 | Atr-ERM99258 |  | | | |  |  |  |  |  |
| 1 | Atr-ERM99259 |  | | | |  |  |  |  |  |
| 1 | Atr-ERM99260 |  | | | |  |  |  |  |  |
| 1 | Atr-ERM99261 |  | | | |  |  |  |  |  |
| 1 | Atr-ERM99262 |  | | | |  |  |  |  |  |
| 1 | Atr-ERM99263 |  | | | |  |  |  |  |  |
| 1 | Atr-ERM99264 |  | | | |  |  |  |  |  |
| 1 | Atr-ERM99265 |  | | | |  |  |  |  |  |
| 1 | Atr-ERM99266 |  | | | |  |  |  |  |  |
| 1 | Atr-ERM99267 |  | | | |  |  |  |  |  |
| 1 | Atr-ERM99268 |  | | | |  |  |  |  |  |
| 1 | Atr-ERM99269 |  | | | |  |  |  |  |  |
| 1 | Atr-ERM99270 |  | | | |  |  |  |  |  |
| 1 | Atr-ERM99271 |  | | | |  |  |  |  |  |
| 1 | Atr-ERM99272 |  | Vvi-Vitvi10g01110\_t001 |  |  |  |  |  |
| 1 | Atr-ERM99273 |  | | | |  |  |  |  |  |
| 1 | Atr-ERM99274 |  | | | |  |  |  |  |  |
| 1 | Atr-ERM99275 |  | | | |  |  |  |  |  |
| 1 | Atr-ERM99276 |  | | | |  |  |  |  |  |
| 1 | Atr-ERM99277 |  | | | |  |  |  |  |  |
| 1 | Atr-ERM99278 |  | | | |  |  |  |  |  |
| 1 | Atr-ERM99279 |  | Vvi-Vitvi10g01098\_t001 |  |  |  |  |  |
| 0 | Atr-ERM99280 |  |  |  |  |  |  |
| 0 | Atr-ERM99281 |  |  |  |  |  |  |
| 0 | Atr-ERM99282 |  |  |  |  |  |  |
| 0 | Atr-ERM99283 |  |  |  |  |  |  |
| 0 | Atr-ERM99284 |  |  |  |  |  |  |
| 0 | Atr-ERM99285 |  |  |  |  |  |  |
| 0 | Atr-ERM99286 |  |  |  |  |  |  |
| 0 | Atr-ERM99287 |  |  |  |  |  |  |
| 0 | Atr-ERM99288 |  |  |  |  |  |  |
| 0 | Atr-ERM99289 |  |  |  |  |  |  |
| 0 | Atr-ERM99290 |  |  |  |  |  |  |
| 0 | Atr-ERM99291 |  |  |  |  |  |  |
| 0 | Atr-ERM99292 |  |  |  |  |  |  |
| 0 | Atr-ERM99293 |  |  |  |  |  |  |
| 0 | Atr-ERM99294 |  |  |  |  |  |  |
| 0 | Atr-ERM99295 |  |  |  |  |  |  |
| 0 | Atr-ERM99296 |  |  |  |  |  |  |
| 0 | Atr-ERM99297 |  |  |  |  |  |  |
| 0 | Atr-ERM99298 |  |  |  |  |  |  |
| 0 | Atr-ERM99299 |  |  |  |  |  |  |
| 0 | Atr-ERM99300 |  |  |  |  |  |  |
| 0 | Atr-ERM99301 |  |  |  |  |  |  |
| 0 | Atr-ERM99302 |  |  |  |  |  |  |
| 0 | Atr-ERM99303 |  |  |  |  |  |  |
| 0 | Atr-ERM99304 |  |  |  |  |  |  |
| 0 | Atr-ERM99305 |  |  |  |  |  |  |
