# Supplementary material for: Genome-wide identification, characterization and gene expression of BES1 transcription factor family in grapevine (Vitis vinifera L.)
Source: Sci Rep. 2023 Jan 5;13:240. doi: 10.1038/s41598-022-24407-y (PMC9816167; doi:10.1038/s41598-022-24407-y)
Supplement: Supplementary file 3 — Supplementary Information. [file 41598_2022_24407_MOESM3_ESM.zip › Vvi_Atr/Vitis_vinifera.PN40024.v4.dna_sm.toplevel.fa.vs.Amborella_trichopoda.AMTR1.0.dna_sm.toplevel.fa.html/Atr-AmTr_v1.0_scaffold00093.html]

|  |  |  |  |  |  |  |  |  |  |  |  |  |  |
| --- | --- | --- | --- | --- | --- | --- | --- | --- | --- | --- | --- | --- | --- |
| Duplication depth | Reference chromosome | Collinear blocks | | | | | | | | | | | |
| 0 | Atr-ERM98778 |  |  |  |  |  |  |
| 0 | Atr-ERM98779 |  |  |  |  |  |  |
| 0 | Atr-ERM98780 |  |  |  |  |  |  |
| 0 | Atr-ERM98781 |  |  |  |  |  |  |
| 0 | Atr-ERM98782 |  |  |  |  |  |  |
| 0 | Atr-ERM98783 |  |  |  |  |  |  |
| 0 | Atr-ERM98784 |  |  |  |  |  |  |
| 0 | Atr-ERM98785 |  |  |  |  |  |  |
| 0 | Atr-ERM98786 |  |  |  |  |  |  |
| 0 | Atr-ERM98787 |  |  |  |  |  |  |
| 0 | Atr-ERM98788 |  |  |  |  |  |  |
| 0 | Atr-ERM98789 |  |  |  |  |  |  |
| 0 | Atr-ERM98790 |  |  |  |  |  |  |
| 0 | Atr-ERM98791 |  |  |  |  |  |  |
| 0 | Atr-ERM98792 |  |  |  |  |  |  |
| 0 | Atr-ERM98793 |  |  |  |  |  |  |
| 0 | Atr-ERM98794 |  |  |  |  |  |  |
| 0 | Atr-ERM98795 |  |  |  |  |  |  |
| 0 | Atr-ERM98796 |  |  |  |  |  |  |
| 0 | Atr-ERM98797 |  |  |  |  |  |  |
| 0 | Atr-ERM98798 |  |  |  |  |  |  |
| 0 | Atr-ERM98799 |  |  |  |  |  |  |
| 0 | Atr-ERM98800 |  |  |  |  |  |  |
| 0 | Atr-ERM98801 |  |  |  |  |  |  |
| 0 | Atr-ERM98802 |  |  |  |  |  |  |
| 0 | Atr-ERM98803 |  |  |  |  |  |  |
| 0 | Atr-ERM98804 |  |  |  |  |  |  |
| 0 | Atr-ERM98805 |  |  |  |  |  |  |
| 0 | Atr-ERM98806 |  |  |  |  |  |  |
| 0 | Atr-ERM98807 |  |  |  |  |  |  |
| 0 | Atr-ERM98808 |  |  |  |  |  |  |
| 0 | Atr-ERM98809 |  |  |  |  |  |  |
| 0 | Atr-ERM98810 |  |  |  |  |  |  |
| 0 | Atr-ERM98811 |  |  |  |  |  |  |
| 0 | Atr-ERM98812 |  |  |  |  |  |  |
| 0 | Atr-ERM98813 |  |  |  |  |  |  |
| 0 | Atr-ERM98814 |  |  |  |  |  |  |
| 0 | Atr-ERM98815 |  |  |  |  |  |  |
| 0 | Atr-ERM98816 |  |  |  |  |  |  |
| 0 | Atr-ERM98817 |  |  |  |  |  |  |
| 0 | Atr-ERM98818 |  |  |  |  |  |  |
| 0 | Atr-ERM98819 |  |  |  |  |  |  |
| 0 | Atr-ERM98820 |  |  |  |  |  |  |
| 0 | Atr-ERM98821 |  |  |  |  |  |  |
| 0 | Atr-ERM98822 |  |  |  |  |  |  |
| 0 | Atr-ERM98823 |  |  |  |  |  |  |
| 0 | Atr-ERM98824 |  |  |  |  |  |  |
| 0 | Atr-ERM98825 |  |  |  |  |  |  |
| 0 | Atr-ERM98826 |  |  |  |  |  |  |
| 0 | Atr-ERM98827 |  |  |  |  |  |  |
| 0 | Atr-ERM98828 |  |  |  |  |  |  |
| 0 | Atr-ERM98829 |  |  |  |  |  |  |
| 0 | Atr-ERM98830 |  |  |  |  |  |  |
| 0 | Atr-ERM98831 |  |  |  |  |  |  |
| 0 | Atr-ERM98832 |  |  |  |  |  |  |
| 0 | Atr-ERM98833 |  |  |  |  |  |  |
| 0 | Atr-ERM98834 |  |  |  |  |  |  |
| 0 | Atr-ERM98835 |  |  |  |  |  |  |
| 0 | Atr-ERM98836 |  |  |  |  |  |  |
