# Supplementary material for: Genome-wide identification, characterization and gene expression of BES1 transcription factor family in grapevine (Vitis vinifera L.)
Source: Sci Rep. 2023 Jan 5;13:240. doi: 10.1038/s41598-022-24407-y (PMC9816167; doi:10.1038/s41598-022-24407-y)
Supplement: Supplementary file 3 — Supplementary Information. [file 41598_2022_24407_MOESM3_ESM.zip › Vvi_Atr/Vitis_vinifera.PN40024.v4.dna_sm.toplevel.fa.vs.Amborella_trichopoda.AMTR1.0.dna_sm.toplevel.fa.html/Atr-AmTr_v1.0_scaffold00094.html]

|  |  |  |  |  |  |  |  |  |  |  |  |  |  |
| --- | --- | --- | --- | --- | --- | --- | --- | --- | --- | --- | --- | --- | --- |
| Duplication depth | Reference chromosome | Collinear blocks | | | | | | | | | | | |
| 0 | Atr-ERM98297 |  |  |  |  |  |  |
| 0 | Atr-ERM98298 |  |  |  |  |  |  |
| 0 | Atr-ERM98299 |  |  |  |  |  |  |
| 0 | Atr-ERM98300 |  |  |  |  |  |  |
| 0 | Atr-ERM98301 |  |  |  |  |  |  |
| 0 | Atr-ERM98302 |  |  |  |  |  |  |
| 0 | Atr-ERM98303 |  |  |  |  |  |  |
| 0 | Atr-ERM98304 |  |  |  |  |  |  |
| 0 | Atr-ERM98305 |  |  |  |  |  |  |
| 0 | Atr-ERM98306 |  |  |  |  |  |  |
| 0 | Atr-ERM98307 |  |  |  |  |  |  |
| 0 | Atr-ERM98308 |  |  |  |  |  |  |
| 0 | Atr-ERM98309 |  |  |  |  |  |  |
| 0 | Atr-ERM98310 |  |  |  |  |  |  |
| 0 | Atr-ERM98311 |  |  |  |  |  |  |
| 0 | Atr-ERM98312 |  |  |  |  |  |  |
| 0 | Atr-ERM98313 |  |  |  |  |  |  |
| 0 | Atr-ERM98314 |  |  |  |  |  |  |
| 0 | Atr-ERM98315 |  |  |  |  |  |  |
| 0 | Atr-ERM98316 |  |  |  |  |  |  |
| 0 | Atr-ERM98317 |  |  |  |  |  |  |
| 0 | Atr-ERM98318 |  |  |  |  |  |  |
| 0 | Atr-ERM98319 |  |  |  |  |  |  |
| 0 | Atr-ERM98320 |  |  |  |  |  |  |
