# Supplementary material for: Genome-wide identification, characterization and gene expression of BES1 transcription factor family in grapevine (Vitis vinifera L.)
Source: Sci Rep. 2023 Jan 5;13:240. doi: 10.1038/s41598-022-24407-y (PMC9816167; doi:10.1038/s41598-022-24407-y)
Supplement: Supplementary file 3 — Supplementary Information. [file 41598_2022_24407_MOESM3_ESM.zip › Vvi_Atr/Vitis_vinifera.PN40024.v4.dna_sm.toplevel.fa.vs.Amborella_trichopoda.AMTR1.0.dna_sm.toplevel.fa.html/Atr-AmTr_v1.0_scaffold00095.html]

|  |  |  |  |  |  |  |  |  |  |  |  |  |  |
| --- | --- | --- | --- | --- | --- | --- | --- | --- | --- | --- | --- | --- | --- |
| Duplication depth | Reference chromosome | Collinear blocks | | | | | | | | | | | |
| 0 | Atr-ERM98101 |  |  |  |  |  |  |
| 0 | Atr-ERM98102 |  |  |  |  |  |  |
| 0 | Atr-ERM98103 |  |  |  |  |  |  |
| 0 | Atr-ERM98104 |  |  |  |  |  |  |
| 0 | Atr-ERM98105 |  |  |  |  |  |  |
| 0 | Atr-ERM98106 |  |  |  |  |  |  |
| 0 | Atr-ERM98107 |  |  |  |  |  |  |
| 0 | Atr-ERM98108 |  |  |  |  |  |  |
| 0 | Atr-ERM98109 |  |  |  |  |  |  |
| 0 | Atr-ERM98110 |  |  |  |  |  |  |
| 0 | Atr-ERM98111 |  |  |  |  |  |  |
| 0 | Atr-ERM98112 |  |  |  |  |  |  |
| 0 | Atr-ERM98113 |  |  |  |  |  |  |
| 0 | Atr-ERM98114 |  |  |  |  |  |  |
| 0 | Atr-ERM98115 |  |  |  |  |  |  |
| 0 | Atr-ERM98116 |  |  |  |  |  |  |
| 0 | Atr-ERM98117 |  |  |  |  |  |  |
| 0 | Atr-ERM98118 |  |  |  |  |  |  |
| 0 | Atr-ERM98119 |  |  |  |  |  |  |
| 0 | Atr-ERM98120 |  |  |  |  |  |  |
| 0 | Atr-ERM98121 |  |  |  |  |  |  |
| 0 | Atr-ERM98122 |  |  |  |  |  |  |
| 0 | Atr-ERM98123 |  |  |  |  |  |  |
| 0 | Atr-ERM98124 |  |  |  |  |  |  |
| 0 | Atr-ERM98125 |  |  |  |  |  |  |
| 0 | Atr-ERM98126 |  |  |  |  |  |  |
| 0 | Atr-ERM98127 |  |  |  |  |  |  |
| 0 | Atr-ERM98128 |  |  |  |  |  |  |
| 0 | Atr-ERM98129 |  |  |  |  |  |  |
| 0 | Atr-ERM98130 |  |  |  |  |  |  |
| 0 | Atr-ERM98131 |  |  |  |  |  |  |
| 0 | Atr-ERM98132 |  |  |  |  |  |  |
| 0 | Atr-ERM98133 |  |  |  |  |  |  |
| 0 | Atr-ERM98134 |  |  |  |  |  |  |
| 0 | Atr-ERM98135 |  |  |  |  |  |  |
| 0 | Atr-ERM98136 |  |  |  |  |  |  |
| 0 | Atr-ERM98137 |  |  |  |  |  |  |
| 0 | Atr-ERM98138 |  |  |  |  |  |  |
| 0 | Atr-ERM98139 |  |  |  |  |  |  |
| 0 | Atr-ERM98140 |  |  |  |  |  |  |
| 0 | Atr-ERM98141 |  |  |  |  |  |  |
| 1 | Atr-ERM98142 |  | Vvi-Vitvi08g00914\_t001 |  |  |  |  |  |
| 1 | Atr-ERM98143 |  | | | |  |  |  |  |  |
| 1 | Atr-ERM98144 |  | | | |  |  |  |  |  |
| 1 | Atr-ERM98145 |  | | | |  |  |  |  |  |
| 1 | Atr-ERM98146 |  | | | |  |  |  |  |  |
| 1 | Atr-ERM98147 |  | | | |  |  |  |  |  |
| 1 | Atr-ERM98148 |  | | | |  |  |  |  |  |
| 1 | Atr-ERM98149 |  | | | |  |  |  |  |  |
| 1 | Atr-ERM98150 |  | | | |  |  |  |  |  |
| 1 | Atr-ERM98151 |  | | | |  |  |  |  |  |
| 1 | Atr-ERM98152 |  | | | |  |  |  |  |  |
| 1 | Atr-ERM98153 |  | | | |  |  |  |  |  |
| 1 | Atr-ERM98154 |  | | | |  |  |  |  |  |
| 1 | Atr-ERM98155 |  | | | |  |  |  |  |  |
| 1 | Atr-ERM98156 |  | | | |  |  |  |  |  |
| 1 | Atr-ERM98157 |  | | | |  |  |  |  |  |
| 1 | Atr-ERM98158 |  | | | |  |  |  |  |  |
| 1 | Atr-ERM98159 |  | | | |  |  |  |  |  |
| 1 | Atr-ERM98160 |  | | | |  |  |  |  |  |
| 1 | Atr-ERM98161 |  | | | |  |  |  |  |  |
| 1 | Atr-ERM98162 |  | | | |  |  |  |  |  |
| 1 | Atr-ERM98163 |  | | | |  |  |  |  |  |
| 1 | Atr-ERM98164 |  | | | |  |  |  |  |  |
| 1 | Atr-ERM98165 |  | | | |  |  |  |  |  |
| 1 | Atr-ERM98166 |  | Vvi-Vitvi08g00913\_t001 |  |  |  |  |  |
| 1 | Atr-ERM98167 |  | Vvi-Vitvi08g00912\_t001 |  |  |  |  |  |
| 1 | Atr-ERM98168 |  | Vvi-Vitvi08g00911\_t001 |  |  |  |  |  |
| 1 | Atr-ERM98169 |  | Vvi-Vitvi08g00910\_t001 |  |  |  |  |  |
| 1 | Atr-ERM98170 |  | Vvi-Vitvi08g04152\_t001 |  |  |  |  |  |
| 1 | Atr-ERM98171 |  | Vvi-Vitvi08g00908\_t003 |  |  |  |  |  |
| 1 | Atr-ERM98172 |  | Vvi-Vitvi08g02107\_t001 |  |  |  |  |  |
| 1 | Atr-ERM98173 |  | | | |  |  |  |  |  |
| 1 | Atr-ERM98174 |  | | | |  |  |  |  |  |
| 1 | Atr-ERM98175 |  | | | |  |  |  |  |  |
| 1 | Atr-ERM98176 |  | | | |  |  |  |  |  |
| 1 | Atr-ERM98177 |  | | | |  |  |  |  |  |
| 1 | Atr-ERM98178 |  | | | |  |  |  |  |  |
| 1 | Atr-ERM98179 |  | | | |  |  |  |  |  |
| 1 | Atr-ERM98180 |  | | | |  |  |  |  |  |
| 1 | Atr-ERM98181 |  | | | |  |  |  |  |  |
| 1 | Atr-ERM98182 |  | Vvi-Vitvi08g02106\_t001 |  |  |  |  |  |
| 1 | Atr-ERM98183 |  | Vvi-Vitvi08g00906\_t001 |  |  |  |  |  |
| 1 | Atr-ERM98184 |  | | | |  |  |  |  |  |
| 1 | Atr-ERM98185 |  | Vvi-Vitvi08g00905\_t001 |  |  |  |  |  |
| 1 | Atr-ERM98186 |  | Vvi-Vitvi08g00904\_t001 |  |  |  |  |  |
| 1 | Atr-ERM98187 |  | | | |  |  |  |  |  |
| 1 | Atr-ERM98188 |  | Vvi-Vitvi08g00903\_t001 |  |  |  |  |  |
| 1 | Atr-ERM98189 |  | Vvi-Vitvi08g00901\_t001 |  |  |  |  |  |
| 1 | Atr-ERM98190 |  | | | |  |  |  |  |  |
| 1 | Atr-ERM98191 |  | | | |  |  |  |  |  |
| 1 | Atr-ERM98192 |  | | | |  |  |  |  |  |
| 1 | Atr-ERM98193 |  | | | |  |  |  |  |  |
| 1 | Atr-ERM98194 |  | | | |  |  |  |  |  |
| 1 | Atr-ERM98195 |  | | | |  |  |  |  |  |
| 1 | Atr-ERM98196 |  | | | |  |  |  |  |  |
| 1 | Atr-ERM98197 |  | | | |  |  |  |  |  |
| 1 | Atr-ERM98198 |  | | | |  |  |  |  |  |
| 1 | Atr-ERM98199 |  | | | |  |  |  |  |  |
| 1 | Atr-ERM98200 |  | | | |  |  |  |  |  |
| 1 | Atr-ERM98201 |  | | | |  |  |  |  |  |
| 1 | Atr-ERM98202 |  | | | |  |  |  |  |  |
| 1 | Atr-ERM98203 |  | | | |  |  |  |  |  |
| 1 | Atr-ERM98204 |  | | | |  |  |  |  |  |
| 1 | Atr-ERM98205 |  | | | |  |  |  |  |  |
| 1 | Atr-ERM98206 |  | | | |  |  |  |  |  |
| 1 | Atr-ERM98207 |  | | | |  |  |  |  |  |
| 1 | Atr-ERM98208 |  | | | |  |  |  |  |  |
| 1 | Atr-ERM98209 |  | | | |  |  |  |  |  |
| 1 | Atr-ERM98210 |  | | | |  |  |  |  |  |
| 1 | Atr-ERM98211 |  | | | |  |  |  |  |  |
| 1 | Atr-ERM98212 |  | | | |  |  |  |  |  |
| 1 | Atr-ERM98213 |  | | | |  |  |  |  |  |
| 1 | Atr-ERM98214 |  | | | |  |  |  |  |  |
| 1 | Atr-ERM98215 |  | Vvi-Vitvi08g00900\_t001 |  |  |  |  |  |
| 0 | Atr-ERM98216 |  |  |  |  |  |  |
| 0 | Atr-ERM98217 |  |  |  |  |  |  |
| 0 | Atr-ERM98218 |  |  |  |  |  |  |
| 0 | Atr-ERM98219 |  |  |  |  |  |  |
| 0 | Atr-ERM98220 |  |  |  |  |  |  |
| 0 | Atr-ERM98221 |  |  |  |  |  |  |
| 0 | Atr-ERM98222 |  |  |  |  |  |  |
| 0 | Atr-ERM98223 |  |  |  |  |  |  |
| 0 | Atr-ERM98224 |  |  |  |  |  |  |
| 0 | Atr-ERM98225 |  |  |  |  |  |  |
| 0 | Atr-ERM98226 |  |  |  |  |  |  |
| 0 | Atr-ERM98227 |  |  |  |  |  |  |
| 0 | Atr-ERM98228 |  |  |  |  |  |  |
| 0 | Atr-ERM98229 |  |  |  |  |  |  |
| 0 | Atr-ERM98230 |  |  |  |  |  |  |
| 0 | Atr-ERM98231 |  |  |  |  |  |  |
| 0 | Atr-ERM98232 |  |  |  |  |  |  |
| 0 | Atr-ERM98233 |  |  |  |  |  |  |
| 0 | Atr-ERM98234 |  |  |  |  |  |  |
| 0 | Atr-ERM98235 |  |  |  |  |  |  |
| 0 | Atr-ERM98236 |  |  |  |  |  |  |
| 0 | Atr-ERM98237 |  |  |  |  |  |  |
| 0 | Atr-ERM98238 |  |  |  |  |  |  |
| 0 | Atr-ERM98239 |  |  |  |  |  |  |
| 0 | Atr-ERM98240 |  |  |  |  |  |  |
| 0 | Atr-ERM98241 |  |  |  |  |  |  |
| 0 | Atr-ERM98242 |  |  |  |  |  |  |
| 0 | Atr-ERM98243 |  |  |  |  |  |  |
| 0 | Atr-ERM98244 |  |  |  |  |  |  |
| 0 | Atr-ERM98245 |  |  |  |  |  |  |
| 0 | Atr-ERM98246 |  |  |  |  |  |  |
| 0 | Atr-ERM98247 |  |  |  |  |  |  |
| 0 | Atr-ERM98248 |  |  |  |  |  |  |
| 0 | Atr-ERM98249 |  |  |  |  |  |  |
| 0 | Atr-ERM98250 |  |  |  |  |  |  |
| 0 | Atr-ERM98251 |  |  |  |  |  |  |
| 0 | Atr-ERM98252 |  |  |  |  |  |  |
| 0 | Atr-ERM98253 |  |  |  |  |  |  |
| 0 | Atr-ERM98254 |  |  |  |  |  |  |
| 0 | Atr-ERM98255 |  |  |  |  |  |  |
| 0 | Atr-ERM98256 |  |  |  |  |  |  |
| 0 | Atr-ERM98257 |  |  |  |  |  |  |
| 0 | Atr-ERM98258 |  |  |  |  |  |  |
