# Supplementary material for: Genome-wide identification, characterization and gene expression of BES1 transcription factor family in grapevine (Vitis vinifera L.)
Source: Sci Rep. 2023 Jan 5;13:240. doi: 10.1038/s41598-022-24407-y (PMC9816167; doi:10.1038/s41598-022-24407-y)
Supplement: Supplementary file 3 — Supplementary Information. [file 41598_2022_24407_MOESM3_ESM.zip › Vvi_Atr/Vitis_vinifera.PN40024.v4.dna_sm.toplevel.fa.vs.Amborella_trichopoda.AMTR1.0.dna_sm.toplevel.fa.html/Atr-AmTr_v1.0_scaffold00096.html]

|  |  |  |  |  |  |  |  |  |  |  |  |  |  |
| --- | --- | --- | --- | --- | --- | --- | --- | --- | --- | --- | --- | --- | --- |
| Duplication depth | Reference chromosome | Collinear blocks | | | | | | | | | | | |
| 0 | Atr-ERN02333 |  |  |  |  |  |  |
| 0 | Atr-ERN02334 |  |  |  |  |  |  |
| 1 | Atr-ERN02335 |  | Vvi-Vitvi17g00845\_t001 |  |  |  |  |  |
| 2 | Atr-ERN02336 |  | | | |  | Vvi-Vitvi01g00697\_t001 |  |  |  |  |
| 2 | Atr-ERN02337 |  | | | |  | | | |  |  |  |  |
| 3 | Atr-ERN02338 |  | Vvi-Vitvi17g00844\_t001 |  | | | |  | Vvi-Vitvi14g01734\_t001 |  |  |  |
| 3 | Atr-ERN02339 |  | Vvi-Vitvi17g00843\_t001 |  | | | |  | Vvi-Vitvi14g01735\_t001 |  |  |  |
| 3 | Atr-ERN02340 |  | | | |  | Vvi-Vitvi01g00698\_t001 |  | | | |  |  |  |
| 3 | Atr-ERN02341 |  | | | |  | | | |  | | | |  |  |  |
| 3 | Atr-ERN02342 |  | | | |  | | | |  | | | |  |  |  |
| 3 | Atr-ERN02343 |  | | | |  | | | |  | | | |  |  |  |
| 3 | Atr-ERN02344 |  | | | |  | | | |  | | | |  |  |  |
| 3 | Atr-ERN02345 |  | Vvi-Vitvi17g00842\_t001 |  | | | |  | | | |  |  |  |
| 3 | Atr-ERN02346 |  | | | |  | | | |  | | | |  |  |  |
| 3 | Atr-ERN02347 |  | Vvi-Vitvi17g00841\_t001 |  | | | |  | | | |  |  |  |
| 3 | Atr-ERN02348 |  | Vvi-Vitvi17g00840\_t001 |  | | | |  | | | |  |  |  |
| 3 | Atr-ERN02349 |  | | | |  | | | |  | | | |  |  |  |
| 3 | Atr-ERN02350 |  | Vvi-Vitvi17g00839\_t002 |  | | | |  | | | |  |  |  |
| 3 | Atr-ERN02351 |  | Vvi-Vitvi17g00837\_t003 |  | Vvi-Vitvi01g00701\_t001 |  | Vvi-Vitvi14g01736\_t001 |  |  |  |
| 3 | Atr-ERN02352 |  | Vvi-Vitvi17g00836\_t001 |  | | | |  | | | |  |  |  |
| 3 | Atr-ERN02353 |  | | | |  | | | |  | | | |  |  |  |
| 3 | Atr-ERN02354 |  | | | |  | Vvi-Vitvi01g00703\_t001 |  | | | |  |  |  |
| 3 | Atr-ERN02355 |  | | | |  | | | |  | | | |  |  |  |
| 3 | Atr-ERN02356 |  | | | |  | | | |  | | | |  |  |  |
| 3 | Atr-ERN02357 |  | | | |  | | | |  | | | |  |  |  |
| 3 | Atr-ERN02358 |  | | | |  | | | |  | | | |  |  |  |
| 3 | Atr-ERN02359 |  | | | |  | | | |  | | | |  |  |  |
| 3 | Atr-ERN02360 |  | Vvi-Vitvi17g00834\_t001 |  | Vvi-Vitvi01g00705\_t001 |  | | | |  |  |  |
| 3 | Atr-ERN02361 |  | | | |  | Vvi-Vitvi01g02052\_t001 |  | | | |  |  |  |
| 2 | Atr-ERN02362 |  | Vvi-Vitvi17g00833\_t001 |  |  |  | Vvi-Vitvi14g01739\_t001 |  |  |  |
| 2 | Atr-ERN02363 |  | Vvi-Vitvi17g00832\_t001 |  |  |  | Vvi-Vitvi14g01740\_t001 |  |  |  |
| 2 | Atr-ERN02364 |  | | | |  |  |  | | | |  |  |  |
| 2 | Atr-ERN02365 |  | | | |  |  |  | Vvi-Vitvi14g01741\_t001 |  |  |  |
| 2 | Atr-ERN02366 |  | | | |  |  |  | | | |  |  |  |
| 2 | Atr-ERN02367 |  | Vvi-Vitvi17g00831\_t001 |  |  |  | | | |  |  |  |
| 2 | Atr-ERN02368 |  | | | |  |  |  | | | |  |  |  |
| 2 | Atr-ERN02369 |  | | | |  |  |  | | | |  |  |  |
| 2 | Atr-ERN02370 |  | | | |  |  |  | | | |  |  |  |
| 2 | Atr-ERN02371 |  | | | |  |  |  | | | |  |  |  |
| 2 | Atr-ERN02372 |  | | | |  |  |  | | | |  |  |  |
| 2 | Atr-ERN02373 |  | | | |  |  |  | | | |  |  |  |
| 2 | Atr-ERN02374 |  | | | |  |  |  | | | |  |  |  |
| 2 | Atr-ERN02375 |  | Vvi-Vitvi17g00829\_t001 |  |  |  | | | |  |  |  |
| 1 | Atr-ERN02376 |  |  |  |  |  | Vvi-Vitvi14g01743\_t001 |  |  |  |
| 1 | Atr-ERN02377 |  |  |  |  |  | | | |  |  |  |
| 1 | Atr-ERN02378 |  |  |  |  |  | | | |  |  |  |
| 1 | Atr-ERN02379 |  |  |  |  |  | | | |  |  |  |
| 1 | Atr-ERN02380 |  |  |  |  |  | | | |  |  |  |
| 1 | Atr-ERN02381 |  |  |  |  |  | | | |  |  |  |
| 1 | Atr-ERN02382 |  |  |  |  |  | | | |  |  |  |
| 1 | Atr-ERN02383 |  |  |  |  |  | | | |  |  |  |
| 1 | Atr-ERN02384 |  |  |  |  |  | | | |  |  |  |
| 1 | Atr-ERN02385 |  |  |  |  |  | | | |  |  |  |
| 1 | Atr-ERN02386 |  |  |  |  |  | | | |  |  |  |
| 1 | Atr-ERN02387 |  |  |  |  |  | | | |  |  |  |
| 2 | Atr-ERN02388 |  | Vvi-Vitvi01g04175\_t001 |  |  |  | | | |  |  |  |
| 2 | Atr-ERN02389 |  | | | |  |  |  | | | |  |  |  |
| 2 | Atr-ERN02390 |  | | | |  |  |  | | | |  |  |  |
| 2 | Atr-ERN02391 |  | | | |  |  |  | | | |  |  |  |
| 2 | Atr-ERN02392 |  | | | |  |  |  | | | |  |  |  |
| 2 | Atr-ERN02393 |  | | | |  |  |  | Vvi-Vitvi14g01744\_t001 |  |  |  |
| 2 | Atr-ERN02394 |  | | | |  |  |  | | | |  |  |  |
| 2 | Atr-ERN02395 |  | | | |  |  |  | | | |  |  |  |
| 2 | Atr-ERN02396 |  | Vvi-Vitvi01g00710\_t001 |  |  |  | Vvi-Vitvi14g01745\_t001 |  |  |  |
| 2 | Atr-ERN02397 |  | | | |  |  |  | Vvi-Vitvi14g01746\_t001 |  |  |  |
| 2 | Atr-ERN02398 |  | Vvi-Vitvi01g00711\_t001 |  |  |  | | | |  |  |  |
| 2 | Atr-ERN02399 |  | | | |  |  |  | | | |  |  |  |
| 2 | Atr-ERN02400 |  | Vvi-Vitvi01g04177\_t002 |  |  |  | Vvi-Vitvi14g01748\_t001 |  |  |  |
| 2 | Atr-ERN02401 |  | | | |  |  |  | | | |  |  |  |
| 2 | Atr-ERN02402 |  | Vvi-Vitvi01g00714\_t001 |  |  |  | | | |  |  |  |
| 2 | Atr-ERN02403 |  | | | |  |  |  | | | |  |  |  |
| 2 | Atr-ERN02404 |  | Vvi-Vitvi01g00715\_t001 |  |  |  | | | |  |  |  |
| 2 | Atr-ERN02405 |  | | | |  |  |  | | | |  |  |  |
| 2 | Atr-ERN02406 |  | | | |  |  |  | | | |  |  |  |
| 2 | Atr-ERN02407 |  | | | |  |  |  | | | |  |  |  |
| 2 | Atr-ERN02408 |  | | | |  |  |  | | | |  |  |  |
| 2 | Atr-ERN02409 |  | | | |  |  |  | | | |  |  |  |
| 2 | Atr-ERN02410 |  | | | |  |  |  | | | |  |  |  |
| 2 | Atr-ERN02411 |  | | | |  |  |  | | | |  |  |  |
| 2 | Atr-ERN02412 |  | Vvi-Vitvi01g00716\_t001 |  |  |  | | | |  |  |  |
| 2 | Atr-ERN02413 |  | | | |  |  |  | | | |  |  |  |
| 2 | Atr-ERN02414 |  | | | |  |  |  | | | |  |  |  |
| 2 | Atr-ERN02415 |  | | | |  |  |  | | | |  |  |  |
| 2 | Atr-ERN02416 |  | | | |  |  |  | | | |  |  |  |
| 2 | Atr-ERN02417 |  | | | |  |  |  | | | |  |  |  |
| 2 | Atr-ERN02418 |  | Vvi-Vitvi01g00717\_t001 |  |  |  | | | |  |  |  |
| 1 | Atr-ERN02419 |  |  |  |  |  | | | |  |  |  |
| 1 | Atr-ERN02420 |  |  |  |  |  | Vvi-Vitvi14g01751\_t001 |  |  |  |
| 0 | Atr-ERN02421 |  |  |  |  |  |  |
| 0 | Atr-ERN02422 |  |  |  |  |  |  |
| 0 | Atr-ERN02423 |  |  |  |  |  |  |
| 0 | Atr-ERN02424 |  |  |  |  |  |  |
| 0 | Atr-ERN02425 |  |  |  |  |  |  |
| 0 | Atr-ERN02426 |  |  |  |  |  |  |
| 0 | Atr-ERN02427 |  |  |  |  |  |  |
| 0 | Atr-ERN02428 |  |  |  |  |  |  |
| 0 | Atr-ERN02429 |  |  |  |  |  |  |
| 0 | Atr-ERN02430 |  |  |  |  |  |  |
| 0 | Atr-ERN02431 |  |  |  |  |  |  |
| 0 | Atr-ERN02432 |  |  |  |  |  |  |
| 0 | Atr-ERN02433 |  |  |  |  |  |  |
| 0 | Atr-ERN02434 |  |  |  |  |  |  |
| 0 | Atr-ERN02435 |  |  |  |  |  |  |
| 0 | Atr-ERN02436 |  |  |  |  |  |  |
| 0 | Atr-ERN02437 |  |  |  |  |  |  |
| 0 | Atr-ERN02438 |  |  |  |  |  |  |
| 0 | Atr-ERN02439 |  |  |  |  |  |  |
| 0 | Atr-ERN02440 |  |  |  |  |  |  |
| 0 | Atr-ERN02441 |  |  |  |  |  |  |
| 0 | Atr-ERN02442 |  |  |  |  |  |  |
| 0 | Atr-ERN02443 |  |  |  |  |  |  |
| 0 | Atr-ERN02444 |  |  |  |  |  |  |
| 0 | Atr-ERN02445 |  |  |  |  |  |  |
| 0 | Atr-ERN02446 |  |  |  |  |  |  |
| 0 | Atr-ERN02447 |  |  |  |  |  |  |
| 0 | Atr-ERN02448 |  |  |  |  |  |  |
| 0 | Atr-ERN02449 |  |  |  |  |  |  |
| 0 | Atr-ERN02450 |  |  |  |  |  |  |
| 0 | Atr-ERN02451 |  |  |  |  |  |  |
| 0 | Atr-ERN02452 |  |  |  |  |  |  |
