# Supplementary material for: Genome-wide identification, characterization and gene expression of BES1 transcription factor family in grapevine (Vitis vinifera L.)
Source: Sci Rep. 2023 Jan 5;13:240. doi: 10.1038/s41598-022-24407-y (PMC9816167; doi:10.1038/s41598-022-24407-y)
Supplement: Supplementary file 3 — Supplementary Information. [file 41598_2022_24407_MOESM3_ESM.zip › Vvi_Atr/Vitis_vinifera.PN40024.v4.dna_sm.toplevel.fa.vs.Amborella_trichopoda.AMTR1.0.dna_sm.toplevel.fa.html/Atr-AmTr_v1.0_scaffold00097.html]

|  |  |  |  |  |  |  |  |  |  |  |  |  |  |
| --- | --- | --- | --- | --- | --- | --- | --- | --- | --- | --- | --- | --- | --- |
| Duplication depth | Reference chromosome | Collinear blocks | | | | | | | | | | | |
| 0 | Atr-ERN01718 |  |  |  |  |  |  |
| 0 | Atr-ERN01719 |  |  |  |  |  |  |
| 0 | Atr-ERN01720 |  |  |  |  |  |  |
| 0 | Atr-ERN01721 |  |  |  |  |  |  |
| 0 | Atr-ERN01722 |  |  |  |  |  |  |
| 0 | Atr-ERN01723 |  |  |  |  |  |  |
| 0 | Atr-ERN01724 |  |  |  |  |  |  |
| 0 | Atr-ERN01725 |  |  |  |  |  |  |
| 0 | Atr-ERN01726 |  |  |  |  |  |  |
| 0 | Atr-ERN01727 |  |  |  |  |  |  |
| 0 | Atr-ERN01728 |  |  |  |  |  |  |
| 0 | Atr-ERN01729 |  |  |  |  |  |  |
| 0 | Atr-ERN01730 |  |  |  |  |  |  |
| 0 | Atr-ERN01731 |  |  |  |  |  |  |
| 0 | Atr-ERN01732 |  |  |  |  |  |  |
| 0 | Atr-ERN01733 |  |  |  |  |  |  |
| 0 | Atr-ERN01734 |  |  |  |  |  |  |
| 0 | Atr-ERN01735 |  |  |  |  |  |  |
| 0 | Atr-ERN01736 |  |  |  |  |  |  |
| 0 | Atr-ERN01737 |  |  |  |  |  |  |
| 0 | Atr-ERN01738 |  |  |  |  |  |  |
| 0 | Atr-ERN01739 |  |  |  |  |  |  |
| 0 | Atr-ERN01740 |  |  |  |  |  |  |
| 0 | Atr-ERN01741 |  |  |  |  |  |  |
| 0 | Atr-ERN01742 |  |  |  |  |  |  |
| 0 | Atr-ERN01743 |  |  |  |  |  |  |
| 0 | Atr-ERN01744 |  |  |  |  |  |  |
| 0 | Atr-ERN01745 |  |  |  |  |  |  |
| 0 | Atr-ERN01746 |  |  |  |  |  |  |
| 0 | Atr-ERN01747 |  |  |  |  |  |  |
| 0 | Atr-ERN01748 |  |  |  |  |  |  |
| 0 | Atr-ERN01749 |  |  |  |  |  |  |
| 0 | Atr-ERN01750 |  |  |  |  |  |  |
| 0 | Atr-ERN01751 |  |  |  |  |  |  |
| 0 | Atr-ERN01752 |  |  |  |  |  |  |
| 0 | Atr-ERN01753 |  |  |  |  |  |  |
| 0 | Atr-ERN01754 |  |  |  |  |  |  |
| 0 | Atr-ERN01755 |  |  |  |  |  |  |
| 0 | Atr-ERN01756 |  |  |  |  |  |  |
| 0 | Atr-ERN01757 |  |  |  |  |  |  |
| 0 | Atr-ERN01758 |  |  |  |  |  |  |
| 0 | Atr-ERN01759 |  |  |  |  |  |  |
| 0 | Atr-ERN01760 |  |  |  |  |  |  |
| 0 | Atr-ERN01761 |  |  |  |  |  |  |
| 0 | Atr-ERN01762 |  |  |  |  |  |  |
| 0 | Atr-ERN01763 |  |  |  |  |  |  |
| 0 | Atr-ERN01764 |  |  |  |  |  |  |
| 0 | Atr-ERN01765 |  |  |  |  |  |  |
| 0 | Atr-ERN01766 |  |  |  |  |  |  |
| 0 | Atr-ERN01767 |  |  |  |  |  |  |
| 0 | Atr-ERN01768 |  |  |  |  |  |  |
| 0 | Atr-ERN01769 |  |  |  |  |  |  |
| 0 | Atr-ERN01770 |  |  |  |  |  |  |
| 0 | Atr-ERN01771 |  |  |  |  |  |  |
| 0 | Atr-ERN01772 |  |  |  |  |  |  |
| 0 | Atr-ERN01773 |  |  |  |  |  |  |
