# Supplementary material for: Genome-wide identification, characterization and gene expression of BES1 transcription factor family in grapevine (Vitis vinifera L.)
Source: Sci Rep. 2023 Jan 5;13:240. doi: 10.1038/s41598-022-24407-y (PMC9816167; doi:10.1038/s41598-022-24407-y)
Supplement: Supplementary file 3 — Supplementary Information. [file 41598_2022_24407_MOESM3_ESM.zip › Vvi_Atr/Vitis_vinifera.PN40024.v4.dna_sm.toplevel.fa.vs.Amborella_trichopoda.AMTR1.0.dna_sm.toplevel.fa.html/Atr-AmTr_v1.0_scaffold00098.html]

|  |  |  |  |  |  |  |  |  |  |  |  |  |  |
| --- | --- | --- | --- | --- | --- | --- | --- | --- | --- | --- | --- | --- | --- |
| Duplication depth | Reference chromosome | Collinear blocks | | | | | | | | | | | |
| 0 | Atr-ERM99818 |  |  |  |  |  |  |
| 0 | Atr-ERM99819 |  |  |  |  |  |  |
| 0 | Atr-ERM99820 |  |  |  |  |  |  |
| 0 | Atr-ERM99821 |  |  |  |  |  |  |
| 0 | Atr-ERM99822 |  |  |  |  |  |  |
| 0 | Atr-ERM99823 |  |  |  |  |  |  |
| 0 | Atr-ERM99824 |  |  |  |  |  |  |
| 0 | Atr-ERM99825 |  |  |  |  |  |  |
| 0 | Atr-ERM99826 |  |  |  |  |  |  |
| 0 | Atr-ERM99827 |  |  |  |  |  |  |
| 0 | Atr-ERM99828 |  |  |  |  |  |  |
| 0 | Atr-ERM99829 |  |  |  |  |  |  |
| 0 | Atr-ERM99830 |  |  |  |  |  |  |
| 0 | Atr-ERM99831 |  |  |  |  |  |  |
| 0 | Atr-ERM99832 |  |  |  |  |  |  |
| 0 | Atr-ERM99833 |  |  |  |  |  |  |
| 0 | Atr-ERM99834 |  |  |  |  |  |  |
| 0 | Atr-ERM99835 |  |  |  |  |  |  |
| 0 | Atr-ERM99836 |  |  |  |  |  |  |
| 0 | Atr-ERM99837 |  |  |  |  |  |  |
| 0 | Atr-ERM99838 |  |  |  |  |  |  |
| 0 | Atr-ERM99839 |  |  |  |  |  |  |
| 0 | Atr-ERM99840 |  |  |  |  |  |  |
| 0 | Atr-ERM99841 |  |  |  |  |  |  |
| 0 | Atr-ERM99842 |  |  |  |  |  |  |
| 0 | Atr-ERM99843 |  |  |  |  |  |  |
| 0 | Atr-ERM99844 |  |  |  |  |  |  |
| 0 | Atr-ERM99845 |  |  |  |  |  |  |
| 0 | Atr-ERM99846 |  |  |  |  |  |  |
| 0 | Atr-ERM99847 |  |  |  |  |  |  |
| 0 | Atr-ERM99848 |  |  |  |  |  |  |
| 0 | Atr-ERM99849 |  |  |  |  |  |  |
| 0 | Atr-ERM99850 |  |  |  |  |  |  |
| 0 | Atr-ERM99851 |  |  |  |  |  |  |
| 0 | Atr-ERM99852 |  |  |  |  |  |  |
| 0 | Atr-ERM99853 |  |  |  |  |  |  |
| 0 | Atr-ERM99854 |  |  |  |  |  |  |
| 0 | Atr-ERM99855 |  |  |  |  |  |  |
| 0 | Atr-ERM99856 |  |  |  |  |  |  |
| 0 | Atr-ERM99857 |  |  |  |  |  |  |
| 0 | Atr-ERM99858 |  |  |  |  |  |  |
| 0 | Atr-ERM99859 |  |  |  |  |  |  |
| 0 | Atr-ERM99860 |  |  |  |  |  |  |
| 0 | Atr-ERM99861 |  |  |  |  |  |  |
| 0 | Atr-ERM99862 |  |  |  |  |  |  |
| 0 | Atr-ERM99863 |  |  |  |  |  |  |
| 0 | Atr-ERM99864 |  |  |  |  |  |  |
| 0 | Atr-ERM99865 |  |  |  |  |  |  |
| 0 | Atr-ERM99866 |  |  |  |  |  |  |
| 0 | Atr-ERM99867 |  |  |  |  |  |  |
| 0 | Atr-ERM99868 |  |  |  |  |  |  |
| 0 | Atr-ERM99869 |  |  |  |  |  |  |
| 0 | Atr-ERM99870 |  |  |  |  |  |  |
| 0 | Atr-ERM99871 |  |  |  |  |  |  |
| 0 | Atr-ERM99872 |  |  |  |  |  |  |
| 0 | Atr-ERM99873 |  |  |  |  |  |  |
| 0 | Atr-ERM99874 |  |  |  |  |  |  |
