# Supplementary material for: Genome-wide identification, characterization and gene expression of BES1 transcription factor family in grapevine (Vitis vinifera L.)
Source: Sci Rep. 2023 Jan 5;13:240. doi: 10.1038/s41598-022-24407-y (PMC9816167; doi:10.1038/s41598-022-24407-y)
Supplement: Supplementary file 3 — Supplementary Information. [file 41598_2022_24407_MOESM3_ESM.zip › Vvi_Atr/Vitis_vinifera.PN40024.v4.dna_sm.toplevel.fa.vs.Amborella_trichopoda.AMTR1.0.dna_sm.toplevel.fa.html/Atr-AmTr_v1.0_scaffold00099.html]

|  |  |  |  |  |  |  |  |  |  |  |  |  |  |
| --- | --- | --- | --- | --- | --- | --- | --- | --- | --- | --- | --- | --- | --- |
| Duplication depth | Reference chromosome | Collinear blocks | | | | | | | | | | | |
| 2 | Atr-ERM99646 |  | Vvi-Vitvi12g02059\_t001 |  | Vvi-Vitvi18g04639\_t001 |  |  |  |  |
| 2 | Atr-ERM99647 |  | | | |  | | | |  |  |  |  |
| 2 | Atr-ERM99648 |  | | | |  | | | |  |  |  |  |
| 2 | Atr-ERM99649 |  | | | |  | | | |  |  |  |  |
| 2 | Atr-ERM99650 |  | | | |  | | | |  |  |  |  |
| 2 | Atr-ERM99651 |  | | | |  | | | |  |  |  |  |
| 2 | Atr-ERM99652 |  | | | |  | | | |  |  |  |  |
| 2 | Atr-ERM99653 |  | | | |  | | | |  |  |  |  |
| 2 | Atr-ERM99654 |  | | | |  | Vvi-Vitvi18g02183\_t001 |  |  |  |  |
| 2 | Atr-ERM99655 |  | | | |  | Vvi-Vitvi18g02184\_t001 |  |  |  |  |
| 2 | Atr-ERM99656 |  | | | |  | Vvi-Vitvi18g02187\_t001 |  |  |  |  |
| 2 | Atr-ERM99657 |  | Vvi-Vitvi12g02060\_t001 |  | Vvi-Vitvi18g02188\_t001 |  |  |  |  |
| 2 | Atr-ERM99658 |  | | | |  | Vvi-Vitvi18g02190\_t001 |  |  |  |  |
| 2 | Atr-ERM99659 |  | | | |  | Vvi-Vitvi18g02191\_t001 |  |  |  |  |
| 2 | Atr-ERM99660 |  | | | |  | Vvi-Vitvi18g04652\_t001 |  |  |  |  |
| 2 | Atr-ERM99661 |  | | | |  | | | |  |  |  |  |
| 2 | Atr-ERM99662 |  | | | |  | | | |  |  |  |  |
| 2 | Atr-ERM99663 |  | | | |  | Vvi-Vitvi18g02230\_t001 |  |  |  |  |
| 2 | Atr-ERM99664 |  | | | |  | Vvi-Vitvi18g02231\_t001 |  |  |  |  |
| 2 | Atr-ERM99665 |  | | | |  | | | |  |  |  |  |
| 2 | Atr-ERM99666 |  | | | |  | | | |  |  |  |  |
| 2 | Atr-ERM99667 |  | | | |  | Vvi-Vitvi18g02236\_t001 |  |  |  |  |
| 2 | Atr-ERM99668 |  | | | |  | Vvi-Vitvi18g02237\_t001 |  |  |  |  |
| 2 | Atr-ERM99669 |  | | | |  | | | |  |  |  |  |
| 2 | Atr-ERM99670 |  | | | |  | | | |  |  |  |  |
| 2 | Atr-ERM99671 |  | | | |  | Vvi-Vitvi18g02240\_t001 |  |  |  |  |
| 2 | Atr-ERM99672 |  | | | |  | | | |  |  |  |  |
| 2 | Atr-ERM99673 |  | | | |  | | | |  |  |  |  |
| 2 | Atr-ERM99674 |  | | | |  | | | |  |  |  |  |
| 2 | Atr-ERM99675 |  | Vvi-Vitvi12g02061\_t001 |  | | | |  |  |  |  |
| 2 | Atr-ERM99676 |  | Vvi-Vitvi12g02062\_t001 |  | Vvi-Vitvi18g02244\_t001 |  |  |  |  |
| 2 | Atr-ERM99677 |  | | | |  | Vvi-Vitvi18g02246\_t001 |  |  |  |  |
| 2 | Atr-ERM99678 |  | Vvi-Vitvi12g02063\_t001 |  | Vvi-Vitvi18g02248\_t001 |  |  |  |  |
| 2 | Atr-ERM99679 |  | | | |  | Vvi-Vitvi18g02249\_t001 |  |  |  |  |
| 2 | Atr-ERM99680 |  | | | |  | | | |  |  |  |  |
| 2 | Atr-ERM99681 |  | | | |  | Vvi-Vitvi18g02251\_t002 |  |  |  |  |
| 2 | Atr-ERM99682 |  | | | |  | | | |  |  |  |  |
| 2 | Atr-ERM99683 |  | | | |  | | | |  |  |  |  |
| 2 | Atr-ERM99684 |  | | | |  | | | |  |  |  |  |
| 2 | Atr-ERM99685 |  | | | |  | Vvi-Vitvi18g02253\_t002 |  |  |  |  |
| 1 | Atr-ERM99686 |  | | | |  |  |  |  |  |
| 1 | Atr-ERM99687 |  | Vvi-Vitvi12g02064\_t001 |  |  |  |  |  |
| 2 | Atr-ERM99688 |  | | | |  | Vvi-Vitvi18g03214\_t001 |  |  |  |  |
| 2 | Atr-ERM99689 |  | | | |  | | | |  |  |  |  |
| 2 | Atr-ERM99690 |  | | | |  | Vvi-Vitvi18g02319\_t001 |  |  |  |  |
| 2 | Atr-ERM99691 |  | | | |  | Vvi-Vitvi18g02322\_t001 |  |  |  |  |
| 2 | Atr-ERM99692 |  | | | |  | | | |  |  |  |  |
| 2 | Atr-ERM99693 |  | | | |  | Vvi-Vitvi18g04729\_t001 |  |  |  |  |
| 2 | Atr-ERM99694 |  | | | |  | Vvi-Vitvi18g02341\_t001 |  |  |  |  |
| 2 | Atr-ERM99695 |  | | | |  | Vvi-Vitvi18g02345\_t001 |  |  |  |  |
| 2 | Atr-ERM99696 |  | | | |  | | | |  |  |  |  |
| 2 | Atr-ERM99697 |  | | | |  | | | |  |  |  |  |
| 2 | Atr-ERM99698 |  | Vvi-Vitvi12g04605\_t001 |  | | | |  |  |  |  |
| 1 | Atr-ERM99699 |  |  |  | | | |  |  |  |  |
| 1 | Atr-ERM99700 |  |  |  | | | |  |  |  |  |
| 1 | Atr-ERM99701 |  |  |  | | | |  |  |  |  |
| 1 | Atr-ERM99702 |  |  |  | | | |  |  |  |  |
| 1 | Atr-ERM99703 |  |  |  | | | |  |  |  |  |
| 1 | Atr-ERM99704 |  |  |  | | | |  |  |  |  |
| 2 | Atr-ERM99705 |  | Vvi-Vitvi12g02106\_t001 |  | | | |  |  |  |  |
| 2 | Atr-ERM99706 |  | | | |  | | | |  |  |  |  |
| 2 | Atr-ERM99707 |  | | | |  | | | |  |  |  |  |
| 2 | Atr-ERM99708 |  | | | |  | | | |  |  |  |  |
| 2 | Atr-ERM99709 |  | | | |  | | | |  |  |  |  |
| 2 | Atr-ERM99710 |  | | | |  | | | |  |  |  |  |
| 2 | Atr-ERM99711 |  | | | |  | Vvi-Vitvi18g02358\_t004 |  |  |  |  |
| 2 | Atr-ERM99712 |  | Vvi-Vitvi12g02115\_t001 |  | | | |  |  |  |  |
| 2 | Atr-ERM99713 |  | | | |  | | | |  |  |  |  |
| 2 | Atr-ERM99714 |  | | | |  | | | |  |  |  |  |
| 2 | Atr-ERM99715 |  | | | |  | | | |  |  |  |  |
| 2 | Atr-ERM99716 |  | | | |  | | | |  |  |  |  |
| 2 | Atr-ERM99717 |  | | | |  | | | |  |  |  |  |
| 2 | Atr-ERM99718 |  | | | |  | | | |  |  |  |  |
| 2 | Atr-ERM99719 |  | | | |  | | | |  |  |  |  |
| 2 | Atr-ERM99720 |  | | | |  | | | |  |  |  |  |
| 2 | Atr-ERM99721 |  | | | |  | | | |  |  |  |  |
| 2 | Atr-ERM99722 |  | | | |  | | | |  |  |  |  |
| 2 | Atr-ERM99723 |  | | | |  | | | |  |  |  |  |
| 2 | Atr-ERM99724 |  | | | |  | Vvi-Vitvi18g02360\_t001 |  |  |  |  |
| 2 | Atr-ERM99725 |  | | | |  | | | |  |  |  |  |
| 2 | Atr-ERM99726 |  | | | |  | | | |  |  |  |  |
| 2 | Atr-ERM99727 |  | | | |  | Vvi-Vitvi18g02361\_t001 |  |  |  |  |
| 2 | Atr-ERM99728 |  | | | |  | | | |  |  |  |  |
| 2 | Atr-ERM99729 |  | Vvi-Vitvi12g02121\_t001 |  | | | |  |  |  |  |
| 2 | Atr-ERM99730 |  | | | |  | Vvi-Vitvi18g02365\_t001 |  |  |  |  |
| 2 | Atr-ERM99731 |  | | | |  | | | |  |  |  |  |
| 2 | Atr-ERM99732 |  | | | |  | | | |  |  |  |  |
| 2 | Atr-ERM99733 |  | | | |  | Vvi-Vitvi18g02380\_t001 |  |  |  |  |
| 2 | Atr-ERM99734 |  | | | |  | Vvi-Vitvi18g03258\_t002 |  |  |  |  |
| 2 | Atr-ERM99735 |  | | | |  | | | |  |  |  |  |
| 2 | Atr-ERM99736 |  | | | |  | | | |  |  |  |  |
| 2 | Atr-ERM99737 |  | | | |  | Vvi-Vitvi18g02389\_t001 |  |  |  |  |
| 2 | Atr-ERM99738 |  | | | |  | | | |  |  |  |  |
| 3 | Atr-ERM99739 |  | | | |  | | | |  | Vvi-Vitvi07g02023\_t001 |  |  |  |
| 3 | Atr-ERM99740 |  | | | |  | | | |  | | | |  |  |  |
| 3 | Atr-ERM99741 |  | | | |  | | | |  | | | |  |  |  |
| 3 | Atr-ERM99742 |  | | | |  | | | |  | | | |  |  |  |
| 3 | Atr-ERM99743 |  | | | |  | | | |  | Vvi-Vitvi07g04772\_t001 |  |  |  |
| 3 | Atr-ERM99744 |  | | | |  | Vvi-Vitvi18g02391\_t001 |  | | | |  |  |  |
| 3 | Atr-ERM99745 |  | | | |  | Vvi-Vitvi18g02392\_t001 |  | | | |  |  |  |
| 3 | Atr-ERM99746 |  | Vvi-Vitvi12g02124\_t001 |  | | | |  | | | |  |  |  |
| 3 | Atr-ERM99747 |  | | | |  | Vvi-Vitvi18g02393\_t001 |  | | | |  |  |  |
| 3 | Atr-ERM99748 |  | Vvi-Vitvi12g02125\_t001 |  | Vvi-Vitvi18g02396\_t001 |  | | | |  |  |  |
| 3 | Atr-ERM99749 |  | | | |  | Vvi-Vitvi18g02398\_t001 |  | | | |  |  |  |
| 3 | Atr-ERM99750 |  | | | |  | Vvi-Vitvi18g02399\_t001 |  | | | |  |  |  |
| 3 | Atr-ERM99751 |  | | | |  | Vvi-Vitvi18g02400\_t001 |  | Vvi-Vitvi07g02028\_t001 |  |  |  |
| 3 | Atr-ERM99752 |  | | | |  | Vvi-Vitvi18g03270\_t001 |  | | | |  |  |  |
| 3 | Atr-ERM99753 |  | | | |  | | | |  | | | |  |  |  |
| 3 | Atr-ERM99754 |  | Vvi-Vitvi12g02126\_t001 |  | | | |  | | | |  |  |  |
| 3 | Atr-ERM99755 |  | | | |  | | | |  | | | |  |  |  |
| 3 | Atr-ERM99756 |  | | | |  | | | |  | | | |  |  |  |
| 3 | Atr-ERM99757 |  | | | |  | | | |  | | | |  |  |  |
| 3 | Atr-ERM99758 |  | | | |  | Vvi-Vitvi18g02401\_t001 |  | | | |  |  |  |
| 3 | Atr-ERM99759 |  | | | |  | | | |  | | | |  |  |  |
| 3 | Atr-ERM99760 |  | Vvi-Vitvi12g02128\_t001 |  | | | |  | | | |  |  |  |
| 3 | Atr-ERM99761 |  | | | |  | | | |  | | | |  |  |  |
| 3 | Atr-ERM99762 |  | | | |  | Vvi-Vitvi18g02404\_t001 |  | | | |  |  |  |
| 3 | Atr-ERM99763 |  | | | |  | | | |  | | | |  |  |  |
| 3 | Atr-ERM99764 |  | | | |  | | | |  | | | |  |  |  |
| 3 | Atr-ERM99765 |  | | | |  | | | |  | | | |  |  |  |
| 3 | Atr-ERM99766 |  | | | |  | | | |  | | | |  |  |  |
| 3 | Atr-ERM99767 |  | | | |  | | | |  | | | |  |  |  |
| 3 | Atr-ERM99768 |  | | | |  | | | |  | | | |  |  |  |
| 3 | Atr-ERM99769 |  | | | |  | Vvi-Vitvi18g02408\_t001 |  | | | |  |  |  |
| 3 | Atr-ERM99770 |  | | | |  | Vvi-Vitvi18g02409\_t001 |  | | | |  |  |  |
| 3 | Atr-ERM99771 |  | | | |  | | | |  | Vvi-Vitvi07g02029\_t001 |  |  |  |
| 3 | Atr-ERM99772 |  | | | |  | | | |  | Vvi-Vitvi07g02031\_t001 |  |  |  |
| 3 | Atr-ERM99773 |  | | | |  | Vvi-Vitvi18g02410\_t001 |  | | | |  |  |  |
| 3 | Atr-ERM99774 |  | | | |  | Vvi-Vitvi18g02415\_t001 |  | | | |  |  |  |
| 3 | Atr-ERM99775 |  | Vvi-Vitvi12g02130\_t001 |  | Vvi-Vitvi18g02418\_t001 |  | | | |  |  |  |
| 3 | Atr-ERM99776 |  | Vvi-Vitvi12g02134\_t002 |  | | | |  | | | |  |  |  |
| 3 | Atr-ERM99777 |  | Vvi-Vitvi12g02135\_t001 |  | | | |  | Vvi-Vitvi07g02032\_t001 |  |  |  |
| 2 | Atr-ERM99778 |  | Vvi-Vitvi12g02136\_t001 |  | | | |  |  |  |  |
| 2 | Atr-ERM99779 |  | Vvi-Vitvi12g02138\_t001 |  | Vvi-Vitvi18g02423\_t001 |  |  |  |  |
| 2 | Atr-ERM99780 |  | | | |  | Vvi-Vitvi18g02424\_t001 |  |  |  |  |
| 2 | Atr-ERM99781 |  | Vvi-Vitvi12g02139\_t001 |  | Vvi-Vitvi18g02426\_t001 |  |  |  |  |
| 2 | Atr-ERM99782 |  | | | |  | | | |  |  |  |  |
| 2 | Atr-ERM99783 |  | | | |  | | | |  |  |  |  |
| 2 | Atr-ERM99784 |  | Vvi-Vitvi12g02140\_t001 |  | | | |  |  |  |  |
| 2 | Atr-ERM99785 |  | | | |  | Vvi-Vitvi18g02427\_t001 |  |  |  |  |
| 2 | Atr-ERM99786 |  | Vvi-Vitvi12g02141\_t001 |  | | | |  |  |  |  |
| 2 | Atr-ERM99787 |  | | | |  | | | |  |  |  |  |
| 2 | Atr-ERM99788 |  | Vvi-Vitvi12g02142\_t001 |  | | | |  |  |  |  |
| 2 | Atr-ERM99789 |  | | | |  | | | |  |  |  |  |
| 2 | Atr-ERM99790 |  | | | |  | | | |  |  |  |  |
| 2 | Atr-ERM99791 |  | | | |  | | | |  |  |  |  |
| 2 | Atr-ERM99792 |  | | | |  | | | |  |  |  |  |
| 2 | Atr-ERM99793 |  | | | |  | | | |  |  |  |  |
| 2 | Atr-ERM99794 |  | | | |  | Vvi-Vitvi18g02429\_t002 |  |  |  |  |
| 2 | Atr-ERM99795 |  | | | |  | | | |  |  |  |  |
| 2 | Atr-ERM99796 |  | Vvi-Vitvi12g02144\_t001 |  | | | |  |  |  |  |
| 2 | Atr-ERM99797 |  | | | |  | | | |  |  |  |  |
| 3 | Atr-ERM99798 |  | | | |  | | | |  | Vvi-Vitvi12g02162\_t001 |  |  |  |
| 3 | Atr-ERM99799 |  | | | |  | | | |  | | | |  |  |  |
| 3 | Atr-ERM99800 |  | | | |  | | | |  | | | |  |  |  |
| 3 | Atr-ERM99801 |  | | | |  | | | |  | | | |  |  |  |
| 3 | Atr-ERM99802 |  | | | |  | | | |  | Vvi-Vitvi12g02758\_t001 |  |  |  |
| 3 | Atr-ERM99803 |  | | | |  | Vvi-Vitvi18g02434\_t001 |  | | | |  |  |  |
| 2 | Atr-ERM99804 |  | | | |  |  |  | Vvi-Vitvi12g02757\_t001 |  |  |  |
| 2 | Atr-ERM99805 |  | | | |  |  |  | | | |  |  |  |
| 2 | Atr-ERM99806 |  | | | |  |  |  | Vvi-Vitvi12g02755\_t001 |  |  |  |
| 2 | Atr-ERM99807 |  | Vvi-Vitvi12g02158\_t001 |  |  |  | | | |  |  |  |
| 1 | Atr-ERM99808 |  |  |  |  |  | Vvi-Vitvi12g02152\_t001 |  |  |  |
| 1 | Atr-ERM99809 |  |  |  |  |  | Vvi-Vitvi12g02150\_t001 |  |  |  |
| 0 | Atr-ERM99810 |  |  |  |  |  |  |
| 0 | Atr-ERM99811 |  |  |  |  |  |  |
