# Supplementary material for: Genome-wide identification, characterization and gene expression of BES1 transcription factor family in grapevine (Vitis vinifera L.)
Source: Sci Rep. 2023 Jan 5;13:240. doi: 10.1038/s41598-022-24407-y (PMC9816167; doi:10.1038/s41598-022-24407-y)
Supplement: Supplementary file 3 — Supplementary Information. [file 41598_2022_24407_MOESM3_ESM.zip › Vvi_Atr/Vitis_vinifera.PN40024.v4.dna_sm.toplevel.fa.vs.Amborella_trichopoda.AMTR1.0.dna_sm.toplevel.fa.html/Atr-AmTr_v1.0_scaffold00100.html]

|  |  |  |  |  |  |  |  |  |  |  |  |  |  |
| --- | --- | --- | --- | --- | --- | --- | --- | --- | --- | --- | --- | --- | --- |
| Duplication depth | Reference chromosome | Collinear blocks | | | | | | | | | | | |
| 0 | Atr-ERN00406 |  |  |  |  |  |  |
| 0 | Atr-ERN00407 |  |  |  |  |  |  |
| 0 | Atr-ERN00408 |  |  |  |  |  |  |
| 0 | Atr-ERN00409 |  |  |  |  |  |  |
| 0 | Atr-ERN00410 |  |  |  |  |  |  |
| 0 | Atr-ERN00411 |  |  |  |  |  |  |
| 0 | Atr-ERN00412 |  |  |  |  |  |  |
| 0 | Atr-ERN00413 |  |  |  |  |  |  |
| 0 | Atr-ERN00414 |  |  |  |  |  |  |
| 0 | Atr-ERN00415 |  |  |  |  |  |  |
| 0 | Atr-ERN00416 |  |  |  |  |  |  |
| 0 | Atr-ERN00417 |  |  |  |  |  |  |
| 0 | Atr-ERN00418 |  |  |  |  |  |  |
| 0 | Atr-ERN00419 |  |  |  |  |  |  |
| 0 | Atr-ERN00420 |  |  |  |  |  |  |
| 0 | Atr-ERN00421 |  |  |  |  |  |  |
| 0 | Atr-ERN00422 |  |  |  |  |  |  |
| 0 | Atr-ERN00423 |  |  |  |  |  |  |
| 0 | Atr-ERN00424 |  |  |  |  |  |  |
| 0 | Atr-ERN00425 |  |  |  |  |  |  |
| 0 | Atr-ERN00426 |  |  |  |  |  |  |
| 0 | Atr-ERN00427 |  |  |  |  |  |  |
| 0 | Atr-ERN00428 |  |  |  |  |  |  |
| 0 | Atr-ERN00429 |  |  |  |  |  |  |
| 0 | Atr-ERN00430 |  |  |  |  |  |  |
| 0 | Atr-ERN00431 |  |  |  |  |  |  |
| 0 | Atr-ERN00432 |  |  |  |  |  |  |
| 0 | Atr-ERN00433 |  |  |  |  |  |  |
| 0 | Atr-ERN00434 |  |  |  |  |  |  |
| 0 | Atr-ERN00435 |  |  |  |  |  |  |
| 0 | Atr-ERN00436 |  |  |  |  |  |  |
| 0 | Atr-ERN00437 |  |  |  |  |  |  |
| 0 | Atr-ERN00438 |  |  |  |  |  |  |
| 0 | Atr-ERN00439 |  |  |  |  |  |  |
| 0 | Atr-ERN00440 |  |  |  |  |  |  |
| 0 | Atr-ERN00441 |  |  |  |  |  |  |
| 0 | Atr-ERN00442 |  |  |  |  |  |  |
| 0 | Atr-ERN00443 |  |  |  |  |  |  |
| 0 | Atr-ERN00444 |  |  |  |  |  |  |
| 0 | Atr-ERN00445 |  |  |  |  |  |  |
| 0 | Atr-ERN00446 |  |  |  |  |  |  |
| 0 | Atr-ERN00447 |  |  |  |  |  |  |
| 0 | Atr-ERN00448 |  |  |  |  |  |  |
| 0 | Atr-ERN00449 |  |  |  |  |  |  |
| 0 | Atr-ERN00450 |  |  |  |  |  |  |
| 0 | Atr-ERN00451 |  |  |  |  |  |  |
| 0 | Atr-ERN00452 |  |  |  |  |  |  |
| 0 | Atr-ERN00453 |  |  |  |  |  |  |
| 0 | Atr-ERN00454 |  |  |  |  |  |  |
| 0 | Atr-ERN00455 |  |  |  |  |  |  |
| 0 | Atr-ERN00456 |  |  |  |  |  |  |
| 0 | Atr-ERN00457 |  |  |  |  |  |  |
| 0 | Atr-ERN00458 |  |  |  |  |  |  |
| 0 | Atr-ERN00459 |  |  |  |  |  |  |
| 0 | Atr-ERN00460 |  |  |  |  |  |  |
| 0 | Atr-ERN00461 |  |  |  |  |  |  |
| 0 | Atr-ERN00462 |  |  |  |  |  |  |
| 0 | Atr-ERN00463 |  |  |  |  |  |  |
| 0 | Atr-ERN00464 |  |  |  |  |  |  |
| 0 | Atr-ERN00465 |  |  |  |  |  |  |
| 0 | Atr-ERN00466 |  |  |  |  |  |  |
| 0 | Atr-ERN00467 |  |  |  |  |  |  |
| 0 | Atr-ERN00468 |  |  |  |  |  |  |
| 0 | Atr-ERN00469 |  |  |  |  |  |  |
| 0 | Atr-ERN00470 |  |  |  |  |  |  |
