# Supplementary material for: Genome-wide identification, characterization and gene expression of BES1 transcription factor family in grapevine (Vitis vinifera L.)
Source: Sci Rep. 2023 Jan 5;13:240. doi: 10.1038/s41598-022-24407-y (PMC9816167; doi:10.1038/s41598-022-24407-y)
Supplement: Supplementary file 3 — Supplementary Information. [file 41598_2022_24407_MOESM3_ESM.zip › Vvi_Atr/Vitis_vinifera.PN40024.v4.dna_sm.toplevel.fa.vs.Amborella_trichopoda.AMTR1.0.dna_sm.toplevel.fa.html/Atr-AmTr_v1.0_scaffold00101.html]

|  |  |  |  |  |  |  |  |  |  |  |  |  |  |
| --- | --- | --- | --- | --- | --- | --- | --- | --- | --- | --- | --- | --- | --- |
| Duplication depth | Reference chromosome | Collinear blocks | | | | | | | | | | | |
| 0 | Atr-ERM98999 |  |  |  |  |  |  |
| 0 | Atr-ERM99000 |  |  |  |  |  |  |
| 0 | Atr-ERM99001 |  |  |  |  |  |  |
| 0 | Atr-ERM99002 |  |  |  |  |  |  |
| 0 | Atr-ERM99003 |  |  |  |  |  |  |
| 0 | Atr-ERM99004 |  |  |  |  |  |  |
| 0 | Atr-ERM99005 |  |  |  |  |  |  |
| 0 | Atr-ERM99006 |  |  |  |  |  |  |
| 0 | Atr-ERM99007 |  |  |  |  |  |  |
| 0 | Atr-ERM99008 |  |  |  |  |  |  |
| 0 | Atr-ERM99009 |  |  |  |  |  |  |
| 0 | Atr-ERM99010 |  |  |  |  |  |  |
| 0 | Atr-ERM99011 |  |  |  |  |  |  |
| 0 | Atr-ERM99012 |  |  |  |  |  |  |
| 0 | Atr-ERM99013 |  |  |  |  |  |  |
| 0 | Atr-ERM99014 |  |  |  |  |  |  |
| 0 | Atr-ERM99015 |  |  |  |  |  |  |
| 0 | Atr-ERM99016 |  |  |  |  |  |  |
| 0 | Atr-ERM99017 |  |  |  |  |  |  |
| 0 | Atr-ERM99018 |  |  |  |  |  |  |
| 0 | Atr-ERM99019 |  |  |  |  |  |  |
| 0 | Atr-ERM99020 |  |  |  |  |  |  |
| 0 | Atr-ERM99021 |  |  |  |  |  |  |
| 0 | Atr-ERM99022 |  |  |  |  |  |  |
| 0 | Atr-ERM99023 |  |  |  |  |  |  |
| 0 | Atr-ERM99024 |  |  |  |  |  |  |
| 0 | Atr-ERM99025 |  |  |  |  |  |  |
| 0 | Atr-ERM99026 |  |  |  |  |  |  |
| 0 | Atr-ERM99027 |  |  |  |  |  |  |
| 0 | Atr-ERM99028 |  |  |  |  |  |  |
| 0 | Atr-ERM99029 |  |  |  |  |  |  |
| 0 | Atr-ERM99030 |  |  |  |  |  |  |
| 0 | Atr-ERM99031 |  |  |  |  |  |  |
| 0 | Atr-ERM99032 |  |  |  |  |  |  |
| 0 | Atr-ERM99033 |  |  |  |  |  |  |
| 0 | Atr-ERM99034 |  |  |  |  |  |  |
| 0 | Atr-ERM99035 |  |  |  |  |  |  |
| 0 | Atr-ERM99036 |  |  |  |  |  |  |
| 0 | Atr-ERM99037 |  |  |  |  |  |  |
| 0 | Atr-ERM99038 |  |  |  |  |  |  |
| 0 | Atr-ERM99039 |  |  |  |  |  |  |
| 0 | Atr-ERM99040 |  |  |  |  |  |  |
| 0 | Atr-ERM99041 |  |  |  |  |  |  |
| 0 | Atr-ERM99042 |  |  |  |  |  |  |
| 0 | Atr-ERM99043 |  |  |  |  |  |  |
| 0 | Atr-ERM99044 |  |  |  |  |  |  |
| 0 | Atr-ERM99045 |  |  |  |  |  |  |
| 0 | Atr-ERM99046 |  |  |  |  |  |  |
| 0 | Atr-ERM99047 |  |  |  |  |  |  |
| 0 | Atr-ERM99048 |  |  |  |  |  |  |
| 0 | Atr-ERM99049 |  |  |  |  |  |  |
| 0 | Atr-ERM99050 |  |  |  |  |  |  |
| 0 | Atr-ERM99051 |  |  |  |  |  |  |
| 0 | Atr-ERM99052 |  |  |  |  |  |  |
| 0 | Atr-ERM99053 |  |  |  |  |  |  |
| 0 | Atr-ERM99054 |  |  |  |  |  |  |
| 0 | Atr-ERM99055 |  |  |  |  |  |  |
| 0 | Atr-ERM99056 |  |  |  |  |  |  |
| 0 | Atr-ERM99057 |  |  |  |  |  |  |
| 0 | Atr-ERM99058 |  |  |  |  |  |  |
| 0 | Atr-ERM99059 |  |  |  |  |  |  |
| 0 | Atr-ERM99060 |  |  |  |  |  |  |
| 0 | Atr-ERM99061 |  |  |  |  |  |  |
| 0 | Atr-ERM99062 |  |  |  |  |  |  |
| 0 | Atr-ERM99063 |  |  |  |  |  |  |
| 0 | Atr-ERM99064 |  |  |  |  |  |  |
| 0 | Atr-ERM99065 |  |  |  |  |  |  |
| 0 | Atr-ERM99066 |  |  |  |  |  |  |
| 0 | Atr-ERM99067 |  |  |  |  |  |  |
| 0 | Atr-ERM99068 |  |  |  |  |  |  |
| 0 | Atr-ERM99069 |  |  |  |  |  |  |
| 0 | Atr-ERM99070 |  |  |  |  |  |  |
| 0 | Atr-ERM99071 |  |  |  |  |  |  |
| 1 | Atr-ERM99072 |  | Vvi-Vitvi08g00935\_t001 |  |  |  |  |  |
| 1 | Atr-ERM99073 |  | | | |  |  |  |  |  |
| 1 | Atr-ERM99074 |  | | | |  |  |  |  |  |
| 1 | Atr-ERM99075 |  | Vvi-Vitvi08g00939\_t001 |  |  |  |  |  |
| 2 | Atr-ERM99076 |  | | | |  | Vvi-Vitvi13g01402\_t001 |  |  |  |  |
| 2 | Atr-ERM99077 |  | Vvi-Vitvi08g00940\_t001 |  | | | |  |  |  |  |
| 2 | Atr-ERM99078 |  | | | |  | Vvi-Vitvi13g01398\_t001 |  |  |  |  |
| 2 | Atr-ERM99079 |  | | | |  | Vvi-Vitvi13g01396\_t001 |  |  |  |  |
| 2 | Atr-ERM99080 |  | | | |  | | | |  |  |  |  |
| 2 | Atr-ERM99081 |  | | | |  | | | |  |  |  |  |
| 2 | Atr-ERM99082 |  | | | |  | | | |  |  |  |  |
| 2 | Atr-ERM99083 |  | | | |  | | | |  |  |  |  |
| 2 | Atr-ERM99084 |  | | | |  | | | |  |  |  |  |
| 2 | Atr-ERM99085 |  | | | |  | | | |  |  |  |  |
| 2 | Atr-ERM99086 |  | Vvi-Vitvi08g00943\_t001 |  | Vvi-Vitvi13g01389\_t001 |  |  |  |  |
| 2 | Atr-ERM99087 |  | | | |  | | | |  |  |  |  |
| 2 | Atr-ERM99088 |  | | | |  | | | |  |  |  |  |
| 2 | Atr-ERM99089 |  | | | |  | | | |  |  |  |  |
| 2 | Atr-ERM99090 |  | | | |  | | | |  |  |  |  |
| 2 | Atr-ERM99091 |  | | | |  | Vvi-Vitvi13g01387\_t001 |  |  |  |  |
| 2 | Atr-ERM99092 |  | | | |  | | | |  |  |  |  |
| 2 | Atr-ERM99093 |  | Vvi-Vitvi08g00947\_t001 |  | | | |  |  |  |  |
| 2 | Atr-ERM99094 |  | Vvi-Vitvi08g00950\_t001 |  | | | |  |  |  |  |
| 2 | Atr-ERM99095 |  | Vvi-Vitvi08g00954\_t003 |  | | | |  |  |  |  |
| 2 | Atr-ERM99096 |  | | | |  | | | |  |  |  |  |
| 2 | Atr-ERM99097 |  | | | |  | | | |  |  |  |  |
| 2 | Atr-ERM99098 |  | Vvi-Vitvi08g00955\_t001 |  | | | |  |  |  |  |
| 2 | Atr-ERM99099 |  | | | |  | | | |  |  |  |  |
| 2 | Atr-ERM99100 |  | | | |  | | | |  |  |  |  |
| 2 | Atr-ERM99101 |  | | | |  | | | |  |  |  |  |
| 2 | Atr-ERM99102 |  | | | |  | | | |  |  |  |  |
| 2 | Atr-ERM99103 |  | Vvi-Vitvi08g00958\_t001 |  | | | |  |  |  |  |
| 2 | Atr-ERM99104 |  | | | |  | | | |  |  |  |  |
| 2 | Atr-ERM99105 |  | | | |  | Vvi-Vitvi13g04451\_t001 |  |  |  |  |
| 2 | Atr-ERM99106 |  | | | |  | | | |  |  |  |  |
| 2 | Atr-ERM99107 |  | | | |  | | | |  |  |  |  |
| 2 | Atr-ERM99108 |  | | | |  | | | |  |  |  |  |
| 2 | Atr-ERM99109 |  | | | |  | | | |  |  |  |  |
| 2 | Atr-ERM99110 |  | | | |  | | | |  |  |  |  |
| 2 | Atr-ERM99111 |  | | | |  | | | |  |  |  |  |
| 2 | Atr-ERM99112 |  | Vvi-Vitvi08g00962\_t001 |  | Vvi-Vitvi13g02315\_t001 |  |  |  |  |
| 2 | Atr-ERM99113 |  | Vvi-Vitvi08g00967\_t001 |  | | | |  |  |  |  |
| 2 | Atr-ERM99114 |  | | | |  | | | |  |  |  |  |
| 3 | Atr-ERM99115 |  | | | |  | | | |  | Vvi-Vitvi06g01144\_t001 |  |  |  |
| 3 | Atr-ERM99116 |  | | | |  | | | |  | Vvi-Vitvi06g01146\_t001 |  |  |  |
| 3 | Atr-ERM99117 |  | | | |  | | | |  | Vvi-Vitvi06g01868\_t001 |  |  |  |
| 3 | Atr-ERM99118 |  | Vvi-Vitvi08g00971\_t001 |  | | | |  | Vvi-Vitvi06g01149\_t001 |  |  |  |
| 3 | Atr-ERM99119 |  | Vvi-Vitvi08g00972\_t001 |  | | | |  | Vvi-Vitvi06g01161\_t003 |  |  |  |
| 3 | Atr-ERM99120 |  | | | |  | | | |  | | | |  |  |  |
| 3 | Atr-ERM99121 |  | Vvi-Vitvi08g00977\_t001 |  | | | |  | | | |  |  |  |
| 3 | Atr-ERM99122 |  | Vvi-Vitvi08g00979\_t001 |  | | | |  | | | |  |  |  |
| 3 | Atr-ERM99123 |  | | | |  | | | |  | | | |  |  |  |
| 3 | Atr-ERM99124 |  | | | |  | | | |  | | | |  |  |  |
| 3 | Atr-ERM99125 |  | | | |  | | | |  | | | |  |  |  |
| 3 | Atr-ERM99126 |  | | | |  | | | |  | Vvi-Vitvi06g01162\_t001 |  |  |  |
| 3 | Atr-ERM99127 |  | | | |  | | | |  | Vvi-Vitvi06g01163\_t001 |  |  |  |
| 3 | Atr-ERM99128 |  | Vvi-Vitvi08g00981\_t001 |  | | | |  | | | |  |  |  |
| 3 | Atr-ERM99129 |  | | | |  | | | |  | Vvi-Vitvi06g01873\_t001 |  |  |  |
| 3 | Atr-ERM99130 |  | Vvi-Vitvi08g00982\_t001 |  | | | |  | | | |  |  |  |
| 3 | Atr-ERM99131 |  | | | |  | Vvi-Vitvi13g04465\_t001 |  | | | |  |  |  |
| 2 | Atr-ERM99132 |  | | | |  |  |  | | | |  |  |  |
| 2 | Atr-ERM99133 |  | | | |  |  |  | | | |  |  |  |
| 2 | Atr-ERM99134 |  | Vvi-Vitvi08g00983\_t001 |  |  |  | | | |  |  |  |
| 2 | Atr-ERM99135 |  | Vvi-Vitvi08g00984\_t001 |  |  |  | | | |  |  |  |
| 2 | Atr-ERM99136 |  | Vvi-Vitvi08g00986\_t001 |  |  |  | Vvi-Vitvi06g01165\_t002 |  |  |  |
| 0 | Atr-ERM99137 |  |  |  |  |  |  |
