# Supplementary material for: Genome-wide identification, characterization and gene expression of BES1 transcription factor family in grapevine (Vitis vinifera L.)
Source: Sci Rep. 2023 Jan 5;13:240. doi: 10.1038/s41598-022-24407-y (PMC9816167; doi:10.1038/s41598-022-24407-y)
Supplement: Supplementary file 3 — Supplementary Information. [file 41598_2022_24407_MOESM3_ESM.zip › Vvi_Atr/Vitis_vinifera.PN40024.v4.dna_sm.toplevel.fa.vs.Amborella_trichopoda.AMTR1.0.dna_sm.toplevel.fa.html/Atr-AmTr_v1.0_scaffold00102.html]

|  |  |  |  |  |  |  |  |  |  |  |  |  |  |
| --- | --- | --- | --- | --- | --- | --- | --- | --- | --- | --- | --- | --- | --- |
| Duplication depth | Reference chromosome | Collinear blocks | | | | | | | | | | | |
| 0 | Atr-ERN00499 |  |  |  |  |  |  |
| 0 | Atr-ERN00500 |  |  |  |  |  |  |
| 1 | Atr-ERN00501 |  | Vvi-Vitvi06g01341\_t001 |  |  |  |  |  |
| 1 | Atr-ERN00502 |  | | | |  |  |  |  |  |
| 1 | Atr-ERN00503 |  | Vvi-Vitvi06g01344\_t001 |  |  |  |  |  |
| 1 | Atr-ERN00504 |  | | | |  |  |  |  |  |
| 1 | Atr-ERN00505 |  | | | |  |  |  |  |  |
| 1 | Atr-ERN00506 |  | | | |  |  |  |  |  |
| 1 | Atr-ERN00507 |  | | | |  |  |  |  |  |
| 1 | Atr-ERN00508 |  | | | |  |  |  |  |  |
| 1 | Atr-ERN00509 |  | | | |  |  |  |  |  |
| 1 | Atr-ERN00510 |  | | | |  |  |  |  |  |
| 1 | Atr-ERN00511 |  | | | |  |  |  |  |  |
| 1 | Atr-ERN00512 |  | | | |  |  |  |  |  |
| 1 | Atr-ERN00513 |  | | | |  |  |  |  |  |
| 1 | Atr-ERN00514 |  | | | |  |  |  |  |  |
| 1 | Atr-ERN00515 |  | | | |  |  |  |  |  |
| 1 | Atr-ERN00516 |  | | | |  |  |  |  |  |
| 1 | Atr-ERN00517 |  | | | |  |  |  |  |  |
| 1 | Atr-ERN00518 |  | | | |  |  |  |  |  |
| 1 | Atr-ERN00519 |  | | | |  |  |  |  |  |
| 1 | Atr-ERN00520 |  | | | |  |  |  |  |  |
| 1 | Atr-ERN00521 |  | | | |  |  |  |  |  |
| 1 | Atr-ERN00522 |  | Vvi-Vitvi06g01345\_t001 |  |  |  |  |  |
| 1 | Atr-ERN00523 |  | | | |  |  |  |  |  |
| 1 | Atr-ERN00524 |  | | | |  |  |  |  |  |
| 1 | Atr-ERN00525 |  | Vvi-Vitvi06g01346\_t001 |  |  |  |  |  |
| 1 | Atr-ERN00526 |  | | | |  |  |  |  |  |
| 1 | Atr-ERN00527 |  | | | |  |  |  |  |  |
| 1 | Atr-ERN00528 |  | | | |  |  |  |  |  |
| 1 | Atr-ERN00529 |  | | | |  |  |  |  |  |
| 1 | Atr-ERN00530 |  | | | |  |  |  |  |  |
| 1 | Atr-ERN00531 |  | Vvi-Vitvi06g01349\_t001 |  |  |  |  |  |
| 1 | Atr-ERN00532 |  | | | |  |  |  |  |  |
| 1 | Atr-ERN00533 |  | Vvi-Vitvi06g01350\_t001 |  |  |  |  |  |
| 0 | Atr-ERN00534 |  |  |  |  |  |  |
| 0 | Atr-ERN00535 |  |  |  |  |  |  |
| 0 | Atr-ERN00536 |  |  |  |  |  |  |
| 0 | Atr-ERN00537 |  |  |  |  |  |  |
| 0 | Atr-ERN00538 |  |  |  |  |  |  |
| 0 | Atr-ERN00539 |  |  |  |  |  |  |
| 0 | Atr-ERN00540 |  |  |  |  |  |  |
| 0 | Atr-ERN00541 |  |  |  |  |  |  |
| 0 | Atr-ERN00542 |  |  |  |  |  |  |
| 0 | Atr-ERN00543 |  |  |  |  |  |  |
| 0 | Atr-ERN00544 |  |  |  |  |  |  |
| 0 | Atr-ERN00545 |  |  |  |  |  |  |
| 0 | Atr-ERN00546 |  |  |  |  |  |  |
| 0 | Atr-ERN00547 |  |  |  |  |  |  |
| 0 | Atr-ERN00548 |  |  |  |  |  |  |
| 0 | Atr-ERN00549 |  |  |  |  |  |  |
| 0 | Atr-ERN00550 |  |  |  |  |  |  |
| 0 | Atr-ERN00551 |  |  |  |  |  |  |
| 0 | Atr-ERN00552 |  |  |  |  |  |  |
| 0 | Atr-ERN00553 |  |  |  |  |  |  |
| 0 | Atr-ERN00554 |  |  |  |  |  |  |
| 0 | Atr-ERN00555 |  |  |  |  |  |  |
| 0 | Atr-ERN00556 |  |  |  |  |  |  |
| 0 | Atr-ERN00557 |  |  |  |  |  |  |
| 0 | Atr-ERN00558 |  |  |  |  |  |  |
| 0 | Atr-ERN00559 |  |  |  |  |  |  |
| 0 | Atr-ERN00560 |  |  |  |  |  |  |
| 0 | Atr-ERN00561 |  |  |  |  |  |  |
| 0 | Atr-ERN00562 |  |  |  |  |  |  |
| 0 | Atr-ERN00563 |  |  |  |  |  |  |
| 0 | Atr-ERN00564 |  |  |  |  |  |  |
| 0 | Atr-ERN00565 |  |  |  |  |  |  |
| 0 | Atr-ERN00566 |  |  |  |  |  |  |
| 0 | Atr-ERN00567 |  |  |  |  |  |  |
| 0 | Atr-ERN00568 |  |  |  |  |  |  |
| 0 | Atr-ERN00569 |  |  |  |  |  |  |
| 0 | Atr-ERN00570 |  |  |  |  |  |  |
| 0 | Atr-ERN00571 |  |  |  |  |  |  |
| 0 | Atr-ERN00572 |  |  |  |  |  |  |
| 0 | Atr-ERN00573 |  |  |  |  |  |  |
| 0 | Atr-ERN00574 |  |  |  |  |  |  |
| 0 | Atr-ERN00575 |  |  |  |  |  |  |
