# Supplementary material for: Genome-wide identification, characterization and gene expression of BES1 transcription factor family in grapevine (Vitis vinifera L.)
Source: Sci Rep. 2023 Jan 5;13:240. doi: 10.1038/s41598-022-24407-y (PMC9816167; doi:10.1038/s41598-022-24407-y)
Supplement: Supplementary file 3 — Supplementary Information. [file 41598_2022_24407_MOESM3_ESM.zip › Vvi_Atr/Vitis_vinifera.PN40024.v4.dna_sm.toplevel.fa.vs.Amborella_trichopoda.AMTR1.0.dna_sm.toplevel.fa.html/Atr-AmTr_v1.0_scaffold00103.html]

|  |  |  |  |  |  |  |  |  |  |  |  |  |  |
| --- | --- | --- | --- | --- | --- | --- | --- | --- | --- | --- | --- | --- | --- |
| Duplication depth | Reference chromosome | Collinear blocks | | | | | | | | | | | |
| 0 | Atr-ERN00799 |  |  |  |  |  |  |
| 0 | Atr-ERN00800 |  |  |  |  |  |  |
| 1 | Atr-ERN00801 |  | Vvi-Vitvi07g00122\_t001 |  |  |  |  |  |
| 2 | Atr-ERN00802 |  | Vvi-Vitvi07g00123\_t001 |  | Vvi-Vitvi05g01885\_t001 |  |  |  |  |
| 2 | Atr-ERN00803 |  | | | |  | | | |  |  |  |  |
| 2 | Atr-ERN00804 |  | | | |  | Vvi-Vitvi05g00486\_t001 |  |  |  |  |
| 2 | Atr-ERN00805 |  | Vvi-Vitvi07g00124\_t001 |  | | | |  |  |  |  |
| 2 | Atr-ERN00806 |  | Vvi-Vitvi07g02133\_t001 |  | | | |  |  |  |  |
| 2 | Atr-ERN00807 |  | | | |  | | | |  |  |  |  |
| 2 | Atr-ERN00808 |  | | | |  | | | |  |  |  |  |
| 2 | Atr-ERN00809 |  | | | |  | | | |  |  |  |  |
| 2 | Atr-ERN00810 |  | | | |  | | | |  |  |  |  |
| 2 | Atr-ERN00811 |  | | | |  | | | |  |  |  |  |
| 2 | Atr-ERN00812 |  | | | |  | Vvi-Vitvi05g00476\_t001 |  |  |  |  |
| 2 | Atr-ERN00813 |  | Vvi-Vitvi07g02134\_t001 |  | | | |  |  |  |  |
| 2 | Atr-ERN00814 |  | | | |  | | | |  |  |  |  |
| 2 | Atr-ERN00815 |  | | | |  | Vvi-Vitvi05g00475\_t001 |  |  |  |  |
| 2 | Atr-ERN00816 |  | Vvi-Vitvi07g00125\_t001 |  | Vvi-Vitvi05g00474\_t001 |  |  |  |  |
| 2 | Atr-ERN00817 |  | Vvi-Vitvi07g00126\_t001 |  | | | |  |  |  |  |
| 2 | Atr-ERN00818 |  | Vvi-Vitvi07g00127\_t001 |  | | | |  |  |  |  |
| 2 | Atr-ERN00819 |  | Vvi-Vitvi07g00128\_t001 |  | | | |  |  |  |  |
| 2 | Atr-ERN00820 |  | | | |  | | | |  |  |  |  |
| 2 | Atr-ERN00821 |  | | | |  | | | |  |  |  |  |
| 2 | Atr-ERN00822 |  | | | |  | | | |  |  |  |  |
| 2 | Atr-ERN00823 |  | | | |  | | | |  |  |  |  |
| 2 | Atr-ERN00824 |  | | | |  | | | |  |  |  |  |
| 2 | Atr-ERN00825 |  | | | |  | | | |  |  |  |  |
| 2 | Atr-ERN00826 |  | | | |  | Vvi-Vitvi05g00470\_t001 |  |  |  |  |
| 2 | Atr-ERN00827 |  | | | |  | | | |  |  |  |  |
| 2 | Atr-ERN00828 |  | | | |  | | | |  |  |  |  |
| 2 | Atr-ERN00829 |  | | | |  | | | |  |  |  |  |
| 2 | Atr-ERN00830 |  | | | |  | | | |  |  |  |  |
| 2 | Atr-ERN00831 |  | | | |  | | | |  |  |  |  |
| 2 | Atr-ERN00832 |  | Vvi-Vitvi07g00131\_t001 |  | Vvi-Vitvi05g00469\_t001 |  |  |  |  |
| 2 | Atr-ERN00833 |  | | | |  | | | |  |  |  |  |
| 2 | Atr-ERN00834 |  | Vvi-Vitvi07g00132\_t001 |  | | | |  |  |  |  |
| 2 | Atr-ERN00835 |  | | | |  | | | |  |  |  |  |
| 2 | Atr-ERN00836 |  | | | |  | | | |  |  |  |  |
| 2 | Atr-ERN00837 |  | | | |  | | | |  |  |  |  |
| 2 | Atr-ERN00838 |  | | | |  | | | |  |  |  |  |
| 2 | Atr-ERN00839 |  | | | |  | | | |  |  |  |  |
| 2 | Atr-ERN00840 |  | Vvi-Vitvi07g02136\_t001 |  | Vvi-Vitvi05g00467\_t001 |  |  |  |  |
| 1 | Atr-ERN00841 |  |  |  | | | |  |  |  |  |
| 1 | Atr-ERN00842 |  |  |  | | | |  |  |  |  |
| 1 | Atr-ERN00843 |  |  |  | | | |  |  |  |  |
| 1 | Atr-ERN00844 |  |  |  | Vvi-Vitvi05g00464\_t001 |  |  |  |  |
| 1 | Atr-ERN00845 |  | Vvi-Vitvi08g01906\_t001 |  |  |  |  |  |
| 1 | Atr-ERN00846 |  | | | |  |  |  |  |  |
| 1 | Atr-ERN00847 |  | | | |  |  |  |  |  |
| 2 | Atr-ERN00848 |  | Vvi-Vitvi08g01905\_t002 |  | Vvi-Vitvi13g01684\_t001 |  |  |  |  |
| 2 | Atr-ERN00849 |  | | | |  | | | |  |  |  |  |
| 2 | Atr-ERN00850 |  | | | |  | | | |  |  |  |  |
| 2 | Atr-ERN00851 |  | Vvi-Vitvi08g01901\_t001 |  | | | |  |  |  |  |
| 2 | Atr-ERN00852 |  | | | |  | Vvi-Vitvi13g01699\_t001 |  |  |  |  |
| 2 | Atr-ERN00853 |  | | | |  | | | |  |  |  |  |
| 2 | Atr-ERN00854 |  | | | |  | | | |  |  |  |  |
| 2 | Atr-ERN00855 |  | | | |  | | | |  |  |  |  |
| 2 | Atr-ERN00856 |  | | | |  | Vvi-Vitvi13g01700\_t001 |  |  |  |  |
| 2 | Atr-ERN00857 |  | | | |  | | | |  |  |  |  |
| 2 | Atr-ERN00858 |  | | | |  | Vvi-Vitvi13g02487\_t001 |  |  |  |  |
| 2 | Atr-ERN00859 |  | | | |  | Vvi-Vitvi13g01702\_t001 |  |  |  |  |
| 2 | Atr-ERN00860 |  | | | |  | Vvi-Vitvi13g01703\_t001 |  |  |  |  |
| 2 | Atr-ERN00861 |  | | | |  | | | |  |  |  |  |
| 2 | Atr-ERN00862 |  | | | |  | | | |  |  |  |  |
| 2 | Atr-ERN00863 |  | | | |  | | | |  |  |  |  |
| 2 | Atr-ERN00864 |  | | | |  | | | |  |  |  |  |
| 2 | Atr-ERN00865 |  | Vvi-Vitvi08g01895\_t001 |  | | | |  |  |  |  |
| 2 | Atr-ERN00866 |  | | | |  | Vvi-Vitvi13g01709\_t001 |  |  |  |  |
| 2 | Atr-ERN00867 |  | | | |  | Vvi-Vitvi13g01710\_t001 |  |  |  |  |
| 2 | Atr-ERN00868 |  | | | |  | Vvi-Vitvi13g01711\_t001 |  |  |  |  |
| 2 | Atr-ERN00869 |  | | | |  | | | |  |  |  |  |
| 2 | Atr-ERN00870 |  | | | |  | | | |  |  |  |  |
| 2 | Atr-ERN00871 |  | | | |  | | | |  |  |  |  |
| 2 | Atr-ERN00872 |  | Vvi-Vitvi08g02394\_t001 |  | Vvi-Vitvi13g02493\_t001 |  |  |  |  |
| 2 | Atr-ERN00873 |  | | | |  | | | |  |  |  |  |
| 2 | Atr-ERN00874 |  | | | |  | | | |  |  |  |  |
| 2 | Atr-ERN00875 |  | | | |  | | | |  |  |  |  |
| 2 | Atr-ERN00876 |  | | | |  | | | |  |  |  |  |
| 2 | Atr-ERN00877 |  | | | |  | | | |  |  |  |  |
| 2 | Atr-ERN00878 |  | | | |  | | | |  |  |  |  |
| 2 | Atr-ERN00879 |  | | | |  | | | |  |  |  |  |
| 2 | Atr-ERN00880 |  | | | |  | | | |  |  |  |  |
| 2 | Atr-ERN00881 |  | | | |  | | | |  |  |  |  |
| 3 | Atr-ERN00882 |  | | | |  | | | |  | Vvi-Vitvi08g01881\_t001 |  |  |  |
| 3 | Atr-ERN00883 |  | | | |  | | | |  | | | |  |  |  |
| 3 | Atr-ERN00884 |  | | | |  | | | |  | | | |  |  |  |
| 3 | Atr-ERN00885 |  | | | |  | | | |  | | | |  |  |  |
| 3 | Atr-ERN00886 |  | | | |  | | | |  | | | |  |  |  |
| 3 | Atr-ERN00887 |  | | | |  | Vvi-Vitvi13g02503\_t001 |  | | | |  |  |  |
| 3 | Atr-ERN00888 |  | | | |  | | | |  | | | |  |  |  |
| 3 | Atr-ERN00889 |  | | | |  | | | |  | | | |  |  |  |
| 3 | Atr-ERN00890 |  | | | |  | | | |  | Vvi-Vitvi08g02393\_t001 |  |  |  |
| 3 | Atr-ERN00891 |  | | | |  | | | |  | | | |  |  |  |
| 3 | Atr-ERN00892 |  | | | |  | | | |  | | | |  |  |  |
| 3 | Atr-ERN00893 |  | Vvi-Vitvi08g01887\_t001 |  | | | |  | | | |  |  |  |
| 3 | Atr-ERN00894 |  | | | |  | | | |  | | | |  |  |  |
| 3 | Atr-ERN00895 |  | | | |  | | | |  | | | |  |  |  |
| 3 | Atr-ERN00896 |  | | | |  | | | |  | | | |  |  |  |
| 3 | Atr-ERN00897 |  | | | |  | | | |  | | | |  |  |  |
| 3 | Atr-ERN00898 |  | | | |  | | | |  | | | |  |  |  |
| 3 | Atr-ERN00899 |  | Vvi-Vitvi08g01884\_t001 |  | | | |  | | | |  |  |  |
| 3 | Atr-ERN00900 |  | Vvi-Vitvi08g01883\_t002 |  | | | |  | Vvi-Vitvi08g01883\_t002 |  |  |  |
| 3 | Atr-ERN00901 |  | | | |  | | | |  | | | |  |  |  |
| 3 | Atr-ERN00902 |  | | | |  | | | |  | | | |  |  |  |
| 3 | Atr-ERN00903 |  | | | |  | | | |  | | | |  |  |  |
| 3 | Atr-ERN00904 |  | | | |  | | | |  | Vvi-Vitvi08g01888\_t001 |  |  |  |
| 3 | Atr-ERN00905 |  | | | |  | | | |  | | | |  |  |  |
| 3 | Atr-ERN00906 |  | | | |  | | | |  | | | |  |  |  |
| 3 | Atr-ERN00907 |  | | | |  | | | |  | | | |  |  |  |
| 3 | Atr-ERN00908 |  | | | |  | | | |  | | | |  |  |  |
| 3 | Atr-ERN00909 |  | | | |  | | | |  | Vvi-Vitvi08g01889\_t002 |  |  |  |
| 3 | Atr-ERN00910 |  | | | |  | | | |  | | | |  |  |  |
| 3 | Atr-ERN00911 |  | | | |  | | | |  | Vvi-Vitvi08g01890\_t003 |  |  |  |
| 2 | Atr-ERN00912 |  | | | |  | | | |  |  |  |  |
| 2 | Atr-ERN00913 |  | | | |  | Vvi-Vitvi13g02505\_t001 |  |  |  |  |
| 2 | Atr-ERN00914 |  | | | |  | | | |  |  |  |  |
| 2 | Atr-ERN00915 |  | | | |  | | | |  |  |  |  |
| 2 | Atr-ERN00916 |  | Vvi-Vitvi08g01880\_t002 |  | Vvi-Vitvi13g01732\_t001 |  |  |  |  |
| 0 | Atr-ERN00917 |  |  |  |  |  |  |
