# Supplementary material for: Genome-wide identification, characterization and gene expression of BES1 transcription factor family in grapevine (Vitis vinifera L.)
Source: Sci Rep. 2023 Jan 5;13:240. doi: 10.1038/s41598-022-24407-y (PMC9816167; doi:10.1038/s41598-022-24407-y)
Supplement: Supplementary file 3 — Supplementary Information. [file 41598_2022_24407_MOESM3_ESM.zip › Vvi_Atr/Vitis_vinifera.PN40024.v4.dna_sm.toplevel.fa.vs.Amborella_trichopoda.AMTR1.0.dna_sm.toplevel.fa.html/Atr-AmTr_v1.0_scaffold00104.html]

|  |  |  |  |  |  |  |  |  |  |  |  |  |  |
| --- | --- | --- | --- | --- | --- | --- | --- | --- | --- | --- | --- | --- | --- |
| Duplication depth | Reference chromosome | Collinear blocks | | | | | | | | | | | |
| 0 | Atr-ERN00327 |  |  |  |  |  |  |
| 0 | Atr-ERN00328 |  |  |  |  |  |  |
| 0 | Atr-ERN00329 |  |  |  |  |  |  |
| 0 | Atr-ERN00330 |  |  |  |  |  |  |
| 1 | Atr-ERN00331 |  | Vvi-Vitvi11g00461\_t001 |  |  |  |  |  |
| 1 | Atr-ERN00332 |  | | | |  |  |  |  |  |
| 1 | Atr-ERN00333 |  | | | |  |  |  |  |  |
| 1 | Atr-ERN00334 |  | | | |  |  |  |  |  |
| 1 | Atr-ERN00335 |  | | | |  |  |  |  |  |
| 1 | Atr-ERN00336 |  | | | |  |  |  |  |  |
| 2 | Atr-ERN00337 |  | | | |  | Vvi-Vitvi04g01901\_t001 |  |  |  |  |
| 2 | Atr-ERN00338 |  | | | |  | | | |  |  |  |  |
| 2 | Atr-ERN00339 |  | | | |  | | | |  |  |  |  |
| 2 | Atr-ERN00340 |  | | | |  | | | |  |  |  |  |
| 2 | Atr-ERN00341 |  | Vvi-Vitvi11g00460\_t001 |  | | | |  |  |  |  |
| 2 | Atr-ERN00342 |  | | | |  | | | |  |  |  |  |
| 2 | Atr-ERN00343 |  | | | |  | | | |  |  |  |  |
| 2 | Atr-ERN00344 |  | | | |  | | | |  |  |  |  |
| 2 | Atr-ERN00345 |  | | | |  | | | |  |  |  |  |
| 2 | Atr-ERN00346 |  | | | |  | | | |  |  |  |  |
| 2 | Atr-ERN00347 |  | | | |  | | | |  |  |  |  |
| 2 | Atr-ERN00348 |  | | | |  | | | |  |  |  |  |
| 2 | Atr-ERN00349 |  | | | |  | | | |  |  |  |  |
| 2 | Atr-ERN00350 |  | | | |  | | | |  |  |  |  |
| 2 | Atr-ERN00351 |  | | | |  | | | |  |  |  |  |
| 2 | Atr-ERN00352 |  | | | |  | | | |  |  |  |  |
| 2 | Atr-ERN00353 |  | Vvi-Vitvi11g00458\_t001 |  | | | |  |  |  |  |
| 2 | Atr-ERN00354 |  | | | |  | | | |  |  |  |  |
| 2 | Atr-ERN00355 |  | | | |  | | | |  |  |  |  |
| 2 | Atr-ERN00356 |  | | | |  | | | |  |  |  |  |
| 2 | Atr-ERN00357 |  | | | |  | | | |  |  |  |  |
| 2 | Atr-ERN00358 |  | | | |  | | | |  |  |  |  |
| 2 | Atr-ERN00359 |  | | | |  | | | |  |  |  |  |
| 2 | Atr-ERN00360 |  | | | |  | | | |  |  |  |  |
| 2 | Atr-ERN00361 |  | | | |  | | | |  |  |  |  |
| 2 | Atr-ERN00362 |  | | | |  | | | |  |  |  |  |
| 2 | Atr-ERN00363 |  | | | |  | Vvi-Vitvi04g00472\_t001 |  |  |  |  |
| 2 | Atr-ERN00364 |  | | | |  | | | |  |  |  |  |
| 2 | Atr-ERN00365 |  | | | |  | | | |  |  |  |  |
| 2 | Atr-ERN00366 |  | | | |  | | | |  |  |  |  |
| 2 | Atr-ERN00367 |  | | | |  | | | |  |  |  |  |
| 2 | Atr-ERN00368 |  | | | |  | | | |  |  |  |  |
| 2 | Atr-ERN00369 |  | Vvi-Vitvi11g00454\_t002 |  | Vvi-Vitvi04g04121\_t001 |  |  |  |  |
| 2 | Atr-ERN00370 |  | | | |  | | | |  |  |  |  |
| 2 | Atr-ERN00371 |  | | | |  | | | |  |  |  |  |
| 2 | Atr-ERN00372 |  | | | |  | | | |  |  |  |  |
| 2 | Atr-ERN00373 |  | | | |  | | | |  |  |  |  |
| 2 | Atr-ERN00374 |  | | | |  | | | |  |  |  |  |
| 2 | Atr-ERN00375 |  | | | |  | | | |  |  |  |  |
| 2 | Atr-ERN00376 |  | Vvi-Vitvi11g01432\_t001 |  | | | |  |  |  |  |
| 2 | Atr-ERN00377 |  | | | |  | | | |  |  |  |  |
| 2 | Atr-ERN00378 |  | | | |  | | | |  |  |  |  |
| 2 | Atr-ERN00379 |  | | | |  | | | |  |  |  |  |
| 2 | Atr-ERN00380 |  | | | |  | | | |  |  |  |  |
| 2 | Atr-ERN00381 |  | | | |  | | | |  |  |  |  |
| 2 | Atr-ERN00382 |  | | | |  | Vvi-Vitvi04g00469\_t002 |  |  |  |  |
| 2 | Atr-ERN00383 |  | Vvi-Vitvi11g04102\_t001 |  | Vvi-Vitvi04g00467\_t001 |  |  |  |  |
| 2 | Atr-ERN00384 |  | | | |  | | | |  |  |  |  |
| 2 | Atr-ERN00385 |  | Vvi-Vitvi11g00451\_t001 |  | Vvi-Vitvi04g00466\_t001 |  |  |  |  |
| 2 | Atr-ERN00386 |  | Vvi-Vitvi11g00450\_t001 |  | Vvi-Vitvi04g01899\_t001 |  |  |  |  |
| 1 | Atr-ERN00387 |  | | | |  |  |  |  |  |
| 1 | Atr-ERN00388 |  | | | |  |  |  |  |  |
| 1 | Atr-ERN00389 |  | | | |  |  |  |  |  |
| 1 | Atr-ERN00390 |  | | | |  |  |  |  |  |
| 1 | Atr-ERN00391 |  | | | |  |  |  |  |  |
| 1 | Atr-ERN00392 |  | Vvi-Vitvi11g00449\_t001 |  |  |  |  |  |
| 1 | Atr-ERN00393 |  | | | |  |  |  |  |  |
| 1 | Atr-ERN00394 |  | Vvi-Vitvi11g00448\_t001 |  |  |  |  |  |
| 0 | Atr-ERN00395 |  |  |  |  |  |  |
| 0 | Atr-ERN00396 |  |  |  |  |  |  |
| 0 | Atr-ERN00397 |  |  |  |  |  |  |
| 0 | Atr-ERN00398 |  |  |  |  |  |  |
| 0 | Atr-ERN00399 |  |  |  |  |  |  |
| 0 | Atr-ERN00400 |  |  |  |  |  |  |
| 0 | Atr-ERN00401 |  |  |  |  |  |  |
| 0 | Atr-ERN00402 |  |  |  |  |  |  |
| 0 | Atr-ERN00403 |  |  |  |  |  |  |
| 0 | Atr-ERN00404 |  |  |  |  |  |  |
| 0 | Atr-ERN00405 |  |  |  |  |  |  |
