# Supplementary material for: Genome-wide identification, characterization and gene expression of BES1 transcription factor family in grapevine (Vitis vinifera L.)
Source: Sci Rep. 2023 Jan 5;13:240. doi: 10.1038/s41598-022-24407-y (PMC9816167; doi:10.1038/s41598-022-24407-y)
Supplement: Supplementary file 3 — Supplementary Information. [file 41598_2022_24407_MOESM3_ESM.zip › Vvi_Atr/Vitis_vinifera.PN40024.v4.dna_sm.toplevel.fa.vs.Amborella_trichopoda.AMTR1.0.dna_sm.toplevel.fa.html/Atr-AmTr_v1.0_scaffold00105.html]

|  |  |  |  |  |  |  |  |  |  |  |  |  |  |
| --- | --- | --- | --- | --- | --- | --- | --- | --- | --- | --- | --- | --- | --- |
| Duplication depth | Reference chromosome | Collinear blocks | | | | | | | | | | | |
| 0 | Atr-ERN00011 |  |  |  |  |  |  |
| 0 | Atr-ERN00012 |  |  |  |  |  |  |
| 0 | Atr-ERN00013 |  |  |  |  |  |  |
| 0 | Atr-ERN00014 |  |  |  |  |  |  |
| 0 | Atr-ERN00015 |  |  |  |  |  |  |
| 0 | Atr-ERN00016 |  |  |  |  |  |  |
| 0 | Atr-ERN00017 |  |  |  |  |  |  |
| 0 | Atr-ERN00018 |  |  |  |  |  |  |
| 0 | Atr-ERN00019 |  |  |  |  |  |  |
| 0 | Atr-ERN00020 |  |  |  |  |  |  |
| 0 | Atr-ERN00021 |  |  |  |  |  |  |
| 0 | Atr-ERN00022 |  |  |  |  |  |  |
| 0 | Atr-ERN00023 |  |  |  |  |  |  |
| 0 | Atr-ERN00024 |  |  |  |  |  |  |
| 0 | Atr-ERN00025 |  |  |  |  |  |  |
| 0 | Atr-ERN00026 |  |  |  |  |  |  |
| 1 | Atr-ERN00027 |  | Vvi-Vitvi14g01006\_t001 |  |  |  |  |  |
| 1 | Atr-ERN00028 |  | | | |  |  |  |  |  |
| 2 | Atr-ERN00029 |  | | | |  | Vvi-Vitvi01g00392\_t001 |  |  |  |  |
| 2 | Atr-ERN00030 |  | | | |  | | | |  |  |  |  |
| 2 | Atr-ERN00031 |  | | | |  | | | |  |  |  |  |
| 2 | Atr-ERN00032 |  | | | |  | | | |  |  |  |  |
| 2 | Atr-ERN00033 |  | | | |  | | | |  |  |  |  |
| 2 | Atr-ERN00034 |  | | | |  | | | |  |  |  |  |
| 2 | Atr-ERN00035 |  | | | |  | Vvi-Vitvi01g00390\_t001 |  |  |  |  |
| 2 | Atr-ERN00036 |  | | | |  | | | |  |  |  |  |
| 2 | Atr-ERN00037 |  | Vvi-Vitvi14g01007\_t001 |  | | | |  |  |  |  |
| 2 | Atr-ERN00038 |  | Vvi-Vitvi14g01011\_t002 |  | | | |  |  |  |  |
| 2 | Atr-ERN00039 |  | | | |  | | | |  |  |  |  |
| 2 | Atr-ERN00040 |  | | | |  | | | |  |  |  |  |
| 2 | Atr-ERN00041 |  | | | |  | | | |  |  |  |  |
| 2 | Atr-ERN00042 |  | | | |  | | | |  |  |  |  |
| 2 | Atr-ERN00043 |  | | | |  | | | |  |  |  |  |
| 2 | Atr-ERN00044 |  | | | |  | | | |  |  |  |  |
| 2 | Atr-ERN00045 |  | | | |  | | | |  |  |  |  |
| 2 | Atr-ERN00046 |  | | | |  | | | |  |  |  |  |
| 2 | Atr-ERN00047 |  | | | |  | | | |  |  |  |  |
| 2 | Atr-ERN00048 |  | | | |  | | | |  |  |  |  |
| 2 | Atr-ERN00049 |  | | | |  | | | |  |  |  |  |
| 2 | Atr-ERN00050 |  | | | |  | | | |  |  |  |  |
| 2 | Atr-ERN00051 |  | | | |  | | | |  |  |  |  |
| 2 | Atr-ERN00052 |  | | | |  | | | |  |  |  |  |
| 2 | Atr-ERN00053 |  | | | |  | | | |  |  |  |  |
| 2 | Atr-ERN00054 |  | Vvi-Vitvi14g02841\_t001 |  | Vvi-Vitvi01g00388\_t001 |  |  |  |  |
| 2 | Atr-ERN00055 |  | | | |  | | | |  |  |  |  |
| 2 | Atr-ERN00056 |  | | | |  | | | |  |  |  |  |
| 2 | Atr-ERN00057 |  | | | |  | | | |  |  |  |  |
| 2 | Atr-ERN00058 |  | | | |  | | | |  |  |  |  |
| 2 | Atr-ERN00059 |  | | | |  | | | |  |  |  |  |
| 2 | Atr-ERN00060 |  | | | |  | | | |  |  |  |  |
| 2 | Atr-ERN00061 |  | | | |  | | | |  |  |  |  |
| 2 | Atr-ERN00062 |  | | | |  | | | |  |  |  |  |
| 2 | Atr-ERN00063 |  | | | |  | | | |  |  |  |  |
| 2 | Atr-ERN00064 |  | | | |  | | | |  |  |  |  |
| 2 | Atr-ERN00065 |  | | | |  | | | |  |  |  |  |
| 2 | Atr-ERN00066 |  | | | |  | | | |  |  |  |  |
| 2 | Atr-ERN00067 |  | | | |  | | | |  |  |  |  |
| 2 | Atr-ERN00068 |  | Vvi-Vitvi14g01023\_t003 |  | Vvi-Vitvi01g00386\_t001 |  |  |  |  |
| 2 | Atr-ERN00069 |  | | | |  | Vvi-Vitvi01g00385\_t001 |  |  |  |  |
| 2 | Atr-ERN00070 |  | | | |  | Vvi-Vitvi01g00384\_t001 |  |  |  |  |
| 2 | Atr-ERN00071 |  | Vvi-Vitvi14g01025\_t001 |  | | | |  |  |  |  |
| 2 | Atr-ERN00072 |  | | | |  | | | |  |  |  |  |
| 2 | Atr-ERN00073 |  | | | |  | | | |  |  |  |  |
| 2 | Atr-ERN00074 |  | | | |  | Vvi-Vitvi01g00382\_t001 |  |  |  |  |
| 2 | Atr-ERN00075 |  | Vvi-Vitvi14g02850\_t001 |  | Vvi-Vitvi01g01967\_t001 |  |  |  |  |
| 2 | Atr-ERN00076 |  | | | |  | | | |  |  |  |  |
| 2 | Atr-ERN00077 |  | | | |  | | | |  |  |  |  |
| 2 | Atr-ERN00078 |  | | | |  | | | |  |  |  |  |
| 2 | Atr-ERN00079 |  | | | |  | | | |  |  |  |  |
| 2 | Atr-ERN00080 |  | | | |  | | | |  |  |  |  |
| 2 | Atr-ERN00081 |  | | | |  | | | |  |  |  |  |
| 2 | Atr-ERN00082 |  | | | |  | | | |  |  |  |  |
| 2 | Atr-ERN00083 |  | Vvi-Vitvi14g01035\_t001 |  | Vvi-Vitvi01g00380\_t001 |  |  |  |  |
| 2 | Atr-ERN00084 |  | | | |  | | | |  |  |  |  |
| 2 | Atr-ERN00085 |  | Vvi-Vitvi14g01037\_t001 |  | Vvi-Vitvi01g00379\_t001 |  |  |  |  |
| 1 | Atr-ERN00086 |  |  |  | | | |  |  |  |  |
| 1 | Atr-ERN00087 |  |  |  | | | |  |  |  |  |
| 1 | Atr-ERN00088 |  |  |  | | | |  |  |  |  |
| 1 | Atr-ERN00089 |  |  |  | | | |  |  |  |  |
| 1 | Atr-ERN00090 |  |  |  | Vvi-Vitvi01g00378\_t001 |  |  |  |  |
