# Supplementary material for: Genome-wide identification, characterization and gene expression of BES1 transcription factor family in grapevine (Vitis vinifera L.)
Source: Sci Rep. 2023 Jan 5;13:240. doi: 10.1038/s41598-022-24407-y (PMC9816167; doi:10.1038/s41598-022-24407-y)
Supplement: Supplementary file 3 — Supplementary Information. [file 41598_2022_24407_MOESM3_ESM.zip › Vvi_Atr/Vitis_vinifera.PN40024.v4.dna_sm.toplevel.fa.vs.Amborella_trichopoda.AMTR1.0.dna_sm.toplevel.fa.html/Atr-AmTr_v1.0_scaffold00106.html]

|  |  |  |  |  |  |  |  |  |  |  |  |  |  |
| --- | --- | --- | --- | --- | --- | --- | --- | --- | --- | --- | --- | --- | --- |
| Duplication depth | Reference chromosome | Collinear blocks | | | | | | | | | | | |
| 0 | Atr-ERN00653 |  |  |  |  |  |  |
| 0 | Atr-ERN00654 |  |  |  |  |  |  |
| 0 | Atr-ERN00655 |  |  |  |  |  |  |
| 0 | Atr-ERN00656 |  |  |  |  |  |  |
| 0 | Atr-ERN00657 |  |  |  |  |  |  |
| 0 | Atr-ERN00658 |  |  |  |  |  |  |
| 0 | Atr-ERN00659 |  |  |  |  |  |  |
| 0 | Atr-ERN00660 |  |  |  |  |  |  |
| 0 | Atr-ERN00661 |  |  |  |  |  |  |
| 0 | Atr-ERN00662 |  |  |  |  |  |  |
| 0 | Atr-ERN00663 |  |  |  |  |  |  |
| 0 | Atr-ERN00664 |  |  |  |  |  |  |
| 0 | Atr-ERN00665 |  |  |  |  |  |  |
| 0 | Atr-ERN00666 |  |  |  |  |  |  |
| 0 | Atr-ERN00667 |  |  |  |  |  |  |
| 0 | Atr-ERN00668 |  |  |  |  |  |  |
| 0 | Atr-ERN00669 |  |  |  |  |  |  |
| 0 | Atr-ERN00670 |  |  |  |  |  |  |
| 0 | Atr-ERN00671 |  |  |  |  |  |  |
| 0 | Atr-ERN00672 |  |  |  |  |  |  |
| 0 | Atr-ERN00673 |  |  |  |  |  |  |
| 0 | Atr-ERN00674 |  |  |  |  |  |  |
| 0 | Atr-ERN00675 |  |  |  |  |  |  |
| 0 | Atr-ERN00676 |  |  |  |  |  |  |
| 0 | Atr-ERN00677 |  |  |  |  |  |  |
| 0 | Atr-ERN00678 |  |  |  |  |  |  |
| 0 | Atr-ERN00679 |  |  |  |  |  |  |
| 0 | Atr-ERN00680 |  |  |  |  |  |  |
| 0 | Atr-ERN00681 |  |  |  |  |  |  |
| 0 | Atr-ERN00682 |  |  |  |  |  |  |
| 0 | Atr-ERN00683 |  |  |  |  |  |  |
| 0 | Atr-ERN00684 |  |  |  |  |  |  |
| 0 | Atr-ERN00685 |  |  |  |  |  |  |
| 0 | Atr-ERN00686 |  |  |  |  |  |  |
| 0 | Atr-ERN00687 |  |  |  |  |  |  |
| 0 | Atr-ERN00688 |  |  |  |  |  |  |
| 0 | Atr-ERN00689 |  |  |  |  |  |  |
| 0 | Atr-ERN00690 |  |  |  |  |  |  |
| 0 | Atr-ERN00691 |  |  |  |  |  |  |
| 0 | Atr-ERN00692 |  |  |  |  |  |  |
| 0 | Atr-ERN00693 |  |  |  |  |  |  |
| 0 | Atr-ERN00694 |  |  |  |  |  |  |
| 0 | Atr-ERN00695 |  |  |  |  |  |  |
| 0 | Atr-ERN00696 |  |  |  |  |  |  |
| 0 | Atr-ERN00697 |  |  |  |  |  |  |
| 0 | Atr-ERN00698 |  |  |  |  |  |  |
| 0 | Atr-ERN00699 |  |  |  |  |  |  |
| 0 | Atr-ERN00700 |  |  |  |  |  |  |
| 0 | Atr-ERN00701 |  |  |  |  |  |  |
| 0 | Atr-ERN00702 |  |  |  |  |  |  |
| 0 | Atr-ERN00703 |  |  |  |  |  |  |
| 0 | Atr-ERN00704 |  |  |  |  |  |  |
| 0 | Atr-ERN00705 |  |  |  |  |  |  |
| 0 | Atr-ERN00706 |  |  |  |  |  |  |
| 0 | Atr-ERN00707 |  |  |  |  |  |  |
| 1 | Atr-ERN00708 |  | Vvi-Vitvi02g00545\_t001 |  |  |  |  |  |
| 1 | Atr-ERN00709 |  | | | |  |  |  |  |  |
| 1 | Atr-ERN00710 |  | | | |  |  |  |  |  |
| 1 | Atr-ERN00711 |  | | | |  |  |  |  |  |
| 2 | Atr-ERN00712 |  | | | |  | Vvi-Vitvi02g04165\_t001 |  |  |  |  |
| 2 | Atr-ERN00713 |  | | | |  | Vvi-Vitvi02g00554\_t001 |  |  |  |  |
| 2 | Atr-ERN00714 |  | | | |  | Vvi-Vitvi02g00553\_t001 |  |  |  |  |
| 2 | Atr-ERN00715 |  | | | |  | | | |  |  |  |  |
| 2 | Atr-ERN00716 |  | | | |  | Vvi-Vitvi02g04164\_t001 |  |  |  |  |
| 2 | Atr-ERN00717 |  | | | |  | | | |  |  |  |  |
| 2 | Atr-ERN00718 |  | | | |  | | | |  |  |  |  |
| 2 | Atr-ERN00719 |  | | | |  | | | |  |  |  |  |
| 2 | Atr-ERN00720 |  | | | |  | | | |  |  |  |  |
| 2 | Atr-ERN00721 |  | | | |  | | | |  |  |  |  |
| 2 | Atr-ERN00722 |  | | | |  | | | |  |  |  |  |
| 3 | Atr-ERN00723 |  | | | |  | | | |  | Vvi-Vitvi15g00650\_t001 |  |  |  |
| 3 | Atr-ERN00724 |  | | | |  | Vvi-Vitvi02g00551\_t001 |  | | | |  |  |  |
| 3 | Atr-ERN00725 |  | | | |  | | | |  | | | |  |  |  |
| 3 | Atr-ERN00726 |  | Vvi-Vitvi02g00549\_t001 |  | Vvi-Vitvi02g00549\_t001 |  | | | |  |  |  |
| 3 | Atr-ERN00727 |  | | | |  | | | |  | | | |  |  |  |
| 3 | Atr-ERN00728 |  | | | |  | Vvi-Vitvi02g00548\_t001 |  | | | |  |  |  |
| 3 | Atr-ERN00729 |  | | | |  | | | |  | | | |  |  |  |
| 3 | Atr-ERN00730 |  | | | |  | | | |  | | | |  |  |  |
| 3 | Atr-ERN00731 |  | | | |  | | | |  | | | |  |  |  |
| 3 | Atr-ERN00732 |  | | | |  | Vvi-Vitvi02g00546\_t001 |  | | | |  |  |  |
| 2 | Atr-ERN00733 |  | | | |  |  |  | | | |  |  |  |
| 2 | Atr-ERN00734 |  | | | |  |  |  | | | |  |  |  |
| 2 | Atr-ERN00735 |  | | | |  |  |  | | | |  |  |  |
| 2 | Atr-ERN00736 |  | | | |  |  |  | | | |  |  |  |
| 2 | Atr-ERN00737 |  | | | |  |  |  | | | |  |  |  |
| 2 | Atr-ERN00738 |  | | | |  |  |  | | | |  |  |  |
| 2 | Atr-ERN00739 |  | | | |  |  |  | | | |  |  |  |
| 2 | Atr-ERN00740 |  | Vvi-Vitvi02g04169\_t001 |  |  |  | | | |  |  |  |
| 2 | Atr-ERN00741 |  | | | |  |  |  | | | |  |  |  |
| 2 | Atr-ERN00742 |  | | | |  |  |  | | | |  |  |  |
| 2 | Atr-ERN00743 |  | | | |  |  |  | | | |  |  |  |
| 2 | Atr-ERN00744 |  | | | |  |  |  | Vvi-Vitvi15g00644\_t001 |  |  |  |
| 2 | Atr-ERN00745 |  | | | |  |  |  | | | |  |  |  |
| 2 | Atr-ERN00746 |  | | | |  |  |  | | | |  |  |  |
| 2 | Atr-ERN00747 |  | | | |  |  |  | Vvi-Vitvi15g00641\_t001 |  |  |  |
| 2 | Atr-ERN00748 |  | | | |  |  |  | Vvi-Vitvi15g00639\_t001 |  |  |  |
| 2 | Atr-ERN00749 |  | | | |  |  |  | | | |  |  |  |
| 2 | Atr-ERN00750 |  | | | |  |  |  | | | |  |  |  |
| 2 | Atr-ERN00751 |  | | | |  |  |  | | | |  |  |  |
| 2 | Atr-ERN00752 |  | | | |  |  |  | | | |  |  |  |
| 2 | Atr-ERN00753 |  | | | |  |  |  | | | |  |  |  |
| 2 | Atr-ERN00754 |  | | | |  |  |  | | | |  |  |  |
| 2 | Atr-ERN00755 |  | | | |  |  |  | | | |  |  |  |
| 2 | Atr-ERN00756 |  | | | |  |  |  | Vvi-Vitvi15g00637\_t001 |  |  |  |
| 2 | Atr-ERN00757 |  | | | |  |  |  | | | |  |  |  |
| 2 | Atr-ERN00758 |  | | | |  |  |  | Vvi-Vitvi15g01469\_t001 |  |  |  |
| 2 | Atr-ERN00759 |  | | | |  |  |  | Vvi-Vitvi15g00636\_t001 |  |  |  |
| 2 | Atr-ERN00760 |  | Vvi-Vitvi02g01706\_t006 |  |  |  | | | |  |  |  |
| 2 | Atr-ERN00761 |  | | | |  |  |  | | | |  |  |  |
| 3 | Atr-ERN00762 |  | | | |  | Vvi-Vitvi15g00614\_t001 |  | | | |  |  |  |
| 3 | Atr-ERN00763 |  | | | |  | Vvi-Vitvi15g00616\_t001 |  | | | |  |  |  |
| 3 | Atr-ERN00764 |  | | | |  | Vvi-Vitvi15g00617\_t001 |  | | | |  |  |  |
| 3 | Atr-ERN00765 |  | Vvi-Vitvi02g00564\_t001 |  | Vvi-Vitvi15g00618\_t001 |  | | | |  |  |  |
| 3 | Atr-ERN00766 |  | | | |  | Vvi-Vitvi15g00619\_t001 |  | | | |  |  |  |
| 3 | Atr-ERN00767 |  | | | |  | | | |  | | | |  |  |  |
| 3 | Atr-ERN00768 |  | | | |  | Vvi-Vitvi15g00620\_t001 |  | | | |  |  |  |
| 3 | Atr-ERN00769 |  | | | |  | Vvi-Vitvi15g00621\_t001 |  | | | |  |  |  |
| 3 | Atr-ERN00770 |  | Vvi-Vitvi02g00565\_t001 |  | | | |  | | | |  |  |  |
| 3 | Atr-ERN00771 |  | | | |  | Vvi-Vitvi15g00628\_t002 |  | Vvi-Vitvi15g00628\_t002 |  |  |  |
| 2 | Atr-ERN00772 |  | | | |  | | | |  |  |  |  |
| 2 | Atr-ERN00773 |  | | | |  | | | |  |  |  |  |
| 2 | Atr-ERN00774 |  | Vvi-Vitvi02g00566\_t001 |  | | | |  |  |  |  |
| 2 | Atr-ERN00775 |  | | | |  | | | |  |  |  |  |
| 2 | Atr-ERN00776 |  | Vvi-Vitvi02g00567\_t001 |  | | | |  |  |  |  |
| 2 | Atr-ERN00777 |  | Vvi-Vitvi02g00568\_t001 |  | Vvi-Vitvi15g00629\_t001 |  |  |  |  |
| 2 | Atr-ERN00778 |  | | | |  | | | |  |  |  |  |
| 2 | Atr-ERN00779 |  | | | |  | | | |  |  |  |  |
| 2 | Atr-ERN00780 |  | Vvi-Vitvi02g00572\_t001 |  | | | |  |  |  |  |
| 2 | Atr-ERN00781 |  | | | |  | | | |  |  |  |  |
| 2 | Atr-ERN00782 |  | Vvi-Vitvi02g04172\_t001 |  | | | |  |  |  |  |
| 2 | Atr-ERN00783 |  | Vvi-Vitvi02g00573\_t001 |  | | | |  |  |  |  |
| 1 | Atr-ERN00784 |  |  |  | Vvi-Vitvi15g00633\_t001 |  |  |  |  |
| 0 | Atr-ERN00785 |  |  |  |  |  |  |
| 0 | Atr-ERN00786 |  |  |  |  |  |  |
| 0 | Atr-ERN00787 |  |  |  |  |  |  |
