# Supplementary material for: Genome-wide identification, characterization and gene expression of BES1 transcription factor family in grapevine (Vitis vinifera L.)
Source: Sci Rep. 2023 Jan 5;13:240. doi: 10.1038/s41598-022-24407-y (PMC9816167; doi:10.1038/s41598-022-24407-y)
Supplement: Supplementary file 3 — Supplementary Information. [file 41598_2022_24407_MOESM3_ESM.zip › Vvi_Atr/Vitis_vinifera.PN40024.v4.dna_sm.toplevel.fa.vs.Amborella_trichopoda.AMTR1.0.dna_sm.toplevel.fa.html/Atr-AmTr_v1.0_scaffold00107.html]

|  |  |  |  |  |  |  |  |  |  |  |  |  |  |
| --- | --- | --- | --- | --- | --- | --- | --- | --- | --- | --- | --- | --- | --- |
| Duplication depth | Reference chromosome | Collinear blocks | | | | | | | | | | | |
| 0 | Atr-ERN00275 |  |  |  |  |  |  |
| 0 | Atr-ERN00276 |  |  |  |  |  |  |
| 0 | Atr-ERN00277 |  |  |  |  |  |  |
| 0 | Atr-ERN00278 |  |  |  |  |  |  |
| 0 | Atr-ERN00279 |  |  |  |  |  |  |
| 0 | Atr-ERN00280 |  |  |  |  |  |  |
| 0 | Atr-ERN00281 |  |  |  |  |  |  |
| 0 | Atr-ERN00282 |  |  |  |  |  |  |
| 0 | Atr-ERN00283 |  |  |  |  |  |  |
| 0 | Atr-ERN00284 |  |  |  |  |  |  |
| 0 | Atr-ERN00285 |  |  |  |  |  |  |
| 0 | Atr-ERN00286 |  |  |  |  |  |  |
| 0 | Atr-ERN00287 |  |  |  |  |  |  |
| 0 | Atr-ERN00288 |  |  |  |  |  |  |
| 0 | Atr-ERN00289 |  |  |  |  |  |  |
| 0 | Atr-ERN00290 |  |  |  |  |  |  |
| 0 | Atr-ERN00291 |  |  |  |  |  |  |
| 0 | Atr-ERN00292 |  |  |  |  |  |  |
| 0 | Atr-ERN00293 |  |  |  |  |  |  |
| 0 | Atr-ERN00294 |  |  |  |  |  |  |
| 0 | Atr-ERN00295 |  |  |  |  |  |  |
| 0 | Atr-ERN00296 |  |  |  |  |  |  |
| 0 | Atr-ERN00297 |  |  |  |  |  |  |
| 0 | Atr-ERN00298 |  |  |  |  |  |  |
| 0 | Atr-ERN00299 |  |  |  |  |  |  |
| 0 | Atr-ERN00300 |  |  |  |  |  |  |
| 0 | Atr-ERN00301 |  |  |  |  |  |  |
| 0 | Atr-ERN00302 |  |  |  |  |  |  |
| 0 | Atr-ERN00303 |  |  |  |  |  |  |
| 0 | Atr-ERN00304 |  |  |  |  |  |  |
| 0 | Atr-ERN00305 |  |  |  |  |  |  |
| 0 | Atr-ERN00306 |  |  |  |  |  |  |
| 0 | Atr-ERN00307 |  |  |  |  |  |  |
| 0 | Atr-ERN00308 |  |  |  |  |  |  |
| 0 | Atr-ERN00309 |  |  |  |  |  |  |
| 0 | Atr-ERN00310 |  |  |  |  |  |  |
| 0 | Atr-ERN00311 |  |  |  |  |  |  |
| 0 | Atr-ERN00312 |  |  |  |  |  |  |
| 0 | Atr-ERN00313 |  |  |  |  |  |  |
| 0 | Atr-ERN00314 |  |  |  |  |  |  |
| 0 | Atr-ERN00315 |  |  |  |  |  |  |
| 0 | Atr-ERN00316 |  |  |  |  |  |  |
| 0 | Atr-ERN00317 |  |  |  |  |  |  |
| 0 | Atr-ERN00318 |  |  |  |  |  |  |
| 0 | Atr-ERN00319 |  |  |  |  |  |  |
| 0 | Atr-ERN00320 |  |  |  |  |  |  |
| 0 | Atr-ERN00321 |  |  |  |  |  |  |
| 0 | Atr-ERN00322 |  |  |  |  |  |  |
| 0 | Atr-ERN00323 |  |  |  |  |  |  |
| 0 | Atr-ERN00324 |  |  |  |  |  |  |
