# Supplementary material for: Genome-wide identification, characterization and gene expression of BES1 transcription factor family in grapevine (Vitis vinifera L.)
Source: Sci Rep. 2023 Jan 5;13:240. doi: 10.1038/s41598-022-24407-y (PMC9816167; doi:10.1038/s41598-022-24407-y)
Supplement: Supplementary file 3 — Supplementary Information. [file 41598_2022_24407_MOESM3_ESM.zip › Vvi_Atr/Vitis_vinifera.PN40024.v4.dna_sm.toplevel.fa.vs.Amborella_trichopoda.AMTR1.0.dna_sm.toplevel.fa.html/Atr-AmTr_v1.0_scaffold00108.html]

|  |  |  |  |  |  |  |  |  |  |  |  |  |  |
| --- | --- | --- | --- | --- | --- | --- | --- | --- | --- | --- | --- | --- | --- |
| Duplication depth | Reference chromosome | Collinear blocks | | | | | | | | | | | |
| 0 | Atr-ERM99316 |  |  |  |  |  |  |
| 0 | Atr-ERM99317 |  |  |  |  |  |  |
| 0 | Atr-ERM99318 |  |  |  |  |  |  |
| 0 | Atr-ERM99319 |  |  |  |  |  |  |
| 0 | Atr-ERM99320 |  |  |  |  |  |  |
| 0 | Atr-ERM99321 |  |  |  |  |  |  |
| 0 | Atr-ERM99322 |  |  |  |  |  |  |
| 0 | Atr-ERM99323 |  |  |  |  |  |  |
| 0 | Atr-ERM99324 |  |  |  |  |  |  |
| 0 | Atr-ERM99325 |  |  |  |  |  |  |
| 0 | Atr-ERM99326 |  |  |  |  |  |  |
| 0 | Atr-ERM99327 |  |  |  |  |  |  |
| 0 | Atr-ERM99328 |  |  |  |  |  |  |
| 0 | Atr-ERM99329 |  |  |  |  |  |  |
| 0 | Atr-ERM99330 |  |  |  |  |  |  |
| 0 | Atr-ERM99331 |  |  |  |  |  |  |
| 0 | Atr-ERM99332 |  |  |  |  |  |  |
| 0 | Atr-ERM99333 |  |  |  |  |  |  |
| 0 | Atr-ERM99334 |  |  |  |  |  |  |
| 0 | Atr-ERM99335 |  |  |  |  |  |  |
| 0 | Atr-ERM99336 |  |  |  |  |  |  |
| 0 | Atr-ERM99337 |  |  |  |  |  |  |
| 0 | Atr-ERM99338 |  |  |  |  |  |  |
| 0 | Atr-ERM99339 |  |  |  |  |  |  |
| 0 | Atr-ERM99340 |  |  |  |  |  |  |
| 0 | Atr-ERM99341 |  |  |  |  |  |  |
| 0 | Atr-ERM99342 |  |  |  |  |  |  |
| 0 | Atr-ERM99343 |  |  |  |  |  |  |
| 0 | Atr-ERM99344 |  |  |  |  |  |  |
| 0 | Atr-ERM99345 |  |  |  |  |  |  |
| 0 | Atr-ERM99346 |  |  |  |  |  |  |
| 0 | Atr-ERM99347 |  |  |  |  |  |  |
| 0 | Atr-ERM99348 |  |  |  |  |  |  |
| 0 | Atr-ERM99349 |  |  |  |  |  |  |
| 0 | Atr-ERM99350 |  |  |  |  |  |  |
| 0 | Atr-ERM99351 |  |  |  |  |  |  |
| 0 | Atr-ERM99352 |  |  |  |  |  |  |
| 0 | Atr-ERM99353 |  |  |  |  |  |  |
| 0 | Atr-ERM99354 |  |  |  |  |  |  |
| 0 | Atr-ERM99355 |  |  |  |  |  |  |
| 0 | Atr-ERM99356 |  |  |  |  |  |  |
| 0 | Atr-ERM99357 |  |  |  |  |  |  |
| 0 | Atr-ERM99358 |  |  |  |  |  |  |
| 0 | Atr-ERM99359 |  |  |  |  |  |  |
| 0 | Atr-ERM99360 |  |  |  |  |  |  |
| 0 | Atr-ERM99361 |  |  |  |  |  |  |
| 0 | Atr-ERM99362 |  |  |  |  |  |  |
| 0 | Atr-ERM99363 |  |  |  |  |  |  |
| 0 | Atr-ERM99364 |  |  |  |  |  |  |
| 0 | Atr-ERM99365 |  |  |  |  |  |  |
| 0 | Atr-ERM99366 |  |  |  |  |  |  |
