# Supplementary material for: Genome-wide identification, characterization and gene expression of BES1 transcription factor family in grapevine (Vitis vinifera L.)
Source: Sci Rep. 2023 Jan 5;13:240. doi: 10.1038/s41598-022-24407-y (PMC9816167; doi:10.1038/s41598-022-24407-y)
Supplement: Supplementary file 3 — Supplementary Information. [file 41598_2022_24407_MOESM3_ESM.zip › Vvi_Atr/Vitis_vinifera.PN40024.v4.dna_sm.toplevel.fa.vs.Amborella_trichopoda.AMTR1.0.dna_sm.toplevel.fa.html/Atr-AmTr_v1.0_scaffold00109.html]

|  |  |  |  |  |  |  |  |  |  |  |  |  |  |
| --- | --- | --- | --- | --- | --- | --- | --- | --- | --- | --- | --- | --- | --- |
| Duplication depth | Reference chromosome | Collinear blocks | | | | | | | | | | | |
| 0 | Atr-ERM98552 |  |  |  |  |  |  |
| 0 | Atr-ERM98553 |  |  |  |  |  |  |
| 0 | Atr-ERM98554 |  |  |  |  |  |  |
| 0 | Atr-ERM98555 |  |  |  |  |  |  |
| 2 | Atr-ERM98556 |  | Vvi-Vitvi04g00341\_t001 |  | Vvi-Vitvi11g00340\_t001 |  |  |  |  |
| 2 | Atr-ERM98557 |  | | | |  | Vvi-Vitvi11g01403\_t001 |  |  |  |  |
| 2 | Atr-ERM98558 |  | | | |  | Vvi-Vitvi11g00337\_t001 |  |  |  |  |
| 2 | Atr-ERM98559 |  | | | |  | | | |  |  |  |  |
| 2 | Atr-ERM98560 |  | | | |  | | | |  |  |  |  |
| 2 | Atr-ERM98561 |  | | | |  | | | |  |  |  |  |
| 2 | Atr-ERM98562 |  | | | |  | | | |  |  |  |  |
| 2 | Atr-ERM98563 |  | | | |  | | | |  |  |  |  |
| 2 | Atr-ERM98564 |  | | | |  | Vvi-Vitvi11g00336\_t001 |  |  |  |  |
| 2 | Atr-ERM98565 |  | | | |  | | | |  |  |  |  |
| 2 | Atr-ERM98566 |  | Vvi-Vitvi04g01858\_t001 |  | | | |  |  |  |  |
| 2 | Atr-ERM98567 |  | Vvi-Vitvi04g00339\_t001 |  | | | |  |  |  |  |
| 2 | Atr-ERM98568 |  | | | |  | Vvi-Vitvi11g00335\_t001 |  |  |  |  |
| 2 | Atr-ERM98569 |  | | | |  | | | |  |  |  |  |
| 2 | Atr-ERM98570 |  | | | |  | | | |  |  |  |  |
| 2 | Atr-ERM98571 |  | | | |  | | | |  |  |  |  |
| 2 | Atr-ERM98572 |  | | | |  | | | |  |  |  |  |
| 2 | Atr-ERM98573 |  | Vvi-Vitvi04g00338\_t001 |  | | | |  |  |  |  |
| 2 | Atr-ERM98574 |  | | | |  | | | |  |  |  |  |
| 2 | Atr-ERM98575 |  | | | |  | Vvi-Vitvi11g00334\_t001 |  |  |  |  |
| 2 | Atr-ERM98576 |  | | | |  | Vvi-Vitvi11g00333\_t001 |  |  |  |  |
| 3 | Atr-ERM98577 |  | | | |  | Vvi-Vitvi11g00332\_t001 |  | Vvi-Vitvi09g00347\_t001 |  |  |  |
| 3 | Atr-ERM98578 |  | | | |  | | | |  | Vvi-Vitvi09g00348\_t001 |  |  |  |
| 3 | Atr-ERM98579 |  | | | |  | Vvi-Vitvi11g00331\_t001 |  | | | |  |  |  |
| 3 | Atr-ERM98580 |  | | | |  | Vvi-Vitvi11g00330\_t001 |  | | | |  |  |  |
| 3 | Atr-ERM98581 |  | | | |  | | | |  | | | |  |  |  |
| 3 | Atr-ERM98582 |  | Vvi-Vitvi04g00337\_t001 |  | Vvi-Vitvi11g00329\_t001 |  | | | |  |  |  |
| 3 | Atr-ERM98583 |  | Vvi-Vitvi04g00336\_t001 |  | Vvi-Vitvi11g00328\_t001 |  | | | |  |  |  |
| 3 | Atr-ERM98584 |  | Vvi-Vitvi04g00335\_t001 |  | | | |  | | | |  |  |  |
| 3 | Atr-ERM98585 |  | Vvi-Vitvi04g00334\_t001 |  | | | |  | Vvi-Vitvi09g00350\_t001 |  |  |  |
| 3 | Atr-ERM98586 |  | | | |  | | | |  | | | |  |  |  |
| 3 | Atr-ERM98587 |  | | | |  | Vvi-Vitvi11g00327\_t001 |  | | | |  |  |  |
| 3 | Atr-ERM98588 |  | | | |  | | | |  | Vvi-Vitvi09g00351\_t001 |  |  |  |
| 3 | Atr-ERM98589 |  | | | |  | | | |  | | | |  |  |  |
| 3 | Atr-ERM98590 |  | | | |  | Vvi-Vitvi11g00326\_t001 |  | Vvi-Vitvi09g00352\_t001 |  |  |  |
| 3 | Atr-ERM98591 |  | | | |  | | | |  | | | |  |  |  |
| 3 | Atr-ERM98592 |  | Vvi-Vitvi04g00333\_t001 |  | | | |  | | | |  |  |  |
| 3 | Atr-ERM98593 |  | Vvi-Vitvi04g00332\_t001 |  | | | |  | | | |  |  |  |
| 3 | Atr-ERM98594 |  | | | |  | Vvi-Vitvi11g00322\_t001 |  | Vvi-Vitvi09g00355\_t001 |  |  |  |
| 3 | Atr-ERM98595 |  | | | |  | | | |  | Vvi-Vitvi09g00356\_t001 |  |  |  |
| 3 | Atr-ERM98596 |  | | | |  | | | |  | | | |  |  |  |
| 3 | Atr-ERM98597 |  | | | |  | Vvi-Vitvi11g00321\_t001 |  | | | |  |  |  |
| 3 | Atr-ERM98598 |  | Vvi-Vitvi04g01855\_t001 |  | Vvi-Vitvi11g00320\_t001 |  | | | |  |  |  |
| 3 | Atr-ERM98599 |  | | | |  | | | |  | | | |  |  |  |
| 3 | Atr-ERM98600 |  | | | |  | | | |  | | | |  |  |  |
| 3 | Atr-ERM98601 |  | | | |  | Vvi-Vitvi11g00319\_t001 |  | Vvi-Vitvi09g00359\_t001 |  |  |  |
| 3 | Atr-ERM98602 |  | | | |  | | | |  | | | |  |  |  |
| 3 | Atr-ERM98603 |  | | | |  | | | |  | Vvi-Vitvi09g00361\_t001 |  |  |  |
| 3 | Atr-ERM98604 |  | Vvi-Vitvi04g00328\_t001 |  | Vvi-Vitvi11g00318\_t001 |  | | | |  |  |  |
| 3 | Atr-ERM98605 |  | | | |  | | | |  | | | |  |  |  |
| 3 | Atr-ERM98606 |  | Vvi-Vitvi04g00327\_t001 |  | | | |  | | | |  |  |  |
| 3 | Atr-ERM98607 |  | | | |  | | | |  | Vvi-Vitvi09g00363\_t001 |  |  |  |
| 3 | Atr-ERM98608 |  | | | |  | | | |  | | | |  |  |  |
| 3 | Atr-ERM98609 |  | | | |  | Vvi-Vitvi11g00317\_t001 |  | | | |  |  |  |
| 3 | Atr-ERM98610 |  | Vvi-Vitvi04g00326\_t001 |  | | | |  | | | |  |  |  |
| 3 | Atr-ERM98611 |  | Vvi-Vitvi04g00325\_t001 |  | | | |  | | | |  |  |  |
| 3 | Atr-ERM98612 |  | Vvi-Vitvi04g00324\_t001 |  | | | |  | | | |  |  |  |
| 3 | Atr-ERM98613 |  | | | |  | Vvi-Vitvi11g00316\_t001 |  | | | |  |  |  |
| 3 | Atr-ERM98614 |  | | | |  | | | |  | | | |  |  |  |
| 3 | Atr-ERM98615 |  | | | |  | | | |  | | | |  |  |  |
| 3 | Atr-ERM98616 |  | | | |  | | | |  | | | |  |  |  |
| 3 | Atr-ERM98617 |  | | | |  | Vvi-Vitvi11g00315\_t001 |  | | | |  |  |  |
| 3 | Atr-ERM98618 |  | | | |  | | | |  | | | |  |  |  |
| 3 | Atr-ERM98619 |  | | | |  | | | |  | | | |  |  |  |
| 3 | Atr-ERM98620 |  | Vvi-Vitvi04g00321\_t001 |  | | | |  | | | |  |  |  |
| 3 | Atr-ERM98621 |  | Vvi-Vitvi04g00320\_t001 |  | Vvi-Vitvi11g00314\_t001 |  | | | |  |  |  |
| 3 | Atr-ERM98622 |  | | | |  | | | |  | | | |  |  |  |
| 3 | Atr-ERM98623 |  | | | |  | | | |  | | | |  |  |  |
| 3 | Atr-ERM98624 |  | | | |  | | | |  | | | |  |  |  |
| 3 | Atr-ERM98625 |  | | | |  | Vvi-Vitvi11g00312\_t001 |  | | | |  |  |  |
| 3 | Atr-ERM98626 |  | | | |  | | | |  | | | |  |  |  |
| 3 | Atr-ERM98627 |  | | | |  | Vvi-Vitvi11g01400\_t001 |  | Vvi-Vitvi09g00366\_t001 |  |  |  |
| 2 | Atr-ERM98628 |  | Vvi-Vitvi04g04072\_t001 |  | | | |  |  |  |  |
| 2 | Atr-ERM98629 |  | | | |  | Vvi-Vitvi11g00310\_t001 |  |  |  |  |
| 2 | Atr-ERM98630 |  | | | |  | | | |  |  |  |  |
| 3 | Atr-ERM98631 |  | | | |  | | | |  | Vvi-Vitvi09g00365\_t001 |  |  |  |
| 3 | Atr-ERM98632 |  | | | |  | | | |  | | | |  |  |  |
| 3 | Atr-ERM98633 |  | | | |  | | | |  | Vvi-Vitvi09g00345\_t001 |  |  |  |
| 3 | Atr-ERM98634 |  | | | |  | | | |  | | | |  |  |  |
| 3 | Atr-ERM98635 |  | | | |  | Vvi-Vitvi11g00309\_t001 |  | | | |  |  |  |
| 3 | Atr-ERM98636 |  | | | |  | | | |  | Vvi-Vitvi09g00342\_t001 |  |  |  |
| 3 | Atr-ERM98637 |  | | | |  | | | |  | | | |  |  |  |
| 3 | Atr-ERM98638 |  | | | |  | | | |  | | | |  |  |  |
| 3 | Atr-ERM98639 |  | | | |  | | | |  | | | |  |  |  |
| 3 | Atr-ERM98640 |  | | | |  | | | |  | | | |  |  |  |
| 3 | Atr-ERM98641 |  | | | |  | Vvi-Vitvi11g00308\_t001 |  | | | |  |  |  |
| 3 | Atr-ERM98642 |  | | | |  | | | |  | | | |  |  |  |
| 3 | Atr-ERM98643 |  | | | |  | | | |  | Vvi-Vitvi09g01597\_t001 |  |  |  |
| 3 | Atr-ERM98644 |  | | | |  | | | |  | | | |  |  |  |
| 3 | Atr-ERM98645 |  | Vvi-Vitvi04g00310\_t001 |  | Vvi-Vitvi11g00306\_t001 |  | Vvi-Vitvi09g00340\_t001 |  |  |  |
| 2 | Atr-ERM98646 |  |  |  | | | |  | | | |  |  |  |
| 2 | Atr-ERM98647 |  |  |  | | | |  | | | |  |  |  |
| 2 | Atr-ERM98648 |  |  |  | Vvi-Vitvi11g00305\_t001 |  | | | |  |  |  |
| 2 | Atr-ERM98649 |  |  |  | Vvi-Vitvi11g00304\_t001 |  | | | |  |  |  |
| 2 | Atr-ERM98650 |  |  |  | | | |  | | | |  |  |  |
| 2 | Atr-ERM98651 |  |  |  | | | |  | | | |  |  |  |
| 2 | Atr-ERM98652 |  |  |  | | | |  | | | |  |  |  |
| 2 | Atr-ERM98653 |  |  |  | | | |  | | | |  |  |  |
| 2 | Atr-ERM98654 |  |  |  | | | |  | | | |  |  |  |
| 2 | Atr-ERM98655 |  |  |  | | | |  | | | |  |  |  |
| 2 | Atr-ERM98656 |  |  |  | | | |  | | | |  |  |  |
| 2 | Atr-ERM98657 |  |  |  | | | |  | | | |  |  |  |
| 2 | Atr-ERM98658 |  |  |  | | | |  | | | |  |  |  |
| 2 | Atr-ERM98659 |  |  |  | | | |  | | | |  |  |  |
| 2 | Atr-ERM98660 |  |  |  | | | |  | | | |  |  |  |
| 2 | Atr-ERM98661 |  |  |  | | | |  | | | |  |  |  |
| 2 | Atr-ERM98662 |  |  |  | | | |  | | | |  |  |  |
| 2 | Atr-ERM98663 |  |  |  | Vvi-Vitvi11g00303\_t001 |  | Vvi-Vitvi09g00339\_t001 |  |  |  |
| 2 | Atr-ERM98664 |  |  |  | | | |  | | | |  |  |  |
| 2 | Atr-ERM98665 |  |  |  | | | |  | | | |  |  |  |
| 2 | Atr-ERM98666 |  |  |  | Vvi-Vitvi11g00302\_t001 |  | | | |  |  |  |
| 2 | Atr-ERM98667 |  |  |  | | | |  | Vvi-Vitvi09g00338\_t001 |  |  |  |
| 2 | Atr-ERM98668 |  |  |  | | | |  | | | |  |  |  |
| 2 | Atr-ERM98669 |  |  |  | | | |  | | | |  |  |  |
| 2 | Atr-ERM98670 |  |  |  | | | |  | | | |  |  |  |
| 2 | Atr-ERM98671 |  |  |  | Vvi-Vitvi11g00301\_t001 |  | Vvi-Vitvi09g00336\_t002 |  |  |  |
| 3 | Atr-ERM98672 |  | Vvi-Vitvi04g00316\_t001 |  | | | |  | | | |  |  |  |
| 3 | Atr-ERM98673 |  | Vvi-Vitvi04g00314\_t001 |  | | | |  | | | |  |  |  |
| 3 | Atr-ERM98674 |  | | | |  | | | |  | | | |  |  |  |
| 3 | Atr-ERM98675 |  | | | |  | | | |  | | | |  |  |  |
| 3 | Atr-ERM98676 |  | | | |  | | | |  | | | |  |  |  |
| 3 | Atr-ERM98677 |  | | | |  | | | |  | | | |  |  |  |
| 3 | Atr-ERM98678 |  | | | |  | | | |  | | | |  |  |  |
| 3 | Atr-ERM98679 |  | | | |  | | | |  | | | |  |  |  |
| 3 | Atr-ERM98680 |  | | | |  | | | |  | | | |  |  |  |
| 3 | Atr-ERM98681 |  | | | |  | | | |  | | | |  |  |  |
| 3 | Atr-ERM98682 |  | | | |  | | | |  | | | |  |  |  |
| 3 | Atr-ERM98683 |  | | | |  | | | |  | | | |  |  |  |
| 3 | Atr-ERM98684 |  | | | |  | | | |  | Vvi-Vitvi09g00335\_t001 |  |  |  |
| 3 | Atr-ERM98685 |  | | | |  | Vvi-Vitvi11g00299\_t001 |  | | | |  |  |  |
| 3 | Atr-ERM98686 |  | | | |  | Vvi-Vitvi11g00298\_t001 |  | | | |  |  |  |
| 3 | Atr-ERM98687 |  | Vvi-Vitvi04g00309\_t001 |  | Vvi-Vitvi11g00297\_t001 |  | | | |  |  |  |
| 3 | Atr-ERM98688 |  | | | |  | Vvi-Vitvi11g01398\_t001 |  | | | |  |  |  |
| 3 | Atr-ERM98689 |  | | | |  | Vvi-Vitvi11g01397\_t001 |  | | | |  |  |  |
| 3 | Atr-ERM98690 |  | | | |  | Vvi-Vitvi11g01396\_t001 |  | | | |  |  |  |
| 3 | Atr-ERM98691 |  | | | |  | | | |  | | | |  |  |  |
| 3 | Atr-ERM98692 |  | | | |  | | | |  | Vvi-Vitvi09g00334\_t001 |  |  |  |
| 3 | Atr-ERM98693 |  | | | |  | | | |  | | | |  |  |  |
| 3 | Atr-ERM98694 |  | | | |  | | | |  | | | |  |  |  |
| 3 | Atr-ERM98695 |  | | | |  | | | |  | | | |  |  |  |
| 3 | Atr-ERM98696 |  | | | |  | | | |  | | | |  |  |  |
| 3 | Atr-ERM98697 |  | | | |  | | | |  | | | |  |  |  |
| 3 | Atr-ERM98698 |  | | | |  | Vvi-Vitvi11g00296\_t001 |  | | | |  |  |  |
| 3 | Atr-ERM98699 |  | | | |  | | | |  | | | |  |  |  |
| 3 | Atr-ERM98700 |  | Vvi-Vitvi04g00308\_t001 |  | | | |  | | | |  |  |  |
| 3 | Atr-ERM98701 |  | Vvi-Vitvi04g01849\_t001 |  | Vvi-Vitvi11g00295\_t001 |  | | | |  |  |  |
| 3 | Atr-ERM98702 |  | | | |  | | | |  | | | |  |  |  |
| 3 | Atr-ERM98703 |  | | | |  | | | |  | Vvi-Vitvi09g00333\_t001 |  |  |  |
| 3 | Atr-ERM98704 |  | | | |  | | | |  | | | |  |  |  |
| 3 | Atr-ERM98705 |  | | | |  | | | |  | | | |  |  |  |
| 3 | Atr-ERM98706 |  | | | |  | Vvi-Vitvi11g00294\_t001 |  | | | |  |  |  |
| 2 | Atr-ERM98707 |  | Vvi-Vitvi04g00307\_t001 |  |  |  | Vvi-Vitvi09g00329\_t001 |  |  |  |
| 2 | Atr-ERM98708 |  | | | |  |  |  | | | |  |  |  |
| 2 | Atr-ERM98709 |  | Vvi-Vitvi04g04066\_t001 |  |  |  | | | |  |  |  |
| 1 | Atr-ERM98710 |  |  |  |  |  | Vvi-Vitvi09g00328\_t001 |  |  |  |
| 0 | Atr-ERM98711 |  |  |  |  |  |  |
| 0 | Atr-ERM98712 |  |  |  |  |  |  |
| 0 | Atr-ERM98713 |  |  |  |  |  |  |
| 0 | Atr-ERM98714 |  |  |  |  |  |  |
| 0 | Atr-ERM98715 |  |  |  |  |  |  |
| 0 | Atr-ERM98716 |  |  |  |  |  |  |
| 0 | Atr-ERM98717 |  |  |  |  |  |  |
| 0 | Atr-ERM98718 |  |  |  |  |  |  |
| 0 | Atr-ERM98719 |  |  |  |  |  |  |
| 0 | Atr-ERM98720 |  |  |  |  |  |  |
