# Supplementary material for: Genome-wide identification, characterization and gene expression of BES1 transcription factor family in grapevine (Vitis vinifera L.)
Source: Sci Rep. 2023 Jan 5;13:240. doi: 10.1038/s41598-022-24407-y (PMC9816167; doi:10.1038/s41598-022-24407-y)
Supplement: Supplementary file 3 — Supplementary Information. [file 41598_2022_24407_MOESM3_ESM.zip › Vvi_Atr/Vitis_vinifera.PN40024.v4.dna_sm.toplevel.fa.vs.Amborella_trichopoda.AMTR1.0.dna_sm.toplevel.fa.html/Atr-AmTr_v1.0_scaffold00110.html]

|  |  |  |  |  |  |  |  |  |  |  |  |  |  |
| --- | --- | --- | --- | --- | --- | --- | --- | --- | --- | --- | --- | --- | --- |
| Duplication depth | Reference chromosome | Collinear blocks | | | | | | | | | | | |
| 0 | Atr-ERM99877 |  |  |  |  |  |  |
| 0 | Atr-ERM99878 |  |  |  |  |  |  |
| 0 | Atr-ERM99879 |  |  |  |  |  |  |
| 0 | Atr-ERM99880 |  |  |  |  |  |  |
| 0 | Atr-ERM99881 |  |  |  |  |  |  |
| 0 | Atr-ERM99882 |  |  |  |  |  |  |
| 0 | Atr-ERM99883 |  |  |  |  |  |  |
| 1 | Atr-ERM99884 |  | Vvi-Vitvi17g00678\_t001 |  |  |  |  |  |
| 1 | Atr-ERM99885 |  | | | |  |  |  |  |  |
| 2 | Atr-ERM99886 |  | | | |  | Vvi-Vitvi14g02023\_t003 |  |  |  |  |
| 3 | Atr-ERM99887 |  | Vvi-Vitvi17g00677\_t001 |  | | | |  | Vvi-Vitvi01g01845\_t001 |  |  |  |
| 3 | Atr-ERM99888 |  | Vvi-Vitvi17g00675\_t001 |  | | | |  | | | |  |  |  |
| 3 | Atr-ERM99889 |  | | | |  | Vvi-Vitvi14g02024\_t002 |  | | | |  |  |  |
| 3 | Atr-ERM99890 |  | | | |  | Vvi-Vitvi14g02025\_t001.1.6037826b |  | | | |  |  |  |
| 3 | Atr-ERM99891 |  | | | |  | | | |  | | | |  |  |  |
| 3 | Atr-ERM99892 |  | Vvi-Vitvi17g00674\_t001 |  | | | |  | | | |  |  |  |
| 3 | Atr-ERM99893 |  | | | |  | Vvi-Vitvi14g02026\_t001 |  | | | |  |  |  |
| 3 | Atr-ERM99894 |  | Vvi-Vitvi17g00673\_t001 |  | | | |  | Vvi-Vitvi01g00073\_t001 |  |  |  |
| 3 | Atr-ERM99895 |  | | | |  | | | |  | | | |  |  |  |
| 3 | Atr-ERM99896 |  | | | |  | Vvi-Vitvi14g02027\_t001 |  | | | |  |  |  |
| 3 | Atr-ERM99897 |  | | | |  | Vvi-Vitvi14g02028\_t001 |  | | | |  |  |  |
| 4 | Atr-ERM99898 |  | | | |  | | | |  | | | |  | Vvi-Vitvi01g00056\_t001 |  |  |
| 4 | Atr-ERM99899 |  | Vvi-Vitvi17g04190\_t001 |  | | | |  | | | |  | | | |  |  |
| 4 | Atr-ERM99900 |  | Vvi-Vitvi14g02046\_t001 |  | | | |  | | | |  | | | |  |  |
| 4 | Atr-ERM99901 |  | | | |  | | | |  | | | |  | | | |  |  |
| 4 | Atr-ERM99902 |  | | | |  | | | |  | | | |  | | | |  |  |
| 4 | Atr-ERM99903 |  | | | |  | | | |  | | | |  | | | |  |  |
| 4 | Atr-ERM99904 |  | | | |  | | | |  | | | |  | | | |  |  |
| 4 | Atr-ERM99905 |  | Vvi-Vitvi14g02043\_t001 |  | | | |  | | | |  | | | |  |  |
| 4 | Atr-ERM99906 |  | | | |  | Vvi-Vitvi14g02042\_t001 |  | Vvi-Vitvi01g00065\_t001 |  | | | |  |  |
| 3 | Atr-ERM99907 |  | | | |  |  |  | | | |  | | | |  |  |
| 3 | Atr-ERM99908 |  | | | |  |  |  | | | |  | | | |  |  |
| 4 | Atr-ERM99909 |  | | | |  | Vvi-Vitvi17g00684\_t001 |  | | | |  | Vvi-Vitvi01g00060\_t001 |  |  |
| 4 | Atr-ERM99910 |  | Vvi-Vitvi14g02039\_t001 |  | | | |  | | | |  | | | |  |  |
| 4 | Atr-ERM99911 |  | | | |  | Vvi-Vitvi17g00683\_t001 |  | | | |  | | | |  |  |
| 4 | Atr-ERM99912 |  | | | |  | | | |  | | | |  | | | |  |  |
| 4 | Atr-ERM99913 |  | | | |  | Vvi-Vitvi17g04192\_t001 |  | | | |  | | | |  |  |
| 4 | Atr-ERM99914 |  | Vvi-Vitvi14g03099\_t002 |  | Vvi-Vitvi17g00682\_t002 |  | | | |  | Vvi-Vitvi01g00061\_t001 |  |  |
| 4 | Atr-ERM99915 |  | | | |  | Vvi-Vitvi17g00681\_t001 |  | | | |  | | | |  |  |
| 4 | Atr-ERM99916 |  | | | |  | | | |  | | | |  | | | |  |  |
| 4 | Atr-ERM99917 |  | | | |  | | | |  | | | |  | | | |  |  |
| 4 | Atr-ERM99918 |  | | | |  | | | |  | | | |  | | | |  |  |
| 4 | Atr-ERM99919 |  | | | |  | Vvi-Vitvi17g00680\_t001 |  | | | |  | | | |  |  |
| 3 | Atr-ERM99920 |  | | | |  |  |  | | | |  | | | |  |  |
| 4 | Atr-ERM99921 |  | Vvi-Vitvi14g02034\_t001 |  | Vvi-Vitvi17g00669\_t001 |  | | | |  | Vvi-Vitvi01g00062\_t001 |  |  |
| 4 | Atr-ERM99922 |  | Vvi-Vitvi14g02033\_t001 |  | Vvi-Vitvi17g00670\_t001 |  | Vvi-Vitvi01g00063\_t001 |  | Vvi-Vitvi01g00063\_t001 |  |  |
| 4 | Atr-ERM99923 |  | | | |  | | | |  | | | |  | | | |  |  |
| 4 | Atr-ERM99924 |  | | | |  | | | |  | | | |  | | | |  |  |
| 4 | Atr-ERM99925 |  | | | |  | | | |  | | | |  | | | |  |  |
| 4 | Atr-ERM99926 |  | | | |  | | | |  | | | |  | | | |  |  |
| 4 | Atr-ERM99927 |  | Vvi-Vitvi14g04696\_t001 |  | Vvi-Vitvi17g01488\_t001 |  | | | |  | | | |  |  |
| 4 | Atr-ERM99928 |  | | | |  | | | |  | | | |  | | | |  |  |
| 4 | Atr-ERM99929 |  | | | |  | | | |  | | | |  | Vvi-Vitvi01g01844\_t002 |  |  |
| 4 | Atr-ERM99930 |  | | | |  | Vvi-Vitvi17g00671\_t003 |  | | | |  | | | |  |  |
| 4 | Atr-ERM99931 |  | Vvi-Vitvi14g02032\_t001 |  | | | |  | | | |  | Vvi-Vitvi01g00064\_t001 |  |  |
| 3 | Atr-ERM99932 |  |  |  | Vvi-Vitvi17g01490\_t001 |  | Vvi-Vitvi01g00059\_t002 |  | | | |  |  |
| 3 | Atr-ERM99933 |  |  |  | | | |  | | | |  | | | |  |  |
| 3 | Atr-ERM99934 |  |  |  | | | |  | | | |  | | | |  |  |
| 3 | Atr-ERM99935 |  |  |  | | | |  | | | |  | | | |  |  |
| 3 | Atr-ERM99936 |  |  |  | | | |  | Vvi-Vitvi01g00058\_t001 |  | | | |  |  |
| 2 | Atr-ERM99937 |  |  |  | | | |  |  |  | | | |  |  |
| 2 | Atr-ERM99938 |  |  |  | | | |  |  |  | | | |  |  |
| 2 | Atr-ERM99939 |  |  |  | | | |  |  |  | | | |  |  |
| 2 | Atr-ERM99940 |  |  |  | | | |  |  |  | | | |  |  |
| 2 | Atr-ERM99941 |  |  |  | | | |  |  |  | | | |  |  |
| 3 | Atr-ERM99942 |  | Vvi-Vitvi14g01414\_t001 |  | | | |  |  |  | | | |  |  |
| 3 | Atr-ERM99943 |  | Vvi-Vitvi14g01413\_t001 |  | | | |  |  |  | | | |  |  |
| 3 | Atr-ERM99944 |  | | | |  | | | |  |  |  | | | |  |  |
| 3 | Atr-ERM99945 |  | | | |  | | | |  |  |  | | | |  |  |
| 3 | Atr-ERM99946 |  | | | |  | | | |  |  |  | | | |  |  |
| 3 | Atr-ERM99947 |  | Vvi-Vitvi14g02946\_t001 |  | | | |  |  |  | | | |  |  |
| 3 | Atr-ERM99948 |  | | | |  | | | |  |  |  | Vvi-Vitvi01g04012\_t001 |  |  |
| 2 | Atr-ERM99949 |  | | | |  | | | |  |  |  |  |
| 2 | Atr-ERM99950 |  | | | |  | Vvi-Vitvi17g04195\_t001 |  |  |  |  |
| 2 | Atr-ERM99951 |  | | | |  | | | |  |  |  |  |
| 2 | Atr-ERM99952 |  | | | |  | | | |  |  |  |  |
| 2 | Atr-ERM99953 |  | | | |  | Vvi-Vitvi17g04200\_t001 |  |  |  |  |
| 2 | Atr-ERM99954 |  | | | |  | | | |  |  |  |  |
| 2 | Atr-ERM99955 |  | Vvi-Vitvi14g02944\_t001 |  | | | |  |  |  |  |
| 2 | Atr-ERM99956 |  | | | |  | | | |  |  |  |  |
| 2 | Atr-ERM99957 |  | | | |  | | | |  |  |  |  |
| 2 | Atr-ERM99958 |  | | | |  | | | |  |  |  |  |
| 2 | Atr-ERM99959 |  | | | |  | | | |  |  |  |  |
| 2 | Atr-ERM99960 |  | | | |  | | | |  |  |  |  |
| 2 | Atr-ERM99961 |  | | | |  | | | |  |  |  |  |
| 2 | Atr-ERM99962 |  | | | |  | | | |  |  |  |  |
| 2 | Atr-ERM99963 |  | | | |  | | | |  |  |  |  |
| 2 | Atr-ERM99964 |  | | | |  | | | |  |  |  |  |
| 2 | Atr-ERM99965 |  | | | |  | | | |  |  |  |  |
| 2 | Atr-ERM99966 |  | | | |  | | | |  |  |  |  |
| 2 | Atr-ERM99967 |  | | | |  | | | |  |  |  |  |
| 2 | Atr-ERM99968 |  | | | |  | Vvi-Vitvi17g00690\_t001 |  |  |  |  |
| 2 | Atr-ERM99969 |  | | | |  | Vvi-Vitvi17g01504\_t001 |  |  |  |  |
| 2 | Atr-ERM99970 |  | | | |  | | | |  |  |  |  |
| 2 | Atr-ERM99971 |  | | | |  | | | |  |  |  |  |
| 2 | Atr-ERM99972 |  | | | |  | | | |  |  |  |  |
| 2 | Atr-ERM99973 |  | Vvi-Vitvi14g01407\_t003 |  | Vvi-Vitvi17g00692\_t004 |  |  |  |  |
| 2 | Atr-ERM99974 |  | | | |  | | | |  |  |  |  |
| 2 | Atr-ERM99975 |  | | | |  | | | |  |  |  |  |
| 2 | Atr-ERM99976 |  | | | |  | | | |  |  |  |  |
| 2 | Atr-ERM99977 |  | Vvi-Vitvi14g01406\_t002 |  | | | |  |  |  |  |
| 2 | Atr-ERM99978 |  | | | |  | | | |  |  |  |  |
| 2 | Atr-ERM99979 |  | | | |  | | | |  |  |  |  |
| 2 | Atr-ERM99980 |  | | | |  | | | |  |  |  |  |
| 2 | Atr-ERM99981 |  | | | |  | | | |  |  |  |  |
| 2 | Atr-ERM99982 |  | | | |  | | | |  |  |  |  |
| 2 | Atr-ERM99983 |  | | | |  | | | |  |  |  |  |
| 2 | Atr-ERM99984 |  | | | |  | | | |  |  |  |  |
| 2 | Atr-ERM99985 |  | | | |  | Vvi-Vitvi17g00705\_t001 |  |  |  |  |
| 1 | Atr-ERM99986 |  | | | |  |  |  |  |  |
| 1 | Atr-ERM99987 |  | | | |  |  |  |  |  |
| 2 | Atr-ERM99988 |  | | | |  | Vvi-Vitvi01g00043\_t001 |  |  |  |  |
| 2 | Atr-ERM99989 |  | | | |  | Vvi-Vitvi01g00048\_t001 |  |  |  |  |
| 2 | Atr-ERM99990 |  | | | |  | | | |  |  |  |  |
| 2 | Atr-ERM99991 |  | | | |  | | | |  |  |  |  |
| 2 | Atr-ERM99992 |  | | | |  | Vvi-Vitvi01g00049\_t001 |  |  |  |  |
| 2 | Atr-ERM99993 |  | | | |  | | | |  |  |  |  |
| 2 | Atr-ERM99994 |  | Vvi-Vitvi14g01404\_t001 |  | Vvi-Vitvi01g00050\_t001 |  |  |  |  |
| 1 | Atr-ERM99995 |  |  |  | | | |  |  |  |  |
| 1 | Atr-ERM99996 |  |  |  | Vvi-Vitvi01g00051\_t001 |  |  |  |  |
| 1 | Atr-ERM99997 |  |  |  | | | |  |  |  |  |
| 1 | Atr-ERM99998 |  |  |  | | | |  |  |  |  |
| 1 | Atr-ERM99999 |  |  |  | | | |  |  |  |  |
| 1 | Atr-ERN00001 |  |  |  | | | |  |  |  |  |
| 1 | Atr-ERN00002 |  |  |  | | | |  |  |  |  |
| 1 | Atr-ERN00003 |  |  |  | | | |  |  |  |  |
| 1 | Atr-ERN00004 |  |  |  | Vvi-Vitvi01g00070\_t001 |  |  |  |  |
| 0 | Atr-ERN00005 |  |  |  |  |  |  |
| 0 | Atr-ERN00006 |  |  |  |  |  |  |
| 0 | Atr-ERN00007 |  |  |  |  |  |  |
| 0 | Atr-ERN00008 |  |  |  |  |  |  |
