# Supplementary material for: Genome-wide identification, characterization and gene expression of BES1 transcription factor family in grapevine (Vitis vinifera L.)
Source: Sci Rep. 2023 Jan 5;13:240. doi: 10.1038/s41598-022-24407-y (PMC9816167; doi:10.1038/s41598-022-24407-y)
Supplement: Supplementary file 3 — Supplementary Information. [file 41598_2022_24407_MOESM3_ESM.zip › Vvi_Atr/Vitis_vinifera.PN40024.v4.dna_sm.toplevel.fa.vs.Amborella_trichopoda.AMTR1.0.dna_sm.toplevel.fa.html/Atr-AmTr_v1.0_scaffold00111.html]

|  |  |  |  |  |  |  |  |  |  |  |  |  |  |
| --- | --- | --- | --- | --- | --- | --- | --- | --- | --- | --- | --- | --- | --- |
| Duplication depth | Reference chromosome | Collinear blocks | | | | | | | | | | | |
| 0 | Atr-ERN00136 |  |  |  |  |  |  |
| 0 | Atr-ERN00137 |  |  |  |  |  |  |
| 0 | Atr-ERN00138 |  |  |  |  |  |  |
| 0 | Atr-ERN00139 |  |  |  |  |  |  |
| 0 | Atr-ERN00140 |  |  |  |  |  |  |
| 0 | Atr-ERN00141 |  |  |  |  |  |  |
| 0 | Atr-ERN00142 |  |  |  |  |  |  |
| 0 | Atr-ERN00143 |  |  |  |  |  |  |
| 0 | Atr-ERN00144 |  |  |  |  |  |  |
| 0 | Atr-ERN00145 |  |  |  |  |  |  |
| 0 | Atr-ERN00146 |  |  |  |  |  |  |
| 2 | Atr-ERN00147 |  | Vvi-Vitvi15g01459\_t001 |  | Vvi-Vitvi16g00504\_t001 |  |  |  |  |
| 2 | Atr-ERN00148 |  | | | |  | | | |  |  |  |  |
| 2 | Atr-ERN00149 |  | Vvi-Vitvi15g00611\_t001 |  | | | |  |  |  |  |
| 2 | Atr-ERN00150 |  | | | |  | | | |  |  |  |  |
| 2 | Atr-ERN00151 |  | Vvi-Vitvi15g00610\_t001 |  | | | |  |  |  |  |
| 2 | Atr-ERN00152 |  | Vvi-Vitvi15g00607\_t001 |  | | | |  |  |  |  |
| 2 | Atr-ERN00153 |  | Vvi-Vitvi15g00606\_t001 |  | | | |  |  |  |  |
| 2 | Atr-ERN00154 |  | | | |  | | | |  |  |  |  |
| 2 | Atr-ERN00155 |  | Vvi-Vitvi15g00604\_t004 |  | | | |  |  |  |  |
| 2 | Atr-ERN00156 |  | | | |  | | | |  |  |  |  |
| 2 | Atr-ERN00157 |  | | | |  | | | |  |  |  |  |
| 2 | Atr-ERN00158 |  | | | |  | | | |  |  |  |  |
| 2 | Atr-ERN00159 |  | | | |  | | | |  |  |  |  |
| 2 | Atr-ERN00160 |  | Vvi-Vitvi15g00601\_t001 |  | | | |  |  |  |  |
| 2 | Atr-ERN00161 |  | Vvi-Vitvi15g00600\_t001 |  | | | |  |  |  |  |
| 2 | Atr-ERN00162 |  | | | |  | | | |  |  |  |  |
| 2 | Atr-ERN00163 |  | | | |  | | | |  |  |  |  |
| 2 | Atr-ERN00164 |  | | | |  | | | |  |  |  |  |
| 2 | Atr-ERN00165 |  | | | |  | | | |  |  |  |  |
| 2 | Atr-ERN00166 |  | | | |  | | | |  |  |  |  |
| 2 | Atr-ERN00167 |  | Vvi-Vitvi15g00595\_t001 |  | | | |  |  |  |  |
| 2 | Atr-ERN00168 |  | | | |  | | | |  |  |  |  |
| 2 | Atr-ERN00169 |  | | | |  | | | |  |  |  |  |
| 2 | Atr-ERN00170 |  | | | |  | | | |  |  |  |  |
| 2 | Atr-ERN00171 |  | | | |  | Vvi-Vitvi16g00479\_t001 |  |  |  |  |
| 2 | Atr-ERN00172 |  | | | |  | | | |  |  |  |  |
| 2 | Atr-ERN00173 |  | | | |  | | | |  |  |  |  |
| 2 | Atr-ERN00174 |  | | | |  | | | |  |  |  |  |
| 2 | Atr-ERN00175 |  | | | |  | | | |  |  |  |  |
| 3 | Atr-ERN00176 |  | | | |  | | | |  | Vvi-Vitvi16g00455\_t001 |  |  |  |
| 3 | Atr-ERN00177 |  | Vvi-Vitvi15g00593\_t001 |  | | | |  | | | |  |  |  |
| 3 | Atr-ERN00178 |  | | | |  | | | |  | | | |  |  |  |
| 3 | Atr-ERN00179 |  | | | |  | | | |  | | | |  |  |  |
| 3 | Atr-ERN00180 |  | Vvi-Vitvi15g00584\_t001 |  | | | |  | | | |  |  |  |
| 3 | Atr-ERN00181 |  | | | |  | | | |  | | | |  |  |  |
| 3 | Atr-ERN00182 |  | | | |  | Vvi-Vitvi16g00463\_t001 |  | Vvi-Vitvi16g00463\_t001 |  |  |  |
| 3 | Atr-ERN00183 |  | | | |  | | | |  | Vvi-Vitvi16g00474\_t001 |  |  |  |
| 3 | Atr-ERN00184 |  | Vvi-Vitvi15g00579\_t001 |  | | | |  | | | |  |  |  |
| 3 | Atr-ERN00185 |  | Vvi-Vitvi15g00578\_t002 |  | | | |  | | | |  |  |  |
| 3 | Atr-ERN00186 |  | Vvi-Vitvi15g00577\_t001 |  | | | |  | | | |  |  |  |
| 3 | Atr-ERN00187 |  | | | |  | | | |  | | | |  |  |  |
| 3 | Atr-ERN00188 |  | | | |  | | | |  | | | |  |  |  |
| 3 | Atr-ERN00189 |  | | | |  | | | |  | Vvi-Vitvi16g00475\_t001 |  |  |  |
| 3 | Atr-ERN00190 |  | | | |  | | | |  | Vvi-Vitvi16g00477\_t001 |  |  |  |
| 3 | Atr-ERN00191 |  | | | |  | Vvi-Vitvi16g00439\_t003 |  | | | |  |  |  |
| 3 | Atr-ERN00192 |  | | | |  | | | |  | Vvi-Vitvi16g01711\_t001 |  |  |  |
| 2 | Atr-ERN00193 |  | | | |  | | | |  |  |  |  |
| 2 | Atr-ERN00194 |  | Vvi-Vitvi15g00574\_t001 |  | | | |  |  |  |  |
| 2 | Atr-ERN00195 |  | | | |  | Vvi-Vitvi16g00437\_t001 |  |  |  |  |
| 2 | Atr-ERN00196 |  | | | |  | | | |  |  |  |  |
| 2 | Atr-ERN00197 |  | | | |  | | | |  |  |  |  |
| 2 | Atr-ERN00198 |  | | | |  | | | |  |  |  |  |
| 2 | Atr-ERN00199 |  | | | |  | | | |  |  |  |  |
| 2 | Atr-ERN00200 |  | | | |  | | | |  |  |  |  |
| 2 | Atr-ERN00201 |  | | | |  | | | |  |  |  |  |
| 2 | Atr-ERN00202 |  | | | |  | | | |  |  |  |  |
| 2 | Atr-ERN00203 |  | | | |  | Vvi-Vitvi16g00436\_t001 |  |  |  |  |
| 2 | Atr-ERN00204 |  | Vvi-Vitvi15g00573\_t001 |  | | | |  |  |  |  |
| 2 | Atr-ERN00205 |  | | | |  | | | |  |  |  |  |
| 2 | Atr-ERN00206 |  | | | |  | | | |  |  |  |  |
| 2 | Atr-ERN00207 |  | Vvi-Vitvi15g00572\_t001 |  | Vvi-Vitvi16g00429\_t001 |  |  |  |  |
| 2 | Atr-ERN00208 |  | | | |  | Vvi-Vitvi16g00427\_t001 |  |  |  |  |
| 2 | Atr-ERN00209 |  | | | |  | | | |  |  |  |  |
| 3 | Atr-ERN00210 |  | Vvi-Vitvi15g00571\_t001 |  | | | |  | Vvi-Vitvi02g01739\_t001 |  |  |  |
| 3 | Atr-ERN00211 |  | | | |  | | | |  | Vvi-Vitvi00g04042\_t004 |  |  |  |
| 3 | Atr-ERN00212 |  | | | |  | | | |  | Vvi-Vitvi00g04041\_t002 |  |  |  |
| 3 | Atr-ERN00213 |  | | | |  | | | |  | | | |  |  |  |
| 3 | Atr-ERN00214 |  | | | |  | | | |  | | | |  |  |  |
| 3 | Atr-ERN00215 |  | | | |  | Vvi-Vitvi16g00381\_t001 |  | | | |  |  |  |
| 2 | Atr-ERN00216 |  | | | |  |  |  | | | |  |  |  |
| 2 | Atr-ERN00217 |  | Vvi-Vitvi15g00567\_t001 |  |  |  | Vvi-Vitvi00g04040\_t001 |  |  |  |
| 2 | Atr-ERN00218 |  | Vvi-Vitvi15g01427\_t001 |  |  |  | | | |  |  |  |
| 2 | Atr-ERN00219 |  | | | |  |  |  | | | |  |  |  |
| 2 | Atr-ERN00220 |  | | | |  |  |  | | | |  |  |  |
| 2 | Atr-ERN00221 |  | | | |  |  |  | | | |  |  |  |
| 2 | Atr-ERN00222 |  | | | |  |  |  | | | |  |  |  |
| 2 | Atr-ERN00223 |  | Vvi-Vitvi15g01202\_t001 |  |  |  | Vvi-Vitvi02g01780\_t001 |  |  |  |
| 2 | Atr-ERN00224 |  | | | |  |  |  | | | |  |  |  |
| 2 | Atr-ERN00225 |  | | | |  |  |  | | | |  |  |  |
| 2 | Atr-ERN00226 |  | Vvi-Vitvi15g00565\_t001 |  |  |  | Vvi-Vitvi02g01779\_t001 |  |  |  |
| 2 | Atr-ERN00227 |  | Vvi-Vitvi15g00564\_t001 |  |  |  | | | |  |  |  |
| 2 | Atr-ERN00228 |  | Vvi-Vitvi15g00563\_t001 |  |  |  | | | |  |  |  |
| 2 | Atr-ERN00229 |  | | | |  |  |  | Vvi-Vitvi02g01778\_t001 |  |  |  |
| 2 | Atr-ERN00230 |  | | | |  |  |  | Vvi-Vitvi02g01777\_t001 |  |  |  |
| 2 | Atr-ERN00231 |  | | | |  |  |  | | | |  |  |  |
| 2 | Atr-ERN00232 |  | | | |  |  |  | | | |  |  |  |
| 2 | Atr-ERN00233 |  | | | |  |  |  | Vvi-Vitvi02g01776\_t001 |  |  |  |
| 2 | Atr-ERN00234 |  | | | |  |  |  | | | |  |  |  |
| 2 | Atr-ERN00235 |  | Vvi-Vitvi15g00562\_t001 |  |  |  | | | |  |  |  |
| 2 | Atr-ERN00236 |  | | | |  |  |  | | | |  |  |  |
| 2 | Atr-ERN00237 |  | | | |  |  |  | | | |  |  |  |
| 2 | Atr-ERN00238 |  | | | |  |  |  | | | |  |  |  |
| 2 | Atr-ERN00239 |  | | | |  |  |  | | | |  |  |  |
| 2 | Atr-ERN00240 |  | Vvi-Vitvi15g04322\_t001 |  |  |  | | | |  |  |  |
| 2 | Atr-ERN00241 |  | | | |  |  |  | Vvi-Vitvi02g01774\_t001 |  |  |  |
| 2 | Atr-ERN00242 |  | | | |  |  |  | | | |  |  |  |
| 2 | Atr-ERN00243 |  | | | |  |  |  | | | |  |  |  |
| 2 | Atr-ERN00244 |  | | | |  |  |  | Vvi-Vitvi02g01770\_t004 |  |  |  |
| 2 | Atr-ERN00245 |  | Vvi-Vitvi15g00556\_t001 |  |  |  | | | |  |  |  |
| 2 | Atr-ERN00246 |  | | | |  |  |  | Vvi-Vitvi02g01769\_t001 |  |  |  |
| 1 | Atr-ERN00247 |  | | | |  |  |  |  |  |
| 1 | Atr-ERN00248 |  | | | |  |  |  |  |  |
| 1 | Atr-ERN00249 |  | | | |  |  |  |  |  |
| 1 | Atr-ERN00250 |  | | | |  |  |  |  |  |
| 1 | Atr-ERN00251 |  | Vvi-Vitvi15g00555\_t001 |  |  |  |  |  |
| 1 | Atr-ERN00252 |  | | | |  |  |  |  |  |
| 1 | Atr-ERN00253 |  | Vvi-Vitvi15g00554\_t001 |  |  |  |  |  |
| 1 | Atr-ERN00254 |  | | | |  |  |  |  |  |
| 1 | Atr-ERN00255 |  | Vvi-Vitvi15g00549\_t001 |  |  |  |  |  |
| 1 | Atr-ERN00256 |  | | | |  |  |  |  |  |
| 1 | Atr-ERN00257 |  | | | |  |  |  |  |  |
| 1 | Atr-ERN00258 |  | | | |  |  |  |  |  |
| 1 | Atr-ERN00259 |  | | | |  |  |  |  |  |
| 1 | Atr-ERN00260 |  | | | |  |  |  |  |  |
| 1 | Atr-ERN00261 |  | | | |  |  |  |  |  |
| 1 | Atr-ERN00262 |  | Vvi-Vitvi15g00548\_t001 |  |  |  |  |  |
| 0 | Atr-ERN00263 |  |  |  |  |  |  |
| 0 | Atr-ERN00264 |  |  |  |  |  |  |
| 0 | Atr-ERN00265 |  |  |  |  |  |  |
| 0 | Atr-ERN00266 |  |  |  |  |  |  |
| 0 | Atr-ERN00267 |  |  |  |  |  |  |
| 0 | Atr-ERN00268 |  |  |  |  |  |  |
| 0 | Atr-ERN00269 |  |  |  |  |  |  |
| 0 | Atr-ERN00270 |  |  |  |  |  |  |
