# Supplementary material for: Genome-wide identification, characterization and gene expression of BES1 transcription factor family in grapevine (Vitis vinifera L.)
Source: Sci Rep. 2023 Jan 5;13:240. doi: 10.1038/s41598-022-24407-y (PMC9816167; doi:10.1038/s41598-022-24407-y)
Supplement: Supplementary file 3 — Supplementary Information. [file 41598_2022_24407_MOESM3_ESM.zip › Vvi_Atr/Vitis_vinifera.PN40024.v4.dna_sm.toplevel.fa.vs.Amborella_trichopoda.AMTR1.0.dna_sm.toplevel.fa.html/Atr-AmTr_v1.0_scaffold00112.html]

|  |  |  |  |  |  |  |  |  |  |  |  |  |  |
| --- | --- | --- | --- | --- | --- | --- | --- | --- | --- | --- | --- | --- | --- |
| Duplication depth | Reference chromosome | Collinear blocks | | | | | | | | | | | |
| 0 | Atr-ERN00093 |  |  |  |  |  |  |
| 0 | Atr-ERN00094 |  |  |  |  |  |  |
| 0 | Atr-ERN00095 |  |  |  |  |  |  |
| 0 | Atr-ERN00096 |  |  |  |  |  |  |
| 0 | Atr-ERN00097 |  |  |  |  |  |  |
| 0 | Atr-ERN00098 |  |  |  |  |  |  |
| 0 | Atr-ERN00099 |  |  |  |  |  |  |
| 0 | Atr-ERN00100 |  |  |  |  |  |  |
| 0 | Atr-ERN00101 |  |  |  |  |  |  |
| 0 | Atr-ERN00102 |  |  |  |  |  |  |
| 0 | Atr-ERN00103 |  |  |  |  |  |  |
| 0 | Atr-ERN00104 |  |  |  |  |  |  |
| 0 | Atr-ERN00105 |  |  |  |  |  |  |
| 0 | Atr-ERN00106 |  |  |  |  |  |  |
| 0 | Atr-ERN00107 |  |  |  |  |  |  |
| 0 | Atr-ERN00108 |  |  |  |  |  |  |
| 0 | Atr-ERN00109 |  |  |  |  |  |  |
| 0 | Atr-ERN00110 |  |  |  |  |  |  |
| 0 | Atr-ERN00111 |  |  |  |  |  |  |
| 0 | Atr-ERN00112 |  |  |  |  |  |  |
| 0 | Atr-ERN00113 |  |  |  |  |  |  |
| 0 | Atr-ERN00114 |  |  |  |  |  |  |
| 0 | Atr-ERN00115 |  |  |  |  |  |  |
| 0 | Atr-ERN00116 |  |  |  |  |  |  |
| 0 | Atr-ERN00117 |  |  |  |  |  |  |
| 0 | Atr-ERN00118 |  |  |  |  |  |  |
| 0 | Atr-ERN00119 |  |  |  |  |  |  |
| 0 | Atr-ERN00120 |  |  |  |  |  |  |
| 0 | Atr-ERN00121 |  |  |  |  |  |  |
| 0 | Atr-ERN00122 |  |  |  |  |  |  |
| 0 | Atr-ERN00123 |  |  |  |  |  |  |
| 0 | Atr-ERN00124 |  |  |  |  |  |  |
| 0 | Atr-ERN00125 |  |  |  |  |  |  |
| 0 | Atr-ERN00126 |  |  |  |  |  |  |
| 0 | Atr-ERN00127 |  |  |  |  |  |  |
| 0 | Atr-ERN00128 |  |  |  |  |  |  |
| 0 | Atr-ERN00129 |  |  |  |  |  |  |
| 0 | Atr-ERN00130 |  |  |  |  |  |  |
| 0 | Atr-ERN00131 |  |  |  |  |  |  |
| 0 | Atr-ERN00132 |  |  |  |  |  |  |
| 0 | Atr-ERN00133 |  |  |  |  |  |  |
