# Supplementary material for: Genome-wide identification, characterization and gene expression of BES1 transcription factor family in grapevine (Vitis vinifera L.)
Source: Sci Rep. 2023 Jan 5;13:240. doi: 10.1038/s41598-022-24407-y (PMC9816167; doi:10.1038/s41598-022-24407-y)
Supplement: Supplementary file 3 — Supplementary Information. [file 41598_2022_24407_MOESM3_ESM.zip › Vvi_Atr/Vitis_vinifera.PN40024.v4.dna_sm.toplevel.fa.vs.Amborella_trichopoda.AMTR1.0.dna_sm.toplevel.fa.html/Atr-AmTr_v1.0_scaffold00113.html]

|  |  |  |  |  |  |  |  |  |  |  |  |  |  |
| --- | --- | --- | --- | --- | --- | --- | --- | --- | --- | --- | --- | --- | --- |
| Duplication depth | Reference chromosome | Collinear blocks | | | | | | | | | | | |
| 0 | Atr-ERM98509 |  |  |  |  |  |  |
| 0 | Atr-ERM98510 |  |  |  |  |  |  |
| 0 | Atr-ERM98511 |  |  |  |  |  |  |
| 0 | Atr-ERM98512 |  |  |  |  |  |  |
| 0 | Atr-ERM98513 |  |  |  |  |  |  |
| 0 | Atr-ERM98514 |  |  |  |  |  |  |
| 0 | Atr-ERM98515 |  |  |  |  |  |  |
| 0 | Atr-ERM98516 |  |  |  |  |  |  |
| 0 | Atr-ERM98517 |  |  |  |  |  |  |
| 0 | Atr-ERM98518 |  |  |  |  |  |  |
| 0 | Atr-ERM98519 |  |  |  |  |  |  |
| 0 | Atr-ERM98520 |  |  |  |  |  |  |
| 0 | Atr-ERM98521 |  |  |  |  |  |  |
| 0 | Atr-ERM98522 |  |  |  |  |  |  |
| 0 | Atr-ERM98523 |  |  |  |  |  |  |
| 0 | Atr-ERM98524 |  |  |  |  |  |  |
| 0 | Atr-ERM98525 |  |  |  |  |  |  |
| 0 | Atr-ERM98526 |  |  |  |  |  |  |
| 0 | Atr-ERM98527 |  |  |  |  |  |  |
| 0 | Atr-ERM98528 |  |  |  |  |  |  |
| 0 | Atr-ERM98529 |  |  |  |  |  |  |
| 0 | Atr-ERM98530 |  |  |  |  |  |  |
| 0 | Atr-ERM98531 |  |  |  |  |  |  |
| 0 | Atr-ERM98532 |  |  |  |  |  |  |
| 0 | Atr-ERM98533 |  |  |  |  |  |  |
| 0 | Atr-ERM98534 |  |  |  |  |  |  |
| 0 | Atr-ERM98535 |  |  |  |  |  |  |
| 0 | Atr-ERM98536 |  |  |  |  |  |  |
| 0 | Atr-ERM98537 |  |  |  |  |  |  |
| 0 | Atr-ERM98538 |  |  |  |  |  |  |
| 0 | Atr-ERM98539 |  |  |  |  |  |  |
| 0 | Atr-ERM98540 |  |  |  |  |  |  |
| 0 | Atr-ERM98541 |  |  |  |  |  |  |
