# Supplementary material for: Genome-wide identification, characterization and gene expression of BES1 transcription factor family in grapevine (Vitis vinifera L.)
Source: Sci Rep. 2023 Jan 5;13:240. doi: 10.1038/s41598-022-24407-y (PMC9816167; doi:10.1038/s41598-022-24407-y)
Supplement: Supplementary file 3 — Supplementary Information. [file 41598_2022_24407_MOESM3_ESM.zip › Vvi_Atr/Vitis_vinifera.PN40024.v4.dna_sm.toplevel.fa.vs.Amborella_trichopoda.AMTR1.0.dna_sm.toplevel.fa.html/Atr-AmTr_v1.0_scaffold00114.html]

|  |  |  |  |  |  |  |  |  |  |  |  |  |  |
| --- | --- | --- | --- | --- | --- | --- | --- | --- | --- | --- | --- | --- | --- |
| Duplication depth | Reference chromosome | Collinear blocks | | | | | | | | | | | |
| 0 | Atr-ERM98881 |  |  |  |  |  |  |
| 0 | Atr-ERM98882 |  |  |  |  |  |  |
| 0 | Atr-ERM98883 |  |  |  |  |  |  |
| 0 | Atr-ERM98884 |  |  |  |  |  |  |
| 0 | Atr-ERM98885 |  |  |  |  |  |  |
| 0 | Atr-ERM98886 |  |  |  |  |  |  |
| 0 | Atr-ERM98887 |  |  |  |  |  |  |
| 1 | Atr-ERM98888 |  | Vvi-Vitvi13g00924\_t001 |  |  |  |  |  |
| 1 | Atr-ERM98889 |  | | | |  |  |  |  |  |
| 2 | Atr-ERM98890 |  | | | |  | Vvi-Vitvi06g01478\_t001 |  |  |  |  |
| 2 | Atr-ERM98891 |  | | | |  | | | |  |  |  |  |
| 2 | Atr-ERM98892 |  | | | |  | | | |  |  |  |  |
| 2 | Atr-ERM98893 |  | | | |  | | | |  |  |  |  |
| 2 | Atr-ERM98894 |  | | | |  | | | |  |  |  |  |
| 2 | Atr-ERM98895 |  | Vvi-Vitvi13g00925\_t001 |  | | | |  |  |  |  |
| 2 | Atr-ERM98896 |  | Vvi-Vitvi13g00887\_t001 |  | | | |  |  |  |  |
| 2 | Atr-ERM98897 |  | Vvi-Vitvi13g00886\_t003 |  | | | |  |  |  |  |
| 2 | Atr-ERM98898 |  | | | |  | | | |  |  |  |  |
| 2 | Atr-ERM98899 |  | | | |  | Vvi-Vitvi06g01476\_t001 |  |  |  |  |
| 2 | Atr-ERM98900 |  | | | |  | | | |  |  |  |  |
| 2 | Atr-ERM98901 |  | | | |  | | | |  |  |  |  |
| 2 | Atr-ERM98902 |  | | | |  | | | |  |  |  |  |
| 2 | Atr-ERM98903 |  | | | |  | Vvi-Vitvi06g01473\_t001 |  |  |  |  |
| 2 | Atr-ERM98904 |  | | | |  | | | |  |  |  |  |
| 2 | Atr-ERM98905 |  | | | |  | | | |  |  |  |  |
| 2 | Atr-ERM98906 |  | | | |  | | | |  |  |  |  |
| 2 | Atr-ERM98907 |  | | | |  | | | |  |  |  |  |
| 2 | Atr-ERM98908 |  | | | |  | | | |  |  |  |  |
| 2 | Atr-ERM98909 |  | | | |  | | | |  |  |  |  |
| 2 | Atr-ERM98910 |  | | | |  | | | |  |  |  |  |
| 2 | Atr-ERM98911 |  | | | |  | | | |  |  |  |  |
| 2 | Atr-ERM98912 |  | | | |  | | | |  |  |  |  |
| 2 | Atr-ERM98913 |  | | | |  | | | |  |  |  |  |
| 2 | Atr-ERM98914 |  | | | |  | | | |  |  |  |  |
| 2 | Atr-ERM98915 |  | Vvi-Vitvi13g00883\_t001 |  | Vvi-Vitvi06g01472\_t001 |  |  |  |  |
| 2 | Atr-ERM98916 |  | | | |  | | | |  |  |  |  |
| 2 | Atr-ERM98917 |  | | | |  | | | |  |  |  |  |
| 2 | Atr-ERM98918 |  | | | |  | | | |  |  |  |  |
| 2 | Atr-ERM98919 |  | | | |  | | | |  |  |  |  |
| 2 | Atr-ERM98920 |  | | | |  | | | |  |  |  |  |
| 2 | Atr-ERM98921 |  | | | |  | Vvi-Vitvi06g01471\_t001 |  |  |  |  |
| 2 | Atr-ERM98922 |  | | | |  | | | |  |  |  |  |
| 2 | Atr-ERM98923 |  | | | |  | | | |  |  |  |  |
| 2 | Atr-ERM98924 |  | | | |  | Vvi-Vitvi06g01470\_t001 |  |  |  |  |
| 1 | Atr-ERM98925 |  | | | |  |  |  |  |  |
| 1 | Atr-ERM98926 |  | | | |  |  |  |  |  |
| 1 | Atr-ERM98927 |  | | | |  |  |  |  |  |
| 1 | Atr-ERM98928 |  | | | |  |  |  |  |  |
| 1 | Atr-ERM98929 |  | | | |  |  |  |  |  |
| 1 | Atr-ERM98930 |  | | | |  |  |  |  |  |
| 2 | Atr-ERM98931 |  | | | |  | Vvi-Vitvi08g01417\_t001 |  |  |  |  |
| 2 | Atr-ERM98932 |  | | | |  | | | |  |  |  |  |
| 2 | Atr-ERM98933 |  | | | |  | | | |  |  |  |  |
| 2 | Atr-ERM98934 |  | | | |  | | | |  |  |  |  |
| 2 | Atr-ERM98935 |  | | | |  | | | |  |  |  |  |
| 2 | Atr-ERM98936 |  | | | |  | | | |  |  |  |  |
| 2 | Atr-ERM98937 |  | | | |  | | | |  |  |  |  |
| 2 | Atr-ERM98938 |  | Vvi-Vitvi13g00859\_t001 |  | Vvi-Vitvi08g02257\_t001 |  |  |  |  |
| 1 | Atr-ERM98939 |  |  |  | Vvi-Vitvi08g01415\_t001 |  |  |  |  |
| 1 | Atr-ERM98940 |  |  |  | Vvi-Vitvi08g01414\_t001 |  |  |  |  |
| 1 | Atr-ERM98941 |  |  |  | | | |  |  |  |  |
| 1 | Atr-ERM98942 |  |  |  | | | |  |  |  |  |
| 1 | Atr-ERM98943 |  |  |  | | | |  |  |  |  |
| 1 | Atr-ERM98944 |  |  |  | | | |  |  |  |  |
| 1 | Atr-ERM98945 |  |  |  | | | |  |  |  |  |
| 1 | Atr-ERM98946 |  |  |  | Vvi-Vitvi08g01412\_t001 |  |  |  |  |
| 1 | Atr-ERM98947 |  |  |  | | | |  |  |  |  |
| 1 | Atr-ERM98948 |  |  |  | | | |  |  |  |  |
| 1 | Atr-ERM98949 |  |  |  | Vvi-Vitvi08g01411\_t001 |  |  |  |  |
