# Supplementary material for: Genome-wide identification, characterization and gene expression of BES1 transcription factor family in grapevine (Vitis vinifera L.)
Source: Sci Rep. 2023 Jan 5;13:240. doi: 10.1038/s41598-022-24407-y (PMC9816167; doi:10.1038/s41598-022-24407-y)
Supplement: Supplementary file 3 — Supplementary Information. [file 41598_2022_24407_MOESM3_ESM.zip › Vvi_Atr/Vitis_vinifera.PN40024.v4.dna_sm.toplevel.fa.vs.Amborella_trichopoda.AMTR1.0.dna_sm.toplevel.fa.html/Atr-AmTr_v1.0_scaffold00115.html]

|  |  |  |  |  |  |  |  |  |  |  |  |  |  |
| --- | --- | --- | --- | --- | --- | --- | --- | --- | --- | --- | --- | --- | --- |
| Duplication depth | Reference chromosome | Collinear blocks | | | | | | | | | | | |
| 0 | Atr-ERM97865 |  |  |  |  |  |  |
| 0 | Atr-ERM97866 |  |  |  |  |  |  |
| 0 | Atr-ERM97867 |  |  |  |  |  |  |
| 0 | Atr-ERM97868 |  |  |  |  |  |  |
| 0 | Atr-ERM97869 |  |  |  |  |  |  |
| 0 | Atr-ERM97870 |  |  |  |  |  |  |
| 0 | Atr-ERM97871 |  |  |  |  |  |  |
| 0 | Atr-ERM97872 |  |  |  |  |  |  |
| 0 | Atr-ERM97873 |  |  |  |  |  |  |
| 0 | Atr-ERM97874 |  |  |  |  |  |  |
| 0 | Atr-ERM97875 |  |  |  |  |  |  |
| 0 | Atr-ERM97876 |  |  |  |  |  |  |
| 0 | Atr-ERM97877 |  |  |  |  |  |  |
| 0 | Atr-ERM97878 |  |  |  |  |  |  |
| 0 | Atr-ERM97879 |  |  |  |  |  |  |
| 0 | Atr-ERM97880 |  |  |  |  |  |  |
| 0 | Atr-ERM97881 |  |  |  |  |  |  |
| 0 | Atr-ERM97882 |  |  |  |  |  |  |
| 0 | Atr-ERM97883 |  |  |  |  |  |  |
| 0 | Atr-ERM97884 |  |  |  |  |  |  |
| 0 | Atr-ERM97885 |  |  |  |  |  |  |
| 0 | Atr-ERM97886 |  |  |  |  |  |  |
| 0 | Atr-ERM97887 |  |  |  |  |  |  |
| 0 | Atr-ERM97888 |  |  |  |  |  |  |
