# Supplementary material for: Genome-wide identification, characterization and gene expression of BES1 transcription factor family in grapevine (Vitis vinifera L.)
Source: Sci Rep. 2023 Jan 5;13:240. doi: 10.1038/s41598-022-24407-y (PMC9816167; doi:10.1038/s41598-022-24407-y)
Supplement: Supplementary file 3 — Supplementary Information. [file 41598_2022_24407_MOESM3_ESM.zip › Vvi_Atr/Vitis_vinifera.PN40024.v4.dna_sm.toplevel.fa.vs.Amborella_trichopoda.AMTR1.0.dna_sm.toplevel.fa.html/Atr-AmTr_v1.0_scaffold00116.html]

|  |  |  |  |  |  |  |  |  |  |  |  |  |  |
| --- | --- | --- | --- | --- | --- | --- | --- | --- | --- | --- | --- | --- | --- |
| Duplication depth | Reference chromosome | Collinear blocks | | | | | | | | | | | |
| 0 | Atr-ERM97760 |  |  |  |  |  |  |
| 0 | Atr-ERM97761 |  |  |  |  |  |  |
| 0 | Atr-ERM97762 |  |  |  |  |  |  |
| 0 | Atr-ERM97763 |  |  |  |  |  |  |
| 0 | Atr-ERM97764 |  |  |  |  |  |  |
| 0 | Atr-ERM97765 |  |  |  |  |  |  |
| 0 | Atr-ERM97766 |  |  |  |  |  |  |
| 0 | Atr-ERM97767 |  |  |  |  |  |  |
| 0 | Atr-ERM97768 |  |  |  |  |  |  |
| 0 | Atr-ERM97769 |  |  |  |  |  |  |
| 0 | Atr-ERM97770 |  |  |  |  |  |  |
| 0 | Atr-ERM97771 |  |  |  |  |  |  |
| 0 | Atr-ERM97772 |  |  |  |  |  |  |
| 0 | Atr-ERM97773 |  |  |  |  |  |  |
| 0 | Atr-ERM97774 |  |  |  |  |  |  |
| 0 | Atr-ERM97775 |  |  |  |  |  |  |
| 0 | Atr-ERM97776 |  |  |  |  |  |  |
| 0 | Atr-ERM97777 |  |  |  |  |  |  |
| 0 | Atr-ERM97778 |  |  |  |  |  |  |
| 0 | Atr-ERM97779 |  |  |  |  |  |  |
| 0 | Atr-ERM97780 |  |  |  |  |  |  |
| 0 | Atr-ERM97781 |  |  |  |  |  |  |
| 0 | Atr-ERM97782 |  |  |  |  |  |  |
| 0 | Atr-ERM97783 |  |  |  |  |  |  |
| 0 | Atr-ERM97784 |  |  |  |  |  |  |
| 0 | Atr-ERM97785 |  |  |  |  |  |  |
| 0 | Atr-ERM97786 |  |  |  |  |  |  |
| 0 | Atr-ERM97787 |  |  |  |  |  |  |
| 0 | Atr-ERM97788 |  |  |  |  |  |  |
| 0 | Atr-ERM97789 |  |  |  |  |  |  |
| 0 | Atr-ERM97790 |  |  |  |  |  |  |
| 0 | Atr-ERM97791 |  |  |  |  |  |  |
| 0 | Atr-ERM97792 |  |  |  |  |  |  |
| 0 | Atr-ERM97793 |  |  |  |  |  |  |
| 0 | Atr-ERM97794 |  |  |  |  |  |  |
| 0 | Atr-ERM97795 |  |  |  |  |  |  |
| 0 | Atr-ERM97796 |  |  |  |  |  |  |
| 0 | Atr-ERM97797 |  |  |  |  |  |  |
| 0 | Atr-ERM97798 |  |  |  |  |  |  |
| 0 | Atr-ERM97799 |  |  |  |  |  |  |
| 0 | Atr-ERM97800 |  |  |  |  |  |  |
| 0 | Atr-ERM97801 |  |  |  |  |  |  |
| 0 | Atr-ERM97802 |  |  |  |  |  |  |
| 0 | Atr-ERM97803 |  |  |  |  |  |  |
| 0 | Atr-ERM97804 |  |  |  |  |  |  |
| 0 | Atr-ERM97805 |  |  |  |  |  |  |
| 0 | Atr-ERM97806 |  |  |  |  |  |  |
| 0 | Atr-ERM97807 |  |  |  |  |  |  |
| 0 | Atr-ERM97808 |  |  |  |  |  |  |
| 0 | Atr-ERM97809 |  |  |  |  |  |  |
