# Supplementary material for: Genome-wide identification, characterization and gene expression of BES1 transcription factor family in grapevine (Vitis vinifera L.)
Source: Sci Rep. 2023 Jan 5;13:240. doi: 10.1038/s41598-022-24407-y (PMC9816167; doi:10.1038/s41598-022-24407-y)
Supplement: Supplementary file 3 — Supplementary Information. [file 41598_2022_24407_MOESM3_ESM.zip › Vvi_Atr/Vitis_vinifera.PN40024.v4.dna_sm.toplevel.fa.vs.Amborella_trichopoda.AMTR1.0.dna_sm.toplevel.fa.html/Atr-AmTr_v1.0_scaffold00117.html]

|  |  |  |  |  |  |  |  |  |  |  |  |  |  |
| --- | --- | --- | --- | --- | --- | --- | --- | --- | --- | --- | --- | --- | --- |
| Duplication depth | Reference chromosome | Collinear blocks | | | | | | | | | | | |
| 0 | Atr-ERM97931 |  |  |  |  |  |  |
| 0 | Atr-ERM97932 |  |  |  |  |  |  |
| 0 | Atr-ERM97933 |  |  |  |  |  |  |
| 0 | Atr-ERM97934 |  |  |  |  |  |  |
| 0 | Atr-ERM97935 |  |  |  |  |  |  |
| 0 | Atr-ERM97936 |  |  |  |  |  |  |
| 0 | Atr-ERM97937 |  |  |  |  |  |  |
| 0 | Atr-ERM97938 |  |  |  |  |  |  |
| 0 | Atr-ERM97939 |  |  |  |  |  |  |
| 0 | Atr-ERM97940 |  |  |  |  |  |  |
| 0 | Atr-ERM97941 |  |  |  |  |  |  |
| 0 | Atr-ERM97942 |  |  |  |  |  |  |
| 0 | Atr-ERM97943 |  |  |  |  |  |  |
| 0 | Atr-ERM97944 |  |  |  |  |  |  |
| 0 | Atr-ERM97945 |  |  |  |  |  |  |
| 0 | Atr-ERM97946 |  |  |  |  |  |  |
| 0 | Atr-ERM97947 |  |  |  |  |  |  |
| 0 | Atr-ERM97948 |  |  |  |  |  |  |
| 0 | Atr-ERM97949 |  |  |  |  |  |  |
| 0 | Atr-ERM97950 |  |  |  |  |  |  |
| 0 | Atr-ERM97951 |  |  |  |  |  |  |
| 0 | Atr-ERM97952 |  |  |  |  |  |  |
| 0 | Atr-ERM97953 |  |  |  |  |  |  |
| 0 | Atr-ERM97954 |  |  |  |  |  |  |
| 0 | Atr-ERM97955 |  |  |  |  |  |  |
| 0 | Atr-ERM97956 |  |  |  |  |  |  |
| 0 | Atr-ERM97957 |  |  |  |  |  |  |
| 0 | Atr-ERM97958 |  |  |  |  |  |  |
| 0 | Atr-ERM97959 |  |  |  |  |  |  |
| 0 | Atr-ERM97960 |  |  |  |  |  |  |
| 0 | Atr-ERM97961 |  |  |  |  |  |  |
| 0 | Atr-ERM97962 |  |  |  |  |  |  |
| 0 | Atr-ERM97963 |  |  |  |  |  |  |
| 0 | Atr-ERM97964 |  |  |  |  |  |  |
| 0 | Atr-ERM97965 |  |  |  |  |  |  |
| 0 | Atr-ERM97966 |  |  |  |  |  |  |
| 0 | Atr-ERM97967 |  |  |  |  |  |  |
| 0 | Atr-ERM97968 |  |  |  |  |  |  |
| 0 | Atr-ERM97969 |  |  |  |  |  |  |
| 0 | Atr-ERM97970 |  |  |  |  |  |  |
| 0 | Atr-ERM97971 |  |  |  |  |  |  |
| 0 | Atr-ERM97972 |  |  |  |  |  |  |
| 0 | Atr-ERM97973 |  |  |  |  |  |  |
| 0 | Atr-ERM97974 |  |  |  |  |  |  |
| 0 | Atr-ERM97975 |  |  |  |  |  |  |
| 0 | Atr-ERM97976 |  |  |  |  |  |  |
| 0 | Atr-ERM97977 |  |  |  |  |  |  |
| 0 | Atr-ERM97978 |  |  |  |  |  |  |
| 0 | Atr-ERM97979 |  |  |  |  |  |  |
| 0 | Atr-ERM97980 |  |  |  |  |  |  |
| 0 | Atr-ERM97981 |  |  |  |  |  |  |
| 0 | Atr-ERM97982 |  |  |  |  |  |  |
| 0 | Atr-ERM97983 |  |  |  |  |  |  |
| 0 | Atr-ERM97984 |  |  |  |  |  |  |
| 0 | Atr-ERM97985 |  |  |  |  |  |  |
| 0 | Atr-ERM97986 |  |  |  |  |  |  |
| 0 | Atr-ERM97987 |  |  |  |  |  |  |
| 0 | Atr-ERM97988 |  |  |  |  |  |  |
| 0 | Atr-ERM97989 |  |  |  |  |  |  |
| 0 | Atr-ERM97990 |  |  |  |  |  |  |
| 0 | Atr-ERM97991 |  |  |  |  |  |  |
| 0 | Atr-ERM97992 |  |  |  |  |  |  |
| 0 | Atr-ERM97993 |  |  |  |  |  |  |
| 0 | Atr-ERM97994 |  |  |  |  |  |  |
| 0 | Atr-ERM97995 |  |  |  |  |  |  |
| 0 | Atr-ERM97996 |  |  |  |  |  |  |
| 0 | Atr-ERM97997 |  |  |  |  |  |  |
