# Supplementary material for: Genome-wide identification, characterization and gene expression of BES1 transcription factor family in grapevine (Vitis vinifera L.)
Source: Sci Rep. 2023 Jan 5;13:240. doi: 10.1038/s41598-022-24407-y (PMC9816167; doi:10.1038/s41598-022-24407-y)
Supplement: Supplementary file 3 — Supplementary Information. [file 41598_2022_24407_MOESM3_ESM.zip › Vvi_Atr/Vitis_vinifera.PN40024.v4.dna_sm.toplevel.fa.vs.Amborella_trichopoda.AMTR1.0.dna_sm.toplevel.fa.html/Atr-AmTr_v1.0_scaffold00118.html]

|  |  |  |  |  |  |  |  |  |  |  |  |  |  |
| --- | --- | --- | --- | --- | --- | --- | --- | --- | --- | --- | --- | --- | --- |
| Duplication depth | Reference chromosome | Collinear blocks | | | | | | | | | | | |
| 0 | Atr-ERM97819 |  |  |  |  |  |  |
| 0 | Atr-ERM97820 |  |  |  |  |  |  |
| 0 | Atr-ERM97821 |  |  |  |  |  |  |
| 0 | Atr-ERM97822 |  |  |  |  |  |  |
| 0 | Atr-ERM97823 |  |  |  |  |  |  |
| 0 | Atr-ERM97824 |  |  |  |  |  |  |
| 0 | Atr-ERM97825 |  |  |  |  |  |  |
| 0 | Atr-ERM97826 |  |  |  |  |  |  |
| 0 | Atr-ERM97827 |  |  |  |  |  |  |
| 0 | Atr-ERM97828 |  |  |  |  |  |  |
| 0 | Atr-ERM97829 |  |  |  |  |  |  |
| 0 | Atr-ERM97830 |  |  |  |  |  |  |
| 0 | Atr-ERM97831 |  |  |  |  |  |  |
| 0 | Atr-ERM97832 |  |  |  |  |  |  |
| 0 | Atr-ERM97833 |  |  |  |  |  |  |
| 0 | Atr-ERM97834 |  |  |  |  |  |  |
| 0 | Atr-ERM97835 |  |  |  |  |  |  |
| 0 | Atr-ERM97836 |  |  |  |  |  |  |
| 0 | Atr-ERM97837 |  |  |  |  |  |  |
| 0 | Atr-ERM97838 |  |  |  |  |  |  |
| 0 | Atr-ERM97839 |  |  |  |  |  |  |
| 0 | Atr-ERM97840 |  |  |  |  |  |  |
| 0 | Atr-ERM97841 |  |  |  |  |  |  |
| 0 | Atr-ERM97842 |  |  |  |  |  |  |
| 0 | Atr-ERM97843 |  |  |  |  |  |  |
| 0 | Atr-ERM97844 |  |  |  |  |  |  |
| 0 | Atr-ERM97845 |  |  |  |  |  |  |
| 0 | Atr-ERM97846 |  |  |  |  |  |  |
| 0 | Atr-ERM97847 |  |  |  |  |  |  |
| 0 | Atr-ERM97848 |  |  |  |  |  |  |
| 0 | Atr-ERM97849 |  |  |  |  |  |  |
| 0 | Atr-ERM97850 |  |  |  |  |  |  |
| 0 | Atr-ERM97851 |  |  |  |  |  |  |
| 0 | Atr-ERM97852 |  |  |  |  |  |  |
| 0 | Atr-ERM97853 |  |  |  |  |  |  |
| 0 | Atr-ERM97854 |  |  |  |  |  |  |
| 0 | Atr-ERM97855 |  |  |  |  |  |  |
| 0 | Atr-ERM97856 |  |  |  |  |  |  |
