# Supplementary material for: Genome-wide identification, characterization and gene expression of BES1 transcription factor family in grapevine (Vitis vinifera L.)
Source: Sci Rep. 2023 Jan 5;13:240. doi: 10.1038/s41598-022-24407-y (PMC9816167; doi:10.1038/s41598-022-24407-y)
Supplement: Supplementary file 3 — Supplementary Information. [file 41598_2022_24407_MOESM3_ESM.zip › Vvi_Atr/Vitis_vinifera.PN40024.v4.dna_sm.toplevel.fa.vs.Amborella_trichopoda.AMTR1.0.dna_sm.toplevel.fa.html/Atr-AmTr_v1.0_scaffold00119.html]

|  |  |  |  |  |  |  |  |  |  |  |  |  |  |
| --- | --- | --- | --- | --- | --- | --- | --- | --- | --- | --- | --- | --- | --- |
| Duplication depth | Reference chromosome | Collinear blocks | | | | | | | | | | | |
| 0 | Atr-ERM97174 |  |  |  |  |  |  |
| 0 | Atr-ERM97175 |  |  |  |  |  |  |
| 0 | Atr-ERM97176 |  |  |  |  |  |  |
| 0 | Atr-ERM97177 |  |  |  |  |  |  |
| 0 | Atr-ERM97178 |  |  |  |  |  |  |
| 0 | Atr-ERM97179 |  |  |  |  |  |  |
| 0 | Atr-ERM97180 |  |  |  |  |  |  |
| 0 | Atr-ERM97181 |  |  |  |  |  |  |
| 0 | Atr-ERM97182 |  |  |  |  |  |  |
| 0 | Atr-ERM97183 |  |  |  |  |  |  |
| 0 | Atr-ERM97184 |  |  |  |  |  |  |
| 1 | Atr-ERM97185 |  | Vvi-Vitvi06g00007\_t001 |  |  |  |  |  |
| 1 | Atr-ERM97186 |  | | | |  |  |  |  |  |
| 1 | Atr-ERM97187 |  | | | |  |  |  |  |  |
| 1 | Atr-ERM97188 |  | | | |  |  |  |  |  |
| 1 | Atr-ERM97189 |  | | | |  |  |  |  |  |
| 1 | Atr-ERM97190 |  | | | |  |  |  |  |  |
| 1 | Atr-ERM97191 |  | | | |  |  |  |  |  |
| 1 | Atr-ERM97192 |  | | | |  |  |  |  |  |
| 1 | Atr-ERM97193 |  | | | |  |  |  |  |  |
| 1 | Atr-ERM97194 |  | | | |  |  |  |  |  |
| 1 | Atr-ERM97195 |  | Vvi-Vitvi06g00008\_t001 |  |  |  |  |  |
| 1 | Atr-ERM97196 |  | | | |  |  |  |  |  |
| 1 | Atr-ERM97197 |  | | | |  |  |  |  |  |
| 1 | Atr-ERM97198 |  | | | |  |  |  |  |  |
| 1 | Atr-ERM97199 |  | | | |  |  |  |  |  |
| 1 | Atr-ERM97200 |  | | | |  |  |  |  |  |
| 1 | Atr-ERM97201 |  | | | |  |  |  |  |  |
| 1 | Atr-ERM97202 |  | | | |  |  |  |  |  |
| 1 | Atr-ERM97203 |  | Vvi-Vitvi06g00009\_t001 |  |  |  |  |  |
| 1 | Atr-ERM97204 |  | | | |  |  |  |  |  |
| 1 | Atr-ERM97205 |  | Vvi-Vitvi06g00012\_t001 |  |  |  |  |  |
| 1 | Atr-ERM97206 |  | | | |  |  |  |  |  |
| 1 | Atr-ERM97207 |  | Vvi-Vitvi06g00013\_t001 |  |  |  |  |  |
| 1 | Atr-ERM97208 |  | | | |  |  |  |  |  |
| 1 | Atr-ERM97209 |  | | | |  |  |  |  |  |
| 1 | Atr-ERM97210 |  | Vvi-Vitvi06g00015\_t001 |  |  |  |  |  |
| 1 | Atr-ERM97211 |  | | | |  |  |  |  |  |
| 1 | Atr-ERM97212 |  | Vvi-Vitvi06g00016\_t001 |  |  |  |  |  |
| 1 | Atr-ERM97213 |  | | | |  |  |  |  |  |
| 1 | Atr-ERM97214 |  | | | |  |  |  |  |  |
| 1 | Atr-ERM97215 |  | | | |  |  |  |  |  |
| 1 | Atr-ERM97216 |  | | | |  |  |  |  |  |
| 1 | Atr-ERM97217 |  | | | |  |  |  |  |  |
| 1 | Atr-ERM97218 |  | | | |  |  |  |  |  |
| 2 | Atr-ERM97219 |  | | | |  | Vvi-Vitvi01g01018\_t001 |  |  |  |  |
| 2 | Atr-ERM97220 |  | | | |  | | | |  |  |  |  |
| 2 | Atr-ERM97221 |  | | | |  | | | |  |  |  |  |
| 2 | Atr-ERM97222 |  | | | |  | | | |  |  |  |  |
| 2 | Atr-ERM97223 |  | | | |  | | | |  |  |  |  |
| 2 | Atr-ERM97224 |  | | | |  | | | |  |  |  |  |
| 2 | Atr-ERM97225 |  | | | |  | | | |  |  |  |  |
| 2 | Atr-ERM97226 |  | | | |  | | | |  |  |  |  |
| 2 | Atr-ERM97227 |  | | | |  | | | |  |  |  |  |
| 2 | Atr-ERM97228 |  | | | |  | | | |  |  |  |  |
| 2 | Atr-ERM97229 |  | | | |  | | | |  |  |  |  |
| 2 | Atr-ERM97230 |  | | | |  | | | |  |  |  |  |
| 2 | Atr-ERM97231 |  | Vvi-Vitvi06g00017\_t001 |  | | | |  |  |  |  |
| 2 | Atr-ERM97232 |  | | | |  | | | |  |  |  |  |
| 2 | Atr-ERM97233 |  | | | |  | | | |  |  |  |  |
| 2 | Atr-ERM97234 |  | | | |  | | | |  |  |  |  |
| 2 | Atr-ERM97235 |  | Vvi-Vitvi06g00018\_t002 |  | | | |  |  |  |  |
| 2 | Atr-ERM97236 |  | Vvi-Vitvi06g00019\_t001 |  | | | |  |  |  |  |
| 2 | Atr-ERM97237 |  | | | |  | | | |  |  |  |  |
| 2 | Atr-ERM97238 |  | | | |  | | | |  |  |  |  |
| 2 | Atr-ERM97239 |  | | | |  | | | |  |  |  |  |
| 2 | Atr-ERM97240 |  | Vvi-Vitvi06g00020\_t001 |  | Vvi-Vitvi01g01023\_t001 |  |  |  |  |
| 2 | Atr-ERM97241 |  | Vvi-Vitvi06g00021\_t001 |  | | | |  |  |  |  |
| 2 | Atr-ERM97242 |  | Vvi-Vitvi06g00022\_t001 |  | | | |  |  |  |  |
| 2 | Atr-ERM97243 |  | | | |  | | | |  |  |  |  |
| 2 | Atr-ERM97244 |  | | | |  | | | |  |  |  |  |
| 2 | Atr-ERM97245 |  | | | |  | | | |  |  |  |  |
| 2 | Atr-ERM97246 |  | Vvi-Vitvi06g00024\_t001 |  | Vvi-Vitvi01g01024\_t001 |  |  |  |  |
| 2 | Atr-ERM97247 |  | Vvi-Vitvi06g00026\_t001 |  | | | |  |  |  |  |
| 1 | Atr-ERM97248 |  |  |  | | | |  |  |  |  |
| 1 | Atr-ERM97249 |  |  |  | | | |  |  |  |  |
| 1 | Atr-ERM97250 |  |  |  | | | |  |  |  |  |
| 2 | Atr-ERM97251 |  | Vvi-Vitvi08g00110\_t001 |  | Vvi-Vitvi01g01026\_t001 |  |  |  |  |
| 2 | Atr-ERM97252 |  | Vvi-Vitvi08g00108\_t001 |  | | | |  |  |  |  |
| 2 | Atr-ERM97253 |  | | | |  | Vvi-Vitvi01g01027\_t001 |  |  |  |  |
| 2 | Atr-ERM97254 |  | | | |  | | | |  |  |  |  |
| 2 | Atr-ERM97255 |  | Vvi-Vitvi08g00107\_t001 |  | Vvi-Vitvi01g01028\_t001 |  |  |  |  |
| 1 | Atr-ERM97256 |  | Vvi-Vitvi08g00105\_t001 |  |  |  |  |  |
| 1 | Atr-ERM97257 |  | Vvi-Vitvi08g00083\_t001 |  |  |  |  |  |
| 1 | Atr-ERM97258 |  | | | |  |  |  |  |  |
| 1 | Atr-ERM97259 |  | | | |  |  |  |  |  |
| 1 | Atr-ERM97260 |  | | | |  |  |  |  |  |
| 1 | Atr-ERM97261 |  | | | |  |  |  |  |  |
| 1 | Atr-ERM97262 |  | | | |  |  |  |  |  |
| 1 | Atr-ERM97263 |  | | | |  |  |  |  |  |
| 1 | Atr-ERM97264 |  | | | |  |  |  |  |  |
| 1 | Atr-ERM97265 |  | | | |  |  |  |  |  |
| 1 | Atr-ERM97266 |  | | | |  |  |  |  |  |
| 1 | Atr-ERM97267 |  | | | |  |  |  |  |  |
| 1 | Atr-ERM97268 |  | | | |  |  |  |  |  |
| 1 | Atr-ERM97269 |  | | | |  |  |  |  |  |
| 1 | Atr-ERM97270 |  | | | |  |  |  |  |  |
| 1 | Atr-ERM97271 |  | | | |  |  |  |  |  |
| 1 | Atr-ERM97272 |  | | | |  |  |  |  |  |
| 1 | Atr-ERM97273 |  | | | |  |  |  |  |  |
| 1 | Atr-ERM97274 |  | | | |  |  |  |  |  |
| 1 | Atr-ERM97275 |  | | | |  |  |  |  |  |
| 1 | Atr-ERM97276 |  | | | |  |  |  |  |  |
| 1 | Atr-ERM97277 |  | | | |  |  |  |  |  |
| 1 | Atr-ERM97278 |  | | | |  |  |  |  |  |
| 1 | Atr-ERM97279 |  | | | |  |  |  |  |  |
| 1 | Atr-ERM97280 |  | Vvi-Vitvi08g00079\_t001 |  |  |  |  |  |
| 1 | Atr-ERM97281 |  | | | |  |  |  |  |  |
| 1 | Atr-ERM97282 |  | Vvi-Vitvi08g00070\_t001 |  |  |  |  |  |
| 0 | Atr-ERM97283 |  |  |  |  |  |  |
| 0 | Atr-ERM97284 |  |  |  |  |  |  |
| 0 | Atr-ERM97285 |  |  |  |  |  |  |
| 0 | Atr-ERM97286 |  |  |  |  |  |  |
| 0 | Atr-ERM97287 |  |  |  |  |  |  |
| 0 | Atr-ERM97288 |  |  |  |  |  |  |
| 0 | Atr-ERM97289 |  |  |  |  |  |  |
| 0 | Atr-ERM97290 |  |  |  |  |  |  |
| 0 | Atr-ERM97291 |  |  |  |  |  |  |
| 0 | Atr-ERM97292 |  |  |  |  |  |  |
