# Supplementary material for: Genome-wide identification, characterization and gene expression of BES1 transcription factor family in grapevine (Vitis vinifera L.)
Source: Sci Rep. 2023 Jan 5;13:240. doi: 10.1038/s41598-022-24407-y (PMC9816167; doi:10.1038/s41598-022-24407-y)
Supplement: Supplementary file 3 — Supplementary Information. [file 41598_2022_24407_MOESM3_ESM.zip › Vvi_Atr/Vitis_vinifera.PN40024.v4.dna_sm.toplevel.fa.vs.Amborella_trichopoda.AMTR1.0.dna_sm.toplevel.fa.html/Atr-AmTr_v1.0_scaffold00120.html]

|  |  |  |  |  |  |  |  |  |  |  |  |  |  |
| --- | --- | --- | --- | --- | --- | --- | --- | --- | --- | --- | --- | --- | --- |
| Duplication depth | Reference chromosome | Collinear blocks | | | | | | | | | | | |
| 0 | Atr-ERM98002 |  |  |  |  |  |  |
| 0 | Atr-ERM98003 |  |  |  |  |  |  |
| 0 | Atr-ERM98004 |  |  |  |  |  |  |
| 0 | Atr-ERM98005 |  |  |  |  |  |  |
| 0 | Atr-ERM98006 |  |  |  |  |  |  |
| 0 | Atr-ERM98007 |  |  |  |  |  |  |
| 0 | Atr-ERM98008 |  |  |  |  |  |  |
| 0 | Atr-ERM98009 |  |  |  |  |  |  |
| 0 | Atr-ERM98010 |  |  |  |  |  |  |
| 0 | Atr-ERM98011 |  |  |  |  |  |  |
| 0 | Atr-ERM98012 |  |  |  |  |  |  |
| 0 | Atr-ERM98013 |  |  |  |  |  |  |
| 0 | Atr-ERM98014 |  |  |  |  |  |  |
| 0 | Atr-ERM98015 |  |  |  |  |  |  |
| 0 | Atr-ERM98016 |  |  |  |  |  |  |
| 0 | Atr-ERM98017 |  |  |  |  |  |  |
| 0 | Atr-ERM98018 |  |  |  |  |  |  |
| 0 | Atr-ERM98019 |  |  |  |  |  |  |
| 0 | Atr-ERM98020 |  |  |  |  |  |  |
| 0 | Atr-ERM98021 |  |  |  |  |  |  |
| 0 | Atr-ERM98022 |  |  |  |  |  |  |
| 0 | Atr-ERM98023 |  |  |  |  |  |  |
| 0 | Atr-ERM98024 |  |  |  |  |  |  |
| 0 | Atr-ERM98025 |  |  |  |  |  |  |
| 0 | Atr-ERM98026 |  |  |  |  |  |  |
| 0 | Atr-ERM98027 |  |  |  |  |  |  |
| 0 | Atr-ERM98028 |  |  |  |  |  |  |
| 0 | Atr-ERM98029 |  |  |  |  |  |  |
| 0 | Atr-ERM98030 |  |  |  |  |  |  |
| 0 | Atr-ERM98031 |  |  |  |  |  |  |
| 0 | Atr-ERM98032 |  |  |  |  |  |  |
| 0 | Atr-ERM98033 |  |  |  |  |  |  |
| 0 | Atr-ERM98034 |  |  |  |  |  |  |
| 0 | Atr-ERM98035 |  |  |  |  |  |  |
| 0 | Atr-ERM98036 |  |  |  |  |  |  |
| 0 | Atr-ERM98037 |  |  |  |  |  |  |
| 0 | Atr-ERM98038 |  |  |  |  |  |  |
| 0 | Atr-ERM98039 |  |  |  |  |  |  |
| 0 | Atr-ERM98040 |  |  |  |  |  |  |
| 0 | Atr-ERM98041 |  |  |  |  |  |  |
| 0 | Atr-ERM98042 |  |  |  |  |  |  |
| 0 | Atr-ERM98043 |  |  |  |  |  |  |
| 0 | Atr-ERM98044 |  |  |  |  |  |  |
| 0 | Atr-ERM98045 |  |  |  |  |  |  |
| 0 | Atr-ERM98046 |  |  |  |  |  |  |
| 0 | Atr-ERM98047 |  |  |  |  |  |  |
| 0 | Atr-ERM98048 |  |  |  |  |  |  |
| 0 | Atr-ERM98049 |  |  |  |  |  |  |
| 0 | Atr-ERM98050 |  |  |  |  |  |  |
| 0 | Atr-ERM98051 |  |  |  |  |  |  |
| 0 | Atr-ERM98052 |  |  |  |  |  |  |
| 0 | Atr-ERM98053 |  |  |  |  |  |  |
| 0 | Atr-ERM98054 |  |  |  |  |  |  |
| 0 | Atr-ERM98055 |  |  |  |  |  |  |
| 0 | Atr-ERM98056 |  |  |  |  |  |  |
| 0 | Atr-ERM98057 |  |  |  |  |  |  |
| 0 | Atr-ERM98058 |  |  |  |  |  |  |
| 0 | Atr-ERM98059 |  |  |  |  |  |  |
| 0 | Atr-ERM98060 |  |  |  |  |  |  |
| 0 | Atr-ERM98061 |  |  |  |  |  |  |
| 0 | Atr-ERM98062 |  |  |  |  |  |  |
| 0 | Atr-ERM98063 |  |  |  |  |  |  |
