# Supplementary material for: Genome-wide identification, characterization and gene expression of BES1 transcription factor family in grapevine (Vitis vinifera L.)
Source: Sci Rep. 2023 Jan 5;13:240. doi: 10.1038/s41598-022-24407-y (PMC9816167; doi:10.1038/s41598-022-24407-y)
Supplement: Supplementary file 3 — Supplementary Information. [file 41598_2022_24407_MOESM3_ESM.zip › Vvi_Atr/Vitis_vinifera.PN40024.v4.dna_sm.toplevel.fa.vs.Amborella_trichopoda.AMTR1.0.dna_sm.toplevel.fa.html/Atr-AmTr_v1.0_scaffold00121.html]

|  |  |  |  |  |  |  |  |  |  |  |  |  |  |
| --- | --- | --- | --- | --- | --- | --- | --- | --- | --- | --- | --- | --- | --- |
| Duplication depth | Reference chromosome | Collinear blocks | | | | | | | | | | | |
| 0 | Atr-ERM97697 |  |  |  |  |  |  |
| 0 | Atr-ERM97698 |  |  |  |  |  |  |
| 0 | Atr-ERM97699 |  |  |  |  |  |  |
| 0 | Atr-ERM97700 |  |  |  |  |  |  |
| 0 | Atr-ERM97701 |  |  |  |  |  |  |
| 0 | Atr-ERM97702 |  |  |  |  |  |  |
| 0 | Atr-ERM97703 |  |  |  |  |  |  |
| 0 | Atr-ERM97704 |  |  |  |  |  |  |
| 0 | Atr-ERM97705 |  |  |  |  |  |  |
| 0 | Atr-ERM97706 |  |  |  |  |  |  |
| 0 | Atr-ERM97707 |  |  |  |  |  |  |
| 0 | Atr-ERM97708 |  |  |  |  |  |  |
| 0 | Atr-ERM97709 |  |  |  |  |  |  |
| 0 | Atr-ERM97710 |  |  |  |  |  |  |
| 0 | Atr-ERM97711 |  |  |  |  |  |  |
| 0 | Atr-ERM97712 |  |  |  |  |  |  |
| 0 | Atr-ERM97713 |  |  |  |  |  |  |
| 0 | Atr-ERM97714 |  |  |  |  |  |  |
| 0 | Atr-ERM97715 |  |  |  |  |  |  |
| 0 | Atr-ERM97716 |  |  |  |  |  |  |
| 0 | Atr-ERM97717 |  |  |  |  |  |  |
| 0 | Atr-ERM97718 |  |  |  |  |  |  |
| 0 | Atr-ERM97719 |  |  |  |  |  |  |
| 0 | Atr-ERM97720 |  |  |  |  |  |  |
| 0 | Atr-ERM97721 |  |  |  |  |  |  |
| 0 | Atr-ERM97722 |  |  |  |  |  |  |
| 0 | Atr-ERM97723 |  |  |  |  |  |  |
| 0 | Atr-ERM97724 |  |  |  |  |  |  |
| 0 | Atr-ERM97725 |  |  |  |  |  |  |
| 0 | Atr-ERM97726 |  |  |  |  |  |  |
| 0 | Atr-ERM97727 |  |  |  |  |  |  |
| 0 | Atr-ERM97728 |  |  |  |  |  |  |
| 0 | Atr-ERM97729 |  |  |  |  |  |  |
| 0 | Atr-ERM97730 |  |  |  |  |  |  |
| 0 | Atr-ERM97731 |  |  |  |  |  |  |
| 0 | Atr-ERM97732 |  |  |  |  |  |  |
| 0 | Atr-ERM97733 |  |  |  |  |  |  |
| 0 | Atr-ERM97734 |  |  |  |  |  |  |
| 0 | Atr-ERM97735 |  |  |  |  |  |  |
| 0 | Atr-ERM97736 |  |  |  |  |  |  |
| 0 | Atr-ERM97737 |  |  |  |  |  |  |
| 0 | Atr-ERM97738 |  |  |  |  |  |  |
| 0 | Atr-ERM97739 |  |  |  |  |  |  |
| 0 | Atr-ERM97740 |  |  |  |  |  |  |
| 0 | Atr-ERM97741 |  |  |  |  |  |  |
| 0 | Atr-ERM97742 |  |  |  |  |  |  |
| 0 | Atr-ERM97743 |  |  |  |  |  |  |
