# Supplementary material for: Genome-wide identification, characterization and gene expression of BES1 transcription factor family in grapevine (Vitis vinifera L.)
Source: Sci Rep. 2023 Jan 5;13:240. doi: 10.1038/s41598-022-24407-y (PMC9816167; doi:10.1038/s41598-022-24407-y)
Supplement: Supplementary file 3 — Supplementary Information. [file 41598_2022_24407_MOESM3_ESM.zip › Vvi_Atr/Vitis_vinifera.PN40024.v4.dna_sm.toplevel.fa.vs.Amborella_trichopoda.AMTR1.0.dna_sm.toplevel.fa.html/Atr-AmTr_v1.0_scaffold00122.html]

|  |  |  |  |  |  |  |  |  |  |  |  |  |  |
| --- | --- | --- | --- | --- | --- | --- | --- | --- | --- | --- | --- | --- | --- |
| Duplication depth | Reference chromosome | Collinear blocks | | | | | | | | | | | |
| 0 | Atr-ERM97018 |  |  |  |  |  |  |
| 0 | Atr-ERM97019 |  |  |  |  |  |  |
| 0 | Atr-ERM97020 |  |  |  |  |  |  |
| 0 | Atr-ERM97021 |  |  |  |  |  |  |
| 1 | Atr-ERM97022 |  | Vvi-Vitvi09g00436\_t001 |  |  |  |  |  |
| 2 | Atr-ERM97023 |  | Vvi-Vitvi09g00437\_t001 |  | Vvi-Vitvi11g00394\_t001 |  |  |  |  |
| 2 | Atr-ERM97024 |  | | | |  | | | |  |  |  |  |
| 2 | Atr-ERM97025 |  | | | |  | | | |  |  |  |  |
| 2 | Atr-ERM97026 |  | | | |  | | | |  |  |  |  |
| 2 | Atr-ERM97027 |  | | | |  | | | |  |  |  |  |
| 2 | Atr-ERM97028 |  | | | |  | | | |  |  |  |  |
| 2 | Atr-ERM97029 |  | | | |  | | | |  |  |  |  |
| 2 | Atr-ERM97030 |  | | | |  | Vvi-Vitvi11g00396\_t001 |  |  |  |  |
| 2 | Atr-ERM97031 |  | | | |  | | | |  |  |  |  |
| 2 | Atr-ERM97032 |  | | | |  | | | |  |  |  |  |
| 2 | Atr-ERM97033 |  | | | |  | | | |  |  |  |  |
| 2 | Atr-ERM97034 |  | | | |  | | | |  |  |  |  |
| 2 | Atr-ERM97035 |  | | | |  | | | |  |  |  |  |
| 2 | Atr-ERM97036 |  | | | |  | | | |  |  |  |  |
| 2 | Atr-ERM97037 |  | | | |  | | | |  |  |  |  |
| 2 | Atr-ERM97038 |  | | | |  | | | |  |  |  |  |
| 2 | Atr-ERM97039 |  | | | |  | | | |  |  |  |  |
| 2 | Atr-ERM97040 |  | | | |  | | | |  |  |  |  |
| 2 | Atr-ERM97041 |  | | | |  | | | |  |  |  |  |
| 2 | Atr-ERM97042 |  | | | |  | | | |  |  |  |  |
| 2 | Atr-ERM97043 |  | | | |  | | | |  |  |  |  |
| 2 | Atr-ERM97044 |  | | | |  | | | |  |  |  |  |
| 2 | Atr-ERM97045 |  | Vvi-Vitvi09g00439\_t001 |  | | | |  |  |  |  |
| 2 | Atr-ERM97046 |  | | | |  | | | |  |  |  |  |
| 2 | Atr-ERM97047 |  | Vvi-Vitvi09g00446\_t001 |  | | | |  |  |  |  |
| 2 | Atr-ERM97048 |  | | | |  | | | |  |  |  |  |
| 2 | Atr-ERM97049 |  | | | |  | | | |  |  |  |  |
| 2 | Atr-ERM97050 |  | | | |  | Vvi-Vitvi11g00398\_t001 |  |  |  |  |
| 3 | Atr-ERM97051 |  | | | |  | | | |  | Vvi-Vitvi04g00436\_t001 |  |  |  |
| 3 | Atr-ERM97052 |  | Vvi-Vitvi09g00448\_t001 |  | | | |  | Vvi-Vitvi04g00435\_t001 |  |  |  |
| 3 | Atr-ERM97053 |  | | | |  | | | |  | Vvi-Vitvi04g00434\_t001 |  |  |  |
| 3 | Atr-ERM97054 |  | | | |  | | | |  | | | |  |  |  |
| 3 | Atr-ERM97055 |  | | | |  | Vvi-Vitvi11g00399\_t001 |  | Vvi-Vitvi04g00431\_t003 |  |  |  |
| 3 | Atr-ERM97056 |  | | | |  | Vvi-Vitvi11g00400\_t001 |  | | | |  |  |  |
| 3 | Atr-ERM97057 |  | | | |  | | | |  | | | |  |  |  |
| 3 | Atr-ERM97058 |  | | | |  | | | |  | Vvi-Vitvi04g00430\_t001 |  |  |  |
| 3 | Atr-ERM97059 |  | | | |  | | | |  | | | |  |  |  |
| 3 | Atr-ERM97060 |  | | | |  | | | |  | | | |  |  |  |
| 3 | Atr-ERM97061 |  | | | |  | | | |  | | | |  |  |  |
| 3 | Atr-ERM97062 |  | | | |  | | | |  | | | |  |  |  |
| 3 | Atr-ERM97063 |  | | | |  | | | |  | | | |  |  |  |
| 3 | Atr-ERM97064 |  | | | |  | | | |  | | | |  |  |  |
| 3 | Atr-ERM97065 |  | | | |  | | | |  | Vvi-Vitvi04g00419\_t001 |  |  |  |
| 3 | Atr-ERM97066 |  | | | |  | Vvi-Vitvi11g00401\_t001 |  | | | |  |  |  |
| 3 | Atr-ERM97067 |  | | | |  | | | |  | | | |  |  |  |
| 3 | Atr-ERM97068 |  | | | |  | Vvi-Vitvi11g00402\_t001 |  | Vvi-Vitvi04g00405\_t001 |  |  |  |
| 2 | Atr-ERM97069 |  | | | |  | | | |  |  |  |  |
| 2 | Atr-ERM97070 |  | | | |  | | | |  |  |  |  |
| 2 | Atr-ERM97071 |  | Vvi-Vitvi09g04146\_t001 |  | | | |  |  |  |  |
| 2 | Atr-ERM97072 |  | | | |  | | | |  |  |  |  |
| 2 | Atr-ERM97073 |  | | | |  | | | |  |  |  |  |
| 2 | Atr-ERM97074 |  | | | |  | | | |  |  |  |  |
| 2 | Atr-ERM97075 |  | | | |  | | | |  |  |  |  |
| 2 | Atr-ERM97076 |  | | | |  | | | |  |  |  |  |
| 2 | Atr-ERM97077 |  | | | |  | | | |  |  |  |  |
| 2 | Atr-ERM97078 |  | | | |  | | | |  |  |  |  |
| 2 | Atr-ERM97079 |  | | | |  | | | |  |  |  |  |
| 2 | Atr-ERM97080 |  | | | |  | | | |  |  |  |  |
| 2 | Atr-ERM97081 |  | | | |  | | | |  |  |  |  |
| 2 | Atr-ERM97082 |  | | | |  | Vvi-Vitvi11g00405\_t001 |  |  |  |  |
| 2 | Atr-ERM97083 |  | Vvi-Vitvi09g00482\_t001 |  | | | |  |  |  |  |
| 2 | Atr-ERM97084 |  | | | |  | | | |  |  |  |  |
| 2 | Atr-ERM97085 |  | | | |  | | | |  |  |  |  |
| 2 | Atr-ERM97086 |  | | | |  | | | |  |  |  |  |
| 2 | Atr-ERM97087 |  | | | |  | | | |  |  |  |  |
| 2 | Atr-ERM97088 |  | Vvi-Vitvi09g00486\_t001 |  | | | |  |  |  |  |
| 2 | Atr-ERM97089 |  | | | |  | Vvi-Vitvi11g00409\_t001 |  |  |  |  |
| 2 | Atr-ERM97090 |  | | | |  | | | |  |  |  |  |
| 2 | Atr-ERM97091 |  | | | |  | Vvi-Vitvi11g00410\_t001 |  |  |  |  |
| 1 | Atr-ERM97092 |  | Vvi-Vitvi09g00488\_t001 |  |  |  |  |  |
| 1 | Atr-ERM97093 |  | | | |  |  |  |  |  |
| 1 | Atr-ERM97094 |  | Vvi-Vitvi09g00489\_t001 |  |  |  |  |  |
| 1 | Atr-ERM97095 |  | Vvi-Vitvi09g00490\_t001 |  |  |  |  |  |
