# Supplementary material for: Genome-wide identification, characterization and gene expression of BES1 transcription factor family in grapevine (Vitis vinifera L.)
Source: Sci Rep. 2023 Jan 5;13:240. doi: 10.1038/s41598-022-24407-y (PMC9816167; doi:10.1038/s41598-022-24407-y)
Supplement: Supplementary file 3 — Supplementary Information. [file 41598_2022_24407_MOESM3_ESM.zip › Vvi_Atr/Vitis_vinifera.PN40024.v4.dna_sm.toplevel.fa.vs.Amborella_trichopoda.AMTR1.0.dna_sm.toplevel.fa.html/Atr-AmTr_v1.0_scaffold00124.html]

|  |  |  |  |  |  |  |  |  |  |  |  |  |  |
| --- | --- | --- | --- | --- | --- | --- | --- | --- | --- | --- | --- | --- | --- |
| Duplication depth | Reference chromosome | Collinear blocks | | | | | | | | | | | |
| 0 | Atr-ERM97434 |  |  |  |  |  |  |
| 0 | Atr-ERM97435 |  |  |  |  |  |  |
| 0 | Atr-ERM97436 |  |  |  |  |  |  |
| 0 | Atr-ERM97437 |  |  |  |  |  |  |
| 0 | Atr-ERM97438 |  |  |  |  |  |  |
| 0 | Atr-ERM97439 |  |  |  |  |  |  |
| 0 | Atr-ERM97440 |  |  |  |  |  |  |
| 0 | Atr-ERM97441 |  |  |  |  |  |  |
| 0 | Atr-ERM97442 |  |  |  |  |  |  |
| 0 | Atr-ERM97443 |  |  |  |  |  |  |
| 0 | Atr-ERM97444 |  |  |  |  |  |  |
| 0 | Atr-ERM97445 |  |  |  |  |  |  |
| 0 | Atr-ERM97446 |  |  |  |  |  |  |
| 0 | Atr-ERM97447 |  |  |  |  |  |  |
| 0 | Atr-ERM97448 |  |  |  |  |  |  |
| 0 | Atr-ERM97449 |  |  |  |  |  |  |
| 0 | Atr-ERM97450 |  |  |  |  |  |  |
| 0 | Atr-ERM97451 |  |  |  |  |  |  |
| 0 | Atr-ERM97452 |  |  |  |  |  |  |
| 0 | Atr-ERM97453 |  |  |  |  |  |  |
| 0 | Atr-ERM97454 |  |  |  |  |  |  |
| 0 | Atr-ERM97455 |  |  |  |  |  |  |
| 0 | Atr-ERM97456 |  |  |  |  |  |  |
| 0 | Atr-ERM97457 |  |  |  |  |  |  |
| 0 | Atr-ERM97458 |  |  |  |  |  |  |
| 0 | Atr-ERM97459 |  |  |  |  |  |  |
| 0 | Atr-ERM97460 |  |  |  |  |  |  |
| 0 | Atr-ERM97461 |  |  |  |  |  |  |
| 0 | Atr-ERM97462 |  |  |  |  |  |  |
| 0 | Atr-ERM97463 |  |  |  |  |  |  |
