# Supplementary material for: Genome-wide identification, characterization and gene expression of BES1 transcription factor family in grapevine (Vitis vinifera L.)
Source: Sci Rep. 2023 Jan 5;13:240. doi: 10.1038/s41598-022-24407-y (PMC9816167; doi:10.1038/s41598-022-24407-y)
Supplement: Supplementary file 3 — Supplementary Information. [file 41598_2022_24407_MOESM3_ESM.zip › Vvi_Atr/Vitis_vinifera.PN40024.v4.dna_sm.toplevel.fa.vs.Amborella_trichopoda.AMTR1.0.dna_sm.toplevel.fa.html/Atr-AmTr_v1.0_scaffold00125.html]

|  |  |  |  |  |  |  |  |  |  |  |  |  |  |
| --- | --- | --- | --- | --- | --- | --- | --- | --- | --- | --- | --- | --- | --- |
| Duplication depth | Reference chromosome | Collinear blocks | | | | | | | | | | | |
| 0 | Atr-ERM97468 |  |  |  |  |  |  |
| 0 | Atr-ERM97469 |  |  |  |  |  |  |
| 0 | Atr-ERM97470 |  |  |  |  |  |  |
| 0 | Atr-ERM97471 |  |  |  |  |  |  |
| 0 | Atr-ERM97472 |  |  |  |  |  |  |
| 0 | Atr-ERM97473 |  |  |  |  |  |  |
| 0 | Atr-ERM97474 |  |  |  |  |  |  |
| 0 | Atr-ERM97475 |  |  |  |  |  |  |
| 0 | Atr-ERM97476 |  |  |  |  |  |  |
| 0 | Atr-ERM97477 |  |  |  |  |  |  |
| 0 | Atr-ERM97478 |  |  |  |  |  |  |
| 0 | Atr-ERM97479 |  |  |  |  |  |  |
| 0 | Atr-ERM97480 |  |  |  |  |  |  |
| 0 | Atr-ERM97481 |  |  |  |  |  |  |
| 0 | Atr-ERM97482 |  |  |  |  |  |  |
| 0 | Atr-ERM97483 |  |  |  |  |  |  |
| 0 | Atr-ERM97484 |  |  |  |  |  |  |
| 0 | Atr-ERM97485 |  |  |  |  |  |  |
| 0 | Atr-ERM97486 |  |  |  |  |  |  |
| 0 | Atr-ERM97487 |  |  |  |  |  |  |
| 0 | Atr-ERM97488 |  |  |  |  |  |  |
| 0 | Atr-ERM97489 |  |  |  |  |  |  |
| 0 | Atr-ERM97490 |  |  |  |  |  |  |
| 0 | Atr-ERM97491 |  |  |  |  |  |  |
| 0 | Atr-ERM97492 |  |  |  |  |  |  |
| 0 | Atr-ERM97493 |  |  |  |  |  |  |
| 0 | Atr-ERM97494 |  |  |  |  |  |  |
| 0 | Atr-ERM97495 |  |  |  |  |  |  |
| 0 | Atr-ERM97496 |  |  |  |  |  |  |
| 0 | Atr-ERM97497 |  |  |  |  |  |  |
| 0 | Atr-ERM97498 |  |  |  |  |  |  |
| 0 | Atr-ERM97499 |  |  |  |  |  |  |
| 0 | Atr-ERM97500 |  |  |  |  |  |  |
| 0 | Atr-ERM97501 |  |  |  |  |  |  |
| 0 | Atr-ERM97502 |  |  |  |  |  |  |
