# Supplementary material for: Genome-wide identification, characterization and gene expression of BES1 transcription factor family in grapevine (Vitis vinifera L.)
Source: Sci Rep. 2023 Jan 5;13:240. doi: 10.1038/s41598-022-24407-y (PMC9816167; doi:10.1038/s41598-022-24407-y)
Supplement: Supplementary file 3 — Supplementary Information. [file 41598_2022_24407_MOESM3_ESM.zip › Vvi_Atr/Vitis_vinifera.PN40024.v4.dna_sm.toplevel.fa.vs.Amborella_trichopoda.AMTR1.0.dna_sm.toplevel.fa.html/Atr-AmTr_v1.0_scaffold00126.html]

|  |  |  |  |  |  |  |  |  |  |  |  |  |  |
| --- | --- | --- | --- | --- | --- | --- | --- | --- | --- | --- | --- | --- | --- |
| Duplication depth | Reference chromosome | Collinear blocks | | | | | | | | | | | |
| 0 | Atr-ERM97096 |  |  |  |  |  |  |
| 1 | Atr-ERM97097 |  | Vvi-Vitvi07g01768\_t001 |  |  |  |  |  |
| 1 | Atr-ERM97098 |  | | | |  |  |  |  |  |
| 1 | Atr-ERM97099 |  | | | |  |  |  |  |  |
| 1 | Atr-ERM97100 |  | | | |  |  |  |  |  |
| 1 | Atr-ERM97101 |  | | | |  |  |  |  |  |
| 1 | Atr-ERM97102 |  | | | |  |  |  |  |  |
| 1 | Atr-ERM97103 |  | | | |  |  |  |  |  |
| 1 | Atr-ERM97104 |  | | | |  |  |  |  |  |
| 1 | Atr-ERM97105 |  | | | |  |  |  |  |  |
| 1 | Atr-ERM97106 |  | | | |  |  |  |  |  |
| 1 | Atr-ERM97107 |  | | | |  |  |  |  |  |
| 1 | Atr-ERM97108 |  | | | |  |  |  |  |  |
| 1 | Atr-ERM97109 |  | | | |  |  |  |  |  |
| 1 | Atr-ERM97110 |  | Vvi-Vitvi07g01766\_t001 |  |  |  |  |  |
| 1 | Atr-ERM97111 |  | | | |  |  |  |  |  |
| 1 | Atr-ERM97112 |  | | | |  |  |  |  |  |
| 1 | Atr-ERM97113 |  | | | |  |  |  |  |  |
| 1 | Atr-ERM97114 |  | | | |  |  |  |  |  |
| 2 | Atr-ERM97115 |  | | | |  | Vvi-Vitvi18g00825\_t001 |  |  |  |  |
| 2 | Atr-ERM97116 |  | | | |  | | | |  |  |  |  |
| 2 | Atr-ERM97117 |  | | | |  | | | |  |  |  |  |
| 2 | Atr-ERM97118 |  | | | |  | | | |  |  |  |  |
| 2 | Atr-ERM97119 |  | | | |  | | | |  |  |  |  |
| 2 | Atr-ERM97120 |  | | | |  | | | |  |  |  |  |
| 2 | Atr-ERM97121 |  | | | |  | | | |  |  |  |  |
| 2 | Atr-ERM97122 |  | | | |  | | | |  |  |  |  |
| 2 | Atr-ERM97123 |  | | | |  | | | |  |  |  |  |
| 2 | Atr-ERM97124 |  | Vvi-Vitvi07g01765\_t001 |  | | | |  |  |  |  |
| 2 | Atr-ERM97125 |  | | | |  | | | |  |  |  |  |
| 2 | Atr-ERM97126 |  | | | |  | | | |  |  |  |  |
| 2 | Atr-ERM97127 |  | | | |  | | | |  |  |  |  |
| 2 | Atr-ERM97128 |  | | | |  | | | |  |  |  |  |
| 2 | Atr-ERM97129 |  | | | |  | | | |  |  |  |  |
| 2 | Atr-ERM97130 |  | | | |  | | | |  |  |  |  |
| 3 | Atr-ERM97131 |  | Vvi-Vitvi07g02667\_t002 |  | | | |  | Vvi-Vitvi03g01562\_t001 |  |  |  |
| 3 | Atr-ERM97132 |  | | | |  | | | |  | | | |  |  |  |
| 3 | Atr-ERM97133 |  | | | |  | | | |  | | | |  |  |  |
| 3 | Atr-ERM97134 |  | | | |  | Vvi-Vitvi18g00810\_t001 |  | | | |  |  |  |
| 3 | Atr-ERM97135 |  | | | |  | Vvi-Vitvi18g00809\_t001 |  | Vvi-Vitvi03g00486\_t001 |  |  |  |
| 3 | Atr-ERM97136 |  | | | |  | Vvi-Vitvi18g00808\_t001 |  | | | |  |  |  |
| 3 | Atr-ERM97137 |  | Vvi-Vitvi07g01763\_t001 |  | | | |  | | | |  |  |  |
| 3 | Atr-ERM97138 |  | | | |  | | | |  | | | |  |  |  |
| 3 | Atr-ERM97139 |  | | | |  | | | |  | | | |  |  |  |
| 3 | Atr-ERM97140 |  | | | |  | | | |  | | | |  |  |  |
| 3 | Atr-ERM97141 |  | | | |  | | | |  | Vvi-Vitvi03g00491\_t001 |  |  |  |
| 3 | Atr-ERM97142 |  | | | |  | | | |  | | | |  |  |  |
| 3 | Atr-ERM97143 |  | | | |  | Vvi-Vitvi18g00806\_t001 |  | | | |  |  |  |
| 3 | Atr-ERM97144 |  | | | |  | | | |  | Vvi-Vitvi03g00492\_t001 |  |  |  |
| 3 | Atr-ERM97145 |  | | | |  | | | |  | | | |  |  |  |
| 3 | Atr-ERM97146 |  | | | |  | | | |  | | | |  |  |  |
| 3 | Atr-ERM97147 |  | | | |  | Vvi-Vitvi18g02690\_t001 |  | | | |  |  |  |
| 3 | Atr-ERM97148 |  | | | |  | | | |  | | | |  |  |  |
| 3 | Atr-ERM97149 |  | Vvi-Vitvi07g01761\_t001 |  | | | |  | | | |  |  |  |
| 3 | Atr-ERM97150 |  | | | |  | | | |  | Vvi-Vitvi03g00493\_t001 |  |  |  |
| 3 | Atr-ERM97151 |  | | | |  | | | |  | | | |  |  |  |
| 3 | Atr-ERM97152 |  | | | |  | | | |  | | | |  |  |  |
| 3 | Atr-ERM97153 |  | | | |  | Vvi-Vitvi18g00804\_t002 |  | | | |  |  |  |
| 3 | Atr-ERM97154 |  | Vvi-Vitvi07g01760\_t001 |  | Vvi-Vitvi18g04190\_t001 |  | | | |  |  |  |
| 2 | Atr-ERM97155 |  |  |  | | | |  | | | |  |  |  |
| 2 | Atr-ERM97156 |  |  |  | | | |  | Vvi-Vitvi03g00496\_t001 |  |  |  |
| 2 | Atr-ERM97157 |  |  |  | | | |  | | | |  |  |  |
| 2 | Atr-ERM97158 |  |  |  | | | |  | Vvi-Vitvi03g01568\_t001 |  |  |  |
| 2 | Atr-ERM97159 |  |  |  | | | |  | | | |  |  |  |
| 2 | Atr-ERM97160 |  |  |  | Vvi-Vitvi18g00799\_t001 |  | | | |  |  |  |
| 1 | Atr-ERM97161 |  |  |  |  |  | Vvi-Vitvi03g00497\_t001 |  |  |  |
| 0 | Atr-ERM97162 |  |  |  |  |  |  |
