# Supplementary material for: Genome-wide identification, characterization and gene expression of BES1 transcription factor family in grapevine (Vitis vinifera L.)
Source: Sci Rep. 2023 Jan 5;13:240. doi: 10.1038/s41598-022-24407-y (PMC9816167; doi:10.1038/s41598-022-24407-y)
Supplement: Supplementary file 3 — Supplementary Information. [file 41598_2022_24407_MOESM3_ESM.zip › Vvi_Atr/Vitis_vinifera.PN40024.v4.dna_sm.toplevel.fa.vs.Amborella_trichopoda.AMTR1.0.dna_sm.toplevel.fa.html/Atr-AmTr_v1.0_scaffold00127.html]

|  |  |  |  |  |  |  |  |  |  |  |  |  |  |
| --- | --- | --- | --- | --- | --- | --- | --- | --- | --- | --- | --- | --- | --- |
| Duplication depth | Reference chromosome | Collinear blocks | | | | | | | | | | | |
| 0 | Atr-ERM97374 |  |  |  |  |  |  |
| 0 | Atr-ERM97375 |  |  |  |  |  |  |
| 0 | Atr-ERM97376 |  |  |  |  |  |  |
| 0 | Atr-ERM97377 |  |  |  |  |  |  |
| 0 | Atr-ERM97378 |  |  |  |  |  |  |
| 0 | Atr-ERM97379 |  |  |  |  |  |  |
| 0 | Atr-ERM97380 |  |  |  |  |  |  |
| 0 | Atr-ERM97381 |  |  |  |  |  |  |
| 0 | Atr-ERM97382 |  |  |  |  |  |  |
| 0 | Atr-ERM97383 |  |  |  |  |  |  |
| 0 | Atr-ERM97384 |  |  |  |  |  |  |
| 0 | Atr-ERM97385 |  |  |  |  |  |  |
| 0 | Atr-ERM97386 |  |  |  |  |  |  |
| 0 | Atr-ERM97387 |  |  |  |  |  |  |
| 0 | Atr-ERM97388 |  |  |  |  |  |  |
| 0 | Atr-ERM97389 |  |  |  |  |  |  |
| 0 | Atr-ERM97390 |  |  |  |  |  |  |
| 0 | Atr-ERM97391 |  |  |  |  |  |  |
| 0 | Atr-ERM97392 |  |  |  |  |  |  |
| 0 | Atr-ERM97393 |  |  |  |  |  |  |
| 0 | Atr-ERM97394 |  |  |  |  |  |  |
| 0 | Atr-ERM97395 |  |  |  |  |  |  |
| 0 | Atr-ERM97396 |  |  |  |  |  |  |
| 0 | Atr-ERM97397 |  |  |  |  |  |  |
| 0 | Atr-ERM97398 |  |  |  |  |  |  |
| 0 | Atr-ERM97399 |  |  |  |  |  |  |
| 0 | Atr-ERM97400 |  |  |  |  |  |  |
| 0 | Atr-ERM97401 |  |  |  |  |  |  |
| 0 | Atr-ERM97402 |  |  |  |  |  |  |
| 0 | Atr-ERM97403 |  |  |  |  |  |  |
| 0 | Atr-ERM97404 |  |  |  |  |  |  |
