# Supplementary material for: Genome-wide identification, characterization and gene expression of BES1 transcription factor family in grapevine (Vitis vinifera L.)
Source: Sci Rep. 2023 Jan 5;13:240. doi: 10.1038/s41598-022-24407-y (PMC9816167; doi:10.1038/s41598-022-24407-y)
Supplement: Supplementary file 3 — Supplementary Information. [file 41598_2022_24407_MOESM3_ESM.zip › Vvi_Atr/Vitis_vinifera.PN40024.v4.dna_sm.toplevel.fa.vs.Amborella_trichopoda.AMTR1.0.dna_sm.toplevel.fa.html/Atr-AmTr_v1.0_scaffold00128.html]

|  |  |  |  |  |  |  |  |  |  |  |  |  |  |
| --- | --- | --- | --- | --- | --- | --- | --- | --- | --- | --- | --- | --- | --- |
| Duplication depth | Reference chromosome | Collinear blocks | | | | | | | | | | | |
| 0 | Atr-ERM96808 |  |  |  |  |  |  |
| 0 | Atr-ERM96809 |  |  |  |  |  |  |
| 0 | Atr-ERM96810 |  |  |  |  |  |  |
| 0 | Atr-ERM96811 |  |  |  |  |  |  |
| 0 | Atr-ERM96812 |  |  |  |  |  |  |
| 0 | Atr-ERM96813 |  |  |  |  |  |  |
| 0 | Atr-ERM96814 |  |  |  |  |  |  |
| 0 | Atr-ERM96815 |  |  |  |  |  |  |
| 0 | Atr-ERM96816 |  |  |  |  |  |  |
| 0 | Atr-ERM96817 |  |  |  |  |  |  |
| 0 | Atr-ERM96818 |  |  |  |  |  |  |
| 0 | Atr-ERM96819 |  |  |  |  |  |  |
| 0 | Atr-ERM96820 |  |  |  |  |  |  |
| 0 | Atr-ERM96821 |  |  |  |  |  |  |
| 0 | Atr-ERM96822 |  |  |  |  |  |  |
| 0 | Atr-ERM96823 |  |  |  |  |  |  |
| 0 | Atr-ERM96824 |  |  |  |  |  |  |
| 0 | Atr-ERM96825 |  |  |  |  |  |  |
| 0 | Atr-ERM96826 |  |  |  |  |  |  |
| 0 | Atr-ERM96827 |  |  |  |  |  |  |
| 0 | Atr-ERM96828 |  |  |  |  |  |  |
| 0 | Atr-ERM96829 |  |  |  |  |  |  |
| 0 | Atr-ERM96830 |  |  |  |  |  |  |
| 0 | Atr-ERM96831 |  |  |  |  |  |  |
| 0 | Atr-ERM96832 |  |  |  |  |  |  |
| 0 | Atr-ERM96833 |  |  |  |  |  |  |
| 0 | Atr-ERM96834 |  |  |  |  |  |  |
| 0 | Atr-ERM96835 |  |  |  |  |  |  |
| 0 | Atr-ERM96836 |  |  |  |  |  |  |
| 0 | Atr-ERM96837 |  |  |  |  |  |  |
| 0 | Atr-ERM96838 |  |  |  |  |  |  |
| 0 | Atr-ERM96839 |  |  |  |  |  |  |
| 0 | Atr-ERM96840 |  |  |  |  |  |  |
| 0 | Atr-ERM96841 |  |  |  |  |  |  |
| 0 | Atr-ERM96842 |  |  |  |  |  |  |
| 0 | Atr-ERM96843 |  |  |  |  |  |  |
| 0 | Atr-ERM96844 |  |  |  |  |  |  |
| 0 | Atr-ERM96845 |  |  |  |  |  |  |
| 0 | Atr-ERM96846 |  |  |  |  |  |  |
| 0 | Atr-ERM96847 |  |  |  |  |  |  |
| 0 | Atr-ERM96848 |  |  |  |  |  |  |
| 0 | Atr-ERM96849 |  |  |  |  |  |  |
| 0 | Atr-ERM96850 |  |  |  |  |  |  |
| 0 | Atr-ERM96851 |  |  |  |  |  |  |
