# Supplementary material for: Genome-wide identification, characterization and gene expression of BES1 transcription factor family in grapevine (Vitis vinifera L.)
Source: Sci Rep. 2023 Jan 5;13:240. doi: 10.1038/s41598-022-24407-y (PMC9816167; doi:10.1038/s41598-022-24407-y)
Supplement: Supplementary file 3 — Supplementary Information. [file 41598_2022_24407_MOESM3_ESM.zip › Vvi_Atr/Vitis_vinifera.PN40024.v4.dna_sm.toplevel.fa.vs.Amborella_trichopoda.AMTR1.0.dna_sm.toplevel.fa.html/Atr-AmTr_v1.0_scaffold00129.html]

|  |  |  |  |  |  |  |  |  |  |  |  |  |  |
| --- | --- | --- | --- | --- | --- | --- | --- | --- | --- | --- | --- | --- | --- |
| Duplication depth | Reference chromosome | Collinear blocks | | | | | | | | | | | |
| 0 | Atr-ERM95983 |  |  |  |  |  |  |
| 0 | Atr-ERM95984 |  |  |  |  |  |  |
| 0 | Atr-ERM95985 |  |  |  |  |  |  |
| 0 | Atr-ERM95986 |  |  |  |  |  |  |
| 0 | Atr-ERM95987 |  |  |  |  |  |  |
| 0 | Atr-ERM95988 |  |  |  |  |  |  |
| 0 | Atr-ERM95989 |  |  |  |  |  |  |
| 0 | Atr-ERM95990 |  |  |  |  |  |  |
| 0 | Atr-ERM95991 |  |  |  |  |  |  |
| 0 | Atr-ERM95992 |  |  |  |  |  |  |
| 0 | Atr-ERM95993 |  |  |  |  |  |  |
| 0 | Atr-ERM95994 |  |  |  |  |  |  |
| 0 | Atr-ERM95995 |  |  |  |  |  |  |
| 0 | Atr-ERM95996 |  |  |  |  |  |  |
| 0 | Atr-ERM95997 |  |  |  |  |  |  |
| 0 | Atr-ERM95998 |  |  |  |  |  |  |
| 1 | Atr-ERM95999 |  | Vvi-Vitvi09g00015\_t001 |  |  |  |  |  |
| 1 | Atr-ERM96000 |  | | | |  |  |  |  |  |
| 1 | Atr-ERM96001 |  | | | |  |  |  |  |  |
| 1 | Atr-ERM96002 |  | Vvi-Vitvi09g00017\_t001 |  |  |  |  |  |
| 1 | Atr-ERM96003 |  | | | |  |  |  |  |  |
| 1 | Atr-ERM96004 |  | | | |  |  |  |  |  |
| 1 | Atr-ERM96005 |  | | | |  |  |  |  |  |
| 1 | Atr-ERM96006 |  | | | |  |  |  |  |  |
| 1 | Atr-ERM96007 |  | | | |  |  |  |  |  |
| 1 | Atr-ERM96008 |  | | | |  |  |  |  |  |
| 1 | Atr-ERM96009 |  | | | |  |  |  |  |  |
| 1 | Atr-ERM96010 |  | | | |  |  |  |  |  |
| 1 | Atr-ERM96011 |  | | | |  |  |  |  |  |
| 1 | Atr-ERM96012 |  | | | |  |  |  |  |  |
| 1 | Atr-ERM96013 |  | | | |  |  |  |  |  |
| 1 | Atr-ERM96014 |  | | | |  |  |  |  |  |
| 1 | Atr-ERM96015 |  | | | |  |  |  |  |  |
| 1 | Atr-ERM96016 |  | | | |  |  |  |  |  |
| 1 | Atr-ERM96017 |  | | | |  |  |  |  |  |
| 1 | Atr-ERM96018 |  | | | |  |  |  |  |  |
| 1 | Atr-ERM96019 |  | | | |  |  |  |  |  |
| 2 | Atr-ERM96020 |  | | | |  | Vvi-Vitvi11g00011\_t001 |  |  |  |  |
| 2 | Atr-ERM96021 |  | | | |  | | | |  |  |  |  |
| 2 | Atr-ERM96022 |  | | | |  | | | |  |  |  |  |
| 2 | Atr-ERM96023 |  | | | |  | | | |  |  |  |  |
| 2 | Atr-ERM96024 |  | | | |  | | | |  |  |  |  |
| 2 | Atr-ERM96025 |  | Vvi-Vitvi09g00018\_t001 |  | | | |  |  |  |  |
| 2 | Atr-ERM96026 |  | | | |  | | | |  |  |  |  |
| 2 | Atr-ERM96027 |  | | | |  | Vvi-Vitvi11g00015\_t001 |  |  |  |  |
| 2 | Atr-ERM96028 |  | | | |  | Vvi-Vitvi11g00016\_t001 |  |  |  |  |
| 2 | Atr-ERM96029 |  | | | |  | | | |  |  |  |  |
| 2 | Atr-ERM96030 |  | | | |  | | | |  |  |  |  |
| 2 | Atr-ERM96031 |  | | | |  | | | |  |  |  |  |
| 2 | Atr-ERM96032 |  | | | |  | | | |  |  |  |  |
| 2 | Atr-ERM96033 |  | | | |  | | | |  |  |  |  |
| 2 | Atr-ERM96034 |  | | | |  | | | |  |  |  |  |
| 2 | Atr-ERM96035 |  | Vvi-Vitvi09g00019\_t001 |  | | | |  |  |  |  |
| 2 | Atr-ERM96036 |  | | | |  | | | |  |  |  |  |
| 2 | Atr-ERM96037 |  | | | |  | | | |  |  |  |  |
| 2 | Atr-ERM96038 |  | | | |  | | | |  |  |  |  |
| 2 | Atr-ERM96039 |  | | | |  | | | |  |  |  |  |
| 2 | Atr-ERM96040 |  | Vvi-Vitvi09g00020\_t001 |  | | | |  |  |  |  |
| 2 | Atr-ERM96041 |  | | | |  | Vvi-Vitvi11g00017\_t001 |  |  |  |  |
| 2 | Atr-ERM96042 |  | | | |  | | | |  |  |  |  |
| 2 | Atr-ERM96043 |  | | | |  | | | |  |  |  |  |
| 2 | Atr-ERM96044 |  | | | |  | Vvi-Vitvi11g00018\_t001 |  |  |  |  |
| 2 | Atr-ERM96045 |  | Vvi-Vitvi09g00021\_t001 |  | | | |  |  |  |  |
| 2 | Atr-ERM96046 |  | Vvi-Vitvi09g00022\_t001 |  | | | |  |  |  |  |
| 2 | Atr-ERM96047 |  | Vvi-Vitvi09g00023\_t001 |  | Vvi-Vitvi11g04003\_t001 |  |  |  |  |
| 2 | Atr-ERM96048 |  | | | |  | | | |  |  |  |  |
| 2 | Atr-ERM96049 |  | | | |  | | | |  |  |  |  |
| 2 | Atr-ERM96050 |  | | | |  | | | |  |  |  |  |
| 2 | Atr-ERM96051 |  | Vvi-Vitvi09g00026\_t001 |  | | | |  |  |  |  |
| 2 | Atr-ERM96052 |  | Vvi-Vitvi09g00027\_t001 |  | | | |  |  |  |  |
| 2 | Atr-ERM96053 |  | | | |  | | | |  |  |  |  |
| 2 | Atr-ERM96054 |  | | | |  | | | |  |  |  |  |
| 2 | Atr-ERM96055 |  | | | |  | | | |  |  |  |  |
| 2 | Atr-ERM96056 |  | | | |  | | | |  |  |  |  |
| 2 | Atr-ERM96057 |  | | | |  | | | |  |  |  |  |
| 2 | Atr-ERM96058 |  | | | |  | | | |  |  |  |  |
| 2 | Atr-ERM96059 |  | | | |  | | | |  |  |  |  |
| 2 | Atr-ERM96060 |  | | | |  | | | |  |  |  |  |
| 2 | Atr-ERM96061 |  | | | |  | | | |  |  |  |  |
| 2 | Atr-ERM96062 |  | | | |  | | | |  |  |  |  |
| 2 | Atr-ERM96063 |  | | | |  | | | |  |  |  |  |
| 2 | Atr-ERM96064 |  | | | |  | | | |  |  |  |  |
| 2 | Atr-ERM96065 |  | Vvi-Vitvi09g00028\_t001 |  | | | |  |  |  |  |
| 2 | Atr-ERM96066 |  | Vvi-Vitvi09g01493\_t001 |  | | | |  |  |  |  |
| 2 | Atr-ERM96067 |  | Vvi-Vitvi09g00029\_t001 |  | Vvi-Vitvi11g00020\_t001 |  |  |  |  |
| 2 | Atr-ERM96068 |  | Vvi-Vitvi09g00031\_t002 |  | | | |  |  |  |  |
| 1 | Atr-ERM96069 |  |  |  | Vvi-Vitvi11g00022\_t001 |  |  |  |  |
| 0 | Atr-ERM96070 |  |  |  |  |  |  |
| 0 | Atr-ERM96071 |  |  |  |  |  |  |
| 0 | Atr-ERM96072 |  |  |  |  |  |  |
| 0 | Atr-ERM96073 |  |  |  |  |  |  |
| 0 | Atr-ERM96074 |  |  |  |  |  |  |
| 0 | Atr-ERM96075 |  |  |  |  |  |  |
| 0 | Atr-ERM96076 |  |  |  |  |  |  |
| 0 | Atr-ERM96077 |  |  |  |  |  |  |
| 0 | Atr-ERM96078 |  |  |  |  |  |  |
| 0 | Atr-ERM96079 |  |  |  |  |  |  |
| 0 | Atr-ERM96080 |  |  |  |  |  |  |
| 0 | Atr-ERM96081 |  |  |  |  |  |  |
| 0 | Atr-ERM96082 |  |  |  |  |  |  |
| 0 | Atr-ERM96083 |  |  |  |  |  |  |
| 0 | Atr-ERM96084 |  |  |  |  |  |  |
| 0 | Atr-ERM96085 |  |  |  |  |  |  |
