# Supplementary material for: Genome-wide identification, characterization and gene expression of BES1 transcription factor family in grapevine (Vitis vinifera L.)
Source: Sci Rep. 2023 Jan 5;13:240. doi: 10.1038/s41598-022-24407-y (PMC9816167; doi:10.1038/s41598-022-24407-y)
Supplement: Supplementary file 3 — Supplementary Information. [file 41598_2022_24407_MOESM3_ESM.zip › Vvi_Atr/Vitis_vinifera.PN40024.v4.dna_sm.toplevel.fa.vs.Amborella_trichopoda.AMTR1.0.dna_sm.toplevel.fa.html/Atr-AmTr_v1.0_scaffold00130.html]

|  |  |  |  |  |  |  |  |  |  |  |  |  |  |
| --- | --- | --- | --- | --- | --- | --- | --- | --- | --- | --- | --- | --- | --- |
| Duplication depth | Reference chromosome | Collinear blocks | | | | | | | | | | | |
| 0 | Atr-ERM97624 |  |  |  |  |  |  |
| 0 | Atr-ERM97625 |  |  |  |  |  |  |
| 1 | Atr-ERM97626 |  | Vvi-Vitvi10g00612\_t001 |  |  |  |  |  |
| 1 | Atr-ERM97627 |  | | | |  |  |  |  |  |
| 1 | Atr-ERM97628 |  | Vvi-Vitvi10g00614\_t001 |  |  |  |  |  |
| 1 | Atr-ERM97629 |  | | | |  |  |  |  |  |
| 1 | Atr-ERM97630 |  | Vvi-Vitvi10g00615\_t001 |  |  |  |  |  |
| 1 | Atr-ERM97631 |  | | | |  |  |  |  |  |
| 1 | Atr-ERM97632 |  | | | |  |  |  |  |  |
| 1 | Atr-ERM97633 |  | | | |  |  |  |  |  |
| 1 | Atr-ERM97634 |  | | | |  |  |  |  |  |
| 1 | Atr-ERM97635 |  | Vvi-Vitvi10g00617\_t001 |  |  |  |  |  |
| 1 | Atr-ERM97636 |  | | | |  |  |  |  |  |
| 1 | Atr-ERM97637 |  | Vvi-Vitvi10g00618\_t001 |  |  |  |  |  |
| 1 | Atr-ERM97638 |  | Vvi-Vitvi10g00619\_t001 |  |  |  |  |  |
| 1 | Atr-ERM97639 |  | | | |  |  |  |  |  |
| 1 | Atr-ERM97640 |  | | | |  |  |  |  |  |
| 1 | Atr-ERM97641 |  | | | |  |  |  |  |  |
| 1 | Atr-ERM97642 |  | | | |  |  |  |  |  |
| 1 | Atr-ERM97643 |  | Vvi-Vitvi10g00622\_t001 |  |  |  |  |  |
| 1 | Atr-ERM97644 |  | | | |  |  |  |  |  |
| 1 | Atr-ERM97645 |  | | | |  |  |  |  |  |
| 1 | Atr-ERM97646 |  | | | |  |  |  |  |  |
| 1 | Atr-ERM97647 |  | | | |  |  |  |  |  |
| 1 | Atr-ERM97648 |  | | | |  |  |  |  |  |
| 1 | Atr-ERM97649 |  | | | |  |  |  |  |  |
| 1 | Atr-ERM97650 |  | | | |  |  |  |  |  |
| 1 | Atr-ERM97651 |  | | | |  |  |  |  |  |
| 1 | Atr-ERM97652 |  | | | |  |  |  |  |  |
| 1 | Atr-ERM97653 |  | | | |  |  |  |  |  |
| 1 | Atr-ERM97654 |  | Vvi-Vitvi10g00627\_t001 |  |  |  |  |  |
| 0 | Atr-ERM97655 |  |  |  |  |  |  |
| 0 | Atr-ERM97656 |  |  |  |  |  |  |
| 0 | Atr-ERM97657 |  |  |  |  |  |  |
| 0 | Atr-ERM97658 |  |  |  |  |  |  |
| 0 | Atr-ERM97659 |  |  |  |  |  |  |
| 0 | Atr-ERM97660 |  |  |  |  |  |  |
| 1 | Atr-ERM97661 |  | Vvi-Vitvi10g00603\_t001 |  |  |  |  |  |
| 1 | Atr-ERM97662 |  | | | |  |  |  |  |  |
| 1 | Atr-ERM97663 |  | | | |  |  |  |  |  |
| 1 | Atr-ERM97664 |  | | | |  |  |  |  |  |
| 1 | Atr-ERM97665 |  | | | |  |  |  |  |  |
| 1 | Atr-ERM97666 |  | | | |  |  |  |  |  |
| 1 | Atr-ERM97667 |  | | | |  |  |  |  |  |
| 1 | Atr-ERM97668 |  | | | |  |  |  |  |  |
| 1 | Atr-ERM97669 |  | | | |  |  |  |  |  |
| 1 | Atr-ERM97670 |  | Vvi-Vitvi10g00602\_t001 |  |  |  |  |  |
| 1 | Atr-ERM97671 |  | | | |  |  |  |  |  |
| 1 | Atr-ERM97672 |  | Vvi-Vitvi10g00601\_t002 |  |  |  |  |  |
| 1 | Atr-ERM97673 |  | | | |  |  |  |  |  |
| 1 | Atr-ERM97674 |  | Vvi-Vitvi10g00599\_t001 |  |  |  |  |  |
| 1 | Atr-ERM97675 |  | | | |  |  |  |  |  |
| 1 | Atr-ERM97676 |  | | | |  |  |  |  |  |
| 1 | Atr-ERM97677 |  | | | |  |  |  |  |  |
| 1 | Atr-ERM97678 |  | | | |  |  |  |  |  |
| 1 | Atr-ERM97679 |  | | | |  |  |  |  |  |
| 1 | Atr-ERM97680 |  | Vvi-Vitvi10g00598\_t001 |  |  |  |  |  |
| 1 | Atr-ERM97681 |  | | | |  |  |  |  |  |
| 1 | Atr-ERM97682 |  | | | |  |  |  |  |  |
| 1 | Atr-ERM97683 |  | Vvi-Vitvi10g00597\_t001 |  |  |  |  |  |
| 0 | Atr-ERM97684 |  |  |  |  |  |  |
| 0 | Atr-ERM97685 |  |  |  |  |  |  |
| 0 | Atr-ERM97686 |  |  |  |  |  |  |
| 0 | Atr-ERM97687 |  |  |  |  |  |  |
| 0 | Atr-ERM97688 |  |  |  |  |  |  |
| 0 | Atr-ERM97689 |  |  |  |  |  |  |
| 0 | Atr-ERM97690 |  |  |  |  |  |  |
| 0 | Atr-ERM97691 |  |  |  |  |  |  |
| 0 | Atr-ERM97692 |  |  |  |  |  |  |
| 0 | Atr-ERM97693 |  |  |  |  |  |  |
| 0 | Atr-ERM97694 |  |  |  |  |  |  |
| 0 | Atr-ERM97695 |  |  |  |  |  |  |
| 0 | Atr-ERM97696 |  |  |  |  |  |  |
