# Supplementary material for: Genome-wide identification, characterization and gene expression of BES1 transcription factor family in grapevine (Vitis vinifera L.)
Source: Sci Rep. 2023 Jan 5;13:240. doi: 10.1038/s41598-022-24407-y (PMC9816167; doi:10.1038/s41598-022-24407-y)
Supplement: Supplementary file 3 — Supplementary Information. [file 41598_2022_24407_MOESM3_ESM.zip › Vvi_Atr/Vitis_vinifera.PN40024.v4.dna_sm.toplevel.fa.vs.Amborella_trichopoda.AMTR1.0.dna_sm.toplevel.fa.html/Atr-AmTr_v1.0_scaffold00131.html]

|  |  |  |  |  |  |  |  |  |  |  |  |  |  |
| --- | --- | --- | --- | --- | --- | --- | --- | --- | --- | --- | --- | --- | --- |
| Duplication depth | Reference chromosome | Collinear blocks | | | | | | | | | | | |
| 0 | Atr-ERM99380 |  |  |  |  |  |  |
| 0 | Atr-ERM99381 |  |  |  |  |  |  |
| 0 | Atr-ERM99382 |  |  |  |  |  |  |
| 0 | Atr-ERM99383 |  |  |  |  |  |  |
| 0 | Atr-ERM99384 |  |  |  |  |  |  |
| 0 | Atr-ERM99385 |  |  |  |  |  |  |
| 0 | Atr-ERM99386 |  |  |  |  |  |  |
| 0 | Atr-ERM99387 |  |  |  |  |  |  |
| 0 | Atr-ERM99388 |  |  |  |  |  |  |
| 0 | Atr-ERM99389 |  |  |  |  |  |  |
| 0 | Atr-ERM99390 |  |  |  |  |  |  |
| 0 | Atr-ERM99391 |  |  |  |  |  |  |
| 0 | Atr-ERM99392 |  |  |  |  |  |  |
| 0 | Atr-ERM99393 |  |  |  |  |  |  |
| 0 | Atr-ERM99394 |  |  |  |  |  |  |
| 0 | Atr-ERM99395 |  |  |  |  |  |  |
| 0 | Atr-ERM99396 |  |  |  |  |  |  |
| 0 | Atr-ERM99397 |  |  |  |  |  |  |
| 0 | Atr-ERM99398 |  |  |  |  |  |  |
| 0 | Atr-ERM99399 |  |  |  |  |  |  |
| 0 | Atr-ERM99400 |  |  |  |  |  |  |
| 0 | Atr-ERM99401 |  |  |  |  |  |  |
| 0 | Atr-ERM99402 |  |  |  |  |  |  |
| 0 | Atr-ERM99403 |  |  |  |  |  |  |
| 0 | Atr-ERM99404 |  |  |  |  |  |  |
| 0 | Atr-ERM99405 |  |  |  |  |  |  |
| 0 | Atr-ERM99406 |  |  |  |  |  |  |
| 0 | Atr-ERM99407 |  |  |  |  |  |  |
| 0 | Atr-ERM99408 |  |  |  |  |  |  |
| 0 | Atr-ERM99409 |  |  |  |  |  |  |
| 0 | Atr-ERM99410 |  |  |  |  |  |  |
| 0 | Atr-ERM99411 |  |  |  |  |  |  |
| 0 | Atr-ERM99412 |  |  |  |  |  |  |
| 0 | Atr-ERM99413 |  |  |  |  |  |  |
| 0 | Atr-ERM99414 |  |  |  |  |  |  |
| 0 | Atr-ERM99415 |  |  |  |  |  |  |
| 0 | Atr-ERM99416 |  |  |  |  |  |  |
| 0 | Atr-ERM99417 |  |  |  |  |  |  |
| 0 | Atr-ERM99418 |  |  |  |  |  |  |
| 0 | Atr-ERM99419 |  |  |  |  |  |  |
| 0 | Atr-ERM99420 |  |  |  |  |  |  |
| 1 | Atr-ERM99421 |  | Vvi-Vitvi09g00004\_t001 |  |  |  |  |  |
| 1 | Atr-ERM99422 |  | | | |  |  |  |  |  |
| 1 | Atr-ERM99423 |  | Vvi-Vitvi09g00006\_t001 |  |  |  |  |  |
| 1 | Atr-ERM99424 |  | Vvi-Vitvi09g00007\_t001 |  |  |  |  |  |
| 1 | Atr-ERM99425 |  | | | |  |  |  |  |  |
| 1 | Atr-ERM99426 |  | | | |  |  |  |  |  |
| 1 | Atr-ERM99427 |  | | | |  |  |  |  |  |
| 1 | Atr-ERM99428 |  | | | |  |  |  |  |  |
| 1 | Atr-ERM99429 |  | | | |  |  |  |  |  |
| 1 | Atr-ERM99430 |  | | | |  |  |  |  |  |
| 1 | Atr-ERM99431 |  | | | |  |  |  |  |  |
| 1 | Atr-ERM99432 |  | | | |  |  |  |  |  |
| 1 | Atr-ERM99433 |  | | | |  |  |  |  |  |
| 1 | Atr-ERM99434 |  | Vvi-Vitvi09g00008\_t001 |  |  |  |  |  |
| 1 | Atr-ERM99435 |  | Vvi-Vitvi09g00009\_t001 |  |  |  |  |  |
| 1 | Atr-ERM99436 |  | | | |  |  |  |  |  |
| 1 | Atr-ERM99437 |  | | | |  |  |  |  |  |
| 1 | Atr-ERM99438 |  | | | |  |  |  |  |  |
| 1 | Atr-ERM99439 |  | Vvi-Vitvi09g01491\_t001 |  |  |  |  |  |
| 0 | Atr-ERM99440 |  |  |  |  |  |  |
| 0 | Atr-ERM99441 |  |  |  |  |  |  |
| 0 | Atr-ERM99442 |  |  |  |  |  |  |
| 0 | Atr-ERM99443 |  |  |  |  |  |  |
| 0 | Atr-ERM99444 |  |  |  |  |  |  |
| 0 | Atr-ERM99445 |  |  |  |  |  |  |
| 0 | Atr-ERM99446 |  |  |  |  |  |  |
| 0 | Atr-ERM99447 |  |  |  |  |  |  |
| 0 | Atr-ERM99448 |  |  |  |  |  |  |
| 2 | Atr-ERM99449 |  | Vvi-Vitvi11g00061\_t001 |  | Vvi-Vitvi09g00071\_t001 |  |  |  |  |
| 2 | Atr-ERM99450 |  | Vvi-Vitvi11g00060\_t001 |  | | | |  |  |  |  |
| 2 | Atr-ERM99451 |  | | | |  | Vvi-Vitvi09g00070\_t001 |  |  |  |  |
| 2 | Atr-ERM99452 |  | | | |  | | | |  |  |  |  |
| 2 | Atr-ERM99453 |  | | | |  | | | |  |  |  |  |
| 2 | Atr-ERM99454 |  | Vvi-Vitvi11g01327\_t001 |  | Vvi-Vitvi09g01501\_t001 |  |  |  |  |
| 2 | Atr-ERM99455 |  | Vvi-Vitvi11g00058\_t001 |  | Vvi-Vitvi09g00069\_t001 |  |  |  |  |
| 2 | Atr-ERM99456 |  | | | |  | Vvi-Vitvi09g00068\_t002 |  |  |  |  |
| 2 | Atr-ERM99457 |  | Vvi-Vitvi11g00057\_t001 |  | | | |  |  |  |  |
| 2 | Atr-ERM99458 |  | Vvi-Vitvi11g00056\_t001 |  | | | |  |  |  |  |
| 2 | Atr-ERM99459 |  | | | |  | | | |  |  |  |  |
| 2 | Atr-ERM99460 |  | | | |  | Vvi-Vitvi09g00067\_t001 |  |  |  |  |
| 2 | Atr-ERM99461 |  | | | |  | | | |  |  |  |  |
| 2 | Atr-ERM99462 |  | | | |  | | | |  |  |  |  |
| 2 | Atr-ERM99463 |  | | | |  | | | |  |  |  |  |
| 2 | Atr-ERM99464 |  | | | |  | | | |  |  |  |  |
| 2 | Atr-ERM99465 |  | Vvi-Vitvi11g00052\_t001 |  | | | |  |  |  |  |
| 2 | Atr-ERM99466 |  | | | |  | | | |  |  |  |  |
| 2 | Atr-ERM99467 |  | Vvi-Vitvi11g00051\_t003 |  | | | |  |  |  |  |
| 2 | Atr-ERM99468 |  | | | |  | | | |  |  |  |  |
| 2 | Atr-ERM99469 |  | | | |  | | | |  |  |  |  |
| 2 | Atr-ERM99470 |  | | | |  | | | |  |  |  |  |
| 2 | Atr-ERM99471 |  | | | |  | | | |  |  |  |  |
| 2 | Atr-ERM99472 |  | Vvi-Vitvi11g00050\_t001 |  | Vvi-Vitvi09g00064\_t001 |  |  |  |  |
