# Supplementary material for: Genome-wide identification, characterization and gene expression of BES1 transcription factor family in grapevine (Vitis vinifera L.)
Source: Sci Rep. 2023 Jan 5;13:240. doi: 10.1038/s41598-022-24407-y (PMC9816167; doi:10.1038/s41598-022-24407-y)
Supplement: Supplementary file 3 — Supplementary Information. [file 41598_2022_24407_MOESM3_ESM.zip › Vvi_Atr/Vitis_vinifera.PN40024.v4.dna_sm.toplevel.fa.vs.Amborella_trichopoda.AMTR1.0.dna_sm.toplevel.fa.html/Atr-AmTr_v1.0_scaffold00133.html]

|  |  |  |  |  |  |  |  |  |  |  |  |  |  |
| --- | --- | --- | --- | --- | --- | --- | --- | --- | --- | --- | --- | --- | --- |
| Duplication depth | Reference chromosome | Collinear blocks | | | | | | | | | | | |
| 0 | Atr-ERN04400 |  |  |  |  |  |  |
| 0 | Atr-ERN04401 |  |  |  |  |  |  |
| 0 | Atr-ERN04402 |  |  |  |  |  |  |
| 0 | Atr-ERN04403 |  |  |  |  |  |  |
| 0 | Atr-ERN04404 |  |  |  |  |  |  |
| 0 | Atr-ERN04405 |  |  |  |  |  |  |
| 0 | Atr-ERN04406 |  |  |  |  |  |  |
| 0 | Atr-ERN04407 |  |  |  |  |  |  |
| 0 | Atr-ERN04408 |  |  |  |  |  |  |
| 0 | Atr-ERN04409 |  |  |  |  |  |  |
| 0 | Atr-ERN04410 |  |  |  |  |  |  |
| 0 | Atr-ERN04411 |  |  |  |  |  |  |
| 0 | Atr-ERN04412 |  |  |  |  |  |  |
| 0 | Atr-ERN04413 |  |  |  |  |  |  |
| 0 | Atr-ERN04414 |  |  |  |  |  |  |
| 0 | Atr-ERN04415 |  |  |  |  |  |  |
| 0 | Atr-ERN04416 |  |  |  |  |  |  |
| 0 | Atr-ERN04417 |  |  |  |  |  |  |
| 0 | Atr-ERN04418 |  |  |  |  |  |  |
| 0 | Atr-ERN04419 |  |  |  |  |  |  |
| 0 | Atr-ERN04420 |  |  |  |  |  |  |
| 0 | Atr-ERN04421 |  |  |  |  |  |  |
| 0 | Atr-ERN04422 |  |  |  |  |  |  |
| 0 | Atr-ERN04423 |  |  |  |  |  |  |
| 0 | Atr-ERN04424 |  |  |  |  |  |  |
| 0 | Atr-ERN04425 |  |  |  |  |  |  |
| 0 | Atr-ERN04426 |  |  |  |  |  |  |
| 0 | Atr-ERN04427 |  |  |  |  |  |  |
| 0 | Atr-ERN04428 |  |  |  |  |  |  |
| 0 | Atr-ERN04429 |  |  |  |  |  |  |
| 0 | Atr-ERN04430 |  |  |  |  |  |  |
| 0 | Atr-ERN04431 |  |  |  |  |  |  |
| 0 | Atr-ERN04432 |  |  |  |  |  |  |
| 0 | Atr-ERN04433 |  |  |  |  |  |  |
| 0 | Atr-ERN04434 |  |  |  |  |  |  |
| 0 | Atr-ERN04435 |  |  |  |  |  |  |
| 0 | Atr-ERN04436 |  |  |  |  |  |  |
| 0 | Atr-ERN04437 |  |  |  |  |  |  |
| 0 | Atr-ERN04438 |  |  |  |  |  |  |
| 1 | Atr-ERN04439 |  | Vvi-Vitvi08g01779\_t002 |  |  |  |  |  |
| 1 | Atr-ERN04440 |  | | | |  |  |  |  |  |
| 1 | Atr-ERN04441 |  | | | |  |  |  |  |  |
| 2 | Atr-ERN04442 |  | Vvi-Vitvi08g01780\_t001 |  | Vvi-Vitvi06g04028\_t001 |  |  |  |  |
| 2 | Atr-ERN04443 |  | | | |  | | | |  |  |  |  |
| 2 | Atr-ERN04444 |  | | | |  | | | |  |  |  |  |
| 2 | Atr-ERN04445 |  | Vvi-Vitvi08g01782\_t001 |  | Vvi-Vitvi06g00086\_t003 |  |  |  |  |
| 2 | Atr-ERN04446 |  | | | |  | Vvi-Vitvi06g00085\_t001 |  |  |  |  |
| 2 | Atr-ERN04447 |  | Vvi-Vitvi08g01783\_t001 |  | | | |  |  |  |  |
| 2 | Atr-ERN04448 |  | Vvi-Vitvi08g01784\_t001 |  | | | |  |  |  |  |
| 2 | Atr-ERN04449 |  | | | |  | Vvi-Vitvi06g00084\_t001 |  |  |  |  |
| 2 | Atr-ERN04450 |  | | | |  | | | |  |  |  |  |
| 2 | Atr-ERN04451 |  | | | |  | | | |  |  |  |  |
| 2 | Atr-ERN04452 |  | Vvi-Vitvi08g01786\_t001 |  | | | |  |  |  |  |
| 2 | Atr-ERN04453 |  | | | |  | | | |  |  |  |  |
| 2 | Atr-ERN04454 |  | | | |  | | | |  |  |  |  |
| 2 | Atr-ERN04455 |  | | | |  | | | |  |  |  |  |
| 2 | Atr-ERN04456 |  | | | |  | | | |  |  |  |  |
| 2 | Atr-ERN04457 |  | | | |  | Vvi-Vitvi06g00083\_t001 |  |  |  |  |
| 2 | Atr-ERN04458 |  | Vvi-Vitvi08g02366\_t001 |  | | | |  |  |  |  |
| 1 | Atr-ERN04459 |  |  |  | Vvi-Vitvi06g01588\_t001 |  |  |  |  |
| 0 | Atr-ERN04460 |  |  |  |  |  |  |
