# Supplementary material for: Genome-wide identification, characterization and gene expression of BES1 transcription factor family in grapevine (Vitis vinifera L.)
Source: Sci Rep. 2023 Jan 5;13:240. doi: 10.1038/s41598-022-24407-y (PMC9816167; doi:10.1038/s41598-022-24407-y)
Supplement: Supplementary file 3 — Supplementary Information. [file 41598_2022_24407_MOESM3_ESM.zip › Vvi_Atr/Vitis_vinifera.PN40024.v4.dna_sm.toplevel.fa.vs.Amborella_trichopoda.AMTR1.0.dna_sm.toplevel.fa.html/Atr-AmTr_v1.0_scaffold00134.html]

|  |  |  |  |  |  |  |  |  |  |  |  |  |  |
| --- | --- | --- | --- | --- | --- | --- | --- | --- | --- | --- | --- | --- | --- |
| Duplication depth | Reference chromosome | Collinear blocks | | | | | | | | | | | |
| 0 | Atr-ERN02948 |  |  |  |  |  |  |
| 0 | Atr-ERN02949 |  |  |  |  |  |  |
| 0 | Atr-ERN02950 |  |  |  |  |  |  |
| 0 | Atr-ERN02951 |  |  |  |  |  |  |
| 0 | Atr-ERN02952 |  |  |  |  |  |  |
| 0 | Atr-ERN02953 |  |  |  |  |  |  |
| 0 | Atr-ERN02954 |  |  |  |  |  |  |
| 0 | Atr-ERN02955 |  |  |  |  |  |  |
| 0 | Atr-ERN02956 |  |  |  |  |  |  |
| 0 | Atr-ERN02957 |  |  |  |  |  |  |
| 0 | Atr-ERN02958 |  |  |  |  |  |  |
| 0 | Atr-ERN02959 |  |  |  |  |  |  |
| 0 | Atr-ERN02960 |  |  |  |  |  |  |
| 0 | Atr-ERN02961 |  |  |  |  |  |  |
| 0 | Atr-ERN02962 |  |  |  |  |  |  |
| 0 | Atr-ERN02963 |  |  |  |  |  |  |
| 0 | Atr-ERN02964 |  |  |  |  |  |  |
| 0 | Atr-ERN02965 |  |  |  |  |  |  |
| 0 | Atr-ERN02966 |  |  |  |  |  |  |
| 0 | Atr-ERN02967 |  |  |  |  |  |  |
| 0 | Atr-ERN02968 |  |  |  |  |  |  |
| 0 | Atr-ERN02969 |  |  |  |  |  |  |
| 0 | Atr-ERN02970 |  |  |  |  |  |  |
| 0 | Atr-ERN02971 |  |  |  |  |  |  |
| 0 | Atr-ERN02972 |  |  |  |  |  |  |
| 0 | Atr-ERN02973 |  |  |  |  |  |  |
| 0 | Atr-ERN02974 |  |  |  |  |  |  |
| 0 | Atr-ERN02975 |  |  |  |  |  |  |
| 0 | Atr-ERN02976 |  |  |  |  |  |  |
| 0 | Atr-ERN02977 |  |  |  |  |  |  |
| 0 | Atr-ERN02978 |  |  |  |  |  |  |
| 0 | Atr-ERN02979 |  |  |  |  |  |  |
| 0 | Atr-ERN02980 |  |  |  |  |  |  |
| 0 | Atr-ERN02981 |  |  |  |  |  |  |
| 0 | Atr-ERN02982 |  |  |  |  |  |  |
| 0 | Atr-ERN02983 |  |  |  |  |  |  |
| 0 | Atr-ERN02984 |  |  |  |  |  |  |
| 0 | Atr-ERN02985 |  |  |  |  |  |  |
| 0 | Atr-ERN02986 |  |  |  |  |  |  |
| 0 | Atr-ERN02987 |  |  |  |  |  |  |
