# Supplementary material for: Genome-wide identification, characterization and gene expression of BES1 transcription factor family in grapevine (Vitis vinifera L.)
Source: Sci Rep. 2023 Jan 5;13:240. doi: 10.1038/s41598-022-24407-y (PMC9816167; doi:10.1038/s41598-022-24407-y)
Supplement: Supplementary file 3 — Supplementary Information. [file 41598_2022_24407_MOESM3_ESM.zip › Vvi_Atr/Vitis_vinifera.PN40024.v4.dna_sm.toplevel.fa.vs.Amborella_trichopoda.AMTR1.0.dna_sm.toplevel.fa.html/Atr-AmTr_v1.0_scaffold00135.html]

|  |  |  |  |  |  |  |  |  |  |  |  |  |  |
| --- | --- | --- | --- | --- | --- | --- | --- | --- | --- | --- | --- | --- | --- |
| Duplication depth | Reference chromosome | Collinear blocks | | | | | | | | | | | |
| 0 | Atr-ERN02880 |  |  |  |  |  |  |
| 1 | Atr-ERN02881 |  | Vvi-Vitvi07g02582\_t001 |  |  |  |  |  |
| 2 | Atr-ERN02882 |  | | | |  | Vvi-Vitvi18g02604\_t001 |  |  |  |  |
| 2 | Atr-ERN02883 |  | Vvi-Vitvi07g01449\_t001 |  | Vvi-Vitvi18g00511\_t001 |  |  |  |  |
| 2 | Atr-ERN02884 |  | | | |  | Vvi-Vitvi18g02603\_t001 |  |  |  |  |
| 2 | Atr-ERN02885 |  | | | |  | Vvi-Vitvi18g00510\_t001 |  |  |  |  |
| 2 | Atr-ERN02886 |  | | | |  | Vvi-Vitvi18g00509\_t001 |  |  |  |  |
| 2 | Atr-ERN02887 |  | | | |  | | | |  |  |  |  |
| 2 | Atr-ERN02888 |  | Vvi-Vitvi07g04600\_t001 |  | Vvi-Vitvi18g00508\_t001 |  |  |  |  |
| 2 | Atr-ERN02889 |  | | | |  | | | |  |  |  |  |
| 2 | Atr-ERN02890 |  | Vvi-Vitvi07g04599\_t001 |  | Vvi-Vitvi18g00507\_t001 |  |  |  |  |
| 2 | Atr-ERN02891 |  | | | |  | | | |  |  |  |  |
| 2 | Atr-ERN02892 |  | | | |  | | | |  |  |  |  |
| 2 | Atr-ERN02893 |  | | | |  | | | |  |  |  |  |
| 2 | Atr-ERN02894 |  | | | |  | | | |  |  |  |  |
| 2 | Atr-ERN02895 |  | | | |  | Vvi-Vitvi18g00506\_t001 |  |  |  |  |
| 2 | Atr-ERN02896 |  | | | |  | | | |  |  |  |  |
| 2 | Atr-ERN02897 |  | Vvi-Vitvi07g01451\_t001 |  | Vvi-Vitvi18g00505\_t002 |  |  |  |  |
| 2 | Atr-ERN02898 |  | | | |  | | | |  |  |  |  |
| 2 | Atr-ERN02899 |  | | | |  | Vvi-Vitvi18g00504\_t001 |  |  |  |  |
| 2 | Atr-ERN02900 |  | | | |  | | | |  |  |  |  |
| 2 | Atr-ERN02901 |  | | | |  | Vvi-Vitvi18g00501\_t001 |  |  |  |  |
| 2 | Atr-ERN02902 |  | | | |  | Vvi-Vitvi18g02601\_t001 |  |  |  |  |
| 2 | Atr-ERN02903 |  | Vvi-Vitvi07g02589\_t001 |  | | | |  |  |  |  |
| 2 | Atr-ERN02904 |  | | | |  | | | |  |  |  |  |
| 2 | Atr-ERN02905 |  | | | |  | | | |  |  |  |  |
| 2 | Atr-ERN02906 |  | Vvi-Vitvi07g01457\_t001 |  | Vvi-Vitvi18g02600\_t001 |  |  |  |  |
| 2 | Atr-ERN02907 |  | | | |  | Vvi-Vitvi18g00500\_t001 |  |  |  |  |
| 2 | Atr-ERN02908 |  | | | |  | | | |  |  |  |  |
| 2 | Atr-ERN02909 |  | Vvi-Vitvi07g01460\_t001 |  | Vvi-Vitvi18g00499\_t001 |  |  |  |  |
| 2 | Atr-ERN02910 |  | | | |  | | | |  |  |  |  |
| 2 | Atr-ERN02911 |  | | | |  | Vvi-Vitvi18g04102\_t001 |  |  |  |  |
| 2 | Atr-ERN02912 |  | | | |  | | | |  |  |  |  |
| 2 | Atr-ERN02913 |  | | | |  | Vvi-Vitvi18g00496\_t001 |  |  |  |  |
| 2 | Atr-ERN02914 |  | | | |  | | | |  |  |  |  |
| 2 | Atr-ERN02915 |  | | | |  | | | |  |  |  |  |
| 2 | Atr-ERN02916 |  | | | |  | | | |  |  |  |  |
| 2 | Atr-ERN02917 |  | | | |  | Vvi-Vitvi18g00495\_t001 |  |  |  |  |
| 2 | Atr-ERN02918 |  | Vvi-Vitvi07g02594\_t001 |  | | | |  |  |  |  |
| 2 | Atr-ERN02919 |  | | | |  | | | |  |  |  |  |
| 2 | Atr-ERN02920 |  | | | |  | | | |  |  |  |  |
| 2 | Atr-ERN02921 |  | Vvi-Vitvi07g04592\_t001 |  | Vvi-Vitvi18g00492\_t001 |  |  |  |  |
| 2 | Atr-ERN02922 |  | | | |  | | | |  |  |  |  |
| 2 | Atr-ERN02923 |  | Vvi-Vitvi07g01468\_t001 |  | Vvi-Vitvi18g00489\_t001 |  |  |  |  |
| 1 | Atr-ERN02924 |  |  |  | | | |  |  |  |  |
| 1 | Atr-ERN02925 |  |  |  | Vvi-Vitvi18g00487\_t001 |  |  |  |  |
| 1 | Atr-ERN02926 |  |  |  | | | |  |  |  |  |
| 1 | Atr-ERN02927 |  |  |  | | | |  |  |  |  |
| 1 | Atr-ERN02928 |  |  |  | | | |  |  |  |  |
| 1 | Atr-ERN02929 |  |  |  | | | |  |  |  |  |
| 1 | Atr-ERN02930 |  |  |  | Vvi-Vitvi18g00486\_t001 |  |  |  |  |
| 1 | Atr-ERN02931 |  |  |  | | | |  |  |  |  |
| 1 | Atr-ERN02932 |  |  |  | Vvi-Vitvi18g00485\_t001.1.6037826c |  |  |  |  |
| 1 | Atr-ERN02933 |  |  |  | Vvi-Vitvi18g00483\_t001 |  |  |  |  |
| 1 | Atr-ERN02934 |  |  |  | Vvi-Vitvi18g00480\_t001 |  |  |  |  |
| 0 | Atr-ERN02935 |  |  |  |  |  |  |
| 0 | Atr-ERN02936 |  |  |  |  |  |  |
| 0 | Atr-ERN02937 |  |  |  |  |  |  |
| 0 | Atr-ERN02938 |  |  |  |  |  |  |
| 0 | Atr-ERN02939 |  |  |  |  |  |  |
| 0 | Atr-ERN02940 |  |  |  |  |  |  |
| 0 | Atr-ERN02941 |  |  |  |  |  |  |
| 0 | Atr-ERN02942 |  |  |  |  |  |  |
| 0 | Atr-ERN02943 |  |  |  |  |  |  |
| 0 | Atr-ERN02944 |  |  |  |  |  |  |
| 0 | Atr-ERN02945 |  |  |  |  |  |  |
| 0 | Atr-ERN02946 |  |  |  |  |  |  |
| 0 | Atr-ERN02947 |  |  |  |  |  |  |
