# Supplementary material for: Genome-wide identification, characterization and gene expression of BES1 transcription factor family in grapevine (Vitis vinifera L.)
Source: Sci Rep. 2023 Jan 5;13:240. doi: 10.1038/s41598-022-24407-y (PMC9816167; doi:10.1038/s41598-022-24407-y)
Supplement: Supplementary file 3 — Supplementary Information. [file 41598_2022_24407_MOESM3_ESM.zip › Vvi_Atr/Vitis_vinifera.PN40024.v4.dna_sm.toplevel.fa.vs.Amborella_trichopoda.AMTR1.0.dna_sm.toplevel.fa.html/Atr-AmTr_v1.0_scaffold00136.html]

|  |  |  |  |  |  |  |  |  |  |  |  |  |  |
| --- | --- | --- | --- | --- | --- | --- | --- | --- | --- | --- | --- | --- | --- |
| Duplication depth | Reference chromosome | Collinear blocks | | | | | | | | | | | |
| 0 | Atr-ERM93961 |  |  |  |  |  |  |
| 0 | Atr-ERM93962 |  |  |  |  |  |  |
| 0 | Atr-ERM93963 |  |  |  |  |  |  |
| 0 | Atr-ERM93964 |  |  |  |  |  |  |
| 0 | Atr-ERM93965 |  |  |  |  |  |  |
| 0 | Atr-ERM93966 |  |  |  |  |  |  |
| 0 | Atr-ERM93967 |  |  |  |  |  |  |
| 0 | Atr-ERM93968 |  |  |  |  |  |  |
| 0 | Atr-ERM93969 |  |  |  |  |  |  |
| 0 | Atr-ERM93970 |  |  |  |  |  |  |
| 0 | Atr-ERM93971 |  |  |  |  |  |  |
| 0 | Atr-ERM93972 |  |  |  |  |  |  |
| 0 | Atr-ERM93973 |  |  |  |  |  |  |
| 0 | Atr-ERM93974 |  |  |  |  |  |  |
| 0 | Atr-ERM93975 |  |  |  |  |  |  |
| 0 | Atr-ERM93976 |  |  |  |  |  |  |
| 0 | Atr-ERM93977 |  |  |  |  |  |  |
| 0 | Atr-ERM93978 |  |  |  |  |  |  |
| 0 | Atr-ERM93979 |  |  |  |  |  |  |
| 0 | Atr-ERM93980 |  |  |  |  |  |  |
| 0 | Atr-ERM93981 |  |  |  |  |  |  |
| 0 | Atr-ERM93982 |  |  |  |  |  |  |
| 0 | Atr-ERM93983 |  |  |  |  |  |  |
| 0 | Atr-ERM93984 |  |  |  |  |  |  |
| 0 | Atr-ERM93985 |  |  |  |  |  |  |
| 0 | Atr-ERM93986 |  |  |  |  |  |  |
| 0 | Atr-ERM93987 |  |  |  |  |  |  |
| 0 | Atr-ERM93988 |  |  |  |  |  |  |
| 0 | Atr-ERM93989 |  |  |  |  |  |  |
| 0 | Atr-ERM93990 |  |  |  |  |  |  |
| 0 | Atr-ERM93991 |  |  |  |  |  |  |
| 0 | Atr-ERM93992 |  |  |  |  |  |  |
| 0 | Atr-ERM93993 |  |  |  |  |  |  |
| 0 | Atr-ERM93994 |  |  |  |  |  |  |
| 0 | Atr-ERM93995 |  |  |  |  |  |  |
| 0 | Atr-ERM93996 |  |  |  |  |  |  |
| 0 | Atr-ERM93997 |  |  |  |  |  |  |
| 0 | Atr-ERM93998 |  |  |  |  |  |  |
| 0 | Atr-ERM93999 |  |  |  |  |  |  |
| 0 | Atr-ERM94000 |  |  |  |  |  |  |
| 0 | Atr-ERM94001 |  |  |  |  |  |  |
| 0 | Atr-ERM94002 |  |  |  |  |  |  |
| 0 | Atr-ERM94003 |  |  |  |  |  |  |
| 0 | Atr-ERM94004 |  |  |  |  |  |  |
| 0 | Atr-ERM94005 |  |  |  |  |  |  |
| 0 | Atr-ERM94006 |  |  |  |  |  |  |
| 0 | Atr-ERM94007 |  |  |  |  |  |  |
| 0 | Atr-ERM94008 |  |  |  |  |  |  |
| 0 | Atr-ERM94009 |  |  |  |  |  |  |
| 0 | Atr-ERM94010 |  |  |  |  |  |  |
| 0 | Atr-ERM94011 |  |  |  |  |  |  |
| 0 | Atr-ERM94012 |  |  |  |  |  |  |
| 0 | Atr-ERM94013 |  |  |  |  |  |  |
| 0 | Atr-ERM94014 |  |  |  |  |  |  |
| 0 | Atr-ERM94015 |  |  |  |  |  |  |
| 0 | Atr-ERM94016 |  |  |  |  |  |  |
| 0 | Atr-ERM94017 |  |  |  |  |  |  |
| 0 | Atr-ERM94018 |  |  |  |  |  |  |
| 0 | Atr-ERM94019 |  |  |  |  |  |  |
| 0 | Atr-ERM94020 |  |  |  |  |  |  |
| 0 | Atr-ERM94021 |  |  |  |  |  |  |
| 0 | Atr-ERM94022 |  |  |  |  |  |  |
| 0 | Atr-ERM94023 |  |  |  |  |  |  |
| 0 | Atr-ERM94024 |  |  |  |  |  |  |
