# Supplementary material for: Genome-wide identification, characterization and gene expression of BES1 transcription factor family in grapevine (Vitis vinifera L.)
Source: Sci Rep. 2023 Jan 5;13:240. doi: 10.1038/s41598-022-24407-y (PMC9816167; doi:10.1038/s41598-022-24407-y)
Supplement: Supplementary file 3 — Supplementary Information. [file 41598_2022_24407_MOESM3_ESM.zip › Vvi_Atr/Vitis_vinifera.PN40024.v4.dna_sm.toplevel.fa.vs.Amborella_trichopoda.AMTR1.0.dna_sm.toplevel.fa.html/Atr-AmTr_v1.0_scaffold00137.html]

|  |  |  |  |  |  |  |  |  |  |  |  |  |  |
| --- | --- | --- | --- | --- | --- | --- | --- | --- | --- | --- | --- | --- | --- |
| Duplication depth | Reference chromosome | Collinear blocks | | | | | | | | | | | |
| 0 | Atr-ERM93892 |  |  |  |  |  |  |
| 1 | Atr-ERM93893 |  | Vvi-Vitvi11g00718\_t001 |  |  |  |  |  |
| 1 | Atr-ERM93894 |  | | | |  |  |  |  |  |
| 1 | Atr-ERM93895 |  | Vvi-Vitvi11g00719\_t001 |  |  |  |  |  |
| 1 | Atr-ERM93896 |  | | | |  |  |  |  |  |
| 1 | Atr-ERM93897 |  | | | |  |  |  |  |  |
| 1 | Atr-ERM93898 |  | | | |  |  |  |  |  |
| 1 | Atr-ERM93899 |  | | | |  |  |  |  |  |
| 1 | Atr-ERM93900 |  | | | |  |  |  |  |  |
| 1 | Atr-ERM93901 |  | | | |  |  |  |  |  |
| 1 | Atr-ERM93902 |  | Vvi-Vitvi11g00722\_t001 |  |  |  |  |  |
| 1 | Atr-ERM93903 |  | | | |  |  |  |  |  |
| 1 | Atr-ERM93904 |  | | | |  |  |  |  |  |
| 1 | Atr-ERM93905 |  | Vvi-Vitvi11g00725\_t001 |  |  |  |  |  |
| 1 | Atr-ERM93906 |  | Vvi-Vitvi11g00726\_t001 |  |  |  |  |  |
| 1 | Atr-ERM93907 |  | | | |  |  |  |  |  |
| 1 | Atr-ERM93908 |  | | | |  |  |  |  |  |
| 1 | Atr-ERM93909 |  | | | |  |  |  |  |  |
| 1 | Atr-ERM93910 |  | Vvi-Vitvi11g00728\_t002 |  |  |  |  |  |
| 1 | Atr-ERM93911 |  | | | |  |  |  |  |  |
| 1 | Atr-ERM93912 |  | | | |  |  |  |  |  |
| 1 | Atr-ERM93913 |  | | | |  |  |  |  |  |
| 1 | Atr-ERM93914 |  | | | |  |  |  |  |  |
| 1 | Atr-ERM93915 |  | | | |  |  |  |  |  |
| 1 | Atr-ERM93916 |  | | | |  |  |  |  |  |
| 1 | Atr-ERM93917 |  | | | |  |  |  |  |  |
| 1 | Atr-ERM93918 |  | | | |  |  |  |  |  |
| 1 | Atr-ERM93919 |  | | | |  |  |  |  |  |
| 1 | Atr-ERM93920 |  | | | |  |  |  |  |  |
| 1 | Atr-ERM93921 |  | | | |  |  |  |  |  |
| 1 | Atr-ERM93922 |  | | | |  |  |  |  |  |
| 1 | Atr-ERM93923 |  | | | |  |  |  |  |  |
| 1 | Atr-ERM93924 |  | | | |  |  |  |  |  |
| 1 | Atr-ERM93925 |  | Vvi-Vitvi11g00730\_t001 |  |  |  |  |  |
| 0 | Atr-ERM93926 |  |  |  |  |  |  |
| 1 | Atr-ERM93927 |  | Vvi-Vitvi07g01612\_t001 |  |  |  |  |  |
| 1 | Atr-ERM93928 |  | | | |  |  |  |  |  |
| 1 | Atr-ERM93929 |  | | | |  |  |  |  |  |
| 1 | Atr-ERM93930 |  | | | |  |  |  |  |  |
| 1 | Atr-ERM93931 |  | | | |  |  |  |  |  |
| 1 | Atr-ERM93932 |  | Vvi-Vitvi07g01610\_t001 |  |  |  |  |  |
| 1 | Atr-ERM93933 |  | Vvi-Vitvi07g01608\_t001 |  |  |  |  |  |
| 1 | Atr-ERM93934 |  | Vvi-Vitvi07g01606\_t001 |  |  |  |  |  |
| 1 | Atr-ERM93935 |  | | | |  |  |  |  |  |
| 1 | Atr-ERM93936 |  | | | |  |  |  |  |  |
| 1 | Atr-ERM93937 |  | Vvi-Vitvi07g01605\_t001 |  |  |  |  |  |
| 1 | Atr-ERM93938 |  | Vvi-Vitvi07g01604\_t001 |  |  |  |  |  |
| 1 | Atr-ERM93939 |  | Vvi-Vitvi07g01603\_t003 |  |  |  |  |  |
| 1 | Atr-ERM93940 |  | | | |  |  |  |  |  |
| 1 | Atr-ERM93941 |  | | | |  |  |  |  |  |
| 1 | Atr-ERM93942 |  | | | |  |  |  |  |  |
| 1 | Atr-ERM93943 |  | | | |  |  |  |  |  |
| 1 | Atr-ERM93944 |  | Vvi-Vitvi07g01602\_t001 |  |  |  |  |  |
| 0 | Atr-ERM93945 |  |  |  |  |  |  |
| 0 | Atr-ERM93946 |  |  |  |  |  |  |
| 0 | Atr-ERM93947 |  |  |  |  |  |  |
| 0 | Atr-ERM93948 |  |  |  |  |  |  |
| 0 | Atr-ERM93949 |  |  |  |  |  |  |
| 0 | Atr-ERM93950 |  |  |  |  |  |  |
| 0 | Atr-ERM93951 |  |  |  |  |  |  |
| 0 | Atr-ERM93952 |  |  |  |  |  |  |
| 0 | Atr-ERM93953 |  |  |  |  |  |  |
| 0 | Atr-ERM93954 |  |  |  |  |  |  |
