# Supplementary material for: Genome-wide identification, characterization and gene expression of BES1 transcription factor family in grapevine (Vitis vinifera L.)
Source: Sci Rep. 2023 Jan 5;13:240. doi: 10.1038/s41598-022-24407-y (PMC9816167; doi:10.1038/s41598-022-24407-y)
Supplement: Supplementary file 3 — Supplementary Information. [file 41598_2022_24407_MOESM3_ESM.zip › Vvi_Atr/Vitis_vinifera.PN40024.v4.dna_sm.toplevel.fa.vs.Amborella_trichopoda.AMTR1.0.dna_sm.toplevel.fa.html/Atr-AmTr_v1.0_scaffold00138.html]

|  |  |  |  |  |  |  |  |  |  |  |  |  |  |
| --- | --- | --- | --- | --- | --- | --- | --- | --- | --- | --- | --- | --- | --- |
| Duplication depth | Reference chromosome | Collinear blocks | | | | | | | | | | | |
| 0 | Atr-ERM93806 |  |  |  |  |  |  |
| 0 | Atr-ERM93807 |  |  |  |  |  |  |
| 1 | Atr-ERM93808 |  | Vvi-Vitvi01g00516\_t002 |  |  |  |  |  |
| 1 | Atr-ERM93809 |  | | | |  |  |  |  |  |
| 1 | Atr-ERM93810 |  | | | |  |  |  |  |  |
| 1 | Atr-ERM93811 |  | | | |  |  |  |  |  |
| 1 | Atr-ERM93812 |  | | | |  |  |  |  |  |
| 1 | Atr-ERM93813 |  | | | |  |  |  |  |  |
| 1 | Atr-ERM93814 |  | Vvi-Vitvi01g00517\_t001 |  |  |  |  |  |
| 1 | Atr-ERM93815 |  | | | |  |  |  |  |  |
| 1 | Atr-ERM93816 |  | | | |  |  |  |  |  |
| 1 | Atr-ERM93817 |  | | | |  |  |  |  |  |
| 1 | Atr-ERM93818 |  | | | |  |  |  |  |  |
| 1 | Atr-ERM93819 |  | | | |  |  |  |  |  |
| 1 | Atr-ERM93820 |  | Vvi-Vitvi01g00519\_t001 |  |  |  |  |  |
| 1 | Atr-ERM93821 |  | Vvi-Vitvi01g00520\_t001 |  |  |  |  |  |
| 1 | Atr-ERM93822 |  | | | |  |  |  |  |  |
| 1 | Atr-ERM93823 |  | Vvi-Vitvi01g00521\_t001 |  |  |  |  |  |
| 1 | Atr-ERM93824 |  | Vvi-Vitvi01g00522\_t001 |  |  |  |  |  |
| 1 | Atr-ERM93825 |  | Vvi-Vitvi01g00524\_t001 |  |  |  |  |  |
| 1 | Atr-ERM93826 |  | | | |  |  |  |  |  |
| 1 | Atr-ERM93827 |  | | | |  |  |  |  |  |
| 1 | Atr-ERM93828 |  | | | |  |  |  |  |  |
| 1 | Atr-ERM93829 |  | Vvi-Vitvi01g00525\_t001 |  |  |  |  |  |
| 1 | Atr-ERM93830 |  | | | |  |  |  |  |  |
| 1 | Atr-ERM93831 |  | | | |  |  |  |  |  |
| 1 | Atr-ERM93832 |  | Vvi-Vitvi01g00526\_t001 |  |  |  |  |  |
| 1 | Atr-ERM93833 |  | | | |  |  |  |  |  |
| 1 | Atr-ERM93834 |  | Vvi-Vitvi01g00527\_t001 |  |  |  |  |  |
| 1 | Atr-ERM93835 |  | | | |  |  |  |  |  |
| 1 | Atr-ERM93836 |  | | | |  |  |  |  |  |
| 1 | Atr-ERM93837 |  | | | |  |  |  |  |  |
| 1 | Atr-ERM93838 |  | | | |  |  |  |  |  |
| 1 | Atr-ERM93839 |  | | | |  |  |  |  |  |
| 1 | Atr-ERM93840 |  | | | |  |  |  |  |  |
| 1 | Atr-ERM93841 |  | | | |  |  |  |  |  |
| 1 | Atr-ERM93842 |  | | | |  |  |  |  |  |
| 1 | Atr-ERM93843 |  | | | |  |  |  |  |  |
| 1 | Atr-ERM93844 |  | Vvi-Vitvi01g00529\_t001 |  |  |  |  |  |
| 0 | Atr-ERM93845 |  |  |  |  |  |  |
| 0 | Atr-ERM93846 |  |  |  |  |  |  |
| 0 | Atr-ERM93847 |  |  |  |  |  |  |
| 0 | Atr-ERM93848 |  |  |  |  |  |  |
| 0 | Atr-ERM93849 |  |  |  |  |  |  |
| 0 | Atr-ERM93850 |  |  |  |  |  |  |
| 0 | Atr-ERM93851 |  |  |  |  |  |  |
| 0 | Atr-ERM93852 |  |  |  |  |  |  |
| 0 | Atr-ERM93853 |  |  |  |  |  |  |
| 0 | Atr-ERM93854 |  |  |  |  |  |  |
| 0 | Atr-ERM93855 |  |  |  |  |  |  |
| 0 | Atr-ERM93856 |  |  |  |  |  |  |
| 0 | Atr-ERM93857 |  |  |  |  |  |  |
| 0 | Atr-ERM93858 |  |  |  |  |  |  |
| 0 | Atr-ERM93859 |  |  |  |  |  |  |
| 0 | Atr-ERM93860 |  |  |  |  |  |  |
| 0 | Atr-ERM93861 |  |  |  |  |  |  |
| 0 | Atr-ERM93862 |  |  |  |  |  |  |
| 0 | Atr-ERM93863 |  |  |  |  |  |  |
