# Supplementary material for: Genome-wide identification, characterization and gene expression of BES1 transcription factor family in grapevine (Vitis vinifera L.)
Source: Sci Rep. 2023 Jan 5;13:240. doi: 10.1038/s41598-022-24407-y (PMC9816167; doi:10.1038/s41598-022-24407-y)
Supplement: Supplementary file 3 — Supplementary Information. [file 41598_2022_24407_MOESM3_ESM.zip › Vvi_Atr/Vitis_vinifera.PN40024.v4.dna_sm.toplevel.fa.vs.Amborella_trichopoda.AMTR1.0.dna_sm.toplevel.fa.html/Atr-AmTr_v1.0_scaffold00140.html]

|  |  |  |  |  |  |  |  |  |  |  |  |  |  |
| --- | --- | --- | --- | --- | --- | --- | --- | --- | --- | --- | --- | --- | --- |
| Duplication depth | Reference chromosome | Collinear blocks | | | | | | | | | | | |
| 0 | Atr-ERN04753 |  |  |  |  |  |  |
| 0 | Atr-ERN04754 |  |  |  |  |  |  |
| 0 | Atr-ERN04755 |  |  |  |  |  |  |
| 0 | Atr-ERN04756 |  |  |  |  |  |  |
| 1 | Atr-ERN04757 |  | Vvi-Vitvi06g01357\_t001 |  |  |  |  |  |
| 1 | Atr-ERN04758 |  | Vvi-Vitvi06g01933\_t001 |  |  |  |  |  |
| 1 | Atr-ERN04759 |  | | | |  |  |  |  |  |
| 1 | Atr-ERN04760 |  | | | |  |  |  |  |  |
| 1 | Atr-ERN04761 |  | | | |  |  |  |  |  |
| 1 | Atr-ERN04762 |  | | | |  |  |  |  |  |
| 1 | Atr-ERN04763 |  | | | |  |  |  |  |  |
| 1 | Atr-ERN04764 |  | Vvi-Vitvi06g04411\_t001 |  |  |  |  |  |
| 1 | Atr-ERN04765 |  | Vvi-Vitvi06g04410\_t001 |  |  |  |  |  |
| 1 | Atr-ERN04766 |  | | | |  |  |  |  |  |
| 1 | Atr-ERN04767 |  | | | |  |  |  |  |  |
| 1 | Atr-ERN04768 |  | | | |  |  |  |  |  |
| 1 | Atr-ERN04769 |  | | | |  |  |  |  |  |
| 1 | Atr-ERN04770 |  | | | |  |  |  |  |  |
| 1 | Atr-ERN04771 |  | | | |  |  |  |  |  |
| 1 | Atr-ERN04772 |  | | | |  |  |  |  |  |
| 1 | Atr-ERN04773 |  | | | |  |  |  |  |  |
| 1 | Atr-ERN04774 |  | | | |  |  |  |  |  |
| 1 | Atr-ERN04775 |  | | | |  |  |  |  |  |
| 1 | Atr-ERN04776 |  | | | |  |  |  |  |  |
| 1 | Atr-ERN04777 |  | | | |  |  |  |  |  |
| 1 | Atr-ERN04778 |  | | | |  |  |  |  |  |
| 1 | Atr-ERN04779 |  | | | |  |  |  |  |  |
| 1 | Atr-ERN04780 |  | | | |  |  |  |  |  |
| 1 | Atr-ERN04781 |  | Vvi-Vitvi06g01354\_t001 |  |  |  |  |  |
| 1 | Atr-ERN04782 |  | | | |  |  |  |  |  |
| 1 | Atr-ERN04783 |  | | | |  |  |  |  |  |
| 1 | Atr-ERN04784 |  | | | |  |  |  |  |  |
| 1 | Atr-ERN04785 |  | | | |  |  |  |  |  |
| 1 | Atr-ERN04786 |  | | | |  |  |  |  |  |
| 1 | Atr-ERN04787 |  | | | |  |  |  |  |  |
| 1 | Atr-ERN04788 |  | | | |  |  |  |  |  |
| 1 | Atr-ERN04789 |  | | | |  |  |  |  |  |
| 1 | Atr-ERN04790 |  | | | |  |  |  |  |  |
| 1 | Atr-ERN04791 |  | | | |  |  |  |  |  |
| 1 | Atr-ERN04792 |  | | | |  |  |  |  |  |
| 1 | Atr-ERN04793 |  | | | |  |  |  |  |  |
| 1 | Atr-ERN04794 |  | | | |  |  |  |  |  |
| 1 | Atr-ERN04795 |  | | | |  |  |  |  |  |
| 1 | Atr-ERN04796 |  | | | |  |  |  |  |  |
| 1 | Atr-ERN04797 |  | | | |  |  |  |  |  |
| 1 | Atr-ERN04798 |  | | | |  |  |  |  |  |
| 1 | Atr-ERN04799 |  | | | |  |  |  |  |  |
| 1 | Atr-ERN04800 |  | | | |  |  |  |  |  |
| 1 | Atr-ERN04801 |  | | | |  |  |  |  |  |
| 1 | Atr-ERN04802 |  | Vvi-Vitvi06g01351\_t001 |  |  |  |  |  |
| 0 | Atr-ERN04803 |  |  |  |  |  |  |
| 0 | Atr-ERN04804 |  |  |  |  |  |  |
| 0 | Atr-ERN04805 |  |  |  |  |  |  |
| 0 | Atr-ERN04806 |  |  |  |  |  |  |
| 0 | Atr-ERN04807 |  |  |  |  |  |  |
| 0 | Atr-ERN04808 |  |  |  |  |  |  |
| 0 | Atr-ERN04809 |  |  |  |  |  |  |
