# Supplementary material for: Genome-wide identification, characterization and gene expression of BES1 transcription factor family in grapevine (Vitis vinifera L.)
Source: Sci Rep. 2023 Jan 5;13:240. doi: 10.1038/s41598-022-24407-y (PMC9816167; doi:10.1038/s41598-022-24407-y)
Supplement: Supplementary file 3 — Supplementary Information. [file 41598_2022_24407_MOESM3_ESM.zip › Vvi_Atr/Vitis_vinifera.PN40024.v4.dna_sm.toplevel.fa.vs.Amborella_trichopoda.AMTR1.0.dna_sm.toplevel.fa.html/Atr-AmTr_v1.0_scaffold00141.html]

|  |  |  |  |  |  |  |  |  |  |  |  |  |  |
| --- | --- | --- | --- | --- | --- | --- | --- | --- | --- | --- | --- | --- | --- |
| Duplication depth | Reference chromosome | Collinear blocks | | | | | | | | | | | |
| 0 | Atr-ERN06985 |  |  |  |  |  |  |
| 0 | Atr-ERN06986 |  |  |  |  |  |  |
| 0 | Atr-ERN06987 |  |  |  |  |  |  |
| 0 | Atr-ERN06988 |  |  |  |  |  |  |
| 0 | Atr-ERN06989 |  |  |  |  |  |  |
| 0 | Atr-ERN06990 |  |  |  |  |  |  |
| 1 | Atr-ERN06991 |  | Vvi-Vitvi10g00753\_t001 |  |  |  |  |  |
| 1 | Atr-ERN06992 |  | | | |  |  |  |  |  |
| 1 | Atr-ERN06993 |  | | | |  |  |  |  |  |
| 1 | Atr-ERN06994 |  | | | |  |  |  |  |  |
| 1 | Atr-ERN06995 |  | Vvi-Vitvi10g00764\_t001 |  |  |  |  |  |
| 1 | Atr-ERN06996 |  | | | |  |  |  |  |  |
| 1 | Atr-ERN06997 |  | | | |  |  |  |  |  |
| 1 | Atr-ERN06998 |  | Vvi-Vitvi10g00765\_t001 |  |  |  |  |  |
| 1 | Atr-ERN06999 |  | | | |  |  |  |  |  |
| 1 | Atr-ERN07000 |  | Vvi-Vitvi10g00767\_t002 |  |  |  |  |  |
| 1 | Atr-ERN07001 |  | | | |  |  |  |  |  |
| 1 | Atr-ERN07002 |  | | | |  |  |  |  |  |
| 1 | Atr-ERN07003 |  | | | |  |  |  |  |  |
| 1 | Atr-ERN07004 |  | | | |  |  |  |  |  |
| 1 | Atr-ERN07005 |  | | | |  |  |  |  |  |
| 1 | Atr-ERN07006 |  | | | |  |  |  |  |  |
| 1 | Atr-ERN07007 |  | | | |  |  |  |  |  |
| 1 | Atr-ERN07008 |  | | | |  |  |  |  |  |
| 1 | Atr-ERN07009 |  | | | |  |  |  |  |  |
| 1 | Atr-ERN07010 |  | | | |  |  |  |  |  |
| 1 | Atr-ERN07011 |  | Vvi-Vitvi10g00774\_t001 |  |  |  |  |  |
| 1 | Atr-ERN07012 |  | | | |  |  |  |  |  |
| 1 | Atr-ERN07013 |  | | | |  |  |  |  |  |
| 1 | Atr-ERN07014 |  | | | |  |  |  |  |  |
| 1 | Atr-ERN07015 |  | | | |  |  |  |  |  |
| 1 | Atr-ERN07016 |  | | | |  |  |  |  |  |
| 1 | Atr-ERN07017 |  | | | |  |  |  |  |  |
| 1 | Atr-ERN07018 |  | | | |  |  |  |  |  |
| 1 | Atr-ERN07019 |  | | | |  |  |  |  |  |
| 1 | Atr-ERN07020 |  | | | |  |  |  |  |  |
| 1 | Atr-ERN07021 |  | | | |  |  |  |  |  |
| 1 | Atr-ERN07022 |  | | | |  |  |  |  |  |
| 1 | Atr-ERN07023 |  | | | |  |  |  |  |  |
| 1 | Atr-ERN07024 |  | Vvi-Vitvi10g00777\_t001 |  |  |  |  |  |
| 1 | Atr-ERN07025 |  | Vvi-Vitvi10g00778\_t001 |  |  |  |  |  |
| 1 | Atr-ERN07026 |  | Vvi-Vitvi10g00781\_t001 |  |  |  |  |  |
