# Supplementary material for: Genome-wide identification, characterization and gene expression of BES1 transcription factor family in grapevine (Vitis vinifera L.)
Source: Sci Rep. 2023 Jan 5;13:240. doi: 10.1038/s41598-022-24407-y (PMC9816167; doi:10.1038/s41598-022-24407-y)
Supplement: Supplementary file 3 — Supplementary Information. [file 41598_2022_24407_MOESM3_ESM.zip › Vvi_Atr/Vitis_vinifera.PN40024.v4.dna_sm.toplevel.fa.vs.Amborella_trichopoda.AMTR1.0.dna_sm.toplevel.fa.html/Atr-AmTr_v1.0_scaffold00142.html]

|  |  |  |  |  |  |  |  |  |  |  |  |  |  |
| --- | --- | --- | --- | --- | --- | --- | --- | --- | --- | --- | --- | --- | --- |
| Duplication depth | Reference chromosome | Collinear blocks | | | | | | | | | | | |
| 0 | Atr-ERN06010 |  |  |  |  |  |  |
| 0 | Atr-ERN06011 |  |  |  |  |  |  |
| 0 | Atr-ERN06012 |  |  |  |  |  |  |
| 1 | Atr-ERN06013 |  | Vvi-Vitvi09g00394\_t001 |  |  |  |  |  |
| 1 | Atr-ERN06014 |  | | | |  |  |  |  |  |
| 1 | Atr-ERN06015 |  | | | |  |  |  |  |  |
| 1 | Atr-ERN06016 |  | | | |  |  |  |  |  |
| 1 | Atr-ERN06017 |  | | | |  |  |  |  |  |
| 1 | Atr-ERN06018 |  | | | |  |  |  |  |  |
| 1 | Atr-ERN06019 |  | | | |  |  |  |  |  |
| 1 | Atr-ERN06020 |  | | | |  |  |  |  |  |
| 1 | Atr-ERN06021 |  | | | |  |  |  |  |  |
| 1 | Atr-ERN06022 |  | | | |  |  |  |  |  |
| 1 | Atr-ERN06023 |  | Vvi-Vitvi09g00395\_t003 |  |  |  |  |  |
| 1 | Atr-ERN06024 |  | | | |  |  |  |  |  |
| 1 | Atr-ERN06025 |  | | | |  |  |  |  |  |
| 1 | Atr-ERN06026 |  | | | |  |  |  |  |  |
| 1 | Atr-ERN06027 |  | | | |  |  |  |  |  |
| 1 | Atr-ERN06028 |  | | | |  |  |  |  |  |
| 1 | Atr-ERN06029 |  | | | |  |  |  |  |  |
| 1 | Atr-ERN06030 |  | | | |  |  |  |  |  |
| 1 | Atr-ERN06031 |  | | | |  |  |  |  |  |
| 2 | Atr-ERN06032 |  | | | |  | Vvi-Vitvi11g00354\_t002 |  |  |  |  |
| 2 | Atr-ERN06033 |  | | | |  | | | |  |  |  |  |
| 2 | Atr-ERN06034 |  | | | |  | | | |  |  |  |  |
| 2 | Atr-ERN06035 |  | | | |  | | | |  |  |  |  |
| 3 | Atr-ERN06036 |  | | | |  | | | |  | Vvi-Vitvi04g01877\_t001 |  |  |  |
| 3 | Atr-ERN06037 |  | | | |  | | | |  | | | |  |  |  |
| 3 | Atr-ERN06038 |  | | | |  | | | |  | | | |  |  |  |
| 3 | Atr-ERN06039 |  | | | |  | | | |  | | | |  |  |  |
| 3 | Atr-ERN06040 |  | | | |  | | | |  | | | |  |  |  |
| 3 | Atr-ERN06041 |  | Vvi-Vitvi09g00404\_t003 |  | Vvi-Vitvi11g00363\_t001 |  | Vvi-Vitvi04g00384\_t001 |  |  |  |
| 3 | Atr-ERN06042 |  | | | |  | | | |  | | | |  |  |  |
| 3 | Atr-ERN06043 |  | | | |  | Vvi-Vitvi11g00368\_t001 |  | | | |  |  |  |
| 3 | Atr-ERN06044 |  | | | |  | Vvi-Vitvi11g00370\_t001 |  | | | |  |  |  |
| 3 | Atr-ERN06045 |  | Vvi-Vitvi09g00406\_t001 |  | | | |  | | | |  |  |  |
| 3 | Atr-ERN06046 |  | | | |  | | | |  | Vvi-Vitvi04g00380\_t001 |  |  |  |
| 3 | Atr-ERN06047 |  | | | |  | | | |  | | | |  |  |  |
| 3 | Atr-ERN06048 |  | | | |  | | | |  | | | |  |  |  |
| 3 | Atr-ERN06049 |  | | | |  | Vvi-Vitvi11g00373\_t001 |  | | | |  |  |  |
| 3 | Atr-ERN06050 |  | | | |  | | | |  | | | |  |  |  |
| 3 | Atr-ERN06051 |  | | | |  | | | |  | | | |  |  |  |
| 3 | Atr-ERN06052 |  | | | |  | | | |  | | | |  |  |  |
| 3 | Atr-ERN06053 |  | | | |  | | | |  | | | |  |  |  |
| 3 | Atr-ERN06054 |  | | | |  | | | |  | | | |  |  |  |
| 3 | Atr-ERN06055 |  | | | |  | | | |  | | | |  |  |  |
| 3 | Atr-ERN06056 |  | | | |  | | | |  | | | |  |  |  |
| 3 | Atr-ERN06057 |  | | | |  | Vvi-Vitvi11g00376\_t001 |  | | | |  |  |  |
| 3 | Atr-ERN06058 |  | | | |  | | | |  | | | |  |  |  |
| 3 | Atr-ERN06059 |  | | | |  | | | |  | | | |  |  |  |
| 3 | Atr-ERN06060 |  | | | |  | Vvi-Vitvi11g00385\_t001 |  | Vvi-Vitvi04g01874\_t001 |  |  |  |
| 3 | Atr-ERN06061 |  | | | |  | | | |  | | | |  |  |  |
| 3 | Atr-ERN06062 |  | | | |  | | | |  | | | |  |  |  |
| 3 | Atr-ERN06063 |  | Vvi-Vitvi09g00420\_t001 |  | | | |  | | | |  |  |  |
| 3 | Atr-ERN06064 |  | | | |  | | | |  | | | |  |  |  |
| 3 | Atr-ERN06065 |  | | | |  | | | |  | | | |  |  |  |
| 3 | Atr-ERN06066 |  | | | |  | | | |  | Vvi-Vitvi04g00376\_t001 |  |  |  |
| 3 | Atr-ERN06067 |  | | | |  | | | |  | | | |  |  |  |
| 3 | Atr-ERN06068 |  | | | |  | | | |  | | | |  |  |  |
| 3 | Atr-ERN06069 |  | | | |  | | | |  | | | |  |  |  |
| 3 | Atr-ERN06070 |  | | | |  | Vvi-Vitvi11g04085\_t001 |  | | | |  |  |  |
| 3 | Atr-ERN06071 |  | | | |  | | | |  | Vvi-Vitvi04g00374\_t001 |  |  |  |
| 3 | Atr-ERN06072 |  | Vvi-Vitvi09g00428\_t001 |  | | | |  | | | |  |  |  |
| 3 | Atr-ERN06073 |  | Vvi-Vitvi09g00429\_t002 |  | | | |  | | | |  |  |  |
| 3 | Atr-ERN06074 |  | | | |  | | | |  | | | |  |  |  |
| 3 | Atr-ERN06075 |  | | | |  | | | |  | | | |  |  |  |
| 3 | Atr-ERN06076 |  | Vvi-Vitvi09g00430\_t001 |  | | | |  | | | |  |  |  |
| 3 | Atr-ERN06077 |  | | | |  | | | |  | Vvi-Vitvi04g00373\_t001 |  |  |  |
| 3 | Atr-ERN06078 |  | | | |  | | | |  | | | |  |  |  |
| 3 | Atr-ERN06079 |  | | | |  | Vvi-Vitvi11g00389\_t001 |  | | | |  |  |  |
| 3 | Atr-ERN06080 |  | Vvi-Vitvi09g00431\_t001 |  | Vvi-Vitvi11g00390\_t001 |  | Vvi-Vitvi04g00372\_t001 |  |  |  |
| 1 | Atr-ERN06081 |  |  |  | | | |  |  |  |  |
| 1 | Atr-ERN06082 |  |  |  | | | |  |  |  |  |
| 1 | Atr-ERN06083 |  |  |  | | | |  |  |  |  |
| 1 | Atr-ERN06084 |  |  |  | Vvi-Vitvi11g00393\_t001 |  |  |  |  |
| 0 | Atr-ERN06085 |  |  |  |  |  |  |
