# Supplementary material for: Genome-wide identification, characterization and gene expression of BES1 transcription factor family in grapevine (Vitis vinifera L.)
Source: Sci Rep. 2023 Jan 5;13:240. doi: 10.1038/s41598-022-24407-y (PMC9816167; doi:10.1038/s41598-022-24407-y)
Supplement: Supplementary file 3 — Supplementary Information. [file 41598_2022_24407_MOESM3_ESM.zip › Vvi_Atr/Vitis_vinifera.PN40024.v4.dna_sm.toplevel.fa.vs.Amborella_trichopoda.AMTR1.0.dna_sm.toplevel.fa.html/Atr-AmTr_v1.0_scaffold00143.html]

|  |  |  |  |  |  |  |  |  |  |  |  |  |  |
| --- | --- | --- | --- | --- | --- | --- | --- | --- | --- | --- | --- | --- | --- |
| Duplication depth | Reference chromosome | Collinear blocks | | | | | | | | | | | |
| 0 | Atr-ERN05977 |  |  |  |  |  |  |
| 0 | Atr-ERN05978 |  |  |  |  |  |  |
| 0 | Atr-ERN05979 |  |  |  |  |  |  |
| 0 | Atr-ERN05980 |  |  |  |  |  |  |
| 0 | Atr-ERN05981 |  |  |  |  |  |  |
| 0 | Atr-ERN05982 |  |  |  |  |  |  |
| 0 | Atr-ERN05983 |  |  |  |  |  |  |
| 0 | Atr-ERN05984 |  |  |  |  |  |  |
| 0 | Atr-ERN05985 |  |  |  |  |  |  |
| 0 | Atr-ERN05986 |  |  |  |  |  |  |
| 0 | Atr-ERN05987 |  |  |  |  |  |  |
| 0 | Atr-ERN05988 |  |  |  |  |  |  |
| 0 | Atr-ERN05989 |  |  |  |  |  |  |
| 0 | Atr-ERN05990 |  |  |  |  |  |  |
| 0 | Atr-ERN05991 |  |  |  |  |  |  |
| 0 | Atr-ERN05992 |  |  |  |  |  |  |
| 0 | Atr-ERN05993 |  |  |  |  |  |  |
| 0 | Atr-ERN05994 |  |  |  |  |  |  |
| 0 | Atr-ERN05995 |  |  |  |  |  |  |
| 0 | Atr-ERN05996 |  |  |  |  |  |  |
| 0 | Atr-ERN05997 |  |  |  |  |  |  |
| 0 | Atr-ERN05998 |  |  |  |  |  |  |
| 0 | Atr-ERN05999 |  |  |  |  |  |  |
| 0 | Atr-ERN06000 |  |  |  |  |  |  |
| 0 | Atr-ERN06001 |  |  |  |  |  |  |
| 0 | Atr-ERN06002 |  |  |  |  |  |  |
| 0 | Atr-ERN06003 |  |  |  |  |  |  |
| 0 | Atr-ERN06004 |  |  |  |  |  |  |
| 0 | Atr-ERN06005 |  |  |  |  |  |  |
| 0 | Atr-ERN06006 |  |  |  |  |  |  |
| 0 | Atr-ERN06007 |  |  |  |  |  |  |
