# Supplementary material for: Genome-wide identification, characterization and gene expression of BES1 transcription factor family in grapevine (Vitis vinifera L.)
Source: Sci Rep. 2023 Jan 5;13:240. doi: 10.1038/s41598-022-24407-y (PMC9816167; doi:10.1038/s41598-022-24407-y)
Supplement: Supplementary file 3 — Supplementary Information. [file 41598_2022_24407_MOESM3_ESM.zip › Vvi_Atr/Vitis_vinifera.PN40024.v4.dna_sm.toplevel.fa.vs.Amborella_trichopoda.AMTR1.0.dna_sm.toplevel.fa.html/Atr-AmTr_v1.0_scaffold00144.html]

|  |  |  |  |  |  |  |  |  |  |  |  |  |  |
| --- | --- | --- | --- | --- | --- | --- | --- | --- | --- | --- | --- | --- | --- |
| Duplication depth | Reference chromosome | Collinear blocks | | | | | | | | | | | |
| 0 | Atr-ERN03633 |  |  |  |  |  |  |
| 0 | Atr-ERN03634 |  |  |  |  |  |  |
| 0 | Atr-ERN03635 |  |  |  |  |  |  |
| 0 | Atr-ERN03636 |  |  |  |  |  |  |
| 0 | Atr-ERN03637 |  |  |  |  |  |  |
| 0 | Atr-ERN03638 |  |  |  |  |  |  |
| 0 | Atr-ERN03639 |  |  |  |  |  |  |
| 0 | Atr-ERN03640 |  |  |  |  |  |  |
| 0 | Atr-ERN03641 |  |  |  |  |  |  |
| 0 | Atr-ERN03642 |  |  |  |  |  |  |
| 0 | Atr-ERN03643 |  |  |  |  |  |  |
| 0 | Atr-ERN03644 |  |  |  |  |  |  |
| 0 | Atr-ERN03645 |  |  |  |  |  |  |
| 1 | Atr-ERN03646 |  | Vvi-Vitvi07g01613\_t001 |  |  |  |  |  |
| 1 | Atr-ERN03647 |  | | | |  |  |  |  |  |
| 1 | Atr-ERN03648 |  | | | |  |  |  |  |  |
| 1 | Atr-ERN03649 |  | | | |  |  |  |  |  |
| 1 | Atr-ERN03650 |  | | | |  |  |  |  |  |
| 1 | Atr-ERN03651 |  | | | |  |  |  |  |  |
| 1 | Atr-ERN03652 |  | | | |  |  |  |  |  |
| 1 | Atr-ERN03653 |  | | | |  |  |  |  |  |
| 1 | Atr-ERN03654 |  | | | |  |  |  |  |  |
| 1 | Atr-ERN03655 |  | | | |  |  |  |  |  |
| 1 | Atr-ERN03656 |  | Vvi-Vitvi07g01620\_t002 |  |  |  |  |  |
| 1 | Atr-ERN03657 |  | | | |  |  |  |  |  |
| 1 | Atr-ERN03658 |  | | | |  |  |  |  |  |
| 1 | Atr-ERN03659 |  | | | |  |  |  |  |  |
| 1 | Atr-ERN03660 |  | | | |  |  |  |  |  |
| 1 | Atr-ERN03661 |  | | | |  |  |  |  |  |
| 1 | Atr-ERN03662 |  | | | |  |  |  |  |  |
| 1 | Atr-ERN03663 |  | Vvi-Vitvi07g01621\_t001 |  |  |  |  |  |
| 1 | Atr-ERN03664 |  | | | |  |  |  |  |  |
| 1 | Atr-ERN03665 |  | | | |  |  |  |  |  |
| 1 | Atr-ERN03666 |  | | | |  |  |  |  |  |
| 1 | Atr-ERN03667 |  | Vvi-Vitvi07g01624\_t001 |  |  |  |  |  |
| 1 | Atr-ERN03668 |  | Vvi-Vitvi07g01625\_t001 |  |  |  |  |  |
| 1 | Atr-ERN03669 |  | Vvi-Vitvi07g01626\_t001 |  |  |  |  |  |
| 1 | Atr-ERN03670 |  | | | |  |  |  |  |  |
| 1 | Atr-ERN03671 |  | | | |  |  |  |  |  |
| 1 | Atr-ERN03672 |  | Vvi-Vitvi07g01627\_t001 |  |  |  |  |  |
| 1 | Atr-ERN03673 |  | | | |  |  |  |  |  |
| 1 | Atr-ERN03674 |  | | | |  |  |  |  |  |
| 1 | Atr-ERN03675 |  | | | |  |  |  |  |  |
| 1 | Atr-ERN03676 |  | | | |  |  |  |  |  |
| 1 | Atr-ERN03677 |  | | | |  |  |  |  |  |
| 1 | Atr-ERN03678 |  | | | |  |  |  |  |  |
| 1 | Atr-ERN03679 |  | | | |  |  |  |  |  |
| 1 | Atr-ERN03680 |  | | | |  |  |  |  |  |
| 1 | Atr-ERN03681 |  | | | |  |  |  |  |  |
| 1 | Atr-ERN03682 |  | | | |  |  |  |  |  |
| 1 | Atr-ERN03683 |  | Vvi-Vitvi07g01630\_t001 |  |  |  |  |  |
| 1 | Atr-ERN03684 |  | | | |  |  |  |  |  |
| 1 | Atr-ERN03685 |  | | | |  |  |  |  |  |
| 1 | Atr-ERN03686 |  | | | |  |  |  |  |  |
| 1 | Atr-ERN03687 |  | | | |  |  |  |  |  |
| 1 | Atr-ERN03688 |  | | | |  |  |  |  |  |
| 1 | Atr-ERN03689 |  | | | |  |  |  |  |  |
| 1 | Atr-ERN03690 |  | | | |  |  |  |  |  |
| 1 | Atr-ERN03691 |  | | | |  |  |  |  |  |
| 1 | Atr-ERN03692 |  | | | |  |  |  |  |  |
| 1 | Atr-ERN03693 |  | Vvi-Vitvi07g01634\_t001 |  |  |  |  |  |
| 0 | Atr-ERN03694 |  |  |  |  |  |  |
