# Supplementary material for: Genome-wide identification, characterization and gene expression of BES1 transcription factor family in grapevine (Vitis vinifera L.)
Source: Sci Rep. 2023 Jan 5;13:240. doi: 10.1038/s41598-022-24407-y (PMC9816167; doi:10.1038/s41598-022-24407-y)
Supplement: Supplementary file 3 — Supplementary Information. [file 41598_2022_24407_MOESM3_ESM.zip › Vvi_Atr/Vitis_vinifera.PN40024.v4.dna_sm.toplevel.fa.vs.Amborella_trichopoda.AMTR1.0.dna_sm.toplevel.fa.html/Atr-AmTr_v1.0_scaffold00145.html]

|  |  |  |  |  |  |  |  |  |  |  |  |  |  |
| --- | --- | --- | --- | --- | --- | --- | --- | --- | --- | --- | --- | --- | --- |
| Duplication depth | Reference chromosome | Collinear blocks | | | | | | | | | | | |
| 0 | Atr-ERN05927 |  |  |  |  |  |  |
| 0 | Atr-ERN05928 |  |  |  |  |  |  |
| 0 | Atr-ERN05929 |  |  |  |  |  |  |
| 0 | Atr-ERN05930 |  |  |  |  |  |  |
| 0 | Atr-ERN05931 |  |  |  |  |  |  |
| 0 | Atr-ERN05932 |  |  |  |  |  |  |
| 0 | Atr-ERN05933 |  |  |  |  |  |  |
| 0 | Atr-ERN05934 |  |  |  |  |  |  |
| 1 | Atr-ERN05935 |  | Vvi-Vitvi06g00403\_t001 |  |  |  |  |  |
| 1 | Atr-ERN05936 |  | | | |  |  |  |  |  |
| 1 | Atr-ERN05937 |  | | | |  |  |  |  |  |
| 1 | Atr-ERN05938 |  | | | |  |  |  |  |  |
| 1 | Atr-ERN05939 |  | | | |  |  |  |  |  |
| 1 | Atr-ERN05940 |  | | | |  |  |  |  |  |
| 1 | Atr-ERN05941 |  | | | |  |  |  |  |  |
| 1 | Atr-ERN05942 |  | | | |  |  |  |  |  |
| 1 | Atr-ERN05943 |  | | | |  |  |  |  |  |
| 1 | Atr-ERN05944 |  | | | |  |  |  |  |  |
| 1 | Atr-ERN05945 |  | | | |  |  |  |  |  |
| 1 | Atr-ERN05946 |  | Vvi-Vitvi06g00407\_t001 |  |  |  |  |  |
| 1 | Atr-ERN05947 |  | | | |  |  |  |  |  |
| 1 | Atr-ERN05948 |  | | | |  |  |  |  |  |
| 1 | Atr-ERN05949 |  | | | |  |  |  |  |  |
| 1 | Atr-ERN05950 |  | | | |  |  |  |  |  |
| 1 | Atr-ERN05951 |  | | | |  |  |  |  |  |
| 1 | Atr-ERN05952 |  | | | |  |  |  |  |  |
| 1 | Atr-ERN05953 |  | Vvi-Vitvi06g00409\_t001 |  |  |  |  |  |
| 1 | Atr-ERN05954 |  | | | |  |  |  |  |  |
| 1 | Atr-ERN05955 |  | | | |  |  |  |  |  |
| 1 | Atr-ERN05956 |  | | | |  |  |  |  |  |
| 1 | Atr-ERN05957 |  | | | |  |  |  |  |  |
| 1 | Atr-ERN05958 |  | Vvi-Vitvi06g00411\_t001 |  |  |  |  |  |
| 1 | Atr-ERN05959 |  | Vvi-Vitvi06g00413\_t001 |  |  |  |  |  |
| 1 | Atr-ERN05960 |  | | | |  |  |  |  |  |
| 1 | Atr-ERN05961 |  | | | |  |  |  |  |  |
| 1 | Atr-ERN05962 |  | | | |  |  |  |  |  |
| 1 | Atr-ERN05963 |  | | | |  |  |  |  |  |
| 1 | Atr-ERN05964 |  | | | |  |  |  |  |  |
| 1 | Atr-ERN05965 |  | Vvi-Vitvi06g00414\_t002 |  |  |  |  |  |
| 1 | Atr-ERN05966 |  | | | |  |  |  |  |  |
| 1 | Atr-ERN05967 |  | | | |  |  |  |  |  |
| 1 | Atr-ERN05968 |  | Vvi-Vitvi06g01682\_t001 |  |  |  |  |  |
| 0 | Atr-ERN05969 |  |  |  |  |  |  |
