# Supplementary material for: Genome-wide identification, characterization and gene expression of BES1 transcription factor family in grapevine (Vitis vinifera L.)
Source: Sci Rep. 2023 Jan 5;13:240. doi: 10.1038/s41598-022-24407-y (PMC9816167; doi:10.1038/s41598-022-24407-y)
Supplement: Supplementary file 3 — Supplementary Information. [file 41598_2022_24407_MOESM3_ESM.zip › Vvi_Atr/Vitis_vinifera.PN40024.v4.dna_sm.toplevel.fa.vs.Amborella_trichopoda.AMTR1.0.dna_sm.toplevel.fa.html/Atr-AmTr_v1.0_scaffold00146.html]

|  |  |  |  |  |  |  |  |  |  |  |  |  |  |
| --- | --- | --- | --- | --- | --- | --- | --- | --- | --- | --- | --- | --- | --- |
| Duplication depth | Reference chromosome | Collinear blocks | | | | | | | | | | | |
| 0 | Atr-ERN04813 |  |  |  |  |  |  |
| 0 | Atr-ERN04814 |  |  |  |  |  |  |
| 0 | Atr-ERN04815 |  |  |  |  |  |  |
| 0 | Atr-ERN04816 |  |  |  |  |  |  |
| 0 | Atr-ERN04817 |  |  |  |  |  |  |
| 0 | Atr-ERN04818 |  |  |  |  |  |  |
| 0 | Atr-ERN04819 |  |  |  |  |  |  |
| 0 | Atr-ERN04820 |  |  |  |  |  |  |
| 0 | Atr-ERN04821 |  |  |  |  |  |  |
| 0 | Atr-ERN04822 |  |  |  |  |  |  |
| 0 | Atr-ERN04823 |  |  |  |  |  |  |
| 1 | Atr-ERN04824 |  | Vvi-Vitvi04g01792\_t001 |  |  |  |  |  |
| 1 | Atr-ERN04825 |  | | | |  |  |  |  |  |
| 1 | Atr-ERN04826 |  | | | |  |  |  |  |  |
| 1 | Atr-ERN04827 |  | | | |  |  |  |  |  |
| 1 | Atr-ERN04828 |  | | | |  |  |  |  |  |
| 1 | Atr-ERN04829 |  | | | |  |  |  |  |  |
| 1 | Atr-ERN04830 |  | | | |  |  |  |  |  |
| 2 | Atr-ERN04831 |  | Vvi-Vitvi04g00132\_t002 |  | Vvi-Vitvi11g00127\_t002 |  |  |  |  |
| 2 | Atr-ERN04832 |  | | | |  | Vvi-Vitvi11g00128\_t001 |  |  |  |  |
| 2 | Atr-ERN04833 |  | | | |  | | | |  |  |  |  |
| 2 | Atr-ERN04834 |  | Vvi-Vitvi04g00130\_t001 |  | | | |  |  |  |  |
| 2 | Atr-ERN04835 |  | | | |  | | | |  |  |  |  |
| 2 | Atr-ERN04836 |  | | | |  | | | |  |  |  |  |
| 2 | Atr-ERN04837 |  | | | |  | | | |  |  |  |  |
| 2 | Atr-ERN04838 |  | | | |  | | | |  |  |  |  |
| 2 | Atr-ERN04839 |  | | | |  | | | |  |  |  |  |
| 2 | Atr-ERN04840 |  | Vvi-Vitvi04g00129\_t001 |  | | | |  |  |  |  |
| 2 | Atr-ERN04841 |  | | | |  | | | |  |  |  |  |
| 2 | Atr-ERN04842 |  | | | |  | | | |  |  |  |  |
| 2 | Atr-ERN04843 |  | | | |  | | | |  |  |  |  |
| 2 | Atr-ERN04844 |  | | | |  | | | |  |  |  |  |
| 2 | Atr-ERN04845 |  | | | |  | | | |  |  |  |  |
| 2 | Atr-ERN04846 |  | | | |  | Vvi-Vitvi11g00129\_t001 |  |  |  |  |
| 2 | Atr-ERN04847 |  | | | |  | | | |  |  |  |  |
| 2 | Atr-ERN04848 |  | | | |  | | | |  |  |  |  |
| 2 | Atr-ERN04849 |  | | | |  | | | |  |  |  |  |
| 2 | Atr-ERN04850 |  | | | |  | | | |  |  |  |  |
| 2 | Atr-ERN04851 |  | | | |  | | | |  |  |  |  |
| 2 | Atr-ERN04852 |  | | | |  | | | |  |  |  |  |
| 2 | Atr-ERN04853 |  | | | |  | Vvi-Vitvi11g00133\_t001 |  |  |  |  |
| 2 | Atr-ERN04854 |  | | | |  | | | |  |  |  |  |
| 2 | Atr-ERN04855 |  | | | |  | | | |  |  |  |  |
| 2 | Atr-ERN04856 |  | | | |  | | | |  |  |  |  |
| 2 | Atr-ERN04857 |  | | | |  | Vvi-Vitvi11g00136\_t002 |  |  |  |  |
| 2 | Atr-ERN04858 |  | | | |  | | | |  |  |  |  |
| 2 | Atr-ERN04859 |  | | | |  | | | |  |  |  |  |
| 2 | Atr-ERN04860 |  | | | |  | | | |  |  |  |  |
| 2 | Atr-ERN04861 |  | | | |  | | | |  |  |  |  |
| 2 | Atr-ERN04862 |  | | | |  | | | |  |  |  |  |
| 2 | Atr-ERN04863 |  | | | |  | | | |  |  |  |  |
| 2 | Atr-ERN04864 |  | Vvi-Vitvi04g01791\_t001 |  | | | |  |  |  |  |
| 2 | Atr-ERN04865 |  | | | |  | | | |  |  |  |  |
| 2 | Atr-ERN04866 |  | | | |  | | | |  |  |  |  |
| 2 | Atr-ERN04867 |  | | | |  | | | |  |  |  |  |
| 2 | Atr-ERN04868 |  | | | |  | | | |  |  |  |  |
| 2 | Atr-ERN04869 |  | | | |  | | | |  |  |  |  |
| 2 | Atr-ERN04870 |  | | | |  | Vvi-Vitvi11g00137\_t002 |  |  |  |  |
| 2 | Atr-ERN04871 |  | | | |  | | | |  |  |  |  |
| 2 | Atr-ERN04872 |  | | | |  | | | |  |  |  |  |
| 2 | Atr-ERN04873 |  | | | |  | | | |  |  |  |  |
| 2 | Atr-ERN04874 |  | | | |  | | | |  |  |  |  |
| 2 | Atr-ERN04875 |  | | | |  | | | |  |  |  |  |
| 2 | Atr-ERN04876 |  | Vvi-Vitvi04g00118\_t001 |  | Vvi-Vitvi11g00142\_t001 |  |  |  |  |
| 0 | Atr-ERN04877 |  |  |  |  |  |  |
| 0 | Atr-ERN04878 |  |  |  |  |  |  |
| 0 | Atr-ERN04879 |  |  |  |  |  |  |
