# Supplementary material for: Genome-wide identification, characterization and gene expression of BES1 transcription factor family in grapevine (Vitis vinifera L.)
Source: Sci Rep. 2023 Jan 5;13:240. doi: 10.1038/s41598-022-24407-y (PMC9816167; doi:10.1038/s41598-022-24407-y)
Supplement: Supplementary file 3 — Supplementary Information. [file 41598_2022_24407_MOESM3_ESM.zip › Vvi_Atr/Vitis_vinifera.PN40024.v4.dna_sm.toplevel.fa.vs.Amborella_trichopoda.AMTR1.0.dna_sm.toplevel.fa.html/Atr-AmTr_v1.0_scaffold00147.html]

|  |  |  |  |  |  |  |  |  |  |  |  |  |  |
| --- | --- | --- | --- | --- | --- | --- | --- | --- | --- | --- | --- | --- | --- |
| Duplication depth | Reference chromosome | Collinear blocks | | | | | | | | | | | |
| 0 | Atr-ERN04335 |  |  |  |  |  |  |
| 0 | Atr-ERN04336 |  |  |  |  |  |  |
| 0 | Atr-ERN04337 |  |  |  |  |  |  |
| 0 | Atr-ERN04338 |  |  |  |  |  |  |
| 0 | Atr-ERN04339 |  |  |  |  |  |  |
| 0 | Atr-ERN04340 |  |  |  |  |  |  |
| 0 | Atr-ERN04341 |  |  |  |  |  |  |
| 0 | Atr-ERN04342 |  |  |  |  |  |  |
| 0 | Atr-ERN04343 |  |  |  |  |  |  |
| 0 | Atr-ERN04344 |  |  |  |  |  |  |
| 0 | Atr-ERN04345 |  |  |  |  |  |  |
| 0 | Atr-ERN04346 |  |  |  |  |  |  |
| 0 | Atr-ERN04347 |  |  |  |  |  |  |
| 0 | Atr-ERN04348 |  |  |  |  |  |  |
| 0 | Atr-ERN04349 |  |  |  |  |  |  |
| 0 | Atr-ERN04350 |  |  |  |  |  |  |
| 0 | Atr-ERN04351 |  |  |  |  |  |  |
| 0 | Atr-ERN04352 |  |  |  |  |  |  |
| 0 | Atr-ERN04353 |  |  |  |  |  |  |
| 0 | Atr-ERN04354 |  |  |  |  |  |  |
| 0 | Atr-ERN04355 |  |  |  |  |  |  |
| 0 | Atr-ERN04356 |  |  |  |  |  |  |
| 0 | Atr-ERN04357 |  |  |  |  |  |  |
| 0 | Atr-ERN04358 |  |  |  |  |  |  |
| 0 | Atr-ERN04359 |  |  |  |  |  |  |
| 0 | Atr-ERN04360 |  |  |  |  |  |  |
| 0 | Atr-ERN04361 |  |  |  |  |  |  |
| 0 | Atr-ERN04362 |  |  |  |  |  |  |
| 0 | Atr-ERN04363 |  |  |  |  |  |  |
| 0 | Atr-ERN04364 |  |  |  |  |  |  |
| 0 | Atr-ERN04365 |  |  |  |  |  |  |
| 0 | Atr-ERN04366 |  |  |  |  |  |  |
| 0 | Atr-ERN04367 |  |  |  |  |  |  |
| 0 | Atr-ERN04368 |  |  |  |  |  |  |
| 0 | Atr-ERN04369 |  |  |  |  |  |  |
| 0 | Atr-ERN04370 |  |  |  |  |  |  |
| 0 | Atr-ERN04371 |  |  |  |  |  |  |
| 0 | Atr-ERN04372 |  |  |  |  |  |  |
| 0 | Atr-ERN04373 |  |  |  |  |  |  |
| 0 | Atr-ERN04374 |  |  |  |  |  |  |
| 0 | Atr-ERN04375 |  |  |  |  |  |  |
| 0 | Atr-ERN04376 |  |  |  |  |  |  |
| 0 | Atr-ERN04377 |  |  |  |  |  |  |
| 0 | Atr-ERN04378 |  |  |  |  |  |  |
| 0 | Atr-ERN04379 |  |  |  |  |  |  |
| 0 | Atr-ERN04380 |  |  |  |  |  |  |
| 0 | Atr-ERN04381 |  |  |  |  |  |  |
| 0 | Atr-ERN04382 |  |  |  |  |  |  |
| 0 | Atr-ERN04383 |  |  |  |  |  |  |
| 0 | Atr-ERN04384 |  |  |  |  |  |  |
| 0 | Atr-ERN04385 |  |  |  |  |  |  |
| 0 | Atr-ERN04386 |  |  |  |  |  |  |
| 0 | Atr-ERN04387 |  |  |  |  |  |  |
| 0 | Atr-ERN04388 |  |  |  |  |  |  |
| 0 | Atr-ERN04389 |  |  |  |  |  |  |
| 0 | Atr-ERN04390 |  |  |  |  |  |  |
| 0 | Atr-ERN04391 |  |  |  |  |  |  |
| 0 | Atr-ERN04392 |  |  |  |  |  |  |
| 0 | Atr-ERN04393 |  |  |  |  |  |  |
| 0 | Atr-ERN04394 |  |  |  |  |  |  |
| 0 | Atr-ERN04395 |  |  |  |  |  |  |
| 0 | Atr-ERN04396 |  |  |  |  |  |  |
