# Supplementary material for: Genome-wide identification, characterization and gene expression of BES1 transcription factor family in grapevine (Vitis vinifera L.)
Source: Sci Rep. 2023 Jan 5;13:240. doi: 10.1038/s41598-022-24407-y (PMC9816167; doi:10.1038/s41598-022-24407-y)
Supplement: Supplementary file 3 — Supplementary Information. [file 41598_2022_24407_MOESM3_ESM.zip › Vvi_Atr/Vitis_vinifera.PN40024.v4.dna_sm.toplevel.fa.vs.Amborella_trichopoda.AMTR1.0.dna_sm.toplevel.fa.html/Atr-AmTr_v1.0_scaffold00148.html]

|  |  |  |  |  |  |  |  |  |  |  |  |  |  |
| --- | --- | --- | --- | --- | --- | --- | --- | --- | --- | --- | --- | --- | --- |
| Duplication depth | Reference chromosome | Collinear blocks | | | | | | | | | | | |
| 1 | Atr-ERN08344 |  | Vvi-Vitvi13g00604\_t001 |  |  |  |  |  |
| 1 | Atr-ERN08345 |  | | | |  |  |  |  |  |
| 1 | Atr-ERN08346 |  | | | |  |  |  |  |  |
| 2 | Atr-ERN08347 |  | | | |  | Vvi-Vitvi06g01658\_t001 |  |  |  |  |
| 2 | Atr-ERN08348 |  | | | |  | | | |  |  |  |  |
| 2 | Atr-ERN08349 |  | | | |  | | | |  |  |  |  |
| 2 | Atr-ERN08350 |  | | | |  | | | |  |  |  |  |
| 2 | Atr-ERN08351 |  | | | |  | | | |  |  |  |  |
| 3 | Atr-ERN08352 |  | Vvi-Vitvi13g00605\_t001 |  | Vvi-Vitvi06g00281\_t001 |  | Vvi-Vitvi08g01038\_t001 |  |  |  |
| 3 | Atr-ERN08353 |  | | | |  | | | |  | | | |  |  |  |
| 3 | Atr-ERN08354 |  | Vvi-Vitvi13g00606\_t001 |  | Vvi-Vitvi06g00280\_t001 |  | Vvi-Vitvi08g01037\_t001 |  |  |  |
| 3 | Atr-ERN08355 |  | | | |  | Vvi-Vitvi06g00279\_t001 |  | Vvi-Vitvi08g01036\_t001 |  |  |  |
| 3 | Atr-ERN08356 |  | | | |  | Vvi-Vitvi06g00277\_t001 |  | | | |  |  |  |
| 3 | Atr-ERN08357 |  | Vvi-Vitvi13g00607\_t001 |  | | | |  | | | |  |  |  |
| 3 | Atr-ERN08358 |  | Vvi-Vitvi13g00609\_t001 |  | Vvi-Vitvi06g00276\_t001 |  | | | |  |  |  |
| 3 | Atr-ERN08359 |  | | | |  | | | |  | Vvi-Vitvi08g02145\_t001 |  |  |  |
| 3 | Atr-ERN08360 |  | | | |  | | | |  | | | |  |  |  |
| 3 | Atr-ERN08361 |  | | | |  | | | |  | | | |  |  |  |
| 3 | Atr-ERN08362 |  | | | |  | Vvi-Vitvi06g00274\_t004 |  | | | |  |  |  |
| 3 | Atr-ERN08363 |  | | | |  | | | |  | | | |  |  |  |
| 3 | Atr-ERN08364 |  | Vvi-Vitvi13g00611\_t001 |  | | | |  | | | |  |  |  |
| 3 | Atr-ERN08365 |  | Vvi-Vitvi13g00612\_t001 |  | | | |  | | | |  |  |  |
| 3 | Atr-ERN08366 |  | Vvi-Vitvi13g00613\_t002 |  | | | |  | | | |  |  |  |
| 3 | Atr-ERN08367 |  | Vvi-Vitvi13g02058\_t001 |  | Vvi-Vitvi06g00272\_t002 |  | Vvi-Vitvi08g01033\_t001 |  |  |  |
| 3 | Atr-ERN08368 |  | | | |  | | | |  | | | |  |  |  |
| 3 | Atr-ERN08369 |  | | | |  | | | |  | | | |  |  |  |
| 3 | Atr-ERN08370 |  | Vvi-Vitvi13g00614\_t001 |  | Vvi-Vitvi06g04095\_t001 |  | | | |  |  |  |
| 3 | Atr-ERN08371 |  | | | |  | | | |  | | | |  |  |  |
| 3 | Atr-ERN08372 |  | | | |  | | | |  | | | |  |  |  |
| 3 | Atr-ERN08373 |  | Vvi-Vitvi13g00615\_t001 |  | Vvi-Vitvi06g00269\_t001 |  | | | |  |  |  |
| 3 | Atr-ERN08374 |  | Vvi-Vitvi13g00617\_t001 |  | | | |  | | | |  |  |  |
| 3 | Atr-ERN08375 |  | | | |  | Vvi-Vitvi06g00268\_t001 |  | | | |  |  |  |
| 3 | Atr-ERN08376 |  | | | |  | Vvi-Vitvi06g00267\_t001 |  | | | |  |  |  |
| 3 | Atr-ERN08377 |  | | | |  | | | |  | | | |  |  |  |
| 4 | Atr-ERN08378 |  | | | |  | | | |  | | | |  | Vvi-Vitvi08g01023\_t001 |  |  |
| 4 | Atr-ERN08379 |  | | | |  | | | |  | | | |  | | | |  |  |
| 4 | Atr-ERN08380 |  | | | |  | | | |  | | | |  | Vvi-Vitvi08g01024\_t001 |  |  |
| 4 | Atr-ERN08381 |  | | | |  | | | |  | | | |  | | | |  |  |
| 4 | Atr-ERN08382 |  | | | |  | | | |  | | | |  | | | |  |  |
| 4 | Atr-ERN08383 |  | | | |  | | | |  | | | |  | | | |  |  |
| 4 | Atr-ERN08384 |  | | | |  | | | |  | | | |  | | | |  |  |
| 5 | Atr-ERN08385 |  | Vvi-Vitvi13g00620\_t001 |  | | | |  | | | |  | | | |  | Vvi-Vitvi06g00258\_t001 |  |
| 5 | Atr-ERN08386 |  | | | |  | | | |  | | | |  | | | |  | Vvi-Vitvi06g00259\_t001 |  |
| 5 | Atr-ERN08387 |  | | | |  | | | |  | | | |  | Vvi-Vitvi08g01026\_t001 |  | | | |  |
| 5 | Atr-ERN08388 |  | | | |  | | | |  | | | |  | | | |  | | | |  |
| 5 | Atr-ERN08389 |  | | | |  | | | |  | | | |  | | | |  | Vvi-Vitvi06g00261\_t001 |  |
| 5 | Atr-ERN08390 |  | | | |  | | | |  | | | |  | Vvi-Vitvi08g01027\_t001 |  | | | |  |
| 5 | Atr-ERN08391 |  | | | |  | | | |  | | | |  | Vvi-Vitvi08g01028\_t001 |  | Vvi-Vitvi06g00262\_t001 |  |
| 5 | Atr-ERN08392 |  | | | |  | | | |  | | | |  | | | |  | | | |  |
| 5 | Atr-ERN08393 |  | | | |  | Vvi-Vitvi06g00263\_t001 |  | Vvi-Vitvi08g01030\_t001 |  | Vvi-Vitvi08g01030\_t001 |  | Vvi-Vitvi06g00263\_t001 |  |
| 5 | Atr-ERN08394 |  | | | |  | | | |  | | | |  | | | |  | | | |  |
| 5 | Atr-ERN08395 |  | | | |  | | | |  | | | |  | | | |  | | | |  |
| 5 | Atr-ERN08396 |  | | | |  | | | |  | | | |  | | | |  | | | |  |
| 5 | Atr-ERN08397 |  | | | |  | | | |  | | | |  | | | |  | | | |  |
| 5 | Atr-ERN08398 |  | | | |  | | | |  | | | |  | | | |  | | | |  |
| 5 | Atr-ERN08399 |  | | | |  | | | |  | | | |  | | | |  | | | |  |
| 5 | Atr-ERN08400 |  | | | |  | | | |  | | | |  | Vvi-Vitvi08g01032\_t001 |  | | | |  |
| 4 | Atr-ERN08401 |  | | | |  | | | |  | | | |  |  |  | Vvi-Vitvi06g00266\_t001 |  |
| 3 | Atr-ERN08402 |  | Vvi-Vitvi13g00622\_t001 |  | Vvi-Vitvi06g04091\_t001 |  | | | |  |  |  |
| 3 | Atr-ERN08403 |  | | | |  | Vvi-Vitvi06g04090\_t001 |  | | | |  |  |  |
| 2 | Atr-ERN08404 |  | | | |  |  |  | | | |  |  |  |
| 2 | Atr-ERN08405 |  | Vvi-Vitvi13g04174\_t001 |  |  |  | | | |  |  |  |
| 1 | Atr-ERN08406 |  |  |  |  |  | | | |  |  |  |
| 1 | Atr-ERN08407 |  |  |  |  |  | Vvi-Vitvi08g01018\_t001 |  |  |  |
| 1 | Atr-ERN08408 |  |  |  |  |  | Vvi-Vitvi08g01016\_t001 |  |  |  |
| 0 | Atr-ERN08409 |  |  |  |  |  |  |
| 0 | Atr-ERN08410 |  |  |  |  |  |  |
| 0 | Atr-ERN08411 |  |  |  |  |  |  |
| 0 | Atr-ERN08412 |  |  |  |  |  |  |
| 0 | Atr-ERN08413 |  |  |  |  |  |  |
| 0 | Atr-ERN08414 |  |  |  |  |  |  |
| 0 | Atr-ERN08415 |  |  |  |  |  |  |
| 0 | Atr-ERN08416 |  |  |  |  |  |  |
