# Supplementary material for: Genome-wide identification, characterization and gene expression of BES1 transcription factor family in grapevine (Vitis vinifera L.)
Source: Sci Rep. 2023 Jan 5;13:240. doi: 10.1038/s41598-022-24407-y (PMC9816167; doi:10.1038/s41598-022-24407-y)
Supplement: Supplementary file 3 — Supplementary Information. [file 41598_2022_24407_MOESM3_ESM.zip › Vvi_Atr/Vitis_vinifera.PN40024.v4.dna_sm.toplevel.fa.vs.Amborella_trichopoda.AMTR1.0.dna_sm.toplevel.fa.html/Atr-AmTr_v1.0_scaffold00149.html]

|  |  |  |  |  |  |  |  |  |  |  |  |  |  |
| --- | --- | --- | --- | --- | --- | --- | --- | --- | --- | --- | --- | --- | --- |
| Duplication depth | Reference chromosome | Collinear blocks | | | | | | | | | | | |
| 0 | Atr-ERN09237 |  |  |  |  |  |  |
| 0 | Atr-ERN09238 |  |  |  |  |  |  |
| 0 | Atr-ERN09239 |  |  |  |  |  |  |
| 0 | Atr-ERN09240 |  |  |  |  |  |  |
| 0 | Atr-ERN09241 |  |  |  |  |  |  |
| 0 | Atr-ERN09242 |  |  |  |  |  |  |
| 0 | Atr-ERN09243 |  |  |  |  |  |  |
| 0 | Atr-ERN09244 |  |  |  |  |  |  |
| 0 | Atr-ERN09245 |  |  |  |  |  |  |
| 0 | Atr-ERN09246 |  |  |  |  |  |  |
| 0 | Atr-ERN09247 |  |  |  |  |  |  |
| 0 | Atr-ERN09248 |  |  |  |  |  |  |
| 0 | Atr-ERN09249 |  |  |  |  |  |  |
| 0 | Atr-ERN09250 |  |  |  |  |  |  |
| 0 | Atr-ERN09251 |  |  |  |  |  |  |
| 0 | Atr-ERN09252 |  |  |  |  |  |  |
| 0 | Atr-ERN09253 |  |  |  |  |  |  |
| 0 | Atr-ERN09254 |  |  |  |  |  |  |
| 0 | Atr-ERN09255 |  |  |  |  |  |  |
| 0 | Atr-ERN09256 |  |  |  |  |  |  |
| 0 | Atr-ERN09257 |  |  |  |  |  |  |
| 0 | Atr-ERN09258 |  |  |  |  |  |  |
| 0 | Atr-ERN09259 |  |  |  |  |  |  |
| 0 | Atr-ERN09260 |  |  |  |  |  |  |
| 1 | Atr-ERN09261 |  | Vvi-Vitvi02g00685\_t001 |  |  |  |  |  |
| 1 | Atr-ERN09262 |  | | | |  |  |  |  |  |
| 1 | Atr-ERN09263 |  | | | |  |  |  |  |  |
| 1 | Atr-ERN09264 |  | | | |  |  |  |  |  |
| 1 | Atr-ERN09265 |  | Vvi-Vitvi02g00684\_t001 |  |  |  |  |  |
| 1 | Atr-ERN09266 |  | Vvi-Vitvi02g00683\_t001 |  |  |  |  |  |
| 1 | Atr-ERN09267 |  | Vvi-Vitvi02g00682\_t001 |  |  |  |  |  |
| 1 | Atr-ERN09268 |  | | | |  |  |  |  |  |
| 1 | Atr-ERN09269 |  | | | |  |  |  |  |  |
| 1 | Atr-ERN09270 |  | Vvi-Vitvi02g00680\_t001 |  |  |  |  |  |
| 1 | Atr-ERN09271 |  | | | |  |  |  |  |  |
| 1 | Atr-ERN09272 |  | | | |  |  |  |  |  |
| 1 | Atr-ERN09273 |  | | | |  |  |  |  |  |
| 1 | Atr-ERN09274 |  | | | |  |  |  |  |  |
| 1 | Atr-ERN09275 |  | | | |  |  |  |  |  |
| 1 | Atr-ERN09276 |  | | | |  |  |  |  |  |
| 1 | Atr-ERN09277 |  | | | |  |  |  |  |  |
| 1 | Atr-ERN09278 |  | Vvi-Vitvi02g00679\_t002 |  |  |  |  |  |
| 1 | Atr-ERN09279 |  | Vvi-Vitvi02g00678\_t001 |  |  |  |  |  |
| 1 | Atr-ERN09280 |  | Vvi-Vitvi02g00677\_t001 |  |  |  |  |  |
| 1 | Atr-ERN09281 |  | | | |  |  |  |  |  |
| 1 | Atr-ERN09282 |  | Vvi-Vitvi02g00673\_t001 |  |  |  |  |  |
| 1 | Atr-ERN09283 |  | | | |  |  |  |  |  |
| 1 | Atr-ERN09284 |  | Vvi-Vitvi02g00672\_t001 |  |  |  |  |  |
| 1 | Atr-ERN09285 |  | Vvi-Vitvi02g00671\_t001 |  |  |  |  |  |
| 1 | Atr-ERN09286 |  | Vvi-Vitvi02g00670\_t001 |  |  |  |  |  |
| 1 | Atr-ERN09287 |  | | | |  |  |  |  |  |
| 1 | Atr-ERN09288 |  | | | |  |  |  |  |  |
| 1 | Atr-ERN09289 |  | Vvi-Vitvi02g00669\_t001 |  |  |  |  |  |
| 1 | Atr-ERN09290 |  | Vvi-Vitvi02g00667\_t001 |  |  |  |  |  |
| 1 | Atr-ERN09291 |  | Vvi-Vitvi02g00666\_t003 |  |  |  |  |  |
| 1 | Atr-ERN09292 |  | | | |  |  |  |  |  |
| 1 | Atr-ERN09293 |  | | | |  |  |  |  |  |
| 1 | Atr-ERN09294 |  | | | |  |  |  |  |  |
| 1 | Atr-ERN09295 |  | | | |  |  |  |  |  |
| 1 | Atr-ERN09296 |  | | | |  |  |  |  |  |
| 1 | Atr-ERN09297 |  | | | |  |  |  |  |  |
| 1 | Atr-ERN09298 |  | | | |  |  |  |  |  |
| 1 | Atr-ERN09299 |  | | | |  |  |  |  |  |
| 1 | Atr-ERN09300 |  | | | |  |  |  |  |  |
| 1 | Atr-ERN09301 |  | | | |  |  |  |  |  |
| 1 | Atr-ERN09302 |  | | | |  |  |  |  |  |
| 1 | Atr-ERN09303 |  | Vvi-Vitvi02g01498\_t001 |  |  |  |  |  |
| 1 | Atr-ERN09304 |  | Vvi-Vitvi02g00663\_t001 |  |  |  |  |  |
| 1 | Atr-ERN09305 |  | Vvi-Vitvi02g00661\_t001 |  |  |  |  |  |
| 1 | Atr-ERN09306 |  | Vvi-Vitvi02g00660\_t001 |  |  |  |  |  |
| 1 | Atr-ERN09307 |  | | | |  |  |  |  |  |
| 1 | Atr-ERN09308 |  | | | |  |  |  |  |  |
| 1 | Atr-ERN09309 |  | | | |  |  |  |  |  |
| 1 | Atr-ERN09310 |  | | | |  |  |  |  |  |
| 1 | Atr-ERN09311 |  | | | |  |  |  |  |  |
| 1 | Atr-ERN09312 |  | | | |  |  |  |  |  |
| 1 | Atr-ERN09313 |  | | | |  |  |  |  |  |
| 1 | Atr-ERN09314 |  | Vvi-Vitvi02g00658\_t001 |  |  |  |  |  |
| 0 | Atr-ERN09315 |  |  |  |  |  |  |
| 0 | Atr-ERN09316 |  |  |  |  |  |  |
| 0 | Atr-ERN09317 |  |  |  |  |  |  |
| 0 | Atr-ERN09318 |  |  |  |  |  |  |
| 0 | Atr-ERN09319 |  |  |  |  |  |  |
| 0 | Atr-ERN09320 |  |  |  |  |  |  |
| 0 | Atr-ERN09321 |  |  |  |  |  |  |
| 0 | Atr-ERN09322 |  |  |  |  |  |  |
