# Supplementary material for: Genome-wide identification, characterization and gene expression of BES1 transcription factor family in grapevine (Vitis vinifera L.)
Source: Sci Rep. 2023 Jan 5;13:240. doi: 10.1038/s41598-022-24407-y (PMC9816167; doi:10.1038/s41598-022-24407-y)
Supplement: Supplementary file 3 — Supplementary Information. [file 41598_2022_24407_MOESM3_ESM.zip › Vvi_Atr/Vitis_vinifera.PN40024.v4.dna_sm.toplevel.fa.vs.Amborella_trichopoda.AMTR1.0.dna_sm.toplevel.fa.html/Atr-AmTr_v1.0_scaffold00150.html]

|  |  |  |  |  |  |  |  |  |  |  |  |  |  |
| --- | --- | --- | --- | --- | --- | --- | --- | --- | --- | --- | --- | --- | --- |
| Duplication depth | Reference chromosome | Collinear blocks | | | | | | | | | | | |
| 0 | Atr-ERN08431 |  |  |  |  |  |  |
| 0 | Atr-ERN08432 |  |  |  |  |  |  |
| 0 | Atr-ERN08433 |  |  |  |  |  |  |
| 0 | Atr-ERN08434 |  |  |  |  |  |  |
| 0 | Atr-ERN08435 |  |  |  |  |  |  |
| 0 | Atr-ERN08436 |  |  |  |  |  |  |
| 0 | Atr-ERN08437 |  |  |  |  |  |  |
| 0 | Atr-ERN08438 |  |  |  |  |  |  |
| 0 | Atr-ERN08439 |  |  |  |  |  |  |
| 0 | Atr-ERN08440 |  |  |  |  |  |  |
| 0 | Atr-ERN08441 |  |  |  |  |  |  |
| 0 | Atr-ERN08442 |  |  |  |  |  |  |
| 0 | Atr-ERN08443 |  |  |  |  |  |  |
| 0 | Atr-ERN08444 |  |  |  |  |  |  |
| 0 | Atr-ERN08445 |  |  |  |  |  |  |
| 0 | Atr-ERN08446 |  |  |  |  |  |  |
| 0 | Atr-ERN08447 |  |  |  |  |  |  |
| 0 | Atr-ERN08448 |  |  |  |  |  |  |
| 0 | Atr-ERN08449 |  |  |  |  |  |  |
| 0 | Atr-ERN08450 |  |  |  |  |  |  |
| 0 | Atr-ERN08451 |  |  |  |  |  |  |
| 0 | Atr-ERN08452 |  |  |  |  |  |  |
| 0 | Atr-ERN08453 |  |  |  |  |  |  |
| 0 | Atr-ERN08454 |  |  |  |  |  |  |
| 0 | Atr-ERN08455 |  |  |  |  |  |  |
| 0 | Atr-ERN08456 |  |  |  |  |  |  |
| 0 | Atr-ERN08457 |  |  |  |  |  |  |
| 0 | Atr-ERN08458 |  |  |  |  |  |  |
| 0 | Atr-ERN08459 |  |  |  |  |  |  |
| 0 | Atr-ERN08460 |  |  |  |  |  |  |
| 0 | Atr-ERN08461 |  |  |  |  |  |  |
| 0 | Atr-ERN08462 |  |  |  |  |  |  |
| 0 | Atr-ERN08463 |  |  |  |  |  |  |
| 0 | Atr-ERN08464 |  |  |  |  |  |  |
| 0 | Atr-ERN08465 |  |  |  |  |  |  |
