# Supplementary material for: Genome-wide identification, characterization and gene expression of BES1 transcription factor family in grapevine (Vitis vinifera L.)
Source: Sci Rep. 2023 Jan 5;13:240. doi: 10.1038/s41598-022-24407-y (PMC9816167; doi:10.1038/s41598-022-24407-y)
Supplement: Supplementary file 3 — Supplementary Information. [file 41598_2022_24407_MOESM3_ESM.zip › Vvi_Atr/Vitis_vinifera.PN40024.v4.dna_sm.toplevel.fa.vs.Amborella_trichopoda.AMTR1.0.dna_sm.toplevel.fa.html/Atr-AmTr_v1.0_scaffold00151.html]

|  |  |  |  |  |  |  |  |  |  |  |  |  |  |
| --- | --- | --- | --- | --- | --- | --- | --- | --- | --- | --- | --- | --- | --- |
| Duplication depth | Reference chromosome | Collinear blocks | | | | | | | | | | | |
| 0 | Atr-ERM95496 |  |  |  |  |  |  |
| 0 | Atr-ERM95497 |  |  |  |  |  |  |
| 0 | Atr-ERM95498 |  |  |  |  |  |  |
| 0 | Atr-ERM95499 |  |  |  |  |  |  |
| 0 | Atr-ERM95500 |  |  |  |  |  |  |
| 0 | Atr-ERM95501 |  |  |  |  |  |  |
| 0 | Atr-ERM95502 |  |  |  |  |  |  |
| 0 | Atr-ERM95503 |  |  |  |  |  |  |
| 0 | Atr-ERM95504 |  |  |  |  |  |  |
| 0 | Atr-ERM95505 |  |  |  |  |  |  |
| 0 | Atr-ERM95506 |  |  |  |  |  |  |
| 0 | Atr-ERM95507 |  |  |  |  |  |  |
| 0 | Atr-ERM95508 |  |  |  |  |  |  |
| 0 | Atr-ERM95509 |  |  |  |  |  |  |
| 0 | Atr-ERM95510 |  |  |  |  |  |  |
| 0 | Atr-ERM95511 |  |  |  |  |  |  |
| 0 | Atr-ERM95512 |  |  |  |  |  |  |
| 0 | Atr-ERM95513 |  |  |  |  |  |  |
| 0 | Atr-ERM95514 |  |  |  |  |  |  |
| 0 | Atr-ERM95515 |  |  |  |  |  |  |
| 0 | Atr-ERM95516 |  |  |  |  |  |  |
| 0 | Atr-ERM95517 |  |  |  |  |  |  |
| 0 | Atr-ERM95518 |  |  |  |  |  |  |
| 0 | Atr-ERM95519 |  |  |  |  |  |  |
| 0 | Atr-ERM95520 |  |  |  |  |  |  |
| 0 | Atr-ERM95521 |  |  |  |  |  |  |
| 0 | Atr-ERM95522 |  |  |  |  |  |  |
| 0 | Atr-ERM95523 |  |  |  |  |  |  |
| 0 | Atr-ERM95524 |  |  |  |  |  |  |
| 0 | Atr-ERM95525 |  |  |  |  |  |  |
| 0 | Atr-ERM95526 |  |  |  |  |  |  |
| 0 | Atr-ERM95527 |  |  |  |  |  |  |
| 0 | Atr-ERM95528 |  |  |  |  |  |  |
