# Supplementary material for: Genome-wide identification, characterization and gene expression of BES1 transcription factor family in grapevine (Vitis vinifera L.)
Source: Sci Rep. 2023 Jan 5;13:240. doi: 10.1038/s41598-022-24407-y (PMC9816167; doi:10.1038/s41598-022-24407-y)
Supplement: Supplementary file 3 — Supplementary Information. [file 41598_2022_24407_MOESM3_ESM.zip › Vvi_Atr/Vitis_vinifera.PN40024.v4.dna_sm.toplevel.fa.vs.Amborella_trichopoda.AMTR1.0.dna_sm.toplevel.fa.html/Atr-AmTr_v1.0_scaffold00152.html]

|  |  |  |  |  |  |  |  |  |  |  |  |  |  |
| --- | --- | --- | --- | --- | --- | --- | --- | --- | --- | --- | --- | --- | --- |
| Duplication depth | Reference chromosome | Collinear blocks | | | | | | | | | | | |
| 0 | Atr-ERN08478 |  |  |  |  |  |  |
| 0 | Atr-ERN08479 |  |  |  |  |  |  |
| 0 | Atr-ERN08480 |  |  |  |  |  |  |
| 0 | Atr-ERN08481 |  |  |  |  |  |  |
| 0 | Atr-ERN08482 |  |  |  |  |  |  |
| 0 | Atr-ERN08483 |  |  |  |  |  |  |
| 0 | Atr-ERN08484 |  |  |  |  |  |  |
| 0 | Atr-ERN08485 |  |  |  |  |  |  |
| 0 | Atr-ERN08486 |  |  |  |  |  |  |
| 0 | Atr-ERN08487 |  |  |  |  |  |  |
| 0 | Atr-ERN08488 |  |  |  |  |  |  |
| 0 | Atr-ERN08489 |  |  |  |  |  |  |
| 0 | Atr-ERN08490 |  |  |  |  |  |  |
| 0 | Atr-ERN08491 |  |  |  |  |  |  |
| 0 | Atr-ERN08492 |  |  |  |  |  |  |
| 0 | Atr-ERN08493 |  |  |  |  |  |  |
| 0 | Atr-ERN08494 |  |  |  |  |  |  |
| 0 | Atr-ERN08495 |  |  |  |  |  |  |
| 0 | Atr-ERN08496 |  |  |  |  |  |  |
| 0 | Atr-ERN08497 |  |  |  |  |  |  |
| 0 | Atr-ERN08498 |  |  |  |  |  |  |
| 0 | Atr-ERN08499 |  |  |  |  |  |  |
| 0 | Atr-ERN08500 |  |  |  |  |  |  |
| 0 | Atr-ERN08501 |  |  |  |  |  |  |
| 0 | Atr-ERN08502 |  |  |  |  |  |  |
| 0 | Atr-ERN08503 |  |  |  |  |  |  |
| 0 | Atr-ERN08504 |  |  |  |  |  |  |
| 0 | Atr-ERN08505 |  |  |  |  |  |  |
| 0 | Atr-ERN08506 |  |  |  |  |  |  |
| 0 | Atr-ERN08507 |  |  |  |  |  |  |
| 0 | Atr-ERN08508 |  |  |  |  |  |  |
| 0 | Atr-ERN08509 |  |  |  |  |  |  |
| 0 | Atr-ERN08510 |  |  |  |  |  |  |
| 0 | Atr-ERN08511 |  |  |  |  |  |  |
| 0 | Atr-ERN08512 |  |  |  |  |  |  |
