# Supplementary material for: Genome-wide identification, characterization and gene expression of BES1 transcription factor family in grapevine (Vitis vinifera L.)
Source: Sci Rep. 2023 Jan 5;13:240. doi: 10.1038/s41598-022-24407-y (PMC9816167; doi:10.1038/s41598-022-24407-y)
Supplement: Supplementary file 3 — Supplementary Information. [file 41598_2022_24407_MOESM3_ESM.zip › Vvi_Atr/Vitis_vinifera.PN40024.v4.dna_sm.toplevel.fa.vs.Amborella_trichopoda.AMTR1.0.dna_sm.toplevel.fa.html/Atr-AmTr_v1.0_scaffold00153.html]

|  |  |  |  |  |  |  |  |  |  |  |  |  |  |
| --- | --- | --- | --- | --- | --- | --- | --- | --- | --- | --- | --- | --- | --- |
| Duplication depth | Reference chromosome | Collinear blocks | | | | | | | | | | | |
| 1 | Atr-ERN08969 |  | Vvi-Vitvi13g00089\_t001 |  |  |  |  |  |
| 1 | Atr-ERN08970 |  | Vvi-Vitvi13g00090\_t001 |  |  |  |  |  |
| 1 | Atr-ERN08971 |  | | | |  |  |  |  |  |
| 1 | Atr-ERN08972 |  | Vvi-Vitvi13g00091\_t001 |  |  |  |  |  |
| 2 | Atr-ERN08973 |  | Vvi-Vitvi13g00092\_t001 |  | Vvi-Vitvi06g00689\_t001 |  |  |  |  |
| 2 | Atr-ERN08974 |  | | | |  | | | |  |  |  |  |
| 2 | Atr-ERN08975 |  | | | |  | | | |  |  |  |  |
| 2 | Atr-ERN08976 |  | | | |  | | | |  |  |  |  |
| 2 | Atr-ERN08977 |  | Vvi-Vitvi13g00097\_t001 |  | Vvi-Vitvi06g00690\_t001 |  |  |  |  |
| 2 | Atr-ERN08978 |  | | | |  | | | |  |  |  |  |
| 2 | Atr-ERN08979 |  | | | |  | | | |  |  |  |  |
| 2 | Atr-ERN08980 |  | | | |  | | | |  |  |  |  |
| 2 | Atr-ERN08981 |  | | | |  | | | |  |  |  |  |
| 2 | Atr-ERN08982 |  | | | |  | | | |  |  |  |  |
| 2 | Atr-ERN08983 |  | | | |  | | | |  |  |  |  |
| 2 | Atr-ERN08984 |  | | | |  | Vvi-Vitvi06g00691\_t001 |  |  |  |  |
| 2 | Atr-ERN08985 |  | | | |  | | | |  |  |  |  |
| 2 | Atr-ERN08986 |  | | | |  | | | |  |  |  |  |
| 2 | Atr-ERN08987 |  | | | |  | | | |  |  |  |  |
| 2 | Atr-ERN08988 |  | | | |  | | | |  |  |  |  |
| 2 | Atr-ERN08989 |  | | | |  | | | |  |  |  |  |
| 2 | Atr-ERN08990 |  | | | |  | | | |  |  |  |  |
| 2 | Atr-ERN08991 |  | | | |  | Vvi-Vitvi06g00694\_t001 |  |  |  |  |
| 2 | Atr-ERN08992 |  | | | |  | | | |  |  |  |  |
| 2 | Atr-ERN08993 |  | | | |  | | | |  |  |  |  |
| 2 | Atr-ERN08994 |  | Vvi-Vitvi13g00098\_t001 |  | | | |  |  |  |  |
| 2 | Atr-ERN08995 |  | Vvi-Vitvi13g00100\_t001 |  | | | |  |  |  |  |
| 2 | Atr-ERN08996 |  | | | |  | Vvi-Vitvi06g01759\_t001 |  |  |  |  |
| 2 | Atr-ERN08997 |  | | | |  | | | |  |  |  |  |
| 2 | Atr-ERN08998 |  | | | |  | | | |  |  |  |  |
| 2 | Atr-ERN08999 |  | | | |  | | | |  |  |  |  |
| 2 | Atr-ERN09000 |  | | | |  | | | |  |  |  |  |
| 2 | Atr-ERN09001 |  | | | |  | | | |  |  |  |  |
| 2 | Atr-ERN09002 |  | | | |  | | | |  |  |  |  |
| 2 | Atr-ERN09003 |  | | | |  | Vvi-Vitvi06g00699\_t001 |  |  |  |  |
| 2 | Atr-ERN09004 |  | | | |  | | | |  |  |  |  |
| 2 | Atr-ERN09005 |  | | | |  | | | |  |  |  |  |
| 2 | Atr-ERN09006 |  | | | |  | | | |  |  |  |  |
| 2 | Atr-ERN09007 |  | | | |  | | | |  |  |  |  |
| 2 | Atr-ERN09008 |  | | | |  | | | |  |  |  |  |
| 2 | Atr-ERN09009 |  | | | |  | | | |  |  |  |  |
| 2 | Atr-ERN09010 |  | | | |  | | | |  |  |  |  |
| 2 | Atr-ERN09011 |  | | | |  | | | |  |  |  |  |
| 2 | Atr-ERN09012 |  | | | |  | | | |  |  |  |  |
| 2 | Atr-ERN09013 |  | | | |  | | | |  |  |  |  |
| 2 | Atr-ERN09014 |  | | | |  | | | |  |  |  |  |
| 2 | Atr-ERN09015 |  | | | |  | | | |  |  |  |  |
| 2 | Atr-ERN09016 |  | | | |  | | | |  |  |  |  |
| 2 | Atr-ERN09017 |  | | | |  | Vvi-Vitvi06g00705\_t001 |  |  |  |  |
| 2 | Atr-ERN09018 |  | Vvi-Vitvi13g00112\_t001 |  | | | |  |  |  |  |
| 1 | Atr-ERN09019 |  |  |  | | | |  |  |  |  |
| 1 | Atr-ERN09020 |  |  |  | | | |  |  |  |  |
| 1 | Atr-ERN09021 |  |  |  | | | |  |  |  |  |
| 1 | Atr-ERN09022 |  |  |  | | | |  |  |  |  |
| 1 | Atr-ERN09023 |  |  |  | | | |  |  |  |  |
| 1 | Atr-ERN09024 |  |  |  | Vvi-Vitvi06g00720\_t001 |  |  |  |  |
| 1 | Atr-ERN09025 |  |  |  | | | |  |  |  |  |
| 1 | Atr-ERN09026 |  |  |  | Vvi-Vitvi06g00722\_t001 |  |  |  |  |
| 0 | Atr-ERN09027 |  |  |  |  |  |  |
