# Supplementary material for: Genome-wide identification, characterization and gene expression of BES1 transcription factor family in grapevine (Vitis vinifera L.)
Source: Sci Rep. 2023 Jan 5;13:240. doi: 10.1038/s41598-022-24407-y (PMC9816167; doi:10.1038/s41598-022-24407-y)
Supplement: Supplementary file 3 — Supplementary Information. [file 41598_2022_24407_MOESM3_ESM.zip › Vvi_Atr/Vitis_vinifera.PN40024.v4.dna_sm.toplevel.fa.vs.Amborella_trichopoda.AMTR1.0.dna_sm.toplevel.fa.html/Atr-AmTr_v1.0_scaffold00154.html]

|  |  |  |  |  |  |  |  |  |  |  |  |  |  |
| --- | --- | --- | --- | --- | --- | --- | --- | --- | --- | --- | --- | --- | --- |
| Duplication depth | Reference chromosome | Collinear blocks | | | | | | | | | | | |
| 0 | Atr-ERN07507 |  |  |  |  |  |  |
| 1 | Atr-ERN07508 |  | Vvi-Vitvi01g00043\_t001 |  |  |  |  |  |
| 1 | Atr-ERN07509 |  | | | |  |  |  |  |  |
| 1 | Atr-ERN07510 |  | | | |  |  |  |  |  |
| 2 | Atr-ERN07511 |  | | | |  | Vvi-Vitvi14g01387\_t001 |  |  |  |  |
| 3 | Atr-ERN07512 |  | Vvi-Vitvi01g00041\_t001 |  | | | |  | Vvi-Vitvi17g00711\_t001 |  |  |  |
| 3 | Atr-ERN07513 |  | | | |  | | | |  | Vvi-Vitvi17g01512\_t001 |  |  |  |
| 3 | Atr-ERN07514 |  | | | |  | | | |  | | | |  |  |  |
| 3 | Atr-ERN07515 |  | | | |  | | | |  | | | |  |  |  |
| 3 | Atr-ERN07516 |  | | | |  | | | |  | | | |  |  |  |
| 3 | Atr-ERN07517 |  | | | |  | | | |  | Vvi-Vitvi17g00712\_t001 |  |  |  |
| 3 | Atr-ERN07518 |  | Vvi-Vitvi01g00039\_t004 |  | | | |  | | | |  |  |  |
| 3 | Atr-ERN07519 |  | | | |  | Vvi-Vitvi14g01389\_t001 |  | Vvi-Vitvi17g00713\_t001 |  |  |  |
| 3 | Atr-ERN07520 |  | | | |  | Vvi-Vitvi14g01390\_t001 |  | | | |  |  |  |
| 3 | Atr-ERN07521 |  | | | |  | | | |  | | | |  |  |  |
| 3 | Atr-ERN07522 |  | | | |  | | | |  | | | |  |  |  |
| 3 | Atr-ERN07523 |  | | | |  | Vvi-Vitvi14g04527\_t001 |  | | | |  |  |  |
| 3 | Atr-ERN07524 |  | | | |  | Vvi-Vitvi14g01391\_t002 |  | | | |  |  |  |
| 3 | Atr-ERN07525 |  | | | |  | | | |  | | | |  |  |  |
| 3 | Atr-ERN07526 |  | Vvi-Vitvi01g01840\_t001 |  | Vvi-Vitvi14g02938\_t001 |  | Vvi-Vitvi17g00719\_t001 |  |  |  |
| 3 | Atr-ERN07527 |  | | | |  | | | |  | | | |  |  |  |
| 3 | Atr-ERN07528 |  | | | |  | | | |  | | | |  |  |  |
| 3 | Atr-ERN07529 |  | | | |  | | | |  | | | |  |  |  |
| 3 | Atr-ERN07530 |  | | | |  | | | |  | | | |  |  |  |
| 3 | Atr-ERN07531 |  | | | |  | | | |  | | | |  |  |  |
| 3 | Atr-ERN07532 |  | | | |  | | | |  | | | |  |  |  |
| 3 | Atr-ERN07533 |  | | | |  | | | |  | | | |  |  |  |
| 3 | Atr-ERN07534 |  | | | |  | | | |  | | | |  |  |  |
| 3 | Atr-ERN07535 |  | Vvi-Vitvi01g00038\_t001 |  | Vvi-Vitvi14g01392\_t001 |  | | | |  |  |  |
| 3 | Atr-ERN07536 |  | | | |  | | | |  | | | |  |  |  |
| 3 | Atr-ERN07537 |  | | | |  | | | |  | | | |  |  |  |
| 3 | Atr-ERN07538 |  | | | |  | | | |  | | | |  |  |  |
| 3 | Atr-ERN07539 |  | | | |  | | | |  | Vvi-Vitvi17g01521\_t001 |  |  |  |
| 2 | Atr-ERN07540 |  | | | |  | | | |  |  |  |  |
| 2 | Atr-ERN07541 |  | | | |  | | | |  |  |  |  |
| 3 | Atr-ERN07542 |  | Vvi-Vitvi01g00037\_t001 |  | Vvi-Vitvi14g01394\_t001 |  | Vvi-Vitvi17g01448\_t001 |  |  |  |
| 3 | Atr-ERN07543 |  | | | |  | | | |  | | | |  |  |  |
| 3 | Atr-ERN07544 |  | | | |  | | | |  | | | |  |  |  |
| 3 | Atr-ERN07545 |  | | | |  | | | |  | | | |  |  |  |
| 3 | Atr-ERN07546 |  | | | |  | | | |  | | | |  |  |  |
| 3 | Atr-ERN07547 |  | | | |  | | | |  | | | |  |  |  |
| 3 | Atr-ERN07548 |  | | | |  | | | |  | | | |  |  |  |
| 3 | Atr-ERN07549 |  | | | |  | | | |  | | | |  |  |  |
| 3 | Atr-ERN07550 |  | | | |  | | | |  | | | |  |  |  |
| 3 | Atr-ERN07551 |  | | | |  | | | |  | Vvi-Vitvi17g00497\_t001 |  |  |  |
| 3 | Atr-ERN07552 |  | Vvi-Vitvi01g00036\_t002 |  | Vvi-Vitvi14g01395\_t001 |  | Vvi-Vitvi17g00496\_t002 |  |  |  |
| 3 | Atr-ERN07553 |  | | | |  | | | |  | | | |  |  |  |
| 3 | Atr-ERN07554 |  | | | |  | Vvi-Vitvi14g01398\_t001 |  | Vvi-Vitvi17g00495\_t001 |  |  |  |
| 3 | Atr-ERN07555 |  | Vvi-Vitvi01g00035\_t001 |  | | | |  | | | |  |  |  |
| 3 | Atr-ERN07556 |  | | | |  | | | |  | | | |  |  |  |
| 3 | Atr-ERN07557 |  | | | |  | | | |  | | | |  |  |  |
| 3 | Atr-ERN07558 |  | | | |  | Vvi-Vitvi14g01400\_t001 |  | | | |  |  |  |
| 3 | Atr-ERN07559 |  | | | |  | Vvi-Vitvi14g01401\_t001 |  | Vvi-Vitvi17g00494\_t001 |  |  |  |
| 3 | Atr-ERN07560 |  | | | |  | Vvi-Vitvi14g01402\_t001 |  | Vvi-Vitvi17g00493\_t003 |  |  |  |
| 3 | Atr-ERN07561 |  | | | |  | | | |  | | | |  |  |  |
| 3 | Atr-ERN07562 |  | | | |  | | | |  | Vvi-Vitvi17g00490\_t001 |  |  |  |
| 2 | Atr-ERN07563 |  | | | |  | | | |  |  |  |  |
| 2 | Atr-ERN07564 |  | | | |  | | | |  |  |  |  |
| 2 | Atr-ERN07565 |  | | | |  | | | |  |  |  |  |
| 2 | Atr-ERN07566 |  | Vvi-Vitvi01g00034\_t001 |  | Vvi-Vitvi14g01403\_t001 |  |  |  |  |
| 0 | Atr-ERN07567 |  |  |  |  |  |  |
| 0 | Atr-ERN07568 |  |  |  |  |  |  |
| 0 | Atr-ERN07569 |  |  |  |  |  |  |
| 0 | Atr-ERN07570 |  |  |  |  |  |  |
| 0 | Atr-ERN07571 |  |  |  |  |  |  |
| 0 | Atr-ERN07572 |  |  |  |  |  |  |
