# Supplementary material for: Genome-wide identification, characterization and gene expression of BES1 transcription factor family in grapevine (Vitis vinifera L.)
Source: Sci Rep. 2023 Jan 5;13:240. doi: 10.1038/s41598-022-24407-y (PMC9816167; doi:10.1038/s41598-022-24407-y)
Supplement: Supplementary file 3 — Supplementary Information. [file 41598_2022_24407_MOESM3_ESM.zip › Vvi_Atr/Vitis_vinifera.PN40024.v4.dna_sm.toplevel.fa.vs.Amborella_trichopoda.AMTR1.0.dna_sm.toplevel.fa.html/Atr-AmTr_v1.0_scaffold00155.html]

|  |  |  |  |  |  |  |  |  |  |  |  |  |  |
| --- | --- | --- | --- | --- | --- | --- | --- | --- | --- | --- | --- | --- | --- |
| Duplication depth | Reference chromosome | Collinear blocks | | | | | | | | | | | |
| 0 | Atr-ERN07646 |  |  |  |  |  |  |
| 0 | Atr-ERN07647 |  |  |  |  |  |  |
| 0 | Atr-ERN07648 |  |  |  |  |  |  |
| 0 | Atr-ERN07649 |  |  |  |  |  |  |
| 0 | Atr-ERN07650 |  |  |  |  |  |  |
| 0 | Atr-ERN07651 |  |  |  |  |  |  |
| 0 | Atr-ERN07652 |  |  |  |  |  |  |
| 0 | Atr-ERN07653 |  |  |  |  |  |  |
| 0 | Atr-ERN07654 |  |  |  |  |  |  |
| 0 | Atr-ERN07655 |  |  |  |  |  |  |
| 0 | Atr-ERN07656 |  |  |  |  |  |  |
| 0 | Atr-ERN07657 |  |  |  |  |  |  |
| 0 | Atr-ERN07658 |  |  |  |  |  |  |
| 0 | Atr-ERN07659 |  |  |  |  |  |  |
| 0 | Atr-ERN07660 |  |  |  |  |  |  |
| 0 | Atr-ERN07661 |  |  |  |  |  |  |
| 0 | Atr-ERN07662 |  |  |  |  |  |  |
| 0 | Atr-ERN07663 |  |  |  |  |  |  |
| 0 | Atr-ERN07664 |  |  |  |  |  |  |
| 0 | Atr-ERN07665 |  |  |  |  |  |  |
| 0 | Atr-ERN07666 |  |  |  |  |  |  |
| 0 | Atr-ERN07667 |  |  |  |  |  |  |
| 0 | Atr-ERN07668 |  |  |  |  |  |  |
| 1 | Atr-ERN07669 |  | Vvi-Vitvi10g00947\_t001 |  |  |  |  |  |
| 1 | Atr-ERN07670 |  | | | |  |  |  |  |  |
| 1 | Atr-ERN07671 |  | | | |  |  |  |  |  |
| 1 | Atr-ERN07672 |  | Vvi-Vitvi10g00944\_t001 |  |  |  |  |  |
| 2 | Atr-ERN07673 |  | Vvi-Vitvi10g00941\_t002 |  | Vvi-Vitvi13g00603\_t001 |  |  |  |  |
| 2 | Atr-ERN07674 |  | Vvi-Vitvi10g01915\_t001 |  | Vvi-Vitvi13g00604\_t001 |  |  |  |  |
| 2 | Atr-ERN07675 |  | Vvi-Vitvi10g00940\_t001 |  | | | |  |  |  |  |
| 2 | Atr-ERN07676 |  | | | |  | | | |  |  |  |  |
| 2 | Atr-ERN07677 |  | Vvi-Vitvi10g01912\_t001 |  | | | |  |  |  |  |
| 2 | Atr-ERN07678 |  | | | |  | | | |  |  |  |  |
| 2 | Atr-ERN07679 |  | | | |  | | | |  |  |  |  |
| 2 | Atr-ERN07680 |  | | | |  | Vvi-Vitvi13g00605\_t001 |  |  |  |  |
| 2 | Atr-ERN07681 |  | Vvi-Vitvi10g00937\_t001 |  | | | |  |  |  |  |
| 1 | Atr-ERN07682 |  |  |  | | | |  |  |  |  |
| 1 | Atr-ERN07683 |  |  |  | | | |  |  |  |  |
| 1 | Atr-ERN07684 |  |  |  | | | |  |  |  |  |
| 1 | Atr-ERN07685 |  |  |  | | | |  |  |  |  |
| 1 | Atr-ERN07686 |  |  |  | | | |  |  |  |  |
| 1 | Atr-ERN07687 |  |  |  | | | |  |  |  |  |
| 1 | Atr-ERN07688 |  |  |  | | | |  |  |  |  |
| 2 | Atr-ERN07689 |  | Vvi-Vitvi10g00914\_t001 |  | | | |  |  |  |  |
| 2 | Atr-ERN07690 |  | Vvi-Vitvi10g00913\_t001 |  | Vvi-Vitvi13g00609\_t001 |  |  |  |  |
| 2 | Atr-ERN07691 |  | Vvi-Vitvi10g00911\_t001 |  | | | |  |  |  |  |
| 2 | Atr-ERN07692 |  | | | |  | | | |  |  |  |  |
| 2 | Atr-ERN07693 |  | | | |  | | | |  |  |  |  |
| 2 | Atr-ERN07694 |  | | | |  | | | |  |  |  |  |
| 2 | Atr-ERN07695 |  | Vvi-Vitvi10g00910\_t001 |  | | | |  |  |  |  |
| 2 | Atr-ERN07696 |  | Vvi-Vitvi10g00908\_t002 |  | | | |  |  |  |  |
| 2 | Atr-ERN07697 |  | Vvi-Vitvi10g00907\_t001 |  | | | |  |  |  |  |
| 2 | Atr-ERN07698 |  | | | |  | | | |  |  |  |  |
| 2 | Atr-ERN07699 |  | | | |  | | | |  |  |  |  |
| 2 | Atr-ERN07700 |  | | | |  | | | |  |  |  |  |
| 2 | Atr-ERN07701 |  | | | |  | Vvi-Vitvi13g02058\_t001 |  |  |  |  |
| 2 | Atr-ERN07702 |  | | | |  | | | |  |  |  |  |
| 2 | Atr-ERN07703 |  | | | |  | Vvi-Vitvi13g00614\_t001 |  |  |  |  |
| 1 | Atr-ERN07704 |  | | | |  |  |  |  |  |
| 1 | Atr-ERN07705 |  | | | |  |  |  |  |  |
| 1 | Atr-ERN07706 |  | Vvi-Vitvi10g00906\_t001 |  |  |  |  |  |
| 1 | Atr-ERN07707 |  | Vvi-Vitvi10g00905\_t001 |  |  |  |  |  |
| 0 | Atr-ERN07708 |  |  |  |  |  |  |
| 0 | Atr-ERN07709 |  |  |  |  |  |  |
| 0 | Atr-ERN07710 |  |  |  |  |  |  |
| 0 | Atr-ERN07711 |  |  |  |  |  |  |
