# Supplementary material for: Genome-wide identification, characterization and gene expression of BES1 transcription factor family in grapevine (Vitis vinifera L.)
Source: Sci Rep. 2023 Jan 5;13:240. doi: 10.1038/s41598-022-24407-y (PMC9816167; doi:10.1038/s41598-022-24407-y)
Supplement: Supplementary file 3 — Supplementary Information. [file 41598_2022_24407_MOESM3_ESM.zip › Vvi_Atr/Vitis_vinifera.PN40024.v4.dna_sm.toplevel.fa.vs.Amborella_trichopoda.AMTR1.0.dna_sm.toplevel.fa.html/Atr-AmTr_v1.0_scaffold00156.html]

|  |  |  |  |  |  |  |  |  |  |  |  |  |  |
| --- | --- | --- | --- | --- | --- | --- | --- | --- | --- | --- | --- | --- | --- |
| Duplication depth | Reference chromosome | Collinear blocks | | | | | | | | | | | |
| 0 | Atr-ERN08280 |  |  |  |  |  |  |
| 0 | Atr-ERN08281 |  |  |  |  |  |  |
| 0 | Atr-ERN08282 |  |  |  |  |  |  |
| 0 | Atr-ERN08283 |  |  |  |  |  |  |
| 0 | Atr-ERN08284 |  |  |  |  |  |  |
| 0 | Atr-ERN08285 |  |  |  |  |  |  |
| 0 | Atr-ERN08286 |  |  |  |  |  |  |
| 0 | Atr-ERN08287 |  |  |  |  |  |  |
| 0 | Atr-ERN08288 |  |  |  |  |  |  |
| 0 | Atr-ERN08289 |  |  |  |  |  |  |
| 0 | Atr-ERN08290 |  |  |  |  |  |  |
| 0 | Atr-ERN08291 |  |  |  |  |  |  |
| 0 | Atr-ERN08292 |  |  |  |  |  |  |
| 0 | Atr-ERN08293 |  |  |  |  |  |  |
| 0 | Atr-ERN08294 |  |  |  |  |  |  |
| 0 | Atr-ERN08295 |  |  |  |  |  |  |
| 0 | Atr-ERN08296 |  |  |  |  |  |  |
| 0 | Atr-ERN08297 |  |  |  |  |  |  |
| 0 | Atr-ERN08298 |  |  |  |  |  |  |
| 0 | Atr-ERN08299 |  |  |  |  |  |  |
| 0 | Atr-ERN08300 |  |  |  |  |  |  |
| 0 | Atr-ERN08301 |  |  |  |  |  |  |
| 0 | Atr-ERN08302 |  |  |  |  |  |  |
| 0 | Atr-ERN08303 |  |  |  |  |  |  |
| 0 | Atr-ERN08304 |  |  |  |  |  |  |
| 0 | Atr-ERN08305 |  |  |  |  |  |  |
| 0 | Atr-ERN08306 |  |  |  |  |  |  |
| 0 | Atr-ERN08307 |  |  |  |  |  |  |
| 0 | Atr-ERN08308 |  |  |  |  |  |  |
| 0 | Atr-ERN08309 |  |  |  |  |  |  |
| 0 | Atr-ERN08310 |  |  |  |  |  |  |
| 0 | Atr-ERN08311 |  |  |  |  |  |  |
| 0 | Atr-ERN08312 |  |  |  |  |  |  |
| 0 | Atr-ERN08313 |  |  |  |  |  |  |
| 0 | Atr-ERN08314 |  |  |  |  |  |  |
| 0 | Atr-ERN08315 |  |  |  |  |  |  |
| 0 | Atr-ERN08316 |  |  |  |  |  |  |
| 0 | Atr-ERN08317 |  |  |  |  |  |  |
| 0 | Atr-ERN08318 |  |  |  |  |  |  |
| 0 | Atr-ERN08319 |  |  |  |  |  |  |
| 0 | Atr-ERN08320 |  |  |  |  |  |  |
| 0 | Atr-ERN08321 |  |  |  |  |  |  |
| 0 | Atr-ERN08322 |  |  |  |  |  |  |
| 0 | Atr-ERN08323 |  |  |  |  |  |  |
| 0 | Atr-ERN08324 |  |  |  |  |  |  |
| 0 | Atr-ERN08325 |  |  |  |  |  |  |
