# Supplementary material for: Genome-wide identification, characterization and gene expression of BES1 transcription factor family in grapevine (Vitis vinifera L.)
Source: Sci Rep. 2023 Jan 5;13:240. doi: 10.1038/s41598-022-24407-y (PMC9816167; doi:10.1038/s41598-022-24407-y)
Supplement: Supplementary file 3 — Supplementary Information. [file 41598_2022_24407_MOESM3_ESM.zip › Vvi_Atr/Vitis_vinifera.PN40024.v4.dna_sm.toplevel.fa.vs.Amborella_trichopoda.AMTR1.0.dna_sm.toplevel.fa.html/Atr-AmTr_v1.0_scaffold00157.html]

|  |  |  |  |  |  |  |  |  |  |  |  |  |  |
| --- | --- | --- | --- | --- | --- | --- | --- | --- | --- | --- | --- | --- | --- |
| Duplication depth | Reference chromosome | Collinear blocks | | | | | | | | | | | |
| 0 | Atr-ERN07573 |  |  |  |  |  |  |
| 0 | Atr-ERN07574 |  |  |  |  |  |  |
| 0 | Atr-ERN07575 |  |  |  |  |  |  |
| 0 | Atr-ERN07576 |  |  |  |  |  |  |
| 0 | Atr-ERN07577 |  |  |  |  |  |  |
| 0 | Atr-ERN07578 |  |  |  |  |  |  |
| 0 | Atr-ERN07579 |  |  |  |  |  |  |
| 0 | Atr-ERN07580 |  |  |  |  |  |  |
| 0 | Atr-ERN07581 |  |  |  |  |  |  |
| 0 | Atr-ERN07582 |  |  |  |  |  |  |
| 0 | Atr-ERN07583 |  |  |  |  |  |  |
| 0 | Atr-ERN07584 |  |  |  |  |  |  |
| 0 | Atr-ERN07585 |  |  |  |  |  |  |
| 0 | Atr-ERN07586 |  |  |  |  |  |  |
| 0 | Atr-ERN07587 |  |  |  |  |  |  |
| 0 | Atr-ERN07588 |  |  |  |  |  |  |
| 0 | Atr-ERN07589 |  |  |  |  |  |  |
| 0 | Atr-ERN07590 |  |  |  |  |  |  |
| 0 | Atr-ERN07591 |  |  |  |  |  |  |
| 0 | Atr-ERN07592 |  |  |  |  |  |  |
| 0 | Atr-ERN07593 |  |  |  |  |  |  |
| 0 | Atr-ERN07594 |  |  |  |  |  |  |
| 0 | Atr-ERN07595 |  |  |  |  |  |  |
| 0 | Atr-ERN07596 |  |  |  |  |  |  |
| 0 | Atr-ERN07597 |  |  |  |  |  |  |
| 0 | Atr-ERN07598 |  |  |  |  |  |  |
| 0 | Atr-ERN07599 |  |  |  |  |  |  |
| 0 | Atr-ERN07600 |  |  |  |  |  |  |
| 0 | Atr-ERN07601 |  |  |  |  |  |  |
| 0 | Atr-ERN07602 |  |  |  |  |  |  |
| 0 | Atr-ERN07603 |  |  |  |  |  |  |
| 0 | Atr-ERN07604 |  |  |  |  |  |  |
| 0 | Atr-ERN07605 |  |  |  |  |  |  |
| 0 | Atr-ERN07606 |  |  |  |  |  |  |
| 0 | Atr-ERN07607 |  |  |  |  |  |  |
| 0 | Atr-ERN07608 |  |  |  |  |  |  |
| 0 | Atr-ERN07609 |  |  |  |  |  |  |
| 0 | Atr-ERN07610 |  |  |  |  |  |  |
| 0 | Atr-ERN07611 |  |  |  |  |  |  |
| 0 | Atr-ERN07612 |  |  |  |  |  |  |
| 0 | Atr-ERN07613 |  |  |  |  |  |  |
| 0 | Atr-ERN07614 |  |  |  |  |  |  |
| 0 | Atr-ERN07615 |  |  |  |  |  |  |
| 0 | Atr-ERN07616 |  |  |  |  |  |  |
| 0 | Atr-ERN07617 |  |  |  |  |  |  |
| 0 | Atr-ERN07618 |  |  |  |  |  |  |
| 0 | Atr-ERN07619 |  |  |  |  |  |  |
| 0 | Atr-ERN07620 |  |  |  |  |  |  |
| 0 | Atr-ERN07621 |  |  |  |  |  |  |
| 0 | Atr-ERN07622 |  |  |  |  |  |  |
| 0 | Atr-ERN07623 |  |  |  |  |  |  |
| 0 | Atr-ERN07624 |  |  |  |  |  |  |
