# Supplementary material for: Genome-wide identification, characterization and gene expression of BES1 transcription factor family in grapevine (Vitis vinifera L.)
Source: Sci Rep. 2023 Jan 5;13:240. doi: 10.1038/s41598-022-24407-y (PMC9816167; doi:10.1038/s41598-022-24407-y)
Supplement: Supplementary file 3 — Supplementary Information. [file 41598_2022_24407_MOESM3_ESM.zip › Vvi_Atr/Vitis_vinifera.PN40024.v4.dna_sm.toplevel.fa.vs.Amborella_trichopoda.AMTR1.0.dna_sm.toplevel.fa.html/Atr-AmTr_v1.0_scaffold00158.html]

|  |  |  |  |  |  |  |  |  |  |  |  |  |  |
| --- | --- | --- | --- | --- | --- | --- | --- | --- | --- | --- | --- | --- | --- |
| Duplication depth | Reference chromosome | Collinear blocks | | | | | | | | | | | |
| 0 | Atr-ERN10941 |  |  |  |  |  |  |
| 0 | Atr-ERN10942 |  |  |  |  |  |  |
| 0 | Atr-ERN10943 |  |  |  |  |  |  |
| 0 | Atr-ERN10944 |  |  |  |  |  |  |
| 0 | Atr-ERN10945 |  |  |  |  |  |  |
| 0 | Atr-ERN10946 |  |  |  |  |  |  |
| 0 | Atr-ERN10947 |  |  |  |  |  |  |
| 0 | Atr-ERN10948 |  |  |  |  |  |  |
| 0 | Atr-ERN10949 |  |  |  |  |  |  |
| 0 | Atr-ERN10950 |  |  |  |  |  |  |
| 0 | Atr-ERN10951 |  |  |  |  |  |  |
| 0 | Atr-ERN10952 |  |  |  |  |  |  |
| 0 | Atr-ERN10953 |  |  |  |  |  |  |
| 0 | Atr-ERN10954 |  |  |  |  |  |  |
| 0 | Atr-ERN10955 |  |  |  |  |  |  |
| 0 | Atr-ERN10956 |  |  |  |  |  |  |
| 0 | Atr-ERN10957 |  |  |  |  |  |  |
| 0 | Atr-ERN10958 |  |  |  |  |  |  |
| 0 | Atr-ERN10959 |  |  |  |  |  |  |
| 0 | Atr-ERN10960 |  |  |  |  |  |  |
| 0 | Atr-ERN10961 |  |  |  |  |  |  |
| 0 | Atr-ERN10962 |  |  |  |  |  |  |
| 0 | Atr-ERN10963 |  |  |  |  |  |  |
| 0 | Atr-ERN10964 |  |  |  |  |  |  |
