# Supplementary material for: Genome-wide identification, characterization and gene expression of BES1 transcription factor family in grapevine (Vitis vinifera L.)
Source: Sci Rep. 2023 Jan 5;13:240. doi: 10.1038/s41598-022-24407-y (PMC9816167; doi:10.1038/s41598-022-24407-y)
Supplement: Supplementary file 3 — Supplementary Information. [file 41598_2022_24407_MOESM3_ESM.zip › Vvi_Atr/Vitis_vinifera.PN40024.v4.dna_sm.toplevel.fa.vs.Amborella_trichopoda.AMTR1.0.dna_sm.toplevel.fa.html/Atr-AmTr_v1.0_scaffold00159.html]

|  |  |  |  |  |  |  |  |  |  |  |  |  |  |
| --- | --- | --- | --- | --- | --- | --- | --- | --- | --- | --- | --- | --- | --- |
| Duplication depth | Reference chromosome | Collinear blocks | | | | | | | | | | | |
| 0 | Atr-ERN12102 |  |  |  |  |  |  |
| 0 | Atr-ERN12103 |  |  |  |  |  |  |
| 0 | Atr-ERN12104 |  |  |  |  |  |  |
| 0 | Atr-ERN12105 |  |  |  |  |  |  |
| 0 | Atr-ERN12106 |  |  |  |  |  |  |
| 0 | Atr-ERN12107 |  |  |  |  |  |  |
| 0 | Atr-ERN12108 |  |  |  |  |  |  |
| 0 | Atr-ERN12109 |  |  |  |  |  |  |
| 0 | Atr-ERN12110 |  |  |  |  |  |  |
| 0 | Atr-ERN12111 |  |  |  |  |  |  |
| 0 | Atr-ERN12112 |  |  |  |  |  |  |
| 0 | Atr-ERN12113 |  |  |  |  |  |  |
| 0 | Atr-ERN12114 |  |  |  |  |  |  |
| 0 | Atr-ERN12115 |  |  |  |  |  |  |
| 0 | Atr-ERN12116 |  |  |  |  |  |  |
| 0 | Atr-ERN12117 |  |  |  |  |  |  |
| 0 | Atr-ERN12118 |  |  |  |  |  |  |
| 0 | Atr-ERN12119 |  |  |  |  |  |  |
| 0 | Atr-ERN12120 |  |  |  |  |  |  |
| 0 | Atr-ERN12121 |  |  |  |  |  |  |
| 0 | Atr-ERN12122 |  |  |  |  |  |  |
| 0 | Atr-ERN12123 |  |  |  |  |  |  |
| 0 | Atr-ERN12124 |  |  |  |  |  |  |
| 0 | Atr-ERN12125 |  |  |  |  |  |  |
| 0 | Atr-ERN12126 |  |  |  |  |  |  |
| 0 | Atr-ERN12127 |  |  |  |  |  |  |
| 0 | Atr-ERN12128 |  |  |  |  |  |  |
| 0 | Atr-ERN12129 |  |  |  |  |  |  |
| 0 | Atr-ERN12130 |  |  |  |  |  |  |
| 0 | Atr-ERN12131 |  |  |  |  |  |  |
| 0 | Atr-ERN12132 |  |  |  |  |  |  |
| 0 | Atr-ERN12133 |  |  |  |  |  |  |
| 0 | Atr-ERN12134 |  |  |  |  |  |  |
| 0 | Atr-ERN12135 |  |  |  |  |  |  |
| 0 | Atr-ERN12136 |  |  |  |  |  |  |
| 0 | Atr-ERN12137 |  |  |  |  |  |  |
