# Supplementary material for: Genome-wide identification, characterization and gene expression of BES1 transcription factor family in grapevine (Vitis vinifera L.)
Source: Sci Rep. 2023 Jan 5;13:240. doi: 10.1038/s41598-022-24407-y (PMC9816167; doi:10.1038/s41598-022-24407-y)
Supplement: Supplementary file 4 — Supplementary Table S1. [file 41598_2022_24407_MOESM4_ESM.docx]

| **Target genes** | **Forward sequence（5’ → 3’）** | **Reverse Sequence（5’ → 3’）** |
| --- | --- | --- |
| *VvBES1-1 (Vitvi10g00636)* | CATACAGCTGATGTTCCAATGG | CAAGTTCATCGGATCCACATTC |
| *VvBES1-2 (Vitvi04g01234)* | ATAGCTGCTAAGATATACGCGG | AACATAGAGCCTTCAAGACCTC |
| *VvBES1-3 (Vitvi19g00061)* | GAAGGAGAGGGAGAACAACAAT | CTTTGAGGACTTCGTTGTTGTC |
| *VvBES1-4 (Vitvi10g01901)* | AAAACCAAGATGAGAGAGAGGC | GAGGACTTCATTAATGTCAGCG |
| *VvBES1-5 (Vitvi18g00924)* | CCATAGCGGCAAAGATATATGC | GAAGTTCCTGCAATCTCAGTTG |
| *VvBES1-6 (Vitvi02g01232_t001)* | TGATATGAATGACGTTTTGGCG | GAAATCAAACCATCTGTGAGGG |
| *VvBES1-7 (Vitvi15g01128_t001)* | GTTGTGATGGGAGATAGAGGAG | GCTTCAATAAACCGTCTGGATC |
| *VvBES1-8 (Vitvi08g00772_t001)* | TGTCACGAAGTATCGCTTCCC | TCAGCATGTTTAGGGAGGTGG |

**Table S1**. The primers qRT-PCR quantification, the sequence information of genes was all acquired in EnsemblPlants (http://plants.ensembl.org/info/about/index.html).
